# Traces of a Primitive RNA Ring in Current Genomes

**DOI:** 10.3390/biology14050538

**Published:** 2025-05-12

**Authors:** Jacques Demongeot

**Affiliations:** Faculty of Medicine, University of Grenoble Alpes, AGEIS EA 7407, 38700 La Tronche, France; jacques.demongeot@univ-grenoble-alpes.fr

**Keywords:** origin of life, evolution, amino acid RNA interaction, nucleotide motifs, evolution

## Abstract

**Simple Summary:**

The search for RNA molecules at the origin of life is a major challenge for understanding the primordial stages of evolution. Here, we propose a small RNA that could have served as a catalyst for the formation of the first peptides and that has left numerous traces in current genomes.

**Abstract:**

(1) Background: Previous theoretical studies have provided arguments for the existence of a circular or hairpin RNA that could have served as a primitive informational and functional molecule at the origin of life. The present article consists of searching in current genomes for RNAs closest to this primitive RNA in terms of the occurrence of similar nucleotide motifs. (2) Methods: In searching for the smallest possible RNA capable of interacting with amino acids in the construction of the peptides of the primitive living world, we found a circular docosamer RNA molecule (length 22), which we called AL (for ALpha or Archetypal Loop). Then, we started to systematically track AL relics in current genomes in the form of motifs like pentamers or pairs of consecutive codons in common with AL. (3) Results: The sequence correspondence between AL and RNA sequences of organisms from different kingdoms of life (Archaea, Bacteria, and Eukarya) was found with high statistical significance, with a frequency gradient depending on both the antiquity of the species and the functional necessity of the genes. (4) Conclusions: Considering the suitability of AL as a candidate for being a primitive sequence, and the evolution of the different species considered, we can consider the AL RNA as a possible actor that favored the appearance of life on Earth.

## 1. Introduction

For 55 years, considerable efforts, both theoretical and experimental [1,2,3,4,5,6,7,8,9,10,11,12,13,14,15,16], have been made to demonstrate that before the emergence of the ribosomal machinery, molecular assemblies involving RNA molecules and amino acids could have given rise to the first peptides. In this article, we focus on an RNA molecule that is a candidate for the role of peptide catalyst at the origin of life. To find it, we selected four criteria from information theory and arrived at a unique RNA molecule, which we called AL (for ALpha or Archetypal Loop), in which we discovered 18 biological properties concerning its fit with the sequences and motifs of current genomes. Considered by Eigen [8,9] as the first “function” of life, proteinogenesis requires adequate production of peptides, an absolute necessity for evolution, as suggested in 1951 by Bernal, who said that this process could be favored on very fine clay deposits such as montmorillonite [10]. As a “polymerization catalyst”, montmorillonite would indeed have the consequence of decreasing the content of free amino acids following their polymerization [1,2,3,4,5,6,7]. In 1963, Ponnamperuma and his collaborators described the formation of ATP under possible primitive terrestrial conditions [15], and in 1995, they proposed the interactions between amino acids and nucleotides as a possible physicochemical basis for the origin of the genetic code [16]. All of these observations form the experimental corpus of the stereochemical theory of the origin of life [17,18,19]. Shapiro [20] admitted that “life began in a mixture of simple organic molecules, with possible participation of minerals”, but with Bernhardt [21] he was critical of the montmorillonite hypothesis, the alternative (or complement) being hydrothermal chimneys, i.e., cracks between tectonic plates with discharges of geothermally heated water [22]. Yarus, for his part, defended the idea of a catalytic role of simple RNA structures (like internal and bulge loops) promoting peptide bonds between amino acids [23,24,25], and recent work has emphasized the role of lipids in the very early stages of life [26,27,28,29]. Section 2 will present the materials and methods, followed by Section 3 with the results obtained and the discussion. The final section will be devoted to the conclusion and outlook.

## 2. Materials and Methods

### 2.1. Theoretical Criteria

Four theoretical criteria for a primordial RNA called AL (for ALpha or Archetypal Loop) to be a candidate for primordial catalysis of peptide biosynthesis have already been identified as optimal combinatorial properties and published [30,31,32,33,34,35,36,37,38,39,40,41,42,43,44,45,46,47,48,49,50,51,52,53,54,55,56,57,58,59,60]. The concept of ring for the structure of AL can be considered as a sort of circular consensus capable of embedding all possible genetic encodings, whose properties can be summarized as follows:

(1) The AL must satisfy the principle “be as short as possible and contain at least one codon per synonymy class of the genetic code”;
(2) The AL codon sequence obtained with overlap after 3 turns of its circular form (the theoretical ring) must begin with the start codon and end with the stop codon;
(3) The AL must have a hairpin configuration in balance with its circular shape, and this hairpin must have a minimum head length (3 nt) and a maximum number (9) of codon pairs;
(4) If multiple rings possess properties (1) to (3), they must have a single barycenter for classical inter-ring distances (circular Hamming, permutation, and editing distances), i.e., the AL ring.

### 2.2. AL-Codon-Counter, an Algorithm for Finding AL Traces in Current Genomes

**Figure 1:**
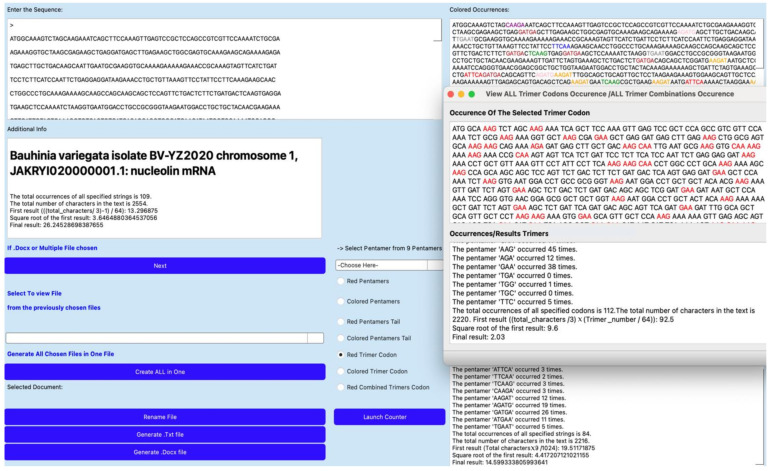
Screenshot of AL-Codon-Counter application showing the processing of the mRNA sequence of nucleolin gene from Bauhinia variegate. It shows an AL-proximity of 14.6 (resp. 26.2) due to pentamers (resp. pairs of codons) common between AL and the mRNA sequence.

The AL-Codon-Counter algorithm detects certain motifs in any RNA sequence that are common with AL, using, for example, a sliding window of five nucleotides to find pentameric motifs [60]. This algorithm systematically analyzes the sequence by shifting the window by one nucleotide at a time, thus capturing all possible pentamers. Once identified, the pentamers are mapped onto the RNA sequence, and the distances between consecutive occurrences of each motif are calculated (see Figure 1).

In Figure 1, the process of calculation includes handling overlapping pentamers and taking into account cases where pentamers are close to each other. The program calculates various distance measures, including the mean, median, standard deviation, and mode of distribution of these motifs, thus providing useful statistical information. In addition, pentamer analysis is extended to the study of evolutionary trends, particularly hypothetical remnants of the ancestral AL. 

The program’s statistical framework allows for comparison of pentamer distributions across multiple species, potentially revealing the evolutionary conservation of identified motifs. The 9 pentamers of the head of the hairpin form of AL all have at least one nucleotide linked to a nucleotide of the AGA head, which explains their fragility and their tendency to detach from AL. This also explains the fact that they are found in RNAs involved in evolution with a decreasing frequency as we move away from the origin of life. These 9 pentamers are AUUCA, UUCAA, UCAAG, CAAGA, AAGAU, AGAUG, GAUGA, AUGAA, and UGAAU. The proximity to AL P_PAL_ of an RNA sequence is obtained by calculating the number n_o_ of these 9 pentamers observed in this RNA sequence, and the expected number n_e_ equal to the expectation of a binomial distribution B(*n*,*p*), where *n* is the size of the RNA sequence minus 4 (i.e., the number of the possible pentamers in the sequence) and *p* = 9/1024 (the probability of observing all 9 pentamers by chance). The proximity P_PAL_ is then equal to twice the number of standard deviations (npq)^1/2^ in the interval [n_e_, n_0_], which is directly related to the probability P to observe n_o_ pentamers by chance. If we use the Gaussian approximation of the binomial distribution, for example, P_PAL_ = 8 corresponds to P < 5 × 10^−5^. More generally, P is obtained thanks to Gaussian Tables or calculated using the classical approximations of the Gaussian repartition function.

Another proximity called P_PAL_ Doublet can be calculated by counting the number of pairs (or doublets) of successive codons in a given mRNA sequence. If the occurrence of a bond between two amino acids is due to their reversible weak link to codons close on AL acting as a proto-ribosome, this pair could occur with a significant frequency in the mRNA. To show this, the number observed along the mRNA of pairs of AL codons (such as ATT, CAA, GAT, GAA, CCA, AGA, ACT or AAT, TAC, or AAG) is calculated, as well as its expected number, and then P_PAL_ Doublet is obtained in the same way as P_PAL._ Both will be calculated for species belonging to the three domains of life, Archaea, Bacteria, and Eukarya.

## 3. Results and Discussion

### 3.1. Presentation of AL

The discovery of the structure of AL occurred in four stages. In 1975, the first 22-nucleotide ring satisfying criteria (1) and (3) was discovered among the 4^22^ possible rings of length 22 [30,31]. This ring was called the cyclic code because it represented a non-degenerate summary of the complete genetic code with 64 codons. The ring had a short hairpin configuration with only six hybridized nucleotide pairs (in red and blue, nucleotides in green being not hybridized):

5′-**GCCAT TCAG A A** **TGGTA TCAG T A**

In 1983, a second ring called C3 (because it presented three zones of hybridization) was published [32] with a longer hairpin (eight hybridized pairs) starting with AUG and having UGA among its codons, but not at the end after three laps:

5′-**TGGTG****AA** **GA C G** **ACCAT AA CT T C**

In 1996, a third ring called AB (for Ancestral Basal) was discovered [33]. It has nine hybridized pairs, but they are not contiguous, and verified criteria (1) and (2):

5′-**GCCATTCAAG A** **TGGTAAGTAT C**

In 2004 [34], it was established that criterion (1) had no solution for a cycle of length 20 or 21, but only for a length of 22, for which there were 29,520 solutions (out of the 4^22^ possible solutions) containing only one repeated codon AXN, with X being G for 52% of the solutions. In 2006 [35], an attempt to explain the degeneracy of the genetic code from a non-degenerate cyclic code was proposed. In 2007 [36], it was finally shown that among the 29,520 solutions, only 25 cycles satisfied criteria (1) and (3) with the existence of a hairpin of nine or more nucleotides, of which only 19 encompassed both a start and stop codon, and 9 satisfied criterion (2). By calculating several distances (e.g., circular Hamming distance, permutation distance, and edit distance), the singular ring called AL (for ALpha or Archetypal Loop) ATGGTACTGCCATTCAAGATGA had a minimal average distance to the other 18, thus acting as their unique barycenter and satisfying all criteria (1) to (4):

5′-**TGCCATTCAA**                                     **G** **CATGGTAAGTA**

**Figure 2:**
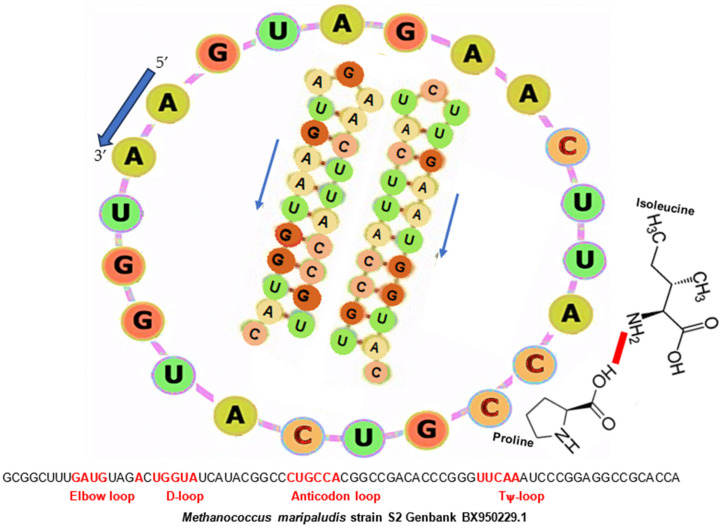
Circular form of AL fitting the loops (in red) of the tRNA-Gly^GCC^ of *Methanococcus maripaludis* (bottom). Inside the ring, the hairpin configurations of AL (left) and anti-AL (right), and outside the ring, a dipeptide whose synthesis could be favored by the proximity to AL of the amino acids constituting the dipeptide. Blue arrows indicate the 5′-3′ sense.

Then, the two configurations (circular and hairpin) of AL are summarized in Figure 2, where two hairpins appear inside the circular configuration, one corresponding to AL on the left and the other to the complement of AL (in the complementarity A/U, G/C), which also has a hairpin form on the right.

### 3.2. Structural Properties of AL

**Figure 3:**
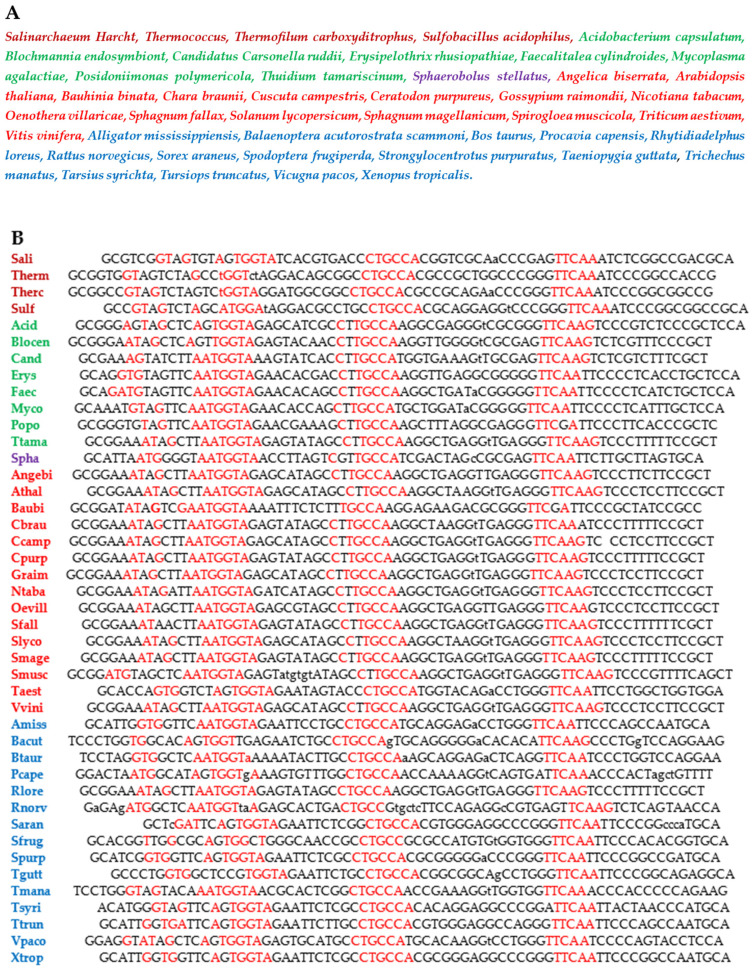
(**A**) List of species from the three domains of life: Archaea (mauve), Bacteria (green), Eukarya with Fungi (violet), Plants (red), and Animals (blue); (**B**) List of short names of the species in the same order as in (**A**) with the sequences of their transfer RNA, tRNA-Gly^GCC^.

In the following, some biological properties of the circular and hairpin forms of AL (Figure 2) will be explored, with reference to current genomes, in which some of these properties persist, such as the survival of common motifs between sequences of these genomes and the AL sequence. This is the case for the loops of the transfer RNA (D-loop, anticodon-loop, and T ψ-loop), which are well conserved during evolution, unlike the branches of the clover leaf structure of the tRNAs, which are very variable. Examples are given for various species of the three domains of life with their full names given on Figure 3A in various colors: Archaea (mauve), Bacteria (green), Eukarya with Fungi (violet), Plants (red), Animals (blue), and short names (in the same order) with the sequences of their transfer RNA tRNA-Gly^GCC^ given on Figure 3B.

**Figure 4:**
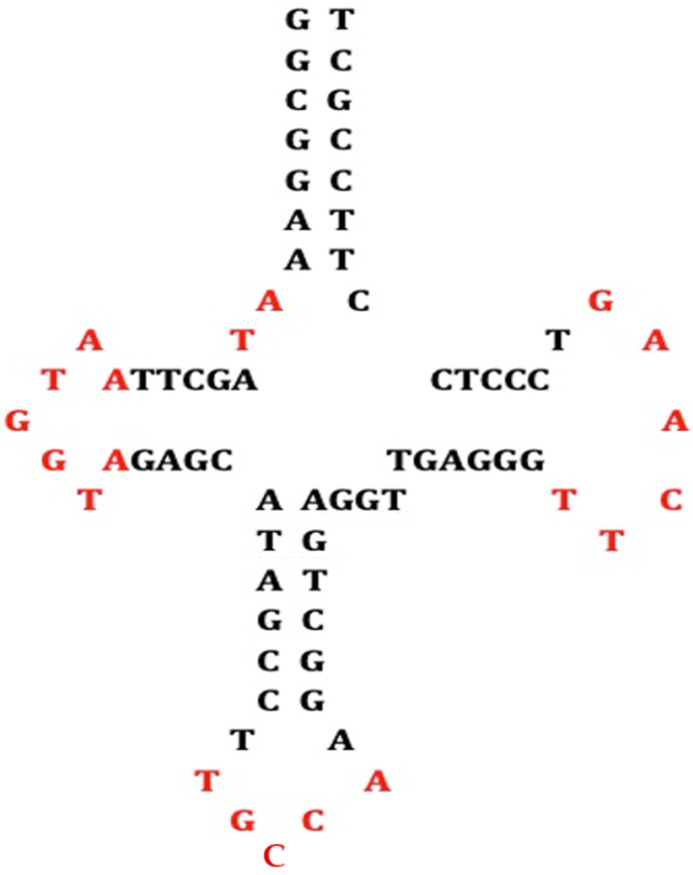
tRNA-Gly^GCC^ of *Arabis alpina* mitochondrion with loops in red [65,66].

**Figure 5:**
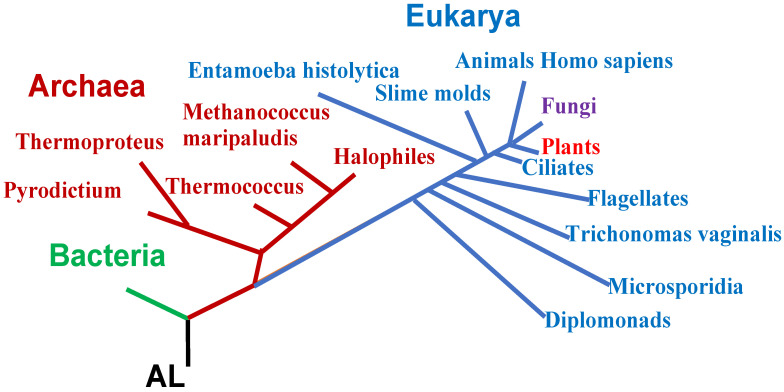
Phylogeny of the 3 domains of life—Archaea, Bacteria, and Eukarya—with indication of some species.

**Figure 6:**
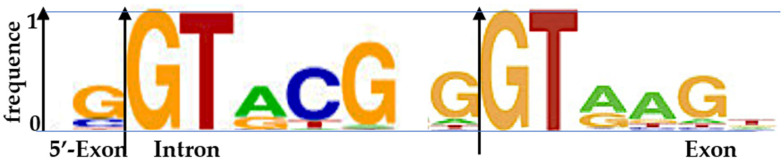
Exon/intron boundary (Left from [67], Right from [68]). The black arrow indicates the splicing site.

The other structural properties of AL correspond to the following optimal characteristics:

(1) All dinucleotides appear in AL, except CG, the least frequent dinucleotide in Archaea [61] and archaeal virus genomes [62]. Among the AL codons, 12 belong to the set of the 20 most frequent codons of chloroplasts [63].

(2) AL fits well the loops of tRNA-Gly^GCC^ of *Arabis alpina* mitochondrion [64], and more generally, the set of the most invariant nucleotides located in the loops (in red) of the tRNAs from the database GtRNAdb (Figure 4).

(3) Among the rings verifying criteria (1) to (4) m, AL is the closest in mean edit distance to all tRNAs of GtRNAdb species [65] belonging to the three domains of life—Archaea, Bacteria, and Eukarya—whose full and short names, along with the sequences of their tRNA-GlyGCC, are given in Figure 2 and the phylogenetic tree in Figure 5.

(4) A total of 50% of the tRNAs of GtRNAdb have the edit distance of their loops to AL less than 4 [37].

(5) The four domains of any tRNA (three loops and one articulation pivot) are ranked in their natural order inside AL.

(6) The average edit distance from AL to 20,000 different randomized versions of randomly repeated microRNAs preserving length 22 and nucleotide composition of AL is significantly larger [37] than the average edit distance of AL to the real microRNAs from the database miRBase [67].

(7) AL has at least 15 common nucleotides with the barycenter of these 20,000 randomly repeated microRNAs of length 22 and with the same nucleotide composition as AL, whose edit distance to AL is less than 7 [37].

(8) AL fragments match exon/intron boundary [67,68] with sequences 5′–3′ GGTAC or 3′–5′ TGAATGG (Figure 6).

(9) AL matches with Hamming and edit distances ≤ 2 with at least 43 tRNA-Gly from GtRNAdb from the three domains of life, Archaea, Bacteria, and Eukarya (Figure 4 and [69,70,71,72]).

(10) In the anticodon position, AL has “GCC” suggested as the first anticodon, because it “anticodes” for the simplest amino acid, glycine.

(11) AL aligns with the main articulation pivot “AUG” and allows the pairing TGG-ΨCA, needed between the D- and TΨ-loops of tRNAs for their 3D folding.

(12) AL matches well with many non-coding genomes from viral origin [38].

(13) AL matches well with many microRNAs [40], IRE and YUNR loops [42], as well as circular RNAs [42].

(14) AL contains all the most unexpected dimers twice, as defined by P.P. Slonimski [73].

(15) There is experimental evidence of direct RNA–amino acid interactions with AL-pentamers GCCAU [74] and AUGGU [75,76,77,78].

(16) The CRISp-R cas9 system shows in the guide RNA sequences the occurrence of AL-heptamers like GAAUGGU [79] and AAGAUGA [80].

(17) Complete genome of one the oldest bacteria, *Cyanobacterium aponinum*, contains a significant proportion of AL-codons from the set {CCA, ATT, CAA, AAG, GAT, AGA, GAA, AAT}, such as the distance between observed and expected numbers of such codons is more than 212 standard deviations (cf. Supplementary Material S1), and complete genomes of *Methanococcus maripaludis* (Archaea), *Dojkabacteria bacterium* (Bacteria), *Clitoria ternatea* plasmid, and *Oenothera villaricae* chloroplast (Plants), and mitochondrion of *Jaculus jaculus* (mammal) have their proximity P_PAL_ Doublets in decreasing order in evolution, i.e., 312.5, 224.1, 93, 92.4, and 4.7 (cf. Supplementary Material S2).

(18) The AL heptamer TCAAGAT is part of the palindromes located upstream of replicase genes in *Rhodobacterales* repABC-9 replicons, and in replication units of the alphaproteobacterial plasmids [81].

(19) Twelve hexadecameric peptide sequences of 16 amino acids from MVLPFKMNGTAIQDEW to IQDEWYCHSRMVLPFK corresponding to 16 successive codons without overlap on AL (see Figure 7 Top) are observed in 332 proteins with a probability of observing that by chance equal to 4 × 10^−12^ ± 3 × 10^−6^, these proteins having been selected by NCBI Blast [66] from 117,262,330 protein sequences with a total number of 42,988,570,095 amino acids. Among these 332 proteins, many come from extremophiles of the *Rhodobacterales* family, like *Roseivivax marinus*, *Ponticoccus litoralis*, *Thiobacimonas profunda*, and *Tropicibacter naphthalenivorans*.

### 3.3. Functional Properties of AL

The circular form of AL hybridizes with its complement anti-AL, which exhibits the same stable hairpin characteristics (identical to AL hairpin, except for the head), in equilibrium with a circular form capable of restoring AL by the same process (Figure 3). The main function of AL could have been that of a “protoribosome” favoring peptide bonds between amino acids interacting with its codons (Figure 7), as predicted by Ponnamperuma [16] and experimented with first by Tamura and Schimmel [75,76,77,78] and Yarus [25].

**Figure 8:**
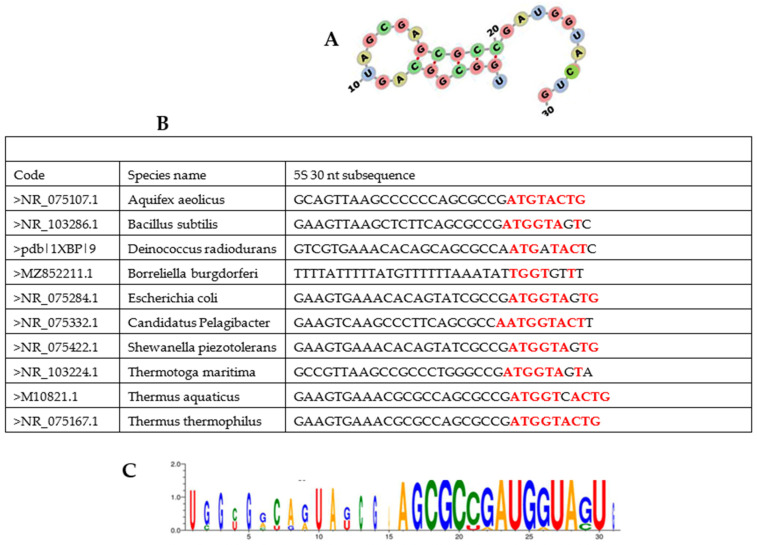
(**A**) Consensus third quarter of 5S rRNA sequences from 10 bacteria with weak nucleotides (A and T/U) flanked by strong nucleotides (G and C); (**B**) third quarter of the sequence of 5S rRNA with fragment of the tail part of AL (in red); (**C**) Consensus sequence calculated for 26 Bacteria and Archaea.

Traces of AL in current ribosomes can be found by considering the 5S rRNA of 10 bacteria (Figure 8 and [82]). The third quarter of the sequence is folded like a hairpin, and the tail of this hairpin is similar to a fragment of the tail part of the AL hairpin (in red in the table in Figure 8). Eigen [8,9] demonstrated that the early genetic code (PGC) consisted of nucleotides with the pattern RNY, where R represents purines (A/G), Y pyrimidines (C/U), and N any nucleotide (A/C/G/U), and that this RNY pattern was frequent in ribosomal RNA subunit 5S (5S rRNA) for more than 200 varied species [83]. On Figure 8, the 30 nt third quarter of the 5S rRNAs of 10 bacteria shows the RNY-encoded pattern, namely on the tail of its hairpin configuration (Figure 8A), which corresponds to the tail part of the AL hairpin, which has also codons with RNY patterns.

### 3.4. Searching for AL Motifs in Current Genomes

The four basic functional activities with their main proteins considered in the following correspond to membrane transport (ATPase, translocase), proteolysis (FtsH), translation (ribosomal RNAs and proteins, and aminoacyl-tRNA ligases), and RNA synthesis (RNA polymerase, helicase, and gyrase) [83,84,85]. The AL RNA is capable of replication as in a “quine” informatics program, leaving functional traces in the current mRNAs of these proteins. The eight pentamers at the head of the hairpin form of AL all possess at least one nucleotide linked with a nucleotide of the AGA head, which causes their fragility and the fact that they are observed in the RNAs of many species during evolution with a decreasing frequency as we move away from the origin of life. These pentamers are the following: AUUCA, UUCAA, UCAAG, CAAGA, AAGAU, AGAUG, GAUGA, AUGAA, and UGAAU. The proximities to AL, P_PAL_, and P_PAL_ Doublet are calculated in [42] as one for tRNA-Gly and mRNA sequences of the genes of gyrase, helicase, translocase, ATPase, RNA polymerase, Gly-tRNA ligase, PFK, FtsH, and rprotein L18 for five species, from the oldest to the youngest: Methanococcus maripaludis (Mm), Trichomonas vaginalis (Tri), Entamoeba histolytica (Ent), Saccharomyces cerevisiae (SC), and Homo sapiens (HS) (see Supplementary Material S3).

Among the five species listed in Table 1—Methanococcus maripaludis (Mm), Trichomonas vaginalis (Tri), Entameoba histolytica (Ent), Saccharomyces cerevisiae (SC), and Homo sapiens (HS)—the most frequent pairs of consecutive codons in the mRNA of their gyrase have been calculated (Table 2). These calculations show that the most frequent are those corresponding to identical or close (but without overlap) AL codons, which correspond to hydrophilic amino acids (GAA-GAA, GAA-GAT, GAA-GAT, GAA-AGA, and GAT-GAA) and a pair of codons corresponding to a pair of hydrophobic and hydrophilic acids (ATT-GAA). This observation reinforces the hypothesis of the primordial catalytic role of AL in favoring peptide synthesis at the origin of life.

**Figure 9:**
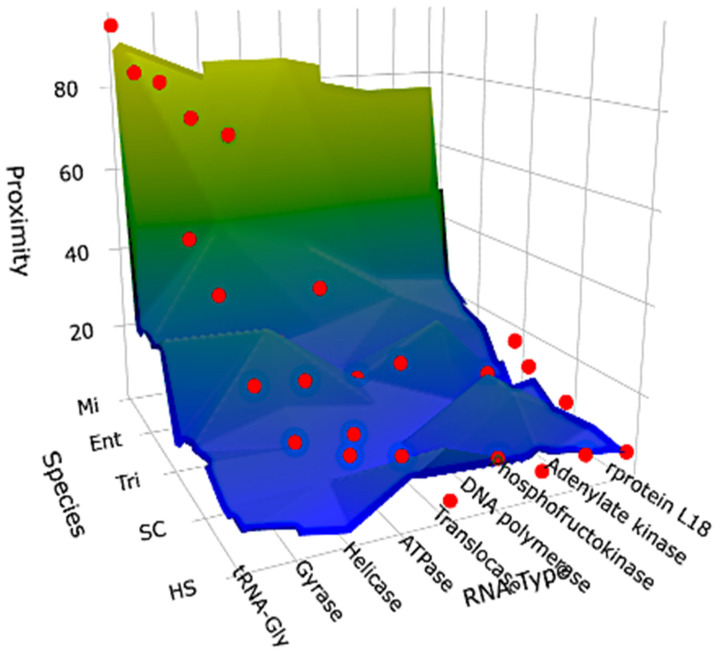
Surface representing the data of Table 1.

From Table 1 and Table 2, it can be considered that AL could belong to a family of ancient RNAs made from diverse RNA types involved at the Origin of Life (OL), these OL-RNAs close to AL, such as riboswitches, ribozymes, rRNAs, tRNAs, circRNAs and mRNAs of essential proteins, that are presumably close to ancestral RNAs. In perspectives, the data summarized in Figure 9 show the double dependency of the proximity to AL on the seniority on the species axis and of the functional necessity on the RNA-axis. The future work would concern more species from the 580,000 species of NCBI GenBank (formally described in October 2024) in order to confirm the tendencies shown in the present paper.

## 4. Conclusions

To support a network view of the origin of life, as discussed in 2018 by Fontecilla-Camps [86], Aguirre et al. [87], and Seligmann and Raoult [88], the AL RNA can be proposed as a key in the primitive machinery for building peptides (Figure 7). In this view, the boundary of this primordial functional « machine » able to build the first proteins could be defined as a peptide gradient boundary, centered on the “proto-nucleus” AL. The amino acids confinement around AL could indeed favor the occurrence of peptide bonds, the machine functioning as a “proto-ribosome” into a “proto-membrane”, close to a “proto-cell” with a network organization, each element favoring the survival of the others. This approach stands as a solution to a variational problem in that peptide synthesis favored by AL was necessary to repair the proto-cell membrane made of hydrophobic peptides and lipids, which reciprocally ensured the integrity of the proto-nucleus, and so protected it against denaturation. This mechanism has been supported for a century by different works, theoretical as well as experimental: for example, in 1926, H.J. Muller suggested that life began not as an enzyme, but as a gene [89]. The four amino acids glycine, aspartic acid, asparagine, and serine have been claimed to have been coded by the first four triplets of the early, evolving genetic code [8,9], constituting the first class of amino acids selected following the min–max principle: “mean mutation error M equals information I”, which uses the notion of information as proposed by Eigen [90,91]. In the theory of autopoiesis [92,93], the first living system is self-reproducing [94,95] and “continuously generates and specifies its own organization through its operation as a system of production of its own components, and does this in an endless turnover of components”. Statistical and theoretical arguments have been made about the role of the primitive RNAs in the progressive constitution of the genetic code [96,97,98,99,100,101,102,103,104,105].

As a singular prototype, this AL sequence should be useful to assess as a model matrix of future applications, ranging from synthetic biology used for producing proteins [106] to DNA computing [107]. As shown in this paper, the sequence AL and pentamers extracted from AL are indeed frequently retrieved as remnants in many genomes, notably in proteins essential for the protein translation and maintenance of the cell integrity (tRNA synthetases, RNA polymerases, tRNA nucleotidyl-transferases, lipids synthetases, CRISPR-Cas 9, etc.), which are considered essential building blocks for cell survival.

Further studies could experimentally investigate the AL RNA as a potential catalyst of peptide synthesis [39] and search for its role in building protein and cell worlds after the RNA world, and its possible role in consolidating the genetic code. This should be carried out in accordance with all the reference works establishing the present knowledge of the field, notably those concerning the evolution of the genetic code and of ancient ribo-nucleo-protein structures like the ribosome or RNAs like the ribozymes [108,109,110,111,112,113,114,115,116,117,118,119,120,121,122,123,124,125,126,127,128,129,130,131,132,133,134,135,136]. In particular, many peptide synthesis experiments have been carried out, with or without RNA template [137,138,139,140,141,142,143,144,145,146,147,148,149,150,151,152,153,154,155,156,157,158,159,160,161,162,163,164,165,166]. Those carried out with RNA template use sequences comparable to AL fragments, but in the reverse 3′–5′ direction [137,138,139,140,141,142,143,144,145,146,147,148,149,150,151,152,153,154,155,156,157,158,159,160,161]. In the future, it will be necessary to systematically compare the efficiency of these amino acid polymerizations: in solution without an RNA template [162,163,164,165,166], in solution with an RNA template (an attempt using microcalorimetry encountered some technical obstacles in the past [39]), and with or without an RNA template in solid substrate (such as montmorillonite, initially proposed by A. Katchalsky [1]). The research presented in these articles is very important for understanding the synthesis of the first peptides, but also the progressive structuring of the genetic code, from its ancient versions to the current genetic code, from biochemical–statistical experimentation [167] to phylogenomic explorations [168,169,170], which help to explain the transition from a possible early ‘operational’ version to the current ‘standard’ genetic code.

## Figures and Tables

**Figure 7:**
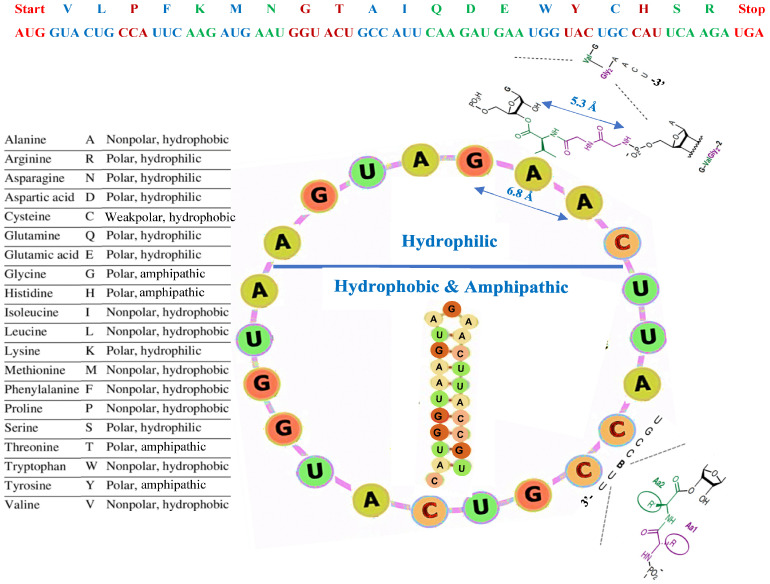
The evolutionary machinery. On top, the succession of codons without overlap and corresponding amino acids of AL. On the left, the amino acid polarities. On the right, AL in the catalytic function of its circular form, where the codons CCA for proline and AUU for isoleucine temporarily attract their amino acids through weak electromagnetic binding, promoting the creation of a strong peptide bond between them. The lower part of AL contains codons (Met, Trp, Gly, Val, Tyr, Thr, Leu, Cys, Ala, Pro, His, Ileu, Phe) corresponding to hydrophobic amino acids (Met, Val, Leu, Cys, Ala, Pro, Ileu, Phe) or amphipathic amino acids (His, Thr, Tyr). The upper part contains codons corresponding to hydrophilic amino acids (Ser, Gln, Lys, Arg, Asp, Glu, Asn), plus START (AUG) and STOP (UGA) codons. The representations of RNA–amino acid complexes are adapted from [6].

**Table 1:** n_o_ (resp. n_e_) is the observed (resp. expected) number of pentamers (in red) belonging to the head of the hairpin form of AL, and P_PAL_ (in blue) is twice the number of empirical standard deviations σ_e_ of n_e_ contained in interval [n_e_,n_0_]. Mean P_PAL_ (in red) is the expectation of P_PAL_ for the 5 observed species. The calculation is identical for the P_PAL_ Doublet (in blue). P_PAL_ and P_PAL_ Doublet are measures of the proximity of RNAs (tRNAs or mRNAs of the 5 observed species) to AL.

Molecule	Species	n_o_	N	n_e_	(s_e_)	P_PAL_ = $\frac{2(n_{0}-n_{e})}{\sigma_{e}}$	Mean P_PAL_	P_PAL_ Doublet	Mean P_PAL_ Doublet
rprotein L18	HS	14	639	5.7	(2.4)	7	10.1	4	8
	SC	19	536	4.7	(2.2)	13.1		7.4	
	Ent	9	536	4.7	(2.2)	4		5	
	Tri	25	562	5	(2.2)	18		7.1	
	Mm	8	227	2	(1.4)	8.4		16.2	
mRNA FtsH	HS	36	1918	16.9	(4.1)	9.3	11.4	1.3	13
	SC	53	2968	26.1	(5.1)	10.5		4.4	
	Ent	21	944	8.3	(2.9)	8.8		27.1	
	Tri	58	1598	14.1	(3.8)	23.4		23.4	
	Mm	23	1457	12.8	(3.6)	5.7		9	
mRNA PFK	HS	43	3036	26.7	(5.2)	6.3	10.6	8.6	13.5
	SC	79	2960	26	(5.1)	20.8		26.8	
	Ent	21	1413	12.5	(3.5)	4.8		2	
	Tri	25	1286	11.3	(3.4)	8.1		9.5	
	Mm	35	1385	12.2	(3.5)	13		20.7	
mRNA Gly-tRNA ligase	HS	34	2230	19.6	(4.4)	6.5	10.8	6.1	19.5
	SC	42	1856	16.3	(4)	12.7		20.1	
	Ent	44	1880	16.5	(4.1)	13.6		36.6	
	Tri	39	1946	17.1	(4.1)	10.6		8.7	
	Mm	36	1721	15.2	(3.9)	10.8		26	
mRNA DNA polymerase	HS	75	3959	34.8	(5.9)	5.5	13.5	8.6	18.9
	SC	30	1316	11.6	(3.4)	14.5		16	
	Ent	72	3194	28.1	(5.3)	10		11.7	
	Tri	29	1040	9.2	(3)	13.2		8.2	
	Mm	45	2351	20.7	(4.6)	10.7		18.2	
mRNA ATPase	HS	78	3414	30	(5.5)	17.5	15.7	19.1	21.6
	SC	42	1850	16.3	(4)	12.8		14.3	
	Ent	52	1832	16.1	(4)	17.9		30.1	
	Tri	35	1366	12	(3.5)	13.2		10.4	
	Mm	98	2978	26.2	(5.1)	28		39.4	
mRNA Translocase	HS	15	1027	9.1	(3)	4	17.9	4	19.1
	SC	133	4856	42.7	(6.5)	27.6		23	
	Ent	106	3002	26.4	(5.1)	31		38.1	
	Tri	33	1066	9.4	(3)	15.4		14.8	
	Mm	20	1325	11.7	(3.4)	4.9		15.3	
mRNA Helicase	HS	73	2716	23.9	(4.9)	20.1	20.5	16.4	19.7
	SC	110	4541	39.9	(6.3)	22.2		16.6	
	Ent	63	2573	22.6	(4.8)	17		30.9	
	Tri	46	1256	11.1	(3.3)	21		11.3	
	Mm	56	2236	19.6	(4.4)	22.2		23.2	
mRNA Gyrase	HS	138	5691	50	(7.1)	24.9	32.5	26.2	29.9
	SC	122	4283	37.7	(6.1)	27.4		34.6	
	Ent	176	4046	35.6	(6)	47		55.7	
	Tri	162	4376	38.5	(6.2)	39.8		33.6	
	Mm	66	1094	9.7	(3.1)	23.2		24	
tRNA-Gly	HS	18	22	0.19	(0.44)	81	84.6		
	SC	17	22	0.19	(0.44)	76.4			
	Ent	19	22	0.19	(0.44)	85.5			
	Tri	19	22	0.19	(0.44)	85.5			
	Mm	21	22	0.19	(0.44)	94.6			
AL		22	22	0.19	(0.44)	100	100		

**Table 2:** Pairs of consecutive codons observed more than 2 times at least once among the 5 species of Table 1. Doublets appearing more than μ+2σ=61.3 times (where µ = 29 is the expectation of the totals for all considered pairs and σ = 5.4 the corresponding standard deviation) are in red.

Doublet	Homo Sapiens	Saccharomyces	Entamoeba	Trichomonas	Methanococcus	Total
ATT ATT	3	8	20	0	5	36
ATT CAA	2	5	14	4	11	36
ATT GAT	3	11	12	0	8	34
ATT GAA	16	17	25	3	27	88
ATT CCA	4	2	2	6	7	21
ATT ACT	1	10	4	3	3	21
ATT AGA	3	5	4	3	1	16
ATT TAC	0	0	2	0	3	5
CAA ATT	6	4	9	0	1	20
CAA CAA	2	5	7	4	0	18
CAA GAT	7	6	4	5	0	22
CAA GAA	11	12	11	6	6	46
CAA CCA	0	0	4	0	0	4
CAA AGA	6	4	9	0	4	23
CAA TAC	2	6	1	0	0	9
CAA ACT	7	0	5	0	0	12
GAT ATT	9	14	21	0	9	53
GAT GAT	19	14	26	15	4	78
GAT GAA	22	23	35	16	26	122
GAT CCA	5	3	4	3	0	15
GAT AGA	5	2	9	1	4	21
GAT ACT	1	6	6	0	7	20
GAT CAA	1	2	6	0	1	10
GAT TAC	2	0	0	5	5	12
GAA ATT	9	6	27	2	7	51
GAA CAA	6	11	12	2	7	38
GAA GAT	20	21	33	14	14	102
GAA GAA	20	33	43	16	37	149
GAA CCA	3	9	11	6	2	31
GAA ACT	8	10	8	1	6	33
GAA AGA	8	10	7	3	7	35
GAA TAC	0	10	0	4	4	18
CCA ATT	2	3	7	4	6	22
CCA GAA	8	6	7	3	4	28
CCA AGA	8	0	3	4	1	16
CCA GAT	8	0	3	0	0	11
CCA ACT	6	0	6	0	0	12
ACT CAA	0	3	9	4	1	17
ACT GAT	4	0	7	0	4	15
ACT GAA	8	0	5	3	6	22
ACT ATT	2	6	9	0	2	19
ACT CCA	7	9	4	0	1	21
ACT TAC	1	0	0	0	2	3
ACT ACT	0	7	4		2	13
AGA ATT	3	5	7	1	5	21
AGA GAT	6	12	11	0	5	34
AGA GAA	13	11	12	9	20	65
AGA CAA	4	9	2	11	0	26
AGA AGA	7	6	7	0	4	24
AGA CCA	0	0	2	0	0	2
AGA TAC	1	0	0	0	4	5
TAC ATT	2	3	0	4	1	0
TAC GAT	1	5	1	0	6	13
TAC GAA	1	0	1	5	10	17
TAC TAC	3	0	0	3	2	8
TAC AGA	0	0	0	3	2	5
TAC CCA	3	0	1	1	5	19

## Data Availability

All data come from referenced public databases.

## Supplementary Materials

The following supporting information can be downloaded at: https://www.mdpi.com/article/10.3390/biology14050538/s1, Supplementary material Biology S1, Supplementary material Biology S2, and Supplementary material Biology S3.
