# Supplementary material for: Traces of a Primitive RNA Ring in Current Genomes
Source: Biology (Basel). 2025 May 12;14(5):538. doi: 10.3390/biology14050538 (PMC12109556; doi:10.3390/biology14050538)
Supplement: Supplementary file 1 [file biology-14-00538-s001.zip › biology-3598710-supplementary/Supplementary material Biology S3.pdf]

1) Methanococcus maripaludis

>NZ_DAIONM010000001.1:c1226615-1226543, Methanococcus maripaludis isolate, tRNA-Gly

GCGGCTTT**GATG**TAG**A**C**TGGTA**TCATACGGCC**CTGCCA**CGGCCGACACCCGGG**T**
TCAAATCCCGGAGGCCGCA

>NZ_DAIONM010000001.1:c934684-932690 Methanococcus maripaludis isolate, gyrase

---> Sequence

ATGGCTGAAGATACTAATGCGGTTGTCGAAGAAAGTTTATTTGACGAATTTAAAGAACACTCAAT
TTCAGAGTTTTTTAGGAAAAACAGGCACATGCTCGGTTACAGCGGTAGATTAAGAAGTATGACTA
CAATTGTACACGAACTTGTAACAAACAGCCTTGATGCATGTGAAGAATCTGAAATTCTTCCTGAA
ATTACGGTTGAAATCAAAAAAATAGGTTCTGAAACATTTGGAGTCAATATTGAAGATAACGGTC
CAGGAATTCCTCCAGAATTTGTTCCAAAAGTATTTGGTAAAATGCTTGCAGGGTCGAAATTACAC
AGGCTGGTCCAGTCAAGAGGTCAGCAGGGTATCGGGGCTGCAGGTGTATTATTATTTGCGCAGAT
GACAACCGGAAAGCCATTAAAAATTACAACTTCAACTGGAAACGGTACTATTTATGAAATGGAAA
TTAAAATGAGTATCGAGAAAAACGAAGGAGATGTAGTTTCAAGAAACACTCGAGAAGGATTTTG
GAGAGGAACTAGGGTTGAAGGAGAATTTAAAGATGTTACATACAATAGAAGAGAGCAAGGACCA
TTTGAATATTTAAGAAGGATTAGTCTTTCAGCACCGCACGCTAAAATTACTTTGGAAGATCCGGA
AGAAACAATTGTTTTTGAAAGAACTGTAAATGATATACCTGAAAGACCTGAAGTAATGAAACCA
CACCCATATGGGTTAACAACTGATGAATTACTTCACATTTCAAGAGTAACCGATTCTTCAAGAGT
TTCAAGCATGTTAAATAGTGAACTTTCAAGAGTCACCATGAAAAGAATTAAGGAACTTGAAGAA
TATGTTTTAAGAGATACCCTTCTTGAAAACTACCGTTCAAGTGTATTTTGGGATACAATTGTAAG
CTGTTACTTAAACTTTGATTTCACGAAATACTTTGATATATACGGCCATTATTTTGATAAAAAAG
AAATTGAAGATGTAAAACAGCTTATAAATGGTCTTCCTGAAGGATTGGGTGAATTAAAAACTTA
CTGTATGAAATATCTTGTAACCCAGTATTTGGTTCAAAAATTGGATGATGAAGAAATAAAAGAG
ATTAAAAATCATTTCAAGAAAAAACCAGAAAATTTCATGGAATACATGGATAAAAAATATTTGA
ATGCATCACTACTTGATGAATTCAGAAGAAAATTGAAAAACATTACAAAATCTCCTGCAGAATTT
GTAAAATCCCTCGTGGACCTTGCAATGGTTTCAGATACTGAGCTTGAAAAATACCGAAAAGCAAT
TAGGGATCTTTTAAAGAAAAATCCAAAAGAAATGACTTGGAAAGATTCTGAAGTCATTGTAAAT
GTTTTACAGGATATGGACTTTATGGCACCTTCAACAGTTGGATTAAGGCCAATTGGTGAGGAAAA
CATTGAAAAATCACTTAATACTTTGGAACCTGAGTTTTTAAAGACCCTGACAAGAAAACCTAAAA
CTTACAAAGGAGGAGTTCCTTTTGCAGTTGAAGTAGGTCTTGCTTATGGTGGAGAAGCTGGAAGA
AGTGGTGATGAGTCACGAAAGATGGAAATCATGAGATTTTCAAATCACGTACCATTACTTTTTGA
TACATCAGGTTGTGGTTTAACAAATGCTGTTAAAAGTGTTAACTGGAGAAGATACGGTCTTAGAA
ATGATGAAGATGCTCCCGTAACGGTATTTGTAAACCTTATTTCAACGCACATTCCATATACTTCA
GCAGGAAAACAGGCAATTGCATCAAGCAACGAAGAAAATGAGGAAATTTTCAACGAGATAAGGC
AGTCTTTAATGATATGTGCAAGAGAGCTTGAAAAGCACATCTCAAAATTAAGAAAAGAAAAAGA
AGAAGAACAGAAACGAAAATATGTTATGAAATATGCGGTAATTTTTGCAGAAGGGCTTGCAAGC
ATTACTGGAAGACCAAAAGAAGAAATAGAAGCAAATGTTGTTAATTTGCTTAGGTAA

---> Sequence Showing highlighted Existant Combined Trimers

ATG GCT GAA GAT ACT AAT GCG GTT GTC GAA GAA AGT TTA TTT GAC GAA TTT AAA GAA CAC TCA ATT TCA GAG TTT TTT AGG AAA AAC AGG CAC ATG CTC GGT TAC AGC GGT AGA TTA AGA AGT ATG ACT ACA ATT GTA CAC GAA CTT GTA ACA AAC AGC CTT GAT GCA TGT GAA GAA TCT GAA ATT CTT CCT GAA ATT ACG GTT GAA ATC AAA AAA ATA GGT TCT GAA ACA TTT GGA GTC AAT ATT GAA GAT AAC GGT CCA GGA ATT CCT CCA GAA TTT GTT CCA AAA GTA TTT GGT AAA ATG CTT GCA GGG TCG AAA TTA CAC AGG CTG GTC CAG TCA AGA GGT CAG CAG GGT ATC GGG GCT GCA GGT GTA TTA TTA TTT GCG CAG ATG ACA ACC GGA AAG CCA TTA AAA ATT ACA ACT TCA ACT GGA AAC GGT ACT ATT TAT GAA ATG GAA ATT AAA ATG AGT ATC GAG AAA AAC GAA GGA GAT GTA GTT TCA AGA AAC ACT CGA GAA GGA TTT TGG AGA GGA ACT AGG GTT GAA GGA GAA TTT AAA GAT GTT ACA TAC AAT AGA AGA GAG CAA GGA CCA TTT GAA TAT TTA AGA AGG ATT AGT CTT TCA GCA CCG CAC GCT AAA ATT ACT TTG GAA GAT CCG GAA GAA ACA ATT GTT TTT GAA AGA ACT GTA AAT GAT ATA CCT GAA AGA CCT GAA GTA ATG AAA CCA CAC CCA TAT GGG TTA ACA ACT GAT GAA TTA CTT CAC ATT TCA AGA GTA ACC GAT TCT TCA AGA GTT TCA AGC ATG TTA AAT AGT GAA CTT TCA AGA GTC ACC ATG AAA AGA ATT AAG GAA CTT GAA GAA TAT GTT TTA AGA GAT ACC CTT CTT GAA AAC TAC CGT TCA AGT GTA TTT TGG GAT ACA ATT GTA AGC TGT TAC TTA AAC TTT GAT TTC ACG AAA TAC TTT GAT ATA TAC GGC CAT TAT TTT GAT AAA AAA GAA ATT GAA GAT GTA AAA CAG CTT ATA AAT GGT CTT CCT GAA GGA TTG GGT GAA TTA AAA ACT TAC TGT ATG AAA TAT CTT GTA ACC CAG TAT TTG GTT CAA AAA TTG GAT GAT GAA GAA ATA AAA GAG ATT AAA AAT CAT TTC AAG AAA AAA CCA GAA AAT TTC ATG GAA TAC ATG GAT AAA AAA TAT TTG AAT GCA TCA CTA CTT GAT GAA TTC AGA AGA AAA TTG AAA AAC ATT ACA AAA TCT CCT GCA GAA TTT GTA AAA TCC CTC GTG GAC CTT GCA ATG GTT TCA GAT ACT GAG CTT GAA AAA TAC CGA AAA GCA ATT AGG GAT CTT TTA AAG AAA AAT CCA AAA GAA ATG ACT TGG AAA GAT TCT GAA GTC ATT GTA AAT GTT TTA CAG GAT ATG GAC TTT ATG GCA CCT TCA ACA GTT GGA TTA AGG CCA ATT GGT GAG GAA AAC ATT GAA AAA TCA CTT AAT ACT TTG GAA CCT GAG TTT TTA AAG ACC CTG ACA AGA AAA CCT AAA ACT TAC AAA GGA GGA GTT CCT TTT GCA GTT GAA GTA GGT CTT GCT TAT GGT GGA GAA GCT GGA AGA AGT GGT GAT GAG TCA CGA AAG ATG GAA ATC ATG AGA TTT TCA AAT CAC GTA CCA TTA CTT TTT GAT ACA TCA GGT TGT GGT TTA ACA AAT GCT GTT AAA AGT GTT AAC TGG AGA AGA TAC GGT CTT AGA AAT GAT GAA GAT GCT CCC GTA ACG GTA TTT GTA AAC CTT ATT TCA ACG CAC ATT CCA TAT ACT TCA GCA GGA AAA CAG GCA ATT GCA TCA AGC AAC GAA GAA AAT GAG GAA ATT TTC AAC GAG ATA AGG CAG TCT TTA ATG ATA TGT GCA AGA GAG CTT GAA AAG CAC ATC TCA AAA TTA AGA AAA GAA AAA GAA GAA GAA CAG AAA CGA AAA TAT GTT ATG AAA TAT GCG GTA ATT TTT GCA GAA GGG CTT GCA AGC ATT ACT GGA AGA CCA AAA GAA GAA ATA GAA GCA AAT GTT GTT AAT TTG CTT AGG TAA

Occurrences/Results 9 Pentamers

The pentamer 'ATTCA' occurred 1 times.
The pentamer 'TTCAA' occurred 13 times.
The pentamer 'TCAAG' occurred 9 times.
The pentamer 'CAAGA' occurred 8 times.
The pentamer 'AAGAT' occurred 9 times.
The pentamer 'AGATG' occurred 6 times.

The pentamer 'GATGA' occurred 6 times.
The pentamer 'ATGAA' occurred 9 times.
The pentamer 'TGAAT' occurred 5 times.
The total occurrences of all specified strings is 66.
The total number of characters in the text is 1991.
First result (Total characters *9 /1024): 17.5341796875
Square root of the first result: 4.187383393898868
Final result: 11.574249538056636

Occurrences/Results TRIMERS Codons

The pentamer 'ATT' occurred 28 times.
The pentamer 'CAA' occurred 2 times.
The pentamer 'GAT' occurred 27 times.
The pentamer 'GAA' occurred 65 times.
The pentamer 'CCA' occurred 13 times.
The pentamer 'ACT' occurred 17 times.
The pentamer 'AGA' occurred 26 times.
The pentamer 'TAC' occurred 11 times.
The total occurrences of all specified strings is 189.
The total number of characters in the text is 1992.
First result ((total_characters / 3) * (Trimer_number / 64)): 83
Square root of the first result: 9.1
Final result: 11.6

Occurrences/Results TRIMERS Combinations

The pentamer 'ATT ATT' occurred 0 times.
The pentamer 'ATT CAA' occurred 0 times.
The pentamer 'ATT GAT' occurred 0 times.
The pentamer 'ATT GAA' occurred 3 times.
The pentamer 'ATT CCA' occurred 1 times.
The pentamer 'ATT ACT' occurred 2 times.
The pentamer 'ATT AGA' occurred 0 times.
The pentamer 'ATT TAC' occurred 0 times.
The pentamer 'CAA ATT' occurred 0 times.
The pentamer 'CAA CAA' occurred 0 times.
The pentamer 'CAA GAT' occurred 0 times.
The pentamer 'CAA GAA' occurred 0 times.
The pentamer 'CAA CCA' occurred 0 times.
The pentamer 'CAA ACT' occurred 0 times.
The pentamer 'CAA AGA' occurred 0 times.
The pentamer 'CAA TAC' occurred 0 times.
The pentamer 'GAT ATT' occurred 0 times.
The pentamer 'GAT CAA' occurred 0 times.
The pentamer 'GAT GAT' occurred 1 times.
The pentamer 'GAT GAA' occurred 4 times.

The pentamer 'GAT CCA' occurred 0 times.
The pentamer 'GAT ACT' occurred 2 times.
The pentamer 'GAT AGA' occurred 0 times.
The pentamer 'GAT TAC' occurred 0 times.
The pentamer 'GAA ATT' occurred 5 times.
The pentamer 'GAA CAA' occurred 0 times.
The pentamer 'GAA GAT' occurred 5 times.
The pentamer 'GAA GAA' occurred 8 times.
The pentamer 'GAA CCA' occurred 0 times.
The pentamer 'GAA ACT' occurred 0 times.
The pentamer 'GAA AGA' occurred 2 times.
The pentamer 'GAA TAC' occurred 1 times.
The pentamer 'CCA ATT' occurred 1 times.
The pentamer 'CCA CAA' occurred 0 times.
The pentamer 'CCA GAT' occurred 0 times.
The pentamer 'CCA GAA' occurred 2 times.
The pentamer 'CCA CCA' occurred 0 times.
The pentamer 'CCA ACT' occurred 0 times.
The pentamer 'CCA AGA' occurred 0 times.
The pentamer 'CCA TAC' occurred 0 times.
The pentamer 'ACT ATT' occurred 1 times.
The pentamer 'ACT CAA' occurred 0 times.
The pentamer 'ACT GAT' occurred 1 times.
The pentamer 'ACT GAA' occurred 0 times.
The pentamer 'ACT CCA' occurred 0 times.
The pentamer 'ACT ACT' occurred 0 times.
The pentamer 'ACT AGA' occurred 0 times.
The pentamer 'ACT TAC' occurred 2 times.
The pentamer 'AGA ATT' occurred 1 times.
The pentamer 'AGA CAA' occurred 0 times.
The pentamer 'AGA GAT' occurred 1 times.
The pentamer 'AGA GAA' occurred 0 times.
The pentamer 'AGA CCA' occurred 1 times.
The pentamer 'AGA ACT' occurred 1 times.
The pentamer 'AGA AGA' occurred 3 times.
The pentamer 'AGA TAC' occurred 1 times.
The pentamer 'TAC ATT' occurred 0 times.
The pentamer 'TAC CAA' occurred 0 times.
The pentamer 'TAC GAT' occurred 0 times.
The pentamer 'TAC GAA' occurred 0 times.
The pentamer 'TAC CCA' occurred 0 times.
The pentamer 'TAC ACT' occurred 0 times.
The pentamer 'TAC AGA' occurred 0 times.

The pentamer 'TAC TAC' occurred 0 times.
The total occurrences of all specified strings is 49.
The total number of characters in the text is 1991.
First result (((total_characters/ 3) -1) / 64): 10.375
Square root of the first result: 3.2210246816812815
Final result: 11.991525622162841

>CP002913.1:446133-447749 Methanococcus maripaludis isolate X1, helicase

---> Sequence

ATGGAAAGTTTTAAAAATTTAGGTCTATCTGACGAGATATTAGAAGCATTAGAAAAGAAAGGCT
TCACAAACCCAACCCCTATTCAGGAACAAGCAATTCCTATTTTAATTGAAGGAAAAAGAGATATT
GTCGGCCAAGCTCAAACAGGTACTGGAAAAACCGCAGCATTTGGTATTCCTATTTTAGAAACAAT
TGATGAACACTCAAGGAACACGCAAGCACTTATTTTAGCACCTACAAGAGAACTCGCAATACAAG
TTGCAGAAGAAATCGATTCAATCAAAGGTTCAAAAAGATTGAATGTTTTCCCAGTATACGGCGGT
CAATCAATTGACAGACAGATAAGAGAATTAAGAAGAGGAGTTCAGATTGTTGTTGGAACTCCTG
GAAGAATACTTGATCACATCTCAAGAAGAACAATAAAACTTGAAAACGTTTCATATGTAGTTTTA
GATGAAGCTGATGAAATGTTAAACATGGGATTCATTGACGATGTTGAAGAAATCTTAAAATCAG
TAAACACTGACAAAAGAATGCTTTTATTCTCAGCAACACTTCCTGATAGTATCATGAAACTTGCT
AAAAATTACATGAGAGAATACGACATCATCAAAGTTAAAAGACAGCAGCTTACAACTACTTTAAC
TGATCAATCATTCTATGAAATTCACTCAAGAGATAAATTTGAATTACTTTCAAGAATCATTGACA
CGGAAAAAGAATTTTACGGTTTAATCTTCTGTAAAACAAAAGCAGACGTTGACGAAGTTGCAAAC
AGATTGAATGAGAAAGGGTATGCTGCAGAAGGTCTTCACGGGGACATGACCCAAGCTCAAAGAGA
AAAAACACTCGACAAATTTAAAGGAAAGAAAATTAACGTTCTCGTAGCAACTGATGTTGCAGCAA
GAGGAATTGATATCAACGATTTAACACACGTTGTAAACTTTGATATTCCACAAAATCCTGAATCA
TACGTCCACAGAATTGGAAGAACAGGCCGAGCTGGAAAACAAGGTTATGCAATTACATTTGTTGA
ACCTTCAGAATTTAGGAAGTTTAAATACATCCAAAAAATTGCAAAAACTGAAATTAAAAGAGAA
GAAGTTCCTGATGTTAAAGATATAATCAGCGCTAAAAAAATAAAAATCATTTCCGGAATAAAAG
AAGTTTTAGAATCTGGAAAATACGCTGACTGCGAAAAAATGGCTTCTGATTTATTAGAAGATGCA
GATCCTCAAGAAGTTCTTTCAGCAGTTTTAAAATATTCTTTAAAAGATGAATTAAGTGAATCAAA
CTACAAAAAAATTGGAAGAGGTTCACAAAGATCTGAAAGATCTGGAAGAAATGACAGCAGAGGA
AGAAGATCTTTCGCACCTGGTGAAAACGTAAGACTCTTTGTAGCTCTTGGTAAACTTGATAAAAT
GAACCCTAAAAAATTAGTGGATCACATTTCAAGAAAATCCGATGTTAAAGGAAGAGACATCGACG
ATGTAAAAGTATTTGAGAAATTTTCCTTTGTAACGGTATCTTCAAGCGATGCAGAAATAATTCTT
GATTCATTCAAGAATGAAAGAAGAGGAAGAAGATCAATAATCGAAGTTGCAAGCGGCAACTAA

---> Sequence Showing highlighted Existant Combined Trimers

ATG GAA AGT TTT AAA AAT TTA GGT CTA TCT GAC GAG ATA TTA GAA GCA TTA GAA AAG
AAA GGC TTC ACA AAC CCA ACC CCT ATT CAG GAA CAA GCA ATT CCT ATT TTA ATT GAA
GGA AAA AGA GAT ATT GTC GGC CAA GCT CAA ACA GGT ACT GGA AAA ACC GCA GCA TTT
GGT ATT CCT ATT TTA GAA ACA ATT GAT GAA CAC TCA AGG AAC ACG CAA GCA CTT ATT
TTA GCA CCT ACA AGA GAA CTC GCA ATA CAA GTT GCA GAA GAA ATC GAT TCA ATC AAA
GGT TCA AAA AGA TTG AAT GTT TTC CCA GTA TAC GGC GGT CAA TCA ATT GAC AGA CAG

ATA AGA GAA TTA AGA AGA GGA GTT CAG ATT GTT GTT GGA ACT CCT GGA AGA ATA CTT GAT CAC ATC TCA AGA AGA ACA ATA AAA CTT GAA AAC GTT TCA TAT GTA GTT TTA GAT GAA GCT GAT GAA ATG TTA AAC ATG GGA TTC ATT GAC GAT GTT GAA GAA ATC TTA AAA TCA GTA AAC ACT GAC AAA AGA ATG CTT TTA TTC TCA GCA ACA CTT CCT GAT AGT ATC ATG AAA CTT GCT AAA AAT TAC ATG AGA GAA TAC GAC ATC ATC AAA GTT AAA AGA CAG CAG CTT ACA ACT ACT TTA ACT GAT CAA TCA TTC TAT GAA ATT CAC TCA AGA GAT AAA TTT GAA TTA CTT TCA AGA ATC ATT GAC ACG GAA AAA GAA TTT TAC GGT TTA ATC TTC TGT AAA ACA AAA GCA GAC GTT GAC GAA GTT GCA AAC AGA TTG AAT GAG AAA GGG TAT GCT GCA GAA GGT CTT CAC GGG GAC ATG ACC CAA GCT CAA AGA GAA AAA ACA CTC GAC AAA TTT AAA GGA AAG AAA ATT AAC GTT CTC GTA GCA ACT GAT GTT GCA GCA AGA GGA ATT GAT ATC AAC GAT TTA ACA CAC GTT GTA AAC TTT GAT ATT CCA CAA AAT CCT GAA TCA TAC GTC CAC AGA ATT GGA AGA ACA GGC CGA GCT GGA AAA CAA GGT TAT GCA ATT ACA TTT GTT GAA CCT TCA GAA TTT AGG AAG TTT AAA TAC ATC CAA AAA ATT GCA AAA ACT GAA ATT AAA AGA GAA GAA GTT CCT GAT GTT AAA GAT ATA ATC AGC GCT AAA AAA ATA AAA ATC ATT TCC GGA ATA AAA GAA GTT TTA GAA TCT GGA AAA TAC GCT GAC TGC GAA AAA ATG GCT TCT GAT TTA TTA GAA GAT GCA GAT CCT CAA GAA GTT CTT TCA GCA GTT TTA AAA TAT TCT TTA AAA GAT GAA TTA AGT GAA TCA AAC TAC AAA AAA ATT GGA AGA GGT TCA CAA AGA TCT GAA AGA TCT GGA AGA AAT GAC AGC AGA GGA AGA AGA TCT TTC GCA CCT GGT GAA AAC GTA AGA CTC TTT GTA GCT CTT GGT AAA CTT GAT AAA ATG AAC CCT AAA AAA TTA GTG GAT CAC ATT TCA AGA AAA TCC GAT GTT AAA GGA AGA GAC ATC GAC GAT GTA AAA GTA TTT GAG AAA TTT TCC TTT GTA ACG GTA TCT TCA AGC GAT GCA GAA ATA ATT CTT GAT TCA TTC AAG AAT GAA AGA AGA GGA AGA AGA TCA ATA ATC GAA GTT GCA AGC GGC AAC TAA

Occurrences/Results 9 Pentamers

The pentamer 'ATTCA' occurred 5 times.
The pentamer 'TTCAA' occurred 6 times.
The pentamer 'TCAAG' occurred 8 times.
The pentamer 'CAAGA' occurred 8 times.
The pentamer 'AAGAT' occurred 8 times.
The pentamer 'AGATG' occurred 3 times.
The pentamer 'GATGA' occurred 4 times.
The pentamer 'ATGAA' occurred 8 times.
The pentamer 'TGAAT' occurred 6 times.
The total occurrences of all specified strings is 56.
The total number of characters in the text is 1613.
First result (Total characters *9 /1024): 14.2119140625
Square root of the first result: 3.7698692367905813
Final result: 11.084757404762302

Occurrences/Results TRIMERS Codons

The pentamer 'ATT' occurred 25 times.
The pentamer 'CAA' occurred 14 times.
The pentamer 'GAT' occurred 26 times.
The pentamer 'GAA' occurred 42 times.

The pentamer 'CCA' occurred 3 times.
The pentamer 'ACT' occurred 8 times.
The pentamer 'AGA' occurred 35 times.
The pentamer 'TAC' occurred 8 times.
The total occurrences of all specified strings is 161.
The total number of characters in the text is 1614.
First result ((total_characters / 3) * (Trimer_number / 64)): 67.25
Square root of the first result: 8.2
Final result: 11.43

Occurrences/Results TRIMERS Combinations

The pentamer 'ATT ATT' occurred 0 times.
The pentamer 'ATT CAA' occurred 0 times.
The pentamer 'ATT GAT' occurred 2 times.
The pentamer 'ATT GAA' occurred 1 times.
The pentamer 'ATT CCA' occurred 1 times.
The pentamer 'ATT ACT' occurred 0 times.
The pentamer 'ATT AGA' occurred 0 times.
The pentamer 'ATT TAC' occurred 0 times.
The pentamer 'CAA ATT' occurred 0 times.
The pentamer 'CAA CAA' occurred 0 times.
The pentamer 'CAA GAT' occurred 0 times.
The pentamer 'CAA GAA' occurred 1 times.
The pentamer 'CAA CCA' occurred 0 times.
The pentamer 'CAA ACT' occurred 0 times.
The pentamer 'CAA AGA' occurred 2 times.
The pentamer 'CAA TAC' occurred 0 times.
The pentamer 'GAT ATT' occurred 2 times.
The pentamer 'GAT CAA' occurred 1 times.
The pentamer 'GAT GAT' occurred 0 times.
The pentamer 'GAT GAA' occurred 4 times.
The pentamer 'GAT CCA' occurred 0 times.
The pentamer 'GAT ACT' occurred 0 times.
The pentamer 'GAT AGA' occurred 0 times.
The pentamer 'GAT TAC' occurred 0 times.
The pentamer 'GAA ATT' occurred 2 times.
The pentamer 'GAA CAA' occurred 1 times.
The pentamer 'GAA GAT' occurred 1 times.
The pentamer 'GAA GAA' occurred 3 times.
The pentamer 'GAA CCA' occurred 0 times.
The pentamer 'GAA ACT' occurred 0 times.
The pentamer 'GAA AGA' occurred 2 times.
The pentamer 'GAA TAC' occurred 1 times.
The pentamer 'CCA ATT' occurred 0 times.

The pentamer 'CCA CAA' occurred 1 times.
The pentamer 'CCA GAT' occurred 0 times.
The pentamer 'CCA GAA' occurred 0 times.
The pentamer 'CCA CCA' occurred 0 times.
The pentamer 'CCA ACT' occurred 0 times.
The pentamer 'CCA AGA' occurred 0 times.
The pentamer 'CCA TAC' occurred 0 times.
The pentamer 'ACT ATT' occurred 0 times.
The pentamer 'ACT CAA' occurred 0 times.
The pentamer 'ACT GAT' occurred 2 times.
The pentamer 'ACT GAA' occurred 1 times.
The pentamer 'ACT CCA' occurred 0 times.
The pentamer 'ACT ACT' occurred 1 times.
The pentamer 'ACT AGA' occurred 0 times.
The pentamer 'ACT TAC' occurred 0 times.
The pentamer 'AGA ATT' occurred 1 times.
The pentamer 'AGA CAA' occurred 0 times.
The pentamer 'AGA GAT' occurred 2 times.
The pentamer 'AGA GAA' occurred 5 times.
The pentamer 'AGA CCA' occurred 0 times.
The pentamer 'AGA ACT' occurred 0 times.
The pentamer 'AGA AGA' occurred 5 times.
The pentamer 'AGA TAC' occurred 0 times.
The pentamer 'TAC ATT' occurred 0 times.
The pentamer 'TAC CAA' occurred 0 times.
The pentamer 'TAC GAT' occurred 0 times.
The pentamer 'TAC GAA' occurred 0 times.
The pentamer 'TAC CCA' occurred 0 times.
The pentamer 'TAC ACT' occurred 0 times.
The pentamer 'TAC AGA' occurred 0 times.
The pentamer 'TAC TAC' occurred 0 times.
The total occurrences of all specified strings is 42.
The total number of characters in the text is 1613.
First result (((total_characters/ 3) -1) / 64): 8.40625
Square root of the first result: 2.8993533761858004
Final result: 11.586635239404222

>NZ_DAIONM010000001.1:1278583-1281564 Methanococcus maripaludis isolate, ATPase

---> Sequence

ATGATTATTAAAAATATCAAAATGGAAAACTTCAGAAGCCACAGAAACACATCAATAAATTTTA
GCAAAGGAATAACTTCAATAATCGGGCAAAATGGAAGTGGAAAGTCGTCAATTTTTTCAAGCAAT
GAATTTTGCACTTTTTGCTCCGCGTGGAAACAATTTTAGGATTGAAAATTTAATGCAACAGGGCG

CTGCATCTTTTTCTGTAGAATTAGAATTTGAAATGATGGGAAATACATACCTTGTAAAAAGAAAA
CGGTTTCAACATAAAACAGATGATAAATTATATGTTAATGGAAAATTAAATGCCGAATCTGCATC
TGAAATTAATAAAAAAATTGAAGAAATTTTAGAAATTGATAACAGCGTATTTTCAAATGCAATA
TACATAAAACAGGGCGAAATTGCAAATTTAATCCAGATGACTCCAAGAGATAGAAAAGAGGTTA
TTGGAAAGCTTCTTGGAATTGAAAAGTACGAAAAAGCCTCTGAAAAAATGAATATTGTAAAAAA
AAGCTACGAAGAAACGCTTTTAAAACTTGAAGGAGAATTAACCCAAGAACCTGAAATTTTAGAA
AATCTTGAAAAATTAAAAAATGAAGTTTCTGAATCTGAAATTTTAAAAGAAGAAATTTTAAAAA
AATATGAAAATTTAGAAAAACTAAAACTTGAAAAAAATTCAGAAATACTCCAAATGGAAGAAAA
ATTTGCTGAAAATAACCAGTTAAAAGAAAATTTAAAGGATATTATCTCAGAAATTAAAAATATA
AATTTAGAAATTCAAAATTTCAAAAATTCACTAAATTTAGTTGCTGAAGAATCAAAAAACATAT
CTGAAAATGAAGAAAATTACAAAAAATATTTAGAACTTGAATTAAAAATTAAAGAATTAAATAA
TAAGTTAATAGGCCATAAATCAAACTACGAATCATATAATAAACTAAAAACGATTGAAGAATCA
TTATTAAAAGAATTAGGTGTATTAAAAGAATCTTTAAAAGATAATAAAAAAAAATCCTGATGAAT
TGAAAGAAAATTTAAAAGAAAATGATGAAAAAATTCTAATTTTAGATAAAATAAAAGAAAAAAT
TAAGGAATTAGAATTTATTGAAAAACAGATTTATGAAATAAAAATCCACAAAAAAACAGTGGAA
ACTCTTTTTGATAGTGTTAAAATTTACGATGACTCGATTAAAACTTTTGAAGAACTAAAAACTAA
AAAAAATAGTTATGAAAATCTTTTAAAAGAAAAATTTGACCTTGAAAAAAAACTTCAAAACGAA
ACTGACGAAAAAACAAAATTAATTTCTGAATTAACTGATTTTGAAAAAATAGAAGAAAAAATAA
ATCTTGAAAATGAATTAAAAGAAAAATATGAAGATTTATCTGAAAAAATAGATAAATTAAATGA
AATCGTTTTGAAAAAAGAAAGTAAAATCTCAGAATATAAAAATTCTAAGGCAGAACTTGAAAAA
ACCAAAGATTCGTGTCACGTTTGTCAGTCGAAAATAACTGAAGAAAAAAAGCAGGAATTGCTTGA
AAAATATAATTCTGAAATTCAAAATGAACAATTATCAACTGAAAGTCTTAAAAAACAGCTTGAA
ATCATATTAAACAAAAAAGAGAAGATGAAAGTTAAATTAAATGAAATTGATTCATTCAAATTAA
AATACGGGGAATTAAAAGAAAAAAAGAATTATTCTTTAAAAGTTGAAGAATCAATAATCGAAAC
TACTGAAAAATTAAACGAATTAACTGGAAAAATTAACGAATATTCTTCATTGAATGACGAAATA
AGTTTAATTGAAAATAAATTAAAAAATTTAGAAAATGATTATAAAAATTGCAATTATTCCTCCC
AATTTTTAACTAAAAATGACGAATCTGAATTTTTAACTAAAAAATTGGAACTTTCAAAAATTAT
TGGAGATTACGATTCTTCAAAAATTGAAAATGAAAAAAAATCCCTTGAAAATTTAAAAGATGAA
TTAAAAAATACTATTTACAATTTAGAAAGAGAAATTAACTTAAAAAAGGAATTAAAAAATATTC
AAAACGATATTTCTTCAAAAATCGGTATTGTTGAATGTTACGTTAAATGGGAAACTGAAAAATC
AGACTTTGAAAACAAATTATCAGAATGTAAAGAAAATTATGAAAAATATATGGAAAGTCTGGCA
GTTCTTAAAAACTATTCAAAAACGTATTCTGTTGAAATAAATAATTTAAACGAGTTTTTAAACCA
AAAAATTGCTGAAAAACAGCAGTTTTGTGAAAAACTCCTTGAAACACGAACCGAAATTGAAAAA
AATATTCAGACTGTAAACTACAATCCAGAATTGCATGAAAACGCTAAAAGACTTTATGAAAATAT
ATTAAATGAATTTAACGACATTTTAAGAACTTTAGAAAGAATAAGCTCCGAATTAAAACTTAAA
AATGAGAATATAAGTTATTTAAATGAAAAAATTCAAAATCTGTCGAATAAAAAAGAAGAAAAGA
AAAAAATTGAAGAATTTAAAGAATATCTCGATAAAATAAAAAGAGAAATCTTTTCAAAAGATGG
TTTCCAAAAATATTTAAGAGAAAAGTATATTCCATTAATTCAAAGGCATACAAACCAGATTTTTC
AGGAATTTGAACTTCCATATTCCCATATTCAACTAAAAGATGATTACAGCTTGATAGTTGACGGC
CTTCCTGTTGAAACATTAAGTGGCGGAGAGCAGATTGCAGTATCTCTTGCATTAAGACTTGGAAT
TTCTAAAGCAGTTTGTAATAATATCGAATGCATTATTTTGGATGAACCAACAGCGTATCTTGATG
AAGATCGCCGTAAAAACCTTTTAAATATTTTCAAAAACATAAAAACGATAAACCAGATGGCAATA

ATTACGCACCATCAAGAACTCGAACAGATTGCAGATAACATTGTAAAAGTTAGAAAAATCGGCGA AAATTCAAAAGTATCTTTAGAATAG

---> Sequence Showing highlighted Existant Combined Trimers

ATG ATT ATT AAA AAT ATC AAA ATG GAA AAC TTC AGA AGC CAC AGA AAC ACA TCA ATA AAT TTT AGC AAA GGA ATA ACT TCA ATA ATC GGG CAA AAT GGA AGT GGA AAG TCG TCA ATT TTT CAA GCA ATG AAT TTT GCA CTT TTT GCT CCG CGT GGA AAC AAT TTT AGG ATT GAA AAT TTA ATG CAA CAG GGC GCT GCA TCT TTT TCT GTA GAA TTA GAA TTT GAA ATG ATG GGA AAT ACA TAC CTT GTA AAA AGA AAA CGG TTT CAA CAT AAA ACA GAT GAT AAA TTA TAT GTT AAT GGA AAA TTA AAT GCC GAA TCT GCA TCT GAA ATT AAT AAA AAA ATT GAA GAA ATT TTA GAA ATT GAT AAC AGC GTA TTT TCA AAT GCA ATA TAC ATA AAA CAG GGC GAA ATT GCA AAT TTA ATC CAG ATG ACT CCA AGA GAT AGA AAA GAG GTT ATT GGA AAG CTT CTT GGA ATT GAA AAG TAC GAA AAA GCC TCT GAA AAA ATG AAT ATT GTA AAA AAA AGC TAC GAA GAA ACG CTT TTA AAA CTT GAA GGA GAA TTA ACC CAA GAA CCT GAA ATT TTA GAA AAT CTT GAA AAA TTA AAA AAT GAA GTT TCT GAA TCT GAA ATT TTA AAA GAA GAA ATT TTA AAA AAA TAT GAA AAT TTA GAA AAA CTA AAA CTT GAA AAA AAT TCA GAA ATA CTC CAA ATG GAA GAA AAA TTT GCT GAA AAT AAC CAG TTA AAA GAA AAT TTA AAG GAT ATT ATC TCA GAA ATT AAA AAT ATA AAT TTA GAA ATT CAA AAT TTC AAA AAT TCA CTA AAT TTA GTT GCT GAA GAA TCA AAA AAC ATA TCT GAA AAT GAA GAA AAT TAC AAA AAA TAT TTA GAA CTT GAA TTA AAA ATT AAA GAA TTA AAT AAT AAG TTA ATA GGC CAT AAA TCA AAC TAC GAA TCA TAT AAT AAA CTA AAA ACG ATT GAA GAA TCA TTA TTA AAA GAA TTA GGT GTA TTA AAA GAA TCT TTA AAA GAT AAT AAA AAA AAT CCT GAT GAA TTG AAA GAA AAT TTA AAA GAA AAT GAT GAA AAA ATT CTA ATT TTA GAT AAA ATA AAA GAA AAA ATT AAG GAA TTA GAA TTT ATT GAA AAA CAG ATT TAT GAA ATA AAA ATC CAC AAA AAA ACA GTG GAA ACT CTT TTT GAT AGT GTT AAA ATT TAC GAT GAC TCG ATT AAA ACT TTT GAA GAA CTA AAA ACT AAA AAA AAT AGT TAT GAA AAT CTT TTA AAA GAA AAA TTT GAC CTT GAA AAA AAA CTT CAA AAC GAA ACT GAC GAA AAA ACA AAA TTA ATT TCT GAA TTA ACT GAT TTT GAA AAA ATA GAA GAA AAA ATA AAT CTT GAA AAT GAA TTA AAA GAA AAA TAT GAA GAT TTA TCT GAA AAA ATA GAT AAA TTA AAT GAA ATC GTT TTG AAA AAA GAA AGT AAA ATC TCA GAA TAT AAA AAT TCT AAG GCA GAA CTT GAA AAA ACC AAA GAT TCG TGT CAC GTT TGT CAG TCG AAA ATA ACT GAA GAA AAA AAG CAG GAA TTG CTT GAA AAA TAT AAT TCT GAA ATT CAA AAT GAA CAA TTA TCA ACT GAA AGT CTT AAA AAA CAG CTT GAA ATC ATA TTA AAC AAA AAA GAG AAG ATG AAA GTT AAA TTA AAT GAA ATT GAT TCA TTC AAA TTA AAA TAC GGG GAA TTA AAA GAA AAA AAG AAT TAT TCT TTA AAA GTT GAA GAA TCA ATA ATC GAA ACT ACT GAA AAA TTA AAC GAA TTA ACT GGA AAA ATT AAC GAA TAT TCT TCA TTG AAT GAC GAA ATA AGT TTA ATT GAA AAT AAA TTA AAA AAT TTA GAA AAT GAT TAT AAA AAT TGC AAT TAT TCC TCC CAA TTT TTA ACT AAA AAT GAC GAA TCT GAA TTT TTA ACT AAA AAA TTG GAA CTT TCA AAA ATT ATT GGA GAT TAC GAT TCT TCA AAA ATT GAA AAT GAA AAA AAA TCC CTT GAA AAT TTA AAA GAT GAA TTA AAA AAT ACT ATT TAC AAT TTA GAA AGA GAA ATT AAC TTA AAA AAG GAA TTA AAA AAT ATT CAA AAC GAT ATT TCT TCA AAA ATC GGT ATT GTT GAA TGT TAC GTT AAA TGG GAA ACT GAA AAA TCA GAC TTT GAA AAC AAA TTA TCA GAA TGT AAA GAA AAT TAT GAA AAA TAT ATG GAA AGT CTG GCA GTT CTT AAA AAC TAT TCA AAA ACG TAT TCT GTT GAA ATA AAT AAT TTA AAC GAG TTT TTA AAC CAA AAA ATT GCT GAA AAA CAG CAG TTT TGT GAA AAA

CTC CTT GAA ACA CGA ACC GAA ATT GAA AAA AAT ATT CAG ACT GTA AAC TAC AAT CCA GAA TTG CAT GAA AAC GCT AAA AGA CTT TAT GAA AAT ATA TTA AAT GAA TTT AAC GAC ATT TTA AGA ACT TTA GAA AGA ATA AGC TCC GAA TTA AAA CTT AAA AAT GAG AAT ATA AGT TAT TTA AAT GAA AAA ATT CAA AAT CTG TCG AAT AAA AAA GAA GAA AAG AAA AAA ATT GAA GAA TTT AAA GAA TAT CTC GAT AAA ATA AAA AGA GAA ATC TTT TCA AAA GAT GGT TTC CAA AAA TAT TTA AGA GAA AAG TAT ATT CCA TTA ATT CAA AGG CAT ACA AAC CAG ATT TTT CAG GAA TTT GAA CTT CCA TAT TCC CAT ATT CAA CTA AAA GAT GAT TAC AGC TTG ATA GTT GAC GGC CTT CCT GTT GAA ACA TTA AGT GGC GGA GAG CAG ATT GCA GTA TCT CTT GCA TTA AGA CTT GGA ATT TCT AAA GCA GTT TGT AAT AAT ATC GAA TGC ATT ATT TTG GAT GAA CCA ACA GCG TAT CTT GAT GAA GAT CGC CGT AAA AAC CTT TTA AAT ATT TTC AAA AAC ATA AAA ACG ATA AAC CAG ATG GCA ATA ATT ACG CAC CAT CAA GAA CTC GAA CAG ATT GCA GAT AAC ATT GTA AAA GTT AGA AAA ATC GGC GAA AAT TCA AAA GTA TCT TTA GAA TAG

Occurrences/Results 9 Pentamers

The pentamer 'ATTCA' occurred 12 times.
The pentamer 'TTCAA' occurred 20 times.
The pentamer 'TCAAG' occurred 2 times.
The pentamer 'CAAGA' occurred 3 times.
The pentamer 'AAGAT' occurred 8 times.
The pentamer 'AGATG' occurred 7 times.
The pentamer 'GATGA' occurred 10 times.
The pentamer 'ATGAA' occurred 24 times.
The pentamer 'TGAAT' occurred 12 times.
The total occurrences of all specified strings is 98.
The total number of characters in the text is 2978.
First result (Total characters *9 /1024): 26.208984375
Square root of the first result: 5.119471103053518
Final result: 14.023131331316652

Occurrences/Results TRIMERS Codons

The pentamer 'ATT' occurred 58 times.
The pentamer 'CAA' occurred 18 times.
The pentamer 'GAT' occurred 29 times.
The pentamer 'GAA' occurred 149 times.
The pentamer 'CCA' occurred 5 times.
The pentamer 'ACT' occurred 18 times.
The pentamer 'AGA' occurred 13 times.
The pentamer 'TAC' occurred 13 times.
The total occurrences of all specified strings is 303.
The total number of characters in the text is 2982.
First result ((total_characters / 3) * (Trimer_number / 64)): 124.25
Square root of the first result: 11.15
Final result: 16.03

Occurrences/Results TRIMERS Combinations

The pentamer 'ATT ATT' occurred 3 times.
The pentamer 'ATT CAA' occurred 6 times.
The pentamer 'ATT GAT' occurred 2 times.
The pentamer 'ATT GAA' occurred 9 times.
The pentamer 'ATT CCA' occurred 1 times.
The pentamer 'ATT ACT' occurred 0 times.
The pentamer 'ATT AGA' occurred 0 times.
The pentamer 'ATT TAC' occurred 2 times.
The pentamer 'CAA ATT' occurred 0 times.
The pentamer 'CAA CAA' occurred 0 times.
The pentamer 'CAA GAT' occurred 0 times.
The pentamer 'CAA GAA' occurred 2 times.
The pentamer 'CAA CCA' occurred 0 times.
The pentamer 'CAA ACT' occurred 0 times.
The pentamer 'CAA AGA' occurred 0 times.
The pentamer 'CAA TAC' occurred 0 times.
The pentamer 'GAT ATT' occurred 2 times.
The pentamer 'GAT CAA' occurred 0 times.
The pentamer 'GAT GAT' occurred 2 times.
The pentamer 'GAT GAA' occurred 5 times.
The pentamer 'GAT CCA' occurred 0 times.
The pentamer 'GAT ACT' occurred 0 times.
The pentamer 'GAT AGA' occurred 1 times.
The pentamer 'GAT TAC' occurred 2 times.
The pentamer 'GAA ATT' occurred 13 times.
The pentamer 'GAA CAA' occurred 1 times.
The pentamer 'GAA GAT' occurred 2 times.
The pentamer 'GAA GAA' occurred 13 times.
The pentamer 'GAA CCA' occurred 1 times.
The pentamer 'GAA ACT' occurred 4 times.
The pentamer 'GAA AGA' occurred 2 times.
The pentamer 'GAA TAC' occurred 0 times.
The pentamer 'CCA ATT' occurred 0 times.
The pentamer 'CCA CAA' occurred 0 times.
The pentamer 'CCA GAT' occurred 0 times.
The pentamer 'CCA GAA' occurred 1 times.
The pentamer 'CCA CCA' occurred 0 times.
The pentamer 'CCA ACT' occurred 0 times.
The pentamer 'CCA AGA' occurred 1 times.
The pentamer 'CCA TAC' occurred 0 times.
The pentamer 'ACT ATT' occurred 1 times.
The pentamer 'ACT CAA' occurred 0 times.

The pentamer 'ACT GAT' occurred 1 times.
The pentamer 'ACT GAA' occurred 4 times.
The pentamer 'ACT CCA' occurred 1 times.
The pentamer 'ACT ACT' occurred 1 times.
The pentamer 'ACT AGA' occurred 0 times.
The pentamer 'ACT TAC' occurred 0 times.
The pentamer 'AGA ATT' occurred 0 times.
The pentamer 'AGA CAA' occurred 0 times.
The pentamer 'AGA GAT' occurred 1 times.
The pentamer 'AGA GAA' occurred 3 times.
The pentamer 'AGA CCA' occurred 0 times.
The pentamer 'AGA ACT' occurred 1 times.
The pentamer 'AGA AGA' occurred 0 times.
The pentamer 'AGA TAC' occurred 0 times.
The pentamer 'TAC ATT' occurred 0 times.
The pentamer 'TAC CAA' occurred 0 times.
The pentamer 'TAC GAT' occurred 2 times.
The pentamer 'TAC GAA' occurred 3 times.
The pentamer 'TAC CCA' occurred 0 times.
The pentamer 'TAC ACT' occurred 0 times.
The pentamer 'TAC AGA' occurred 0 times.
The pentamer 'TAC TAC' occurred 0 times.
The total occurrences of all specified strings is 93.
The total number of characters in the text is 2978.
First result (((total_characters/ 3) -1) / 64): 15.515625
Square root of the first result: 3.9389878141471826
Final result: 19.671138540136837

>AP011526.1:1574724-1576052 Methanococcus maripaludis KA1 DNA, translocase

---> Sequence

TTGGAAAGTTTTCTATTAAAAATAAAACCCATACTTGAATTAATTCCAGAAGTAAAAAGACCACT
AAGAGAAATTTCATTTAAAGAAAAATTACAATGGACTGCACTTGTGCTTGTACTCTACTTTATTT
TGGGTACAATCGATATTTACACGGGCGGTTCAGAAATGCCTGCAATATTTGATTTTTGGCAGACC
GTTACGGCTTCTAAAATGGGTACTTTAATTACACTTGGTATCGGACCAATTGTAACTGCAGGAAT
TATCATGCAGCTTTTAGTTGGATCGGAGCTTATAAGTCTGGATTTATCAAAACCGATGAATAGGG
CATTATTTCAAGGTTTACAAAAGTTATTTGGTATAGCATTATGTTTCCTCGAAGCTTTGATGTTT
GTTGGAGCAGGGGCATTTGGTGCATTAACCCCACTAATGACAGCAGTGTTAGTATTCCAGCTTGC
ACTTGGTGCGATATTAATTATTTATTTGGATGAAATTGTTTCAAGATACGGTATTGGATCAGGTA
TCGGTCTTTTCATTGCAGCAGGAGTCTCACAAACAATATTTGTAGGAACCTTTGGTGCAGAAGGA
TATTTATGGAAATTCTTCACTGCAATGACTGCAGGAAGTTTATGGACTGCATTAGAATATATCTT
ACCAATTTTAGGTACAATACTTGTATTCTTAGTTGTAGTTTACGTTGAAAGTATCAGAGTAGAAA

TTCCACTAGCACACGGAAGAGTAAAAGGTGCTGTTGGAAAATACCCAATAAAATTCATCTATGTT
TCAAACCTGCCAGTTATCCTTGCTGCAGCACTCTTTGCAAACATACAGCTTTGGGGAATGTTTTT
AGAAAAAATGGGTTTCCCAATTCTCGGACACTATACGAGTGGTAGGGCAGTTGATGGACTTGCAT
ACTACTTCTCAACACCGTACGGAATTTCAAGTTTGACTGCAGATCCGCTTCATGCAGTATTTTAC
ACTGTAATGATGGTAATATTCTGTATATTATTTGGTTTATTCTGGGTTGAAACCTCAGGACTTGA
CGCAAAATCGATGGCTAAAAAACTCGGAAATCTTGATATGGCAATTAAAGGATTTAGAAAAAGC
CAAAAATCAATTGAACAACGTTTAAAAAGATACATCACACCAATTACCGTAATGGGTTCAGCATT
TGTTGGATTCTTAGCCGCAGCTGCTGACTTTACAGGGGCCCTTGGTGGAGGTACTGGAGTATTGC
TTACAGTATCGATCGTATACAGATTATACGAACAACTCGTTCAAGAACAGCTCTCTGAACTTCAC
CCATCAATTGCGAAGTTCATAAGAAAATAA

---> Sequence Showing highlighted Existant Combined Trimers

TTG GAA AGT TTT CTA TTA AAA ATA AAA CCC ATA CTT GAA TTA ATT CCA GAA GTA AAA
AGA CCA CTA AGA GAA ATT TCA TTT AAA GAA AAA TTA CAA TGG ACT GCA CTT GTG CTT
GTA CTC TAC TTT ATT TTG GGT ACA ATC GAT ATT TAC ACG GGC GGT TCA GAA ATG CCT
GCA ATA TTT GAT TTT TGG CAG ACC GTT ACG GCT TCT AAA ATG GGT ACT TTA ATT ACA
CTT GGT ATC GGA CCA ATT GTA ACT GCA GGA ATT ATC ATG CAG CTT TTA GTT GGA TCG
GAG CTT ATA AGT CTG GAT TTA TCA AAA CCG ATG AAT AGG GCA TTA TTT CAA GGT TTA
CAA AAG TTA TTT GGT ATA GCA TTA TGT TTC CTC GAA GCT TTG ATG TTT GTT GGA GCA
GGG GCA TTT GGT GCA TTA ACC CCA CTA ATG ACA GCA GTG TTA GTA TTC CAG CTT GCA
CTT GGT GCG ATA TTA ATT ATT TAT TTG GAT GAA ATT GTT TCA AGA TAC GGT ATT GGA
TCA GGT ATC GGT CTT TTC ATT GCA GCA GGA GTC TCA CAA ACA ATA TTT GTA GGA ACC
TTT GGT GCA GAA GGA TAT TTA TGG AAA TTC TTC ACT GCA ATG ACT GCA GGA AGT TTA
TGG ACT GCA TTA GAA TAT ATC TTA CCA ATT TTA GGT ACA ATA CTT GTA TTC TTA GTT
GTA GTT TAC GTT GAA AGT ATC AGA GTA GAA ATT CCA CTA GCA CAC GGA AGA GTA AAA
GGT GCT GTT GGA AAA TAC CCA ATA AAA TTC ATC TAT GTT TCA AAC CTG CCA GTT ATC
CTT GCT GCA GCA CTC TTT GCA AAC ATA CAG CTT TGG GGA ATG TTT TTA GAA AAA ATG
GGT TTC CCA ATT CTC GGA CAC TAT ACG AGT GGT AGG GCA GTT GAT GGA CTT GCA TAC
TAC TTC TCA ACA CCG TAC GGA ATT TCA AGT TTG ACT GCA GAT CCG CTT CAT GCA GTA
TTT TAC ACT GTA ATG ATG GTA ATA TTC TGT ATA TTA TTT GGT TTA TTC TGG GTT GAA
ACC TCA GGA CTT GAC GCA AAA TCG ATG GCT AAA AAA CTC GGA AAT CTT GAT ATG GCA
ATT AAA GGA TTT AGA AAA AGC CAA AAA TCA ATT GAA CAA CGT TTA AAA AGA TAC ATC
ACA CCA ATT ACC GTA ATG GGT TCA GCA TTT GTT GGA TTC TTA GCC GCA GCT GCT GAC
TTT ACA GGG GCC CTT GGT GGA GGT ACT GGA GTA TTG CTT ACA GTA TCG ATC GTA TAC
AGA TTA TAC GAA CAA CTC GTT CAA GAA CAG CTC TCT GAA CTT CAC CCA TCA ATT GCG
AAG TTC ATA AGA AAA TAA

Occurrences/Results 9 Pentamers

The pentamer 'ATTCA' occurred 1 times.
The pentamer 'TTCAA' occurred 5 times.
The pentamer 'TCAAG' occurred 4 times.
The pentamer 'CAAGA' occurred 2 times.
The pentamer 'AAGAT' occurred 2 times.
The pentamer 'AGATG' occurred 0 times.
The pentamer 'GATGA' occurred 2 times.

The pentamer 'ATGAA' occurred 2 times.
The pentamer 'TGAAT' occurred 2 times.
The total occurrences of all specified strings is 20.
The total number of characters in the text is 1325.
First result (Total characters *9 /1024): 11.6806640625
Square root of the first result: 3.41769865004216
Final result: 2.4341923584741374

Occurrences/Results TRIMERS Codons

The pentamer 'ATT' occurred 20 times.
The pentamer 'CAA' occurred 8 times.
The pentamer 'GAT' occurred 7 times.
The pentamer 'GAA' occurred 18 times.
The pentamer 'CCA' occurred 11 times.
The pentamer 'ACT' occurred 9 times.
The pentamer 'AGA' occurred 9 times.
The pentamer 'TAC' occurred 12 times.
The total occurrences of all specified strings is 94.
The total number of characters in the text is 1325.
First result ((total_characters / 3) * (Trimer_number / 64)): 55.2
Square root of the first result: 7.4
Final result: 5.22

Occurrences/Results TRIMERS Combinations

The pentamer 'ATT ATT' occurred 1 times.
The pentamer 'ATT CAA' occurred 0 times.
The pentamer 'ATT GAT' occurred 0 times.
The pentamer 'ATT GAA' occurred 1 times.
The pentamer 'ATT CCA' occurred 2 times.
The pentamer 'ATT ACT' occurred 0 times.
The pentamer 'ATT AGA' occurred 0 times.
The pentamer 'ATT TAC' occurred 1 times.
The pentamer 'CAA ATT' occurred 0 times.
The pentamer 'CAA CAA' occurred 0 times.
The pentamer 'CAA GAT' occurred 0 times.
The pentamer 'CAA GAA' occurred 1 times.
The pentamer 'CAA CCA' occurred 0 times.
The pentamer 'CAA ACT' occurred 0 times.
The pentamer 'CAA AGA' occurred 0 times.
The pentamer 'CAA TAC' occurred 0 times.
The pentamer 'GAT ATT' occurred 1 times.
The pentamer 'GAT CAA' occurred 0 times.
The pentamer 'GAT GAT' occurred 0 times.
The pentamer 'GAT GAA' occurred 1 times.
The pentamer 'GAT CCA' occurred 0 times.

The pentamer 'GAT ACT' occurred 0 times.
The pentamer 'GAT AGA' occurred 0 times.
The pentamer 'GAT TAC' occurred 0 times.
The pentamer 'GAA ATT' occurred 3 times.
The pentamer 'GAA CAA' occurred 2 times.
The pentamer 'GAA GAT' occurred 0 times.
The pentamer 'GAA GAA' occurred 0 times.
The pentamer 'GAA CCA' occurred 0 times.
The pentamer 'GAA ACT' occurred 0 times.
The pentamer 'GAA AGA' occurred 0 times.
The pentamer 'GAA TAC' occurred 0 times.
The pentamer 'CCA ATT' occurred 4 times.
The pentamer 'CCA CAA' occurred 0 times.
The pentamer 'CCA GAT' occurred 0 times.
The pentamer 'CCA GAA' occurred 1 times.
The pentamer 'CCA CCA' occurred 0 times.
The pentamer 'CCA ACT' occurred 0 times.
The pentamer 'CCA AGA' occurred 0 times.
The pentamer 'CCA TAC' occurred 0 times.
The pentamer 'ACT ATT' occurred 0 times.
The pentamer 'ACT CAA' occurred 0 times.
The pentamer 'ACT GAT' occurred 0 times.
The pentamer 'ACT GAA' occurred 0 times.
The pentamer 'ACT CCA' occurred 0 times.
The pentamer 'ACT ACT' occurred 0 times.
The pentamer 'ACT AGA' occurred 0 times.
The pentamer 'ACT TAC' occurred 0 times.
The pentamer 'AGA ATT' occurred 0 times.
The pentamer 'AGA CAA' occurred 0 times.
The pentamer 'AGA GAT' occurred 0 times.
The pentamer 'AGA GAA' occurred 1 times.
The pentamer 'AGA CCA' occurred 1 times.
The pentamer 'AGA ACT' occurred 0 times.
The pentamer 'AGA AGA' occurred 0 times.
The pentamer 'AGA TAC' occurred 2 times.
The pentamer 'TAC ATT' occurred 0 times.
The pentamer 'TAC CAA' occurred 0 times.
The pentamer 'TAC GAT' occurred 0 times.
The pentamer 'TAC GAA' occurred 1 times.
The pentamer 'TAC CCA' occurred 1 times.
The pentamer 'TAC ACT' occurred 1 times.
The pentamer 'TAC AGA' occurred 1 times.
The pentamer 'TAC TAC' occurred 1 times.

The total occurrences of all specified strings is 27.
The total number of characters in the text is 1325.
First result (((total_characters/ 3) -1) / 64): 6.90625
Square root of the first result: 2.62797450520358
Final result: 7.646097768533492

>NZ_DAIONM010000001.1:c336864-334510 Methanococcus maripaludis isolate, DNA Polymerase

---> Sequence

ATGGAAAGCCTGATTGATTTGGACTACAATTCTGACGACTTGTGCATATACTTATATTTAATAAA
TAGTATCATAAAAGAAAAAGATTTCAAACCTTATTTTTACGTAAATTCAACGGATAAGGAACAA
ATCCTCGAATTTTTAAAAGATTACGAAAAAAAACATAAATTGGATAGTGAAATCAGTAAAATGA
TTGAAAACATCGAAACTGTTAAAAAAATAGTTTTTGATGAAAATTATCAGGAAAAAGAGCTCTC
AAAAGTTACTGTAAAATACCCAAATAACGTAAAAACAGTTCGAGAAATATTGATGGAATTTGAA
AGGCTCTATGAGTATGATATTCCATTTGTAAGACGTTATTTAATAGATAATAGTGTTATTCCTAC
GTCCACATGGGATTTTGAGAACAATAAAAAAATAGATAATAAAATTCCAGATTTTAAAACTGTT
TCTTTTGATATTGAAGTTTACTGCAATAAAGAGCCAAATCCAAAAAAAGACCCTATAATAATGGC
AAGTTTTAGTTCAAAAGATTTTAACACCGTAGTTTCCACCAAAAAATTCGACCACGAAAAATTAG
AGTATGTAAAAGATGAAAAAGAGCTTATAAAACGAATTATCGAGATTTTAAAAGAGTATGATAT
AATTTACACATATAATGGGGACAATTTTGATTTTCCATACCTTAAAAAAAGAGCTGAAAGTTTCG
GGCTTGAACTAAAGCTTGGGAAAAACGATGAAAAAATAAAAATCACGAAAGGCGGAATGAATTC
GAAAAGCTACATCCCTGGAAGAGTTCATATCGATTTATACCCGATTGCAAGACGGTTATTAAATT
TAACAAAATACCGACTCGAAAATGTAACCGAAGCTCTTTTTGACGTGAAAAAAGTAGATGTTGGT
CATGAAAATATTCCAAAAATGTGGGATAATTTAGATGAAACACTTGTAGAATATTCACATCAGG
ATGCATATTATACTCAAAGAATCGGGGAACAGTTTTTACCTCTTGAAATAATGTTTTCTCGAGTT
GTAAATCAATCTTTGTACGATATAAACCGTATGAGCAGTAGCCAAATGGTAGAATATTTACTTTT
AAAAAATTCTTACAAAATGGGAGTTATAGCCCCAAACAGGCCGTCTGGAAAAGAATATCAAAAA
AGAATCCGAAGTTCATATGAAGGAGGATACGTAAAAGAGCCATTAAAGGGAATTCATGAAGATA
TCGTTTCGATGGACTTTTTAAGCCTTTACCCATCAATTATAATGAGCCACAACTTAAGCCCTGAA
ACAATAGACTGTACTTGCTGCAGTGATGAAGAAAATGGCGAAAATGAAGAAATATTGGGCCACA
AATTCTGTAAAAAAAGTATTGGAATTATCCCAAAAACTCTCATGGATTTAATAAATAGGCGAAA
AAAAGTCAAAAAAGTTTTAAGGGAAAAAGCTGAAAAAGGGGAATTTGACGAAGAATACCAGATT
TTAGACTACGAACAAAGATCTATCAAAGTTCTTGCAAATTCACACTACGGATACCTCGCATTTCC
AATGGCAAGGTGGTATTCGAGAGATTGCGCAGAAATTACAACTCATCTTGGAAGGCAGTACATTC
AAAAAACGATAGAAGAAGCTGAAAATTTTGGATTTAAAGTAATTTATGCAGATACGGATGGATT
TTATTCTAAATGGGCCGATGATAAAGAAAAACTGTCAAAATATGAACTTTTAGAAAAAACCCGCG
AATTTTTAAAAAATATAAACAATACATTGCCGGGAGAAATGGAACTTGAATTTGAAGGGTATTT
CAAAAGGGGAATTTTTGTAACCAAAAAGAAATACGCACTTATTGACGAAAATGAAAAAATTACG
GTAAAAGGCCTTGAAGTAGTAAGGCGGGATTGGTCCAATGTTTCAAAAAATACGCAAAAAAACG
TATTGAACGCACTTTTAAAAGAAGGTAGCGTTGAAAATGCAAAAAAAGTGATTCAGGATACCAT
TAAAGAGTTAAAGGATGGAAAAGTAAATAATGAAGATTTATTGATTCATACTCAGCTTACAAAA
AGAATTGAAGACTATAAAACCACAGCCCCGCATGTTGAAGTTGCAAAAAAAATATTGAAGTCTGG
AAATCGGGTAAATACTGGCGACGTTATAAGTTATATTATAACAAGCGGAAATAAATCGATTAGT

GAGCGGGCGGAAATACTGGAAAATGCAAAAAATTACGATACAAATTATTATATCGAAAATCAGA
TTTTACCCCCCGTTATACGATTAATGGAAGCTTTAGGAATTACTAAGGACGAATTAAAAGACTCT
AAAAAGCAGTACACGTTGCACCATTTTTTAAAGTGA

---> Sequence Showing highlighted Existant Combined Trimers

ATG GAA AGC CTG ATT GAT TTG GAC TAC AAT TCT GAC GAC TTG TGC ATA TAC TTA TAT
TTA ATA AAT AGT ATC ATA AAA GAA AAA GAT TTC AAA CCT TAT TTT TAC GTA AAT TCA
ACG GAT AAG GAA CAA ATC CTC GAA TTT TTA AAA GAT TAC GAA AAA AAA CAT AAA TTG
GAT AGT GAA ATC AGT AAA ATG ATT GAA AAC ATC GAA ACT GTT AAA AAA ATA GTT TTT
GAT GAA AAT TAT CAG GAA AAA GAG CTC TCA AAA GTT ACT GTA AAA TAC CCA AAT AAC
GTA AAA ACA GTT CGA GAA ATA TTG ATG GAA TTT GAA AGG CTC TAT GAG TAT GAT ATT
CCA TTT GTA AGA CGT TAT TTA ATA GAT AAT AGT GTT ATT CCT ACG TCC ACA TGG GAT
TTT GAG AAC AAT AAA AAA ATA GAT AAT AAA ATT CCA GAT TTT AAA ACT GTT TCT TTT
GAT ATT GAA GTT TAC TGC AAT AAA GAG CCA AAT CCA AAA AAA GAC CCT ATA ATA ATG
GCA AGT TTT AGT TCA AAA GAT TTT AAC ACC GTA GTT TCC ACC AAA AAA TTC GAC CAC
GAA AAA TTA GAG TAT GTA AAA GAT GAA AAA GAG CTT ATA AAA CGA ATT ATC GAG ATT
TTA AAA GAG TAT GAT ATA ATT TAC ACA TAT AAT GGG GAC AAT TTT GAT TTT CCA TAC
CTT AAA AAA AGA GCT GAA AGT TTC GGG CTT GAA CTA AAG CTT GGG AAA AAC GAT GAA
AAA ATA AAA ATC ACG AAA GGC GGA ATG AAT TCG AAA AGC TAC ATC CCT GGA AGA GTT
CAT ATC GAT TTA TAC CCG ATT GCA AGA CGG TTA TTA AAT TTA ACA AAA TAC CGA CTC
GAA AAT GTA ACC GAA GCT CTT TTT GAC GTG AAA AAA GTA GAT GTT GGT CAT GAA AAT
ATT CCA AAA ATG TGG GAT AAT TTA GAT GAA ACA CTT GTA GAA TAT TCA CAT CAG GAT
GCA TAT TAT ACT CAA AGA ATC GGG GAA CAG TTT TTA CCT CTT GAA ATA ATG TTT TCT
CGA GTT GTA AAT CAA TCT TTG TAC GAT ATA AAC CGT ATG AGC AGT AGC CAA ATG GTA
GAA TAT TTA CTT TTA AAA AAT TCT TAC AAA ATG GGA GTT ATA GCC CCA AAC AGG CCG
TCT GGA AAA GAA TAT CAA AAA AGA ATC CGA AGT TCA TAT GAA GGA GGA TAC GTA AAA
GAG CCA TTA AAG GGA ATT CAT GAA GAT ATC GTT TCG ATG GAC TTT TTA AGC CTT TAC
CCA TCA ATT ATA ATG AGC CAC AAC TTA AGC CCT GAA ACA ATA GAC TGT ACT TGC TGC
AGT GAT GAA GAA AAT GGC GAA AAT GAA GAA ATA TTG GGC CAC AAA TTC TGT AAA AAA
AGT ATT GGA ATT ATC CCA AAA ACT CTC ATG GAT TTA ATA AAT AGG CGA AAA AAA GTC
AAA AAA GTT TTA AGG GAA AAA GCT GAA AAA GGG GAA TTT GAC GAA GAA TAC CAG ATT
TTA GAC TAC GAA CAA AGA TCT ATC AAA GTT CTT GCA AAT TCA CAC TAC GGA TAC CTC
GCA TTT CCA ATG GCA AGG TGG TAT TCG AGA GAT TGC GCA GAA ATT ACA ACT CAT CTT
GGA AGG CAG TAC ATT CAA AAA ACG ATA GAA GAA GCT GAA AAT TTT GGA TTT AAA GTA
ATT TAT GCA GAT ACG GAT GGA TTT TAT TCT AAA TGG GCC GAT GAT AAA GAA AAA CTG
TCA AAA TAT GAA CTT TTA GAA AAA ACC CGC GAA TTT TTA AAA AAT ATA AAC AAT ACA
TTG CCG GGA GAA ATG GAA CTT GAA TTT GAA GGG TAT TTC AAA AGG GGA ATT TTT GTA
ACC AAA AAG AAA TAC GCA CTT ATT GAC GAA AAT GAA AAA ATT ACG GTA AAA GGC CTT
GAA GTA GTA AGG CGG GAT TGG TCC AAT GTT TCA AAA AAT ACG CAA AAA AAC GTA TTG
AAC GCA CTT TTA AAA GAA GGT AGC GTT GAA AAT GCA AAA AAA GTG ATT CAG GAT ACC
ATT AAA GAG TTA AAG GAT GGA AAA GTA AAT AAT GAA GAT TTA TTG ATT CAT ACT CAG
CTT ACA AAA AGA ATT GAA GAC TAT AAA ACC ACA GCC CCG CAT GTT GAA GTT GCA AAA
AAA ATA TTG AAG TCT GGA AAT CGG GTA AAT ACT GGC GAC GTT ATA AGT TAT ATT ATA
ACA AGC GGA AAT AAA TCG ATT AGT GAG CGG GCG GAA ATA CTG GAA AAT GCA AAA AAT

TAC GAT ACA AAT TAT TAT ATC GAA AAT CAG ATT TTA CCC CCC GTT ATA CGA TTA ATG GAA GCT TTA GGA ATT ACT AAG GAC GAA TTA AAA GAC TCT AAA AAG CAG TAC ACG TTG CAC CAT TTT TTA AAG TGA

Occurrences/Results 9 Pentamers

The pentamer 'ATTCA' occurred 7 times.
The pentamer 'TTCAA' occurred 6 times.
The pentamer 'TCAAG' occurred 0 times.
The pentamer 'CAAGA' occurred 1 times.
The pentamer 'AAGAT' occurred 7 times.
The pentamer 'AGATG' occurred 3 times.
The pentamer 'GATGA' occurred 6 times.
The pentamer 'ATGAA' occurred 13 times.
The pentamer 'TGAAT' occurred 2 times.
The total occurrences of all specified strings is 45.
The total number of characters in the text is 2351.
First result (Total characters *9 /1024): 20.6982421875
Square root of the first result: 4.549532084456598
Final result: 5.341594995126324

Occurrences/Results TRIMERS Codons

The pentamer 'ATT' occurred 30 times.
The pentamer 'CAA' occurred 8 times.
The pentamer 'GAT' occurred 36 times.
The pentamer 'GAA' occurred 67 times.
The pentamer 'CCA' occurred 12 times.
The pentamer 'ACT' occurred 10 times.
The pentamer 'AGA' occurred 9 times.
The pentamer 'TAC' occurred 23 times.
The total occurrences of all specified strings is 195.
The total number of characters in the text is 2355.
First result ((total_characters / 3) * (Trimer_number / 64)): 98.1
Square root of the first result: 9.9
Final result: 9.8

Occurrences/Results TRIMERS Combinations

The pentamer 'ATT ATT' occurred 0 times.
The pentamer 'ATT CAA' occurred 1 times.
The pentamer 'ATT GAT' occurred 1 times.
The pentamer 'ATT GAA' occurred 3 times.
The pentamer 'ATT CCA' occurred 3 times.
The pentamer 'ATT ACT' occurred 1 times.
The pentamer 'ATT AGA' occurred 0 times.
The pentamer 'ATT TAC' occurred 1 times.
The pentamer 'CAA ATT' occurred 0 times.

The pentamer 'CAA CAA' occurred 0 times.
The pentamer 'CAA GAT' occurred 0 times.
The pentamer 'CAA GAA' occurred 0 times.
The pentamer 'CAA CCA' occurred 0 times.
The pentamer 'CAA ACT' occurred 0 times.
The pentamer 'CAA AGA' occurred 2 times.
The pentamer 'CAA TAC' occurred 0 times.
The pentamer 'GAT ATT' occurred 2 times.
The pentamer 'GAT CAA' occurred 0 times.
The pentamer 'GAT GAT' occurred 1 times.
The pentamer 'GAT GAA' occurred 5 times.
The pentamer 'GAT CCA' occurred 0 times.
The pentamer 'GAT ACT' occurred 0 times.
The pentamer 'GAT AGA' occurred 0 times.
The pentamer 'GAT TAC' occurred 1 times.
The pentamer 'GAA ATT' occurred 1 times.
The pentamer 'GAA CAA' occurred 2 times.
The pentamer 'GAA GAT' occurred 2 times.
The pentamer 'GAA GAA' occurred 4 times.
The pentamer 'GAA CCA' occurred 0 times.
The pentamer 'GAA ACT' occurred 1 times.
The pentamer 'GAA AGA' occurred 0 times.
The pentamer 'GAA TAC' occurred 1 times.
The pentamer 'CCA ATT' occurred 0 times.
The pentamer 'CCA CAA' occurred 0 times.
The pentamer 'CCA GAT' occurred 1 times.
The pentamer 'CCA GAA' occurred 0 times.
The pentamer 'CCA CCA' occurred 0 times.
The pentamer 'CCA ACT' occurred 0 times.
The pentamer 'CCA AGA' occurred 0 times.
The pentamer 'CCA TAC' occurred 1 times.
The pentamer 'ACT ATT' occurred 0 times.
The pentamer 'ACT CAA' occurred 1 times.
The pentamer 'ACT GAT' occurred 0 times.
The pentamer 'ACT GAA' occurred 0 times.
The pentamer 'ACT CCA' occurred 0 times.
The pentamer 'ACT ACT' occurred 0 times.
The pentamer 'ACT AGA' occurred 0 times.
The pentamer 'ACT TAC' occurred 0 times.
The pentamer 'AGA ATT' occurred 1 times.
The pentamer 'AGA CAA' occurred 0 times.
The pentamer 'AGA GAT' occurred 1 times.
The pentamer 'AGA GAA' occurred 0 times.

The pentamer 'AGA CCA' occurred 0 times.
The pentamer 'AGA ACT' occurred 0 times.
The pentamer 'AGA AGA' occurred 0 times.
The pentamer 'AGA TAC' occurred 0 times.
The pentamer 'TAC ATT' occurred 1 times.
The pentamer 'TAC CAA' occurred 0 times.
The pentamer 'TAC GAT' occurred 2 times.
The pentamer 'TAC GAA' occurred 2 times.
The pentamer 'TAC CCA' occurred 2 times.
The pentamer 'TAC ACT' occurred 0 times.
The pentamer 'TAC AGA' occurred 0 times.
The pentamer 'TAC TAC' occurred 0 times.
The total occurrences of all specified strings is 44.
The total number of characters in the text is 2351.
First result (((total_characters/ 3) -1) / 64): 12.25
Square root of the first result: 3.5
Final result: 9.071428571428571

>NZ_DAIONM010000001.1:c178586-176862 Methanococcus maripaludis isolate, Glycyl-tRNA ligase

---> Sequence

ATGGATAAATACGATAAAATAATTGATTTAACTAAAAGAAGAGGATTCTTATGGAATTCTTTTG
AAATATACGGCGGTATTGCAGGATTTTTTGACTACGGACCACTCGGTGCAATCTTAAAAAACAAT
GTTATAAACACGTGGAGAAAACACTATATCGTAAATGAAGGATTTTACGAAATTGACAGCCCAAC
TGTAACTCCTTACGAAGTTTTAAAAGCATCCGGACACGTTGAAAACTTTACAGATCCTCTAGTTG
AATGTAAAGACTGTCTTGAGTCTTTCAGAGCTGACCACATCATCGAAGAAAATGTTGATGTAGAT
ACTGAAGGTAAAACTTTACAAGAACTTCAAGAAATGATTGAAAAAAATAACATTCGATGTCCAA
AATGTGGCGGAGAATTTAAAGAAGTAAGCACATTTAATTTAATGTTTGCAACTTCAATTGGCCCT
GGTGGAAAAAGAGCTGCATTTATGAGACCTGAAACCGCTCAAGGTATTTTTATACAGTTTAAAAG
AATAAGCCAGTTCTTTAGAAACAAACTTCCATTTGGTGCAGTTCAAATTGGAAAAGCATACAGAA
ACGAAATTTCCCCTAGACAGGGAGTAATAAGATTAAGAGAATTTACACAGGCTGAAGGTGAATTT
TTCATTGATTCAAGGAAAAAAGAGAACTTTGAGAAATTTGAAAGCGTAAAAGATATGGTTTTAC
CATTACTTTCAGGTAAAAATCAGGAAGACGAATCATTAAGTTCTGAAGAAAAGTTGTTAAAAT
GAGTCTTTCTGATGCTGTTAAAAACGGAATCATCGCACACGAAGCAATTGCATACTATATTGCAG
TTACAAAAAAATTCTTGATGGAAATTGGAATCGATGAAACAAAATTAAGATTCAGACAGCACCT
TCCAAACGAAATGGCACACTACGCTGCTGATTGTTGGGATGCGGAATTATATACTGATAGATACG
GCTGGATTGAGTGTGTAGGTGTTGCTGACAGGACAAATTACGATTTATTGGCACACATGAAAAAT
AGCGGTGACGATTTATCAGTATTTGTGGAACTTGACGAAGAACATGAAATCGAAGCTTACGAAAT
TGAATTAAACTACAAATTAGTCGGTAGAACATTTAAAGGCGATGCAAAAGTTTTAGAAGAGTCT
TTAAAAGAATTGGACGACAAAAAAATGGAAGAATTGGTAGAAGCTCTTGAAACAGAAGGAAAAT
ATGTTTTAAAAACATGCAAAAGAGACTTCGAAATATTAAAAGAATATTTAACTGCTAAAAAAGT
TAAAAAGATCGTTAAAGGGGAAAAAATAATCCCTCACGTGATAGAGCCTTCATATGGTATCGACA
GGATTACATACTGCGTTATGGAGCATGCTTTCAAAGAAGATGAAGATAGAACCGTAATGGGCTTT

TCAAATGCAGTTTCTCCGATAAAAGTCGGCGTTTTCCCACTTGTTAACAAAGAAGGAATGCCTGA
AATTGCAATGGATTTGAAAAATAAATTAAGAGAAAACGGCCTAATTGCAGAATACGACGACAGC
GGTGCAATTGGTAGAAGATACATGAGAATGGACGAGGTAGGAACTCCATTCTGCATAACTATCGA
TGGGGAAACCTTAAAAGACAGATCAGTCACAATTAGAGAAAGAGATTCAAGAGAACAGTTCAGA
ATCCCAATTAACGAAGTTGTGCCATACATTAAAGATAAACTCTAA

---> Sequence Showing highlighted Existant Combined Trimers

ATG GAT AAA TAC GAT AAA ATA ATT GAT TTA ACT AAA AGA AGA GGA TTC TTA TGG AAT TCT TTT GAA ATA TAC GGC GGT ATT GCA GGA TTT TTT GAC TAC GGA CCA CTC GGT GCA ATC TTA AAA AAC AAT GTT ATA AAC ACG TGG AGA AAA CAC TAT ATC GTA AAT GAA GGA TTT TAC GAA ATT GAC AGC CCA ACT GTA ACT CCT TAC GAA GTT TTA AAA GCA TCC GGA CAC GTT GAA AAC TTT ACA GAT CCT CTA GTT GAA TGT AAA GAC TGT CTT GAG TCT TTC AGA GCT GAC CAC ATC ATC GAA GAA AAT GTT GAT GTA GAT ACT GAA GGT AAA ACT TTA CAA GAA CTT CAA GAA ATG ATT GAA AAA AAT AAC ATT CGA TGT CCA AAA TGT GGC GGA GAA TTT AAA GAA GTA AGC ACA TTT AAT TTA ATG TTT GCA ACT TCA ATT GGC CCT GGT GGA AAA AGA GCT GCA TTT ATG AGA CCT GAA ACC GCT CAA GGT ATT TTT ATA CAG TTT AAA AGA ATA AGC CAG TTC TTT AGA AAC AAA CTT CCA TTT GGT GCA GTT CAA ATT GGA AAA GCA TAC AGA AAC GAA ATT TCC CCT AGA CAG GGA GTA ATA AGA TTA AGA GAA TTT ACA CAG GCT GAA GGT GAA TTT TTC ATT GAT TCA AGG AAA AAA GAG AAC TTT GAG AAA TTT GAA AGC GTA AAA GAT ATG GTT TTA CCA TTA CTT TCA GGT AAA AAT CAG GAA GAC GAA TCA TTA AGT TCT GAA GAA AAA GTT GTT AAA ATG AGT CTT TCT GAT GCT GTT AAA AAC GGA ATC ATC GCA CAC GAA GCA ATT GCA TAC TAT ATT GCA GTT ACA AAA AAA TTC TTG ATG GAA ATT GGA ATC GAT GAA ACA AAA TTA AGA TTC AGA CAG CAC CTT CCA AAC GAA ATG GCA CAC TAC GCT GCT GAT TGT TGG GAT GCG GAA TTA TAT ACT GAT AGA TAC GGC TGG ATT GAG TGT GTA GGT GTT GCT GAC AGG ACA AAT TAC GAT TTA TTG GCA CAC ATG AAA AAT AGC GGT GAC GAT TTA TCA GTA TTT GTG GAA CTT GAC GAA GAA CAT GAA ATC GAA GCT TAC GAA ATT GAA TTA AAC TAC AAA TTA GTC GGT AGA ACA TTT AAA GGC GAT GCA AAA GTT TTA GAA GAG TCT TTA AAA GAA TTG GAC GAC AAA AAA ATG GAA GAA TTG GTA GAA GCT CTT GAA ACA GAA GGA AAA TAT GTT TTA AAA ACA TGC AAA AGA GAC TTC GAA ATA TTA AAA GAA TAT TTA ACT GCT AAA AAA GTT AAA AAG ATC GTT AAA GGG GAA AAA ATA ATC CCT CAC GTG ATA GAG CCT TCA TAT GGT ATC GAC AGG ATT ACA TAC TGC GTT ATG GAG CAT GCT TTC AAA GAA GAT GAA GAT AGA ACC GTA ATG GGC TTT TCA AAT GCA GTT TCT CCG ATA AAA GTC GGC GTT TTC CCA CTT GTT AAC AAA GAA GGA ATG CCT GAA ATT GCA ATG GAT TTG AAA AAT AAA TTA AGA GAA AAC GGC CTA ATT GCA GAA TAC GAC GAC AGC GGT GCA ATT GGT AGA AGA TAC ATG AGA ATG GAC GAG GTA GGA ACT CCA TTC TGC ATA ACT ATC GAT GGG GAA ACC TTA AAA GAC AGA TCA GTC ACA ATT AGA GAA AGA GAT TCA AGA GAA CAG TTC AGA ATC CCA ATT AAC GAA GTT GTG CCA TAC ATT AAA GAT AAA CTC TAA

Occurrences/Results 9 Pentamers

The pentamer 'ATTCA' occurred 3 times.
The pentamer 'TTCAA' occurred 7 times.
The pentamer 'TCAAG' occurred 4 times.
The pentamer 'CAAGA' occurred 3 times.
The pentamer 'AAGAT' occurred 8 times.

The pentamer 'AGATG' occurred 1 times.
The pentamer 'GATGA' occurred 2 times.
The pentamer 'ATGAA' occurred 5 times.
The pentamer 'TGAAT' occurred 3 times.
The total occurrences of all specified strings is 36.
The total number of characters in the text is 1721.
First result (Total characters *9 /1024): 15.1611328125
Square root of the first result: 3.8937299357428476
Final result: 5.351903581244227

Occurrences/Results TRIMERS Codons

The pentamer 'ATT' occurred 22 times.
The pentamer 'CAA' occurred 4 times.
The pentamer 'GAT' occurred 22 times.
The pentamer 'GAA' occurred 56 times.
The pentamer 'CCA' occurred 10 times.
The pentamer 'ACT' occurred 10 times.
The pentamer 'AGA' occurred 27 times.
The pentamer 'TAC' occurred 16 times.
The total occurrences of all specified strings is 167.
The total number of characters in the text is 1725.
First result ((total_characters / 3) * (Trimer_number / 64)): 71.9
Square root of the first result: 8.5
Final result: 11.2

Occurrences/Results TRIMERS Combinations

The pentamer 'ATT ATT' occurred 0 times.
The pentamer 'ATT CAA' occurred 0 times.
The pentamer 'ATT GAT' occurred 2 times.
The pentamer 'ATT GAA' occurred 2 times.
The pentamer 'ATT CCA' occurred 0 times.
The pentamer 'ATT ACT' occurred 0 times.
The pentamer 'ATT AGA' occurred 1 times.
The pentamer 'ATT TAC' occurred 0 times.
The pentamer 'CAA ATT' occurred 1 times.
The pentamer 'CAA CAA' occurred 0 times.
The pentamer 'CAA GAT' occurred 0 times.
The pentamer 'CAA GAA' occurred 2 times.
The pentamer 'CAA CCA' occurred 0 times.
The pentamer 'CAA ACT' occurred 0 times.
The pentamer 'CAA AGA' occurred 0 times.
The pentamer 'CAA TAC' occurred 0 times.
The pentamer 'GAT ATT' occurred 0 times.
The pentamer 'GAT CAA' occurred 0 times.
The pentamer 'GAT GAT' occurred 0 times.

The pentamer 'GAT GAA' occurred 2 times.
The pentamer 'GAT CCA' occurred 0 times.
The pentamer 'GAT ACT' occurred 1 times.
The pentamer 'GAT AGA' occurred 2 times.
The pentamer 'GAT TAC' occurred 0 times.
The pentamer 'GAA ATT' occurred 5 times.
The pentamer 'GAA CAA' occurred 0 times.
The pentamer 'GAA GAT' occurred 2 times.
The pentamer 'GAA GAA' occurred 4 times.
The pentamer 'GAA CCA' occurred 0 times.
The pentamer 'GAA ACT' occurred 0 times.
The pentamer 'GAA AGA' occurred 1 times.
The pentamer 'GAA TAC' occurred 1 times.
The pentamer 'CCA ATT' occurred 1 times.
The pentamer 'CCA CAA' occurred 0 times.
The pentamer 'CCA GAT' occurred 0 times.
The pentamer 'CCA GAA' occurred 0 times.
The pentamer 'CCA CCA' occurred 0 times.
The pentamer 'CCA ACT' occurred 1 times.
The pentamer 'CCA AGA' occurred 0 times.
The pentamer 'CCA TAC' occurred 1 times.
The pentamer 'ACT ATT' occurred 0 times.
The pentamer 'ACT CAA' occurred 0 times.
The pentamer 'ACT GAT' occurred 1 times.
The pentamer 'ACT GAA' occurred 1 times.
The pentamer 'ACT CCA' occurred 1 times.
The pentamer 'ACT ACT' occurred 0 times.
The pentamer 'ACT AGA' occurred 0 times.
The pentamer 'ACT TAC' occurred 0 times.
The pentamer 'AGA ATT' occurred 0 times.
The pentamer 'AGA CAA' occurred 0 times.
The pentamer 'AGA GAT' occurred 1 times.
The pentamer 'AGA GAA' occurred 4 times.
The pentamer 'AGA CCA' occurred 0 times.
The pentamer 'AGA ACT' occurred 0 times.
The pentamer 'AGA AGA' occurred 2 times.
The pentamer 'AGA TAC' occurred 2 times.
The pentamer 'TAC ATT' occurred 1 times.
The pentamer 'TAC CAA' occurred 0 times.
The pentamer 'TAC GAT' occurred 2 times.
The pentamer 'TAC GAA' occurred 3 times.
The pentamer 'TAC CCA' occurred 0 times.
The pentamer 'TAC ACT' occurred 0 times.

The pentamer 'TAC AGA' occurred 1 times.
The pentamer 'TAC TAC' occurred 0 times.
The total occurrences of all specified strings is 48.
The total number of characters in the text is 1721. N,
First result (((total_characters/ 3) -1) / 64): 8.96875
Square root of the first result: 2.9947871376777346
Final result: 13.033063188012164

>NZ_DAIONM010000001.1:c32337-32107 Methanococcus maripaludis isolate, rprotein L18

---> Sequence

ATGGCAAAAATCGTAAGAATAAAAGGAGAAATCGTTGGAAAAGATGAACCAATGGTATTTACAA
AAGAATACAACGTTTTAAAAGAAAATGATGCTTTAGAAACTATGTATTCAGAAATTGGCAGCAA
ACATGCTGTAAAAAGAGCAAATATCAAAGTTGTAGAAATTTCAGAAATTTCTGTAGAAGAAATT
CAAAGCCCGATTTTGAAAAAAACTCTTGAAATGAACTAA

---> Sequence Showing highlighted Existant Combined Trimers

ATG GCA AAA ATC GTA AGA ATA AAA GGA GAA ATC GTT GGA AAA GAT GAA CCA ATG GTA TTT ACA AAA GAA TAC AAC GTT TTA AAA GAA AAT GAT GCT TTA GAA ACT ATG TAT TCA GAA ATT GGC AGC AAA CAT GCT GTA AAA AGA GCA AAT ATC AAA GTT GTA GAA ATT TCA GAA ATT TCT GTA GAA GAA ATT CAA AGC CCG ATT TTG AAA AAA ACT CTT GAA ATG AAC TAA

Occurrences/Results 9 Pentamers

The pentamer 'ATTCA' occurred 2 times.
The pentamer 'TTCAA' occurred 1 times.
The pentamer 'TCAAG' occurred 0 times.
The pentamer 'CAAGA' occurred 0 times.
The pentamer 'AAGAT' occurred 1 times.
The pentamer 'AGATG' occurred 1 times.
The pentamer 'GATGA' occurred 1 times.
The pentamer 'ATGAA' occurred 2 times.
The pentamer 'TGAAT' occurred 0 times.
The total occurrences of all specified strings is 8.
The total number of characters in the text is 227.
First result (Total characters *9 /1024): 2.0302734375
Square root of the first result: 1.4248766393972496
Final result: 4.189644490926113

Occurrences/Results TRIMERS Codons

The pentamer 'ATT' occurred 5 times.
The pentamer 'CAA' occurred 1 times.
The pentamer 'GAT' occurred 2 times.
The pentamer 'GAA' occurred 11 times.
The pentamer 'CCA' occurred 1 times.

The pentamer 'ACT' occurred 2 times.
The pentamer 'AGA' occurred 2 times.
The pentamer 'TAC' occurred 1 times.
The total occurrences of all specified strings is 25.
The total number of characters in the text is 231.
First result ((total_characters / 3) * (Trimer_number / 64)): 9.6
Square root of the first result: 3.1
Final result: 5

Occurrences/Results TRIMERS Combinations

The pentamer 'ATT ATT' occurred 0 times.
The pentamer 'ATT CAA' occurred 1 times.
The pentamer 'ATT GAT' occurred 0 times.
The pentamer 'ATT GAA' occurred 0 times.
The pentamer 'ATT CCA' occurred 0 times.
The pentamer 'ATT ACT' occurred 0 times.
The pentamer 'ATT AGA' occurred 0 times.
The pentamer 'ATT TAC' occurred 0 times.
The pentamer 'CAA ATT' occurred 0 times.
The pentamer 'CAA CAA' occurred 0 times.
The pentamer 'CAA GAT' occurred 0 times.
The pentamer 'CAA GAA' occurred 0 times.
The pentamer 'CAA CCA' occurred 0 times.
The pentamer 'CAA ACT' occurred 0 times.
The pentamer 'CAA AGA' occurred 0 times.
The pentamer 'CAA TAC' occurred 0 times.
The pentamer 'GAT ATT' occurred 0 times.
The pentamer 'GAT CAA' occurred 0 times.
The pentamer 'GAT GAT' occurred 0 times.
The pentamer 'GAT GAA' occurred 1 times.
The pentamer 'GAT CCA' occurred 0 times.
The pentamer 'GAT ACT' occurred 0 times.
The pentamer 'GAT AGA' occurred 0 times.
The pentamer 'GAT TAC' occurred 0 times.
The pentamer 'GAA ATT' occurred 4 times.
The pentamer 'GAA CAA' occurred 0 times.
The pentamer 'GAA GAT' occurred 0 times.
The pentamer 'GAA GAA' occurred 1 times.
The pentamer 'GAA CCA' occurred 1 times.
The pentamer 'GAA ACT' occurred 1 times.
The pentamer 'GAA AGA' occurred 0 times.
The pentamer 'GAA TAC' occurred 1 times.
The pentamer 'CCA ATT' occurred 0 times.
The pentamer 'CCA CAA' occurred 0 times.

The pentamer 'CCA GAT' occurred 0 times.
The pentamer 'CCA GAA' occurred 0 times.
The pentamer 'CCA CCA' occurred 0 times.
The pentamer 'CCA ACT' occurred 0 times.
The pentamer 'CCA AGA' occurred 0 times.
The pentamer 'CCA TAC' occurred 0 times.
The pentamer 'ACT ATT' occurred 0 times.
The pentamer 'ACT CAA' occurred 0 times.
The pentamer 'ACT GAT' occurred 0 times.
The pentamer 'ACT GAA' occurred 0 times.
The pentamer 'ACT CCA' occurred 0 times.
The pentamer 'ACT ACT' occurred 0 times.
The pentamer 'ACT AGA' occurred 0 times.
The pentamer 'ACT TAC' occurred 0 times.
The pentamer 'AGA ATT' occurred 0 times.
The pentamer 'AGA CAA' occurred 0 times.
The pentamer 'AGA GAT' occurred 0 times.
The pentamer 'AGA GAA' occurred 0 times.
The pentamer 'AGA CCA' occurred 0 times.
The pentamer 'AGA ACT' occurred 0 times.
The pentamer 'AGA AGA' occurred 0 times.
The pentamer 'AGA TAC' occurred 0 times.
The pentamer 'TAC ATT' occurred 0 times.
The pentamer 'TAC CAA' occurred 0 times.
The pentamer 'TAC GAT' occurred 0 times.
The pentamer 'TAC GAA' occurred 0 times.
The pentamer 'TAC CCA' occurred 0 times.
The pentamer 'TAC ACT' occurred 0 times.
The pentamer 'TAC AGA' occurred 0 times.
The pentamer 'TAC TAC' occurred 0 times.
The total occurrences of all specified strings is 10.
The total number of characters in the text is 227.
First result (((total_characters/ 3) -1) / 64): 1.1875
Square root of the first result: 1.0897247358851685
Final result: 8.086904618937302

>AP011526.1:1464873-1466261 Methanococcus maripaludis KA1 DNA, PFK1

---> Sequence

ATGGAACTGACTAATCATTTTAAAGACTTTTCAAACGTTTCTATTTTTTTAGCATATAATGTAAA
TGTCGATGCATTGAAATATCTGGTAGATCCTTCAAATATCGAAGAATTAAAAGAAAATTTTAGCG
ATTCAGAAATTAAAACAAAAATTGAAGAATACCCTCGAACAATTGAAAAACCGATAGACTTTGT
TGCAAGATTAATTCATGCCATGAAATCTGGAAAACCTGCAGAAGTTCCATTAAAAAATAATTTAG
AAATTGATACTTTTTTAAACAGATTAACATACAATGAAGAAAGAATTGGGGGGCAGGTTGGAAT

AATCTCAAATTTACTCTCAATTTTAAATTTAAAAAAAATAATTTTTTATTCTCCAATTTTGGCAA
AAAAACAGGCTGAAATGTTTGAAAATAATGAAAATTTAGTATTTCCAAATATTACGAATGGAAA
ACTGGTTTTAAAAAAACCAATTGAATCTTTTAAAAACGATGAACTTAAAATAAATCGGATTTTT
GAGTACAAAGAAGATATTGAATTTTATTTAGAGAATGAAAAAATCACAACTCCTCAAAGTAACC
GATTTATCGTTGCATCTCGACCGGAAAATTTACGAATTGAAATAAAAGAAGAATTAAAAAATTA
TATGCCTGAAATTGGAAAATTAGTTGATTGTGCAATAATTTCAGGGGTTCAAGCGATTAAAGAA
GAGTATTCTGATGGAAAAACTTCAGAATACTACTTAAATCAGGTTAAAGAAGATATAAAATCGT
TAAAAAAAGAAAATAAAGACTTAAAAGTACATTTTGAATTTGCATCGATTCAAAATACGGAAAT
GAGAAAAAAAATTGCAGAATCAATTTTACCTGAAGTCGACTGTGTTGGAATGGATGAAACTGAA
ATTGCAAACATAATTCATGTTTTAGGTTACGAAGAGTTAAGCGAAGGAATATTAAAACACAGTA
AAATTGAAGATGTGCTAAAAGCTTCAAAAATACTTCTTGAAAAATACAATTTAGAGGGAATGCA
GGTGCATACAATGTACTACATAATGTATCTCTGTAAAAAAGGCGGCATTTTAAGCGACGAATCTT
TGGAAAAAACTCTCGAATTTGCAACAGTTTTGGCTTCAACAAAAGCAGCTCTCGGGCAGATTTCA
AGTATTGAGGATTTAAAAACCGGTCTTAAAATACCACATAATAAACATGGTGAATTACTAAAAG
AGATAGTTGAAAACATTTCAAAAGAAAAAGAATTAGAGGGTTACAAAATTATTTTAGTTCCTTC
AAGAATCGTTGAGAACCCAAAAAGTACCGTTGGACTTGGGGATACAATTTCAGCAGGTGCTTTTG
TAGGTTATGTTTCAGAACTAAAAAAAAATTAAAAAAATAA

---> Sequence Showing highlighted Existant Combined Trimers

ATG GAA CTG ACT AAT CAT TTT AAA GAC TTT TCA AAC GTT TCT ATT TTT TTA GCA TAT
AAT GTA AAT GTC GAT GCA TTG AAA TAT CTG GTA GAT CCT TCA AAT ATC GAA GAA TTA
AAA GAA AAT TTT AGC GAT TCA GAA ATT AAA ACA AAA ATT GAA GAA TAC CCT CGA ACA
ATT GAA AAA CCG ATA GAC TTT GTT GCA AGA TTA ATT CAT GCC ATG AAA TCT GGA AAA
CCT GCA GAA GTT CCA TTA AAA AAT AAT TTA GAA ATT GAT ACT TTT TTA AAC AGA TTA
ACA TAC AAT GAA GAA AGA ATT GGG GGG CAG GTT GGA ATA ATC TCA AAT TTA CTC TCA
ATT TTA AAT TTA AAA AAA ATA ATT TTT TAT TCT CCA ATT TTG GCA AAA AAA CAG GCT
GAA ATG TTT GAA AAT AAT GAA AAT TTA GTA TTT CCA AAT ATT ACG AAT GGA AAA CTG
GTT TTA AAA AAA **CCA** ATT GAA TCT TTT AAA AAC GAT GAA CTT AAA ATA AAT CGG ATT
TTT GAG TAC AAA GAA GAT ATT GAA TTT TAT TTA GAG AAT GAA AAA ATC ACA ACT CCT
CAA AGT AAC CGA TTT ATC GTT GCA TCT CGA CCG GAA AAT TTA CGA ATT GAA ATA AAA
GAA GAA TTA AAA AAT TAT ATG CCT GAA ATT GGA AAA TTA GTT GAT TGT GCA ATA ATT
TCA GGG GTT CAA GCG ATT AAA GAA GAG TAT TCT GAT GGA AAA ACT TCA GAA TAC TAC
TTA AAT CAG GTT AAA GAA GAT ATA AAA TCG TTA AAA AAA GAA AAT AAA GAC TTA AAA
GTA CAT TTT GAA TTT GCA TCG ATT CAA AAT ACG GAA ATG AGA AAA AAA ATT GCA GAA
TCA ATT TTA CCT GAA GTC GAC TGT GTT GGA ATG GAT GAA ACT GAA ATT GCA AAC ATA
ATT CAT GTT TTA GGT TAC GAA GAG TTA AGC GAA GGA ATA TTA AAA CAC AGT AAA ATT
GAA GAT GTG CTA AAA GCT TCA AAA ATA CTT CTT GAA AAA TAC AAT TTA GAG GGA ATG
CAG GTG CAT ACA ATG TAC TAC ATA ATG TAT CTC TGT AAA AAA GGC GGC ATT TTA AGC
GAC GAA TCT TTG GAA AAA ACT CTC GAA TTT GCA ACA GTT TTG GCT TCA ACA AAA GCA
GCT CTC GGG CAG ATT TCA AGT ATT GAG GAT TTA AAA ACC GGT CTT AAA ATA CCA CAT
AAT AAA CAT GGT GAA TTA CTA AAA GAG ATA GTT GAA AAC ATT TCA AAA GAA AAA GAA
TTA GAG GGT TAC AAA ATT ATT TTA GTT CCT TCA AGA ATC GTT GAG AAC CCA AAA AGT
ACC GTT GGA CTT GGG GAT ACA ATT TCA GCA GGT GCT TTT GTA GGT TAT GTT TCA GAA
CTA AAA AAA ATT AAA AAA TAA

Occurrences/Results 9 Pentamers

The pentamer 'ATTCA' occurred 4 times.
The pentamer 'TTCAA' occurred 9 times.
The pentamer 'TCAAG' occurred 3 times.
The pentamer 'CAAGA' occurred 2 times.
The pentamer 'AAGAT' occurred 4 times.
The pentamer 'AGATG' occurred 1 times.
The pentamer 'GATGA' occurred 2 times.
The pentamer 'ATGAA' occurred 6 times.
The pentamer 'TGAAT' occurred 4 times.
The total occurrences of all specified strings is 35.
The total number of characters in the text is 1385.
First result (Total characters *9 /1024): 12.2080078125
Square root of the first result: 3.493995966297042
Final result: 6.523187893561048

Occurrences/Results TRIMERS Codons

The pentamer 'ATT' occurred 32 times.
The pentamer 'CAA' occurred 3 times.
The pentamer 'GAT' occurred 13 times.
The pentamer 'GAA' occurred 47 times.
The pentamer 'CCA' occurred 6 times.
The pentamer 'ACT' occurred 6 times.
The pentamer 'AGA' occurred 5 times.
The pentamer 'TAC' occurred 10 times.
The total occurrences of all specified strings is 122.
The total number of characters in the text is 1385.
First result ((total_characters / 3) * (Trimer_number / 64)): 57.7
Square root of the first result: 7.6
Final result: 7.148

Occurrences/Results TRIMERS Combinations

The pentamer 'ATT ATT' occurred 1 times.
The pentamer 'ATT CAA' occurred 1 times.
The pentamer 'ATT GAT' occurred 1 times.
The pentamer 'ATT GAA' occurred 6 times.
The pentamer 'ATT CCA' occurred 0 times.
The pentamer 'ATT ACT' occurred 0 times.
The pentamer 'ATT AGA' occurred 0 times.
The pentamer 'ATT TAC' occurred 0 times.
The pentamer 'CAA ATT' occurred 0 times.
The pentamer 'CAA CAA' occurred 0 times.
The pentamer 'CAA GAT' occurred 0 times.
The pentamer 'CAA GAA' occurred 0 times.
The pentamer 'CAA CCA' occurred 0 times.

The pentamer 'CAA ACT' occurred 0 times.
The pentamer 'CAA AGA' occurred 0 times.
The pentamer 'CAA TAC' occurred 0 times.
The pentamer 'GAT ATT' occurred 1 times.
The pentamer 'GAT CAA' occurred 0 times.
The pentamer 'GAT GAT' occurred 0 times.
The pentamer 'GAT GAA' occurred 2 times.
The pentamer 'GAT CCA' occurred 0 times.
The pentamer 'GAT ACT' occurred 1 times.
The pentamer 'GAT AGA' occurred 0 times.
The pentamer 'GAT TAC' occurred 0 times.
The pentamer 'GAA ATT' occurred 4 times.
The pentamer 'GAA CAA' occurred 0 times.
The pentamer 'GAA GAT' occurred 3 times.
The pentamer 'GAA GAA' occurred 4 times.
The pentamer 'GAA CCA' occurred 0 times.
The pentamer 'GAA ACT' occurred 1 times.
The pentamer 'GAA AGA' occurred 1 times.
The pentamer 'GAA TAC' occurred 2 times.
The pentamer 'CCA ATT' occurred 2 times.
The pentamer 'CCA CAA' occurred 0 times.
The pentamer 'CCA GAT' occurred 0 times.
The pentamer 'CCA GAA' occurred 0 times.
The pentamer 'CCA CCA' occurred 0 times.
The pentamer 'CCA ACT' occurred 0 times.
The pentamer 'CCA AGA' occurred 0 times.
The pentamer 'CCA TAC' occurred 0 times.
The pentamer 'ACT ATT' occurred 0 times.
The pentamer 'ACT CAA' occurred 0 times.
The pentamer 'ACT GAT' occurred 0 times.
The pentamer 'ACT GAA' occurred 1 times.
The pentamer 'ACT CCA' occurred 0 times.
The pentamer 'ACT ACT' occurred 0 times.
The pentamer 'ACT AGA' occurred 0 times.
The pentamer 'ACT TAC' occurred 0 times.
The pentamer 'AGA ATT' occurred 1 times.
The pentamer 'AGA CAA' occurred 0 times.
The pentamer 'AGA GAT' occurred 0 times.
The pentamer 'AGA GAA' occurred 0 times.
The pentamer 'AGA CCA' occurred 0 times.
The pentamer 'AGA ACT' occurred 0 times.
The pentamer 'AGA AGA' occurred 0 times.
The pentamer 'AGA TAC' occurred 0 times.

The pentamer 'TAC ATT' occurred 0 times.
The pentamer 'TAC CAA' occurred 0 times.
The pentamer 'TAC GAT' occurred 0 times.
The pentamer 'TAC GAA' occurred 1 times.
The pentamer 'TAC CCA' occurred 0 times.
The pentamer 'TAC ACT' occurred 0 times.
The pentamer 'TAC AGA' occurred 0 times.
The pentamer 'TAC TAC' occurred 2 times.
The total occurrences of all specified strings is 35.
The total number of characters in the text is 1385.
First result (((total_characters/ 3) -1) / 64): 7.21875
Square root of the first result: 2.6867731575255847
Final result: 10.340005788053007

>CP026606.1:c683483-682023 Methanococcus maripaludis strain DSM 2067 chromosome, complete genome FtsH 1

---> Sequence

ATGGAAGAGTGGGTTGAAAAATACAGGCCAAAATCATTAAATGATGTCGCAGGGCACAATAAAA
CTAAACAAACACTTGTTGAAT**GG**ATAGAATCTATTATAGGCGGTCAAAATCAAAAACCAATACTT
TTAGCAGGGCCTCCTGGATCGGGTAAAACTACTTTAGCTTACGCGATTGCAAACGATTATGCTTT
TGATGTAATCGAACTTAACGCGAGTGACAAGCGAAATAAGGATGTAATTTCACAAGTTGTTGGAA
CTGCGGCAACTTCAAAATCACT**TACTG**GAAGAAGGACGTTAATCGTTTTAGATG**A**GGTTGACGGG
CTCTCTGGAAATGATGACCGAGGAGGGGTAGCTGAAATAATAAAAGTTTTAAAAACAGCAGAAA
ACCCCGTAATTTTA**ACTGC**AAACGATGTATACAAACCTGCTTTAATGACGCTTAGAAATTCTGTA
AATTTGATAAATGTCGGTTCAGTTCACACAAACTCGATTCCACCGGTTTTAAGAAGAATTGCACT
AAAAGAAGGTTTTGAAATCGATGA**A**AAAATAATTAAAATGATTGCAAGTCACTCTGGCGGAGAT
TTAAGGGCTGCAATAAATGATTTACAGTCATTAGCAACGGGCGGATCGATTGAAATCGAAGATGC
AAAAGAACTTCCCGATAGGGACAGTGAAAAGAGTATTTTTGATGCAATGAGAATAATAATGAAA
ACAACCCACTACGACATTGCAACAAGTGCTACAAGGGATGTAAAAGAAGATATCGGAACCATTGA
AGAGTGGATTTCTGAAAATTTACCAAAAGAATATTTAAAATACAAGGATCTTGCAGAAGGGTAT
GACTACCTTTCAAAATCAGATGTATTTTTGGGAAGGGTTTACCGAAGACAGTATTTTGGACTTTG
GAGATATGCTTCAGCTTTAATG**ACTGC**TGGA**ACTGC**TTTAGCAAAAGAAGAAAAGTATCGAGGA
TTTACCCGATATGGCCACCTGCTATCTTTACAAAATTAAGCAGGACTAAGGGTAGCAGACAGAA
AATGAA**AGAT**ATTTTGAAAAAAATAGCTTTAAAGACCCATACTTCAACAAAAAGAGCAAGA**A**AT
ACCGTGGATTATTTGACTGTAATTTTTGAATCAAATCCGGAAGTTTCTGCAGAACTTGTGGAATA
TTATGAACTTACAAAGGATGA**AATGG**AGTTTTTAACCAACAAAACAATTACAAAAAAAATACTT
TCAGTAATTGC**TGGTA**AAAAACCAAAAGTTAAAAAAGAAACCCCGAAGAAAAAAGAAACTCCCA
AAGAAGTAATGCCCGTTATTCCAAAACGTCCTAGAATTTCAGAAACTCCAAAAGAACCTTTAAAA
GAGGTAATCGAAGAAACCGTTCAACCTTCTGAAAAAGTAAATAAAAAAGAAGAAGAAAAGAAAA
AAGATCCAAAAAAACAGGCAACTTTGGACAGCTTCTTTTAA

---> Sequence Showing highlighted Existant Combined Trimers

ATG GAA GAG TGG GTT GAA AAA TAC AGG CCA AAA TCA TTA AAT GAT GTC GCA GGG CAC
AAT AAA ACT AAA CAA ACA CTT GTT GAA TGG ATA GAA TCT ATT ATA GGC GGT CAA AAT

CAA AAA CCA ATA CTT TTA GCA GGG CCT CCT GGA TCG GGT AAA ACT ACT TTA GCT TAC GCG ATT GCA AAC GAT TAT GCT TTT GAT GTA ATC GAA CTT AAC GCG AGT GAC AAG CGA AAT AAG GAT GTA ATT TCA CAA GTT GTT GGA ACT GCG GCA ACT TCA AAA TCA CTT ACT GGA AGA AGG ACG TTA ATC GTT TTA GAT GAG GTT GAC GGG CTC TCT GGA AAT GAT GAC CGA GGA GGG GTA GCT GAA ATA ATA AAA GTT TTA AAA ACA GCA GAA AAC CCC GTA ATT TTA ACT GCA AAC GAT GTA TAC AAA CCT GCT TTA ATG ACG CTT AGA AAT TCT GTA AAT TTG ATA AAT GTC GGT TCA GTT CAC ACA AAC TCG ATT CCA CCG GTT TTA AGA AGA ATT GCA CTA AAA GAA GGT TTT GAA ATC GAT GAA AAA ATA ATT AAA ATG ATT GCA AGT CAC TCT GGC GGA GAT TTA AGG GCT GCA ATA AAT GAT TTA CAG TCA TTA GCA ACG GGC GGA TCG ATT GAA ATC GAA GAT GCA AAA GAA CTT CCC GAT AGG GAC AGT GAA AAG AGT ATT TTT GAT GCA ATG AGA ATA ATA ATG AAA ACA ACC CAC TAC GAC ATT GCA ACA AGT GCT ACA AGG GAT GTA AAA GAA GAT ATC GGA ACC ATT GAA GAG TGG ATT TCT GAA AAT TTA CCA AAA GAA TAT TTA AAA TAC AAG GAT CTT GCA GAA GGG TAT GAC TAC CTT TCA AAA TCA GAT GTA TTT TTG GGA AGG GTT TAC CGA AGA CAG TAT TTT GGA CTT TGG AGA TAT GCT TCA GCT TTA ATG ACT GCT GGA ACT GCT TTA GCA AAA GAA GAA AAG TAT CGA GGA TTT ACC CGA TAT GGG CCA CCT GCT ATC TTT ACA AAA TTA AGC AGG ACT AAG GGT AGC AGA CAG AAA ATG AAA GAT ATT TTG AAA AAA ATA GCT TTA AAG ACC CAT ACT TCA ACA AAA AGA GCA AGA AAT ACC GTG GAT TAT TTG ACT GTA ATT TTT GAA TCA AAT CCG GAA GTT TCT GCA GAA CTT GTG GAA TAT TAT GAA CTT ACA AAG GAT GAA ATG GAG TTT TTA ACC AAC AAA ACA ATT ACA AAA AAA ATA CTT TCA GTA ATT GCT GGT AAA AAA CCA AAA GTT AAA AAA GAA ACC CCG AAG AAA AAA GAA ACT CCC AAA GAA GTA ATG CCC GTT ATT CCA AAA CGT CCT AGA ATT TCA GAA ACT CCA AAA GAA CCT TTA AAA GAG GTA ATC GAA GAA ACC GTT CAA CCT TCT GAA AAA GTA AAT AAA AAA GAA GAA GAA AAG AAA AAA GAT CCA AAA AAA CAG GCA ACT TTG GAC AGC TTC TTT TAA

Occurrences/Results 9 Pentamers

The pentamer 'ATTCA' occurred 0 times.
The pentamer 'TTCAA' occurred 4 times.
The pentamer 'TCAAG' occurred 0 times.
The pentamer 'CAAGA' occurred 1 times.
The pentamer 'AAGAT' occurred 4 times.
The pentamer 'AGATG' occurred 3 times.
The pentamer 'GATGA' occurred 4 times.
The pentamer 'ATGAA' occurred 5 times.
The pentamer 'TGAAT' occurred 2 times.
The total occurrences of all specified strings is 23.
The total number of characters in the text is 1457.
First result (Total characters *9 /1024): 12.8408203125
Square root of the first result: 3.5834090350530734
Final result: 2.8350600191388793

Occurrences/Results TRIMERS Codons

The pentamer 'CAA' occurred 5 times.
The pentamer 'GAT' occurred 21 times.
The pentamer 'GAA' occurred 38 times.

The pentamer 'ATT' occurred 19 times.
The pentamer 'TAC' occurred 7 times.
The pentamer 'AGA' occurred 11 times.
The pentamer 'ACT' occurred 15 times.
The pentamer 'CCA' occurred 9 times.
The total occurrences of all specified strings is 125.
The total number of characters in the text is 1461.
First result ((total_characters / 3) * (Trimer_number / 64)): 60.9
Square root of the first result: 7.8
Final result: 8.2

Occurrences/Results TRIMERS Combinations

The pentamer 'CAA CAA' occurred 0 times.
The pentamer 'CAA GAT' occurred 0 times.
The pentamer 'CAA GAA' occurred 0 times.
The pentamer 'CAA ATT' occurred 0 times.
The pentamer 'CAA TAC' occurred 0 times.
The pentamer 'CAA AGA' occurred 0 times.
The pentamer 'CAA ACT' occurred 0 times.
The pentamer 'CAA CCA' occurred 0 times.
The pentamer 'GAT CAA' occurred 0 times.
The pentamer 'GAT GAT' occurred 0 times.
The pentamer 'GAT GAA' occurred 2 times.
The pentamer 'GAT ATT' occurred 1 times.
The pentamer 'GAT TAC' occurred 0 times.
The pentamer 'GAT AGA' occurred 0 times.
The pentamer 'GAT ACT' occurred 0 times.
The pentamer 'GAT CCA' occurred 1 times.
The pentamer 'GAA CAA' occurred 0 times.
The pentamer 'GAA GAT' occurred 2 times.
The pentamer 'GAA GAA' occurred 3 times.
The pentamer 'GAA ATT' occurred 0 times.
The pentamer 'GAA TAC' occurred 0 times.
The pentamer 'GAA AGA' occurred 0 times.
The pentamer 'GAA ACT' occurred 2 times.
The pentamer 'GAA CCA' occurred 0 times.
The pentamer 'ATT CAA' occurred 0 times.
The pentamer 'ATT GAT' occurred 0 times.
The pentamer 'ATT GAA' occurred 2 times.
The pentamer 'ATT ATT' occurred 0 times.
The pentamer 'ATT TAC' occurred 0 times.
The pentamer 'ATT AGA' occurred 0 times.
The pentamer 'ATT ACT' occurred 0 times.
The pentamer 'ATT CCA' occurred 2 times.

The pentamer 'TAC CAA' occurred 0 times.
The pentamer 'TAC GAT' occurred 0 times.
The pentamer 'TAC GAA' occurred 0 times.
The pentamer 'TAC ATT' occurred 0 times.
The pentamer 'TAC TAC' occurred 0 times.
The pentamer 'TAC AGA' occurred 0 times.
The pentamer 'TAC ACT' occurred 0 times.
The pentamer 'TAC CCA' occurred 0 times.
The pentamer 'AGA CAA' occurred 0 times.
The pentamer 'AGA GAT' occurred 0 times.
The pentamer 'AGA GAA' occurred 0 times.
The pentamer 'AGA ATT' occurred 2 times.
The pentamer 'AGA TAC' occurred 0 times.
The pentamer 'AGA AGA' occurred 1 times.
The pentamer 'AGA ACT' occurred 0 times.
The pentamer 'AGA CCA' occurred 0 times.
The pentamer 'ACT CAA' occurred 0 times.
The pentamer 'ACT GAT' occurred 0 times.
The pentamer 'ACT GAA' occurred 0 times.
The pentamer 'ACT ATT' occurred 0 times.
The pentamer 'ACT TAC' occurred 0 times.
The pentamer 'ACT AGA' occurred 0 times.
The pentamer 'ACT ACT' occurred 1 times.
The pentamer 'ACT CCA' occurred 1 times.
The pentamer 'CCA CAA' occurred 0 times.
The pentamer 'CCA GAT' occurred 0 times.
The pentamer 'CCA GAA' occurred 0 times.
The pentamer 'CCA ATT' occurred 0 times.
The pentamer 'CCA TAC' occurred 0 times.
The pentamer 'CCA AGA' occurred 0 times.
The pentamer 'CCA ACT' occurred 0 times.
The pentamer 'CCA CCA' occurred 0 times.
The total occurrences of all specified strings is 20.
The total number of characters in the text is 1457.
First result (((total_characters/ 3) -1) / 64): 7.59375
Square root of the first result: 2.7556759606310752
Final result: 4.502071425393156

2) Trichomonas vaginalis

>JAOSJJ010000002.1:2976314-2976384 Trichomonas vaginalis G3 chromosome V, tRNA-Gly

GCACC**GC**T**G**GTCT**AATGGTA**GAATTTCACCT**TGCCA**AGGTGGGGTCCGGG**TTC**G**A**TTC
CCGGGCGGTGCA

>XM_001324229.1 Trichomonasvaginalis G3, DNA topoisomerase II (TVAGG3_0240260)

---> Sequence

ATGAGCCGCTCTGACTCTCAAGAGAAACTCTCTGAGTCAGATGAACAATATGTTAAGCTCACACA
CCGCGAGCACGTCCTTACTCGCCCTGATTCTTACATTGGTTCCATTGAGCCACGTGAATGCAAGAT
GTGGGTTTGCAACGAAGACGGAAAGTTCGAATTCAGAGAAATATCCTATGTTCCAGGTCTCTACA
AGATTTTTGATGAAATTCTTGTTAATGCTGCCGATAACAAGCAGAGAGACCCTCCATTAACAACG
ATCAAAGTTGAAGTTGACCCAGCTCAAAACAGAATCTCAGTATGGAACGATGGTAAAGGTATCTC
CACCCAAAAACAGTTCAGCCCTAATGATAATTGCGAATATTACATTCCAGAGTTCATTTTCAGCC
AGCTTTTGACTTCATCCAACTACAATGATAAGCAAGAGAAAGTTACAGGTGGCCGTAATGGTTAC
GGTGCTAAACTTGCCAACATTTTCTCTAAAAAGTTCCATATCAACATCTACAATGCTGATGAAGG
AATCAACTACCAGCAGACAATTACAGACAACATGACCAATATCCAACCTCCTAAGCTGAAGAATG
GAAAAGGAAAAGGTTCATACACAGAAATCTCCTTCTGGCCAGATCTCGAGCGTTTCAACTTACAG
ACGATCACAAAGGATCACGTCGACCTCATCAGACGTCGTGTTTACGATCTTGCCGGTATTCTTCCA
AAAGTCAAAATTTTCTGGAATGGAAACCAAGTTCCAATCAAGAAATGGGAAGATTACTGCAAGG
TTTACCTCAAGGAAGATGTCAAGCCATTAGTTTGTGAAGTAAAGAACGAGAAAGGCTACACATGG
AAGATCGGTGTTGCTCCATCAAAGAGCCGTGAATTCCAGCAGATTTCCTTCGTCAACTGCGTCGCC
ACATCCAATGGTGGTACACACGTCGATGTTGTCGTTAACCAAATCACAAAATACATCACAGATTA
CTTGAAGAACAGAAGAAGTGGCAAAGTCGAGACAAAGCCAAGTGTTGTCAAGAACTTCCTCTACG
TTTTCGTTGATTCTCTCGTTATCAACCCATCATTCGATTCCCAGACCAAAGAGAGATTAACACTCG
ACCAGAAGAAGCTCAAAGACGATTGCAAGCTTCCAGACAACTTCCTCGACAAGATCCTCAAGACA
GACATCGTTGCTCTTGTTACAGAATACGCCAATTTCAAGGAACAGCGTAAAGTTGCCAACATGAA
AGGCACAAAGAAAGGCCGTCTCATCATCAACAAGCTTGAAGACGCGAACATGGCCGGAAAAGCCG
AATCTCTCAAATGCACTTTAATTTTAACCGAAGGTGACTCAGCTAAAGCAACCGCCGTTACAGGC
CTTGCCACAATTGGTCGTGATTACTACGGTGTCTTCCCATTGAGAGGCAAACTCCTTAATACTCGT
GATATTTCCGCAAAGAAGCTTTCCGAGAATGATGAAATCCAGAACATTATCAAGATCATGGGTTT
GGATCCAATGGAGAAGTACGAAAACGATGATTCCATGAAGAGATTGAGATACGGCTCAATCATGA
TCATGGCCGATCAAGATGTCGATGGTTCCCACATCAAAGGTTTGATCATCAACTTCGTACACTCA
ATGTGGCCGAACTTACTCAAAAAGAGGAACTTCATCACAGAATTCATTACTCCTATCGTAAAAGT
GACAAAGGGGAACAACAAGAAAGGTGCCATTTCCTTCTTCACCATTCCAGAATTTTCACAGTGGA
AAGCAGCCAACAACAATGGCAAAGGCTACAAGATGAAGTACTACAAAGGTCTTGCTACATCCACA
GCAGAAGAAGCCAAAGAATACTTCTCAAACATCTCCAAGCACAAGAAAGTTTTCATTTACACGAA
TGAAAACGATGAAGAAAGGATCAACTTGGCATTCGCAAAGAAGAGAGCTGATGACAGAAAGAAC
TGGCTCGCACAACTTGATGCAAATGAAACATATCTTGGTACAAAAGATACACACATCAAGTACAG
TGATTTCGTCGATAAAGAACTCATTCTTTTCTCTTCTTACGCGAACTATCGTGCAATTCCTTCATC
AATCGATGGTTGGAAGCCAGGCCAGAGAAAGATCTTATGGGTCTGTCTCAAGAACAACATCAAGA
ACGACTTGAAAGTTGCTCAGTTATCAGGTAAAGTATCTGAACAAGCAGCTTATCACCATGGCGAA

GCATCACTCAACGAAACAATCATTGGCATGTGCCAGGATTTCGTTGGCTCAAACAACATCAACGT
TTTCCAGCCAATCGGTATGCTCGGTACAAGACTTGCTGGTGGTGAAGACTCTGGTTCTCCACGTT
ACGTATACACCGCTCTTGAAAAGATCACGAGAACAATCTTCATGAAGGAAGATGATGACTTGTTG
ACATACAATACTGATGAAGGTCTTCCAATCGAACCAGTCACTTACATCCCAATCATCCCAATGGT
TCTTGTTAATGGCGCTAAAGGTATCGGTGTCGGTTGGTCAAGTGAAGTTCCTCAGTATTGTCCAC
AAGACATCATCGACAATCTCAGGAGAAAGATGAATGATGAACCAATGGTTGAAATGACACCATGG
TACTCAGGATTCACAGGAAAGATCATTCCAATCCAAGTAATCAAAGACGGTGTCAAACAGCCAAT
TGTTAAATGGGAATCTCGTGGTATTGTCAAGAAGATTGATGATACAACAATCGAGATTACTGAAT
TACCAATTGGTATTTGGACATACGATTACAAGAAATTCTTGGAAGGTTTGACTGTCGGAGAATCA
TTGAATCCACACAAGAAACTCGCAGACAGAAAGGCAGCTGCAGCAAAGGCAAAGAGAACAAAGA
AGAAAGCTGCTGCAGGTGATGACCCAGTTGAACAGGCCGATGTCGAAGAAAAAGTTAATCTCGGT
GGTCCAAAGATCTCCGAATTCAGAGAATACCATACAAACGTTTCTGTTCACTTCATCATTACTGT
TGATGATTCTCAGATGAGAGAAATCGAGTCTGTCGGCTTGAGAGAATTCTTCAAACTCAAGTCTT
CAATCACTGCCACAAACATGACACTTTTCAATGCACAGAACAAGATTGTTCAGTTCAACTCTCCT
TTGGACATCATCAACGACTACTATCCAGTGAGAATGAAGTACTACGAAGAGAGACACAGATATTT
AATCGACGCTCTCCAGTTGCAGTACAAGAAACTCTCAAACCAAGCTCGTTTCATCAGAGAAGTTA
TTGCAGGAATCATCAAAGTCAACGGTGTTCCAAGAGCAACAATTCTCGCTGCTTTGGAGAGAGGA
AACTACGATCTCTATGATGATAAGGAACTCACGAAACGCATCAAGTTCGCTGATGAAGTTATCCA
ACAGCACAATGAAGAAGAAGAAGAACAGAACGAGTTCAAGAGTGAATCGAATGCAGATCATTTG
GCATTGCTTGAGAAAGGTTATGATTACTTGCTCGCAATGAAGATCTGGACATTGACAAAAGAAAA
AGCAGACAAATTGGATGACGAAGCCAAGAAGAAGAACCAAGAATTGGAAGAAATGCTCAAGAAG
AAGCCAATCGATTTCTACAAAGAAGATCTCGATAAATTCGAAGTCGAATGGAAGGCTTTCGAGGC
AAAGAGAGAAAAGACTCGTCTCGAAAACGTCGCTGATGCAGAGAAAGTCAATAGGAAGTCAATC
GCAAAGAACAAGAAGGCTGCAGACCAGAAGAAGGCCAAACAACAGAAGAAGGATGCAAAGGCGA
AACAAGAAGCAGCTGCTTTGCAAACAGCAAATGGAGAAGAGCTCAAACAAGAAGTCGAAAACCA
GATGCCAACGAAAGAAGAAACAGTTGTTCAGGAATCAGATGATGACATCATCGTAATCAATGAA
GACGTCAAGAAACCAAAGAAAGAAAGAAAACCAAAGGCTGAGACAACAACACCTACAAAAGAAG
GCGAAGAAAAGCCAAAGAAGGAAAGAAAACCAAGAGTTAAGAAAGAAAAGAAAGAAGGTGAAGA
AGAAACAGAAGAAAAGAAACCAGCAGCAACAAAGGTAAAGAAAGAAACAACTGAAACAGAAACA
AAGAAGAAATCGAAGACAACAAAGAAGTCAAATAAGTCTGCTTTCGAAGATGAAGACGAAGATG
ACGATGTCGATGTAGATTTAGAGGATTCTGATGAAGAACCAGTAGAAACAATCGCTTCAAGACTC
GCGTCAAGAAGAAAGACTGAACCAAAGAAACAGGCAATTTTGGAGGAATTCTTCTCTGGAAAGA
AGAAGGACGGTGAAGACGGCGAAGAAGAGGAAGACGAGGATGATGACCAGGATGAAGATGATGA
CGGAGATGACGCTTGGGAAGATGATGAGTAA

---> Sequence Showing highlighted Existant Combined Trimers

ATG AGC CGC TCT GAC TCT CAA GAG AAA CTC TCT GAG TCA GAT GAA CAA TAT GTT AAG
CTC ACA CAC CGC GAG CAC GTC CTT ACT CGC CCT GAT TCT TAC ATT GGT TCC ATT GAG
CCA CGT GAA TGC AAG ATG TGG GTT TGC AAC GAA GAC GGA AAG TTC GAA TTC AGA GAA
ATA TCC TAT GTT CCA GGT CTC TAC AAG ATT TTT GAT GAA ATT CTT GTT AAT GCT GCC
GAT AAC AAG CAG AGA GAC CCT CCA TTA ACA ACG ATC AAA GTT GAA GTT GAC CCA GCT
CAA AAC AGA ATC TCA GTA TGG AAC GAT GGT AAA GGT ATC TCC ACC CAA AAA CAG TTC
AGC CCT AAT GAT AAT TGC GAA TAT TAC ATT CCA GAG TTC ATT TTC AGC CAG CTT TTG

ACT TCA TCC AAC TAC AAT GAT AAG CAA GAG AAA GTT ACA GGT GGC CGT AAT GGT TAC
GGT GCT AAA CTT GCC AAC ATT TTC TCT AAA AAG TTC CAT ATC AAC ATC TAC AAT GCT
GAT GAA GGA ATC AAC TAC CAG CAG ACA ATT ACA GAC AAC ATG ACC AAT ATC CAA CCT
CCT AAG CTG AAG AAT GGA AAA GGA AAA GGT TCA TAC ACA GAA ATC TCC TTC TGG CCA
GAT CTC GAG CGT TTC AAC TTA CAG ACG ATC ACA AAG GAT CAC GTC GAC CTC ATC AGA
CGT CGT GTT TAC GAT CTT GCC GGT ATT CTT CCA AAA GTC AAA ATT TTC TGG AAT GGA
AAC CAA GTT CCA ATC AAG AAA TGG GAA GAT TAC TGC AAG GTT TAC CTC AAG GAA GAT
GTC AAG CCA TTA GTT TGT GAA GTA AAG AAC GAG AAA GGC TAC ACA TGG AAG ATC GGT
GTT GCT CCA TCA AAG AGC CGT GAA TTC CAG CAG ATT TCC TTC GTC AAC TGC GTC GCC
ACA TCC AAT GGT GGT ACA CAC GTC GAT GTT GTC GTT AAC CAA ATC ACA AAA TAC ATC
ACA GAT TAC TTG AAG AAC AGA AGA AGT GGC AAA GTC GAG ACA AAG CCA AGT GTT GTC
AAG AAC TTC CTC TAC GTT TTC GTT GAT TCT CTC GTT ATC AAC CCA TCA TTC GAT TCC
CAG ACC AAA GAG AGA TTA ACA CTC GAC CAG AAG AAG CTC AAA GAC GAT TGC AAG CTT
CCA GAC AAC TTC CTC GAC AAG ATC CTC AAG ACA GAC ATC GTT GCT CTT GTT ACA GAA
TAC GCC AAT TTC AAG GAA CAG CGT AAA GTT GCC AAC ATG AAA GGC ACA AAG AAA GGC
CGT CTC ATC ATC AAC AAG CTT GAA GAC GCG AAC ATG GCC GGA AAA GCC GAA TCT CTC
AAA TGC ACT TTA ATT TTA ACC GAA GGT GAC TCA GCT AAA GCA ACC GCC GTT ACA GGC
CTT GCC ACA ATT GGT CGT GAT TAC TAC GGT GTC TTC CCA TTG AGA GGC AAA CTC CTT
AAT ACT CGT GAT ATT TCC GCA AAG AAG CTT TCC GAG AAT GAT GAA ATC CAG AAC ATT
ATC AAG ATC ATG GGT TTG GAT CCA ATG GAG AAG TAC GAA AAC GAT GAT TCC ATG AAG
AGA TTG AGA TAC GGC TCA ATC ATG ATC ATG GCC GAT CAA GAT GTC GAT GGT TCC CAC
ATC AAA GGT TTG ATC ATC AAC TTC GTA CAC TCA ATG TGG CCG AAC TTA CTC AAA AAG
AGG AAC TTC ATC ACA GAA TTC ATT ACT CCT ATC GTA AAA GTG ACA AAG GGG AAC AAC
AAG AAA GGT GCC ATT TCC TTC TTC ACC ATT CCA GAA TTT TCA CAG TGG AAA GCA GCC
AAC AAC AAT GGC AAA GGC TAC AAG ATG AAG TAC TAC AAA GGT CTT GCT ACA TCC ACA
GCA GAA GAA GCC AAA GAA TAC TTC TCA AAC ATC TCC AAG CAC AAG AAA GTT TTC ATT
TAC ACG AAT GAA AAC GAT GAA GAA AGG ATC AAC TTG GCA TTC GCA AAG AAG AGA GCT
GAT GAC AGA AAG AAC TGG CTC GCA CAA CTT GAT GCA AAT GAA ACA TAT CTT GGT ACA
AAA GAT ACA CAC ATC AAG TAC AGT GAT TTC GTC GAT AAA GAA CTC ATT CTT TTC TCT
TCT TAC GCG AAC TAT CGT GCA ATT CCT TCA TCA ATC GAT GGT TGG AAG CCA GGC CAG
AGA AAG ATC TTA TGG GTC TGT CTC AAG AAC AAC ATC AAG AAC GAC TTG AAA GTT GCT
CAG TTA TCA GGT AAA GTA TCT GAA CAA GCA GCT TAT CAC CAT GGC GAA GCA TCA CTC
AAC GAA ACA ATC ATT GGC ATG TGC CAG GAT TTC GTT GGC TCA AAC AAC ATC AAC GTT
TTC CAG CCA ATC GGT ATG CTC GGT ACA AGA CTT GCT GGT GGT GAA GAC TCT GGT TCT
CCA CGT TAC GTA TAC ACC GCT CTT GAA AAG ATC ACG AGA ACA ATC TTC ATG AAG GAA
GAT GAT GAC TTG TTG ACA TAC AAT ACT GAT GAA GGT CTT CCA ATC GAA CCA GTC ACT
TAC ATC CCA ATC ATC CCA ATG GTT CTT GTT AAT GGC GCT AAA GGT ATC GGT GTC GGT
TGG TCA AGT GAA GTT CCT CAG TAT TGT CCA CAA GAC ATC ATC GAC AAT CTC AGG AGA
AAG ATG AAT GAT GAA CCA ATG GTT GAA ATG ACA CCA TGG TAC TCA GGA TTC ACA GGA
AAG ATC ATT CCA ATC CAA GTA ATC AAA GAC GGT GTC AAA CAG CCA ATT GTT AAA TGG
GAA TCT CGT GGT ATT GTC AAG AAG ATT GAT GAT ACA ACA ATC GAG ATT ACT GAA TTA
CCA ATT GGT ATT TGG ACA TAC GAT TAC AAG AAA TTC TTG GAA GGT TTG ACT GTC GGA
GAA TCA TTG AAT CCA CAC AAG AAA CTC GCA GAC AGA AAG GCA GCT GCA GCA AAG GCA

AAG AGA ACA AAG AAG AAA GCT GCT GCA GGT GAT GAC CCA GTT GAA CAG GCC GAT GTC GAA GAA AAA GTT AAT CTC GGT GGT CCA AAG ATC TCC GAA TTC AGA GAA TAC CAT ACA AAC GTT TCT GTT CAC TTC ATC ATT ACT GTT GAT GAT TCT CAG ATG AGA GAA ATC GAG TCT GTC GGC TTG AGA GAA TTC TTC AAA CTC AAG TCT TCA ATC ACT GCC ACA AAC ATG ACA CTT TTC AAT GCA CAG AAC AAG ATT GTT CAG TTC AAC TCT CCT TTG GAC ATC ATC AAC GAC TAC TAT CCA GTG AGA ATG AAG TAC TAC GAA GAG AGA CAC AGA TAT TTA ATC GAC GCT CTC CAG TTG CAG TAC AAG AAA CTC TCA AAC CAA GCT CGT TTC ATC AGA GAA GTT ATT GCA GGA ATC ATC AAA GTC AAC GGT GTT CCA AGA GCA ACA ATT CTC GCT GCT TTG GAG AGA GGA AAC TAC GAT CTC TAT GAT GAT AAG GAA CTC ACG AAA CGC ATC AAG TTC GCT GAT GAA GTT ATC CAA CAG CAC AAT GAA GAA GAA GAA GAA CAG AAC GAG TTC AAG AGT GAA TCG AAT GCA GAT CAT TTG GCA TTG CTT GAG AAA GGT TAT GAT TAC TTG CTC GCA ATG AAG ATC TGG ACA TTG ACA AAA GAA AAA GCA GAC AAA TTG GAT GAC GAA GCC AAG AAG AAG AAC CAA GAA TTG GAA GAA ATG CTC AAG AAG AAG CCA ATC GAT TTC TAC AAA GAA GAT CTC GAT AAA TTC GAA GTC GAA TGG AAG GCT TTC GAG GCA AAG AGA GAA AAG ACT CGT CTC GAA AAC GTC GCT GAT GCA GAG AAA GTC AAT AGG AAG TCA ATC GCA AAG AAC AAG AAG GCT GCA GAC CAG AAG AAG GCC AAA CAA CAG AAG AAG GAT GCA AAG GCG AAA CAA GAA GCA GCT GCT TTG CAA ACA GCA AAT GGA GAA GAG CTC AAA CAA GAA GTC GAA AAC CAG ATG CCA ACG AAA GAA GAA ACA GTT GTT CAG GAA TCA GAT GAT GAC ATC ATC GTA ATC AAT GAA GAC GTC AAG AAA CCA AAG AAA GAA AGA AAA CCA AAG GCT GAG ACA ACA ACA CCT ACA AAA GAA GGC GAA GAA AAG CCA AAG AAG GAA AGA AAA CCA AGA GTT AAG AAA GAA AAG AAA GAA GGT GAA GAA GAA ACA GAA GAA AAG AAA CCA GCA GCA ACA AAG GTA AAG AAA GAA ACA ACT GAA ACA GAA ACA AAG AAG AAA TCG AAG ACA ACA AAG AAG TCA AAT AAG TCT GCT TTC GAA GAT GAA GAC GAA GAT GAC GAT GTC GAT GTA GAT TTA GAG GAT TCT GAT GAA GAA CCA GTA GAA ACA ATC GCT TCA AGA CTC GCG TCA AGA AGA AAG ACT GAA CCA AAG AAA CAG GCA ATT TTG GAG GAA TTC TTC TCT GGA AAG AAG AAG GAC GGT GAA GAC GGC GAA GAA GAG GAA GAC GAG GAT GAT GAC CAG GAT GAA GAT GAT GAC GGA GAT GAC GCT TGG GAA GAT GAT GAG TAA

Occurrences/Results 9 Pentamers

The pentamer 'ATTCA' occurred 4 times.
The pentamer 'TTCAA' occurred 8 times.
The pentamer 'TCAAG' occurred 21 times.
The pentamer 'CAAGA' occurred 34 times.
The pentamer 'AAGAT' occurred 24 times.
The pentamer 'AGATG' occurred 15 times.
The pentamer 'GATGA' occurred 28 times.
The pentamer 'ATGAA' occurred 22 times.
The pentamer 'TGAAT' occurred 6 times.
The total occurrences of all specified strings is 162.
The total number of characters in the text is 4373.
First result (Total characters *9 /1024): 38.4697265625
Square root of the first result: 6.202396840133659
Final result: 19.916538174109796

Occurrences/Results TRIMERS Codons

The pentamer 'ATT' occurred 34 times.
The pentamer 'CAA' occurred 20 times.
The pentamer 'GAT' occurred 75 times.
The pentamer 'GAA' occurred 110 times.
The pentamer 'CCA' occurred 43 times.
The pentamer 'ACT' occurred 14 times.
The pentamer 'AGA' occurred 34 times.
The pentamer 'TAC' occurred 42 times.
The total occurrences of all specified strings is 372.
The total number of characters in the text is 4377.
First result ((total_characters / 3) * (Trimer_number / 64)): 182.4
Square root of the first result: 13.5
Final result: 14

Occurrences/Results TRIMERS Combinations

The pentamer 'ATT ATT' occurred 0 times.
The pentamer 'ATT CAA' occurred 0 times.
The pentamer 'ATT GAT' occurred 1 times.
The pentamer 'ATT GAA' occurred 0 times.
The pentamer 'ATT CCA' occurred 3 times.
The pentamer 'ATT ACT' occurred 3 times.
The pentamer 'ATT AGA' occurred 0 times.
The pentamer 'ATT TAC' occurred 1 times.
The pentamer 'CAA ATT' occurred 0 times.
The pentamer 'CAA CAA' occurred 0 times.
The pentamer 'CAA GAT' occurred 1 times.
The pentamer 'CAA GAA' occurred 3 times.
The pentamer 'CAA CCA' occurred 0 times.
The pentamer 'CAA ACT' occurred 0 times.
The pentamer 'CAA AGA' occurred 0 times.
The pentamer 'CAA TAC' occurred 0 times.
The pentamer 'GAT ATT' occurred 1 times.
The pentamer 'GAT CAA' occurred 1 times.
The pentamer 'GAT GAT' occurred 9 times.
The pentamer 'GAT GAA' occurred 11 times.
The pentamer 'GAT CCA' occurred 1 times.
The pentamer 'GAT ACT' occurred 0 times.
The pentamer 'GAT AGA' occurred 0 times.
The pentamer 'GAT TAC' occurred 5 times.
The pentamer 'GAA ATT' occurred 1 times.
The pentamer 'GAA CAA' occurred 2 times.
The pentamer 'GAA GAT' occurred 8 times.

The pentamer 'GAA GAA' occurred 12 times.
The pentamer 'GAA CCA' occurred 4 times.
The pentamer 'GAA ACT' occurred 0 times.
The pentamer 'GAA AGA' occurred 2 times.
The pentamer 'GAA TAC' occurred 3 times.
The pentamer 'CCA ATT' occurred 2 times.
The pentamer 'CCA CAA' occurred 1 times.
The pentamer 'CCA GAT' occurred 1 times.
The pentamer 'CCA GAA' occurred 1 times.
The pentamer 'CCA CCA' occurred 0 times.
The pentamer 'CCA ACT' occurred 0 times.
The pentamer 'CCA AGA' occurred 2 times.
The pentamer 'CCA TAC' occurred 0 times.
The pentamer 'ACT ATT' occurred 0 times.
The pentamer 'ACT CAA' occurred 0 times.
The pentamer 'ACT GAT' occurred 1 times.
The pentamer 'ACT GAA' occurred 3 times.
The pentamer 'ACT CCA' occurred 0 times.
The pentamer 'ACT ACT' occurred 0 times.
The pentamer 'ACT AGA' occurred 0 times.
The pentamer 'ACT TAC' occurred 1 times.
The pentamer 'AGA ATT' occurred 0 times.
The pentamer 'AGA CAA' occurred 0 times.
The pentamer 'AGA GAT' occurred 0 times.
The pentamer 'AGA GAA' occurred 6 times.
The pentamer 'AGA CCA' occurred 0 times.
The pentamer 'AGA ACT' occurred 0 times.
The pentamer 'AGA AGA' occurred 2 times.
The pentamer 'AGA TAC' occurred 1 times.
The pentamer 'TAC ATT' occurred 2 times.
The pentamer 'TAC CAA' occurred 0 times.
The pentamer 'TAC GAT' occurred 3 times.
The pentamer 'TAC GAA' occurred 2 times.
The pentamer 'TAC CCA' occurred 0 times.
The pentamer 'TAC ACT' occurred 0 times.
The pentamer 'TAC AGA' occurred 0 times.
The pentamer 'TAC TAC' occurred 3 times.
The total occurrences of all specified strings is 103.
The total number of characters in the text is 4373.
First result (((total_characters/ 3) -1) / 64): 22.78125
Square root of the first result: 4.772970773009196
Final result: 16.806880623202474

>XM_001323658.2 Trichomonas vaginalis G3, helicase (TVAGG3_0283960)

---> Sequence

AATGGCCGCTCCCGCCAAACTCGATGATCTTGAAATGCAGCTATTTTTCAGACCTCATGATTTGA
AAGTCATTCAGTCTGATAAAACATCACCATATTCTGCTCCACAAGATTGGGAAGATCTTTTTGAT
TATGATACGTATTCAGACCTTCATCAGAAGCTTATTGATAATAACTTCGAAAAACCTTCTTCCAT
TCAAGCTAGCTCCATTCAGATCGCTAACGGCGAAGACCATCCAAGTATTTTAGGTCAAGCTCAAA
ACGGTTCTGGTAAAACCCTGGCTTTTGTTGTCAATTCGCTTTTACGCGTTGACCGTAACTCTAGCA
AAGTCCAGGTTGTTATTTTAGTTCCAAATATGGAATTGGTCGATCAAACAGAAGAATATTTCCAG
AAATTAGCACCAGAGGGCGTTACATATACGAAAATTGATAAGAATGGTGCTGATATTGATCACTT
AGGTCAAATTGTTGTTACATCACCAGGTTTATTCTTCAACGCTGCAAATGCGATTCCCCAATTGA
AAGATATTGATGTTTTCGTTGTAGATGAATGTGATGAAGTTATTACAAATAAGATTTACCACGA
AGATTTAATTCAAGTACGCTGGTGAGATTCAAGAATGCACAGTTCCTCTTATATTCGGCCACTCTTG
TTGATAAACTTAACGATTTCATTAATACGTTCAGACCAAATATGCAAAGAATTATTTTACCACAA
AAGAACGTAATTAACAGGACAAACAAGCACTTCTTCATCGATTGCAGGAAAGGAATGGATAAGA
TTGACGCAATTAATATTATGTTCCAATATTTATTCCAATTCCAAACCTTCATTTTCTTCAATACA
AGAAAATACTTGCAAAAAGTTCAGAAACAACTCGAAGCTCTTAAGTTGGAATGCGATTGGTGCTC
AGGTGATCGTACAAAAGATGAAAGACGTGATGTTGTCAAAAAATTCAGAGAACAGAAAATCAAA
GTATTACTTACAACAAACTTACTTGCAAGAGGTATCGATATCCCTTCATGTAAGGTTGTTATCAA
CTTTGATATGCCAAGAAAAGAGAGGGGTGAACCAGATTATGAGTCATACTTGCATCGTCAAGGAA
GATGTGGCCGTTTCGGTAAGGAAGGTAAAGTATTCAACCTCATAAGAGATGATGAAGATATGAA
AATGATTGAGTACATCAAGAAGACATATTCTGTCGAGATTACTCAAATCACTCCACAGGATGTTC
AGAAGTTCGCAGAAAAGAACAAATAG

---> Sequence Showing highlighted Existant Combined Trimers

AAT GGC CGC TCC CGC CAA ACT CGA TGA TCT TGA AAT GCA GCT ATT TTT CAG ACC TCA
TGA TTT GAA AGT CAT TCA GTC TGA TAA AAC ATC ACC ATA TTC TGC TCC ACA AGA TTG
GGA AGA TCT TTT TGA TTA TGA TAC GTA TTC AGA CCT TCA TCA GAA GCT TAT TGA TAA
TAA CTT CGA AAA ACC TTC TTC CAT TCA AGC TAG CTC CAT TCA GAT CGC TAA CGG CGA
AGA CCA TCC AAG TAT TTT AGG TCA AGC TCA AAA CGG TTC TGG TAA AAC CCT GGC TTT
TGT TGT CAA TTC GCT TTT ACG CGT TGA CCG TAA CTC TAG CAA AGT CCA GGT TGT TAT
TTT AGT TCC AAA TAT GGA ATT GGT CGA TCA AAC AGA AGA ATA TTT CCA GAA ATT AGC
ACC AGA GGG CGT TAC ATA TAC GAA AAT TGA TAA GAA TGG TGC TGA TAT TGA TCA CTT
AGG TCA AAT TGT TGT TAC ATC ACC AGG TTT ATT CTT CAA CGC TGC AAA TGC GAT TCC
CCA ATT GAA AGA TAT TGA TGT TTT CGT TGT AGA TGA ATG TGA TGA AGT TAT TAC AAA
TAA GAT TTA CCA CGA AGA TTT AAT CAA GTA CGC TGG TGA GAT CAA GAA TGC ACA GTT
CCT CTT ATA TTC GGC CAC TCT TGT TGA TAA ACT TAA CGA TTT CAT TAA TAC GTT CAG
ACC AAA TAT GCA AAG AAT TAT TTT ACC ACA AAA GAA CGT AAT TAA CAG GAC AAA CAA
GCA CTT CTT CAT CGA TTG CAG GAA AGG AAT GGA TAA GAT TGA CGC AAT TAA TAT TAT
GTT CCA ATA TTT ATT CCA ATT CCA AAC CTT CAT TTT CTT CAA TAC AAG AAA ATA CTT
GCA AAA AGT TCA GAA ACA ACT CGA AGC TCT TAA GTT GGA ATG CGA TTG GTG CTC AGG
TGA TCG TAC AAA AGA TGA AAG ACG TGA TGT TGT CAA AAA ATT CAG AGA ACA GAA AAT
CAA AGT ATT ACT TAC AAC AAA CTT ACT TGC AAG AGG TAT CGA TAT CCC TTC ATG TAA

GGT TGT TAT CAA CTT TGA TAT GCC AAG AAA AGA GAG GGG TGA ACC AGA TTA TGA GTC ATA CTT GCA TCG TCA AGG AAG ATG TGG CCG TTT CGG TAA GGA AGG TAA AGT ATT CAA CCT CAT AAG AGA TGA TGA AGA TAT GAA AAT GAT TGA GTA CAT CAA GAA GAC ATA TTC TGT CGA GAT TAC TCA AAT CAC TCC ACA GGA TGT TCA GAA GTT CGC AGA AAA GAA CAA ATA G

Occurrences/Results 9 Pentamers

The pentamer 'ATTCA' occurred 6 times.
The pentamer 'TTCAA' occurred 4 times.
The pentamer 'TCAAG' occurred 6 times.
The pentamer 'CAAGA' occurred 6 times.
The pentamer 'AAGAT' occurred 9 times.
The pentamer 'AGATG' occurred 4 times.
The pentamer 'GATGA' occurred 5 times.
The pentamer 'ATGAA' occurred 5 times.
The pentamer 'TGAAT' occurred 1 times.
The total occurrences of all specified strings is 46.
The total number of characters in the text is 1254.
First result (Total characters *9 /1024): 11.056640625
Square root of the first result: 3.3251527220565373
Final result: 10.508798330739005

Occurrences/Results TRIMERS Codons

The pentamer 'ATT' occurred 10 times.
The pentamer 'CAA' occurred 14 times.
The pentamer 'GAT' occurred 7 times.
The pentamer 'GAA' occurred 15 times.
The pentamer 'CCA' occurred 8 times.
The pentamer 'ACT' occurred 5 times.
The pentamer 'AGA' occurred 17 times.
The pentamer 'TAC' occurred 10 times.
The total occurrences of all specified strings is 86.
The total number of characters in the text is 1258.
First result ((total_characters / 3) * (Trimer_number / 64)): 52.4
Square root of the first result: 7.2
Final result: 4.6

Occurrences/Results TRIMERS Combinations

The pentamer 'ATT ATT' occurred 0 times.
The pentamer 'ATT CAA' occurred 1 times.
The pentamer 'ATT GAT' occurred 0 times.
The pentamer 'ATT GAA' occurred 1 times.
The pentamer 'ATT CCA' occurred 2 times.

The pentamer 'ATT ACT' occurred 1 times.
The pentamer 'ATT AGA' occurred 0 times.
The pentamer 'ATT TAC' occurred 0 times.
The pentamer 'CAA ATT' occurred 0 times.
The pentamer 'CAA CAA' occurred 0 times.
The pentamer 'CAA GAT' occurred 0 times.
The pentamer 'CAA GAA' occurred 2 times.
The pentamer 'CAA CCA' occurred 0 times.
The pentamer 'CAA ACT' occurred 1 times.
The pentamer 'CAA AGA' occurred 0 times.
The pentamer 'CAA TAC' occurred 1 times.
The pentamer 'GAT ATT' occurred 0 times.
The pentamer 'GAT CAA' occurred 1 times.
The pentamer 'GAT GAT' occurred 0 times.
The pentamer 'GAT GAA' occurred 0 times.
The pentamer 'GAT CCA' occurred 0 times.
The pentamer 'GAT ACT' occurred 0 times.
The pentamer 'GAT AGA' occurred 0 times.
The pentamer 'GAT TAC' occurred 1 times.
The pentamer 'GAA ATT' occurred 1 times.
The pentamer 'GAA CAA' occurred 1 times.
The pentamer 'GAA GAT' occurred 0 times.
The pentamer 'GAA GAA' occurred 0 times.
The pentamer 'GAA CCA' occurred 0 times.
The pentamer 'GAA ACT' occurred 0 times.
The pentamer 'GAA AGA' occurred 1 times.
The pentamer 'GAA TAC' occurred 0 times.
The pentamer 'CCA ATT' occurred 2 times.
The pentamer 'CCA CAA' occurred 0 times.
The pentamer 'CCA GAT' occurred 0 times.
The pentamer 'CCA GAA' occurred 1 times.
The pentamer 'CCA CCA' occurred 0 times.
The pentamer 'CCA ACT' occurred 0 times.
The pentamer 'CCA AGA' occurred 0 times.
The pentamer 'CCA TAC' occurred 0 times.
The pentamer 'ACT ATT' occurred 0 times.
The pentamer 'ACT CAA' occurred 0 times.
The pentamer 'ACT GAT' occurred 0 times.
The pentamer 'ACT GAA' occurred 0 times.
The pentamer 'ACT CCA' occurred 0 times.
The pentamer 'ACT ACT' occurred 0 times.
The pentamer 'ACT AGA' occurred 0 times.
The pentamer 'ACT TAC' occurred 1 times.

The pentamer 'AGA ATT' occurred 0 times.
The pentamer 'AGA CAA' occurred 0 times.
The pentamer 'AGA GAT' occurred 0 times.
The pentamer 'AGA GAA' occurred 0 times.
The pentamer 'AGA CCA' occurred 1 times.
The pentamer 'AGA ACT' occurred 0 times.
The pentamer 'AGA AGA' occurred 1 times.
The pentamer 'AGA TAC' occurred 0 times.
The pentamer 'TAC ATT' occurred 0 times.
The pentamer 'TAC CAA' occurred 0 times.
The pentamer 'TAC GAT' occurred 0 times.
The pentamer 'TAC GAA' occurred 1 times.
The pentamer 'TAC CCA' occurred 0 times.
The pentamer 'TAC ACT' occurred 0 times.
The pentamer 'TAC AGA' occurred 0 times.
The pentamer 'TAC TAC' occurred 0 times.
The total occurrences of all specified strings is 21.
The total number of characters in the text is 1254.
First result (((total_characters/ 3) -1) / 64): 6.53125
Square root of the first result: 2.555631037532609
Final result: 5.661517561615302

>AF145283.1 Trichomonas vaginalis, calcium motive P-type ATPase

---> Sequence

GGATCCATTCCTCCGTGGTGGTGGTGCTATTGAGAACGGTATCGGTACAGCTCTTGTCGCCGCTGT
TGGTCCAAACTCTCAGTACGGTGTTACAATGACCACAATCACAAACCTTGGTGCAACAGAGACAG
AAACCCCACTTCAGAAGAAGCTCAACAAACTCGCTGTCCAACTTCTTTATGTCGCTGTTGTTTGCG
CTTCTGTTACATTTGTTGTTGTTATTGGTGAATGGGTTGCTCATCTCGTTAAGGCACTCAAGAGC
AAGACATTCAACAAGACAATCATCCAGGATCTCCTTAACAGAATCATGACCGTGATCACAATCTT
CCTTTGCTGCGTTCCAGAAGGTCTTCCACTCGCTGTCACACTTTGCCTTTCATTCTCAATGAAGAA
GATGATGAAGGACCAGAACTTCGTCCGCCACCTTTCTGCTTGCGAAACAATGGGTGGTGCTACAA
CAATTTGCTCAGATAAGACAGGTACCCTCACACAGAACAGAATGACAGTCGTCAAGTTCTGGATG
GATGGTGTCGAACAAGATGGTCATCCAGATCTTATCGAAGAAGTCAAGGAGAGATTAGCCGAATC
AATCGCTATTAACTCTACAGCTTCCCACACACTCAAGGAAGGCACAGACGAGGTCGTCTTTGTCG
GCTCATCCTCTGAATGCGCTCTTCTCAAGATGATCTCAGATCTTGGTAAGGATTACATGGAAATC
CGTGAACTCAACCCAATTCTCTACCTCAACGAATTCAACTCCGCCAGGAAGAGAATGTCTACAGTC
GTATCATCTGAAAACGGATGCCACGTCTACTTCAAGGGTGCTCCAGATTTCTCACTTCCACTCATT
TCTCACTACCTCGCTAACGATGGCTCTGTCAAGGAGGCCGACCAAGCCTTCAAGGCATCCGTCCTC
GCCAAAGTCAACGACTTCGCTTCTCAGGCTTACAGAACAATGCTTATTGCATACCGTGAAATCGT
TGGTGAAGAATCCCAGCAGTGGTCCGATCCAAACTTCGTCGAATCAAATCTTACAATGATTGCCT
TAGTTGGTATTCAGGATCCACTTCGTCCAGAAGTTCCATTGGCCATCCAGCGCTGCAAAGAAGCT

GGTGTCGTTGTTCGTATGGTTACAGGTGACTTCATTGCTACAGCTCGTGCCATTTCCAAGCAATG
CGGTATCATTTCCAGCGAAACAGATATCGTCATGGAAGGTGCAGAATTCGCTTCCCTCTCCAAGA
CACAGCTCATCGACAAGATCGACAACCTCAGAGTTCTCGCTCGTTCCTCTCCAACAGATAAGTACC
GCCTTGTTTCTCTTCTCATGGAATGCGGTGAAGTCGTCGCTGTTACAGGCGATGGTGTCAACG

---> Sequence Showing highlighted Existant Combined Trimers

GGA TCC ATT CCT CCG TGG TGG TGG TGC TAT TGA GAA CGG TAT CGG TAC AGC TCT TGT CGC CGC TGT TGG TCC AAA CTC TCA GTA CGG TGT TAC AAT GAC CAC AAT CAC AAA CCT TGG TGC AAC AGA GAC AGA AAC CCC ACT TCA GAA GAA GCT CAA CAA ACT CGC TGT CCA ACT TCT TTA TGT CGC TGT TGT TTG CGC TTC TGT TAC ATT TGT TGT TGT TAT TGG TGA ATG GGT TGC TCA TCT CGT TAA GGC ACT CAA GAG CAA GAC ATT CAA CAA GAC AAT CAT CCA GGA TCT CCT TAA CAG AAT CAT GAC CGT GAT CAC AAT CTT CCT TTG CTG CGT TCC AGA AGG TCT TCC ACT CGC TGT CAC ACT TTG CCT TTC ATT CTC AAT GAA GAA GAT GAT GAA GGA CCA GAA CTT CGT CCG CCA CCT TTC TGC TTG CGA AAC AAT GGG TGG TGC TAC AAC AAT TTG CTC AGA TAA GAC AGG TAC CCT CAC ACA GAA CAG AAT GAC AGT CGT CAA GTT CTG GAT GGA TGG TGT CGA ACA AGA TGG TCA TCC AGA TCT TAT CGA AGA AGT CAA GGA GAG ATT AGC CGA ATC AAT CGC TAT TAA CTC TAC AGC TTC CCA CAC ACT CAA GGA AGG CAC AGA CGA GGT CGT CTT TGT CGG CTC ATC CTC TGA ATG CGC TCT TCT CAA GAT GAT CTC AGA TCT TGG TAA GGA TTA CAT GGA AAT CCG TGA ACT CAA CCC AAT TCT CTA CCT CAA CGA ATT CAA CTC CGC CAG GAA GAG AAT GTC TAC AGT CGT ATC ATC TGA AAA CGG ATG CCA CGT CTA CTT CAA GGG TGC TCC AGA TTT CTC ACT TCC ACT CAT TTC TCA CTA CCT CGC TAA CGA TGG CTC TGT CAA GGA GGC CGA CCA AGC CTT CAA GGC ATC CGT CCT CGC CAA AGT CAA CGA CTT CGC TTC TCA GGC TTA CAG AAC AAT GCT TAT TGC ATA CCG TGA AAT CGT TGG TGA AGA ATC CCA GCA GTG GTC CGA TCC AAA CTT CGT CGA ATC AAA TCT TAC AAT GAT TGC CTT AGT TGG TAT TCA GGA TCC ACT TCG TCC AGA AGT TCC ATT GGC CAT CCA GCG CTG CAA AGA AGC TGG TGT CGT TGT TCG TAT GGT TAC AGG TGA CTT CAT TGC TAC AGC TCG TGC CAT TTC CAA GCA ATG CGG TAT CAT TTC CAG CGA AAC AGA TAT CGT CAT GGA AGG TGC AGA ATT CGC TTC CCT CTC CAA GAC ACA GCT CAT CGA CAA GAT CGA CAA CCT CAG AGT TCT CGC TCG TTC CTC TCC AAC AGA TAA GTA CCG CCT TGT TTC TCT TCT CAT GGA ATG CGG TGA AGT CGT CGC TGT TAC AGG CGA TGG TGT CAA CG

Occurrences/Results 9 Pentamers

The pentamer 'ATTCA' occurred 3 times.
The pentamer 'TTCAA' occurred 4 times.
The pentamer 'TCAAG' occurred 8 times.
The pentamer 'CAAGA' occurred 7 times.
The pentamer 'AAGAT' occurred 4 times.
The pentamer 'AGATG' occurred 3 times.
The pentamer 'GATGA' occurred 2 times.
The pentamer 'ATGAA' occurred 2 times.
The pentamer 'TGAAT' occurred 2 times.
The total occurrences of all specified strings is 35.

The total number of characters in the text is 1366.
First result (Total characters *9 /1024): 12.041015625
Square root of the first result: 3.4700166606228278
Final result: 6.616390242598757

Occurrences/Results TRIMERS Codons

The pentamer 'ATT' occurred 8 times.
The pentamer 'CAA' occurred 24 times.
The pentamer 'GAT' occurred 8 times.
The pentamer 'GAA' occurred 9 times.
The pentamer 'CCA' occurred 9 times.
The pentamer 'ACT' occurred 11 times.
The pentamer 'AGA' occurred 16 times.
The pentamer 'TAC' occurred 11 times.
The total occurrences of all specified strings is 96.
The total number of characters in the text is 1368.
First result ((total_characters / 3) * (Trimer_number / 64)): 57
Square root of the first result: 7.5
Final result: 5.2

Occurrences/Results TRIMERS Combinations

The pentamer 'ATT ATT' occurred 0 times.
The pentamer 'ATT CAA' occurred 2 times.
The pentamer 'ATT GAT' occurred 0 times.
The pentamer 'ATT GAA' occurred 0 times.
The pentamer 'ATT CCA' occurred 0 times.
The pentamer 'ATT ACT' occurred 0 times.
The pentamer 'ATT AGA' occurred 0 times.
The pentamer 'ATT TAC' occurred 0 times.
The pentamer 'CAA ATT' occurred 0 times.
The pentamer 'CAA CAA' occurred 2 times.
The pentamer 'CAA GAT' occurred 2 times.
The pentamer 'CAA GAA' occurred 0 times.
The pentamer 'CAA CCA' occurred 0 times.
The pentamer 'CAA ACT' occurred 1 times.
The pentamer 'CAA AGA' occurred 1 times.
The pentamer 'CAA TAC' occurred 0 times.
The pentamer 'GAT ATT' occurred 0 times.
The pentamer 'GAT CAA' occurred 0 times.
The pentamer 'GAT GAT' occurred 2 times.
The pentamer 'GAT GAA' occurred 1 times.
The pentamer 'GAT CCA' occurred 0 times.
The pentamer 'GAT ACT' occurred 0 times.

The pentamer 'GAT AGA' occurred 0 times.
The pentamer 'GAT TAC' occurred 0 times.
The pentamer 'GAA ATT' occurred 0 times.
The pentamer 'GAA CAA' occurred 0 times.
The pentamer 'GAA GAT' occurred 1 times.
The pentamer 'GAA GAA' occurred 2 times.
The pentamer 'GAA CCA' occurred 0 times.
The pentamer 'GAA ACT' occurred 0 times.
The pentamer 'GAA AGA' occurred 0 times.
The pentamer 'GAA TAC' occurred 0 times.
The pentamer 'CCA ATT' occurred 0 times.
The pentamer 'CCA CAA' occurred 0 times.
The pentamer 'CCA GAT' occurred 0 times.
The pentamer 'CCA GAA' occurred 1 times.
The pentamer 'CCA CCA' occurred 0 times.
The pentamer 'CCA ACT' occurred 1 times.
The pentamer 'CCA AGA' occurred 0 times.
The pentamer 'CCA TAC' occurred 0 times.
The pentamer 'ACT ATT' occurred 0 times.
The pentamer 'ACT CAA' occurred 3 times.
The pentamer 'ACT GAT' occurred 0 times.
The pentamer 'ACT GAA' occurred 0 times.
The pentamer 'ACT CCA' occurred 0 times.
The pentamer 'ACT ACT' occurred 0 times.
The pentamer 'ACT AGA' occurred 0 times.
The pentamer 'ACT TAC' occurred 0 times.
The pentamer 'AGA ATT' occurred 1 times.
The pentamer 'AGA CAA' occurred 0 times.
The pentamer 'AGA GAT' occurred 0 times.
The pentamer 'AGA GAA' occurred 0 times.
The pentamer 'AGA CCA' occurred 0 times.
The pentamer 'AGA ACT' occurred 0 times.
The pentamer 'AGA AGA' occurred 0 times.
The pentamer 'AGA TAC' occurred 0 times.
The pentamer 'TAC ATT' occurred 1 times.
The pentamer 'TAC CAA' occurred 0 times.
The pentamer 'TAC GAT' occurred 0 times.
The pentamer 'TAC GAA' occurred 0 times.
The pentamer 'TAC CCA' occurred 0 times.
The pentamer 'TAC ACT' occurred 0 times.
The pentamer 'TAC AGA' occurred 0 times.
The pentamer 'TAC TAC' occurred 0 times.
The total occurrences of all specified strings is 21.

The total number of characters in the text is 1366.
First result (((total_characters/ 3) -1) / 64): 7.109375
Square root of the first result: 2.666341125962693
Final result: 5.209620353804031

>AF145277.1 Trichomonas vaginalis, translocase (APLT-1)

---> Sequence

GACAAGACAGGCACACTCACAGAGAACAAAATGAACTTAGTTTCTTTTGTTGATCATTCTGGAGT
TCATTCTCAGTATTTCTCGCAGTTTTCCGCTGAAGATAAGGAGAAAAGCGCTGATATGTTACTCA
GTTTTGTCCTTAATAACTCTGTAATTGTCTACAATTCTCCAAATGGAATTGAATACAACGCAGAA
TCCCCTGATGAAGCAGCGGGCGTCAAATTCGCTTCTGAATGCAACTGGGTTTTGAAAGCAAGAAA
TCCAGCAAGAATTGCTATTGAAGTTAATGGAGTTTACCAAGTTTATGACATAATTGCTGTTTTCC
CATTCAATTCAACAAGAAAGAGAATGACAGTTGTTGTCAGGAAACAGGGCGAAGAAGGTCTTTTA
GTTCTGACTAAAGGAGCTGATAACATCATCTACCCAACATGCAGAGAAGTATATTTCAGACAGGA
GCTTAATGATTATGCAATCAGTGGCTACAGAACTCTTGTTTTCGCTATGAAAGAACTTTATGGAG
AAGAAAAACAGAAATTCATGGAAAATTTGAACAAAATCACTCTTTCTATGGACAAAGTTGATGA
GAAATTGTTGGAATTCGCTGAATCTGTTGAACCAAGACTTAGAATGCATTGGAATTGCAGCAATTG
TTGACAAATTACAAGAAGGAGTTCCAGAAACAATCGAATGGCTAAGAAGAGCAGGTATCCATGTT
TGGGTATTAACAGGAGATAAGCTCGAAACAGCAATTGAAATTGGAAAAACATCAAAAGTTATCC
CACATGGTTCAGATGTTTTGATTGTAGGTAACAGTGACAAAATCGTTACATCATTAGACCTTGGA
AGATACATTGATGAGTTTGACAACTTCAATGATCCTGTATTAGTCATTACAGAGGAAGTTTTGGA
ATTCTGCTTAAGTAATCAGTCTTATCTCTTCTTCAAGCTGGCAATGAAGTGCAAATCTGTCATCT
TTTCAAGAGTCTCTCCATACATGAAGGCTAAGATTGTTAATTTGGTCCGAACCAAGAAATAATGCG
ATCACATTAGCAATCGGGGACGGCGCCAACGA

---> Sequence Showing highlighted Existant Combined Trimers

GAC AAG ACA GGC ACA CTC ACA GAG AAC AAA ATG AAC TTA GTT TCT TTT GTT GAT CAT
TCT GGA GTT CAT TCT CAG TAT TTC TCG CAG TTT TCC GCT GAA GAT AAG GAG AAA AGC
GCT GAT ATG TTA CTC AGT TTT GTC CTT AAT AAC TCT GTA ATT GTC TAC AAT TCT CCA
AAT GGA ATT GAA TAC AAC GCA GAA TCC CCT GAT GAA GCA GCG GGC GTC AAA TTC GCT
TCT GAA TGC AAC TGG GTT TTG AAA GCA AGA AAT CCA GCA AGA ATT GCT ATT GAA GTT
AAT GGA GTT TAC CAA GTT TAT GAC ATA ATT GCT GTT TTC CCA TTC AAT TCA ACA AGA
AAG AGA ATG ACA GTT GTT GTC AGG AAA CAG GGC GAA GAA GGT CTT TTA GTT CTG ACT
AAA GGA GCT GAT AAC ATC ATC TAC CCA ACA TGC AGA GAA GTA TAT TTC AGA CAG GAG
CTT AAT GAT TAT GCA ATC AGT GGC TAC AGA ACT CTT GTT TTC GCT ATG AAA GAA CTT
TAT GGA GAA GAA AAA CAG AAA TTC ATG GAA AAT TTG AAC AAA ATC ACT CTT TCT ATG
GAC AAA GTT GAT GAG AAA TTG TTG GAA TTC GCT GAA TCT GTT GAA CAA GAC TTA GAA
TGC ATT GGA ATT GCA GCA ATT GTT GAC AAA TTA CAA GAA GGA GTT CCA GAA ACA ATC
GAA TGG CTA AGA AGA GCA GGT ATC CAT GTT TGG GTA TTA ACA GGA GAT AAG CTC GAA
ACA GCA ATT GAA ATT GGA AAA ACA TCA AAA GTT ATC CCA CAT GGT TCA GAT GTT TTG

ATT GTA GGT AAC AGT GAC AAA ATC GTT ACA TCA TTA GAC CTT GGA AGA TAC ATT GAT GAG TTT GAC AAC TTC AAT GAT CCT GTA TTA GTC ATT ACA GAG GAA GTT TTG GAA TTC TGC TTA AGT AAT CAG TCT TAT CTC TTC TTC AAG CTG GCA ATG AAG TGC AAA TCT GTC ATC TTT TCA AGA GTC TCT CCA TAC ATG AAG GCT AAG ATT GTT AAT TTG GTC CGA ACA AGA AAT AAT GCG ATC ACA TTA GCA ATC GGG GAC GGC GCC AAC GA

Occurrences/Results 9 Pentamers

The pentamer 'ATTCA' occurred 3 times.
The pentamer 'TTCAA' occurred 5 times.
The pentamer 'TCAAG' occurred 2 times.
The pentamer 'CAAGA' occurred 8 times.
The pentamer 'AAGAT' occurred 3 times.
The pentamer 'AGATG' occurred 1 times.
The pentamer 'GATGA' occurred 3 times.
The pentamer 'ATGAA' occurred 5 times.
The pentamer 'TGAAT' occurred 3 times.
The total occurrences of all specified strings is 33.
The total number of characters in the text is 1066.
First result (Total characters *9 /1024): 9.404296875
Square root of the first result: 3.0666426063367735
Final result: 7.694311386740304

Occurrences/Results TRIMERS Codons

The pentamer 'ATT' occurred 14 times.
The pentamer 'CAA' occurred 3 times.
The pentamer 'GAT' occurred 11 times.
The pentamer 'GAA' occurred 24 times.
The pentamer 'CCA' occurred 7 times.
The pentamer 'ACT' occurred 3 times.
The pentamer 'AGA' occurred 12 times.
The pentamer 'TAC' occurred 7 times.
The total occurrences of all specified strings is 81.
The total number of characters in the text is 1068.
First result ((total_characters / 3) * (Trimer_number / 64)): 44.5
Square root of the first result: 6.7
Final result: -5.3

Occurrences/Results TRIMERS Combinations

The pentamer 'ATT ATT' occurred 0 times.
The pentamer 'ATT CAA' occurred 0 times.
The pentamer 'ATT GAT' occurred 1 times.
The pentamer 'ATT GAA' occurred 3 times.
The pentamer 'ATT CCA' occurred 0 times.

The pentamer 'ATT ACT' occurred 0 times.
The pentamer 'ATT AGA' occurred 0 times.
The pentamer 'ATT TAC' occurred 0 times.
The pentamer 'CAA ATT' occurred 0 times.
The pentamer 'CAA CAA' occurred 0 times.
The pentamer 'CAA GAT' occurred 0 times.
The pentamer 'CAA GAA' occurred 1 times.
The pentamer 'CAA CCA' occurred 0 times.
The pentamer 'CAA ACT' occurred 0 times.
The pentamer 'CAA AGA' occurred 0 times.
The pentamer 'CAA TAC' occurred 0 times.
The pentamer 'GAT ATT' occurred 0 times.
The pentamer 'GAT CAA' occurred 0 times.
The pentamer 'GAT GAT' occurred 0 times.
The pentamer 'GAT GAA' occurred 1 times.
The pentamer 'GAT CCA' occurred 0 times.
The pentamer 'GAT ACT' occurred 0 times.
The pentamer 'GAT AGA' occurred 0 times.
The pentamer 'GAT TAC' occurred 0 times.
The pentamer 'GAA ATT' occurred 1 times.
The pentamer 'GAA CAA' occurred 1 times.
The pentamer 'GAA GAT' occurred 1 times.
The pentamer 'GAA GAA' occurred 2 times.
The pentamer 'GAA CCA' occurred 0 times.
The pentamer 'GAA ACT' occurred 0 times.
The pentamer 'GAA AGA' occurred 0 times.
The pentamer 'GAA TAC' occurred 1 times.
The pentamer 'CCA ATT' occurred 0 times.
The pentamer 'CCA CAA' occurred 0 times.
The pentamer 'CCA GAT' occurred 0 times.
The pentamer 'CCA GAA' occurred 1 times.
The pentamer 'CCA CCA' occurred 0 times.
The pentamer 'CCA ACT' occurred 0 times.
The pentamer 'CCA AGA' occurred 0 times.
The pentamer 'CCA TAC' occurred 1 times.
The pentamer 'ACT ATT' occurred 0 times.
The pentamer 'ACT CAA' occurred 0 times.
The pentamer 'ACT GAT' occurred 0 times.
The pentamer 'ACT GAA' occurred 0 times.
The pentamer 'ACT CCA' occurred 0 times.
The pentamer 'ACT ACT' occurred 0 times.
The pentamer 'ACT AGA' occurred 0 times.
The pentamer 'ACT TAC' occurred 0 times.

The pentamer 'AGA ATT' occurred 1 times.
The pentamer 'AGA CAA' occurred 0 times.
The pentamer 'AGA GAT' occurred 0 times.
The pentamer 'AGA GAA' occurred 1 times.
The pentamer 'AGA CCA' occurred 0 times.
The pentamer 'AGA ACT' occurred 1 times.
The pentamer 'AGA AGA' occurred 1 times.
The pentamer 'AGA TAC' occurred 1 times.
The pentamer 'TAC ATT' occurred 1 times.
The pentamer 'TAC CAA' occurred 1 times.
The pentamer 'TAC GAT' occurred 0 times.
The pentamer 'TAC GAA' occurred 0 times.
The pentamer 'TAC CCA' occurred 1 times.
The pentamer 'TAC ACT' occurred 0 times.
The pentamer 'TAC AGA' occurred 1 times.
The pentamer 'TAC TAC' occurred 0 times.
The total occurrences of all specified strings is 23.
The total number of characters in the text is 1066.
First result (((total_characters/ 3) -1) / 64): 5.546875
Square root of the first result: 2.3551804601770967
Final result: 7.410525560613568

>JAOSJJ010000002.1:101575-102618 Trichomonas vaginalis G3 chromosome V, polymerase

---> Sequence

ATGAAATCAAACAATCTACCAGTTGACCAGAAACAGAGGAAATTAACTCAGTTAGCATCAACACA
CAACCAATATCAACCATTTTCTTCTTATTATCTTTGGTATCTAATAGACAGATTTCATTTTATCA
TTGATGACATTCAATCAATTTTAACATTTACGAAAAATACTTGTTTTAATGAATTTGCGAATGAA
TTTATGAACGAAAGACAAAAGGCTGAATTAGAAGGAAATAAAGGAAAAAGTTTATTTTGCAAGA
TTTCACTTAATGGATCATATGGTTACGATGCAATGAATACACAAAATTACGCAAAGACTAAAATA
ATGAATGCACAGAAGGCACGCGTTGCATGTATGTCAAATAAGTTTAAAAACATAAGAGAAATAG
GAGAGGATACGTATCAAGTGATGCTTAAAGATAGATTTTATAGATGTGATACATGTTTACAAGA
GGCATTCTTCACTTTAGATAACGCAAAATACTGGTATTTGGTTTTCATTTATGATTTTATGTATA
AATGCATGGATGTTAATCGTTTTCATTTCATTGAAGGTGATACTGATTCATCTTATTGGGCAATC
GCTGGCGACCCAAATCTTCCTAACACTCAAGCATTTCAAGCAATTGTAACCGATAAAAAATTTTA
TGATAAAAACATTTACAAATTCGCACCTTTTGATTTCTTCTGTTTTAATGAGAAATTTAAACCTA
AATTAAAGAATAAAGCTGAAGAAAAAGCACATGAGAAGAAGTTATTGGGTTTAGCAATTGAGAA
ACAAGGTGATAACATGGTTGCTTTATGTCCTAAGTGTTATACTTCATTCAACGGTTCAATTGATG
GAAGTGATTTTAAAAAGATTGCACAAAAAATGAAAGGAGTTAGTTTACGACAAAATAAACAATT
AACACCTAAAAATTATTTGGATATAATAAATGATAAAGTGATATTTGATGGTCAGAATATTAAT

TTACAACTTAAAAACGGGTCAATGACTCGCTTAACAATCGGTAAGACTGCATTAACAGGAGCACA
CACTAAGGCT

---> Sequence Showing highlighted Existant Combined Trimers

ATG AAA TCA AAC AAT CTA CCA GTT GAC CAG AAA CAG AGG AAA TTA ACT CAG TTA GCA TCA ACA CAC AAC CAA TAT CAA CCA TTT TCT TCT TAT TAT CTT TGG TAT CTA ATA GAC AGA TTT CAT TTT ATC ATT GAT GAC ATT CAA TCA ATT TTA ACA TTT ACG AAA AAT ACT TGT TTT AAT GAA TTT GCG AAT GAA TTT ATG AAC GAA AGA CAA AAG GCT GAA TTA GAA GGA AAT AAA GGA AAA AGT TTA TTT TGC AAG ATT TCA CTT AAT GGA TCA TAT GGT TAC GAT GCA ATG AAT ACA CAA AAT TAC GCA AAG ACT AAA ATA ATG AAT GCA CAG AAG GCA CGC GTT GCA TGT ATG TCA AAT AAG TTT AAA AAC ATA AGA GAA ATA GGA GAG GAT ACG TAT CAA GTG ATG CTT AAA GAT AGA TTT TAT AGA TGT GAT ACA TGT TTA CAA GAG GCA TTC TTC ACT TTA GAT AAC GCA AAA TAC TGG TAT TTG GTT TTC ATT TAT GAT TTT ATG TAT AAA TGC ATG GAT GTT AAT CGT TTT CAT TTC ATT GAA GGT GAT ACT GAT TCA TCT TAT TGG GCA ATC GCT GGC GAC CCA AAT CTT CCT AAC ACT CAA GCA TTT CAA GCA ATT GTA ACC GAT AAA AAA TTT TAT GAT AAA AAC ATT TAC AAA TTC GCA CCT TTT GAT TTC TTC TGT TTT AAT GAG AAA TTT AAA CCT AAA TTA AAG AAT AAA GCT GAA GAA AAA GCA CAT GAG AAG AAG TTA TTG GGT TTA GCA ATT GAG AAA CAA GGT GAT AAC ATG GTT GCT TTA TGT CCT AAG TGT TAT ACT TCA TTC AAC GGT TCA ATT GAT GGA AGT GAT TTT AAA AAG ATT GCA CAA AAA ATG AAA GGA GTT AGT TTA CGA CAA AAT AAA CAA TTA ACA CCT AAA AAT TAT TTG GAT ATA ATA AAT GAT AAA GTG ATA TTT GAT GGT CAG AAT ATT AAT TTA CAA CTT AAA AAC GGG TCA ATG ACT CGC TTA ACA ATC GGT AAG ACT GCA TTA ACA GGA GCA CAC ACT AAG GCT

Occurrences/Results 9 Pentamers

The pentamer 'ATTCA' occurred 3 times.
The pentamer 'TTCAA' occurred 4 times.
The pentamer 'TCAAG' occurred 3 times.
The pentamer 'CAAGA' occurred 2 times.
The pentamer 'AAGAT' occurred 3 times.
The pentamer 'AGATG' occurred 1 times.
The pentamer 'GATGA' occurred 1 times.
The pentamer 'ATGAA' occurred 7 times.
The pentamer 'TGAAT' occurred 5 times.
The total occurrences of all specified strings is 29.
The total number of characters in the text is 1040.
First result (Total characters *9 /1024): 9.17578125
Square root of the first result: 3.0291552040131586
Final result: 6.544471119781515

Occurrences/Results TRIMERS Codons

The pentamer 'ATT' occurred 12 times.
The pentamer 'CAA' occurred 14 times.
The pentamer 'GAT' occurred 19 times.
The pentamer 'GAA' occurred 9 times.
The pentamer 'CCA' occurred 3 times.
The pentamer 'ACT' occurred 10 times.
The pentamer 'AGA' occurred 5 times.
The pentamer 'TAC' occurred 4 times.
The total occurrences of all specified strings is 76.
The total number of characters in the text is 1044.
First result ((total_characters / 3) * (Trimer_number / 64)): 43.5
Square root of the first result: 6.6
Final result: 4.95

Occurrences/Results TRIMERS Combinations

The pentamer 'ATT ATT' occurred 0 times.
The pentamer 'ATT CAA' occurred 1 times.
The pentamer 'ATT GAT' occurred 2 times.
The pentamer 'ATT GAA' occurred 1 times.
The pentamer 'ATT CCA' occurred 0 times.
The pentamer 'ATT ACT' occurred 0 times.
The pentamer 'ATT AGA' occurred 0 times.
The pentamer 'ATT TAC' occurred 1 times.
The pentamer 'CAA ATT' occurred 0 times.
The pentamer 'CAA CAA' occurred 0 times.
The pentamer 'CAA GAT' occurred 0 times.
The pentamer 'CAA GAA' occurred 0 times.
The pentamer 'CAA CCA' occurred 1 times.
The pentamer 'CAA ACT' occurred 0 times.
The pentamer 'CAA AGA' occurred 0 times.
The pentamer 'CAA TAC' occurred 0 times.
The pentamer 'GAT ATT' occurred 0 times.
The pentamer 'GAT CAA' occurred 0 times.
The pentamer 'GAT GAT' occurred 0 times.
The pentamer 'GAT GAA' occurred 0 times.
The pentamer 'GAT CCA' occurred 0 times.
The pentamer 'GAT ACT' occurred 1 times.
The pentamer 'GAT AGA' occurred 1 times.
The pentamer 'GAT TAC' occurred 0 times.
The pentamer 'GAA ATT' occurred 0 times.
The pentamer 'GAA CAA' occurred 0 times.
The pentamer 'GAA GAT' occurred 0 times.

The pentamer 'GAA GAA' occurred 1 times.
The pentamer 'GAA CCA' occurred 0 times.
The pentamer 'GAA ACT' occurred 0 times.
The pentamer 'GAA AGA' occurred 1 times.
The pentamer 'GAA TAC' occurred 0 times.
The pentamer 'CCA ATT' occurred 0 times.
The pentamer 'CCA CAA' occurred 0 times.
The pentamer 'CCA GAT' occurred 0 times.
The pentamer 'CCA GAA' occurred 0 times.
The pentamer 'CCA CCA' occurred 0 times.
The pentamer 'CCA ACT' occurred 0 times.
The pentamer 'CCA AGA' occurred 0 times.
The pentamer 'CCA TAC' occurred 0 times.
The pentamer 'ACT ATT' occurred 0 times.
The pentamer 'ACT CAA' occurred 1 times.
The pentamer 'ACT GAT' occurred 1 times.
The pentamer 'ACT GAA' occurred 0 times.
The pentamer 'ACT CCA' occurred 0 times.
The pentamer 'ACT ACT' occurred 0 times.
The pentamer 'ACT AGA' occurred 0 times.
The pentamer 'ACT TAC' occurred 0 times.
The pentamer 'AGA ATT' occurred 0 times.
The pentamer 'AGA CAA' occurred 1 times.
The pentamer 'AGA GAT' occurred 0 times.
The pentamer 'AGA GAA' occurred 1 times.
The pentamer 'AGA CCA' occurred 0 times.
The pentamer 'AGA ACT' occurred 0 times.
The pentamer 'AGA AGA' occurred 0 times.
The pentamer 'AGA TAC' occurred 0 times.
The pentamer 'TAC ATT' occurred 0 times.
The pentamer 'TAC CAA' occurred 0 times.
The pentamer 'TAC GAT' occurred 1 times.
The pentamer 'TAC GAA' occurred 0 times.
The pentamer 'TAC CCA' occurred 0 times.
The pentamer 'TAC ACT' occurred 0 times.
The pentamer 'TAC AGA' occurred 0 times.
The pentamer 'TAC TAC' occurred 0 times.
The total occurrences of all specified strings is 15.
The total number of characters in the text is 1040.
First result (((total_characters/ 3) -1) / 64): 5.421875
Square root of the first result: 2.3284920012746446
Final result: 4.1134455238655825

>JAOSJJ010000003.1:c11389053-11387104 Trichomonas vaginalis G3 chromosome II, glycyl-tRNA ligase

---> Sequence

ATGACGAAAGACACTCGCGCCACCGCAGAAGATCTTGAAGTTTCTGGTTTCTTCTGGGTTCCATC
ATTCGAAATCTATGGCTCTGTTGCAGGCATTTACGATCTTGGCCCAACAGGCTGCGCAATCGAAC
GCAACTTCCTCCAGAAGTGGAGAGATCATTTCGTTCTTGAAGACGACATGCTCGAAGTTCGCTGC
TCCGCTCTTACCCCACGTCCAGTCTTAGATGCATCTGGCCACACAGAAAAATTCAATGATCTCATG
CTCACAGACATGACAACAAAGGCACTTTACAGAGCTGATCAGTACATCGCTGCTTACCTTAAGGA
GCGTGCTGAGAAGGAAACAGACCACGACAAGAAGAAGCAGTACGAGAAGGATGCCGAAGATGTC
GATGGCATGACAAAGGAGCAGATGATGGCTCTCATGGCCAAGTACAACATCAAGTCTCCAGAAGG
CAACGAGTTCTCTGAACCAGCCCCATTCAACCTTATGTTCAATACACGTGTTGGTCCAGGCGCCCG
CTCCATCGAGGCATTCCTCCGCCCAGAAACAGCTCAGGGCATCTTCGTTAACTTCACACGTCTCCT
CAATGCCAACCGCGGATCTCTTCCATTCGCTGCTGCCCAAGTCGGCGCTGGTTACCGTAACGAAAT
TTCCCCACGTAACGGTCTTGTCCGCTGCCGTGAATTCCAGATGGCCGAAATCGAGCACTTTGCTGA
TCCAGAGCAGCTCAACAACTTCCCTAAGTTCGAGACAGTCAAGAACCTCAAGGTCAAGCTCTTCCC
AGCCAGTATCCAGGAATTAGAAGACGAAGAAAAGCGTATCCCAATCGAGATCACCCTCGAAGACG
CTATCGCACAGCACGTCGTTTCCCACAAGACACTCGGCTACTACATTGGCCGTGTCTACTTATTCC
TCTGCGAAATCGGTATCCAACCAGATACAATTCGCTTCCGTATGCACCGCAAGAACGAAATGGCC
CACTACGCTCGCGAATGCTGGGATGCCGAAATCTACACAAAGACACTCGGCTGGCTCGAGTGCGT
CGGCATTGCTGACCGCCAATCATGGGATCTTTCTCGCCATGCTAAGTACACAACAAAGAAGGGCG
ATGCTGAATCATCACCACTCTACCTTTCCGCTCCACTCGATACACCAATCCACCAGACAAAGGTTG
AAGGCGAAAAGTCCGCAATCGGCAAGATCTTCCGCAAGGATGCCAAGGAGATCATGGATGCCCTC
GCAACCATCCCAGCCGACCAGGTCGAAGCCCTCCGTGTCAAGGTTGCTGAAGCTGAGAAGCTCTTC
GGTGCTGAGAAGCCAGCCAAGAAGAACATCGCCAAGGCCATCGCAGCCCTTTCTGCTGAAGACAA
GAAGAAGTTCGAGGAACTCACAAACATCACAGTTTGCGGAGACAAGACAGTTACATACGAAATGT
ACAACATCAACGATACAGTTGTTACAACACGTAAGTTCTTCCCGAACGTTATCGAGCCATCGTTC
GGTGTCGGCAGAATCATGACATGCCTCTTCGAACAGGCATTTTATGTCCGCGAAGATGGCGAACG
CCGTGTTCTCAGATTGAAGCCATTCATGGCACCATACAAGGTTAACCTCATCCCAATCAACCAAGA
AGAACGCTAAGGATGAAGATATCCACAACATCCGTACAAACCTCCGCAAGAAAGGTCTCGCTAAT
ACAATCGATGCTGCTGGTGTCTCAATCGGTAAGCGCTACGCTCGTTCTGATGAAATCGGTACCCC
ATTCTGCATCACAATGGATGACGTCACAAACGAGGACGGAACAGTCACACTTAGAGAGCGTGACT
CAACAAAGCAGGTCCGTGGAAAGCTCGAACAGATCATCAACGCTATCTGCGAAATGGTCGACGGA
AAGTCTTGGGAAGACGCTACAAAGGGCTACGAAACAGTCACACGCCCAGAAAACTAA

---> Sequence Showing highlighted Existant Combined Trimers

ATG ACG AAA GAC ACT CGC GCC ACC GCA GAA GAT CTT GAA GTT TCT GGT TTC TTC TGG
GTT CCA TCA TTC GAA ATC TAT GGC TCT GTT GCA GGC ATT TAC GAT CTT GGC CCA ACA
GGC TGC GCA ATC GAA CGC AAC TTC CTC CAG AAG TGG AGA GAT CAT TTC GTT CTT GAA
GAC GAC ATG CTC GAA GTT CGC TGC TCC GCT CTT ACC CCA CGT CCA GTC TTA GAT GCA
TCT GGC CAC ACA GAA AAA TTC AAT GAT CTC ATG CTC ACA GAC ATG ACA ACA AAG GCA
CTT TAC AGA GCT GAT CAG TAC ATC GCT GCT TAC CTT AAG GAG CGT GCT GAG AAG GAA
ACA GAC CAC GAC AAG AAG AAG CAG TAC GAG AAG GAT GCC GAA GAT GTC GAT GGC ATG

ACA AAG GAG CAG ATG ATG GCT CTC ATG GCC AAG TAC AAC ATC AAG TCT CCA GAA GGC AAC GAG TTC TCT GAA CCA GCC CCA TTC AAC CTT ATG TTC AAT ACA CGT GTT GGT CCA GGC GCC CGC TCC ATC GAG GCA TTC CTC CGC CCA GAA ACA GCT CAG GGC ATC TTC GTT AAC TTC ACA CGT CTC CTC AAT GCC AAC CGC GGA TCT CTT CCA TTC GCT GCT GCC CAA GTC GGC GCT GGT TAC CGT AAC GAA ATT TCC CCA CGT AAC GGT CTT GTC CGC TGC CGT GAA TTC CAG ATG GCC GAA ATC GAG CAC TTT GCT GAT CCA GAG CAG CTC AAC AAC TTC CCT AAG TTC GAG ACA GTC AAG AAC CTC AAG GTC AAG CTC TTC CCA GCC AGT ATC CAG GAA TTA GAA GAC GAA GAA AAG CGT ATC CCA ATC GAG ATC ACC CTC GAA GAC GCT ATC GCA CAG CAC GTC GTT TCC CAC AAG ACA CTC GGC TAC TAC ATT GGC CGT GTC TAC TTA TTC CTC TGC GAA ATC GGT ATC CAA CCA GAT ACA ATT CGC TTC CGT ATG CAC CGC AAG AAC GAA ATG GCC CAC TAC GCT CGC GAA TGC TGG GAT GCC GAA ATC TAC ACA AAG ACA CTC GGC TGG CTC GAG TGC GTC GGC ATT GCT GAC CGC CAA TCA TGG GAT CTT TCT CGC CAT GCT AAG TAC ACA ACA AAG AAG GGC GAT GCT GAA TCA TCA CCA CTC TAC CTT TCC GCT CCA CTC GAT ACA CCA ATC CAC CAG ACA AAG GTT GAA GGC GAA AAG TCC GCA ATC GGC AAG ATC TTC CGC AAG GAT GCC AAG GAG ATC ATG GAT GCC CTC GCA ACC ATC CCA GCC GAC CAG GTC GAA GCC CTC CGT GTC AAG GTT GCT GAA GCT GAG AAG CTC TTC GGT GCT GAG AAG CCA GCC AAG AAG AAC ATC GCC AAG GCC ATC GCA GCC CTT TCT GCT GAA GAC AAG AAG AAG TTC GAG GAA CTC ACA AAC ATC ACA GTT TGC GGA GAC AAG ACA GTT ACA TAC GAA ATG TAC AAC ATC AAC GAT ACA GTT GTT ACA ACA CGT AAG TTC TTC CCG AAC GTT ATC GAG CCA TCG TTC GGT GTC GGC AGA ATC ATG ACA TGC CTC TTC GAA CAG GCA TTT TAT GTC CGC GAA GAT GGC GAA CGC CGT GTT CTC AGA TTG AAG CCA TTC ATG GCA CCA TAC AAG GTT AAC CTC ATC CCA ATC AAC AAG AAG AAC GCT AAG GAT GAA GAT ATC CAC AAC ATC CGT ACA AAC CTC CGC AAG AAA GGT CTC GCT AAT ACA ATC GAT GCT GCT GGT GTC TCA ATC GGT AAG CGC TAC GCT CGT TCT GAT GAA ATC GGT ACC CCA TTC TGC ATC ACA ATG GAT GAC GTC ACA AAC GAG GAC GGA ACA GTC ACA CTT AGA GAG CGT GAC TCA ACA AAG CAG GTC CGT GGA AAG CTC GAA CAG ATC ATC AAC GCT ATC TGC GAA ATG GTC GAC GGA AAG TCT TGG GAA GAC GCT ACA AAG GGC TAC GAA ACA GTC ACA CGC CCA GAA AAC TAA

Occurrences/Results 9 Pentamers

The pentamer 'ATTCA' occurred 3 times.
The pentamer 'TTCAA' occurred 3 times.
The pentamer 'TCAAG' occurred 5 times.
The pentamer 'CAAGA' occurred 10 times.
The pentamer 'AAGAT' occurred 5 times.
The pentamer 'AGATG' occurred 5 times.
The pentamer 'GATGA' occurred 4 times.
The pentamer 'ATGAA' occurred 2 times.
The pentamer 'TGAAT' occurred 2 times.
The total occurrences of all specified strings is 39.
The total number of characters in the text is 1946.
First result (Total characters *9 /1024): 17.138671875

Square root of the first result: 4.139887906091178
Final result: 5.280657017991858

Occurrences/Results TRIMERS Codons

The pentamer 'ATT' occurred 5 times.
The pentamer 'CAA' occurred 3 times.
The pentamer 'GAT' occurred 24 times.
The pentamer 'GAA' occurred 42 times.
The pentamer 'CCA' occurred 26 times.
The pentamer 'ACT' occurred 1 times.
The pentamer 'AGA' occurred 5 times.
The pentamer 'TAC' occurred 19 times.
The total occurrences of all specified strings is 125.
The total number of characters in the text is 1950.
First result ((total_characters / 3) * (Trimer_number / 64)): 81.25
Square root of the first result: 9
Final result: 4.85

Occurrences/Results TRIMERS Combinations

The pentamer 'ATT ATT' occurred 0 times.
The pentamer 'ATT CAA' occurred 0 times.
The pentamer 'ATT GAT' occurred 0 times.
The pentamer 'ATT GAA' occurred 0 times.
The pentamer 'ATT CCA' occurred 0 times.
The pentamer 'ATT ACT' occurred 0 times.
The pentamer 'ATT AGA' occurred 0 times.
The pentamer 'ATT TAC' occurred 1 times.
The pentamer 'CAA ATT' occurred 0 times.
The pentamer 'CAA CAA' occurred 0 times.
The pentamer 'CAA GAT' occurred 0 times.
The pentamer 'CAA GAA' occurred 0 times.
The pentamer 'CAA CCA' occurred 1 times.
The pentamer 'CAA ACT' occurred 0 times.
The pentamer 'CAA AGA' occurred 0 times.
The pentamer 'CAA TAC' occurred 0 times.
The pentamer 'GAT ATT' occurred 0 times.
The pentamer 'GAT CAA' occurred 0 times.
The pentamer 'GAT GAT' occurred 0 times.
The pentamer 'GAT GAA' occurred 2 times.
The pentamer 'GAT CCA' occurred 1 times.
The pentamer 'GAT ACT' occurred 0 times.
The pentamer 'GAT AGA' occurred 0 times.
The pentamer 'GAT TAC' occurred 0 times.

The pentamer 'GAA ATT' occurred 1 times.
The pentamer 'GAA CAA' occurred 0 times.
The pentamer 'GAA GAT' occurred 4 times.
The pentamer 'GAA GAA' occurred 1 times.
The pentamer 'GAA CCA' occurred 1 times.
The pentamer 'GAA ACT' occurred 0 times.
The pentamer 'GAA AGA' occurred 0 times.
The pentamer 'GAA TAC' occurred 0 times.
The pentamer 'CCA ATT' occurred 0 times.
The pentamer 'CCA CAA' occurred 0 times.
The pentamer 'CCA GAT' occurred 1 times.
The pentamer 'CCA GAA' occurred 3 times.
The pentamer 'CCA CCA' occurred 0 times.
The pentamer 'CCA ACT' occurred 0 times.
The pentamer 'CCA AGA' occurred 0 times.
The pentamer 'CCA TAC' occurred 1 times.
The pentamer 'ACT ATT' occurred 0 times.
The pentamer 'ACT CAA' occurred 0 times.
The pentamer 'ACT GAT' occurred 0 times.
The pentamer 'ACT GAA' occurred 0 times.
The pentamer 'ACT CCA' occurred 0 times.
The pentamer 'ACT ACT' occurred 0 times.
The pentamer 'ACT AGA' occurred 0 times.
The pentamer 'ACT TAC' occurred 0 times.
The pentamer 'AGA ATT' occurred 0 times.
The pentamer 'AGA CAA' occurred 0 times.
The pentamer 'AGA GAT' occurred 1 times.
The pentamer 'AGA GAA' occurred 0 times.
The pentamer 'AGA CCA' occurred 0 times.
The pentamer 'AGA ACT' occurred 0 times.
The pentamer 'AGA AGA' occurred 0 times.
The pentamer 'AGA TAC' occurred 0 times.
The pentamer 'TAC ATT' occurred 1 times.
The pentamer 'TAC CAA' occurred 0 times.
The pentamer 'TAC GAT' occurred 1 times.
The pentamer 'TAC GAA' occurred 2 times.
The pentamer 'TAC CCA' occurred 0 times.
The pentamer 'TAC ACT' occurred 0 times.
The pentamer 'TAC AGA' occurred 1 times.
The pentamer 'TAC TAC' occurred 1 times.
The total occurrences of all specified strings is 24.
The total number of characters in the text is 1946.
First result (((total_characters/ 3) -1) / 64): 10.140625

Square root of the first result: 3.184434800714249
Final result: 4.352224450282803

>BQ640942.1 TVEST017.G09 Tv30236_PT cDNA Library Trichomonas vaginalis, ribosomal protein L18

---> Sequence

CGCGTTCCTCGCAAGCAGAGACAAGAACCAAAGTCAGAAGATGCACAGATGAGGATGCTCCATAA
CCTTTATGCTTTCCTCGCTCGCCGCACAGGAAATGATTTCAATGCTAAGATTGCTCNGCCGCTTAT
GCATTTCACGCACAAACAGACGTCCATACTCTCTCAGCCGCCTTGCTACAGACCTCAAGGATAAGG
AAGATGAGACAATTGCAGTTCTTGTTGCTAAGATCACAAACGATGAACGCCTTCTTGAAGTTCCA
AAGATGAAAGTTTGCGCTCTCAAGTTTACAGAAACAGCTCGTGATCGTATNCCTTGCTGCTGGTG
GCGAATGCATCACATTCGACCAACTTGCTCAGCTTCGCCCAACAGGTGACAAGTGCTTGCTTTTAG
AAGGTGATCGTACACACCGCCTTGTTAACAAGCATTTCGGAGCAGCTCCAGGCGATGACAATTCT
AAGACTCGTCCAAAGATTGCTTCACACGGACGCAAGTTCGAAATGGCTCGTGGCCGCAGAGCATC
AAGATGGTACCACGCTTAAACCTAAAAATTCTATTTTTTTAAA

---> Sequence Showing highlighted Existant Combined Trimers

CGC GTT CCT CGC AAG CAG AGA CAA GAA CCA AAG TCA GAA GAT GCA CAG ATG AGG ATG
CTC CAT AAC CTT TAT GCT TTC CTC GCT CGC CGC ACA GGA AAT GAT TTC AAT GCT AAG
ATT GCT CNG CCG CTT ATG CAT TTC ACG CAC AAA CAG ACG TCC ATA CTC TCT CAG CCG
CCT TGC TAC AGA CCT CAA GGA TAA GGA AGA TGA GAC AAT TGC AGT TCT TGT TGC TAA
GAT CAC AAA CGA TGA ACG CCT TCT TGA AGT TCC AAA GAT GAA AGT TTG CGC TCT CAA
GTT TAC AGA AAC AGC TCG TGA TCG TAT NCC TTG CTG CTG GTG GCG AAT GCA TCA CAT
TCG ACC AAC TTG CTC AGC TTC GCC CAA CAG GTG ACA AGT GCT TGC TTT TAG AAG GTG
ATC GTA CAC ACC GCC TTG TTA ACA AGC ATT TCG GAG CAG CTC CAG GCG ATG ACA ATT
CTA AGA CTC GTC CAA AGA TTG CTT CAC ACG GAC GCA AGT TCG AAA TGG CTC GTG GCC
GCA GAG CAT CAA GAT GGT ACC ACG CTT AAA CCT AAA AAT TCT ATT TTT TTA AA

Occurrences/Results 9 Pentamers

The pentamer 'ATTCA' occurred 0 times.
The pentamer 'TTCAA' occurred 1 times.
The pentamer 'TCAAG' occurred 3 times.
The pentamer 'CAAGA' occurred 2 times.
The pentamer 'AAGAT' occurred 7 times.
The pentamer 'AGATG' occurred 5 times.
The pentamer 'GATGA' occurred 5 times.
The pentamer 'ATGAA' occurred 2 times.
The pentamer 'TGAAT' occurred 0 times.
The total occurrences of all specified strings is 25.
The total number of characters in the text is 562.

First result (Total characters *9 /1024): 4.974609375
Square root of the first result: 2.230383235006935
Final result: 8.978452810571692

Occurrences/Results TRIMERS Codons

The pentamer 'ATT' occurred 4 times.
The pentamer 'CAA' occurred 6 times.
The pentamer 'GAT' occurred 5 times.
The pentamer 'GAA' occurred 3 times.
The pentamer 'CCA' occurred 1 times.
The pentamer 'ACT' occurred 0 times.
The pentamer 'AGA' occurred 6 times.
The pentamer 'TAC' occurred 2 times.
The total occurrences of all specified strings is 27.
The total number of characters in the text is 564.
First result ((total_characters / 3) * (Trimer_number / 64)): 23.5
Square root of the first result: 4.8
Final result: 0.7

Occurrences/Results TRIMERS Combinations

The pentamer 'ATT ATT' occurred 0 times.
The pentamer 'ATT CAA' occurred 0 times.
The pentamer 'ATT GAT' occurred 0 times.
The pentamer 'ATT GAA' occurred 0 times.
The pentamer 'ATT CCA' occurred 0 times.
The pentamer 'ATT ACT' occurred 0 times.
The pentamer 'ATT AGA' occurred 0 times.
The pentamer 'ATT TAC' occurred 0 times.
The pentamer 'CAA ATT' occurred 0 times.
The pentamer 'CAA CAA' occurred 0 times.
The pentamer 'CAA GAT' occurred 1 times.
The pentamer 'CAA GAA' occurred 1 times.
The pentamer 'CAA CCA' occurred 0 times.
The pentamer 'CAA ACT' occurred 0 times.
The pentamer 'CAA AGA' occurred 1 times.
The pentamer 'CAA TAC' occurred 0 times.
The pentamer 'GAT ATT' occurred 0 times.
The pentamer 'GAT CAA' occurred 0 times.
The pentamer 'GAT GAT' occurred 0 times.
The pentamer 'GAT GAA' occurred 1 times.
The pentamer 'GAT CCA' occurred 0 times.
The pentamer 'GAT ACT' occurred 0 times.

The pentamer 'GAT AGA' occurred 0 times.
The pentamer 'GAT TAC' occurred 0 times.
The pentamer 'GAA ATT' occurred 0 times.
The pentamer 'GAA CAA' occurred 0 times.
The pentamer 'GAA GAT' occurred 1 times.
The pentamer 'GAA GAA' occurred 0 times.
The pentamer 'GAA CCA' occurred 1 times.
The pentamer 'GAA ACT' occurred 0 times.
The pentamer 'GAA AGA' occurred 0 times.
The pentamer 'GAA TAC' occurred 0 times.
The pentamer 'CCA ATT' occurred 0 times.
The pentamer 'CCA CAA' occurred 0 times.
The pentamer 'CCA GAT' occurred 0 times.
The pentamer 'CCA GAA' occurred 0 times.
The pentamer 'CCA CCA' occurred 0 times.
The pentamer 'CCA ACT' occurred 0 times.
The pentamer 'CCA AGA' occurred 0 times.
The pentamer 'CCA TAC' occurred 0 times.
The pentamer 'ACT ATT' occurred 0 times.
The pentamer 'ACT CAA' occurred 0 times.
The pentamer 'ACT GAT' occurred 0 times.
The pentamer 'ACT GAA' occurred 0 times.
The pentamer 'ACT CCA' occurred 0 times.
The pentamer 'ACT ACT' occurred 0 times.
The pentamer 'ACT AGA' occurred 0 times.
The pentamer 'ACT TAC' occurred 0 times.
The pentamer 'AGA ATT' occurred 0 times.
The pentamer 'AGA CAA' occurred 1 times.
The pentamer 'AGA GAT' occurred 0 times.
The pentamer 'AGA GAA' occurred 0 times.
The pentamer 'AGA CCA' occurred 0 times.
The pentamer 'AGA ACT' occurred 0 times.
The pentamer 'AGA AGA' occurred 0 times.
The pentamer 'AGA TAC' occurred 0 times.
The pentamer 'TAC ATT' occurred 0 times.
The pentamer 'TAC CAA' occurred 0 times.
The pentamer 'TAC GAT' occurred 0 times.
The pentamer 'TAC GAA' occurred 0 times.
The pentamer 'TAC CCA' occurred 0 times.
The pentamer 'TAC ACT' occurred 0 times.
The pentamer 'TAC AGA' occurred 2 times.
The pentamer 'TAC TAC' occurred 0 times.
The total occurrences of all specified strings is 9.

The total number of characters in the text is 562.
First result (((total_characters/ 3) -1) / 64): 2.921875
Square root of the first result: 1.709349291397168
Final result: 3.5558121623181727

>JAOSJJ010000002.1:c32934930-32933329 Trichomonas vaginalis G3 chromosome V, FstH

---> Sequence

ATGTTTGTCTTAATTGTTTTCTTCTGGTACAGGATGATGCAACAAACTGGTGGCGGAGGAAAGA
TGAGTTCTTTCGGCAAGAGTAAGGCAAAATTAATTAACAAAGAAGATGGCAAAAATTTAGTTAA
GTTTAAGGATGTTGCAGGCTTAGAAGAAGAAAAGGAAGAACTGGAAGAAATTGTTGACTTCTTA
AGAGATCCTAAGAAATTTATAGATATGGGAGCCAGAATACCTAAGGGAGTCCTATTAGTAGGTCC
TCCAGGAACTGGTAAAACTTATCTTTCAAAGGCTGTTGCTGGCGAAGCAGGAGTTCCTTTCTTTA
TAATGAGTGGATCTGACTTCGTAGAAATGTTTGTTGGTGTTGGTGCATCAAGAGTAAGAGACCTT
TTTGAATCTGCAAAGAAAAATGCGCCTTGTATAATTTTTATTGACGAAATTGACGCAGTGGGAAG
AAAAAGAGGGACGGGTCTTGGCGGAGGCCATGATGAAAGAGAACAAACTCTTAATCAACTTCTT
GTTGAGATGGACGGTTTTGGAACTAACGAAGGTGTTATAGTTATGGCTGCAACAAATAGGGCAGA
CATACTTGACCCAGCAATCCTAAGACCAGGTAGATTTGACAGGACAGTTTATGTTGGCAAGCCGG
ACGTTCGTGCAAGAAAGGCAATACTCAAAATTCATTCTAGGGACAAGAAGCTTGCTGATGACGTA
AATCTTGAAGTAATTGCAAAGAGAACATCAGGCTTTACTCCAGCAGATCTTGAAAATCTAATGAA
TGAATCAGCCCTACTTGCTGCAAGACGTGGCGAAAATGCAATCTCGATGGAAGATGTTGATGAAG
CATCAATAAAGGTCCAAGCAGGTCCTGCAAAGAAGTCTCGTGTTGTTTCAGAGAAGGAAAGAAAG
CTAACAGCAGTTCACGAATCTGGTCACGCAATTGTATCAAGACTTCTTCCAGAAGAAGATTCAGT
TCACATGATTACAATTATTCCAAGGGGAATGGCTGGTGGTTTTACTGCGTACCTACCTGAAGAC
GATGTTTCTTTTATGACAAAGAGGAAGATGGAAGCTTCCATTGTATCTCTTCTTGGAGGAAGAGT
TGCCGAGTCCCTTGTCCTTGATGATATTTCAACTGGTGCATCAAACGACATTGAGAGGGCTACAA
AAATTGCTCGTGCTATGGTAACCCACTATGGAATGAGTGAAAAGCTTGGAACAATTAATTATGA
TTCATCAGAAAATGAAGTTTTTATAGGAAGGGATCTGGGACGTTCAAGAGATTATTCTGAAAGA
ACTGCAGCAGAAATTGATGATGAAGTTACAAGAATTATAAACGAGGCTTATACAAAGTGTAAGA
AACTTTTAAGTGATAACTTAGACAAGTTGCTTGCATTATCAGATGCCCTTCTAGAAAAGGAAACA
ATTTACTCCAAGGACTTTGAAAAAATTTTTAATGGCGAAAAGCTTGATGAAGAATCAATTGACCT
TGATGACACGATAAATGAAGATGACTTATCCGATGAAGCGAAAGAACTCATCCACAAGAATGAT
AAGAAGGATGAAGTGAATAAGGAAGATTCAGAAGATAGTGAAAGTTTATAA

---> Sequence Showing highlighted Existant Combined Trimers

ATG TTT GTC TTA ATT GTT TTC TTC TGG TAC AGG ATG ATG CAA CAA ACT GGT GGC GGA
GGA AAG ATG AGT TCT TTC GGC AAG AGT AAG GCA AAA TTA ATT AAC AAA GAA GAT GGC
AAA AAT TTA GTT AAG TTT AAG GAT GTT GCA GGC TTA GAA GAA GAA AAG GAA GAA CTG
GAA GAA ATT GTT GAC TTC TTA AGA GAT CCT AAG AAA TTT ATA GAT ATG GGA GCC AGA
ATA CCT AAG GGA GTC CTA TTA GTA GGT CCT CCA GGA ACT GGT AAA ACT TAT CTT TCA
AAG GCT GTT GCT GGC GAA GCA GGA GTT CCT TTC TTT ATA ATG AGT GGA TCT GAC TTC

GTA GAA ATG TTT GTT GGT GTT GGT GCA TCA AGA GTA AGA GAC CTT TTT GAA TCT GCA AAG AAA AAT GCG CCT TGT ATA ATT TTT ATT GAC GAA ATT GAC GCA GTG GGA AGA AAA AGA GGG ACG GGT CTT GGC GGA GGC CAT GAT GAA AGA GAA CAA ACT CTT AAT CAA CTT CTT GTT GAG ATG GAC GGT TTT GGA ACT AAC GAA GGT GTT ATA GTT ATG GCT GCA ACA AAT AGG GCA GAC ATA CTT GAC CCA GCA ATC CTA AGA CCA GGT AGA TTT GAC AGG ACA GTT TAT GTT GGC AAG CCG GAC GTT CGT GCA AGA AAG GCA ATA CTC AAA ATT CAT TCT AGG GAC AAG AAG CTT GCT GAT GAC GTA AAT CTT GAA GTA ATT GCA AAG AGA ACA TCA GGC TTT ACT CCA GCA GAT CTT GAA AAT CTA ATG AAT GAA TCA GCC CTA CTT GCT GCA AGA CGT GGC GAA AAT GCA ATC TCG ATG GAA GAT GTT GAT GAA GCA TCA ATA AAG GTC CAA GCA GGT CCT GCA AAG AAG TCT CGT GTT GTT TCA GAG AAG GAA AGA AAG CTA ACA GCA GTT CAC GAA TCT GGT CAC GCA ATT GTA TCA AGA CTT CTT CCA GAA GAA GAT TCA GTT CAC ATG ATT ACA ATT ATT CCA AGG GGA ATG GCT GGT GGT TTT ACT GCG TAC CTA CCT GAA GAC GAT GTT TCT TTT ATG ACA AAG AGG AAG ATG GAA GCT TCC ATT GTA TCT CTT CTT GGA GGA AGA GTT GCC GAG TCC CTT GTC CTT GAT GAT ATT TCA ACT GGT GCA TCA AAC GAC ATT GAG AGG GCT ACA AAA ATT GCT CGT GCT ATG GTA ACC CAC TAT GGA ATG AGT GAA AAG CTT GGA ACA ATT AAT TAT GAT TCA TCA GAA AAT GAA GTT TTT ATA GGA AGG GAT CTG GGA CGT TCA AGA GAT TAT TCT GAA AGA ACT GCA GCA GAA ATT GAT GAT GAA GTT ACA AGA ATT ATA AAC GAG GCT TAT ACA AAG TGT AAG AAA CTT TTA AGT GAT AAC TTA GAC AAG TTG CTT GCA TTA TCA GAT GCC CTT CTA GAA AAG GAA ACA ATT TAC TCC AAG GAC TTT GAA AAA ATT TTT AAT GGC GAA AAG CTT GAT GAA GAA TCA ATT GAC CTT GAT GAC ACG ATA AAT GAA GAT GAC TTA TCC GAT GAA GCG AAA GAA CTC ATC CAC AAG AAT GAT AAG AAG GAT GAA GTG AAT AAG GAA GAT TCA GAA GAT AGT GAA AGT TTA TAA

Occurrences/Results 9 Pentamers

The pentamer 'ATTCA' occurred 4 times.
The pentamer 'TTCAA' occurred 3 times.
The pentamer 'TCAAG' occurred 3 times.
The pentamer 'CAAGA' occurred 9 times.
The pentamer 'AAGAT' occurred 8 times.
The pentamer 'AGATG' occurred 7 times.
The pentamer 'GATGA' occurred 12 times.
The pentamer 'ATGAA' occurred 9 times.
The pentamer 'TGAAT' occurred 3 times.
The total occurrences of all specified strings is 58.
The total number of characters in the text is 1598.
First result (Total characters *9 /1024): 14.080078125
Square root of the first result: 3.7523430180355315
Final result: 11.704666035034677

Occurrences/Results TRIMERS Codons

The pentamer 'CAA' occurred 5 times.
The pentamer 'GAT' occurred 28 times.

The pentamer 'GAA' occurred 46 times.
The pentamer 'ATT' occurred 22 times.
The pentamer 'CCA' occurred 6 times.
The pentamer 'ACT' occurred 9 times.
The pentamer 'TAC' occurred 3 times.
The pentamer 'AGA' occurred 18 times.
The total occurrences of all specified strings is 137.
The total number of characters in the text is 1598.
First result ((total_characters / 3) * (Trimer_number / 64)): 66.6
Square root of the first result: 8.2
Final result: 8.6

Occurrences/Results TRIMERS Combinations

The pentamer 'CAA CAA' occurred 1 times.
The pentamer 'CAA GAT' occurred 0 times.
The pentamer 'CAA GAA' occurred 0 times.
The pentamer 'CAA ATT' occurred 0 times.
The pentamer 'CAA CCA' occurred 0 times.
The pentamer 'CAA ACT' occurred 2 times.
The pentamer 'CAA TAC' occurred 0 times.
The pentamer 'CAA AGA' occurred 0 times.
The pentamer 'GAT CAA' occurred 0 times.
The pentamer 'GAT GAT' occurred 2 times.
The pentamer 'GAT GAA' occurred 6 times.
The pentamer 'GAT ATT' occurred 1 times.
The pentamer 'GAT CCA' occurred 0 times.
The pentamer 'GAT ACT' occurred 0 times.
The pentamer 'GAT TAC' occurred 0 times.
The pentamer 'GAT AGA' occurred 0 times.
The pentamer 'GAA CAA' occurred 1 times.
The pentamer 'GAA GAT' occurred 6 times.
The pentamer 'GAA GAA' occurred 5 times.
The pentamer 'GAA ATT' occurred 3 times.
The pentamer 'GAA CCA' occurred 0 times.
The pentamer 'GAA ACT' occurred 0 times.
The pentamer 'GAA TAC' occurred 0 times.
The pentamer 'GAA AGA' occurred 3 times.
The pentamer 'ATT CAA' occurred 0 times.
The pentamer 'ATT GAT' occurred 1 times.
The pentamer 'ATT GAA' occurred 0 times.
The pentamer 'ATT ATT' occurred 1 times.
The pentamer 'ATT CCA' occurred 1 times.
The pentamer 'ATT ACT' occurred 0 times.

The pentamer 'ATT TAC' occurred 1 times.
The pentamer 'ATT AGA' occurred 0 times.
The pentamer 'CCA CAA' occurred 0 times.
The pentamer 'CCA GAT' occurred 0 times.
The pentamer 'CCA GAA' occurred 1 times.
The pentamer 'CCA ATT' occurred 0 times.
The pentamer 'CCA CCA' occurred 0 times.
The pentamer 'CCA ACT' occurred 0 times.
The pentamer 'CCA TAC' occurred 0 times.
The pentamer 'CCA AGA' occurred 0 times.
The pentamer 'ACT CAA' occurred 0 times.
The pentamer 'ACT GAT' occurred 0 times.
The pentamer 'ACT GAA' occurred 0 times.
The pentamer 'ACT ATT' occurred 0 times.
The pentamer 'ACT CCA' occurred 1 times.
The pentamer 'ACT ACT' occurred 0 times.
The pentamer 'ACT TAC' occurred 0 times.
The pentamer 'ACT AGA' occurred 0 times.
The pentamer 'TAC CAA' occurred 0 times.
The pentamer 'TAC GAT' occurred 0 times.
The pentamer 'TAC GAA' occurred 0 times.
The pentamer 'TAC ATT' occurred 0 times.
The pentamer 'TAC CCA' occurred 0 times.
The pentamer 'TAC ACT' occurred 0 times.
The pentamer 'TAC TAC' occurred 0 times.
The pentamer 'TAC AGA' occurred 0 times.
The pentamer 'AGA CAA' occurred 0 times.
The pentamer 'AGA GAT' occurred 2 times.
The pentamer 'AGA GAA' occurred 1 times.
The pentamer 'AGA ATT' occurred 1 times.
The pentamer 'AGA CCA' occurred 1 times.
The pentamer 'AGA ACT' occurred 1 times.
The pentamer 'AGA TAC' occurred 0 times.
The pentamer 'AGA AGA' occurred 0 times.
The total occurrences of all specified strings is 42.
The total number of characters in the text is 1598.
First result (((total_characters/ 3) -1) / 64): 8.328125
Square root of the first result: 2.885849095153799
Final result: 11.667926454139655

>XM_001329178.2 Trichomonas vaginalis G3 pyrophosphate-dependent phosphofructokinase TM0289 type family (TVAGG3_0696580)

---> Sequence

ATGTCTACTGAAGCTCCAGTTCTCGGTATCATCATTGGCGGCGCCCCAGCTCCAGGTCTTAATGGT
GTTATTGCCTCTGCTACATTCTATGCTCGTCAGCTTGGCTGGAAAGTCATTGGTTTCCACGATGG
ATATTTACATCTTGCCACTGGCTCATTAGAAGAAGTTAAGGCTAACACACTTGAACTTAACGAGG
ACTTTGTTGCACCACACCTCTCAACAGGTGGCTCAATCATTCGCCCACACAGATATGATCCAACAA
AGTCAAACAAGGAAATTCACAATGTCCTTAGAAATCTTAAGGAATTCCACATTCGCTACCTCATC
ATCATTGGTGGCAATGATAAAATCGCTACAACACATATCATTACATCTGGCCTTGATCCAGCTCA
GATGCAAGTTATTGCTATTCCAAAGACAATCGATAACGACATTTCACTTCCTTACAACACCGATA
CATTCGGCTTCCATTCTGCTCGCAAGTTCTGCTCAGAACTTGTTATGAACCTTGCTGTTGATGCCC
GCTCTGCTCCACGTTGGTTCATCATCGAGACAATGGGTCGCCGCTCCGGCCACCTTGCTCTCTCCG
TTTCTGAGGCTACAGCTGCCCATCTTTGCATCATCCCAGAAGATTTCAAGGGCAGACAAGTCACA
CTTAAGGAGATCTGCGACGTCTTCGAGGGCGCAATCCTTAAGCGTTATCTTGCTGGAAAACCATA
CGGTGTTTGCATCATCACAGAAGGTTTGATCCATTACCTTCCAAAGGAGGAGATCGAATCCCTCT
TCAAGGATGGCATCGTCCAGTACACAGCTGAAGGCCAGCTCAACCTCGATGAGGCTGAAATTTCC
CGCGCCATCAGAAACGAGATGAACAACAGACTTTCCAAGATGGGACTCGACGTCAGAGTCAATCC
AAAGAAGATCGGTTACGAACTTCGTTGCATGGACCCAATCTCTGCAGATATGTGCTACACAAGAG
AACTCGGCGCTGCTGCAATCGAAGGATTCCTCAACAACCACTCCAACGTCATGGTCGTTTGGGAG
AATGGTAACGCTACATACGTTTCATTCCGCTCTCTCATGAACGAGGAAGACGGACAGATCTACCC
AAGACTCGTCGACACAAAGTCACAGAACTACCGCATTTCACGCGAATACGGCTGGCAGGTCAAGA
AGTGCGACCTCGACGACACTGAGAAGGTCGAGAAACTCGCAAAGATTGCAAAGATCACTCCTGAG
GAATTCCACAAGAAGTTCGACCCAATTATGGATGATCCAGCTAAATACTAA

---> Sequence Showing highlighted Existant Combined Trimers

Occurrences/Results 9 Pentamers

The pentamer 'ATTCA' occurred 1 times.
The pentamer 'TTCAA' occurred 2 times.
The pentamer 'TCAAG' occurred 3 times.
The pentamer 'CAAGA' occurred 5 times.
The pentamer 'AAGAT' occurred 5 times.
The pentamer 'AGATG' occurred 3 times.
The pentamer 'GATGA' occurred 3 times.
The pentamer 'ATGAA' occurred 3 times.
The pentamer 'TGAAT' occurred 0 times.
The total occurrences of all specified strings is 25.
The total number of characters in the text is 1286.
First result (Total characters *9 /1024): 11.337890625
Square root of the first result: 3.3671784367627446
Final result: 4.057435515099982

Occurrences/Results TRIMERS Codons

The pentamer 'ATT' occurred 15 times.
The pentamer 'CAA' occurred 2 times.
The pentamer 'GAT' occurred 13 times.
The pentamer 'GAA' occurred 18 times.

The pentamer 'CCA' occurred 17 times.
The pentamer 'ACT' occurred 4 times.
The pentamer 'AGA' occurred 8 times.
The pentamer 'TAC' occurred 12 times.
The total occurrences of all specified strings is 89.
The total number of characters in the text is 1286.
First result ((total_characters / 3) * (Trimer_number / 64)): 53.6
Square root of the first result: 7.3
Final result: 3.5

Occurrences/Results TRIMERS Combinations

The pentamer 'ATT ATT' occurred 0 times.
The pentamer 'ATT CAA' occurred 0 times.
The pentamer 'ATT GAT' occurred 0 times.
The pentamer 'ATT GAA' occurred 0 times.
The pentamer 'ATT CCA' occurred 1 times.
The pentamer 'ATT ACT' occurred 0 times.
The pentamer 'ATT AGA' occurred 0 times.
The pentamer 'ATT TAC' occurred 0 times.
The pentamer 'CAA ATT' occurred 0 times.
The pentamer 'CAA CAA' occurred 0 times.
The pentamer 'CAA GAT' occurred 0 times.
The pentamer 'CAA GAA' occurred 0 times.
The pentamer 'CAA CCA' occurred 0 times.
The pentamer 'CAA ACT' occurred 0 times.
The pentamer 'CAA AGA' occurred 0 times.
The pentamer 'CAA TAC' occurred 0 times.
The pentamer 'GAT ATT' occurred 0 times.
The pentamer 'GAT CAA' occurred 0 times.
The pentamer 'GAT GAT' occurred 1 times.
The pentamer 'GAT GAA' occurred 0 times.
The pentamer 'GAT CCA' occurred 3 times.
The pentamer 'GAT ACT' occurred 0 times.
The pentamer 'GAT AGA' occurred 0 times.
The pentamer 'GAT TAC' occurred 0 times.
The pentamer 'GAA ATT' occurred 2 times.
The pentamer 'GAA CAA' occurred 0 times.
The pentamer 'GAA GAT' occurred 1 times.
The pentamer 'GAA GAA' occurred 1 times.
The pentamer 'GAA CCA' occurred 0 times.
The pentamer 'GAA ACT' occurred 0 times.
The pentamer 'GAA AGA' occurred 0 times.
The pentamer 'GAA TAC' occurred 1 times.
The pentamer 'CCA ATT' occurred 1 times.

The pentamer 'CCA CAA' occurred 0 times.
The pentamer 'CCA GAT' occurred 0 times.
The pentamer 'CCA GAA' occurred 1 times.
The pentamer 'CCA CCA' occurred 0 times.
The pentamer 'CCA ACT' occurred 0 times.
The pentamer 'CCA AGA' occurred 1 times.
The pentamer 'CCA TAC' occurred 1 times.
The pentamer 'ACT ATT' occurred 0 times.
The pentamer 'ACT CAA' occurred 0 times.
The pentamer 'ACT GAT' occurred 0 times.
The pentamer 'ACT GAA' occurred 1 times.
The pentamer 'ACT CCA' occurred 0 times.
The pentamer 'ACT ACT' occurred 0 times.
The pentamer 'ACT AGA' occurred 0 times.
The pentamer 'ACT TAC' occurred 0 times.
The pentamer 'AGA ATT' occurred 0 times.
The pentamer 'AGA CAA' occurred 1 times.
The pentamer 'AGA GAT' occurred 0 times.
The pentamer 'AGA GAA' occurred 1 times.
The pentamer 'AGA CCA' occurred 0 times.
The pentamer 'AGA ACT' occurred 0 times.
The pentamer 'AGA AGA' occurred 0 times.
The pentamer 'AGA TAC' occurred 0 times.
The pentamer 'TAC ATT' occurred 0 times.
The pentamer 'TAC CAA' occurred 0 times.
The pentamer 'TAC GAT' occurred 0 times.
The pentamer 'TAC GAA' occurred 1 times.
The pentamer 'TAC CCA' occurred 1 times.
The pentamer 'TAC ACT' occurred 0 times.
The pentamer 'TAC AGA' occurred 0 times.
The pentamer 'TAC TAC' occurred 0 times.
The total occurrences of all specified strings is 19.
The total number of characters in the text is 1286.
First result (((total_characters/ 3) -1) / 64): 6.703125
Square root of the first result: 2.5890393971509975
Final result: 4.7495897565450695

3) Entamoeba histolytica

>BK005662.1:1-71 TPA_exp: Entamoeba histolytica, tRNA-Gly GCC

GCACTTG**TG**GTCC**AATGGTA**TGACGTATCC**CTGCCA**CGGATGAGACCCGGG**TTC**G**A**
TTCCCGGCAAGTGCA

>AY504965.1 Entamoeba histolytica, topoisomerase II gene

---> Sequence

ATGAGCAAAGAGAAAGAAAAACTTGAAGATATTTATGTAAAGCTTTCACATAAAGAACAAATTT
TAACCAGACCTGATACTTATATTGGAAGTGTAGAACGAAATGATGAACAGGTGTGGATATATGA
AGAACCATCTGAACAATATAGTGAAGGAAGAATGATTCAAAAAGTTATTTCATTAGCACCTGGAC
TTTTTAAAATTTATGATGAAATTCTTGTTAATGCAAGTGATAATTTCCAAAGAGATCCTAAAATG
ACTACTATTAAAGTTACTATTGAAGGTAATTCAATAACAGTTTATAATAATGGGAAAGGTATTCC
AATTGAAATTCATAAAAAGGAACATATTTATATTCCTGAATTAATTTTTGGACATCTTTTAACAT
CCTCAAATTATAAAGATGATGATAAAAAAGTGACTGGTGGAAGAAATGGTTATGGAGCAAAGTT
AGCTAATATTTTTTCTACTAAATTTTCAATAGAAACTTGTGATGGAAAAAAAATTTATAAACAA
ACATGGAAAAACAATATGTCAGATAAAGAAGAACCAGAAATTGAAAGTGTTAAAAAAGTAGAAG
AATATACAAAAGTTACTTTTACTCCAGACTATAAAAGATTCCAAATGAAAGGAATGGAAGATGA
TGCTAGAATGTTATTAATAAGAAGAGCATATGATATTGCAGGATGTAATCCAGGTAAAAGGATA
TGGATTAATGGTAAAAAAGTTTGTTTTAAAAATTTTGCAGAATATGCTCGAATGTATGTTGGTG
ATGAACCAATAGTTTCTGAGAAAGCTGGAGAAAGATGGGATATTTGTGTATGTGCAAGTAAAGG
TGAACCTATGCAAATATCATTTGTAAATTCTATTAATACATCACATGGAGGAACTCATGTAGACT
ATGTTACAGGATTAATGACTAAATATCTTGCTGAAAAGATGAAAAAGTTAAATAAGAAAGGTGC
TGAGATTAAACCATTTCAAATCAAAAATCATCTTTTTGTATTTGTCAATTCATTAATAGAAAATC
CTGCATTTGATTCTCAAACAAAAGAAACTTTAAAAACACAAGTTAATAAATTTGGATCAAAACCA
TCGTTAAGTGATAAATTTTTCAAAGAATTAGGGAAATTATCTGTTGTTGACGATATTCTTGCATG
GGCTATTAAGAAAGGTGAAATGGATTTAAATAAGTCTGGAGGAAAGAAAACAGCTAGAATTACT
GGAATTCCAAAATTAGATGATGCTAATAAAGCTGGAACTAAAGAAGGAAATTTATGTACATTAA
TTCTTACAGAAGGAGATTCAGCTAAGACTTTAGCCGTATCAGGATTATCTGTTGTAGGAAGAGAT
TATTATGGAGTATTTCCATTAAGAGGAAAACCATTAAATGCTAGAGAAATAGCACCATCAAAAGT
AAAAGAGAATCATGAATTTGAAAATATTGCAAAAATTATGGGACTTAGATATGGTAAAGTTTAT
ACTTCAATTGATGAATTAAGATATGGTAGTGTTATGATAATGGCAGATCAAGATTTTGATGGTTC
ACATATTAAAGGTTTACTTATCAATTATTTCCATACTTTTTGGCCTTCATTATTAAAAATTGATA
ATTTCTTAGTTGAATTTATTACACCAATTGTCAAAGTTAAACGTGGGAAACAAGAAATTTCATTC
TTTACATTACCACAATTTAATGAATGGAAAGATGCAAGAATTCAAGAAGGTCAATTAAAAAAAT
GGGAAATTAAATATTACAAGGGTCTTGGTACTTCAAAAGACTCTGATGCTAAAGCATATTTCAGT
GATCTTGATACACATAAAATTAAGTTTGCATATGATGGAGCTGGTTCAGATGAAGTAATAGATTT
AGCATTTAATAGAAAAAGAGCAGATGATCGTAAAGAGTGGTTAAAAGGATATGATCCAAATACA
TATCTTGATCAAGATGTTGATAGTATTAGTTATGAAGATTTTGTTCATAAAGAATTAATATTAT
TCTCATATGATGATTGTGAAAGGTCTATTCCTTCTGTTGTAGATGGATTAAAACCATCACAAAGA
AAAGTGTTATGGACATGTTTAGAAAAAAATATTATGAAAGAATTAAAAGTATCACAATTATCTG
GACTTGTTTCTGAAAAGTCATCATATCATCATGGAGAAGTATCTCTTCAAGCTACCATTGTTAAT
ATGGCACAAAACTTTTGTGGATCAAATAATGTTAATTGGTTATTACCATCTGGTCAATTTGGTTC
AAGATTAGGAGGAGGCAAAGATCAAGCAGCAGCAAGATATATTTATACACGATTATCAAGTATT

AGTAGATATTTATGTCCTAAAGATGATGACCAAATATTAGAATATCTTATTGATGAAGATAGAA
GAATTGAACCAAAATATTATTTCCCTATTATTCCTACAGTATTAGTTAATGGAGCTGATGGTATT
GGAACAGGATGGAGTACTGCCGTTTCAAATTATAATCCTCTTGATCTTGTAGATAATTGTAGAAG
ATATTTAAATGGAGAGGAATTTAAAGAAATGAAGCCATGGTTTAGAGGATGGACAGGACAATTA
AGAAAGAATAAAGATGGAAGTGGTTATGATTGTTATGGAAAATGGAGAAGATTATCTGAAGATA
AAATTGAAATTACTGAATTACCTATTCAAATATGGACTGATTCATATAAACAACATTTAGAAAA
GTGTATTGAAGCAAAGATTGTAAAAGGATATTCAGAATATCATATGGTTAATACAGTGCATTTT
GTTATTGATTTAGTCCAACCAATGAGTGATGAAGATATTTGGACTAATTTAAAATTAGTTAGTA
GTGTTAAGACAACAAATATGCATTTATTCAATTCTGAATATAAAATCACTAAATATCATACTGTT
CTTGACATTTTATCAGAGTTTTGTGATGTTAGAATGAAAGGTTATGAAGAAAGAAAGAAAGCTA
TTATTGAAAGTATGGAAAGACAATTAATGATATTATCAAATAAAGCAAGGTTCATTAAAATGGT
TATTGAAGGAGAATTAATTGTTAACAACAGAAAGAAAAATGAGTTATATAAGGATTTAATTAAA
TTTGGATTTGACCAAATAAGAGACCCCAAAAAAGCAAATAAATCAAAACCTATTGGTGAATCTGA
AGATGATGAAATAAAAGAAGAAGAGGAAGATGATGAAGTTGGTGGATATGATTATTTGTTGAGT
ATGAAGATTTGGACATTAACATATGAAAAAGCAAAGAAATTGATGGAAGATTGTGATGACTTAA
AGACACAAATTGATACAATTAAAACAACACCAGTAAAAGATATGTGGTTGAAAGATTTAGATGC
ATTTGTAAAAAAATATCCACAATGGTTAGAAGAAGAAGATATTTTGTTAAAGAAAGGAGACAAA
AGTACAAAAAGAGTTATTATGGGAAAGAAAGGAAAATCAACTAAAAAAACAAGTAGAAAAGTAG
CATTAACAACTTCTATAATGGATAAAAGTCCTAGTGAATTAGCTAAAAAATATAAAGGAAGATT
AGAAGATTATGAATGGAAAACAATGCCAGATGAAAATACTGAGATTAAGAAAAAAACAATTAAA
AAAGTTACTACAACACGAAAACGAAAAGATGAAGATTCAAGTATGAGTGGTGATGAAAAAGTAA
AAAGAAAAAGAACAACGAAAAAATCAACAACTAAGAAAACTACGAAAAAAAGAACAACTAAAGC
TTCAAAAGAAGAAGAAAATGATACTGTTGATACTCCTGCTAAAACATCAAGAAAAAAGAAGACT
AAGAAAGTGACTGTTAAAGAAGAAGATGATGATGAAAGTGATTCAGTTTTTATTGATGATGATG
AAAATGATAATGGATCAGTAGAAGAAATACAAAGTGATTCTACTGATGAAGAAATGAGTGAAGA
AGATGAAGAAATGTTTGAAGAAGATGAAGAAGATGAAGATCCAAAACCAAAAAGAAAAACAAGG
TCATCAAAAAAAGAAAAGAGTGATGACAGTTTCATTGTTGATGATGATGAAATATGGTAA

---> Sequence Showing highlighted Existant Combined Trimers

ATG AGC AAA GAG AAA GAA AAA CTT GAA GAT ATT TAT GTA AAG CTT TCA CAT AAA GAA
CAA ATT TTA ACC AGA CCT GAT ACT TAT ATT GGA AGT GTA GAA CGA AAT GAT GAA CAG
GTG TGG ATA TAT GAA GAA CCA TCT GAA CAA TAT AGT GAA GGA AGA ATG ATT CAA AAA
GTT ATT TCA TTA GCA CCT GGA CTT TTT AAA ATT TAT GAT GAA ATT CTT GTT AAT GCA
AGT GAT AAT TTC CAA AGA GAT CCT AAA ATG ACT ACT ATT AAA GTT ACT ATT GAA GGT
AAT TCA ATA ACA GTT TAT AAT AAT GGG AAA GGT ATT CCA ATT GAA ATT CAT AAA AAG
GAA CAT ATT TAT ATT CCT GAA TTA ATT TTT GGA CAT CTT TTA ACA TCC TCA AAT TAT
AAA GAT GAT GAT AAA AAA GTG ACT GGT GGA AGA AAT GGT TAT GGA GCA AAG TTA GCT
AAT ATT TTT TCT ACT AAA TTT TCA ATA GAA ACT TGT GAT GGA AAA AAA ATT TAT AAA
CAA ACA TGG AAA AAC AAT ATG TCA GAT AAA GAA GAA CCA GAA ATT GAA AGT GTT AAA
AAA GTA GAA GAA TAT ACA AAA GTT ACT TTT ACT CCA GAC TAT AAA AGA TTC CAA ATG
AAA GGA ATG GAA GAT GAT GCT AGA ATG TTA TTA ATA AGA AGA GCA TAT GAT ATT GCA
GGA TGT AAT CCA GGT AAA AGG ATA TGG ATT AAT GGT AAA AAA GTT TGT TTT AAA AAT
TTT GCA GAA TAT GCT CGA ATG TAT GTT GGT GAT GAA CCA ATA GTT TCT GAG AAA GCT

GGA GAA AGA TGG GAT ATT TGT GTA TGT GCA AGT AAA GGT GAA CCT ATG CAA ATA TCA
TTT GTA AAT TCT ATT AAT ACA TCA CAT GGA GGA ACT CAT GTA GAC TAT GTT ACA GGA
TTA ATG ACT AAA TAT CTT GCT GAA AAG ATG AAA AAG TTA AAT AAG AAA GGT GCT GAG
ATT AAA CCA TTT CAA ATC AAA AAT CAT CTT TTT GTA TTT GTC AAT TCA TTA ATA GAA
AAT CCT GCA TTT GAT TCT CAA ACA AAA GAA ACT TTA AAA ACA CAA GTT AAT AAA TTT
GGA TCA AAA CCA TCG TTA AGT GAT AAA TTT TTC AAA GAA TTA GGG AAA TTA TCT GTT
GTT GAC GAT ATT CTT GCA TGG GCT ATT AAG AAA GGT GAA ATG GAT TTA AAT AAG TCT
GGA GGA AAG AAA ACA GCT AGA ATT ACT GGA ATT CCA AAA TTA GAT GAT GCT AAT AAA
GCT GGA ACT AAA GAA GGA AAT TTA TGT ACA TTA ATT CTT ACA GAA GGA GAT TCA GCT
AAG ACT TTA GCC GTA TCA GGA TTA TCT GTT GTA GGA AGA GAT TAT TAT GGA GTA TTT
CCA TTA AGA GGA AAA CCA TTA AAT GCT AGA GAA ATA GCA CCA TCA AAA GTA AAA GAG
AAT CAT GAA TTT GAA AAT ATT GCA AAA ATT ATG GGA CTT AGA TAT GGT AAA GTT TAT
ACT TCA ATT GAT GAA TTA AGA TAT GGT AGT GTT ATG ATA ATG GCA GAT CAA GAT TTT
GAT GGT TCA CAT ATT AAA GGT TTA CTT ATC AAT TAT TTC CAT ACT TTT TGG CCT TCA
TTA TTA AAA ATT GAT AAT TTC TTA GTT GAA TTT ATT ACA CCA ATT GTC AAA GTT AAA
CGT GGG AAA CAA GAA ATT TCA TTC TTT ACA TTA CCA CAA TTT AAT GAA TGG AAA GAT
GCA AGA ATT CAA GAA GGT CAA TTA AAA AAA TGG GAA ATT AAA TAT TAC AAG GGT CTT
GGT ACT TCA AAA GAC TCT GAT GCT AAA GCA TAT TTC AGT GAT CTT GAT ACA CAT AAA
ATT AAG TTT GCA TAT GAT GGA GCT GGT TCA GAT GAA GTA ATA GAT TTA GCA TTT AAT
AGA AAA AGA GCA GAT GAT CGT AAA GAG TGG TTA AAA GGA TAT GAT CCA AAT ACA TAT
CTT GAT CAA GAT GTT GAT AGT ATT AGT TAT GAA GAT TTT GTT CAT AAA GAA TTA ATA
TTA TTC TCA TAT GAT GAT TGT GAA AGG TCT ATT CCT TCT GTT GTA GAT GGA TTA AAA
CCA TCA CAA AGA AAA GTG TTA TGG ACA TGT TTA GAA AAA AAT ATT ATG AAA GAA TTA
AAA GTA TCA CAA TTA TCT GGA CTT GTT TCT GAA AAG TCA TCA TAT CAT CAT GGA GAA
GTA TCT CTT CAA GCT ACC ATT GTT AAT ATG GCA CAA AAC TTT TGT GGA TCA AAT AAT
GTT AAT TGG TTA TTA CCA TCT GGT CAA TTT GGT TCA AGA TTA GGA GGA GGC AAA GAT
CAA GCA GCA GCA AGA TAT ATT TAT ACA CGA TTA TCA AGT ATT AGT AGA TAT TTA TGT
CCT AAA GAT GAT GAC CAA ATA TTA GAA TAT CTT ATT GAT GAA GAT AGA AGA ATT GAA
CCA AAA TAT TAT TTC CCT ATT ATT CCT ACA GTA TTA GTT AAT GGA GCT GAT GGT ATT
GGA ACA GGA TGG AGT ACT GCC GTT TCA AAT TAT AAT CCT CTT GAT CTT GTA GAT AAT
TGT AGA AGA TAT TTA AAT GGA GAG GAA TTT AAA GAA ATG AAG CCA TGG TTT AGA GGA
TGG ACA GGA CAA TTA AGA AAG AAT AAA GAT GGA AGT GGT TAT GAT TGT TAT GGA AAA
TGG AGA AGA TTA TCT GAA GAT AAA ATT GAA ATT ACT GAA TTA CCT ATT CAA ATA TGG
ACT GAT TCA TAT AAA CAA CAT TTA GAA AAG TGT ATT GAA GCA AAG ATT GTA AAA GGA
TAT TCA GAA TAT CAT ATG GTT AAT ACA GTG CAT TTT GTT ATT GAT TTA GTC CAA CCA
ATG AGT GAT GAA GAT ATT TGG ACT AAT TTA AAA TTA GTT AGT AGT GTT AAG ACA ACA
AAT ATG CAT TTA TTC AAT TCT GAA TAT AAA ATC ACT AAA TAT CAT ACT GTT CTT GAC
ATT TTA TCA GAG TTT TGT GAT GTT AGA ATG AAA GGT TAT GAA GAA AGA AAG AAA GCT
ATT ATT GAA AGT ATG GAA AGA CAA TTA ATG ATA TTA TCA AAT AAA GCA AGG TTC ATT
AAA ATG GTT ATT GAA GGA GAA TTA ATT GTT AAC AAC AGA AAG AAA AAT GAG TTA TAT
AAG GAT TTA ATT AAA TTT GGA TTT GAC CAA ATA AGA GAC CCC AAA AAA GCA AAT AAA
TCA AAA CCT ATT GGT GAA TCT GAA GAT GAT GAA ATA AAA GAA GAA GAG GAA GAT GAT
GAA GTT GGT GGA TAT GAT TAT TTG TTG AGT ATG AAG ATT TGG ACA TTA ACA TAT GAA

AAA GCA AAG AAA TTG ATG GAA GAT TGT GAT GAC TTA AAG ACA CAA ATT GAT ACA ATT AAA ACA ACA CCA GTA AAA GAT ATG TGG TTG AAA GAT TTA GAT GCA TTT GTA AAA AAA TAT CCA CAA TGG TTA GAA GAA GAA GAT ATT TTG TTA AAG AAA GGA GAC AAA AGT ACA AAA AGA GTT ATT ATG GGA AAG AAA GGA AAA TCA ACT AAA AAA ACA AGT AGA AAA GTA GCA TTA ACA ACT TCT ATA ATG GAT AAA AGT CCT AGT GAA TTA GCT AAA AAA TAT AAA GGA AGA TTA GAA GAT TAT GAA TGG AAA ACA ATG CCA GAT GAA AAT ACT GAG ATT AAG AAA AAA ACA ATT AAA AAA GTT ACT ACA ACA CGA AAA CGA AAA GAT GAA GAT TCA AGT ATG AGT GGT GAT GAA AAA GTA AAA AGA AAA AGA ACA ACG AAA AAA TCA ACA ACT AAG AAA ACT ACG AAA AAA AGA ACA ACT AAA GCT TCA AAA GAA GAA GAA AAT GAT ACT GTT GAT ACT CCT GCT AAA ACA TCA AGA AAA AAG AAG ACT AAG AAA GTG ACT GTT AAA GAA GAA GAT GAT GAT GAA AGT GAT TCA GTT TTT ATT GAT GAT GAT GAA AAT GAT AAT GGA TCA GTA GAA GAA ATA CAA AGT GAT TCT ACT GAT GAA GAA ATG AGT GAA GAA GAT GAA GAA ATG TTT GAA GAA GAT GAA GAA GAT GAA GAT CCA AAA CCA AAA AGA AAA ACA AGG TCA TCA AAA AAA GAA AAG AGT GAT GAC AGT TTC ATT GTT GAT GAT GAT GAA ATA TGG TAA

Occurrences/Results 9 Pentamers

The pentamer 'ATTCA' occurred 12 times.
The pentamer 'TTCAA' occurred 15 times.
The pentamer 'TCAAG' occurred 9 times.
The pentamer 'CAAGA' occurred 8 times.
The pentamer 'AAGAT' occurred 38 times.
The pentamer 'AGATG' occurred 21 times.
The pentamer 'GATGA' occurred 31 times.
The pentamer 'ATGAA' occurred 32 times.
The pentamer 'TGAAT' occurred 10 times.
The total occurrences of all specified strings is 176.
The total number of characters in the text is 4046.
First result (Total characters *9 /1024): 35.595703125
Square root of the first result: 5.966213466261495
Final result: 23.53323387923951

Occurrences/Results TRIMERS Codons

The pentamer 'ATT' occurred 74 times.
The pentamer 'CAA' occurred 32 times.
The pentamer 'GAT' occurred 101 times.
The pentamer 'GAA' occurred 109 times.
The pentamer 'CCA' occurred 25 times.
The pentamer 'ACT' occurred 36 times.
The pentamer 'AGA' occurred 43 times.
The pentamer 'TAC' occurred 1 times.
The total occurrences of all specified strings is 421.

The total number of characters in the text is 4050.
First result ((total_characters / 3) * (Trimer_number / 64)): 168.75
Square root of the first result: 13
Final result: 19.4

Occurrences/Results TRIMERS Combinations

The pentamer 'ATT ATT' occurred 2 times.
The pentamer 'ATT CAA' occurred 3 times.
The pentamer 'ATT GAT' occurred 6 times.
The pentamer 'ATT GAA' occurred 8 times.
The pentamer 'ATT CCA' occurred 2 times.
The pentamer 'ATT ACT' occurred 2 times.
The pentamer 'ATT AGA' occurred 0 times.
The pentamer 'ATT TAC' occurred 0 times.
The pentamer 'CAA ATT' occurred 2 times.
The pentamer 'CAA CAA' occurred 0 times.
The pentamer 'CAA GAT' occurred 2 times.
The pentamer 'CAA GAA' occurred 2 times.
The pentamer 'CAA CCA' occurred 1 times.
The pentamer 'CAA ACT' occurred 0 times.
The pentamer 'CAA AGA' occurred 2 times.
The pentamer 'CAA TAC' occurred 0 times.
The pentamer 'GAT ATT' occurred 6 times.
The pentamer 'GAT CAA' occurred 3 times.
The pentamer 'GAT GAT' occurred 11 times.
The pentamer 'GAT GAA' occurred 19 times.
The pentamer 'GAT CCA' occurred 2 times.
The pentamer 'GAT ACT' occurred 3 times.
The pentamer 'GAT AGA' occurred 1 times.
The pentamer 'GAT TAC' occurred 0 times.
The pentamer 'GAA ATT' occurred 6 times.
The pentamer 'GAA CAA' occurred 2 times.
The pentamer 'GAA GAT' occurred 17 times.
The pentamer 'GAA GAA' occurred 14 times.
The pentamer 'GAA CCA' occurred 4 times.
The pentamer 'GAA ACT' occurred 2 times.
The pentamer 'GAA AGA' occurred 3 times.
The pentamer 'GAA TAC' occurred 0 times.
The pentamer 'CCA ATT' occurred 2 times.
The pentamer 'CCA CAA' occurred 2 times.
The pentamer 'CCA GAT' occurred 1 times.
The pentamer 'CCA GAA' occurred 1 times.
The pentamer 'CCA CCA' occurred 0 times.

The pentamer 'CCA ACT' occurred 0 times.
The pentamer 'CCA AGA' occurred 0 times.
The pentamer 'CCA TAC' occurred 0 times.
The pentamer 'ACT ATT' occurred 2 times.
The pentamer 'ACT CAA' occurred 0 times.
The pentamer 'ACT GAT' occurred 2 times.
The pentamer 'ACT GAA' occurred 1 times.
The pentamer 'ACT CCA' occurred 1 times.
The pentamer 'ACT ACT' occurred 1 times.
The pentamer 'ACT AGA' occurred 0 times.
The pentamer 'ACT TAC' occurred 0 times.
The pentamer 'AGA ATT' occurred 3 times.
The pentamer 'AGA CAA' occurred 1 times.
The pentamer 'AGA GAT' occurred 2 times.
The pentamer 'AGA GAA' occurred 1 times.
The pentamer 'AGA CCA' occurred 0 times.
The pentamer 'AGA ACT' occurred 0 times.
The pentamer 'AGA AGA' occurred 4 times.
The pentamer 'AGA TAC' occurred 0 times.
The pentamer 'TAC ATT' occurred 0 times.
The pentamer 'TAC CAA' occurred 0 times.
The pentamer 'TAC GAT' occurred 0 times.
The pentamer 'TAC GAA' occurred 0 times.
The pentamer 'TAC CCA' occurred 0 times.
The pentamer 'TAC ACT' occurred 0 times.
The pentamer 'TAC AGA' occurred 0 times.
The pentamer 'TAC TAC' occurred 0 times.
The total occurrences of all specified strings is 149.
The total number of characters in the text is 4046.
First result (((total_characters/ 3) -1) / 64): 21.078125
Square root of the first result: 4.591091918051739
Final result: 27.863061180941138

>Entamoeba histolytica HM-1:IMSS, helicase (EHI_012470), partial mRNA

---> Sequence

ATGACAGACAAGAAGCAACTCCTCAATATCCCTAGTCAACGTGGGATAATAAGAATACCACCTCC
ATCACAAAGAGGTTCAACTCAAACAATTCATCGTTCTTCAGTAGAAAATAAAACCATATCTTCTC
AAACAAACTCTACTTTTAAAACCCCAACAAAAAGTGATGATTTCAATATAGATGCTACTTTAGCA
CTATTTGATAATGAAAAACAACAAACTAAAATTCCTACTCCGATAACTATTAGTCAACGAAAGTC
TACAGAGAAGAACCCTATTTTGACACACACAGCTTCAAGTCATAAGATCATTTCTATTCCAAAGG
CTTCTCAAGTTCAGTTAAACCAACGTCACAATACTCAAACTTTACAAAAACAACTCTCCCATACA

ACAGTTGTTATACCATCTAAACCATCTCCAGTTATCACACCAATAAAAACAACACCAAATAAAGG
ATTGAACCACACAACTCAATTTTCTTCACAAAAAACAATTGTTATTAAGCCAACTGGGTCTTCTC
AAAAAATTAGTCAACCACAACAACAAGTTGAATGCAAAACGAAGTGTATAGAAGAAGAAAATTT
TTCGTTGTCTCATCAAACAACTCCAACATTAATTCCCTCTGCTTCAGCAAGTTCGTCTGTTGTAAT
TCAACAAAAAAGTTCAAGTTCAACGTTACCAGCTAAATCAAATTTACACGAACGAGGAGGGAGAG
TTGGAATGACAAATGTTGTATTTACTGGAGAAGGAGATAATGAAGTTCATTTAAATTTCTCTTCT
AATAGATTTATGGATGTTAAAGAACAATTTAAAGAAAGTCATATTAATTTTGAGCTTCTTGGAA
AAGAAAAGAAAGAAATGAAAATTTCATTTAAGGATTATCAAAGTGCATTGGATATTTGTCGTAA
AAGACCATTATTAACATGTGAACCAATTACAAAATGTGTAAGAGATCTTCTTGTTTTAGATGGAA
AAGAAGAACAAGGAGATCTTGATAGATATGAAAGTATTCCAAGTAAATTACGAAATACAATGTT
TGAATATCAGAGAATTGGAGTACAGTTTGGGTTAAGAAAGAAAGGAAGACTTTTGATTGGAGAT
GAAATGGGACTAGGAAAAACCTTGCAAGCATTAGCACTTGTGTCAGCATATCCAGAAAATGAGCA
CATATTAGTTATTACTCCTAACAGTCTTGTGTATCAGTGGTGTGATCAAATCCAACGATGGCTTG
ATGTTGATCCAAATGACATTGCCATATATAAACCAAAAGACATAGAAGTGCCGAATACTAGGTTT
GTAGTTATTTCATACAACTCTATGGCAAATACACAAGGGAATATGTTTTCTCACGCATTTCCAAT
GGTAATTTGTGATGAATGTCATTTTATTAAAACAGACTCAAGCAAACGAAGTAAAGAAACGTTA
GATGTTTGTAAGAAAGCAAAACAAGTTATCTTCTTATCAGGAACACCAGCATTATCTAGGCCGAT
GGAATTATATAATATTTTAAGTGTACTGATAAAAGACATTGGAACAAAAGAGTCATTTGGTAAA
CGGTATTGTGAAGAAACAGGAACTAGGTATAAAAATTATCTTTCTATGAAGAATGAGAAAGAAT
TGAAATACTTGCTCACTACAGTTATGATTAGAAGACTCAAACATGATGTTTTAAAGGAACTTCCA
CCAAAAATAAGAGAAAAAGTATATCTTGGAGAATTAGATAAAAATGAGTATAGAGAATGCCTTG
AAAAGATGAAAAAAGATTTAGACAATGCTCGAGGAGTAAAACAAAAAAGACAACAAGTATTTGA
ATTACATAGATCAACAGGACTAGCAAAAATACCACTGATACAAGCATATCTTGATGATGTATTAG
ATTCTGGAATAAAAAAAGTTGTAGTATTTGCACATCATAGAGATGTTCTTGATGGAATCGTATAT
AACTTACAAAGAAAAAAAGTACAGTTTATTCGAATTGATGGTGAGACAAAGTCAGAAAACAAGA
AAGAACTTGTTGATATTTTTAGAGATGATGATAATTGTCGTGTTGCTGTATTAAGTATATTAGCA
GCAAATTGTGGATTAGAATTTCAAAAAGCTGCGTTGTGTATATTCGCAGAGATGACATTTGTTCC
TGGTGAAATGTTACAAGCTGAAGATAGAATACATAGAATTGGACAACAGGCTGACTCAGTTAAG
ATAGAATATTTAATTGCTAATAAGAGTTATGATGAACAAATATGGAATACTATTGAAAAGAAAT
TGGATGTTGTCGGTAAAGTATTAGATGGGAAATCAAGAGAACTTGATCATTGTACAAAAGAAGT
AGATATTGGAGAAGAAGAAATGGGTGAATTTGTTAAAGGGATGATGGAGGTTATTAAAAGCTAT
GATGAAAGAAAAAAGTTAAGAGAAGAAGAAGAAGATAGAATAGAAAAAAGGAAGTTAGGGAAA
GAAGAGTTAGTTCAAAACAAAGATTTACAAAATGAGATTGACTTACTGGATGATATTGGTGATG
ATAAAACTTGTAGTAAATCATTAAGTAAATTCAGTAAATTTCAATTTGAAAAGAAATAA

---> Sequence Showing highlighted Existant Combined Trimers

ATG ACA GAC AAG AAG CAA CTC CTC AAT ATC CCT AGT CAA CGT GGG ATA ATA AGA ATA
CCA CCT CCA TCA CAA AGA GGT TCA ACT CAA ACA ATT CAT CGT TCT TCA GTA GAA AAT
AAA ACC ATA TCT TCT CAA ACA AAC TCT ACT TTT AAA ACC CCA ACA AAA AGT GAT GAT
TTC AAT ATA GAT GCT ACT TTA GCA CTA TTT GAT AAT GAA AAA CAA CAA ACT AAA ATT
CCT ACT CCG ATA **ACT ATT** AGT CAA CGA AAG TCT ACA GAG AAG AAC CCT ATT TTG ACA
CAC ACA GCT TCA AGT CAT AAG ATC ATT TCT **ATT CCA** AAG GCT TCT CAA GTT CAG TTA
AAC CAA CGT CAC AAT ACT CAA ACT TTA CAA AAA CAA CTC TCC CAT ACA ACA GTT GTT

ATA CCA TCT AAA CCA TCT CCA GTT ATC ACA CCA ATA AAA ACA ACA CCA AAT AAA GGA TTG AAC CAC ACA ACT CAA TTT TCT TCA CAA AAA ACA ATT GTT ATT AAG **CCA ACT** GGG TCT TCT CAA AAA ATT AGT CAA CCA CAA CAA CAA GTT GAA TGC AAA ACG AAG TGT ATA GAA GAA GAA AAT TTT TCG TTG TCT CAT CAA ACA **ACT CCA** ACA TTA ATT CCC TCT GCT TCA GCA AGT TCG TCT GTT GTA ATT CAA CAA AAA AGT TCA AGT TCA ACG TTA CCA GCT AAA TCA AAT TTA CAC GAA CGA GGA GGG AGA GTT GGA ATG ACA AAT GTT GTA TTT ACT GGA GAA GGA GAT AAT GAA GTT CAT TTA AAT TTC TCT TCT AAT AGA TTT ATG GAT GTT AAA GAA CAA TTT AAA GAA AGT CAT ATT AAT TTT GAG CTT CTT GGA AAA GAA AAG AAA GAA ATG AAA ATT TCA TTT AAG GAT TAT CAA AGT GCA TTG GAT ATT TGT CGT AAA AGA CCA TTA TTA ACA TGT GAA **CCA ATT** ACA AAA TGT GTA AGA GAT CTT CTT GTT TTA GAT GGA AAA GAA GAA CAA GGA GAT CTT GAT AGA TAT GAA AGT **ATT CCA** AGT AAA TTA CGA AAT ACA ATG TTT GAA TAT CAG AGA ATT GGA GTA CAG TTT GGG TTA AGA AAG AAA GGA AGA CTT TTG ATT GGA GAT GAA ATG GGA CTA GGA AAA ACC TTG CAA GCA TTA GCA CTT GTG TCA GCA TAT CCA GAA AAT GAG CAC ATA TTA GTT **ATT ACT** CCT AAC AGT CTT GTG TAT CAG TGG TGT GAT CAA ATC CAA CGA TGG CTT GAT GTT GAT CCA AAT GAC ATT GCC ATA TAT AAA CCA AAA GAC ATA GAA GTG CCG AAT ACT AGG TTT GTA GTT ATT TCA TAC AAC TCT ATG GCA AAT ACA CAA GGG AAT ATG TTT TCT CAC GCA TTT CCA ATG GTA ATT TGT GAT GAA TGT CAT TTT ATT AAA ACA GAC TCA AGC AAA CGA AGT AAA GAA ACG TTA GAT GTT TGT AAG AAA GCA AAA CAA GTT ATC TTC TTA TCA GGA ACA CCA GCA TTA TCT AGG CCG ATG GAA TTA TAT AAT ATT TTA AGT GTA CTG ATA AAA GAC ATT GGA ACA AAA GAG TCA TTT GGT AAA CGG TAT TGT GAA GAA ACA GGA ACT AGG TAT AAA AAT TAT CTT TCT ATG AAG AAT GAG AAA GAA TTG AAA TAC TTG CTC ACT ACA GTT ATG ATT AGA AGA CTC AAA CAT GAT GTT TTA AAG GAA CTT CCA CCA AAA ATA AGA GAA AAA GTA TAT CTT GGA GAA TTA GAT AAA AAT GAG TAT AGA GAA TGC CTT GAA AAG ATG AAA AAA GAT TTA GAC AAT GCT CGA GGA GTA AAA CAA AAA AGA CAA CAA GTA TTT GAA TTA CAT AGA TCA ACA GGA CTA GCA AAA ATA CCA CTG ATA CAA GCA TAT CTT GAT GAT GTA TTA GAT TCT GGA ATA AAA AAA GTT GTA GTA TTT GCA CAT CAT AGA GAT GTT CTT GAT GGA ATC GTA TAT AAC TTA CAA AGA AAA AAA GTA CAG TTT ATT CGA ATT GAT GGT GAG ACA AAG TCA GAA AAC AAG AAA GAA CTT GTT GAT ATT TTT AGA GAT GAT GAT AAT TGT CGT GTT GCT GTA TTA AGT ATA TTA GCA GCA AAT TGT GGA TTA GAA TTT CAA AAA GCT GCG TTG TGT ATA TTC GCA GAG ATG ACA TTT GTT CCT GGT GAA ATG TTA CAA GCT GAA GAT AGA ATA CAT AGA ATT GGA CAA CAG GCT GAC TCA GTT AAG ATA GAA TAT TTA ATT GCT AAT AAG AGT TAT GAT GAA CAA ATA TGG AAT **ACT ATT** GAA AAG AAA TTG GAT GTT GTC GGT AAA GTA TTA GAT GGG AAA TCA AGA GAA CTT GAT CAT TGT ACA AAA GAA GTA GAT ATT GGA GAA GAA GAA ATG GGT GAA TTT GTT AAA GGG ATG ATG GAG GTT ATT AAA AGC TAT GAT GAA AGA AAA AAG TTA AGA GAA GAA GAA GAA GAT AGA ATA GAA AAA AGG AAG TTA GGG AAA GAA GAG TTA GTT CAA AAC AAA GAT TTA CAA AAT GAG ATT GAC TTA CTG GAT GAT ATT GGT GAT GAT AAA ACT TGT AGT AAA TCA TTA AGT AAA TTC AGT AAA TTT CAA TTT GAA AAG AAA TAA

Occurrences/Results 9 Pentamers

The pentamer 'ATTCA' occurred 3 times.
The pentamer 'TTCAA' occurred 9 times.
The pentamer 'TCAAG' occurred 5 times.
The pentamer 'CAAGA' occurred 3 times.
The pentamer 'AAGAT' occurred 7 times.
The pentamer 'AGATG' occurred 9 times.
The pentamer 'GATGA' occurred 12 times.
The pentamer 'ATGAA' occurred 10 times.
The pentamer 'TGAAT' occurred 5 times.
The total occurrences of all specified strings is 63.
The total number of characters in the text is 2573.
First result (Total characters *9 /1024): 22.6494140625
Square root of the first result: 4.759140054936395
Final result: 8.478545592632129

Occurrences/Results TRIMERS Codons

The pentamer 'ATT' occurred 36 times.
The pentamer 'CAA' occurred 43 times.
The pentamer 'GAT' occurred 44 times.
The pentamer 'GAA' occurred 55 times.
The pentamer 'CCA' occurred 24 times.
The pentamer 'ACT' occurred 18 times.
The pentamer 'AGA' occurred 25 times.
The pentamer 'TAC' occurred 2 times.
The total occurrences of all specified strings is 247.
The total number of characters in the text is 2574.
First result ((total_characters / 3) * (Trimer_number / 64)): 107.25
Square root of the first result: 10.36
Final result: 13.5

Occurrences/Results TRIMERS Combinations

The pentamer 'ATT ATT' occurred 0 times.
The pentamer 'ATT CAA' occurred 1 times.
The pentamer 'ATT GAT' occurred 1 times.
The pentamer 'ATT GAA' occurred 1 times.
The pentamer 'ATT CCA' occurred 2 times.
The pentamer 'ATT ACT' occurred 1 times.
The pentamer 'ATT AGA' occurred 1 times.
The pentamer 'ATT TAC' occurred 0 times.
The pentamer 'CAA ATT' occurred 0 times.
The pentamer 'CAA CAA' occurred 4 times.
The pentamer 'CAA GAT' occurred 0 times.

The pentamer 'CAA GAA' occurred 0 times.
The pentamer 'CAA CCA' occurred 1 times.
The pentamer 'CAA ACT' occurred 2 times.
The pentamer 'CAA AGA' occurred 2 times.
The pentamer 'CAA TAC' occurred 0 times.
The pentamer 'GAT ATT' occurred 4 times.
The pentamer 'GAT CAA' occurred 1 times.
The pentamer 'GAT GAT' occurred 5 times.
The pentamer 'GAT GAA' occurred 4 times.
The pentamer 'GAT CCA' occurred 1 times.
The pentamer 'GAT ACT' occurred 0 times.
The pentamer 'GAT AGA' occurred 3 times.
The pentamer 'GAT TAC' occurred 0 times.
The pentamer 'GAA ATT' occurred 0 times.
The pentamer 'GAA CAA' occurred 3 times.
The pentamer 'GAA GAT' occurred 2 times.
The pentamer 'GAA GAA' occurred 6 times.
The pentamer 'GAA CCA' occurred 1 times.
The pentamer 'GAA ACT' occurred 0 times.
The pentamer 'GAA AGA' occurred 1 times.
The pentamer 'GAA TAC' occurred 0 times.
The pentamer 'CCA ATT' occurred 1 times.
The pentamer 'CCA CAA' occurred 1 times.
The pentamer 'CCA GAT' occurred 0 times.
The pentamer 'CCA GAA' occurred 1 times.
The pentamer 'CCA CCA' occurred 1 times.
The pentamer 'CCA ACT' occurred 1 times.
The pentamer 'CCA AGA' occurred 0 times.
The pentamer 'CCA TAC' occurred 0 times.
The pentamer 'ACT ATT' occurred 2 times.
The pentamer 'ACT CAA' occurred 3 times.
The pentamer 'ACT GAT' occurred 0 times.
The pentamer 'ACT GAA' occurred 0 times.
The pentamer 'ACT CCA' occurred 1 times.
The pentamer 'ACT ACT' occurred 0 times.
The pentamer 'ACT AGA' occurred 0 times.
The pentamer 'ACT TAC' occurred 0 times.
The pentamer 'AGA ATT' occurred 2 times.
The pentamer 'AGA CAA' occurred 1 times.
The pentamer 'AGA GAT' occurred 3 times.
The pentamer 'AGA GAA' occurred 4 times.
The pentamer 'AGA CCA' occurred 1 times.
The pentamer 'AGA ACT' occurred 0 times.

The pentamer 'AGA AGA' occurred 1 times.
The pentamer 'AGA TAC' occurred 0 times.
The pentamer 'TAC ATT' occurred 0 times.
The pentamer 'TAC CAA' occurred 0 times.
The pentamer 'TAC GAT' occurred 0 times.
The pentamer 'TAC GAA' occurred 0 times.
The pentamer 'TAC CCA' occurred 0 times.
The pentamer 'TAC ACT' occurred 0 times.
The pentamer 'TAC AGA' occurred 0 times.
The pentamer 'TAC TAC' occurred 0 times.
The total occurrences of all specified strings is 70.
The total number of characters in the text is 2573.
First result (((total_characters/ 3) -1) / 64): 13.40625
Square root of the first result: 3.6614546289692025
Final result: 15.456630147000526

>>BDEQ01000001.1:c809290-807455 Entamoeba histolytica DNA, contig: contig1, strain: AAA family ATPase putative

---> Sequence

ATGTTTAGAAATAAACCAAATTCACTTGATCAATTCTTTCTTCCTAAACAAAAGAAAGAACCAAC
TACAACCAGTAGTGTAGATGATCGACAAGAAAAGTCTCCTCAAGAGATTAAACAAATTTATATCC
CATTGGCTGAAAAACAACGACCAAAAAACTTAGAAGATATAATTGGACAAGAGGATATTCTTGC
AATTGGGACACCTTTTAATACTATGATTAAAAATGATAAGATTCAATCTACCATTCTCTATGGAC
CACCTGGTTGTGGTAAAACCACAATTGCAGGTATTATTAAAAATAATAGTAAGTCTACATTTATT
TCTATGTCTGCTGCTACAAGTAAAAAAGAGGATTTTAAGAAAGTGATTAATGACGCTAAACACAG
AAAACGTATGGGAATAAATACCATTTTATTTCTTGATGAAATCCATAGTTTAAATAGATTGCAGC
AAGACACATTTTTACCTGCTATAGAAAGTGGAACTATTATTCTTATAGGTGCAACTACTGAAAAT
CCATCGTTTGAATTAAATAATGCATTAATGAGTCGTTGCCAACTTGTAACATTAAAGAAATTAAC
TGATGAAGACGTTGTTAAAATAATGAGAAAGGCTATTGATGAGGAATATTGTAATTCAAAAATT
GATATTGACAATGAAGGACTTCATTTTATTGCAGCTATTTCTGATGGAGATGCAAGAAGTGCATT
AAATACATTAGAAAAAGTATTTGTTCATTTTAACCTCATGAGTGAAAAGGTTTTAAAAAATGAA
ATTAATGAAAAAGGTGGAATAGACATTGAGGTAAGAGAGTTAGAAGAAAAGGTTAAGAAAGGAG
AGATTAAAGTATTAAGAATTAATGGAAAAATAGTCTCAATTATTGATAACAAAAACGAAAAACA
AAAAACAGAAGAAATTGAGAAAGGGGTAATAGACAAAACAGACTCAGTCATAACTATTTCTGAT
GATATTAGTGATGTTGTTATAGTTCAACCTGAACCAAAAGAAATAACTCAAACAGAAGGAGATG
TTTTTAATGAAAATATTGATTCTCATATAAAAGAAATAACTAAACCAAATGAAAAGTTAAAGAT
TAAAAAAGTGGAGAAAAAATATATTTTAACATATAAACTTATTGAAAATTACTTACAAAGAAAT
ATTTACCAACCTAAAAATCAAGGAGAAAGATCATTTTAAGGTTTTAACTGCTTTTCAAAAAAGTAT
TACTCATTCAGATGAAGAAGCAACAATATATTGGTTACAACGATTGATAGAAATGGGAGAAAAT
CCAAAGTACATAGTAAGAAGAATGATTAAAATATCTAGTGAAGACATTGGACTTGCAGATGTAG
AAGCGTTAAATATTGCAGTCAAAACATTTCAAATTGTTTCTTCTTTAGGATATCCAGAAGCAGAC
ACTGCGTTATATGAATGCGCTTTATATTTAGCCAGAGCACCAAAGTCATGTGCTGTTTTTGAAAC

AATGAAATATACTGAAGAAATTATAAAACAAACAGGATCATTACCTGTCCCACTTCATATCAGAA
ATGCACCTACTTCATTAATGGAGAAGATGGGATATTCTGTTGGTTATAAATATCCACCAAATTAT
AATGAAGTTTTAGATCAAACTTATCTCCCAGAAAAACTGATTAACACAAAGTTAGTTTTTTATGA
TCGTTTTGTTAAGCCTATTAATAAAGATGATATTATAATGAAGGCAGAAGAAAATGATTTAAGT
TCAGGACAAGTTCAACGAAGTTATCAATTTAGAAATTTTGATGTTGATTATTGCAAAGATAAAA
AAGAAATAACTCAAATTGTTAATAATTAA

---> Sequence Showing highlighted Existant Combined Trimers

ATG TTT AGA AAT AAA CCA AAT TCA CTT GAT CAA TTC TTT CTT CCT AAA CAA AAG AAA
GAA CCA ACT ACA ACC AGT AGT GTA GAT GAT CGA CAA GAA AAG TCT CCT CAA GAG ATT
AAA CAA ATT TAT ATC CCA TTG GCT GAA AAA CAA CGA CCA AAA AAC TTA GAA GAT ATA
ATT GGA CAA GAG GAT ATT CTT GCA ATT GGG ACA CCT TTT AAT ACT ATG ATT AAA AAT
GAT AAG ATT CAA TCT ACC ATT CTC TAT GGA CCA CCT GGT TGT GGT AAA ACC ACA ATT
GCA GGT ATT ATT AAA AAT AAT AGT AAG TCT ACA TTT ATT TCT ATG TCT GCT GCT ACA
AGT AAA AAA GAG GAT TTT AAG AAA GTG ATT AAT GAC GCT AAA CAC AGA AAA CGT ATG
GGA ATA AAT ACC ATT TTA TTT CTT GAT GAA ATC CAT AGT TTA AAT AGA TTG CAG CAA
GAC ACA TTT TTA CCT GCT ATA GAA AGT GGA ACT ATT ATT CTT ATA GGT GCA ACT ACT
GAA AAT CCA TCG TTT GAA TTA AAT AAT GCA TTA ATG AGT CGT TGC CAA CTT GTA ACA
TTA AAG AAA TTA ACT GAT GAA GAC GTT GTT AAA ATA ATG AGA AAG GCT ATT GAT GAG
GAA TAT TGT AAT TCA AAA ATT GAT ATT GAC AAT GAA GGA CTT CAT TTT ATT GCA GCT
ATT TCT GAT GGA GAT GCA AGA AGT GCA TTA AAT ACA TTA GAA AAA GTA TTT GTT CAT
TTT AAC CTC ATG AGT GAA AAG GTT TTA AAA AAT GAA ATT AAT GAA AAA GGT GGA ATA
GAC ATT GAG GTA AGA GAG TTA GAA GAA AAG GTT AAG AAA GGA GAG ATT AAA GTA TTA
AGA ATT AAT GGA AAA ATA GTC TCA ATT ATT GAT AAC AAA AAC GAA AAA CAA AAA ACA
GAA GAA ATT GAG AAA GGG GTA ATA GAC AAA ACA GAC TCA GTC ATA ACT ATT TCT GAT
GAT ATT AGT GAT GTT GTT ATA GTT CAA CCT GAA CCA AAA GAA ATA ACT CAA ACA GAA
GGA GAT GTT TTT AAT GAA AAT ATT GAT TCT CAT ATA AAA GAA ATA ACT AAA CCA AAT
GAA AAG TTA AAG ATT AAA AAA GTG GAG AAA AAA TAT ATT TTA ACA TAT AAA CTT ATT
GAA AAT TAC TTA CAA AGA AAT ATT TAC CAA CCT AAA AAT CAA GGA GAA GAT CAT TTT
AAG GTT TTA ACT GCT TTT CAA AAA AGT ATT ACT CAT TCA GAT GAA GAA GCA ACA ATA
TAT TGG TTA CAA CGA TTG ATA GAA ATG GGA GAA AAT CCA AAG TAC ATA GTA AGA AGA
ATG ATT AAA ATA TCT AGT GAA GAC ATT GGA CTT GCA GAT GTA GAA GCG TTA AAT ATT
GCA GTC AAA ACA TTT CAA ATT GTT TCT TCT TTA GGA TAT CCA GAA GCA GAC ACT GCG
TTA TAT GAA TGC GCT TTA TAT TTA GCC AGA GCA CCA AAG TCA TGT GCT GTT TTT GAA
ACA ATG AAA TAT ACT GAA GAA ATT ATA AAA CAA ACA GGA TCA TTA CCT GTC CCA CTT
CAT ATC AGA AAT GCA CCT ACT TCA TTA ATG GAG AAG ATG GGA TAT TCT GTT GGT TAT
AAA TAT CCA CCA AAT TAT AAT GAA GTT TTA GAT CAA ACT TAT CTC CCA GAA AAA CTG
ATT AAC ACA AAG TTA GTT TTT TAT GAT CGT TTT GTT AAG CCT ATT AAT AAA GAT GAT
ATT ATA ATG AAG GCA GAA GAA AAT GAT TTA AGT TCA GGA CAA GTT CAA CGA AGT TAT
CAA TTT AGA AAT TTT GAT GTT GAT TAT TGC AAA GAT AAA AAA GAA ATA ACT CAA ATT
GTT AAT AAT TAA

Occurrences/Results 9 Pentamers

The pentamer 'ATTCA' occurred 4 times.
The pentamer 'TTCAA' occurred 6 times.
The pentamer 'TCAAG' occurred 2 times.
The pentamer 'CAAGA' occurred 5 times.
The pentamer 'AAGAT' occurred 7 times.
The pentamer 'AGATG' occurred 7 times.
The pentamer 'GATGA' occurred 7 times.
The pentamer 'ATGAA' occurred 12 times.
The pentamer 'TGAAT' occurred 2 times.
The total occurrences of all specified strings is 52.
The total number of characters in the text is 1832.
First result (Total characters *9 /1024): 16.13671875
Square root of the first result: 4.0170534910553535
Final result: 8.927757952403581

Occurrences/Results TRIMERS Codons

The pentamer 'ATT' occurred 45 times.
The pentamer 'CAA' occurred 25 times.
The pentamer 'GAT' occurred 30 times.
The pentamer 'GAA' occurred 44 times.
The pentamer 'CCA' occurred 15 times.
The pentamer 'ACT' occurred 16 times.
The pentamer 'AGA' occurred 13 times.
The pentamer 'TAC' occurred 3 times.
The total occurrences of all specified strings is 191.
The total number of characters in the text is 1836.
First result ((total_characters / 3) * (Trimer_number / 64)): 76.5
Square root of the first result: 8.75
Final result: 13.1

Occurrences/Results TRIMERS Combinations

The pentamer 'ATT ATT' occurred 3 times.
The pentamer 'ATT CAA' occurred 1 times.
The pentamer 'ATT GAT' occurred 4 times.
The pentamer 'ATT GAA' occurred 1 times.
The pentamer 'ATT CCA' occurred 0 times.
The pentamer 'ATT ACT' occurred 1 times.
The pentamer 'ATT AGA' occurred 0 times.
The pentamer 'ATT TAC' occurred 1 times.
The pentamer 'CAA ATT' occurred 3 times.
The pentamer 'CAA CAA' occurred 0 times.
The pentamer 'CAA GAT' occurred 0 times.

The pentamer 'CAA GAA' occurred 1 times.
The pentamer 'CAA CCA' occurred 0 times.
The pentamer 'CAA ACT' occurred 1 times.
The pentamer 'CAA AGA' occurred 1 times.
The pentamer 'CAA TAC' occurred 0 times.
The pentamer 'GAT ATT' occurred 4 times.
The pentamer 'GAT CAA' occurred 2 times.
The pentamer 'GAT GAT' occurred 3 times.
The pentamer 'GAT GAA' occurred 3 times.
The pentamer 'GAT CCA' occurred 0 times.
The pentamer 'GAT ACT' occurred 0 times.
The pentamer 'GAT AGA' occurred 0 times.
The pentamer 'GAT TAC' occurred 0 times.
The pentamer 'GAA ATT' occurred 3 times.
The pentamer 'GAA CAA' occurred 0 times.
The pentamer 'GAA GAT' occurred 2 times.
The pentamer 'GAA GAA' occurred 5 times.
The pentamer 'GAA CCA' occurred 2 times.
The pentamer 'GAA ACT' occurred 0 times.
The pentamer 'GAA AGA' occurred 0 times.
The pentamer 'GAA TAC' occurred 0 times.
The pentamer 'CCA ATT' occurred 0 times.
The pentamer 'CCA CAA' occurred 0 times.
The pentamer 'CCA GAT' occurred 0 times.
The pentamer 'CCA GAA' occurred 2 times.
The pentamer 'CCA CCA' occurred 1 times.
The pentamer 'CCA ACT' occurred 1 times.
The pentamer 'CCA AGA' occurred 0 times.
The pentamer 'CCA TAC' occurred 0 times.
The pentamer 'ACT ATT' occurred 2 times.
The pentamer 'ACT CAA' occurred 2 times.
The pentamer 'ACT GAT' occurred 1 times.
The pentamer 'ACT GAA' occurred 2 times.
The pentamer 'ACT CCA' occurred 0 times.
The pentamer 'ACT ACT' occurred 1 times.
The pentamer 'ACT AGA' occurred 0 times.
The pentamer 'ACT TAC' occurred 0 times.
The pentamer 'AGA ATT' occurred 1 times.
The pentamer 'AGA CAA' occurred 0 times.
The pentamer 'AGA GAT' occurred 0 times.
The pentamer 'AGA GAA' occurred 0 times.
The pentamer 'AGA CCA' occurred 0 times.
The pentamer 'AGA ACT' occurred 0 times.

The pentamer 'AGA AGA' occurred 1 times.
The pentamer 'AGA TAC' occurred 0 times.
The pentamer 'TAC ATT' occurred 0 times.
The pentamer 'TAC CAA' occurred 1 times.
The pentamer 'TAC GAT' occurred 0 times.
The pentamer 'TAC GAA' occurred 0 times.
The pentamer 'TAC CCA' occurred 0 times.
The pentamer 'TAC ACT' occurred 0 times.
The pentamer 'TAC AGA' occurred 0 times.
The pentamer 'TAC TAC' occurred 0 times.
The total occurrences of all specified strings is 56.
The total number of characters in the text is 1832.
First result (((total_characters/ 3) -1) / 64): 9.546875
Square root of the first result: 3.0898017735770686
Final result: 15.034338253428194

>BDEQ01000001.1:c8139836-8136831 Entamoeba histolytica DNA, contig: contig1, strain: HM1:IMSS clone 6, translocase

---> Sequence

ATGATAATTAAATTAAAACAATCTGATATTGTTCCAGCAGATTGTATTCCTTTATTATCAAGTAA
TCAAGATGGAATTACATTTATTGAAACTGCTGCATTAGATGGTGAAACTAATCTTAAACAAGTCT
TAGTTCCTAATTATTTTATTGGTAAAACAATTAATGATATTAATGAATTGAAAGGAACTTTATT
ATGTGAATATCCACAACCAAAATTTGATCAATTTAGAGGGTCAATAACAATAGGAAATGATAAA
ATTTCAATAAATGAAAAAAATTTATTAATGCAAGGAACAATAATAAGAAATACTAATTTTGTTT
ATGTTCTTATTTGTTATTGTGGAATTCATACTAAGTTAAGTCTTAATCAAACTCCACCAAAATTA
AAAAAGTCAAATATTGATACAAAATTTAATATTTTTGTATTTGTTATGATTATTATTCAATGTG
TTATATGTTTAATTCTTGCTATTTTATCAGGACATAGTCATTCAATAATAAATGATCCAAATGAA
GGGTTTTGGTATTTACCTAAAGATGATATTAATAACAAATTTTATGGATTAAAGAAATTTTTTG
GATATTTTACGTTAATTTCTTATATAATTCCTATTTCGTGTCAAGTTTCTTTAGAATTAGCTAAA
TTTGCACAAGGTATTTTCTTTGAACAAGATGATGATATGAAAATTAAACAAATAAATAGTAATG
GAAAAGAAGAAATTGTTGGAATGAATGCTAAATCTACTGGTCTTAATGATGAACTTGGAATGGT
AAAATTTGTTCTTTCAGATAAGACAGGTACATTAACAGAAAATGAAATGAAATTCAAAAAGTGT
GCAATAAAAGAAAGGGTTTATGATATTTCAAAATTACAAGAAATTTTAAATATATCAAATAATA
AAATTAAGAAGAATAAAGAAATAAATGAAAATACAAATAATTTTAATAATAATAATTCTAAAGA
AATAAATCATTCATGTTCTTTTATTATTAATTCTAATGAACAAGAAAATCAAGAAGAAAGTAAT
AAAAACGATATAATAAATTTCTTAAAATGTATGGCATTATGTAATACTGTAAATATAAATGATG
AAAAATTTTCATCTCAAAGTCCTGATGAAGAAGCATTATGTTTGGCAGCAAAAAATTGTGGAATT
GAATTAATTTCAAGAAATCAAAAAAGTATTGAATTATTAGAATTTGGAATAAATAAAAAGTATA
ATATTCTAAGTACTTTTGAGTTTAATTCAGATAGAAAAAGAATGAGTGTTTTAACAAGAGATGA
AAATGGTATAATAACACTTTGGTGTAAAGGAGCAGATAATGTAATGAATGAAAGATGTAATGAA

GAAGGAAAAGAATGTATGAAGTATCTTAATGAATTTAGTTCTGTTGGATTAAGGACATTAGTTC
TTGCAATAAAAACAATTAATGAAGATATCTTTAGTAAATGGTATGAAAAATATGATGATGCAAT
AAATTTACTTGAAGGAAGAGAAGAAGAAGTAGAATTATTACAAAATGAAATGGAAAAAGATTTA
CAAATTATAGGAATCAGTGCAATTGAAGATAAATTACAAGAAGGAGTTCCAGAAACGATTGAAA
TGTTATTAAGAGGAGGAATTAAAGTATGGATGATTACAGGAGATAAAATGGAAACAGCAATAAA
TATAGGAAAGTCATGTAAATTAATAAATAATACATATTATTGTATTAATGATGATACACTTAAT
CAATGTAAAACTACTTTAAATGAAATTAAATTAAAAATTAATCAACAACAACATAATTTTAGTT
TAATTATTAATGGAAAAAAATACAGATTGGTGTGTTCATGAATTAAAAAATATTTTTAAAGATAT
TGTATTACTTTCTTCAAGTGTTATTTGTTGTCGTGTAACTCCAAAACAAAAAGCTGAAATAACTA
ATTGTGTTAAAAAAATTACAAATAAAATTGTTTTAACTATTGGAGATGGTGCAAATGATGTTCC
AATGATTAATACTGGAAATGTTGGTGTTGGAATTTATGGAAAAGAAGGAAATCAAGCTGCAAGA
GCATCTGATTTTGCAATAAGAAAATTTAGACATTTAGCAAAATTAATTTTATATCATGGAAGAAC
TTCTTTATTAAGAAATTCTGAAATTATTAAAATATGTTTTTATAAAAATGCATCATTCTTTTTAA
TATTATTATGGTATTCTTTTATAAGTAATTTTACTTGTCAAGTCGTTTTTGATGATTATATTATG
ACTTTCTTTAATATTTTATTTACTCAAATTCAACCAATTCTTATTGGTATTTTTGATAGAGATTT
ACAATGGCAAACAATTCAATTATTCCCTGAAGTTAATAAAGAAATTCATAAATCATTACGTGGAA
AAGTTATCAATTTTATTCTTTGGTTCTTTTATGGTATTTATCAATCTCTTATATTCTTTTTCGTA
TTCTTTTGGTTTATATCACCTTCAGATATAACTGGTAAAGATGGATTAAATGGAGGTATTATTTA
CACTTCATTAACAATTACATTTTATTCTTTATTTACTATAATTGTCACTTTAATTATTGAAACAA
AAACATGGAATTGGATATTAGTTATTGGTCATATTATTTCTATTATTTTTATCTTTATTATCTAT
GGATTAACTTGTTATATTCCTGGCTATTCTACTTATGACATTTCTTGGAATGGATTTACTTATGT
TTTCCAATCATTTAATTTCTATTTAATTACTATCTTTACAATAATAATAAGTGTTGCTCCATTAA
TTCTCAAAAGATTTATTCAAAGATATTTCTTCCCTGATATGTATCATGTCGTTCAAGAAATTCAA
ACATCTCTTGAAACTAATTATGGTAAGAAGAGAAATTTCTTCTTTGAAGGAAGTAATGAATTAA
AACAATTAATTAATCATCGCCTCCCTTTAATCTCAAAAGATATTTCTTTTAATAGTACCTCTTCT
AACAGCCAAATAAATGAAATTGAATTGGTTGAAGAAAATTGA

---> Sequence Showing highlighted Existant Combined Trimers

ATG ATA ATT AAA TTA AAA CAA TCT GAT ATT GTT CCA GCA GAT TGT ATT CCT TTA TTA
TCA AGT AAT CAA GAT GGA ATT ACA TTT ATT GAA ACT GCT GCA TTA GAT GGT GAA ACT
AAT CTT AAA CAA GTC TTA GTT CCT AAT TAT TTT ATT GGT AAA ACA ATT AAT GAT ATT
AAT GAA TTG AAA GGA ACT TTA TTA TGT GAA TAT CCA CAA CCA AAA TTT GAT CAA TTT
AGA GGG TCA ATA ACA ATA GGA AAT GAT AAA ATT TCA ATA AAT GAA AAA AAT TTA TTA
ATG CAA GGA ACA ATA ATA AGA AAT ACT AAT TTT GTT TAT GTT CTT ATT TGT TAT TGT
GGA ATT CAT ACT AAG TTA AGT CTT AAT CAA ACT CCA CCA AAA TTA AAA AAG TCA AAT
ATT GAT ACA AAA TTT AAT ATT TTT GTA TTT GTT ATG ATT ATT ATT CAA TGT GTT ATA
TGT TTA ATT CTT GCT ATT TTA TCA GGA CAT AGT CAT TCA ATA ATA AAT GAT CCA AAT
GAA GGG TTT TGG TAT TTA CCT AAA GAT GAT ATT AAT AAC AAA TTT TAT GGA TTA AAG
AAA TTT TTT GGA TAT TTT ACG TTA ATT TCT TAT ATA ATT CCT ATT TCG TGT CAA GTT
TCT TTA GAA TTA GCT AAA TTT GCA CAA GGT ATT TTC TTT GAA CAA GAT GAT GAT ATG
AAA ATT AAA CAA ATA AAT AGT AAT GGA AAA GAA GAA ATT GTT GGA ATG AAT GCT AAA
TCT ACT GGT CTT AAT GAT GAA CTT GGA ATG GTA AAA TTT GTT CTT TCA GAT AAG ACA
GGT ACA TTA ACA GAA AAT GAA ATG AAA TTC AAA AAG TGT GCA ATA AAA GAA AGG GTT

TAT GAT ATT TCA AAA TTA CAA GAA ATT TTA AAT ATA TCA AAT AAT AAA ATT AAG AAG AAT AAA GAA ATA AAT GAA AAT ACA AAT AAT TTT AAT AAT AAT AAT TCT AAA GAA ATA AAT CAT TCA TGT TCT TTT ATT ATT AAT TCT AAT GAA CAA GAA AAT CAA GAA GAA AGT AAT AAA AAC GAT ATA ATA AAT TTC TTA AAA TGT ATG GCA TTA TGT AAT ACT GTA AAT ATA AAT GAT GAA AAA TTT TCA TCT CAA AGT CCT GAT GAA GAA GCA TTA TGT TTG GCA GCA AAA AAT TGT GGA ATT GAA TTA ATT TCA AGA AAT CAA AAA AGT ATT GAA TTA TTA GAA TTT GGA ATA AAT AAA AAG TAT AAT ATT CTA AGT ACT TTT GAG TTT AAT TCA GAT AGA AAA AGA ATG AGT GTT TTA ACA AGA GAT GAA AAT GGT ATA ATA ACA CTT TGG TGT AAA GGA GCA GAT AAT GTA ATG AAT GAA AGA TGT AAT GAA GAA GGA AAA GAA TGT ATG AAG TAT CTT AAT GAA TTT AGT TCT GTT GGA TTA AGG ACA TTA GTT CTT GCA ATA AAA ACA ATT AAT GAA GAT ATC TTT AGT AAA TGG TAT GAA AAA TAT GAT GAT GCA ATA AAT TTA CTT GAA GGA AGA GAA GAA GAA GTA GAA TTA TTA CAA AAT GAA ATG GAA AAA GAT TTA CAA ATT ATA GGA ATC AGT GCA ATT GAA GAT AAA TTA CAA GAA GGA GTT CCA GAA ACG ATT GAA ATG TTA TTA AGA GGA GGA ATT AAA GTA TGG ATG ATT ACA GGA GAT AAA ATG GAA ACA GCA ATA AAT ATA GGA AAG TCA TGT AAA TTA ATA AAT AAT ACA TAT TAT TGT ATT AAT GAT GAT ACA CTT AAT CAA TGT AAA ACT ACT TTA AAT GAA ATT AAA TTA AAA ATT AAT CAA CAA CAA CAT AAT TTT AGT TTA ATT ATT AAT GGA AAA AAT ACA GAT TGG TGT GTT CAT GAA TTA AAA AAT ATT TTT AAA GAT ATT GTA TTA CTT TCT TCA AGT GTT ATT TGT TGT CGT GTA ACT CCA AAA CAA AAA GCT GAA ATA ACT AAT TGT GTT AAA AAA ATT ACA AAT AAA ATT GTT TTA ACT ATT GGA GAT GGT GCA AAT GAT GTT CCA ATG ATT AAT ACT GGA AAT GTT GGT GTT GGA ATT TAT GGA AAA GAA GGA AAT CAA GCT GCA AGA GCA TCT GAT TTT GCA ATA AGA AAA TTT AGA CAT TTA GCA AAA TTA ATT TTA TAT CAT GGA AGA ACT TCT TTA TTA AGA AAT TCT GAA ATT ATT AAA ATA TGT TTT TAT AAA AAT GCA TCA TTC TTT TTA ATA TTA TTA TGG TAT TCT TTT ATA AGT AAT TTT ACT TGT CAA GTC GTT TTT GAT GAT TAT ATT ATG ACT TTC TTT AAT ATT TTA TTT ACT CAA ATT CAA CCA ATT CTT ATT GGT ATT TTT GAT AGA GAT TTA CAA TGG CAA ACA ATT CAA TTA TTC CCT GAA GTT AAT AAA GAA ATT CAT AAA TCA TTA CGT GGA AAA GTT ATC AAT TTT ATT CTT TGG TTC TTT TAT GGT ATT TAT CAA TCT CTT ATA TTC TTT TTC GTA TTC TTT TGG TTT ATA TCA CCT TCA GAT ATA ACT GGT AAA GAT GGA TTA AAT GGA GGT ATT ATT TAC ACT TCA TTA ACA ATT ACA TTT TAT TCT TTA TTT ACT ATA ATT GTC ACT TTA ATT ATT GAA ACA AAA ACA TGG AAT TGG ATA TTA GTT ATT GGT CAT ATT ATT TCT ATT ATT TTT ATC TTT ATT ATC TAT GGA TTA ACT TGT TAT ATT CCT GGC TAT TCT ACT TAT GAC ATT TCT TGG AAT GGA TTT ACT TAT GTT TTC CAA TCA TTT AAT TTC TAT TTA ATT ACT ATC TTT ACA ATA ATA ATA AGT GTT GCT CCA TTA ATT CTC AAA AGA TTT ATT CAA AGA TAT TTC TTC CCT GAT ATG TAT CAT GTC GTT CAA GAA ATT CAA ACA TCT CTT GAA ACT AAT TAT GGT AAG AAG AGA AAT TTC TTC TTT GAA GGA AGT AAT GAA TTA AAA CAA TTA ATT AAT CAT CGC CTC CCT TTA ATC TCA AAA GAT ATT TCT TTT AAT AGT ACC TCT TCT AAC AGC CAA ATA AAT GAA ATT GAA TTG GTT GAA GAA AAT TGA

Occurrences/Results 9 Pentamers

The pentamer 'ATTCA' occurred 11 times.
The pentamer 'TTCAA' occurred 12 times.
The pentamer 'TCAAG' occurred 9 times.

The pentamer 'CAAGA' occurred 10 times.
The pentamer 'AAGAT' occurred 12 times.
The pentamer 'AGATG' occurred 8 times.
The pentamer 'GATGA' occurred 10 times.
The pentamer 'ATGAA' occurred 24 times.
The pentamer 'TGAAT' occurred 10 times.
The total occurrences of all specified strings is 106.
The total number of characters in the text is 3002.
First result (Total characters *9 /1024): 26.419921875
Square root of the first result: 5.1400313107023
Final result: 15.482411159500643

Occurrences/Results TRIMERS Codons

The pentamer 'ATT' occurred 87 times.
The pentamer 'CAA' occurred 39 times.
The pentamer 'GAT' occurred 44 times.
The pentamer 'GAA' occurred 64 times.
The pentamer 'CCA' occurred 11 times.
The pentamer 'ACT' occurred 28 times.
The pentamer 'AGA' occurred 18 times.
The pentamer 'TAC' occurred 1 times.
The total occurrences of all specified strings is 292.
The total number of characters in the text is 3006.
First result ((total_characters / 3) * (Trimer_number / 64)): 125.25
Square root of the first result: 11.2
Final result: 14.9

Occurrences/Results TRIMERS Combinations

The pentamer 'ATT ATT' occurred 8 times.
The pentamer 'ATT CAA' occurred 5 times.
The pentamer 'ATT GAT' occurred 1 times.
The pentamer 'ATT GAA' occurred 7 times.
The pentamer 'ATT CCA' occurred 0 times.
The pentamer 'ATT ACT' occurred 1 times.
The pentamer 'ATT AGA' occurred 0 times.
The pentamer 'ATT TAC' occurred 1 times.
The pentamer 'CAA ATT' occurred 2 times.
The pentamer 'CAA CAA' occurred 1 times.
The pentamer 'CAA GAT' occurred 2 times.
The pentamer 'CAA GAA' occurred 5 times.
The pentamer 'CAA CCA' occurred 2 times.
The pentamer 'CAA ACT' occurred 1 times.
The pentamer 'CAA AGA' occurred 1 times.

The pentamer 'CAA TAC' occurred 0 times.
The pentamer 'GAT ATT' occurred 6 times.
The pentamer 'GAT CAA' occurred 1 times.
The pentamer 'GAT GAT' occurred 5 times.
The pentamer 'GAT GAA' occurred 4 times.
The pentamer 'GAT CCA' occurred 1 times.
The pentamer 'GAT ACT' occurred 0 times.
The pentamer 'GAT AGA' occurred 2 times.
The pentamer 'GAT TAC' occurred 0 times.
The pentamer 'GAA ATT' occurred 7 times.
The pentamer 'GAA CAA' occurred 2 times.
The pentamer 'GAA GAT' occurred 2 times.
The pentamer 'GAA GAA' occurred 6 times.
The pentamer 'GAA CCA' occurred 0 times.
The pentamer 'GAA ACT' occurred 3 times.
The pentamer 'GAA AGA' occurred 1 times.
The pentamer 'GAA TAC' occurred 0 times.
The pentamer 'CCA ATT' occurred 1 times.
The pentamer 'CCA CAA' occurred 1 times.
The pentamer 'CCA GAT' occurred 0 times.
The pentamer 'CCA GAA' occurred 1 times.
The pentamer 'CCA CCA' occurred 1 times.
The pentamer 'CCA ACT' occurred 0 times.
The pentamer 'CCA AGA' occurred 0 times.
The pentamer 'CCA TAC' occurred 0 times.
The pentamer 'ACT ATT' occurred 1 times.
The pentamer 'ACT CAA' occurred 1 times.
The pentamer 'ACT GAT' occurred 0 times.
The pentamer 'ACT GAA' occurred 0 times.
The pentamer 'ACT CCA' occurred 2 times.
The pentamer 'ACT ACT' occurred 1 times.
The pentamer 'ACT AGA' occurred 0 times.
The pentamer 'ACT TAC' occurred 0 times.
The pentamer 'AGA ATT' occurred 0 times.
The pentamer 'AGA CAA' occurred 0 times.
The pentamer 'AGA GAT' occurred 2 times.
The pentamer 'AGA GAA' occurred 1 times.
The pentamer 'AGA CCA' occurred 0 times.
The pentamer 'AGA ACT' occurred 1 times.
The pentamer 'AGA AGA' occurred 0 times.
The pentamer 'AGA TAC' occurred 0 times.
The pentamer 'TAC ATT' occurred 0 times.
The pentamer 'TAC CAA' occurred 0 times.

The pentamer 'TAC GAT' occurred 0 times.
The pentamer 'TAC GAA' occurred 0 times.
The pentamer 'TAC CCA' occurred 0 times.
The pentamer 'TAC ACT' occurred 1 times.
The pentamer 'TAC AGA' occurred 0 times.
The pentamer 'TAC TAC' occurred 0 times.
The total occurrences of all specified strings is 91.
The total number of characters in the text is 3002.
First result (((total_characters/ 3) -1) / 64): 15.640625
Square root of the first result: 3.9548230048890938
Final result: 19.055056296283816

>Entamoeba histolytica, DNA polymerase 1 (odp1)

---> Sequence

CCCCCCATGGATCCCCCTGTAAGCCCCTCTAGGAGTACCCCTATGAGTCTCCCTGAAGAAACCCCC
TTTAATTCCCCACCTATTGTTCCACCTATTGAACCACCTAATGATGCCCAAATGATGGAGGAGCCC
TTAACTGATGGTGAGGTTGTTGCTCTACTAGAGGAAATAAACCATGAAGAGGATTATACCCCCTT
AGATATCCTCACTCTAGATAGAGTACCACCTACAGAAAATGAAATACAACTCCTCCAAATAGCAA
GAGAAAATACTACTGATGATGAGATTGAATTTACGGTCATCAGAAAAAGAGATTTTGGGCTAGA
AAATACTGCGCAAGCATGGCGTGGTATGTTAGGGGAAACCTATAGAATAAGTCAATTAATTAAG
GAATATGGCTTAATTCCCCTAGTAGAATTAGTATTTGGTGGAAGAACGCGGCGTTTTATTGTTAA
CTTAGAAAATATCGAACCGCAAGAATTTCTTAATCGGTGTGTGGGGTTTGATGTTGGTATAGAAG
AATTTTGGAGGAGTTATGATGATAGAGAAAATGAATTAAATATCCCTCCTGGAAAATTAGTAAA
TGAAATCATCATAACAATATTGAAACCTCCTCAACGCCAACGAATAGAAGGTGCGTTATTTAATT
ATTATTTGGTTGAACATTGGGCACCTCTAGAACATATCCTCAAAAAATATCAAATCTATAGTAAA
GAGGATATAGTACAAATAGAAAACTGTTTTATTCATTGTTTAGAAAACAGTCATACCTTAACAAA
GACTGAAATGGATAAAATCAGAACAAAGTTAAAGGGGAATACTGTGACTAAGAAGACTATGAAA
CGTATAGCCGAAGAATTTCACCTTAATATAATTCTTCGATATTATAATGGAGAAAAAATAGTCAC
AAATAATATTAAACATGGAGAAAGAATAATTAAAATATTTTTAATACGATGGGAGAATAATTTC
CACTATGTCCCTGATGAAAAAGTACCATTAACTACATACTTTATTCATCATTATGAAGAAATAGT
AAATTATTGTAATGAAAATGGAAAGGACATAGAAAAATTCTTTAATGTGACTAAAAAAGAAGGG
GAAATATATAAACACTCTCTTCAAAATTATATCCCAATATATAAATGTCTTAAAGAATTAAGAGA
GGTTGGGGCTATAAAAGAAATTGTAAGAAATGATATGATTAAGAAAAAATATTATGACTCTTTT
CTATTCTCACCAGAAAATATTTCTTTAACATATGAAGAGTCAAAATTAATTGTAGAAGATAAAA
AGAGTGAAACTACTAATACATTGTTGTTTGCTGATTTTGAATGTTTCACCTCTGGTGATTATCAC
AAGTCATATTGTATTATTGTTATGAATGAAGTTGGGGTATGGAAGAAATTTTATGGTATGAATT
GCGCAGATAAATTTATAAATTATCTTCAGACAATAGAGTCCCCCCTTTGTTACTTCCACAACTTA
GGATATGATGGAAGATTTTTAGCAAAATATGGAATTATTAATATGGTTAAAAAGGGGAAGATGA
TATATAAAATGACAATAAAAATAAATGGAAAGAAGATAATATTTAAAGATACTCTGGCCTTAAT
ACCAACAAGCATATCTAATTTTAAAACATTCTTTAAATTAGATGGAAAATATGAAAAAGAAATA
TTTCCTTATAACTATTATAATGAGGAAACAATGAATATTGGAGTTATTGAGAATTGTTGGAATA

AAGAAACACCTAGTTGGTCATTAGAAAAAATTGCACAATTTAAAGAGAATTTAATAAAAAATAA
GTGTATGATAAATGAAACACTATTTAACACAGAAAAATACTGTGAATATTATTGTTTAAGGGAC
GTGTTAGTGTTAAGAGAAGGATTTTTAAAATATAAGAAGATGATGAAAGAAAATTTAAATTTGG
ACTGCACCCAATTTTCCACCTTAAGTGCATTATCTTATTATTACTTTAAAAATAACTGCTTTGTA
AAAGATTTCTTATTTGAATATACTGGTAATGTAAGAGAGTATATTAAAAAAGCAGTATATGGTG
GTAGAAATATGTTAGGGGAAAATAAAAAACATATGGTGGATAAGGAAATAGTAGATTTTGACGC
GTGTAGTCTATATCCTTCAGCAGTGGCAAGACTATTTCTTCCCACAGGCGCACCTCGGGTCATGAA
TAAACCACTCCAGTGGTATATGGAGCATCTAATGGGTGAACAGCAATATGAAACCACCCAAGAAA
GATATATCTCATACTTTATTGTCACTATTGAGATAACAAAAGTAAATAAAAAAAGAAAGATGCC
CATTATTATTAAGAAAATAAATGGCATAAATCAATATGTAAATGAGCCATCCGTTATGACAGTA
GATTCAATTTATTTGGAGGATCTACTGAAATACCAAGAAATTGAATTTAATGTTAAAGAAGGAA
TTTATTGGGATGGGGGAAAGGCTAGTTTATTTAAAGAAAAGATTAAAGAAATTTATGATATAAG
AAAGCAAAAGAAAGCAGAACATGACCCAAGTGAAGTAATATTTAAATTAATAATGAATTCTTGT
TATGGTAAAACTATTCAAAAACCCATAACAGAAGAAATTAAATTATTTAGAACAAAACATAAAA
TGCTTTCTTATTGGAAAAGAAATTTAGAAGATATCCTGTCAGGAGAACAAATATATGATTCTGAC
ATTTGGATAGTAAATATTAAGAAGCAACTGGACGAATTTTTCGTTCCTAATATTATTGGGGTTCT
TATTCTCTCCATGTCCAAGAGAATAATGAATGAATTAATTTATTTATGTGAAGATTTGGGCATTT
ATGTATATTATCAGGATACTGATAGTATACATATAGAAAAAGATAAATTAAGCCAATTGAGAGA
CTCCTACTATAGAACATATAATAGGGAACTAGTAGGAACTAATGTTGGCCAATTCCATTCTGACT
TCCCTCCGGTGAATGGAAAAGAAAGTTGGTCAATTAAATCCATCTTTTTAGGTAAGAAGTCTTAC
CTTGATGTTCTCACGAATGAAGATGGAGACATCGATTATTTAATTAGAATGAAAGGCATCCCCAA
GGAAGTAATACTTGGTACTGCAAAAGAAAAATTTGAAGGGGATGTGGTAGCTTTGTATGAACAC
CTTTACACAGGCTACCCACTTAGTTTTGATTTATCA

---> Sequence Showing highlighted Existant Combined Trimers

CCC CCC ATG GAT CCC CCT GTA AGC CCC TCT AGG AGT ACC CCT ATG AGT CTC CCT GAA
GAA ACC CCC TTT AAT TCC CCA CCT ATT GTT CCA CCT ATT GAA CCA CCT AAT GAT GCC
CAA ATG ATG GAG GAG CCC TTA ACT GAT GGT GAG GTT GTT GCT CTA CTA GAG GAA ATA
AAC CAT GAA GAG GAT TAT ACC CCC TTA GAT ATC CTC ACT CTA GAT AGA GTA CCA CCT
ACA GAA AAT GAA ATA CAA CTC CTC CAA ATA GCA AGA GAA AAT ACT ACT GAT GAT GAG
ATT GAA TTT ACG GTC ATC AGA AAA AGA GAT TTT GGG CTA GAA AAT ACT GCG CAA GCA
TGG CGT GGT ATG TTA GGG GAA ACC TAT AGA ATA AGT CAA TTA ATT AAG GAA TAT GGC
TTA ATT CCC CTA GTA GAA TTA GTA TTT GGT GGA AGA ACG CGG CGT TTT ATT GTT AAC
TTA GAA AAT ATC GAA CCG CAA GAA TTT CTT AAT CGG TGT GTG GGG TTT GAT GTT GGT
ATA GAA GAA TTT TGG AGG AGT TAT GAT GAT AGA GAA AAT GAA TTA AAT ATC CCT CCT
GGA AAA TTA GTA AAT GAA ATC ATC ATA ACA ATA TTG AAA CCT CCT CAA CGC CAA CGA
ATA GAA GGT GCG TTA TTT AAT TAT TAT TTG GTT GAA CAT TGG GCA CCT CTA GAA CAT
ATC CTC AAA AAA TAT CAA ATC TAT AGT AAA GAG GAT ATA GTA CAA ATA GAA AAC TGT
TTT ATT CAT TGT TTA GAA AAC AGT CAT ACC TTA ACA AAG ACT GAA ATG GAT AAA ATC
AGA ACA AAG TTA AAG GGG AAT ACT GTG ACT AAG AAG ACT ATG AAA CGT ATA GCC GAA
GAA TTT CAC CTT AAT ATA ATT CTT CGA TAT TAT AAT GGA GAA AAA ATA GTC ACA AAT
AAT ATT AAA CAT GGA GAA AGA ATA ATT AAA ATA TTT TTA ATA CGA TGG GAG AAT AAT

TTC CAC TAT GTC CCT GAT GAA AAA GTA CCA TTA ACT ACA TAC TTT ATT CAT CAT TAT GAA GAA ATA GTA AAT TAT TGT AAT GAA AAT GGA AAG GAC ATA GAA AAA TTC TTT AAT GTG ACT AAA AAA GAA GGG GAA ATA TAT AAA CAC TCT CTT CAA AAT TAT ATC CCA ATA TAT AAA TGT CTT AAA GAA TTA AGA GAG GTT GGG GCT ATA AAA GAA ATT GTA AGA AAT GAT ATG ATT AAG AAA AAA TAT TAT GAC TCT TTT CTA TTC TCA CCA GAA AAT ATT TCT TTA ACA TAT GAA GAG TCA AAA TTA ATT GTA GAA GAT AAA AAG AGT GAA ACT ACT AAT ACA TTG TTG TTT GCT GAT TTT GAA TGT TTC ACC TCT GGT GAT TAT CAC AAG TCA TAT TGT ATT ATT GTT ATG AAT GAA GTT GGG GTA TGG AAG AAA TTT TAT GGT ATG AAT TGC GCA GAT AAA TTT ATA AAT TAT CTT CAG ACA ATA GAG TCC CCC CTT TGT TAC TTC CAC AAC TTA GGA TAT GAT GGA AGA TTT TTA GCA AAA TAT GGA ATT ATT AAT ATG GTT AAA AAG GGG AAG ATG ATA TAT AAA ATG ACA ATA AAA ATA AAT GGA AAG AAG ATA ATA TTT AAA GAT ACT CTG GCC TTA ATA CCA ACA AGC ATA TCT AAT TTT AAA ACA TTC TTT AAA TTA GAT GGA AAA TAT GAA AAA GAA ATA TTT CCT TAT AAC TAT TAT AAT GAG GAA ACA ATG AAT ATT GGA GTT ATT GAG AAT TGT TGG AAT AAA GAA ACA CCT AGT TGG TCA TTA GAA AAA ATT GCA CAA TTT AAA GAG AAT TTA ATA AAA AAT AAG TGT ATG ATA AAT GAA ACA CTA TTT AAC ACA GAA AAA TAC TGT GAA TAT TAT TGT TTA AGG GAC GTG TTA GTG TTA AGA GAA GGA TTT TTA AAA TAT AAG AAG ATG ATG AAA GAA AAT TTA AAT TTG GAC TGC ACC CAA TTT TCC ACC TTA AGT GCA TTA TCT TAT TAT TAC TTT AAA AAT AAC TGC TTT GTA AAA GAT TTC TTA TTT GAA TAT ACT GGT AAT GTA AGA GAG TAT ATT AAA AAA GCA GTA TAT GGT GGT AGA AAT ATG TTA GGG GAA AAT AAA AAA CAT ATG GTG GAT AAG GAA ATA GTA GAT TTT GAC GCG TGT AGT CTA TAT CCT TCA GCA GTG GCA AGA CTA TTT CTT CCC ACA GGC GCA CCT CGG GTC ATG AAT AAA CCA CTC CAG TGG TAT ATG GAG CAT CTA ATG GGT GAA CAG CAA TAT GAA ACC ACC CAA GAA AGA TAT ATC TCA TAC TTT ATT GTC ACT ATT GAG ATA ACA AAA GTA AAT AAA AAA AGA AAG ATG CCC ATT ATT ATT AAG AAA ATA AAT GGC ATA AAT CAA TAT GTA AAT GAG CCA TCC GTT ATG ACA GTA GAT TCA ATT TAT TTG GAG GAT CTA CTG AAA TAC CAA GAA ATT GAA TTT AAT GTT AAA GAA GGA ATT TAT TGG GAT GGG GGA AAG GCT AGT TTA TTT AAA GAA AAG ATT AAA GAA ATT TAT GAT ATA AGA AAG CAA AAG AAA GCA GAA CAT GAC CCA AGT GAA GTA ATA TTT AAA TTA ATA ATG AAT TCT TGT TAT GGT AAA ACT ATT CAA AAA CCC ATA ACA GAA GAA ATT AAA TTA TTT AGA ACA AAA CAT AAA ATG CTT TCT TAT TGG AAA AGA AAT TTA GAA GAT ATC CTG TCA GGA GAA CAA ATA TAT GAT TCT GAC ATT TGG ATA GTA AAT ATT AAG AAG CAA CTG GAC GAA TTT TTC GTT CCT AAT ATT ATT GGG GTT CTT ATT CTC TCC ATG TCC AAG AGA ATA ATG AAT GAA TTA ATT TAT TTA TGT GAA GAT TTG GGC ATT TAT GTA TAT TAT CAG GAT ACT GAT AGT ATA CAT ATA GAA AAA GAT AAA TTA AGC CAA TTG AGA GAC TCC TAC TAT AGA ACA TAT AAT AGG GAA CTA GTA GGA ACT AAT GTT GGC CAA TTC CAT TCT GAC TTC CCT CCG GTG AAT GGA AAA GAA AGT TGG TCA ATT AAA TCC ATC TTT TTA GGT AAG AAG TCT TAC CTT GAT GTT CTC ACG AAT GAA GAT GGA GAC ATC GAT TAT TTA ATT AGA ATG AAA GGC ATC CCC AAG GAA GTA ATA CTT GGT ACT GCA AAA GAA AAA TTT GAA GGG GAT GTG GTA GCT TTG TAT GAA CAC CTT TAC ACA GGC TAC CCA CTT AGT TTT GAT TTA TCA

Occurrences/Results 9 Pentamers

The pentamer 'ATTCA' occurred 4 times.
The pentamer 'TTCAA' occurred 3 times.
The pentamer 'TCAAG' occurred 0 times.
The pentamer 'CAAGA' occurred 6 times.
The pentamer 'AAGAT' occurred 14 times.
The pentamer 'AGATG' occurred 5 times.
The pentamer 'GATGA' occurred 5 times.
The pentamer 'ATGAA' occurred 22 times.
The pentamer 'TGAAT' occurred 13 times.
The total occurrences of all specified strings is 72.
The total number of characters in the text is 3194.
First result (Total characters *9 /1024): 28.107421875
Square root of the first result: 5.30164331834951
Final result: 8.279051510893511

Occurrences/Results TRIMERS Codons

The pentamer 'ATT' occurred 44 times.
The pentamer 'CAA' occurred 23 times.
The pentamer 'GAT' occurred 41 times.
The pentamer 'GAA' occurred 84 times.
The pentamer 'CCA' occurred 12 times.
The pentamer 'ACT' occurred 20 times.
The pentamer 'AGA' occurred 25 times.
The pentamer 'TAC' occurred 10 times.
The total occurrences of all specified strings is 259.
The total number of characters in the text is 3198.
First result ((total_characters / 3) * (Trimer_number / 64)): 133.25
Square root of the first result: 11.54
Final result: 10.9

Occurrences/Results TRIMERS Combinations

The pentamer 'ATT ATT' occurred 4 times.
The pentamer 'ATT CAA' occurred 1 times.
The pentamer 'ATT GAT' occurred 0 times.
The pentamer 'ATT GAA' occurred 3 times.
The pentamer 'ATT CCA' occurred 0 times.
The pentamer 'ATT ACT' occurred 0 times.
The pentamer 'ATT AGA' occurred 1 times.
The pentamer 'ATT TAC' occurred 0 times.
The pentamer 'CAA ATT' occurred 0 times.
The pentamer 'CAA CAA' occurred 0 times.
The pentamer 'CAA GAT' occurred 0 times.

The pentamer 'CAA GAA' occurred 3 times.
The pentamer 'CAA CCA' occurred 0 times.
The pentamer 'CAA ACT' occurred 0 times.
The pentamer 'CAA AGA' occurred 0 times.
The pentamer 'CAA TAC' occurred 0 times.
The pentamer 'GAT ATT' occurred 0 times.
The pentamer 'GAT CAA' occurred 0 times.
The pentamer 'GAT GAT' occurred 2 times.
The pentamer 'GAT GAA' occurred 1 times.
The pentamer 'GAT CCA' occurred 0 times.
The pentamer 'GAT ACT' occurred 2 times.
The pentamer 'GAT AGA' occurred 2 times.
The pentamer 'GAT TAC' occurred 0 times.
The pentamer 'GAA ATT' occurred 4 times.
The pentamer 'GAA CAA' occurred 1 times.
The pentamer 'GAA GAT' occurred 4 times.
The pentamer 'GAA GAA' occurred 5 times.
The pentamer 'GAA CCA' occurred 1 times.
The pentamer 'GAA ACT' occurred 1 times.
The pentamer 'GAA AGA' occurred 2 times.
The pentamer 'GAA TAC' occurred 0 times.
The pentamer 'CCA ATT' occurred 0 times.
The pentamer 'CCA CAA' occurred 0 times.
The pentamer 'CCA GAT' occurred 0 times.
The pentamer 'CCA GAA' occurred 1 times.
The pentamer 'CCA CCA' occurred 0 times.
The pentamer 'CCA ACT' occurred 0 times.
The pentamer 'CCA AGA' occurred 0 times.
The pentamer 'CCA TAC' occurred 0 times.
The pentamer 'ACT ATT' occurred 2 times.
The pentamer 'ACT CAA' occurred 0 times.
The pentamer 'ACT GAT' occurred 3 times.
The pentamer 'ACT GAA' occurred 1 times.
The pentamer 'ACT CCA' occurred 0 times.
The pentamer 'ACT ACT' occurred 2 times.
The pentamer 'ACT AGA' occurred 0 times.
The pentamer 'ACT TAC' occurred 0 times.
The pentamer 'AGA ATT' occurred 0 times.
The pentamer 'AGA CAA' occurred 0 times.
The pentamer 'AGA GAT' occurred 1 times.
The pentamer 'AGA GAA' occurred 3 times.
The pentamer 'AGA CCA' occurred 0 times.
The pentamer 'AGA ACT' occurred 0 times.

The pentamer 'AGA AGA' occurred 0 times.
The pentamer 'AGA TAC' occurred 0 times.
The pentamer 'TAC ATT' occurred 0 times.
The pentamer 'TAC CAA' occurred 1 times.
The pentamer 'TAC GAT' occurred 0 times.
The pentamer 'TAC GAA' occurred 0 times.
The pentamer 'TAC CCA' occurred 1 times.
The pentamer 'TAC ACT' occurred 0 times.
The pentamer 'TAC AGA' occurred 0 times.
The pentamer 'TAC TAC' occurred 0 times.
The total occurrences of all specified strings is 52.
The total number of characters in the text is 3194.
First result (((total_characters/ 3) -1) / 64): 16.640625
Square root of the first result: 4.07929221802018
Final result: 8.668017173126447

>NW_001914886.1:33724-35607 Entamoeba histolytica HM-1:IMSS scf_1104750517413

genomic scaffold, glycyl-tRNA synthetase

---> Sequence

ATGCAAAGACCAGCTACCAAATTTAATAAAGAAAAACTTCTTATTAATAAGGCTAAATTAGATG
AAATTCTTAAACAAAGAAATATGGTTATTCAATCATATGAAATTTATGGTGGTATTGCTGGTTTA
TATGATATGGGACCTCTTGGATGTGCTTTAAAACAAAATATACTTCAATTTTGGAGAAAACATTT
TACTACATATGAAAATTTCTTTGAAGTTGAAGGACCAATTTTAACACCAAAATGTGTTTTAGCAG
CATCAGGACATACTGCTAAATTCAGTGATTATATGGTTAAAGATTTAAAAAATGGATGTTGCTAT
AGAGCTGATCATTTACTTAAAACATTCTATGAAAATAAAATGGAAGACCCAGCTACTTCTGCAGA
ACAAAAAGCTACTTATGATGCTGAAATGAAACTTGTAGATAATTTAACTCCTGAAGAACTTTCTG
CTGCTATTCAAAGAAATGGAATTAAAGCACCAGATACAGGTAATGATCTTTCAGAACCATTAGCT
TTTAATTTAATGTTTGCAACTGATATTGGACCAGCTGGAGATCTTAAAGCCTTTTTAAGACCAGA
AACAGCACAAGGAATATTTACTATGTTTAAAAGAAATCTTGAATTTAATGGAGGAAAAGTACCA
TTTGGTGTAACTCAAATTGGTAATGTTTTTAGAAATGAAATTGCTCCAAGAAATGGTTTATTACG
TGTTCGTGAATTTACACTTGCAGAAATTGAATATTTTGTCTTACCAGATAAAAAGACTCATTCTA
ATTTCTCTGATGTTGAAAATCTTTCTGTTCAATTATATCCAAGAGAACTTCAACTTGAAGATAAA
GAAGCTGAATATATTACTCTTGGTAAAGCTGTTAATGACGGAATTATTAATTCACAATTATTAGC
CTATTTTATGGGAAGAACATTTAAATTCTTAATTGAACTTGGAATTCCAGCTGAGCATATTAGAT
TTAGACAACACTTAAAAACAGAAATGGCACATTATGCTAAAGATTGTTGGGATGCTGAAATTAG
ATTATCATATGGTTGGGTTGAATGTGTTGGTCATGCTGATAGAGGAGATTTTGATTTATCTAATC
ATGCTCGTTGCTCTAAGGTTGATCAGTCTGTATTCATTGCTTACGATGAACCAAAAGAAGTTAAA
GATGTTACTCTTTCTTTCAATAAAGGAGTTATGGGAAAGAAATATAAGAAAGACTCTCAAAAAT
TATTTGCTTATGCATCTGGTTTAGATGAAGCTGCTAAAGAAGCAGTTGCCAAAGAAGTTGAAGAA
ACTGGAATGTGGAAAGTTACTGTTGATGGTATTAACTTTGAAATTGAAAAAGCTAATATCACTA

TTAAAATTGGAACAAAGAAAGTATATGGTGATAATATTATTCCAAATGTTATTGAACCATCTTTT
GGTGTTGGACGTGTATTAACTGCTGTTCTTGAACACTCATTCTGGGTTAGAGAAGATAATGAAGC
TAAATCTGTTTTATCAATTCCTGCTTCTATTGCTCCAGTCAAAGTTGGTTTATTCCCACTCTTAAC
TAAACTAGAATTTAACAATAAAATCGCAGAAATTGAAAAGATTTGTAAGAATGGTTTCCTTTCA
TTCAAATCTAATACTACTGCTGTTGCTATTGGTAAGAAATATGCTCAAGCTGATGAAGCTGGTAT
TCCATTTGATGTTACAGTTGATTATACTTCTCTCTCTGATAATACTGTCACTCTTAGAGACAGAG
ATACTACAAAACAAATTAGAATTCCAATTGACAAGTTAGTTGAAACAGTGCATGCCCTTACTCAA
CTTCATCCAACTACAACTTTTGAGAAACTTATGACAATTTATCCAGTAGAAGAAGTCAAAGAAAA
TTAA

---> Sequence Showing highlighted Existant Combined Trimers

ATG CAA AGA CCA GCT ACC AAA TTT AAT AAA GAA AAA CTT CTT ATT AAT AAG GCT AAA
TTA GAT GAA ATT CTT AAA CAA AGA AAT ATG GTT ATT CAA TCA TAT GAA ATT TAT GGT
GGT ATT GCT GGT TTA TAT GAT ATG GGA CCT CTT GGA TGT GCT TTA AAA CAA AAT ATA
CTT CAA TTT TGG AGA AAA CAT TTT ACT ACA TAT GAA AAT TTC TTT GAA GTT GAA GGA
CCA ATT TTA ACA CCA AAA TGT GTT TTA GCA GCA TCA GGA CAT ACT GCT AAA TTC AGT
GAT TAT ATG GTT AAA GAT TTA AAA AAT GGA TGT TGC TAT AGA GCT GAT CAT TTA CTT
AAA ACA TTC TAT GAA AAT AAA ATG GAA GAC CCA GCT ACT TCT GCA GAA CAA AAA GCT
ACT TAT GAT GCT GAA ATG AAA CTT GTA GAT AAT TTA ACT CCT GAA GAA CTT TCT GCT
GCT ATT CAA AGA AAT GGA ATT AAA GCA CCA GAT ACA GGT AAT GAT CTT TCA GAA CCA
TTA GCT TTT AAT TTA ATG TTT GCA ACT GAT ATT GGA CCA GCT GGA GAT CTT AAA GCC
TTT TTA AGA CCA GAA ACA GCA CAA GGA ATA TTT ACT ATG TTT AAA AGA AAT CTT GAA
TTT AAT GGA GGA AAA GTA CCA TTT GGT GTA ACT CAA ATT GGT AAT GTT TTT AGA AAT
GAA ATT GCT CCA AGA AAT GGT TTA TTA CGT GTT CGT GAA TTT ACA CTT GCA GAA ATT
GAA TAT TTT GTC TTA CCA GAT AAA AAG ACT CAT TCT AAT TTC TCT GAT GTT GAA AAT
CTT TCT GTT CAA TTA TAT CCA AGA GAA CTT CAA CTT GAA GAT AAA GAA GCT GAA TAT
ATT ACT CTT GGT AAA GCT GTT AAT GAC GGA ATT ATT AAT TCA CAA TTA TTA GCC TAT
TTT ATG GGA AGA ACA TTT AAA TTC TTA ATT GAA CTT GGA ATT CCA GCT GAG CAT ATT
AGA TTT AGA CAA CAC TTA AAA ACA GAA ATG GCA CAT TAT GCT AAA GAT TGT TGG GAT
GCT GAA ATT AGA TTA TCA TAT GGT TGG GTT GAA TGT GTT GGT CAT GCT GAT AGA GGA
GAT TTT GAT TTA TCT AAT CAT GCT CGT TGC TCT AAG GTT GAT CAG TCT GTA TTC ATT
GCT TAC GAT GAA CCA AAA GAA GTT AAA GAT GTT ACT CTT TCT TTC AAT AAA GGA GTT
ATG GGA AAG AAA TAT AAG AAA GAC TCT CAA AAA TTA TTT GCT TAT GCA TCT GGT TTA
GAT GAA GCT GCT AAA GAA GCA GTT GCC AAA GAA GTT GAA GAA ACT GGA ATG TGG AAA
GTT ACT GTT GAT GGT ATT AAC TTT GAA ATT GAA AAA GCT AAT ATC ACT ATT AAA ATT
GGA ACA AAG AAA GTA TAT GGT GAT AAT ATT ATT CCA AAT GTT ATT GAA CCA TCT TTT
GGT GTT GGA CGT GTA TTA ACT GCT GTT CTT GAA CAC TCA TTC TGG GTT AGA GAA GAT
AAT GAA GCT AAA TCT GTT TTA TCA ATT CCT GCT TCT ATT GCT CCA GTC AAA GTT GGT
TTA TTC CCA CTC TTA ACT AAA CTA GAA TTT AAC AAT AAA ATC GCA GAA ATT GAA AAG
ATT TGT AAG AAT GGT TTC CTT TCA TTC AAA TCT AAT ACT ACT GCT GTT GCT ATT GGT
AAG AAA TAT GCT CAA GCT GAT GAA GCT GGT ATT CCA TTT GAT GTT ACA GTT GAT TAT
ACT TCT CTC TCT GAT AAT ACT GTC ACT CTT AGA GAC AGA GAT ACT ACA AAA CAA ATT

AGA ATT CCA ATT GAC AAG TTA GTT GAA ACA GTG CAT GCC CTT ACT CAA CTT CAT CCA ACT ACA ACT TTT GAG AAA CTT ATG ACA ATT TAT CCA GTA GAA GAA GTC AAA GAA AAT TAA

Occurrences/Results 9 Pentamers

The pentamer 'ATTCA' occurred 6 times.
The pentamer 'TTCAA' occurred 7 times.
The pentamer 'TCAAG' occurred 1 times.
The pentamer 'CAAGA' occurred 2 times.
The pentamer 'AAGAT' occurred 6 times.
The pentamer 'AGATG' occurred 3 times.
The pentamer 'GATGA' occurred 4 times.
The pentamer 'ATGAA' occurred 10 times.
The pentamer 'TGAAT' occurred 5 times.
The total occurrences of all specified strings is 44.
The total number of characters in the text is 1880.
First result (Total characters *9 /1024): 16.55859375
Square root of the first result: 4.069225202664507
Final result: 6.743644031308839

Occurrences/Results TRIMERS Codons

The pentamer 'ATT' occurred 37 times.
The pentamer 'CAA' occurred 17 times.
The pentamer 'GAT' occurred 31 times.
The pentamer 'GAA' occurred 49 times.
The pentamer 'CCA' occurred 22 times.
The pentamer 'ACT' occurred 25 times.
The pentamer 'AGA' occurred 19 times.
The pentamer 'TAC' occurred 1 times.
The total occurrences of all specified strings is 201.
The total number of characters in the text is 1884.
First result ((total_characters / 3) * (Trimer_number / 64)): 78.5
Square root of the first result: 8.86
Final result: 13.8

Occurrences/Results TRIMERS Combinations

The pentamer 'ATT ATT' occurred 2 times.
The pentamer 'ATT CAA' occurred 2 times.
The pentamer 'ATT GAT' occurred 0 times.
The pentamer 'ATT GAA' occurred 5 times.
The pentamer 'ATT CCA' occurred 4 times.
The pentamer 'ATT ACT' occurred 1 times.
The pentamer 'ATT AGA' occurred 3 times.

The pentamer 'ATT TAC' occurred 0 times.
The pentamer 'CAA ATT' occurred 2 times.
The pentamer 'CAA CAA' occurred 0 times.
The pentamer 'CAA GAT' occurred 0 times.
The pentamer 'CAA GAA' occurred 0 times.
The pentamer 'CAA CCA' occurred 0 times.
The pentamer 'CAA ACT' occurred 0 times.
The pentamer 'CAA AGA' occurred 3 times.
The pentamer 'CAA TAC' occurred 0 times.
The pentamer 'GAT ATT' occurred 1 times.
The pentamer 'GAT CAA' occurred 0 times.
The pentamer 'GAT GAT' occurred 0 times.
The pentamer 'GAT GAA' occurred 4 times.
The pentamer 'GAT CCA' occurred 0 times.
The pentamer 'GAT ACT' occurred 1 times.
The pentamer 'GAT AGA' occurred 1 times.
The pentamer 'GAT TAC' occurred 0 times.
The pentamer 'GAA ATT' occurred 7 times.
The pentamer 'GAA CAA' occurred 1 times.
The pentamer 'GAA GAT' occurred 2 times.
The pentamer 'GAA GAA' occurred 3 times.
The pentamer 'GAA CCA' occurred 3 times.
The pentamer 'GAA ACT' occurred 1 times.
The pentamer 'GAA AGA' occurred 0 times.
The pentamer 'GAA TAC' occurred 0 times.
The pentamer 'CCA ATT' occurred 2 times.
The pentamer 'CCA CAA' occurred 0 times.
The pentamer 'CCA GAT' occurred 2 times.
The pentamer 'CCA GAA' occurred 1 times.
The pentamer 'CCA CCA' occurred 0 times.
The pentamer 'CCA ACT' occurred 1 times.
The pentamer 'CCA AGA' occurred 2 times.
The pentamer 'CCA TAC' occurred 0 times.
The pentamer 'ACT ATT' occurred 1 times.
The pentamer 'ACT CAA' occurred 2 times.
The pentamer 'ACT GAT' occurred 1 times.
The pentamer 'ACT GAA' occurred 0 times.
The pentamer 'ACT CCA' occurred 0 times.
The pentamer 'ACT ACT' occurred 1 times.
The pentamer 'ACT AGA' occurred 0 times.
The pentamer 'ACT TAC' occurred 0 times.
The pentamer 'AGA ATT' occurred 1 times.
The pentamer 'AGA CAA' occurred 1 times.

The pentamer 'AGA GAT' occurred 1 times.
The pentamer 'AGA GAA' occurred 2 times.
The pentamer 'AGA CCA' occurred 2 times.
The pentamer 'AGA ACT' occurred 0 times.
The pentamer 'AGA AGA' occurred 0 times.
The pentamer 'AGA TAC' occurred 0 times.
The pentamer 'TAC ATT' occurred 0 times.
The pentamer 'TAC CAA' occurred 0 times.
The pentamer 'TAC GAT' occurred 1 times.
The pentamer 'TAC GAA' occurred 0 times.
The pentamer 'TAC CCA' occurred 0 times.
The pentamer 'TAC ACT' occurred 0 times.
The pentamer 'TAC AGA' occurred 0 times.
The pentamer 'TAC TAC' occurred 0 times.
The total occurrences of all specified strings is 67.
The total number of characters in the text is 1880.
First result (((total_characters/ 3) -1) / 64): 9.796875
Square root of the first result: 3.1299960063872287
Final result: 18.275782104280133

>AK421490.1 Entamoeba histolytica cDNA clone: Xeh-013E10, ribosomal protein L18, putative, complete cds, strain: HM-1:IMSS

---> Sequence

GCAGAACAAAGCGATGGCCATTGATTTAGACAGACATCATGTTCGTAAACACGTATCAAAGACCG
CACGTGGAAACAACGCTTATATGAAACTTCTTGTTCGCCTTTATGGTTTCCTTGCCAGAAGAACA
CAATCTAAATTTGCTAAAACAATCTTACACAGACTTTGCTTAAGTCGTGTTAATAGACCAATTGT
TTCAACTAGTAAATTAGCATGCCTTATGAAGAAACACCCAGAAGAAACTGCAGTTTGTGTCAATA
CAGTTACTTACGACAGTAGATATCCAGTTCCAAAAATGAATGTTTGTGCTCTTAAATTCACCAAA
ACAGCAGAAGCAGCCATCACTAAAGCTGGTGGAAAATGTTTAAGATTTGATGAACTTGCACTTAA
AGCACCAACTGGAAGAAAGACTGTCCTCATTAGAGGAAAGAGAAATGTCAGAGAAGCACTCAAAC
ATTTCGGTAAAGTTTGTGCTAAGAAGAACCCAGCTAAGCAATATAAGGGAAAACAAACTAAATA
ATTGCAATGGATTTGAGTTTG

---> Sequence Showing highlighted Existant Combined Trimers

GCA GAA CAA AGC GAT GGC CAT TGA TTT AGA CAG ACA TCA TGT TCG TAA ACA CGT ATC
AAA GAC CGC ACG TGG AAA CAA CGC TTA TAT GAA ACT TCT TGT TCG CCT TTA TGG TTT
CCT TGC CAG AAG AAC ACA ATC TAA ATT TGC TAA AAC AAT CTT ACA CAG ACT TTG CTT
AAG TCG TGT TAA TAG ACC AAT TGT TTC AAC TAG TAA ATT AGC ATG CCT TAT GAA GAA
ACA CCC AGA AGA AAC TGC AGT TTG TGT CAA TAC AGT TAC TTA CGA CAG TAG ATA TCC
AGT TCC AAA AAT GAA TGT TTG TGC TCT TAA ATT CAC CAA AAC AGC AGA AGC AGC CAT

CAC TAA AGC TGG TGG AAA ATG TTT AAG ATT TGA TGA ACT TGC ACT TAA AGC ACC AAC TGG AAG AAA GAC TGT CCT CAT TAG AGG AAA GAG AAA TGT CAG AGA AGC ACT CAA ACA TTT CGG TAA AGT TTG TGC TAA GAA GAA CCC AGC TAA GCA ATA TAA GGG AAA ACA AAC TAA ATA ATT GCA ATG GAT TTG AGT TTG

Occurrences/Results 9 Pentamers

The pentamer 'ATTCA' occurred 1 times.
The pentamer 'TTCAA' occurred 1 times.
The pentamer 'TCAAG' occurred 0 times.
The pentamer 'CAAGA' occurred 0 times.
The pentamer 'AAGAT' occurred 1 times.
The pentamer 'AGATG' occurred 0 times.
The pentamer 'GATGA' occurred 1 times.
The pentamer 'ATGAA' occurred 4 times.
The pentamer 'TGAAT' occurred 1 times.
The total occurrences of all specified strings is 9.
The total number of characters in the text is 536.
First result (Total characters *9 /1024): 4.74609375
Square root of the first result: 2.178553132241672
Final result: 1.9526291037129062

Occurrences/Results TRIMERS Codons

The pentamer 'ATT' occurred 5 times.
The pentamer 'CAA' occurred 5 times.
The pentamer 'GAT' occurred 2 times.
The pentamer 'GAA' occurred 7 times.
The pentamer 'CCA' occurred 0 times.
The pentamer 'ACT' occurred 5 times.
The pentamer 'AGA' occurred 5 times.
The pentamer 'TAC' occurred 2 times.
The total occurrences of all specified strings is 31.
The total number of characters in the text is 540.
First result ((total_characters / 3) * (Trimer_number / 64)): 22.5
Square root of the first result: 4.74
Final result: 1.8

Occurrences/Results TRIMERS Combinations

The pentamer 'ATT ATT' occurred 0 times.
The pentamer 'ATT CAA' occurred 0 times.
The pentamer 'ATT GAT' occurred 0 times.
The pentamer 'ATT GAA' occurred 0 times.
The pentamer 'ATT CCA' occurred 0 times.
The pentamer 'ATT ACT' occurred 0 times.

The pentamer 'ATT AGA' occurred 0 times.
The pentamer 'ATT TAC' occurred 0 times.
The pentamer 'CAA ATT' occurred 0 times.
The pentamer 'CAA CAA' occurred 0 times.
The pentamer 'CAA GAT' occurred 0 times.
The pentamer 'CAA GAA' occurred 0 times.
The pentamer 'CAA CCA' occurred 0 times.
The pentamer 'CAA ACT' occurred 0 times.
The pentamer 'CAA AGA' occurred 0 times.
The pentamer 'CAA TAC' occurred 1 times.
The pentamer 'GAT ATT' occurred 0 times.
The pentamer 'GAT CAA' occurred 0 times.
The pentamer 'GAT GAT' occurred 0 times.
The pentamer 'GAT GAA' occurred 0 times.
The pentamer 'GAT CCA' occurred 0 times.
The pentamer 'GAT ACT' occurred 0 times.
The pentamer 'GAT AGA' occurred 0 times.
The pentamer 'GAT TAC' occurred 0 times.
The pentamer 'GAA ATT' occurred 0 times.
The pentamer 'GAA CAA' occurred 1 times.
The pentamer 'GAA GAT' occurred 0 times.
The pentamer 'GAA GAA' occurred 2 times.
The pentamer 'GAA CCA' occurred 0 times.
The pentamer 'GAA ACT' occurred 1 times.
The pentamer 'GAA AGA' occurred 0 times.
The pentamer 'GAA TAC' occurred 0 times.
The pentamer 'CCA ATT' occurred 0 times.
The pentamer 'CCA CAA' occurred 0 times.
The pentamer 'CCA GAT' occurred 0 times.
The pentamer 'CCA GAA' occurred 0 times.
The pentamer 'CCA CCA' occurred 0 times.
The pentamer 'CCA ACT' occurred 0 times.
The pentamer 'CCA AGA' occurred 0 times.
The pentamer 'CCA TAC' occurred 0 times.
The pentamer 'ACT ATT' occurred 0 times.
The pentamer 'ACT CAA' occurred 1 times.
The pentamer 'ACT GAT' occurred 0 times.
The pentamer 'ACT GAA' occurred 0 times.
The pentamer 'ACT CCA' occurred 0 times.
The pentamer 'ACT ACT' occurred 0 times.
The pentamer 'ACT AGA' occurred 0 times.
The pentamer 'ACT TAC' occurred 0 times.
The pentamer 'AGA ATT' occurred 0 times.

The pentamer 'AGA CAA' occurred 0 times.
The pentamer 'AGA GAT' occurred 0 times.
The pentamer 'AGA GAA' occurred 0 times.
The pentamer 'AGA CCA' occurred 0 times.
The pentamer 'AGA ACT' occurred 0 times.
The pentamer 'AGA AGA' occurred 1 times.
The pentamer 'AGA TAC' occurred 0 times.
The pentamer 'TAC ATT' occurred 0 times.
The pentamer 'TAC CAA' occurred 0 times.
The pentamer 'TAC GAT' occurred 0 times.
The pentamer 'TAC GAA' occurred 0 times.
The pentamer 'TAC CCA' occurred 0 times.
The pentamer 'TAC ACT' occurred 0 times.
The pentamer 'TAC AGA' occurred 0 times.
The pentamer 'TAC TAC' occurred 0 times.
The total occurrences of all specified strings is 7.
The total number of characters in the text is 536.
First result (((total_characters/ 3) -1) / 64): 2.796875
Square root of the first result: 1.6723860200324565
Final result: 2.513250499378384

>U12513.1 Entamoeba histolytica PPi-dependent phosphofructo-1-kinase (PFK) gene,

---> Sequence

CACTTCCTTCATTAAAAATAGAAGAAGTTGGA**GAATG**TACTATTGACAATATTTATGCATCACCA
GAACCATTTGTT**AATGGTA**TGACAATGAAATTATCAGCAGTCAAAAATCATGGAATAGAAAGAG
ATAGTGGAGAAGTAGAACTAGCAGGACC**AATGG**AAAAGATATTTTACAATCCTGAAACAACAAA
AGT**TGCCATT**GTAACATGTGGAGGGTTATGTCCAGGATTAAATAATGTTATCAGGGGGTTAGTA
TTGAATTTATATAATCGTTACCATGTAAACAATATTTTTGGACTAAGATGGGGATATGAAGGACT
TGTTCCAGAATTGTCTGAAGTACAAAGACTAACACCAGAAATAGTTAGTGATATTCATCAAAAAG
GAGGAAGTATATTAGGAACTTCAAGAGGGGCACAAAGTCCAGAGGTTATGGCACAATTTCTTATA
GACAATAACTTTAATATTTTATTTACACTAGGAGGAGATGGTACATTAAGAGGAGCAAATGCAA
TCAATAAAGAATTAAGAAGAAGAAAAGTTCCAATTACCGTTGTTGGTATTCCTAAAACAATTGA
TAATGATATTTGTTATACTGATTCTACATTTGGATTTCAAACAGCTGTTGGACTTTCACAAGAAG
CTATTAATGCTGTTCATAGTGAAGCAAAATCAGCAAA**GAATGG**GATTGGAATAGTTAGATT**AAT**
GGGTAGAGATGCTGGATTTATTGCATTATATGCATCATTAGCT**AATGGT**GATGCTAATTTAGTA
TTAATTCCAGAGATTGATATTCCAATTACACAAATCTGTGAATTTGTTGGTAAAAGAATAATGTC
AAAAGGACATGTTGTTATTGTAGTAGCAGAAGGTGCATTACAAAATCAAAAACCTAAAGATCTT
GATTTAGGAACAGATAAATCAGGAAATATTCTTCATTGGGATTCAATCAATTATTTGAGAGATTC
TATTACAAAGTATTTAAAGTCTATTGGAATTGAAGAACATACAATTAAATTTGTTGATCCTTCAT
ATATGATTAGATCAGCTCCTTGTAGTGCTGCTGATGC**CCATT**TTTGTATGTGTTTAGCTAATGCA

GCAGTTCATGTAGCTATGGCAGGAAAAACAGGATTAGTTATTTGTCATCACCATAATAATTTTGT
TTCAGTACCAATTGATAGAACTAGTTATTATATAAACGAGTTAATACCGATGGA**CCATT**ATATAC
TATGATGACAGCTATTGAAAAGCCAAAGTAAATCAATAAAATTACATAATAAATTTTTGTCTAA
ACTTCTTATTTATGAATCTTAAAATCTTATTATTTCATTAATTAAAATAAAAAAAAATTACAAA
AGAAGAAAATTATAAATTATTATTTAAAAATTAGTTTTGTTTGTACACAAATTCTTCATCTT

---> Sequence Showing highlighted Existant Combined Trimers

CAC TTC CTT CAT TAA AAA TAG AAG AAG TTG GAG AAT GTA CTA TTG ACA ATA TTT ATG CAT CAC CAG AAC CAT TTG TTA ATG GTA TGA CAA TGA AAT TAT CAG CAG TCA AAA ATC ATG GAA TAG AAA GAG ATA GTG GAG AAG TAG AAC TAG CAG GAC CAA TGG AAA AGA TAT TTT ACA ATC CTG AAA CAA CAA AAG TTG CCA TTG TAA CAT GTG GAG GGT TAT GTC CAG GAT TAA ATA ATG TTA TCA GGG GGT TAG TAT TGA ATT TAT ATA ATC GTT ACC ATG TAA ACA ATA TTT TTG GAC TAA GAT GGG GAT ATG AAG GAC TTG TTC CAG AAT TGT CTG AAG TAC AAA GAC TAA CAC CAG AAA TAG TTA GTG ATA TTC ATC AAA AAG GAG GAA GTA TAT TAG GAA CTT CAA GAG GGG CAC AAA GTC CAG AGG TTA TGG CAC AAT TTC TTA TAG ACA ATA ACT TTA ATA TTT TAT TTA CAC TAG GAG GAG ATG GTA CAT TAA GAG GAG CAA ATG CAA TCA ATA AAG AAT TAA GAA GAA GAA AAG TTC CAA TTA CCG TTG TTG GTA TTC CTA AAA CAA TTG ATA ATG ATA TTT GTT ATA CTG ATT CTA CAT TTG GAT TTC AAA CAG CTG TTG GAC TTT CAC AAG AAG CTA TTA ATG CTG TTC ATA GTG AAG CAA AAT CAG CAA AGA ATG GGA TTG GAA TAG TTA GAT TAA TGG GTA GAG ATG CTG GAT TTA TTG CAT TAT ATG CAT CAT TAG CTA ATG GTG ATG CTA ATT TAG TAT TAA TTC CAG AGA TTG ATA TTC CAA TTA CAC AAA TCT GTG AAT TTG TTG GTA AAA GAA TAA TGT CAA AAG GAC ATG TTG TTA TTG TAG TAG CAG AAG GTG CAT TAC AAA ATC AAA AAC CTA AAG ATC TTG ATT TAG GAA CAG ATA AAT CAG GAA ATA TTC TTC ATT GGG ATT CAA TCA ATT ATT TGA GAG ATT CTA TTA CAA AGT ATT TAA AGT CTA TTG GAA TTG AAG AAC ATA CAA TTA AAT TTG TTG ATC CTT CAT ATA TGA TTA GAT CAG CTC CTT GTA GTG CTG CTG ATG CCC ATT TTT GTA TGT GTT TAG CTA ATG CAG CAG TTC ATG TAG CTA TGG CAG GAA AAA CAG GAT TAG TTA TTT GTC ATC ACC ATA ATA ATT TTG TTT CAG TAC CAA TTG ATA GAA CTA GTT ATT ATA TAA ACG AGT TAA TAC CGA TGG ACC ATT ATA TAC TAT GAT GAC AGC TAT TGA AAA GCC AAA GTA AAT CAA TAA AAT TAC ATA ATA AAT TTT TGT CTA AAC TTC TTA TTT ATG AAT CTT AAA ATC TTA TTA TTT CAT TAA TTA AAA TAA AAA AAA ATT ACA AAA GAA GAA AAT TAT AAA TTA TTA TTT AAA AAT TAG TTT TGT TTG TAC ACA AAT TCT TCA TCT T

Occurrences/Results 9 Pentamers

The pentamer 'ATTCA' occurred 2 times.
The pentamer 'TTCAA' occurred 3 times.
The pentamer 'TCAAG' occurred 1 times.
The pentamer 'CAAGA' occurred 2 times.
The pentamer 'AAGAT' occurred 3 times.
The pentamer 'AGATG' occurred 3 times.
The pentamer 'GATGA' occurred 1 times.
The pentamer 'ATGAA' occurred 3 times.
The pentamer 'TGAAT' occurred 3 times.

The total occurrences of all specified strings is 21.
The total number of characters in the text is 1413.
First result (Total characters *9 /1024): 12.4541015625
Square root of the first result: 3.5290369171347584
Final result: 2.421595080518017

Occurrences/Results TRIMERS Codons

The pentamer 'ATT' occurred 15 times.
The pentamer 'CAA' occurred 18 times.
The pentamer 'GAA' occurred 15 times.
The pentamer 'GAT' occurred 9 times.
The pentamer 'TAC' occurred 7 times.
The pentamer 'CCA' occurred 1 times.
The pentamer 'ACT' occurred 1 times.
The pentamer 'AGA' occurred 3 times.
The total occurrences of all specified strings is 69.
The total number of characters in the text is 1413.
First result ((total_characters / 3) * (Trimer_number / 64)): 58.9
Square root of the first result: 7.67
Final result: 1.3

Occurrences/Results TRIMERS Combinations

The pentamer 'ATT ATT' occurred 1 times.
The pentamer 'ATT CAA' occurred 1 times.
The pentamer 'ATT GAA' occurred 0 times.
The pentamer 'ATT GAT' occurred 0 times.
The pentamer 'ATT TAC' occurred 0 times.
The pentamer 'ATT CCA' occurred 0 times.
The pentamer 'ATT ACT' occurred 0 times.
The pentamer 'ATT AGA' occurred 0 times.
The pentamer 'CAA ATT' occurred 0 times.
The pentamer 'CAA CAA' occurred 1 times.
The pentamer 'CAA GAA' occurred 0 times.
The pentamer 'CAA GAT' occurred 0 times.
The pentamer 'CAA TAC' occurred 0 times.
The pentamer 'CAA CCA' occurred 0 times.
The pentamer 'CAA ACT' occurred 0 times.
The pentamer 'CAA AGA' occurred 1 times.
The pentamer 'GAA ATT' occurred 0 times.
The pentamer 'GAA CAA' occurred 0 times.
The pentamer 'GAA GAA' occurred 2 times.
The pentamer 'GAA GAT' occurred 0 times.
The pentamer 'GAA TAC' occurred 0 times.
The pentamer 'GAA CCA' occurred 0 times.

The pentamer 'GAA ACT' occurred 0 times.
The pentamer 'GAA AGA' occurred 0 times.
The pentamer 'GAT ATT' occurred 0 times.
The pentamer 'GAT CAA' occurred 0 times.
The pentamer 'GAT GAA' occurred 0 times.
The pentamer 'GAT GAT' occurred 0 times.
The pentamer 'GAT TAC' occurred 0 times.
The pentamer 'GAT CCA' occurred 0 times.
The pentamer 'GAT ACT' occurred 0 times.
The pentamer 'GAT AGA' occurred 0 times.
The pentamer 'TAC ATT' occurred 0 times.
The pentamer 'TAC CAA' occurred 1 times.
The pentamer 'TAC GAA' occurred 0 times.
The pentamer 'TAC GAT' occurred 0 times.
The pentamer 'TAC TAC' occurred 0 times.
The pentamer 'TAC CCA' occurred 0 times.
The pentamer 'TAC ACT' occurred 0 times.
The pentamer 'TAC AGA' occurred 0 times.
The pentamer 'CCA ATT' occurred 0 times.
The pentamer 'CCA CAA' occurred 0 times.
The pentamer 'CCA GAA' occurred 0 times.
The pentamer 'CCA GAT' occurred 0 times.
The pentamer 'CCA TAC' occurred 0 times.
The pentamer 'CCA CCA' occurred 0 times.
The pentamer 'CCA ACT' occurred 0 times.
The pentamer 'CCA AGA' occurred 0 times.
The pentamer 'ACT ATT' occurred 0 times.
The pentamer 'ACT CAA' occurred 0 times.
The pentamer 'ACT GAA' occurred 0 times.
The pentamer 'ACT GAT' occurred 0 times.
The pentamer 'ACT TAC' occurred 0 times.
The pentamer 'ACT CCA' occurred 0 times.
The pentamer 'ACT ACT' occurred 0 times.
The pentamer 'ACT AGA' occurred 0 times.
The pentamer 'AGA ATT' occurred 0 times.
The pentamer 'AGA CAA' occurred 0 times.
The pentamer 'AGA GAA' occurred 0 times.
The pentamer 'AGA GAT' occurred 0 times.
The pentamer 'AGA TAC' occurred 0 times.
The pentamer 'AGA CCA' occurred 0 times.
The pentamer 'AGA ACT' occurred 0 times.
The pentamer 'AGA AGA' occurred 0 times.
The total occurrences of all specified strings is 10.

The total number of characters in the text is 1413.
First result (((total_characters/ 3) -1) / 64): 7.359375
Square root of the first result: 2.7128168017763383
Final result: 1

>BDEQ01000001.1:9849783-9850730 Entamoeba histolytica DNA, contig: contig1, strain: HM1:IMSS clone 6, cysteine proteinase 2

---> Sequence

ATGTTTGCTTTTATTTGTTTACTTGCTATTGCAAGTGCTATTGATTTCAATACATGGGCTTCTAA
AAACAATAAACACTTCACAGCAATTGAAAAGCTTAGAAGAAGAGCTATCTTCAATATGAA**TG**CT
AAATTCGTTGATAGTTTCAATAAAATTGGTTCATTCA**A**ATTATCAGTAGATGGA**CCATT**TGCTGC
TATGACTAATGAAGAATACAGAACTCTTCTTAAATCTAAAAGAAC**TACTG**AAGAA**AATGG**ACAA
GTTAAATATTTGAATATCCAAGCACCAGAATCAGTAGATTGGAGAAAAGAAGGAAAAGTAACTC
CAATTAGAGATCAAGCACAATGCGGATCATGTTATACATTTGGTTCACTTGCAGCTCTTGAAGGA
AGATTATTAATTGAAAAAGGAGGTGATGCTAATACACTCGATCTTTCAGAAGAACATATGGTTCA
ATGCACAAGAGATAATGGAAATAATGGATGTAATGGAGGACTTGGATCAAATGTCTATGATTAC
ATTATTGAACACGGAGTTGCTAAAGAAAGTGATTATCCATACACTGGAAGTGATTCTACATGCAA
AACTAATGTAAAATCATTTGCTAAAAT**TACTG**GATATACTAAAGTCCCAAGAAACAATGAAGCT
GAACTTAAAGCTGCACTTTCACAAGGTCTTGTTGATGTTTCAATTGATGCATCATCTGCTAAATT
CCAATTATACAAGAGCGGAGCTTA**TACTG**ATACTAAATGCAAGA**AT**AACTACTTTGCTTTGAAT
CACGAAGTTTGTGCTGTTGGAT**ATGGT**GTTGTTGATGGAAAA**GAATG**TTGGATAGTTAGAAACT
CATGGGGAACAGGATGGGGAGATAAAGGATACATTAAT**ATGGT**TATTGAAGGAAATACCTGTGG
TGTTGCTACAGATCCACTTTATCCAACTGGCGTTCAATATCTTTGA

---> Sequence Showing highlighted Existant Combined Trimers

ATG TTT GCT TTT ATT TGT TTA CTT GCT ATT GCA AGT GCT ATT GAT TTC AAT ACA TGG
GCT TCT AAA AAC AAT AAA CAC TTC ACA GCA ATT GAA AAG CTT AGA AGA AGA GCT ATC
TTC AAT ATG AAT GCT AAA TTC GTT GAT AGT TTC AAT AAA ATT GGT TCA TTC AAA TTA
TCA GTA GAT GGA CCA TTT GCT GCT ATG ACT AAT GAA GAA TAC AGA ACT CTT CTT AAA
TCT AAA AGA ACT ACT GAA GAA AAT GGA CAA GTT AAA TAT TTG AAT ATC CAA GCA CCA
GAA TCA GTA GAT TGG AGA AAA GAA GGA AAA GTA ACT CCA ATT AGA GAT CAA GCA CAA
TGC GGA TCA TGT TAT ACA TTT GGT TCA CTT GCA GCT CTT GAA GGA AGA TTA TTA ATT
GAA AAA GGA GGT GAT GCT AAT ACA CTC GAT CTT TCA GAA GAA CAT ATG GTT CAA TGC
ACA AGA GAT AAT GGA AAT AAT GGA TGT AAT GGA GGA CTT GGA TCA AAT GTC TAT GAT
TAC ATT ATT GAA CAC GGA GTT GCT AAA GAA AGT GAT TAT CCA TAC ACT GGA AGT GAT
TCT ACA TGC AAA ACT AAT GTA AAA TCA TTT GCT AAA ATT ACT GGA TAT ACT AAA GTC
CCA AGA AAC AAT GAA GCT GAA CTT AAA GCT GCA CTT TCA CAA GGT CTT GTT GAT GTT
TCA ATT GAT GCA TCA TCT GCT AAA TTC CAA TTA TAC AAG AGC GGA GCT TAT ACT GAT
ACT AAA TGC AAG AAT AAC TAC TTT GCT TTG AAT CAC GAA GTT TGT GCT GTT GGA TAT
GGT GTT GTT GAT GGA AAA GAA TGT TGG ATA GTT AGA AAC TCA TGG GGA ACA GGA TGG

GGA GAT AAA GGA TAC ATT AAT ATG GTT ATT GAA GGA AAT ACC TGT GGT GTT GCT ACA GAT CCA CTT TAT CCA ACT GGC GTT CAA TAT CTT TGA

Occurrences/Results 9 Pentamers

The pentamer 'ATTCA' occurred 1 times.
The pentamer 'TTCAA' occurred 7 times.
The pentamer 'TCAAG' occurred 1 times.
The pentamer 'CAAGA' occurred 4 times.
The pentamer 'AAGAT' occurred 1 times.
The pentamer 'AGATG' occurred 1 times.
The pentamer 'GATGA' occurred 0 times.
The pentamer 'ATGAA' occurred 3 times.
The pentamer 'TGAAT' occurred 3 times.
The total occurrences of all specified strings is 21.
The total number of characters in the text is 944.
First result (Total characters *9 /1024): 8.33203125
Square root of the first result: 2.886525809688872
Final result: 4.388655977881394

Occurrences/Results TRIMERS Codons

The pentamer 'CAA' occurred 8 times.
The pentamer 'GAT' occurred 17 times.
The pentamer 'GAA' occurred 18 times.
The pentamer 'ATT' occurred 13 times.
The pentamer 'TAC' occurred 6 times.
The pentamer 'CCA' occurred 7 times.
The pentamer 'ATC' occurred 2 times.
The pentamer 'AGA' occurred 11 times.
The total occurrences of all specified strings is 82.
The total number of characters in the text is 948.
First result ((total_characters / 3) * (Trimer_number / 64)): 39.5
Square root of the first result: 6.3
Final result: 6.8

Occurrences/Results TRIMERS Combinations

The pentamer 'CAA CAA' occurred 0 times.
The pentamer 'CAA GAT' occurred 0 times.
The pentamer 'CAA GAA' occurred 0 times.
The pentamer 'CAA ATT' occurred 0 times.
The pentamer 'CAA TAC' occurred 0 times.
The pentamer 'CAA AGA' occurred 0 times.
The pentamer 'CAA ACT' occurred 0 times.
The pentamer 'CAA CCA' occurred 0 times.
The pentamer 'GAT CAA' occurred 1 times.

The pentamer 'GAT GAT' occurred 0 times.
The pentamer 'GAT GAA' occurred 0 times.
The pentamer 'GAT ATT' occurred 0 times.
The pentamer 'GAT TAC' occurred 1 times.
The pentamer 'GAT AGA' occurred 0 times.
The pentamer 'GAT ACT' occurred 1 times.
The pentamer 'GAT CCA' occurred 1 times.
The pentamer 'GAA CAA' occurred 0 times.
The pentamer 'GAA GAT' occurred 0 times.
The pentamer 'GAA GAA' occurred 3 times.
The pentamer 'GAA ATT' occurred 0 times.
The pentamer 'GAA TAC' occurred 1 times.
The pentamer 'GAA AGA' occurred 0 times.
The pentamer 'GAA ACT' occurred 0 times.
The pentamer 'GAA CCA' occurred 0 times.
The pentamer 'ATT CAA' occurred 0 times.
The pentamer 'ATT GAT' occurred 2 times.
The pentamer 'ATT GAA' occurred 4 times.
The pentamer 'ATT ATT' occurred 1 times.
The pentamer 'ATT TAC' occurred 0 times.
The pentamer 'ATT AGA' occurred 1 times.
The pentamer 'ATT ACT' occurred 1 times.
The pentamer 'ATT CCA' occurred 0 times.
The pentamer 'TAC CAA' occurred 0 times.
The pentamer 'TAC GAT' occurred 0 times.
The pentamer 'TAC GAA' occurred 0 times.
The pentamer 'TAC ATT' occurred 2 times.
The pentamer 'TAC TAC' occurred 0 times.
The pentamer 'TAC AGA' occurred 1 times.
The pentamer 'TAC ACT' occurred 1 times.
The pentamer 'TAC CCA' occurred 0 times.
The pentamer 'AGA CAA' occurred 0 times.
The pentamer 'AGA GAT' occurred 2 times.
The pentamer 'AGA GAA' occurred 0 times.
The pentamer 'AGA ATT' occurred 0 times.
The pentamer 'AGA TAC' occurred 0 times.
The pentamer 'AGA AGA' occurred 1 times.
The pentamer 'AGA ACT' occurred 2 times.
The pentamer 'AGA CCA' occurred 0 times.
The pentamer 'ACT CAA' occurred 0 times.
The pentamer 'ACT GAT' occurred 1 times.
The pentamer 'ACT GAA' occurred 1 times.
The pentamer 'ACT ATT' occurred 0 times.

The pentamer 'ACT TAC' occurred 0 times.
The pentamer 'ACT AGA' occurred 0 times.
The pentamer 'ACT ACT' occurred 1 times.
The pentamer 'ACT CCA' occurred 1 times.
The pentamer 'CCA CAA' occurred 0 times.
The pentamer 'CCA GAT' occurred 0 times.
The pentamer 'CCA GAA' occurred 1 times.
The pentamer 'CCA ATT' occurred 1 times.
The pentamer 'CCA TAC' occurred 1 times.
The pentamer 'CCA AGA' occurred 1 times.
The pentamer 'CCA ACT' occurred 1 times.
The pentamer 'CCA CCA' occurred 0 times.
The total occurrences of all specified strings is 35.
The total number of characters in the text is 944.
First result (((total_characters/ 3) -1) / 64): 4.921875
Square root of the first result: 2.218529918662356
Final result: 13.557682836269953

4) Saccharomyces cerevisiae

>CANBMX030000004.1:80603-80673 Saccharomyces cerevisiae genome assembly, chrIV, tRNA-Gly

GCGCAAG**TG**GTTT**A**G**TGGTA**AAATCCAACGT**TGCCA**TCGTTGGGCCCCCGG**TTC**G**A**TT
CCGGGCTTGCGCA

>WMJW01000001.1:1574641-1578927 Saccharomyces cerevisiae strain FDAARGOS_613 tig00000001_pilon, gyrase

---> Sequence

ATGTCAACTGAACCGGTAAGCGCCTCTGATAAATATCAGAAAATTTCTCAACTGGAA

CATATCTTAAAAAGACCAGACACTTATATCGGTTCTGTTGAAACTCAAGAGCAGCTG

CAATGGATATACGATGAAGAGACCGATTGCATGATTGAAAAAAATGTCACAATTGT

ACCAGGGTTGTTCAAAATCTTTGATGAAATCTTAGTCAATGCGGCAGATAATAAAGT

TCGTGATCCATCGATGAAACGAATCGATGTAAACATACATGCTGAGGAACATACTA

TAGAAGTGAAAAATGATGGAAAAGGTATTCCCATAGAGATTCATAACAAGGAGAAT

ATTTATATTCCTGAAATGATATTTGGTCATTTGTTGACATCATCCAATTATGATGATG

ATGAGAAGAAAGTCACTGGTGGTAGAAACGGTTATGGTGCTAAGCTTTGTAATATA

TTTTCCACTGAATTCATATTGGAAACTGCAGATCTAAATGTTGGCCAGAAATATGTT

CAAAAATGGGAAAATAACATGAGCATTTGCCACCCCCCAAAAATAACATCTTACAA

GAAGGGTCCATCATATACAAAGGTGACATTTAAGCCGGATTTAACCAGATTCGGAA

TGAAAGAGCTAGATAATGATATCTTAGGAGTGATGCGAAGAAGAGTTTATGATATC

AATGGTTCTGTTCGTGACATTAATGTCTATCTGAATGGCAAGTCCTTAAAGATAAGA

AATTTCAAAAATTATGTTGAACTCTACTTGAAATCACTCGAAAAAAAAAGACAACT

AGATAACGGTGAGGACGGTACCGCTAAGTCTGATATCCCGACTATTCTTTATGAGAG

AATAAACAACAGATGGGAAGTTGCTTTTGCGGTTTCTGATATCTCTTTTCAACAAAT

TTCTTTTGTGAATTCCATTGCAACTACCATGGGTGGTACCCATGTCAATTACATAACA

GACCAAATTGTAAAAAAAATTTCAGAAATTTTGAAGAAAAAGAAGAAAAAAAGTGT

GAAGTCTTTTCAGATTAAAAATAATATGTTCATTTTCATTAATTGTTTGATTGAGAAT

CCTGCATTTACCTCACAAACAAAAGAGCAACTGACAACAAGAGTCAAAGATTTTGG

GTCCCGTTGTGAGATTCCTCTTGAATATATTAATAAGATTATGAAAACTGATTTGGC

TACAAGAATGTTTGAAATTGCCGACGCAAATGAAGAAAATGCGCTAAAGAAGTCTG
ATGGTACAAGGAAAAGCAGAATTACTAATTACCCTAAACTGGAAGATGCCAACAAA
GCCGGTACAAAAGAAGGCTATAAATGTACTTTAGTTCTGACAGAAGGGGATTCCGC
CTTGTCATTAGCTGTTGCAGGTTTAGCTGTTGTTGGTAGAGATTATTATGGTTGTTAT
CCACTTCGTGGTAAAATGCTGAATGTTAGAGAGGCTAGTGCTGATCAGATACTAAA
AAACGCGGAAATTCAAGCCATTAAAAAAATTATGGGGTTACAACATCGCAAGAAAT
ATGAAGATACAAAATCTTTAAGATATGGGCATCTTATGATCATGACCGATCAAGATC
ATGATGGTTCGCATATTAAAGGTTTAATTATAAACTTTTTAGAAAGCTCATTTCCTGG
TCTTTTGGATATCCAAGGTTTCTTACTTGAATTCATAACTCCGATCATCAAAGTTTCC
ATCACTAAACCAACAAAAAACACTATTGCATTCTACAATATGCCGGACTATGAAAA
ATGGAGAGAGGAAGAATCGCACAAATTTACTTGGAAGCAGAAGTATTATAAAGGAT
TAGGGACTTCTCTAGCACAAGAAGTCCGAGAATATTTTTCGAACTTGGACAGACATT
TGAAAATATTCCATTCTTTGCAGGGTAATGATAAAGATTACATTGATTTAGCTTTCTC
CAAGAAAAAGGCAGATGACCGTAAAGAATGGCTGAGACAATACGAACCTGGTACT
GTTTTAGACCCTACTTTAAAAGAGATTCCAATTAGCGACTTCATTAATAAGGAATTA
ATCCTTTTTTCTTTGGCCGATAATATACGGTCGATTCCCAATGTTTTAGATGGATTTA
AACCTGGCCAAAGAAAAGTTCTTTATGGTTGTTTCAAAAAAAATTTAAAGTCGGAAC
TGAAAGTAGCTCAACTTGCACCATACGTGAGCGAATGTACGGCATATCACCATGGT
GAGCAGTCATTGGCACAAACTATTATTGGGCTAGCCCAAAGCTTTGTTGGGTCCAAC
AATATTTACTTGCTATTACCTAACGGTGCTTTCGGTACAAGAGCCACTGGTGGTAAA
GATGCAGCGGCAGCGAGATATATCTACACAGAATTGAACAAATTAACTCGTAAGAT
ATTTCACCCTGCTGATGATCCATTATACAAATATATACAAGAAGATGAGAAAACAGT
GGAGCCAGAGTGGTATTTACCAATTCTTCCTATGATTCTTGTTAACGGTGCTGAGGG
TATTGGCACTGGCTGGAGTACTTACATTCCTCCATTCAACCCATTGGAAATTATAAA
GAATATAAGACATTTAATGAACGACGAGGAGCTTGAGCAAATGCATCCGTGGTTTA
GGGGATGGACAGGTACTATTGAAGAAATTGAGCCTCTGCGTTACAGAATGTACGGT

AGGATTGAACAAATTGGAGATAACGTCTTAGAAATAACTGAGTTGCCAGCCAGAAC
TTGGACATCGACTATAAAGGAGTACCTACTTTTAGGTTTAAGCGGTAACGATAAAAT
AAAACCCTGGATCAAAGATATGGAGGAGCAGCACGATGATAACATCAAATTCATAA
TCACGCTATCACCTGAGGAAATGGCTAAAACAAGGAAAATAGGTTTTTATGAAAGA
TTTAAACTAATTTCGCCTATAAGTTTGATGAATATGGTCGCATTTGATCCTCACGGG
AAAATCAAGAAGTACAATTCCGTGAATGAAATATTAAGCGAATTTTACTACGTCAG
ACTAGAATACTATCAAAAAAGAAAAGACCATATGAGCGAAAGGTTACAGTGGGAG
GTAGAGAAATACTCTTTCCAAGTAAAATTTATTAAAATGATTATTGAAAAGGAGTTA
ACAGTCACCAATAAGCCTAGGAACGCTATTATCCAAGAACTTGAGAATTTAGGGTTC
CCCAGATTTAATAAGGAAGGTAAACCATATTATGGAAGTCCTAAGGATGAGATAGC
TGAACAAATTAACGACGTAAAAGGCGCAACTTCTGATGAAGAAGATGAAGAAAGTT
CACACGAAGATACTGAAAATGTTATAAATGGTCCTGAAGAACTATATGGCACATAT
GAATATTTATTAGGAATGAGAATATGGTCATTGACCAAGGAAAGATATGAAAAGCT
GTTGAAACAAAAACAAGAAAAGGAGACAGAGTTGGAAAACTTGTTAAAACTTTCCG
CGAAAGATATATGGAACACTGACTTGAAGGCTTTTGAGGTGGGATATCAAGAATTTT
TGCAACGAGATGCAGAAGCTCGCGGTGGTAATGTTCCCAATAAAGGGAGCAAAACA
AAAGGTAAAGGAAAAAGAAAGCTTGTTGACGACGAAGACTACGACCCATCAAAAA
AAAACAAGAAAAGTACTGCTAGAAAGGGCAAAAAAATTAAGTTAGAGGATAAGAA
TTTTGAAAGGATTTTGTTAGAACAAAAACTAGTAACCAAAAGCAAGGCGCCTACAA
AGATTAAAAAAGAGAAAACGCCTTCTGTTTCAGAAACAAAAACAGAAGAAGAAGA
GAATGCTCCTTCTTCCACGAGTTCTTCTTCTATTTTCGACATAAAGAAAGAAGATAA
AGATGAGGGCGAACTGAGTAAGATTTCGAACAAGTTTAAAAAAATTAGCACGATTT
TTGACAAAATGGGTTCAACTTCCGCTACATCGAAGGAAAATACACCAGAACAGGAC
GATGTAGCCACTAAAAAAAATCAAACAACCGCTAAAAAAACAGCTGTGAAACCTAA
ATTGGCCAAGAAGCCAGTCAGGAAACAACAAAAAGTTGTGGAACTATCTGGTGAAA
GCGACCTAGAAATTTTAGATTCATACACTGATCGGGAAGATAGCAATAAAGATGAA

GATGATGCTATACCACAACGATCAAGGAGACAAAGATCGTCGAGAGCTGCGTCGGT

TCCTAAGAAATCTTACGTTAAAACTTTAGAATTATCTGACGACAGTTTTATCGAAGA

TGATGAAGAGGAAAACCAAGGATCAGATGTTTCGTTCAATGAAGAGGATTGA

---> Sequence Showing highlighted Existant Combined Trimers

ATT;CAA;GAT;GAA;CCA;ACT;AGA;TAC

ATG TCA ACT GAA CCG GTA AGC GCC TCT GAT AAA TAT CAG AAA ATT TCT CAA CTG GAA CAT ATC TTA AAA AGA CCA GAC ACT TAT ATC GGT TCT GTT GAA ACT CAA GAG CAG CTG CAA TGG ATA TAC GAT GAA GAG ACC GAT TGC ATG ATT GAA AAA AAT GTC ACA ATT GTA CCA GGG TTG TTC AAA ATC TTT GAT GAA ATC TTA GTC AAT GCG GCA GAT AAT AAA GTT CGT GAT CCA TCG ATG AAA CGA ATC GAT GTA AAC ATA CAT GCT GAG GAA CAT ACT ATA GAA GTG AAA AAT GAT GGA AAA GGT ATT CCC ATA GAG ATT CAT AAC AAG GAG AAT ATT TAT ATT CCT GAA ATG ATA TTT GGT CAT TTG TTG ACA TCA TCC AAT TAT GAT GAT GAT GAG AAG AAA GTC ACT GGT GGT AGA AAC GGT TAT GGT GCT AAG CTT TGT AAT ATA TTT TCC ACT GAA TTC ATA TTG GAA ACT GCA GAT CTA AAT GTT GGC CAG AAA TAT GTT CAA AAA TGG GAA AAT AAC ATG AGC ATT TGC CAC CCC CCA AAA ATA ACA TCT TAC AAG AAG GGT CCA TCA TAT ACA AAG GTG ACA TTT AAG CCG GAT TTA ACC AGA TTC GGA ATG AAA GAG CTA GAT AAT GAT ATC TTA GGA GTG ATG CGA AGA AGA GTT TAT GAT ATC AAT GGT TCT GTT CGT GAC ATT AAT GTC TAT CTG AAT GGC AAG TCC TTA AAG ATA AGA AAT TTC AAA AAT TAT GTT GAA CTC TAC TTG AAA TCA CTC GAA AAA AAA AGA CAA CTA GAT AAC GGT GAG GAC GGT ACC GCT AAG TCT GAT ATC CCG ACT ATT CTT TAT GAG AGA ATA AAC AAC AGA TGG GAA GTT GCT TTT GCG GTT TCT GAT ATC TCT TTT CAA CAA ATT TCT TTT GTG AAT TCC ATT GCA ACT ACC ATG GGT GGT ACC CAT GTC AAT TAC ATA ACA GAC CAA ATT GTA AAA AAA ATT TCA GAA ATT TTG AAG AAA AAG AAG AAA AAA AGT GTG AAG TCT TTT CAG ATT AAA AAT AAT ATG TTC ATT TTC ATT AAT TGT TTG ATT GAG AAT CCT GCA TTT ACC TCA CAA ACA AAA GAG CAA CTG ACA ACA AGA GTC AAA GAT TTT GGG TCC CGT TGT GAG ATT CCT CTT GAA TAT ATT AAT AAG ATT ATG AAA ACT GAT TTG GCT ACA AGA ATG TTT GAA ATT GCC GAC GCA AAT GAA GAA AAT GCG CTA AAG AAG TCT GAT GGT ACA AGG AAA AGC AGA ATT ACT AAT TAC CCT AAA CTG GAA GAT GCC AAC AAA GCC GGT ACA AAA GAA GGC TAT AAA TGT ACT TTA GTT CTG ACA GAA GGG GAT TCC GCC TTG TCA TTA GCT GTT GCA GGT TTA GCT GTT GTT GGT AGA GAT TAT TAT GGT TGT TAT CCA CTT CGT GGT AAA ATG CTG AAT GTT AGA GAG GCT AGT GCT GAT CAG ATA CTA AAA AAC GCG GAA ATT CAA GCC ATT AAA AAA ATT ATG GGG TTA CAA CAT CGC AAG AAA TAT GAA GAT ACA AAA TCT TTA AGA TAT GGG CAT CTT ATG ATC ATG ACC GAT CAA GAT CAT GAT GGT TCG CAT ATT AAA GGT TTA ATT ATA AAC TTT TTA GAA AGC TCA TTT CCT GGT CTT TTG GAT ATC CAA GGT TTC TTA CTT GAA TTC ATA ACT CCG ATC ATC AAA GTT TCC ATC ACT AAA CCA ACA AAA AAC ACT ATT GCA TTC TAC AAT ATG CCG GAC TAT GAA AAA TGG AGA GAG GAA GAA TCG CAC AAA TTT ACT TGG AAG CAG AAG TAT TAT AAA GGA TTA GGG ACT TCT CTA GCA CAA GAA GTC CGA GAA TAT TTT TCG AAC TTG GAC AGA CAT TTG AAA ATA TTC CAT TCT TTG CAG GGT AAT GAT AAA GAT TAC ATT GAT TTA GCT TTC TCC AAG AAA AAG GCA GAT GAC CGT AAA GAA TGG CTG AGA CAA TAC GAA CCT GGT ACT GTT TTA GAC CCT ACT TTA AAA GAG ATT CCA ATT AGC GAC TTC ATT AAT AAG GAA TTA ATC CTT TTT TCT TTG GCC GAT AAT ATA CGG TCG ATT CCC AAT GTT TTA GAT GGA TTT AAA CCT GGC CAA

AGA AAA GTT CTT TAT GGT TGT TTC AAA AAA AAT TTA AAG TCG GAA CTG AAA GTA GCT CAA CTT GCA CCA TAC GTG AGC GAA TGT ACG GCA TAT CAC CAT GGT GAG CAG TCA TTG GCA CAA ACT ATT ATT GGG CTA GCC CAA AGC TTT GTT GGG TCC AAC AAT ATT TAC TTG CTA TTA CCT AAC GGT GCT TTC GGT ACA AGA GCC ACT GGT GGT AAA GAT GCA GCG GCA GCG AGA TAT ATC TAC ACA GAA TTG AAC AAA TTA ACT CGT AAG ATA TTT CAC CCT GCT GAT GAT CCA TTA TAC AAA TAT ATA CAA GAA GAT GAG AAA ACA GTG GAG CCA GAG TGG TAT TTA CCA ATT CTT CCT ATG ATT CTT GTT AAC GGT GCT GAG GGT ATT GGC ACT GGC TGG AGT ACT TAC ATT CCT CCA TTC AAC CCA TTG GAA ATT ATA AAG AAT ATA AGA CAT TTA ATG AAC GAC GAG GAG CTT GAG CAA ATG CAT CCG TGG TTT AGG GGA TGG ACA GGT ACT ATT GAA GAA ATT GAG CCT CTG CGT TAC AGA ATG TAC GGT AGG ATT GAA CAA ATT GGA GAT AAC GTC TTA GAA ATA ACT GAG TTG CCA GCC AGA ACT TGG ACA TCG ACT ATA AAG GAG TAC CTA CTT TTA GGT TTA AGC GGT AAC GAT AAA ATA AAA CCC TGG ATC AAA GAT ATG GAG GAG CAG CAC GAT GAT AAC ATC AAA TTC ATA ATC ACG CTA TCA CCT GAG GAA ATG GCT AAA ACA AGG AAA ATA GGT TTT TAT GAA AGA TTT AAA CTA ATT TCG CCT ATA AGT TTG ATG AAT ATG GTC GCA TTT GAT CCT CAC GGG AAA ATC AAG AAG TAC AAT TCC GTG AAT GAA ATA TTA AGC GAA TTT TAC TAC GTC AGA CTA GAA TAC TAT CAA AAA AGA AAA GAC CAT ATG AGC GAA AGG TTA CAG TGG GAG GTA GAG AAA TAC TCT TTC CAA GTA AAA TTT ATT AAA ATG ATT ATT GAA AAG GAG TTA ACA GTC ACC AAT AAG CCT AGG AAC GCT ATT ATC CAA GAA CTT GAG AAT TTA GGG TTC CCC AGA TTT AAT AAG GAA GGT AAA CCA TAT TAT GGA AGT CCT AAG GAT GAG ATA GCT GAA CAA ATT AAC GAC GTA AAA GGC GCA ACT TCT GAT GAA GAA GAT GAA GAA AGT TCA CAC GAA GAT ACT GAA AAT GTT ATA AAT GGT CCT GAA GAA CTA TAT GGC ACA TAT GAA TAT TTA TTA GGA ATG AGA ATA TGG TCA TTG ACC AAG GAA AGA TAT GAA AAG CTG TTG AAA CAA AAA CAA GAA AAG GAG ACA GAG TTG GAA AAC TTG TTA AAA CTT TCC GCG AAA GAT ATA TGG AAC ACT GAC TTG AAG GCT TTT GAG GTG GGA TAT CAA GAA TTT TTG CAA CGA GAT GCA GAA GCT CGC GGT GGT AAT GTT CCC AAT AAA GGG AGC AAA ACA AAA GGT AAA GGA AAA AGA AAG CTT GTT GAC GAC GAA GAC TAC GAC CCA TCA AAA AAA AAC AAG AAA AGT ACT GCT AGA AAG GGC AAA AAA ATT AAG TTA GAG GAT AAG AAT TTT GAA AGG ATT TTG TTA GAA CAA AAA CTA GTA ACC AAA AGC AAG GCG CCT ACA AAG ATT AAA AAA GAG AAA ACG CCT TCT GTT TCA GAA ACA AAA ACA GAA GAA GAA GAG AAT GCT CCT TCT TCC ACG AGT TCT TCT TCT ATT TTC GAC ATA AAG AAA GAA GAT AAA GAT GAG GGC GAA CTG AGT AAG ATT TCG AAC AAG TTT AAA AAA ATT AGC ACG ATT TTT GAC AAA ATG GGT TCA ACT TCC GCT ACA TCG AAG GAA AAT ACA CCA GAA CAG GAC GAT GTA GCC ACT AAA AAA AAT CAA ACA ACC GCT AAA AAA ACA GCT GTG AAA CCT AAA TTG GCC AAG AAG CCA GTC AGG AAA CAA CAA AAA GTT GTG GAA CTA TCT GGT GAA AGC GAC CTA GAA ATT TTA GAT TCA TAC ACT GAT CGG GAA GAT AGC AAT AAA GAT GAA GAT GAT GCT ATA CCA CAA CGA TCA AGG AGA CAA AGA TCG TCG AGA GCT GCG TCG GTT CCT AAG AAA TCT TAC GTT AAA ACT TTA GAA TTA TCT GAC GAC AGT TTT ATC GAA GAT GAT GAA GAG GAA AAC CAA GGA TCA GAT GTT TCG TTC AAT GAA GAG GAT TGA

Occurrences/Results 9 Pentamers

The pentamer 'ATTCA' occurred 7 times.
The pentamer 'TTCAA' occurred 9 times.

The pentamer 'TCAAG' occurred 6 times.
The pentamer 'CAAGA' occurred 16 times.
The pentamer 'AAGAT' occurred 26 times.
The pentamer 'AGATG' occurred 13 times.
The pentamer 'GATGA' occurred 17 times.
The pentamer 'ATGAA' occurred 19 times.
The pentamer 'TGAAT' occurred 9 times.
The total occurrences of all specified strings is 122.
The total number of characters in the text is 4283.
First result (Total characters *9 /1024): 37.6787109375
Square root of the first result: 6.138298700576569
Final result: 13.73691525545665

Occurrences/Results TRIMERS Codons

The pentamer 'ATT' occurred 61 times.
The pentamer 'CAA' occurred 38 times.
The pentamer 'GAT' occurred 67 times.
The pentamer 'GAA' occurred 90 times.
The pentamer 'CCA' occurred 20 times.
The pentamer 'ACT' occurred 36 times.
The pentamer 'AGA' occurred 35 times.
The pentamer 'TAC' occurred 24 times.
The total occurrences of all specified strings is 371.
The total number of characters in the text is 4287.
First result ((total_characters / 3) * (Trimer_number / 64)): 178.5
Square root of the first result: 13.36
Final result: 14.4

Occurrences/Results TRIMERS Combinations

The pentamer 'ATT ATT' occurred 2 times.
The pentamer 'ATT CAA' occurred 1 times.
The pentamer 'ATT GAT' occurred 1 times.
The pentamer 'ATT GAA' occurred 4 times.
The pentamer 'ATT CCA' occurred 1 times.
The pentamer 'ATT ACT' occurred 1 times.
The pentamer 'ATT AGA' occurred 0 times.
The pentamer 'ATT TAC' occurred 1 times.
The pentamer 'CAA ATT' occurred 4 times.
The pentamer 'CAA CAA' occurred 2 times.
The pentamer 'CAA GAT' occurred 1 times.
The pentamer 'CAA GAA' occurred 5 times.
The pentamer 'CAA CCA' occurred 0 times.
The pentamer 'CAA ACT' occurred 1 times.

The pentamer 'CAA AGA' occurred 2 times.
The pentamer 'CAA TAC' occurred 1 times.
The pentamer 'GAT ATT' occurred 0 times.
The pentamer 'GAT CAA' occurred 1 times.
The pentamer 'GAT GAT' occurred 5 times.
The pentamer 'GAT GAA' occurred 6 times.
The pentamer 'GAT CCA' occurred 2 times.
The pentamer 'GAT ACT' occurred 1 times.
The pentamer 'GAT AGA' occurred 0 times.
The pentamer 'GAT TAC' occurred 1 times.
The pentamer 'GAA ATT' occurred 6 times.
The pentamer 'GAA CAA' occurred 3 times.
The pentamer 'GAA GAT' occurred 9 times.
The pentamer 'GAA GAA' occurred 7 times.
The pentamer 'GAA CCA' occurred 0 times.
The pentamer 'GAA ACT' occurred 2 times.
The pentamer 'GAA AGA' occurred 2 times.
The pentamer 'GAA TAC' occurred 1 times.
The pentamer 'CCA ATT' occurred 2 times.
The pentamer 'CCA CAA' occurred 1 times.
The pentamer 'CCA GAT' occurred 0 times.
The pentamer 'CCA GAA' occurred 1 times.
The pentamer 'CCA CCA' occurred 0 times.
The pentamer 'CCA ACT' occurred 0 times.
The pentamer 'CCA AGA' occurred 0 times.
The pentamer 'CCA TAC' occurred 1 times.
The pentamer 'ACT ATT' occurred 4 times.
The pentamer 'ACT CAA' occurred 1 times.
The pentamer 'ACT GAT' occurred 2 times.
The pentamer 'ACT GAA' occurred 3 times.
The pentamer 'ACT CCA' occurred 0 times.
The pentamer 'ACT ACT' occurred 0 times.
The pentamer 'ACT AGA' occurred 0 times.
The pentamer 'ACT TAC' occurred 1 times.
The pentamer 'AGA ATT' occurred 1 times.
The pentamer 'AGA CAA' occurred 3 times.
The pentamer 'AGA GAT' occurred 1 times.
The pentamer 'AGA GAA' occurred 0 times.
The pentamer 'AGA CCA' occurred 1 times.
The pentamer 'AGA ACT' occurred 1 times.
The pentamer 'AGA AGA' occurred 1 times.
The pentamer 'AGA TAC' occurred 0 times.
The pentamer 'TAC ATT' occurred 2 times.

The pentamer 'TAC CAA' occurred 0 times.
The pentamer 'TAC GAT' occurred 1 times.
The pentamer 'TAC GAA' occurred 1 times.
The pentamer 'TAC CCA' occurred 0 times.
The pentamer 'TAC ACT' occurred 1 times.
The pentamer 'TAC AGA' occurred 1 times.
The pentamer 'TAC TAC' occurred 1 times.
The total occurrences of all specified strings is 104.
The total number of characters in the text is 4283.
First result (((total_characters/ 3) -1) / 64): 22.3125
Square root of the first result: 4.723610906922796
Final result: 17.29344385251567

>CAKOBR010000005.1:c394423-389879 Saccharomyces cerevisiae genome assembly, contig: chromosome_IV, helicase

---> Sequence

ATGACCACATCTCGTAAATCGCATGCGAAAGACAAAAAGGCTGGTGGTGAACAGGATCTGGCGGA
TTTGAAGTTTAGGTATGATCTTCTGACGAATGAACTGTTCCATTTAAGAGAGTTTGTTTCATTGG
TAGATTATGATCCAACCCATTTTAATGATTCTGAAAGTTTTCAGAAGTTTCTCAAGGAAACACAC
TTGTCGTTAGAGGAGAGAGGAGAAAACTTCACTGATGACGTAGCGAAGAAAGGCACGAATGGTG
ATCTTACTCGCAGAAGGCGAAACCTCAGGACATCGACAGTTGTTAGTTCAGAAACGACTAATGAG
AAAAAGGGTGATATAGAACTGAAGTTAGAAAGCATAGCACCATTAGTACGAAATAAATGTGAGG
AGCTAAAATACAAACTAAGTGATCATTCAAATAGGAAATCTATGGTACCGCAAAAAAGACCAAT
ACAGCATTTGAAGAAGAGAGAAGCAGCGAAGTCCTTGAAGTTCAAAAGTGAAAGAAAGGAAAAT
CCACTGCCATTGCATGAACATAAAGCTGAAGAGAGATATGATCACATCGCAAAAGTTGAAGAACC
CTCTGAAGCCTTCACTATAAAGTGTCCTTCTGATGATAGCTCCTTTGAAAATACTAGTGAACATT
ATAGTGATAATTTTTATTTCACCACATCATCCGAAGAGGAAGATATAAAGAAAAAAAGAGGTAG
AAAGAAAAAGAAGCCTAGAATAAAACTGGTAGTCCATCCTCCAAAGCAAACCATAACGAACCCGT
TGCATGTAGTCAAGCCGGAATACGAAAGCTTGCACGAATACATCGCTTCCTTTAAGTCATTAGAA
GACGATTTAACGCTGGAAGAATATAATAAATACATCGACGAACAAAGACGGCTTTTATCGAGACT
CAAAAAAGGTATTGAAAATGGCGCATTGAAGTATGACAAAGAAACGGACTCACTGCAACCTATA
ACATCCAAGGAAATCAAGACTATCATAACATATAAACCAGACCCGATTTCATATTTCTATAAACA
ACAAGATCTGCAAATCCATACCGATCACTTAATTAACCAAGGTATTCATATGAGTAAGCTGTTTA
GAAGTTCAACAAAGGCAAGAATAGCAAGAGCCAAAAAGGTTTCTCAAATGATAGAACAGCATTT
TAAACACGTAGCCGGAGCAGAAGAAAGAAAGGCAAAGGAGGAGGAGAGGCACAAAAAGTCGCTA
GCCAGGTTCGCAGTTCAAGCTGTTAAAAAAAGATGGAATATGGCTGAGAAGGCTTATAGGATTTT
GAGAAAAGATGAAGAAGAACAATTAAAAAGGATTGAAGGTAAGCAACACCTTTCTAAAATGTTG
GAGAAAAGTACACAATTGTTGGAAGCTCAGCTAAACCAAGCAAATGACGATGGGAGAAGCAGTA
CCCCGTCTTCTGACTCAAACGATGTATTGAGTGAAAGTGACGATGATATGGATGACGAATTATCA
ACATCTTCCGATGAAGACGAAGAGGTCGATGCTGATGTGGGGCTAGAAAATAGTCCAGCATCGAC
GGAAGCTACTCCAACAGACGAATCATTAAATCTGATACAATTGAAAGAAAAATACGAGCATTTTA

ATGGTTCATCGACAGTATATGACTCAAGAAATAAAGATGAGAAGTTTCCGGCATTGGATAAACAT
GAAAGTTCAAGTAGTGAAGGTTCTGTAATGACTGGAGAGGAGTCCTCCATATATTCATCCTCTGA
AAATGAAAGTCAAAACGAAAATGATCGAGAATCGGACGACAAGACTCCATCTGTGGGTTTAAGTG
CATTATTTGGTAAAGGTGAAGAATCGGATGGGGATTTAGACCTTGACGATAGTGAAGATTTTACG
GTTAATAGCTCATCTGTAGAAGGTGAAGAACTTGAAAAAGACCAAGTTGACAATTCAGCTGCTAC
ATTTGAAAGTGCAGGTGATTTTGTTCACACTCAAAATGAAAATAGGGACGATATAAAAGATGTC
GAGGAAGATGCTGAAACAAAAGTACAAGAAGAACAGTTATCAGTGGTGGATGTACCTGTGCCATC
TTTATTAAGAGGAAACTTAAGAACGTACCAGAAACAAGGTTTGAATTGGTTAGCCTCTCTTTATA
ACAATCACACAAATGGTATCCTGGCAGACGAGATGGGGTTAGGTAAAACTATTCAGACAATTTCT
CTGCTAGCATATTTAGCTTGTGAAAAGGAAAACTGGGGGCCACATCTTATTGTTGTTCCTACATC
AGTTCTGTTGAACTGGGAGATGGAATTCAAAAGGTTCGCGCCTGGGTTTAAGGTTTTAACATATT
ATGGTTCTCCCCAGCAACGTAAAGAAAAAAGGAAAGGTTGGAATAAGCCTGATGCTTTCCATGTT
TGTATTGTTTCTTACCAACTTGTAGTTCAAGACCAACATTCCTTTAAGAGAAAGAGATGGCAATA
TATGGTGCTGGATGAGGCACACAATATCAAAAACTTCAGATCTACAAGATGGCAGGCTCTATTGA
ATTTCAACACACAAAGAAGGCTTCTGTTGACTGGTACACCTTTACAGAATAACCTTGCTGAGCTG
TGGTCATTATTATATTTCTTAATGCCCCAGACTGTTATTGACGGGAAAAAGGTTTCTGGATTTGC
TGATCTTGATGCATTTCAACAATGGTTTGGACGCCCTGTCGATAAAATTATTGAAACGGGTCAGA
ATTTTGGACAGGACAAGGAAACGAAGAAGACAGTTGCCAAGTTGCATCAAGTTTTACGTCCATAC
CTCTTAAGAAGGTTAAAGGCCGATGTTGAGAAACAAATGCCAGCGAAGTATGAGCATATTGTTTA
TTGTAAGCTATCTAAAAGACAAAGGTTCTTATATGATGATTTTATGTCTAGAGCTCAAACGAAAG
CAACACTAGCCAGTGGAAATTTTATGTCAATTGTCAACTGTTTGATGCAATTGAGAAAGGTGTGT
AACCATCCCAATTTATTTGAAGTGAGGCCAATATTAACATCATTTGTCCTTGAACATTGTGTAGC
TTCCGACTACACGGATGTAGAAAGGACTGTGCTTAAATTGTTCAAAAAAAACAATCAAGTTAACC
GGGTTGACTTGGATTTCTTGAACTTGGTGTTTACGCTTAATGACAAAGATTTAACTTCTTATCAT
GCCGAGGAAATCTCAAAACTGACGTGTGTAAAAAACTTCGTTGAAGAAGTCAATAAATTGAGGG
AAACCAATAAACAGCTTCAGGAGGAGTTTGGAGAGGCGTCTTTTTTAAATTTTCAAGATGCAAAT
CAATATTTCAAGTATTCTAATAAGCAGAAACTTGAAGGGACAGTTGACATGCTAAACTTTCTAAA
GATGGTAAATAAACTTAGATGCGACAGAACACCCATATTCGGAAAGAACCTTATTGACCTATTAA
CGAAAGATAGAAGGGTAAAATATGATAAATCAAGCATTATTGATAATGAACTAATAAAACCCTT
GCAAACCAGGGTATTGGATAATAGAAAGATCATAGATACCTTTGCAGTGTTAACCCCAAGTGCCG
TCTCACTAGATATGAGAAAGTTGGCACTGGGATTAAATGATGACAGTTCGGTAGGCGAAAACACA
AGGCTAAAGGTAATGCAGAACTGTTTTGAAGTATCCAACCCATTACACCAACTGCAAACCAAATT
AACAATCGCATTCCCGGACAAATCGCTGCTTCAATACGATTGTGGTAAATTGCAGAAGTTAGCAA
TTTTATTACAGCAGCTGAAGGATAATGGACATAGGGCACTTATCTTCACACAAATGACAAAAGTT
TTGGACGTTCTGGAACAGTTTTTAAACTATCATGGTTATTTGTATATGAGGCTAGATGGTGCCAC
AAAGATCGAAGACCGTCAAATCCTAACAGAGAGATTCAACACTGACTCAAGAATAACGGTATTTA
TCCTATCAAGTAGATCAGGAGGGCTAGGTATCAATCTGACTGGGGCAGATACAGTTATTTTTTAC
GATTCAGATTGGAATCCAGCCATGGACAAACAGTGTCAGGATAGATGTCACAGAATTGGACAGAC
TCGAGATGTGCATATTTACAGATTCGTCAGCGAACACACCATAGAAAGCAATATCTTAAAAAAAG
CCAACCAAAAAAGACAGTTGGATAATGTTGTTATTCAAGAGGGTGACTTCACAACAGATTATTTC
AGCAAACTTTCGGTGAGAGATCTATTGGGATCCGAATTGCCCGAAAATGCTTCAGGTGGAGATAA
ACCACTAATAGCAGATGCAGATGTGGCAGCCAAAGATCCCAGACAACTGGAAAGACTTCTTGCGC
AAGCGGAAGATGAGGATGATGTTAAAGCTGCAAACTTAGCCATGAGAGAAGTCGAAATTGACAA

TGACGATTTCGACGAAAGCACAGAAAAAAAAGCAGCTAATGAAGAGGAAGAAAATCATGCTGAA
CTTGATGAGTATGAGGGCACTGCCCATGTTGACGAGTACATGATCAGGTTTATTGCCAACGGTTA
TTATTATTGA

---> Sequence Showing highlighted Existant Combined Trimers

ATG ACC ACA TCT CGT AAA TCG CAT GCG AAA GAC AAA AAG GCT GGT GGT GAA CAG GAT
CTG GCG GAT TTG AAG TTT AGG TAT GAT CTT CTG ACG AAT GAA CTG TTC CAT TTA AGA
GAG TTT GTT TCA TTG GTA GAT TAT GAT CCA ACC CAT TTT AAT GAT TCT GAA AGT TTT
CAG AAG TTT CTC AAG GAA ACA CAC TTG TCG TTA GAG GAG AGA GGA GAA AAC TTC ACT
GAT GAC GTA GCG AAG AAA GGC ACG AAT GGT GAT CTT ACT CGC AGA AGG CGA AAC CTC
AGG ACA TCG ACA GTT GTT AGT TCA GAA ACG ACT AAT GAG AAA AAG GGT GAT ATA GAA
CTG AAG TTA GAA AGC ATA GCA CCA TTA GTA CGA AAT AAA TGT GAG GAG CTA AAA TAC
AAA CTA AGT GAT CAT TCA AAT AGG AAA TCT ATG GTA CCG CAA AAA AGA CCA ATA CAG
CAT TTG AAG AAG AGA GAA GCA GCG AAG TCC TTG AAG TTC AAA AGT GAA AGA AAG GAA
AAT CCA CTG CCA TTG CAT GAA CAT AAA GCT GAA GAG AGA TAT GAT CAC ATC GCA AAA
GTT GAA GAA CCC TCT GAA GCC TTC ACT ATA AAG TGT CCT TCT GAT GAT AGC TCC TTT
GAA AAT ACT AGT GAA CAT TAT AGT GAT AAT TTT TAT TTC ACC ACA TCA TCC GAA GAG
GAA GAT ATA AAG AAA AAA AGA GGT AGA AAG AAA AAG AAG CCT AGA ATA AAA CTG GTA
GTC CAT CCT CCA AAG CAA ACC ATA ACG AAC CCG TTG CAT GTA GTC AAG CCG GAA TAC
GAA AGC TTG CAC GAA TAC ATC GCT TCC TTT AAG TCA TTA GAA GAC GAT TTA ACG CTG
GAA GAA TAT AAT AAA TAC ATC GAC GAA CAA AGA CGG CTT TTA TCG AGA CTC AAA AAA
GGT ATT GAA AAT GGC GCA TTG AAG TAT GAC AAA GAA ACG GAC TCA CTG CAA CCT ATA
ACA TCC AAG GAA ATC AAG ACT ATC ATA ACA TAT AAA CCA GAC CCG ATT TCA TAT TTC
TAT AAA CAA CAA GAT CTG CAA ATC CAT ACC GAT CAC TTA ATT AAC CAA GGT ATT CAT
ATG AGT AAG CTG TTT AGA AGT TCA ACA AAG GCA AGA ATA GCA AGA GCC AAA AAG GTT
TCT CAA ATG ATA GAA CAG CAT TTT AAA CAC GTA GCC GGA GCA GAA GAA AGA AAG GCA
AAG GAG GAG GAG AGG CAC AAA AAG TCG CTA GCC AGG TTC GCA GTT CAA GCT GTT AAA
AAA AGA TGG AAT ATG GCT GAG AAG GCT TAT AGG ATT TTG AGA AAA GAT GAA GAA GAA
CAA TTA AAA AGG ATT GAA GGT AAG CAA CAC CTT TCT AAA ATG TTG GAG AAA AGT ACA
CAA TTG TTG GAA GCT CAG CTA AAC CAA GCA AAT GAC GAT GGG AGA AGC AGT ACC CCG
TCT TCT GAC TCA AAC GAT GTA TTG AGT GAA AGT GAC GAT GAT ATG GAT GAC GAA TTA
TCA ACA TCT TCC GAT GAA GAC GAA GAG GTC GAT GCT GAT GTG GGG CTA GAA AAT AGT
CCA GCA TCG ACG GAA GCT ACT CCA ACA GAC GAA TCA TTA AAT CTG ATA CAA TTG AAA
GAA AAA TAC GAG CAT TTT AAT GGT TCA TCG ACA GTA TAT GAC TCA AGA AAT AAA GAT
GAG AAG TTT CCG GCA TTG GAT AAA CAT GAA AGT TCA AGT AGT GAA GGT TCT GTA ATG
ACT GGA GAG GAG TCC TCC ATA TAT TCA TCC TCT GAA AAT GAA AGT CAA AAC GAA AAT
GAT CGA GAA TCG GAC GAC AAG ACT CCA TCT GTG GGT TTA AGT GCA TTA TTT GGT AAA
GGT GAA GAA TCG GAT GGG GAT TTA GAC CTT GAC GAT AGT GAA GAT TTT ACG GTT AAT
AGC TCA TCT GTA GAA GGT GAA GAA CTT GAA AAA GAC CAA GTT GAC AAT TCA GCT GCT
ACA TTT GAA AGT GCA GGT GAT TTT GTT CAC ACT CAA AAT GAA AAT AGG GAC GAT ATA
AAA GAT GTC GAG GAA GAT GCT GAA ACA AAA GTA CAA GAA GAA CAG TTA TCA GTG GTG
GAT GTA CCT GTG CCA TCT TTA TTA AGA GGA AAC TTA AGA ACG TAC CAG AAA CAA GGT
TTG AAT TGG TTA GCC TCT CTT TAT AAC AAT CAC ACA AAT GGT ATC CTG GCA GAC GAG

ATG GGG TTA GGT AAA ACT ATT CAG ACA ATT TCT CTG CTA GCA TAT TTA GCT TGT GAA AAG GAA AAC TGG GGG CCA CAT CTT ATT GTT GTT CCT ACA TCA GTT CTG TTG AAC TGG GAG ATG GAA TTC AAA AGG TTC GCG CCT GGG TTT AAG GTT TTA ACA TAT TAT GGT TCT CCC CAG CAA CGT AAA GAA AAA AGG AAA GGT TGG AAT AAG CCT GAT GCT TTC CAT GTT TGT ATT GTT TCT TAC CAA CTT GTA GTT CAA GAC CAA CAT TCC TTT AAG AGA AAG AGA TGG CAA TAT ATG GTG CTG GAT GAG GCA CAC AAT ATC AAA AAC TTC AGA TCT ACA AGA TGG CAG GCT CTA TTG AAT TTC AAC ACA CAA AGA AGG CTT CTG TTG ACT GGT ACA CCT TTA CAG AAT AAC CTT GCT GAG CTG TGG TCA TTA TTA TAT TTC TTA ATG CCC CAG ACT GTT ATT GAC GGG AAA AAG GTT TCT GGA TTT GCT GAT CTT GAT GCA TTT CAA CAA TGG TTT GGA CGC CCT GTC GAT AAA ATT ATT GAA ACG GGT CAG AAT TTT GGA CAG GAC AAG GAA ACG AAG AAG ACA GTT GCC AAG TTG CAT CAA GTT TTA CGT CCA TAC CTC TTA AGA AGG TTA AAG GCC GAT GTT GAG AAA CAA ATG CCA GCG AAG TAT GAG CAT ATT GTT TAT TGT AAG CTA TCT AAA AGA CAA AGG TTC TTA TAT GAT GAT TTT ATG TCT AGA GCT CAA ACG AAA GCA ACA CTA GCC AGT GGA AAT TTT ATG TCA ATT GTC AAC TGT TTG ATG CAA TTG AGA AAG GTG TGT AAC CAT CCC AAT TTA TTT GAA GTG AGG CCA ATA TTA ACA TCA TTT GTC CTT GAA CAT TGT GTA GCT TCC GAC TAC ACG GAT GTA GAA AGG ACT GTG CTT AAA TTG TTC AAA AAA AAC AAT CAA GTT AAC CGG GTT GAC TTG GAT TTC TTG AAC TTG GTG TTT ACG CTT AAT GAC AAA GAT TTA ACT TCT TAT CAT GCC GAG GAA ATC TCA AAA CTG ACG TGT GTA AAA AAC TTC GTT GAA GAA GTC AAT AAA TTG AGG GAA ACC AAT AAA CAG CTT CAG GAG GAG TTT GGA GAG GCG TCT TTT TTA AAT TTT CAA GAT GCA AAT CAA TAT TTC AAG TAT TCT AAT AAG CAG AAA CTT GAA GGG ACA GTT GAC ATG CTA AAC TTT CTA AAG ATG GTA AAT AAA CTT AGA TGC GAC AGA ACA CCC ATA TTC GGA AAG AAC CTT ATT GAC CTA TTA ACG AAA GAT AGA AGG GTA AAA TAT GAT AAA TCA AGC ATT ATT GAT AAT GAA CTA ATA AAA CCC TTG CAA ACC AGG GTA TTG GAT AAT AGA AAG ATC ATA GAT ACC TTT GCA GTG TTA ACC CCA AGT GCC GTC TCA CTA GAT ATG AGA AAG TTG GCA CTG GGA TTA AAT GAT GAC AGT TCG GTA GGC GAA AAC ACA AGG CTA AAG GTA ATG CAG AAC TGT TTT GAA GTA TCC AAC CCA TTA CAC CAA CTG CAA ACC AAA TTA ACA ATC GCA TTC CCG GAC AAA TCG CTG CTT CAA TAC GAT TGT GGT AAA TTG CAG AAG TTA GCA ATT TTA TTA CAG CAG CTG AAG GAT AAT GGA CAT AGG GCA CTT ATC TTC ACA CAA ATG ACA AAA GTT TTG GAC GTT CTG GAA CAG TTT TTA AAC TAT CAT GGT TAT TTG TAT ATG AGG CTA GAT GGT GCC ACA AAG ATC GAA GAC CGT CAA ATC CTA ACA GAG AGA TTC AAC ACT GAC TCA AGA ATA ACG GTA TTT ATC CTA TCA AGT AGA TCA GGA GGG CTA GGT ATC AAT CTG ACT GGG GCA GAT ACA GTT ATT TTT TAC GAT TCA GAT TGG AAT CCA GCC ATG GAC AAA CAG TGT CAG GAT AGA TGT CAC AGA ATT GGA CAG ACT CGA GAT GTG CAT ATT TAC AGA TTC GTC AGC GAA CAC ACC ATA GAA AGC AAT ATC TTA AAA AAA GCC AAC CAA AAA AGA CAG TTG GAT AAT GTT GTT ATT CAA GAG GGT GAC TTC ACA ACA GAT TAT TTC AGC AAA CTT TCG GTG AGA GAT CTA TTG GGA TCC GAA TTG CCC GAA AAT GCT TCA GGT GGA GAT AAA CCA CTA ATA GCA GAT GCA GAT GTG GCA GCC AAA GAT CCC AGA CAA CTG GAA AGA CTT CTT GCG CAA GCG GAA GAT GAG GAT GAT GTT AAA GCT GCA AAC TTA GCC ATG AGA GAA GTC GAA ATT GAC AAT GAC GAT TTC GAC GAA AGC ACA GAA AAA AAA GCA GCT AAT GAA GAG GAA GAA AAT CAT GCT GAA CTT GAT GAG TAT GAG GGC ACT GCC CAT GTT GAC GAG TAC ATG ATC AGG TTT ATT GCC AAC GGT TAT TAT TAT TGA

Occurrences/Results 9 Pentamers

The pentamer 'ATTCA' occurred 9 times.
The pentamer 'TTCAA' occurred 15 times.
The pentamer 'TCAAG' occurred 15 times.
The pentamer 'CAAGA' occurred 12 times.
The pentamer 'AAGAT' occurred 17 times.
The pentamer 'AGATG' occurred 18 times.
The pentamer 'GATGA' occurred 13 times.
The pentamer 'ATGAA' occurred 9 times.
The pentamer 'TGAAT' occurred 2 times.
The total occurrences of all specified strings is 110.
The total number of characters in the text is 4541.
First result (Total characters *9 /1024): 39.9462890625
Square root of the first result: 6.32030767150619
Final result: 11.083908344102104

Occurrences/Results TRIMERS Codons

The pentamer 'ATT' occurred 25 times.
The pentamer 'CAA' occurred 46 times.
The pentamer 'GAT' occurred 78 times.
The pentamer 'GAA' occurred 98 times.
The pentamer 'CCA' occurred 19 times.
The pentamer 'ACT' occurred 19 times.
The pentamer 'AGA' occurred 47 times.
The pentamer 'TAC' occurred 13 times.
The total occurrences of all specified strings is 345.
The total number of characters in the text is 4541.
First result ((total_characters / 3) * (Trimer_number / 64)): 189.2
Square root of the first result: 13.76
Final result: 11.33

Occurrences/Results TRIMERS Combinations

The pentamer 'ATT ATT' occurred 2 times.
The pentamer 'ATT CAA' occurred 1 times.
The pentamer 'ATT GAT' occurred 1 times.
The pentamer 'ATT GAA' occurred 3 times.
The pentamer 'ATT CCA' occurred 0 times.
The pentamer 'ATT ACT' occurred 0 times.
The pentamer 'ATT AGA' occurred 0 times.
The pentamer 'ATT TAC' occurred 1 times.
The pentamer 'CAA ATT' occurred 0 times.
The pentamer 'CAA CAA' occurred 2 times.
The pentamer 'CAA GAT' occurred 2 times.

The pentamer 'CAA GAA' occurred 1 times.
The pentamer 'CAA CCA' occurred 0 times.
The pentamer 'CAA ACT' occurred 0 times.
The pentamer 'CAA AGA' occurred 2 times.
The pentamer 'CAA TAC' occurred 1 times.
The pentamer 'GAT ATT' occurred 0 times.
The pentamer 'GAT CAA' occurred 0 times.
The pentamer 'GAT GAT' occurred 4 times.
The pentamer 'GAT GAA' occurred 2 times.
The pentamer 'GAT CCA' occurred 1 times.
The pentamer 'GAT ACT' occurred 0 times.
The pentamer 'GAT AGA' occurred 2 times.
The pentamer 'GAT TAC' occurred 0 times.
The pentamer 'GAA ATT' occurred 1 times.
The pentamer 'GAA CAA' occurred 2 times.
The pentamer 'GAA GAT' occurred 4 times.
The pentamer 'GAA GAA' occurred 9 times.
The pentamer 'GAA CCA' occurred 0 times.
The pentamer 'GAA ACT' occurred 0 times.
The pentamer 'GAA AGA' occurred 3 times.
The pentamer 'GAA TAC' occurred 2 times.
The pentamer 'CCA ATT' occurred 0 times.
The pentamer 'CCA CAA' occurred 0 times.
The pentamer 'CCA GAT' occurred 0 times.
The pentamer 'CCA GAA' occurred 0 times.
The pentamer 'CCA CCA' occurred 0 times.
The pentamer 'CCA ACT' occurred 0 times.
The pentamer 'CCA AGA' occurred 0 times.
The pentamer 'CCA TAC' occurred 1 times.
The pentamer 'ACT ATT' occurred 1 times.
The pentamer 'ACT CAA' occurred 1 times.
The pentamer 'ACT GAT' occurred 1 times.
The pentamer 'ACT GAA' occurred 0 times.
The pentamer 'ACT CCA' occurred 2 times.
The pentamer 'ACT ACT' occurred 0 times.
The pentamer 'ACT AGA' occurred 0 times.
The pentamer 'ACT TAC' occurred 0 times.
The pentamer 'AGA ATT' occurred 1 times.
The pentamer 'AGA CAA' occurred 2 times.
The pentamer 'AGA GAT' occurred 1 times.
The pentamer 'AGA GAA' occurred 2 times.
The pentamer 'AGA CCA' occurred 1 times.
The pentamer 'AGA ACT' occurred 0 times.

The pentamer 'AGA AGA' occurred 0 times.
The pentamer 'AGA TAC' occurred 0 times.
The pentamer 'TAC ATT' occurred 0 times.
The pentamer 'TAC CAA' occurred 1 times.
The pentamer 'TAC GAT' occurred 2 times.
The pentamer 'TAC GAA' occurred 1 times.
The pentamer 'TAC CCA' occurred 0 times.
The pentamer 'TAC ACT' occurred 0 times.
The pentamer 'TAC AGA' occurred 1 times.
The pentamer 'TAC TAC' occurred 0 times.
The total occurrences of all specified strings is 64.
The total number of characters in the text is 4541.
First result (((total_characters/ 3) -1) / 64): 23.65625
Square root of the first result: 4.86376911458593
Final result: 8.294750233725807

>Saccharomyces cerevisiae S288C, translocase ULS1 (ULS1), partial mRNA

---> Sequence

ATGGCAGCAGTACCCACCATCGACCTTACACTAGCCGATTCAGATAATGAAGACATATTCCATTC
TTTTTCGTCTTCAACAAGTGTGGATAAGATAGACATTAGGAAAGAAAATGGGAAATTAAGAATG
GCAGGCTTGGAAGTCGCCCAATCTAATGACGATGCAGCACGTCAAGCATTCCATGTTTTCAAGAC
GAACATATCAAATAATGAAACCTTCGATACTATTCTTTCAAAGAGCAAGACAATCACAGACAGTA
CCTTCAACAATGAAAAATCTTCCAACGAAGTAAAACAGCAGCAAGTCTTGAAGGAAGAAACGATG
GGATCTTCCAACGATGAGAAGAAAACACAAGAGAGCAGCCCAAGCGCAGAGATGATAAAACTTTT
TTATGAAAACGATGATGTGCCGTTATCCGATAGTTTTAAACAAAAAGAGGAAGGTAAAAGAATA
AATCAGGATGAGCAAGTGAAGGAAAATATCTGTGGCATTTCTTCCTCATACGTCTCCAAAGACTA
CGATGGCGTTGAAGATGATTTCGAGCCTAATACTTGCCAGGACAGCAATTTGGATTTTCAAGAGG
AGAAGTTAAACTTGAATAACAAACCGTCACAGCAACAGTTCAGCGACCCAGAAACAAAAGATAAC
AGTTTGAAGAGCGAAAACAAAGACCAAATAAAAGGAGTCACAACAACAAGTTATCGTGATTTAC
CTATTGAATCTAGTGCTTTCCAGGATTCTGAAACTCAAAATAACTCCAAAAACACTATACCTAAC
ATCGTAAATGAAAAGCGAACGCCAGCATTACCGTCCAATCTAAGCTCTGTTGAGAGTTCCCTTAA
AAACGAGACGGCCAAAGTAGAGGGGAAGACGACCGTTCGTCTTCCAGGACTTCAAAATAATGTTG
CCTTACTTGAGCAAGAACAAAGTGAACTATTCAAGCACTTTAGTGAACAACCTGTCGATATTAGC
GATTTTGGTCGTAAGATTAAAAGGAAGCATAGCGGAGATTTTGCAGATAACAAGATACTTAAAA
GACCGATATTGCCCTCAAAAAATATGGACCACACAACACATAATTCACATGATTCCGAACAGAAA
AATAGCTCGATTATTATTCTTTCAGATGAGGACGAATCTGGTGCCGGTATCAATGATATAGAAAG
TCCTTTGAAAGTTTCTGAACCAAATACAGCTGATGCGCTTAGGAGCAGTGTACCAGAAGTCATAT
CTTTACTTGACCTACCCAATATTGACTTGAATAACTCAGTAATAAAAGAAGCAAGTGGCAGCAAT
TCAATTCCTACATCAGAAACAGACGCACAATCCTCGAGTTCGTCAGTTCTTCAAGGAACTATCAT

GACAGAACAAGCTACTCAATCCTCTCAACACGAATGTAACAGTTCACTTGATACTCTAAAAAAAA
ATCATCAAAAATTATTGAAGGATTTGAATTCTAGAGAGTCTGAACTACGTAATGCTCTCAGTTGC
TGCAAGACAAACTCTGAAATTTTAAGAAGGAAACTAAGTAGAAGAGAAAAGGAGGTCTCTGATG
CTGAAAAACATTGGCAGTTATTGTTGACATCGATGGCTAGAGGGGGAAGAACTATCAGCTCAACT
CAGCAGATATTAGTTGATGAAGCTGAGAACCAATTAAACAAACTGAAGGAAAAAGACAGTTGA
CAAAATCGAAGCTAGATTCAATCAATTTGAAAATGTATAATTATAACGAACAATGGAAGTCCTTT
GTGCATTCGAAAAATATAAACCTGCAGAAATCTTTGGCTGCCCTTGAGCGGTCAGCCAGGGATAG
TAAAGCTTCTGCGACCGTAAATAAAAGAAACGAATGCCTGGCTGAGAAAGAAAAGCTAGACCAAA
TGTTAAAAGAAGGAACCCTAAGCTTCAGTACCTACAAGCAGTTAACAGGGGAGATCCAGCAGAAA
CTGAATGACTTGAAACTGGGGGACCAACGCACAACTGATATCAATAGTGTATTGCCAATAGTACG
GCAACCTCTCGCCAAAAGAGATTTATTTATTAAGTCCATTGATACTGCAAAAGATTTATTAGCCA
AGAACACATCCAGGACAGAAATGACGAAGAGAATTCTTTATAGACATTTGGACAACTTGGTCTCT
TATAAAAACTTTTTTGAAGACGGAAGATCCTTAATAGATATAAACAGAAGACACGTTGCACATGA
ATCGGCCCAAATATTGTTCACCAACGGTGTAAAGATGCCCATTGTTTTTGAAACATTACAAGATT
ACGGCATCAAGTTTTCTAATCCAGCCATTGTCAATCCTGACAGAAGAGCCCAATATTTTAAAAGC
ATCGAAGTTGCACGCGATCTCATATCAAAGTCTACTAGATCAGAGGATGCTAAACGAAAGATTAC
TCGATTTTTGAATATAATTGAAGAATTTCGTAAAGATATTGACACCGGATTTCCACCTACTCCTT
TAAAAAGGGAAGGCGTCGGAAAAGCTGTTGTTGGGCTAAGACAACAAGGACTTAAGATGGACAG
ACTATATGAAAATTTGAGAAGATATAAGATTCCAATAACAAGTGAAGAATTACTGCAGCAAAGC
TATTTATTTCCGGTAAACGCAGATCAACGACCTCCTTCAAACTGGAACATCGTGGAAAATACCGA
AGATACTAGTTCTACAGCCAATGATCTATCGATGCAAGATGAATTTCACATCTCTAATATGCATG
CAGCAGAAGATCAAGAACAGATTAGGGCTCTTTTAGAGAATGTCAAACAATCTGAATCCATTATA
GACGGCGAAGCACTTACTCCCGAGGATATGACCGTAAACTTATTAAAACACCAGAGGCTGGGTCT
GCATTGGTTGTTACAAGTTGAAAACTCCGCAAAAAAAGGGGGTCTGCTTGCTGACGATATGGGCC
TGGGTAAAACGATTCAAGCTATTGCATTGATGTTAGCAAATCGCTCAGAGGAATCCAAATGTAAA
ACCAATTTGATCGTCGCACCAGTCTCAGTCTTGAGAGTTTGGAAAGGTGAATTAGAAACGAAAGT
AAAAAAACGCGCAAAATTTACTACATTTATTTTTGGAGGATCAGGTAACGGAAAAGTTAAGCACT
GGAGGGATTTGGCAAGATATGATGCTGTTTTGGTATCTTATCAAACTCTAGCAAACGAATTTAAG
AAACATTGGCCGAAAAAACTGGATGGTGAACAAAATCAGCTACCAGCTGTGCCTCATATACAAGC
TCTTAATAGGTTGAAAACCTCAAATGAATACTACTCTCCATTTTTCTGCAATGATTCAACGTTTT
ACAGAATTTTACTGGACGAAGGCCAAAACATCAAGAACAAAAATACAAGGGCTTCTAAAGCGTGC
TGTACTATAAATGGAATGTATAGGTGGGTCTTGTCTGGTACGCCGATTCAAAATAGTATGGATGA
GCTTTATTCCTTGATAAGATTTCTAAGAATTCCACCCTACCACAAGGAGCAACGGTTTAAATTAG
ACATTGGAAGGTTTTTTCAGAGAAATAAGCAATATCAATATGATAACGAGGATAGAAAAAACGC
ATTAAGAAAGGTTAGAGTCCTTCTAAATGCCATTATGCTTCGTCGGTCTAAGGCTGATAAGATCG
ACGGCAAACCTTTACTGGAGCTCCCTCCTAAAATTGTGGAAGTAGATGAATCACGCCTGAAGGGA
GAGGAATTAAAATTTTACACAGCTTTGGAATCAAAAAATCAAGCACTCGCTAAGAAATTGTTGAA
CAATTCTACGAGGGGGAGCTACTCTAGCGTTCTCACATTACTACTTCGCTTAAGGCAGGCTTGTT
GTCATTCAGAATTAGTTGTGATGGGAGAAAAAAAAGCGGAAGGTACAAAGGTAGCTAACGGCAA
AAGCTTTGAAGATGACTGGCTCAGGCTCTACTATAAAATCACTCACATGAGCGGAGAAGCGCAGG
CTCAGGTTATTACTTCAATGAATTCTATGACATGTTTCTGGTGCATGGAACAATTAGAACCAGAA
GCGATGTCCGTTCTGACAGGTTGTGGTCATTTGATTTGCGACACTTGTATCGAGCCCTTCATCGA
AGAGTCATCCATGCTACCTCAGGCCAAGAAAACCAAAGGTGGGGCATTTGCTATCCCATGCAAAG

ATTGTCAACGTTTGACAAACGAGAAAGATATTGTTTCGCACAAGTTATACGACCAGGTAATAAAC
CAGGGATTTACTGAGGAAGATTTGCATGCAGAGTATTTAAGTGAAATGGAGAAACAAAAAATAC
AGCAAAAAAATGTATATGTGCCAAATTTTGAGAGTTTAGAACCATCTACGAAGATAGAGCAATG
TATACAGGTTATTCAAAGAGTTTTTGATGAATCAGCAACCGAAAAAATTATTATTTTTTCCCAGT
TTACAACATTTTTTGAGATTCTCGAACATTTTCTCAAAAATAAACTGAATTTTCCCTACTTGAAA
TATATTGGATCAATGAACGCTCAGAGAAGATCAGATGTTATCAACGAATTTTATCGTGACCCTGA
GAAAAGGATACTTCTGATTTCCATGAAAGCCGGTAACTCAGGACTAACATTGACATGTGCGAACC
ATGTAGTTATAGTTGATCCATTTTGGAATCCTTACGTCGAAGAACAAGCTCAAGACCGTTGCTAT
AGAATTAGTCAAACAAAAAAAGTCCAGGTGCATAAATTGTTTATAAAGGACTCTGTTGAGGATC
GTATTTCTGAATTACAGAAGAGAAAGAAGGAAATGGTCGATTCTGCCATGGACCCAGGCAAGATA
AAGGAAGTGAATAGTTTAGGGCGTCGGGAGTTAGGATTTTTATTTGGTTTAAACGCTCTTTGA

---> Sequence Showing highlighted Existant Combined Trimers

ATG GCA GCA GTA CCC ACC ATC GAC CTT ACA CTA GCC GAT TCA GAT AAT GAA GAC ATA
TTC CAT TCT TTT TCG TCT TCA ACA AGT GTG GAT AAG ATA GAC ATT AGG AAA GAA AAT
GGG AAA TTA AGA ATG GCA GGC TTG GAA GTC GCC CAA TCT AAT GAC GAT GCA GCA CGT
CAA GCA TTC CAT GTT TTC AAG ACG AAC ATA TCA AAT AAT GAA ACC TTC GAT ACT **ATT**
CTT TCA AAG AGC AAG ACA ATC ACA GAC AGT ACC TTC AAC AAT GAA AAA TCT TCC AAC
GAA GTA AAA CAG CAG CAA GTC TTG AAG GAA GAA ACG ATG GGA TCT TCC AAC GAT GAG
AAG AAA ACA CAA GAG AGC AGC CCA AGC GCA GAG ATG ATA AAA CTT TTT TAT GAA AAC
GAT GAT GTG CCG TTA TCC GAT AGT TTT AAA CAA AAA GAG GAA GGT AAA AGA ATA AAT
CAG GAT GAG CAA GTG AAG GAA AAT ATC TGT GGC ATT TCT TCC TCA TAC GTC TCC AAA
GAC TAC GAT GGC GTT GAA GAT GAT TTC GAG CCT AAT ACT TGC CAG GAC AGC AAT TTG
GAT TTT CAA GAG GAG AAG TTA AAC TTG AAT AAC AAA CCG TCA CAG CAA CAG TTC AGC
GAC CCA GAA ACA AAA GAT AAC AGT TTG AAG AGC GAA AAC AAA GAC CAA ATA AAA GGA
GTC ACA ACA ACA AGT TAT CGT GAT TTA CCT ATT GAA TCT AGT GCT TTC CAG GAT TCT
GAA ACT CAA AAT AAC TCC AAA AAC ACT ATA CCT AAC ATC GTA AAT GAA AAG CGA ACG
CCA GCA TTA CCG TCC AAT CTA AGC TCT GTT GAG AGT TCC CTT AAA AAC GAG ACG GCC
AAA GTA GAG GGG AAG ACG ACC GTT CGT CTT CCA GGA CTT CAA AAT AAT GTT GCC TTA
CTT GAG CAA GAA CAA AGT GAA CTA TTC AAG CAC TTT AGT GAA CAA CCT GTC GAT ATT
AGC GAT TTT GGT CGT AAG ATT AAA AGG AAG CAT AGC GGA GAT TTT GCA GAT AAC AAG
ATA CTT AAA AGA CCG ATA TTG CCC TCA AAA AAT ATG GAC CAC ACA ACA CAT AAT TCA
CAT GAT TCC GAA CAG AAA AAT AGC TCG ATT ATT ATT CTT TCA GAT GAG GAC GAA TCT
GGT GCC GGT ATC AAT GAT ATA GAA AGT CCT TTG AAA GTT TCT GAA CCA AAT ACA GCT
GAT GCG CTT AGG AGC AGT GTA CCA GAA GTC ATA TCT TTA CTT GAC CTA CCC AAT ATT
GAC TTG AAT AAC TCA GTA ATA AAA GAA GCA AGT GGC AGC AAT TCA ATT CCT ACA TCA
GAA ACA GAC GCA CAA TCC TCG AGT TCG TCA GTT CTT CAA GGA ACT ATC ATG ACA GAA
CAA GCT ACT CAA TCC TCT CAA CAC GAA TGT AAC AGT TCA CTT GAT ACT CTA AAA AAA
AAT CAT CAA AAA TTA TTG AAG GAT TTG AAT TCT AGA GAG TCT GAA CTA CGT AAT GCT
CTC AGT TGC TGC AAG ACA AAC TCT GAA ATT TTA AGA AGG AAA CTA AGT AGA AGA GAA
AAG GAG GTC TCT GAT GCT GAA AAA CAT TGG CAG TTA TTG TTG ACA TCG ATG GCT AGA
GGG GGA AGA ACT ATC AGC TCA ACT CAG CAG ATA TTA GTT GAT GAA GCT GAG AAC CAA
TTA AAC AAA CTG AAG GAA AAA AGA CAG TTG ACA AAA TCG AAG CTA GAT TCA ATC AAT

TTG AAA ATG TAT AAT TAT AAC GAA CAA TGG AAG TCC TTT GTG CAT TCG AAA AAT ATA AAC CTG CAG AAA TCT TTG GCT GCC CTT GAG CGG TCA GCC AGG GAT AGT AAA GCT TCT GCG ACC GTA AAT AAA AGA AAC GAA TGC CTG GCT GAG AAA GAA AAG CTA GAC CAA ATG TTA AAA GAA GGA ACC CTA AGC TTC AGT ACC TAC AAG CAG TTA ACA GGG GAG ATC CAG CAG AAA CTG AAT GAC TTG AAA CTG GGG GAC CAA CGC ACA ACT GAT ATC AAT AGT GTA TTG CCA ATA GTA CGG CAA CCT CTC GCC AAA AGA GAT TTA TTT ATT AAG TCC ATT GAT ACT GCA AAA GAT TTA TTA GCC AAG AAC ACA TCC AGG ACA GAA ATG ACG AAG AGA ATT CTT TAT AGA CAT TTG GAC AAC TTG GTC TCT TAT AAA AAC TTT TTT GAA GAC GGA AGA TCC TTA ATA GAT ATA AAC AGA AGA CAC GTT GCA CAT GAA TCG GCC CAA ATA TTG TTC ACC AAC GGT GTA AAG ATG CCC ATT GTT TTT GAA ACA TTA CAA GAT TAC GGC ATC AAG TTT TCT AAT CCA GCC ATT GTC AAT CCT GAC AGA AGA GCC CAA TAT TTT AAA AGC ATC GAA GTT GCA CGC GAT CTC ATA TCA AAG TCT ACT AGA TCA GAG GAT GCT AAA CGA AAG ATT ACT CGA TTT TTG AAT ATA ATT GAA GAA TTT CGT AAA GAT ATT GAC ACC GGA TTT CCA CCT ACT CCT TTA AAA AGG GAA GGC GTC GGA AAA GCT GTT GTT GGG CTA CAA CAA GGA CTT AAG ATG GAC AGA CTA TAT GAA AAT TTG AGA AGA TAT AAG ATT CCA ATA ACA AGT GAA GAA TTA CTG CAG CAA AGC TAT TTA TTT CCG GTA AAC GCA GAT CAA CGA CCT CCT TCA AAC TGG AAC ATC GTG GAA AAT ACC GAA GAT ACT AGT TCT ACA GCC AAT GAT CTA TCG ATG CAA GAT GAA TTT CAC ATC TCT AAT ATG CAT GCA GCA GAA GAT CAA GAA CAG ATT AGG GCT CTT TTA GAG AAT GTC AAA CAA TCT GAA TCC ATT ATA GAC GGC GAA GCA CTT ACT CCC GAG GAT ATG ACC GTA AAC TTA TTA AAA CAC CAG AGG CTG GGT CTG CAT TGG TTG TTA CAA GTT GAA AAC TCC GCA AAA AAA GGG GGT CTG CTT GCT GAC GAT ATG GGC CTG GGT AAA ACG ATT CAA GCT ATT GCA TTG ATG TTA GCA AAT CGC TCA GAG GAA TCC AAA TGT AAA ACC AAT TTG ATC GTC GCA CCA GTC TCA GTC TTG AGA GTT TGG AAA GGT GAA TTA GAA ACG AAA GTA AAA AAA CGC GCA AAA TTT ACT ACA TTT ATT TTT GGA GGA TCA GGT AAC GGA AAA GTT AAG CAC TGG AGG GAT TTG GCA AGA TAT GAT GCT GTT TTG GTA TCT TAT CAA ACT CTA GCA AAC GAA TTT AAG AAA CAT TGG CCG AAA AAA CTG GAT GGT GAA CAA AAT CAG CTA CCA GCT GTG CCT CAT ATA CAA GCT CTT AAT AGG TTG AAA ACC TCA AAT GAA TAC **TAC** TCT CCA TTT TTC TGC AAT GAT TCA ACG TTT **TAC** AGA ATT TTA CTG GAC GAA GGC CAA AAC ATC AAG AAC AAA AAT ACA AGG GCT TCT AAA GCG TGC TGT ACT ATA AAT GGA ATG TAT AGG TGG GTC TTG TCT GGT ACG CCG ATT CAA AAT AGT ATG GAT GAG CTT TAT TCC TTG ATA AGA TTT CTA AGA ATT CCA CCC TAC CAC AAG GAG CAA CGG TTT AAA TTA GAC ATT GGA AGG TTT TTT CAG AGA AAT AAG CAA TAT CAA TAT GAT AAC GAG GAT AGA AAA AAC GCA TTA AGA AAG GTT AGA GTC CTT CTA AAT GCC ATT ATG CTT CGT CGG TCT AAG GCT GAT AAG ATC GAC GGC AAA CCT TTA CTG GAG CTC CCT CCT AAA ATT GTG GAA GTA GAT GAA TCA CGC CTG AAG GGA GAG GAA TTA AAA TTT TAC ACA GCT TTG GAA TCA AAA AAT CAA GCA CTC GCT AAG AAA TTG TTG AAC AAT TCT ACG AGG GGG AGC TAC TCT AGC GTT CTC ACA TTA CTA CTT CGC TTA AGG CAG GCT TGT TGT CAT TCA GAA TTA GTT GTG ATG GGA GAA AAA AAA GCG GAA GGT ACA AAG GTA GCT AAC GGC AAA AGC TTT GAA GAT GAC TGG CTC AGG CTC TAC TAT AAA ATC ACT CAC ATG AGC GGA GAA GCG CAG GCT CAG GTT ATT ACT TCA ATG AAT TCT ATG ACA TGT TTC TGG TGC ATG GAA CAA TTA GAA CCA GAA GCG ATG TCC GTT CTG ACA GGT TGT GGT CAT TTG ATT TGC GAC ACT TGT ATC GAG CCC TTC ATC GAA GAG TCA TCC ATG CTA CCT CAG GCC AAG AAA ACC AAA GGT GGG GCA TTT GCT ATC CCA TGC AAA GAT TGT CAA CGT TTG

ACA AAC GAG AAA GAT ATT GTT TCG CAC AAG TTA TAC GAC CAG GTA ATA AAC CAG GGA TTT ACT GAG GAA GAT TTG CAT GCA GAG TAT TTA AGT GAA ATG GAG AAA CAA AAA ATA CAG CAA AAA AAT GTA TAT GTG CCA AAT TTT GAG AGT TTA GAA CCA TCT ACG AAG ATA GAG CAA TGT ATA CAG GTT ATT CAA AGA GTT TTT GAT GAA TCA GCA ACC GAA AAA ATT ATT ATT TTT TCC CAG TTT ACA ACA TTT TTT GAG ATT CTC GAA CAT TTT CTC AAA AAT AAA CTG AAT TTT CCC TAC TTG AAA TAT ATT GGA TCA ATG AAC GCT CAG AGA AGA TCA GAT GTT ATC AAC GAA TTT TAT CGT GAC CCT GAG AAA AGG ATA CTT CTG ATT TCC ATG AAA GCC GGT AAC TCA GGA CTA ACA TTG ACA TGT GCG AAC CAT GTA GTT ATA GTT GAT CCA TTT TGG AAT CCT TAC GTC GAA GAA CAA GCT CAA GAC CGT TGC TAT AGA ATT AGT CAA ACA AAA AAA GTC CAG GTG CAT AAA TTG TTT ATA AAG GAC TCT GTT GAG GAT CGT ATT TCT GAA TTA CAG AAG AGA AAG AAG GAA ATG GTC GAT TCT GCC ATG GAC CCA GGC AAG ATA AAG GAA GTG AAT AGT TTA GGG CGT CGG GAG TTA GGA TTT TTA TTT GGT TTA AAC GCT CTT TGA

Occurrences/Results 9 Pentamers

The pentamer 'ATTCA' occurred 10 times.
The pentamer 'TTCAA' occurred 16 times.
The pentamer 'TCAAG' occurred 11 times.
The pentamer 'CAAGA' occurred 16 times.
The pentamer 'AAGAT' occurred 27 times.
The pentamer 'AGATG' occurred 9 times.
The pentamer 'GATGA' occurred 12 times.
The pentamer 'ATGAA' occurred 15 times.
The pentamer 'TGAAT' occurred 17 times.
The total occurrences of all specified strings is 133.
The total number of characters in the text is 4856.
First result (Total characters *9 /1024): 42.71484375
Square root of the first result: 6.535659396725016
Final result: 13.814238284088274

Occurrences/Results TRIMERS Codons

The pentamer 'ATT' occurred 44 times.
The pentamer 'CAA' occurred 55 times.
The pentamer 'GAT' occurred 65 times.
The pentamer 'GAA' occurred 90 times.
The pentamer 'CCA' occurred 20 times.
The pentamer 'ACT' occurred 23 times.
The pentamer 'AGA' occurred 38 times.
The pentamer 'TAC' occurred 14 times.
The total occurrences of all specified strings is 349.
The total number of characters in the text is 4857.
First result ((total_characters/3) * (Trimer_number / 64)): 202.4
Square root of the first result: 14.22
Final result: 10.3

Occurrences/Results TRIMERS Combinations

The pentamer 'ATT ATT' occurred 2 times.
The pentamer 'ATT CAA' occurred 3 times.
The pentamer 'ATT GAT' occurred 1 times.
The pentamer 'ATT GAA' occurred 2 times.
The pentamer 'ATT CCA' occurred 2 times.
The pentamer 'ATT ACT' occurred 2 times.
The pentamer 'ATT AGA' occurred 0 times.
The pentamer 'ATT TAC' occurred 0 times.
The pentamer 'CAA ATT' occurred 0 times.
The pentamer 'CAA CAA' occurred 1 times.
The pentamer 'CAA GAT' occurred 2 times.
The pentamer 'CAA GAA' occurred 2 times.
The pentamer 'CAA CCA' occurred 0 times.
The pentamer 'CAA ACT' occurred 1 times.
The pentamer 'CAA AGA' occurred 1 times.
The pentamer 'CAA TAC' occurred 0 times.
The pentamer 'GAT ATT' occurred 3 times.
The pentamer 'GAT CAA' occurred 2 times.
The pentamer 'GAT GAT' occurred 2 times.
The pentamer 'GAT GAA' occurred 4 times.
The pentamer 'GAT CCA' occurred 1 times.
The pentamer 'GAT ACT' occurred 4 times.
The pentamer 'GAT AGA' occurred 1 times.
The pentamer 'GAT TAC' occurred 1 times.
The pentamer 'GAA ATT' occurred 1 times.
The pentamer 'GAA CAA' occurred 7 times.
The pentamer 'GAA GAT' occurred 5 times.
The pentamer 'GAA GAA' occurred 4 times.
The pentamer 'GAA CCA' occurred 3 times.
The pentamer 'GAA ACT' occurred 1 times.
The pentamer 'GAA AGA' occurred 0 times.
The pentamer 'GAA TAC' occurred 1 times.
The pentamer 'CCA ATT' occurred 0 times.
The pentamer 'CCA CAA' occurred 0 times.
The pentamer 'CCA GAT' occurred 0 times.
The pentamer 'CCA GAA' occurred 3 times.
The pentamer 'CCA CCA' occurred 0 times.
The pentamer 'CCA ACT' occurred 0 times.
The pentamer 'CCA AGA' occurred 0 times.
The pentamer 'CCA TAC' occurred 0 times.
The pentamer 'ACT ATT' occurred 1 times.
The pentamer 'ACT CAA' occurred 2 times.

The pentamer 'ACT GAT' occurred 1 times.
The pentamer 'ACT GAA' occurred 0 times.
The pentamer 'ACT CCA' occurred 0 times.
The pentamer 'ACT ACT' occurred 0 times.
The pentamer 'ACT AGA' occurred 1 times.
The pentamer 'ACT TAC' occurred 0 times.
The pentamer 'AGA ATT' occurred 4 times.
The pentamer 'AGA CAA' occurred 1 times.
The pentamer 'AGA GAT' occurred 1 times.
The pentamer 'AGA GAA' occurred 1 times.
The pentamer 'AGA CCA' occurred 0 times.
The pentamer 'AGA ACT' occurred 1 times.
The pentamer 'AGA AGA' occurred 5 times.
The pentamer 'AGA TAC' occurred 0 times.
The pentamer 'TAC ATT' occurred 0 times.
The pentamer 'TAC CAA' occurred 0 times.
The pentamer 'TAC GAT' occurred 1 times.
The pentamer 'TAC GAA' occurred 0 times.
The pentamer 'TAC CCA' occurred 0 times.
The pentamer 'TAC ACT' occurred 0 times.
The pentamer 'TAC AGA' occurred 1 times.
The pentamer 'TAC TAC' occurred 1 times.
The total occurrences of all specified strings is 83.
The total number of characters in the text is 4856.
First result (((total_characters/ 3) -1) / 64): 25.296875
Square root of the first result: 5.0295998846826775
Final result: 11.472706840106934

>CAKOBR010000005.1:c1058845-1056986 Saccharomyces cerevisiae chromosome_IV, Gly-tRNA ligase

---> Sequence

ATGCATAGCGATACTAACGGTCGAACAAAGAGCAATAATAGCCCATCAGACAATAATCCAAATGA
GACAGTTATATTAATAGATAGTGATAAGGAAGAAGACGCTTCTATTCGCGAGGCTAACCTTCCTG
TGAGGTTGTACCCAGACAGACGGGTAGGGAGACGACGTGATGCTTTAAATAGGTTTGTGAGGTCA
GATTCAAGGAGCAGGAATTCTCAACGGACTCACATAACAGCAAGCTCTGAGCGCCCTGATTTTCA
AGCTAATAACGACGATATCACTATAATACGAGAAGTTGGACGGTTTTTTGGAGACGATGGACCTA
TTGATCCTTCTGCACATTACGTCGATCTTGATCAGGAGCCAGGTTCTGAAACGTTAGAAACTCCA
AGAACAATACAAGTAGATAACACAAACGGGTACTTGAATGATAATGGTAATAACAATGAAAGTG
ATGATGGCCTGACAATAGTTGAAGAGAGAACAACACGACCCAGAGTGACACTGAACCTGCCAGGC
GGGGAGAGGCTTGAGGTAACTGCGACGACAACAGACATACCGATAAGAAGGTCATTTGAATTCCA
AGAAGATCTAGGCGCATCACGCAGGCAGTTACTGAGAAGGAGTGCTACAAGAGCCCGTAATTTAT

TTGTGGACCGGTCTGATGAAAACGATGAAGATTGGACAGATGATACCCATAATTTACCAGAAGCT
ATTCAAAGGGCTCGTAGGGAGAGCCGTATGCGAATGAGCAGACGGATTGCAGAAAGACAACGTAG
GGTACAACAGCAAAGGGTGTCCAGTGACGAAAATATAAGTACATCTATTAGGTTACAATCAATCA
GGGAACGAATACAATCATATACGCCTGATATTCGTAGTGCTTTTCATCGTGCAGAGTCGCTGCAT
GAATTCAGGTCTATTTTACAAAATGTTGCTCCAATTACTTTGCAAGAATGTGAAGAAGAACTAAT
GGCACTCTTCACTGAGTTCAGAAATCAACTGCTGCAGAATTGGGCCATTGATAGGGTCAGAAATA
CTCAGGAAGAGGCCTTGAGACTTCACCGAGAAGCATTGGAAAGACAAGAAAGAACGGCAGGAAG
AGTTTTCCATCGTGGCACATTACGTGAATCAATCACAAACTATCTTAATTTCAATGGAGAGGATG
GGTTCTTAAGTCGTTTGTGGAGTGGCCCAGCATTGAGCGATGCTGATGAGGAACGACACACTCAA
AATATCATTGATATGATCCAAGAGAGAGAAGAACGAGAGCGGGATGTGGTCATGAAGAATCTAA
TGAACAAAACCAGAGCCCAACAGGAAGAATTCGAAGCTAGGGCAGCCAGCCTACCCGAAGGTTAT
AGCGCGTCATTTGATACTACGCCAAAAATGAAATTAGACATTACGAAAAATGGTAAAGAAGAAA
CCATCATTGTGACAGATGACGATTTAGCAAAAACACTCGAGGATATTCCTGTTTGTTGCTTGTGT
GGCGCAGAATTGGGGGTCGGAATACCGGATGATTTTACTGGTATCAGTCAAAAGGATCGTGGTGT
TTCATTTGAAGGGTTAGTGTCAAAATACAAATTCCACTGCCCTTACCAAACCCTAGCCAGACCAT
CAATGCTTGATAGAGATTTATCAAAACGAACGTTTATTGCATCGTGTGGTCATGCATTTTGTGGT
AGGTGTTTCGCCAGGATTGACAATGCAAAAAAAAAATCTAAGATGCCCAAAAAGAAACTAGCTCA
ACTAAAGGGTTCGGCTCATCCGGATAATTATGGCCCCAAATTATGTCCCGCAGATTCTTGTAAGA
AGCTTATTCGTTCAAGAGGCAGGTTGAAGGAGGTTTACTTCTAG

---> Sequence Showing highlighted Existant Combined Trimers

ATG CAT AGC GAT ACT AAC GGT CGA ACA AAG AGC AAT AAT AGC CCA TCA GAC AAT AAT
CCA AAT GAG ACA GTT ATA TTA ATA GAT AGT GAT AAG GAA GAA GAC GCT TCT ATT CGC
GAG GCT AAC CTT CCT GTG AGG TTG TAC CCA GAC AGA CGG GTA GGG AGA CGA CGT GAT
GCT TTA AAT AGG TTT GTG AGG TCA GAT TCA AGG AGC AGG AAT TCT CAA CGG ACT CAC
ATA ACA GCA AGC TCT GAG CGC CCT GAT TTT CAA GCT AAT AAC GAC GAT ATC ACT ATA
ATA CGA GAA GTT GGA CGG TTT TTT GGA GAC GAT GGA CCT ATT GAT CCT TCT GCA CAT
TAC GTC GAT CTT GAT CAG GAG CCA GGT TCT GAA ACG TTA GAA ACT CCA AGA ACA ATA
CAA GTA GAT AAC ACA AAC GGG TAC TTG AAT GAT AAT GGT AAT AAC AAT GAA AGT GAT
GAT GGC CTG ACA ATA GTT GAA GAG AGA ACA ACA CGA CCC AGA GTG ACA CTG AAC CTG
CCA GGC GGG GAG AGG CTT GAG GTA ACT GCG ACG ACA ACA GAC ATA CCG ATA AGA AGG
TCA TTT GAA TTC CAA GAA GAT CTA GGC GCA TCA CGC AGG CAG TTA CTG AGA AGG AGT
GCT ACA AGA GCC CGT AAT TTA TTT GTG GAC CGG TCT GAT GAA AAC GAT GAA GAT TGG
ACA GAT GAT ACC CAT AAT TTA CCA GAA GCT ATT CAA AGG GCT CGT AGG GAG AGC CGT
ATG CGA ATG AGC AGA CGG ATT GCA GAA AGA CAA CGT AGG GTA CAA CAG CAA AGG GTG
TCC AGT GAC GAA AAT ATA AGT ACA TCT ATT AGG TTA CAA TCA ATC AGG GAA CGA ATA
CAA TCA TAT ACG CCT GAT ATT CGT AGT GCT TTT CAT CGT GCA GAG TCG CTG CAT GAA
TTC AGG TCT ATT TTA CAA AAT GTT GCT CCA ATT ACT TTG CAA GAA TGT GAA GAA GAA
CTA ATG GCA CTC TTC ACT GAG TTC AGA AAT CAA CTG CTG CAG AAT TGG GCC ATT GAT
AGG GTC AGA AAT ACT CAG GAA GAG GCC TTG AGA CTT CAC CGA GAA GCA TTG GAA AGA
CAA GAA AGA ACG GCA GGA AGA GTT TTC CAT CGT GGC ACA TTA CGT GAA TCA ATC ACA
AAC TAT CTT AAT TTC AAT GGA GAG GAT GGG TTC TTA AGT CGT TTG TGG AGT GGC CCA
GCA TTG AGC GAT GCT GAT GAG GAA CGA CAC ACT CAA AAT ATC ATT GAT ATG ATC CAA

GAG AGA GAA GAA CGA GAG CGG GAT GTG GTC ATG AAG AAT CTA ATG AAC AAA ACC AGA GCC CAA CAG GAA GAA TTC GAA GCT AGG GCA GCC AGC CTA CCC GAA GGT TAT AGC GCG TCA TTT GAT ACT ACG CCA AAA ATG AAA TTA GAC ATT ACG AAA AAT GGT AAA GAA GAA ACC ATC ATT GTG ACA GAT GAC GAT TTA GCA AAA ACA CTC GAG GAT ATT CCT GTT TGT TGC TTG TGT GGC GCA GAA TTG GGG GTC GGA ATA CCG GAT GAT TTT ACT GGT ATC AGT CAA AAG GAT CGT GGT GTT TCA TTT GAA GGG TTA GTG TCA AAA TAC AAA TTC CAC TGC CCT TAC CAA ACC CTA GCC AGA CCA TCA ATG CTT GAT AGA GAT TTA TCA AAA CGA ACG TTT ATT GCA TCG TGT GGT CAT GCA TTT TGT GGT AGG TGT TTC GCC AGG ATT GAC AAT GCA AAA AAA AAA TCT AAG ATG CCC AAA AAG AAA CTA GCT CAA CTA AAG GGT TCG GCT CAT CCG GAT AAT TAT GGC CCC AAA TTA TGT CCC GCA GAT TCT TGT AAG AAG CTT ATT CGT TCA AGA GGC AGG TTG AAG GAG GTT TAC TTC TAG

Occurrences/Results 9 Pentamers

The pentamer 'ATTCA' occurred 3 times.
The pentamer 'TTCAA' occurred 5 times.
The pentamer 'TCAAG' occurred 3 times.
The pentamer 'CAAGA' occurred 7 times.
The pentamer 'AAGAT' occurred 3 times.
The pentamer 'AGATG' occurred 3 times.
The pentamer 'GATGA' occurred 7 times.
The pentamer 'ATGAA' occurred 7 times.
The pentamer 'TGAAT' occurred 4 times.
The total occurrences of all specified strings is 42.
The total number of characters in the text is 1856.
First result (Total characters *9 /1024): 16.34765625
Square root of the first result: 4.043223497408967
Final result: 6.3445277676188025

Occurrences/Results TRIMERS Codons

The pentamer 'ATT' occurred 16 times.
The pentamer 'CAA' occurred 20 times.
The pentamer 'GAT' occurred 39 times.
The pentamer 'GAA' occurred 36 times.
The pentamer 'CCA' occurred 11 times.
The pentamer 'ACT' occurred 11 times.
The pentamer 'AGA' occurred 21 times.
The pentamer 'TAC' occurred 6 times.
The total occurrences of all specified strings is 160.
The total number of characters in the text is 1860.
First result ((total_characters / 3) * (Trimer_number / 64)): 77.5
Square root of the first result: 8.8
Final result: 9.4

Occurrences/Results TRIMERS Combinations

The pentamer 'ATT ATT' occurred 0 times.
The pentamer 'ATT CAA' occurred 1 times.
The pentamer 'ATT GAT' occurred 3 times.
The pentamer 'ATT GAA' occurred 0 times.
The pentamer 'ATT CCA' occurred 0 times.
The pentamer 'ATT ACT' occurred 1 times.
The pentamer 'ATT AGA' occurred 0 times.
The pentamer 'ATT TAC' occurred 0 times.
The pentamer 'CAA ATT' occurred 0 times.
The pentamer 'CAA CAA' occurred 0 times.
The pentamer 'CAA GAT' occurred 0 times.
The pentamer 'CAA GAA' occurred 3 times.
The pentamer 'CAA CCA' occurred 0 times.
The pentamer 'CAA ACT' occurred 0 times.
The pentamer 'CAA AGA' occurred 0 times.
The pentamer 'CAA TAC' occurred 0 times.
The pentamer 'GAT ATT' occurred 2 times.
The pentamer 'GAT CAA' occurred 0 times.
The pentamer 'GAT GAT' occurred 3 times.
The pentamer 'GAT GAA' occurred 2 times.
The pentamer 'GAT CCA' occurred 0 times.
The pentamer 'GAT ACT' occurred 2 times.
The pentamer 'GAT AGA' occurred 1 times.
The pentamer 'GAT TAC' occurred 0 times.
The pentamer 'GAA ATT' occurred 0 times.
The pentamer 'GAA CAA' occurred 0 times.
The pentamer 'GAA GAT' occurred 2 times.
The pentamer 'GAA GAA' occurred 5 times.
The pentamer 'GAA CCA' occurred 0 times.
The pentamer 'GAA ACT' occurred 1 times.
The pentamer 'GAA AGA' occurred 3 times.
The pentamer 'GAA TAC' occurred 0 times.
The pentamer 'CCA ATT' occurred 1 times.
The pentamer 'CCA CAA' occurred 0 times.
The pentamer 'CCA GAT' occurred 0 times.
The pentamer 'CCA GAA' occurred 1 times.
The pentamer 'CCA CCA' occurred 0 times.
The pentamer 'CCA ACT' occurred 0 times.
The pentamer 'CCA AGA' occurred 1 times.
The pentamer 'CCA TAC' occurred 0 times.
The pentamer 'ACT ATT' occurred 0 times.
The pentamer 'ACT CAA' occurred 1 times.

The pentamer 'ACT GAT' occurred 0 times.
The pentamer 'ACT GAA' occurred 0 times.
The pentamer 'ACT CCA' occurred 1 times.
The pentamer 'ACT ACT' occurred 0 times.
The pentamer 'ACT AGA' occurred 0 times.
The pentamer 'ACT TAC' occurred 0 times.
The pentamer 'AGA ATT' occurred 0 times.
The pentamer 'AGA CAA' occurred 2 times.
The pentamer 'AGA GAT' occurred 1 times.
The pentamer 'AGA GAA' occurred 1 times.
The pentamer 'AGA CCA' occurred 1 times.
The pentamer 'AGA ACT' occurred 0 times.
The pentamer 'AGA AGA' occurred 0 times.
The pentamer 'AGA TAC' occurred 0 times.
The pentamer 'TAC ATT' occurred 0 times.
The pentamer 'TAC CAA' occurred 1 times.
The pentamer 'TAC GAT' occurred 0 times.
The pentamer 'TAC GAA' occurred 0 times.
The pentamer 'TAC CCA' occurred 1 times.
The pentamer 'TAC ACT' occurred 0 times.
The pentamer 'TAC AGA' occurred 0 times.
The pentamer 'TAC TAC' occurred 0 times.
The total occurrences of all specified strings is 41.
The total number of characters in the text is 1856.
First result (((total_characters/ 3) -1) / 64): 9.671875
Square root of the first result: 3.109963826156182
Final result: 10.073469259197326

>DB661503.1DB661503 strainS288C Saccharomyces cerevisiae cDNAcloneY070_L18_F.ab15', mRNAsequence

---> Sequence

AACCATAGCACAATGTTTACTGGTATTGTAGAATGCATGGGGACTGTTTTGGAAAACAACCCATA
TGATGACTCTGAAAGTGGAGGTCAAGGAGTTTCTATTACTATTGGCAATGCGGGGAGTATTCTCA
CCGATTGTCACGTTGGAGATTCAATAGCCGTAAATGGGGTATGCCTTACTGTGACCGAGTTTAAT
AACGACTCCTTCAAAGTTGGGATATCACCAGAAACTATAAAACGAAGTAATGTCGCTTCCTGGAT
TCAAGGCACCCAGGTCAACTTGGAGAGAGCGGTATCTCAAGACGTTAGGTTCGGTGGTCATTATG
TACAGGGTCACGTAGACACTGTTGCTAATATTGTCTCAAGAAGACCTGAGGGGAATTCAATTATT
TTTGGGTTTCAGTTAAGAGATCAAGAGTACTTTAAATACATAGTAGAAAAGGGATTCATTTGTA
TAGATGGAACTTCCTTGACCATAATCAAGGTTGACCCACTTTCGCAAGGTGGAGCCTTCTATATT
AGTATGATAAAGCACACCCAA

---> Sequence Showing highlighted Existant Combined Trimers

AAC CAT AGC ACA ATG TTT ACT GGT ATT GTA GAA TGC ATG GGG ACT GTT TTG GAA AAC AAC CCA TAT GAT GAC TCT GAA AGT GGA GGT CAA GGA GTT TCT ATT ACT ATT GGC AAT GCG GGG AGT ATT CTC ACC GAT TGT CAC GTT GGA GAT TCA ATA GCC GTA AAT GGG GTA TGC CTT ACT GTG ACC GAG TTT AAT AAC GAC TCC TTC AAA GTT GGG ATA TCA CCA GAA ACT ATA AAA CGA AGT AAT GTC GCT TCC TGG ATT CAA GGC ACC CAG GTC AAC TTG GAG AGA GCG GTA TCT CAA GAC GTT AGG TTC GGT GGT CAT TAT GTA CAG GGT CAC GTA GAC ACT GTT GCT AAT ATT GTC TCA AGA AGA CCT GAG GGG AAT TCA ATT ATT TTT GGG TTT CAG TTA AGA GAT CAA GAG TAC TTT AAA TAC ATA GTA GAA AAG GGA TTC ATT TGT ATA GAT GGA ACT TCC TTG ACC ATA ATC AAG GTT GAC CCA CTT TCG CAA GGT GGA GCC TTC TAT ATT AGT ATG ATA AAG CAC ACC CAA

Occurrences/Results 9 Pentamers

The pentamer 'ATTCA' occurred 4 times.
The pentamer 'TTCAA' occurred 4 times.
The pentamer 'TCAAG' occurred 6 times.
The pentamer 'CAAGA' occurred 3 times.
The pentamer 'AAGAT' occurred 0 times.
The pentamer 'AGATG' occurred 1 times.
The pentamer 'GATGA' occurred 1 times.
The pentamer 'ATGAA' occurred 0 times.
The pentamer 'TGAAT' occurred 0 times.
The total occurrences of all specified strings is 19.
The total number of characters in the text is 536.
First result (Total characters *9 /1024): 4.74609375
Square root of the first result: 2.178553132241672
Final result: 6.542831588106882

Occurrences/Results TRIMERS Codons

The pentamer 'ATT' occurred 10 times.
The pentamer 'CAA' occurred 6 times.
The pentamer 'GAT' occurred 5 times.
The pentamer 'GAA' occurred 5 times.
The pentamer 'CCA' occurred 3 times.
The pentamer 'ACT' occurred 7 times.
The pentamer 'AGA' occurred 4 times.
The pentamer 'TAC' occurred 2 times.
The total occurrences of all specified strings is 42.
The total number of characters in the text is 540.
First result ((total_characters / 3) * (Trimer_number / 64)): 22.5
Square root of the first result: 4.74
Final result: 4.1

Occurrences/Results TRIMERS Combinations

The pentamer 'ATT ATT' occurred 1 times.
The pentamer 'ATT CAA' occurred 1 times.
The pentamer 'ATT GAT' occurred 0 times.
The pentamer 'ATT GAA' occurred 0 times.
The pentamer 'ATT CCA' occurred 0 times.
The pentamer 'ATT ACT' occurred 1 times.
The pentamer 'ATT AGA' occurred 0 times.
The pentamer 'ATT TAC' occurred 0 times.
The pentamer 'CAA ATT' occurred 0 times.
The pentamer 'CAA CAA' occurred 0 times.
The pentamer 'CAA GAT' occurred 0 times.
The pentamer 'CAA GAA' occurred 0 times.
The pentamer 'CAA CCA' occurred 0 times.
The pentamer 'CAA ACT' occurred 0 times.
The pentamer 'CAA AGA' occurred 0 times.
The pentamer 'CAA TAC' occurred 0 times.
The pentamer 'GAT ATT' occurred 0 times.
The pentamer 'GAT CAA' occurred 1 times.
The pentamer 'GAT GAT' occurred 0 times.
The pentamer 'GAT GAA' occurred 0 times.
The pentamer 'GAT CCA' occurred 0 times.
The pentamer 'GAT ACT' occurred 0 times.
The pentamer 'GAT AGA' occurred 0 times.
The pentamer 'GAT TAC' occurred 0 times.
The pentamer 'GAA ATT' occurred 0 times.
The pentamer 'GAA CAA' occurred 0 times.
The pentamer 'GAA GAT' occurred 0 times.
The pentamer 'GAA GAA' occurred 0 times.
The pentamer 'GAA CCA' occurred 0 times.
The pentamer 'GAA ACT' occurred 1 times.
The pentamer 'GAA AGA' occurred 0 times.
The pentamer 'GAA TAC' occurred 0 times.
The pentamer 'CCA ATT' occurred 0 times.
The pentamer 'CCA CAA' occurred 0 times.
The pentamer 'CCA GAT' occurred 0 times.
The pentamer 'CCA GAA' occurred 1 times.
The pentamer 'CCA CCA' occurred 0 times.
The pentamer 'CCA ACT' occurred 0 times.
The pentamer 'CCA AGA' occurred 0 times.
The pentamer 'CCA TAC' occurred 0 times.
The pentamer 'ACT ATT' occurred 1 times.
The pentamer 'ACT CAA' occurred 0 times.

The pentamer 'ACT GAT' occurred 0 times.
The pentamer 'ACT GAA' occurred 0 times.
The pentamer 'ACT CCA' occurred 0 times.
The pentamer 'ACT ACT' occurred 0 times.
The pentamer 'ACT AGA' occurred 0 times.
The pentamer 'ACT TAC' occurred 0 times.
The pentamer 'AGA ATT' occurred 0 times.
The pentamer 'AGA CAA' occurred 0 times.
The pentamer 'AGA GAT' occurred 1 times.
The pentamer 'AGA GAA' occurred 0 times.
The pentamer 'AGA CCA' occurred 0 times.
The pentamer 'AGA ACT' occurred 0 times.
The pentamer 'AGA AGA' occurred 1 times.
The pentamer 'AGA TAC' occurred 0 times.
The pentamer 'TAC ATT' occurred 0 times.
The pentamer 'TAC CAA' occurred 0 times.
The pentamer 'TAC GAT' occurred 0 times.
The pentamer 'TAC GAA' occurred 0 times.
The pentamer 'TAC CCA' occurred 0 times.
The pentamer 'TAC ACT' occurred 0 times.
The pentamer 'TAC AGA' occurred 0 times.
The pentamer 'TAC TAC' occurred 0 times.
The total occurrences of all specified strings is 9.
The total number of characters in the text is 536.
First result (((total_characters/ 3) -1) / 64): 2.796875
Square root of the first result: 1.6723860200324565
Final result: 3.709146647781482

>CAKOBR010000005.1:c39213-37894 Saccharomyces cerevisiae genome assembly, contig: chromosome_IV, RNA polymerase II-associated protein ---> Sequence

ATGGACTTACTGGGCGATATAGTGGAGAAAGATACATCTGACTCCGTTGAAAGTAATGACAATGG
CACTCTTAGTACCAACAACTGTGGAACGGGATTTCCCGAGTTGTACAAACCCAAGAAAATATCAT
CATGGAAGGAAAGACTAAGAGAAAAAAGAGCCCAAAAGAAAAAAACTAGTGGCAAGGATGCTGA
AAAGCAACAAACGAGTACAGACGCCCCACTATCGGAGGCAAAATCTATCCATAATGAAAATATTA
AAGTTCTGCAGGGAATGAGTGATGAACAAATCGTGCAAGAACGTGAGGATCTGTATAACTCTTTA
GATCCCAAACTGATTGCCAAGCTATTGAAAAACATAAATAAAAGAGCGAAGAACGAAAACAACA
CTCCATTATTTGCAGAAATAGAAGGCGCTTCTGGTACCTGGGTAGGTGGCAACAAGCAAGGCATA
TACGATCTACCACCGTTAGACGATGAAAATGTAGACGTTGCCTTAGAAATTAGGCCTAAGTTAGG
CAAAGATACAAAACATGTTCAATTTGAAGAAGCAGGAAAAGAAAAAGACGTGGAAGAAGAAGCA
AAAACTAATGATGACGTCGATGATATCGCACCCTTAGATTTCCAGATGGCACAATGCATTGATCA

TATGAAAATGAAGAGCTATTCAAAGACGTTCATTTTATCAAAGAGGAGAGCCAAAATGAAATC
AATTTGGAGAAACTTGATATAAATGATCCTAATTTCAACGATAAATTGCACGAAAAATACTTCCC
TGATTTGCCTAAAGAGGTCAACAAATTAAAATGGATGCAACCTGTTCAGCAAAAAACAGACAAGA
ATTACATCATTGAAGACGTATCTGAATGTAGGTTTGACTTTAATGGCGACCTTGTTCCACCCACA
AGACAAATAGACTCTACCATTCACTCAGGGTTGCACCATCACAGCGACTCGCCTGAACTCGCCGGT
TACACCATAGTGGAGTTAGAACATTTAGCAAGATCCACTTTTCCTTCTCAAAGGTGCATTGCTAT
ACAAACTTTGGGGAGAATACTTTATAAGCTAGGCCAAAAAAGCTACTACCAGCTAGTGCCAGAAA
TAGATGCAGACACATACAAGGAAGACGGAAGCATATCGAACGTTATGGATAAGATCTATTCCATG
TTTTGGGACTTGATTAAAGACGGAAAAGTCATCGAATCCTTAGAAATCGCCTCTGATGAGAAATT
CACTAGAAATTTGTCAGTCAGAAATTATGCTATTGATGCACTTTGGTTATGGAAACAAGGCGGTG
GGGACTTCCGAACCAAGAAATAG

---> Sequence Showing highlighted Existant Combined Trimers

ATG GAC TTA CTG GGC GAT ATA GTG GAG AAA GAT ACA TCT GAC TCC GTT GAA AGT AAT
GAC AAT GGC ACT CTT AGT ACC AAC AAC TGT GGA ACG GGA TTT CCC GAG TTG TAC AAA
CCC AAG AAA ATA TCA TCA TGG AAG GAA AGA CTA AGA GAA AAA AGA GCC CAA AAG AAA
AAA ACT AGT GGC AAG GAT GCT GAA AAG CAA CAA ACG AGT ACA GAC GCC CCA CTA TCG
GAG GCA AAA TCT ATC CAT AAT GAA AAT ATT AAA GTT CTG CAG GGA ATG AGT GAT GAA
CAA ATC GTG CAA GAA CGT GAG GAT CTG TAT AAC TCT TTA GAT CCC AAA CTG ATT GCC
AAG CTA TTG AAA AAC ATA AAT AAA AGA GCG AAG AAC GAA AAC AAC ACT CCA TTA TTT
GCA GAA ATA GAA GGC GCT TCT GGT ACC TGG GTA GGT GGC AAC AAG CAA GGC ATA TAC
GAT CTA CCA CCG TTA GAC GAT GAA AAT GTA GAC GTT GCC TTA GAA ATT AGG CCT AAG
TTA GGC AAA GAT ACA AAA CAT GTT CAA TTT GAA GAA GCA GGA AAA GAA AAA GAC GTG
GAA GAA GAA GCA AAA ACT AAT GAT GAC GTC GAT GAT ATC GCA CCC TTA GAT TTC CAG
ATG GCA CAA TGC ATT GAT CAT ATG AAA AAT GAA GAG CTA TTC AAA GAC GTT CAT TTT
ATC AAA GAG GAG AGC CAA AAT GAA ATC AAT TTG GAG AAA CTT GAT ATA AAT GAT CCT
AAT TTC AAC GAT AAA TTG CAC GAA AAA TAC TTC CCT GAT TTG CCT AAA GAG GTC AAC
AAA TTA AAA TGG ATG CAA CCT GTT CAG CAA AAA ACA GAC AAG AAT TAC ATC ATT GAA
GAC GTA TCT GAA TGT AGG TTT GAC TTT AAT GGC GAC CTT GTT CCA CCC ACA AGA CAA
ATA GAC TCT ACC ATT CAC TCA GGG TTG CAC CAT CAC AGC GAC TCG CCT GAA CTC GCC
GGT TAC ACC ATA GTG GAG TTA GAA CAT TTA GCA AGA TCC ACT TTT CCT TCT CAA AGG
TGC ATT GCT ATA CAA ACT TTG GGG AGA ATA CTT TAT AAG CTA GGC CAA AAA AGC TAC
TAC CAG CTA GTG CCA GAA ATA GAT GCA GAC ACA TAC AAG GAA GAC GGA AGC ATA TCG
AAC GTT ATG GAT AAG ATC TAT TCC ATG TTT TGG GAC TTG ATT AAA GAC GGA AAA GTC
ATC GAA TCC TTA GAA ATC GCC TCT GAT GAG AAA TTC ACT AGA AAT TTG TCA GTC AGA
AAT TAT GCT ATT GAT GCA CTT TGG TTA TGG AAA CAA GGC GGT GGG GAC TTC CGA ACC
AAG AAA TAG

Occurrences/Results 9 Pentamers

The pentamer 'ATTCA' occurred 3 times.
The pentamer 'TTCAA' occurred 3 times.
The pentamer 'TCAAG' occurred 0 times.
The pentamer 'CAAGA' occurred 6 times.

The pentamer 'AAGAT' occurred 4 times.
The pentamer 'AGATG' occurred 2 times.
The pentamer 'GATGA' occurred 5 times.
The pentamer 'ATGAA' occurred 6 times.
The pentamer 'TGAAT' occurred 1 times.
The total occurrences of all specified strings is 30.
The total number of characters in the text is 1316.
First result (Total characters *9 /1024): 11.6015625
Square root of the first result: 3.4061066483596782
Final result: 5.401603472651207

Occurrences/Results TRIMERS Codons

The pentamer 'ATT' occurred 9 times.
The pentamer 'CAA' occurred 16 times.
The pentamer 'GAT' occurred 22 times.
The pentamer 'GAA' occurred 29 times.
The pentamer 'CCA' occurred 5 times.
The pentamer 'ACT' occurred 7 times.
The pentamer 'AGA' occurred 9 times.
The pentamer 'TAC' occurred 8 times.
The total occurrences of all specified strings is 105.
The total number of characters in the text is 1320.
First result ((total_characters / 3) * (Trimer_number / 64)): 55
Square root of the first result: 7.4
Final result: 6.74

Occurrences/Results TRIMERS Combinations

The pentamer 'ATT ATT' occurred 0 times.
The pentamer 'ATT CAA' occurred 0 times.
The pentamer 'ATT GAT' occurred 2 times.
The pentamer 'ATT GAA' occurred 1 times.
The pentamer 'ATT CCA' occurred 0 times.
The pentamer 'ATT ACT' occurred 0 times.
The pentamer 'ATT AGA' occurred 0 times.
The pentamer 'ATT TAC' occurred 0 times.
The pentamer 'CAA ATT' occurred 0 times.
The pentamer 'CAA CAA' occurred 1 times.
The pentamer 'CAA GAT' occurred 0 times.
The pentamer 'CAA GAA' occurred 1 times.
The pentamer 'CAA CCA' occurred 0 times.
The pentamer 'CAA ACT' occurred 1 times.
The pentamer 'CAA AGA' occurred 0 times.
The pentamer 'CAA TAC' occurred 0 times.

The pentamer 'GAT ATT' occurred 0 times.
The pentamer 'GAT CAA' occurred 0 times.
The pentamer 'GAT GAT' occurred 1 times.
The pentamer 'GAT GAA' occurred 2 times.
The pentamer 'GAT CCA' occurred 0 times.
The pentamer 'GAT ACT' occurred 0 times.
The pentamer 'GAT AGA' occurred 0 times.
The pentamer 'GAT TAC' occurred 0 times.
The pentamer 'GAA ATT' occurred 1 times.
The pentamer 'GAA CAA' occurred 1 times.
The pentamer 'GAA GAT' occurred 0 times.
The pentamer 'GAA GAA' occurred 2 times.
The pentamer 'GAA CCA' occurred 0 times.
The pentamer 'GAA ACT' occurred 0 times.
The pentamer 'GAA AGA' occurred 1 times.
The pentamer 'GAA TAC' occurred 0 times.
The pentamer 'CCA ATT' occurred 0 times.
The pentamer 'CCA CAA' occurred 0 times.
The pentamer 'CCA GAT' occurred 0 times.
The pentamer 'CCA GAA' occurred 1 times.
The pentamer 'CCA CCA' occurred 0 times.
The pentamer 'CCA ACT' occurred 0 times.
The pentamer 'CCA AGA' occurred 0 times.
The pentamer 'CCA TAC' occurred 0 times.
The pentamer 'ACT ATT' occurred 0 times.
The pentamer 'ACT CAA' occurred 0 times.
The pentamer 'ACT GAT' occurred 0 times.
The pentamer 'ACT GAA' occurred 0 times.
The pentamer 'ACT CCA' occurred 1 times.
The pentamer 'ACT ACT' occurred 0 times.
The pentamer 'ACT AGA' occurred 1 times.
The pentamer 'ACT TAC' occurred 0 times.
The pentamer 'AGA ATT' occurred 0 times.
The pentamer 'AGA CAA' occurred 1 times.
The pentamer 'AGA GAT' occurred 0 times.
The pentamer 'AGA GAA' occurred 1 times.
The pentamer 'AGA CCA' occurred 0 times.
The pentamer 'AGA ACT' occurred 0 times.
The pentamer 'AGA AGA' occurred 0 times.
The pentamer 'AGA TAC' occurred 0 times.
The pentamer 'TAC ATT' occurred 0 times.
The pentamer 'TAC CAA' occurred 0 times.
The pentamer 'TAC GAT' occurred 1 times.

The pentamer 'TAC GAA' occurred 0 times.
The pentamer 'TAC CCA' occurred 0 times.
The pentamer 'TAC ACT' occurred 0 times.
The pentamer 'TAC AGA' occurred 0 times.
The pentamer 'TAC TAC' occurred 1 times.
The total occurrences of all specified strings is 21.
The total number of characters in the text is 1316.
First result (((total_characters/ 3) -1) / 64): 6.859375
Square root of the first result: 2.6190408549696205
Final result: 5.39916167140662

>CAKOBR010000005.1:c523839-520828 Saccharomyces cerevisiae chromosome_IV, ATPase

---> Sequence

ATGAGAGAAGTGATACTTGCTGTACATGGAATGACATGCAGCGCCTGTACTAACACAATCAATAC
GCAGTTACGAGCTTTAAAGGGTGTAACAAAATGTGATATTAGTTTAGTGACTAATGAGTGCCAGG
TGACATACGATAACGAGGTTACCGCCGATTCTATCAAGGAAATTATAGAGGATTGTGGATTTGAC
TGTGAGATACTAAGAGATTCTGAAATTACAGCCATAAGTACGAAGGAAGGACTACTGAGTGTACA
AGGTATGACCTGTGGGTCTTGTGTTTCTACAGTCACCAAACAAGTGGAAGGCATTGAGGGTGTTG
AATCGGTAGTCGTTTCCTTGGTAACGGAAGAGTGCCATGTTATTTATGAACCGTCCAAGACAACG
CTAGAAACCGTCAGAGAAATAATTGAAGACTGTGGATTTGACTCAAATATTATTATGGATGGCAA
CGGGAATGCAGACATGACCGAAAAAACGGTGATCTTGAAAGTAACTAAGGCTTTCGAGGACGAAT
CCCCACTCATACTTTCTTTAGTAAGCGAAAGGTTTCAATTTTTGTTAGACCTAGGTGTAAAATCG
ATAGAAATTTCTGATGATATGCACACACTCACCATAAAATACTGTTGTAACGAACTCGGCATTAG
AGATTTATTGAGGCACCTTGAGAGGACCGGATATAAATTCACTGCATTTTCCAATTTAGATAATA
CTACCCAATTAAGGCTTCTCTCTAAAGAGGACGAGATAAGATTCTGGAAAAAAAACAGCATAAAA
TCCACCCTTTTGGCTATAATATGTATGCTATTGTATATGATTGTCCCTATGATGTCGCCAACAAT
TGTTCAGGACCGCATATTCCCTTACAAAGAAACCTCCTTTGTTAGAGGTCTGTTCTACAGAGATA
TTTTGGGTGTAATATTGGCAAGCTATATTCAGTTCAGCGTTGGTTTTTACTTTTACAAGGCAGCA
TGGGCATCTTTCAAGCACGGTTCAGGAACCATGGATACACTAGTTTGTGTTTCCACTACTTGTGC
ATACACATTTTCTGTGTTTTCTTTAGTTCACAATATGTTCCATCCCTCAAGTACTGGCAAACTCCC
AAGGATCGTTTTCGACACATCAATCATGATCATTTCATATATTTCCATCGGGAAATATTTGGAAA
CTTTAGCTAAATCACAAACATCAACCGCACTTTCTAAATTAATTCAGCTCACTCCATCGGTGTGT
TCAATTATATCAGACGTGGAGCGGAATGAAACCAAGGAAATTCCCATAGAATTATTGCAAGTGAA
CGATATAGTGGAGATTAAACCAGGGATGAAAATTCCGGCTGATGGCATCATAACCAAGAGGTGAA
TCTGAAATTGATGAGTCGTTAATGACAGGCGAATCCATTTTGGTGCTCAAGAAAACTGGTTTTCC
GGTTATTGCTGGTTCTGTTAATGGACCTGGACATTTCTACTTCAGAACTACTACCGTTGGGGAGG
AAACTAAATTAGCAAATATTATCAAGGTAATGAAAGAAGCACAATTGAGTAAAGCTCCCATTCA
GGGGTATGCAGATTATTTAGCCTCTATTTTTGTTCCGGGGATCCTAATTTTGGCAGTATTGACTT
TCTTTATTTGGTGTTTTATTTTAAACATCTCGGCTAATCCTCCCGTCGCTTTCACCGCAAATACTA
AGGCTGATAATTTTTTTATTTGCTTACAAACTGCTACTTCTGTTGTCATTGTCGCATGCCCATGT

GCATTGGGACTTGCCACGCCGACTGCTATAATGGTGGGTACAGGGGTTGGAGCTCAGAATGGTGT
CTTAATAAAGGGCGGAGAAGTATTGGAAAAATTCAATAGTATTACTACTTTTGTTTTTGATAAA
ACAGGTACCTTAACTACAGGTTTTATGGTTGTGAAAAAATTCCTTAAAGATTCAAATTGGGTTGG
AAACGTGGATGAAGACGAAGTTCTCGCCTGTATAAAAGCAACGGAATCCATTAGTGACCATCCAG
TTTCGAAAGCAATTATACGTTATTGTGATGGTTTGAACTGTAATAAGGCTTTAAATGCCGTTGTT
TTAGAAAGCGAATACGTGCTTGGAAAGGGAATAGTCTCAAAGTGTCAAGTTAATGGAAATACTT
ATGATATTTGTATTGGTAACGAGGCGCTGATTTTGGAGGATGCATTAAAAAAATCCGGATTTATT
AACAGTAATGTCGACCAAGGAAACACTGTATCATACGTATCAGTAAACGGGCATGTGTTTGGCTT
GTTCGAGATTAATGATGAAGTTAAACATGATTCTTACGCAACAGTTCAGTATCTACAAAGAAATG
GATATGAAACGTATATGATTACTGGCGATAATAACTCAGCAGCAAAAAGAGTCGCTAGAGAAGT
AGGTATAAGCTTCGAAAATGTTTACAGCGATGTGTCACCCACAGGCAAATGTGATCTCGTGAAAA
AAATTCAAGATAAAGAAGGTAATAACAAGGTTGCTGTTGTTGGCGATGGCATTAACGACGCTCCC
GCTTTAGCACTGAGTGATCTAGGTATTGCCATTTCAACAGGTACGGAGATTGCTATTGAAGCAGC
TGATATTGTTATACTGTGCGGTAATGATCTGAATACTAATAGTTTGAGAGGACTGGCCAACGCCA
TCGATATTTCACTAAAAACGTTCAAAAGAATAAAGCTGAATTTGTTCTGGGCACTTTGCTATAAT
ATATTCATGATTCCGATTGCTATGGGTGTGCTCATTCCTTGGGGCATAACTCTTCCTCCAATGCTC
GCCGGTTTAGCGATGGCGTTCAGCTCGGTCAGTGTCGTTCTAAGTTCATTGATGTTGAAGAAGTG
GACTCCACCGGATATTGAATCACATGGGATTTCAGATTTCAAGTCCAAATTTTCGATTGGGAATT
TTTGGTCAAGGCTTTTTTCTACGCGGGCTATTGCAGGTGAGCAAGACATAGAATCACAGGCCGGA
CTAATGTCAAACGAAGAAGTCTAG

---> Sequence Showing highlighted Existant Combined Trimers

ATG AGA GAA GTG ATA CTT GCT GTA CAT GGA ATG ACA TGC AGC GCC TGT ACT AAC ACA
ATC AAT ACG CAG TTA CGA GCT TTA AAG GGT GTA ACA AAA TGT GAT ATT AGT TTA GTG
ACT AAT GAG TGC CAG GTG ACA TAC GAT AAC GAG GTT ACC GCC GAT TCT ATC AAG GAA
ATT ATA GAG GAT TGT GGA TTT GAC TGT GAG ATA CTA AGA GAT TCT GAA ATT ACA GCC
ATA AGT ACG AAG GAA GGA CTA CTG AGT GTA CAA GGT ATG ACC TGT GGG TCT TGT GTT
TCT ACA GTC ACC AAA CAA GTG GAA GGC ATT GAG GGT GTT GAA TCG GTA GTC GTT TCC
TTG GTA ACG GAA GAG TGC CAT GTT ATT TAT GAA CCG TCC AAG ACA ACG CTA GAA ACC
GTC AGA GAA ATA ATT GAA GAC TGT GGA TTT GAC TCA AAT ATT ATT ATG GAT GGC AAC
GGG AAT GCA GAC ATG ACC GAA AAA ACG GTG ATC TTG AAA GTA ACT AAG GCT TTC GAG
GAC GAA TCC CCA CTC ATA CTT TCT TTA GTA AGC GAA AGG TTT CAA TTT TTG TTA GAC
CTA GGT GTA AAA TCG ATA GAA ATT TCT GAT GAT ATG CAC ACA CTC ACC ATA AAA TAC
TGT TGT AAC GAA CTC GGC ATT AGA GAT TTA TTG AGG CAC CTT GAG AGG ACC GGA TAT
AAA TTC ACT GCA TTT TCC AAT TTA GAT AAT ACT ACC CAA TTA AGG CTT CTC TCT AAA
GAG GAC GAG ATA AGA TTC TGG AAA AAA AAC AGC ATA AAA TCC ACC CTT TTG GCT ATA
ATA TGT ATG CTA TTG TAT ATG ATT GTC CCT ATG ATG TCG CCA ACA ATT GTT CAG GAC
CGC ATA TTC CCT TAC AAA GAA ACC TCC TTT GTT AGA GGT CTG TTC TAC AGA GAT ATT
TTG GGT GTA ATA TTG GCA AGC TAT ATT CAG TTC AGC GTT GGT TTT TAC TTT TAC AAG
GCA GCA TGG GCA TCT TTC AAG CAC GGT TCA GGA ACC ATG GAT ACA CTA GTT TGT GTT
TCC ACT ACT TGT GCA TAC ACA TTT TCT GTG TTT TCT TTA GTT CAC AAT ATG TTC CAT
CCC TCA AGT ACT GGC AAA CTC CCA AGG ATC GTT TTC GAC ACA TCA ATC ATG ATC ATT
TCA TAT ATT TCC ATC GGG AAA TAT TTG GAA ACT TTA GCT AAA TCA CAA ACA TCA ACC

GCA CTT TCT AAA TTA ATT CAG CTC ACT CCA TCG GTG TGT TCA ATT ATA TCA GAC GTG GAG CGG AAT GAA ACC AAG GAA ATT CCC ATA GAA TTA TTG CAA GTG AAC GAT ATA GTG GAG ATT AAA CCA GGG ATG AAA ATT CCG GCT GAT GGC ATC ATA ACA AGA GGT GAA TCT GAA ATT GAT GAG TCG TTA ATG ACA GGC GAA TCC ATT TTG GTG CTC AAG AAA ACT GGT TTT CCG GTT ATT GCT GGT TCT GTT AAT GGA CCT GGA CAT TTC TAC TTC AGA ACT ACT ACC GTT GGG GAG GAA ACT AAA TTA GCA AAT ATT ATC AAG GTA ATG AAA GAA GCA CAA TTG AGT AAA GCT CCC ATT CAG GGG TAT GCA GAT TAT TTA GCC TCT ATT TTT GTT CCG GGG ATC CTA ATT TTG GCA GTA TTG ACT TTC TTT ATT TGG TGT TTT ATT TTA AAC ATC TCG GCT AAT CCT CCC GTC GCT TTC ACC GCA AAT ACT AAG GCT GAT AAT TTT TTT ATT TGC TTA CAA ACT GCT ACT TCT GTT GTC ATT GTC GCA TGC CCA TGT GCA TTG GGA CTT GCC ACG CCG ACT GCT ATA ATG GTG GGT ACA GGG GTT GGA GCT CAG AAT GGT GTC TTA ATA AAG GGC GGA GAA GTA TTG GAA AAA TTC AAT AGT ATT ACT ACT TTT GTT TTT GAT AAA ACA GGT ACC TTA ACT ACA GGT TTT ATG GTT GTG AAA AAA TTC CTT AAA GAT TCA AAT TGG GTT GGA AAC GTG GAT GAA GAC GAA GTT CTC GCC TGT ATA AAA GCA ACG GAA TCC ATT AGT GAC CAT CCA GTT TCG AAA GCA ATT ATA CGT TAT TGT GAT GGT TTG AAC TGT AAT AAG GCT TTA AAT GCC GTT GTT TTA GAA AGC GAA TAC GTG CTT GGA AAG GGA ATA GTC TCA AAG TGT CAA GTT AAT GGA AAT ACT TAT GAT ATT TGT ATT GGT AAC GAG GCG CTG ATT TTG GAG GAT GCA TTA AAA AAA TCC GGA TTT ATT AAC AGT AAT GTC GAC CAA GGA AAC ACT GTA TCA TAC GTA TCA GTA AAC GGG CAT GTG TTT GGC TTG TTC GAG ATT AAT GAT GAA GTT AAA CAT GAT TCT TAC GCA ACA GTT CAG TAT CTA CAA AGA AAT GGA TAT GAA ACG TAT ATG ATT ACT GGC GAT AAT AAC TCA GCA GCA AAA AGA GTC GCT AGA GAA GTA GGT ATA AGC TTC GAA AAT GTT TAC AGC GAT GTG TCA CCC ACA GGC AAA TGT GAT CTC GTG AAA AAA ATT CAA GAT AAA GAA GGT AAT AAC AAG GTT GCT GTT GTT GGC GAT GGC ATT AAC GAC GCT CCC GCT TTA GCA CTG AGT GAT CTA GGT ATT GCC ATT TCA ACA GGT ACG GAG ATT GCT ATT GAA GCA GCT GAT ATT GTT ATA CTG TGC GGT AAT GAT CTG AAT ACT AAT AGT TTG AGA GGA CTG GCC AAC GCC ATC GAT ATT TCA CTA AAA ACG TTC AAA AGA ATA AAG CTG AAT TTG TTC TGG GCA CTT TGC TAT AAT ATA TTC ATG ATT CCG ATT GCT ATG GGT GTG CTC ATT CCT TGG GGC ATA ACT CTT CCT CCA ATG CTC GCC GGT TTA GCG ATG GCG TTC AGC TCG GTC AGT GTC GTT CTA AGT TCA TTG ATG TTG AAG AAG TGG ACT CCA CCG GAT ATT GAA TCA CAT GGG ATT TCA GAT TTC AAG TCC AAA TTT TCG ATT GGG AAT TTT TGG TCA AGG CTT TTT TCT ACG CGG GCT ATT GCA GGT GAG CAA GAC ATA GAA TCA CAG GCC GGA CTA ATG TCA AAC GAA GAA GTC TAG

Occurrences/Results 9 Pentamers

The pentamer 'ATTCA' occurred 8 times.
The pentamer 'TTCAA' occurred 9 times.
The pentamer 'TCAAG' occurred 9 times.
The pentamer 'CAAGA' occurred 5 times.
The pentamer 'AAGAT' occurred 3 times.
The pentamer 'AGATG' occurred 0 times.
The pentamer 'GATGA' occurred 5 times.
The pentamer 'ATGAA' occurred 7 times.
The pentamer 'TGAAT' occurred 5 times.

The total occurrences of all specified strings is 51.
The total number of characters in the text is 3008.
First result (Total characters *9 /1024): 26.47265625
Square root of the first result: 5.145158525254591
Final result: 4.767072507019858

Occurrences/Results TRIMERS Codons

The pentamer 'ATT' occurred 56 times.
The pentamer 'CAA' occurred 13 times.
The pentamer 'GAT' occurred 36 times.
The pentamer 'GAA' occurred 43 times.
The pentamer 'CCA' occurred 9 times.
The pentamer 'ACT' occurred 28 times.
The pentamer 'AGA' occurred 14 times.
The pentamer 'TAC' occurred 12 times.
The total occurrences of all specified strings is 211.
The total number of characters in the text is 3012.
First result ((total_characters / 3) * (Trimer_number / 64)): 125.5
Square root of the first result: 11.2
Final result: 7.6

Occurrences/Results TRIMERS Combinations

The pentamer 'ATT ATT' occurred 1 times.
The pentamer 'ATT CAA' occurred 1 times.
The pentamer 'ATT GAT' occurred 1 times.
The pentamer 'ATT GAA' occurred 3 times.
The pentamer 'ATT CCA' occurred 0 times.
The pentamer 'ATT ACT' occurred 2 times.
The pentamer 'ATT AGA' occurred 1 times.
The pentamer 'ATT TAC' occurred 0 times.
The pentamer 'CAA ATT' occurred 0 times.
The pentamer 'CAA CAA' occurred 0 times.
The pentamer 'CAA GAT' occurred 1 times.
The pentamer 'CAA GAA' occurred 0 times.
The pentamer 'CAA CCA' occurred 0 times.
The pentamer 'CAA ACT' occurred 1 times.
The pentamer 'CAA AGA' occurred 1 times.
The pentamer 'CAA TAC' occurred 0 times.
The pentamer 'GAT ATT' occurred 6 times.
The pentamer 'GAT CAA' occurred 0 times.
The pentamer 'GAT GAT' occurred 1 times.
The pentamer 'GAT GAA' occurred 2 times.
The pentamer 'GAT CCA' occurred 0 times.

The pentamer 'GAT ACT' occurred 0 times.
The pentamer 'GAT AGA' occurred 0 times.
The pentamer 'GAT TAC' occurred 0 times.
The pentamer 'GAA ATT' occurred 5 times.
The pentamer 'GAA CAA' occurred 0 times.
The pentamer 'GAA GAT' occurred 0 times.
The pentamer 'GAA GAA' occurred 1 times.
The pentamer 'GAA CCA' occurred 0 times.
The pentamer 'GAA ACT' occurred 2 times.
The pentamer 'GAA AGA' occurred 0 times.
The pentamer 'GAA TAC' occurred 1 times.
The pentamer 'CCA ATT' occurred 0 times.
The pentamer 'CCA CAA' occurred 0 times.
The pentamer 'CCA GAT' occurred 0 times.
The pentamer 'CCA GAA' occurred 0 times.
The pentamer 'CCA CCA' occurred 0 times.
The pentamer 'CCA ACT' occurred 0 times.
The pentamer 'CCA AGA' occurred 0 times.
The pentamer 'CCA TAC' occurred 0 times.
The pentamer 'ACT ATT' occurred 0 times.
The pentamer 'ACT CAA' occurred 0 times.
The pentamer 'ACT GAT' occurred 0 times.
The pentamer 'ACT GAA' occurred 0 times.
The pentamer 'ACT CCA' occurred 2 times.
The pentamer 'ACT ACT' occurred 3 times.
The pentamer 'ACT AGA' occurred 0 times.
The pentamer 'ACT TAC' occurred 0 times.
The pentamer 'AGA ATT' occurred 0 times.
The pentamer 'AGA CAA' occurred 0 times.
The pentamer 'AGA GAT' occurred 3 times.
The pentamer 'AGA GAA' occurred 3 times.
The pentamer 'AGA CCA' occurred 0 times.
The pentamer 'AGA ACT' occurred 1 times.
The pentamer 'AGA AGA' occurred 0 times.
The pentamer 'AGA TAC' occurred 0 times.
The pentamer 'TAC ATT' occurred 0 times.
The pentamer 'TAC CAA' occurred 0 times.
The pentamer 'TAC GAT' occurred 1 times.
The pentamer 'TAC GAA' occurred 0 times.
The pentamer 'TAC CCA' occurred 0 times.
The pentamer 'TAC ACT' occurred 0 times.
The pentamer 'TAC AGA' occurred 1 times.
The pentamer 'TAC TAC' occurred 0 times.

The total occurrences of all specified strings is 44.
The total number of characters in the text is 3008.
First result (((total_characters/ 3) -1) / 64): 15.671875
Square root of the first result: 3.958771905528279
Final result: 7.155786106403559

>L14616.1 Saccharomyces cerevisiae putative ATPase (YME1) FtsH gene

---> Sequence

T**TGGTAC**CTCTTTCTTCGAGGAG**TGGTA**GCTCCTGTACGCTGTAGCGGTAGTGAAAAGGCAATA
TATCGGAGTTGGTGAAGTGAGTTAAATCTCTACCTTGCGTTTTTGATAAGTAAAAAGCAAGCACA
GCTTAAAGGATAAACAGGATTTTATTGAAGCAATTTTTAATTATAATACATTGTGGATAGAACG
AAAACAGAGACGTGATAGATG**AA**CGTTTCAAAAATACTTGTGTCGCCCACGGTCACGACAAATGT
TTTACGCATATTTGCTCCCAGGCTACCTCAAATCGGTGCTTCTTTGTTAGTTCAAAAAA**AATGG**G
CCTTAAGATCAAAGAAGTTCTACCGTTTTTATTCTGAAAAGAATAGCGGTGAAATGCCTCCTAAG
AAGGAAGCTGATAGCTCTGGAAAGGCATCCAATAAATCCACGATATCTTCAATCGACAATTCGCA
ACCACCACCTCCATCGAACACTAATGATAAAACCAAACAAGCGAACGTAGCTGTGTCACATGCTA
TGCTAGCAACTAGAGAACAAGAGGCCAATAAAGACTTAACGAGTCCTGATGCACAAGCAGCCTTT
TACAAACTTCTCTTACAATCAAACTACCCGCAATACGTGGTCTCTAGGTTTGAGACCCCCGGTATT
GCGTCATCGCCTGAATGCATGGAACTGTACATGGAGGCCCTGCAGAGGATAGGCAGACACTCGGA
GGCTGATGCCGTTAGACAAAACC**TACTG**ACAGCCAGCTCTGCTGGCGCAGTTAACCCATCATTGG
CGTCATCTTCATCAAACCAGTCAGGTTATC**ATGGTA**ACTTTCCATCGATGTATTCTCCGCTTTAT
GGATCTCGCAAAGAGCCACTACATGTTGTCGTATCTGAATCAACTTTTACTGTTGTATCGAGATG
GGTAAAGTGGCTGCTTGTTTTCGGTATCTTAACCTACTCTTTTTCTGAAGGTTTTAAATACATCA
CAGAAAATACAACGCTACTAAAGTCGTCAGAAGTAGCCGACAAATCAGTTGATGTAGCTAAGACA
AATGTTAAATTTGATGATGTCTGCGGTTGTGATGAGGCCCGTGCGGAATTGGAAGAAATTGTTGA
TTTCCTGAAAGATCCAACTAAGTACGAGTCCTTGGG**TGGTA**AACTACCAAAGGGT**GTACTG**TTG
ACTGGACCTCCTGGTACAGGTAAAACTTTGTTGGCTAGGGCCACTGCCGGAGAGGCTGGTGTAGA
TTTTTTCTTTATGTCAGGTTCTGAATTTGATGAAGTTTACGTCGGTGTTGGTGCTAAACGTATCC
GTGATTTGTTTGCTCAAGCGCGTTCTCGTGCACCAGCTATTATTTTTATTGATGAATTAGATGCC
ATCGGTGGTAAGCGTAATCCAAAGGACCAAGCTTACGCCAAACAAACGTTGAATCAGTTATTGGT
CGAATTAGATGGTTTCTCACAAACAAGTGGAATTATTATTATTGGTGCCACAAATTTCCCTGAGG
CTTTAGATAAGGCGTTAACTAGACCAGGTAGATTTGATAAGGTTGTGAATGTGGATTTGCCAGAT
GTTCGTGGTCGTGCTGATATTTTAAAGCATCACATGAAGAAGATTACATTGGCAGACAATGTGGA
TCCAACTATTATTGCGCGTGGTACCCCCGGTTTATCAGGCGCTGAGCTGGCAAATTTAGTCAACCA
AGCAGCAGTTTACGCGTGTCAAAAAAATGCTGTTTCCGTTGATATGTCCCACTTCGAGTGGGCTA
AGGATAAGATATTGATGGGTGCTGAGAGAAAGACTATGGTCTTAACAGATGCAGCCAGAAAGGC
CACTGCTTTCCACGAGGCTGGACATGCCATTATGGCCAAATACACCAATGGTGCTACCCCGCTATA
CAAGGCCACGATATTGCCTAGAGGTAGGGCATTGGGTATTACTTTTCAATTGCCAGAAATGGATA
AGGTCGACATCACCAAAAGGGAGTGTCAAGCCAGACTGGACGTGTGCATGGGGGGCAAAATTGCA
GAAGAATTAATTTATGGTAAAGATAACACCACAAGTGGTTGTGGGTCTGACTTGCAAAGCGCCAC

CGGCACAGCAAGGGCTATGGTTACTCAATATGGTATGAGTGATGATGTAGGTCCCGTTAACTTGT
CAGAAAATTGGGAATCTTGGTCTAATAAGATTCGCGATATTGCTGATAATGAAGTGATTGAACTT
TTGAAGGACTCCGAGGAAAGAGCAAGAAGACTATTAACTAAGAAAAATGTTGAGCTACATAGAC
TTGCGCAAGGTCTTATTGAATATGAAACTCTAGATGCCCACGAAATCGAACAAGTTTGTAAAGGT
GAAAAACTGGACAAACTGAAAACTTCCACCAATACAGTCGTAGAAGGACCAGACAGTGATGAACG
TAAAGATATAGGCGATGATAAACCCAAAATTCCTACAATGTTAAATGCATGATTTTATTCTAAGA
AGTTAAACGTATGAAGGAACCTACCTCAAGACCGAGAATGCTTTGTTCTTTTCTGGTCGGTTTCG
CTTTTGCTTCATAACACCAGCAATGAAGCATGATCGCGGGAAACTTTCAGAATCTACATCTTCTC
CTGGTCAGCTTGAAACTTCAGATTATTCGTAAGGAGAAATTATAATATTATTTCTGTGATAAGAA
AAGAGGCATTAAGCTTGGGTATTTGCGACAAAATACTTCAGGATAACCGATGATCGCAAGTGGGT
TATTAATTTCTTTCATCCCTTAACCAACCGTCGCTTTCCCCCAGTGACAAGCCTTAAAAAAGTCAC
ACCATGGAACGGAGTATCGTTGATGATTAAACCAATCATTAATCTTTCATAAAAGTTAAGATAAA
AGCACTTGTTGATATATATAGGTACATGTGTATCAATAC**GCCAT**ACATTGTAAATAGAATATTCA
ATGGATAAACGTGTGTAGCAAAAATAATCACATTGTAGCGCTAGTTAG

---> Sequence Showing highlighted Existant Combined Trimers

TTG GTA CCT CTT TCT TCG AGG AGT GGT AGC TCC TGT ACG CTG TAG CGG TAG TGA AAA
GGC AAT ATA TCG GAG TTG GTG AAG TGA GTT AAA TCT CTA CCT TGC GTT TTT GAT AAG
TAA AAA GCA AGC ACA GCT TAA AGG ATA AAC AGG ATT TTA TTG AAG CAA TTT TTA ATT
ATA ATA CAT TGT GGA TAG AAC GAA AAC AGA GAC GTG ATA GAT GAA CGT TTC AAA AAT
ACT TGT GTC GCC CAC GGT CAC GAC AAA TGT TTT ACG CAT ATT TGC TCC CAG GCT ACC
TCA AAT CGG TGC TTC TTT GTT AGT TCA AAA AAA ATG GGC CTT AAG ATC AAA GAA GTT
CTA CCG TTT TTA TTC TGA AAA GAA TAG CGG TGA AAT GCC TCC TAA GAA GGA AGC TGA
TAG CTC TGG AAA GGC ATC CAA TAA ATC CAC GAT ATC TTC AAT CGA CAA TTC GCA ACC
ACC ACC TCC ATC GAA CAC TAA TGA TAA AAC CAA ACA AGC GAA CGT AGC TGT GTC ACA
TGC TAT GCT AGC AAC TAG AGA ACA AGA GGC CAA TAA AGA CTT AAC GAG TCC TGA TGC
ACA AGC AGC CTT TTA CAA ACT TCT CTT ACA ATC AAA CTA CCC GCA ATA CGT GGT CTC
TAG GTT TGA GAC CCC CGG TAT TGC GTC ATC GCC TGA ATG CAT GGA ACT GTA CAT GGA
GGC CCT GCA GAG GAT AGG CAG ACA CTC GGA GGC TGA TGC CGT TAG ACA AAA CCT ACT
GAC AGC CAG CTC TGC TGG CGC AGT TAA CCC ATC ATT GGC GTC ATC TTC ATC AAA CCA
GTC AGG TTA TCA TGG TAA CTT TCC ATC GAT GTA TTC TCC GCT TTA TGG ATC TCG CAA
AGA GCC ACT ACA TGT TGT CGT ATC TGA ATC AAC TTT TAC TGT TGT ATC GAG ATG GGT
AAA GTG GCT GCT TGT TTT CGG TAT CTT AAC CTA CTC TTT TTC TGA AGG TTT TAA ATA
CAT CAC AGA AAA TAC AAC GCT ACT AAA GTC GTC AGA AGT AGC CGA CAA ATC AGT TGA
TGT AGC TAA GAC AAA TGT TAA ATT TGA TGA TGT CTG CGG TTG TGA TGA GGC CCG TGC
GGA ATT GGA AGA AAT TGT TGA TTT CCT GAA AGA TCC AAC TAA GTA CGA GTC CTT GGG
TGG TAA ACT ACC AAA GGG TGT ACT GTT GAC TGG ACC TCC TGG TAC AGG TAA AAC TTT
GTT GGC TAG GGC CAC TGC CGG AGA GGC TGG TGT AGA TTT TTT CTT TAT GTC AGG TTC
TGA ATT TGA TGA AGT TTA CGT CGG TGT TGG TGC TAA ACG TAT CCG TGA TTT GTT TGC
TCA AGC GCG TTC TCG TGC ACC AGC TAT TAT TTT TAT TGA TGA ATT AGA TGC CAT CGG
TGG TAA GCG TAA TCC AAA GGA CCA AGC TTA CGC CAA ACA AAC GTT GAA TCA GTT ATT
GGT CGA ATT AGA TGG TTT CTC ACA AAC AAG TGG AAT TAT TAT TAT TGG TGC CAC AAA
TTT CCC TGA GGC TTT AGA TAA GGC GTT AAC TAG ACC AGG TAG ATT TGA TAA GGT TGT

GAA TGT GGA TTT GCC AGA TGT TCG TGG TCG TGC TGA TAT TTT AAA GCA TCA CAT GAA GAA GAT TAC **ATT** GGC AGA CAA TGT GGA TCC AAC TAT TAT TGC GCG TGG TAC CCC CGG TTT ATC AGG CGC TGA GCT GGC AAA TTT AGT CAA CCA AGC AGC AGT TTA CGC GTG TCA AAA AAA TGC TGT TTC CGT TGA TAT GTC CCA CTT CGA GTG GGC TAA GGA TAA GAT ATT GAT GGG TGC TGA GAG AAA GAC TAT GGT CTT AAC AGA TGC AGC CAG AAA GGC CAC TGC TTT CCA CGA GGC TGG ACA TGC CAT TAT GGC CAA ATA CAC CAA TGG TGC TAC CCC GCT ATA CAA GGC CAC GAT ATT GCC TAG AGG TAG GGC ATT GGG TAT TAC TTT TCA ATT GCC AGA AAT GGA TAA GGT CGA CAT CAC CAA AAG GGA GTG TCA AGC CAG ACT GGA CGT GTG CAT GGG GGG CAA AAT TGC AGA AGA ATT AAT TTA TGG TAA AGA TAA CAC CAC AAG TGG TTG TGG GTC TGA CTT GCA AAG CGC CAC CGG CAC AGC AAG GGC TAT GGT TAC TCA ATA TGG TAT GAG TGA TGA TGT AGG TCC CGT TAA CTT GTC AGA AAA TTG GGA ATC TTG GTC TAA TAA GAT TCG CGA TAT TGC TGA TAA TGA AGT GAT TGA ACT TTT GAA GGA CTC CGA GGA AAG AGC AAG AAG ACT ATT AAC TAA GAA AAA TGT TGA GCT ACA TAG ACT TGC GCA AGG TCT TAT TGA ATA TGA AAC TCT AGA TGC CCA CGA AAT CGA ACA AGT TTG TAA AGG TGA AAA ACT GGA CAA ACT GAA AAC TTC CAC CAA TAC AGT CGT AGA AGG ACC AGA CAG TGA TGA ACG TAA AGA TAT AGG CGA TGA TAA ACC CAA AAT TCC TAC AAT GTT AAA TGC ATG ATT TTA TTC TAA GAA GTT AAA CGT ATG AAG GAA CCT ACC TCA AGA CCG AGA ATG CTT TGT TCT TTT CTG GTC GGT TTC GCT TTT GCT TCA TAA CAC CAG CAA TGA AGC ATG ATC GCG GGA AAC TTT CAG AAT CTA CAT CTT CTC CTG GTC AGC TTG AAA CTT CAG ATT ATT CGT AAG GAG AAA TTA TAA TAT TAT TTC TGT GAT AAG AAA AGA GGC ATT AAG CTT GGG TAT TTG CGA CAA AAT ACT TCA GGA TAA CCG ATG ATC GCA AGT GGG TTA TTA ATT TCT TTC ATC CCT TAA CCA ACC GTC GCT TTC CCC CAG TGA CAA GCC TTA AAA AAG TCA CAC CAT GGA ACG GAG TAT CGT TGA TGA TTA AAC CAA TCA TTA ATC TTT CAT AAA AGT TAA GAT AAA AGC ACT TGT TGA TAT ATA TAG GTA CAT GTG TAT CAA TAC GCC ATA CAT TGT AAA TAG AAT ATT CAA TGG ATA AAC GTG TGT AGC AAA AAT AAT CAC ATT GTA GCG CTA GTT AG

Occurrences/Results 9 Pentamers

The pentamer 'ATTCA' occurred 1 times.
The pentamer 'TTCAA' occurred 5 times.
The pentamer 'TCAAG' occurred 3 times.
The pentamer 'CAAGA' occurred 3 times.
The pentamer 'AAGAT' occurred 8 times.
The pentamer 'AGATG' occurred 7 times.
The pentamer 'GATGA' occurred 10 times.
The pentamer 'ATGAA' occurred 9 times.
The pentamer 'TGAAT' occurred 7 times.
The total occurrences of all specified strings is 53.
The total number of characters in the text is 2968.
First result (Total characters *9 /1024): 26.12109375
Square root of the first result: 5.110879938914628
Final result: 5.2591543083103875

Occurrences/Results TRIMERS Codons

The pentamer 'CAA' occurred 25 times.
The pentamer 'GAT' occurred 13 times.
The pentamer 'GAA' occurred 17 times.
The pentamer 'ATT' occurred 25 times.
The pentamer 'TAC' occurred 11 times.
The pentamer 'CCA' occurred 7 times.
The pentamer 'ATC' occurred 22 times.
The pentamer 'AGA' occurred 29 times.
The total occurrences of all specified strings is 149.
The total number of characters in the text is 2970.
First result ((total_characters / 3) * (Trimer_number / 64)): 123,75
Square root of the first result: 11.1
Final result: 2.3

Occurrences/Results TRIMERS Combinations

The pentamer 'CAA CAA' occurred 0 times.
The pentamer 'CAA GAT' occurred 0 times.
The pentamer 'CAA GAA' occurred 0 times.
The pentamer 'CAA ATT' occurred 0 times.
The pentamer 'CAA CCA' occurred 1 times.
The pentamer 'CAA ACT' occurred 2 times.
The pentamer 'CAA TAC' occurred 2 times.
The pentamer 'CAA AGA' occurred 1 times.
The pentamer 'GAT CAA' occurred 0 times.
The pentamer 'GAT GAT' occurred 0 times.
The pentamer 'GAT GAA' occurred 1 times.
The pentamer 'GAT ATT' occurred 2 times.
The pentamer 'GAT CCA' occurred 0 times.
The pentamer 'GAT ACT' occurred 0 times.
The pentamer 'GAT TAC' occurred 1 times.
The pentamer 'GAT AGA' occurred 0 times.
The pentamer 'GAA CAA' occurred 0 times.
The pentamer 'GAA GAT' occurred 1 times.
The pentamer 'GAA GAA' occurred 1 times.
The pentamer 'GAA ATT' occurred 0 times.
The pentamer 'GAA CCA' occurred 0 times.
The pentamer 'GAA ACT' occurred 0 times.
The pentamer 'GAA TAC' occurred 0 times.
The pentamer 'GAA AGA' occurred 1 times.
The pentamer 'ATT CAA' occurred 1 times.
The pentamer 'ATT GAT' occurred 1 times.
The pentamer 'ATT GAA' occurred 0 times.

The pentamer 'ATT ATT' occurred 1 times.
The pentamer 'ATT CCA' occurred 0 times.
The pentamer 'ATT ACT' occurred 0 times.
The pentamer 'ATT TAC' occurred 0 times.
The pentamer 'ATT AGA' occurred 2 times.
The pentamer 'CCA CAA' occurred 0 times.
The pentamer 'CCA GAT' occurred 0 times.
The pentamer 'CCA GAA' occurred 0 times.
The pentamer 'CCA ATT' occurred 0 times.
The pentamer 'CCA CCA' occurred 0 times.
The pentamer 'CCA ACT' occurred 0 times.
The pentamer 'CCA TAC' occurred 0 times.
The pentamer 'CCA AGA' occurred 0 times.
The pentamer 'ACT CAA' occurred 0 times.
The pentamer 'ACT GAT' occurred 0 times.
The pentamer 'ACT GAA' occurred 1 times.
The pentamer 'ACT ATT' occurred 1 times.
The pentamer 'ACT CCA' occurred 0 times.
The pentamer 'ACT ACT' occurred 0 times.
The pentamer 'ACT TAC' occurred 0 times.
The pentamer 'ACT AGA' occurred 0 times.
The pentamer 'TAC CAA' occurred 0 times.
The pentamer 'TAC GAT' occurred 0 times.
The pentamer 'TAC GAA' occurred 0 times.
The pentamer 'TAC ATT' occurred 1 times.
The pentamer 'TAC CCA' occurred 0 times.
The pentamer 'TAC ACT' occurred 0 times.
The pentamer 'TAC TAC' occurred 0 times.
The pentamer 'TAC AGA' occurred 0 times.
The pentamer 'AGA CAA' occurred 1 times.
The pentamer 'AGA GAT' occurred 0 times.
The pentamer 'AGA GAA' occurred 0 times.
The pentamer 'AGA ATT' occurred 1 times.
The pentamer 'AGA CCA' occurred 0 times.
The pentamer 'AGA ACT' occurred 0 times.
The pentamer 'AGA TAC' occurred 0 times.
The pentamer 'AGA AGA' occurred 1 times.
The total occurrences of all specified strings is 24.
The total number of characters in the text is 2968.
First result (((total_characters/ 3) -1) / 64): 15.453125
Square root of the first result: 3.931046298379097
Final result: 2.1741985088102793

>CAKOBR010000010.1:c986085-983122 Saccharomyces cerevisiae genome assembly, contig: chromosome_VII, PFK1

---> Sequence

ATGCAATCTCAAGATTCATGCTACGGTGTTGCATTCAGATCTATCATCACAAATGATGAAGCTTT
ATTCAAGAAGACCATTCACTTTTATCACACTCTAGGATTTGCAACTGTGAAAGATTTCAACAAAT
TCAAACATGGTGAAAATAGCTTACTATCTTCAGGGACTTCCCAAGATTCCTTGAGAGAAGTTTGG
TTAGAATCTTTCAAGTTGAGTGAGGTTGATGCTTCTGGGTTCCGTATACCACAACAAGAAGCTAC
TAACAAGGCTCAAAGTCAAGGTGCTCTATTAAAGATTCGTTTAGTGATGTCTGCTCCAATCGATG
AAACTTTCGACACCAACGAAACCGCCACAATCACTTATTTCTCTACTGATTTGAATAAAATTGTC
GAGAAATTCCCAAAACAAGCCGAAAAATTGTCCGATACCTTAGTGTTTTTGAAAGATCCAATGGG
CAACAACATCACCTTCTCAGGCTTAGCTAATGCAACCGATTCCGCTCCAACTTCCAAAGATGCTTT
CTTAGAAGCTACCTCCGAAGACGAAATCATCTCTAGAGCTTCTTCCGATGCTTCTGACTTACTAA
GACAAACATTGGGCTCTTCTCAAAAGAAGAAGAAGATCGCTGTCATGACTTCTGGTGGTGATTCT
CCAGGTATGAATGCCGCTGTTCGTGCTGTTGTTCGTACAGGTATACATTTCGGCTGTGATGTTTT
TGCTGTTTACGAAGGTTACGAAGGTTTACTAAGAGGTGGTAAATATTTAAAGAAAATGGCTTGG
GAAGATGTCAGAGGTTGGTTAAGTGAAGGTGGTACTTTGATTGGTACTGCTCGTTCCATGGAATT
CAGAAAGCGTGAGGGTCGTAGACAAGCTGCAGGCAATTTAATTTCGCAAGGTATTGACGCTTTGG
TTGTTTGTGGTGGTGATGGTTCTTTAACCGGTGCTGATCTTTTCAGACACGAATGGCCATCTTTG
GTTGATGAATTGGTTGCAGAAGGTAGATTCACTAAAGAAGAAGTCGCCCCATACAAGAATTTGTC
CATTGTTGGTCTTGTCGGTTCCATCGATAATGATATGTCTGGTACTGACTCTACCATTGGTGCTT
ATTCTGCTTTGGAAAGAATCTGTGAAATGGTTGACTACATTGATGCCACCGCTAAATCCCACTCC
CGTGCCTTTGTTGTTGAAGTTATGGGTAGACATTGTGGTTGGTTGGCCTTGATGGCTGGTATTGC
TACCGGTGCCGATTACATTTTTATTCCAGAAAGAGCTGTTCCTCACGGAAAATGGCAGGACGAAT
TGAAGGAAGTGTGCCAAAGACACAGAAGTAAGGGTAGAAGAAATAACACAATTATTGTCGCTGA
AGGTGCTTTAGATGATCAATTAAACCCTGTTACTGCCAATGACGTCAAGGATGCTTTGATTGAAT
TGGGTCTAGACACCAAGGTAACCATTCTAGGTCACGTTCAAAGAGGTGGTACAGCTGTTGCTCAT
GACAGATGGTTAGCTACTCTACAAGGTGTCGATGCTGTTAAGGCCGTTCTGGAATTTACCCCTGA
AACTCCTTCTCCATTAATTGGTATTTTAGAAAACAAGATAATTAGAATGCCATTGGTTGAATCTG
TGAAGTTGACTAAATCTGTTGCCACTGCCATTGAAAACAAAGATTTCGATAAGGCAATTTCTTTA
AGAGACACAGAATTTATTGAACTTTACGAAAACTTCTTATCCACTACCGTTAAAGATGATGGTTC
CGAATTATTGCCAGTATCTGACAGACTAAACATTGGTATTGTCCATGTTGGTGCCCCATCTGCTG
CTTTGAACGCTGCCACCCGTGCCGCAACCCTATACTGTTTGTCTCACGGCCATAAACCATACGCTA
TCATGAATGGTTTCAGTGGATTGATTCAAACCGGTGAAGTGAAGGAACTATCATGGATTGATGTC
GAAAACTGGCATAACTTGGGTGGTTCCGAAATCGGTACAAACAGATCTGTTGCTTCAGAAGATTT
AGGTACCATTGCTTACTACTTCCAAAAGAACAAGCTAGACGGTTTGATTATTCTTGGTGGTTTTG
AAGGTTTCAGGTCCTTGAAGCAATTGCGTGACGGTAGAACCCAACACCCAATCTTTAACATTCCA
ATGTGTTTGATTCCAGCCACTGTTTCTAACAACGTTCCAGGTACTGAATACTCACTTGGTGTTGA
TACCTGTTTGAACGCATTAGTCAATTACACTGATGACATCAAACAAAGTGCTTCTGCCACAAGAA
GAAGAGTCTTCGTCTGTGAAGTCCAAGGTGGTCACTCTGGTTACATCGCTTCTTTCACCGGTTTA
ATCACTGGTGCTGTTTCCGTGTACACTCCAGAAAAGAAGATCGACTTAGCTTCTATCAGAGAAGA
TATAACTCTATTAAAAGAGAACTTCCGTCACGATAAAGGTGAAAACAGAAACGGTAAGCTATTGG
TTAGAAACGAACAAGCTTCTAGCGTATATAGCACTCAATTGTTGGCTGACATCATCTCTGAAGCA
AGCAAGGGTAAGTTTGGTGTTAGAACTGCTATCCCAGGCCATGTTCAACAAGGTGGTGTTCCATC

TTCTAAAGACCGTGTCACCGCTTCCAGATTTGCTGTCAAATGTATCAAGTTTATCGAACAATGGA
ACAAGAAAAATGAAGCTTCTCCAAACACTGACGCTAAGGTTTTGAGATTCAAGTTCGATGCTCAC
GGTGAAAAGGTACCAACTGTTGAGCACGAAGATGACTCTGCTGCTGTTATCTGTGTTAATGGTTC
TCACGTTTCCTTCAAGCCAATTGCTAACCTTTGGGAAAACGAAACCAACGTTGAATTAAGAAAGG
GTTTTGAAGTTCACTGGGCTGAATACAACAAGATTGGTGACATCCTGTCCGGTAGATTAAAGTTG
AGAGCTGAGGTAACCGCTTTAGCCGCTGAAAACAAATGA

---> Sequence Showing highlighted Existant Combined Trimers

ATG CAA TCT CAA GAT TCA TGC TAC GGT GTT GCA TTC AGA TCT ATC ATC ACA AAT GAT
GAA GCT TTA TTC AAG AAG ACC ATT CAC TTT TAT CAC ACT CTA GGA TTT GCA ACT GTG
AAA GAT TTC AAC AAA TTC AAA CAT GGT GAA AAT AGC TTA CTA TCT TCA GGG ACT TCC
CAA GAT TCC TTG AGA GAA GTT TGG TTA GAA TCT TTC AAG TTG AGT GAG GTT GAT GCT
TCT GGG TTC CGT ATA CCA CAA CAA GAA GCT ACT AAC AAG GCT CAA AGT CAA GGT GCT
CTA TTA AAG ATT CGT TTA GTG ATG TCT GCT CCA ATC GAT GAA ACT TTC GAC ACC AAC
GAA ACC GCC ACA ATC ACT TAT TTC TCT ACT GAT TTG AAT AAA ATT GTC GAG AAA TTC
CCA AAA CAA GCC GAA AAA TTG TCC GAT ACC TTA GTG TTT TTG AAA GAT CCA ATG GGC
AAC AAC ATC ACC TTC TCA GGC TTA GCT AAT GCA ACC GAT TCC GCT CCA ACT TCC AAA
GAT GCT TTC TTA GAA GCT ACC TCC GAA GAC GAA ATC ATC TCT AGA GCT TCT TCC GAT
GCT TCT GAC TTA CTA AGA CAA ACA TTG GGC TCT TCT CAA AAG AAG AAG AAG ATC GCT
GTC ATG ACT TCT GGT GGT GAT TCT CCA GGT ATG AAT GCC GCT GTT CGT GCT GTT GTT
CGT ACA GGT ATA CAT TTC GGC TGT GAT GTT TTT GCT GTT TAC GAA GGT TAC GAA GGT
TTA CTA AGA GGT GGT AAA TAT TTA AAG AAA ATG GCT TGG GAA GAT GTC AGA GGT TGG
TTA AGT GAA GGT GGT ACT TTG ATT GGT ACT GCT CGT TCC ATG GAA TTC AGA AAG CGT
GAG GGT CGT AGA CAA GCT GCA GGC AAT TTA ATT TCG CAA GGT ATT GAC GCT TTG GTT
GTT TGT GGT GGT GAT GGT TCT TTA ACC GGT GCT GAT CTT TTC AGA CAC GAA TGG CCA
TCT TTG GTT GAT GAA TTG GTT GCA GAA GGT AGA TTC ACT AAA GAA GAA GTC GCC CCA
TAC AAG AAT TTG TCC ATT GTT GGT CTT GTC GGT TCC ATC GAT AAT GAT ATG TCT GGT
ACT GAC TCT ACC ATT GGT GCT TAT TCT GCT TTG GAA AGA ATC TGT GAA ATG GTT GAC
TAC ATT GAT GCC ACC GCT AAA TCC CAC TCC CGT GCC TTT GTT GTT GAA GTT ATG GGT
AGA CAT TGT GGT TGG TTG GCC TTG ATG GCT GGT ATT GCT ACC GGT GCC GAT TAC ATT
TTT ATT CCA GAA AGA GCT GTT CCT CAC GGA AAA TGG CAG GAC GAA TTG AAG GAA GTG
TGC CAA AGA CAC AGA AGT AAG GGT AGA AGA AAT AAC ACA ATT ATT GTC GCT GAA GGT
GCT TTA GAT GAT CAA TTA AAC CCT GTT ACT GCC AAT GAC GTC AAG GAT GCT TTG ATT
GAA TTG GGT CTA GAC ACC AAG GTA ACC ATT CTA GGT CAC GTT CAA AGA GGT GGT ACA
GCT GTT GCT CAT GAC AGA TGG TTA GCT ACT CTA CAA GGT GTC GAT GCT GTT AAG GCC
GTT CTG GAA TTT ACC CCT GAA ACT CCT TCT CCA TTA ATT GGT ATT TTA GAA AAC AAG
ATA ATT AGA ATG CCA TTG GTT GAA TCT GTG AAG TTG ACT AAA TCT GTT GCC ACT GCC
ATT GAA AAC AAA GAT TTC GAT AAG GCA ATT TCT TTA AGA GAC ACA GAA TTT ATT GAA
CTT TAC GAA AAC TTC TTA TCC ACT ACC GTT AAA GAT GAT GGT TCC GAA TTA TTG CCA
GTA TCT GAC AGA CTA AAC ATT GGT ATT GTC CAT GTT GGT GCC CCA TCT GCT GCT TTG
AAC GCT GCC ACC CGT GCC GCA ACC CTA TAC TGT TTG TCT CAC GGC CAT AAA CCA TAC
GCT ATC ATG AAT GGT TTC AGT GGA TTG ATT CAA ACC GGT GAA GTG AAG GAA CTA TCA
TGG ATT GAT GTC GAA AAC TGG CAT AAC TTG GGT GGT TCC GAA ATC GGT ACA AAC AGA
TCT GTT GCT TCA GAA GAT TTA GGT ACC ATT GCT TAC TAC TTC CAA AAG AAC AAG CTA

GAC GGT TTG ATT ATT CTT GGT GGT TTT GAA GGT TTC AGG TCC TTG AAG CAA TTG CGT GAC GGT AGA ACC CAA CAC CCA ATC TTT AAC ATT CCA ATG TGT TTG ATT CCA GCC ACT GTT TCT AAC AAC GTT CCA GGT ACT GAA TAC TCA CTT GGT GTT GAT ACC TGT TTG AAC GCA TTA GTC AAT TAC ACT GAT GAC ATC AAA CAA AGT GCT TCT GCC ACA AGA AGA AGA GTC TTC GTC TGT GAA GTC CAA GGT GGT CAC TCT GGT TAC ATC GCT TCT TTC ACC GGT TTA ATC ACT GGT GCT GTT TCC GTG TAC ACT CCA GAA AAG AAG ATC GAC TTA GCT TCT ATC AGA GAA GAT ATA ACT CTA TTA AAA GAG AAC TTC CGT CAC GAT AAA GGT GAA AAC AGA AAC GGT AAG CTA TTG GTT AGA AAC GAA CAA GCT TCT AGC GTA TAT AGC ACT CAA TTG TTG GCT GAC ATC ATC TCT GAA GCA AGC AAG GGT AAG TTT GGT GTT AGA ACT GCT ATC CCA GGC CAT GTT CAA CAA GGT GGT GTT CCA TCT TCT AAA GAC CGT GTC ACC GCT TCC AGA TTT GCT GTC AAA TGT ATC AAG TTT ATC GAA CAA TGG AAC AAG AAA AAT GAA GCT TCT CCA AAC ACT GAC GCT AAG GTT TTG AGA TTC AAG TTC GAT GCT CAC GGT GAA AAG GTA CCA ACT GTT GAG CAC GAA GAT GAC TCT GCT GCT GTT ATC TGT GTT AAT GGT TCT CAC GTT TCC TTC AAG CCA ATT GCT AAC CTT TGG GAA AAC GAA ACC AAC GTT GAA TTA AGA AAG GGT TTT GAA GTT CAC TGG GCT GAA TAC AAC AAG ATT GGT GAC ATC CTG TCC GGT AGA TTA AAG TTG AGA GCT GAG GTA ACC GCT TTA GCC GCT GAA AAC AAA TGA

Occurrences/Results 9 Pentamers

The pentamer 'ATTCA' occurred 9 times.
The pentamer 'TTCAA' occurred 9 times.
The pentamer 'TCAAG' occurred 8 times.
The pentamer 'CAAGA' occurred 9 times.
The pentamer 'AAGAT' occurred 16 times.
The pentamer 'AGATG' occurred 6 times.
The pentamer 'GATGA' occurred 7 times.
The pentamer 'ATGAA' occurred 6 times.
The pentamer 'TGAAT' occurred 9 times.
The total occurrences of all specified strings is 79.
The total number of characters in the text is 2960.
First result (Total characters *9 /1024): 26.05078125
Square root of the first result: 5.103996595806075
Final result: 10.374070153868846

Occurrences/Results TRIMERS Codons

The pentamer 'ATT' occurred 33 times.
The pentamer 'CAA' occurred 27 times.
The pentamer 'GAT' occurred 38 times.
The pentamer 'GAA' occurred 61 times.
The pentamer 'CCA' occurred 24 times.
The pentamer 'ACT' occurred 29 times.
The pentamer 'AGA' occurred 36 times.
The pentamer 'TAC' occurred 16 times.
The total occurrences of all specified strings is 264.
The total number of characters in the text is 2960.

First result ((total_characters / 3) * (Trimer_number / 64)): 123.3
Square root of the first result: 11.1
Final result: 12.7

Occurrences/Results TRIMERS Combinations

The pentamer 'ATT ATT' occurred 2 times.
The pentamer 'ATT CAA' occurred 1 times.
The pentamer 'ATT GAT' occurred 2 times.
The pentamer 'ATT GAA' occurred 3 times.
The pentamer 'ATT CCA' occurred 3 times.
The pentamer 'ATT ACT' occurred 0 times.
The pentamer 'ATT AGA' occurred 1 times.
The pentamer 'ATT TAC' occurred 0 times.
The pentamer 'CAA ATT' occurred 0 times.
The pentamer 'CAA CAA' occurred 2 times.
The pentamer 'CAA GAT' occurred 2 times.
The pentamer 'CAA GAA' occurred 1 times.
The pentamer 'CAA CCA' occurred 0 times.
The pentamer 'CAA ACT' occurred 0 times.
The pentamer 'CAA AGA' occurred 2 times.
The pentamer 'CAA TAC' occurred 0 times.
The pentamer 'GAT ATT' occurred 0 times.
The pentamer 'GAT CAA' occurred 1 times.
The pentamer 'GAT GAT' occurred 2 times.
The pentamer 'GAT GAA' occurred 3 times.
The pentamer 'GAT CCA' occurred 1 times.
The pentamer 'GAT ACT' occurred 0 times.
The pentamer 'GAT AGA' occurred 0 times.
The pentamer 'GAT TAC' occurred 1 times.
The pentamer 'GAA ATT' occurred 0 times.
The pentamer 'GAA CAA' occurred 2 times.
The pentamer 'GAA GAT' occurred 4 times.
The pentamer 'GAA GAA' occurred 1 times.
The pentamer 'GAA CCA' occurred 0 times.
The pentamer 'GAA ACT' occurred 2 times.
The pentamer 'GAA AGA' occurred 2 times.
The pentamer 'GAA TAC' occurred 2 times.
The pentamer 'CCA ATT' occurred 1 times.
The pentamer 'CCA CAA' occurred 1 times.
The pentamer 'CCA GAT' occurred 0 times.
The pentamer 'CCA GAA' occurred 2 times.
The pentamer 'CCA CCA' occurred 0 times.
The pentamer 'CCA ACT' occurred 2 times.
The pentamer 'CCA AGA' occurred 0 times.

The pentamer 'CCA TAC' occurred 2 times.
The pentamer 'ACT ATT' occurred 0 times.
The pentamer 'ACT CAA' occurred 1 times.
The pentamer 'ACT GAT' occurred 2 times.
The pentamer 'ACT GAA' occurred 1 times.
The pentamer 'ACT CCA' occurred 1 times.
The pentamer 'ACT ACT' occurred 0 times.
The pentamer 'ACT AGA' occurred 0 times.
The pentamer 'ACT TAC' occurred 0 times.
The pentamer 'AGA ATT' occurred 0 times.
The pentamer 'AGA CAA' occurred 2 times.
The pentamer 'AGA GAT' occurred 0 times.
The pentamer 'AGA GAA' occurred 2 times.
The pentamer 'AGA CCA' occurred 0 times.
The pentamer 'AGA ACT' occurred 1 times.
The pentamer 'AGA AGA' occurred 2 times.
The pentamer 'AGA TAC' occurred 0 times.
The pentamer 'TAC ATT' occurred 2 times.
The pentamer 'TAC CAA' occurred 0 times.
The pentamer 'TAC GAT' occurred 0 times.
The pentamer 'TAC GAA' occurred 3 times.
The pentamer 'TAC CCA' occurred 0 times.
The pentamer 'TAC ACT' occurred 2 times.
The pentamer 'TAC AGA' occurred 0 times.
The pentamer 'TAC TAC' occurred 1 times.
The total occurrences of all specified strings is 68.
The total number of characters in the text is 2960.
First result (((total_characters/ 3) -1) / 64): 15.421875
Square root of the first result: 3.927069518101252
Final result: 13.388641264853813

5) Homo sapiens

>NC_000001.11:c16546009-16545939 Homo sapiens chromosome 1, GRCh38.p14, tRNA-Gly

GCATTGGTGGTTCAGTGGTAGAATTCTCGCCTCCCACGCGGGAGACCCGGGTTCAA
TTCCCGGCCAATGCA

>NM_001067.4 Homo sapiens, DNA topoisomerase II
---> Sequence

AACCGACGCGCGTCTGTGGAGAAGCGGCTTGGTCGGGGGTGGTCTCGTGGGGTCCTG
CCTGTTTAGTCGCTTTCAGGGTTCTTGAGCCCCTTCACGACCGTCACCATGGAAGTGT
CACCATTGCAGCCTGTAAATGAAAATATGCAAGTCAACAAAATAAAGAAAAATGAA
GATGCTAAGAAAAGACTGTCTGTTGAAAGAATCTATCAAAAGAAAACACAATTGGA
ACATATTTTGCTCCGCCCAGACACCTACATTGGTTCTGTGGAATTAGTGACCCAGCAA
ATGTGGGTTTACGATGAAGATGTTGGCATTAACTATAGGGAAGTCACTTTTGTTCCTG
GTTTGTACAAAATCTTTGATGAGATTCTAGTTAATGCTGCGGACAACAAACAAAGGG
ACCCAAAAATGTCTTGTATTAGAGTCACAATTGATCCGGAAAACAATTTAATTAGTA
TATGGAATAATGGAAAAGGTATTCCTGTTGTTGAACACAAAGTTGAAAAGATGTATG
TCCCAGCTCTCATATTTGGACAGCTCCTAACTTCTAGTAACTATGATGATGATGAAAA
GAAAGTGACAGGTGGTCGAAATGGCTATGGAGCCAAATTGTGTAACATATTCAGTAC
CAAATTTACTGTGGAAACAGCCAGTAGAGAATACAAGAAAATGTTCAAACAGACAT
GGATGGATAATATGGGAAGAGCTGGTGAGATGGAACTCAAGCCCTTCAATGGAGAA
GATTATACATGTATCACCTTTCAGCCTGATTTGTCTAAGTTTAAAATGCAAAGCCTGG
ACAAAGATATTGTTGCACTAATGGTCAGAAGAGCATATGATATTGCTGGATCCACCA
AAGATGTCAAAGTCTTTCTTAATGGAAATAAACTGCCAGTAAAAGGATTTCGTAGTT
ATGTGGACATGTATTTGAAGGACAAGTTGGATGAAACTGGTAACTCCTTGAAAGTAA
TACATGAACAAGTAAACCACAGGTGGGAAGTGTGTTTAACTATGAGTGAAAAAGGC
TTTCAGCAAATTAGCTTTGTCAACAGCATTGCTACATCCAAGGGTGGCAGACATGTT
GATTATGTAGCTGATCAGATTGTGACTAAACTTGTTGATGTTGTGAAGAAGAAGAAC
AAGGGTGGTGTTGCAGTAAAAGCACATCAGGTGAAAAATCACATGTGGATTTTTGTA
AATGCCTTAATTGAAAACCCAACCTTTGACTCTCAGACAAAAGAAAACATGACTTTA
CAACCCAAGAGCTTTGGATCAACATGCCAATTGAGTGAAAAATTTATCAAAGCTGCC
ATTGGCTGTGGTATTGTAGAAAGCATACTAAACTGGGTGAAGTTTAAGGCCCAAGTC
CAGTTAAACAAGAAGTGTTCAGCTGTAAAACATAATAGAATCAAGGGAATTCCCAA
ACTCGATGATGCCAATGATGCAGGGGGCCGAAACTCCACTGAGTGTACGCTTATCCT
GACTGAGGGAGATTCAGCCAAAACTTTGGCTGTTTCAGGCCTTGGTGTGGTTGGGAG
AGACAAATATGGGGTTTTCCCTCTTAGAGGAAAAATACTCAATGTTCGAGAAGCTTC
TCATAAGCAGATCATGGAAAATGCTGAGATTAACAATATCATCAAGATTGTGGGTCT
TCAGTACAAGAAAAACTATGAAGATGAAGATTCATTGAAGACGCTTCGTTATGGGAA
GATAATGATTATGACAGATCAGGACCAAGATGGTTCCCACATCAAAGGCTTGCTGAT
TAATTTTATCCATCACAACTGGCCCTCTCTTCTGCGACATCGTTTTCTGGAGGAATTT
ATCACTCCCATTGTAAAGGTATCTAAAAACAAGCAAGAAATGGCATTTTACAGCCTT
CCTGAATTTGAAGAGTGGAAGAGTTCTACTCCAAATCATAAAAAATGGAAAGTCAAA
TATTACAAAGGTTTGGGCACCAGCACATCAAAGGAAGCTAAAGAATACTTTGCAGAT
ATGAAAAGACATCGTATCCAGTTCAAATATTCTGGTCCTGAAGATGATGCTGCTATC
AGCCTGGCCTTTAGCAAAAAACAGATAGATGATCGAAAGGAATGGTTAACTAATTTC
ATGGAGGATAGAAGACAACGAAAGTTACTTGGGCTTCCTGAGGATTACTTGTATGGA
CAAACTACCACATATCTGACATATAATGACTTCATCAACAAGGAACTTATCTTGTTCT
CAAATTCTGATAACGAGAGATCTATCCCTTCTATGGTGGATGGTTTGAAACCAGGTC
AGAGAAAGGTTTTGTTTACTTGCTTCAAACGGAATGACAAGCGAGAAGTAAAGGTTG
CCCAATTAGCTGGATCAGTGGCTGAAATGTCTTCTTATCATCATGGTGAGATGTCACT

AATGATGACCATTATCAATTTGGCTCAGAATTTTGTGGGTAGCAATAATCTAAACCTC
TTGCAGCCCATTGGTCAGTTTGGTACCAGGCTACATGGTGGCAAGGATTCTGCTAGTC
CACGATACATCTTTACAATGCTCAGCTCTTTGGCTCGATTGTTATTTCCACCAAAAGA
TGATCACACGTTGAAGTTTTTATATGATGACAACCAGCGTGTTGAGCCTGAATGGTA
CATTCCTATTATTCCCATGGTGCTGATAAATGGTGCTGAAGGAATCGGTACTGGGTG
GTCCTGCAAAATCCCCAACTTTGATGTGCGTGAAATTGTAAATAACATCAGGCGTTT
GATGGATGGAGAAGAACCTTTGCCAATGCTTCCAAGTTACAAGAACTTCAAGGGTAC
TATTGAAGAACTGGCTCCAAATCAATATGTGATTAGTGGTGAAGTAGCTATTCTTAAT
TCTACAACCATTGAAATCTCAGAGCTTCCCGTCAGAACATGGACCCAGACATACAAA
GAACAAGTTCTAGAACCCATGTTGAATGGCACCGAGAAGACACCTCCTCTCATAACA
GACTATAGGGAATACCATACAGATACCACTGTGAAATTTGTTGTGAAGATGACTGAA
GAAAAACTGGCAGAGGCAGAGAGAGTTGGACTACACAAAGTCTTCAAACTCCAAAC
TAGTCTCACATGCAACTCTATGGTGCTTTTTGACCACGTAGGCTGTTTAAAGAAATAT
GACACGGTGTTGGATATTCTAAGAGACTTTTTTGAACTCAGACTTAAATATTATGGAT
TAAGAAAAGAATGGCTCCTAGGAATGCTTGGTGCTGAATCTGCTAAACTGAATAATC
AGGCTCGCTTTATCTTAGAGAAAATAGATGGCAAAATAATCATTGAAAATAAGCCTA
AGAAAGAATTAATTAAAGTTCTGATTCAGAGGGGATATGATTCGGATCCTGTGAAGG
CCTGGAAAGAAGCCCAGCAAAAGGTTCCAGATGAAGAAGAAAATGAAGAGAGTGAC
AACGAAAAGGAAACTGAAAAGAGTGACTCCGTAACAGATTCTGGACCAACCTTCAA
CTATCTTCTTGATATGCCCCTTTGGTATTTAACCAAGGAAAAGAAAGATGAACTCTGC
AGGCTAAGAAATGAAAAAGAACAAGAGCTGGACACATTAAAAAGAAAGAGTCCATC
AGATTTGTGGAAAGAAGACTTGGCTACATTTATTGAAGAATTGGAGGCTGTTGAAGC
CAAGGAAAAACAAGATGAACAAGTCGGACTTCCTGGGAAAGGGGGGAAGGCCAAG
GGGAAAAAAACACAAATGGCTGAAGTTTTGCCTTCTCCGCGTGGTCAAAGAGTCATT
CCACGAATAACCATAGAAATGAAAGCAGAGGCAGAAAAGAAAAATAAAAAGAAAA
TTAAGAATGAAAATACTGAAGGAAGCCCTCAAGAAGATGGTGTGGAACTAGAAGGC
CTAAAACAAAGATTAGAAAAGAAACAGAAAAGAGAACCAGGTACAAAGACAAAGA
AACAAACTACATTGGCATTTAAGCCAATCAAAAAAGGAAAGAAGAGAAATCCCTGG
TCTGATTCAGAATCAGATAGGAGCAGTGACGAAAGTAATTTTGATGTCCCTCCACGA
GAAACAGAGCCACGGAGAGCAGCAACAAAAACAAAATTCACAATGGATTTGGATTC
AGATGAAGATTTCTCAGATTTTGATGAAAAAACTGATGATGAAGATTTTGTCCCATC
AGATGCTAGTCCACCTAAGACCAAAACTTCCCCAAAACTTAGTAACAAAGAACTGAA
ACCACAGAAAAGTGTCGTGTCAGACCTTGAAGCTGATGATGTTAAGGGCAGTGTACC
ACTGTCTTCAAGCCCTCCTGCTACACATTTCCCAGATGAAACTGAAATTACAAACCCA
GTTCCTAAAAAGAATGTGACAGTGAAGAAGACAGCAGCAAAAAGTCAGTCTTCCAC
CTCCACTACCGGTGCCAAAAAAAAGGGCTGCCCCAAAAGGAACTAAAAGGGATCCAG
CTTTGAATTCTGGTGTCTCTCAAAAGCCTGATCCTGCCAAAACCAAGAATCGCCGCA
AAAGGAAGCCATCCACTTCTGATGATTCTGACTCTAATTTTGAGAAAATTGTTTCGAA
AGCAGTCACAAGCAAGAAATCCAAGGGGGAGAGTGATGACTTCCATATGGACTTTG
ACTCAGCTGTGGCTCCTCGGGCAAAATCTGTACGGGCAAAGAAACCTATAAAGTACC
TGGAAGAGTCAGATGAAGATGATCTGTTTTAAAATGTGAGGCGATTATTTTAAGTAA
TTATCTTACCAAGCCCAAGACTGGTTTTAAAGTTACCTGAAGCTCTTAACTTCCTCCC
CTCTGAATTTAGTTTGGGGAAGGTGTTTTTAGTACAAGACATCAAAGTGAAGTAAAG
CCCAAGTGTTCTTTAGCTTTTTATAATACTGTCTAAATAGTGACCATCTCATGGGCAT
TGTTTTCTTCTCTGCTTTGTCTGTGTTTTGAGTCTGCTTTCTTTTGTCTTTAAAACCTGA
TTTTTAAGTTCTTCTGAACTGTAGAAATAGCTATCTGATCACTTCAGCGTAAAGCAGT
GTGTTTATTAACCATCCACTAAGCTAAAACTAGAGCAGTTTGATTTAAAAGTGTCACT
CTTCCTCCTTTTCTACTTTCAGTAGATATGAGATAGAGCATAATTATCTGTTTTATCTT
AGTTTTATACATAATTTACCATCAGATAGAACTTTATGGTTCTAGTACAGATACTCTA
CTACACTCAGCCTCTTATGTGCCAAGTTTTTCTTTAAGCAATGAGAAATTGCTCATGT
TCTTCATCTTCTCAAATCATCAGAGGCCGAAGAAAAACACTTTGGCTGTGTCTATAAC

TTGACACAGTCAATAGAATGAAGAAAATTAGAGTAGTTATGTGATTATTTCAGCTCT
TGACCTGTCCCCTCTGGCTGCCTCTGAGTCTGAATCTCCCAAAGAGAGAAACCAATTT
CTAAGAGGACTGGATTGCAGAAGACTCGGGGACAACATTTGATCCAAGATCTTAAAT
GTTATATTGATAACCATGCTCAGCAATGAGCTATTAGATTCATTTTGGGAAATCTCCA
TAATTTCAATTTGTAAACTTTGTTAAGACCTGTCTACATTGTTATATGTGTGTGACTTG
AGTAATGTTATCAACGTTTTTGTAAATATTTACTATGTTTTTCTATTAGCTAAATTCCA
ACAATTTTGTACTTTAATAAAATGTTCTAAACATTGCAA

---> Sequence Showing highlighted Existant Combined Trimers

AAC CGA CGC GCG TCT GTG GAG AAG CGG CTT GGT CGG GGG TGG TCT CGT GGG
GTC CTG CCT GTT TAG TCG CTT TCA GGG TTC TTG AGC CCC TTC ACG ACC GTC
ACC ATG GAA GTG TCA CCA TTG CAG CCT GTA AAT GAA AAT ATG CAA GTC AAC
AAA ATA AAG AAA AAT GAA GAT GCT AAG AAA AGA CTG TCT GTT GAA AGA
ATC TAT CAA AAG AAA ACA CAA TTG GAA CAT ATT TTG CTC CGC CCA GAC ACC
TAC ATT GGT TCT GTG GAA TTA GTG ACC CAG CAA ATG TGG GTT TAC GAT GAA
GAT GTT GGC ATT AAC TAT AGG GAA GTC ACT TTT GTT CCT GGT TTG TAC AAA
ATC TTT GAT GAG ATT CTA GTT AAT GCT GCG GAC AAC AAA CAA AGG GAC CCA
AAA ATG TCT TGT ATT AGA GTC ACA ATT GAT CCG GAA AAC AAT TTA ATT AGT
ATA TGG AAT AAT GGA AAA GGT ATT CCT GTT GTT GAA CAC AAA GTT GAA AAG
ATG TAT GTC CCA GCT CTC ATA TTT GGA CAG CTC CTA ACT TCT AGT AAC TAT
GAT GAT GAT GAA AAG AAA GTG ACA GGT GGT CGA AAT GGC TAT GGA GCC
AAA TTG TGT AAC ATA TTC AGT ACC AAA TTT ACT GTG GAA ACA GCC AGT AGA
GAA TAC AAG AAA ATG TTC AAA CAG ACA TGG ATG GAT AAT ATG GGA AGA
GCT GGT GAG ATG GAA CTC AAG CCC TTC AAT GGA GAA GAT TAT ACA TGT ATC
ACC TTT CAG CCT GAT TTG TCT AAG TTT AAA ATG CAA AGC CTG GAC AAA GAT
ATT GTT GCA CTA ATG GTC AGA AGA GCA TAT GAT ATT GCT GGA TCC ACC AAA
GAT GTC AAA GTC TTT CTT AAT GGA AAT AAA CTG CCA GTA AAA GGA TTT CGT
AGT TAT GTG GAC ATG TAT TTG AAG GAC AAG TTG GAT GAA ACT GGT AAC TCC
TTG AAA GTA ATA CAT GAA CAA GTA AAC CAC AGG TGG GAA GTG TGT TTA ACT
ATG AGT GAA AAA GGC TTT CAG CAA ATT AGC TTT GTC AAC AGC ATT GCT ACA
TCC AAG GGT GGC AGA CAT GTT GAT TAT GTA GCT GAT CAG ATT GTG ACT AAA
CTT GTT GAT GTT GTG AAG AAG AAG AAC AAG GGT GGT GTT GCA GTA AAA GCA
CAT CAG GTG AAA AAT CAC ATG TGG ATT TTT GTA AAT GCC TTA ATT GAA AAC
CCA ACC TTT GAC TCT CAG ACA AAA GAA AAC ATG ACT TTA CAA CCC AAG AGC
TTT GGA TCA ACA TGC CAA TTG AGT GAA AAA TTT ATC AAA GCT GCC ATT GGC
TGT GGT ATT GTA GAA AGC ATA CTA AAC TGG GTG AAG TTT AAG GCC CAA GTC
CAG TTA AAC AAG AAG TGT TCA GCT GTA AAA CAT AAT AGA ATC AAG GGA ATT
CCC AAA CTC GAT GAT GCC AAT GAT GCA GGG GGC CGA AAC TCC ACT GAG TGT
ACG CTT ATC CTG ACT GAG GGA GAT TCA GCC AAA ACT TTG GCT GTT TCA GGC
CTT GGT GTG GTT GGG AGA GAC AAA TAT GGG GTT TTC CCT CTT AGA GGA AAA
ATA CTC AAT GTT CGA GAA GCT TCT CAT AAG CAG ATC ATG GAA AAT GCT GAG
ATT AAC AAT ATC ATC AAG ATT GTG GGT CTT CAG TAC AAG AAA AAC TAT GAA
GAT GAA GAT TCA TTG AAG ACG CTT CGT TAT GGG AAG ATA ATG ATT ATG ACA
GAT CAG GAC CAA GAT GGT TCC CAC ATC AAA GGC TTG CTG ATT AAT TTT ATC
CAT CAC AAC TGG CCC TCT CTT CTG CGA CAT CGT TTT CTG GAG GAA TTT ATC

ACT CCC ATT GTA AAG GTA TCT AAA AAC AAG CAA GAA ATG GCA TTT TAC AGC CTT CCT GAA TTT GAA GAG TGG AAG AGT TCT ACT CCA AAT CAT AAA AAA TGG AAA GTC AAA TAT TAC AAA GGT TTG GGC ACC AGC ACA TCA AAG GAA GCT AAA GAA TAC TTT GCA GAT ATG AAA AGA CAT CGT ATC CAG TTC AAA TAT TCT GGT CCT GAA GAT GAT GCT GCT ATC AGC CTG GCC TTT AGC AAA AAA CAG ATA GAT GAT CGA AAG GAA TGG TTA ACT AAT TTC ATG GAG GAT AGA AGA CAA CGA AAG TTA CTT GGG CTT CCT GAG GAT TAC TTG TAT GGA CAA ACT ACC ACA TAT CTG ACA TAT AAT GAC TTC ATC AAC AAG GAA CTT ATC TTG TTC TCA AAT TCT GAT AAC GAG AGA TCT ATC CCT TCT ATG GTG GAT GGT TTG AAA CCA GGT CAG AGA AAG GTT TTG TTT ACT TGC TTC AAA CGG AAT GAC AAG CGA GAA GTA AAG GTT GCC CAA TTA GCT GGA TCA GTG GCT GAA ATG TCT TCT TAT CAT CAT GGT GAG ATG TCA CTA ATG ATG ACC ATT ATC AAT TTG GCT CAG AAT TTT GTG GGT AGC AAT AAT CTA AAC CTC TTG CAG CCC ATT GGT CAG TTT GGT ACC AGG CTA CAT GGT GGC AAG GAT TCT GCT AGT CCA CGA TAC ATC TTT ACA ATG CTC AGC TCT TTG GCT CGA TTG TTA TTT CCA CCA AAA GAT GAT CAC ACG TTG AAG TTT TTA TAT GAT GAC AAC CAG CGT GTT GAG CCT GAA TGG TAC ATT CCT ATT ATT CCC ATG GTG CTG ATA AAT GGT GCT GAA GGA ATC GGT ACT GGG TGG TCC TGC AAA ATC CCC AAC TTT GAT GTG CGT GAA ATT GTA AAT AAC ATC AGG CGT TTG ATG GAT GGA GAA GAA CCT TTG CCA ATG CTT CCA AGT TAC AAG AAC TTC AAG GGT ACT ATT GAA GAA CTG GCT CCA AAT CAA TAT GTG ATT AGT GGT GAA GTA GCT ATT CTT AAT TCT ACA ACC ATT GAA ATC TCA GAG CTT CCC GTC AGA ACA TGG ACC CAG ACA TAC AAA GAA CAA GTT CTA GAA CCC ATG TTG AAT GGC ACC GAG AAG ACA CCT CCT CTC ATA ACA GAC TAT AGG GAA TAC CAT ACA GAT ACC ACT GTG AAA TTT GTT GTG AAG ATG ACT GAA GAA AAA CTG GCA GAG GCA GAG AGA GTT GGA CTA CAC AAA GTC TTC AAA CTC CAA ACT AGT CTC ACA TGC AAC TCT ATG GTG CTT TTT GAC CAC GTA GGC TGT TTA AAG AAA TAT GAC ACG GTG TTG GAT ATT CTA AGA GAC TTT TTT GAA CTC AGA CTT AAA TAT TAT GGA TTA AGA AAA GAA TGG CTC CTA GGA ATG CTT GGT GCT GAA TCT GCT AAA CTG AAT AAT CAG GCT CGC TTT ATC TTA GAG AAA ATA GAT GGC AAA ATA ATC ATT GAA AAT AAG CCT AAG AAA GAA TTA ATT AAA GTT CTG ATT CAG AGG GGA TAT GAT TCG GAT CCT GTG AAG GCC TGG AAA GAA GCC CAG CAA AAG GTT CCA GAT GAA GAA GAA AAT GAA GAG AGT GAC AAC GAA AAG GAA ACT GAA AAG AGT GAC TCC GTA ACA GAT TCT GGA CCA ACC TTC AAC TAT CTT CTT GAT ATG CCC CTT TGG TAT TTA ACC AAG GAA AAG AAA GAT GAA CTC TGC AGG CTA AGA AAT GAA AAA GAA CAA GAG CTG GAC ACA TTA AAA AGA AAG AGT CCA TCA GAT TTG TGG AAA GAA GAC TTG GCT ACA TTT ATT GAA GAA TTG GAG GCT GTT GAA GCC AAG GAA AAA CAA GAT GAA CAA GTC GGA CTT CCT GGG AAA GGG GGG AAG GCC AAG GGG AAA AAA ACA CAA ATG GCT GAA GTT TTG CCT TCT CCG CGT GGT CAA AGA GTC ATT CCA CGA ATA ACC ATA GAA ATG AAA GCA GAG GCA GAA AAG AAA AAT AAA AAG AAA ATT AAG AAT GAA AAT ACT GAA GGA AGC CCT CAA GAA GAT GGT GTG GAA CTA GAA GGC CTA AAA CAA AGA TTA GAA AAG AAA CAG AAA AGA GAA CCA GGT ACA AAG ACA AAG AAA CAA ACT ACA TTG GCA TTT AAG CCA ATC AAA AAA GGA AAG AAG AGA AAT CCC TGG TCT GAT TCA GAA TCA GAT AGG AGC AGT GAC GAA AGT AAT TTT GAT GTC CCT CCA CGA

GAA ACA GAG CCA CGG AGA GCA GCA ACA AAA ACA AAA TTC ACA ATG GAT TTG GAT TCA GAT GAA GAT TTC TCA GAT TTT GAT GAA AAA ACT GAT GAT GAA GAT TTT GTC CCA TCA GAT GCT AGT CCA CCT AAG ACC AAA ACT TCC CCA AAA CTT AGT AAC AAA GAA CTG AAA CCA CAG AAA AGT GTC GTG TCA GAC CTT GAA GCT GAT GAT GTT AAG GGC AGT GTA CCA CTG TCT TCA AGC CCT CCT GCT ACA CAT TTC CCA GAT GAA ACT GAA ATT ACA AAC CCA GTT CCT AAA AAG AAT GTG ACA GTG AAG AAG ACA GCA GCA AAA AGT CAG TCT TCC ACC TCC ACT ACC GGT GCC AAA AAA AGG GCT GCC CCA AAA GGA ACT AAA AGG GAT CCA GCT TTG AAT TCT GGT GTC TCT CAA AAG CCT GAT CCT GCC AAA ACC AAG AAT CGC CGC AAA AGG AAG CCA TCC ACT TCT GAT GAT TCT GAC TCT AAT TTT GAG AAA ATT GTT TCG AAA GCA GTC ACA AGC AAG AAA TCC AAG GGG GAG AGT GAT GAC TTC CAT ATG GAC TTT GAC TCA GCT GTG GCT CCT CGG GCA AAA TCT GTA CGG GCA AAG AAA CCT ATA AAG TAC CTG GAA GAG TCA GAT GAA GAT GAT CTG TTT TAA AAT GTG AGG CGA TTA TTT TAA GTA ATT ATC TTA CCA AGC CCA AGA CTG GTT TTA AAG TTA CCT GAA GCT CTT AAC TTC CTC CCC TCT GAA TTT AGT TTG GGG AAG GTG TTT TTA GTA CAA GAC ATC AAA GTG AAG TAA AGC CCA AGT GTT CTT TAG CTT TTT ATA ATA CTG TCT AAA TAG TGA CCA TCT CAT GGG CAT TGT TTT CTT CTC TGC TTT GTC TGT GTT TTG AGT CTG CTT TCT TTT GTC TTT AAA ACC TGA TTT TTA AGT TCT TCT GAA CTG TAG AAA TAG CTA TCT GAT CAC TTC AGC GTA AAG CAG TGT GTT TAT TAA CCA TCC ACT AAG CTA AAA CTA GAG CAG TTT GAT TTA AAA GTG TCA CTC TTC CTC CTT TTC TAC TTT CAG TAG ATA TGA GAT AGA GCA TAA TTA TCT GTT TTA TCT TAG TTT TAT ACA TAA TTT ACC ATC AGA TAG AAC TTT ATG GTT CTA GTA CAG ATA CTC TAC TAC ACT CAG CCT CTT ATG TGC CAA GTT TTT CTT TAA GCA ATG AGA AAT TGC TCA TGT TCT TCA TCT TCT CAA ATC ATC AGA GGC CGA AGA AAA ACA CTT TGG CTG TGT CTA TAA CTT GAC ACA GTC AAT AGA ATG AAG AAA ATT AGA GTA GTT ATG TGA TTA TTT CAG CTC TTG ACC TGT CCC CTC TGG CTG CCT CTG AGT CTG AAT CTC CCA AAG AGA GAA ACC AAT TTC TAA GAG GAC TGG ATT GCA GAA GAC TCG GGG ACA ACA TTT GAT CCA AGA TCT TAA ATG TTA TAT TGA TAA CCA TGC TCA GCA ATG AGC TAT TAG ATT CAT TTT GGG AAA TCT CCA TAA TTT CAA TTT GTA AAC TTT GTT AAG ACC TGT CTA CAT TGT TAT ATG TGT GTG ACT TGA GTA ATG TTA TCA ACG TTT TTG TAA ATA TTT ACT ATG TTT TTC TAT TAG CTA AAT TCC AAC AAT TTT GTA CTT TAA TAA AAT GTT CTA AAC ATT GCA A

Occurrences/Results 9 Pentamers

The pentamer 'ATTCA' occurred 8 times.
The pentamer 'TTCAA' occurred 9 times.
The pentamer 'TCAAG' occurred 6 times.
The pentamer 'CAAGA' occurred 16 times.
The pentamer 'AAGAT' occurred 22 times.
The pentamer 'AGATG' occurred 22 times.
The pentamer 'GATGA' occurred 25 times.
The pentamer 'ATGAA' occurred 22 times.
The pentamer 'TGAAT' occurred 8 times.
The total occurrences of all specified strings is 138.
The total number of characters in the text is 5691.

First result (Total characters *9 /1024): 50.0537109375
Square root of the first result: 7.074864729272214
Final result: 12.430808563536587

Occurrences/Results TRIMERS Codons ATT;CAA;GAT;GAA;CCA;ACT;AGA;TAC

The pentamer 'ATT' occurred 47 times.
The pentamer 'CAA' occurred 33 times.
The pentamer 'GAT' occurred 81 times.
The pentamer 'GAA' occurred 103 times.
The pentamer 'CCA' occurred 41 times.
The pentamer 'ACT' occurred 33 times.
The pentamer 'AGA' occurred 38 times.
The pentamer 'TAC' occurred 18 times.
The total occurrences of all specified strings is 394.
The total number of characters in the text is 5691.
First result ((total_characters / 3) * (Trimer_number / 64)): 237
Square root of the first result: 15.4
Final result: 10.2

Occurrences/Results TRIMERS Combinations

The pentamer 'ATT ATT' occurred 1 times.
The pentamer 'ATT CAA' occurred 0 times.
The pentamer 'ATT GAT' occurred 1 times.
The pentamer 'ATT GAA' occurred 5 times.
The pentamer 'ATT CCA' occurred 1 times.
The pentamer 'ATT ACT' occurred 0 times.
The pentamer 'ATT AGA' occurred 2 times.
The pentamer 'ATT TAC' occurred 0 times.
The pentamer 'CAA ATT' occurred 1 times.
The pentamer 'CAA CAA' occurred 0 times.
The pentamer 'CAA GAT' occurred 2 times.
The pentamer 'CAA GAA' occurred 2 times.
The pentamer 'CAA CCA' occurred 0 times.
The pentamer 'CAA ACT' occurred 3 times.
The pentamer 'CAA AGA' occurred 2 times.
The pentamer 'CAA TAC' occurred 0 times.
The pentamer 'GAT ATT' occurred 3 times.
The pentamer 'GAT CAA' occurred 0 times.
The pentamer 'GAT GAT' occurred 9 times.
The pentamer 'GAT GAA' occurred 12 times.
The pentamer 'GAT CCA' occurred 2 times.
The pentamer 'GAT ACT' occurred 0 times.
The pentamer 'GAT AGA' occurred 2 times.
The pentamer 'GAT TAC' occurred 1 times.
The pentamer 'GAA ATT' occurred 2 times.
The pentamer 'GAA CAA' occurred 4 times.
The pentamer 'GAA GAT' occurred 10 times.
The pentamer 'GAA GAA' occurred 5 times.
The pentamer 'GAA CCA' occurred 1 times.
The pentamer 'GAA ACT' occurred 3 times.
The pentamer 'GAA AGA' occurred 1 times.
The pentamer 'GAA TAC' occurred 3 times.
The pentamer 'CCA ATT' occurred 0 times.

The pentamer 'CCA CAA' occurred 0 times.
The pentamer 'CCA GAT' occurred 2 times.
The pentamer 'CCA GAA' occurred 0 times.
The pentamer 'CCA CCA' occurred 1 times.
The pentamer 'CCA ACT' occurred 0 times.
The pentamer 'CCA AGA' occurred 2 times.
The pentamer 'CCA TAC' occurred 0 times.
The pentamer 'ACT ATT' occurred 1 times.
The pentamer 'ACT CAA' occurred 0 times.
The pentamer 'ACT GAT' occurred 1 times.
The pentamer 'ACT GAA' occurred 4 times.
The pentamer 'ACT CCA' occurred 1 times.
The pentamer 'ACT ACT' occurred 0 times.
The pentamer 'ACT AGA' occurred 0 times.
The pentamer 'ACT TAC' occurred 0 times.
The pentamer 'AGA ATT' occurred 0 times.
The pentamer 'AGA CAA' occurred 1 times.
The pentamer 'AGA GAT' occurred 0 times.
The pentamer 'AGA GAA' occurred 3 times.
The pentamer 'AGA CCA' occurred 0 times.
The pentamer 'AGA ACT' occurred 0 times.
The pentamer 'AGA AGA' occurred 2 times.
The pentamer 'AGA TAC' occurred 0 times.
The pentamer 'TAC ATT' occurred 2 times.
The pentamer 'TAC CAA' occurred 0 times.
The pentamer 'TAC GAT' occurred 1 times.
The pentamer 'TAC GAA' occurred 0 times.
The pentamer 'TAC CCA' occurred 0 times.
The pentamer 'TAC ACT' occurred 1 times.
The pentamer 'TAC AGA' occurred 0 times.
The pentamer 'TAC TAC' occurred 1 times.
The total occurrences of all specified strings is 101.
The total number of characters in the text is 5691.
First result (((total_characters/ 3) -1) / 64): 29.640625
Square root of the first result: 5.444320435095642
Final result: 13.10712252350121

>Homo sapiens mRNA, helicase

---> Sequence

GCTGTCCGCAGGGAGATTTATGACAAAGTCCAGTTTTCAACAATGCTGAAATCAAATG
ATTGCCTGTTTTCTTTGGAAAATTTGTTTTTTGAAAAACCAGATGAAGTTGAAAACCAT
CCAGACAATGAAAAGTCATTGGATTGGTTTCTCCCTCCTGCTCCATTGATTTCAGAAAT
TCCAGATACTCAGGAGTTAGAGGAAGAATTAGAAAGTCATAAACTGTTAGGTCAGGA
AAAGAGGCCAAAAATGTTAACATCAAATTTAAAGATAACTAATGAAGATACAAATTA
TATTTCACTAACACAAAAATTCCAGTTTGCCTTTCCTTCTGATAAATATGAACAGGATG
ATCTAAATTTAGAAGGGGTAGGTAATAATGACTTACCACATGTTGCTGGCAAGCTGAC
ATATGCTTCTCAGAAATATAAAAATCACATTGGCACTGAGATAGCACCTGAGAAGAGT
GTTCCTGATGATACAAAATTAGTTAATTTTGCAGAAGATAAAGGAGAGAGCACATCAG
TATTCCGGAAAAGATTATTTAAAATATCTGACAATATACATGGGAGTGCTTATTCTAAT
GACAATGAATTGGACTCTCACATTGGCTCAGTGAAAATTGTACAAACAGAAATGAACA

AAGGGAAATCAAGGAACTATAGCAATAGTAAGCAGAAATTTCAGTATTCTGCAAATGT
GTTTACAGCAAATAATGCTTTTTCTGCTTCTGAAATCGGAGAAGGCATGTTCAAAGCA
CCATCTTTTTCAGTTGCTTTCCAACCTCATGATATTCAAGAGGTAACAGAAAATGGTTT
AGGTTCCTTGAAGGCTGTCACAGAAATTCCGGCAAAATTTAGAAGTATTTTCAAAGAA
TTTCCATATTTCAACTATATACAGTCCAAGGCCTTTGATGATCTTCTTTACACAGATAG
GAATTTTGTGATTTGTGCTCCAACTGGTTCTGGAAAAACTGTAGTGTTTGAACTAGCTA
TAACAAGATTGTTAATGGAAGTACCATTGCCATGGTTGAATATTAAAATTGTTTACATG
GCACCAATAAAAGCCTTGTGCAGTCAGCGTTTTGATGACTGGAAAGAAAAATTTGGAC
CAATAGGATTGAATTGTAAAGAACTTACTGGAGATACAGTAATGGATGATCTATTTGA
GATTCAGCATGCCCATATTATTATGACAACTCCAGAAAAATGGGATAGCATGACTAGG
AAATGGAGAGACAACTCTTTGGTTCAGCTGGTTCGACTGCTTCTCATTGATGAGGTAC
ATATTGTAAAAGATGAAAATCGTGGTCCAACTCTTGAAGTTGTAGTTAGCAGAATGAA
AACTGTACAGTCTGTTTCTCAGACTTTAAAAAATACCAGCACTGCTATTCCAATGCGAT
TTGTAGCTGTATCTGCAACAATTCCAAATGCTGAGGATATTGCAGAATGGCTTTCAGAT
GGTGAAAGACCGGCTGTGTGTCTGAAAATGGATGAGAGCCATAGACCAGTGAAACTT
CAGAAAGTGGTCCTTGGATTTCCCTGCAGTAGTAACCAAACTGAGTTTAAGTTTGATTT
AACCCTCAACTACAAAATTGCCAGTGTTATACAAATGTACTCTGATCAGAAACCCACA
CTTGTGTTTTGTGCAACAAGGAAGGGTGTGCAACAGGCTGCTTCTGTTCTTGTGAAAG
ATGCTAAATTTATTATGACTGTGGAACAGAAACAGAGGTTACAGAAGTATGCATATTC
CGTAAGAGATTCAAAACTGAGAGATATCTTAAAAGATGGTGCTGCTTATCATCATGCT
GGTATGGAGCTGTCAGATAGAAAAGTAGTTGAGGGAGCTTTTACTGTTGGAGATTTAC
CAGTTCTTTTTACTACCAGTACTTTAGCTATGGGAGTAAATTTGCCTGCTCACCTAGTA
GTTATAAAATCTACAATGCATTATGCTGGAGGACTGTTTGAAGAGTACAGTGAAACAG
ATATTCTACAGATGATTGGTAGAGCTGGTCGACCTCAATTTGACACTACAGCTACTGC
AGTTATCATGACTCGATTAAGCACAAGGGACAAGTACATTCAGATGTTAGCTTATAGA
GACACTGTAGAAAGCAGTTTGCACAGACATCTTATTGAACATTTAAATGCAGAGATAG
TACTGCATACCATCACGGATGTGAATATTGCTGTGGAATGGATACGATCAACTCTGCTT
TATATCAGAGCCTTGAAAAATCCATCTCATTATGGTTTTGCATCTGGATTGAACAAAGA
TGGAATTGAAGCAAAATTACAAGAATTATGTTTGAAGAATCTGAATGATTTATCATCC
CTGGACTTAATAAAGATGGATGAAGGTGTTAATTTCAAACCAACTGAAGCAGGAAGAT
TGATGGCTTGGTATTATATTACATTTGAGACAGTGAAGAAATTTTATACAATCAGTGG
AAAAGAAACCTTATCAGATCTGGTTACATTGATAGCTGGCTGCAAGGAATTTCTAGAT
ATACAGTTAAGGATAAATGAAAAGAAAACACTGAATACTTTGAACAAAGATCCAAAT
CGGATAACTATCAGATTTCCAATGGAAGGAAGAATTAAAACAAGAGAAATGAAAGTG
AATTGTCTTATTCAGGCTCAACTAGGATGCATTCCCATACAAGATTTTGCTTTGACACA
AGATACCGCAAAGATTTTCAGACATGGCTCCCGAATTACAAG

---> Sequence Showing highlighted Existant Combined Trimers

GCT GTC CGC AGG GAG ATT TAT GAC AAA GTC CAG TTT TCA ACA ATG CTG AAA
TCA AAT GAT TGC CTG TTT TCT TTG GAA AAT TTG TTT TTT GAA AAA CCA GAT
GAA GTT GAA AAC CAT CCA GAC AAT GAA AAG TCA TTG GAT TGG TTT CTC CCT
CCT GCT CCA TTG ATT TCA GAA ATT CCA GAT ACT CAG GAG TTA GAG GAA GAA
TTA GAA AGT CAT AAA CTG TTA GGT CAG GAA AAG AGG CCA AAA ATG TTA ACA
TCA AAT TTA AAG ATA ACT AAT GAA GAT ACA AAT TAT ATT TCA CTA ACA CAA
AAA TTC CAG TTT GCC TTT CCT TCT GAT AAA TAT GAA CAG GAT GAT CTA AAT
TTA GAA GGG GTA GGT AAT AAT GAC TTA CCA CAT GTT GCT GGC AAG CTG ACA
TAT GCT TCT CAG AAA TAT AAA AAT CAC ATT GGC ACT GAG ATA GCA CCT GAG
AAG AGT GTT CCT GAT GAT ACA AAA TTA GTT AAT TTT GCA GAA GAT AAA GGA
GAG AGC ACA TCA GTA TTC CGG AAA AGA TTA TTT AAA ATA TCT GAC AAT ATA
CAT GGG AGT GCT TAT TCT AAT GAC AAT GAA TTG GAC TCT CAC ATT GGC TCA
GTG AAA ATT GTA CAA ACA GAA ATG AAC AAA GGG AAA TCA AGG AAC TAT
AGC AAT AGT AAG CAG AAA TTT CAG TAT TCT GCA AAT GTG TTT ACA GCA AAT

AAT GCT TTT TCT GCT TCT GAA ATC GGA GAA GGC ATG TTC AAA GCA CCA TCT
TTT TCA GTT GCT TTC CAA CCT CAT GAT ATT CAA GAG GTA ACA GAA AAT GGT
TTA GGT TCC TTG AAG GCT GTC ACA GAA ATT CCG GCA AAA TTT AGA AGT ATT
TTC AAA GAA TTT CCA TAT TTC AAC TAT ATA CAG TCC AAG GCC TTT GAT GAT
CTT CTT TAC ACA GAT AGG AAT TTT GTG ATT TGT GCT CCA ACT GGT TCT GGA
AAA ACT GTA GTG TTT GAA CTA GCT ATA ACA AGA TTG TTA ATG GAA GTA CCA
TTG CCA TGG TTG AAT ATT AAA ATT GTT TAC ATG GCA CCA ATA AAA GCC TTG
TGC AGT CAG CGT TTT GAT GAC TGG AAA GAA AAA TTT GGA CCA ATA GGA TTG
AAT TGT AAA GAA CTT ACT GGA GAT ACA GTA ATG GAT GAT CTA TTT GAG ATT
CAG CAT GCC CAT ATT ATT ATG ACA ACT CCA GAA AAA TGG GAT AGC ATG ACT
AGG AAA TGG AGA GAC AAC TCT TTG GTT CAG CTG GTT CGA CTG CTT CTC ATT
GAT GAG GTA CAT ATT GTA AAA GAT GAA AAT CGT GGT CCA ACT CTT GAA GTT
GTA GTT AGC AGA ATG AAA ACT GTA CAG TCT GTT TCT CAG ACT TTA AAA AAT
ACC AGC ACT GCT ATT CCA ATG CGA TTT GTA GCT GTA TCT GCA ACA ATT CCA
AAT GCT GAG GAT ATT GCA GAA TGG CTT TCA GAT GGT GAA AGA CCG GCT GTG
TGT CTG AAA ATG GAT GAG AGC CAT AGA CCA GTG AAA CTT CAG AAA GTG GTC
CTT GGA TTT CCC TGC AGT AGT AAC CAA ACT GAG TTT AAG TTT GAT TTA ACC
CTC AAC TAC AAA ATT GCC AGT GTT ATA CAA ATG TAC TCT GAT CAG AAA CCC
ACA CTT GTG TTT TGT GCA ACA AGG AAG GGT GTG CAA CAG GCT GCT TCT GTT
CTT GTG AAA GAT GCT AAA TTT ATT ATG ACT GTG GAA CAG AAA CAG AGG TTA
CAG AAG TAT GCA TAT TCC GTA AGA GAT TCA AAA CTG AGA GAT ATC TTA AAA
GAT GGT GCT GCT TAT CAT CAT GCT GGT ATG GAG CTG TCA GAT AGA AAA GTA
GTT GAG GGA GCT TTT ACT GTT GGA GAT TTA CCA GTT CTT TTT ACT ACC AGT
ACT TTA GCT ATG GGA GTA AAT TTG CCT GCT CAC CTA GTA GTT ATA AAA TCT
ACA ATG CAT TAT GCT GGA GGA CTG TTT GAA GAG TAC AGT GAA ACA GAT ATT
CTA CAG ATG ATT GGT AGA GCT GGT CGA CCT CAA TTT GAC ACT ACA GCT ACT
GCA GTT ATC ATG ACT CGA TTA AGC ACA AGG GAC AAG TAC ATT CAG ATG TTA
GCT TAT AGA GAC ACT GTA GAA AGC AGT TTG CAC AGA CAT CTT ATT GAA CAT
TTA AAT GCA GAG ATA GTA CTG CAT ACC ATC ACG GAT GTG AAT ATT GCT GTG
GAA TGG ATA CGA TCA ACT CTG CTT TAT ATC AGA GCC TTG AAA AAT CCA TCT
CAT TAT GGT TTT GCA TCT GGA TTG AAC AAA GAT GGA ATT GAA GCA AAA TTA
CAA GAA TTA TGT TTG AAG AAT CTG AAT GAT TTA TCA TCC CTG GAC TTA ATA
AAG ATG GAT GAA GGT GTT AAT TTC AAA CCA ACT GAA GCA GGA AGA TTG ATG
GCT TGG TAT TAT ATT ACA TTT GAG ACA GTG AAG AAA TTT TAT ACA ATC AGT
GGA AAA GAA ACC TTA TCA GAT CTG GTT ACA TTG ATA GCT GGC TGC AAG GAA
TTT CTA GAT ATA CAG TTA AGG ATA AAT GAA AAG AAA ACA CTG AAT ACT TTG
AAC AAA GAT CCA AAT CGG ATA ACT ATC AGA TTT CCA ATG GAA GGA AGA ATT
AAA ACA AGA GAA ATG AAA GTG AAT TGT CTT ATT CAG GCT CAA CTA GGA TGC
ATT CCC ATA CAA GAT TTT GCT TTG ACA CAA GAT ACC GCA AAG ATT TTC AGA
CAT GGC TCC CGA ATT ACA AG

Occurrences/Results 9 Pentamers

The pentamer 'ATTCA' occurred 5 times.
The pentamer 'TTCAA' occurred 7 times.
The pentamer 'TCAAG' occurred 2 times.
The pentamer 'CAAGA' occurred 6 times.
The pentamer 'AAGAT' occurred 15 times.
The pentamer 'AGATG' occurred 9 times.
The pentamer 'GATGA' occurred 11 times.
The pentamer 'ATGAA' occurred 11 times.
The pentamer 'TGAAT' occurred 7 times.
The total occurrences of all specified strings is 73.

The total number of characters in the text is 2716.
First result (Total characters *9 /1024): 23.90625
Square root of the first result: 4.889401803901986
Final result: 10.040849978993492

Occurrences/Results TRIMERS Codons

The pentamer 'ATT' occurred 35 times.
The pentamer 'CAA' occurred 12 times.
The pentamer 'GAT' occurred 43 times.
The pentamer 'GAA' occurred 45 times.
The pentamer 'CCA' occurred 23 times.
The pentamer 'ACT' occurred 25 times.
The pentamer 'AGA' occurred 19 times.
The pentamer 'TAC' occurred 6 times.
The total occurrences of all specified strings is 208.
The total number of characters in the text is 2721.
First result ((total_characters / 3) * (Trimer_number / 64)): 113.4
Square root of the first result: 10.65
Final result: 8.9

Occurrences/Results TRIMERS Combinations

The pentamer 'ATT ATT' occurred 1 times.
The pentamer 'ATT CAA' occurred 1 times.
The pentamer 'ATT GAT' occurred 1 times.
The pentamer 'ATT GAA' occurred 2 times.
The pentamer 'ATT CCA' occurred 3 times.
The pentamer 'ATT ACT' occurred 0 times.
The pentamer 'ATT AGA' occurred 0 times.
The pentamer 'ATT TAC' occurred 0 times.
The pentamer 'CAA ATT' occurred 0 times.
The pentamer 'CAA CAA' occurred 0 times.
The pentamer 'CAA GAT' occurred 2 times.
The pentamer 'CAA GAA' occurred 1 times.
The pentamer 'CAA CCA' occurred 0 times.
The pentamer 'CAA ACT' occurred 1 times.
The pentamer 'CAA AGA' occurred 0 times.
The pentamer 'CAA TAC' occurred 0 times.
The pentamer 'GAT ATT' occurred 3 times.
The pentamer 'GAT CAA' occurred 0 times.
The pentamer 'GAT GAT' occurred 4 times.
The pentamer 'GAT GAA' occurred 3 times.
The pentamer 'GAT CCA' occurred 1 times.
The pentamer 'GAT ACT' occurred 1 times.
The pentamer 'GAT AGA' occurred 1 times.
The pentamer 'GAT TAC' occurred 0 times.
The pentamer 'GAA ATT' occurred 2 times.
The pentamer 'GAA CAA' occurred 0 times.
The pentamer 'GAA GAT' occurred 2 times.
The pentamer 'GAA GAA' occurred 1 times.
The pentamer 'GAA CCA' occurred 0 times.
The pentamer 'GAA ACT' occurred 0 times.
The pentamer 'GAA AGA' occurred 1 times.
The pentamer 'GAA TAC' occurred 0 times.

The pentamer 'CCA ATT' occurred 0 times.
The pentamer 'CCA CAA' occurred 0 times.
The pentamer 'CCA GAT' occurred 2 times.
The pentamer 'CCA GAA' occurred 1 times.
The pentamer 'CCA CCA' occurred 0 times.
The pentamer 'CCA ACT' occurred 3 times.
The pentamer 'CCA AGA' occurred 0 times.
The pentamer 'CCA TAC' occurred 0 times.
The pentamer 'ACT ATT' occurred 0 times.
The pentamer 'ACT CAA' occurred 0 times.
The pentamer 'ACT GAT' occurred 0 times.
The pentamer 'ACT GAA' occurred 1 times.
The pentamer 'ACT CCA' occurred 1 times.
The pentamer 'ACT ACT' occurred 0 times.
The pentamer 'ACT AGA' occurred 0 times.
The pentamer 'ACT TAC' occurred 0 times.
The pentamer 'AGA ATT' occurred 1 times.
The pentamer 'AGA CAA' occurred 0 times.
The pentamer 'AGA GAT' occurred 2 times.
The pentamer 'AGA GAA' occurred 1 times.
The pentamer 'AGA CCA' occurred 1 times.
The pentamer 'AGA ACT' occurred 0 times.
The pentamer 'AGA AGA' occurred 0 times.
The pentamer 'AGA TAC' occurred 0 times.
The pentamer 'TAC ATT' occurred 1 times.
The pentamer 'TAC CAA' occurred 0 times.
The pentamer 'TAC GAT' occurred 0 times.
The pentamer 'TAC GAA' occurred 0 times.
The pentamer 'TAC CCA' occurred 0 times.
The pentamer 'TAC ACT' occurred 0 times.
The pentamer 'TAC AGA' occurred 0 times.
The pentamer 'TAC TAC' occurred 0 times.
The total occurrences of all specified strings is 45.
The total number of characters in the text is 2716.
First result (((total_characters/ 3) -1) / 64): 14.140625
Square root of the first result: 3.760402239122831
Final result: 8.206402676538774

>L34673.1 Homo sapiens, ATPase (HIP116)

---> Sequence

ATTCCCGGGGTCTGACTGGACTCGCGGCGACTTACCTTTCAGTCGTGCGCTCCTGATCC
GGCGCTCGGAATTTGTCCCCGGCTTCAGGGCTGCGGGGCCTGGAAGGAGGCGTATCGA
GGCGGCTCGAAAACGATCCAGGGGAGCCGAGGCGCTCCTCTTGTCATCCCACTCAGCG
CCATGTCCTGGATGTTCAAGAGGGATCCAGTTTGGAAGTACTTGCAGACTGTCCAGTA
TGGAGTTCATGGAAATTTTCCACGCCTCTCATATCCAACTTTCTTTCCACGTTTTGAATT
CCAAGATGTTATCCCTCCAGATGACTTTCTAACTAGTGATGAAGAAGTAGATTCCGTTT
TATTTGGAAGTTTGAGAGGTCATGTGGTTGGACTACGCTATTACACGGGAGTAGTTAA
TAATAATGAAATGGTTGCATTACAACGAGATCCTAATAACCCTTATGATAAGAATGCA
ATTAAAGTAAACAATGTGAATGGAAATCAAGTTGGCCATTTAAAGAAAGAGCTTGCA
GGTGCTTTGGCCTATATCATGGACAACAAATTGGCACAAATTGAAGGGGTAGTTCCTT

TTGGTGCAAACAATGCTTTTACCATGCCTCTGCATATGACTTTTTGGGGAAAAGAAGA
AAATAGAAAAGCGGTTTCAGATCAGTTGAAGAAACATGGATTTAAATTGGGTCCTGCA
CCAAAAACTTTAGGATTCAATTTGGAAAGTGGTTGGGGCTCTGGAAGAGCTGGACCAA
GCTATAGTATGCCAGTGCATGCTGCAGTACAGATGACAACTGAACAGCTTAAAACAGA
ATTTGACAAATTGTTTGAAGATTTAAAAGAAGATGATAAAACCCATGAAATGGAACCA
GCTGAGGCTATTGAAACACCACTGCTTCCACATCAAAAACAAGCTCTAGCTTGGATGG
TGTCACGGGAAAATAGCAAAGAACTTCCACCATTCTGGGAACAGCGAAATGACTTATA
CTATAACACAATAACAAATTTTTCTGAGAAGGACCGACCAGAAAATGTCCATGGAGGA
ATTTTAGCTGATGATATGGGTTTGGGTAAAACTCTTACAGCCATTGCAGTAATCCTTAC
CAACTTCCATGATGGCAGACCTCTTCCTATTGAAAGAGTTAAAAAGAATCTACTGAAG
AAGGAATATAATGTTAACGATGACTCTATGAAACTTGGAGGAAACAATACCAGTGAA
AAGGCAGATGGACTAAGCAAAGACGCATCTAGATGTAGTGAACAACCCAGTATTTCA
GATATCAAGGAGAAGAGTAAGTTTCGCATGTCAGAATTGTCTACGTCCCGCCCCAAAA
GAAGAAAAACTGCTGTCCAGTACATAGAAAGCAGTGATTCAGAGGAAATTGAAACAA
GTGAATTGCCGCAGAAAATGAAAGGCAAACTGAAAAATGTACAGTCTGAAACTAAAG
GCAGGGCGAAAGCAGGATCTTCTAAGGTTATAGAAGATGTGGCATTTGCATGTGCATT
AACTTCATCTGTTCCTACAACAAAAAAGAAAATGTTGAAAAAGGGAGCTTGTGCAGTG
GAGGGGTCAAAGAAAACTGATGTTGAGGAGAGACCAAGAACAACACTGATCATCTGT
CCGCTTTCTGTGTTAAGCAACTGGATTGACCAGTTTGGACAACATATAAAATCAGATG
TACACTTGAATTTTTATGTTTATTATGGTCCTGATCGTATTAGAGAACCGGCCTTACTTT
CAAAACAGGATATTGTTTTGACTACGTATAATATTTTAACTCATGACTATGGAACTAAA
GGAGATAGTCCATTACATAGCATAAGGTGGCTAAGAGTGATCCTGGATGAAGGACAT
GCCATACGAAATCCAAATGCTCAGCAGACAAAAGCTGTACTTGACTTAGAATCAGAAA
GAAGATGGGTTTTGACAGGTACTCCAATCCAGAATTCTTTAAAGGACTTGTGGTCTCTT
CTTTCCTTTTTAAAACTTAAACCATTTATTGATAGAGAATGGTGGCATAGAACAATACA
GCGTCCTGTCACAATGGGAGATGAAGGAGGACTTAGGCGTTTACAGTCCCTAATTAAA
AATATTACACTTAGAAGAACAAAGACAAGCAAAATTAAAGGAAAACCTGTTTTGGAG
TTACCAGAACGTAAAGTATTTATTCAGCACATTACACTTTCAGATGAAGAGAGAAAGA
TTTATCAGTCTGTGAAAAATGAAGGCAGAGCCACTATTGGAAGGTATTTTAATGAAGG
GACTGTCCTGGCACATTATGCAGATGTCCTGGGTCTTTTGCTTAGACTGCGGCAAATTT
GTTGCCATACTTACCTTCTTACAAATGCAGTGTCTTCCAATGGCCCCTCAGGAAATGAT
ACACCTGAAGAACTGAGAAAGAAGTTAATAAGGAAGATGAAGTTAATTCTGAGCTCA
GGTTCAGATGAGGAATGTGCAATTTGCCTGGATTCTTTAACAGTTCCTGTGATAACACA
TTGTGCACATGTATTTTGTAAACCCTGTATTTGCCAAGTCATTCAGAATGAGCAGCCAC
ATGCTAAATGCCCTTTATGCAGAAATGATATACATGAAGATAATTTATTAGAATGTCCT
CCAGAAGAATTAGCACGTGACAGTGAGAAAAAGTCTGATATGGAATGGACATCCAGT
TCAAAGATTAATGCGCTAATGCACGCATTGACTGACTTAAGAAAGAAGAATCCCAACA
TAAAAAGTTTGGTTGTTTCTCAGTTTACAACATTCCTGTCTTTAATAGAAATACCACTT
AAAGCCTCTGGATTTGTGTTTACTCGTTTGGATGGTTCCATGGCCCAAAAGAAAAGAG
TTGAATCAATTCAGTGTTTTCAAAACACTGAAGCAGGATCTCCAACTATAATGCTTCTG
TCCTTAAAAGCAGGTGGAGTTGGTTTGAATCTGTCTGCAGCTTCTCGAGTGTTTTTAAT
GGATCCAGCCTGGAATCCTGCTGCTGAAGATCAGTGCTTTGACAGATGCCATAGACTT
GGTCAGAAGCAAGAAGTTATCATCACAAAATTCATTGTAAAGGACTCTGTTGAAGAAA
ATATGCTGAAAATACAAAACAAAAAGAGAGAACTTGCAGCAGGAGCCTTTGGAACTA
AAAAACCAAATGCTGACGAAATGAAACAAGCCAAAATTAATGAAATCAGAACATTAA
TTGACTTATAATTTGTGGGATTTTAGTAAGAAGACTACTATATGTGAGAGGCGTGATAT
CTGGATGGAAGTTGGGCTGGATGATCTCCAAAGTCGTTTCAACTCTTAAAGACATCTT
AATCCTGAATGTAAACAATTGTTATGTGTTTAGAATCAGAATTTGATTTTGAACTTGAG
TAATTCATCCTTACAGCTATCTGTAGAATTAGTCATCTTTTTTCTT

---> Sequence Showing highlighted Existant Combined Trimers

ATT CCC GGG GTC TGA CTG GAC TCG CGG CGA CTT ACC TTT CAG TCG TGC GCT
CCT GAT CCG GCG CTC GGA ATT TGT CCC CGG CTT CAG GGC TGC GGG GCC TGG
AAG GAG GCG TAT CGA GGC GGC TCG AAA ACG ATC CAG GGG AGC CGA GGC
GCT CCT CTT GTC ATC CCA CTC AGC GCC ATG TCC TGG ATG TTC AAG AGG GAT
CCA GTT TGG AAG TAC TTG CAG ACT GTC CAG TAT GGA GTT CAT GGA AAT TTT
CCA CGC CTC TCA TAT CCA ACT TTC TTT CCA CGT TTT GAA TTC CAA GAT GTT
ATC CCT CCA GAT GAC TTT CTA ACT AGT GAT GAA GAA GTA GAT TCC GTT TTA
TTT GGA AGT TTG AGA GGT CAT GTG GTT GGA CTA CGC TAT TAC ACG GGA GTA
GTT AAT AAT AAT GAA ATG GTT GCA TTA CAA CGA GAT CCT AAT AAC CCT TAT
GAT AAG AAT GCA ATT AAA GTA AAC AAT GTG AAT GGA AAT CAA GTT GGC CAT
TTA AAG AAA GAG CTT GCA GGT GCT TTG GCC TAT ATC ATG GAC AAC AAA TTG
GCA CAA ATT GAA GGG GTA GTT CCT TTT GGT GCA AAC AAT GCT TTT ACC ATG
CCT CTG CAT ATG ACT TTT TGG GGA AAA GAA GAA AAT AGA AAA GCG GTT TCA
GAT CAG TTG AAG AAA CAT GGA TTT AAA TTG GGT CCT GCA CCA AAA ACT TTA
GGA TTC AAT TTG GAA AGT GGT TGG GGC TCT GGA AGA GCT GGA CCA AGC TAT
AGT ATG CCA GTG CAT GCT GCA GTA CAG ATG ACA ACT GAA CAG CTT AAA ACA
GAA TTT GAC AAA TTG TTT GAA GAT TTA AAA GAA GAT GAT AAA ACC CAT GAA
ATG GAA CCA GCT GAG GCT ATT GAA ACA CCA CTG CTT CCA CAT CAA AAA CAA
GCT CTA GCT TGG ATG GTG TCA CGG GAA AAT AGC AAA GAA CTT CCA CCA TTC
TGG GAA CAG CGA AAT GAC TTA TAC TAT AAC ACA ATA ACA AAT TTT TCT GAG
AAG GAC CGA CCA GAA AAT GTC CAT GGA GGA ATT TTA GCT GAT GAT ATG GGT
TTG GGT AAA ACT CTT ACA GCC ATT GCA GTA ATC CTT ACC AAC TTC CAT GAT
GGC AGA CCT CTT CCT ATT GAA AGA GTT AAA AAG AAT CTA CTG AAG AAG GAA
TAT AAT GTT AAC GAT GAC TCT ATG AAA CTT GGA GGA AAC AAT ACC AGT GAA
AAG GCA GAT GGA CTA AGC AAA GAC GCA TCT AGA TGT AGT GAA CAA CCC
AGT ATT TCA GAT ATC AAG GAG AAG AGT AAG TTT CGC ATG TCA GAA TTG TCT
ACG TCC CGC CCC AAA AGA AGA AAA ACT GCT GTC CAG TAC ATA GAA AGC AGT
GAT TCA GAG GAA ATT GAA ACA AGT GAA TTG CCG CAG AAA ATG AAA GGC
AAA CTG AAA AAT GTA CAG TCT GAA ACT AAA GGC AGG GCG AAA GCA GGA
TCT TCT AAG GTT ATA GAA GAT GTG GCA TTT GCA TGT GCA TTA ACT TCA TCT
GTT CCT ACA ACA AAA AAG AAA ATG TTG AAA AAG GGA GCT TGT GCA GTG
GAG GGG TCA AAG AAA ACT GAT GTT GAG GAG AGA CCA AGA ACA ACA CTG
ATC ATC TGT CCG CTT TCT GTG TTA AGC AAC TGG ATT GAC CAG TTT GGA CAA
CAT ATA AAA TCA GAT GTA CAC TTG AAT TTT TAT GTT TAT TAT GGT CCT GAT
CGT ATT AGA GAA CCG GCC TTA CTT TCA AAA CAG GAT ATT GTT TTG ACT ACG
TAT AAT ATT TTA ACT CAT GAC TAT GGA ACT AAA GGA GAT AGT CCA TTA CAT
AGC ATA AGG TGG CTA AGA GTG ATC CTG GAT GAA GGA CAT GCC ATA CGA AAT
CCA AAT GCT CAG CAG ACA AAA GCT GTA CTT GAC TTA GAA TCA GAA AGA AGA
TGG GTT TTG ACA GGT ACT CCA ATC CAG AAT TCT TTA AAG GAC TTG TGG TCT
CTT CTT TCC TTT TTA AAA CTT AAA CCA TTT ATT GAT AGA GAA TGG TGG CAT
AGA ACA ATA CAG CGT CCT GTC ACA ATG GGA GAT GAA GGA GGA CTT AGG
CGT TTA CAG TCC CTA ATT AAA AAT ATT ACA CTT AGA AGA ACA AAG ACA AGC
AAA ATT AAA GGA AAA CCT GTT TTG GAG TTA CCA GAA CGT AAA GTA TTT ATT
CAG CAC ATT ACA CTT TCA GAT GAA GAG AGA AAG ATT TAT CAG TCT GTG AAA
AAT GAA GGC AGA GCC ACT ATT GGA AGG TAT TTT AAT GAA GGG ACT GTC CTG
GCA CAT TAT GCA GAT GTC CTG GGT CTT TTG CTT AGA CTG CGG CAA ATT TGT
TGC CAT ACT TAC CTT CTT ACA AAT GCA GTG TCT TCC AAT GGC CCC TCA GGA
AAT GAT ACA CCT GAA GAA CTG AGA AAG AAG TTA ATA AGG AAG ATG AAG
TTA ATT CTG AGC TCA GGT TCA GAT GAG GAA TGT GCA ATT TGC CTG GAT TCT
TTA ACA GTT CCT GTG ATA ACA CAT TGT GCA CAT GTA TTT TGT AAA CCC TGT
ATT TGC CAA GTC ATT CAG AAT GAG CAG CCA CAT GCT AAA TGC CCT TTA TGC

AGA AAT GAT ATA CAT GAA GAT AAT TTA TTA GAA TGT CCT CCA GAA GAA TTA GCA CGT GAC AGT GAG AAA AAG TCT GAT ATG GAA TGG ACA TCC AGT TCA AAG ATT AAT GCG CTA ATG CAC GCA TTG ACT GAC TTA AGA AAG AAG AAT CCC AAC ATA AAA AGT TTG GTT GTT TCT CAG TTT ACA ACA TTC CTG TCT TTA ATA GAA ATA CCA CTT AAA GCC TCT GGA TTT GTG TTT ACT CGT TTG GAT GGT TCC ATG GCC CAA AAG AAA AGA GTT GAA TCA ATT CAG TGT TTT CAA AAC ACT GAA GCA GGA TCT CCA ACT ATA ATG CTT CTG TCC TTA AAA GCA GGT GGA GTT GGT TTG AAT CTG TCT GCA GCT TCT CGA GTG TTT TTA ATG GAT CCA GCC TGG AAT CCT GCT GCT GAA GAT CAG TGC TTT GAC AGA TGC CAT AGA CTT GGT CAG AAG CAA GAA GTT ATC ATC ACA AAA TTC ATT GTA AAG GAC TCT GTT GAA GAA AAT ATG CTG AAA ATA CAA AAC AAA AAG AGA GAA CTT GCA GCA GGA GCC TTT GGA ACT AAA AAA CCA AAT GCT GAC GAA ATG AAA CAA GCC AAA ATT AAT GAA ATC AGA ACA TTA ATT GAC TTA TAA TTT GTG GGA TTT TAG TAA GAA GAC TAC TAT ATG TGA GAG GCG TGA TAT CTG GAT GGA AGT TGG GCT GGA TGA TCT CCA AAG TCG TTT CAA CTC TTA AAG ACA TCT TAA TCC TGA ATG TAA ACA ATT GTT ATG TGT TTA GAA TCA GAA TTT GAT TTT GAA CTT GAG TAA TTC ATC CTT ACA GCT ATC TGT AGA ATT AGT CAT CTT TTT TCT T

Occurrences/Results 9 Pentamers

The pentamer 'ATTCA' occurred 7 times.
The pentamer 'TTCAA' occurred 6 times.
The pentamer 'TCAAG' occurred 3 times.
The pentamer 'CAAGA' occurred 4 times.
The pentamer 'AAGAT' occurred 10 times.
The pentamer 'AGATG' occurred 15 times.
The pentamer 'GATGA' occurred 12 times.
The pentamer 'ATGAA' occurred 14 times.
The pentamer 'TGAAT' occurred 7 times.
The total occurrences of all specified strings is 78.
The total number of characters in the text is 3414.
First result (Total characters *9 /1024): 30.041015625
Square root of the first result: 5.4809684933413
Final result: 8.750093059878786

Occurrences/Results TRIMERS Codons

The pentamer 'ATT' occurred 34 times.
The pentamer 'CAA' occurred 16 times.
The pentamer 'GAT' occurred 41 times.
The pentamer 'GAA' occurred 62 times.
The pentamer 'CCA' occurred 28 times.
The pentamer 'ACT' occurred 23 times.
The pentamer 'AGA' occurred 30 times.
The pentamer 'TAC' occurred 6 times.
The total occurrences of all specified strings is 240.
The total number of characters in the text is 3414.
First result ((total_characters / 3) * (Trimer_number / 64)): 142
Square root of the first result: 11.9
Final result: 8.2

Occurrences/Results TRIMERS Combinations

The pentamer 'ATT ATT' occurred 0 times.
The pentamer 'ATT CAA' occurred 0 times.
The pentamer 'ATT GAT' occurred 1 times.
The pentamer 'ATT GAA' occurred 4 times.

The pentamer 'ATT CCA' occurred 0 times.
The pentamer 'ATT ACT' occurred 0 times.
The pentamer 'ATT AGA' occurred 1 times.
The pentamer 'ATT TAC' occurred 0 times.
The pentamer 'CAA ATT' occurred 2 times.
The pentamer 'CAA CAA' occurred 0 times.
The pentamer 'CAA GAT' occurred 1 times.
The pentamer 'CAA GAA' occurred 1 times.
The pentamer 'CAA CCA' occurred 0 times.
The pentamer 'CAA ACT' occurred 0 times.
The pentamer 'CAA AGA' occurred 0 times.
The pentamer 'CAA TAC' occurred 0 times.
The pentamer 'GAT ATT' occurred 1 times.
The pentamer 'GAT CAA' occurred 0 times.
The pentamer 'GAT GAT' occurred 2 times.
The pentamer 'GAT GAA' occurred 4 times.
The pentamer 'GAT CCA' occurred 2 times.
The pentamer 'GAT ACT' occurred 0 times.
The pentamer 'GAT AGA' occurred 1 times.
The pentamer 'GAT TAC' occurred 0 times.
The pentamer 'GAA ATT' occurred 1 times.
The pentamer 'GAA CAA' occurred 1 times.
The pentamer 'GAA GAT' occurred 5 times.
The pentamer 'GAA GAA' occurred 5 times.
The pentamer 'GAA CCA' occurred 1 times.
The pentamer 'GAA ACT' occurred 1 times.
The pentamer 'GAA AGA' occurred 2 times.
The pentamer 'GAA TAC' occurred 0 times.
The pentamer 'CCA ATT' occurred 0 times.
The pentamer 'CCA CAA' occurred 0 times.
The pentamer 'CCA GAT' occurred 1 times.
The pentamer 'CCA GAA' occurred 3 times.
The pentamer 'CCA CCA' occurred 1 times.
The pentamer 'CCA ACT' occurred 2 times.
The pentamer 'CCA AGA' occurred 1 times.
The pentamer 'CCA TAC' occurred 0 times.
The pentamer 'ACT ATT' occurred 1 times.
The pentamer 'ACT CAA' occurred 0 times.
The pentamer 'ACT GAT' occurred 1 times.
The pentamer 'ACT GAA' occurred 2 times.
The pentamer 'ACT CCA' occurred 1 times.
The pentamer 'ACT ACT' occurred 0 times.
The pentamer 'ACT AGA' occurred 0 times.
The pentamer 'ACT TAC' occurred 1 times.
The pentamer 'AGA ATT' occurred 1 times.
The pentamer 'AGA CAA' occurred 0 times.
The pentamer 'AGA GAT' occurred 0 times.
The pentamer 'AGA GAA' occurred 3 times.
The pentamer 'AGA CCA' occurred 1 times.
The pentamer 'AGA ACT' occurred 0 times.
The pentamer 'AGA AGA' occurred 3 times.

The pentamer 'AGA TAC' occurred 0 times.
The pentamer 'TAC ATT' occurred 0 times.
The pentamer 'TAC CAA' occurred 0 times.
The pentamer 'TAC GAT' occurred 0 times.
The pentamer 'TAC GAA' occurred 0 times.
The pentamer 'TAC CCA' occurred 0 times.
The pentamer 'TAC ACT' occurred 0 times.
The pentamer 'TAC AGA' occurred 0 times.
The pentamer 'TAC TAC' occurred 0 times.
The total occurrences of all specified strings is 58.
The total number of characters in the text is 3414.
First result (((total_characters/ 3) -1) / 64): 17.78125
Square root of the first result: 4.21678194835825
Final result: 9.537782719748803

>BC000882.1 Homo sapiens translocase of outer mitochondrial membrane 20 homolog (yeast), mRNA (cDNA clone MGC:4905 IMAGE:3462090), complete cds

---> Sequence

CTGTGTTCCTGGCCCGCGGCCGTCGGGTGTGAGCTGCGCCGACCGCTCTGAGGGTTCGTGGCCCAC
CGCTCCTTCGCGGTCCCTGCCGCCACCGTCCACGCTCAGCGTTGTAGAGAAGATGGTGGGTCGGAA
CAGCGCCATCGCCGCCGGTGTATGCGGGGCCCTTTTCATTGGGTACTGCATCTACTTCGACCGCAA
AAGACGAAGTGACCCCAACTTCAAGAACAGGCTTCGAGAACGAAGAAAGAAACAGAAGCTTGCCA
AGGAGAGAGCTGGGCTTTCCAAGTTACCTGACCTTAAAGATGCTGAAGCTGTTCAGAAGTTCTTC
CTTGAAGAAATACAGCTTGGTGAAGAGTTACTAGCTCAAGGTGAATATGAGAAGGGCGTAGACCA
TCTGACAAATGCAATTGCTGTGTGTGGACAGCCACAGCAGTTACTGCAGGTCTTACAGCAAACTC
TTCCACCACCAGTGTTCCAGATGCTTCTGACTAAGCTCCCAACAATTAGTCAGAGAATTGTAAGT
GCTCAGAGCTTGGCTGAAGATGATGTGGAATGAGAAACAAATGTCAACATAATAAAATCTCAGTT
AAAAATATTTTAAAAATTCTTGGTAGTTGAGCAGCTCTGGGGGAATAAGGGCAAATATGCTTGTT
ATGAACTACACTGAAATCTACCAAAGTTAATGTTTACTTTGTGTAGATCCATTTGTCTATTTTAT
TTATTTTTCCCAGTGAAAAGTGTATTTTGATAGAGAACTTTTCATTCTATAAATACACTATGAGT
TACTAAAATATCATGGATTTTGTTTATTCCTGAAACATAGTTACATAGTTAAACTGTACATATGA
CATGGCTTATGTTAAAAATACCCAGTGCTCAGTTTTGAAAGATAGGCAAAAAAAAAAAAGTATA
GGAGAAACTGAAGAATGTACACTTTTTAGAGGGCACATTTTGCTGTAAATCTGGAAATTTGATA
GACTTGACTGTGTTTGTGAAAACTGAGCATTAAAGGTTTTGATTGATCCTTTAA

---> Sequence Showing highlighted Existant Combined Trimers

CTG TGT TCC TGG CCC GCG GCC GTC GGG TGT GAG CTG CGC CGA CCG CTC TGA GGG TTC
GTG GCC CAC CGC TCC TTC GCG GTC CCT GCC GCC ACC GTC CAC GCT CAG CGT TGT AGA
GAA GAT GGT GGG TCG GAA CAG CGC CAT CGC CGC CGG TGT ATG CGG GGC CCT TTT CAT
TGG GTA CTG CAT CTA CTT CGA CCG CAA AAG ACG AAG TGA CCC CAA CTT CAA GAA CAG
GCT TCG AGA ACG AAG AAA GAA ACA GAA GCT TGC CAA GGA GAG AGC TGG GCT TTC CAA
GTT ACC TGA CCT TAA AGA TGC TGA AGC TGT TCA GAA GTT CTT CCT TGA AGA AAT ACA
GCT TGG TGA AGA GTT ACT AGC TCA AGG TGA ATA TGA GAA GGG CGT AGA CCA TCT GAC
AAA TGC AAT TGC TGT GTG TGG ACA GCC ACA GCA GTT ACT GCA GGT CTT ACA GCA AAC
TCT TCC ACC ACC AGT GTT CCA GAT GCT TCT GAC TAA GCT CCC AAC AAT TAG TCA GAG
AAT TGT AAG TGC TCA GAG CTT GGC TGA AGA TGA TGT GGA ATG AGA AAC AAA TGT CAA
CAT AAT AAA ATC TCA GTT AAA AAT ATT TTA AAA ATT CTT GGT AGT TGA GCA GCT CTG
GGG GAA TAA GGG CAA ATA TGC TTG TTA TGA ACT ACA CTG AAA TCT ACC AAA GTT AAT
GTT TAC TTT GTG TAG ATC CAT TTG TCT ATT TTA TTT ATT TTT CCC AGT GAA AAG TGT
ATT TTG ATA GAG AAC TTT TCA TTC TAT AAA TAC ACT ATG AGT TAC TAA AAT ATC ATG

GAT TTT GTT TAT TCC TGA AAC ATA GTT ACA TAG TTA AAC TGT ACA TAT GAC ATG GCT TAT GTT AAA AAT ACC CAG TGC TCA GTT TTG AAA GAT AGG CAA AAA AAA AAA AGT ATA GGA GAA ACT GAA GAA TGT ACA CTT TTT TAG AGG GCA CAT TTT GCT GTA AAT CTG GAA ATT TGA TAG ACT TGA CTG TGT TTG TGA AAA CTG AGC ATT AAA GGT TTT GAT TGA TCC TTT AA

Occurrences/Results 9 Pentamers

The pentamer 'ATTCA' occurred 0 times.
The pentamer 'TTCAA' occurred 1 times.
The pentamer 'TCAAG' occurred 2 times.
The pentamer 'CAAGA' occurred 1 times.
The pentamer 'AAGAT' occurred 4 times.
The pentamer 'AGATG' occurred 4 times.
The pentamer 'GATGA' occurred 1 times.
The pentamer 'ATGAA' occurred 1 times.
The pentamer 'TGAAT' occurred 1 times.
The total occurrences of all specified strings is 15.
The total number of characters in the text is 1027.
First result (Total characters *9 /1024): 9.0615234375
Square root of the first result: 3.0102364421254353
Final result: 1.9727608367889615

Occurrences/Results TRIMERS Codons

The pentamer 'ATT' occurred 7 times.
The pentamer 'CAA' occurred 8 times.
The pentamer 'GAT' occurred 5 times.
The pentamer 'GAA' occurred 13 times.
The pentamer 'CCA' occurred 2 times.
The pentamer 'ACT' occurred 6 times.
The pentamer 'AGA' occurred 8 times.
The pentamer 'TAC' occurred 3 times.
The total occurrences of all specified strings is 52.
The total number of characters in the text is 1027.
First result ((total_characters / 3) * (Trimer_number / 64)): 42.8
Square root of the first result: 6.54
Final result: 1.4

Occurrences/Results TRIMERS Combinations

The pentamer 'ATT ATT' occurred 0 times.
The pentamer 'ATT CAA' occurred 0 times.
The pentamer 'ATT GAT' occurred 0 times.
The pentamer 'ATT GAA' occurred 0 times.
The pentamer 'ATT CCA' occurred 0 times.
The pentamer 'ATT ACT' occurred 0 times.
The pentamer 'ATT AGA' occurred 0 times.
The pentamer 'ATT TAC' occurred 0 times.
The pentamer 'CAA ATT' occurred 0 times.
The pentamer 'CAA CAA' occurred 0 times.
The pentamer 'CAA GAT' occurred 0 times.
The pentamer 'CAA GAA' occurred 1 times.
The pentamer 'CAA CCA' occurred 0 times.
The pentamer 'CAA ACT' occurred 0 times.

The pentamer 'CAA AGA' occurred 0 times.
The pentamer 'CAA TAC' occurred 0 times.
The pentamer 'GAT ATT' occurred 0 times.
The pentamer 'GAT CAA' occurred 0 times.
The pentamer 'GAT GAT' occurred 0 times.
The pentamer 'GAT GAA' occurred 0 times.
The pentamer 'GAT CCA' occurred 0 times.
The pentamer 'GAT ACT' occurred 0 times.
The pentamer 'GAT AGA' occurred 0 times.
The pentamer 'GAT TAC' occurred 0 times.
The pentamer 'GAA ATT' occurred 1 times.
The pentamer 'GAA CAA' occurred 0 times.
The pentamer 'GAA GAT' occurred 1 times.
The pentamer 'GAA GAA' occurred 1 times.
The pentamer 'GAA CCA' occurred 0 times.
The pentamer 'GAA ACT' occurred 1 times.
The pentamer 'GAA AGA' occurred 0 times.
The pentamer 'GAA TAC' occurred 0 times.
The pentamer 'CCA ATT' occurred 0 times.
The pentamer 'CCA CAA' occurred 0 times.
The pentamer 'CCA GAT' occurred 1 times.
The pentamer 'CCA GAA' occurred 0 times.
The pentamer 'CCA CCA' occurred 0 times.
The pentamer 'CCA ACT' occurred 0 times.
The pentamer 'CCA AGA' occurred 0 times.
The pentamer 'CCA TAC' occurred 0 times.
The pentamer 'ACT ATT' occurred 0 times.
The pentamer 'ACT CAA' occurred 0 times.
The pentamer 'ACT GAT' occurred 0 times.
The pentamer 'ACT GAA' occurred 1 times.
The pentamer 'ACT CCA' occurred 0 times.
The pentamer 'ACT ACT' occurred 0 times.
The pentamer 'ACT AGA' occurred 0 times.
The pentamer 'ACT TAC' occurred 0 times.
The pentamer 'AGA ATT' occurred 0 times.
The pentamer 'AGA CAA' occurred 0 times.
The pentamer 'AGA GAT' occurred 0 times.
The pentamer 'AGA GAA' occurred 1 times.
The pentamer 'AGA CCA' occurred 1 times.
The pentamer 'AGA ACT' occurred 0 times.
The pentamer 'AGA AGA' occurred 0 times.
The pentamer 'AGA TAC' occurred 0 times.
The pentamer 'TAC ATT' occurred 0 times.
The pentamer 'TAC CAA' occurred 0 times.
The pentamer 'TAC GAT' occurred 0 times.
The pentamer 'TAC GAA' occurred 0 times.
The pentamer 'TAC CCA' occurred 0 times.
The pentamer 'TAC ACT' occurred 1 times.
The pentamer 'TAC AGA' occurred 0 times.
The pentamer 'TAC TAC' occurred 0 times.

The total occurrences of all specified strings is 10.
The total number of characters in the text is 1027.
First result (((total_characters/ 3) -1) / 64): 5.34375
Square root of the first result: 2.311655251113366
Final result: 2.014249312373635

>NM_001303269.2 Homo sapiens RNA polymerase II subunit B (POLR2B), transcript variant 3, mRNA

---> Sequence

ACTTCGTCTTTAGCTCCTGGCGCTGCTGGCTTCTGGGCGGTTTTTGTCTTTTGATTTCA
AGAGTTAGGAGCTCGAGAACCGTTTGGCAATATGTACGACGCGGATGAGGGCTGGA
GTGCAGTGGCGCGATCTCAGCTCCTGCGAGGAGGCGGACCGCCCATTCCGTGCCCGG
TAGCCGCGAGGGACCGGTGGTAGCCGCGAGGGACGGGCGGCGGGCTCGGTGCTGTG
GCGAGGCGGGGATGGCACTCCCCCCCGCCGCCCCGTGGGGCGGCCCCGACTTTCGGG
CGATATGCAATATGATGAGGATGATGATGAAATCACCCCGGATTTGTGGCAAGAAGC
ATGCTGGATTGTAATCAGTTCCTATTTTGACGAGAAAGGCTTGGTTAGACAACAGCT
GGATTCTTTTGATGAGTTTATTCAGATGTCTGTTCAAAGAATTGTGGAAGACGCTCCT
CCTATAGACCTACAGGCTGAAGCTCAGCATGCTAGTGGAGAAGTTGAAGAACCGCCA
CGATATTTGCTGAAGTTTGAACAAATTTATCTTTCCAAGCCTACCCATTGGGAAAGAG
ATGGTGCTCCTTCACCAATGATGCCCAATGAAGCTAGATTAAGGAATCTCACGTATT
CTGCTCCGCTTTATGTTGATATAACAAAAACAGTCATTAAAGAAGGTGAAGAACAAC
TTCAGACTCAGCATCAGAAAACTTTTATAGGAAAAATTCCAATTATGTTGCGGTCAA
CTTACTGCCTTTTGAATGGCTTGACAGATCGTGATCTTTGTGAGTTAAATGAATGCCC
TTTGGATCCTGGTGGCTATTTCATTATTAATGGATCAGAAAAGGTTCTGATTGCCCAA
GAGAAAATGGCAACAAACACAGTTTATGTGTTTGCCAAAAAGGATTCTAAATATGCC
TACACAGGAGAGTGTAGATCATGTCTTGAGAATTCTTCCCGACCCACCAGTACTATA
TGGGTTAGCATGCTGGCAAGAGGAGGACAGGGTGCCAAGAAGAGTGCTATTGGTCA
GCGCATTGTGGCAACTCTACCATATATCAAGCAAGAAGTTCCCATCATTATTGTGTTC
AGAGCATTAGGTTTTGTGTCCGACAGAGATATTTTAGAACATATTATTTATGATTTTG
AAGATCCAGAGATGATGGAAATGGTTAAACCTTCTCTCGATGAAGCTTTTGTCATCC
AAGAACAGAATGTTGCACTAAATTTCATTGGTTCAAGAGGAGCAAAGCCTGGTGTTA
CTAAAGAGAAAAGAATTAAATATGCAAAGGAAGTTTTACAAAAAGAAATGCTCCCT
CATGTTGGTGTCAGTGATTTTTGTGAGACCAAAAAAGCCTATTTCTTGGGATACATGG
TTCATAGGTTACTTCTGGCAGCTTTGGGTAGAAGAGAACTAGATGACAGAGATCACT
ATGGAAACAAGAGATTGGATCTTGCTGGGCCGCTGCTTGCATTCTTATTTAGAGGTAT
GTTTAAGAATTTGCTTAAAGAAGTGCGGATCTATGCACAGAAATTTATTGATCGAGG
AAAGGATTTTAACTTGGAGTTGGCAATTAAAACACGGATCATATCTGATGGCCTAAA
ATACTCTTTAGCTACTGGAAACTGGGGTGATCAAAAGAAAGCTCATCAAGCCAGAGC
TGGAGTATCTCAGGTGTTAAACCGCCTGACTTTTGCGTCTACTCTTTCTCACCTGCGT
CGTTTAAATTCTCCTATTGGTAGAGACGGCAAGCTAGCAAAACCAAGACAGTTGCAT
AATACGTTGTGGGGAATGGTGTGTCCTGCCGAGACCCCAGAGGGCCATGCTGTAGGA
CTTGTGAAGAATTTAGCCTTGATGGCGTATATTTCAGTTGGATCTCAACCATCTCCAA
TTCTGGAATTTTTAGAAGAATGGAGTATGGAAAATTTAGAAGAAATTTCTCCTGCAG
CTATTGCTGATGCAACCAAGATTTTTGTTAATGGCTGCTGGGTTGGAATACATAAAG
ATCCCGAACAACTTATGAACACCCTAAGGAAATTGAGACGTCAGATGGACATCATTG
TGTCTGAAGTTTCTATGATCAGAGATATTCGAGAGAGGGAGATTCGGATCTATACGG
ATGCAGGCCGTATTTGTAGACCACTTCTGATTGTGGAAAAACAAAAGCTACTTTTGA
AGAAGAGGCATATTGACCAATTGAAAGAGAGAGAATATAACAACTATAGTTGGCAG
GATCTTGTGGCCAGTGGGGTAGTGGAGTATATTGATACCCTGGAAGAAGAAACAGTG
ATGCTTGCAATGACTCCAGATGATTTACAGGAGAAAGAAGTAGCTTATTGTTCCACA

TATACACACTGTGAGATTCATCCCTCAATGATCCTTGGTGTCTGTGCATCTATTATTC
CCTTTCCTGATCATAACCAGTCCCCTAGAAACACATACCAGTCTGCTATGGGTAAGC
AGGCTATGGGAGTTTACATCACCAACTTCCATGTTCGCATGGACACATTGGCCCATGT
TCTCTATTATCCTCAAAAGCCACTTGTGACTACACGGTCTATGGAATATCTACGATTT
AGAGAGCTGCCAGCAGGCATCAACTCAATTGTGGCCATTGCATCATACACTGGATAT
AATCAGGAAGACTCTGTTATCATGAATCGTTCAGCTGTAGACCGCGGCTTCTTCAGGT
CTGTTTTCTATCGCTCATACAAAGAACAGGAGTCTAAAAAAGGATTTGATCAAGAAG
AAGTTTTTGAGAAGCCTACACGTGAAACATGCCAGGGCATGAGGCATGCCATTTACG
ACAAGCTGGATGATGATGGTTTGATAGCTCCAGGGGTTCGTGTATCAGGAGATGATG
TTATTATAGGCAAAACAGTCACCTTGCCTGAAAATGAAGATGAATTGGAGAGCACCA
ATAGACGCTATACCAAGAGAGACTGTAGCACTTTTCTCAGAACTAGCGAGACGGGCA
TTGTGGATCAGGTTATGGTAACTCTCAATCAGGAAGGATATAAATTTGTAAAATAA
GGGTACGCTCTGTTAGGATTCCACAGATTGGAGACAAATTTGCTAGTCGACATGGTC
AAAAGGGTACTTGTGGTATTCAGTATAGACAAGAGGATATGCCTTTCACCTGTGAAG
GTATCACCCCTGATATCATCATCAATCCCCATGCCATCCCCTCTCGTATGACTATTGG
TCACTTAATTGAATGCCTTCAAGGGAAGGTATCGGCTAACAAGGGTGAAATTGGTGA
TGCCACTCCATTTAATGATGCTGTTAACGTGCAGAAGATTTCTAATCTTTTATCTGAT
TATGGCTATCATCTCAGAGGAAATGAGGTCCTGTACAATGGGTTCACTGGTCGAAAA
ATCACATCACAAATATTTATTGGCCCCACTTATTACCAGCGTTTGAAGCATATGGTGG
ATGATAAGATTCACTCTCGTGCTAGGGGACCTATTCAGATCCTCAATAGACAGCCCA
TGGAGGGTAGATCTCGTGATGGTGGCCTGCGTTTTGGAGAAATGGAACGAGATTGTC
AGATTGCCCATGGAGCAGCCCAGTTTTTAAGGGAAAGATTGTTTGAGGCATCAGATC
CATATCAGGTTCATGTTTGCAATCTTTGTGGAATAATGGCGATTGCCAACACCAGGA
CCCATACATATGAATGCAGGGGCTGCCGCAATAAAACCCAGATTTCTTTGGTGCGAA
TGCCTTACGCATGCAAACTATTGTTTCAGGAACTTATGTCTATGAGTATTGCACCGCG
AATGATGAGTGTTTAGCTATTTTACAGGAGTCAACAAGATAATTAAATATCTTGGTGT
CTTGTTTCTATTGTGTGGCTTTTTAAAAATGACAAATATGTACTGTGTTGTGATAAAA
AGTATTTTATTTGTTTAATGATATGCATGCTTTTCTTCTGTAAATATATAATAAATTTT
TGTAGATAGTC

---> Sequence Showing highlighted Existant Combined Trimers

ACT TCG TCT TTA GCT CCT GGC GCT GCT GGC TTC TGG GCG GTT TTT GTC TTT
TGA TTT CAA GAG TTA GGA GCT CGA GAA CCG TTT GGC AAT ATG TAC GAC GCG
GAT GAG GGC TGG AGT GCA GTG GCG CGA TCT CAG CTC CTG CGA GGA GGC
GGA CCG CCC ATT CCG TGC CCG GTA GCC GCG AGG GAC CGG TGG TAG CCG CGA
GGG ACG GGC GGC GGG CTC GGT GCT GTG GCG AGG CGG GGA TGG CAC TCC
CCC CCG CCG CCC CGT GGG GCG GCC CCG ACT TTC GGG CGA TAT GCA ATA TGA
TGA GGA TGA TGA TGA AAT CAC CCC GGA TTT GTG GCA AGA AGC ATG CTG GAT
TGT AAT CAG TTC CTA TTT TGA CGA GAA AGG CTT GGT TAG ACA ACA GCT GGA
TTC TTT TGA TGA GTT TAT TCA GAT GTC TGT TCA AAG AAT TGT GGA AGA CGC
TCC TCC TAT AGA CCT ACA GGC TGA AGC TCA GCA TGC TAG TGG AGA AGT TGA
AGA ACC GCC ACG ATA TTT GCT GAA GTT TGA ACA AAT TTA TCT TTC CAA GCC
TAC CCA TTG GGA AAG AGA TGG TGC TCC TTC ACC AAT GAT GCC CAA TGA AGC
TAG ATT AAG GAA TCT CAC GTA TTC TGC TCC GCT TTA TGT TGA TAT AAC AAA
AAC AGT CAT TAA AGA AGG TGA AGA ACA ACT TCA GAC TCA GCA TCA GAA
AAC TTT TAT AGG AAA AAT TCC AAT TAT GTT GCG GTC AAC TTA CTG CCT TTT
GAA TGG CTT GAC AGA TCG TGA TCT TTG TGA GTT AAA TGA ATG CCC TTT GGA
TCC TGG TGG CTA TTT CAT TAT TAA TGG ATC AGA AAA GGT TCT GAT TGC CCA
AGA GAA AAT GGC AAC AAA CAC AGT TTA TGT GTT TGC CAA AAA GGA TTC TAA
ATA TGC CTA CAC AGG AGA GTG TAG ATC ATG TCT TGA GAA TTC TTC CCG ACC
CAC CAG TAC TAT ATG GGT TAG CAT GCT GGC AAG AGG AGG ACA GGG TGC CAA
GAA GAG TGC TAT TGG TCA GCG CAT TGT GGC AAC TCT ACC ATA TAT CAA GCA

AGA AGT TCC CAT CAT TAT TGT GTT CAG AGC ATT AGG TTT TGT GTC CGA CAG
AGA TAT TTT AGA ACA TAT TAT TTA TGA TTT TGA AGA TCC AGA GAT GAT GGA
AAT GGT TAA ACC TTC TCT CGA TGA AGC TTT TGT CAT CCA AGA ACA GAA TGT
TGC ACT AAA TTT CAT TGG TTC AAG AGG AGC AAA GCC TGG TGT TAC TAA AGA
GAA AAG AAT TAA ATA TGC AAA GGA AGT TTT ACA AAA AGA AAT GCT CCC TCA
TGT TGG TGT CAG TGA TTT TTG TGA GAC CAA AAA AGC CTA TTT CTT GGG ATA
CAT GGT TCA TAG GTT ACT TCT GGC AGC TTT GGG TAG AAG AGA ACT AGA TGA
CAG AGA TCA CTA TGG AAA CAA GAG ATT GGA TCT TGC TGG GCC GCT GCT TGC
ATT CTT ATT TAG AGG TAT GTT TAA GAA TTT GCT TAA AGA AGT GCG GAT CTA
TGC ACA GAA ATT TAT TGA TCG AGG AAA GGA TTT TAA CTT GGA GTT GGC AAT
TAA AAC ACG GAT CAT ATC TGA TGG CCT AAA ATA CTC TTT AGC TAC TGG AAA
CTG GGG TGA TCA AAA GAA AGC TCA TCA AGC CAG AGC TGG AGT ATC TCA GGT
GTT AAA CCG CCT GAC TTT TGC GTC TAC TCT TTC TCA CCT GCG TCG TTT AAA
TTC TCC TAT TGG TAG AGA CGG CAA GCT AGC AAA ACC AAG ACA GTT GCA TAA
TAC GTT GTG GGG AAT GGT GTG TCC TGC CGA GAC CCC AGA GGG CCA TGC TGT
AGG ACT TGT GAA GAA TTT AGC CTT GAT GGC GTA TAT TTC AGT TGG ATC TCA
ACC ATC TCC AAT TCT GGA ATT TTT AGA AGA ATG GAG TAT GGA AAA TTT AGA
AGA AAT TTC TCC TGC AGC TAT TGC TGA TGC AAC CAA GAT TTT TGT TAA TGG
CTG CTG GGT TGG AAT ACA TAA AGA TCC CGA ACA ACT TAT GAA CAC CCT AAG
GAA ATT GAG ACG TCA GAT GGA CAT CAT TGT GTC TGA AGT TTC TAT GAT CAG
AGA TAT TCG AGA GAG GGA GAT TCG GAT CTA TAC GGA TGC AGG CCG TAT TTG
TAG ACC ACT TCT GAT TGT GGA AAA ACA AAA GCT ACT TTT GAA GAA GAG GCA
TAT TGA CCA ATT GAA AGA GAG AGA ATA TAA CAA CTA TAG TTG GCA GGA TCT
TGT GGC CAG TGG GGT AGT GGA GTA TAT TGA TAC CCT GGA AGA AGA AAC AGT
GAT GCT TGC AAT GAC TCC AGA TGA TTT ACA GGA GAA AGA AGT AGC TTA TTG
TTC CAC ATA TAC ACA CTG TGA GAT TCA TCC CTC AAT GAT CCT TGG TGT CTG
TGC ATC TAT TAT TCC CTT TCC TGA TCA TAA CCA GTC CCC TAG AAA CAC ATA
CCA GTC TGC TAT GGG TAA GCA GGC TAT GGG AGT TTA CAT CAC CAA CTT CCA
TGT TCG CAT GGA CAC ATT GGC CCA TGT TCT CTA TTA TCC TCA AAA GCC ACT
TGT GAC TAC ACG GTC TAT GGA ATA TCT ACG ATT TAG AGA GCT GCC AGC AGG
CAT CAA CTC AAT TGT GGC CAT TGC ATC ATA CAC TGG ATA TAA TCA GGA AGA
CTC TGT TAT CAT GAA TCG TTC AGC TGT AGA CCG CGG CTT CTT CAG GTC TGT
TTT CTA TCG CTC ATA CAA AGA ACA GGA GTC TAA AAA AGG ATT TGA TCA AGA
AGA AGT TTT TGA GAA GCC TAC ACG TGA AAC ATG CCA GGG CAT GAG GCA TGC
CAT TTA CGA CAA GCT GGA TGA TGA TGG TTT GAT AGC TCC AGG GGT TCG TGT
ATC AGG AGA TGA TGT TAT TAT AGG CAA AAC AGT CAC CTT GCC TGA AAA TGA
AGA TGA ATT GGA GAG CAC CAA TAG ACG CTA TAC CAA GAG AGA CTG TAG
CAC TTT TCT CAG AAC TAG CGA GAC GGG CAT TGT GGA TCA GGT TAT GGT AAC
TCT CAA TCA GGA AGG ATA TAA ATT TTG TAA AAT AAG GGT ACG CTC TGT TAG
GAT TCC ACA GAT TGG AGA CAA ATT TGC TAG TCG ACA TGG TCA AAA GGG TAC
TTG TGG TAT TCA GTA TAG ACA AGA GGA TAT GCC TTT CAC CTG TGA AGG TAT
CAC CCC TGA TAT CAT CAT CAA TCC CCA TGC CAT CCC CTC TCG TAT GAC TAT
TGG TCA CTT AAT TGA ATG CCT TCA AGG GAA GGT ATC GGC TAA CAA GGG TGA
AAT TGG TGA TGC CAC TCC ATT TAA TGA TGC TGT TAA CGT GCA GAA GAT TTC
TAA TCT TTT ATC TGA TTA TGG CTA TCA TCT CAG AGG AAA TGA GGT CCT GTA
CAA TGG GTT CAC TGG TCG AAA AAT CAC ATC ACA AAT ATT TAT TGG CCC CAC
TTA TTA CCA GCG TTT GAA GCA TAT GGT GGA TGA TAA GAT TCA CTC TCG TGC
TAG GGG ACC TAT TCA GAT CCT CAA TAG ACA GCC CAT GGA GGG TAG ATC TCG
TGA TGG TGG CCT GCG TTT TGG AGA AAT GGA ACG AGA TTG TCA GAT TGC CCA
TGG AGC AGC CCA GTT TTT AAG GGA AAG ATT GTT TGA GGC ATC AGA TCC ATA
TCA GGT TCA TGT TTG CAA TCT TTG TGG AAT AAT GGC GAT TGC CAA CAC CAG

GAC CCA TAC ATA TGA ATG CAG GGG CTG CCG CAA TAA AAC CCA GAT TTC TTT GGT GCG AAT GCC TTA CGC ATG CAA ACT ATT GTT TCA GGA ACT TAT GTC TAT GAG TAT TGC ACC GCG AAT GAT GAG TGT TTA GCT ATT TTA CAG GAG TCA ACA AGA TAA TTA AAT ATC TTG GTG TCT TGT TTC TAT TGT GTG GCT TTT TAA AAA TGA CAA ATA TGT ACT GTG TTG TGA TAA AAA GTA TTT TAT TTG TTT AAT GAT ATG CAT GCT TTT CTT CTG TAA ATA TAT AAT AAA TTT TTG TAG ATA GTC

Occurrences/Results 9 Pentamers

The pentamer 'ATTCA' occurred 5 times.
The pentamer 'TTCAA' occurred 4 times.
The pentamer 'TCAAG' occurred 6 times.
The pentamer 'CAAGA' occurred 15 times.
The pentamer 'AAGAT' occurred 8 times.
The pentamer 'AGATG' occurred 8 times.
The pentamer 'GATGA' occurred 14 times.
The pentamer 'ATGAA' occurred 9 times.
The pentamer 'TGAAT' occurred 6 times.
The total occurrences of all specified strings is 75.
The total number of characters in the text is 3959.
First result (Total characters *9 /1024): 34.8310546875
Square root of the first result: 5.901784025826428
Final result: 6.806237764160666

Occurrences/Results TRIMERS Codons

The pentamer 'ATT' occurred 21 times.
The pentamer 'CAA' occurred 29 times.
The pentamer 'GAT' occurred 30 times.
The pentamer 'GAA' occurred 27 times.
The pentamer 'CCA' occurred 16 times.
The pentamer 'ACT' occurred 14 times.
The pentamer 'AGA' occurred 54 times.
The pentamer 'TAC' occurred 15 times.
The total occurrences of all specified strings is 206.
The total number of characters in the text is 3963.
First result ((total_characters / 3) * (Trimer_number / 64)): 165
Square root of the first result: 12.85
Final result: 3.2

Occurrences/Results TRIMERS Combinations

The pentamer 'ATT ATT' occurred 0 times.
The pentamer 'ATT CAA' occurred 0 times.
The pentamer 'ATT GAT' occurred 0 times.
The pentamer 'ATT GAA' occurred 1 times.
The pentamer 'ATT CCA' occurred 0 times.
The pentamer 'ATT ACT' occurred 0 times.
The pentamer 'ATT AGA' occurred 0 times.
The pentamer 'ATT TAC' occurred 0 times.
The pentamer 'CAA ATT' occurred 1 times.
The pentamer 'CAA CAA' occurred 0 times.
The pentamer 'CAA GAT' occurred 1 times.
The pentamer 'CAA GAA' occurred 1 times.
The pentamer 'CAA CCA' occurred 0 times.
The pentamer 'CAA ACT' occurred 1 times.
The pentamer 'CAA AGA' occurred 1 times.

The pentamer 'CAA TAC' occurred 0 times.
The pentamer 'GAT ATT' occurred 0 times.
The pentamer 'GAT CAA' occurred 0 times.
The pentamer 'GAT GAT' occurred 1 times.
The pentamer 'GAT GAA' occurred 0 times.
The pentamer 'GAT CCA' occurred 0 times.
The pentamer 'GAT ACT' occurred 0 times.
The pentamer 'GAT AGA' occurred 0 times.
The pentamer 'GAT TAC' occurred 0 times.
The pentamer 'GAA ATT' occurred 2 times.
The pentamer 'GAA CAA' occurred 0 times.
The pentamer 'GAA GAT' occurred 1 times.
The pentamer 'GAA GAA' occurred 2 times.
The pentamer 'GAA CCA' occurred 0 times.
The pentamer 'GAA ACT' occurred 0 times.
The pentamer 'GAA AGA' occurred 2 times.
The pentamer 'GAA TAC' occurred 0 times.
The pentamer 'CCA ATT' occurred 1 times.
The pentamer 'CCA CAA' occurred 0 times.
The pentamer 'CCA GAT' occurred 1 times.
The pentamer 'CCA GAA' occurred 0 times.
The pentamer 'CCA CCA' occurred 0 times.
The pentamer 'CCA ACT' occurred 0 times.
The pentamer 'CCA AGA' occurred 2 times.
The pentamer 'CCA TAC' occurred 1 times.
The pentamer 'ACT ATT' occurred 1 times.
The pentamer 'ACT CAA' occurred 0 times.
The pentamer 'ACT GAT' occurred 0 times.
The pentamer 'ACT GAA' occurred 0 times.
The pentamer 'ACT CCA' occurred 0 times.
The pentamer 'ACT ACT' occurred 0 times.
The pentamer 'ACT AGA' occurred 1 times.
The pentamer 'ACT TAC' occurred 0 times.
The pentamer 'AGA ATT' occurred 0 times.
The pentamer 'AGA CAA' occurred 1 times.
The pentamer 'AGA GAT' occurred 1 times.
The pentamer 'AGA GAA' occurred 2 times.
The pentamer 'AGA CCA' occurred 0 times.
The pentamer 'AGA ACT' occurred 1 times.
The pentamer 'AGA AGA' occurred 4 times.
The pentamer 'AGA TAC' occurred 0 times.
The pentamer 'TAC ATT' occurred 0 times.
The pentamer 'TAC CAA' occurred 1 times.
The pentamer 'TAC GAT' occurred 0 times.
The pentamer 'TAC GAA' occurred 0 times.
The pentamer 'TAC CCA' occurred 1 times.
The pentamer 'TAC ACT' occurred 0 times.
The pentamer 'TAC AGA' occurred 0 times.
The pentamer 'TAC TAC' occurred 0 times.
The total occurrences of all specified strings is 32.
The total number of characters in the text is 3959.

First result (((total_characters/ 3) -1) / 64): 20.625
Square root of the first result: 4.541475531146237
Final result: 2.5046925656624706

>AB528358.1 Homosapiens, glycyl-tRNA ligase

---> Sequence

GCGATCGCCATGCCCTCTCCGCGTCCAGTGCTGCTTAGAGGTGCTCGCGCCGCTCTGC
TGCTGCTGCTGCCGCCCCGGCTCTTAGCCCGACCCTCGCTCCTGCTCCGCCGGTCCCT
CAGCGCGGCCTCCTGCGCCCCGATCTCCTTGCCCGCCGCCGCCTCCCGGAGCAGCAT
GGACGGCGCGGGGGCTGAGGAGGTGCTGGCACCTCTGAGGCTAGCAGTGCGCCAGC
AGGGAGATCTTGTGCGAAAACTCAAAGAAGATAAAGCACCCCAAGTAGACGTAGAC
AAAGCAGTGGCTGAGCTCAAAGCCCGCAAGAGGGTTCTGGAAGCAAAGGAGCTGGC
GTTACAGCCCAAAGATGATATTGTAGACCGAGCAAAAATGGAAGATACCCTGAAGA
GGAGGTTTTTCTATGATCAAGCTTTTGCTATTTATGGAGGTGTTAGTGGTCTGTATGA
CTTTGGGCCAGTTGGCTGTGCTTTGAAGAACAATATTATTCAGACCTGGAGGCAGCA
CTTTATCCAAGAGGAACAGATCCTGGAGATCGATTGCACCATGCTCACCCCTGAGCC
AGTTTTAAAGACCTCTGGCCATGTAGACAAATTTGCTGACTTCATGGTGAAAGACGT
AAAAAATGGAGAATGTTTTCGTGCTGACCATCTATTAAAAGCTCATTTACAGAAATT
GATGTCTGATAAGAAGTGTTCTGTCGAAAAGAAATCAGAAATGGAAAGTGTTTTGGC
CCAGCTTGATAACTATGGACAGCAAGAACTTGCGGATCTTTTTGTGAACTATAATGT
AAAATCTCCCATTACTGGAAATGATCTATCCCCTCCAGTGTCTTTTAACTTAATGTTC
AAGACTTTCATTGGGCCTGGAGGAAACATGCCTGGGTACTTGAGACCAGAAACTGCA
CAGGGGATTTTCTTGAATTTCAAACGACTTTTGGAGTTCAACCAAGGAAAGTTGCCTT
TTGCTGCTGCCCAGATTGGAAATTCTTTTAGAAATGAGATCTCCCCTCGATCTGGACT
GATCAGAGTCAGAGAATTCACAATGGCAGAAATTGAGCACTTTGTAGATCCCAGTGA
GAAAGACCACCCCAAGTTCCAGAATGTGGCAGACCTTCACCTTTATTTGTATTCAGC
AAAAGCCCAGGTCAGCGGACAGTCCGCTCGGAAAATGCGCCTGGGAGATGCTGTTG
AACAGGGTGTGATTAATAACACAGTATTAGGCTATTTCATTGGCCGCATCTACCTCTA
CCTCACGAAGGTTGGAATATCTCCAGATAAACTCCGCTTCCGGCAGCACATGGAGAA
TGAGATGGCCCATTATGCCTGTGACTGTTGGGATGCAGAATCCAAAACATCCTACGG
TTGGATTGAGATTGTTGGATGTGCTGATCGTTCCTGTTATGACCTCTCCTGTCATGCA
CGAGCCACCAAAGTCCCACTTGTAGCTGAGAAACCTCTGAAAGAACCCAAAACAGTC
AATGTTGTTCAGTTTGAACCCAGTAAGGGAGCAATTGGTAAGGCATATAAGAAGGAT
GCAAAACTGGTGATGGAGTATCTTGCCATTTGTGATGAGTGCTACATTACAGAAATG
GAGATGCTGCTGAATGAGAAAGGGGAATTCACAATTGAAACTGAAGGGAAAACATT
TCAGTTAACAAAAGACATGATCAATGTGAAGAGATTCCAGAAAACACTATATGTGGA
AGAAGTTGTTCCGAATGTAATTGAACCTTCCTTCGGCCTGGGTAGGATCATGTATACG
GTATTTGAACATACATTCCATGTACGAGAAGGAGATGAACAGAGAACATTCTTCAGT
TTCCCTGCTGTAGTTGCTCCATTCAAATGTTCCGTCCTCCCACTGAGCCAAAACCAGG
AGTTCATGCCATTTGTCAAGGAATTATCGGAAGCCCTGACCAGGCATGGAGTATCTC
ACAAAGTAGACGATTCCTCTGGGTCAATCGGAAGGCGCTATGCCAGGACTGATGAGA
TTGGCGTGGCTTTTGGTGTCACCATTGACTTTGACACAGTGAACAAGACCCCCCACAC
TGCAACTCTGAGGGACCGTGACTCAATGCGGCAGATAAGAGCAGAGATCTCTGAGCT
GCCCAGCATAGTCCAAGACCTAGCCAATGGCAACATCACATGGGCTGATGTGGAGGC
CAGGTATCCTCTGTTTGAAGGGCAAGAGACTGGTAAAAAAGAGACAATCGAGGAAG
TTTAAAC

---> Sequence Showing highlighted Existant Combined Trimers

GCG ATC GCC ATG CCC TCT CCG CGT CCA GTG CTG CTT AGA GGT GCT CGC GCC
GCT CTG CTG CTG CTG CTG CCG CCC CGG CTC TTA GCC CGA CCC TCG CTC CTG
CTC CGC CGG TCC CTC AGC GCG GCC TCC TGC GCC CCG ATC TCC TTG CCC GCC

GCC GCC TCC CGG AGC AGC ATG GAC GGC GCG GGG GCT GAG GAG GTG CTG
GCA CCT CTG AGG CTA GCA GTG CGC CAG CAG GGA GAT CTT GTG CGA AAA CTC
AAA GAA GAT AAA GCA CCC CAA GTA GAC GTA GAC AAA GCA GTG GCT GAG
CTC AAA GCC CGC AAG AGG GTT CTG GAA GCA AAG GAG CTG GCG TTA CAG CCC
AAA GAT GAT ATT GTA GAC CGA GCA AAA ATG GAA GAT ACC CTG AAG AGG
AGG TTT TTC TAT GAT CAA GCT TTT GCT ATT TAT GGA GGT GTT AGT GGT CTG
TAT GAC TTT GGG CCA GTT GGC TGT GCT TTG AAG AAC AAT ATT ATT CAG ACC
TGG AGG CAG CAC TTT ATC CAA GAG GAA CAG ATC CTG GAG ATC GAT TGC ACC
ATG CTC ACC CCT GAG CCA GTT TTA AAG ACC TCT GGC CAT GTA GAC AAA TTT
GCT GAC TTC ATG GTG AAA GAC GTA AAA AAT GGA GAA TGT TTT CGT GCT GAC
CAT CTA TTA AAA GCT CAT TTA CAG AAA TTG ATG TCT GAT AAG AAG TGT TCT
GTC GAA AAG AAA TCA GAA ATG GAA AGT GTT TTG GCC CAG CTT GAT AAC TAT
GGA CAG CAA GAA CTT GCG GAT CTT TTT GTG AAC TAT AAT GTA AAA TCT CCC
ATT ACT GGA AAT GAT CTA TCC CCT CCA GTG TCT TTT AAC TTA ATG TTC AAG
ACT TTC ATT GGG CCT GGA GGA AAC ATG CCT GGG TAC TTG AGA CCA GAA ACT
GCA CAG GGG ATT TTC TTG AAT TTC AAA CGA CTT TTG GAG TTC AAC CAA GGA
AAG TTG CCT TTT GCT GCT GCC CAG ATT GGA AAT TCT TTT AGA AAT GAG ATC
TCC CCT CGA TCT GGA CTG ATC AGA GTC AGA GAA TTC ACA ATG GCA GAA ATT
GAG CAC TTT GTA GAT CCC AGT GAG AAA GAC CAC CCC AAG TTC CAG AAT GTG
GCA GAC CTT CAC CTT TAT TTG TAT TCA GCA AAA GCC CAG GTC AGC GGA CAG
TCC GCT CGG AAA ATG CGC CTG GGA GAT GCT GTT GAA CAG GGT GTG ATT AAT
AAC ACA GTA TTA GGC TAT TTC ATT GGC CGC ATC TAC CTC TAC CTC ACG AAG
GTT GGA ATA TCT CCA GAT AAA CTC CGC TTC CGG CAG CAC ATG GAG AAT GAG
ATG GCC CAT TAT GCC TGT GAC TGT TGG GAT GCA GAA TCC AAA ACA TCC TAC
GGT TGG ATT GAG ATT GTT GGA TGT GCT GAT CGT TCC TGT TAT GAC CTC TCC
TGT CAT GCA CGA GCC ACC AAA GTC CCA CTT GTA GCT GAG AAA CCT CTG AAA
GAA CCC AAA ACA GTC AAT GTT GTT CAG TTT GAA CCC AGT AAG GGA GCA ATT
GGT AAG GCA TAT AAG AAG GAT GCA AAA CTG GTG ATG GAG TAT CTT GCC ATT
TGT GAT GAG TGC TAC ATT ACA GAA ATG GAG ATG CTG CTG AAT GAG AAA GGG
GAA TTC ACA ATT GAA ACT GAA GGG AAA ACA TTT CAG TTA ACA AAA GAC ATG
ATC AAT GTG AAG AGA TTC CAG AAA ACA CTA TAT GTG GAA GAA GTT GTT CCG
AAT GTA ATT GAA CCT TCC TTC GGC CTG GGT AGG ATC ATG TAT ACG GTA TTT
GAA CAT ACA TTC CAT GTA CGA GAA GGA GAT GAA CAG AGA ACA TTC TTC AGT
TTC CCT GCT GTA GTT GCT CCA TTC AAA TGT TCC GTC CTC CCA CTG AGC CAA
AAC CAG GAG TTC ATG CCA TTT GTC AAG GAA TTA TCG GAA GCC CTG ACC AGG
CAT GGA GTA TCT CAC AAA GTA GAC GAT TCC TCT GGG TCA ATC GGA AGG CGC
TAT GCC AGG ACT GAT GAG ATT GGC GTG GCT TTT GGT GTC ACC ATT GAC TTT
GAC ACA GTG AAC AAG ACC CCC CAC ACT GCA ACT CTG AGG GAC CGT GAC TCA
ATG CGG CAG ATA AGA GCA GAG ATC TCT GAG CTG CCC AGC ATA GTC CAA GAC
CTA GCC AAT GGC AAC ATC ACA TGG GCT GAT GTG GAG GCC AGG TAT CCT CTG
TTT GAA GGG CAA GAG ACT GGT AAA AAA GAG ACA ATC GAG GAA GTT TAA AC

Occurrences/Results 9 Pentamers

The pentamer 'ATTCA' occurred 5 times.
The pentamer 'TTCAA' occurred 4 times.
The pentamer 'TCAAG' occurred 3 times.
The pentamer 'CAAGA' occurred 7 times.
The pentamer 'AAGAT' occurred 3 times.
The pentamer 'AGATG' occurred 5 times.
The pentamer 'GATGA' occurred 4 times.
The pentamer 'ATGAA' occurred 1 times.
The pentamer 'TGAAT' occurred 2 times.

The total occurrences of all specified strings is 34.
The total number of characters in the text is 2230.
First result (Total characters *9 /1024): 19.634765625
Square root of the first result: 4.431113361786178
Final result: 3.2419017980639944

Occurrences/Results TRIMERS Codons

The pentamer 'ATT' occurred 20 times.
The pentamer 'CAA' occurred 8 times.
The pentamer 'GAT' occurred 22 times.
The pentamer 'GAA' occurred 30 times.
The pentamer 'CCA' occurred 10 times.
The pentamer 'ACT' occurred 8 times.
The pentamer 'AGA' occurred 8 times.
The pentamer 'TAC' occurred 5 times.
The total occurrences of all specified strings is 111.
The total number of characters in the text is 2232.
First result ((total_characters / 3) * (Trimer_number / 64)): 93
Square root of the first result: 9.6
Final result: 1.87

Occurrences/Results TRIMERS Combinations

The pentamer 'ATT ATT' occurred 1 times.
The pentamer 'ATT CAA' occurred 0 times.
The pentamer 'ATT GAT' occurred 0 times.
The pentamer 'ATT GAA' occurred 2 times.
The pentamer 'ATT CCA' occurred 0 times.
The pentamer 'ATT ACT' occurred 1 times.
The pentamer 'ATT AGA' occurred 0 times.
The pentamer 'ATT TAC' occurred 0 times.
The pentamer 'CAA ATT' occurred 0 times.
The pentamer 'CAA CAA' occurred 0 times.
The pentamer 'CAA GAT' occurred 0 times.
The pentamer 'CAA GAA' occurred 1 times.
The pentamer 'CAA CCA' occurred 0 times.
The pentamer 'CAA ACT' occurred 0 times.
The pentamer 'CAA AGA' occurred 0 times.
The pentamer 'CAA TAC' occurred 0 times.
The pentamer 'GAT ATT' occurred 1 times.
The pentamer 'GAT CAA' occurred 1 times.
The pentamer 'GAT GAT' occurred 1 times.
The pentamer 'GAT GAA' occurred 1 times.
The pentamer 'GAT CCA' occurred 0 times.
The pentamer 'GAT ACT' occurred 0 times.
The pentamer 'GAT AGA' occurred 0 times.
The pentamer 'GAT TAC' occurred 0 times.
The pentamer 'GAA ATT' occurred 1 times.
The pentamer 'GAA CAA' occurred 0 times.
The pentamer 'GAA GAT' occurred 2 times.
The pentamer 'GAA GAA' occurred 1 times.
The pentamer 'GAA CCA' occurred 0 times.
The pentamer 'GAA ACT' occurred 2 times.
The pentamer 'GAA AGA' occurred 0 times.

The pentamer 'GAA TAC' occurred 0 times.
The pentamer 'CCA ATT' occurred 0 times.
The pentamer 'CCA CAA' occurred 0 times.
The pentamer 'CCA GAT' occurred 1 times.
The pentamer 'CCA GAA' occurred 1 times.
The pentamer 'CCA CCA' occurred 0 times.
The pentamer 'CCA ACT' occurred 0 times.
The pentamer 'CCA AGA' occurred 0 times.
The pentamer 'CCA TAC' occurred 0 times.
The pentamer 'ACT ATT' occurred 0 times.
The pentamer 'ACT CAA' occurred 0 times.
The pentamer 'ACT GAT' occurred 1 times.
The pentamer 'ACT GAA' occurred 1 times.
The pentamer 'ACT CCA' occurred 0 times.
The pentamer 'ACT ACT' occurred 0 times.
The pentamer 'ACT AGA' occurred 0 times.
The pentamer 'ACT TAC' occurred 0 times.
The pentamer 'AGA ATT' occurred 0 times.
The pentamer 'AGA CAA' occurred 0 times.
The pentamer 'AGA GAT' occurred 0 times.
The pentamer 'AGA GAA' occurred 1 times.
The pentamer 'AGA CCA' occurred 1 times.
The pentamer 'AGA ACT' occurred 0 times.
The pentamer 'AGA AGA' occurred 0 times.
The pentamer 'AGA TAC' occurred 0 times.
The pentamer 'TAC ATT' occurred 1 times.
The pentamer 'TAC CAA' occurred 0 times.
The pentamer 'TAC GAT' occurred 0 times.
The pentamer 'TAC GAA' occurred 0 times.
The pentamer 'TAC CCA' occurred 0 times.
The pentamer 'TAC ACT' occurred 0 times.
The pentamer 'TAC AGA' occurred 0 times.
The pentamer 'TAC TAC' occurred 0 times.
The total occurrences of all specified strings is 22.
The total number of characters in the text is 2230.
First result (((total_characters/ 3) -1) / 64): 11.609375
Square root of the first result: 3.4072532926097527
Final result: 3.049560483964314

>NM_000979.4 Homo sapiens ribosomal protein L18 (RPL18), transcript variant 1, mRNA

---> Sequence

CTCTTTCCGGACCTGGCCGAGCAGGAGGCGCCATCATGGGAGTGGACATCCGCCATAACAAGGACC
GAAAGGTTCGGCGCAAGGAGCCCAAGAGCCAGGATATCTACCTGAGGCTGTTGGTCAAGTTATAC
AGGTTTCTGGCCAGAAGAACCAACTCCACATTCAACCAGGTTGTGTTGAAGAGGTTGTTTATGAG
TCGCACCAACCGGCCGCCTCTGTCCCTTTCCCGGATGATCCGGAAGATGAAGCTTCCTGGCCGGGA
AAACAAGACGGCCGTGGTTGTGGGGACCATAACTGATGATGTGCGGGTTCAGGAGGTACCCAAAC
TGAAGGTATGTGCACTGCGCGTGACCAGCCGGGCCCGCAGCCGCATCCTCAGGGCAGGGGGCAAG
ATCCTCACTTTCGACCAGCTGGCCCTGGACTCCCCTAAGGGCTGTGGCACTGTCCTGCTCTCCGGT
CCTCGCAAGGGCCGAGAGGTGTACCGGCATTTCGGCAAGGCCCCAGGAACCCCGCACAGCCACACC

AAACCCTACGTCCGCTCCAAGGGCCGGAAGTTCGAGCGTGCCAGAGGCCGACGGGCCAGCCGAGGC
TACAAAAACTAACCCTGGATCCTACTCTCTTATTAAAAAGATTTTTGCTGACA

---> Sequence Showing highlighted Existant Combined Trimers

CTC TTT CCG GAC CTG GCC GAG CAG GAG GCG CCA TCA TGG GAG TGG ACA TCC GCC ATA
ACA AGG ACC GAA AGG TTC GGC GCA AGG AGC CCA AGA GCC AGG ATA TCT ACC TGA GGC
TGT TGG TCA AGT TAT ACA GGT TTC TGG CCA GAA GAA CCA ACT CCA CAT TCA ACC AGG
TTG TGT TGA AGA GGT TGT TTA TGA GTC GCA CCA ACC GGC CGC CTC TGT CCC TTT CCC
GGA TGA TCC GGA AGA TGA AGC TTC CTG GCC GGG AAA ACA AGA CGG CCG TGG TTG TGG
GGA CCA TAA CTG ATG ATG TGC GGG TTC AGG AGG TAC CCA AAC TGA AGG TAT GTG CAC
TGC GCG TGA CCA GCC GGG CCC GCA GCC GCA TCC TCA GGG CAG GGG GCA AGA TCC TCA
CTT TCG ACC AGC TGG CCC TGG ACT CCC CTA AGG GCT GTG GCA CTG TCC TGC TCT CCG
GTC CTC GCA AGG GCC GAG AGG TGT ACC GGC ATT TCG GCA AGG CCC CAG GAA CCC CGC
ACA GCC ACA CCA AAC CCT ACG TCC GCT CCA AGG GCC GGA AGT TCG AGC GTG CCA GAG
GCC GAC GGG CCA GCC GAG GCT ACA AAA ACT AAC CCT GGA TCC TAC TCT CTT ATT AAA
AAG ATT TTT GCT GAC A

Occurrences/Results 9 Pentamers

The pentamer 'ATTCA' occurred 1 times.
The pentamer 'TTCAA' occurred 1 times.
The pentamer 'TCAAG' occurred 1 times.
The pentamer 'CAAGA' occurred 3 times.
The pentamer 'AAGAT' occurred 3 times.
The pentamer 'AGATG' occurred 1 times.
The pentamer 'GATGA' occurred 3 times.
The pentamer 'ATGAA' occurred 1 times.
The pentamer 'TGAAT' occurred 0 times.
The total occurrences of all specified strings is 14.
The total number of characters in the text is 639.
First result (Total characters *9 /1024): 5.6513671875
Square root of the first result: 2.3772604374573687
Final result: 3.5118713460900373

Occurrences/Results TRIMERS Codons

The pentamer 'ATT' occurred 3 times.
The pentamer 'CAA' occurred 0 times.
The pentamer 'GAT' occurred 0 times.
The pentamer 'GAA' occurred 4 times.
The pentamer 'CCA' occurred 13 times.
The pentamer 'ACT' occurred 3 times.
The pentamer 'AGA' occurred 5 times.
The pentamer 'TAC' occurred 2 times.
The total occurrences of all specified strings is 30.
The total number of characters in the text is 639.
First result ((total_characters / 3) * (Trimer_number / 64)): 26.6
Square root of the first result: 5.2
Final result: 0.7

Occurrences/Results TRIMERS Combinations

The pentamer 'ATT ATT' occurred 0 times.
The pentamer 'ATT CAA' occurred 0 times.
The pentamer 'ATT GAT' occurred 0 times.

The pentamer 'ATT GAA' occurred 0 times.
The pentamer 'ATT CCA' occurred 0 times.
The pentamer 'ATT ACT' occurred 0 times.
The pentamer 'ATT AGA' occurred 0 times.
The pentamer 'ATT TAC' occurred 0 times.
The pentamer 'CAA ATT' occurred 0 times.
The pentamer 'CAA CAA' occurred 0 times.
The pentamer 'CAA GAT' occurred 0 times.
The pentamer 'CAA GAA' occurred 0 times.
The pentamer 'CAA CCA' occurred 0 times.
The pentamer 'CAA ACT' occurred 0 times.
The pentamer 'CAA AGA' occurred 0 times.
The pentamer 'CAA TAC' occurred 0 times.
The pentamer 'GAT ATT' occurred 0 times.
The pentamer 'GAT CAA' occurred 0 times.
The pentamer 'GAT GAT' occurred 0 times.
The pentamer 'GAT GAA' occurred 0 times.
The pentamer 'GAT CCA' occurred 0 times.
The pentamer 'GAT ACT' occurred 0 times.
The pentamer 'GAT AGA' occurred 0 times.
The pentamer 'GAT TAC' occurred 0 times.
The pentamer 'GAA ATT' occurred 0 times.
The pentamer 'GAA CAA' occurred 0 times.
The pentamer 'GAA GAT' occurred 0 times.
The pentamer 'GAA GAA' occurred 1 times.
The pentamer 'GAA CCA' occurred 1 times.
The pentamer 'GAA ACT' occurred 0 times.
The pentamer 'GAA AGA' occurred 0 times.
The pentamer 'GAA TAC' occurred 0 times.
The pentamer 'CCA ATT' occurred 0 times.
The pentamer 'CCA CAA' occurred 0 times.
The pentamer 'CCA GAT' occurred 0 times.
The pentamer 'CCA GAA' occurred 1 times.
The pentamer 'CCA CCA' occurred 0 times.
The pentamer 'CCA ACT' occurred 1 times.
The pentamer 'CCA AGA' occurred 1 times.
The pentamer 'CCA TAC' occurred 0 times.
The pentamer 'ACT ATT' occurred 0 times.
The pentamer 'ACT CAA' occurred 0 times.
The pentamer 'ACT GAT' occurred 0 times.
The pentamer 'ACT GAA' occurred 0 times.
The pentamer 'ACT CCA' occurred 1 times.
The pentamer 'ACT ACT' occurred 0 times.
The pentamer 'ACT AGA' occurred 0 times.
The pentamer 'ACT TAC' occurred 0 times.
The pentamer 'AGA ATT' occurred 0 times.
The pentamer 'AGA CAA' occurred 0 times.
The pentamer 'AGA GAT' occurred 0 times.
The pentamer 'AGA GAA' occurred 0 times.
The pentamer 'AGA CCA' occurred 0 times.

The pentamer 'AGA ACT' occurred 0 times.
The pentamer 'AGA AGA' occurred 0 times.
The pentamer 'AGA TAC' occurred 0 times.
The pentamer 'TAC ATT' occurred 0 times.
The pentamer 'TAC CAA' occurred 0 times.
The pentamer 'TAC GAT' occurred 0 times.
The pentamer 'TAC GAA' occurred 0 times.
The pentamer 'TAC CCA' occurred 1 times.
The pentamer 'TAC ACT' occurred 0 times.
The pentamer 'TAC AGA' occurred 0 times.
The pentamer 'TAC TAC' occurred 0 times.
The total occurrences of all specified strings is 7.
The total number of characters in the text is 639.
First result (((total_characters/ 3) -1) / 64): 3.328125
Square root of the first result: 1.824314939915803
Final result: 2.012741835118374

>NM_001354747.2 Homo sapiens phosphofructokinase, muscle (PFKM), variant 17 ---> Sequence

AGAGATCACGTTAGGGTACACCAAGAAAGAGACGGTTCAAGGGAATTACCAGGAGGCGATGAGCC
AGAGTGGATCATGACCCATGAAGAGCACCATGCAGCCAAAACCCTGGGGATTGGCAAAGCCATTG
CTGTCTTAACCTCTGGTGGAGATGCCCAAGGGTTATCAAGGCCTGGTGGATGGTGGAGATCACAT
CAAGGAAGCCACCTGGGAGAGCGTTTCGATGATGCTTCAGCTGGGAGGCACGGTGATTGGAAGTG
CCCGGTGCAAGGACTTTCGGGAACGAGAAGGACGACTCCGAGCTGCCTACAACCTGGTGAAGCGT
GGGATCACCAATCTCTGTGTCATTGGGGGTGATGGCAGCCTCACTGGGGCTGACACCTTCCGTTCT
GAGTGGAGTGACTTGTTGAGTGACCTCCAGAAAGCAGGTAAGATCACAGATGAGGAGGCTACGAA
GTCCAGCTACCTGAACATTGTGGGCCTGGTTGGGTCAATTGACAATGACTTCTGTGGCACCGATA
TGACCATTGGCACTGACTCTGCCCTGCATCGGATCATGGAAATTGTAGATGCCATCACTACCACTG
CCCAGAGCCACCAGAGGACATTTGTGTTAGAAGTAATGGGCCGCCACTGTGGATACCTGGCCCTT
GTCACCTCTCTGTCCTGTGGGGCCGACTGGGTTTTTATTCCTGAATGTCCACCAGATGACGACTGG
GAGGAACACCTTTGTCGCCGACTCAGCGAGACAAGGACCCGTGGTTCTCGTCTCAACATCATCATT
GTGGCTGAGGGTGCAATTGACAAGAATGGAAAACCAATCACCTCAGAAGACATCAAGAATCTGGT
GGTTAAGCGTCTGGGATATGACACCCGGGTTACTGTCTTGGGGCATGTGCAGAGGGGTGGGACGC
CATCAGCCTTTGACAGAATTCTGGGCAGCAGGATGGGTGTGGAAGCAGTGATGGCACTTTTGGAG
GGGACCCCAGATACCCCAGCCTGTGTAGTGAGCCTCTCTGGTAACCAGGCTGTGCGCCTGCCCCTC
ATGGAATGTGTCCAGGTGACCAAAGATGTGACCAAGGCCATGGATGAGAAGAAATTTGACGAAGC
CCTGAAGCTGAGAGGCCGGAGCTTCATGAACAACTGGGAGGTGTACAAGCTTCTAGCTCATGTCA
GACCCCCGGTATCTAAGAGTGGTTCGCACACAGTGGCTGTGATGAACGTGGGGGCTCCGGCTGCA
GGCATGAATGCTGCTGTTCGCTCCACTGTGAGGATTGGCCTTATCCAGGGCAACCGAGTGCTCGTT
GTCCATGATGGTTTCGAGGGCCTGGCCAAGGGGCAGATAGAGGAAGCTGGCTGGAGCTATGTTGG
GGGCTGGACTGGCCAAGGTGGCTCTAAACTTGGGACTAAAAGGACTCTACCCAAGAAGAGCTTTG
AACAGATCAGTGCCAATATAACTAAGTTTAACATTCAGGGCCTTGTCATCATTGGGGGCTTTGAG
GCTTACACAGGGGGCCTGGAACTGATGGAGGGCAGGAAGCAGTTTGATGAGCTCTGCATCCCATT
TGTGGTCATTCCTGCTACAGTCTCCAACAATGTCCCTGGCTCAGACTTCAGCGTTGGGGCTGACAC
AGCACTCAATACTATCTGCACAACCTGTGACCGCATCAAGCAGTCAGCAGCTGGCACCAAGCGTCG
GGTGTTTATCATTGAGACTATGGGTGGCTACTGTGGCTACCTGGCTACCATGGCTGGACTGGCAG
CTGGGGCCGATGCTGCCTACATTTTTGAGGAGCCCTTCACCATTCGAGACCTGCAGGCAAATGTTG
AACATCTGGTGCAAAAGATGAAAACAACTGTGAAAAGGGGCTTGGTGTTAAGGAATGAAAAGTG
CAATGAGAACTATACCACTGACTTCATTTTCAACCTGTACTCTGAGGAGGGGAAGGGCATCTTCG

ACAGCAGGAAGAATGTGCTTGGTCACATGCAGCAGGGTGGGAGCCCAACCCCATTTGATAGGAAT
TTTGCCACTAAGATGGGCGCCAAGGCTATGAACTGGATGTCTGGGAAAATCAAAGAGAGTTACCG
TAATGGGCGGATCTTTGCCAATACTCCAGATTCGGGCTGTGTTCTGGGGATGCGTAAGAGGGCTC
TGGTCTTCCAACCAGTGGCTGAGCTGAAGGACCAGACAGATTTTGAGCATCGAATCCCCAAGGAA
CAGTGGTGGCTGAAACTGAGGCCCATCCTCAAAATCCTAGCCAAGTACGAGATTGACTTGGACAC
TTCAGACCATGCCCACCTGGAGCACATCACCCGGAAGCGGTCCGGGGAAGCTGCCGTCTAAACCTC
TCTGGAGTGAGGGGAATAGATTACCTGATCATGGTCAGCTCACACCCTAATAAGTCCACATCTTC
TCAGTGTTTTAGCTGTTTTTTTCATTAGGTTTCCTTTTATTCTGTACCTTGCAGCCATGACCAGTT
CTGGCCAGGAGCTGGAGGAGCAGGCAGTGGGTGGGAGCTCCTTTTAGGTAGAATTTAACATGACT
TCTGCCCCAGCTTTATCTGTCACACAAGGCTGGGCACCTCTAGTGCTACTGCTAGATATCACTTAC
TCAGTTAGAATTTTCCTAAAAATAAGCTTTATTTATTTCTTTGTGATAACAAAGAGTCTTGGTTC
CTCTACTACTTTTACTACAGTGACAAATTGTAACTACACTAATAAATGCCAACTGGTCACTGTGC
TTTTGCTTCTCCTGTTATCATCTTCCTAAGTGGAATGTAATACTGTCAGCCCCATGTATCAGACAC
TTGTCTGATGAAGCAGTAAAGACGTTAAGGGTATCACAGGGGGTGGAGGAAGGGATTATCTCTA
GTACACTACTTGCTGGCTGTCTGAAAAATTGTCACTGCCAAACTCTAAAAACAGTTCTAAATAGT
GACTGAGAAGGTTTGTTGCTGGAGTCAGGGAATAAGGCAGCCAAATACTCTTTGCACAGTTCTTT
AGTGGGAAGAGAAATTAACAATAAATATCAAGCACTGTG

---> Sequence Showing highlighted Existant Combined Trimers

AGA GAT CAC GTT AGG GTA CAC CAA GAA AGA GAC GGT TCA AGG GAA TTA CCA GGA GGC
GAT GAG CCA GAG TGG ATC ATG ACC CAT GAA GAG CAC CAT GCA GCC AAA ACC CTG GGG
ATT GGC AAA GCC ATT GCT GTC TTA ACC TCT GGT GGA GAT GCC CAA GGG TTA TCA AGG
CCT GGT GGA TGG TGG AGA TCA CAT CAA GGA AGC CAC CTG GGA GAG CGT TTC GAT GAT
GCT TCA GCT GGG AGG CAC GGT GAT TGG AAG TGC CCG GTG CAA GGA CTT TCG GGA ACG
AGA AGG ACG ACT CCG AGC TGC CTA CAA CCT GGT GAA GCG TGG GAT CAC CAA TCT CTG
TGT CAT TGG GGG TGA TGG CAG CCT CAC TGG GGC TGA CAC CTT CCG TTC TGA GTG GAG
TGA CTT GTT GAG TGA CCT CCA GAA AGC AGG TAA GAT CAC AGA TGA GGA GGC TAC GAA
GTC CAG CTA CCT GAA CAT TGT GGG CCT GGT TGG GTC AAT TGA CAA TGA CTT CTG TGG
CAC CGA TAT GAC CAT TGG CAC TGA CTC TGC CCT GCA TCG GAT CAT GGA AAT TGT AGA
TGC CAT CAC TAC CAC TGC CCA GAG CCA CCA GAG GAC ATT TGT GTT AGA AGT AAT GGG
CCG CCA CTG TGG ATA CCT GGC CCT TGT CAC CTC TCT GTC CTG TGG GGC CGA CTG GGT
TTT TAT TCC TGA ATG TCC ACC AGA TGA CGA CTG GGA GGA ACA CCT TTG TCG CCG ACT
CAG CGA GAC AAG GAC CCG TGG TTC TCG TCT CAA CAT CAT CAT TGT GGC TGA GGG TGC
AAT TGA CAA GAA TGG AAA ACC AAT CAC CTC AGA AGA CAT CAA GAA TCT GGT GGT TAA
GCG TCT GGG ATA TGA CAC CCG GGT TAC TGT CTT GGG GCA TGT GCA GAG GGG TGG GAC
GCC ATC AGC CTT TGA CAG AAT TCT GGG CAG CAG GAT GGG TGT GGA AGC AGT GAT GGC
ACT TTT GGA GGG GAC CCC AGA TAC CCC AGC CTG TGT AGT GAG CCT CTC TGG TAA CCA
GGC TGT GCG CCT GCC CCT CAT GGA ATG TGT CCA GGT GAC CAA AGA TGT GAC CAA GGC
CAT GGA TGA GAA GAA ATT TGA CGA AGC CCT GAA GCT GAG AGG CCG GAG CTT CAT GAA
CAA CTG GGA GGT GTA CAA GCT TCT AGC TCA TGT CAG ACC CCC GGT ATC TAA GAG TGG
TTC GCA CAC AGT GGC TGT GAT GAA CGT GGG GGC TCC GGC TGC AGG CAT GAA TGC TGC
TGT TCG CTC CAC TGT GAG GAT TGG CCT TAT CCA GGG CAA CCG AGT GCT CGT TGT CCA
TGA TGG TTT CGA GGG CCT GGC CAA GGG GCA GAT AGA GGA AGC TGG CTG GAG CTA TGT
TGG GGG CTG GAC TGG CCA AGG TGG CTC TAA ACT TGG GAC TAA AAG GAC TCT ACC CAA
GAA GAG CTT TGA ACA GAT CAG TGC CAA TAT AAC TAA GTT TAA CAT TCA GGG CCT TGT
CAT CAT TGG GGG CTT TGA GGC TTA CAC AGG GGG CCT GGA ACT GAT GGA GGG CAG GAA
GCA GTT TGA TGA GCT CTG CAT CCC ATT TGT GGT CAT TCC TGC TAC AGT CTC CAA CAA
TGT CCC TGG CTC AGA CTT CAG CGT TGG GGC TGA CAC AGC ACT CAA TAC TAT CTG CAC
AAC CTG TGA CCG CAT CAA GCA GTC AGC AGC TGG CAC CAA GCG TCG GGT GTT TAT CAT

TGA GAC TAT GGG TGG CTA CTG TGG CTA CCT GGC TAC CAT GGC TGG ACT GGC AGC TGG GGC CGA TGC TGC CTA CAT TTT TGA GGA GCC CTT CAC CAT TCG AGA CCT GCA GGC AAA TGT TGA ACA TCT GGT GCA AAA GAT GAA AAC AAC TGT GAA AAG GGG CTT GGT GTT AAG GAA TGA AAA GTG CAA TGA GAA CTA TAC CAC TGA CTT CAT TTT CAA CCT GTA CTC TGA GGA GGG GAA GGG CAT CTT CGA CAG CAG GAA GAA TGT GCT TGG TCA CAT GCA GCA GGG TGG GAG CCC AAC CCC ATT TGA TAG GAA TTT TGC CAC TAA GAT GGG CGC CAA GGC TAT GAA CTG GAT GTC TGG GAA AAT CAA AGA GAG TTA CCG TAA TGG GCG GAT CTT TGC CAA TAC TCC AGA TTC GGG CTG TGT TCT GGG GAT GCG TAA GAG GGC TCT GGT CTT CCA ACC AGT GGC TGA GCT GAA GGA CCA GAC AGA TTT TGA GCA TCG AAT CCC CAA GGA ACA GTG GTG GCT GAA ACT GAG GCC CAT CCT CAA AAT CCT AGC CAA GTA CGA GAT TGA CTT GGA CAC TTC AGA CCA TGC CCA CCT GGA GCA CAT CAC CCG GAA GCG GTC CGG GGA AGC TGC CGT CTA AAC CTC TCT GGA GTG AGG GGA ATA GAT TAC CTG ATC ATG GTC AGC TCA CAC CCT AAT AAG TCC ACA TCT TCT CAG TGT TTT AGC TGT TTT TTT CAT TAG GTT TCC TTT TAT TCT GTA CCT TGC AGC CAT GAC CAG TTC TGG CCA GGA GCT GGA GGA GCA GGC AGT GGG TGG GAG CTC CTT TTA GGT AGA ATT TAA CAT GAC TTC TGC CCC AGC TTT ATC TGT CAC ACA AGG CTG GGC ACC TCT AGT GCT ACT GCT AGA TAT CAC TTA CTC AGT TAG AAT TTT CCT AAA AAT AAG CTT TAT TTA TTT CTT TGT GAT AAC AAA GAG TCT TGG TTC CTC TAC TAC TTT TAC TAC AGT GAC AAA TTG TAA CTA CAC TAA TAA ATG CCA ACT GGT CAC TGT GCT TTT GCT TCT CCT GTT ATC ATC TTC CTA AGT GGA ATG TAA TAC TGT CAG CCC CAT GTA TCA GAC ACT TGT CTG ATG AAG CAG TAA AGA CGT TAA GGG TAT CAC AGG GGG TGG AGG AAG GGA TTA TCT CTA GTA CAC TAC TTG CTG GCT GTC TGA AAA ATT GTC ACT GCC AAA CTC TAA AAA CAG TTC TAA ATA GTG ACT GAG AAG GTT TGT TGC TGG AGT CAG GGA ATA AGG CAG CCA AAT ACT CTT TGC ACA GTT CTT TAG TGG GAA GAG AAA TTA ACA ATA AAT ATC AAG CAC TGT G

Occurrences/Results 9 Pentamers

The pentamer 'ATTCA' occurred 1 times.
The pentamer 'TTCAA' occurred 2 times.
The pentamer 'TCAAG' occurred 6 times.
The pentamer 'CAAGA' occurred 4 times.
The pentamer 'AAGAT' occurred 4 times.
The pentamer 'AGATG' occurred 7 times.
The pentamer 'GATGA' occurred 9 times.
The pentamer 'ATGAA' occurred 8 times.
The pentamer 'TGAAT' occurred 2 times.
The total occurrences of all specified strings is 43.
The total number of characters in the text is 3036.
First result (Total characters *9 /1024): 26.71875
Square root of the first result: 5.169018282033833
Final result: 3.1497760525609673

Occurrences/Results TRIMERS Codons

The pentamer 'ATT' occurred 8 times.
The pentamer 'CAA' occurred 31 times.
The pentamer 'GAT' occurred 24 times.
The pentamer 'GAA' occurred 31 times.
The pentamer 'CCA' occurred 19 times.
The pentamer 'ACT' occurred 14 times.
The pentamer 'AGA' occurred 22 times.
The pentamer 'TAC' occurred 16 times.

The total occurrences of all specified strings is 165.
The total number of characters in the text is 3036.
First result ((total_characters / 3) * (Trimer_number / 64)): 126.5
Square root of the first result: 11.24
Final result: 12.3

Occurrences/Results TRIMERS Combinations

The pentamer 'ATT ATT' occurred 0 times.
The pentamer 'ATT CAA' occurred 0 times.
The pentamer 'ATT GAT' occurred 0 times.
The pentamer 'ATT GAA' occurred 0 times.
The pentamer 'ATT CCA' occurred 0 times.
The pentamer 'ATT ACT' occurred 0 times.
The pentamer 'ATT AGA' occurred 0 times.
The pentamer 'ATT TAC' occurred 0 times.
The pentamer 'CAA ATT' occurred 0 times.
The pentamer 'CAA CAA' occurred 1 times.
The pentamer 'CAA GAT' occurred 0 times.
The pentamer 'CAA GAA' occurred 4 times.
The pentamer 'CAA CCA' occurred 0 times.
The pentamer 'CAA ACT' occurred 0 times.
The pentamer 'CAA AGA' occurred 2 times.
The pentamer 'CAA TAC' occurred 2 times.
The pentamer 'GAT ATT' occurred 0 times.
The pentamer 'GAT CAA' occurred 0 times.
The pentamer 'GAT GAT' occurred 1 times.
The pentamer 'GAT GAA' occurred 2 times.
The pentamer 'GAT CCA' occurred 0 times.
The pentamer 'GAT ACT' occurred 0 times.
The pentamer 'GAT AGA' occurred 1 times.
The pentamer 'GAT TAC' occurred 1 times.
The pentamer 'GAA ATT' occurred 1 times.
The pentamer 'GAA CAA' occurred 1 times.
The pentamer 'GAA GAT' occurred 0 times.
The pentamer 'GAA GAA' occurred 2 times.
The pentamer 'GAA CCA' occurred 0 times.
The pentamer 'GAA ACT' occurred 1 times.
The pentamer 'GAA AGA' occurred 1 times.
The pentamer 'GAA TAC' occurred 0 times.
The pentamer 'CCA ATT' occurred 0 times.
The pentamer 'CCA CAA' occurred 0 times.
The pentamer 'CCA GAT' occurred 0 times.
The pentamer 'CCA GAA' occurred 1 times.
The pentamer 'CCA CCA' occurred 1 times.
The pentamer 'CCA ACT' occurred 1 times.
The pentamer 'CCA AGA' occurred 0 times.
The pentamer 'CCA TAC' occurred 0 times.
The pentamer 'ACT ATT' occurred 0 times.
The pentamer 'ACT CAA' occurred 1 times.

The pentamer 'ACT GAT' occurred 1 times.
The pentamer 'ACT GAA' occurred 0 times.
The pentamer 'ACT CCA' occurred 0 times.
The pentamer 'ACT ACT' occurred 0 times.
The pentamer 'ACT AGA' occurred 0 times.
The pentamer 'ACT TAC' occurred 0 times.
The pentamer 'AGA ATT' occurred 1 times.
The pentamer 'AGA CAA' occurred 0 times.
The pentamer 'AGA GAT' occurred 1 times.
The pentamer 'AGA GAA' occurred 0 times.
The pentamer 'AGA CCA' occurred 1 times.
The pentamer 'AGA ACT' occurred 0 times.
The pentamer 'AGA AGA' occurred 1 times.
The pentamer 'AGA TAC' occurred 1 times.
The pentamer 'TAC ATT' occurred 0 times.
The pentamer 'TAC CAA' occurred 0 times.
The pentamer 'TAC GAT' occurred 0 times.
The pentamer 'TAC GAA' occurred 1 times.
The pentamer 'TAC CCA' occurred 0 times.
The pentamer 'TAC ACT' occurred 0 times.
The pentamer 'TAC AGA' occurred 0 times.
The pentamer 'TAC TAC' occurred 2 times.
The total occurrences of all specified strings is 33.
The total number of characters in the text is 3036.
First result (((total_characters/ 3) -1) / 64): 15.8125
Square root of the first result: 3.9764934301467165
Final result: 4.322275467550779

>AF070656.1 Homo sapiens FtsH homolog mRNA, complete cds

---> Sequence

CGACGTCTCCAGTCTACCTCCGAGAGATTAGCTGAAACACAGAATATAGCGCCATCATTCGTGAA
GGGTTTCTTTTGCGGGACAGAGGATCAGATGTTGAGAGTTTGGACAAACTCATGAAAACCAAAAA
TATACCTGAAGCTCACCAAGATGCATTTAAAACTGGTTTTGCGGAAGGTTTTCTGAAAGCTCAAG
CACTCACACAAAAAACCAATGATTCCCTAAGGCGAACCCGTCTGATTCTCTTCGTTCTGCTGCTAT
TCGGCATTTATGGACTTCTAAAAAACCCATTTTTATCTGTCCGCTTCCGGACAACAACAGGGCTT
GATTCTGCAGTAGATCCTGTCCAGATGAAAAATGTCACCTTTGAACATGTTAAAGGGGTGGAGGA
AGCTAAACAAGAATTACAGGAAGTTGTTGAATTCTTGAAAAATCCACAAAAATTTACTATTCTTG
GAGGTAAACTTCCAAAAGGAATTCTTTTAGTTGGACCCCCAGGGACTGGAAAGACACTTCTTGCC
CGAGCTGTGGCGGGAGAAGCTGATGTTCCTTTTTATTATGCTTCTGGATCCGAATTTGATGAGAT
GTTTGTGGGTGTGGGAGCCAGCCGTATCAGAAATCTTTTTAGGGAAGCAAAGGCGAATGCTCCTT
GTGTTATATTTATTGATGAATTAGATTCTGTTGGTGGGAAGAGAATTGAATCTCCAATGCATCCA
TATTCAAGGCAGACCATAAATCAACTTCTTGCTGAAATGGATGGTTTTAAACCCAATGAAGGAG
TTATCATAATAGGAGCCACAAACTTCCCAGAGGCATTAGATAATGCCTTAATACGTCCTGGTCGT
TTTGACATGCAAGTTACAGTTCCAAGGCCAGATGTAAAAGGTCGAACAGAAATTTTGAAATGGT
ATCTCAATAAAATAAAGTTTGATCAATCCGTTGATCCAGAAATTATAGCTCGAGGTACTGTTGG
CTTTTCCGGAGCAGAGTTGGAGAATCTTGTGAACCAGGCTGCATTAAAAGCAGCTGTTGATGGAA
AAGAAATGGTTACCATGAAGGAGCTGGAGTTTTCCAAAGACAAAATTCTAATGGGGCCTGAAAG
AAGAAGTGTGGAAATTGATAACAAAAACAAAACCATCACAGCATATCATGAATCTGGTCATGCC
ATTATTGCATATTACACAAAAGATGCAATGCCTATCAACAAAGCTACAATCATGCCACGGGGGCC

AACACTTGGACATGTGTCCCTGTTACCTGA**GAATG**ACAGATG**GA**ATGAAACTAGAGCCCAGCTGC
TTGCACA**AATGG**ATGTTAGTATGGGAGGAAGAGTGGCAGAGGAGCTTATATTTGGAACCGACCA
TATTACAACAGGTGCTTCCAGTGATTTTGATAA**TGCCA**CTAAAATAGCAAAGCGG**ATGGT**TACC
AAATTTG**GAATG**AGTGAAAAGCTTGGAGTTATGACCTACAGTGATACAGGGAAACTAAGTCCAG
AAACCCAAT**CTGCCAT**CGAACAAGAAATAAGAATCCTTCTAAGGGACTCATATGAACGAGCAAA
ACATATCTTGAAAACTCATGCAAAGGAGCATAAGAATCTCGCAGAAGCTTTATTGACCTATGAGA
CTTTGATGCCAAAGAGATTCA**A**ATTGTTCTTGAGGGGAAAAAGTTGGAAGTGAGATG**A**TAACTC
TCTTGATATGGATGCTTGCTGGTTTTATTGCAAG**A**ATATAAGTAGCATTGCAGTAGTCTACTTTT
ACAACGCTTTCCCCT**CATTC**TTGATGTGGTGTAATTGAAGGGTGTGAAATGCTTTGTCAATCATT
TGTCACATTTATCCAGTTTGGGTTATTCTCATTATGACACCTATTGCAAATTAGCATCCCATGGC
AAATATATTTTGAAAAAATAAAGAACTATCAGGATTGAAAAAAAAA

---> Sequence Showing highlighted Existant Combined Trimers

CGA CGT CTC CAG TCT ACC TCC GAG AGA TTA GCT GAA ACA CAG AAT ATA GCG CCA TCA
TTC GTG AAG GGT TTC TTT TGC GGG ACA GAG GAT CAG ATG TTG AGA GTT TGG ACA AAC
TCA TGA AAA CCA AAA ATA TAC CTG AAG CTC ACC AAG ATG CAT TTA AAA CTG GTT TTG
CGG AAG GTT TTC TGA AAG CTC AAG CAC TCA CAC AAA AAA CCA ATG ATT CCC TAA GGC
GAA CCC GTC TGA TTC TCT TCG TTC TGC TGC TAT TCG GCA TTT ATG GAC TTC TAA AAA
ACC CAT TTT TAT CTG TCC GCT TCC GGA CAA CAA CAG GGC TTG ATT CTG CAG TAG ATC
CTG TCC AGA TGA AAA ATG TCA CCT TTG AAC ATG TTA AAG GGG TGG AGG AAG CTA AAC
AAG AAT TAC AGG AAG TTG TTG AAT TCT TGA AAA ATC CAC AAA AAT TTA CTA TTC TTG
GAG GTA AAC TTC CAA AAG GAA TTC TTT TAG TTG GAC CCC CAG GGA CTG GAA AGA CAC
TTC TTG CCC GAG CTG TGG CGG GAG AAG CTG ATG TTC CTT TTT ATT ATG CTT CTG GAT
CCG AAT TTG ATG AGA TGT TTG TGG GTG TGG GAG CCA GCC GTA TCA GAA ATC TTT TTA
GGG AAG CAA AGG CGA ATG CTC CTT GTG TTA TAT TTA TTG ATG AAT TAG ATT CTG TTG
GTG GGA AGA GAA TTG AAT CTC CAA TGC ATC CAT ATT CAA GGC AGA CCA TAA ATC AAC
TTC TTG CTG AAA TGG ATG GTT TTA AAC CCA ATG AAG GAG TTA TCA TAA TAG GAG CCA
CAA ACT TCC CAG AGG CAT TAG ATA ATG CCT TAA TAC GTC CTG GTC GTT TTG ACA TGC
AAG TTA CAG TTC CAA GGC CAG ATG TAA AAG GTC GAA CAG AAA TTT TGA AAT GGT ATC
TCA ATA AAA TAA AGT TTG ATC AAT CCG TTG ATC CAG AAA TTA TAG CTC GAG GTA CTG
TTG GCT TTT CCG GAG CAG AGT TGG AGA ATC TTG TGA ACC AGG CTG CAT TAA AAG CAG
CTG TTG ATG GAA AAG AAA TGG TTA CCA TGA AGG AGC TGG AGT TTT CCA AAG ACA AAA
TTC TAA TGG GGC CTG AAA GAA GAA GTG TGG AAA TTG ATA ACA AAA ACA AAA CCA TCA
CAG CAT ATC ATG AAT CTG GTC ATG CCA TTA TTG CAT ATT ACA CAA AAG ATG CAA TGC
CTA TCA ACA AAG CTA CAA TCA TGC CAC GGG GGC CAA CAC TTG GAC ATG TGT CCC TGT
TAC CTG AGA ATG ACA GAT GGA ATG AAA CTA GAG CCC AGC TGC TTG CAC AAA TGG ATG
TTA GTA TGG GAG GAA GAG TGG CAG AGG AGC TTA TAT TTG GAA CCG ACC ATA TTA CAA
CAG GTG CTT CCA GTG ATT TTG ATA ATG CCA CTA AAA TAG CAA AGC GGA TGG TTA CCA
AAT TTG GAA TGA GTG AAA AGC TTG GAG TTA TGA CCT ACA GTG ATA CAG GGA AAC TAA
GTC CAG AAA CCC AAT CTG CCA TCG AAC AAG AAA TAA GAA TCC TTC TAA GGG ACT CAT
ATG AAC GAG CAA AAC ATA TCT TGA AAA CTC ATG CAA AGG AGC ATA AGA ATC TCG CAG
AAG CTT TAT TGA CCT ATG AGA CTT TGA TGC CAA AGA GAT TCA AAT TGT TCT TGA GGG
GAA AAA GTT GGA AGT GAG ATG ATA ACT CTC TTG ATA TGG ATG CTT GCT GGT TTT ATT
GCA AGA ATA TAA GTA GCA TTG CAG TAG TCT ACT TTT ACA ACG CTT TCC CCT CAT TCT
TGA TGT GGT GTA ATT GAA GGG TGT GAA ATG CTT TGT CAA TCA TTT GTC ACA TTT ATC
CAG TTT GGG TTA TTC TCA TTA TGA CAC CTA TTG CAA ATT AGC ATC CCA TGG CAA ATA
TAT TTT GAA AAA ATA AAG AAC TAT CAG GAT TGA AAA AAA AA

Occurrences/Results 9 Pentamers

The pentamer 'ATTCA' occurred 2 times.
The pentamer 'TTCAA' occurred 2 times.
The pentamer 'TCAAG' occurred 2 times.
The pentamer 'CAAGA' occurred 4 times.
The pentamer 'AAGAT' occurred 2 times.
The pentamer 'AGATG' occurred 8 times.
The pentamer 'GATGA' occurred 4 times.
The pentamer 'ATGAA' occurred 8 times.
The pentamer 'TGAAT' occurred 4 times.
The total occurrences of all specified strings is 36.
The total number of characters in the text is 1918.
First result (Total characters *9 /1024): 16.892578125
Square root of the first result: 4.110058165646808
Final result: 4.648941962599459

Occurrences/Results TRIMERS Codons

The pentamer 'CAA' occurred 20 times.
The pentamer 'GAT' occurred 5 times.
The pentamer 'GAA' occurred 18 times.
The pentamer 'ATT' occurred 10 times.
The pentamer 'TAC' occurred 4 times.
The pentamer 'CCA' occurred 16 times.
The pentamer 'ATC' occurred 13 times.
The pentamer 'AGA' occurred 13 times.
The total occurrences of all specified strings is 99.
The total number of characters in the text is 1920.
First result ((total_characters / 3) * (Trimer_number / 64)): 80
Square root of the first result: 9
Final result: 2.1

Occurrences/Results TRIMERS Combinations

The pentamer 'CAA CAA' occurred 1 times.
The pentamer 'CAA GAT' occurred 0 times.
The pentamer 'CAA GAA' occurred 0 times.
The pentamer 'CAA ATT' occurred 1 times.
The pentamer 'CAA TAC' occurred 0 times.
The pentamer 'CAA AGA' occurred 1 times.
The pentamer 'CAA ACT' occurred 1 times.
The pentamer 'CAA CCA' occurred 0 times.
The pentamer 'GAT CAA' occurred 0 times.
The pentamer 'GAT GAT' occurred 0 times.
The pentamer 'GAT GAA' occurred 0 times.
The pentamer 'GAT ATT' occurred 0 times.
The pentamer 'GAT TAC' occurred 0 times.
The pentamer 'GAT AGA' occurred 0 times.
The pentamer 'GAT ACT' occurred 0 times.
The pentamer 'GAT CCA' occurred 0 times.
The pentamer 'GAA CAA' occurred 0 times.
The pentamer 'GAA GAT' occurred 0 times.
The pentamer 'GAA GAA' occurred 1 times.

The pentamer 'GAA ATT' occurred 0 times.
The pentamer 'GAA TAC' occurred 0 times.
The pentamer 'GAA AGA' occurred 1 times.
The pentamer 'GAA ACT' occurred 0 times.
The pentamer 'GAA CCA' occurred 0 times.
The pentamer 'ATT CAA' occurred 1 times.
The pentamer 'ATT GAT' occurred 0 times.
The pentamer 'ATT GAA' occurred 1 times.
The pentamer 'ATT ATT' occurred 0 times.
The pentamer 'ATT TAC' occurred 0 times.
The pentamer 'ATT AGA' occurred 0 times.
The pentamer 'ATT ACT' occurred 0 times.
The pentamer 'ATT CCA' occurred 0 times.
The pentamer 'TAC CAA' occurred 0 times.
The pentamer 'TAC GAT' occurred 0 times.
The pentamer 'TAC GAA' occurred 0 times.
The pentamer 'TAC ATT' occurred 0 times.
The pentamer 'TAC TAC' occurred 0 times.
The pentamer 'TAC AGA' occurred 0 times.
The pentamer 'TAC ACT' occurred 0 times.
The pentamer 'TAC CCA' occurred 0 times.
The pentamer 'AGA CAA' occurred 0 times.
The pentamer 'AGA GAT' occurred 1 times.
The pentamer 'AGA GAA' occurred 1 times.
The pentamer 'AGA ATT' occurred 0 times.
The pentamer 'AGA TAC' occurred 0 times.
The pentamer 'AGA AGA' occurred 0 times.
The pentamer 'AGA ACT' occurred 0 times.
The pentamer 'AGA CCA' occurred 1 times.
The pentamer 'ACT CAA' occurred 0 times.
The pentamer 'ACT GAT' occurred 0 times.
The pentamer 'ACT GAA' occurred 0 times.
The pentamer 'ACT ATT' occurred 0 times.
The pentamer 'ACT TAC' occurred 0 times.
The pentamer 'ACT AGA' occurred 0 times.
The pentamer 'ACT ACT' occurred 0 times.
The pentamer 'ACT CCA' occurred 0 times.
The pentamer 'CCA CAA' occurred 1 times.
The pentamer 'CCA GAT' occurred 0 times.
The pentamer 'CCA GAA' occurred 0 times.
The pentamer 'CCA ATT' occurred 0 times.
The pentamer 'CCA TAC' occurred 0 times.
The pentamer 'CCA AGA' occurred 0 times.
The pentamer 'CCA ACT' occurred 0 times.
The pentamer 'CCA CCA' occurred 0 times.
The total occurrences of all specified strings is 12.
The total number of characters in the text is 1918.
First result (((total_characters/ 3) -1) / 64): 9.984375
Square root of the first result: 3.1598061649411346
Final result: 0.6378951412791961
