# Supplementary material for: Traces of a Primitive RNA Ring in Current Genomes
Source: Biology (Basel). 2025 May 12;14(5):538. doi: 10.3390/biology14050538 (PMC12109556; doi:10.3390/biology14050538)
Supplement: Supplementary file 1 [file Supplementary material Biology S1.pdf › biology-3598710-supplementary/Supplementary material Biology S1.pdf]

>CP138348.1 Cyanobacterium aponinum AL20115 chromosome, complete genome

---> Sequence

ATTAATAAGAGTTTAAACACATCACCAAAGTAAAATAGCAATCCAAAAGGACGTTTCAATCCCTA
AGAGGTATTAATAAGAGTTTAAACATGAACTCTTCTCTAAATTAGCCAACCTCAACCAAGATATG
TTTCAATCCCTAAGAGGTATTAATAAGAGTTTAAACAGATTTTGTTCAGTTTAGGCAACGATACT
GAAGCAAGTTTCAATCCCTAAGAGGTATTAATAAGAGTTTAAACCGCCCACGATCGAAGCCTTTG
ATATATAAAGTTTTCAAGGTACAATTGCGTAAGCCTATCCATAATTATAAATGAAATCAATACCA
ACGTCCAAAAAAAATAACGAAAACCCTTGTGAACAAAAGAGCGTAAGGCTATAAAAATTTGAAA
TTGGTAGAAACTAGACTATATATAGAGTTGGGAGGTAAATCTTCTCAAGTCTAATTTTACACCCT
ATCCCTTACGCATATCTTATATTGCTGATTGCCTATGTTTCTATATAGTTATATTTTTATACTCCC
TCAAATAAAAGAGTTATAATCAGAAAATAAAAAGATAGGACAATCAAGATATGACTCAAACTTT
AACAGCAGAACAACTTTTTCGCAATGCTTACGAAAATCGCTATACATGGGATGAGAATTTTCCGG
GTTATACCTGTGACATTACCTATAAAACCCCTACAGCTACCTATACAGCCAAGGGTAAAATAGAA
TCTAATCTACAGTTTAGCGTTTCTGACATCGAAGATAGTTCCGCTCAAAAAGCAATTCAACAACA
GTTATGGGAGATTACCATTCACCGAGTTAATCATAGTTTTGAAAAATCCCACGGAGAAAATACTT
TCAGCTTTGGAGAAACTGATGCAGATGGTGCGGTAGAAATTCTTGTGGGCGGTGCTTCTCAAGGC
AATAAATATAAGGTAAAAGATAATGTTGTTACCCTTGTTTATCGCAAAATCCGTGATATTTTCGT
TACCATCAACACTTTTGAGGTTTTACACACTGACAAAGGCTATTTATCTTTAGGTTATGACTCCA
TTTACGCTGATGCTCAAACTGGTGAACAAAAAGGGGCTAAATCTATTTTTCATGACAAATTTGAG
AAAGTTGGTGATTACTACGTTTTAACTAATCGTCAGATTATTACGGAAGAAAATGAGCAACCAAC
AATTACCGAATTTAGTTTTTCTAATATCGTTTTTAATTAATGCCAATTAATAATTAACCTGAGTT
TGGGATAAATTTTCATGTCTGAGTGATGAGAAAAAGGACAAAGGGCAAACCCTCCTTTATCTCCC
CTAAATAGGGAGGAGAGTAAGAGGCAAAAGGAAAAGGTGTTTAATAAAATTTCCTCTCTCCCCTC
ATCCCCGTAGGGGTTGAATATATTCAACCCCTACCACCTGCAACCTGAAACCTTATCCCATATTTT
TAAACCAAACTGAGATGATAATTATGTTGTAAAATTATTTTTAATAAAATCAATAGCAGTCTTT
TTATCATTTATTTTACCTTCGATATAGGCAATTTGTATTTCTGTTAAGATTTTACCAATTAAAGG
GCTAGGGGCAATATTCAAATATTTCATAATATCTTTACCTGATAATAGTGGTTGAGGATGTGCGA
TCGCATCTTCAGGATTTTGGTATAATTCCATAAGCTGAAGAATTAAATCTTTGGATACATTGTGA
GCAAGAGCAAAAACAGCTAAATTTGGAAAATAATCCCCTACTGACTGGAAGAAAAAATATTGTT
TTCTTTTATTTTTTTGGAAATCTATTTCTGTTAAATTATTAGTGAATTGTAGTATAGTCAAAACT
AATTTTATTTCTTGACGGGAGTATTTTAAATTAATTAATTCTGTTTCAGCAATCGAAGGATTTTG
GTTAGTTAAACAAGCTAATTTTGCTTCTTTATACCATGAATAATTATCAGGAAAATTTGACCTAT
ATTTACTGCTTAAAAACTGGGCAGATTCATCAATGTTTGCTAAGAGATTGACCATTTTTTCATCA
GCAGAAGGAAAACAAAGGTCGAGTAAACCATCTTTAAAGGCTTCTTTTAACCAATAACTACCTTT
TTTTGTCTCAAATAAATAAGCTAATTCCTGATTAACCCTCTCCATTGCCACATTTTTTAATAATT
CTTTTAAGTCTTTAATAGTGGCACGGGTAAAAGATTCAATGGTAAAATCTAGTTGGGAAGCCTGT
CGATAACCTCGCATTAATCTTAAAGGATCATCCGCTAAATTATCACGACTAATCATCTTTAATAT
CCCTTTTTTTATATCTATTTCTCCCTGAAAAGGATCAATAATTACACCCTCTCGACAATGAAATG

CGATCGCATTTATGGTATAATCTCGCCTAGCCAAATCCTGATAAATTGAATCTCCTTCTTGCCCTG
CAAAATCAATAGTTGCATGGGGAAACACTACCCTAGCAATATACCTTACAGGATCTAAAACAACA
AATCCTGCTTGATATTCACGGGCGATGATTTTTGCTGTTTCTATTGGCTTATGGGGCAAAACAAA
ATCTAAGTCTAAATAACTCCTTGAACGTTTTAATAATATATCCCTAACTGCACCACCAACTAAAT
AACAATTATCAGGTAATAAATTGACATCAAAAGGTAAATTTTGCTTTAAAAAATTATGTAATAT
CATTAAAAATATTTTTACGTAATTATTATATTTTAATTGAATAATTATACTGTTTATTTGATGGT
TATGTATAATTTCAATTCACGATAAAAGTTTATAAATTATTATTTAACAATTGCCTATTGCCTTT
TGCTTACCAAACATCAATAATTCCCATACTCAAAGTAATAGAGCCAACTATTTAAACCCAAATTG
TGCTTTTAAATTAGGATCTATTTTTGCATAATTACTTAAATCTTCTCCTCTTTCAATAGCTTCTT
TTAAGGCTTTTTTATCAGCACTGTAGCTTATTTTTTCAAGACGATATTCTTCTGGTAATTCTTCT
GTTTTTACTTTTATATCTACTTTAGGATACTCCCTAAATAATAATCTTCTTTCTTCCCCTTCTTCT
TTTTTATTTAATGAACCTTCTTGATATAAATTAACTAATGTTTTTTGTAGTAATTCTTGTCCTTT
TTCTAATTGTTGAACCTGATTTTTGAGCATTTCATTAACTTTTTTTTGTCTAGCTTTAACAGATG
CGATCGCAATTTCATATTGATCATTAATCCATGCAAAACCATCTATATCTAAACCTAATTTTCTT
AGTAAAATAATAATTTCTTCCTCATCTTCAAGGTTAACTAAATCCTGCCAAATAGTCGCCGCTTT
TTCATGATAATTTTGATGTAAAGAAGTAACAATTTCTTCATTAATAACGACAGACTTTTGAGAAT
TAATTTGTTGAGTAATATTTTCCAATGGTAATTGATAATCATAACTATTGTTTTCTTGCTTCTCT
TTCCATCGATTATTAATCCATTCAGTTTCAAAACGATTCAAAGAATCTTGATAAAGATGCAAAAT
TTCTACCGCCGAAGACATACTTCTTTTTTGATAAATTTTGACTAAATCTGATAATAAACTCTGAC
GGTGATTAATTTCTTTTTTTAGCTCAATATATTGTTTAAAACTTAGGTAAAATCGATATAGTTTT
TCATCAGTAATATTTTTATTTTGATTATAATTTTGTAAAATAATTTTAACAGAATTTAATTTAGT
ATTTCCTTCTAAATATTTAACTATTTCTTGGGCAAATATCAAACCGATTTCATCGACTTCAACAG
TAGAATTAACAGGAGAAAGAAAAGGAAAAGCAATAGAATTTCTTCCGCTACTATCAGATAAGTT
TAAACAACGACTAATAACTGTACTAATTAGATTTCTATCCGTTAAACTATGATTATGATAATCAT
AAATAACTGCATGAAAAATTTTATCCGCTCTTAATTGACCTCCATTAGTTATCAAAATTTGCCCT
AAGTTTTTGACTTGTTCCTGTTTTAATTCCCGATTAATATTTTTTCCCCCTGCCTCTGAAATAATC
CTATTAATATCCTCCGCAAAAGATAAATCTTTATCGATATAATTAACTATTACTTCTGTATTTGT
TTCCGTCAAAGATTCATATTCAATTATCAACTGAGTTTGATTAATCAGAGTTTCTAAAACTAAAT
GTTGGAAATTTTCAGGAGGTTTAATTGTTTTAATACTAATTGGAGCAAAATCAAGACCTTGTAA
ATTCTGCCAAGTCAAGGGTAAACTAGCAGGATTTTGACAAATAATCGGCAACCAAGTAGCACAGG
GATATTCGTTTTCAAGTTCTTGGAGTCTTTCCCTAGCTTGTCTCACTGCCAGATAAAGAGTTTGA
TTTTGAGCAAAATTAGCTAGAAATTCTTTGATGAAAACATGGGCAACTTTATCGGGTATCATTTC
TCCCATAACAATGGTTTGAGGAAGATGTAAAGCGGCTAAATCTTTGGCTAACCCTAACCCATCAC
AAGAGTTAAAAATTGCCAGTCTTAATCCTTTTTGAATAGACTTCATTATCGCTTTATTAAATTCT
TCAATAGTAATAATATCTTCTTGATTTAGATATAATTGCCCTTGTTGAGTTAGTGGATTTTTTCC
GCTATGTCCTGCAAAAAAAAATAAGTCCCATTCTTCTTCATAAAGATGAGCAAATAATTCTTTTC
TACAAGGATTAATTAAAGTTTTAATTTCTGCTTTTTCTAAGTTATTTAATGTCTCTAAATCTGGC
TTTAAATTAATTCCTTCATCATTACCTAACACCGCTAATATTCTTATATTTTGTCTCTGTTTTTG
AGGAGTATTTAAACTCTTATATTCAATAGGTGCGATCGCAATTTCAGCTTGAGGATATTTAGATA
AAAAAGGTTCAAAAAATAAATGCCAAGGAAGAAAAATCAACTCAGAAATTTCCGTTTGAATTAA
AAGACGCACAGATTCATCTCTATTTAATTCTGACAATAAATTTTCTCGAACACGGGCAAAATCAG
GAAAAGAAGACTTATACCAATCACTTAAAGTTTTTTTAACCGGGAAGCTGACTGACGGCAACTA
TCACTAATATGAGTTATTTGTTGAGGAGAAGACCCTAATCTCACTTTTTGATTTAAACTGCGATA

ACTTTCTTGCCATTGTTGATAATGTTGACGAATCGGTTGACAAGGGGGAAGTTTCCCTGTAAAAG
TAGCAAAAGGATGAGCGATGCGATCGCAACTATCTTCACAAATTTGTAAAGTTAAATAAAAACCT
GTCTGAAAACTACCTGCTTGAATAGTAAAAAGAATGAGCTTACCCATAATTTAATTAGGAATTGG
TAATCAGTAATTAACAATTAAAACAAGGCAAAGTTAAAAGGGCAAAAGGCAAAGGTAAATAGTG
AATAGTGTTGGGGTGAATAGTTGATAATTACTCGTTACTCATTACTTTCTTCTAAAACCTGAAAC
CTGAAACCTGACACCTTATCTCCCTCATCCCATCATCTCCCCATCCCCTAAACACCCTGACACCTG
CAACCTGCAACCTGAATGCAACCTGACACCTATTTCCGAATTCCAAACTTAGATTAATTGACCTT
GGTTTCCGTTTCCGTTGTTGGTACAGGCTCTTTAAATACTAACTTAAGTAAAGGCGGAGCAACAA
AAGTTGTAACAATAACCATCACAATAATTGCCGCATCCGTTGCAGGAGAAAGAGCGCCACTAGCC
GCCCCCACACCAGCAAACACTAAACCAACCTCACCTCTAGGAATCATACCCACACCGACTGCGAGC
TTATTAACTTCAGGTTGTCCCCAAATAGTAAAACCAGCCACAACTTTACCGATAATTGCCACCAC
TATCAAGAAAGAAGCAATAATCAATCCTTCCCTATTACTAGGCACAGCAGGATTTAAAACACTTA
AATCAGTTTTTGCCCCAACACAAACAAAGAAAATAGGGACAAACATATCAGCCACAGGAATAATT
TGTTCTTCTAATTCTTTTCTTTTCTCCGTCTCTGCCAAAATTAGACCCGCCGCAAATGCTCCTAAA
ATCGCCTCTAATTGAATGACATTAGCAATATATGCCAGAGTAAAAGCAAAAATCAAACCAGTAAT
TAGAATATCTCCTCTCGTCTTCATCTGGTTCACTAAACCTACTAACCAAGGACTGATTAAACGTCC
AATGAGAATTGCACCGATTAAAAATGCACCAGCACTAATAATGAGATAAACAATTTGACTAATTT
GGATTTCTCCAGTTTTAGCTAAACTTGCAACCACTGCCAAAACAATAATACCTAAAATATCATCT
AAAACCGCCGCCCCGATGATAATTTGACCTTCTTTTGAGCTTAACTGCCCTAACTCAGCTAATACT
TTCGCCGTAATACCAATACTGGTAGCAGTCAAGGCCGCACCGGCAAAAATAGAAGGAATTAAGGG
TAGGTGGAAAAAATAATCTAAACCAAAAGTACCTAGGGCAAAAGGAACAACAACCCCTGCCACAG
CTACAACAGACGCAGAAGGTCCCACTCTAATTAATTCTTTTAGGTCAGATTCTAAACCAATTTCA
AATAAGAGAATAATCACCCCTAACTCAGAAAAAACAGAAATCACCTCACTCACGGTGTCAAAAAC
CTTCTCTGCTGATGAGGGTTCTAAATTAGCTGTAGTTTCCAAAAATTGAATAATTAAAGACTCAT
CTCCTGACAATCCACCTTCAGGAAAAATAACTAACTTAAGGGCAGATACCCCGATCACAACCCCA
CCAACTAACTCCCCTAAAACCGCAGGAAGACCAATTCTTGAACATAACTCCCCCCCGACTTTACTT
GCAAAGTAAATTACGATTAATGTTAATAATACCCCAGCCAGAATAAGGGCAGGACTGGCTGTTTC
TGTTGATGTTGCTGTTGCGAAAACAGGATAAAACCAAGCCATATATTTTTTGATTCTATTGTTAA
AGTTTACTTTCTTTTTTTGATTGTTTACACTTTAACCTATCATGACCTCTGTTCGAGCTAAAATT
ATGGGGCAAAAAAGGGGATGGTGTATAGTGAAGAATTAAATTAAATATTTATAGGAGCTATCAT
TGATGAAAATCGCAATTACGGGAGCAACAGGTTTTATTGGAGAAAAATTAGTCGATAAATTAGC
CGTTAAACATCAAATTGTCATATTTACCCGTAATATTGAAAAAGCAAAATCAGCTTTTAAAGCTA
GTCTTTTATCTAACTTAGAGTTTGTCTCTTATACTCCCAAACAAGCAGGAGAATGGCAAAAAAAA
ATAGATGGTTGCGATGCAGTAATTAATTTAGCAGGTGCTCCCATTGCCGAGAGATGGACGACAGA
TTATAAGCGAGAAATATTGGAGAGTAGGCAATTAGGTACAAGGATGATTGTAGAAGCAATTTCT
CAAGGGGAAAATAAACCAAAAGTTTTAATTAATGCTTCTGCCATTGGTTACTATGGCACCAGTGA
AACGGAAACTTATACAGAAATTAGCCCTGCAGGAAATGATTTTTTAGCCGAAGTTTGTCAAAGTT
GGGAAAATGAAGCCCAGAAAGTGACCTCAGAGGGGGTAAGATTAGTTATTTTTCGTTTAGGCATT
GTTTTGGGTAATGGGGGAGCATTAGCAAAAATGATTCCACCTTTTAAAATTTTTGCCGGCGGTCC
TATTGGTAGTGGTAAGCAGTGGTTTTCTTGGGTGCATATCGATGATGTGGTAAATATGATTGAA
AATGCTTTAACGTCTGACAACATTGAGGGAACATTCAATGCTACTGCTCCTTATCCTGTAACCAT
GAATCAATTATGTGAAACCCTAGGAAATGTTTTAAAACGTCCTTCTTGGTTGCCTGTGCCGAGTA
TTGCCCTAGAGCTATTGTTAGGAGATGGAGCAAAAGTGGTATTAGAAGGACAAAAGGTTTTACC

ACAAAAAACCCAAGAGGTGATGAATTATGATTTTCAGTACTCTAATTTAAAATCTGCCCTAGAAG
ATATTGTCGGGTGAGAGGGGGAAGTAAGGGCGAATGGCCATTCGCCCGTACAGGAGTTCGGAGTT
ATGAAAGAGGTCTCAGGTTTCAGGTTTCAGGTTTCAGGTTTTAGAAGAAAGTAGTGAGTGTTCAG
AGTTTTTCATTCTTAATTTTTAATTATCAACTATTCACTATTCACTATTCACTAATTGCCCTTTTC
TTAATTGCTAATATCTCTAATGGCTTTCGAGATAAATTCTGGCATGGTCAAGTGCGATCGCGATC
GAAGGAGAAAACATACTCATGAAATCTTCCATATCTTCGTTGAAATGCCTTGGTTTGGGGTGAGT
TAAAGTAAGAATACCTAAAACAAGACTACCCCTTAAAAAAGGAACACAAAGGGCAGAACCAACCT
CATAGGGTTGATTCTCAAAATTTAACCATCGCTCATCCTGTTGAGTATCCTTGACTAAAGCCATA
CGACGATAACGATAAACCCAACCTGCTAAACCTCGATCTAAAATAGTGCCAATCAGGTGAGTTTT
TTCTTCTTGAATTGTCGGGCCTCTGGCTAAAATACTTTCTGTAATAACTCCTTTCGGGTTAAGAA
TAAATAAACTAGCATCCTGTGCTTTTAATAAATCTCTGGTTACTTCGGATATTTCTAGTAAAATA
GAGCGTAGCATCAATTTCCCACTCGCCACATTACTCATCATTAAAATAGAACGAAATAACTCTTT
TTGAGCTTCCATCGCTCGTTTATATTTACTCAAATCAACTACCAATTTTTTTAGAGAGGCAACCT
CCAATGCTAAGGCTTCAGAAGAATGTTGACGGGGGGAATTCATGATTGTTATTTTTTCAGATGAG
ATTCTGTCAAGCTAATTATAGAACAATCTACAATTTGAGAGTAGAGTTATTTTTTTTACCTCAGT
TTGGCTTATTTCGCTCTTACATCCCCTTCCGTTGCGGACAGTTTTAAGAATATCTGAACTCAAAG
AAAAAAGTATTAAAAAATACCAAAAATTTCATATCTCACATTCAGAAAGAAGAAAATATCATTA
CATATAATTCATTCTCATTTCAAATATAAAAAACCTGATTTAAACTAATTTTTAATCAAAATAAT
GCTTAATAACGTTAGTAACGATAGAGATATAACCGCATTTGTGAAAGTTAGCTCAAATGAATGA
GTATAAATTTATCTTGGAAATAAATAAAAAAGTAGGCAAAAATAAATATTAAGTGTCAAAAAAT
AAGTAGTTTTACTCTTTATTTAAAAAAAGTTATTTTCCTCATTTAAGAATAACAATTTTTTTAGT
GCGGAATTTGCATTTTACTATTTAATAATCTCCTGTTGCCTGTTGCTCATTGCCTACCTTAACCAA
TAATAATTTACATACTCAAAGTAATAGAGCCATTAATTTTGAGTATTTGTTTCTATTTTCTGAGA
AGGAGTTTGTTTCTGCTGTTGCATTTCTAAAATTCTCTTTTGCTGTAATTGTTTGAAATTCTCCG
CTTCTGAATTGATGTTATTTTCTGTATCTTCTGCAAAGTCTGAGCCACTACGACTATTGAAAAAA
TTAGCATTGTGAATTATGTCAAGGGGATTAATGCCGTCACCATAAAAAGGGTTTCTCTCATTACT
TTGAAAAGGTTCATTTTTGCCATTAATGTTGTTACTTTGAGCATTAGCAGGAGCAAAAAAACTTG
CCAGTGCAATGAAAGCAAAGGAAATGGCTAGGGTTTTATTTTTGTTAATAATCATAAAATATAC
TGTTTAATATCTATCTGTTTTTATACTTGTTAGACGGTGAAAAGTTAATCGAGTTTCCGTCTCAA
TTACTATTTTAAACCTGATTAAAATTGATTGGGGTTTTTATGACGTTAGCCCAACGAAGATCAAC
ACTTTCCATGATAGCATGATCAAAATTACACATAAAAATGTCTGCTTTTCCTAGATGGGTATTTA
CTAACTTGGCATAGGAAAAAGAAGTCTTGTAAATTTTGCTTTCTCCTAATTCCGTCTCATAAAAA
ATTGCCTTGCTGAAGTCTGATTCACAAATAAAACTTTGCACCATATTCGCTTCACTTAAATTGCT
ACGATGTAAACGAGCTTTAAATAAGACGGATTGTCTCATGTTAGCAAGTCTTAGGTTAACTCCAA
TTAAGTTACTATGACTTAAATCTGCACTACTCAGGTTAGCAAATCGTAAATTACTACCAATAAAA
TTGACATGACTTAGATTGCATCCACTAAAATTTATTGCTTCTAAATCATAACTATTCAAATTGAT
ACCAGATAAGTCGATGGTTTCTTCTGGATTTAATTCACGCCAGTGATTCCATTTTGGTACATGAT
GTAATAAGTCTAACCAATATTCTCTTTTTTTCATATGTATTTGTTTTATTTTTAATTAAGATAAA
ATCTATTAGGGTTTTCTTATGGGACTATTGTAACTTTTTTACTCTCATTTATTATTCTCATTATT
ACTATATCTGTTGGTTAGCAAGTAATAAACTAAATATTTCATAAGGTTTTATTAAAGTATCTTCG
ACATTCGGTGCATCATTTTCTAAGACATTAATTCTTTTGATTATCTTTAAATCAAATTCATTTTT
AAAAGTTAATAGACTTGACTTTCCTTCACATTCATAACCACGAATAATCATTTGAGAAGGATTTT
TTTCTGCTTGTTTAAATGTCATTAAAACAAAATTATTTTCCTCAATATTTATAAATTCATGATTA

GTCTTTAAACCTAAATCTTTTTTCTCTCTTTTTTCTGTTAATAAAGCTAATAAAGATATATTAAA
TTCATAGCCCTTTTGTACTGTTTTTCCTTCTTTCCAACCCTGTTTATGTCCATAAATACCATAACT
AAATTCGTGAAAGCCTCGATCGCATTTTGGATCTGGCCATTTCGGACTTCTTAATAAACTCAAAC
GTAATTGATTAGGTTTAGCAGAATAACCATATTTACAATCATTGAGTAAACTTACCCCATAATTA
TCATTATTATCGGTTAAATCTGCCCAGTGATGGCCATAAACTTCCCATTGTGCCTTTTCCTCATCT
GTCTGAGGTTTAGTAGTGCGTTGGATGTTGCCACAGGCGATTTCATAGGTGACATAATCACTTTC
TAAATTGAAAGGAAAACTTACTTTTAAGAGGGTATAATCTTCTTGCCAATTAACCTTATTTTTTA
TTTTTAAAATCGGTGACTGAAAATCAAGAATATAGTCTTGGATAAACTCTGATTTGTTATAAGTT
TTAATAACTCTAATTCTCTGTCTTAATATGCCTTTTTCTAACCAGTTAATTGACACTAATTGAGG
GTTATCTAATCGATAATTTTCATAATTAGGATCTATATTCCAAGCATCCCAATATTGCCCTTTAT
CCTCGAATAATTGTAACTCGTTTCCCTCTCCTTTAATAATTTCTCGTTGGTTGACTTTGTCAAATA
TCCCGTCAATATTACCTGTTTTTTTATCAATAATTACCCGTAAAGAATCATTCTCTAAAATATAA
GTAATATCTTCTTCAATTATTTCCATTCCATTGTGTGGAACAGGCATCTTACCCCTATTTATATCC
TCTTTTAGGATAGTATCATGATCCTCTCTAAAACCCCTTAATAAGGGAAGGATTTTATTGGTTAA
ATAAAAGGATTTATAGCCAACACTAGGGACATTTTCAGCAAGAAATAACAACTTACCTTCATGGC
TTATTTGTGTTAATAACTCTTTTCCTTCACCATCATAAATTTTATATTTTTCTCCCTCTATATCCA
ATTCAACTATTTCTGACCTTATCCAATTCAGGCTATTAAAAATTGTTACTTCATAATCATATTCT
AAATTAAAATTAGGTAATTTAATATAAGATGCGATCGCATTTAAGCTGTTAGATAAAATATTAT
TAGTAGAGGTAATTATTTCTTGATAAAGTTGATTAGCTTCTGTAAAAACAGGAGTTATAGAAGT
GCCGGGTAAAATATCATGGAATTGATTTAATAATAGTTTTTGCCAATTAGCCTCTATTTGAGATT
GTATGTGATTATCAACTTGATACTCTGGAATAAATTGATTATGTAAAATAGTGGCAATAGTAGA
GAATAATTCCGCTTGATATAGTAACTTTTCACAATAACGATTTTGATATTTTTGATCTCCATGAG
TAGTATAACAACCCCGATGTAATTCTAAGTAAAGTTCACTATTCCACACAGGATAATTTTGAGGA
GAAATAGTACTAATTTTGTTTAAGTAATTTTCCGCAGTGCTAAACTTAATTTTAGGGAAAAAAG
GAGAGTCATCATAACGTTGGGCAACCTCTAGCATATCACGAGTGGGTCCTCCGCCGTGATCTCCA
ACCCCGGGCAACCAGAAAATATCTTTTAATCCCGTTTGTTTTTCCCAGTCAATACTGTAGTTAGT
CATGGTGGTAGGGTTTGTATCCATAACCCCCGTGACATTGGGCGGAGACATAAGAGTGAGAATTT
GACTACCATCAGGAGATTGCCACCAAAAGCAACCATAAGCAAATTTATTCGTATCATTCCAATGC
AATTTACCTGTCACAAAATATTCAATTTCTGCTTGTCTCAAAATCTGCGGTAATTGCCACGGAAA
ACCAAAGGTATCAGGAAGCCATGCAACTTTATTATATTTCCCAAACTTTTCCTTAAAATATTTTT
GTCCATATAATAACTGTCTAACTAAAGATTCTCCGCTAATTAAATTAACTTCAGGCTCAATCCAC
ATACCCCCCAATATTTCCCATTGAGAAGATGCGATCGCATTTTTTATTTGATTAAATAATGATAA
ATTATGAGACTCAATCCATTGATATAGATACGCAGTGGTATGACCAAATGTTAAGTATTTATATT
CTTTTTGAAGATTTAAAACAGAATTAAAAGTTCTTTGAGCGACTTCATAAGTTTCCTCAAGAGTC
CATAACCAAGCCATATCTAAATGAGCATGACCTAAAGGATAAAAACATCTTTCTTTGATATAATT
ACTTAGGGGTAAAAGTTTATCTCTTAGATTAGCTAATTCTTGATGAAAAAGAGACTTATTATCAA
CATACTCCCAATTTAAATCTTGAATTGCTAGATCTAAAAATGGTTTTTTGTCAGGGTGAAAATTA
TCAAGATATTGACTTAATACGGTTAATTCATTTGCTACAAAACTGGATCAATTTCTCCTTTTTT
TGCTTCATAAACACATCGAGAAATCATCAAACCACCAATATCATGATTAGGACTAATTAAGCGAA
TTGTGATTAAAAATTCTTGGTGAGGCTTAACATTTTCTGTAACTAAAAGACGGCAAGAAGAATCA
AATAAATCCCCTTCAACTTTTAACTGACCATTAACAAAAATTTGAGCAGATTCTGCCCACCATGT
CAAGAAAAGTCTTAAATCCAAACCTTCGAGAGGATAATTGCTATCTAAACTATGGGGAATAATTA
TTTTTTGAGCAAACCAACGAACTTTATTACCTTTTTCCCATACTAAATAGCCTTTCTCATTGATA

ACTGCATTTTGCCAATTTTGCTCAATTATTTTTGGTGATTGTGCATAGTCTTTATCACTAAAATA
CCAGTTATTTTTTATATCTAATTCGGTTAATAATCGTAATTTGTTAATCATGGATATCTAAATTA
AATTTGAAGATAGAGATTCCTTTTCTATCATCAATGACAGTATTTATATACAGTTATTCAATATG
TAAATCTTTGTTTTAGTATAAAATAAAAGCTAGTGATAAAAACATAAAAATTTAACTATGAGAA
TTATTGAAACAAAAATAAATATAACCGACGAACATTTATTAAATATTAAACTCCCTGAAGATAT
TACAGTGGGGGAGTATGAAATTGCAATTATTATTAATCCTGTTACAAAAAGTACCAACTCTCATC
CTATCAATCAATTGGCAGGAAAAGTCAAAGCATTTAAAAATATTAACTCGGTAGAATGGCAAAA
GGAAATTCGGGGAGAGTGGGATGAAATTAGATTATCTGATAGATACTAATATATTTATTTGTTT
AATTAATGAAGAATTAATAGAAGCAATACCCAACGGAATTTTAGGTTATTCTATCATTACCCAGA
TTGAATTACTTTCCTTTCCATTATTGGAAAAAGAGGAGGAAAACTTGATAAAAAGGTATCTTAA
TACCTTATCTGAAATTCCTTTAAACAAAATTATTGCCGAAAAAACTATTTATTTACGTCGCAAAT
ATAATTTACAAGTACCAGATGCTATTATTTTGGCAACGGCGTGGGAATCTGAGTCAATATTATTA
AGTTAATGATCAAAAATTAACTAACGTAAAAGAGGTTTCTGCAATCTCATTAAAAATGAAAAAC
TAGCCAGAGGAAAATTAAAAGATGAAAACTAGACAACTCGGCAAATCCGACATTTATATCACTCC
GATTATAATGGGTACATGGCAAGCAGGAAAAAGGATGTGGGCAGGTATTGAAGATAGTGAGAGT
ATCAGTGCCATTCGTCAAGCTGTGGAATCTGGTATCACGACTATTGATACAGCAGAAGTTTATGG
AGAAGGGCATTCAGAGAGAATTGTGGGAGAAGCCTTAAAAAGTGTTAGAGATAAAGTAATTTAT
GCCAGTAAGGTGTTTGCTAACCATCTCAAATATGATCAGGTGATTAGTGCTTGTCATAATTCCTT
AAAAAATCTACAGACAGATTATATTGATTTATACCAGATTCATTGGCCTTCTGGTAGTTGGAATA
GTGAAATTGTACCCATAGAAGAAACCATGAGGGCATTGAATGATTTAAAAAGAGAAGGTAAGAT
AAGAGCGATCGGAGTTTCTAATTTTTCTCAGCAACAGTTAGCCTCTGCCCTTGAATGTGGACAAA
TCGATAGCATTCAACCACCTTATTCTTTGTTTTGGCGCATTGTAGAAAAGGAAATTCAACCCTAC
TGTGTAGAAAACAATATTTCCATCCTTGCTTACTCATCCCTCGCCCAAGGTATTTTAACGGGAAA
ATTTGGAGATAATCCCACATTTGCTGAAGGTGATCATCGCAAAAATAATCGCTTATTTCAGCCCC
CTCACTGGGAAAGAGTGAAACAGGCTTTATCTCAATTACAACCCTTTGCCGATAAATATAATTGT
ACTTTAGCTCAAATTGCGATCGCATGGCTAATACAACAACCCCAAACCAATGCTATAGTTGGGGC
AAGAAATGCTCAACAGGCGAAAGAAAATGCCCAAGCAGGAGAAATAGAATTAACAATAGAAGAT
ATTAAACAAATCAGTAATATAGGAACAAATGTAACTAAAGACTTAGACGATAATCCCGTAATGT
GGAATTTTTCTTAAAGAGGGATAAATTATGACAATAATGGGAAAAATTAATGTTATAAAAACAA
TCCTTAATTTAAAAAGAAAAATCAATATAATAAAAAAACAAAAGTTTCTCAGTAACTATGAATC
AAAGGGATTTATCGGAAGGAAAACTATTAGTACAGCGCTATCTCCTTCGAGAAGTAGTGGGCGTT
GGCGGCATGGGTACAGTATATCGAGCCCAAGATATCGCCTCTAAAAACCATAATGTGGCAGTAAA
AATATTATCCCGTTCCCTAGACAACATGAAAATGATTAAACAATTTCAACGGGAAGCCACTTTTA
GTGCTTTACTCTCAGAAAGAAGCCAAAACATTGTCAAAGTCACAGACTACGGAGTCGATGAACAG
AAAGTTCCTTTTTACGTGATGGAGTTTCTCGAAGGGGAAAATCTTGATGACATGATAGAAGTTCA
CGATATATCCCTACCCCAATTTTTTGACTTTATTTGTCAAATTTGTCGAGCAATGGAAACAGCCC
ATAATGGCATATTTTTTGAAGGGGAAATTTATCCAGTTATTCATAGAGACTTAAAACCCAGTAAT
GTTTTTATCATTGAAGATGATACGGGAAAACAAATAATTAAAGTCTTAGATTTCGGCATTGCAA
AATTAGTTAAGGATGATCCCATTGATACAGAAAATTTTATGGGAACTCCAAAATATTGCTCCCCA
GAACAGCTACAAGGTAAAGACTTAGATAATCGTTCCGATATTTACAGTTTAGGAATGATTATGTA
CTTAATGTTATGCAAAAAACTCCCTTGGAACTTAGAAGTAGAATCCGTAGGTTTGTGGTATAAGG
CACACACTGAACTATCGCCTAAACCCTTTCCTCCAGAACTAAACATATCTTCAGACTTAGAAAAA
TTAGTTTTACAATGCTTAGAAAAATCTCCTTCTAACCGCCCTCAAAACGTGGGAGAAATTATTCA

ACGACTAGAATCTATTGCTAAGAAATTAAAAATAGTCAAGAAAACCATAAGTGAAGGTCAAAAT
CATTTACTGAATATTAATAATAACTTCGTTGAGAGTGACGAAAATACTAATCCCAAAGAAGATTT
TTTATTATCTCATCCATGGCCGAAGAATAAACCCAAACAAAAAATAGTTTTTCCTCGTGTTGTTT
TTTATCAAAATTCTTCCTTACCAACAATTTGCACAATGTTAGAAACGGAAGATATACGAAAAAGA
AAAAATAATATTCGCTACAGTCAATTTCTGTTCCAAAGTTACCCTCATCCAATGATTTTATGGAT
AACTCTACTTTATAGCCCTAAACATGAACCCAGATGGTTTCCCTGCTACCTAGATTTAAAAGCAA
AAATAGGTCAACAATTAGTAAGCACTTTAAGTGAATCAAAGGAATATTTTGTTCTATTTTATTCC
TTAGAAAAACCCCATAAATGTATTGATTTACAACCCTTTAAAATCATGCTGAAACAGAGAACTAG
CCTAAAGCAATGGATGTCTGTTAGTAATATGATTAGTGTTAAGCATGACGATGAGGTTTTATTTA
GTCGGCGTAAACTAAAACAAGACTTAGAAGCATTGAAGCCAAAAATTATAATCGATCTTGAAAA
AACAAATACTTTAGAAGTTTACGAATAAAAAATGTTTATCTATTGTTAAAAAACTATATTGACG
AATACAAGTTAAAAATTAAGTTGCAATCTAACGTTTTTATTTTTATTTAATAAAAAGTAGCAAT
ATGGTTTTTATTTTTAATTACTAGCAGTTAATAGCTAATTACTAATTACTAATTACTAATTATTC
GTAACTTTTTATGTGGGATTTAATAAAGTTATAAGGTGCAGTTTTCTATGCTATTTAATTAGTGT
GCTGTTGCCTATTTACTATTTCCCGATACGGATAAGTAGTGTTAATGTAAAACACCATATCTTGT
TGTTACTGTAGCTAATTTTTTACAGGAGCTAATTTAATATTTTTAGCTAATCCAAAAAATGCTAA
TAACTTAATGGTCATCCATGTTAAATCAATTTCCCACCACTGCAAACCATGACGAGCAGAGTATT
GAAAAGCATGATGATTATTATGCCAACCTTCACCAAAAGTTAATAAAGCCACCCACCAACAATTA
CGAGAGTGATCATTTGATTCATGACTTTTGTAACCAAATTTGTGAGTAGCGCTATTAACAAACCA
AGTCACATGGAAAACTAAAACTAAACGAAGAAATATACCCCAAACGACGAAAGACCATCCTCCCC
ACCAAAAGAGCAATAATCCTAAAACAACTTGAATAGGAATAAAATACTTTTCGCAGAAAACATA
GAAAGGATCATTTTGTATATCCTTGGTGTATTTAGGGACATCGTTATTCGCAGGATTTTTGACAA
ACATCCATCCCATGTGACTCCACCAAAAACCCTTATTGGAGTCATGGGGATCACCCTCATGATCAG
AATATTTATGATGGATACGGTGCAATCCTACCCAGGAAATAGGACCACCTTCACAGGCTAAAGTA
CCACAAAAAACAAGAAAATATTCTAGCCATTTGGGAGTTTCAAAACTGCGGTGAGAAACTAGACG
ATGATAGCCTAAAGTAACTCCTATACAGGCTGTCAGCCAATAGAGAAAAGCTGTTATTCCCACTG
CTCCCCAACTAAAATTGCTGGGTAAAAAAGCTAATAAAGCTATTAAGTGGATACTTGTCATATAA
ATGATAACACTCCATGCTGGAGCAAGTTTTTCGGTTGTTGTTGTTGCTGTCATTACTTACGGTAA
ATTTTTTTACAAAACTTCAAATTGTTCAGATTAAGATTGCCTAAATCTGAGCTGTTTTTAGGTTG
ATAATTAAAGGTCAGTTTTAAAGATCAAAAAAGAAAAAGGTTTATAATTCTGCTCTTACAAAAA
AGAGAGATTTAACTTTGATTTTGAACTTAATAGATCACCGAGTTTAAATTATAAACTATAAACA
GTGATCAACAAATTATAGTTTACCATCATCATTTAATATAATTATCTTAATTGTTATTAAATAAA
GTTTAAAAAAAATTTATTATATCAGTAACGCACAATATAGTTGACATACCCGAATGAATAATAA
CTTTTCCGTCCCTTCTTTAGAAACAATCAAAAAACAACTCCATAATCAAGACTTAGAATTCATCG
AAATCTTTGATTGGGGGCAAGAATCTCCTTGGAAAGAGTGTATTGAGGCATCTGAATTAAAAAA
TGGTCTTCCTCGCTACATAATTGTCCCGAATACTAACAAAATGCTATCACAGTTTTTAAAGATTT
GTTGCAAAAATAAGTGGCGGATATTACCCCTTGGCAATGGCTCAAAATTACATTGGGGACAGTTA
CCTTCTGGGGCGAGTGTTTTAGTTAGTACGCATAAACTAAATAAAATTATTGAACACGCTCAAGA
TGATTTAACTATTACGGTTCAATCTGGAATGAAAATCAAAGATTTACAGGATTTTTTAGCTTCTT
TTAATCAGTTTTTACCAATAGACCCTATTTATTCTCAATTTGCTACGGTTGGAGGGGTTGTTGCT
ACTGCTAATACTGGTAGTTTAAGACAAAAATATGGAGGAGTTAGAGATTTAATTTTAGGGATTT
CTTTTTTTAGAAGTGACGGAGAGGAAGCAAAAGCAGGGGGAAAAGTGGTCAAAAATGTAGCTGG
TTACGATCTAATGAAGTTATTTACGGGTTCTTATGGTAATTTGGGTATTATTACGGAAGTTACTT

TTCGTTTATTTCCAGTTCCTTCTGATTCCATAACTTTGCTTTTGACGGGAGGTCAAGAAAAAATT
AATCAGTTACAAAAAAAGATTTGTGCTTCTGCTTTAACTCCTACATCGGCGGATTTATTATCTGA
ATCTTTGATCAATGGTTTAAATTTAAATTTTTCTTTGGGATTGGCGTTGCGTTTTCAGGGTATAG
AAGAGAGCGTTTCACAACAGAGTGCAAGAGTAAAACAGTGGGCGGAAGATATTGGTTTATTAAT
CCATCAATGGGATTCAGAAAAAGAGTTAGACTTATGGACTCGTTTAAAGGATATGATATTCCAAG
GTGCTAATAACAGAGGAATTATTTGTAAGGTTGGCATTTTGCCCACTAAAATAGCGGAATTGTTT
GCCTTAACTCAAGGTTATGGTTTTGTTAATATGGGTACTGGTGTCGGTTATGTTTGTTTGCCAAA
AGGGATAAAAAATCATCAAGTTAGAGGTATCAGAAATTTTTGTCAGGATAATGAGGGTTATTTA
ACAGTTCTTGAATCTGGGGCTTCTGCCATTAAACAAGAAATTGAACCATGGGGCTATGTTGGTAA
TGGATTGGAGATGATGAAAAAAATCAAGTCAAATTTTGACCCTTTTAATATTTTTGTTGATAGA
TTATAGGTTATAAGCTATTGTTTCCATATTCCAATAAAGCCCAGTATCGGGAATCTTGAGGAAAT
TTGGCATAAAGAATAATATCTCCATTGCCCACGTTAATATTTCCAGCAAAAACATTCCCGTAACG
TTCTAAATCACGCCATTGATTCGTTGATTTGTTAACTGCTTTTACTTCTAAGGCTTGATCTATTT
TTAACTTAAAATAAGTATTTTGGTTGGGATTTAAGCTGTTATTTAAGGGGGTTTCTAAATAGCCA
TTGTATTCGTTAAAGTGTTGGAATGTTTTAGGAAATTGATTACCATTACCTGAAGCTAATATGTT
GTAGCTGACTACTAAAGGATAAGAATTAGTCTCATCTTTTGGTTTCGCAAATATATCCAATTTAT
ATTCCCCTTTTTCGGGAAATGAGGCGTTGATGAGTATATTTTCTCCTTGTTTTTGAACGAATGTA
TAATTGTCTTGCAGTTGTTTTTCTTCAAATTTCAAAGTTGCGATCGCAACTACATTTTTAGGAAC
TTTTAGGGCAATATTAATATTATTTTCATCGGTACTAATATTCACATTAGGATGACTTAATAATT
GAATATCGTATTCAAATAAAGTAGGGGAAACATTAGCCAAACTATCAAATTCAGAACGAGACAT
AATAGGATTTAATAATTGCCATTTATCGTTTTCTGGGAAATGAGTATAGATAAATTCTCGTGGAT
TTGTGGCAAAATAAAAAGGATTAAATTGAGCATTAAAACCATTATTAGTTACAGTTCCAGCTCCC
CATGTTGTATCAATTAAGTACCAATTGCTATCTATTTTTACTGCATTCCAAGCATGATTTACATT
ATTATCTAAACCTACAATATAATTTTCTCCTTTAGCATATCCTAAAACAATAACAGATTTTAAAC
CCATTTTTTTTGCTAATTGCTGGTATAAATTAGCATATCCTGAACAAATTGTTGAGCGAGTATTT
AAAACTATTTCGGTTCTAACATCGGGATAAATATTATTATTAAATAAGTCGTTTAATGCGCCTAC
ATCGTAACTAATATTATACGTTATCCAAGTGTAAATAATTCTAGCTTTATCCGCTTCTGTTTTGG
CATACCTAGACAAAATTTGACTTAATTCTTCCACAGAATTACCAGTATATTTTATGCTTTTAGCT
TTTCTGTCAATATCACTAAAATTTCTTTTTTCTAATAATTGATAATTTTCAGTGATAACCACTGG
CTCATCAATTTCTGTAATAAATTCTTCAGTGAGAGATTTAAGAGAATTAACTCGAAAACGAAAGG
CATTAAAAAAACTGGATAGATTAGGCGATTCTAACTGATTTTGTAAATAATCTATTATTTGAGG
ATTACTTTTAATCGTTACCCCCAGAGAAATAGCAATGATTAAAAATACAAAAAAGCCAAAATTAC
TTTTTTTTCTTTGACGGCGTTTAACTTGTTTATTTCTTTGTTTATGCCATTCTCGATATTCTCTTT
CTGTAGTCATAATAAAAACTAAAAGTTTATAAATTATTATTTAGTAATTGTCTTTTGCCTCTTGC
CTACCCTAACCAATAACTCACATACTCAAAGTAATAGAGCCTTATTTTATACCATTGCCCATTCCC
TATTCCCCGATACCAATAGTTTTTGTATCTCATCATAGTGAGAGATGCTATATTTCGTTGAAAAT
ACTAACTTAAAATTTATTAAATTTTCCCTAGTTGAAACATCATTAATTAAACGCAATGCCGAAGA
CACAGCTAACGCTGTTCGCAACTTTAATGAACTAAAACAATATAAGCTATCTATATTAATTGACT
AAAAATAGGTTCACTAATATTTGCTAACTCATCCAAATTCAGAGAATTAACATCCCCCAAGTAAG
GAAAATAACCCAAAACGGAAATACCAGTTAAAGACTCAATTAAACCCTTGGGTGTCCATTGATTT
ATTTGCGTAGGAGTGTAATCCTGAACACAATTAAAAATAATACCCGTTAACTTTATATTATACTG
TTTCGCTAAAGCTACATTTGCCACCGTGTGTGCGATCGCACCCAATTTTACAGGTACAATCAAAA
TAGTATCCAATACCCAGTCATGGGCAATATCAGCAATAATTAATTCTTCTGTAATTGGTGAACCT

AACCACCTAAAGCCTCAACTAAGACAAAATCATGACTTTTTTGTAAATCTAGTAAACGATTCCT
GACAATATTTAAATCAATAGATTTACCCTCCAACTTAGCAGACAAAGGAGGAGCAAGGGGGGCTT
GATAACGTAACGGAATAACAGCATCATCAAAATACTGACCATAAAATTCTTCATCTCCTATTTCT
CCTGTTTGCAATAACTTCATTAAAGCAGTAACTAAATGAGATTTTTTTCTCTGCAAATAAGCTCT
AAAAGCAGTGGTGATAATGGTTTTTCCCGCATCAGTATCCGTTCCTGCGATCAATAAAGTTTGCA
TATAGTATTGTTGACTCAATATAAAAAATACAAAAGATTAATAAAAAATTAAAAATAGTTAACT
AAAAAAGCGGTTTATATAGTAAATTATAATGTTAAGCTAAGAGTATCCTATGCAAAAAAAAGGA
AACACATTAACACACATAAACCAGTATTAAATATCAAGAGGTTGAGAATTTGCCGTGAGTCATTC
TGCCACCTTAACAGTGAGTTCAATCTTACCACAACAGGTATCGGACAACAACCGTTTACGCCTAT
TTTCTGGCTCAGCCAACATTGGCTTAGCCACTGAAGTAGCCCGTTATTTGGGCATGGATTTAGGT
CCGATGATTCGGAAAAGATTTGCTGATGGAGAATTATATATTCAAATTCAAGAATCGATCAGAG
GTTGTGATGTTTACTTAATTCAACCCTGTTGCCATCCAGTGAATGATAATCTGATGGAGTTGCTG
ATTATGATTGATGCTTGTCGTAGAGCATCAGCACGGCAAATTACAGCAGTAATCCCTTATTATTC
CTATGCCAGAGCGGATCGTAAAACCGCAGGGAGGGAATCTATTTCAGCGAAATTAGTCGCAAATT
TAGTCACCACTGCCGGGGCTCATCGAGTCTTAGCAATGGATTTACATTCCGCCCAAATTCAGGGC
TATTTTGACATTCCCTTAGATCACGTTTATGGTTCACCCGTAATTTTAGATTATTTGAATCATAA
AAATATTGAGGATTTAGTTGTTGTATCTCCTGATGTTGGGGGAGTAGCTAGAGCGAGAGCTTTTG
CAAAAAAATTAAATGATGCCCCTCTTGCCATTATTGATAAACGTCGCCAAGCTCATAATGTAGCT
GAAGTGATGAATGTTATCGGTGATGTTAAGGGTAAAAATGCTGTATTAGTCGATGATATGATTG
ATACTGCTGGAACATTGTTAGAAGGTGCAAGGTTGTTGAAAAAAGAGGGTGCAAACAAAATTTA
TGCTTGTGCAACCCATCCCGTTTTTTCAGGCCCTGCTATTTCCCGTCTTTCCAGCGATGTCTTTGA
GGAAGTAATTGTAACAAATACGATTCCTTTACCAGTAGATAGTTATTTTCCCCAATTAAAAGTGT
TATCCGTAGCTAATTTATTGGGTGAAACTATTTGGCGTATTCACGAGGATAGTTCTGTTAGTATT
ATGTTTCGTTAACTAAGGGTTGCCGAGAAAGTGGTATTATGGGGATAGGTGTCAGGTAATTTCAA
TTAGTAATTAGCAATTATTAAGATAAGAGAATAAGCAACGGGCAACTTCGCATCTGGCAATAGTT
TTTTATTAGCTTTCCCCTTATACTTCGATTTAGTATCTAGCCAAAAATGGGCTGAGAAAGGTAAG
GAGCTGGGAAAAAGAATAAGAATTGATGAAAAACTTATTAAAGTTGATTTTAAATACTTCATGT
TTAGCCATGATGTCCTAACGTATTTTCTCAGTAATATCTTAGCTTTGAAATCCTGATTTCTGCCT
ATAACTCCACTCAACCTTTATACCTAAAAATCAACCCAAATCTTATCTTTTTATATCGAAAAGAA
TTAAAATTTATGGTTCTTCAGAGTGTGGTTATGATAAATTAAGAGAAACAGGACTTCCATAACTT
TTATTTAATAGTGTTTGTAGTAAAATAAAAACTAAAAGTTTATAAATTATTATTTAATAATCCT
CTATTGCCTCTTGCCTCTTGCCTCTTGCCTTTCAGCCTACCCTAATTAATAATTTACATACTCAAA
GTGATAGAGCCAAATTTATTTTTTGATGAATACTTGCCTCTAAAACTCCGTTAAATTTCCTTGTT
TGCTCACAAACTTAACCAAGTGTAAAATCAATTGTTTTCGTCCTTTCTTAGAAAAAGAGCATTAG
ATTGAGCAACGGCTCTAGTAGTGTTAAAATGGCTACTTAGAAGTAAAAGTAGAGTTACATAATC
ACATTTTAATCCGAGTCAAATTTTGCTACAATAAAGGTCATTTAAGGAGGATAGTTATTTGTCAA
ATCAAGTTAGGGTTGCCATTTTAGGAGCAACAGGTGCAGTTGGAACAGAACTAATAAACTTATTA
ATAGAGCGTAAATTTCCCTTAAAAGACCTAAAATTATTAGCTTCTCCTCGCTCTGCTGGAAAAAA
GGTAACTTTTCAAGATCAAACCTTAGAAATTGAAGCTGTTGACGATAATTCTTTTGATGATGTTG
ATATTGTTTTAGCCTCTGCTGGTGGTTCAACTTCAAAACAATGGGCAGAAAAAATCGTTGCGGCT
GGAGCGGTGATGATAGATAACTCTAGTGCTTTCCGCATGAATCCTGACGTGCCTTTAGTTGTACC
TGAAATCAATCCTAATGATGTTCATAATCATAAAGGAATCATTGCTAACCCCAACTGTACAACTA
TTTTGATGGGGGTTGCTATCTATCCCTTACATCAAATTCAACCCATTAAAAGAGTAGTGGTTTCT

ACTTACCAATCTGCAAGTGGTGCAGGGGCAAGGGCAATGGAGGAAGTAAAAACTCAATCAGAAGC
CATTTTAAAAGGGGAAAATCCCCAACCTGAAATTTTACCTTATCCTTTAGCTTTCAATCTATTTC
CTCATAATTCACCATTGATGGATAATGGTTATTGTGAAGAAGAAATGAAAATGGTTAATGAAAC
TCGCAAAATTTTTGGAGATGATGATATTCGTATTACTGCAACTTGTGTGAGAGTACCTGTATTAA
GAGCTCATTCAGAGGCGGTTAATATCGAATTTGCCCAGCCTTTTCCCTTAAATAAAGCCCGTGAT
ATAATTGCCCAAGCGCCCGGGGTGAAATTAGTGGAAGATTGGCAAAATAATTATTTTCCTATGCC
AATGGATGCAACAGGAAAGGATGAGGTATTAGTGGGTAGAATTCGTCAGGATTTATCTAACCCT
AATAGTATAGAATTGTGGTTATGTGGAGATCAAATTCGCAAGGGTGCGGCTTTAAATGCGGTGCA
AATTGCTGAACTATTGGTTCACGATAATTAACCAAGAGCGTTTTTTGATTTTTCTTTTATGGATT
TTGCCCACAGCTTTTATTTTAACAACTCTATTTATTTGGTAACAGGATTAATTATTGAGCGTGAT
GAGTAATTATATTTTTGGTCGAGTTTTAACCGCTATGGTAACACCTTTCAATGGTGATGGTTCAG
TGAATTATGGTGTTGCAGAAAAGTTAGCTGACCATTTAGTTAATAATGGTAGTGATGGTTTAGT
TATTTGCGGTACAACGGGAGAGTCTCCTGCTTTGGAGAGTGACGAAAAATATCAATTACTAAAAG
TTGTTAAAAATGCTGTGGGCGATCGCGCTAAAATTGTTATGGGTACAGGTAGTAACTCCACTCAA
AAAGCCATCACAGAAACAAAGAAAGCCTCTCAGATTGGTATAGATGGAAGTTTACAAGTAGTTCC
TTACTATAATAAACCCCCCCAAGAAGGATTATATCAACATTTCGGTGCGATCGCACGTTCTTGTC
CTGATGTTCCCATAATGTTGTATAATATACCCGGACGTACTGGTAAAAACTTAGAAGCGGAAACT
ACCGCCCAACTAGCACAAGATTTTGATAATATTGTGGCAGTAAAAGAAGCTAGTGCAGATTTAGA
TCAAACTGCTAAGATAAATAGAATAGCACCGCCAAATTTTTTAATTTACTCAGGAGAAGATTTTT
TAACTCTACCAATGATGACCGTGGGATGTGTAGGAGTCGTCAGCGTCGCTAGTCACCTAGTAGGT
AAACAAATGCAAATGATGATTCAATCCTATGAAAAAGGAGATAATTTCCAAGCTCAAAAAATTC
AAAAACAACTCTACCCTTTATTTAAAGTTTTATTTTGCAATACTAATCCAATTCCTGTAAAATAC
GCTCTGCAATTGCAAGGTTGGGATATTGGCGGTTTCAGACTTCCCCTGTGTAATCTCCCCCCTCAT
CAACAAAAAGAAGTAGAAGAAGTTTTAAATAATTTAGAATTAATTTAAAAGAGCCAAGACTCAT
ATTAAAAAAAGTATTTCAATGATTTATTTAATTCGACACAAATATAAGTTTACCGAGTATTGAT
AAAAGTTTATTCAAGTTTTTTTATTCATTAATTACTGTTGAGGAGGTGAAATTGTGATTTTAAAC
TTATGACATTTAGATTCCCCAGATACCTATAAAAAATTAAAGGTAAAATCCACCGAATATCTTAA
TAAAAGTCAACGTAATGATATTTAAGCATTATTTTAGGGGGGCTTTCTTATCAAAAAATATCTCG
TTGTTGCGGGGAAAACGAATCTATTTGACTTATTAACTATTTATTCTTAAAGAAAAACTTAATAA
GTAATTATTTAACTACCCTTAAAAATCACATTGTATTCATTTTATTCTAGTCAAAAAAATCAAAC
CAATATGAAAGACAAAAAAGTATATACCATTAAAGGCAGACGCACCAGAAAAACCATAACCACA
GAAAACATTGCTCCTACCCCTGCGGAAAAAAGCAATTCTAATAATCAACCCCATGTAAAAATTGT
TCCTTTAGGGGGATTACACGAAATCGGAAAAAATACCTGTATCTATGAATATGAAGATGAGATG
ATTTTAGTGGATGCGGGTATCGGTTTTCCTACGGATGGAATGCACGGTATTAACGTGGTTTTACC
TGATATGACTTATTTAAAGGAAAATAACCACAAAATTAAGGCAATGATTGCCACTCACGGACACG
AAGATCATATCGGTGGTATCCCCTACCATTTAAAACAGGTGGATTTTCCCTTGATTTATGGTCCT
CGTCTAGCCATGTCTTTACTTTCTGATAAATTAGAAGAGGCTGGACTTTTAGAAAAAACTAAGAT
TCATACTGTTTCCCCTAGAGAAGTAGTTAAAATTGGTAAACATTTTCGAGTGGAATTTATCCGCA
ATACCCACTCCATCGCTGATAGTTTCACCTTGGCAATTCATACCCCTATCGGGGTTTTAATCCACA
CAGGAGATTATAAAGTAGATCATACCCCCGTTGACGGAGAATATTTCGATTTTCACCGTTTAGCC
CAATTAGGAGAAGAGGGAGTATTATGCTTACTGAGTGACTCTACTAACTCCGAAGTACCCGGATT
CACCCCCTCAGAAATGTCGGTTTATCCCGGTTTAGATCGAGCCTTCACTCAAGCTCAAGGGCGTTT
AATGGTGACAACCTTTGCCACTTCCGTTCATCGTATCAATATCATCCTTGCTTTAGCCATGAAGC

ATAACCGTAAAGTAGGAGTAGTGGGGCGATCGATGCTGAATGTGATTGCCCACGCCCGTAAATTA
GGTTACATCAAATGTCCAGATCATATATTTGTACCTTTAAGAACTTTACGCTCTTTACCCGATGA
CCAAATATTAATATTATGTACAGGGTCTCAAGGGGAAAAATTTGCGGCGATGACTCGTATTTCAC
GGGGAGAACATCCCCATATTAAAGTAAGAGAAGGAGATACGATCGTCTTTTCTGCTAATCCTATT
CCCGGTAACACCATTGCCGTAGTAAATACAATTGACCGCCTTATGATTCAAGGGGCAAACGTTAT
TTATGGTAAACAAGCAGGAGTACACGTTTCAGGTCATGGTTGTCAAGAAGATCAAAAATTGATG
TTAGCTCTAACCAAGCCGAAGTTTTTTGTGCCTGTCCACGGTGAACATAGAATGTTAGTAAAACA
CTCAGAAACTGCTCAAAGTGTAGGAGTGCCTAAAGAAAACATTGTCATCATCGATAATGGTGATA
GCATCGATCTAACGGTAGATAGCATTGCTAAAGGAGAAAAAGTACCTTCAGGTATTCAATTAGTC
GATACTGCTGGGGTTGTTCATGAACACGTAATGGAAGAAAGACAACAATTAGCTGAAGATGGTG
TTATTACTATTGCCACAGTGATTGACTCTAAAGGTAATTTATTAGTAGAGCCTCAAGTTAATTTA
CGGGGGGTTGTCTGCACGATGGAAGTACCCAGTTTAGAAAGGTTGATTACTAGAACAGTGGATCG
TACAATAAGCGATCGCATCAAAAGTAACTTACTCCTAAGTGAAGGAGACAATATCAACTGGAATA
GTATTCGTATTGAAGTCGAAACTAACCTCAGTCGTGTCATTCAAAGAGAAATCAATAGCGAACCT
TTAGTTATTTTTATCTTACAAGTCCAAGAAGGGATAACTAATAACAACGGACAAATAAACAATG
AATCTCTTTCTGATAGTGATGATAATGACGAAGGAGACGGTAAATTTACTCGCCGTCGTCGCAAA
GCTACTGCTAATGTCTAGGTTAGGAGTTTGGGGTTAGGACTTCGGAAATATGTATCAGGTGTCAG
GTTGCAGGTGTTAGGGGATGGGGGGATGAGGTGATGAGGGGAGAGGGAGATAAGGTGTCGGGTT
TCAGGTTTCAGGTTTTAGAAGAAAGTAATGAGTAATGAGTAACGAGTAATTATCAACTATTCAC
TATTCACTATTTAATATCAAATCCGTTTAATTACCCACACAAAAAGATATTAATTAGGCCACCAA
TAAAAATTGGCTATTCCTTTAACTATTTCTTTTTCTTCCATAATTTTTTTGCATCTACTAATAAC
TACCTCTTCTAACTCTTCTAAATTTTTAAATGTCCTATTAGCGATGGCCTCATTTACTAATGGCC
ACAAACGTTCTGCCGGTTGTAATTCTGGAGAGTGAGAAGGCATCAGCATAACGTGGATTCCCTCT
GGTATTTCCGTCGTTTCTTTAATCGGATGCCAACCAGCTTGATCCATTGCTAAAATCACCCTTTTG
TTTTTACCTATTTCATAGTGCTGTGCAAAATCTTTTAAGACTCGATTAAACAATTCAGTATTTAC
ATATGGTAGTAGCCACCAGTATGTCTCTCCTGTATTTGGTTCCACAAAGCCATATAACCACAACC
ATTGAAAACGCCAATTGACCGTAGCTATCGGTTGATAACCTTTTTCTACCCATACTCTTCTTAAT
ACTGGTTTGAGTCCTAAACGATGCTCATCCTCAGCCCACACTTGAACGTCCGCGTCGGGATATTCT
TGTTGAATCTTTTTTTTTTTCTGCTAGTTGATTTTTCCATTCATTTTGTTCAAGGCAGCTAGAAT
TTTGATGATTTGGACGAGGACATCGGAGACGATATTCTAATCCTTTGAGATATTCCCATCCTCTT
TGAGGAGAAATAGTACGATCTAGAAGTTGAGTGAGCCATTTTGCTACTTTGACACCATTCCAAAG
TCCTTGATCATCGGGGGGTGATTGTAATCTTTGATATAGCAGAGCTAATTGTTGATCGTCTAATA
ACGGTTTTCTACCCCGATTATGTTGTCTTTGATCCCCCAACATTTTTGCACCCGAACGATTATAAC
TACGAACAAGTTCGTAAATCCAACTTAAGCTATATCCTGTAACGTGGGAAACATAAGAGGTTCGT
TTCCCTTGTGCTAATAACCAAATCACTTGGTAGTGAATTTTTTCAATCCCTTGTTTGGCTTTTCG
ATACCCTTGTTCTAATTCTTGTAAACTTAAATGGGGTTCAATTTTGATTCTTGAAGGCATTGGTC
AAAATCTACGACAACAAATATACATCCTCTTGATTATACCATGTGCAATTAAGCGGATTTGATAT
AACCCTTTTTTGTCATGTTCCGAGATAAAATAATAGAAGAAAAAGAGAAATGGAACTTGGGAGG
CTTAGATGATAATGGATGTAGCAGAGCAAGTTAGTAAACATTTGCCTCGAACTCCAGAACCCACT
ATACCTTTAGTGGATCAATATTGCAGTTACTATCAGGATTTATTTCCTGATGTAAGAAGTTATGA
ATATTTTAAGTTATTACATTTAGGAATAATCTCTAATCTTAAACGAAAATCATTACCAGAATTAG
AAAAAGTACTAGGAGTATCATCACAATCCTTACACCATTTTCTCACATCATCACCTTGGTTATTA
TCGGAACTAGACCAAAGAAGATTAAATAAATTAGTAGAGATATTGCAGGGGAAAAAAATCATTC

TCATAGTAGATGAAACAGGAGATAGAAAAAAAGGTAATAAAACAGACTATACTGCTCGACAATA
TTTAGGAAATGTGGGGAAAATAGATCAAGGAATAGTTTCAGTTAATGTCTATGGTGTTTATCAG
AATATAACCTTTCCATTAATGACAAAAATATATAAACCAAAGTCAAGATTAAAAGTAGGAGATA
AATATAAAACAAAGACAGCAATAGCAGGAGAAATGGTAAGAGAATTAAAGGAATTAGGATTAGA
AATAGAAATAGTGTTAGCAGATAGCTTATATGGTGAAAGTAGTAGTTTTTTGGAAGTATTAGAA
GAGTTAAAACTGGAATATGTGGTATCGATTAGAAAGAATCATGGGGTGTGGATGGGAGCAAATG
AAAGAGTTAGAGCAAATAAATGGTGTAAATTTACTAGGACATTTAGGGATGGAAAAAGAGAAAC
AAGATACATAAGAGAAATAATTTATGGAAAAAAAAGGAGAATAACATACTGGGAAATGACCACA
GATACAGAAAATTTACCGAAAAATTCAACAAGTTTTGTGATGACAAATTGTCAGGGAAATTTGA
AGAAAAGATTAGGAGATTTGTATGGTCAAAGGACATGGGTAGAATATGGATTTAGACAATGTAA
ACAAGAATTAGGATGGAAAGATTATCGACTAACAAAAATAGAAAGTATTGAGAAATGGTGGGGA
CTAATAAATAGTGTTTATACCATGGTAAGTTTACAAACAAAACCATTGAGAGAATTACTATTTTC
AGAAGAAGAAAAGAAAGAGGAAATAGAAGAAAAAGAGCCAATAAATAGTTGGAAAACAAGTTTA
AAGAAAATAATGATGATGATTGAGCCATTAATCTTATTTTGGTTAATAACACCATGGTTAAAAA
TGGTAAATTCATCAGGATTATTAGAAGGATTGAATAATTTATTATCGATCACTCATGATTCTAAA
TTATATTTTCCGACAGGATAATTGTTTCTATTTTTTGATAGTTGATTCGGAGTTTGACAAAAGAG
GGATAACCTGAGTTTTGGATAAGCTGAAAGCATTATTTTCTCGTAGTCAGAAAACCTTATAGCTT
CTTCTTAGAAATAACGACAAAATTGCCTTAACCCGAACTGAAGTTAAATATATTCAACCCCTGCC
ACCTGCAACCTGACACCTGACACCTAACCTTATTCAGATATTCTTAAACCGAACTGAGGTTATTT
ACCTTTACCCCTTGTCCTTTCAATTTTAACTGCGATGAAGAGGATTTAAACGCTCACTTAATCCCT
CTCCTAATAAAGATAAACCCGTTACCATAGTTGCCATTGCCAAGCCGGGGAATAAGGCATTCCAC
CAAATACCAGTGGGTAAACCGTCTAATGCTAATTTTAGATCATGACCCCATTCTGGGACATCTTC
AGGTAAACCTAAACCTAAAAATCCTAAGCCCCCTAAAATTAAAATGGCATCGGCGGCGTTAAGGG
TAAATAAAACAGGTATGCTTTGGACTACATTAAAAAAGAGATATTTATAAATAATACGAGTGGG
AGATGCCCCCATAGCTTGGGCGGCTTCGATGAATAATTCTGTTTTAACGCTAGTAGTATGATTTC
TGACTACTCGATAATATTGAGGAATATAAGATATAGATAAGGCAAGGGCGGCATTTATTACTCCC
CTACCAACGACAAAAGCTAAAGTAACTGACAATAATAACCCCGGTAAGGTATAAATAGTATCCAT
TAAAAAGAGTAAAATTTTGTCAATTTTTCCTCCAAAATAACCACTAATTAAACCTAATGGAACAC
CGATAATTAAACTTAAAAATGTAGCAGTAATAACAACTTTTAAAGCCGCTTGTGAGCCAAAAATA
GTTCGGGAGAAAACATCATAACCCTGTCCTGTTGTACCAAACCAATATTTACTATTAGGAGGTTG
GTTAATGGGATTACTAAGAGATTCTAAGGGATCTTGAATAATGCCTATATTTTCTAATAAAGGA
GCAGAAAAAGCAAAAAATATAAAGGTTAAAGTGATAATAATACCCACCATCAGCAAAACATTTG
ATAACTGTTTAAAAGTATTTTTTTTTCTTGAAAAACTACGAGCAATCATTTTTATTAGACCTTTT
ATAAAAATAAAAATAGCAGAAAATTTAAATGCTTTAATTAACCTTACTTTGGAGAATTGAAGAG
TAATTAATAAGACAGAGGTCTCAGACGGTAGAGATTTAATATATTTAACTCATAATATGTGGGA
AAGTTATATACAACTTTTGTACTAGGTATTAGAAGTTTTTAATTATTCACTATACACTATACACT
ACTCATAAACTCTCCCACGCCATTGACTAGGTTTTGTTAATGCCGAAAGGATAATTCTCGTCACT
GCTAATATATCGGCAAAAGGTGAAAGAATAAAGGTTAATTCTGATATTGATTTATGGAATTGAT
AGGAAGATGCGATCGCACCTAGGAGGGCAAAACGGATAATAACGAGAAAAGTATTTAGGGCAAA
TAATAACTTGAAACTAAGAAAATCAAAGTGGGGATAGAAAAATAAGCAGAGGAAAAAGAGAGGT
AAAGGTAAAGCCTGAATTAGTAGCAAAAAAAGACATTCCCCCCATAATTGTAGCTTTGAGGACGC
ATCTTTAAGATCTAATGATCTACCCCAACCCTGCCAAGTATCTTGTAAACCTTCATACATCCGCAC
CTTAATAATTTTACTGCCATCCGCAAACCCGACTTTATAACCTAAACTTGCCACATAACGAGCTA

AAGTGACATCATCACAAAAGGAATTCGCCGCATAAGTATAACCGTTAATTTCTGTTAGGATATTA
CGTTTAATTAAAAAACATTGACCATTCGCCATGATTGTACTGGGGTTGTTCGTATTCACGCCAGA
GGATTCAAAGCGAAATAACAAAGTCATTAATAAAGAAGGTTGTAGCCACCATTCCCCCATAGACT
TTAAAATAAATTGAGGGGAAAAAGAGACTAAATCATAATCCTGTTGAATGGCAAAATCTAAGAC
AGTGGCAACGAGGTTTTTATGGGGAATAGTATCCGCATCAACTCCTAAAATCCATTCGCTATTTT
CAGAACTATTTAAGAAGCCGTTGTGTAATGCCCAAGGTCTGCCTACCCAATTTTTAGGCAAAGGT
GGATCTGTAATTAAATGAATACGAGGGTCTTTTTTGCTATAGTTCTCAATTTTTTCCTGAGTGCC
ATCAGTGGAATTACTATCAACAAAAATAATTTCTCTAGTTTCGTAGCTTTGACGAGTAATACTTT
CTAATAAAGGAGAAATGCGATCGCACTCATTTAAAGTAGGTATAATGACACTTACTTTGCCAATT
AAAGCTAAATAAGGAAGTTTTGGAGTTAAAGGAGGAATCCTTCTTGCCCCTCGAGCCAAGCGACT
TAAAAGAATAAAAGTACAAACTCCTTGAAATAAAAGTAATTTAAAGCTCAGAATGGCAATAATA
ATAGTCATAATAAATCAAACTAAGGGTTAAATTGACACCAAGTATATTTAACAATCCTTAATTA
ACATAGCATCTCCAAAAGAATAGAAACGATATTTTTCTCGAATTGCTTCCTCGTAAACCATCATT
AATCTTTCTCTACCAATTAAGGCACTAACTAGCATTAGTAAACTCGACTTAGGTAAATGAAAATT
AGTAATTAGTCCATCAATAACCTTAAATTCATAACCCGGATAGATAAATAGATTTGTTTTGCCCT
CAAAAGCCTGTAAATCTCCACATTCTTTACAAACTCCTTCTAATGTTCTTACAACCGTAGTTCCCA
CCGCAATTACTTTATTTCCCCTTGCTTTAGTTGCCCGAATCAAATTAACCGTTGCTTCATCAATTT
CAATCCATTCTTGGTGCATATCATGGTTAACAATATCTTCTACCTCCACTGGGCGAAACGTACCA
ATCCCCACATGGAGAGTTACTTCTGCTTTATTAATTCCTTCTGCTTTTAATTGTTCAAATAGTCG
ATCTGTAAAATGTAGTCCTGCTGTCGGAGCTGCGATCGCACCTTGTTTTTGAGCATAAACCGTCT
GATACTGGCTCGGATTAGCCGATGACTCAGTGACGTAAGGAGGAAAAGGAATATTACCCACCTTC
TCCAACAAATGCCAGAAAGACTCCCCCTCTTGCCAAGAAAATTCTAAATATCTTCCCCCCGTTGCC
TCATCCTTATCAACAACCGTCGCCTTTAATAAACCATCCCCAAAATAAATTTCACTGCCCACAGAA
AAACGCTTTCCGGGCTTTACCAAAGCTAACCAACAATTATCCCTCGTTTCCTCCACTAATAAAACC
TCTACCTGAGCGCCAGTGCTTTTTTCTCCATATAAACGGGCAGGAATAACACGAGTATTATTAAC
AACTAATAAATCTCCCGCCGACAAAAACTGAGGTAAATCAGCAAAAATTTGATGCTTAGGGCTAT
TTTCTCCCTCAATTACTAATAAACGGGAACTATCTCTAGGAGTAACAGGATTTTGAGCAATTAAT
TCTGAAGGTAAATAATAATCGTAGCTAGATAATTGTTGATCAGGACTCATAGTTTATAGTGTAT
AGTTAATAATGAATAGTTGATAATTACTCGTTACTGGTTACTCATTACTCCCTTAAACCTGACAC
CTGCCACCCGATACCTGCCACCTCTTGTTGCTTCTAGGGTAAAAATTTTGTCACAATTGTGAAAG
AAACCCTTTATCAAGAGATTTTATGGACAGTATTCAGGCAATTAGAGGAACAAAAGACATTTTAC
CTGAAGAAATAGTTTATTGGCAGTATTTGGAAAAAACCGCCGCCGATATTTTAACTAAAGCGGCT
TATAGAGAAATTCGTACCCCTATTTTTGAGCAAACAAATTTATTTGAAAGAGGCATTGGTGAAGC
CACGGATGTTGTAGGGAAAGAGATGTACACATTTACTGATAGAGGCGATCGCAGTTTAACTCTAC
GGCCAGAAGGAACAGCAGGAGTAGTAAGATCTTATATACAAAATAAATTATTTGCCAGTGGCGG
AGTAGAGAGACTTTGGTACACTGGAGCAATGTTTCGTTATGAGCGCCCCCAAGCAGGACGACAAA
GACAGTTTCATCAAATAGGTTTGGAGTTATTGGGTAGTAAATCTCCCCGGGCAGATGTGGAAGTG
ATAGCCATCGCTACAGATATTCTCAAAGCATTAGGTTTAAAAAACCTTAGCCTACAACTAAACTC
CGTGGGCAATCAAGAGGATAGACAAAAATATCGTCAAGCCTTAGTCGATTATCTAACCCCCTATA
AAGCTGATTTAGATCCAGATTCTCAAGACAGATTAACCCGCAATCCCCTACGCATCCTCGATAGT
AAAGATCAAAAAACCCAAGAAATTACAGAAAATGCCCCGAGCATTCTTGATCATCTAGGAGAAGA
TTCCCGTTACCATTTTGAGCAAGTTTGTGGATTATTAACAGAATTAGGAATTGATTATCAACTGA
ATGCCCGTTTAGTCAGAGGATTAGATTACTATACTCACACTGCTTTTGAAATTCAATCAGCAGAT

TTGGGGGCTCAAGCAACGGTTTGCGGAGGAGGTAGATACGATGGGTTAATTTCTCAATTGGGGGG
GAATGAAACCCCTGCCATTGGTTGGGCAATGGGTATGGAAAGGTTAATTTTATTACTGCAAAATC
TCAATCCTTTAACTCCTCAATGTCCTGATATTTATTTTGTTTCCAGAGGAGAAAAAGCGGAAAAT
CAGGCTTTAATTATGGCTCAAAAACTTCGTCACAACGATTTTAATGTGGAGTTAGACCTCAGTGG
TAGTAATTTTGGTAAACAGTTCAAAAGAGGCGATCGCAGTGGTGCTAAAATTTGCTTAATTTTGG
GTGATGAAGAAGTGGAAAATAAAACCATTCAAATTAAGCATCTGGTTACAGGAGAACAAAAAAC
AGTGTCTCAAGATGAATTAATTAATGTTCTAAAAAAATAAAGTGAAAAACTATTTCAAGTATTT
ATTTCTAATCATAAAGTAGTTTTAAGACAAAAACAACCTATGTTTAATATTTATTAATGCTTGTT
TCTTAGTAACTATGGCTACAATTTCGGTTATTAATAACTATCTTGAATTAGATGTCATGAAAAAA
TATATTGCCGAATTTTTTGGCACTTTTTGGTTAGTGCTTGGAGGTTGCGGTAGTGCCGTTTTAGC
GGCCAATTTTGGAGGAGAAGGCAATCCTCTGGGGCTAGGATTTTTGGGGGTTTCTCTTGGCTTTG
GTCTAACGGTTCTAACTATGGCTTATGCTGTGGGACATATTTCAGGAGGTCATTTTAACCCGGCG
GTTTCCTTTGGTTTGTTTGCTGGAAAACGTTTTAGTGGTTCTGACTTACTACCTTATATTATTGC
TCAGGTTTTAGGTGCAATCCTTGCAGGGGCTGTTTTATTTATCATCGCCAGTGGTAATGGTGCTT
TAGACTTAAGTGGCTCAAATCCCTTAGCTACTAACGGTTATGGTTCTCACTCTCCCGGTGGTTAT
AATTTATTTGCCGCTCTGATTACAGAAATTATCATGACCTTTATGTTTCTTGTGATCATTATGGG
TGCAACTGATCGCCTTGCAGTAGGAGGATTTGGCCCATCTGCCATCGGTTTAGCGTTAACATTAA
TTCACCTCATCAGTATTCCTATAACGAATACATCCGTCAATCCGGCAAGAAGTACAGGAGTAGCT
TTATTTTGCGGTAATATGGAGATTATTGCTCAACTATGGTTATTTTGGTTCGCTCCGATTGTTGG
TGCTGTTCTCGGAGGCTGGTTTTACTATCAATTTTTAGAAACAGGAATTGAAAGTCGCCCCTTGC
AACCTATCGAATCTGAACCCAGAGACTAAAGAAAGAATCAAACATTAGGAATTAGGACTTAAAT
TATTTATTATTCACTCCCGAAAAAATGCGATCGCCCATAGAAAGAATCAAACTATCAAAAAAGGC
TAAAGATCAATTAATTAAATTAAGAAAAGTTACAAAAATAGAACAATGGAATATTCTCTGTCGT
TGGGGATTTTGTCGCTCTCTGCGTGAAAAACATACTCCCGCCCCCTATCCAATTCCTGCCGATAGT
AATGTGGAAATGAGTTGGAACACCTTTGGAGGGGAAATAGCAGAGATTTTACTCTTGGCTTTAAT
TCATCGTTGTCATCAAGATGGTTTTGAAACCAATGCAGAAACCCTTAATCAACAATTTCGCTTAC
ATCTTCACCGAGGAATTAATTACCTAGTCACAGATTTAGAATCCAAAACTCTCTCTGATTTCGTT
GCCCTAGGCATCAAAAAAGATGACTCTCAATAAAAAAGACCGCTAAAAACTAGCAGTCTGAAAAG
AATAATTATTTTTTATTTTTAGCTTTTGCTATTTAGACTAATCACGAGGTTTAGCTTTATTTACT
TTGATTTGGCGTCCCATCCACTGAGCACCATCCAAAGTTTCGATCGCTTTTATTTCCTCATCGTCA
TTGGACATTTCCACAAAACCAAAACCACGTTTTCTCTTGGTTTCACGATCAAGGGGAAGATAGAC
ACGCTTTACTTCACCATAATCGGCAAAGACTGATTGTAAATCATTTTCTGAAATATCGTAGGATA
AATTCCCAACGTATATGGACATAAACTGTTCCTCAATTTTTTACTTTTGTATAAGATAATTGAGA
CGACCGACACGAAGCATTCCATATAGAAGGGATAATCCTGCAAAACTGGAAGCAAACACTTTTAA
CGGAACCGAGACTCAAATTAACATTATATATATAATATCATACTTAGGCTTTGAAATCTGATAAG
TTATAGAAAAAAACTTCATCAAGAGAAAATATTTTCACAATATCCAACAGAGGGTTAGAATTAA
GTTTCAAAGCGAAAAGAGTTCTAAATTCTCAAGAGGTGTAGGCGGTGAGTGACAACTTCAAGGCA
ACGGAGAACAATTCTCGTCATGAATTACGGGATTTAGTACGTTCTCAATTACAAATATTATTAGA
CCAAAATAACCTAAAAGGTGCAAAATCTTTATTAATCCCCGTGCAACCCGTGGACATTGCAGAAG
CGATCGCATCTTTATCAGAATCAGCTCAAGCGATTGCTTTCCGTCTTTTAGATAAAAACAAAGCC
ATTGAAGTGTACGAACATTTAGAGGCAAAAATTCAGGGTATTCTCATTGAGGATTTTAAACGCCA
AGAAGTCATTGATATAGTTGATAAGATGTCTCCTGATGATCGCGCTCGCTTATTTGATGAACTTC
CCGCCACTATTGTTAGTCGTATCATAACTCAATTGAGTGATCAAGAAAGACAAGCCACTAATTTA

CTGTTGGGTTATCAGGAGAACACAGCAGGAAGAATTATGACTCCTGAATATATCTTCGTCAAAGA
AACTTTTAATATAAATGAAACCTTTGCTAAAATTCGTTCTCTGGCCAATACTTCTGAGTTAATTT
ACTACGTTTATGTCGTTAATGAAACAAGACAATTAACAGGGGTAGTATCTTTTCGAGATTTAGTC
ATTCATGGGCGACAAACCATAGTTAAAGATATTATGACTCAAGATATTATCTATGTGGAAACTGA
TACAGATCAAGAAGAAGTCGCTAATTTAATCAAACGCTATGATTTTTTCGCCCTGCCAGTAGTGG
ATCGAGAATTACGAATCGTTGGCGTTGTCACCGTTGATGATGTAATTGATATTTTACAAAAAGAA
GCCACTGAAGATATATACGCCCTTAGTGCTATTCAAGGAGAAGGAGAAAATTATTTTAAAACAAA
TCTATTTACTATTACTAAAAGGCGAGTAGGTTGGTTATTGGTTTTATTATTAACTAACACGGTAA
CAGGGGGAATTATTGGAGCTCAAGAAGATATAATTGCTCAATATACGATCCTTGCCGCATTCATG
CCTCTTTTGGCTGACACTGGGGGAAATATCGGTGCTCAATCTTCCACTGTTGTAATTAGAGGTAT
TAGTACGGAAGAAGTTAGAGAGTTAGGTACTACTAAAGTTATTATTAGAGAAGGCTTAGCCGGA
TTGCTTTTAGGATTCGTGTTAGGTCTTGTGACTATGATATGGGCTTATTTACTACCTAGTAATGA
TAACAATCTGATCGTTGCTTTTGCTGTGGGTATTACTTTAACTGCTATTTGTACCTTAGCTTCTA
TTGCTGGTTCTGCCTTACCCTTCCTTTTTCGCCGTTTGGGTTTAGATCCAGCTTTAATGTCTGGTC
CTTTTATCACCACAGCAGTGGATGTTTTAGGTATTCTGATTTATTTTAATATTGCCCGTGCAATG
TTAGGATTTTAGAACCCACTATGACTAAATTGACTAAGATTTTTGATATTCCCAATTTCTAATTG
TGATCAAGCCTGATGAGGAGGTAAAAGCGTGTCTGAAAATACTGTTACTGTAAAAAATGATTCCC
GTCATGAGTTGAGAGAATTGGTTTCTTCTCAATTACAAATACTTTTAGAGCAAAATAATTTAGAG
GGTGTTAAGGCTTTATTATTACCTGTTCAGCCGGTGGATATAGCAGAAGCGATCGAATCTTTACC
TTCCCAAATGCAGTTAATTGCTTTTCGTTTATTAAATAAAACAGAAGCCATCGATGTTTACGAAC
ATTTAGACTATACCGTCCAACAGTCTTTAATTGAACAGTTTAAAAGGCAAGAGGTAATTGATATA
GTTGATAAAATGTCTCCTGATGATCGCGCTCGTCTGTTTGATGAACTTCCTGCCAGTGTAGTTAG
TCGCATTTTAGAACAATTAAGCCCAGAAGAAAGACAGGCGACTAATCTAATTTTGGGTTATGAAG
AAGATACCGCCGGGCGAATAATGACCCCAGAGTATATTTCTTTGAAACAAAATTTAACTGTAGAA
CAAACTCTCGAAAAAATACGCTCCCTTGCTCCTAACTCTGAATTAGTTTATTACATGTATGTGGT
GGATCAATCTCGTCATCTGGTTGGTATTGTTTCTCTAAGAGATCTCGTACTCCATGAACCACAAC
AAACTTTAGAAAATATTATGACTAAAGATGTAATTTATGTAAAAACAGACATGGATCAAGAAGA
AGTCGCCCGCTTGATTCAACGTTATGATTTACTCGCTTTACCTGTTGTGGACAAAGAATTACGTC
TGGTGGGTATTGTCACAGTTGATGATGTTATTGATGTTTTGGAACAAGAAGCAACCGAAGATATT
TACGCTTTAGGGGCGGTTTCCTCTGACGGAGATAATTATTTTCAAACAAACTTATTTACCGTTGC
CCGTCGTCGGGTTACGTGGTTGTTGGTATTATTACTAACCAATACCGTCACAGGAAGTATTATCG
GAGCTCAAGAAGATGTTTTAACCCAAGTAACAGTTCTAGCGGCTTTTATCCCCCTTCTCATCGGT
ACAGGAGGAAATATTGGTACTCAGTCCTCCACTGTGGTAATTAGAGGTTTAAGCACTGATGAAAT
TAGCGATTTAGGTCCTGCTAAAGTTGTTTTTCGAGAAGCGATCGCAGGTTTGTTATTGGGGTTGA
TTTTGGGTTTGTTGGCTACTATCTGGGCTTATCTTTTACCCCAAACCGACAAAAATATAATCGTT
TCTCTCTCTGTGGGTATTAGCTTAATTGCAATTTCTACTCTTGCTTCTATTTCTGGTTCAGCTTTA
CCTTTTATATTTCGCCGTTTAGGATTAGATCCTGCTTTAATGTCTGCACCTTTTATTACCACTGCG
GTGGACGTTTTAGGCGTTTTAATTTATTTTAATATTGCCCGTTTAATGTTAGGAATTTAACTATA
AAAATATTCAATCACGGTTTTATATTATTATCTGATGTTACGCAAAAATATAATCAATTGTCGAT
AGAAGGTGACAGGTAGCAGGTTAAGAGCAGATGTAAGTATTTTCGGGGGACTGTGTTTGGATTT
CATATTCAAACTTTATCTTGTTGACGTAAGACTCCCATTAAATAACGAGTTTGAGTAGAGTTACC
GTAGGTGACGGCAAACAATGTTATTAATTGGTGAGATGTAGGGGTAGGGGTCAACGGCCGTTGAC
CCGTACAACGCCAACGTTATGATATAATCAAATTAAGTCGTTGACTTTGGGAACGCAGGAAACAA

GAGACACTCCGTAATCCCTAAATCTCTAGCTGTCAATGGTAGAGTCCACACAGTCAGAGCGTTAC
AGCAATTAAATACGGTGGGCACATCGAAATTCACGCTTGGGGAGTAGCTCCGTTAAGTGGCATA
AGTAGCGATATTTGTGTCGGACTAGCATGAATGTGTAAGACCAAAATCAACTCTTGTTGGTGGAG
ACAGTATCGGTTCCCAAGAATCCCCCGTTACACCGCAAGGTTAACGGTGGGAGTTGTCAAAGTTT
CCCCCTATGCTTGAGGTAAAAATAATCATGTTTTTGCACAATGGCTTTAACTCAAATCTTATACT
TCGTTTAAAATTAGCTTCTTTTTGATGGGCAATTGGCAATGATGACAATATTTTGATGTTTCAAT
TTTTGGCATGATTTCAGTACATTTCATCTCATAATTAACCTATGCCTTAAAATTGATTTTAACAA
GTTTTTTATCAATTCTTATTATTTTACTGTTTTTTGTTCCCCTTCTCCCCTTAAAAGGGGAGACAC
AGAGGGGTGTCTCCTGTTTCTTACTTTCACCAGACAATTTTATATCGAACTCAGGTTAAGTACGT
AACGTCAGTTATTATCAAAAAAGCAATAGACTAGGATGAAAATTAAAAATTTATCTTTTACTAT
TTAAGAATTCCCTGTTGCCTGTTAGTAGATGCGAATTTGCCTACCTTAACTAATAATTTACACAA
CAAGAACTAAGAAAGCTATATTTCTTAAGTGACAGTGAAAGATTTGACAATAGTTTTAAAACGA
TTTTCCACATTTTCCCAGCGAGATTCGGTAGTAGAGATATTAAAAGTATAAAGTTTTCCATTTTT
TGTCACCACACTAGCTAGATTATGACGATATTGATTGTCTCCTAATTTAACCTTATACTCTAATA
GATAGTAGTCTTCTAAATTCTGTTCTCTTTTCTCAGCAGAAATTAATTCAGCTTCTCTTTGATTA
TTAGTATCTTGATTTACTATTTGCATAAAACGATAGCCTACATCAGTGGGGCTACCTAAATCAGA
CAAATCCATATCTTTATTAACATCACTGATAATTACGGATAAATTTTCACTCCGTTCGATAAAAT
CTCGAAAAACTACATCAACCCCTTCTGAAGCGTCTTTAACGTCAACAGCCATCCAACCATTAGGA
TATAAAAATTTATAACCATCTGCACCATCAACGTAGGGATTTAAGCCACCAGTGGGAGAAACACA
AGCGGAAAGAGTTACTGTAAGAATAATAACGATAAAAGTTGCGATAGACTTTAACATAGCTAAG
ATGTTTAATATTTTTACTCGCTTATTATCCCATTTTTGTAGAGAATAATCAAAATTCCTGAAACT
GAAGAGTATTAATTTTATTTGAGTTCCCCTCCCGAAGGAAGGGGGGAATAGGGAACGGTTTCACC
CATTACCCATCACCCATTGCCCATTGACTTCTAATATAGCTAATAATTGACCTATATTTCAATAG
TTTATAAGTTTTTCTTAGTATTGCTACTTACTTACGGCTGTGTGCTTGAATTTCATCAATGGTAA
AAATACTTGCAAAACTCAAACCGTGAGATTGATACAATTCTCGTCCCCCAGAAAGACGATCTACT
AAAGAAATGACCTGATTTACTTTATAACCAGCATCTGTTAATCTTTCCACGGCTAACATTGCCGA
TTTTCCTGTCGTCACCACATCTTCTAATACCGTGACTAATGCCCCTTGTTTTAAGGTCGGTCCTTC
AATATAAGCCTTTGTTCCATGGCCTTTGGCTTCTTTACGAATAATTAAGGCAGGGATTGGTTTAT
TTTCGTAAGCAGAAACAACACTTACCGCCGAAACCATTGGATCTGCTCCCAGAGTTAAACCTGCG
ATCGCATCTGTGTCATCAGACAACATACTAAAAATTAATTTTCCCACCAGTAAAGCCCCCTCTGCC
CTGAGCGTCACCTGCTTGCCATTGAGATAAAAACTACTTTTTTGCCCCGAAGATAAAGTAAAATC
CCCTTCCTGATAAGCATATTCCACTAATAAATTTAATAATTGTTGGCGCCAAGCAGAGTTATTAT
TTTCCATAAATATTTTTCAAAATAGATAAGAATAGATCCGTTACAATTAAAGCAACATAGATTTT
ACCAAATATAAACTTATGTTATCAAAATTCTCTCGTTTTCTAACTATTGCCGCTCTTACTACTTT
AACTTGTGTGATTACCCATCCTAGTTCTGCTCAACCTGAAACCTCAGAAATGGGGGAAGAAATTG
AAATTGTAACTGTCCCACTCACCATTAACCAACTTGTTAATGAAGCATTTTGGGAAAACAGTGGT
GATTTTTTCGAACAAGCCAGTATGGGCGGACAACTAAATACTATTTTTGGTTGGCGTAAATTCCC
TCAAGGTTCATATCCCGAAAATAATATTACGGAAGATGGTTTGCTTCTGTACGCTATTTTAAGTG
ACTATTTTCGTCAGCTTCAAGAAAGAGAACCTATTATTAGAACTAGAGACTTAGCGAACCCTTTT
GACACCTCTCTAAGAGAAAATCCTCAATATCAATAAACAATCACAAACATCTCAAAAATCCAGTT
TTGTCCGCAGTTATAGAACGAAATTCAGTTAATGAAAAACCCTCGAAACTGGATAATATTTTTAT
CAACATTTAGTAGCAATATTGAGAAGCCCACGCTTACACGAAGTGAAGCGATGGGAGTAGTCACT
AACTTATCCAGGGTACAGGAGTTGAACCTGTCTAGGGCGAATTATGAGTTCGCTGCCTCAACCGC

TCGGCCAACCCTGGTCACTTAGGGCTTAATTTTTGCCCTTTATTATTATAAAATATATAATCCCT
TATTTGTCAAATTACTTTTATTAAAAATCTTTATTTTTATATGGGAATCATGTCCAATTCCACGA
TCTTAGCTAGTAGTTTAGTCATTCTTAGCAGTTTAACCATTGGTGTTGCTAGTGGTTTGATTTCA
TTTTATGTCGGCAGTGAATCCCTTAAAAGTGTTAATACTCCCCCTGAAAATCCTACTCAGAAAAT
TAACGAACAAGAAAAAGAAGTTCAAGACAATAAAAAATTCTCTATTATTCCTGAACAAAAAATT
CTTGTTAAGGTTTATGACTATGTTCACAAACAGCAAGAGGCGAGTAAAGTGAAACAATCTCAGAG
TAATTCAAATCCTCAATAATTTATTTATGGGTATAGACGAAAATCATAACTGTGGTTTCTTTTTT
GGGGATGTCCAAGGAGTTGGCATCAGTTTTTAATGAGATTATTTGCCATTTTTCTTTTGATCGTA
ATCAATGCTTTTTTTGTCACCGCAGAATTTGCGATTGTTTCTGTGCGAAAATCAAGGATTGATCA
TTTAGTCATGGAAGGGGATATTCAAGCCTCTACAGTCCAGTCTCTACAAAAAAGTCTTGATAAAC
TACTTTCTAGCACTCAATTAGGTATCACCCTTTCTAGTTTGGCTTTGGGATGGATCGGAGAAGGT
ACGATCGCACTTTCTGTGCAGTATGCTATTTCTTCTTTGCCTGTTTCGGAAAATATTTCTACTACC
CTAAGTCATTCTTTTGCCATTCCTATTTCTTTCTTTTTATTAGTTTACTTACAAATAGTATTGGG
AGAATTGTGTCCTAAATCCTTAGCTTTACTATATCCCGAACAATTAGCCCGTTTTCTTGCCCCTCC
TATTAGCGTTATCAGCACTATTTTTAAACCTTTTATTGATATTCTTAATCTTTCCACTCGATTTT
TATTAAAACTTATTGGAGTTGAATACACAGGGCAAGGGTGGTATAAACAAGTTACTCCTGAAGA
ATTGCAATTAATTATTAGTACGGAAAGAGATTCTTCTGGTTTAGAAGCCGAAGAAAGAGAATTA
TTAAGCAATGTCTTTGAATTTGGAGATGTGGAAGCATCAGAAATTATGACTCCTAGAGTTAATAT
TAAAGCCCTTAATCTCCATTTAACTTGTAGAGAATTATTTCAAGAAATAGTCAAAACAAAGCATT
CTTTTTATCCCGTCATTGGAGATTCTCTGGATGATATTCGAGGAGTTATTGATTTTAAAGATTGT
CTTTCCATTTTAAATGAAGATCCTATTAACTTAGATATACTCCTAGAAGATTTTATTAAACCTGT
ACGTTTTTTACCTGAATCAACTCTATTGAGTGAATTACTCGCTATAATGCAACAATCACGATCAA
AATTAGTCATCATCGTTGATGAATATGGCGGCACTTCTGGATTAGTCACAATGCAGGACTTAATT
AATGAAATTTTAGGGGGTGATGATAGTGAATATACTGAAGACCAATTTAGTATTACAGTAATTG
ACGAGCAGAACTTTCTCGTATCTGCCCAAATAAACTTAGAAGAATTGAATGACTTATTAGATTTT
GATTTACCCCTAACAGACGACTATCAAACCTTAGGAGGATTTCTTGTCTATAATTGGCAGAAAAT
ACCCAAGCAATACGAAGTATTTAACTATGATCATTTTCAATTCACAGTCATAGATACAGATGGTC
CTCGTATCAACAAAATAAAAATCACCTTGAAAGAACAATAATCAAATAGTAGCGGGTTGATTAAC
TTTCTTTAGATACGGAGCGAAAAAGAGAAGATCAAAAATGTAAGAGAATGAGAAAAAATAATTA
CTCATTACTCATTCCTAATTGCTAATTGAAATAACTTTTACTGGTTATTTTGAGAATCAGATCTA
GTCTCTGTGGTATCATTATCATCGGATTTAGAATTATTACGGTTAAACCCTTTATTATTACGAGA
AGAATACCGAGAAAACTTCTTCCTAAATTGTCTTGCATAAGCCTTATTACGCTCTGCTTTTTCTT
TTTTGGGATTGCGACGTTTAGACATTCATTACCTCATCTATAAAAAATACAAAATGCCCTCGAAT
ACTCACAAGTAAGAGACTAACTCACCTCAGTAACCGAAAGCATTAACAAAAATAAGTTGATAAAC
TCTTGTTTGATTTGTTCAAACAATTAAGTTAGAAGCGCTGAGAAAAGCGTTCTTTTCTTTGACCA
CCACCACGATTGAAAGAATCTCTTTTTTCACGGGGCTTAGCTTTGTTTACTCTGAGTTCCCGTTTC
ATCCATTCCGCTCCATCGAGAGCATCGATCGCACTTTCTTCTTGATTATCGTTTTCCATTTCCACA
AAGGCAAAGCCTCTCATACGACCAGTTTCACGGTCTGTGGGAATGTGAACACGGCTAACAGTTCC
ATACTCGGCAAAAACTTCCATCAGATCTTCCGATGTAACCTCGTAGGGGAGATTTCCTACATATA
TTGACATAGCTTGAATGATTCTTTTTTAATTGAACTTTGTAGAGAGCGAAGATCTGGGAAATAAG
TCTGCCAATACTTTAATAGTCGATTCTATCAATACTAAAAACAAACGTTGTCACTAAATCTTTAT
TCTCGAGTTTAATTATAGCATCTTTTTTTCCAAACACATAACCCTCTTGCACAAAAAAAATAAAA
TCACAAAATTTTTCCCATTCAACTTAGGTTGTTTTTTTGCTACCAGAAATCCCCTGTTTTCCTCGC

TACAAACTCAATTCACCCACAAAGACTACACCCATTAAACTCTCGGATAGCTTTAATGAAAATAA
AACTAAATATTTCTTAAAATTAAATATTTTCCTACAACTAACACCTGATACCTTTTTATCTTTAT
CTCGAATTCAGTTAGGTGGAATGTTAACTCAAATTAGTGTTAGGTAAGCAAACTTTTGTTCCCCC
TAAACCACAATAACCATTAGGATTTTTAGCAAGATACTGTTGATGATAACCTTCGGCAAAGTAAA
ACTCTGGGGCATCAATAATTTCTGTGGTTATTTCTGTATAACCTGCTTTACTTAATTCTTCCTGA
TATAAGGCTTTTGATTTTTCTGCTAAGGCTTTTTGTGTATCAGAAAAAGTATAAATACCTGAACG
ATACTGAGTACCTTTATCATTGCCCTGACGCATTCCCTGAGTAGGATCATGATTTTCCCAAAATA
CTTTTAACAGAGTTTCATAACTGATAATTTTAGGATCGTAAACCACTAAAACTACCTCATTATGA
CCAGTTAAGCCGCTACACACTTCTTCATAAGTAGGATTAGGAGTTAAACCAGCACTATAACCCAC
CGCAGTGATAAAAATACCTTTTTCTAACTGCCAAAATTTCCTTTCAGCCCCCCAGAAACAACCCAT
CCCAAACATCGCTTTTTCCATAGTTTCAGGATAAGGCGGTTTCATCGGATTACCATTAACATAGT
GTTTACTAGGAATGGGCATCTTGTCTTTTCTGCCGGGTAATGCTTCTTCTGGGGTGGGCAGAGTT
AATTTCTTATTTCCTAAGCCAAATAAAAACATAATAATTACTGAATTTTAATATTTCTTCATATT
CAATTTTATACCCAAGATTTTGGAATTGAGAGTTTTTGGCTAATTGGAAATAATGTTTCTAAACA
GCTAATAATTCTTGCCAATCTTTTGCCGGAGCGGACTCTGCTTGTATAATTTCTAGGATGCGATT
ATTCGTTTCAGGATAAAAAATGGATTCTATACAAACTTTTGCCACTTCTTGACGAGAGATTGAAC
CCTCAAATAAAGTATCTGCTTCACCTACAATCAAAGAGTATTGATTATCTTCATTCCTTAAACCC
CCGGGACGAATAATAGTATAAGTTAAACCACTATCGATTAAATAATTTTCAGCTTGTTTTTTCCA
AAAAAGTACTAAGCCGAATAAATTGAGGGGATGAAAAAAGCGAGACACACACAAAGAAGTAACA
AGAATAAATTTTTCGATTTGTTTTTTCTTTGCCACGTTAATTAAATTTTTAGTTCCCTCGTAATC
CACCCAATAAAAAACTGTTGGGTCTAAACTAGGGGTTGCACCGGCGGCACAAATAACAATATCGC
ATTCGTCCATTGCAGAAGCAAAGGAGGAGGGATTCAAAATATCTGCGATAATTAAATCCACACAG
GCAGGTAAAACATCTCTAGCTTTAGCTTTATCTCTTACCATTGCCATGACGGGTATTTTTCGCTT
TACTAATTCAGCAACGATTCTTCTACCTGTTTGCCCTGTTGCCCCAACAACTAATATTTTCATCGA
ATATATTATTTATATTTTTCTTGATTATATTGCTTACCTTCTCCTCTTGAGCAGAGTTAATCAAA
GCAAAATATTAGGCAATGCAAAAATAATTTAAGATGGGTGAAGGAAAAATTAAGATTAAGATTA
ACAAAAATCTCATGTCAGCGATTGTTATTTTTGATATTGATGGTGTGATTCGTGACGTTACTAAT
TCTTATCGTCGTGCTTTAGCGGATACGGTAGAACATTTTACGAATAATCATTATCGCCCTTCAATT
GGAGGATATTGATAGTTTGAAGGCGGAGGGAATTTGGAATAATGACTGGTTAGGTTCACAAGAG
TTGATTTATCGTTATTTTGAGCAGATGGGGAAATCCAGAGATGAGATTAACCTTAATTATGAGG
AAATTGTGGATTTTTTTCAAAGACGTTATCGAGGGCAAAATTTAGAACAACCAACAAGCTGGGAT
GGTTATATTTCTTCTGAGCCTCTTTTGGTGAGTAGGGAGTATTTCGAGATATTGGATAAAAATAA
TTTATATTGGGGCTTTTTTAGCGGTGCAACAAATGGTTCTGCCCAATATATCTTACAACGTCGCT
TAGGTTTAGAGAATCCCGTGTTAGTGGCAATGGATGACGCTCCTAGTAAACCTGATCCTACTGGT
TTGTTTTTAGCCGTCAATTTATTAGAAGAAAAACTTTCTCTTGATAATTCTTTACCCGTAATTTA
TTTAGGGGATACTGTAGCAGATATTATTACTGTTATGAAAGCAAAAGAAATAAAACCTGAAAGA
GAATGGATTGCAGTGGGAGTTTTGCCTCCCCATGTTAGTAATGATAATGAGAAAGGAGATAAGT
ATCGTCAACAGTTAATCGATAGCGGTGCATCTTTTGTAGTTAATCAAATTACTGATTTTTATGAT
TTTTAAATTTCATATTCAAGAATAACGCCAATTTTGGAAGTTATCTTTTGATTTACTCATCCATA
ATAGTTTTTTGGCTCTAGTTATGGCTACATATAGTAATCTTAATTCTTCGGCTTGTTTTTCTTGG
ATGGCTTTTTCATGGGCTTGATGAAGGGATAAGGGTTCTATTTTTTTACCTAATCTTTGTTGATG
GATTTCTTGTCTTAGTTGTGTGCGAATAATATCATTTAAGTCACATTTGCCCAAAAATTTAAGAC
TTCCGTTAATTTTTTTTTCTGAGAAAATATTTTCTTTGTCATAAAACGGTAAAAAGACATAATCC

CATTCTAAACCTTTAGATTTGTGCATGGTCATAATGGTAATTTGACCTTTTTTTGTATAAATATC
TTCATTCTCTGTTTCTACTCCTTCAAACCTTTCTGTATTATTAATATTAGTTAATGTTTCTATCAT
TGCTTTTAAGGACATTTTTCCTTGATTTTCTTTTTGGATGCGATCGCACAATTTTTGAGCAGTAG
CAAGGGCTAATTGATCATAATTTAAAACAGAGCAAATAAAAGGAATTAGTTGATAGGAAGATAA
TTCAATACTAGCTTGGAGAAATTTGAGGCAAAATTTTCGAGCTTTTTCCTGTTCAGGAGTTAGGG
CATCATCTAAAGGTGTTGGATATAAAAATTTTTCAGGATAGATACTTAATTGATCAAAATCTTGG
TTACTAATAATTTCCTGTTGTGCCAAAATTTTCAAAATATTATAGACAAATTTGGAAGAATGAGG
AAAAGCAATAAACTGTAAAACTCCTAGTATATCTTTCACTATATCACTGTCAGAGATGCGATCGC
CTATTAGTTTTAAGGGAATATCATAGGCAGATAATCTTTTTTGTAATTCTTGAAAAATAAAACTT
CCCTGCTTATTAGCTCTGACTAAAATGGCACAATTAGTATTAATTTCTTGTTCTTTTTTGTCAAA
TAAATGAATGATTCTTTCACTCATTAAATCTAAAACTTGCTCAAAGGTTTCAGGTTCATAAAATT
CTACCCCTTTCCCCTCTGCCAAAGGATTAGAGTCTGGTTGAGAATCATTTTCTCCAACAATTTTTA
TCTCCTCTTTTTCAATTAAATTTGCTTCTACCTTTTCCGCCATCCAAGCCAAAGTTTCATTGGCAT
AGTTAATAATATTTTCATTACTTCTTCCTGCTTGTTCCATTAAAAATAATCGATTTTTTTTGCGA
CAATCCTCACAAAATTGATGAAAATAAAAGGGGTCTGAAGCGGTATAAGTGGAATTAATTGCTT
GATTGGGGTCCCCCACCCTCACTAAATTAGGTTCGCTACAATCCTTATCTTGGGCTAAGATACTG
ATTAAATCACCCTGCAACGGTGTAGAGTCTTGGGCTTCATCTTCAAATACTCCTGCAATTTCGTG
TTGTAGTGTTTGTCTTGCATCTTCATTATTCAGAACGTTTAATGCCCCAATAATTAAGTCATCAT
AGTCTATTACCTGATATTTATGAATTAGTTTTTGATATTCTTGATATAGTTCAGAGGCTACCGTG
CCTAAAGGATAAATACCGGAATTATCTAAATTTGCTAGATTGTCAAGGTTATCAATATTCCAACC
AGAACTTTTAAGGGTACTAATGACAGTATGAGTAAATTTGGGTAAAACTTCCGTACGCAGTACCG
ATTCTCTACGCAAAACTTCGCTTTCTTCATAATTAAATGACTTTTCATTTTCCCCCCTTAACAAGG
CATCAAAAGCAGGAGAATGAAGAGACATCCAATTGTCCACCGTTTCTAGGATTAAGGGATGATTG
CTAGACACATCGACTAAATTACTGGTTTCTAAATTTAATCCCGACAAAAAAGGATAACGATTGAC
AATACTTGACGCTAAAGCATGAATCGTTTGTACCATAAAACCCACAGGAGGCATCTCAAATTCTT
CAGTTAAAATCGTCCTCATTTTGTCTTTAATACTAGCGGCGGCGGAACGAGTATAAGTTACAACT
AATAGATAGCGATTCGGATTTAGTTTTTCTTGACAAACCAATATTGCACCAGCAACGGCTAATGA
AAAAGATTTTCCTGAACCCGGCACCGCAGAGACTGCCATTTTACCACCCTGCCATTGACTTAATTC
TATTTGACCCGGACGCAGTTTTTGTCTGAATTGGGCGATTGTTTCTTCTATTCTCCTATGGTTGT
TCATGCTTATCATTAGGGATTGCTAAAATTATATCAACAATCTTTCAATTAGTAATTAACTTATA
ACTCCTGATTTCTACTTATTATTCATTACTCATTAATTTCACTAAACACCAGTCTTTCATTTCTCG
ATAAATTCGGTTATGATAGAGGGTGTGTCAATTTTTATTGTAAATAATTAAACAAAAGTTGAGT
AAGACATCGCTTAACTATAAAAGGCTTTGGTATCCATTCCAAAGAAGCCTACATCATAATTAGGT
AAAATGGACAAAGTCCCGCCTCTGATGTGATGATAGGGTTATGTCACTGTAAATAAATATATAAG
TATTAATTTAAATGGCTAAAAAATCAATGATTGCACGGGAAGTAAAACGTGCTAAATTAGTGGC
TAAGTATGCCCATAAAAGAGCAGAATTAAAAGAACAAATTCGCACCGCAGAAGATCCTGCAGAAA
GATTCGAGTTACAACGCCAATTACAAAGATTACCTCTCAACAGTTCTCCTTATCGTCAACGTAAT
CGTTGTTGGGTAACTGGAAGACCTAGAGGTTATTACCGTGATTTTGGTTTATCTCGTAATGTATT
GCGTGAATGGGCGCATCAAGGTTTATTACCCGGTGTGGTTAAGTCTAGTTGGTAGGTTGAGCCAA
CTTTAAATAAGTGGGATGAGTAGGGTGTGTTAAGGATTATATAACGCACCTTATTATCTATTCTT
TTTAATTCTTATTTTCGGTTATAGTCTGATTTTCCTCCCGTTTTGCTGACTAACTTGATGTCAGT
GATGGTAATAGATTTATCTATGGCTTTGAGCATATCATAAAGGGTCAAAGCGGCGATCGATACAG
CCGTTAAAGCCTCCATTTCTACCCCTGTTTCTGCGTTGGTTTTGGTAGATGCAGATATTTCATAAC

AATAATTGTCTTGATTTAGGTGAATATCAATGGCAATTTTCTGTAACGGTAGGGGATGGCATAGA
GGAATTAGAGAAGATGTTTGTTTTGCTCCCATAATACCGGCTATCTTGGCGGTAGCGATCAGATC
TCCCTTGGGGTTTTTCTGTTTTAAAATAGTTTCTAAAGCAGTGGGATTTAATAACACTTTTCCTG
ATGCGATCGCAGTTCGAGAGGTGACGGATTTAAGAGAAACATCCACCATTTGCACCTCTCCTTGG
GAATTGAGATGAGATATTTTTTGTTCTGTCATTCTTGATTATGAGTATATAAACAGTCTGAACTG
TGATTAGGGATTAAGTAAAACTTGCCCTTCAATTAATTTTTCTGCCGCCCTTAATAACTCTTCTT
CAAGATAAGGCTTGGTAAAATAGGCTTTTGCTCCCAAATCAGCTGCAATTTTACGATGTTTTTCC
GCTCCCCTTGATGTTACCATTGCCACAGGTAACTTAGAAAGGTTCTCATCCTGTTGCATTTTCGCC
AATAGTTCTAAACCATTCATTCTCGGCATTTCAATATCACACAGTAACAAGTCACAAGGCAAACC
ATTAGATATTTGTTCCCATGCGTCTTGTCCATCTCTAGCTTGTTCTACCTGATAACCTGCTTTTTT
AAAGGTCATAGATAACATTTCTCGTACTACTACCGAATCATCAATAATTAAAACCATACGGGCAG
ATTTACCTTTTGTTTTTCCTTGAGTAACGAACCCAGAAGATCTAATATTAGGATTTGTGGCTTTA
TTAATCTTAACTTTGGGAACTTTACGACTACCTGCAAGTAAAACTTCACCCTTAATCATTCTTTC
CGCAGAATCTAATAAATCTTTTTCCACATAAGGTTTAATTAAATAAGCCGAAGCTCCTAATTCTG
CCGCAATACGTTGATGTTTTTCCGCCCCCCTAGAAGACAAAATGGCAACGGGGATGTTTTCTAAA
GACTCATCTTCTTGCATATGTTGAAGAAGTTCTAAACCATTCATCCTCGGCATTTCAATATCGCA
GAAAACAAGATCACAAGGAAGACCCGATCGCAATTTTTGCCATGCTTCCTGTCCATCTCTTGCCT
GTTCTACCCGATACCCTGATTTACTGAAACTGATAGATAACATCTCTCTAACCGTAATCGAGTCA
TCCACAATTAAGACTAATGGTTGATTGTTAACGGGAATATCATAAATGGTTTCATGGTTACTAAC
CACATCAGGAAGATTTTGAGGTAAGTCAAGGCTTAAATTACCGTTGGCAATTTCAATTAATTCGA
TGACATCTCCAATAGGCATGACAATACCATCACTGCGCACCGTAGCCCCAGCAATTCCCTTCGGTT
TTGGTAAAGGTCCTGATATTTGCTTGATAACAATTTCTTCCTGCCCTAAAACCTGATCTACTTGA
ATTGCCAAAAGGTTGTTACCCCCCCTGAGAATGATAATGGGTATAGTTTCATCTTCATTATTGCT
TGTGTAAATAATGCTTCGGGTAATCTGACGGTTATATCTCAAGAGAGTACTTAAGGGACGGAAAG
GTAATAAATTATTTTTCCAGAGAATACATTTTTGACCTTGAGCATTAATTTTTATTTCACTAGAT
GTAAAATCTTTCGTATCTTCGATCGCATCCATGGGGAAAGCAATACGAGTATTATCATTAAGGCA
ACAAAGCGCTTTACCAACAGTAACCGTTAAAGGCAAACGAATGGTAAAAGTTGTACCCTGACCTA
CAACAGAATCAATACTGACAATCCCTCTAATCTCATTTAATTTACTGCGAACAATATCTAAACCA
ACTCCCCTTCCTGCGTGGCTGTCTGCTTTTTCCTTCGTGGTAAAACCGGCATGGAAAAGAAAGTC
ATAGACTTCTTGAGGCTTTAAATTTTTTGCCTGTGCAGGAGTGATTAATTTATTTTGAATTGCTT
TTTTCTTAATTTTTTGAGGATCAATTCCTCCCCCATCATCCGAAACAGAAATAACTGTTTGCTGA
CCCTGTAAAAATGCCCTAACGGTGATTGTACCGACCGGCGGTTTACCCATTTCTTCCCTTTCTTCA
GGTAATTCGATACCATGAGTGACCGAATTTTTCGTAATTTGTAAAAGAGGATCCCAAATATGTTC
AAGGATCATTTTATCGATTAAAACATCTCTACCCTCTAACTTCAGTTGTACCTGTTTGTTATAAC
CTTCTGCTATTTTACGAATTGGTAAAGGAAGGCGATCGGCGTTTTGGGCAAAAGGAACCATGCGA
GACTTATTAATTCCTTCTTGCAGTTGAGTTGTAACTTCTCTTAGGTTTCTTCCTAACTGATCTGT
TTCATCCACTAGGAATTGAATATCAGAGGCTGATTCTCTTACCTTAACAATTAACTCTATAATTT
CCTGAGATAGCAAATGAAAACCCGTGAAGCGATCGAGTTCTAGCTCGTCTAATTCAGGAGTTTCT
AACTTAGAAGTATTTGTGTTACTATTGCTACTAATATAGGTCGATCGTCCTTCAGTCCTTGCTAA
CTGCGCTTTTACCGTTTCTTGATTCTTACGACGACTGTTCATTAACGCTCCTTCTAACAAACTACG
CTCGTAAAGATCTTGCATTCTTGCACTGACATCACTTAAATTTTGTACCTGATACAGTAAATTGT
CGAGGAACTGTCTTAACTTTTCTTGGTCTTCTTCTAAGCGATTTCGCCTTACAACCATCTCCCCAA
TCAAGTTATTGAGATTATCTAACTGTTTTACTGGCACACGCATACTTTGTTCAAAAACTTTCGTC

TGAGAACTAGATTTAGTATGGCCTTGACGGGAAGTGCGACTTTTAGGAGATTTTCCCCTGTCTTT
CTCTGCTTGTGCTAATAATTGATCTAAATCATTAAACGGAGACGGTTGAGGTTGCCTTGTTTGTA
CTCCCGCATTAAAACCATTATCAACAGTTCCAAACTCCGCTTCAGGCAAATCTTCAATAATATTA
CTTAGTTGTTGCCAATCAACTTCTTCTAAGTGACGGGGTACAGAATTAACTAAACGTTCTAATTC
ACTCTGTTGATCATAGTAGTTGATTTTGTTGTTGTCTAAAATTAATTCTAAATCCTGCCAAGAAT
CACTTTCTAACCAAGATAAATTAGATGACAAAACATTGTTTAATTCAGAAAACTCGTCATAAAAA
ACGATCTTGACTTCCGAAGATTTTACGGGCTGTAGAATTTGAAATGTATTTGCACTGCCCATGCT
AGGGAAATCATCTTCTATTTCTAAATCATCTAAACTGTTAATAATATTTTCTAATTCTTCTTCAG
GGTCATCAATAATAACAGTACTTCTTTCTGTTTCTGTGAATAATTGATCTAAATCATCTATATCA
AGATTATCCTGACTAATGGTCACTTTATAATCTTCTTGCTCTACTTCTGAAGAATTATCCTCTTC
TACTAAATCTGATAAATCATCGACAAACGAATCATATTCAGAGTCATCAGTATCCTCTATGGGGA
AAATAGAATCTTCTTCTTCTAGTCCTAACGACTCCCATTCTAAAGAATCTTGGGCTTCTAGTTCT
TCTGTCTCTTCTAGTGAAGACCATTGGTCTATACTAATATTCTCATCTTCACCTTCAAATAAACT
GGTAAAAGCATCAACTTCTTCAGAAGAATCATGATTGGTGGTAAAAGATGTGTCATGAAGAAAA
ACAGAACTAATTTGAGGATTATCATCTGCAAAGGAATCATCTTCAAACCAGTCTTCCAGTAAATT
ATCGTCTTCTCCTAAATCTCCAATTCCTTCAAAAATATCTAAATCTTCTTCATTGTCATCATGTTC
TGAAGGGTGACTATTTTTTTCTTCTGAACTATGGGAAACCTCAAATAAGGTTTCTTCCCACAAAT
CTTTGTCTAATGTGCTTTTAGTCACTGCCTGAGATGGTGTAGCCGTCGCCTGTGCTTCTATTTCTT
CCATGGAAGGGATTTCATCTGCAAAAACTGTTTCTACATCCATCAATCTTGCTTTTGCTTGGGGT
GCTAAATCTTGTAATTCATCACTCACACAAATAGAATTAATTTCTTGTTGAAGAAGAAGCTCACC
AGCATTTTTAATTTCTTTAATAATTAATTTGGCGGTAGAGCTGTACTCATTATCTGGTCGAGAGA
TGGCTATTTTTGCCGTTTCCATGATTTCCACCCAGCCGAACAAATTTTGTTTTTTCCCCTGAGTTG
ATAGGTAGTCGCAATGATTTTTTAAAATTTCTCGATTACGACTATTATCCCCCTGTTTTAATATT
TTTAACATTTCTCCTAAGCGTAGGAGAGTTTGCTTGTATAATTTTTGGGCTTGGTGGGCTTGTTC
ACTATTAATCATAGACCTGCTCTAATTTTCAACTCTCAAACTCAAGATTTTACATTAGTACTAAA
GGCAATGGACCCATGACAAAATATTTACCACTGAAACTGTAATGTTATAACCTTGTCATTAAGTT
TTTTTTAGTATAACTCAATGATAACTTTTTCTTTCTATTCATCTTCAGTTTTTGACATAAAACAA
TCGTGAACGCAGTTTTAATTGTTTCAATTAGTTAATTTTCAAGGCATTAACCGGTACATCATCCC
AAGATTCTACGGTTATTAAGCTCACTTCCCACCCCTGTTGTTTTTGTTGAGGCGTTAATTTTGCT
AACCAATCGGCATGACAATCTTGCGCGGCTTCTTTATTTTTTGTAGCGATACAGGCTTGAAGAAT
TCCACATCCATCTTTTTGTTGATTTACCCAAATAGTCATGATCTTTAATACGTAATTAATTACTA
ACAATAATAATATAGTTTACCTCGATATTTTGTAACTCCAAGACCTCTTCAGATTAAAATTAAAA
TAAATTTCTAGATCAAAGTGATCAATTTTTTCACTACTGCTTCTTGCCTTTCACCTTTTGCTTGA
GAAAGCAAGAACTCTCATGACTATGTATTAGAAGTTACATATATAAAATTTAGATTTGAGTTAT
CCTTATCTATAGAACCTCTTTTTAATCACTCTTTATCTTTAGTGAAGGAACAGTTATGATTTCAT
CGACTATAGCCAATAATTTACCTCAAGACCAAGAAATTTGGAACAGTTTACAAAAAGCCATTGCT
AAAAGTTCTGGTTTTCAAAGATGGCAAAAAGAAAACAAGTTTGATGGCACTGTGGAAGAACAAG
TCACTCAGTATTTACGTTCTACGTTAGAAACATTAGCTTATTAACATTTACTGCATCTAAAAGGA
ATATTACAAGAATGTTGGTGAGCAAGGGCTTTTGTTGGCAAATATCACTTATTTTTAATTTCTAA
TGGGGGGAGTCCTATCCCCTCCCAAGTTTTCCAGAATGACTGAGGTGCAATAAAATACCAAGCTA
CGCCGATGGTAGTTATCAGGGTAGCAACAGTTAGAAATCCTGATTTGATAATACTGAAAATGTTA
TCTCCTGTGTTGCGTTGTAGATTCCTTTGGGATTGCACTTTTCTTGAGTTTAAGTTTTTTGCATT
GTTGGTCTTGCCCAATGAAGAACTAATTCCCTTTAATTTAGATGTTTTTAAGGAATTATGGGAAG

ATACCTGATTGTTTTCCTGAAAGGATAAGTTTTCCATAACATCTTGAACTGCTTGAGGGGTTTCA
TCCGCTCGAATCCACTGTTTCAACTGGGATGAACTGATGATTGCTAAATTAGTTTGTCCAATTTG
ATTTAACTCTCGCTTCAAATTACCGTCATTAGTGACAAAAATAACTATTTGCTCAGAATGTCTTA
AAGCTAAATCATATACTGATTCTGCGATCGCAATTGATAAACGAGCTTTTTTACTAATACTTTCT
CCATCTTTGGCAGTTAAAGCAGGATGAGATGACATCGTTGAGGTTATTTGCCAACCGCTATCAGG
GAAAAAACGACTAAATTCCCTTGCGATTTTCTCATCTTTGGGAGTGATTGCCCTTTGAGTTAAAA
AATTTAATTCCTGTAAAACAACTTCAGGTAAATAGCATTCACCTTTGTCACTAAATTTAGTCCAA
TCACGGATACATCCTGATAATATTGTGGTTAGTTCAAAAACAAGTATCATAGATAAATATATATT
TAATAGCTTTATTTTTTATTAATAAATCCTATACACTAACTAATGTCTATTTCTACTATACCCAC
TCAGGATAATTTACAAGTCGTTAATCCTTACGAGCGTACAGCTTCTGTTAGTCGTACGACAAAGG
AAACTGATATTAATGTCACCGTAAATCTTGATGGTACGGGAAAATGTAATGTTAATACGGGTATT
CCTTTTCTTGATCATATGCTACACCAACTATGTTCCCATGGGCTTCTTGATTTAGATATTCAAGCC
ACTGGAGACATTGAAATTGATGATCATCACACTAATGAAGATGTTGGTATTACTTTAGGACAAGC
CATCGCCCTTGCTGTGGGAAATCGTAAAGGAATCAATCGTTTTGGTCATTTTATTGCCCCTTTGG
ATGAATCTTTAATTCAAGTGGCTCTTGATTTTTCTGGTCGTCCTCACTTAAGCTACGGGTTAGAT
ATTCCTACTCAAAGAGTTGGGAATTACGATACCCAATTAGTAAGAGAATTTTTCGTTGCATTAGT
CAACCATAGTCAAATAACTCTACACATTCGCCAACTAGATGGTATTAATTCTCATCATATAATTG
AAGCAACCTTTAAGGCTTTTGCCCGTGCCATGCGTATGGCTTTAGAAATTGATCCCCGTCGCATCC
ATGAAATACCTAGTTCTAAAGGAGTTTTATAACAATTAACAATTTAGACAAAAAATTTAAACTG
TGTATTTCAGATTAGATGTCGCTGGTTTTATTGTAATTAGTAATTAGACAGAAGGTTTTAATCCC
TTCTTAAAGTAATGAACAATTGATTCATTCGCTATTAACTATTCACCACTAACTATTAACTATTC
ACTATTATGGTTTCTTCTCAATCAAAACATTTATTAATTGCTTCTAGTGAGCCTTATACTGGAAA
AACTGCCACAATTTTAGGTATCGCCCATCAGCTACAAAAGAAAGGAGTAAAACTAGGTTATGGTA
AACCCATTGGCACTTGTTTTAATCCTGATGATGGAATTCAAGATGAGCAAGATTTACAATTCATT
AGTGAAATTCTCAGTTTATCCCCCGAAAGAGTCAAAGCACCTTTATTATTTTTGGATGAGAAAAC
TATTACAGATTATCTAAAGACAAGTAATGACTATCAAGATACATTAAAGGAATATTGTGATTCT
ATTGAGGGAGATTTAATTTTATTAGAGGGTGCAGGAAATTTAACTCAGGGTAAGCTATTTAATC
TTTCTGTACCAGAAATAGTGGAAAAAATTTCAGCAGAAGTTTTATTAGTGGTCAAATACGATGG
ATTATTAATAGTTGATCAAATTCTTCATGCAAAAGACGTTTTGGGCGATCGCCTCTTAGGTGTTT
TAATTAATAGTATCCCCAATGATGCCTTAGATCATATTGAAAGTGATCTGAAACCCTTTTTTCAA
AGTAGAGGGATTGATATATTGGGAATGTTACCCACTGACAGACTATTACAAAGCGTTAGCGTTAG
AGAATTAGTCTCCCAACTAAATGCAAGGGTATTGTGTCGAGAAAATCGCCTTGATTTAATGGTGG
AAAGTTTAACGGTAGGGGCGATGAATGTTAACTCTGCTTTAGAGTATTTCCGTAAAGGACAAAAT
ATGGCTGTAGTTACAGGAAGCGATCGCACTGACTTACAATTAGCCGCCCTAGAAAGCTCTACAAA
CTGCTTGATTTTGACAGGACACACCCCACCTCAACAACTCATTTTAGCAAGGGCTGAAGACCTAG
AAATTCCCATTCTGATGGTAGATTGCGATACCCTTACCACAGTGGAAATCATTGATAGAGCTTTT
GGCAAAGTCAGACTTCATGAGACCATTAAAGTAGAATGTATGCAGAATCTTATTGAAGAACATTT
CAATGTAGATTTATTGTTAAAAAAACTAGGCTAGTTGGCACTTTAAAACAGGCGTTTTGCCTGTA
TTTATTGGGTGTCTATTTTTAGGTTGATGAACTATTAGACTCGAAATATAACTTTGATTTTAACG
GATCAGGAATCATGGTTTTGTCTCCTTCCTGCCAATCAACAGGACAAACTTCGTTATCATGAGAG
TGAACGTGTTGAATGGCTTTTAGATTTCTCAACGTCTCATCTACACTGCGTCCGCAAGAAAGATT
ATTGATGGTAATTTGTTGTATGTTACCCTCCGTATCAATAATAAACAATCCCCTAGCGGCAACCC
CTGCACTTTCGTCTAAAACTTCATAGGCTTTACTAATTTCTTTCGACAAATCAGAAATTAATGGA

TAGTTAATATCTCCTATTCCACCCTGTTTTCTTTCTGTTTGAATCCACGCTAAATGAGCGAATTCA
CTATCAACAGAAACCCCTAAAATTTCTGTATTTAAACTGCTAAATTCTTCGTAACGTTCACTAAA
AGCAATAATTTCTGTGGGACAAACAAAGGTAAAATCAAGGGGATAAAAAAATAAAACCACATAC
TTTCCGAGATAATCAGATAGTTTAACTACTTTAAATTCTTGATCAATAACGGCAGTTGCGGTAAA
TTCTGGTGCTTTTTGTCCTATTTTTAACATCTTTCTATAGGTATTTTTATACATTAATAGTTTTT
AGGAAAATACAGGCAAGATGCCTGTTTCACGATGAAAAAAATCGTAAGTTAATTTGGATTTAAG
TTATTTTGTGATTATGGGAGAAAAGCTATTGGTACAGGAATTTTCAATTTTTATTTTTAATTCTT
AATTCTTTCCATGACTAATTGCTAATATTACTGATAGCTAATTCTTTATCTCCTTCATGGACTGT
TTTAACCCATTTTAAACGTTTTTGACGAATAGACATTCTTCCAGTGGTAACAGGCATTACAATAA
ACCAATGAGTCATATATATCATGCCTAGGATACTCTTTTGTATCAGTTGTAAGTAATCAGCAAAT
TTGTATTCTTTCTCTGGGTTAGTACGTTTTAAGCCGATAATCATACCCCAAAAGGCAAGAAAGAG
GGTTAAACTACTCAAAGGTGCAATTAAGGGATAAGTATGATGAGTTGTCACCATAATTAAATCG
GGAATGGCGGCGGTAGGAAAGATATACTGCACCATGAAAAAATATAGCAGATCGAATTTTTTTT
GCCAGTTGAGGCGGTTACTGATAATAAAGCGCCAATAGTCAAGATAGCGTTGATAGCCCCCTTCT
GCCCAACGACTTCTTTGATGCCAAAGTGCGATCGCACCTTTAACTCCTTCTTCTTCTACGGCAGGA
TGTTCTAAAATACCTATATCCCAATGATCAAGGTGTAAACGAATAGTTAAGTCTAAATCATCGGT
GATGGTTTCCTCGTTCCAACCGCCACAACTTTCTAAGGCTAATCGACGCACAAACTGCCCATTTCC
TCTTAATTCTCCCATACCACCACAAGCAATTCTTTGTTGCTGAAAGTAGCTATCTAAAGCCATTT
CCACCGATTGCCCTTTTGTCCAAAAATTATCGTTACTATTAGAAATAGATTTTCTGACTTGAATC
GCCCCTATTCTTTCCTCGGTAAATAAAGGTACAGTTTTTAAGAGAATATCACGAGGAATAATAGC
ATCAGCGTCAAATACTCCCACTATTTCCCCTTTGGTTTGTAAAAATGCCTGATTTAAAGCTCCAG
ACTTGCCTCCTTTGGCACTAGCATCACGGTGCAAAACTTTTAATTGGGGATATTCTTGCTTTAAT
TCATCTAAAATAATACCAGTGCGATCGCTACTATTGTCATTAATTACCCAAAAATCGTACAAATG
GTGGGGATAGTCCACATTTGCCAACATTTTCACTAACTTAGCCACCACTGCTTCTTCATTCTTTGC
CGATACCATTAAAGAAACATAGGGTAATTTTTCCAACTCAGGGGTTTTTATCGTAGGAGAAGCCA
CAAACATTAACCTTAAAGCCTGAATGGTGACAAAAATCCCTAAAATAGCGATGATCCAGACACTC
CAAGAAACAAGATGGAGAGTTACTGTCACAATCCAGATAACACCCAAAGCAAAAGCGGCTTTCTT
TCGCCTTCCCGATAATCCCTGAAAAAAGTCACTTCTAAATTCCTCTTCTTCCATTTCTGGGTCAGA
AAACTCATCTATAAAGGAAGTGATGGGATCTAATTCTTGATTTTCGTAGTTATCTTGGTGATTAT
TTGGCATCATTGTTTGAGTAATGAGTAATGAGTAATGAGTAATGAGTAGGAAGTGGGAAAATTA
AATAAAATTAATTATTAACCTGACACCCGAAACCTGAAACCTCTATTCTTAATTCTTTAAATGTT
TTTCATTCTGGCACTAGAGCGCGATCGCACGGAGGGGGTTTTCAGGGGTTGATTACCGCCTTCTG
GAGTCTTCTGTTGTTTTGCTTTTGCCGCACTACGTCTTAAAGCCTGTAAACGCTCTTCATATTTCT
TGCGTTGACTACGGTTAGGAGTTTGCTCTATTAAAGTTTTAAAAGCACTTCCTAGACTTTGATAG
GCATTTTTTAAGGTGTATCCGAAACGAGTGGCAATTTGAATCGCTTTTTCATCGGCTTCAATAGC
TTCTTTTAGGTTTTTCTCCCCATTATTTTTCTGCCAGAGACGATAACCAGAAATACCACACAAAG
CCAAAGCTAAAAGCAACAATAAACCATCTTGTACCCATAACTCCCCTACAGCACCGCCTAAACCA
ATAGCCAACGCCGCCATTTCCCATCCTTCTTTGGGAATCGTATCATTTTGAATTCGGGCTACTTCA
TGCCAGAATAAGAGATTACGTTGATCTATCGCTAAGTTTTCCCATTTTGCCAAATCAATTTGAAT
TTCTACCTCATCCTTCCCTAATTCTTCTGTGCGAACTAAAGGAGGATTGACTTCTGTAGAGGCTTC
AATCATCACCCAACTTTGTAACTCAGGGGGTAGTAACGTTTTCAACCTTCTCAGTTCATTCATTT
CAGCCCTTGCGGAGGAAGTTGTATAAGAAGTCATATAAATATTTACTCTCTAAAATATATAATTT
TCGATAAACTATGATTTTACTGACTCAGGGTCAATTTTTAATTATTTCCTGAGATAAAATCAATT

TTTATTCTATGCTAGTCCTAATAAAGTTTAATTGCCAATTTTAACATGGAACGTGGTTTACTCTG
GTTGCCGTTACTCGTGCTATTCTTTTGGTTAGCGTGGAGTGGTAAAAAAGAATATGATAAAGTTC
AGGCTTATCAAGCATGGGCAGAACAATTTGATAAGTCCAAATATGACATTTATTCAGTGTTGGGG
AAAAAAGGTGATTTAATCACTATTGGTATTCCCACTAACCAAGGAATAAAAGAGCAAAAAACTT
TTTCTCTACAGAATTTATCCCAATTGAATTTATTAATTAAAAATCAAGTCGTTGATGTAGATAAT
CTCCCAGAAAAAGGAGAAGCAACTCTCCAGTTTAGTTTAAAGGAAAATCAAACTATTGATGTACC
CTTTACCGATATTAATATTGCCAGTCAGTGGTTTAAGTATTTAAAAAAATCAATTATTTAAGGGT
GTTGGTGGGGAATGTTTTTGAGGAGATGAGGAAAAGTAGATAAGAAACATAAATAAAAATAATA
ATCTATTACTATGGATAAAAAAATCATCGTTATCGGTGCAGGATGGGCAGGTTTAGGGGCAACTT
ATCACCTTGTTAAACAAGGTTTTGATGTTACTTTACTAGAAGCATCCTCCCATGCAGGAGGATTA
GTAGCTGGTTGGAAAACCGCACAGGGTAAGAGTGTAGAAGCTGGAATACACGGCTTTTGGTATCC
CTACCGTAATATTTTCCATCTGGTGAAAGAACTAAATTTAGAGCCTTTTACCGCCTTTACTCAAT
CTAATCAATATTCTCCCTACGGCTTAGAAACCACTTCCCCTATTTTTCAAAATCAACCCTATTTGC
CCTCCCCTTTAGGTACTTTTGTCTATCCCCAATTCCATCGTTTACCATTGTGCGATCGCATCTCTG
CGTTACCTTTGATGTATGCAGTAATAGATTTTGATAATTCAGAAGAAGCATGGAGAAGATACGA
TAAAATCACCGCAAGGGAGTTATTTAGACAATACGGAGTCTCATCCCGTTTATATCATGAATCCT
TTGAACCAATGTTGTTGGTAGGTTTGTTTGCACCCGGAGAGCAGTGTAGCGCGGCGGCGGCGTTG
GGGATGTTGTATTATTTTATCTTGGCACATCAGCCCGATTTTGATGTGGTTTGGTGTCGTGGCAC
AGTGGGAGAGAAAATTTTTCAACCTTGGATAGAGGAAATTGAGAAATTAGGAGGAAAAGTCGTC
ACGAATCAGAGAGTAACCGATATTCTAGTTGACGAGCGGGGGTTAGCCACTGGGGTAGTTTGTTC
TGACAACGTCTATGAAGCTGATAGTATTATTTCTGGGGTAAGTGTTTCGGGTATCAAAAAAATCG
TTAGCAGTAGTAAGTCTCTTAACCGTTATCAGCAGTTTAGAAACCTTAATAACTTAGGTGGCATT
GATGTTTTAGCCGTCAGACTGTGGTTTGATAAAAAAGTGAATATTCCTCAACCTTCTAACGCTTG
TTTTGGGTTCGATCGCACTACGGGTTGGACATTCTTCGATCTTAATGCTCTTCATGATCAATATC
AAGGGGAAAAAGGAAGCGTTGTAGAAGTAGATTTTTACCACGCCAATCAGTTTTTATCTATGGCA
GATGATGCCATTGTCGAAAGAGTACATCAATATCTAAAAACTTGTTTATCAGAATTTGCGATGGC
GGAAATTATCGATAGTAGCGTCATTAGGATACCTCAAGGAGTGACTCATTTTGCTCCCGGTAGCT
ATCAACACCTTTTAAAAGTGCAAACAGCTATTCCCAATTTATTTATGAGTGGTGATTGGATTGTC
ACTAATCATGGTTCTTGGTCACAGGAAAAAGCCTATGTAACAGGTTTAGAAGCGGCAAATAAAGT
AATAGAATATTTCCATCAGGGTAAACAAGCTGATATTATCCCTGTTCAAAAAGACGAACCTCATA
TAGAAATAGCTCGTATAATAAATAAAAATCTACGCAATGTGACTGAAAATATTTTTTCTAACTTC
TCCCTTTAATTTTTAACTTAGTAAATTTTTACTAATATTGCATAATCAACTTATTCTAAATAGGT
ATGAACTATGTTAAATTTGAATGGGTTACAGACTTATGCTATCAGACTATCGAAAAGGATTATTT
GGTTGACCAATACGAAAAGTTAATCGAAAAACGTTATCTGAGAGGGAACGTTGATTTCCTAAAAT
ATCCAATCCATAATTATATTCATAATTAGTCCAAGAAAAAATTAATTGAGGCGTGTTATAGTCTA
GGTCTAAATTCAAATTTAGGTTCATACCATAACGAGTACGACTAGAATTTTGATCATAGGGAGCA
AAATAGCCGCCTATATATAAATAATTTGTAACGTTAATTCCTATATTAACTAGCATTTTAGAACG
GGAATTATTTTCTAAATTGTCTAAACCCCAAAAATTAGTTATTCCTATCATCAATTTTGGAACAT
TTAATCTTGTCCCCAATGATATTTCACTTAACGGGGTTACGCTTATCGTATCGCCAATATAAGTG
GTGTTTTGCAAAGCATATTGAAAAGCTGTATAGAAAGTTAGGGTTGAATTTTCTTCTAAAGTTAC
TTGATAGTTTATATTTCCATTGATTTGACTATAATAATCTTGATCGGAGTTAAGGTAGCCAACTC
CTCCAATTCGATAGTTCCAATCATTGTAATTTTTTGTGTAATCTAACCCCACGTAAGGCTTTAAA
TCATTTGCAAAAATAGCACCGAAATAGTAACGAAAAATAGAGCTATTATTTGTAATATTGCCTGT

AAAACTTAAATGAGGATAGTAGTTAATTTTTTCTGAATAGATACTTTTGACGGCAACCGTTGCTT
CCTGATTAAAAAAGGTAAAATAAACTTGTAAATATGGGGATGTAAGGGTGTTATTAGAATAAAA
ATCATCATTCACAACAGAACTAAAATCAATATTATCAATATCCTCTCCTCCTTCTCCACCACCACT
TTCTACCACCTCTATTGGTTTCATTATTTCATAACCTCCCTCGAATACTTCACTAAAACGGGCAAT
AACTGGAGAAAGTCCAAACCAGATATTATTGAAATAGGCTGGTGGAATTTCGTCTGGGGTTTTTA
TTCCATCAGGAGTATTCATAGCTTCCCCTTTTCCCGCTTGATAAATCACAAAACTAGATGATGGT
AAACGAGAGTTGTTAGCGGCAAAAAATAAATTACGATTAGCACTACTTTTAATTGGTTGTGTATT
TTCACTCGCTTTTAAGTTTGGTTCTAACTCAGGTAAAATTAAATTTAAAAAAGATAATCCTGAAT
ACAATAAATTATTGTCTTGATTACTAATAATCGGTACTCCTTTTACTGTACGTGCGATCGCAGCT
TTTTCTCCGTTAGCCTGAACAATTTGGCGTATCTTGATATAACTACCAGCTAAAGCATTAGTAAA
TTCTTCTTTACGAGTTAAAATAATATTGCTTCCTTGGCTATTGCCTGTTACCACCCCTGAATTGG
GAAAATAGAGATTTAAACCGATTTTTTGATTATCTGCCCCCTGTTGAAACCCTGGTTGTGCGATC
GCATCTAAAGATTTAGGGGTAACAATATCACCAAAATTAGAAGTAGTATTAATATTCAGAGGAA
CAAAAGACTTAGGATCAATACTATTACGATCAGTTTCTAAACCGGGAGTATAAGTACAAATAGA
ATTAGGAGGGGCATCCGATGCAATACATTCACCAGTTAAAGACATTTGTAATTCTTGCCCAATAA
GAGTTACGGGAGTTACTTGGGTTGTGATTACTTGACGAGTATCCCGAACTGTACCTAAGTGCAAA
TAACTTCCCACCTGATCTACTGTAAAAACTCTATCTTTTCTTAAACTTTCTCTAACTTCACTTTTT
AATTTCCATATACTATTGAAATTAGGACTACTTATGGTTTCCTCTCCCCATGAATAACCGCCTTG
AAGTTCCCATTCACTGCGATGGGTTAGCAAAAAATCATTAGTGAAAATTCTTGTTGTTAGAGGAT
TTATCTTTTTCGGATCTGCCAAAGTTGGGGCATTGATATAAATAGAAGTATCTGGTGGGGAATTA
TCCCCTAAAAAAAAAGAAGGAACAGTCCTCACTTCGTTAAGAGTATCCTGTGGTAAGTTGCCATT
GTAAAAAAACTCAGAGGGAAGTAAAAGATCTTCTTGCAACGAATAATCAAAATTGAAATTACTA
TTTACTGGTACATAATTAGGTATTTGATGCTCTCCCCAATACCAACCTCCATCCAGTTCCCCGATA
AAATCTACCCTTGGCTTCTTTGTCTCAGCCATTAGAGGGTTTTCCCATACTACAGTATTGAGAAT
AATTGCTAATGAACTTATAAAAAAAGTGTCGAATGTAATCCTAGTCAGCTTTAATAAACAGGAG
TAAAAATCAGTCATTAAAAGTTGTTTTCCAGGCTATCACTAACTGTATTTTATGTAAGAAAATGA
GCAATTTACCATAACTTTAATAAAACTTTAATAATAATATTTCTGAGTTCAGTTTTATCACGTAA
AAGGATTTTTACAATTTTCCGCATATAAAAAAATAAGCAAACTAATTTATAATATTTTTTTACTT
TATTTTTTGCAAAATTATAGAATAAAATATTTTTAACGGTATAAAAAAATAACTACATTGCTTA
CTATACAAGCAACTAGCTCATTAAAATTGATATTTTAAGAAAATCATATCCTGATATTTGTTGAA
TCACATCCTATAAACTTTAGTGATATTATCATAAAATGCAGGAACTTATTTACACAAAATTTCTT
AGGTAAACTCAATAAATATCGATACATTTACTTAAATTTAGATAATGAAAAAATAGGTAGTTAT
ATCGATAACAGTGATATATTGATTTGGTTAAAGTATAAATGTAAAAGAAAAAATGTTTTACATC
GATAGCAAAAGATTTACAAAGTAATAGATTTTGAATAACTAAACAATAGTGAGTCAAAAACTAG
ATTCCGATAGCAGTCTATTCTTTGTGAGTCTATTAAAAACAATAAAAAGGGGTGCTAACTATGAA
AAATATACATAGAGTTTTATTGGTTTCAGGAATGGCGATCGGTTCATCTACTTTATTTGGTGCAT
CAGCTTTGGCTGGTGGTGGTATGGCTGGTTCTGCGGCTTTTAATCTTATTAGTGATGGACAAGCT
ACACCCGCTCAAGTAGTTTCAGGGGTTGCAACGGCGGCGGCAGTTGGACAAAATGGAGCTTCTGC
TTGGTCTTTTCAAACGGGTACAAATAATTCAGCAGGTGCGATCGGTTCTGCTGGGGTTATAACCG
TTGATACTTTTAGTGCAAGTAGTGTTACTAGTGTTTCAGATACAGTACCAGCTAGTTATACTGAT
AATACCAATACTTTAACTAATAATGCTGATATTCAGTTAGGAACAACTTCTGGTGACGCTATTAT
CAACTCCCCATAGGTATCTTAAATCGGTAATGGTAATATCTCAAGTTTCAACTATTTTGGGCAAT
AGATTTATTGAATAAATAGGAGGTCTAATAATGATCAATTTACATAGAATTAGTCTATTCACAG

GAGTTGGGGGCGTACTTATTTCCCTTTTAAGTTTTTCTCCTACTGCCTTGGCGGGTTCAGGAGGC
ATGGCAGGTTCTGCCGCTTTCCAAGTTGATACAGTCGTCACAGGAGTAGGCGTTTCAGCGGCTGT
GGGAGAAAATGGTGCTTCTGCTTGGTCTTTTAATGACTCAACAAATGGTTCTGAAGTAAATTCTG
CTGGTTCTCTCGGTTCTGGTGGTTCAATAACTATTGGTGGAGCAGCGGATCAATTCAACGCATCT
GGAGTGGGGCAAGTTGTTGATAGCACAAATAGTGCTGAGGTAAACTCAAACACTTTAAGTAATA
ATGCAGATCTCGAATTGGGTACTGGTAGCAATAATATAATTGTTGATTCTCCCCCTGTTAATTAA
TTATTAGTAAAAAACTAATAACTAAGTTTAGCCTAACTTTGGTTTCACTGAGGTTAGGCTTTGTA
TATTCTGAATTTTATGGTAATAAAACTTTAGTTAAAATTCTACTAACATAACTTTAATTTTGTAC
TTATGAATCAACTATCAATTTTTAAAGTTTTTCATTGGAAGCCTATTTTTATCACTTTCTGGGTA
TGTTTCTTAGGTATCATTACTGAGAATGTCGCTATTCAAGCTGGAGAAGCGAAAATAACTGGCTC
GGCTGGTTTTCATATAGATGCAGATAATAATGTTAAAGGTGTGGTTGTGGGTATCGCTGTAGGTA
AAAATGACGCTTTTAGTCGTGCATTTTATGATCCAAATACAAATCAACTATCAACTGTTTCTATT
GGTTCGGGGGGATTGATCAATATTATGGGTGAAACAAGTATCGGTATTGACTCAATTAAAGATA
CTTTAGACTCCAATCCTAGTCAACCTCAAACAACTAGCGTTTCCGATTCAGGTCAATTACAAATC
AATAACAGCAGTGGCACTGCTAATATACAATTAAATGCGCAGTAATAAGTGAATAGTCAAAAAA
GCGAAAAAGAAGAGCCTTGCTATTCTTACATCTTGCTATAATTTTATTTATTATTAGAAACTCTC
CAAATATAGCAATGTTTAGTAAAAACAACCCTGATAAAAACCCAATGGCAACCTCTGAAATAGAA
AACCTAACTAATAAGAATAATGAGGATTTATTTGATACGGAATTAGAAGAAATCGACTCTCCACC
ATTGGTTAAATTAGTGGAAAAAATCAGTGGTATTTTATCACCCTATTTTATTGTAATAGTTGGTT
TATATTTATATGATAATAACTTTTTATTTGGTTCTATTTTAATCTTGATAGGTGTTTTATCTTTG
CTGAAAATTTCCTATGAAGATGTTTTGGCATGGGTCGAAAAAATTAAGGGGATATTTAAAAGTT
AGTTTTCTGAGTAATCAAAATTATGCTCTATCAATTTGAGTATGTAAATTATTAGTTAAGGTAGC
CAATAGGCAATAGGGGATTATTATTAAATAATAATTTATAAACTTTTAGTTTTTATTTTACTAT
AAACACTATTCAATAAAGGTTATGGAAATCCTGTTTCTCTTAATTTATCATAACCACAATCTGAA
GAACCAAAATTGTTTTTATTCCCCTGTTTAATTATGTTATGTTGGGCTTTGATTTATTCAACCCA
ACCTATTTTAAGTTACTTTATTCCACTGAAACTGCATCCACATCGACAGTTTTTTCTGCTGGTTTT
TCTTCTTCTTTTTTCTGACTATTAAATACCATTTGGGTAACTTCGATCGCCATTATTGATAGTAG
TAAAAAATCATCAATTTGTCCTGCTAAGGGGAATATATCGGGGGAAATATCAATAGGACTTAAA
ACATAAATTAATGTGCCAAGAATAATCCAATTACGATATTTAGGGTTACGAATAGCATTGCTATA
AAGACTATAAATTGCTTGAGGGGTAATTTTCATAATATTTTAAAAGCCAGAAAAATTTTATTTCC
ATACTTCTATCATATATTAATCTCAAATCAGTTGGACAAAATGCGATCGCTCTTAAATGTAATAA
TTAAAGTATTTATAAAAATTACAAGGAACAAACTGAGAATTTTTGGAATCTTATTATTAATTGT
CAGACAAGAATAAAGAAACATAATGAATATTGCCCTACCTTATTTTAAGAATTTATGGACACATC
ATCGTAATGTTGTTCCGATTTCCGAACCTCAAATTAAGATTAATCAATATTTTGCTGTTAAAAAA
GAACAAATATTTCCTAAAAAATTAAAAATAGTTCTTTATTCTCATGACACGATGGGTTTAGGGCA
TAAAAGAAGAAATCAGCTAATCGCTCAAACTTTAGAAGAATCCGGTATTTCTGGCAATATTTTAA
TAATTAGTGGTATAGGAGAGGGTAATCAGTTTTTTTCTTCTTCTGCTATTGATTACTTAACTCTT
CCTGCCTTATATAAGTCTAGTGATGGGGAATATCAGGCTAGGCATTTTGATATGTCTTTGAAACA
AATTATTAAGATGCGATCGCAACTTATTTTAACAGCAATGAAGAATTTTCAACCAGATGTTTTTA
TTGTTGACAATGTGCCTAGGGGTGCAATCAATGAGTTGAATCCTATCCTTAAATATCTAAGAAAA
AAGACAAAAACTAAATGTATTCTGGGTTTAAGAGATATTTTAGATGATCCTGATGTTATTAAAA
ATAGTTGGCAAAAAAATAATTATGAAGATGCCATTCGCAAATATTATGATCAAGTATGGATTTA
TGGGGATGAGCAAATTTATGACTCGATACAAGAATATACTTTTAGTCCTGATATAGTCAGTAAGT

TGCACTATACAGGTTATTTAGATCAAACTGCTCGTATTCAATGGTATCAAAAACATCATCATAAA
ACAGATTTACATTTTCCGTCAAACTCTCTCGCTTTATGTATGGTGGGAGGAGGGCAAGACGGTGG
AAATTTAGCTTTAGCTTTTGCTCAAAGTAACTTACCAAAAAATACTTATGGTATTATTGTCACAG
GTCCCATGATGCCTCCTTCTGTGCGGCAACAAATTCAGGAATATTGTGAAATGCCATCGCAAAAT
GGTGCAGTGCGATCGAATCTACAGGTATTAGAATATGTTAATGAACCCACCTTGTTGCTAAAAAA
AGCCGATTGGGTAGTGTCAATGGGAGGATATAATAGCACCTGCGAAATACTTTCTTTTCAAAAAC
GGGCTTTAATAGTGCCACGAATTACCCCCCGCAAGGAACAATTAATTAGGACAGAAATACTAAAC
AAACTAGGTTTTGTCGATATGTTGCACCCTCAACAATTGAGTGCCGATGCCTTAAGTCAGTGGTT
TAGACAAGAAAAAAAACACCATCCACAAACAGTAAATATAGATTTAGATGGGTTAAAAAAAATT
CCGCAATTAATCACAGAAATTGTTAAAAAGAAGAAAAATTCTGCCAAAATTCTCTTAAAAACTTG
AATAAATTCCCAATTTCTCATTGCTAATTAGGATTTGCTGAATAATTCAAAATCTTTACCTCAAG
AGTTTTTTACCGTGAAACAGACATCTTGCCTATAAAAATTAAAATTATTATTATCAAATTTATTA
ACAAATGTAGCAAAATAGCCATTTTTAAAGAATAAAAAGGCTACAATAAAGGCAAAATCAACCT
AAAATAATCGAAAAGGGGGGAATCATGCTAGTAATTCTCCAAAAAGAACAGATTTTAAGCACGG
ATAGTGTGTGTCAAAATTGCTTATGGGCTAATCAACAAGGAAGCCCTCGTTGGCAAGGGGGAAAA
CTCAGTTGTGGTCATTGTTTAGGTACATCAGAACCAAGACAACCTCAATTATATGAATGTCAGAT
GGGGTTTCGTCTTGTTAATATCGTATAAATAAACATTGGTTAAAATCAAACACCTAATCTTAAAT
TTATGAATTGGCGAACCTCATCTACTGACTGGAAAGATAGACTTTTTGCTTCTCTTGTTTATCTT
TTCCCTCTTTACTACGCCCTAGAGTTTGGCAGTTTTTTATTTGGACAATTCCCTTTTTTACAATTA
ATTATCATACCTCTTTATCCTCTAATTTTGATCAATCAAATACCCTTTGGCGGTTTTATTCTATT
TATTGTTTTATTTGCCGCCGTGGTGCGAAATGCCCGTATCAGTCATTTTATTCGCTTTAATACCA
TGCAGGCAATTTTAATCGATATTCTACTGATTCTCGTCGGTTTGGTTTTCAATGTCATTTTTAGA
GGTTTAGGGACATCCTTGCTCACAGAAACTATCTCTAATGCACTATTTTTAGGTACTCTGGTAGC
CTGTGGCTATGGTATTTTCCAGTCTGCTTCAGGTAAATATGCAGAAATGCCTACTATATCTGAAG
CCGCCTATTCTCAAGTACCTTGGTAATGAGCTTAAGGTTGGGAGTTGCATTCAACAAATATTGAA
GAAAAAATTTCTAAAATAAGGGCTTTAGCCCTCACTATAAATCTATATTAATAGATTAGTAATTA
TTTACTTTATTTCTTTATTCACTCCGTTGGACTAGCTTCAATAAAGTTGGTTAACTTGCCAATAC
TTTCAATTTCTACTGTTACTGAATCTCCCACTTGTAGAGGGGCAATGCCTTCTGGAGTACCTGTT
AAAATCACGTCTCCGGGCAATAGAGTCATAATTTGAGAAATATAAGCAACTAATTCTTCAGGTGA
AAAAACCATATCAGATAATAAAGCGGATTGCTTCGGCTCGTTTTCATCTTCATTGATATATGTTT
GTATTCTTGCTCCTGAGGTCAATTCTCGCACAATCCAAGGACCTAGAGGGCAAAATGTGTCAAAT
CCTTTCGCTCTACTCCACTGTCCATCTTTTTTCTGTAAATCTCTAGCCGTTACATCATTGGCAATG
GTATAACCCCAAATTTTATCTTTTGCATCTTCAATATTACAATTTTTTGTGCGATCGCCTATAAT
TAAAGCCAACTCTCCCTCAAAATCAACTCTTTGAGACTGTTTAGGATAAATAACCGCCTGTTCAT
GAGCAATAATAGCAGAAGGGGGCTTAAGAAAAATCAGAGGTTCTTTTGGTATATCTGAACCCATT
TCTGCCGCATGAGCAGAATAGTTTTTGCCCACAGCCACAATTTTAGAGGGAGCACAGGGGGGCAG
TAACCGATAATCGGATTCTTGTAAGATTAAATCTGTTACTTTTCCATTTAACCATGGTGGAGCAT
CTAAAACTTCTACAGTACGATTCAGTTGTAATCGACCATAATAGGTTTGTCCAGTAATGGTTTTT
ACCCTGACATAACGTTGTGCCATAATCGAAAAATAAAATGAATTAAAATTATATAATGAGAATG
ATTGTGCAATAAATTCTTTTCTGCTATAAATATATAAGCACTATTGTCCGAAAGTATCAATAAAA
ATATTTTTCAGATAGTAAATAGGACAACAAAAAACCTTGCTTCTGGTAATAAAAAGAAGCCTTTA
CAGTCCAAAAGGAGAAACTTACAAAAATGACCAATACCTATGAAATGATGTTTGTCTTGCGTCCT
GATTTAACTCAAGAGCAAGTCAACGGGCAGATGCGCAAATATCGTGATTTCCTCAAAGAAAATGG

AGCAGAAAAAGTATCCCTTGAAGTTTGGGGCAAACGTCGTCTAGCTTACCCTATTCAAAAATTTC
AAGAAGGAATCTACATTCTAACCTACTATACAGGTGATGGTTCTCAAGTTGCCTCCATAGAAAGA
GATATGCGCTTAGGAGAGGAAGTTTTACGTTATCTTACTATCAAATTAGATTCTGATTTCGACTT
TGAAGAAAAAGATATTCCTTCCGCCCCTTCTACCGCTTCTGAATCACCCATTGAGTTACCAACTCC
TGCGGTGGCTGATTCTTCTCCCACTGAAGAAAGTAGTGAGGAGGAAGTGGAAGTAGAAGAAACA
GAAACACCTGTAGAAGCGTAATTTTCTCTTAGAGGATAAATTTCTTGATTAAACAAGGTAAAGG
ATGGCGTATTGGTTGGAAACCAAATCCTTGCACTTATCAAGGATTATTAGGGGGCGAAGATTGGG
CGATGGAGCTAACTTCTGCCGAAATGGCTGATTTTTGTCGTCTTTTACCAGAAATTAATCAAGCA
GTAATTGATATAAGCGAACATCTCATGGATGAAGAAAAAATCGTCTGTGAGATGGAAAGTGAAC
TTTTGTGGTTAGGAGCAGAAGGATATCCTCAAAATTATTCCTTACGAGTTATTTTATCTCAAGGT
AGATGTTGTGAAGGAACTTGGTCTGTCGAAGTTCTACCAGAGCTTTTAAAGGCTATACAGTCTTT
TCACTTATTCTAACTTGACGACTTGTTCTTTTTTTATTATAATAGGATAATGTGATTAAACGGGG
CGTAGCGCAGCTTGGTAGCGCACCACTTTGGGGTAGTGGGGGTCGTGGGTTCAAATCCCGCCGCT
CCGATTTTTTGTTTTTAAGACAACCTAGCTTTTTAAGGTGGGGTTCAGCTTAGGAATATCAGATA
AACTTGAGTTATTGGGGTTGAACATTGTTCAATAAATCCCCTGATAACCTGCAAAAATTAAAGAG
AATCAGTATTGCTGATACTGTCTTCTAAATCCTTTCTTTTTTCCATCTGGCAAAGAAAATAACCA
GTCATCATCGCCGAAGCTAATAAATTCGCTAAATACTGTCTATCTGTGACAATTTGTACGTTAAA
TCCATCAGAGGGTAAAACTCCTAATAAACCTTGTACATTGTGAGTGATAATTTCTCTTACTTCTG
GAGTTGCCGATTGGGCAACTCTTTCTAATGTTTCTGGATGTTGTTTTTGTAAATATTCAACTAAT
GTATTTTTGAGTGTTGTTTCTGATTCGCGCTGAAAAAAATTAGAATCAAATACCATTATTAATTT
CCTCTGTTGGAATTTACGCCGTTACTCTTTGCTTTGTTAACAATATGATAATCTTTTTTTCTCTAT
TCTGTCGGCTAAATATATATCCACAAATTTATTTTTACTGGTTTATGATGAAGATATTGTTACAA
TAGTTAACAAAACTAGGCTCTCGACAAGATCTTGATCTTTCATGAGATATTCTAGCTCAGTTATT
CTAATTGTTCTTGACCAAAGAGTATAGTAGAAACTTTTATGACTCAAACCATCACCCGTCCAGAA
AGCAAAGAAAATCTTGATAATATTGATGACAATATCGTTAACATTCATGATAATCCTGTTGTTAT
CCCCGTAAAACATAAGGAAGATTTAGGTCCAGTGAGTGATATGTCTCCTGATAGTTGGCGGTATC
ACCCAGATATGATTATTGAATATTATCGTCAACGCCCTTTACAAGTATTCGGTAGATTATTAAAT
ATTCTGTTTCCATTTCTGTCTTTCTTGTTGGATGGTTGGTGGGATGGTTTTTGGGGAAATACGAA
AAAAAATGAATTGAAGCGAGCGGTAAAATTAAGAAAAATTCTGACAAAATTAGGTCCTGCTTAT
ATAAAAATAGGCCAAGCATTGTCCACACGCCCTGATTTAGTTCCTCCTAAATATTTGGATGAATT
AACCAGATTACAGGATCAGCTACCTCCTTTTCCCAATGAAATTGCCTATCAGTTTATCGAGGAAG
AATTGGGGGCAAAACCTCAAGAAGTTTATGCGGAAATTTCTGAACATCCCATCGCCGCCGCTTCT
TTAGGACAAGTATATAGAGGAAAATTACATTCAGGGGAGGAAGTAGCGATAAAAGTACAGCGCC
CTGATTTAGTAAGACGTATCACCCTTGATATTTATATTATGAGGTCGATCGCATCTTGGATCAAA
GAAAATGTCAAAAAAATTCGCTCAGACTTAGTCGCAATTACCGACGAGTTAGCCGCCCGTATCTT
TGAGGAGATGAATTATTTACAAGAAGGGGAAAATGCGGCTCGATTTAAAGAATTATATGGACAT
ATTCCCGAAATCTACGTACCGAAAATTTATTGGGAATACACCGGGCGCCGAGTCTTAACGATGGA
ATGGATTAATGGCACAAAATTAACCAAAATCAAAGAAATTGAAGCTCAAGGCATCAACGCCACAG
GATTGGTAGAGATAGGGGTACAATGTTCCTTACAACAACTATTAGAACATGGATTCTTTCATGCT
GACCCTCACCCCGGTAATTTATTAGCAATGGAAGACGGTAAACTGGCTTATCTCGATTTTGGGAT
GATGAGTACGATTTTACCCTATCAACGATATGGCTTAATTGATGCGGTGGTACACCTTGTAAATC
GTGATTTTGAAGCTCTCGCTCAAGATTATGTAAAATTAGACTTTTTAACTCCCGACACGGATTTA
ACGCCCATTATTCCAGCCCTAGCAAACGTTTTTAACAATGCCCTTGGTGCTAGTGTGGCAGAACTC

AATTTTAAGCGAATCACAGACGAAATGTCGGCAATGATGTACGAATTTCCTTTCCGAGTACCAGC
TTACTATGCTTTAATTATTCGCTCAATGGTGACATTGGAAGGTATCGCTATTAATATAGATCCCA
ATTTTAAAGTATTAAGTAAAGCCTATCCCTATGTTGCCAAAAGACTATTAACAGATCAATCTCCT
GAGTTAAGAAAATCCCTGAAAGATTTGTTATTTAAAGATGGTAGTTTCCGTTGGAATCGTCTGGA
AAATTTATTAAGAAATGCCAAAGATTCCCCTGATTATGATTTTGACAGAGTAATTAACCAAGGCT
TAGATTTTCTGTTATCGGAAAGAGGTGAGTTTATCAGAGAAAGATTAGCCGATGAAATAGTTAA
TTCTTTAGATAACTTTGGACAAAAAACATGGATTAATATTTCTACCACCGTGCGAGAAACTCTTG
GTTTCAATAATGGCTCATCGGTAAATCATAATAATGGTAATGGTAGAAACGGGAATCATAATAA
TGGCAGTAATGATGAATCTTTTGAAAGATTGATGAATATTGTTAAAATCCTTCAAGAAACTGAT
GGATATGATCCTTTAAAATTAATTCCTGTAGTCACGAATATTATTCAGAATAAAGAAACTCAAAA
ATTGGGACAGAAAATAGCTGGAGGTTTAGCAGAAAAAGCAACGGCAAGGTTAATTCGTAGTTTA
TTACTAGAGTCGAATAATGGTAATAACTATAATGGCAATGGTAAAAATGGGGTTAAAAATAATC
CTCAATATTCTTTACCTAGTGCAAGAAAATAAAGACTCTCTTACTTTGAGTAAGTTAGGATATAT
CCAAATCATAAGGGGCGGTAGGTAGCTAGTTAACCTGAGTTTTGGATAAGCTGAAAGCATCCCAA
CTCTTAGTCAGAAAACCTTATAGCTTCTTAGAAATAACGATAAAATTGCCTTAACCCGAACTGAA
GTGAAATATATTCAACCCCTACCACCCCAAAACCCTAAACCCCTACTTCCCCCTCTCACCCTCTCC
CCTCATCTCCTCATCACCCTAACACCTGCAACCTGACACCTGACACCCAACCTTATCGGATATTCT
TAAACCAAACGAAGGTTAGTTATCGGAATAAATCCGCTAGCAAAACCAAAAAATTTACACTGAAC
TAGCTTTAATTTTACTCAACTTGTTGAATTTGAAAATCTATATTTTTTTGCTGTTCAACTTTACT
AATTAATTTATTTATAGATTGGCTTGATATTCCCCAACTTAAATTACTCATATTTTTTACTTGAT
TTTCGGGGATGTCATTTCCATTTTGATAAATATAAGGATTACCTAAAGGGGGATATTTTCCTAAA
CCATTAATACCTATTAGTTGACCTTGTAAATTGAGAATTGAGCCTCCACTCATACCACTATGAAC
ATTATTTGTATAACCAATTTGGTAGCCTCCGATTAAAGGCTTTGGTAAAACTTGGCTTAAATTTC
CGAAAGTGTATTTAATGTTTTCTGATTGTTTTAAGTTGTCTTGAAAAGGAAATCCACTAGCTAAA
ATTAATTCATTTTTCTTAGGTATATAATTATTTTTGATATTGATAGTTTGATATTGTTCTTGACT
TTCAAAAGTTAATAAAGCTAAATCATCAACTATTAAATTATCATTATTTTGCCAAATAACTTCCC
CTTTATAAATTTTTCCTGTGTGAGTTTGAATTTGATAATTAATTTCTTTGTTATCAACTACATGG
TTGTTTGTTAATACTAAATAACTATTTTTTCTTTGACCAATTATTACTCCTGAACCACCTATTTC
ATGGTCATCAATATTTGCAAAAACTCTAACATTTACTAATTTTGCTATTTCTTTTAATTCTGTTT
GATTAAGACTTTTTTTTGGTATTTTACTATTTTTACCTATCTCTTTTATTTTCGTTTCGAGGGTT
TTATGTTGGGTAATTTTCTGCTCTTGGATAATGAAAGTGAGGGTAAAGAAAAGGAAAAATGCGA
TCGCCCATAACTGCCATTTTTGACGAAATACCTTAATAAATAGCTGGTTTACGGGCAACATAGGG
GGAGTTTAACCAAAAAAGATAATGCCATTTTAACATTGTAAACTCATCTTAAACAGAGAACCCAT
CTCAGCAAAAAAAACTTAATTTGCCCAATTCCATCAAAAGACTTTTTCAGAAAACCCTCAACATA
TCTTATTCATAGCATTATATTCTCGTAGATTTTAAATAATGCAACAGATGAGTTATAACCCTAAT
CTTAGAATGATGATTTAGAATAAAATTAGTCTAAAGTAATATAGAAAATATAGTTAAAATAAAG
GTCAAAGTAATTATGGGAATTTTTGATCGCATCAGTAGGGTAATAAGAGCAAATGTCAATGATA
TGATTGATAAAGCTGAAGATCCCGAAAAGGTTTTAGAGCAGAGTATCCGTGAAATGGGCGATGAC
CTCGTTAAAATGCGTCAGGCTGTAGCACAAGCGATCGCCTCTCAAAAAAGAACAGAACAACAGTA
CCAGAAAAATCTAAGCGAAGCCAATACATGGCAACAAAGGGCGCAATTAGCTTTATCAAAAGGAG
AAGAGGGTTTAGCAAGAGAAGCGTTAGTGAGAAAGAAAACCCATGCTGAAACTGCCGCTACTTTA
AAAGCACAATTAGATAGTCAAGTGCAACAAATTGATGCCCTTCGTCGTAATTTAACCGCTTTAGA
GAGCAAAATTTCAGAAGCAAAAACGAAGAAAGATATGCTTAAAGCTCGTATTAGTGCGGCTAAA

GCAAATAAACAGCTACAAAGTACCATCAGCAATATTAACAGTAGTTCCGCCATGTCTGCCTTTGA
GCGCATGGAAGAAAGAGTAATGCTAGAAGAAGCAGAAGCAAGTGCTGTAGGAGAATTAGCGGGA
ATGGGAGAGGATTCCAAATGGGCATCCTTAGAAGGTGGCACGGCGGTAGATGATGAATTAGAAG
CCTTGAAACGTCAATTATCTGGTACTCCTGAACCCGGTACTGCTTTACCTGCTGGTAATAATGAT
GGCGAGATTATTGATGGTGAAACAGTTTCTAGCAATACACCTCCCATTGATGATGAGTTGGAAAA
ATTAAGAGCGGAATTAAGAAAAAGCTAATTATTTTATGGGGTTTTAAGGGATTGGGAAGAAATA
AGTTAAGTAATGAGTTCGGGGTTTGGAGTTAGGTAGGGGCGAATGGCCTTCGCCCGTACATTAGA
AGTTATTAATTATCAACTATTTACCTTTGCCTTCCCCCATATTTAGGAGGGATAGAGGGGGATTC
ACCTTTTATCCTTTCTAATTACAACTATCTATAGCAAAACAAGTCAATGATTAAACTTTATCTTA
TACTATGGAATAGTTAAAAATATTGATGGCTTTTATATGACGATTTTAAATAAAAATAAGGGAA
AAAATCATTGGTTATTAAGACTTAATAAACTTTTCTTGAATATTGCAATATTAACTATTATTCCT
TTTTTATTTCAGACTTTGCCTCTTTTAGCATTGGATCTACAAATTGGTATTGTGCAAAGATTTGG
AGAAGAATTAGAAGATGAAATAACTTTAACAAGTATTGGTGAGGATAATTTAAAACTAACTTTT
ACAGATAATCTCACTGGGGAAAATAAGTCTTTAACTACTAAGCAATTAAAATTAGCCATCATACC
CCAGCCTTTACCCCACAAATACATTGAAGAAAGATTAATATTAGGAGATCATGGTACGTTTGAAA
CAGCAGAAGATGATGCCAATAATTGGAGGGAATTAGGTATTGATGTGGAGATAACTCAACCCGG
GCGTTGGCAGGTATGGGCAAAGCGTGGCACTTACAACACTCCTTTTTTGAGACGATTATTATTAG
AACAGTTACAAGAAGCAGGTTATAGTAATGTCTATATTGAAACAGCTATTTTATTAGAAAAGCC
AGAGGTTTCTTTTTCGGTGGGGGAGAATACCTATCAAGTAAATTTTTTAAAAATCAGTAGCAATC
AAGGATTAATAGAAATTAAGGAAGGAAAAGACGGCAAAAATCGCAAATATGGAGGCAATTTTAC
ACTTCAGCCCAATTCCTATGGTAATTATACTTTGGTTAATAAAGTTGATATAGAAACCTATTTAC
GGGGAGTTGTGCCTCACGAAATCGGTAGCAATGCACCCTTAGCCGCCGCTCAAGCTCAAACTATT
ATTGCTCGAACTTACGCCCTACGCAACACAAGAAGGTTTGCGGCTGATAATTATGAACTTTGTGC
AAATACCCATTGTCAAGTTTATTATGGTTTAAGTGGTACATCCAAAATAGCAGATCAGGCGATCG
CATCTACAAAAGGATTAGTATTAACGTATGAAAATGAATTAGTGGATGCTTTATATTCTTCTACC
ACAGGGGGAATAACCTCTAATTTTACAGATATTTGGAATGGAGAAAATCGCCCTTATCTAAAATC
CGTGATTGATTCACCGCAACAAGTGTGGAATTTAAGTCAACAAAGTCTTGAGTCTGAAGATAATT
TTCGTAAGTTTATCTCTTTAGAAAAAGGCTTTAATGAAACGGGTAGAAATTTATTTCGTTGGCAA
AAACAAAGTAGCATCACAGATTTAACTAAAGATTTGCAAAAATATTTAACCCGAATAAAACATCC
TCTTGTAGATATAAAAGAAATTCAATCCATGAAAATAATAGAGCGATCGCCCTCTGGTAGAATCT
TAAAGTTAGATGTAAAAACTGACTTAGGAGTTATTACCCTCGAAAAAAATGAAGTCAGAAGTGC
CTTTGCTCCCCCTAGAAGCACCTTTTTTTACTTAGAACCTATTTATGATAAAAACAAAAAATTAA
CAGCCTATCAATTCATAGGCGGTGGTTTTGGTCATGGAGTAGGACTAAGTCAATTTGGCAGTTAT
AATCTTGCTAAACTAGGTTGGACTGCAGAAAAAATATTACAATTTTATTATCCCGGAACACAAGT
TCAACCTTTAAAAGATAATCAGGCAATCAACAGCAACTAAATTAAACCAGAAACCTATCTAAATC
GCATATTCTTATATCGAACTTAGGTAATCTCAATTCTGGATAAATTTTTATAGAGAGTGATTGAG
AAAAGGGCAATGGGCAAACCCCCCTTTATCCCCAAGGTTTTTTAATTCTCAAAAATGTCAATTTT
TGAAACTAACAAATAGTAACAGATACAGGAGTTTTTAAGTATTAATTAATCAATTATTAAGAAA
AAACGCTCCCCTCTACTGTGGGAGAGGGGTTGGGGGTGAGGGCAAGATAATTTCGCCCTGACTTT
GAAAAAACCCTGCTTTATCCCAGGGCTGTTTCATACTAAAATCTTTGTTTAAGAGGGAGTAATAA
TTAACGAGCAATGAGTAATGAGTTTTGATAATTTATTATGATTAATTGATTGTTTCAAGATTTT
TTATTCTGACTTAAAATCTGCAAGTCAAAAATCTCCCCATCTCCCTCTCCCCTTATCCCCCGCTCT
TTTTTCTTTCTCGAAAATGAAACAGCCCTGCCCTTTATCCCCCATCCCCTAACACCTGCAACCTGA

CACCTACTCTTATCTGATATTCTTAAACTGAACTGAGGTTAAGTAGGTAAATTAAAGACAATTTT
TGTCATTCATGAAACATTACAAAGATTTATAATCCTTTACCTTAAAGCAAGAAAAGAAAAATTA
AGACAAAATAAAGGAAAGTAGTCTTAACAATCTGACATATTAATCAATATATATTATAGGGAAA
AAAGAATAATGCACATAAGTGAAATAACCCATCCCAATCAACTACATGGTTTATCAATTCGGCAG
TTAGAAGACATCGCGCAACAAATCAGAGAAAAGCATTTAGAAACCGTAGCGGCAACAGGAGGAC
ATTTAGGACCTGGTTTAGGGGTTGTGGAACTAACCATTGGCTTATATCAAACCCTAGATTTAGAT
CATGATAAGGTGATTTGGGATGTGGGACATCAGGCTTATCCCCATAAAATGCTCACAGGAAGATA
CCATGATTTTCATACTTTAAGACAAAAAGATGGCATTGCAGGGTACTTAAAACGCTGTGAGAACA
AATTTGACCATTTTGGTGCTGGACACGCCTCTACAAGCATCTCAGCAGGGTTAGGAATGGCTTTA
GCAAGAGATAGCAAAGGGGAAAATTTCAAAGTAGTTTCTATTATCGGTGATGGGGCGTTAACTG
GAGGTATGGCATTAGAAGCCATTAATCACGCTGGACATTTACCGAATACTAACTTAATGGTAGTC
TTGAATGATAACGAAATGTCTATTTCTCCTAACGTGGGGGCGATTTCTCGTTATTTGAATAAAGT
ACGCCTATCTGACCCCATTCAGTTTATCACAGGTAATTTAGAGGAACAATTCAAGCATTTGCCTT
TATTTGGTGATGGAGAAAATTTATCTCCTGAAATGAAAAATCTGAAAGAGGCGATGAAACGTCT
AGCAATGCCCAAGGTTGGTGCAGTCATTGAGGAATTAGGCTTTACTTATTTTGGTCCCATTGATG
GTCACAATTTGAGCGAATTGATTGATACTTTCAATAAAGCCCATAAAGTGAAAGGTCCTGTTTTA
GTTCATGTGGCAACGGTGAAAGGCAAGGGTTATGCTTTGGCAGAAAAGGATCAAGTGGGTTATCA
TGCTCAAAATCCCTTTAATTTAGCTACAGGGAAACCAATCCCTTCTAATAAACCCAAACCCCCTA
GTTACTCGAAAGTTTTTGCTCATACTTTAACTACTTTGGCGGCAAATAATCCCAAAATTGTAGGT
ATTACGGCGGCAATGGCTACGGGTACAGGTTTAGACAAGTTACAACAAAAATTACCTGATCAATA
TATTGATGTAGGAATTGCAGAACAACACGCTGTTACTTTAGCCGCCGGTTTGGCTTGTGAGGGAA
TGCGCCCTGTCGTTGCTATTTATTCTACTTTCTTACAACGGGCTTATGATCAAATTATCCATGAT
GTGTGCATTCAAAAATTACCTGTATTTTTCTGTTTAGACAGAGCGGGTATTGTGGGTGCAGATGG
GCCTACTCATCAGGGTATGTATGATATTGCTTATTTACGTTGTATCCCCAATATGGTGATTATGG
CACCTAAAGATGAAGCGGAAATGCAACGAATGATTGTCACTGGTATTAATTATACTGAAGGTGCG
ATCGCAATGCGTTATCCTCGTGGTAGTGGTATTGGTGTACCTTTAGCCGAGGAAGGTTGGGAAGA
ATTGGAAATTGGCAAGGGCGAAATTTTACGTCAGGGAGATGATATTTTATTGCTTGGTTATGGTT
CAATGGTAAATACTGCCATGCAAACTGCTGGTATTTTAAGTGAACACGGAATCGAAGCAACTGTG
GTTAATGCACGTTTTGTTAAACCCTTAGACAAAGAGTTAATTTTACCTTTAGCTGAAAAAATTGG
TAAAGTTGCCACCCTCGAAGAAGGTTGTTTGATAGGTGGTTTTGGTTCAGCAGTGGCAGAAGCCC
TAACAGAAGCGGAAGTTATGGCAAAATTAAAACGTTTTGGTGTACCTGATATTTTAGTAGATCAT
GCTAAACCCGATCAATCTTTTGCTGATTTAGGCTTAACTGCACCTCAAATTTCTGAGCAAATTCT
CTCCTCTTGGTTTGCGGAAAAACCTGCAGTTGTTGCTAGTTAATCTTTATTAGAGGTGTTGAAAA
AGTGTTTTGGTGAGGATAGGTGGCAGGTTGCAGGTTTCAGGTGTCAGGTGTCAAGTTTGACTGTT
TTAATTAATTACTAATTACTAATTACCTATTGCCTATTGCCTATTGCCTATTGCCCTTTTGAAAT
CTATTTACTATTCACTATTGCCTTATCCTACATTCCTAATTCCTTTAGCTAATTGTTTTGTGGATT
ACAGTTAATTTCTGGGTGCTAGAGGATAATTTTGATCAGGTTAATAGAGGTTTGATAAAAAATT
AGCAGATAGGGCAATGAAACCACTTATTATAGTTTCGGGTTTAGGGCGTACAGGATACAAAATTT
ATCAGTTGTTAAAGCAACAGGGTGCCGAGGTTGTGGGTTTAAGCGATCGCACCTTGGTAGTTCAA
GAAGATGATAGAATTATTGTGGGAGATTTATGTTCACCACAAGTATTAATAGAAGCGGGAATAA
AAGAAGCAACCACGATTGTTTTAGCTAGTAATGATGATGCTTTAAACATAGCTATATTAACTCAA
GCAAAGACTTTAAACCCAAAAATTCGCATCGTTAATCGTCTTTATAATCAAACTCTCGGACAAAG
ATTAGATTATACCGTTACCGATCATTTAACCATGAGTGTTGCTCGTTTAGCCGCCCCCATTTTCGC

TTTTTCGGCATTGGGAAGTAAAACTATTGGACATTTACGACTATTTGAGCAAACTTGGTCATTAC
ATGAAGAAATTATCGACTCTAATCATCCTTGGTATGGTGTCAGTTTGTCTCACTTATGGGAAAAT
CTTGAACAAATGTTAATTTATTATCTGCCCAAAATAGGGGAGATGGACTTAGTAACAGGGGTAA
AAACAGAGCAAAAATTACAATTAGGGGATCATTTGATTGTTGCTCATAAAACTAAATTTAAGCG
CAAATCTCGTTTTTTATGGAAGCAGTGGTTCAAATTTTTTTTCAATTTAAGCACCTATAAGCAAT
ATGTGCGCCCTGTCATCATGGTGACATTAATTTTGATAATTCTCATTGCCCTTGCAAGTATTATT
TATTTAATGGTTAATTGGAATATTTCTCCAGTAGATGCTCTTTATTTTAGTGTGGGAATGATTAC
AGGAGCAGGAGGAAAGGAGGAAGTAGCGGAATCCGCCCCCGATGGGATTAAAATTTTTACGGCTG
TCATGATGATTATTGGGGCGGGAATTGTGGGTATTTTTTATGCTTTACTCAATGATTTTGTTTTG
GGTAGCCGTTTTAAACAGTTTTTAGATGCGGCAAGGGTAGCAACGGTTAATCATTATGTTGTTTG
TGGTTTGGGTAATGTCGGTATGGAGGTAATTCGGGAATTAAAACAGCAAGGACATGATATAGTG
GTTATTGAGTCTGATCATAGTAATCGTTTTTTACACTCGGTGCGATCGCTCGGTATTCCAATTAT
TCTCGAAGATGCAACCCTAGCAGAAACCTTACAATCTGCCCATATTGAGGGAGCAAATGGCATCA
TCGTCGTTACTAGCAATGATATGGTAAATGTGGAAATTGCCTTAACAGCAAAAGCCCTAATGCCG
AAATTACCCGTAGTGTTACGTATCCAAGACAATCAATTTACTGAATCTGTGCAGGATGTCTTTCA
ATTTGAAAATGTTTTTTCTCCTAGTGAATTAGCAACTTATTCTTTTGCGGCGGCGGCTCTTGGCG
GTAGAATATTAGGTAATGGTATGACTGATGATTTATTATGGGTTGCGATCGCAACTTTAATTACT
CCGAATCATCCCTTTTGTGGGAAAATCGTTGAGAATATCGCACCAGATGGAGATTTTGTTCCCTT
ATACATTGCCAGAGAAGATAAAAATATTCACGGTTGGAATTTACTCACCACAGAATTAAAAAATC
AAGATGTATTATATTTGACAGTCCCAGCTACAAAATTAAAACAATTATGGCAAAGCAATTCAAAT
CAAATTTTACAGATGATCTACTAATATTGTGTAATATGTGAGTGACAAGCAAGTTTCAGAAGTTA
GGGTATTAGGGTATTGGGGTGTTAAGGTTATTATTAATTAAACACTTTTGCCTCTTGCCCTTTAC
CTCATCTTCACCAAAACATTTTTTCGGCAATCATCGAGTAAAAAGTGTAAAGAGATGTAACAAAA
AACTACGGATAACCGTAAAAAAAGACTTCATCTAGGTAAAAGAGCTTATTTTCATGGTAAAAAC
AATAAAGAGCCTATTATTGGGCAAGTCATTATTTTTTCACATACCTAGTATAAAAATCATATAAC
ACGGAGTTTTCTGCTTAATGTTTACTAACGTAAAATCAGCTATCCGACATATAGCACCAGAAGAT
TTACAAGGACGTGCTTTAATGAAGGTAGTTTATGTGGTACTAGAGTCACAATATCAAAGTGCCAT
TTCTGGAGCAGTTAAATCCATCAATGCTCATAATCCCAATCTTGCCATTGAGGTAAGTGGTTATT
TAATTGAGGAGTTGAGAAGCCCAGAAAATTACGAGGAATTTAAAAAGGATGTAGCAGAAGCAAA
TATTTTCATTGCCTCTCTTATCTTTATCGAAGAATTAGCTCAGAAAGTTGTAGATGCGGTAACAC
CCTATCGAGATAACTTAGATGTTGCGGTGGTTTTCCCTTCTATGCCTGAAGTGATGCGCCTTAAC
AAAATGGGCAGTTTCAGTATGGCACAGCTAGGACAAAGCAAGAGCGTTATTGGCGACTTCATGAA
AAAACGTCGTCAAAAATCTGGTGCTGGTTTTGAGGATGCTATGCTCAAATTATTACGCACTTTAC
CTACGGTATTAAAATATCTCCCCGTAGAAAAAGCACAGGATGCGCGTAACTTTATGCTGAGTTTT
CAGTATTGGTTAGGTGGTTCATCTGATAACCTTGAAAACTTTTTCTTGATGCTAGCAGACAAATA
CGTATTCACGGGGGAAAAATCTTTATTAGCTAGTAAAGACTACGCAGAGCCTATTGTCTATCCTG
ATATGGGTATTTGGCATCCTCTCGCCCCAAAAATGTTTGAAAACATCAAGGATTATCTAGCATGG
TATAACAGCCGTGATGACATCAACGAAGATTTAAAAGATCCTCTTGCTCCTTGTGTAGGTTTAGT
CTTACAAAGAACCCATTTAGTAACTGGGGATGATGCTCACTATGTGGCGATGTTACAGGAGTTAG
AATATCGTGGTGCGAGAGTTATTCCTGTGTTTGCTGGTGGTTTGGATTTCTCGAAACCTGTGGAT
GAGTATTTCTGGGATAAGCCTGTAGAGGGAGTTGAGCCTTTACCTATCGTTGATGCTGTAGTCTC
TTTGACTGGTTTTGCATTGGTAGGAGGTCCTGCTAGACAAGATCATCCCAAGGCAGTGGAATCCT
TAACCCGTCTCAATCGTCCCTACATGGTAGCTTTACCTTTAGTATTCCAAACTACGGAAGAATGG

GAAGAAAGTGATCTCGGGTTACACCCTATCCAAGTTGCATTACAAATCGCTATTCCTGAATTAGA
TGGTGCGATCGAGCCTATTATTTTATCAGGGAAAGATGGCAACACGGGTAAATCTATCACCCTTC
AAGATAGAGTAGAGGCGATCGCAACTCGTGCTTTAAAATGGGCAAATTTACGCAAAAAACCCAAA
TTAGAGAAAAAATTAGCAATTACCGTCTTTAGCTTCCCTCCCGATAAAGGAAACGTGGGTACAGC
CGCTTATTTAAACGTGTTTGGTTCAATTCATGAAGTGTTAAAAGGCATGAAAAACAACGGCTATG
ACGTGCAAGACGTGCCAGACACCCCCGAAGAATTGATGCAAATGGTAATCCATGACGCACAGGCT
CAATATTCTAGCCCCGAATTAAATGTTGCCTATCGTATGAGTGTGGAAGAATACGAGCGTTTAAC
CCCCTACTCCAAAAAATTAGAAGAAAACTGGGGCGCACCTCCGGGGCATCTGAATAGCGATGGAC
AAAATCTGTTAATTTACGGCAAACACTTCGGCAACGTTTTTATCGGTGTACAACCCACCTTCGGT
TACGAGGGTGATCCGATGAGATTACTTTTCTCTCGCTCTGCGTCTCCTCATCATGGTTTTGCGGCT
TACTATACTTATCTTGAAAAAGTCTGGGGTGTTGATGCTGTATTGCACTTTGGCACACATGGTTC
TCTCGAATTTATGCCAGGTAAACAAATGGGTATGTCTGGGGAATGCTATCCTGACAGTTTAATCG
GTAATATTCCTAATATCTACTACTACGCCGCTAACAACCCCAGTGAGGCAACCATTGCAAAACGT
CGTAGTTATGCCGCTACTATCTCCTATTTAACCCCTCCTGCGGAAAATGCTGGATTATACAAAGG
TTTAGAGGAATTAAGCGAGTTAATCGGTTCTTATCAAAGCCTTAAAGACGGTGGGCGAGGTGTGC
AAATTGTCAATACCATCGTGGATAAAGCCCGTATCTGCAACCTAGACAAAGACATCCCTGAAATT
GCACCCGTAGGGGCGAATGGCATTCGCCCAGAAGCAGAAACCGATTTATTTGATTCTAGCACCCT
TTCCCAAGATGAAAGAGATCACATTGTCGGTGCAGTCTATCGCAAGTTGATGGAGATTGAGTCTC
GTCTTTTACCCTGTGGCTTGCACATCATCGGTAAACCTCCTACCGCAGAAGAGGCGATCGCAACTC
TTGTCAATATCGCTAGTTTAGACAGAGAAGAAGAAGGCATTTGGGCATTACCAACCATTATCGCC
AATAGTATTGGACGCAACATGGAGGAAATTTACCGCAACGCTGATAAAGGTGTCTTAGCGGATGT
GGAGTTGTTGCAACAAATCACCGAAGCCACCAGAGCCGCAGTAAGAGCTTTAGTAGAGGAGCAAA
CCAACGCAGAAGGGCGAGTTTCCTTTGTTTCTAAGCTCAACTTCTTCAATATGGGCAAAAAAGAA
CCTTGGGTAGCCAAATTACACGAATTAGGTTACACCAAAGTGGATGAAAAAGCCATCAAACCCTT
ATTTGAATACCTCGAATTTTGCTTAGAGCAAGTTTGTGCCGATAATGAATTAGGCGGATTATTAA
AAGCCCTTGAGGGTGAATATGTGCTTCCTGGACCCGGAGGCGATCCCATTCGTAACCCCAATGTA
TTGCCCACTGGTAAAAATATTCATGCCCTCGATCCCCAAAAAATACCTACTTTAGCGGCAGTACA
ATCAGCCAAAATCGTTGTTGACAGACTCTTAGAGCGTCAAAAGATAGACAACGGAGGAAAGTACC
CAGAAACCATCGCTTGTGTATTATGGGGAACAGATAACATCAAAACCTACGGGGAATCCTTAGCT
CAAATTATGTGGATGATTGGGGTTAAACCCGTGCCTGATGCTTTAGGGCGTGTCAACAAACTCGA
ATTAATCCCCTTAGAGGAATTAGGCAGACCTCGTATCGACGTAGTCGTTAACTGTTCGGGTGTAT
TTCGTGACTTGTTCATCAATCAAATGGCATTATTAGATCAAGCGGTGAAAAAAGCCGCCGAAGCC
GATGAGCCTTTAGAAATGAACTATGTACGCAAACACGCCTTAGAACAAGCGGAAGAAATGGGCGT
TAATATTCGTGAAGCGGCAACCCGTGTCTTTTCTAATGCTTCTGGTTCATATTCTAGTAACGTTA
ACCTAGCGGTAGAAAATAGTAGTTGGGAAGAAGAGCAAGAATTGCGGGATATGTACTTAAACCG
TAAATCCTTTGCCTTTAACTCCGATAATCCGGGGGTAATGGACGAAAACCGAGCTATTTTTGAGA
AATCCTTGAAAACTGCCGATGCTACCTTCCAAAACCTAGATTCTTCTGAGATTAGCTTAACGGAT
GTATCCCACTACTTCGACTCCGATCCTACCAAAGTAGTTGCATCTTTGCGCGATGATGGTAAAAA
ACCCTCTGCATATATCGCAGATACTACTACCGCCAATGCCCAAGTGCGTACTCTTTCCGAAACCGT
GCGTTTAGATGCTCGTACCAAATTATTAAATCCTAAGTGGTATGAGGGTATGTTATCTAACGGTT
ATGAAGGGGTAAGGGAATTATCCAAACGCCTTGTAAATACGATGGGATGGAGTGCTACTGCCGAC
GCTGTGGATAACTGGGTTTATGAGGATACCAACACCACATTTATCAAAGATGAAGAGATGTGTAA
ACGGTTGATGGATTTAAACCCCAACTCATTCCGCCGTATGGTAAGCACCCTCTTAGAAGTTAACG

GGCGTGGTTACTGGGAAACCTCAGAGGAGAATATTGAAAGGTTACAACAACTCTACCAAGAAGCA
GAAGATCGCATCGAAGGCATTGAATAGTTAATATAGATTTATGGGTAATAAGTTTTAACTTTTA
TGAACAAAGGTTTTTTCTTATTACCCGATTTATCTTGATAAAAATATTAATTTTATGACTACCAC
AACAGATAGACTCCCAAAAAAATTGATCACAGAAAGAGTAGAAAATATCTATGCTCCATCTGAAA
TCAATATGTGGGAGGAAAATAGACAATTTTCGACACCTAATGCACCAAAATTAAGAATTTTAACC
GAATTTAATCGTGAAAGATTACCCGCCTTTGTCGAAACCTTAAAAAAAGATAATTATTTAAAAAT
ACAACCGTTTTATCAAAGAAGAAAACGATGGACAAATAAAGATAAATCACTTCTAATAGAATCA
TTTATTATTAATATTCCTATACCTCCCGTTATTTTTTATGAAAAATCATATAGTTCATACGAAGT
AATTGATGGTCAACAAAGAATTAATGCAATTCAAGAATTTTATAGTAATAAATTTAAATTAACA
GGTTTGGATTTATGGAAGGACTTAAATGGTTATACTTATGAATCATTACCATCGGATATTAGATC
TGAAATTGATAGAAGATCAATTCCTATAATAGTTGTAATAACAGAATCTGCCAAAAGAGAAGAG
GATAAATTTTATTTAACACAACTTGCTTTTGAACGTCTTAATAGGGGTGGAATTGATATGAAACC
TCAAGAAATTAGAAACTGTTTATATCATGGTAAATTTAGTGAATTATTATCAGATTTAGCGAAA
AACACTATTTTTAGAAAAGCATGGGATCTTCCATTAGATGAAACAAAGTTGGAAAAAAATATCA
TGTACAAAAAAATGGAAGATATAGAGTTAATTCTTCGATTTTTTGCTTTGCGTCATCTTGATGAT
TTTAACGGAGATATTAAAAGATTTTTAGATAGTTATATGTTAAAAGCGTTAAATTTTAATCGGG
ATGATATTAATTATTTAGAAACTGTTTTTAATCAAACTATTGAGCTTTGCTTTTCTATTTATGAA
GAAAATCTTTTTAAACCTTTTGATATAAAAAATAATGATTGGGGTAAAAAATCTCATAAAGTTT
ATTATGATGCAATTATGGTTGCATTTAGCTTTAATTTAGACAAAGCAAATATTATTCTTAATAAG
AAAGAATTAATAATTCAAAAGACTAAAGAGATATTTTATGGAGAAAAATCTAAGCTCTTATCAT
CGGGAAAAACTAGCAAATCAGAAACACAGGAAAGAATCAAAATAGTTAATGATATGTTAATAGG
AATTGTTAAAAACTGATATTAGATGAATAAATTTATTCAAAAACTACAAAACCTTTTAGATAAA
GTTGATAATAATATTCAAAGTATAAAGGAAATTACCACAAAAAATCAGAAGTTAACGGACATAA
TATTTGGTAATGATTCTACTGTAAAAAATATTTTGAAAGAAAATAATCAATTGGATTTAACTAA
AAGTAGTGTAGGATTTGATCAATGGAAACGTTATCAACATTGTTCTGTCATTACGCAATTATATG
CTATTTATGAAGATTTTGTTGAAGAGTTGATTTCAATTTGGATTGATAATCTACCAAATATATTT
GAGAAATACTCAGATTTAGGAATGGATTTTCAGAAACAATATATTTTAGGAATTGCTAAATTAA
TAAAAAGATTAAATAGAAATAAATATCCAGAATTAAAACTAGAAAAATTAATCAACATTCCTAA
AAATACAAATATTAATTATAAGTTAGTTCAAGAAGTTTTTACAGATCACCAATATAATTTGCGA
ATGAGTGAATTAAATAAATTATTAGACAATGCAGGAATAAAAAATACATCGGATTGGCTACAAA
AAAGTAACAGTATTCAGCAATCAATATCAAGTATTAATAGTAATCAAACTACTGTTGAAAAAGA
ATTACAGAGGTTTATTAATTATCGAAATGATGCTAGTCATAATATTCAACAAGATAATATATTA
AATTTTAATGAGTTAATTGATTTTTGTAACTTTATAAATACTATATGTTTTTCTCTTCATGATTT
GATTTTATATCAAACTCTTGAAAGAATGATTGCTTTAAATAAAATAATCACTTTAGGCCAAATTA
GACAATATCGAGATAAATTAAAATCTGCAAGACTAAAAGTAAATAATAGTATTTTATTATCAGT
CGGTTTACCTACTCTATTAGTCAAGAAAGAAACATCTATTTGTAAATTTGCAAAAATAGAAAAA
ATAAGTATTGTATCGAATGACCAATATATAGAAAAAGAAGATATTAAAGTTCCTGAAAAAGAAC
AAGATATTTGGATTCAATTTAATATTGATGCAAGAAAAAATTGGACAGTATATATATTTAATTT
TGATAAACTAGATAGTGGAACATTTTAATGATATACTACAATCATATTTGCTACCTATTTAATCT
AAGTATTCGTATCAAAAAAACATTAATACTCTTTTATCAATAAACTTGATTAACTTTACATATTA
TATGTAGTTAGTAAAAGATATTTTAGTGGAAAACCTCAGAGAAGAATATTGATCAAATGAATCT
CAATTATTATACTAAGAAGGATGTGATCGATGATGAAGTGGTGCTGTGCGATCGAATTTAAGA
CATTGAGTAATCATTAGAGTTGTTGCAAAATAACGAAAAAAACGCCTGAAAACTTTACTGAGTA

TAAGTTGTAGCCATTTGACGCTAGAAGTGTTTGTAAATCTTATATAGCAAGGATTTCGAGCATTT
TAAGACATTATTTCACAACAAGTTTATTAATAATAGGGTGGGAAATGAGTACCTTTTTCATTTAA
TAAAAAAAATTAACTTGAAAATATTGCTTTATTTTTAATCATTAATATCAGGTTTTTAAAAA
AATATTGTATATTTGGTAAATTCTGAAAATGATCATCGGATGTTATAATTTGCACTTTTTCTTGT
ACGGCAGTGGCATAAATAATTGCATCGGCAAGAGTTAGCTTAAATAAGGCTCAAATTTATCCGCC
AAAATCCTGTCTGTTCGCTATTCAATCCATCCACCTTCATTTAATTAACGGAATCGTATCGCAAA
CCACTTTACAAAAAATCATATTTTTATAAAAAAATTAGTCTCCATAATGACTTTATTTTGTTTTA
AAGTAACTGCTTCTTTTTGTGCAGTTTCAATTATCTGATTAAAGTTTTTATGGGCATATTCTATG
GAAAATTCTTGCCGATGAGGGATATTTACAAAAAAACAATCGCATTTTGCTTGATCTTAACAATT
ATTTAACCATTAAATGTTTAAAGATTTTCATTAACAAAACGAGGTAAATCTGATAGATTATTAA
TAAATTCAGCATTTAAGGGTTTACCAATAAAAGCCACATCAAAAAATTTAAACATTCCACGATGT
TGAGGATTATTAATTTCATCATAAAAATTTACCGTTGCAAACCCAACTAAAGGCATATTTTTATG
TTCATAACTAATGTTATATTTTTCTTTAATGGGATTATCAGAAGTAGCTATTTCTAATAATAATT
TCCATTTATAAGTTTGTCCTGCACGTTCTCTTAAAGATGTTTTTAATGATAGGATTAAACATTTA
TTTAACTCATTTTCTTTGTTGATGCTATAGATTGCTAAATCCATATCTGGTTTTTGAGTTTCATC
ATCAACTTTAATTGTGACTATATCACTAATTAATGTATGTTGTTTAGGATTAGAGGTAACAAAA
ACATTAGATTTAATACATTTATTTTCCTTCAAAGTTAGAAATAAATATTTAATAAGAAGAGAAA
AAGCATTTCCTGCAATAGATTTTCTGGTCTGAGATTTATCTTTTATTTCTCCAGCTAAAATTCTC
TTATCTAATAAAATTTCAACATCATCTTTTGCCAAATTTAAAATAGTATTGAGATAATCTAGTGA
TTTTTCTAAACCATATTTATCAATATACTCATCAAGTTTTCTTAGAATGGGAATCATATAATAAG
TATTTTTGATTTTTGATAAATCTGCTAACTCATTTTTATTTAAAATATTCATTTTTTATCTTACT
AAAATAAGGATAATTGCTTTTGTCGTGATTCACCTAAATTATATTTTTCTACCGTGATTTTAATT
TGTTTAATAATCGCCTCAACAACTGGTACAATAACAGAATTACCAAATTGTCGGTAGGCTTGAGC
ATCAGATACTGGAATGATAAAATTATCTGGAAAACCTTGCAATTTTGCACATTCACGAGGTGTTA
GTCTACGAGGGTTTTTATTAGTTTGTTCAACCAATATTTCTGAACCATCCTTATAATACCTAGCA
CTTAAAGTTCTAGTATATTCTGATTCTATATTAATAATTCCATAACCAAAACCGTTTCCTTTTGC
TTGACTATTTTTTTTATGTTGTTGTAAATAATTCCACAATTTATCCGATAATGTATATTTATCAT
CAACATTTTTCTCCAGAATATCTTTTAATTCATAAAGTCTTTTTACGGGTAAACTAGGAAATTCA
AAATTAATATTAGGAAAGACATTTTTATTAAAACAAACAATAAATATTCTCTCTCTATGTTGAG
GAACATAATATTTCCCGTCAATTATCTCTGAAAAAATCTCATAATTTCTAATCAACAATTCATGT
TTTATTACTTGCCAAGTATTTCCTTTATCATGACTTTTCAAATTTTTTACATTTTCCAAAAAGAT
AATTTTAGGTTGATGATAATCAATTATCCTTAAAATCTCAAAAAATAAATTACCTTGTTTTTCAT
CTTCAAATCCATGTTTTTTTCCTAAAGATTTCTTTTTACTTACACCTGCGATCGAAAATGGTTGA
CAGGGAAAACCAGCACATAATAAATCAAAGTTAGGGATATTTTCAGCAGGAATATTGTTTAAAT
CTTCACAAAATATTTCCCCAAAATTAGCTGAATATGTTTGACAACTATATTTGTTATAATCATTA
GAATAAATACAATTTAAACCATTATTTTCTAAGGCAATTCTAAAACCACCTATTCCTGCAAATAA
ATCAATGCCAGTTTTTATATCTATTGTGTTCAAGGCTCTTAACTAATATGTGTGGTAAGGAAATT
ATAACGTAGGGTGGGCAATGCCCACCATCAGGAAAAATTAAATTATACAAAATAATCCTGTAAAG
CCTTAACCTGTAAAGGTTCGGACTGGAGTGATCGAATCGCTTCCACCGTTGCTTTAGCCCCTGCAA
TCGTTGTAATAATCGGTAACTTGTAATCTAAGGCCGCCCGACGAATTTTACGCCCATCAGTTTGG
GTTTCCTCCTCATTGCTAGGAGTATTGATAATAAACTGAATTTGCTTATTTTTAATCCAGTCCAC
TACATGGGGACGACCTTCATGAAGTTTTAAGACTAAATCCACATCCTTAACCCCATTCTCTAACA
GAGTTTGACGAGTGCCAGAAGTTGCTACAATCTTGAAACCTAGAGCCTGTAAGTCTTTCGCCACA

GATACGATCGCACCTTTATCTCGGTTATTCATGGAGATAAATACTGTACCCTTAGTATCTAAGAT
TACACCCGCTCCGATTTCCGCCTTAGCGAAGGCTTTACCAAAATCGCTATCAATGCCCATTACCTC
GCCCGTGCTACGCATTTCAGGACCTAATAAAGTATCAGTACCCGGAAACTTACTGAAAGGCAATA
CAGCCTCTTTAACCGCCACATGACGGGGAATTATCTCCTCGGTTACCCCTAATTCTTCGAGGGTTT
TTCCAGACATGACGAGGGAAGCGACTTTTGCTAATGGTCTGCCTGTCGCTTTAGAAACATACGGC
ACAGTACGAGACGCACGGGGATTAGCTTCGAGGATATAGACAGTTTCTCCTTGTACGGCGTACTG
GATGTTCATTAAGCCAATTACTTTCAAAGATTGGGCTAATTTGATAGTTGCACTGCGAATAGTGC
TTAAAACACCATCTGAGAGGGATGTATAAGGAATTGAACAAGCAGAATCTCCAGAATGTACACCA
GCTTCCTCGATATGTTCCATGATACCACCGATAACCACTTGTCCTGTGAGATCTGCGATCGCATCT
ACATCAACCTCAATGGCATTTTCAAGGAATTTATCAATTCAAGATTGGATGATCAGGCTCAACATC
TACTGCATAGGTCATATAATGCTCTAATTCCTGCTCATTATAAACGATACGCATAGCTCTGCCTC
CGAGTACATAAGAAGGACGCACAACTACAGGATAACTAATGCGATTAGCAATGGCTAAAGCCTCC
TCAAAACTCCTTGCGATACCGTTGGGAGGTTGTTTGATGTCTAACTCTTTCAAAATTGCTTCAAA
TTTTTCTCTATCTTCGGCGGTGTCGATAGAATCAGGAGAAGTTCCCCAAATTTTGGTTTTAACGG
TAGTATCAGGATGAGCGAGGTAATTTTTCAAAGGTACAGCTAATTTTAAGGGAGTTTGTCCCCCA
AATTGAATAATTACACCGTCAGGGTTCTCTGCTTCGATGATATTTAATACATCTTCTTTGGTTAA
AGGCTCAAAATAAAGGCGATCGCTTGTATCATAATCAGTGGAAACAGTTTCGGGATTGGAGTTAA
CCATAATTGTTTCATAACCTGCATCTGATAAAGCAAAAGCCGCATGACAACAACAATAATCAAAT
TCTATACCCTGTCCGATACGATTAGGGCCTCCGCCTAAAATCATTACCTTCGGTTTATCAGTGACA
TTGTTTTCACTTTCTCCCGATTCATAGGTGGAGTAATAGTAGGGAGTAAAGGCTTCAAACTCGGC
AGCACAAGTATCCACCAATTTATAGACAGGAGTGATACCTAATGATTTACGATATGCTCTTACTT
CATCTTCTGTCGTCTTGGTTGCAAAAGCAATTTGGCGATCGCTGAATCCATGTTGTTTAATGGTA
AGCATATCATCGGCTTTAATATCCTTTAAAGAAGTCCGTTTCATAAACTTCTCTATCTCTACTAA
TGACTGCATTTTATCCAAAAACCAAGGGTCGATCGCAGTTAACTCAAATACTTGTTCTACGCTCA
TCCCCAATTTAAAGGCATGATAAACACTAAAAATCCTTTCTGGGTTAGGGGTGCGTAAATGAGAG
CTAATTTGCGAGAGAGTGGGTAAAGTCTCGTTTTTATCACAACCAAAGCCATAACGTCCCGTTTC
TAAGGATCTTAACGCTTTTTGGAAAGATTCACAGAAAGTACGTCCGATGGCCATTGCTTCACCCA
CAGACTTCATTTGAGTGGTTAAGACAGGTTCACTACCGGGGAATTTTTCAAAAGCAAAACGAGGA
ATTTTGGTCACTACATAATCGATGGTAGGCTCAAAAGAAGCAGGAGTTTTCTGGGTAATATCATT
AGAAATCTCATCAAGGGTATAACCTACCGCCAATTTTGCCGCAAATTTAGCAATGGGGAAACCCG
TCGCTTTTGATGCCAATGCTGAAGAGCGAGACACACGGGGATTCATTTCAATTACAATTACTTCC
CCATTAACAGGATTAACGGAAAACTGAATATTAGAGCCTCCTGTTTCTACTCCAATCTCTCGGAT
AATAGCTTTTGCGTAGTCTCTTAATCGTTGATATTCTTTATCGGTTAAAGTTTGAGCAGGGGCAA
CGGTGATAGAATCTCCAGTATGTACCCCCATAGGATCAATATTTTCGATGGAACAGATAATAACC
ACATTATCCGCTAAATCCCGCATTACTTCCAGCTCGTATTCCTTCCAACCGATGAGGGATTTTTCG
ACCAAAATCTGCGACACAGGAGAAGCATCAATCCCAAACTTTGCCATTTCCTCATATTCTTCTTG
GTTATAGGCAATACCGCCACCAGTACCGCCCATAGTGAACGCAGGACGTATAATTAAAGGATAAG
AACCAATTTCTTCCGCTACGGCTTTGGCTTCTTCCCAGTTACTAGCAATCCCTGAAGGGCATACAG
GCACACCGATACGAGCCATCGCTTCTTTGAATAATTCCCTATCCTCTGCCATTTTAATAGCAGGTA
ATTTCGCTCCAATTAACTCAACTCCGTATTTATTTAATACCCCTGATTCTGCTAAGGCAACAGCA
GTATTAAGGGCGGTTTGTCCCCCCATTGTGGGTAATAATGCGTCAGGTCTTTCCTTGGCGATTAC
TTTCTCGACTATTTCAGGGGTAATGGGTTCAATGTAGGTACGTTCTGCCGTTTCGGGATCAGTCA
TAATAGTAGCGGGGTTGGAGTTAACTAAAACTACCTCATAACCTTCCTCTCGTAGCGCTTTACAG

GCTTGTGTACCAGAATAGTCAAATTCACAGGCTTGTCCAATAATAATAGGTCCTGAACCTAATAG
TAATATTTTATTTATATCTTCACGACGTGGCATAGATTTTATGGCTTAACAATATTCAATTTTAT
ACATCGATCCCGATCATTTTAATCTACATAGCGGTTATTTCTCTATCTCTTTATCAAGATTTACTC
ATAAATAATCTTTGATATACTCACTGTTGAGCATAACTTGAGCTAGGTGAGGGTAAGCAATAGGT
TTTCCCCCCTAAATAAACAGGGTTTTTTCAAAGTAGATATTAAAACGCATTTGTTTTGGAAGGGG
AGAAATGAATAGAGGGGGAAAGGGGTATTTTTTTCTAATATTTTTTCTAATAGATTAATAGTTA
AAAATTTTTGTATTTAACATTATCTCTTAATTTTAGAAATTGACATTTTCGAGAATAAAAACGCC
CTGCTTAATAAAAAGGCAACAGGCAACAGTTTTTTGTAGTATTAAATTATAACATTGGATAGTA
TATTAAATTCAAATATGATTTTTAGAGAAAGTGACCAGATTTTTGATTTAAATTTGAAAATAAA
TATTTTAAGTTCAATTTAATTATTCTTATTTTTCTCATCCAGTCTATTAATTAAAATTCAACTGG
AAAAATACTTTTTATTGTTTGAACTACCTACGATTATCTATTGCAAATAATTATAAATAATTAGG
AATTAAATAGTGATTCTCTGACTTCTAGTTTTGATAAATTCTTTTTTAAAAAGATGTAGGGTATT
TTCTTTTAGGTATTAGTTTTAGGAATTGATATTAAATATATCTTAATCAATTTTCTTATTCTCAA
TTTTTAGATTTATTAATTCAAGATTTCAATGGTTTTCAAGCTGAAACCTGCTAAATCATAACTGAT
AACTGTCCCAATCTGATACTTTTGTTTCGAGCTGAGTTTAAAACAAAGTGGGAAAAATATTTTAT
ATCCTCTGTAATATTTTACATTTTTTAATGTTATTCAGTTCAAATCATTACTTACTTAATAGATA
CTTAATGTTATTAGTTAACTTAATCAAAAATTAAAATAAAAATGCGTATTATTACTGACATTTTC
CCCTCTAAAAAGGGTTAAAATTTAGAGTAAATTCTGGTTTTATACTTAGATATTTTTACATGGGG
GTAGGGGGCATCAATGGAAAAACAAGGAAAAAAAACAAAATTTTTAAACAACTTTATACAATCT
TTAAAAACCTCAAAAAACTATGGTTATACTTTGGTGGAGGTTCTAACGGTTTTAATCATTTTGGG
GATTATGGGCGCATTGATGGGGCCTCGTCTTTTTTTCGGGGATTCTAGTAGTGCTAGTGGTGATG
CTTATAACTTGGCTAAATCTTTAGTGGGAGTTACCCGTCAACGGGCTATATCTCAAACTTCGGCG
GCAAGGTTAAGTTTTGATCCGAATCAATCGGATAAATTTTTGATTGAAATTGCTAACACTAGGGG
TTGTGAAGCCTTAACCAAGATTAAGTGAAGCTGCGGTTAGTTCTCAAACAGATATTAAAGTTTATT
CGACAACTGGTTTTCAAATAGGAGATAAGCTTAAAATTGGTAGTGACACTACTAGCAATGAAATT
ACGGGTAAAGATAATACAACAATTACTTTAGGTGTGGCTTTGGGTACATCTCAAAATAAAGATA
GCACTGTGGAGTTAGTAGAAAACTGGAAGGAAGATAGTGCTTTAAATCTGAAAAAAAATATCGG
AAATGGTAAGTATATTTATGAGTATTTGCAATTACCTGAAAATGTGACTGTGACATCAAACATA
ACTAATTGGACTCTGTGTTTTAACAGTCGTGGTATAGCTACTATTTATGATAATTCTGGAAATTC
TCAAGCTAAGTTGGAACTTACTTTCAAGGACGGTTCTAGCAATGAGGAAACTTTAACTATTTTAA
AAGGAGGTGCGATCGCAACTAATTGATTGACAAGAAAAATACTGATTTGATTTTTTACAGGCAA
GATGCCTGTTTCACAAAAAAGATTTCTATTCACTCACTTATCTAAATCCTATGAATAATTTACTC
AAACACTGGCTAATTAAATACCGAAATCGAGAGGAAGGATTTACTTTTCTGGAATGTTTAGTTGC
GATCATCATTCTAAGTATGGCTTTTGCTTTTAACGGACAAATGGTTCTGATGTTAAAAATGCAAA
ATTTAAAACAAGAAATTGAAAGTGCCGCCGTGGCGGTAGGGAAAGATGTTTTAGATGATCTACG
TTTTCAGCTAGGTAAAAACATTAATAACGTATCTGTGACAGGTAACACACCTACCACTCTGACCG
ATCGCATCAGTTTTGGTCATACCTTTGACGCTGATGTATATGTATGTACTGATCCGCCAACGGTG
GAAACAGATACAGATAATCCCAATCAATTAAAAGTCAGTAATTGTCCATCTGGTTCAACGGATGT
CTTAATTCGTTACATAGTTGTTCAAATTATCGATAAAAAAAGAGACAATGAAAAAATCTACACT
GTACAGACTAATTTTGCACAACTGCAAAGATAAAAGCAGTCTCGAAAAGGGCTTTAGTTTAATTG
AAGCAATTTCAACTTTACTTATGGGTAGCATCATTCTTGGTGTTGCCCTTTCTGGATTTTTCCAA
ATTAAAGAATTTTTTGGTAAAGATAAATTGAGTGTGGATGTTAACCAAAGATTGAGAACTGCTT
TTCAAACTATTGGGCCAGATTTACAACAAATGGGGGAAAATGTCAGTAATATAGAATTTCCTGCC

GTTGCCCTTAGCACCAATAACGGAACATCAGAAATAACTATTCGCCGAGGTGGTTTAGAGCCTTT
AACCCTCTGTGCTGACATAAGTGCTAACAGTACTGACTCTGTTACTGTGCTTGATAAAAATTTTA
CCGCTTCTCCTGCTTGTATCTCCGTTAATGACGATGATGGAGATGGCTGGCCTGACACGGTGAAA
GAATGGCAAAATTTTCGTAATGGCAATACCATAAGAGCCTATATTGTCGATACCTCGACCAATAA
AGGAGAATTCTTTGAATACAAAGGAGAAGAAACTCTTGACAGTGGGGGGGCGACGATGACACCG
TCTGGGGGTACAGAACCTTATGTTGTTAATTTGACTACAAATACTCATACTTGGGCAAATGATTA
TTCGGCGGCGACAACGGCAATTTATTTATTTGATGAGAGTACATACAAGGTTACGAATAACACTC
TGCAACTAATTAGGAATGGGGAGGTTTTTGATTTAGTAGAAGACATTGAAAAATTAGATGTTAC
AGCGATTCTTCAGGAAAATCCCACCGCTACAGAGTACGAATGCAAGACTATAAATTTAACTGAGG
TGGACTGTGATCCTACTTTTTCCATAGATGATTATACGTGGAATCTAATCAAGTCTCTTGATGTT
GAAGTGACGGCTTTACCCCCTCAAGACAAGGGTAATTTTGCGAAATTAACGGAGGATGATTTAAC
TTTATCACAGCAATTTCTCCCTCGTAACCGTTTAAATTTTTAAATTTATATTTCTTACTATTAAC
CAATGTTAAACATTTATTTTATTTATTTAATGTCATGAAAAATAACGCTAAAAAACAATTAATTC
TTCAAAAAGATAGTTTATCCTCTATTAAATGCTGGTTTCTATCAAGGTTACTCAATACTGATTCT
GAAAAGGGATATGTTTTGGTAATTGCGATCGCACTTTTATTTGGTTTGGCTAGTTTAGTTTTAGT
TTATGCCCGAAGTAGCCGTATAGAACAAGTAAGTAATACTGCGGCAGTGGATAGCACTAGTGGTT
TCTACGGAGCCGAGGCGGCTTTGAATGAAAGAGCCAATGAAATTACGAAAATTTATCAAAGTAAT
AGCGTTCCCAGTGGTACAAGTCCAGAAAGTACAATGGCTTGTTTTGATAATAGTATCAGTGATGG
TACAGGGGATTATCAATGTAAAACCTATCAATTTTCAGGGGGAGATAGTGAGCGTAGTGGTTATT
CTGCTACCACTTATGTTTTAGAGAAAAATGAGGGTGTCGGCAATGTGGGTAATGTTCCGCCGGGG
GATAACCATGCTGGATTGAATATGATTGAGTATGGACACTCTATTATTGCTTTAGCCTTTAAAGA
TAGTGATGTACAACAAAGACAACAACAGGGAAGTGGTCAAGGAAGAGGTGCGATCGCAATTCTGC
AAATGGACATCAAAAACCGCCTGATTCCTCTGTTTCAATTTGCCGCTTTCTATAAAGATGACTTA
GAAATCTTCCCCGGTGCAACCATGACTCTGAGCGGACCAGTTCATACTAATGGAGATTTATATAT
AGGAAATCAATTAGCAGTAGATGGAGACGTAACCACCGCAGGAGAAGATTTATTTGCAAGTAAA
GGAAATGACAGTGCGAAAAAAGGAAATAATCCTTCTCCCGTTAGCCCTAGTAATTACAACAGTCA
AGTAAAAACACCTTTTACTATTACTGATCTTAGAGTAGGAATAGATCCTTTAGGACTGCCAGAAC
CGGACTTCTTAAACTCATCAGGGGAATATTTTACCAAAGCTGATATTAGAATTAAATTTACCCCC
ACAGGCACAAATCAATCAACATACGGTGAAGACATACCTTTCACTATTGAAGTCATTGAACGAAC
CCAAGCAAACAATAGTCCCGTGTCAGCTCCTGTCGCTGTTTCCCTCACTTCTGCTCAAAGACACAG
TTTAAGACAACCTATATTAGTGAGTGAACAATTAGCTTCTATTCCCAGTAGTAGCGAATCTTTTA
GTAACAGCACAAGTAACAAATTCTATTCTAAGGCACAAAATCCAGATTTTAGTACTTGTAAACCC
ATTTATAAAGCTGATTTTTCCGACATGAGGAAAAATATTAAGTCCGCCTTTCCTAGTACTTGGAA
TAGCTTAACCCCTCAACAAAAGAGAGATGCGGCACAATTTGCCCATCAATTGTTAGTAACTAGAG
TTCAAGAAGAAGAAAGTCCTCTTAAATTTACCGATTTAGACTTAGTTGGCAACAATGGGGTTGAT
GGTTTAACTTTTACTAATATTCCCAATATTCCTTCATCATTACAAACTAATTTAGTAGCAAAATT
AAATTCTCTGACGGCGAATGAAATTGCCGCTCTTCCTTATCCTGATGCCAGTGGAAATACAGTGC
AAAGATGTTTTGCACCAGCTCCCCTAACTGATATTGGCAGAGATGCGTCTAATCATAATAAGCCC
CCATTTCGATTTTTTAATAATCGAGAGAATGATAATAATGGTAGGGATATGCGGTTGCTACAAAT
TAACATAAAAAGTCTCACCATCTGGAATTATGAAGGAATATATTTAGATGGCTCTCAACTCAAAT
CTGCTAACCAATTACTATTCAAAACAGCACCTGCTGATAACAAAGCCCCCGCAAATTCATTTCAG
GCTTTAGGTATGGCGGCAAGAGACACTTCCGATGATGGTTTAGTCATGTATTTGACTATTGATAG
TAACTCTTATACCAATGCAGGAGGAAATACTAGCCCCTACGGTTTCGCTATTGTGGAAGGAAATC

AGTTACCCGGTTTAGGTTCAGATAATGCTTTTAATCTTAATGATCCCACAGGGTTAACCATTGCT
AGTGACCAAGCGGTTTATCTTCAAGGAGATTATAACACTAGAAATAAACAACCTGCTTCTATTCT
CAGCGATTCTTTAAATGTTCTTTCTAATAAATGCTTTGACCCTAACAAAGTTATAAACAAAGAAA
ATAACATGAACTGTAACGATAGCACTAAAAATAATAATAATAGTTTAGGTAATGCACTTCAAAC
AGTAATGAATGTGGCGTTTTTAGCGGGTAATGATGAAACTAAAGGTAGTCAATATAATGGAGGT
TTAGAAAATTATCCTCGTCTCCATGAGAGTTGGAGCGGTATTCCTTTACGTTATAGAGGTTCTTT
TGTCAGTTTGGGAACTCCAAATCATGTCAACGGTAAATGGCCTGGCACTGGGGGCAAGTTTATA
ATGCACCCAATAGACAATGGGATTATGATACTGAATTTAATGATGCAAAAAATCTACCGCCTTTA
ACTCCCCGATTCAGTGCGATGAAACAAGAATCCTTTATCCGTAGCTTTGATCAGTAGTTTTGATC
GTTTCCCAATAATTTCCTGCCCTCACCCCTAACCCCTCTCCCACGGGGCGAGGGGAGATTTTTTA
TCACTATTAATTGATAGAACGACTTTACCTAACTATCTTAAATGTCAAAAGAGGACTCCCGTTGC
ATTTGACAAGAAAGAAATACACTACAAATCCTTTAGTCCTGACGAAAGGAAGGAACTGGGAGAT
GAATTTTGACCAACAAAAAAAAATAAAACGAAAATCAATGATTAATTGATTGAGTTTTTTCCTC
TTAAATGTTATGATGCTTTCCAGCAGTAATGTGCGTAAGGTATTCGGGGGAGTGACTTCCCTAGC
TTCTTTCGTCGGAACAAATATCGCCCAAGAAAATGTAATGGTGACGCGTTTGGTAAGTTAAAGCA
TTGATAGCAAAGTTAAAAAATTAAACAACTAAAGTTTTAAATCCTTCGGCGGTAGGGCATACCGT
AGAAGTAAAAGTAAAATGCTTGTGGATTCGGTCTGACGGGGGCATCATTAGTGATAATGATGCCT
AGTTAAGAGGAGTAGAAGCAAGAATCACCTGTTACAATCTTTGATTTAACGGGTGAGTGTCAAA
TTCAACTATTGTTTTCTAACTCTGATAACTCTAACCATTTTTCTGTTTTACTATCTATTTCCTTGA
TTATTTGGTTTAATTCTTCTGTTAAAATATTTAATTGCTCATAATCTTTCCCGCCATTTTCATAA
ATTATTTTTTCTAAATTGGCTTTTTTTTCCTCTAATTCAGGTATGATTACTGTTTCTAATTTTTC
TAATTCTCTTCTTTGAAAGTTAGATATTTTTCTTTTTTTCTGTTCAATATTATTCTGTTTTTTTT
CTTTCTTTTCTACTGTTTTAGTTTCTTTTACTTCATTTATTTTTTCTACTTTGTTTTCTTTTTCTT
CTCTGGTTTTATATTCTAAGTAAAGAGAGTAATTTCCCGGATATTCTCTAATTATTCCTTCTCCT
TCAAAGGCAAAAATTGTATCCACAGTGCGATCGAGAAAATAGCGATCGTGAGATACAATAATTAC
ACAACCCTTAAAAGATTCGAGATATTCTTCTAATACGCCTAAAGTTTGTACGTCTAAATCGTTGG
TGGGTTCATCAAGAATCAAAACGTTGGGATTCCCGATGAGAATTTTAAGCAGAAAAAGACGACGT
TTTTCCCCTCCTGATAGTTTTTCTATGGGTGCATATTGCTGATTTGGGGTGAATAAAAATCTTTC
TAAAAGTTGAGAAGCACTGATTTGCTTTCCATCTCCTGTTTCAATATAACTAGCAACGTCTTTGA
TATATTCTATAACTCTCTGTTGATTTTCTGCGGCAGTAATTAGATTTTCTGAGTATTGATCAAAA
TAGCCGATTTTTATGGTGCTACCGATTTCGATTTTACCTTCATCTGGTTCAATTTTACCCATTATT
AAATTCATCAGGGTTGATTTTCCTACCCCATTACCGCCAATAACGCCCACTCTATCATCGGGTTCA
AAAAAAATAGGTAAAGTCTTTAATGAGAGGATAACCCTCCCAGAATTTAGAAATATTATGTATTT
CAATAACTTTTTTCCCGATGCGACGGGAGGGAGTATCAATTTCTACTTTTCCTTGTTTTGCCTTA
AATTCTTTGTTTTGCATTTGCTCAATGCGTTGAATACGGGCTTTTTGTTTTGTGCTACGGGCTTT
TGCTCCTCTTTTGAGCCATTCTAATTCTTTTCTGAGGATTCCTTTAAGTTTTTGTTGACTACTTGC
TGTAGATTCTTCTGCTAGGGCTTTTTTCTCTAAATAATAACTATAATTGCCACTATAATTGTACA
TATCACCGCGATCGATCTCTAAAATACGATTAGTAACTTGGTCTAAAAAGTAGCGATCATGGGTT
ATTAATAAGATAGCACCAGAAAACTTTTGCAGATAGGTTTGTAACCATTCCACTAATTCTGCATC
AAGATGGTTTGTAGGCTCATCCATTAATAATAAATCAGGTTCAGCCATCAGGACGGCGGCTAAGG
CGACTCTTTTACGATAACCCCCCGATAAGCCTCCCATTTTTAATTCAAAATCCTGAATACCCAATT
TATCAAGGACAATTTTAGCGTTGGTTTCTAAATCCCAAGCATTTTCTTGGTTAATTTTTTCTGTT
ACCGTTGCCAATTTTGCCATTAACTCCTGCTGTTTTTCTTCCCTTACACTTGCCAATCGATGAGAT

AAGTCTTCATATTCAAGTATCAACTGCATTTGTTCGCCACAATAGGCTAAAACCTGCTGTAAAAC
TGTATTTTCTGGGTCTATTTCTGGTTGTTGAGGTAAATAGATAATTTTTGCCCCAGATTTTACCT
GCATTTCTCCCCCATCATTAGCTTCCCATCCAGCCAACATTTTGAGGAGAGTAGATTTTCCACTGC
CGTTAACTCCAATTAAGCCAACTTTATCACCTTCTTCGATACTGAAATTTGCGTCTTTTAATATT
TCTTTGATTCCAAAGTCTTTTTTGAGAGATTTAACGGTTAATACGGTCATTATTTTTAATAATTC
AGTTTAGATAAATTTATTCTTTGATTAACAATACTACTGCATAGGCACAAATAGCTTCTTCTCTA
CCCACAGGACCCAATTTTTCGTTAGTAGTAGCTTTAATACTAATTTGTTCAGGTGCGATCGCAAT
TTTATCAGCTAAAGACTTGATCATGGCAGGTAAATGGGGCTTTAACTTTGGACGTTCTGCGACAA
TAACACTATCAATATTACCAATGCTCCATCCTTTATCTTTAATCAACTTATCGACTTGATACAAT
AACTCCATGCTATCAGCACCAGCCCATTTTGGATCGGAAGGGGGGAAATAATGTCCAATGTCACC
TAAACTTAAAGCCCCTAATAAAGCATCCATAATTGCATGAGTTAAAACATCCGCATCACTATGAC
CCAATAAACCAAGATGATGATCAATTTCTACACCACCAAGAATCAGTTTTCTACCCTCAACTAAC
CTGTGTAAATCGTAGCCATTGCCAATTCTAATCATAATTTAAGTATATGCAATTTTTTTACTTAG
TTATAGGTAAATAATTATAATGGAAATAATCCTTTCAATAAACCAACCTCATACTGATATTTTTG
AGAAGAGAAAAAAGAAAGAAATATTTTAGCTAACCACATTATGTAAAAATATTATATCTATCAA
ATTTGTCGAGTTACTAATTGATACTATTAATTTTAAAATATATAGCAAATAAATGAACAAATCG
AAAGCAAAACCTTTTTTAAAATGGGCGGGAGGAAAAACTCAATTATTAGAAATAATTACCTCAA
ATTTTCCATATAAAAATGAAGATGAATTTATTTATGTTGAACCATTTGTTGGTAGTGGTGCTGTT
TTATTTTGGGTTTTAAATAATTATCAAAATATAAAAAAAGCCATTATTAATGATATAAATTTAG
ATTTAATTAATGCTTATAAAATAATCAAAAAAAATGTAGAAGATTTAATTTTCATTTTAGAAAA
ATGGCAAAATGAATACTATTCTTTAGAGCATGATCAAGAAGACAAAAAAAAATATTTTTATGAA
AAAAGAGATTTATTTAATCAAAGAAAATCCAACCAAATCAAACATACAGCTTTATTTATTTTTCT
AAACAAGACTTGTTTTAATGGGTTATATCGTGTTAATAAAAATAATCTATTTAACGTTCCTATAG
GTAGTTATAGAAAACCTGTTATTTGTGACAAAAATAACCTGCGACTTGTCAGCAATTCCCTTAAA
AAAGTTGAAATTATTCACGGTGATTTTGAAAAAACTTTGAGGTATGCCGAGAACAAAAGTTTAT
TTTATTTAGATCCCCCTTATAAACCATTAAATAAAACATCAAACTTTAATTCTTATGCTGACCAA
AAATTCAATGATACCGAGCAAATAAGACTAAAAAAATTTTGTGACAAATTAGACCAATTAGGTT
GTAAGTGGATATTAAGTAACTCTGATGTTAGAGAGGCTAATTCCGAAAATATTTTTTTTGATGAC
CTATATAAAAAATATCAAATTCATCGAGTTCTCGCCAGAAGAAATATCAATTCTAATGGTTTAAA
AAGAGGTTTATTAAATGAATTATTAATAACGAATAATCGTAAATAAAGTTAACTCTTAATGGGT
ATTTTACCGTAGCTATGATAATTTAATAGAAAATAAGAGCTTCAAATTATTATCACAAAAGCAT
ATAGATTAAAATGAAAATTTGATCTTTTACTATATTAAAAATCGCCCGTTGCCTATTAACCAATA
ATTTACACAACGGGAAGTAAAAGAGCCTTCTTAATTCCTAATTCCTGATACTTTCTTATTCTTTC
CCCGCCATAGATAAAATAAGGTCTATCATTCGATTAGAATAACCCCACTCGTTATCATACCAAGA
AACTACTTTAAAAAAGTGAGAATTTAATTCAATTCCAGCACCAGCATCAAAAATACTTGAATGGG
CATCAGAGATGAAATCAGAAGAAGCAACAGGCTCATCGGTATAACCTAAAACCCCTTTTAATTCG
CCCTCAGAAGTCTCTTTCATCCTCAGACAAATTTCTTCATAACTGGTAGGTTTTTCCGTCCTAAAA
GTTAAATCTACCACAGATACATTAGGAGTTGGAACACGAAAAGCCATTCCCGTAAGTTTTCCTTT
CAGTTGGGGTAACACTAAAGCTACTGCTTTTGCCGCCCCCGTAGAAGCAGGAATAATATTTTGTG
CCGCACTACGCCCTCCCCGTAAGTCTTTTTTACTCATACCATCTACAGTCGGTTGAGTGGCAGTCA
TAGCGTGAACTGTAGTCATTAATCCTTCTGCTAAACCAAAATTATCATCTATAACTTTAGCAATG
GGTGCTAAACAATTAGTTGTACAACTGGCATTAGAAACAATATCATCTTTTGTGGGATCATAATT
ATGATGATTAACTCCTACTAAAAGAGTCGGTATTTGTTCTGGAGTTTTTGTTGGAGCGGAAATAA

CTACTTTTTTTGCCCCCGCAATTAGGTGTTTTTTTGCTCCTTCATAATCAGTGAATAAACCTGTTG
ATTCAACAACATATTCTGCTTGGTTATCTTTCCACGGTAATTCTTCAGGATTGCGTATGGAGTAA
CAAGGAATTAGCTTATCATCAACTAAAATACCTTCTTCTGTGGTTTCTACTTTTCCCTGAAAACG
TCCATGAGTAGAATCATATTTAAGTAAATAGGCAATATTTTTAGGCGGTACAAGATCATTTATAC
CAACAAATTCAATATTAGGGTTAGCTAATCCTGCTCGAAAAACTAATCTTCCAATGCGTCCAAAT
CCGTTAATAGCTACTTTAACTTTTGACATAGATGACTCCATTAATTATGGGTATTGAGAGTTTTG
ATGTGTTAAACTCCTTACTTCTAATTGTGTTGATTTTAGAGCAAAGCAAATCTTAATTTTTTGGG
AATTTTTTATAATCGAGTATTGGGTTTTGTCCTAGCAACCAACCGAAAACCGCACTTGTTACAAA
CATTATTAATGAATATATTGCCGCCGGAATTGCCATATTAGGCATATTTAATAAAGTTGTAGCAA
TGGCAATGGCTAATGTCCCATTTTGAATACCAACTTCCACTGTAATAGATTTACGACTGGATTGA
TCAAGGTTAGAAATAGTTGCTAGAGTATATCCTAAGATCATAGTTAATATATTAAGAGTCAAAG
TTACCCCCCCTACTTGTACAAAGAAGTTACTGACATTTTCTTTCTCTTTAAGCAATAAACCAAAG
ATAATTATTCCTAAAAAAAACAAGGAAAGCCATTTTACTCCTTTGTCACAAACTGTTGCTAATTT
AGGTGAATAGTAATGTATTAACATACCTAAAGAAATAGGTATAAGGGTGATTACAGCTATTTGA
AGAATAGTTTTTCCGACAGGCAGTTGTAGTGTTACTCCTTCTCCCATAAACTTGGTCATGGAAAG
ATTAATTAGTAAAGGGATAGTAAATATTGTAACTAGGCTAGTTATTGCTGTTAAAGTAATGGAT
AAAGCAACATTTCCCTTTATTAGGTAAGTAATCATATTAGAAGTTGAACCTCCGGGGCAAGTAGC
CAATATCATCACTCCGACGGCTAATTCTGGGGCAAGTTGAAAAGCTAATGCTAAAAAATATCCAA
TAATTGGTAACATGACTAATTGGGCAATTAAACCAATGATGACTGCTTTTGGTTCAACAAAAACT
CGTTTAAAGTCATCTAATGTTAAACTAAGTCCCATGCCCAGCATGATAATAAACAAGGCTAAAGG
TAGAAAAATAGCTGTTAAAAAATTTGATTCCATTACTTTAAAAACTAAATAATATTGAAAATTG
TGCTTATTTTACTTCATTATTCTATTTTTATTAATGGTTATTTATTGAGATAATATTATGAGATT
TATTTATTGCTCTCAAATCACTAATTTCTTGAAAAAGAAAAAATACAAGAGTTGATCAGGGTTAA
TTAAGATTAATTCCCAAAACTTGCGTATAAGCTCCTCTGGCTTGTGTCACTCCAATAGTTCGTTG
TGCCGCTTCAATCATTGGTCTTCTTAAACTAACTACAATAAATTGAGCCTCTTTTGCTTGTTGTT
GAATCATTTTAGATAGTTTTTCCACATTCGCACCATCTAAAAACATATCCACTTCATCAAAGGCA
TAAAAAGGAGAAGGGCGGTATCTTTGCAAAGCGAAAATAAAACTTAATGCCGTCAAAGATTTTT
CTCCCCCTGACATAGAACTTAATCTTTGAACGGCTTTTCCTTTAGGGTGAGCCACGAGGGTTAAA
CCACCATTAAAAGGGTTATTTTCATCCTCTAATTTTAAGTAACCATCTCCATCTGAAAGGGTAGC
AAAAATGTTTTTAAAATTTTCATTTACTGCATCAAAGGCTTCTTTAAATGCTCTCAATCTTAAAG
TAGTAAAATTTTCGATTCTTAATAGTAATTCTGTCCTTTCTCCCTCTAAGGTTGTTAGTTTTTCT
GATAATTCTTCTAGTCTTTCTTGATTCTTTTCAAACTGTTCTAATGCTAACATATTAACAGGCTC
TAAAGCCTCTAATTTTTTCTCTAGTTTACTTATTTCTAGTTTAATTTGTTCCAATTGTTCCTTAA
GATTAGCAAAGGTTATTTTTTCTCCCATACCTTCTTTTAAATCAGTATTAACTAAAAAGGGAATT
TCGGGTAAAGGATTAGGTAAGTTAGTCTCTATCTCTTGAATAAGAGAGTTTAATTCACTGATTTT
AGTTTGATACTCTTGATGTTGTAGAACAAATTTTTCTAACTTCCATTGGAGGTTTTTCTGCTCAT
TTTCAATTTTCTTTAATTCTTGCTCTTGTTTATCTCTTTTTGCTTTCGCTTGTTCTAATTTTTGGG
CTAATACTTGAACTTGTTGCTCAAATTGAAATATATTTTGGCTGATTATTTCTGACTCTTGGTTA
AGATTAACTATTTCTTCTTTTTGTTTCTGTTGATTATGTAATAAATCATTAATTTTATCCTCATT
AATTTGTAATTCTTTCTCTAATTGACTCTGTTTGTTAACTAAAGTTCTTAGTTTTTCTTTATTTT
CACTTAAAAGTAATTGTCTTTCTTCTAACATTGATTCTTGCTCATTAATTAACTGTTGTAATTGT
TGCCATTCTTCGTTATCAAAGCTAGATTCTAAATCTTTTAATTGTGCTTGTTTTTCTTCTAATTC
TCTTTGCAATTGAGGTAATTTTTCTAGCAGACTATTTAACTCTTGTGTGCCTATTACTAATTGTT

GATAGTTATTAGCGTTACTCTCAATAATATTTTCTTTTTCTCTTTCTAAACGGCTAATTTCTCCCT
GATTTTGCTCTAACATTAATTGTTGTTTTTGTCGATTTTGTCTTGCTTGATTTAATTCTTCTGAT
AAGTTCTTGACTTTTTCTCTTTTTTGAGATATTTTGATGGTAAGCTGAGGCTTAATGTTTTCAAT
TTCAGAAATACGATCGCGCCATTCCCTAAATTCTTCATTCTCAGAAGCGGAAACCTTACCAAAAT
GTAGGGATGATTTTTGTGACACACTACCCCCCGTCATCGCGCCACTGATTTCCAATAATTCACCAT
CAAGGGTGACAATGCGATGTTGCCCAATCAAATTTCTAGCGGTTTCTAAATCTTCAAATACCATA
GTATTGCCAAAAACATAGGCAAAAATAGGTTGATAAATAGGCTCAAAATCCACTAAATTAAGAG
CTAAATCCACAAATCCTTTTGTTTTTAGTAAATTATTAGGCAAATGAATGCGAGGGGCGTTAATC
TTATTTAAAGGTAAAAAAGTTGCCCTTCCTGCCCTTTGTTGTTTTAATAGCTTAATCGCCATAGA
AGCGATACTGTCATCCTCTACCACAATAAAACCGAGACGACTTCCTGCGGCAATTTCCAACGCCA
ATTGATACTGCGATTCTACTTCTCCCAATTGGGCAACCAAACCGCAAACCCCGGGTAAATCAGAG
TTAAGGATAATTTTACTAGCATAAGTGCCTTGGGCTTCCTGTTGTGCCTGACGAGTGGCTTCTAG
TTTATCTAATTGTCTTTGTTTGTCTCTTTGCTCATTTTCGAGACGGGTTAAGGTTTCCTGATTTA
AAAATATTTCCGCTTCAATTTTTGCCAATTTTTCGGCAATATTCTGAATATTTGTCTCTGATACT
GCTATTTGCTGAGTTAAACTATTAATTTCTTCATTTTTGGCAATGATAGTATCTTCTAAATGGGC
TAGTTGTTGGTTTTCCTGTTGAATAGTTACTTGTAATTGACTACATCTTTCTGTTAATAATGCCT
GTTTAGTTAATTGAGGATTTAATACTCCCTGCAAAGCATTTATTTGTTGATTTAATTCTGTTTGT
TGGTTAACCCATGCTTGGGATTGAGAGGCAATTGCTTGGGCTTTTTTCTTAGTTTCATCTAAGGT
TTGTTTGCTTTCATTTTTACTATTTTCTAACTGAGGAATAAGGTTATTTTCTAAGGATTCAATTT
CTTTGTTTAACGCAGAGATTTCCTGTTGATTCTGTTTAATATTCCGTTGATATTCTTCCTGTTTA
ATGGTTGCTTCTTTTTGAAGGTTAACTAACTCCTGTTTCCGTTTGACTAGATTTTCTTGTTTCAG
TTTCTCCGTAGCTAGTTGGGAAGCAATGCTGATTTGTTCATCTTCACCTAATGCTTTTACCTGTT
GATTAAGGGTGACTAATTCTCCTTGAGTTATATCAATTTTTTCTTGAATTTTGACCAAATTTGCT
TGAGTTTTTGTTTGTTGAATATCTATCTCATTTAATTGCTTTTGATAGTCTTCTTTTTCTTGTAA
TAAGCAACGCCAGTTAATCACTATTTCCCATTGTTTTTCTCCTGTATTTGGGCTTTAATTTTCTG
ATACTTTGCCGCTTTTTCGGAATCCTGTTTTAATCTGGCACTATTTTGGATTAATTCTTCTTGGA
TAATATGACATTTTTCCTCTCTCTCTCTCACCGCCTCTAAAGTGTTTTTGGTTTGCTCAATTTTGC
GATCGAACTCTGCAACACCCGCTAATTCATCAATAATTTCTCGTCTTTCTTTACCATTCATGGTAA
TGATACGGGTAACATCTCCCTGTAAAACCACGTTATAACCTTCGGGATAAATGCGAAGGCGACTC
AAATGCTCATGTAATTCTGTAGCTGTACAAGGCACATCATTAATATAGAAGGTAGAAGCATAGCT
TCCCCCTTTAGTGACTCTTAGGCGACGGGTAATTTTTAGTTCTGTTTGACTGTCAAGGTTAAATT
TTGTCTCTGATTCTTCCTCACTATTACTATTGTCATTATCTGCTAATAAAGGATTATTTAAACTA
CTTAAATCCTCTAAATCAAACACCACAGAAACCACAGTTTCAATACTCTTACTATTACTTTTTG
ACTATTATTAATCAAATCTGGCAGTCTTTCCGCCCTCATTCCTTTTGAACTAGCCAAACCTAAACA
GAATAATAAAGCATCTAAAATATTTGATTTTCCTGAACCGTTAGGACCTGAAATAACTGTAAATC
CAGTTAAAAAAGGAACTATATTACTACCACCAAAAGACTTGAATCTTGACAATTCAACTTGTTTA
ATATGCACCATATAGCAATTCGATTCGGAATATAGAAATTGAGACGAGGAAAACTTAACTTCGAT
TTATGGTTAGCAATTTACTACTAACATCAAAATTTTCAAAGATGTATAAATATATCATAAATAGC
GGTTGATGAAAAAGTTTTGTCTAGGTATCAGGTGTCAGACAGGTAGTAGGGGTTGAATATATTC
AACCCTTATGGTGATGAGGGGGGAGAGAGAAACAAGTAATTAGTTAGGAGTTAGTTAGGGGCGA
ATGGCTATTCGCTCATACAGGAGTTAAGAGTTTTTAATTCTTAATTTTTAATTATCAACTATTAA
CTATTAACTATTAACTATTAACTATTAACTATTAACTATTCACTATTCACTAATTGCCCTTTGTT
AATACAATTCATACTTTAATAAGGTGCAATCTATGCCACCATTATTAATGGCAATACGACGGGAA

GTCCTCAAACCAACTTTTTTAGTTAATAATTTATTACCTGTTAAGATATAAGCTGTCCAACCTTT
AAAACGTTGCTTTAAAATATCTCCTAAAAGTTTATATAAAGGAAATAAGGCTTCAGTATCTTCAA
GGCGTTTACCGTAGGGGGGATTACAAATCAAAATTCCAGACTCGGCAGGAGGAGTAGCATCCACT
AAGTGACGTTGGAAAAATTCGACGTGATTAGCAATTTGGCAGGTTTTAGCGTTATGTTGAGCCTG
TTTAATGATACTACTATCAATGTCTGTGCCGACAATGGGGGTAAATCTATTTTTTGACTTTTTC
TTGCTTCATCAATCAAATTTTGCCATAAATTATGATCATAGTCTTGCCAATTTTCAAAGCAAAAT
TGATGACGATATAATCCCGGTGCAATATTTAAACCCATTAATGCCCCTTCAATTAGGATAGTACC
TGAACCACAAAAAGGATCATATAATGGTATGCTTGAATCCCATTCTGCTATGTATAGTAACGCCG
CCGCTAGAGTTTCTTTTAGAGGTGCTAATCCCACTGCAGGGCGATAACCTCGTCGATGCAGACTT
TCTCCTGTGCTATCTAAGCTAAGGATACATTCGTCATGGTGAATATGGGCGTTAATGACTATATC
AGGGGAATTAGTATCAATGTTCGATCTCACTTTATGATATTTTTGTTGTTGTTCAACGATGGCAT
TTTTGATTTGAATAGCAGTAAAATGGCTATGATTTAGTTGATCATTTTTACCTGTACAACGAATA
GCTAAAGTTTGTTGAGGTTGTAAAAATTCTGACCAATCGATTTTTTGTACATTGCGGAATAACTG
TTGTGGAGTTTGACTTTTTATTGTAGCGATAGGTTTCAAAACACGAAAAATAGTTCGTCCCCAAA
GATTTACTCGGTATAATAATTCTTTATCTCCCTGAAAATGTACTCCTGTAAAGTCGGTTTTAACT
TCCTTCGCCCCCAATGTGGTTAATTCTTGGGCGGTAATTTCTTCCAACCCTCTGGAAGTAGTGGCA
AAATAAGATGTAGTCACTATGATAAATACTAATTGATATATTCCTATTATTTTATAAAAAAATG
AAGGTCTGGCAAAATTTTTGGTGGCAAAGATTAATTATAATTGTCTCGATCGCAGCTTGTTGTAT
ATTATTAATGGTAAGAATTTCAGGACTGGAATTATTAGGAGTTACCCCCAATTGGCTTTTAATCT
GGCTTGTTACATGGAGTGTCAGACGTAATATTTGGCAAAGTGCGATCGCATCTATCTGTTTAGGG
TTAATATGGGATAGCTTAACGGGAGTATTTCCTTCTCATGTTTTAGGTTTATTGGTAGTGGGAAT
ATTTACCAGCAATGTTTATGGTAAAGAATATATTAAAGAAGACTTAATTTCGATTATGTTGATA
GTCTTTGGCATGGCAATTATCAACGAAACTATTACCGCCCTTCAATATAGCATACAAACTCAAGT
ACCTTTAACCGATATATGGTTAAACTACCAGAAAAACTCTCTTACTTCCGCTATTATAAGCAGTT
TATGGACTCCTTTAGTTTATTACCCCTTAAATTATCTCGTTAATAAGTAGATGGAAGATTGGGGA
AATAGGGAGATAAGGTATCAGGTGTCAGGTGGTAGGGGTTGAATATATTCAACCCTTACGGTTTT
AGGGGAAAACTTCTTTTTTATATTAACTCCGAACTCAAATTATTGCCTCTTGCCCTTTGCCCCTTA
CTCTATTCTTTCGTTATGCCTTGAAAACTTTTGATCCGTTGTCTCTATTTTGCTATAATTAGCATT
CTAAAAGTTAGAGATACCAATAAATTCTAAGATGATTAATAATTCTAAATTAGCTCAAGCCTTCG
CCTCAAAAAAAGCTCTAAAAGTGATTAGCGGTTTAAATAACTTCGATGTTGAAAATGTAACTGCT
GTATGTAAATCTGCTGAGTTAGGTGGTGCAACCTTTGTCGATATTGCGGCGGATATGGATTTAAT
CAAAACTGTACGTCAGTTAATCAGTCTTCCTATCTGTGTTTCTGCCGTTTCTGCTGAAATTTTAG
CCCAAGCAGTAGATAATGGAGCAGATTTGGTGGAAATCGGTAACTTTGATAGTTTTTATGCTCAA
GGTATTACTTTTTCCGCAGAAGATGTTTTAAACTTAACCCGTGAAACCCGTAAATTATTACCTGA
AACAACCCTTTCTGTTACTGTACCTCATATTTTAGCCCTTGATGAACAAGTTGAGTTAGCGCAGG
CTTTAGTTGCTTCTGGTGCAGATATTATTCAAACAGAAGGTGGCACAAGCTCCAACCCCAATCAT
AGCGGTATTTTAGGATTAATTGAAAAAGCATCTCCTACCCTCGCCGCCGCTTATGCCCTATCCCGT
GCGGTTGAAGTACCTGTTATGTGTGCTTCAGGTTTATCTGATGTTACTGCTCCTATGGCGATCGC
ATCTGGTGCTTCTGGTATTGGTGTTGGCTCTGCTATCAATAAATTAAATGACCAAGTTGCCATGA
TTGCTGTAGTACGCAGTTTAGTTGAGGCTTTAAATACTGCTTCTGTAAAAGTATCTATGTAACCC
AAGTTATAAGTTCGGAGTTGGAAATTGAGAGTTAAAATTAACAGATTTTGACAAAGAAAAGAAA
TAATGGGCAATAAAAAAACTTTAATTCAGATTATAACCATTTATATCTTATTAATAATAATTGCC
CTAGCAATGTTATTTCCGTTATTTTGGTTGTTAAGTACCTCTTTTAAGTCTGCAGGAGAAGATAT

TTTTGCATTTCCTCCTCAATTTATTCCGCAACAATTTACCCTTGAAAATTTTGTTTTAGTTTGGC
AAAATTACCCCTTTGGTAAGTATTTATTAAATAGTAGTATTAGTGCGGTTTTAACTGTTAGTTTA
AACTTATTATTTTGTGCTTTAGCCGCTTATCCTTTAGCCAGATTAGAATTTAAAGGTAGAGATTT
TATTTTTGTCTTAATTGTGGCAACTATCATGATTCCTTTTCAAATCGTGATGATTCCCCTATATA
TTTTTGCTGTTAATCTAGGTTTAAAAAATAACTATTTAGGTTTGATTTTGCCACAAATTACCTCA
GCTTTCGGTATATTTTTACTAAGACAAGCATTTCAGGGAGTACCAAAAGAGTTAGAAGAAGCAGG
AAGAATGGACGGTTGTAGTGAATTGGGTATTTGGTGGCATATTATGTTACCTGCGATTCGCCCTG
CAATTATTACCCTTGCTATCTTTGTTTTTATTGGTTCTTGGAGTGATTTTCTCTGGCCTCTTATTA
TCCTCGATCGCCCCGACTACTATACTTTACCATTAGGAGTTGCATCTTTAGCTAGTTCTTTGGACT
TAAATTGGCGTTTTATTGCCGCAGGCTCGATTATTTCGTTAGTTCCAGTTTTAGTTTTATTTATT
ACTTTACAACATTATATTATTCCTAGTGATGCCAATACAGGAGTGAAGGGATAAAGAGTAATTA
GGAGTTATATTAGTTTAGATTGCGGAGTTAAAGTTAATAGACTTCTTGCAGAAATCAGGTAACA
GGCAACGATAAAAAATGTTTCTATTACTTTGAGTATGTAAATTATTGGTTAGGAAAAGGTTGCA
GGTGGCGGGTTTGACGGTTAAATAAAAAATAGAAAAAAAGACAGTTAATTAATAGTAAATAGCT
CATAATGAATAGATTTCAATTAAGAATAAAGAAAAAGTAGTGACACACTAGATAAAAAAAGAAG
AATGAAAATAGTTAACTCCTAATTTTAATGCCTGATACCTGAAACCAGAAACCTACCTAACATTA
TTGATATTATTACGTCGAATTGAGATTTTTTTATGCGTGCAGATAACATACAATTACCATTAATA
CCCATTATTAATGAATTAATTATTAATAACCCGAATACTATCTCTTTAGGGCAAGGAGTTGTATT
TTATCCCCCTCCAGATGAGACTTTCAAAGAGATTGATAAATTTGCCGGTAATCCTCAAAATAATT
TATATCAATCTATCAATGGTATCAATAAATTATTAGAAGAAATAGAAACTAAATTAGCTCAAGA
TAATCAGATTAATTATAGTCAAAATAGGTCATCTATTTTCGTAACTGCAGGGTCAAATATGGCGT
TTATGAATGCAGTTTTAGCTATCACTAATCCAGAAGATGAAATAATTATTTTAGCCCCTTATTAT
TTTAATCATGAAATGGCGATTAGATTGGCTAATTGTAAACCCATAATTGTCGATACAAATAATCA
ATATCAACCAGATATAGATCAAATTATTAATGCCATAACACCAAAAACAAAAGCAATTGTAACTA
TTTCTCCTAATAATCCCACAGGGGTTGTTTACTCAAAAAAAGACTTATTAGCTATTAATATAATA
TGTAAGGAAAAAGGAATTTATCATATCAGTGATGAGGCTTATGAGTATTTTACTTACAATGGAA
TTAAACATTATTCTCCCGCCTCTTTTGTAGATAGTGATAATCATACAATTTCTTTATTCAGTTTA
TCGAAAGCCTATGGTTTTGCCAGTTGGAGAATTGGCTATATGGTGATTCCAAAACATTTAGAAAT
GGCTGTTAAAAAAATTCAAGATACCATTCTAATTTGTCCTCCTGTAATTTCTCAATATGGTGCTT
ATGGTGCTTTAAAAATCGGCAAAAATTATTGTCAAAAATACTTACTAGAAATAAATAAAGTTAG
AGAAAAAATGCTTGATTCTTTAGCAAAAATTTCTAATATTTGTGAAGTGAGTAAATCTGATGGG
GCATTTTATTTGTTTTTAAAAATTAAGAGCGATATGAATTCTTTTGAATTAACAAAAAAACTCAT
TGAAAATTATCGAGTGGCGGTGATTCCCGGAACAACTTTTGGTGTAGAAAATAAGTGTTGTTTAA
GGGTTGCCTATGGAGCGTTAAATTATGATACTGCATCTATAGGTATTAATAGATTGGTGGAAGGT
TTATCACAACTTATCGAGGTTTAAAGCGGGGGAGAAAGCAAAGTTAAAAGGGCAATGGGCAATA
ATTTGAGTTCGGAGTTAAGATAAAAAAGAAATTTCCCTCTTTTTATCCCCTCTCTCAAGGCAGGG
TTTTTTCAAAGTCAAGGCAAAATTATCTTGCCCTCACCCCCAACCCCTCTCCCACAGTAGAGGGGA
GCGTTTTTTCTTAATAATTGATTAATTAATACTTAAAAACTCCTGTATCTGTTCTGACGGGAGAC
AATGAAACTGCAGTGAGGGTAGGTTTTAGGTTTCAGGTTGCAGGTTTTAAATACATAAACAGTA
TTTTGTATTTACGAAATAAATAGCCAGACATATTATCTCAAAATGTTGATAATCCAATAGTTTTG
CTTATTATTTGAACCATTGAGTAAATAGAGATAATGATAAATATTAGTGGAGACAAAACCTAAC
ACCTAACACCTGCTACCTGCTACCTATAAGGTTTTTAGAGTTATTGTCCCCCCTTAAGGTATCTGT
TACTATTTGTTAGTTTCAAAAATTGACATTTTTGAGAATGAAAAAACCCTACTCTCAAGGCAGGG

CTGTTTCAAAGTAAAAAATTAGGTTAACTGAGATTAAGTGAGAGTAAGAGATACAAGGATTTTT
TTCTATTAGTTAAAACTTTATGAACAAAAATCCCCCATCTCCCTCTCCCCTCATCACCTCATCCCC
AAATATTCCAATACCCTAAAACCTGACACCTCTTTCTCAACTCTGAACTTTCCTAATTCATAGGTT
TTGTAATCAATCTACCTACGGTTTTATTGTCGAAGGTAAAAGTATTTTGAGCAACCCTTTGAATA
TCTTGGGCAGTAACGGCAGAAATTTTGTCTAAACTTAAAAACAAATTACGCCAATCTCCTGTTTT
CGCTTGATATTCTGCTAATAACTTCGCCATACCTGCATTAGAGTTAAGACTACGCAGTAAACTAG
CTCTAGCTTGAGTTTTTACCCTTTGTAATTCTTCTTCTGTGACTAAGTCTGATTTTAATTTTTCTA
TCTGTTCGTGTAAAGCTACTTCTAAATCTTCCAATTGTCTTTCAGGAGCTAACATGGCATAAAAT
AACATTAAATTGGGGTATTTGTCTCCGGGAAAACCGTTAAACCCTGCGGCCGAAAGAGCTATTTT
TTGTTCATCTACTAAGGATTGATACAACCGGGAAGTGCGTCCGCTACTCAAAATAGAACTGATAA
TATCATAAACCACATAATCAGGATCGTTAATGTTAGGGATGTGGTAGCCTTCTAAATACCAAGGT
TGTGAAGGATATTCGATCACAACTTCTCTTGTTTCAGTTTGTTTTGGTTCGACTATATTTAGTTT
AGCGGGTTTGGTGCCAGAAGGAAAACGCCCGAAGTATTTATTAGCCATTTGCTTAACTTCTTTGG
GGTCAACATCTCCGACAATGGCAATAGTAATATTATTACCCCCATAGTAACTATCAAAGAAATTT
TGTACATCCTCTCTGGTTAAGTTAATAATATCTTCTTCATAACCGATGACAGGGCGTTTATAAGG
ATGAGTAGTAAAAGCGCTATCTAAAAAGGCTTCTACCATTTTGCCTACAGGAGAATTGTCTGTAC
GCAATTTTCTTTCTTCAAGAATTACTTGTTTTTCCTGATAAAATTCCCTAAAGACTGGATTCAAG
AAGCGATCGGATTCTAAATACATCCATAATTCCAATTTGTTAGAGGGAAAATTGTAAAAGTAAA
CCGTTGAATCTGCCGAAGTTGCTGCATTTAGACCAACTCCACCTTCCAGCTCTATTATTTGTCCAA
ATTCATTTTGCTTAACTAAATTATGGGCTTGATTATTTAACTGTTCAAATTTATTTTCTAACTCG
GTTAATTGTTCTTGATTATTCTCTTTTTTAGCTTCTTTTATCTGTTGAAAAGTTTTATCTAATTC
TTCTAAAATCTGTTTTTCTTTTTCATAGTTGATCGTACCAATTTCCTTCGTACCCTTAAAGGCTA
AATGTTCTAAAAAATGAGCGACTCCTGTTTTTCCTTCTGGTTCATTAGCACCACCAACATCCACA
TAAGTGTTAAAAGAAATAACGGGGGCTTGGTGATTTTCTAAAACGATAAAATGAATACCGTTGC
TAAGGGTAAATTCTGTTATGTCTTCTTTTACTTGGTTAAGATAAGGTTCTATGGATTGATTGCTT
TCATTTAGGGATATTTTAGCTAAAGCTAGTTCACAATTATTTGTCCAAGCAAAAAAGATTATTAA
TCCGAAAATAAAGGCTTTATATAAGGGTCTAAAAAACATATAAATTTAGTTTTCAAATACTATT
TTGATGAAAATTATAGATAATTATTTATTACTAAAATTATTAAACTAATCTTCTTTTTTAACTAC
TCTTTTGATTTCGGAATAAAAGCAAGTCTGCCGTACTTGATTATTGTGTTTGAAAATAGTATTTC
TTAGGCGTAAATTATCACTGGCAAAATTAATTCTTTCCTCAAGGTATTGATCATCTTTTATTATT
CTTAAAATTAATGACTCATCCTCTGCTAAAACATATTCTCCTTTAACTATTTCCGATGAGTTAGT
AATTCTGACAATATTACCCTTATTGAGATCATTTTCAGAACGGAATATCAACATTAAACTTTCTC
CCGTTTGTTTTGGTTTTCCCCAATCAGGTGAATTATCCCAACTAGATGCGATCGCACCCAAACTTA
AATCGAGATTAAAATGATATTGGTTAGCTAACTGAGTTATTTTGCTATCATCAGCAGTTAAAAGA
GTAAGAGTAACATTAGCTTTTGCATTATCTACTTGATTTTGAGGAAGATGATAAGTAGTGCGTTG
AGAAAACCAATCGCCAACAAATTTATCTAAAAAATTATTAATATCCATTCTTATAAAAAAATCAT
CATATTAAGCAATGGTAGTAATTATAACCCGACATCGTTCCAGATAATTAACCTGAGTTCGGGAT
AAATTTTCATCTATGAATGATGGAAAAAGGGCAAGGGGCAAAGGTAAATAGTGTTGGGGTGAAT
AGTTGATAATTACTCGTTACTCGTTACTCATTACTCCCTTAAACCTAACACCTGCAACCTGCAACC
TGAAACCTGACATCTATCCTTATCCAATATTCTTAAACCGAATTGAGGTTAATTAGTGAACTTGG
GTTCGGCGATTTCACTGCACTCATTCTGCAAAGGGTACAAAGTTTGGAAATAAGTTTCAGGTGTT
AGGAGGCAGGTTTTAGGTTTTAATTACTAATTGCTAATTCTTCAGTGGATTTATGTCAACGCTTC
AAAAGCTACCCCTTCAAGGGTAAATTGAGGGAAATTTTCTAAGATATTATTTTTTTCTGTTTCTG

TAACATAAATATAAGTACCGGGTTGTTGGGTATTTTGAAAACGGTAAAAGTCCGTCGCCATATTA
GCATCTGCGTCATAGGCATAAAAAGCGATTCCTTCTTCGACAAAGTTAGGATTATTTTCTCTAAT
GCTAATGCTTTCTTCCTCGCTAGCATAAAGATAAGTTCCGGGTACATCCGTATTTTGAAAGCGAT
TCAAACGAATTAAAGTATCATTTTCTGTGCTGGAGGCTTTAAACGCAAATCCTTCTTCGACAAAG
TTGGTAAAATCTCGGCGGATAACTTCGCTTTCGGTTTCGTTAGCATATAAATAAGTGCCGGGGCG
ATCGCTATTTTGAAAACGATTAAAGGTAGTGGTTAAAAGGTTTTCAGGTTTATCTGCTGGATCTA
CTGGTGTACCAATTAAAGGAGGCTCAACGGGATCTGCTAAACCAACAAATTGTACAACTCCATCA
CCACCAACTATATTTTCTGGATCTGACACAGAACTTTCTCTTGTCCAGTTGTTAGCTAACCAAAT
ATTACCACTAGGGTCAACTTGTACGGCTGTAATATGCTGTAGAGCTTCACTTTCAAAAACAGTGG
AATTTCTGGAATTTTCTCGATTGAGGAGTACTAAACTAAAATTATCAAAACCCCCTGCCCAAACT
TGATTTTCTGAGTCCACAGATAAGCCCCACAAGCCAAAATTTTCTAAACCAAGGGGAAATTCTTG
AGTTTCTTTTGTCTCTTTATCAATGAAAGTTAAACTAGAAGTTGTCCAGTTTGTAACCCATGCGT
TGCCCTCTGTATCGAAAGCAACGGTTCTCGGTGTGCCAAAGGTGTGGGGAAAAGGAGGGGATGTA
GGGGGTGCGTTGGTGGGAATGTTGTCGATTAATTTGATTTCTCCATCTTCATAGTCAAGGATAGT
AACACTACTATTTCCTGCTTGATCATTTGTTACCCATGCCAATCCTTCGTTATCAATGGTAATGTT
AAAAGGTTTACCGATGACATCATTATTGGTGAAAGTAACCGCCTGAGATGGATTACCTTGAGGAT
AAACGGTGACTGAGCCTAAATCATCTAAATCCGCAAAGCCTCCACTATTGGGAATCCAAAGATTG
CCTTCTTGATCAATAGCAATTCCTTGGGGGTTATCATTTTCTTGGATACCTGCACTACTCCACAGG
TCTAAATTAGCCGTGTTATTTTCAAAGTCAGGGGTAAAACGAACAATAGAACCGTTACCATAGTT
GGCAAACCACGCTTTATCATCTGCTCCTACCGCCACTCCGTAACCTACCCCATTAACTAAACTGGT
GTCGGGAATGGGACTGTCAATAATAGGACTGCCTAAAATTGGCTCTCCTGCTGGATTTAAAACTG
TTATCTGACTGGCAGGGATGCGTGCTGCGCAGCAGATCCCTTCGGGATCGCCTTCTCCATTAAGA
AAAGACTGATAATCCCAATTGTTAGCAATCCAAGCGTTACCCTGACTATCAAAAGCCATTCTACC
TACCGCATACATCCCACCTGCGGTATAGTGAAGGGGAATAGGTAAAGTGGTGCGAAATTCCTCTA
ACATAGGTAAAGGATTAGAGGCAGATTCTGCTAAACGGAAAAAACTTCTAGCATTGGCTTCACTC
AGAGTTTGGTTTAATTCTTCTGCTAAATTAAATAATGCCTCAAAAGTGCTTTCAGGGCGATCGCC
GTCTGGTAAAGTAGTTATATCAAAAAGAGTATTGCGATTTTGCTCAGTAGCGATACTAGCAGATA
AAGTATTAACTAGAGTATTAAAAATTAACAGGGCTTCTTCATTATCTTCATTGGCAATAATTACT
CCTGCCTCACCCGTTTCAATATTAGCAATATTAGGCACAATGGAAAAAGCATTATCCAACCCCGG
AGAAGGACCAAAAATAAAACCTTCATCGCTGATAAACTGAGCTAAAGCATAAGTGGAGGCGATA
GTAGTTCGTTCATTGATGGTTACATTTTGACTATCTGAATTAGGGTTTACATTACCAATGGCATT
GATTAGGATAACTTCACTATCTTCAGCATTACTTTCAGAAGCAACAATATAAAAAAGATCATCTT
CACTAAAATTGTCTAAATTGAATTGAAATGAACCACTCGCATTAGTAGTAGTTTGTCCAACCAAGA
TTAGCCTCTTTTAAATCTGATTTATATACCAATACCTGTCCGAATCTGATACCCTCGCCATTGGCA
ATAACTTGCCCATTAAGTGTCGTCATGATCAAAAATTACTGGTCTTTAGTTTTGCATTGACCTAA
TTCTAAGTATTTTTATCCATAAACAAAGATATTCTTAATATTCCTTAACCTATTAAAGTAAAGGC
AATTATCCCCTTTAGGATAAGATAGCAATCGGATAATTAAAATATTGAGAAGCTGTTAACTTTTC
ATCTTCAGGAAAAGGTAAATCAGCAACCTTTAGACCTTTTTTTAACTTCGACTGAAAATCTAAAA
CTTCAATACCAACAATATTATCATTTTTATCATAATCAATGATAATGCCGTCTTTTATCTCATCG
GAGTCATAAGAGAGAATATCTTTTAAATCAAAATAAATTGCGTCTATGTCGTTGTCATATCTAAC
TTTCATATTTTCCTTAATTTGAGTAGTTCAACAACAATAGTGAAAACCTATATTTTTATGCCGAT
AACAATACCGATAGTTATACCAATAACTAATATATCTTATCCCGATAAACAATAATTTTTAAACC
GATTGTTGTACCAACATAACTAAATTTACTGATAAGATAAGAGTAGCTAACCGATAACAATACCG

ATTATTATACCGATATGGCGACTAAAAGAGTTAATAATGAGTTTCATCGTTTGGATGTTAAATTG
CCCATTGATGTTTATGAGCAAATATCCAAAATAGCGGTTAATAGTTTTTCTGCAAGGATTCATCA
CATCAGTGGAAAACCAGAAATAACACAGACTTTAATTTATCTTTTAGAGCTAGGGATACAATCTT
TTCTCGATGGAAATGAACTGATAAAAAAGTATGATACCGATAAATATACTGATACCATACCGATA
ACTGAGAAAGGGGTAAAGCAATTAATTAAGGAGGAGCTAGTACCCATTCAATCTAGGCTCAATG
AGCTATCAGAGGTCGTTCATCGGTTAACTTATACCGATAATAATACCGATATTCTATCCATAGCG
GTGAATGAAGGAACATTGGCAAAAATAACCGATAACAATGCCGATAAGTATGCCGATAAAATAG
AAATAGATACCGATAATATTACCCCTTCAGAATCGCTTAAAGGTTCTTAGTGAACCTCTTTCCTA
ACTTCGTTCGAAGGGAGTGCATGAGCGTTTTTTGCGTTCTCCTCTAGCTCTAATTCTCATTACTCA
TTATCCATTTTTTATCAATTCTAATTAATTTGTAGGCTTTTTTTCTGATAAAATATCGTAGTTT
ATATTGTTAATTTCACTGTTATTTAAGTAGTTAGATTCTCCCAGAATAATTATAGTGTCACTAGG
CATTGTTTGAGTTTTCAAAAACTCTTTTAGTTCTGATGTGTCATAAATAAAATTAATGTTTTGGT
TACTATAATAAACGGTACTATAACGAGTAAAACCGAGTAAAAACACTTCTTCTTCTGGTTTGATT
ACTTGGGAAATTTGTTTTGATATTTCTCTAAATTCTAGTTGTCTTTCTCGATCTAAAAAAGGAAT
TAACGGAGGAAAAGTTAGGGTAATAAAACTGAGAAAAGCGATAAAATTAGCACTCCAAAGCCAT
TTTCTTAGCCTTTGTTTAAATAATAAATAAATACCGAAGACAGCCCCTGTTCCCCAAGAAATTGC
CGCCATAATGGGGATTCCTGATTTTACTAAATTTTCTGCTAAGGTGGGGGCGGTGGGATCTTCTC
CTACTAATTTACCACTAATTCCAGATGCGATCGCAAGTATAATAAGAATTAAAATATTAACAATA
CTACTAATTATAAATAGCCAATTATTTTTATTTTTATGGTTTTTATTTTCCCTAAGTTTTTCGCC
CCAAAATAAAGCTAAAATAATTACCACTGCGGGAGTAATAGGAAGAGTATAACCCTGTAATTTA
GTATCGGCGGAAGAGAAAAAAACGAAAGTGGTAATAAACCACGCAAAAGCATATATTCCTAAGT
GAGAGTTACGGGCAGATTGTCTTACAGCTTGAATACGCCAACAAGATAATTGAATGACGGCGATG
GGGAAATAAATTGACCAAGGTAACATTAATACCGTAGCCCAAATAATGTAATAGTACCAAGGTCC
TGGATGGTTAAAAACAACTTCTGTAAAGCGTTGAAAGTTACTTTCTAGGATAAATTCATTGATAA
AAATTTCTCCGTCAAAAATGGTAGCCATGATATACCAAGGGAGGGCAACGAGCAAAAATAGAGTT
AATGCACGAAAAGGCTTTATTTCCCAGAAAATTTCCCAAAATTTGCCCACATAGATTAAAAAAGC
CCCTATTCCTAACACTGGTAAAATAACACCAATGGGACCTTTGATTAAAACTGCGATCGCAGCTA
AAATGGGAGAGAGACAATAATAAACCGTTTGTTGCTTTGGTTTCTCAGGTTGGGCATAACCCAGA
AAAAAAGATAGCATGGACAAGCCAACAAAACTAGATAGCAACATATCTGACACTGCCACCCTACC
CCAAGCTATCCAATAGGGATTTAATGCCATAATACCTGCACCAATCCAAGCGGTTAACCATAACT
GTGGCGAATTAATTTCTGGTTTTTCACCAATAAAGCCAAAATAACGTAAAGTATAAAATACCACA
AATACAGATGCGATCGCACTTAAAGCTACAGGGAATCTAGCCGCCCATTCACTGATACCAAAAAT
TTTAAAACTTAGTGCCAGAAACCAATATCCCCCCGCAGGATAAGAATAAAAATAATCTCCATTCC
AATGGGGGCTAATCCAATTACCTGTAAGCACCATTTGACGGGCAACTTCTACATATAAAGCCTCT
GTTTTGTCGATTAAACCAATGTTTCCTAAACCCATCAAAAAAGCTAACCAACAAACAATACCGAT
ACAGATAAGAGAAACTATCCATAATTTTTGTGATTGTTTATGCCATAACTGCTGAAATTGCAAAG
ATTTATCCTCCCTGTAAGTTAATTTGCATCTAAACTAAAAATCATAGCTTAATTCTTTAACGCTC
AAATTTTCTCCCCTGTTAAAAAGATTGCCAATTTTCAGCAATAAATTTTTCTGCTTGAATTAAAG
TTTCAGGTGTACCAATATCTAAAAAAGGAGCAGAAGTAATATGAACTTTTATCTTACAACCTTGA
CTTAGTAAATAAGGAAAAATATCATATTCAAAACTCAATTTATTATTTTGAGGAAACTTAGCTA
AAACATCATTTTTTTAAAAGATATATTCCGCCATTAATTATACCTTTACCGTCTTTTTTTTCGGCA
AAACTAATTAAATTATAATCATCATCAAATTTTAAAGAACCATAACGGGAAGCATCTTCTACGGT
TAAACCTAAAATAACCCCTTCTACAGTGTCATCATTTAAACATTCAAAAAAAGGTTTTAAATTGC

TAAAAATAAGAGAATCTCCGTTTGCTACCAACCAATAATCTGTATAACTTTTATCTTGTTTAACT
GCATTAATAAAGCCGCCTCCAGTACCTAAAGGCTCTAACTCTTCATAGCATTTGATAGATAAATT
ATCAAATAAACTTGTATTAAAATAATTTTTAATTATTGTTGATAAATACCCTGTAGAAAGAAGA
AAATTATTGATATTTTGTTGATGTAAATAACGAATAATCCACTCAAGAAAAGGCTTATCTAAAA
CCTTTGCCATCGGTTTAGGAATATCTGGTAATAAATGTTTTACCCTTGTGCCAAATCCTCCTGCT
AAAATAACAGCAGTAATTTTTTTTTCTTGGTTCACAAATTTATTATTCATGTTAATAATATATTG
AGTAGAAATAAGTGATGATTGGTGCGGATAGTTAAGGTTTTAGGGGATTGGGGTATTAGGAAGT
ATTTTATTTCATCTGACACCTGACACCTGCAACCTGACACCTTTTCCTAAGCAATAATTGACACG
ACGAGAAGTGACAGAGCCACTATTATCGATAATTAAAACCAATTAAACCTAAATCTAAATATTTT
TTGATTCGTAAAATTTTGCCCTCTAAACAAGCACTAAAAGTAAAAAAATGTCTAATTTGATTATT
GGGAATTTTGCCTTTTAATTCTAGTTTAAAATAATTAGGTCTTTGGCGAGATTGACCTTGATTTA
TTTTGACAATAACTTTTTCTTTATACTTAGAAGAATAAGCAATATCCCCTCGAATAGAAAAATAA
TTATCAGGTAAAATTTCTGGAATATTTGTTTGAGGTTGATAGATTCCTGCTATTTGAAAATGAAT
ATTATCTGTGTCTGGAATAGTATGAGGATAGACAACCCAATTTTGTACTTGGTCTAAATCCACAT
GATTTTTCACCGCACCAATGGTTTTTCCTAATAGTACAGTATTAAAAGTTTGATTGTCAGCAGTA
GTAATAATTCCTTTAGTTAAAGCATTTTGCGAAGGTTCATATATTCCTTGAATGACACCTATTGC
TCTATATTGTAAGGGATGATCGGGAATACCAATAGCAGGATGATCTAGGAGAAAAGTAGAATCG
GGGTTAGAATTAGCAGAAAAATCAGAAGAATTAATGACGCTCACACTCTCTATATCAGATGAAA
AAGTTAAGATGAAATTAGTAGGATAAAATCAATCGCCTCTATTGGCAACGGTGAAAAACCCTTTT
CCTAGAATATAACAACTAGATTGAACATAATCAATTTAAACATCAATTAAAGATAACAAATCAA
AGTGGGGGGCAAATGAACACGGCAGAATTATTAATCAAATGCTTGGAAAATGAAGGAGTTGAAT
ATATTTTCGGCTTGCCCGGAGAGGAAAATCTGCATATTCTTGAAGCCTTAAAGGAATCCTCCATT
CAATTTATCACCACTCGCCACGAACAGGGAGCGGCTTTTATGGCTGATGTATATGGACGTTTAAC
GGGCAAAGCAGGGGTTTGTCTTTCCACCCTCGGACCAGGGGCGACAAATTTAATGACGGGGGTTG
CTGATGCCAATTTAGATGGAGCTCCTTTGGTAGCAATCACAGGGCAGGTGGGGACTGATCGTATG
CACATCGAGTCACATCAATACCTCGATCTTGTGGCGATGTTTGCTCCTGTCACTAAATGGAATAA
GCAAATTGTACGACCGGGTATAACTCCTGAAGTGGTAAGGAAGGCTTTTAAGGTAGCGGAGAAGG
AAAAACCGGGGGCGGTACATATAGATTTACCTGAAAATATTGCGGCGATGGAGGTTCAAGGTTCT
CCTTTACGTATCGATAATCGAGAAAAAATCTATGCTTCTTTTAAAAGTGTTAATAGTGCGGCAGT
GGCGATCGCAAAAGCTAAAAATCCGTTAATTTTGGCAGGAAATGGCACAATTAGAGCAAAAGCGG
CGGATGCGTTAACCCAGTTCGCTACAGAGTTAAATATTCCCGTTGCGAATACGTTTATGGGTAAA
GGTACAATTCCCTATCTTCACCCTCTTGCATTATGGACGGTAGGTTTACAACAGAGAGATTTTAT
TACCTGCGCCTTTGAAAAAAGTGATTTGGTGATTGCGATTGGTTATGATTTAATTGAATATTCTC
CAAAACGCTGGAATCCTGATGGCACAATTCCCATTGTTCACATTGCTGAAACTTCTGCCGAAATT
GATAGTAGTTATATTCCTACGGTGGAAGTGATTGGGGATATTAGCGATGCTTTAAATGAAATTA
TTAAAAGATGCGATCGCACTTCTAAACCTTTACCTTTTGCGGCTACTCTCAAACAGGAAATTCGG
GAAGACTACGAACAATATGCCCATGATGAGGGATTTCCCATCAAACCGCAAAAAATTATCTATGA
TCTAAGACAGGTAATGGGTTCTGAAGATATAGTTATTTCTGATGTGGGAGCTCATAAAATGTGG
ATGGCAAGACACTACCACAGTGAAGCCCCTAATACTTGTATTATTTCCAACGGTTTTGCCGCTAT
GGGTATCGCTATTCCCGGTGCGATCGCCGCTAAGTTAGTTCACCCTGATAAAAAAGTAGTGGCAG
TAACGGGAGATGGGGGCTTTATGATGAATTGCCAAGAATTAGAAACCGCCTTAAGGGCAAAAACT
CCCTTTGTGACACTGATATTTAACGATAATGGATATGGCTTAATTGGTTGGAAACAAATGAATCA
ATTTGGTGCGACAAGTTTTGTTGATTTTGGTAATCCAGACTTCGTTAAATTTGCCGAGAGTATGG

GTTTAAAAGGCTATCGTGTGGAATCCTCCGAAGACCTCATTCCTACCCTTAAAACTGCCCTTGAA
GACAATGTTCCTGCGGTGATAGATTGCCCTGTGGATTATCGTGAAAATATCCGTTTTTCTCGTAA
GTCAGGGGACTTAAGTTGTCCGATATGGGAATAAAAATAACCTCTGTTCGGAGTTAAGGTATTGA
GATATTTGGGGATGAGGTGATAAGGAGAGAGGGAGATGAGGGGAAGGGGTGAGAGGGGGAAATG
AGAGTTCGCTGTGCTTGTAAACTCCCTTCAGTCGTGAACGGAGTTTTTAATTATTGACTATTCAC
TATTCACTATTTACCTTTGTCCCTTGCCTCTTGCCTCTTGCCTCTTGCCTCTTGCCTCTTGCCTCTT
GCCTCTTGCCCCTTGCCCCTTGCCCCTTGCCCCTTGCCTCTTGCCTTTTGCCTCTTGCCCCTTGCCT
CTTGCCTCTTGCCTCTTGCCTCTTGCCTCTTGCCTCTTGCCTTGTCTTAACTACTAATTACTAATT
ATATTCTCTTGCCGAGTATCGTAATAGGACAATTTGCCTAAACATCATACACACTATACAATAAA
AACTTTGAAACTTATGTCTAAATCAATAGGGTTTTATCTTCCCTTAAGGTAATTGTGTCAATAAA
TTGACAAATAATAGTTATTTTGATGGTGAAGCTAACTCCTTATTTACTTCTATAAGGCTTGGACA
TAGGATAATTAAAAGAAATTGATAATATTAATAACTAATAGAAAGTAAAGTGAGACAAATTATT
ATTGCTGGTAACTGGAAGATGCACCAAGACTCAAACAGAGTCCTTAGAATTTTTACAGGGTTTTCT
TTCTCATTTAGAAGATACCCCCGAAGAAAGAGAAACAGTGTTATGTGTACCTTTTACCTGCCTAA
ATTTCATGTCGAAAAATCTTCATGGCAGTCGCGTTAAGTTGGGAGCTCAAAATATTCATTGGGCA
GATCAAGGGGCATTTACTGGAGAAATTTCTGGGGAAATGCTGAAAGAGTTTGGTATTAACTATG
TCATTGTTGGTCATAGTGAACGTCGTCAATATTTTGGTGAAACTGACGAAACAGTTAACGCTCGT
TTATTAGCGGCTCAAAAACACGGTTTAACCCCTATCTTATGTGTGGGAGAAAGTAAGGCTCAAAG
AGATGCAGGGGAAACAGAGGCGGTAATTTCTGCTCAAATTGAGAAAGATTTGGTGAATGTGGAT
CAAAATAATTTGGTAATTGCCTATGAGCCTATCTGGGCAATCGGAACTGGGGATACCTGTGAAGC
GGTAGAGGCTAACCGAGTTATTGGTTTGATTCGATCGCAACTCACTAATAAAAACGTTACGATTC
AGTATGGCGGATCTGTGAATCCTAAAAATGTTGATGAAATCATGGCACAACCTGAAATTGACGGT
GCATTAGTTGGAGGAGCAAGTTTAGACCCTGAAAGTTTTGCTCGTTTGGTTAACTATCAATAGGT
AATGGGTTCGGGGTTAGAAAAGAGGTGTCAGGTGTCAGGTTGCAGGTGAGGAGGAGATGGGGAG
ATGAGGGGATGGGGGGAGAGGTGGCAGGTATCAGGTGGCAGGTGTCAGGTTTAAGGGAGTAATG
AGTAACCAGTAACGAGTAATTATCAACTATTCACTATTCACTATTCACTATTCACTGTTAGATGA
CTCAATCTTTAACTATCCGCAATTATCATTTTGAGTGGGGTAAACGTACCTATTTAATGGGCATT
CTCAATGTTACTCCCGACAGTTTCAGTGATGGGGGAAATTTTTGTTCTGTGGATAATGCGGTTAA
ACAGGCGTTAAAAATGGTCGATGAAGGTGCGGATATTATTGATATTGGGGGGCAATCCACTCGCC
CAGGGGCAAAACAAATTACTCTCGAAGAAGAATTGAATCGGGTTATTCCTGTGGTTCAGGCTTTA
CGGCAAAAATCTGATATTCCTATTTCCGTTGATACCACTCGCTCTGAAGTTGCAAAAGAAGCGAT
CGCATCTGGTGCGGATATAATTAATGATATTTCAGGGGGAACTTTTGATCAGGAAATGCTACCCA
CAATCGCAAAATTAAATGTACCGATTATTTTAATGCACATTAAAGGTACTCCTGAAACTATGCAA
AGTTTAACAGATTATCAAGATTTAATAGGAGAAATACAAGACTTTTTACAAAAACAAATTAATC
AAGCAACTAACTTAGGAATAAAAAAAGAACATATTATTATCGATATTGGTATTGGTTTCGCAAA
AAACTATGAGCAAAATATAGAATTACTAAGAGCAATTAGTCAATTTAAATTAATGAATTTTCCCC
TGTTAGTGGGAACATCGAGAAAAAGTTTTATCGGCAAAATTTTAAACCAAGACGATCCCAAAAA
ACGAGTTTGGGGAACTGCCGCCAGTTGTGCTTATGCTATTACTCAAGGAGTAGATATTCTCAGAG
TTCATGACGTTGCACCTATGTACGATATTGTCAGAGTAACAGATGCTATATCCCGAAGACTAAAT
TAACTGAAGTAACGAGTAATGAGTTTTTAGCTATTAATTAACCTGAGTTCGGGATAAATTTTCAT
CTATGAATGATGGAGAAAAAAAGGCAAGAGGCAAGAGGCAAGAGGCAAACCCCCCTTTATCCCCC
CTCGAGAGGGGGGAGGGCAAAGGAAAAATTAAGAATTAGGAATTAAAAACCCCGAACACTCATT
ATTTGTTACTCATTACTTTCTCTCAACACCTGCTGTTCCTGCAACCTGAAACCTGACACCTACCCT

TATCCAATATTCTTAAACCGAACTGAGGTTAATTACAATTATCAACTATACACTATTCACTAATT
GCCCTTCTCGTTCTAAAGGTGATGTTATAATGTTGAAAAATGAAACTTTTATGAGTACAAAAAAA
GTATCAGATAATCAGCAAACATCAGAAAATTATCAACAAGTAAATCAACATCGCCCTCAATTTAG
TTTTCGGCAAATATTGAATATGAATCTAGGTTTTTTAGGTATTCAATTTGCTTGGGGGTTACAAA
TGGCAAATATGAGTGCTATATTTGAGTATCTAGGGGCAGACGCTCATCAGTTACCTATTCTTTGG
ATTGCCGCACCTTTAACGGGTTTATTAGTTCAACCAATTATTGGTAACTTAAGTGACTATACATG
GACTCCTTTAGGTAGGAGAAGACCTTATTTTTTAGGAGGAGCGGTTTTAGGTGCGATCGCACTTT
TATTTATGCCTCATTCTCCCAGCATTTGGGTGGCGGCTATTCTACTATGGCTATTAGATAGTAGT
GCTAATATTAGTATGGTTCCTTTTCGAGCCTTTGTTGGTGATTTACTGCCTCAAGACCAACGTAC
AAAGGGATTTGCGATACAAAGTGTTATGGTGGGTATGGGGGCAATTTCAGCTTCTGCCCTGCCTT
GGATTTTAAGTCACTTTTTTCAAATAGATAACACCACTAATGCTCTTCATCAAATACCCTTAACC
GTTGAATATTCCTTTTATACTGGTGCAATTCTCTTTTTAGGAACAGTAATTTGGACTGTTATCAC
AACTCCAGAATATCCTCCTCAAAACTTAGAAAAATTTGAACGCCTACAGGAACAAAGAGGAGGAA
TTATGAGTAGTATAAGAGAAAGTTGGCAATCATTGCAAAATATGCCTTCTACTATGACTTATCTA
GCCAAAATTCAATTTTTTACATGGTTAGGTATATTCTGTTTTTTTCTTTACTTTCCCCCAGCCGTA
GCTAGAAATATTTTCGGTGCAACTAATCAAGGTTCACTGCTTTATAGTGATGGAATTGAATGGGC
TGGAGTCTGTTTTGCTCTATTTAATGGTGTATGCGTTATTTTTTCCTTTTTGTTACCTAACTTTG
CCAAAAAAAATAGGTCGTCAAACAATTCATTGTTTATGCTTATTCTGTGGTGGTATTAGTCTTATC
GCTTTACTATTTATCCACGACAAGTATATATTACTATTCTCCATGTTTGGTTTTGGTATCGCTTG
GGCCAGTGCTTTAGTTATGCCCTATGCCATGTTAACCGAATCAATTCCCCCTCAAAGACGAGGCA
TTTATCAAGGAATCTTCAACTTTTTTATTGTTTTACCAGAAATTGCGGTTTCTCTGTTTTTTGGC
TGGATTATGAACTATTTTCTCCATGACAATCATCTTTTAGCCGTTGTTTTAGGAGGAGTTTTTCT
GATTATTGGTGCTGGTTTTACTCTACCTAGAGTGGAAGGGTTGAAAGCTATTTTTCCGCAAAGTT
CCAATTAGGAGTAACGTCAGTTCGGGTTAAGGCAATTTTATTCTTATTTCTAAGAAGCTATAAGG
TTTTCTGACTAGGAGAAAATAACGCTTTCAGCTTATCCAAAGCTCAGGTTATATTAACTCAGAAC
CCCGAACTTAGGTTTATTCACTATTCACTTTAGAGAGAGTAGCTACTCCTACCGATAGTAATTTG
TGTTTAGTTAAAACTTGTCGGGCTTCATTAACGGTTGTGCCTGTGGTGTAAATGTCATCTACAAT
TAGGGTTGGGTAACTTTTATTTATTGTGGCTAAATCCTTCCCTATTTCAAAAGCCTTTGAGATAT
TTTTCTTTCTAGCAGAGGGATTTAAACCAAACATTGCTGTTGTATCTTTCACTCTTTTTAAAAGA
TTTGGCTTAAGAGAATAACCTGTAATTTGAGTAAAACCACGAGCGATTAATTCAGCTTGATTGAA
TCCCCTTTGTTTCAATTTTTCAGGGTGTAGAGGAATCGGAATTACTGTTAATTTTTTATATTTCT
CTTTGTTTCCCGAATCAAGCCATTTTTTTCCTAACCATTGCCCCATTAACACCCCTAAATTTCTAT
TGCCATTATACTTTAGAGATGCGATCGCTCTTTTTAATGTACCATCATATTTTCCCCATGCAAAA
ATAAGAAAATTATCAGATGTTAATTCTTGGGAAGAGTCCAGTTGACAAAGATCTATTTTTTGTTG
ACAATAAGAACAAATAATCTTTTCTGCTGTGCGTTGACAAAGGGGACAATTATCCTTTAAAAATA
ACGACAAAAAAATGATTTAACATTACCTTAATTTTTACTGATTCTTGGCAAATAAAAACTTATTAA
AAAATGGAAATAAACTTCAGAGAATTTAACCCTTTCGATTTATGGATTTGGTTAGAGTTTGATCA
TGTTCCCTCAGAAATGGAAAAGCAATATGTAGAAGAATTATTTAATTCGTGGTTTTATATTGGTA
AATTGGGGGGCTTTAACGCAGAAAATTTACAAATTCAAGATACAGGAATTGATATTAGTTACCT
ACAGTATGATTATGATTTTGCGGACAACGCCATGATGTCTCCTATGCACAATATGGGAGAATTTG
AGTATGTGGGTAATTGGGGAAGATGTTGGTTTGATTTAGGCACAAGTGACTTAATTGCTCTTGAT
GTTTTAATCAATTCTCTCCGGGAATTAGCAAAAGAATATGTTGGCATTATTCAGTTAATTATTGG
CGGTGAAAATGAAGATTGGACAGTAGAAAAAAGAAATGATTTTGATGATTAAATTATCTTTTTT

TATGCCTTAAAAGTTATTCTTTACTTAATTTTTCTCCTACTTTTTTGATCAACCAACTTAACAAT
GCACCTGTGAATAAGATTCCAATGGCTGGATTGAAAACAGGTTGCCAAAGTTGATACCAAATATC
CCAACCCAAAGAAATTCCAAAAGAACGTCCCATAAGTGCGATCGCAAACCAAAAAAAGCCAATAA
TCACAAGAAATACATCGGCGATTAAAAAGCGATCTAACCAGAGTAACAATTTTTCTTTCATAGTA
AAATTTAGGGTAAAATCTTAAATCCTAAAGGTCATTGTATAATTCTGGTTAAAAAGAATACTAG
ATTAAAAGCAATAAAAATTACTTATATAAAATCAAAAACTATGGACTTAAAAGCCTTAATCCGA
GATATTCCAGATTTTCCCCAGCCCGGTGTCATTTTTAGAGATATTACCACATTATTAAAAGAACC
AAAGGGCTTGAAATACGTCATAGACTCCCTTGCATTGGAGTGTGAAAAATTAGAGAACACTCCCG
ACTATATTATCGGCATTGAATCAAGAGGATTTATTTTTGCTCCCGCTTTAGCTTATCACTTAGAC
GCAGGATTCGTTCCCGTACGTAAAAAAGGTAAACTGCCTGCAGATGTACATTCCATCGAATATCA
ATTAGAATACGGTACAGATACATTGGAAATTCACCAAGACGCTATACCATCCGGCTCGAAAGTGA
TGATTGTAGATGATGTGATTGCAACAGGTGGTACAGCGAAAGCAACGTCAGATTTAGTTCAACAG
ATTGGTTCAGAACTTGTAGCCTTTGGTTTTATTGTAGAATTGACAGGATTAGAGGGTAGGAAAA
AATTACCTGATTTACCCTGTATTAGCCTTGTTGAGTATTAAAAAAATTAACAATCAAGTTTTCTC
CCTAATCCTAATCCCCCAAGTCATCAACACTCCTTATCCTTAACCCCGAACTCTCCAAATTCCCTT
AAACTCTACCTCTCAACATCATTGCCATTTCATTGCGAGTGGGGGAATGTTCAAGGGCGGTTTCT
TCGGTGATTCTTCCCTCTTGATAGAGATTAAATAAAGATTTATTCATCGTCACCATTCCATCATA
TTCTCCATCTACCATTAATTGAGCAATTTCTTCATATTTGTTGCCGGTAATGTAATCTTTTACTG
TTTCCGTATTGATAAGAATATCGTGGTAAGCGGCTCGTTTGCCGTCAGTAGTGCGACATAGTCCC
TGTGCAATAACGCACACTAAAGACTCTGCGATCGCAGATCTAATGGCTGGTTGTTCCTCGGCAGT
GTATAAAGTGAAAATACGTTCTAAGGTTTTGATAGCACTGTTGGTATGTAAAGTACCCATAACTA
AGTGACCTGTTTGGGCGGCTTTAAGTGCCGTGTTAACGGTAGATTTATCCCGCATCTCACCCACT
AGGATGATGTCAGGATCTTCCCGAAGTGCCGCTTTTAGGGCATTATCAAATAATAGAGTATTCAT
TCCTACTTCTCTTTGCTTAATCAAAGACTTACGACTTTTATGGACAAATTCCACAGGATCTTCAA
TGGTAATAATGTGCTTAGGAAATTCATTGTTGATATAGTCAATCATTGCCGCTAGGGTGGTGGAT
TTACCCGAACCCGTAGGACCTGTGATCAAAATTAAGCCTTTATGAACCTCACACACATCTCTGAA
AACAGGAGGAAGGCGTAATTGTTCCATAGAAAGAATTTTTAAAGGAATTAAACGCAATACTAAA
GCAGGACCAGTTAAAGTATCAAAAACATTAATCCTTACCCTGGCAAAGTCATATTGAGTCGCCCC
GTCAAATTCTAATTCCTTCTTAAATCTCTGGATTTCCTCCTCTCTCATAATCTCTCTTAACCAACT
CATAAAAGTATTAAGGTCGATTTCTGGGTATTGCTCCAAAATTATCATCTGACCTCGATCGCGCA
TTCTGGGTAATTCACCCACCCCTAAATGCACATCAGAATAGCCATTATCAAAAGCCAACTTAACC
AATTCTTGTAGAGAAGGTTGTCCGGGGGATCTCCCCACCGGTGCTGAAGGGCGAACCCCTGCCTG
TGGCCCCGGTCTTAAATTAGCTTTCTGTACTGTGCCACCTCGGCGTACATCTGTTTCTCCCGGTCT
AGCTGTGCTACGAGGAGGACTTAAAGGAGGGGGAGTTAAAGGGGGAGGGGTTAGGGGAGGAGGA
GCAGGGCGAACATTTTTTGCCGATGGGGAAGGATTACTAGGGGGTTGAGAAGAATTAGTATCTTT
TCTTAACCCCGGTGGTGGTGGTAAATTGGGGCTTTTTCTTCCCAAAGGAGGCATCATATCAGGAG
AGGTGATTTGAGTTTGATCTTCAGATTCAAAATCGTCATCATCGTCAAAATCAGGGGCATCAAAG
TCAAGAGATTGAGAAGAAAGGCTTTGGCTATCATCCTCCAGAGAGGGAAATTTACGATTACTCAT
TCTGGTTTCATCATCAGTTTCTGTTATACCCTGACGTTGAGTTGGGGGTATGATGGGAGGAAAAG
GTAAAGGCTGTTTTTTAGATCTATCAGAGGATTGGGTCATAGCTATTTTTCTCTCAGCTTAGAAT
ATCTCTTTTTCTCAATTCTACTGACATATTCTCTGAATTTAAAGTAAATCTTAACAATACTATAG
TAGTTGCAAGGAGCAAAGAGTAAAGGGCAACGGGCAATGGGCAAACCCCCCTTTATCCCCCAGGG
TTTTTTCATTCTCAAAAATGTCAATTTTTGAAACTAACAAATAACCTCAGTTCGGTTTAAGAATT

TCCGATAAGGTTAGGTGTCAGGTGTCAGGTTGCAGGAACAGCAGGTGGTAGGGGTTTTTAGCAAG
GGGCTTAAGCCCCTTGTTTAAGTCAGTTCGGATTAAGGCAATTTTATCGTTATTTCTAAGTTATT
TCTAAGAAGCTATAAGGTTTTTTGACTACGAGTTGGTCTGCTTTCAGCTTATCCAAAACTCAGGT
TAACAAATAGTAACAGATACAGGAGTTTTTAAGTATTAATTAATCAATTATTAAGAAAAAAACGC
TCCCCTCTACTGTGGGAGAGGGGTTGGGGGTGAGGGCAAGATAATTTTGCCCTGACTTTGAAAAA
ACCCTGCTCGAGAGGGAGGGGGGCAAAAGTAGGGGCGAATGGCCATTCGCCCCTACCTAACTCCT
AACCCCGAACTCATTACTTATTGTTGATTCCCTTGTTATCATAAATGTGCAGGAATAAGAGATGA
GATTAAAATTTTAATGAGGTAGAAAAATGAAAAATAACAATAATCCCTATTTCCTCCGAGGGAT
AAGTAAATTATTTGTTGCAGTTACAAGTATCATGGTTATCTTGCCTGTATCTGTCAACGCCCTTG
AATTTCCTAGCGCTCCTGAGAGAAATCCCCCTAAAAGTACGGCGGCGGGTGGACGTAGGGGCGGT
TGTGTTCAAGGTGATTTACCGATAAAAGCTATTACCCCCGGAGACGATAACAAAATTAAAACTAT
ATCTTCTCAGCCTCCCTTTTATATTTATATTCCTAAAACTAAGGCAGATTCTGCACAATTTCTAA
TTAAGAGTGAAGATGGAACTCAATTAGATTTTCAGGAAATAGCCATTGAAAATGGAGATTACAT
TATTGAAATTAATTTATCTGACCAAATAAATATAACAGAGGGTCAAACCTATACTTGGGAAATA
TCATTAGTCTGTAATCCCATGTTTATTAATAGTGGTAATTATACAAAGGGTACTATTGAAAGAGT
ATCCCTAGATGAGTTAACACAAACTCAATTAGAAATGACAGGGGATACCCTAGATAAAGCCCAAA
TTTTTGCCAATGCTGATATTTGGACGGAAACTTTGATTAATGTTATTGCTGTTAAGGAGTCACAA
CCCCAAGAGTGGATTGAGTTGCTCACTTCTGTTGGTTTGCATGATTATGCTGAAAAAAGTTTTGC
TACAAATTAAAGGAAATAAGGGTTTCTTCGCCGTTGACTAAATAGTCGGCAAAGTATCGGGCAAG
GGGAGGCACGAACACAAATGGACTGGTAAACCCAGAAAATATAGACAATCCTTCTATTTGTCCGA
TTTTACCCACTAAAAAAGGCATTCCTTCTGTAAAGGCAACTTGGCAATTATGCCATTTTCCCGAT
AGGTTAGCCAAGGAAGGTAAAATAGATGCGATCGCATCTCTGAGTCTTTTTTCACTGATGACAGA
ATCAATGGGAGAATTAAGATTAGGAATTATTTGACTGATTTGCCCCAAACAAAAGCTACCATCAA
GAAATTGAATTGCTCCAGCTTCTAACACATCTCCATATAGAGTATTACTTTCTTTTTGCCATATC
TTTTCTGCTGATTGAGCCACTTGTTTTTCTGTATCAAGACGTTTCATTGTACAAGGCATAACCAA
CGTTTTTAGTTTTACGGGAGCAGGAGGAGTCATAATTAACTGGGCATGACTAAAATAAAGAGGT
AAATTTATCCCTAAATTTTGCAAAATAGAACGAGAAAAAGCTCCTGCACAAAGAATTATTTGACT
AGCAGAATAACTATTTTTTTCGGTTTTTACCCCTATAATTTTATCTTGGTTTTTCTCTACAGAAA
TTACTAATTCTTGCTCAACTTTTCCTCCTAATCTCATCAACGCTTTTTGATAAGCAAATATCATTT
TCTCTGGATGAACATGACCTTGAGGAAATCTTAAACTACCTGAAATAGCATCAATATTAATTAAT
GGTTCAAGGGAATTAGTTTCTTTTTTATCTAATAATTTTGGGGCAATATGAAATTTTTGATATTG
TTGAAAAGTGCTTTCAGGATCTTGATTTTTATCAATAGTAAATAGTAAATCTAATTCTCGAAAAT
CAATGTTATAGTCTAGCTCTTCTGTTAAATTACGATAAATATTAATACCTTCTTGGCATAATTTA
ATAGTTGTTTCATCTGTTCCGCACCAATAAGAAATTCCTCCATAACTATAAACTGTAGCATTATT
AAAAATGGAATCTTTTTCTAATAGCAAAACTCGCAGATTTTTTTTAGCTAGTTCATAACTTAAAA
CAGAGCCAGTTAAGCCCCCACCAATAATGATTACATCATAATTATTCATAGAAAATAACAGTAAA
AATATCTTTAGACTTTCAGCATATATTATATATAACCTCCGCTCGAAATGAAATTTTTTTGGCTA
AAATAAAATGTAAAACAGTAGGTATTGAATGATATTCAATCTATACAGGCTAAAGAATTATATA
AAACTTATCTTCATGGATTTTTGTTTCTTTTAACACATTGATTAATCCCTAAAATTTTCATTGAT
TAAGAAAAAAACGTGTCATAATTTGAGCAAATCTTGAAAATCACTATCAATCCTGTGATGAGTTC
CGAAAAAATTAATTTACTCCTGATTGAACCACAGAAAGGTAATTATAGTTTAATTCGCAAATTAC
TCACACAAATAAAATATGTAGATTACAGTTTAAAATGGTTTAAAAATATTCAAGAAGCTGCGGT
GATCGCATCTCAATTTTATGACTATGATGCTTATATAATCAACTCATCAGCAAATAATATCAAAC

AATGGTGTAAACAACTTTTTCCCTCTCCAATAATTTTACTCACCGAAGACATCGCAACAGGTAGA
AATTTTTTACATCAAGGTATTAGCGATTATTTAGTAATTAATCAATTAAATTCCTTTTTACTAGA
ACATTCTCTCCGTTTATCTCTTGAAAATAGCCGTTATCAAAAACAATTAAAACAAAATACCATAA
ACTATCAAACTACTATTGCTCAAGAAAGAGAAAAACTAAAACAGATTATAATTTTTCACGAAGA
TACAACCCTACGCAAACAAACTCAATTAGAATTAGAAAGCCAAAAAAAGAAATATAAAACAATA
CTCAAAACCGCCATGAATGGTATTTGGATTGTTGATGTTGCCACAAATCCTCCTCGTCTAGTGGA
AGTCAATGATGCTTACTGTTATATGAGTGGTTATACCAAGTCAGAATTACTGTCTATGTCAATTT
TAGACATTGAATATGATCTTAATCCTAAAACCCTTCAAGAAAGGACGGAAATGATTATCAAATCC
CAAAAACTTTGTTTTGAAACTAAACACCGACGTAAAGACGGCACAATGATGGATTTGCTAGTTAG
CGTAAACTATATTAAAGAATTTAACTTAGTCGTTGGTTTTCAGCAAGACATAACAGAAAAAAAT
AAAGTCCAAAATCAACTTTTAGAAACAAATGCAAAGATGAGTGCCATCTTTGACGCACTGCCAGA
CTTATTCTTAACTATTTCTTCTCAAGGAATAATTATTGACTATAAATCTTCTTCTCGTTTTAGTA
ACTTATATCTATCGGCTGAAGTATCTTTATCTAAATCGATTATGGATGTTTTTCCCGATTACTTC
TGTCAAAAAATATTTGATGCCATAAAGTATTGTCAACAAACAAAAGAAGTAGTTAAATTTGACT
ATTCCATACTTAGAAACGGAAAAATAAACTATGAAGAAGCAAGAGTAGTTGCTTTAAATAACCA
AGAATTTTTAATAAATATTCGTAACATCACTCAAACCAAATTAGCTGAAATTGCACTTAAAGAAA
GCGAAAATCGTTATGCAGAGTTAGCAGAATCTTTACCTATAGGTTTATATCGCAATAATACAGAG
GGTCAATGTATTTATATTAATCAGAAAACCAGTGAACTTTTAGGCATTAGTTTTGAAGAATGTTT
AGGAGAAGGGTGGGCAAAACGACTTCATCCAGAAGATGCCCAAAGAATTTATGAATCGTGGATG
AAAGCCTTTAAAAGTAAATCTTATTGGCAAGAAGAATATCGTTTTTTACATCCTAACGGTAAAGT
GGTATGGGTGATGGCAAATTGTACTTTTACCTACAATGAAAAAGGGGAAAATACTGGCTCAATTG
GAGGATTAACAGATATTACGCCCCTAAAAGAATTAGAGCAAAATTTACAAAAATCACAAAAATT
TATCGAAACTATTATTAACACTATCCCCTTTCCCATTTTTTGGAAAGACAGAAATAGTATTTTTC
TTGGCTGTAATCAACTACTAGCAGATATTCACGGTTTATCATCAACCAACGAAATAATCGGAAAA
ACCCCTTTTGACTTTGCTGTTACAGAGGAGGAAGCCCTAAAGTACATTGAAGATGATAGAGAAGT
GATAGAATCCAATCAAGCAAAATTATTTATTGAAGAAACATTCACTTTAGATAATGGTGAGCAA
CTTTGGATAGAAACTCATAAAGCCCCTTTGCCTGATGAAAAGGGAAATATTATTGGCTTAGTGGG
AATGTTTAAAGATATAACAGAGCGTAAACAAACAGAAATCGAATTAAAAAAAGCAAAAATTAAA
GCCGAAGAAGCCTCCCAAGCAAAAAGCCTTTTTTTGGCAAATATGAGCCATGAGTTACGCATTCC
CCTTAATGCAATTTTAGGCTTTACTCAACTAATGAGTCTTGATGATGATATTTCCCAAAGACATA
AGCAATATATCAACACTATTAGTAAATCTGGGGAACATTTATTATCACTTATTAATGAAATAATA
GATCTATCAAAGATAGAATATGGTAAATTAAAAGTCAATAAATACTCTTTCAATTTTCTGGATTT
TCTTAATAACCTCGATGAAATTTTTACCCTAAAAGCTCGAAACAAAAATATCGATTTTATTTTAC
ATAAACAGTCTGGTTTGCCCGAATATATAAAAACAGATCAAGGAAAACTGAGATCTATTATTACT
AATTTGATTAATAATGGGATTAAATTCACTCGTCAAGGTTCAATAACACTCACTGTTAAAATAAT
TAATGACGATAATAGTCAACAAGAAGATAATCAAAATGTTAATATTTATTTCTCTGTTGAGGAT
ACTGGGGCGGGTATTCCCGAAAATGAACTAGAAAAAATCTTTAAAGTTTTTACTCAGGGAAATAG
GGGGCAAAATTCAGGAGAAGGAAGTGGTTTAGGGTTAGCTATTTGTAAAACATATACTAATTTA
TTGGGGGGACATATTAAAGTTAGCAGTAAACTCCATTATGGTAGTAAGTTTGAATTTAACATTAT
ATGTCAAAAAGTAAAATTTACTGATTTAGTTAAGGGCAATAACATTGTTAAGATCGTTAATAAT
GACTCTTTTATCTCAAAATATAGAATATTACTACTAGAAAAAAACTCCGTAGATATTCAAATTAT
TCGTCATCTTTTTAGGGAAGCGAATTTTGAGCTTAGAGAGGGCAAAAATAGACAAGAAATTCTCG
ATATTTATCAGCAATGGCAACCAAGTCTAATTTTAATTTCCGTTTCGAGTTTAAATAGTAAAAAT

ATAGAAACAGTAAAAATAATTAGAAGGTTAGAATTCAATTATTCATATCAGACTAAAATTGTCG
CTTTAACAACTTTATCGTCAACTAATGAACTTTTAACGGCGGGTTTTGATGATTTTATCTTAAAG
CCTTTTGATTTTACTAATTTTTGGCGAAAAATAGAATTATGTTTGAATATAGAACTTAAGTGGCA
AAATGAAATTGTTGATCATGAAAATAATCAAAAGACGATTCGATTAAAACCTGAAGATTTAGTC
TTTATGGGCTATTCTTGGCGCAAAAAACTTTTTTATAATTCTTTAAGTGCCCGAAGTAGTAAAAT
ACTAAAGACCATTGAAGAAATTCCCTATAACTATCAACAAATAAGAGAGTCTCTAAAATTATTGA
CTGATCAACTTAACTTTGAAGAGTTAATGTATTTGAGTAACATAAATGACTACTGATAGTGGTGC
GAATACAAGTGACGGCGGAGATAACCAAAATTCAAAAAGAGGTGATATTTTAATTGTTGATGAT
CAAATTGAAAATATCCAATTTCTTTCCACGATGTTGATGGATAATGGTTATGAGGTGAGACAAGT
ATTAAGTGGAAAACAGGCGTTATATCAAATCCGCTTAATTGCACATGGTATAATCAAGAGGATGT
ATATTTGTTGTCGTAGATTTTGACCAATGCCTTCAAGAATCAAAATTGAACCCCATTTAAGTTTA
CAAGAATTAGAACAAGGGTATCGAAAAGCCAAACAAGGGATTGAAAAAATTCACTACCAAGTGA
TTTGGTTATTAGCACAAGGGAAACGAACCTCTTATGTTTCCCACGTTACAGGATATAGCTTAAGT
TGGATTTACGAACTTGTTCGTAGTTATAATCGTTCGGGTGCAAAAATGTTGGGGGATCAAAGACA
ACATAATCGGGGTAGAAAACCGTTATTAGACGATCAACAATTAGCTCTGCTATATCAAAGATTAC
AATCACCCCCCGATGATCAAGGACTTTGGAATGGTGTCAAAGTAGCAAAATGGCTCACTCAACTT
CTAGATCGTACTATTTCTCCTCAAAGAGGATGGGAATATCTCAAAGGATTAGAATATCGTCTCCG
ATGTCCTCGTCCAAATCATCAAAATTCTAGCTGCCTTGAACAAAATGAATGGAAAAATCAACTAG
CAGAAAAAAAAAAAGATTCAACAAGAATATCCCGACGCGGACGTTCAAGTGTGGGCTGAGGATG
AGCATCGTTTAGGACTCAAACCAGTATTAAGAAGAGTATGGGTAGAAAAAGGTTATCAACCGAT
AGCTACGGTCAATTGGCGTTTTCAATGGTTGTGGTTATATGGCTTTGTGGAACCAAATACAGGAG
AGACATACTGGTGGCTACTACCATATGTAAATACTGAATTGTTTAATCGAGTCTTAAAAGATTTT
GCACAGCACTATGAAATAGGTAAAAACAAAAGGGTGATTTTAGCAATGGATCAAGCTGGTTGGC
ATCCGATTAAAGAAACGACGGAAATACCAGAGGGAATCCACGTTATGCTGATGCCTTCTCACTCT
CCAGAATTACAACCGGCAGAACGTTTGTGGCCATTAGTAAATGAGGCCATCGCTAATAGGACATT
TAAAAATTTAGAAGAGTTAGAAGAGGTAGTTATTAGTAGATGCAAAAAAATTATGGAAGAAAAA
GAAATAGTTAAAGGAATAGCCAATTTTTATTGGTGGCCTAATTAATATCTTTTTGTGTGGGTAAT
TAAACGGATTTGATATTAAAAGTAGTTGATTATGATCCTCCTGAATTAATTTTGTTAGATATAAT
GATGCCTGAAATAGATGGTTATGAGGTTTGTCGTCAACTAAAGAGTAATTCTCGTACCTGTCAAA
TACCCATTATATTTCTTAGTGCTAAACAACAATTATCGGAAAAAATTAAGGGTTTTAAAGTGGGG
GGGGTTGATTATATAACTAAGCCTTTTGTGGTGGCTGAAGTAGTTTGTCGTTTGGAAACTCATTT
AAAAATTTATCGTTATCAAAATCTTTTAAATCAGGAAATAGTTGCCCGAAAAAAAGTTGAACAA
CAGTTATTAATTGCAAATCAAAAACTGGAACAAATTGCTAATATTGATGGTTTAACGGGGGTTT
ATAATCGTCGTTATTTTAATGATTTATTATCAAAAGAATGGTATCGTTTATGTCGAGAAAAGCA
ACCTTTATCAATGATTATGGTTGATATTGACTGTTTTAAGGAATATAATGACACCTATGGACATT
TACAGGGTGATGAAGTTTTAAAGGCGATCGCATCAACCCTAAAGTCTAGTTTACAACGCTCCAGT
GATTTTGTTGCCCGTTATGGGGGGGAAGAATTTGTGATTCTCCTACCTAATACTGATTTACAGGG
TTCAATCACGATATGTGAGAATATTTCTCTAAAAATAATGGAATTAGCTATACCCCATCGGGGTT
CTAGTGCCAGTAATAGCATTACGATTAGTATGGGCGCTCATTGTCTTATTCCCTCTGCAAAAATT
AATCCCTATACTTTAATAGATAAATCAGATGATGCTCTTTATCTGGCAAAAAAAGAGGGTAGAA
ATTGTTTTCGATATAGTGTTGACTTAGAATAATATTTAACCTCAGTTCGGTTTAAGAATATCGGG
TAAGGGTAGGTTTCAGGTTTCAGGTTTCAGGTGTTAGGATGTTTAGGGGATGAGGAAGAAACAA
GTAATGAGTGATAAGTGTTCAGAGTTTTTAATTCTTAATTAAGGGGCTGTCTTTTGCCTTTTGCC

TCTTGCCTCTTGCCTTTTGCCTATTCCCTACCTAAATGAGAAATCATAGTCTAAATACTTCTTTGC
CGTCCGCAATCACTACTAAGTTAATTTGTAATGGTCCTGATGTATAGGTTTCACTGGCGGCGACA
GTTAAAATTTCGTCAATAGTTTGGTCTGAAGACTGTAATTCTTCGATAAAGTTAACAAGGGAGGT
TATTTTGCCATCCCCGATAAAAATTTGTCCTAATATTCCCTCTCTACGAGCTCGGAATTGGCTAAC
GGAAATGAGAAAGTCTAGTTTTTCTTGTAATTCGCCACTGTTTAAATCTTGTTCATCAATAGAAA
TAGATGCGATCGCTTCATTTTTTTGGTAGATTATTTGATTAGGGGTAACATCTCCAAAAATACGG
ATTCTTTCTTCTCCTTGCACATAATTACCAGCCGACAAAATTCTCACTAAATACTGCCCCGGATTG
GCTAATTGTTGTTTCATCTGTTCTACTTGCCCCCTAGTTATCTGTACAAACCTTTCTTTGGGGGGC
TGATTACCATAACCAATAATCTGCATCACATTGCGATTGGCTTGACTTAAAATAGCATCTACTAA
CTCGCTGATGTCTGTATCTTTTTTGATTTGTACCAAAGTAACGCTTAATACTTGTCCTTTAATAA
CAGCAATGGGTCTTTCTCTTAAATCTTGATAAGTTTGGTAGTATTGTTCGAGAATTTCCACTTCT
TGGCGGTAAAATTCCAGTTGACCTTCTAAAGAAGCTAATTCCCGCTCTTTTGCGTCTAATATATC
ATCCGTTTGAGAAATGAGGCGATCGAGTTCTACTATTTGCTCATCTCGGCTACTTATTTCTGATT
CTAACTGTTTTTGTCTATTTTTTAACTGTTCAAGGCTGATTTGTTGTTGTTGTAAAATTGCCTCT
TGCTGTTCAATTTGTCCTTGTTTTTCTCGTAATTCTTGATCTCTTTGGGCGATGGATTGTTCTAAC
TTTAAGCTCTGTTGTTGAATTTCATCTTTTTCTATTAATAAATCTTCTTTTTCTTTAATAATTCT
TTCTACTTCTTTTCTTAGTTTTTCTGTTCTACTTTGAATTTCTCTAACTTTTTTTTGAGTTGTTGA
CAATTCATTTTCTTTAACATTCAACTGATTTAAGACAGTGTTTTGTCTATTTTTAGCCTCTAGCA
GAGCTTCTTCTACCCTTTCTTTTTCCGCTTTTACCGTATCTAATTGTTGAGTAATTTCTCGTCTTT
TTTTGAGAATTTCATCTAGTTGAAAAACCCCCTGACGCAAAGATGCACTTAAACTAAATAAAATA
ATTAAAGTAGAAGCAGAAATTAATGTTCCTGTACCGATGGTAATTAAAACAGCAGTTTGTTTGG
GTCTTAATTGAAAGAGTCTTAATCTTGCTTTTCCAACCCTTGTACCAATGCGATCGCCCAATGCT
GCGATTAGTCCCCCCAAAAGTAACATCGCTATGATTAAAATATAAGCGCTAGTCATTAGTTAATA
ATTTGTGTAGTTAAAAATCAAAAAAAATGATTATTTTTTGTTAGTAGAGGTGGGAGGAAATAGAG
GAAAAACTTATGCAAATTGCTGACTAAGGGCAACAGGATTATGTACTGTGATTTTTTTCTTATTG
ATAGTAATTAAATTTTCTTGTCTTAATTCTCCTAATAAACGAGTCACCGTTACCCGTGTTGAACC
AATAGCCTCTGCGATCGCCTGATGAGATAATTTTAAATCAATAGTAATACCGCTAGGGCTAGGAA
TACCAAAATCTCGACAAAGAATCAATAAAAAACTAACTAAACGAGAAGCCATATCACGATGAGCT
AAAGTTTCAATCATCATCTCTGTTTGCAATATGCGAGACGACAAGCCTTGTAACATCAATCGAGA
CAACTCAGGATTTTCTTTTAAAGAACGCTGAAAATGCTCGATAGGAGCGGAAAGTAATTCCACAG
GAGTAAAAGCCACCGCATGATAGAAGCGATCGGATTTTTGCCCAGTAATCAAAGACAAGACCCCA
AAAACACTATTTTCTCGCAACAAAGCCACGGTAATCTCTTCCCCTGCCTCATAAACCCGTGACAAC
TTGACAGCACCTTTCATTAAAAAATATACTCTTTCCGCAGGGTCACCCGGAAAAAAAATTGTTTT
ACCTCGTTCAAATTTTTCCACCACAGGGGTATAAATTCCCGCCCCAATTTGACGAAAAACTGAAG
CTAAGGGTTGTTCAGACATAGACAATGATTGCATAGGGATAATTAACTTAAAAATAAAATTAAA
AATGGCTCGATAAACGACCCTTTCTATAAGAAAATAGGAATAACGAGAAAACCGTCTATAACTTG
ATCAGTTTAGCGAATAATTGTCCTTAAATTACAGAAAATTAGATTCAAAATCAAGAATATGTTA
GATTTGACAGGAAAAAACGCCTTAGTAACAGGTATTGCTAACAATAAATCCATTGCTTGGGGTAT
TGCTCAACAACTACATAAAGCCGGTGCAAATATTGGTGTTACCTATTTACCCGATGAAAAAGGAC
GTTTCGAGAAAAAAAGTTGGAGATTTAGTAGAACCTTTAAACCCCAGTGTTTTTCTTCCTTGCAAT
GTGCAAGATGATGCTCAAATAGATTCTACCTTCGATCAAATCAAAGACCAATGGGGTCATATAGA
CATTTTAATTCATTGCCTAGCCTTTGCGCAGAAAGAAGACCTATCGGGAGAATTTAGTAATACTT
CTCGTAATGGTTTTCAAACAGCCTTAGATGTCAGTGCCTATTCACTAACCCGTTTAGTGCAATCA

GCGAAACCCTTATTTCGTGAAGGTAGTAGTATCTTAACCCTTAGCTATCTTGGGGGTGTAAAAGT
TATCCCTAACTATAATGTCATGGGTGTTGCTAAATCCGCCTTAGAAATGAGTGTTCGTTACTTAG
CCTCCGAATTAGGCCCTATGAATGTGCGAGTAAATGCGATTTCTGCAGGCCCCATTCGCACTCTTG
CCTCTTCTGCAGTAGGAGGAATTCTTGATATGATTCACCACGTGGAAAAAGTTGCACCCCTCAGA
CGCACTGTTACTCAAACTGAAGTAGGGAATACCGCCGCTTTTTTATGTAGTGACCTAGCTAGTGG
TATTACAGGACAAATTATTTATGTGGATGCAGGTTATGAAATTATGGGCATGACAGAGTAAGTA
TTGAGTTAGAGTGTTAGGGGATTGGGAGATTGGGGGATTTTTGTTCATAAAGTTTTAACTAATA
GAAAAAAATCCTTATATCTCTTACTCTTACTTAATCTCAGTTAACCTAATTTTTTACTTTGAAAC
AGCCCTGCCTTGAGAGAGGGGATAAAAAGAGGGAAATTTCTTTTTTATCTTAACTCCGTTCACGA
CTGAAAGGAGTGTAAGAGCCGAAGGCGAACTCTCCGAACTCCGAACTCAAATTATTGCCCATTGC
CCTTTTAACTTTGCTTTTTATTTATTATCTTCCTGAGTATAATGCTATTTTAAGACCCGATCGCG
GATGTAAAGATGGAATACGATCTAAAGATAAATCCTGATTTGGTTCTAATTGCCAATGATAGTTT
CTCAATAATAGACTAGCAAAAATTTTCATCTCTAATTGGGCAAAAGCGTAACCCAGACAAAATCT
TGCACCACCGCCAAAACCCACTAAACTAAAGTCAGTCTTTTTATGTTCCATTCTCTGACTATTGA
ATCTTTCAGGATCAAATTTTTTCGGTTCAGTATAGATGTTAGGATCTTTATGAGTCCTTTCTATT
CTATACAATACTTGCCATCCTTTCGGCACATAGTAGCCACTATAAACAAAAGGCTTAACTACTCC
TCTAAAACCACCAGCAACGGGAGGATATAACCTTTCTACTTCTTTTAATACTTGGTCTAAATAGG
TCATTTTTTTGATCTGTTCCATCGTAAAATCAGACTCATCCATCAGAGTTTTTTGTTCTAAAATA
GCCTTTTCCCGCACTTCGGGATTTTGTGCCAAAGCCATACAAAAAGAAGTTAACATGGAGGTTGT
AGTTTCATGTCCTGCGAATAGCATTAATAGGGCTTGAACTTTGATTTGGGCTTCAGAAAAACGGT
TGCCTTCTTCATCTTGGCTCTGAAGTAGTAGGCTTATGGTATCTTCTTTTAGGTTATTTTTCCGTT
TCTCGATTTCTTTTTCTATGTGTTGCAAAAGAAAATCCCTAGCTTTTAGAGCTTTACCATAGTTA
GTAAACGGTAGATTAAAGGGGAAAGCAAACAAGCCTTTAGTTAATTCGCCAAACTTTTGAGAAA
GTAGCTTAATTTCTTCCCGATTGTCCATTTCACTGCCCAACAATAGCACACTAGCAATCTCAAAG
GTCATTTCTTTCATTTCTGGAAATAGGGTCAATTTCCCCATTTCTGCCCATTTTTTCAAGGTGCGA
TCGATGATTTCTTTCATGGTGTTAAAATATTTAGTCAATGCCACCCCATGAAATGCGGGCATCAG
CAAACGTCTATTTTTTTGATGAATCTCTCCATCTTGAAGAAATAGGGATTCTCCCAATAAAGTAC
GGAAATTTTCAGGCCATCCTTCTCGCCATGAAAAGTGATCGAAATGAGTTTGTAAGATAAAATGA
TTCGCTTCCGCACCAACCATAAATACAGTGGGTTTACCGATAATATTGGTTTTGAATATTGAGCC
ATATTTGGCTTCTTTTTTATTCCCGAAATTTGGGTCTTTTATAAAGCTGAGGGTTTCACCGATTA
AGGGTAAACCAAAACTACCGGGGGGAAGCGGATATTCTTGTTTTTTCTTTCGATTAATAGTCATT
TTTTTCTGAATAATTTGCTAAAGTTTGAGAATAAATTTACCTTATTTCACATTATGACTTTTCAA
CTTAACAAACTAGATAATCTTGACTTTGATGAAGGACAGGAAATCATTGAAGATTACATAGAAG
ATGCTATTTGGGATTTTGTTGATTCTCCCGTAGGACAAGAATATATAAAAACAAGAGAGCAAGG
AGGTTTTTGGATTCACAATTTTATTGAATTTGGTTATATGTATGAGGGTTATACCCTTTCAACCCA
TGACTGTTAGCGATGTAAAAACTATCATGGAATATATCCTCCCCCGTAAATTGATGATTTTGAAT
GAAGAAGAAGTGGAAGATGCGATTCCTGAATTAATTGCTTTTTGGCAATATTTGGGAGAAACCCA
TAAACGAAAAACGGCAAAAGGGATTATCAAATATCTAAAGACTTTGGAAAATAAATTTACCGCT
ATGATGACCGATGATAGCTCTGGTAGTTTTGTCAAATCAATGTTGATGAATGCCCATAAATCAGA
ATTAGATATGACATCAGAAGAAGCATTAATAAAATTTCTTCAAGGTCAAAGTCAGAATAAAATC
TCATCTGATAAAAATTCTCAAGTTTCTCAACTACTTGAGAAAAGCACCATTCCCCCCTCCATGAA
AAATAAATATGATGAAATAACCAATTTAACAGATGATTTTTGTCGCAATTATCTTAATGACGAA
TATTTAGAATTTTCAAGAAAATTAACCACCGTTTTATGTAACTATCAACCATCCCCATTAAATAA

AGGTAAGGCAAAATCTTGGGCTTGTGGTATTATTCATGCTTTAGGGATGATCAACTTTTTAGCAG
ATTCTTCTTTTGAACCTTATATGAAATCTTCAGAACTTTATCAACGTTTCGGGGTTTCAGAAAGT
ACAGGTTGCGGAAAATCAAAACAAATTCGTGATTTACTGGAAATGAGCAGATTGGATCAAGAAT
GGATGATGCCTAGTGTTTCTGAAAGTCATCCAATGTCGGCAATTGCTAATATAGCAAGCACCTTG
AAGCAAATCTTGGATTGGGAATAAATAAAGAATCATTCATGTCTAACTCTGTAATTACTTTAGTT
AGTTGACCCAAATAATTACGGTGACGAATATATCTATCAGGGTAATTTGTAGAACGTAATGATAT
AACGTTACCTGATTTTAACAGTTGACGTTGTTTATTATTCGCCAAAGATTTACCAGTAATACTTA
GTAGAACTAGTATAAAAAAACTTAATCCTAAAAATGAAAAATAATTTGTTTTGTTCTTCACTAA
TATTATAATTGTTTTTGAGAATAAAGTTTAATTTTTATTTATTTTAAATTCCTAATTCCCTATGA
ATAATAATTAAATTTCTATTTTTATTTCCTTTTTATTAAAAGGTAAATCTTCATTAATTGCCCTT
ATTTCTTCCTCCGTTGCCATATTTATTTCATCAATATAATGATTTAAAATAGTATTCATTCTGGG
GTGATAAAAACGGTGTAAACTATAGTTAGGAGTGGCAGGAAAACCTCGCCTTCTCTTATGACTTC
CTCCTGCCCCCGGGTCATACATTTTTACTCCATGTGCGATCGCCCATTCAATAGGTTTGTAATAAC
AAGTCTCAAAATGCAAACTGTCATACTCTTCTAAACAACCCCAATAACGCCCATATAAATTCTCT
CCTTTCCGCACACAAAAAGACATAGCAACAGGATGATGAACGTCATTTTCTCTATGCACAACCGC
TAACATTAAACGATGGGAATAATTAGGATAAAGTTGCTCAAAAAACTTCTTAGTTAAATACTTA
CTGCCCCAATAAAATTTATCACAGGTGCTACTATAAAAACGATAAATATAAGGATATAAATAAT
GGGGAATTTCATCTCCTACTAAGGTTTTTAAAATTAATCCAGCTTTCTCAACAACTTTCCTTTCT
CTTTTGATATTTTTCCTTTGATTGGACTTAAATATTTTTAAAAAATCTTCAAAATTATGGAAGTT
TTGGTCAGACCAAATATAACTATGGTGCATCCATGCACTATATCCTAATTGAGAAATAAGCTCTT
TCCATTGAGGATCAACAAAGAGAAAATTACAACCAGACAATTTATTATTAATACAAAAATGATC
GATCGCATTCACCATCAACTCAGTAATTTGTAACTCATTTTCTCCCTCAGCAATTAAAAAACGAT
AACCCACCGCAGGAGTGAAAGGAGTCATGCCCAACAATTTGGGATAGTATTGTATTCCCAAACGA
TAAGCCAAATCAGCCCATTGATGATCAAAAACAAACTCTCCATAACTATGCCCTTTTATATATAA
AGGAGCACCAGCAACCAAATTATTATCCCGCCATACCGTTAAATGACAAGATTGCCAACCAGCAC
GAGGTTTTACACTACCCGAAGTTTCTAAGTTGTGTAACCATTCCCACTCTAAAAAAGGAGTTTTC
AACGGTAAAGCCATTGCATTCCAAGCTGATTGAGGAATATCTGCCATTTTTTCACACCAACGAAT
CGTATAATAAGGATATTGCTTCTGTGCTAAAATTTGCTCTTTCATAACCTCAATCTTTTTGATTT
TTTAAACACCTTAGACGTACAATATTGCCATTATCTTAGCTTAATTTTATGAGACTTCTTGCACA
AATCAAACAAGAGAAAAGAGTTAATACAATTTCTCAGGTGTACAAAATCTCACTTATCTGAGTTT
AGAGTTCAAAGTTAGGAATTAGGAGTTAAAACTAATAGATATTGATGAAAAAAAAACATAAATT
GATTTTTTTATTCTCAACTCTTTGATGGATTAATAAATGTTTAGAATATTAAAAACCGTCAAACT
CACCATCTACCCAGATTAGATATTCTTGCCAAATGACCAAGGTTAAGTAATCTAAGTCAATCCTA
TTTTTTAGAAAAATTATCAGTTTCCTGATAAAATCTGATTCTCAACAAAAATAAATTTATAACTT
GCACAATTTTAAGATGAAGATATATTTAATTCGTCATGGTATCGCTGAATCAAGAGCGATGGAAA
AAAATGATTCTCAAAGAATACTTACTCGCAAAGGCATAACTAAAACCCAAAAAGTTGCCCAAAAA
TTAACAAATTTGGATATAGAATTTGATGTTATTATCACAAGCCCCTACACTAGGGCAAAAGAAAC
CGCTATTATTTTGCAACAGGCAAAAAGAAGTTTAAATATTGTCGAACATTCTGCTCTAATACCCC
AAGGAAATATTCTGGAATGGTTTAACTGGTTACAAACTTCTCCTTATAAATCTGCGGGAAACATT
GCCCTAATTGGACACGAACCAGATTTAAGTGAATGGGCTCAGTTACTTATTTGGGGAAAAACTGA
AGATAAATTAATCCTCAAAAAAGCTGGTATTATTGGTTTGGAAGTAGCTGACTTAAGCAATCCCC
TTGGTACTGCCCAATTATTCTTGTTAGCTAGTCCAAAATGGATTAGTTGCTAAAGTATTAGTCGG
CACTTTTATCATCATAAATTTTATAGTATATTAACTCCTTTGACCTCTAATAAGAGTAATCATAA

ACTTTCCCCATGATTTATTTTTAATTCAAAAACTCACGGGCAAACCAAAATATAAGTGAATGTTA
TTTTTTTTAGAGGTGAATTATTTTGTTACACCTTTGGTGAGGGCTCTTTCCTATTTTCCCCATAA
TATGGGTATGCAAATCTATTAATTTTTCCATCAATTTTGATTTTATTATATTTTTCTTGAGGTAT
AAACATGACAAAGCCTTCTTGCGAATATCAACCCGGATTAGAAGGAATCCCCGTTGCTCAATCTA
GTATTAGTTTCGTGGACGGCAAAAACGGTATTTTAGAATATAGAGGTATAAACATTGAAGAGTT
AGCGCAAAAAAGCACTTTTCTGGAAACAGCTTATCTATTAATTTGGGGTAAATTACCCACGAAAG
ATGAACTTGCTAACTTCCAAGATGAAATTACACGCCATCGTCGTGTTAAATACCATATTAGAGAT
ATGATGAAGTGTTTTCCCGAAAGTGGTCACCCCATGGATGCCTTACAAACTTCTGCTGCTGCTTT
GGGCTTATTCTATTCTCGTCGTGCTTTAGCTAAAGAAGAATATATTAGAGATGCTGTAGTTCGCC
TATTAGCCAAAATACCCACAATGGTGGCCGCTTTTGCACAAATGCGCATGGGCAATGATCCAGTA
CAACCAAGCGACAATCTAAATTATGCGGCTAATTTCCTCTATATGTTGACAGAAAAAGAACCCGA
ACCCCTAGAAGCGAGAATTTTTGATGTGTGCTTAATGCTTCATGCAGAACATACTATGAATGCTT
CAACGTTTTCTGCCATGGTAACAGCTTCAACTTTAACTGATCCTTATGCCGTTGTTGCTTCGGCGG
TGGGAACATTAGCAGGTCCTCTACATGGTGGAGCAAATGAGGAAGTGTTAGTGATGTTAGAAGA
AATTGGTTCAGTGGAAAATGTACGTCCCTATATTGAAGATTGTATTGAAAATAAAAAGAAAATT
ATGGGCTTCGGTCATAGGGTTTATAAAGTAAAAGACCCTCGTGCAAAGATATTACAGCAATTGGC
AGAGAGATTATTTGAGCTAACCGGTCATGATGAATATTATGATATTGCCTTAGAAGTGGAAAAA
GTGGTTACTGAAAAACTTGGTCATAAAGGTATTTACGCTAATGTTGATTTTTATTCGGGTTTAGT
TTACCGTAAGTTGGGCATTAAGCCAGATTTATTCACTCCTATGTTTGCAATTTCTAGGGTTGCCG
GTTGGTTGGCTCACTGGAAAGAGCAATTAGCAGTTAATCGTATTTTCCGTCCTACTCAAGTTTAC
ATCGGCAATAGAGGTATTCCTTATGTAGCGATCGAAGATCGGTAAAAGTTCGATGAGTTTATATG
GATGAATTAGGGTATTCGGGTATTGGGGAGATAAGGTGTCAGGTTTCAGGTTTCAGGTTTTAGA
AGAAAGTAATGAATGTTAGGAGTTTTTAATTCTTAATTCTTAATTATCAACTATTCACTATTTAC
CTTTGCCCTTTATCTTTTGCCTTTTTTTTATTATTCAACAGTAATTCTTCTAATGAAGAAGGAAC
AGGATCATAACCGCCGGGGTGAAATGGATGACATCGCAAAATTCTTTTGATTGCTAAATAACTGC
CTCTCCAAACTCCAAAAGTAGAAATAGCTGCGATCGCATACTGAGAACACGTTGGTTGAAAACGA
CAAGAAGGAGGGAATAGAGGGGAAATATATTGTCGATAGATATTGATTAAAAATATTAGGATTA
CTTTTCCCATTGTTCAGTTATTTTAATACCATTAAGTCATAAACTTCTATAGTGCTGAAAAATCT
ACTATACAAAAGATTATAAAGCCCTCATAGTTTCACAGAAGAAAAGAAGTTAAACCCCCCTTAAC
CTTAAAGGTTTATGATGGACGGTGAAAAAGCACACCATTATTTTTCAGCTACTTAATAGTTTTTT
TCTCATTTACTATATAATATCTATTTGATGACAAAAAAATGAAAAAGTTGTTATCTTAGTTACAG
AAAAAATGGCAAAAAAGTAAATCTTTGTGCTATTAATTATTAAGAGACTGATATTCAAGGTGTG
AGGAAACTTAAAAGAATTATGTTTAAATTAGTCAAAAACTATACAATCGCAACTCCCGCATTGAT
CGCAATGGCGATCGCGTCTAATGGTTTAGCTCCCGTATCTGCCAATGCGAACGAAGTAGATACAG
CTACTATTGACCAGATTTCTCAGTACAATGAAACCCAGCCTATGGGACAGGTAACATCTGTATCT
CAGCTAAGAGACGTTTCTCCTACTGACTGGGCATTTGAAGCATTAAGAAGTCTTGTTGAACGTTA
CGGCTGTATTGTTGGTTATCCTGATCGTACTTACCGTGGTAATCGTGCTTTAAGTCGTTATGAGT
TCGCCGCAGGTTTAAACGCTTGTATGCAACAAATGGAGCGTTTAATTGCTTCTAGCGAGTCTGTA
CTTAGAGAAGATATTGAAAAATTAAAACGCTTAATGTCTGAGTTTGAAGCAGAATTAGCGGCTT
TAGGTGCAAGAGTTGATAACTTAGAAGGTAGAGTAGCTTTCTTAGAAGATCACCAATTCTCCACC
ACTACCAAGTTGAAAGGGGAAGTTGTTATCGCAGCAGTTGGTCAAGGAGGCGGTACTGACGGTTT
AGGTCTTTTCAATGAAAACTACTTAACTGGTGCTGGTGAAAGATTAAGAGACCTTCCTGCTGATT
CCATTGATGACCAATTTACTTTAAGTAATCGTGTACGTTTGACTTTAGATACTAGCTTTACTGGT

AAAGACACCTTAAGAACTCGTTTAGCCGCTGGTAACATTGTTCGTTTGGGTAACGCCTTCGGTAG
CGATATGGCTCGTCTCAACTTTGAACAAAATACTGGTAATGACATCATTCTTGACCGTGTATATT
ACAAAACCAAGCTAGGTGATAAAGCAACTTTCTGGGTAGGTACTGAAATGCCCATCGAGGACATC
TTTGAAACCTTTAGCCCTTACACCGAAAGTAGTGGTACTGGTGCATTATCTCGAGTAACCCGTTT
CAACCCCTTCATTTATCGTCAACCCGGTAGTGCAGGTATCGGTTTAAAATATGCCTTCAACAAAA
ACTTTGACATTACTGCTTCTTATTTAGCAGATAATGCGGCGGATGCTTCTCAAGGAAATGGTTTA
TTCAATGGTACTTATAGCACCGGTGTTCAGTTAGGGGTTCATCCTACTGATACTCTTAGCTTTGG
TTTAGCTTATGTACACAGCTATTTTTCTCAGGATGATGTTAACTTAACTGGTAGTACTGGAAGTC
GTTACACTGATGGTATCTCCAGTCAAATTGCGGCATTAGGTTCTGGAAACAGAGACGGTGGTTTT
GCTGGAACTAGTGATCCTTTCAGTGGTGCGGCTACTTCTTCTGAAAACGTTGGTTTACAAGCAAC
TTGGCGTGTAGCTGAAAAAGTAAACCTCGCAGGATGGGTAGGTTGGTCTTTCGCTCAAGGTCATT
CTCATGATAGCACTGGTGCTAACCGTAACGGAGATAACGCAGACTTATTTACTTGGAATACCAAT
GTTTCTTTCATCGACTTAGGTAAAGAAGGATCTGTATTAACTATTGCGGGTGGTCAAGTTCCCCG
TGCTGGAAGTGTTGATGGTACTATTGGACGTGACAGAGGACAATCTTGGGTTGTTGAAGGTCAAT
ATAAATATCCTTTAACCGACAATATTTCCTTAACTCCCGGTGTTTACGCTGTTTTCAATCCTAAC
AATAGTGACGACAGCAGAAACCAAACCGTTGTTGTTGGTGTTCTTCGTACCGTATTCAAATTCTA
AGTGTAGTTAGTTAATAATAATTACCGCAAACTCTCGAATTTTCGGGAGCTTTAACTAAATAATT
CGCCTAGCCCTATGGGCTAGGTTTTTTTTTGATTTAATTAAATCCAGCGACTGGCAATAGGCAT
TTTCCAACCAGTGCCAAAAGCTCGATCAGTAATTTTTAGACCGGGAGGGGCTTGTTTTCGTTTAA
ATTCTGCTCTTACAACTAACTTGATTACTTTATTGATGGTATCTAAATCATGACCTGAATTTTCT
ATTTCTGTAACGGATTGATGATGGTTTATGTATCTTTCTAAAATATCATCTAATATTTCGTAAGG
GGGTAAAGAATCTTGGTCTTTTTGATCTGGTTTTAGTTCGGCACTAGGAGGTTTAGTGAGAATAT
TATGAGGTATGATTTCTTTTTCTTGATTAAGCCAATGACACAAGGAAAAAACTTGAGTTTTCGGT
AAATCTGCAATTACTGCTAGTCCGCCATTCATGTCGCCGTATAGGGTGCAATATCCTACTGCCAT
TTCTGATTTGTTACCTGTTGATAATAGTAGATAACCAAATTTATTTGCGATCGCCATTAATAATG
TTCCACGAATACGAGACTGCAAATTTTCTTCTGCGATACCAAATTCTGTATTTTTAAAAAGAGGG
TGTAAAGTAGAGTCATAACTTTTCATTACTTCTTGAATGGGTAATTTTTCGCTTTTTATACCTAG
ATTATTGACCAGTTGTTCTGCATCTGTAATTGAATGGGCTGAACTATAGGGCGAAGGCATTAAAA
CCCCTAAAACATTGTCTTTACCTAAAGCAGAAACGGCAATAGTTGCCACCAAAGCAGAATCTATT
CCACCGCTTAAACCGATAATTGCTTGTTTAAAACCACATTTTTTTGCATAATCTTTTACACCTAA
AACTAAGGCTTGATAAATTTCTTCTTCTTTTGTTATATTTACTAAATTTATCTTATTTTCTGAGA
TTAAATCTTGATTTTTTTTGCAATAATCTACATAAATTAAATCCTGTTGAAAGTTTTTGGCTTTA
TTAATAATATTGCCTTTTTTATCAACAACAAAACTATTACCATCAAAAATTAAATCATCATTTCC
TCCCACTTGATTCACATAAATAAGAGATTTTTTGTATTGTTTAGATAAATATATCAACATTTTTT
CTCTAACTTGTTGTTTCTGTACAACATAGGGAGAAGCGGAAAGATTAACAATCAAATCAACGTCA
AGATTAACTAAATCTTTTACGGGGTTAGTAGTATAGTTTTTCTTACCCCAAAAATCCTCTTGATT
CCACAAATCTTCACAAATTGTAACGCCTATTTTGACATCATCAAGATAAAAATAATTAGCCTCAT
TTCCTGCACTAAAATAGCGATCTTCTTCAAATACATCATAAGTCGGTAAAAGACGTTTATGAAAA
AAAGTTTCAATTTTTCCATTTCTAATTAAAGCAGAACTGTTATAAAGGGTTTTTTTGCCTTCTTT
TTCTGCATTATCATTTATGACAGCAATTCCGATTAAAACCGATATTTGAGGATTGATTTCTTGAG
CAAATTGTTCTAATTCTTTTTTCATTTGCCCTAAAAAATTTTTATTTAAAAGTAAATCTTTAGGA
GGATAACCACATAATGATAATTCAGGAGTAAGAATTAATTTTGCTCCTTTTTTTTTCAGCAATATT
AACAGTTTCTTTTATTTGTTTAAGATTGTTTTTTATGTCTCCGATAGTCGGATTAAGTTGTGCGA

TCGCAATTTTCATGATGATAAGGAGTAATAAATAGGGAGATGGGGATTCAGAGTTCGGGGTTTG
AAGTTAGGGGCGAATGGCCATTCGACCGTACAGGAGTTTTTAATTCTTAATGTTTTACTCTTGCC
CCTTGCCCTTTTTACTTTTTCTAATTATTTAAGATTTCTTGATCAAGGCTAAAATCAACATCGAC
TTTATCTCTACCATTAGCTAAATAGTCATTTTCAAAAGACTCTTTTAATCCAGAAATTAAATCAT
ATTTTGGTTGCCAATTTAGTTCTTTTTGAGCTTTGGAAATATCGGCAAAAAAATGTTGAACTCTA
ATGGGAAAAGCCTTTCTTTTTCCTAAGTTAAATTGAGCAGGATCATAATATTTGATCTCAATTTC
TTCTGGGTTTTTTCCCACCGCTTCGGCACAAGCTAAGGCTAGTCCCTTAAAAGTAACATAGCGAT
CGCCTGATATATTGTATATTTGTCCTACCGCTTGAGAATTTCCCAAAACCAGACTCATGGCAGTT
GCTAAATCTTCCACATGACCAAATTGAGTAAAATGTAATCCATGATAAGGTATTAAAATAGGCTG
ATTACGAAGGATGCGATCGAAAAACCATGCTTCTAAATCATTATAGTTGCCAGAACCATAAATAT
AAGTAGGACGAATAGAAGTGAAAGGCACACCCTCAGCTTTTAAGTAGGTTTCCGTTTCATATTTA
CCCTTGTGGCGACTTTGTGGGTCAACGGGATCATCTTCTCGATGGGGCATTTGTTCAGATGGTAA
ATACACCCCCGCCGAGCTAACATAAACGAAATGGGATATTTGATCTTTGAATAACTCTACTAAAG
GCTTTGTATCTTTTAATTCTCTACCATTATTATCGAAAATTGCATCAAATTTTTGTCCTGCTAAC
TTATCTTTTAACTCTGAAGCATTGGTGCGATCGCCGTGTATTTGTTCAATGCCGTTAACTGGTGC
TGGTTTATTACCTCGATTAAAAAGTACTACTTCATGACCTTGAGCAACTAAAATTTTAGTTAGAG
AAACCCCTATAAAACGAGTTCCTCCCATAATTAAAATACGCATATACTAATTGATTATTATATTT
TTGATCTTGCTCTTACTAATGTACCTGAGTTCGATTGCTTTTGTGATAACAGTTTTTGTTGCTCT
TATTTTCTATTAATTTATCATAATCACCGGGTCAGAGCCTTAATTTTTTAGGCATCTTGTTTATA
AACATAAATTGTTTGAGTTTCTAATTTTGGGGTTAACTCTTGAATAGAAGAATAATTAGAAATA
TTTTCTCTTTGTAATCTTACCTGTAATCCCAACATAGGATCATCCCCATCAGCTAGTAAAAATTG
CACAGAATCAATATTTTCCCAAGTAAGGATTTTATCAATTATTTCTAAAATTCCATTCAAATAAG
GATGATTTTTATTCTTATACCAGTTAGATTCAACCATTTTAGGTCTATCAAAACCCAAGTGAAAA
ATCATTGATGGTTGACTAAATAAAGCAATAGAAACTGGGCGCATTTCTTCCTGTAAAAGTAAATT
ATTACTACGCACCAAATAACGTATATTTTCTAGTTTTTCATAGCGCTTAGTGGCAGAGTTAAGGG
GATTTTTCCATGTAACTGCAACGGTGCAAAGATAAGTTTGTAAGAGTAAATGTCCTAACTTTTCT
GCTTTTGTGGCTAAATAATTAGAATTATAGTCATTGGCTTCAATAAATAAGGGTAAACGCTCCAC
AATTTCAATACCATAACCCTTTAAACCAGCAATTTTACGAGGATTATTGGTAATTAAGCGAATTT
GTTTAATGCCTAAATCATTAAGAATTTGCGCCCCCATACCGTAATCCCTTAAATCCGCAGGGAAA
CCTAATTTTTCATTGGCTTCAACGGTATCAAAACCCTTATCTTGTAAAGTGTAGGCTTTAAGTTT
GTTAACTAAACCAATGCCCCTACCTTCTTGACGTAAATAAACAACTACCCCCAATCCTGCATTTTC
AACCATTTTCAATGCCGCTTGTAACTGCATTCGACAATCACAACGTAATGAGCCTAAAGCATCCC
CAGTCAAACATTCTGAGTGCATTCTCACCATGACTGGTTGTTGGGAAAATTCGTTTATATCTCCT
TTAACAATGGCTAAATGTTCCGTATTATTTAAAGCATTATGGTAGGCATAAATTTGAAAATCTCC
AAATTGACTGGGAAACTTACAAATTGTCTCTCTATATACGAAGCGATCGTATTTTAAACGATAAC
TAATTAAATCGGCAATACTAATTAATTTTAATTGATGAGTTTGGGCATACTCATATAATTGAGGC
AAACGAGCCATACTACCATCAGGATTTTGAATTTCACAAATAACTCCTGCTGGGTATAAACCAGC
CAGTCGAGATAAATCTACCGCCGCTTCAGTATGGCCAGCCCTTTTCAAAACCCCACCCTTTTTCGC
TCTTAAAGGAAAAATATGTCCGGGCCGTGCTAAGTCTTCTGGTTGAGTTTGAGGATTAATAGCTA
TTTGTATCGTTTTTGCTCTATCATCGGCAGAAATACCTGTTGTTACTCCAAGATGTTTTGCCCCAT
CAATGCTAACAGTAAAAGCGGTTTGATTACTGTCAGTATTTTTTGTCACCATCAAAGGTAAATCT
AGCTCATCGAGTCTTTCCCCTGTCATTGCTAAACAAATTAAACCCCTTGCTTCTACCGCCATAAAA
TTAATCATATCGGGAGTGGCAAATTGGGCGGCACAAATTACATCACCTTCATTTTCCCTATTTTC

ATCATCCACTACTACAATCGCCTTTCCTGCTTTTATATCTGCTAAGGCGGATTCAATACTGTCAAA
TTGGATATTTTCGCTCTTCACAACTGCTGATTTACTTTTTATAGCTTTTTAGTAATTATTTGTTA
ATTTATTATTTTAACATTTCCTATCCATAGAGAAAAATGTATTAAACCTCCGTTTGGTTTATGAA
TATCGGATAAGGCTAGGTGTCAGGGTGTTTAGAGGATGAGGAGATGAGGAGATGAGGAGAGAGG
GAGATGAGGTGTCAGGTTTCAGGTTTCAGGTTTCAGGTTTTAGAAGAAAGTAATGAATGCTCGG
AGTTTTTAATTCTTAATTCTTAATTCTTAATTCTTAATTATCAACTATTTACCTTTGCCCTCCCTC
CTCTCGAGGGGGGATAAAGGGGGGTTTGCCCTTTGCCCATTCCCGAATACGGATAAGTGATGTGC
CTCATAGTAATGAGAATTGCTATAAAAAATCATCGATAGATTTTGCAGAGATGGCTTTTTTCAAT
AAATCGGCTATTTCACTTAAATTTTTTATTTTATGAATACTGACATTTAATTGGGAGGTAATTTG
TCCAAAACGAGTAGTTAAAATTTCTACTACCGTATCTTGTAAAATTTTTATTTCTTGATTATGGT
TTTCTGGAGTTTCACCACTTAAAACTTGTAGTTGTTCCTGTTGATTATTTATAATTGTAATCATC
TCTTGAAGTGCTTGAGCAAGAATCCCTAATTCATCGGGGCGATCGCAAAAAGCATCAAAATCATC
AGGGGAAAACTGTTTATTTTCTACAGCTGAAACGGCTGTTGCCGTCACTGCATATAATTCTTCTA
AAGACTCCATATTTTGACGGTGATTTTGTTCAAGAGCATCCATAACTTGATTGTAACGAGTAGCA
ATATGACCCACTTCTGTAAAAGATTCTACCGGCACTCGCAAGGTTAAATCGTGGGTTTTTGCCTG
TAAATCCATAACGTTTAATAATTCATATGTATCTGTTTTTGCTTCATGTTCAGCTACATTTAAAC
CTAATTCTTCTGCTTCAAGAGAGACTCGGAGGGGAAAAATCTGATTTAAAGGTTTCAAAATTAAC
CAAGTTAAACCAAACGCCCAAACCATAGCGATGACTATACCTAATAACTGAATTAACAATAATTC
TATTCGACTGATAGGCATCTTCATCAAAGCTAAATCACCAAATAACGCTACGGAAATAGTACCCC
ATAAACCAGCACCACCATGTACTGCTACAGCATCCACCGCATCATCAATTTTGGCTCTAATTAAG
GCTTGTGAAACAAATTTAGCCATCGCCGCCCCTGTCATGCCCACTATAATCGCCAACGGATTAGGT
ATTATATGACAACCCGCAGTAATTGCCACTAAACCAGAAATTGAACCGTTGATTAATTCTTCCAC
CTCAACCCGTTTTTCTTTTACTTGAGAAAAAATCATTGCCATTATCATCCCTCCCACTCCTGCCAA
TACAGTGTTAAGAATAATTTTTGGTACTTGATTGTTAAAAGCGAAAGTGCTACCGCCATTAAATC
CTAACCAACCTAACCATAAAAATAAAGCACCTAACACGGCAAAAGGTAAATTTGAGCCTTGAATT
TTATTAACCTCTCCTTTATCGCTAAATCTACCCTGACGAGAACCAATGATAATCAGAGTAGCGAA
TCCTACCCATGCACCAATACCATGTACCACAGTGCTTCCAGCAAAATCGATAAAACCTAATTGTT
TTAACCAACCACTACTATTCCATACCCAATGTCCATAAAAAGGGTAAATTAAACCTGAGACTAAG
ATGACAACTATAGTATAAGCTAAGAATTTAATTCGTTCTGCGATCGCACCTGAAATAATAGTTGT
TGCCGTACCACAAAACATGGCTTGAAACAGAAAAAAAACTGTCATATCTCCTTGGGTATCCGCAG
AGAAAAAGAAATTATCCCCACCAAACCAACTTCCTTGAGAAGCACCAAACATAATACCATAGCCA
AAAGCCCAAAATAACAGTACAGAGATACCAAAATCTGCAAAATTTTTAACAGCGACATTGATACT
ATTTTTAGAGCGAGTTAATCCTGACTCAAGGCACATAAAACCCGCTTGCATCAAAAAAACTAACC
CCGTACATAACAATACCCAAAGTTTATCCATGTTGATTAGTCGATAAAAAGCGATTAAGTCGATA
GTAACGGGCAAATTGAGGCAAATCCGCTTGATGCCCTAAAACAATTAAAATATCTCCCCTATTTA
AGATTAGAGTTGGGGCAGGATGACTAATTAAGTCTCCACTGTAACGCCTAATAGCCACCACTAAA
AATGACCCCTTTCCACGAATTTCCAACTCTTTAACAGTTTTGCCCACCAAAGGAGAAGTATCAGC
AAGGGATAACTCCTCTATCTGCACCTCTAACTGTTTAAGTAAATCATTGAGATAACTTTGCTCTT
CGGGATGTTGAAAAAAGTCATTGGTGTTAGGGGAAGTAATCAAATTCGCCATTCTCATGCCACTT
ACTGAAGCAGGTAAAACCACATGATTCGCTCCAGCTAATCGCAATTTTTTTTCCGTGCTAGGAAA
CTCACCCCTTGCGATAATAATTAGGTTATGATTCAATTCACGGGCGGTGAGGGTAATAAAAACAT
TACTAGCATCACTAGCTAATACTGTTACTAAGACTCTGGCTTTATCAATACCTACTTTCGTTAAA
ATATTTTCATCTGTTGCATCTCCCTCTTCAGTTAAGTAGTTACGACTTTTAGCTAATTCAATGAT

TTCATGGTTATTATCAATTACTACAAAATTCTCCGCTACTTCTTCTAGTTGTTGAGCCAAGACTT
GTCCAATATGCCCAAAACCACAGATAATAACGTGATTTTCTAAGTTAGAAATTGTTTTCCTTTGT
CTTTGAGCCTCTAAAGCTCGATTAATTTCTCCTTCTGTAATCATTGAAATAAATCCCCCCAAACCA
TAAATAGCTGAAGATGTCCCTGCAATTATCACACTAATAGTAAAAATTTTTTCTGCGGGGGTTTG
TAACGGTTTTACTTCTCCATAACCGACTCCAAAAATAGTAATTACAACCATGTAGGTTGATTCTA
ATAATGTCCAGCCAAAAGTCATGTAGCCAGCAATAGCCACAACAATGGTGACACAAAAAAAGACT
GAACCTAGAATAATTCGATTAAGAGAGTTTTGCATTCATTTATTAAATCCCCTCTTAAAAAAAAA
TAAAGTCAAATTTAAACCATTTTTTCAAGTTAAGATTCAGAAAAATGCGATCTATATTCACTTAA
CAAAAAATATTGTGCAAAAATAGAATTAGTTTATTAATGTGAAGTGTCAGAAAATTAAAAGACT
TGTATTTTAAAGAATACCTTTTATAATTTCTTCTAGTCGAGATTTATCCAATTCTGTGATCACAA
AAACTTCTTGTACACTTTTCCCACATCTAGCAATCAGCTTAAACCCTCCCCGAATCGGCACGGATA
TTTTTAACTTCATATGGGGAGAGTGCGATCGCGATCTACTTAATACGGCTGGAGTAACGGTTTTA
ACGCCTTCATAAGTAATCAATTTTTCTAAAATGGGAATTAACCCGTCTAAATGGGTAGAATGATT
CCAGACAATTCTCCCCTCTGAAGATTTACTCATACTGCCTCCAAGGGTGCCATGGTTAAACCTTCC
CTTCTTAACTGTTGATGATATAACTCTGCTTGTTCCAATGGACCACTCCACACTAAAGCCTGTCCC
TCAAAATGTACTTGATTAGTTAACTGCCAAGCATAATCAGGAGTCATATTAGGAATATACTTGAT
TAAACAATTAGCTACATGATCAAAGGTGTTAAAATCATCATTAAGGACGATAACTTTATAGTTTG
GATATGGTTTGCGAGTAACTTGACTCGATTTATTCGGAGCAACAATAGTACTAGCTAAAATGGTT
GAAGTCATATTATTGCAGATAGGAAATTTTTGTTTATTTTTTAGGGAACATATTTAATTATACTC
AATTTTGCTTTTGTTTATAATTTTGTATTGTTTATACATATTCTTCAGAGACTATCCAAACTATC
TCGATAGCTACTTACAAAACAAAATCGGCTAATATTATTAGTATTTTATTTCCTTACGAGTACTG
TCTATAAATTTGACTATTTAATCATAATTAATAATTACAATAAATATATATATATAATCTAATGAT
TTGCTGAATAAAAGTTTTTTTCGATATAATTTAAAAGCATAATTAATAACCTACAAAATTATAA
GCAAAAATGGCAAGAATAATTAATATTTTGAGAAACGGAAAAGCTCAAGAAGAAATAGAAGTAA
AAGGTTGGATTAGAACTAAAAGAGAGTTAAAAGAATTTTCTTTTATAGAAATTAATGACGGTTC
TTCCCTTGGGAATTTACAAGCAGTTTTAAACTCAGATTTACCAAACTATCAAGATATTATTAAAA
GATTAAATACAGGGGTTTCTGTTAGCATAAAAGGCATTTTAGTTGATTCTCCTGCCAAAGGTCAA
AAAGTAGAATTACAAGCTCAAGAAGTAATCATTTTTGGTGATTGTGATCCTGAAACTTATCCTTT
ACAAAAAAAACGTCACTCTTTTGAATTTCTTAGAACTATTGGACATTTAAGAGGTAGAACAAACA
CCATTGGAGCAGTCATGCGAGTAAGAAATGCTTGTGCGAATGCTATTCATCAATTTTTTCAAAGT
AAGGGTTTTTTATGGGTTCATACTCCTATTATTACCGCCAGTGATTGCGAAGGAGCAGGGGAATT
ATTTACTGTAACAAATCTCTCTTTAGAAAAAATCATTCAAGATAAAAAAGATGAAATTAATTAT
GAAAATGATTTCTTTGGCAGACAGGCTTACTTAACAGTTAGTGGTCAATTAGAAGCTGAAATTAT
GGCGATGTCTTTTCAAAATGTTTATACTTTTGGCCCTACTTTTAGAGCAGAAAATTCTAACACTT
CTCGACATTTAGCAGAGTTTTGGATGGTTGAACCAGAAATTGCTTTTTGTGATTTAGAAGGAGAT
CAAAATTTAGCAGAAGAATTTTTAAAATATGTCTTTAAATATGTTTTAGATCATTGTCCTGAAGA
TATGGCTTTTTTCAACCAAAGAATTAATCAAAATGTACTTATTAATGCAGAAAATATCATTAATA
ATGAGTTTGCAAGAATCACTTATACAGAAGCAATAAATCTATTAGAAAAAAGCAAGAAAAAATT
TGAATTTCCTGTTGAGTGGGGGATAGATTTACAATCAGAACATGAAAGATATTTGGCTGAAGAA
TTATTCAAAAAACCCGTCATAGTTACTGACTATCCAAAAGAGATTAAAGCCTTTTATATGCGTCT
CAATGATGACCAAAAAACTGTAGCGGCGATGGATATTTTAGCCCCGGGCATCGGCGAAATTATAG
GCGGTTCACAAAGAGAAGAAAGATTAGATGTATTACAATCTCGTATTGTTGAATGTAATGTTAA
TGGCGATGAACTTTGGTGGTATTTAGATTTACGTAGATATGGCACTGTTCCTCATGCTGGTTTTG

GTTTAGGATTTGAAAGATTAGTTCAATTTATGACAGGAATGGAAAATATCAGAGATGTAATTCC
TTTTCCTCGTACCCCAATGAATGCAGAGTTTTAACATAATTAACCTGAGTTCGGGATAGATTTTC
ATCTATGAGTGATGGCAAAAAGGGCAAAGGGCAAACCCCCTTTTATTCTAGATCTGTTTCATACT
AAAATCTTTGTTTAAGAGGGAGTAATAAGTAACGAGTAATGAGTAATGAGTTTTGATAATTTAT
TATGATTAATTGATTGTTTCAAGATTTTTTATTCTTACTTAAAATCTGCAAATCAAAAATCCCCC
CATCTCCCTCTCCCCTTATCTCCCGCTCTTTTTTCTTTCTCGAAAATGAAACAGCCCTGCCCTTTA
TCCCCCATCCCCTAACACCTGCAACCTGCAACCTGAAACGTGACACCTACCCTTATCCAATATTCT
TAAACCCAACTAAGGTTACTTAAATGCAGACTGGCTTATAATAAAAACATTCATTGTTAATCACT
ATAAGGCTATGCGTGAACTACCCAACACCAAAATCAAAGATTTATGGTGTGGGCTTCATCTCCAA
CAGATAGCCTAGTAGTGGCTTTCTTACGTCCACCCTTACTAGGTCTTACATCTGGGCAAGATGCT
GACTCGCTAGTTCCTAACGACAATATTCGCAGTCCTTCATCCCTGATATTACATGATGCGTTCAT
ATCCCTGTCGTGTTTGGTTTTACATCTAGGACATTCCCACTGTCTAATATCTAAAGGTAAGCTAT
CTACAATATTCAAACAATGATTACAAGTTTTGGAACTAGGAAAAAATCTATCTATTTCAAGATAA
ATCTTACCTTCTTGTTCACATTTATACTTGAGCATTGTCTGAAATTGTCCCCAACCTACCTGTTGT
ATTGACTTAGCTAGACAATGGTTTTTTACCATCCCTTTGATGTTTAGATTCTCACATATTACAAC
TTGGTTTTCGTTTACTATCCTGCGTGATAGTTTATGGTGAAAATCCTCACGAGTGCGAGATATTT
TATTATGAACCTTTGCTACTTTAATTCGGGCTTTATTACGGTTATTTGAGCCTTTTTGTTTACGA
GATAAATCTTTCTGTTTATTTTTTAAGTTGCGTTCATGTTTTTCTAACCATCTAGGGTTATCAAA
CTTAGAACCATCACTGGTGACACAAAAATCTTTTATCCCCATATCCAATCCGATTGCTTTACCTTC
AGTGTTTGGTAATGGTTGTTCTTTTCCATCTTCAAATAAAATAGAAGCATAATACTGCTCCCCTT
TATTTTTAGAGATAGTTACAGTCTTGATTTTACCATCTATAGAACGATGAATCTTAGCATAAATC
TCTCCTAACTTCGGAAAAATAATCATATCTCCTTTGATTTTGACATTTTGAGGATAGCTAAGAGA
TTGCTTACCATGCTTAGACTTGAATCTAGGAAAAGAAGCCCGTTTCTCAAAAAAGTTGATAAAAG
AACGACTTAGATTGAGACAAACCACTTGTAAACATTGGGAGTACGTTTCTGTTAGCCATTCATAT
TCTTTTTTAAGGGAAGGTAATTGCTTTTGCAAGTCATAACGAGATAACCCTTTACCTGTTTGCTT
GTATGTTTCATTAGTCAAAGCTAGAAAACGATTCCACAACCAACGGCAAGAACCAAAAGCCTTAG
CTAACGATTCTTTCTGTGGATTTGTAGGATATAATCTTACTTTGACTACTTTTAGCATTATCAGA
AACTAGGTTATTGATAAATAGATTCTAACAGAGATTTTTCATTTTGTGTGGACTCCCTACGGTCG
ATTTTATTTACTGGTCGAAATTCATCCATACGCCTGTTTAACTGTCTTTTTTATGGAAAACTAGG
CGTGGGACTTCTTTCGACATTAAGTTAAAATTTCGGAGGATGTTGTTAATCCTATTGTTGAAAAT
GAAATCCAAACAAATATACGCTCCCATGAATCATTTTTAAGGGGATATGCTCCTGAAGATGAGGG
GCTGTATGATGACTATTAAAGCCGGGGATTTTTGGGTTGCCAAGATCATGCCAAGATCAACTTTA
CCGATGGTATTAGTTTCAAAAAACGCCCAGTTTTAATTTTGTGGTGTGATGGAGATGATGTTATT
GTGGCAGTCGTTATTTCTGCTCAACCTAGATCTAATACTGATATAGTTTTAGAAAAATGGTGAGA
AAGTGGATTAAAAATACCTTCTACGGTACGCTTATCTCGGTTAGATTCGCTGGAAAAATTCCTAT
TGATAGCAAAAATTGGTCATATTGACACCAAAGATGCTCAAAATATTCTTAAGACTTAGAATCAA
TATATAAAACCCCAATTTTTAGATCGAACTGAACAGGGGTAATGATATAATTTGCCCTTGGACAT
GAAAAGTATTGTTCATCATTAGTAAAATCTAATTCATATATCTTCTAACAGAATCATGACTAAAA
AGCGTGTTTTATCAGGAGTTCAACCCACAGGAAATTTACACATCGGCAATTATTTAGGGGCAATT
CGTAACTGGGTAGAAATACAACCTAATTATGATAATTTTTTCTGTGTTGTTGATTTACACGCTAT
TACAGTTCCCCACGATCGCACTTCATTAGCAGATAATACTTATACTATTGCCGCTTTATATTTAG
CTTGTGGTATTGATTTAAACTACTCCACTATTTTTGTGCAGTCCCATGTAAAAGCCCATACAGAA
TTAACATGGTTGCTTAATTGTGTTACTCCATTGAATTGGCTAGAAAGAATGATTCAGTTTAAAGA

AAAAGCGATTAAACAAGGAGAAAATGTCAGTGTGGGTTTACTAGATTATCCCGTACTAATGGCGG
CAGATATTCTATTATATGATGCAGATCAAGTACCTGTAGGAGAAGATCAAAAACAACACTTAGA
ATTAACTCGTGATATTGCTATTCGTGTCAATGACAAGTTTGGTAGTGAAGATAAACTCGTTTTTA
AAGTGCCTGAACCAATGATTAAAAAGGAGGGGGCTAGGGTTATGAGTTTAACTGACGGTACAAA
TAAAATGTCAAAATCTGATCCTTCTGAATTGAGTAGGATTAATTTGTTAGATAGCCCTGATGAAA
TTAGGAAAAAGATTAAAAAATGTAAAACTGATCCTATTATGGGTTTAAGTTTCAGTGAAGATCG
TCCCGAATGTAATAACTTATTGGGATTATATAGCATTTTTAGTGGTAAAAGTAAGGATGAGGTA
AGTGACGAGTGTGGGGATATGGGATGGGGTAAATTTAAGCCTTTATTGACGGATACGGTGATTG
AAGCATTACAACCTATTCAAAATAAATATGCAGAAATTATGGCAGATAAGACTTATCTCGATTCT
GTGTTAAGGGATGGTAGGGAAAAAGCGGAAAATGTCGCCAATGCAACTCTAAAAAGAGTTAAAG
ATGCTTTGGGTTATTTACCACCGATTTATTAATAGGGTGTTGGGGTATTGGGATTAGTGTTAATT
AGCAAGGGGCTTAAGCCCCTTGTTACTCCTCGCCTCTTGCCTTTTGCCTTTCGCCCTTAACCTAAA
TATCCAATTAATTTTTCCTACCCACTTATAATGACTAAACTAGAAAATGCGATCGCATCTAAAGA
ATTCATCATCACCGCCGAGGTGACCCCCCCTAAAGGTGGCAATCCTGAACGGATGCTAGAAGTTG
CCCGTTTACTCAAAGATAGGGTACACGGAGTTAATATTACAGATGGTAGTCGTGCCGTATTGCGG
ATGTCTTCTATTGCTTCTTGTGTGTTGTTGCAACGAGAAGGAATTGAGCCGATTTGTCAAATGAC
CGGGCGCGATCGCAACTCCATTGCATTACAAGCAGATTTAATGGGGGCTTATAGTTTAGGAATTA
GAAATATTTTAGCCCTAACAGGAGATCCTTTAAAAGCAGGAGATCACCCTCAAGCAAAAGCAGTT
TTTGAGTTAGAATCAGTCCGTTTATTAAAGTTAATTGGACAATTAAACGAAGGTTTTGATCTGAA
TAATAAAACCCTACCCGATGGAAATTTAAACTTGTTTGCTGGTGCGGCGGTTGATCCTCAACTAA
AGAGTTGGTCAGGGTTACAAAGACGTTTTGAACAAAAAATTAAAGCAGGAGCAAAGTTTTTTCA
AAGCCAATTAATTACAGATTTTGATAAATTAGATCGCTTTATGAATGAAATTGCTAAAGGATGC
GATCGACCTATTTTGGCAGGAATATTCTTGTTAAAAAGTGCTAAAAATGCTCGATTTATTAATAA
AAATGTGCCGGGAGTAGAAATACCAGAACATATTATTAATCGCCTAGAAAAAGCAGAAAACCCAT
TACTAGAAGGCATGACTATTGCCGCCGAACAAATCAAAGATGCCCAACAAATTTGCCAAGGAGTC
CATCTAATGGCGGTTAAAAGAGAGGATTTAATACCACAAATATTGGATTTAGCAAAGATTCATCA
CTAAGAGGTGTCAGGTTTCGGGTTGCAGGTGTCAGGGTGTTTAAGGGATAGGGGGAAACCAGTAA
TAAATAATAAATAAGGAATAAGTGTTCGGAGTTTTTAATTCTTAATTATCAACTATTCACTATTC
ACTAATTACCTTTGCCCCTAAAATTATCTATTGACAAGATTGGATGTAAAGATAGACAATAAGAA
AACCTGTAAATATTTTCTAACAATCATCAATAAAATAAATATATATGGTGGAAACTGTTCCCTCT
CCTGTTGAAATTGCAGAAGCTCTTACCATCCCTGCTGATTTAGAGTTTAATTTACCCGATCCAGA
AGATGATCAAATTGCGGAGGAGGATTTTCTGCAAAAGGTTGATGATGTTTGGAAAGTGTGCGAT
CGCTTCGATTTACAGACAGATATTTGGCGTGGTAGAATCTTAAGGGCTGTTCGGGATCGAGAAAA
AGTCGGCGGAGAAGGCAGAGGTACAGGTTTTTTAAATTGGCTAAAAAATAGGGAAATTACCAAA
AGTCAAGCCTATGCTTTAATTCAACTTGCCAATAGTGCAGATACCCTCTTGGAAGAAGGAAATTT
AGAACCTACCGCTATTAATAATTTCAGTAAACGGGCATTTGTAGAAACCGCCAAAGCAGAGCCTG
AAATACAAAAATTAGTCAGCGAAGCGGCACAAAAAGGCGATCGCATCACCCGTCGAGAAGTAAAA
CAGTTGACGGATGAATATACAGCGATGAACTCGGATTTAATTCCTGAAAGTGTAAAAGAGAAAG
CCGTTGATGGTTCATTACCTCCCCGTCATTTAGCACCTTTAGTCAAAGAATTAGAAAAACTACCA
GAAGAACAAATTAGCGAAATTCAAAAAGAAATTGAAAGTAATCCTGATGTGGACACAGTTAAGC
AGATGACTTCAACGGCGAAAAGTTTAAGTAAATATCTCGATTCTGCCGCCCAAGTAGAAACCCTG
AGAGATTCTGCTGTAGATATTGAGTCTGCTTTACAAGAAGCCCTGAGATTAGATTGTTTAAGTCT
GACGGCTGATTTAGTCAAACAAGCAACCAATTTAGAGCAGAATGTGGGTAAACTCTATACTCTCT

GGAAAAAATTAGGTAGTATAGCTGATCGCCTTTATGTGGAAACAGGAAGTAGTACCCCAAGTTTG
CGATCGCTTCTTAGTTGTTTGGAAAAATTAACCAGTGAAGTCATCGAGTTACGTCTTGATGATGA
GGGAGAAAAAATGATTCGTTTGCGTATTATGACAGATGAGTAATGGGTTCGGAGTGACAAAATA
AACATTGTTCCCTGTTCCCTTTCTCCCCTTAAAAGGGGAGACACAGAGGGGTTTCTCCTTTTTCTG
ACCCTAACCGAAAATTTTACATCGAATTCAAGGGAATTCAAGTTAAATACTAAATAAACCAGACG
GATTAACAGATAATAAATCTCGTCGTTGTAAACCCTCTCGATAAAGAATAGCATTAGCAGACAAC
AAACCACTACTAATTGCCCTCTCCATTAAACCACAGGGAAAAGGCATTTTGACCCAATCTCCTGC
AAACATTAAATTACTAATTTGAGACTCGGTAGAAGGACGCATTTTAAAGCTATTGGGAGGATAA
CCAGAAAAGTTCTTTTGGTTGACTAATTGCTGATGTAAAATGTTCGCATCGGCTAATTCCGGCAC
AATCTCAATCAATTCTTCTCTGAAGGTATTTAAAATAGCTTCTTGGGTAGGAAATTCTTTTTCTT
TATAACAATAGGCGTGTAACTCTACCACACTGCCCCCCGTTTTTTCTGCCCATGCTTTATATTCAT
TTTGAATGCGATGATAAAGGGTAATACTATCAGTGAGACGATAGCCTGATAAGGAAGCAAAATT
ACTGTGTTGCCACTCAAAATCTTTATCTAACCAAAAACGCCCAACGGCAAAGGGGTCAGCTAATG
CTAAACTTTCTATTTGTGCTTTAGTTTCGGGATAACTTTTTGTGTCACCTTCTATGAGATTAAAT
AGGTGTTTTGTTCCTACCACATCAGTAGCAAAAACAAAATAATCCGCTTCAATGGTTTTAGGTTT
ACTGGCGGTTTTTTGATTTGCAACTAATTTGACTTGATTCTCTCCTCTTTCGATTACCTTATAAG
TTTTTAAGTTACGTTTTGCAGGAGGATTTAGCACTTTCCCTTGTTGATCATATACTGCACCGTGA
CAAGGACAAACAAATTTTCCGTCTTCTTGTTTATTAACAATACAACCTTGATGAGTACAGCTTAA
AGAAAAGGCTTCATCGGTATCTTTAACTACAGCAAAGAGGCGATCGCCTCCGCCATAATAGCTAT
AGTGTTCATCTTTTAAGAGGGGATTGAGATCTACCCAAAAAGGTACATTATTTTCCTCATCCCCT
TGATAATATTCAATAGAAGTAATTTTCCCAGAACGGCAATTAATTTTACTAATATTGGCTTCAGT
GATGATTTTACCTTGATTATTGACGACGTGAGCGGCGATGGGTTCAACTAAACTTCTACCCATAT
CATCTACTGTGCCGTTAAAAGCCAACCCTTCAGGATTACCGAAAAAGTAAAAGTGGAAAAATTGC
ATTAGTTCACCTGTACTGAGAACATCAGGGGCATTAAGAGAAGATTTTGCGAAGGGTAAAAAGT
ACAAATCGAATAAACCTTGGGGAATACCTTTTGCCGCCCAGTCTGCCACAGAAATATTGTCTAAG
TCATAGTAGGTGTTAGGAATTTTAAAGCTCGTAATAGCGTGAAAAACTCGCCAATGGGCAGGATT
TGTTAAATTAATACCCCAATGAAGAGAATTGCGAGAAGAAATAGCTAAATCAACGATATTCCAA
GGGAAAGAAGAATGAGAAGGACGAAAATTTTCGGGTTTATATTTATCTCCTTTAAACACCACCGA
ATAATATTCTAATGATTGGAAATTAGATTCGATCGCAACCTCTTTTACAATGCTTTTAAGATTAT
AGTATTGGGGAAAAAAGCCATGAAAACCATGTTCCATCCTAAAACTATCATTTCCCACTTGAATG
TCCCAACTAGCAATTTTACCGCCTAACTGGGGAGATTTTTCCAATAAAGTAACTTCAAAACCCCG
ACTACTCAACTCATAGGCAGAAGCCAATCCTGCCAAACCACCGCCAATAACTACCACTTTCTTCGG
CTTACTTAAAAAGCGAGGTAATTCTAAAGTATCCCATTTATGAATTGTCGGCTGAGGCTTAACAA
CACGGGAATAGCCGAGAATACCAACAATGCCACTAATAGCGGATAATTTTAGCAAACTGCGACGG
GATATATTATTCATGCAACAATTAGGATAAGATTATGGCAATTTTAATGATTTTAATAATACTT
AACATAAGTTAGCACGAATTTATAGAGCCTGAACGATGAGTATTTTCTTCATTCAACCCCTACCA
CCCCAAAACCCTAAGCCCCTACTTCCCCCTCTCACCCTCTCCTCTCATCTCCCTAACACCTGCAACC
TGCAACCTGACACCTGAAACCTACCCTCATCCAATATTCTTAAACCGAACTGAGGTTAAAATTGA
TTTTGAAGACCTCACATTTTAGTTTTAATGTACTAATATTGGTTCTCAGTTAACGTCAAACAAAG
GTAGATTTAGCAGTAAAACCGATATGTCAGTAAAATATAGAGATTATAAATAAATAACCAAAAA
AAGAATAGAGAAGGAGATCACTTTGGTAAACACAGTATCATTCAAAACTACTAAATCCGATGAA
ATCTTTGCCGCCGCCCAAAAATTAATGCCGGGAGGAGTTAATTCTCCTGTAAGAGCCTTTAAATC
TGTCGGTGGACAACCTATTGTTTTTGACCGTGTAAAAGGTGCATATATTTGGGATGTGGACGAAA

ATAAATATATTGACTATGTTGGGACTTGGGGACCTGCAATTTGTGGTCATGCTCATCCTGAAGTC
ATCAAATCTTTAAGTGAGGCTTTGGAAAAAGGTACAAGTTTTGGCGCTCCTTGTTATTTAGAAAA
TATCCTCGCCGAAATGGTGATTGATGCAGTACCAAGCATTGAAATGGTGCGTTTTGTTAACTCTG
GTACAGAAGCCTGTATTTCTGTTTTAAGATTGATGAGAGCCTATACAGGTAGAAACAAAATCATC
AAGTTTGAAGGTTGTTATCATGGCCATGGCGATATGTTTTTAGTTAAGGCAGGTTCTGGAGTTGC
TACCCTCGGCTTACCAGATTCTCCCGGAGTACCTAAAAGCGTTACATCTGATACTTTGACTGCACC
TTATAACGATTTAGAAGCGGTGAAAAAATTATTTGCCGATAATCCTGATGAAATTGCGGGGGTT
ATTCTTGAGCCTGTAGTAGGTAACTCTGGTTTTATTGTCCCTGATGCGGGTTTTTTAGAGGGTTT
AAGATTAATTACTGAGCAGAATGGTGCTTTATTGGTATTCGATGAAGTAATGACTGGTTTCCGCA
TATCTTATGGTGGCGCTCAAGCTAAATTCGGTATTACTCCCGATTTAACAACTTTAGGAAAAGTT
ATTGGCGGTGGTTTACCTGTGGGTGCTTATGGTGGTAAAAAGGAAATCATGGCAATGGTTGCTCC
TTCTGGCCCCATGTATCAGGCTGGTACTTTGTCTGGTAATCCTTTAGCTATGACAGCAGGAATTA
AAACCCTTGAATTATTACAAAAACCCGGCACTTATGAATATTTGGAAGAAATTACCCATAAACTA
ATAAGTGGTTTATTAACTATTGCTAAAAATACTGGTCATAAGGTTACTGGCGGTAATATTAGTGG
TATGTTTGGCATGTTTTTCACTGGTGAAGAAGTTCATAATTATGATGATGCGAAAAAAGCTGACA
CCAGTAAATTCGCCCGTTTTCATAGAGGAATGCTTGAGAGAGGAGTATATTTAGCACCCTCCCAG
TTTGAGGCTGGATTTACTTCTATTGCTCACACCGAAGAGGATATAAACCAAACTTTAGCTATGGC
GGAAGAAGTTTTGAAAGAAATTCCTGCCTAACTAACAGTAAATAAGTCCATTAGAGAGGTTGAC
AATTAAAAACAAAAAATAGATAATGATTGACTGAATGCTGATAGCTGAAAGCTAAACAGGGGGG
TTATCCCCCTTGTCACCTCTTCATTTATTAAATAAATAAATATTATTAAAGAAATATTGTATTAA
GCTGCCTTCACCGAAGGTGCGCCTGCGTCGGTGGAATCGTCATTTCGTAATTGTTGCCTATAATCC
TTGTCTCTTATGCCGATAACCTTATCTAAACGGCTAATTTCAAATATCATTCTGACGGAAGGGGA
AACATTGAAAAGATATAAGTTTTTCTCCTGTTTTTTCGCTTCTTGATAAACACGCACCAGAGCAA
TTAAACCACTAGAATCAACAAACTCAACACCCTTAAAGTCAACAACAACATCCTTTTCCTTTGTG
TTGTCAATAAATTTTAATAACTGTTCTTGAAATAGACTTTCATTGTTGTGGTCAATTATTCCTTG
CGGTGTACAAATTTGCTGATTATTCATAACTGGTAGAACTCCTCATTATAGTATGACGATCAATT
CTAAATTTTATTGCTAATAAAACTAGGATATTATCATAGTGGTAATGATAATATGTTTTGGAAA
ATTTTGCTTAATAATTTATCATTCTAGGACAAAAACTTTAAATAAAATTTAATAAGAGTTAAAG
TTTTGAGAAAAAACAAAGTAATAATAATTATTTCACACAAAAAAATGATTTACTATGAAAAATA
AATATATTGTTATAGCAGTTTAATTTTATATTTTTATATCAAAAATTATTTATACTTAGAAAAG
TGTAATTTAGAATACATTTTTCAAATTAAAAGATATTAATACTAAGATAATCATTAAAAAAATC
TGAAAGTAAGATCAATTATTAAATTCAACTATTTTTCTTAGATTTATTTGATGTCTTTTATGTTC
CCACAATGATTTTTTTGCTAAAGCTACACTAAACAATCTGAATTCTGAGTTCAGGGTTTTTAATT
TCTAATAGGTGGAACATTTTGCCCTTATTCCTTGCCTCGCCTTTTCCTCACTTTACCCAAACACTT
TTAAGCAATCCCTAATTTAATAGACACTGACTGAATTTAAAATAAAAATCAATTCTTGTTAAGAA
AACGCCAATATTAACCCTGTTACCCATTACTTGTTCCCTATTGCCTGATTTTTGCAGAAGTTAATC
TATTTTTTCGATATTACCTGCTTTAACCCAGCCCTCTTGCCCACTGCTAGGAATACGAATTTTTTG
CCAACCGCCATCGTCACTTGTTCCAAGAATGATAATTTCCCAGTTATAGCCCACTCCTCCAATTCT
TTCTGAATCGATTCCGGGTTCTGCCCTTAAACTTAAACCAGTAGACCATATTACTTTACCCCTATA
AGCTCCTTCTGGTAAATCTTCTGTTTCTTCATTGGTTGTGGTTTCTTGTGTCTCTTTGGCTGTTTC
TTCCGTTGCTGTTTCTGCTTGATTGGAAGATTCATCAGGTTGTGGGGTTTCTTCTACGGCGGAGT
TTTCTTCTGGGAAACTCGGTTTAGGGGGATTGCCTGAGACTTGGGTTAGGAAAAAATATGCGCCT
CCTGCAATTCCTGCGGTGAAAAGAATTATTCCGAGGAAAAAACCGATTAAAAATTGGAACAAGCT

CGATATACTCATCTTTAATGATTGGAAAATGTGAGATTTTAATAGTAGATATTAGCAAATTATT
TATAGAAGGGAAAAATTTTATCTAAATTCATTACAGGGTTCATATGTATTGCCTGTAAACCGTAT
TTCTTTGCTTCGATCACATTATGATAATTATCATCAATAAATAGGGTACGAAGGGGATTAAGTTT
TGAGGTTTTGACAACATAATCATAGATTGCTTGATCAGGTTTATGTAAACTTATTTCATGGGAAT
AATATATCTTCTCAAACAAGGATTCAAAAGTATATTCTTTTACACTTTGGGCAAATCTTTTTTTT
ACTTCTCGGATATGAATGGGATTAGTATTACTTAGAATAAATGTCCTTTTTTCCTGTTTAATTTT
AGCTAGTAATTTTCCCATATATTCAGGAACATAGTTTAGCATTTGATTCCAAATATTTTCTATTT
CTACTAAATCTAAATCAATTTGTGCTTTTTTTTTGATGGCAGATAAAAAGGTTATATCTTCTATT
TTTCCTATTTCATACTCATAGGCTATTTGTTTTAATTCAGACAGTAATTCTTGCTCATTTCTAGG
GCTAATTTTTAATAGTGTTTGATAAGGGCTTTCCATTTCTACACCTATAATTACACCACCTAAAT
CAAAGATTATTGCCTCAATATTATCTAAGTTGATTTTGTTTTCCATGACACCTTTTATAACTTAT
AAATCAAAATTTCAATTCTATTTTTTCCGTTCCTTTTTTTTCCTAATTTTACCAAGAAACCTTAA
TAAAAAACTAATTATTTTGGCTCTTCAGAGTGTGGTTATGATAAATTAATAGGAAATAACTTCT
ATAACTTATATGGAATAGTGTTTATAGTAAAATAAAAACTAAAAGTTTATAAATTATTATTTAA
TAATTACCTATTGCCTGCCTTAACTAATAATTTACATACTCAAAGTGATAGAGCCATTATTTCAC
TGCATATAAATCACTACTTATACCCCATGCAAATTTCCATGAGTTAGCTATATTTTTGTTACTAA
AGCCGATAAAAATTAACAAAGCTGCGATCGCCATTAATGCTGTCACACCAAAGAGTACACGATAG
CCAAAATCAAGAATTAAGCTACCTAAAACCGGTCCACCGAGAGCAACCCCCACATCAAAACCACT
AACACATACTGCGAATACTTTACCACGTTCGATCGCACTGCAACGATCTGAAATTAAAGCAAGGG
TAATCGGAACTAATACACCTGCACCTATTCCTTCTAATATTGCCGAAAGAATCAACATCATATTA
TCTTGAACAAAGGTGAGGAAAATCATAGATAAGATATAGCAAATTAAGCTACCACTGATGAATA
AACCTCTACCATATTTATCAGATGCTCTCCCTGAAAGAAAACGTACTGCAAAAGATGCGATCGCA
GCCGCAGTATAAAAGAAACCAACATTAAAGTTGAGTCCCAAATCTCGAATATATAAGGGTAAAA
ATGTGACTAGAGTACCAAAAAGACAACCAATCAAGAGTAAAACTAAAGCAGGTACTGCAAAAGA
AGCACTAAAAATTAACTCTTTAAAATCACGGTTAATCAAAGACTCTTTACTTAATATTTCCCCTG
ACACGGAAATATTTTGATTTTTATCAACTTGAGAATCTAACTCTCGAATCTGACAAGCCAAAATT
AACGCCATTAAACCGCAACTTGCCGAAAGGAGAAATAGAAATTCATAACTGGTGTAAACCTCTAA
AAATCCCCCCATAGCAGGACCTATAGCCATTCCAATCGGCACAGCCAAACTCATATAACCAATTA
ACTCCCCCTTTTGTTTCGGGGGTGATAAATCTACCACTAAAGCACTGTAACCCGTAGTAAAAGCC
GCAATACTCACTCCATGAAAAGCACGATTAATCATTAATTCTGATAAAGAATCAAAAAATAAAT
ATCCTAAAGGTGCAATAAAAGCGACAATTGTACCAATGATAATTACAACCTTACGACTAGGATAA
TAAACTAATTTCCCCAAGAAGCGACGAAAAAAACGGAGAATAAAATTATTAATCCCAGAAGGAA
AAGGAAGATAATTAATAAATTTTTGTAAACCTTCGTCTGCTAATTTTCCTAACCATACACGAGAT
AAAAGTAAACCAATGGCAAAACAGCCCATAATGTAACCTACTTGCTTCACCGTTGCCCCTATATT
CTGAAGATAAGAAGGCAGAGTAGGAATTAGGCTAGTTAAACTCATCCAAAAAAACAAAGCACAG
AAAAAAAGAATAAATAAATTAACTAACTTATCTCTTTCAACGGCTTTAAAGGTATTGATAAAAC
TCACAATAGTTAAGAAGGTTAATTTTGTTACAAAACTTTATCTTCCATTGTAGATTGCATTTTCA
ATAATTTTCAGACTAGCTTTAACAGAATTGAGGAAATTACCAGAAATATTAGCGGTTTCTTTGCC
CGTAGAATCTTCCCAATTAATCATAAAGGAATAGTAAGCCCATGTATCTGCAGAAAAATTAACTC
CCCCTGCCAAACATAAAGCGTATTGGGGTTGTAAATCGATGCTTCCCCCTTTAACATAACCAATC
GCCTCAGAAGGAATAAATTTATCTAAAATGGCGGGAAGTCTTAATATACGTTTAAATTCCGTTAA
GGTTTCTTCTTGTTTAAAAAACTCTCCCTGTAAAGCACGACTATAAAAATTAACAAAATCAGTAG
AAGATGCGATCGAGCTTTGTTCTTGATTAATAATACTTCTAGCGGGTAAATTAGGACTATTCATC

TCTTGATTTATCTTTTCCCATCCCCAATCCTCGCCATTTTTCGCCCCTAAAACATAAGAAAACATC
ACACTAATGCTATCGGGAATTAAAGTATTTGTTAAACCGATTTCCGCAATAAATTCTCTTACCGC
TTGAGGAGTGACGTAAGCCATAATCAAATCTGTTGCGGTATTATCACTGTGCATCATCATCGCCT
CTAAGGCAATCAAAGCAGTGGTTTTGCCCGATACACCCCCAAAAACCGGACTAGAAAGCACCCTC
ACATCATCATTTATCACCAACTCCTCATCAAAATTAACCTTTCCTTCCTCCATTTGTCGCAAAAAT
ACTGTTAAAACAAAAACTTTGAAACTACTACCACAAAAAAGAGGAATATCACTGTTAAGAGAAA
CATCCATAGAATTACCTTGGCTTACTGCTTTTATCTTCATAGTTGATTTACCCGGTAAATTCGTA
AATAAAGGAAAAATTTTTGCTTCTAAATCATCAGAATTAATTGCTTTACTTATCATAGGAGTAA
AAAAAGTGCCTGCAGAAGCTAAAGTCAAAATAGAGATAAAATCACGACGAAACCACTTAACCAT
AATCAACCTCTGACAAAACTAAAATTAAAATCAATTTATATGACCTGAGTTTGAGATAAATTTTC
ATCTATAGAGTGATGGTGAAAAGGGTAAAGGGCAAACCCCTCTTTATCCCCAAGGCTGTTTCATT
TTCGAGAAAAAAAAAGTGGGAAATAGGGGGATTTTTATTCATAAAGTTTTAACTAATAAAAAAAA
TCCTTGTATATCTTACTCTCCCTTAATCTCAGTTAACCTAATTTTTTACTTTGAAACAGCCCTGCC
CTTTATCTCCCCTAAATTAGGAGGGAGGGCAAAGGTAAATAGTTGATAATTAATAATTAATAACT
TCTAACTCTTAACACAATACCCCTAACTCATTATTTTTAGAGTTACTTTCATCTTAATTGCTCATC
GACGTAAAATTTATTGAGAAAAATAACGAAAAATAACAATGACAAGCAACAATTATCAATCTAT
CTCTTTATCAGACACAACCATTCCGAAAGTTAATACTCTAACTATGTTTCGCCTAGGTTTATTTA
ACTTAGGTATTGGCTTAATTTCCGTATTAACCTTAGCAGTCTTAAATCGAGTCATGATTGCAGAG
TTGGGTATTCCTGCCTCTATTGCCGCAGGGGTATTAGCGATTTCTCAATTAGTGTCTCCTACTCGC
ATTTGGTTAGGGCAATTATCCGATGGCAAAAAATTATTTGGTTTTCATCGCACAGGATATATTCG
TTTAGGAGTCATTATTTCAGGTATTGCTATTTTCTTCGCTATTCAACTGGTATGGTTATTGGGGA
GTAATATCATCATTAATAATGGTTGGCAGTGGAATAATGTGACGATTTTTGTTAGCATTCTTTTG
GCAATTGTTTTTGTTGTTCAAGGTATTGCAACGGGGGCTTGTTCAACTCCTTTCACTGCTTTGTT
AGTGGATATTTCCGATGAAGATAATCGCTCAAAAATTGTGGCTACCATTTGGTCAATGTTGATGG
TAGGTATTGTTATTGGGGGTATATCGGGAAAAATTTTGTTAAATAATTTGTCGGGGGTAGAAGG
TGAAGGGATACCTTTAGCAACTCTGCAAGCTCCCATTAACGGTATTTTCCTTGTTGTACCGATAA
TTGTGGTGATTTTGACTTTGGTAGCAACTTGGGGAGTGGAGAAAAAATATTCTCGTTTTCGGCAA
CGCTCGTCTTTTCAAGATAGAGAGGAAGGAGTTAGTTTTAAAAAAGCTCTCAAGATATTAACTTC
TTCTCGTCAAACTGGTATTTTCTTCTCTTTTTTGGTGATGATTACTCTTAGTTTATTTATGCAGG
AAGCGGTTTTAGAGCCTTATGGGGCAGAGGTTTTTAATATGCCCATAGGAGATACTACTTTATTA
AACTCTTTTTGGGGAGTGGGTATTTTATTGGGATATGGCACAACAGGTTTTTTTGTTGTTCCTCG
TTTGGGTAAAAATAATACTACTCGTTTAGGTTGTATTTTAGTAGCTTTATCTTTTGGTTTGATTA
TCTTTTCCGGTTTGACTCAGACTCCTTCTGTATTAAAAGGGGCGATGGTGTTATTTGGTATTGCG
GCTGGAATTACGACTATCGGGGCGATTAGTTTAATGCTTGATTTAACGGCGGCTGAGACGGCTGG
AACGTTTGTGGGGGCTTGGGGGTTAGCTCAATCCTTATCCCGTGGTATCGCCATCGCTGTGGGGG
GATGGATTCTCGATTTAGGGCGTTTTTTGTTCAACAATCCTTGGTTAGCTTATAGCTTGGTTTTT
GTTTGTGAAGCCTTATGTATCATAGGTGCGATCGCACTTTTAAATCGGGTTAATGTTCAGGAATT
TCAAGCCACTACCCGTAAAGCAACAGCTATGGTTATGGAAGGAGATTTAGATTAGTTAACCTTCG
TTGGGCGCAAGAATATTGGGCATTGGCGAATTAAGCCATGATTTTTCGGTTTAGATTGGGAATTA
GGAATGGAGAATAGGCAATGGTAATAATAGACACAAATGCAAAATTAAAATTAAAATTAATTTC
CCTAGAGAAATAGTCAATTTTTTATCGTTGCCTGTTGCCTGATTTCTACAAGAAGTCTAATTAAG
AAATAAGTAAAGATTTAGGTTGATAAATTGACTCTACTAAAGGCTTAAAACAGAAATTAAATT
CTCTTTTGCCACTGCTAAACAAGGAAAACTGATATTACCATAATTGACTCCTTTAGTAAGAGGAA

AAGCATTTTCTGGATCGATATGGACAAAATTTCCCCCTGCGGCTAAAAGTATAGGATAAGCACCA
GCAATATCCCAAACTTTCGGACTAGCTTCGATCGCTCCCAATGTAGCACCACAAGCGACAGAAAT
AAGATCGTAACTTGTCACACCAGTAATTCTAACTTTGCAGGGAAAGTCGTGTTTGAGAATATCTA
AACTGCGGTTGCACATTGTCACCACATGATTCATGGTAGGAGATGCGTCACTGGTAGAAATAAGA
TTATTGTTGAGAAAAGCACCTTTGGGTCCAGTTAATCCCGTATTACCATAATAATAACCATGATA
AGTTTGTTGTACTTGAGGAAAATGAACAAAACCGAAAACAGGAATACCTCGATAAAGTAAGCCG
ATGGATATACCCCAAATCGGAATCCCACGGGTAAAATTAGTTGTACCATCAATAGGGTCAATTAC
CCAACACCATTCATTGGGGGGTAAAATATGCTCGGTTTCCTCCGTCAAAACCCCATGGGTGGGAA
AACATTGCTTAATTCCCAGTCTTAACTCCTCATCCGCCCATTTATCCGCCTTTGTTACTAGACTAC
CATCGGCTTTCCGAGTTGGTTCTAATTGAGCAAAATCCGCTAGTAAACTTTCCCCAATGCGGTGG
GTAATCTGGTGACAGAAATTATATACTTGATGCCAGAAATAAATCATTTTTTCCTCAGTGAAATT
GCTTGGACGAATAAATAAAGAGTTTGGAGTTTGAAGTTAGATAATAATTATGGTCTATTATTTA
TTTCTTAATTTTTAATTCTTGAATTATTAATTCAAAACTCTATCCTTTACAAAGTATAATTTATT
ATTCCGAACAATTGATAATTTATATTTATAGATATTCGTGTCTGTGAAACAACCTCGTTTACTCA
TTGCCGCCAGTGGCACAGGAGGGCATTTATTCCCTGCTTTAGCTGTAGCTCAACAGTTACCTGAT
TATCACATTCAATGGTTAGGTGTGCCTAATCGCTTAGAAACTACCCTTGTGCCTTCAGATTACCG
TTTACATACCGTTGATATTGAGGGTTTTCAAACCAGTTTCGGCATAAAAACTATTTTTGTCATGG
GTAAAATGCTCAGTGCGATCGCACGTACTTATCAAATTATCAAAAAGAGTAAAATAGAGATAGT
TTTCACCACAGGGGGTTATATTGCCGCTCCTGCCATTATTGCCGCTAAATTAGCCAAAATTCCCGT
TATTCTACACGAATCTAACTATATACCCGGGAAAGTAACCAAGTTACTCGGAAAATGGTGTAAAT
TAGTGGCTATTGGTTTTCGAGGTACAGAAAAATATTTACCCAAAAGTAAAACCCAATGGGTAAGT
ACACCCGTTAGAGAGGAATTTTTACAACCCCAAACTTTGCCGTTAGATATTCCTGACGATGTTCC
TCTAATTGTCGCTATGGGGGGTTCACAAGGGGCAGTAGCCTTGAATAAATTGATCAGACGATCAG
CACCTCAAATACTGGACACAGGGGCTTATATCGTCCATTTAACAGGCAATCAAGACGATGAAATC
GGAACATTTACCCATCCTCACTATTTAGAAATGCCTTTTTATGATAAAATGGGGGCATTATTACA
AAGGGCAAATCTGGCCATTAGTCGTTCGGGTGCTGGAAGTTTAACGGAATTAGCCATTACTAAAA
CTCCCTCCATTTTGATTCCTTATCCTTTTGCCGCAGAAGATCATCAATTTTTTAATGGTCAAGAAT
TTGTTAACACTGAGGCTTCTTTGATGTTTAGGCAAGAACATTTATCCCCAGAACTATTAACAGAA
ACTGTACTAGATTTGTTAAATAATCATGATAAATTGATGAAAATGAGTTCCCATGCTCAACAGTT
AGCTGATAGAAATAGTGCAAGTGCGATCGCACATATAATTATGAATGAATTAACTTATACTCGGC
GAGAGAATAAAAAATATTGATGAAGACGAGGCAAGAGGCAAGAGGCAAAAGGCAATGGTAATTA
AAAATTAGTAATTAGTAATTAATTAAAACCTGAAACCTGACACCTGAAACCTATTTTATAACTCC
TAACCAAGAAATCAGTTTAGACTATGTCTTAACGTCCAATGACGAGGAGAAGACTGATATACTTT
AGCTGAGTGCCATTCCATTTCATGGGCTAAATCTTTTATTTCATCAACGGTTAAGGCCGCCCGTA
AGGAATCTTTAAATAGTTGTTTCTGTCGAGGACTATAATCTAAGTTAGCTTCTTGAACAATTTTT
TCTATATCATCCTCCGATTCAGGGCGGAATAAATCTCTAATTAAAATAGCTCCATTTGTTTTTAC
AACCCGATTAATTTCTTTGAGCAATTCCAAAGGTTGCGGTATGTGATGAATCAAACTGTTAGAAA
TAATTAAATCAAAACTATTGTCAGGATAGGGCATTTTTTTGCCATCCACTAACTGTAAAGAAATT
TGGGATGATTTATTTTTCTCCTGAATATTTTTTTCCGCTATTTTTAACATAGATGAGGCTAAATC
AATAGCCGTAATGTGCCATTGAGGGCAAAAATCAGCTAGTATTAGAGGTATTCTGGCTGTACCAG
TACCAACGTCAAGCACTTTACTTTGTTCTTTTCCTAATTTACTAGCAAGAAGGGCGAAATCTGTG
TTAACTTCTGTGAAGTCCATTTGATCATATTCTTGGGCTTCTAAGTCGTCGTTCATGACTTCAGG
TTCTAAAATACGCTCTATCATTGATAAATTGGGTTCTCTTACTTTGGATATAATAAATTATCCCT

AAAAAAAGGTTTCAGGTGGCAAGTTTTAGGTTGAATTAAAATTGTTAATTTATGAAGAATTGCA
TTGTTTACTTGAACAAATCCTTGTCAGTTAAAGTTTGTACCAATTATTTTATAGTAATTTGTCAT
ACTTAATTTGTCATACTTACTGTATAAAAGCCATAAATTGCTTTATTTATTGTTAATAACATTAG
CAACAATTTGACTTATTTTTTCACTAGCTCCAGCACTTCCTCTGAGTTTCTGCAAATCCGATTTTA
TTTGCTTCAATTTATCCTCATTATTCAACAAATCTAAGACTAAATTACCCACTTCTTCTACTTTCA
AATGTCCTATTAATTCAGGGACAATTTCTTTTTTTGCCCAGATATTAGGCCAAGCATAAAGTTTT
TTATTTTTCACCGTATATTGAATAACTATCCAATTAATTAATTTAGCAAAATAATCACCAATAAT
TGGTAAGTTTGCCAATAACCCTAAGATTCCATCCCATGATTTCATTGCTGTTAGTTGATAGGTAG
GAATTAAAACAATCATCGGCATAGTTAATGAGCCTAGTTGTGCGGTGTTTGCCCCTACGGTTGTT
AAGCATAATTGACATTTTGTTATTTCTTCATAACAGGGAAATTTAGAGATTAATTTTATTTTTAT
TCCGTCAGATGTTTGTAAATAAAGTTGTTTATTAATAGTAATTAATTCCCCTGTAATATGACCTA
ATTGTTCAATTAAATTATTATTCTCTTTTTTCCCGTAACTAGCTAATATTTCTGGAGTAATAGTT
GGAGCAACAGGAATGATAAATTCCACATTATTATTATATTTATATATATAAGATGCGATCGCCAT
TAAAAAAGGTACACCTTGGGTTAATTTTGCAGATTTAGAACCGGGCAAAAGTCCGATTTTAATTG
TTCCATTACTTCCTATGTTTTGTAGGTTATTAGATGTTATATCCGCCATTAAATCCCCAACCAAA
TAGCATTTACCATGAAAATAGCTGGGTATTTTATCCAAAATAGATTGTTGCATCAAAGCAAAATA
ATCAATATACCTATACCATCTTGCATCCCATTCCGCATAAACTAAACTTAAAAAACCTAATCTTT
TCGCAATAACAACAGTAAAAAATTGATCTCCTCCTAGAAATAAAACCAATCCTCGTTGATACCAC
TCCCAATTATCTATTGTTTTTCCCCATAATAAAAATGAGAAAAAATTTTCAGCTCCCAAAACACG
GTTAATTTCAGGCAATTTACGGGCAATATTAACCTCATTACCCGTTGCATGAGGACAAGGAGATA
AAATTAGAGAAATACGCAAATTTTCTTGATTAATAGTGGATAAAGAACGAATTTGCTTAACAAT
AGGTAAAACCCATGTTGTAATTTCCCCTGGCCCATTAGATAAAATTACTAAATCAAAATATGAGT
CCACAATTTCTTAATCTTCCCAAGTTTCAGGTTGAATTAAATCAATAATATGATCTCTCAATAAA
TAACCATTAAATTCTAATTCTATTTCAATTGTGTGCCATTCTCCCGCAGGAATAAAATTACATAG
AGTATAAATAGGTTGTTGTCTACTAATTAATCCTTTCGAGACTAATTCTTTCGCTTTTTGTTTAA
TAATACCCACAGAATAAATAGTTTTAACACTCATAATTTTATCCCATTAATAACATCTATTTCAA
TACAACTCAAAGAGAATTCATCTGGAAAACTATCTGGTAAAAATTAAGATTGACTGATATTTATC
AACAGTATAAATAATTCATATTGATAATATAAGATAACAAAGAATCTATTGATTATTCTTGATG
AGCTTTACCGAAAGCATATATAAAAACAGTCAATTTTTACTAAAAAACCTGCAAAAATTCTGTTG
ATAAATTCCCAAGTATCTTTTTTTTACTACCTCCTAAACAAATAAGTATTTGTAAAAAAGTTAGA
GGGTTATTACTCATTATCTCAGATAAAAATACTTTTACTAGACTGAAAAAGTAATCTTAATCTTT
TCTTTAACTTATGAGCTAATCAACATTAACTGTTAGAATTCCTTTTATTTGGTAAATTATCAATC
AGTAATTTGTAGTTAATTAAAATCTGAAACCTGCAACCTTACACCTAAAACCTGCCCCAGTTAAA
TATTCTTGTCATCAAACTGAGATTATAAAAACTTCTGACTTTTAACTACCACCTAATTTATCTCC
CAAACCTGTCAGATAACTGATATAACTAAGCTAAATAATAAACCTGATTAACAAAAATGGGTCGC
AAAGCATTATTATTGATTAATCGTCATTCCCGTAAGGGTGAAAAGTCTTTACCTTTAGCTGTAGA
GTATTTTTATGCCCATGACTTGGAATTGATTATTAAACCAGTCAAAGATGCCCAAGAGTTGGGAA
TTGTAGTCAGAGAATACCATCATCAAATTGATTTTGTCATTGTGGGCGGTGGTGATGGAACTTTG
AATGCGGTAGTTGATAGCTTAGTGGAAACTAATTTACCCCTAGGTATTTTACCATTGGGAACGGC
AAATGATTTAGCTCGAACCTTAAATATTCCTTTAGCTATGAGAGAGGCTTGTAATGTTATTGGTA
ACGGTAAATTAAAGTCTATTGATTTAGGGTGGGTTAACGGCAAGTATTTCTTTAATGTCGCCAGT
ATGGGCTTAAGTGTTGATATTACAAAAAAGTTGTCTCGTGGTGTGAAGCGTCGTTGGGGAGTTTT
TGCCTATGCCATCACAGCATTACAAGTTATCAGCACTGCTAGGCGTTTTTCTGCTACTATTAAGG

TGGATAACGAAACTTTACCCGTTAAAACCGTCCAAATTGCGATCGGTAATGGTAGATACTACGGA
GGTGGAATGGCGATCGCAAATGATGCCGCAATAGATGATCAAAGACTTGATTTATATAGCATTGA
ACTACAACACTGGTGGCAAATATTCCCTTTAATTTGGCATCTACCAAAAGGAGAACATCATCAAT
TACAATGGGTAAGGACGATAGAAGGCAAAGAAATAGAAATCCACACTTATAAATCTTATAGTGT
CAACACCGATGGTGAAATTACCACTATTACCCCAGCTAAATTTAAAGTTATTCCCCATGCTTTAA
AAGTTTTTGTACCCTAAATATTATGGCTCTTCAGAGTGTGGTTATGATAAATTAATAGAAAAGA
ATTTTTGTAATGCCTGAATACTATTCATGGCAGAATATAAGCTAAAAATTTAGGTTCTTCAGAGT
GTGGTTATGATAAATTAATAAAAAATAACTACTATAACCTTTATTGAATAGTGTTTATAGTAAA
ATAAAAACTAAAAGTTTATAAATTATTATTTAATAATCCTCTATTGCCTATTGCCTCTTGCCTAC
CCTAACTAATAATTCACGTATTCAAAGTAATAGAGCCTAAAATATCTTTGCCTCTTAACTTTTTC
CTCTCTCCATAAGTGTTTTCTGATTATGAAAACTATTGATTATTTCACTGGGGGCTTGTCAAAAA
AGGGGTCAATATAAGATAATAGTAGATTGTGTATAAAATTACTCCCATAGTAACTTAAGCTAAA
AAAATGGCAAGTAAAAAAGGTGTCCGTATTATCATCACTCTTGAATGTACAGAGTGTCGTACAAA
CCCTGACAAACGTTCTAAAGGGGTTTCTCGCTATACTACCATGAAAAATAGAAGAAACACCACTG
GTAGAATGGAAATTAAAAAATTCTGTACCCATTGCAATAAACATACTATTCACAAAGAAATTAA
ATAGTTTTATTCGGTACAATTTTCAAAAAATTAAAGTAAAAAATTAAAGGTAAATTATGGCTTA
TTTTCGTAAAAGATTATCTCCTATTCCTCTAAATCAAAAAATAGATTATAAAGATTTAGAATTAC
TCCATAAATTTGTAACAGAAAGAGGGAAAATGCTTCCTCGTCGTATCACTGGCTTAACCGCTCGT
CAGCAAAGAGATTTAACCAATGCTGTAAAAAGAGCTAGACAATTAGCTTTATTACCTTTCTTAAA
CGTGGAAGGTTAAATTTAATTTGAAGGTTTAGTCGCTAATATTCTAAATCACTTTAATGTGTAAA
AAAGACTCCCGTTAACTTTTCCCAAACGGTTATTATTCTTTTCAATTAACGGGAGATATATAGCA
ATTAAATTTAAGTTCTGAGATAGATTTTTATTAAAATAATGTAACGTTTAGTGTTTTTATTGGA
AAATTATTGATTGAGCTTTTCGAGGCTTTAATAAATACAATAAAAAATATCACAACCTATTGAG
AACTGCTATACTAAACTAAGTACCATCATTACTTTAAAAATTGGGTGCGTAGCTCAGTTGGATAG
AGCACCAGATTCCGGTTCTGGGTGTCGGGGGTTCAAATCCCTCCGTACTCGTAGCTATTTGGTTG
ACAAGCAAATGAAAATTAGCTAGAATCATTTAATGTCTTTAAACTAATTTATAATTTCTTTCTAA
ATTATATTTGGCGACAGCCGAAGACAGCAGGGGCAATAGCTTAATCATCCTGCCTAGGTTGATGC
GAATTACAGATCAAACATAAGGTCTGTCTCAAGATTAACTAACTATAGTAAAAATCTACCGCGAT
ACATCAACCTAATGCCGTGCAAATTGTAGCAAGGCTTTTTTTATTGTCTGTTGGTTAATAAATCA
AAAGTTTAATCACTCCCTTGAGTTAATCAATGGCGCAAAAAAGAACATAAAACACCCGAGGAGGT
GAGAGTCATTTCTAAGTCATTAGAGAGTAAAAAACAAGAAGTAACTGAAATCAAACAGTTGCTA
GAAAAATCTCGCTTGGCGGTGGTAATAGACTATCAAGGGCTGACTGTTGCTGACATCACTAATTT
GCGTAATCGCTTAAGAGAAGGCGGAACTATCTGTAAAGTAACTAAAAATACTTTAATGGGTAAA
GCCATCGAAGGTGATGAAAATTGGCAACCGATGACCGAGTTTTTGAAGGGGTCTTCTGCGTTTAT
TTTGGCGGATGAAGAAAACATCGGAAGTGCAGTTAAAGCCTATCAAGCCTTTGCGAAAGAGAGCA
AAAAAACAACCCTTAGAGGTGGTGTGATGGAAGGTAAAGCCCTCAGTGAAGCTGAGGTTAAGGCT
TTAGCAGATTTACCCACCAAGGAACAACTTATTGCTCAAATTGCAGGTTCGATCAATTCTATTAC
TGCAAAAATTGCGATCGGAGTCAAGGAAGTACCTGCTTCTATTGCTCGTGGTATCGAAGCCTACA
GAGCCAAGCAAGAGGAAGAAGCGGCTTAAGAGTCGTAATTTCAGTGTCGATTTTTTAGTGTTTAC
ATAGTTTATAGGAGATTACATTTAATGTCTGATAAAGTTGCAAATATTGTTGAAGAGTTAAAGA
CTTTAACCTTATTAGAGGCTTCCGAGTTAGTTAAACAAATCGAGGAAGTATTTGGTGTAAGTGCC
GCCGCTCCTGCTGGTGGTATGATGATGATGGCTCCCGGTGCGGCCGCTCCTGCAGAAGAAGTTGA
AGAGAAAACCGAATTTGATGTTATTCTCGATAGCTTCGGTGATGCTAAAATGGGCGTTCTCAAAG

TCGTTCGTGCTATCACTGGTTTAGGCTTAAAAGAAGCTAAAGAGTTAGTTGAATCTGCTCCTAAA
GCTATCAAAGAAGGTATTAACAAAGAAGAAGCTGAAAACATCAAGAAACAGTTGGAAGAAGCTG
GTGGTAAAGTAACCGTTAAATAGTTACTTGGCGTTAGGTTAAGGTGTTAAGAGTTGGGAAATTT
CCCAATTCTTCTTCTCAAGGTTAAAAAATATAAGGTTAAAAAATTTTCCATAGGTCAAGATTTAG
TTCACGTCGCTCTACAGATACGTCACTTAAGTAGCTTTTTCTCAGCATTAATTTTTACCATAACTC
CAAAAGTAGATTATTCATCTTTTTTTGAAAGGAGTAATTGATCTGAATATTTTTTCTATATAATT
CTTCAAATAAATTTTAGGAAATATTGAACTCAGGCGATTTTCTTAGGGGAAAAAAGAAGAATTT
ATTAGCATTCATTGCAATATTTCTCATATCCACAGCCAACTTGTTTGTGAAAATAAGTTCAACTA
CTATCTTGTCTCTCTTGAGAAATAAGCCTTAGCCCAGTCAAAATCACTATATGAGCTTAATAAAA
ATGGCGGGAGGCGGATTTGAACCACCGACCTTCGGGTTATGAGCCCGACGAGCTACCTGACTGCT
CTATCCCGCGACGCATCTACTATGTTAACGTCTTTTCTTAACAAATGCAACCTTAATTTGAATAT
TTTTAAATAAGAGGGAGTTTTTGGTATTTTAGATAAAATCCTAGGATGATGAAAAGTGCGATCG
CACTTGCACCTAAACCGATATAATTAGGAAGCCAGAAGACAGCTTGTAATTGATTGATTTGCCCT
GCGGATAATTGAATATTGTATTGATTGTTGGGTAGTTTTTCCCATTGGGCAAATTCGTTGGTAGT
GAGTTTAGCACCCAAAGGAAAATCAAACTGAATTTGTAGGTTAATTAAGTCTCCAGAAGAAATG
ATGATGTTACCTTCACTGGATACGACTCCCAAAGGAGTTAAATCTGCATCAAAATTAAGGGTGTT
TCTCTCCAGAAACAATAGATTATTTTGTTTAATGGACATCTTTGCAGATAAATCCAAAAGACTAT
TAGAATTATTATCTGAGATTTTTTGAGAAGATTTATCACCCAATGTCGGAATAAAAAACTGGTTG
AATTTCTTAACCAAATCTTGACCATTACTAAAAGGAATTGTAACCACTAATTCATCTTGAGAAAG
TCGTTTACTCTTTCCTTGTAATTTCAATGCACGACGTTCAATACTATTTAACCACGCATTCCCTTC
TTTTTCGCTGAAGTTGGTTAGTTGTTCACCCAATTTGATATGTTGAATCATTGTACCATGATTAG
CCTGTGGGAATTGAATGCCGACATCATAGCGAACACATCCAGTTAAGCAAAGACATAAGGCTAAA
AAAATAAATATCTTTTTCATGGAGATAAATAGCATTATTAATAAATTAAGTTACAGTTATTGAC
GACAATATTATCTATTCTTCTGAAGAATTAGAAGAGGCAAAAGATTGAACAAAAATTTCCCTTGT
TATTGGTTGTTCTATAATTAATCCTTCTCCTCCTAAAACATCCAGAGGTTGCCCCTCTACACTAAT
CCAAACACTGGCATTAGGATCTAAACTACTAGCAGTATAAATGACTTGTCCTAATCTGGAAATCA
TTGACTCACTACCCCCACCGAGAGTAAATTCTTCTGATAAGTTTAGGTGAATACCATCTTCTTTT
ACTTCTAAATTCTCTAATCTTGTATCAGAGGGGATTGCACTATCCTGACTTTCTTCTTCAGATAA
TAACTGATTAAAAGCTGTAGTCAGGGCTGACTGAGGATTTTCTTCTTTTGTCAGGGGAATATTTT
TGGGAGTTAATTGGAGATTTTCATCTAACAGGTATATTGCGACTTGCTGTTGTTGATTATTGGCT
GGATTGTCAGGAATTACTTCTACATCTGTAGGAGCGGTGTCATCTGGTTGACTAACTTGATTTTT
CGACTCTAGGTTACTATAAGCATACCATGCGGCAAAAGTCCCTACTGCTAAAACTGTAGTGGCAA
TGGAAGCAATAATTTTAGTCGAGAAAAAAGAATTTGGTTTTTGATCTTTTGCCATAATTGTTACT
GTAATTGATAGTGACTACTTAGTTATTTATTTTATGGGGATTGGTTATCAAGATGAACAAATCCT
GCGATTTCCAATTTATTATCCTCTATCTTAAATTTTAATAGCCTTGCAATAACGCCGTATTTTTC
TAGCTTTTGTAAGGTTAACTGAGTATTTATGTTATCAGCAAATCCTTGTAATAGTTTAGATGATA
GTTGTCTGTTGTTTAATGTTCCCTTAACATCTGTAATTCTTAGTTTATGACCCTTAATTAAGTCT
AGGGTAAATTCTAAATTAATATTTAACAATTCTTCTTTTCTAATGGGTAATTTGGCTTTAGTATC
GATACGAATACGGTTATTTGTTTGTAAATCTAATTTTAGATCCATAATTTGAACATCGGATTCTT
CTGCTAATTTAGCCATAATTGATTTTGCTTGAGAAGATGTAAGTATGTTGTTCAAATCATCTTGA
TTAAGAATCATTCTTCCTGCTAAATTAAGAGGTGAATTTAGTATTTTTTTCCATTCATTTTGTTC
TATTTTCTTGAGATTTCTTAACTGAGATAAGTTAAGGTTGATTTCATCGGTTTCTATTTCGACTA
ATTCCAAGGAAATATTCTCACGAGGTTGCCATTCTTTGAGACTAATTTGAATAGAATCTGCTTTT

CCTTTTAAAAGTTGGTGAGTGGGAACACTGTTAACTCTAACATCTATTTGTTTTACTTCTTCTGA
GTTTTTTTCAATTACTTTGGTTAAAGAATTATTAACAAATAATCCCCCGAAAGATAAGCTCGATA
CTATAATGGTTAGTATAGTAGTAAAAATTTCCATATTATTTATATTTAGTCAACTTTTAGATAT
TTTTATTTATTAGCGTTAATATTCTACATCTTAATATTATTTTTTTGTCCAATATTCGTATTTTT
CTATTTAATCTTGGAAGTATTAAGTTAATAAAGAAATGTATTTATTGCTTTGTTTATAGAGTGA
TAATTTATACAGTAAACTCGACAATAAGTGTTTGATGGTTTTTTATTACCACTTTTGAAATTTGG
AAAATGAGAGTTAAGTTACCACGGAATGAGGGAGATAAATTGATTGATGAAATCGCACCGACTA
AAGAAGATTCTTTTTTAATTTATATTGTTGATGATGATAGAATAAATTTGAGTTTAATTGGTCG
TTTTCTTCATAAAAGTGGTTTTAATGTTGTAACTAACACAGATATTTTTCAAGCAATTAGAGAAA
TTTGTCAATTAGTCCCGGATTTAATTTTATTAGATATTTTGATGCCTGATATAAATGGTTTTACG
GCTTGTTCTTTACTAAAAAAATCAAGTAAAACTAAAGATATTCCCATCATTTTTTTAACTGCTTT
AGATAGTTCAGAAGATAAAATTAAAGGATTAGAATTAGGCGCGATCGATTATATTACAAAACCA
ATTCATTACCCTGAACTTCTATCAAGGGTAAATACTTGTTTAAAAATTAGTAATTTAGCGAATAG
TTTAAAGACACAAAACAGGCTTTTGAAATCAGAAATAGAAGCGAGAAAAAATGCCCAAATCTCA
CAGCAAAAAGCGGAGAAAAAACTGAAAACAATTATTAACAATCATTTTCATAGTATGGTTGTTT
TAGATTTAGATGGAAAGGTTTTATTTCTTAATGAAGAAGCAGAAAAATTATTCAATCGTACTCA
TGAGCAAATGGTGGGGGATACTTTAGGTATTCCCTTAGAATTAAATAAAATTAGTGAGTTAGAA
ATACTGCGATCGCAATCTGAATTAATTACAGTAGAAATGAGAGCAGTACCAATTATTTGGAATGA
TGATAATGCTTATTTACTATCCTTTATTGATATTAGTGAAAAGAAAAAAATGGAGCAAGAATTA
AAGATATTATATCGTGCTTCTGAACAAAGTCCAGCTTCAATTGTTATTACTAATGTGGAGGGAAA
TATACAATATGTTAATTCTAAATTTGAAACTGTTTCAGGATATAAAAAAGAAGAAGTAATAGGT
AAAAATCCTCGTGTTTTAAAGTCGGGTTATACCCATGAAACCGATTATAAAAATCTGTGGAAGAC
AATTAGTAATGGCAAAGAATGGCAAGGAGAATTTCATAATAAAGAAAAAACGGAGAATTATAT
TGGGAGAGAGCGCTAATTTCACCAATATTTAATTCTGTAGGGGCAATAACCCATTATATTGCAGT
GAAAGAAGATATAACAGAAGAAAAACAGCAACAACTGCTTTTAAAATATCAAGCAAAATATGAT
TATTTAACAAAAATTCCTAATCGTAATTATGCGTTAGAAAAAGTACATAAACTGTTAAATCAAGC
TGAAGAAAATAAATATAATACTGGTTTAATGTTTATTGATTTAGATCATTTTAAAGAAGTAAAT
GATACTTTAGGTCATGATTTTGGAGATGAATTGCTAGTAGAAGCAACCATGAGAATGAAAGGAG
AATTGCGTAGCACTGACTTATTAGCCCGTTTAGGGGGAGATGAATTTTTTATTGTTATTCCCATT
GTTCAAAGTCGTCATGAATTACAAATTATTGCAAATAAAATTATTCAAATATTAAAAAAGCCTT
TTTATATTTTTCAAGAAAAAATTTTTATTTCTGCCAGTATTGGAATCACTATTTTTCCCGAAGAT
GGACAGGAATTAAAACAACTTATTCGCAATGCTGATTTAGCAATGTATCAATCAAAGAAAAATG
GAAGAGATCAATTTCAATTTTATCAGTATGATATGAACAAAGTTGATATTGGAAAATCCACTTT
AGAAAAAAATTTTTTGAATGCCATTAGTAATGATGAATTTAAAATTGTCTATCAACCAGTCATA
GATATTTCTACGGGAAAAATTGTTAGTGCAGAGGCTTTAGTGAGATGGGAAAATAAAGATTTAG
GTTTGATATATCCAGAAAAATTCATTCCTTTTTTAGAGAAAAATGGCTTAATTTTAGTTTTAGAA
CAATGGATATTAGAAACTATCATCAATGATTTAAAATTTTGGGAAAGATACGAAGATTTACTTA
TAAGTATTAACTTATCTGAATATCAGTTTAAAAATGCAAAAATGGTTAAGAATATTAAAGATTT
GACAGGAAAAAAACAACTACGACAATGTAATTTATGTATAGAAATAAAAGAAGAATATTTAGAG
GAAAACAAAAAATTTATTGATAGTATCTTCAATGATATTAATTCGATTAATATTGAGTTTTGTT
TAGATAATTTTGGTGCAGGACTAACATCAATTTCTAATCTTAGTAAGTTTCCTTTTAAACACTTA
AAAATAGATCGGAGCTTAGTTCAATCTTTACCTAGAAATTCTCAAAATAAAAATATTATTAAATC
AATTATTGCTGTGGCTAATATTTTGAATATTAAAGTTACCGCCAAAGGAATAGAAAGTAAAGCA

CAATTAGATTCTTTGAGAGCTTTAGGTTGCCATTATGGACAAGGTTATTTTTTTTCTCAACCATT
ATCAGCTCTTAATTTTATCACTTATTTAACCAAAAATACTAATTGAATATATTGGTTTAATAATA
GAAATATTTATCCCTAAATATCTATTGGTGGGCTATGCCCACCCTAATTTTTTCCATATAGACTA
ATTTACCAAGAAATATTAGAAGTTACGGTTAAATCTAAATCTTCCTGAGTGTTAATGGAAATCAA
TTCTCTGTCTCTTGTACCACCAAATAAAAATCCAGCTAAAGCACCAAAACCAGCACCACCTAAAA
CTTCTTCAGTTGCGATCGCTTTATCTCCTGTTACTGCCGATATAAGGGTGGCGGCGGCACTTCCTG
CCAATGCACCTTGCCAAATAGCGTCAGTGTTTCTACCATCAGATATTTTTTCTGTGCGAGTTACA
ATCTTAGAGTTAGCCTGAATAGAATACTGCTCACCATTAGGTAAAATAACAGTATTACCAACAAA
TCTTGAACCACTACCTGCAGGGCGAACTTCCCCAACAACCTCACTACCACGGGGAATAACAACTCT
TCCTGAATTATCTTTTACATCACGAGTAACCACTAAGGTAACAGGTAAAGTTTCATCTTTAGTGA
CAAGTATTTTTTTCGCTGACGGTAAAGTAGTAGGAATAACAGTACCTGAATTTAAGTTAGCAGAA
TAATAGGTAGGATTTTTTCTATTTTGATTTACTTCGTTTTTGCGTTCCCATCCCGGAGGAGCGGA
TCTATAGTATTTGTCGGGTCTAGCTTCCACTGAAGCAAAACTACCCACCAAAGTAGTTAAAGAAA
GACTTAAAACCATAAAACGAAGAGTTTTTGAAGTAAGAGAAATAGGTTTAAACATAACTAAATT
ACCCGAGGTAACGATAAACTATTTTTTTTGACGATAGCGGTTTCAATTTGTTCCTATTTTAGAGT
TGCTGATTTTAGATATGATTTCTAAATTTAGGTGGGGAATGGGCAAAGGTTTGATAATTAAGAA
TTAAGAATTAAGAATTAAGAATCCTGTTCACGATTGAAAGGAGTGTACGAGAGCAAAGGATTTT
CCGAACTCCGAACTCGTCTTTCCCCCAATACCCTAATCCCTAGCTTTTTTTAGAAGGTAGTATGTG
AAATATAAAGTAATCATAAATTATCGTCAGGCAAAAAATCAACTGAGGTAAGCTGAAAGTAAGG
ACTAGAAAATAATATCTGTAAGAGCGATCCCGAAGGGGCTACGCAGTAAGCATCTCATTGCAAAA
AAATAAGCCAACACGAGGAATAATTATGGCAACAGAAAAAGCCCACGACATCATGCGGTATCACC
ACAATCCCTTAAACTTCATGTTTGCACCCAAATCTGTGGCGTTAATTGGGGCTTCAGAAAAAGAA
GGCAGTGTCGGGCGTACATTACTTTGGAATTTAATTACCCATCCCTTTGGTGGCACAGTTTTTCC
TGTCAATCCCAAGAGGAAAAGTGTTATGGGAATTAAGGCTTACTCCTCTATTCAAGATGTTCCTG
AAAATGTTGATTTAGCAATTATCGCAACTCCCGCAGTAACCATTCCCGACATCATTAGAGATTGC
GTTAAAGCGAAAGTGAAAGGGGTAATAATTATTTCCGCAGGATTTAAGGAAATTGGAGCAGAAG
GTTTAAGATTAGAAAAAGAAGTTCTTGCAGAGGCGAAAAAAGGTAACTTAAGAATAATTGGTCC
TAATTGCTTAGGTTTAATATGTCCTCCCACGGGATTAAATGCTAGTTTTGCCAGTGCTAGTCCAA
AAACGGGAAATGTTGCTTTTATCAGTCAAAGTGGGGCTTTTTGTACGGCAATTTTAGACTGGAGT
TTTGCGGAAAATCTTGGTTTTAGTGCTTTTATCTCCATCGGTTCAATGTTGGATGTGGATTGGGG
CGATCTAATTTACTATTTTGGGGATGATCCTTTAACTAAAAGTATCGTAATTTACATGGAATCCA
TTGGTAATGCTAGATCCTTTATTTCCGCCGCAAGGGAAGTGGCTTTATCAAAACCGATTATTGTC
ATCAAAGGAGGGCGTACCCAAGAAGCGGCTAGGGCGGCGGCTTCCCATACAGGGGCTTTAAGTAG
TAGTGATAAGGTATTAGCGGCGGCATTTCGCCGTAGTGGGGTTTTACAAGTTGATACGGTGAAAG
AAGTCTTTAACATGACTGAGATTTTGGCAAAACAACCTCGCCCAAAGGGTAGGAATTTGACTATT
TTAACTAATGCAGGAGGTCCGGGGGTTTTAGCTACGGATGCACTAACGTGGGGAAAAGGAAAACT
AGCCAAATTAAGCGACAATACCATCGAAGAGTTAAATAAAATTCTTCCTCTCCATTGGAGTCATA
GTAACCCTATTGATATTTTAGGGGATGCTAATCCTGAACGTTATGCTAAAGCCCTTGAAATTGCG
GCAAATGATCCCAATAGTGATGCATTATTAGTCATTTTAACTCCTCAAGACATGACTGATCCTAC
TAAAACGGCAGAGGCATTGGTGAAAGTGGCACAAAAGATTAAGGATAAACCGGTTTTAGCTAGT
TGGATGGGAGGAGAAACCGTTGCTAAGGGTTCAGAAATTCTTAATAATGCTCATATTTATACCCA
ACCTTATCCCGATGATGCCGCCCGTTTATTTAATTTGATGTGGCGCTATAGTTATAATTTAAGGG
GTTTGTATGAAACTCCTAGTTTTGGTGAGTCCGATGATTTTATTAATCGTGACATTGCGGAAAAT

ATCATCAATCATGCCCGTGAGCAAAATCGCACTCTTTTAACGGAATATGAGTCTAAGCAATTGTT
AAAGGCTTATGGTATTCCTATTATCGAAACTATTATCGCTGAAAATGTGGAAGATGCGATCGCAG
TTTCCGAAAAAATAGGTTATCCAGTGGTGTTAAAACTCCATTCCGAAACCATTACCCATAAAACC
GATGTGGGAGGAGTCAAACTTAACCTGAAAAATGGCGATGAAGTGAAACAAGCCTTTTTGGAGA
TGCAAAGTCGCATATCAGCTAGTGACTTTTTAGGGGTAAGTGTGCAGAAAATGGCTAACCTTGAG
GGTTATGAATTGATTTTAGGTAGTACCATTGATCCTCAATTTGGGGCAGTAATCTTATTTGGCAC
TGGAGGACAATTAGTAGAAGTGTATCAAGATCAGGCGATCGCACTTCCTCCCCTCAATACAACTC
TTGCCAAGAGGTTAATGGAACAAACCAAGATATATACTGCTTTAAAAGGAGTTAGGGGGAGAAA
AAGTGTTGATTTAGAAAAATTAAAACAGATTTTAGTTTTATTCAGTCAATTAGTTCTCGAACAAC
CTTTAATTAAGGAAATTGACATCAATCCCCTATTAGTATCAGAAAATCAACTCATCGCTCTTGAT
GCAAGGGTACTATTATACAATTCGAGTATTTCTAAGGAGGATATAATTCCCCCTGCCATTCGCCC
TTATCCGAATCAATATCTCTCCTATAGTCAGTTAAGCAACGGTAGAAAAGTTATAATTCGCCCCA
TTCGCCCGGAAGATGAACCCGAAGCAGTCAATTTTTGCCGAGATTTGTCTCAAGAAAGCGTTTAT
CTGCGATATTTTCACTCTATTAGCCATAAATCTCTTATTTCCCACGAACGTCTTGCTCGTATTTGT
TTTATTGACTATGATCAAGAAATTGCTCTGGTGGCAGAATCATTGCAACCTGATAGTAAGGAAAA
AGAAATACTTGCGATCGCCCGTTTAAGTAAGGTACACGGTACACCTGAATCTGCTGAATTAGGGA
TGTTAGTGAAAGATAAGTGTCAAAAACAGGGCTTAGGTAGTATTTTAGGGCAAAAACTTATCGA
AATAGCTAAACAGGAGGAAATTAGGTCAATTATTGCGGAAATCCTCCCTGAAAATATAGGGATGC
AAAATCTTTGTCAAAAGTTAGGTTTTCGGTTAGACAAGCATTCCGATGTGGTTAAGGCAGTTTTA
CAATTAAACTAGGAAATATTTAGGTTTATTGATTGAGATAGGGGAAAAGAGAAACCCCCCTTTA
TCCCCAGGGCTGTTTCATTTTCGAGAAAGAAAAAAGAGCGGGAGATAGGGGCGTAGAGCCGAGAT
ACGAGCAAAATCGGACACCAATGTCAACGAACCATGACATTTACGATTGAAACGCTTATTCCCGT
TAGGTTTACGAAAATCTATTTTAACAAAACTAAATACTACATAACTGATTTAATCAACAGAATAT
TTCGTCAAGAGCATTTAGATAACTAAATAAACTACAGACGGAAGAAACATTGCCGAGTTAATTAT
ATTTTCGGCAATGTACAATAGAAAGTAGTATGATACTTATCAGCATTTAGAATTAAATCCTTCTG
CTAGAGAAATTGTTTTAAATTGTTGTTTATTTTTCGATGAAAGTCGTCATCAAAATGATGAAAG
ATACCATACTTTTACTATAGTAATTATGCCGGATCATGTTCACTGGTTAATGCAACCTTTACCTA
AGTCTAATGGCGAATATTGGTCTCTTAGTAGTATTCTTCATAGCGTTAAGAGTTATAGTTCCAAA
CAAATTTCGAAAGTGATGAATCATCGGGGTATAGTATGGCAAGATGAAAGGTTCGATCGCATCAT
GAGAAATGAAAAGGAATTTTTGAATACTTGGCAATATATTCAACTAAATCCTGTTAAAGCAAAT
TTGGCAAAGACTCCAGAAGAATATCCTTTTTTTTGGCAGGAAACTCATACAGGCTAGAAGCCCGT
TTCACGATAAATTTAGTTAGTTGATAATTTAATTTTTATTTTGTTATTGTTTCTATGGTATTTGG
GAGTTTCTGGGAAGTAATGCCCCATTTATCTTTACTGCTTTTGGCGGTTTCTTTGTCTAATTCTTC
CAATAAAATGGCTAATTCTACTTGTTTTTCCAATAAATCTGTATAAGAAACCAAATTACTTAAAC
CATCTACTAAATTCTTTAAATTGCGTCTAAATTGGGGATCTCCTGTAAATTGATCTATATCTGAT
GTAATTTTAGCGGTATTAGCAAAGGTAACTCTAGCAGAGTCTAGGGTTTGTTGTAGGGTAGCGAG
GCTTGTTGGGTTATTTAATTCATCGGCAATGGTTTTTAAATCACTACTAATTTGAGCAAGGTTTT
CACTGGTTGTGCCTAGGTTTTGAGTAATTTTTTGTACATCTTTTTCATCTATTTTTGCCACTAAG
TTATTAGTTGTTTGAGCAACCTGACTAATATCAGTAGCTGTTTGACTCAAGGATGCGATCGCATT
TTGAAAATCAGTTTTATTATCAGCAATAATTTGATTAAGATTAGAGGCCAAAAGATTAATTTGA
ATAGCGGTATTAGTATATTCAGACCCTAATTTCTCAATTTGAGTGGTAGCAGTATTAGCGGTATT
TGACAAAGTATTGGCGGTGTTTGATAAGCTATCGGCAGTCCTTCCAATACTGGTAAAAGTATTCG
AGATAGTATCCATTTCCTTTTGAACTCTGTTAGTAACTCCTGTAACTTCTTGAGTTAAAATAATA

AACTGCTCTGTAGCTTTGTTGGTATTATCAACAGCAGTATTCAGATTCTCAAAAAAAGTTTCATC
ATCAAAACTATCTGCCAATCGAGCTAAACTTTCGATCAAATCAGGACTAGCTTGAGCGGGTATTT
TCTCGTTATTACACAATATCAACTGTTTTGCTCACATTCTTTTCCTAAAGGATTAATATCTTCT
CCTACGTTGCTTAAAACCCCTTGAGGAATAATATTAACACTCACTTCTCCCAATAAACCAGAGCG
AGTAGTAGAAACAATTACATCTCTGGGGATGGGTAATTTATTATTAATTTCCGTCCACACTTCCA
CCCCATTACTTGTGGGCTGAATCGAAGCCACTCGCCCCACTGCTACTCCCCGAAAAAAGACCCTTG
CACCTTCCCTTAAACCTCCAGCATTTTCAAACATCAATTTTATTTGATAATTACTGCCCCTTAATT
GATCTTTTTTCAAGAAAAAAACTATACCCCCAAAAACCAGTAAACCGAGCAATAAAAACAAGCCC
ACAGAACCTTCTCTCATTAACCTTGAACGAATCATTAATAACCCCCAATTTATTGACTAATAACT
TTAATCGGTCCTTGTGTGTTAGCACTAAAAAACTGACGTACTAAAGGATTCTCGGTAGTATCAAT
GTCTTTGACATTACCTTGCCATTGAATTTTACCATCATATAGGAAAGCGAGGCGATCGCCTGTTC
TGCGAATCGTACTTTCTTGGTGAGAAACCATGACATAAGCACAAGATTCACAAACACTACTTTTA
AGAGTACGCACAAGATCTTCTACCACCGTTGAAGCAATAGGATCTAATCCTGCGGTAGGCTCATC
ATACAAGATCACGTCAGGTCTTTCAAAGGGTTTTTCTGGATTGTAAATAACAGCCCTCGCAAAAC
TAACTCTTTTACGCATTCCCCCCGACAATTCTGCTGGAAAGAGATTCCCCGTAGAAGGAGGTAAA
CCCACCATTTCTAAAGCTCGATCGACCAATTGACTGATATAGTCATAATCAAGGTCAGAATGTTG
ATAAAGGGAAAAACCGACATTTTCTCTTACACTCAAAGAATCAAAAAGGGCGGCTTGTTGGAAAA
CCATGCTTATATGAATAGGATAATCCCCATCATCAATTAAACTGACTCTCTTTTCCCCTTTAATA
TAAATCTCTCCCGCATCGGGTTGCATAAAACCTGCGATTAATCTTAAGATGGTTGACTTGCCCGT
GCCACTAGGGCCAATTACCACTAAAGCATCTCCCTCGTAAATTTTCAAATCGGCATCTTTGAGAA
TAACTTTATTCCCGAAAGCCTTGCTAACTCCTCTTAATTCTATTAGTGGTTCTTGTGCGGTCATTC
CATTACTCTTAAATTCTTGACAACAACTAATTAATAATAAATAAAAAATACTTTTACAGTCTTAA
TTTTATACATCTTTTGAAATTTGTATCACTTTAGATTTTAATATTTATTTGTTTAGCAAGCTCTT
AATGGTGAATAGTTTATAACTTATAATGGTTTTAGAATGGAAAAGGAATAAGTTATCATGACTA
GAAGTATAAAACTTTAACATACAAGGTATGAACCGCAGACCAAAATCTTCCTATTCCCGTTATTC
TGAACCCCCAAGCCGTAAAAGTACAAAATCATCTCCTTTGGAAAATATTAACTATACTCTTGCCG
CTATCTGTGCAGGTATCTTTATATTGGGGGTAGGAGTTGGTATTGCCTTAAGTTCTGGCCAAACT
ATAAGTACTCAAAATGTAGCATCAAGGGAGGTGATCGATCGCAGCGCTCCGAATCCCGAAATTTG
TGTACAATTTGGAGCAAGTGCCATTGTTTCCGATTTAAGGGTATTTGTCACTCTAAATCCCTTTA
ATGTGTATGTAACTCAACCTAATATGCGTCCGGGGTGTGTTTTGAAAAGAAATAACTGGTCTATT
TTAGAGCAAAGACGCTTAGTCAGTGATAATGATGTGAGACAGTGTAAAAATAGAATGAATACTT
TTGGTTTCACTGGTACATTAGAAAGCTCTCCCAAAATCGACTGTATTTATCAGAATGACAGTGCC
GGGAATCTATTTTTGAATCCAGAAGGGGCAGTTAATCCTCAAGGAGTTCAAGATTTTTAAATTGT
CAAAAAAATCCTCGATCTCAATAATGCCCCACAGAATAATAGTACAAACAGCCAGTTTAATTAAT
TCTTTCAATACTATCACTCAAGCGTTGTTGTACTTTTGCCATCTCTGTTTCGTATTTAGAGGTAG
CTTTAAAAATATCGTCATTAAATTGTTTTTGTGCTTCCATAAATGCCTTCTCAAATTTATCTTTT
GCGGTAACAATATCATTTTCTAAATTGGTTGTTAGTTTTTCCACATCTGTTTTGGATTTATCAAT
TACTGACTGTAAATTTTCAATTTGATTGATTTTTTTTTCTAGTTCATTAATTTTATAAAGTAAAA
AGGCATTACTTACTAGAAGGAGGACTAAAATTATCGAGAATATATTTAATAGTGTTCTTTCCGTT
TTGTTGGATTTCGTCAATGATTTCTCCGAATCCGTCAATGATTTTGTTGATATTTGAGAAGTCTG
TTTTTTCGTCTTTTTTTCATTTACTACAGGTTTTTTATTTTTATTTTCTAAAATAATCCATCCTTC
ACTAGAAATATTATTAGATAATCCACGCCACGGCTTTGCTTCCTCAAAAGTTTTTTGTGATTTGT
TGTCAGCAATAAACACTAATAAACCCTTTTCCTTCGGTAAGCTACAACTTTTTATCTCATCAATT

AATTCTTGTTTATACTTGTCTAAATTACCAATTTTACAAATCAACCTTGCAGAATTCAAAGCGTT
ACTTTCAAGATTATTATCACCAATACTCTCAATTTTTTGGTAATCAATTTTAATTTCTGGTGTCT
CCGTAATGGCGTCAGCAATATCTTTTTCTAATCCATCTTTAGCTGTTAAGTATTCATAAATAATC
GCCCTGATTTTAGCTTCTTCATCATTATCGGCAATCCAAATTAAAGAATTACGAATGGGACGAGT
ACGGTTATCAATTCTACCGCTTTTTAAAGCAGTTATTGCCAGAATTAATTGCCTTGATTCACGAT
ACAAAACTAGAGATTCGAATTCAGTGTCTGCTAATTTTTTTACTAATTCAGGAAGTTGAGGTAAT
TGTTTTTTATTAACTTCTAAAGCCTCAAACCAGTTATAAACCTCAGATATTCCACGAGTTTCAAC
AAAAATTTTGCTCATAATATTTACTTTAATTTATCATTTAATTAATAGTTAATAAGTTTGCTCCT
TGAAAAATGGTAAATGCTCCATTATTTCTACACCCGTTAGAAAATTCACTCATAGCATTGAGAAA
AGTTTTGTCTCGATCGAGCATCATAAAAATTAATTCGAGAAAAGACATTTTACGTTTTTGTAGGT
GAAGTTTTAATAAAAATCTGAGTAGATAACGGAGGGGTTGATCACTATTTTTTGGCTCATAAAA
AAGGTTGGGATTCGGCTTCCGAAAATAGAAAATGGGTTGATGATTTTGATCTTCTTCTACCCTAG
AAAAATCAACACTACCCAATGTTTCTACAGGTGTCATAACTACTGCAACATGAGGAACGAGAGCA
TCACTACGGAAAAAACTGAGCAAGTTACTATATTTTTCTTGTATAGTTGTTACTAATTGGTTTTC
TCCTGTGCTTTCTCGTAAATATTTTTCACATTTAACAGGAGCAAAAATTACCAATTTAGGGGAAT
CAAGATCGGCAAAACTACTTTTAAATAAATCATAAATAACTTGAGGTTGATTTAATTCCTCATGC
CATTGACTATTATTTTCTATTAAAGCAGGAGTATCGATGGCAATAATAATAGCTACAGATTCCTT
GATAAATTTTTCTACTTCCTGTAAATTTTCATTTACACAATAGTCACCAGGATAGTCTTGAAATT
GCAACTCAATGGAAGGAGTAACTCCTGTTTTTCCCAAATCGAAATTATAGGTTCTTGCTGTTGTG
GTGGATTCTAATCCTCCTCTAATTTTAATGCTATTTTGTTGGGCGGATTCTTTTAATTGCTTTAA
ACGTTGATCTAAAATAGTTTTGGTATCATCATCAGGAGTTAATTGTAAATTAGTTTTCCCAATAA
TCTGGTCAAACTGATCATACATGGCACATAAAAGACTTGTTTTTCCCACTCCCGCATGACCTAGC
ATTGTTACTTTAAGAGTCTGTTTTTTGATTAGGGGTTGCAATATTCTTGAAAGCATAATTTTTGT
TCCTTAATTAATCATGAGTTATTGAAGTAAAGAAGAATTGATACTTTCTGTTTCTTCTATTAATT
GAATCCACTGTTGACGTAAACGGGTATTATCTCCCAAAATTTGAAACGTATCAGTCCAAATATCC
GCTCTGTAAGGCAATAAAAACTTACGCCATTGTCTTTGAGCATCTTTACTTCTCAAAACTAAATC
AACAAATTCCTCCACGATGGCAAAAGCCGCTTGACTTGGTACTCTTAAAAAATTATTATCAAGAG
CGTTTTTACACTGATAAACTGTTTCTTTGTATAGTTCTTGCAGATTATCTAAAACATCTTGCTCT
TTTCCTCCTTTTGGTAAAGGACTATTATCCTGTTTTAAATTATCCAAATTTCCTCGAATATGGTA
TTGAATTAAACCTCGATAAGATAACTCAAAATTAGCCAGAACAGTAAAACCTTCATGTAAAGTTG
AAGATTCTTGAGGAATTAAATGTGCAATATCTGCTAAAAATTTACTACCACGATGAGAACTTAAC
CCCTTTAAATTACATTTATCAGCTAACAAATTTGTTATTTCTATTTTTAATTCTTCAATGGATTT
TTTTAACCCATCATCTAAGTTAAGAAAACGACGAGACAAACCAGTTCTAACCTCATCAATATAGT
AACCATAGGCACGGGCATAATCCCCTTCTTTATTTTTTCTCCGTTTTATTTCTTCTAAACTAGGAA
AGATACTTTGATCTTTTTGGCACTCTTGAAAAACAGCTTCTACTTGTTCTTTAAAATAATCATCT
TGATCATTTCTTTTTTCTCTCAAAACCTCTAACAAATCAGTTAAATCATTGGTTATGTTATCCCA
AAAATCATAAAAAAGATCATCAAATTTTGACTCAGATTCAACACTGTCAACTTTATTTAATGTCT
GTTTTGCTTTCTGTAATTCTCCTTTAATTGTTAACTGTAAATCAGCTAGTTGATCATAAAAAACT
GTGACGTATTTTTGATCTAACTGGTTAATATTGTTACTTAAATAATCAAGAGTTTTTTCTAAAAT
TTTCTCGTTTACTTCTTCAGGATTCGCACAGTTTGTAATGATATAATCACTAACGTGAATGTGTT
TTTCTGTCATGGATTCAATTAAATCTTGGCAGTTAGCGGAATTATCACCATACTTTGAATCCTTA
TTAGTTTGATTTAACACCATAAATGACCATAATTTTACGGGCAAATCAACTAAAGAAGCATTGAC
AGTATCGTAAAGTTTAACATCCACATCTGCCCAATAATCTCCCGAAGGTTTAGGCATTTTCACAA

ACAAGACAAAATCTACGTCTTCAGCTAAAGTTTGAATCATTCTTGTTTCATCACCAATGCCAGTA
TCTCCTAACCCCGGCATATCCACTACGGCAATTTGCCCTACTTCTTTGTTAGGAAATTGACAAACT
ATTTTGACTTCTTTAACAGCTAAATAGTTGAAATAAATACGCTCTCCGTTTTCGTCATCTTGGGC
TACATATTGGCGAATTTCCTCTTGACGGATATTGCGAGGAGAAGGGGATTTTAAGAGATGACTAT
ACTCGGAAAAATGGATGTGATAACGTTTTAAGTGTTCGTATTTAGCTTGATTTTCAGAGCCTTCT
AATCTCAGTGTCGGTAAGGGATTATTGGCAAAATCTTGAATGGAAATGGGACTTTTTCCTAAATC
TAAGGTTGTATAGTAAGGATAGATAATTTCGTGAAGAAAACTGCGTTCTGTATAAAACCATACT
TCGCCGTAGGTTTCCACATGGGGATTATGATGAATAGTACTACGAACACCTGTACAATGTTGGCG
ATCACCATCGGGTATTTCTCCTGAGACTAAACCCGTGATACTTTGTAATAATCGACTTTTACCTT
GTCTAGCACGTCCTACTACCCCTATATTGAGAGTTTCTCTTTGAAAACGACTAGATAGTTTACTG
AGTTTTTCTAACAATTCCAGTATTTTTGTTTTGGAGTCACTGAAATTGATTTCTTGTAATTTACT
CAATACTTTATCGTCTTGAGTTTTTGCTTGTAACTGATTACGATAATCTTCGAGTTTGTGCCAAG
TTATCTGTAAGTGATTCAGGTTTTCTTCTACTGTCGTCAATTTTTGAGCGAGAGGTTGACGCAGA
GAGATTATTTCTTGGATTTTTTGACCTTTATTCATGAGCATTCTTGAGTCATAATAAAAAATTAT
ACACTGTTTTGTATAATTAATATTTCATTTTGTTAAATGTTCCATTCTAAAGTTGAGTTAAATAA
TTAAGTTAAATAATTAAGTAAAATAATTAATGACTTTACTAAGAGAGCAACCGAAAGCAAAAGA
ACTAAATTTCAAACAAAAATGTTTTGGATTAATGAGTTATAGTCAATTGTTTATGCTTATAATGT
ATCAAAATTAAGATTATTGGGATAAAGTTATGTGTGGAATTGTTGGTTATATAGGAACACAAGG
CGCGATCGAAATTTTAGTGAGCGGTTTAGAAAAACTAGAATACAGAGGGTATGATTCTGCGGGA
GTTGCAACAATTGTCGATGGGGAAATTCATTCTATTAGGGCAAAAGGAAAACTATATAACCTCCG
CACAAAACTAGAAAAAGAAACCAATATCTCACAAATTGGTATAGGGCATACAAGATGGGCAACTC
ATGGTAAACCAGAGGAATATAACGCCCATCCCCACCGAGATAATAAAGGTAGATTCGCAGTGGTG
CAAAATGGCATTGTCGAAAACTATCAAGAATTGAAGCAAGAATTAATTAGTAAAGGACATGAGT
TTATTTCGGATACCGATACAGAAGTTATCCCTCATCTTATTGCCGAATATTATAACCCTGAAAGT
AATGATCCTTTTATGGATGCAGTGAATAAAACTGTTACCCGTTTAGATGGAGCTTTTGCCATTGG
GGTGTTATGTGCAGATTTTCCCGATGAATTGATTGTAGCCCGTCAAAGCGCACCTTTAACCATTG
GATTTGGGCAGGGAGAATTTTTCTGTGCTTCTGACGTTACAGCCCTTGTGCATCACACCAACACG
GTTTTATCCTTAGATAATGGGGAAATTGCCCGTTTAACGCCATTAGGGGTAGAAATTTACGACTT
TACAGGCAAACGCTTACGGAAAAACCCTCGTACCCTCGATTGGAGTCCTGTAACTGTTGAAAAAC
AAGGTTATCGCCACTTTATGCTCAAAGAAATTCATGAGCAACCTTCAGTAGTACGTACCTGTTTA
GAAGCCTACATTAATCCCGACTGGCATTCCCAAAACAGTGACGAGGAAAACCCCATTACCCTCAA
TCTCAAGCCTGAAATTTATGAAGATTTAGAACATATCCAGATTGTAGCTTGTGGCACTTCTTGGC
ACGCAAGTTTAGTGGGTAAATATTTATTAGAACAAATCGCACAAATTCCTACTTTTGTCCAATAT
GCTTCAGAATATCGTTATGCCCCTTCTCCAATGATGCGTAATACAATTACAATCGGTGTTACCCA
GTCAGGAGAAACGGCTGATACCATTGCGGCTCTTGAGATGGAGCGTCAAAGACGTTCTAGTTGCG
ATCGCACCTATCAAGCTCGTATTTTAGGTATTACAAATCGTCCTGAAAGTACGTTAGGGGGCATG
GTAGATCAAGTAATTAACACTTATGCAGGTATCGAGATTGGAGTAGCGGCAACTAAAACTTTTGT
GGCTCAAGTGATTGCTTTTTATATCCTTGCTTTAGATTTAGCGGGGCGAAGAAAAACCATTACTC
CTTCACGCATGGAGGAGATATTAAAAGGATTACTGCAATTACCCACTCAAATCGAGCAGATTATC
CACTCAGAAGAGCCAAAAATCAGAGATTTAGCCCATCAGTTCCCTGATACCAAAGATTTTATCTT
CCTCGGTAGAGGTATCAATTTCCCCATTGCCTTAGAAGGTGCGTTAAAACTCAAAGAAATTAGCT
ATATTCACGCTGAAGGCTATCCTGCTGGTGAAATGAAACATGGTCCAATCGCACTTTTAGATGCT
CATGTACCTGTAGTTGCGATCGCAATGGGGGAAGTGTTTATGAGAAGGTGATTTCCAACGCCCA

AGAAGCGAAGGCAAGGGATGCTCGTTTAATCGGAGTTTTACCCCAAAACCATCAAGAAGATATGT
TTCAAGATACTTTAATTGTGCCTGAAGTGGACGAAATCTTGTCTCCTATCTTGGCGGTGATACCC
TTGCAATTGTTATCTTATTACATTGCATCCATGCGCGGTTTAGACGTGGATCAACCTCGCAACCT
CGCAAAATCAGTCACTGTAGAATAATTTTAGGGGCGGTGCGATCGCCCCTGTGATAAGGGAATAA
AGTTGTATAATTTTAAGTAAAGTCGTAGATTTAAAAATAAAGCTGTACATTTTAATTATATATT
ATAACAATTATATATTATAACAAGTTTAAATAAATCACAAGGTGAAGAAAATTACAGAAGTCAC
TAGAAGATAAATTGCTAATTTATTTTACAAAGAATTCAATTATTGGGGGGATTATGGAGAAGAT
GATGAGGAAATTAAATTTTTAGAAAGACTGTATGATTTAGAAAAATTACCTTCAACTGATGGTA
GATTCAAAGATGCTAAAGGTGATATTTGGCAACATCGAATCAATAACTGGGATTGGCAAAATAA
TAATTGGATTTTTAAAGATGAAGAAGATCGATTTAATCTAAATGATGGTAATGATGACGAGTAT
CTATTAAAATTTCTGTGTGAAACATTAGATCCTGCGGTTAGAGAACCTGAAAACGTGCAAGAATT
ACTTAAGAAATATAATAATCTTTTGTCAGTGGATGGTTATAAAATTATAGAAATAAATAAATTA
TCAAATCGTCCAGTTTACGGATTTTGTGAAATAAATAATCAAATATATTTTTCGAGTAATATATT
AATGAAACCAATTCAAAAAATTCTTTTTGGCAGTCCGGGTACAGGTAAAAGTTATCAAATTCGTG
AAATTGCTCAACAACAATTAAATATTCAGATTGATGAATTTACCAAAACGTCTCTCAATATCGTA
AAAGCTGTTTTTCATCCTGAATATAGTTATGCTGATTTTATGGGTAAATTACTACCTTTTACTCA
GGGAAATTCAGTAATTTATAAATTTTATCCCGGACATTTTTTGAAGATTTTAGGAAAGGCTTATA
AATCTCTAATTGAGGGTAAAAATGAGAATTACTTATTAGTAATTGATGAGTTAAATAGAGGAAA
TAGTGCAGCTATTTTTGGTAATATTTTTCAATTACTAGATCGTGAAAGTGATGGATGGTCAAGTT
ATGAAATCGATATTTCTGAGATGGAGTTAGTCGGTTTATTTCAAGCTATGAATATAAAAGTTGA
GATTTCTACACATAATGAAATTAACGTACAATTTTCAGATATTGGAAAAGATAGTTACGATAAA
GTATTAACTAAAGAATCGGAAAATTTTAGGGATCAAAATAATTATGAAGGTTTTTTAATTTGTA
ATCTTTTACGACAATGTCAAATAAAATTACCTCCTAATTTATCAATTATTGGTACAATAAACACC
TCTGATGAATCAATTTATTATCTGGATACTGCTTTTAAAAGACGTTGGGACTGGGGTTCAGTAAA
TGCACCTGTAGGAGAATTTTCTAACAACGATATTCCCCAAGAATTATTAGATGTGAAATTAGTTA
TTAATGATCAAGAACAAGAATATTGGTACTATTATATAGTAGGCATTAATGAGTTTATTAAATC
TAATTATCAAACTATTAGACGTATTGAAGATAAGATGATTGGATGGTGGTTTATTAAGCCAGAA
AATGATAAAATCGAGCTTCAAAAAGTTAAAGATAAGTTAATGTTTTATTTATGGGATAGTGTAT
TTGCTAGAGATAAAAAACCTTTAGAGATTTTCCTGAGTGAAGATGATAATACCCTTGTAAAACTA
GCACTTTATTCAGATTTTTTAGCCTTCACAGAAAGATTTTTAGAGAAAGTTTATCAGTGGGGAGA
AAAACAGTATCAAGAAAAGCAACTTTTTTAAAAATACTGATAACAATTCAATTATTATTGCTAA
GTAAAAATATGTTTTTATACAATGATTAATTTTAACTCTTTACAATTAGTTATTAATAATAAGT
ATTCTTTTGTCGGGATTGATAAACAAGAAGATAGTTCACGTTTTTGTTTACCTAAAGGTTTTAAT
TCACAAAAATCTCAATTAGATAATTTTAATAGCAAAAGAGATTTATTTTTTCTTTTCTATAAAAT
TTTGACAACTTTTAAAAGTATTTGCTTAGAGAGGGGTTATTTAGATAATAATAGTAAGCAGTTA
ACAAACGATCGAGATGGAGTTGTAAAAACAAAAATTGGTTCTAATATCGAAGATGATGGAGAAG
ATAACGAAAATATTTTTTATTCTAAAATTGATATTCTTGGTAAATTATTAGATGCGTATAATGA
GCCAAAAATTTTAAGTTTAGCCTCTCGATTAGGTAAAACAGATAAATTTGATTTAAGCAAAATTC
ATAAATATTTACATCAAGGAGTTTATTTACCTAATAATGCTATTTATGTAGATCAAATGCTTCTC
CCCAAAAAAGTAGTTCAATTTAAAACTACTGATATAGTTGCAATGTATTGTTACTTGTTTTGTGA
GATAAAACAGCAATTAAAAGAAACAATAAATCCAAAAATTATATCTTTAGGTGAAGATTTTAGA
GAACGTTATTTAAACTCTCAAGATAGTCTTTTTGATGAGAATACTTACGAATCTGTTTTAAATTT
ACTCAAAGATGCTTTAGAAACGATCGACAATTATACAGCGATAAAAGATGCTGATTATTGGGAA

TATTATGATGCGATCGAACTATTTTTATATGGTGACTTAAATCAAGCTGAAGAAGGAGAAATTT
GGGGTATAAAGAATTTTCATACGGTTTGGGAATCAATGTGTTTGACTTACTTAGCAGAAAATAA
TGATTTATCTTGTTTATTACATTGTGATACTCGGTATTTAAGTTCAAGAATTATAGATAAACTGA
AAGCATCAGAAAAAGTTATTGACATAGACAATGTTTTTAAAATAAATGGTGTTTGTTTAAATCC
TGATGCTATTTTATTTAATTTAATCAAGAGTAATTTCAGACAAAAAACAACTTATGAAATATTC
ATAAGTCGTCATGATAACTATGGTTATAAGACAAATTTTAGATATGATAATGGTTTCAAAACTT
ATCAAAAAGGTAAATCAATATTTATTAACATTGCTTACCCTGCTCAAAAACAAAGCATTTATACT
GCTCAAGAATTAGGAAAAATATATCAAAATCGAAACAATCAATATTTAATTATCAATTCTCCTTT
GCCTAAAAATTTTTATTCGTTTTGGGCAATTCCAGAAATAGTAAATAATGAACATCTTAATTTAA
TGAGTTATTTTAATCATTTTTTTTATTTAGCTCTAAAAAATAAAATATTTACTTGGAATAGTTTT
TATGAAAAATTTTTAAAACCATTAAATATTAATTTTGGTGATGATGATGATTTTAATGAGTATA
ATGTATTTACTAGGTCACTTTTCAGAGATAAATCAGAAAAATATATAAGCAGTCAATTTAATCA
ATTTATGACTCAAATATTTAATAGTCAAAATACTTCGGTAAAAATTATTGACATAAAATATCAT
GATTCTGATTACTTTTTCAATACTAATCATAAGCAAGAAAATAAAGAAAAAAATGTGAGAAAGC
AATTTGTTTATGAGTATTTATTACAAAAGAGATTGAAACAACATTTTGATAGTAACTATTTGCCG
AATATTGAAAGTAGTTTTTGGATTCCTGATTATCGTTCTGATGATTCAAATTTATTTAAAAAAGA
TCAAGTTTTTCTTGATGATTACATTGAGTTAAATAAAGTCAACTTCTTATTACTTGCTGAAAATT
ATAGTGCTTATTAAATAAGAATTTTAGAAGTATGGATCAATAGGAATAGAGAAGATACTATAAT
CAGGAATAATGGTAGAAAAACTTTTTTCCAGAGACAAAGTACACTAGATTTTCATGAAAGGTAA
AATACAACCTGCCCCGGGTGCAAGAGTAGTTATTAGAGATGCAGAATGGTTAATCCAAAGGGTAG
ATCGCACCGTCACTGGTAAGATGGTATTGCAGGTAGTGGGTGTCAGTGACTTTATCAAGGGCAAA
TCGGGCAAATTTATTGATGAGTTGGAGAAAGACTTAGAAGTATTAGATGCCAAAACCACGAAAT
TGGTTACGGATGACTCATCAGGATATAGTAAATCTTTACTGTTTATTGAATCTAACTTGAGAAAT
ACTGCCCCTACCACCCCTCATTTATATGTCGGCAATAAAGGGGCTATGGATATTTTACCCTTTCAA
CTCGATCCAGCCCTAAAAGCCCTGAAAATGCCCCGTCAAAGGCTTCTCATCGCCGATAGCGTAGGT
TTAGGCAAAACCCTCGAATCAGGAATATTAGTAACGGAATTAATGCGCAGGGGTAAAGGAAAGC
GAATTTTGGTTGTAACTACCAAAAGTATGTTAACTCAGTTTCAAAAGGAATATTGGTTTAGGTTT
ACTATTCCTCTGGTGCGTCTCGATTCTGTGGGGATTCAAAGATTGCGTAATCGCATCCCAGCTAA
TCATAATCCTTTCCATTACTATGATAAAGCGATCGTATCTGTGGATACCTTGAAACAAGATCGGG
AATATCGTACTTATTTAGAACAGGCATACTGGGATATTATCGTGATAGATGAATGTCATAATGTG
GCAAAAAGAGGGCAGGGTAAATCAGCATCGTTACGATCGAAATTAGCAGAAAGATTGTCTAGTC
GCTCTGATACTCTCATTATGTTGTCTGCTACCCCCCACGATGGTAAAGCCGAAAGTTTCGCTAGTT
TGATGAAAATGTTAGATCCAACGGCGATCGCCTCTGAGTCGGATTACACTAAAGATGATATTCGA
GATTTATATGTGCGCCGATTTAAAAAAGACGTTGCCCATCAATTAGCAAAGCATTTTCCAGAGCG
TCAAGTGTTGCCTGTAGAAGCCATAGCTACCCCTCAAGAAGAAAAGGTTTTTTCTCTTCTAAATG
AGCTTAAACTAGCCTCCATAGACGCTAAAGCTACCGCAGGGAAGTTATTTAAAACTACCCTCCTC
AAAGCTATGCTATCAAGTCCCATGGCTTGTTTAGAAACCGTCAACAATCGCCTGAAAAAGTTAGA
AAAAAGCGAAGAAAAGGAATTTAGAGCGGATTTTCAGGAATTACAAGCCCTAAAATCTGCTTTA
GAAGTGATTACTCCTGATAACTTCGCTAAATATCAACAGTTACTAAAATTGATTACATCAAAGGA
AAAAGGCGGTTTTGCTTGGAATGGCAAGGATTCAAGAGATAGATTAGTGATTTTTACCGAGAGA
TTAGAAACCATGCGTTTCTTACAGTCAAATTTACAACGAGATTTAAAACTCAAAGAAGAAGCCAT
TGCCACTTTAGATGGAAGCATGGCGGATGTGGAATTAAATCAAGTTATTGAGTCATTTGGGGATG
AAAAATCGAAATTAAGGTTAATTATCGCTACTGATGTGGCTTCGGAGGGGATTAACTTACATTTC

CTCTGTCATCGTTTGATTCACTTCGATATTCCTTGGTCATTGATGGCATTGCAACAACGTAATGG
TAGAATCGATCGCTATGGGCAGAATGATCAACCCTTAATTCGTTATCTGTTGACTCGTAGCGAAA
ATAAACGTATGGATGAGGCAGAAAGGATTATTAGGGTATTATTGACTAAAGATGAACAAGCAAT
CAAAAATATTGGTGATCCTTCCGTTTTTATGGGAGTATTTGACATAGATGAAGAAGTGAGAATT
ACCGCCAGTGCGATCGAATCTGGGGAATCGGCGGAAGATTTTGATGCCATGTTAACCCCTGATAC
TAGGGATGATGAGTTTGATTTCTTTGCTTTATTGGATGCGGAAACAGAAGTAAGTCAAGAGGAA
GAAGTAGAAAGCCAAATACAACGATTACCGCAACTATTCGACGATGATTTTCATTACACCCTCAA
ATCCCTAGAAGTGGTGAATCAGAAAACATCATTGCAATATCAAGTAATAGAAGAACAATCATTA
ATAGAGTTAACCTTCCCTCATGACTTGAAACGCCGTTATGAGAGACTACCAAAAGAGATTCAACC
TGCACCCTCCGCACCATTAAGACTTTGTGCCGATAAAGAATTAGTCAAAAAAGACTTAGAAGAAT
CTCGCAAAGCGGAGGAAAGATGGACTCCTGTACAATATTTATGGGAATTACATCCTGCCGTACAA
TGGTTAAATGATCTGAATTTAACTGCCTTTGGCAGACATCAAGCACCTGTTTTGACAATACCTTC
TCTGAGGGATAATGAGGGGGTATTTGTGATGACAGGGTTAATCCCGAATCGGCGAGGGCAACCTT
TACTCAATGAGTGGTTTTCGGTAGTATTTATTAATGGTAAGTTTAGCCATATTGAAACCTTTGCC
GACACGGTAAAACGTACTGGTTTAGGGAAAAATCCCATTCCCAATCAAATGTTACCCATACCCGA
AATTTTACTAGATTTAAGGGAAAAGGCGGTGGATATTGCCCGTAGGGAAATGTTGAAGAAACGC
CAAGAGTTTGAAAGTAAAATCAACAAAGAATTACAAGAACAACTCGATCGCCTCGAAGCCTTGAA
AAGTGAGCATAATCAACAATTAGAGCTAAAATTTAACGATGGTACTCCCCTAGTAGAAGAAAGA
AAACAACGGGAAAAAAATCAAATAGAGACTATATTTGATCAATATTTTCATTGGGTTGAAGAAA
GTATGACAACTGAACCTGAACCTTATATTAAAATTATTGCCGTTTTTAAGCAATAGACATCTAAT
AACAATACAGGCAAGATGCCTGTTTTACCGTAGTTAGTAGGGTGGGCATTGCCCACCATTTCCCC
ATAAACAAAATAAAACTTAACCCGTAGGTTGGGTTGAGGAAACGAAACTCAACATCCCCTAATTT
TGCAGTTTAAGATTAAAATTAAAAATAAAGCAAATCTCTAAATGGAAATTTATCGCTATGATAA
AAACCATCGATGTTAGTCATTTAAACTCAAGACAAATAGAAGAAATTAGTAACTTAATTAAACT
ATTTGAAAATGAGAATCAATTAGAAAATAGTCAATCAAAACATAAAAACTATTTATCAGACAGC
GAACATAAATCAATCATAAACAATGATAACCTTGCTGAAAGAATAAAACAAAGATTCAAAGATA
TAGATAATGTAGAAATACCAATTATTGAACGTGATCAAATACGTCCTTTACCTGATTTTTCTTGA
TGATTATTTTAGACACCAATATTATTTCTGAATTAATGAAGGGTGATCGCACTGATAAAAATGTA
TATAATTGGATTGCAAATCAAGAGATAAAATCATTATACACAACCACTATCAGCCAAGCAGAAAT
TCTCTTAGGTATTGCTATTTTGCCAGAAGGAAAAAGACGAATAGAACTAAATAAATTAGCTCATT
CAATGTTTACTGAAGACTTTAATAATCGTATTCTTTCCTTTGATGAAAAATCAGCTATAGCTTTT
GCACAAATAGTTAGTAATAGACGTAAAAAAGGTCAGCCAATTTCCCAGGCAGACGCTCAAATTGC
CGCAATTACTTATACTAATAATGGAATTCTTGCCACTCGTAATATTAAGGATTTTATTAATTGTA
ATATCGAAATTGTTAACCCCTTTTATAATTAATTACCCCGTAGGCTGGGTTGAGGAAACGAAACC
CAACATCCCCTAATTTTGCAGTTTAAGATTAAAATTAAAAATAAAACAAATTTCTAAATAGGAA
TTTATAGCCATGATCAAAACCATCGATGTTAGTCATTTAAACTCAAAACAAATAGAAGAAATTAG
CAACTTAATTAAACTATTTGAAAATGAGAATAAATTATATAAAAAAGATAGCAAAGAATCAAAA
ATTTTACTAAAAAGTAAAACTTTTAGAGATTTATTAGAAAATCAACAATCTACACATCAAAATT
ATGATGTTGAAGAAGCCAAATGGAATTATTTAAAAGAAAAATATGATTTATGAAAGTATTAATT
GACACTAATATAATTGGCTCTCTCACTTTGAATATGATAAATTATGTGTTGAGATAGGGCAAAGG
GCAAAGGGCAAAGGGCAAAGGGCAAAAGAGATTCTAAAACTTGAATTTTATAAACTTTTTATTT
TTATTTAACTGAAACCTTTGTTATCTAATAGTTTTAGTCACTATTTACTATTAATTTGTCATAAC
TACTGTAGAAGAGCCATAATTTTTATAAAGATTTTGAAGATGATATTCAATATTATTCAGCAAT

AACTAATAATCTTGATGCTATAGTTACTCGTAATGTACAAGATTTTCCCCAAAATAATTTAACCA
TTTTACAACCATCTGAACTGATTAATATTTTTAAAGATTAGTCCTTTTTATTTTTTATTTCAAGG
TTTCCTTTCTAATTCTATATCCTATGGGACAATTAACAGGTATTACCAACTACAACGAATTTTAC
TCTAACCACTATCTCGATGAAATTCTCTCCAATGACTTAAAAGACATCGTGAAAAAGTGGCAGGA
AGAAGCTAACGCCAATGATACCAAAACGCCCCCAGATTTAATTAAATCCCTGGCTAAATCCTACT
TTAAAATCCTCAATCAATGGGATGATGATAACGATAAAGTCGAATTATTAGCCACCCAACAGGAA
TGGTTACAGGAATTTTTAGAGATATTGGGTTATGAATTTAAACCCCAAGTTAAACCCCTCGATAA
CGAGCAAATTTTACCGATTATTGCCGAAGTCAAAAAAGAGAATGGTATTCCCTTACTGTGGATTG
TCGAAACTTTAACCGATCGCACCGACAATGACGATATATTAAACCTAACTTTACATCCGAGTCAA
TATCCTAGCCTTTCCTTTGACCATGATCCATTAACAGGGATACCCCTTGAAAATGTTGTCTCAGA
TGGTATTTTTGGGCTTGATAACCCCCCTCGTTGGATAATTTTGATTAATTTAACCCAAATCGTGC
TTATTGACCGTTTTAAATGGAATAGCTCCCGTTTTCTTCGTTTTGACTTGACGGAAATTTTGGGC
AGAAAAGATAATGATACCATCGAAGCCACTGCCATTCTTTTACATAGAGAGCATACTTGTCCCTC
GGAGGGTAATACTTTATTAGATCAACTTGACGAAAATTCCCATCGTCATGCCTTTGCGGTATCTG
ATGATTTAAAATATGCCCTCAGAGAATCCATCGAACTTTTAGGCAACGAAACCATCTATTATTTA
AAAGATATAGCCAGAGAGGGAGTATATAACGATAAAACAGACGAAAATCAGCTAACTATTGAAT
GTTTGCGTTATATGTATCGACTGCTGTTTATCTTCTACATCGAAGCGAGACAGGAGTTAGGTTAC
GCACCCATGAAAGACGAAACCTATCGGGATGGTTATAGTTTAGAGTTTCTCAGGGATTTAGAACA
AGCAGACTTAAACACGGAAGAAGACAGCGAAAGTTATTTTATCTCCCTTTCCCTCGACAAGTTAT
TTACCCTTATTTGGGAAGGTTATCCCCACACTAAAAATCAGCAATTAGCCCTTTTAGAAAAACAG
ATTTTCAGCGATACCTTTCAATTAGCCCCCCTCAAAAGTCATTTGTTTGATCCCCAGCGCACCCCA
ACCCTCAATAAAGTCAAATTCCGTAACCGAGTATTAAGAAGAGTTATTGAGTTAATGTCTCTGTC
ACGGGAAGGCAGTGGCAAAAGACGGGGTAGAATTTCCTATGCTCAATTAGGCATTAACCAACTAG
GCTCAGTATATGAAGCCTTACTCTGTTTTCGGGGATTCTTTGCCCAAGAAGACTTATACGAAGTC
AAAAGAGAAGATGATGATAACCCCAATCCCCTCGAAGTTGCCTATTTCGTCACCTTAGCAGATTT
AGATAAATATACCGAAAAAGAACGAGTTTTCAACCAAGACGGCACACCGAAAATGTACCCCAAAG
GTAGTTTTATTTACCGTTTAGCAGGGAGAGACAGACAAAATTCCGCCTCTTATTACACTCCCGAA
TCTTTAACCCAATGTTTAGTAAAATATGCCCTCAAGGAGTTACTTAAAGACAAAACCGCCGATGA
TATTTTAACCCTGAAAATTTGTGAACCTGCCATGGGTAGCGCCGCCTTCCTCAATGAAGCAGTTA
ATCAATTATCGGAAGCCTATTTGCAACTCAAACAAGAAGAATTAAACATCAGAATACCCCACGAC
AGGTATCAACTGGAAAAGCAAAAAGTCAAAATGTATCTGTGCGATCGCAATGTGGTGGGTATTG
ACTTAAATCCTGTGGCGGTGGAGTTAGCGGAAGTTTCTTTATGGTTAAATAGCATCTATAGCCCA
GAAAATGGCGAAGCCTTTATTCCTTGGTTTGGGCATCAACTATTTTGCGGTAATTCCCTCATTGG
TGCAAGAAGACAGGTATATCATAAATCCTTAATTCCCACTTCTAGTAAACACAAAAAGGGTAATT
TCTGGTATGAACATCAACCACAAAAAGTCCCCCTTTCTAAGGGGGATTTAGGGGGATCTAGCGTT
GGGATTGATCCCCCCCAGCCCCCCTTGATAAGGGGGGAGAAAGAAGAGGAAAATACCCCCCTTGC
GAAGGGGGGATTAAGGGGGGATACTATCTTCCATTTTCTCTTACCCGATCCGGGTATGGCAGACT
ATAGCGATAAAGTCATCAAAACCCTAGCACCCGATCGCATCTCTCATATTAAACAATGGCAAAAA
GACTTTATTAAAACGCCCTTTGAATCGGAAGACGTAGAAAAATTGATCGAACTCTCAAAGAGAAT
TAATGAACTGTGGTTACACCATGCCCAACAACTGCAACGCATGAGACAACAAACCACAGATCCCC
TTACCATTTGGGGACAAGAAGAAGAAAATGCCCCCCTTGTAGGGATTGATCCCCCCCAGCCCCCCT
TCGTAAGGGGGGAGAAAGAACAGGAAAAAGTCCCCCTTATTAAGGGGGATTTAGGGGGATCTACC
ACCCTAGAGATGAAAGACAAAATCTATCAGCAAGAAAAACTCTCCGAAGGGTTAGGTAACTCTAG

CGCCTATCGCCGTCTCAAGTTCATTATGGATTATTGGTGCGCCCTGTGGTTTTGGAAAATCGATC
GCGCCGACTTATTACCGACACGCAGTGAATTTTACCTCGAAATCAGTATCATTCTAGGGGAATTA
GAAATGATGTTTGAAGAAACCAATCAATTACCCCTTTTCCCCGAAACCGACACCGATAACGAGAT
GTCATCATGGGTGCAAGAATATGGCTTAGTTAACCTTGATAAACTCATCGAGAAATATCCCCGAC
TGGCATTAGTGCAGGAAATAGCCGAAGAAAAACGCTTCTTTCATTGGGAGTTAGAATTAGCCGAT
ATTTTCATGGAAAAAGGCGGATTTGACCTGTTTTTAGGGAATCCTCCGTGGATTAAGGTGGAATG
GAGCGAAGGGGATGTATTAGGGGATGCTGAACCATTATTTCAGTTACGCAAATTTTCCGCTAGTA
AACAAAGTAAATTAAGGGAAGAAGTTTTGGTTAATTATCCTCAGTTAAAAAACGATTATTTTCA
TGTTTATGAAGGGTTTGCAGGGAGTCAAAATTTCCTCAATGCCTATCAAAATTATCCCCTCTTAA
AAGGCATTCAAACTAATTTATATAAATGTTTTATTCCTCAATCTTGGGCTTTTTCTAACCCCACA
GGAGTTCAAGGTTTTATTCATCCTGAAGGCACTTACGATGATCCTAAAGGCGGTATTTTTCGAGA
GGCAATTTATCCCCGTTTAAAATCTCATTTTCAGTTTCAAAATGAATTTCAATTATTTGTAGGTA
CAAATGATCATGGGCGTTTAAGATTTGGTAGTCATATTTACGGAGAAAAAAAAGAAAATAATAG
TTTTTATAATATCAATAATTTATTCACTCCCAAAACCGTTGATGAATGTTTTGAGCATCATGGCA
AGGGTAAGGTAGGAGGCATCAAAAACGATGAAAATAAATGGAATGTGGTGGGACATCGCCAAAG
AATTGTCACCGTTAATGAAGAAGTTTTAGCACTTTTTGCCACCCTTTACGATGAAGAAAATACCC
CTGCTTTACAGGCAAGATTACCCGCCATCCATTCCTCACAACTGTTAACGGTATTGGCTAAATTT
GCCCAACAAAAACAACGGTTAGCAGATTTACAAGGGCAATATTATTCTACAGTATGTTTTGATGA
AACTTACGCTCAAAGAGACGGCACTATCAAGAGAAATACTTGTTTTCCTGATACTGCTAAACAAT
GGATTTTATCAGGTCCTCATTTTTTTGTGGGTAATCCTTTTAACAAAACACCGAGAGCAATTTGT
ACCGCCAATGGGCATTATGATGTCATTGATTTAACCACCATCCCCGATGATTATTTACCCCGTACT
AACTATCTTCCTGCCTGTGATGAAGCAGAATATCTTAACCGTATTCCTTCCGTTTCTTGGCTGGA
GGAGGGGGAAGGTAAGGCGAAAAAAGTTACGGAATATTATCGTTTAGTTTTTCGTGGAATGGTT
GGCTCTGCTTCAGAAAGAACTTTAAGCGGTGCAATTATACCTAAAAATTCTGCTCATATTAATGG
AGTGAGAAGTTATGTTTTTTCTGATCAATTACAGTCAAAATATTTAGTTTTAAGTGCTTTAACTT
TTTCTATTCCTTATGACTTTGTATTAAAAACAACAGGAAGAACAAATTTACATCAAACCTTAGAT
GATTTTCCGATTATTTACGGCACAAAATACGACAAAGCGTTAATAATTCGTTGTTTAACCCTAAC
TTGTTTAACCAACCATTACGCCGAGTTATGGGAAAGTAGCTACACCCCAGAATTTAACGAAGATA
GTTGGGCAACCAAAAAAGAAGTCCCCCTTGCTAAGGGGGATTTAGGGGGATCTAGCGTAGGGATT
GATCCCCCCCTGCCCCCTTTAATAAGGGGGGAGAAAGAAGAAAAAGTGCTAAACCCTGATTTTTT
CCGCAACCTAACGCCCCATTGGCAAAGAAACAACGCCCTCCGCACGGATTATGAACGCCGACAGGC
ATTAGTAGAAATTGACGTATTATCAGCGATGGCATTAGGATTAACCCTCGATGAATTAATCACCA
TTTACCGTGTGCAATTCCCCGTGATGCGTCAATATGAACAAGATACATGGTATGACCAAAACGGG
CGTATTGTCTTTACCGCCAGTAAAGGGCTGGTAGGGGTTGGTTTCCTCGGAAGGGGAAAAATGG
GGAAATTGGCTGGGAAGACATCAAAGAGATGAAATCAGGCACGGTAGAACGAACAATTATAGAC
GATACCACCCCGTCAGGCAGGGTTGAAAGGGTAATTAGTTATCAAGCGCCCTTTTTCAAAGCGGA
TAGAGAAGAAGATTATGCGATCGTCTGGGCAGAATTTGAGAGGAGGTTTAATTAACCCTCACCCC
CCCAACCCCCCTCTCCCAAAGGCGAGGGGGGAGAAAACCCCACCAAAACGCCTCTTATTCCCCTCT
CCCAGTGGGAGAGGGGCTAGGGGTGAGGGCGAGAATTTATGGTTATGGTGGAGGGCTCGATTCGG
GGCAAAGAAAAAATTGCGATCGAAATTAAAAATTTTCGCAGTAAATCACCTATTGTCGATTTAG
AAGGGGCGATCGGTCAATATGTTTTATATCAATTACTTTTACAACAAATTGAACCAGAAAGAGTG
ATTTATTTAGCTATTACTCAGGAAATGAAAAATGAAATATTTAGCGAACCATTATATGATGGTA
TAGAAAGAGGAATTACAGAAGAATTAATTAATTTAGGTATTGTTGAGGAAGATATTGTTTTAGC

TTTTCAAGACTTAGAAAAAGAATTAATATTATCCTAGTTTTTTGGGGAGGTGCGATCGTCCTTTA
ATTGTAGGGGTTGAATATCATTCAACCCACCCACACCCCACATTCAACCCAACACAAATTTTAGG
GAAGGTTCGATCGACAGCAGATATGCTATCATTTGGTTATGACAGTTAAAATAGTAGTTAATACC
AACATTTTTATTAGTGCAATTATTAGTAAATCTGGGGCATCAAGAAAGGTTATTCGGGGATGTTT
AGAGGGAAAATATTTACCGTTAATGGGCAATAGTTTATTTGCTGAATATGAATCTTTAATAAAT
AGAGATGAAGTTAACAAAAAATCTCCTCTAACTGTAACTGAAATAGAACTTTTATTGGCTTCTTT
TATAAGTGTTTGTAACTGGATTTCGATTTATTATTTATGGCGACCTAATCTTAGGGATGAAGCCG
ATAATCATCTAATAGAATTAGCTTTGGCAGGTAATGCTCAATTTATAATTACTAATAATATTAAA
GATTTTCGAGGAGCAGAATTACTGTTTCCCCAATTACAAATTATTCAACCTGAATATTTTTTAGA
GGTATAAATTTATGGCCACTTTGACGATTCGACTACCTGATGATAAACACGAAAGATTGAAACAG
TTAGCACAAACTAGAGGAGTTAGTGTTAATAAATTAATGGAAGAATTTTCCACTAAAGCTATAG
CTGAATTTGATGCTTATAATCGTTTTCAAGTTATGGCAAGTAAAGGTGATATTCCGCAAGGGTTG
GCTTTATTAGACAAGTTAGACGCTAGTTTTTAGATGGTGCTTTTCATGTTCGATCACAATATTAC
CGTAGATTGGATTGAGGTAACGACACCCAACACAAATTTTAGGGAAGGTTCGATCGTGTAATGCT
ACTTGGTGATTGAGGTAAAATAAATTATGCTATTTGTTAAAATTACCCGTAATCAAAAATTAGG
ATTTTATGAAGGGAAAACGGAGGTGATTAATCATACCTGCTAAAGATATTTACCATGATGCGGTG
AAAAATGCTTTAATCAAAGATAGTTGGGAAATTACAAAAGATCCCCTTGTTTTAGAATGGGGGG
AAAAGATTTATTTGTGGATTTAGGAGTGGAAAAACTAATTTCTGCGGAAAAAGGAAATAAAAAA
ATAGCTGTAGAAATAAAAAGTTTTCTCGGTACTTCTCAAGTTAATGACTTAGAAAAAGCAGTTGG
ATAATATATTGTATATCGCAATATTTTGGAAGAAGTAGAACAAGACAGAATATTATATTTAGCT
ATTACCAAAAAAGCATTTAAAGATGTTTTCAATGAGGCTTTTGGTAAATTAATTCTCAGCAAAAA
TAACATTTTATTGATCGTGATTGATCCACAAAAACAGGAGATTATTCAATGGATACCTTAAATCA
ATATCGGCAAATTATTGAAGAAGTTTTAACCGATTACTCTCAATTACCCTATTCCTATGGCGACT
TAGAAAGAGGATTTATCATTGGGTGCGATCGCAATAGTTATTTATTATTAACAGTAGGATGGCA
ACAAGAACAAAGAGTACATGGTTGTTTAATTCATATAGAAATCAAAAATTAATTCTCGCATTTTT
TTTGGTTCGATCGCAAAATTTTTTAATCGTAGGAGTTGAATATAATTCAACCCACTCACACCAAA
AATAAAGGGTTAAAACCCTTACTACGAACCTATTAAAAAAATTTTTCTCATCCCTTGCATAATTT
GCGAAAATGAAACTGATAGATAGATAGAGAGACAAAATCCTCCTGCTGGGTTTTTGCCTCAACCA
CCACCTAATATCTTTTGATTATGATTCCTTCCCTTGTAGCTAAAGAACTTCGATCGAGTATCGCT
GACTATCTCCGCACAGAATTTAGCCCCACAACTCCTAGTTTTACCAACTTAATCGAAAATTTCTT
ACAAGAAGAAGATAGTATTTTCAAAGGTTGTTATCTATCCATTGGTTTACCCTTCCGTCAAGGCA
ATGTCGGCAAAGATTTTTTCCCCGCAGTGCCGATGAAATTTACTCCCCATCTTCATCAAGAATCT
GCTTTTCATCGCCTCAAAAGCCCTAATTATCAGTCAACCCTAGTGGCAACGGGTACGGGTTCAGG
AAAAACAGAATGTTATATGATACCCATACTTGATTATTGTTATCAAAACTATAGAGAAAGAGGG
ATCAAGGCTATTTTAATTTATCCCATGAATGCTCTTGCCACAGATCAAGCTAAACGTTTAGCAGG
TTTAATTTGGCATAATCCTCACCTCAAGGGAAAAGTTACCGCAGGGTTATATGTCGGAGATACCG
AAAGAGAACCAAAAACCACCATGGGAGAAGATTTTGTTATTACCGATAAAAATTTATTAAGAAA
GTATCCCCCCGATATTCTCTTGACTAACTATAAAATGCTTGATTATTTATTGATTCGTCCAAGTA
ATCAAACTATTTGGGGGAAAAATACCCCTGAAACTTTGCGTTATTTGGTAGTGGACGAAATTCAT
ACCTTTGATGGGGCGCAGGGTACGGATTTAGCTTGTTTAATTCGCCGTCTCAAGGCAAGACTGAA
AACCCCTCATCAACATTTAACCTGTGTAGGTACTTCTGCTACTTTAGGGGGTCAAAATAGTAAGC
AGGAAATGTTACACTATGCCCAAACTATTTTTAACGAGTCTTTTAATGAGTCTGCCATTATTGAA
GAAGATCGTTTATCCTCAGCAGAGTTTTTGGCTAATGCTTTAATTGATTATCCCCTATTACCCAC

TAGTGAGGATTTATTACGACTCCATACTGCTGATTATCCTAGTTTAGCGGACTATATTCGATCGC
ACTTTCAATTATGGTTTCAGGAAGAATTAAAAGATGACATTGAGGATTTATCATGGCGAGTGAG
GTTGGGCGATCGCCTCAAGTCTTTATTTATCATGCAAAATTTGCTCAAGATATTAGACAATCATC
GGGTAATTTCGTTACGGGCATTATGGCAGGAGTTGAGCGAAAACAACAGTTTAGGGCAAAGATAC
AACGATGAAGAAGGGGAAATTATTTAAATAGTTTATTAACATTAGGTGCGATCGCCCGTAATG
AGCATGGTAGGGCGTGGGTAAATATCAGATTGCAATTTTGGTTAAGAGAATTAAGAAGAATGGT
GGCAACGGTGAGTAAGCAACCGAGATTAGTTCATTATGACGACTTAACCCGTGAAATCCAGAGAA
AAACCTTACCTGTTATCCATTGTCGCCACTGCGGAAGTACAGGATGGGGAGGATTAAGAAGGCAA
ACGGCAGATAATAAGATAAGTTGCGATGTCAAAGATTTTTATGTGGGTTATTTTTCCCAAAATCC
GTTGATTACCTTTGTTTTTCCCGATGAGAAACCTCAGTCCTCCCTCATCCCCAGCCCTTCTCCCAC
AGGAGAAGGAACAATATCCCCCCTCGCCCCTAGGGAGAGGGGGTTAGGGGGTGAGGGCATACATC
ATCGTCTTTGCAGTCAATGCCTAACTTTAAATAAAAAAGATGCGATCGCCTGTAATCGTTGTGGT
AGTGGGGAGTTAATTTCAGTTATCGAACCAAGTAACCTAATCCAAACCACCTCCAAAAAAAATGA
GACATTGAGAAAAATAAATAACGATTGCCCTTTATGTGGTAGCAAAGATGGTTTAGTGATATTA
GGTTCAAGATCAGCCAGTTTAGCAAGTGCTGCGATCGGAACTTTATTTACTTCTAGCTACAATAA
CGATCCTAAGTTAATCACTTTTTCTGATTCCGTACAAGATGCCGCCCATCGTGCAGGATTTTTTG
AAGCGCGCACCTATAGAATGACGATGCGATCAGCTATTTGTCAATATTTGGAACAGATGTCATTC
CGAGCAACACCGAAAGAATCTCAAAATCTCGAACAAATTAGACAAGAATTTGCTACTATGATGCG
ATCGCACTTTAATCATGATGCTGATTATGTAGCAACCTTTATGCCTTCAGATTTGGAATGGTTAA
AAGAATGGGAATCATTGCAACAAGGGGGAAAACTAGACCCTTACTTAGTAAAATTAATTAATCA
GCGTTTAGATTGGGAAATTATCGCCGAATTAGGCTTATATAGCAAAAAAGGCGGATCATTAGAG
AAAAGTTATAGTTGCAGTATCGGCTTAAATCAGTCATTGTTGACTGACAGTCTAAATCTCCTACA
TCAAACCTTAACTAATGAAATTGGTGGTTTAGAAAACCTTACCCTTGACAGCTTACAAAAATTAA
TTGTCGGGATGGTTTATTACCTGCGACAACAGGGAGGCATTTTACACAGTGCCACGAAAGGCTAT
ATCGAAAGTGGAGGCAATACCTTTGTTTTGCAATATCTCCCCTTTATGCCTCGCTTTGGTAGTGC
TTCCCTTAAGCCTACCTATTTATCACAAAAAAATTTACCCTTCTTTGAAACGTTACTTAAATCCA
GTGGTAATCCCAGTTGGTACGAAGTTTGGGCGTATAAATTATTGGGCAACTATTCTCCTTTAATT
ATCAGTCAATTAGAATCAATCTATGGCATCATTCTTCATCGTTTAGTAGAAACGGGAATTTTTGG
GCTAAACTTGACTAACAAAAATATCAAAGTTTGGGGTATCGAACAAAAAGCCTTACAAGTTGAT
ATTAATAGCCATAAATTCAGATGTAATCATTGTTCCCATGAGGTATTAAGCACCCCTCAAGATGT
GCAACTATGGGAAAATATGCCCTGTTTACGCCCTCAATGTCATGGGTTTTATCAAGAAAATGTAA
CCCTAACTTCTAACTTTTATCGTAATCTTTACCGTCAAGGTAATTTACGCCGTATTTTTGCTAGA
GAACATACTGGTTTACTATCAAGGGAAGTAAGAGAGGAATTAGAAAATAGATTTATTCAAGGCG
ACAGAAAAACTGATCCTAATTTGCTTTCAGCCACTTCTACCCTCGAAATGGGTATTGATATTGGG
GATTTATCCTCCGTTTTATTATGTTCTATTCCCCCTAATCAAGCTAACTATCAGCAACGGGTAGG
GCGTGCGGGAAGACGTGACGGTAATGCCCTCATCAGTGCGATCGCCAATGGCACCCCCCATGATCT
CTATTTTTGGGCAAATCCCTTAAAAATGCTTGATGGTGGTGTAAATCCCCCCGGTTCTTATTTAG
ATGCGTCTGCTATCTTACAAAGACAATTAACCGCTTATTGTTTAGATCGTTGGGTAGTAGCCGAT
AATAAATCTATACAAATCTTAGAAGAATTAGGCAAGGTTTTAGGTGCGATCGAATCTAAAAATT
TATCTCGCTTTCCTTATCCTTGGTTAGACTATATAGATCAGCATCAACAGGAGTTACTACAAGAT
TTTATCGACTTATTCAGTCATTTTGTACCCAGTAAAGGAATCAAAGATACTGATGAAGGTAGGCA
ACAGAATTTTTTGCAAAATTCAAATTATGATAATGAGCAAAATATTACCAATCAAGGGATAATT
TCTGATTCAACTATCGAACAATTAAGACTATTCATTGACAAAGGGCAAGATAACGAAGGGGGGTT

AACATGGCAAATTCTCAATCGTTTGCAAGAGGTAGTGGAAGAGAGAAAAAGATTGAAAAATCAA
ATTGATACCTTAAAACGTCGTCTAAAAAAGAAAGAAACAGAAGCGAAATCCCAACATTATGACG
AGGAAGTCGCCGAGTTAAAAAGAGAGCGAGATGGTTTAATGGAGTTAGTCAAACAGATTAATCA
AAAAAATATCCTCAACTTTCTCACCGATGAAGGATTATTGCCTAATTATGCCTTTCCTGAACCCG
GAGTAACCCTTAAATCGGTGATTTGGCGTAAAAATAACTCTCAAAACAGTAAATATGATACTAAA
CCCTTTGAATATGAACGTCCAAGTGCGATCGCTATTAGGGAATTAGTACCAAGTGGGGTGTTTTA
TGCTGAGGGAAGACAGGTAAAAATTGATCAAATCGATCTTAGTCTCTCCAAAGTGCAAGAATGGA
GACTATGCCGTAATTGTTCCTTTGCCACCGAAGCCATTAGAGACATTGCCCATCAAGCCACTTGTC
CCCGTTGTGGTGATATAATGTGGTCTGATGAGGGCAGAAAACGCAAATTACTACGATTAAGACAG
GTAATGGCTACCAGTAGCGCCAAAGATAGTGCAATTAGTGACGATCGAGATGAGAGAAATACTGC
CTTTTTTACCCGTCAAATGTTAGTAGATTTTGAACCGAATTTCTGTGAAAATAGCTACTTAATCG
ATGATGAAACCTTCCCCTTTGGCTTCGAGTTTATCAGTAATGTCACCTTTCGAGAGATCAATTTT
GGTGAACCTTCTCACCTAGCGGATAATTTTGAGGTAGCAGGAGAATCTTTTCCCCGCGCAGGTTT
TAAAATTTGTCGTCACTGTGGCAAAGTACAAAATAGTCGGGATGAGAAAAAAAATCATACCTTT
ACTTGCACCACTCATAATAAAAAGCAACGGGAAGAAGATATTGTCGATGTTGTCTATCTCTTTCG
GGAATTTTCCTCCGAGGCGATTCGTTTTTTAATGCCCATAGATACCCTCAACTCAGATCAACAAT
TACATTCTTTTATCGCCAGTTTACAATTAGGCTTAAAACTATACTTTAAGGGCAATGTAGATCAT
CTCAAAACTTTAATTAATCAAGAACCTTCCTTTAATAGTCCTCGCTTACTACGAAAACCCTTTTT
ATTCCTTTATGATACCATCCCCGGCGGTAGTGGCTATTTAAGACAATTACTGAGAAATCCCGATA
ACTTTTTTCAACTATTCCAACAGGCTTTGATGGTGTTAAAAACCTGTGAATGTGAGGATGGTTGT
TATAACTGCCTCTATGGTTATCGCAATAGTTTTGACAGAGATTATACCAGTCGCACCGTTGCCCG
AAATTTACTCAATAGTATTCTTTCCCGTCAATGTCACCTCAAACCCCATAAATCAAATCTTTCTCA
AGTTCCCCTTAATGCCTTATTTGACAGTGTTTTAGAACAAAGATTCATTGAAGCCTTAAGACATT
ATCGCCATAATGATCAACCTACCATAGTTAGGAAAGAAATCATCAACGGTAAAGCGGGTTATTTT
GTACAAATAGGCGAAAAGTCATGGAATATTGAGCCACAGGTGGAATTAAGCACTAATCAAGGGG
TAAATATCCCCAGTAGAGCGGATTTTGTTTTTTATCCCACGAAAAAGGAACAAAGTAAACCCATT
GCTATTTTTACTGATGGTTGGCAATATCACTGCGATCGCATCTCCTCTGATTTTAGTCAACGGAT
GGCAATAAATAAAACAGGGGAATATTATATTTGGTCATTAACGTGGCAAGATGTAGAATCAGAG
TTCAACGGGAACAAAAATCAAGATTACTATTATAATTTTATCGAAAATCAAAGCAATAAAACCT
TTACAGACAATAAAAACCGCCTTTACCAACAGTATCAATGTACAGAAGCCTTAGAGTTAATTAAC
AAAAATAGCTTTCTCTGGTTAATGAATTGGTTAGTCAACCCCGATGAGCAACTCTGGACAAAATT
CTCTCTAGTCAACACTTTAGCTTATATTAATCCTTTTATTGCTAAAGACACCGAGAAAAAAGCCC
AATGGTATCAAGATTTAGCCCGATATATTTCCCCTCATCTTTTCCTTGAGTTAGAAAAACAAATC
AAAATATCTATTTATGGTGATTTTTGCTATTATTCCTCCGATAATGTTTTATTAGTGGACATAAA
AAGTTCGATCGCTCCTAAACTTCATCAAGAAGGCAATCCGCAAAGTGTATTTAGTTTAATCACCT
TAAACCTGAATGAAGAAGAAACCGAAGAAGAAAAACAAAAACTCTGGAATGGCATCTTAAGACT
ATTTAACCTGTACCAATTTTTACCCTATAACTACTTAATAACCCCAGATAATCAACTAGATGCGA
TCGTTGAATCTGATATACAAAAACATTCCCCCCAAGTTACTGTTACCACATCAAAACAATGGTTA
GCCATCGAAAAATTTGCCTTAGAGGAAGTATATCCCCTCATTGCTCACATGAAAGAAAATAACTG
GCAACTACCAGAAACAGGATATGAATTAACCAACGATATAGGCACAGTGATAGCAGAAGCGGAG
TTAGCTTGGAGTGATTATCAATTTGCTATAGTCATAGATGAATCAGAAAAACAACCTTTCAGCCA
ATATAACTGGGTTGCCGTCACCCTTGAGGAAATCTTGACGAATCCCCAATCTTATTATGAACAAT
ATTTAACCAATACCACATGACCTGTAGATTGTGTTAACGACAGCGTAACCCGACAAAAATATCCA

GTAATTAAAAGGGATTAAAAAGGGCTAAAGCCCTTACTACGAAAAAACAAAACTTTTATTGTTG
GGTTTCATTTTTCAACCCAACCTACAATTAAAAAATTTTGGATAAAGAAATTAAATTACAATTAA
GTAGGATGAAATTTATCCTAGAGGAGGTTTTATGATAGCTTATCAAGAAATTTTAGAGGCGATC
GCAGTATTATCGGAAGAAGATCAAGAGGAGTTATTTGAATTAATTCGTAAGAGAAGAATTGAGA
ATCGCCGATCTCAAATTGCTACTAGTGCTCAGGCGGTGCTTCAGGCAGTAGATGAGGGAAAGGTT
GTTAAGGGCAGTTTTGCTGATATTAAGGCTTATTTATTGAAGGATGAAGAAGAATGATTGTTAT
TGCTGAAAAGACTTTCAAAAAGAGTTTTAAGCGATTGATCAAAAAAAAGTCCTCACTTACAAGCC
AAAATTTTAGCAGTTTTAGAGCTTTTAGCAGAAGAACCTTTTTCTCCTTCGTTAAAATCTCATAA
ATTAACGGGACAATTACAAGGTTTATGGTCTTGTTCTGTTACTTACAGCTATCGAATTATTTTTA
CATTTAGACGAGATCCAGAACTAGAGGAAGATGTCATTGTCTTAATTGAGATTGGTGATCATGAT
GAGGTTTATTAGATTTCTCCCGAAATTATTTTTTACTTAAGATTGAGACAAATTTAATAATTTTG
TAGGGTGGGCATTGCCCACCAGATAAAACTTTTGATTAATTATTAGTAATAGAAAACTGTATCAT
TTTTATTTAAAACGACTATATTGTTGGGGTTCATTCTTCAACCTAACCTACAAATTTTATTCATT
ATTGATTAATTGTTATGATTCCTTCTCTCAAAATTGCTTTTGGTGCTGATTTTTTACAATCTTTT
AGCAATCTACCACTGAAAACCCAAAACAAAACCCGTCGATTTATTGATAAATTTAAAGAAAATCC
AACTCTATCAGGACTAAACTACGAAAATATCACCAATGCTAGGGATTCTCAGCTAAAATCCGTTC
GAGTTGATGATAACTACAGGGCGATCGTCCGTAAACCAGAAAAAGGTAATACTTATTTACTATTA
TGGGTAGATAAACACGATCGCGCCTATGAATGGGCGCAAAATCGAGTCTGTAAAATTAACCCCGA
ATCAGGGGCAATACAAATCATTGACATTGAGCAAGTCAAGAAAATTGAGGAAGATATTAATAAA
GATAAAACAGGAATAGAAATAGGTCGATTTAAAGATATTCCCGCCAAAGATTTATTGAGATTAG
GTATCCCCGAAGAATATTTAACCGCCGTTTATGCAGTGGTGACAGATGCAGACATTGATCAACTA
TTACCCGAATTGCCAGAAGAAGCCGCCGATGCCGTGTTAATGTTAGGAGCAGGTTATACCCTCAA
TGAAGTTTATCAGGAAATACAAAAAACCCCTAATGAAGATATTGACACGGAGGATTTAGACACG
GCTTTAACCAGAGAAGATAGTAAACGGCGTTTTTATCTCATTGAGGATGATTCTGCTTTAGCGGC
GATTTTAGATGCACCCTTAGAAAAATGGCGAGTATTCTTACATCCCACTCAACGGCGTTTAGTGG
AAAGGGATTGGAATGGACCTGTAAGAGTTTTGGGGGGGGCTGGTACAGGTAAAACTGTGGTAGC
CATGCACCGTGCGAAATGGTTATTGCAAAATCGCTTCACCGATGAAAATGATCGCATCTTATTTA
CTACTTTTACCAAAAATTTAGCCATTGATATAGAGGCTAACTTGCGGAAAATCTGTTCTTCTGCC
ATGATGAGACGGATTAAAGTAATCAATATTGACGCATGGGTGGTTAATTTTTTGAAAAGTGAGG
GGGTTAATATCCGCATTGTGTACGATGAAAAATTACAAGAATTATGGCAAAAGGCATATTCCATC
GCACCTTCTTCTCCAGATTTACCCTTATCCTTTTATCAAGACGAATGGAAAGAGGTAATCCAAGC
CAATGACATCAAAACCGAAAAAGACTATCTGAGAGTATCGAGAAAGGGTAGAGGTACAAGTTTA
AAACGTGCTGAAAGGGTAGCTATTTGGGCAGTATTTGAGGAATATCGCACTTTACTTCAAATTAA
AGGTTGGAAGGAAATTGAAGACGCTCTCAGGGATGCTCGTAGCCTTTTAGAGAAAAATCCCGACA
TGATCCACCCCTTCAAATCTATCATTGTTGATGAAGCCCAAGACATGAGCGAGGGAGTATTCCGT
TTATTACGCATCCTTATCCCTCAAGAAAGAACAAATGATCTATTTATTGTGGGTGATCCTCATCA
ACGCATCTATGGCAATAAAGTGGTTTTAAGTCAATGTGGTATCGAAATTCGGGGGCGATCGAAAA
AACTACGCATTAATTACCGCACCACTGAGGAAATCCGTCAATGGGCAACCGCTATCTTAGAAGGA
TTAAATTTTGATGATCTCAACGGTAATGAAGATAATTTAAAGGAATATCGATCGCTTCTGAGAGG
TGAAAAACCAATTATTCAACACTTTACCAGTGAAACGGAAGAAATTGATTTTCTCATCGCCAAAA
TTAAAGAATTAAATAATAATGGCATACCTCTCAAAAGTATCTGCTTAACCCTCAGAACAAATACA
TTGGTTAACACTTATGAGGCTATACTCAACCAACATCAAATCAAAACCACAAAAATCAGTAAAGA
TGAAGGAGATAATCAAGCCATCGATGGTTTAAGGGTTGCCACTATGCACCGAGTGAAGGGATTAC

AGTTTGATTATATGTTAATTCCTTGTGTCAATTATGACATTATTCCTTTACAATCTGTCTTGGGT
AAATTGACGGATGAAACCGCTAAAAATAATTTTATTCAACAAGAACGCTCTTTACTTCATGTTGC
TGTCACTAGGGCTAAACGAGGTGCGATCGTATCTTCTTATGATCAATGTAGCTTTTTGATTACTA
ATAGTTTAACTTAATATCGAAAGAAGTCCCACGCCTAGTTTTCCACAAAAAAGACAGTTAAACAG
GTGATGGGGACGGGGCTTTTAAAACACAAAAACTATTCATCAGACAGCGAACATAAATCAAGCAT
AAACAATGAGAACCTTGCTGAAAGAATAAAACAAAGATTCAAGGATTTTGACACTCCCACCGACA
AATCAAAGATTATGTCGGGGGATTCTAGCTTCACAGATTCGTGGCTAGACGAATAAACTCGCCCC
CGCTGCGACCATCTCTACTAGCTTTTTCTTGGACGGGCTGCCCGACCGCCACTAAATCTAAACCAC
GATTCCTAATTACTAAAGCAGAAGCATGATCTCGCCCTGTAATGTAACCACACTCAGGACATTGG
TGTACTCGTTCTTTTAAGTCTTTCTTCCCTGTTCTCGCACCACATTCAGGACACTCTTGACTTGTG
TAACGGTGATCTACTTTGAACAAATAAGTATCTGTTCGGAAACAAACCCATTCCAAAATGTCAAT
AAACTGTCCAAAACCAAAATCGGCACTAGATTTTGACAAGAATGACCTTTGCCACATTCGGAAAT
TTATATCCTCGACAAATATCATCCCTGCATCCTTTACCAAGTGGTGGGAGAGTTTGAAATGGTAA
TCTTTACGATGGGAAGTAGTCTTAAAATGTAAACGAGCGATACGTTTTTGTAACTTTTTCCATCC
ACAAGACCCTTTCTGTTTCTTTTTCAGCATTCTTTGTAGTCTTTTCAACTCCCGTGAATACTTATT
TAAGAACCTAGGTCTAGGGATGGATTCATTGTCTGATGTCACCACCGCGCAGATAAAGCCGATGT
CCAAACCTTTAGGATGCCCATGAGGGATTGGCTGGGGAATGCCCACATCTGACTGTAATGACAGT
ATCACAAAATACCCTGTCGCTTTTCTCACTATCCGAGCTTGTTTTACCAAGAAACCATCGGGAAT
GGGGCGAGATTGTCTAAATTTAACCCAACCTAATTGGGGTAACTTAATTCGATTTCCTTTAATGC
AATCTTTAAGCATTTGAGGAAAAACGAATGACCGCAGACGACTATGGTTTTTGAAACGAGGATAC
CCTAATCCCTTACGTTCACGGTCTAAAAAAGCTCTTTCCAGAGTCCTTAAGACTTGTTGCTGTACT
TGTGCATTTACAGAAGCTAGTCTAGGGTTTTCCTTTCTAGCTTTAGTTAGTGCCTTAGCTTGAAT
ATGGTAATTGGGGTAGGGAGCAGGCGCAGGTATTATATACTCCTGTTGAATTGAACAGGCGTTGC
TCCTTGATTTTCGGGAATTTGACCAATCCATCCTCTCCCTTAACGCAAAATTCCAAACCGAACGAC
ACACCTCAAGGATATGCTCTGTTTGCTCGATCTGGATTTTTGTTAAACTAAGTTTGTATTCGTAG
GAAAGGTTTAGCATGATTTTAATCAATTACCTTACTATCAACCTTAAACTATCTACAGGATTCTG
TCAAGTGTGTAGATGTCACTTTCGTTCCTTTTTCTTTGCTGGTCGTCATTCATCTCCCGTTAATTT
CCGTGGGCTTTTCTTTTGTTCGTGAAATTTAACGGGAGTCCTGTGACGACATTTCAGGATAGATA
ATGTAGAAACACCTATTATTGAAGGTGATCAAATACGTCCTTTACCTGATTTTTCTTTATGATTA
TTTTAGACACTAATATTATTTCTGAATTAATAAATGGCGATCGCACTGATAAAAATGTATATAAG
AGGTAGGTAATTATAGTGAACTACTCACATTCGCTACGCTAAAATGTGAACTTCTTACCCAACAG
ATGGCGTTACAGTAGATTTCTTTCGTCCTTTGATAAAAATAGCTTTGACAGGGGATGAAAGTTTC
ATTTTTATTAGTTATTAATAACTATTATTTTTATAAATAAATTGATTTAATTTTTTTTCTATTTC
TCCTAAATGCGGTGATTTAAGTCTTTTATTCGGTGGTTTAGATAAATTTTGAGCGGTTTCCGTTA
ATTGTAACTCTAAAATAATTCTCTCCAGTTGTTGAGTATCAATGGGAATTTCATTATTGACGGGG
ATTGTTTCGGAAATAGGAGCTTTTTTCTCTATTATCCTCATTAATGATTTCCGCTTCTATCTTCAC
AGGTGGATTTTCTGGTGGAGGAACATTAGTAACATTCGTATTATTAATAGTGTTGTTGTCAGAGT
TAATTTTTGTATAATTAGCGGAGAATTGAGAAGGATTAGGGGAAGGAGTGGTTTCTGGGGTGGG
TTGAGAAGGGATTATTTCTCCTTCCTGATATTGATTAACTAAGGCTTGGATTGTGGGTAAATCAA
TCTTGCCATTCTTTTTCGTCTCCTTCTGGTAAAGGGTAATTAATATTTCCTGTTCCCATGTCGTTA
GGTTCATATTCATGATAGTAGCTGTTTTCTAAGGATTGAATGGTGGGATCGATTTCGGGGATAAT
ATGGGTGTCATTGGCTTGGGCGCTCATCAAAGCCTGACGCATTTCTTGAAAGTAGTCATCAATAA
TGATTTGTAGTTGGGGATCGTACGAGTTTTGCACATCTTTACTGAGGGCGTGGGTTAATGCCATC

ACGCAGGAGGCTATACTGGTAATAGCTGATAAGATAAAGATGAAGTAAGAAAGACCTAATTCTG
TCCATTTTCCGCTAGAGATTTTATTAAATACTTCGGTAGTGGCTAACCCCACAAGGATTGTTCCC
GACTTGATCTCTCGTTGATTTGTAAAATGTTCGTTAAAGACTTGGGGGGCAAATTTTTCTAAAAA
GACTAAATCTGATCCTGTTTGACTACGTTCGGTTAGGAGGTTTTGATAAGATAAAATCGCAGTTT
CTAATTCTTGATTTTTTTCGTTTTCTAAACGGGTAACTTGACGACAAACGGGTAATTTTTCAAAG
GGTAAATTGTTCCAGTTAGTATCTTTTTGGGCAAAAGCACCGTATAAACTCACATATAAAGCATC
TCGGTTAGGATGGCTAGGGGGAAGGGTATTAAATTGAGTTGTGCCGACATCGCATCTTTCTTTAA
CATTAAGGTAGATGGGGTTTTCTGATTGTTGTAGGTAGGTAATTTGATTTTCTTTATCGGTTAAA
TAATTATTGGCGATTTTAGTTGCATAGATGCGGTTCAATTCAGAGCGGTTATTCATCATTTCTAC
ACCGATACCAGTAGCCCCTGTTTGTATGACGTTTAAGCCCACTAATGCCAGCAAACCACCCCATAA
TGACCAACTACGATTACCTTTGCGACATTTCGCCACACTTGCACCAAAGATGTTCCCAATCCCTAA
AACTCCCCCTGTGGCTAACATTGCCACCATTCCTGCAAAAATAGGGAAAAATGTACTTTCCAATA
AGAAGAAAAATAGGGGAAAACTAAAAATACCATTAATCCAGTTAGCAATAGTGATGGAACTAGC
GGTGAGGTGCGATCGAGATTCCACATTATCCGTTACATATACTTTACCATGTAAATCGTTAGGGT
TAACAATAATTAAATTAAGGAGACAACGAAGAAAGGTGAGCTCATCTTTATTAAGAGAATCATC
TTGATTCGGATTGTATTTACCTTGAGCAATTAGTTTCGCCTTTGCTACCAAATCAGGGCGAGTGG
AAATTATCCAAGTAATATCAGAGCGAACCTTATCGAAATTATTAGATGGATTCATAGATTTAATC
AATACAACGTGAAGGGATATAATTTTTCAATTGTTAAACCGTTTTTAACAGATTTAGATTTGTCG
GGGGATAAGCCTCCTATTTGATGATGAGAAATAAATTATGAGTCGTTATTATAAGTCGTTTGATT
GAATTTTTCTCCGTGCTTTTTTGATGGCGAGACTGTTTTCAACAGTGGCGATTAAACTGGCAATA
GGTGATAAAACTTGTCCTCTCCTAAAACTTTCCTCAAAAGATTCACTCCCCGCCGATACAAACATC
ATCAAAATAAATGCAGTAAAACTAAATATGCCCATTTGCTTCCATCTTCTGGTATGAATATAACT
AGCTAAAAAGGAAATAAACATCGATAATAAAATGTTTGCCCAAATAGCCCTACGGGATAAATAA
ACAGTGTGTTGAATTTCTTTCTGTTCTTTTTCTAGGTTCATTTGTTTTGCAACTTGCTGATACTT
GTTATTTTGATCATTCATAATCCCTTGATTTTGAGATGCGTACTACGTAGCAGTTTATTTGTCTT
AGGTTACGCTATTGATCCTCTAACCTACGAAAGCGATGAAAGTTTTACTTACAAAAGTCTGGTTT
CTACCTGTTTAGAATGATTGATTTCTTGGTATTAATTTTCTAAGTTTGTCTCAACAGTCACTATT
GTTACCCATTCCTAAACAAATAACTCTCATAAGCTAATATTCATTAAAAGATTTTTTTTTCTCAA
AACTTCTTATTTATAAATCTTTTATCAGATATGAACTAAAATGATTAATATTAAATTTTTCAAAA
TTCTATGGCAAAAAAACCAACTCTACATCCCCATAGCGATTTAAAATCCTTTCAACGATTAATGT
TATTAATCCTCACTATCATTAATCATCCCAACCTCGGCACAGAAGAAGACTCCAACACCAACGTT
ATCCATCAACTGCAAGAAAAAATCATCTCCATTGCCCAACAATATCATATTGACATTCCTCCTCC
AGCCATTGCCACTCTCAGAAAAGATTTAGAAACTCTCCGAGAATACAATATTTTAGAAAGAAGAA
TGTATCGTTGGGGTTACTATTTCGGTACTGGCGTTATGACTAAACCCGAATTTAAAACCGCTTTT
GATGCTTTAGAGTCTATGGCAAATTATCAAGGAGATGCCACAGCCAAAAAAATTTATCAACAATT
AGAAAAAAGAATTAGAGGATTAGAGTTAAAGACAAAGGATGATTTTTTCTATCCCATCAAAAGA
AATTATAACCGAGCCGTTAACTATACAGATCCTAAAGAAATGATTTTAAAGGGAAATAATCAAA
AGACTTTATATCATCATATACCATCTTTAGAAGAAGCTATTATCAAAGGACAAGCCATCGAAATT
AGTCGCAAAAAAGATTTTTATAATCAAGGAAATATTGGTATAGAAATTATCATTCCTATTCAGTT
AATTTATTATAATATTGCTTGGTATTTAGTATATGAAAACTGTGCTAATGGTCATTTAATTATAG
GAAGATTAAACCGTTTTAGTGATTATTTTCAATTATTATCCCCGGCAGGTAGAAATATTCAAGCT
CAAAAAGAATGTCTGAAAAATGTTGAACAGTTATTAACTAATGGTTGGGGATTAAACTTAGGTA
ATTTAGAGGAACAACAGTTAGAATTAAAAGGAAAACTGACACTAAAAAAAATTAAAGTCAGGTT

TTATCCTCCTATTTCTAATTTTATTTTGGAAGGAGATTTAAGACACTCTAAACAAAGAATAAAAG
TTACTAAAGATAACTATAATAATCAAATTTTATTTCTCGATTATTTTATAAGTCTTCCTCCCCGT
TCTCTTAATGAATTTTTATTTTGGTTACAGAGTTATGGTAGTTGTGTCGAAGTTATTTACCCTGA
AGATTTACGGGATAGACATAAAGAAGGTGCGATCGCACTCACTCAACGTTATAACAATATTTCTT
AAAAAATAATATTTTATGGACAATTTGTCTATTTTGTCTATAATATGGTTAATACCCCTAAACAA
AGGTGTAATTATGCAATTCGTTACCGCAACCGAAGCCAAACAAACCTTTGCCTCAGTTATTGATA
AAGCTCAACGTGAACCTGTAGTTATTCGGAAACAAAACCGTGATGTTGCGGTTATTATGTCAATG
GAAGACTATAACCGTATCACTCGCACTAATATTCAGGAATTTCAACAATTTAGGGCAAATATTGG
ACGTAAAGCCCAAGAGAATGGCTTAACCGAAGACATCTTGAATGAACTTTTAAAAGATGACTAAT
TTTTCCATACGCCTCGTTGTTGATACAAATGTTTTAGTTAGTGCTATTTTATCCCCTTATTCCATT
CCTGCAAAAGTTTTAAACTGGGGAGAGGATAATGGAATTATTTTATATTCTACTGACACTTTAGC
TGAACTTTTATCGGCTTTAGCTCGATCAAAATTTGCCAAATATATTCAACCTGATGACATTGAAG
GATTATCTTTACGCATCAAGGCTACATGGTTTTATGTGCCAATATTGAAACGAGTTAATTTATGC
CGAGATACAAAAGATGATAAATTTATTGAATTAGCTTTAAATGGTAACGCTTCACATCTGATTAC
TGGAGATACAGATTTACTGATTTTACATCCTATTCAAAATATTCCCGTTATTAATCCCCGTACTT
TTTGGGATTTTATTAATCAAGAAATTTCCTAGATTTCCTAAAGTGAGTGCAGGGTAGCTGTGTCG
AAGGCATTGCACTCTCCCAACGCTATGATTTATTATAATTTTCTTAATTTTCTGCTAATTTAATA
ACTAATTCTTTTTGATTCTAACGGTATCTGTCTTGGATTTGCGAGGATGTTCGATTCTTTTTGCT
GTTTTGAAAAAATCTTCATTAATTTCAGGTATATCGCTAAAATCAATATCCTCATCAGACATTTC
CCAGACAATTTTTTGTCTTTCTTCTTTTGACATATCAGAAAAATTATTCTTACTCATATAGTTTT
CTTTCCTGTTTAGAGGCTTTTCTCGCTGAAATAGTCCGAATTTTGTCTGCTCTTTCAGTATAAAC
GACAATACCTAAAATATACTGATGTTTCCATTCAAGTAGTTTCCCCCATTAAGGGAGAGTAAAAA
CAAAATTGACGACTACTTACTATAAATGACAATCCCCTAGGGTTATATGCTCTAAGGGATTTGCT
TTATCTATGGCATAGCTCCTTTAGGTACTTGAGGATTGTATAACTTAACTTTAGGAAATTCAAAC
TCAGGAAACTTCATTTTAACATTGTGTAAAAAATCCACCCACAAACAATAATCTATGTTAATACC
ATTCCCATACATCCCTCTAGCTCCATCTTGTAGGATGTTTTTTAATGCCATAATATCTTTTTTGTC
ACCATAGTATTTTGGAGTGACATAGGATTTTGCTTCATGGTCTGCTTCGATTATAACGTTGTTGA
TTTTAATTTTCATGATATTAATACTTAATTAGCCTATATACTGTTTCCATTCAATTAGTTTCCCC
CATAAAGGGAGAGAGAGCCAGTTTTACAGCACTTATCTGATAATAATTTCAGAGGCAGTTCCCGT
TCTGTAACAAAAATGTAACCAGTCAACTCTATTATTGTAAAAGAAAAGCCCCTGAAAGCTCATCT
GGCAATATATTGAGGGATAAAACGAGATAATCTGTATTTGAGGTAATTTATCAGATCGATCACT
AAGTATTTTTGTAATTTGTCTTATTTACTTAGGCTGATACCGTTGAATTAAATCTTTAATTTCCT
TGTGATACTCTGCCATGAAACTAGCCTTTTCTAGGCTAGGATAATATTGTCCAGAAAGAAAGTTC
AAATAATATAAAACCCTCTTCTTCCATAGTTGAACATCTTGTACTTCCCAGCAAACATTAAATAA
ATCACCCTTTTCTAAATGTTTTAAGCGGTGAAAAATGCGATTACGCCATGGTTTAATGTGAACAA
AAAAACGTTTCCATTGATATTCATTTTTTATCTGTGGTAAAGCAATCTCTACTAAAGAATCCAAA
CCACTACCATACAAATAAACTCTACCATCTTGATTAAATAAGCCACGTTGTTTTTTAAATTCTGG
AAATGGGCTATTTTTATCGCAAATACTCGACTTTAAATAGGGAGTTTCACCCCGAGGAAAAATTA
CATAATTTTTATATTTAAAATTAATTAACTCACTCATTAAACCTTCGATCGCACGAAAAGCATGG
AAAAAAGCCTCAATATTATTTCCCTGTTTAAAACGTACAAAACCAAGATAAGCAGACTCATAACC
CTTCCACCACCAATTATCATTTGGTATCTGTTGTAAATCTTTAATAAAAGTATCAAAATCAGTGA
TATTCCACTTAATTGCTTTACCTAAATAAAGTTTTAATTTGCGAATATACTCATTATTACCATCT
TGATAATAGGGTTTAAGAATCGAATTTACTCCCTCATAATCATGTCTTTTTAATAAACTTAAAGC

CTCCGCTTTTTTTCGACTCCAAAGATAATTGACTCCTATGAACGGTTTTGTATAAGGAGATGGTA
TTCCTGCTAAATTATTCTGAGTATTTTCTTCAAAGTCATAAAAAATTACTTTTTCTTCAAATCTG
GTAATAGAGGTAATTCTTGCCGCTTCTGAGGACTGATTAACCCCACCTTTTAAACATAAAATTAA
GTTTTGATTGTTTTTATTCTTAGGTGCGATCGCATCCCAAAATTGTTCCCACCAAAGAAACATTT
GTTCAAAATTAGCGGGATTATTATTAGGTTCATTTCCTTGAATAAAAACCTTAGTTTCAAACTTA
TAATGTTTCTGTAGATATTTTTGGATTATATGACCAGAATATAGAGTATCATAGCGATAGTATTG
AAGATTAGTAGTGTTAATTTGATCGGTAACAATGAGATAAACCGTTTTAATAGTATCTTTTTTAT
CTTCAATTAATTTCCCGATAATAATAGGCTTAATTTTATCTTGATATTGTTCCCAATTTTCCAAT
AAATATTTAGTGACATTACGAAAAGTATCAGAAATACCTAATTCATATTGAACCCTTACTGCTTG
GCTATCTTCTCCGTCAACATTAGATTTAGAGGCATTCCCAATATTAAGCCACTGTTGACTATATT
CATCACAATATAAAGCTAAATCACGAGTACCGATGGTAGCAACTAAAATAGTTTGAGTCATAATG
AATTAAAAGTTTTTTATGAATATTTAAAAGGAATTACATTAAATCCTGCTTGGGTAAAATGTTT
ATTGAAAGTTAAGACATCAAATATATTTTTAGAACGCATAATCATAAAACACGACTTAACGCACT
ATGAGTAGAAGGTCTCATTGCTTTTCTTTAATTTGATAAAATCATTAAATTCATGATATATACTG
ATTGGAGACTTATATATCTCTATCTAATATTCATAGATTTTGCAGTTAGCTCTTTCAAAGGTTGA
TTATAAATACTTTGCTTTATTATTATTTATTTATGTAGTAAATATCATTTATTTGTAACTTTTTT
TATATCTATACCATACTCCTATCTTTTGAATTTTTTTATTATAAATTATTACAAAAAGTAAAAAT
CATAGCATTTGAGGGTAAAACGAATAACTAATCAATAGTTACAAGACTAACTCGATCGCTTCAAA
ACTTTAAAAAGTTAGTCTAATTAACAATTAGAGTTAGAATTTAATGATGATAGTTCCTTAATAA
AGGAAACAACGGGAAAAAATTAAATTGAGGTGATTTTTGATCGGTATTTCCATTGAGTTGAAGA
AAAGCATGATAACAGAATCTGAACTCTGCATTAAAATTATTTAAGAACCCTCTTTTCCCTACTGC
CTAAAGAAAAGAAGCCAAATTTAAATGGGTTGCAAGATTTAAGTTAATTAAAAAATAGTGCAAT
TAGAGCAAAATTTTACAAGAATTTTCCTTAAGCCAAAAAAAAAATCTTAAGCTATAATCAAGCGT
ATAACTAATTATTAAGAGTTAGTCAGCCTAGTTCCTTAATACTAATCATTTTTCGATTATTAAAT
AGGAAGCGGGGGAACCAATTTAGGGGCTTATTTTTGGAGTGTTTCCAGAAAAGAACATCTCTCAG
TTCTCGTCCGTCAGCTAACCTCGTAGGCATTGAGAGGAGACTAAAGAATAGGTATTTGTAAAAAC
CTCCAATTCATTAGGTATTCCTAATTGACAAAACTCCTCATCTATAAAATAATCCTTTTTGAATT
TGGAGCAATTCAGATTATGAGTGTGAAATATATTAAATAGAATTTGTGATTGATTATGATTAAT
TAAACGTGAAATAGTAAAGGGTTTGTAGTGAGGGCTTTAGCCCTTATCTTTAAAAGATTTGAAG
TCCCCAAAAGGGCAAATACAAACTTTTGCTTATTTAATTTGAGCTACTTAATAGTATATATTTTT
GACTCTTTTTGTCCATTTTTATTAAGTGTTAATTTGTAAAAAAAACCCTTTTCAGAAGAGAAATA
TCAAGTATTATTTTGAGCTTATCAAATTCTAGTTAAGCTGAATAATTGATTTTCAGATTAAAAAA
TAAAGTTTCGATCAAGAAAAAGTGGCCTTTTTTAACTGTTAATTATCCACTTATTTACTCTCAAA
CTGTATTTTATTTCTTCATCAAAACCTAATCCTCAGAACAAACAAAAATATCAAACATGAGCGAA
CTTTCTAGTATTATTTCCGCCTCAGTTTTTGTTATTGTTATTATTCTGATTATGTCAGAAAAATT
GCATTTGACGATCGCAGCTTTATTAGGAGCATTAATTCTGATTTTTACCCATACAATTACTCTTA
CAGAAGCCGTTGAATTTATCAGTAAAAGCCATGCAACTTTAGTTTTATTTTTTGGTGTAATGGTA
TTAATTAGAGCATTTGAACCGACCAAAATTTTTGAATATATTGCCACTAAAATGGTAATTATTGC
TAAAGGTAGAGGAAAAGTCCTCATATTAGGAATCATTGGAATAACTACCCCCATTTGTGCAGTGC
TTCCAAACGCCACAACGGTTATGCTTTTAGCTCCTTTAATCCCTCCCCTAGCAGAAGAAATAGGT
GTTAATTTTGTTCCTCTATTAATTCTTATGGTATTTGTAGCTAATAGTGCAGGTTTAATTACATT
AGTAGGAGATCCAGCTACTTTTATTGTGGGAGATGCCATAAATATGACATTTCTCGAATATCTAC
AAAAGTTAACCCTCGGAGGTATTATTGCCGTCGTCAGTGTAGCTATCGTTGCACCTCTCTTATTCC

GCAAAATTTGGAATACAAAATTCACCCACCTAGAAGATTTACCTCACCCTCAAGTTAATCATCCT
AGAATGTTAATCCTTGGGGCTTTGATCATGGTTTTTGTACTTACATTCTTTGTTATTGGGGATTC
TCTTGCCGTACCGATTTCCCCTGCTTCAGTGGCACTACTGGGGGCAACTTTGGCGTTACTATTAGC
CCATCACAGTAAAATTGACACAGTACATAATATTCTCAGAGATGTAGATTGGAGTACCCTTATTT
TCTTTATGTCCATTTTTGTTTTGATTGGCAGTTTAGAAAAAACAGGGGTAGTGAGCCATTTATCT
GGAATATTAGCATTAGTTTTGGGAAAAAATATTTTTGTTGGTTCTATATTATTAGTTTTCGTGGT
GGGCATACTATCAAGCGTTGTCCCCAATATTCCTTTAGTGGTTGCGATGGTACCTTTATTAAAAG
AATATTTGGTTAATGTTAATTTAGTTGACTCATCAATTCTTGATCCTAACTATACAGGACAACTG
CCTCCGGAAGTTTTACCTTTATTTTATGCCATGATGTTTGGAGCAACTTTGGGGGGAAATGGTAC
TTTAGTTGGTGCTTCTTCTAATATTGTGGCGGCGGGAATTGCAGAACAACATGGAAAGATAATTT
CTTTTCAAACTTTTTTAAAATATGGATTACCAATAATGACAGTACAATTATTTATTTCTTCAATT
TATATTGGATCATTTTGGCTATTTAACTAGGAAAAGGTAAAGTTAAAAGGACAAGGGTAAATAA
TTAAAAATTAAGAATTAAGAACTTCTAACTCTCATTACTCATTACTTATTTCCTCCTCTCACCCCT
TCTCCTGATCACCTCTTCTCCTCAATACTCGGAACTCATTTCCCCATGGTGTGACACTCAAATCAA
AAGTCTGTTATTGACTTTCAGATGAAATATTACTATGATAACAATATCCCCCCTATGGGTATATA
AAAATATGAGCGATATTAGTTAAAAATCAAATTTGGATTTTTTTTCTGAAAATATTTTAAGATT
AAGTAAAGATAAGTAAAGAAATTATAAGCAATTTTGTTAAATCATACCTTGTTATCTCAATTAA
CCACCTTGTTGCCATCTGTAGGGGAAATTATCTTTGATTTTCTCGTGGGTTGGTGGTGGGACAAA
GAAAAGCGTAAAAGGATTCTTCTTTTTTCTTCATCGGCTTTTCTAGTTTCTATTCCTGTGTTTTT
CCAAGCTCCTTTGGTGAGGATTTTTCCTTGGTTGAGTTTATTATTAACTTTTGTGTGGGTTGCTT
TAAGTTTTCACTGGCGTAAACAACCTGAAACTTACATCTGGGGAGATTTACTCTGGGGTTTTAGT
TGGAGTTGGTTGTGTGGTTCAATTTATTGGGGATGGTTAAGATATTATCCTTCTATTCATATTCC
GATCGAGGCTATCGGTTTACCTTTCGCTATATGGGGAATTCGTAAAGGATGGGGATTAATAGGTA
ATTATTTTTATCTTGGTTCTTTAATTGGTACTGCTATTACTGATTTATATTTTTATATTGATGGG
TTAATTCCCTACTGGAAAGAGTTAATGGTAACTGATCCTACTCTCGTTAAACCTCTTTTACAAGG
TGCGATCGCACAGGTTCAAACAATTTGGGGTATAAGTTGGGCAATTCTATTAGCGAACACTCTTT
TAGGAATTAGTTTGTGGGGATTAGTAAAGAAAAAGACTCATCATATAGTTTTTGCTAGCGCCGTT
TTGAGTACTATTTTTACTGATGCTTTATTTTTAGTCGCCGCTTATCTGGGTATTAGTTAACTGAG
GTTTGATATAAACTTATCGGTTAAGGTAGGCAATAGACAATGTAAAAAGGGCAAAGGCAATTAA
AAATTAGTAATTAGTAATTAAGAAAAGGGCAATTAGTGAATAGTGAATAAATTTCAGTTAAAAA
TTAAGAGTTTGTTTTCCCCTCTCACCCCTTCCCCTCATCACCCCTTCCCCTCATCACCTCATCACCT
CATCACCTCATCACCTCATCGCCTCTAAACCCTATACCTGTGTTTATCTAATATTTTTATATTGAA
ATGAGTCAATATTAATCCTTTATGAAAGTATATATTGAGATCCACAACTATTGAGTTAAAATGA
GAAGGAAATATGCAAACATCTAAAACCTATACGAATAGGTGCTTTATTATCTTTATATCCATAAG
TAATAACACTTAGAAATAACCACTATTAGTAAACTATCTGAGGAAAGTTCCATGACTCAAACTTC
CCCCCAAGCTGACCGTCAAGAGCAAAAAAAGGCTAATCAAAATCAAGAATTATCTCCCTCTCCAT
CGACACAACAACAACCACCAAAACGCCCGACAAATAATCAATCATCTCAAAGTTTAATTCAATGG
TGGCAAAGTAAAAATCTTAGGTTTAAAACTTCCTCGATCGCGATCGCATTGGGTTTATTACCCAT
TATTATTGTTGGGAGTGTAATTGGTGTCACATTAAACCAAGTATTTCGAGCAGAAATCAAAAGA
ACACAAGAAGAAGAAGCCCAAATCATTAGTCAAGATTTTGATTTAATCATTGGAGACCGTTTTGC
CCAAGTTCAGGGTATGGCGCAAGTAATACCCGAATTGTCTTCTTATCAACAACAAGATAAAGCAA
GATTACAAGAACGGTTGAATGACTTTCAAACTTTATATAAATACTTTGACAGTATTGCAGTATTT
GATGCACAAGTCAATGCACTAGCAGAAAGCAATGGACGTTCCATGCCTAACCACAGTGAAAGAAC

CTATATTCAACAAGCGAGACAGGCAAAAGGAACTATTGTCAGTGAACCAATTATTTCTGAATGGG
ACGGTAATTATGGAATTTATTTTGCTTCCGCCCTTATAGATCCTAATACTAATCAAATTACAGGC
TATTTAAGGGCAAGAGTCCCCGTGGAAGAACTACAAAAGTTTATTGCGAGAAATGAAACTGTCGA
AGGTAGCGGAGAAAATATTTACCTTGTTGACGGGAATAATCAAATATTTTTATCCTCTGATACTA
TCTACTCGACTAACGTTTCTACCACAGGAAAAACCACCACGGAAAGTACAACAGAATCTGTAAAA
TCTCTCGAACAAGTATTTCCGCAATTGAGATGGCAAGATTCATCTGATCAAGGAACTACCGTCGA
AGATAGGAATCAAATTACAAAAACCAATCAGGTAGTTACCTATGCTCCTATTGAGGTTGATTTAG
GAACTAATCCTTTAAATTGGGGATTGGTTTTGGCAGGAGATACAAGTGTTATCCTCGCTCCCGTT
AATCGCATTACAACTTTCATCTTTCTGGGGATTCTCATCTCTGGGGGGGTAGTTGGTTATATTGC
CTATACCAGTGCAGGATTAATTGTTAATCCCATTGAAAAAACTTCAAAAACAATTAAACAGATTG
CGGAAGGGGATTTAGACGCATCTTTGGATATTAAAGGAGATGATGAGATTGCCCAGTTAGGTAA
TAGTGTCAATAACATGACCTATCAGTTGAAAAACTTACTTGTAGAGCAACAAATCTTAGCCAGTC
AGTCTAATTTACTTAAGAATATTACCCTGGAGATGACAAAGGCTTTTAATGTCAATGAAGTTTTT
GAAATTGCTGTTAGAGAGATTCGTCAAGCACTACAGAGCGATCGCGTCATTGTCTATAGTTTTGA
TGATAATTGGCAAGGAACAATTATTGCAGAGTCAGTCAACTCTAACTACCCTCAATCATTAGGGG
CAAAAATTCATGATCCTTGTTTTGAGGATAAATACGTTGATAAGTATCTACAAGGCAGAGTTCAA
GCAACCCCAGATATTTATAAAGCTGGACTAACAGAATGTCACCTTCAACAATTAGAACCCTTTGC
CGTAAAAGCTAATTTAGTTGCTCCTATCATTGTAGAGAATAGACTTCTTGGCTTATTAATTGCCC
ATCAATGTTCAAGCCCTAGAAATTGGACATCTCAAGAAATTGACTTTTTCGGACAAACCGCAACT
CAAATGGGACCTGCTTTACAAAGGGTTATTTTATTAGAATCTCAGCAACTAGATACTTTGTTATC
TCAACAATTAAAAGACATTACTTTCAAAGTAGCCGAAGCGTTAAATCAAGATAAAGTGTTTGAA
GTGGCAACAAATATGGGTAGAGAAGCCCTAAATGCCAGCCGAGTAATTATTTACACTTTTGATGA
GGATTGGAAAGGTACAGTTATTTTTGAATCTGTTGATCCAAAATATCCCAAGGCACTAGGATCAA
AAATAAAAGATCCTTGTTTTGCTGATAAATACGTTGACAAATATAAACAAGGTAGAGTTCAAGC
CACCAACAATATTTATACAGCCGGTTTAACCAGTTGTCATATTCAACAGCTAGAACCTTTTGAAG
TAAAAGCTAATTTAGTTACCCCGATTATTGTGGATGGTGAACTACTAGGCTTATTAATTGCTCAT
CAATGTGATAAACCTCGTCAATGGACTCCTTCTGAATGCAATTTCTTGTCTCAATTAGCAACTCA
AATTGGCCCTGCTTTAGAAAGAATCAGACTTTTAAATAAGCAACAAGAATCTGAGTTAGAACAA
AGAAAAGCACGGGAAACCATGCAACAAAGAGCATTAGAATTATTAATGCAGGTTGATCCTGTTTC
TCAAGGGGATTTAACCATTCGGGCAACAGTAACAGAAGATGAAATCGGTACGATTGCTGACTCTT
ATAACGCCACCATCGAAAGTTTGAGAAAAATTGTAACTCAGGTACAAGGAGGTACAAAGGCAGTT
AATCAGGTTGTTACGGAGCAAAATGATTACGTAGAAAATCTAGCACAAGAGGTAATTCGTCAGGC
GGATGATATTAACGTTGCCTTGGAAAAAATTCAGATTTTGGCTTTTTCGGCTCGTACAGTATTAG
ATAATGCAGAACAAGCAGAAGAGGCAGTAAAACAAGCAAACCGCAGTGTAGAAGAAGGAGAAGT
TGCCATGAACCGCACAGTGGACGGTATTTTGGCAATTCGAGAAACAGTAGCTGAAACGGCGAAAA
AAGTAAAAAGATTAGGAGAGTCAACCCAAAAAATTTCTAAGGTAGTTAACCTGATTGGTAGTTT
TGCGGATCAAACTAACCTATTGGCGTTGAATGCTTCCATCGAGGCGGCACACGCCGGGGAAGAAG
GACGAGGTTTTGCGGTGGTAGCCGAAGAAGTTCGTTCCCTCGCTCGTCAGTCTGCTGAAGCGACG
GCGGAAATTGAAAAAGTGGTTGCGGAAATTCAAGAAGAAACCAACGAAGTTGTAAAAGCGATGG
AAACTGGTACTGAGCAGGTAGTCGAAGGGACAAAACTGGTGGAAGAAACCCGTCAAAGTCTGAAC
GATATTACAGCCGCATCTGCTTTAATTAGTCAACTGGTAACTGCGATCGCAGCTTCGGCGGCAGA
ACAATCTCAAACCTCTGAATCCATTACCGAAACTATGGCAGAGGTAGCGGGAATTTCCAATGATA
CTTCTAATGCGGTGACTCAAGTGGCTCGTAGTTTTAAAAAGTTACAAAAAGTTGCTGATAGTTTA

CAAGACACCATTAGTAAGTTTAAGGTTAGCTAGGGGTTAGGATTTCGGAGTTTTTTAAAGTAGGG
GAGTACGGAGTACGGAGAATTAAAAATTCAGAAATTTTCTAACACCCGAAACCTGAAACCTGACA
CCTATTTTCTACATTACTCATTACTCATTACTTTCCATGACTCAAGCACCAGAAAAACCAAGTTT
ACAATCAACCACTCAGGAAAAAGGAAATACTTCTCCTGTTTATGCCACAAGAAATCAAAAAACCC
CTCCTGTGAATACAACCCCCCCGTGGTGAAATGGTTTTATGATTTATCCATTCGTAAAAAACAA
TTGTTAGTAGTAGTAGGAGCTCAAGCCATTTCTTTTGCCGTACTTTTAGGAGTAGGTGTTTTAGA
AATTGTTACAGGTGGTAGAGAGCAACTTATTAACCAATCAATTTCTGAGCTAGAAGCCACCAAGA
TTAACTACGACATTAAAATTAATCAGATGGGCTTCGGTTTTCGAGGACAATCGGATAACGTTGCA
ATTATCAACGCCGCTTTAACTCGTGCTAATGGTAATAGTTTGACTCCCACTCAACAAAGTTTAGT
GAAACAAATTCTTCAGAATGAAATTACTGCTCGTAATATTGAGTATGCTACTTTAGTGGGAAGAG
ATCAGAGAATAATTGTTAGTGCTAATAATAATCGGGCAGGGGAGGTTTTTGATCCCAATGGCTTA
GTTTCTCAAGTAATTAATAATCCTCAACAAATTAAAACCACTGAAATTGTTTCTTGGCAAGATTT
ACAAGCAGAAAATCCTCCTTTGTTGCCTCGTCTAATTCCTAATCAAGATGTGGTTATTCGTTATA
CTTTTACTCCTGTTTTTAACCCCAACAATCGGGATCAAGTGGTAGGGGTGTTAGTTTCTGGAGAC
GTGGTTAACGGGAAATATTCCATTGTATTTGATACGATTAATGAGTTTAAGGGGGGGTATAGTG
CGGTTTATCAGGTTAACTCCGAAGATAATATCTCTTTAGCTAGTTCTGCTACTAATAGTGAAGAT
ACAACGAATCAGCTAATTGCTAGTCAAAAATTATTGGAGTTAGCATTAGAAAATCCCAATCAAAT
TGTTTCTTTCAGAGATAAAGTAGGGGACGAAACTTTTACTTTTACTGCCCAGACCATTCAAAATA
AAGCCGGTGAACCTGTGGCCATTTTAGTACGGGGGACTCCTGAAAAAGCACTTAATCAAATTATT
GCCAATAGCCTTAAAACTCAAGGACAAACGGCACTAATCGTATTAATTCTTAATTTTATTCTGAT
AATTGTTTTAGGAAGAGTTATTGCAAATCGTTTAGAACAGTTACAAAAAACGACCACTCAATTTG
CTGAAGGTAACTATCGAGTAAGGGCAAATATTGTTGGACAGGATGAAATTGGTGATTTAGCGGA
TACATTTAATTTACTCGCAGAAAATATTGAAAGTAATGAAACTTTACTTTTATTAGATGCAGAA
AAAGCCAGTTTATTCCAAGAAATTACGGGGGCAAGGGTAGTTGATGAAGATGATATTAATTCAAG
TGTTCGATCGCAGTTTACCAAAAGCAAAAGAAATTTTAGAAGTTGATCGTCTTGTTATCTATCGC
TTTAAGCCTAATTGGAGTGGTTATATTAGCAATGAAGCAGGAGATCAAGATTTACCCAGTGCTTT
GAGTGAGCAGTTAAATGATCCTTGTATTCCTTTATCCTTAAGGGATTCTTATATAAACGGTAGAG
TTGTGGCAACAGAAAATGTTTATACCGCAGGGTTTGCTCCTGAACATGAAGCCTTGATGCACCGC
TTACAAATCAAATCCAATTTAGTTATTCCCATCATCAGTCAAGGACAACTATTTGCTTTATTGAT
TGCCCATCATTGTCGTCAACATCATCAATGGCAAGAAAAAGAAATCTCTTTCTTAAGTCAAATTG
CTTTACGATACGGGGTAATTTTAGACCGGGTTAATATCTTAAAAGCCCAAATGACTTCGGCAACT
CGTGCAGAACAACTCAAAGAAATAACCCTTAATTTAGCTGAAGGTTTAAGTCCCCAAGAGGTAAT
GAATACGGCAGTTTTAGAGACTCGTAAAGTATTGAAATGCGATCGCTCTATTGTTTATGAATTTG
ATGAAAAATGGCAAGGTACAATTATTGCTGAATCTGTAGCAGAAGGCTATCCTCAAGCCCTCGGT
GCGAAAATCCATGATCCTTGTTTTGAAGAAGAATATGTAGAAAAATATTTGCAGGGCAGAGTTC
AAGCAACTCCAGATATTCACAATGCAGGATTAACTTTATGTCACCTAAAACAATTAGATCCTTTT
GAGGTAAAAGCCAACTTAGTTGCCCCCATTATCGTTAACCGTAAATTAATCGGCTTATTGATTTG
TCACCAATGCTCAAACATAAGAGAATGGGAACTATCAGAAATTGACTTTATGACTCAAGTTGCCA
CTCAAGTAGGACTAGCTTTAGAAAGAGTCAAATTGATTGACTTACAGTTACAGGCAGAGCAAGAA
CAAAGACAGGCAAAAGAGTTATTACAACAAAGAGCGTTAGAACTATTAATGCAAGTTGACCCTGT
TTCTAAAGGGGATCTAACCATTCGTGCCACTGTTACGGAAGATGAAATCGGTACGATTGCTGACT
CTTATAACGCCACCATTGAAAGTTTAAGACAAATTGTATCCCAAGTACAAAATGCGGCGTTAGAA
GTAGCTCAAACTACCAGTAATAATGAAAGTGACATGGAAATTCTGCGCTTAGAAATTGCTGAACA

GGTAGAAAATATTGCTCAAGCCTTACAAACGGTAAATGCTATGAGTCGTTCTAGTAAAATTGTGG
CAGAAAGTGCGGAACAAGCGGAAGAAGCCCTAATCAAAGCTCAAGAAAGCGTTGCTGTGGGAGAT
TCAGCAATGAATAAAACCGTGCGCTCAATTATGGATATTCGTGCCACTGTACAACAAGCCGCAGA
TCAGGTTAAAAAACTAGGGGATACCACAGAAAATATTTCCAATTTCGTGTCTCTGATTGGCAGAT
TTGCCGCTCAAACACACCTTTTGGCTTTGAAAGCCTCCATTGAAGCGGCACGAGCTGGAGAGCAA
GGACAGGGCTTTGCGGTGATTGCTGATGAGGTTCGGGCTTTAGCGACTCAGTCTGCTCAGGCAAC
GGCTGACATTGACAAGTTAGTGACGGACATTCTTTCAGAAACAAAGGTGGTGGTAACTTCCATGG
AAGAGGGCAATGAATTAGTTGTTGAGGGTAGTAAACTGGTAGAGGAAACCCGCCAGAGTCTTAA
CCAAATTACCGCCGCTACTGTACAGATTAACGAGTTAGTAGAAGCGATCGCGGCTGCTGCTTTTG
AACAGTCAGAAAACTCGGAGGAAGTTAGTATCACAATGGGAGATGTGGCAAAGGTAGCTGAAAA
AACTAATTCTTCGGTAACTCAACTTTCTCAATCCTTTATTCAGTTAAGGTCTCTTGCTAGAAAAT
TAGAGTCTGATGTGGCTAAATTTAAGGTCAGTTAGAAAAGGGTAAGGGGCAAAGGGCAAACCCCC
CTTTATCCCCCCTCTTGAGGGGGGAGAGCAAAGGTAATTGATTAGTTTCTCCCCAATACCCCAATC
CCCTACTTCCCCCTCTCCCCTCGTCTCCGTAAGGGTTGAATATATTCAACCCCTATCACTTGCAAC
CTCTTTCATAACTCCTGTACGGGCGAATGGCCATTCGCCCCTAACTAACTCCTAATCCCGAACTCA
GGTAATTAATGATTTGGTTAATTGGTGGCACATCTGAAAGTGTAATTATCGCTCGTTTATTGTCT
GATTTAAAGCAAAATTTTGTGGTGACTGTCACGACGAAGTCTGCTTGTCAGTTATATGAGAATAT
TAATAATATTCAAGTTGTTGTCACTAAGTTATCTTTGGCACAGATGAGGGATTTTATTTCCCTTT
GGCAAGTTTTTGCTATTATCGATGCTTCTCATCCCTTTGCTCTTGAGGTTTCCCAAAATGCAATCC
ACTTGGTCACATCTTCTCAAGACTTATGTTTACTTTATCTTCGTTATGAAAGAGAAATGGTAACT
ACCAACAACCACAAGAATATTTTTTATTATCCAGATTTACAGTCTTTATTGTCAGATAATTGGAT
TTTAAAGCAAAAAAAGGTTTTATTAACTATAGGAGCAAAATATTTACACTTATTTAAACCTTATC
ACCATCAGGCTCAATTTTATGCTCGAATCCTTCCCTATCAATCTTCTTTAGAATTGGCTGAAAAA
GCTGGTTTTGGGTGCGATCGCATCATAGCTATTCGTCCTCCTCTGAGTTTAGAATTAGAAAAAGC
CTTGTGGCAATTATGGGATATTGAGATAGTAGTTACCAAAGCAGGAGGAAAAGCAGGAGGAGAA
GACATAAAATATCAGTTAGCGGAAGAATTAAAGATTCCTCTGGTAGTGATTAAGCGCCCCTCCCT
TAATTATCCTTTAATGGCTAAATCTGTTGAAGATGTCAAGGAGTTTTTAAAGAATTTTTGAGAA
AATCTGTTAGTAATCTAATTTTGTTCGATAGATGTCGATTGATAGGATAAAGTGCGAATAAACTT
AGGGTTTTAGGAGAGTAATCGGGTAGGATTCTTCTTAATTTCCCTTCTTGAAGATAAGATTCGAT
AATAAATCTTGGTAATATTGTTATACCTAAATCTGCGATCGCACCTTTTGCGAGAATTTCCCCAT
TATTAGAACATAATCGACTTTTAATTTTGACGATTTTTTCTTCATCTCCTTTTGTTAATCTCCAT
AGGTGAGGGGAATTAAAATAACCATATTGTAAACAAAAATGATTCTCTAATTCTTCAGGGGAAT
TTGGTTCACCATAATTTTGGATATATCCTTCCGAAGCACATAAAATTAACTCAATCTCAGCTAAT
TGATGAACTATTAAGCTAGAGGACTCTATTTTATCGGTAATTCTAATTACCATATCATAACCTTC
GGCAATGGGATCAACAAAACGATCATCTAAACTAAGTTCCACTTTTAAAAGAGGATATTCTGCCA
TAAAAGATGCGATCGTATCTCCTAAATTAATTGTGCCAAAAGTCATGGGAGCATTAATCCTCAAT
TTACCTTGAGGACGTTGTTGTAAATTAGTAACCGCTATTTCCGCTTCCTCTAAATCCGCTAAAAT
TCCCGTGCAACGTTCATAAAAAGCTAAACCCGTTTGAGTAGGAGTGACATTACGAGTATTGCGGT
GTAATAACTGCACCCCTAAATAAGTTTCCAAATTAAAAACCAACTTATTCACTGCCGAACGAGAT
ATACCCAAAATGCGAGAAGCCTGAGCAAAACTACCCGACTCCACCACTAACACAAAACCTCTAAT
CTGATTAAATTTATCCATTTGTCAACTTTTATTCAACAATCAGTTAAATATAATATACATTGTCA
ACCAAAAAATAGAGAAGTATCATAGAAGATAAGAAAACAAATGGAAAAAACGATTAAAACCAT
GATTACTATTCGCCCAAGTGAAGAAAGAGGAAAAGCCAATTTAGGATGGCTTGACAGTAAACAT

ACTTTTTCTTTTGGTAGTTACTATGATCCTAATTACATGGGTTTTGCCAGTTTACGAGTAATCAA
TGAAGACAAAATTGCACCCACTCAAGGCTTTAACACCCATAGTCATCAAAACATGGAAATAATTA
CCTATGTTTTAGAAGGAGAACTAGAACATAAAGACAGTATCGGTAATGGTTCAATTATTCGTCCT
GGTGATGTACAAAGAATGTCTGCCGGTAGAGGAATCGCCCATAGTGAATTTAATCCCTCATCTCA
AGAAACAGTCCATCTACTACAAATTTGGATTCAACCAAATCAAATAGGAGTTGACCCCAGTTATG
AACAAAAATACTTTGCCGATGATGAAAAACAAGGAAAATTAAGACTAATTGCTTCTGAAGATGG
TAGAGAAGGATCTGTCAAAATTCACCAAGATGCGGATCTTTATGTAATGCTTTTGGGTAAAAATG
AAGAAATTAAGCATAATATTCCAGAAAATCGCTCTTTATGGTTACAAATAGTTAGAGGTTCAGG
AAAAATTAACGGTCATATCGTTAATGTTGGTGATGGTGTTGCTATCACTCAAGAAAAGAAATTA
GACTTTATAGCTACTGAAGATAATACAGAAATTTTATTATTTGATCTAGCCTAGAATATTTCCAT
CCAATGACTTTCAAAAAGGAGAATTAAGAATCCAATGAATACATTAGAAGCAATCTATCAACGTC
GCTCCGTTAAAGCCTTTGATCCTAATCACAAAATTACCCCAGAAGAAGAAAAACAACTGCTAGAG
GCAACAATCCAAGCCCCAACTAGTTTTAATATTCAACATTGGCGTTTTGTAATTCTCCGAGATCC
TCAATTAAGACAAAAAATTCGTCAAGAATATGGTAACAATCAAGCTCAAATGACCGATGCTTCTC
TTTTAATTCTATTTACTGCTGACGTAAAAGCATGGGAAAAAAATCCTTCTCGCTATTGGCAAAAT
GCTCCCAAAGAGGTGGCGGATTTATTGGTTAACTGGATTGCACCTTTTCACGAAGGTAGAGAATG
GCTACAAAGAGACGAAGCCCAACGCTCCATCGGCATGGCAATGCAAAATTTAATGTTAGCGGCAA
AAGAAATGGGTTACGATTCTTGTCCGATGATTGGCTTTGACATTGAGAAAGTGGCAGAGTTAGTC
AATTTACCAAAAGATTATGTTATTGGCCCTATGGTAGCAATTGGTAAAAAAATTAAAGAACCCTG
GCCCAAACCCGGACAAATTCCCTTGGAGGATTTAATAGTAGAAAATTCTTTTTAAGGGAAGTTAT
TTAACCCGACTTCAGGATAAATTTTTATCTATGAGTGATGGTGAAAAGGGCAATGGGCAACCCCC
CTATATCCCCCCTCGAGAGGGGGGGCAAAGGTAAATAAGAATTAAAAACTCTGAACACTTATCAC
TCATTACTTGTTTCCCCCTATCCCCTAAACACCCTGACATCTGTAACCTGTAACCTGACTGAGGTT
ATAGTAGTTTTAATTAAGACTGTAACAATAATATAATTAAATAAATATTGATTTCTCTAACAAC
GTTAAACTATGCCTTACAGTCGTGAATCAAGTATGCGATCGCAAAGATTGAAGCAATACACCTCG
CACCACTGTAGAAGTTTTAAAAAGATTAGTTATTGTTGTAGTAAAGCAAAAAAGAGCTTATAAA
CCCCTTGATTTAAGTGAAGATTATTTCAATGGACAAGTACAATTTTATGGCACACAACAACATCG
CATCAACTTAGCAATACAAACATTAGAAAAATTACCAACCGTTTATAATCATCCTCAAGGAAAAC
AAATAGTTAGATTGTTAGCCATGTTTCCTGATGGTTTATCTGAGGATTTATCTGAGGATTTATCT
GAGGATATGACAGAAAGTTTAGGCATGAATAAGAGTCTTAATATTATCCATAGCAGGTACAAAT
CAAGTTGACTTTAATTTAAAATTAAATAATGGTTTTGCTAATGGTTATCCTCTCTCTTTTGATTT
ATTAAGTAACCCGAATTTGGGATAAAAAAATACAACCTGAAACCTGTCTATTAAGCCAAACTGAC
GTTTATTAATTGGTTTCCACCGTCAGGATTTGCGGAGGAGTAGCATCAGGAGGATAAAGAAAGTC
TATGGATACTAACTTTTGTCCTTGAGGAGGTATCGTCACTGTTACTAAATTTTCTCCTTTTTGTC
CACGATTCTGTACTAAATGAAAATAACGCCAACGGGGTACATTTTGATTATCATTATATTTGACT
TGTACTAATCCTCGGAAAAAGACTTGATTTGGTGGGTTTTCAAAAAAATTAATTTCTGGAGTCTC
TTGCTTAAGGGGAGTAGAAAGGGCAATATTAACAGTCTTACTTTCATTGCTAGGATTAAATAAA
GGCAAAGTCAAACTATACTCAACACCATAATTTCCATGGGCGCTATAGGCGGTATCTGGGTATCT
CACGGTTAAAGGTGCAGTTTGTACTTGATTAAGTCCAAAAGTTCCCCCTACTAAAGTGCTTAAGC
CATAGGAGAAAGCCTTTCCTGATTCGGGAATAGTCAAAATTGTGCTATTAACAGAGTCAACTAAA
TCTGTTTTCCATTGATTACCAATAGACACTCCGGCTACCCTGCCATAAATCCTTTTTCCAGTGGCA
TCGGGAGGAGTTGGTGCTAAATCTCTGGGGGCGGACAATTCGCCGTTATTTAATAAATCTTGCCA
TTGAGACAAGCTAGGGGGGTTATTTTCTTCGTCTCGATACATAGCTAAACTGGCTACATAGACTT

CTCCGTCGCTTAATAACCTCATATAAGTTGAGCGCCCGTTAATTTTTGGTTCAAGGGTTTGTACT
GGAATAGGGGCATTTAACAACATTTTACTTTCCCCTGCTTTCAAGGTAATTTGAGCAGGAAAAAT
ATCTTGGCGTTTTTCTTGTAATATTTCTCCCATCACTCTACTACCCGGTCCTGCGTAAGTGTTACT
GCTTTCTACCAGAGAAGGTAATTTAATGAAAGGAGCATCAGGTTGGCTTAAATAACTCGATCCCT
GCAAAATATTGATAGTAACAGTTTCATTAGTGGGATTTTGAAGGATTATACCTAAATAAAGAGT
TCTAGTATCATTTTCATCAACTGCCTTGGCAACATGATGGGCAAAAATATCGAATCTTCCTTGTA
AGGGGTAGTTTAAATGGGCTGTAGGGGTATTTTTATTGTCGGGAGGAAAAGTAGAAAGTAAAAT
TCCCTCTCCTACGATTAATTCAGGGCTATTACTGTTAAATACGGGTATTTGGTTTAATTCCCCTG
TGAGGGGGCGAATAGTATTATTTTCTTCGGTGACTATGGCAGGGGTATTATTTTGAGCCAGAAGA
ATAGAAGGTGATGGTTGATTGAAGAAGAATGATATTAGCTCTAACATAAATTAATTCTTAATAA
ATAATTGTGTGAATAGACTCAATCCACTAAGATAGGTTTCTCAACAAATTAAATAATGTGTTGAG
GTTACCATAAAATTTTAAATAATTATTATCAATTATTATGATGAATAGTGAAGATTTAGCTAAA
TATATTGAGCAAAGCCAAGGAATATCAAAACCTTGGTTGTTAATACAGTTAAGACTCAAAAAAT
TGGAAGAAAGTAAAGATTCTCTTTCTGAAACGGAATATATCAATCAATTAATGGACATTCATCAA
GATTTAATGAAGTTAGGGGAATGGTGGATAGGCAGAGAGGATGAAGTGTTTGGCAGTTCTTTAT
AATATTTTTTTTATAGACAATAATTGTTGAAATATAGTTGAAAATGAAGAACTTGAGCTAAGGT
GATAGTTAGGATACATTAGCCCCTGAAAAAGTTATGTGTATTTGTGTTAATTGTTTGTATGTCGA
TCGCTGCAATACTTATTATGCGGTAGAAGCTCAACATCAACAACCCCATTTGACGGAAAGTCCAG
ATTTTGAACCCGTTGAACCTTCTATAAACGTTAATATTCGCCCTCAAGAGGATTATATTGAGATG
GAGTGGGATGTAGTAGGATGTGCGAGTTTCACTGAGGATATGGGTAGATGGGCAAGATTACGCCC
CGGAGAGGCTATTCCTACTTAGATTATATCAAAAAGGAAATATTCTCTTATTTCTCCCAATAATT
TATGAGATTTTATCTTGAGCCCCGATAAATTCTTTCTGCTTGTTCTTGGCTAAGACGGTTAGAGC
GAGGTAGATTAGGATTTCTATTGTTTTGGGTATGGGTGGCGGTGTGGGCTATTTGGTGGTGGGTA
ATGTTGTGATTATTGTTATCTTCTTCGATACCATAGGTTCGACAACCTTCTGCTTGAGAGACAGA
ACAACTAGGGTATAAATTATAGATATACTCTGGTTCTGGTGAATTGTTTCTCTGTTCTAGGGTGA
CGAAATCATGAATTACTGGATCATAGGTTAATACTTCTCCCCTTTCAATGTTATAAACCCATCCG
TGAAGAGATAATTTGCCTTGATGTAATCTACTGCGAATAATGGGATAAGTGCTAAGATTTTCTAG
TTGAGTTAGGACGTTTTCTGCAACGGTAATTCTCAATAAGTTTTCTTCGGGAACATGACTATAAT
TTTCTTTGATGAGACGGCGGGTAGCTTCTGCTTGTTTTAACCACTCGTAAACTAAGGGCATATCT
TCTGCTAACTTAGACATTTTTAATAATCCTTTCATTGCACCACAGTGGGAATGACCACAGACGAT
CACTTGTTCTATCCCTAAAGCTGCGATCGCATATTCTACGGCCGCTCCTTCTCCACCATTTGTAGC
TCCGAAGGGGGGAATAATATTACCTGCATTACGAATAACGAATAATTCCCCGACATTTGCTTGAG
TGATTAAGTTTGGGTCAATACGAGAATCAGAGCAAGTTATAAATAGAATACGGGGGTTTTGCCCG
TGGGATAAACTTTCAAATAAATCCTGATGACTTTTAAAATAACCTGCTTGGAATCGGTGTAATCC
TTCAATAAGTTTTTTCATAACTATAGCTTGATGTCTCGTCTAAGGAAATAATTATACTAAGTAGA
GGTACTCAAATCAAGGGAGAAAAAAATTAAATTTTCGTTGCCAATACAACACTATAATCCGAAAC
CTCATGATAGCCTAGATGAAATAAAAGTAAGACTAATAGCAAAGATAATTATGACTGAATTTAA
GGATTTTGATCAGCAATTACTAACACAAATTAAAATTCTTTTAGAAAAATTGGATAAAAAGTCA
GAAAACAGAAAAAAATTACAAAACATACTCAAAATACTTAAAAATCAAGATAATCTCCAAGATA
AAGAAGGTGGGCATTTAGAGAAATTTGATCAGAAAAAATTTGATAATCAAAAATATTTTAATTT
AAAAATTAAGCCTTTATTAGATGTTTTATATACACCAGAAATAAGCGAAAATTTATTAGGGATA
ATTTACAATTTAATAGAGGATAAATTTGCTGAATCTTTGACGATTAATTGGCATAAATGGAGCG
AAAATAATGTACAGTTAATCACCTATGGGGATACTATAAAAGACAGTAAATCTTCAGAAAAACC

TCTAGTTACTCTTGCTAATTTTTTGGAGGATTATCTTCAGGATACTATTACAGGAGTTCATATTC
TACCATTTAATCCGTACAGTTCTGATGATGGTTTTGCTGTGATTGATTATTTACAAGTGGACGAA
AAATTAGGCGATTGGGATGATATAAGTGCGATCGCACTTCAGTTTAATTTAATGATCGATTTAGT
GATTAATCACGTTTCGAGCCAACATCAATGGTTTAAAAATTTTCAAGAAGGAAAAGAACCTGAAT
GTAACTATTTTATAACCGTTGATCCTGATACTGACTTATCCTCCGTTGTTCGTCCTCGTAGTACGC
CATTGCTAACGAAAGTAGAAACCAAAACAGGAGAAAAATATGTTTGGAGTACTTTTAGTGCTGA
TCAAATAGATGTTAATTTTGCTAATTCTGACGTATTACTGGAGTATATCAAGATTATTATTTTTT
ATATTGAACTAGGAATTAAATACATTCGTCTTGATGCAGTGGGATTTCTTTGGAAAAAAATTGGC
ACAACTTGCATTCATCTTCCTGAAACTCATACTATAATTCGCCTGATTAGGGAAATTTGTCAAAT
GCTAGATCCCACCATTGCTATTATTACAGAAACAAATGTTCCTAACTGGGAAAATTTGAGCTATT
TTGGGAACGGCAATGAAGCCCACATGATTTATAACTTTAGTTTGCCTCCCCTATTATTAAATGCT
TTAATACAAGGAAGATCAGACCATCTAAAAACATGGATGATGAGAATGCCCCCTGCCCCTAAAGG
ATGTGCGTATTTTAACTTTACGGCTTCCCATGACGGTATTGGTTTACGCCCGGCAGAAGGGTTAT
TAAGAGAGGAAGAATATCAAACCTTATTGACAAAAATGCAGGAATTTGGCGGGAAAATCAGCTA
TCGTCAACAGCCTGATGGTAGTGAATCCCCCTATGAAATAAATATTTCTTGGTTTTCTGCCATGA
AAGGTACAATGGAAGGGGAAGACCAATGGCAGATAGAAAGGTTTTTATGTTCTCAGACTATCAT
GTTAGCTTTGGAAGGAATTCCAGCGTTTTATATTCATAGTTTGTTAGCAACAAGCAATAATGAAG
AGAATGTGGTCAAAACGGGGCAAAATCGTGCTATCAATCGTCATAAATGGGATTATCAAGAGTT
AAAAGCACAATTAGAAAACCCTCAATCTGACCAAAGTATTGTTTTAAACAAATTATCTCGTTTAA
TCAAGATTCGTCGTCAACAACCTGCTTTTCATCCTAATGCAACCCAATATACTTTACATCCCTTAA
ATCGGGCATTATTTACATTCTGGAGACAAAGTAGCGATCGCGCCCAGAGTATTTTCTGCATTCAC
AATCTTAGCAAACAAAGACAAAATCTAATCCTATCTCAACTTAACCTGATTGATACAGAAAAATG
GTTCGACTTAATTAGTCATAGTGCGATCGAAAATATCCATGATAAATATACTTTACAACCTTATC
AATGTCTTTGGATTACAAACTATAGAGCCTATAAATAACCTGAGTCTGGATAAATTTTCAGCAGA
TTTAGAGCAAAGGGCAAACCCCCCTTATCCCCAGGGCTGTTTCATTTTCGAGAAAGAAAAAAGAG
AGGGAGATGGGGGGATTTTTGTTCATATAATTCTTGCTTTTTAAGATAGCTTAATCGTCCTACAA
TCATCAAATGAGTTTCTAGTTCTTTGAGTGAGCTTTTTCCCAACCCTCACCAATCCCTATTAACCT
CCGAACACCTGAAACCTCATTCCAAACTTCGCCCCCCTTTTACATCACTAAAAATTTCGGATCAAA
AACTTAGTGCTATTAATTTGCAATACTGTTAAAATAGATTTTCAGTTAGTAAAAAATCTAAAGT
ATAAACATATACAAAAGTAGAAAATGAGTAAAATTACTAGAAGAGTTTTTTTAGGCACAAGTGC
CGCTATTGGTGCGGTAGCGCTAGGGGAATTAACTGGAATTAAAAGTTCTTCCGCCCAAGCAAAAC
AAATTAATCTTTATTCTTCTCGTCACTACAACACAGATCAAAGACTCTACACTGATTTTGAAAAG
CAAACAGGTATTAAGGTTAATTTAGTAGAAGGTAAAGCAGAACAACTATTAGAAAGAATTAGAA
GTGAGGGCAGAAACTCCAAGGCTGATGTATTCTTGACTGCTGATGCGGGTAATTTATGGCAGGCA
CAACAAGCAGGGATTTTTAGCCCTGTATCTTCTTCAGTTTTAACTCAAAAAATCCCTTCTTATTT
TCGAGATCCTAATAATAATTGGTTTGGTTTTAGTAAGCGTATTAGGGTAATTATGTATAACAAG
AGTAAAGTTAACCCTAGTCAATTATCTACTTATGAGGATTTAGCTAATTCTAAATGGCGCAAAAA
AATTGCTATTCGCTCCTCTAGTAATATTTATAATCAATCTTTAGTTGCTTGGTTAATTGCTATTA
ATGGGCAACAAAAAACAGAGGAATGGTGTCGGGGGATTGTAGCTAATATGGCTCGTCCACCCCAA
GGAGGCGATCGCGATCAAATTGAAGCAGTAGCGGCGGGAATTGCGGATTTAGCGGTAGCCAATAC
TTATTATTTAGCTGGTTATGGAGCGTCTAATGATCCTCAGAAAAAAGCTATTTTCAATAAAGTAG
GTGTTTTCTTCCCAAATCAAAAAGGTAGAGGAGCTCATGCCAATATTAGCGGTGGTGGGGTACTT
AAAAACGCTCCAAATAGAGCCGGTGCTGTTAAATTCTTAGAATATTTAGTTAGTCCTAGCGCCCA

AAGATTTTTCGCTCAAGGAAATAATGAATATCCTGTGGTTGAAGGTATTGCGTTAGATCCTGTAT
TGGCTAGTTTTGGTAGTTTCAAGTCCGATGTTACCAGTGTTGCTAAATATGGTCCTAATTTAGCT
AATGCTGTGAAAATTATGGATCGTGCAGGATGGAAATAGTCAAAAAATTGATTTATTTATACTC
GGTGAGAGAATAAAAGATATTAGTAATCAGCAATTAGCAATTAGCAATCTTCTGCATTCATCATT
ATAAAATTATTGACAAAAACTCAGTGACTGGCTTTCTTTAAATCTATTTTCACATTAATTAAATC
TTCAATCGTTGTCACAATTTTATGTAATTGTACTGGTTTCCATGAAGTGCCACAGTCGAGACAAA
CGATTGCTTTTCGGTCATCCCCTGTCACAGCACCTACTGCACCACCGACAACTCCAAATAAAGCCC
AACCAAGGGCAGCTCTTCCCCAATCCACATTTGTTTCTGTGGTTAATTGAATATGCTCAGAACCA
CAATTAGGACAATTTGCCATAGCCAAATCTCAAGAAAATTCCGTTTTCGACAATAATTTTACTTA
ATAGGTATGTTTCAAGATTGTTAATTTTTATTATGAAGTAATTTCATGACAAAAATGTGTGTAG
AGTGCGATCGCTTTTTCAATTACTTATGATTTAATTTAACAATAAGCATAATAAGCTAAAGTAGT
TAATTAACATAACTTTATAAAACTTCTCATAATCAATTAGCTGAACATGAAAAGCCGATTTTTAG
AAAGACTACACAGCAGCGATCGCCCCGTGCTAGTATTTGATGGTGCGATGGGTACAAATTTATAG
TCCTTAAATCAATTCCTTGTCAATTTATAGTTAAGATTAGATTATTATTGTAAATTAATCAATTC
AACACTCTTAGAATGAAATATCAACAACTAACGGCAATTATTGAAAAAGAGGGAGATAGTTATG
TTTCCCTTTGTCCTGAATTAGATGTTGCTAGTCAAGGTGATACGATCGAAGAAGCTAGAAATAAT
CTTAAAGAAGCCTTAGAATTATTTTTTGAAACTGCATCGGCTCAAGAAATAGAAAAAAGATAAC
ATAATGAGATGTATATAACCCGTTTGGAGGTTGCCGTTGGGTAAATTACACAATTAATTCCTTGC
ATAATTTTATCCTTCCTTAATATAATGGCTCAAAAAAATTGATAAATTTTAAATATCAATAGTTT
TTAGAAAATCGATATTATTTCTTTGATTAACACAATAAATATTATATTAATAATAATTCTGTAT
ATTTGTTATTACCATTAGAGTACATAGTAAGTATTTTCTTGACAGAAGAAGATGGTAATTTCTCT
GGATTATTTTCTCCATTAGTATTAGATACCAGCCGATGATCACCATAGTTATTTTCTGACAATTT
TAAAAATAATGTTTCATTATCTTTATCAGAAAGTGCAAAACAATAAGCCGAAATTAATGAATCT
AAATCATTCTGATGTATATTGGCAGTATAGTAATTAAAATTTTTGGGTTCAGGCTCAAAACCAAT
AGCCTTACGGAAAATGTTATTTTTAACTAGATAAATCGAAACGGTTCCAATATTACTTCCTATAT
CAATAAGGCAATTATTGTTTTTTGAATTTATCTTTCCCAAAGTTTGTAATAATCGTATTGTATTA
TCAATACTAGATTTTTCAAATAAACGCCAAGTATATAAACTTTTTCCAATAATATTATCTTTGGA
ACTAAAACTATATTTTCCTTGCAAAGTATCACAAGTGATAGTTTGTTCACTTCCTTGATGAAATA
ATGGCCATATTATTGTTCTTATATTACCCATTATTTTTCGCTTAAGTCCGATGATTTTTCTCCCA
ATAATGTTGCTCATGAAAATCATAGTTAGTCAATACTCCTGTTTATTAAAATTTATTTGCTCAAT
TTTTTCACGGATAAGTTCTAACTCATCACTTAAAAGTTTAGGTGTTTCATTAAAAATATTAGCTA
TTTTTAGAAACCGCCAACGCCAATCAAGTTTTTTTAAAGCATGATAATAGTTATTTTGTGCTTTC
TCTGGAGTCCAAGATTGAACTGCATCAATTATAACTTTTAGTCCTTCATTACGATCGATGGTATT
CAAATCCAATAGCGAACCTTCCCATAATAATTCTTGGGTGCAAAAACAGTTTGGAGGAATACCGG
CTACGGTTGCTCCACAAGCAAGAGCATCAACCCAACGAGCAGTGATATATTCTTTAAGGGGATGA
GTATAATTACTAGGACTTGCTAAATTGGAAAAAGCTAATAAAAATTTACTTTGAGAATATATTT
CCATAATCTTTTGCTGATTAACTAAAGCATCATCATAAGGCTTAGGTCTTCCTTGAAATTTAAGA
TTAAATTTTTGACACTCAGACAAAGTTGCTTTATCATCTTCCCATTGTGGGGGTTGTCTTCCCAC
TCTGGTCAAATCGACGATTCTATCAGAATTATCAGAACCTAAACGAAGAACATCAGCTCCCCATG
GTAAGTACGTTGTTGGAGTTTTTGTTTGTTGTTCCCATTCGTTAACATCTTCTTTCCTCATCACA
AAGAAGTGGTCAAAATGTCTTGTCATTTTTATCGATTTAGGAATACGATCAACCCAAAAAGAGTC
GATTATCCAAGCCGCTATAAAACGAAATCTTTTACGCCAATTCATAATTTCCATTATTGATAGTA
AAGAAGTGGGTGAAGGACAAATCAACAAACAAGATTCTTGAGACAACTTACTATTTAAGCGAGG

AAAAAAGATTTTTAATTGGTCAATTTTGCTAAAACTTTTATCCGACGAAACTATTAACAAATCCG
CCTCTAATAATTCCTGAGCTAATTTGACCATATAAGTGATAACAGACCAACCGATACCTGAAGGC
ATAGAATACACAATTAACATAAATTGATAAATTTAATTTAAAGTCTTTGTCTCCCATATTATCTG
AATCTTAAATAAGCTAAAGTAATTAGTTAATATAACTTTATAGACATTCTCATAATCTCAATCAT
CTATGAAAAGCCGATTTTTAGAAAGACTACACAGCGATGATCGCCCCGTGCTAGTATTTGATGGT
GCGATGGGTACGAATCTGCAAGTGCAAAACCTCACAGCCGAAGACTTTGGCGGTGCAGAATACGA
AGGATGTAACGAGTATTTAGTCCATACTAAACCTGAAGCCGTAGAAACCGTGCATCGAGGCTTTT
TGGAAGCAGGGGCAGACGTTATCGAAACCGACACCTTTGGGGGTACTCCCTTAGTTTTAGCAGAA
TATGACTTAGCCGATCGCTCCTATTATCTCAACAAAACCGCCGCCCAACTAGCCAAAAGATTAGCC
GATGAATATTCAACCCCAGAAAAACCCCGTTTCGTGGCAGGAAGCATGGGACCTGGGACAAAATT
ACCAACACTAGGACATATCGACTACGACACCCTCAAAAACGCCTATGTAACCCAAGTACAAGGGT
TATACGATGGTGGGGTTGACTTATTATTAGTCGAAACCTGTCAGGATGTCTTACAAATCAAAGCC
GCCCTTAACGCCATTGAAGAAGTATTTGCCGAAAAAGGAGAAAGACTCCCTCTCATGGTATCTGT
GACGATGGAAACAATGGGTACAATGTTAGTGGGTACAGAAATTAACGCCGCCCTCACCATTTTAG
AGCGCTATCCTATCGATATTATGGGGCTAAACTGTGCCACTGGTCCTGATTTGATGAAGCCCCAC
ATCAAATATTTGAGCGAAAACTCTCCTTTTGTTGTCTCCTGTATTCCCAACGCAGGATTACCCGA
AAACGTGGGCGGACAAGCTCATTACCGCTTGACACCTATCGAACTACGCATGGCGTTAATGCACT
TCATCGAAGACTTAGGGGTTCAAATTATCGGCGGGTGTTGTGGAACCCGTTACGAGCATATTAGA
GCCTTAGCAGAAATCGCTAGTACATTAAAACCCAAACAAAGACAATATCATTATGAGCCTTCGGC
GGCATCCATTTACACCACTCAACCCTATCAACAGGATAACTCTTTCTTAATTGTAGGGGAAAGAC
TCAATGCCAGTGGCTCGAAAAAATGCCGTGATTTACTCAATGCCGAAGATTGGGATGGTTTAATT
TCCCTCGCCAAAGCCCAAGTAAAAGAAGGGGCGCACATCCTCGATGTTAACGTGGATTATGTGGG
GCGCGACGGGGTAAAAGATATGCACGAATTGGCTTCCCGTCTGGTGAATAATGTTACCCTCCCCT
TGATGTTAGACTCCACTGAGTGGCAAAAAATGGAGGCAGGTTTAAAGGTTGCAGGGGGTAAATG
TATCCTCAACTCTACCAACTACGAAGACGGAGAAGAACGATTTTTCAAGGTGTTGGAATTAGCCA
AGGAATATGGTGCAGGGGTGGTTATTGGTACTATAGATGAGGATGGTATGGCACGCACGGCGGAT
AAAAAGGTGGAAATAGCCCAACGTGCTTATAACGATGTAGTTAAATTTGGTATTCCCGCCCATGA
AATCTTTTTTGATACCCTTGCTTTGCCCATTTCTACGGGGATTGAGGAAGACAGAGAAAACGGTA
AAGCAACGATCGAATCTATCCAACGCATCCATGCAATGTTCCCCGAATGTCATTTTATGTTAGGG
GTGTCGAATATCTCCTTCGGGTTGAATCCTGCGGCGAGACAAGTGTTAAACTCGGTGTTTCTCCA
TGAAGCCATGCAGGTAGGGATGGACGGTGCGATCGTATCTGCTAGTAAAATATTACCATTGGCAA
AAATAGACGAAGAAGCGTTAAAAGTATGCCGTGATTTAATTTATGATTTAAGAGAATTTGACGG
TGATATTTGTACCTATGATCCATTAGGAAAATTAACCACCTTATTTGAGGGTAAAACCACTAAAA
GTAATAAGAAAAATGTGGCAGATTTACCCATCGAAGAAAGACTAAAACAACATATTATTGAAGG
GGAAAGAATCGGCTTAGAAGATGCGTTAAAAATAGCCTTAGAAAAGTATCCTCCCTTAGATATTA
TCAACGTCTTTTTATTAGATGGGATGAAAGTTGTGGGAGAATTGTTTGGTAGTGGACAAATGCA
ATTACCCTTCGTTTTACAATCTGCTCAAACAATGAAAGCCGCCGTTGCTTTTCTTGAACCATTTAT
GGATAAAGCGGATAGTGATGGTAGTGGAAAAGGTACATTTTTAATCGCTACGGTGAAAGGAGAT
GTTCACGATATTGGTAAAAATTTAGTGGATATTATTCTGTCTAATAATGGCTATAAAGTGATTA
ATTTAGGTATTAAACAACCCGTAGAAAACATTATTCAAGCCTACCGTGAATCGAACGCAGATTGT
ATCGCTATGAGTGGTTTATTAGTAAAATCCACTGCTTTTATGAAAGACAATTTAGAGACATTTAA
CCAAGAGGGAATTACTGTGCCTGTTATCTTAGGGGGAGCGGCATTAACTCCAAAATTTGTCTATG
AAGACTGTCAAAAAACCTATAAAGGAAAAGTAGTTTACGGTAAAGATGCCTTTGCTGATTTGCAT

TTTATGGATAAATTAATGCCCTCTAAACATAGCAATCAGTGGGATGATTTTGAGGGATTTTTAGG
AGATTTTGCCGAAGAAAATCATCTCTTTAAAGGGAGAAATTTTGATGAATCAGGGGAAGAAAAA
GAAAAAGTTAAAAAGGATTCTAGCCTGTCAGAAAAAATAGAAATTGATACCCGAAGAAGTGAAG
CGGTTGACCCAAATATTGAACGTCCAACTCCTCCTTTTTGGGGAACAAAAATCATGTCTCCTGAT
GAGCTTAATTTAGATGAAATTCTCTGGTATTTAGATTTACAAGCACTGTTTGCAGGGCAATGGCA
ATTCCGTAAACCTCAAGGGCAACCTAGAGAGGAATATGATCAGTTTTTGCAAGAAAAAGTTTATC
CTATTTTAGAGGAATGGAAAGCCAAAATTAAAGCGGAAAATTTATTACATCCTACTCTAGTTTAT
GGTTATTTTCCTTGTCAATCGGAGGGGAATACTTTATATATTTATAACCCGGAAAATCCAGAAGC
GAAGGAAGAAATTGCCAAGTTTGAATTTCCCCGTCAAAAGTCTGGTAAAAGGTTGTGTATTGCGG
ACTTTTTCGCCTCTAAAGATTCGGGCGTAATCGATGTTTTCCCTCTCCAAGCAGTGACAGTGGGA
GAAATTGCCACAGAATACGCTCAAAAACTGTTTGCCAATGATGAATATACCAATTACCTCTATTA
TCATGGTATGGCAGTACAAACGGCGGAAGCGATGGCGGAATGGTGTCATGCAAGGATTCGCCGTG
AGTTAGGTTTTGGACATTTAGAGCCTGACAATATCAAAGAAATCTTTAAACAACATTATCAAGGT
TCACGGTATAGTTTTGGTTATCCTGCCTGTCCGAATATGCAAGATCAGTATGTACAATTAGATTT
ACTAGACGCTAAACGGATTGGTATGTATATGGATGAGTCAGAACAAATATACCCCGAACAGTCCA
CAAGTGCGATCGTTTGTTATCATCCTGTAGCTAAGTATTTTAGTGCTTAGAAGTTTTGGTGGGCA
ATGCCCACCCTACATGAAAAATAAATATAATTAAAGATGAAATTTGAATGGGATGAGAATAAAC
GTCAAACAAATATTACTAAGCACGGTATAGATTTTATCGATGCTCGAAAAAATATTTGATTATGAC
ACGGTTACAATAGAAGATAATCGATTTAATTATGGTGAACAAAGATTTATTGCCATTGGCTTGTT
AAATGGTAAAACCATTGTTGTTGTTTATACAGAGATAAGTAATAAAATTAGAATTATATCAGCA
CGAAAGGCAACGAAAAATGAGCAACAAATTTACTTCAATGAAATCTATGACTGATTGGGAAAAA
CTAGAATCAATGAGTGATGAAAATATTGATTTTTCCGATTGCCCTGAAATTACTCCAGAAATGGC
TAAAAATGCTGTCATTAGACGTGGTTTAAAATCTCCAGAAAAACAATCTTTAACGCTTTTTCTTG
ATGCTGAAATTGTGAATTGGTACAAAGAAAATGAGATTAAGCATCAAACAGAAATTAATGATTT
ATTGAGAGATTATATTAAACCTCATTAAATGGGTCTGTTATCATCCTGTGACGAAGTATTTTAGT
GCTTAAGTTCGTAGTAAGCCCTTTAGGGCTTTTTAAGAGCTAAAGCCTTCATTATGAACCCGTTT
GTAGTAATCTCTTTAGAGGTTTTAAAGGGCTAAAGCCTTCACTACGAACCTGTTTGTAGTAACCT
CTTTAGAGGTTTAATCATCCCATCCTTTACCACAGTCTCTTACAGGGTATTTCACCCAACTATCCC
TTAACCACTCTCTACCTTTCTCCTCTAAATACCAATGTACACTACTTTCTAACCAATCATAGGGAG
AATTGACGTAGTTATGTTTCACGGGATTATAATGTATATAATTTAATGTTGTATAGTAATGTCTT
TCGGAACGAATAGCTCGATCGCTAAAACGATACCATACTCTACGCCCTCGTTGATTATCTTCTAA
ATTCCATTGATAGGAAGTAACACCGTGAATAGCTTTAAAAATTGAAGTGAGAATTTATAAATTA
GACAAATATACCAATAAATGATAATGATTCACCAAAATTACCCACCCCATAATATCTATTACTTC
ATGGATACATTTTTCAACAAGAGGTTTAATAATTGTTGACGGCGAGAAGAATTATTTAAAATC
TTTTGATGTTGATAACAGGCAATTGTAAGCAAATAAAAACCCTCAATTTGTATGGGATGAGGAG
GGGAATGGGAGGGAAATCCCCTTTTAAGTCTCTCTTGTGCTAATTCTAATCTTTGTTGAGGAGTT
AATTTATGATACTCATACATAATTCTCGTTCGTAGTAATGGCTTTAGCCTTCATTCTACCGATGA
TAATGCCACTAAAGTGGCAACTACAAACTTTTATTCTTTATTTTCAACTTTCTCAATTAGAAGTT
TCGATCGCCCAAGCCCAACCCACCGCAAGTAATCCTATGATTGATTTATTAACAAAGGCTATACA
ACAAAATTAACTCCTGAATTTAAAATCTCTATTTCAAAATGAAAAAACAAGAATTATTTTAGGA
GATGCTATCACATTATTAAATGAATATATTGAAGATGAATCGATTTAATATTTGTCGATCCTCCT
TATAATATCGGCAAAAAATTCGCCTAACTTTCAAGATAGACGGGAAGATGAAGGAAACTATATA
TTATGAAGTTATCATTCTTTATTAAGAGATTATATTAGAAATAACCAATACATTAAAAGATTAT

TTAGGGTAAGATTAATATGCCAGTACCAGCTGAATACGCAAGAGCTTCTGACAAGTTTTATGATT
ATCTTATAGATGCAAGGGATACCGCAGGACTCTGGAGTACTCATGTTACATACACCATGACCCAA
GCCGTTTTTCAGGTGTTTCGTCGTCGGCTTTCCACAAAAGATTCGATAGCATTTGCAAATGTTCT
ACCTATTTGTCTTCGAGCCTTGTTTGTCACGGATTGGGATATTAACGAGGAAAAAAAACCATTTG
AAAACCGTGACGTTATGACAGAAGAAGTGAAATCATTACGTAAAGACCACAATTTTTCCACAGAC
ACCGCAATTCAAGATGTAGCTAGGGCATTACGAAGACACGTGGACGAAGAGGCATTCGATAAATT
ACTAAAACAACTACCCAAGGGGGCAATTGAATTTTGGAAACCATAATCAAAACCTTACTTTCCCC
AAATAATCAAAATCAAAACTTGACAGGTGGCGGGTAATAGTTGATATTTGAAGGTTGTTAATTC
CTACTCATCTTGACTCCTAAATATTTATGGAAACCCTTGAATTATCTATCATTATGCCTTGTTTG
AATGAGGCGGAAACCCTTGCTACTTGTATTGAAAAAGCCCAATGGTATCTTCAAGAGTATCAAAT
TCAGGGGGAGGTGTTAATTGCGGACAATGGAAGCACGGACGGCTCTCAAGATATTGCAAAAAGGA
TGGGAGCGAGATTAGTTAATGTCACCGAAAAAGGTTATGGAAGTGCTTTGATGGGGGGAATAAT
TGCGGCTAAAGGTGAGTTTATTATTATGGCGGATGCTGATGATAGTTATGATTTTAGGAGTTTAA
ATCCTTTTGTAGAAAAGTTGAGAGAAGGCTATGATTTAGTTATGGGTAATCGTTTCAAGGGGGG
AATCAAACCGAATGCGATGCCTTTTCTTCATAAATATTTAGGTAATCCTGTCTTAACTTGGCTAG
GTAAGCTATTTTTTAAAAGTCCTTGTAATGATTTTCATTGTGGCTTGAGAGGATTTCGTAAACGG
GCAATTATGAGCTTGAATTTACGCACCACAGGTATGGAATTTGCTTCAGAAATGGTTGTCAAAGC
GACTCTTAACGGCTTAAAAATAACTGAAGTACCAACTACCTTATCACCTGATGGACGTTCTCGTA
AACCCCATTTACGCACTTGGCGTGATGGTTGGCGCCATCTTCGTTTTTTGTTGTTATATAGCCCCC
GTTGGTTATTTCTTTATCCCGGTTTTTTCTTGATGATTATGGGATTAATTAGCACTATTATTCTT
TTAGCTAGTCCAAGAGTTCATAGTTTATTATATTCAGCCACAGCTATCATTATTGGTTTTCAGCT
AGTTAATTTTGCAGTTTTCACTAAAATTTATGCTATTCAAGAAGGCTTATTACCTAATGATAAAA
GAGTACTTAAATTTAAGGATTTATTTAGTTTAGAAATTGGTTTAATTATTGGTTCAATTTTATTT
ATTGCTGGTTTAATTACTTCTATTTTTGCTCTTTTTCAATGGGAAGCTGTGGGTTTTGGGGCATT
AAACCCTGTCCAAACTATGCGTTTAGTTATCCCCTCTGTAACTGCGATCGCACTCGGGTTACAAAT
TATTTTTGCCAGTTTTTTCCTTAGTGTTTTCCAGTTAAAAGTAAAATAGTTAACAGATTTTTATT
AGGAATTAAGAAAAGGGCAAGAGGTAAACCCCCCCCTTTATCCCCGGGGTTTTTCATTCTCAAA
AATGTCAATTTTTGAAACTAACAAATAACCTCAGTTCGGTTTAAGAATATCTGATAAGGTTAGGT
GTTAGGTGTCAGGTTGCAGGTTGCAGGTTTAGAAGAAAGTAATAAGTGTTCGGAGTTTTTAAG
TATTAATTAATCAATTATTAAGAAAAAACGCTCCCCTCTACTGTGGGAGAGGGGGTGGGGGTGAG
GGCAAGATAATTTTGCCCTGACTTTGAAAAAACCCTGCTCTCGAGGGGGAGGGCAAAGGTAAATG
GTTTATAGTTGATTATTGATAATAAATAATAAAAAACATCTGCAATCTGAAACCGGAAACCTGAC
ACCTCTTTCTGAACTCCTAACGTCTTAATCATTGACTTTGTAGATATTGCAAAAACTCAATTAAT
TCTTGATCTGATAATAGATGGCGAGATTTTTTACCGTAGGTTTCTAATAGATATTTTTTACCTTG
ATCTTGAGTCCATCCTAAACGCTTCATTTCAAAAGTAGTTTGATCTATAATTTGACTGAAATCCA
TAGTTTCATTCGTATCTAAAGATAAAGGTAGAGTGGGGCTTTCTTCAATATCATTTGTTGGGGCA
GAAAGTATTTCCTCTTTGCCATTGTGATTTTCTGGGATTTCTAATGGTAAAGTTTCATCACGATG
ATTTATGGGAGGAGGGGTTATTTCCTCTGGTGGACTTTCATTTTGAAGGGGAGGAGGAGTTATTT
CCGATGGTTTTGTTTCCTTTTGTGGAGACATCGGGGGTTTAATCGGTGGTTTTTGAGGAGGGGCA
GGAGGATGGGTAATATTTTCAGAAAGATTCGTCTTTGTGGAGGGGGAATTTGTCGGAGGTTTCGA
TTTTTGTTCCACAGGAGGAGGAGAGGTTACTTTATTGGTAGTGGAAACGGGAATATTCTGCACAT
TATGCACATTAACAGATTCATTGGCAAAGAGAATCGCTCTTTTTCTGGCATTGTCTTCCGCTTCT
TCTACGGTATAACCTTCTGAGTGGGCAGTGCTAATAATTTGCTGATTTTGATGGACACACACTTT

CACAATGTACTTACCATGCTTAATGTTTACTAATTCACTGGTCAAAATAAAGGGATAGTTAGCCA
TATAAAATATTATTTATCAATAAACTAATACAATTTTACTATATATTAAGGAATAATTGGAAGA
AAAGACAGAATAGGTTATATTTATAGATTAAGACTAAAATCTGTTGAGTCAAGATTAGTTAAAC
CAATTTTAAATACTTTTAGAGATAAGAATGAATCCTTTGATAGATCATTTCTGTTCACAACTAAT
AATCTTTATTACACTTAGTTTTTGTTCAATGGATACAAAGAAATAATAACCTTTGCAAGTTAAA
TTGGTGTAATCGAAGCACAGTTTTCTAAGGGTGAATAATGTTTTATCTCTATCAACTCCTGAAAA
TTGGTCAAAACTAATACAGGGGGAGGCATCTTCTCACTGACCTCAATTTTTAGATATAGGTTCAT
GCTATCTCTCTCTTTGTACTTGACTCAATGTTCATTGACATTACCATTTTAGATATAGAAAAGGA
TATTATAGTAAATGGATTTTTCAATCGCTACAATTCTTTCCCTTTTGAGTCCCGATAAATTAGTG
GCGGGGAAAGTAATCGAAAAAAAATTAGGCTGTGAAGACGAACATGAAATCGAAAAGTTACAAA
TCGCCCTAGATATTCTTGAAAAAATAGGGGTTGTAACGAAGGAATTTGGCAAGTATCGCCGTGTC
ATTGAAGAAGATGTGGTGGAAGCCAAGTTACGTTGCTCTAGTAAGGGTTTCTGTTTTGCTATTCA
AGATGAGGAAGATGCTGACGATATTTACATTCGGGAAAGTCATCTTAGCAATGCTTGGAATAGCG
ATCGCGTCTTGGTTAAAATTATTAAAGAAGGTACGAGAAGAAGATCTCCTGAAGGAGAGGTAAA
ATTAATTTTAGAACGGGCAAACCCTTCAGTTTTAGCCAGAGTGGTGGAGGATGAATCGGGGGATT
ATTTAGCTGTTCCCCTAGATGATCGCCTCTTGTTTGAGTTACAGTTGAAGCAGAATGGGCAAACT
CTTGATGATGCGATTAATCATCTTGTCCATGTTAATGTTTTAAGATATCCCATCGGACAAAACCC
CCCAGTGGGAAAAGTTGTCAGAGTGTTGGGTAGTGATGCAGAAGCCGCCGCCGATACTGATATTG
TTTCTTCAAAACATGATTTACCCCAAGAATTTCCTGAAAGAGTCCTAAATCAAGCTAACCAAATT
AGTCCAGAAATCAGCCCCGAAGAAAGAGAAAAAAGAGTTGATTTAACAGACCTTCTCACGGTTAC
TATTGAGCAAGATATTGTTCAGGAAAATAATCTTTTAAAAGAAACGGCATTAAGTCTGGAAAAA
ACAGAAGCTGAAAATTGGTTGTTGGGGGTTCATATTGCCGATGTTGCCCATTTTGTGCCAGAAGA
AACCCATTTAGACCGTGAAGCGAGAAAAAGAGGCACTACGGTTCATTTAGGAGACAAAATTATTG
ATTTACTCCCCCCTAGTATCTATGAATGTTGTTCTCTAATTCCTAATAAGGAACGTTTAGCAATT
TCTATTTTTCTGACTCTTGATGATAAGGGGCAATTATTAGGCTACGAATTTAAACAAAGTATCAT
CAAGGTTGATCATCAACTTACCTACAAAGAAGTACAAATGATGATTGGTGCAGGGAGTACCAAAC
CTGAGTTAGAGTCAACTCTGGAGATGTTAAACAATCTTGTTTTTAGTATTACTCCTCTGATCAAA
GCTAGACGTTTACAGTTAGGCGGTTTTGAGATTAATTTAGATGATATTACCTCTTATTTTAAAGA
TGAAGGAAGAATAGGTGCGATCGCATCTTATTCCACTTTGCCTGTACTATCTCTAATGACTGAGT
TAATGACTCTTGTGGGAAAAGTAGTTGCAGAACACCTTAACGAATTGCAAGTCCCTGCTATTTAT
TGCACTCAGGGTAAACCCGATTGGGATGAATTGGAAGATTTACTTAAATTAGTTGCCAATCTCAA
ACTAGACTTTAAACTGCAATCAGAAGACGAAATTCAACCCTTAGACTACTATCATTTAATTCAAG
AGTTTAGTAAATCAGATGATGAAAAAGTATTACATTACCTCTTACTTAATTCTCTAAAACCCAAC
AAATATACCCAACATCCAGCCCCTCATTTTGGTTTAGCCTATCCTATCTATACTCACTGTACTTCC
CCCGGACAGAGATATATTGATTTGCAAATCCAAAGAATCATTAAAGCCGTATTTGAGCAAGGACG
GGATAGACGTTCTAGTCGTGTTACAAAGGGAGTAGATTTATTTAGTAATAGCTGTCATGGACAAA
TTAACTGGAATGTTTTACCTCCGGCGGTACAATCCAACTTAGAGGCTGATTTACATTCTCTGATT
ACCCATTTAAATGAAAGAGAAAAAAATTGCCCAAGATGCGGAAACAGACTTAGCAGGATTGAAAA
AAGCGGAAAAAATGAAAGACTGTACAGGGCAAATCTTTAATGGTTTGATAACTGGAGTACAATC
CTATGGTTTCTTTGTACAAATAGAAGATACTTTAGTTGAAGGCTTAGTTCATGTTAGCTCACTCA
AAGATGATTGGTATGAATATCGACCCAAACACACTTGTTTAGTAGGCAGAAAAAACCGCACTGCT
TATCGTCTCGGAGATAAAGTGGAAGTGGAAGTTAAAAGCGTTGACTACTATCGTCAGCAAATTGA
TTTAGTCACGGTGCGTGGGGGTAGTGCCGCCGTTGATGAGGATTTTGAAGACTAAGTAACCTCAG

TTCGGTTTGAGAATATCGAATAAAGGTAGGTGTTAGGTGTCAGGTTTGACAGTTTCAGGTGGTAG
GGGTTGAATATATTCAACCCTTATGGTGTTTAGGGGATGAGGACAGATAAAAATTTATCCCGAAC
TCAGGTTAGCCTAAGCAATTCTAATCAAAACGCACTCTAGGATCAAGCCAAGAATAAGTTAAATC
AGCAATTAAGTTGAAAATGACGATCAAAATTCCATAAATCATCGTAATTGCCATAATCACAGGGG
TATCACTACGGTAAATAGATTCGATTAACAAAGCACCAATACCGGGGATACGAAAGATTTGTTCT
GTTACCAATGCTCCTGTAAAAATACTTGGTATGTCTAAAGCTATTAGGGTAATGACGGGAATTAA
GGCGTTACGCAATACATGATTGATAACTACTTGATAAGGATTTAAGCCTTTACTATAGGCGGTGC
GTACATAGTCATGATTTAATTCTTCTCTAACGCTCGATCGCATGAATCGCATTAATACGGCGGTT
TGATATAATGTTAGGACGATAATAGGCATAATTGACTGTTTAATTTGAGCGATGAAACTAGCCCA
ACTGTTAACTACCAAGTTACTGTCATAAATAAAGGGTAGCCATTGCAATTTAACACTAAAAATAA
TTATTAATAATAATCCCGTGAAAAAAGGCGGAATCGAATAACCGAAAAGTGCGATCGCATTACTG
AATTTATCAAACAAAGAATGACGTTTCAAAGCAGAAAATATACCCAAAGGAATAGCAATTACAA
CACTTAATAAATAAGCTAAACCGACAATAGATAAAGTATTAGGTAAACGTTGCCAAATTAAATC
AGATACAGGGCTTCTACTGGTAAAAGAATAGCCCAAATTTCCTTGTAAAAATGCTGTTAGCCATT
TAATATAACGTATATAAATAGGTTGATCCAAGCCAAAGGATTTACGGATATTTTCCCTAACCTCT
GCCGTTATATTTGGATTTGAAGCAAACTCACTTAAAGGATCTCCCGGTGCTAATGCGAGAATAAT
AAAAACAATAATACTAATGGCTAATAGAGTAGGTAAAGAAATCAATATTCTTTTGCCCAAATAA
ATCATATATAGTAATCCTAAATTATGAGTGATGGCAAAAAGGGCAATGGGCAAGGGGCAAAGGT
AAATAGTGAATAGTGAATAGTGAATAGTGAGTAGTTGATAATTATTCGTTACTCATTACTCATT
ACTTTCTTCTAAAACCTGAAACCTGAAACCTGAAACCTGACACCTTATCTCCCTCTCCCCTCATCC
CCCCCATCTCCTAAACACCCTGACACCTGCAACCTGCAACCTGAAACCTACCCTTATCTGATATTC
TTAAGCCGAAATGAGTTTAATTACGTCCGCTAGACTGAGCAAGTCTAATCATTTCTGGATTATCC
ACATCAATTTCATATACATAACCCACACTTTGGGCCACACGGCGACGAATGCCATAGGCAGGATA
TAATTCCATGGGGAGTAAGCTAGTAGCCCTAACATTGGCAATATCTACCAACTCTAACATTACAT
CACGACTTATTTGATTACCAAAAACCTCTTTTACGTTTTGTAAACAAGAGTTATCACCCACAGGA
TTTCTTCCTTCATATAGTAATAACAGCGCATCTAAGCAATTGTTATAGCCATAAATATCGACAAA
ATCAATGACGACATCAGCGATACGAGGGTCTGCATTACTAGGATTAGGATTAACGTAACCCTCGG
TAACGACTTCTTCTATATTTTGACTTGCACCACGAGTATTGGTTTGACTAACATTAGCTGTATTG
CTATCAACATTAGTGTCAGGGCGAATTTGATTGGTTACTCTATCATATACATAATTACCTGAACT
TATTTGTTGATTTGGCTTAACACAAGCTATTTCTTGAGTGTTTAAATCTCTGATACTAACCACAT
TACCATCGCAATTGCTAATAGAGAAACCCCAGTCTATAAGTTTTTGTTGAAAGGGTGAATTATTA
TTATTAATAATAGTGGGAAAGGATGAGTTTTGAGCTTGTAAAGGACCAGAAAAGAGAGTAAATA
GAGATATATTTGTTATTAGTAAATGTTTAATTTTCATGAATCTTGTTTATTTTTTACTGGCAAAA
AAACTAATATTAATTACTAATCACTAATTACTAGATTTTTATTACTTTAAATAAGTTAATGGAAT
TGCGAATTTGAAATATATATAGCAATTCTCGTTATGGTGAGGTACAAAACTAATTGGTATTAGA
GAATAGGAAATCTGAACTTTAGCAAGTTTTAGGTGCAAATTAAATGACAAATGAAAAAAGAATT
AGTTTCTCCCTAAACCTCCAATCCGCCAAAAACCCATTTCCCAGACACATCATACTTTAATTCAAC
AACCCCATCTTAATCAAGAGAAGGTATTTCTAAAGAAAAGTTCCCAGAAGGATGTTTACTGTTTC
CTTCTGCACACATTACATATCGATTACTCAATGTGATAGTAACTTGATGGCGATCGCTTCCACAA
GGGGCTTCTTTAAAATTGTATCCTGCAATAAAAGAACGTAGTAAAGAAATTTTACGAGGGGTTA
GGTTTGCACCAGTTAAATCCTGAAAAGGACTGTTCGAGCGATTATTAATTGCTACACTATCATTA
TTGAGGGGCTGAGGTTTTTTCTCTTCCACCACAGGAGGGGTAATATTTTCTTCTTTCCTCGTGAT
ATTTTGATTATTATTAGATGAAGGTTTTTCTTGACTACCTGAGAAAAAGAAGTCTCCAATTAAAG

AAGAAGTAGTCCAAGGCACTTGAGCATTATCGGTTTTATTCGCCACATCAATTCTCACCTGTTTA
AATATTTCTTCCACATTTTGATTAGGATTTTTCAGGTTTTTGAGCAAGGCTTCTGTAAAAGTTCC
ATTTCTACCTGTACCATCTTGAGCGACATTCCCCGGCCCTGTGGCATAAGCAATAAAAGTACCTG
TCGCACTATTTTGAATAGCGGCTAATCCTTTATTATTAGAAGAACGAGTCCAGCTACGACCGAAA
GGATTATCACGACAAGCATCAAGAATGACGATATTGACATTATTATCCGTTTCTTCCATTGCCGC
TAAAAGTTTTCCTAAAGGTAAGGTTTCATAGTTAACTTCGCTTTCCCTGTCTAAACGAGCATCCA
CTGGAATTAAGTAATTTTCCCCATTGACTTGAATACCATGACCTGCATAATAAAATAAACCGACT
GCTCCTTTCTCTAATTCTCGGTAAAATCTTTCTAAACTTTGATCCATTGATTTTAAGTTACCATT
GGTGAGCAGAATAACCTCAAAATTTAAGTCTCTAAGACTTTTCGCTACATCGTTAGCATCATTAA
CCGCATTACGCAATTTACTACCGTGTTGATAGTTAGAGTTACCAATCACTAGAGCAACTCTCTTT
TCTGATGTTTGTCTCTGGCTGATTAAATATGCTTGTTGACTAAGGGAGAAAGATTCTCTTATTTT
TTCATTTATTGAAATCTGATAATCTTGAGAAAAGGCAGAATTACTCATACCTAATATTAATCCGA
AAGATGCGATCGCATAAGGTGTGGCCATACCGAGTGCAGTCGGTATCATCATGGCGAAATTACCA
ATAAAATTAAATTTTTTCATGATCAATAAATCCTTAATCTACGAAATATTTGACTGATATTTTGC
ACTAACTTAACCTCAGTTCGGTTTAAGAATATCGGAGAAGGTTAGGTGTCAGGTGTCAGGTTGCA
GGTTGCAGGTGTCGGGGATAGGAGGATGAGGGGATGAGGGGATTTTTGTTCATAAAGTTTTAAC
TAATAGAAAAAAATCCTTGTATTTCTTACTCTTACTTAATCTCAGTTAACCTAATTTTTTACTTT
GAAACAGCCCTAGGGGATAAAGGGGGTTTGCCCCTTGCCCTTTACAACCCATTAGGGACATAAAA
TGGGTGGACAAGAGGGATTATCTTTGGGTTTTTGATTAGTTTCAGGATTAGAAATAATGGTAGG
AGGGGGAGAGTTATTATTAGATGTGGGTAAATTATCGTTATTATCACTGGAAATAGTGGAAGCA
GTAGCCAAAAAAGAATCTAAATCTAAGACTCCCCCACGAGATAAACCACCAGTGGAAGTACGGGC
AGACATATTGAATAAATCAGTTAACACTTTGCTAGGATTATCTTTCGGTTGAAGAGTTAAAAGTA
AACCGTCTCCCCTACACTTAAAACCTGATGATTTTTTTTCCGCCACACAAATAACGGGCTGTTGAT
TCATTGTACCAGTTGCAATATATCTTAAACTACCACTGTCAGAAAATTGTTGAAATCTTTTTGTT
ACCTCATTACATCTTTTTTGGGGAGTCCACCCAGAATCACGGAAAAAGTCGCTTTGCCAAACTAT
TAGTTGGATTCTTCCCCTTGCAGTATCTACGATTGTTGTGGGTTTACCATTGAGTTGAATACACT
TATAACTATTTTTAGTTTGTGCCAAGGAATCTTGAGAAAAAAAGCCATTAGCAATAGTCAATAA
ACCAAAAATAAAAAATTGAATAAAAAATTGATTTTTCATAATATTTAGAAGTGTTTATCTTCAC
GAAATTTTTAGCCTAAACCTAAGCCTAATTAATTACTCAGGACATAAAATCGGGGGGCAAATAAT
TACATTATCAGGAAGTTCTTGATTATTTTGATTATCTTGATTATTACTAGAATTATCGGTGCTAT
TATTAGTATTAATGTTACTTTCAATACCCTGATCCTGAAAACATTTTTCAGCTTGATCAACTATT
TTTTTCCCTTCTAATAACCAATAATTTGAGCCTTCAATTTTACTCACAGTTTCGATCGCACTTTGA
CAGTTACCATATTCTAAGTCTTTTTGTGCCTGATTAATTAAATAACTATTCGTACGATATTCTTC
TTGCCACTGACTAACAACTATTAAACCTGCTTGACGTACAGGATTATCAGGAATTGTATCAATTT
CCTTTAAAGCATCTTCCAGCTTTCCCTCCTCAGTATATAAATGCTTTGCCTTATCAAAAATAGCCC
TACTCCAATCCTCCATCATAATTTGAGCCTGATTATGATATTGATTCTGGGTTGGAATTTGTTTC
GCCGTTGCTACAGCCTCCGCATAATTGAGAAATTGAGCTTTTTGTTTTGCTTCTTCTAAACGACA
TTTCCCGATATATTCCCCTAACTCTTGAGCAGAGATATGATTATTCTCATCTTGCAATCTTTCCTC
TGCCTTTTCAAAACACTCCAAATAATTTTGATTATTATAAGCAGTATCTAAAGAGGTCAAAAAAT
CAGCTTTTTCTTTCTCAATTTGAGCTTTTTGTTGTTGATTCATCACATATACCCCCCCCGTTGCAA
TAACACCAATCGTTAAAGCAGTAGTCAAAGTTGAACCAAAAGGAGACTTTAACCAATCACTAAAA
GAAAATTTTTCCGAGGTGTCTTCTTGATTTTTCAATGATTGATTTGTTGAGGAAGAAATTGAATT
TATCTTAGAGGTAGAGGTATTAGAATAATTATCTTCTATGGAAGAAGGTTTATTTTTCTCCTCCA

AAATTTGAGTAGGGGGGCAAGACTGTTGCTTTGCATAAATATAAGCGTGTAAATCCTTAATCACA
TCATCGGCTGACTGATGACGCAATTTATGTTGATAGCGAGTCATTTTCGTGATAATTTCGCCTAA
TTTGTCATCAACAGTAACTAAATTACGCCAAAGAATTTCTCCTTCTTCATCCTCCTCTAATTCAA
GGGGATTTTTCCCCGTCAAAGCCTGAATAGCTATAACCCCTAAAGCATAAATATCACTATTTTTT
CTAGGTTTTCCCCTCGCTTGTTCTGTGGGCATATAGCCACGAGTTCCCATTGCTATGGTAGGATCT
AAAAGGCGACTTTGTTCTAAATTAAACTCTTTTACTGCCCCAAAATCAATCAAGACTAATTTTTG
ATCCTCTTGACGACGAATAAAGTTATCAGGCTTGATGTCTCTGTGGATAATGCCTAAATTGTGAA
TATCCTTAAGAATTCCTAATCCCTCTAATAATAATTTAATGGTCGCTACTTCCGTCCAACATTTT
CCAGAAGGTAACTCATGACGTAGAGTTTTTCCTTCGATATACTCTTCGACTAAATAAAATTCTCC
TTTATCTTCAAAATAATCAATTAATTTAGGAATTTGAGGATGATTTAGTTTTTTAATATCTCTG
CTTCTTGGCGAAAAAGTCTTCTTGCCTGTTTGATGAAATTGGGTTGCTCTGAAGAGGGGTGTAAC
TGCTTAATTACGCATTGTTGTTGATTTGTGGTATTTATAACTAAATATACTTTGCCAAAACTGCC
GTTACCCAACTTTTTGATTACTTGGTAAGGAAAAGGTATTAGTGGAAGTTGAGCGGCAGGGTTAT
CCATAAGTCATTTAGGGGTTGATGAAAAAGTGAATTTTTAATTATTAACTATAACCTCAGTTCGG
TTTCAGAATATCCAATAAGGTTAGGTGTCAGGTATCAGGTATCAGGTTGCAGGTGATATTTTTGC
TGAAAAGCTGAGGTATTAATCACATAAAATAGGGGGACATTCGATAGTTGTACCTGATTGGGGCG
GTTTTTTTTCTTCTGGTATTAAGGGAGGTTTTTGAATGGGGGAAACAGCATCCATTAACGGGGCA
ACAGCAAAATATTTGTCTAAATCTAATACTGCTTGACCTCTTCTTATGGGCATTGCTCCGATTTT
AACTGCATCCTCAAATAATTGTTTCATAACTTCTACAGGTTTATCCTTTGGTTCAAGAGTCATCA
ATAAGTTTTGCTCTTGACAAACTATATTTGTAGATGGAGGGGGAGATTGAGGAGATACACAAAT
TACTTGATATTTTCCAATCATGCCATTGGCAATAAATCTTAGTGTACTATTATCGGAAAATTGTT
GAAATCTTTTACTTACTTCTTGACAGCGTTTTTCTGGAGTCCACCCAGAGGCTCTAAAATAGTCA
CTTTCCCAGACAATTAATTGAATTCTTCCCCTTGTGGTATCAACCACCGTCATGGGTTTACCTTCA
TGGTCTATACAACTATAAGCATTTTTTTTACTATCTGCTTGAGCAAAGTTAACAAATATCAGAGA
GATAATTTGTCCAAATATCAAACTTAATATCAAACTAAAAACTAGAGCAAATCGAGGTTTCATGG
TTTTTACCTCATGGATAAATAAGGTTTTAATCACATAATAGGGGTGAGCATTCAATTAAAGGAGG
TTCACTTTTTATTGCTTTAGACTCGGAGGTAACTTCTGTTGTCACTTCTGTTTCCTCCACTTTAAT
ATCAGCAATGTCTGTTTCTGTCATAATTGGGGCATTCTCAATGAAATTAGACATGGGGAAAATAT
ATTTTCCTGTAGGCTCTCTAGTAACGGGAGTGCCTCCAACTTTAGCGGCATTGGTAAAGAGGTTT
TCTAAAACTTTATTCGGATTGTCGTTTTTTTGTAGAGTTATGAGCAATCCATCTTTTTGGCATTG
ATAACCGCCTCCCGGCACTTGTGTGCCCACACAAATAACAGGATATGAATTTATTTGCCCAGTGG
TTACATATTTAAGAATACCTTTGTCAGAAAATTCTTGAAATCTTTGTGTTACTTCTTGACATCTT
TTGTTCGGAGTCCAACCAGAATTTTGGAAATAATCGCTTTCCCAAACTATTAATTGAATTTTTCC
TCTGGAAGTATCCACAACGGTGGTTGGTTTGCCTTTGTAGTTGATGCAACTATAAGTATTTTTAC
TTTGAGCAAGGGTATTTGGGGATAGGCAAGATACGAGAGCAACTGAAGTAACTATACTAGAAAA
AAAAGAGGACTTTTTCATTGTTTTCGGGGGAAAGCTACACTTCCATTATAACTTTTACTCAACCT
GAGTTCGGGATAATTATCAATAGGTGATGGCAAAAAGGGCAAAGGTAAATAGTTGATAATTAAG
AATTAAGAATTAAGAATTAAAAACTCGGAACTCCTGCACGGGCGAATGGCCATTCGCCCCTACCT
TCTCATCTCCCGAAACTGTTTTAATAAACCGCTACTAAAGAAGGCTTGGGAAAAGCATTTTTATC
TAAACTAGGCCAGTTTGAACCTTTGGGAATAGTTCTAGTTTGGGTAAATTCTTCGCCATCAGTGG
TAAGGATACTTATTTCTCTGGTTTCTTCTGCCATATTACTTCTAGTTCCATTTTTCTCTCCCGGAT
GTTGAATCGCCAAAAAGAGAGTATTTTGTTCTTGGTTGAAGCATAAACCACAAGTTTCCGTTTCC
ATCGGTGCAATACCAAATGGATAGGCTTTTCCTGCATTTTCTCCTGATAAGGGTATATACCAAAT

ACTATTATTTCCTAATGCTCCCGTAATATAAGGATTATCTCCCTGACGCACTGGTTGATTGTGTA
ATCCTGTCGGCATATCTGTCACCATCCAAAGATTTCCCTGTTTGTCAAACTCAAGGTTATCTGGA
TTCGCAAAGCCTAAACCTCCTGATGCTGGTTCACCACCAACTGCGATCGCATCCCATGTAAAATTA
AGGGCTGAGGGGTTATTTTCATTTTCTCTAAGGCGGATTAAACAGCCATACTCATATATATTTCC
TTGTTTATCGCTAAAAACTCGTTTATCTGGACTACCATCATTTCCTGCCGCTCCAGAGGTAAAGG
CAATCACTAAAGATTGATCCACAGGATGTAAAGCCACATCTTCAGGACGAGCAGTACAAGTACCT
CCAATAATATTACCGGCAAAATGAGCATCAATTAAAATCGCTCCTTGAATTTCTTCGGCGGTGTC
TCCTATGTATAAGTCTCCGAGGGTTGAATATTTTTCTTGAAATTGGTCTAATTCTTCTTGTTTAG
TAACGGTGACAATACCTCCTTTTTCTCTATCTGGATTGGGAATGGTTAGTAATTCCCCTCGCAAA
TTGCTTAAGGCAGTGGGATTAATGGCAGTGTCGGCTTTTAAGGGTATCCATTCGCCACTACCATC
GGGATTCATTTTGGCTACATATAACATTCCCTCTTGTAATAATTTCGAGTTGCCTTTATCTTGGG
GGTCAACAACTGTGCCTGTGGAGATAAACTTATACAAATGTCCACCAGTGCGATCGCATCCTGAG
TAAACCGCTAAAGGTTGATTCGCTTCGGCTCGAATGGCAACAGCTTCATGACGAAAACGCCCTAA
CCATGTATGTTTTGTACCATAGTCATTTTTATTAGCAGGATCAACTTCTACCATCCAACCATACT
TATTACCCGCCAAACCAAAGGGATTACCCTGTCCACTAAAACCATTGATGGGTAAAGACTCTGGA
GGAAAAGATGTGCCATCTGCCATTACAACTTCTGGTACTTGTGTTTGGAAATTTTCTTCAGCACT
AAATACTGTACCCCAAGGCGAAACACCCCCCGCACAATTAGCAAAAGTTCCAATGATTAGCTCTC
CTTTATCGTCAGTGTAACCCAAACCCTTCTTTTTCAGAAAAACACTTTTAGCAGGTCCAGTTACCT
GTAAATATCGATTATCTCTCCATCCTGATATACCTGTAATACGACGATCTTGCTCAGAGTAAGTC
CGCTCCCACTTACCATCACCATTGCGACGAATGGAAATAACCCCCAAACCTTGATCTGTTAACAAC
TCCCCAAAAAACTCTTGAAAAAGAATCTTAGCAGAATTATCATCAGGCAAAGCAAAAATATTTAA
TCCTTGTCGATTTTCCCTTGCTGAGTGTAAAGCTGATAAAACCTCTTGAGGCAATGATTTTTGAA
GCACCTGCTCATAACCTTGTAGCCAAGGAGTGGGACTGACATATTCAAAATTGACCACTAAATAA
CCCTCATTGGGATTAGTCTCCACAAAAGCAACATAGTCGTTGTTATAACCAAAACGAGAATCACC
CACTTTATCTCCCCAAGAAGCAATCACATCATAGCTAAATCCTTCGGGTAGAATTAAATCGTCTT
GTACTTGATAAGAATTAAATAACTGTTTTTGTTCTTGTGAATTAAGGTTATTTATTTCCAAAGGA
ATAGGATATTGAACAGGTTTAAAATTAGCAAATTTAAAAATTTCCTGCTCAGTATTTCCTTGGGC
ATGAGAATAATTTTTAAAAACAGAACTACAGGCGGTAGAAGCAACACTGACTCCCAAAAATGTT
AAAAAATCCCTACGTTTAAATTTCATATATTATCTTAACCCTTAACTGAATTTCTTTAAATTATT
TTTATTTTATATTACTATCAATAAATGCGAATCTACTGCTTATATCTAAATAATTAATATTAAGA
ATTGGTTAAATTTTAATTATAATTAGTAAAGAAATAAAAGATAATGGTAAATATGTAGAAATGA
ATAGGAATAAACCAAGCAATGGAATGATGAAAAATATTTCCCACTGATTGTTAGTTCTTAATTCT
TAAGTTTAAATTTTTAGGTTTTTCAGAGTGTGATTATGATAAATTAAGAGAAACAGGACTTCTA
TAAGCTTGATTAAATAGTGTTTAGTAGTAAAATAAAAACTAAAAGTTTATAAATTATTATTTAA
TAACCGCCTATTGCCTCTTGCCTACCCTAACCAATAATTTACATACTCAAAGTGATAGAGTTAAT
TTTTAATCCTTCCTTTTCTCTATTTATTTAAACTTAAACTAACTAATTTAGAAATGAAAATAGCA
GGAAAAAGTTGTCCTAGTAAAGCCTCTATGTTAGATAACATCATTCCCACGCTATTAAGAGGACT
TATATCTCCATATCCCAAGGTTGTGAGGGTGGTAAAACTAAAATAAAATAATTGATATTGTGGAC
TTGCATCTATAAATATACTTAATTCAAAAGAGTTAGCATTTATATTAACCAGAATTTTATAAAA
AATACCCCAAAGAATGCCAATCATTAAATATATGCAAATTGCCCCTTTAATTACATCTCCATTGA
TTTCTGTTTCCGAAAGAATCCGAAGAGCAATTGCAACGATAGATAAAATCATAAATAAAGCACTT
AATACATCTGAGAAAATTTCAATAGTATCTATATGATTAAGCATTTGAAGGTGACTAATGATAG
AACTTAGATAGGCAAAAATAGCTATTCCACGAAAAATATAAATAAACTTTTTCGAAAAACCAAG

GGTATTAATTGCTAAAATAATGGTAGAAAAAAAGATAATAGGAAACCAAATATTAAACCTATCA
AAATATTGTAAAAAAGGTGTAACAACCATAAAAATTAACAAATTATAAAATAGGCGATTATATG
TGGTTTTAGTAGAATAAAATTGGCTCATAAAGGTTTTTTAAGTCTTCTAATGTGTTAAAAAGTCT
GATTTTCAAAGGGCTTAGAATTGTTGAGCAAACCTTCGTTAATTACAAAAAAAGGGGGCTTTAAC
CCCCATATTAAATTACTATTTTGTATTTATCTATAATGTTCCTAAATCGTTGATGAAAAATCAAT
TATTAGAAAAAACATAGAATTGCGATAGATACTTATTAAGCAGTAGCCAATTCAGGTTCAGGGCG
TTTGCTGTTACGGATACCTTCGATCGCATCTGCATAACTAGGAGCTTTAAAGACAGCAGAACCAG
CTACAATAGCATTAGCACCAGCTTCTAAAACTTGCCAAGTATTGTTAGGTTTTAAACCACCATCA
ACCTCAATCCAAGGATCTAAACCTTTCTCATCGCACATTTGGCGTAAAGCCTTGATTTTAGGCAC
AACAGAGGGAATGAAACTTTGTCCGCCAAAGCCGGGGTTAACGCTCATGATTAAGACTAAATCGC
AAACGTCAATTACATACTCGATTAACTCTAAAGGAGTAGAAGGATTTAAAACTACCCCAGATTGT
TTGCCTAACTCACGGATTTGACAGAGAGTGCGGTGTAAATGGGGGGAAGCATTGTGTTCAGCGTG
AACAGAAATAATGTCAGCACCTGCCTTGGCGAAGTCTTCTACATACTTCTCAGGTTCAACAATCA
TTAAATGCACATCTAAAGGCTTTTTGGTGTAAGGGCGAATCGCCTCTACAATTAAAGGACCAATG
GTAATATTAGGTACAAAACGACCATCCATTACATCCACATGAATCCAATCTGCACCGGCTTCGTC
AACGGCTTTGATTTCTTCTCCTAAACGACTGAAATCCGCAGATAAAATGGAAGGAGAAATAACGA
CTTTTTTATCACTCATAATAATTATTAATAGATTAACTTTTGTTGTGTTAATTGTAACAAAATGT
TTAATTTCTCTATAGTGTATCAGCTATTGACAAAATCGAGAATATGATGAGCTGTAATTTCTGAT
TTTTCTAAATGCGGCACATGACCGCAATTATCAATCCAAACTAACTTACTTTGAGGGATTAACTC
TTGGAATTGACTAGCGGGTTTTATGCCTAAGATTTTGTCATTTTTTCCCCATAAAATCAGGGTTT
CTGCTCTAATTTGTCCTAATTCTTCTGCAAATGAACCATAACCGCCACTCTTGGTAAAAGAAATT
AACGCTTTGCTCCAATTCTCACATTGTAGATGTAACGCCGCACATCTTAAGGCGTCTTCACTGGCA
AACTCTGGATTAAAATAAGCCGTTTTACTGATACTCTGTCTTACTTTCAAATTCCGTAAAAATTC
CGTAGCTAAATAACCTAAAGGGGGAAATAAAAACTTCCCGATTATCGGTTGCTTAGTCAAACCAC
CGCTATCTAACAACACTAATTTTTCCACTGCTTGGGGATAACTTAAGCAAAAGTCAATGGCACTT
GCCCCTCCCATAGATGCACCCACTAAAATCATGGGTTTGTTAATCATGGTTGACCAAAAATGATA
GAGATGAGTTCTAATACTATGAGGAGAATAGTTTAAATTAGCTTGTCTTTGGGTAAAACCGAATC
CTAGCAAGTCTAAAGCCCAAACCTCGTATTCTTTCTTTAAAAATGGCAGTAAACGGCGATATTCA
AACACAGAACTATCAAAACCGTGCAATAATAATACTGGTGTTTTTCCTTCTCCCTTTTTTATATA
ACTTGTGGCGATTTCCTTTGAACTTAAGGGAGTGTTAATTTTTGTAAACTGGATTTGCTCAAAAA
GTGCGATCGAATCTTTTTCTATAAGTTTTTTTGCTTCAGTGACAATTTCATTAGTTAACATGAGG
AATTGAGAATTTAGAGAAATTTATTCAAGTTTATCGGTAAATTATGGCAGTTTATTATAGATTA
ATCTCGTTCCCCGTGTCTTTATCTCTATCAAACAAATCTTCATGATCATCTATTTCTGATGTGAGA
AGATAATCTGATAATTTATCTTTTTTTTCTTGATTTGAGTCGGATTGAATGAAATGAATACAATT
TCTTCCTTTACTTTTGGCTAAATATAAGGCTTGATCCGTTGTTTTAATAAAATCTTCTATGGTGT
TATTGTGGTTTGGAATCACGGTAAAAACTCCTAAACTTAATGTAACATGGTCGGCAATTTCTGAG
CGGGGATGATTAATTTTCAAAGATGCGATCGCATCTCGCATTTGTTCAGCAATGGTTATACCACC
TTGAATATTGGTATTAACAAGGATTACAGCAAATTCTTCTCCCCCATAACGGGTAGTCAAATCAC
TCGAACGATGGGAAATAGATGCAATAGTTTGAGCCACTTTTTTCAAACACTCATCACCCTGCTGA
TGACCAAAATGGTCGTTATAGCGTTTAAAATAGTCAATGTCTGTCAAAATTAAAGTTAAAGGTT
GTTTTTCCCTTAAATGTCTTTGCCATTCTGACTTTAAGTATTCATCAAAACGACGACGATTAGCA
ATTTGCGTTAAACCATCAAGATTAGCAATTAATTCTAGTTTTTCATTGGCAATTGTTAGTTCTTC
TGTTCTTTCCTTTACCTTATTCTCTAAAGTTTCAAAAGCATTTTCTAATTGATTTGCCATGAAAT

TGAAAGAGTTAGCAAGGTTCTGTAATTCCGTAATTTGCGTCATAATAGCAGTTGATGTCATTTTT
CCTGACGCTATGTCGCTACTAGCCTGATTTAAACGCTCAATGGGTTGACTAATATAATGGGCAAT
GGCAATGCCAACCACAATAGACAAAATAAAAGCTCCAATACACAGAAGCCAAGTGATTTTTCTAT
TCTCATCTATTTCTTTCATAAAATCAGAGCGAGGAATAATTGTCACTAGCAACCAATGCAAACCA
TACTTATCTTTATAAGGTTCAACTTGGGCGAATAAAATATTATTTTTGACATCTACTCGTAAATT
AATCCTTTCAGAAATACTGCTTAGGTTTGAATATTCCTGTTCTAACTGAGATGCGATCGCACTTA
TAATCGGATTATCGCTATTTTTCGCATTTAAAGGGGTTAAATAATTGCTCTGATTTCGATAGGGA
GCATCTTGAGTGGATGTAGCAACTAAATTACCGTTTAATTCCATAATAAAAGTTTGTCCAGAAGG
AGAAAACTTCAGTTGACTCAAAAAACTGCCAATACTTCTTAATCTAACTCCACTATGGAATACCC
CTAATAAATTATTTGACTGATCGTAAATGGGGTATAAACTATTAATAACTAGAGTAGGCACAGT
TTGATCGATAAAAATCGGAGACCAAGTTTGTTTTTTTTTCTCTCTAGCTTGTAAATACCAATCCG
TTGTACGTATATCAAGGGGAACTGTTTTTGCTGATAAAAGTTGCTCTTTTTGTCCTCGATTATCT
AACCGATAAAATTTATTGCTGAAAATATCGGGTAAAGTAATCTCTCTAACAATAATATTATCTTC
TTCTGGTAGGGACATATAAATAACATTTTCCCCCTTACTATTCGCAAATCCCGTCACAAAAGGAG
GAGAAGCTAAAGTTGCCTGTTGCCAAAGATGCGATCGAACCTCATTCAAATTTGTTACATCCACA
TGACCTTCTTGAAACGCTTTATAATTAACAGCAATGGTTTCTTGCTTAATTTGCAAATAATGATT
AAGATGATCCCGAACACGATTAGAAGTTTGAGTCATCAATTGATTCGCTAAATTCTCCACAGCAC
TTCGCCCAGTACGATAAGACAAGTACCCCACTAGGGTGACAGTACCAACAATTTGCACAATGAAA
GGGATAATTAAGACTAACTTCAGAGGTAGTTTTTTATTTAAAGAAGTTTTCACAATATCGAGTTA
TTCTACTACTCTAGAAAGATAAATTAGTTAAATATTATTAATAATTACAATTAGAAAAAGATGA
AATAAATTCATTATCTGCGTTAGTAATTTACTGATTTAAGCAATATTACCTTATATTAGAATAAC
TAATAAAAAATAATTGCCATAAGCTATGTAAAACAAGAGATTAACGATAAAAAAAATTAATTGT
TATAAATTAACATTTATTATTTAACCGTCAAACCTGCCACCTGACACCTTTTCCTAACCAATAAT
TTACACGACGAGCAGTAAGAGAGCCAATTTTGTATCGAGCTTAGACAAAACTTAAGATTTGATTG
GTCGTTTTTATTAGACAATAAGTATGTGATTGAATATAATCACACTAAAGTCATTTTACATGAGG
TTATATATTATGCCATATACAACAGAAGACGGCGGCAGATTAAACAACTTTGCTAATGAGCCTAA
AGTGTATCAGGCTGACCCCCCTAACAAAAACGAAAAGCGTAACTATATCATTGGGGGTGCTTTAG
CCATTATCCTTTTAGGTGGGGTAGTTGCGATCGCATTTTATGCTTCTAACATCTAAGTAATCACG
AAGATTCTATCAGGAATTAGGAATCGAACTGAGTTAGAAATTAAAAATCAAGAATGAGGAATGA
GGAATTAACTATTTTCCCTTCTTCCCCCTCTTCCCTACTCTTTCCCCTTCAAATAACCTCTACAAT
CTCCGTATATTCAAACTGATTTTTACTTCTTTTCACCAGTGGATATTCTTGATTATTAAGAATAA
TTACATCTTCTTGACAATCATCCTTAGATGATACATAAGTCAAAACATACTGTTTATTAATATAA
CTATTCATCTCTGTTTCAACCTCATTACGCCATTGAGTTTGAGTCACTAACACAATCAATTGATT
AGCTAACTTAGGGATAATTTTGGCTACCTGACGACGATATACTTCATCTAAACTGCCAAAAGGAG
AATCCATAACGATAGGAAAATTACTTGAGTTGAGATTAACTAAACGACTTTCCTTCGACCATTTT
CTCACCATATCAATAATTGCACCAATAAAAGAAAGACTCAAAATTTGATTTTCTCCAGTAGAAGC
GGCAACGGGTAAAGGAATACCCAAAGTATTTTCTACTAAATTTAATTCGTAGTTTTCATTTAATA
AAGGTTGATATGGAGTGAAAGAAATGGAGGCAAAAATTTCTTGTAATCTTTTTTCTAAGGTTAA
ACGGAATTGATTTTCTAATCTTTTTTTAACCTCTAAAATACAATCAATCGCTTTTTGGGTTGCTT
CAATTCTTCGTTTAATTAAATTCTCTTTTTCTGCTTTTATTTCTTGCTTAATAATCTGTTTTTCTA
ACTTACTTAACTCTTCCTCTAAGTTATCTAATTGAAGGTTAATTTCTCCCTGATTCAAAATTACT
TGACGGATATGATTTTCTATATTATCCAGTTGCTTTTGCATCTGTTGAATATTTTTATCAGGATA
TTTTCTTAGTTGCTCATTCAGATTATCTAATTCATTTTCAATTTGGTTTAAATCCAAACGACAAT

TATTAATTATATCTTGATATTCATTGATATTATTCCAAAAATTTTCTCTCAATTCTAATAAATTA
CTCACACTACTTTCTAAGCGAATTAATATTTCTTCTAATTCGCTACTACCTGTTTTTTGTAATAA
TTTTTCTACCTCTTGATAACTAAAACTATCAGGAATTAATTGATTACCACATAAGCAACGATTTT
GTTTTAATAATTGCTCGATAAATTCTTTTTTAATTCCGATGGGTAATTCTCCTTTTTCTCTCATC
TGATTAACTATTTCTTGAAATTCTGTAAAAGTCTTAGATAAAAATACTAAATAGCTTTTACTGCT
AATCTCTTTTTTGATGCTTTGTTGAGTATCAACTAAAGTTATTCTTAATTCTTTTTCTCGCTGTA
GCAATTTCTGTTTTAATCGCTGTAAGTTTTCTATGCCACTTATTTCTAACATTTGTTGAGAAATA
TTATCTTTTTCTGCTTGTAATTTAGCTAATCTTTCTTTAAGTAATTCAAAATCATTTTTTAGTTT
TTCTTTTAAATTGCACTGCTTTTCTCTTTCTTTCAAAAGGCTTTTTATCTGTATATCTCCTAAGG
ATTCTAATTCTTCATGAAAATGTTTTTTGGCATTTTTTAAATGATTTATCGCCCTTTCTAACACT
TTTACTCCAATTAACTCTTTGGTATCTTCAGCAATTTTTTGTCTTTCTTGACTTCTAAATAAATG
ATCAATATGTTCGCCATCAAAGAAAAAAATATCCGTGTAAACTTTGAGGTAAAATTTTTTCAATAA
TATCTTTTGGTTGCTGTAAAGGATGTTGCCATCTACCATCATCTCCTGTAATCATCATGAATAAT
TGAGGAGTTGTTATTTGGATTTGATTTTGTTTATTTCTACTACCAAAACATTTTCTTTTTAACTG
ATAAGTTTTATAATCGTGTTCAAATATTACCTCAACAAAACACTCTACAGAAGCTCCTTCTTCTA
CTTCTTGAATTGCTTTTTTATTCACTAAGGATTGAGGTTCAGAAAAAGCCCCAGTAAATTTTTCG
TAAAAAACCCAGGTAAAAGCATTAAGAATGGCTGTTTTTCCCGCTCCGTTATTACCGTGAATAAC
TGTGGTATTTTTTTTACCATAGCTAAAATTAATAATGGGAGTTTTACCATAGAATTGGCGAAAAT
TTGCTAGTTGTAAGCTGATAATTTTCATTGAATATTGTTTCTAATAATATTTAAAATATCTTCAT
TTATATAACGACTATTTTTTTGAGATAGCTTATCTTTTTCTAGTTGTAGAACTTGCTCAACAATG
AGTTTTATTTCTGGTTCTGCCTTGTTAATTGGTTCTGTGATATGTTCTAATTTATAGTTATTCTG
ATAAATCATGAAAATTATTATCAATAAACTTTGTTTTGATTATAAAGTATTTATGTAATGAGCC
TATTTTTTTTGACATATTTTTTACATAATTTAATTTTTTTCTCATGAGCTAAGACTATAATAAAT
AGAGGATAATAGCTGGTTTGTTATTTTTTATCATCAATTAATAAATGAAAAATGGTTCAAGATA
ATCGCAAAAAAGTTCGGCAGGTTCTAATCATTACATTAATATTAAATTTAGTTGTTTTATTATTA
AAAGCAGTTGTTGGTACAATAACAGGTTCATTAAGTTTACAAGCAGATGCTTTACATAGTGTAAC
TGATAGTGCTAACAATATTTTAGGCTTAGTAACAAATCAACTTTCATCTCCCATACCAGATAGAG
ATCATCCCTATGGACATCAAAAATTTGAAGCGATTGGTGCTTTGGGGATTGCCGCTTTTTTGGGG
ATTGCTTGTTTTGAAATTATTACTGGTGCTTTAAATAGAATTATTTTTGGTGGTGATCCTGTTAA
AATTGGTGGTGGTGAATTATGGCTTTTAATTATTGTTTTAGGTATCAATATTTTTGTTGCTTTTT
ACGAGCGAAAAGTCGGGTATAAAATAGGCAGTAAAATTTTAATTGCTGATGCTACCCATACCATG
AGTGATGTGTGGATAACAATTAGTGTATTGTTTGGTTTAATAGGTGTTTGGCAAAGTGAAAGAT
TAAATTTACCTCAGCTACAATATTTAGATATATTATTAGCTTTTCCTGTAGCTTTTTTAGTTTTA
AAAAGTGGTTGGCAAGTTTTAACAAGCAATCTTCCTTGGTTAGTGGATCAAGTTGCGATCGCACC
TGAATTAATACATAAAATAGTAACAGAAGTACCGGGGGTAATTAACTGTCATGATATTGCATCA
AGGGGTTTATTAGGAAGACAAATCTTTATTGAAATGCACTTAATTGTTGATACTGAAGATGTAA
AAATTGCCCATGATATAACAGAAGAAATAGAGAGAAAATTAGAAGAAAAATTCGCTCCCGTCAG
GATTTTAATTCATATTGAACCCCCAGAATATAAATCTCCTAAAATTAGTTATGGATAATTAAGAA
TTAAAAATTAAAAATTAAAAATTAAAAATTAAAAATTAGGCTCTTCTACAGTTGCTATGGTAAA
TTAACAAAAAATAATTGCTATAAGCTATGTAAATTAAGAGATTAACGATAACAAAAAATTAATT
GTTATAAATTAATATTTATTATTTAACCTGACACCTGCCACCTGCCGCCTGACACCTTTTCCTAAC
CAATAATTTATATACTCTCAGTAAGAGAGCCAAAAATTAAAAACTCCGAACTTAGATTATCTAGT
TATCTAATAAGTCTTCTGCCCAATTTTGCTTTCAATTCCCCTTTGATGCGGTTTAAAATCGAGGT

TTCGTCAAGGGAATTAATTATGTTTTCTACTACTGCGTGAGGTCCATTTTCTCCCCATGATGCCCC
ATTTGCTAAATAAACTTTACATATTTGTCCAATTGTATAAGTGGAAACTCCGGCAAATGCCCCTT
GTGCGATCGCAACTGACATATAAGGCACAACGGTTAAACCTCCAGAAACAGGGGTTGTTAATCCT
AAAATACCTTTAAGGGAACTTAAACCAAAGGTTACTAACACATCACTAGCGGTAATTCCTCCTAA
ACTGATGGCTATTTTTTGCAACAATTTCACCGCACTTTGTTGGGTCATGGCGATATTATAAAGTT
GTGATAACCTTAAAATCATAACCACGTCGATGATTGCCCCTGTAAATAAATCTATAGCTGTGACA
GGATTAAGAGCGATCGCACCTGCTTTAGTCATCATAGTTTGTTGTATAATCTTTTCTGCCTCTGC
ATCTCGATAAACAAGTTTTTGTTGAATTAAACGCTCATTAATTTGTCCAGTGGATAACATGGTAT
TTAAAGCAACCAGAGATTTACCCTCTTGATGGAGAATTTCTAATATTTTTAACTGTAAATCTCCT
ATTTGTGGCTTTCCCCGTTGACGTATTATTTTCACTTTTCCCTCTTTATCCTTTACCGCCACTGTT
TCTAAAGGAGAAGCCGAAACCATGACAATTTCATCCGGTGACAATAATTCCCTTACCCTAACATC
CCTAATCGTAGAATAAATTTCGATGCGATCGGACTGAGGATATTGATCAATTTTATTAAATACTA
AAATCATTGGTTTGCCCACCTCTCGCAATTGGCATAAAGCCTGATACTCTACCCTCGTAATATCTC
CTGCAATCACAAACAAAATTAAATCTACCCTAGAAGCAATATCATGGGCTAACTTTTCCCTCGTT
TCCCCATCAATTTCATCAATCCCCGGAGTATCTATCAACTGAATTTGGACATTTTCTGAACCCTTA
ATCAATCGTTGAATATTTTGCTCTCCATTACCAATACTCTCCTTACTTAAACGCCAATTAGTTTG
ATCAATTTCTCTTGTCACTCCATGAAGTGGTCCAGTAATAAAAATGTCTTGACCAATTAAAGCAT
TCAAAACACTAGATTTTCCCTTTCCCACCATGCCAAAAGCCGCAATTTGAATCACTGAATCTTCTA
ACTTTTGTAACATTTGATTCAAGTGATCAATTTCTGTCTCTAATCCCTGCTTCTCTTCCTCTTTCA
AATCCAGATTTAATAATAAATTCCGTAAAGATTCCTGTGCCTTATGATAATTTATTTCTCCCTGC
CAATGAGCAAAATCAATAATCGCATTTTCCCAATCTTCTTCCCACATAACATTATTAACTCAAGT
TATAGCCTTAAAAGGATTATTCTTATATTTTAAAATGTTCTCGGTAATTACAATTCATTTCACCA
CAAGAATCATTAACAACCATGAGGGTTGAAAAATTTTCAAATCCTATGCTGAGGTTAATTTTTAA
TTTTCAAAATGTTGTACAATAAAATGTACAATTTAAAAGTTAGACAAAAATAGTAATGAAAATA
ATTAATATAAGTACTGCAAGAAATAATTTATTCAGTCTAATAGAACAAGTGAATCAAGATCATC
ATCCTCGGATTATCAGCAGTAAAAAAGGGGATGTCGTAATCTTATCAAAAGAAGACTGGGACAGT
TTACAAGAAACCTTGTATTTACAATCTATTCCTAACTTAGTCTCATCCATAAAAACAGCAGAAAA
AGATAATGAATGGGTATCAGAGGAGGAATTTTTGGGGGTTTTAAATGACTTGGAAGATTGAATT
TAGTCGAAATACTCTCAAAGATGCGAAAAAACTCAAATCAGCAAAATTAGACGGTAATCTCAAAG
AATTACTCAACATCTTAAAATCAAATCCCTATGAGCCTCCTTACGAAAAATTATCAGGTAATTTA
AAAGGTTACTATTCAAGGAGAATAAATATTAAGCATCGCCTAGTTTATCGTGTTGATGACAATAA
TAAAACCATCAAAATTCTTTCTGTCTGGTCTCATTACGAATAGATTTTATCGAAACTCCAATTAC
AATTATTTCCGACTGAGGGTTAATTCTTCTTGTAGTAACTTCTTATATACTTTGGGCAAAAATTT
CGTCATATTATTATCCTCTATGCCATAACCCAAACTCGTCCAAGTGGGAGATAACATTCTTAACA
CATCATCTATTTTTTCTTCTCTCAATAACTCTGCTATATCTTTTTCCCATGCCGTAGTATCCTTTA
ACCAGTTGTAGAGAACTTTATCTTGATACTCAGGCTCAAAACTGTAATCTTCCCACACCAGAAAC
TTAGAAGGATGAGGATGATATTTTGCGGCTTTTTCTGCCCAGGTGGTATTTAAAAATTGATAACG
TCCAGAGGCAGTGCTACATTTACCCTTATTAGGTCCATTAACAATGGTCATGCAAATATTCGGAT
GTTCGCTTAAATCGTCCACATATTCGCCACTATAAATTACATAGTAGGGGTTCAAATAATTAGAT
TCACTAAAACTAATAGTACGCATCAACGCCCTAATGTAAGGATCTCCCCCTTCCATCACTAATGG
AGGACTCCCATATATACTCGAAATTAAATGTTGTCCTGCGGTTTTTATTTCCGCTTTTTTTTCCCG
AAAAATAACGTTGCCGATTCCAGTTAGTATGATTAGTAAGATAATCAGTTTAAAAAGTTAACA
ATTTGTGTACTAAAGGAGTGCGATCGATTAATGTTGGTCATACTACCAGAAACAAATAACTAGAT

TAATTCAGTATTAGTTACTGTTCCTATCATATAGTAATCGGATTCTAGTGAGCACCTTTACGCAA
TAAAACCACTTTTACTGTGGCGATAATCAAAGCTAAATCGTACCAAATAGACCATTTTCTCTGAT
ACTGTAAATCTAAAGACACTATTTGCTCAAAATCATCTACCAAGGAGCGTCCATTAACTTGCCAT
TCTCCAGTAATACCGGGCTTAACTCTCAATCTCAACCAATGACGTGAACTATAGTGACTAACTTC
ACAAATTGTTGGGGGGCGAGTTCCCACCAGACTCATATCTCCTAACAAAACATTCCAAAACTGGG
GAAACTCATCAAGGCTAGTTTTCCGTAAAAATGCCCCTACTCTCGTAATTCTGGGATCTTGGTCA
TTTTTGAATATATGACCTTTGGCTTTATTTTCCACTAAATGCTTTTTTTGATCTGCATCGCTATA
CATGGAACGAAATTTCCAGATGCGAAAAGGCTTACCATTCAAACCGCAACGAATTTGACTATATA
ACACCGGTCCTTGACTATCCCACTTAATTGCGATCGCAATAGGAATAAATATCAACGCAGTAATC
ACTAAGCCCACGAAACCACCAAGAATATCAATATATCTTTTAAAAGTGCTATAAGCAGATACATG
AATAGTAGTATTTATTCCTTGAGGGCAAGAAACTATATCTGGGGATGGTAATGGATTATTGAGA
AATCGGCTGTCAGAAAAAAACATGATTTTTTTAATGGTACTAAACAACGCTATCAATATTTATA
CGCAATTTAAGGTTTTTACTTGTTACTCACGGTTGCGTACTTACACTTAAGTAAAATGCAGTAGT
AATTAAATATAACATTTAATACTTAAGTCTATAATTTTTTTTTGCAAACGTCACTTTTTTTTCTT
AAAGATTATATAAAGTCTATAATATAAAGATTTTATTAAATTTTGTTACGTGAAAAACAAGGAT
TTTTGTAAAGATTTTATGAAGTTAACAATACTTAGTTGATTTTTTAGAAAAAACTGTTATAGTT
AGTTTTATCCAAGAAAATACGTAAGTATAAATTTTATTAGAGAGAGGAAAATACTCATGAATAA
ACTTTGGTCTTTCTCCCTTGTACCCTTAGCTGTCACTGCTACGGTTGTAAGTTTCGCTTTCACTCC
CGACTCCGCCCAAGCTATCGGAATCGGAACAGTCGCTATTAGTGGCACTACTAATTGGTTTGGAG
ACACTGCCAGTTTTACCTCTGTTACTGTTGGTTCACTCCCCACTAGTGCAACAGGTATGTTTGATG
GTCTTGAAAGCACCGTTGTAGCAGTAAAAAATATTAACTTTACTCCCATTCCTAGCTCTCAAGCT
ACCTATGCGGCTATTAGCGATTTTTTAACTTTTACTAATGGTCTTAAATTTAATTTAAACGCTGG
AACTGCAGAGTTTTATGACGCTGGTCTTGCTTTTGAAGGGGACTTTACAGGGTTATTTTATGATA
CGAATGGCTCAATTGTAGGTAAAGGTAACTTTGGAATTACAAATAGTGGTTCTTCTACTACTTTC
AGTTTATCTGCTCAAGCTGTGCCTGAACCTTTAACTATTCTTGGTACTGGTTTAGCTTTAGGTTT
GGGGGGTTTATTCAAGTCTAAGCAGTCTAAAAAACAAGCTGTTGAGGTTTAATTGTTTGTATATT
TAAGTGGAAGTTACCTCAAATACTTTTCTATTTATTTTGTATTGGTTTTATATAAATAGTTTGTT
CTCGCAATTTTACTGGTTTATTGTCCCCCATTTTTATCCCAATTATGTATGGGTTATGGGGGATT
TTAATTTTTTGGAGTTGGTGAATTAAGCTATGACTCTTAGTTAAGATCAGAAGTAAATAATAGG
CATTCTTACGATAGTAATAATATTTTGAGTGTTAGATAATTGGAGTAATTCAATTATGTTTTATG
TCACAATTAATTAACCTCCTTTTTTTGGTTTATAATCGTCACATATTTTTATTGCAATATAATTA
TTGACTTATGTATTTCAACTGACACTACTTTTTTATTAACCCTTCACTAATTGATAAAGCCTTTT
AAATTCTCTTTGTTGTTGACTATGATCAACTATGGGTAAGGGATAACCACAACTACTCGCTTCGA
GAGGAGATATTTTTCCTGTCACCAAATCCTTAGTATCCAAATGTCTAATTTCTGGTAGCCATGTG
CGAATATACTCAGCTTCTGGGTCAAATTTTTGAGCTTGAGATGCGGGGTTAAAAATTCTTAAAGG
TTTGGGGTCCATTCCACTAGAAGCACTCCATTGCCAACCGCCATTGTTTGCGGCTAAATCGCCATC
ATAGAGTTTTTGCATAAAATATTTTTCTCCCCACCGCCAATCAATTATTAAATCTTTGGTAAGGA
AACTGGCCACAATCATCCGACAACGGTTGTGCATCCATCCTGTTTCATTGAGTTGTCTCATTGCCG
CATCAACTATGGGATAACCTGTTTTACCTTCACACCATGCTTGAAATTTTTTTTCATCATTTTCCC
AAGGGAATTGTTTAAATTGTTCTCTATATGCTCCTTCTGCTAATTGAGGGAAGAAGTATAAGCAA
TGTTGGTAAAATTCTCGCCACGCAATTTCTTTCTGCCATGCAATAATATTTTCTCTAGCTTCGTCA
CTATTACAATTTGTTAATTCTGCTTGACTTTTTTTCCAAATAGTTCTTACAGATATTGCACCAAA
TTTTAGGGCGGCACTTAGTTGAGATGTGCCGTCAAGAAAGGGATAATTGCGATCGCACTCATAAT

TATAAATAAGAGATTGACAGAAATACTCTAACCTTTCGAGGGCGGATTCTTCTCCGGGCGACAAA
GGCAAATCTGCATCCCATGGGAAACCTAAATCATTAAGAGAAGGTATATTAATAGTGCCTAATTC
TTTGACAATGGTTTTTTCTAAATCATTTAAACCTGTGAGTTGTTGAGGTGAGTCAACGGGAGATG
ATTTAGGCTGTGCTTCCCAGTTGCGCCAGAAGGGAGTATAAACAGTATAAGCCGTTTGATTACTT
TTAGAGAGAATTTCCCCGGGAGAATGCAAAAGTTGATCCCAAAAAGTTTGAGTTTCGATGGATTT
TTCTTTCAATGCTTGACTAACATTTTTATCTCTGTGGCGAGAAAAAGGTTCAACGTCTAAATTCC
AGTAAACCGCATCAGCTTTGAGTTTTTCTGCAAGGGAAGGAATAATCTCTTCTGGGGTGTTATGA
AAAATTAATAAATCACTACCGAGTTTTTGATATTTAGTTTTTAATGCCTCTAGGCATCCTAATAA
GTATTTAACTCTGGCAGGTGCGATGTCATCTCTATTTAAAATATTAGGGTCTAAGCAAAATACAC
CGATAACTTTTTTATCTCTTGTGTAGGCTTTTGCTAAACCGATATTATCGTTAATTCTTAAGTCT
CGACGATGCCAGAAAATAATCATAAATTAAGGATTTAGGGTTCGGGGTTAGGAGTCAGTTAGGG
GCGAATGGCCATTCGCCCGTACAGGAGTTAAGGGATAAGAGTTGAAAGATTCATGAATGTTTATT
CAAGTCCAATTTCACTGCTACGTCTGTATTGACGGTAAGCGGCGAAAAATAAACCAATTAAGGTT
ACAGGAATTAAACCTAAAACAATACCAAGTAATAAAGGCTCTACCATTTATTTTTTTCCTCAATA
AAAATATTTCTTAAATTATCTATTAGAAAGTTTATCAAAAATATGACTGGAAAAATGGATTTTT
TCTGAATCTATCGCTGAAAAAGTTGATTAATAGTCACTTTCAGAAAATTTGTGCTACCATCAGCA
ATAAAATATTTAAGAAAATACGTTTATGGGTAAGGTTTTAGTCTTGAATGCCTCCTATGAACCGC
TTAATATTACTAACTGGAAAAGAGCGGTTATTTTATTACTCAAAGGAAAAGCCGAACAGTTAGA
ACATTATGAAAATTTTATTTATCCGACTTTTCCCTTTCCTTCAGTTATTAGGTTACGTCACTATG
TCAAAGTACCTTATAAAGATATTCCTTTAACTAGAAGAAATATCCTAGAGCGCGATCGCCATTCT
TGTCAATATTGTGGTTACAGAGGAGATCAATTAACCTTAGACCATATTCTACCTCGTTCTCGTGG
AGGTGCAGACAGTTGGGAGAATCTTGTTACCGCTTGCGTGCGTTGTAATATTAAAAAAGGAAATA
GAACTCCAAAAGAGGCTCATATGCCGTTACACAAATCACCGAGAAAGCCTTATAGTAGTCTTCAC
TTTGAAATTGTTAAATGTACCAAAGATAATCTTAACCATGAATGGCGTAAGTATGTTATAGGTA
TCTAAAGTAAGGCAAAGGCAATTAAGAATTAGTAATTAAGACAAGTAAGACAAGGCAAAAGGCA
AGAGGCAAAAGGCAAGAGGCAAAAGGCAAAAGGAAACTCCTCTGTGTCTCTCCTTAAAGGAAAC
CCCCCTTTATCCTCCCTCAAGAGGGGGGAGGGCAAAAGTGTTTAATTAACCTCTCCCTCTCTCCTC
ACCCCCCTGTCTTCACCTGTAAATAGAAGAGCCTAATTTATAAAGATGGAAATTTTAATTGTTGA
AGACGAAAGGGAAATCGCCCAATTGATTAATAGTTGTTTAAGTCGAGAGGGTTTTACTTGTCATC
ATGCTTATGATGGAGTCACTGCCCTTCAACAGGCTCAAACTATTCAACCAGACTTAATTATTCTC
GATTTGATGTTGCCCCAATTAGATGGATTGGAAGTGTGTACTCGCATTCGTAATCAAGGCATGAC
AAAAGACCCCTATATTCTCATGTTAACTGCAAAAGGAGAAGAGCTCGATCGCATCATTGGTTTAT
CGACAGGGGCAGATGATTACTTAGTAAAACCTTTTAGCCCCAGAGAATTAGTTGCCAGAGTTAGG
GCATTGCTACGCCGTAGTTTACGCCACGAAAAACAGTCACAAATATATGAAACTCCCCACTTTAT
ACTCAATTTAGACGAACATATCGCCACCCGTAAACTAGAAAATCAACCCCAAGAAGAATTAGACT
TAACCGCCCTTGAATTTAATCTACTTTCTGCCTTCATTAGTTATCCCGGAAAAGTTTGGAGTAGA
GAGCAATTAATTGATAAACTATGGGGAGCAGATTTTTTCGGAGATGAAAGAGTCGTTGATACTC
ACATTCGCCGTTTACGCAAAAAAATTGAACCTAATCCTGCTCAACCTTCTTTTATTAAAACCGTT
GTCGGTGTAGGCTACAAATTTGAAGATAATTAATCATAATTAGTGTTAAGTGACAATATTTCTAC
TAACCAACTCCGAACTCCGTTCACGACTGAAAGGAGTGTACGAGCGCAGCGAACTCCCAGAACTC
AAATCATGCAACCTTTTGACTACACAACCCTGAAAGCCGTTTGTTACTCCCTCCAAGAAAATTAT
ATTCCCTCTCGCTTAGAACAAGTTTACCAGTGCGATCGCACCTCTATTAGTCTTTGTTTGAGAAA
TATTGAGAAAAAATCATGGTTAACCATCTCATGGCATCCCCAAGCCGCCAGAATTTGCGTTGGTA

ATCCGCCTCCCCGTGGTAAAGACACTTTTACTTTTAGCGATCAACTCAGACACTTAATAAATGGT
TATGCTTTGATAGGGATAGAAATAGTGAGTGATTGGGAAAGAGTTATTGATTTTCAATTTGCAC
AACGCCCCAATGAATCCCCCTTAAATCATCTCTACGTAGAAATTATGGGCAAATATAGCAATGTT
ATTCTCACCAATGCCGACAATCAAATTATCACCGTTGCCAAACAAGTTACCGCCGACAAATCCACC
CTGCGCACCGTCGAAACCAGTCAAATTTATCAACTACCCCCACCATTAACAGGAAATATTCCCAA
ACTAGAAGAATCCCAAGAGACATGGCAAGAAAAAGTTAGTTTAATACCCGGAAGAATAGATAAA
CAATTAATAAAAAGTTATCGAGGAATTAGTCCGAATATTGCCAAGGATTTATTGCACAAAAGTG
ACATAGATTTAAGTAAAACTAATCAAGAATTAACACAAAATGAGTGGAAAGAATTATATAAAAA
TTGGCAAACTTGGTTAACATCTATAGAAAAAAATCAGTTTTATTCTCATTTAACATCCGAAGGAT
ATACAGTTTTAGGAGAGCCAAAGCAAGCAGAAAATATTAATTTTGTTATTAACGAATACTATAA
CCAAAAAAATATATCAAGAAAATTTTATTCAACTTAAACAACAACTACAACAAAAAAATAAAAAAT
ATATTAAAGAAATTAAAAACAAAAAAAAATAAATATTTAGAAAAAATAAAAGAATCTGATAACT
GCGAAATATATAGTAACAAAGCTGATTTATTAATGGCTCACCTAAATCAATGGCAAGTAGGAAT
GAAAGAAATTACTCTTAACGATTTTACCACTGGAGAACCCGTAAAAATTAACTTAGCTCCTGACA
AAAATATGATTCAAAATGCCCAAATATTGTATAAAAAATATCAAAAATTAAAAAGAGCAAAACA
AGCAGTAAAGCCCTTATTGGATGAAGTGAATGAAGAAGTAAATTATTTAGAACAAATAGCAATA
AATTTGCAACAATTAGACTATCAAGAAAAAGACGATCTAACAACTCTTCAAGAGATTAAATCAG
AATTAATCAATCAGAAATATATAGAAGATAAACAGTATCGAAATAATCAAAACATTGAAGAAGA
ATCTCAACCGAGACGCTATCAAACACCATCAGGTTATGAAGTTTTAGTCGGGAGAAATAATCGTC
AAAATGACATTCTAACCTTCAAAACTGCTACTGATTATGATTTATGGTTTCATGCACAAGAAATC
TCAGGCTCTCATGTTTTATTGCGCTTAAACGCTGGAGATGCACCAGAAGAAAAAGATTTACAATT
CACTGCCAACTTAGCCGCTTATTACAGTCAAGGTAGAGAAAGCGAACAAGTGCCTGTAGTTTACA
CTAATCCCAACCATGTTTATAAACCAAAAGGAGCTAAACCCGGAATGGTTATCTATAGCAAGGAA
ACTGTTATTTGGGGTAAACCATTATTCAACTAAGCAGTTAGTTAGCAATTAAAAATTAGTTTCCC
CGCAATTCCCCAACTTCTCAATCCCCCTAAAATAACCATTTTCTGGCAGTTTAAAATTTTTTGCGT
TTAAAAAATTGAACTCAGGTTAAAGCTGATCGACATGGGGTATGATGAAAAATGTAAACTTTTA
TTGAGCATAATTGCTAATGGTTGCAACACCAACAAAAATTAATTACGACATTAAGGACATTAACT
TGGCTGGTGTCGGTAAGCAGAGAATTGAATGGGCGGCAAGAGAAATGCCCGTTTTAGCTCAAATT
ACCGAGCGTTTTCGTCAAGAAAAGCCTTTTGCTGGTATCCGCTTAGTGGCTTGTTGTCACGTTAC
TACTGAAACCGCAAATTTAGCGATCGCACTTCAAGCAGGTGGTGCAGATGCTATTTTAATCGCTA
GTAACCCTCTTTCTACTCAGGATGACGTTGCCGCTTGTTTAGTTAAAGACTACGGTATTCCCGTA
TTTGCTATCAAAGGGGAAGATAACGCTACTTATCATCGTCACGTACAAACCGCATTGGATCATAA
ACCCAATATTATCATTGATGACGGTTCTGACGTAGTAGCTACCCTTGTTAAAGAAAGACAACATC
AACTAGCGGACTTAATTGGTACTACTGAAGAAACCACCACTGGTATTGTCCGTTTAGAGGCAATG
TTTAAAGATGGTGTACTAACCTTCCCCGCCATGAATGTCAATGACGCAGAAACCAAACATTTCTT
CGATAACCGTTATGGTACAGGTCAATCTACCCTTGATGGTATCATCCGTGCAACTAACGTTTTAT
TAGCAGGTAAAAACCTTGTGGTTGCTGGTTATGGCTGGTGCGGTAAAGGTGTCGCCATGCGCGGG
CGTGGTTTGGGTGCCAATGTTATCGTAACTGAAATTAACCCCGTAAAAGCGATCGAAGCTGCAAT
GGATGGTTTCCGTGTAATGCCTATGGCTGAAGCCGCTACCATTGGTGATGTTTTCGTAACCGTAA
CAGGTAATAAACATATCATCAGAGAAGAACATTTCGCCGTAATGAAAGATGGTGCAATGGTTTGT
AACTCTGGTCACTTTGACATCGAAATCGACTTAGAAGCCTTAAAAACTCTCAGCACAGAAATCAA
AGAAGTTCGTAACTTTACTCAACAATATATCCTCAAAAACGGTAAATCCGTAATTGTTATTGGCG
AAGGACGTTTAGTCAACTTAGCCGCCGCCGAAGGACATCCCAGTGCGGTAATGGATATGAGCTTT

GCTAACCAAGCCTTAGCTTGTGAATACTTAGTGAAAAATAAAGAGACTTTACAACCCGGTATTCA
TTCTATTCCTACCGAAGTGGATCAAGAAATTGCCGCTTTGAAATTACAAGGAATGGGTATTAATA
TCGACTCTTTAACCCCTGAACAAATTGAGTATATGAACTCTTGGACAGTGGGAACTTAATTTGAG
ATGTTAGGTAGGGTGTTAGGGAAGTAGGGGCTTAAGGTTTTGAGGTATTAGGGTTTTTAGGTGT
CAGGTTTCAGGTTGCAGGTGTCAGGAGAAAGTAATGAGTAACGAGTAAGGAGTAGAGAGAAACG
GTGATGAGGGGAGAGGGAGAAGTAGGGGCGAATGGCCATTCGCCCGTACAGGGTTTCAGGTTTCA
GGTTTTAGAAGAAGGTAATGAGTGTTCAGAGTTTTTAATTCTTAATTCTTAATTTTTAATTCTTA
ATTATCAACCATTCACTATTCACCCCAACACCATTCAATATTCACCATTGCCCATTGCTCTTTTAT
TTTTTTCGCACTTATTTATTTAGCTAGAAAAATTCATTATGAAAGTCTTTTCTTGTATAGTAGGA
TTTATTTTATCAATACTATTATTATTAACTCCTAATCACGTTCTTGCCGCAAGTTCATCGGCTGT
TACTTCTACCATTGATAATAAAGATTTGAGTCAACAAGATTTTTCCTCTCAAAATTTGCAATCGA
TGGAGTTTAGTAACGTGAAATTAAACGGTGCTAATTTTAGTAATAGTGATTTAAGAGGCGCTGT
TTTTAATGCCGCAAGATTAGAAGAAGCTAATTTTCACGGAGCTGATCTTACCAACGGTTTTATTT
ATGTTACTAGCTTAAATAGAGCCGATTTAACCGATGCTATTTTACGAGAAGCGATTATGAAACGC
ACTACTTTAAAAGGTGCTAATGTTGATGGTGCTGATTTTACTTTTGCAGTTTTAGATAATGAGCA
AGTAATCGAACTTTGTAAAAATGCTCAAGGTATTAACCCTGTAACCGGTGCTAGTACCCGTCAAT
CATTGGGCTGTCCTTAAATTAATCTCCTCAGTTGGGCATAAGCATCTCAGATTTAGTTAGGTATC
AGGTGTCACCTCAATTAGATATAAGAATATTTAATTTCGGTAGGTATTAGGTTTTAGGTATCAGG
TGGCAGGTTTTAGATTTTTATTCTAATTCATTACTAATTATGGTTCTTCAGAGTGTGGTTATGAT
AAATTAAGAGAAACAGGACTTGCATAACCTTTATTGAATAGTGTTTATAGTAAAATAAAAACTA
AAAGTTTATAAATTATTATTTAATAATCCCCTATTGCCTCTTGCCTACCCTAATTAATAATTTAC
ATACTCAAACTGATAGAACCCTAATTATTAATTAAAATTAACTTTACAGCACTAGACATTTTATA
TCAAACTCAGATTAAGTATAAAATAAAACTCTCTTCAAATTTTTTTACCTCTTAAATTAAAAATA
TTTCTTTGCTTATTATTAAAAAATAATACGTTTTTATATTTACCAATTTTTGATACTGAAAAGCT
ATAATATTTTCATTTCTAATTCCCTGTAATTTGATGTTATTAATTAACCAACAATCTTTAACATA
GATTTTACGTAATAATAAAGCATTGATAATGTCTATATTTTCTTTGGGAATTAAAGTCTATAGAT
AATCATTTGTATATACTATTCTGCTTTAATTATCTAGTCTGAGTCTTTGATTTAAAAAAAACTAA
AGCATTATCTTGAGTGAATAAACATTCTTTCATAATGGTTTTCTAATAACTTAGCAATTATATAT
ATTTTTAATATAATGTCTATTAATAATGTCAATTCACAAAGTTTGACTCTATTGTTTAACCCTTA
TTTTCCCAGAAATACTCAAAAAATTATCTCTGAAAATAAGCAAAAATCAGTGAATAACAATTTTT
TAAATCAATATCAACTTTCCCTAATTGATAATTTTGTGGATGGGATTGCTTTGTTAATAGATGAA
AAATTCGTTTATTTAAACCAAGCCCATTTGAGTTTATTTGGCTATAATCAAGAAGAGTTATTAGG
GCGATCGTGGCTTTCTCTTTACAATTCGGCAGAAGGTTTGAGAATACAAGAAGAAATTTTCCCGA
TTTTGAGAGAAAAAGGTTTTTGGCGAGGAGAAGCAAAAGGTTTAAGGAAAGATAAAACAGAATT
TTGGCAAGAAGTTTCCCTTCAGTTAATTGATAGTCAAACACTAATTTGTAATTGTCGAGATATTA
CCGAAAGAAAAATCATGGAACAAGATTTAAGTCGTAGCCATGATTTATTAACTATTATTGAAAA
TGCCCAATCTCAATTCATCGCCAACGGAGACAAAGGACTGCTATTTGATCATCTTTTAGAAAATC
TTTTAACCCTGACTGGTAGCGAATATGGTTTTATCAGCGAACTAGACTATAACACGGATGGAAAA
ATAGAGTTCGGTGATAGCTACATGAAAAATATTGGCAAACCTTTTCTTAAAGTCCACTCCATCAG
TAATGTGGCTTGGAATGAAGAAACGATCCGTTTATATGAGGAAACCCAGGGTAGGGGCATGGAG
TTTCATAATTTAAATACCCTATTTGGGGCAGTAATTTACACTGGTAAACCAGTTATTTCTAACCA
TCCCTCTAAAGATAAACGTAGCGGTGGTACACCCCAAGGACACCCGCCCTTAAATTCTTTTTTAG
GTATTCCTTTTAAGTCCCATGGACAATTATTAGGCATGGTAGGTATAGCTAATCGAGAAGGCGGT

TACAGTGAGGAGTTAGTGGAATATTTACAACCTTTTGTTTCTGTCTGTAGTAACCTGATTGATGC
TTATAAAATAGAAAAACGTCGTCTTGAATCGGAAAAGCAACTAAGAGAGAATCAAATTGCCCTTC
GACAACAAGAAAGAGTCATTAAAAACTTATATCAGATTTGTTCTTCTCCCCAACTCAATTTTAAA
CAAAAGCTACAAGGTATTTTCACTCTGGGGCGTAAGGCTTTTAATTTGGATGTAGCCATGTTAAC
TAATATCGATGATAGCTATTGTCGCATTGTACAGTGGCAAAGCAGTCCTAAATATAGGGAAATTT
TTAATCGAGAAATAACATTGAATTTAGAAGATAGTTTTTGCTTTTTGGCTTATCAAAAAAAAGA
GCCTTTTAGTATTGATCATGTGAGTAATTCCAGCTACATTCGCCATCCTGCTCACGTTAATTTAA
GTATAGAATCTTATTTAGGTACAAGAGTAGAAGTTTTTGGTAAAAGTATTGGCACTTTGTGCTTT
TTTTCCCTTGATTCTCATGGTGTGAAAATAACTAGCATCATGAAAGAACAAATTAAATTAATGGC
ACAATGGATTGGTTATGAGTTGGAAAAAGAAAAATCAGAGGCATTGATTCAACATCAATTCAAG
CAAGAAATTCTACTGAAAAAAATTACTCAGGAAATCAGGCAAACTCTCAACTTCGATCAATTATT
TGACACTGCGGCTAAAACTATTCTCAATGCTTTTCAGGTAAATCGTTGTCATTTGTTTACTTATG
ATAAATCTCAGTCTTCTTGTTTAACCATGGTAGCAGAGGCAATAACAGGCGATTTTCCGCCTATG
TGGGGAGCAAATATAGATCTTTCTGAAGGTAATGACCATTTTAATCAGGTGATTATGAGCGATCG
CGCCTTAGTAAACAATAATGTTTATCAAGACCCCCTCCTTCAACCGATGACTCCTTTTTGTCAGGA
AATTCATCTTAAATCAATGTTGTGTGTACGCACCTCCTATAAAGGCGAAGCCAATGGTATTATTG
GACTACATCAGTGCGATCGCTTCCGCCAATGGACATCAGAAGAAATAAATTTATTACAAAGTGTC
GCCGAGCAATTAGGTATTGCCATTAGTCAAGTAAAACTATTAGAGCAAGAAAAACAACAAAAAC
AACAACTAGAAAAACAAAATCAAGCCCTCTTAAAAGCTGAAAAACAAGCAAAAGAAGCCAGTGA
GGCAAAAACAGAATTTTTGGCTTCCATGAGCCATGAAATTAGAACTCCTATGAATGGCATTATAG
GCATGACAGAGTTGCTACTAGATACCCAATTAAATTCCGAACAGAAAAACTTTGTAGAAATTATT
AACCATAGTAGTAATACTTTGTTAACCATAATTAATGATATTTTAGACCTATCGAAAATTGAATC
AAAAAAAATCGATTTAGAATCAACCACCTTTAATATTCATGAATGCCTAGAGTCTGTCATCAGTC
TTATGGAATTACAAGCAGAAAAAAAAGGCATTAATTTAATTTACATAGCCAATCCTCAAGATAA
TTATTGGTTTAAAGGTGATGTGACCCGCTTAAGACAAGTTGTTTTAAACCTAGTTAGTAATGCAG
TCAAATTTACTCATCAAGGGGAAGTTATCGTGCGTTTAAACGTAGCATCACAGGAACTTGACTCT
TGTTATCTAAAAATCATTGTCGAGGATACAGGTATTGGCATACCCCAAGATAGGAGAGAATCCAT
TTTTCAGGCATTTTCTCAAGTGGATGCTTCTACAACCCGTCAGTATGGTGGAACTGGCTTAGGCT
TAACCATTGCCCGAAAGTTAGTAGAATTAATGGGGGGAAGTTTAACAGTTAGTAGTATTGTCGGT
AAGGGATCAAAGTTTTGTGTTTCCCTAACCCTAGAAAAAACAAATGATGAAAACGATCAATATCT
TTCTTTAAAACAACAAAATCATTTAAGAGGGAAAAAAGCCCTAATTATTTCCGATTCCTCTGTCA
TGGCGGAAATGCTTAGTTTACAAGCCCTCGCCCTAGGCTTAGAAAGTCAAATTTGTCATAGTGCT
AATTTTTCCACAGACCAACTACATCAAGTCGCCGTTGTCATTGCCGATTATCCTCTACAACATCTC
AATCGTTTTGACTTAGCAGAATCTCTTCGATTGTCTCATCCTAACTTAGCCCTCATTTGGTTAACT
CCTCTTAGTCTTGTCACCAAAGAAAATTTAGCTCAAAGCTGCCCCTTTGTAACAGTTATTACTAA
GCCTGTTAAACAATTCCAATTATGCGACACGATTCAAAAACTATTAATAAAAAATGAGATAGTAT
CAAACAATAGTATTTTCATATCTCCAAAGCAACAAAATCTTATTCACAAAGATATTTCTATATTG
CTAGTAGAAGATAACTTGGTAAACCAAAAAGTTGCTCGTTTGATGCTAAATAAACTAGGCTACCG
ATGGATTGAAATTGCCAATAATGGTTTAGAAGCCATTAAAATGATTCAAAATAAAACCTATCAA
TTAATTTTTATGGATATGCAAATGCCGTTACTAGATGGTGTTGCCACTACCAAAGAAATTAGAAA
GTTAGGAAACCAGATTCAACAACCTTGGATTATTGCCATGACTGCTTCTGCTTTATCTACCGATA
GAGACAGTTGTTTAGAGATTGGCATGAATGATTATTTAAGTAAGCCCGTAAAATCAGATACTATT
ATTCAAGCCTTAAAACGATTTGAGTCTTTATCTTGTTTAGCTTCTTGAATCCAACTTTGAACTAG

AGAAAAAGAGGGACATAAATCCCGACGACGTAAGTTTGCAACCTGTAATCTTAAAATTTGTTTTT
GTAATTGAGGTGCATTACATTGGCTCAATTGAGCAACGGAAATAATACCAGAATGTAAAATTAA
ACCGCAATATTCAACCCCAACACTAGGTAAACGAGCTAAATCTGCCAAAGCAAACCATTTATGAA
CATAACGGGGGCTAACACCCATTTTTTGTGCTAATAATTCTCTATCCTGTTTATTTTTACCCATA
ACTAGCAACTGTTGTATATTAGTGATTCCTAGATTTTGTAATCTATCAATATCCGTTTTTTGCAT
ACCGGGTAAGTCAGAAATTGCGATCGACATTTTTAATTCAAATATTATTTAGTCACGACTCTTTT
ATCCTCTAAGCTCAGAACCAGAAGTCAATAAAGGGCAAGGAGCAAAGATAGATAATGGTATATA
GGGTTGGGGTGAATAATTAATAACTTCTAACTCTGAACTCCTAACTCCCGACTCTTGCCATTATT
TCTAAGAAAAGATAGAATTTTTAACATAAAATTAATACTTTAGGGATATTCTATCAAAAAACCA
TAAAAGCGAAAACCCAAAAAGGTTATACTAAACTAGATCTATCCCCAAATTTAGAAAAAAAAT
TAAATCAAATAGGAGGAGCGTAGTCGATGGGACTACCCTGGTATCGAGTACACACAGTTGTAATT
AACGATCCCGGTCGCCTGATTGCGGTACATTTAATGCACACCGCACTAGTCGCAGGTTGGGCGGG
ATCTATGGCGTTATATGAATTAGCCGTTTTTGATCCTAGTGATCCCGTGTTAAATCCCATGTGGA
GACAAGGGATGTTTGTATTACCCTTCATGGCACGTTTAGGAGTCACTGGCTCTTGGGGTGGCTGG
AGTGTAACTGGCGAAACCGGTGTTAATCCCGGTTTTTGGTCTTTTGAAGGAGTTGCGATCGCACA
TATCGTCCTCTCTGGATTACTTTTCTTAGCCGCCGTATGGCACTGGGTATTCTGGGATTTAGAAT
TATTTACCGATCCCCGTACAGGTGAACCCGCTTTAGATTTACCCAAAATGTTTGGGATTCACTTA
TTCTTATCTGGCTTACTCTGTTTCGGTTTTGGTGCATTCCACCTTACCGGATTATGGGGACCTGGA
ATGTGGGTATCAGACCCCTATGGACTAACAGGACACGTTCAACCCGTAGCACCTGAATGGGGACC
TGCAGGATTTAACCCCTTTAACCCCGGTGGTGTTGTTGCCCACCATATTGCCGCAGGAATCGTTGG
TATCATTGCCGGATTATTCCACCTCAGTGTTCGTCCCCCCGAAAGACTTTATAAAGCATTACGGA
TGGGTAACATTGAGACAGTTCTTTCTAGCAGTATCGCCGCAGTATTTTTTGCCGCTTTCGTCGTG
GCAGGTACAATGTGGTATGGAAATGCTACCACCCCTGTAGAATTATTCGGACCTACTCGCTATCA
GTGGGACCAAGGATACTATCAACAGGAAATTCAACGTCGTGTACAAGCTAGTATTGCCGACGGAG
CAACCCCCGAAGAAGCATGGGAAGCAATTCCTGAAAAATTAGCTTTCTACGATTACGTTGGAAAC
AGCCCCGCAAAAGGTGGTTTATTCCGTACCGGTGCTATGAACAAAGGTGACGGTATTGCTCAAGG
TTGGTTAGGACACCCTGTCTTCCAAGATAAAGAAGGACGTGAGTTAACTGTGCGTCGTCTTCCTA
ACTTCTTTGAAACCTTCCCAGTTGTCTTAACTGATGCTGACGGCGTAGTTCGTGCAGATATTCCTT
TCCGTCGTGCAGAATCTGCTCTTAGTATCGAGCAAACTGGCGTAACTGTTAGCTTCTTAGGCGGT
GAATTAGACGGTCAAACTTTCTCCGATCCTTTAGATGTAAAACGTTATGCTCGTAAAGCACAATT
AGGTGAACCCTTTGAGTTTGATACAGAAACTCTCAACTCTGATGGTGTTTTCCGTACCAGCACTC
GTGGTTGGTTTGCTTTTGGTCATGCTTGTTTTGCCCTCTTATTCTTCTTTGGACATATCTGGCATG
GCGCTCGTACTCTATTCCGTGACGTATTTGCAGGTATTGATCCTGACTTAGATCCTGAACAGGTA
GAGTGGGGTATGTTCCAGAAAGTTGGTGACAAATCCACTCGTCGTGCAGAATCTGCATAATTTTT
GGTTTTTATAGTAGGATGGATAGAGACAAGTTAATCTTATTTTCTTTGTCATTTATCCTCCTAAA
TGTAAACTCAGTGCATTTTCATCTTGTCCAATTCAGATAATATAGAGGAATAAAAATGGAAAGTG
TAGCTTATATTTTAGTGTTAGCGATGATGATCGCCGTCTTATTCTTTGCAGTAGCGTTCCGTGAA
CCTCCCAAGATTCAAAAATAAATCATCTGTGAAATGGGACGTTTTCACAAACTTATAATCAAACT
TTTTAACTTTAGAGAAGTCTTTTTCATCTATTCCTGACTATGGAGTAGAAAGAGAAAGGCTTTTT
TCATTAAATGTTGAAAAAATAACAACTGCAACCTACCTGAGTTCAGACTTTAGATTTCGGAGTTA
AAGTTAATAGATTTTTATCAAAATAGTCGATAAAAGGACTCAGTCGTAGGTAGCTAATACCTCAA
GATGGAAAATTAAGCATTTATTTAGCACAAAAGAAGCCATTTATTATGACTCCTAAATTTTATTG
ACAAATTGATTTTCTTGTTTTCAATATGAGTGAGTAATAAGTCTATCTTTAAAAAATCAAGTCTG

AAAACTACTCTTATCCGATATTCTCCTAAGCCGAACTGAGGTTATTTAAGCTAAAATAGTTCATC
TTTATCTTTTTAAAATTTAACTCATTAATTAAAAACAATTACATAAATAATAATAATAATTAAC
AACAATAAATATAGTTATTACTAATATTTTAACTTTATTATTTGTTTCGATAAATTTATTCACGT
AAAAAAATCCATAAGGGGTGTTGAAAACGTAATTTTCTTTTATCTAATAAAGTGACGGTAAAAT
AAATTATTTTGTCTGTGAGATAGAAGCAAAGAGACTTGAACAAGTAGTAAAAACAATAATAAAC
TATATATTCTTTTCCGAAAGCTAATGGATATTTTAGCTCAACTTTCTATTTTTACTCATAAAAAA
TGATACATAAATTTTTTAATATTACTTTTTAATTATTATTTTTCTCTACTGGGAATGAGTTTGAT
ACCCCCTTGCTAATCATCAACAATTTTATTGCGATTTTGTCATCTAATTAAAAATTATGCAATGT
CCTTCATGTCATTTTAATCAAACAAAAGTAATAGAGTCACGAACTACGGAAAAGGCACAAAGCAT
CAGACGTAGAAGAGAATGCCTCCAATGTCATCACCGCTTTACAACCTATGAAAGAATAGAATTTG
TAGCAATTACAGTGATCAAAAAAGATGGTAGTACAGAATCTTTCGATCGCTCTAAAGTTCTTAGA
GGAATAGTAAGAGCTTGTGAAAAAACGGGCATTGAGTCAGAATTACTAGACTCTATTGTGGACA
ACCTAGAATCAGAAATCGAAAATCAATTTCCGAGAGAGATTACCAGTAAACAAATTGGGAACTT
AGTATTAAAACAACTGAGCCAAATTTCCCAAGTAGCATATATTCGATTTGCATCAGTTTATGAAA
ACTTTACCAGTATTGAAGATTTTGTGGCAACCCTGAAAAAATTGCAAAATTCACCAGAAAATTCA
GGAAATAACCCTGAGCCTCAATCTCCCATTTCAGAAAATGAAAACAAAAAGAATCAGACAACAAA
TCAATAATGATTAACACTTGTCAAAATCTCAATTATAGTGTAGAGTAAAAATTCTGTATAATAA
TACTATTAATTAAAGATTTTTTATCTTTATATAAGTATTCGGTAGGGAACGACAAATCCCAAACC
AATTCCGAGAGGAGTTAAAGGCTTAGGGAGAGAGGCTTTCAGGAAGTTAAAGCAAACGCCTTAGC
TTTGATGAAACAACTCGCGCAACTAAGAATGTCCTAACTTTTTTAGTAGGGCTTTGTCTAAATTA
ACCCTCTAGTGTTTTACTAGAAGGCTTTAAACAAAACTTATTGAAAAACGATAGAGTAGTAAATT
TTATATTTAATTCCTATATCTATCTAATCCTGTAAACTTGAAATTTTTTAAGTTTCTGAGGAGGG
ATCAGAATTATCGCAATAGTAATTGAAAATCAAAAAATAGAAGAAATGCCTATGGTCAATCAAA
CAGAAACCCTAAATAGTAATGTTAGCTTTACCCATGAGGAGTTTGCTGCCCTCTTAGATCAATAT
GACTATCATTTCAGTCCTGGGGATGTAGTTCCGGGGACTGTTTTCAGTATTGAGCCTAAAGGTGC
TTTGATTGATATAGGTGCAAAAACCGCCGCTTATATTCCTGTACAGGAACTTTCCATCAATCGTG
TGGATGATCCTAGTGAAGTCTTACAAGCTAACGAAACCAGAGAATTTTTTATTCTCACTGACGAA
AATGAAGATGGACAACTAACTCTTTCTATCCGTCGTATAGAATATATGCGTGCATGGCAAAGAGT
TCGTCAGTTACAAGAAGAAGATGCTACCGTTTATTCTAACGTGTTTGCTACTAATCGTGGTGGTG
CATTAGTGAGAGTGGAGGGTTTAAGAGGGTTTATCCCCGGATCTCACATCAGTGCTAAAGACACT
AAAGAAGATTTAGTTGGTCAAGATTTGCCCCTGAAATTCTTAGAAGTGGATGAAGAGCGCAATCG
TCTAGTTTTAAGTCATCGTCGTGCGTTAGTTGAGCGCAAGATGAATGGTTTAGAAGTTGGACAAG
TGGTTATCGGTTCTGTACGTGGTATTAAACCTTACGGTGCGTTTATCGATATTGGTGGCGTTAGT
GGTTTATTACACATTTCCGAAATTTCTCACGATCATATTGATACTCCTCATAGTGTCTTTAATGT
CAATGATGAATTGAAAGTGATGATTATTGACTTAGACGCAGAAAGAGGTCGTATTTCTCTCTCTA
CCAAGCAGTTAGAACCCGAACCGGGCGATATGCTCAAAAACCGTGAGTTAGTCTTTGAGAAAGCA
GAGGAAATGGCTCAGAAGTATAAAGAAAAATTATTAGCTGGTGATGCTGAAGAAGCTAATATTG
ATTCTGATTCTGTGCAAGAAGTAGCAGAAGGAGAAGAGGTTACAGCAACTGAGACAACTGAAAC
TTCAGATATTGAAAATCAGTCTGAGGAAGAATTAACCACTACAGCAGTAGAGTAAATTACTTTCT
AGTCAGTCAGTGGACTTTCAGTTCGTTAAATTTACCTGAGAGTGTAGCTGACTGTTATGAGTAAA
TTGGAGCATAAATTTAATGCTTTTTATCAATCATCAACTATTATTAAGCAAGGGGTTTACTTTTT
TATCCCTTGTTTTTTATTGTCTGTAAAAGATGACAAAAATCTGATAAATTATGATGATGGTTTAT
GGCTTCTTCAAAAATGTTAACGACTAAAAAAGAAACTGTTAAGATTGTTCCTCTGCCTGAATTAA

CTTCTGAAGAAAAACTGTTTCAAAAACAAAGGATGAGGGATGTTTATTTGTTGTTGGAAAATTT
AACTTTAAAAGAAGAAGCAACCATTAAATTAATTATCGATTGTTTATATGATGTAGGTATCATC
AATTTAATTAATAAAAAAATACAGAATCGTCCTTTAAATAAATTAGCGAAGATGATGGCAAGTA
CCCCCAAACCAATTTTTAAAATTCTTGCTTGGCGGTGGGTGAGGAAAAATTTATCGAATAAAGTG
ACAAATTGGCTTAATAATAAAGTGAAATTTAAGTAAGAAACTGAGTTCAAGATAATATTGTTGG
CTATAAGTCGGCAATAAGAAAACCCCTCTGTGTCTCCCCTTAAAAGGAAAGAAGGGCAAGGATGC
ACCGAGGTAACAGTAATTAAGAATTAAAAACACTTGACACCTGCCACCTGAAACCTACCCAAATC
GATATTCTTATATCAAACTAAGGTAAAATAAGGGTTTAGTGCCATAAAGGTTGCGTTAATAGAA
TTACTATATATATTTATTTAGATTAAGATTATTATGTTTGACGCTTTAGCAGAACGTTTAGAAGA
TACATGGAAAAAACTAAGAGGACAAGGCAAGATTAGTCAATCTAATATACAAGAAGCCTTACAG
GAAGTTAGAAAAGCCTTATTAGAAGCAGATGTTAATTTTCAAGTCGTCAAAAGTTTTGTTGCCGA
GGTAGAAAAAAATGCTCTGGGTGCGGAAGTTATTTCTGGGGTTAATCCTCAACAGCAATTCATCA
AGATTGTTTATGATGAGTTAGTGAAGGTGATGGGGGAGAGTAATGCCCCTCTTGCGGAGGCTGAT
AAACCTCCTAGAGTGGTTTTGATGGCTGGTTTACAGGGTGCTGGTAAAACTACGGCAACGGCAAA
ATTAGCTTTATATCTACAAAAACAAAAGCGTAGTTGTTTGATGGTGGCAACGGACGTTTATCGCC
CTGCGGCTATTCAGCAATTAATTACTTTAGGGACGCAAATTTCTGTTCCTGTGTTTGAATTAGGC
ACTGGGGCGAATCCTGTGGATATTGCCCGTCAAGGTTTGGAAGAGGCAAAAAATAAAGGTATTGA
CACTGTTATCATTGATACTGCAGGTCGGTTACAAATTGATCAGGAAATGATGGCAGAATTAGCTC
AGGTGAAAGATGCTGTTAATCCTGATGAAGTGTTATTGGTGGTTGACTCCATGACGGGGCAAGAA
GCGGCTAATGTTACTCGCTCTTTTCATGAGGAGATTGGTATTACTGGGGCTATTCTCACGAAGAT
GGATGGGGATAGTCGTGGAGGGGCGGCTTTATCTATTCGTACTATTTCTGGGCAACCCATTAAGT
TTATTGGGGTTGGAGAGAAGGTTGAGGCTTTAGAGCCTTTTTATCCTGATAGGATGGCTTCTCGT
ATTCTCAATATGGGGGATATTGTCACATTAGTGGAAAAAGCTCAAGAGGAGTTGGATATTGCCGA
TGTGGAGAAGATGCAGAAGAAGATGATGGAGGCAAAATTTGATTTTAATGACTTCCTCAAACAG
ATGCGTCTTTTGAAGAATATGGGGTCTTTTGCCGGGGTAATGAAGTTGATTCCGGGGATGAATAA
GTTGGGAGCGGGAGCATTAGAGCAAGGAGAAGTACAATTAAAGCGTACAGAAACAATGATTAAT
TCCATGACGAAGGAGGAAAGGGCTAATCCTCAATTGTTGGCTCAGTCTCCTAGTCGTCGTCGCCG
GGTGGCAAAGGGTTCAGGCCATCAAGAAAAGGATGTTTCTCGTCTTGTTAGTGATTTTACAAGGA
TGCGATCGATGATGCAACAAATGAGTATGGGCGGTGGTTTACCCGGTATGGGAGGTATGCCGGGC
ATGGGCGGTTTAGGTGGCTTATTGGGGGGGGGAATGCCTCGCCCGGGTTATCGTAATCAACCGAA
GAAAAAAGCGAAAAAAACTAAGAAGAAAAGAGGTTTTGCTGATTTATAAGTAATTAGGAGATTA
GGAAATTGGGGTGTTGGGGGGATGAGGTGTCAAGGAAGACAAGTTAGGAGTTAGTTAGGGGCGA
ATGGCCATTTGTCCGTACAGGGGTTCGGGGTTTTTAATTCTTAATTCTTAATTCTCAACTATTCA
CCATTGCCTTTTACTCTCTTTGCCCGTTGCTCTTTTTCTGACAAAATTTATGGTTTAAAAATGAA
TAAATCTCATAAACATATAGGTGTTGCCATTATTATTAATGATAAAAAGGAAATTCTAATTGAT
CGTCGTTTATCTACGGGGGTTATGGCGAATTTATGGGAGTTTCCGGGGGGAAAAATAGAAAGGGG
AGAAACGCCGGAAGATTGTATTATTCGAGAGATAAAAGAGGAGTTAGGGGTTGTGGTGAGATGC
GATCGCATCTTTGCTGAGATTACTCATGATTATGAACAATTTACGGTGACTCTATATGTATATCT
TTGTCATATTTGTCAGGGAAAACCTCAACCTTTAGAGTGTGCAGAAATATTATGGGTAAAATCTC
ATGAATTAAGCCAATTTGAATTTCCCTCAGCTAATCAAGAAATAATTAGTAAGTTGCAAAAAGA
GTTTTCTTGATGTGATAATTTTTGGGGAAATAATGAAGTCAATTTTAAGTAAAATAACGGCGAA
AACGAGGGTCAAATTTTGCTTTTTTCTTTTGGTTACATTGATAGCAGAGAATTTGTAAGTTACTT
AAATCATTACTTCCCCCTTTTGCCAAAGGAATTATATGATCATTATTTAATGTAAAACTGGTGTG

TTTCTCTGACATAGCGATCGCACTCTGAGGTTGTAACGGCTTTTAATGCTTGATATAGCTTGTTT
TTGAGAGCTGATAACTCATCATAAATTCCTGTATTGCCATCCACGTTAAATTTTGGAAAGAGACT
GAGAAGAAGGCACTTCCTGCCAAAATAAAGGTATTGCAATCAATCCTGAAATCAGTTGAACTAAT
CCGAAAACAGCAAGGGAAATACCAAAACCAAAGTTGAGTAGCTCACCAAAAATTAGACTGCCAAT
CCCAAAACCAGTGAATAAAAGAAACACTTTTAAACCCATAGTTTGCCCCAATTTATCATCATCAC
CCAAATCAGTCACAATTCCTACAAATAAAGGCTGAGTTAGATCATAACCTAAAGATAAAACTAAT
ACTGCCATAGTCGTAACATAAGGAGGAATTTGTAAAATCATTAATAATCCAGCCAAAGCCGCCAT
AATTAACCCTGCGGGAATTAACCAACGTCTCCCCCACTTATCCACAGCTTTCCCGATGGCAGGACT
TAACAACAATCCGGGGATACCATAACCTAAAATAGTTAAACCAATATTTAAGGCATCCATATTAT
AGCGTTGAGACAAATATAATCCTAACCAAGTATAAACCCCTGAATGATAAATACCATTCCAAAGT
ACATAACTATAAGTTCTTTTACCCCGAAATGTTACTAATACTTGAGCATAACCCCTAAAAATTTG
GCGAATAGAAGGAAGTTTATCCACTTTTGCAGTATCAAATAACGCACCATAGGGGTTGAGTTGCC
AAAGTACAATCCCCGCCATAATTGCCGTACCAAGAAAAAGCCAACGCCATCCGATATAAGGTTCG
AGAATTGCCCCTCCGGCTGAACCTGCGGCCATTCCTCCTTCCATTGCGGCAAATACTAATCCTAAT
TTTACCCCTCTTTGATCAAAAGGGAATAAATCTCCAATAAGGGCAAAAGTAAGAGGTATTACACC
ACTAGCTCCAATTCCTGTTAATAAACGCCATGTTGCCATTTGACTAGCAGTTTGAGAAGTTGCAG
TCAAAGCAGTACACACTACAAAAATAATAAGAGAAAGGCGAATAATTGACCAACGACCAAAACG
GTCGGATAATAGTCCGTAAAATAATGCCATTAGTGCATAAGAAAGCATATAGGCAGGAACAATA
AAACCAATCTCCTGCACCGGCACATTAAACACTTCTGAGAGTCTGGGTATAAGAGGGGCTATCAT
ATAGCCTTGTAAAAAGATTAAACCTGCCGCAATTGAAAGTAAATTAAATAATTCCTTATGTTTTT
TTAGCCTTCGGGCTTTGTCTGCTTCTTCGATATGCTGTAGTTGTTTTTTGCTCATCTCCGTTTTTA
CGATGGTAAACCCTCTATATTTTACATCCTCTCATTTCTCTTTATGCAAGAATTGCATTTTACTTT
CTTTGTTTCGATGGAAAAATATGCGATAATTCACTACCATTCACCTTTGCCCCTTGCCCTTTATCT
TGTTTTAATTGCTAATTGCTCATTGTTGATTGCCATTGGTTTATAATTAATTTTTGTACAGGTGC
GATCGCACCATTTAGTTCATAATTACGCCGTAAAGGATTACGATTAAAAGCCTCTTGTTCTTGTT
TTATCATTGCAATATCCTGCTCTACCAATCCATCTAATAAACCCTTTGCCGCATTAAATAAACTA
TTTTTCACAAAACGACGAAACCACACGGGTAACTTATGTAAACGCCAAAAAGCCTGTAAAGAAGT
AAAATGAATTAGATAAGCCTTCGTAGTAGTTTCATCAATGGGGCAGAATAAACAATAAATTTTG
AAATCTTCCCCTAAACTTGAACTCCAATGAGGATAAACATAACTTACCCTTAAAGGTTCAGGATG
TAAACGTCTTAGGGCAGGAAAAAATAATTGAGAAACAGACCATATTTTATCAATACGATAATAA
CTTTGAGCAGTATATAAAACATCTATGCGTTGAGAAGTTGTCTCAATTTCCTTTAAATTCGCTTC
TGCCCATGCCTGATAATTGTCATGTAAATGCCCGTGATACATATCCATTAAATTTTCGATTAAAT
AAGAAAAATGTCCGGGGCAGTTAATAGTAGTTACACTAGCAATATAGTTAAGATGCTCCCATTCA
GGCAAACCCGTAGGCTTAATTTTTTCCGAGTCACCATCACCTAAAAACAACCAAATAAAACCATC
TAATTCCTGTACGGGATAAGCCTTTAACTTACAACTTGGCAACCTTTGATTTTCCTGTAAATAAG
GTACAAAAGAGCATTTACCCTCCCCATTAAACCGCCAACCATGATAAGCACATTCAATATCATCA
CCCACTACTACACCATCACTTAACCTTACCTGACGATGGGGACAACGGTCTTCTAAAGCCTTAATT
TTTCCTTGAGCGTTACGAAAAATTACAATATTTTCATGCCAAAGAGTCACACAATAGGGTTTATC
TGTTACTTCCACACTTCTAGCAACCACATACCAATGATTAGGATTAATTCCACAAGTACGAATAT
TTGGCTTAACTTCTAGTTGTCCCATACAATATTGAATATTCAATTATTATCTTTAATTCTCTTAC
AAATATTATCGGAAAATCTCAATAATTCTCCTTAAAATCTCAAGAAAGAGAAATGAGTTAGGGG
ATTAGGGTATTAGGGGCTTAGGATATTTGGAGGGGGATGAGGTGATGAGGGGAGAGAGGGGATA
AAGGGGGGTTTGCCCTTTGCCTACTGCCCTTTTTTGTTCTAATCAATAATTCAAATATACTCAAA

GCAAGAGAGTCAAAATATGCCCTTATTAAGTTGGAATGAAATTAAAAATAGAGCCTTAATTTTT
AGTCAAGAATGGCAAGGAGAAATATCAGAAAAAGCTGAATCGCAATCATTTTGGAATGACTTTT
TTAATGTGTTTGGTATTTCCCGCCGAAGGGTGGCAACTTTTGAAGAGTCTGTGAAAAAATTGGGC
AATAAGCAGGGATTTATTGATTTATTTTGGAAAGGGGTAATTTTAATTGAACATAAATCAAGAG
GTAAAAATTTAGATAAAGCCTATCAACAAGCCATTGATTATTTTCCTAATTTACAAGAATTTGAG
TTACCAAAATATATTTTAGTTTCAGATTTTGAGCGGTTTAGATTATATGATTTAGATAGTAATA
ATAATCATGAGTTTTTGCTTAAAGATTTTATTAATTATGTTCACTTATTTGATTTTATAGCAGGA
TATAAAAAACGAGAATATAAAGAAGGAGATCCAGTTAATATTCAAGCGGCGGAGTTAATGGGAG
AATTGCACGATCGCCTCTTAAATATTGGTTATGATGGACATAATTTAGAAGTATATTTAGTGCGT
TTATTATTCTGTTTATTTGCCGATGATACGGGCATATTTAATAAAGGCATTTTTCAAGAATATAT
AGATTTATATACGAAAGAAGATGGTAGCGATTTAGCTATGCACTTGGGTTATATTTTTTATATTT
TAAATCAACCAGAAAATAAAAGACTGAAAAATTTAGATGAAAATTTAGCTCAATTTCCCTACAT
TAATGGTAAACTTTTTGAGGAAGTTTTACCCCCTGCTTCCTTTGACAGTGAAATGCGTCAAATGC
TTTTAAAAGCCAGTGCTTTAGATTGGGGGAAAATATCTCCTGCAATTTTTGGCTCAATGTTTCAG
GCGGTGATGAATCCCCAACAAAGACGCAATTTAGGGGCGCATTATACCTCAGAGAAAAATATTCA
GAAAGTAATTAAACCTCTTTTTTTAGATGATTTATATGAGGAGTTTTATAAAATCAAAAATAGC
CAGAATAAGTTAAAAGAATTTCAAGCAAAATTAGCTCAATTAAAATTTTTAGATCCTGCTTGTGG
TTGCGGTAACTTTTTAATTGTTTCTTATCGGGAGTTACGAGATTTAGAAATTTTAGTCTTAAAAG
AATTACAAACAAAAGGACAATTAGAGTTAGATATTAGTAATATTATTAAAGTTGATGTAGATCA
ATTTTATGGCATTGAATATGATGAATTTGCCGTTAGAGTGGCAGAAGTTGCAATGTGGTTAATTG
ACCATCAAATGAATATGAAAGTTAGTAATGAATTTGGTCAATATTTTGTCCGTTTACCTCTGAAA
AAGTCGGCTAATATTATTCATGGTAATGCTTTACAAATTGACTGGCACTCTCTCTTAAATCCCCC
TCCTTTGAAGGAGGGGTTAGGGGTGGTAGAATCTAAATCTTTAAAGGATGGATTAGGGGTATTT
GAATTAACATCTGGAAAAGAAACAACAGAAAATAATTATCCCACTTATAAATCAATAAGCAACC
TTAAAGATAAAAAAGAGTTGAGAAAATCTCTAAGAAACAATGCAACTTCAGCCGAACAATTACT
TTGGAAAGCCTTACAAGGAAAGCAATTAGATGGTTTTAAATTTCGCAGACAACATTCTATTGGTA
ATTACATCTTAGACTTTTTCTGTCCCAGTGTTAGTTTAGCCATTGAATTAGACGGTGAAACTCAT
TATACCCCTGAAGCACAAGAATACGATCGCATCCGAGATAATTTTCTTGCCAATGTCGGAATTAG
GGTAATTCGTTATCACAATCATGATGTATGTGATAATTTGACGGGGGTTTTAGAGGATATTCGGC
AATATCTTTATAATCCTGATGCTTCTTTCAGTCAACGTAAATCTATCACTTCCAACCCTTCTAGCA
TAGGAGAATCTAACACCCCTAACCCTTCTAGCATAGAAGAATCTACCACCCCCAACCCCTCCTACA
TAGAAGAATCTACCACCCCCAACCCCTCCTACATAGGAGGGGAGAATAATGGGCTAGGGGAAGTA
AATAACTTTGATTTTATTTTTGGTAATCCTCCTTTTGTGGGTAAAAGTTATCAAAATGCTCAACA
AAAAGCTGATATGAGTTTAGTTTTTAATGGAGTTAAAAATGCAGGTGTTTTAGATTATGTGTGT
GCATGGTATTTAAAAGCCTCTCAATATCTCAACCCCCCTCAATCCCCCCTTAGTAAGGTGGGAGGT
TTTACCGTTAATTCCTCTCAATCTTCGATAAATTCCCCTACTTTGAAGGAGGGGTTAGGGGTAGT
TAAAACCCGTTGTGCTTTTGTTAGTACAAGTTCTATTTCTCAAGGTGAGCAAGTAGGGGTTTTAT
GGCAGGAATTATACACTAAATATAAAATTAAAATTCAATTTGCCCATCGGACTTTTTCTTGGAGT
AATGAAGCAAAAAATAACGCAGGAGTCCATTGTGTTATCATCGGTTTTGGGTTAGAAAATATAG
AGAATAAGCGACTTTTTTCCTATGAAAATATTAGGGGTGAAGCTACTGAGATTAAGGTTAAAAA
TATCAATCCCTATTTAGTGGCAGGAAATGATTTAATTATATTAAAAAGAGCTAAACCTATATCTA
ATATTCCACAGATAATGTACGGTAGTAAACCCACAGATGGAGGTAATTTATTAATTGATGAAACT
GAAAAAATGGAATACCTAAAAATAGAAAAAATCGGAGAAAAATTTATTAAATCTTTTATTTCTG

CTGATGAATTTATTAATGGAAAAAATCGTTACTGTTTTTGGTTAGTAGATATTCAACCTAGTGAA
TTAAAACAATTACCTTTACTGTTAAAAAGGGTTGAAGCTGTGAAAAAAATGCGTCTAGCTAGTAC
AAAAAAAGCTACTCAAAAATGGGCAGAATTTCCAACTCTTTTTGCTGAAAATAGACAACCTAAAA
GCGATTATATTCTTATTCCTTTACATTCTTCTGAAAATAGAAAATATATTCCTATTGGTTTTTTT
AATAAAGATAATATCGTAGCTAATAGTTGTAGTTGTATTCCCAATGCTGATTTATTTTTATTTGG
AATATTAACCTCAGAAATGCACATGACATGGGTTAAATATGTGTGCGGAAGATTAAAAAGTGAT
TATAGATATTCTAATTCTATTGTTTATAATAACTTTCCTTTTCCTGAAAATGTGACGGCAAAACA
GAAAGAAAAAGTAACTAATTTAGCTCAAAAAATCCTCGATATAAGAAATAAATATACCACCCCT
AACCCCTCCTTAAAAGGAGAGGAAAAAGGTGCTAGTTTAGCGGATTTGTATGATCCTTTAACCAT
GCCTCCTGATTTATTAAAAGCTCATCAAGCCCTTGACAAAGCAGTAGATTCATGCTATCGAAAAC
AGCCTTTTTCCAGTGAGTTAAATCGCATTGAATTCTTATTTAATTTATATGAAAATCTTACTAAT
CCTCTCCTGAAATCACAAGGCAAAAAGAGCAAAAAATAATCCTAAAACTTCTCCTCTTTTAAGGG
TAGATACAGAGCAGGGTTTTTTCATTCTCAAAAATGTCAATTTTTGAAACTAACAAATAATCTCA
GTTCGGTTTAAGAATATCTGATAAGGTTAGGTGTCAGGTGTCAGGTTGCAGGTTGCAGGTGGTAG
GGGTTTTTAGCAAGGGGCTTAAGCCCCTTGTTTAAGTCAGTTCGGATTAAGGCAATTTTATCGTT
ATTTCTAAGTTATTTCTAAGAAGCTATAAGGTTTTTTGACTACGAGTTGGTCTGCTTTCAGCTTA
TCCAAAACTCAGGTTAACAAATAGTAACAGATACAGGAGTTTTTAAGTATTAATTAATCAATTA
TTAAGAAAAAACGCTCCCCTCTACTGTGGGAGAGGGGTTGGGGATGAGGGCAAAATAATTTTACC
CAGACTTTGAAAAAACCTTGGATACAAAGGGGTTTCCCTTTTTTTTACCTCAATGAAATTTATTG
CACTCAAAGCAAGAGAGTCAAAAATGTTAAATTAGCAATGTTTAGTTTTATTAAATTTATTTGCC
ATTATGCTTACTTCTAATTATTCCATTGCTTCTCTACTAGATAGACAAAGACAGTATTTTGCCAC
AGGGGAAACCAAGTCTGTTGATTTTCGTTTACAACAGTTAAAAAAATTAAAGAGTGCGATCGCAT
CTCGTCAAGATAAGATATTAGAGGCATTAAAAGCGGATTTAGGTAAACCTACCTTTGAGGGATGC
TTTGAATTAGCAGTTTTACAAGATTTAACCTATGCCATTAAAAACCTGAAAAAATGGGTTAAACC
CCAGAAAGTAAAAACAGGGCTTGAACTTTTTCCCGCCCAAGCCAAGATTCATCCTGAACCTTTAG
GCGTAGTATTAATTATAGGTCCTTGGAATTATCCCTTTTCTTTGATGATTTCTCCCCTTATTGGC
GCGATCGCATCTGGTAACTGTGTACTGCTAAAACCCTCGGAAATCGCCCCTCATACCTCTGCTTTA
CTCACAGAATTAATTAGGGATACATTCCCCTCCGAATATATTTGTATCCAAGAAGGTGGCGTAGA
AGTAGCTCAAGAATTATTAGCCTGTAAATTTGACCATATTTTCTTTACTGGGGGTACAAAAATTG
GACAAATCGTCATGGAAGCGGCGGCGAAACATTTAACCCCTGTCACCCTCGAATTAGGAGGAAAA
AGCCCTTGTATTGTCGATACACAAATCAATCTCACGGAAACCGCAAAACGCATCACTTGGGGTAA
ATTTATTAACGCAGGACAAACCTGTATCGCTCCTGATTATATCCTTGTGGATGAGAAAATCAAAG
CAGATTTAATTAAGGAGATGAAAAAATGTGTTGAAAGCTATTTTGGTGATAATCCTGCTGATAG
TCCCGATTTTGCCCGAATTATTAATCATAAACAGTTCGATCGCCTTCAAAATTTATTAACCGATG
GTAAGATTATTATTGGTGGTAACTATAACCGTGAAGAAAAATATATTGCTCCCACCATCTTAGAT
GAAATTTCCCCTGACTCTCCCATCATGCAAGAGGAAATTTTTGGACCAATTCTCCCTGTTTTATCC
TATCAAACCCTAGAAGATGCGATCGCGTTTGTCAACAGCAAACCCAAACCCCTTGCCCTTTATTTC
TTCTCCCATAACAAAGACAAACAGCAGAAAATCTTGAGAGAAACCACATCAGGGGGATTATGCTT
TAACGAAACAATTATGCACGTAGGAGTCACCGAATTACCCTTTGGAGGAGTAGGAGATAGCGGTA
TTGGTGCATACCATGGAAAAGCAACCTTTGATACTTTTTCCCACCATAAAAGCGTTTTATCCCGC
CCATTTTGGGGAGATTTAAACTGGCGTTTTGCTCCCTATAGCGAAAAAAATGTCAAGTTATTTAA
ACAAATATTTACAAAATAACCTGAATCCAGAAACTTATCGGATAATAATGACGGAAATCAATGCT
AATTACGATGAACCTTGGAAAGAAGCCATCGGAGAATACTTCGATCGCTTCTTGGAGTTCTTTTT

TCCACAAGTATATCACTTAATAGACTGGTTAAAACCACCAATTTCTTTAGACAAAGAATTACAGA
AAATTACTGCCGATTCTGATGATAGTAAAAGATTGGTAGATAAGTTGTTTCAAGTATGGCTAAA
AAATAATCAACCAGTGTGGATTTTGGTTCATATCGAAGTACAAAGTCAATATGATAGCGATTTTA
ATCAACGAATGTTTATCTAACCTGAGTTCGGGATAAATTTTCATCTATGAATGATGGAGAAAAAA
AGGCAAGAGGCAAGAGGCAAACCCCACTTTCTCCCCCCTCTCGAGGGGGGAGGGCAAAGGAAAAA
TTAAGAATTAGGAATTAAAAACCCCGAACACTCATTATTTGTTACTCATTACTTTCTCTCAACAC
CTGCAACCTGCAACCCGAAACCTGACACCTACCCTTATCCAATATTCTTAAACCGAACTGAGGTTA
TCTATCATTATCGCACCTTTGATTTATACCGAAAACCCGTAATTAGCCTTGCTATACTAGGAGAC
GAAAAAGAAAAATGGCGACCATCGAATTATGGCTATGATTTAGGTGGATGTAAATTAAACTTAG
ATTTTCCCGTAATTAAACTATTAGATTATCAAGAAAAATGGGAAGAATTAGAAAGAGATTTAAA
CCCCTTCGCCATAATGATAATGGCACATTTAAAAACCAAGACAACCACAAGTAATTTAACAGAAA
GAGAACAATGGAAATGGTATTTAATCAGAAGTTTATACGATAAAAATTTCAGTAAATTGGAAAT
AGTAAAACTGTTTAAATTTATTGATGCGATGATGACATTACCACCATTGTTACAACAAAGTTTAA
ATAAAAAAGTTATTGAATACGAGGAGGAAAAAGTGATGCCCTTAATTAGTCCTTTCGAGCAAAT
GGCGGAAGAAAGAGGTTTAGCGAAAGGTTTGGAAAAAGGTTTAGAGCAAAGAATTGAAAAGGAA
AAAGAATTGATTATTCGCTTATTAACACGTAAATTGGGAACAATAAATACTGATTTACAAACTCA
AGTAAAAGCTCTAAATATCGAAGATTTAGAAACCTTAGCAGAGGATTTATTTGATATGAATTCC
ATTGAAGATTTACAACAATGGTTAAGTAATTACTAATTTTTATATCAGACAAAAATCTTGATAG
TAGCTTGAAATATAGCTACTTTTTTTATTTCAAAATAATTAAGTAAGCTAATGTTTTTGGTCAAT
TTTCTCACCTGACACCTAACACCTAACACCTTTTCAACAACGATACTTTTATAAGCTAAGACGCA
GTATAACTCTCCATGCCTACACAAGAACATACCAAATTGCGATCGCCCTTAGCATTATCAATACG
AGCCACAGAAGTCCAAAATTTATGCTCTTTAGTCCATTTATCAGGATAAGCGGCCATTTCTCTGG
TGTAAGGGTGATTCCACTTAGATTTAAGCAACATTTCGGCAGTATGGGGGGCATTTTTTAAGAGA
TTATCATTTTTATCCATTTCCCCTTTTTCAATGGCTTCAATTTCCTTACGAATGCTAATCATAGCA
TCACAAAAACGATCTAATTCGGCTTTTGATTCACTTTCTGTAGGTTCGATCATCATTGTACCTGC
AACAGGCCAAGACATGGTAGGAGCATGGAAACCATAGTCCATTAATCGTTTTGCCACATCTTCCA
CGCTTATATCCGCAGTTTTACGAAAATGACGTAAATCAATGATACACTCATGGGCAACTAAACCA
TCTTTACCAGTAAATAGAATAGGATAGTAAGGGGCGAGACGATGTGCCATATAATTAGCACTGAG
AATGGCTATCTTCGTGGCATGGGTTAACCCTTCTCCTCCCATCATGGCAATATACATCCAAGAAA
TGGGCAAAATACTCGCACTTCCCCAAGGGGCGGCAGAAATCATACCAATAGACTCCTCCACATTT
CTTTCCTCATTAAGGATATACTCCTCCGCATCTCCCCCCTTATTAAGGGGGGTTAGGGGGGTTAA
GGAAGTAGAAGGTAAAAAAGGTACTAAATGAGAGGCGACACCAATGGGGCCAACCCCGGGGCCAC
CCCCACCATGAGGGATACAAAAGGTTTTATGTAAATTGAGGTGACAAACATCGGCCCCAAAATCT
CCCGGTTTACATAAGCCAACTTGGGCATTCATATTCGCACCATCGAGATATACTTGCCCACCATA
ACTGTGGATGATACCGCATATATCCTTAATCCCTTCCTCAAATACCCCATGAGTAGAAGGATATG
TCACCATTAATGCCGCTAATTTATCCTTATGTTTCTCGGCTTTTGCCTGTAAATCAGCAATGTCA
ATGTTGCCTTCCTCATCACATTTAACCGCAACCACTTTTAAACCACACATCACCGCACTAGCAGGA
TTTGTACCGTGAGCAGATTCTGGAATAAGACAAATAGTGCGATCGCCCTCTCCTCGACTGTCATG
ATAACGACGAATAACTTGTAACCCTGCATATTCCCCCTGAGAACCAGCATTGGGTTGTAAAGAGA
CTCCCGCAAAGCCCGTAATTTCTGCTAACCATGTTTCTAACTGAGAAAAAAGAGCCTGATAACCC
TTTGTTTGAGACAATGGTGCAAAAGGATGAATATTATTAAATTCAGCCCAAGTAATAGGTAACA
TTTCTGAAGTCGCATTTAACTTCATTGTACAAGAGCCTAGAGGAATCATGGAAGTTGTTAAGGAT
AAATCTTTGCTTTCTAAACGGTGTAGATAGCGTAATAATTCCGTTTCGCTAGTGTGTTGATTGAA

AACTGGTTCTGTGAGAAAACGACTTTGACGCTGTAAAGAAACAGGAATCCCTAAACGGCTAATAT
TATCATCTCCCTGATAGATAGAAGCATCCAATAAAGGAGATTTATTTTCTAATAAGCGAATATAG
ACAGTATTTTGAGCAAAAATTGCACAAATCTGATTAACTTCTGTGAGAGTGGTTGCCTCATCCAG
ACTAATACCAACACCATCACGGTAATAGCGGAAATTAATCTGCTGATTTTCTGCTAATTCCCGAA
TAGAGGCAATTAATTCATTATCATCGATTCTAACCTTGATAGTGTCGAAGAAATGTTCAGATTGT
AACTGATAACCTAATTTTTCTAAACTATTGGCTAAAATACGGGTTAATTGATGTACTCTGGTGGC
AATATTTTTGATCCCTTTTTCCCCGTGATAAACCGCATAACTAGAAGCAATTACCGCCAATAAAA
CCTGTGCTGTGCAAATATTACTGGTAGCCTTATCTCTGCGAATGTGTTGCTCTCTAGTTTGTAAT
GCTAATCTTAAAGCAGGTTTACCTTGGGCATCTACCGAAACTCCCACTAAACGCCCCGGTATCTGT
CGTTTATATTCTTCTTTCGTGGCAAAATAACCAGCATGGGGACCACCATAACCTAATGGTACACC
AAAACGTTGACTATTTCCCACTGCAATATCAGCATTTAATTCTCCGGGGGATTTTAACAATGCCA
ATGCTAATAAATCGGCGGCTAGAATCACTAAGCCTTTTTCTTGATGAATTTGCTCAATAATGGCT
TGATAGTCGTAAATAGTGCCGTCAGTGGCAGGATATTGAAGTAAACAACCGAAAACGGCGTTTGT
AAAGTCATAAGTAAAGGGATTGTCAATAATTAACTCAATATCTAAATATCTCGCCCTTGTTTTAA
TTACTTCAATGGTTTGAGGATGACATTTTTCATCAACAAAAAACAGCTTTGACTTACTTTTACTT
GCTCCATAACTCATCGTCATGGCTTCTGCGGCGGCAGTGCCTTCGTCTAAGAGGGAGGCGTTAGC
AATTTCTAATCCCGTTAAATCTATTACCATTGTCTGAAAGTTTAACAAGGCTTCTAATCTTCCTT
GGGCAATTTCAGGCTGATAGGGAGTATAAGCAGTGTACCAGTTGGGGTTTTCTAAAATGTTGCGT
TGAATCACCGCAGGGGTGACACAATTATAATAACCCATACCAATAAAAGAACGATATACCTGATT
CTCAGATGCGATCGCACTTAATGTTTTTAATGCCTGAGTTTCAGTTTGAGGAGAAGGTAAATTAA
AGGGTTTAGAGAAGCGGATAGATTGAGGTATCGCCCTATCAATGAGAGAGTCAAGGCTATCATAA
CCCAGTTGTTTCACGATCGAATCTTCTTCATAACTATCGATACCGATATGACGATGAATAAATTG
ATCTTCCCTAGGTATGCTAACATCACTATTGACTTTCTGCTCACTATTTTCCGAGACAAAAGAGG
AAGCAATATTTTCTAAGTCAAGCATATTTTTGCAGAGGAAATAAATCTCATATTTACATATCTTA
ATGTTAACGAAATCTTAAGAGATTCGAGCAATGGATTTTCTTCGCTACCAGGAGAGGGTATTGGG
GGGGAGAGGGAGACAAGGTGTCAGGTTTCAGGTTTCAGGTTGCAGGTGTTGGAGAAAGTAATAA
GTAACGAGTAATGAGTAATGAGTTTTGATAATTTATTATGATTAATTGATTGTTTCAAGATTTT
TTATTCTTACTTAAAATCTGCAAGTCAAAAATCCCCCCATCTCCCTCTCCCCTTATCCCCCGCTCT
TTTTTCTTTCTCGAAAATGAAACAGCCCTGGGGGATAAAGGGGGGTTTGCCCTTCCCCCTTGCCCT
TTCCCGAGTATAATTAAAGATTAGAGCTTAAAAAATATCAGCAGGATCAGCAGATTTTAATTTAT
TAACTGCGATCGCACCTGAGAAGCCACACATAAACAAAGTTAACAGAAAAACAGAAATAATTAA
AGACTTACTTAAAAAAATAGGTAAGCCCGTAGCTTTAGCAGTAACAGAGTAAAGAAAGGCAGAA
AATCCTAAGCCGGGTAAAAATCCAATACAAGCGAGAATAATTGCCTGTTGAAAAACCAACACTAA
CAGATAATTGTTGGTATATCCCATGGCTTTGAGAGTGGCATATTCGGGCAAATGATCCGCAACAT
CTGTGTAAAGTATTTGATATACAATTACCGTTCCCACAATTAACCCCATTACCGTGCCAAGGGTG
AAAACAAAACCCACGGCAGTAGTATTTTCCCAGTAACTACGCTCATAGTTAACAAATTCATCTCT
GGATAAAACTAAAACATCTCCGCTATTGAGTTTGTTTTTGAGAGTTTTATTACAGCCTCTGTAT
TGGCATCTTCTTCAAGGTTAATTACCCCTACATCAATTAAACCTTTATCTCTTTGGGCAAATATT
CTTAAAAAGTTTAAATCGCTGGTGATAATATTTCCGTCTGCTCCAAAAGTTGTACCAATGGTAAA
TAATCCAGCTACTTCAATTTGACGATTATTTATTTCTGTTTTAACTTCTTTTCTCTCTTGAAAAA
GCTGAGGAATAGGTCCAAATTCTGGGCGAGAATCTGTATCAAATAGTACCTGATCTGGCTGTTTT
AATTTATCTAAGTTTTTTTGTACTCCTGATAAGTTAAAAATTGCTTCTTTGGGATTAAATCCCAT
AATCATTATCTGTCTAGTTTTTTGATTAAGGGGATTTTTCCACGTAGTAAAACCTATATATAAAG

AGTTGATTGATTTTACTTCTTTAATAGCTAAAGCCTCATAAAGGCGGCGACTAGAAAAACTTTTC
ATGCCAATTAAAGCATCAGATTGAGGGCTAATTAAAAATATATCTCCTTTTATATTTTCATGAAT
TCTTACTGCACTATTTAACAAAGCTGTCTTAAATCCTAACTGCATAAACATCAAAATATCAGCAA
AGCTAATACCTGCGATCGCAATTAAAAGGCGGATTTTTTCTTTACTTAATTGTAGCCATGCAAGG
GGAGTTTTCATAATTAATTTTTTATCTAAAATTATTAATAATGGTAAAGAAATAAGGAATAAAA
ATTTTGATAATTGTCAGTTATTATATTAATTAAGTACAGTTTTTATTTATAAGAGAATATTCACA
ATGACTTGGGAATAAATTAGGTTTTTTATCTTATTATTATCTTCTGGATTAACCGCAATTTTTAC
CTCTACTACCCTGGCATCAACGCTGGCGGCTGGATCGGTTTCTAAAACGTCTTTTTTACCTATTTT
AGAGCTTATTTCCGCAATTTTGCCCGTAATTATACCATCAAAGCTATTATTCTCACTCCTTATCTG
GGTTTCTTGCCCTAACTTTACTTTACTAATATCGCTCTCATAAATTTCTGCGACAACTATCATCTC
CTGAGTATTGCCTATTTCTACTATCCCTTGGTCACTACTAATATTTTCTCCCGGATAAGCATTTAT
ATCGATTATTTCCCCATCTATGGGGGCTTTAATGATAGTCAAATCTAAATCGACTTCTGCTTGAT
TAACCAGTGCGATCGCACTCTCTACTTGTGCTTGAGCTTGTGCTACATCCACAGGGCGAATTTCCG
AAATTTCATTTAAAGTAGCTTGGGCAGAGGCTATCTGTTTATTTAGGGTATTAACTCTTTGAGTG
GCAAGGGCTTGAGCCTCGTTAATTTCCTCCGACAGGGTGCTAATGGTTTTTCGATAAATAGCTAA
GGCTTCTTGATAACGTTTATCGGCTGTCTCAAGGGTTAATTGACGATCATCTAATTCGGATGCAG
AAATTACCCCCTCTTTTGCTAATTGTTCATATCTATTTAATTGGGCTTGAGCATTATTTTTTTCT
GCTTGTAATCGGTCTATGGTAGCTTGTCTTTCCTCCCTTTCAGAAGCTAATTGGGCTTGTAAACG
ATTGATTTTAGCTCGATCAGTTGCGATCGCACCTTGTAATTCAGCTTGAAGTTGAGAAACAATAG
CTTTTTGAGCATTGATTGAACCTTCCTTTGCTCCAGCTTTAACGATATTAAGATTTTCTTGGGCA
ACAGTTAACTCTTTTTTTGCTCGTTCTAATTCTGCTTTTTTCAAATCATAATCGGAAGTATAGGC
AATAATATCACCCTTTTTTAGCCAATCTCCTTCTTTCACTAAAAGAGTTTTGACCCTTGCCCCTGC
CAAACTAGGACTAGGGGAAACATTGATAACCTCTGTTCGGGGTTCTATTCTACCTAAACCACCGA
TGGAAGTAATAGGAACAGGTTGGCTAATCTCATTTTCTGAGGAAACATTCGGCTTTCTTTGACTA
ACCACATAAAGACCTAGACCAACCATCATTATTACCCCAAATGCCGTTGCAATTATCGTAATGGA
TTTGCCATATTTTTCCATTATTGAAATTTTAGCTTCCCCCATATTCCTTTTTATGATTTAATGAA
TGTATAGATCAGAATGGCTTTTCTACCCTGTTATCATACTAGACGGTCTAGTCTAATCTTGTCAA
GACAAAAATATTGTCAAGAAAATCAAGAACTTTAAGAAGAAAAAGAAAAGAATGGAAGAAAAGG
CGGAAAAAATTTTGCAGGGTGCATTATCACAGTTTTTATCTTATGGTTATGCAGGTACGACCATG
GATAAAATAGCTAAATCATCTGGAGTTTCAAAACAAACTTTATATAGTCATTTTGGAGATAAAG
AAGGATTATTTTATGCCTTAGTTAGTGACATGGCTTCCCGTAAATTTAAGTTAGTCTGGGAAAAG
CCCTTAGAAGGAAAACCACAATTAGTATTAAAAAACCTAGCAGAAACCATCATAAAAGAAGTTA
GTGATCCTCTTTATTTAGATTTTATTCACCTCATTGTTACGGAAGTAAAAAACTACCCAGAAGTT
GCTCAATTATGTTTTAAAAACCTTGCTAAACCTGCCATCAATCAACTTACTAACTATCTACAAAA
TAGTCCAGATTTAACCTTAGATGACCCAGAAGCGATCGCACATATTTTTGTCAATAGCATCTTAC
ACTATGTTTTAACTCAAGAAAAACTACAGGCAAAAAAAGTCATTCCTTTAGAGCAAGAGCGTTTA
ATCAATAATTTAATTACTCTCATCGTTACACCAAAGGGCAATGGGTAAGAGGCAAGGGGCAAAGG
TAAATAGTGAATAGTGAATAATTAATAATTAATAACTCAGAACACTCATTACTCATTACTCGTTA
CTTATTACTTTCTCCAACACCTGCAACCTGAAACCTGAAACCTGACACCTTGTCTCCCTCATCCCC
CAAACACCCTGATACTTAATCTTATCCGATATTCTTAAGCCGAAATGAAGTTAATTATTCTTTGT
TGAAGATTTTTAGTATAATTAATCACATTTGCAAAGAAAGCCCAAACCTTAATTAAATCAACAAA
GGGAGAAATAGTCAAAAAAAAATTTTACAAAAATTAAATCTATGCTTTATGATGATATAGGGTC
AACTTTTCTATTATGCCGTAAGAAAAACAGATAAGACAAGGTTGACTCATTGTATTTGAACTATT

GTTCAGCATAAAACCTAAAAAAATTGCTGACTTTTGCACTTTATTTTGTATAATTTTAATTCTCG
GAAATATTATTAACTGAAATATATAAAAAAAAATAGAACAATTATGCAAGCACTACCTAAAAAT
ATTGATACGATTAGAGTATATCTCAAGGAAATAGGCAGAATCCCCCTTTTAACTAAAGAGGAGGA
AATTGTTTTAGCCAAACAAATTCAAGAGTTAGTTGCGTTAGAAAAAAAACGAGAAACTTTGAAG
AAAAAGTTGGAAAGAGAACCTTCTAAAAAAGAGTGGGCAGAAGCGGCAAAAATCTCTGAAAAAA
ATCTCGATATAAAAATTAGTCGAGGAGAAAAAGCCAAACGTAAAATGGTTGAAGCGAATCTTCG
TCTTGTAGTTTCTATTGCGAAAAAGTATCTTAAACATAATTTAGACCTACTCGATTTAATTCAAG
AAGGTACTATTGGTATGCAAAGAGGGGTAGAAAAATTCGATCCCAAAAAAGGTTATCGTTTCTCT
ACTTATGCTTACTGGTGGATTCGTCAAGCTATTACTCGTGCGATCGCAGAAAAAAGCAGAGCAAT
TCGTTTACCCTTACATATTACAGAAAAATTAAATAAAATCCGTCAAACTCAAAGAAAATTGTCTG
AGGAAAAAGGTCGTCCAGCAACCATTGCTGAATTATCCGAAGCCTTAGAGTTAACTACAGAGCAA
GTGAGAGACTACTTAGGTAAAGCTCGTCAACCCCTCTCCTTAGATTTAAAAGTAGGAGACAATAA
CGATACTGAATTAGGAGAGTTATTAGAAGATAAAGGGCAATCTCCCGAAGATTTTACTGACTACT
CTTGTCTTCAGTTTGATTTGCGTCGTATGATGTCCGATTTGACTCAACAACAACAAGATGTCATC
AGACTTCGTTATGGGTTAGATGATGGAAAACCTTTGACCTTGTCAAAAATTGGTACTATCCTCAA
TATTTCCAGAGAAAGAGTTAGACAAATTGAAAGAGAAGCCTTAACCAAATTGCGTAAACGGAAA
GAGGTTATTGAGGAATATATTGCTAGTTAGTTTAATTAGGAATTAGGAATTAGTAATCAATCAT
GTTTTTTGTTAAAAATTAATCTATACAATTAACCTAAATACAATAAGGGAAAAAGTTAATGCAA
GATAAATTAATGTTAATGATTCCAGGTCCTACTCCCGTGCCTGAGAGGGTATTATTAGCCATGGC
ACAGCACCCAATAGGGCATCGTAGCGGGGATTTTAGCAAAATCATTGCGGAATTGAATGAAAATT
TGAAGTGGCTACATCAAACTAAAAACGAAGTTTGTACCCTTTGTGCTTCTGGTACTGGAGCGATG
GAAGCTGGTATTATTAATTTCTTGAGTAAAGGCGATCGCGTTTTAGTCGGAAATAATGGTAAATT
TGGTGAACGTTGGGGTAAAGTAGCAAAAGCCTTTGGTTTGGATGTCGAAGAAATTACCGCCGAAT
GGGGCAAGGGCTTAAATCCCGATGATTTCAAAGCAAAATTGGAAGCCGATACAGAGAAAACAAT
TAAAGCAGTAATCGTTACTCACTCTGAAACTTCCACAGGTGTTTTAAACGACTTAGAAACCATTA
ACAAATACGTTAAAGCCCACGGAGAAGCCTTAATCATTGTCGATGCTGTTACCAGTTTAGGAGCT
GTAAATGTACCTGTGGATGAATGGGGCTTAGATGTAGTTGCCTCTGGTTCTCAAAAAGGTTTCAT
GATTCCTCCCGGTATGGGCTTTGTTGCAGTGAGTGAAAAAGCATGGAAAGCCTATGAAACCTCAG
ATTTACCTAAATTTTATTTAGATTTAGGAGCATACAAAAAAGCCAATGCCAAAGATAGCTCACCT
TTCACCCCTCCTATTAATCTCATGTATGGCTTAAAAGCCGCTTTAGAGATGATGAAAGAGGAAGG
TTTAGAAAATATTTTTGCCCGTCATAAATATCTTACTCAAATTACCCGTGAGGGAGTTAAAGCCT
TAGGATTACCTTTATTTGCCAGTGATAACGATGCGGCTTTAGCAATTACTGCTGTCGCACCAGAT
AACCACGACGCAGAAGCAATTCGTAGCGCCATGAGAAAAAGATTTGATATTGCTTTAGCGGGTGG
ACAAGATCACTTAAAGGGTAAAATCTTCCGTATTGGGCATTTAGGCTTTGTTTCCGAAAGGGATG
TTTATACTGCACTATCTGCCTTAAAAGAAGTAATTTCATCTCTCTAGTACATTTTTGGGACTAGG
TTTTGGGGATTGGGGTGTTAGGGAGATAAGGTGTCAGGTTTCAGGCTTCAGGTTTCAGGTTGCAG
GTTGCAGGTTTCAGGGGGATGAGGGGAAAATTAATTAAACATTGTTGCCCTCCCCCCTCTCGAGG
GGGGATAAAGGGGGGATAAAGGGGGGTTTGCCTCTTGCCTATTGCCTTGTATTAATTACTAATTG
CTAATTACCATTGCCCTTTGAACTTTGCCCCACTGCAAAAAGTTTATTAATCAAAAGGTTGAACT
ATCCAAACCGAAGCAGGTGTTATCTCACCAAAGTAATCTGCCTGACATTGACTATGGGATTTTTG
CCAAATAACTGCGGTATCCACCTTGACTTGCCATTGATTTTCTAGGGCTTTTTTCACTGCTATCAT
TGTTACTCCCGTATCTAATAAGTCATCTACTAATAAAACTCTGTTGGTGGGAGGCTCAATACAAG
ATAGTTGTCCCAATAATAAATTTCCTTGTTGATTCTGTTCAGAATAACTTTTCGCTACAATAGCC

GATAAGGGGATATTTAACCTTCGGGAAAGATAATCTCCTAAATAAAAACCTCCTCTTAAAATAGA
GATAATTTGAGAATATTCTTGGTTTTCTTGTATGATTTTTTCGGTTACTTTCTGACAATATTGCC
AAAATTCATCCCAAGTAAATATCAGTTTTTGATTTTCTTCTAACATTTTTTCTATATCAATGTCC
TTTGAATAATATCACTAGAGCTAAAATTTTATTATTAACCCTGAAATTTAAATTAAATTGTAATT
ATTTTTTTCAGTAAACTACCTAATTCAAATTTTATTATATTTTTTGAATAACCTTTGATGACAAC
GATTAAGTCCCCTTTTAATATCTTATTTTTTGCAGTATTTAGTCTATTAATAATTTGGATTATTC
TTTTATTAATTAGTTATTTTACTGTTAATGTTCTTTGGTTTGAAGAGTTAGGCTATCCGCAAACT
TTTTTAACTCAATTTCTAAGTAAGTTTACTATTGGGGGTCTTGCTTTTATTATTTCCACTTCTTT
TTTATGGTTTAATTTAGCGATCGCATCTAGGCAAAGGAAGCAACAAGAACAATTTTTTGCTAAGG
GAAAAGATAAATTAACTCCTCAATCTCCTTCCTTAAAATTACGTTTATTATTACCAATAGTAATC
AGCTTTTCTGGTCTAATTAGCTTAACTTTATTCTATTATACAAAGTTTTTCACTCAAATTTCTGG
GGCTAGTTTATTATTACCAAATTTAAGATCTCCTTCTTCACTACCTTTACAATTAGCTATTTTTA
CCGATATTGCTAATAATTTACCTCTGAGAATATGGCAGATTATTTTAATAATACTTGTTACTTTA
TTTTTAATATTTAAACCTAGACAATGTTTTAAATTTATCTGTATTTATTTTAGTATCATAACTAG
CTTAATTATTGGTGATTATTGGTCAAGATTTCTTCATTATTTTTATAGTATAAATCTAGGAAAAA
CCGATCCTATATTTAATATTGATTTGAGTTTTTATTTATTTAAAATTCCTGCTCTTCAGATTCTT
GCACTGTGGTTACAGAGTTTATTTATTTTTACAATTTGCACTGTTACCTTAACTTATTTACTATC
TAATAAAAGTATTTCTGAGGGAAAATTTGAAGGTTTTTCTCGCTATCAATTAAGGCATTTATATG
GTTTAGGTGGCGGTTTAATGATTATTTTAGGGGGAATTCACTGGCTAAATCGTTATTTACTCTTA
CTTTCTCCCGATGGAGTTGTTTATGGTGCGGGTTATACAGATATTCATATTAATTTGCCAAGGGA
AACTATTGCTACAGCTTATGCTTTATTAATGGGCATTTGGTTATTAACTAAGTCTTTTACTGGTT
CAGGAAAAAGAAATCATCCTCATTTACAAGCAGAAAAAAAAATATTCTTTACCTTTTTCGACTTTA
CCTTTTATTGGTTATTTCGTCTTATATTCTTTCGGAATTTTATTGGCTGTGATGGTGCAAAATAC
CATTGTCCAACCCAATGAATTAGCCCTAGAAACTCCCTATATAATTAATAACATTAAAGCCACAA
GATCTGCTTTTAATTTAGATGAAATAGAAGTAAAAACGTTTAATCCTGAGGGGAATTTAACCGCC
GAAGACATTAAAAAAAATCACCTTACTATTGATAATATTCGTCTTTGGGATACCCGCCCTATTTT
ACAAGCTAATCGTCAATTGCAACAGATTCGTCCTTATTATGTGTTTGCGGATGCGGATATTGACC
GCTATCAGTTAAAGAGTAGTATTAATAGTGATATTAACGAAAATACTCAAGTTATTCTCGCTCCT
AGAGAGTTAGACTCCCAACTATTGCCGAATCAAGCACAAACATGGGTAAATCAACATTTAGTTTA
TACTCATGGCTATGGATTTACCATGTCTCCTGTTAATAGAGTGGCACAGGGAGGACTACCTTATT
ATTTTATCAAAGATATTGGTACGGCTCAGGATCCGGGGGAATTAAATACTTCTTCAGCAAGAATT
AGAGAGAGTATCCCCTTTTCCCGCCCTCGTATTTATTACGGGGAGGTAACAAAAAACTACGTCAT
GGTAGATACGAAAGTGCCTGAATTTGATTTTCCTAGTGGGGAAGATAATGTTTATCATACCTATA
CTGGCGATGGGGGAATTAAAATTGATTCTTTTTGGCGCAAGTTTATTTTTTCTGTCTATCTTCGG
GATTGGCGGATGTTGTTAACTCCCAATTTTACCCCTGATACTCGCATTCTTTTTCGCCGTAATATC
ACAGAAAGACTAGGTGCGATCGCACCTTTTTTATATTATGATCGAGATCCATACCTAGTAATAGC
ACAAACAAACCCTGAAGATAAAAAATTCCCTACATTGGCTTATAGACGGTTATACCATATCTGACC
ATTATCCCTATTCTGACGGTGGAGAAAACCAATTCAACTATATTCGCAATTCAGTAAAAGTCTTG
ATTAATGCGGAAAATGGCAATACCCATTTTTATGTGAGTGATTCCACAGATAGAATTATTCAAGT
ATGGCAAAAAATATTTCCCGAATTATTCAAACCTCTTTCGGCAATGCCACCAACTATTAAACAAC
ATATTCGTTATCCCACTGACTTGTTTAATACCCAATCAGAAAGACTTTTAACCTATCATATGATA
GACCCTCAAGTATTCTATAACAGAGAAGACCAATGGCAAGTACCCGAAGAAATTTATGGCAATGA
AATATTGTCCATGCAACCTTACTATCTAATTATGAAACTGCCCATCGGCAAACAAGAAGAGTTCA

TTCTCCTTCATCCTTATACCCCTATTTCCCGTGCCAATTTAATTGCGTGGTTAGCGGCTCGTTCTG
ACGGAGAAAACTATGGTAAACTTTTACTATACCAATTTCCAAAACAAAAATTGATTTATGGACCT
GATCAGGTAGAAGCCTTGATAAATCAAGATCCTGTCATTTCTGGTCAAATATCTTTATGGAATCG
TCAGGGTTCAAAAGCTGTTCAAGGACACCTGTTAATTATACCCATCGAACAATCACTATTATATG
TTGAGCCTGTTTATTTAGAAGCGGATAAAAACAGCGTACCAACTTTAGCAAGGGTGATTGTAGTT
TATCAAAATAAAATTGTTATGGCAGATAGTTTAGAAAATGCGATCGATAATATTTTTTCCGATG
GAAAGGAAGATAGTTCAGCTATTATTCGCCTTTTGGAAGATGCAGCAATTCCGCTATTAGAACCA
AACTTGAATAATACTAACCAATGAGAAAGCAAAGACCTTTTTTTGTTAAGATCCTGCTTTATCGT
ATAATATAGTTAAATGATTTAAAGTAAAAGGTAGAGTCTTATTTCTCCCCTATTTATCTAACTAG
CGGAAAAGAAATGGGTGAAGAGTCTCTACCGTTTTTTGTTGTTTGATCAGCCTGACAGTCGGGGC
AAGGTACAGGATAATAATCAAAAGAGTTAACGTAGTCAGACAATCTTTGCTCGAGAATTCTACCA
GTGTTTTTACATTTTAAACAAAACTTCATGATTCCACCTCGAATTTAATAAATGAATAAATGAAT
ATTTGCATCAGACATTTCATATTGTAAATCTTTTGGCTGTAACTATGATCGTTGATATTACTTAA
TTTTAGACATTTTTAAATGTTAATTTATGTTAAGTATTTCACGATAAATTATAAAAAAAATATT
AAATTAATATAATAATTTTGCAATTTATATTGCCTAATTTTTCTTTTGGTTTCCTCAGATACATT
TTCATCACGTAATAGTCAGTAAATTTGCTTAATTTTTGATTTTTATTTATCAGTTTATTTACAGA
AATTCATCTTTATAAATACAGATCATTATTTGTGATAAATGATTCTTAAAGATCATCATCTTCAC
CTAAATTAGCTGATAACTTAAGAGAAGATTCATCAAGGACTATTCCCGATGGTCTTAAATCGTCA
ATAATTTTTTTGATGACACTAGCTACAGGTACAGCTAAAAATATGCCTAACAAACCACCTATTTT
TGCCCCAAGAAATAAAGAAATAATCAACCAAATAGGATTTAAACCAATTAACTCTCCCATTAAAC
GAGGTGCAACTACATTATCGGTAACTTGGTCAATAATAGTAGCTGAGACAAAGAAAAATAAAGC
TAATTTACCACTCTTAAAAACAATAATTAGAGTACATAATATAGTCAAAAATCCTCCTAAAAGAG
GTACTAAACTAGCAATACCAATACCAAAAGCAAATAATAAAGCCGAAGGTACACCTAATAATATA
AAGACGACAAGACGAACCACACTAGCGATGCCCACTAAAATTAAACGGCTAAAAAAGTAATCTTT
AAAAGTCTGACGAAAATAGCGGGGAATTTTCACATCCCAAGGTTCAGGGAAAAAACTATATAAA
CCCTGCCAAAATTTATCCCCTCCTATCAATAAAAATATGGTAAAAATAACAATAAATAAAACATC
AAAAACGCTATTAATTGTAGTTACAACAATATTTAGAGTTTGAGTTCCTAAAGTCTTAAGAATA
GAAGATATTTTATTTGTTATTTCTTGAATAATACCGTCTAAATTAATGGAAAACTTTTCAAATAT
GGGTAAATCAGAAAGAGATATTAAATATTGATTAGCCTGTTTTATCCAAGTGGGAGAGTTGATA
ATTAGCTCATTTAGCTGTTGAATTAAAAGAGGAATTAAGATAAAACTATTAACAATGAGGAATA
TTAAGGCAGATAACAAAACGATGGTAATAGCGATCGAGCGATTTACACCTTGTTTGATTAATAAT
TTAATGACTAATTCTAGTAAAAAAGCAATAATACTAGCAATAATTAAAAAACTAACTAAAGGCT
GTAGATAATCAATAAGAACAAACAGTAAAAAACCGTTAAGAAAAAGAAGAGGAAATAGAAAAAA
TATTTTTAACCATTGGGGTATTTTATTCCACCATTCAAAAAATTTAAAGTTATCTTTCATTAGGG
AAATAATCTAAAGATTTTATGTTTTAGGGAGTGAAAATTGGAAACTTTAATATATAACCTGAGT
TACTTATTACTTTCTCCAACACCTGCAACCTGAAACCCTGTACGGGCGAATGGCCATTCGCCCCTA
CTTCTCCCTCATCACCTCATCCCCCCAACACCCTCACACCTGCAACCTGACACCTTTTTTTAGGGA
TAATTTATCATATCCAAAGCAAGAGAGCTATTATCTTTTTCCTTGTCTAATTCTTAAACCTTCTT
GAAAACCTCGATCATAATCAGGGGAATTTCTCGGATTATCATATCTTAAACCGTTAATACCATCA
CTACGTCCTCGAAAATACTCTTGCTCAGGTGGTACAGGGCGATACCTGTCTTCATTGGCACGACG
GTTATTGTTAATAATTACACTTGTACCAACTCCTGCACCTACTCCTAACATAAAATCACCAAAAC
TAGCCTGTGCAGGTTTAATATTTATAGTACCAAAAGAGGCAAAACTGGTGGCACAAATACTGAAG
GTAATTAGCTGATTTTTTAAACTGTATTTTGACAAATTTTTCATGGCTATTTAATATGATTTATT

GACTTAAAATTGAGCTTGATCGGAAATAGGTTCTAACCATCCAGAACTTTCTATGTTGATAATTT
TTGGTTGATTATCTATCATGGCAAAACGAATTTTATCGGTGCTTAAGGATAGGTAGCGATTTCCC
GTTTCGGTATCAACTTCTCTGGCTCTTACTCTGGTTAACAATGCCATTTCTCCACTATCATTAATT
TTTGTGGTGGTATAAGAAGAAATTACTTTTTTTCCTTTCACTTTTTTGAAACTATCGTTTAAAAA
GCCCTCATGATCGTCTTTTCCTTCTACGAAAGTAGTAATTTTTTGATTATCTATTTCCACTGTGA
TAGAAGTAATAACATAGGGAGCAAGATATTCTAAAATACCTGCAATATTTTCTTCTGCTTCCGCT
TGTTCTATTTCTGTCATAATTTGAGTTAAAGTGTCCTCTGTTAGCCCTGTTTGTGTTTCTTGAAC
TAACAACAATGGTTTTTTCTCTGATACAGTTTCGGCTTTAATAGAATCAGAGAAGGGTATTAATT
GACTGGCAATAAAACCCATAATTAAAACACTGATTAAATTATTTTTTTTCACTTTTTGTTACTTA
TTTTTATTGATTTTGAACTCCTAGAAAATTATATCTTTTATTATGATGATGGAAAGAAATTATA
ATATTTATTTAAAAATGATAATTATGGAAAAGTAGATGTATAATATTATGTCTATGTTTATTAA
AGGTTAAATTATGGATCATTCTTCCTCTTCTTTTATTATTTCTCCTTATCCCCAGTCCATTAAGTT
AGATAAAACCCTTAAATTAGGGGTTATGGCTTCGGGTAGTGGCAGTAATTTTGAAGCGATCGCGC
GTTCTATTGATGAGGGAGAGTTAAATGCTAAAATTGAATTGTTGATTTATAACAATCCTGAAGCT
ACGGTAAAAAATAGAGCTCAAAAGTATCAAATTCCTTCACTTTTATTGAATCATCGGGATTTTCC
CCACCGTAAAGCCTTAGATAGGGCAATAGTAGAAGAATTTAAAGCCCGTGATGTGGAATGGGTAA
TCATGGCAGGTTGGATGAGAATTGTTACAGAAGAATTGTTAAAGGCTTTTCCTCAGAGAGTGATT
AATATTCATCCTAGTCTCTTACCTAGTTTCAAAGGGATTAACGCCATTGAACAAGCATTAGACGC
ACAGGTGAAAATAACTGGTTGTACTGTACATTTAGTGGATTTAGAGGTGGATAGTGGGCCTATTT
TAGTTCAAGCCGCCGTACCAATTTTACCCAATGACACCCCTGAGACTTTACATCAACGAATTCAG
ATTCAAGAACATCGTATCATAACTCAAGGAATTGCGATCGCGAGCGCGCTTCAGCGAAGCTGAGA
TTGCACATCACCACTTCGTGAATACAGCTAGTGCTGATAGAGAGTAAACTATACAAAATTCCAAA
GTTATCTTTTATTTGTATCCGATAAAAATGAATAAATATTGGAAATTATCTCAAAGTCAACTCAA
TTTACTGCAAACTTGTCCTCCCCAATTTCAAAAAGTTTATATAGAAAAATATACGTCGATTCCTA
ATCTTAGCAATGAAGAAAAAACTGTTTGGGGAAAAAAATTTCATTATCTAATGCAACAATATCA
ATTAGGAGTAAATATAGAATTATTAAAAGATGAAAATCCTGATTTAATTAGAAGTGCAAAAACA
TTAATCGAGGCAACAGAAGATATTTGGAGTAATCCTAAGATAAAGCTAAGAGAAGCAGAATATC
AAATTAATTATCAACTAGATAAATATCTATTTACTGTTATTTATGATTTGTTAGTTTTTTATGAT
AATAAAGCTATTATTTTTGACTGGAAAACTTATCCATTACCAGAAAACAATAATAGGATATTACA
TAATTGGCAAACAAAATTATATTTATATGTTTTAGCAGAAAATAGTAATTACTTACCTGAGCAA
ATTTCCTTTACTTATTGGTTTGTTAAACAGTCTGATAAGCTCCAAAATTTAACTATTAATTATGA
TCAAAAACAGCATTCTAGCACAGAAAAAGAATTAAAAAACCTCTTAGTTAATTTAGAAAAATAT
TATCAAGATTATCGAAATAATAATATAGATTTTCCTCATCATTCAAAATGCGAAACTTGTTTTTA
TCGGAAAAATTTTGCTCATTTATTTGTTTCTAATAATGAGTTTATTCCTCAATCTCTCGATGATA
TTTTTTAACCTGAGTTCAATATAAAATTGTCAGTTAACTATTAACTATTAACTATTAACTATTAA
CTAATTGCCCCCTGAGTGTTCAGATTAAGGCATTTGACGATCGCATTTACACCTAAACTAGCCCA
AGTTTGTGCCATAGCCTGAGTAACAGTTTCTTGATGTTGCTCACTGGTAAGAGCCAGTAAAGTAG
GCCCCGCTCCACTAATTACCATACCATATCCCCCTGCTTTGACAACGGCTTGATAAATCTCATCAT
AGCCTTTGATTAAACTTTTACGATAGGGCTGATGAAGTTTGTCTTGTAGTGCTTCTCTCAACCAA
TCTTCGTTTCCTGTCTCTAAGGCTTTAATTAATAAGCCTAAATGGGCTATGTTATAAACAGCTTC
ACTATAAGTTAAATGGGGAGGTAAAACTTTTCTAGCGGATTCGGTAGATAATTCAAAGTTGGGA
ATAGCAACTATAAATTTGATCTTTTTATGGCAGTTAATAGGGACAAAATGCCAATTTCCTTTTTC
TCCTACAGATAAAATACAATTACCGAGGAGTGCAGGTACAACGTTATCGGGATGTCCTTCTAGTG

CGATCGCCATTTCTAGTAATTCTGTTTGGGTTAGTGAATTAGAAGAAAAGTGATTTGCCCCTAAT
AACCCCGCAATAATAGCTGTTGCAGAACTTCCCAAACCTCTGGCTAAGGGTACATTGATTTTAAT
AGAAATGTTAACTTTAGGAGGAGTTAAATTAAGTTGTTGATAATAGTAAAGAAAAGACTGATAT
AAAAGGTTATTTTCATCTAAAGTAATTTTATCTGCCCCTTCCCCTTCAACGGTAAAGGTTGTATC
ATCTTGACTCAAACTAAATGCAAACTCATTATAAAATTCTAGTGCCGCCCCAATACAGTCAAAAC
CTGCCCCTAAATTAGCGGTGGTTGCAGGTACTTTTACAACAATAGAATCAGGGTTACTCATGCAA
TAATCTCTCCAAAAGCAAACATAAATTATTGTACCCTTAAAGATGTTAGTTGTCTTTTAAAGCCT
CGATAGCATCTTCGGGAATAAATACCGAATACATACCTCTTAAATCTCCCACATGGAAATTATAG
GCTAAATCTTGAGGATATTTTTGTTGAACAAAGCTAGGACGCTCATTTTTTAAGCCATGACAAGC
TAAACAACTTGATTCTACGTTAATACGGCGATAATATTTTGTGCCTTTGACTCCATCTACAATTT
CTGGTTGCCAAAATCCTTTTAGATTTTCGTCTTGTTCAAATTTGGCTAATGCCATGACTTCCTGT
AAATTTTGGGGAGCATGGTTAGGATTACGATATTTTTTCGCTATTTGCTTTGTTTGCCAACCATT
TTCTTCCCCTAATTGTTTCATCTTCATACCCACTGGTTTACACACTTGTTTAAAAGTATCTGGAGT
TGGTTTGTCTGGGGTTGATTCCAAAAAACTAGCAAGATGCGATCGCATTTCATCCAAATTTTCAA
TTTCTGTCACTGCTTTTCCCAAATCATTGGGAAGGGTATCCGCATGAAGTATCTTATTCCCACCAC
TAAACCAAATTAGAGAAACACAAAGAATGCTCAACACCATTTTCCATAATTTTTTCATCATAAAG
ACCTCAAAATTTAAAGCTACAACATCAATTATATAACTCAATAACCATATGGTTTTAATTAAACA
TCTATTCATCTGAGCTAAGACACATTTAATTTACTTTCTTTTACTCTCTCTTTTTAAGGAGAAAT
ATAAATGGCTTTCTTTGCTTCCCTAAGTAGCTCCACTTAATTAAACGTAAAATAGGCTCGGTTAG
TAGTAAGGGCTCGGTTCCATTATTTCAAGAACGTTTAAAACCTCTAAAGGGGTAACTACAAACTT
TCTTTTATTTAATTGGCTCTACTTATCATCGCCAGTAAACTAAAATGCACTCCTAGCTTATCTAT
ATTTTTTATTTACCTAAAATTTTCTGAAAAATCTCAATATAATATTGCTATCAAGATAAATTTAA
CTGAGCCAATGTTTAAGAAAAAAATATCTCATTTAATAGTTTCTGTTTCTTTACTTTTTCTATTA
ACATTAACTGCTTGTTCTCCTCAACCTCCTTCTCGTTTTGAACAAGCACAACAAGATAGTATTGCC
GCAGGAAATAAAAACACGGCGGTGAGTGAAGATGCTGTTAAAGGGGCTAGTTTTAATCAATTTT
TTCCTGTTAGTAGTGGTGATTATGAACGGGTTTTCACTCAAGAAAAAACAGGTTTTGTTCAAGCA
AAATTAAAATTTCAAGGGCAAGATGTTGCTACCTTAGCTATTTTTGATACCATCAGCAATCCCAC
TGCTAAAAACGATTTTGCCAATGCCACAGAAACAATTAAGGGCTTTCCCACTGTGGAAAAAGGCT
CTAATAACACTGCTGTTTTAGTAGGCGATCGCTATCAAGTAAGTATCAGATCCAGTGATCCTAAT
TTTACCGCAGAAGAAAGAAAAGAATGGTTAAATAAATTTGATTTAGCCAATTTAGCTCAACAAA
AATAAACAAAAAACAGCATTTTTTTATATTCAAAAAAAAATATCATGAGCAAAGCAATTTACGA
ATTAGTTAACGAATTACCCCAAAATAATATTACGACTTTAGCTCTAAAAAGTCTGGATAAATTCA
TCCCCGGACAATGGGAAAATATTACGAATTTTGAAGAAATGATTCGTCAGGTAACAGGGGAAAA
CGATGAAGAAATGATCCAAAAAATAGGCGATCGCGCTGTCTGGTTATTTAACGATAAGACTCAAG
GGTATCAAAACGCCCTTTGGCTATATCAAACCGTAGATAAAACCGATTACGCTTTAGGGGCGGCG
GCATTAGCCAATAAAGTAGGAGAAAAAATCAAGTTACTAGGTTTCCTTAACCGTCTCACCCCCAA
TAACGAAAAAGCCCAAGCCATTGATTTAGCCATTAAATTAGTGGTGGAAGTGGTGGCATTTTGTC
AGATTAACGGTTTACCCGGAGATAGCATTAGTGACTTTGTCAAAGCATTAGGAGAATACGAAAA
AGAAGCCCTAATTCGTATGGGAGGGTTAGTTTGTTTTGATGGTTTAATTCCCTTTGGAGGAGATT
TTATCCTGCAAATCGAGTCAACCTTGAATAATCTTTCAGGAAAAGAATTGAGTAATAATTCCTCT
TTTTCTCAAGTTAGTCGTTTAATTCCGGGAGATAATCCCGCAGGACAATTACAATTTATTCACGA
TAGCTTTTCGGCGGTAAAAGGTTGGATGACAGGATTTGTCGCCAATCATAGCCTAACCAGAGATA
AACTCTTAAGCAGTCTTCATAATTTTATTGAATTTTCTGAGGATCAATTTGACTATGTGGCCGCC

TTTCTTGATATGTCCACTAATTATTTTGAACACACAGGCACTCAATCCATTGCTAAAAGTTTAAT
TTCTCGTGCCATGTTTGAAGTTTAATGCTTTGGCATAGCAAAGGGCAGAGGGCAAACCCCCCTTA
ATCCCCCCTCTCGAGGGGGGAAGGCAAGGTAAATAGTTTATAATTAAAGATTAAGAATTAAAAGC
TCCTAAACCTAGCACCCTAAGTCCTTATCTAATTAAATTTCTTCCCAACCAGCAACTCCAGAAGAC
ATCACATAGCTAGTAACACTAGACTCAAAGAAATTAGCTTTAGTATTACCATCTTTTTTCGTGTC
TGAGAATTTTTCTAAATGGGTGTAAGGACTTTTGCTATACTCAGGATCTGTATAGAGAGGATCAA
GTCCGATCGCTCTTAGTCTAATATTTGCTAGATATTTTGTATAGCGTTCTGTGCTTGATTGGGTT
ATTCCTAGGATATTATCCCCTACAATGTGATTAGTCCAACGACATTCATGCTGTACCGCAGTATC
AAACATTTCATAAATTTGATCAAGAGAATGTGGAAACGATCGCATTGCTTCAGGGATCAATCTTT
GATATAGTCTAACGTGAGATAATTCATCACGATTAATCATTTTGAATATATCTGCACTCCCCGGC
ATCAACATTCTAGCGGCGAGATTATAAAAATAAATAAAGCCGTTATAGAAATAGAGACTCTCTA
ATAAATAATCAGCTAACAAGGCGATAAAATAATTCTCCTCAGTGGGATGATCAATGTATTTTTGA
TACATCCGAGCAATAAATTCACACCTACCTGCCAATACTTTGTCCGTACGCCAAAAATCATATAC
CTTATCTCTCTTTTCACTGGGAATAATGGTTTCAATCATGTATTGATAAGCGTGATTGTGCATCC
CCTCTTGAGAAATTTGTTCTGCCATACATAAGCTGATTTCAGGGGCAGTAATGGAACTTTTTAAG
TGGGGAATATTGCAGGTTTGAACAGAGTCTAAGAAGGTTAAATATGATAAAATCCCCTCAAAGG
CACGACGTTCATCATTAGTCAAATTCCAGTAATCTGTGACATCTTGTGTAATATCTAGTTTCTGG
GGAATCCAAAAATTTTCCCGCATCTGTTGATAAAGTCCTATTGCCCATGAGTAGCGGACATCATT
AAGTTGCATCAAGTTTGTAGTGTTACCGAACCAAATAGTTCTGTTTTCGATGCGATCGTCCCCTT
GGGGGTTAAAAATCGGTGTTAGGGGCATTCTATGTTTATTTTCTGGGAATATGGCGACCATAAAT
AAATGTTTCAAAAATATAATTGCGGTGTAAATTAAAATTATAACTTTCTATGACTTGTAATAAA
AACAATTTACAAATCTCTATGTTTTGTAATATATATAATGTCATTATTACATGATAAACTCAAAA
TTTACGTCGGTTCGTTGTGAGATAAAATCCGTGCAAAATTCACACTACAGAATAGTTGGTTTGGC
AGGAGAAGGACAATTTGGGAAAGTCTATGCCGCTATACATCGTCAAACAGGAGAATTAGTGGCAC
TGAAAGAATTAAATCCTTTTAAATTCTCGACAAAAAAATTCTTAAGAGAGATTCGCATTTTGCTA
AGTTTAGATCATCCTAACATTATTCGTTGTTATGGAGTAGAGCATTATCAGGACAAAAGATATTT
AGTCACAGAATATTGCGATGGAGGCACTTTAAGAGATTTACTCGCTACAAATAATAATAGGGTGA
ATATTGAATATAAGCTAAAAATCATTATCGATATATTAGAAGGTTTAAGTCATGCTCACAAAGA
GGGAATCATCCATCGAGATTTAAAGCCAGAAAATATTTTACTCTCCGTTACTTCTCAGGGATGGA
AAGCTAAAATTTCCGATTTTGGTATCGCTAAAATTGAAGTTGAAGATGCGATTGCAAACATTAGT
AATATGGGTGATACTGGTTCTCCTGCCTATATGGCACCAGAGCAATTTTACGGCAAATATTCTTA
TGGGTCTGATATTTATTCTGTGGGTATTATCTTATATGAGTTAATTACAGGAGTTCGTCCTTTTT
CTGGCACTCCTTCAGAAATAATGTTTGCCCATTTAAACAGACAACCCAAATTTCCTAAAAATTTA
TCTCCCAATTTGCGTTTAATTTTAAGAGAAGCCTTACAAAAATTACCACAACATCGTTTTCGCAC
CGCCTCAGAAATGAAAGCACAAATTTTACAATGTGTTTTAGATTTAGAAAATAAAGATGTTGGT
TTCTATGAAGAAATTAGAAAAAAGCCCCTTTATTTAGAATTAATACAAGAAGAATTAAATGATA
AAGAAAACATTATTTGTTTAAAAACTCAAGGCAACTTATTATATCAAACCAGTGCAAAAAAAGT
AACGGTTAAAACATTTAATTTTCATCATCAAAAAGTTAATTTTTGCTTTAAAAATAACTATCTTT
TTCCTGAGAATATTATTGATTTAAAAATCATTAATAATGGCTGTGTTGTTGTTACCAAAGATGGC
AAGGAAAGAGATGTATATAAACTCCATTTATGTCAAGATAAAAAAAGAAGTTTAATTCATATAA
AAAGTGATTATTTTCATTATGGAATAGATAGTAATTGTCGTTGGTTTGCGGTGACGAAAACTGA
GGGTAATGAGCAGGGCTTTCAAATTATCAAAACACAAGATTTATCCCCGATTACTCCTTTAATTA
CTGATTTTATTCCCTCAGAAATTATTATTCTTGATTCCTGTCATGGAATTGTTATTTATACTCAA

AATGATATTGATAAAAACCGAACTTATTTTAGATTATTCAACCGTCGAGGAGTTTGGTATGATAC
TTATACCGTATTTTTACCATTAAGAAGAATCGTTTATCACTCCCGACATTCTAAGACTTTATTGG
CAGGAGAAAGAAAAACAAATAATCTAATTTTGCTTAAGTTTTATCCCTTTTCTGTTAAGCGCATT
CCCCTTCATTTTTACCCAGATTTTTATTATCCCATGACCACCGGCTTTATTTGTGCTAGTACTTCA
GGAAATGTTGCTTTATTAGATTTAGAGGGGAACTTACTAGGTTTATCCCATTTTCAACATAATAT
AAGAGCGATCGCACCTTTAAATGAAGATACCATTATTACTATCACTGGGGATAATAGTGAGCAAA
AACAACTAATTTACCGCTTTAAAACCATTAAAAATAATAAAGTTATTCCTATTAATTTTTCTTAG
CATAAATACAGTTTTTACTAAGCAAGACTAATAAGAAACTGATGAGCGAAATTGTGGATATTAT
GAATATTGCGATAGCTTTCCTGCATTTTTTTAACTTGCATCTCGTTTCCTGACAATGAAAAACT
TTTTCTATATGTTGCCACACAGATTCAGGATTTTCCCAACTAATTATTAAATTATCTAAACCAAA
TTTTAACAATTCCCTTGCCATCCAAGTATTCGTAGTTGTTAAAGGAATATTTCCCGCCATAACTG
CCTCAGTGAAAACACCTGATGTTCTTTCTTGATATGCGATCGCATCATAGGGTATCAACATAATA
TCAGTTTTTTGCAACCAATGAATATAATCGAGACGAGACAAATTATCATCAGTAGTAAGAACTTT
TACTCCCCCATTAATAGAGATTAAATCAGCACTTTTTGCTGTAACTAAAACGAAATTATCGCCAT
TTTTTATCGGATAGTCTGCCAAACTTCTGATTACTTGCCATCCTTTCTCTTCACGGGGAGGACCTG
CCCACCAACAAATGATAGAATTATTTTCAAGATCCCTATTTTTATCCACAAATTCTGTATGAGGA
ATAGGCATTACCGTAAAACTTTCATTAAAATACTTCTCCATAGAATTAGCCAATAACTCGCTGTC
ACTAAAAAGATGGAAATGGTCTTTTTTTACAGACTTTTTGAGAAGTTTATTTAATAGTTTATAT
ATGCCTTTAGTTTTATGCTGATGAAAATTATGACGATATAAAACCCAAATATATAAATTATCCTT
TGGCAAATTATTAATAGCTAATAACAAAGAAAATAATTGTAAATGAATAAACCTTTCAACAAAAA
ATAATTATCGGTTGATTACTATTAGAAATTAGCCTTTGTTCCAGAAAAATTTTGATAGTTTTAGC
AAATAAATCTACATCCTTAATAGTTTGAAAAATATTAGCTTTTTCTTCTAGTTGATTACCCTTAA
CACAACCCTGCCAATGAGAAGGCAAAGAAGGTAAATTATCCTCCGTTGAATAAATAACTTCATGG
TGCCACCCCAAAATATTCATCGCCTTAGTAATCGACTGATGATAGGGGATAATATGCCCTTCCCC
TCCCATTAAATTGGGTATTAAAGAAATAAACAGAGGATTATTTTTTGTTTGGCTATTAATCATAA
AGTTGAGTTCAGAATTGGGAGTTAAAATTAAGTATAGAATATCCCCTTTAACAATTATATATAG
ATTGTCTATTATGACTCTCTCCGTAGAACATCAAATCATTTATCCCGAAAGCGATGGGCAACCGA
TGGCAGACAATACAAAACAATTTCGCTGGATTGTATTTTTGAAAGAAAATTTGGAATGTTTATTT
GCTAACAACTCTAATGTATTTGTCGCAGGTGATTTACTCTGGTATCCCCAACAGGGAAAACCTGA
TATTCGGGTTGCCCCCGATGTTATGGTAGTATTTGGTAGAGCTAAAGGTGATAGAGGTTCTTATC
GTCAATGGGAAGAAGATAATATCGCCCCTCAAGTGGTATTTGAAATCATCTCACCCAGCAACACC
ATGAAAGAAATGTTGAAAAAACAGCAATTTTATGAACGTTATGGGGTCGAAGAATATTACATCT
ATGATCCTGATCGCCATGACTTCACTGGATTTCAACGAATCAATGATAAATTAACCCTCATCGAT
CATATTAATACTTGGATTAGTCCGAGATTGGGGATAAAATTCGTCTTCACCCAAGAAAATTTAAA
AGTCTATTACCCCGATGAGAGTCCGTTTTAACCACCATTGAATTAAAACAAAAGGCAGATGCGG
AAAAACAACGGGCGGACATGGAAAAACAACGGGCAGATTCAGCCGAGTTGGAATTAGAAAAATT
AAAACAGTTTTTGCAACAACAAGGTATTGATTTACCCAAATCTGAGTAACCTCAGTTCGATTTAA
GAATATCCGATAAGGTTAGGTGTTAGGGTGATGAGGGGAGAGGGTGAGAGGGGGAAGTAGGGGC
TGAATATATTTTTAACGTCAGTTCGGGTTAAGGCAATTTTATCGTTATTTCTAAGAAGCTATAAG
GTTTTCTGACTAGGAGAAAATAACGCTTTCAGCTTATCCAAAACTCAGGTTGAGCAAAAAATAAT
GGTGGGCAATGCCCACCCCACAATAAAAAATAAAAGTGAAACAGGCATCTTGCCTGTATTATCCA
GAGATTTATCTTTTAGCTAATTTCTTACTAGGTAACTTGCGTAAACGAATAGACTCAGGAGTAAC
CTCCACTAACTCATCAGGTCCAATATATTCCAACGCCCTTTCTAAAGTCATTTCTACAGGAGTTTG

CAACTGCACTAATTCATCCCCTGTGGCAGAGCGATGGTTAGTTAACTGCTTAGTTTTACAAACGT
TTAACTCTACATCTTGGGCACGGTTACTTTCTCCGATAATCATACCCTTATAAACTTTAGTACCA
GGGTGAATGAAGAATACTCCTCTGTCTTCAGCGTTTTTCATAGCGTAGAAAGTAGAAACACCCTC
TTCAAAAGCGATTAAAACACCGTTGTAACGGGTTTCTAACTCACCAACTAAAGGACGATATTCAT
GGAAACTATGGTTCATGATACCTTCACCCTTAGTCATGCGAATGAACTCACCACGGAAACCAATT
AAACCCCGTGCAGGAATAATAAATTCCATCTGAGTACGTCCATTGCCACCAGACTGCATATCTTG
CATTTCAGCTTTTCTTTGACCTAAACGCTCAATACAGCTACCCTGAGCCTCTTCTGGAACGTCTAA
AACGAGATATTCAAAGGGTTCGTAGGGTTGTCCATTGACTTCACGGTAAATAACTTGAGGCTGAG
ATACTTGGAACTCAAAACCTTCACGGCGCATATTTTCGATGAGGATACCTAAATGTAACTCACCA
CGTCCAGAGACGATAAATTGTTCTGCAGATTCACCATCTTCTACCCTTAAAGCTACGTTGGTTTC
TAACTCACGGGTAAGTCTGTCTCTGATTTGACGAGAAGTAACGAATTTACCTTCTTGCCCTGCAA
AAGGAGAGTTATTAACAGAGAAGGTCATTCTTAAGGTAGGTTCATCCACCTTAATTAAAGGTAA
GGCTTGAGGTTCATTGGGGCAAGTGATAGTTTCACCGATATTTGCATCAGAAAAACCAGCAACCG
CTACGATATAACCTGCACTAGCTTTTTCTAATTCAACTCTTTGTAAGCCTTCAAAGCCTAATAAT
TTGCTAATTCTGGCTTTAACCATGCTACCATCTTCTTTCATTAAAGCCGCTTGTTGTCCTGCTTCA
ATGGTTCCATTATGGATTCTGCCAATGACAATACGTCCTAAATATTCAGAATAGTCAAGGGTGGT
AACTTGTAATTGTAAAGGCTTTTCGGGATCTCCTGCTGGGGGTGGAACATGGTGTAAAATTGCCT
CAAATAGAGGTTGCATATCCACTTCCTCATCATCGAGGTTCGCTTTGGCAAATCCTGCTAAACCA
GAGGCATAAAGGGTAGTAAAATCGCATTGATCATCATCTGCACCTAGTTCGACAAATAAGTCAAA
TACTTTATCTACGGCAGTATCAGGTTCAGCACGGGGGCGATCGATCTTATTAACAACAACAATAG
GACGTAAACCTTTTTCAAGGGCTTTTTTGAGTACAAAACGGGTTTGAGGCATAGGTCCTTCATTG
GCATCCACAATCAAGATACAGCCATCAACCATTCCTAAAACTCGTTCTACTTCACCACCAAAGTCA
GCGTGGCCGGGGGTATCAACAATATTAATTAAAGTATCTTTATAACGTACTGCGGTGTTTTTAGA
TAAGATGGTGATTCCTCTTTCTCTTTCAATATCATTGGAATCCATCACGCAGGTGGGTATTTCTT
CTCCTTCTCGGAAGATACCAGATTGTTTTAATAAAGCATCAACTAAAGTGGTTTTGCCGTGGTCA
ACGTGGGCAATAATCGCAACATTGCGAATGGGGAGAGACATAAATATTATTTAACTTAATGTAA
TGATTATTCATATATCCTTTTCATCTTAACCGATCATTGTTATTTTTTGTTAAATTGTTTACCTA
TTTTGACTTGTCTAAGCTAGGACTCTCAAAAAAAGTCAGGATGAGTTCACCTGATTTAAGTAACC
TTAGTTCGGGGTTAGGAGTTCGAAGGAGGGGGAGAGGGAGAAGTAGGGGCGAATGGCCATTCGC
CCGTACAGGGTTTCAGGTTTCAGGTTTTAGAAGAAAGTAATGAGTAATTATCAACTATTCACTAT
TCACTCAGAAATGAAAATTTATCCCGAACTCAGGTTAAGTATCTCGAAAAATAGCCATCACTCTC
TCATATTGCTAAGATAATATTAAGTTTTGTAAAGACAAGAGGCATGATTTTGAAAACGGAAAAC
ATCGTAACCTCATGGCATAAATTGGAGAATATTTGGCAAGGAGGAGAGGAATTAGTCAAGATAG
GTTTACCTCATAGTCAACTTGCACCCCCATGGCAGATACTTTTATTGGGGGATGGTTCACCCACTC
GACACTTAAGACTTTTAACCAGTGAAACCATTAAAGTTGACATCATTGATATGTCATTGGTGGGT
AATAGTCATGATAATGCACCTGCTGATATAGAAAAAGTACCAGGTCCTAGACTCAGAAGGCAAGT
ATGGTTAAAAACTGCTAGTGGTAAGAGATTAGCTTATGCCTCTTCTTGGTGGGAAGCTAGTCAGG
TAGATGATTATTTACAAAATCGCAATTTACCTATTTGGGAGAGTTTATCTAGCCTACACACTGAG
TTATACCGAGACATTCAGGGGATTTATTATGGTCATAGTCAAGCCTTAGAAGAAGGTTTTGGGGA
AAAAGGCGCATTTTGGGGGCGACATTATCTTTTTTGGCACGCTAGAAAGCCTTTAACCCTAATTT
ACGAAGTTTTTTCTCCTTATCTTACTAAATATTTAGGAGACATGAATTATAGTTAAAGTATTGTT
TAATTCCAATCTAACTAAGTTTTGTAACAAAAATAGTAGTGATTATTTTAATTTATGAGAAATG
GACAAACCACCCTTAATAATTTGCTTTAAGATAGGTTTTGCCATTTTTGCTGTGGCTGGAATTAC

TACTGTGCCTACTAAAATTGCTCCTAATCCTGCTAAAGCGGTGCGATCGCCTCTATTATGATCTTC
AATAATTTCTTTTAAATGTAAACTCAAAACCGATTTATCCCAATGAACAACAGACATTAGCTATT
CTCCTAATTAATAAAACTTATTATATATTGATTTTATTATTGCATAAGATTTGCATTTGTAAAGA
GGATAAGGTTAAATTTTATCAACAGGAGTATTTTCCTCTGCTTGTTGTCTTCTGCCTTTTGCTTA
ACTCTTCCTGATGATTTAGATGGTTTCTAGCTTAATAATTAAGTTAAATAATAATTAAGTTAAAA
CAGACATATTGCTGTGTAATTTAAAATCTTTTGCAAAACTAAACTGTGTTATTCCATATATTTAG
TAATTATCATTAAGAAAAACTTTGATTATGCGATTAATTCTATGAACTTTGAAATAAAAAGTCT
TTAGAAAAAGACACAAAATCAAAATAAGGAACTATAATGATAAGTGTAAAGGTTAAACTCCCTA
AGAATGCTAAATAGTGGGAAAACCAAAATTAGTTTAAGAATAAGGAGATATTCATGATGAAAAG
AAGAGGTGGATTTCGCCAAGGTGCAGGACGTAAACCCATGTGGCAAAATGGAGAAACTCAAACTA
TTCGTGTGCCTGTCGCTCTTAAAGATGATTTAATTACTATTGGTAGAGAATTAGATCAAGGGCGA
GGGGTGATAATGGGAAAAACCTGCTCCCAGTTGGAAAATATCGTGGAACGATGGGAGGCTAAGT
GCGAAGAAAATCAGGGTCAGGAATGGGATAATATTCGGCAGTTAATATTGGAAATCAAAGAGGT
TTTAGCTCAAAGACAGAATAAGGGTAGAATTGGGAACAGACATCAACATCAACAAGGACAATGT
CATAAAAAACAAATGAGAAGGGGTAATCCCAATTGGGTTGACACAGAGGCGGATATTGTTGACT
AAAACTATTCGTTATTGTTGAGTTAAGGAAAGCGACGATTTCTAACATCTTCGGCAAAATAGTTC
ACTGATGTTAGGATAGTTTCTCTTAAATTGGCGTAGGGTTTGGCAAAGGGAGGCAGTTTATCGGA
TAAGCCAAGTAAGTCAGAAGTGACTAAAACTTGTCCATCACAGCTATTTCCTGCACCAATTCCAA
TGGTGGGGATAGTTAACTCTTTAGTTATCTTTTGGGCTAAATCTGCGGTAATGTGTTCTAAAACA
ATGGCAAATGCACCGGCTTCTTGAAGTGCGATGGCTTGATGAAAAATTTTTTCCTGTTGTTCGGG
GGTTTTTCCTTGTTTTTTATAGCCTAAAATCCTTACTGATTGAGGAGTTAAGCCCACATGAGCCA
TAACAGGAATGCCAACTTCTGTCAAACGGGTTACAGTATTGATCATGGCAGGATAGCCCCCTTCT
AATTTAACTGCTCCTGCGTTGGTTTCTTTGATTATTTTTCCTGCGGATTCTATGGCTTGACTTAC
ATCTCGTTGATAGGTTAGAAAGGGTAAATCACATACCACTAAAGCCCGATTAACTCCTCTACAAA
CAGCTTTTGTATGATGGATCATTTCTTCTAGGGTGACGGGCAGGGTACTAGAATGACCTAGTGCT
ACCATACCCAGTGAATCCCCTACTAAGATTAAATCAACTCCTGCTGAATCTAATAATTGTGCGAT
CGCATAATCCCAAGCAGTTAAAGAAACAATAGGGCGTTTTTCCCCTTTCCATTGTTGTAAATTTT
GAATTGTTACCTTCATTTAAGAAATTAGAAATTAAGAATTAGATTTCAATTAGGAATCAGGAAT
TAGGAATAAAAAACCCTCGTCTCTTGCCTTTCCATAAGTGCCGAATATCTCTAGTATCTTTTATT
TTGTTATAGAGTAAACCATTATATATCCCTCATTCTACATTTTACATTAGACCTTTGCTTAAGGG
GTTTGCCAAATACGTTCATGATAAGCAGTCGGATCTATTAAGGGATCGTTATCATGATGATGGTG
ATGATGCCCATGGGAATGATCATGGTGGTGATGGTGATGATCATTATTTTGTAAAGCATTGAGAC
GGAATTTGCACATTTCACAATTCATCTTTACTTCTCCTAATTGGGTTTCTATTTCTCTTTCTCTCA
CCAATTGCAATAATTCTGGTTGAATACCCATTTCTGGTAAGCATTTAATTTCTATTTCAGGATAA
GTTGTTTGATGCTCTTGTGTAATAGTAAATATTTTTTTCATCAATGCACCCATAAAAAGAAAGTA
GGGTAAAACAATAATACGCTTCGGTTGATAGAGTAAGGCACGACGGAAACCTTCTTCCAGACGAG
GATGAGTAATGCCAATAAAACAGGTTTCTACAGTTTTGTAGCCACTTCCTTCCCACAAAATACGG
GCTAATTTATAAACGTCTCCGTTGGCGTCTGGATCACTTGAGCCTCTACCCACAAATAGTAAAAC
CGTATCTTCTCTGGTAATTTGATGAGGGTTATATTCCTTTGAGTCTAATTGATTTAAACGCTCTT
GCCACAAATTGATAATACTGGGGTTTATACCAAAATGTCTGCCATAGTGGAAAGTTAGTTGAGGA
TATTTAAGACGGGTGCGATCGAGTTCATTTGTAACATCAAATTTATTATGACGAGCGGCAAAAAG
CAATATCGGTAAAGCAGATATATCTGTGTATCCTTGCTCTACACAATAGTCAACTCCTGTCATAA
TATTGGGTTCAGTTAATTCTAAAAAACAAGGAATGACAGGACGAGATGTGTCTAATTTTTGATA

AGTTTCTACAAATTCGAGAAAGATTTGTTTACCATGAGGATCTCTTGTACCATGACCAATCATTA
GAAGCGGACGTTGATAAGGTAAAGGAGTTAAGGTAATTTCTTGCTCTTGTCTGGGTAATTGTTCG
ATAATTGCAGTTTGCATTAATTAAATTTTTTAATCCATGAGATAAATAAAATTCCTTTTAAATTA
TAGCCATACCCTGAAATGACGGTAAATGCCTTCAGGGGGGGATTAAGAATTAATAATTGAGATAT
TTCTAGGTTTTCAGTTCATTTATACTCTTGTCAATATTATTGTCTCTGTGCCATTATTAAATTAA
TTGGCTTAAAGCCATTTTAAGAGAGGAGCAAAAAATGCCTATTTTTATCAATGATGAGCAGATTT
TTTCCTTCGTTTTTCCTGCAGGAGAGTCTCACGTCAGGATTTCCCCCGAAATGATTAAACCCATAA
CGGTGATAGAGGCATGGTTGTATAAAGCAGAGGATATTATGGCTTTATTATTATCTGTTGATGCG
ATTCATCGTTTTTCTTTGGGTGTCAAAATTCATTTAACTATTCCCTATTTTCCCTATGCTAGACA
GGATCGAGTTTGTAATCAGGGAGAGGCACTCAGTGTTAAGGTAATGGCAAATTTAATTAATAGT
CTCAGGTGCGATCGCATCTTAGTTATTGATCCCCATTCTGATGTAACCCCTGCCCTATTAGATAAC
TGTCAAGTCAAAACCCAAGCAGATATTATTTTTGATAGTGTTTTAAAAAATATCTTAATTGAAGA
TAATTGGGCGTTGGTTGCTCCCGATGCGGGGGCAGAGAAAAAAGTTTTGACAGTGGCACAACGTT
TAGGATTAAGAGATGTTTTCTGTGCAAGTAAAATTCGTGACTCAAAAACAGGAAAAATAACTGA
CACCACTTTTCATGATGAGGTTAAAGGGAGGAAAGTCTTAATAGTGGACGATATTTGTGATGGCG
GTAGAACTTTCATTGAATTGGGTAAATTATTACGAAAAAAACAAGCAGAAACAATTTATTTATA
TATCACCCACGGTATTTTTTCTAAAGGCTTACAGGCTCTAAATCCCTATTTCGATCATATCTATT
GCTATCACTCTTTTTTACCCATAACAGAAAAAAATCAAGATTTTATAACAGTTATATCGTCTCAT
GACAGGTACTCTCAGCCATCATAAAAAAACCTCAGCTTGATAGCAAGAATATTTAATTGAGACAGG
TGTCAGGTTTCAGGTTTCAGGTGTTTTTAATTATTAATTACTAATTGTTAATGGGTATGAGGTTA
AAGAATTTAGAAAAAAGTTTGAATCATTAAAAGGAGTAAAAGCCATGAATATAAATCCCCTTCA
CGCCATTGATTTTTATAAAACCGATCATCGTCGTCAGTATCCTCAAGGTACAACGGAAGTCTATG
CTAACTTTACCCCTCGTAGTGGACGTTTAGCTAAAGTTTTAGATTCTGATGATGTCAGTGTTATT
TTTTTCGGGTTACAGTATTTTATTCAAGACTATCTTATCGATTGTTGGAATGAAAATTTCTTTAA
GCAAGACAAGCAAAAAGTAATCTCAGCTTATAAAAGACGCATGGATAATTCCCTTGGTGTGGGTG
CAATTCCAACTAATCATATTGAAGCCTTACACGATTTGGGTTATTTACCCCTAAGAATTAAAGCC
TTACCTGAAGGAAGTAAAGTTGATATTGGTATCCCCTTACTAACAGTGGTTAATACTCATCCTGA
TTTTTTCTGGTTAACCAACTATATTGAATCAGTTATGTCAACTTATCTTTGGAAACCAATTACTT
CGGCAACTACTGCTTTTCAATATAAGAAACTGCTGACAAGATATGCTGTAAAAACAGGTACATCC
CTAGATTTTGTTCCTTTGCAATGTCATGATTTTTCTTTTAGGGGTTTATCTGGCATTCAAGATGG
AATCATTAGTGGTTCGGCTCATCTTACCTCATTTTATGGCACAGATACGGTACTTGCGATCGATC
TTTTAGAAAATTATTATCATGCTGATGTAACTAAAGAAATAGTAGGTGTTTCTGTTCCTGCAACG
GAACATTCAGTCATGGCAATGGGTTTACAAGATGGAGAATTAGAGACATTTCGTCGTTTAATTGA
AGATTTATACCCCAGTGGAATTATTTCCATTGTTTCGGATACATGGGATTTTTGGAAGGTGGTAA
CAGAATATACTGCTATCTTGAAAGAAAAAATTTTAAATAGAGATGGCAAAGTTGTTTTACGTCCT
GATAGTGGCGATCCTGTTAAAATAATTTGTGGGGATGAAAACGCACCCCCCTACACCCCAGAAAA
TAAAGGGGCTATACAATGTCTGTGGGAAATTTTTGGCGGTACAATGACCCCTCAAGGTTATAAAA
TGCTTGATTCTCATATTGGACTTATCTACGGAGATTCTATTACTCTCGATCGCGCTTCACAAATA
TTATCTAAATTAGAAGAAAAAGGATTTGCATCAGGAAACGTAGTATTTGGCATCGGCTCTTATAC
TTATCAGTATGTAACCCGTGATAACTTCGGTTTTGCCGTCAAAGCCACTAGCGGAGTAGTCAACC
ATCAACGCCGTAATATTTTTAAGAACCCAAAAACTGATAATGGGGTGAAAAAATCGGCAAAAGG
ATTACTAAGAGTTGAGCAAGAGGGGGATAGATTTGTGTTATACGAAGAACAAACAGAAGCACAA
GAAAAACAAGGGGTATTGGATACGGTATTTTTGAATGGAAAACTAATCAAAAGTCATAGCCTTG

CAGAAATTCGTCAAAGAATATATGAACTATCTTCTTGCTTAACAGACAATTATCTCTGAATAACT
AAAAAATATCGTACCTGAATGGTCAATAGTTTGTTATTCTTGGTGTGAGCAAGTAAACATAGTA
ACGGTGGGTTAAAATTTGATGTTTGGGTCGGGTTTTCCTGACAATTTATCTTCTTTTATTGTTTT
TTTCCGTCAATTTCTCGATTTAGGTTTTGCAGTTTTCTCTTTTTACTATATTTCAGTTTTAATTA
GAGAACGTCGTGCACAACGGATGATTCGAGGATTATTCTTCCTTTTTATCATTTTTTATGTGAGC
GATCGCATCTTTCAGTTAAAGTTATTTAGCTTTTTCTTAGAAAGACTAATTTTAATATGCTCGTT
GTCACTGGCATTAGTATTCCAGTCCGATTTCCGCCGTTTTTTGGAACAAATAGGCAGATGGCAAT
TTGATTTTTCTTCTTCTAATTCTTCACTACCAAAAGAAGATAGTGCCATTGATGAAATGGTAGAA
GCAGTCAAAGACCTTTCTCAGAATAGGGTTGGAGCATTAATTATTGTAGAAACATTTAATACTCT
TGAAGAAAGAATTTTTGTCAATCCGGGGGTACAGTTAAACGCTATTGTCAGTAAAGAACTATTAC
AAACTATCTTTCAAACAAAAAGCCTTCTCCATGATGGCGCAGTATTTGTCAGAGGAAATGAAGTG
GTTTCCGCAGGGGTAATATTACCATTATCAGATCGTAGTGCTTCTCGTCAATTAGGAACTCGTCA
TCGTGCGGCAATGGGAATCACGGAAAGAGTTGACAATTGTATTTGCATTGTAGTTTCGGAAGAAA
CAGGATCGATTTCTTTGGCGGAAAAAGGTACTTTAAATCGTCCTTTAACCAGTACAAAATTAAAA
GAGTTACTAGAGGAAAAATTATCTCAACCTTCCGAAGGAGAAGTTGTCACGGGAGTTAGTCGTTT
AAGTAAAAAATTGAGTTTTGGCGGTCGAAAATTTTTGGCAAATCTGTTTAAATTCGGGAAAAAT
AAAAAGTTACTTGAACAACAAAAAAAAAGAAATTAAGACAAGGCAATAGTGAATAGTGAATAGT
GAATAGTTGATAATTAAAAACTCCAAACTCGCCTCTGTTTCCCCAAACTTAAGTAACTTTCTTGC
ATCGTATCGTAATTAGTCATTACACTATTAAACTTATATCTAAAAAACTTTTTTTTACAACAAAA
ACCAATCAGAAAAGTATTATATTGACATATAATGAACCAACAAGAAAAACGAGGGAAACTAATT
GTTATTACTGGACCTAGCGGTGTGGGAAAAGGAACAATAGTTAAGCAATTACTGGCTAAACACTC
ACAAATTTTTATTTCGACTTCGGCTACGACTCGTAAACCCCGTGAGGGAGAAATTCATGGTCAGG
ATTACTTTTTCCTCTCTCAAGATGAGTTTAAACAGATGATTGATAACTCTCAATTGTTGGAATGG
GCAGAATATAACGGTAACTATTATGGGACTCCGAAACAACCAGTTATTGAACAAATTGAACAAGG
GAAAATCGTTATTTTAGAAATTGAGGTTTTGGGGGCAAGACAAGTTAAGGAAACTTTTCCAGAT
GCTCTCAGAATCTTCATTTTACCTCCTTCAGAAGCGGAATTGGAGCGAAGATTAAGAGGTAGAGG
AACAGACTCAGAATCGGCGATTATAAAGCGTTTAGAAAAAGCTAAATTTGAGTTATCCACCTGTA
ATGAATTTGACTATCAAGTTATTAATGATCAATTAGAAAATGCGATCGCATCCGTTGAGAAAATC
ATAACAGGAGTTCGGGGTTCGGAGTTTTTTAATTCTTAATTCTTAATTCTTAATTACTAACTCC
TATCTAAATCAAGACACTCTTGCTGAACAAATGAGATTAAATTATGTTTAATTATTTTAAGGGAA
TAGTTAATAGTATAAATGCTAACAATAATCGTTATTTTTTAATATTAGAAGTGAATAATATTGG
CTATGAAATTCAAGTTCCTAGTCGCTTTACCAGACAATTAGAGTCAAATTCAGACCAGATTATTA
GTGTTTTTACCCATTTTCAAGTAAGAGATGATCAATATATACTATATGGTTTTGAAAATTCTGCT
CAACGAGATTTATTCCGACAATTAATTAGTGTTTCTGGGGTTGGTTCTCAAACTGCGATCGCACT
TATTGATACTTTAGGATTAGAAGAATTAGTTCAAGCTATTGTCACGGGAAACATTCGTTTATTAT
CAAAAACTCCCGGTATCGGACAAAAAACCGCCGAAAGAATAGCCTTAGAGTTAAAAACGAAACTT
GCCCAATGGCGGGTTAATGCAGGGGTTAGAGTTGACAGTGGTAATTTTTTACCTAGTGAGGAAAT
TATGGCAGATTTAGAAATGACTTTATTGGCACTAGGATACACCAACAACGAAATTCAACAAGCGA
TTTCTGTTATCAGTCAAGATAATTTATTACAGAAAAATCCCCATGTAGAAGAATGGATTAGAAGC
GCGATCGCATTTTTATCCCTGAATTGATAAATCTTGATTTTTATTTAAAATCTAACAGAGAAACT
GGTTCTAAAAGAAAACGACTGGCATTAGAAGCGATAGCCTCCCTAACATTCATTTGTTTTAAGGT
AACTAAATTATCAGCAACAAAAGCATCTCGCACTAATAACTTATTTCCTGAATTAAAATTAGTTT
CGTAAAATACTTCTCTAATTCCTGCTGAAATAATTAATTTCAACAAGCAATACAGGGTTCAAGA

GTTACGTAAATAGTTGCCCCTTGGGTAGCAATACCGTGTCTAGCCGCCTGTGCGATCGCATTTGCC
TCCGCATGAACCGCCCGAGAAGGTAGAGTTTTACTTGCATCACAACTGCTCAAGCCCTCATAACA
ATAACCCTGTGCAGTACAATGAGCAGAGCCTGATGGTGAACCATTATAACCTGTAGCTAACACTT
GACGATCCTTAACAATTACCGCACCAACGGGAAAAGCAAGACAAGTTGATCTAGTCGATGCTAAC
TTAGCCATCAACATAAAGTATTCATCCCAAGTTGGGCGATGTATTTCTCTTTCTAATGTGGACAT
GATTTTTAGTTCAGAGTTCGGAGTTGGGAAAGAGGTTTCAGGTTTCAGGTGACAGGTTTCAGGTG
TTTTTATTATTTATTATCAACTATAAACTATTCCCTATTGCCTATTGCCTCTTGCCTATTGCCTTG
TTTTAATTACTAATTACTAATTTAATTTTTAATTACCTTTGCCCATTGCTTTCCCCCCTCTCGAGG
GGGGATAAAGGGGGGTTTGCCCTTTGCCCATTCATTGCCCATTGCCCTTTATCAATTATTTTTCCT
ACCATAAAGATAAGTTAAATAACGAAAATGACCAGTAACATCAAGGTTTAAAGCCCCTTCAGGAA
AACGCCAAGTCCAATTATTCTCTATAGTGCCGGGGGTGTTCATTTTTGCATCTGAACCTAAGCCT
AATAAGTCTTGTAAGGGGAATATTGCCCAGTTAGCAACAGAGGATAAGGCTAATTGAATCATACT
CCAGTGAATACCGCGATCGCAAACTGCCCCTAAATAATCAATTACTTTTTGTTTATCTTCGTGGG
AGCGTTTTTCAAACCAACCGATAGTCGTATCATTATCGTGAGTACCAGTATAAACAACGCAATTG
TTTTGGTAATTGTAAGGTAAAAAAGGATTATCACGATCAGAATCGAAGGCAAAATGTAAAACTT
TCATTCCGGGGAAACCATATTTATCCCTCAAAGCCTCCACTTCAGGAGTAATAACCCCCAAATCTT
CCGCCACAATGGGTAAAGTACCTAATTCCTGCTTTAACACCGCAAAAAATTCATCTCCGGGGGCT
TCCTTCCACTCTCCACTCATAGCAGTGGTTTCCCCTTGAGGTACAGCCCAATAAGCCTCAAAACCC
CTAAAATGGTCAATGCGCATTACATCCACATATTCCAACATTCCTTTAATGCGTTTAATCCACCA
TGCAAACTTAGTTTCCTGGAGCTTTTGCCAATTATAAACAGGATTACCCCAAAGCTGACCAGTAG
CACTAAAATAATCTGGTGGTACACCTGCCATCGAAGCCGCTTCTCCTGTTTCTTCATCTAAACAA
AAAATACTTTGATTAGCCCATACATCGACGCTATCGTGAGCAACGTAAATAGGAATATCGCCAAA
AATATGGATTCCTCTTTCATTGGCATAGTCTTTTAAATTACGCCATTGACGAAAGAAGCTAAACT
GTAAAAATTGATGGAAAAGCATTTCTTCTGCCAGTTTTTTCTGCCAAGTTTTAATGGCTTCTGGT
TTTCTTCTGGCAATATCTTCATCCCACTGAAACCAAGGTTTATCTTGGTAGTAATCTTTTAAAGC
CATGTACAGGGAATAGTTTTCTAGCCAGTCTTTATGAGTTTGGCAAAAATCAATAAATTCTTTGG
TGTATTCGTCTTTTTCTCTTTCCTGAAAGGCATTAAAGGCTTTGCGTAAAATAGGATACTTAGTT
TGAATGACTAAATCATAATCAATATGAGTAGGGGAATGAGAAGCGAAAGCATTGCTAAGACTAT
CTAAATCTTCTTGAGTTATTAAGCCATCCCCAAATAAAATATCAACACTGATTAAAAGAGGATTC
CCCGCTAAAGCAGAATAAGCTAAGTAAGGTGAATTTCCAGCCCCCGTAGGACCTAACGGTAAAAT
CTGCCATACCCCTTGATCACTTTCGTTCAAAAAATCGACAAAACGATAAGCACTATTTCCTAAAT
CTCCAATACCGTAAGCATTGGGCAAAGAGGTGGGATGGAGCAAAACACCGCTCAATCTACGATTT
AACATAAAAACTATATCCTAAAAATGTTGTCTATAAGCCTGTGTACGTAACTTGCAGGGGGAATG
AAAAATAAAAACAAAGAATATTCTGACTCTACAAAGGGGTTTTGTGTTGTCAACTGTCAAGGGTC
AATTGATAGTAATAATTACTCGAATTATCGTAATTTAGAGATCTCCACGCAAAGCATAAAGAATA
AACACAATTACAGGACCGGCAATTAAGATAAAACTAACAGAGGTCAATTGTAAAATGACTTCTA
AGTTTAGATTACTAAAAAAATTGCTAATAAAATCCATAGTAACTCCTAGCTTTTTAATTGATAAT
TTTAAATTTTATTTCTTAGTCTCACCAGAATATATCATACTAGGAAAAGTTCTCCTATTCATCTG
TCTGTTTAGGGAAAATTTATTAAGTTTTGTTACAGATTTTATCTTCTATGAATCAATGGCGTTGT
ATTGAAAACTGTGGTGCGTGTTGTAATTTAGATCCGAGCGATCGCCCCGATTTAGAGCAGTATTT
AACTCAGGAGGAATTAACCTTATATTTGAGTATGGTGGGAGAAGATGGTTGGTGCATTAACTAT
AATCATGAGCAACGTAGGTGTAATATTTACGATGATAGACCTCGTTTTTGTCGTGTAAAACCTGA
TATTTTTGAGCAGATGTATGACATACCCTTAGAAGAATTTGAAGAATTTGCGATCGATTGTTGCC

ATCAACAAATTGAGGCTGTTTACGGTGAGGAAAGTGAAGAAATGGAGAGATATATTAGTCAAGT
AGGTTAGAAGATGCTTAAAAACTATATTTTTGCTGTTGCCCCACCCTGTAATAAATTATAAAACA
GAGTTTGTAGTTACCCCTTTAGGAGTTTCAAAGTTTCCGAGAATAAGGGTACTAACTGAGCCTAT
TCTACGTTATTTAACCCCTACCACCTGACACCTGTTTCGTAACCCTGAACTCCGAACCCGGAAAAC
CCCTAATTAATTGACTGAATGTACTTATCTAACCTATTAATTCCTTTTTCAATGGTTGTCATATC
TGTTGCATAAGAAAGACGGATACAATTATCATCGCCAAACGCAATACCGGGAATAGCGGCAACTT
TTTCTTGTTGTAAGAGTTTTTCGCAAAATTCGAGGGATTTTAAGCCTGTTGAGCTAATATCCACA
AATAGATAAAATGCTCCATAGGGTAAAGGGCAACTAATACCCTGAATAGATTGTATGGCAGAGT
ACATAAATTTTCTTCTTTCGGTGAAGGCTTCTAGCATTTCTTTCACGCAGTCTTGGGAAGATTCT
AAAGCTGCGATCGCACCATATTGAGCAAAGGTACAAACATTAGAGGTGGTATGACTTTGAATCGT
TGTCATGCCTTTTATTATGTCCACGCTACCTGCAATATAACCCACACGCCAACCAGTCATAGAATA
GGCTTTGGCAAATCCATTACAAACAATAGTGCGTTTAAAAACCTCTTCGCTGACAGCACCGATAC
TCAGATGGGTTGCTCCTTCGTAAAGGATTTTTTCATAAATTTCATCTGAGACTACTAATATATCG
TGTTTGATCACAATTTCTGCAAGAGAGTGGATTTCTTCGGGAGTATAAACTGCCCCTGTGGGATT
AGAAGGGGAATTGAGAATTAAGAGTTTGGTTTTAGGAGTGATAGCCGATTCTAATTGCTCAGGA
GTAATTTTATAATTGTTTTCTGCGGAGGTAGTAACAAAAATGGATTTTCCTCCCGCTAACGTGAC
CATTTCAGGGTAACTTAACCAATAGGGTACGGGGATTATAACTTCGTCTTCAGGCTCAATTAGGG
CGAATATAAGGTTAAATAAGGCGTGTTTTCCCCCATTGGTAACAATGACATTTTCCGCTTGATAG
TTTAAATTATTATCCCTCTGTAATTTAGATGCGATCGCATTTCTTAAGGCTAATTCTCCAGCCGC
CGCTCCATATCTAGTTTTACCCTCATCTAGGGCTTTTTTCGCCGCTTCTTTAATATGATTGGGGGT
ATCAAAATCTGGTTCTCCTGCACTAAAACTACATACGTCTAAGCCCTCAGCTTTCATTGCTTTAGC
TTTTGCAGAGATGGTTAAGGTAATAGAAGGAGTTACTTGGGAGATGCGATCGGCTAATTGTGTC
ATGGAATTTATTATAAGAAAAAAATTATCTATTAGTAAATAATAATTATTTAATCTTGATCAGT
AAAAAACATTACCATAATTTGCTAAAAAAGTATTAGCCATGTTCAGGATTTGGGCTTTGGAAAG
AGGTGTCAGGTTTCAGGTTTTACTGTTGCAGGTGTTAGGGTGTTTGGGTGTTAAGGTTAGTGTTA
ATTAACCACTTTTGCCCTTCCCCCTCGAGAGGGAGGATAAAGGGGGGTTTATTTTAAGGGGAGAT
ACAGAGGGGTTTACCTCTTACCTCTTGCCTCTTACCTCTTGCCCTTTCGCTGTTAGAATCTTACAA
ATTAATCAAATCAATCTCTAATTAATTCATGTTAAATGTTAATGAAGTAGAACAAATTATTTCTG
ATTTAATTAAACCCATTAAAGATATAGAATTAGTTTCTCTCTCTTCAGTTGATAATCGTGTTTTA
GCTCAAGATATTACCTCTGATTTAGATTTTCCCTATTGGGATAACTCGGCAATGGATGGCTATGC
AGTTAAATATAGTGATGTTGCCCTTACATCTGAAAAAAATCCCGTAATTTTAAAAGTAATAGAA
GAAATTAAAACAGGATATAGTCCCCAAAAAAAACTAAAAATGGGAGAATCTGCCCGAATTTTTA
CGGGGGGGATGTTACCTCAAGGGGCGGATACTATCGTTATTCAAGAAAATACCCTCCGTCAAGGG
GATAATGTCTTAATTACTCATCCCCCTCTCAAAGGAGAATTTGTAAGGCATAAAGGTTCATTTTA
TCAGGCAGGAAAATGTTTAATTCCCGCAGGAAGTCGTATTAATGCCCCTGAAGTTGCCATTTTAG
CCACAGCACAATGTTTAGATATACCTGTAGTGCGATCGCCTCGTATTGCCATTATTTCCACTGGA
GATGAATTAATTAGTCCTCAAGATACCCTAAAAACAGGGAAAATTATCGACTCTAATCAATACTT
AATCGCCAGTTTCTTGAAACAAAATGGGGCAATTCCTTTACCATGGGGTATTATTCCCGATGAAT
TAGATGCCCTTGAAACCGAGGTAAAAAAAGCCTTAGCCGAAGCTGACTGGATTATTTCCACTGGG
GGAGTATCTGTAGGAGAATATGACTATGTGGACAAAGTGATTGAAAAATTAGGAGGAGAAATCA
AGGTTAAAAATGTCAGAATGAAACCGGGTAAACCCTTAACTATAGCCACATTTGGCGAAAATAAA
CTATATTTTGGTATTCCGGGAAATCCTGTTTCCACCATGGTTACTTGTTGGCGTTTTCTAAAAAG
TGCGATCGCAAAATTATCAGGGGAGATAAATCCTTCTTCTTTACCCATTGTTAAAGGTATAACTT

GTAATCATTTGCGTTCTCAAGGAACAAGAGAAACTTATTTATGGGGCTATACTAATCTTGTGGAT
GGTTATTATCAATTCACTATGGCAGAAGGTTCTCATAATTCTGGTAATTTAATTAATCTGCAAGG
AGTAAATAGTTTAGCCATTATTCCAGTGGGGGTTAATTTAATTGAAATGGGAGACGATGTTTCTA
TATTTCTTATAAAGTAAAAGTTATTTTTTGATACTACAAAAAGCAAAGGTGAATAGTGAATAGT
TGATAATTAAAAACTCCTAACTCCTGTACGGGCGAATGGCCATTCGCCCCTAACTGACTTCGAAC
TTATGAGATTGCGGCCACTTTTTGATTACTCTTAAGAATCGCTCTCGCCGCTTGTAATCCAGAAA
TTGTCGCTCCTTCCATACTGTCGATATAGTCTTGTTGAGTATAACTACCTGCAAGGAAGAAATTA
GAAATAGGTGTAGCCTGAGAAGGGCGATAAACATCCATCCCCGGAGCTTCACGGTAGAGAGATTG
AGCTAACTTAACCACGCTATACCAAGTCATATTTAATTCACGAGAAGAAGGAAATAACTCATGAA
CTTGTTTTAAGACATGATTAGCAATATTTTCATTGCTTTCTTTGATAAAAGGATCACCGGGAGTT
AAAACTAATTGTAATAATGATCCTTGTCCCTCTCGATAATAATCTGCAGGACTAGATAAAGCTAA
ATCAGAAAAACAAGAAAAATCAGCATCCGCAGTATAGAGTAAATTATCAATCCCCTCTGCTTTCT
CTAATTGACGGCGTTTTTGAGGATCATTTAATTCTGTTACCCAACCATCAAATCTTAGTTGTACT
GTAGCTACTGGTACAGCCGTGAGCTTGTAGATATTATCAAACTCTGACCACTTACGCCAACCATC
AGGAATTAATTTTTGAATACCCGGTACATCACAAGCACAAACGTAAGTATCTGCTGTAATAACTT
CTTCGGTTTCTCCTTTAGCAATTAATAAACCTGTGACAGTGGTATCATCTCCTTCTTGATATAAA
ATTTCTCTAACACGACGACGGGTATGAATTTTTACGCCCCTCGATTCCAAATAGTTAATAATGGG
TTTATGTAAATATTCGTGGGGTGAACCTTCTAACATTCTTAATACAGAAGCCTCAGTTTTTGCCG
CAAAAAATTGGAAAATGGTTAACATACAACGGGCAGAAATATTTTCTGTATCGATAAATCCTAA
AGCATAGGCGATGGGATTCCACATTTTATCCAAACTGCCTTGATTACCCCCGTGTTTACGAAACC
AATCAGCAAAACTAATACTGTCTAAATTTCTAATTTGACGCATTGCCCCATGAAAATCAATCAAA
CCTCTAACAATAGGGCTAGTGCCAAGGGCTAGAGAATTGGCAATCTTATCCACCGTTGAGAGTTG
AGAAGTGGTAAAAAAGGCTTTTAAACCATTAAATGGAGCACCAGTGGGAAAACGAAAATCTAAT
TCCCCGACTCTACCACCTTGATTAATAAAAGTGTGAGTATGCTCTTTTAAACGCAAATTTTCGAT
CGCACCTACTTTTTCCATTAAAGCAAAAAGATTATAGTAACAACCGAAAAAAACGTGTAAGCCCA
TTTCAATATGATTACCGTCATTATCAACCCAACTGCCGACTTTTCCACCAACAAAAGGGCGAGAC
TCAAATATTTCCACTTCACAACCTGCATCAACTAATTCAATGGCTGTGGCTAATCCTGCTAATCC
AGCACCTACTATGGCAACTCGCATTCTCTATCCTAAATATTTGCTTAACTTTTCTTTACGTATTTT
ACTCGATTGTGGGGTCATTCGGAGTTCAGAATTAGGAGTTATAAAATAGGTTTCAGATTGCAGGT
TGCAGGTTGCAGGTGGTAGGGGTTGAATATATTCAACCCTTACGGAGATGGGGTTAGTGTTAATT
AAGCACTTTTGCCGTTTGTCTCTTGTCTCTTGCCCCTTACCCTTTTCACTTTGCCTTTTTTTCTGG
ATAGTCTATGAGGTAGGCTATATTGAGAAAGGTTTGTCCATTTTCAATCACTACCATAGTTAATA
ATCTTATATGAATACTAACCAGCAAAAAACATCGGGTTTAGGCACTTTTGGCGGTGTTTATACCC
CCTCTATTCTCACTATTCTGGGGGTAATCATGTATCTGCGTTTTGGTTGGGTAGTGGGAAATGTT
GGTCTATTCGGTACTTTTATTATTGTCACTCTTTCTAATCTCATCACCTTTCTAACCGCTTTATCT
GTATGTGCGATCGCAACTGACCGAGTAGTTAGGGCAGGAGGGGCATATTATATGATAAGTCGTTC
TTTAGGCATAGAAACAGGGGGAGCAGTGGGTATTCCTTTATATTTTGCTCAAGCCTTATCCGTTG
CCCTTTATACCCTCGGTTTTGCTGAAAGTGTGGTGCAAATTTTTCCTAGTTTGGAAGCCTATGAA
GTTTATATTGCCTTAGCGGTGACGATTGGGGTGGGAGTTTTAGCTTTAACCAGTGCAGAAATTGC
CATTAAAGCCCAATATTTTATTATGGCGGCCATTGCACTTTCTTTAATTTCTTTCTTTTTAGGGC
ATCCAGTGGAACAAACCCACGTTGAGTTATGGAGAACCACAGATGTTTCTTTTTGGCAAGTATTT
GCCGTCTTTTTTCCCGCCGTTACGGGTATCATGGCGGGGGTTAATATGTCTGGAGATTTAAAAGA
CCCCACAAAAGCCTTACCCATTGGTACTTTAGCCGCCGTTGGGACTGGTTATGTTATTTATATGG

TTATTCCCTTATTTCTCGGTTTACGGGCTGATGCTCAAACCCTTGTAGATGAACCATTTATCATG
GCTAGAATGTCTTTTTGGGGAGGTGCGATCGCTCTTGGAGTATGGGGAGCAACTCTTAGTAGTGC
CATAGGTAGTATTTTAGGAGCTCCCAGAGTTTTACAGGCATTAGCAAGGGATGGAATTTTGCCCA
ATCAACTCAATTTTTTAGGGCAGGGAAACGGTAGAAATGACGAACCTCGTGTCGGTACAGCAGTA
ACTTTAGGGGTTGCCATTGCGGCAGTCTGTTTAGGGGATTTAAACTTAATTGCCCCGGTATTGAC
AATGTTTTTCTTGACTACTTATCTAGTTTTAAATGCTTCGGCGGGAATTGAAAGTTTTTTACAAT
CTCCTTCTTTTCGTCCTACTTTTAAAGTAAATTGGTTTCTTTCTTTTTTGGGGGCAGTGGGATGT
TTAGCCGTAATGTTTTTGATCAATGCGATCGCAACTATTGTGGCAGGTATAATTGTTTCAGGAAT
TTTTATCTATTTACAAAGACAAGAATTAAAAGTAACTTGGGGAGATAGTCGGCGAGGAATGTGG
ATGGCATTTTTACGCACGGGTATCTATCAATTAGACCAATTAACCGATCCCAAAAATTGGCGCCC
TCATATTATTGTCTTTTCCGCTTCCCCTCATAAAAGTTGGTCATTGATTGAATTAGCAGACAGTT
TTAACCACAAAGGATTATTGACTGTAGCCAGTATTGTACCCGAAAGGAGAGAATACGAAAGCAAA
AAAAATCTCGAAAGTACCATCAAAGACTATCTCAGTAAAAATAATGTCCAAGCCTTAGTCAAAAT
AATTCGCTCCAATCAAAATTACGCCGTTATTCCGCAAGTGGTAGAAACTTATGGTATTGGTGCAT
TAATTCCTAACACTGTTTTACTCGGTAATAGTAATGCTAGTTTCAATTCAAGATTGGGAAAGCCAT
AAACAATATTGTCAAACCATCGCCCAACTACATCAACTCAAACGCAATATTATTATTCTAAAAGA
AAATAGCGATCGACAAAGTCGTACTTTCGGTGAACACAAATTTGGCAACTATCAACGAATAGATA
TATGGTGGAGTGGTATTCAAGCTAATGGTAGTTTTATGTTACTCCTTGCCTATTTACTCAAAAAC
GATTGGCAATGGCGTAAAGCAAATATCTTTGTCAAACTGGTTATAAAAAATCCTCACGCCTCAGA
AACGACTCAAAGCAATCTTAGTAATCTCATCGAAAAACTAAATATAGATGTTATTCCTCAAGTTA
TCGTCTCGGAAGAAACCTCTTTTGAAAAAATCTTGCACCAATATTCAGATAAGGCGGATTTAATC
TTTTTAGGATTAGCTGAACCTCAAGAAGATTTTGATCAATATTATTATCAATGGCAACAAAAAAC
AAAAAATTTACCCAGCGTTGCCTTTATGATGGCGGCTAATGATTTTCCCTTTGAAGAAGTGTTGC
AAAAAGACTAATGAGTAATGAGTAATGAGTAATGAGTAATGAGTAATGAGTAATGAGTATTTGGGGTTTTT
AATTCTTAATTCTTAATTATCAACTATTCACTATTCACTATTCACTATTCATTATTCATTAAATA
TTACCAAGCTAAATAAGGTAATTTTTCCGCAGTATTTTTGATTTGGCTAATATTTTTAATTAATA
AACTGCAAGTATCCCATTTTCCAGTAACGAGCATATTTCTAGCACCAGAATCAATTTTTATTGCA
CTGCTATAGTGACCACATTTAACCTTTAAACTTTCCAAATCTAAAGTCATTTGAATATCTGGATT
TTCTTGTAATAAATTCTGAATACTTTTGATATTTTCTGCTGAAGAAGTTACACAAGGAATGCCCA
TTGCTAAACAATTACCAAAAAAAATCTCGGCAAAACTTTCACCAATTATTGCATCAATGCCCCAA
CGTGCGATCGCTTGTGGTGCGTGTTCTCTACTAGAGCCACATCCAAAATTACCATTGACAACTAA
TATCTTGGCATTTTGATACTGAGGTAAATCAAAGGGATGTTGACCATTTAAAGCCTTTCTATCAT
CGGCAAAAACCTGCTCTCCTAAACCATCAAAAGTAACGCAACGTAGAAAACGGGCTGGAATAATG
CGATCAGTGTCAATATCATTACCAACTAAAGGAAGACCTTTTCCAGTGATAACAGTTCTTTCACT
CATACTCTTTTATGTGTAGAAAATAATTTGATTTAGCAACCCTTATTTTACCTCAGTTCGATGCC
AGAATGTATGATTTAGCAGGTTGTAGGTTTCAGGTTGCAGGTGTCATCTTTCTCCAACATTAATG
GTTGAACAAAAAAATAGGCGTTTATTGTTTGTAAGTGATTAGTATTTCTAAGTCTTATTTTGGGC
AAAATTGGGAAAATATACGTACTTTTTCGATAATGTAGTGTACTTTAATTCTTTTTTGCCTTATG
ACTAATTCGGCAGAAACTATATAATTAATATCTAATAATTACAAAGATTGCGATCGCTTCTTTAG
TAAGATGTATCTATTTTTTGATTCATGCAACAAAGTCTAAAAATATTTTAAATCAAGTTATGAA
AAAAACTATAGTTATTACAGGAGTTAGCAAAGGTTTAGGACGAGCTTTAAGTGAAAAATTTATT
GCTTTAGGTCATACTATTATTGGTTGTTCCCGCTCTCAAAATTCCATCAAAGATTTACAACAAAA
ATATCCTCAACATTATTTTAGTACCCTTGACGTATCCAACGATGAACAAGTAAAAACATGGATTC

AATCTTTTCCTAACATTCCCGATTTAGTGATTAATAATGCGGCGGTAATTAATTATCCAGCCCCC
CTATGGGAAATTAGTGCCGAAGATTTCTCTTATTTAATTGATGTTAACATCAAAGGAACAGCAAA
TGTGATTCGTCATGTTGTTCCCGTAATGATGAAAAGGGAGCAGGGTATCATTGTTAATCTTAGCT
CAGGTTGGGGGCGATCGACCTCTCCTGAAGTAGCTCCCTATTGTGCCTCAAAATGGGCTATAGAA
GGATTAACCCAAGCATTAGCCCAAGAATTACCATCTGGTATCGCAACCATTGCCCTTAACCCCGGT
ATTATTCATACCCAGATGTTAGATATTTGTTTTGGGGATGAAGCCCAATACTACACACCCATTAA
CCAATGGGTAGAAAAAGCTGTACCCTTTTTACTGAAATTATCCACAAAAAATAATGGACAATCTT
TGACTGTATCTTAATTGAAATTATTAGCCATAATACATAGTAATTTTCGCTTTGCTGAGGTACAA
GGCTTATTGGTATCAAGAAATAGGGAATAGGCAATGGGCAATAGTATAAAAAACTATAAAGTAT
AGTTTCAATAAATGCTAAATATTCTAAATTATAGAATCATTTAAAATGAAACAAGCATAAAAAG
ATGTAAAACTCTATAATATAGACCTTTAAACGTTTAAAATTCATTTTAATTTTTATTTTATATAA
TATTTAATATTTCCTTTTCTATTTTTAATTTTTAGTGCCACCGAAAAAAATAAATGGCTCTTCTA
CAATTATTGTGATAAATTAGGGCTTTTATCTTTATCTGAAAAATGAAACAGCCATGTAAAATAGC
TTACATCTTAAAACGTTATCCCCGTTACTCAGAAACCTTTATCGTCAATGAAATCTTGGCCCATG
AATCCGCAGGTTTTGCCATCGATATTTTCGCCTTACGCCCTCCTAGCGATACTCATTTTCAAAATA
TTATTTCCCAAGTTAGGGCTTCCGTTACCTATATCCAGAAACCAACTCAAGGTAGAAAAAGTAAT
TTTCTTAATACCCTTTCCCCCACTCCCGCCAGTTACTTTTGGGCAGAATTACAAGAAACCGCTCAA
ATCATACCCGATATTTGGCAAAAACTCCCCTTAGCCACAGGAGAAAGGGCAAGTGTTGTCTATCA
AGCATTATGGTTAGCCAGAGAAGTGCGTCAGAGAAATATTACCCATTTACACGCTCATTTTGCTT
CGGTTGCTACTAGCGTCACTCGTCTAACCTCTCATTTTACTAATATTCCCTACACTTTTACCGCCC
ACGCCAAAGACATTTATCATCAAAGTGTTGATTTTAACGATCTCCAGAATAAAATCAAAGATAGT
AAAATTGTTGTTACTGTCAGTGACTATAACCGTCAATATTTGCAGGAAAATTATGGAAAAGTAGC
TCAATCAGTTAAAAGAATTTACAATGGCATTGATTTGGCTAAATTTACCTATCTTTCCCCCGAAA
ATCGTCCACCAAAAAATTATTTCAGTGGGGCGTTTAGTTGAAAAAAAAGGATTATCAATCTTAATT
AAGGCTTGTGATTTACTCAAACAATGGGGATGTCATTTTTCCTGTCAAATAGTGGGAGGCGGAGA
GTTAGAGACATCCCTAAAAAATCAGATAGAGGAACTAAATTTATCAGATAAAGTCATTTTAATC
GGTTCACGCCCTCAAAATGAAGTTTGTGAATTAGTACAAAATTCCGCAGTATTTGCCGCCCCTTA
TATTATTGCCAAAGATGGTAATAGAGACGGTTTACCAACGGTGTTATTGGAAGCAATGGCATTAG
GCACTCCCTGTATCGGTACGAATGTAACTGGTATTCCTGAACTAATTAAAGATGGAAAAACTGGT
TTGATTGTACCTCAAGAAGATGAAAAGGCTTTAGCAGAAGCATTAAAACAACTTCTATACAAATC
TGATTTAAGGGTGAAATTAGCTCAATCAGCAAGGCATTTACTCGAATCAGAATTTGATATTCACA
AAAATACTGCTTTATTGAGGAATTTATTTTAAGTGTTGGGAATTGGGAAGATGGGGAGGCATAG
GAAGTATGGGAAGCATGGGGGAGTTGTTTATTAGTTTTTAAAAATGACTCAAACGCTTACCTCTT
GCCTCTTGCCTTTTGCCTCTTGCCTATCTCCACCAATAGACTTAGATGCAAACTAGCTTACAACAG
GGGGTTTAATTTGAAAGTTGCTTATGTCTGTGCTGATGTGGGTATTCCCGTATTTGGGCAAAAAG
GATGCGCAATTCATGTACAAGAAATAATCCGCTCATTCTTGGCGAAAAATATCGAAATTTCCCTA
TTTACCCCTCGTTTAGGGGAAAAAAAACCCCGTGATTTTGAAAAGATTAATATTTATCCTCTTCC
TCCCATTCCAAAGGTAGAAAAAGGTGAAAGGGAGAAAATAGCTTTAAGTATTAACTCTGATTTA
GATTCCTTATTAACTTTAACTCAACCTTTTGATTTTATTTATGAGCGTTATTCCTTGTGGAGTTA
TGCAGGAGTAGAATTTGCTCAACAAAGAGGTATTCCCAGCATTTTAGAAGTTAATGCTCCTTTGA
TTATAGAACAAGATCGACATCGGGGTTTGGTTCATCGTCAGGAGGCGGAAAAAATCGCTCAAAGG
GTATTTGAGTCGGCAACTGTCATTATTGCTGTGTCAAAGGAAATTAAAAGTTATGTTAGTCAATA
TGTAAGCAATCCAGAGAAAATTACAGTTATTCCTAATGGGGTTAATGCTCAAAGATTTTCTGATG

ATGTCATTAAAAATAGCCATAAAGAGTCCAATTATCCTTTTACTGTGGGTTTTGTTGGTTCTTTG
AAACCTTGGCATGGTTTACCAATATTAATCGACAGTTTTGCTCGTTTTAATCGAGATTACCCTGA
AAGTCGTCTTTTAATTGTGGGGGATGGCACAGAAAAAGATTTTTTAGTGGAAAATGTTGAGCAA
CGTCAATTACAATCTGCGGTGCAATTTGTGGGGGCGGTATCTCCTGACATGATTCCTTATTGGTT
AGGGAAAATGGATGTGGGTGTTGCACCTTATCCTTCTTTGGATAATTTTTATTTTTCTCCTCTCA
AGGTTTATGAGTATATGGCGGCAGGTTTACCTGTAATTGCGAGTAATATTGGGCAAATTAAGGA
ATTGATAGAGGATGGGGTTGATGGTTTACTGTGCGAGGCTGGAGATAGTCTTGCTTTAACTAATG
CTTTAATAACTTTAGCTCGATCGCATCTTCTTCGTGATAAACTGGGTACATCTGCTAGAGCAAAG
ATTTTAAGTCATTATACATGGGATAAAGTCGTTGATAAAATTTTAGCCTGTGTGGAACAATCTAG
GTTAAGGATAAAGGAGGGTAATTCATGACATCCGCAAAATCCTTGACAAAAACTCTGCCCATTCT
CTCAGAATTATTAAGCTATTTTTCCCCCTATCTACATCAACAAAAATGGTTAGTGATTATAACCT
TTATCGCTATCGTGGCAGAAGTTGGTTTAAGAGTTTTAGAGCCTTTACCCTTAAAATTTATATTT
GACTACGTTTTAGGAGATACTCACACAACTCCTCTCATTCTGAATCAAATCTCACCTCTTTCTCTA
TTAACCTTAAGCAGTCTCTCTATTTTACTGATTACCTCTTTAAGAGCTTTTGCAGGGTACTGGAG
TACTATAGCTTCTGCCATAGTTAGCAGTAGAGTTATGACACAAGTAAGAGATGATTTATATTGCC
ATCTACAACAACTTTCTCTCAATTATCACAATCAATCCAGAAGTGGAGATTTAATTATTCGAGTT
AGCAGTGATGCTAGTCGGTTACAAGAGATTTTGCTAACAGCTACTTTACCCCTGATTATCAGTAG
TTTAACCTTAGTGGCAACCCTTGGGGCAATGTTGTGGATTGATGTTAAGTTGACTTTACTGTCTT
TATTAACAGTACCTTTGTTCGTATTAGCTGGAAATCTTCTTAGTTCTAAGATACAGGAATCTTCT
CTTAATCAACGGAAAAAAGAGGGGTTAGTTGCTTCTACGGCGGCGGAGTCGATGATGGCAATTAA
ACTGATTCAATCTTTGTCTTTAGAAAATGCCTTTGCCCAAATATTTTCTCATCAAAATCAGGCTA
GTTTAACAAAAAAAGTTGAAACTCAACGTTTATCGGCTTCTTTAGAAAGGATTGTCGATATGATA
ATTGCTTTGGGCATTGCTTTGGTTTTATGGTATGGTTCTTATTTAGTGTTGCAAGATAATTTGAC
GGCAGGGGATGTAATTGTTTTTTTTACCTATCTCAAAAACGCTTTTAAACCAATCCAAAATTTTG
CTAAATATACTGGGCGTTTAGCTAAGGCTTCGGCTTCTGGAGAAAGAATTTTAAATATTTTCAAG
GAAACTCCCGAAATTCGAGATTTACCCCATACTATCAATGCTTCTCCTCTACAAAGAGAAATAAC
TTTCAAAAATCTTAGTTTTACTTATCCGTCAGGGAAACAAACCCTTAATAATATCAATTTAACGA
TAAAAGCAGGTCAATTTGTTATGATTACGGGGATTTCTGGGGGCGGAAAATCTACCTTGATGAGT
TTATTATTACGTCTTTATGAGCCTACTTCTGGGGCAATTTTAGTCGATGGTGAGGATATTAGGAA
TTATACCCTTAAATCGTGGCGATCGCAAATTAGTGTAGTTTTACAAGAAAGTCTTTTGTTTGGGG
CGACTATCAGAGAAAATATTGCTTACGGGGTGAAGAATGTTACTGATAAAGAAATTGTTGAAGC
TACCAAATTAGCCAATATTCACGACTTTATCTCCAATTTACCCCACGGATACGATACCATGGTCG
GAGAAAGAGGTACAACCCTTTCAGGAGGGCAAAGACAGCGAATTGCGATCGCACGAGCGGCTATT
CGACAAACTCCCATCTTAATTTTAGACGAACCAACCACAGGACTTGATCGTCACAATGAAGAAAT
TGTTATGGAATCCCTACAAAAATTAGCTCAAAATCGCACTACTTTTTTAATTACCCATGATTTAT
CTTTAGCAGAAAAAGCGGATTTAATTCTTTATTTAAACAACGGAAAAATAGAAATTTTATCTAA
TTGTGAACATCTAAATAAATTAGTTAAATTATAAGATTATTCAAAAAAAATAATGAATTGTACT
ATTATTAATAATTATCAAATTCATCCAAAAGATAAACTAATTAACCATATTAAAAATGCTATTA
ATCCTCATCAAGTAATCAAAATTTTTAGCGAAAATATATCAATTCTTCAGGAAGATAATTATAAA
TTAATTAAAATCGAATTAATACGTTACAAACCACAAAAACGCTGTTTAATTGAATATCATTTTCA
AGGTAAAAATAATTTAATTTTAATAGGTAAAATTATGGCAAAAAAAACAGATTTTCATAGTTTT
CAATTACAAAAAAACCTCTGGCAAAATGGTTTTGATGAAAATAGCCAAGATAATATTTCTGTCCC
TGAAGTTGTTGGTATAGTTACTCAATGGCAAATGTGGTTACAAAAAAAAGTGTCAGGGCAAATA

CTAAGTCAATATTTAACAAAAAATCAACCCATTAAAATTTATCAAAAAGTTGCTCATATTGCTCA
CAAATTGCATAAAACAAATATTCCCACCAATCGTAATCATACTATTATAAATGAATTAAATATTT
TGCATGAAAAATTACCATTACTAACGAAATATTATCCCCATTGGCAAAAAAGAATTGAAAACTTA
CTAAATCAATGTGATATTTTAGCCTCAAAAGTTATTGAAACAGAATTATGTGGTATTCATAGAG
ATTTTTATTTTGATCAGATTATTATTGATAATAACCGCTTTTACTTATTAGATTTAGACTTATAT
TGTCGAGGAAATCCTTGTTTAGATATTGGCAATTTTATCGGACATGTTAGTGAATATAGCCTACG
TCAATTTGAAGATATATCTGCGTTAAAATATCAAGAATTAGCATTAAAAAATGAATTTATAAAT
ATTCATAAAAGGGAAAAGCAAGACCATTTATCCCTATCTGAATTAAAAGATTTAAAAACTATTA
TTAACAACAATATTGATATTTATAGCCTACTAACTTTAGTGCGTCATATTTATCTTAGTACCCAA
TTTATTGAACGTCATACTTATACAAAAAAACTATTTGATTTCTGCGAGGAAAAAATAAAACTAGC
TTTGACTTTCTCACTTTCTTCGTCATTATAGGGAAAAGGGAAAAATATTTATAATTGACATAAAC
TGCTGTTCAACTTATTTTAAGTAGTGAATAATGAACAGTTAATAATTAATAATTAAAAACTCCTA
ACCCTGAATTTCTCTCTCCTCATTTTCCTTTTTATCATTACTCCGTTAAACCGAACACTCGCAACA
ATGAGGGTTGAACAATGTTCAACCCCTACAACCTGCCCTTATTGCATATTCTTAAACAAAACTGG
GGTTAAATAGGTTGATTTATAAGCACTTCCATTGATAAGTACTTCTCCTAACTCCTAACCCCTAA
ACCTTCACTCTTAAGCGATAGTATTAATTGCCTGGGTTACTTTTTGAATAACATTATCAGGTAAG
GCAACATAACCTAACTGCTCACTATATTTTTGTCCGCCCGTTTTGCTAACGCACCACTTGAGAACA
TTTTTTAAAGCGGCGGCTTTTTTAGCATCAGGATATTTTTTATAAGCTAAAATCCAACTATAGGA
AACAATAGGATAAGAACCATCTCCAGAAGGATCAGGAATAAATGCTCTTAAATTAGCAGGTAAA
GCAACTTGAGATAAACTTATACTTGCATTTTGTAAGGTGGGAGTGATATATTTACCAGCTTTGTT
TTGTAATTGAGCAGTTTTCAAATTATTATTTTTGGCGAAACCATATTCTACATAACCGATGGCAC
CATCAGTTTGCATAACGGTAGCAGTAACGCCCTCATTACCTTTTGCACCAACACCCACAGGCCACT
GAACAGATTTTCCTTCTCCAACTTTACTTTTCCACTCACTGCTAATAGCAGATAAATGCTTAGTG
AAAACCCCAGTTGTTCCACTACCATCGGAACGATAAACTACTCTGATAGGCTTACTTTTAGAAGC
TCCTAATTGCTTCCAATCTTTAATTTTACCCAACAAAACATCAACCAATTGCTGACGAGATAGCT
TAAGAGTACCAACATCTTTGTTATTGTAAGCCATAACGATACTACCTGCAGTTACGGGTAACAAA
ACAACTCCACGGGAAACCTTAGAAATTTCCTCATCTTTCATGGCTACATCACTAGCACCAAAATC
CACAGTGCCACCCATAAACTGTTGTACACCAGCACCACTACCCACAGACTGATAGTTTACTTGCAC
ATTTCTATTTACTTTATTGTATTCTACGAACCAGCGTTGATATAAAGGAGCAGGAAAAGATGCAC
CAGCACCATTTAATCTCATAGCTTGAGCAGAAGCAGACTCAAAAGGTAACTGACTAGCAATAACG
CTACCAGATAAACCGGCTAAACCGTATAAAAAATCTCTTCTTCTAATTTGAAAATCAGTCATTAG
ATTAATGCTATACCATAAAAAACTATTTACAGCTTAAATTCTAAAACAAATTGCCCTATTTTTAA
TCTTTTTAAATAATTGTTTTTTCTTATCTTTTTTTACTATGACAATTATGATATTTTTCTTCTTT
TCATGTTATCTATAACACTAAAATTTTGCTAGGTTTAAGAATAAATTAAAACAAGGTTAATCGA
AGTTTAAGACTTTTGAGCGGAATATAAAATATTTTTTATCCAGAACACGAATAAGGCTATTAACT
ATTTCTCTTTTGCCAACTGGAGGAATAATTTAATATACCGATGAAGTGTAATCCAACTAGAAAA
TAACTTTATATGTTTGAAAATTAAATAAAAATTAAGGCTCTATTTACTTCTCGTCGTGATAAATT
GTTAGTTGAAAAGGGGAATAGTTGATAATTACGAATTAAGAATTAAAAACTCCGAACTCTTATC
CTCATTACTTATTACTTATGGTTCTTCAGAGTGTGGTTATGATAAATTAAGAGAAACAGGACTTC
CATAACCTTTATTGAATAGTGTTTATAGTAAGAGAAAAACTAAAAATTTATAAATTATTATTTA
ATAATCCCCTATTGCCTCTTGCCTCTTGCCTTTCAGCCTACCCTAACTAATAACTGTAGTTTAGAC
TAAATGTAGAGAAAAATGAGGAAGGACAAGACAAAAAAGAAAAATAGAGAGAAAATAAAATAA
TCACAAATTAAAATCTCTCTACATGAACAAATATAACAGACTAAAAAATTTTCGTCATGATACCT

ATGAAATGTTAGTCAAAAGTAAAGATGCCACATTTGAGCTAATGGACAGCATCATGACCAGAGA
AAATGCTCGTAGTTTGGCAGAATTTTCTTTATCGCCTTTTTTCCAGAGGCAATGGTGTAGCACCT
ATGAAGCGATAGAGGATAGTCGTCCCAATGGCAACAAATTGATGAAACGATACACTCAAGAAAT
AGACACTTTAGAATATACGTTATTAGGCATAGATCATACCCAGTGGGAATGTAAAGATAGTCCCA
CGATGAAAGATAGAGGTTATCAATATTCTCATTCATCCCTAAATTCATCAGTAGTAGGACAAGGA
TATAGCACCATAGCATGGTTACCACCATTAAAAGAGAAAGGAAGTTGGGCATTACCTTTAAGACA
TGAAAGAATAACATCCTTTGAAACTCCTTTAAGTAAAGCCCGATGGCAACTCAAACAAGTGTGTC
GAGAATTGCCAGAAAAGAGACGGAAATTAGTGGTCTTAGATTGTGAATATGGCAATGGCAAATT
TATCCAACAGACGGCGGACATTGAGATAAGTAAGTTGATTCGAGTACGTTCTAATTTATGCCTTT
ATAGCAAGCCAGACCCTTATAGCGGTAGAGGAAGACCGAAAAAACATGGAGCAAAGTGGAAGTT
AAATCAATGTGATGATTTTCCTCAGAGTAATAAAGTATTGGAAATGGAAGATTCATCCCTAGGAC
TCATACGCATCAGTAAATGGACGGAACTTCATTTCTATAATGCCCCCACACAACAATTAACTTTA
TTCAAAATTGAAAGATTAAAGGAGAAAAAAACAGGGAGTTTACATCGCCCACTTTGGTTAATCT
GGGTAGGAGAGGAATTTTTATCTTTGGAGTATATTTGGAGTCAATATAAGCGAAGGTTTGGATT
AGAACACTGGTATCGTTTTGCCAAGCAGAGGTTGCATTGGACTTTGCCAAATTTTCGCACTCCTG
ACAGGTGTCAAAGATGGAGTAATTTAATCGTCAATATCACTTGGCAATTATGGCTCTCAAAGGAT
TTAGTCCAAGAACACCATTTGCCGTGGCAAAAACCACAACAGAATTTAACTCCTGGAAGGGTTGC
TCTATCTATGACTTCATTATTGATAGAGATTGGAAGTCCTACCCTTTCTCCTAAAAGTCGGGGAA
AATCCCATGGCTGGCCTCAAGGGCGAAAAAGAAACAAAGCTAAAGTTTATCCCACCATTAAAAAA
CAGCAATCCAAAAGTAAAAAAAGGCGAAAAACCTAACTTTTAAGGAATAGTTAACTCAATTTAT
TTTCTCCGTAAGCTAATTTTTGTTAGCTTATTGATTGTCTTGCAATTTTTTCTTAGTCTAAACTA
CAGTAATAATTTACACGACGAGAAGTGATAAAGTCTTACTTATTACTTATTACTTGTTTTTCTCT
CATCTCCTTATCACTCTATACTTCTGACTTTTGAGTTATTTTGTTGCCAGTAGCCAATTAATTTA
TAGATTAGTTTAGCCGCAGTAAATTCAGAAACAACGGAATCTTTTGTTGGTGCTAATTCCATAAC
ATCACAGCCAATTACATCAAAGCTCGAAAAAATTAAATTCATAAAAGCTAATAGTTGATACCAGT
CCATGCCACCGGGTTCAGGCGTACCAACTCCGGGCATTAAACTAGGGTCAATTCCGTCTAAATCA
ATGGTAATGAATACTTTTTTCGTTTTTATGTTACTGATAGCTTCTGCCATCCAATGGGGATTAAA
ATAAATGTTTCTCGCCCACATTACGGGTATATTTTTTTCTTTGATTAAATCAGCTTCCTCTTGAC
AAATTGCTCTGATTCCCACTGGTAATGTCGGCAAGTCTAATTCTAATATTCTTCTCATAACACAA
GCATGATTATGAATTGACCCCTCAAATTCGTGTCTCATATCTCCATGAGCATCTATCTGTATTAC
TGTAAAGGGTTCATCAAGGGTTTGTTGATAGGCTTGAACTACTCCTGTCGTGATAGCGTGTTCTC
CGCCAACGGCAATGACAAATTTATTATCTTTGATCAATTTTGTAACGGTTTCTGTTGTGACTTTC
AACATATCATCTGCCGTTAGATTTTTGTTGATGCGGGTATCGGCGATATAGTCATGGGTAAAAAT
AGGGGCATGAAAACAAGTTTCCATTCCTAATTCTTCGTCATAACATTCTAGTTGATCAGAGGCAG
TTAATACGGCATCAGGTCCATATTCGCACCCTTTACGATAGGTAGTAGTAATTTCATAGGGGATT
GGTAAAATAACTATTTTTGCTTCTTCATAAGTAGAGATAATTTCTGATTCTAAAAACTGTTTCAT
TGCTTATATTTTTATGAGTTAGAAAAGATGATGTTTTTTTATTTTTAGGATTAATAAAATTTTA
AATCCTTAAGATTTATAAAACTATAACATCTAAAAATTTTACAAGATTTAAGTCTTTTTGTCTAG
TTAGTTTTTTCTTTATTGCTATTTAATTTTTTCAAATAAAAATATATTATCTTGATTATATTTCA
ACTGAAATTCTCTATTTTTTTCTAAAAAGTTAATTAGATTTTTAATATCTTCTTGAGAACTATAT
CCAGTGGGGTTGTCTAGGTTTAAGAGTACATAATGAAATTTATTAACTTGATTATAATCAATAG
AATCGATCGCAAGATCTAAAGTTAAACGATGAGTTAAATGAGGGGCAAATTGAGGAGCAGTAAA
AACACTTTTTGAGGTATTTATCAGACTAATAGCTTCATTACTAGCTTTCCATGTATGTAAGTTTT

TTAAATATAAGCCATTGGGGAAAAAATAAACATATTTAGCTAAGGCTAAAAATAAAATTAATGA
CCAAATTCTAATTTTTATATTTTGATAAAACCATGTTTATGATGAGCCAAACTAGCAATTATAG
TAAAGATTAAAAAAGGAATTATCGGTAAAGAATATTGATGAACTAAGTCTTTTTGAGGTTGATA
TTCAGTAAGAAGATTGAGAAACAGAGTGGGAATAGCGGGGATTAGATTAGGTATTTCTTGCCAA
GCTAAAACAGGAATTACTGGTAAGAATAAAAGTAGTAAATATTCTGCATTAGCTAAAGTAAAAA
GATGAGATAAAATAATTTGGGGTTTAATAATTAAATTCAGGGCTATTTCACTAACAGAATCTCCT
AAAAAAGCATAACGACTGACTGCGGCGGCTTCTTCTCCGCTAAAAGCAGGAATAATTAATTGAGT
GGCAATAATAAACCAAAAAATACCACTAAAAATTGAGATCGCACCATAGTTTTTTTTACGATAAA
TAAGAATTAACCATACCCCCATCATTACTACATTTAAAGCTAAAACTGCTTTACATCCTAAAATA
AGTAAAATAGAGACAATAAACAAAATAATTTTATCTAATTTAACAGCTAAAATAGCGATAAAAA
ATAAAGGTAAAGTAATAACTTCTGGATGAAAATCAAATAGATTAACATTGAATATTAAGGGATA
TAAAATATAAATTAAGCAGATAGTCTTAGCTTTAGAATCACTTAAATTATTTAATTTACTTAGTT
TCCAAATAGGTATAAGTGCGATCGCACTTGCTAGAGATTGAATAAAAAAGAGCCAATAAACAGA
AGGAAAAAGAAAGTAAAAAGGAGCGATAAAATAAAGAATCCAAGCGGCGTGATCCCCTAAAATA
TGTAATTCTCTGAAAGAAACATAAGGATTTTTTCCTTGAGAAATAAGATAAATTCCATTATCAAA
AATACCTAAATCAAAAGCATGGGAATTAAATAAAATATGACGAAAGCTACTAGCAATAAATAAA
AGAAAAAAACTAATTAATATATATGTTATTATATTGTTATATTTTGCAAAAAAAATTTTCATTA
AATTAACGATAAAAAATATTTATTGATATTAAATTAAGCTATGAAATATAAGTATATTTTATTT
TATAAACCCTATAATGTTCTATGTCAATTTACCGATGATAGTCCCCATAAAAACCAAAGACAAAC
TCTCAAAGACTATATTAATATACCCCATATTTACTCAGTGGGAAGACTGGATTTAGATAGCGAAG
GATTACTTTTATTAACAAATAATCCCAGAGTAAAACACCGTCTTTGTGAACCTAGTTTTGCTCAT
CCTCGCACCTATTGGGCACAAGTGGAAAATATCCCTACCGCAGAATCATTAAAACAATTAGAAGA
AGGAGTTATTATCAAAGGGAAAAAAACAAAAAAGGCAACAGTATCGATGTTAAAATCAGAGCCA
GAATTGCCCCCTCGAAATCCCCCCATTCGTTACCGTCAAAATATCCCTACTGCTTGGTTAGAGTTA
ACTTTAACAGAAGGTAGAAATCGTCAAGTAAGAAGAATGACTGCGGCGGTAGGTTATCCTACTTT
ACGATTAATAAGAGTAAAAATCGGCATTACCCAACAACTATCTTTAACTTTAGAAGGTTTGAATG
TAGGAGAATACAGAGAATTAACTACCTCAGAAATAAAAATATTAGACAATCTATAAATAACCTG
AGTTCGATATAAAATTGTCAGTGAAGGTAGGCAACGGGCAACAGGTAGGGGTTTAAATCATTTT
TAAGAATTAGTAAAAAGGTAAGTTTAATGGGTTTTAGCTTATAGTATTTGTAACTTGTAATATT
ACTTAGGAATTTAACTTTTTCTGCCAAAATAATGCCCTGCCCATGTCGGGATTCAACTGATAACC
GTCAAAGCCAAAAGCCTTATAAGCCGCTTGAGCGACTTGATTACCTTCTAATACTTCTAGGGTGA
GTTTACAGCATCCTAACTCTAGGGCAATGTCTTCTATTCTCTGTAACAATAACTTACAAATACCC
CGACGACGATAAGCAGAAGCAACCACCACATCATGAATATTTAATAACGGTTTACAAGCAAAAGT
TGAAAAACCTTCAATGGCAATAATTAACCCTGCTGGATTATCATTCACAAAAGCTAAAACAGTAT
GAATATATTTTCTCTGTGCTAATTCATTAGCTAAATTGTTTTTGGCAAAATCTGATAATTCTTCT
CCTCCTCCCATATAATCAAGGGCATAGGTATTCATCAACTCCACTACGGCTTCTCGATGAGCGGAT
ATATTCAAATCTGCGTCAATAACTTTTATCATTTAGATTGGTTTAATTTGGCGAAAAATGTTCTT
ATTTTACTTTTACCTCAAATTTTTGAAACTGATTCAAACTCTTATATTACTTCAGTTCGCTATAA
AAATATCCCATTTGGATAGATTTCAGGTGATGAGTTTGACGGTTTGATTTTTTGAACATTAACTA
TTTTTGTCAAAATCTATTAACTTTAACTCTGAAATCTCAACTCAGGTTTATATTTCCCTTTTGCT
TACCTCATCAGTAAACTTAGATGCACTCAAGGCTCATAAAAATCAAAAATTTTTGCCTTATTTAC
TTTAACTTTTGTTATGAATATAATTCTTCAATCTGTTTTACCTGTTGCTTTTATTATTATTATTG
GTTATGTTGCAGGAAAAAAATTGAGTTTGGAGCGCTCTACTCTTTCGCAATTAACGGTTTATATT

CTCGCACCTGCTTTAGTTACAGATAGCTTTTATCGCAATTCTATGGGGGGAGATGTTACTTTACG
CTTTTTATTAGCCTATGGTCTTATTTCTGTAATTTTGTATGGTGGGATAATCTTAATTGGTAAAG
TGATTAAGTTATCGTCGGAAAATCATCGCAGTTTATTCGCAATTTTGTTATGTCCTAATAATGGT
AATATGGGGCTTTCAATTACCGCATTTGCTTTAGGAGAAGGGGGATTAGAAAGAGCAGTAATTT
ATATGATTGGTTCAAGTATTGTCTTATTTGGTGTTTTACCTGCGGTTTTGAAGGGTGAATCAATT
ATCAAGGGTATAAAAATGACTTTTCGCCTTCCTTTGATTTGGGCAATGATATTAGGAGTCTCTCT
TCATTTAGCTCATATTACTCTGCCGTTTAATTTGGGGAAAAGTATTGAATGGTTAGGGATGTCTT
CTATTCCCATCGCTCTTTTAATTTTGGGAATGCAGTTAACAAATACTCGTTTTCAATTTGATTTT
ATTCACTTTTTAATCAGTTTAGGTAAACTTGCGATCGCACCTTTAATTGCTTATGGAGTGGGTAA
AATAATGGGTTTAGAGGGCTTAGATTTACAAATATTGGTTTTACAAACTGCCATGCCTACGGCTA
TTAATACGGCGGTGATGATGCAAGAATTTGGAGGGGATATAACAATGGTATCTAGGACGATAAT
TCTTTCCACTCTGATGAGTTTTATTACTATACCAATGGTTATTTTTCTAATAATTTAATCTATTT
GTAATCTATATAGTCATTAGAGAGATAACTTTGATGTCTGTCACTCGATGGAGAGGTTTAGTCGG
TTTTTCTGTGGTAATAAATTCCTCAGCATTGACAGAAATACCACTAGCTATTTGTAAAGCATCTA
AAGCATTTAAACCATATTTACTGGCTAAATTTTCTGCTAAGTTAATAATAGTATTTAAGTCATTT
GCCCATACATTACACATATCAAAAAAAGTATTATAAAAGTCCACTTCATCTATTTGTTTATGGAA
TATTGCTTTTGATAAAGTCTCTAATTTAACAAAGTCACTACTCGCAAATTTTCTGGTACAATCAT
CCACTATTTCTTGTGCTTTTAAAGCTAAATTATCACTAGCTCTAAATAAAGTAATTAGTACTCCA
GAGTCAAGATAGGTTAAAGTCATTCTCTAATACCCCCTCTTCTATCAGCTAACTCTTTTTCTACTT
CATCCCAATTATTTAATAATGGTTGACTACCTAAAGATTTTTGTCTTAAAGCCCATAACTTTTTA
CCTAATTCCGTTTTTGGTTGATAGTTTAGTGGCTCACTATCATTTTCCTCATTATCCTTAATTAA
ATCATCTATTAAAGCCAAAACTTTAGCTTTTTTTTCAGTATCTAAGGATTGCCATTTTATTAATA
ATAATTGTTCTGTAATCATCATCTTATACCTCTATTTTTTACATAAGAGTAACCTCAGTTCGGAT
TAAGGCAATTTTATCGTTATTTCTAAGTTATTTCTAAGAAGCTATAAGGTTTTCTGAGTACGAGA
AAATAATGCTTTCAGCTTATCCAAAACTCAGGTTAAGAGTAGAAAAACAACAGTTTTTAGTAAA
ATACTTCTAATGATATAGAAAATAACTCCTCTTACCAATATTTGACTTTTTTCACTGGCAATTAG
CAATTAGTTAATAATAAATAATCAATCGTTAATAATAAAAAACTCCGAACTCCCAACTCATTACC
TACTCAGAGACTTTAACGACGACGACGGCTACCGAATCCCCCACTTCTTCTAGCGGGAGTAGTTCT
TCTATAACCACTTGATTTATTACTGCCAAAGCTATTTTTGCTAGGCTGTTTATATTTGCTCGAAT
TACCACTGTTTTTCAGATCACTTGAGCCAAAACCTGACCCAGTAGCTTTACTATTGTTACTATTG
ACCCGATTAATCGTATTACCAGAACTATTTTTAAGAGTACCAGTGGAGCGGAAAGATTGACGATT
TTTTTCTACTGTGGGGACAGAATTATAGTTACTACGATAGCTTTGCACTGCCTGATTATAAGTGC
TTCCATAGCCACCAATACCTGATAAATTGCCACCGGAATAGACAGGAGGAACATAATATCTAGGA
GTAAATAATAAACTACCTAGGGCTTGTCCTGCCATCGCCCCTGCAAAAGGAGTCCAAAAAATTGGA
TTCTCGTCTAACAATAACGGTTTCTTGTCTGCCTGTATCTGGATTGGTGACAGTTTGAGTTTCGT
TGTGGACATATTCAATGCGAAAATCCTCTGTGAGGTGCATTATTGCTTCGTTACCCTTAATTTCA
GCGTAGGTACTTTGTCCTTCTTTGATTTCTTCATCCGTTAAACGAGCCATTTGTAAGTTAGTGGT
ACGAAAAACAGGACTGCTACCGGCTGGAGTATTTAACAACATGAGGGTATATTCACCGTCTATAT
CATTAAATGTTGCCTGTTGTACGGGATATTTACCACTAGCAATGTTATTACTAGGAATATTGGTA
TTATTATTAAAATTGTTGGAGTTACTAACGGCTTCACTATTAGGTGCGCCACAAGCAAAAGTCCC
AAAAGAAAGGAAGATTGTTAAAAATAGGGCTACAATTCGCTTAAACATCGTTTTTTATGGTGTT
ATTTTTTCTTGACTAGAGGGGGATTAATTCAGAAAAATTAATTAAGATTTGCTCAGAATTAAGT
TGTTCTCCCAGTCTGGCAGGAGAGAAATGAATTGCGATCGCTCTATATCTTTGTTTATAATGTAA

GCTAATTAGGGCTTTCAATCCATCTTTTAAACTATCTCTCTGGGCTAAATCCGTTTCAATTTCTG
GCACTTCTCCCTCAAAAGCTACTGTGATCATAACAATAATATTATCGGTTACGGGGAGGGATAAA
CCGTCATTTTCCTCATCGTCTTCTAAGTCTGGTTGGCTAAGATACCGTTGAGCAGAATCAGTAAA
TAACTCCGTTACATAATCTCCTGCATCTCCCTCATTCCAAAATACATCCCCTTCATTAGAAGCACT
TTTCCAGTAAAATTCGTATTGCAATAAACCTTCGCAAATGTTGACTAAACATTCTCCCATAGTGT
CCATTTCACCATCGGCATCTACTACCCTACGGGCATTTTGGTTTAAAATACCCAAAAAAGGAGCT
ACTTCTTCTCCCTCAAGATGAACAAAGATACGGGAAACCACATAGCGAGTTTTGCCGATAAATTC
ATTAATGCGATCGCCTATACTCATGATAATTTTAAATTTGGAAGGAATTACGAGTTATTAATTTT
ATATATTATTATTTTATAATCACTAATTACTAATTGCTAATTTCTGCTTAAAAGTGAGTTTACTT
TTAATTTATTTTCTGACTAATAAAACAGGGCAATTTGCATTAACCCTTACATAATCAGATAAAGA
AGTACCTAATAAACGGTCTAAATCTACTAAACTTTTAGCAATAGAAGGACGACGATCAGGAGATC
CTAATAAGAGTAAATCAATATTATTATCATCAGCTAATTTGCAAATTTGTTGTGCAGGTTTTCCG
CCTTTAACAATACATTTAGCAGAAATTCCCATTCTTTTCGCTTGTTGTAAAGCAGGAGCTAAAAT
CGGATTTTGCTCCATTTCCGCCGTCGATAATTCTAAATCTGCATTCATATCAGGATTAACTCTGA
CTAAATACAACTCTGCCTCACGATAATCTCTGAGAAGATATAACGCCATTTCAAGGGCATAGGAA
GAAGACTCGGATTTATCCAAGGCTAACATTACTTTTTAATTTTTCTTACATATATATCATCTTT
CACAAGTAACATGGGGCGATCGCTCAATTGGAATACATACTGGCTAACAGAGTTTTCCAAAAAGG
ACTCTAAACGTTTCAAACCACGAGAACCCATTAAAATTAGGTCTGCATTAATTTCCTGTGCCGTA
TTACAAACCGTATCTTTAGGATCGCCTTGTTTTAAAATAGTAGCAACCTTAGAAGGATCAATATT
AACATTTTTGACGATTTCAGAAAGTATTTTCCCACCTTCTTCCCATTTTTCAGCTAAAGCATCCGC
AGAAACTTGAGGGGGAATAACATGAAGAATATTTATTTCACTGTTTTTAAATGCTGGTATATCA
AGTAAAATTTTCAGCATTTCTTGAGTGTGACCAGTACCAGAATCGGCATATAAAATTTTATCTAA
CATATTCTACAACAGTATTTTAAATTAAACTCTTTATCTCTAAAATACAGCGATTAGGTACAGCA
ATATCTAACATTTTGTAAATAGGGTGTTTGGGATTCGGGGTTCGGGGTTAGGTGTCAGGTGGCAG
GTGATGAGGGGAGAGGGAGATAAGGTGTCGGGTTTCAGGTTTCAGGTTTCAGGTTTCAGAAGAA
AGTAATGAGTAACGAGTAACGAGTAATTATCAACTATTCACTATTCACTATTCACTAATTGCCTT
GTCTTAATTACTAATTGCTAATTGAACCTGAGTTCGGGATAAGCTGAAAGCATTATTTTCTCCTA
GTCAGAAAACCTTATAGCTTCTTAGAAACAACGATAAAATTGCCTTAATCCGAACTGACTTAAAC
AAGGGGCTTAAGCCCCTTGCTAAAAACCCCTACCACCTGCAACCTGCAACCTGCAACCTGACACCT
AACCTTGTTGGATATTCTTAAATCGAACTGAGGTTAATTGAATTAAAAAGCGACATTCTGAGAAA
TTACTTCTCCAATTTTTAAAGATGCTGTAGCCGCAGGAGAAGGAGCATTACAAACATGAAGGGCA
TTTTCTGTCGGTAATAAAGCAAAATCTTGTACTAAAGTACCATCTTGATTTAATGCTTGCGCTCT
GACCCCCGCAGGAGAGGGAATAATATCATCTTCTTGTACTTCCGGAATTAGGGTTTGTAAACTTC
TAACAAAAGCTGTTTTACTAAATGAGCGGATAATTTCCTCTATTCCTTCCTCATAGTATTTACTC
ACTAATTTCCATAATCCAGAGTAAGTAACTGTTTCCCAAAATTCTCCCAAAGCAAAATCTGTTTT
TTTGTATCCTTCTCGTTTTAATGACAAGACAGCGTTAGGCCCTGCGTGAATATTGCCGTCAATTA
AACGGGTGAAATGCACTCCCAAAAAGGGAAAATCTGGGTTAGGCACAGGGTAGATTAAATGTTT
AACTAAATACCGTTTTTCTGGCTTTAGTTCGTAGTATTCTCCCCTAAAAGGATAATTTTCATTT
TTGGATTGATACCGCCTAATATAGCGATTTTGTCACTGTATAACCCTGCACAGTTAACTAGATAT
TTTGACGTTATTTCCCCTTGATTTGTGATGATAATATGTTGAGAATCTTGACATTTAAGGTTAAC
TACTTTTGTATTTAAGCGAATTTCTCCGCCAATATTACAGAATATTTCTGCGTATTTTTTTGCTA
CTTCCTTATAGTCAACAATTCCTGCTGTAGGTACTTTTATGGCTTGAATACAATTAACATAGGGT
TCAATTTCTTTAGCTTCTTCCCTTGAAATTTTAGTGACGGCAATGCCGTTTTGTAATCCCCGTTGA

TAGAGATTTTCTAATTGAGATAATTCTTTTTCTTTACAGGCAACAATTAATTTTCCACAGACATC
ATATTTGATATTGTGGGTTTGACAAAATTCAACTAAGGCTCGATTTCCTTGAGTGGTTAACTTTG
CTTTGTAGCTACCGGGTTGATAGTAAACTCCCGAATGAATAACTCCGCTATTATGGCCAGTTTGA
TGAGTTGCACAATGATTTTCTTTTTCTACAAGGATAATTTTTTTCTGAGGATATTTTTGTTGTAA
ATTCATGGCAGTGGCTAAACCAACTATGCCGCCTCCTATAATTGCTATATCGTAAGTCATGTTTA
ATTTGTTATTCTGGTAATTTCTCTTTTAAAGAATAATATTGCGAGGTTTCTGATTGAATCTCTAT
TTCTTTTAAATTATTGCCTAATTTTTCCTGTATTGCTTTAGTTAAGGTGATGGGAAAATTAATAT
TAACTTGTTTTTCTTTGATCAATTTCACTAATTCTTGTAAATCTGCTTTGATAGTTTTTTTGAG
GTTGATTGTATTTTAAAACATTTGTTTTCCTTAATATTTTGAGAAATCATTGACTGTTTTATTCT
TTCTTTTAAAAAGCCTATTTCTGAAGTTAATCTTAGATAAATATCTTCTATTTCTTCATATCTTT
CTAACAAATTTTCAATATTTTCGGTTTGGGGATCATATTCTAATTGTTTACTTATTTCTGTTAAT
TTATAATAAACTTGAGCTAAATAAACACAGTCTTTTTTCGCATATTCTAGCTGATTTGTGCTTAG
TGGTCTTATACCCCAATCTCCACTTTGTTCTTGTTTATCAACTTTAAAGTCTGTCAAATATTCTGT
TAAATATTTTAAATTATATCTTTTTACAGGTAGTAAATAGTAAGGTATTTTTCTGGCAAATTGAT
AAGTACAAAATATATTTTTAGCTCTTTTTTTATCAAAAAATCTTAAATCATAATTAGCGTTGTGG
AAAACTTTTTTAATGTTTTCGTTTACCATAATATAATCAATAAAAAATTCTATTATTTCCGGTTT
TTCTAATACATCAATTATATAAGTGCGATCGCCCCTTGTGTCTTGAGGATAGGCTAAAACTTGAA
TAAGAGATAAGCGAGGATGAGAAGTTTGATAATCGGCTACTTCAGTATCTACCCAAAGAATATCA
GTATCTGTTAAATCAAGAATAATCTCTTTTATTTCTTCAACTTCCGTTAAATAAACCATTTATTT
TTTATTTTAATTTTCGTCTCTTTTAACACCTAGATTAGTCATTAATTCTCAAATATTAAATCTAA
ATAATTAGGGTTTTACCTACGTTTAAATTATTGCTGATTATTGGGGTTATTTGTTTCTTCATCTT
TAGGTAATTTATCTTTCAATATAAACAAATGATCGTAAACTTGTTGATATACTTTTTTCTCATCA
TTAGTGATACTATCACTAACTAGATATTCATTTAGTACATTTAAACATTTTTCCAAGCCCGTCAA
AGTATTTTTACCTTCATTTTTACTAATCAATAAATAGGTGGCACGAGAATCTCCAGCACGAGAAC
GAATATAATAGTAGCGACAACGCTTGGCTAATACTGAAAGCGTGTCGGAAGATTGGTTGTCTTGC
GCAACGGAAAAATTCATTAAACCAGTAAATAGACAAATCAACATTAGTACAAAAGGACTAAAAA
CAAGTCTATAATGAGATCCCACAGTGCCACTTTGTATTCTTATTCGTTGGGCTTTTAAATTAGGT
TTCATAATAATACAAAAAAAGAGATTTTTCGATTAGATTTTTAGATTCAAGATAATAATTTTAC
TTCAGACTAAATGAAACATAAATTAGAAAAAAATGCTTTTAGAGAAAATAAAGAGCAAATAAAA
ATAACCATAAATTTTATTTTTTATCTTATGAATAAATATAGCAAACCTTAAAATTTTCCGTCTGA
TAATCATTCAATATTTGTAAAAAGTAAAGATTCATAATTCTATTCATTAGGGTTTATTGATATTC
AAAGATAGCTTTTACAATAAACAAAGAGAATTTAAGAGTAATTGGATTACATGACTAAGTTAGT
AGTATTAAATTTAGGCGCAGGAAACTTAAACGATGGATTTCCCCATATTATCGCCCGATTAAGAG
CTAACCAAGGCACAATAGAGCAACAATTTGTTGGTAGTTTACCCCCTGCTTCAGAATTAGAGAAT
CTTAACCACCAATGGCAATCCCTTTACTATGCTTTACATCAACGTTTTGATGTGGCTAAACGCAG
ACTTAGGCTTTTTGAGGTGGAAACTCAGGAAATGACGAATGTTTCTGTCGTAACTTTTCAAGAGC
TTTGTCAAGAAGATGTTATCTACAAATATCAGAAAAAGTTAAAAGAAATTATGGATAATTATAT
GATTAATATTGATAATAAATAACTCCTAACTCCTAACTCTCTATTCAGTACCAAAAGCGCGATCG
CCTGCATCGCCTAAACCGGGTACAATAAAACCATTATCATTGATCTGCTCGTCAATCATGGCACT
ATAAATATTAAGAGTAGGATAATTGGCACTTAATTTTTGTAAAGCAGGGGGGGCAGATACCACG
GAGATAATTCTGGTGAGATCAGGATTTCCACCTCGTTTGATAATTTCTTCCATCGCTAATATAAT
TGTACCGCCAGTGGCTAACATGGGGTCAAGAATTAAAATATGGGTATCAGCAGTAAATTGTGTTG
GTAATTTGTTCAGATAACAGGTTGGATTAAGTGTTTTTTCATCTCTTATCATTCCAAGATGATAA

GTGGAAGCTAAGGGTAATATAGTTTGACTACCTTCGGCAAGGGTTAAACCTGCTCTCAATATAGG
GATAATAGCTATTTTTGTTTCAGGATTTAGAAAAGTAGCTGGTGCAAGGGCGAGGGGAGTTTCA
ACTGTCACATCCATTGTCGGCAACCATTCTCTAATTGCTTCATAGGTTAACCAACGTCCCAATTCC
ACCATAGCGGTTTTAAATAGAGTAGAAGGTGTATTTTTATCTCTTGCAACTCCGAGCCAATGTTT
GATTAGAGGATGAGGGGGGACATATATTTTTAATTGGGGCGCCATTCAACTTTATTCTAATAAAT
TATATAAGAGTTTAATTTCGTGTTTAATTATACCCTAACCTCAGTTCGATCTAAGAATATCTATC
AACTAACCTGTAGGTTTCAGGTATCAGGTTTGACGGTAAAGATTTTCTGAACATTTGTTAGTGGG
TCAAAGAAGTAATAAAAGCTCGAAGCTCTTATTTTTTTTGTTCTTCAACAGTGGTTATGATAAAT
TAATAGAAAATAACCAATAACTTCTATAACCTTTATTGAATAGTGTTTATAGTAAGAGAAAAAC
TAAAAGTTTATAAATTATTATTTAATAATCTCCTATTGCCCTTCTCCCCTTAAAAGGGGAAACAC
AGAGGGGTTTGCCTCTTGCCTTTTGCCTACCAAACACTAATAATTTACACGACGAGAAGTGATAG
AACCAAATTTATTTGATTTTTAACCAAGAAAATATATGGTTAGAAGTTATGGATAAATCCATAT
TTTCGATAATAGGTAATGTCTCATCTTGACGATAAATTTTAAGGGGTTTTTCTGGTTGGAATACC
ATAATTATTTTTTCTTGACTATCAATTAACCATCCTAATTGTGAGCCGTTTTCTAAACAGAATAA
AATATTATCAATTACTCTAGTTGGTGTTTGATTCGGTGATAAAATTTCTATAGTCCAATCTGGAT
AAGAGTTAAATTGATTAGCAATTTCTCCTTGATTGTCTAAGGGTATTCTTGCCCAATAAAAAACC
GCAATATCTGGTACTATAGAACGATTAGAAAAACTGCAACGCAATTCGGGTAAAGCGTAAACAAT
TTTCTCTTTTTCAGATAATTGATTGATTGTTTCACAAATTTTATACTGAATTTGACTATGTTTTC
CTTGAGGCATAGGTTTTCTGATTATCTGATTATCAATATATTCATAAGCAGGGGATTCATCAATA
TAATCTTGTTGCAAAAATTCTTCTAAGGTTAATCCTTTTATTTGAGTAGATGCAATTGTCATAAA
CTTTACTGAATAATGGTTTCTTTTATTTCTGTAATTACTCTAACTCATCAGAATCGAGATTTTGA
AGGTTATATAAGTCTTCTAATTTTTGATTACTCACTTTACGGGAAACTCGACGATATTTCTTACT
TTCTAGCTTTGGCTCGGTATATTTTTTCCCGTTGGCTTTAAATTTCGTTTTTACTGTGGATTCCTC
ATCCTTTTTATGGGCAGTAGCTTCTTGAAATGCGATCGCTTCTTCTAAAAATTTAAGATAATATG
GATAACGTTCCCAATTTCCTTTAACCGCACAATCAGGCTCATCTTTATGAATGCAGTTATGGAAT
TTACAAGTATTTTCCGCTAATCTTGCCCTTGCCTCGGGAAAATAGTTAACTAAAGATTCTACACT
AAAATCAAAATGAGGTTGATTAAAACCCGGACTATCTGCGATGTATCCCCCAGAAGGTAACTCAA
ATAACTCTACATGACGGGTGGTATGTCTGCCTTTTTGCAACTTTCCTGACACTTCCCCTACCCTCA
CCTCAATTTCGGGAATTAACAAAGATGTTAAACTAGATTTACCTACTCCACTAGGACCTGCTAGG
ATAGTAATTTTGTTCGCTAAAACTTTTCGTAAAGATTGGAGTCCTTGACTGGTCTGCACACTAAA
AAAATAAGGCTCATATCCCCACGCACAAAGGCGTTCTTGCCATGCTTTTTGCTTTTCAGGGCAAA
TTAGATCAACTTTATTTAAACCCAGTAATAGTTTTAATTTTGTGGACTCAGCTTTAACTAAAAAA
CGGCTTAACTGTACTGGATCTAAAGTGGGTTCTTCTAGGGCAAATACTAACAAAATCTGTTGAGC
ATTGGCAATGGGAGGACGTTCTAATTCTGTCTCTCTTGGTAAAACAGATGCGATCGCACCTCTTT
CAAATTCCGTAGATTTCACCAAGACGCGATCGCCAACAAACACCGATTGACCGATTTTTTTAAC
CGTGTGGGGCGTGTACATAAAAGACTTTCGTTACTATCAAGACGAACCTGATAAAAATTAGCCTG
AGAAGCGATAACAGTGCCTTGAATTAAATCAGATTCAGTAATATTTGCAGAATTAGAATTAACC
ATAGAAATTAACTGAGTTCGGAGTTCGGAGTTCGTCGTTAAAAATCATAGTTATTAACAAAAAA
GCTGAATAAATTAATTTCATACGAGTTTTTCTAAGTTTAATTATTTTTTCTGGTGAAATTTGCT
CGGTGATAACTCTCAACGCCTTATTTTTCAAGACCTTTTTCATCGAACTCAGATACATTAGGCAC
GAACAGATAAAACGAAATATTCTCCCTTGTCTTCAATGGCTTCAATACTATAACCTTCCATGGAT
AAACTATTAGGAACTTGCTCAATAGGTTCACCACCATCAAGATGAATTTCTAAAACATCACCCTG
AGACATTTTTTCTAACTGTAGTTTACTGCGAATAAAATTAATGGGGCAAGGAGTGCCACGTAAAT

CTAATAACTGAGCCATATAAAAAAGAAAAAGTAAGTTTTAAACAAGAAACACGGTACATTCACT
TTTCCCCAACACTTTCCCTAATACCTTAACTAATTGCTAACCAAATAAATAACTACTTTCTTACTC
AAATTCCCTAACTTAAGGAAAAGTCGCTATTTATGAAAAATACTTTCAAGGAAACCCTCAAAACC
TTTCTTAGAAGTATGTTCACCCTTAATTTGAGCTAATTTTTCCAGTAAGTCTCTTTCCTCACCACT
AATTTTAGTAGGAATGTCAACTTTTACCGTTAATAAATGATCCCCACGACTGACAGAATTACCTA
ATTTTGGTACACCTTTATCCTCTAAAGTCAAAACAGTATTTGGTTGTAAACCAGCAGGAATTTGC
ACCTCAAAATCGCCATCAATAGTAGGCACTTTTATCTTACAGCCCAAAATTGCTTGTAAATAACT
AATACTAATCTCAGATAAGATGTTAATACCCTCACGACGGAAATGAGAGTCAGATTCCACCGTCA
AATAAACATATAAATCTCCCGCAGGACCTCCACGAGTGCCGGCATCCCCTTCAGAACTCACTCGCA
AACGAGTACCATTATCAACCCCTGCCGGAATAGTAATTTTTAATTTTTTGTTAACCTGTTTTCTT
CCTTGACCACCACAGGAATCGCATTTCTCCTCAATGATTTGCCCTTCACCATTACAAGTAGGACAA
GTTGACACTTGAGCAAAACTACCAAAGGGAGTACGAGTAGCCCTACGTACTTGACCAGTGCCGCC
ACAAGTTCCACAGGTTTTTGCACTACTACCCTTTTTTGCCCCAGAACCATTGCAACTACTACAGGT
TTCTAAATGGGGAATTTTGATCTCTTTTTCACCACCGAAAACCGCTTCTCTAAATTTCAAACGTA
AGTCAAGCCGTAAGTCATCTCCTCTTGTCGGTCCGCGCCTACGGGTTTGGGTACTTGCACCCCCGC
CAAAACCACCACCAAAAAAAGTTTCAAAAATATCGGCAAAACCGCCCATATCAGTGGGATCAAAA
CCTACGCCGCCACTTCCGGCACCACCAACTCCCGCCTCTCCGAAGCGATCGTATCGAGCCTTAGTT
TCAGGATCTGATAATACCTCATAGGCACGGCTAATTTCTTTAAAACGTTCTTCTGCCCCCGGATCT
TGGTTAACATCTGGATGATATTTGCGCGCCATTTTGCGATAGGCGGATTTGATTTCATCTTTACT
TGCATTCCGAGAAACACCTAGTATTTCGTAATAGTCACCTGCCATATTTCTTGATAATAATTTCC
TTACTAATCTGCTCTATACACTATGGTTTAACATTCTATTCTATTAGGTTAACATTTTTTTACGA
ATCTTTAACTTTAAAAGACTTTAAGGAAATCGAGCGATTACTTTACCAACTATCAAACTCTGACT
AATTAAGCCAAAGTGTCTGCTATCAGTACTAAAATGTTGATTATCACCAATAATAAAGTATTTAT
CCCCAATAATTTGATTTATTCTTTTAACTATTTTGAGTTGAGATTGTAAAGGATGCTCAACTATA
ACAATATCATTAACTTGAGGAGAAATATTACGGTAATAGTAAGGATCAATAATAATTTCCTCTTC
GGGAGAAAGTAAAGGAAGCATAGATAGACCTGTCACCCTAAATCTACGCCTCCTCCTAGTCACTA
GCAGTAAGAAATCAATAAAACTGCAATGAGGTATTTCTGACTGCATTATTTTTTTATCTACAAT
CAAGATAAAATCATTAACTAGCTTTGTAGAAAGTCACATCACGCTCTTTAGAAGCCCAGAACATA
TTATGGATTTTTTCCACTGCCGCCATTAATTCTTCAGCGTGTTGGAGACTAACTTCTACTTTACA
AGCAGAACATAATTTAGCTGCCTTCCAGAAAGTATCGTGTAAGTCAGGATATTTTTCTAAATGCA
CAGGTTTAAAGTAATCAGTCCACAAAATTAATAAATCTTCTTTGGTTTTCTGTGCCTGTTCCTCT
TTAATAGCGATATAGCGAGAAAGAGTATTGTTATATGCTACAACCTCTGCAGGACTAGCACCAGG
TGCAGGTGCATCTAAGGCAAGAATTTTTTTGGTCATGGAAAGTACAGCTTCGGCGGTGATTCTAG
CGGAAGCGGGATCATAAACTCCACAAGGACCATCACAATGAGCAGAGACGGTTTGTGCAGGAAAC
CAATTCTTGATTTGTTGAGCAATAGCTTGAGATAACTTCATCATAAATTGCGAACTTGATCTTAT
TTCTTCACTTTCTCACTTTACTATATAGGAGTATATTTTGCTAGGGGAGGGGATCAACACCTCTT
CGCTAATACCTATTTTCTGAGCTTTGGGTATTGGGGCATTGGGATATAAGAAACAATGGGAGATA
GGGAAAGAAGAGAGTGAGATTAATTTTTGCAAAAAAAAGTCTAAAATTATTATGAATTTACCAT
TACGATAAAAATATGTCTGTCCCCACTCAAAACACGGAATCAACTCCAAATAACGTTGTAACAAA
ATCACAAAAATCTCCTGCTAAACCTTTCAAATTATGGCAGGATATTCAGAGTCATCAAGAGGTAA
GAATTGAGATTGTACCTATGATTGACGTTATTTTTTGTATTTTGACATTTTTCATTTTGGCGGCG
GTGGGTTTTTCTCGTCAACAAGCGATTAGTCTGAATCTGCCTAAAGCGGTTACAGGTACTCCTCA
AATGCGAGAAATTTTAGTGGTGAGTTTAGATAATCAAGGTCAGCTTTATTTGGAAAAACAACCT

GTTAGTCAGGTACAGTTGTACAGTGCTATTAAAAATTATCATAGTCTTAACCCCACGGGTTTAAT
GGTTCTTCATGCTTCCCAAGATGTCCGTTATAGTGAAGTAATTGAAGTGCTAGATATGTTAAAAG
AAGTAGGAGGCGATCGCGTTGCTCTAGCTACCCTAGCCGGTAAGTCTAATGCCCCAACTCAAACC
GTTGATCCTTATTCTGGTTTTAATCCTTACTATCCCAGTCCTAACCCTAGCAATCCCACAGCCCCC
CCAATGCCTTCTCAACCTCCTTCTCCTTCCGCACCTCCCTCTCCTGAAGGTTTTAACAACTAACCC
TAATCCCCTAATACCCCAACACCTGCAACCGTCAAACCTGACACCTCTTTCTTAACTCCGAACCCC
TATCCTCTACATCCAAAACCTTTAACTTTTACCCCGAACGGCTATAAAATGTAATAATATTAACT
GTTCATTCACACTGCATCCATGAGTCAAAAATTCAATTCCCGTGTTTATGTCACCCTCAGACCATC
TGTATTAGATACTGCTGGTACGGCAGTGGAGTCTGGATTACATCAATTAGGTTATCAGGGGGTAG
AAAATGTGCGTATTGGGAAATATATAGAATTGAGTATTACTGCCGCCAATAAAGAAGAAGCGGA
TCAACAATTACACGCTATGTGTGACCAACTCTTAGCTAATCCAGTTATTGAAAACTATTGTTTCG
AGTTAATCCCCGTCGAAGAATAAAATCAAGAGGAAAAAGATAAATGAAATTTGGTGTAATAGTG
TTCCCCGGTTCCAACTGCGATCGCGATGTTGCCATGGTAACAGAAGGAATTTTAAATCAACCTAC
CCGTATGGTATGGCATCAAGAAACAGATATTGATGATTTAGATGTAGTCATAATTCCGGGAGGAT
TTAGTTACGGAGATTATCTTCGTTGTGGTGCGGTAGCCCGTTTTTCTCCAGTGATGAAAAGTGTA
ATTCAACACGCAGAAGCAGGAAAATATGTCTTAGGAATTTGCAACGGTTTCCAAGTTTTAACCGA
ATCAGGCTTATTACCCGGGGCGTTAATCCGTAACCGAGATTTACACTTTATTTGTGATCAAGTTC
CCGTAAGAGTAGAACATAATAACTCTGTGTGGACAAAAAATTATCAATCATCTCAAGTAGTTTGT
TTTCCCATTGCCCATGGGGAAGGGAACTATTATGCTGATGATGATACCCTCAAAGAATTGGAAGA
TAACGGACAAATATTATTTCGCTATGCCAGTGAAAAAGGAGAAATAAACTCCGAATCTAATCCTA
ACGGATCATGTAAAAATATTGCAGGAATTACTAACAAAAAAGGTAACGTTTTAGGTATGATGCC
CCATCCTGAAAGAGCAAGTGATTCCGCCTTAGGCTTTGTGGATGGTAAAGACTTATTTACAAGTT
TATTAGGTTAATTAAACGGGAATAAGGAATAGGAAATCATACGAATAAATTAAACATCGGCAAG
TTATTAAAATTTGTTTATTTTTGTTAAAAACTTAAATCTAAATGAAGAATGCAACCCATTGAAA
ATTTAATAGATCAAATTCAAAAAGAAGCTCAAAGAGAAGAATTTCCCATAGATATTCCTGTATA
TAAAAATTGTGAAAAAGATCCCACTAAGCCAATTTTATACTATGGCAATTTAAAGAGTAATATT
TGTTTTTTCGGTAGAGACTTAGGTAGGGATGAAGTCAAAGCAGGACAACCTTTGATTGGAGCGGC
GGGGCAATTGGTGAGACAGGGTTTTTATAAGGCTATTTACCAAGAAGAATTAATAAACGAAGAA
AAATTAGCATCTATAAAAGAACGTTTAATCTTGACCAATACAGTCCCCTATAAACCTCCAGAAAA
CAAAGCCTATGCGATGAAAGTTAAAAAAAGATTTAGACCTTTTATTGAGCAGTTATTAGTTATTT
ATTGGCAAGGAACACAAATAATTACTTTAGGTACAGAAGCATTCAAGTGGTTTGAATATTATAG
TAATAAAGACAAATTTAATGCCTTTTGGTCAAAAGGTGATTCTCGTTATGAAAGTAGCTTAAATG
TTACCATTTCTACCATGGGAGATAAGGGAAAAACCTATCAGAAAAATGTTACTATTTATCCTCTA
CCTCATCCTTCCCCATTAAATCAAAAATACTATAATCGTTTTCCTTCAATGTTAGAAAATACATT
GTCCCGATTAGCGTTTTAAGAAAATGAATAATAGTTAAAATGCTTAAAATAATTAGTTTTCCCCT
AGGGATAATTTTTGGCTTAATAATATTGGGTGGTTGTCATAATTCTCCACCTAAATTGCCACCTT
TAGCTCAAGAGGAAAGCATTCAAGTTTATTTTAATCACAATCAAGCTAAAGGGAAAGAATACGA
AGAACCCTATCGTAAAATTAAAAGATTTGGCGATGATTTAGAAACAGTTTTAATTGAGCAAATT
AACAGTGCAAATCAAACTATTGATATAGCAATTCAAGAATTAAATTTAGCACGATTAGCAAGGG
CGATTGTAGAGAAAAAAAAGCAAGGTGTAAAAATAAGAATTGTCACGGAAAATAATTATAATAA
ACCTTTAAATCAGTTGCCTAATAATCATGGTTTAGCAATTTTGAAAAGAGCTAATATACCTATCA
TTGACGATACGGAAGATGGGAGTAAGGGTAGCGGTTTAATGCACCATAAATTTGTTATTATTGA
TCAGAAAAAAGTTGTAACCGGATCAGCAAATTTTACTCTTAGTGGTATTCATGGAGATTTTGATA

ATCAAGAAACAAGGGGAAATACAAATCATTTAATTGTATTTAATAGTCAAGAAATGGCTAAAAT
TTTTACTCAAGAATTTAACTACTTATGGGGTGATGGAGTCGGAAAAAAGAAAGATAGCTTATTT
GGTTTAAATAAACCAGAAAGACGCACAAAAATAGTAAATATCGGTAGTAGTTCTATTGTGGTAA
AATTTTCTCCTAATAGTCGATCAACTCTTTGGGAAAATAGTAGTAATGGTATTATTGCTAGGGAA
TTGGCAAAGGCGAATAGCTCCATCGATTTAGCGTTATTTGTTTTCAGTGATCAAGAAATAGCAAA
TACCCTTGAAAAAGAATCATTATCGGGGGTTAAAATAAGGGTTTTAATCGATCCTAATTTTGCTT
ATCAATATTATAGTGAAGGGCTTGATTTATTAGGTGTTGCAATACCAAGAAAATGTGTTTATGA
GAATGATAATAATCCTTGGCAACAACCCCTAAAGACTGTTGGCATTCCAGCTCTACCATTAGGAG
ATAAATTACACCATAAATTTGCTATAATCGACCGTTACATAGTTATTACAGGTTCTCATAATTGG
TCTAATTCTGCCAATCATATTAATGATGAAACTTTATTAGTTATTTATAATCCTCTAATCGCTCA
ACATTTTCAAAGAGAATTTGATTATCTTTATCACGATGCAATATTAGGAGTTTCTTCGGAGTTAA
AAAAGGAAATTGATCAAGAAAATTCAAAATGTGGGCTATCGAATTAATAAGCAACCAATAAATC
AAAAATCTATGTTTTTGTTAATTATTTTCTCTGAATTGGTTAATTATGACTCCTGACTCCTACTA
TTCTCATTCAAATGTTCCGTAGGTGCAAGATCTGATTTTTCGTTATAAGATGATGATTGATTCAA
AAAGCGAGAAGATAAACCAAATGGGGTAGGGGAATATGTGGAATAGAATTCGTGACTTTATCAG
GGTATTTTTTGCAAAATCGAGAAAAATTGAAGACGAACCTATAAATAAAGTTAGTTTGATTGTA
ATTATTATCATCGATTTATTTATACTAGGTAACGTTTTCTATGGTTTAGATGATATTAGTCGCTG
GCCTTTATCTCCTACTCAGGCTTATCCTTGTTATCAATCTTGGCAAAATTATCAAGAAGACTCTTC
TTCCCCAGACAACTATCAATTCATTCGGGATTTTATTGGTGAAAATCAAGACTCATTATCTTTTA
ACTCTGTAGAAGAAAATCATCTGGGAAAAGTATCACCCCTATGTTTACAGTATGAAAGTTTAACT
CAGGCTTTAAATAATAACACCAATCAGAATATTGTCAGAAGAATTGATGAAAAAAATACTCAAA
TTAGACTATTAGAAGATAAAAATAAACAAATTCGTCAACAATACGATTCTACGTTGCTCGAAGAA
ATTGCAGGGCAACCGAGAGAATTATCTATCAATGAAATTGAGGCAAAAACAGCCAAAGAAGAAT
TAACTAATAATCAAAGTAATATTAATTTATTAACTGGAGAAATTAAAACACTTAAGGGGGAGTT
ATTAAATAAAAATGAGTCTGTTGCCTTGCTAAATTTTGTTAATTCTCAGGATAAATTTAGTCAAA
TTAAGAGTAGTTGGGAAAGAGCTAATTTTTGGTATCCAACAATTCAATTATTGTGGCAAATGGT
ATTTTTAGTACCTTTAATTATTATTAGTTCGCTAGTGCATAAATGGTCAGATAACAAGGGTTACG
GTTTAGTATCCCTAATGAGTTGGCATTTGTTGGTTATCTTTTTTATTCCCTTACTGATTAAATTA
TTTGAGTTTTTACAGATTGGATTTATTTTTGAGTCGTTATTAAATATTGTTACAATTCTTTTCGG
GGGTTTACTATTTTTAGTCTCTTATTTATATATATTTATTATTCCCTTAGTGGGCTTTTTATTAA
TTAAATTTTTTCAAAAGTTTGTTTTTAATCCTTATACTCAAGCTAGTAAAAGAGTGGAAAAATCA
AGGTGTTTGAGATGTGGTAAAAAAATTAATCATGACACTGCCTATTGTCCTCATTGTGGTTTTTA
TCAATATCAAGAATGTAGTAATTGTCATAATTTAACTTATAAATATTTATCTTATTGCTATCATT
GTGGTACTAAGCAAAATAACTAGCAAAATAACTAAACTTAACAGTAAAAAACAGTAATTTCTAT
TAATTTTATTCGAGAGAAAAATCATGATTTCGTCGTTGAAAATAACGATTTTAGTGGAAAATAC
CGCAGGGGGACGAGGTTTATTGGGTGAACATGGTCTTTCTTTTCTGATAGAGGCGGATAACTCTC
GAATTCTTTTTGATACTGGTCAAGGGATGACTTTAATTCACAATGGAGATATATTAGGAGTTTCT
CTGAATAATTTAGATGCGATCGCACTTAGTCATGGACATTATGATCATAGCGGAGGGTTAGTTCA
TTTATTACCTTTGTGCAAAAATACTGACTTATATCTTCATCCTAATGCCTTGATGCCCAAATATA
GCCCCAGAGGTGATATTGGCTTCAGCCTAGACGAAGAAACTTTGAGCAGAAGTTTTCAGCGTGTG
ATTAAAACAGAAAAACCTACCGAAATTGCAAAAGATATTTATATCACAGGTAGTATCCCCCGAAA
TCATCCCCTAGAAGACACAGGAGGCTTGTTTTGGCACGATCAAAATCATCATGAGGTTGACCCCA
TTCTCGATGATCAAGCCCTTTATTTAGACACATACAAGGGTTTATTTGTGATTTTAGGGTGCGCT

CATGCAGGAGTTATCAACACTTTAGATTATATTAGTCAAATTACTAATAAGAAAGAGATTTATG
GTGTGATAGGAGGAATGCACCTCTTACGAGCAAGTCAAGAAAGATTAATGACAACGGTAGAAACC
CTCAAAAAATATAATATACAATTATTAGGAGCAAATCACTGTACAGGTATAGATGCGATTTCGTT
TTTGTGGCATGAATTAAAATCCATTGATTGTAGAGATTGTCGAGTCGGGACTATTTTGGAGATTG
AATCTATGGGGAAATAGTTTAATTTCCTTTCTCTAGCCATTCATCAAAGTTTCTTTCCAAAGCAT
ATTGAAAACTTTTAGAAACTAAATAAATCTGCGGTTTTATCTTTTCTTTTTCTGGAAAAATTACC
TCTGCATAAATTTGAGTATCATCAAAGTTAGACTTACCTAATATCATCTGATAGCGATCGCTGTT
TTTTAGGGTTATAGTGATAGTGGGAGAGTTGTTGTTAATGCCATATTCTTTTCTTTGCTCATCTG
TTGCTAATAATTCTATTTTTTTGTTGGCACTGGGAAAAAGATTCAGTAAAAATGCGATCGCAGCA
TCACTCGCCTTGGTGTTTTCTGGTTGTTTCATCATCCAAGATAGTGGCTCATTACCAGTTTTTTCA
AAAGCTATAAACTGATCATCTATTTGAATCTCTAAGGCTTGTATTTCCTCAGTCTTAAAAGGAAA
AATAGACGGAATATTATCCTCTTGTTTACCGACGGGAGAAATATTAAAACCGTCTTTCCTGATTT
CGGTTAAAAACACCAATCCCATTAAACTAAATGCAGTGATAAATAAAATTATTGTATTGCGGTTT
AAGTTCATCTCCCTTTCTTTTTGCCAGAGCCTGATGTTTGTAGCTTTTTAAAATGGGTTATTTTA
ACCAAAGATTTGTAAAACAAAATAGAGGTTAGGTAAACAATTATGGTAAATATTAACATAGTTA
GTAAATCAGTTTTTTATTTCATCATTGAAAATAAATCCCGTGCAAGTTTTTAAGGAAATTATCGC
CCGTTGGTGGTCAGAATTTACATTACAAACTAAATTAATGGCGATCGCTACTTTGGTAGTATCTT
TGTTTATGAGTAGTATAACTTTTTGGGCAGTCAACTCCATTCAACAAGATGCTCAAATGAATGAC
ACTCGTTTTGGCAGAGATTTAGGATTGCTTTTAGCCAGTAATGTCGCCCCCTTATTAGCGGAAGA
AGACTTAACCCAAGTCGCCAGATTTTCAGGGAGATTTTTTCACAGCACTTCCAGCGTTCGTTATA
TCATCTACAGTAATAAAGAAGGGGAAATAATTTTCGGCATACCTTTTTCTCAACCAAGAAGTAAAA
AACTCATTTACCCTCCAACGCAAACTACAATTACCAGAAGAATATAACCCAGAAGTAAATATCCC
CGTTGTTAGACAACATCTCACCCCTGATGGAAAAGTAACTGATGTTTTCGTACCCATTTCCAGCA
ACAATGAATATTTAGGGGTTTTAGCCATTGGAATTAATCCGAATCCCACCGTTGTAGCGTCTTCC
AATTTAACCAGAGATGTTACTATTGCAGTATTTATCTCCATTTGGGCAATGGTAATTTTAGGAGG
AGTATTCAATGCCCTAACCATCACCCAACCCATAAAAGAATTACTTATGGGGGTGAAAAACATCG
CCGCAGGAAACTTTAAACAGAGAATTGACTTACCCCTCGGAGGAGAATTAGGGGAATTAATCCTT
AGCTTTAACGATATGGCGGAGAGACTAGAAAAATATGAAGAACAAAACATCGAAGAATTAACCG
CCGAAAAGGCTAAATTAGAAACTCTTGTGGCAACTATTGCCGATGGTGCAATTTTACTCGACTCA
CAGTTAAAACTTGTATTAGTCAATCCTACCGCTAGAAAAATGTTTGGTTGGGAAGAAACCCACAT
TCTGGGAGAAAATCTTTTAGAATATCTCCCCCAAGAAGTTACTCAACAACTGCAACAACCCCTAG
AAAACATTTCCCATGGAGAGCATCCCGAAGAAGAAAACAATAACAATAGTATCCTGCCCACTAGG
GGAGAATTTACGGTTACGATTACCAAACCTCAACATCACACTTTTCGCATTCTCTTAACTCAAGT
ATTAGACCAAAATAGGGAAAATATTAAGGGTATAGTCATGACAGTACAAGACATTACCAGAGAA
GTGGAATTAAATGAAGCAAAAAGCCAATTTATTAGTAATGTATCCCACGAATTGAGAACTCCCTT
ATTCAATATCAAATCTTTCATTGAAACTCTTTTTGAATACGGAGATGATTTAACAGAAAGCGAAA
AACTGGAATTTTTACAAACTGCCAATCACGAAACAGACAGACTAACACGCTTAGTTAATGATGTT
TTAGACCTATCACGCCTTGAATCTTCCCGTAGCTATCAATTCAATTCTCTTGATTTAGCTCAACCC
ATAGAACAAACCTTAAGAACATATCAACTCAATGCCAAAGATAAAAATATTGAACTTGTTCAAG
AAGTAGAGCCTGATTTACCATCTATTTGGGGACATTACGATCTTTTATTACAAGTATTTACCAAT
TTGGTGGGCAATGCCCTTAAGTTTACCCCAGAAGGTGGAAAAGTCACAGTGAGGGCTTACGGAGT
TAAAGGAGACCCAGAAAATAGTAATGATATAACTCATGTTAGGGTTGAAGTCGCAGATACAGGC
ATTGGTATTGCACCTGAAGATCAAGAGGCAATATTCGATCGCTTCTTTCGAGTAGAAAACAGGGT

TCACACTTTAGAAGGGACAGGTTTAGGCTTATCCATCGTCAAAAATATCACGGAAAAACATAATA
GTCGTATTCATCTAATCAGTGAAGTGGGAGTGGGTACAACTTTTTGGTTTGATTTATTGATTTAT
CAACCAGAAACAAAATTGAATAATCACCAAGAAGAATTAAAAACTCTTACTAACTAACCTGAGTC
TGCGATAATTATCGGTGAGTGATGGCAAAAAGGGCAATGGGCAAAGGAAAAATTAAGAATTAAG
AATTAAAAACTCTTGTACGGGCGAATGGCCATTCGCCCCTACATCCTCTCATCCCCCTATCCCCAA
CACCTGACACCTACCCTTAACCGAACTGAGGTGAATTAATGAAAAACTTATTAAAAAGGTATATT
ATCTAAATTTTTACCATCTATTTCCCCATCATCAAAACTATAATCATCAACATCTTCATTGGATT
GATAACTATTTAAACTAACGACATTATCGGGATTATGAGGAGGATTACTACCCGAAAGAGGATA
AATATGAGAAAGAGTCAACTCGGCAACTTTTTCTTTATAATTTTGTCTCTCTACTAAATCCATTT
TCAAACGCCCCGTCACTACGACTTCATTACCTTCTATGTAATTTTTTTCTATATCCAAAGCTAAAT
TACCCCACGCTACCACTTTTAAGGTATTAGGAGGACTATTGGGCGATATATTTTCAAATTCTACC
ATCATCTCTGCGATCGCCAACTGATTGTCCTGAGTATATCGTAATTGAGGACTACGAACAATTTT
CCCCATTAAAACACAACTATTCATAAATATATCTTTTATTTTTGCTAGTTAACTATCTTTATTTT
GCCACAATCTTTCAACAATAGTAGTTTAAAAATACTAATATTAACCTGCATTCGCTGTAAAAATA
TTATTCTCTAATAAATTTCAGGCTTTAAACTTTAAGTTAATTTGTCTTAACTTTTTCAATTAAAT
ATCACTAAGAGTGCGATTTTTTATTATTTTACAAGGATTTCCCCAAGCTATTTTTTGGGAGGGAA
TACTTTTTAAAACCGTGCTTCTAGCACCAATAATTGTATTTGCACCTATTTTCACTCCAAGACTA
ATAAAGCAGTCACTAGCAATCCAAACACCATTACCAATAAATATGGGGGCGGTAGTTAGCTCAAA
ATTAGCATTCTTAATATTATGACTACCTGTACACAGATAACTTTTTTGAGAAATTACAGTATGAC
TACCAATTTTTACATTATCTAGGTTGTAAATATACACATTCTCTCCAATCCAACTATAATCACCT
ATTTCTAACTTCCAAGGAAAATAAACCCTCGCACTAGCCCTAATTACCACTCCTTGACCAATTTTT
GCACCGAATAATCTTAATAAGGAACAACGTAAACCACTAAAATTATGAGGAGTTAAGGGAAATA
TTAAACCTTCTAGCAACCACCATAAAAAAATAAACCACTGAGGCTTACCTCTATCTAGTTTTGCT
TTATATCCCTTTAAGTTTATCCATGGTTTTTCTTCGAGAATAATCGAATCTTGTTGTGCTTTTAT
GTTATCCTTCTCAGTCATAATTTAGAGATGATTCGTTAATAATCTTTAATAGATGCTTAATCTTA
TTAGTTTAATTCTTATTTAGCAAATGTCTATAACTCTAGTTACCGGTGCGGCAAATTCTGGGAAA
AGTGAATGGGCGGAATATTTGGCTTCTCAATGTCAAAAGCCTGTGATTTATATTGCGACAGGACT
TAAAAATGATCATGATATAGAATGGCAAAAAAAAATTGAGCAACATCAAATCAGAAGACCAAAA
AACTGGCAAACATTAGAAATTCCATATAATTTAGTAGATGCGATCGCACTTAATTTTTCTGATAA
CTGTATTTTAGTTGACTCTTTAGGAACATGGGTTGCTAATTATTTAGATAAAAACAATGATGATT
GGCAGAAAGAAATCAATCATCTTTTGGAAACTTGCCGAGAATATAAGGGGGAAATAATCTTTGT
GGGAGAGGAAACGGGGTGGGGAGTTGTACCTGCTTATGAGTTAGGGCGATTGTTTCGCTCTCGTT
TGGGTAATTTGATTCGTTTGGTGGGAGGAATCGCTAATTCAGTTTACTTGATGGTGGGCGGTTAT
GCAGTAGATGTGGCTAAATTGGGAATTAATCTTACCTCAGTTCAAGATAAAATTATCGGTAGCCA
ACAAGATAAAAAATTTCCTAGTTGATTTTTTCAGGGTATAAACTGAAAATTCCCTCTTTTGATGC
TTGACAAATTCCCCTTTCTGTGATAAGCCCCGTAATTAATCGAGCAGGTGTGACATCAAAACCGT
AGTTAGCCGCCGGGCTGTTTTCAGGAGCAATCAAAACTTCTTTTATTTTTCCCTCATGTAAACCT
TTGATATATTTTACTTCTGTTGCGTCTCTTTGCTCAATGGGAATCTGTTTCACTCCGTCACTGATA
TTCCAGTCAAAGGCAGAATAAGTCAAAGCCACATAGAAAGGCACATTATTATCTTTGGCGGCAAC
GGCTTTTAAATATGTACCAATTTTATTGGCGACATCTCCAGTGTAAGTGGTGCGATCGCTTCCTA
CGATTACAATATCAACCAATTCGTGTTGCATGAGATGTCCCCCGGCATTATCAGGGATAATAGTA
TGATTTATGCCCTGTTGTCCTAACTCCCAAGCAGTAAGGGTTGAGCCTTGATTACGGGGGCGAGT
TTCATCCACCCACACATGAACTTTTATACCCCTGCGATGGGCTTCATAAATAGGAGAAGTGGCAG

TGCCATTATCCACACAACCCAACCAACCCGCATTGCAGTGGGTAAGAATATTAACGGTTTCTCCT
TGTTTGCGTTGACTAATTTCCTCAATCAACCTAACCCCATGTTTACCGATATTTTGGCAGAAGTC
CACATCTTCATCAGCGACAGTCCTAGCAATTTGCAAAGTTTTGGTAATAATTGCTTCAATATCAT
CTTTTAAAGGAATAATTGCCTCTAATTGACGATTTACCGCCCAAGATAAATTCACTGCCGTGGGG
CGAGTTGCCTTTAATTTCTCACTGGCAGAAATCAAAAAAGACTGAACACCGCTAATATTATTTTT
TTTCGCTTCTAAACTAGCAATATACATTCCATAAGCACCAGAAGCACCGATTACTCCTGCTCCTCT
AACGTGCATAGATGCGATCGCATCACAAACATCCTCTAAGGTGACTAAATCTTCAATGACAAACT
GATGAGGTAAAAAACGTTGATCAATAACTTGAACAATCTCAGGGTTATCTGACTTGAGCCAAATC
GTGCGATAAGGCTTACCATTTACATCCATGTAGCGATTTTGAGTAATGAGTAATGAGTGGGAAGT
AGTGCAGGTGTGGGAAGCGTGGGAAGATGGGGAAGAAAACCTAACACCTGCCACCTGCCACCTGC
CACCTGAAACCTTTTAATTCTTAATTCTTAATTTTTCTTTACTTAGCCTCCAGCAACAGCAGGTAC
AATACTTACCTCATCACCATCAGATAAAGGAGTAGCAGTATTGTCAAGAAAACGAATATCCTCGC
TATTAACGTAGAAATTGAGGAAGCGACGGGGCGCTCCTGCCTCGTCACAAAGACGAGCTTTAATA
CCGGGGCAATTTGCCTCTAAAGCGTCAATTAATTCTTGAATGTTGGAAGCAGAACACTCGATGGT
TGCTTGATCTTTGGTAAATTTTTGTAAAGGAGTGGGAATTAATACTTTTACGGACATATTTTCTT
GAACTTAAATTTATTTGTGTATTTTTTTAGTAGTGGAATATCTTAAAATAATAGCTCTTTTACTT
TAAGTATGTAAATTTTAAGTTAGGGTAGGGAACAGGAAGACCCCCCTATTTCCCCCCTTTTAAGG
GGGGAGAGGAACAGGGAACAGAGAATTATTTATTTTTAACTCCGAACTCTTATACTAATACTTGT
TGCCATTCTAAACGATCAAGGGTGCGAGAACGCTCTAAAGCTCTTTCAAAACTGTCTAATTTAGG
CTCAATGATTAAAGGTTCGCTGATGTATCCTTGTACAGCTTCTTGCGTTTTCAAGCCATTACCAG
TGATATAAACAACGGTGCTTTCTTCAGGATCAATTTTTCCCGCTTCTACCAATTTTTTCAATACG
GCAACGGTTGTACCCCCTGCAGTTTCAGTAAAGATACCTTCAGTTTCTGCTAAGAGTTTGATCCC
TTCAATAATTTCTGCATCGGTGACAGATTCGATATTACCGTTAGTTTTACGAGCAATATCTAACG
CATACATACCGTCAGCAGGATTACCAATGGCGATCGACTTAGCAATGGTATTGGGTTTAACAGGG
GTGACAAAATCTCTGCCTTCTCTGAATGCTTGAGCAATAGGAGAACATCCTTCCGCCTGAGCACC
ACTGAAACGCACTGCTTTATCATCCACTAAACCGACTTTGACGAATTCTTGGAAACCTTTATAAA
TCTTGGTGAAAAGAGAACCAGAAGCTAAAGGTGCAACGATGTGATCAGGTAATTTCCAGCCTAAT
TGTTCTGCTACCTCAAAACCGAGGGTTTTTGAACCTTCAGAGTAGTAAGGACGTAAGTTAATGTT
AACAAATCCCCAACCATAGGTGTTACCAACTTCACAACAGAGGCGGTTAACTTGATCATAGTTAC
CTTTAACAGCCATGACAGTGGGGTTATAGATTAATGTACCTAAAACCTTTCCTGCTTCTAAATCA
GAAGGAATGAAGACACAACAGTCTAAACCAGCATGAGCAGCGATCGCAGCCGTAGAATTGGCTAA
ATTACCAGTACTAGCACAGGAAACGGTGGTAAAACCTAACTCTCTAGCACGGGTTAGAGCCACGG
AAACGACTCTATCCTTAAAGCTGAGGGTAGGCATATTAACAGCATCATTTTTAATATAAAGATTT
TTCAGACCTAAACGACGAGCCAAGCGGGTGGATTTGACCAAAGGAGTCATACCTGTACCCACATC
AATAGGATTCTCACTTTCCACGGGTAAAAATGCTTTGTAACGCCAAATGGAATTAGGTCCGGCGG
CGATAGATTCACGACTTACTTGAGCTTTGATGGCATCATAGTCATAGGCTACTTCAAGGGGTGCA
AAGGTTTCCTCACAAATATGAATCGCTTTGAGAGGATATTCTGCACTTCCTTCTTTAGATACTAA
TTTGGTGAAAGTGGGTTTTAATTTATTGTCTGTGGTACGTGCGATCGCTTGTGTCATTTTTTACT
CTCCTGTGCTTTACGAGTGATAAATCTGAAAATTGATCTTTAAAATGATTGCTTTTTCAAAAGTA
TAACAGCAATAAAATTTGTTGGCAAATATATCCGACTAAAATAGTCGGGATTAAAGTAAAAAGG
GCAAGGGGCAAAGGACAAAGGTAAATAGTAAATAACCTCAGTTCGGTTTAATAATATTGTATTG
GGTGAGGGTAGGTTTCAGGTGTCAGGTTGCAGGTTGCAGGTGTTAGGGGTTGAATATATTTAACG
TCAGTTCGGGTTAAGGCAATTTTATCGTTATTTCTAAGAAGAAGCTATAAGGTTTTCTGACTACG

AGTTGGTCTGCTTTCAGCTTATCCAAAACTCAGGTTAAATAGAGAATAATAACTCCGAACTCGAA
ATGTTGAGATATTTAACTAAATTGTCAACTAATAATAAATTTTGGGCATACTGAAATTAGGAGT
ATTAAGTGTGAATATTCTTGTTTTTGCACTAAATGGCTTTTTTTTGACGAATTTTCCTTGTTGTC
TCACATTGAGAATATTTGCCAAATTATTATGTAAAAGCACTTTGGTGATGAGGTCGTGAATCGAA
ACAAAAATTAATTTCTGGATTCATCAAATAAGGAGGTATGTACCATGATGTATAGTTTTCGCAGA
GTTACTCATGCGGTTTGGAGTTATCTTAATCAATCTATTACTCCCACAGAAGAAGAATATTCCGT
TTGGAAGCCGAGTAGATTTTGGTATATTTACAAGATTAGAATTTTAGAGAAGTGTTGGTTACAG
AAGTCTTTTATACCTGCCCATGAGATTCGTGGACAAGACGAGTACAGAGCGGGAAATTAATTGCT
AACATTATGCACTTACCTTGTCATGTCAAATTGGGGTAGGTTTCAGGTTTCAGGTAGCAGGTTTT
CTCTTTCTAGCACTTAGTTATAAAGGTGAAGTACTTACATTCGTCGATTTTTTTACTGTTGTCTG
TTACCTGATTTCTGCTATAAGTCTATATAAAAAGAAGAAAATCAATAAACATACATTTTTTGTAA
ATTATTGTTACTTTTTCCTCTTTCCTTGTTTTCGACATCTTTGTAAATGACTAAATGGTACACCA
AAAGAGGTTTTTACTGGTGACACCTAAAGAGTCAAGAAGCGATCGCATCTTTTGTGAACATTCCC
ACCAATAATCATGATTATCATACATCCAATCGGGATTGTAATAGATATTTTCGTGTAAAACTCCT
TCTAAATCGGAAAACTCCTGAGAATCTTGTTCCGACATTATTAATAAACGAAAGGTTTTTCCCTG
ACATTTTTCCTTTAATTTGCCCATTATATCGATGACTTGTTCTCTATCTGCTCTACCAGTACGAAT
AAAAAGCACTTCATCACACTTATCAATAGTAGCCCAAAAACGGTGCGATCGCCCCTGATAACGTT
TTTTCATTCTCTTGAATACGGGAGTCATATCATTAATGGGGTCATCTGTATTTTCTACTTCATGG
GCAAAAGATAATCCTGTCCATTTACTGTGATATATTCGACCATGCTCGTCATTATAGTGGAGATA
TTCAGGATTCCACATATCTTCAAAACCTGTGTCAATAATGTCCGCCACATCACTTAAATTAGTGG
TACGAGTTAAATCGAAAGGATAAGCAGGCCCATCATACTGAATTTTATGTAACAACATTCTGACG
GCACAGCGATCGCCCAATGGTAAAATAGCAATGCGTTTTAATTCTGATAGGGGGCATTTATACTG
AAAAGGTAAAGCTGACTTCGGAGGTACTTTAATACGACTTTCTAACCCCGTTTCTCCCAACATCA
TACGGCGAAAAGGAATATCAGGATGAAGCAAATCATCATAAACCCCTTGTTTCATGTCCCGTAAA
GCCTCAGAAACCTCTTTCCACATTGGTTGAACATTATATTCATGTTCTGATTCATTAATCAAATA
GAGATTATTTTCATCTATTTTTAAAATTCCTAATGAACCATCATAGAATAACACCTCGCTCTTAT
CAGGAGAAAAGAAAGCCGACAGGGGTAAATATTCTAAAGCATAAGAAGGGGGGATATTGACAAT
CGTATCAATCGTTTTCTTCTCCCCCAACCAGAGGGAAAAATTCGCTACCCTTCTGGTATTAGCCAA
ATATATTCCCTTTTCTAATAATTGATTATTCTGTAAGCAATCTTGTAATTCTAGGCGATAAGGAT
TAATTTGAAAATCAACTTTACTATTGTTTGTGAATAAGAGGCTATTATTTTTTTCTCTCCAAGCT
ATTTTAAAACCCTTTTGGTCATCAAATAAGCAAACATCTATCGGCTTAAGAGATAGTTTTTTTTT
TCCTGCTTCCTCTTTATTAAGATCAAAAATAGGTACATTAATCGCCGAAAACATTACCCGATGCC
CCTCACTATTGGGGTGAGCTACATCAAAAGATAAACCTTCAGCAAAACGCCCGTTACCATCATTC
AATTCCGTTAACCAATCCAATACAGGTACATCCCAACTTAACATACGGCTATGGGTATCGTATAA
AAACCAATTATGTTCAGCACTATATTCACCGTGAGGATAAACTGAGCCTAACATAGGAATTGCAC
CTATTTCCTTTGTCATCTGGATTAACCGTTGTAATCCGCTTTCAAACCTTTGTTGTAATGCTTTTC
TTTCGTGGGGTTGGCAATAAGCAAAACCTTCATTACCGAGAGAAAGGGCAATAATCACAATATCG
GGTTTTTGAGGAATAACTACCGATTCAAATCTTTTAATAGTAGTAGAAACACTCGCTCCCAATTC
AGATACATTAATTAATTGATGACCATATTTCTCTGCTAAAGTCTCTTTTAATAATTCTGCCCATC
CTTTCAAGAGCCAAGCACTACAGCCCATAGCAACAGAACTACCGATAACTAAAATTCTTAAACCA
TTTTTATCCTTGAGGGGAGGATTTTGAATCAAAGGTTTTTCCCAATAGCCATAGGGATAGGGTTG
TAAGTGTCCAAACCAACCATCTTCCACAATTAAAGTGGCTTCTTTTGCTTCTTTTTCAAGGGGTA
TCCAACGATTATCATTACCTAAAGATTCCCAAATCACTTCTCCTTCGGCAGAGAATTTGAGATAT

TTATATTCTATTTTTGAGTCAGTATAGGGAATGTCAACATCTGCCCACCATAAAGGATAGCGATC
GCTCTTTGTTTGTAATGGGATACAATTAGAAATGCTCCAAGATCCTAATTCAGCCGTTGAACCAA
GAAGGGCAATTGATTCTCCCATATTAGTATGGGCACTTACCTGAAAACGATACATAAAATCCCCA
GCCTCTGACGAGTAAAATCTAGTTTATGCCCTAATTTTTAGCACGAATTGTCCCAAAAGTTTCAA
TTACCTTGCCTGAATTCGATATAAAATTATCGGTTAAGGTAGGTTTCAGGTTTCAGGTTTTACTC
ATTTCCTAGCTTTCCGATGATGTGGTGAAGTTGCTTAATATTAGGTAAAAATAAACTATTAGTTT
CAGATTCAACTACTAATAAGCCTTTGGTTTCTAGTTTTTCAACAATGTAATCTAATTCTTCTAAA
CTAAGTTGAGCTAAATCGGCTAAATCTTTGCGACCAAAATTAAATAATTTGATGCCTTTTTCTGT
AGCTTTACCATATCTATCAGCTAAAGAAATTAATATTGTTGTCAATCTAACTTTTGGGGTTTGAC
GATGAAATTGACAATATTTTTGATATTCTGCCACTCGTTTAACCATCATTTTCAGTAAACGATTT
TGAATTTGAGTATCCCGATAGAGAATTTGTAAAAATCTTTGGGCAGAAATACTTAATAATTCCAC
ATCGGAAATGCAAGTGATACGACTATTGGTATTGACACTATCTAAAATACCCGCTTCTCCTACTA
AACCGCCTTTGCCTGTAACTTCTACGGTGATACTTTCTTGATGATGATAAATATTTTCTAGTTTT
ACCCATCCTGAGACAATAAGATAAATTGCTTTACCCCAATTATCCTCGGAAATAATTACTTCGTT
AGCACTGTAACTTTCATTTTCTGTCATGGATAAAACCCATTCGAGAGTTTCTGTGTTGAGATTGT
GAAATAAGGAGAAAGTGTCGCTAAATTCCTGCGTCTCCATGAATTATTAACTTAAGAATTGCTTG
ATTTCATATTTTAGATTACTGCATTTTACTTGATCCTGTCATGATTTTCAATTAATTTTTGAAAA
TGAGAACACACAAAAACATATATAAAGCAAATTTCAATTCTCTTTCTCGGCAATTTAATTATAGA
CTATTTTCTATTTCATGATATATAATGATTGACTATTAACTATAAAACTTTTTTTCTTAACAAGG
GTCAATGATACTGCATCTTATATTTCGGCTAGGAATATGGTTTTTACTGACTTCTGATTTGAGTC
CGGCAAATATTATGATTGGAGTTGCGATCGCATTTATCTTACCAAAGCCGAGAATTGGTTTAGCA
GATATAAAAGAATGGTTAAAGATATTAGCGAAAATGTTGATCGTTTTTCCTCAAGCCTACTGGGA
AGCTCTGGAAATTATGCTTTTTCCCCATCGTTATGAGGGATTAAATATGGAAAAAGTACCTGCGG
GGCGATCGCCTTTACTAATTTTTTTGGACATATTTTTTATTACTTTTACGCCCAAAACTGTTGTT
GTCAAATATCACAAAGAAGGATGGTACGAAGTTCACAGAATTGACAGAAAACAATTATTGACCG
ATTAATATTTACTCAATTTATTATTTTATTTAAGAGATGAATATTGTTATTATTTCGATGTTTTT
AATCCTATTAATACCCATTTATCAAGCATGGCAAAATGAGGATATATGGCAAAGAATGTTAGCGG
TTAGTAGCATTTCCACTAAAGCATCTATCATGATTTTAGCTATTTCTGTGTTTAAAGATGATTGG
ATGATGGGAGTTGTTGGCGTTCTTATTTTAACTATAGGTAATGCTGGTTTAATGTTATTGGCTCA
TCTTTTAAAACGTTTGAAATTATAAAAAAAATAGGCTCTCTTACTGTGAGTATATAAATTATTGG
TTAGGAAAAGGTGTCAGGCGGCAGGTGGCAGGTTAAATAATAAATATTAATTTATAACAATTAA
TTTATTGTAAATTATTTTAATCTTTGTATATATTTGGGTAATTTAATTTTCTGATTCTTTCAATC
AATTATTTCTATTTTTCATTGTTACTTAATTCATTTAAACTATATTTAAAATCTGTATTATTATC
AATAAAGTATGGACTTTCTTACGTATTTTTTTATAGTAATTGGTTTAGTTTTTTGGTTATGGGGA
ACAATTCAAATATTAGATAAACGTTCCGTTTTGTATAAATTACATAGTTTATCTGTAGCTGACAC
TTTAGGTTCAATTTTAATTGTTTTCGCCTTAGCTTTGAAAATTCCTAGCGAATTACCATTATTAA
TTTTAGCAATAATTTCTTTAACTTTATGGAACACAATGCTCGGTTATGTCTTAGCTTATTGTAGT
CGAAATCAAGAGGGGAAAAAGTTAAATGGATAATTTATATATTTACTTAATTATGGCTTTACTG
CCCATTTCTGCATTAATGTTAATGATTCAAGTTAATCCTTATCAAGCCTTAGTAATTAGAGGAAT
TTTAGGTGCGATCGCAGTTTTGAGCTATATCATTTTAGGGGCGGCAGATGTAGCTTTAACAGAAG
GTTTAATGGGAACAATGTTAGGGGTTGCTTTATATATTATTGCTGTTAGATCATCTTTTGTGATG
CGTTTAGGAGTAATTCAGGAGGATAAAAAAAATACAGAAAAAGACCCTAATTTTGACAATATAA
AAAATCAAATTAAACAAGTTATTAATAATTTTTATTTACGATTAGAATTAGTTTATTATTCTGA

GCAGGAACTATTAAAAAATTCTTTTAATGATAAGAAAATTCATGGTTATATTTTAACAAGTAAT
CAATTAGATAAAAAATATTTGCTAAATATACGCACAAAAAGACTATTTGATATTCTTCAACAGG
GAATAAAATCAGAGCAATTACTAATTGGCTATCAAATGGATATAAAAGAAAACCAAGATCAATA
TTTAGAATACGTAAAAAATAACGCTAATTAATATTTATTAAAACTATTTAAGCAAGGGTAAAAA
CCATGAAAATACTATACATAATTGCTAGTTTGGCAATGTTTACAAAAATCTTAGTTATGCCCAAT
ACCATGGCGGAATTGTCGGAAATGTCCATTGTTGAAATGGTTGTTGAAGATACAGGTGTACCTAA
TGCTGTATCAGGAATTATATTTCGCAACCGTCTTTACGACACCATTTTTGAAGTGATTGTATTTA
CTATTGCTATTTTAGGAGTCGGGTTTTTGCTGTCTAATGAACAGCCCACTGCTGTAGTTTATCAA
TTTACAGATCAACCATCTATCGTTTTAGCCAGATTAGGGGCGACTATCACCGCCCTTGTCACCACG
GAATTAGCGATAAGAGGTCATTTAAGTCCGGGGGGCGGATTCGCCGCAGGAGTGGCAGGAGGCAC
AGCTATTGGCTTAATAGCGATTACATCTTCATCTCAAACGATGGAGAAAGTTTATAACCGATTTT
CTGCCGCAACCTTAGAAAAAGTATCTGTTTTATTATTTATTATTCTTGCGATCGCAACTTTAACT
GGTATAGAATTACCTCATGGTAACTTAGGTTATCTTATTAGTGGAGGCTCGATCCCTCTTTTAAA
TATTATTGTAGCTTTAAAAGTTGCCTTAGGTTCATGGGCGGCTATTCTCTTATTTGTACGTTATC
GAGGGCTTTTTTGATCTCAAAAAGTCAAAAGAAAGGGCAAGGGGCAATAATTCGAGTTCGGAGT
TAGTTAGGAGCGAATGGCCATTCGCCCGTACAGGAATTCGGAGTTAAGATAAAAAAAGATAAAT
AAATTCTCCCCTAAGACCTTAAATACCCCAATCCATAATAAATTGAATCCAGTTACCAGACAAGA
TGGCTCATTTGGAAATAGAAGATTTAGGTTTATTACCTTACAAAATTGCATGGGAATATCAACAA
AGTATCGTCCAACAAAAATTAAATAATCCTTCTTTCCCTGATAAGCTAATTTTAGTACAACATCC
CCCTGTCTATACATTAGGAACGGGAGCAACCACAGAAAACCTCAAATTTGACCTAAATTCTTTTT
CAGGGGAAATACATCGCATCGAAAGGGGCGGAGAAGTAACATATCACTATCCCGGTCAAATAGTT
GCTTATCCTATTTTAAATTTACGTTCCTATCAACAGGATTTACATTGGTATTTAAGGCAATTAGA
AGAAGTTATTATTTGCTTATTGGCAAAGTATGATGTAAAAGCAGAAAGAATAAAAGGTTTAACG
GGTGTTTGGGTTAATAATACAAAAATAGCCGCGATCGGAATTAAAGTCAAACGTTGGGTTACGAT
GCACGGTTTCGCATTAAATGTTAACGGTGATTTATCGGGATTTAATCAGATTATTCCCTGTGGTA
TTAAGGATAAAGGAGTAACCAAATTAAGTAATTTTTTACCCAGTATTTCCGTTGAAGATGTTAA
ACCAGAATTAGTTAAAACTTTTATCAGTATTTTTGAATGTTCGTAACAAATCTTAACTCAAGTTT
GTGAGGGGTGCAAGTTGAGAAACTGTCACTATAGCGGTTTAAAAGTTTGATAAATGCCTTTAAGC
TAGAAATATAAGAAGTTTAGTATTTAATTACATAAAGTTCCATGGTGACAAAAACAGAATCCCCC
ATAACCAAAAATGAAAATCAGCACATTATTAAGTCTGAAATTTCCCCTTGGTTAATTAGGTTAGT
TTATCCTTTAGCTAACAGAATAATTTTTCCTTTCTTTTTCAAAAATATCCATATTGAAGGCAAAG
AGAATGTCCCAAAAAGTGGGGCGGTAATCATTGCACCAACTCATCGCTCCCGGTGGGATGCTTTA
ATTGTGCCTTATGCCACTGGTAGAATGGTAACAAATAGAGATCCTTTTTTTATGGTATCAGCAAA
CGAAATGAAAGGGTTTCAAGGTTGGTTTATCCGTCGCTTGGGGGGGTTTCCCGTTGACACAGAGC
GCCCAGGGTTAGAAAGTTTATATCATAGTGGAAAATTATTATCTGAAGGCAAGATGGTTGTTAT
TTTCCCTGAAGGAAATATTTATCGCACTGTTAATACAGTTAATCCATTGAAAAGAGGTATTGCCA
AAATTGCCTTGGAAGTGGAGGCTAGTAATCCAGAAGTCGAAGTAAAAATTTTACCTGTTGCGATT
AAGTATAGTGAGGTGATTCCTAGTCGTGGCTGTAGTGTTAATGTGAAAATAGGTTCTTGTTTAAG
GGTTAATGAATATCTTAATGGTTCAATTAGGGAAAATAGTATTCGTTTAACCGCAGATTTAAAG
ACTTCTTTACAGAATGAATTAAATTTATTATCGTGAACTACCCAACACCAAAATCAAAGATTTAT
GGTGTGGGCTTCCTACCCAAAGAAACGCTTTTTACTTCGAGAAGTAACGAGTCTTACTTTCTGGC
GATAGGCTTTACCCCTCGTTCCGAAGGTCATTACACGGTATCATAGAATTATGTTTATGTATATG
GCTTGGTTATCGCCATTATCCGCCCGTGTGCGGTTGTCCTCATCTTATCAGAATTTCCTGAATTGT

GACAAGATAGAACGTTTCTTTTCTGTAAAAATATATATAATTTCTGATGAAAGGGAAACGTTAT
GGGAATAGCCTTAAAAATATCGAAAATAATAGACAATATAAGTGAATGGTGTGGCAAGATTAGC
TATGGCTTAGTATTAGTAATGATTGCTTTGGGAGTGTGGAATGTTTTAGGTAGATATATTGGTA
AGATTTTTGGTGAAAATTTGAGTTCTAATAGCTTTATTGAATTGCAGTGGTACGTATTTGATATT
ATCTTTTTTTTAGGGGCGGCTTATGCTTTAAAGGTAGATAGCCATGTTCGGGTTGATCTTTTTTA
TAAAGATTTTTCTCCGAAATTAAAGGCTTTAGTTAATATTTTAGGGGTTGTTTTATTTGTTTTTC
CTTTTTGTGGGGTAGTAATTTATTTTTCTTGGCAATATGTAGCTAATTCTTGGCAGATATGGGAA
ATTTCTGCTGATGAGGGGGGTTTACCTCGTTATCCCATCAAAACAATGATTCTGGTTAGTCCTTT
TTTGCTTATCCTACAAGGAATTTCCGAAATTATTAAAAATTTGGATATTTTTCTCAATAATTCTT
CGGTTAAAGTCAATGAATCATGACATGAGTTTATTTTTATAAACTTTATACTTAATATTTTTTAG
CAAAAATTTGTCATAGTTAAGATTCTATTTGGGTAGTCTTGAAATATAGCGGCAGAAAGTCGGA
GCTAAGATGATGAAAGACTACCATCGTTCGCTTTTAAAAAAAGTAATCTCAAATTTACCGACAAA
TTATCACTATTTTGGGCAAAAAGGCTTTAATTTGCTTGAATTGTTGATTGTAACTTTTATTCTCG
GCATTTTAGCTGCGATCGCAGTTCCTAATTTATTGAAACAAGTAGATAAAGCCCGTTATACTCAA
GCGATAATGGATATGGACTGTATAGCAGACGATGTCAGGACGTATCACTTAGAAAACGGAGTTTT
TCCGAAAGACGTAGGCAGTAACACGAAACCATCTGGGGTTAATTGTTTTCCCCGTCGGAATATCG
ATAAAATTCCCTTTAATTCTCGCTACGATTATGAAAGTTGGAATGGTGGTTCGCAAAAGTGTTAT
ATTCAAATAACTTTTTTTGGTAAAAACGGAGTGCGCAACAGTCCTACAAATCGGGTTGTTTTTTC
TGAACCCGGTCTATATTATGACTATGATGATGACTTAATTTTAACTTTGGGTATATTTGATACCC
CCTGCCAGTAAAATCATTAGACTTTTTACTAAGTCTGGCAATAGGTAAAATCCACTTTATCCTCT
ACAGAAATGATTTATGAGCAATTATACAAGCATTAAGCACTGAAACTGTATTAAAATTCTGACTC
CTGATTTCTACCCAACCGCCATATCTTGATATATTTTATTTTTTACTTTGCAAAAGGTCTATTAA
ATTGTATTCAATTCTCGCTCTAATATTGTCTTAAATTCATGTTAACTATTCTTTCCGTATTGCTT
TTTCTGCTTAGTTTTATTCACTTCTGTTTAGCAGTAACTTTGATGGGTGAATGTCTGGCTTCTGC
TTTAAACATAAAAAAAGACGAGAATTTTAACGATAAATGGTTACATACATCTGTTAGCGTTTTA
ATTCCTGCTCATAATGAAGAAAAAGTGATTTCTCAAACTTTAAAAACGATTATTCCCCAATTAAA
ATCAACAGACAATATCATTGTAATCGCAGACAATTGTACAGATTGTACTGTGGCAGTGGTAGAAA
ATATGGGGGTAAAAATTATTGAGCGTCAAAATGATTTATTAAAAGGCAAGGGTTATGCTTTGGA
TTTTGGGGTTAACTATTTGAGAGAAAATCCCCCAGATGTAGTTATTTTTATAGATGCAGATTGTA
ATGTTGAACAAAATGCGATCGCATTTTTAGTAAAAAAAACATTGATGAGCAAAAAACCTGTACA
AGCATTATATCTAATGGAAAAACCAATGCAACCCTCTGGAAAAGATCAAATATCCGCTTTTGCTT
TCAAGGTAAAAAAATTTAGTTCGCCCTTTAGGTTTATATAATCTTAATCAACCCTGCTTGTTGACT
GGCACAGGAATGGCTATCAGGTGGGAAGTATTAAAAACAACTAATTTAGCCAGTGGAGAGATAG
TAGAGGATATGAAACTAGGTTTAGATTTAGCCATTGCATCTTATCCTCCTATTTTTTGTCCCTCG
GCTCATATTACAGGAAGATTACCCCAAAAAGAACAAGCGGCAACGACTCAACGAACTCGTTGGGA
ACATGGTCACTTACAAACAGCTATTAATTATGTTCCTTTACTTATCAAAGAGTCAGTCAAACAGA
GAAGGTTTGATTTATTTACTGTAGTTTAGACTAAATGTAGAGAAAAATGAGGAAGGACAAGACA
AAAAAGAAAAATAGAGAGAAAATAAAATAATCACAAATTAAAATCTCTCTACATGAACAAATAT
AACAGACTAAAAAATTTTCGTCATGATACCTATGAAATGTTAGTCAAAAGTAAAGATGCCACATT
TGAGCTAATGGACAGCATCATGACCAGAGAAAATGCTCGTAGTTTGGCAGAATTTTCTTTATCGC
CTTTTTTCCAGAGGCAATGGTGTAGCACCTATGAAGCGATAGAGGATAGTCGTCCCAATGGCAAC
AAATTGATGAAACGATACACTCAAGAAATAGACACTTTAGAATATACGTTATTAGGCATAGATC
ATACCCAGTGGGAATGTAAAGATAGTCCCACGATGAAAGATAGAGGTTATCAATATTCTCATTCA

TCCCTAAATTCATCAGTAGTAGGACAAGGATATAGCACCATAGCATGGTTACCACCATTAAAAGA
GAAAGGAAGTTGGGCATTACCTTTAAGACATGAAAGAATAACATCCTTTGAAACTCCTTTAAGTA
AAGCCCGATGGCAACTCAAACAAGTGTGTCGAGAATTGCCAGAAAAGAGACGGAAATTAGTGGTC
TTAGATTGTGAATATGGCAATGGCAAATTTATCCAACAGACGGCGGACATTGAGATAAGTAAGT
TGATTCGAGTACGTTCTAATTTATGCCTTTATAGCAAGCCAGACCCTTATAGCGGTAGAGGAAGA
CCGAAAAAACATGGAGCAAAGTGGAAGTTAAATCAATGTGATGATTTTCCTCAGAGTAATAAAG
TATTGGAAATGGAAGATTCATCCCTAGGACTCATACGCATCAGTAAATGGACGGAACTTCATTTC
TATAATGCCCCCACACAACAATTAACTTTATTCAAAATTGAAAGATTAAAGGAGAAAAAAACAG
GGAGTTTACATCGTCCACTTTGGTTAATCTGGGTAGGAGAGGAATTTTTATCTTTGGAGTATATT
TGGAGTCAATATAAGCGAAGGTTTGGATTAGAACACTGGTATCGTTTTGCCAAGCAGAGGTTGCA
TTGGACTTTGCCAAATTTTCGCACTCCTGACAGGTGTCAAAGATGGAGTAATTTAATCGTCAATA
TCACTTGGCAATTATGGCTCTCAAAGGATTTAGTCCAAGAACACCATTTGCCGTGGCAAAAACCA
CAACAGAATTTAACTCCTGGAAGGGTTGCTCTATCTATGACTTCATTATTGATAGAGATTGGAAG
TCCTACCCTTTCTCCTAAAAGTCGGGGAAAATCCCATGGCTGGCCTCAAGGGCGAAAAAGAAACA
AAGCTAAAGTTTATCCCACCATTAAAAAACAGCAATCCAAAAGTAAAAAAAGGCGAAAAACCTA
ACTTTTAAGGAATAGTTAACTCAATTTATTTTCTCCGTAAGCTAATTTTTGTTAGCTTATTGATT
GTCTTGCAATTTTTTCTTAGTCTAAACTACAGTTATTTATTCTTGCCCTTGATTTGGCTATTCCCC
CCCTCTCTTTATTTGTTTCTTTTAGTATCGCTTTAACTTTAATTTCTTTTTTATCTCTATACTATG
GAATATTCTTTCCTTTTTTAGTAAATGCGATCGCATCTTTACTGATATTTTTCGGGATATTTATT
GCGTGGGTGAAATTTGCACGGGAAGAATTAACCTTAACAACACTAATGAAAATTCCTTTATATAT
ACTTTGGAAAATACCTCTTTATTTTAAGTTTTTATTTAAGCGAGAAAATCAATGGATTCGCACGG
AAAGAGATGACATTTAGTTATCAGTAATGAGTAATTAACCTCAGTTCGGTTTAAGAATATTGGA
TAAGGGTAGGTGTCAGGTTTCAGGTTGCAGGTGTTGAATATATTTTTAACGTTAGTTCGGGTTAA
GGCAATTTTATCGTTATTTCTAAGAAGCTATAAGGTTTTCTGACTACGAGTTGGGATGCTTTCAG
CTTATCCAAAACTCAGGTTAATTATGAACTATTTACCTTTGCCCTTTGCCCCTTGCCCCTTGCCCT
TTCACTATAAAAACAATAATCCTGTAAGAGTTAAACCAGAAACAATTAAACTTAACCAAATAAA
ACGATTATCTTTAGTATTTATTTTGCTGAAATCTTCTAAAACAATCTGAGGCGGAGGTTTAATTT
TTTTTCCGTTACTAGGAGGATTATATTTAATTAAATTGTTAGTATCAGCGTTTAAGTCTGGAATT
TTTGTCATCCATTCTTCTGGTCTTTTTAATTGAGGTGCTTTCAGGATATACAATAATCTTAGAGC
CTGTTTCTTTGTTTCTAAGTGAGGATGTGCTGTTAATTCCTCACAAAGTGCGATCGCTTTTTCTG
TTTCTCGTCCTGCTTCATAGGCATTTACTAACCAAATTCTTGCTTCTCCTCCCACACGAGTATAAA
AAGAAGCTAATTGACAGGCTTTCTCCAAATTTTCAATACTGAGACGATAGCACCCTTTTTCAAAA
GCTAATTTTCCCGTTTGATATAAATTGTTAAATTCTGACTCAGAAATCGGCTTCACGATCGAATT
TTTGACTATTTAATAATTTCTTTACTATTCATAATATCTCAAAAGACTTCTATCTTTTTAAGTAA
ACTACCTCCTCTAAGAATAAACACTTATTGACTATATTCCTTTTTAGTTATAAAATGGCATTTAA
AGTTACTCAAAATATTTGCAATCATTCTACAAAACATAGTGTATTTCCTTGTATTAAGCGTATTG
TGTCCTGTTAGATAATGTAGGAAAGATTGTGAGTTGATAGGTGATAAATACATAACTCATTAGA
CAACAAGATAAAGTTGTAGGAGTTCTAAATTATGGCAAACATAGCTCAAAATAGTTCCAGTTTA
AACTATGATTCTCTTTGGGATCGTTTCTGTCGATGGATTACTAGCACTAATAACCGTATCTATCT
AGGATGGTTTAGTGTGTTAATGATTCCCACTGCCTTCGTCGCTGCGATCGCATTTATTATTGCCT
TTGTTGCCGCCCCCGGAGTGGATGTAGATGGTATTCGTGAACCTGTCATGGGTTCGCTTTTAAGC
GGTAATAATATTGCAACCGCCGCCGTTGTGCCTACTTCTGCCGCCATTGGGCTACATTTTTATCCT
TTATGGGAAGCAGGTTCGATCGATGAATGGCTTTATAATGGTGGTACTTACCAGATGATTATATT

TCACTTCTTAATCGCTGTCTGGGCTTATTTAGGCAGACTTTGGGAATTAAGTGAGCGTCTTGGTA
TGCGCCCTTGGATTGCTATCGCATTTTCTGCCCCTGCGGCCGCCGTAACTTCCGTATTGTTAGTTT
ATCCCATTGGGCAAGGTAGTTTTTCTCAAGGGATGCCTTTAGGAATCGTGGGGACATTTAATTTC
ATGATTGTGCTACAAGCAGAGCATAATATTTTAATGCACCCATTCCATATGTTAGGAGTCGCAGG
AGTTTTAGGTGGTGCGTTATTGAGCGTCATGCACGGTTCATTAGTAACCTCTAGTTTAATTCGGG
AAACCACAGAAGATATATCTCAGAATGAAGGTTATCAATTTGGTCAACAGGAAATGACTTATAA
TCTAATAGCTGGTCATGTGGGTTATTTAGGACGATTATTAATTCCCTCTCTTGCTTTTAATAATA
GTCGTTCTGTTCATTTCCTCTTAGCGATTTTTCCCACTATTGGCATTTGGTTTGCGAGTTTAGGTA
TCAGTAGCGTTGCCTTTAACCTTAATGGATTCAACTTTAACCATTCTATTTTAGATAGTAACAAT
CATGTGATTCCTACCGATGCAGATATGATTAACCGTGCCAATTTAGGCTTTGAAGCTATGAATGC
ACCGAATACTCATAACTTCCCAAATTTGATGTAGCTACATCTTAACTTAAATTACTACTCAATTT
TTATCAGGCTAGGATGATTTATTCTTAGTCTGATTTTTTTTAGCATTTATCTAAATACTGAGGTA
TATCACTTATTTGTATTCGGAGTTAGGAGTTAAAATTAACAGATTTTGACAAAAACAGTAAATA
AATTGATTTTCTTATTTTCCATTTTTGACTAATGATTCAACATTCAAAAAATCAAACTGTCAAAT
CTGCTCTTATCCGATATTCTTAAACTTAACTGGGGTTATCTTTTAATTTGCCCATTATTGATAAC
AAATATACTAAAATGTCTCAAGAAAAACTAATCCAAAGAATGCAACAGGCTAATTTTTATCATCA
CCCCGTAAAAGAGCCTATAGAAATAATACAAACACACTGCTCGATCGTATTTTTAACGGGAAACT
ATGCCTATAAAATGAAGAAAATAGTTGATTTTGGTTTTCTTGACTATTCTACTTTGGCAAAACGC
CAACATTTCCTAAGTGTTGAGTTAGAAATGAATAAGCAGATTGCCCCTGATTTATATTTAGAAGT
CTTACCCATCTACGAAGAAAATAACAATTTTTATTGGCAAGAGCAAGGGCAAATAAAAGAATAC
GTCTTAAAAATGAATCAGTTTCCCCAAGAAAATCTATTACTAAATATCTATCAACAGGGAAATTT
AACCTCTCAACACATGAAGGAATTAGGGAAATTAGTGGCAAAATTCCATCAAAACACCGTTACCA
ATGAGTATATAACCAGTTTTGGTACTCCTGAAAAAATAGGCAAATCCATCAGTCAAAATTATGAG
CAAACGCAAAAATATATCGGTATTGCCCAAAATCAAGAACAATATGAACAAACTAAAGCATTTAC
AGATAGTTTTCTCCATAATTATCGAGATGTTTTAGAAAAGCGTCAAACTGACCATAAAATCAAAG
AATGTCACGGGGATTTACACCTTAAAAATATTTGCATATGGCAGGATAAAATTCAACTTTTCGAT
CGCATCGAATTTAATGAACCTTTCCGCTTTGTGGATGTTATGTATGATATTGCCTTTACGGTGAT
GGATTTAGACTCTAAAGGAGAAAAAACCCTTGCTAACGTCTTTTTAAATACTTATTTAGAAGAAA
CGGGGGATTGGGAAGGAGTGCAGGTTTTACCGTTATATTTGAGTCGTCAGGCTTATGTGAGGGCA
AAAGTTACCTCTTTATTGTTAGATGATAGAGCCATATCGGAAGAAGAAAAAGTAAAAGCTAAAG
AAACCGCCGCCGCCTATTATCATTTAGCTTGGCAATATACCCAACCATCTCAAGGGAAAATTATA
ATGATGTCGGGGTTATCAGGCTCAGGAAAGACAACCATTGCCACCAAAAAAGCACGGGAGTTAAA
TGCTATCCATATCCGCACTGATGCAGTGAGAAAACATCTTGCAAATATTCCCTTACAAGAAAAAG
GCGAGAAAGAAATTTATTCTCAGGCAATGACGGCAAAAACCTATCAAAAACTCCTAGATTTAGCT
AAAATTCTTACCATTCAAGGGTTTACGGTGATTCTTGATGGAAAATTCGATCGCATCTGTTGGCG
ACAACCCGTACTGGAATTTGTCTTAAACCATAACATCGAATTTGAAATTATCTATTGCCATGCAC
CATTAGATGTTTTACAAAATCGCCTTCGCCAGAGAAATAGCGATATATCTGATGCCACACCAGAA
TTATTAAAACAGCAACAGAAGCAACAAGAAGCCTTTACGGAGAAAGAAAAACTTTTCTTAAAAC
AAGGTTAAAGACGAAAAAATCAATTTATCTCATTTTTTCCTTATTTTAACTCTGAAATCTGAACT
CAGAACTCCGAACTTTGATCCGTATTTAACTGAGAAAAAGTAACAAATCTTTACACAAAAGCAAA
AAAATGTAAAAAATTCGATAAAAACTATAATTTTTCGACATTTCAGCGTTGAATCATGTAATATC
AGATCTAGTGTCAAAACACGAAAACACTTAGTGAGTGGTAATTTGAGCAAATCTTTACCATAAAC
TTTGTTTTATGACAATCATTTTCAATCAATTAGGAGATTATTTTATGAACAAAGGTGAATTAGTT

GATGCAGTAGCGGCAAAGGCAGAAGTAACCAAAAAACAAGCTGATTCCGTAATCAGTGCTACTAT
CGAGGCAATTATGGAAGCAGTATCCTCTGGCGACAAAGTAACTTTAGTTGGTTTTGGTTCTTTTG
AAAGCCGTCACAGAAAAGCAAGAGAAGGGCGTAATCCGAAAACTGGGGAAAAAATGGAAATTCC
CGCTACCAATGTTCCTGCGTTTTCTGCGGGTAAACTATTTAAAGAGAGTGTAGCAAAAAATAACA
AGTAATTTGGCGAAATTCATGCGTAGCAAAGATCGACCTTTTTCGGTTGGTTTTTGCCTTGTATT
TTTGTCAAAGAATAGTGGCTTTAAAGCCCTGTTTTTTTAGAAAAGTAATTTTTGGTGTATAGTCA
ACCTTTGCCCTGTACATCCTTTTACTTTGCCCCTTTTATCTTATCTTTGGGTGATTGATTTGAAGC
GACCAATTCACGGAGGAAATCTTGATTGGGCTATGAGCCTGATGAATCATGAAACTGCTACTCAG
GTAGTGGATTTTTCTGCTAGTATCAATCCTTTAGGTCCTCCCCAAAGTGCGATCGCATCTTTAAA
AGCTGGTATAGTTGAGTTAAATCACTATCCGAATCCTGACTATCCTCAATTTCGCCTTGGGGTGG
CAAATCATCACCATCTTGACGAGAAATATATCTTACCGAGTAATGGGGCGGCGGAGTTACTCACT
TGGATAGCATGGGAATGTCACGAGTTAGAAGGAGTTTTACTTCCTTCTCCTTGCTTTGCAGACTA
TAAAAGGGCATTAAAGACATTTGGGGTGAAATATCAATTTTATCCCTTGGATGATTTAGAAAAG
GGTATTAATTCCTCTCAATCATCCCGTCTGGCAATTTTGATTAATAATCCCCATAATCCCACAGG
AAAACTTTGGACTCGTCTTAGTTTACAGCCCTATTTAGAAAAATTTGCTTTAGTCATCGTTGATG
AAGCGTTTATGGACTTTCTGCCCCCTCAAAACCAAGAAAGTTTGATCGATTTTATCAAAGATTAT
GATAATTTAGTAATATTGCGATCGCTCACTAAATTTTATTCACTCCCCGGCTTAAGAATCGGTTA
CGGTATTAGTAACCCCCAAACAATTGCTAAATGGCAAAAATGGCGCGATCCTTGGAGTGTTAATA
GTCTTGCGGAAATAGCCGCCGTCGCCTGTTTACAGGATAAAGCATTTCAAGAAAAAACATGGCAA
TGGCTACCCCCAACCCGTGAATCGTTAAAGCAAGGTTTAGATAACATTGAATTCTTACAAGTTTT
ACCCAGTCAAAGTAACTTTCTTTTAGTTAAAACCCAAATCCCAAGCACTCAGTTACAGCTAAGAC
TGCTAAGACAAAAACAAATCCTCATCCGTGATTGTGTCAGTTTTCCTGAGTTGGGAGAAAAATAT
TTCCGTGTCGCTGTTAAAACCCATATTGATAACCAAAAACTCCTAGATGAACTAAATAAACTATC
TGAAGCCATTGTCGAAAACCAAGAATGGTTATCTTGAAGTGTGAGTGAAAGACCCATTAGAAGA
TGGTAGAGAAGCGGGAAGAGTTTTTATAATCGATATGAACTGCTTAATGATTGTTTTGGGGAAA
ATTTTGTCGATCAATAGTCTCAACTCCTCATTTTCCAAAAGGTTTTTCATCGAACTCAGGTGATT
TTGGTTGACATCGGTTATAAATCCTTCTGAAGATAATCCACTAAAGCCCTTAAACCCAAACGATA
GCTATTCGCACCAAAGCCACTTATTTGACCAATTGCGACAGGGGCAATATAGGAGTGATGACGAA
ACTCCTCTCGACGATAAATATTACTTAAATGAACTTCAACGGCAGGAATTGCTACACCCGCTAAA
GCATCTCGAATAGCAACACTGGTGTGAGTAAATGCCCCTGCATTAATCAAAATTCCCTGATATTT
ATTTAAAGCCTGATGTATTTGGTCAACTAAAACACCCTCATGATTAGACTGCACACAAGTAACAC
TTACCTGCATTTTTTCACCGTCTAATCTCAAAATATCGTTTATATCTTCCAGAGTTTGATGTCCA
TATATGCTAGGCTCTCTAAGACCCAGTAAGTTAAGATTCGGACCATGCAATACTAAAATATTATG
ATTGGACAATTTATCTAACTATCTAAACAATATTAGTGAACTATTTAAGTCTTTATAAGAATTAT
TATTAATATCGTCTAGGATAATCTTTAACTGGAATGGGGATTAACTCCGCCTCTGGTTGACTTTC
TGGACCGAACAAGGCTTCTATAACTTTTTGAGTCCATTCTTTGAGTTTTTCTAAGATTTTATCTA
CTTGCTCCATTGATCAAGAAGACTCCCATTTTTATTTGTTCAATATAACCATCATAGCCCTTCATT
TCGTGAAATGCAACTTTTCTTAAACTTTGATTCGAGCAAAACTATTTCTCATCATTTAATTTTCT
TTTCGTTATTATTAAAAAAAATTTTTATCCACTTACCCTCAACATTAATAACTGAAATGACTGCA
ACTGCTCATCCAAAAAACAACACAAATCAATACATTCCCAAAAATAAAAAAAAAGTTTTATGTAT
TTTTCCTCAATATAGTCGTTCATTTGGCACTTTTCATCATGCGTATCCTCTGATGCCTAGGGTAA
AAGGCTTTATGCCTCCTCAAGGAATACTTGTGGTTGCTTCTTATTTACCGCAAACATGGAAGTA
AGATTAATTGATGAAAATATTAAATCAGTAACCAAGACAGATTATCAATGGGCAGATGCCATCA

TAGTTAGTGGAATGCACATTCAAAAACCTCGAATCAAAGCAATTAACGAACTAGCCCATAAATTT
GATAAAATCACCATTCTAGGTGGCCCTTCTGTCTCTGGATGTCCAGAATATTATCCTGATTTTGA
TATTCTTCATTTAGGAGAATTAGGAGATGCAACAGATAAAATTATTGAATACATTGATAATCAT
CAAAAAAGACCTGAAAAACAGATTATTTTTGAAACTAAACATCGATTACCTTTAGATCAATTCCC
GATTCCTGCTTATAATTTAATTGATATATCACAGTATTTTATTGCTAACATACAATTTTCCAGTG
GTTGCCCTTATAACTGTGAATTTTGCGATATTCCAGAATTGTACGGAAATTCCCCTCGTTTAAAA
ACCCCTCAACAAATAATAACGGAATTAGATGCTATTATCGCCTCTGGAAATCCGGGGGCGATTTA
TTTTGTAGATGATAATTTTGTGGGTAATCGTCGTGCCTTAATGGAATTATTACCCCATTTAATCA
AATGGCAAAAAGAAAATGGCTACCCTATAGAGTTTACCTGTGAAGCCACCCTCAATATTGCACAA
AGCCCGAAATTATTAGAAATGATGAGGGAAGCGTATTTCACTACGGTTTTTTGTGGCATAGAAAC
TCCTGAAGTATCAGCATTAAAAGCTATTTCTAAACAACATAACTTAAGTATGCCTATATTAGATG
CTGTAAAAACCCTCAATAGCTATGGTTTAGAGGTTGTTTCTGGTATTATTATCGGTTTCGACACA
GATACACCACAAACGGCGGACAATATTCTTGAGTTTATTAATGCTTCTCAAATTCCTATTTTGAC
AATTAATCTACTTTATGCTCTGCCAAAAACTCCCTTATGGAATCGTTTAAAAGCTGAAGGGCGAA
TTATTGAAGATGAATCAAGAGAATCTAATATCGATTTTTTGATGCCCTATGAGGAGGTGTTGAAT
ATGTGGAAACGCTGTATTTCAACTGCTTATGAGCCAAAATATTTATATCAAAGATTTGCTTATAA
TGCTGAAAATACTTATCCTAATCGTATCGAAGTTCCAAATAGTCCAGAAAGGGTTTCTTGGGCAA
ATATTAAGAAAGGATTGCATATAATGGGCAATTTGGTGTGGCAGGTAGGTTTAAAAAGTAGTTA
TCGTCAAATTTTTTGGGATATGGCTATTCCTGCACTAAAAAAATTAGATATAGAGACTATCATTC
ATATTGGCTTAGTTAGTCATCATCTAATTGAATTTACTAAAGAATGTACCCAAGGTAAAGAATCA
GCTTCTTTTTACTCTCAAAAGAGAAAAGAATTAGTTGCTAGTGCTAATCAATAGTTTTAATTTAT
TTTTAGTTTTTTGGGCTAAAATTGAGAAATGAGGAATTCACGATAAATCAAATATTTAATTGAT
ATTTTGTGTTTGAGTTAATCAAAAATTATCAATTTTTTTAAGTTGGTAATGGATATAATATTTTT
TGTATAATGAAGGAAGATAATTTAAGAAGCAGAAAAAGAAAACCTCGCAATCTATCAGAAAAAC
TGGATGCTCAGGTAGAAAAAAATTGTCTATAAAGTAATGACAAATGAGGGTTTAGAAGAAACAGT
AAATACTGCCATTCAAAAAGCATTAATTAGTCTTATTATTCGTTATTCGGCATTAATTATTATAG
GGTTATTATTGGTACTGGCTTTGCAAAATCTAATTTTAGTCATAATTTTAAAAAGTTTTTGGGAA
TAAATTCGTTCTTTGCAATTTATTTTTCAAATTAAAATATAGTTTGTAATTCATAATTTAGTTTT
GTATTTATTTGTTATTTTTAATTAGTAATTGATTTGTCAAACATAATTTAGTATTATTTTATTAT
AATTATAAGAAAATTTTTATATTTTTTATCATCATAATATGAGTATTTTTTCACAAGAAAAATT
ATTATTTACTCCCACGTCTAAAGATATTAATGCTATTCCTTTAATTTTTGCTTTTCCTAATGAAT
ATACTGTCGGAATAACGAGCTTAGGTTATCAATTAGTATGGGCAAATTTTTGCTCTCGCCCTGAT
GTGGATGTTAGTCGTTTATTTACAGATATTCATGAGAGTTTACCCCGCAATCCTGAATTGGTTGG
TTTTTCTTTTTCTTGGGAATTGGATTATGTTAATATCCTTAATTTGTTAGAGTTTTTAGGTATTC
CTATTCATGCTAAAGATAGAAATGAAAATCATCCATTAATTTTTGGAGGTGGCCCTGTTTTTACT
GCTAATCCTGAACCTTTTGCAGATTTTTTTGATGTTATTCTTTTAGGTGATGGAGAAGTTTTATT
GGATAGTTTTATTAATTCTTATCAGGAAATAAGACAAGATAGTCGTTTTTCTAAGTTACATTATT
TGGCAAAAAATAGAAGGTATTTATATCCCTAATTTGTTTGAGGTTAATTATCAACAGGAGGATGG
AAAAATTGATAGTATTAAACCTCTTAATTCTGATATTCCTTCTTTTGTGGAAAAGCAAACTTATC
GGGGAAACATTTTGTCTGCTTCTACGGTGGTGACGGAAAAAGCCGCTTGGGAAAATATTTATATG
GTGGAGGTGGTGAGAAGTTGTCCAGAAATGTGTCGTTTTTGTTTAGCGAGTTATCTAACTTTGCC
CTTTCGCACGGCTAGTTTGGAAAAGTCTTTAATTCCTGCCATTGAAAGGGGTTTAAAAGTAACTA
ATCGTCTAGGTTTATTGGGGGCTTCTGTGACGCAACATCCTGAATTTTCAGGGTTGATTGATTAT

TTAGGAAAATCGGAATTTGATGATGTTAGGGTGAGTATTGCATCGGTAAGAACTAATACTGTAA
CGGAAAAATTGGCAAATTTTCTGAAGAAGCGAGATACAAAATCCATCACGATTGCTATTGAAAGT
GGAAGCTCTAAAATTCGGGAGATTATTAATAAAAAGCTAGAAAATGAGGAAATTATTGAGGCGG
CAATCAATGCTCAGGCAGGAGGATTAAGGGCGATTAAATTTTATGGAATGGTGGGTTTACCCCAT
GAGGATTACGGTGACTTAGATGCAACTATTGAGATGATGAAGGCGGTTAAAAAAGCCGCTCCTAG
ATTAAAAATAACCTTGGGATGCAGTACTTTTGTTCCTAAGTCTCATACTCCTTTTCAGTGGTTTG
GAGTCAATCAGGATAGTAAAAAGCGATTACAATATCTACAGAAAAATTTGGGCAAAATTGGGGT
TGATTTTCGTCCTGAAAGTTATAATTGGTCTATTATTCAAGGTTTAATTTCCAGAGGCGATCGCC
GCTTGAGTAAATTGTTAGAATTAACTCGTAATTATGGGGATACTCTCGGTAGTTATAAACGGGCA
TTTAAGGAGTTAAAGGGGCAAATTCCGCCGTTAGATTATTATGTTCACCAAAATTGGCAAGTAGG
GCAGGTTTTACCTTGGCAACACTTAAAAACGGCGTTGACGGAAGAGACTTTAGTAAAACATCTTG
AATCAACGGGCATGAGATAAGTTCGAAGTGTTGAGGGATGAGGGGAGGGAGTGATGGGGTGAGC
GGGAGAAATTAACGTCAACATCTTAATTCAATTTATTGAACGAACTACTATTAGCTGTGTATCAT
TCATTACACGGTGAAATAACTTTTAATCAATCCCTAGGTAAAGTAAATCTAATTCTTTTTGCATG
GTATTGACGACTAAATCATAACATTCCTGAACATAAAGCCGAGCGCAACTTGCTTCTTCTCCGTA
GCGAGGGAAATAGATGGGAGGGCAAACCCTCGTGGTAATCTGTAAGGGTAAGGGAATATTGGGT
AGGGGGCCAACAGCTAATCCCCAAGGCAATCCGAAGTATATAGGAAACACTTGAGGATCAATATC
AAACATCCATGGAAATAAACCCTGTTGATTTAATTTTTTGATTTCTTTGTATATGTTGGCAACCA
CAAAAATAGTTTCATGAGCTCCTTTAGCAATAAGAGGTACAATGGGAACTTGATATTTTAAGGCT
AATTTAATAAATCCTTTTCTACCAACAAATTTTATTTTCTGTCTTTCTGTATAGGGACGAAATAC
ATCATCTGCACCCCCCGGATATACTAAAACACTGGCTTTTTTCTCCAAAGCTGCGATCGCCATTTT
GGGATGAGCTCGAATAGCCCCTGTTTGGGCGGCTAAACGAGTAAGACTGCCATAGGCACTCCACA
CGTTACGGTGCATTAAGCCATAAATGGGGCGTTGAGTGCCAAAACGACGAAACCAATCATACATC
ATCATTGTCATGTCAGGAGCGGCTAAACCACCATTATGAGAACCAACAAATAACACTGGCCCTTC
TGGAATATTTTCCCAACCACTGGTTTTTACCTGAAAATAATATTTATACAAAAAACCCCATAAAG
GCATTAATTGTTCAATGACTTCGGGGTTTCTCGCTGTTAATGACCAGCCTGAATATTGATTTGAC
ATATACAATGAATAATTACTGAATAGCAATCTCCCATTTTAATTCTTAATTTCACCCAATGACTA
ATTAGGATTTACTGAGTCAAATCATAGCTTTAATAAAAAATCTTGGTAAGGCTAACATTCTACGC
CAACGTGATGGTTCTTGATAAAGGCGGTATAACCATTCTAAATGATTATTGCAGAAAAATTCTGG
GGCTCTTTCTTTGACTCCTCCCCAAATGTCGAAACTTCCTCCCACTCCAATCCATACAGCATTCGG
GGCTAAGTGACGATGATTCATGATCCATAATTCTTGTCTTGGTACGCCTAAACCAACTAAAATTA
ATTTAGGTTGCACTTTTTTGATGGTTTCGCACCAATCATCTAATTCTTGTCCAGATAAGTAACCG
TGATTGTAAATGATATGGGCAGAAGTTACTTTTTCTTTCCATGCTTGGGCGGCTTTTTCACTTAC
TCCTTTTGCACCTCCATAGAAACAGATAGGGTAGTCTTTCCCCTTTTTGCCAATTGCTTCAATGAG
GGAAGCGGCTAATTCAATCCCTGCAATTCTTTGTTGTTTTTGTCCTCTACGTTTTAAATAAAATA
TCACTCCTGAACCATCAGGCACAACTAAATCCGCTTTATTAATGGCAGTGGCTAAATCTGTGTTT
TTTTGTGCCATCATCGCCATTTCTGAGTTCATGGTAACAACGTGAGTGGGAGTATTATTTTCGAT
ACGACTAATTAATAAATCTTGATAGTTTCACTCAAATGTATAGGTAAACCTAAAACAGGAAAA
GTCTTCATAAAATAGCAATTTTAAATATTTAGGAGAAATAATAATTTAAGCTAATAGGATTTTG
AGTCACTATTCTATCATTAGAATGGAAAAACTGTTTTTTATATACTCTTTGCAAAACTAAAATTA
ATATCAATTTCCATGGAGAAATCGTCAATTTTTTTACTGTTACCTGTTGCCCACCTTAACCAATA
ACTTACATACTCAAAATAATAGAGCCATATTAATCACTAATACTGAGTTCCTGCTCTAATACATT
AATAATTAATAAACTAAGCCCAGAAAATCAATTTAGGGACTGTTTTTATTAAATATTATAATAT

TGTCTATTGAAAATTGCCTACTCTCACAAACTAATTTTAAATGAGATGCAAGATTTAAGTGATAA
CTGAATCTATTAATTGCTGATGTTTAAGGGCATCCTCTGCTAATTTTTGTATTTGATGAGGGGAA
ATCGGATTAGGATTGTTATAGGTGTTTAAACATTCTTGGACAATTTCGTCGATATTAGTTGGTAA
TTTTTCTAAATCGTATCCTTTGATATTTATTTCCCTCATTAAATTGCTTACTTTGGGATCATAAC
TAAGAGCAAAACATTTACAATTTTCACTTCCAGCCATGATTAAGCTATGAAGACGCATTCCTATG
AGCATATCTATCTGAGAAAATAATCCTTTTAATTCTTGGGCATTTTCTAAGGTTAAAATCTGATA
GTTAGTTTTTAATTGCTGGGCTATTTTTTCACAAACTTCTAAATCTTTTGATATTTGAAAAGGAA
TTAAAATTATATTTGCATTAATTTTTTCTTTTATTTTAATTAAAACTTGGGATATAGTTTCTATT
TTGGCAATAGTTAAGCTAGAATGAGAACGCAAATTAACAGCAATTCTAGGAGTAGGTAAAAGTT
CTATTTTTTTTGTTATTTTACTAGGCAAAGCCCACACTGGATCAGGTGCAATTAAGCATTTTATT
CCCCATTTATCCAACAGTTTAGCTGATTTTTGATCTCTGACGCTGATGCGATCGCATCTTTTCAGT
ACAATCTTAGTTAACCATTGAATGAGGGGAGTTTTTATCGGTCCAATTCCTTGAGCATAGGCAAT
AGTTTTAACTCCTTTCAATTGTGCTAATGCCATTAAGGTTAAATAATAAATAGCACTTTTAACAC
TGGTAATATCTTGGATTAAACTTCCTCCTCCCCAGATAAATAAATCTTTTTTTGTTAGTTTTAAT
ATTTCTTTGATAATTTGAAAACGAGAATAGCTAATTACTCCATAATCTTGATTTGTTTTTTGCGG
ATTACCAGACAAAATAATGGGAGTAATATTATTATTTAAACGGGATAAAATAGCCAGCAATAGG
GCTTCATCTCCTGCATTTCCCTGACCATAATATCCACAAATAATTGCTTTTCTCATTGACTATAAG
TTATTTTTTATTAGTAATTAAAATGCGATCGCCTACAATCTTACCATTATAAATTAAATCGTGAT
GATTATTGGTATTAAGAGTAACTTCAATTTCATTGTCCTTTTCAGGAAGTTCAGATATATAGTAC
CAATTACTGTAAATTCTACTTACTTTTACTCCGTTAATATACAAATGTGCATGACCTTGATTGGG
GTTATTATTTTCTAAATTAGGAGAGATAAATTCAAAATTTTTAGTATTTAAGTATAGATTCCATC
CTTTAATTTTATCTGGAAAAATCTCTATTTCTATTGATGGATTTTCATTAAGATTAGATACATCA
AGAGTTTTATGAGCGTGTCCATTTTCGGAAGACATATTCATTCCGTGTTGATGGGAAGAGTCGGC
TTTAACTTGAACTAAAAAGCTATTTAATAATGTATTTAATCCAACAAATACACTAAGAAAAATA
ATAGAATTAAAGTTTAAAAATTTATTATGTCTAATTTTCATTGGCTATCTAAATGAATTATTAA
TGAGTTAAATAGTTGATCATGTAGAATCTTTTATGAATAGTCAAGATTTACATTTAAGGGTATT
GTTTATATATCTCAAATAAATGTTTAAATCAATAGACAAGTTGTCAAAGATAGCCATCTCAATTA
GTACAAAGGGCAATTAGTGAATAGTGAATAGTGAATAGTTGATAATTACTCGTTACTCGTTACTC
ATTACTCATTACTCATTACTTTCTTCTAAAACCTGAAACCCGACACCTTATCTCCCTCTCCCCTCA
TCACCTCATCACCTCATCCCCTAATACCCTAATACCTGAAACCTAACCCCTAACTCCTAACAATTC
CTAATTACTTAACAATTTCCGTCTCAATTGATCAAGGGCAAAACAACAGGTAAGATAACGAATAG
TCTGTCTATCCCGATTAATGCCCCATTTTAATTCATAGCTAATTGTATCGGATGATGGAAATGCG
GTCACGAAGTGGGGCTGTGCGATCGCAACATACACTAATCCGACAGGTTTGGTTTCAGTGCCACC
ATCAGGGCCTGCTATGCCCGTAATTCCAATACCCCAATCGGTTTGTAATCGGTTTTTAACTCCCTT
TGCCATCTGTTGGGCTACCGTTTCAGAAACTGCACCAAACTGCTCTATATCCGCCTCTGATACTCC
CAATTGACGAATTTTGACTTGATTGCTATAGGCAATGATACCCCCATAAAAATAAGCAGAACTTC
CCCCGCAAGCTGTAAACATAGCCCCCAAACCACCGCCAGTACAAGATTCCGCAACGCTCACTGTTT
CTCCTCTTGCAGTTAACAATTTACCCACAACTCCTTCTAAGGTATCTTCGTCTGAGCCATAGTAAT
CTTCAAGGGCGATCGCTTTTATTTCTTCTACAATAGGATTAATTATTTTTCTCGCCTCAGCTTCAT
TTTTTGCTTTGGTAGAAACCCTTAATTTTACCTCTCCATGGGAGGCGTAAGGGGCAACGGTAGGA
TTCGTTAAGGCAAAGAGATGATTAACTTTTTCTGCCAGTGCTGACTCTCCTATCCCCCGAAACCGC
ATCATTTCACTGACAATAATTTCTTTCCCCCATCCCTGACTCTGTAAAAATGGTACAGCAGTATC
TCGCCACATCTGCTTCATTTCTGAAGGCACTCCGGGAAAGGTTAAAATTGTTAATTTTTGTTGCG

GATGGTTGATATTATTTAAAAAAGGTTGCCAAATCATACCCGGGGCTGTTCCTGTTAAATTAGGT
AATATGTCCGCTCCTTTTGGTAATAAAGCCTGTTTACGGTTATTATTTGTCATTTTTCGCCCTCTT
TGGGCAAATTTTACCTCAATATCTTCAATAATCTCTGATTTTTCTTCTAAAGTCCCCTGAAAAAA
AGATGCGATCGCTTCTGTGGTAAGATCATCGGGTGTAGGGCCTAAACCCCCCGTAAAAATAAGAA
TAGAAGAACGTTGGGACGCAATGTTAATCACCTCATGAATTCTGCCTAAGTTATCTCCAACAACC
GTTTGAAAATAATGGGGAATACCTAAAATAGCTAATTCTTGGGCTAAAAATTGAGAATTGCTAT
TCAAAATGTCACCTAACAATAGTTCTGTCCCGATACAAATAATTTCTGCACTCATAATAATAAAA
AAGTAGTAATTGGTTATAAATAAATTTTATGTTTAAGAATAATTTAATGGTAAAATATAATTTT
AAACTTTAATTGCAATTATTCAACATTTTAAAAATAAAAAGTGAACCAATCTTATTCTATAAAT
ATCAATACAAAAGAAGAAAATAAGCAAAAAATAGATGTTGCAATTATTTTTTATAGAGCTCATT
TTTTGTCGGGCAACACAAAACCCATTGATTCTTTATCCTCTGCATTAAAAAAACAAAATTTAAGT
ACTATCGCTATTTATTTATCATCCCTTAGAGAAGCCGATATTCAAGCTGAATTAATTGAAATTTT
ACAAAAATATCAAGTAAATTTAATTTTAAATACTACCAGCTTTTCAGTAGCAAAAATAGGTTCAG
AATCTAATACAAAAATATGGGAAACCCTAGATATTCCAGTGCTTCAAGTAATATTAAGTGGAAG
TACAGAAGAATATTGGCAAAATAGCTTTCAAGGTTTATCCCCCAGAGATGTTGCCATGAATGTTG
CTTTACCAGAAGTGGATGGAAGAATAATCACTAGGGCAATTTCTTTTAAGTCAGTGGCTACTAAA
CACCCTCAATTAGAAACTGACATTGTTATTTATCAACCTCAAGAAAATAGAGTTAATTATATTGC
TGAGTTAGCTAAAAATTGGATAAAGCTAAAAAAGAAGAAAAACTCAGAAAAAAAGGTAGCTTTA
ATCTTAGCAAATTATCCCAATAAAGATGGAAGAATTGCGAATGGAGTGGGGTTAGATACTCCCGC
TAGTTGTATTAATATTTTACAGGCGTTAAAAGTAAATAATTATGATATTCAAAATATACCTGAA
AATGGAGATAAGCTAATTTTTCAGTTAATTGAAGGGATAACAAATGATTTAGAAATGACATCTC
AAAAGAAAATTAATCAATATTTATCTTTAGAAGAATATCAAAAATATTTTCATACTTTACCCTTA
GAAATTCAACAAAAAATTACTAATCGATGGGGAAATTACAAGCAAAATGCCTGTTTCACGGATG
AAGATGAAATTCAGGAGCAAAATTTATTTATTAACCAAAATTATATTCCTGTTTCAGGGATTCAA
TTTGGCAAAATATTTGTCGGAGTACAACCATCGAGGGGTTATGATTTAGATCCTAGTCTTAATTA
TCACGCTCCAGATTTAGAGCCAACTCACCATTATTTAGCTTTTTATTATTGGTTAAAACACATTT
TTCAAGCAGATGTAATTATTCACGTTGGTAAGCACGGAAATTTGGAATGGCTACCGGGTAAAAGT
TTAGTTTTATCTAATAATTGTTATCCCGAAATTGCTTTAGGCACACTTCCCCATTTTTATCCTTTT
ATTGTCAATGATCCGGGGGAGGGATCACAAGCAAAAAGAAGGGCAAATGCGGTAATTTTAGATC
ATTTAACTCCCCCTTTAACCCGTGCAGAATTGTATGGAGAATTATTAGAATTGGAGGCTTTAATT
GATGAATATTATCAAGCTCAAAGTCTCAATCCTAGTAGGTTAAAAATAATCTGCGATCGCATTTC
AAATTTAATCAAAGAAACTAAGCTAAATGAAGATTTAGGTATAAAAGAAGTAAATAGAGATAGT
TTAACCGAATTTTTAACGATTGCTGATGGTTATTTATGTGAGTTAAAAGAAGCCCAGATTAGAGA
TGGTTTACATATTTTTGGACAATGTCCAGAAGGAAGACTATTAAGAGATTTAATAAGTGCGATCG
CACGTTTTTCTAGTTATAATAACTTAGGATTAATAGAAGCTATCAAAGAAGACTTAAACCACAAT
CAAATTCCTCCCTTATTAAGGGGGGTTAGAGAGGATCAAAACAATAACTTATTAAGGAAAGCTAA
GGAGGATAAAAATAATGAGAGCATAGTAGAAAAAGATGAATTAGAAAACATAGCCAATGAATTA
ATAGAAAAGCTGATAAAAGGAGAAAAAACCGAGGAAATAAAACTAGGAAAACAGACAGAAAAGT
CTCTTAACTGGATCAAAAATAAACTCCTACCTAACTTAAAAAAAACAGAGCAAGAAATAGATAAC
CTTATCAGAGGCTTAGAGGGAAAATATATTCCCAGTGGCTCATCAGGCGCACCCACCAGAGGCAG
AAGCGATGTTTTACCCACAGGCAGAAACTTTTATTCCGTTGATATTCGAGCTATTCCTAGTGAAA
CCGCTTGGGATGTAGGCAGAAAAGCCGCCGAAACTCTTATCGAAAGATATACCCAAGAAAATGGA
GAATATCCCCGCACCCTAGCAATTTCCGTTTGGGGTACTTCTACCATGCGCACAGGAGGAGATGA

TATTGCTCAAATAATGGCATTAATGGGAGTTAAACCAATATGGGATGGGGTAAGCCGTAGAGTT
GTGGATTTTGAGGTAATTCCCCTCGAAGTATTAGGCAGACCTAGAATTGATGTTACTGTGAGAGT
TTCTGGGTTTTTTAGAGACGGATTCCCCAATATAATGGAATTACTTTATAAAATTAATCAATATT
TGTCAAGCCTCAAAGAAGAAAAAGAAAATAATCCCCTTGCCCAACAGGTAGAAAAAGATACTCAA
TATTGGATTAAACAAGGATTAGAGAAAAAACAGGCAAGAGAGAGGGCAACCTATCGGCTTTTTG
GTTCAAAACCGGGGGCTTATGGGGCTGGATTACAAGGACTTATCGAAGCCCAGAATTGGCATAGT
GATGAAGATTTAGCAAGGGCATATTTAAACTGGAGTAGCTACGCCTATACAGGCACAAAAGGAGT
ATCTCAACCCGAAGCCTTTGAACAAAGACTGAAACAATTACAGATAGTTTTGCATAATTCAAGATA
ATCGTGAACATGACCTATTAGACTCCGATGATTATTACCAATTTCAAGGGGGATTAACCGCCGCC
GTCAGACATTTACAAGGGGAAAACCCAACGGTTTATTTTGGTGATAATTCCCAACCAGATAACCC
TAAAGTTAGACATTTACAAGAAGAGATTGCCAGAGTATATCGCAGTAGAGTTATTAATCCTAAA
TGGATAGCAGGGGTAATGAGACATGGTTATAAAGGAGCATTTGAAATGAGTGCAACAGTGGACT
ATTTATTTGCCTATTCCGCTACAACTAAGTCTGTACCCGATTTTATGTTTACAGGAGTTGCTCAA
AGTTATTTATTAGATGAAAAAGTCCGACAATTTATTCAAGAAAAAAAATCCTTGGGCTTTAAGAG
ACATGGGAGAAAGGCTCATTGAAGCACATCAGAGAAACTTATGGATGAATGTAGATGCGAATTT
ATTGGATGAACTAAGAGGTATTATTCATCAATCGGAGAGGATTATTGAGGGATTTGGAGAATCA
TCATTATAACCTCAGTTTGGTTTAAGAACATCAGATAAGGGACAGAAGACGATAGTTTGCGGCAC
TCGCTTCGCAAAGCGGTACGGGGTTAGTTAGGGGCGAATGGCCATTCGCCCGTACAGGAGTTCAG
AGTTTTTAATTTTTAATTATCAACTATTCACTATTCACTAATTGCCCTTTTCGCCATTACTTATA
CATGAAAATTTATCCTGATCTCAGGTTACAATAATTTACCAGAAGAATGGCAAAAAAACTTATAG
ATGCGATCGCACAGGTTCACGGAAAGGCGATGTTAGTAAAATTACCGTTGATTAGGATGAAGAA
ATAGGGAACTAAACTAAAGCATCTTATAATTTAATAACTAACATTTTAGCTGATTTTGATTAATT
ATGAACAAAGATGATCCTCAAATCACTTCCCCAATGATAATAGCTCATCGAGGTTCATCAAAAGA
AAGCCCAGAAAATACACTTTCCGCTTTTCAGTTGGCTATATCTCAAGGAGCAGACGGTATAGAAG
GGGATTTTCATTTAACCAAAGACAAGCAGGTTGTTTGTATTCACGATTCCAATACTGCCAGAGTT
GCTAATACTAAGTTAATTGTTAAAAATTCTACCTTAGCAGAATTAAAAAAACTTGATGTAGGAT
ATTGGTTTAATTCAACTTTTAAAAACGAAAAAATTCCCACTCTTAATGAAGTTTTAGAAATCTTA
CCTTCAGATAAAAAGTTATTCTTAGAAATAAAATCAGATCCCGATATTGTACCTTATCTATTAAG
TATATTAATTGATAGTAAAATAAATTTAAACCAGTTAGTAATTATTTCTTTCAATAGCAGAATT
TTAAGAACGATAAAACAGCAGAAACCAGAGTTAAAAACATTATTTTTAGTTAGTTTCAAAACTT
TTAATCGTATTAAACTATCAAATTTTTTAGTTAAGAAATTATTAAATAAATTAGAAGATATTAG
TGCAGATGGAATAAGTACTTCTATCTCTAATTTTCTCAATGAAACTTATATTCATCAATTTATTT
CAAAAGGATATGAATATCATTTATGGACTATAGATAACGAAAAAATAGCCGAACAATTTATCAA
AATAGGGGTAAACTCTATCACAACAAATACACCGAAAATAATTAAACAAATCAAATATTTCCAA
ACTAAAAGTTAAAAATCACCCAAAAACCACGGTACGATTTCCATAAACTAAAACTCGATTTTGTA
AATGTAATCTCACCCCCCTAGCTAAAACAATTTTTTCTAAATCTTTCCCTTTTCTAATTAAATCTT
GTACTGTATCTCTATGACTAATTCTTACTACATCTTGCTCAATAATCGGTCCTGCATCTAAATCT
TGAGTTACATAATGGGCAGTAGCACCAATAATTTTTACACCTCTGGAGTAGGCTTGATGATAAGG
TTTTGCTCCTATAAAAGCGGGTAAAAAAGAGTGATGAATATTAATTACTGCGGGAAATTCTTTGA
TAAAGTCTGAACTTAAAACTTGCATATATTTAGCTAATATAACTAAGTCTATTTCTGCATTTTTT
AGTAGTTCTAATTGTTTAAGCTCTTGCTCTTTTTTATTCTCTTTCGTAATAGGTAAATGATGATA
TTCAATATTAAATTGTTGAGCAATAGGGTCTAAATCTGGATGATTACTAATAATTAAACCTACTT
CTGCTTTCAATTCTTTACTTTTTATCCTCCATAACAAATCATATAAACAATGATCTTGTTTACTC

ACCCAAATAGCAACTTTGGGAACTGTATCAGAAAAATGTAATTTCCATTTAGCTTGTAGAGGAGA
TGCGATCGCACCAAAGGCTTCATTAATCAGATGCCGAGGTAAATTAAAACCTTGTAATTGCCACT
CAATACGGGAAAGAAATAAACCAGCTTCTAAATCCGTATGATGATCCGCATGAATAATATTACCC
CCATTAGCATAAATAAAATTAGCAATTTTTGCTACTAAACCTTGTTGATCTGGACATGATACTAA
TAAAGTAGCGGTATCTACAGCCATAAATTAATTCCCAACAATACCCGAATAGATTGCACACATTA
AATCTCGAATATCTTATCATTATTTTGAACTATAGAGCCGATAACCTTGAAAAATTGGCCTTTAT
CTAAGTTAGGAAAAAGGTGTCAGGTTTCAGGTTGCAAGTTTCAGGGTGTTAGTGTTAATTAAAC
ACTTTTGCCCTTAATAAAACTCAGGTTTGCTTCTCGTAAAATTCCCTTACCACTTCCCATTGAATC
CTATTAATAGGAATATCTAATTGACGGGCAATGGTTGCACAATCTTCATATTCAGGATGGATATT
AACAACTTTTCCCTGTTTTTTTGCAATTTTTAATCTTATATCACCATATTTACTCTTAATAGTCG
TCATTTCTCGCTCTAGTATCGATCTTTGTTGAATTTGTCTGCGAATACCTAAAGTAGTAGTCTCT
CGGAAAATAAGATTTTCACATATATTGATTTTGTCAAGATCACAAATTACTGTCAACAAAACCCC
AAGGCGACTTTTTTTCATCATCACCGGTTGAGTAAAAACATCTTTTGCACCAATATTTAATAATT
CTTCTAAGACATAAGCCAAAATTTGGGGGTTACAATCATCAATTTGAGTTTCTAAGACTGCTACT
TCCTCTAAATTAGATAATTCCATTTCCCCTATCCAAAGACGCAAAATATTAGGTAGAGATAAATC
TTGACTTCCGGCACCATTGCCTATCTTCTCTAAACTCATTTTCGGGGGTTGTCCAAAACTTTCCGT
TAACGTCGCCACAATTGCCGCCCCTGTAGGAGTAACTAATTCTTTTTCGATACCGTTGCTATATA
TTGGGATTTTACGGCTTTGTAACAACTCTAGCACGGCGGGTACTGGTACTGGTAATATTCCGTGA
GCCACTTTGACTGTGCCTCCTCCTGTGGGTAAAGATGAGCAGTATAATTTGTCTATCCCTAAATA
ATCTAATCCGATACAAGTACCAACAATATCAACAATGGCATCTACTGCCCCCACTTCATGAAAAT
GGACTTTATCTATGGTTGTATTATGAACTTTTGCTTCTGCGATCGCAAGTTGACGAAAGATAGCT
AAACTATTAGCCTTAATTTGAGGGGCTAAATTACTCTGATTAATAATATGTTCAATATCTGCTAA
ATGTCTATGATGATGGTGAGAATGAGATGTTAAATGAACATGAATTTTTGTCGCACCAAGCCCAT
TTCTAGTTACTGTTTCTTCTTGTAATTTATACTCTTGAATAATATTTAAACTCTCTAAACTTTTC
TCTAAATATCCTAATGGCACACCCGCAGAAACTAACGCCCCTAAAAACATATCCCCTGCTATTCCT
GTAGTGCATTGTAAGTAAGCGATTTTACTCATATAAGATACAATTAATTAATAAGTTTTATAGCT
TTTAGTTATTAGATTAAAAATAAATTATTTATTTATAGTTATGGCTATTTTATACGAATTAAAT
CCTGATAATCCTCAAAGCAGAAGTATTGAGCAAATAGCATCAGCATTGAAAGATGGAGCAATCAT
GCTTTATCCCACGGATACTGTTTATGCCATTGGTTGTGATATTAATGTTAAAAGTGCCGTAGAAA
AAGTGAGAACTTTAAAACGTCTTGCCAATGATAAACCATTAACCTTTCTTTGTTCTTCTTTGTCG
AAAATTTCTGAATATGCGATCGTGACTGATGAAGCCTACTATATCATGAAAAGATTAATACCCGG
TCCTTACACTTTTCTTTTACCTGCCACGAAATTAGTGCCAAAACTGGTTATGTCACCAAAACGCA
AAACCACTGGTATTCGAGTGCCAGATCAAAACGTTTGTCAAGCAATTTTAACAACTCTTAATAAT
CCAGTTATTTCCACCTCTGCTCATATCAATGATGAAGAAGGATATTCTCCCTCTGATGACTGGGA
AAAGGCAAAATTATTTGATTACTTTGATAAACTGGTTGATATTATTATTGATGACTATACAGATC
CGGGGTATCAAGTTTCAACAATTTTAGATTTTTCCACAGAAAATCCCGCCGTAGTACGACAAGGT
TTAGGGTGGGAGGAACTAGAAAAATGGTTTAACTTCAATAATTAGGAAGAAAAATGGCAAAGCA
GAGAAAAAAAATCTGATTTCCCCACCAAAATCTGTCCTGTTTGTGGTTTATCTTTTACATGGCGCA
AAAAATGGTCTAAATGTTGGGATGATGTTAAATATTGTTCTGAAAAATGTCGTCGCCAAAAATA
AACAAAAGTTTGTAGTTACCCCTTTAGAGGCATTAAGCTATAAGTTAAATAGGGGTAATATTTGT
TACAGATTCGATCGCACGCTACTTGAAAGGATTTTGGGCTGATATATGTGAGATTGAGGACAAGT
TGAGAGGTGAGGAGATAAAAGCTATCAGATATTTTTGAGGATTAGGGTAAAATTGGGGCAGGAT
AGGAGCAAGGCTAATACTTCTATCCTCTGGGGGGAAAACACGGTTAACAACTTATTGAAGATTTT

AACATGGGTAGATAACAAGTTAAATGATATGAGAGTAGAAATAGCCTCAATTAAGGGTAATCAA
GAAGGTTTTAGCAAGAGACTTGATAATCTTGAGTTTACTAGCAGAGGTATTTTTATTATTAACTA
AGAATTAAAAAGCCGCCTCAAAAGCAATGCCCTCTTCGGAAAAATTGGAATAGTTTTGAAGAATG
CTTTGTCTTTCATTCTGCCCTACAAATAAATATGTACCGGGTAAATTATCGTTTTGGAAACGATA
TAAGCTGTCGGCTTGATTAGCATTAGCCCCAAAAGCATAAAAAGCTAATCCCTCTTCTCGGAATT
GAGGAAAACTTTGTAAAATACTTTGTCTTTCCTCCTGCCCTACATAAAGATATGTTCCTTGTATT
TGGGTGTTTTGGAAACGGTAAATGGGAATTAATCCATCTCCCGGTTGGGTAGCAACACTAAAAGC
AAATCCTTCTTCTTCAAATTTGGGATAATTATTTAAAATGCTTTGTCTTTCTTCTTCTCCCACAT
AAAGATATGTGCCGGGTATTTCTGTATTTTGGAAGCGATATATGGGATTATTTAAGGCAGAGCGA
GGAGTTGCACCGCTTTCTGTACTACCTATATCCACTGTACCTCCAATTATACGGGCATAACCTCTT
TGGTCTGAATTGCCCACTTGAGACGAAGCAATTTCGTTATTTCCTGCATTTATGACAGGGCTACC
TCTGAGGGGCAAATGGGTAGGAGTATAACCACCATTATCTTGTAAATCTCCCAACAGAGGATCTA
ATGGATTAACGATGTCTCCGACAATATTATTGTTAAATCCATCTAAAATAATACCGTTACCATCG
CCAATAAGGTTATAAGAGCTATTTTCGGTAAAAAATCCCTCAATATCACTACTATCTAAGTTTCC
TGCAACAATACTGTTTTGCAGTAAAAGAGGGTTATTTGCTCCTAAAACGGTAATACCTGAGCCAA
TTTGAGAGCTATTATTCGTAATAGTTGAATTGATAATAGAAACGGTGGCATCTTGACTGAGAAT
ACCTCCCCCTGCGTCTCCTGTAATATTACCAGAGATAGTGCTATTTAGTATTTGTACTGTTCCTCC
GAAGGTGTAAAGCCCTCCCCCTCCTGCTTCAATCACTTCTACGGCGGCGGCATTGGCGAAGTTACC
CGCTATGGTGCTATTACGAATATTTAATGTTCCTTCGTTATAGATACCCCCTGCGTCATCTCCTGA
GAGGTTGCCTGAAATAATGCTGTTAATTATTTCTGTATCTCCGAAGTTGGCTATACCCCCTCCAT
TATTATTATCTGTGTCGGTGTTAATGGCAGTGGAATTATTTTTGATAATACTGTTATTGATAATA
GCGGAAGCCCCTGTGTTAATGCGAATACCTCCGCCGCTACTACCATTTTCATCAAGGGCGTTTTCG
GCAATTCCATCCTCGATAGTAACTCCACTAAGGGAAAGTAATCCTCCTTGTCGAACATCAAAAAT
GCGATCGCCTAGAGCCGTACCATTAATGGTAGTTGAGCCTGAGCCGACTCCCACAATGGTAACTC
TGGTTTCAACATCTAAATCTCCTGTACGAGCTACGATGTCATCTACTTCACCGGGTAAATTAGGA
TTGTCACTGGGAAATAAGGAGTCTTCGTTACCCTGAATAGACAACTCGTAAACCCCTGCAGGAAG
GTTGATAACATATTCTTTGCTAGGGTCTGCTTTTGCCCTTAAAATAGCATCTCGAAGGGATAAAC
CATTTCTGTCTCCGCCGTCATTTTGATCCTCGAAAGTAGTTACAGTCAGAAAAACTTTTTCTAAT
CCACCACCACCACCATTAAGTTCTTGTAGGATAATGGAATTATCTTCTATCCATCTTTCTGTATCT
TCCACAAAACTATCGGAAGGAGTACCAAAAGAGGCAACGATGCGATCGAATTCTGCTTGGAGGGC
TTCTATATCAATTTCAAACATTTTTGCTTTTTATTTAATTATTTTATTAGTTAATATTAAGTTTT
AATGATAAATCACACTTCCTTATTCATAGATTTTTTTTTATCAAAATTATCTCGTTGACGTAAGA
CTCCCATTAAATAACGAGTTTGAGTAGTTCACCATCTATTCCCTTAAACTTTACATATTGATTGA
CTTTGATAAGCCTTGAGGGTTGCATTGGCTACTCTTAGAGCATAAACACTGGCTTTATTTTCAAT
ATAAAGAGGTTTTCCTGTAGTTAAATAATCGATAACTGCTTCTGTATCTTTAACAAAGAGTCCTC
GCCTTGTACCAACTTCGATCGCATTTTCTTCTCCATCCCTTATAATCTTACCTGTTTCCCCCTCGA
ATCTTAACGTCCCTTGAGTACCATAAATTTCTAAGGTGCGATCGCTGCTTTTGAATTGTTCACCC
TTGCCATAAGTTAAACAAGCTAATAAACCATTCTTGAAAGATAACTGTGCTTGAGTATAACAAGC
AGAAAAATAACCAACCTTTTCAGCATCCCAAAAACGCCCATAACAACTTACCGAATCCACTTCTC
CAAATAAATCAGTAAAACGATTAATGCGAGAAAGAGCCGCTATAAAAGGGAAACCATAGTACTC
ATAGTTAAAAGTCCACTTATTCATTAAACTACTCTTGGGCAAAATAGTCGTATAACGAGCTAAAA
AAGGATCGCCAATCGCTGATAAATTAGCCCTAATCGCTTGATGCACTCCTCCCAAAATTTCGATA
TGTTCTACATGAAGTAACTTATTTTTTTCTTGTGCTAAACTTAGCAACTCCTCCGCCTCATCAGG

ATTAATAGTTAAAGGGAACTCAACAATAACGTGTTTATCAGCTAATAAAGCACCTCTTACAATCA
TTCCATGATCCCGATTAATATTAGAAATGCAAATTAAATCTAAACTAGAATCATTTACTAACTGT
TGCCAACCAGAAACCGAGGGAATGCCATAGGTTTCACAAAAACTAGCTGTTGTTTCCACACTGTT
ACCCACCACCGCCACCAACTCAGTTAAAGGATTTGCTTGAAAAGCCTCTGCCCGTCTTTGAGCCGC
ATATCCCGTACCAACTAAACCAACTTTAATCATTCTTTTTACTGATTATTTTCGCTTTATTTTCA
GTCTTGCTATTTTATCACCACCTGAGTTTGATATTGAAAGAATGGAAACAAAAATACTATTTGTA
AAGTCATTTTTTCTGCTTAAATATTCCTAACAAAAAAAACAACACTCGCAGAAATTAAGTAAGAT
TCAGTTAACAATCATCAAAAAACGAATTACGGTGTGAGATTATGAGTAAAAAATTAATATTACA
ATGGTTGGGTAAGTTTAGCTTATTATCTTTACTCTGGTTAATCTTTAGTCCATCTGCCCAAGCCG
CTTTAGAATTAAGAGTTGCAATCAAAAAAGGTGCTAATTCTTTACAGGTAGGTAGTTCAACCCCA
GCAGTTGTGAAAGATAATGCAGGGCGGGTTGTAGGACAAGTTGCAGGGATGAATTCTTTGTATC
CAAAGTACAAGGAGGTAATGTTAATCTTAGTCAATGGAAATCCAATCGTTTATGGTTAGAGCCTA
GTGGCGATGGGGTTGTCTGGATTGGCGATCGCTGGTATCGTGGAAGAGTGCTATTAGTACCTCAA
GGTAAAGGAATTACAGCAATTAACTATGTGGATTTAGAAGATTATCTATATAGTGTTGTCGGTG
CAGAGGCAATTCCTAGTTGGCCTAAAGACGCATTGAAGGCTCAAGCCGTAGCCGCCAGAACTTAC
GCCCTATATAAACGAAATAGTGGACGTAATCAACTTTTTGATTTAGATACAACTACCGCTACTCA
GGTTTATAAAGGTTTAAGCAGTGAATATACCACTACCCATCAAGCAGTGAGGGAAACTATGGGGG
AGGTAATGACTTACAACAACCAAATTATTTTAGCGGTATTTCATTCTTCTTCTGGTGGTCATACG
GAAAATGTCGAAGATATTTGGGTATCTCCCTTACCCTATCTTAGAGGAGTAGTCGATTACGATCA
AAACTCCCCTGTATTTAGTTGGAATAAAAGCGTTTCTCCTTCTACCATGAATAGAATTGTTGGTG
GTGTCGGCAACATCCAAGCCCTAATCCCCCAAAAAACCACTCCCCAAGGCAGAATCGTAACCATG
AAAATTGTAGGCGATCGCACCACTAAAAACGTCGCAGGTAGTGATTTACGCAAAGCTCTTGATTT
AAGAAGCACATTATTTCGTGTCTCTTTAGGAGGAAATAACTTACAAGTATCAGGTAGAGGATTTG
GTCACGGCTTAGGGTTAAGTCAGTGGGGAGCTCAATATTTAGCCCAAAATGGTGTTAACTATCGT
CAAATTTTAGCTCATTATTATCGTAATGCGAAGTTATCTACTTTGAAAACACAATAGAATCAATT
TATTAATGGGCAAGGGGGAAACCTCCCTTTATCCCCCCTCTCGAGGGCGGGGGGCAAAGATGAAT
AGTAAATAATTAAGTCTCTATCACTTCTCGTCGTGTAAATTATTAGTTAGGGTAGGCAAGAGGCA
AGAGGCAAGAGGCAATAGGCAATAGGGGATTATTAAATAATAATTTATAAACTTTTAGTTTTTG
TTTTACTATAAACACTATTCAATAAAGGTTATGGAAGTCCTGTTTCTCTTAATTTATCATAACCA
CACTCTGAAGAACCATAATTAATACAACCTCCAACTCCTGTACGGGCGAATGGGCATTCGCCCCT
AACTAACTCCGTACTGCTTTGCGGAACGAGCGCAGCGAACTCTCCGAACCCTTATCCAATATTCTT
AAACCCAACTGAAGTTAATTAATTACCTTTTTTTGTAAGTACCTTAATATCTTCACTGCAAATAT
TCAAATGCAACTTTTGCATAAAAACAATGTTTTTTTAGAATCATGCAATTCCCGCAAATGATATT
ATAAAAACTGATAAAAAATAATCTATCTGTTTAGATTAACTAATCAAACATAGCTAGATAACTA
TTTAGAAAAATAATAATCATGAGTCAAATTTGTCCTAAATCTGTCAATATAATTCCTCAAATCAT
CGATGTTTTAAATAAAGGAGAAATTGTCATTTTACACACAGATATTGTCTATATCTTTTTAGCCA
ATGGTTTAAATGAAAATTCAGCAAATAAGATTCATGAGTTAAAACGATGGAATCCCAGAAAACC
TCTAGTTTTGCTTTCCAATCAAGAGAGAATTTCTGAATATAGCAATTTAAGTAAAGATGCTCAAA
TATTAGTTAATCAATTTCCTTACCCGATTAGTGTGATTATTCCTCACCGCAATAATTTACCTGAA
ACAGTAACTGCTGGACATAATACTATTTTCGTTTCTTGTCCTGATGATTTTATTTACAATCTAGT
GAATAGATGTCCTTTTCCCATTGTCTCTGGCACAGCAAGTTTGGGGGGTGACTATAGAGCAACTA
ATGCAAAAACTGCACAAGATTTATTCGGTAAAGATGTTCGTTTAATTGTCGATGGGGGTAAACCT
AAATATGGCATTAGAACCACTTTAATTGATTGTAGTTTGCCGCTACCAACGATAATGAATTTCGG

CATTATTTCCTATGATGATTTACGTCCTTTACTACCCCACATCGAGTTACCTTCTCATTTACGTAA
ATAGAAATTGCTCAAAAGTGTTTTTGTGAAGGTAAGAAAGAAGCAAAAGGCAAAGGGCAAGGAT
TAATAATATAACCCGAGTTCAGGATCAATTATCGATGAGTGATAGCGGAAAGGGCAATGGGCAA
ACCCCCCTTTATCCCCCTCGAGAGGGGGGAAGGCAAAAGAAAATAGTTGATAATTGATAACTTCT
AACTCCTGTACGAGCGAATGGGCATTCGCCCCTAACTAACTCCGTACCGCTTTGCGGAACGAGCGC
TCGAGCGAACTCTCATTTCCCCCTAAACACTACCCTTATTTGACGTTCTTAAGCCGAACTGAGGTT
AAAATATTATTCACAGACATCATTATTGCTAATAAATCTGTTATCATGGTTAAGTTGTGAAAAAT
GTTGTTAGCAAAATCAAAATAGAGCTAAATCGTAAATATGGGCAATAAGCCAACCATCGCCGTTT
CTCATTTAGGCTGTGAAAAAAATCGCATTGATTCTGAGCATATGCTCGGTTTATTGGCAAAATCA
GGCTACGGCATCGATAATACCGAAGAATTAGCCGATTATGTTATTGTAAATACTTGTAGTTTTAT
TGAGTCCGCTCGTCGAGAATCTGTAAGAACTTTAGTAGAATTAGCGGAAAACCAGAAAAAAATA
ATTATTGCTGGTTGTATGGCACAGCACTTTCAGGAGGAATTGTTAGCAGAATTACCAGAAGCTGT
TGCGTTAGTTGGAACTGGTGACTATCAAAATATTGTCGATATTATTGAACGGGTAGAAAAAGGT
GAAAGAGTTAAGGCGGTTTCTGCTGAACCTACTTTCATTGCGGATGAAAATATACCTCGTTATCG
CACTACCACTGAAGGGGTGGCTTATTTAAGAGTCGCCGAGGGTTGTGATTATCGTTGCTCTTTTT
GTATAATTCCCCATTTACGTGGGAATCAAAGATCCCGTACCATCGAATCTATTGTAGCAGAAGCC
CAGCAGTTAGCAGATCAAGGAGTGAAGGAATTAATCTTAATCTCCCAAATTACTACTAATTATGG
TGTTGATCTATACGGTGAACCTAAGTTAGCTGAATTATTAAGGGCGTTAGGAAAAGTTGATATTC
CTTGGATTAGAATTCATTATGCTTATCCGACAGGATTAACCGATAAAGTTATAGATGCGATCGCA
TCTACAGATAATATATTGCCCTATTTAGATTTACCTTTACAACATTCTCACCCCGAAATTCTTAA
AAGCATGAATCGTCCTTGGCAAGGGAAAGTAAATGATGAAATTATAGAACGCATCAAACTAGCCC
TTCCCGAATCGGTTATGCGAACAACATTTATTGTGGGATTTCCCGGAGAGACTGAACAACATTTC
CAACATTTGTTACAATTTGTTAAAAGACATCAATTTGATCATGTGGGTGTATTCACTTTTTCTCC
CGAAGAAGGCACTAAGGCTTATGAATTAAGTAACCAGTTGCCCGAGGAAGTAAAGGAGGAAAGA
CGCAATCAGTTAATGGAAATACAACAGCCCATTGCCGCTATGAAAAATGAGGCAGAAATAGGTAA
AGTTGTTCCAGTATTGGTTGAGCAAGAAAATCCCATGACTGGTGAATTGATTGGGAGATCCCCCC
GTTTTGCCCCCGAAGTAGATGGATTAGTCTATATTAAGGGTTCAGCAAACTTAGGTGAGATTACC
CCTGTTCGCATCACCTCAGCCGATGTCTATGATCTCTATGGAGAAACAGAGATAAATTAGTAATT
ATCCTAGATTAAGTAGGTCTTATTCAGTGGCTACATTCTGATAATCATAGTAAGGATAGTTGTCA
CGAAAAATAAAAAAGTAAAAAAACTTAATCTTCTTACTCAATTGTCTCTCGGAAATCGTTTCGTG
CTTAAGTAAAAGCGGAATGTAAGGGCTAATGGCATCATAATCATTAGCTATAAAGATAACGGTA
GTAAAAGCAGTTATTAGATAGGAATAAAGAACTAAAAATTCTAAAGTGTAGCAAACTTTAACCG
ATGATTTTTAGCTTCTGCTCACCAATTCCTCATTCTTAATTAAATTCCCCCTTACTGTTACCAGTT
TTGCTTCACGGGATTGTTTCCTTTGATCTGTGGAGAGTAACACAGTAAACATTCACAAATAACAT
TACAGATGACAATTTCATTTTCCAGTTTAGGACTATCCGAATCCTTAGTTAATCAACTAGAAAAA
ATCGGTTTTGTGACTCCCACCAAAATTCAATCATTAGCAATTCCCGAATTATTATCAGGGCGTGA
CGTAGTAGGGCAATCGCAAACTGGTACAGGTAAAACAGCCGCTTATTCTCTACCTATCTTAGATA
GCATTGATCGCAGTTCCAAAGATGTTCAAGCCTTAATTTTGACTCCCACCAGAGAATTAGCTCAA
CAAGTAGCTGAATCTTTAAAGGATTTTATCGCCAGAGGTGACAGGACTTATGTTCTAACTGTTTA
TGGTGGTCAATCCATAGAGCGTCAAATTCGCTTTCTCGAAAAAGGTTCTCACATTGTTGTTGGTA
CTCCGGGGCGGATTATCGATCTACTCAACCGTAAAAATCTTACGTTAGATTCTCTTCGCTATGCA
GTTTTAGATGAAGCGGACGAAATGTTAAGCATGGGCTTTATTGACGATGTAAAAGAGATTCTTTC
TCAAACTCCTAAATCTCGTCAAACCATTTGTTTCTCGGCAACTATGCCCAAAGAGATTCAATATC

TGGTAAAAGAGTTCATGAATGATCCTATCAATGCTACAGGAGAAAAACCTCAAGATACACCTTCT
CGCATTGACCAGTGTGTGTACATGGTGCCTCGTGGTTGGTCTAAAATTAAAACTATTCAACCAAT
CCTTGAAATTGAAGATCCTCAAAGTGGCATTATTTTCGTGAGAACTAAGCGCACTGCGTCTGAGC
TAACGACTAAATTACAAGAAGCGGGTCAAAGTGTAGATGAATACCATGGCGATTTAAGCCAATCT
CAACGGGAGCGTTTAATTCAACGCTGGCGTGATGGAAAAATTAAGTTGGTAGTGGCTACAGACAT
CGCCGCCAGAGGTTTGGATGTATCTGATTTAACCCACGTTTTCAACTTCGATTTACCAGATAATA
CCGAGACTTATATTCACCGTATCGGGCGTACAGGAAGGGCAGGAAAAGAGGGTAAGGCTATTGCT
TTAGTTGAACCAAGTGATCGCCGTTTTTTACGTCAAATCGAGCGTCGTTTAAATCAAACCATTCG
GGTGGAAAATGTCCCAAATCGCTCTACGGTAGAAGCGAAAAGAATGGAGAAACTTACGGAACAA
ATTAAAGAGTCTTTAGCGGGTGAAAGGTTAGCTTCTTTCTTACCTTTAGTTAAAGAACTACATGA
AGATTATGATCCAACTGCGATCGCAGCCGCTACGTTACAAATATTATATGATCGAGACTGTCCTA
GTTGGCTACAGCAAGATTGGGATGTTCCCCCTCCTGCAACCCCTAAACCTTATATCAAAAAAGGT
AAAGGAGGACGTACTAAATATGGTAGTCGTGGTGCTGATAGCTTCGATCGTCAAAATAAATACA
AAGGTAAACCTAAACGTAGTTATAACCGCTCAGTAAAGCTAAATAACCGATAAGATTTAAGTCTT
AGGAATTAGGCAAGGGGTTCATTCCCCTTGTTAGGAATCATAGGAAATATAAAATAGTTGCAATC
AAAAAGATTATTTTCATTTATATAAAGTTTTATCTTACTGAAACTACCTGAGTTAGCTTAAGCTC
AGAGTTAAGACAAAAAAGAGGTTTCTGTTTCTCCCCACAATTCCCTAATCAATTAATAATAACTC
CGCTAAACCTCATACCTGTCATTTAAAATCACGAGATTTGAATATTTTTTAAACCCCACAAGTTG
AGCTTATGCAATATTCTTTATGCAGAACCTAGGTTAACCTCAGTTCGGTTTAATAATATCGGATA
AGAGTAGGTTTCAGGTGGTAGGAGTTGAATATATTCAACCCCTACGGTGTTAGGGATTTCGGAGT
CAGGAGTTAAGGGTTTTTAATTCTTAATTATTAACTATTTACCTTTGCCCTCTCCCATCTCGAGG
TGGGACAAAGGGGGGGTTGCCCATTGCCCTTTGCTCAATCAATCTCCATAAAAATTTATCCAGAA
TTGAGGTGAGGTTGAATAGGATTGCTCAAAAACTGTGTATGTCAGTATGATAATTTTAGTTTAA
AATATTATTAACTTTTGTTAAAAATATAAAAAATACATATCAAAAATTTAATTTATAATGACTT
TTTTTAAGCCTAGACAACCTATTTTACAACGAAAGCAATCTTCCTCTGATACTCAGCAGTTTTTA
TCAATGAAATGTTTAAGAATAAAGTCTGCAGAAAAATTTTTTCTCTCAGGAATATATACTTGTGT
TGATCAGGCAATGTTAGTTTGGGGAGTGGTGGCGGCAATAATTTTTTTCAGTGCTCAATTTTTAT
CTGTTAGTTGGGTTGATCAAGCGTTTTTTTGGTCTGTATTTACCATTTTTGCCATCATTGTCATG
ACCAGCTTAACTTATTCTTGGGCAGTATGGGAAAAAGTTTCAGGTTTACTTTATGGCTGGATTTT
TTTGATGATTTTTGGTCTGATTATTACCGATAGTGCGATCGCATTTTCTTGGGGTTATATTCTCG
GTCATTTATGTGAGTTGTGGCTAATTTTAAGTGCTTTAGGTTATTTCCTCACAGGTTGGATGATG
CGATCGAGGGCTTTTTTCTTAGCAGGTATAATTCATGGTAGTATGGTTTTAGTATTACCCTTATT
TGCAGGATGGCAATTTGCAACCACAGGATTAGTCATGATGAGTAATCTGTTTTTATTCTCCGAAG
GTCAATGGGATATGCTTTTGCCAAGAGAATTAAAAGAATATTCAGTACAAGAAGTTAATCATGT
ATCCTTAACGAGAAAATCAGCTCATCAAGATTCAAGTACCAGAAATCAGTTATCTATTATTAGCT
TTTAATTTTTCTTTTAATATTGTTTTTAGCTCTTTTACGGCAATTATGATAAATTATAAATTAAT
TAGCAATTTTCCTAAACTATTGCTCATCAGCTATTCCTAACTTTCCCTAAACACTTTTTCAGCTCC
CCCTATTAAAGTAAGAGAGCCTTATTTTTCTCGATTCTCCACACTTGAGATGAATTTATAGGTTA
TCAACCACCCTTAGCAAAAGAAATCATTACCAGAAAAGAAAGTAATAATCCGGGTGCTAACAGAG
TTACTAACATTAAAAATTGATCACCAGTCATAATAATATTGCTAAAAAATAAGATCACCTTTAAT
TGTAAAAGAAGATTAAAAAATTAGAACATTGTATTCTCTGTGATTAGTCCATTTCATGAGATGA
TATTCTTAAAAAGATAACTTGTTTTTAATATATATATATTTTTAGTTTTTCTTAAGATTTTCTAT
GTACTTATTAGAATGCCACAATCAATGTCCTGATTGTTTCTCTGCTGATTTAAAGTTAATTCCTG

TTGCTGATAAACAAGGAGAATATGAGTTTTATTTTAATGCTAAAGTAAATGAACAACAAGAGAG
TATCTTAGATGGAAAAGTTAAATTTAGTCTCAGATTAGCAAAATTATTAGTAACGTTTAATGACC
TTGATATTTTAGAAATTTATGACATTAATAAGGATTACATTCAAATAAGAAAAAATACGTCAAA
AAATCATCAAGAGTGGTTAATCAAACCAAATTTACTCAAGGATAACTCTTCCGTTAAATTAGCAA
AACTCAAAGCTGTGGGAGATGAAAGTAATTTAACTGCTCAAATTGTAATAGATAAATCTAATGT
TGTTTTAACTGATATAGAAGGATTATGGGTTCATAATATAACCCCCAATAAACACGCTATTTTAG
AAAGAAAAGTAGCCGAATTTATTGAAAAAGTCTATCTTAGTCCTTATGTTAGTAGAGCAATTTTT
TCCTATCAAAAATCTGAAACAACACCTAATTTCCATCACAAAAATAATCAAGAAATTGAACAAAA
AATAAGTAGTTTAAAAAATGTCATTCAAGGAATTTATCAAAATCCTCAAGAAGACTTTAATACT
TTAGCTAAAAAAGCTAATCTTAATCCTTTGATAGATTTTGTTGGTGCTAATTTGATTGGTGTCAA
TTTAAGTGGTTTGAATTTGAGTAGTGCCAATTTTCAGGATGCAAATTTGCGAGGTGCTGACTTAA
CAGATGTTGATTTAAGCGAAGCTAATTTACAAAATACAAAACTTAATGGTGTGGATTTAAGTGG
AGCTTACCTAGAAGGTGCAAATTTAACTAATGCCAATTTGACTAATGCTAGTTTAGCTTTAAGTA
ATTTAATTGGGGCAAATTTAACCAATGCCAACTTAACTAATACCAATTTACAAAATACTAGCCTA
GGTCAAACAATTGTTAAAGGGGCTATTTTTGCTAAAAATCTCGGTTTAAATGAGGAGAAAAAAC
AGGAGTTAATAAGTAAGGGGGCTATTTTCTAATTTCTTTCAGGTGTTGGATAATTAAGCTATAGC
TTTTAGTTTAGATAGGGGATAGGGGATGGACAAAGGGCAAGAGGCGAAGTAAAAAGGGCAAACC
CCTCTGTGTCTTCCCTTAAAATGGGAGAGTGGCAAAGGTAATTAATTCATTTACCTCCAAACACC
CCAATCCCCCAACAACCTAACAGAGGTTAATCAAGAATCTACTACTACAAAATAATTAAACGACA
ATCAGAACTACTGGAATCATCGTCTATTTCTCTGTACAGAATATCAATCCATTCATCTTCTTCAG
GAAAGTTTTTCCATTTTACAGCGATAACCCAGTGTTGATCAATATTTAATTCTTGACGAATACTG
GCACGTTGCTTACTTTTTAAATCATAAAAAATACTTTGAGCTTTGCTCTCACTTTCTCTTAGGGT
AATAGCGACTCTCAAGGATTGACCTTCTTGATTTTCTAGGGTAATTATATTATCGATGCCGATAA
TTTCCCAGAGTATGAGATAGTCACTCAAATCATAACGTTTACCCTTTGCCCAACGTAATTTTCCCT
GACAGTATTTCGTTGCTACTTCAAGCAAGTGTTCTTCGTTCAAATCTGGGGCGTTTAAATCAGAA
ATTTTCAGTTTTGAAGCTACTGTTTGAAATCTTAAACGTAAACTAAACAAGAGTTCCTCAACAAT
GATTTGGCTACAATAGTATAACCAGATATGGGTACTTTTGACAACGATTTACTTATCAATGATCA
CGATTCAAGAAATAGAAAAATATAGTGAAAATCATCCTCAAGAGGTATTAATCTTAAGGGCTAG
AGATAATCAAGAGGAGGTAGAAATTATGATTTTTAAAGGATTTTCCAGCAATTTAACTGGTTCA
ACTGCTTTTGATCCTGATATGCCTATTTTATCTCCTGATGGAGAAATTATAAGCTGCGATCGCAT
CTTAAGTCCTTATAATCCAAATAATCCCCAATATATTGAAAAAAATATATCTCCGTCAGAATTGC
TAAAATTACTAAGGGAAAACGAATCCAAATAATACTTATGACTCAAACCCTAGAAACATCAGTTC
AACAACTAATTAACGCTGTCAACGAAGCAGATTCAGCCGTCAAACTACTTCAGGCAGTACAAAAA
TTAGCCGCCGCTCAATCTCCCGAAGCCATACCCACCCTAATTCAAGTATTAGGTTTCAATAACCCC
GGTGCCGCCGTTGCCGCCACAGAAGGCTTAATCAATCTCGGAGAAATTGCGATCGCACCTCTGAT
GAAAAACCTTGATGATTATAATTATGGGGCAAGAGCATGGGCATTAAGAGTATTTGCAGGAATT
GGGGATATTAGAGCCTTAGACTTACTAATTAGTGCCGCTACTACAGACTTTTCCCTGAGCGTGAG
AAGAGCGGCAACAAAAGGATTAGGAAATATTCAATTGTCTAGCTTACCCCCAGAAGATATTGCCC
CAACCCAAGAAAAAATTTTCCGCACCCTTTTCTTAACTATTCAAGATGGAGAATGGGTAGTTCGT
TACGCCTCCATTGTTGCTTTAGAAAACTTAACACAAAGCCTAAAAGCCTTTAATAACAATTATTT
AATACCTCAAATTGTTGAACAATTAACATTACTATCTCAAAAAGATGATGAGATCGGGGTTCAAA
CCCGTGCTAAACTAGCATTGACAAAAATCAAACAGGAAGTATAGCAATTTTTTAAGAATATGAGT
AAATCAACAGATGTAAAAGGATTAGCCCGATTAATGGCGGCTGACTTCAGCAATCAACAACAGGC

ATGGGATAACCCCCCTTTTTTCGCTCATATTCGAGTTTGTATGCGTCCCCTCCCCGAATCTCTCCT
CGGTGGTACAAGTCTTTTTCTTGAACAAGCCTATGATTTTCTCTTGAATCAACCCTATCGTTTAA
GAGTATTTAAAATTCAAGCAGTGGATGATCATTTAGAATTAGAACACTACAAACTGAAAGAAGA
AGCCGAATTTTATGGAGCTTCCCGTAATCGAGAAAAATTGAAAGAATTAACTATTGACCATTTAG
AAAAAATGAATGGTTGTGATATGATTGCCGAATGGAATGGAAGTCATTTTAAAGGTTACATCAA
ACCGGGTAAAGCCTGTATTGTAGTTAGAAAAGGAAAAGAATCCTATTTAGATAATTCCTTTGAA
ATTGATGAAGGTAAACTAATTAGTTTCGATCGAGGTAGAGATTTAGTTACAGATGAATTATTGT
GGGGTTCTGTTGCCGGACCATTCCACTTTACCAGAATTGAAAGTTTTGCCCATGAAGTTTAATTT
ATAGCAATGACCGACTTTTCCACTCATAAATACTGATAGAAACGTCAATTACTCAGTAAAGACAT
AAAATATACTTCCTTTTTCATTTTTTCCCTTTTCAACCCCCAAATTCATTCATCTAACCTATGAGA
CAGTTAAGAGAATTATTATTTCCTAGCCAAAACAGCAAAAAAAATCCCCCCAAAACAAAAGGACT
TCCGCCAATAAAAAAAAGTCGCAGGGGAATAGAAATAAAAACTGAAGCTGAAATCGAAATCATG
AGGGTTTCAGGGAAAATAGTGGCAACAGTATTAAAAGAAATTCAAGAAATTGCCAAACCCGGAA
TGACAACGGCGGATTTAGATGCCTATGCAGAAAAGCGTATTCGAGAAATGGGAGCAACTCCTAGT
TTTAAAGGTTATCATGGCTTTCCCGCCTCCATTTGTGCTTGTATTAATGATGAAGTAGTCCATGG
CATTCCTAACCCCAAAAAAGTCCTGAAAATGGGTGATATTGTCAAAGTTGATACCGGGGCTTTTT
ATAATGGCTATCACGGTGACTCCTGTATTAGTTTCCCCATTGGCAAAATCAAAAGACAAGGAGTT
AAGTTATTAGAAGTAGCTGAAAAAGCTATGTATCAAGGAATCGAGCAAGTAAAAGCCGGTAATT
ATTTAATGGATATAGCAGGAGCAATTCAAGATTATGTAGAAAAAAATGGGTTTTCTGTGGTAGA
AAATTTTGTGGGACATGGTGTGGGTAGAAATTTACATGAAGAACCTTCTGTATTTAACGTTCGCA
CTAGAGATTTACCCAATGTAAAATTGCGCAGTGGAATGACTTTAGCCATTGAACCCATTGTTAAT
GTGGGTAGTAATAAAACTCGTACCCTTAAAGACCGTTGGACAGTCGTAACCATTGATAAATGTCT
TTCAGCCCAGTTCGAGCATACTGTTTTAGTAACTGATGATGGTTATGAAATTCTAACTTGGCGAG
ACTAAGTACTTTAATTGATCTCAGTTAGGCTTAGGAATATCGAATAAGTGTAGGTGTCAGGTTAC
AGGCTTCAGTTGGCAGGTTTGACAGTTTGTTTTTTTGAATATTAACTTATTAGTCACTTTTCCTC
TTTTTCTACCATAACTAGGAAAATTTATCCCCAACTCTGGTTTACTTAATAAGTAGCTCCCGTTA
AATAAAACTAAAGTTCGTAGTTACCCCTTTAGGGGTTGCAAAAGTTATGAGAATAAGGGCTGAA
GCCCTCACTACTAACTGACCCTATTTTACAACTTCCTCTAAACGTTTTAAAACAAAATCCTTATCT
AAATAAGAAAATAAATAACCAGCTTTAGGTCCTCTTTCCTTACCTAAAAACGAATAATAAATGGC
AGGAAAAGCAACACTAGGACTAATCCCTAATTGTTTAGTGGCAGTAAATAAAGCAGTTTGCAATT
CTTCCCCTTCCCATTTTTCCAAAGCGATTAAATTTTCTTTTAATTTAGTCAAATATTGTTTTTGT
TCATCGCTCAACCCAGAGGCGGTTTCAGGAATATTATCAAAATAAATTACCAGTTTTTCTTCCTC
ATCGGCATAATCTTCTAACCATTTTTTCGCTACGGCAATACGTTCGTTTATTTTCTCCCAATCATA
GTCGGTTAATTCTGTTTCTGCTCTTTTTTCTATTTCCGCTTTTAAGTCTAAATGGGGTACTTGTA
ATAAGGAAATGAGAGTACTAATGTCGAAAGTATAATAAGGTTTAATTTCCTCCTCTAATTGAGCA
TAGAATAGAGGTAACAAAGTTCTGTCTAATTCTACATTTTCATTTTCCTTTTGCAGTTGAGCATA
TTGATTTAAAAGATTATCATAATCTCTAAATAAACGAGTGATCGTCTCATAATTAGGAGCAAAAT
TAATCACCGTTTTTGGTTGAGTTCTTAACATTAAAAAGCGTAATAACTCTGGAGGCAACAAATCT
GCCATATCTTTGGCACTTGAACCCACCCCCTTAGAAGAACTCATTTTTGTACCGTTAACCAAAAT
AAATTCATAGGGAGAATGGAAAGGGGGTTTTTATGTAAAACTTTACGACAAATAGCATTTGCT
ACATCCCTTGAGCCACCTTTTTGAGAATGGTCTTTCCCCGCCATTTCCATGGTTACACCAACTAAA
TCCCACTTGGCAACCCATTCAACTTTCCACGGAAGTTTGCCATTACCGTCAAAAGGAGAATCCA
GCCTTGATGTCCACATCCTTTCACCCAGTCAGTGGCATCTTCCTTACAGGTAAAAAATACTTCTGA

ACCATTATAATCAGTTACATTGGTAGTTGCTATCTTGCCACAATTAGGACAGATTACTTGAAAAG
GATACCAATTATTAGCGCGATCGGCTTTACTTACATCTCGATAAACTTCTCTAACATCCTGAGCA
TGATTGAGAAATTTGTCGATATAGGGGTTTAATTTTCCTGAACGATATAAGTCTCTCAAATAGTA
AATTTCGGCTTTCACCCCTAAATATTCAAATACTTCTAAAAATTCACCCATAAAATATTTGGCAT
AATCAGGGGCATTTTCGTCGGGAGAAGGAACATTACAAAGAGGATAACCTAAATAAGGAGAAAA
TTTTTCTTTATCTAAATAATGGGGGACTGTATCTAAAGCGTCGTAATCATCAACTCCATAGGTAA
ACTTTACCGGCTTTCCTGCTCTTTTCAAAGCCCGATACATAACATCATGAATAATCACCCCTCTAA
CAGAACCTACATGAACTCTACCTGAAGGGGTTTTGGAATCGTTTACTAATTCTTCTTGGTGTGAG
GAAGCCGCTATTTTATCCGCCCAAAACATATTTATAATTTATTGTTCACTATCTTTAAACTGCAG
AATATCCATTATATCTTACTTTCGCTATCTAACCTCAGCTCGATCGAAGAATATCGGATGAAAGC
AAGTTTTAGGTTGCAGGTATTTTTTAATTATCAACTATTTATTTAACCCTCTTTTGTCAAACTCC
GAATCAACTATCAACTCGTCACCATTGCCCCTTGTCCATTGCTCTTTTGCCCATCACTCAGAGATG
AAAATTTATCCTGAACTCAAGTTAATTAAACTATCTTGACATTTTTAAGCAAACATTATATAATT
GCCCCAAAATGTAGTAAAAATAACATTTAACTTATGAAGGAGGACATAAGGTTGGCAAGAAAAA
GAAAACGTAAAAGTCGCCGCCGCCTTGAGGGACGCAAAATATTAGAGCTAGTGCCTCACTATCAT
ATAGAAAGTGGTGAAGATAAACCAGTCACCGCCGCTCGTAAACATATAAGAGCAAATGGAATTAC
ACCTCCTGCTGTTTTAGTTGTCAAAAGAAATGAACACACAACTGATCGCTATTTCTGGGCAGAAA
AAGGTTTGTTTGGTGCTCAGTATGTTGAGGAAAATCATTTTCTTTTTCCTAGTTTGAGGATTATT
AAACCCCAAGTTAAGGGAACTGCTCCTATTTTATCTACCACTCATTAAGCTAATACTTTTTATTT
ATTGGGGCTCGGAATAAATTTTAATATTAGTTTTTTAGTCTTGAGATTATAGTTAACTAAATTTA
TTTTTTTCAACTGATTTTTTAGACTAACTTAAACTAGCTTTAAGATTTATTTCTGAGCTTCTTTT
TATTTGACAGTGCTTTCTGGCTCATTAATTTGATTGCTATTTATCTTAAACTTTATAATTTTGAG
GAGGAGTTAAACTTTAAGATTACACATTTATAAGTTTAGAATTGTCAGTATTTTGATAATTATT
AATTTCTTTAAAAATCTTATTTCATATTTTGAAGAAAAGATATTTATAAAAAAACCAATTTTTTC
AATTTTTCACCCCGGCAACCCCAGCTTATTTCTTTTAGAACCAAGCAAGAGAGGGAGGCTGATAT
AAAAGATAGATGAATGAATTTAATGATGGCAGGAAAAATTTATTCTAAACCCCATTGATGTTTT
AATGTGTCTTTATAAAGATTTTCCCTAACAAGCATTTCTGCATAAAGTTTAACTAAAAAGTCTTG
TGCTTGTTCTTTTGTCATTTTCTCTACCTGAGTTTCAAAAGAACGAAGATTGAATTGTTGTTCTA
AAGACAAACCGACTGAATTAGACATAATCTCATATTCTCCTTTACTTTCAAAGTAAAAACTGCTA
TCCTTGATAAGGGTAAAATTTTGCGTTTTACCTAGTGTCAAATCAATTATCTTAATGAGTAACAT
AACCAAAATTGTTAAACTGCTCATCTTGAACAATAAGCATAAATACTTATAGTAAAAAAGTTCGT
CAGTTAACTGAGGTTAAAAAAAGTTAAAGATATTTCCTATTATACTTAACAGAAATTAATAACT
GTGATAGGATCTAAGGGAATAAATAAGAACTGTTAGTAAAACAAATACCAATACTTCAGATTAT
TCTCGATAAAGATAAAATAAAGTTCTAAACTAAGTTGATAATAAACAGTCAATGATCTTAATAG
CTTCATCTGACAGACAAAATAAAATAAAACATCAGCGATTAAGAGTTAGTTATGCCTTGTAGGGT
TAAACTAGCTCATTTGTTGTGAATAGAAAGAGTTAATCTGTGGTAATTTTATAAATTTTACGAA
TAATCAACCAAATATTTAACATTAATGGAGGAAATCTAAATGTCTGTACGTCTATATGTGAAGTT
ACCAAAGGCGGAAATAGAGAAAGAAGGTTTAGAAAAAATGTTTAGTGATTTTGAGCCTTCTTTT
ACCACTAAGTTAATAAAAGAAAGAAAGAAAAAAGAATGTCGTGGTTTTGGTTTTGTAACTGTCC
CCAGTGATGAGGCGGCGGAAGATTTTATTAACAGATACAATGAAAAGCCATTAGTTTATAATGG
TGAAACCTATAAGGATGAAGAGGGTAATGATTTCCTTTTAGTTATAGAAAAAGCGTTACCTCGTA
ATAAGGGTAGCAAGGAAGAAGGGGAACAACAAGAAGAAGCTGAAACCACTAATAATAATCAGGT
AGAAGCTAACACCGATAATAAGCCTTCTCGTCGTAATAATAACAATAAAAAACCGAAGAAAAATC

GTAAAGGTGGTAAGGCTAAAACTCAAAAAGCCGTTTCGGTTTCTGAATCTATTCAACCAGATCCT
CGTTGGGCAAATGAACTCAATAAGTTAAAAGAAATGTTTGCCGCTCAGACTTCTAATTAGGATTT
GACATTGTTATAAAACAAATTCCTTCATCAGCTCTACTTTATGATTTATTGTAGGGCTTTTTTCT
TTTTTGAAGGGAGTTACAAAGGAGAGATTGTTCTGATAAGGTTGATAACTCTTAGCAAAAATAT
TTTCTGGCGTATATCGGAACTTATTTGAGGTGGATATTTACTATTTGCTTGTTTTTCTGTGAGGG
TCACATATTCCTCTGTTGTAAGGGGTTCATTATTAAGAGGCGATCGCGCTTCTGTAATAATTTCT
GTTGCTAAAACTTCTTCCGGAATATCATCGGGAGGAGGTAAGGCAATAGCTGAAAAATGACCAAA
AAAGAATAAAAACCACAATAAAGAGAATCTGCCCAAAAAAGAACTCATAAAAAAGATCCTAACG
AAAAAAATTAAGCGAATTGAGAAAAAAACAGACAGAAAAAATTAATTGACCCAAATAATAGGCT
ATATTATACTATTAACCGATCATCGCTATTTTAACAACTACGAGAAGTAAGGAGGTTGCTATTTC
CGAACAACTAAACCTGACTGTTAGTCTTAGAGGGACTCGTGAAGTCAGAAATAATTGTCAAATCT
TTCATCTCATTGGTCAACTAGACGCATTTTCTGAGCCAACTTTTCAGAAAATCATTTCTGGGTAT
ATTTCAGATAAAGCTAATAAAATTGTCCTTGATTTATCTCAAATCGATTTTATTGATAGTTCTGG
ACTAGGTGCGTTAGTACGCATTGCCAAACAAGTTGAGAATGTGAAAGGAAAAATGCAAATTGTT
ACTAATCCTAGAGTTACCCAGACTGTAAAAATGGTAAGATTAGAGAAGTTTCTTAAGTTACAGAA
CTCTCTCGATGAGGCTTTGAAAAATGTTTAACTCATGTCTTGACTATTACAATCAAAAATGGATT
TGTGAGGATAGGAGTTTGACAAGGACGTAGAAAACTAACTACGTCCATGACCAATTGATAAGTT
AGTAATAAATATTCGAGTTTGACTACGGTTAAATCAATTAATTTTAGTCAACCATTACGTTTGTT
CTAACAATAAAGTCTCAGAATAAATTTAAACTACAATCTTGATTTACGGATTTGTCTAAGAAAAA
AACACAACTTTCATTTTCTCCACCACCTCACTCTGAAACTCGGAATGAGGTAAAAGTCTTGTCTA
AATTGGTGTTTAAAAGTGATGACATTTCTAAAGTATTGTCGCAAGTTTCTCCTGATGCTCTCGCT
TATATTGGCGATGCAGTTTATGAGCTATATATTCGTACCTCCTACTTATTGCCCTCAAAAAAAAT
AGCGGACTACCATCATGTTGTTGTCAGCAAAGTCAGGGCGGAGTCTCAGGCAACATATTTACAAA
ATATTTATCCTCTTCTCAACGACATAGAGAAAGATTGGGTGCGTAGGGGTAGAAATGCGGTTAAT
AAATCTCCTCGTCGTTTACCGTTACAAACTTATCAAGCGGCTAGTGGATTTGAAACTCTCTTGGG
ATACTTGTATATTTCTGAGCCAGAAAGGTTAGAGTTTCTTTTATCTCATTTAACGGACGTTACAC
AAAAATATAATTAACCTCAGTTCGGTTTAAGAATATTGGATAAGGGTAGGTGTCAGGTTTCAGG
TTGCAGGTTGCAGGTTGCAGGTGTCAAGGTTTTAAGGGATGGGGAGATGAGGGGATGGGGGGAT
GAGGGGAGAGGGAGAAGTAGGGGCGAATGGCCATTCGCCCCTACCGGAGTTCAGGGTTTTTAATT
CTTAATTCTTAATTCTTAATTTTTCATTTGCCCCTTGTCCTTTTGAGATTTATTCATGATTAATA
ACTATTCACCCCATCACTAAAATCGCTATACTGATTATCAATAGAGCGACATAACAAATGCTGAA
TGATTCTGAGTTATTGCCTAAATCCCGATTGTTCTCTGCCACAAAACCCATCTAATTTAAAGATT
TGTCAAAATTGTGGTGGCGATTTAATTTTACATGGGCGTTATCGTGCGGTTAAGAAAATTGGTAA
GGGTGGATTTGGGGCAACTTTTTTGAGTATAGATTTATCTCTGCCGGGTAATCCCCTATGTGTAG
TGAAACAATTAAGACCTTCTGCGAATGACCCTGATGCTTTTGAGATGGCATTAGATTTGTTTGAG
AGAGAGGCAAAAACTTTAGGAAAATTAGATCATCCTCAAATACCTAGATTGTTGGATTACTTTGA
ACAAGATAAGAGATTTTATTTAGTACAAAATTGGGTCAAGGGTAAGACGTTGCAACCAAGAGGTG
AAAAAAGATGGGATTTTTAGTGAAACCAAGGTGAAAAAATTTTTGTTGGAAGTTTTAGAGATAC
TTAAATATATTCATTCTTTAAAGATTATTCATCGAGATATTAAACCCGCAAATATTATTCGTCGT
GAGCAGGATGGGAGACTTGTTTTAATCGATTTTGGAGCGGTAAAGGATCAAGTTAATAGTCATTT
AGCTAAAACTTATGGACAAACGGCTTTAACGGAATTTGCTGTGGGTACAATGGGATTTGCCCCCC
CTGAACAATTAGCAATGCGTCCTTTTTATGCTAGTGATATTTATGCTTTGGGGGCAACGTGTCTT
TATCTGATGAGTGCGAAGTCTCCAAAAGATTTTCCCATTGATGAGTTAACGGGAGAATTAGCTTG

GCAGAGAGAAATAAAGGTAAGTAAAAGTTTTGAAAAAGTCCTTTCTCGAATGTTGGAAATGAAT
TTGCGCGATCGCTATAAGTCGGCGGATGAGGTAATTAAGGATTTAGATATAATGCCCTATGCAAA
GGAATTAGAGGAAGGAATGTTGAATGCTCAAGGGAAAGATAAAAAAGAAATAGAGGAAAAAGAT
AATACATCCCAAAATACTCATTTATCAGCAACTTCTCGTTTAGCCATGGCAATTAGAGCGAGAAA
ATCTCGTCAGGGAAAAAGTAAGTATATAACTCAGGTTAACCCTCGTACTGTTTTAAATGCGTATC
TCAATGGGAGAAGGGATTTTAATGGACAAAATTTCAATCAATTTAATTTTGCTCAAGGGGAAAT
ACCTGATATTAATTTTCGTAACTGTCAGTTAATTAGAGTAAATTTTGAAGAGGCTAACTTAGAAG
GAGCTAATTTTTATTGTGCGAATTTAGCAGGAGCAAGATTTTTTAAAGCCAATCTAACTGGAGTT
TATTTTTTTAAAGCTGATTTACAATCAGCCGATTTGCGCAATGCTAATTTAAAATCTGCTAATTT
GGAAGGTGCTAATTTGGAAGGAACTAATCTTTGCGGTGCAAACTTAACGGACGCAAATATAACAG
AGGAACAAATACAACAAAGTCAAACTAATTGGGCTACCATATTTCCAGATGGTAAGAGACATATC
TGGTAAAACCTCAGTTTAGGTTAGAATTGATTAAAACCTATTGGAAATATAGTCACTGATTGAGC
CTTGATTGAAAAAGAGACTTTAAAACTACTAGAATGGCAAAGGTTATGTCAACATCTTTCCACTT
TTGCCTCAACAAAGTTAGGTGCGATCGCATCTCAAAATTTAACCATTCCTTCTACCCTTGCTCACA
CGAGGTTATTATTAGCACAGACTCAAGAAGTTGTCACATTAGAAACAGATATTAACTGTAATTGG
TCTTTTCAGGGTATTCATGACGTGGGTGATGGTATTGAAAGGGCAAAAATGGGCGGTATTTTATC
AGGGGAGGAGTTGCTCAATTTAGCGACAACTTTGGCAGGGGTGCGTAAATTAAGAAGAATTATT
GATGATAAAAATGACTTAATTACTCTCCAAGAATTAGTTGCGAATATTAGAACTTTTCCTGAATT
AGAACAAGAAATTCATTATTGTATAGATGATAGGGGCGAAATCACAGAAAGAGCAAGTCCCCAA
CTAGCAGAAATTAGACAAAAAATAAAGTCCTTACGCAGTAAAATTCAACAAACTTTACAAGGTA
TTATTCAACGTTATGGCAATGCCTTACAAGAATTAGTCATCACCCAAAGGGGCGATCGCTTCGTG
CTACCAGTGAAAGCGGATCATAGTGGTATAATTACCGGTATTGTCCATGATACTTCTAGTACAGG
CTCAACTTTATACGTTGAACCGAAAGCGGTGGTTGAATTGGGAAATAAGCTACAAACTAGCCGTA
ATCAAGAAAAAAGAGAAGAAGAAAAGATATTAAGAGCTTTATCGGAGAAAGTAGCCGAAAATTG
GGAAGACTTAGAAAAATTACTCGCTGTGGCAACCACTTTGGATTTAGCAACCGCAAGGGCAAGAT
ATAGCCTATGGTTAGATGCTTATCCCCCTGAGTTTGTGGATTTTGCTGACGGAGAAAGCATCACT
TTAAGACAGGTTAGACATCCTCTATTAATGTGGCAAGAAAGACACGAAGAAGGAAGTCAAGTAA
TTCCCATTGATGTGCGTATTTCCCCAGAAACAAGGGTTGTCGCCATTACAGGACCGAATACAGGG
GGTAAAACTGTCACATTGAAAACCATCGGTATGGTGGCGTTAATGGCAAAAGTAGGTTTATTTAT
TCCTGCAAAATCCCCCGCAAGAATTCCTTGGTTTAATAATGTTTTAGCCGATATTGGTGATGAGC
AGTCCTTACAACAAAGTTTATCTACTTTTTCAGGGCATATTCGTCGTATTGTCCGTATTTTAGAT
GAAATTAAAGAAAATAACTCGTTAGTATTGTTAGATGAAGTTGGAGCTGGTACAGATCCCAATG
AAGGAACTGCGATCGCAATTTCTATTTTGAAATATTTAGCTAGTAATAACCTCTTGACCATCGCC
ACTACCCACTATGGGGAGTTAAAAAGCCTGAAATATAGCGATTCCCGCTTTGAGAATGCGTCAGT
GGAATTTGACGATGTGAGTTTACAGCCTACCTATCGTTTATTATGGGGCATTCCGGGGCGCTCTA
ATGCCATTACTATCGCTCAAAGATTGGGGTTAAATGATGACATTGTTGCCGAAGCAAAAGAATTA
GTCGGAGGCTTTTCTGCGGATGTCAATGAATTGATTTCTGCCTTAGAAAAACAGAGGAAAGAGCA
AGAAGATAAGCAGAAACAGGCACAGGATTTGTTAACCAAAACGGAGTTATTCTATCAACAGGTA
GAAGCAAAAGCCATCTCTTTACAGGCAAGAGAGAGAGATTTAAAGAAAGAACAAGAACAAGAAG
TTCATAAGCTCTTATTAGACGCTAAAAGCCAAATTAACCAAGTTATTAAGGGGTTAAAGCAAAAA
GGAAGTCCCACCGCCCAAGATGCACACCAAGCAACGGAAAATTTAAGGAAGATTGGCGATCGCTT
TTTGACTCCTATTCAAAAAAGTAAGCCCAAATCTAGTTATAAACCCAAAGTGGGAGAAAGGGTCA
GAATTTTGAGTATTGGGCAGGTAGCGGAAGTGTTGGGGGTAGATGACAGCAACGAGCAAATCAG

CGCCCGTTTTGGCTTAATGAAAATGGTTATCCCCTTCTCTGATTTAGAATCCCTTGACGGTAAAC
GATGGGAAAAACCTGAAACTCCCGTTAAAGAAAAACCCCCCGTTACCCCTGGGAAAAATCCTGTC
AAAACGGAGACGAAAAGCCCCCCTACAGCTATACGTACTTCCCGCAACAGTGTTGATATTCGAGG
TAAAAGAGTACATTTAGCCTATCCTATCTTAGAAAAAGCGATCGCATCTGCAACGGACATGGGTA
CAGTGTGGATTGTGCATGGTAAGGGGACAGGAAGCCTACGACAAGGGGTACACGAATTTTTAGAT
CGTCATCCCCAAGTTAGTCATTATGAACTAGCACCCCAAAAAGAAGGAGGGGCAGGGGTTACGGT
TGCTTATTTGAATTAAGAAAAGGGCAAAGGTGAATAGTGAATAGTGAATAGTTGATAATTAAAA
ATTAAAAACTCCGAACATTTGTACGAGGGAATAGCCATTGCCCCTACTTCCCCCTCATCACCCCTT
CCCCTCATCACCTGATACCTTTTTCCTAATCCATTACTTCCCCTTAAAGCACCGATTAAAGTCTGG
TTTTTTCCGCCCAAACACGGGAGGACAATCCCCAAATATAAATGAATCCCTCGGCGGCTTTATGG
TCAAATTTGTCGTCACTACCATAGGTTGACAAATCAGGACTATAAAGAGAATAATGAGATTTTCT
GCCCACAATGGTAGCATTACCCTTGAAAAATTTAATTCTCACAATGCCACTTACTCTATCTTGAG
TTTGATCAATAAAAGCGTCTAACGCAGATTTTAACGGGCTATACCATAAACCCTCATAAACCAAA
CGAGTGTACATTAAGTCAATTTGAGCTCGTTTATAATGGGTAACATCGCTGGTTAAAGTGAGGCT
TTCTAAATCTTCATGGGCTTTAATTAAGACCAACAATGCAGGAGCTTCATAAATTTCTCTCGACT
TGATACCCACAACTCTATTTTCAATCATATCCAATCTGCCAACACCGTGATTACCAGCAATTTCAT
TCAATTTAGTAATCAGTGCCACAGGGGATAAGGCTTCTCCATCTACGGCTACGGGAATACCCTTT
TCAAAAGTAATTTCTACATATTGAGGCTCATTAGGGGCATCAGTAATAGATTTAGTTAAAGCATA
AATTTCTTCTGGGGGTTCTGTCATCGGATCTTCTAAAGGTCCTGCTTCTATACTACGTCCTAATA
AGTTGCGATCGATGCTATAGGGAGAAGATTTTTTGACAGGAGACTCAATACCAAATTTTTCTCCA
TAGGCAATAGTTTCTTCTCTACTCATTCCCCACTCTCTAGCAGGGGCTAAAATTTTCAAATTAGG
ATTTAAAGCAGTAATTCCTAAGTCAAATCTGACTTGATCATTTCCTTTTCCTGTGCAACCATGAG
CAACAGCATCCGCTCCATATTTTTCAGCCGCCTCGACTAATAATTTTGCAATTAAAGGACGAGCT
AAAGCAGTAGATAGAGGATAACGATTTTCGTATAAAGCATTAGCTTTAATGGCAGGGAAAGCAT
AATTAGTGACAAACTCTTCCGTTGCGTTTTCAACCTAAAGACTCAACAGCACCACATTTGAGGGCT
TTTTCTTGAATAGGAGCTAATTCTTCTCCTTGCCCTAAATCTGCCGCTAGGGTGATAACTTCTTTT
ACACCCCATTCGTTTTTGAGGTAGGGAATACAGACGGAGGTGTCAACCCCTCCAGAATAAGCTAA
AACTACTTTATTGGCACGACTCATATAATTTACATTACCCAACATTATGACTAACAGGTTATTGT
AACAATAAGAGCATACCAAACCCAATTGCAAGTCAAGATTTGTCTATTTTTATCGAAAAAATTTT
TTGTTATTTAGCCTTGACTTTTTTTTTTTCTTTCGTTAACATTGAATATGTCAGTCAAATGGGAT
TGTAGCTCAGTTGGTTAGAGTACCTGCCTGTCACGCAGGGTGTCGCGGGTTCGAGTCCCGTCAGT
CCCGTAACTTGAAGGCTTCTATCGGAGTCTTATTGAGCATTATTGAGGTATTTTTTTAAGTATGT
CATCATGAAATAAAAATAGGTCTTGTATTTCTGAGAAAGTAGTGTTTTACTTTTAGGGATTGAT
AATTTTAATATGAAATATTATCTTGTTGACGTAAGACTCCCATTAAATAACAAGTTTGAGTAGAG
TGAGATGTAGGGGTCAACGGCCGTTGAACCGTACAACGCCAACGTTATGCTATAATTAATGATGT
GGAGTTAACGTCCACCTACGGGGAGTCCCTCCGAAAGTTACGGCTAACCATGAATTGGCATGGTG
TCTAGGTTTCCTAGATGGATGTATGATCCAGTAAGGTAAGTACCAGTCAGCCTAGCTGTTCCTCG
TGAGAGAGGAGGGAATTGCAAGTAATTGCAGTTCAGAATCTCCCATTACAATCTATTGATTTAAT
GGTGAGAGTCTTCAAGGAATATTCGTCAAACGAACCGAGGTTGCCTACTTCGTTATATTGAAAGT
CAGGATTCCTCGCTCGTTTCATATCATTAAATAATTGCGCTTCTTTCCAGAATAAGCAACTCTCTT
AGTTGATCGGTGTCAAGGTTAGTTAACCAATCTTCTCCGCTATTAATTGTTTGTTCTGCTAACTG
TTGTTTACTTTCCAAAATATCATTGATTTTTTCTTCCAAAGTACCACTGCAAACAAATTTATGCA
CTTGAACATTTTGTTTTTGTCCAATACGATATGCTCTATCTGTTGCCTGATTTTCTACTGCAGGA

TTCCACCATCTATCATAATGAAAAACATGATTTGCTCTGGTTAAATTTAAGCCTGTTCCTCCTGC
TTTTAATGATAGTATAAATATCGGAGGCCCTTGAGGATCATTTTGGAAGCGATCGACCATTTCTT
GGCGCTTTTCTGTTTTTGTACTACCTGATAAAAACAATACTTCAACCCCTAACTTTTTACGCAAA
TAGGGTTGTAATAGATTTCCCCACTCTGCAAATTGAGTAAAAATTAAACTGCGATCGCCCTCTGC
TACCACTTCTTCTAACATCTCTTCTAGGCGTAACAATTTACCAGAGCGAGAGGAAAAATTGAGCT
TTTCTTCTTTTAATAAATGAGCAGGATGATTGCAAACTTGCTTTAATTTCATCAGCAAAGTTAAT
ACTAAACCATGACGTTTAATTCCCGTAGTTTCGTTAATTTTTTCTAAGGATTCATCCACTAAATT
TTGATATAATTGAGCTTGTTCACTAGACAATCCACAATAGACATTCATTTCCTGTTTTTCAGGTA
AATCTTGAATAATATTTTTGTCTGTTTTTAAGCGTCTCAAAATAAAAGGTTGTACTAAAGAGCGT
AGGGTTTGTAAGGAATGTTGATCCCCATATTTTTCAATAGGCAAGGTAAAACGCCGTTGGAAAAA
TTGTTGTGTCCCTAAATAACCGGGGTTTAAAAACTCTAAAATTGACCATAATTCTGAGAGTCTAT
TTTCTACGGGTGTACCCGTTAAAGCAATTCTAAATTCAGAATTTAATTCATGAACTGCTTGAGTT
TGTTTTGCTTGAGGATTTTTGATATTTTGTGCCTCATCGAGAATAATTCCTTGCCATTCTACTAA
GTTTAACGTATCAAAATCTCTATAAACTAAAGAATAACTGGTGATGATAATATCTAATTTTTTGC
TAGTCTTAACAAACTCTTTTTCCTTACTACGATTATCCCCATGATGAATTAAGGCATTTAAAGAA
GGAGCGAATTTTTTTATTTCCCTTGCCCAGTTATTTAAAACCGAAGTTGGGGCAACAATTAAACT
AGGTTTATTTAGCTCTTTTTTCTCTTTTAAAGTTAAGATAAAAGCAATTAATTGTATCGTTTTTC
CTAATCCCATATCATCGGCTAAACATCCTCCTAAATTCCATTTTTGTAGAAAATATAACCAGCTA
AATCCTAATTCTTGATAAGGGCGCAAAGTACCTTGAAAATCTTTTGGTGAATTCAATACTTCTAC
TTTTTGATTGTCATTAATACTATTGATTAAATCTGCCAAAGCACCGCTACTTTCAAAGGCGACAA
CGGGTAATTTGGCGATGGTTTCACTGTTCCCGCTACTCAAGCGTAGAGCATCTTCTACGGTTAAA
TTAGTGCCAGAATCAGTATTATTTAAAATGTCTTCTGCGGCTTTTACATCGGCGGGTTGTAAGGC
TAACCATTGTCCGTCAATTTCTACAATAGGAGATTTTTGTGCTAGTAAATTTTGAAACTCTTTTT
TGGTTAAAGTTTTATCTCCTACGGCAACTTTCAAATTATAATTGAGTAGAGTATTTAAGTTTAAT
CTTTCACCTTTTTTAAGGTTAACTTTGGCTTCTATTTTTACTCCCAAACGTTTTTCATCAACACCA
CTATTTAAACCATTGGGTAAAATTATTCCTAATCCATTATCTTGTAATTGCCATACCGCACTGCG
AATAAATTGATAAGCAGAAATTGCATTTAAAGAGCAATAATTCGGCATTGATTCTTGTAAACTT
TCCGCTATCGGTTCATAGAATTTTGCCGCTAATCCTAACCCCTTCAAAATGATTTCTTGAGGATT
ATCAATGATGCGATCGCCTATTTCTAATTGGGGGTGAGGATGTAACCAAATTTGCTCGGTAGGAA
TTAAAAAATCAGGATTATCTATCGCTTGTAAATAATAATCTAGTTTCCAATTATCGTAATTATCT
GTTTGATTTTGCCCACTAGGAGGCTGTAATTTAAAACAAAGTCTATATTGTTTTTTGACCAATTG
ATAATCAGAAGAAATTAAGTTTTCTTGAATCGGTAAAGTCCAATTATCAAAAGCCATTTGTAGTC
TTTTATTATCTAAAGAAGATAAATGACATTTACTTTTTTCCTCACTATTCAATGATTCTAACCAA
GTTTGCACCATTAATTCATTATTAACAGGGGTAGAAATATTTGTTAATAAACATCTTATTTGAGC
ATCTAAAATAGTAGCTAAAAATTCTAAAATTAACTTTTGTTGAGGAATATTAGGATCGTCTTCAT
TTTCTACATAACAACGACAAATAGAGGGCATTAATTGTATAAAATGAGCTAATCGACCCTGATCA
ATATTACTATCTAATAAAGGTTGCCAAAAACAGTTATTTTTCTCATCAATACTAGGAATAAATTT
TTGACGACAAATTAAATCTAAAGACCAACGATAAACATGACTCCAAAATCTGAGATCAGGGGCTA
TATAATCTACTTTATTTAAGGGTAACTGTTGTAATAGCCTTAAGAGTTGATCCATGTTTAACTTA
ACTCCGCACACATCCCAAGGATAGAGAATAACAGAACTTAATTTATTGCTAATACCCAAATAATG
CTCATAAAGGAGAGGTATAACCGTTTTTGATTTTGGTTTTTTCTGAACAGGAAATAAAGCCATTT
CCCACTGCCAAAAAGATTCAGGATCTTTATTATTTATAGCTTCATCAGAAATTATACCGCCCAAA
AAATTCTGTTTTAAAAGCTCACCTAATTCTTGCTTATCTTCAATTAAAAAAGGATAAATATATAA

AAACTCTGCCTCTAAATCAAACTTTTCTTTCTCTATATTTCTCCACTTTTCTCCCCAAATAATAAA
GTATTTTTCTTCTTTTTTAATTAAAAAACTTCCATGTAAAATTGACATATTTAATTTTAGTAATT
ACAATGAATGAGGATAAATAAAATAATAAAATGTTTAATTTAATATATAGAAAATATTGTGTTT
AATTGACAAAAACTAAAGACAATTAGTAGAAAAGTGATAATTTAATCCTAATGAACTCACTATA
CATGATGGATATATTTATTTTAAATTTTAAGAAATTTATTAATATTTTTACGATAATTTACTAAC
TGATGATTATAAAAAAATATAGTTAGTCTCGATAAAAAATAATTCTAATAATAATTAATGTTAAT
TGATTTAAAATAAGAAGTAAGATATAAAAAATTTATTTATAAATAACAATAATCATTGTAGTTA
TCAACATTTATTTTTCTAATTATTAGTTTTGTGAAAACCGATGACATATATTCATGAGAATAAGA
TTTAATATAAAATGACAATGCCATCGAATAAAATATCATGGTAAAAAATAGACACAAAGTATAA
CTATATAGCAGAAAAACCAACAAAAAATTAGTGAAATTATACTTCTGCCGGTGAATTAAAGTTA
TTTAAGACTATTTTTCATCTGAGATAAGGAACTAATAGTAAGGTAACATAGCAATGTTGATGAT
TTGTTATATTATCATCTTTGGTGAATTTTCCATAGTAGGGAACTTAATTTCTTAGATTGAAGGT
AGATAACGTGAGTAATGACCAAAATACTCAACCTTTCTCTTTCTTCTCTTAAAAGGGTAGATACA
GAAGGGTTTGTCTTTTCTCTAACCTAACCAACCTCAGTTCGGTTTAAGAATATCCGACAAGGTTA
GGTGTCAGGTTGCAGGTTGCAGGTGGTAGGGTTTGAATATATTTAACGTCAGTTCGGGTTAAGGT
AATTTTATCGTTATTTCTAAGAAGAAGCTATAAGGTTTTCTGACTGCGAGAAAATAATGCTTTCA
GCTTATTCAAAACTCAGGTTAACCAGTAAAATTAGATGAAATTCTAGCTTATCTGTCCTTCGCTT
TTTTACCTTTCCTTAATTTTTACGAATAATGCGGCGAGAATCACATTTTTCTCTTACTTCTTCTTC
ACTGAGATTAAATTCGGCTTTCACAGAGTAATTTCTTCCTTCATGGAAACTGGAGCTATAAAGAA
AAGTTAGAGGAGTAAAACCAACTAAACAAACTTCTTCATTTTCTTCTATATCTGCGATAATTTCT
TGATCTACTCGACTACTCATGAATTTTTCTAATGACATAGCATTAGGATAAGTAAAGTCCCAACG
AGTTAATATAGCAAAAATCATTAACACCATTGCGGCAACATAACCATAATTTTCAGGTTTTAGCC
ATTTATTTTTAGTGAAGGAATAATGATTAATTAATAAGTATAAATTTAAGGAAATTAATATTAT
TATCCAGTACATATAGTAACGTAATTGATAAGAAAAAGGCATAAATGGTGTTATCAAACTTAAA
ACAATTATAAATATTAAAGCTATTCTAGTTTCAGGGTTATTTTTCTTACATAAAAAAGCAAATA
AAACCACATTAAATATTACATAAAAAGCAAAATATCCCCCCATACCAAAACTATCCGCTTCTAAA
GGCACATAGTCCATTGCAATAGTCCAAGGCCAAGGGCGACGCTCATCAAAAGCACCTATTTCTAA
TAGAGAAAATATCCATCTTTGAATAGGAGACATAGATGCGATCGCATCTGCCATATAGGTAGAAC
TAGGTACAACTGTGTGATTTAACTCATAACCAAAAAGAGAAACTTTTAAAGGATAAAAAGGATT
TTGATAAAACCATAAATTTTTAACCTCTGTTGCAAAAATTAATAATCCAGAGGTAAAGCTAATTA
TTAATAATTTAACAGTATTTTTTACTTTATTAACAGGATTGAAGCGATAAAAAATTAACCACAA
AATTCTACCCAAAACTACTAAAATTAATAAAAATAAAGGTGGTACTAAAAGATACTTACTGTTA
GCGGCAATAAATCCACCAATGATAAAAATAATAACGTTTTTCGCTTTAATAAAGTCTTCCTTAAT
AAATAATAAATAGGTAATGATAATAGTGATAGAAAAACCAATATTAGTAAATAAATCTACATAA
GCACTGGTAACAGCAATATGAAATAAAGGAACAGCTAATAAAGCTAAAGATGATAAATAGTAAG
GAATTTTCAGATAATGTTTAACAAAAATAAGATAACCAATGAAGCTAAAAAAACTAACTAAATT
TGCCCCTTGAGGACGCTCTAAGCCAAACAGCCACCAAAAAAAACCCTGCAAAATATTGGGTAACA
TGGTCGAAGTATTATAAAAAGGTTCTCTTTCAACTTCAAAAACATATTCTTGAGGAGAAACCATT
CCCCAAAAACGAGCGGCAAAAGGTAATTGATACATCCAACTATCACCATTAGGCCAATCCACCGC
CACAATACTTTTTAAAGCTAAAAAACTGACAATTATTCCCGCAAAAACTTGTAAAGCCTGAGAGT
TATTAATAGTAATAATTCTTTTAAAAAAAGGATTGATATTAGGGAATAAATTTATGTTTTTTGC
TAAAGGTATTTTTACACAAATTAAGCCCACAATTATTATTAATAATCTGATGTAGCGAAAATTAC
CACCAAGTTTTAACCAATATCCTACCAAGCCTAAAAATAAAGCAAAAGCTATAAGATTTAAGCCG

ATGAAAATTAGTTTATTTTTTTAACTACTTTGGATAAATTATCAGAAATAAAAGGAAATTCAC
CTTTTAATAAATAAAAACTAGCAACAGAAATTATGATGGCAAGAATCGCACTAACTAGAGGAAC
AAACATTATAGTTATAGAATTAGTTTCAGCTAATTGCTAAATTATTTTAACCGTTTTACCGAAAA
AAAATAAAAATAAAATGAGATTATTGAATTGGGGTAGTGAGGGCAGTGTTGCAAAAATTAACGC
AACTTATATTTATATTTAGTGATACTTATACACCGAAAAATCCGGCAGTAAAAATAATACCCATA
CCGCAGATAAGATAACCTACAAATTTGAGATTCAAGGCAGATGAAGAATTCTTTTGCCAATATTT
TCCTAATTGATAAGCCGTGAAACTGATAAAACTTTGAGTAATAGCAAAACCTGCTAAATAAGATA
TTAAGGGAGTTGTTTGCGCCCCAATAATACTTTCTCCGTAGGCGTAACCATGAAAAATACCTGCA
ACCACAGCTAAACTTATCACCACAAATTGAGGTAAAATTCTGTCTCTGGTTAAAAGAATACCTGT
CACAAAAACAGATAGAGAAATAATTAATTCTGGCAAAGGTAAATCAATGTTCATTAAATGTACA
ACTGTACCCAAAATGGAAGTTAAAACAAAACTTAAAGGTATTGTCATACCCCAAGAAAAGAAACT
AGCCAAAAATCCTACCCCTATTACAAAGGCTAAATGATCTAAACCAATAATAGGATGAGCCAAAC
CTGAAATCAATCCAGCAAGAAAACTATTGGGTGTTTCTCCTCCCATCGCATGGTGAGCATATGCA
GGAGTTGCAATTAATAGAAGTAAAATTGTCGATAGAATTAATCTACTGTTTATTGATAAACTTT
TCTTCATTTAGACTGCTCCAAAAAATTGTGAATACCTAATCATCATATCTTTAAACAGTCTTTTC
TCTTTACCCATAAGGGGGAATAATAAGTAATGAGTAAGATGTTATTGAACTTAGGTTGTTTATT
ACAGCCAAAATGGTACACTACCATCTGTCGCTTGAGGACGTAATCCCCTTGAGGCTAGAGTAGAG
ATTTTATCTGCATAATAAGTCGTAACGGGTAGTCTGGGAGGACGTTCTGAACCATAATGGAGTAA
AGTTAAAGATAAAACAGAATGCAGGGCTGATTCTAGGGGAAAAGGAGGATAAGTACGAATTTTT
AACGGGCGTGGTGTGCCCATATTAGCCGAGAAATCTGATGAGACTAGTAAAGCCTCTGTTTCACT
TAATTTAAAAATACTGCCTTTTGGAGCGTTTTTTGTTTTACTATCTTCCCTAGCATATAAGCGAG
GATTACCATTTTTAATAATTTCCACAAAATAAAAAGTGGCGTTAATATCCTTCCCCCATGCTAAT
AATGATTCTTTTTCCTGAAGAGGTAGTCTGCCATCACGATGAATGATAATTGTTTTATTAGAAAA
TTCTTTAACGGGAAACATATCTTGTAGGATATGACTAGGAATTATTTCTCCTTCTAGCATCGCAT
CACGGATACTATATCGTAATAATTCACCATTATTAAAATAAATTCTCGCCATGGCAGCACTGTTG
ATAGTGCCTGACAAAGCACGTTTTTTTTGACGAGAAACATCTAAACCTATAACAAAATCTGCATA
TTTGAGTGGATGAGCCAAAATATAGGGTATCGTTCCTGTTTTGGCTAAAATTCCTAGTAAAATGT
TTTTCAAAGCAAATTGATTGTTGATAGTTATTGGTCTAATTACCTGACTAGGAATGTCATTTTTT
AAGGTAAGATGTTTAAACTCTTCGTAAAGAGTCCGCTCATTATTTTTTCCTTCTATTTTTCCTTT
AATAATTGCTAGAATAATATTCGGTTTTCTTTCTTTTAATCTCTTGATCGCACTTTCTAAATTAA
ATCGACTAAGACTATGTATATTTTCTTCCCCTGCTAAGTTTAACTCATAGCCTAAGCCCTCTTTTC
CCAAATTTTTTCTAATATTACTTCTTAAATCATCTAAGTTCAAATTAGGTACAGTATTTAAAATT
CCAATTCTTATCTTCTGATTTTGAGAAGGTTTATAAACTCCAAATTTTTTCACAGAATTAAAGAC
ATTTTTGTTATCTTGATGATTATTAGCAAAAAGTAATTTAGGAGAATAATTGAGCTTGTCTTGA
GTGAGAAATAAATGCTGATGACGACTAGAAGCGTAAGCGTTGCCCACCAAATTAGTTTTTTTAAT
TAGTTCAATAATGGGAATAAGAAATTCTATTCTTTTATCCGAAGTAATTTGCAACTTTTCACTAA
TTTTTAGTCGTTCGTAATCTTGATTATAAACTCTAATTTTTAAAGCATTTACTACATAATGATAA
ATATTATTGGTTTCCACAATTAATTCTTGATCATTAGCTTCTGTAATAATACGCCGCATTTTATC
GCTGATGGAATAACTTAAGAGTCTTTGTCGATTATTATTTTCTCCTAATCTCCCGATGATAGAAG
TTATTGTCATGGCAGATTGAAAATCTGGTTTTGTGATGTCTGTGACGTGAAGTCCTAATATTTCA
TTTTTATTCTCGATCTTGCTTAGGTAGTCTTTTAGTGTTTCTTTATAGTCTAGTCTTGATTTAAG
AGAAATAGAGACAGAGGGATGATTATTGACAACATAACCATATTTCTCACATTCATACGTAATAG
TATATTTGCCTTTATTTTGAATAAATTTCTTTAGGATTTCTCTGATATTAGGATTAATATCCGCT

AGTAAGCCATGGGCTACGAAGTCAGCTATACCTTGATGGGATGGACTTATTTTATCATTAGGGAC
TATTGACTGTAGAGAGTTTTTAAAAATATCATCTTTACTTTGCCAAAGTAGAGCAATGAAATCAT
TAAATTTAGCCTCCTGTTGAGGAGAATCCGTAATTAAACATTTATTTACTCTATCCCAATACCAA
TGACCAGAGAAATTTTTTTGTAATTGATATTGAAGTTTTCTACCCACTTTATCTTTATTTTCTGA
CGTAATAGTTGCGATTGAATAAGTGGAAAACTTGGGTAAAGATTTTAGTTGAATAGGGAAAATT
TCAGTAAATAATTCCATCGGCAATTATTAATATTTAGACTACGTACTACTCTGATAAAAGTATTT
TAAACTATAGCGATCTTATTAAAGTTATGAGATTATTATTGGGTCTTTTTAACTAGATTGAATGA
AACATTCTGTAACCTTATCAAGTCTTGATTTTAAGAATAAAACAAGCATAGAGTACGGAAAATAT
TAATATTCTAATTTTCTCAGAACTAAGATAAAAAAGTAAATAATTCCACTTAGAATAGATAGAA
ACTTATCTGTGTCTCTAATCCCGAAAATGTTTCTTAATCAAAAACCTGTCTGGCGATTAATCCCCC
TGTTTTGTGCTAGTGGGAAACTACAAATGGCGATCGACCTTTGGTTGCTTAAGCAACATAAAGAA
AAAAATCATCCTCCATCTCTCCGGTTTTATACATGGAATCCTCCAGCTATTTCTATTGGGGTTAG
CCAAAAAAGACATATTCCTCCCCATTGGCAAAATTTAACCTATGAAAATCAACCTTTAGAAATTG
TTAAACGCCCCACAGGAGGTAGAGGGGTTTTACACCAAGGGGATTTAACCTATAGTGTTGTTACG
TCTTATTCCCAAGGCACTGTCACAGAGACTTATGAGGAAATTTGTCAATTTCTGATTTTAGGTTG
GAAAAAGTTGGGTTTATCTCTTCATTTAGGAAAGCCAAAACAAAAATACTTAAATTCAGCAAATT
GTTTTTCCTTAGCTACTAATGCTGATTTAATGGATGATAAGGGTAATAAATTTATTGGAAGTGCT
CAGTTAAGACAAGGAAAATATATATTGCAACATGGTTCAATGATGTTAAAATGCAATCGCACAA
CATCACTTGGCGATTGCACTCTATATGAGCAAGTATTTAATTCATCTCCGCCTCAAATCAATATA
CCCAGTGGCTTAATTTTGCCCAAAATAGTCAATGTTTTGGTAGAAGCGGCCGCCGAGTGTTTTCA
ATGTCGTTTTGAGATACAACCTCTTTCGGTAGAAGAAATTACAGAAATCACGGAGACAATTTTGA
TTTAGGTTATCGTGTCTTTGATTATTAAAGCTGTGATGGGGCTATTGACATCCTCCCGTGCCTGA
AGGCGACGGGATTCCTACTGGAATAGACTTAAGTGCTTAAATCTAATCTCTCGGTGGGTTGACGC
TTCATCGCCTACTGCTAACTTTATGCTAGTCTTACGCCCTGCTCCACGTCCGTTTAAAGTCTCTGA
ATGCCCTCCAGCGACTAAGATATTTTTACTTGCATTAATATCACGATCATGCACTACACCGCAAT
TAATGCAAGTCCATTCTCTTATATTTAATTCTTTTTTACCGCCATTAAATCCACAATTAGAACAT
ACTTGAGATGTTGGTATCCACCGATCAATCACAATAAACTCTCTACCATACATTACAGATTTTGC
TTCTAGCAGAGTCCTAAATGTACCCCATCCCAGATCGCTAATTGCTTTACTTAATTTACGGTTTT
TTACCATGGCACTGACGTTCAAATCTTCTAATACTATCGTTTGGTTTTCACGAATAATATCCGTT
GACAATTTATGTAGAAAATCAGTGCGAGTGTCTTTTATTTTACTGTGAACTTTAGCTACTCTTTT
TCTTGCCTTTTCATCTCTTTTACTCCCTTTTTTCTTTCTACTTAAATTTCTCTGGCAACGTCTCAG
TTTCCTTTGATATTTCTTTAATGGTTTAGAAGATTTTATCTTTTGCCCATTGCTTAGAGTTGCAA
AAGTTTCAATTCCTAAATCAATCCCCACACTTTGACCATTGTCAGGCAATTTTTCAGGGATTGTC
TCCACCACAAAAGAAATAAAATACCGACTGGCAGAATCTTTGATCACTGTTGCCGATGATGGTGG
GTTAGGCAATTTTCTTGACCAGATAATTTTTACTTTACCTATTTTTGCCAACTTAATTTTATGTT
GCCCAACTTTAAAACCACCACGAGTGAACCGTGCTGATTGATTTGATTTTCTCGACTTAAACTTA
GGCGGTTTTACTTTTTTTCCCTTTCTTTTTCCTTGACAGGAATTAAAAAAGTTCTGATAGGCGAT
GTTAAAATCATTTAGTGATTGTTGCAAAGGGATTGCACTAACCTTTGACAACCATTCTCTTTTTT
CAGTTTTCTTGGCTTGGGTGATTAACAGTTTTTGAAGTTCACTATTACTTGGTTTTTTACCTCCT
TCTTTATAGATTTGCTGACACAACGCTACCCCATCATTATAAACCACACGACAACAACCAAACAG
TTGAGCTAACAACTGTTCTTGCTGTGACGTAGGATAAACTCTATAGTTATATCGTTGTTTCATGT
TAGACTAACACCGTATATATTGGTATGATAGCGAAAAAAAAGAGAAATAAACCCACAAAATAAA
AAAGTTCATCTAACGATTTTGATAACAATAGAAGAAAAAAAAGCTCAAGTTATATTGTGAGCAA

ACATGGAGGAATCAAACAGAGGTAGTGAGAGATTTTATCAGGAGGATAAGATTAAAGGAGTAGG
CTCATCGCCTTTTATTGGTTAAAGTCGCCCTAAAAGGGCGAGGCTTTTGACCCATTATTTTGGTA
AAGAATCATTACGATAATTTCTCATTGCTAGAATAATATTTTGTCGGGTTCTAATTTTTTAATTA
TAAGAACAGATTTCCCTTCAGGACGTAGTATTTTCCCGTCAAGGATAACACCATGGGAACTTGAG
CTAAACTACAGAAAGTGCGATCGCGCAGCACCACTTTGTGATCGCTTTTTATAACACGGATATAT
TTAAAATAAGACGACTTCATCATTGTCCTCGGTATTCTAACTGAAAATCGCCGTCAAACAAATAA
AAATCAGGTACGATAATAATATTAGAATTGTTACAAGAAATTAAAGAACAAAATTAATTATAAA
TTAATTTATTTTTAAGCCTTTTTAAATCTAACTTTTTTAAATCTCTAAATTAATGATGAATGAAA
AAATAAAACAAGCCCTATCTTTACTTAAAAGTTATAGTTGCCTACAATTAAAAACCATCAAATCA
GAACAAGAAAAACAGGAATTAGAGTCAAGTATAAAACTGATTGTCAGTCTTTCTGATAACCAAA
ATTTTGGCATTTGTGCATCTAATTCCCATGAAGCCTTTAATACTCTTAAAAATTATTTGAAAGCC
CTAGGCTATAAAGAAAATATAGATACAGAAAAAGTAACAGAAAATAAACCCGTTTATTTAAAGT
TTAGTACCGAAAGAAAGTCCTATCATCTTAGTGATTATTCAGGAGATTATAGAGGAGTTTTGATT
ACCATTTTTGCTGATTTTAATGAAGAAATTATTGGTACTTATGGACATTTTCCCCTTAATTTATT
CAATTGATTAGGGATGAATGAAAAAGTGGTATCCCTGAAAGTAAGCAATTAACAATTAGTAATT
AACCTGAGTTCGATGAAAAACCATTGAAAAATAAAGAGTTGAGACTTATAGTTAACAAAATTTT
CCCTGAAAAAAATCATTAAATTTAGGCAAAATTTATTTAAAATCAACTTAAACTCAGTTTATGTC
AATTAAAAACACTGTTGGCTATCTTCATCGACAATTCTATATCGAACTCAGGTTCCATCAAGTAT
CAAAAGTTTTTCACACAAGGTTAATTAATTATCCGCTACAATGTAGAACTCTCCCCAATCGTTAT
CTTCAATAGTAATATTTCTATAGTTTAGTTGACCCTGTATAGTGACATCTTCTCCTCGTTCAAAT
TGTTGACTGGTTGTAACAACCCATATTGAACCACTTTCATCCTTAACCTTATAGGCATATGAATT
AAGTAAAGGAATCGATTTTTCAATTTTTCCTTGTATAGTTACCGATTGGGTTAAATTGGTTTCGT
TTTTAATTTGAGTAATTTCTTGTATTTGAGGAGAGGCTATACCCCAATTAATTAAATTAGTACAT
CCTGTTATTCCCCAAAAAAAAAAGTTAAAGGAATTAAATATAAACTACGCTTAATTAAATTCATG
ACAGAGAATTGAAATAATATTATTTGGTTATTTATTGTAATTCAATCACGAAATAAAAAAACTG
ACCAACTTCGCAATCAAAAGTCAATCAGTTTTGGTTAAATAGTTATATACAGTGAAAAAATTATG
GGTTTAAGATTTTTTCTAAACAGTAAGTAATTAAACTAGCTCTAGCTCAAGGCTTTCTTTTTTCA
ACATTAACAACAGTGTCTCATTGTTTTCCTCTTGAGCGGCTTTGATTCTTCTTTCTAAATTACGA
CGCATATTTTCAAGATGGATGCTATGTATTTTCTCGCAAACTATTACCTTTCTTGATTTGTTATT
GATTGGGGCGACATTGGTGTTGCTATCAACGGAGTTAGACATTGTTGGATTAGTACAATAACTTA
CACCACGATATTGAAGACCTAATTTGGGTCTTAATTGTGTTACGTGTCTAGGCAATTTATGATTC
CACTGACAACCACGATATTTACCACCAACTATATCGGTTTCTTTTACTTCCAAAAAGTTGTTTTC
TGATTTATAGACGTTTCCACGATATTTCATATTCACTCTCTTAACTCCTCTGCTGATATATAAAT
AAGATTCTTATGATGTCAAAATTAAATAGATGTTACTTAATGATGTCTATTGTTTATCTCCCTGT
TTATCGTTGCTTTTCTTAACAAAATTATGACTTTATTTGATTTGGGTATAAATAGATTAGTCTAT
CGATCATCATTCTCTTAAATATCCACCTTATTTTTTATTGTGTTTAAGGTCATTACTTTTAAAGG
CTAACGATTGTAATCTATACCACATAAAGTGTACTTTTTTAAGAAATTGATTTTCAAAGAGATTT
ATTTTTTTATTTCTGTATCTATTTTTACATTTTTCCTAGTTTCTGTAAAGTGTACTTTATAAAGA
TTTAATATTTGTTCGTTTATTCTTGTTTATACTTCAGTTTGATCTAAAATTATCGGTGGAGGTGA
AAAATAGGGAAAAGAGAAGAAAGTTTATCTCAGAGCTTATATGTACTATCAAAAAACAGTATTC
ACTAAGAAATGATAAGCGCAAAATTTTTAGTTTTTGCTTACCTCCATCAAAATACTTTTTCAGCT
AACCTTAATTATCTTTGAGAAATAGATAGAAGGTACTTGGTCAACTAACCATATGTTATTCTCGG
AAAGATAAAATTTATATCCGTCTTCTGTCATTTTCTTGGTGTCAATTACTAATATCATTACTTTT

CCGTGTCTGCTACCTACTTTTTTGGCTGTTTCAATATCTGAAGAAAGATGCACATGATGACGATT
CATTTTTAGTAGTCCTTGTTTTATGATTGAATCTTTGAATTTAATTGTTGTACCGTGATAGAGAA
TATCGGGAGGATTTGCACATTTGAGATTTAATGAAACTTTAATAGAATGTCCTTGATTGGCTTTG
ATTTTGTCGGTATATGTACAAATAGTAAATCTTTTTTTATCATTAGTTTTTACTATTTCTTCTAT
CTCCTCTCGACTTATTTTTATTCCTTTTTTTTCGCAACCTGATAAAAGTTCATTTATAGAAATCC
ATCCTTTTTCATCTAATTTTATATCTATATATTCTGGTTGATGTCTAAGTATTAAACTCAAAATT
TTTGATTTTTTGATATGTTCTTTTTTCATTTTTTTTGATATTAAAGTTTATTTTTATAAAATATT
TTGATTATGATATTATAGGTTATAGTTTTATATTAAGATATTTTATTTGTTAAATAAGATAGTT
TATGTTTAAATATAATTTCCCATTCTTTTTTCTTATTATTATAATTTTTGAAATAATCAAAATTC
CTGTTCCTTTTATTTCTTTTGTTAAGTCTGCAAAAGCTGAAAAATTTAAGTCTAATATATGTGAA
AGTTCCCAATTAGAGGCTAATTTTGAGTTAAAGGAATCTAGTATTTATATCTGTACAAATAATGA
AGAAAAACAGTTGATTCAAATAAATACTAATACTCATGAAAAAATACTCAGCTTGAGGGCTTTT
GGTTCTTTTCCTACTTATGGGGCAACAGAGGGTAATTATGATGATCCTGATAGCAAAATATACAA
TATTAGTCCTTTTGATTTTAAGGTTATTCAAGCCAGTATTATTACTAAAATTGAACCCGTAATTT
CGACCCAGTATTTACCAAAAGAATTAGAAATAACTATTTTACAGGGATCATTGGAAGAAAATGCG
ATCGCATCTTGTCAACCCAGAAAACCAGTGGCAGTATTTGAAACGGAACAGGATAATATCTATAT
CTGTATTGAAGAAAAAGATAATGTTAATGCCATTGAGATGAATTATTTGCAAATTTCAAAAAAA
AGCCAAGAGGAAATAAATAACATTCCTGCTCATTTGACTAATAATTTTAGCTATGAAACTGAAAC
TAAAAATAATAAAAAATATGTTGTTAGTTATCGTGGATTAAAGATATATGAAAATCAAAAAAGT
ATTGAAGAAATACCCATAAAAAATATTTATTTATCTCATCCAGACTATACCGAAGAAGATACTCA
TTAAAGTGATAATGGATAAAGAAAAAAAACAGCTAAGAAAAAAATATTTGCAGGAAAGATTAAC
ACTATCACCGCTTCAATGGCAAGAAAAAAGTAAGCAAATTTGTCAGCATTTATCAGAAAGTTTAT
TGATTAGTAATAGTAAAATTATTCTAAGTTATTTAAGTTTTAAGCAAGAACCAGACTTAAGCCTA
TTGCACCATGATAAAAGAAAAATTTGGGGTTTACCTCGTTGTCAGGGAAATGAATTAATTTGGCA
TCAATTTACTTCTGTTAATGACTTAGAGATTGGAAAATATGGAATTCTTGAACCTTCTACTCACT
CGCCCTTGATTTACCCTCATCAGGTAGATTTAATTTTAGTTCCAGCAGTGGGGTGCGATCGCTTC
GGTTATCGGTTAGGATATGGAGGAGGATACTACGATCGCCTCTTTGCTCAACCACTATGGCAAAA
TATAGTTAGGATGGGCATTGTTTTTGACTTTGCTTATGTTGATAAACTAATAAAAGAATCCTGGG
ATAAACCCTTAGATTATATCTGTACAGAATCAGGAATAAAAACCCCTATCAAGGAACAAAAAGAG
TAAGAAATACAGTCCATAGAAATAGTTTGTTACATTATCCATTAGCCCTTTAAACTAAAAGAACT
AAACATATACTAAACCATGACTAATTTATTTAAACTCAATAGAAAAAGTATTTTTGCCACCATCG
CCTTAAGTGCAACCCTAGTAACAGGTTTACCTTACCTTGTATTTGCTCAAAGTAATCCGGGGTTA
ACTATTTTTAGTGGAGTTGATCGTAAAGATATACTAAATTATCGCTTGGATTTTGGCGGCAATGC
GGGACAATGGGATCGTTATCGTTTGAGAATTCCGGGGAAAAAACTAACTCAAGGAGTAGATAAA
TTTTTCATCAGTTATCCAGACTATTTTGATGGTAAATTTGATGTCAATAAAATAGAAGTCAGGGC
GAAAAATACCACCTTACCAGTTAGGGAAATCATTTGGGATGAAGAAAGTCGTATCATTGAAATTG
CTTTGGAAGAACCTTTACTCGAAAGTAATCAAGTGGAGTTAGTTTTCTCTAACGTTAAAAATCCT
GATAACAGTGGCACGTTTTATTTTCAAGGACAAATACTCACTCCGGGGCAAGTACCTTTAAGATT
AAGTGTTGGTACGTGGATTTTGAGTATCAACTAATATAACCTCAGTTTGGTTCAAGAATATCGAA
CAAAAGCAGGTATCAGGTATCAGGTAGGAGGCTTAGCAGAGTAATGAGTAAAAGTTACTGAGTC
AGAAACAAAAGTTTTTGGTTCTTCAGAGTGTGGTTATGATAAATTATAGAGAAACAGGACTTCT
ATAACCTTTATTAAATAGTGTTTATAGTAAATTATTATTTAATAGTCGCCTATTGCCTACTCCCT
CTTGCCTACCCTAATTAATAATTTACACGACGAGAAGTGATAGAGCCAAGTTTTTTACTATTAAC

TATTCATTATTCGCCTTTGCTCTTTCCCCAATCATGCTCAAGAAAATTTATCCAGAACTTGGATT
AGTTCGGATATACTTAAAACCTATCTTAAATGTCAAAAGAGGACTCCCGTTGCATTTGACAAGAA
AGAAATACACTACAAATCAACGGGATATGAATTTTGACCAACAAAAAAAATAAAACGAAAATCA
ATGATTAATTGATTGAGTTTTTTCCTCTTAAATGTTATAACGGTTTCTAGCAGTAATGTGCGTAA
GATATTCGGGGGAGTGACTTCCCTAGCTTCTTTCGTCGGAACAAATATCGCCCAAGAAAATGTAA
TGGTGACGCGTTTGGTAAGTTAAAGCATTGATAGCAAAGTTAAAAAATTAAACAGCTAAAGTTT
TAAATCCTTCGGCGGTAGGGCATACCGTAGAAGTAAAGGTAAAATGCTTGTGGATTCGGTCTGAC
GGGGGCATCATTAGTGATAATGATGCCTAGTTAAGAGGAATAGAAGCAAGAATCACCCGTTAAA
TCAAAGATTGTAACGGGTGAGTGTCAATTAAGCGACATATTCTTAGTAGAGAGAAGATATAGTT
CTAATCGTAATTATTACTCGACATCAATCGCAGATATAAAAAATCACAACTATTAATAAAGATAA
AAATAAGTTATAAAGAATTATAATTTGTTACTGCACAGCACTTAGAAATACAATACCTATTTAG
GAAGATGGGGGCGGAGAGACTTGAACTCTCACGACCGTTTAAGGTCAACGGATTTTCATTCTTCT
GCAACTTTCGTTACTGCTAAGTCTAGCTTTAAGAATTGGACTCTCTCTTTACCCTCGGCTTGAACC
GTTAGGGTAGTTCCCGTCGAGTCTCTGCACCTTCCGATTTCTCGGCTTGGCTCAGGATTGCCCTAT
CAAAAAAAGATGACTTAGGTTTCCCTGAATTTGAGAACTTACACTTATAAGGTTTCCCTCACAAG
GCTCAATTTTCCTTAAGTCCGTAGCGTCTACCATTCCGCCACGCCCCCAGGTGAATTGCTCACATT
TATATATTCTAGCGAAGGCATTTTTAAAATGCAAGGTAAAAAGAAAATTTTTTTTATTTTTTGGC
AAAAACTCCGCAAATGCGGCATTGGATTATATATTTACCCGTTATTGTTGAGGAATTGTAACTTC
AAGTTAAACCTATCTGTGCATTTTGATACAATAAGCTATGTTAAAAAATCTCAAATAATAAATTC
AGATGAATAAAACTGTTGGCGAAGTGATGACTCCTAATCCCATCACTGTGAAACCTGAAACTCCG
TTGAAAGAAGCGATCGCACTTTTAGTAGAGCATAAAATTAGTGGTATGCCCGTGATAAAAGAAG
GTGGAGAGTTAGTCGGGATATTATCCGAAAGTGATTTAATGTGGCAAGAAACAGGGGTTGAACC
ACCGCCATATATTATGATTTTAGACAGTATTATCTATCTACAAAATCCTAATCGTTACGATAAAG
AAATTCATAAAGCATTAGGGCAAACAGTGGCAGATGTCATGAGTGACAAACCTATTACTATTTCT
AGCACTAAAACCATAAAAGAAGCGGCTCAATTACTTCATCAAAAGCAAATTCGTCGTTTACCCGT
AGTAGATTCAGATAAAAAAATAATTGGTATTTTGACTCAGGGCGATATTATTCGTGCTATGGCTG
ATGAATAAGGTATTCTGCATTAATTATAAATTTTTATTGATATGACTATAACACCCGAATCTGTA
AAACAATTACTTTACTCCGATAATTTTGGCGATCGCATTAAAGGTATAAATGAACTAAAAAAATT
AGGAAGTGCGATCGCATTTTCACTGATACAACCAATCCTCAAAGATGAAAATGTGAGAGTGCGCT
ATGCCGCAGTATCCCAATTAGACAGTATCGGAGATGCTAATCGAGAAGAATCTTTAGAATTACTA
CTAGACACATTATATAATGATCCCGAAGCCGATGTGAGAGCCGCCGCCGCCGACGCTATTGCAGG
ATTAAAACTACAAGAAGCCTTCCCTGATCTTAAAAAAGTATATCAAGAAACCAACGATTGGTTAA
TACAATTCAGTATTATCGCCGCTTTAGGAGAATTGGGGCATCCCGAAGGATTTGATATTCTCAGC
GAAGCCCTTGCTGGAGATAACTCCCTCTTACAAACTACCGCTATTAGTGCATTAGGTGAATTAGG
AGATAAACGTGCCATTGATTTACTTTTACCCTTCGTCAACAACGAAGATTGGCAAATTAGACATC
GTTTAGCCCAAGCATTAGGAAAACTAGGGGGAGATAAAGCCCAAGAAATTTTACAAAAATTGTC
ACAAGATGAATTAGATATAGTTGCCCAAGAAGCAAAAACATATCTCGTCTAAATTTTTAACAATA
AAAGGGCGGGGAAAGGGGCAAAGGGCAAAACCCCCTTTATTCTCCCTTTCGAGGGAGGAGGGCAA
CAGTAAATATTGTTGGGGTGAATAATTAAGAATTAAAAACTCCGTTCACGACTGAAAGGAGTGT
ACGAGCGCAGCGACTCTCTTAACCCTGAACTCCTGTACGGGCGAATGGCCATTCGCCCCAAACCAA
CTTCTAACTCATTTCTCCCCAATACCCCAATACCCCAATCCCCCAACACCCCAATTGCCTATTACT
CCATCTCTTTCCACTCACTACCCATTAATTTTAATAGTTCTTCAGAGTGTGGTTATGATAAATTA
AGAGAAACAGAACTTTCATAACCTTTATTGAATAGTGTTTATAGTAAAACAAAAACTAAAAGTT

TATAAATTATTATTTAATAATCCCCTATTGCCTCTTGCCTACCCTAACTAATAATTTACACGACG
AGAAGTGATAGAGCCATTTTAATTTTATGTTACTCCTCAATAATTACTTGCCAATCTTCGTTGGA
TAAATCACAGGTTTCAAGAATTTTTTGACAAAAAGAATAAATGTCTTGGGCGGATAATTGTACTT
CTATAAAAATACCATTTTTAAGCTGACGAGTTACTCTTAAATCTCCTCTATTTTTAGATAGAAAG
CGAGGAAAAGTTAAGATTAATTCTTCAAATTTATCCGCTTCTACCTCAGAGATAATGTTTAAAGT
TGTTTCTAAAACGTCTCGCCAAGTTTTAACGGAATATTCTTCATCAAATATTTTTAAATTTCTGG
GAGTAGTGCCAGTTACACCCTTTCTTCTCTTTTGACTTTTATAATTTTTATCACCAAAATAACTCC
ATATTGTTAAAGCCATATCAGCTAATTTTTCTGCTCGATTGTCAATTTCTTCTCTACGCCATTGG
CTAATATTTTGAAAATATTTATTAATTTCTAAGTGACTATTTTTTAATTCTTTTTGTTTATTGAT
AAAACTATCATTAGATAATTCAGAATTATAAGCAGTTAAGGTTAAATTTCCTAGGGAATGTAAC
AATAATTCGTGGGTTATTTCGTAATCTTCTCCTAAATGATTTTTCCACCATTCATTTAATCTTTG
AGGCATAATATGTTCAATGGTTAGATTCTCAAAAGATACTTTTTCTTTGTGTCCAAAAGATTCTT
CCATAGATTCTAAAATTAATCGGGCTTTTTCTGTGCGATTACTACCATATAGTTTTACTTGTTTT
ATTTTTTCTCTAAATTCTTCGTCTTGGGGATAATCTTGGGTTTGTAAATGTAGTTTTAATTCGTC
TAAAAAATTGATATTTTCTAAGTCAATATTTTTACTTACTTGAGTATATAAAAGAGCAAAAATG
CGATTTAATCCCCTAGTTTGTACATTACATACAAATCGTCTGATAATAAAATTCTCTATAATTTT
ACATACATCAATAAATTCAGTTTCGCTTAACTTATTGGTTTGCCAATCATCATAACAATTCAATA
AAAAAGGATAAACTGTTTTTATATCTAAACTTTTAATTCTTATTAAATATTTTCTAACTTTAATA
TTATTTTCTTGGTTAGGATCAAGAAATCTTGAATAGTAATTAGCAAATTTATAAATATCTTTTAA
ATAATCACTAACATTATTTTTTATAGTTTGTTCTTTTAATTCAAAATAAACTTGATTCTTTTTTA
CATCTTTGCCTTTTTTTGTGAGATAGTATCTAAGAAAATCCGTGAGATTATCTTGTAATAAGTTT
TCCATCGGCAACCAATATTGTTGATACATTTTATCTTGTTTTTCTCCTTGAATACCCATGAATAA
ATAATTGCGAATTAAATCTGCTTGGGTTAAAGGTTGCCCTTTTGCGTTCAAACTTTCAAAGACTA
AATAGGGGTTATCATCTTTACTTAAAACAATACTTATAACTATTAAATAGCCTCCGATAATATTA
ATTAATTTTTTGTATTCAGTTTCGGGAAATCGTTTAATTTTACGAGTAAAAAATTGATAACATTC
AGCTATGAGATTTTTTTGTTGTTTGATATGATTATACTCTTCATTAATAATTTGATAAAAAGATT
GTCTATCTATTTGTGTTGGTAAAAGTTTATAAAAATTTTCTCCTTCATCATAGGCATTAATCAAA
TATTTATCATTAATTTCTTTAAATAATTTTAGATTATGAATTATTTTGGCTTGATCTCTAATGGT
TGCTAAAAGTATTAAGATAGTTGTTAATCTTTGTTGTCCATCTATCAATAAAAATTTGCTAACTT
CTTCGGGAAGAAATTCCATTTGCATTGTGACAATTGAACCGAAAAAATGAGGGGAATTAGATTCA
TTTTCTAATAATTCGACAATATCACTCCATAGTTGTTCCCATTGAGGTTTACCCCAACTATAAGA
ACGTTGAAACAAGGGTACAATGTATTGTTTCTCACCTTGTATGATTTCTTTTAATGTGGTTTCTC
CTGCTTGCATTGTGTTTGATTCTTATTATTTTCCTCAAATAATTGTATCATGTTTTATTTGTATA
GCAGTACTGAATCATTAATGTCAAAAAAATTCGCTCAAAAAGCTAATAAGTAATAAGCTATAAG
TAATAGTAGTAATATCTACAATGTTTTTTCTAATAATTTATTTATTAGTAATCATTGAGGATTAT
TATAATTAATTACATTTTAATAAATATCAATAAAAAATATTATTTTATGAGGAATATACAAAAT
TAAAGATAGTTAATTTAAATAATAAAATATCTCTGTTTTAATATTAATAAAATATCTCAATTAA
ACTAAAAATATAAAATTATTAAAGATGATAGTATAATCTTAAATTACCTAGAGTTTATATCAAA
TTAAATAAATTAGTAGTCAACCTCATAAATAAAATTAATGATTATAATTGGCTTAATGAGTGGC
ACTTCTGTAGATGGCATTGATGCTGCTATTGTTAATATTGAAGGACAAGGACTAGATTTAAAAGT
AGATTTATTAGTCGGTCAAACTTTTCCTTATTCTCCCTCTTTAAGAGAAGAAATTTTGCAGGTTT
GTGCTGGTAAACCTCTATCAATGGAAGAACTTAGTAAACTAGATAATGCGATCGCATCTGAATTT
GCACAAGCAGTTACAAGCATAATTCCAGACGATTTAAAAGTTGACCTAATCGGTTCTCATGGTCA

AACAGTATATCACCAACCACCACAAAAAAATCAATTAGGCTACACCTTACAATTAGGAAGGGGGG
ATTTAATCTCTTACTTAACGGGTATTAATACAGTTAGTAATTTTAGAGTAGCCGACATCGCTTTG
GGAGGGCAAGGGGCGCCCTTAGTCTCAAAAATAGATGTATGTTTATATCGCCATCCCCATCATCA
TCGTTGTTTACAAAATATTGGCGGTATTGGTAACGCCACTTATCTTCCATCGAGTCATACACCCC
AATGGCAAGAGAAAATTATGGGTTGGGATACAGGCCCCGGAAATGTTTTAATCGATTTAGCGGTA
CAAGAATTAACCAATAATCAACAAAAATTTGATTTTAATGGTGAATGGAGTCGTCAGGGAAAGC
CCTGTCAAGTCTTAGTCAAAAAATGGTTAAATCAAGAATTTTTCAAGCAAAAACCACCAAAATCT
ACGGGCAGAGAGCTTTTTAGTCCCCATTACCTTCAACAATGTCTAAAAGATGCTCAGACTTATAA
TCTTTCTTCGGCGGATTGCCTTGCTACTCTCACAGAATTAACCGTTGCTTCCATTGCCGATAGTTA
TCATCGTTTTTTGCCATCTCCCCCCCATGAAGTGGTTTTAGCAGGAGGAGGAAGTCGTAATAGTT
ACTTAAAGGAGAGATTACAAGCACATTTACCCCATTCCCATCTTCTCAATAGTGACGATTTGGGT
ATTAGTAGTGAATTTAAAGAAGCGATCGCATTTGCAATTTTAGCCTATTGGCATATTAACAACTT
TCCGGGTAATCTACCACAGGTAACAGGGGCAAAAAAAGAGGTTATTTTAGGACAGCATTACCTGA
GTTCGAGATAAAATTTTCAGTTAAGGTAGGAAAAAGAGAAAAACCCTCTGTGTCTCCCCTTAAAA
GGATAGGACTGTTTCATTGTTAATCAAAGTAGTATGATTAATCCCTTTTATCCTCAATTTACACG
ACGAGAAGTGATAGAGCCAAAAATATTACATAAAAGGTGGGAAATACCCACCTAAAAACTGTTA
TACAGTGGTTAAAGATGCTAAAGGTTCAGGAATGTCTGCTGAAGGTGCATCAAATTTACCAGTAA
TAACATACTCTAAACGCAATTTTAAAAAAGTAATCCATTGCTCATTTAGAGACAAAATCGCCGCC
GCAGGTTGAGGGCATTTTGCTTTAATTTCTTTAAATTCTGGAGCATCTAAAAATGCGGGTTGTTT
GATAAACCAAAAATCTTTTTCTTTATTTTTTTCCTTGTAGTCTCTTTCTTTTTCTTCTAAGACTTC
ATGGAGATTTTCATTGTCACTGGTTAAGAATTTTTCACTGGCAACAAGATAGTAATAAGTAGTCA
TAAATTTATCTATTTTCGAGTTAATTATAAAATTTGTTGGCTATTAGAAAGGTTATTTTGACACG
GTTGTTAATTATATCTTACTTAATAGGAATAAGCTGAAACATCTTAATTAAAGTTGATTTTTCAT
CGCTAGCTTCATTTCTTTAACCGCTCTTTCTAATCCTACTAACACAGCACGGCTAATGATACTATG
ACCAATATTCAATTCTTCCATGCCTTCAATACAAGCAACAGGATACACATTCCAATAGGTTAAGC
CATGGCCTGCATTTACTCTCAAACCCAACTCTAAAGCCTGTTTAGTACCTTTTTTTAAAGCGCTTA
ATTCCTGTTGACGGGTTGACTCATTTTCCGCATCCGCATATTTCCCCGTGTGTAATTCGATAAAG
ATAGCCTTAGTTTTTGCCGCCGCTTCAATTTGTGCCTCATCCGCATCAATAAACCAACTCACAGGA
ATATCCGCCGACTGCATTTTATCGACAATGGCGGTAAATTTTTCGAGGTTATTAACAATATCAAT
TCCCCCTTCTGTTGTTACCTCTTCTCTTTTCTCAGGAACTAAAGTAACATAGTCAGGTTTCAAAGA
AAGTGCGATCGCAACCATTTCTTCTGTGGGTGCCATCTCTAAATTCAAATGAGTACGCACAGTTT
CTCTTAAAATACGCACATCTCGATCTTGAATATGACGACGATCTTCTCTTAAATGGGTAGTGATA
CCATCCGCACCCCCCAACTCTGCTAAAACCGCAGCTGCAACGGGGTCTGGTTCAACAGTACGCCTT
GCTTGACGAATAGTTGCTACGTGGTCTATATTAACTCCTAATGTTAACAATCTTTTCCTCCTAAC
AGACAAGTATTCAATATAGTTTACTCAAGGGGCAAAGAGCAAGGGTAAATAGTGAATAATGAAT
AGTTGATAATTACTCGTTACTCGTTACTCGTTACTCATTACTCCCCTAAACCTGACACCTGAAACC
TGAAACCTGAAACCTGAAACCTTATTTCTTGGCAGTTTTAATTTGTTGTAAGAGCAACTCTAATT
CAGCTTCTAAGCTCATATATTTAACTTGTTGGTCTGCTTGATAGGCTTTTTGTAACTGTTCTAGG
TTATAGTTAGTGGTTTTTAATTCGGTTTTCATAAGTATATATGCTTAATTATATTTTTATTAAAT
AATTGATTTTAACTCAAATCTAATCATAGACGATATTTTAAGAAATGTAAACGAGAAAAATTGA
GCGAGTCTCCCGTCAAATCTTAATTTTTTTCAGTCTTAAAGCATTTGTAACTACGGAAACGGAAC
TAAAAGCCATTGCACCCCCTGCAATAATGGGGTTTAATAACAGTCCGAAAATAGGGTAAAAAATT
CCTGCGGCCACTGGAATACCTATCACATTATATATATATGCAAAGAAAAGGTTTTGTTGAATATT

TCTCATGGTAGCTTTACTCAATTTGATGGCAGAAACAAGGGTTTGCAAGTCTCCTGAAATCAGAG
TTATATCACTAGATGAGATCGCAACATCTGTACCCGTACCAATAGCAAAGCCAACATCAGCTTGA
GCAAGAGCAGGAGCGTCATTAATACCATCCCCCACCATTGCCACTAATTTCCCTTGATTTTGTTGA
ATTTCCTTAATTTTTGCCGTTTTTTCATCAGGGCGAACTTGAGCATAAAATCGTCTAATCCCTGC
TTGTTGAGCAATTTTCTCCGCTGTTTGTTCATTATCCCCCGTTAACATGATTACTTCTAAACCCAT
TTTTTGTAATTTTTCCACTGCGGATTTAGATGAAGACTTGAGACTGTCAGCAAGGGCTAATAAGC
CTACTATATCGCTCTCTATGGCTATCCAAGCGTTAGTTTTAGCAAAAACTTCGTTATTACATAAA
CTCTCTAATTTACCCGTATTAATGCCTAATTCCTGAAACCATTTTTTTGTACCCATGCGTACTAAC
TTGCCTTCGATAAATCCTTGCACACCGCAACCAGAAACTGCCTCAAAGCTACTTACTTCTAATGG
ATTTACCCCCTGTCTTCGGGTATATTCGATAATAGCTTCAGCAATGGGATGTTCAGAATTTGCCT
CTAAACTGGCAACGTAGGTTAATATCTCTTTCTCTGTTTTAGTACCATTAACCGTGATAAAGTCC
GTTACGACTGGTTTACCTACTGTTAAAGTGCCTGTTTTATCCAAGACGATAGTTTTGATTTTGTG
GGCTTTTTCCAAACTACTAGCATCCTTAATCAAAATACCGTGACTTGCCCCTAACCCTGTGCCAAC
CATAATGGATGTAGGGGTAGCTAATCCTAATGCACAAGGACAAGCGATAATTAAAACATTGATA
GAAGCGATTAAGGCTAAAGTAAAATTACCCCCTATCAACCACCAGATAAGAAAAGTAACAAGGGC
AATAATTATAACCACAGGTACAAACCAACCCGTAATTTGATCAGCTAATTTTTGAATCGGTGCTT
TACTCGATTGGGCTTGTTTCACTAATTCGACGATTTGAGCTAATACCGTATCTTTGCCGATTCTT
GTGGCTTTAAACTGAAAACTACCTGTTTTATTAATAGTCGCTCCAATTACTTCATCTCCCACTGT
TTTTTTTACTGGTTCAGACTCTCCCGTCACCATACTCTCATCAATGGAAGACTCGCCTTTAATTAT
TTCTCCGTCCACTGGTATTTTTTCTCCCGGGCGTACTATAATAATTTCATCAATTTTAACTTTCAC
AATGGGGATTTCCTGCTCATTTCCGTCTCGAATGACTCTAGCAGTTTTTGCTCCTAGCTGTAATA
ATTTCTTAATGGCTTCAGAAGTCTGATTTTTAGCCCGATGTTCAAAAAATTTCCCCAAGAGAATT
AAAGTAATAATCACTGCCGCCGATTCATAGTAAACACTAACTTGTAAACCCTGATTTTCAAGCCA
CTGAGGGAAAATAGTTACGACTAAGGAGTAGAGATAAGCCGCCCCCGTACCCAAAGCCACAAGGG
TATTCATGTTAAAACTACGATGTTGAAGAGCAGAAATTGCCCCAGTAAAAAATGATTGTCCACAC
CAAAACATAACGGGAGTAGTTACCAACAACTGTAACCAAGGATTATGTAGCCACATAGGAATGAA
AGGAATATGTAAACCTGTCATCATGGGCAGTGAGGTAATAACTAAAAAAGCACTTATTACTCCTC
CGAAGATTAATTTATTGATGAAATCTTGGCTTTCTTGCCGTTTACTTTCTTCTTCATCGGTGAAA
AGATTATCTTCTTCATCAACAAAGGCTTGATAACCAATATTTTCTACTACTTGCTCAATGGCTTC
AATATTTGTTGATTGAGGATTATAAGTTACACTAGCTTCTTCTAGGGCATAGTTAACGATACATT
CTTCCACCCCTTCTATACGACTAATGGCTTTTTGAATACTGTTAGCACAAGCCGCACAACTCATGC
CCCTTAATTGTAGAGTTTGTTTTTCTAATGGTTGGGTTATCATCTTTTTCTTTGGATATAGTTAG
TTATACCGAAGGCAATATCAATATTATTTTTAGCTTAAACCTTCTAGTTAACTAGAATGTCCATC
AATAATTATTTAGATATGGTTGAAACAAAAATTAATTAACTCGATCGAAAAAAATTTCTACACT
ATTGCATAAATGGGTAAAAAGTAACTGATAAATAAGTAAGAAAGTTTTAGGAGTATAAAATATG
ATTGATAAACAACAATCTCAACCCAAATTAGAACAAGAAGAAGATATTAATTGGGATGATTACC
TTGATGAGGAAGAGGAGGAAAGAGAAACTGAAGATTAAAGAGAGTTTGCTGGAAAAATATAGCT
TTGTCGTAGTTTTCAGGTTTTAGGTTTTGGGTTTCAGGTTGATTTTCTTCTTTACCTATTTTTTA
TTCGTGTATTTTTATAGGATAGCAGATGTATAATTAACTAAATATTCCCCTTCCTGCTTGGATTC
ATGCTATTACAACCCCATCAAAGAACTAAATTAGATCAAAGTGATGATAATTTATTTTACTCTTT
CCCTCGTTTTGTCACCCATGTGGATGAGAATTTTATTAGTCAGTTAACGGATTTATATAGGCAAC
TTTTGCAACCACAGACAAGAATTTTGGACTTGATGAGTAGTTGGGTATCTCATTTACCTCCTGAG
ATGGAATTTGCTCATGTGGAAGGTCACGGAATGAATGAGGAAGAATTAAGAAGGAATCCTCGTT

TAAATAGTTATTTTATCCAGAATTTGAATGAAAATCCTAAATTGCCGTTAAATGATGCTGATTTT
GATGGGGTTTTGTGTGCGGTTTCTGTACAGTATTTACAGTATCCTGAAGCCATTTTTAGCGAAAT
TGCCCGTATTCTTAAGCCCAATGGGGTGGCAATTTTTAGCTTCTCTAATCGAATGTTTTATCAAA
AAGCGATCGCAGTTTGGAGGGATGCAACAGATAGACAGAGAATTCATCTTGTTAAATCTTACTTC
CAATCGGTTAAAGAATTCAATACTCCTCAAATAGTTGCTACTCAACCTGAATTACCTGCTATTTT
ACAAATGTTCGGAATTGGAGGAAAAGATCCTTTTTATGGAATTTTTGCTAGAAAAAATTAAGTT
TTTATTTGTGGTTATAGATTTTTTTAAATAAGGCTTTACTAATTAATTTTAGAAATATTATACTA
TTTTAAATAGAAAAATTGATGATTTATAAATATTAGTAATTTAGAAGATGAAGTCCTATCAAAA
AATTATCATTCAAGAATGCGGTGAGCCATTGGTGCAGATTCCTGAGCAGGATTTTTATTTGGAAA
CACCTTCTGCTTACGCTAGGTTGGGGGCAGATTATCAAGGAAAGTCTCCTTATTTTCTTCGTGCA
GGAGTATTAGATAAGTTATTGATAGCAAAAGAAGAGTTAAAAAAAATAAATCCTCATTGGAAAA
TTAGAATTTTTGATGCTTATCGACCTGTTTCAATTCAAAAGTTTATGGTGGAATATACTTTTAAT
TCTATTTGTAAAGAAAGAGAAATTAATGTTGATATTTTAGGAGAAGAAGAGAAAGAAAAAATTT
ATCAAGAAGTATTTAAAATTTGGGCAATTCCTAGCGATAATCCTCTTACTCCTCCTCCTCATAGT
ACGGGAGGTGCGATCGACCTTACAATTATGGATGAGAATAAGCAATCGATTCCGATGGGGGGGGA
AATTGACGAATTATCTCTTGTATCTTTGCCTGACTATTATCTTAATGCCCGAGATGAACCAGAAA
AAACCTATCATCAACATCGACAAATACTCTTACAAATTATGACTAAAGCAGGATTTCACCGTCAT
CGTGAAGAATGGTGGCATTTTTCTTATGGTGATCAAATGTGGGCATGGTTAGAAAATGAAGCTA
ATCCCCAGAGTCATTGTATCGCTAAATATGGAAAATACGAACAATGATCGCTATATAATGATTTT
GAGATATAAATAAGAGCTTATAAAAAGCAAGGCGTTTCTTTTCAACTATGAATTATAAAATATC
GGAGGGTAAAACCTATCCCCTCGGTTCAAGGGTAGAAGATGGGGGGGTGAATTTTTCTCTCTTTT
CTAAACACGCTGAAAGAGTAGAATTATTATTATTTGATCATCCTGATAGTGAACCTAGTCACGTT
ATTCCTTTAGACCCTAAACTTAACAAAACTTATCATTATTGGCATATATTCGTCCATGGTATTAA
GGACAAACAAATTTATGCTTATCGAGTTTACGGGGCTTTTCAACCGGAGAAAGGACATCGTTTTG
ATGGTTCAAAAGTATTATTAGATCCCTATTCTAAGGATGTGGTAAATACAGAAAAATTCGATCGC
AATTTAGCGAAACAATATGGGGTTGATAATTGTTGTCAAGCCTTAAAAAGCGTGGTGGTAGATA
CTCGTCATTATGACTGGGAAGGAACAACTCATCCTCGTATTCCTTTTGCTTCCACAGTAATTTAT
GAGATGCACGTTGGTGGTTTTACAAGAAGGGAAAATTCGGGAGTAAATCCGAAAAAACGGGGAA
CTTTTGCTGGATTGGTGGAAAAAATACCTTATTTAAAAGATTTGGGCATTACTGCAGTGGAATTA
TTGCCGATACATCAATTTGACGTGAAAGATGTTGTGAAGCCTGAGTTGGAGAATTATTGGGGTTA
TAGCACTATCAGCTTTTTTGCACCCCACCGTCAATATAGCCATAACCAAAGTTTAACTGGTCCTGT
GGATGAATTTCGAGACATGGTTAAAGCCTTCCACAAAGAAGGAATTGAGGTTATTTTAGATGTG
GTTTTCAACCCATACTGCGGAGGGGGATGACACTGGCCCTACTTTTTGCTTTAAGGGCTTAGATAA
CTCTATTTACTATCTTTTAGAGGATAATCAGGCAAAATATAAGAATTTTAGTGGTTGTGGTAACA
CTTTCAAGGCAAATCAACCCGTTGCTCGTAATTTAATTATAGATTCCCTCCGTTATTGGGTATCA
GAAATGCACATTGATGGTTTTCGTTTTGACCTTGCTTCTATTTTAGGGCGAGATCATCTTGGTGA
ACCCATGTTAATTACTTCTGTTTTAGCGGAGATGGAATCTGACCCCATTTTAGCTGGTACTAAGT
TGATTGCAGAGGCTTGGGATGCGGCGGGATTGTATCAGGTGGGAGAATTTATCAATCATGGGGAT
TGGTTTGCAGAATGGAATGGCCCTTTTCGAGATGATGTACGGCGTTTTGTGCGAGGGGATGAAAA
AACAGTTAAACTTCTAGCGGCAAGGATTTTGAGTAGTTCTGATATTTATACTAAACCTGATCGAG
AGCCTAACCGTAGTATCCATTTTGTCACCTGTCATGATGGTTTTACCCTTGCTGATTTAGTTTCCT
ATAGCAAAAAAAAATAACTATGCCAATGGGGAAGATAATCGAGATGGTTGCAGTAATAATTTTAG
TGCCAATTATGGGGTAGAAGGTTATACCAATGACGGGCATATCAATCGCCTAAGAGAAAGACAGA

TGAAAAACTTTTGGGTGATTCTACTTTTAGCTCAAGGAACACCTATGATTACAATGGGTGACGAA
GTAGGGCGATCGCAACACGGAAATAATAATGCCTACTGTCAAAATAATGAACTCAGTTGGTTTAA
TTGGGATGATGTTGAGAGAAATAAAGGCTTGTTGCGGTTTGTGAAAGGTTTAATCAAATTGATT
CAATCCCTTGATGTGTTTCAGCAGGAGAGATTACTTAAAACCGAGGAAAATGGAGGTAATCCTTA
TTTAATTTGGCATGGTACACAACTTTATAAGCCTGATTGGAGTGATTATTCTCATAGTATTGCTT
TCACTTTACATCATCCTCAAAAGGGGGAATTTCTCCATGTAATGTTTAATGCTTATTGGGAATGT
CTTGATTTTCAGATTCCTCAACCACCGGATGGTAAAAAATGGTTTCGCATTATTGATACTTCCGC
CCCTCCTAATGATGATTTTTATGAGCTTAAAGATGCGATCGAAATTAATGGACATACATATTTAG
TACACGATCGCACTTCTGTGGTTTTAATTGCAAAATAGGTTAGGGATTGAGTGAGGTGTTAGGGT
GTTGGAGTGTTGGAGTGTTAGGGAGAAACAAGTAACGAGTAATGAGGATGAGAGTTAGGAGTTT
TTAATTCTTAATTTTTCACCTTTGCCCTTTTATATACATTCGGCGGCGGCACGATGAATCAGGGA
ATGACGATAAACTAACTCATCTAAGTCTTTGCAATATCCTCCTCCAATGACTCCGGCGACGGGAT
AACCTTCTGCTAAACAGGTACTTAAAACCATCATTTCTCGTCGATAAATTCCTGTATCCGTTAAG
GATAATTTACCGAGGCGATCGCATCCTGAAACATCTACTCCAGCATCATATATGACAATATCTGG
TTTAACTTGTTTGAGTAAATCTGTTAAATGAAAGGCTAATATTTGCAAATAACCATCATCATCTA
AGCCAATGGGTAGGGGTATATCTAAATCGCTCTTTTGTTTACGGTAAGGAAAATTCGCTTCGCAG
TGCATGGAAAAAGTAAACACTCTTTCATCATCTCTAAAAATAAAAGCTGTGCCGTCTCCTTGATG
AACATCTAAGTCAATTATCAAAATTTTATTGACTATCTTTTCTATTAATAAATAGCGAGATGCGA
TCGCAATATCATTGAAAATACAAAAGCCTGAACCATAATCAGGAAAAGCATGATGAGTACCTCCA
GCGAGATTACAACAAATTCCATGCTCTAGTGCTAATTGTACAGTTAAAATAGTTCCCCCCACCGC
AGTGCAAGTTCTTTTTACTAATCCCTCACTCCAAGGCAAACCAATACGGCGCTTACTTTTATCGTC
TAAAGTACCATCAAAAAATTGACTTACATAAGTTTCATTATGTACTAGTTGTAATAACTTATCAT
TAGCGATAGTTGGTTTATAGGTAGATTCTTGAGTGATAATTTGATCCTTTAACAACAACTCATAG
AGAAGTTTAAACTTCGGCATCGGAAAGCGGTGTTGTTCAGGCAATGGAGTAACATAGTCAGAATG
ATAAACAACAGGAAGATTCATTCAAATTGAGCTTGATATAATTTTATGTAAAGAAATTAAATAA
TAAAAAGATAACTATTTAATCTAAGTTATATAATTTTTTGCTAGGTAATTGCTTATATATATTG
ATATTAATATTATATTATATAACAGTATATAAAAAATTAAAACAAAATATTATTGATTCTATTA
AATCTCAGAAAGACAATATTTCAATTCTTAATTTTCTTCCCAAAAATAGGGAATTATTAGAAATT
TATAAAATAGCTTATAAATAAAAATGAAAGTGAAAAATATGCCATTCAAAGATTAGCAGGTAAA
TCATTTTGGTCTTTGATAGACTTAGAATATAATTTTATTTTAAGTAAGTTCGGTGAAACTATATA
TAGAATAGATCAAAAACTTTTTTCTTAAACAAAAGCCTACTTTATTTGATGAAAATATATGTACT
GTATTTAATTATGTCAACAATTAATCTTTCTGGTAGCTATCAAATTTTACAGAGTAAGAATAAAT
CTAAATTCGAGGTGATAGAACTATTTGCAGGGTGTGGAGGTATGGCTTTAGGTTTTGAAAATGCT
GGATTAAATACTAAATTATTAGTAGATATAAATAAAAATAGTTGTGAGACTTTACAAAAAAACC
GTCCCAACTGGAATGTTATTAACCAAGATATAACTCAATTAGAATTTATTGAATATGCAAATCAA
ATTGATGTTGTTGCAGGAGGGTTTCCCTGTCAAGCCTTTAGTCATGCGGGGGAAAGAAAAGGTTT
TTTAGACACTAGAGGAACATTATTTTTCGATTTTGCTCGATGTATTAAACAAGTTCAACCAAAAA
TCGTTATTGCAGAAAATGTTAAAGGTTTAATTACTCATAAAAAAGGAGAAACCCTACAAATAAT
TCTAAATACTTTAGATGAATTAGGTTACTATAGCAGTTATAAATTGTTGAATGCTCAATATTTTG
ATGTACCTCAAAAAAGAGAGAGAGTTATTATTATTGCTGTGAGAAAAGATTTAGATATAAAGAT
AGTTTTCCCTCAAGAAAAAAATTATATTATCACTTTAAAAGAAGCGTTAAAAAACTGCCCAAAAT
CAAAAGGAATTATTTATAATCAACGAAAGAAAAATATTATGCAATTAGTTCCAGAAGGTGGAAA
TTGGCGTAGCTTACCTGAACAAATTCAAAAAGAATATATGAAAAATAGCTATTATCAGGGGGGT

GGTAGAACAGGATTTGCAAAAAGATTGGCTTATGATGAACCTTGTTTGACCCTTACTTGTAGTCC
TTCTCAAACTCAAACAGAAAGATGTCACCCCATTGAAACAAGACCTTTAACAATAAGGGAATATG
CCAGAGTTCAAACTTTCCCTGATGATTGGGAATTTGCTGGTTCGTTGACTTCTCAATATCAACAA
ATTGGGAATGCTGTGCCTGTTAATTTAGCTTATCATTTAGGTTGCAGTTTGATTGCAATGTTAGA
GGGGAAGACTGGTTTATGTTCAGAAAAAGTCACTCAACTATCGCTATTTTAAATTTGACTATCTT
CATTTACAGCCTTTTGTAGTTGTTCTCGCAAATCGGGCATAACAGCCAAAGCCCTTGTCCAATCA
GGGATTAAAGCACCGGAAGGATCAGCAAAAAAGTATTCTCCAGATTCTCCTATCACTTGTTGGAA
AATTTCGATTATGACTTCTTCTTGATGACGATCGTAGCTTAAGCCATTAAAACGAGCATCGAGGG
AATATTGACGAGTGTAGTCTTGAGCTTCCCGACGATATTTGATTCTGAGGGCATGAATATGATCT
CTGGTAATTATAACTTGTTCTGTTTCAGTTAGAGTACGAAAGAGCGATCGCAGTATATCTAAACA
CATTTTCTGTAAACCTTCGTTAGTGGATTTACCAATTTTTTGATGTTTATGGTCAAATATACCTA
AATCAATTTGAGAGATTCTTTTTTCTGCTACATTGCGATAAACTTCTGCTAACAAACCGATTTCT
AAACCCCAATTGGCAGGAATACGAATATTTAAGGCTAAGTCACTATTTAAGGCAAATTCTCCTGA
TAAAGGGTAACGAAAAGCATTAAGATAGCGTAGATAGTTTTGGTAACCAAACATATCCGTTAAG
GCGCTTAACAAAGGAGTTACGAACAAACGTACGGCTCTACCATTCAAATTACGAGAGTTTTCCCC
AATACGGGCATAATAGGCTTTATTAAAAGCAATACCAAATTCTTTTTCTACCAGAGGATAAAGGA
GTTTGAGAGGGTAAGATTTATCATAGGTAATTATATCAGCATCATGGAGTGCGATCGCTTCTCCT
GCTAAGGTAGCCAATCCTAACCCTAACCATACAGCTTTACCTTTACCTTTATATCTCATTAAATTC
AAGGTCTTTTGCTCGTAAACATTCGAGAATATTGGTGATTCTTTGACCATTTTCCCAGAGAATAT
AGGTTTTTTGGGGTAAGGATTGAAAAAACTGTACTGTTTCTCTGTATTCTTCTATGGTTTTAGCA
TAGACACAGATAATGACTGTTTGTACAAATTGACATTTTCCTAATTCCTGACGAATAATGTGTAA
AGCAGGGCGTTTTATTTCTTCATATAAAGCAGGGATTAAAACAACTGTTTTTGTTTCTTTTGTTA
AGTCAATTAATTTTAATTCTAGCTCATTAATATCACAACCAAAATCGTGAATTGTGGTGATTAAT
TCTTGTTTGTAATCCATTTTTTTGAGAAATTAATTCCATTTTTATGATTATTTTTTAGTAATAAT
GCTAACTCTGAAATCCTAACTCCTAACTCAGATTAGTTTCTAGCAATTAAAACTCCGTAAGCATC
AAGAGAAAGATATTTAATGGCAGTTTCTTGGATTTTTTCTACAGTAATATATTTTACTTTTTCAC
TATATTCTAAAGCAGGGGTAATAGTTCTTAATTGAGAATAATAATAACCATATAAATTAGTGCG
ATCGCTTGGTTTTTCACTTTGAAAAATAAATTGACTAGCAACATTTTGTTTAACTCTATTTAACT
CTGCTTCAGTAACTCCAAACTGCTGAATTTCTTGAATATGTTTAATAATTTCTGCTTGGACAATT
TCTATATTTTCTTGAGATAATTGAGCAGAAACATAGAATCCCCCTTGAATTTTATAAGTGGTATT
AGATGCGGATATGCGGGTAACTAATCGACGATTTTCCCTTAAATCACGAAATAAACGAGAAAGTT
TACCTCTACCTAAAATTACCGCCAACACATCTAAAGCAATGGTTTCCTCCAGATGATTCAAACCC
GGCACACGCCACAACATCATCAAACGTGCTTGTTGTAAATTACTGTCTGTATATTCTTCGGTAAT
TATGTCTGTAAATGGCAATTCAGGGGAATACTCAGGGGAAGGAGGTTGAAGTTGATTAGATTTT
AAAGAAAGGGTATTGAGGATATTTTCGGTTAATTCTTCCACAGGCAAATTACCCACCGCCACAAT
AGTCATCCCTGAAGGTTGATACCATGAATGGTGAAATGACTGCATTTGTTCGAGTTTCAAATTTT
CTATAATTTCCTCTGTACCTAAAATGGGGCGATGATAAGGTAAATTAGGGAAACAAAGATTCATC
ATTTTGTCAAAAACACGGCGACGGGGGTTATCATGGGAGCGGCGAATTTCCTCCAAAACAACTTT
TTTCTCCCGTGTAAATTCTTCCGGGGGTAGGGAAGGGTTAGAAACTAAATCTAGTTGTAATGGTA
ATATATCGACAAAATCTTGGGGGGCGCAAGTAAAGTAAAAATGGGTATATTCTTGGCTAGTTGCC
GCATTTGTCATCGCACCTCTAGCTTCTAATAAACGCTCAAATTCCCCTAAACCTAATTTTCGGCTA
CCTTTGAAGATCATGTGTTCTAAAAAATGTGCCATGCCATTGATATTATCTGCTTCTACTGCCGA
GCCCATATTAAACCAAATATTTAATGTTACAGCCGCAACGGGTATTTGTTCAGCGATAATAGTTA

GATTATTCTCTGGCAGAGTGGTAATGGTTGGCAGATGAAGGGATTGAGGTTTATTTAGAGTTAT
AGTCATGGAGTTGCGATCGTCAATAGTTGAGTAACAATTCTTATTGTAAATGCGATCGAGTCTCT
TAATTTATTAAATTCATCACAAAACAGCTAAATTTTTCAACATTAAGCCTTAAAGATTTTGATTC
TTCGGCTTTGGCTATGATAAATTGCTAGTTGAAATAAGCCAGTTTGCATTTAAGTTATCTGGTTA
AGATAGAGAAAAGGCAAACCCCCCTTTATCACCCTTCTCGAGCAGGGTTTTTCATTCTCAAAAA
TGTCAATTTTTGAAACTAACAAATAACCTCAGTTCGGTTTAAGAATATCGGAGAAGGTTAGGTGT
CAGGTGTCAGGTTGCAGGTTGCAGGTGGTAGGGGTGAATATATTTAACGTCAGTTCGGATTAAGG
CAATTTTATCGTTATTTCTAAGAAGCTATAAGGTTTTTTGACTACGAGTTGGTCTGCTTTCAGCT
TATCCAAAACTCAGGTTAACAAATAGTAACAGATACAGGAGTTTTTAAGTATTAATTAATCAAT
TATTAAGAAAAAACGCTCCCCTCTACTGTGGGAGAGGGGTTGGGGGTGAGGGCAAGATAATTTTG
CCCTGACTTTGAAAAAACCCTGCTTCTCGAGGGGAGAGGGTAAGAGGGAAAAGTTAAGACTTTGA
GTCATTTATTTTCACAAATATAAGAAAAAATGTAAGTTAAATGCGTCTTTGCTTCACGGATTTGG
GACAAAAGAAAAAAATTAATTATCATGAAAAAATATGGCTCTATCACTTCTCGTCGTGTAAATT
ATTAGTTAGGGTAGGCAAGAGGCAAGAGGCAAGAGGTAATAGGCAATAGGGAATTATTAAATAA
TAATTTATAAACTTTTAGTTTTTATTTAACTATAAATACTATTCAATAAAGGTTATAGAAGTTA
TTTTTTATCAATTTATCATAACCACACTCTGAAGAACCAAAAATATTTTCAATTTTAATATCTAT
CAGGCAAAATTTTCTCTAAATTTCTTAATCGAGATAAACGATAAAAAAACAAAGTAATAATAAT
TCCGCAAGCTGAAAATAAAGCATATTGTAGAGCCATTCCTGCCCCCTCTTCATTACCAAAAATAG
GATTGAGAAAAGAAAAATATGAGATGTTTTTTTCAAAAAAATTATCTGCTAAAAAACCAGAAAT
CAATAAACCTAAAGGTGACATAACTTGAGCAATAAAATAGCGAGAAGCAAATACTCTTCCTTGAA
TAGATGGATCAATTTTTGATAACCAAATAGACTGATTTGAGCTACCAATTAAAGGAGCAAAAAA
AGACATAAAGAAACCAGATAACATCCATAAATAAGGTATTTTAAATAAACCGATTAAACAACTA
CTAAACTCCGTTAAAATATTTCCCAATAATAAACCATTAACTCGATTTTTAAAACCGCCCCAAAA
AGTAAAAGCAATACCGCCGATAACTCCTCCTAAACCCATCGCCCCCTGCACATAAGCTAAAATTTC
TCCCGAATTATTACTTCTCGCTAAAATCATAGCGGGGAAAATTCCCCAAGCAATATTACTGACAA
AATTAGCACTTAAAAGGAATAATAATAAACTCAATAAACTAGGATGACTAAATATATAACGAAA
ACCAAAAGTTAATTTATTATAGATAGATTCTCGATGATTGACTGTTGCTTGAGGTTGAGGCACTT
TAACAATAATAACTGTAAAAATAGCACATAAAAAAGTTAATAAATCAATAATTAAAACACCCTT
TAAATTGAATTGATAATAAATAAAAGTTGCTAAAAAAGGGGCTAATATTTCTCCTCCAGAGTAA
GTTAAATAATCTTCCATAACAGTGGCTCTAGTATAATGTTCTTTTGGTACAATCATCGTCATAGA
AGTAGAAAAAGCTAAATCTTGAAAATAGCTAAATAAGCCATTAATTGCCCCAGTTAAATATAAA
TGCCAAATTTGTAAATTATTGGTTAAAAATAAACCTAAAATTATCACTGTAGAAATAGCAGAAA
CACTATCACCCAACATCATTAAATATTTACGATTCCAACTATCAACAATCACCCCTGCAAATAGA
GAGGCGATAATTCTCGGTGTTTGAGTAAAAAATAAAATTAAGGCTAAAGGAGTGGCTTTTTCTG
TGGCTTCCCATGCCCATAATGTTACGGCAAAATTGGTCATTTGTGAGCCAATAGTTGACAGGGTA
ATTCCTATCCATAAAATTAAAAATGTGTTGAGATTTTTTGCTTTATTCACTAATAAAATGAAGAA
TTAAAAATTAAGAATTAAGAATGATTTGAGTTCGGAGTTCGGTATTAAGATAAAAAAGAAGTTT
CTCCCTAATACCCCAATACCCCTTTAGAATGTAGAATTAACTTCTCCTCATTCCCTTATCACCTCA
TCCCCTATCCCCCCATCTACTTATAGGTATCAACGGCAGGGATTCTAACGAGGAGTTTTTCTTGTA
AAATTTCAGGTACTTTTGCAGGTAATATATAGCCGTTTTCACTGTCTGCATATAACTCTCCAAAT
TCGAGCAAGGCTTGATGATATTTTTCATCTAAGGGAGGAATATTGACAAAATGATAGGTATATT
TATTACTAGCTAAAAAAGATACTGCACAGGGTTGATTACAAGTCCATAAACATCCACTTTTTACT
ATTTCTATGGATGAGTTTTCTGCATTTTTGATTAAATTTTGATGGAGATAATAGCCCCCTGATTT

ACCTTCTAAGATTTCTTGATTAACGGAAATTTGACAAGATTTACAAATAAATAATTTTTCTTTTT
TCATGTTTTTAAAGTTTATTTATTACTATTTTTTTGTTCATCAATTAAATACTTAAATAAATCAT
CTAAGACTAGGTTTGCGGCAATTAAACCGCTTCCAATCCAATAACTAGGCACTTGATAAACCCTG
CCATTTTGGACTGCTTTTAAATTTTGCCAGAGGGGATCTTTTTGCAGTGCTATTATTTTTTGTTG
TAATTCTTCGTTGATTTTTGGGTCATTTTCCCCTGTCCAAATAAAGATTACATCGCCATCTGCTTT
AGTTAATAACTCCTTACTAATCGTCATTTGAATCTCATTCCCCGCAATTTTTTGCGCTTCTTCAGC
AGAGAGATTTTGACTTTCTGGGCGTGGTAAACCTGCATCGGCTAAAATTGTGCCAACAAAGGAGT
CTCGCAAATACAGATTAATGCTATCGGGATAAATCCTCACTACAGAAACTACTGGGGGATTATCA
CCTATTTTTGCCCTAAATTCTGCGATTCTTCGGTCATATTTCGCCATTACTTCTTCTCCTAGCTCT
TTTTTGCCTATGAGGGTGCTAAACTCTTGAAATACAGCCTTCCAATGACCGCTATGGGAGAATTT
AATCAGGGCTGTAGGGGCAATTTGAGACAGTTGAGGGTAGATATTTTCTTGGCCATCGATACCGA
CAATTAAATCGGGCTTCAGTGCTAATATTTTCTCGATATTTGGTTCGCCCGTTATGCCAATATTG
TTGACATTGGTTAATCTATCCTCTAAATGTTTAGATATGCCCGAAAAAGTTGTACCTAATGGCGT
TATACCAAGCGCGATCGCACCTTCGGCTGTAACAGAATCGAGGGCGACTAAGGTTTGCCAATCAT
TGGGAATACAAGTAACTCCTTGAGAGTGGTTAACTTGATGACAATTTTGATTAGTGATAACAGGG
GGAGAATTAGCACTTTGACAACCCCAAGAGAATAAGCTAATTAGAGAGAGACAGAGAAATAATT
TGGAAAAATGAATATTCATGGGATTTGATTAAGAGGCATTGAGATTGTGTGGAGTGGGTATATT
GTCCGTTGTTTTTAAAATAAGGGCTAAAGCCCTTACTACAAACCTAAAAACGGAAAGAAACCTTA
CCCGAAACAGCAAAAGGCTTACCTACAGATGCCCCTAAAAATTCATCCGCCGCAATAAAATAAGT
GTTATCAAACAGGTTTTCGATATTTAGCTGTACATCCCAATTATCTCGTTTATAAAATAACGCCA
TATCCGTACGAAAATAACTAGGAATGCTGTAACTATTACTAATATCCCCTTGTCTATCACCTACA
TAAAATAAGCCTAAACCAAAGCCTAAACCTTTCAAATCTCCCTCTGGGACTTCATAAGTTGTCCA
TAAGCTAAACTGGTTTTCTGGTACATTAGGTAAACGATTATCCGTTAAGCCAGTGGTATCCTTAC
TCACAAACGCATCTAAATAAGTGTAACCTGCGGTTAAATTCCACCCCGGCAAGATTTCTCCTCCT
AAATACAGTTCAAAACCTTTGCTGGTTTGTTCCCCTGTTTGAATAGAGAAAAATCGATTATTGGG
ATCTTCTACGGCGATATTCTGCTTTCTGATGTCAAAAAGTGACGCAGTAATGCTAAAACTCTCAG
ATATGTCTGTTTTTACCCCAATTTCCCACTGTTGCCCTGTTTCAGGGTTAAAAGTTGAACCGTCA
AAGTTACGACTGGCGGCAAAAGATGGATTAAAGGAAGTCGTATAAGAGGCATAAAGGGAAACAT
TAGAAACGGGTTGATAAATTAAACCGATACGGGGAGAGACTTTACTATCATTTTGCTCAAATTCG
TCACGAGGATCACCGACAAATCCTGCGGTGCGATTTTGTTGTGCATAATCATAACGAAATCCTGC
CAATAGCTTAAAATTAGGGCTAAATTCTACTTGATCTTGAAAATAAAAGGCAATTCTTGAGATGC
GATCGTCTCTAAAAAATTCTGTTTTTCTGGCAAATGGGCGCCCAATATAAACAGGATCAAAAACG
TTAATAGATGGATATAAGTCACTAAACTGAAATCCGGGGCGTTCGGAATTATAACGGTATTCTAC
ACCTGCATAGAGTTTATGTTTAACCGAGCCTGTGTAAAAATTTCCTGTAAATTTAACATCAGTAA
AAAAGCGATTATAACTGCCGTCAGCCGAGTAAGCAAGGCGAAAAAGTTCCCCCGTTTCTTCGTCA
AAGAAGACAAATAAAGGGGCGTAACGCAAAGGTTTATAGTTGAGATATTGTAGGTTATGTTGAA
ATGACCAATTATCGCTGAATTCGTGCTTAAAGTTATAACCAATCATATATTGATCCTGTTCAAAC
TTAGCAAAATCTTCTCCTAAAAACCGCTCACGGGGTAAATTCAGTATTTTGCCGTTACTCGCAGG
TAAACCTTCATCTATCGTTTCTCGACTACCTACATACTGCCCATAAAAGTTTAAATTCGTTCTCTC
TCCCATCTGCCAAGTAATTATTGGAGATACAAGGAGGCGATCGCCATCCACAAAATCACGGAAAC
TACCATAATTTTCATAGGAAAAATTCAAACGATACTTGATACTTTTATCCTCGTTTAAAGGGGCG
GATAAATCTGTGGTTACTTGATAGGTGTTAAAACTGCCAATGGTGGCGGAAACTTCATAATAAGG
CTCATCTAGGGGTTTTTTAGAAGTTAAGTTAATAATTCCCCCCGGCTCACCCGCACCAAACATCAC

CGAAGCAGGACCTTTTAAAACTTCGATCGACTCTATATCTGTAGTGCTAATAGGGGCAAGAGTAA
AATAAGGAATACCATCCCGAAAGACACTCTCATTACTAGGAAAACCCCGAATAATAAAACCCGGT
CCAAATACACTTGTCCCCCTACCGCTACCCTCTACAACCCCTGTCACGGTGCGTAAAGCGTCCCCT
AATTCCCTCACATTGCGATCTTCTAATACTCTTTGAGGTACAACTTGAATGGATTGAGGCACATC
TAATAAACGAGTATTTGTACCTGTGGCAGTGGATGATGAAGGCACAAAATAAGTGTTATTACTTT
GATTTTCTTGGGTTGCTAAGATCTCGATTTCATTATTTTCAGAGACTACGGTTTGCCCTTTTGTA
ACATTTAAGACTAGATTATCCGATGATGGTATCACTTGGGCGGTGGAATGCCCTCTTTTCCCGT
GATGGTGATACGTACAAAAGTTGCTCCAGGAGAGGTTACTTGAATTTCGTTAATGTCATCGGTGG
GGTTAAATTCAACAAATTCTGAACCTTGAGGAATGTCTAATTGAGTGTTTAGTAATTCAATAACT
AATTGATTATTATTAGTAAATATTAGTGGTTGAATTTGATCTTGTTGAGGGGATTGCAAGATAA
TTTCTAGCCCGTTGTCAGTGGGTTTAAAGTCTAATTTTGTAATAATTATAGACTCTTGGGGATTT
TGCCCTAACAGTTGAGCTGAGGTGTTAAAATTTCTACATCTGTGATTCTTTGGGCTTGTTTAAC
GGTAGTTTCAGCATTTACAGCAGAGATTAACCAGAGATTTTGTAGGGTTATTCCCCACCATAACC
AAGATGATTTCATTTTTAGTTTACTCCTTCACACTATTGCATATTTTTTGCAGTTAACTTCATAG
TGTAAAGAATCTTGATTTTTTTTTCTTATCCCAAAAGGATTTTATTTATCCCAAACGGAGTTTTT
TTGTTAGTGTTTAGTAGTAAAATCGAATTAAATATTAATCAATAATGAATTTCACTTAGTATTTC
CATATGTACGGGAATCTCTACAAAAGTATGTATTAGTTTACAAAGTCACTTATAAACAAACTTGC
TAGTATAAACTTATCAAATACAATTGTTAATAAAAAGAATTGATTGAGTTTAGTACCGATATTA
ATTATAAGTAATACCTATCTTATTTTTTTATTTTCAAAGATACAACAAAATAATGTAATAGCATT
GCAAATTGTGAGAAAATAAATTTTTTCCATGAAAAATAAAAAACAAAAACACAGAGTAAAAAAA
TTTATAAAACAAGATAAATACTGTAATATTTGTGAGCAAAAACATGATAAATTATCGGCAGATC
ACGTTCCTCCGAAATCTTGCCCTCCAAAAAAAGAACGTTTTATTTCTCAGCTTAGATATAAACTT
CTAGGAGATAGTTCTTTTCAATTAAGAATTTCACAAAGTGGAGTAAAATATGAAACTATATGCA
ATCAATGTAATAATAATTTAGGTTCAAAATATGATTGGGCTTTAGTAGATTTGAGCAAAAAAAT
TGAAAGTTTGACTAATTCTAATATTATTCTTCCTGATTCATTTGAAATAGAATGTTATCCTAATG
CGATTATCAGGTCTGTTTTGGGACACCTTTTAGCGGCAAAAACCATAACAGATAAATCTATTATT
GATTCTTTAATAAGACCTTGTATTTTAGATGAGTCTATTCCTATTGATAAAAACATTCATGTTTT
CTATTGGATATATCCTTACGATAAAATAATTATACTTAGAGATGTGGGAATGCCAAGCATAAGA
AATAAATTGACGAGTGAAATCTGTATATTCAGTATAATCAAATTTTTTCCTATCGCTTTTTTAGT
CACATATCAACTTGATAAGTACGAAAATCTACAAAATTTATATCAATTTAACAATTTATCTATTA
AAGATAAAGCCAATTTACAAATTAACTTAAAAAATATTAAAAAAGCCACTTGGCCAGAAGAATG
TATAGGATTTGAGAATTTTCTGCTTATTGGACAAAGTTTTGATGACTCTGTGTACTCCGTACCCC
GTAAATAGTTTACTAACTATTTAAACAATAACTTTCTATGACTATTTATTATCTTAATTTTTTCT
CGCACTGGTAGTCAAAAACTAGATTATCATATTATTTTACTTTTTTTGCTAACTTTATTGTCGCT
ATTTTGTTATTTAATTATCATAATTATATCCTTTAGCTTGAATATTTTTAGTTAATTAATAATTA
CTTGATAATTACAAAAAAAAAGAAAAGAAATCAATACTTTTAAAAACTAAAAATTATTAACTAT
ATCATTAAAAAAGATCATCGCCATACATAAAAATCGATCGCATGATAAGAAAAAATAATTCTTT
ACCAAACAAATTTTAATTTTCATTGACAAAACGCAATAAAACTTTACGAAATTCTAGGGAAAACT
GCACGGAGTTTGCACAAAGATTCTGTTTAAATAATCACCAAAGTTGAACAAAGTAGAAACAACAT
TTAAAAATTCAGTAATTGCAACCACTGAAAAACTTTTTTAACAAATAAAACAAAATTCATTAAA
TCACATAATCAATCATGAAAACACCTTTAACCGAAGCAGTATCCAGTGCTGATTCTCAGGGACGC
TTTTTAAGCAGTACCGAAATTCAAGTAGCTTTTGGACGTTTTCGTCAGGCTACCGCAAGTTTACA
AGCCGCTAAGTCTTTAACTGAAAATAGCGACAAATTAATTAAAGGAGCGGCTCAAGCCGTATATC

AAAAATTCCCTTATACCACCCAAATGCAAGGAGCAAACTTTGCCGCCGATCAAAGAGGGAAAGAT
AAATGTGCAAGAGATATTGGCTACTATCTTCGTATGGTGACATACTGCCTTGTTGCTGGTGGTAC
TGGTCCTATGGATGAATACTTAATTTCTGGTATTGAGGAAATTAATCGTACTTTTGATTTATCTC
CTAGTTGGTACATTGAAGCTCTCAAATATATCAAGGCAAATCATGGCTTAAGTGGTGATCCTGCG
GTAGAGGCTAATTCTTACATTGATTATGCTATTAACGCTTTAAGCTAAAGCTAATTCATGTCTTT
TTAATTAATTTATTTAATCCCAAATAAAACAAATAACGGAGGAAAAAACATCATGGCTATTACT
GCCGCTGCATCTCGTTTAGGTGTATCTGCATTTAACGACTTCAAACCCGTAGAATTGCGCCCTAAT
TGGACAAAAGAAGAAGCACAAGTTGCTATTAATGCTGTTTATAAGCAAGTTCTCGGTAATGACTA
TATTATGAAATCCGAGCGCCTTTCTAATGCAGAGTCTTTATTATGTAATGGTTCAATTACTGTTC
GTGATTTTGTGCGCATGGTTGCGAAATCTGAACTTTACAAAAATAAATTCCTTTATAACAACTTC
CAAACCAGAGTCATCGAACTCAATATTAAGCACTTATTGGGAAGAGCTCCTTATGATGAGTCTGA
AGTGATTGAGCATTTAGACTTATATCAAAATGAAGGTTATGAGGCTGATATTGATTCTTACATCG
ACTCTGAAGAATATAGTCGCAGTTTCGGGGAAAATGTTGTTCCTTACTACAGAGGATTCAATACT
CAAACTGACCAAAAAACTGTTGGTTTTACCCGTATGTTCCAATTATATCGTGGCTATGCTAACAG
CGATCGCGCCCAAGTTGGTGGTAATAAATCCCGTTTAGCTTCCGAATTAGGACGTAATGGAGTTT
CTGCGGTAGTAAATCCCTCTGGTAGCGGTGAAGGTTGGGCATATCGTCCATCTAGTAAGGGTGTT
ACCCCCAATCAAACCTTCAGAAATTCCACCCGTGAAGGACGTGTTTATCGTATTGAAGTAGCGGC
GATGAATTTACCCCGTTATCCCAAAGTACGTCGTATTAGCCGTGAGTGGTTAGTACCTTACGAAG
ACTTATCCACCACTTTACAAAAAATCCAAAAAATGGGTGGAAAAGTAGCTAGTATTACTTTAGCG
TAATTATTCAACTATCATGGAGGTTTAAAAAATGTTAAGTACATTAGTTAGTGGTAGTTCCTATT
CTCCTGCTAAAAATCGTGTTTTCGTCTATGAAGTCACAGGCTTAAAGCAAAGTGGAGAAAACGAC
AAAAATAACTATGCTTTCCGCAAAAGTGGCAGTGTGTTTATTCAAGTGCCTTATGCTCGTATGAA
TGAAGAAATGCAACGCATTAACCGCATGGGAGGCAAAATTATCAATATTAGTCCCCTTGTTATCT
CTGAAGGCGCAAGTGGTGATTCCGAAGAATAAATATCAATAATTTATCTCGAATAAATTTAAGA
TTAGCATGGGCTTTTTGTCCGTGCTATTTATGTTTAAATGATGAATTTTTCGCTGAAAATTTCTG
AAATTTCCCATTTTTTGGGCAATAATGAAAAAGAAAAAAATCAATAATCGAATATTAAGAGTAAT
GAAAGTAAAAGCCGCAGTCGCCCTAGAAGCGGGAAAACCCCTCACCATTGAAACCGTTGATTTAC
AACCTCCTCAAGCAGGAGAAGTGTTAGTAGAAATTAAAGCAACAGGAGTTTGTCATACTGATGCT
TATACTTTATCAGGAAAAGACCCTGAAGGATTATTCCCTAGTATTTTAGGTCATGAAGGGGCTGG
GGTTGTTGTGGAAGTAGGCAAGGATGTCAAAAGTTTAAAGGAGGGAGATTGTGTTATTCCTTTA
TATATCCCTGAATGTCGTGAATGTGAATATTGTCTCAGTTTTAAAACAAATCTTTGTCAGGCAAT
TCGAGTAACTCAGGGTAAAGGAGTAATGCCCGATGGTACTTCTCGTTTTTCCCTTGATGGTAAGC
CTTTGTATCATTATATGGGAACTTCTACTTTTGCTAATTATACTGTTGTCCCTGAAATTGCTTTA
GCCAAAATTCCTTCTGATGTACCTTTTGAAAAAGTTTGTTATATCGGTTGTGGTGTCACTACAGG
TATCGGGGCGGTGATTAATACTGCGAAAGTGGAAACTGGAGCGAATGTGATTGTATTTGGTTTGG
GGGGTATTGGTTTAAATGTAATCCAAGCCTGTCGCATGGTGGGGGCGGATATGATTATCGGGGTG
GATATTAATAATAGTAAAAAAGCGATCGCATCTAAGTTTGGTATGACTCACTTTGTCAATCCGAA
AGAAATAGAAGGGGATTTAGTCTCTTATTTAGTGGAATTAACCAAAGGCGGTGCAGACTATACTT
TTGAGTGTATTGGTAATGTTAATGTTATGCGTCAAGCCCTAGAATCTTGCCATAAGGGTTGGGGA
GTTTCTACTATTGTTGGTGTGGCAGGGGCTGGAGAAGAAATTAGTACAAGACCTTTCCAATTAGT
TACAGGTAGAGTCTGGAAAGGAACAGCTTTTGGAGGTGCAAAAGGACGAACAGATGTACCTCAG
ATAGTTGAATGGTATCAGTCAGGAAAAATAAATATTGATGATTTAATTACCCATGTAATGCCTTT
AGAGGAAATTAACACAGCATTTGATTTAATGCACAAAGGGGAGTCTATCCGCAGTGTTATTACTT

TCTAAACCTTTCTAGGGTGGGTTATAACCCGCCTTTTTTGTTTTCCTATTTTTATGCCGAGCTGAG
ATTATTTAATATTGAATTAAAAATGCGAGCGCAGCCTTTTCCTCCAGAAATGGAAATCCAAGAAG
AAGACTACTTCGATTATTACTATGATGAACCGGGTAGTGAACCCGGTAGCATTATAATCGATCCA
GAGGCTTTACCCACTCATATTAATCTGATTGAATATAATCCCATTAAGGCTTATAAAAAATTTGA
TATTCAACCGAAAGATTGTCAATCTTGTTTGAATAATGACTTAGTTTCTTGGGCAGATATTCAGG
GTTTGGGCAGTGAAAAGATTTTACGTGAAATTGCTGAGGTTTTTAGATTACATCCTTTAATTCTT
GAAGATGTGGTCAATGTGCCTCAAAGACCAAAATTAGAGGATTGTCAAAATCAATTATTGATAA
TTTTACATTGTATTAAACCTAATTCCAATGAAATGGGTTTACTTCTGAACAAATTAGCCTTGTT
TTAGGAAAAAATTATTTATTAACTTTTCAAGAAGATGCCATAGATGATTTTTCTAAATTGCGCGA
TCGCATCAAAAATAATCAAGGAAAAGTTAGAAATTTAGGAGCTGATTATTTAGCATATTTAATA
TTAGATACATTGATTGATAATTACTTTCCCGTATTAGAAGATTATGGAGAAAGGATAGAATTAT
TAGAGGATTTAATCGTCGCAGATCCCACTCAAAAAACTCTCAAAGAAATCTATAATATTAGGAGA
GAATTATTAGCTTTAAGACGTTCTATTTGGCCTCTAAGAAATTTATTTAATCAACTGACAAGAGA
AGAAAATGAACTTATCAGTAAAGAGGTTTACATCTATTTTCGAGATTGTTATTACCATACCATTC
AAATACTAGATATATTAGAAACTTACAGGGAATTAGCCTCTAGTTTAATGGATGTTTTAATGTCC
TCTATGAGTAATAAAATGAACGAAGTTGTAACGTTATTAACTATTATTTCTAGTATTTTTATCCC
TCTGACTTTTATCGCTGGAATTTATGGTATGAATTTTGATTTTATGCCTGAATTAGAGTGGCATT
GGGGATATTTTTTATGTTTATTATTAATGTTAAGTATTGCTTTATTAATGCTTTTCTTTTTCTGG
CAAAGAGGATGGTTTAAGTTCTATTTTTCCAAAAAAACTAAGTGGTAATTTCCAGTCACTTCTAT
AAAATGTTTCTCATTGGTTAAATAAGCTAATATTTGTGTAACTGGTCACAAGTTTAATTACTAAT
CTATAAGGACATGAATCCCGAAATCACTTTAACTTGGAACATCCTAGAGGAAGCCTTTGAAATCG
TCAACATTAGTTCCATCACTGTTAATCCTCAAGATGCGATCGCAACTTATAAGAGTGCAGATGAT
CCGAATCAAGAGGGAGATAATGAAATCCCTGACGAAGTTTGGGTAGATTATACTCCTCCTCTTCC
TTATGTTAAAAATACTACTCGCAACTTACAATTCAATGAATCTCAATTTCTCGCTTCTCCGGGGG
AAGAAATCGGCGTAACAACTTATGTAACTACTTCAGACGGTTATACATGGGCGGCGATGTCCACT
GCAATTAATGCCATGTGGCCTTATGATGCTACTGATTATTCTGGTATTGCCAGTCAAAGTCCCTA
TTATGCAGGAAATATCGTGATTACTCCCCCTGAAGGTGTGGTAAAAGTCACCGCAAACTATAAAG
GGCAAAATATGAAGTTTTGGGCGAATGAAGATGGCTTAACTTCCGATAATCCTGAAGCTATCAAA
CTCGATCGATATTTTGTTATCGATGAATGGGGTAATGAATATATTATGCACGCATCAGGACAATT
AGAACAAAGTCAGGTAGCTACTGCTTTTGAGGAAGCCATATTGCCCGAAGGTTGGACAAAAGAAA
CTCGTCAATTATCAGAAGATTTAATTTTAACTCCCGCCGAAGGTGCCGATGGTAGTTTTCATTAT
CTAGTATTTCGTGATAGTGCTGATAACACTTATCATCAAACAAAATGCTCTGATACAGGCTCACT
TTCTGCTCAAATAGAGGATTTTCCCATTTGGGGAGGGCAAGATGATAACATCCTTTCAGGAGATG
TTAACGGTGAAATTAGAGACGATTTAATTCACGGTGCGGGAGGTAATGATACTATCATTCCGGGA
TTAGGTAATGATGAAATTTGGGGAGATGCTGACATTGATACCGTAATTTTAACAGGAGACAGTTC
TGATTACTCGATCGAAGAAATCTCCTCTGAGGAAATAAATACCTTTACAGTGTCAGGATTTGGTT
ATACCAAAACTCTTTATGATGTGGAAAATTTACAGTTTGATAATGAAACAATTTCCCTAAATAAT
AACGATAGCCTACTTAATACACAAATATATCGCTTCCGTACGGGAGAGGGAACTTATCTCTATGT
AGCTGACGAAGAACGTCAAGCTATCCTAGCAAATAACTATAATTTTGTAGAAGAAGGAGAAGTT
TTTCAAGTATCTATGGAAATGGAAGACGACTTAATTCCCATTTATCGTTTTCGTAATACTAATGT
TACGGGAGCTTATTTATATGTAGAGGAAGAAGAACGTCAAGCCATATTGCAAGGCGACTACGGTT
TTGCCGAAGAAGGTTTAGCTTTTTATACTTATGGTGCTATGAGTGAGCAAGGGCAAGAAATTTAT
CGATTCCAAACAAACCCCGGTAGTTATATTTTTGTGGAAGAAGAAGAAAGGCAAAATATCTTACA

AAATTATTCTAGTTTTACAGAAGAAGGAATTGCTTTTAACGTAGCATAACCTTATTTAACTTTAT
TTCGACACTTCGACTCAGATAGGCAACAGGCAAGATGCCTGTTTCACGATTGATAAATCTAGTAG
GGTGGGCAATGCCCACCATTAGGATTAATTGAATTAAATAGCTTCTAGGTTTTTTTCGCCAGTTC
TAATACGGATGGTTTCATCCACGGGAGAGATAAAGATTTTACCGTCACCGATTTCCCCTGTACGA
GAGGCTTGAACGACTTTATCTACAACCATGTCCACTTGATCATCTTCCACGACAATTTCCAATTT
GAGCTTTTGTAAAAACTCAACGGTGTATTCTGAACCACGATAGCGCTCAGTTTGTCCTTTTTGAC
GACCAAAACCTCTGACTTCAGAAACTGTCATTCCGACTATTCCAGCGTTAACCAGAGCAATTTTG
ACTTCATCTAGTTTAAAGGGACGAATAATGGCTTCTATTTTTTTCAAATTAGTATCTCCTTTCTA
TTTTAATTTCCTTTATATACCTTATACAATTTTCCTGTTATTTTTTAACACTCTTATTCAACGAG
TTTTTGTAAAAATAAGTACATAAATGATATTACCTTATGGGAGTTCGAAATTCAGAGAGTTTGCT
GCGCTCGTACACTCCTTTCAGTCGTGAACGGAGTTCGGGGTTTTAAGTAATCTGAGTTCAAGATA
AATTTTCATCTATGAGTGATGGCGGAAAGGGCAAGGGGCAAAGGAAAAATTAAGAATTAAGAAT
TAAAAACTCCGAACACTCATTAATCATTACTTATTTCCCCTCATCTCCTCATCCCCTAGACACCCC
GACACCTGACACCTTCTTTTTTAATTTTTCGTTCTTAATTGTAAATTCTTTTATTGAGTCGAGAT
AGCAAATTTTTGTTAACATTATTGGGTACATTTTTACAATTAAATTAATTATTTTTGTTTTGCTA
TGGCGCTAATTGTCCAAAAATACGGTGGTAGTTCTGTTGGTTCAACCGATCGCATCAAGTCTGTG
AGTAAGAGAATTTACAAAACAGTACAGGAAGGAAATCAAGTAGTGGTGGTGGTTTCGGCAATGG
GCAAGACTACTGACGGCTTGGTCAAATTAGCCCACGAAATTACATCAAACCCCTGTCGTCGGGAA
ATGGATATGTTACTCTCTACAGGAGAACAAGTGTCTATATCTTTGTTAAGCATGGCATTACAAGA
AATTGGGCAATCTGCGATTTCCCTCACAGGCGCACAGGTAGGAATTTCGACGGAGGCAAACTATA
GCAAAGCCCGTATCTTAGAAATTAAGACCGATCGCATCAATCGTCATTTAGAGCAAGGGCAAGTG
GTGGTAGTGGCTGGTTTTCAAGGCATTATTCACGGAGAAGAATTAGAAATCACGACATTGGGTAG
AGGGGGGTCTGATACTTCAGCTGTGGCTTTAGCGGTGGCATTACAGGCAGACTGTTGCGAGATTT
ATACCGATGTCCCCGGCATCCTCACCACTGATCCACGCATTGTGCCTTCGGCTAAATTAATGGATG
AGATTACGGCGGATGAAATGCTTGAGTTAGCTAGTTTAGGGGCAAAAGTATTACATCCTAGGGCG
GTGGAAATTGCCCGTAATTTTGGAATGCCGTTGGTGGTAAAATCCAGTTGGACTGATGATCCGGG
TACGAGGGTAATTTCTCCCCCTAATCAGGGTAAATCTTTAGTCGGTTTAGAAATTACTAAAGCAG
TGGATGCGGTACAATACGACGCAGACCAAGCTAAGGTTGCCCTCTTGCGTGTTCCTGATCGCCCCG
GGGTTGCCGCTCGTTTATTTGGGGAAATTGCCCGTCACGATGTGGATGTGGATTTAATTATTCAG
TCTATCCATGAGGGTAACAGTAACGATATTGCTTTTTCTGTGGTGAAAGGAGTTTTACCAAAAGC
TGAAGCCGTAGCAATGGCGATCGCTCCTATCCTAGGAGATTCAGGCAACACCGATATTTTAGTAG
AGAAGAAAATTGCAAAAATTGCGATCGCAGGGGCAGGAATGATAGGCAGACCGGGTATTGCGGC
TCAAATGTTCCAAGCCTTGGCAAAAGCAAAAGTTAATATTCAGATGATTTCTACTTCTGAGGTGA
AAGTTAGTTGTATCGTAGCAGAAGATGAATGCGATCGTGCGATCGCTGCTTTATGTGAAACTTTT
AATATAGCTAGTTCTAGCGTCACTTTGAACGATAATAGTAAAGATATAGCCAATCTTGTACCAGT
GAGAGGAGTTGCTTTAGATCAAAATCAAGCCCAGTTAGCAATCTTATTTGTACCAGATGTACCGG
GAATGGCGGCGAAAATCTTTACCGTCTTAGCAGATGCAAATATCAGCGTTGACATGATTATTCAA
TCCCAGAGATGTCGTTTAGTAGATGGAATTCCCATGCGTGATATTGCCTTTACTGTTGCTCAATC
TGATGCAGAAGAAGCGAAAAAAGCCCTCTATCAACTGCAATCAGAAATTAAATTCAAAGAAATA
GTAGTTGAGCCTCATATCGCTAAAGTAAGTATAGTTGGATCAGGGATGATTGGTTCGCCCGGAGT
AGCGGCTAAATTTTTTAGTGCTTTGGCACAAGAGAAGATAAACATTTCCATGATTACCACCTCTG
AAATAAAAATTAGTTGTGTTGTGGATGAATCCCAAGCTATTCAAGCCTTAAAAGCTGTCCATGAA
GCCTTTGGTTTAGGAGGGGAAACTTTATTGGAAGTTCCTTCAAAGGAGGGATAATTTTAAGTAA

AGGACAATTAAAAATTAGTAATTAAGGCAATGGGCAATGGGCAATAGGCAAGAGGCAAACCCCC
CTTTATCCCCCCTCGAGAGGGGGGGAGGGCAAAAGTGTTTAATTAATTTCCCCCTCATCCCCTCAT
CACCCTGACACCTGCAACCTGAAACCTGAAACCTGAAACCTGAAACCTGAAACCTGAAACCTGAC
ATCACTTTTTCATCAATCCCTATTTATTATTATGATTGCTAGTAACCAAAAGCGTTCGATCGAAA
TTCAGTATTTAGAAAACATTTTACCCATAGCAAATGATGGTCAAGATGTAATTGAGGGATTGAGT
TCAACACCAAAAACAATACCCGCCAAATATTTTTATGACGCTTATGGTTCAGAATTATTTGAACA
AATTTGCCTCCTACCTGAATATTATCCCACTCGCACCGAAACAGAAATTTTACAACAAGCTAGTG
CCGAAATTGCTGAACTCACAGGAGTTTGTGAATTAGTGGAATTAGGCAGTGGTAGCTCCACAAAA
ACCCGTCTGTTACTATCTGCTTATGAAAATTTAGGGCAACCGTGGCAATACGTGCCGATTGATGT
TAGTAGCGAAATCCTCAAAGAAAGTGCTTTGCAACTCCATGGCGAATATCCCCATTTATCAATTC
TTGGTTTGGTAGGTACTTATGAACAAGCCTTATTGCAACTCCCTGCCTCTTCTTTGCCTAAACGT
ATGATCGTTTTTCTCGGTAGTACACTAGGTAATTTTACTCAAGAAGAGTGCGATCGCCTCTTTAA
TGAAGTAAGAGATATATTACAAGTAGGGGATTATTTTTTATTAGGTATAGACTTGCAGAAACCG
AAGGAGATATTAGAAAAAGCCTATAACGATAGTCAAGGGGTGACAGCAAAATTTAACTTAAATA
TGTTATCTCATCTCAATCAACGTTATGAGGGTAATTTTGATCTCGATTCATTCCAACACCATGCT
ATTTATAACCAAAATGAGCAACAAATAGAAATGTACTTAAGGGCAAAACAAGTGCAGAGAATAC
AATTGAAAAAACTAGATTTAAAGATAGAAATAGAAGCAGGAGAAAGCATCTTAACAGAAATTTC
CCGTAAATTTAATTTAGAGCAAATGGAAGAATTTTTAGCCCAACATCGTTTAAAACCCATCAAAT
ATTGGACGGATGAAAAACAGTGGTTTGGTTTAATTCTCTCACAATTAAAGGAGTCATGAATTATG
TTACGTTTTACAGACTCAGTTCCTTAAGATTCCAATTAAATTTCTAATGTTGATTAAATCTAATC
ACTCAACCCTATCAATGAATTACAAAGAGCTATTAATATCGATATATCAAGAAGTTATTGAAGGA
AAATCATATTCATCAAAGATAAATAATGGTACACTTAAAAACATTGAAATTATTGCCAAAAAAT
GTTTTTCTCAAAAGGGTGTTTACACAGTTCTTGTCACTTTAATTATTTACAAAATTTTGCACCCA
GAACAAGACATTAGAAATCATCAAGCTCAAATTGAAGGAGGTTTTTCTGGTAGAGTAATTGATA
CTCAATATATATCACCAACATTAAGAGAATTAGGTTTACCAGCAATGGCTGAAAGCGGTTGGACA
ACTCGTTCATTAGAACAACCTTATCCTTATACGTTAGATTATCAAGGAAGAATAGGAAATAAACA
AGTTAAAAAGGCATTTTTAGAATTACTTGATACTATCCAAAATCAAAAAATTAATCCTAAATAT
ATACTTCATGAACTGCTAACACAAATAATAGCTATTCAACAATTAAATAAAGTAGTTATTCAACC
ACTAAATAATGCTGAAAAATTAACAATTAATAAAATAATAAAAGTTTTAGATTCCCAATTTTCC
TTTAATTATAAAATTTTCGGAGGTTCTAAATTACCAGTATTAGCATTTTATTCTCTATATCAAGT
AATTATTACAGAAATTTCAAGATATAATAATTGTCAATTAAAACAACTAGGAAGTCATACAGCT
TCTGATAAAAGATCAAAAACTGCTGGAGATATAGAAATATTTAATAATGATAAATTATTTGAAG
TTTTAGAGATCAAGTTAGATAAACAAGTCGATCTTAATATTATTAGAATTGCCAGAGAAAAAAT
TATTAAATATAATCCTCAAAGATATTATGTATTATCATATTTAGATGTAAAAGAAGAAGAAAGA
AAAGAAATTGATGACATAATTGAAAAAGTAAAATATGAGCATGGGTGCCAAATGATTGTTAATG
GAGTGATTCCCACGTTAAAATATTACCTCAGATTAATTTCCAAATTGGATAATTTTATTAATGCT
TATTCTCGTTTAATACAAGATGACTTAGAGTTAAAGTCTGTTCATAAACAAAAGTGGATTGAACT
TTTAAAAGAGTTAGAAAATGACTTATAAATTTATAGATTTATTTGCTGGAATCGGCGGTTTTAG
GTTGGGCTTTGAAAAGGCAGGTTTTAACTGTGTTTTTAGTTCAGAAATTGATATTCATGCACGAG
AAATGTATTTAGCAAATTTTCAAGAAAAGCCAGATGGAGATATAAGAAAAATTAACCATAAAAA
TATACCTAATTTTGATGTTTTATTAGCAGGGTTTCCCTGTCAGCCTTTTAGTATTGCGGGGGAGA
AAAAAGGATTTAATGATATTAGAGGAACTTTATTTTTTGAAATTGCTAAAATAATTGAATATCA
TCAACCTAAAGTAATTGTTTTAGAGAATGTTAAACACTTTAAAAATCATGATAATGGTAAGACT

TTAATCACTGTATTAAACACTTTAAATAATTTAGGTTATACCACCAGTTGGACATTATTAAATGC
CAAAGATTTTGGTGTACCTCAAAATAGGGAAAGAACTATTATTATAGGCTGTTTGGAGCAAATCC
AATTTGATTTTGATAAATTAGAAACAAAACCACAAAAAAAATTGAAAGATATTTTAGAAAATGA
ACATGAAAAATTAGAATATTTACAAGAAGATGAATATACTTTGATTGAGAATCCTAAACAGCAA
TTATCAGGTCTTATTTTTGCAGGTTATCGCAATAAAAATATCAGAAAAAAAGGCACTCGCCCTAA
TACGGAACATTTATCAAGAGTACATAAACAACCTAACAGAATTTACTCATCTGAAGGCACTCATC
CTACATTATCTTCTCAAGAATCTAGCGGTAGATATTATATTTATCACAATGGAAAAGTTAGAAAA
TTAACTCTCAGAGAATGTTATCGCTTAATGGGTTTTCCTGATAGCTTTAAACTAATTGGCTCATC
TTCTAAATTATACAATCGTGTCGGTAATTCCATCGTTATTCCAATGGTGGAGGAAATTGCTCAAC
AGGTCAAAAAACAATTATTAGAAAAAAATTTTGATTCTCCTACTCCATTATTTAAACCTCGTCAA
ATGTCTTTGTTTGATTTTAATTTATTTGTAAGCTAGAAACAAAAATTGATCGTATTTAGAATATG
ATCAATTTACTCCATTTGTGAGTTTCCGTATCTTAAAAAGCTAAAATGATATTACCTTTCCTCTC
CATAAGGCCAAATATTATCCCGACTCTTCATTTAAAATAATACTCCCTTGATGTAGAATAAATCA
TTGCTGATCAATATGTTGGGGCATACTCAATCTATTTATTTGTTAAGAGAGATTTAATATACTTA
TGAAAACCAGTTGGATTAATCGTGCTAATCGTTTAAGTGCCTTACCCCCCTATGTTTTTGCCCGT
TTAGATGAATTAAAAGCCAAAGCCAGAGAACAAGGTTTAGATTTGATAGACTTAGGGATGGGTA
ATCCAGACGGTTTCGCCCCAACTCCTGTAATTGAAGCGGCAAAAGAGGCGTTAAATGTCGCAAAA
TATCACGGTTATCCACCTTTTGAAGGAACTGCAAATTTTAGAAATGCGATCGCATCTTGGTATCA
AAGACGCTATCAAGTAGAATTAAATCCAGATAGTGAAGCCTTACCATTACTAGGATCAAAAGAAG
GATTATCCCACTTAGCCTTAGCCTATGTTAACCCCGGTGACACTGTTATTGTACCAAGTCCATCTT
ATCCTGCTCATTACCGAGGGCCCGCCATTGCTGGAGCGGACATATATGCGTTGAAACTGAGTGCT
GAAAATGATTGGTTAATTGATTTAACCCAAATTCCCGAAGATATAGCCCGTAAAACAAAAATTCT
TTATTTCAACTATCCTAATAACCCTACCACCGCCACCGCACCGAGGGCATTTTTTGAAGAAGTGG
TGTCATGGGCAAAACATTATGAAGTGATGTTAGTTCACGATTTAGCCTATGCGGAATTAGCATTT
GAAGGCTATGAACCTACTAGCTTACTGCAAATTAAAGGTGCAAAGGAAATAGGAGTTGAATTTC
ACACCCTTTCTAAAACCTATAATATGGCTGGTTGGCGTGTTGGTTTTGTGGTAGGTAATAGCGAT
ATTATTCAAGGTTTGCGCACCCTGAAAACTAATCTCGACTATGGCATTTTTAGCGTGATTCAGGC
GGCCGCACAGACAGCTTTACAATTGCCTGATAGTTACATCCATGAGGTGCAACAACGGTATCAAA
AACGCCGTGACTTTCTCATCGAAGGTTTAAATAAAATGGGATGGAATGTAAAACCCTCTTCCGCT
ACGATGTATTTGTGGATACCAGTACCCCGCAATTCTAATTCTACTGACTTTGCCCTTGACTTACT
ACAAAAAACAGGAGTTGTTGTCACCCCGGGTAATGCTTTTGGTGATGGTGGAGAAGGTTATGTGA
GAGTTAGTTTAATTGCAGATTGCGATCGCCTAATGGAAGCCCTACAACGTTGGGAAAAAGCGAAT
ATCCGATATTAAATTAATAATTTAATAATTAATAAGTTTTTCATTTCTATCGCCTGACTCCTATT
TTTTTCGTACCTAATTTTTTATTCAAGAATAATGGAAACCCCAAAAAAAATATTTCTTCCTGATG
AGGAATCCACAAAAAAACTGGGTAAAGAATTTGCTCAAAAATTAACCCCTAATTCCGTCTTGTTA
TTACAAGGTAATTTAGGGGCGGGAAAAACCACTTTTATTCAAGGTTTAGGGGAAGGCTTAGGTAT
AAGTGAACCTATTGTTAGTCCTACTTTTACCCTCATTAACGAGTATTTAGAAGGAAAAATCCCCC
TTTACCATATTGATTTATATCGCTTAAATCCAGAGCAAGTAAAACATTTACATTTACAAAACTAC
TGGGAAGGTATAGAAGTAGAAGCAGGTATTACCGCCATCGAATGGTCTGATTTACTACCCCAACT
GCCACCACACTATTGGCAAATTATTTTGGAATTAACCCCAGATTCTAGCAGAAACGCCTTTATTA
CTTATATTTAACAGAAAAAGGTAAGGTATTGGGGTATTAGGGTATTAAGGAGAAACTAATTAAC
CTCAGTTCGGTTTAAGAATATCCGATAAGGTTAGGTGTCAGGTAGCAGGTAGCAGGTAGCAGGTA
GCAGGTATTAGGGGATGAGGGGATGAGGTGATGAGGGGAAAGGGGGAAGTAGGGGCTTAGGGTT

TTGGGGTGGTAGGGGTGAATATATTTTACTTCAGTTCGGATTAAGGCAATTTTATCGTTATTTCT
AAGAAGCTATAAGATTTTCTAACTACGAGAAAATAATGCTTTCAGCTTATCCAAAACTCAGGTTA
ATTACCTTTGCCCCTTGCCCTTTGCCCCTCGCCCCTTGCTTTTTGCCCAATATTACGTCTGATTTC
AGGAAATTGTGATCAGTTTCACTAACTATCAATCTATTTTCGCTATTATTAAATAATTCTAAAAT
CCTGTATATCAGCCCTGGAGGGATATTTTATGAAATTACGTCAATTATTAGCTGATTTAAACTAT
TTAACCCCTCTTACGAATCATCCTAACTGGGATCAGGAAGTTAAAGGTATTACGACAAATTCTCA
TAGTTGTGGTATTGGTGATGTTTTTATTGGTATGCCCGGTACTAGAGTTGATGGAGGTGAGTTTT
GGCAAAGTGCATTGGCAGAAGGTGCGATCGCATCTATTATTAGTCAAGAAGCAAGTCAGAAAATC
CCCCCTACGGATAATGATTGTGTAATAGTTGCCGATGATATACCTTCAATCTGTGCAGACATAGC
CTCTAAATTTTATGGTTATCCTAGTGAAAAATTAAAGATGGTGGGGGTTACAGGTACTAATGGA
AAAACTACCACCACTCATTTAATCGAATTTTTTTTGAATCAGGCTGTTCAGAAAACCGCCCTTTT
CGGTACTTTATACGCTCGTTGGCAGGATTATCAGAAAACCGCAACCCATACCACTCCTTTTGCAG
TAGATTTACAAGCACAGTTAGCAGAAGCCCTAGAAGCAAAGAATGAATATGTAGTGATGGAAGT
AAGTTCTCATGCTCTAGCCCAAAAAAGAATCAGAGGTTGTCATTTTGATGTAGCTGTGTTCACTA
ATTTAACTCAAGATCATCTTGACTATCATAAAGATATGGAAGATTATTTTGAAGCCAAAGCCTTA
CTATTTTCTGAAGACTATTTAAGGGGGAGAGCAATTATTAATTATGATGATGATTATGGACAAA
GATTAATTCAGCGTCTTGCTCAAAAACAAGTTTGGAGTTACAGTGTCAACAACTCCTCGGCAGAT
TTATACACCAGTGATTTAACTTATCAGGCAGATGGGGTTAAAGGGATTTTGCATACTCCAGAGGG
TAATATCAATTTTAGTTCTCCTTTAGTAGGGCAATTTAACCTCTCGAATTTACTTGCCAGTGTCG
GTGCAGTATTATCTTTAGGGGTGAGTTTGGAGGTGATTACAGAAAACCTCTCAGCTTTTGCCGGT
GTGCCAGGAAGAATGGAAAGGGTGCATTTTTTACCTCATCAAGATATAAGTGTGATAGTGGATT
ATGCTCATACCCCTGATAGTTTAGAAAATCTTTTACAAGCCGCTCGTCCTTTTATTTCTGGTAAG
ATGATTTGTGTATTTGGTTGTGGAGGCGATCGCGATCGTACCAAACGCCCTATAATGGGTAAAAT
AGCCGCTCAGGGTGCTGATATAGTGGTAGTAACTTCAGATAATCCTCGCACAGAAAACCCAGAAC
AAATCCTTGCAGATATTCTTACAGGTATTCCCGAAGATATTAAACCTATTGTACAGTGCGATCGA
GCTTTAGCAATTCAAGAAGCAATTTCTATGGCACAATCAGGAGACGGAGTTTTAATTGCAGGAAA
AGGCCATGAAGACTATCAAATCCTTGGTACGGAAAAAATCCATTTTGATGATAGAGAAGAAGCA
AGAAAAGCTCTAAGTCAAAAGGGCAATTAAAAATTAGTAATTAAGGCAAGAGGCAAGAGGCAAA
AGGCAAACCCCTCTGTGTCTCCCCTTTCAAGGGGAGAAGGGCAAAAAAAGTGTTTAATTAATTTC
CCCCTCATCATCTCATCCCCTAATCCCCTCATCACCCTAACACCTGAAACCCGAAACCTAATACCT
ACCCTTATCTAATATTCTTAAACCAAACTGAGGTTAAGAATTAAAAACTCCCAACTAAAGCCATA
GTTAAACTATATAAACAGGTCAACAATAAAGCCATTATACTTAAACTACTAATGCCAAAGCCAAC
CATTCTTTTTTTCGCTTCTTGATAGTAACCCCAAGCATATAAAACCCTTCCTAAAATCCAAACTCC
GCCAATAATTGCTCCCCATAATTCACTAACATAATAAGAAAAAATCCACATTATAGGCAGAAACA
AAACAAGTTGCTCTAACATATTTTGTTGTACTCTCAATGCCCTTTCAAAGTTTTCATCTCCCGTCA
TTGCAGGAGGCATAACTTTATATTTTGCCCTTGCCCTACCAACATTGATGATTGTGACAAAATAA
ACTAATAAAGCCGAAACTGTAATTAAACTTGCACCCAACAACATTTGTTTGACTCCTTGAAAAAA
TAAAAGCTCTATAAATAAATAATAAGCTAGTTTACATTTAAGTTGTCTGGTTAAGACAGGCAAT
AGGCAATAGGTAAAGGTAAGCGTTGGAATTATTTTCAAGAATTAAGAAAAAATGTAAGTTAAAT
GCGTCTTAGCTTAGTGGTAAGTTTTTTTCCTAACAAGTCAAGAAACTGTTAACCTTTCCAATTAT
TCTCAGCTTGTTGTAAAACATACTCGGCAACTTCCTTAATTTCCCTTTCACTCAGACGATTAGAA
AAAGCAGACATATTATTTTTACCATTACTTACTATTTGAGTAATAGCTTCTACTGAATCATAACC
GTTACGATGAAGGGCTTTTAATTTTAAATTTTTTCCCCGTCTAATAATATTATTGCCATTGGGAT

GACATCCTGCACAATTTAACTCGAATATTTTTTGGGGCTGGTTATTGATAACTGTTTCTGCATTA
ACAGGGAAAATAAATAAAAGATTAATTGTCAAAATTAAAGAAATAATAATAGTTAAATATTTTG
TCATGGTTGATTAAGGTTGAAGAAAAAAATAACTTAATTATCATAACATCAGGAATTTGGCATT
TTTTCTGGTTTCAATATCAAGGTAAAATTAAATTTTCTATCTCAAAAGTAGAATCACTTATATTC
CAAAAGGATAGAGAAAAAATTGTGTATTTTTAATTTGGAAAAATAATTGCAATGACTAAAAAAT
ATGACGTTTATGGTATGGGTAACGCCCTAATGGATATGGAATTTTCCGTTACTCCTGAATTATTA
GCTCAACTCAACATTGATAAAGGGGTAATGACATTAATGGATGAAACCCAACAAAAAGAAGTCTT
AACTCATCTTCCTAACCCTTGTAAACAATCCTCTGGTGGTTCTGCGGCCAACACATTAGTGGCAAT
TAGTCAACTAGGTGGTAAGGGGTTTTATTCCTGCAAAGTGGCGGCGGATGAAGTGGGTAAAGCCT
ATCTTGAAGATTTGGTGAATTGTGGTTTAGATACTAATTTAGCTTTAGACAATCGCCCAGAAGGT
ATTACGGGTAAATGCTTAGTTTTAGTTACCCCAGATGCCGATCGCACCATGAATACTTTTTTAGG
TATTACAAGTGATTTAGGTTTAACAGAAATAGATGATGAAGCCTTAAAAGACTCTCAATATCTTT
ACATAGAAGGTTATTTAGTTTCCTCTCCTGTGGCAAAAACAACGGCAATTCATGGCAAAAAAGTA
GCAGAGTCGGCAGGGGTAAAAACTTCTTTTTCTCTTTCCGATGCTAATATGGTGGATTTTTTCCG
TGATGGTATCCTTGAGATTATCGGTGATGGAGTTGATTTATTATTTTCCAATGAAATTGAAGCTC
TAAAAATGGCTAATACAGATAAATTAGAAGATGCGATCGCATTTTTACAAACCCTAGCCAAAACC
TTTACTATCACCCGTGGCAAAGAAGGCTCATTAATTTTTGATGGTGAAAAAATCATTGAAATTGA
ACCTTATCCAGTCAATGCTGTCGATACCGTTGGTGCAGGAGATATGTATGCAGGTTGTCTGTTAT
ATGGTATTACCAATGGTTTAGGTTGGGAAAAAGCAGGAAAACTAGCTTCCCTCGCTTCCTCCAAA
TTAGTTACCAGTTTTGGAGCTAGATTAGACACCAGTGTTTTACAATCTTTGCTTGAGCAAATTAA
GAATTAGATTTCAAAAGGGGGCAAAGGGCAAAGGTAAATAGTGAATAGTTGATAATTACTCGTT
ACTCGTTACTCATTACTCCCCTAAACCTGAAATCTGAAACCTCTTTCCTAACTCCTAACCCCGAAC
TAATCTCCCTCTCTCCTCATCTCCTCATCACCCCTTCTCCTCAATCCCTCAAAAACCAAACCCAGA
TCATATACAATTACCTATTCAAATGTAATTAATTATTTATTGGACTGAATGACAAAAAAATGTA
ACAATGAATGAAAGATTGCAGTAAATAAGAACATAAAGCGAACTTAATATGACAACTAGCCCAT
TTTATCAAGGTATCCAATATTTTAAAGAAGAATTACCCTATTTTAACGAATACGGTCAAGAAAGT
GCGATCCCGAAGGGATCTGCTACGCAGCACGCACCCAATGAAAAAGCCATTTCTAGCACAGGTGA
CAAAAAAGCCATCTATCAAACCCTACTAGGTGCAGACGCTTTAAGGTATTTAACCCTTCACATTA
CCGCTAGTAAAGAATCAGGACACCCCGGCGGTTTCGCTAGTAGTGCAGATGTTATCTCCGCCCTA
GTCATGTTAGGACATAAAAACCTGATTACTGAAGTAGGACATCACGCTCCGGGTTACTACAGTAC
TGTATTTCTGGACAAATCCCTTGAAAAAATGGGTATTCATACCGTAGAAGACATGAGTGAGCGTT
TTCGGGAAATGCACGGGCTTTTAGGTCATTTATCAGGACAAATTCCCGGATTACTCAATCCTGCG
GGACCTCTTGGGCAAGGACAACATTTTGCGATGGCAGGAGCAAAATTACATCCCAACGTTTTATT
TCCTGTTACCATCGGTGACGGTGGTTTAGGTGAACCCTATGTTATGAGTAGCTTTGGACACTTCA
ACACTGCCTATCCTCAAGTGACCAACTTCTTACCCATCCTAGTATGGAATGGTTACTCCCAAGAGC
ATCATAGTATGGTATCCACCAAAACCAACGAAGAAATGATCGCCTATTGGCAAGGTAACGGTTTT
AAAGAAGTCATCCTCGTTAACGCTAAAGACTTCGACGACGCAAACCAAGAGGGTGATTATGTTGA
TAGCACCAGATTCTCCTTTGAAAAACGCTTAGAATTTACCCAAGCCGTCTTAGAAGCCACCGACA
AAGCCGCTAAATCAGCATTAGGAGGCACATTAACCGTCTTAATCATTAAACAACTCAAAGGGGCA
GGAGTCCACAAAACAGGAGCAAAATCCCATAACCTCTACCCAGGAGATACCCTTGATAAAGATTA
TATTGCCTCCGCTTTGCAAGAAAGAGCCTTAACTAAAGAAGCATGGGAATTAGTACGCACCAACT
TTGTTCGCTCTAACGGCGGTTCAAACGTAGATGTAGCCGTTACTGAAAAAGAATTACCCTTACCA
GACTTAGGCAAATTACCTCTAACTGAGTATGAAGTAGGGGGAGAAAAAAAAGTTGCCACTACCGC

TATGGGTGAAATTGTTGTTCATGTCGGTAAAAATGACCCCAATTTTGTCGTAACAAATGCTGACG
GTAATGCCGCATCTGGTATCAATAACATCAATATCGGCTTAAACATTGTCCATCCTACCACCGAT
GAAACCTACTTCCAACAACCCCAAGGGCAAGTATATGAGCCTTTAAGTGAGGATGCCTGTGCAGG
TTTAGCCGTTGCTTTATCTTTATTTGGTGCTCGTACGCTATGGTGTTCTTATGAATCCTTTGCAA
TTAATGGCTTACCCATTTGGCAAACCGTCACCCAAGCAATGGCAGAATTGAGAAGGGAAACCCCT
TCTACTATCACTTTATTCACCGCCGGGGCATTAGAGCAAGGGCGTAATGGTTGGACTCATCAACG
CCCCGAAATAGAGAATTACTTTGCCGCCATGATGCGTAATGGTAATGTTTTTCCTCTCTTTCCCTG
CGATGCTAATAGTATTCAAGCCTGTTATGAATGGGCATTAAGTACCAAAAATAAAGGTATCACTA
TTACTGCGAGTAAATCTCCTTTACCGATTTTAACTACCTTTGAGCAAACCAGAGAAGCCTTAGAA
AAAGGTGGAGTTGTCTTACGTGATTCTGAAGGTAGCAAAAAAATCGTCTTTGCAGTTATCGGTGA
TATGACTTTAATTCCTGTGTTTGAAGCGGCGAAAACCTTAGAAAGCGAAGGTTATGGAGTGCGTA
TCGTTTCTGTTATCAATCCTCGTCGTTTATATCGCCCCACTGATGTGGCTTGGGATACTTGCACTG
CGTCTGATGGTAATTTCTTAGATGATGCAGGTTTTGAGGCTTTATTCGGTGGTGATGCAGTCATT
GGGGTAACTGGTGGCACAAGTGCGATGTTAGAGCCTATTATGTTGCGTAGTAATGCGAAACGAGA
TACCTTTGCATGGAAACGGGGTGAAACTACCGCTAGTGCAGGGCAATTAATGGAATTTAACGGTT
TAACCGCTTCTGCTTTAGTTTCCAGAGCGAAACAATTAGCATAGCACGTAAGTTCGGAGTTAGTA
AGGGGCGAATGGCCATTCGCCCATACAGGAGTTAGAAGTTATTAATTAACCTGAGTTTTGGATAA
GCTGAAAGCAGACCAACTCGTAGTCAGAAAACCTTATAGCTTCTTCTTAGAAATAACGATAAAAT
TGCCTTAACCCGAACTGACGTTAAATATATTCACCCCTACCACCTGCTGTTCCTGCAACCTGACAC
CTAACACCTAACCTTATCAGATATTCTTAAACCGAACTGAGGTTATTTATTACTTGTTTCCCCCTT
TCCCCTCATCACCTCATCCCCCCATCACCTCATCACCCTAACACCTGCAACCTGACACCTAACATCT
AACCTTGTTGGATATTCTTAAACCGAGCTGAGGTTAAATTGAGTTAAAGATGAGTTTGGTATTTG
TTATAAATTTCTAAACCCGGAATATCGGCTTTAAATATATCAGGTTGTTTATATTCACTGGGGGG
ATATTCTTTTGCGATCGCTATTATGGGATTATAGAAGTTTTCCCACTGTCTTAGTAAACCTAATA
GTTGTTGAAACTGGGCAAAAGAGTTTTGGTGACGATTTCGTAAAGCAATATATTGTAGAGTTAT
AGCATAGGTTTCAGGGACATTTTTCTTTTGAGGTACTTGGATAATTTGACCTAATTTTAAGAAAA
TATCCGCATTAACTTGCACGACAACCATAAATTCTTTAAGAGAATCTAAATCTTTATCATAAAAA
CTATCTGCAATCTCACCCACCGTGCGCAGTAAGGAGAAATTGCTCATGTCATACTGTTTAAACAA
GTAGAGTAATTTTAAACCTAATTTATCTTTACAAACCGTTTCTAGGTGATTAACATAGGATTGAA
TTGCCGCTTTCCCTTCATCACTTTTAATTTTAGGAATAACTCTTACTAACTGTTTTTGTATTGCTT
CCTGAAACTGAGCAACTTCCATATCTTTAACTAGCAAATTATAAACATAATCGTAAAATTCCTGT
TGAGAAAAGCTACGATAACGGGTTTCTGTTGCCTCAATTTTCAAAAAACTTTCTTTTGTTTCTAA
AGCTACCCTTAATAGTTCGATACTATTTCTTAAACCTTCATAAGCTCCTGCATTTGACTCAAATT
GACGATTGAGCATTAAAAAGTTGATAAATTCTTTACTGGTAAATTTTTCTTTATCAATAGTACGT
GCGATCGCAGCTATATTTTTTAATTGACCAAAAGAGTTTGTATATAGTTGTATCGTACTTTCTGG
GATTTCCTGCTTAGATTGAAAAGAAAGCACTGTTTTTAACCATTGTAACAAAGACTGATTGCCCA
ATAAGGGACGATTCCACCAAGGTTGAGTCGGAGTTTGAACTCCCGGAATACCGCTTTTCATGACA
ATATCTATAGGTAATGAGCCTAAATGTTTGTTAAAATTAACTCAAGTATTACATAAAAAACTTA
AAACAGATCCATCGATTTTGATAAATTAAAGAAAATTATTATTTTCACCAAAAGCACACATATAT
GAAAATATCCTTAGCTGATTTAAGAAAAAACTATACCCAAGGAGGTTTAACAGAAGAAGACATT
AGCCAGAATCCCTTTGAGCAATTCCGCCATTGGTTTGAACAAGCATTGGAGTCACAAATCTTAGA
ACCAAATGCCATGACTCTTGCCACTGTTAATGAAGCGGGAAAACCCACAGCTAGAATAGTTCTCT
TAAAAAATCTTGATGAGAAAGGATTTGTTTTCTTTACCAATTATGAAAGTAAAAAAGGTAAAAA

TATTGCTGTTAATCCCTTTGGTTGTTTAGTGTTTTTGTGGGGGGAATTAGAAAGACAAGTGAGAG
TTGAAGGAAAAATAGAAAAAATTACCCCAGAAGAATCAGATCAATATTTTCATTCTCGCCCCATC
GGTTCACAGTTAGGGGCGCATATTTCCCCCCAAAGTCAGATTATCCCTAACCGAGAATATTTGGA
TAACCGTTTACAAGAAGTAACTCAACAATATCAAAATCACACCATTCCTCGTCCTTCCCATTGGG
GAGGTTATAGATTAGTACCAGATGCGATCGAATTTTGGCAGGGCAGGGAAAACCGTTTGCACGAT
CGCCTTGAATACCGTTTAAATGATAATAAACAATGGCAAATAGTGCGTTTAGCACCATAAAATAT
TTGCAAGTATATATTGAATGAACCACAAGCCCAGGCAATAGTAAGTTGATGAGGATGATTTTTTC
CAAAAATCTGTGGTTCTAAAGCCCTAAGCCCCTACTTCCCCCTCTCACCCTTTCCCCTCATCTCCT
CATCACCCTAAGGGTTGAATATATTCAACCCCTACCACCTGCAACCTGACACCTGACACCTAAACT
TATCGGATATTATTAAACCGAACTGAGGTTAATTATCTTCGCCCTTTCTTTAATTGCCAATCAAA
TATAATAAAGCCATTCTCACTGCTACCCCATTTGTCACTTGTTCCCCGATTAAACTATACTTACCA
TCATCCATTAATTCTGAGCTTAGTTCTACCCCTCTGTTAACAGGTCCTGGGTGCAACACTTTCACC
TCTGGATTACACCATTGTAAACGCTCATGGGTAATGCCAAATAATTGATGATATTCTCTTAAACT
CGGCAATAAATAGTCTGTCATCCGCTCTTTTTGTAATCTTAAAGTCATAACAAAATCAGCATTCT
CTAAAGCCGGTTTTAAATGCCAATGAACAAATAGGCGCTCTTTTTGGCTACTATTTATCGTCTCC
AAAAACAAAGAAGGTAATAAAGTTGGAGGGGCGGCCAAATGAACTTCACATCCTCCTGCTAATAA
ACTATTAATATTAGAACGAGCTACCCGAGAATGAAGTATATCTCCCACTATCGCCACTTTTTTTC
CTTTTAATAAGGATAACTGAGGATTTTTTTCATCTAATAACGAACAAATAGTAAATAAATCTAA
TAACGCTTGAGAAGGATGTTGGTGTAAGCCATCTCCCCCATTAAAAATGCTTACTCCAGAATTTA
AACGATCCATTTCCATTGCAACATTCAAGGGGACACCAGAGTTACTATGACGAATGACCATGATA
TTAGCACCCATCGCCCAATAAGTTTTTGCAGTATCTAAAATTGTTTCCCCTTTAGTTAGAGAAGA
AGTGCCGGGGGCAAAATTGAGAATATCTGCCGAAAGCCTTTTTGCGGCTAACTCAAAACTGCTAC
GAGTACGAGTAGAAGGCTCAAAAAACATATTTGCTACTACTTGCCCTTGTAATGCGGGTACTTTT
TTGGTGCGACTTTTGAGAACAGATTTGAAACTAGATGCCGTCGTTAAAACTGCATCATATTCATC
TATCGTAAAATCTTCCAGAGAAATAATGTGTTTTCTAGTCCAAGGTGTAGCAGTCATTTTTATAA
TAATTAACCAAAAAAATTAGAGTTTTTTACGTTCAGAATAAAACTTAATAACTCTTGCCTTAAAT
ATCATAACCTGAAATGATATATTAATTAACAAGGGTGACAATATCTAAATAAACTTTATATGAT
AGATTGGCATAACTTTTTTTTGGGGATTTTACTTTGGTTAGGAGGTAGCGGTTTTTTACTAACTT
TAATGAGAGATAAATCTTCCTCAGAAATGTCATCAACGGAATCTTTATTAATCTCTCGCAATGAA
AGAATTTTAGAGCTGGAAAAACAGCTTTTAGAAGCAAAAGAGGAGTTAAATTCCGCTAACCAGA
GAATCGATACTCTTTGTGCTGAGTTAGAAAGAAATAGTCAAGAGCAAAATCAATTAATACAGGA
ATTAGAGTTAGAAAAACAAGAATTGCGATCACAAAATGCCTCTGTGCGATCGCAACTTAGGAGTT
TGGAAAATCAACTAAAAGAAAAAACCAATGAGTTAGATAATGCCTATACAGAAATAGAATTATG
GCACCAACAAGTAGAAAAAATAAACACAGACCTTCCCGATAGAGAAGATGATAGTCAAACACAA
ATAGAAATATTAGAAAAACAACTAAAAGAGCAATTAAACAACTTAGAAGAAAGTAGAAATAATA
TTAAAGAAATTGAACAAGAATTAGCACAAACAAATGATGAATTGGAACAAACCCAAGATGAATT
AAGTTTTTCTCAAAAAGAATCAAAAGAATTAAAAAAACAAAATTCAAGAATTAAATTCTCAATGT
GAGCAATTAAATCAAAAACTAGAAAAACTCCCCTTAGAGTTAGAAGCAAATTGGCAAGAACAAT
TATTTAACTCCTTACAATATCTTTTTGTAAATTATCCCATCGCCAAAATGGTAGCAAAATTAAAA
CCAAATACCAACATAAAAAATTTTATCACCCTGCTAAAACCTATTGATGATTTGTTAAAGCAATG
GCAAATTGAACCCATTGGTAAACCATGGGAAAAAGTATCTTTTAATCCTGAAATTCACTACAATG
AAGAAGAAAAATTAGTGACAGCAGAAGAAGTTTATATTCGTTTAGTGGGCTATAAACAAGGGAA
AAAAGTTCTCACTAAAGCTAAAGTCAGTCGTCAATTGCCGGGGCAAAATTAGAATAAAAGTTTAA

ACTTAACTAAGATTCTCTTTACCCCTAACAAATTTATTTGTTTGAAGAAACTTTAACCTTCAATT
TGTTTAAGGTGGATATTTTTTCTACCAAGGATAACTTCAAACTCATCCCCCGGTTTTAACCCCAT
TTTTTCAGTGTAGGCTTTTCCAATGGAAAGATTACCATTTGATTGGACATTTATACGATAATTGG
CACTTCTTCCTCTTTTTTTGCTTTCTGTGGGCAATTCATTATCAAGACTGATTCCTTCTGCTTCAA
TTAGAGCATTGAGAAACTTAACCATACTAACTCTTTTTTGTCCATTATCTGTGAGGGTATAGTAA
CCACATTCAATGGCTTTTTCTTCACGAGAAACGTTACCTAATTTTTTAACTTTTTTTAGTAAGTC
TTTTCCTGTTAATGGCTGTACTTTTTTATTTCGCATTTACTTTTATTCTACTAAATTTTTAAACA
GTCTATTTTGAGCAGATATACAAAACAGTTTATCAGTTTTTGTTCATCAACTATAATATACTAAA
ATAGCGAAATTTTATTTTTTTATAAAGATAATCTAAAAAAACTAGACAAAAAACAAATCATTAA
AAGTCTCAGGAAATAGATTGTATTTTATATTTATGTTAAGAGAAATATTTTTGACCTAATAAGA
ATCATTATGTACCTCTCTTTTATCTCTTAGCTTGTAAGTTTTAATAGTGAATAGTGCATCGATTT
TAATTAGGAATTAGCCATTAGGATAAGAGCAAAGTTAAAAGGGTAAAACCAAGCCCCTTTCCGTA
GATAGAGGGGAAGGCAAAGGTGAATAATGAATAAGTAAATATTGACAACTCCCACCGTTAAGCT
AAGACGCAATTAATTTCGATTTTTATTAATTTGTGCAAATGACTCAAAGTCTTAACTCTTCCCCT
CCCCCCTCTCGAGGGGGAATAAAGGGGGGTTTACCTTTTCCCTACCATCACCAGTAAACTTAAAT
GCAAACTAGCTTAATCAATCTTTAAATAACTTATGGGCAAACTTTATGATAATGTATAGAAATAT
AAAGCCATCTGCTTAAATTATCTCCGTGTTAAAGATTATCACTTATAATAGTGCTGTTGCTATAG
AACAAAATTCTCTACAAAACATTCATCGGGCTATTGCCCTTGGTAACTTTGATGGTGTGCATCTG
GGACATCAGGAAGTAATTAAGCCTATACTGAAAGATAATATGGCAAAAAATCTTGTTCCTGCTTT
GGTCACATTTATCCCCCATCCCCAAGAGTTTTTTAGCGGACAAACCAAAAAATTACTGACTCCTA
TCAAGGAAAAAGCCCAAATTTTAGATAAATTGGGTATTCAAGAGTTAATTTTACTACCTTTCGAT
CGCGAATTAGCTAATTTAACTCCTTTGGAATTTGTCGAAGAAGTTTTACTCGAAAAAATCAAGGC
TAATTTTGTCAGTGTGGGAGAAGATTTCTGTTTTGGATATAAAAGACAGGGAAAAGCTAAAGAT
TTACAATCCCTTGCTAATCAATATCAAATTGAAGTTAATATTACTCCAGAGCAACATTTTCTTGT
CAATCAACAAAATATACGTATTAGTAGCTCTTATATTCGTCAATCTCTCAGTGAAGGAAAAGTCG
AATTAGCTAAAGATATGTTAGGCAGAAATTATCAAATACAAGGAAAAGTCGTCGAAGGGCAAAA
ACTTGGTAGAGAGTTAGGTTTTCCTACGGCTAATTTAAAAATACCACCAGAAAAATATTTACCTA
AACAGGGAGTTTATGGTGTAAAAGTAGATATACCTCAACAAAAGACATATGATTTATCCGCCGTT
ATGAATGTGGGCAAACGCCCCACTGTAGGAGGAGAAAATATTATTGTAGAGGTGCATTTATTAA
ATTGGCAAGGAGACTTATATGGGCAAGATTTAGTGATTAAATTAGTTAATTTTATTCGTCCAGAA
AAAAAATTTACTTCTTTAGATGAGTTGAAGAATCAAATAACTCTCGATTGTCAATTATGTCTTTA
AGTCTTTAATTTTATTTTCTTAGTAATCTAAATAACTATCAACTATACACTATTTCTGTAATGTC
TATATTTTTCAAAAAGAAAAAAATTTTGGTCTTTAAGAAGTGCGGAAGTGACGACTAATGCTTTA
ATTTCAACTTTCTCAGTAATTATCATATTAGTATTAGTTAATTTTTTAGGCATTAAATATTCTTG
GAAATTTGATTTTACAGAGAATAAATTATATACTCTTTCTCCTCAAACTCAAGAAGTAATAAAAA
ATCTTCAAGAGCCTTTACAGGTCTATGTTTTTGATTCTCCACCAAATAATTTTGATCGCCAATTA
TTAAATAATTATGCTCGTTATAATGACCTATTTTCCTTTCAATTTGTCGATCCTCAAGTCAATCT
TAATCTAACACAAAAATTCCAAGTTGATCGCATCGGTGATGTTTATTTGGAATGGCGCGATCGCG
TTCAATTAGTACAAACTGTCTCTCCTGAAAGTAGATTAACAGAAGAAAAATTAACCAAAGCCATG
GTTAAAATACAAAAACAAACTCAAGCAGTAGTGTATATAATACAAGGACACGGAGAAGCAAATA
TCGAAAATCAAGGACAAAGTAGCTTTTCTCAAGCAGTGAGGAATCTTCAGGATATGGGCTATATT
GTCAATCCCCTTAACTTGGGAGAATCTCCTCTAATTCCCCCTGATGCAGATGTATTAATTGTTAG
TAGTAGTGATAGAGAATTATTATCAGGAGAAATAAAAACAATTAAACAATATTTAGAGCGGGGA

GGCAACTTGATGGTTATGTATAATGCTCTAAGCCCCATCAGTTTAGCAAGTATCTTGGAAGAATG
GGGTATAACTTTTAATGATACTCTAGTGATTGACTCATCAGGTGCAGGAGAAATTCTAGGCTTAG
GCCCCTCCATCACTATCATCACCGATTATGGGCAACATCCCATAACCCAAGATTTTAACAATGGTT
TGACTATTTTTCCTTGGGCAAGACCCATTATTACAGAAAACAAAGATAATATTGAGACAATACCT
CTATTAATTACTAATACTCAATCTTGGGGAGAAAGTAATTTAGAAGGGGAAACAGTGGAGTTTG
ATCCCACAAAAGACATACAAGGGCCTTTAAATATTGGTGTTGCCTTAAGCAAAAAAAATGATGTT
ATCGCCGATAACCCTAACAACATCACGGAAAATTCTACTCCCCCTCAAGAATCCGGCTTAAATCCA
GAGGAAGAATTGCCCAAGATAGAAGGAGAAACGGATTTACCTATTCCACCTACCATGAAAACTCC
TTCCCCTATTACCAATAATAATGATGCAAATCGCACAAATAAAATGTCTAGGGAGACGAGACTAT
TAGTTATTGGCAATTCCAATTTTGCAACAGATGGTTGGATTTCTCAGCAACTTAACAGTGATTTC
TTTCTTAACAGTGTTGCTTGGTTAGCTAATGAAGATGAAAATAATTTATCTATTCGCCCGAAAGA
ATCTGTTAATCGTCGTCTTAATCTCACCCCTTTACAAGCCAATTTGATTTCATGGTTAGCCATATT
TATTATACCTGCCCTCGGTTTCATCACTGCGATCGCACAAAGGCAACTTCGTCAGTGATTGCAACT
TGGCGGCTAAGAAATAATTGACACTCACCCGTTAAATCAAAGATTGTAACGAGCGATTCTTACCT
CACAGACACTTAACTAGACTAATCAAATCTTGACTAATCCCCGTCAGTACGTCTCCGTAAGCATT
TTGATTTACGGTATGCCCTACCGCTATTAATCCTCTATTTTTAATTACCTCTGCACTCACAACATC
TCTTGGTGCTGTATAACCACAATATTGACAATGGTGTATTCTTTCTTTTAAATGCTTTTTGCCAG
TATGATTCCCACAGTTAGAGCATTCTTGAGATGTTCCATCTTTATTTACTTTCAGATAAAACTTA
CTTCGTTTCCAACATACAAACGGTAATATTTCGTTGATAAATTGCCCTATTCCTGAGTCTAAAGA
TTGCTTTCTGACAATCCCACGACTCCAAGAAGTGAAGTTTATATCTTCAACCAAAGATATTATCGG
TTAATTCACAGAGGTAATTTGCCAACTTGAAATGCCAGTCACGGCGAGTGTTCGCTACTTTTTCA
TGAAGTTTGGCTATTTTATTTTGGAGTTTTAACCAATTATTAGAGCCTTTAATCTTATGTTTCAA
ACGTCTTTGTAGCAATTTCAGCTTACTCTGTGCCTTTAACAAAAACTTAGGGGCTTCGATTAATT
CTCCTCTGTCGGTAGCTACGAACGATTCAATCCCTGCGTCAATACCTAAACTTGTTTTTCCTACAG
GTGCTGATGGGCAAGATTCTTGAGATTGAAAAGCAATCATCAAATAATATCCTGTTGCCTTTTTA
ACAATACGAGCTTGTTTTGCTTCAAAACCAGTGGGATAATCTCTCGACTGCTTTATCTTTAGCCA
ACCTAATTGTGGTAACTTTACTTTCCCTTCCGCTAAACAATTTTTCAGCATGGCCGGGAAGACAA
AACTTTTCATTTGCTTCTTAAATCTAGGGAAGCCAAAACCTTTAGACTTCATGTCGGAGAAGGCT
TTATCTAAAGTTTTTAGAGTTTGCTGTAACACTTGAGCATTAACAGATTTTAAGTCTTGGTTCGT
CTTCTTGGCAATGGTTAAGTTTTTTGCTTGATTATTGTAGCTAGGGTAAAGTGCGTTAGCAGGAA
TAATGTACTCAGAAATTAAGGAACAACTACTAACAGGCGATTTACGACTATTATACCAGTGCTTA
CGTTCAGCTAAAGCGTAATTGTAAACAGATTTACAGACGCTCAAGATATGTTCTATCTCTTGAGT
TTGTTGTCTATTTACTTTGAGCTTGAACGGATACGTCAGAGTAATCACTTGTTGAACAATTAATT
ATTTGCTAAACTTTAATTATATATTTAGCGATTTTAAATGTCAAGCAATTATAATAAAGGCTTTA
GGTCTGTATATAAACTTACCGCTCATGTTGTGTTAGTCACCAAATATCGTAAAAAAGCAATCTCA
GAAAAAATATTGTTGAGGTTGAAGGAAATCTTCACAGAAACTTTAATTAAATGGGAATGTGATT
TAGTTGAATTTAATGGGGAGTCAGACCATGTACATTTACTGATTGATTACAAACCTGATATATCT
TTATCTAAATTGATTGCTAATCTAAAAACTGTTAGCAGTCGATTGATTAGACGAGATTTTCCAGA
GTTGGCTAGTAAATATTTCGACAATAAACCGTATTTTTGGACGGGTGCTTATTTTGTGGCAAGTT
GTGGTGGTGTCACTGTCAGTCAGCTAAAAAAATATGTGGAAACCCAAAAAACTCCTCAAGAATAA
GGATGTCCTACAGACATTTTTTTTGGTTGGTCAAAAATTCATCTCCCAGTTCCTTCCTTTCGTCAG
GACTAAAGGATTTGTAGTGTATTTCTTTTTTGTCAAATGCAACGGGAGTCCTCTTTTGATATTTA
AGATAGAATTTTTGGAACTACATTCCCCTCAAAGCTAAAGTAATAATACCTTGTTGACCTAGTAA

AATTTCTCGTAACAGAGTGATAATTCCCAACCAAATAACAGGGCTGATGTCAATACCCCCCAAAG
GAGGCACTATTTTTCTGGTAAAAGCTAAAAAAGGCTCAGTAGGTATATAAGCTAAACTGTAAGG
AAATTGAGTTAGATCAATATTAGGATACCAAGTGAGAATTATGCGGAAAATAAACAACACCGTC
ATAATTAGTAAAAGAATACCGAGAATCACACTGGCAATGATAGCAATATTCATCAGCTTTTCTTG
TCGATAATTTCACTGTTTTAGCCTTTCCATCCTAGCATACTTGTGAACTCCAAACTTAACTTACA
GAAGCTAATTTCAAGGGTGACACCAAACTACTAGCTATTTCAACCCCCTTCCAAAGTAAATCAGG
GTAATTTTCGATTTGATTAGTCGTCGAAATATTTTCTAGCTCTTGCAACACATCAGCAATAATTT
TTCTTCCTTTACTGCCAAAAGCAATTCCTGCCACCTTAGAAGTTAAATTTAATTCAGACAATTTT
TTGATAGTATATTCGTTTGTCCCTCCTGCTAACTGTATAAATCCTTTCAGATTGCTATTCATTAA
TTTCTGAGCATATTTAATGGTTAAATGAGTTGTGCCTTCTCCTATATCGCCACTCATAGGGCGCC
CATCCGTCTGCCAAATTAAAGGAATTTCTAAGGGAGAAATAAAATCAGAAATCTTTTGTAAATA
ATCCATTATATTGGATGTATAAGGACAACTAATTGCAATTATTTTTAATTTAGATAAATGAGGCT
GTAATTTTTTCCAAACAGATATAAATTGAGAAAAATGACCTTCCTGAGTATGAATTTCGATCGCA
TCTATAGGTAAAATATCTAACCATTCCACAATGGTATCAATAGTAATAAAAATAGATTCAGTTTG
GATTAAGTTAAGAGGACAAATCGGTAAACAACGCCCACAACCATAACATAAATCTGACTTTACCC
CCCGATAAATAGAAGAAAATGTAATAGCATCCGCCGGGCAAATTGACTCACAAGGGCGAGGACAA
TCTGTCGGACAAAGGTTAGGATTGAAGTAGGCTTTGCGAAAATGGGGGTCTTCACCATCATTAAT
ACTAACCATTACAAAGGGAGAAAAATGGGAATCATAGCCTTGATTTTTAGCCTCTACAAATAAAT
CCTGCGCTTTCTTAATTCCCTCCAATGCACTATGAATCACAGCCCTATCCGCCGCCATATCAATAC
AATCCGCCCCTGCAAGAGTATAAATCAGTGCTAAATTACGAATAGAAGGCAAATGTTGATAACTT
GCACCACAAATCAACTTAAACCAATTACCTTCTTGTAAAGAATAAAACGGAGAATAAGACATTAA
TTATACTTGGTAGAAAAATATAAAGATATATAGTCAGGCTATGGAAACAAGAAAAATTTTTTAA
AACACTCAAATTATGATCTCAAATTCTCTTTCAATTATCATATTTGCCCATAATTCGCAAAGATA
AATTAAAATAAAATATAGGTATAGTTAATAAACAAGATGATCAATTTATTTTTTATTTATATTT
AGTTTGAATCATCAAGAATTAATAGTATAAAAAATCTTCAAATATAACCTAATTATTCACCTCTG
CCATTTCAATGACTCGATTATCCCAATCTTTTACTAAAAAATTAAGAGGTTTTTCACTGCGAATT
TTATATTTCAAACGACGGGCTTGTACTCTTAGTAACAACGCTTCTAAACATTCACCATCGAAACA
TACATGACGTTGACAATTTTTATAACCTAAACTAGCACCGCCAATAATATGTAATTGAGTATTTT
TTTTCAATTGATACCATAAACCATCCTTCGGCTTAACCATAGTATCATTAAAACCAGAACCCATA
TAAATAGGATCAAAAGCACTTGCTCCTAAACTTTGCTCATAGTTATAGTAATAGTGTAGAGGAAC
ATCTGCAACGGGTAAACCTAATAAATTTTCATAAAATTCCTGAGCAATCACCAAATCAGACACCA
TTACAGTATAAACTTTTGGCGCGCTAGTCAAAAACAGCCACATCGCACCGCCATAAGCAACTAAC
AGCATTACCATAATGCCCTGAGTGGAGAAAAGAGTATCTGTTAATTCTGGGAAAAATGCCGCTAA
AGAAATATTGGTTAAGGTCAAACTCCCCTCTATAATCATATCAGTCTAATTTTTTAGGTTTTTCT
ACCATTATAAAAGGCCTAAGTATTAACTACAAGCATGACAGATAAAGAGTAATTATTATAATTC
TTAGAAAAATAATGTTATCAAAATTAAAAATTTTATATTTTAAATCATCAATTCACTATATCTTT
TACTTTTTATTTTAAAATTATTTCATATAATAAACGTAATTTAAGACTAAAATGTATAGCTATTT
AAAAATATTTTTTAGATCACAATAAATCAATAAACTTGTATTTTTACACTATTATGAATGGTAA
TAATATATTTTTTTCAGACCTCAATCATGAATTGTTAATTCCCTTACATAAAGAATCAGATATTT
TTTTGTGGAAGAAATGGCAAAGTAATCCGAGTAAGGCTAGATATTTAGTGGAAATTTTTATCCGC
TATCATAAGCTAATTAATGACTCCCAAGAAAATGATATTGATAAAGTTACCTTAGATAAATATT
ATAAACAAATATGGTTTTATATTATTGATAATTTACTTGAATATCCTATCAGTAAAGATACCATT
CTTAGAGATGTTATTTTTACTTTAAGCAAAAATTTTTTTCAAGAACATAATGTTATTTATAAATC

TTTTTATCTTAGTCCTATTGATTATGATTTATTAAAAAAATATATTCCCCTGATTTATTTCTTAA
ATAAGAGCTTAAATAAATTAAATTCCCTAGAAAGACTAACCATAGTTGCCATAGACAAATTTGGG
TGGCAAGAAGAAAAACTGATAGAGTATTTGAGACAACATCAACAAAATTTAACCATTCCTGAAT
TAAAAAGTTATTACACAAAGGGTTACAGTAATTTAATTAATAATCTGCCTACAGACATAATTGCT
ATTTATCTTCAAAATGATGACTAAAGCTCACCCAATCTAAAAGAGTAGATGGTTTGAAAATATAG
TAATCTAATAAGATAGAACAAAACCTTAACGATAAAACTATATAGATAATGGAAAACTTATTAA
ACTTAGAAAATATTGACTTAGAAACCATCATTACCGCATCACTATATCTAACTTTAAGCGGCTTA
TATCTATTAATCATTCCTGCAGGAGTTTACTTTTATCTTAATCAAAGATGGTATGTTGCTAGTTC
TTTTGAAAGAGCTTTTATGTATTTTATGGTGTTCTTTTCTTTCCCCGGAATGCTTTTATTAAGTC
CTTTTCTGAATTTCCGTCCTCGTCGTCGTGAGTTAAGCTAACACAAAAATAATTAATTTATGAGA
AGAATAGATGCTATTTTAATTGCCTTTGCCGTGTTTGTTGCGGGGGGCATTATTTATATAATATT
TCAATATCTTGGTTTTGATGCCTATGATGCGGGTATTTGGAGTCAAGTTTTATTAGTTTTAGGTT
TAATTGGCTGGGTAGTCACTTATTTATTTCGGGTTTTCACAAATAACATGACTTATCATAAGCAG
GTTAAAGATTATGATGACGCTTTTTTTGCTAAACAGTTAGAAAAAATGTCTCCCGAAGAAATTGA
AAAATTAATGGCGGAAAAAGATAGTTAAACACTGAAAATTAACGTTTTTTTTCTGTAAGGGGCG
ATAGGTAAAAGCAATAAAAAATTGGTTTCTCTCTGACACCATAAGGGTTGAATATATTCAACCCC
TAGCACCCTAATGCCCTAAAAACCTATTTCACTTTCTCACTCCCTCCCCTCATCACCCTTTTTTTA
TACTCATTACTTTTTAGCTACAAAAAAAATAAATATTTTTAACTATGTCTTCTATTTCTCAACGT
TTTGCTGAACTAAAAAAACAAAATAAATGTGCTTTAATTCCTTTTATTACTGCAGGTGATCCAAA
TTTGGCAACCACTGAAAAAGCTATTCAGATTTTGGTAGAAAAAGGAGCTGATTTTATTGAATTGG
GTGTTCCTTATTCTGATCCTTTGGCTGATGGTCCTGTGATTCAAGCGGCGGCTACTAGGGCTTTG
AAAAAGGGTGTTTGTTTGGAAGATGTTTTAAGTATCGTCAGAAACTTAAAGGGAAAAATTGAAA
TCCCTATTATTCTTTTTACTTACTATAACCCCATTTTTTACCGAGGAGCAGAAAATTTTTTGGGA
ATGATTGCAGAGGCTGGTGTTAAAGGTTTAGTTGTGCCTGATTTGCCTTTGGAGGAAGCAGAAGG
CTTTTTAAATACTGCACAAAAAAAAGGCATTGAAGTTATCCTTTTAGTTGCTCCTACAAGTCCGA
TGGAACGCATTAAGCATATTGCTGAGAAATCTCAAGGGTTTATCTATTTAGTGAGTGTCACTGGG
GTAACTGGGGTACGTTCTGAAGTACAGAGTAGAGTTAAGGATTTAATTGCTCAATTAAAAACAG
TAACAGATAAGCCCATTGGGGTTGGTTTTGGTATTTCTGAGCCTTCTCAGGCAACTCAAATTAAG
GATTGGGGGGCAGATGGAGTTATTGTTGGTAGTGCTTTCGTGAAAAAGTTGAATAATGATAATC
AAGAAGAAGGCTTAAGTGCGATCGCATCTTTATGTTCTGATTTGAAACAAGCCATCATTTAAGAA
TTGGTATTAGGTGTTAGACTCTGGATTTTAGTTTACAACTATTAATTTATAATTAATTAACCTGC
TTTTTGTGAAATAATTGAAGATGAAGAATTCATAACAAAATAAAACCAATAACCATGAAACTTA
ATTGGTGGAAACGTTTATTAAAAAATGCGATCACGAAGTGGCGCTGCGCGATCGCTACACTTTTA
ATTGCGATAATTACTATTATTAATCCAGTGGGATTACATCCTGCTCAAGCAATTTTAGCTCAAGG
AGATGCTATCACCGATCCAGAGGCAATACTTCGTTATGCTTTACCCATAGATAATGATATTATCA
GACAAATTCAAGGAGATATTGAAAAAATATCCCGAAATTTGAGAGCAAAACGTTGGGCGCCAGT
AGAAAAAGAAGTTCGTAATGCGGCTTTCTTGCTCAAACTTCATCAAGATGATTTAGTACAAAGTG
TACCTGAAGAACTACAGCCCAAAGCCAAGGAATTAGTTACTGCTATTACTGAAGATGTGGCAAAA
CTTCAAGAATTGGTAGAAAAACAAGATAAAGAGCAAGTATATCTGACCAGAGCCAGAATTCTGG
ATAATATAACTGAATTAGAAGAAGATATGGTAACAGGCTACCCTTTTACTGTACCTGAAGAATAC
GCCAATTTACCTCAACTTTTAGGAAGAGCTACAGTAGAAGTTGAAACGACTCAAGGCAATTTAAC
CATTGTTGTCGATGGTTATAGTGCTCCTGTGACGGGAGGAAATTTTGTTGATTTGGTAGAGCGTA
AATTCTATGATGGTTTACCCTTCATCCGTGCAGAAGATTATTATGTGTTGCAAACAGGAGATCCA

GAAGGCGAAGAAGTTGGTTTTATAGACCCAGATACTAACAAATATAGAGCTATTCCCCTCGAAGT
TTTAGTGAAAGGAGAATCAGAACCTCTTTATGGTTTTACCACTGAAGATGTAGGAATGTACTTAG
CTCAACCTGTATTACCATTTAACGCTTATGGTGCGGTAGCTTTAGCTCGTCCTAGTACAGATCCT
AATGGGGGTTCATCTCAATTTTTCTTCTTTAAGTTTGATACAGAAGTAACACCACCGGGATACAA
TCTAATGGATGGACGTTTTGCTGTATTTGGTTATGTTACTGAAGGGGCAGATGTATTAGAGAAAT
TAACCGCCGAAGATAAAATTATTTCTGCCCATGTCATAGAAGGCAAAGAAAATCTAGTTAAACCT
CAGCAGAGTTAAATTTACCCTTTTCCCCTCCCCCCTCGAGAGGCAGAGCTGTTTCATTTTAAAATT
TTTGGTTAAGAGAGAGTAATGAGTAATGAGTAATAAGTAATGAGTTTTGATAATTTAAAATGAT
TAATTGATTGTTTTAAGATTTTTTACTATTACTTGAAATCTGCAAGTCAAAAATCCCCCCATCTC
CCCCTCCCCTTATCTCCCGCTCTTTTTTCTTTCTCGAAAATGAAATAGCTCTGGGATAAAGGGGGG
TTTGTCTCTTTTTACAACCTCTAATTATCAATACTGATTTGCCCTTGACGAAGAATTTGCCAATC
TGAACCTGTCCATTTAACTACTGTGGAAGCAATACCAGAAATTTTCAGGTTTTTCACCTCTAAGG
CATAAACAGAAGGAAATTTATCTGCAATATCATACATATCTGTTAACGCTGATTCTCCTGAGAGA
TTGGCACTGGTGGTAGCTAATGCCCCTGTTTTTCCTAATATTTCCTGTGCGATCGCATCTTTGGGA
ACTCTAACACCAATACTATGAGGATTAAGAGGATTCATGGTGGGAGGAATCTTGTCTGATGCAGG
TAAAACCATGGTTAATGCACCCGGTAAGTGTTGTTGAGCCAAATTTTGCCAAATGTTTAATTCTT
CAGGTGTGCCTTTTACATATTGCCAAATTTCTTCAATACTGCTTGTCATTAAGATTAAAGGTTTT
TCTAAACTCCTTTGCTTTAATTCAAAAATTAATTCACTATGACTAGGTTTGACCGCTAAAGCTGG
TAATGTATCGGTAGGAAAGCTAACTACTTGAGAGGCGATCGCCTTTTCTATTAAAGTGTCAAGAT
TAACTAAAGGCATAATCAAGATTTGAAATTTGAAGTTTTAGGTAGTGAATAATAGTTGAGAATT
TTATTTTTAATTCTTCATTAATTTACCATCTTAGGTTAATCTTTTCTAGGGAAAGATAATTTATA
TATAAATTCAGGAGAATCTCGTGGAACGTACTTTTATTATGATCAAACCCGACGGCGTACAACGT
AACTTAGTTGGGGAAATTATCAAACGTTTTGAGCAAAAAGGCTTTACCCTCGCAGGATTAAAACT
CATGCAGGTATCCCGTGAGTTAGCCGAAAAACATTACGATGTTCACAAAGAAAGACCCTTTTTCA
ATAGCTTAGTGGAGTTTATTGTTTCCTCTCCCGTAGTAGCAATGGTTTGGCAAGGAGAAGGGGTT
GTGGCTTCTGCTCGTAAATTAATTGGTGCAACTGATCCTTTAGCCGCCGAACCCGGTACTATTAG
AGGTGATTTCGGGATTGAGATTGGACGTAATATTATTCACGGTTCAGATGCCATTGAAACCGCCC
AAAGAGAGATTAGCTTATGGTTTAGTGAAGAAGAAATTTGCTCTTGGGAAAACTCTATGAAACC
TTGGTTAAAAGAGTAGTTAATTGAGTAGTAAGTAATAAGTAATGAGAAAGGAAATGGGAGAAAT
GAGGAGATAATTAACCGTTTTAAATTCATCATTATTTGGGCTAATTTTAAGATAAGCTAAGGGG
AAGATTAAAAGGAGATAAATATTTTATGAGCAAAATTGAAGAAGTCAAAGCCCAAATCAGACAA
GGAAATTTAGAAGAAGCAATGGCGATCGCTATTTCAGAAGCCATGAAAATAGAGATTATGACGG
CTAATATCAACGGTGATGACTCCACAGCTTGTCATAGTTTAATCGATTTATTAGAAAATGAGATC
GAGCATCAATTAGGAGACAAAAGTTTAGAAAATATCCACTTTGAAGAACTAGAAAATGCTCATA
ATCATATTTTGCAAAACGTGCAAAGTTTACAACAAATGTTTGTAATATTAAAGCAAAATTATCA
AGAATTATCTTAATTATTATTATTTATAGTTACTATTGGGTTTGCGAATAAATCTTAGAAAAAA
GGCGTATTTACTATGAAAAATTATGAAAAGATGAAGGAATTAATCAGAGAAAATAAATTGCAAG
AAGCTCTACTTTTGGCTTTTAGTAATAGTTTAAAATTGAAAATAACTACTAAATCTAAGGGCAAA
AAAGAAGATGTTATCGAAACTTTGATTAATCTTGTTGGGGGAATAAGTAATAAAGTTAGTGATC
CAGACTTAGTTAATAATTCTACTTCAGATAATTCAGATACAATTAATATTGTTGATTTTCATGAA
AGACAAAGACAGTTAGCCTACGAAACTTGGCACAAAAATAGGGAAACCCTGATCGGAATTTTACA
AATAATTTCTGGCAATTCTACTAGCATTAATAAGTTTAAAAAATCAGAAAATAATGTTATTTCCA
TTAAAGGAAAGACAGAACAAAAAGAAACTGATTTCCAAGATTTTGATTTTGCACAAGAAGAATT

AGACAACCACCAACCCCAAGAAGAAGAAGGAAATTGGGTAGATAACATTGTTGATAGCATTGTTG
GTGGAGAAGAAGACGAAATGTCTCATACGGAAAACTCAGAACAAAACTGGGGTGACTTTATCAC
AGAAGATGAGGAAGAAGAGTCCGCAGAAGTTATTATTAATTCCGAAGAAGAAAACTGGGATGAA
TTTATCGATAACGATTATTCAGAAGACTCTATTGCTAATAGTAATGGAGCTAACATAGATAAATC
TTCTGATGGAGGAGAGTGGCAAGAATGGTTAGAGGAAGATTCTGTATCCTCGGAAAATGAAGAA
GTAATTAATAATGCCGTGACAGATAATCCATCAAACCTAGAAGTTGGAGAAGAATGGCAAGAAT
GGTTAGAAAATGAAGATAATTCAGATGATGAGATTATAACTCATGACGAAATGGAAGAAATTGA
TTGGAATGATGAAGACTGGCAAGAGGAAAAAATTAATTGAAGAAGGCAAAAAGCAAAGGGCAAA
GAGCAAAACCCCCTTTATCCCTCTTAAATAGGGGGAGAAGGGCAAAGGTAAATAGTGAATAGTGA
ATAGTTGATAATTAAAAATTAAGAATTAAGAATTAAAAACCCCAAACACTTGTACGGCGAATGG
CCATTCGCCCCTACTTCCCCCTCTCCCCTCATCCTCTCATCCCCCTATCCCCAACACCTCCAACCTG
AAACCAGATACCTACGTTTATCCGATATTCTTAAACCGAGCTGAGGTTTAGTTAAGATTTAGTAA
ATTTTAAACTACCCCATGTTAATTTTTGTTGCAAAGTCTTGACATCTCCTGACTTTGCTGCTTCTT
TTACTTCTTGAATATACTTAGCATTTGCGATCGCATCTTCTTGAGAACGAGAACTGGTTTCAAAG
CATTTTTCAAGGGCTTGATAGATACCGACACGACGAACCTTAGTAAAAAATTGTCTTTCAATATC
AAGTAACTTGGTCATTAATTCCAAGTGCATAGCATCTCCATCGCACAATTCCTCTAGCAATTGCC
ATTCTTCACTGCCCAAAAGATTATTTCCTGCCCCAACACGAGGATCAAAAAATTCTTTCCCTGTT
ACTTCTTGATAAATGCGGGGTAAGCTATCCTCAAATTCGTGCTTTTCCTCCAACCAAATACGACG
AATTTCACTTAACTCCTCTGTGGTAATTAACTCTAAATTCTGGAATTGAGGGGGGGCATTTTTCT
GAACACTTACCTGTGCTTCCAATAATTTTCTTAACCAATATTCCCGCCACTGTTTGAGATAAGGA
CCATGAATCGGTTGTATAGATTCTTCTTCTCCATTATTACGGGTAAATAATTCTACTTTGCCATA
AATACGCCGAAAATCTCGTTTATCTTTATCATCTCGAATATCTAATTCATTACGCAAATCTAGCA
AAGGTTGTAACCAATCCTTATCTTCATCGTTTTGAATCATTGCTTCCATGGATTTATCTTTACTT
ACCATCGTGCATACCCAACAACCAAAGCGAGAATCACCACAACTAGGAGTAGAAGTATCCACCAC
TAAGGGGCATTCATTATCGGCAGTAGCACCTCGATAGAGAGTAAATAAATCTTGATTATCTCCTC
CCCAAGGATTTTGATATTGCAATAAATATAGCCAAACTTCATCGGTACGCCAATCTTCAATGGGT
GTATAAATATAGGAATTGGGCAAACGAGAGTTAATATTCAAACGTTCTCTAAATCTTCCCTTGGC
GTGTTTTTCCATACTTCTCGCCCTAGTAATACTTTCCGCTTTACGAGTGCCTAAAACTAAAATTAC
CTCTCCATAGCAACGCACATTTTCTTTGATAAAATTTTCCGCAGGTTGGATTTTCATTCTTTCTGT
ACACCAACGAAAACCATTACGAGGAGCAGGGTAGCCTTTACCAATTAAACAAACCCAAAAAGTGT
TTTTTACCGCAGGATATATTAAATGAGGCTCGAAGGGCATATTTTTTTCTTGTGCAGTTTTTTTG
ATCCTTTCGAGACAATTCTTCACCCAATGGGCAACCACTGGATTCTCTACCTGTGTATCTGTTGTA
ATTATATAGATTTTTTTGTGGCGTTTATTTTCTGGTAAAGATGCGATCGCATTCCAGATTAGTTG
TAAAATACAGGAACTATCTTTGCCCCCGCTATAACCAATAATCCAAGGAAGAGAATCTTGACAGT
ATAGCTCTTGAATTTCCGCCGTCAACATTTCAATATCATTTACCAATTCCTCTAAACTGCGAGGG
GGAAAAAGTGGAATTTGTTGAGTATTCATGGCAAAAATTGAGAAAAAGGAACAAAGGATAATTA
CAATAGTCTTAACCAAGAGTTTAACAAGATATTAGCTATCATGGGAGTAAACTAATTCATATTTT
TTGGCAAATTACTATTACTTCTAATTGTTATACTAATAATATTCCTTGTTTTTTTATCTTAGGAA
TTCTTAATTATTTTACAATTAATTTTACCATAAATACAGATTTTTATTAAATCAAAACATGAATT
TTGTTCATAAAAATAAAACTTCTAATGTAAAACAAAAATTGACAAAAAATTGCTACTATTCTAA
AACAAAAAAATTGAATAAAAAGCAATTTAGAAAAAAAGATTCTCTATGGAAAAGATATGGAAAA
TATTATAAATGGAAGTTACTAAGATTAAGGGAACATCCCCATAAAATTGCGAGAGGCTTTGCGGT
AGGAGTCTTTTCCGGATGCTTACCCCTAATGGGTTTGCAATTTTTAATAAGTCTTTTATTAGCTT

TTATAGTCAGAGGAAATAAATTCACAGCTTTAATGGGTACTTGGATTAGTAATCCCTTTACCTAT
GTCCCTTTATTTATATTTAATTTTCATGTGGGTAAAATTATCTTAGGTTTTTTTATCAGTAATCA
AGACTTACAATTTAATTGGCAATCATGGCGTGAATTTGCACAAATGGGAACGGAAATAACTGTCA
CTCTATTATTTGGATCTGTCATTGTAGGAATAGTTTTTGCTACCATTGCTTATCATTTTATATTA
CATTTACTATATCATTGGAAAAAAAACAAATTATAATTTGACGACCTCCACATTTTCTAGATAGA
GTATTTCTGTTTCCACTTCCCTGAAAATGCGTTTATAACAACTTTTTTCCCGCTCATTATATGCTG
ATTTTAGACACAAAATTGGTTCAATAAAAGATTTTTTTAAAGCACAATTACCGAGATATTTCTCA
ATTTCATTAACTATATCTTCTTGATTATTTTTGTCTTGCTTAAACCAATTAAACAATAAATTATT
TTCAGTTATTTTCCCTATCAATATAGAGTGATGACTTTTATCAAAAGAAGTAATGATATTTTTTA
ACCAGCTATCTTTTGGATAATATTCAATCTTTAATAAAATAAGGACATCTCCCTTCAAATTACCC
AACAAGTTATTTATTTTATTTATTTCAGAAAAGCTAATAATTGTAGAATCTAAAAAATTAATAT
TAATTTCTATCTGATTTTTAAAAAATTCTAAACATTGACTCTTTTGCAATTCCAACTCTCTATTT
AGCTTCTTCAAATTGACTCGCTTATTATTGCCAAATACCACTAAAGAGTAATATTTATTCATCAT
TAAATTATTAATCAAGTAATTTTATTTAATTGCTTTTATTTTTCCTCGGTAATTTGCTCTTTTCT
TTGATTCTGTAATAAAGGAATTTTTCTTTGTGCAGGAGGAAATTCAGAGGTATCTATTTCTTCCT
CCTTTTGAGCATCTGCGGTATTTTGAGAATTTGATGTTGCGTTTCCAGTATTTGCAGAGTTTTCT
GGGGGTGTTGTGTTAGTTTGATTTGGATTTACGTTAGATTGACCATTCTGAGGCGAAGCAGGAGG
AGTAGGAGGTTGTCTTTCTTGAATAATAGCAGTTTTTTTAGATTGACCACAAATACCCGATAAAT
TGATTTGATTTCCATTCGCATCAATAAGATAACATAATGGTCTAGCAGAAATACTCTTAGTTGTT
ACCGTTAAAAGAAAAAGACTTGATAAAAATAGTAAGGAAAAAAGAGATTGATGAAGAGATAAAA
TACTTTGTAATAGTTTTTGACAGTTATTCATCTTGGAAAAATTAAAAATAAAATTATTTTTCAAT
CATAATTATAATCTCATCTAATAGCAATTTATTTGATTTGATACAAGTCTCCCCAAACCCCGTTT
TTAAGCAGAGTTTTTCACAGTCGACATCAAAACGTTTGACCGTCATCACCAACTTTTCAAACTAA
ATTAACCCCAATTTCGCCTAACAATATCAGATAAGATCAGGTTTCAGGTTCTCTGATTTTAACTT
AACATTTAGTAAGAAAGATTAAAAAAAAGAGGCTTGAGTGCCAGTGAATGTTGGGAATTTGAAG
ATATTAAAGAAACACCACTAGAGCATGACGAAAACTATACTGGTCTAAAGTACAGCTATAGACCA
GAAAATCACCACTTAACTTATAGAAAGTGATGATTCAATGGAAGATAGAATTGTAAAGGAAGAA
TGTTAAAGCTGGCATGGAACTATTGTCCCACTCGGTCTCCCAAGCAGTATCGTCGCCGCTACTATG
TTTCACCCACGAGTTCGGGATGGTTCGTAGTGGTTCCATAGTGCTAGTCACACCAGCTATACTCTC
GAAGGAATAACCTTCAAGACTGCATAGAATTTCATAGTTCTTTAGGTCAAGATGACGGTCTATTA
GTACACTTCGGCTTCATACATTACTGCACTTCCACCTTGTGCCTATTAACGGGTCTTCTTCCCGTG
ACCTTACACCAGAACACTCATCTTGAGGTGGGCTTCCCACTTAGATGCTTTCAGCGGTTATCCTCT
CCGCACTTGGCTACCCTGCGTTTACCGTTGGCACGATAACAGGTACACCAGCGGTGCGTCCTTCCC
GGTCCTCTCGTACTAAGGAAGGCTCCTCTCAATGTTCTCACGCTTACACCGGATATGGACCGAACT
GTCTCACGACGTTCTGAACCCAGCTCACGTACCGCTTTAATGGGCGAACAGCCCAACCCTTGGGAC
GTACTTCCGCCCCAGGTTGCGATGAGCCGACATCGAGGTGCCAAACCTCCCCGTCGATGTGAACTC
TTGGGGGAGATCAGCCTGTTATCCCTAGAGTAACTTTTATCCGTTGAGCGACGGCCCTTCCACAC
AGCACCGTCGGATCACTAAGACCGTGTTTCCACCCTGCTCGACTTGTTGGTCTCGCAGTCAAGCTA
CCTTCTGCCTTTGCACTCTTCGACTGATTTCCAACCAGTCTGAGGTAACCTTTGTACGCCTCCGTT
ACCATTTTGGAGGCGACCGCCCCAGTCAAACTACCCACCTGAAACTGTCCTCATTCGAGTTAGAAT
TCTAGCTAGCAGAGAGTGGTATCTCACCGTTGGCTCTGTATCCCCCACAAGGAATACTTCTACGCC
TCCCACCTATCCTGCGCACTGCCAGCCCGAACACAATTCCAAGCTATAGTAAAGCTTCATAGGGTC
TTTCTGTCCAAGTGTAAGTAGTCCGTATCTTCACAGACAATCCTATTTCGCCGAGCCTCTCTCCGA

GACAGCTCCCAGATCGTTACGCCTTTCGTGCGGGTCGGAACTTACCCGACAAGGAATTTCGCTACC
TTAGGACCGTTATAGTTACGGCCGCCGTTCACCGGGGCTTCAGTCGCTAGCTTCACTTTCGCTGAC
CAACTTCTTTAACCTTCCGGCACTGGGCAGGCGTCAGCCCCTATACCTCCTCTTTCGAGTTTGCAG
AGACCTATGTTTTTGCTAAACAGTCGCCTGGGACTCTTCACTGCGACCACCTCTCGGTGGCACCCC
TTCTCCCTAAGTTACGGGGTCATTTTGCCGAGTTCCTTGGAGAGAGTTACCTCGCTCCCCTTCGTA
TTCTCTACGTGCCCACCTGTGTCGGTTTCGGGTACCGGGACTTAATTCTCAACATTACTACAGCTT
TTCTTGCCACTATCTTTCCACACTCACGATTACTCGCTCCCAATCCACTCAGGGTGTGCTTAGTTT
TCATGGGTCCCTGTAGCAACTCCAATTAAGCCACGCAGGAATATTTACCTGCTTGCCATCGACTAC
GCTATTCGCTTCGCCTTAGGTCCGGGCTAACTCTCCACGGACGAACCTTCTGGAGAAACCCTTGGG
CTTTCGGGGTTAGGGATTCTCACCCTAATTTTCGCTACTTAAGCCGACATTCTCACTTCTATCTCG
TCCACATCTGCTTGCCGCTAATGCTTCACCCTTAATAGAACGCTCCCCTACCACTACAATGTAGTC
CATAGCTTCGGTGACTAACTTAGCCCCGTTCATTTTCGGCGCAGGAGCGCTTGACCAGTGAGCTA
TTACGCACTCTTTCAAGGATTGCTGCTTCTAGGCAAACCTCCTGGTTGTCTAAGCACTCCCACCTC
CTTTCTCACTTAGTTAGTACTTGGGGACCTTAGCTGATGGTCTGGGCTGTTTCCCTCTTGACGATG
AAGCTTATCCCCCACCGTCTCACTAGTGCTACCATTCATCGGTATTCTGAGTTTGTCTCATCTTGG
TACCGGTCTCCCAGCCCGCAACGAAACAGTGCTTTACCCCCGATGACCTTTCACACCGCTGCGCCT
CAACACATTTCGGGGAGAACCAGCTAGCTCTTGGTTCGATTGGCATTTCACCCCTAACCACACCTC
CTCCGCCAATTTTTCAACATTGGTCGGTTCGGACCTCCACTACCTGTTACGGTAGCTTCATCCTGG
ACATGGTTAGATCACCAAGGTTCGGGTCTACAAACCGTGACTTCTCGCCCTTTTCGGACTCGGTTT
CCCTTTGGCTTCGACTTTTCGTCTTAACCTGCCACGACCTGTAAGTCGCCGGCTCATTCTTCAACA
GGCACACGGTCATCCCTTCTAATGGGACTCCCATTGCTTGTAAGCCGATGGTTTCATGTTCTTTTT
CACTCCCCTCCCGGGGTTCTTTTCACCTTTCCCTCGCGGTACTTTTCTCTATCGGTCACACAGTAG
TATTTAGCCTTACCACGTGGTCGTGGCTGATTCACCAGGGATTCCACTTATCCCTGACTACTCGGG
ATTCACCTATGCTTCGTCGGTTTTCAATTACAGGACTTTCACCTTCTTTGGTCTAGTTTTCAGCTA
GTTCATTTAACCTTCTCTGTCATGTTTTGGTGTCCCACTACCCCATTCTCCTTAGACAATGGTTTA
GGCTTTTCCCGCTTCGCTCGCCGCTACTAAGGGAATCACTTTTGTTTTCTCTTCCTCTGGCTACTA
AGATGTTTCAGTTCACCAGGTTCGCTCATCACCTCTTTTAGGTGTGTTTTCAGGGTTGCCCCATTC
GGACATCCTCGGCTCTACGTCTGCTTCCGACTCCCCGAGGCTTTTCGCAGGTTGCTACGTCCTTCT
TCGCCTCTGTGTGCCTAGGTATCCACCATCGGCCCTTCTTTTCTTGACCTTAATCTTCCCTATTTT
TCTATGCAGTTTTCAAGGTTCTCACTGAACTTTTTGTTCAGCAGTAGTGACACCTCACCGTGCTA
AACTCGTTCTTCTGGAGGTAAGCGGACTCGAACCGCTGACATCCTGCTTGCAAAGCAGGCGCTCT
ACCAACTGAGCTATACCCCCAATGGGCCATCCTGGACTTGAACCAGGGACCTCACCCTTATCAGGG
GTGCGCTCTAACCACCTGAGCTAATAGCCCCAACACCTCTTTCTTTATTTTTCTAGCCCTCCCCAT
TTCCTTGCTGACTTCTTCTCGAAGTCTCCCTTAAAGGAGGTGATCCAGCCACACCTTCCGGTACGG
CTACCTTGTTACGACTTCACCCCAGTCACTAGTCCCACCTTCGGCGTCTCCTTCCACTTGGGTTAG
AATAACGACTTCGGGCGTGACCAACTTCCATGGTGTGACGGGCGGTGTGTACAAGACCCGGGAAC
GGATTCACCGCAGTATGCTGACCTGCGATTACTAGCGATTCCTCCTTCATGCAGGCGAGTTTCAGC
CTGCAATCTGAACTGTGGCTGGGTTTGATGAGATTCGCTCCACCTCGCGGTTTCGCTCCCCTTTGT
CCCAACCATTGTAGTACGTGTGTAGCCCAAGACGTAAGGGGCATGCTGACTTGACGTCATCCCCA
CCTTCCTCCGAGTTCTCCCCGGCGGTCTCCCTAGAGTCCCCAACTTAATGCTGGCAACTAAGGACG
AGGGTTGCGCTCGTTGCGGGACTTAACCCAACATCTCACGACACGAGCTGACGACAGCCATGCACC
ACCTGTCTCTGCGTTCCCTTAGGCACTCTCAGATTTCTCCAAGATTCGCAGGATGTCAAGCCTTGG
TAAGGTTCTTCGCGTTGCATCGAATTAAACCACATACTCCACCGCTTGTGCGGGTCCCCGTCAATT

CCTTTGAGTTTCACACTTGCGTGCGTACTCCCCAGGCGGGATACTTAACGCGTTAGCTTCGGCACA
GTCCTTCTAACAGCACTACGCCTAGTATCCATCGTTTACCGCTAGGACTACAGGGGTATCTAATCC
CTTTCGCTCCCCTAGCTTTCGTCCCTCAGTGTCAGTATATGCCCAGTAGAGCGCCTTCGCCACTGG
TGTTCTTCCTAATCTCTACGCATTTCACCGCTACACTAGGAATTCCCTCTACCCCTACTATACTCT
AGCTCTTCAGTTTCCACTCCCTTTCCGAAGTTGAGCTTCGGTCTTTGAAAGCAGACTTGAAGTGCC
ACCTACGGACTCTTTACGCCCAATGATTCCGGATAACGCTTGCATCCTCCGTATTACCGCGGCTGC
TGGCACGGAGTTAGCCGATGCTTACATTAGGTACCGTCATTTTTTTCTTCCCTAAGTTTTGAGGT
TTACAACCCAAGAGCCTTCCTCCCTCACGCGGTATTGCTCCGTCAGGCTTTCGCCCATTGCGGAAA
ATTCCCCACTGCTGCCTCCCGTAGGAGTCTGGGCCGTGTCTCAGTCCCAGTGTGGCTGCTCATCCT
CTCAGACCAGCTACTGATCGTCGCCATGGTAAGCCTTTACCTCACCATCTAGCTAATCAGACGCGA
GCCCCTCTCTAGGCGATAAATCTTTTACCTATTCGGCTCATCCGGTATTAGCAGTCGTTTCCAACT
GTTGTCCCCGACCTTGAGGCAGGTTCTCACGCGTTCCTCACCCGTCCGCCACTAGCTCCGAAGAGC
CCGTTCGACTTGCATGTGTTAGGCATACCGCCAGCGTTCATCCTGAGCCAGGATCAAACTCTCCAT
AGTGGTTGTTACTTACAATTACTTGCTTTCACTACCTGATTCCTGTTTTTGTTTTTCAATTAAAT
GAATTGAACAAGGATTTGTATTTGGTTATGATGGCTAGAAAAATATTGAATTATCGAGGTTCGT
GGTGTGTCAGAGTTGAGCGTGTGGCTCAATCGCACAAATACTAATATAACTAACCTTTTTTAAAA
TGGCAACTTTTTTTCTACATTTTTTTCTTTTTTCTTGTTCCTTATTCGCTAAAACCTATATATCGC
AACTGTTTAAAGTTTTTAAGATTTTTCTTGTTTTTTTGAGCCAGGAACGTCGATGAGTGTTTTTG
GGGGGTGTAAGGATTAAATCGGAGAGGTACAGTATTGGTAAAGTTGTTTTGTTTTATTCAGAAT
GACAGTTCAAAGACTGAAATAATGTTCTAACCTCATTTCAATGTCGGCTTTTATAGAAAATCATA
AATAGGAGTTGATAAAAAATGGTGTCATCGAGGATGGGCATGGGCTTTAAGGTTTATTAATTGA
GGAATTTGGCATAATGATTTTCTTGATTTTGGACGGACGAATGCTTGATTTTTAGGGGAAAAAG
AGTATTTCAATAATATGTTTTAATGTTTAACAGCTTAAAATTTTAACTTTTATAGGCTTTGGACA
AGTGTAAACGCTAAATAGAAAAGTGTGCATATTATCTACCTTAGGTTATTCGCTTTTTGGTTGTT
GGCTAGGCGGAAGGTCAATGAATACTTTTTGTCCAGGCATTACACCGCTTAACACTTGAGTTTTG
TCGTCAACGGATGCTCCGATAGTTATAGGTTGAAATTCGGGTTCGTTTTCTTGGTTAGGTATCAT
GACTCCTGTTTGCCCTTTTTCTGTAACAATTGCCACTGTCGGTACAACTAATACATTATCTAGTT
GCCTACCAATAAAGTTAACTTCCACATTCATTTTGGATAGTAATTTTTCTTTTCCCGTAAGAATA
TCTATTTTGACTTCAAAGGAAGTGACGTTTTGTTCGATGATGGCTTCAGGGGCGATCGCTCTTAC
TACTCCTTGAAATGTTTGATTGGGATAAGCATCGGCGACAATTTCGACGGGTTGACCTATTTGAA
TTTGATTGAGGTCTATTTCTGGGACTTTAGCGACAATTTCTAATCCTCTAGCTAAGGCAACGATA
GATGTAGAAGTGGCAGAGGCAGTACTCGATGCTGATGTGGTAGGAGTAACGAAAGCACCTTCAGT
GGCAAATCTTTGGGTGATAATACCGTCAAAAGGGGCAGTAATAAAGCTATCTTGATATTGAATTT
TTGCGATTTCTAAATCAGCTTTTGCTGATTCCACATTAGCTTTTAGCTTTTCAATTTCTGCTTGTC
TTCTTACTTGTCTTTCTTCAAGGGCGATTTTGACTTCACCAACAGATGCTTCTAAACGTTTTATT
TCTGGATCTTCTGTGGCTTGTAATTCTTGCAACCTTTGTAAAACCTCTTGAATGTCGGCTTGGGC
TACTAAATATTGATTCGCTAGTTGGTCAAATTGATCTTGACTGATTGCACCTTCTTTTAAAAGAT
TTTCGTTTCTTTTGAGTTGGGTTTCTGCTAGTCTTAATCGTGATTCAGCTTCTTTAAGTTGCGAT
CGCACTTGATCCATTTGTTTGGGTATTCTTTGTTTTGATTCTTCTAATCGTGCGACTGCTTGGGCT
AATTGAGCGGATAACGCTTGTATTTCACCTTGTATTCTTATGTCCGTTTCTTGCAAGGCGGATTG
AGCTTCCTTAAGTCTGGCTTCCGCTTGAATTCCTTGAACGTATAATTCTTCATTTTCCATGACCGC
TATTGGTTGACCTTTTTTGACAATCACCCCTTGATCAACTAATAATTTAACAATGCGTCCGGGAT
TTTTGGGACTGATATTGACGCTCTGAATAGGTTCTACTGTACCACTGGCTTGAATTTCCAGAGCA

ATATTTTCTCTTTGAACAGTAACAGTATATTGATCAATGATATTGGAAGTTTTAGGACGATTTAT
CGAGGAATAGGCAATCCCTACTATTAATAAAATACCACCAGTCATTATTCCAAAAATCCAAGGAA
GAGGATTTTTTTGTTTTTCTGAGTTACTCACGGTCATAAATTGGCTTACACCGAAATCAATAGGT
CATAATCTTTTTCTACAAAGTTAACAAATTTAAGCTCAGTTGTGATGTGGAGAATCAACTAAAAG
AATTAAGACTTTGTAGTGTTGGGTTTAATAACCCAACAAAATACATTTTGAAGGAAGGATTAAA
TTATAGACGTTTATCTCGATCACGACGGCGGCGATCGCGCCATTCAAGGGTGGTAAGATATAGGA
TGCCACCACTAACGAAAACTAGCAAGGCGAGGGCAACGATAAATAAAACATTAAATAAGGGACT
AAATTCCACGATTTAAAAGCCTTTACGCCCCCATACTACCATTGCGATCGACCATGTGAAAAGAG
ATAAAACGCCTACCCATCCTAATGTAAGAATATCCATATTGTTAATTTTTTTATACTTAATTTAG
AGCTTTAACGGTCATATATCATATCATTTCCTTGTCCGATACATCAACTATCCTGATTTGAGTTA
TGGGTTCAAAGTTTAAATTAGTAGAGATTGAATGAAAAACTGCCACAAATCTATTTATATCAAT
AATTTAATAAACTTATTTACTCAAAATTTTGAAAACATTTTATTAACTCTTAATCGTAGATTGGG
ATTACTAATCCAAAGGTTATAAAAATAGACGAATAAAATGACTAAAAAACAAATAAAACCTAAA
GTATTATAAGTGTTAATAATTCGTTCAATACCCCAATCATGTTGATTTCTTAAAATATTCAAATC
TCCACCAGAATCACCTCTACCACTCCAAAAAGTCCCCCAAGGAATTTGACTTTTACCAACTTTTAT
TTTTTGCCATCTTGTCCAACTCCCCCAGAAAGTGATCATTCCCCAAATAAGGAAAATATATCGCC
AAAATTCCCAATAAAAACGTTGAGGTAAATTCCAATAAAAACTGATAATGAAAAGAGTACTTAA
ATAAAATTCCCCTCCAATACCAGCAAAAGCTAATAACATTCCATAAGTAGAGCGGGATATAGTCC
AAGTCAAAATAAACTGTAAAAAGATTAAAAATACAAGAAAAATTATGATATATTTTTTGTTTTC
TTTCCAAGTACTATAGATAGTTAAAAGTAACAAAAATAAGATACCAAAATAAACAAATAAAGAA
TGCTCGAAAGAAACGATAGTTGCCGCAAATGTTACCATCGCTCTATAACCTGAAAACCATGCTAA
AGTTGCGTGTCCTAACTCATGAATCCAGACACTAATAAACCATACTAACGGATCGATAAAATTGA
TAGCAAAGGCAATAAAAAAATGCGATGGGTAAAGCATAAGTATTAGTTTTTATTTCCGCTTCTGCA
AGGAAAATATCTTGATTATAATTAGCAAGTTTATCGGGACTAATATTTGGTAGTATTTTAAATT
CTGTTTGCCAAATAATCTGATTTTTATGACCAAAATTTACTAAAACTATTTTGGTTTCTTCCTTA
ATTAATTTTGTTAAATAATCATAAACTAATTTAACTATTTGTGATTTTTTTATTTTAGTCAAATT
CCTAAATTTCAAGAGAATATGATCAGGATATGATTCAACTTTTATTCTTACCCCTAAATACAGTA
ACTCTTTATTTAAAGTTTTAGTTAAGTAAATGTCAAATTGTTTAGTTTGGGTTTTTTCTTTATTT
TTCTGCTCTTTCTGTTTATATTCTGTTAATTGTAGGTATGCCTGACGAATACTAACTAATTCTTC
TTTTTCTCCAGACTTTAACTTTTCCGCTACTAATGAATAGTAATGTTTATCGATCTCAGCAAGAG
TAAGATCATTATCTATGTTTAATATTTTATAGCATTGCTCAATGCTTAAGTTATCCATATCAAGT
TAAAGTAGAAGATAAATAGTGAGTTGATTGACCCATAAATTTATCTCCCTTATATTTTTAGTTTT
AATTTGTGTTGACTAGGTACATAGTTTTTCTTTCTTGACAGCCTCATTTGCTCGAAAACCAACTA
ATACAGCCTGATTATTTTTCACAAATAAAGGTCTTTTCAATAGCATAGCATCTTTGGCAAATGCC
TTAATCCAATCTGCTTCTGTCCAAGAGTTTTTTTCTTCGCCTAATGCCCGATAAGACATCCCCGAT
GTGTTACGCATGGACTTTACACCTAAAGTATTTACCCATTCTTTTATCATTTTTTCTTGGGGAGG
ATTTTCTTTAGTATTAATGAACTGGTAAGTAACATTATTTTCCTCTAGCCACTGTATGGCTTTTT
TACACGTTCCACAATTGGGAATACCATATACATAAAGGGTCATATTTATTGCTGAAATCTGTTGT
TAACTGACAAACAATAAATATTCTACCTTTAATTTTCATTAATAGTTGAGGATTTAATTTTTGCA
TTTTAAATATTCTTTCACTGCTTATAAATCAATTAAACTAATGCTATATTGATAGAATATTAATT
CTTTAATTAAGTTGTATTTAGTTTATCAAATCATTACTATTAATCAGTGAATAAAGTCGATTATC
TTCGTATTAGTCTTATCGATAGGTGTAATTTTCGTTGTCAGTATTGTATGCCTGAAGGAGAAGAA
TTAAAATACCTTTTATCTTCAGAGATTCTTTCGTGTTCAGAATTATTAATATTGTTAAAAGAGGT

ATTTATTCCCTTGGGTTTCACTAAATTTCGTTTGACTGGGGGAGAACCTTTATTGCGCCCAGATT
TACGAGAAATAATCGAAGAAATTAATCTTTTACCTGAGACACAGGATATTGCTTTAACTACTAAT
GGTTATTTATTAACAGAACAAGCACAGATTTTGTATGATGCTGGTTTAAGGAGAATTAATATTA
GTTTAGACTCTCTTAATCCGAAAAATTTTGATCTTATTATTGGGAGGCAAAATCGTAATTTTTGG
CACAAAACGTGGTTAGGAATACAGAAGGCTTATGAAGTAGGATTTAACCCTTTAAAGTTAAATG
TGGTGATTATTCCTAATATTAATGACCATGAAATTTTAGATTTAGCTCAATTAACCATTGATAAA
AATTGGCACGTTAGATTTATTGAATTTATGCCCATTGGCAATGAAAAATTATTCACTGAAAAAGC
ATGGATTCCCTCAGAGGATATTAGAGAAATGGTCAGGAAAAAATGGGGTTTAGAAGAGGCAAAT
ATTTTGGGGCATGGTCCAGCAGATGTATTTAAAATACCTAATGGTAAGGGGACATTAGGTTTTAT
TTCACAAATGTCAGAGTGCTTTTGCGATCGCTGTAATAGAATTAGGCTTTCTGCGGATGGTTGGT
TAAGACCTTGTTTATTGAATGAAACTGGACAAATTAATTTAAAAAATGATTTGAGAAAAGGAAT
TACAACGGAAGAAATAAGAAAAAAAGTAGCTGATTTATTATTAATAAAGCCTGAAATAAATTTT
AAAGAAAGAAATTCTGGTACTGATAGTGGTATTTATAAACGTACGATGTCTCAAATAGGAGGTT
AGTTTTATTTTATCGAATAACCCTCTTTTGTCAAACTCCGAATCAACTATCAAAAAATAGAAACA
ATTATCCTGTCGGAAAATATAATTTAGAATCATGAGTGATCGATAATAAATTATTCAATCCTTCT
AATAATCCTGATGAATTTACCATTTTTAACCATGGTGTTATTAACCAAAATAAGATTAATGGCTC
AATCATCATCATTATTTTCTTTAAACTTGTTTTCCAACTATTTATTGGCTCTTTTTCTTCTATTTC
CTCTTTCTTTTCTTCTTCTGAAAATAGTAATTCTCTCAATGGTTTTGTTTGTAAACTTACCATGG
TATAAACACTATTTATTAGTCCCCACCATTTCTCAATACTTTCTATTTTTGTTAGTCGATAATCT
TTCCATCCTAATTCTTGTTTACATTGTCTAAATCCATATTCTACCCATGTCCTTTGACCATACAAA
TCTCCTAATCTTTTCTTCAAATTTCCCTGACAATTTGTCATCACAAAACTTGTTGAATTTTTCGGT
AAATTTTCTGTATCTGTGGTCATTTCCCAGTATGTTATTCTCCTTTTTTTTCCATAAATTATTTCT
CTTATGTATCTTGTTTCTCTTTTTCCATCCCTAAATGTCCTAGTAAATTTACACCATTTATTTGCT
CTAACTCTTTCATTTGCTCCCATCCACACCCCATGATTCTTTCTAATCGATACCACATATTCCAGT
TTTAACTCTTCTAATACTTCCAAAAAACTACTACTTTCACCATATAAGCTATCTGCTAACACTAT
TTCTATTTCTAATCCTAATTCCTTTAATTCTCTTACCATTTCTCCTGCTATTGCTGTCTTTGTTTT
ATATTTATCTCCTACTTTTAATCTTGACTTTGGTTTATATATTTTTGTCATTAATGGAAAGGTTA
TATTCTGATAAACACCATAGACATTAACTGAAACTATTCCTTGATCTATTTTCCCCACATTTCCT
AAATATTGTCGAGCAGTATAGTCTGTTTTATTACCTTTTTTTCTATCTCCTGTTTCATCTACTAT
GAGAATGATTTTTTTCCCCTGCAATATCTCTACTAATTTATTTAATCTTCTTTGGTCTAGTTCCG
ATAATAACCAAGGTGATGATGTGAGAAAATGGTGTAAGGATTGTGATGATACTCCTAGTACTTT
TTCTAATTCTGGTAATGATTTTCGTTTAAGATTAGAGATTATTCCTAAATGTAATAACTTAAAAT
ATTCATAACTTCTTACATCAGGAAATAAATCCTGATAGTAACTGCAATATTGATCCACTAAAGGT
ATAGTGGGTTCTGGAGTTCGAGGCAAATGTTTACTAACTTGCTCTGCTACATCCATTATCATCTA
AGCCTCCCAAGTTCCATTTCTCTTTTTCTTCTATTATTTTATCTCGGAACATGACAAAAAAGGGA
TAAGTGCGATCGAAGAAAAATAATAGAATGAACAATTGATGAATAATGGTTTGTTTTTAAGATT
AATAAATTTTTAAGAACCTTAATTAATAGCTAGTAAAAACTATTATCTGTAATTTCTTTTATTGA
GATAAAAAGCCAGTGAGCAGTAAGGGTATTAAAATTAACAGAAATATAATTAAAAAAGCATTTT
CTGGAGCTAGGGGAATTTCATTTTTTTCTCTTTTTACCATAATAAAACCTCTTGATATTTTTGTT
TAATATAAATTTCTAGCAGTAAAATAAAATTGTAGTGATAAGAAAAAAATTAGTTATTCGCATT
TTTTATTAACTTTTAACTTTCAATCAATTAAATGATACCGCTAGTGATGTTTTGATTATATCAAT
TACAAACTTTTATTTAGTTATTTGTAAAAAAACTCATCAAAGCTCGATGATTGCTGATAGCTGAC
AATGAGAAAATATTGCTTAGATAATTAAATTCCTAATCTTTCATAAATAACATCAAGATTTGCTA

AATGTTGATTAGGGTCAAAGCAGGTATTAATATCTTCTTGTGATAATAATTCTGTGACTTCATTA
TTATTCTCAATCAGTTTACGGAAATTACCGTTTTCAGTATTCCATGCTTGATGAGCGCATCCTTG
AACTACTCGATAAGCATCTTCTCTACTCATGCCTTTGCTTACCAATGCCAAAAGGACTTTTTGCG
AAAAAATAACCCCACCATAAACATTCATATTTCTTGCCATATTCTCAGGATAAACTAGGAGATTT
TGCACAAGATTAGTGGTTTCTTTGAGCATAAAATGAGTCAAAATACAAGTGTCAGGAAGAATGA
CTCTTTCTACTGAACTATGAGAAATATCTCTTTCGTGCCATAAAGCAACGTTTTCTAGGGCCGCA
ACTGCATTTCCTCTGATAATTCTTGCCATACCCGTAATTCTTTCACTACGAATGGGATTACGTTT
ATGGGGCATTGCCGATGAACCTTTTTGCCCTTTGGAAAAGTATTCTTCTACTTCTAAAACATCGG
TACGTTGTAGGTTACGAATTTCTACGGCAAAACGTTCTAAGGAAGCCGCTAATAATGCTAATTGC
TGTACAAATTCCCCATGGCGATCGCGCGATATAACTTGAGTGGAAGCGGTGTCAGGTTGTAATCC
TAGTTTAGAACAGGCGATCGCTTCTATTTGGGGATCGATGTTTGCATAAGTTCCCACTGCTCCCG
AAATTTGTCCTACAGCAACATCCTGACGTAATTTGACGAGGCGATCGCGGTGACGACGCATTTCT
GCTAACCAACCAGCCAATTTAAAGCCAAATGTAATGGGTTCAGCATGAATACCATGGGAACGCCC
CACCATGATAGTATAACGATGTTTTTGGGCTTGATAACGAATAGCTTGGATCGTTTCTTCCACAC
ACTCCAAAATCAAGTTTAAACTTGCTACCATCTGTAATGCTAAAGCAGTATCTAGCATATCAGAA
CTGGTCATGCCCAAATGAATATATCTTCCCGCATCTCCTACATATTCATTTACATTAGTCAAAAA
AGCAATTACATCGTGGCGAACTTCCGCTTCAATTTCCAATACTCGCTTTACATCAAAGTTAGCTT
TTGCTTTAATTTCCTCTACCGCCTCCGCAGGAATTTTACCTAATTCTGCTTGAGCTTCGCACACGG
CAATCTCTACTTGTAACCAAGTTTTCAATCGATACTCGTCTGTCCAGATAGCCCCCATTTCGGGCA
AAGTATAGCGTTCAATCACTGCAATAATTTCGCTTTTCTTGCAAATACAACTGTTATATTCTACC
GAAAAAAAGACAAGGGGCAAAGATTTCAACTATTCAGTTTTTAAGTTTTATTGCATTATCTAACC
TCAGTTCGGTTTAAAAATATCGGATAAGATTAAGTGTCAGGTTTCAGGTTGCAGGTGTTGAGGTA
TTAGGGGAATGGGCAATAGTGATAATATTTTGATGTCTCAATTCCTTATTTCTCAATGGTTTTTT
CCTTGAACTCAGATAGGGTATAAATAAGAGACAAAACCTTGTTTAAAAATTAGTTTGTTAATGA
AAAAAAAATTAATTATCTTCACTCGTTATCCAGAAATAGGTAAAACAAAAACTAGATTAATTCC
AGCAATTGGAGCAGAAAAAGCCTGTTTTTTCCATAAGTCAATGACAGAAATGACGGTAAAAAAA
GCTGACAAATTAAAAGAAAAAGTTGATGTGGATGTTAGTTTTTATTTTGCTGGTGGTAATCAAA
CTTTAATGAAAGCATGGTTGGGAAATAATTATAATTATTATCCTCAAAACGGCGATAATTTAGG
AGAAAAAAATGTATTCTGCTTTTGCCGACAGTTTCAAAAATAATTTCTCTCATGTGGTTTTAATTG
GAACAGATTGTATTGATTTGGATGAAAATATCTTAAATGATACTTTTAATCAACTGAATTTTTAT
GATTTAGTCATAGGAAAAGCTGAAGATGGTGGCTATTATTTAATTGCTTTAAAACAGCTAAAAA
AAGAATTATTTACTAATATAAAATGGGGGACTGATTCTGTTTTTGAAACCACAGTTGAGACTGCA
TCTCAATTAAAATATAGTATTTATCAATTACCTACTTTAAGAGACATCGATCGCCCCGAAGACTT
ACAATATTTAGAGAGTAAGATTATCAGTGAGGAAAAATGACTAACTCCAGAAACGTCTATGGAA
AACCCTTAAAAACTTGTTGCACATCACCAATGACAGGATTTTATCGTAGCGGTTGTTGTGACACA
GGATTAGAAGATAGAGGTTTACATACCGTATGCGCACAGGTAACAGAAGAATTTTTGCTTTACAG
CAAGAAACAAGGTAATGACTTAATTACCCCCGTGCCGATGTTTAATTTTCCCGGTTTAAAAGCAG
GGGATAAATGGTGTTTATGTGTTAGTCGTTGGGTAGAAGCCCTAAAAGCAGGATTTGCACCCCCA
ATTATCCTTGAGGCTACCCATGAAAAAACCCTTGAGTTTGTCCCATTAGAAATATTACAGGAATA
CTCTATAGATTAACAGGAATTATTTTTAAAACATCTTCTACCTGACGGTAAAATTCCACAAGGCG
ATCGCCGTTCAGGTGTACTTCATTCGTAGGATAATTATTTAATACTTCTATAATACTAATATCAT
CATTTTCTAGGGCAGAAGTAACTAAGGCCCCCCTCAAAGATTCTCGACTGGCTTTTTTACTAGGG
GTAGTAATCACCTCACCAAATAAATCCAAAATAAAACCACCCACAGGATTATTCAGCATTTTAGA

AAGAGTTACCCCATTAACCGCAATCGGTTTACATAAAACATTATTGATGCGATCGGAACTTTGAT
TCGATAAGCGGATATAATAACTTAAAGTAGTAGAAATTTCCCCAGTATCACAGAACACCGTCATA
TCGGAAACAGGGAGAGATTCTTGCCACATTCCATATTTAAGGATAACTTTTTCTGAGGCATTTAC
AGGGAAAACAAACCCTAACATAGTGGAAGAAAAAGCAACAATCCCCAAACAACGAAAAAAAAGC
AGTTGCATTGAAGATAATCGATATTTGCTATTAGTGCGAGATGTTTTGATATATTTTATATCCAT
TCTTATAGTATTTTCCTCTCAACCTCAAAGTATTATCTTAAACCGAGGTTAAAATAACTTTCATT
AATTAACTATATCCATCAGACGCACAAGTTAAGCAAAAGTTCAATCTTAATCACCTGAATTCTAC
ATAACATTGTCGGTTAAGGTAGGCAAGAGGAAAAACCCCTCCGTGTCTCCCCTTAAAAGAGGAGA
AGAGCAAGAGTCAAAAGTGTTTAATTAACTTCTCACCCCAACACCCTAATCCCCTAATCCCTTGA
CACCTGACACCTTTACACCACCAAAAATTTTCTATTGAATTCAGGTTAATTAAATTATTTCCCCA
TACCTAATTGTTGGGCTTTTTGATAAACTTTACCTTCTGTTAATAAAGAAGGTGCGATTACCACT
TCAACTTCCTGCATTTCTTTTATATTTTTAGCCCCTAAAGTACCCATACTGGTTTGTAATGCACCT
AAAAGATTATGAGTACCATCATCTAATTTTGCAGGACCTCTAAGGATTTCTTTAATTGTACCAGT
AGTACCAACATTAATTCTAGTACCACGAGGAAGAACAGGGCTAGGAGTTGCCATACCCCAGTGGA
AACCACGACCAGGGGCTTCTTCTGAACGAGCAATGGGTGAACCAATCATCACAGCATCGGCACCA
CAAGCGATACATTTACAAATATCCCCTCCAGTAATAATGCCACCATCAGCAATAATCGGCACATA
TTTACCTGTTTCTTTCTCAAAATCATCCCTAGCGGCGGCACAGTCAGCCACAGCAGTTGCTTGAGG
TACACCAACACCAAGTACTCCTCTTGACGTACAAGCGGCACCAGGACCAATTCCCACTAAAACACC
TGCCGCACCTGCTTTCATCAGATTTAAAGCAACATCATAAGTAACACAGTTACCAAGAATGACGG
GCATAGGCATTTCGGCACAAAAACCTGCTAAATCAAGGGGAGTGATAGATTCAGGGGATAAATGG
GCTGTAGAAACTACTGTTGCTTGAATGAAGACTAAATCGGCTCCAGCTTCTGCAACTACTTTTCC
GTATTGACTAGCACCGGCAGGGGTGAAACTAACGGCGGCAATCCCTTTTTGTTCTTTGATTTCAA
TAACTCTTTGTTTGATTAATTCCGGTTTAATTGGCTCACTATATAATTCTTGCATTAAGCCCACA
AATTCTGATTTGCCAACAGAGGCAATGCGATCGAGAATGGGGTTAGGATCTTCATAACGGGTTTG
AATACCCTCTAAATTAAGGACTCCTAAAGCTCCTAATTCAGAGAGTAATACTGCCATTCTTACAT
CAACAACACCATCCATCGCACTGGCAATAATGGGAATTTCTCTTTCAATACCCCCTAAATTAACA
CGGGTGTCTGCTAAACTAGGGTCGAGGGTTCTATTTCCCGGTGCTAATGCAATTTCGTCTATACC
ATACGCTCTACGAGCGGATTTGCCACGTCCTATAATAATACTCACTTTATTCTTTCTACTATAAG
TTATTAGTTTAGATTATCAAATTTTGAACTATCGTGGCAATTTGAGCAATACTTTTTCTATGTAA
TTCTTAATAGCAGAGTTGATCAAGTTATGGCTGATATTATTGATTTATTTATAGTTAATTCTGCC
CCAGACAATATTATACAAGGGTAAACTGCTTGACCCCAAAGAGGATGAAATAAACCACATTCTTT
TGTTTTCACCTTAAATGATGGCAAATATCTTTTGACTAATTGCTGAGTGATAAATATTTCTTTTC
CTTTGTTTGAATAGATGGGAAAACCTGTTTTTGGACAAATTACTTCGGATAATATTTTTTGATTT
TGACTATGATAATAAAACTTTTTTCCTAATGTGTAGAACTCTAAAAGTAGTCTTTCTTTTTCTAT
TTCTTCAGATTTTAAATTATTACCATTTAGGCTATGTTGACAACGAAAAAATAAGAAAATAATT
GTTTTAGGTACTTGTTGCCAGTCTGGTAACAGTTTTTCTTTATTAAGAATAATATAATCATTTAT
ATTAATAGTTTTTACAAAAATTTCTAGGTTTTTACTATTCATTAATACGACAAATAATGAAAAA
TATTAATTATTAATTAACTAAACAATCCGAGTTATTTTTGCAGTTCAAATTATAGGGAGAATAA
TAAGTTTTTTCCCTACCACCAAACCATTGATAACGATTATCTCTAATTTTTAGATAGCTTTTATC
TCCTATGGATTTAAGATTAGGGATAGAACGATAAGCGTTAATAAATAATTGACCATTAGGTAAT
AATTCTATAATTTTTTCTGCGGCTTCGCTTCCTTGCCATATTAAACTATTATCTTCGATGTTTTTC
AATATCATCCCCTGTTCACAGTCTTGGGGAGTAATATTTAAGGTTTCTAAAATTGCGGTATTTTG
CATGGGAATATATCTAAATAATTTACCGCGATCGAATCTTTCTAATAATTTAGTAAAATTGACAC

ACAAATTGCAGTTTCCGTCGTAAATAATTATATAATTGGCTGAACTCATTTTCTGATTAATACTT
ATCTTAATAAAACATTTTATCAGAGATAATTAGTAATTACTAACTGCTAATTTTTTATTCTTAAA
AACTACCAAGTAAAATTAGGGTACAATCATTGAACCAATACCATCATCTGTAAAAATTTCCAATA
GTAACGCATGGGGAATACGTCCATCAATAATATGAGCCGCCTTTACTCCCTGAGCGAGCGATCGC
ACACAACAGTTTACTTTTGGTATCATGCCACCACTAACAATATCATTATCGATTAAATCACGGGC
TTCTTGAATCGTTAATTTATAAAGGAGAGTGGAGTGATCATGATAATCTTTAAGAATGCCGGGG
GTATCGGTCAACAAAATTAATTTTTCTGCTCCCAACGCCGCCGCTATTTCTCCAGCAAAAGTATCC
GCATTAATATTATGGGCTTGTCCTTCATTATCTGCCGCCACACTAGAAATAATCGGCACATATCC
ACTTTTAACAAGGGCATCTACCAAACTAATATCGACATTAGTAACTTCTCCCACAAAACCAACAT
TTTCTTGATCTACTGATCTTGCTTTAACTAAATTACCATCTTTTCCACAGATGCCAACAGCGCTTC
CTCCAGCCCGATTAATCATAGACACTAACTCTTTATTCACCCTACCCACCAAAACCATTTCCACAA
CATCCATCGTAGTAGCATCCGTAACTCTTAAACCATCTTTAAATTGAGGTTCTATATTTAATTTT
GTTAACCATGTATTGATCTCTGGTCCACCACCATGTACAACTACTGGATTAACCCCCACAGAAGC
GAGAAAAACAATATCTCGAATCACGTATTCCTTGAGAGTACCATCTTTCATCGCCGCACCGCCAT
ATTTCACAACAATAGTACGTCCTGAAAATTTTTGAATATAGGGCAAGGCTTCGCTTAAAATTCTG
ACTCTGGTTGCTTCTTGTTCTCGATAATATTCACTCTCAACGATTTTTTTGGACATAGCAATGAT
TATTTTTACTTTTATGTATTTGTAATTACTTATTGAATAGATTTTAAGGGAATCTTGTCAAAAGA
AAGTTTGTCACGAACTGTTATACTTAACAATTATTTAAGTTATTAGAAAAAAATAAGCTAAGAA
ATTAAAGCGTTAGCGGTTCGGAGTTTAGAAAGAGGTGTTAGGGAAGCGTGTTAATTAAACTCTTT
TGCCCTCCCCCCTCAAGAGGGGCGATAAAGGGCGGGGCTGTTTCAAAGTAAAAAATTAGGTTAAC
TGAGATTAAGTAAGAGTAAGAGATACAAGGATTTTTTTCTATTAGTTAAAACTTTATGAACAAA
AATCCCCCCATCTCCCTCTCCCCCTATCTCCCGCTCTTTTTTCTTACTCGAAAATGAAACAGCCCT
GGGGGATAAAGGGGGGGTTTGCCTTTTGCCTCTTGCCCTTTGACCTTTTTTAATTTATTCACCAG
TTAAATTCTATTCTTTACATTTATATTTATTTTGAGCAAGGAAAAAATGGTACACAATAGTAATA
TTTCTTTGGCAATGATCGTCAAAAATGAGGCTCATAACCTTCCTAGATGCTTAGATAGCGTTAAA
GATATTGTCGGGGAAATGATTATTGTTGATACTGGTTCAACCGATAATACCAAAGAGATAGGGA
TTAACTATGGAGCAAAAGTTTATGATTATCAGTGGAATGATGATTTTGCCCAAGCAAGAAATTTT
GGTTTACAGTATGTTTCAGGAGATTGGGTTTTAGTTTTAGATGCGGATGAGGTTTTTAACCCAAA
AATTATCTCTCAGATAACCCCTTTGATGGAAAAAGATGACTGTTTAGTTATTAATCTTATTCGCC
ATGAAATTGGCACGGTTTCTTCTCCTTATTCTTCTCTTTCTCGACTTTTCCGTCGTCATCCCCAGA
TTAGTTTTTCTCGTCCTTATCACGCCCTGATTGATGATTCTGTCATTGCTTTACAAGAAAAAGAG
AAACAATGGAAAATATTTGATTCTAATGAAGTTGCGATCGCACATTATGGTTATCAACCAGAAGT
TATTGCCCAACAAGAGAAAGCCCAAAAAGCTCGTAAAGCCATGGAAAGTTATTTGGCACAAAATC
CCCATGATGCCTATGTTTGCAGTAAATTGGGGGCTTTGTACGTTTCTTTGGGAGAGATCAAAAAA
GGTCTAAAATTGTTAAAAACGGGTTTAAAATCGAGTCTAGCGACCTCTACAACACTATTTGAACT
GCACTACCACCTTGCTAATGCTTATGTGAAAGAAGGGCAAATTTCCTTGGCAGTTAAACATTATC
AAAAAGCGATCGCATCTCCCATCTTAGGTAAATTAAAACTAGGAGCTTATCATAACTTTGGTAGC
CTTTGTTATCAAAGAGGGCAATATCAACAAGCCTTAAACCTTTATCGACAATGTATCATCATTGA
ACCTCACTACGCCTTTGCTTACTATAACTTAGGCTTAACTCACCGAGCGATGGGTAATATTTCTT
TAGCCATGGAAACTTACCGTCAATCAATAAAGTTAAATCCCGAATATCCTTGGGCGTATCAAAAT
TTAGGAGTCTTACTACTTCAACAAGGATATATGGAAGAAAGTGCAGAGGTCTTTCAAAAAGCGTA
CAATTTGCATAAACAACAAAACCGCCCTGTTGCCGAAAACTTGAAAGAAGAATTGAAATTAGTAG
GATTAAATTTAAACGATTAAGCAGAAATGGAGCAAAAACAAGCTATTTACCGCATATTAGATGC

TAATTTAGATCGAGCCAGAGAAGGATTAAGAATCATTGAGGAATGGTGTCGTTTTGGTTTGAAT
AATCAAACAATGGCAGAATCTTGTAAACAAATGCGTCAAGAGTTGTCTCAATGGCATAGTCCAGA
AATTCGTGCCGCTAGGGATACCGTTAATGATGTCGGTACAACTTTAACTCATCCCAAGGAAGAAA
AGAGAGAAAATTTAGACAGTGTATTATTAGCTAATCTATGTCGTATTCAAGAAGCCTTAAGAGT
TTTAGAAGAATATAGTAAATTGATTAACGGCAAATTTGCCCTTACGATGAAACAAATGCGCTATC
AGGTTTACACTTTAGAAAGTCAACTCATGAATAAAAACAACCATCAGTTATTATCGCAATCTTCT
CTATATTTAGTTACTTCCCCGTGGAAAATTTATATGAAGTGGTAGAATCTGCCCTACAGGGAGG
CTTAAAATTAGTTCAATATCGCCATAAAGATGAAACTGACGTTGTGAGATATGAACAAGCTAGTA
AATTAAAACAACTGTGTCATAAGTATAATGCTTTATTTTTAGTAAATGATCGCATCGACTTAGCC
TTAGCTGTAAACGCCGATGGTGTACATTTAGGACAAACAGATTTACCTATTTTCGTAGCCCGTCA
ATTATTAGGCAAAGATAAAATTATTGGCAAATCAACAACTAATCCCGAAGAAATGAAAAATGCG
ATCGCAGACCAAGTGGACTATATTGGGGTGGGGCCTGTTTATGAAACCCCAACCAAAGCAGGAAA
AAAAGCCGCAGGTTTAGAATATGTGCGTTATGCCCAAGAAAATGCCTCTATTCCTTGGTTTGCCA
TTGGTGGCATCGATGAAACGAATATTGAAGACGTTGTGAAGGCAGGTGCAACAAATGTTGCCGTA
GTTAGGGCGATTATGGTTGCCGATAACCCCAAAGAAGTAACACAAAATCTCTTGAAGAAACTTTC
GTGAACTACTACCCGACCGCTATCGCTGAGTCGGGAGCTTCATCTTTCACAGGTTCGTGCCTCGAA
ACGGATTTACGTCCGCCTTTTGGTCTTACCGATCCTCCAGATGCAGAGACGGCTGTTCCATCCGTC
TGTACTTTTCTGATACCTTCTGCTCTAATATTTTTACTTGCGTTCTCATCCCTATCATGATGACTA
TCACAACTAGGACAAGTCCATTCTCTTATCTCTAATGGCATTTCAGACATTTGATAATGACAATG
AGAGCAAGTTTTAGAGCTAGGAAACCATCTGTCTATCTCCACTAATAACCCACCTTTTTGCTTGA
GTTTATAGTCTAGGAAGTTGATAAATGTTCCCCAACCGACATCAGTTATTGCTTTTGCTAATTTG
TGGTTACGAACCATACCCTTGATATTCAGTTGTTCAACGACTACCACTTGATTATTATTCACAAC
TTTGGTGGAGAGTTTGTGTAGGAAGTCTTGACGGGTATTGCTTGTGCGTTCGTGAACTTTAGCGA
CTAGCTTTTTGGCTTTTTCTCTATTCTTAGAGCCTTTTTTCTTACGAGCTAACTTTTGTTGTTTCC
GAGCTAAGTTTTTTTCATGTTTTTTGATGTGTCTAGGGTTAGCATACTTACTGGTTTTTTTGCCA
TCATGAACAATAGCAAAATCCTTAATTCCTAAGTCTATACCACAAATTTTCCCTGTCATATTTAC
TAATGGTAATTCTTGTTGGGTATCGAATAGGATACTAGCAAAATATTTGCCAGTGGGCGTTTTGC
TTACTGTTACAGTCTTAATCTCACCTTCAAATATCCGATGAATAACTGCTTTTACTAAGCCTAAT
TGCGGAATTTTGAGACATCCATCAACTATCGAGACATTTTGGGGATATTGACAAGACTGTTTTCC
GTGATATGACTTGTATCTGGGGTATTTGGCTCTACCTTCAAAGAAGTTTTTGAAAGCCTTAGTCA
GATTAAGAGTCGTTGATTGCAGACATTGTGAGTAACAATCTCCTAACCAAGCCGTATCTTCCTCT
TTTTTTAGCTTAGGGAGATACTTATTAAGGGTTACTTGCTTTAGTGCCTTACCTGTAGCTTTATA
AGTGTCAATACACAAGTTTAGAGCGTAGTTATACCACCAACGACAACAACCCATAACCTGAGATA
ATTTCTTGGCTTGGGTTTTGTTGGGATAGATTCTGACTTTTATCGCTCTGTGCATTGAGGTTTAT
TGCTAACTTAGGTTGCCAATAAATTTATTATAACTAATGATTTTGGTTTTGTGTCGAATCCTACG
GATATTTTTTTATCGAGGCACAATTCATCCGCTAATCCGCTATGCTGAGATTAGGGACTTCTTTT
GCCATTTACGTTAAATTAATAGAAAAGAATTTCCGTAATGCCTACTAAATAGTATTCATCGCAAA
ATACAAGCTAAAAATTTATAAATTATTATTTAATAATTGCCTATCGCCTATTGCCTACCCTAACC
AATAATTTACACGACGAGAAATGATCGAGCCTTTTATTTTAATGGTTAATTTTGATCTGAAAAAT
TATGGGGAAACTTATTAAAGAAAATTCCGTCTAATAAAATATAAGAGTTATGACAGATTCTACT
TGAAAGTTTATCATTTATCGAAATATTTATGATTGATTTAATTAACTCTCAAAATCAAAAATTTA
TTGTTGTTTAATTATATTAATTATTGCCGTGTTTAAATATTATCGAAAGTTAGTTTATCTACTCA
CATCCATCTTTGTTGCTTCTAGTTTAATTATCACTGATGTGCAACCTAGTAATGCTATTCCTTGG

CAGGAATTAATTTTTAGAGGTATTCAGCTTATTCAGATCAATAATATCAGTGATCAACAAGAGAG
AGAAATTGGTGGTCAAATTCGCCAACAACTAATATCTCAAGGCAAAGTTAAGTTGTACAGAGATG
AAAATTTATCCGCTTACGTTAATCAAATTGGACGCAGATTAGTGGCTGTCAGTGGCAGACCAAAT
ATACCTTATCGTTTTGAAATAGTTGATAATCCTCAAGTTAATGCTTTTGCCACTATGGGAGGCTA
TATTTATTTACATACAGGCTTAATTACCGCCGCAGATAATGAAGCAGAATTAGCCAGTGTCATTG
CCCATGAAATTGGTCATGTTGTCGCTCGTCACTCTCAAAAACAAATGCAACAACAAGCAGTAACT
CAGGGTTTATTAAGTGTAGCAGGTTTAGATCGCACTCAGGTAGTACAGTTAGGGGTAGCATTAGC
CGTCGATTTGCCCTATTCTCGTCAAGACGAATATGAGGCGGATACTGTGGGGCTAGAAATGTTGA
AAGGGGCAGGTTATGCTCCTCAAGCTATGGTTGATTTTATGCGTAAGTTAGCCAAAACAGGGGGG
CGAACTCCTACTTTACTTAGTACACACCCTGCTTCTGGAGATCGTGCGATCGCGCTTGAACAAAA
AATTCCTGCTTCGAGGGGTTATCAAGGAGATGGTTTAGACGAACAAGCCTACCGCTATCAAATTC
GCTCTTTACTATAACTTCGGTTTAAGAATATTTGATTTGGGTAGGTTTTAGGTATCAGGTTTCAG
GTGTCTGGTTTAACGTAAATGGCAAAAGAAGTCCCTAATCTCAGCATAGCGGATTAGCGGATGAA
TTGTGCCTCGATAAAAAAAATATCCGTAGGATTCGACACAAAACCAAAATCATTACTTATAATAA
ATTTATTGGCAACCTAAGTTAGCAATAAACCTCAATGCACAGAGCGATAAAAGTCAGAATCTATC
CCAACAAAACCCAAGCCAAGAAATTATCTCAGGTTATGGGTTGTTGTCGTTGGTGGTATAACTAC
GCTCTAAACTTGTGTATTGACACTTATAAAGCTACAGGTAAGGCACTAAAGCAAGTAGCCCTTAA
TAAGTATCTCCCTAAGCTAAAAAAAGAGGAAGATACGGCTTGGTTAGGAGATTGTTACTCACAAT
GTCTGCAATCAACGACTCTTAATCTGACTAAGGCTTTCAAAAACTTCTTTGAAGGTAGAGCCAAA
TACCCCAGATACAAGTCATATCACGGAAAACAGTCTTGTCAATATCCCCAAAATGTCTCGATAGT
TGATGGATGTCTCAAAATTCCGCAATTAGGCTTAGTAAAAGCAGTTATTCATCGGATATTTGAAG
GTGAGATTAAGACTGTAACAGTAAGCAAAACGCCCACTGGCAAATATTTTGCTAGTATCCTATTC
GATACCCAACAAGAATTACCATTAGTAAATATGACAGGGAAAATTTGTGGTATAGACTTAGGAA
TTAAGGATTTTGCTATTGTTCATGATGGCAAAAAAACCAGTAAGTATGCTAACCCTAGACACATC
AAAAAACATGAAAAAAACTTAGCTCGGAAACAACAAAAGTTAGCTCGTAAGAAAAAAGGCTCTA
AGAATAGAGAAAAAGCCAAAAAGCTAGTCGCTAAAGTTCACGAACGCACAAGCAATACCCGTCAA
GACTTCCTACACAAACTCTCCACCAAAGTTGTGAATAATAATCAAGTGGTAGTCGTTGAACAACT
GAATATCAAGGGTATGGTTCGTAACCACAAATTAGCAAAAGCAATAACTGATGTCGGTTGGGGA
ACATTTATCAACTTCCTAGACTATAAACTCAAGCAAAAAGGTGGGTTATTAGTGGAGATAGACAG
ATGGTTTCCTAGCTCTAAAACTTGCTCTCATTGTCATTATCAAATGTCTGAAATGCCATTAGAGA
GAAGAGAATGGACTTGTCCTAGTTGTGATAGTCATCATGATAGGGATGAGAACGCAAGTAAAAA
TATTAGAGCAGAAGGTATCAGAAAAGTACAGACGGATGGAACAGCCGTCTCTGCATCTGGAGGAT
CGGTAAGACCAAAAGGCGGACGTAAATCCGTTTTGAGGCACGAACCTGTGAAAGATGAAGCTCCC
GACTCAGCGATAGCGGTCGGGTAGTAGTTCACTATCACCATTGTAGAAAAGGCAGAAAATCAATT
AACACAAATTTCCTGTAAATTATTATGTATGATTTTCCCCTTCTCCCCTTAAAAGGAGAAATAAA
AAACTATATCCCCCAACCACCGATAAGCATTGTGAGAGAAAAATCATTTAGGTAGGATCAATGCT
GTAACCGATAAAGATAAATAAATAATTATGGCATATCAATTACCAAATCTCCCCTATGAATACAA
TGCTTTAGAACCCTATATTTCTAAAAGTACCTTAGAATTCCATCATGATAAACACCATGCCGCCT
ATGTGAATAAATTTAATGATGCCGTTGCTGGTACAGAATTAGACAACCAACCCATAGAAACTATT
ATCAAAAAATTTGCTGAAGACACTTCCAAACAAGGAATTTTTAATAATGCCGCCCAAGCATGGAA
TCACAGTTTTTATTGGCAATGTATGAAACCTAATGGTGGAGGTAATCCCACTGGTGTTTTGGCAG
ATAAAATCAACACAGATTTTGGTTCTTTTGAAAAATTTATAGAAGCCTTTAAAAATGCCGGTGCT
ACTCAATTCGGTAGTGGTTGGGCATGGTTAGTGTTAGATGGAGACACTTTAAAAGTTACAAAAAC

ACCAAATGCTGATAATCCTTTTACTAAAAATCAAATTCCTCTTTTAACAATGGATGTTTGGGAAC
ACGCTTACTATTTAGATTATCAAAATCGCCGTCCTGACTACATCAATGATTTTATAGAAAAATTA
GTTAATTGGGATTTCGTTGCACAAAATTTCTCTGAGGCACTTTAGTTGAGTTTGGGGTTTAGAGT
TCTGAGTTATGAAAGAGGTTTCAGGTTGCAGGTGTCAGGTTTCAGGGTGTTGGGGGATTTGGGTA
TTGGGGTGTTAGGGAAGCGTGTTAATTAAACACTTTTGCCCTCCCCCCTCTTGAGGGGGGATAAA
GGGGGGTTTACCCTTTGCCCTACCTTAAAATACCTTAACCCTAGAGTTAATCTAACTCAAAATTA
GCGATGATAATCGCTGTAAAAGCAAAAGCTAAGACTACAGGCATGGGACAATTTAAAAAATAAC
TGAAAACTCCTCCCGCAATACTAATCGCAACACTAATTGACAACCATATTTTTTCCTCTGGGGAA
AAACCTTTTTCTGCCTTAATTTCCATTAAAGTTTCTTTGGGAATGGGTAAAGGCATTACACTGTG
GGGGGGAAAAGCCCAATAATCACGACACTCCAAAAACCAGTCTGTCCAACCATTATGATCACACC
ACTCTTGAATCCAATCATCACAAAAATGCTTCATCATCCCTGAAATTTATCTTTTCTACAAATGA
TTTCACCTTATCAAATCTTTTTTTTAAGAGATCATTTTCTTTATCAAATTTAATATTAAAAGTTA
TAAGTATATTTACTTATTTGATTTTTAGTATTCATTAGACGTCTCCAACAATTCTCAAAATAAGG
GAACCAAGCCCTAACTACGAGCCTATATTAATATTTTATGGAGATGTCTATTGTTTCCCTGTCTT
GGCTTCACTCAAATCTTAAAAACCTACCCTTGTAAGCCCTCGAGAAGAGGGAGGAGAAGAGGGAA
GAGTTAAGACTTTGAGTCATTTTCACAAATTAATAAAAAATGTAAGTTAAATGCGTCTTAGCTTA
TTATTACTGAACGACTGAGATTAATGAGAGCAATATACTTTACAATACTTAATGTTGACCGTTAA
TCAAAATCGAGTAATCATGAGTGCAAAAGTAGAGATTTATACTTGGAGTAGTTGTCCTTTTTGTC
TCCGTGCAAAGGCTTTACTAAGAGAAAAACAGATAGAATTTACCGAATATTGTATTGATGGAGA
TAACGCCGCTAGAATGGCAATGACGGAAAGAGCAAATGGGCGTAGTAGCTTACCTCAAATTTTCA
TTGATGATCAACATATTGGTGGTTGTGATGATATTTATGCCCTCGATCGCGCTGGAAAGTTAGAC
CAAATTTTGTATAGTTAAAATGAAAAAGTTTCTTTTTATTATAGATCCGATCGCACAATTAGATC
CTACTCATGATAGTAGTGTGGCAATGATGGAATCTGCCCAAGTTTTAGGGCATGAGGTTTATATT
ACTACCATTGATAAATTAAGCGTTGTAGGTGGTAAAGCCTATGGTCATATTTCTCCTGTCACCTT
AAAACCTGTACAATTAATTGAAGGAAAGTGGATTGCTGAAAAAGAATGGTATCAAGTAGGAGAT
GCTTTTTTTTCTCCTCTGGAATTTTGTGATGTAGTGTTAATGCGTAAAGATCCGCCTGTTACAAC
TGCTTATCTTTATGCTACTTACATCCTAGATTTAGTTGATTCTCAAAAAACAAAGGTTGTTAATT
CTCCTCAAGGAATTCGTGGGGCTAACGAAAAAATGTACGCTCTACAATTTACCGACGTTATTCCT
GAAACCATTGTTACTCAAAGTAAGAAAGTAATTGCCGACTTTTTACAAGAAAAACAAGCCTTAAT
CCTTAAACCCTTGGGAGGCAAAGCGGGAGAAGGCATCTTATTTATGCAAGAAGGCGATCGCAATT
TTAACTCCTTAATTGAAATTAGCACCAAACAAGGACAAGAACCAATTATGGTTCAAGAATACTTA
CCATCGGCAAAAGAAGGGGATAAAAGAATTATTCTCTTAGGGGGTAAACCCATTGGAGCAGTTAA
TCGTATTCCTACGGGTAAAGAATTTAGAGGTAATATGGCTGTAGGTGGCAGAGTAGCTCAAACAG
AAATAACTCCGAGGGAATTGGAAATTTGTGATGCGATCGCACCTACTCTTCTTGATGATGGTTTA
ATTTTTGTTGGTATAGATGTAATTGGCGGTTATCTCACGGAAATCAATGTTACCAGTCCGACTGG
TATTAGGGAAATAGACCGTTTAGACCATGTTAATTTAGGACAAGAAACGATTAAATTCCTTTTAG
AAGTTGAATAAGTTAATAAGCAGAAGTCGGGAGTCAGAAGTCTCCCCTCATCTCCTCATCATCGT
AAGGGTTGAATATATTCAACCCCTAACACCTGCAACCTGACACCTAACCTTATCGGATATTCTTA
AACCGAACTGATGTTAATTATTAATTATTTATCGGTTGGTCATCTTTCCAATTGCCATCAATTAC
CGTGCCATCAGCAAAATTATAAACTCCTCTGCCATTTTTCTTCCCATTAACAAACTCTCCTTTATA
GGTGTCTCCATTGGCATAATCGCAAATGACATCGCCATTTAATTGATTATTTTTAAATGTGCCTT
GACAGACATCCCCATTCGCAAAAGTAAAAATTCCTTCTCCCTCAAATTGTCCATTCACAAATTCGC
CTTCGTAACGATTACCATTGGCAAATTTATATATACCCTTACCAACTTGTTTGCCGTTTTCAAAA

TCTCCCTCATATACTCCCCCATCAGCAAATTTATAAACACCCTTACCATGAGGAATACCATTTTTT
ATTTCCCCCTCATAACTATCTCCATCAGCATATTTACGCACACCTTTACCACTGATTCTTCCTTCT
TGAAAAGTACCTTGATAAATACCGCCATCAGCAAAAGTATAGATTCCTTCTCCTTGAGGTTGACT
ATCCACTAATAATCCTTCATAGCGATCGCCATTTTCATAAGTACAAATACCCTGACCATTAATTT
TACCGTCAGTTACCATCCCAGTACAACTATCCCCATTGCTGTAGATAAATGTACCTTCTCCTTGGG
GCAAACCATTGACAAAATTTCCCTTATAGCGACTACCATCATTAGAGGTATAAACTCCTTTCCCT
TCTGGCTTACCTTCTTTAAACTCTCCCTCATATTTATCCCCTTCGGGAAAAACTCTTACACCTTGC
CCCTCAAATTGGCTATTTTGAAATTCACCTTCATAGTAACCACCATTGGCAAAAGTATATTTTCC
TTTACCTTGCTTTTCTCCATCGACAAAATTACCCTCGTAGCGATCGCCATTACTAAAAGTACATTT
GGCTTCACCGTTAAGATTACCCCCTGAAACCTCACCTTCGCATTTACCGCCATCAGGAAGAGTAAT
AGTATTAGCAAAAACAGGCGATGATGTCATCGTCATGTTGTTGAGAATAACACTTAATAGAGAT
ATTAATAAGAGATTTTTCGCTTTCATTGTAATGATTTCTGCTTTTTTGATGTGATTTTTAAGACT
AATGCAGAGATGATTTATTGATTTAATTATCAACTTAATATCTTATATTAATTAATAATTATTA
GCAAGTAATAGCCAAAAAAATCGAAAATAAGGGGTTTTAGAATTTAAAAGTTAGTTTATAGTTG
AATTAATTTCTGATTAATTAAGAACTTTTGTAACTTTATTGCTAATTAAACTAATCATTACAGCA
TTTATTGCCATTAAGAAAGCCATACCACTACCTGAAGGTAAGGAATATTGTGATTGTAGTAAGGA
TTCAAAAATGCGATCGCATTTTGTGGCTATATCAATTAAGGTAATACCAAGATAAATTAAACGCT
TTATAAGATGAACAATTCCCCGATGATGAGATAGAAATTCCATCAGTGGACTGTTTATGGGCGTG
TAGTCGTGATATTGAAAGTTTGAAATATGAAGATTGGGAAAATTGAGTTTTTATTTACTAACAA
TCTCTAAACTAAAGGATTTTTCAAGAAGTCATTGATAAAAGAGCTAAGTAATCTACTTAGAATA
ATAATTAATTGTTCTGACAATAACATCAAAAATTTTCTTACTTATTCTAATTATGTCGTCAAAAA
ATCATCCCCTTTTCAGACTATTAAAATATAGTAAAAACTATCAAAATAAAATATCGATCGCAACT
ATTGCCACTATTTTAAATACTATATTCGATCTTGCCCCTTCCTACTTGATTGGTGTAGCAGTAGA
TATTGTGGTGTTAAAAGAAGATTCGTTTATTGCTCAATTTGGTTTCACTAATACCATTACTCAAT
TAGCCATTCTCTCTTTGATTACTCTATTGGTATGGAGTTTAGAGTCACTGTCAGAGTTTATTTAT
GAGCGTCTGTGGCGTAATTTAGCTCAAACTTTACAACATGAATTAAGATTTGATGCCTATAGTCA
TTTACAAGAATTAGAATTAAGCTATTTTGAAGAACGATCAACGGGTACGCTATTATCTATTTTAA
ATGACGATATTAACCAACTGGAAAGGTTTTTGGATTCGGGTGCGGAAAGTATTATTCGCTTTATC
ACCACCATAATTTTTGTAGGTGGTTCTTTTATACTAATTGCTCCCCAAGTGGCATGGTTAGCTAT
TTTACCTATTCCTTTTATTTTATGGGGATCATTGGCATTTCAAAAACGACTTGCCCCTCTTTATGC
AGATGTAAGGGATAAAGCAGGATTGATTAATACTCGTCTAGCTAACAATATTTCGGGCATTGCAA
CCATCAAAAGTTTTACCGCCGAAGTTTATGAACAATCGAGGGTAAAAATGAAAGCGAAGCCTAT
CGCCGTAGTAACCGAAGTGCGATCGCCCTTTCTTCGGTTTTTATTCCTGCTATTCGTTTAATCATC
GTCATGGGGTTTATCGCAACCTTATTTTTTGGGGGGGTTGCTGTCAGTCAGAATCAATTAACCGT
AGGCACTTACGGCTTTATGGTGTTTATCGTTCAATATCTTTTATGGCCTTTTGCTACTTTAAGCG
AATTGATGGATCAATATCAAAGGGCAATGGCTTCTATTCGGCGAGTCATGGGTTTACTTGATACT
CCTATTAGCATTCATTCAGGAAATCAAACCTTCTCCACACCAATAAAGGGGGAAATTTCCATCGT
CAATGTGAATTTTGCTTATGACAATGGGGTAAAAGTGCTAAAAAATTTATCCATGGACATTCGAG
CTAAGAGTAATATCGGTATTGTCGGGGCAACAGGTTCAGGAAAAAGCACTTTAGTTAAATTATTA
CTAAGATTTTACGAAATTGATCACGGAGAAATTACCATTGATGGTGTTAATATCCAAGAATTATA
TTTACATGATCTCAGAAAGGCGATCGCATGGGTAAGTCAGGATGTGTTTTTATTTCATGGCACGG
TAGCTGAAAATATCGCTTATGGTAACTTTGACGTTACTTTAACAGAAATCATCCAAGCGGCTAAA
TTAGCAGAATGTGATGACTTTATTCAACAATTACCCCAAGGATACGATACCATCGTGGGAGAAAG

GGGGCAAAAACTCTCAGGTGGACAAAGACAACGAATTGCCATCGCCCGTGGTATCCTGAAAAACC
CGCCTATTTTAATCTTAGATGAGGCTACCTCCGCAGTGGATAACGAAACCGAAGCCGCTATTCAA
AAATCGTTGCAAGTGATTACTAAAGACAGAACAACTATTGCCATCGCCCATCGTCTTTCGACTAT
CAGGAATTGCGATCGCATTTATGTCATGGATAAAGGGCAAATAGTCGAGCAAGGAAAACATGAA
GAATTATTAGCATTAGAAGGAATTTATCATAGCCTTTGGGCGGTGCAGTCGGGAATAAATGAAT
AACTGATGTTACGCAAAAAGATAATTAGTTGTTGTTTTGAGGTATCAGGTGTCAGGTATCAGGTG
TCAGGTTTAAAGTCAGAAAAATTCACCCTTTTTTATTTTGACTTAGTTTTTCGATTAAACTATGT
AACATTCTGCCAATTTCCTCACATTTGTTTAAAGTAACTTTTAATTCTTGCTCTTTAAGATAGCC
TAATCGTCCTACAATCATCAAATGAGTTTCTAATTCTTTGAGTGAGCCATTTGCAATAATAAAGA
TGACGAATATAATCACCCAAATGATTTCTCCCTTTTCCTTCTGCGATGTTGGCAGGAATTGAAAC
TGCGGCTCTTTGTATTTGACTACTTAAACCATCAATTTCTGTTTTGGGAAATAGAGAAGTTAATT
GATAACATATTACTACCAAATCCATTGATTTTTGCCAAACCGTTAAATCTCGATAACTTTTTAGT
TCAATCATTTTTATTATTTCCTGAAACCTGAAACCTGAAACCTGAAACCTCCCTTAACTTAACAC
ATCAATAAGTGCGTAACGTCAGTGAATAAGTTAATTTGCATCTAAGTTTACTGATTAGGATAGAG
AATAGCGAACAAAGGAAAAGTATTTATAATTGATATAAACTGTAGTTAAACTAATAATTTTAAG
TCATTGCTAAAGGCTTCAGGGTTAGATTTGAGAATAATTAACATGAGTTTTGGATAAGCTGAAA
GCATTATTTTTTCGTAGTCGGAAAACCTTATAGCTTATTAGAAACAACAATAAAATTGCCTTAAC
CCGAACTAACGTTAATTAGGCAAAAAGATAATTGTATTGAGGTCAAATTGAAAAGAATCATCCA
TGTCATATACAAGAATATTCCTAATTATTGTAACGGGGGATTATCGGAATCATTTTCCTCAAGAT
TAGTCTCTTTTGTTGGTGTTTCAGAAGGTAATGTTTCCTGTTGATTTTGTTCTAATTCCTGCTGT
TTTCTCAATTCTTCCTCTCTTTTTTCTCTTTCAATTCTTGCTTGTGCTTCCCTTTCCTGTCGCTCT
TTTTCAAGCTGTCTTTGACGCTCTAATCTTTCTTTTTCAAGCTGTGCTTGTCTTGCTTGACGCTCT
TTTTCTTGCTCTAATCTCGCTTGTCTTTCTGCCGCTTCTTTTTTCTGACGAGCGATGCGATCGAAT
TCTGAACCCGCTACGGTAAAGTTAATACTAACCTGCACCGAAGCATTATTCACACCGGGAGAAGA
AAAACGCATTTTTCTCGCCGCTAGCAATGCCTGTCGATTAACCTCTCCGTTACTATGAGAACCAAC
CACACTAACCCCTAAAACATTACCATTGCTATCAAGGTTAACTTTAACAGTTGCTTTACCCTCCAC
CCCTTGTAATGCAGAAGGATACTCAGGATTACAATTACTAATACATTTAATACTTTCAGGCGGGG
GAGGAGTCGGTTTTGCTTGGGGGCGTGATGGTACGGATTGACTAGTAGCGGCAATATTACTGGGA
ATGCCAGATACAGTATTTTGGGAGTTATTATTGCTGGTTTGACTACTGCCACGACTTAAACTATT
TCTTAAATTACCGACTTCTTCTCCTTGACTACTACTAACGGCACTGGGAGTATTGTTACTACTAC
GAGATAATCTTCCACTGGAAACGGGGGTGTCTTGGGTAACACGGGCAACGGTGGAAGACTGACCA
TTATTACGACTTTCTGCCCTTGGGGATGAGTTTAAACTACTACTTAAAGAATTACTATTATCGTT
GATGGGTTTTACTGCACGGGGAATATTATTTTTGACTGGTGCATTACTGGTTAAAACCTCTTGAG
GTGGAGAAATAGGGGATTCAGGCGATGGAGTGATTGAAGGTTGAGGAGTTGTTTCCGTAATCTG
CTCTGGTTTTGGGTTATTTTCAATTGCTTCTGATGTTTTTTGAGGTTGCGGAGTAGGATTTACTC
TAGCAACTTCTTGCATAGGAGGATTTGGTTTTGTTTCTCTCTTATTCACCGTTGGTTGCGGTGGC
ACTTCCCTTGTTTGAGGTAAGGGGGGTTTTACAGTTTCTAATTTTGGTTGAGGTTTTTCTTCTGG
CTGGGGTTTTTCTTCTGGCTTAACTTCAGGCTCTTGTACAACGATAAATTCAATAGGCTTAGTTT
CTTCCGTTAAGGGTTTTTCGATCGAGTTTAGAACTCTAAACATAATACCATGAAGTGCGATCGAA
CCTAAAACCCCAATGGCAAGAAGTTTTCTTGTTTTTTTTATTTCCTGTTGACGTTGCTCAATACA
AGTTACTGAATAAGTCATGATGAATTAATTATTTTAAAGTGAATCAGGCTGTTTTATGACGAAG
AAGGCTTTTTTTACTAACCCTCATTTTGCTAATCTTTTTTAACTTTATCGATATATCTTTGCGTT
AAAATCTAAACTTTGTTATAGTAAAATATTAAATTAATTAATAATAATTTGCAACTATTTAAAG

CAAGTCAATGTCCACAATTGAACTATTAAAAGCAGGAGGTATTGTTTCAATTCCTCTATTGGTCT
TTTCTGTGATCACAATAGCTTTGATCATAGAACGTTTTTGGTTTTGGCTGAGGATAAAGTCGAGA
GAAAGAGTTTTAGTGAAAGAAGTATTAAAAATTTATCCCTCAGATTGTGTAACAGTGATTAATA
AATTAAGAAAAAAATCTTGATTTACCCATTGCCAGAATCTTTTTAGAATCTCTTGAATTAGAAAAT
GCTAATGCTACTGAGTTTCGTTTAGCTTTAGAGACTGCTACCCAAGCAGAGTTGCCCTTATTGAA
AAGATTTAACACTTTTTTTCAAACAGTCATTACTGTTGCACCATTGCTAGGATTACTCGGCACAA
TATTAGGTTTAATTCAATCTTTTGCTTCCCTAGATTTGGGCAATACAGGAGGCACAAACACCGCA
GGAGTTACAGGGGGTATCAGTGAAGCATTAGTTTCTACTGTAATGGGCTTAGTGGTAGCTATTGT
CACTTTGATGTTTGCCAACGTCTTTCGTTCTTTTTATTTACGCCAAATCGCTTTAATTCAAGAAT
ATGGTGGACAGTTAGAACTTTTGTATCGTCGTTTCTACGAAAAAGGAGGAGAAGAGAAAGCCTA
TGCGAATACCTGATGAGCAAGATGTGCAAGGGGAAATTAACATAGTACCAATGATTGATGCTATT
TTTTCAATTTTGGCATTCTTCATTGTATCTAGTATTTCTTTAATTAGATCAGAAGGTTTGCCCGT
TAATTTACCTTCCGCAAAAACAACGGAATCTCAAAAAGTTGCTCAAATCAATGTTACTATTCAGC
CTGATGGCAAAGTTTTTTTAAACAAGCAACCGATAGAAGTTAATGGTTTGCAAGGGGCAGTTACA
AAACTAATCCCCAACAGTGAGCAAGGCATGGTAATTATTAACGCCGATGAAAAAGTATCTCATGG
AATAGTTGTATCGGTGATGGATGAATTAAGACAAATAAAAGGGGTAGGTTTAGCTATTTCCGCA
AAGAAAAAAATAAGGTTTTTAAATAGGGATTGATGAGAAAGTGGTGTCAAGAGTTTAAGCAACAG
TCCAAAATATCTATAAATCAGAGACTTCAGTAAGGATAATGATTTTTTATAAATTGCTATTTTTA
TCAATTATTTTGATGCAGATAAGGGTAGGTGTCAGGTTTCAGGTTGCAGGTGTTAGGGATAGGGG
GATGAGGGAATGAGGGGAGAGGGGGAAACAAGTAATTAGTTCAGAGTTCGGAGTTTTTAATTCT
TAATTCTTAATTTTTCCTTTGCCCTTTTACTTTGCACATTGCCCTTTTTGTTATCACTCATAGACG
AAAATTTATCCTTAACTCAGGTAAATATAGATTTTTAGGATTTTATTGCATTTAAAATCTTTGTT
TGAAAAGGGTTTCGATTGTCAACAGTAAAGCCTAGATAGTTATTGTGGAATAAAAAGCCGTTTTT
GAGTTAGCTGTAGGATAAACGCTCACTAACCGCTATACGGCACAAAACACAAATACAAATAATAT
TCTTAGTGTACATGATTTGACTTCTACTGTTAGATTCGAGACTTGCCAAATTTTTTTCGCTTATA
CTAGCAAATATTTGCAATAACTGCTATTTTAGTATAGGTATTCTAAACAAAACACAAATACAAAT
AATATATAAAAATGAATCATAGATTATTGTTAGGTAGTTCAGCCCTTGCATGGGGTTTAATTATG
TTATCTAACCCTCAAGCTCAGGCTCAAACATCTACTGATAAATATTATAATAATTCTCAACAATC
ATCACTTACTGTTAAAGATTTAAGTCAAGAAAAAACAGAAGCCAAATGGTTAGTACAAAATAAT
ATTATTACCATTACAGATATTGAAATAAACAATACAGATTCTGGATTACAATTAATCATAGACGG
CGATCGCACCTCTGAATTACAACCCTTAATTTCCCCTTTGAAAATACTCTAATTGTAGAATTTA
TCGAAGCGAGATTAGAGACAGATTTTCGAGAGACGGATATTAGCGAGGAAATTACCGCCATTGAA
GGGGTGAATTTAGAAAATGGAGGCGTAAGAATCACTGTTACGGGTAAAAATAACCCTCCCTCCGC
TCAAATTATCCCTTCTGATAGTAAATTAGTATTATCTGTTACCCCTGAAAGTAGTGTAGCAAGTG
AAGAAGGAGAAGAAGAAGCCCAAACCATAGAATTAGTGGTAACGGCAAATAGAGCGCAAGAGGA
AGAGCAAAAAGTTCCTCGCTCGATTACAGTAATCAATCGTCAAGAAATTGAACAACAAAGTAAAA
TAAGCAGAGATTTAGGGGAAATTTTGCCTAAATTAGTGCCGGGGTTAGCTCCTTCTACCCAAACA
TCAAGTATTTTTGGTCAACCTTTACGAGGAAGAAATGTTGGGGTTTTAATTGATGGTGTACCTCA
AAGTACTAATCGTAATGCTCAAAGGGATTTACGCAATATTGATCCTAGTTCCATTGAAAGAATTG
AAATTTTGCGTGGGCCTAGTGCTATCTATGGGGATGGTGCAACAGGGGGTTTGATTAATATTATT
ACTCGTCAAGCAGAAGATCAGCCTTTTGTTGCTAGAACCAATATTGGTTTTGACAGTATTTTAAC
TAATTTTGCTGACAGTTTTGGCTTTAATGTTGAGCAGTATTTTGGTGGTAAAAGTGAGGGCTTGA
GTTACGTTTTCAACACCTCCTATAGCACTACGGGAGGATTTTTTGACAGTGAGAACGATCGCATC

CCTCCAGATCCTCAAGGGCAAGGGGGTTTAGCGGATTTGAACACCTTTAATATTTTGGGTAAAAT
CGGTTATGACATTGATGAAAACCAAAAAATACAGTTTAGTATCAGTTACTATGATGATCAACAA
AATAGTAAATATACATTAGATCCCACTCCTTCAGGACGTAAAGCAGAAGCGAGAAGGGGTTTAAG
ATTGCCAAATCAGCCGAATACAGAAAACTTGGTTTTAAACCTTAGCTACAATAATCAATCCCTTT
GGGGAAGTCGCCTTTCTTCTCAGCTATATTATCGGGATTACTCAACCCGTTTTTCCCATTTGATT
TACGCAGTAGTGCCGCCTTCGGCAACAATGTCTTACAATCAGAAGTACAGTCAGAAAAAATTGGT
ACTAGATTCGACATTGACAGTCCTATTTTGCCCGATGATCAACTAAATATTCTTTGGGGTTTAGA
CTATTTTCACGAAGACTCATCCCAACCTGCTGATATATTTGATTCCCAAGTTTTTGCCCGTAGTG
ATGGTTTAGTATTCCAAAAAACTGGTTCTGGCTTTTTATCCCCTCCTACAGGACAGGATAATATT
GGTTTATTTGCACAATTAAGATGGCAACCCATCGAGCAATTTTCCCTTAGCGGTGGTATTCGTCA
AGAATTTGTTGATGTGAGCGTTAATAGTTTCACTACTTTGGGAGGTAATCGAGTACAAGGGGGTA
ACTTGAATTATGATGCGACTCTCTTTAATATCGGTGGAGTTTATGCTTTCAATGACAATATCAAT
GTCTTTGCTAACTTTGCTCAAGGTTTTTCCATTGCTGATGTGGCGAGGGCTTTGCGTACTGCGCCT
AATGGTTCAAGGGTTACACAATTAAATCCTGCCGCCCAAAAAGTAGATACCTATGAGGTAGGAAT
TAGAGGTAATTGGGATAATGTTCAAGCCTCTTTAGCTTATTTTTACAGTTACTCTGATTTGGGTA
CAACTTTTGATGCTGATTTTGATATTATCCGAGATCCTCAAAGAATACAAGGTATTGAAGGAGAT
ATTAACTATGATATTAATGAGGATTGGAGTATTGGCGGAACATTGACATGGACTACTGGATGGCG
CGATCCTGACGGTAATGGAGATTTTGATACTCCTTTGGGTAATACCCTAATTCCCCCTGTGAAGC
TAACAGCTTATGTTGAAAATCAAACTACTGATACATGGCGTAATCGTTTACAATTCTTGTATTCT
GGCGATCGCAATCCGTCAGGAGAAGGTTTTGATCTCGATCCTGTATATAGCTATTTTACTGCGGA
TTTCATTAGTAGTTTAAAACTCGGTAACGGGGAATTAAGCGTCGGCATTGAGAACTTATTTAATA
CGTTTTACTATCCTAATATTGCTCAAATTTATGGCGGCAGAAGTCAGAGTGCGGCCAAGGGTATG
AGCTTAACGGTGGGTTATCGCTTTGAATGGTAACTTCCTGTAGGATTTGAGGGTATTGAGGGGAT
AAGAAAAAGGGAAGATGGAAGAAGTTTTTTTATTGCTAATTCCTCATTCTTTTCCTAGAGTACCA
TCCCCACAAACCTACAAAAAACATTCCTACAGAACATAAAACGTACAACAAGCTCATTCCCGCAC
CCTTTTGCGTACCGAAAAGAGGGCTAAAAATATTATCCGTCATCATCCAAGGCTCAAAAATGCGA
TCGGCTAAAATACCTCCTAATAAAGCCGAAATAGCACCAACTCCCAACCTTAACCAACTACTAAA
AGCAAAAATTCTTCCTTGTAAATTAGATGGAATGAGATCTAAATAAAGACCTTGACGGGTACTAT
AAATTAAAGGAAAATTTAAACTAGAGCAAAACTGCAATGGTAGCCAAATTAATATAGATTGACC
AAAACCAAAGATTAATTTACAGATACTAGCTCCCATTATTCCTATAATAAAACCCTTAAATCTAT
TTTTTATTTTTCCCCAAAAAGTAATAATTAATCCGCCCATTACTCCCCCAATTCCTGCCGATGCAC
TTATGCTACCCAAAATAAATGTATTTCCATCGGTGCGAGAAAGAATTAATGGTTTAAAAATACTT
CCTCCAATATCGTGGATAAACCAGAATAAAATCTCGATTGTTATTATTGTCAAAAAGTATTTGTT
TTCTTTTAAATAAATAAATATTTCTGTTAAATTATCTGTAATTTTATTAGAATGCAATTTTATCT
TTTTTTCTTTAATAGGAGTAATAATAACTGTAAAAATAGCCAAAATTAAGGTAAATAAATCTAT
AAATAAAATACCTGTTAAACTAATTATGGGATATAAAAAACCTGCTAATGCCGGAGCAACAATTT
GTGAACCGTAATTTAGAATTGAGGTTAAACTACCTGCACGGATATAATTTTCTGGGGTAACTAAA
TCAGCCGTAATGGTTTCACTGGCTAAGGATTGCAGTTGAGAAAAGGGAGAAGTTATCGCAATTAA
TAAATAAATATGCCAAATTTCTAAACTAGAAAAAGTGTAAATTAATAATAAAATAACACTAACT
GTTCCCGTTATTATATCTCCTAAAATCATTAACAATTTTCGCTCAAATCTATCAACAATAACACC
CATAAATAATCCTGTAATTAAATTTGGGGCAAGAAAAGCAAAAGAAATAAAAGCTAGTGCAGTG
GCAGTGCCTGTTTTTTCCCATGCCCAAATAGTTAAGGCAAAATTAGTCATATTACTACCAATAGT
TGATAATAATTGACCACTCCAAATAATTAAAAATTTCCTCACAATTAATTTACTAAAATTTTTTT

CTTTTTCTTAATATCTTAAAATAACTCAAATCTACTCCATATTTCTCTTGTAGATGTACTTTTTT
CTCTTGTAGAGGTACTTTTTATTTACTCTCTCAGATAGTTTACTGGGATAGTTGATTATTCTTGG
TTGATAAATACCAACTCCATAAACCAATTAAAAAAAGTGCGATCGCACTTAGGGTGTATAATAAA
CTTATTCCTGCACTTTTCCCCATACCAAAAACAAAGCTAAATATATTTTCTTGAGTCATTAAAGG
GACAAAAAAATAATCAGCTAAAAAACCACCTAAAATGTAGGCAATCAAAGAAAAAATTAAAATA
CCCATTCGTTGAAAAGCTAAAATTTTACCTTGACTATTTAAAGGAGTTTCTCTTAACCAAATAGT
GGTTTCTGCACTTCCTAAAATAGGAAAATGAAAAGAAGAAAAAAAATTGGGCTATTATCCAAATA
AAATATACATTACTAAATCCAAAAACTATTTTACTTACTCCTGCTCCAATCATACCAATAAAAAC
TCCCTTCATCGGGTGAGAAAAACCCCCTTTAGTGACAATAAATAAAGCACCTATTACTCCGCCAA
TACCAGCAGAAGTAGCGATATGAGAGTAAATTATTTGATCATTATTGGTTCGAGCTAAAATTAA
AGGTGCATAAATAGAATCTCCCAAGTCATGAAAAAACTGGAAAAGACAAGTTATTATTAGGAGA
TTTTTTAAGAGCTTATTATTTTTTATATACTGATAACTACTTTTTATTTCTGTAAATAAAATAGC
ATTTTTATGATAATTAGATGTAACTAAATTTTCTTGATTTTTATGGTTATTAGAAGATGGTATTT
GAACAAAATAAATGGTTATTAGAGCCAGAAAAAAAGTACCTATATCCACCATTAAAATTCCTTGT
AAATTAATTAAAGGATATAATATTCCTGCTATTGCAGGGGCGATAATACGAGAGCCATAGCCAGA
TAAAAACTCTAAACTGTTGGCTCGATCGAACTGATGAGAAGGTATCATTAAGGGAATAGTTGTTG
TATAGGCTAATTCTTGAATTGAACCAAAACAATTATTAATAAAAGTGGTGATATATAAATCTGT
GATGGTTAATTGTTGATTTAATTGCTGAAAAAGCAGAAAAATAGTACTTAAAACGGCAACAAGA
TCCCCTAAAATCATTAATAATTTGCGTGTGCGTAGTGTGCCTTTAACATCGCACCTATCTACTAA
GTAACCTGCTAAGGGAGCAACAAAAATTTGCGGTAATTGTTGAAAAAACCAGAGTAAAGAAAGG
CTAGAGGCTTGAGAAGTAGAATCCCATGTCCAGAAAATAAGGGCAAAAGTAGTGATACGACTGCC
AATTAAAGAAAAACTTTGACCAAGCCAAAGAATAATAAAAGAATAAATACTATTGTTTTTCATC
CACTAAATAACGAAATAAATCATCAATTACTGCATGGGCGGCGATTAAACTAGGTACATTCCAAT
AGGGGGGAATTTGATATACTTGACTTGTTTTAACTGCTTCGAGTTGTTGCCAGAGAGGATCTTTT
TTTAATTTTTCTAATTGTGCTATGGCAGAGTTTTCCATCTGTTCACTAACTCCATAAGTCCATAA
AAAAATAACATCCCCATCGGCTAAGGACATTTGTTCTCGACTAATCATAATTTGAAACGGAGATT
TACCTTTTATCCCCTGATTTTGAAAGGCTGGACGAGATAAACCTGCATCCTCTAAAATACTCCCT
GCAAAAGAGTTATTTAGATAAAGGCTTATTTGTTGAGGATAAACCCTTACTAAAGATACTTGCGT
TTGAGATAATTTTGACCCCATTTTCTGTTTAAATTCGACTAATCGCCCTTGATATTTTTGCAATA
ACTGTTTTATTTCCTCCTGTTTTCCCAAAACTTTTCCGTAAAAAAGAAGGTTTTTCTGCCATTCTC
CATTTGTTTCCCCCTCATCTAACACCGTAGGGGCAATTTGACTCAATTGATTGTAAATGCGACTA
TCACTGTAAACACTACCTAATATTAAATCAGGTTGTAACTTTGCCACCATTTCTAAATTAACCGT
GCCATCTAAACCAACTTTGGGAATATGAGTGACTTTTTCAGCTAATAAGTCTGGTACATTGGTAA
TAACTCCCACTGGTTGAATATCTAAAACTAACAGGCTATCGATTGCTGAACCATTGAGGGCAATT
ATTCTTTGAGGGTTATTAGGTACACAAGTTTCTCCTACACCGTGAATGATAGTATGACAATCAAT
GGAAGATACGAGAGTTTGTTTTTGACAAGCAATGGTTAAAGTTAACAGAATTATTAAGTACAGT
TTTTTCATTAAGCTAATTTTAAGAATTAAAAATTAAGAATTAAGAATTAAAAACCTCGTTCACG
ATCGAAGGGAGCAACTCTAACTCCTGTACGGGCGAATAGCCATTCGCCCCTACTTCCCCCTCTCTC
CTTATCTCCCTAACACCTGCAACCGTCAAACCTGACACCTAATCTTATCGGATATTCTTAAACCGA
ACTGAGGTTAAATTGATTACCAAGAAATACCATACTTAACACTAATGGTTCGCCCACTTCCTGCA
CTGTAAAAGGTGTTAGAAAAGCCTTGAGTCCTTTGGGAGTAAAGGGGAAAATAAAAGGTATCTA
ATAGGTTTTCAATGCCAATTTGTAATGTACCTTGTCCTAATTTTATGCTACTAATTAAGTCTAAG
AGAAAGTAACTATAAACGGGACTAGGATCGACTCCTTCATCAAACGCAGAACTGCGATCGCCTAT

CCATGTGGCTTGGAGACGATTACTCCAATTTGGTAAGGTTTGATTCTGTACATAGGCAGTAAATT
TGATGGGACTGATACGGGCGCTACTGAGGGGTAGATAATTACCATCGTTAGCTAAGTCAGCTTCT
CCGTCAGACCAACTAAAAGTACCGCCCAAATTCCAAAAATCGTTAATTTGAGTATCGATGGTGGC
TTCTATACCATAAACCCTCTCTGGGGCTCGAACAATATCAAAAAACCCTGTATCGTTATTAAATT
CAAAGGCTGAACCAAAATCGGAGTAGTTGTAAAAACCGCTTAAACTAGCTTGAATATTATTCCAG
TTACCTCTTAATCCTAGTTCAAATTCATTGACTTTCTGGGGTTGAGTTATATTGACATCCTGCCC
TACTTCAAAACCGGGATCGGGAAAACCAAAGACAATACCAAAATCGGGTACGGAAAAACCTTGAG
AGAAGTTGGCAAAAAGGGTTACTTCTTCGGTGACGTTGAAACTTGCTCCAACGTTGAAAAGAAAA
TCATCAAAAGTATCGCTACCTCCTTTGATAAAGTCGTCAAAAAATGTTGTATAGTCACCCACGGA
AACTTTTATATTTTCGTAACGCAACCCACCGCTAAGGGTAAAGTTTTCACTTACATCCCATTGCA
CTTGTGCTAGTAATCCTAAACTGTTTAAATCATAGGCGGGGACTAATATTCTTTTCTCGGTTAAA
CGGTTAACTTTTCCGTTAGTGGCATCAAATATGTCCCCGTCAAATAGGTTAAAGGTATTAGAAGA
CGTTTCTTGATCAAAGTCAAAACCCCATAAAATATTAAATTGAGGGCTAACAGGAGTATCAACAC
TGAACCTAGTTCCCCAATTTTGGGAGTTTAATTCCCCTTGAAAGATACCGAAGGCACCGGGGCGG
CGATCGCGCGGATCACTACGACTGACGTTATCCCGATAGTAAATTTGAGTATCCAGACTGCTCCCC
CATAAATCGTTATGACTGTAACTTAAGTTGAGGAGAATATTTTGATCCATTTGAGGACCACCGCC
ATTAGGAAATTCTAAAGTACCAACCCGTAAGGCTCTTGCTTTCCGTCTTCCTTCTATATCATCTAC
AGCAGGATCTGAAATAAATGGACTTTCTCGGCGATCATAAAAATAGTTAGCAGTGAATTGGAGTC
TTTGATTTTCGTCAAATTGAACCCCTAATTTACCTAAAAGGTTGAAGGTTTCGCTTTCATCTGTT
CCTTGAATAGTGGGAATGCGATCGCCCTGTGCATCAAAAGAAGCCCCAGAATCTTTACGAGATAA
ATCAATGAGAAAATCAAAATCGTCTTTCTTCCCTGAAATACCATAGCGAAGAAAATTCCCAAAAC
TTTCTCCCTGCAATTCCCCTAGTGCGGCATCTATACCTATTTCCGCCGTAGAAACAAAACTTTCTT
CCGTTGGGCGACGAGTAATGATGTTAATAATTCCTCCTGTTGCTTCCCCACCATATAAAGCCGAA
GGTCCTCTTAATACTTCAATCCGTTCAATGGCATTGGGGGAAATGGTTTGTAATTCTCGATCGAA
GTCTCGGTTATTGACGTTTAAAGGTACACCATCAATTAAAATAGAGGCATTTCTACCCCGCAAAG
AAGAAGCCGTAAAAGCTCGATCGGATGGAGGACCAAACCCCGGTACTAACTTCCCTAAAATCGTG
CCTAAATCCTCTGTTAAAGCCGTTTGCTCTTCTATTTGTTGACGATTAATCACTGTTACAGAACG
GGGAATACGTCTTAATTCGTCAGGTTTTCTTGTGGCAGTGACAATTAAATCAATGGTTTCGGGAA
TTTCTTGATATTGTTGTGTCTGAGAAAAATCTGATACTGCCTCTGCTTTCAAGGTTAATATTTTG
TCTCCCTGAATAATATTTACTTTGGGTAGATTATCTTTTCCTGTAATTCTAATTTGCACATTGCG
ATCGACTTTTTCTGCTTCCACCGTTGTAATTGCCTCATCAGGAGTAGATAAAATCACATTATTAT
CGTTGGGCAAACCTAAATCTACATCTAAAATTTCAATAATAATGGTATTTCCTTCTCTGTATTCT
TGAATAAGTAATGGCTCATCTGTCAAACTTTTCATTTGTACATCTACCCCCGTTGTTGTTGCTTG
GGCTTGAATATCAAAAATTAAATTGACTACCTCTTGAGCTTGAACTAATTGTATAAATGCGATCG
GCAATAAACCTGCCATTAAACAAGTGGAATTTATAAAACGTTGCATTATTTGTCCTCACACCATT
AATCGTTAAAAAGTAATTGATAAAGTTTCTTAAAAAGACTTTGAAATAATATGGTAGAGGTAAA
GTAAAGAGAAAATTTCTTAGTAACGGATTATTTTTTCTCTCAAACGGATTTTTTCAGAATTAAGA
ATTAAAAATTTTAAAAATCACCCTCATCACCCTTTCACCTCATCACCCTTTCACCTCATCACCCCT
TCACCTCATCACCCTATCAACCTATCCTCTTATCACCTCATCACAAACTGCTTTGCTTTTGATATT
GTTTCGGGGAAACCCCAAATTCCGCCTTAAAAGCCGAAGAAAAACTGCCCAAACTTTTATAACCA
ACTTGGGAAGCAACCCCAGCAATATTATAATTGTAATCTTTAATTAATCTTTGGGCTTCTAATAA
ACGATAATGACGCAAATAACCGAAAACCGTCATTTGAAAACAATGTAGAAAACCTTTTTTTAATT
TATAATCATTTAAGCCCACTTGTCGTGCTAAATTGATTAAAGAAGGTGGATCTTTATAATTATTA

ATCAGAATATTTTTTGCTTGATAAATCCTTTCTATATCATCACGATTAAAAGATAATTTTTGTTC
TATTTCTAATGTCAAAATTTGTTCAAACTGAAGCCTTAATAAATCCATTATTTTACTTTCTAAAT
ACATTTGAGCTAAACAGCCTTGATAGGGACAATCAATAATTTGTTTTAAAAGTGTTTCTAAGTGA
GCAGAGGTTTGACAGTTATATAAAAAAGGTTCTATTTCTTGGTCTTTTCTCCATCTTTCTAAATG
CTGAAAAAGAGGATGATTTGATTCAAAGCATAGATGATTTAAAAATTCTTTATCGATAATTAAT
TTGATCGCTAAAATAGGTTTGTTTGCTGGTATTTCGTCCTTTTCGATCGTATCTTTAATTAAAAA
TAATTGATTATTTTTTGGAGTAACTTCACGGGATAAAAATAAATTATCTACAGTAGAAGTATAA
CCACCTTTAAGATAAAAACTAGACACTAACTGAGACGAGGATTCAACTTTATCTATTTCAATAAT
TTTTTGATGGTAACAATAATCTTGTATTAAAACTGTCAAGCCATTTCTTAATTCTATTTCCCGCC
AAAAACCTGTCCCTAAATAGGGGGGAAAAGGAGTAATTGTTTCAGTCTTACTTCTATATTCTTTG
ATTTCTCCTTTCTCAATGCTTTCTTGCCATATTGCTTTAAATTCTTCTGTGGAAATAGAAATAGT
CACTATTTTCAGCTTGCTACTTATTGATAATAATTAGCATTATATTAGAAATTATATTAATAAAC
AAGATTGATAAAAAATAGTTATCCAAAACTCGATTATTGATTTTTTGGTGTTGGTAAATTAAGCTC
TTAATATGTTGGGGTGGTGGGGTTGAATATATTCAATTCTTATGGGAAATTTCTTTTTTATCTTA
ACTTCGTTCACGACCAAAGGGAGTGTAAGAGATAAAGGCGAACTCTCCGAACTCAGACTAAAATT
GATTTAAAGGGTTTCGCCTTTTATTACTACTAAACTGATGTAAGATCTCAATTTATGAAGATTTT
AAAATTTCGTTTATAGCGTCAACTATAGGATTAACAAAATCTCGATCTGTCCAATACTGTCCATG
ACTTAAAGGAGTCCAACTGGAGAAGACATTTCCTGTATCAATTTCAAAATCTTCCACTAATTTAT
TATAGTCTTCGGAAAGAGGTTGCAAAGGCCAACCTAAACAATCGTCTTTGTCGTAATAATTTTTC
CATTGGAATTCTGAATTAGGTTTTTTAAATGCTTGAATACTATCAAAACCTGCTACAAAAATCGG
AATATTACATCCTGAAGTGAATAAAAATTTCATTGTTTTCAACTTCAAAAAATCAATCTCATCGT
CGTGGGGATTTATATAATCAGAAGAATTCTCATCCCAAATAGTTTTATCCTTATCTTCTTTTTGA
GAGTCCCAAATATAATTTGAAATGACTTGACAACCAAGAGATTGAGCAATAACAATTAAAGGGC
ATTGGCTATTTTGAAGATTATTTTTTGTGGAACGTAAACTCTCCACGATAATTTTTTGAGTTTGT
TTATATACACTATTTTCTTCTTTGGGTTTATGTTCAAGACTTACGGCATCGGAAAAAGCAAACAA
GAAAAATTTTCTCAGTTGAAGCCAACGAAGTTTCTCAGAAGGTTCACACCGCATCTTATTCCAAA
TATTGTACTGATTATCTTGTAAAACTGGTTGATAATAAATTGATTCAAAATGAATTTCCTTCTCT
ACATCAACAGAAAGTTTTTTTAGTCTTATTTTTAGTAGATTTTCCAATTCACAAGAAAATGTAGA
TTCAGTTTCTCCCATACCATGAATTGTTAAGATACCCAATTTTTTTGTTTTTATCATAATTTTTA
CTATTCATGAAAATTCTACTATTCTAATTGATGATTTTTTTTAAGTATATTAAATAAAAATAAA
GAACAAAATGAGGGACTTTTTCTTGGCTTTATTTTATATTAAAAACCTTGATCTATTTGGATATA
TTTTGATTAATAATTTAACAAAAACTCTCTTAATTGCTCTAAAATATACTCAGATGCGATCGCGC
CATTTAAACCATTCCACAAAGCATAACTAGCAAAATAAACTTGATTATTAAGAGTAGCAGGTAGA
GATTTTGTGAGAGAATTTTCTTGCCAATCTTGTTTTATCGTTGCGGTTTGATTAGCTAAAATTTC
TGAATTACTTTTATTTTTTACGTCAGTATTATAACCTAAAATAAAAATCCAGTCCGCTTGTTTAC
CAATATCAGGTAAAGATTCCAAGGAAATGGGCATGGTAACACTAGAATTATCATTAGAAATAGT
AATTATTTCAAAACCTAATTGACTAAAAATATTACCTAAATCACTATTTTTACTAAGCATAAATA
AACTATCATTTAAACCATTTCCTGCTAAAAATAAAACTTGATTTTTATTATTAATAATTTCGGTA
AAATCTGCCTTAGTTTGACTTAATTGATTTTCATAGTTATTGATAATTGTTTGAGCTTTTTCCGT
TTCATTTAAAGCTGTTGCTAATGTTGTTAAATCTTGTTGCCATTTTCCCTTAATGGTACGATCTT
TAAATAATAAAGTAGGAGCAATTTGTGACAAAAAATTATACTGATTTTGATTCCGAGTTTCTCCC
AAAATTAAGTCCGGTTTAATTTTAGATAACACTTCTAAAGAAGGATTATTAGCATTACCTAAATT
ATAAGGCTCAGTGGTGATTAAATTTCCTAAAATGGGATCAATTTTTTTTACATTAGTAATTTTTT

CCCCTTTTTCAAATCCAAAATAACCACCAAAGCCATAGGGTTGATAACCTAAAGAAAGAATTTGA
GCAAGATTATAACTACCTAGCACAGCGATTTTCTCTATTTTTTGACATATTTCAGTTATACCAAA
ATCATGGCTAATATTTTGACAATTATTGGTATCATTAGCTGATTGATTATTACCATTTTGACAGG
CAATAATGAGGAATATTGTGCAAAAAAAAAGGATAAATTTAATTGAGATTTTTCTCATAATATA
TTTAGTTAGTAGTGAGGGCTTTAGCCCTTATTTTCAGAATATTTAAAACCCTAAAGGGTTTACTA
CAAACCTATTTAAAATTTGACGGAAATTGAACCAATAACCGTAAAAGGTTCGCCCGGATAGATTT
CATTTACCCTTCTATTTTCTGAAGCTCGAATATAATTGACATCAAAAATGTTTCTAAAATTAAGA
GCAACACGCCAATTATCTCGGTTATAGAAAATACCTGCATTAGTGAGAAAATAACTATCAAGGGT
AAAACTATTGGCATTATCTCCAAAACGTTGATCAACATAATTGAAACCTAAACCAAAGCCTAAAC
CTTGTAAATCTCCCTGTTGAATTTCGTAACTTGTCCAAAAATTAAAGCTATTGGGTGGCACACTA
TAAAGTTTATTTCCTTCTAAACCTCGATTATCTTGGGTTATTTTGGCATCAATATAGGCATAGTT
AGCAACCATATTCCAACCCGGGAGAATCTCTCCGATTAAGTCTAACTCGAAGCCTTCGCTTTTTTG
CTCCCCTGTGGCAACGGAAAAGTTAGGAAAATCAGGATCAGGAGTGGCTACATTTCTCTTGGTAA
GATTAAAATAAGATGCGGTAAAAGATAGTTTTTTGTCTAGTAATTCTGTTTTAATACCGAACTCA
AATTGTTCTCCTTCTTCGGGATCAAGTAAGTTACCATTGACTGTAATGGCACTATTAGGTACAAA
TGAGCGACTATAACTACCGTATAAGGAAATAGGCTCTATGGGTTGATAAACTAAACCAATACGAG
GAGTAAACGCATCATTGTTTTGACTATCAGTTGAGCCTTCTGGGCTAAATACTGAAGGGCGATTG
GTGTTACTTTGATCGATATTATCATAGCGAAATCCGGCTAATAATTTAAGGTTATCAGTAAAAGC
GATTTGATCTTGTAAAAAGAAGCCAAAAGCGTCAACGGTGGTATCTCCATCAAAAAAAACAGGCT
CTTCTTGAAAGTTAGGACGAGGTACAGTATTGTAAATGGGGTTAAAAATATTGATGGGTATATT
GGTAAAAAAGTCTCCTCTACCAATGTTATTCAAATCCTGTCTTTTAAAAAAGTCAATTCCTGCTA
ATAGTTGATGTTCGATCGCACCTGTGTTAAATTTACCCACAGTGTCCAATCTAATTTCATAGGTT
TCGCTAGGTTGATCTAAAGAGATAAAATTACGGCTCAATATACCTGTTTCTTCGTTCAAACTTCC
AGCATTTGCTGTCTCAAAATAAGAATCATAAGAAAGATAGTTAAAAGCATGACGAATTTTCCAA
TTATCACTGAAATTATACTCAAAACGATAGCCTAGTCTTAATTGCTCAATTCTAGCAATATCATT
AGGTTCACCTAAATTACGCTCTCTGGGTATATTAGCAACTTTATCACCGAGGGCAGTTAAACCAA
AATCATTGGGGCGCTCATCATTCAAATATTCCACATTCACCAGTAAGTTGACTTTTTCCGTTTCCC
AACTAAAAACAGGGCTAATGAAAAATCGTCGAATATCCGTGTCAAAATCTCGAAAAGAGGCTTCA
CTTTGATAGGACGCATTAACACGATAGAATAAGTTACCCTCACCATCAGGATTAAATGGTCCTGA
AATATCAAAACTCGGACGAATTAAACCTCGATTACCTACTTGCAATTGTGCCTCGTAAAAGGGTT
GCTTTAAGGGTTTTTTGGTGACTAAATTGATAACACCTCCCGGCTCAACTACCCCGAATAGAATG
GATGCAGGACCTTTTAACACCTCAATTCGCTCTAAATTACCTAAGTCAGGAAAGCCGATGTTACC
ATTACGTCCGAAGGTTTGACGAAATCCATCCCTTAACACAGAGGCATCGCTGAAACCCCTAATTT
GAAACCTTTGCCCACGGGGATCAGCCGAAGTTTGTTGTACTCCGCTCACATTGCGTAACGCATCTC
CTAAATCAAGTAATTGTTGATCTCTGATGATTTGTTCAGGTATAACTTGAATAGATTGGGGTACA
TCTCGAATAGGAGTATCAGTAAGAGTTGCGGTAGTGGCATCAGGGATGAAATAATCTTCTTCTTG
TACTTCTTGAGTAGCGACAATTTCGATTTGTTCTTCAGTTTGGGCTATGTCTGCGATGGTGATTC
CAAAAACTAAGTTATCATTGATTGATGTCACCTCAACGGTAGGTAATTGATTATTTCCTGCGATG
GTAATACGCACACCGCCCTCAACGTTAATCGCCTGAATCTCCTGAATATCATCGGTGGGATTTAA
TTGTAAAAATTCTTCTCCTTGACTAAGGCTTAATTGAGTGTCTATAAATTCAATGATTAAGTTGT
TTTTATCAGGAAAAATAAGCGGTTGTAAACTGTTAAGATTATTTCCTTGTAAAATCAATTCTAAT
TTATTATCAGTCTCTCTTAACTCAATATTAGTAATGATAGAATCAATAGTAACCTGAGCAAATAA
ATGATTATCTTTATTAATTATTAAATTCTCAGCTTTAACATTATGTTGATTAATTAAAGGAATTA

ATCCTGTTAAAGTCATGAAAAATAGCAATTTGTTTGTTTTCATCTCCCCACACTATTAATATATT
TTCTCAACTAAAAATTAAAGATAACCTGAGGTGCGATCGAATTTCTCTTGCAGAGGTACTTTTTT
TCTCTTGGAAAGGTACTTTTTCAAAAAAGAAGGGAATAAAAATTGGCTCTATTACCTTGAGTATG
CGAATTATTGCTTAGGGTAGGCAAGAGGTAAGCGTTTGAGTCATTTTTAAAAACTAATAAAAACC
GAAATTAATTGTGTCTTAGCTTATAAAATATCTAAAGTAAAAAATATTATTAACCTTTAGACTT
AATTTGTTATAACTATCGTATGAGAGCGATACTTTATTTATTGTGATTATGCTACTAAAATTTCA
GAGAAATAATATTGAAAATTTATGTTTATTCAAATACAAGAAAAAATTAAGCAAGTAAAAGAAA
AAAAATTAAAAGTATTAGACTTAAGTTATGATTGCGATCGAGAAGAACAAAAATTAGAGAAAAT
ACCCCCAGAAATTTTCGACTTAAAACAATTAGAATATCTCTCTTTAAGGGGTCATAGTTTAAAGA
AAATTCCCCAAGAAATTAATAATCTTATTAATTTGCAATATCTGGATTTATCATCAAATCAATTA
GATGAGATACCATCAGAAATGGGGAGTCTTATTTCCTTAGAACATTTAGACTTATCAAGGAATCA
ATTATTTTGTTTACCTACTGCTTTTGTTAATTTAAATAGTCTGATTTATCTTGATTTATCTATTA
ATCAGTTTTCAGAATTTCCTGAATTTTTAGGAGAAATTGCTAGTTTAAAATATTTATCTTTTGCA
GTAAATCGTCTTTATAGTCTGCCAGAATCGATTATCAAATTAAGTAATCTTAACCATTTAGATTT
ATCTAAAAACAAATTTGTTAGATTTCCCGAATTTATAACCTCTTTAAAAAAATTAACTTACTTAG
ATTTGTCGGTGAATCAACTAACCAGCCTGCCAGAAAATATTGATCAATTATCTGAGTTAAGAGAT
TTAGGTTTATTGAATAATCAAATAAAAACTTTACCAACTTCTATGATTAACATGACTAATTTAGA
ATATATGGAATTATGGGATAATCCTTTACCGAAAAATTTGTCAAGAGAAATAGGATTTGAGGAT
ATTAGAAAATATTTTGATTCTTTAAGATAAGGTGTTTGTTTTTTAATTCATAAAGTAAAATGCT
GAGAAAAGATTAACTATGGTCAAACTCCAAATAAAACTATGGTCAGCATTAGCAAAAAAGATTT
ATTTCAAGCTAAATTAACGGGTAATTTGATCAAATTAGAAAATCAATTACAAAACTATTTTACT
AACTCAAATAACTTATCCGATTTTACTCAGGATGAAACAAAAAATAATCTCCATACTTTAACAAA
AATTTATTTATATCAAAGAAAATACGACGAAGCAAAACAACTTATTAACTCGATACTAGAAGCG
AAAATAACACTATTAGGAGAAGAAAATCATGAAGTAGTTTTTATTTTAGAAAATTTAGCTTTTA
TTACTTATTTAGAAACTTATTATCCTTTTTTTCAAGACGCTACAGAAGCAAAAAATTTATATCTT
AAGTTATTAGCAATTCAAAAACAATTATATGGTCAAAATAGCCTCAAAATAGTTCAAACTTTGAT
AGTAATAGGTGTTATTAGCACTCAGCAAGAAGCTCCAGCATTTCTCGCTCAAGCCCTTGATATAT
GTGATAGTACTTTAACAGATAATGACATAGAATTGGCTTATTTTTTATGGCAAATAGGTTCAGCT
TTATTTGATAAATGTTATTTTTTTGGTGCTGAATCTTCCGAGTTAGAATATATAGAAGTTGTTTT
AACAGAATCCTTAGAAATTTGCCAAAAATACCTGCCTGAGTCTCATCCTTTATTTATTTCTTGTA
TGATTAATTTAGCCGAAGCTAAACATCATTTAACTCATTATGAATCTGCCGAAAAATTACTAAGA
AAAAGTCTTGCTGAATATGAAAAAAGGTTCTATCCTTTTCATCCACAAATATTCAACTTACGTTA
CAATTTAATAGTTAATTTGCAAATGCAGAGCAAACACGAAGAATTGGTAGAATTAAAAAAAATA
CAAGATAATTATCGAGAATATCACGAAAAAAGAATCAAAGGAATTGTATTTAGTGATCCTGATG
TTGATACCACCTTATATCCTATCGAAGATATTTTAACAGAAGAAGATCAAGAGTTAGATTTTGAT
TTTGCTAATGAGTACGAAAAAATGATAGAAAAACAACGAGAATATTTATCTCAAGATAATTTTT
ATGTTGCTAATTCTTTGGAAAATTCAGCTTATGAAAATTTAAGAAAATCCGAATTACTTTTATTA
AATGAAGTTGAAACTTTATTTTTAAGAGCTTTACAGATTAAAGGTAAAATTTTAGGTCATAATC
ATGACGAAGTTTTAGATATATTAGCAAGTTTAGCTTATGTTTATGAGTTACAACAAGATCAAGA
TTCTTATGAACTTTGTTCTCAAGAAATAGCTAAATTAAAAATGACGAAATAATCATATGGTTAA
ACCTTCTACTTTCTTTCTAAGTACATTTTTATTTCCCCTACAAAAATTTCAGCCTGTTTAAATAA
TTCTTCTGTTACAGGTAAATCAGGCTGCAACATTTCTCCGTAATCATGGGCTTTTCTTTCATCACT
TGAGATAATAGGGGATTTGCCCCTACTGCTCCATATTTAATTTGTTCTTCTGTAACTACAAAAGT

AGATATGATTCGATCATGTTGAAATCCTATTTCCCAAGCTAAATCATAAATCATTTCTCGCAATG
ATCGATCTAAAAAATTAACTTTAATAAAGACATCTAAATCAGAATCAGGTTGTGCATCACCCCTA
GCACGTGAGCCAAAAATACGCATATCGACAACAGGAGTTATAGATTCGAGACGACAACGAAATTC
ATTAGCTATTTGAAAATCCAAATTTTCCATAAAATTTAAGAATTAAGAATTAACACATATCACCT
CATCACCCTATTAGAATTTTAGACCATAACTTAAACTAATAGTTATATTTTGTCTTGTATAAGTA
ATATTCAAGCTAAAAAGAATAAAAAATTAATAAATCAGGGGCATAATTTGATCTAAAAAAGCAG
ATAATGATTTTGTACCTGTAGTTAAACCTCCTACTTTTTCTTCCTCTAAAGGATAAACTTGATTA
TTTTTTACTGCTTTTAATTCCTGCCAAAAACTATCTTTTCTTAAATCTCCTAATAAATTTGAAGA
AGGGGATTTTACTAAAACAAAAATAATATCAGGATTTATCGTTAAAATTTCTTCTGAAGATAAA
CTAACCCAATTAATGGCACTGGGATTATTCCCAGAAATTGACCAAGGATAGTTCGTTAATTCGTC
TAGCACTGAGCCTAATAATGATTCTTTAGTGGCAATAAAAAAGTTTCCTTTATATCCATTAGTCA
CCAAAACAATTTTCTCATTATTAGCTTGTTTTTTATAATTATCTAATTTAGCGGTAAATTTATTT
TTAGCAACTTTAAATTCTTCATCTTTATTGAGTAAATTAGCAAATTTTTCTAAATTATCGATCGC
ATCTTCGTAGGTTTCTATTTCAATTAAAAATACAGGTGCGATCGCATCTAAGGTATTCCTTTGAC
TACTATATCCTGCTAGATGTCCAACAATTAAATCTGGTTTAAGTTTTAATAACTGCTCTAAATTT
GGTTCTCCATAGCCCGAAATGGGTATTATATTTTTACCTTTTTCTCCGAAATAAATATCACTTTT
TGCTAAATTTAATAACATATTATGCACAGCGATGGGAGGATGATTTAATTCTGCTAAAATATCAA
TACAACTAAGATGTAAACAAACTATTTTTTCAGGAATTTTACTAAATTTGAGCTCATGATTAGAC
GCATCAGTAACAGATATTAAGTAATCATTATTGATTATATTATTCTCAACAGTTAAAGATTGTTT
TTGACAACTGTTTAAGGGAATTATTAAGCCCAAAAAAAGGAGAAAAGAGAATAGTTTTATCATG
ATCTAGTAATCTTCAATATTGTGTAAAAAATTAATAATTAGGAAAAATCTCAAAAATACTAACT
AGCTCTCCTTCCTCCTTCCCCTTATCCCCCCTTCTCCTCATCCCCTCATCCCCCCTTCTCTTCATTC
CCCATCACACTACTTTAAAAGTTAATTTTGTAACCTACCCTTAAATTAATACCACTAGCGGCGGC
GTTGAAGATTTCGTTGAAACCACTCAAAAATTGTGATTGCACTGGGGCATATTGATTATTGAATA
AGTTTTGAATACCTATTTGTAATGTACCTTGTCCTAGTTTGACATTACTAATATAATCTACAGTA
AAATAATCTGTAACCGGTACTGCATCCACACCATCTTTAAAAGCTCGATCGCGGTTTGCTACATA
AAGAAATTGTAAGCGATTATTCCAACTGGGGGTAGTTTGATGCTCAATATATCCCGTTAATTTGA
TTGGTTGAATGCGAAAAGTAGAGATAGGTAAATAACTACCGTTATCATCTTCGTCATTTTCTCCC
TCAGTCCAACTAAATGTACCACCCAATGACCAATAATCGTTAAGTTGCGTGTCGAGGGTGGCTTC
TATGCCGTAAATACGTTCAGGCGCTCTTACTAATTGGGAAACACCATCATTAAAAGCATAGTCTT
CTCCTAAATCTGATGTATTGTAAAATCCTGAAATGGAGGCTTGAATATTATTCCATTGCCCTCGC
AAACCGATTTCATAATTATTGACTTTGATAGGTTCAGTTAGCTGTAAGTTTTGATTAATATTGGT
GAAATCTGGGGGAGGAGTACGCAATACACCACCAAAGGCAGGTACAGAAAAACCTTGAGCAAAAC
TAGCAAAGATATTGAATTGTTCAGTAATTTTATATACAGTACCAAGATTAAATACGGTGGCGTTA
AAATCAAGGTTTCCTCCCTTAATGGCTGTACCAGAAAAAGTAGTATAATCATCTACTTCTAAACC
GATTCTCTCATGACGCAATCCGCCACTGATTAACCAGTTTGGGTTAACATCCCAGCGCACTTGTCC
AAATAGTCCTAATTGTCCTAAAGTATGCAGTGGTACAAAAGTTCGCTCATCAATTTTCCGTAAAG
TGCCGTTATTATCAAAAGAAACGGGATCAAATACTTCAAAGGGTGCTTGATTGTCCTCTTTCACA
TAATCCAAACCCCAAACAACGTTAGATTCTCCTCCCATAAATGTTAAAGGGGTATCTATTTGTAA
TCTTGTCCCCCATTTGTCACCTGTTGCTCTCTGACGGACGATCGCATCAAAGAAACCTCCCCGAAA
ATCTCCCGCCCCTACAATGGAGGTATAATCACGATAATAAACTTGGGCTAGGGCTTTACTACCCA
TTAAATCATCATGGGTGTAGGTAGCAGAAACGAAAATATTTTGATCTCCGGCTAAATCTGTTCCC
TTTGTGTCAGAGTTAACTCTTAATGCCCTCGCTTTTTGAATGCCGTCAATATTCCTAATGGCAGG

GTCTGAAATATAGTTAGTATCTTGCCTCCCATCATAGAAATTGAAAGTTAACTGTAATTTTTGGT
TTTCATCAAAATAAAAGGCACTTTTGGCAAGAATGCTGTAAGCAGTGGCATCAATAACTCCTCCT
CCGCTAGTTTCAGGAATGCGATCGCCCTCAGCGTCAAAAAAAGCACCGTTATCTTCCCGTTTAAC
AAATAAGAGATAATCTACCTCTCCAGATTTAGAAGTACCAGAAACCAAACCCCCAACCCGATTAT
AAAAACTATCGCCGGGGTGAGTTAAAGACAAACCCAAACCTGCGGATAATTCCGTTTCCAAAGTT
TCAGGGGAGGGAAAACGAGTAATAATATTAATTAAACCTCCTGTGGCTTGTCCGCCATAAATCGC
TGTCGGACCTCGTACAACTTCGATTCGCTCAATAACATCAGGATCAATGGTTCTTAATTCCCGAT
CTAAGCCATAATTGGAGGTTAACGGCACACCATCAATTAAAATTAAAGGACTTCTTCCCCGTAAA
TTTTGAACTATAGAGCGAGGAGTGCTTTCACTAGGAGGAGGGCCAAAACCGGGGACTAAATTCCC
TAAAATAGTTCTTAAATCGTTGGTAATATTTGTTTGTTCTTCAATTTGAGGACGATTAATTATTG
TCACAGAACGAGGTATATTTTCCACTTCTTCTTCACTACGGTTAGCTGTTACCACTAATTCGATG
GTTTCTTCTTCTTCCTCTGGATTGGTGGCAATATCGGAATCAGATACTGGAGTGACAGATAAGAT
AAAACTATTGTCATCGGCAGGGATAATTTGAGCAGAAGGGGCTTGATTTTTGCCTTTAATGGTCA
TTCTGACTACATTTCCCTCCAAAGGCTCTACCGTAATGGCGGTAATTTCTTCGCTGATATTTTCCT
GTTGCAATCCCTCAAATCCTAGTTGAGTCTCTAAAAATTCAATAACTAAGTTATTCTCATTAGGA
AAAATTAACGGTTGCAAGGGTTCTGAAGAATCATTTTCGACTAAAATTTGTAATCCTGTTTCTGT
GGGTTCAAAAAAAATGTTGTTAATAGTAATAATTTTCGTTTCTTGAGTTAGTAAAAGGTTCGAT
CGCAAATCAGAATCAATAGTTTCTGCTCTTACTATCATGGGATTTGCACCCAAAATCATTAAGCC
TGTTAAAGATAAAAAGTAAATTTGTTTTTTCATTCCTTCACACCAAAATTTTAAAGAAATATATC
GCAATCTTCGATATTTATAAAACCAGAGTAAATAAAAAATGTTCTCAATAACTCTTAAATCACAG
GATAAAGAATTTTGATAGGCTTTTTTTGCTCGAAAATTACTTTTTTTTGCTGTAGAGGAACTTTT
CTTTGCTCATGAGGCTTGTAGTGATACCTTTAGCCATTGATTATCTTTACTCATGAGGTTTGTAG
TGATGGCTTTAGCCATTAATTAAAAGCGGATTAAATTGAGTTGGTGAATTATAGCTAAACATCA
ATTTTGATCTAAATTTCAAGTATTGTTTCTGGCTAATCCATCTGATCAATTAGGATAGTAGCTGA
TAAATTTTCGGTGTCACACCAAATTGTTTCTGAAAAGCCTTTGTGAACGCACTATGACTACTATA
ACCCACAAGAGAAGAAATTAGAGAAATATTCGTCTTTTTTTCTCTTAATAATTCTCCAGCTATTT
TTAAACGATATTGACGTAAATAACCGAATACTGTGGTGTTAAATACTTGATGAAAACCTTGCTCT
AATTTGCGATTACTTAATCTTACCTGTTGTGCTAAATCAGATAAAGTAGGAGGATTATCAAAATT
TTTTTCGATGATTTCTTTTGCTTGATAAATTGCCTCCACATCACTAGAATTAAATGAAGATGTTT
GAGAAAAATAAGGGCGATCGCACTTAGTTAATAAATCAAAATATAAAGCAATTAATTCTAAAGA
TTTACCTTCTAAATAAATATTTTTAATTAATCCTTGATAAGGGCAATTAATAATTTGTTTGATCA
CTAATTCTATAGCGGAACTGAATTTTAAAGGATAACTTAAGGAAAAGTTTTTTTGTTCTTGTGAT
AAAATTGACTTGAGACAAGGAGGAAAATTTTGTTCGTAACTATTTTCTAAAAAATCAGAAAACT
TATTCATATCTAAATGAATATCAACTCCTTTATTTACACTATTTCCTTGATATTTTACGATCGCA
CCTTGACGATGTTTGCCAATAATATAAGAATATTTATTAAAATTAAAATAATGGTTATTAATCT
GACAATCAGACTTTAATTTAAAAGCAAATTCAATACAATCCGTTTCTTCATTGCTATTATCTATA
ATTAAATCTTGATTTAATTGATAACTATGAATAGTTAATTTTATTCCGCTATTTAACCAAATATG
TTGTTTATAACCCTCACCTAATAAAGGAGGATATAACATTGTTATTTCTGTATTTTCATCATTAA
ATGATTGTGGATTTTCAGCAAGGGTGTAGTTTTCTAATAAATCCCAAAAATCTAGGCTAGAAAGA
TTAATTGTCATGATTCAAAAATTTAAAATTAAAGTATAAAAAATTATCGAAATACCATTTTTTG
TATTAACTCACCTGAGTTGAAGACTCTGATATTAGCGTTAAAATAGCTAAGTATAGACAAAAAG
AATAGATAAATTGATTATGTTTAGATGTAAAATCTATTCCCAGAAAGCTGAAATAGTAACTGAA
TTTAGTTAAAAATTAGCTAAAATTCATAGACTAATGTTTTCCATTAATTCTTATTTAATAATCCC

CTCTTGCCCTTCTCCCCTTTTAAGGGGAGACACAGAGGGGTTTGCCTACCCTAAATAATAATTTA
CACGACGAGAAGTGATAGAGCCAAAATTCATTGTTTAACGATGAGCTAATTGAAAAAGTTTAGG
AGTAACGCCGAATTGTTTTTGAAAAGCGGCATTAAATGAGGTGGCACTGGCATAACCAATTGTTT
GAGCAACAACCCCCACATTTTTCTGTTTTTTGAGCAAATTCTGAGCTAAATTCATTCGATAATGA
TATAAATAATCAAATACCGTAGTATGAAATATTTGTCGAAAACCCTCTTTTAATTTACGGGTGTT
AATGCCCACTTGACGAGATAATTCTATTAAAGATGGTGGATTGTTATAGTTATCAACTATAATTT
TTTCAGCATGATATATTGCTTCTATATCCTCTTTTTTTAGTTTAATTTTGATTTGTTTTACCTGT
TCAATTTGTCTTAAACTATCAATTTTTAAAGCTAATAATTCCAGCGTTTTTGCTTCTAAATATAT
TTGTTTTGTTATACCTTGAAAAGAACAATTTATAATTTGTGAAATTACCATTTTCATTTGAGGTG
AAATAATTTGCGGTAAGGAAACAGGTAGATTATTATTACCAAGAAAAAAAGGTTTTAAACTTTG
GGGTAAACTGTCATCATAATCATTAATTAACGATTCTACTAAAGATTCGTTTAAGTGAATATCTA
CTGCTTTAGAAGGAATATTTGCTAATTCTTGCCATATATCTCCTTCATTATTTTTGCCAATTACA
TAGCAAAATCCGTCCTTTACACTAATATTAATATTGGGTGGAATGTACTGGGGCAAACTCAATTT
AAAAGCGATCTCTAAAGTTTCATCTATATTTTCTCGATTATCAATGATGACATTTTCAAATAGTT
GGTAATTATGAATAATTAATGAAATTCCGTTACGTAATACAATCTCTCTTTTATATCCTTTTCCT
AGCCATTCAGGGGCTAAATAAGTGCGATCGCACTTATCCTTAATTAAATTAGGGTTGATGGTGGA
AGTATATTGTTGAGATAACTTTTTTAACTCATGATTGTCAAACTTGATGGTCATTATCGATAATT
TCTATTATTACCACTATCTTTAATTGCTAGTAATTATAAATTAAATTTAAAATATTGGATACTAT
CCGACTTGTAAATGTTATTCTCCTCCCTCATAATTTTTCCTAAAGCATAAAAATATACTCTGCTT
CCCCAATCTTTTCATAAATAATTTCTGATTGCTATATATAATATAGTTATTGCAAAATTTTATTA
AAAAGTGCATCATTAAAGATGCGATCGAAAAATAAGGGAAGTAGTTTATGTTGTTAGAATCACC
TTTAACCCTAGATGAGTGCCAATTTTGTAACCTTGTCTCCCAAAATAATGGCGAAGACCCATTAG
GGAGTGCTGGATGCTATGATCAATGGTTAATGATGGAATTACCTCAACCATGGGAAGCCAAATTC
TGGACTAGCAGAGAAGAATTAAAACCCTTCGTTTCTCTCATGTCTGAATTAATCTCTCAGGGAAA
ATTAAATAAATTTCGTCCTCTAGCGATCGCACCTGATAAAATATACTCAGAAAAAGGCTTAACCC
GTGTAATTTATTATTATCGCCCGGGGGAATTATTTACTAACCTAGAAAAAAGAGAATATCTCTTA
CCCCTTAATAAAATCAACGACTTAGCTATTAGCTTAATTACATCATCAAATCTGGATGAATTTGA
AACATATAGAAAAAATTCAGATAATATCAGAGAAATTTTAGTTTGTACTCATGGCAATGTAGAT
GTAGCCTGTAGTCGTTTTGGTTATCCTATTTATGAAAAATTGAGAAAAGAATATAGCGATGAAA
ATTTGAGAGTATGGCGTTGTAGTCATATTGGCGGCCATCGTTTTGCACCAACTTTAATTGACTTT
CCCAGTGGTAGATATTGGGGACATTTAAACTTAGAAATATTAGAAACATTAATTAATTCCCCAGA
AGATATTAACAAATTGCGTCCTTATTATCGAGGCTGGAGTGGGGTATCATATTTAGAACAAATTT
TTGAAGCTAAACTGTGGCAAGAAATAGGCAAAAAATGGTTAACTTATCCAAAAACAGGTAGAGT
TATTGCACAAGAAAAAGTAGAAAACGAAGAAGAAGATGAGCCAAATTGGGCTGAAGTTGAAATA
AGTTATTTAGAATTAGAAAATCAAAAAGTAATTACTCGTCAAGGAAAATTAGAAGCCAAAAAAG
AAATATTGACATTGGCTAAATCGGGTAACAATGAGTTATTTCCTGTTAAACAATATCAACTTAAT
GTCAATTTTTGAAACTAATAAATAGCAACAGATACAGGAGTTTTTAAGTATTAATTAATCAATT
ATTAAGAAAAAACGCTCCCCTCTACTGCGGGAGAGGGGTTGGGGGTGAGGGCAAGATAATTTTGC
CCTGACTTTGAAAAAACCCTGCTTTCTTTCTCTCCTTAATCATTTTGCGTTAATTGTGCCAATTGT
GTTAAGGTATCTTTCACTTTTGTCATACTGCCCGGATTAACTAAGCCATAATAGTCAAAAGTATA
AATGCGATCGCGCTTTACTGCATTTAATTCACTCCAAAAATCCTCTTGTTTCAACTGTTCTAAAA
TTCCTTCTCTAGCAGGATCAACTATAATAATTACCTCGGGATTTGTCTCTAAGATTTTTTCAGGA
GAAAGGGTCACATATCCACTAAATTGACCACTTCCCTGTAAATCTGCTACCACATTATTAACCTT

AAATTGAGTTAATAAATCTCCCGTCCAACTATTTTTATTCGGAGCTAAAATCGGCTGACGACTCA
CTAAAACCAATGTGGAAATATTTGACTCTAACTTTGCGGGTAATAAACTTTGATAACTATCTAAT
AACGGTTGTGGATCAGCATCGATCGCACCTGCTAAGATTTTAATGGTATCCTGTAGAGCCAACCA
ACTATCAACTTTTGTGGAAACAGTATTAACTCCCAACTCCTCTAATTTAGCCAAAGTTTGATCAC
TAAAACCATCTGCACCCACTACCAAATCAGGCTTCAGCGCCATTATCTTTTCCAAATCAGGCTGCG
ATCGCCCTTCACTTACAGTTGTAATATCTTGAAAACGGGGATCTTGTTTAATTAAACTGCTACCT
GCAACCCCCACCAACTTAGAAGCATCTAACTGATGAATTATATCCGCACTAAGGGAAGTAAGGGC
AACCACTCGCTCCGCTTTACTAATTGGCTCTTGCTTACTAATTATCTCTTGGGAATTATTATTAG
TTGTAGTATTAGTCGGATTAGTGCAACCATTGATACTTAAACCGAAGAGAAACACTAAAGATAAT
TTTAAAATTAATTGAGATGCGATTGAATTTTTCATAATAAAAACCAAAAAATTAAAAATTAAAA
AATTATCATCTTCCTATGGTGAAACAGGCATCTTGCCTGTAACTCAGTCGATGTCTATTAAACCA
GTCAAAAACGGAAAACAAAATTAACAGCCGCATTAAACCCCGGAGGAGCAAAACGCTTTAAATTA
GGATCATCATTAGTTAAACCCCGAACATCTGACCAAACGTAGTATTCTGAGTTAAAGAGATTAAA
TAAACCCACATTCAAAGAAGCATTTTTAGAAAAATTGTAATAACCCAACAAATCCAATAAAAAAT
AACCATTGGGAGCAAATAAATCATCCCCATCAATGCGACTCCTATTTTTTGCCGAGACAAAAGTA
CCAATTAAATCAGTACCCCATCTATCTTCAAGAGCTTTATAACGCAACCCCAATACCGCCTCAAA
AGGATTAACAGAGTTCAAAGGTACTGAGCCACTGCCTGAATCATTATTTCCCTCAGCCCAAGCTA
AACTAGCAATAATACTGACACCATCTTCTCTAGTGGGGTTAAAGAAATATTCCGCTTTTGCTTCC
ACACCATAAATTTCTGCACTGTCGATATTTTGACTTTGAAAAAGAGAAAAACCATCACTTTCACG
AGTGCCAACACTAACATTATCAATAAAATCTTGATAATCGTTGTAAAATCCAGCCAGACTAAAAT
CAATAGAAGGATAAGCGCCTCGAATACCTATTTCAAAACTATTGCTTGTCTCAGGCTTTAAATTA
GCATTGGGTAACACTGCATAACCAAAGGCAAAATTAGTAAAACCGATATTAGCGTCATCATAAGG
AGGACTGCGAAAACCCCTTGCATACTGCCCATATAAAGCTAATTCAGGAGTAGGCTTATAAACAA
TCCCAATTTTGGGAGAAAATGCCGAATCATTACGATTTTCTACCTTAAAGTTATCCACATTGATA
CGGCGAAAATCATCATCTTCATTAGCATTTAAACTGAAATAATCCCAACGAATCCCCGGAATAAT
AGAAAACTGACCTATTTCAATCTCATCTTGTACATATAACCCTAATCTTAAAGTATCCGTATCAG
GAAAAGTTTTATTGGGAAACTCTTCTCCTATCACAAACTTACTAATACTACCATCAGGAAAAATT
AAAGTATTATCTCTAGGGCGAGAAGTGCTAGTGTTAAACACCTCTAAACCATAAACTAGGCGATG
GGAAGCACTACCCGTCATAAAATCACTTTGTAATTGTACCTCACCGCCGATGACATCCTGCTCAA
ATTTATTTACTTCGTCTCGTACCACAGGAATAAAACTAGGTCTGCCTCTCCCGAAACTTTGTTGA
ATACCCTCCGAGATTTTTTCTTCTTTAATCTCCGCATTTTGATAGTAAAATTTAGCGTTGAGATT
TTGTAACCATCCCGTTTCATTTTCATAGTTATAATCTAAGCTAAAACGGTTACGATTACGAGTAT
CAGTAGCAGTGACATTTTGGCGATCGAAAAATACTAGATTAGGAGGACGAACATTAAAAGGGGT
TGTGCCAATTTCATTTAAGATAATAGTGTCCGTTGTTCCTTGTAAAAATTCCCCTGTTAGTTTTA
AGCTATTATTGTCATCAAAATTATAAACTAACTTACTGGTAATACTATAATCAGTAATATCTTGG
GGATTAACTTGCACCCCTGAAGCAGTCTTTAATGCTGAACCTCCTGAATAGGTGGCAACGGTAGA
AAATTGTAATTTACCCTCCTCATCTTCTCCAGCAATAACCCCTGTCACCGATACGCTTTCATCCGC
AGTATCATAGGTAACTTGTCCACTGGTATAAAAAGAATTACCGAAAGTATCTAAATAGTCTTGAG
GGTCTTTTGTGATAAAAGAAACCACACCCCCAATGGCATCACTACCGTATAAAGCGGAAGCAGGA
CCTTTAATAATTTCCACTCTTTTGATAGTCTCTAAGTTGAAATAATCTCTACCTCTACCGACATA
GTTATCTGGTACACGAATACCATCAACTTGCAGTAAAACCCGATTACCGTCAATTCCCCTAATGT
TGAAATCTTGATTACCAAAGCGATTGGCGTTTCTCCCCACAGATACCCCCGGCTCATATCGAACTA
AATCTCGAATATCTTGGACTAATTGTCTTTCTATCTGTTGAGAATCAATTATGGTGATGGAACTG

CTAGAATCAAGTAATCTTCTAGGAGTGCGAGTGCCTGTAATGGTAATAGTTAAGTCACTAATATC
AGGGAAATTTTGAGCAGTAGTGGCATCAGTTTCCCTTATGGCAAAGGTTAAATTATTATCTGTGG
GAATAATCTCTGCTTGGGGTATGGTGTTATTTTTACCTGTAATTACCATTCTCACGAAGGAATCG
TTAACGGTTGTGGCGGTAATTTGAGTAATATTTGCTGTGGGGTTTATTTCGTTTAATTCATAATT
TATTTTTGCTTCTAATAATTCGATAATCAGGTTATTATTTTCAGTTAAAATAATGGGAACTAATG
ACTTACCTGATTTGGTTAAAATAGTAATTTCTAATCCTGTTTCTGTTTCATTAAAAATAATATCG
GTTATTTCTTCTAAGTTTTCATTGTTTTGAGCTATTACAGAATTATTATGATTATTTAAAATTAG
TCTATTTTCTGCTTTTACAGGCTCAATAGTAAAAAAAATTAGAGGTATTAATAAAGTATATTTA
ATTATTTGTATTTGTGTTTTTAACATAGTTAAAAAATTAATAATGAAGAATTAATAATTAGTAA
TTAACCTCCCCCTCTCACCCTTTTCCCTCATCATCTAACAGCTAAAGAAGGAGTAAGAGGACAAAT
TTGCTTCCCAACGGGGGTATTGAGAATCATTACTTCTACGTTGAAAACCTCGGCGATATTGGTTT
CCGTTAACACTTCTTCAGTTTCACCCACTGTGTATAATCTTCCCTGTCGCATAAAAGCAATGCGAT
CGCTGTATCGGGCGGCAAGGTTAATTTCGTGTAAGACGGTAATGATGGTTAAACCCTCAAGATGA
AGTCGTTTGAGCAGTTCTAACAGTTGTAATTGATAGTGAATATCAAGATAGGTAGTCGGTTCATC
TAATAGCAACACTTGGGGATTTTGGGCTAAAGCTAGGGCTAAAAAAGCCCTTTGTCTTTCCCCCC
CTGACAGTTGAGATACAGGTCGATCAGCATAGGATTCTAACTCAGTTTGAGCTAAGGCTTCATTC
ACAGCGATGCGATCGCACTTTGTTAAATCCCACTGATACCATGATTGATGGGGAGTACGCCCTAA
ACTGACTAATTGTCGTACGGTTAAACCAGAGGGAATAATTTGTTGTTGAGGTAAAATAGCGATAC
GTTTTGCTACGGTTTGAGGGGGCAAACAATGAATTTCCTTACCATCAAGTAAAACTTTCCCCGTG
ATAGGAGGAAGAATCCGAGCCAAAAGTTTGAGAAAAGTAGATTTTCCCGAACCATTCGCCCCCAC
TAGACTTAACCATTCCCCCTGTTGTAAATCCAAATCGAGATGATGAACAATAACACGACTGTCAT
AGCCACCACTCAGGTTATAACTATTTAAAGGCATGATAGGAATTAAGAATGAAAAATGAAAAAT
GATTAATCAACTTTCCCCTCTCCCCTCATCACCCTATCCCCAATCTCAAAAAGAGCGTTTATACAA
CAACCAAACAAATAAAGGCGAACCCACCAAAGCAGTTACTGCACCGACAGGTAACTCCACCGCAC
CGATACGGGAAAGCAAATCCGCCCATGATAGTACCATTGCACCCCCCACCGCCGATAAAGGAATC
AACAATCGATAGTCATTACTTTTGAGTAAAAACCGCATAGAATGGGGTACAAGTAAGCCCACAAA
TCCAATTAAACCCGCTATACTCACAGCACTCGCCGCCAATAGAGTTGCCGTGCCTCCAATTAATAA
GCGAGATCGCGCCAACGGAATCCCTAACCCTACCGCTAACTCATCTCCCAAATTGAGAACATTAAC
AAAACGAGCTAACAAACAAGCGATAATGAGAGCTATGAGAATATAAGGAGTAGCAGTATTTAAC
TCTGTCCAACCTCGTCCATTGAGACTACCGACAATCCAGTTTAAGGCTTTTTGAATCCTGCCATCC
TCCGATAGTATTAATAAAGTAGTTTGGATAGCACCAAAAAGGGAAGAAACCGCTACACCGCCAAG
AATGAGACGCTCTACATTTATGTCATTATTCTGCCGTGCTAAAAAGTAAACTAACATAGTCGTCA
AAACAGCCCCTAACCAAGAAGCTACAGGTATGAGAAATAAGAATAAGTTAAAGGTAATCAGAAC
CACAACTACTAAACCAGCACCAGCAGAAATCCCCAACAAAAAAGGAGTAGCTAAAGAGTTGCGTA
ACATTCCCTGCAAAAGCGCACCAGAAGTGCCTAAAGCAGACCCCACGACAAACGCCGCAAGGATG
CGCGGTAAGCGTAAATCCCAGACAATCACTTGATTTAACTCTGCCCCTCGATGCCATAGTGCTTG
ATAAATTTCCTCTAAACTGAGAGAAACAGAGCCTATTGACAAAGATAAGCAGAAAGTAATTAGT
AGCCCTAAACTAATAGAAATAATTACTATTAATGTTCTTCTATTCTCCGAAGGTATTTCTTTGAG
AATAGACGAATCGACAGAATTAGCCTCCATTAATTTAGTTTTGTTTAGTGAATAATAAAAGAGG
TTCAGAAAATCCCTTAGAAATAAAAGGAACTGTCTCTGCTAGGAGTGAGCAGAGCCTGTAAGCTC
CCTCTGGTGTGCGTCCAATAGTTTTCTATGGCATCATCTATTTCTGAACAAGACTTTCAAAACCT
TAGATAAGGTTATAACAGATTTTTGAAATTATTGCAAATAATATGCAATTATATTGATTAATAT
TTTCAACTTAGTTATAATTCAAATCATAAAAGTTATAAATATAACTTCAGTTCGGTTTAAGAATA

TCAGATAAGGGTTCGGAGTTAGTTAGGTGATGGGGGGATGAGGGGATGAGGTGTCAGGTTTCAG
ATTTCAGGTTTTAGAAGAAAGTAATGAGTGTTCTTAATTCTTAATTCTTAATTCTTAATTCTTAA
TTCTTAATTCTTAATTTTTCCTTCGCCCCTTGCCCCTTGCCCTTTTTGCCACCACTCATAGATAAA
AATTTATCCCGAATTCAGGTTAAATATAAGGAAGATTGATGACTCAAATACAAACCGTTAGTGCA
GAAGATTTGTTTCGTAGAACCTACCAGTCGAATTATGGAATCTCATTAAGACGGTAAAATAGATT
CTGTTGAGTTTCATTTTTCTAATATTTCTTTGATTTCATAGCTTGAGAATAACCATATTGTTGGG
TTACACTTTATGTTTCCCAACCTACCCACCATTAACGATGAAATATTTATTTTTTTATTTAACAA
TTATTTTCTTTTCTCTCACTTTCACTGGATGTAACTATAGCCCCACCGAAGATTTAACGGCAACG
ATGTCATCCGAAAAAACATCTGTTAACCAAGATGAAAAGATGAGGTCTATCTCTCATAGTATGGG
TGTAACCCAAGTTGTAGAAAATCCTCAACGGGTTATAATTCTCACTAATGAAGGTACAGATATAT
TATTAGCATTGGGAGTTAAGCCCATAGGTGCAGTAAAATCTTGGCAGGGAGATCCCTTTTATGAT
TACATTGAAAATAAAATGACCGATGTTATGGTGATTGGGGATGAATTTAAGCCTAATTTAGAGT
TAATCGTTTCCCTCAAACCTGATTTGATTATCGGTAGTAAGGTGCGCCAAGAACAATTATATCCC
CAATTAAGTGCGATCGCACCTACGGTATTTTCGGAGACTATTGGAGTAACATGGAAAGATAATTT
AAAACTCTATGCCCAAGCCGTTAACAAAGAAGCAGAAGCGGAAAAACTTTTACAGGAATGGGAAC
AGAAAGTAGCCACATTCAAAGAGAAATTAGGAGATAATCCTCCCACCGTATCTTTAGTGCGTTTT
ATTGCAGGAAATACCCGTCTTTACTATGAACAATCATTTCCCGGGCAAATTGTCGCAGAATTGGG
ATTAAAGCGCCCTCCTGCTCAACAAAAAAATGATTTTGCTGACGAAATTGCGATCGAAAATATCC
CTTTATTGGATGCAGATTACTTATTTTATTTTACAGATAGTTCTCAAGGAGAAAAAGGAAAAGCA
TCGGAAGAAAAGTGGTTAAATCATCCTTTATGGCAGAATTTAGAAGTAGTAAAAAATGGACAAA
TTTATCAAGTAAGTGATGCACATTGGACGAGTTCAAGTGGTATTTTAGCCGCTCATAAAATATTA
GATGATTTAGATAATTTTATATTCAAAAAAATGGAATAAAAAATAGAATAGTTTTTATACTCAT
ATCTTGACTGATAAAGGTGCAAAACAGGGAAAAGTAATAAAAATTGATAAAAAAATATTAACAT
TGATTTTAAAGATTTAGCTTTATAGTATTAATATCCTTGTGCAAGATTTCAGTTCTAAAGTGGTT
ATGATAAATTGATAGAGAATAACTTGTATAACATTTATTGAATAGTTTTCATAGTAAAATAAAA
GTCTATAAATTATGGTTCTTCAGAGTGTGGTTATGATAAATTAATAAAAAATAACTCCCATAACC
TTTATTGAATAGTGTTTATAATAAAATAAAAACTAAAAGTTTATAAATTATTATTTAATAATCC
CCTATTGCATATTGCCTCTTGCCTTTCAACCTACCCTAACTAATAATTTACATATTCAAAGTAAT
AGAGTCCAAAGTTTTTTTTGATAAATTACTTAATTCGACTTAATACGTAATCTGTTAGCTCTCTT
AAAGAATCAGTGGCAGGGCAAGAAGGTAAATATTGCAACTCTTTTAAGGCTAACTGACTATGCTG
ATGTGCTAAATCTCTCGCTTTTTCAATACCGTTACTATCGTAAACTAAAGCTAAAGCCTGATCTA
AATCTCCTTCTTCACTAAATTCCCTCTCAATAAGGATACTAAGGGAAGGTTTTTCTTCCATAGCA
AATAATACAGGGGCAGTTAAATTACCACAGGCTAAATCTGAACCTGCTGGTTTACCCAATACTTC
TGTAGGAGAGGTAAAGTCAAGAATATCATCTACTATTTGAAAAGCTAAACCGATATTACGTCCGT
AACTATAAATTTGATTTGCAATTCCTTCTCCTGCATCACTCAATATAGCGGCGGCTTTTGCACTA
TTGGCAATCAAAGAAGCTGTTTTAAAATAACTTTTCTGTAAATATTTTTCTAAACTAACATTAGT
GTCAAAACAACTTAAACCTTGTTGAATTTCCCCTTCCGCAAAATCCCTAATTACCTCTGACAATA
GCTTGACTACCTGTAGATTATCTAAATTAGCCAAATACCATGAAGACTGCGCAAAAAGAAAGTCC
CCAGCTAATACTGCTACACGATTACCAAATAAACTATTGACGGTTGCTACTTGTCTTCTTAACTC
TGCTTCATCTACTACATCATCATGAACTAAACTGGCAGTATGAATCATTTCTGTTATTTCTGCTA
AACGACGATGACGAGGAGTTAAGTCATCTCCTTTCATTGTTGCTCTTGATACTAATAGCACAATA
GCGGGACGAATTCTTTTTCCACCAGCCGTGAATAAATGCTCTGCCGCCGCCCCTAAGATGGGATG
TTGGGCTTGAACTAGGCTGGTTAAGTTATCAATCAGAATTGATAAGTCTTTTTCTACAGGGGTGA

ATATCGAGGTTGCAGTTGTCATTGATGAGTAGAATAGCTAAAAATAGTTTACGAAAGTTTACAT
ATCTTATTGTTATTTTAAGCTATGGTTTCCCCTGTTAGATCTACTTAACCAAGATTTTTTCTTGTT
TTTTTTAGTGCTACTATGGTGGAAATGCTTATATAATAATGTTTTGAGGATTTGTAATTTTTTTT
TAATTTTTCCCCATTTTTTTTTAATCTATGTTATTCTATGAAATAGAGGGGAGTTTGTCCATAAA
GTCGCAATTTCCCTAGATTACTTGAAAGAAAGAATCCGAATTCTCCATCGATTGAGAAGGGCTTG
ATTGGAAATTTCTTGTTACAGTCAAAAAATAATTTGGAGAAGAATAAAGATATTTTCGTTTTAT
TTGATTTTTATTCCTCTCTAATGGTTAGATATAGTTTTTTGACTATTAGCATTATTTTTTAGATT
AGCCAGAAGATGGATGAAACTCTGTAAGTTGTTTCTGAAATAATTAAGTTAATTCAAGGGCGATC
GCACTTTTAGTGATAATTAATAATTAAGTATAGTAACAAATGCTTTGAAGAAGATAGACAATAT
TTTTAGTGGTTGATAAGTAGTTGAAAAAGCTAGTTATTTACTACAAAAACAGTCCAAAGGGCAA
AGCTAAATGATGTAATGGGCAAGGTGATTTCTGCAAGTAGCTTGATACAGCAAAAATTACTCGTC
TCCAAAGACAGAATTTTAAACCCATTATTTTTGATAGAACAATATAATCATTTCAGCTTTGGTCT
AAGTAAAAAATATACATACTAATTATTAAGCAGACAAAATTATCCCCCGAAATCTACCTCAGTGG
GTTTGGTTTTTTTGATTTTGCCCAGAGGTCTATCTTATTGAGTTTAGGACAGTTTTCTATTGTTA
TAAAACTAAGTCCTAGTCGGTTTGGTATCAATTTATTCTTTTTCACTTATTGAATCAAAGATGGA
AGGATTCTTGATTAATATTCACCATTTACTTGAACCATTACCATGGGGGTTTATCTATGCTAATA
CCATTAATTGCTTTGTTGTATATCGTCTTAGTTTATTTTTTGCTAACTACTATTTCCCACTGGGA
GAAAAAAGGTAGTCAGAAAAAGATTTTTTCTCGTTTTTATCGTTAGGGAAACCAAATCAAAAAT
TAGGAATTAGTCTTATTAATTTTAAACAAATATAAAGGCTTCCTCCCTTTCAAGAAGAAGCCTCG
GTTTTTTATATAGACTTATTTATGTTTAAGGTCTTTATTAAGGCTGACCTCATTTTATCTTCAGG
AAATATATTTACACGTTTTTGAATTTATATGGAGTTTCTTAATATTTTTTTTAGATTTTATAAAT
TTATATCGCCTTTTGTAAATAAATATTAATTTGTTTTAATTTTTATGAAGAGAAACTCCTAATTG
TTGGGAAATCCAAACCTCAAAATTGCGGATAGAAAATTTTTTCGGAGCATGGGGAGAAATCATTG
GCTCTACTAAAATAGTAAAAACCCCTAGTCTATTACCTGCTAATACATCAGTAAAGAGGCGATCT
CCCACCATAGCAATTTGTTCTAAGGGTAGATTCATGGCTTTAACGGCTTCTCTAAGTTTGCGACG
AGAAGGTTTACCTGCACCATAAATGAAGGGCAAATTAAGGTATTTTGCGATGGTTGCAATACGAT
TTTGACTAAGATTGTTACTTACAAGCCAAAGATCTACATAAGGGCGTATTTCTTCCACCCATGTT
AATAATTCAGGGGAAATAATCCGCTCATTCCAAGGAACTAAGGTTTCATCCACATCGAGAATTAA
GCCTTTCAAATTATGTCTTTGGATCACATCTGGGGTGATATGAATAACTATATCTCCCAATACAA
GGTCTGGTTGTAAAATTTTGGCTTTAGACATTTAATTCAATTAACAACTAATAATCAACAATTGA
GAGGTGTTAGGTGTCAGGTTTTAGGTTTTAGGTATCAGGTGTCAGGTTTAACGGTTTTAATTAAT
TACTAATTACTATTTCCCCAGTAGTAGGTTCAAAAAGATAAATTTGCTCTAAATCTAGTTGGAGT
TTCAGTTCATCACCCATTTTCCCTTGCCACTGAATGGGTAATTGTAAATTAATGTTAATCTCTGA
GTCAACTATCTTAGCATTAACTAAAACTTCTTTTCCTAATGGTTCGATAACTTCAACTGTTGTCA
CAAGAAAATTATCAAGAGCATTTTCTTGATCCTTTACTAAGGTAATATATTCTGGGCGAATACCT
AAATCCCAACCTTGTCCGTTATGAAAATTGTATTGCTTGAGAAAAGGAGGAGGTAATCGGAGAGA
TTGTTGTTCATTAATCCAAAATGCTCCATCGTGGTAGGTAACAGGGATGATATTCATGGACGGTG
TGCCTAAAAAAGTAGCTACCATGCGATTGACTGGGTTTTGATAAATTTCTGCTGGTGTACCTATT
TGCTGTATTCTACCCTGATTTAAAACCACGATGCGATCGCCTAATGTCATAGCCTCAACTTGATC
ATGGGTAACATAAACTGTAGTAATACCCACTTGAGAATGTAATTTTTTTAATTCTGTCCTTGTTT
GATCCCTTAATTTTGCGTCTAAATTAGATAAAGGCTCATCTAACAAAAATACCTTTGGTTGTCTA
ATAATCGCCCTACCTAAAGCAACTCTTTGTTGTTGTCCTCCCGATAGCTGTTTGGGTTTACGCATT
AAAAGATGACCAATATCAAGAATTTCTGCCACTTGTTTAATTTTATTATCTATCAGCGACTTTTT

TTCTCCCCGCATTCTTAAGCCAAAACCAAGATTATCAGCAACAGTTAAATGAGGATATAAAGCAT
AATTTTGAAAAACCATCGCCACATCTCTTTCTCTAGCGGGAATTTCATTCATTAAAACACCATCA
AAATAGAGATTTCCCTCACTAATAGACTCTAATCCTGCAATACTTCTCAAAATCGTTGACTTACC
ACAACCCGAAGGACCTACTAAAACCCAAAATTCTCCATCAGGTACTTCAAAGGTTATATTGCTAA
TAACTTTTACTTGATTATACGAACGGGCAATGTTTTGTAGTGTAACCGTTGCCATAGTAAAAAAT
TAGGAATTAGTAATTATGAATTAAGAAAAGGGCAAAGTTAAAAGGACAACGGGCAAAGGTGAAT
AGTGAACAGTTAAAAACTCTTAACTTTTAAGTAGCTCCACTTAATTAAACGTAAAATAGGCTCGG
TTAGTAGTAAGGGCTTCAGCCCTTATTTCAAGAACGTTAAAACCCCTAAAGGGCTAACTACAAAC
TTTCTTTTATTTAATTGGCTCTACACTTGTTTCTCCCTCTCTCCTTATCCCCCCAACACCCTAACA
CCAGCAACCGGCAACCTGAAATCTCTTTCTGAACTCCGAACTTATTTTTAGTTATCAATGAGAGA
TAAATTACCCCATTTGGGATCAGAAAATGCTTGAATTAAAATATGACCAGCAACGGCTTTAATAT
CATCCTCACTATAGTTTCTGATTTCGGGCCAAAGGTCAGGGCGTTCTGTATTTACGTGTAATAAA
GTAAGATCTTCTTCCCCGTCATAGCTAGTAGGATGCTCGATATAGTCCATTAAACCAACTAAGTT
GTCTCTTGCAGGTAAAGCATTAGCTAAAGCCTCTTTACTTAAACTGACGTTAGGATTAGTTTTGG
TTTTACCTTGAATGTGACATTGAGCACAATTACTTACAAATAAACGAGAACCTTTTAACGCCTCT
TGGTTAGATAAAGTAATAGTTTCTCCAGACTCATTCAGGGGAATAGTACGAATTTCTTCACTTAA
ACTTAAAGCATTTGCGCTACCGACAAAGGATTGCCATGCAAAAATAACCCCGACAATGAAAATTA
AAACAAATTTTTTCATAATTTTGAAACAAATATTTTTTCTTAGTATTTTTGAGGTTCTGATTTAT
ACCCTAATTATTTTCTCTCAAAATCGGTTCATCTTTGAGCAATTATTGTAAAGTTTTGTTAATAA
TATTGTGTTGAAAGGCAATAAAAAAGGATAGAACGACTGTCCTACCCTCATTTTTTCTGAACTTA
ATAAATAACTTAAAAGTTACTGCTAAATAATAAATCTACTTAGTTGAGTTACACTTCTGAGCCAA
CTTCATCGCTATAGCTAGAAGAAAAGCCTCCAGAAAGAATATCAGCTTCTTTGACAAAGCCACTG
TAAGCCTCCATACCATGTTCGCCAATATCCAAACCTTTGACTTCTTCTTCGCCAGAAACTCGAATA
CCAACGGTTGCTTGGAGAGCGTACCAAACGATGAAGCTGAAAGCGACGGTAAAACCACCAACGGA
GAAGATACCAATAATCTGATTGATTAAAAGAGCTATTCCGCCTCCTGTTAACAATCCGCCCTCAG
TGGCAAAGATACCCACCGCTAAAGTTCCCCAAATACCGCAAACTAAGTGAACAGAGGTTGCACCT
ACAGGGTCATCAATTTTTAATACTTGGTCAAACATGGCAACGGAATAGACGACGATTATACCTGC
GATCGCACCAATGATGATAGAACTGAAATAACCAACACTAGCACATCCTGCAGTAATACCGACTA
AACCAGCTAAGATACCGTTAATAATCATAGAAAGATCGGGTTTGCCGTCTTTTAACCAAGAAGTA
AAAGTTGCGGTGATACCACCAGCGGCGGCGGCTAAGTTAGTAGTAACAGCGATATAAGGAACGTT
AGCAGTAGCGGCTAATTCAGAACCGGGGTTGAAACCAAACCAACCAATCCAGAGAATTAAACATC
CGAGAGTAGCAATACTCATATTGTGACCGGGGATAGCACTAACTTTACCATCGGAGGAATATTTC
CCTTGACGAGGTCCTAATACTGCGGCACCCACTAAAGCGGCCCATCCACCAACGGAGTGAACCACT
GTTGAACCAGCAAAGTCAGAGAAGCCTAATTCACTTAACCAACCACCATCCCAAACCCAATGCCC
TGTAACGGCGTAGGAAATGGCTACTAATAACGCACTGAAGATTAAAAACGCATCGAATCGAACCC
TTTCAGCTACAGCACCAGATACGATGGTAGCGGCAGTGGCGGCGAAGGCTACTTGGAATAAGAAA
GAGATAGCCGCAGGTACTGCTTCTGGATAATTTGCATCACCGTAGGGAGTACCATCACCATTGAA
GAAGAAGCCTTGTAAACCGATGAAACCGTTTCCTTCTCCGTACATCAATCCAAAACCAATTGCCC
AATAAGCAATAGTTGCGATCGCAAACACGATCAAGTTTTTCGCTAGAATATTAACAGCGTTTTTT
TGGCGACAAAAACCAGTTTCTAACATTGCGAAACCAGCATTCATGAAAATAACTAAAACAGAACA
AAACAGTAGAAACACTGTATCCAAAACGATTTTCACTTGTTCTAAGTCTTCGGGGCCATCGTAAC
CCTGTGCCATTGCCGCACCATCCCACACCGCAACAATAATCGCCGTCAATGGGATACAAGCCAACC
AATAAGGAGAAATTCTGCTAGTTATTTTAGAAATAAAATTAAGAATAATACTATCTTGATTTTC

TTTTAAAGATCTTCTTAGACCTCTTTTTCTAGGGATCGTCACCATAATTATTAATTCAATTTACT
CGTGAGATAACCGTTAATAAATAAATAATTGATTAACCTATCAATTAACTGTTAAATATTCATAT
GTTTTATTAGCTCTTTTCTGTATCAAACAATACAATAAAGCCCTGTAATTGCTTAAATATGCTGA
AAGAATTTTGGTTTAGTCTCAATCCCCTATATATATTCATCCTTCAGCACTTAGGTGTCTATTTA
GGTGGATTTTTATTAATTTATGGTTTTTTGTTCTTTCTTTTGCGTCCTTTATTTCGCAGTTTAGA
AAGAGATATTGCTTTAGTTACCCTAAATATTTCTGGTTATCCTTTACTCAGTATTTTTACTTTTT
TTAATCTCAAATTTACCCTTTATGATTTGGAAAATTTTGTTGGCAAGGATATATTACAAAAGATT
TTAAATGCTTGTCTAATTGTCGTAATTAGTTATTGGCTAGTGGCAATTATCAAAAAAGTTATTAT
TTATTATCTTAAACAATATACAAAGCAAAGTGAAGTCATGTGGGATGATGTGCTTTTACCACTGG
TAGAAGCAGTTTTACCTGTCATGGTTTTTTTATTGGGGGGTGTTTTTGTTTTAAACAATTTCGGG
GTGGATTTAACTGGTATTTGGGTGGCTTTGGGAGGAGCAACTTTTATTATTGGTTTTGCCTTAAA
AGATATATTGGCTAATTTTTTTAGCGGTATTGTTTTGTTGATTGATACTCCTTTTCAATTCGGAG
ATGTATTGCGATTGGAAGATGGTTCTATCGGGATGTTGTGCCGTATCGGAGTTAGAGTGACCCAA
ATTTACTTATTTAATCATCATTGTGATATTTATATACCTAATAGTGTTTTGCAAAATCAAAATAT
TACTAATCTCAGCCGCCCTACATCCTATTATTATTATTCTACAACCATTGAAATACCTGCGGAAT
TAGAAGTAGATTATCTAAAAAGTTTAATTACTGAAACCGTCTTAGCACATCCTGATACTTTAGCC
GATATTGAAAAGAAAATAGCTTTGATTGATCGTTTTTATCTATGTGAAGAAGAAACAAATAACA
GTTTGACAGAACAACAAATAAGTGGAAAAAAATAGGCTTTTAGCAGAACAAAAAGTTAATATTAA
ATTGCAAGAAATTGAAGAATTTTTAGAGGCTTTAGTAATTACTTTACAGTTTGCAGAAAAGGGT
GGTTTAACATCGGAAGAACTAGAAAATATTTATCAAGAATATCAAGAAATATTAGTTTTAATTG
GTTTAAATATTAACTCGGAAATTAATACTGATAATCTCTTAAATACCACTTTTGCTGAATCTCAA
GCTCCAGATGGTTTGATAGAATTAGTAAGAGAATGGTATCGTTTTTGGCTTAAAGACCCTAATTT
AAAAGAAGAAGACTTATATTTAATTCCTCAAGAATGGGAAAGAAAAATTACTTTATTAAAGAAA
AGAACTTATCGACTTTATCAAAAAATTACTAATCCTCAATTAGAAGAAACTCGATTAGATGATTA
TGTTTTGGAGTTAAGTGATTGGTTTAAATCTCGTTTTAAAGAGCGTAAAAAATGGCAAGATCCAC
AAATTTGGATCACTGCTTTAAATCATGATGAGGGTTTTCTTTATTATGAATTTAAAATTAATTTT
TTTGTAGATGATATTAAATTAGAATACGGCAAAAGAGGCGATCGCATCGCCAGTCAGATTAACCA
AGAAATCCTCAGAATTGTAAAGCCATATTTAAATATTCCTTTAAGAAATGCTTAAAATACCTAGT
AATTATTATTTTTCTTTTTTCAGTAAAAATTCTGATTTAAATTTTTAGTTAATTTGTTAGATTGC
AGTTGTTGAAGTTTTGTTAAAAACCGTACTATTTTGTCACAGAATATTGTTAATATGAAATAAA
TATAAGAAAAAAAATATATCTAAATATTAACAAATTTAACATAAGCAAAAATTCCTAAAAATAA
ATTAAAAGGAAAAATTGTTCAGAAAAATTTGAAATTAAGGTAGGAAAATAAATGCTAGTAAACA
CCGCATTAAGCGACATTCAAGGGCAATATAACTCAGGGGAAAGAAATTTTCCGAAATTACAGTTG
AGAAGAATTGATTTAAGAAATGCTCAACTAAAAGGAGTCAATCTCGAAGGTTCTGATTTAAGCT
ATGCTGATTTAAGAGATGCTGATTTAAGCGGTGCTAACTTAAGTAAATGCTATCTAAATGAAGCA
AATCTTAGTGGAGCAAATCTCACGGGGGCAAACTTAACAGGAGCATATCTAATTAAAGCCTACCT
AACTAAAGTTAATTTCCAAAAAGCAATTGTTAAAGAGGCTTATTTTACCGGTAGTTTTCTAACTA
GAGCAAACTTTTATAAAGCTGATTTATCCGGGGCATTCCTCAATGGTGCTCATTTGAATGGAGGT
ATTTTTAGAAACGCATCCTATGATAATACTACCCGTTTTGATAAAGGATTTGACCCTGAATCTTT
GGGAATGCAAAAAGTTTCTTCTTTTAACCTCGCTACTAGCAAAAAAGTAACCATTGGAGAAATCA
TCACTAATTTTGAAACCATCGCTTCTATTACCAGTAAGTATTTAGGTGGAACTATAACCGTTAAA
AATTTTGAAGACACTCGCCCTGATGTGGAGTGGTTAAGAGAATTTTCTATGGACAAAAATGGTAA
GGTAAATTTTAATGGTTCTCTATCTCATCAAGCTACTACGATACAATTGAAATGGTTTGAAAAAT

GGACTAATGCTTTTGTGAAAAAATCGTCCATGATTATTCAAGACTTGCCCAATATTATCGAAGAA
AAACATTTAACCGTGGACTATTTAATCAAAAGAGGTGTTGCTTAATTAATTTATCTAAGCAACGA
AAAATTATAAAAATTATAATTTCAAAATTTTTTTTCTTATTTAAAAGTTGATTATTCTTAACAA
AATAGTCAACTTCTTCTATCTTTATTTATTCAATTATGAATAATATTTAAGAAATCAAAAGATTG
TTAAAAAAAGTGAGCAAAAAATCAAAATTACTAATAACATAATAAGAGAAGGGTTAGGATTTTG
ACAAATTAAGTCTTTCACTTTTAGAAGTTAATTAATAATCTCTTAATTAAATTTTATTTTAGAGG
AAATAAAAGCCCATGACATTCACACCAACAAAAAGCGATCGCATCGTTGAGAAAATTAGCAAAAA
TCTCTGGAAGAGCAAATATCCCAAAAAACATGATCACTACACTTTGGAAGACTTTTTTCATTTCG
ATACCAACCAGGGGGATATTACCGATTGGAATGAGTCCAGAAACGTTCTTGTAACCGAAGATTTT
ATCGTTGGTTTAATAGAAGGATTAGAAGAAGAAGTCGGTTCTGCCTCCAGTGTGGTTATGTACAA
CATCGGTAAAGCATGGGGTGTTAGAGACGCAGAATTTTTTGAAAAATGGTTTCAAGAAGAATAC
GAATACCCTAAAAATATCCACGACATGAACTTACTCTATGTTTTAGAAGCATGGTGGTGGCCTTT
TACAACTCAAGGATGGGGCAACTGGGATGTGGACTTAAGTGAACAGAAAAACGGCTTCATGTTTG
TTAATATTTTCGATTCCGCCGTCGCTCGTACCCTCGGAGATGTGGGGAAACCCGTTTGTCATATT
TATGCCGGTCTTATGGCTGGTTTTTTTAGTCGCTTTATCAATAAACCCCTTAACTGCATTGAAAT
TCAGTGCTATTCTATGGGTGAAACCTATTGTAAATTTTTGTTAGGAAAACAAGATCGCATCGATG
CGGCAACATTTTGGCAAAACGAGGGGGCGGGGGCTAAAGATATTCAGAAAAAATTAGTTAATGG
AGAATATCTCAAATGATGTTATCAACCTCTACCGTTGAGGATTTCTTTCAAAGAGCAAATTGGCA
GGGATTAAAACAAATTCCAGTCTTCGAGGAAGAAGTTACTTCGGAAGTTTTTACGGGAGAAGAG
TCTTTAAAACCAGAATTAAACTTTACCGTTGAAAAATTCTTTGCTCTCAATAATTGGTCTGGCAA
AACGCAGGTTTTTACTAAGTCTATCTCCGCAGAAGAAGAATTGGAACTATCAGAACAAGGTACTT
ATAAACCTGTTTACTCCTTGAAAATGACTGTTGCTGAGTTTTTTCAGCGTATGGTATGGCAGGGG
CAAGGTAAAAAGAGACAATTAAATATAGCTTCCATACCGGCTATGGATCAAACTCCAATATCTGA
ACCTGAAAAAAGACAAATTAACGTCAAAGATTTATCCGATTTGTTATAAGGAAAAATTATGTAT
CCAGAACTACAGGCATTAATTCACGAATCAGAACATCAATATCTACAACAGTCAGACTTAGATAA
ATTAACCCAAGAAGTCTCGACATTGAAACAAAGATTAGCAGTTTATCGAGTTTTAAGAGATCAAG
AAATTAGCATTTTTCAAGCAATAGCAGATAAGTTATTGGAAAATCTCCCCCAAGAAAAGACCCGC
AAAATAGAAACTTGTGTGCGTCACTGGTTATTAGTTACTCGTTATGGTGCGATGGCGATGTTATT
AAATAATCCTGAATTTTTAGAGCATCGTTTATTAGAGTGGTTGACGGATATTGTTCAAGCCCACG
AATATCAAGTCATCTCAGAAAACTTACATTCTTTACTAATCAAAAAATTAGAAAATGTATTAGA
AGATGACGGTATTAAATATATCCAACCATTTTTAGATCAAGCTCAACGTCATATTACTGAAGCAG
TTTGTATCTAGGTTGATTGGTGAGAGAAGGGAAAAGGCAACAGTGAAAATAAGGGCTAAAGCCC
TCACTACAAGCTAGTTTAGTTGTTCTAAGAAATTCTTTAGTAAGAGGAAAAATCAATGATTTCTG
TAGCGGATTTAGTCAGAGAAAAACCTTTAAAAGGTAATTATTTCGCTCCTTCAGCCTATGTGCAG
GGAGATTTTGAGTTAGGGTTGCTTGAAACTCGTTTAGGCTCTCGTTTAATTGCTTTGCCAGAAGT
TTTTTTACAGGGGGTTTATGAGGGTTTAAATGAAGAATTAGGACAAGCGTCAGGTTTAGTGCTTT
ACAATTGCGGGAAATGGTGGGGTAAAAGTTTTTATCGTCGTTTTTTGCAAGAGGTGAGTGAATA
TTATGAAACTCCTTTGGCACAAATGGAAATGGTGGAGTTTTTGAGTTGTTTAAAACAATGTTGGA
AAACTCACGGTTGGGGAGTGATTGAGTTTAATTTTGACTATTATTCTCAAGGTTTTATCGTGGTT
AATACGGTTAATTCTCCTTTTGCCCAAGCCGCCCCCCAAGGTGAAGGTTTCGCTTGTCAACCTGAA
GCAGGTATTTTAACCTCTTTCTTTAGCCAATTAACAGGAGAGGACTTATTGGCTCTGCAAACTTC
CTGTGAGTCTATGGGTGCAAGTGCTAACTATTTCATTATTGGTTTAAATGAAAGAATGAAGTCTG
CAAAAGCGTGGTTAGAGGAAGGGCATGATCATGAAATGGTAATCAATTTACTTTGTAGCGGTTCA

TCTGAGGCTTAAAGGTGATTGAAAATCAAGTTTATTTCTCGTTCATATATCAGTTAACCTGAAGG
TAGTAGTAGATTATTTAAGGTAAGGATAATGGCAAAAACGATTAGATTAGACCCTCTTGGTAAA
GAAACGGCAATAAAAACTAACGATAATCTTTTGTCGGGTTTACTGAAAAATGAATTAAATGTCA
TGCAAGAATGTGGCGGTAGGGGAATGTGTTCTACTTGTCATGTTTACATCAAAGAAGGGATGGA
AAGTCTTTCTCCTCTCAATCGTCGGGAAAAAAGAACTTTAGAAGTTATTACTACTTGTAAGATGA
ATTCTCGTTTAGCTTGTCAAGCGAGGGTGATTGGAGATGGGGTTGTGGTGGAACTTCCTTCGGGG
ATGTATTTAAGTCAGATTGATGATGTTGAGTCTTTGATTGGTAGAAGAGCTCAGCAAAATATCCT
TCATCCTATTTCTGGAAAGATTTTGGTGGAGGAAGGTAAGTTAATTACTCGCTCGATGATTACTC
AGTTAAGTGATACCAAGGGTGAAGTATCTAAGTATTTAGCTCAAAGTGTTGATGCTATTTAATTT
CTTTAATAAGGGAGTTTTAAATTTATGTTAAGACAATTTTCTGATTTAACTTTAGAAGTGGATG
GACGTTATGCTCAAGATTATGAGCTACAGTTTTTAGAAGATTATCTCGATTCTCATCAGTTAAGA
ATGAGTGCTTATATTAAAATTAGAGATAATGCTCAGGCAATTTGCGATCGCGTCAAAGAAGAAA
AACAAAAACTTCATCCTAACTGGAAAGACTATTATGTCACTTGTTTAAGAGATATGGTGGATTTA
TTGCGTTACAGTGCCACTGCTTTATTATTTGATGACATGGAAAGATTACGCACGAATATGTTGTT
ATGGTTTCAAACTATTTCCCGTGCCTATAATTTTACTGATGATAATATTGAAACCTATGATTTGT
TATTGAAGGTTGTACCTGAGTTTCTTACGCCTGAAGAAGCTAAATATGCGATCCCTGCATTTGAG
CTTAATTGTGCAGTTTTGGTTTAGTCAGTCAGAAGGTATTTCCGTTTTTTGGCGGTGCGAGGGAA
AAGAAAAGATGAATTTGGGAAAAGGGCAATAATTAGATTATTTTCCTCTATTAACTATTAACTA
TCTCAATGGAGTATTTATTTAATCATGATGGAAAAGCAACAAGACATTCTCGGCTATGTAGGCTT
AGGTATTTCGGCGGTAGGTGCGATCGCATCTATTGCAACAGCTAACGTGGCTTTTGCTTCTGCTG
GTTTAACGGTGGGGGTAGGTTGTAATGTATTTAGTCGTAAACAACAGTATGAGGGATTGGTAAA
TGCCTACAATCAACAACAGGAAACTATTAATGAACTAACTCAAAAAATTGAGAGCAATCACGTTG
AATTAAATCAAACTTTGATGGATTATAAAGCGGATTTAGGCACAAGCATGGAAAGGCTACAGCA
TTCTATTCAAGAACTTCTTAATAGTCAAAAAGCAGACATTGCTCAAGAGATTAAACGCCTAGATT
TACAACATCAAGAATTAAATAATGTTGTTAGCAATCTCAAAGAAGTGGAAAATCTTTCCCAAGAA
TTGCGTAATAATGCTGATTCTGCTGATTTCTTTTATCAACGGGGTGTGGGTTATGAAAAATTAGG
TAATATCACAGGTGCGATCGAAGATTACACAGAAGCCATTAAACGAGATGGGAATATGGCAAAA
GCCTACCATAAAAGAGGGGTTTTATATCTCGATAGTAATATGAAACAAAAAGCAGTGGATGACTT
GCGCAAAGCGGCATTATTATATTTGAACAGGGTGATATAGACAGCTATCATCAAGCCAGAGAAA
TGAGTCGCAATATTCATGACTTGAAGGTGGAAGCTAATGGTAAAGCAACAGAGATGGTTGTTGG
TTCAGAATTGTTTTCTTAAAATTTCATTTAGTTGCCAAAATAAGAACTAAAGTTCTAATGGGTTG
AATATCATTCAACCCCTACCAGTCAATTAAAAATATCTTTGGCTCTATTACTTCTCGTCGTGTAA
ATTATTGGTGTTTGGTAGGCACTCTCGCCTTTAATTTATCATAACTATTGTAGAAGAGTCATATC
TTTTGAGGAAGTTTGATCAATAAAAACTGGGAGAAATAAAAATCAGTGGTAAATTAAACTTAAA
TGTGACAAATCACCATTATTTTGATTTATGACTATTAATATTGATCCTATTGATAATATTTCCGT
TGACATTAGCAATATTAGAACGGTTGTTAATTTATTTGATAATTTTGACGATCCTTTTACTACAG
GAAAAGTCGCCAATTTTAATTTGACAGATAATTCCATTGGTAATGGAGAAATCAATATTGTTTTA
TTTGACCAAACGGGAGAAGGAGCCCCAATTACTGTCAATAATTTTATCAATTATGTCAATGGTGG
TCGCTATGATTACTCTATTATTCATCGTTCCGTAGTTACCCCTACTCCTTTTGTCATTCAAGGAGG
AGGTTTTACTGTCAATAACTTGACTCTTAATCGTGTGCCGACAAATTCTCCAATTCAGAATGAAT
TTAGTGAAGAGCGTTCTAATGTTAGAGGTACAATTGCCATGGCAAAGCTGGGAGATGACCCCAAT
AGCGCTACAAGTCAATGGTTTTTTAATTTGGGGGATAATTCTGGTAATTTAGATAATCAAAATGG
TGGGTTTACGGTTTTTGGACAGGTTTTATCTCAAAGAGATTTGAATACTGTGGATGCGATCGCAT

CTTTGCCAGTGGTAAATGCTTCTAGTCTAAATTCTGCTTTTAATACGTTACCTGTTAATCCGAAA
AATGTTGATGTTAATAATCCTAGCCTCAATCAAGATGAAGACTTTATTCGTTTTGAAAGTATTAC
GGTTCAAGATGTAGCTGAATTAACTTTTACCGTAGAAAGAAATACTAACCCTGAGGTTGTCACCG
CCACTATTGACACTGAGGGAAACCTCATTTTAGACTACGGTAGCTTTTTAAAAACTCCCCTCTAT
CGCTTCCAAAACCAAGATTTACCCGGCACATATTTATTTGTGGGAGAAGAAGAAAGACAGAATAT
TCTCCGTAATTTCCGCAATTTTAAGGAAGAAGGATTAGCTTTCAAAACCGCTAGTCAAGCCGATG
GAGATACTATTGCAACAGGGGAAACAGATATTACCATCAAAGCCACCAATCTTTTGGGAGAATCT
GTTAGAGATACTTTTAATATCAATGTGACAGGGAATATACCAGAAAATGAAAATCAAGATCAAT
TAATTCGTTTCAATCGTTTTCAAAATAGAGATATACCCGGTACTTATCTTTATGCGGCAGAGGAA
GAAAGTCGTAGTATTCGTCAAAACTATACCAATTTTATTGAAGAAGGTATCGCTTTTTATACTTA
TGGAGCAGATGCTAATTTAGGACAAGATATTTACCGTTTTCAAAATACGAATCAACCCGGTACAT
ATTTATTTGTAGGAGAAGAGGAGAAAAATAGTATTCTTGCTAACTATCCTCAATTTGTTTTAGAA
GGGGTTGCTTTTGAAGTAGCAATTTAGAGGCAAAACTCTTGTATGATGATGAGTAGTTAATCTAT
ATTCTCACAAGTTATCATACAAGACACCCATGACAATTAACATCACTCCCATTAGCAATGAATCT
TTCAATCTTAACGAAACCAGTAGCACTACCATTAATTTATTGAATCATTTTGATGATCCTTTAAC
CACTGGAAAAGTAGCTAATTTTAATTTAGAGGATAATTCTTTAGGTAGTGGTGAAATTAATATT
GTTTTATTTGACCAAACAGGAGAGGGTGCGCCCATTGCAGTCAATAATTTTGCTAGTTACGTTGA
TGGTGGTAGTTATAACAACTCCATCATTGATCGTTCTTTTTCTACTTCTCAATCCAAATATATTT
TAGGGGGGGCTTATACTGTCGAGAATTTAGAAGTTAAACGTATTACTCCCAATGCGCCTATTGAG
GATGAATTTAGCGCCCAACGGTCTAATAAAGTTGGTACGATTGCTATGTATAAGCTAACAGATGA
AGCCAATAGTGCCAGAAATCGATGGGTTTTTAATGTTGGTGACAATCCTGATTTTGATACCCGTG
GTGGTGGTTTGACTGTTTTTGGTCAAGTTTTATCCCCAGAAGATTTGAATACCTTAAATGCGATC
GCATCTTTACCCATTACCAATACTTCTTTGCCGGTAATTAATCCAGTCACCTCTTCTCAATTTTCA
ACTCTTTTTTCTCGCCTACCCGTTAATGATAATACTATTGCCAACGATTTTAATTTTCCCTATCCC
TCTTATCCATTCACCGAAGATAATCAATTTGTCCGCTTTGAAAATATTACCATTGACAATGTACT
CGAATTCACCTTCACTGTAGAAAGTAATACTGATCCTGATGTTGTTAGTGCTAGTTTTGATAATC
AAGGAAACCTCATTTTAGATTACGGTAGCTCTTTAAAAACTCCCCTCTATCGCTTCCAAAATCAA
GATTTACCCGGTACATATTTATTTGTCGGTGAGCAAGAAAGACAAAGTATTCTCAGTAATTTCCG
CAATTTTAAGGAAGAGGGATTAGCCTTCAAAACCGCTAGTCAAGCCAATGGAGATACCATTAAGA
CAGGAGAAACGGATATTACCATCAAAGCCACCAATCTATTGGGAGAATCCGCCAGACAGAGTTTT
AAAGTCAGTGTGACAGGAGATGTACCAGAAAATGAAAATCAAGATCAATTAATTCGTTTCAATC
GTTTTCAAAATAGGGATATACCCGGTACTTACCTTTATGCGGCAGAGGAAGAAAGTCGCAGTATT
CGTCAAAACTATACCAACTTTATCGAAGAAGGTATCGCTTTTTATACTTATGGAGCAGACGCTAA
TTTAGGACAAGATATTTACCGTTTTCAAAATACCAGTCAACCCGGTACATATTTATTTGTAGGAG
AAGAAGAGAAAAATAGTATTCTCGCCAACTATCCTCAATTTGTTTTAGAAGGAGTTGCTTTTGAA
GTAGCAATTTAGGTGCAAAATTATTGTATGATAATAAGTAGTTAATTTATGTTATCACAAGTTA
TCATACAAGACGCCAATGACGATTAACATTACTCCTATTAACAATCAATCTTTTAATCTTGAAAC
CAGTAGCACTACCATTAATTTATTGAATCATTTTGATGATCCTTTAACCACTGGAAAAGTAGCTA
ATTTTAATTTAGGGGATAATTCTTTAGGTAGTGGTGAAATTAATATTGTTTTATTTGACCAAACA
GGAGATGGGGCGCCCATTGCTGTCAATAATTTTGCTAGTTACGTTGATGGTGGTGACTATAACAA
TTCCATCATTGATACTAACAATTCCATCATTGATACTTCTGTGAAATCTAAATCTAAGTATCTTT
TAGGGGGGGCTTATACTGTCGAGAATTTAGAGGTGAAGCGTATTACCCCTAAAGCACCTATTGAA
AATGAGTTTAGTGCCCAACGGTCTAATAAAGCTGGTACGATTGCTATGTATAACCTAACAAATGA

CCCTAATAGTGCCAGAAATCGATGGGTTTTTAATGTTGGTGACAATCCTGATTTTGATACGTTTA
ATGGTGGTTTGACTGTTTTTGGTCAAGTTTTATCTCCAGAAGATTTGAATACATTAAATGCGATC
GCATCCCTGCCCACTATTGTTACTCAGTTACAAGTAAGATCCTCCAGTAATAATAGTAATTCAGT
ATTATCAACGCCCTTCCGTAATCTACCTGTAAATGATAGTAATGCAAACGATTTTAATTTTCCCT
ATTCCTCTTATCCATTCACCGAAGATAATCAATTTGTCCGCTTTGAAAATATTACCATTGACAAT
GTACTCGAATTCACCTTTACTGTAGAAAGTAATACCAATCCTAACGTTGTTAGTCCTAGTTTTGA
TAATCAAGGAAACCTCATTTTAGATTACGGTAGCTCTTTAAAAACTCCCCTCTATCGCTTCCAAA
ACCAAGATTTACCCGGCACATATTTATTTGTGGGAGAAGAAGAAAGAAAGAATATTCTCCGTAAT
TTCCGCAATTTTAAGGAAGAGGGATTAGCCTTCAAAAGCGCTAGTCAAGCCAATGGAGATACCAT
TAAGACAGGAGAAACGGATATTACCATCAAAGCCACCAATCTATTGGGAGAATCAGCCCAACAGA
GTTTTAATGTCAGTGTGACAGGAGATGTACCAGAAAATGAAAATCAAGATCAATTAATTCGTTTC
AATCGTTTTCAAAATAGGGATATACCCAGTACTTACCTTTATGCGTCAGAGGAAGAAAGTCGCAG
TATTCGTCAAAACTATACCAACTTTATCGAAGAAGGTATCGCTTTTTATACTTATGGAGCAGACG
CTAATTTAGGACAAGATATTTACCGTTTTCAAAATACTAGTCAACCAGGTACATATTTATTTGTA
GGAGAAGAAGAGAAAAATAGTATTCTTGCTAACTATCCTCAATTTGTTTTAGAAGGGGTTGCTTT
TGAAGTCGCTGTTTAAACTTATCTAAACTAACTGACGTTACGCACTTATTGATGTGTTAAGTTAA
AGGAGGTTTCAGGTTTCAGGTTTCAGGTTTCAGGAAATAATAAAAATGATTGAACTAAAAAGTT
ATCGAGATTTAACGGTTTGGCAAAAATCAATGGATTTGGTAGTAATATGTTATTAATTAACTTCT
CAATTTCCCAAAACAGAAATTTATGGTTTAAGTAGTCAAATACAAAGAGCCGCAGTTTCAATTCC
TGCCAACATCGTAGAAGGAAAAGGGAGAAATCATTTGGGTGATTATATTCGTCATCTTTCTATTG
CAAATGGCTCACTCAAAGAATTAGAAACTCATTTGATGATTGTAGGACGATTAGGCTATCTTAAA
GAGCAAGAATTAAAAGTTACTTTAAACAAATGTGAGGAAATTGGCAGAATGTTACATAGTTTAA
TCGAAAAACTAAGTCAAAATAAAAAAGGGTGAATTTTCTGACTTTAAACCTGACACCTGATACC
TGACACCTGATACCTCAAAACAACAACTAATTATCTTTTTGCGTAACATCAGTAAACTAACTAAA
ATCTTAAAGTCACATCTTTAACTTTTTCAATAATTTTTAAGGGGTTAAAGGGCTTTGTTAAATAA
GCATTTGCCCCTAAATCCATGGCTTTTTGTCGGTGTAAATCATTGTCACGGGATGTAAGTATAAT
CACAGGTAAATTTTTCCATGCCTCCGATGCTCGAATTAACTGTAATACTTTGTAACCATCAAATC
CGGGCATTTCTAAATCGCAAATAGCCAAATCAAAGTGCTCTGATGTACTTTGTAAGGTATTCCAT
GCTTCCTTACCATCTCGACATTGAGTAACGTTATAACCCACTTGAGTTAAAGTACGATTCAGGGT
TCGTCTGACAGCCACAGAGTCATCAATAATCATAATTGATAATTCATCCGTCTTAACCGTTAGAG
TCTCTTTCTCTATTTCCTTTAACTGTCGTTTGCTTTCTCTTTGAATGAGTAGGGATTTAAAGTAA
TCAGGAACGAAAATAGGAACAACAGTTCCACTACCTAAGATTGTACAACCTCCTAAATAAGGGGG
AATTGCCACAGTATCATCCAAACTTTTACTGACTAAGGGTTTTTCATCCACTACTGCATCCACTGC
CATAACGACTTTTTCTCCCCCAATTTGCAATAGTAAACCTGCATAGGGTTGTCCCATGTCAACGG
ATAAATTAGGTGCATTGTAGGGTAATATTTGTGCGAGATTATAAAAAGAAATTCTTTCATCTTCC
CATAATATTTGATTATCTTGAATTTCGTGGTCAGAAAGACGAATTACTCGCAAGACATTTTCGGA
AGGAATCGCCACTGTATGAGAACCAATTTTAACTAGGAGCAAAGAAATAATATTCAATGCCATAG
GAATTTTTAGCTGAAAGATTGTGCCTTTTCCTATTTCTGTCTTTACCTCAATAGTACCTCTTAAT
TTTTCAATTTCCGCTTTAACAATATCCATACCCATCCCTCTCCCCGACAAGTCACTAACTTGAGAG
GCAGTGGAAAATCCGGGGGAAAAAATAGTTCCTAAAATTTCCTCTCTAGGGAGTTGATCAAATTC
TTTATTTTGAGGAAATAAGCCAAGCATTTTTGCTTTTTCATAGACTTTTTTAATATCTATTCCTT
GCCCATCATCAGCAATAGTTAAAATCACATTATTTCCTTCTAGTTTTGCACTTAATTTAATTTGT
GCGGGGTTCGATTTCCCTTTTTTTTCTCTTATATCTGGCATTTCTATACCATGATCAAAAGCATT

TCTGATAATATGATTAAAGGGTGTTCTGAGAGTTTCAATAATATTTTGGTCAATTAAAACTTGTT
CTCCTTCTATGATTAATTGAACAGATTTATGGTAATCTTTATTTAATTTTTCGAGAGGTTTAACA
AAACTTCCTGCTAAGTTAATAAAAGGAACTAAGCGCACTTCTCTTAATTCTTGATCAAGGGTATC
AAGACATTTTCTCATATCAATCAAAGTCTCTTGAAATTCCCTATTAACTAAGTCAAAATCTTCTC
TTAATTCTTGGATTTGAATAATCAATTCTGTTAAACTTTGTAGGGTTGTGTGAATTTGGGTATAT
TCATCAAATTCTAGGGTGTCAAACTCTATTTTCTCTTCATTTTTCGCTTTTGAATTATGACCATT
ATGATTAAAATTTTCTTTTTCTCTAACGCTGAATTGATCATACAGATATTCCAGTTGTTCTTTTA
AAGGAGATAATAGCCTTGTCCTTTTTTTCAGACTTAAACTCCCTCTTTTTATTTGATTTTCATAG
AGGCTTAATTGTTCGTAAAAAACTAATAGTTGTCCTGTTAAATCACCCATTTTATTAACCTTATT
AATGGGGATTCTCAAATTACGATTATTACTAGGTAAAGGCGGCTTTTCTAAATTAAATTTAGGTT
TATTTTCTGCTATTTTTTGCGGCGTTACTACTGCTGGTTGTTGTTCTATAATTAGTTGTTCAATT
TTTTCTGGAGCATCCCCTAATAATAATTTTTGAAAGTAGTCAGAATATTGAGGAAGTTGAGGATT
AGTTTCGACAAGATATAGTTGTTTATTCTTTTCTATAAGTGCGATCGCTTCTTTGGTAAAATTAA
TAATATCATCCTCAGAAGCTGATTTTAACTGTTTAATTAATTGACTAATTTCTACCAACCAATGA
CAATTAAGAGCTTGACCTAATAATTCACATTCTTCTAATAGAGTATCAATACTATTTTTGACATT
TTTAGGGGTAGCTAAATCCTCACTTTCAATAATTTCTTGCACTCTTTGAATGCAATCAGATAAAT
CTGACTCTAGGGCAGTTTTGATAAAACTATCATCAGCAAACCCGATTTCCGCCAGTTCATTACTG
GGGTTTAAACCTTTTAAAAACTCATCTAATGCAATAATTAATTCATCAGCAGAATGGGTATCTAA
TAAGCAAGAATTTTTCTCTAATTCCACTAAATTTTCTAATTCTTCAATACCCAAAACTAATAACC
CTAAAGTTGTATCTAAATCTTCCACTCTATCTTGTTGTAGGGCTTCAAATAAATCCTCTATTCGA
TGAGCCAATTTACTAACTAAAGGCATTTCTGCCATTCCTGCACCCCCTTTAATGGAGTGGGCGGC
ACGACCTAATTCTTTATAAACATCATGTAAATTCTGATTATTTTTAGACTCATTTAAAATCTGTT
TTAAATCTTCAATGCCCCTCGTTAAAATCTCTAAAAATTCGGGGGCATCTTCTTCTAAAAAACAA
TTTCTGGCTTCAAGGGCGATCGCAGCTAAACTATTGGCATCTAACATGGCTTAATGGTGAATAAT
GAGTAATGATAGGGGAGTAGGGAGAATTAAAAATTAAGAATTAAGAATTAAAAAATTTCCTAAC
AACTGACACCTGATACCTAATCATTGGTTCATTGAAGAATTAGTTGACTTGGAAACGAGATGCAG
ACTGTTGCATTTCCATCGCTACTTTTACCAACTCTTGTAAAGATTCTGATACTTGATTGGCTTCTT
GAGCGGTTTCTGTTGTCACCGTTGCTACTTCTCGCATTTTCTCGTTTACCTTCTGAGAGGTGTTTT
TCTGCTCTGTAATACTATTGGCGATGGATTCTAATAGGTTGTCAATATCATTACTGATTTGAGCT
AATTTTTGCAGAGTTTCTTTGGTATCTTTGACTAATTTTGTCCCTGTAACCACCTGAGTGGTGCT
TTCTTCCATCATCCTCATCATTTCGCTAGTTTCCTCTTGAATCGTTACTACTAATTGCTCAATTTC
TTGGGTTGAAAATGTCACCTGTTCTGCTAGTCTGCGTACCTCATCTGCTACCACTCGGAAGCCCTG
ACCGTTTTCTCCCGCCCTTGCCGCCTCAATGGATGCGTTAAAAGCGAGAAGGTTGGTTTTTTCAG
AAATACTAGAAATGATACTCACAATTTTAGAAATTTCTTGGGATGAGTCGGCTAAACGTTTGGCT
TTTTTAGCGGTGTCGGCTACGGTTTGACGAATTTGATAAATACTGTTTACAGTTTCATCGATGAT
TTTTTCCCCTTCTTGGGCAGTGAGACGAGATTCTCTGGAGATAGAAGCGGCGTTTTGGGTTGATT
CGCTAATTTTCTCACTTTCTTGCGCAATTTCCGCAATAGAAGCCCCAATGGCTTGAATGGACATA
TCTTGTTTACTGGAGTTTTGGGCTAAATGACTGACTGACTGCCCATTGGCTAAAGCTGTTTCATG
AACTTGATTAGCAGAGGTGACAACTTGTTGTACCAATCGCTGAAGATTATTTAAGGTGGAGTTAA
ACGCATCAGCAATAGAGCCTACTTCCCCTGATACTACTTCCGCTTGTACCGTTAAATTACCCTTAC
TGGCTTCTTCGATGTCGAGGAGGAGGTTAATTACGTTTTTCTGGAGGTTTTCCCGTTGTTGTTTC
TGGAATTGGGCTTCTTTTTCCCGTTGTTGGGCAATGTCTTCAATGGCTTCTGTATAGTCTTCAAT
TCTTTCTGCCATTTCGTTAAAGGTTTTTGCTAATTGTCCCACTTCATCCTCGGAGGTAATTTCTGC

TCTGACATTGCGATCGCCTTTTCCGATTCTTTGGGCAGATTTTTGTAATCTTTGAATGGGTTTAG
TTAAAGCACGTCCAAAGATAATCGCTAAAATAATTGCCACGATTAAGGAAGCAACTCCAGCTAAA
ATTTGATAGGTTAAACTTTGACTGAGTAACCTTTGTAACTGTACTTCAGGAGTACCTCGCACCAC
AAAAGCAATCTCTTCCCCTCTGAAGTTGGGCAAGGCTTTTACCGCCACGGTAAACCATTGATTTT
CAATCAAGAGGCGATCGAAAATATTGCCTCCTACCCCTGTTTCGGCGGTTTGAATTAAGTTTAAA
TCGGGTAAAGGAATGTTATTTTGAAACTCATCGCTTCCTGCTTCTTGTAAACCAGATGTGACTAA
CTCAAATCTTCCGTTATTAAACATATAGATTGCCCCGTATCCCCCCCCAACTGCGTCTAAAGTTCG
TTTTAAAAAGAGGGTTTTACCATTGACAATTTCCCCCGCCATTAAGATAGCAATAACTTGCTGAG
ATTGAGGATCAGTAACAGGAGTAAAGCTAAAGTTAACTAAGACATCTTGATCAGAAATACTACT
AGGTAAAGGAGGTTTTTCTTTTTCAATTTCTGCCCAAGGAATAATCGCATTAGTTTGCATTCTTC
TCGGAAATTGTACTACTTCAGAAACAAGATTTTTGGGGTCAAAAATTTCTCCTCTACGGTCATCA
TTAGCGTTGACAATAATTCGTTTATCTAAGCCAATTAAGGTAGCATATTCAATTTGTCTGGCATT
AGTTTCGTTGCGTAAAATTTGCCTAACAGTATCTCTTAAATTACTAGGAACTTGTCCATTTTGTT
GATAATTTCTAGCGGCTTCAATAATAGCAATATTATCACTTTGTCCCCTGAATCCTGACTCCATC
TCACTAATGCGAGAATTATAATTAATCACTGACACGGCTAACTCCGCTATGGACTGGTTTTCTAG
CTGACTTCGCCCTAAAACTACGGTAATTAATCGCCCGGCTAACACCACCCCAAAAACGGAAATAG
AACTAGAAATAAATAGAGCAATAAATTGTTTATAAGCAATGGGTAAATTAACTAATCTTTGCCA
TAAATTAACTTTCTTTTTCTTAACTTTTTTCTCTTTTTTGTCCTTATCTAAAACAATATCTTCTTT
TGTTAATTGTTCTTTTTGAATCTCCGCGATCGCAACCTTAGCACTTTCGGCAAATACTTCCTCGTT
GTCTTGTTCAATAATTTGTTCATATACTGCTACTGCTTCGTTGTAATATTTTTCTTGTTGAAACG
CAGTGGCAAGATTCAGTTTTGCGATTAAATCTTGAGGATTTTCCTGAACCTTTTTTTGAAGATTA
ATAATATTATCAGAAAATTCTTTGGTTTTGGTTTTTTGTTCCTGTAAATTTGCCATGAGAGTAAC
TCCGTATAAAGTAAAATATTGATAATTAAGTAAAACTTTAAAGATAAAAAATAATAACTAATTA
CTATTTAAGTAAGTTTGAATATTATTCATATTTAAAATTGGTAATAACTCTGTCTCATCCTGAAT
AAAACTGTCCACATATTCACTTAAAAGAGGAATAGGTAAACTTTGCCCTAAATTATCTTGAGTAA
TTTTATCCTGCAAAAAGTCTTTAATTTCTCCTAATTTCTTGACGATCAAACCCACATTATTTTTA
CCTAATTTAGTAACTAAAATAGTACTAGAACTAAGACGATTAGAAAAAGAAGGAATATTTTGCA
ATAGTAAAGATAAATCTAATAACCACAGTAAAATACCCCTTTGACTAATTAACCCCAAAAGACTA
TTTTTCACCCCCGGAATGGGACAAATATCAGCATAATTAACCGTTATCACCTCCTGTATTTGTTC
TAGGGGAATAGCGATTTTTTTTTGATTGGTCAATTCGACAATAAAATAATCCATAGAAGAATTA
AGAATTAAAAATTAAGAATTAAAAAAAGGAGTCAGGTAATTTCTCCCCACTCATTACTCATTACT
CATTACTCATTACTCATTACTTAATCAAAGATAATTCTTGACAGTATTCAACAAATCAGTGGGAC
TATAGGGCTTTGTAATATAAGCAGTACCTCCTTGTCTTAATGCCCAAAATTTATCAAAATCTTCG
TCTTTGGTAGTTACGAAAATAATGGGAACGGTTTTATAATTCATTTCCTTACGAATATGACGACA
AACATCTAAACCGCTCATATCCGGCATAACAATATCCATCAAAATTAAATCTGGTTGTCCATTAT
TGGTTAACCATTTCAAAGCGGATTCTCCATTGTCGGCTAACTCTACTTCCACGTCCAAAGTTTGT
AATAAACCATTAACTAGGCGTTGTTGGGATTTAGAATCATCGACTACTAAAATTTTTTTCATACT
TTTGAAAATTATTTTTAAAATTGATTAAACAGCTTTTTGAATGTTATCTTGTATTGATTGTACTA
ATTCTTGTGGATTAAAGGGCTTAGAAATGTATCCTACAGCACCAACCATTTTTGCTTTAACCCTA
TCTAAAACTCCATCTCTACCTGTTAACATCAAGATAGGAATATCTTTGAGTAAAGCTGATTGACG
TAGCATATAGGCTAATTTATAGCCGTCAATATCAGGCATATTGATGTCCATAAGAATTAGATCCG
GTTTTTGTCGGACAAAGCTACTCATAGCTTTAGCAGGTTCGGTAATGCTTACCACATCAAATCCT
CCTGCAATTAAAGTCATTTTGACTATCCTCTGAATAGCTTGACTGTCATCAATACAAGCAATAAG

GGGTTTGTCTTCAGTCTCAGTGGGTTCTTGAAAAGGACTGATGGTGATAATATCTGATTTGATTA
GGGGATGTAATAGTAAGGCTAAATCTAAAACAGAATGACGGGAATAAGCGGCAATATCATACAA
GGTACGACGATCATCTAAGTATGGTTTTAATTGTTCAACTTCTTGATATTCCGCCATTCTATTAC
CAAAATGGGTTTTTAAAATATCCCAATTATTCACTCGAGGACGAACAAAAGGAGAAGAAATATG
TTCTCTGAGAGTAACCCATGCCCTTACTTTCGATTTTACAGGTTCAATCAGAGATTTTAAACTAA
GGTTGAGAATTAAAGGGTCTAAACCAACAATTTTTTCAAAGGAAATCGTAGCACGAGGTAAAGC
TAAACAAAGTGCGATCGCTTCTTGAGTCATATAACTAAGGATGGTGCGAATCTTCTGCATATCTA
AATCACCATCACGCCAATGCTGACAAATAAAACCATAATCATCTTCCAATTCTTGAGGTATATAA
AAACCATGATCAGGATAACACTGAGATAAAAGGTATCTAATTCTTTCTCTTTTACCATTAACACT
ATGGGCATAGTGAATCTTTCCCTCACCTAGATAAATCAGCCACCTTACCGATTCATCATTGGGAT
CTTGAATCATTAATTTACCCGAAACTTTTTTTGCTAATAATCCCTGTAAGGCTTTATAGGGAATG
GCAACTATTTCCTTAACATTAGGAGAAGTAACAGTCTTTCTGGTCATGGTCGCAATCATAATATT
ACAGTTTAATTCAATTAAAAATTACTATCTTTAAATACAGTCTTTATTCCCACTTTGAATAAGAT
TTTTACTCAGAAAAAAACCTTTTTATTTTCACCATTTACCCTTAACTTTGTCTTAACCTAAGAGA
GAATAAAGTTATAGATGATTCCTTAATTAAAAGCATAACAAAAGATGTTTTTTTTGAGATAAAG
ATGTATTCCAGAACACAATAAGTAAAAGAAAAAGTATATTCTTTAACCAAAAAGTGATCTTCAA
AAAATAATAAAAATTGTGGGTTAGTTAACAAACAAGAAAAAACAAATAACTCAGTAAAAATACT
GAATTTTTATAAACAATTAATAATGTTAGAAGATAGAGAGTGGAGAAAAACTTATAGAGGTAGA
TTTTTGACAACAAAATCATTGAATTCTGGTTTATTTTTATCTTAAAGATCCTGCAAAATGGACTG
GTGAAGGTAGGGAATAGGGAAAACCTCTCTGTGTCCCTCCTTTTAAGGGGAGAAGGGGAACAATA
ATTGAGAAAAACTTATCAAAATTGATTTTAAAAATTTTGTTTTTCAGTATTACTGTACTGGTGCA
AGAACTCAGTTTATCCTATTTTCTGGGTCTTGGGACATCTTTTTATTGCTTAATAAGTATATAGT
GATTTATATTGTTAAAATAATTGTTGATCAGAATAAAAACATCATTTATGATTAATGATCTTGA
TTTTAGGAAAAAAATATGATACTCTTAGAGCAAAATTTTCGTCCTAATTAGCAAAGATTCGGTTAA
GATTACCTAAACAGTATTAATATTGTCCAAGATAACGGGCGTGAGGAGCAAATATCGATGAATTT
GAAAAAGTCTGCGTTGAAAACCCTAAAGGAAACCAGTCGCACGTTTTATATCCCCATTAGTCGTT
TACCTGATAGTTTGCAAGATGCAGTGACTTCTGCTTATTTATGTATGAGGGCGATTGATGAGATT
GAAGATCATCCCCATATTGAGAATAATATAAAGAGGGAAATTCTTACTCAAATTAGTGCTAGTTT
GCAGTCTATTAGTAATGATACTGCGGTAGAAGATATTACTGCTTATATTGAACCTTACAAACATT
TATTGCCTGAAGTTAGTTATCGTTTTGCTGAATGGGCAACTCTTGCTCCTGTCTCTATTGCAGGT
CGAATTTGGGAGGCAACGGCGGCAATGAGCGATCGCATGGCTTATTGGGCTGACAAAAATTGGGT
TATAAAAACTAAGGCGGATTTAGATCAATATACTTTCAGTGTGGCGGGTTCTGTGGGTTTACTAC
TTTCTGATTTATGGGCATGGTACGACAACACAAAAACAAATAGAGAAGAAGCGATCGCATTTGGG
AGAGGTTTACAGGCAGTCAATATTTTACGTAATCATCAAGAAGACAAATTAAGGGGCGTAAGTT
TTTTCCCCGAAGGATGGCAGTTAGAAGATATGCACCAATATGCCCGTTATAACCTTACCTTAGCA
GATTCTTACACTAAATCTTTACCTAAAGGTCCTGCTTTACAATTTTGTCAAATTCCCTTAACTTT
AGCCCATGCAACCCTAGATGTTTTAATTTTAGGCAAACCAAAACTTACTCGTAATGATGTTATGG
CTTTAGTGGCACAGGTGTGCGGTTAATGTTAAGAATTGGCTATAATTGAGCGAGAATTTTGTAGA
AATTAGAACCCTTAGAAGAATAATTGATAACTGAGAATAAAGAGGGGTTTAAGCCCCTTCTCAAA
GGCTTAATTACATAAGGATTTTTGTTGAATTCGTCATTATTTAGTGTGAGGAGGAAGACCATTGA
ATTTTTGGGTAGAAGAAAGATTACAATGTTTACAACAGGCTTTTAATTCTGAATATTGTCAAGCA
GTTGAGCAAGTCAGTCATTTATTAGCTAATCGTTTTCAAGCAGGACATACTCTGTTAATTTGTGG
TAACGGCGGTTCAGCCGCAGACGCTCAACATATTGCCGCCGAATTTGTCGGAAGATTTCAAATAG

ATCGACCCGGATTACCTGCGATCGCATTAGGCACAAACCCCGCAACTCTTACAGCATGGGGCAAT
GATCACGAATTTGAAACTATTTTCGCCCGTCAAGTACAATCCTTTGGCAAAGCAGGGGATTTATT
ATGGGTATTGTCCACATCGGGGCGATCGCGCAATATTCTTCATGCTTTGAAAGTAGCTAAAGAAA
AAGGTTTAATTACCATTGGTATGGCAGGAAACAATGGCGGAATGATGAGTAGCTTAATTGATTA
TCCCCTTTTTGTGGCAGAAAAAAATACTCCTTATGTTCAAGAAATACATTTAATGACATATCATC
GTATCTGCGAACAGGTTGAAGCCATGTTGTTTGCGGAAAACATCGTCGAAAAGAAAATAGCAGTG
TAGGACATGACCTGAGTTTGGCGTTCGGAGTTAAAATTAATAGATATTTAACCTCAACTCAGGTT
AAGCAAATGATGAATTCTTCTTTGATAAATTGGCAAAAATTAGCAGAAATTAAAGAGCTAAAAG
ATTACTTTAATGATGATTTTCAAGAATTTAAAAATAGCATCTGTCATTACTTAAAAATAATGGCA
AAAATGACCTCAACCAGTATTCAAGAAATCGCTATTATTAGGGCTTTAGAAGTTACTAACGGCTG
TACCCAACACTCTTACCGCAGAAATGATTCAGACTGCCTATCTGTGGAGCAAACCAGAGAATGTA
TGAAGTTATCAATTTCCTCTATTCGTAATCAGGAAATCATCTTAAAAAATGGAGATGTCTTGGAA
TTTTCTCCTGAAACAAAAGAATTGATGACTCATATTAAAACCTTATATATGGATGCTTTTAAAAA
TAATATTGCGTCTCAAGAAAAAGAATTTTATGCCTTTTCAACAGCTCAATTTTTAGCTTGTGGAA
AAGAAAAAAATAGATTATGGTTTTCAAGTTGTTAAGGATAATTATCAAGATTTATTTACTGATAC
ATTTATCAATAAAGGGATTAAATATATAGAAAAATATTTAGAAGCAATTAAAAATTAAATGAGC
TCTATCACTTCTCGTCGTGTAAATTATTAGTTAGGGCAGGCTGAAAGGCAAGAGGCAATGGGCAA
TAGGGGATTATTAAATAATAATTTATAAACTTTTAGTTTTTATTTTACTATAAACACTATTCAAC
AAAGATTATGGAAGTTATTTTCTATTAATTTATCATAAGCATTGTAGAAGAAACATTAAATGTT
AGTTCTTTTAAAGAGAAAAGGAATTTATTTTTTGCCACCATAACAAACTGTTTCTAACTAACCTC
AGCTCGGAGTTATTAATTATCAACTATTTATCTTTGCCTTTCCCCCTCGAGAGCAGGTTTTTTTC
ATTCTCAAAAATGTCAATGTTGGAAACTAACAAATAGTAACAGATACAGGAGTTTTTAAGTATT
AATTAATCAATTATTAAGAAAAAACGCTCCCCTCTAGTGTAGGAGAGGGGGTGGGGGTGAGGGC
AAGATAATTTTGCCCTGACTTTGAAAAAACCCTGGGGATAAAGGGGGGTTTGCCCATTGCCCTTT
TCGCCATTACTCATTGCTGTTAACTTACCTCATAATTATAAGAAATGCTATAAATTTAAAAAAAT
GACTAATAACTGGTTCTCAGGCACTGTAAACACCGTCATGGGTGTAAAAAAAGAAAGCCAAAATA
ATAATCAAGAAAACATCCCTTTAAACGGAAAAAAAATTGTTAGCTATGTTGCTGGAACACTGGCT
CAAATTGGCTTAATGACCTTGACTCTATGGCTAGTAAATAAAAGTTTTGAGAGTTTAATTAAATA
CAATTTGGATGAAAAAATTAATATATCAATAGTATGTCTTTTTCTCACTTTTTTTACTATTCGTT
CCCGATTTTTCTCCCCTCTTGATAATACTCGATCGCGCCGTCAATATGATCAAGTTATTCGTCCAA
AATGGGCGCCTCCTCCATTCGTATTTCCCATAGTGTGGATGAGTATTGGGGTATTAAGAATCGTC
TCATCTTATCTAGTGTGGCAAAAATTAGACCAAAATTTTCTTGCTATTCCCTTAATTGTCTTTGC
TATTCATTTAGCCTTGGGAGATACATGGAATACTATTTTCACCGTTGAAGGTAGATACGCCTTAG
CTGTACCTGTTGTAATATTAGGTCCATTACTATCATCTATTATTCTTGCGGTGGTTTATGGGCAA
ATAATACCTTTAGCAGGGTGGCTAATATTTCCTTCTTGCGTTTGGTTAGTTGTTGCCTCCGCCTT
AGTAACCCGTATCTGGCAGTTAAATAGGGGTTAGAAAGAGGTGTTAGGTTTCAGGGTGTTGGGG
GATGAAGGGGAAACTAATCCTTAAATTGCTAATTGTTAATTGCTCTTTTCTTGCATTGAATAACC
ACGAAAATAAAGTTTAATTGATAAAAATTATCAGATTTTAGGTCATTTAATATAAACTGAAGAA
TAATGACTTAACGCCCGAAAAAACTGGGATTTAATTATGGCTCTTAATCTTGCATCCAATCGCAC
CGCTACATCCGTTGACCCTTCTACTGGTATTAATCACATTGTTTGGGCAGATAATGAGTTTATAT
ATCATGCTACCTATGATGAAAATTCAGGTACTTGGGAAAATGCAAATGCGATCGCATCTTTAGGT
AATCAAACTATTAGTAATCTTAACTTAGTCATTAATGATGGTTTAATTAGTGAAGGGCAAAGTC
AATTGCCCGGGTTAGTGGTAGTATGGCAAGAGGGACAAAAGAACGACAGCGAATTATACTATAC

AGCCGCCCAATATGATGAGGTTCAAAATTTACAATGGTTAGACACTCCCCAACCTTTAACTGCTG
ATGAAGTAGCAGATTTAGAGCCAAAAGTTATAGTAAAAGATAATGAGATAATAGTAGTTAGTAC
GAAGGTTGATATTAATAATGTTGAAAATTTATCCATAAAAGAAGATACAGACGTTTATTTTCAA
CAATTCACCGTCGATAGCAGTTTATTCACATCTCCACAAAATGTAAGTGTTACCAACGCCGTTTA
CTCTCCCCGTCTTGTTAATAATGGCGTAATTAGTACAGGAGTCGTTAGAAATGCCTCTTATAATC
CATTCACCGAGAACGAAATTTTAGCTTTTAATGAAACTATTTTAGCCTCTGAGGATAGCTCATTT
GAAGGTTTACTTGGCTCGTGGAGTGCCCAAATTTATTTTTCTGGTAGCTTATTAGACTCATGGAA
ATTAATTGATAGTACTCCTAATGATAGCTTTTTGCGATCGATAATCAAACCCTTTTTCGAGATTT
TTGAAATTGAAGGAGTGTTAAAAAGTGGGGTATCTCCAGAGTGGCTTGGTAATCAGGAGCGATC
ATTATTTGTGGAAACTGTAGCTAATTTAAAGGTAAAAACACCCCTTGAATTAACTGCCCCTTGGA
AAGTCAAAAATGATGCAGGAGGAGAAGAAGATTCAAAATCTCCTATTAGTATTGGTGCTTCCATC
GATTCTAAATACAGTTTTGAGCCTAATTCACCCTTCGATTTAACTCAAATATTAGATAAAATTAC
CGTAAGTGCATCCTTAAAGTTTCCCGTTATTTCCGCCGCCGAAACCGCAGGATTTTTCACCCTTGA
TTTAACTGGTAGCATTGGTGCAGACTGGGAAGTTACGGGGACTCCTGATCCCATTGGGGGAGATT
ATTTCCCTGATAATTTTGGGGCGGCTTTGGGGGTTTTAGGTGCTGGTGCTTTAGCTCAAGGTACT
ATCTTATTATTAACTTTAGATAACAGTAGTAATCTATCTAGTGCCATCCTTGCAGATTTGGCGAT
TAGTTTAGGAGAAACCTTTGGTGCTTATATCAATGGTATTGCAGAAGGGCTAGACTTTAGTAGTA
CGATCGGATTTCCCATTATTAGTGCAGGGTTGAGTGGAGAAATCAAAATTCCTGTTATCCCTATC
TTTAGTGCAGGAGTCGATATAGGTACAACTGCCGCCGTTAACTGGGGTTTAGGTGATACAGATAC
AACTTTTACCCTTAACTTTCCCATTGATTTTAATGTCAAATTAGGTCCTCTCGGCTTTGGCTTAG
ACTTTTATCCCGGTTGGTCATGGGCTATTTTTGACAATAACAATACTTCAAATGCTGTTACTGAG
AATCTGGTTTTAGCTAGTGAGTCTCAAGCTCAAGTGATAGGCTCATTATTATATGTGGATTTGGG
GGCAACTTTTACAGATTTACCCAATCCTAATGATTTTACTATCACTGTCACGGATATAAGTGGAA
ATGAAGAAACAATCGAAGTTTTTAACGTTTTACAAGGAAATTCTGATACTTCCGTCATCTTGCAG
TTAAAAAATGTTATCCCTCCTTCTGCTAATGCTAATTATTTAACCTCAGACAATCCCGACACCAC
AGAAAACCTCATTGTTTTAAGTTTTACGAATAATGAAGGTTTAGTGGATACTAACGGGAATCTTA
TCCCCAATATCAACAATTTATCGGTAGAAAACAATACCCCTGATACTTTTAACTTTACCTTTAAC
CCCACTACGGGCAATAGTCAAAATTACAGTAATTCTTTAGCCATTCAAACTCAATCAGCATTAGA
AAATATTGAGGCGGATTTAAGTCAAGATAGTTTTCTCAATCTTAGTTTAACCAGTGGTGGTTCGG
TTTTGGCGACTTGGGTGGCGGATGTGCCTCCTATAAAGCCCATTGCAGGTTTTGTCAATGGCAGT
GTTATTACTCTCAATTTTGTGGATACTTTGGGTAATTCTGGGGGTAATGAACCAAATCTTAATCA
ATTTACCGTCACAGATGAAGATAATAATAGTTACACCGTTGAGAATATTAGTAACTTAGGTAAT
AGTATCACCTTAACCCTCGATCGCGCCGTAGCCCAAGATACCGCATTAACCATTGATTATCAAATC
GCTTCTACCACTAACAATAACGAAAGTCTTTATATCCTCAATAGCACCACTAACACTAAACTATG
GATTGATAATTTTGAAAGTCTTGCTTTAACCAATATTACTGGTACTAACAATGCTCCTATTTTAC
TAGGTATAGGTTCGATCGTCGAAAATATTAACAGTAATAAAATTACCCTTGTTTTTGACCAAAAT
TTGAGCGGAAATCCCATCAATAGTCAATTTGAGGTTATTAACAATGGAGTTACTTATGATATTAA
CCCAACTGTAACCATTAATAATAATACCGTTATTTTGACCGTTGAACCCCCTACAGGCTCGAATT
TGATTGGTATTGGGGATTTAGTGACAGTCAGTTACACGGGGAGTAATACTCAAACTCAGCAACCG
TTAACGGGTAGTGGTGGCGTGGTAGCGGATTTTAGTAACGCTCCTGTTATCACCTCTGATGATAC
TCTCACCACAGTTATTAAATATGGTTTCGGTCCTTCGGGAGATAATTTATTATCTAATATTACTA
GCATTCCCGGTTCTACTGGTTTAAATTTATATCCTGTTACCGCTTTAGATCAAGAGGGAATAGT
GTCATTGTTTGGGCAAATGTCGATAGCACAAATATTCCTGTTAATTTAACTCCGGGGGAATACTA

TACTCAAGAACAGTTTGAGAATATAACCGATATTTTAGCTACTTCTGATCTGTACTACTCTATTT
ATAATCCCAACAGTGGAGAATGGACTATTGCAGAAGCGATCGAAACTTTAGCAGGAACAGATAGT
AAAATAGCATTAGGGCAAGGGGTTGATGGCAATTTGATGGCAGTGTGGTTAAATACCAACAACG
GAGAAACTAATATTTATTGGTCAACCCTAACCTATAATAACGGAACACCTAACTGGAGTACCCCT
CAAATACTATCAGACAATGCCAAACCTGATCCTCTTACTGAATTAACTATAACCACCTTAAATGG
TCAACCTGCGGTATTTTGGACAGAAACTCAACCACCATCCTACTATGAGTTAACCACTTCTGAAC
AACCATTAATTTATTATCGTCTGGCAGAAACCGAGGGCATTGCCCTAGTTAATGAAGGAATTTTA
GGGGCTGGAGGTAATGGCACTTATAGCGGTGATGATGTGACTTTTAATCAACAAGGTGCATTAGA
AAACACTACCACTGGTAAAGGAGATTCTAATACTTCAGTCTGGTTTAAAGCAGGAAACAATCAAA
TTTCTAGTGCCACATCTGGGGTAATTCCCTTATCGGGTAACTCTTTCAGTCTTGAATTTTGGTTT
AAGCCTACCGCCGTTAATGAGAATCAAAGTGTTAATCTTATTGCCTTAGAAAATATCCTCAATTT
TTCTGCCCGTGTCAACTCTGGTGATGATTCATTCTTTACTCTCAATATCAATGGTGAGACTCTGC
AAACAAGAGACGGATTTCAAATCTCCCCTAGTGATTGGTCTTATATTGTTTTAACTTATGATGAA
ACTAATGGTTCGATCGAACTTTACCTTAACGGTGAATCTTTGCAAGTATATTCGACTAATACAGG
GGAAAATGTCGATACTCTGAATTTAGATATTCCTGATAACACTACCATAACCTTAGCTGATCCGA
ATAACTCTAATGGTATTTATCTTGACGAAGTAGCTTTTTATAACAAGGCACTCAGTTATAGTGAT
ACGCCAAGCAATTTTAATGATATTGAAAATTTAACAGGTTCAGATGTTGCCCAAATTCTACTCAA
TACTAACGACATTGGCAATAAATATAACTCTCAATATATCACTCCAACCCCATCGGGCGCGAAAA
GTTATTATGTCACCTATGACTCAGAATCAAGTATCTGGGAAAATCCTGAACAAATTGAGCCAGAA
TTTGAAGTTATCCCTACTCAATTATCCTCTGCTAATAAGCCTGAATGGGATATAGTTTCTGCCAC
TTCTGCTAACAATAACGGTAACATTACCCCCAATGGCACACCTGATATTTATTTACCTCTCAACCT
TACTAATCAAACTATCAACACGGTAATTAATTCGATCGAAGTTACTGCTCAAACAGGCTCAGGTA
CTACTTTAACATGGAGTATCGGCACAGGGGAAGGTAATCAATTAGGGGTTGTACAAGACAATAA
ATTACTTAATCCTTTAAATCCTGATGAGAGTTTTGCATATACTGTTCTCAATCCTACCCTTGATT
TTGATTTATTTGTCGATGCAGGTTCGATCGATCCTTCTACCTTATCTAACTTTCAATATTCCATCA
ATGGTGGTAATCCTGTCAACTTCTCTCCTGCGGATGTAACCAGTGAACCCACAAACGTTAACTCT
AATCAAGTGATGGGCATTGCAACTGTTACCGAAGCTAATGATTCATCTCTTACTTTAATTGATAG
TGGTTTTATTACTAATACCGATAATGTAGCCATAGGTTATGTCATTACTAACGGCGATTTAAACA
ATGACGGCAACACTGATGTTGTTATCGGCAATCGGGGTTATACGGATACAGAAGGCAATGTGTTA
GAAAATGGCACAATACAAATATTATTCGGTGGCGGTAGTGTATTAAGTAACAGTCAAACCAATCC
TTTAACAACTACTGATTTAAGTGGTAATCCTGACGGTGTTTTGATTAAAGGTATTGCAGATGGTG
GCGATGGTAACGGTGATTATTCCCTCAGTTTAGCGACTGGGGATGTTAATGGTGATGGTTTTGAT
GATTTGGTTATTGGTGCACCCAATGTGAATAATGGACAAGGTGCAGTTTATGTCATTTATGGTTC
AGCTAGTTTAGCAGGTAAAACTATTGATGTTACTAATTTAGGCTCTCAAGGTTATACTATCATAC
CGAATATCTCAGGTTCTATAGCCTTTGGTTATTCCGTAGCGGTGGGAAATTTCAACGGTGGAAGC
AGTGCAGATATTGCTATCGGTACACCGGGGGCTAATAATGGTAACGGAGAGGTTTATGTTGCCTA
TGATGGAAATAGTAACTATTCCACTGTCTATAGTTCACAAATTGAGGGAGAATTAGCAGGTTATG
CTCTTGGTGTGTCTGCCTATAAAGGAACGTCTAGCCCTAGTACTTTTACTAAAAATACCTCCGAT
GATCTTATTATAGGTGCGATCGGGTATAACGGAAATGTCAATAATGATTGGGCAGGGGCAGATG
ATCTTCCTGATGGTACAAATGACCTAGATAGTTATCCTAGTTCTTCCTCCGTGCAGTTGGGTGCA
GTGTATGTATTTCGGAGTAATGGCACAACGAGCAATACTTTTTCCTCAACTCCTTATGTTAGTTA
CACGGGAGCAAATATAGCTAACAGTAACGGTACAGCTAACAATATTTTAGCAGGAAGTGCTTTAG
CTAGTGCTGATTTAGATGGTGATGGTACTCAAGATTTGGCGATCGCATCTGAGGGGGCAAACGAT

AATAATGGAGGAGTATATGTTATCAAAGGAGGGGTTTCGAGTAACAGTAATTATCAAAACCTTG
CTACCGTTGCAAATCTGAATATTGTGGGAGGCATTGCCAAAAGTGAAACAGGGGCGGTGATTACC
TCCGCAGGAGATTTAAACCAAGATGAGTACGAAGATTTTTTGATTACCGCACCCCAAGCAGGAAA
CGGCACAGGGCAAAGTTATGTGCTTTTTGGGCCATTAGATTTAAGCCAAATCAGTAAAACATTTG
ATCTTAATGTCACCGCCAATGACAGCAAAACCACTTTCTTACTTAATGGTAGTCTGCCCTATCAG
TTAGCAGGTACAGCAGGAGGAAGTATCGGTGATGTAAATAATGATGGTGTCAATGATTTAATGA
TAAGCGCCCCCAATGCAGGACAAATTTATAGTCTTTATGGACATCCTTGGTTAGCGGATGATGGT
AGCATCAAACTAGCGAATATTACCGCCGATAATGGCTTTGTTATAGATGGCAACTCTTACAGTAG
CTTTGATATTGGTGGAATTCCCGTTTCTCAACAACAACTAAATACCTCTTCTGCCTTAGCAACCA
ATAATGGGGTTGTTTATCTTGCCTATAAAGGTCAAAGTAACAATAATTTATTCCTTACAACCAGT
AGCAATCAAGGACAAACATGGAGTAATTTTATCAGTCTTCCTGGTGGCATGACAACGGATAAACC
ACCTTCGATCGCATTTTATAATGGTGTTTTATATTTAGCTTATAAAGGGGTAGGAAATAATCAAA
TTAACATCACCTACAGCACAGATAATGGTCAAACTTGGAGTAATCAGTATCAAATTTCAGGACAA
TCAACCCCAGATACTCCCACTTTAGTCGTTTATCAAGGAGAATTACTGGTTTTATTTACCTCTAA
TGATTCTCAAAGTCGCCTTATTTATACTCATTCAAATAATCCTCAATCGGGCAATTCTTGGTCAT
CCAATCAAACTATATTGACATCATCGGGGGGTAGTAATCAAACATCGGCTAACGCCATGAGTGCT
ACGGTTTTAAATGATAATCTTTACATTGCCTATGAGGGAGGAACAAAAAGTAGCCCTTCTACTAA
CTATTATATTACTTGGACTGATGACACAGATTTGAATAGCCTCTCTTGGAATCTTCATCAAGAGG
AGAATATTAAAGCTACAGGTGCAGTGGGATTAACTTCTGATCAAAATAGTCTTTACTTAACTTAT
CGAGATAGCAACAATAATATTTATTTAGGTAATTCTAATAATGGCAATAATTGGTCTGAATTTTC
CCTTATTCCTTCTCAGGATGCCATCGCAACTCCCACTCCCATTATTTCAGGAGAAGAATTAATTAT
TAGCTACCCTGATCAAAATAATGCCATAGATGTTATTAGTTCTGATATTCCTTATCTAAGTGCGA
TCGAAGCTGGAAACATAAATATAATTTTAGGGGATGTTAATGGAGATGGTTTTGCGGATATTCT
TTCAGGAGGAGAAGGGGGATTGGTTATTGTCTTTGGTGGTAGCACCCAAGATTTATTAGACGGTG
CTAGTGGAAGTCAAGATTTAATTATCACCCTAGAAAATGATTTAATTGTAGATGCGGTAAAACTA
GGAGATTACAACGGTGATGGTTTAAAAGATTTCGGTGTTTTAACGGAAAATGGCGATTTTTACTT
GATTTTGGGTAATACTAACCTTGCAACTCTTACCAATTTATCTCTCACAGAATTTGCCATTAATA
ATACCGTCAACGTTAATACCATAGGAGACTATAACGGTGATGGTTATGATGATTTTGTAGCCGAG
AAAGACGGACAAATTCAACTATTTTTAGGTAATGCGGAAGGAAGTATCACCAATAGCTCGATGGA
AATGAGTTTAGAAAACTTCACCATTGGTGGCTTGGGAGATTTCGACGGCGATGGTTATGATGATT
TTGGTTATGGACTTCCTCAAGCTAACATTATCGATGATAATAACCCCATTGGTAATGGGCAATTT
ACCTCCTATCTCGGAAACAAAGACTCTCAATTTAACAATCCCGTCACTATCAATCCTTTTGAGGCT
TCTCTTTACTCAGGGGCGTTAACATCAAGTCAATGGGGGATTTATGGCTCAACTGATAGTAATTC
CGTTGTTAATATTAATTCAACTATATCGACTCAAGGAGTGGATTTATCGCCCTCCATCGTGGCTT
ACAATGGCTATTTGTATATGGTGCGTAATGATAATAATAATAATGACCTTTGGGTACAAAGAAG
TCGTGATGGTTATAACTGGGAAGGAGAAGTTAATTTAGGTAGTAATTTTCAAACAGATAAGAAA
ACTTCTTTAGCTGTCTTTAACAATACTCTCTATTTGGGTTTTAGAGATGCCTCAGAAAGGGTTAT
GTATGCTTCTGCGGTGGAAGATGACAGTAACGTTGCAGGAATTCGTTTTAATAATCCCATTCAGG
TAAGTGGGAATAATCAGTTTCAGGAAAGTGTTGATGGGCCTTCTTTGATTGAATTTCAAGATCAA
TTATATGTATTCTTTATTTCCACTGATGGCACTAGGGAAATTATTTATTCTGTTTCTGATACCGC
TGACAGTGGCAGTTGGAGTGCCAAAAAGAATGTATTTGCTAGTAGTTTTAATCAATCTAGCGATC
ATCGTGTGAGTACAACTATTTTCAATGATCAATTATATGTAGCTTTTACAGGTAATGGTAATAAT
TCTCTCAATGTAGCTTCTAGCGGTAATGGTACAGATTGGAGTCGTACAGTATTGTCACAGTTTGC

TAGTTCTGAAGGGCCTTCTATCGTAGCAGGAAATGATCAGCTTTATTTATTCCATGAGGGAGCAA
CTTCTAGCGGTGGCAATGATTTTCAGGTATTTTATCCTACCTCTACCGATGGAGTTAACTGGACA
AATGCGATCGCACTTGCTGATAATGTCATTGATGATGCACCCTATGCCACATTTTTTAATCAAAG
TATTTATGTGGCAATGGCAGGGTTAAATGATGATTCTCTCAATGTTTATGTATCTGATCCATTTT
ATGCAAGTAATCAAACTCAACAATTAGGTAGTCAACTCCAAAGTATTGGGGATTTCAATGGAGAT
GGCATTGAAGATTTTGCCGTCTTAGCAGAGGGTTATATTACTGGTTTAGGGGTTTTAGATGATTT
AGCCCAGGCAAAAATTAATCAAGGTGCATTATTCATCTACTATGGTAAAACTACAGGCATCACTA
ACACTTCTCTCCCCGACGTAGTTTTAGGTTATCCGACGGTTTCTGATGGTGATGATCTTTATCAG
ATGACTAATTTTAGCGCGATCGGTGATACTAATGGTGATGGTTTTGACGATATTGCCATTTCCTC
TCCTAATACTTCTCTCACCAGTGACAAAACAGAAGATGGAGTGGTAACGGTGGTATTCGGTGGCA
CGAGTTGGGGAAATCAATACTCGAAAGATAATCCCTTTGACTTAAGAAGCCTGAGTAATAATCAA
AATCCCACTAATAACTCTAATGATAACGGTTTTACCATAAAAGGTTTAGCCAGTTCTCAAGCAGG
TGTTGCCCTTGACGGAGGCGATGACGTTAACGGTGATGGATTTAGTGATTTTGTTATCGGGGCGC
CGGGGAATGATGATAATTTGAGTTTTGTCTTGTTTGGTAGTGACTTCACCAAAAAAATTAATCAA
ATAGGTTCGATCGGCGATGATATAATGATTGGTACAGCCACAGGAGAAACATTTATTGGTAGCCA
AGGAGAAGACCAAATTTATACCAATGGTGGCGTTGATGTTGTTTATAGTGGCAATGATGATGACT
TTATCACCGTTAGTGATACTTATTTCCGCCGTATAGATGGTGGCACGGGAGTAGATATATTAAAA
TTTGCTGGTTACAATGGTCAGGATTGGGATTTAACCAAATATTCTGGGCGTTTACGCAACCTAGA
AATTCTCGTCATAGAAGATTATGGGGCAAATACCCTCACCCTCAACTCTCTCACCGTTAATCAAA
TCTCAGACAATAACACCATTACAGTGGTGATGGATAAAAATGATACTCTCAATCTAAGTTCGGAT
TTTGCAGAAGATGGATTCGTTAATCAATACAATCAAAGATTTATCCGCTATACTTCCAATACCTC
ATCAGCGACAGTTTTAGTCAACAGTTTTAACGATGTCAATTACTCTCCTATCGATTTCTCCTTAT
ACAACACAGGAGTAGATGATTCAGGGGTTAAAAAACCCAATGGTACTATTGAAGATATACACTA
TGAATTAACATCATTCCCCTCAACCAGTGTCGGAGAAATTATTATTACCGATGGCAATACCACCA
TCTCAAGTTTTATCCAACCTAATCAACGTGATAGTAATAATAGCCTAAGCACGGCACCGGGAGAC
TATACTTATAAAACCTATTTGACAATTCCTGAAAGCGTTAATCCCAATGGTATATCTATCAATGG
AAAATGGGCTACTGATGATGAAGGTATAACGATAAAAGTAAACGGAGTTGCTCAAAATCTATCG
GTAAATCCTAATTTTAATACTGGTTTTGTGCCTTTTACTCTGGATGAAGGATTTATTTCAGGAGA
GAATGCGATCGAGTTTACTGTCAATAACGCAGGTACTACCAATAATCAAACCTACTTGCAAGTAC
AATTTGAGAATTTTTTTGTTGCTAACGGCAATGCAAGTATAAAATTTAGTGCACCTACCACTAAC
TCTCCTGAAGTAATTACCATTGCTGAAATTAGCGAAAGTGCGTCTGTTTCTGCTCAATTAAATCA
AGGAATTTCTAGTGATATTTTCACCGCCGATGATGTCGATGATAGCCAAACTCGTTTATTTGTTT
CTAATCCCACTGCTAACGAAGCGAGTGGAGAAATAGAGTTTACTATTCAACGCACAGGAAACTTA
AATAAATATGTCCAAGCTCACTATATCAGCACCGATGGAAGGGCAAAAGCAGGAAGCGACTATAA
TCCCGTGCTAGGAAGATTAATATTTGCCCCCGGAGAAACAACTAAAACTGTTTCCGTGCCTTTAC
CTTTAGATGATGTTTATACTGGCACAAGGGAATTTGGTTTACTGGTAACTCTCGAAAAAGAAAGT
AATACTCCCCTTAATGATCAATTTTCCATTTATGTTGATCCCACAGATGGACAAATTCGTAATTG
GAATCATATCATACTCGATCGCCCTTTGAGTGTTATGAATGGTGATTTAGAATTTAGAGTGACAG
CCAATGAGGGAAAAGCCGAGACAAAAATTTACTTTGAAGGAGACAGTAATTTTAATAGTTACTA
CATTTTTAATCTCAAAACCGAAAAATATGAACCCTTTATTCTTAACGGTGATTTTGGGGCAGAAT
TATTTGACGAAAATGGAGACGGTAAAAATGACGGTGTTATTCTAAATCTCCAAGATGGTAGCGAC
TACGACTTAGATGGTGCAGAAAATGGAATCATTCATAAAAGAGGATTTTTCGCCGATGGCGATGC
CCCAGAAATCATTTTAGACACTGCCATGTATCGTTTTCGCAGTTTAAACACTGAGGGCAGTTATC

TTTATGTGGGCGAGGAGGAAAGAAACTCTATTTTAACTAACTACACAAATAGCTTTATTGAAGAG
GGATTAGCCTTTTACGTCTCTGATACCCCTGAAGATGGTTTAATCGCCTTCAATCGTTTTCGCAA
TACTAACCAGACAGGAGGATATATTTACGCAGGAGAGGAGGAAAGTCAGTCAATACAAGCAAAT
TATCCTAATTTTGTCTTTGAAGGCATTGCTTTTTATGCTTATCCTCAAGGCTCACAAATGGCAAA
TGATATTACTCGCTTTCAAAATAGTATAGGAGGGGCATATATTTATACAGCCTCTCCAGAAACAC
AAACAGTTATTACCAATTACGCAGATAGTTTCAATCTTGAAGGTTTAGCTTTTGAGGCTTTAGTT
TAACCTCAGTTTGGTTTAAGAATACCCGATAAGGTTAGGTGTCAGGTGTCAGGTTGCAGGTGTTA
GCGAATGGGGGGATGGGGGGATGAGGTGATGAGGGGAAAGGGGGAAGTAGGGGCGAATGGCCAT
TCGCCCGTACAGGTATTCGTGGTTTTTCATTCTTAATTCTTAATTCTTAATTTTTAATTCTTAAC
TATTTAACGTCAGTTTGCATTAAGGCAATTTTATCGTTATTTCTAAGAAGCTATAAGGTTTTCTG
ACTACGAGAAAATAATGCTTTCAGCCTATCCAAAACTCAGGTTAGTTTGGCTAAGGTGTTAGGTT
TCAGAGTGTTAGGGTGTTAGGCGATTGGGGTATTGGGGTATTAGGGAAGCGTGTTAATTAAACA
CTTTTGCCCTCCTCCCTCTCGAGGGAGGATAATAAAGGGGGTTTACCTCTTGCCTTTTAACTTTGC
CCCTTGCCCTTTGCCCTTTTAACATTAATTCCTAATTCCTAATTCAATTAGTTTATTTGTATAACT
CTGTAGCTAAACGAAAAGCTAAAATACCGGGGATTAAAGCAATAACCAAAGCTACGAATACTTGA
GTTTCAGAAATAGACATAAGAAAAAACTCCTGTAATAAAGGTAAATTGATACAATTTGTAAATT
ACTATTTAATGTGCCTTAAGATGGTAATAATTGCATGAGTTTGTAATAATTTGCAACAGAATTGA
GTAAGTTATGGTTTTATCTCGTTGGTTTGCTTCGGCTAATAAAATAAATATTCACAAAAATGCTC
TTGCATCTAAAATAAAACAAGATCCTTACTATCGCTTTCAATCAGACTTAGAAATCGAGATTGGA
GCGCAATTGGGAATAAAAATAGATGTGAATATTGCTTCTGTAGATGATTGGCTAAGACTACCGGG
TATTTCCATTACTCAGGCGAGGAGTTTAGTAGAAATCACTAATTCTGGAATAAATTTTCTCTGCT
TAGAAGATTTAGCGGCGGCGTTGGGAGTATCAGTTTTAAAAATACAATCGTGGCAACCTATTTTA
TATTTTGCCTATTATGCTGATGATAGTTTTCATGCACCAGCAAAAGTTAATCCTAATAATGCTAC
GCTTAACCAATTAATAAAAATTCCTAATTTAAGCCAAGATATAGCTCAACAAATCCTGATTGATA
GAGAAAGTAATGGTAAATATCGTCATATTGCTGATTTACAAAAAAGACTTAATCTAACTCCAGAT
TTTGCCTATCACTTAATGAATTATTTTCAGTTTTCTAATTAGGATTTAAGGGCAAGGGATAAACG
ACAATGGGCAATAGGCAATTAGTGAATGGTGAATAGTGAATAGTTGATAATTAAGAATTAAGAA
TTAAGAATTAAGAATTAAAAACTCCAAACACTTGTGCGGGCGAATGGCCATTCGCCCCTACTTCT
CCCTCTCCCCTCATCACCTTATCACCTCATCTTCTAAACACTTTAACACCTGCAACCCGAAACCCG
ATACCTGATACCTACCTTTATCCGATATTTTTAAACCGAACTGAGATTATTATTATTATTATTGA
TTGAAAGAAATCCCCTAGTCTCTAATTTGCACAGGCATAATTAAATAAGTCATTTGAGCGCCACT
GAGAGGGGTAGCAATAACGGGTTGGGTTGCACCATTAAATTGAAACTTGATTTCATTGGCAGATA
GGGCTTTTAATCCATCCATCAAGTAGCGGATATTGAAACCTATTTCAAAGTTATCCCCGTTAATT
TCGGCACTAATAGACTCTTTGGCGTTACCTAATTCTTTTGCTTCTACGGATAAGGTTACTTGCTC
GTTATCAGAATTCAAGGTAAACTTGACGAGATTATTTTTTTGATCACTTAACACAGAGACTCTTT
CTAAACTCTCAATCATCCGTTTGCGATCAACTACCATTGTACGCTCAAAATTGTTAGGAATCAAC
TGGTTATAGTTGGGATATGCACCGTCTAATTTTCGACTGGTAAGGTTTTGTTGCCCTAATTCAAA
GACTACTTGCCCTTCATCGACAAATAAGGCAATGGATTCTCCTTCTTTTGCCATGGAAAGGATTT
TTTCTAATTCTCTCAAAGCACGGGCAGGAATGGTTATAGTGAAATTTTGTGCTTCGGTAATGGGG
GGATGAGAGTTAATTTCTTCTTCTTCTGATTCTTTTGTCTCCTCAATGGCACTGGTTTTAACAAC
AGCCAAACGATGGCCATCAGTGGCGGCAAACTCGAGGGTATCAAGATTGCGGGTTAAATGTACTC
CTGTTAGAATTTGTTTAGTTTCATCGTTACTTGCGGCAAATAATGAGCCTTTTAAACCTTCATTT
AAAGCGGCTACGGGTAATAAAAAGGCTTCTTCTCTTTCCACAACGGGTAATTCAGGATATTCTTC

TCCTTTGACTCCTCTTAGCTGAAATTTTCCTGAGAGGGAGTTTATGGTAACGAGGGGATTTTCTT
CCATTTCCTCTTCTTCATAGGAAATAGTTACTTCTCCTTCTGGTAAACGGGCAATAATATCATTT
AATAGTTTTGCAGGTAGGGTGATTACTCCTTCTTCTTCTACTTCGGCACAAAAGCAACTGCGCAT
TCCTAAACTCAAATCAAAGCCTGTAAGGGTGACTTGATTTTTTGTCTGATTTGCCACTAATAATA
CATTAGCTAAAATCGGATGAGTAGGACGAGTTGGCACAGCACGGCTAACTAATGATAAATTATTT
TTGAGATCAACTTGAGAGCAAACTATTTTCACGGTTTTTTGATGGGCTTAACTACTTAGGACTAT
TGTACTCAAATGTTTTTAATTTAAACACAATCTTTCATTTTACCTCAGTTCGATGCAAGAATATC
GGCTAAGGATAGGTTTTAGGTTTTAGGTGGCAGGTGTCAGGTTTGATTTTTTAATATTGACTCAT
TACTCACTCATATTAAAAACAAAAAAATCAATTTATTAACTGTTATTGCCAAAAGAGGTTAAGTT
TAACTCCAAATTTTAAGCTATGAACACCAGTTTATTCTAATTTATTTGTAAAGAAGTTGGCTAAT
GGCAGTTAGTTTTTGTTTTAGTAGTGCAAAATTGATAGGTTTACTCATATAATCATCCATTCCTG
CATTTAAACATAAGATGCGATCGCTGTCCATGGCGTTTGCTGTCATTGCCATAATATAAGGTTGT
TTTTCTGGGAGTAGATTTTGCCTAATCCATTTAGTGGTAGCGATACCATCCATTTCAGGCATCTG
CATATCCATCAATATTAAGTTATAGTCTTTCTGTTGAAGAATTTGAATTGCATCTACGCCGTGAT
TAGCAATATCTGCGTGATAGCCTAACTTTTTTAGTGTTAGTGATATGACTTTTTGATTTACTAAA
TTATCTTCAACAATGAGAATTTTGAGAGAAGACTGACGAGTTAAAGAAGAAACAGAAGTCGAAA
CAATAGAAGAAGTTTGGGGATTGTTACTGACGGATAAAACAACCCTAAAATAAAAACTAGAACC
TTTATTTTCTTCGCTTTCTAACCAAATTTCTCCTTCCATCATCTCTACTAAGCCTTTACAAATAGC
TAATCCTAAACCTGTGCCACCATACTTGCGGTTAATAGAAGCATCTAATTGGGAAAAAGGTTGAA
ACAATTTTGATTGTTTATGAAGGGGAATGCCGATACCTGTATCTTGAACTATAAATAAAATTTGA
TAGGCTGATAGTTTCTCATTGGGGTTAATTTTTTCACCTTGAACAATTATTTTAACTTCCCCTTG
TCTGGTAAATTTTATGGCATTTGCGACTAAATTAAGTAAAATCTGCCTTAAACGAATTATATCTC
CTAAAAAGTAATCGGGAAGAGAATCATTAATCTCAAAAGTTAAGGATAAATTTTTCTCTTGGGCT
TGAAATTGTAGTAAATCAATAACCTTTTTTACACACTGTCTCAGGGAAAAATTTTTTGTTTCTAA
CTCTAGTTTGTTTGATTCAATTTTCGAGAAATTAAGAATATCATTGATGATTGTCAATAAACTTT
CTCCACCGTCACGAATGGTGAGTAAAAATTCTTTTTGAATGTTGTTTAAAGGAGTATCTTCTAGG
AGATTAACCATACCTAAAATACCATTCATGGGAGTGCGAATTTCATGGCTAATCATCGCTAAAAA
CTGACTTTTGGCTAAGTTTGCTGTTTCAGCCTCTTGCTTGGCTTTAATTAATAGTCGATTACGTT
GTTTAAATTCAAATAAAAGATAAACGCTCCATGCTAATAAACCAGCTAAGATTAAACCAATTATT
AATATTATATGAGGGAGTATTGAGCGGTACTTTTTCAGAAAAGAGTTATTGGGAATAATCGTTA
TTTTCCAGTCGATGCCTCTATATTGTAATTCTGTGGATTGTTGCCATTGTAAATTGTCTTTAGGA
GGTGCATAGGTGCTACTGTAGATTAGTTGAGAATCTTCATAAATAAAAATTTGATAACCTTTCCA
TGCCTCTTGACGCAAAAGACTGTAGAGAAAACTATTAAGTTTAACAACGCTAATAAGAAAACCGT
CAAATTGAGGAGGTTTGTTTGCTTGGGGAATCAAAAGAGGAATATAAATAATAAATCCTTTATC
TCCTTGAATTAAATTGAGTGTGGGACTAATATAAATTGTATTGTTGGCGATAGAAGTTGCGATCG
CATCTTTTCGTTTATTTTCAAATTTGATATAAAGATTTTTAACATCTTCATTACCTTTTTCAGGT
ACTAACCAAGTGACATAATATTGAGGATTAACCCATTGAATCGCCTGATACCCAGTATAGTCCTC
TATATACTCAAGGGCATCTTTTTCCCATCTTTCCTTGGGAGTGCCTTTTTCGTTTTGCCATCGTTT
CTGCATTCTTTCTAAAGCTAATACTCTTGTTTTTAACTCTAACTCTGTTTTTTGCCGTAAAGAAA
TTAACTGAGTTTGTACTAAATTTTCTACCTGACGGTGATTATATTCTACGAGAAAACTAAAGGTT
ATAATAGTAATTATCATTACTGTTATCCCTATGGATATGGATATGATTGTCTGAGGTTTACGCCA
CAGAAGATTAAACATTTTCATCGACATATAGGGATGAGTAATGAAATTGAGGTGCTGAATTAAA
TATTGGGGAGAAATTAATGGCTCTCTGATTGTGAGTATGTAAATTATTAGTTAGGAAAGGTATC

AAGTGGCAGGTTTGACGGTTAAATAATAAATATTAACTTATAACAATTAATTTTTTTTTGTTAA
TTCCTTATTTTACATAGCTTACAGCAATTATTTTTTATTAATTTATCATAGCTACTGTAGAAGAG
CCAAATTAATTATTAACTGAGATATTTTCTTGAGTAGTCAACTGAAGGAAAACTTCCTCTAAAGA
GGCTTTTAATCGTCTCATTTCATAAATACCCACTCCTTCATGAACTAACATAGAGGCTATTTCTT
TGCCCACTTCAGAATCGTTTTTCAACGACAATTTTATTATAAAACGATTATCAATTCCTGTTATG
GGTTCAATGATAATTTTGTCAATTGCTTCTATTTTTTCTAGTTTAGAGGTTAAACCATTAACTTC
GCCTTCTACCTCTAAATGATAACCCTTATTATTTAGTTGTTGTAAAAGTTCCTCTGTTTTTCCTGT
AGCGATTACCATACCCTGATTAATTATAGTGACTGTATCACAAGTCATGGTTACTTCAGGCAAGA
TATGGGTTGATAAAATAATGGTTTTTTCTTTTGCCAAACTTTTAATTAAGTTACGCACCTCAATA
ATCTGACGAGGATCTAAACCAACGGTAGGTTCATCTAAAATAATAACAGGAGGATCATGAATTAT
GGCTTGGGCAATACCCACTCTCTGACGGTATCCCTTTGATAATTTACCAATAATACTATTGGCAC
GATCTTCTATTTGACATAATTCTATCGCTTTTTTGAGGGATGAAGTGCGATCGCATCTAGGAATA
TTTTTAATTTTCCCGACAAAATCGAGATATTCTGTCACCGTCATTTCTGGATAGAGAGGGGGATT
TTCGGGTAGATAACCAATTCTTTCTCTAACAGCAAGGGGATTTTCATGGACTTCAAAACCATCAA
TGATTGCTTTGCCGGTAGTCGCAGGGATATAACCACTCAGAATGCGCATTGTGGTAGTTTTTCCT
GCCCCATTTGCCCCCAATAAGCCTAAAATTGAGCCTTTTTCTACGGAAAAGGAAATATCCTTGAT
TGCTTGGGTTGAACCGTAGTTTTTACTTAATTTTTCGACAGTAATCATTGTTTTTTAACTTCAGT
TCAGTTTAAGAATATAGGATTAAGGTTATGTGTCAGGTTTCAGGTGGTAGGGGATGAGGGGATG
AGGGGAAACAAGTAATGAGTTAGGAATTAGTTAGGGGCGAATGGCCATTCGCCCGTACAGAAGT
TATTAATTATCAACTATTTACCTTTGCCCTCCCCTCTATTTCGGAGGGATAAAGGGGGGTTTGCC
CATTACCCTTTTCTTAAAAGTTATAGCAAACTATCCATCAACTCCATGATTTTGACTGCTTCAGG
ATGAATTGAGTCTTTTTCCTCTGTTTCCTTGGCGATAGCAGACTCTAATAACCCTTTCATGGCAA
TTAACTTAAGGCTATTTTCTGCAAAGGTAGCACTAATAGCGTCAGCAGAGCTAGTATAACCGATC
GCACCTAAGTCCATCAATGCGGATCTTCTCAATTGTAGCTCTTTTCCTTGCAAAGCAGTAACAAG
AATTTCCCCATAACTATCTTCTTCTGTTAGTTGATACATTGCCCTTGCCGCACCATAAGCCACTTT
AGGAATAGGATGTTTAAGAAAAGGCTTAATTAAGTTTAGGGCATTTTTTGCCTTTAAAGTACCTA
AAGCCTCAATAATCGCCTCATAGGGTTCATGGGGATGAGGTTTTCCTAGGGGTGTACCCTTACCA
TATTCTGTTAATTCTTCCCCGTCAACCGCCGTTTCTAATAATTTCATCAAAGGTGCGATCGCTCTT
GTATCATTTAACATTTCTAGGGCTTGGGCGGCAGATTCACGGACATAATAATCATCACAATCCAA
GCATTTAATGAGAGGTTCAACAGACCGATTAAAACCTAATTTCCCTAGGGCTTTAGCGGCATTTC
TACGCAGGGGAAAACCACCATCAGGGGCGCGATCCTCTTTATCTTCTAAAGCCTGAATAAGAGCA
TTTACTGCCTCATCTGTAGCAACTCTGAATTTGCCTAACCACCAAGCGGCATAGTACCTCATACCG
AGGTCTTCTTTTTGTAGTAAGTTAGCTATTGCTTTTTCTGGAGTTAAATTTGCTTCTGTCACGGT
TTTTTCTTAGTTAATGAATAATTAATGAGTTCGGTGTTATGAAAGAGGTTTCAGATTTTAGGTGT
CAGGTTTCAGGGTTTTCATTAATTACTAATTCCTAATTACTAATTGCCTTTGCCCCTTGCTCTTTG
CCCTTTTCTATTAGTATCTCTTATTCTCTTCAGGAGTATAATTAATTTCTCTTGTCAGGAAATAA
ATCATCAAGGGATTCGATGGTATCTTCTGGGGGAATTTCCTTTTCTTTCTCTTCCTCTTGAGTGTC
TGTCTTAGGTGATATTTGCTCTTCTACAACGAGATTTTCGGCTTGAGAAGTCACAGTGGTTTCTA
TTTCAGATTCAATTTTTACATTCTCCTCCATGACTTCTGTATTTTCTTCTGTTATTTTGCTTTCTC
CCTCCTCAACATCATTTTCTTTTTCCTCTTCCACAACTACTGATTTATCTTTTATGTCCTCGTTGG
CTTCTTCTTGAGAATCGCTAATTAATTCTTCTTCTATGGCTTTTTCCCCATCACTCTCTTGAGAAA
TTTCGCTTACCTCCTGATTATCTTTTTCTTCTTCTTGTGAGGATTCTGTAACATCTAAGTTTGCGA
TCGCTTCTTCGACTTCTACGGCGGTGGATTCAGGGTCAATTTCTTCCTCTAAATCGTCGATTTCAT

CCAAAAGGGATTCTGGTAATTCATCATTAACCGATTTGGAAGAAGTTTCGGCGGGTGATTTGCGG
AAAATATTTCTAATTCGACCAATTAAGTTTATTTTTGGCTTATTTTCTGCTTTTTCTGTTTCATT
ATCTGGGGTTGTATCTGCATTATCTATTTCTATCTCCTCATCCTCTGAGGTAGTATCTATTTTTTC
TTCTTCTTTACCCTCATCACTAATTGCTATGTCTTTTTCCAACTGAGAGGGAGTAGTTACTTCTTC
TATGGTGTCTCTAATAACTTCTTCCCTATATGCTTTAATTTCCGTTTCTGTAGGCTGATTTTTAA
TTTGAGTTTCTTCTTTTGTGGTTGAAGCGACTGTGGAGGAAATTTCTTCTTTTTCCTCTGGAGGA
GTGGTGATAGTTTCTGATGGTTTTTCTGGTGGGCTAGTTGTAACAACGGGTTTTTTCTTACCTAA
CTGGGATACCTTTGCCAACAGTTGAGCAATAAAAGTTAGAACTGAAAACCCTTCTAAGGCTTGAT
TTGATTGAGATTCTGCGATTAGGGCTTGTCTTAATTTACTATTTTCCCAAATAAATGCGATCGCT
ACTGCAACTCCTGCACATTGTCCTAGTAATACAGATCCAGTTATTCTACCTGCACAAATCCATAA
CACAAGGGCATAAAATAAGCCTAAACCACTCCATAAAAAATCATCTTTCCTATGTATTTTGGGAC
GAATAAAAGCATTTATATATAGAGATAAACTGGTTATTGCCACAAGAATTGCCAGAAAATAAGC
TAACATCGCTTCTTTACCTTTTATAGATTATTTCTGTGATTATTATAAGTCATTAGTTCAATTAA
CAATTAGGAAAAGTTAAAAGGAAAGAGTTAAAAGGCAAGAGGCAAAAGGCAAACCCCCCTTTAT
CCCCCCTCGAGAGGGGAGAGGGCAAAAGTGTTTAATTAACACGCTTCCCCAACACCTCAATATCCC
TGACATCTTTTTCCGTAATCATCTCCAAACTAGGGTCATGAGAAAATAATCGTTACATTTCTTTT
CGTTAGTTATGAAGTGTATAACTCTGGTGATAAGCTAAAAAATGCACTAAATTTAAGAAACAAA
TATATAATGAGCGAATTGCCTTGTCTATTACGGGTTGCCCGTGGTGAAGTTTTAGATCGTCCTCC
TGTATGGATGATGCGTCAAGCTGGTAGATATATGAAAGTCTATCGTGATTTGAGAGATAAATAT
CCTAGTTTCCGTGAGCGTTCAGAAAATCCTGATTTAGCTATTGAAATTTCCCTTCAACCTTGGCG
CGCTTTTCAGCCTGATGGGGTAATTATGTTTTCTGATATTTTAACTCCTTTACCCGGTATGGGTA
TTCCTTTTGATATTATTGAAAGTAAAGGTCCTATTATTGATCCTCCTATTCGTAGTTTGGCTCAA
ATTGAGCAGTTACATCCTCTACAACCAGAGGAGTCTTTACCCTTCATTAAAACTATCCTTAATAC
TTTACGTCAGGAAGTTGGCAATAAATCAACGGTTTTAGGGTTTGTGGGTGCGCCTTGGACTTTGG
CGGCTTATGCTATTGAGGGTAAAAGCTCTAAAAACTATGCTGTAATTAAAAGTATGGCTTTTTCT
GAGCCTGATATGCTTCACAAATTATTAGGTAAAATTGCTGATGCGATCGCAACTTATGTGCGTTA
TCAAATCGACTGTGGAGCTCAAGTAGTTCAGATGTTTGATTCTTGGGCAGGAGAATTAAGCCCTC
AAGACTATGAAACTTTTGCTTTACCTTATCAAAAACGAGTGGTAGAACAAGTTAAAGCTACTCAT
CCTGATACCCCTTTAATTCTTTATATTAGTGGTAGTGCAGGAGTTTTGGAAAGAATGGGACAATC
AGGGGTTGATATTGTTAGCGTCGATTGGACTGTGGATATGGCAGAAGCTAGACAAAGACTCGGTA
AAGAGATGAAGGTGCAGGGAAATATTGACCCCGGAGTTTTATTTGGCTCACCGGAATTCATTAGG
GAAAGAATCCATGACACTGTTGCAAAAGCAGGAAAAGGTGGACATATTCTTAATTTAGGCCATGG
TGTCTTAGTGGGGACTCCTGAAGATCATGTTAGAGTTTTCTTTGAAACTGCTAAATCTATTAGAT
ATTAATTGATATAAGAAAGGGCGGGGGCAGAGTAAAAAGGCAAGAGGCAAGAGGCAAACCCCCC
TTTATCCCCCCTCTCAAGGGGGGAGGGCAAAAGTGTTTAATTAACCTCCTCCTAATACCCCAATCC
CCGAACCCCGAACCCCGAACTCCTAACTCAGATGTTATCTTTGAAAAAAGAGTTTACCCCTTACTT
TTGAATGTTGTTACCTTCTTGGCTAGTAATAGGTGCGATCGCACTTTTGTTGAGTATTGTTATTA
ATCGCATACCTACCGATGACTTACGTTGGTTTTTCGGCTTAAGCGCCCCTCATGGTTAACTTTTG
AGTTTCTTATCCCCTTTATCTGGATTTTTATTTTTGTCTGTTTAGTCTTATCTGCTAGTTTGGTTT
GGGATAGCAATATCAATCTTTTTCCCAAAATAATGCTTATGTCTTCCTACGCCATATTACAAGTG
TCAATACTGGCTTATACAAGAGTGATGTGTGCTGTGCGTAGTTTAATGGTGGGAACTGCGATCGG
AGCTTTTGGCTTTTTTATTGGTTTAATTTTGGCTTTTTTCGTGGCAAAAGTCGATTCTTCTGCAT
TGGCTCTATTGATGCCATATCTTTTATGGAGTCCTATTGGTACATATGTTACTTGGGCAATGATT

CCTTTAAATCGCAATGATATATAGTTACCTGAGTTCGGCGAAAAAAGTATTGAAAAATAATGAG
TAGAGGCTTAGAATCGGCAAAATTTTCCCCGAAAAAATCAATTTTAATAATTTTTTATCAATTCT
TATCACTTTTCCTTATTATTTTCTCCACTTTTAAGGGGAGACACAGAGGGGTTTTCCCTATTCTT
TACCTTAACTTGCAATTTTATATCGAACTCATGTTTTCGTTAAATAAAAATTTTTCCATCGGGCA
TCGTTGCACCCTCTAAAATCGCCTCTGACAAATTAACTCCCGATAAGTCTGCATCCCTTAAACTAG
CACCACTTAAATTGGTATCTTTTAATATCGCCCCATTAAGACAAGCACCCCAAAGATAAGCACCT
CGCAAGTTTGCCCCTGTTAAATCTGCACCTCTTAGGTTAACTAAGTTCAAATTGGCAAAACTTAA
GTCCGCATTGCGTAAATCTGTTTCACTGAGATTACTTTTCATTAAGTTAGCTTGTACTATTTCTG
CTCCTTTCATTTGCCCATGATACAATTGGACATTTTTTAAATTGGCTTTGTGCAAGACGCTACGA
CGGAAAAAAGCACCAGTGAGATTACTTCCTGAAAAATCCGCTCCATAAAGTTTGTACCACTTAA
TTTTGCTTCTTCTAAATCCATCCCGCTAAAGTTCAAACCGTGTAAGTCTAAACCGTTAAGAAATG
CCCCTTTTAATTTTATGCCGTATAAGGTTGCCCCCCTTAAGTTTGCACCGCTCAACCTCGCATGGT
TTAAATTTGTCCAATTTAGATTTGCCCCTTGTAAATTTGCCCCTCGAAGATTAATTCCATCGAGA
TTTGCACCACATAGGTTAACCCCGGGTAGATTTGCCATGTAAAGACTCACCCATGTTAAAACTGC
ACCGCTTAATGTTGCTCCCTGTAAATCTGCTTTATGTAATTGAGCGCCTCTTAAATCTGCCTTGGT
TAAGTCGGCTTCTATCAGTTGAGCTTCTTTTAACTTAACAAAACTTAAATTTGCCCCATATAAAA
ACGCTCCTCTTAAATCTGATTCTAAAAGAAAACAACGCTCTAAATCTGACCATGCAAGATGGGTT
TCAATCATTTTGGAGTAAGAAAAGTTAGAATTACTCAGTTTTATTCCACTTAAATTACTTTTGCT
TAAATTAGTGTTGCGAAAATCTCGATCGCCCTCTTGATAGCGTTTGATAACATCGGTAACAGTCA
TTTCCTTTTCTCCTTAAAAGATAGCGAAATGGGGTATTAAATCATTTTTTATTAACTAATAATAT
TAAATGAAAAAAACAACCTCTCCCTATATTTTATATTCTTTGATTTATCTCTCATTTTTTATCAA
CAAATTCACATTAAATTTATCGAAACTTGATTTTTATTAATAAAAATACACATCATCTGAGTTTG
GAGTTCAGAGTTTGTGATTATAATTTTTTCTTAATAGAATAAGTTACTCCCAACAATAAAATCAA
AGCACCGATGACCACTAACAACTGAGGTATTTCTTGAAAAAACCACCATGCAAATAAACTGGAAA
TAACAGGCTCAAATAAAATCAATAAAGAAATAGTGGTAGGAGAAAAGTATTTTAAAGACCAATT
TAAACTTGTATGACCAATTATTTGAGAGATAATAGCCATTAAAATTAAATATATATAAATATTT
AGGGAATGACCTCCATAGCTAGTTCCGAAAAGTGGAGGTAAAGGTAATAAACATAATGCTCCTGT
GAAGTAAGCAATAATAACGTATTGTTTTGTGGTAAGTCCTTTCTGTTGTGCCAATGTGCCTAGTA
GGATATAGCCACTAGCAAACCAACTACCCATTAATGCCAATATTGCACCTAATAAAGGATTTGAG
CTAAAATTATTACCCCCATGGGCAAAGGCAATCAATACACTACCTAAGACTGCGATCGCAATACC
GATAATGGTTTTTCGAGTTAATTTTTGTCCTAAGCACCACCAAGAAAGCAAAGCCACCCATAAGG
GATTAGTAGTGACGAGACTCACGGAAGCGGCAACGGAAGTAAAACCTAATGAGCTTATCCATGTG
GCAAAATGTAGCCCTAAACAAATACCAGCCCCCAAACCGTAATAAACACCAGAAGAATGATTCTT
TTTGATTGTCCATAAATCACCATAAGAGGGAATTAAGAGAAGGGTAGAAATTATTAATCTACCT
GCGGCGATGAATAAGCTAAATCCAACGGTAACTTCATTTATTTCCCGTTGAGATAAACGCACAAA
AATAGCGGCTAAAGAAACGCAGATAATGCCAACTACTAGAATAGTTCTTTTTTGCCAGTCTGATG
GAATGTTCACTTATGAAAAATTGTAGGTATATTCTTTTTATTTTTACGATGAATGATATAAACAA
AAATTAATAATAATTAACAATAATTAATAATATATTATCTTTCTGTCTTTTATTTCCTTACTGAA
GTATAGTAAATTTAGATATTCAATGATATTTTGGGGAGTTCAATATGGATAATTATTCTCCTGCT
CACGTTACATTAATTAATAGTAAATTAAAAGAAAAAGTAGAGTATATTCTTTCTAGTCAATCCCT
TAATTTAATGGGCAGATCTCCTGATTGTCAAGTAGTTTTAGATCCTCAAGAATATATTACTGTAT
CCCGTTATCATGCACAAATTCAATTAATAGAAAAAGATGATAATTTTTATTGGCAAATAGAAGA
TAAAGGAACAACCAATGGTACTTTTATTAATGGGGAAAAAATCGTTTCTCCTTATACCCTGAAAA

GCGGCGATCGCATCTTACTAGGATTAAAAGGACCAGAATTTATTTTCACTACAGAAATATTGAAT
CCCACGGTATTAGTTGAAGATTCTCCCTCAGAGATTAATCAAGATAAAGATAAATTAGAAAATCA
AGATATAAATACCGAGACGCTAAAAGAAGATATAACAAACAAGAAAACTAATAAAGAAAAAATA
GTTACTAATAAAATTGAAAAAGAACAAGAAAATAATCAAAATTTACCTCAAGAAAAAAGAACGG
ATAAACCGAAAATAAAAAAAGAAAAAATTAACCTTGATACTTATTTAAAACCAATTAGTATTTC
TGAAAAAAGTCTTTTCAATTTATTGAATTTTCAAGACATAGCTACTTTAGAAATAGATAGCAAA
GAAGTTCAATGTTGCGTATTTAATCCTCAAAATCAATTAATTGCGATCGCACTTTCTAACAAAAA
CATAGAAATATGGGAATGGCAAACAAAAACAAGAATTTTAACCCTAGAAAATGCCCATAGAATT
AGTGTAAATAGCTTAAACTTTAGCCATGATAGTAAAAAATTAATTAGTGGTGGTAGCGATAAAA
TAGTTAAAATTTGGGATATAGAAAATCAAGTAGAAATAGCATCCTTGTCAGGGCATAAAATGGC
AATAAATACCCTCTGTTTTAGTCAAGATGATAAATTGTTAGCTAGTAGTGGCAGTGATAAAATA
ATTAAAATTTGGAATGTAGAAAATCAAGCAGAAATAGCATCCTTATCAGGGCATAAAATGGCTG
TAAATAGCCTTTATTTTAGTAATGATAACCACTATCTAATTAGTGGAGGAGGAGATAAATTAAT
CAAAATTTGGAATATAGAAAAACAAGAAGAAGAAAAAAATATCAAAATGGAAAATAAATCTCCT
ATTCAATCCTTAACTTTTAGTCCTGATAATAAATTAATTATTTGTTTACTTCAAGATGGAAAAAT
TTGTATTTATGACCGAGAAAAAGAAACAGAATTAATCACTTTAACATCTCCTGATATATGGGGAG
AATTAATTGCAATTAATCAAAATGGAAATTTATTCATCAGCAAATCCCCAGATAAAGGCATTGTT
ATTGGGCAAATTTAACCCTGTTGTTTCCTGTTGTTTACCCTCGGCAACACCACTTTTTCATCAACC
CTCAATTAAAACCATCAAATCTGTTATCTGAAACCCGAAACTTAACCAAATTCAATATCCTTATG
CCAAACTAAGGTTAATTAATTCTCGAAGTTGGTAAAAATAAAAAATTATATATTTCTGCTATATT
TGCTAATATTAGTTAATTCTAATCAAAATCTCCCAATGACTTATGTTAGCCCACGATACCTTACC
AAAAAGAAATACTAATGCTGTCGTAAAAAATAAATCCCTTAATCAAAGAGATTTTCGCCCTAAAT
CTAGTCATTCCCATAATCAAGAACAAACCTTAAATAAAAACCCTAGGCGCGTTCAAGAAAGAAAT
AGTCATACTTCTCAATCTTCAGAATCAATACAGTATCCTCTATGGTTAAAAAGTTTTATCGTCCT
TAATCATTTTTCATCTGTTATTTGTGGTCTGAGTGTTGGTACGGCATTGGTAATGTATGGTATGA
CAGTTTATGCCCCAAAGCAGTGGACTAATAAATATCATCAGTTACAAAATTTGCAAAAACAAGAA
AGACAATTTACTTTTACTGATGAAGTTCTGAAGGATACATTGGCAGAATCCGCCCAACAGCAGGG
ATCTGGTTTTGTAAATCCAGATCAGAGTAAGCCCCCTATTTTCTTACCAGAAACTAATGCCACCG
CCATTACCCAAAAATCAATTAATCCAGTCAAAATTAAAAAAGTCAACCCTATTTATCCTGTTGCC
TATTAACATTAAACTGATTGGTTCTGTTGCTTCTCATTGTGGTAAATTGTTGATTAGGACAAGGA
TGAGTAAAAGCCCTCTGTATTTCCCCTTTTAAGGGGATAAGGTAACAAGAAAATTGTCAAATGAT
AAATAATAATTTATAAACTTTTAACTTTATATTTAACCTGAAACTTTTTAAAGGTTAATTTATCA
TAACTACTGTAGAAAAATCAATTTATTTCTTTTTAGTCAATTGTTTGTAATAATGCTAAGTTATT
TAACTTCCCCAAGATGAATGTTTTTAAACTAAAATCTGACAATGATCACGTTGGTAACTCTCAAT
CTAACAGTAAATTGAGCAATCTCAAATTAGCTTTTACTCAGTTAAATTTTGATACATGGATTAGA
CTATTACTGATTTGGTTGTTTTTGGTTTTTGGAGCGACTGGTTTAGGATGGCGACTATATAAATT
ACAAATAGTCAAGGGAGATGAGTTAGTTAAACTGGCAAAACAGCAACAAACATATAACTTTCGTC
CATATATTCCCCGTCGCTCGGTTACGGATGCACGGGGTAATGTTATTGCCCTTGATAAAGTGGTT
TATAAGGTTTATGTTCATCCTGTGATGTTAAATGAGGATAAGGATGTAGTTGCTGAAAAATTAG
CAACCATTCTTACAGACAAGGACAAAAATGAGATTTTAAAAGTTTTAAATAGTGGTGATACTGGC
ATCGTTTTAGCTCGAACTGTAACGGAAGACCAAGGAAATAGTATTAAAGAATTGGGATTTGAAG
GAATTGACCTAGAAAAACGCTATGCTCGTTTCTACCCTCAAGGGGAATTGTTTGCTGACATAATC
GGTTATGTGGATACAGAACATAGTGGACAGGCGGGAATAGAATATAGTCAGCAAGATTTATTAG

AACGAGATTTAACTCAACTAGATTTCAAATCGATGATGACGGTAAAAAAAGATGGTGAGGGGGC
TATTATTCCTGCTTCTTTGCCTGATGGTATCGTCCAATTAGATGATTTACAACTACAACTTACCCT
TGATACAAAACTACAAAGGGCGGCAAGGGATTCTCTCAAAGCACAAATTAAAAAATTTAATGCG
AAACGAGGGGCAGTTATCGTCATGAATGTGCATACAGGGGAGATAGTTTCTCTAGTGAGTGAACC
TACTTACGATCCTAATAATTATTCGAGCTATGATTTTGCCCTTTACAAAAATTGGACTGTAACAG
ATAGTTATGAGCCGGGTTCAACTTTTAAACCCATAAATATTGCGATCGCACTTGATGAGGGGGTT
ATTGATTCTAACTCAAGAGTATTAGACGCTCCCAGTGTGGTAATTGATGGATGGCCTATTTCTAA
TGCTTCTAAAACGGGAAAAGGGTGGGTGAGTATCACCAAAGCCATGGAAGATTCTAGTAATACAG
CTATGATTTATACCATGAAAAAACTAAGTCGAGAAAAATACTACCAAAGATTAGAAGAATTAGG
CTTAGATAAATTAATGGGAGTTGATTTACCTTTTGAAGCCACAGGATATATAAAGCCGAAAAAT
ATTTTCACCGCCAGAGACATCGAAACAGCAGTATCTGCCTTTGGACAAGGATTATCTCTCACTCC
TTTAAAATTAGTACAACTTCACGCGGCCATTGCTAATGGAGGAAAGCTAGTTACCCCTCACGTTG
TCAAAGGTTTAGTTAATGCAGAAGGAGAAATAGAATCGAGTGCTTCATTAAAAACCAAACAGGT
ATTTTCCCCCGAATCTGCCCGTAGTGTCAACCTTATGATGCAGTCAGTAGTTGCCAATGGTACAG
GTAAAGCCGCTCAGATAGAAGGCTATCACATAGGCGGTAAAACAGGAACAGCCCAAAAACATGAT
GGTAGAGGAAGATATAAAGCAGATGCTAAAATCACCAGTTTTATTTCCATTTTACCCAGTGATGA
TCCTCAATATATTGTTTTTGCCGTAGTAGATGAACCAAAAGGAGCAAATACTTATGGCTCAACTG
TTGCCGCTCCTGTAGTTAAAGATGTTATTAAAGCCATTATTAGGACTTATGGAATTCCCCCTTCC
TATACTCCCTAAAGGCTAAAAGTTTTTTATTTTCAATTTATTTTTATCTTTTAACGTGAAGTTAT
TTAGCTGTAATTACTTCCACATAACGTAATATTTTTTCTTTTATTTTAAAGCCTTGACGGACAAT
TTTTGTGATGGTTTGTTCGGGTATATCATCACGAATTTCTCTATCTACCACTTTACAAACACTAA
AATCAGGGGTATCTTCATCAAAATCAATTCTTTCTACTTCTCTACGGGATAAAATAGTTAACAAT
TTCTCTTGCATAGTGTTGACAAATTTAGGTAATCTTTTAAAGGCTTTGGGGTTAAAATTTTCCTC
TTGTAAATTATTATCTAAGTAATCAATTAAAAATTCTAAACTATCAAAAATATCTAATAATTCAC
TATATAAAGCCTCTTCTTTTGCTTCACTTTCTTGTTGTTGAATTTTGACTAATTGTTGTAATTTA
GTTTTTTCTTTCAACAGGTTTTGATTTTCTGATAGTAGTTTGTCTCTTTGAGTTTTTGTGATAAT
TAACCCGTCTTTTTCTGGTTGATTTTTCTGGGATGAAAAAATATTTTTCCACAAATTAATACTAT
TCATAATTTTATATTTATTTAGAGAGTTAAATAAGTCTTAAGAAAATAGTTGTTTGCCTAAAGT
GCGATCGCGCTCTGAAATTATTTCACTTTCCTGATAGGCTTGATCTAAATTATCAAACTCAGGAA
CTAAATTAATTACGGGAATAGGTTCATTTAAAGCAGATAAATCATGATTTTTAAACCTTTGAATT
AACGGTAAATTAGGTCTAAGAAAATCTGCAATTAAACGCTGTTGATTATCCATTAACTGCTTTCT
AGCTTCGGCAATTTGTTTAGGATTATACTTCTTTTTTTTCATTGCCATGGCAAAGGCTTTGACTA
TTTCAGCATGAGAAGCGGCAGGAGATACTTCTAAAACATCATAAGGATTAGGTATTATTTTATCA
TTTTTAATCATATTTAAACCTCTTAATTAAATAATTTTTAATGCACGATAAACCTCTCGAAAATT
AGGCTCATGAGGACAAATTTGATAAGCCCATCTTCCTAATTGTTGTAAAGTTTCCCAGTCTAATT
CCCTCGCTTCTGCACCCTTTAATGCAATTTCTACACAAATAGAAGCTACTAAATATTTAATTTCT
TGATTGGAAGAATATCTTGCTTTTCTTACGGCATCTTCAAAGCGATTTTGTTTGAGTAGTTTATC
AATTTCTTCTGCTTCTCGACTATATTCTAATCTACTCAGTAAATCTATAACTAAAGGGTTATTTC
TATCAATATTTTGTACATCTTTTAGTTTATTAATAGCTTGGGAAAAAGATATTTTTTCATCGGCT
ACTTGTTGAGAAATTTCTCTCGCTTTAAACTCCGCTAAATAGCTGGCAGATTGATTTGATTTTAA
TAAATCATACCAGAATTGAGCAAATTCTAAATGCTCTTTTAAATCCTCTATTTTTTGTCTTTGTT
TTTCACATAATTTATTAATATTTTCTACCCAATCATGATTATTTTTTATTTCTGTTTGAGAAATT
TCTAAGGGATTTTTTGCTTTTCTCCAGCTATGATGTTGTAGATAATAACACCCCTCATAATAGTT

GATTAAATGATAGGCTAGTTTTTCTACTCCCGATGAATGTTGTTGAGATGGTTTAATTTTAATCG
CCCTTTCTGTGTCGTTTTTCAAACAAGCGGCAACGGCTAAACCATAATTAGTAAATAAGGTTGAC
AGTTTATCTGTTTTTAATTGGATACCTTTTAACTGTTTTTGATGTTGTAAATAAAATCCGGGAGT
GATAAATATTTGTTTGATTTTTATTCCCGTCTGGGGTGGATTGCCCATTAATTCTAAGGCTAGAT
TTTCTCTTCTCAATACATCTCGAAGCTGATAGTATTTATCTAGGTTATTATCTTTATATTTGTCA
ATCGCACCTTCTACTATTTTAATCAAATCTTCTCTTATTTTTTCATAGTTAATCTCATTGATATTC
AACCAAGATATATTCTGGAAAACCTCACTTTTGTTAATATTTAACAATACTGTCATCCAACAATT
AATCCATCTTTCTATATAGTTTCATCTAGTTGGGCTTGATAATAAAGTGCGATCGCCATATTAT
GTAAAGTAGTTAAATTCGGACTCTGTTTAAATTCTTGCTCTAATTGATTTATAATCTTAATTAAA
TTTTGAGATTGCCATATTTGATGTTGTAAAGCTGGTTGGATATGTTTGTTTAAATTATGATCAAT
TTTGGGATCATAACCGTATTGTTTAAGGAAATTTTGACAGTAATTTTTTGCCTCTTGATATTGAG
ATTCTTTAACTAAATCTTCAATTTTTTTAATGATTTGTATTTTTTCTCTTTCAATTAAACTTTTT
AATGATTCTTTGCTAGTATTAATGGTTGGATTATTAACTAATTTCCATTCTTTGACTGCATTTTG
ATAGTCATTTTTTTGTAGATAAATAAAACCCCGTAAATAACTTGCCCTTTCTGTCTGAAAATTTT
TTAAATAAGATAGAGCAGAGGTAAAATTATGTTGTTTGATGTCAATCAATGCTAATCGATAAGA
AGCATCTTTTAATTGGGGTAATTTAGATAAAGCAGATAAATACTGTTTTTTAGCAAGAGTTAGA
TTATCAATTTTTTCAGCCATTAAACCTTGTCGATAGTCAGTTTTCGCATCAATAACTATCCGCAA
TTTTTCTTTTAATTCATTCCCATCTTGACGCTGAAATTTATCTAAGGCAACTTCTAATTGATTAT
AGGCAGAAATGAAATTGCCTTGTTGAGCAAGGGATTTAATAATTTTTAAGTCATTTTCATATTGA
ATCTGTAGTTTTATTTGATATTCACATTCAATAATTTCTTTATCTAAATCAGCAACTCTATATAT
TTTTTGTGCATCTAATAAGTAGGGTAATCCTTCTTTGAAAAATCCCGTATCAACTAAATTTTTCC
CTTTTTGATACAAAGACTGAAATTTATTTCTACTATTAATTTTATCCTTTAAAACTTGAGCCGTA
ACAGTAGATTCAGTATCATACAAAATTTCATTAGATTTTTCTAAATATTCTAGGGCTTTAGTTAA
AAAACTATTATCAAAAGGATCATTTTTATCTTGTTCAATTAAATTTTTTCCTTGATTTATATAGT
GCCTAGCTTTATTCAGATTTAATCGCCATTCTGTTAATTTTGTTTTGACATTTTCTAATAAATAA
CCCCCAGACATTTCTCTAACTATTTGCTCACGCCAAGAGGGATTTTCTTTCCATAAAGATAAAAT
TTCTTCCCCTAGCTTAATCGCTTCCTCCAGTTTTTTTAACTTAGCTAATTCCTTCGCCTCTAAAAA
CTTTTTCTGACTTTTCTCTGAATTTTCAAAAACTGCTTTTGTTTTAGCAATAAAATCAAATAAAC
CCATCGATATTACCTCTGCATATAAGATTGATTAGTTTGACATAATTCTGTTAAATTAGGAATTA
AAATGACTTGTCCTATTTGTAAATTTTGGGGATTATTAATAGTCGGATTCGCTTGAAAAATTAGT
TGCCAACCGTCTCCATCACCATAAAAACGTAACCCTAACATATAAAGAGAATCACCAGATTTAAC
TCGATAATTAAAGCCTCCACACCAATTTAATTCAGGCTTAATTGCTTCTGATTTTTTTGCTACTT
CTGTGGATTGATTTTTGTTTTGATTGATAGCTTGTAAACGGTTATTGAAGTTATTTTTTGAAACA
AAATAGCCAGTCAAATTACCTACTAATAATCCCATTATTAACACGCTAGCTAGAGTAATGATAAA
TAACTTTTGTGACTTTTTATTATCTTTTTCTTCTACTTGTGGTAACTCTTTTAAAAGTTTGATAG
TAGATAAATCTGTCTCACAATTAGGACAAATATTCGTTTCAATTTCAGTGCGATCGCACACTGGA
CAAGTTAATTTTTCAGTCATTTTGTTATATATTTAAAAAATAAAATTAAGCTCCAAAAAAATTGA
TTAAAAAAGCTAAAAATAGATGTTACTATCTCGCAAAGAATATCGATAAATAAACCAAAAAAGT
TAAATTTATGACGTTGTTTTCTTTTTCTCTTTTTATCAGTTTTATCTGAATTTTCTTGTTTCTCT
AAATCAGTAATTTTTTCAGTCATAAAAACTCCATAATTTTTCTAGGTTGGGCTACACTTTGTTAA
CCCAACACATTTTTTTGACAATTAACGAGTCAAGCCAGTGGCAACCACTCCTGTGGGTAAATCTTG
ATCAATATACTCTCGTATTTCCTCTAGTAATTCACGATAGAGGCGATCGCTTGTATTGCCATCTC
CTCTTTCCATCGCATCGAGAAGATTGATAAACTTAGTTTCTAAAACCTTACCGTGAGAGGGTGCA

ATACGACAGGCGCGAGCGACACCAAAACGAATTAACAAGAGAATACGCACTAAATCAGGCAAATT
ATCAAATTCTCGACTACCATCTTCTGCCAATCTCTGCATCGCTGAAATATTATTGCTATCAACCGC
CTTTTGCATTTCCGACACTAAAATTTTGAGGCGGTTAACTTGATCCTCGTGCATAATATGAGAAC
AGAATTCTAAAGCAAAATTGAAATCTCTCAAATACATCAATATGGTATCGCGATCGCCACAAGCA
AAAGCCTCTAATTCTTTAAGTTTTTCTTCCGCAACCTGTTGTCTATCTCGCTGTAAAACTCCATTG
AGATATATTTGATTTGATGCTCTTACAATCTCACCCGCTAATTCATTAGCATTTTGCACCCCATA
TTCCGTTAAGTTACCCTCTTGATTAAGTTTATGAATTAACTCCTCAACTTTTTGATTAATTTGTT
CATCATGATTACCCCTAGAAAAACTACCACTAACCCGAATCGAAGGATTATTTTTCAGACAAGCA
GTCATGGTTAAAGCAGAATTTTGCTCATCTAACTCTACCATCACATCAACTTCCGTGCCTCTGGG
GTAAGGTTTATCTAACGCTAACCACATATCCCCAATACGCTCATCATTTTTGCGTAAATCAATGC
CTTCCCTTACCTCATCAGGGCTAAAAAACTTAAAATGGATTAACTCTTGCCCTTCCCCTTCAGTTT
TGAAAGTATGATTCTTTTGAATGGGTAAAACTTCCCCTTGCTTAATTAATTGATAACGAGGATCA
TCCACTAATTGGATACAATAATCACGAGATACGGTGCCAACCACTTCCGTGACACCAGCAGACAC
AATACCCGCACCTTCAGCAACAGCATACATGGGGCGAGGATGCACCACCACTTTATCATTTCCAA
AAGCCTTTTTCACCTCCTCTTGTACCAATGGTATCTGGCTTGAACCCCCCACCATTAAAACAATAT
CAATCATATCCGTAGGATAGTCCGAATACTTAATGGCATCGTGACAAATTTCGATACTACGCTTC
ACCAAAGGCAAAATCATCGCCTCAAATTCTTTCCTTGTGATAGTTATATCAATGGGGATTGCCAT
ACCAAACTCGTCAAGGAGGGGAGTAGAGGGATTAACTTTGGCTTCGGTTTTACTGCTTAAATCAA
TTTTTGCTCTTTCTGTGGCAATTTTCAAATCCGCTTTAAATCTTACTTGTTGGTAGTAAGGCATA
TTAGCAATTAAGGTATCAATACTATCTAATCCTTCTTCCTTCGCTACCTTCTGCTTAACTAATTCA
GCTAACTTGTTATCAATATCATCACCCCCTAACCATAAATCTCCGGCTTTACCCGATTCAATAAA
ATGACTTCCTGCGGTAGTAATCAAAGATGAGTCAAAAGTACCACCACCAAAATCATATACCAAGA
TAGTTTTAACATCATCGCTATTGGGCTTAAAACCGTAGGAAATAGCCGCCGCAGTGGGTTCAGGT
AATAAAACAGGTTTTCCTAAACCAGCCCTTACAGAAGCGATTTCCGTGGCGTAGCGTTGTTTATC
ATTAAAGTAAGCAGGTATAGTGATAACGGCTTTAGTAATATTGCCCGTTTGCCCCATATTTTCCT
GATAGGCTTGAGCATTTTCTACCACTTGTTTGAGAATTTCTGCACTAATGTCTTCGGGTTGATAC
TCTTTGCCTCCCAACCACACAGAGAGACTGTTTTCTGTGCCTTCGCTGGATTGAGTAATTTTATA
ACCGAAGTTTTTTAACTGCTGTTGCACCACATCATCTCCAAAACCACGCCCCATTAAACGTTTGAT
GGAGATAATAACGTTTTCTGGATCTTGTAATAGTTGATTATAGGCTTCTTTACCGACTATTAAAC
TATTATTAGAAAAGGATACAACAGACCTAACTAAAGTACCATGTTTTGGATCATCAACAACTTGT
GCTTCTGCAAATTTGAAAGCGGCAACGGAGTTGGTTGTGCCTAAGTCAATTCCGACTATATTTCC
CATAATTGATAGTTAATTTTTTCTTTGCTATCTACTTATCAGTAGCAAAATGTGAATTTATGCAA
ATTAGGGAAATTTTTTTGAAATTTAAGAATTAGTAATTAGCAATTAAGATAAAGCAACGAGCAA
AGGGCAAACCCCCTTTATCCCCCCTTGAGAAGGGGGAGGGCAAAGGTAAATAGTTGATAATTAAG
AATTAAAAACTCCTAACTCCTGTACGGGCGAATGGCCATTCGCCCCTAACTAACTCATTTCCCCCC
AACACCCCGCAACCTGCAACCTGACACCTACTCTTATCCGATATTCTTAAACCTAACTGAGGTTAA
TTATTTGTACCCTCTCTCAAATATGCTTCAATAAAAGCCTCTAAATTACCATCTAAAACATCATC
GACGGCGGTTGTTTCCACGTTGGTGCGTACATCTTTAACTAATTGATAAGGATGAAACACATAAT
TTCTTATTTGATTACCCCAAGCGGCTTCTACTATATCCCCTCGAATATCTGCGATCGCTTTTGCCC
TTTGTTCCTCTAAAATAACTAAGAGTTTACCCTTTAAAATAGCTAAGGCTTTTTCCTTATTTTGA
AGTTGCGATCGCTCCTGAGTACATCTGACCGCTATACCCGTGGGAATATGAACAATTCTCACCGCC
GTTTCCACCTTATTAACATTTTGTCCTCCTTTACCTCCTGCCCTAGAGGTAGTAACTTCTAAATCT
TTTTCAGGAATATCAACCTTAATGGCTTCCTCTCCCAAACTGGGCATCACATCCACCCCAGAAAAG

CTAGTTTGACGCTTTCCATTCGCATTATAAGGAGAAATTCTGACTAGACGATGAGTGCCTTTTTC
TCCCTTAAGATAACCAAAAGCATATTTCCCATCTATCTCTAAAGTTGCCGATTTAATCCCCGCCTC
ATCCCCTTCGGAAAGTTCCGTCAAAGTGACTCGATAATTCTGGTTTTCCGCCCAACGAGTGTACA
TTCTCAATAACATTTGCGCCCAATCCTGAGCATCTGTACCACCAGCACCAGCATTAATCGTTAAA
ATAGCACCTTTTTGATCATAAGTTCCACTCAATAATTGCTGTAATTCCCATTTATCTAAATCTTG
GGCAAGAGATTTTAAATTACATTGAGCCTCGTTAAATAATTCTGAGTCTAATTCCATCTCTAATA
ACTCAACAATCGCCTTCGTGTCTTCCAAACTACTTTGCCATTGATGAAATGTATCCCATAAAGAC
TTGAAATCATTTAATTGCTGTAGAGTTTCCTGTGCCTTTTCCCCTGCTTCCCAAAACTCTGGCTGT
GCGGCAACTTGTTCTAAATCCCTAATTTGTGCGTTGAGATTAGGTAAGTCAAAGATAGTCCTGAG
TTTTACCCAGGCGCTCAGAGATTATGCTTATATCTTTCTTTAGTTCTGATAATTCCATTGCTATT
GAATAATTGTGAAAAAAATTACCGACAACACCAGATATTATCAAAAAAACTGACTATTGTCGAA
TCTCAACGTCTCTATTGCTTCTCGTCATGTAAATTATTGCTTAGGGGAGACACTCTTACACTCAT
TAAATAATATTTAAACACTATTGCCCTCCCTCTATCTCCCGCTCTTTTTTCTTTCTCGAAAATGAA
ACAGCCCTAGGGATAAAGGGGAGTTTGCCTCTTGCTTTTTATTTATCATAACCACACTCTGAAGA
GCCATCTCAACAAACTTCAAGAATCAAAAATATCAAATTTATTCAACTTATAAGTAAAGTTACTT
TTCGATTGGGAAATATCTTTATAATTTGCGTTAAAATTGTGAGAAAAGACTAACTCTATGACCAA
TCATTAGTAATTAATAAATTCAAAGATTAAAAAATTTTTGATGAAAAATTGGTCTGTTTTCCGT
TTATCTGCAAATTTGTTGTCATATTACTCACATAGGGAATGCCCATAGAATTTGATTATTGGTGG
CGTATTATTCTCCCTCCTGTTGCTGGGGGAGTAATTGGATATTTTACTAACGATTTAGCCATAAA
AATGCTATTTCGTCCTTACAAACCTATTCATTTATGGGGGAGAAGATTACCTTTTACTCCCGGAT
TAATTCCCCGTAATCAAGAAAGGTTAGCTAAAAGAGTTTCCGACACAATTATGAATTCTCTTTTA
ACCCCAGAAGAATTACAAAAACTAGCAAAAAGACTTTTACAAATTGATAGAGTTAAATCCGCTAT
TTTATGGTTATTGCAATTAGCTTTAAAACAAATCAAAGAGGATAAACAACAAAAAACAGCTAAA
ATCTTAGCAGATATTTTAAGAGATTTATTTGGCGATTCAATCCCTAAACTCCTAAAAACTTTATC
CAGAAGAGGGGATTTTTTAGAAACTCAAATTAATCAAATATTCGATCGCATCTTATTAGAATTGA
GAATGAATGAAAGTCAAGCGAGACAACTATCAGACTGGCTTTTAAAGGTGGTGTTTTCCCCTGAC
TTAATCAGACAAGCATTGATTAATTTTTTGACAGATAGAAACATAAATGTTATTGATGAAGGAT
TTAAAGAAAAAACCAGTGGCACTTATTGGGTAGTAGCCAATTTATTCGGAGTAAAAAATGCCCTA
AGTCGTTTAAGAAGTTTTTGTTTAGATGATAAAGAAACCGCTAATACAAGAATTCAAGAACTAC
TATTATCCTTAGAAATTAGAACAAGATTAAAAGAATTTTTCCTCGATTTATCTTTACAAAATTTA
CCCGTAGCCACCGTTAAACAATTAAGAGAAACGACTAATAATGCTGTTAGGGATTATCTTCAGGA
AAAAGGAGCAGAATTACTTCATGAATTTAGTTATAGTATTGATTGGGATAAAATTTCAGTATTA
ATCGTTAATCGCTTACAATCTTCTAGTGTGGTTAACAGTTCATTAGAAGTAATCAGTGAAGAACT
AGCCTTAATTTTAGAGCGTTATTTAGAAGAAGATTTAGAAAAAAATTGTCACCGAAGCTATCCCGA
TTTTATCCATTGATCAGGTTATCATTGATAGAGTTAATGCTACAAGTCCTGAACAATTAGAAGTA
GCAACCCAAAGTATAGTTAAGAATGAGTTACAAGCCATTGTTAATTTAGGTGGTGTTTTAGGAGT
TTTTATCGGCGCATTGCAGAGTTTGATATTATGGTTTAGTAATTAGGTTACTCACTTATTTCCAA
TGTTAAGTTGAGGTAAGTTTTGGGAAGAAAAGAGGATAAGGTGATGAGGGGAGGGGGTGAGGGG
GAAATGAGTTAGTTAGGGGCGAATGGCCATTCGCCCGTACAGGAGTTGGGAGTTTTTAATTATCA
ACTATTCACTATTCACTATTCACTATTCACTATTCACTATTCACCTTTGCCCTTTGCCCCTTTAAC
TTCTAATTACTAATTGAATTCGTGGCTAATTTTTTGACTCTGTTAATAATATTCAGTTTTGGTGC
ATCTATTGGTAGTTTTCTTAATGTGGTAATTTATCGTATCCCAGAAAAGATTTCTCTTGTTTACC
CTCCATCTCGATGCCCAAAATGTAAACATCCCCTAGGCAAAACTGAGAATATTCCTGTGGTTGGT

TGGCTATGGTTGAAGGGTAAATGTCGTTGGTGTCATACCCCTATTTCTCCTCGTTATCCTTTGAT
TGAAGCAATGACGGGTTTTTTCTTTGTTTTGATTTTTCAACATTTTGGTTTTTCTTGGTTAACTC
TGGGTTACATGGCTTTTATTAGTTGGTTAATTGCTTTGGCGTTAATCGATTTTGATACCATGACT
CTACCTAATTCTCTCACTCAATCAGGATTGGTTTTAGGTTTATTTTGGCAAGGATGGTTTGGTTT
TAACCTCAGTGATGGAAATGTCAAAGGTTTCGTTTTATCTTTATTTTCTGCTATCGTTGGCGGTG
TAGTGGGAATTTGGTTATTTGATACTATTTTAATTGTTGGTAGTATCGTGTTTGGTAAGGCGGCA
ATGGGAGGCGGTGACCCCAAGTTAGCCAGTATGATAGGAGTATGGTTAGGATGGCAAGGAGTTTT
AATAACTGGCTTTTTGGCTTGTGCTGTAGGTACAATTGTGGGTATAGGTGCGATCGCATTTCGGT
TGATGAAAACAGGACAACCCATGCCTTTCGGGCCTTATCTAGTAATAGGTGCAATTATTACGATG
TTTTGGGGAGACTATCTAATATCTAGCTACTTAGAATTAGTCGGTTTCTGACACTGCCCCCCCTG
AAGTTGTCTGTTGGAAGAAATATTAGGAGTGAGAGGAAGAAATTGTAATCAAAATTGCCATAGC
TGAAAAATATCTCTAATTTTGCTAGGATTATTTCAGCTAATTAGACGACAAATATTGCCATTAGT
TTTCATTTATCCATAACTTACTTATTTCTCTAATAAGATTTTCTCACCCTAATCCAATCACAAAA
AAGCTCAATGATTAATTTTTCCAGACGCAATTTTCTACAAGGAGTAGGATTCTCTCTCGCCTCTC
TATATATGTGGAATCATCAAGCAACTACAGCCTATGGTAAAACTTTAGCTGAATCTAATACTCGT
AAATTAGCCTTGTTGGTGGGTATTAATCACTATGCCCATGGGGATAGTCTTCATGGTTGCGTCAC
GGATGTTGAGTTACAAAGACAATTGCTAATTCATCGTTTTGGCTTTTTTCCTGAAGATATTGTTG
TTTTAACCGATAAACAAGCTACTAGAGAAAATATTTTAACAGCTTTTCAAGAACATCTTTGCCTT
CAAGCTAATGCTAATGACGTAGTTGTTTTCCACTTTAGTGGCTATGGCAGACAAGTTAAGTTATT
TTCCAGTGATGATAATCGCAATTTAGTCGATAGTTTAGTCGTTTATGGTAGTTTACGCTCCGATT
CTGAGTTTTCCCATCCATTTGTCGATGATATTTTATTAGATACTTTAGTTAAATTAGCAGAGTCA
TTAAAAACAACTAAATATACCCTTGTTTTAGATACCAGTTTTATTCCTTCTCCCCTGACAATTAG
GAATAAATTATCTTTGAGATCTTATGATTTTCAACCTAACTTAATTATTAGTGAAAAGGAGCAAG
ACTTTTATCAAAAATTGGTCAAAAGTAATAGTAATTCAATTATTAAAAACAACCAAAACAAATC
TTTGCAAGGGCTAATTTTATCTCCTTCTCAAGATAGTATTTCTGTCGAAATAAATAGTCTCAATT
TTAAATCAGGGCTATTCACATACAACCTAACTCAATCTCTTTGGCAAGAATTTCCTTCCACGGCT
AACTTAGCCCTAATGAAAAATATTGCATCTCAAATGGCTTTATATCACGGTAATAAAGAAAAGAT
AGATTTTACTACTGATAATAAAAGCGACGTTTTATCTTATCATTTACCCCTTGAAATTCAGAATC
GTGGTTCAGCAATTGTAACTAAAATTATTGAATCAAACGTCATAGAATTAGAACTACTAGGACTT
CCCTTATTAGTATTATCTAACTATTGTTTAAATTCTTGTTTTAGGGTAGATAATGATGAGCAATT
TGTCGTTGTACAAATTAATTCTTTACAAGGAAACAAGGCAAAAGGAATTGTGATTCAAGGAGAT
AAAAATTTACTTAAAATTAATACTGTTTTGCAGGAATATATTAGAGTGATAAATCAGGATACGG
GTCTAAATATTGCTTTAAATGATACCCTTGAAAAAATTGAAAAAGTTGATGCTACAAGTGCTTTA
AGTGTGATTGACAATGTTGTTTCTATTAGTAATATTGGTGAAACTTTTGCCGATTGTATCCTTGA
TAAACTAACCTCAGAAGATGGTAATATTGATGGCTATGGCTTATATTCTCCCACAGGGGTTTTAT
ATCCCAATACAACACCTCAATCTCCCCACGAAGCAGTTTCTACTGCGGTTAAACGCTTAACTAAAC
CTTTAAAAACTACTCTTGCTTCTAAATTATTTAACCTTACATATAATCAATATTCTTCTACTTTA
CCAGTGAATATTGCCTTAGAAATTACTCATGATAACATTTCTTCAACCCCATTCAAAATATACTTT
TAATAGTAAGTTAAATAAACCCCATGAATCAGCTAATGTTCACCCAGATAATTTATTAATTAATA
TTCCTTTAGGCAGTCAATTTACGATCAACATTAATAATGAAACTGAACAAAATTTATACTATTTA
CTCTTAGGAATTAATTCTTCCAGAGAAGCGATCGCATATTTTTCTCCTCATTCTACCTTAATCTCC
CCCCAAGACAAAATTTCTATACCTGAAAATGCTAGTCCTTTAAAATGGATTGTTAACGCAGGTAA
AGGCATAGGAGAATTAATCATTATTGTTAGTAAATTTTCTTTTGAAAATACCCTAAAAAATATCG

AAAAAATGATAGAAATGAAAAAAGATATGGAACAAATAATAATTTTAGAAAATCCCGAATTAGT
TGCTAAATCTATACTGCAGGACATCCATCAAAATAGTGATATTAATCCACAATTATTTAATAACT
ATAATGATATTTATGCTCTTAATATGAATAACTGGGCAACTTTTAGATATGTATATGAAATTAA
TTAGAACCAGAGATTAGCCTTCTTGGTTTTTTTGTTGTCTAACTAATTCAACCCCCTTAGAAGTG
CCTAAACGAGTCGCTCCGGCATTAATAAGTGCGATCGCATTTTCTAAGGTTTTAATACCTCCAGA
GGCTTTTATTTCAATCCTGCCCTTTGTAATTTCTTGGAGAAATTTAACATCCTCCACCGTAGCACC
CCCAAACCATCCCGTGCTAGTCTTGAGAAAAGAAACCCCCGCATCCATACAAATTTCAGCCGCTAA
ACGCTTTTCAGTTGGAGTTAATACAGAAGTTTCTAAAATAGCCTTGATAACTTGTCCTGTTTCTT
CCACAATGGAAGCCATCTCTTTATAAATTAACTCGGAATTACCCTCCCTTAACCATCCAAGATTA
ATAACAACATCCAACTCCTGCACCCCATTTTCCACCGCTTCTTGAGCTTCAAAAACCTTACAATTA
GTCGTGCTAGCCCCAGAAGGAAAACCGATAACAGTACAAACCTTTGATTTTTGATTATGAAGTAA
CTCCGCCGCCACTTTCACTGCCGAAGGATAAACGCACACGGAGGCAAAATTGTACTGTATTGCCA
CTTTACAAGCATTTTCAACGTCTTTTCTCGTCGCTGTTGGTTGTAAAAGAGCAAAATCAATATAA
TCCGCAATATCAATATCAGTCGCCGAAATAAAATTACTTCTCATTATCAATTTTTAATAATTAAA
TCTTTATATGTAATTTTCAAGCAAAGCTAGATTCTTAACTTGAGTTTGATGAAAAAAGTATTGAA
AAATAAGGAACTGAAGCCTATTAATCAAGGAAATTTCCCCTGAAACAATCAGTTATTAAATTGAT
AAAAAATGTATTTAAAATCAATTTCAACAAGTTTTTGTGTCAATTCTTGTTGCTCTTCTCTATTT
GCTATTTCCTATTTCCTAACTTCTCCCGTCAATTTTTGGCTAGGAGCAAAATCCGAGTATTACCTA
TTTCCGAATACAAATGATGTACCCTAGCATTTAGATAAACTCTATACCTGATCCAATTAAAATTT
AAATTTGCTTTCAATTAACTTTCATCTTCCGTAGGGCGAGTTTGACTAGGAGATGACTCATAAAG
AGAATCTAATTTTTGTCTTGCATCTTCTACAGATACAGAACGAATTACCAATAGTGGTTCATTAA
TCGGTTTGCCTTGCTCATCATATAATTCAGGATGACTAGCCTGATATGGAGAATTTTGGCGGGAT
TTACTAGGTCTAGGAGTATCAGTCCAAGTACCTCTTTGAGTATCAGCACTAATGGTAATCAAACT
GCGAATCAAGTTGCTGATAGCAAAAAAAGCAATGACCGTAAAAGCTAAAATATAAATAAGATGT
AACATTTCAACCTCTTATCAATAGTTACTATATAGTTTTTATATATCGAAAATTTCAGAAAAAAA
GACCAACCTGATTTTTAATCAATCATTCACCTTTTTGTTTTACTTCTCGCCACCTTCGAGCAACAT
CCCAACATTCCGCTACTAACTGATGCCAAGGTAATAATACTCTAGTATCAACTCCTGCTTGTCCAT
CGGTGACATTAAAAAGCATCCTTGCCGTTTCAACCTCTCCTTGAGCTTGTTTTAATCTGTTTAAT
AGCCCCGTTTGTTCTTCTTGAGAAAGAAAAGGCATATTTTCCGACTCCAACAAGGAAGAAGAACG
AGAAAACCAGTATTTAAAATCCTCTAGTAGAGGTAACAAGATTTTCCTGAGTAGTTCTTTTTCAG
TAGGTTGAGATGGTTGCATGGTGGTATTTTTTCTTATACTTATTCTACTATTCTAGTTGTTATCT
TAACTAAATTTACAAAATTTTACAGATTTCTCATTATCTGTCAAAACGAAATTTAGTAAACCAAT
TTTCTATTTTTTGGTTAGCTAAAGATACTCGTCAAATTCAAACCCGTATAAACTGAGATAATGTT
AAAAAGTGATTTATTAATTTACCGTTATAGCGGAGAAACTATTGTTCCTAAAAAAATTACCTTTAC
AGCCTCGTTTTATTGATTTAGCGTGGGAACAAATAGATTGTTTTGAGAGCTATCTTGGCAAAACT
CAGGGGGAATTAGACGCAAAACTCCAAGAATTAGAGGGGGATAGCCCAGACTATCGAGTGAAGA
GAGGGTTAGCTCATTTACTCAAAAGTAATTTTTCTCAATTTAACATAGTCAGTCCCCTTGAACCT
CAGATATTACGAAAAAAAGTATTTGAAGCGGCGGCAAAATTACCATCTATTCCGCAAAATCGCAG
TAAAACCCTAGATGCGATCGCATCTTCTTTAACCGCAGAACTACATAGAGAAGTTTTACCCCAAG
AGATAGAAAAAGGACTTTATGCTGACTTACTAGAAAACAGAATTTTAACTGAATTTGATGCTCCC
TCCCCAGAAACTTTAATACATCGCTATAACCTCTCCCAAGTACAAGGAATTTTTTACCGTGCTAG
TCATATTCTCATTCATGCCCACCGTAATGACCCCGGAGAATATAAACTTTTGTTTCGTTATCTAA
AATTATTTCAATTAATGGCATATATCGAAGGAGATGCAGACACAGGATTTACCATTACTATTGAT

GGGCCTACCAGCTTATTTAAAGTCAGTAATCGTTACGGTTTAGCCCTAGCAAAAATGATACCAGC
CTTACTCCATGTAAGCAAATGGAAACTAGAAGCCAAATTGAAAATGAAAGACTTTTACAGTAAAC
GAGAAAAAAATAGGTCATTTTTATTTAGACGATAACTGCGGTTTAGTAACTCACTATGCCAACAAC
AAAACCTATGACAGTCTATTAGAAGAATCCTTTGCTAAAGGATGGGAAAAACTAAAAACCCCATG
GCGTTTGGAAAGAGAAGTCGATTTAATTCCCCTGCCCGGTAGTGTCATGATTCCCGATTTTCGTC
TTGTGCATCCTGATGGGAGAGATGTCTTACTAGAAATAGTGGGTTATTGGCGCCCTGAATATCTA
CAAAAAAAGTTTTATCAAGTACGTCGAGCAGAAACCAACAACTTAATCCTCGCAGTATCCGAGCG
TTTAAACCTAGCTAAAGCAGGAGTCAATTTTAAAGATTTACCCCATAAACTTATTTGGTTTAAAA
ATAAATTGTCTCCTAAAGAAGTCCTAAACATATTGCCAGATGAAGTGTAATTCATTGCAAAATAG
CCAAAGTCTCCTGAGTCCAATATAACCTAAGTTCAGGCTAAATTTTCATCTATGAGTGATGATGA
AGAGGGCAAGAGGCAAAAGTGTTCAATAAACTTCTCCCTAACACCTGCAACCTGAAACCTGACAC
CTGAAACCTGACACCTGAAACCTAATCTTATCCGATATTCTAAAACCGAACTGAGGTTAAATAAA
AATATTAACCCTTACTCCTTGTGTTTCCCCATTAAAATATATTTCCTTTGACCTTGCCCTTTGTCC
TTTGTCCTAATCTCTTTCGCTATTTTTCCTATCTTCACCAAAATATATTAAAGGAAAATTGTTAA
AAAGTATTAAGAAAATGAGGCGAAAGGTAGCATCATATCGCAAAATAATCACCGAAGAGATGTT
AAGCCTCTGACAAAAAATAATCAGTAAAAGTAACCTCAAAAGGTTATACTGATTAAAAGAAAAA
ACTCAGCAAGAAATTGAATAGGGTTTTTCAAATCTCCAGACCAGCTTAACGGAATTTCTGGTTTA
ATTAATAAATAGTATTTAACAAATTATTTGGAGGAATCCATAAATGAGTATTGTCACGAAATCC
ATCGTGAATGCTGACGCAGAAGCACGTTACTTAAGCCCCGGTGAATTAGACAGAATCAAAGCATT
CGTTACCTCCGGTGAAGCTCGTTTACGCATCGCTGAAACCTTAACTGGTGCTCGTGAACGTATCGT
TAAAGAAGCTGGCGATCGCCTCTTCCAAAAACGTCCTGATGTTGTTTCTCCCGGCGGAAACGCTT
ACGGTGAAGAAATGACTGCTACCTGTTTACGTGATATGGACTACTACTTACGTTTAATCTCCTAT
GGTGTAGTAGCTGGTGATGTTACCCCCATTGAAGAAATCGGTTTAGTTGGTGTAAAAGAAATGTA
CAAATCTTTAGGTACTGACGTTGGTGCAGTAGCTCAAAGTGTACGTGAAATGAAAGAAGTAGCCA
CCGCTTTAATGTCCGCAGAAGACGCAGCAGAAGCTGGTACTTACTTCGACTACGTTATCGGTGCT
ATGCAATAGGATTGAGATTTTATTTCGTTCTCTCCTATTTAACCAATAATATTACTCTGCTAAAA
CAGTAAGCAAAAGTTCATTTAAGTAAAAAAGGAAATAACAATGCAAGACGCAATTACTGCCGTA
ATCAACTCTTCTGACGTACAAGGTAAATACCTCGACGCTGGTTCTTTAGGCAAACTCAAAACCTA
CTTCCAAACCGGACAACTCCGTGTTCGTGCAGCTAGTGTAATTAGTGCTAACGCTGCTACCATCGT
TAAAGAAGCTGTAGCTAAATCTTTATTATACTCCGATGTAACTCGCCCCGGTGGAAATATGTACA
CCACCCGTCGTTACGCCGCTTGTATTCGTGACTTAGACTACTATCTCCGTTATGCTACCTATGCAA
TGTTAGCTGGTGATGCTTCTATTCTCGATGAGCGTGTATTAAATGGTTTAAAAGAAACCTACAAC
TCTTTAGGTGTACCCATTAGCTCCACCATTCAAGCTATCCAAGCAATGAAAGAAGTTACCGCTAG
TTTAGTCGGTGCTGACGCTGGCAAAGAAATGGGTGTTTACTTCGACTACATCTGCTCTGGCTTAA
GCTAATTTGACTAGAAACTTCAGTCACCATTAACTGCTTAAGGCTTAACTATTGAGGTTAGGAAT
GTCCAGAGTGAGTGCTAGTTTAATTGAAACCTAATTTTGATAATTTTGGCTTAGTTGATTTAGCA
TTGACTCTTGACTCCTGACCATCAAATGATATAAATAAGATTTTAGTTTTAAAATTACTAATCCA
GAAATAAGGCTGGAATCAAATTTTTTTTAAGATTATAGTTTCGGGTTTTTAATAACTATTTAAG
GAGCTTTGCCCCATGAGAATGTTTAAAGTAACGGCTTGTGTCCCAAGTCAAACCAGAATTAGAAC
TCAAAGAGAATTACAAAATACTTACTTTACCAAATTAGTTCCCTACGAAAATTGGTTCAAAGAGC
AACAAAGAATCCAAAAAAATGGGTGGTTCTATTGTTAAAGTTGAGTTAGCAACTGGTCGCCCCGGT
ACTAATACTGGCTTAAAATAGATTTATACCATAGGTAGATTTGATTATAACCTAAATTAATAAT
GAGGGTGGAAAGTTATTATTCTGCTCTTATTTTTTTGTTTACCTCTTTATCTTCATCTTCTAAAA

ACTTATTGCAACGAATACGAAATAATTAAAATATTGTTAAATTTCTAAAATAAAGTACCCAAAT
GTTATTTTTTTTACACAACTAACTAATAGTTGGTATATAATTAGATTTAGGTGAGGAGTGAAAT
AAGTAAAAGTCCTTGAGGTCGAAACATGGCAAGACTCTCGGGGGCTTTTTCTTTTTGATTTGAAG
ACAAATCACATTCAATCAAGTTAGATGAATCATGAGTATTAATTCTAATTTTCCCGAATCTGCAT
CATCAGCAAATATTAGAACTCCCCCTTGGGGTATGGTTGCCGTTTTAGAACAAGGTAAAAATTAC
CGTATTAACAAGATTACCGTTAAGCCGGGACATCATATCAGTACTCAACTTCACTATCATCGTAG
CGAACATTGGGTCGTAGTTGCGGGAACAGCAAAAGTAACCTTTAATGGAAAAGAGCAACTTTTAT
TGCCTAAACAGTCAACTTATGTTCCTATGAATACTCGTCATAGAGTAGAAAATCCGGGGGTTGTG
CCTCTGGTAATGATTGAAATTCAAAATGGGGAATATTTAGGGGATGATGATATAATTCGTTTTCC
TGACGAAGAATGATCAAATATTTATATATAAGAAAGAATGCGTATTGTTTTTTTGGGACACCT
GAATTTGCAGTTACTACTTTAGAAAAATTAATTATTACAGACAATCACGAAATAATCGCCGTTGT
AACCCAACCAGATAAAAAAAGAGGTAGAGGAAGTCAAATGATTCCTTCCCCAGTGAAACAGGTAG
CGTTAAAAAATGGTTTACCAATATGGCAACCGTCAAGAATAAAAAAAGATCAAGAAACTTTACA
GCGACTCAAAGATAGCCAAGCAGATGTTTTTGTAGTTGTCGCTTATGGACAAATTCTTTCCTCGG
AAATATTAGCAATACCAAAATTTGGTTGTGTCAATGTTCATGGTTCTATTTTGCCTGAATATAGA
GGAGCCGCCCCTATTCAATGGAGTCTCTTTGATGGGAAGAAGGAAACTGGTATTACCACCATGTT
AATGAATGAGGGTATGGACACAGGAGATATGCTACTAAAATCTTATACTCCCATTAATTTATTTA
CCAATTTTAATGATCTATCTGTTCAACTAGCAAATCAAGGTGCTGATTTATTGTTAGAAACTTTG
GAGAAAATTAGTTTAGGACAACTTACCCCTATTCCTCAAAATGATACAAAAGCAACCTATGCTAG
ATTAATAAGTAAAGAAGACTATACCATTGACTGGTCTAAATCTGCCTTAGAAATTCATAATCAAA
TTCGTGGATTTTATCCTAATTGCGTAACAATATTTCGGGGACAAAATCTGAAGATAACTCAAAGT
TTGCCTTTGGGAGAAATTGAAAACTTAGAATTACCCCCTCAATTTCAAAAACTCCCTAATTATCT
ACAAGGCATCAATGAAATTTCAGGCAATGTTGGGGAGATCGTAAAAACTATTAAAAATTTTGGT
TTTGTGGTGCAAACGGGAAACGGTTTATTACTGATTTTAGAAGTACAGTTAGCGGGGAAAAAGT
TACAGTCAGCATGGAATTTTATTAATGGGACAAGGTTAGAGGTTGGAGAGAGTTTAATTTCTGA
AGTTAGTTAAAGATATGACATTTTCAACAGTGTAAAGGTTATTTCTCGAAGCACTGTTGCCCATC
AGCCGATCTGAAGTTAGTTAACGTCAGTTCGGGTTAAGGCAATTTTATTGTTATTTCTAAGAAGC
TATAAGGTTTTCCGACTACGAGAAAATAATGCTTTCAGCTTGTCCAAAATTCAGGTTACTTATTA
TTTCTCTAAACTGATAAGCAAAAAGAGGATAGCTGAAGAAAAATAAATTGCTGAAATTGTTCCC
ATAATTTAGGATTACGCCACCCTTTGATCACTTCTTCTTCAAGAATGTCTAGGGCAGATGAAGGA
GTGTAAGTGGTTTTGGACTGACGATTTTTGGTTAGAGCATAGTATATATCAACTAATTGAAAAA
TTTGTGCAAGATAAGGAATTTGTTCCATAGTTAAACCATCTGGATAACCACTACCATCGTATTTT
TCGTGATGATGACGAATTATTTTTAACAAATTAGGACGATTGTGTAAAGGTTGGCAGATTTGTTC
TCCGATAACACAGTGTTGATGAATTATTTCTTTTTCTTCTGATGTTAATGCTCCATGTTTGTGGA
AAATATAATCGGGAATACTCACTGTTCCTATATCATGAATATAAGCGGCGAATACTAAATCTTCT
ATATCGCTATGAGGTAATTCTAAATACTCAGCAAAACCTCTAACTAAATTTGCTAACTGAAATGA
TGATTGATAGTCATAACCAGATTTTGCTTTTATTAAAGTGGCGAGATTGAGCAAAACTTTTTGAG
TTTGATTTAGAACTTCATTGAGGCGTTTTTGTTGAGCGAGATTTTTGAGTTTTGGGTACAAAACA
GTATGATCGAGAGGTTTTAATACTACATCTTCTGCTCCTGCTTTCAAGGCTTGATGACGCAGTTG
GGATTCTTCTGTAACAGACATCAATAAAACAGGGATTGATTGAGTATGAGGTTCGAGTTTTAGAT
GCTTAGTCAATTCCACACCGTTAATTTCTGGCATTACTACATCGGCAAGAATAACGTCGGGTTGT
TCTTCAAAAGCAATTTTTAATGCTTGACGACTATCATTAACTTCTACCACTCTATAACCTTGATA
TTGCACTAAATCAACGGCGTGAAGTAAACTCAAAGGTTGATCATCTACTACCATAACCTTACTCG

GTGTAGAAACTGTGGGAAGGGTTATTTCCTGTGTGCTGAGTTCTTGCACTTGTTTACATTTAATC
ATAATTAATCACAACCCTCTATATTTTTTATTTAATTTATATAGCAATCCTATTTGAATCACTGC
ATTTACTCATTTTCACAAAGAGCAATGAAAGGAATTTTATTTACAAATGATTTCGGAATGCTACA
CAAAAATCGAAGAAATATTAAATTTTATTTTTTCTATTTTATTGATACTTCTATTGTTAGATATT
TATGATGGATTGTCATCAGTTAATTTACGCAATTTTTTCGGATTAAACCATCTTTTTTTCCACTT
ACTTGTTGATTCTAAATTACTTAATAGTTGCTCTTTTTCTCTTCTTTTTTTGATAATTTCTGCTG
TATCCCTTAAGGATAAATCTCGGAAGGGAATTGATGCTCTCGTTTGTAAATGTTCTAATTCCATT
AATGATAGAGGGGGCAGTATTCTTTCATTGTAACTAGCTAAAGTTCCGGGCGATCGCCTCCCGAT
ACAAAAGTTTGCACCCAATTTTAATCCTGCTAATTGTAAAGCTGTGCGTACTGCGGCAGAGCAAG
AATAAGTCGCTAAAATACCTTCTTGATTAAGACATTTTGCCACTAAAGATAAAAATTCTACAGTC
CATAATTGAGGGCATTTAGGGGGGGAAAAGGGGTCAAGAAAAATAGCATCTCCCTGCCAATTTTG
TTCTACTAAAGATGCGATCGCATCTCTTGCATCTCCAATTAATAAACTTAATTTAAAGTTAGGTG
AAATTACTTGTTTGTTAATAATTAATTCTTTTAAAATATCAACAATTGCTGGTTGCCAATCATTT
AGTAGATTATTATTTAAGGCTTGAATGGGAACATTTTCATCTAATTCTAAACCAATGATTTCTAT
AAAGCACTGAGGATTCTCTTTCAGAATATAGTCTATAGCCGCCGCCGTATTATAACCTAAACCAA
AACAAATATCGAGAATGCGTAAATGATTTTGATACTTAATTTTTTCTGGTATCTGACAACCTTTT
ATATAAGTTATTTCTGCTTCTGTTTTTGCTCCATATTTAGTATGGAATGTTTCTCCAAACTCTGA
CGAGTAAAAAGTTTTTGAACCATCTTCCGTGATTCTTGTTTCAAAAGATGTCATGATGTCATAAA
GAAAAAAAGTTAAGAATTAGGATTTAAGTTTGGCTCTATCAGTTTGAGTATGTAAATTATTAGT
TAGGGTAGGCAATAGGAGATTATTAAATAATAATCTATAAACTTTTAGTTTTTATTTTGCTATA
AACACTATTCAATAAAGGTTATGGAAGTCCTGTTTCTCTTAATTTATTATAACCACACTCTGAAG
AGCTTTAAGTTTTTTAAAATTAAGGAATTAATTATAGAAAAAATCATTAATAGTTTGAGCAACA
AATTCTATATTTTCCTGAGTCAATTCTGGGTAAATTGGTAAACTTAAAACTTCTTGCGCTAACTT
CATTGAAATAGGATTTGGGTAGTATTTATCTTTATAAACAGGTAATTGATCTTGAGGAATGGGA
TAGTAAATCATTGAGCCTATGCCTTGTTGAGCTAAATATTCTTTCAATTGATCTCTTTTTCCATT
TATTACTCTGATGGTATATTGATGAAAAACATGACCTTTACTAATATTAGGAGTAATGATATTTT
CGTTATTTGCTAATAGGTGATTATAAATTTCTGCAACTTTTTGTCTTGTTTGATTCCATTGATTT
ATATACTTTAATTTTACTCTTAAAATACAGGCTTGTAAACTATCAAGGCGTGAATTATAACCTAA
CAATTCATTGTGATAATTCTTTTTTGAGCCGTGAACTCGCAACATTTTCGCTAACTCAGCAAGTT
CATCATCATTAGTGACAATCATTCCTGCGTCTCCGTATGCCCCTAGGTTTTTGGTGGGATAGAAA
GAATAAGCACCGAAATCTCCTAAATTACCCACGTATTTACCAACTAGGGAATCCCTTAAGGGTTT
TTGACAGTTTTCCTGACAATCTGGACAATCACCATAGTATATTGCACCAAATGCTTGAGCACAAT
CTTCGATAACCTTTAAGTTATGTTGTTTGGCGATTTCCTGAATTTTCCCCATAGCGGCAGGTTGT
CCATAAAGATGTACAGGTATAATTGCTTTGGTTTTTGGGGTAATTTTTTCTAAAATTGAGTTGGC
ATCGATGTTGAAAGTGGTGGTAGAAATATCAGCAAAAATGGGTGTCGCCCCGACAATACTGATAG
ATTCAGCTGTAGCATAAAAAGAAAATGAAGTGGTAATAACTTCGTCTCCCGCTCCTATATTTAAA
GCTCTCAAGGCAATAACTAAGGCATCTGTTCCAGAATTTACCCCAATAGCGTGTTTCACACCTAA
ATAATCGGCTATTTCTGTTTCTAGGGCTGTTACTTCTGAACCTAGAACAAATTGAGTTGATTCTA
ATACAGAGGCGATCGCACTATCGATTTCCCCCTTTAAGGATTGGTATTGAGGCTTTAAATCAAGA
ATGGGAATTTTCATGAATGACTACTAAGAGGATGATTAATCTAAGAGTTTACACTATTTTTTCAT
CGGAAAAAACCATCATCATTCAGAAGATTGAGAAGGAGGATTTGGGGTGTTTGTAGATGGATTA
TTTTGCTGTGCTAAACCTTGTTTAATAAAACTTAGGGCTTCTTGATAACTTTGATTTTCTGGTTG
AAGACTTACTAATTTTTCCATTAGGGGTAGGGCAGAAGCTAAATCTCCACCAGATAGATAAATTT

GAACTAGCTTTTCTAAGGCAAAAATGTTATCAGGTTCTCTTTGCAAAACCAATTTATAACCCTGA
GCCTCTTTTTCTAAAATTGCTTGAGCCGAATCAGTTTCTTGTTGTTGAGGTTGATTGGCATTACT
AGGACTTTTCAACATTTTTACAACGCCAAAAATTGAAAAAGAGAAAAAAGAAAGCCCAGATAAA
ATTACTAAAACCCTACGAAAAATTTGTTTAGGAGTGGTTTTTTTATTTATGTTAACCATTAGTTA
TAGAAATATTTTAAGCAAGTATCATTAACTATTTTACCCTCGACAAATCCAAGACCATGCAACAT
TTAGGGTAGAATTATTTTTATACTCGTAATGTGATTGATGTTTATAACCCATGATTTTTCCCGAT
TTTTCTGCATTTTCTAACCTTGTTCATCAGGGAAATTTTATTCCTGTTTATCGAGAATTGGTAGC
GGATTTAGAAACCCCTGTTTCTGCTTGGTATAAAGTTTGTGCAGACCAGCCCTATAGTTTCTTGT
TAGAGTCAGTAGAAGGTGGGGAAAATTTAGGAAGATATAGCTTTTTAGGTTGTGATCCTGTTTG
GATACTAGAAGCAAGGGGAAATCAGACCAAACAAGTTCATCGTGACGGTAAAATCGAAACTTTTT
CAGGAAATCCCTTTAACATCCTCTCTCAATGTTTAGAAACTATTAAACCTGTTAAATTACCAGAG
TTGCCCCCCGGTATTGGCGGACTATTTGGCTATTGGGGTTATGAGTTAATTTCTTGGATTGAACC
ACGAGTCAACGTTTATCCTGCCTCTGCGGAAAGTTTACCTGATGGTATTTGGATGCAGGTAGATA
ATTTATTGATTTTTGATCAAGTTAAAAGAAAAATATGGGCGATCGCATATGCAGATTTAAGACA
GGAGCATTTAAGCCTAGAAGAAATCTACAATCAGGCTTGTGACAAAATTTCTAAATTAGTTTTAA
AACTACAATTACCTTTACCAGTATCCGCAAAACCCCTTGAATTTAACCCCCCGCCAAAAACTCAAA
AAAATCATCGCATTCAATACAAAAGCAACACCAGCAAAGAGGCATTTTGTGCCAATGTGGAAAAA
GCTAAAGAATACATTAAAGCAGGAGATATTTTTCAAGTAGTCATTTCTCAGAAATTACAATCTCA
TTATCAAGGTCATCCTTTTGAGTTGTATCGCTCCTTACGTTTGATTAACCCTTCCCCTTATATGGC
TTACTATAACTTTCATGGATGGCAGTTAATCGGCTCTTCTCCCGAAGTAATGGTTAAAGCAGAAA
AAACTAATGATGGTAAAACCAAAGCCGTAGTCCGTCCCATTGCAGGAACACGTCCAAGGGGCAGA
AACTATCAAGAAGACCAATTTTTAGCCCAAGACTTACTCAATGATCCCAAAGAAAGGGCAGAACA
CGTCATGTTAGTAGATTTGGGCAGAAATGACTTAGGCAGGGTCTGTGTAAAAGGAAGCGTGGAA
GTCGATGAATTGATGGTTATTGAACGTTATTCCCACGTAATGCACATAGTCAGTAACGTTGTGGG
AGAGTTAGCAGGGGATTACTGTGCATGGGATTTACTAAAGGCGGCTTTCCCCGCAGGTACGGTAA
GTGGAGCTCCTAAAATTCGAGCAATGGAAATTATTAATGAACTTGAACCCGAAAGGAGGGGACCT
TATTCTGGGGTTTATGGTTACTATGATTTTGAAGGACAGTTAAATACTGCTATCACAATTAGAAC
GATGGTTGTGGAAACCCTTTCTGGGAAGGAAAATCACTTGGTATCCGTTCAAGCTGGAGCAGGCT
TAGTCGCTGACTCAGATCCAGACAAGGAGTATCAAGAAACTTTGAATAAAGCTAGAGGTTTGCTA
GAGGCGATTAGTAGCTTAAGCTAAGTGAAAATTCGTAATAAAAAAGAGCAAATTTTTGTGACTT
TAAACCTGACACCTGACACCTGATACCTGACACCTGAAATTTATTTTTGAACTCCGAACCCAAAT
AATGATTCAGGTTGCTTAAACTCAGAGAATCATTAATAATTGATTCTTGTTAACTCAATTGTCAT
TTTTAACATGGGTGTTGAACTCCGAAGCTATGTCTATCTCGATCGATTACAACCTCAACACGCTT
CCTATATAGGTACGGTTGCTCTAGGATTTTTACCTTTACCCGGTGATGCTTCTTTATGGATTGAA
ATATCCCCCGGAATTGAAATAAATCGAATTACTGATGTGGCTTTGAAGTCTGCTGTAGTCAGACC
GGGAGTTCAATTTGTGGAACGTCTTTATGGTTTATTGGAGATTCATTCTAGTAATCAAGGGGAAG
TGAAAACGGCTGGAATGGCGATTTTAGATGCTTTAGGTGTGAGTGAAAAAGATTCTCTTAAACCA
GAGGTGGTTTCTAGCCAAATTATTCGCAATATTGACCCTTATCAAGCCCAAATCATTAATCGTAA
TCGTCGAGGACAAATGTTGTTAGCAGGAGAAACTCTTTATGTGTTGGAAGTTAAACCAGCCGCTT
ATGCGGCATTGGCGGCAAACGAAGCGGAGAAGGCGGCGTTGATTAATATTTTGACGGTTCAGGCG
GTGGGTAGTTTTGGACGACTTTATTTAGGAGGTGCTGAAAGGGATATTAAGGCAGGGGCGGCGGC
GGCTTTAGATGCGATCGCAAATATCGGTGGGAGAGTAAGTGTTTCAAAAATAAATGAATAAAAA
AACTCTTTGTTTATGGGAATTATAAATAATCCTCATCCTCAGATAAGTTAAGACAAACCTATAAT

GGTTTAATAAGATAAAAAATTACTATTAAAGAGGATTTAATTTATGGTAGCGACCACCGTTCAA
AAAACCTCTCATCGTTTAACTACGCAAATTGATATTATTGCTAAGGATACTACCGCAATTCGTTC
TTTAGATTGGGATCGCGATCGCTTCGATATTGAATTTGGTTTGCAAAACGGCACAACTTATAATT
CCTATATTATCGAGTCAGAAAAAAAGGCTTTAGTTGATACCTCTCATCTTAAATTTAAACAGTTA
TACTTTGACGCATTAAATCAGGTTATTAATCCCCAAGATCTTGATTATCTGATTATTTCCCACAC
TGAACCTGATCATAGTGGCTTAGTTAAAGACTTGTTAGAAATTGCTCCTCACATTACGGTAGTGG
CTTCTAAAGTTGCCATTCAATTTTTAGAGGGATTTGTTCACCTCGATTTTAAAAAACAGATTGTC
AAAAATGGCGATCGCCTCGATTTAGGTAATGGTCATGTAATCGAATTTGTTAATGCTCCTAATTT
GCATTGGCCCGACACCATTTTTAGCTACGATCATGGCACTGGTATTATGTTTACCTGTGATGCTT
TTGGGATGCACTATTGTAGTGATTATCTCTACGACGAAAATTTACAAGACATTGAGCCAGACTAC
CGCTTTTATTATGAGTGTTTGATGGCACCGAATGCCCGTTCGGTTTTATCCGCCATGAAAAGAAT
GGACGCATTGGGAGAAATTAACCTTGTAGCTAATGGTCACGGACCTATTCTCAAGTATAATGTCA
AAGAATTGCTCGATCGCTATCACCGTTGGAGTAGCGAACAAAGTAAAGGGGAAAAAACGGTTGCA
GTATTTTATATTTCTGATTATGGATACAGCGATCGCATCTCTCAAGCTATTGCCAAAGGAATTAC
CAAAACTGGGATTACAGTAGAAATGTTCGATCTCAATGGCATAGAAATTCAAGAAATTCCCGAAA
TAGTAAGTTCTAGTGCGGGAATAGTCTTGGGAATGCCCCCCTATCATCTCAAGCCTTAAACAAC
AAAGTTGGCACAATTTTAGCTACAGTTAACAGCAAGCAATACTTTGGCTTATACGAGTCTTATGG
TGGTGATGACGAACCTATCGATCCTTTAGGGACTAAACTAAGAGATTTAGGCTTAAATGAAGCCT
TTGCCTCTATTCGGATTAAAGAAACTCCCCTAGAAACCACTTATCAAATATGCGAAGAGTCTGGA
GTTGACTTAGGACAGTTTTTAACCAAAAAAGCAACCATTAAGCAAAGAAAAGCCCTTGATAGTGA
CTTAGATAAAGCCATTGGGCGTATAAGTGGCGGTTTATATATTATTACAGCCCAAAAAGGTGAAA
CTAACGGAGCTATGTTAGCTTCTTGGGTAACACAGGCTAGTTTTGACCCCCCCGGATTTACCGTT
GCCGTTGCTAAAGATCGTGCGATCGAATCTTTACTACAAGTAGATGATACCTTTGTTTTAAATAT
CCTTGAAGAAGGAAACTATCAAAACTTAATGAAACACTTTTTAAAACGTTTCCCCCCCGGTGCTG
ATCGTTTTGCAGGAGTAAAAGTTCAAACAGCCAATAATGGTTCACCCATTCTCATTGATGCCTTA
GCCTACTTAGAATGTCAAGTCGTTAGTAGAATGGATTGCGGGGATCATTGGATCGTTTATAGTAA
AGTTACAGCAGGTAGAGTTTCAAAATCAGACGCTTTAACCGCCGTACATCATCGCAAAGTCGGTA
ATTACTATTAAGAATAGGTAATCTCGGTTCAGAGTTCGGGGTTCGGAGTTCGGGGTGTTCACTGC
ACTCGTACACTTCTTTCAGTCATGAACGGGATTTTTAATTATTAATTCTTAATTATCAACTATTT
AACGTCAGTTCAGGTTAAGGCAATTTTATCGTTATTTCTAAGAAGCTATAAGGTTTTCTGACTAC
GGGTTGGGATGCTTTCAGCTTATCCAAAACTCAGGTTATTTACCTTTGCCCTCCCCCCTCTCGAGA
GGGATAAAGGGGGGTTTGCCCCTAGCCCTTTTTATAAAAAAAGAATTAAAAGCTAATCAGAAAT
GAAAAAAATAGGCAGTTTAGGAGAGAAAATAATTGCCCAGTGGTTAATAAATAAAGGTTATTCT
GTTATAGAATCAAATTGGCATTGTCGTTGGGGTGAAATTGACCTTATAGTAATAAATAAAAGTT
TAAAAGAGATTATATTTGTTGAAGTTAAAACTCGCAGTATAAAAAAATTGGGATGATAATGGGCT
ATACTCTATTAATAACAAAAAACAAGAAAAACTCTGGTTAACAGCTAGTTTATTTTTAGAAAAA
AATCAAATATTTACTGATTGGAACTGTCGTTTTGATATTGCTTTATTAACTTACAAAAAATTAAC
TAACTTTGGGACAGTATTTTCTGTTAACGATAATTGTTTAAGACCAAAGTTTAACTATGAAGGCT
ATCAATTTGAAATTGTTGATTATTTGGAAAATGCTTTTTAATCTGATCTTCTGCACGTCAAATAG
TATTCTACAAGACAATTCAGAATTTAGAATATATTTTTTGGCGTTGCTTAACTATGGTATGAAAT
TTACTGAAATTATGCCAAAAATTGAGACATCAAAATATTATCATTACTGCAAGAATGCCCATTTC
CCCAACCCCACCTAAATGAGAAATCATATACAAAATTAGTAAAGCCTATTTTTTTGTTACGAGAT
TATCCCCTAACTTTCTATTGAGGAATCTTCAATTGTTTATCAATGCTTTTTCTGCTATAATTGCA

CTCATGGTAAGGATTGAAAACCTAAATTTTAAGGTCAATTATCTTAACTTAATCGATAGTTGTCT
AAGTATAACTAATTGTTATAGTTCATGAAGATCAAAATATATTGCATTTTCCGCAGTATTGACAG
AAACAGAAGTTAGTAATAAAGCAACACTATGGGTATCAGAGAAATAGTTGATAATTCAAGCGAT
TTAGAATTTAGAATCGTTAGATTGATGCTAATGCCAATAGAAATAGAATTAACTTTTATTAAAG
AAATAGCTACTATTCCTTTAGTCTCAGCTCAAAATTAGTGGACTCTCTACTAAATTCTGTTTAGG
ATTGCCTTGTTAGTTAATAAATTCGCTATTCTCCTGATTATTTTTGCGAGGAGAAAAGTCAAGTA
TTTACTTTTTTAAAATTATGTATAAAGATATAATTAGTCACCGATTAAAACTTTCATTTTAAGAT
GATATGAGTCTAATTGTGAACATGGAAAAGTCGGGTTTTTTAAATTTTACAACTTTTAGGAAACT
GAAAATATAAAGTATGAAAAACTTTATTTATTCCTGAAGTCAATAGGACAAAGTTCGATGGGCA
AAGGGCAAAATGATTTTGCAAGTACCTTGACAAACCAAAAATTACTCGTCTTAAAAGACTGAGTT
TTAAACCCATTGTTCTTTGATAAATGATAAAGAAACGATAGGATAAGTTAATAAAAAATGATTT
TCTGGGAATCACAAGAATTTATGAAGAGATATAAAAAAACTTTCCTAAGTTAAGAAAATTAAGA
AAAACTAATCCATGAGACTACATAAAAACAAGGCAAAATTTTAAGATTGAACTAACATTAATAA
TTGATTTCTTTACCATAATTACTTTGTCTTTTCATAACTAATTGTGATTAAAACAGTTGAAAGTC
AGACTCTTTTTATTAGCTTTATATCGTTAACTAAACATTATCTGATTGTGACAAATTTAAGCAAA
ATATTTTCAAAATTATCAACACAGAAGCGAAAATTTTGTGAGGGAATTGAATGCCAAAACAAAT
AATAATTGCAGAAAAAAATCATATAGCGGCAGTATTTGCTGAAGATCAAATTCAAGAATTAGTG
GTGGCTACCGGAAACCAACAGGTAGGAGATATTTATTTAGGAACAGTCGAAAATGTTATTTCGGG
TATTGATGCGGCTTTTGTCAATATAGGCGATGCGGAAAAGAATGGTTTTATCCATGTTACTGATT
TGGGTCCTTTACGATTAAAAAGAAGCGCAGGGGCAATTACTGAATTATTAACTCCTCATCAATCC
GTATTGGTTCAAGTAATGAAAGAACCTACAGGAACAAAAGGACCTCGTTTGACAGGTAATATTA
GTTTACCGGGGCGTTATTTGGTGTTAATGCCTTATGGAAAGGGAGTTAGTCTTTCTCGACGCATC
AAGAATGATAATGAGCGTAATCGCCTTAGAGCATTAGCTATTCTTTTGAAACCTGCGGGGATGGG
TTTGTTGGTTCGCACAGAAGCTGATGGTAAAGGAGAAGATGCGATTATTGAGGATTTGGAGTTTC
TCAAAAAACAGTGGGAAAAAATCCAACAGGAAGCGAATACCTTAAATCCTCCTGCGTTGTTGAAT
CGAGATGATGATTTCATTCAACGGGTTTTGCGAGATATGTATAGTGACGATGTCAATCGTATTGT
AGTTGATTCTGACGATGCGGTAAGAAGGGTTAAAAAACAGTTAAATAATTGGGGAAGTGGTAAA
AATCCTCATGGTTTATTAATTGATTCTCATCAGGATAGCCGTTCTATTTTAGATTATTTCCGAGT
TAATAGTGCCATTAAAGAAGCATTGAAACCGAGGGTTGATTTACCTTCTGGGGGATATATTATCA
TTGAACCCACAGAAGCATTAACGGTAATTGATGTTAACTCTGGCTCTTTTACTCATTCAGCGACT
TCCCGAGAAACGGTTTTATGGACTAATACTGAAGCTGCGATCGAAATTGCTCGTCAGTTAAAATT
ACGTAATATTGGTGGGGTGATTGTGGTTGACTTCATTGATATGGATTCTCGCCGAGATAAATTAA
AATTGTTAGAGCATTTTAACCGAGTTTTACAAAGCGATAAGGCTAGACCTCAAATCGCACAGTTA
TCAGAATTAGGATTAGTGGAACTAACTCGTAAGCGTCAGGGTAAAAATATTTATGAATTATTTA
GTCAACCCTGTAGTCATTGTGGTGGTTTAGGCTTAGTGGGTCATTTCCCCGGGCATTATGATTTA
GAAAGTCTTACTTCCAATAATTATTATCAACCTCCTCAAATTAGCAAAACAGAGAAGCCTATTCT
CGATGAAAAAATTATTTCTCTTGATGATTTTAGTAGCGATAATGATGAGTTAGATTTAGTTAATC
ATCCTAGTTATCAAGAAACGAACAATGGTAATAGTCGTCGCCGTCGTCGTCGTCGAGAAAATGGG
GTGAAAGAAGAGCCTATTTTAGCGGGAAGTAATGGTAATGGGGGAGAGACTGAAAGTAAGCCTG
AAAGAAAAGAGCGTAGTTTATTATCTCGTTCTTTACGCAGAGATGAAAGTCAGTGGGAAAAAGT
GGTAGTAGAAATGACTCCTGCGGAGCAAGATGTTTATGCTTTTATGGGGGTTTCTCCTTTACTCC
GTTTAGAACAAGAATTTAAAGACCCTAAATCGGTTTTAGTTTATGTGCGTAATCCAGAGGAGCAG
GAAAATATAGAAAATATAGATACTGCCATGGAAAAAAATCAGCAAGAGGATATAGAAATAGCTG

ATGATGAGGACAAAAATGCGATCGAGGTAGCTCCCGCCGCCGAGGTTATTTTCCCCCAAGGGAAG
GAAACGATGGTAAATGAATTAGAAATTGATGTACAAGTGGGAGAAGTGGATGAGCAAATAGAAT
TAGAGGAAGATGACCACTTTGAGGAAAGTTCCAAAGAAGTCTTTGATGGTGATAATAGTGAAAC
TGCTGAAGAAACTATTACTGATGCCATTATAAAAGAGGCTTTAGAGAGCGATCGCCCCGTTGTCA
GAACTCGTCGTCGTCGCACTTCTCGTAATTCTTGATTTTAGTTAACTAACCTCAGTTCGGTTTAA
GAATATCCGATAAGGTTAGGTGTTAGGTGTCAGGTTGCAGGAACAGCAGGTGGTAGGGGTTTTTA
GCAAGGGGCTTAAGCCCCTTGTTTAAGTCAGTTCGGATTAAGGCAATTTTATCGTTATTTCTAAG
TTATTTCTAAGAAGCTATAAGGTTTTTTGACTACGAGTTGGTCTGCTTTCAGCTTATCCAAAACT
CAGGTTAACTAGGAATTGGTAATCAAAGGGTAAGGGGCAAGGGGCAAAGGTAAATAGTGTTGGG
GTGAATAGTGAGTAGTGAATAGTGAGTAGTTGATAATTACTCGTTACTCATTACTTTTTTCTGAA
ACCTGAAACCTGAAACCTGAAACCCGACACCTTATCTCCCTCTCACCTCCTCCCCCCATCACCCCCT
ATTCTCATTATTCCTTAACTTCTATACTCGTGAATAACGTAAGGTAAACTAATTACTCTCCCCTC
TGCTTTTCCTATTTTCACCTGTAATCCTTCTCCTCCTGCTTTGGTGCGAATATAACCAAGGGCAAA
AGGTTCATTTTCATAATTAATGAAACTGGTAATTCTTCCTATTTTTTCTTCTCCTAAAAAAATAG
TATTTTCTATGTCTGGATTAATTTCTTGGTTTAATTTTATACCCCAGAGTCTTTGTTTAACTCCT
TTATAAGTATTTAATCTAGCAATGGTTTCCTGTCCAATATAACAGCCTTTACTAAAGGAAATTGA
GTCCCATAAGCCTGTTTCTAAAGGGTTAAAATCTTCTGTTAATTCTTTTTCTGGGGCTGGTCTGC
CTCTTAATACTCTTAGGATTTCCCATTCTTTACTACCTATTAATTTAGGTTTTTTTTCGATTAAT
TTCTGCCAAATAATGTCTGCTTGCTCTTGGTTAACGATTAAGTTATATCCTTTTATTTTTAAGTT
GCATCCCACAGTTAATAAAATTTCTATACCATCTATGGTTAAATTTTGGTGATAAAATTCTGGTT
TTTCTAATATTTCTTTATCTACCCAATTACTTAATAATTCTTGACTGTTATTACCGAATATTGTG
AATATTTTATAATCTGAGGAAATATCCTTTAATTCTACTTTATCAAAAGGAAATATATAACGATC
CATCCATTGGTATAATTTCTGATTTTGATTTGGTGATGTTAATAATAAAACTTCTTCCTCTTTAA
AGTAAACACTAACTAAATCGATATTTCTACCAGTAGAATTGACAAAAACTGTATCACATCCTTGC
CCAGATTTTAAAGATTGAATATCATTTGTGCTTTGATTATGTAAAAACCTAAGACGATCGCATCC
TGTAACTTTTAATAAACCCCAATCACTGCGATCGCACAAAAAAACGTTATTTTCCCATTGAGAAA
GGGAAAAATTTTGATTGTTAAAAGTCAATGGTACTTCAAGATTTTCTGCAAAAACTGCCCCTGAT
TGTGTCTGTAATTCCTTTAATTTATTCATAATATTATGGTCAAGCCAATGAAAACTTGTGATCAA
TGATACTAAATATAATCTTCTTTCATGAGCATAATTAATGCCATAAATCATAATTGATATAAAAA
TGTTACCTTTAATTGAAGCTATTTTAAATGGTATTAGCATTGGCTCAGTCTTGCTCATCGCCGCT
TTAGGGTTAGCGATTGTTTTCGGTTTGATGGGGGTAATTAATCTCGCTCACGGTGAATTAATGAT
GATTGGTGCTTATACCACCTTTGTGATCCAGAATATATTTAAGCCTTTAGGTGAACCTTTTTTCG
ATTGGTATATTATCGTTGCCATTCCCGTTGCTTTTATCGTTACAGCATTAGCAGGACTAATTTTA
GAAAAAGGAGTCATAAAATTTCTCTATGGCAGACCTTTAGAAACTCTTTTAGCAACATGGGGAGT
TAGTTTAATTCTGCGTCAATTTGTGCGTAGCGTTAACGGCTCAATGGCTATCGGTTTAACTATTT
TTTCCCTTCTCTTTTTCGGGGCGATGTTTTTCTTTTCCCGCCGTCAAAATTGGTTACAAATTCGCA
ACCTTTTCCTTTATATTACCTTACCTCTCACAGGTGCGATCGCAATTACCACAGGAATTTTAATCA
ATCAATCTCAAAATAAATCCCTAACAACTCCTTGGTTTAGTGCCAGAAACGTGGATGTAACCGCC
CCAGAATGGTTAAGAGGAGGAATACCTTTAGGAGACTTTCAATTTCCCACTGCCCGTATTTTTAT
TATCGTTTTAACCTTACTTTGTTTAGCAGGAGTATATTGGTTTTTAAACGTCACAGATTGGGGCT
TAAAAATTCGTGCTGTTACCCAAAACCGTACCATGAGTTCTTGTTTAGGTATTCCCACCGCTACC
GTTGACGCTCTTACCTTCGCCCTCGGTTCAGGTTTAGCAGGGATTGCAGGAGTCGCCATCAGCCTT
TTAGGCTCAGTGGGACCAAACACAGGACAAAACTATATTGTTGATGCGTTTATGGTGGTGGTAGT

AGGAGGAGTTGGTAATCTTTTAGGTTCAATCATCGCCGCCTTTGCCATTGGCACTGTTAGTTATA
TTGTCGGTTCTGGCACTCTTGGTTTAATAATTATGTCTCAAGAATCCTTAACCCCTTTATTAGAC
ATCGTCAACTTTTTTGCAACCACAAGTATGGCGAAAGTTCTTGTTTTCGTAATTATTATTGGTTT
TCTGCAAGTTAAACCCTCTGGTATTTTCCCTCAAAAAGGACGCACCGCAGAATTATAAGCTGACG
TTACGCAATTAATTCAGAGTTTTATTAGGTTTCAGGTTGCAGGTTGCAGGTTGCAGGTGCTAAGG
TGATGAGGAGATGAGGGGAGAGGGAGAAACAAGTAATGAGTAATGAGTAAGGGGTAATGAGTTA
AGGGTTTTTAATTCTTAATTCTTAATTCTTAATTTTCAACTATTCACTATTCACTATTTGTCTCA
TTCTTCTATCACTGAGGGCTTAAGATTCATCGGTAAATAAAAAGTAAAACAACTCCCCTGATTTT
TTTCTGATGTTAGAGTAATATCCCCCCCATGAAGCCTTGCTAATTCACGAGAAATTACTAACCCT
AAACCGCTACCTCTATGTTTTTTTTGCAGATGAGTTTGTAATTGAGAGAAAGGTTTAAATAATTT
ACTTTGAGATTCTTTATCTATGCCAATACCTGTATCAATAATTTGAAAAATTAGATTATTTTTTT
CTTTAGTAACTTCAAGAGTTACAGAGCCTTTTTCGGTGAATTTTACAGCATTGGAAAGAAGATTA
ACTAATATTTGTTTTATACGTCTTTGATCTGCAAAACAATAACTAATATCTGAACTTATTTCCAA
ATTTAAGTTTAAATTTTTTTCATCAGCCTTAGTTTTTATCAGTGCTAAAGATGATTCACAAAGCT
CATATACTAATATTTGTTCAATAAATAATTCTTCTTTTTCTGCTTGTATTTTACTGATGTCTAGC
AAATCACTAATTAATTCTAAGAGATATGTTCCCGACTGATAAATTGCATCTATATATTGAGCTTG
TTTTGGGTTTAAATCTCCATATAATCTTTGCTGTAACATTTTTGCAAATCCAATTACTGCGGCGA
TGGGAGTTCTTAATTCATGATTCATGTGGGAGATATATTGACTCTGATTTCTTTGATTTTCTTTG
AGTCTTTCTTCTTCTTTTTTTAGGGTTTCTTGTTTTTCTTCAAGGGTTTGATTATAAAGATATAA
GGTAATATATTTAGCAATAGTATCAATTTCTTTTATTTTGTTTTTTAAAAATTTTTTATTGGATT
CAGTCTCTATGTATAAATAACCCAGATGATACTTTTTAAAAATTAAGGGAATAATGTATATATTT
TCACGATTTTTCTGCTCCTCATTTTTGGGAAAATTGCTGTAACAACTATAATTTGAATTTAAAAT
TAATTCATCTTTAATTCTTTTTTTGATAACTTCTAATTTATTTTCTCTTTCTCCCGTTAATATTCG
ATCATATATAATTTCTATCTCATCTTCGTTTTCTGCTAATTGTTGACAAACAATAATATTTTTTA
CTTCAATTAATTTAGAAATAAAAATCACACAAGAGTTGCAAAAGTCTTCTAAATTTTCTGCTGTT
TCTAGCTGGAACAATAAAGAATTATTTTTATCTAAATTTTGATTGTTATCTATGACATCATTATC
TTCTACGACAATATTATAGGACATAATTTTCCATGATTTATTCAGTTAAACATCTAAGGTAAATG
GTTAAACGAAGTATATAATTTATACATCTACACTCCTAGTTTTTTCTAGGTTTTAACCTTCATTA
CTGTTTAAAACTGCCTCCGAGAAAAAATTAAAATACTAATTGTTAACAGTAAAACAATATATAA
AATTGCATAAACAACACTACTTAATAGTTCTCCTGTAGAAGGCAAAATGTTATAAACAGCCTCAT
TTTTTAAATTGAGTCTCTCCAGATCTGGTAAGATTAAAAACAATGCTTTTGTAATTTTTTCGATG
CCGGGATTCTCAGTGATTCTACCTAATTCTAATAGATCTCGGCTCAAATGTCCCATAATATAGAC
CCCAAAACTGAGGAGAGTTGCTAAAATCGAGCTAGTAAAAACCCCGAAAGTCATGGCTACTGCGG
TTAAGAGGGCTAATTCTAGTAAAATAAATGCGATCGCAACCAAAAGACTAACTAAAGGGAAAGC
AATACCCAACATTGCCATAAGAGCGAAATAAGTAATTGTCATTACAGCAACTAACACCAAAAGCA
CCGTAGATAAACCTAAGTGTTTTCCGACAATAAATTCTGTATTCGTTATGGGCTTAGGCACAAGA
ATTAAAACAGTTTTTTTCTCAATCTCCTTATTTATTAATCCAGTACCTACAAAGATAGCTACGAT
CGCACCTAATAAACTGATACCACCGATACCTAAATCAAGGAAAATTTTTTCATCCGCACTAACAG
CAATGGGGGGAAGAATGCGAGACGCTAAAACCAAAATGAGGACAAACAAACCGATAACATATAA
AATACGATCTCTAATTACTTCACGAAAACCATTAGTTGCGATCGCAAAAATACGGCTTATATTCA
TGATTAGTTCATTCTCTCATCAACAATAAAATACTTTAAATAGCAATCCTTTAAGGAATACCATA
AAGCAATTTTAACAAAAAAACGCCCCTCGCCTATGGGAGAGGGGTTGGGGGTGAGGGAAAATTA
ACCTACAAACCACCCTCAACTATGACAATCTCTTCCGCCATCAACCCATACAACTTATAAACCATT

TCATCTATCTTTCTCTCTAATTTCTCTATATCTGCTTCAGGATTTTCTTTCTTTTTCTCTAATATT
TTTTCCACCATTTCTTTAAATGGTTTTTCTTCTTTATTGTTTATTTGTTTAACAGGTATTTTTTCC
ATAAATACTTTTCTTAATTCTCTTGTACCTCCCAATAATTCTGGAAAATTATCGCTAAAACAATA
TTTAAAAAGACTAGAGTTTAAAAAACTAACTAACCAATAAATATTATGACCAACTATATGAAAA
GATTTATCATTATGGTAATAATGCTTAGATAAATCTATAGTAAAAGGTAAAAATTTAGTCATAT
TTGGATATATGATTTTAGGTTTTTCAAAATCTTCCCAATAAGCAATATTATCAGAAGTTTCAAAC
CATTTATTATGAGTTTTTTTTCTGCTTCCTTTTTTGCCTGATTGTTCCAATCTTTCCCCAAAACTG
CTTAAATATTCTTTAATAGCAGAAAAATCATTAATATCATATTTTTTAGATGGAAAAAGGGCAA
TTAACCACAAATCTTGAAAATTAGAATAATATTTTTGAATATCTCTACCACGCAATAAAGGCTTA
ATTACTTCTGCCGATTTACTATCTTGATTAATTAAATTTTCTCTGGTTTTACCATCAATAATAAA
CGCTTCATTATAACCAGTTTTAATACCATAATTGATAGTAATATCCCAGTTTTTTAAAGGTGTGC
CTTGAGTTTCAACTTTTCTTTTAATTCTTAATACTTCTGAAGATTCAAAACTCCAACTATCTATA
GTTAAATTGCTTTGTAAAAAATCTCGCTTAACCTCTGCTAAACATTGCCTAAAATTATTAATATT
GAGATGATAACTGGCAAAATTGCCGTATAGAAAATGATGATTATTAATTTTATTTTTCACAAAA
CCCACAATACAAGCATCTACCGTCGCTTCCTCGAACACTGATAAACCACTAAAATCAATAAAATG
AGTTAAAGCAGTATTATCTAATAAAAATTGACGCATTTTTTGCCCATAATTAGCACGAGTAAACT
TGTTAGAAATGATAAACTGAAAAACACCATTAACTTTAAGCAAATTAAAACCTAATTCCACAAA
ATAAGTAAGCAAATCAGCTATAGAATGATAAATTTGATATTTACTTTTTAAAATAGGCTTTAAT
GCAGAAAATTCCTCTTGACGAATATAGGGAGGATTACCGATGATGATGTCAAAACCGAGAAAATC
CCCATTGTCATCGAGAATTTCGGGAAACTCAAAACGCCACTCAAAACCATTTTGATAAATACGAT
TATCCCTTTTATCTTCTCTCTCCAACTGTAATTTATTAATCTCTTTTTCTAACTGTTTTTGTTTTT
TCTCCCTTGCCTTTTTCTCCTTCGCCGTTTCCGCAAACAAAGAAGTTTGATTCACTAAACCATTTA
ACTCCCCTTCCAACTGCCGTAAACGAGTAGAAATAGCATCAACTCCCGTAATCTCACTGCTAAGAC
TTGCCTTAATTTCATTGATTACCCTTTCAACTCTTTGTTTTTCCTGCTTACTCTCAGGGTTGCGAT
AGGCTTTCACCGCCTCCTTATATTCAGCAATTTGACGCTTTACCTTCGGTGAGGAGATACTATCCT
TTAAATCAAAACGACTAATTAAAGAATTACCACACTTAATATTAATATCGATATTCGGTAAAGTT
TGCAGAGGAAAAAAAGAAAAACCACCGCCTCTTATTCCCCTCTCCTGTGGGAGAGGGGCTAGGGG
TGAGGGTAAATTCTCCTGTGGGAGAGGGGTTAGGGCTGAGGGTAAATTCTCCTGCGGGAGAGGAG
TTAGGGATGAGGATAAATTCTCCTGCGGGAGAGGGGCTAGGGGTGAGGGTAAATTCTCCTGTGAG
AGAGGAGTTAGGGATGAGGATAAAACCCCATTGTCAGGAATAATATAATAGGCATTTTTTAACA
ACTCAATCCAAAGACGCAAACGGCATATTTTCACCGAATTAATATTTATATCTACCCCAAATAAG
CAATTTTCAATAATCACTTGCTTTTCGTGAAATAAAGCCTCTTGAATCCGTCTTGATTCTCGATT
TTTAGGATTATAAGCAAAAGGTTTATTATCTTCATCCGTTACTATTAACTCATCATTTTCCACCG
TGACAAAATAATCAGTTTTTTTAATAGTTTTCCCTTGCCTATCAAGCAAAATATTCAACTCACTT
TTGAGGGCGATAATTTCATTTAAAGCCGAAACTAAAAAATGTCCACTACCCACCGCAGGATCGCA
TATTTTGATACTATTAATAATCTGGTTAGCTTCTTGTTTATCCTCTATTTTTTCATACAAATCAT
CAATAGTTTGACAATTCCATCCTTTAATTTGATTAAACTTATCGACAATAGCACGGCGAATAGTT
TCCCGACACATATACATAGTAATGAAACCGGGGGTAAAATAAGAACCATCTTGATAACCGTTAAT
TTTCTCAAAGATTAACCCCAATACCGAAGCATTAATGAGAGTTTTATTATCTTCTTGAATTTCCA
AACTGCCCTCACTACTAAAATCATAGGCAGAAAGAAAGTTAAATAAATAATCAAGGGTTTTTTGC
GGTTGAGTAAGAGGTTTTCCTTGACTATCTTTTAAAACCGTATGGGAATATAAATCTAATTCTAT
CTGATAATTAAGGCTACTAATACCAATAGTTTGTCTTTCAATAACTGTTACTTCAAAAAGTGAAC
TATTAAGATAAGGAATATGGTTAAATTTAGCTTGAATATCTGCTTTTCTTTCGTGATTTTGTTTT

GCTAATACTTGAAAAAAGAGACATTCTAACTCGTGAAAATTAGTGATTATTTTGCTATTAAGAA
AATTAAATCCCCTTAACTCATCGTTACTAAAGTCTCTCCTCGCCTTTGGGAGAGGGGTTGGGGGT
GAGGGTAAAAGAGGGGTTAGAGGGGAGTTATAGCGAATTAGTTGCGCCTCAAGAAGTTTAAGAA
ATAAAATGCGGTTAACCCATGTAATAACTAATTCTAAAGCGATATTAAAGAGTCTTTGTTGATAA
TTTTCGCCGTATTCTTCGGGATTTTGCAGATAATGGACTTTTTCGCTAATATCTAACTGATTGAT
AGTATTTTCTAATAATGAAGCGGAGTGCCGTTGATTTTCGGGTAATCTTTGAATAATTTTCTTGT
TTCCCTGTTTAACTTCTGCTAATCCGATAATGTGTAATAATTCGCTATAAAAACCTTGATCTAAA
CTATTACTATCGTTAGCAAAAGGTTGTTTTAAAAGGTGTTGAGGAGAGAAAAATTTATATAAAA
CAATTAACTCTTTTTCATCTTCTTTTTTATAGAAATTATTTAAAGTAAAATGAATACAGTTATCT
TTTAACTCATTTTCTACCTGATTAATAGCAGGTTTAGCAATTTCTTGATAGAAAAAATTACGATT
ACTACTAGATAATAATTTATTATTAAAATCATCAAATAATTTAATAAGTTTTTTATCTTGATAA
AATAATTTTTCAAATAAAGTGGCAGGGAAAACATAAAAATCAAGAGAGTTAGTAATAATTAAAT
GCTTTAATTCTAGGTTTTTATTAATAACTCTTTCCTGCAAATAGTAAAAAAGAAGTTGCTGTAAT
GATTTACTATTAAACTTCTGCTCATTAATCATTTCTTGGGTGTTAGTGGGGCTTTTAGTTTCGAT
AATTACCCCTACAGATGATTTGGCATTTTTACCATTATAAATAACTAGATCATGGCTTTCCTTAG
TATTTATATAATAGTTAGGATGGTAATAAGTCTGTTTAAAAAAGTTAGCTAAATTGTTTTTATG
ATATTCTTCTGTTTCGCTTAAATTAGTGTTGATTTGAGTGTATAATTCTTGTAAATTCTGTTTAA
ATTGATTAATTTCTTGTCGTGAGGGTTTGACTTTTAAAAAAGCTTTATTCAGACTATGAAGAATC
GTTTTCATTGATTAATCATGGGTGAGGATTTAAACTATCAATCTAATTGTAATATATAACTCACA
TCTTGCACAAATACACCGTAGCTATTTCCAAAAATTGGGGTCTTATGCTCGAAATACGGTGCAAA
AATCCCCATTTAATTTTAACCCCGAACTCCTGTATGGGCGAATAGCCATTCGCCCCTAACTAACTC
CGAACCCATTTAATCCCCTTGGTGTAAATACCGAATTGTCACCAAAATGAGAATTATGTTAAGTT
TAGTTAAGCAATAGTTGTTACAATAAATAAATATAAAAAGCAGAAAAGATAAAATAAGTTAACA
ACTTATACCCTTTTGCAAACAAACAAAATAAGAAAATATGAAATTATCGTGGCGTACAGTCCTAT
TATGGACGATTCCTTTTTTAGTAATAGGATTTTTCATTTGGCAAGGAAGTTTTGCGACTAATGTG
GGTAACACTGCTAATACTGCTAGTACTCGCATGACCTATGGTAGATTTTTAGAATACTTAGATAA
GGGTAGAGTCAGCAGTGTTGATTTGTATGAAGGTGGTCGTACTGCAATTGTAGAAGCGATCGATC
CTGAGTTACATCAAGTACAAAGATTAAGAGTTGACTTACCCGGTACTTCTCCTGAGTTAGTTACC
AAGTTAAGAGAATCTGGGGTTAATTTTGATTCTCATCCCGTTCGTAATGAAGGTGCTATTTGGGG
CATTTTAGGTAATTTAGTCTTCCCCGTATTGTTGATTGCTTCTCTATTTTTCCTTTTCCGTCGCTC
CAGTAATATGCCCGGTGGCCCCGGACAGGCGATGAATTTTGGTAAGTCCAAAGCCCGTTTTATGA
TGGAAGCCAAAACTGGTGTTATGTTTGATGATGTGGCGGGTATTGATGAAGCAAAAGAAGAATT
ACAAGAAGTTGTTACTTTCTTAAAACAGCCTGAGAAGTTTACTGCTGTTGGTGCAAGAATTCCTA
AAGGTGTTTTATTAGTAGGTCCTCCCGGAACTGGTAAAACTTTATTGGCAAAAGCGATCGCAGGA
GAGGCTGGTGTTCCTTTCTTCAGCATTTCTGGATCTGAGTTTGTAGAAATGTTTGTGGGTGTAGG
TGCTTCCCGTGTCCGTGATTTATTCAAAAAAGCGAAAGAAAATGCTCCCTGTCTAATCTTTATCG
ATGAGATTGATGCTGTGGGCAGACAGAGAGGCGCAGGTATCGGTGGTGGTAACGATGAAAGAGA
ACAAACCTTAAACCAATTATTAACAGAAATGGATGGTTTTGAAGGTAATACTGGTATTATCGTCA
TTGCCGCTACTAACCGCGCTGATGTTTTAGATAGTGCTTTGATGCGTCCGGGACGTTTTGACCGTC
AAGTAATGGTTGATCCCCCTGATTTCAAAGGTAGAGTTGGTGTTTTAGAAGTTCACGCTCGTAAT
AAAAAAATCGCCCCCGAAATTTCCATAGAAGCGATCGCACGTCGCACCCCCGGATTCAGTGGAGC
AGATTTAGCTAACTTACTCAATGAAGCGGCAATTTTAACCGCCCGTCGTCGTAAACCTGAAATTA
CCATGGCAGAAATTGATGATGCGGTGGATCGGGTTATTGCAGGTATGGAAGGCACTCCTTTAGTG

GACAGTAAGAGTAAACGTCTGATTGCTTATCATGAAGTAGGTCATGCGATCGTCGGTACACTACT
GAAAGATCACGATCCTGTCCAGAAAGTAACCTTAATTCCTCGTGGACAAGCTCAAGGTTTAACAT
GGTTTACCCCCAACGAAGAGCAAGGATTAATTACCAAAGCTCAATTAATGGCTAGAATTGCTGGA
GCAATGGGAGGCCGTGCCGCCGAGGAAGAAATTTTTGGCGATGACGAAGTAACCACTGGTGCAGG
AGGTGACTTACAACAGGTTACAGGAATGGCTCGTCAAATGGTAACACGTTTCGGCATGAGCGAGT
TAGGACCTCTTTCTCTGGAAGGACAAGGAGGAGAAGTTTTCTTAGGGGGAGGCTTAATGAACAGA
GCTGAATATTCCGAAGAAAGTGCCGCTCGTATTGACGGTCAAATTAAGATGATTGCCGAACATGG
TCATAAGATGGCAAGGCAAATTATCCGTGATAACCGTGAAGTGATCGATCGCCTTGTGGATTTAT
TGATCGAAAAAGAAACCATTGATGGGGAAGAATTCCGCCAAATCGTGGCAGAATACACCGAAGTT
CCTGAAAAAGAACAGTTTATTCCTCAACTGTAAAAATTTACATTATCTCTCGATAAAATAGATAA
AATTTAGGGGTTTTAGCTTTTCTAAGACCCTTTTTTTGTGCTTAACCTGCAACCTGCAACCTGCA
ACCTGAAACCTGACACCTAACCTTATCGGATATTCTTACTTAAACCAAACTGAGGTTTCTTAAGT
AATTAAAGGTCGAATTTGATCAACTATTTCCCGACTTTTTGCATCGGGTGTAATGAACTCTATTT
GTTCACTTTGACCTATTCCTAATCCTAATTCAACGGCTCTTTGAATTTGCTCTAACTCCCATATTG
ATCCATTAGATACCTGATTTGTTGTACCTAATAAACGAAGTAGGGCGATCGCAACTACATCTATT
GCAACTCGATCGACTCCTGCTAAAATGACGTTAGAGCGGATTTTTTTCCAGACGCAGGTCCCCC
ATTAACAAAGGCTTCCACCCCGTCTAAAAGAACTAGAGAAGGCTTATAGGCTTGATTAATTTCCG
CAATCATTAACCGTTGATGGGCAGAAGAATGTAAGTCTCCCATATAGTTAAAGGAGTCTCCCGGT
ACGTATTTTGCTACCATTCCCACCGAATTTTTGAGAGAAAGGGTAAAATGTCCCCCATATTGATG
AGTTTTTAAGCAACAGGTATTGACAATTGCTTCTGAGTCTAAAATAGGACGAGCAAAGGCAAAGC
CCTTCTGCCAGTGTGTTCCTTCCGCAGAGATATATTGCCATTTTTCGGCTTCCATTTCATCAAAAA
CAAGGGTTTTTACACCGTATTTTTCTCCCAATTGAAAAACCCCTTTTTGCTCCATCGCTTGGCGGG
TATCAGCCATTCCAGAGCGATCGCCAATGGTCATTTCTCCTACTTCAGCGCCTTGAAATTCTTGAA
TCAAGGTTTCTAAGAGTTGACTATCAGTAGCCGCAGGGGCAGGATCACCAGTGTTATAGTTAGGC
TTAAGAAAAACAGTTTTACCCTTTAATCCAGTGGGTTGTAAAAGATCCAATGCCTGACGAGTACC
AGCAGAACGATCCTCATTATAAACCAGTACTACTTTACTTATTTGAGGCGGATTTAAAGGGGAAG
ATGAGGCAGATTGAGCCGTTACCTTATTATCGGCAACAGTTGAATTAATAGCACGACAGGCGGAG
GGAAATAAAGATGCACCAGTGGCTAAAGTTGCTAAACGTAAGACTTGTCTTCTTTGTAAAGGTCT
ATCTGAAGGCATAATTGAAGAATTAGAAACTTTTTTCTAGGATAACGAACATTATTTGAAATCTT
GGATGGATTGGAAATTTATCGGGCAAAAGCAAAAGTTAATTAAATTCGTGGGAGAAAATTTCTA
AATTACTGCATAAATCAGAAAAAATGAGACTGATAGATCAATAAGAGCTAAAAGAATTTACTATC
TATGTCAAAGAAAAACATTGTCTGTTTCTTATTGATTTTATTGCTTTGTTTTATACCTGTTCAAG
AAGCCCATGCTGGTAATTTATCTTTGTTTTCTTCTCTGGAATGGAATCAAGAGCAAACCAATTTC
AGCAAAAATCCTCAAAATTTTTCTCAAGAGATTCAAAACCGCCTCAACAGTACCAGAGATAGTAT
TGGTCAATTAGCTTTATTAATATCACATCCTTGGGGTACTTATGATTATACTAGACCCATTTTTG
TTGCTCCTAGAGATGGAGGTGGAATTATTTTTGAAGTTGATATACACTGGCATCGAAATTTAAAG
TTAATTTCCCGCAATCATGTTACCAGAATAGCTTGGGAAATAGTTAATAACAAACATTATAAGGC
TTCTGTTATTTGGGATGACTCTACTTTTAGCCCAATTCATGAGGAAGAAGTTAACGATTTTTTCC
GACAACTTTTACCAAATTAAATTTATTTTTTCTTTTATATATTCAATTATATTGAACTAAAAATT
ATGTTATCAAATATTACTAATTACACCATAAATAACAGTTTAGAAACGGGTAAAAATATGCTTA
AAGAATTAGGAGATCGAGCTATTAATAGTTCTAATAATTTGACTGATGCAATGTCAAAAAATGT
TCAATCTAGCCTAGAGTCTTTGTGGAATAATTGGATTGGAAATCATCCTCTCATTGGATTTTTTG
TTTCTCATCCTCTTATTTTTATTATTGTCATGATAACTTTAGTTTTAACTATTTGGGGAGTAATT

CAGATGATTCCCTCATTATTAGTACGACTGTGGTTATCTGTGTTTAAATCACCTTTTATCTTAGG
AAAATCATTACTGAAAAATAATCAGCAAGATACTCTTAAAGAAGAATTTTATCAACTAATTTTA
AACAAGTTAGATGCGATCGAATCTAAGCAAAATCAATTAGAACAACAGTTATCTCAATCAAAAA
ATCAACAACCTGATTAACAGTTATTATATAACCGATGCTATTTTTTTGTAATAACATCAAGACTC
CATTAATTTTCTTGATTTACCTTCTAGTTTGAAATTGAATATGAACAAATTTATCGATACTTTTT
CATTAGTTTAATCTTAATTTCCAACGGGACTTTTTTCCCTTAAAATTATGACTGTTAAATATTAA
TGAGAATTAACCAATGAGTGAATATCAATACTATGAATTTCAAACATTAGATCAACCTTTAACAA
AGCAACAACAGCAGAAAATTTCCCAGTTATCTAGTCGAGTTAGGTTAAACTCAAGAAAGGCTATT
TTTACTTATAGCTACGGTGATTTTCCTGCAAATGAAGACCAGATTTTAGCTGATTATTTTGATGC
TCTATTTTACATAGCGAATTGGGGAACAGTGAAATTAATGTTTCGTTTCCCTATCTCTTTAGTTA
ATATTAACGAATTAAAAAAATACACTTATGATGATTTTATTACTATCGATAAAATAGGTAGTTA
TTTAATCCTCAAAATAGAAATTAATGACGAGGAAAGAAGTGACTGGATAGAAGAAGATAACAGT
TATTTAGATGATTTAATAGAGCTTAGACAACAAATTTTAGCACAAGATTATCGAGTGCTTTATCT
AGCATGGTTAAAAGCGATCGAATTGAGTGAATTATACGATGATACCCCATTGAAAGATATTTCAG
AACCCCCCATTCCCCATGGTTTAAATCAACTATCAGAATCATTAAAAATCTTTGTTGAACTATTT
GAAGTTAATCAAAATTTGATAAAAGTAGGAGCAACTAATAGTTCTAAAATTACGCCCATACCAG
ATAATATTTTTTTAGAAAATATCAGTAAACTATCTAATATTGAAAAAGATGATTTTTTATCGAG
ATTACTACAAGAAGAACCAAATCTTAGCTTAAAATTAAAGCAAAAATTATTAGAATTAAGTGAG
TTAACTAATAATAAAACTACCATCGAAAAACGTACAATTAAGCAGTTATTCACAGAAGCGGAAA
AAATTGCACAATCAGAGGAAAAAAGACAAAAAGAAGAAGCAAGGCAGAAAAAAATTAAGGAATT
AAAGGCTTTTGCTAATCAAGAAAATCAAGCATGGCTAAATGTTGACAATCTTATTCAAAAATCAA
ACCCCAAAGCCTATGATGAAGCGAAGATGATATTAATAAAGCTGAAAGAATTAGCTATTTATCAG
GGAAAAGAAGTGGAATTCTATCATAAATTAGAAGATATAAAACAAAAGTATTCCAGAAGACCAA
CATTTATTGCACGTCTAAATAATCTATAGTATTTCTCTTTATTTTTTGCTGAAAAATCTACAACT
AAGCAATTAATTATTAGCAGAAACGAAACAAATTGCTCAATTAGAACATTACAAAAAAGATAAA
AATAGAAGTTAAGACAGGAAAAATCAAAAAAATTAATTTTGCTTTTCAAACTGCATTTTCGTTTAC
TTTTTTCTTTAACCTTTCCAACACCTAAAAAACAGGCTATGAATTTTATAGCCAAGTATCAATAG
TTATTTTCTTATAAGTCAAGGCGCATTTAATTTGACATTTTCATTAGCTTTTGAGAATTACTATA
ACTCTCTATTATTGTCTTTTGCTCACCTTTATCGGCAAACTTTCGATGCGTCCTAAATTAACTTTA
GTTAATCAGATTCCCAATTTTCACAAGCAATTAAATCACCAATTTGATCTCTTAATAAATAATCG
CATTGTTCTAATTCACATTCAACACAAACCCATTCCCTAGCGGGGATGTATTGACATAAAATGTA
AATGGGTTGATGACGACTAACAACTCCTTTTTCCACTAAATGACGTGCCTCGTCTCTAATCATCT
CAATTGAGTAATCCGTGGCGTTTTTGGTTAAAGTTTTCATCATTTTTGCCCCGTTAATTTTTATC
TTGTTTCAATACTTAATATAGTCTAACCCAAGGGAATTGATTATAGTTACTTAATGTATCTTAAA
TTACATTTTTCTAAAAAAAAATCAGATATTCTAAAGGGTTGCTATAAGCCTAATAGAGAAAAAA
TACTATAAAAAATCCCCTTTAACAATGTCAAACTTTCCTGACAAATTAAATAGAGATTTTAGGGC
TAATTCTTGGGCAATAGGCATTTGTCCATAGGAAAATACCCAAGGTATAGATTTTGGCATATGGG
ATAAAAACCAATTTTTAACATAAGGGCTACCATAAATAACTACCCCTTGAAGATTATTTTCGGCG
AATAATTTAAGATAGAATTTTTGACTTTCAGGGGTTAAACCTGCCCTTCCTCGAAAAGGATTTCC
TCGTAAAAAAACCTGTAATAGTAATGATTGTCGAGTATTTTGTACTGAAAATAAATTATATTCA
TCAAAAACCTGACGATGATAACCTCTTGTTTGTGGTATTGTAATAGCAGGTGAGCTGCGATCAAG
GTATTCACAATTTAATAAATCTTCCACTACAATTAAATTTATTCCCTCAGAAGCATTCTGAATGG
GTATATTACCCCCTTTACTCATACTTTGGGCTAAAATTTCATTCACTGCTTGACTGGCTTCTAAAC

TGTAAATATCACTCATAGAATGAGATTGTGAATTCACACTTTCACCAAATAGTTTTTGTTTAGCA
CAAGTAACCCGTGAATAACTTTCATCAATGCGACTTTCCGATAAAATACCCGAATCTATGGCTTT
AATTACCGATTTAATTGCTATCTCTGGATTTTCAGGCATGAGTAAAATATCCGCCCCTGCCTGTA
GCGCTTTAACGGCGATTTCTTCTGGGGAAGCATATTTTTTTACTCCCCCCATAATTAAAGCATCG
GTAATAATCAAACCCTCAAAACCCATTTTTTTCCGTAACAAATCCATTAACACCTTTGGTGAAAG
AGTCGCAGGATTGTAGGAGTCTAGGGCATTTACCAACAAATGGGCAGTCATAACGCTATCAACTT
TTTCCTTAATAACCCTTTCAAATGCAACTAATTCAAGGGATTGTAAACGCTCTAAACTATGATTA
ATAATGGGTAAATCAAGATGAGAATCTGTTGCTGTGTCACCATGACCGGGAAAATGTTTTGCACT
TGTTAAGATAGGATAATTATGACAACCCCTAACAAAAGCAGAAGTTAAATCTTCCACTGTTTTTA
ATTTATCTCCAAAAGCACGAACATTAATAACGGGATTATCAGGATTGTTATTAATATCTGTCACA
GGGGCTAAAATCCAGTTAATACCAATGGCAAGGGCTTCTAGGGCGGTAATTTCTCCCATTTTTTC
GGCATATTTAATCGCCAAACTCGGATTCTCATTGTAAATTTCCGCTAATGCCATCGGAGGAGGAA
ACCAAGACGCACCAGAAAATCTTTGCCCTACTCCTTCCTCTATGTCGGCGGCAATAAATAAAGGT
GTTTTTGCCCATGTTTGCAATTGTTGAGTGCGATAGGCGATTTCTGCACAGCTACCTCCTAATAA
AATAACTCCACCTAAATTCAATTCTTCTATACATTTTTTTAATTGGGGTTGAGTGGCTTCCCATA
CGGGATAACGTATTTGATGATCAAAAATATAACCTGTGGTGCGTACAATAATTAATTGTCCAATT
TTTTCAGGCAGAGAGAGTTTTTCAACTGGTAACATAGCTTAATCAATAAAAAATTAAAAATTAT
TTAAAATTTAGTATTTACTATACTCAGTAAAGGAATAAAGTATTAAGGGTTCGCATAAAAACGT
TTTACCCTCATCCCCAACCCCTCTCCCACAGGGCGAGGAGAGAAATTTTTCCGAATATATTAATTA
ATAGTTAGTGATCCCTGTATTTGTTACTATTTATTGGTTTTAGAAGTTAATATTTCTGAGAATTA
AAACATCCTGCTTTTTGAGCAATCCCCAATTATTATTGATTATTTTCTCGAAAACAGTAAGCACA
TATTCCCCAACGGTTTAATTCTCCCTCCGGGCAAAAACTTTGACAACAGGGGCAAGTAGAGTTAT
TTTTTGCTCTTTGTTTAATAATTTCAAACCATTGTTGCAGGGCTTCTTGAGGAGTTTCTGTAGGC
TGTGCGATAAACTCCGTCGATTCGACTAAACTAGGATGACTTAAGTTTGTTTCTGCTTCTGGAAT
GGGGGGAGTGTTTTGACTATACCATTTTCCAAAAACAAAATATATGTCTTTGATGGGATTCTGAA
TACGGCGATTAATTTTGATAATCAAAGTGCGTCTTTGTAAGGATAATTCTTGAGCCCATACTGCA
CTTTGAGTGGCGATGAAAAATACTTCTTCTTTAAGATAAATAGGAGATGTATGTTGTGCTATCTT
TTGATTAACAATTTCATACCAGATTTTTTTTAACTCATAGTATCTTCTTTGTTTTTCCCATTGTG
GTTGAGAGAGAATAGAATTTAGTAATTTATCGATCGAATTTAAGGTCATTAATAGATTATGAGT
AAAGTACCTATGTCAGTTCATCCCCTGATAAAAAAGATTATACAAAATTTAGATTTAGATCTTGG
AGCAGAATTAAGACGCTATGAAAAGAATACAGTAGATAGCAATATTCTAATTCCTGAATTAATA
GAAACTGAATCTATTGACTTGCCCTCGAATTCTAGTTCTTTAGTTTATCATCTCCCTCTTGATAAC
TACGATGAAGATGATGAAGATGATGAAGATGATGAAGATGAGCCAATACCCGTTGCTTCTCGATT
GATAAGCCGCGATCGAGCTTCCTTACTAGATTTAATCTTAACTCCTTGGGGAATTTTTGGAATAA
TTATGTTTTTCGGAACGAATATTTTGATATTTTTAGGAATCAATAACTTTAATTTCAACAGTTCA
AATCAAAAGGCAGAAATAGTTGATCAGAGTAGAAAAGATCAACAAATCATTAATGAAGTGACAC
CAGAAATAGAAAAAAAAGATGAGCAAATAATAGAAGAAAATGAACAAAAATCTCCTCCTTTACC
CTCATCTCTCCCAAGTGTAGATAATGCAAATATTACAAATAATATTCCCGCATCTCCCTATCCTG
ATTTAAAAACAGCTTTAATGAAGGAAATTAATAATAACGAATTAAGTGAATCCTCTTTACCATT
ACCTCCCCCTTTATCGGAAGAAAACTCAAATACATTACCGAATCAAGATGAGCCTATAGAGGCTA
ATTCCTCCTCTCAAAATAATCAAAAAAAAGAATATATTATATTAACTAATTATGAAAATATGAC
TCAATTTAATAATATAAAAAAAGAAACTCCTAAAGCTTTTATAACTAATATTGAAGGAGAAATG
AAAATTCAGCTAGGAGTTTTTAACACAGAAATAGAAGCTAAAACAAAATCTCAGCAATGGCAAA

AACAAGGTATTAAAACTTTCTTGAAGTAATTTATTTACCTTAGAATTTTTAATATTAACTATCCT
AATTTGACATAATTTTTATACTCTAATACTCTTAAGGGCAAATAAAAAATAGAGAGATCTTTAA
ATAGGCTTCTTAGAGAAAATATAATAAAACACTCATATAATCATCTTTTGTTTAATTTGTAGGAG
TATTAATTAAATTCATAATTGTGTCATACTTGGGTGAACTATCTTGATAAGTTGATTCTAATAAA
CCCCAAGAACCATATTTGCTTGAAGGGCTAACATCATTAAAGTGCATAAATAAATCTCCACCCAT
GTTTCTCCATTGAGTCATTAATTGTTCATAAATTTCACCCATACGAGGATCTCTGTTAGCTTCGA
TCATAAGATTATTAATAACTTCGTCGGTAAAACTAGTAACAGCAGTACTTGCGGCTAAATGTTGT
CCTCCTTCATAAGCAACTAGTGATAATCCTTCTGCTTTGGCTGTATCAAGTGCTTTTTGCCAGTCA
GAGTAAGTCTTGGGCAAATCTGTGTTATTAATTCTATTAAATAAACGGTCAAAGCCACCGTCAGA
TTCCTGAGTCCAAGTTAATAATTGATCTTGAGTGACATCTTTTCCTAATCCGACATAACCAGCAA
TAGACAGAACATCAATACCGTAGTCTTTATGGGATTTGTTTTCTGATGACCATTTATAGTTAATA
GCTTGTTCCAGAATCCACGGATTAGCTGCTTGTCCTGCCATTACTCCGATAACTCTTTCTTTATCA
CCTGTTTGAGCAAATACTTCATCCCAAATTTGCATAACTTGAGTGGTTCTGCGACTATACCAGTC
AACTTGTCTCATACCTTCCGTTTGTGCCTGTTCATTAATCCAATGCCATTGTTGAAATTGAGGAT
TCCATGCTTCGTTGGAATATTCTACATATATTTTGAGTTCAGGATCTAAATTATCCCTTACATAA
GTCGCAAATTGACGAACATAATCATCACTAACCATATGGGGCATATTAAACCAAGGATCAGAATC
AAGATGATTAACTAAATCAACCATAATTTCTACGGGAGCACCGATTTTATGCCAAGTGGCATCTT
CAAGGGAGGGGCGATCGCTCCATTCTTGTTGAGTAGAATCATTAGTTTGCATCCAATCCATGAAA
CGGAAAGCACCAAAAGGTTCTATTTTGTTCAACCAGTCGGGATTAAATACTTCAGTCTGATAGGT
ATTTTCGTCGGCTTTGGGAACAACTCTGATATTGCGAATATAATCACCAGTTTGATTGGGATCTG
TTTCAGTTATACTTAACAATACTCCACGATCAGAAGGAGTTATATTAATGACATCTCTACCTTGA
GAAGAAGAAACAAACTTCGCATCAAAGCCATAATTAATAGTTCCTTCGCCATCATAAAGAACCAC
ATATTCTCCACTCGGATAATTACCATTTTGTCCACGGAAAAATAATGTTTGTACATGGGTAAAAT
TGACGTTATCCTCCGCCGTCGGTAAGGAACGAACCCATCCATTTTCATCTAAATCTAATTTATCTC
TTTCGTTTGTACTCCAAACACCTTTTTTGGTTGTTATCCATTGATCAGCAGTTTGGAAAGCATCA
ATAAAAGGTAATTGAGTTGACCAATAACTAATTCCGTTCAAATTACTGCCAATTTCAAAAGAGGG
AGTTTTGACGGTGATATTAACTGTAGCGGTATCTTTTCCTCCGTGTTCGTCAACTATGGTATAAG
TAAAACTATCTTGACCAGTAAGATTATTTACAGGTGTATAAGTAAAGCTACCATCTTGATTTAAG
GCTATACTTCCGTTAACAGGCTGAGTAAAACTATCTAAACTAAAAATATCTCCTTCTGGATCGGA
ATCATTGCTTAAAACATCTATTTTGATAGAGTTATTTCTGGTCGTAGAAGCAATATCATTATTGG
CTTCAGGAAAGTTATTATTGTCATTGGCAGGAATAGCAGATAAATCATTTGCGATCGTGAAATTG
TCGATCGCCATGAATTCATTACTTTCAACACCATTGGTTGTAAATCTAACCGCAATATATTCATA
ACCAGATCCAAAATCTACTTCATTACTGAAATTTGTCCAGTTAAATTCTGCTGTAGCCACATTAC
CCGTATCTAATAAAGTTTTAACGGCGATCGGTTCGAGAGAATTACTAACAGGATCTTTGGTATCC
CAATTACTAAATTTGAAATTACCATTAACTCCATAGACCTGCAATCTTAACGTATTGCCAACTCC
ACGATTAATACCATCAAAACTAATGAATTGTGAGCCTTGAGTTATATAGTCATTTTTGATAACTT
GAGTTAACCCACCCGCACCGCTATTATCCGCATAGGCATATTCATTGGCTTTATCTCTATTCCATT
TACTTTTATCTCCCCCAGAAATAAACCACCCTTTATCCGCATTAGAAGCAAAAGTAGTCCCCCAGT
TTGTATTAAAATCATTGACAGATAAATCAAATTGTGAATCGATTATAGATGTAATTTCAATTTCT
TTTTTCTGAGTAATACTAACATTGTCGATCGCCATAAATTCGTTAGTTTCAACACCATCAGTAAC
AAAACGAATAACTACATATTCATAACCATTACCAAAATCCACATCTTGGCTAAATGTTTTCCAGT
CAAATTCTTCTGTTGCAACATTACCAGTATCTAATAAAGTTTTATATTCTATGGAGTTGCTATTC
GCACTAATCGGATCTTTTGTATCCCAATTACCTAATTTGAATTGTCCATTAATTCCATAAACCTG

TAATCTGAGAGTATTTCTCCCGCCTAAATTTGTTGCATCAAAATTAATAGTTTGCAATCCAGTAC
TGACAAAATCACTCTTGATAACTTGAGTTAAACCACCAGCACCGCTATTATCAGCATAAGCATAC
TCTTTTGCACTATCTTTTACCCATTTATGATTAAATGGTCTTAACCAACCTTTATTCGCATCAGAC
CAAAAAGTTGTACCCCAGTTTTGATTAAATGTATCTTGAGGAAAATTAAAAAGAGAATCTTGAA
TGATATTAGTGGATAACATAGTGGGAACTCCTGAATAAACTAAAGAAAATAAAGAAATAATAAA
AATAAGAAAATAACTAATAAAATTCCGTTTCTGCACTGTTAAATAACAAGCAAAAGTTTTAATA
TAAAAAGATAAGAGCTTAATGCTGATTAATTTTTTAAGGTGTTTAGCAATTAAATTTTTGTTTT
TCTATACTTATATTCTTCATCATTTCTTTAAGAAAAAAATCCGATAAATTATCTAACTTTTAAAT
TCACTTATTAAGGTAAATGCAAATTTTTATCTAGGTGAAAATATCAGTATTTAGTCCTGAATCAT
TATTTTGTAAAGATTGTAAAGATTTTCTTTAAATACTTAGGGAATCAAAAAAATTGAACAATAA
CTTTAGCTCTTTTTCTCAAAAATGCTAAAAACCGTTTATATATGCGAATTTGAAGATTTGTAAAA
TATCAATTGCTAGAAGTCTAATTTTTTCTGGATGAAGAGTATTTTGTTTATTAAGAATAAAAAA
TTCAGATAGACTCGTATTTATACTTAAATTAAACACAAACTTAACAATATATTCAGTTGCAAAAG
CAATTAAGATATTAGATAAAATGTCATCATTAAAACAATTAGCCATAAAAGGCACAATGTGGAC
TCTAATCGGGTACGGAGGTAGTCAAGTTTTAAGATTAGGAAGCAATTTAATTCTTACTCGTTTGT
TAGTGCCTGAATTATTTGGTTTGATGGCATTAGTCAACACCTTTATTACTGGTTTAAACTTATTT
TCCGATATAGGTATAAATCCAAGTATTGTTAGAAGTGAGAGAGGAGAAGATCCTGATTTTTTGA
ATACGGCTTGGACTTTACAAGTAATTAGAGGATTTGGACTCTGGATTGGGTGTTGGTTAATTGCT
TTTCCGGTATCTCAATTTTACGATGAACCTAAATTAATATGGTTAATAACTATCGTAGGTTTTAA
TACAATTCTTTCAGGGTTTAATTCCACATCCCTTGCCGTTTTAAATAGAAAAATAGAAATAGGCA
AATTAACTCTAATTGAAATTACAACTCAAATAACCTCCTTAACAGTAATGATTATTTGGGCTTTT
TTTCAGCCAACTATTTGGGCTTTGGTTATTGGGACTCTTATGTCTAATATAGTTAAATTATCTTG
GAGTCACTATATTTCGCCCATTCATCATCGTTTTACTTGGAATCAAGACTGTTTAAAAGAATTAG
TTACTTTCGGACGTTGGATATTTATTTCTACTGCCATGACATTTTTAGCCTCTCAAGCCGACAGA
TTAATATTAGGGAAATTAGTTTCGATCGAAATGCTTGGAGTTTATACTATTGCATTGACTTTTGC
TGACATACCGAAACAAGTAGCGGGAAAAGTTAGTGACAAAATTATTTTCCCCCTGATTTCCCAAA
ATATTTTTTTACCTCGTAAACAACTAAAAGCTAAGATTTTAGATAAACGCTTTTATCTATTACTA
ATATCTGCCATCTTGATTGCGATTCTATTCTCTTTCGGCGACGTACTAATCAATATTCTTTATGA
TGAAAGATATATTTCAGCAAGTTGGATGTTACCTTTTTTAGCGTTGGGTTTATGGCCACTCATAC
TCTCTTTGACTATTGATAAAATATTATTTGCTTTAGGTAATCCTAAATTTGTCTCTTTAGGTAAT
ATTGTAAAGTTTATATATATGATTGTCCTTTTGCCTTGGATGATAAAAACTTATGGCATATTAGG
GGGAATTATTGTTATCGCTTTTAATGACATTCCATTTTACTTGGTTGTTACCTATGGACTATACA
AAGAAAACTTCGTCAGTTTATTACAGGATTTAAAGACAACTATAATTTTAATTATGCTTTCATTA
ATTTTATCCTTGGGTCGATTTATCTTTGGCTTTGGTTTCTCCCTAAGTACAATCACAAATCTTCA
ATGATTATAAACAAGATGATGAAGATAGGTAAAAGTGCATCTCGACTTGTTGATGAACGTAGTC
GAATTACACTTAATAACTGGGAGAAGAGGAAAGAGCCGTTTTTGAAATACTCAAATTATGACCTT
GTTAAGTATATAAAGAGACTGACAAAAGTTTTCAAATTTGTCGATCTCCATATATTCTAAAGCT
CTTTTTAAAGATTCTATATCTATTTTAGTCGGTGATGTTGCAAAACTTCTTGCCTTATTTTGAAA
ATCAAGATTTCTAATGACATTTTCTGTTATTTGACCTGTTACACCACACCAACTTCCACCCATGGC
GGAAGTCATTTGTCTAAAAGCATGAGTAACATAACCGGGGTGAAATAGTTCTATCACTTTCAATC
TATTTTGATTAAAAACCATACTTGCGAATGCGGCACCATGTAAACCTACTATTATTTCGGCATTT
TTGCTAATTGACCATTGTTCATGTATAGGTATATCTTCATAATAGAATTTTTGGAATCCATATTC
GTGTAAAATCATTTCGACTTCTGATTCATTAATTAAACTTCTTGGTCCTTTTCTCGAAATAAAAA

CCCTTTTTGGCGTATTTTCTTTATATCCTTCAAAAGTTAATTTGCCAAAAAGTTGATCATAATGA
CACTCATATTCTCCAAATCTACCAGTGGGAGCTTTAATAATTGTACCCATAACATTTTTATCTGT
ACATAAAACAGAAAATCCTAAAAGTTTATAAGCTGTTTTCGCCATCGCCGAAGCATTAGCTTTTA
AAATAACTGTTATGTGAGGTATTTCTGGTGGAATTTTCTGAGCGGCCAAGAGTCCAGGTGAAATA
TTCTTTATTATGTGTGCAATATTTTTGTCTGTTTCATATCTTGTATCTAGGATATACTGATCTCC
TAAATCAATGGGCTTTGTTATTTTTTTGTAAATTCTTTTCCATATTCTCTGGGATCGAATATTCA
AAGATTCTGTGGAACCTTTTTCTATCTTGATTTCTATCTCATTACGATTAATTTCATAAGCTGGG
ATATTTATTTCTAATATGTCTGCTTTCCACGCTGCCGCTAGTCTTGTGGGGTAGGAAACTATTTT
ATATGGTCCTGCAAAAATAATTCCACTCTCTAACACCTTTTTTTGAAACATAATGATAGTTTTTT
ATTCAAAAAGTAAAACCGCAGACAAAGATTAGCACTACAACAGATTGAAGTCTTTTTCTTTGGAA
AAGAGTGTTGTGTGCAAATTATTACTACACTTAACTACTTTATCAAGTTAGGATTTAGCATATCA
TAAAATCCTATTTTAGAAGAGCAATATCAATTTTCAGTAAATTTATGAAAATTACTTTTATTACA
CCCTATTTATCTATCAACGGTGGTAATCGTGTTATTACCATTTACGGTCAAAAACTGCAACAACT
GGGGCATGATGTGCTGATTGTTTGTCAACCCCGAAGAAAAATTACCCTTAAACAAAAAATTAAGT
CTATTATTAAAAGGGATCTGTTATTTTTAGATCAAAATAAGACATTTTCCTTTTACGATAGTTTA
GATGTTCCATATAAAATTTTAGAAACTAACCGCCCTGTCATTGATAGTGATGTACCCGATGCAGA
TGTTGTGATTGCGACATGGTGGGAAACAGCAGAATGGGTTGCGAATTTATCTCCTTCTAAAGGTG
CCAAGGCTTATTTTATCCAACATCATGAAATTCACGATTATTTACCTAAAGAAAGAGTTAAGGCA
ACATACAAACAATGTTTACATAAAATAACTATTTGTGAATGGTTGAAAAATATTCTTGAAACAG
AATATTCGGATACAAGTGTTTCTTTAGTTCCGAATAGTGTTAATATACAGCAATTTAATAGTATT
GCAAGAAAAAAACAAGATGTTTGTACTCTTGGAATGCTCTATTCATCTACTTATTGGAAAGGTTG
CGATATAAGTATAAAAGCTTTTGAAATAGCTCAAGAGAAAATACCCAATTTACGTTTAGTCGCTT
TCGGAAGCAAAAAACAACATCACGATTTACCTTTGCCAAAAAACACAACTTACTTTAAGCAACCT
ATACAGAATGAGATAAAAAATATTTATGGACAGTGTAATGCTTGGCTTTTTGGCAGTCGTGCCGA
GGGTTTTGGTTTACCTATTTTAGAAGCAATGGCTTGTCGCACTCCCGTTATTGGAACTCCAGCAG
GGGCAGCACCTGAATTATTGGCAAAGGGGGGTGGAATTTTAGTTAAACCAGAAGATCCAGAAGA
TATGGCAAAAGCAATTATCAGAATAGCTCAAATGTCTAATGATGAATGGAAATTGATGTCAGAT
AAGGCTTATGAAACTGCCACCAGTTATACTTGGGATGATGCAGCAAAATTATTTGAACAGGCTTT
ATATCGGGCTATAGAAAGAACGAAAAAAGGTGAATTATAATTAAATAGCAAAAATAATCTTTTT
AAACTTATGTCACTTCAAGCCCAATTAGCCATGATTTTATGGCTACCCATTGTTTTATATTTATT
CAATAAATATCCTCCGAGAAAGGCTGTGATTGTCGGTTTTTTGGGGGGATTATTGTTTTTACCTC
AAAGGGCAGGTTTTAATTTGCCTTTAATTCCAGACTATCGGGGCATGGTTGCTACTTGCTACGGT
ATTTTTATTGGCATTATCATTTATGATTCGGAAGTCTTTAATCGATTTGAACTTAAGTGGATTGA
CTATCCCATGATTTTATTCGGGATCGCACCCTTATTTTCTTCATTAACTAATGGTTTAGGGTTAT
ATGACGGCATTAATCAATCTATTAGTCAAACTGTTCAATGGGGCATGCCTTATTTTTTAGGACGA
TTATATCTCAATAATTTAGCAGGATTAAAGGAATTAGCCATAAATATAGTTAAAGCTGGATTAT
TATATGTACCTTTATGTCTTTATGAGGTGCGCATGAGTCCTCAATTGCATAATATGGTTTATGGT
TATTTTCCCCATTCCTTTGCTCAAACCATGAGATTCGGAGGGTGGCGCCCTCAAGTGTTTATGCA
ACATGGTTTAATGGTAGGTTTATTCATGATGACGGCGACTCTGATAGCGATTTGGTTATGGCAGG
GGCGAGTAGTAGCTAAAATTTGGCGTATGCCGATGGAGTGGGTAGTAGTAATTTTGTTTGTGACT
TTTATTCTGCTTAAGTCAACGGGAGCTTATGCTTTAATGGCTATAGGCTTAATTATTTTATTTTC
TGCTAAATGGTTTCGTCTGAATTTGCCTTTAATTTTAGTTTTGTTAGCAATAGTTGGTTATTTAG
TGGTTGCCTCTACTGGTAATTTACAAACAGATAATATCATTAGTTCTTTACAACAAGTTTTCCCT

GAAGATAGAGTTGCATCCCTAGAATTTCGTTTTGACAATGAGGAATTGTTAGGGGAAAAAGCCA
GAGAAAGAGTCTTATTTGGTTGGGGAGGATGGGGACGAAATCGTGTTTATGCAGAAAACTGGGC
GGGGGAAATAGAAGATATATCGGTGACAGATAGTTTATGGATTATTGTTTTTGGTATCAATGGC
TTGTTTGGTTTAATTAGTTTATTTGCTTCACTAATATTGCCAGTGGTTTATTTGATTATTCGTTA
TCCTGTTAATACATGGTTAATTCCAAAAGTTTCATCTGCTATGGTTTTGGGTGTATGTTTAACTT
TATTTGTAGTTGATTGTCTAGTTAATTCAATGTTTAATCCTATTTTTCCTCTTATTGGTGGCGGT
TTATCTGGATTATTTGTTTATTCTCCTCAAATTACAAATAAAACTATGAAAAGAAATATACTGAT
TCACCAAAAGAGTCAGAAAAAATCACTTAATAACTATTCTATTTCTAATCAAAATCGATAACAGA
ATTTTTTTGACATAAAATTATTAAACAGCAAAACATAATTGATAAAAAGCCTATATGATCAGAA
AACCTTTTTTTCTCGTTGGTGCGGAAAGATCTGGTACGACTTTACTACGTTTGATGTTAAATCAT
CACCCTCAAATTTTATGGGTACAGGAATTTGAATATTCTGTGGACTTGATTACAGATGATCGAAA
ATTTCCTTCTTTAAATATTTACTATGATTGGTTAGAAACTCATCGTATTTTTCAATCTATGGGGT
TTTCCATCGACAAAAATTTAGATTATCCCCAATTAATTGATAGTTTTCTCTCACAAAGATATAAT
CCTCGTGAACATAAAATTATTGGTGCAACGGTACATCGTCACTTCGATCGCATCTTGTCTATTTG
GGATGACGCTTGTTTTATCCATCTGATTCGAGATCCTCGGGATGTAGCCCGTTCTTGCATTGGTA
TGGGCTGGAGTGGTAATGTATGGTATGGGTGCGATCGCTGGTTAGAAGCGGAAATACTTTGGGAT
AAATTAAAAAACAAGATTTCTCCTGATCGATATATTGAAATTCATTATGAAGACTTAATTAAAG
ATCCTGTTAAAATTCTTACATTCATTTGCAATTTTATAGGGCTTGATTATGATGAGAAAATGTTA
AGTTATGGTAAAAATACCACCTATAGCACCCCTGATCCTAAATTGATAGAACAATGGAAAAAGAA
ACTCTCTTATTTTGAAATTCAATTAGTTGAATCTAAAGTAGGTTCTTTATTAACTGATAGAGGCT
ATGAATTAAGTCAATTACCAAATATTAATCCGAATCAATTTCAGCGTCAACAATTAAAAATAGA
AGATTGGTGGTATCGAGTTCAATTTAGAATAAAACGCTTAGGATTATCTTTATTTATATCTGACT
TCTTATCTCGTAAATTGGGTTTAAAATCATGGCAAAAAGAGATTAAATTAAAGATGAATGAGAT
TGAGAAAAAACATTTAAAATAATTGTCAAAAAGTTTTATTGATGACTATCTAATTTTAATTCTT
TTACCAATAATTTATAATTATCGCAATTCAACTCAACTCCTAACCATTGTCCTAATATTTGCAAA
TCTTGGTCAAAAATTGACTTTAAATATTTTCTACTTTCCTGTGTTAATTGTGGTTTTTCTTTCAT
CATCCAAAGGCTTTTAATCCAATCACGAAAATCTTGAGGAATAAATTTTTTTCTGATTTGCTTTA
AAATAGGTGCTTCTACAAAAAAATCACGCCACTCACTTTTACGCATTCTTTCTTGAGAAACATTT
ACCGCTGATAAATCATGCCATTTAACAGAAGATTTATAACCGATAAAATTCGCAATTCTTTCTAA
TTCTGTTTGAGGATACTGTAAAAGTCTTTCAAAAAATACCAGCAAAATTCTTTCTCGATCAAAAG
TTTCTAAAAAGGGTTGTAATTGCATAGTATATTGACTATAACTAATCAATTCAGGATAATCATAA
ATTGCCTGATTAATATTGATATTAATAACTCTCTGAGTCCACTCGTGAATATACTGGGAAATGAG
GCGATCAATGGGATGACGCATCACATAAATAAATTTAGCATTGGGTAAATGGACTTTTATGCGAT
CGACCGTTTGCGGATATGTGGGTAACTTAGTATAATGAGTGCTAGATTCTCCACAAAGATCCGTT
AATTGTGCACCCTGAAATTGGGACAAGTACCATGTCATACCCTGTTTGTATATTTCATCATTACT
GAAAAAATTAGGTTCTTTTAACTTCGTCATAAAAATGCCCGATTGTAAGGCTAACTGTTCATGAA
GGGTGCTGGTGGCACATTTCATCGCCCCAATAATTAAAAAATCAGGCTTTTTGTTCATCTTTTTG
ATCTTCTTGCAAATAAGGCTTTAAAGGATTATACCAATTTAACCAAATATCTCTTGCTTCTAATG
TACAAGTCTGAGGAGGTTTTCTTTCAAAAATTTCTCTTATGTAAGAGACACTTCTCCCCACTAAC
CATAATAAATTTGCAATCCATAAGCCCAAAATCCCGTAAAATTTGGCAAAATATCGAGAACGAGA
GGCATAAAGATAAGCAGGAGGACGTTTTCTAGTGGCAATGTCTTTTTTCACCGAACCACTTCCCC
CCCTGAGATGAACCACTCTTGCAAAAGGATAATGAATTATTTGCCAACCTGCCTCCCTTGTGCGA
CGACAATAATCTATGTCATCAAAATACATAAAATAATCTTCATCCATCAAGCCTATTTTTTCAAT

CACTTCTTTTCTGATTAAAACACAGGCAAAACTTGTCCATTCTACCTCCATGACTTGATCCGTAA
GAGGTAACGGCACATCATACTTATTCAAGATTTAGTGATAATTCCCGTTTTCGCTGAGTTTATC
AATTCGTTAATTGGAGAACGATTACGAAAACAACTGATTTGAGGTTCACCATCTAACCATTCTAA
ACGAGGACTGAAAATACCCCCTTCTGGATATTTAGTTAATGCGTCCAATAAAGTTGCGATCGCAC
CACTATGAATAATCGTATCACTATTTAAAAGTAAATAATTATCGGCATCAATTTTTTTGATACCA
AGATTATTGCCATAGGAAAAACCACCATTCACTTCAGATTTTAATAACTCCACCCAATCTTGCCA
ATGAGAAGACGAGATCGCATCTTGTATTAAATCAACAGAATCATCTCCTGAATTATTATCGACAA
TGATAACTTTGCTTGGCGGTAAGCTAGAAATTTCTGAAACCAAGGAATTTAAACAATCAATGACT
AACTTAGGAGTGCGATAATTAACTATTATAATACATAAGTAAAGGGAATTTTTTTGTTGAGACA
CGATAAAGAAGTCAAATCATGGCTATAAAACTGATCTTAATAATAACCTGAGTTCGGGATGAATT
TTCATCTATGAGTGATAGCGAAAAGGACAATAGGCAATGGGCAATGGTGACAATATTTTGATGTC
TCAATTTATGGCATAATTTCAATACATTTCATACCATCATTAAGTAACGTCTTAAAATCAATTTT
AATAAATTTTTTATCAATTACTATTATTGTTCCCTATTCCCTGTTCCCTACCTTCACCAGACAATT
TAGGTTAGTCATTAATTCTTCATAGATAAACCGATACGTTTTAATTTTTCGTTAACCTCTAACAC
TCTTACTTTTACAACCTGCCCGACTCTGACAACCTCTGTAACATCACTCACGAAGCGATCGCACAT
TTGGGATATATGTACTAAACCATCTTGATGCACACCTATATCCACGAAAGCACCAAAATTAACCA
CATTAGTGACAACTCCCTCTAAAATCATACCTTCTTTTAAATCGGTAATTTCCGTGACACCCTCCT
TAAAAGTAGCATACTGAAATTCAGCACGAGGATCGCGCCCCGGTTTTTCCAATTCTTGAATAATA
TCTTGTAGGGTAGGTAAGCCAATTTCGTCAGTGATATACTGCTTTAAATCTAGTTTTTTCAGATG
TTGATTAAGTTGATTTATTTCCGTTAAAGGTACTTTTAAATCACTAGAAATTTTTTTAACAATGG
GATAACTTTCTGGATGCACAGCAGTATTATCCAAAGGATTGTCACCGTCTCGAATACGTAAAAAA
CCTCCTGCCAATTCAAAAGTTTTTGACCCTAATTTCTTAACTTTTAGTAATTCTTTTCTATTTTTA
AATACCCCTTTTTCATTGCGATACTGCACAATATTATTCGCAATAGTGCTATTAATTCCAGAGAC
AAAAACTAACAATTCCTTAGAAGCAGTATTCAAATCAACTCCCACATAATTAACACAACTTTCGA
CAGTTTCTTCTAATTTTTGTTTCAATAATTTTTGATCCACATCGTGTTGATACTGCCCTATTCCA
ATGGATTTTGGGTCAATTTTCACCAATTCGGCAAGGGGATCTTGTAACCTCCTACCAATACTAAT
CGCACCTCTGACGGTGACATCCAAATCAGGAAATTCACTAATCGCCACATCACTAGCGGAATAAA
TAGACGCACCAGACTCATTCACCATCACCTTGATGGGTTTAGTGTCAATCTCCTTTAACACCTCCG
TGACAAAATCATCGGTTTCCCGGGAAGCAGTGCCGTTACCAATGGCAATTAAAGCAATATGATAT
TTTTGGATTAGTTTTTTTAAGCTATTTTTGGCAAGATTTCTTTTTTCTGACCCATTATGAGGAAA
TATTGCCTGATACTCTAAAAATTGCCCTGTTTCGGAAAGAATCGCCACTTTGCAACCCGTACGAA
ACCCCGGATCAATACCCATTGTCGGTTTCATCCCTGCAGGAGGAGATAATAACAATTCTCGCAAA
TTTGCCTCAAAGGTTTTAATTGACTCTGTATCTGCCCATATTTTCATTTCTGAACGCACATCTCGC
AGTAAAGCTGGTTTTAATAAGCGATTAAAGCTATTTTTAATAGTTTCTTGGTAAAATTGCTTAAT
TTCAGGGTTTTTGGTTTTAATTATCTTGGACTCTAAATAGTTAATTGCCCTTTCTTCCTCAAAAT
CAACGTCTAAACTGAGGATTTTTTCTCCTTCTCCTCGGTATAAAGCAAGAATGTTATGGGGGGCG
ATTTTGGTTAATTTACAGGTAAAACTACGATACATCTCATACTTTGTGCTTCCTTCGGGGTAATC
TTTTTTTATCTGAGAGACAAAAGTACCGTTATTCAAAATAAATTCTCTCAAATAGGCTCTTAATT
CAGCTTTTTCGGCGATTTCTTCCGCTAAAATATCCTTTGCACCGCTTAAGGCTTCTTCACTGCTGT
TAACACTATCAGAGACATATTTTTCGGCTTCTTGACTCAAAGAAATTACCTCTTGCCTCTTGCCTT
TTGCCTTTTGCCCATCTTCCGATTGATTTTGATTTTTAACCCACTCTGCTAAGGGAGTTAAACCTT
TTTCTCTGGCAATAGTAGCACGGGTTCTCTTTTTAGGTTTATAAGGCAAATAAAGGTCTTCGAGT
TCATTTTTTTGTAAACAAGCGGTAATTTGAGCCTTTAAGGGTTCACTGAGTTTGTTTTGAGAGGC

GATCGCATCTAAGATAAATTGCCTTCTTTGCTCTAATTCTTGGAGATAATTAAAACGCTCTTGAA
TGTCTCTTAATTGTATCTCATTGAGTGACCCAGTGCGTTCTTTGCGGTAACGAGCGATAAAGGGG
ATGGTTGCCCCTTCATTAAGCAGCTCTAAGGCACTTTCTACGTTTTCGAGATTGAGGGATAATTC
AGAAGCTATAACTTGAGGAATATTGAGCATAGGCTAATAGGACTTTTTGCTAAAATTTTACACTA
AAACGAAAACTTGAGTTCAATGTTCCGAGTTCAGGGGAGGTGATTATGAGGGGAGCGAGTGAGA
GGGGGAAGTAGGGGCGAATGGCCATTCGCCCGTACAGGAGTTCGGAGAGTTCGTTGAGCTCGTAC
ACTTCTTTCAGTCGTGAACGAAGTTTTGAATTATCAACTATTCACTATTCACTAATTGCCCATTG
CCTACCCTAATCAATAATTTAGATACCTAAAGTAAGAGAGCCTTATTTCTTTACATTTGTTAAGA
GAAAATATAGAAAAGTTTACATTTTTTTTTATGTTTAAGATAAATAAAATATTCTGGTAAATTA
ATCAAAATATAAGCTACTGTGAAAATATGAGAACCATAAAAAAGATAAATAATAATTAAATTCT
CAGAGAAAAAACTGTCTAATTAATGTCTCGTTGAAAAATAGAAAAGGAGTATAAATTATGAATG
ATAGGGTAGAAACTCCTGCCAGAAATGTGGCAATTGTCGGTCCTTATTCTAGTGGGAAAACCACT
TTGTTAGAAAGTTTATTATTCGTTTCAGGAGCAATTAATCGAAAAGGACGTGTGGAAGATGGTA
ATACCATTGGGGATAATTCCATCGAAGCAAAAAACAGGCAAATGAGCGTGGAAGTCAGTTCAGGT
TATACGGAATATGAAGATATAAATTTCACCTTTCTCGATTGTCCGGGATCCGTGGAATTTATGCA
AGAAACCTATAATGCTTTAGTGGGAGCAGGTACGGCAGTGGTTGTCTGTGAACCTTCTGTAAGTA
AGGTTTTAACTCTCGCACCTTTGTTTAAGTTCCTTGATGATTGGCAGATTCCCCATCTAGTTTAT
ATCAATAAGATGGATCGATCGCAAGATGATTTTATGGATGTTTTAAATGCCATTAAAGATGTATC
TAGTCGTCCTCTGGTACCGCAACAGTATCCCATTAAACAGGATCATGATATTATCGGCTATATTG
ATTTAATTACAGAACAAGCATACCATTATCATCAAGGAGATAAAGCAGATTTAGTTCCTTTTCCT
GAAAATCTTAAGGGGTTAGAAACCCAAGCAAGACAGGAAATGTTAGAAACCCTTGCTGATTTTGA
TGATCATTTATTGGAAGAGTTGATTGAAGATATTGAACCCTCTAAAGAGGAAATTTTAAAAGAT
TTAAAGCAGGACTTAAGTGCAGATTTAATTGTACCTGTTTTCTTTGGTATGGCAAAAGAGGATTA
TGGGGTGCGTCCACTACTAGATTGTCTCATCAAAGAAGCCCCATCTCCCAATATTACTGCGGAAA
GGAGAGGATTAAAAACCGTTGCCCATAGCCCTGTTATTGCTCAGGTATTAAAAACTTATTACAGT
CCCCAAGGAGGAAGGTTATCTTTGGTACGGGTATGGCAAGGAAAAATTACAGATGGCATGACTCT
TAATGGGGTCAGAGTGGGCGGTATTTACCGTTTAATGGGAGGACAACAAAAATCTCAAAATATA
GCAGAAGTGGGAGAAATCGTTGCTCTAGCAAGGTTAGAAGGAATAAAAACAGGAGACACTCTTA
CCGATTCTGACAAAGAAATCAAATCCTTACCAAAAGCAAATTTGATACCCCCAGTATATGCCCAA
GCAGTGACAGCCGAACATCGTCGGGATGAAGTGAAAATTTCAGGTGCGATCGCAAAAATCTTAGA
AGAAGATCCCTCTCTCAAATGGGAACAAGATGAAAACACCCATGAAGTAATACTTTGGGGACAAG
GAGCAATTCATTTGCAGGTAACACTAGAGCGACTAAGCAATAAATATAACCTGTCTATGGTGGCA
CAAAATCCCAAAATTCCCTATAAAGAAACTATCCGCAAATCCACACAAGTTCATGGACGTTATAA
GCATCAAAGTGGTGGCCATGGGGCGTTTGGAGATGTTTACTTAGACATCAAACCTCTAGCAAGGG
GCGAAGGTTTCCAATTCCATGAAACCATTGTGGGGGGAGTTGTTCCCAAACAGTATATTCCGGGG
GTAGAAACAGGAGTTAGAGAATATTTACAACAAGGTCCTTTAGGTTATCCTGTGGTGGATGTGGA
TGTAACCCTTACCGATGGCTCTTATCACTCTGTAGATAGTTCTGAACAGGCTTTTAAACAAGCCG
CTCGTATTGCCATGACAGAGGGAATTGTACAGTGTGATCCTATTTTGCTTGAACCTGTTTTATTG
GTGGACATTTTCACTCCCAATGAGTTTACATCAAAGGTTTTGCAACTGGTTTCTGCCCATCGGGG
ACAAATTCTTGGTTATGAGGGTGTAAGGGATTGGAAAAATTGGGATCAAGTCAATGCTTATCTTC
CCCAGGGAGAAATGCAAGATTTTATTATTGAATTGCGATCGCTTTCTTTGGGAGTAGGCTTTTTT
AACTGGCAATATGATCACTTAGAAATAGTCCCTGAGAAGGTTTCTCACCAAGTCTTAGCGGCTTA
AAAACGGGTAGGTGTCAGGTTTCAGGTGGTAGGGTGTTTAGGGGATGAGGAGATAAGGGGATGA

GGGGAGAGGGAGAAACAAGTAATGAGTTAGTTAGGGGCGAATGGCCATTCGCCCGCACAGGAGTT
AGGGATTTTTAATTCTTAATTATCAACTATTCACTATTCATCTTTGCCCTCCCCCCTCGAGAGGGG
GGATAAAGGGGGGTTTGCCCTTTTAAAATCTATTTACCCTCCGCCTTTTTTTCCCAATCTGCAAT
ATTCCATGTTTTAAAATCCCAAGGAGTTAAATTCACACCAAATAAATTATTACTAACTCCCACCA
CATCCGCACGGTGAATGAGAGGAATTACCGCCGCTTCTTCTACCAATAAATCATTCATCTGTTTA
AATATTTCCGCCCTTGTCTGTGGATTTAATTCTTTGGTTGCCTTCTGCCAGAGTTGATCATATTC
AGGATTGCAGTAGCGAGAGTAATTATCCCCAGACCAATTATTGCTTTTTTGAGGAATATTATCAC
AAGTATGATTTTTTAAATAAGCGATCGGATCTGGGCTTAAGTTACCTGTTGTAAACATTTGAACA
TCAGCAGAAAATTTTTCCACAGTATCATTATTACTAGGATCACTAGAAAATAAGACACTAGCATC
AACACTTTTTAATTCCACTCCCACCCCTAAAGATTGCCAGTTTTGTTTAACAATAGCTTGAGTTT
TTTGCCGTAAAGGATTAACAGAGGTTTGAAAAACAATCTTCATTTCTACACCATTTTTATCTCTG
ATGCCATCCCCATTGGTGTCTTTCCATCCTGCTTCATCTAACAGTTGAGACGCTTTTTCTGGATTA
TACGTCATTGAATTTTCTGAGGATACATATTCTTCTGGTGCAACAATATAGTTATTCGTTGCTTT
TCCCGTCACACCATACAACTGGTTAGCAATAGTTTCTTTGTCCACTGCCATTGCTAGGGCTTGTCT
CACTTTTACATCAGAAAAGAAAGGATGAGGATATTGTAAACTCGATTTTTCTCCTTCATCTGTGC
TACGATTTGGATCGGTAAAATTAAAGATTATTCTTTCACTCAAAGACCCAAAATTAGCCACCAAT
TGACCTTTTCCACTCTTTTGCAGACTTTCGAGAATATTCGCCTCAACCTGTAAATTATGGGCAAA
ATCAGCCTCTCCCGTTTGTAATACAGCCCTAGCGGCAGACGTGGCATCCCCTCCCCCTTTAATTTC
AATTTGTTCAAAATTCAATTTTTCTGGTTGACGGTAGTAGGAGTTAGGCTGATAAATAACAGAAT
CTCCAGGCTTGAATTGTACTACTTTATAAGCTCCCGTTCCCACTGGCATAAGGTTATAAGGAGCT
TCTCGCACATTGGCACCGTTATATTGAGCATATAAATGTTGAGGTAAAATTAAACCTGCATTTCC
CACAAACACTGAATACCAAGCTGGGGTAACTTCTTTAAATGAAACTTTGACGGTATAGTCATCTA
ATGTTTCTACTTTATCAACGATGGCATAATCTCCAGCATTAACACTACCCACTTGAGGATTAGTA
ATGAATTCGTAGGTAAAAACGACATCTTTAGCGGTAAAAGGTTGACCATCAGACCATCTTACATC
TTTTCGTAATTTCCAGACTACAGATTTTCCATCCTTGGCAATACCTCCATTCTCCAGAGTTGGTAT
TTCTTGAGCTAGGATAGGTATAAGTTTACCTTCTTTGTCAAAAGATGCTAGGGGTTCAAGAGTAA
GACGACTAGCTTCAGAATCTTTAAATCCTGTGGAGAGATGAGGATTAAGAATTGTCGGTGCTTGC
CAATAAAGTAATTTTGCCTGTTTCAAATTATTGATATTGGAAGACTGAGAATCGGAGTTACAAGC
AATTAGGGCTAAACTGCTCAGACAAATAGTAAGATAACCAGAAAGTCTTTTCAATTTCTTCATAA
TCAATAACTAAGCAAAATTTTTACCCCATTTTAATCAGATTTTGTTTTATTGCTTAAAAAAGGTT
TCAGGTGTCAGGTTAAATAAAAATTAAGAGTTTATTTTTGCCATGCAGGATGAGAAAGTAACGC
ATCCATTACTCTTACACCCCTAAGTTGATTTGATAGGGGTAAATGCCCGATAGGCGCAGACAAAT
CCCAGATGAATTCTTGAGGATATTTTGTCCATTTGTTGCCACTTTTCCATTTTAATTTAGTCCAA
AGTTGAGTATAGTCTTTGCCTAAAGATAACCACAATTGACGTTGAATTTGATAACCAAATTTGCC
TTCAGAGTAGGTTTGCCAAAGAGTATCAATGGTCAACAAATCTGTTTCTGGGAATTTTTCTACTT
CGGTGAAATATACCCATTTCCTTCTAATGGCTGTTTCTCCTGCTAATTCACAAAGTTTACTCTG
GTTAAACTATCTGCTTCTTGATATTTTTTTGCTATCAATAATTCTTGTAATTCACTGTAATCAAT
ATTCTTTTCTGAGGATAATTGAACGATTCCTTGGGGAAAATGTTGATTTAAAAAGGTCTCATTTT
CTGGATGATTACTATGTTTTAATACTTGATATATTTTACCTTTTAGTGGTGATACTTCTTCGGGA
TTAGATAATAAAAAATCTCTTAAAAATTGGTAATTTTCTTCTCCTTTTTCAACAATCTCATCAAT
TAAAGCTATTTTATTTTTATCAGAAGTTTTGGCAAATTTATCTTTAAGGGTTTCTAATTCATTCA
TTGTTGATTATTTGTTATTTTAAAATATTCTTTTTAAGATAATTTCTATTACCTCTAATTCAAGA
TTTTATTTTGAATAATGGCTAATAGTTTTTTATATTTTTAGAAATTAGTTAGATAGTTATTATA

ATTTTATGAGTTAAACAAAGAAACAATAATAAACTAAATTTATCAATTATTATTTTTTCACCCCT
ACCATTTTACCATTATGGTCTTGAGAAAGAAAAGGAGAATTGAGGGGAATCTTTAATAATTAGG
TAAAAGTAACATGATAAAGAAGCAATAACATAGTGGCAATAACTGCACCAAGAAAAGTATTAAT
AATATTAACGATTTCATTGGTTAGCCAATCTATTTTTTCCTGTAAAGTTGCCCCAATAACGCTTT
CTAGGTTAGTAGCGATGAAAGCTGATATAAGGCATATCACAATACCAATCGGATTGATTAAATTT
GTAAGATAACCCAAAAGTGCGATCGCACCACTGGCAGTTATACCAGCTATAGTACCTTCTAAACT
AATAGCACCTTCTGTACCTCTAGCCACTGGTTGTAAAGTAGTAATTAAAAAAGTGCGTTTACCGT
ATGCTTTACCAATTTCACTCGCGCAGGTATCGGATAACTTAGTCGCAAAACTAGCCACATAAGCG
ATGAGAAAAAAATCATCTCCAGAATCAGTGAAACCATAACCCAACGCACAAATAAAGGCAATCAA
AGCACTACCCCATACATTTTCTACACTTCTGACTCCTTCTCTTTTTTCTGCAATTCCTTCCGCCAT
TTTCTGTGCCATACCTACTTTTGTAATTGCAGAGCCTACTAAAAAATAGAACATAACGACAAGAT
ACCCTTGCCACTGAAGACAACCCCAAACAACAACCCCCAAAATCCAAGCATTGAGATAACCCATA
ACTGTTAGTAACTTTTTGGGGAGCAAAAGTGCGATCGCTAGTAATATTGTATTAATAGTAATCCC
CAGTAGCCATGGGTTTTGCAAAATCACGTTAATGATATTCATTGTTTTATTCTCAACCCTTAATA
ATATTTACCTAACACCCTAATTCCTCAGCTATTGTCAAAATTTATGTTTAGTTTATGTTTACAAA
TTTATTGACTCTTAAGCGCTAACTGGTTGAGAAAGATAACCTAATTTTTCTAAACCTTGTAAAAC
TTTGTTAACACTTTCGTCTAATTCTTCAAGATCTGTGCGACACTCAATTTCAGGATTAGTAGGAG
GTTCATAAGGGTCACTGATACCAGTAAACATCTTGATTTCACCTGCACGGGCTTTTTTGTATAAT
CCTTTAACATCTCTATCTTCACAGACGGCTAGAGGTGCATTTACGAAAACTTCAACAAAGTTGCC
TATTTTAGCCCTAACTTCTTCTCTGATAGCACGATAGGGAGAAATGGCAGAAACAATTACGATTA
CACCATTACGGGTTAATAGTTGAGAAACAAAGCCTATACGGCGGATATTTTCGTCTCTATCTTCT
TTACTGAATCCTAATCCTTTGGTTAAATTAGTACGTACAATGTCACCATCAAGCACTTCTATTTT
ATAACCTTGTTCTCTTAACTTTGCTTCTACCTGTTTACTAATGGTTGTTTTACCTGCACCACTTAA
ACCAGTAAACCAAATCGTTACGCCCTTATGTTCCATATTTTTCAATAGTATTTTTGTCTATAAGT
TAATATTCTTTTGAGTATTGTACAAGTGCCTGTTACCCCAGACAAGAAATAAATGAGAATGGTGT
GTTAGGGTATTAGGGGAATTTTTGACTTGCAGATTTCAAGTAATAACAAAAAATCTTGAAACAA
TCAATATCTAGCTATACATTATAAAAATTTATTTTTTAAATAAATTTAGACCTTATTTTCTACTT
CCTAAAAAATTTATAATTAAAGGGGATATTTCTTCAGAAAAATGTTCTTGAGGATAGTGTTTTGC
TTCTGGCAATGGTGTTAATTTTAGTTTATTATTCGTTTTAATTAAGTTTTCTACAGAGTCAATTT
CTAGCCAAGGGTCTTCATTTCCCCAAATAATTAAACAAGGTTTTTCCCATGTTTTTAAACCTGTT
TCTATTTCTTTTGTTGTTTGTTTTAATTGTAAATTTTTGACTATGGTTACTAACATTCTTCCTGC
GGCGGATGATGTAACAATAGGTTTTCGATATAAGGCTAAATTATTATCAGAAATCACGAATCCTG
AACCTTTTTCTAAGGTGCGATCGACTATTAAAGGATCTTGTGTTACCATATCTCCTAAAAGAGGA
AATCCGCATTGAGCAATCTGCCAAGGTAATTTTGCCTCAGTAGTAATAGGTGCGTTTAATATTAC
CAAATGGTCTATTTTTTCTGGATATTTAAGGGCGTATTGGATGCCAATTGTGCCGAGAAAACCTT
GAATAATTAAGGATACTTTTTCTAATTCTAACGTTTCAATAAAATCTTTTAAAGCCTCAATAAAA
TTATCTGGTTTGTAACCAAATTCTCTTTTTTCTGGTATGTCTGAAAATCCCCATCCTAACCAATC
AGGTGCGATCGCACGATAGCCATATTCTGCTAAACTAGGCATCATTTCCAACCAACTAAAACTTT
GAGAGAATAAACCATGTAATAAAATTACTGGGACTTTATTATTCTCTTGAGGTGGTAACATTTCC
CGATAAAACCATTCAAAATTGCCGACTTTAATTTTCTTTTCTTGTATTTCCACAGATTTTTATTA
TTATTAAATATTTTTAACCAAATTACAGCATTTGCTGTTATAGGATTAATTAAACTATTTTTTGT
TCTTTTACTCAAATTTTTCTTACTTTCAACGCTATTGTGCATATACAAAAAGGCAAAGTTAAAA
GGGTAAGGGGCAAAAGTAAATAGTGAATAGTGAATAGTGAATAGTGAATAATTCCGAACACTCA

TTACTCATTACTCGTTACTTATTACTTATTACTTATTACTTTCTCCAACACCTGAAACCTGAAATC
TACCTCAATCAGACTATAATAAATATTGTTATAAAGTTATATGATTAATTAATTATTGGTATTA
TGACGATCGCATCTAGTTGGATTTATGGGTTAATGGGAGGTATTCTCATTGGAGTAAGTGCCAGT
ATTTTACTTGCATTTAATGGGCGTATTGCAGGAATTAGTGGCATGATTAACGGAGTTTTGGAATT
TTCGCCTACTCAACGATGGCGTTGGTATTTTTTGGGAGGAATGATTGCAGGGGGATTTATTTATG
AGTACATTTTACCCCTTCCTTACACTCCTGCCTCTGGTGTCGCAGTTATTCCCATGATTTTGGGCG
GTTTATTAGTTGGTTGGGGGACAAGAATGGGGAATGGTTGTACTAGCGGTCATGGTGTTTGTGGT
TTGGGTAGGTTATCGATGCGATCGCTTGTATCGGTTATTAGTTTTTTGGCATCAGGAATGATAAC
TGTTTATATCACTAGACATTTAATCAATTAGAGTAGTTTCGTTTCTTCTTTCTTTAATTCATCAC
CCTGTTTTCTATTTCCATCAGTATTTTTATTGTCTTACTAAATATAACTCAAATTAAATTGAAAA
AATGATGAAAAAACAAAGTGTAATTGCCCTAATTAGTGGATTATTATTCGGTTTAGGATTAAGT
ATTTCTCAGATGGTTGACAGAGAAAGAGTCTTAGGATTTTTAGATGTGGCGGGTAAATGGGATG
CAACTTTAATGTTTGTCTTAGGGGGTGCAGTGGGAGTAACTTTGATTACTTTTCGTTTTATTCTC
AAATTAAAACACCCTCTTTTTGAGCAAAAATTTTATTTGCCTAGCCGTCAGGATATTGATCTAAA
ATTAATTTTGGGTGCGGTTATTTTTGGTATCGGTTGGGGTATTGGCGGTTATTGTCCAGGACCTG
CTGTTACTTCCTTAGTACAATTGAGTGTCAACCCGATTATTTTTATTATTGCTTTTATCATTGGC
TCATTAGGATATGAAAAAATCATGGGTAATGAGTAATTAAAACAAGACAACAAGCAATGAATAG
TGTTGGGGTGAATAGTAAATAATAAAAAACTCCTAACTCTCATCCTCATTACTTGTTTCTCCCTG
TCAACCCCTCCCCCTCATCTCCCCATCCCCTTAACAACTAATCCCCCTAATACCCTAAATCCTTTA
ATCCTCTAGTATCCGATTATGGAAAGTAAATTACTGCGAGTTGAATGTTTATTAAAAGGAGTGGC
TGATGCTACAAATTATTTACTTACGGTAAAAGATTATAATGAGGGTATTAATCAGGCTTTAGAT
ACTTTAGGAAAAGCAACAAAACTCGATCGCATCTATATATTTAATGTACACCCTCATCCTCAATG
GAAAAAACCGGCTTATAGTCAACTATGGGAATGGGTAGCCGAAGGAATCACTCCAGAAATAGAT
AATCCTTATCTACAAAATATGCCTTTTGATGACGTTATACCTCGATGGTATGATATATTGTCGTC
AGGTGGCACTGTCGCAGGAAATGTCAGGGATTTTCCTGAATTAGAAAGAATTGTTCTTCAACCTC
AAGGTATTTTATCTTTGTTAGTTGTACCTATTCTTATTAAAGATGAATTTTGGGGTTTTGTCGGT
TTTGATGATTGTCATTGTGAACGAAATTGGACTGAAATTGAAATTTCTACCTTAAGAGCGATCGC
AGGTAGTATTGGGGGTATAATTGCAAGAAATCAGGCAGAGCGAGAATTACAAAATCTTAACCAA
GAATTAGAAAAAAGAATTGAAACTAGAACCCAACAACTATTAATAGCAAAAGAAGAAGCCGATA
AAGCCAATCAGGCAAAAAGTGAATTTCTAGCAAGAATGAGTCATGAATTACGTACTCCATTAAAT
GGAATTTTAGGGTTTTGCCAACTACTAAATAGAGATGAAAATTTAACTTCTTTACAAAGAGAAA
ACTTAAAAATAATTAATAGTAGTGGAGAACATTTACTTAACCTTATTAACGATATTTTAGAATT
AACTAAAATCGAAGTGGGTAAAATATCTCTTAATATTACTGACTGTGACTTAGATAAATTACTAG
ACAGTCTGAGAGATATGTTTTATCTAAAAATCTCAGAGAAAAATTTAGAATTAATAATTACTCG
TGATGTCGATATACCAAATCATGTTCGCTTAGATGAAAGAAAACTCAAACAAGTATTAATTAACT
TACTTCATAATGCTATTAAGTTTACTCATAAAGGACAAATTAGTCTCAAAATACAATCTATAAAA
GATAATAAAAATATAGAAAAAACTAATTTAGTATTTATAGTAAAAGATACAGGAGTAGGCATTA
CTCCAGAAGAACTAAAAATTATATTTAAACCTTTTGAACAAACACAATTAGGAATTAAATCTAAT
CAGGGCAATGGTTTAGGTCTTTCTATCTCTAAGAAATTAGTAAAATTAATGGGAGGAAATTTACA
AGTAAAAAGTAGCTTAAATCAAGGCTCTTCTTTTTATTTTCAGATTCCCGCAACCATCATTAAGA
AAAATAAAAATATTGCAAAACACTCTTTTGATGATAGATTAAATTTAAAACCATCTCCAGAAAA
AAATAAAATTTTAATAGTAGAAGATATAGAAATAAATCGTCAATTTTTAATTAAATTATTAAGT
CCGATGAGCTTTAATATTTATATTGCAGAGGATGGAGAAAAAGCTCTTGATTTGTGGGAAAAAC

ATTTACCAGATTTGATATTAATGGATGTACAGATACCTATTTTAAATGGTTTAGAAGTAACACAA
ATTATTAGAGAGAAAAAAAATTTACCTCAGCCTAAAATTATCATTGTTTCTGCTAGTGCCTTTGC
TAACGATGTTGAAAAAGCTACTTGTCAAGGTTGTGATGATTTTGTAATGAAACCTATTATCGAAA
ACATATTATTGAGCAAAATAGCCCAACATTTAGGCATAGAGTTAAGTACAAAAATATCAACTAA
TGAGACAAGAGAAACTTTAGAGGAAAAAGAAAAATTAATTAAAAGCAACTTACAAAAGATGCCT
TCAGATTGGGTTAAAAAATTAAATAAAGCCGCAAAAGAATTAAATAGAGACAAAATATCTTCAC
TTCTTCAATTAATTCCTTCTGAATATGATCCACTAGCAAGAATATTAAATAAAATGATAAAAGAG
TATGATTTTGAGCAAATAATCAGGCTAACTCAATAATGATCAATGAATATTTTTCCTCAGCAAAA
CTCATTAGGCACGGTTTTAATTGTTGATGATGTTTTAGAAAATCTTGATTTATTGTCCCAAACGT
TGACTTCTCAAGGTTATCAGGTGCGTTGTGCAAAAAATGGCTATTTGGCTTTGATGAGTATTAAG
CATGAATTGCCAGATTTAATCTTATTAGATATAAAGATGCCGGGGATGAATGGTTATCAGGTTTG
TCAAAATTTAAAAGAGAATTATAAGACGAAAGATATACCGATAATTTTTTTGAGTTCAGCCTAT
GAAACAGAAGAGAAAATACAAGCCTTTGCGGTTGGAGGAGTTGATTATATTACTAAACCTTTTG
AAGTTGCGGAGGTATTAGCCAGAGTTAACACCCAAATGGGTTTATTAAAGACACAACTAGAATTA
AAACAATTAAATCAAGAGTTAGAGAAAAGGATAAAAAGAAGAACTGATGAACTAGAAAGATTAA
ATCAAGAATTACGGTTAGAAAATGAAGAAAGAAAAAAAATTCAAGAAAAGTTAATCTATGATGC
TTTACATGATTCTTTAACTAATTTACCTAATCGTACTTTATTTATTGATCGAGTCAAGATGTTGA
TTCGTCATTGTCATCGTTTTCCTAGTTTCCAATTTGCTATTTTATTTATAGATTTAGACCGCTTCA
AGTTAATTAATGATAGCTTAGGACATCATGCGGGCGATCGCCTTTTAGTTTTTTGCGGACAGTTA
TTCCAAAAATGTGTAAGAGAAAGTGATACGGTTGCCCGTTTAGGAGGGGATGAATTTGGTATTTT
ACTAGATAATATTAGCGATGTTACCCATGCCATTGATACAGCAGAAACCATCAAAATGCAGTTGA
GTAAATCATCTCCCTTATTTAATCATCATTTTAGCCCCTCTGCCAGTATTGGTATTGTTATTGGC
AATTATCAGTGTCAAAATGCCACCGATTTATTACGCAATGCTGATATAGCTATGTATCGTGCTAA
AGAAATGGGGAAAGCTAGATACGCTATTTTTAATCAAGAATTACATCAACAAGCAATCCAACGTT
TAGAAATAGAAAGTGATTTAAAAAAAGCACTGGCATTCAACGAGATGCTCGTATATTATCAACC
AATAGTTGAATTACAAACAAAGCGTTTGAAAGGATTTGAATGTTTAATTAGATGGCAACACCCCA
AAAAAGGTTTAATTTCTCCCCTTGAATTTATTCCCATCGCCGAAGAATGTGGTTTAATTTCTTCT
CTAGGAGACTGGGTATTACAGGAATCCTGTCGTCAATTAAAGCAGTGGCAAACAAGATATAGCCA
TCACTCGGAATTAAGCATCAATGTTAATTTCTCCCCCAAACAATTTGAAGATATTTATTTACCCC
AAACAATTCATAATGTTTTACTCGACAATGAATTAAAGCCTCATCATCTAAACATAGAAATTACG
GAAAATGTATTTTTGTCCAACAACATTACGGTGAGTAAAATTTTAAGCCAATTGGATAAATTACA
AATACCCATAAGCCTAGATGATTTCGGCACGGGTTACTCTTCTCTTAGTTATCTTCATCGCTTCCC
CATTAGTGTTATTAAGATAGATGGCTCATTTATTCGACAAATGCAAAACCATAACAAATATTTAG
AAATAGTGAAAACCATTACGACTTTAGGACATACTTTAGGAATGAAAATTATTGCCGAAGGAAT
TGAAACGGAGAGACAACTACACACACTGCAAGAATTAGGATGTGAATTTGGTCAAGGATATTTTT
TTGGTAAACCCCTTCAAGCCTCTGTCGCAGAGAAATTAATTCAAATATCGAATAAAGATTAAACT
TATGTAACTCGATCGCATCTTTATAAGAATGATTGCATAAAATGAAAAATGGAGACTGATATAT
AACAAGAAACCACAAGAGAAAAACTTAAATCATGGCAATTTCATTACAAAAAGGGCAAAGAGTC
TCACTAGATAAAGTTGCCCCCGGATTAAATGCGGCTTTCATCGGCTTAGGTTGGGATGTAAATAC
AACAGATACAGGAGGAGATTTTGACTTAGATGCCTCTGTTTTTCTGCTCAATAGCAATGAAAAGC
TAATTTCTGACCAACATTTTATCTTTTATAATAATCTCACAAGTCCAGACCCTGAAAAATCTATT
AAACACATGGGAGATAATCTCACAGGAGAAGGAGACGGAGATGACGAAGTTATTATCGTTGACT
TAAGAAAAGTACCAACAGATGTCAATCGTATAGTTGTAACTGTAACTATTTATGATGCAGATAA

GAGAAAACAAAATTTTGGTCAAGTCAGAAACGCCTTTGTCAGATTAGTAAATGTAGAAACCAAA
GAAGAAGTTTTACGCTATGACTTAGAAGAAGACTTTTCCACAGAAACCGCCTTAATTATGGCAGA
AATTTACCGCAAAGATGGTGAATGGCGTATGAATGCCGTTGGGGCTGGTTATCAAGGAGGTTTAC
AGGCTTTACTCAATCGTTATCAATAAGAAGCAAAGGGCAAAGGTGAAGGGCAAGGGGCAAAGAT
GAATAATGAATAGTGAATAGTTGATAATTAAGAATTAAGAATTAAAAACCCTTAACTCCTGTAC
GGGCGAATGGCCATTCGCCCCTAACTAACTCATTACTTATTTCTCCCTCTCCCCTCATCCCCTTAT
CTCCTCATCCCCAAAACACCCTAACACCTGAAACCTGCTACTTGATACTTCTGTAATACTTATTGC
TCACTATTTATTACTTATTAATCATCATGGGAATTAATTTACAAAAAGGACAAAGAATTTCACTA
AAAAAAGAAGCCCCTAAATTAGAACAACTAATGTGTGGTTTAGGTTGGGATGTCGCCAAGAAAA
AAGGCGGTTTTTTAAGTGGTTTGTTTACCACAGATTTTGATTTAGATGCTTCGGTTTTATGTTTG
AATCAAGATGGTAAAATAAAATCAAATAGTGAAATCGTTTTCTTCGGCAATTTACGTCATTATTC
TGATGCGATTAATCACATGGGAGATAATCTGACTGGCGCCGGAGATGGTGATGATGAACAAATAT
TAGTCAAATTACCTTTGATTCCTCAAAATATTCACAAATTAGTTTTTGTGGTTAATATTTATAAT
GCCTTAGAAAGAAGTCAAGATTTTTCCCAAGTAGAAAATGCTTTTGTGCGTTTGGTTAACTTAAG
CAATAATCAGGAAATCGCTCGTTATACTCTCTCTGGAAATGGTTATCAAGGAAAAACAGGGATGA
TTATGGCAGAAATTGCCCGTGTAGGTGACGATTGGGAAATGATGGCAAAAGGTGAAGGTTTTCA
GGTAAAAAGTTTGGGTGATGTAATGAAACTTTATAGTTAATAGTTTTATAGTGAATAGTGAATA
ATGAATAGTGGATAGTCAATTGATTTCAATTTGGAATTAGGAAAGGGCAATTAATAATTAGGAA
TTAGTAATTAAAGCCTGAAACCTGACACCTAAAACCTGAAACCTCTATCTTATTCATTACTCATT
ATCCTTCAACTCCTAATTCTTAAAATCTATGTTTAAACCTCTATCTGATTTAGTTAAATCTTTCG
GTAAAGACTCCGGCAATTTATTAGCAAATCGCTATCAGTTAGTCAGTCAAATAGGTAGGGGGGCA
ATGGGGCAGGTTTATAGAGCCATTGATACTCAATCAGGTGACAATCTTGTTGCTATTAAATTTTT
GTCTCAAGCCTTATTAGATGATAAAATGCGTTCTCGTTTTGAGAATGAAGCAAAAATAAGTGCTT
TATTAGGGGAACAAAGTCAGCATATTGTTAGGGTTAAAGATTATGGAATTAGTGAAAATCAAGT
TCCTTTTTATGTAATGGAGTACCTTGAAGGTGATGATTTAGACAGCCTCATCAAGAAAAAAACTA
TTTCTGTAGGTAGATTTTTATATTTAATTAAACAAATTTGTTTAGGGTTGGAATGCGCCCATAAT
GGTATTTTAGTCAATGGACAATTAGCCACTATCATTCATCGTGATATTAAGCCTAGTAATATTTT
CTTAGCCAAGACTGGGGAAGGGGATGTCATCGCTAAAATTCTCGATTTTGGTATTGCTCAAATTC
AAGAACCGCAACAGGGTTCAACTCAACAACGTTTCATGGGTACTCCAGAATACTGCTCTCCTGAG
CAAATGGCGGAAGATGAGTTACAGCCTACATCAGATATTTATAGTTTAGGGGTTTTAATGTACCA
AATGTTAACTCAAAAAACTCCAATTACTGCAGATGGCAAAACTTTTCAGGCATGGTTTAAGGCTC
ATCATGAGCATATTCCGAAACCTTTACCTCGTTATTTGGCGTTACCTCCTGAGTTACAGGATTTA
ATTATGAAATGTCTATCTAAGTCAAGATGCGATCGCCCTCAAAATATAGGTGAAATTTTAAAGAT
TATTACTCCATTAGAGAGAGAATATAACAGAAAAAATAATTCTGAAATTAATAGTAATCCCGAC
TCATTTGCCAAAAGAAAAACAGTATCTAGTGAAAATTTACCATTACTAACAGTTTATGCTCAAAG
TAATTGGCCTAAAGATAAACCCCAGAAAAAAATAGTTTTTCCCTGTTTAACGGAAGCTCAAGAAG
GGAGTTTTTCCAGTTTATGGACTATGTTAGAGTCAGATGAAGTTAATCGTTTTATTCCTAAGTCA
ACTTTTTGTTTTAATCATTTTCTATTTCAACTAAATCCTCATCCGATGATTTTATGGGCTAATTT
ATTGTATCGCCGTAATTATGAACCTAAATGGCTACCTTGCTATTTAGATTTAAAAACAGAAATTG
GCTCTCAAATAGTTAATAATTTAATTAGTAAAAATCTCTATTATATTCTTTTATTTGAATTAGAA
AAACCTCATAAATATAAACAGTTATTAACGGTAAAAATTAATGAAGATAAAGCAAAAAAAATAG
AACAATTTTTGAGTAAAATGACTCTTTCTCCTTCTTCTCGGTCTCAAGCACAAGTAAGCAAAATA
GCTTTGAAAAAACAGTTTGAATCTGTTAAAGATACAATTATTGCGGCAATTAGTCGTAGTAAAT

AACCTCAATTCTGTTCCAGAATATCGGATAAGGGTAGGTTTCAGGTGTTAGGGTATTGGAATATT
AGGGGTAATTTTATTAAACTCTTTTGCCTTTTGCCTTTTGCCCTTTTAACTTTGCCTCTTGCCTAC
CCTAACCAAAATTCCCATACTCAAAGTAATAAAGCCATAATTTAGGTTAATTACTGACGTTACGC
ACTTATTGATGTGTTAAGTTAAGGGAGGTTTCAGGTTTCGGGTTTCAGGTTTCAGGAAATAATAA
AAATGATTGAACTAAAAAGTTATCGAGATTTAACGGTTTGGCAAAAATCAATGGATTTGGTAGT
AATATGTTATCAATTAACTTCTCAATTTCCCAAAACAGAAATTTATGGTTTAAGTAGTCAAATAC
AAAGAGCCGCAGTTTCAATTCCTGCCAACATCGCAGAAGGAAAAGGGAGAAATCATTTGGGTGAT
TATATTCGTCATCTTTCTATGGCAAATGGCTCACTCAAAGAATTAGAAACTCATTTGATGATTGT
AGGACGATTAGGCTATCTTAAAGAGCAAGAATTAAAAGTTACTTTAAACAAATGTGAGGAAATT
GGCAGAATGTTACATAGTTTAATCGAAAAACTAAGTCAAAATAAAAAAGGGTGAATTTTTCTGA
CTTTAAACCTGACACCTGATACCTGAAACCTGAAACCTCAAAACAACAACTAATTATCTTTTTGC
GTAACATCAGTTAATTATTTTGATTATTGCCACAACAAGTATCTATACCTAAACTTTGTAAGATA
ATATCACTTACTGCATTTGCGATCGCAAACTCACTGTAAAAACTTTTCTGAAAATCAAAAGATTG
CATTTCTGTAACAATAGCAATAACTAAGGAGCCAAGGTCATTTTCTCCCTCTAAACGATGACGAA
TAAAAATTTGAGCGGCACGGTTAGCAATGGTTTCGTTAACTAACTCAGGAATGAATTCATTATCT
AGCCATTGATGTAAAGATGTTTTTAACCAACTACGCTCTTTTTCAGGATTTTCCACGGGAGGTAA
AACAATAGGTGGAATTGGTTGAGACATGATTAAATATTAGTATATGTTTCCTTCTTTAATAGAA
ATTATATACATTTGGTTGTGCACATGATCGATGTTCCCTCCATCATTCAGGGATATGCCCACGGT
AATTTTTTAATGGCGGATGATAAACAACAATTAGGATGGTATTCTAGCAATCAAAGAGCCTTAAT
CCCTCTTGATCATCAATTTCGCTATCCCAAATCCTTACGGAGGGTAATCAATCAAAATCGCTTTTC
CGTAGCCATTAATCAAGATTTTTTAGGAGTATGTTGTGGTTGTGCGCAAAGAGAAACCACATGGA
TTTCATCAGAATTAATTCAAATTTACTTAGAATTGAATAAAGCAGGATGGGCTTACAGTTTTGAA
ACTTGGCAGGATGGAAAACTAGCAGGAGGAATTCTAGGTATTGTAATTAGAGGGGCATTTATTG
GAGAGTCAATGTTTTACCATGTACCAGATGCTTCAAAAGTGGCAATGGTCAAATTAGTGGAACAT
CTACGACAAAGAAATTTTATTCTCTTTGACGCTCAGATGCAAAACCCCCACTTAGAAAGATTTGG
TTCTTATATCATCGATAATCGTAAGTATATGGGTTTATTGGCAAAAGCCCTTGAGGCTAATTGTG
ATTTTATCAAAATGTGAGTAGAATACCCTATCTTTTTACAAAGTCGTGTTTTTTTACTAGCCAAC
CTCTCCTAACCTCAGTTCGGTTTAACAATATCGAATAAGGGTAGGTGTCAGGGTTCGGAGTTAGT
TAGGGGGGAATGGCCATTCGCCCGTACAGGAGTTCGGAGTTATTATTCAGCCCAATACTATTTAT
TTTTGTCTCTTGCCCCCCGCCTCTCGAGGGGGGTTTTTTCAAAGTCAGGGCAAAATTATCTTGCCC
TCACCCCCAACCCCTCTCCCACAGTAGAGGGGAGCGTTTTTTCTTAATAATTGATTAATTAATAC
TTAAAAACTCCTGTATCTGTTACTATTTGTTAACCTGAGTTTTGGATAAGCTGAAAGCAGACCAA
CTCGTAGTCAAAAAACCTTATAGCTTCTTAGAAATAACTTAGAAATAACGATAAAATTGCCTTAA
TCCGAACTGACTTAAACAAGGGGCTTAAGCCCCTTGCTAAAAACCCCTACCACCTGCTATTCCTGC
AACCTGACACCTGACACCTAACCTTATCAGATATTCTTAAATCGAACTGAGGTTATTTGTTAGTT
TCAAAAATTGACATTTTTGAGAATGAAAAAACCCTGCCTCTCGAGGGGGGATAAAGGGGAGTTTG
CCCTTTTCTTAATCACTCCCCATGAAAATTTACCCTGAACTCAGGTTAGTTTATTTTTTTTAGGTG
TGGGGCTTCTCAGCAATCTAAAATGATTAGTTTAGAAAAAGCCCGAATTAGAGACTTCTGAGCCA
TTGAGTAGTCCTAAACTGTTCTCAATGAATTGATCCAGAAATGTTTGAGCTTCATTCATATTACC
AATATAGCCAACTCCCGCTACTTGTTGATTAAAGGCTTGAAAGCGATCGCTCGATGAACTTAAAC
CCTGTCCAAAAGAACCAAAAAGATTAGCATTGTTAGCCCATTGTGCGTCACTCCACTCTGCGGAA
GAAGCAAGAAAACTAACCCGAGTGCGATCGTCCACATTAAAACGAATAATTGCATCATCATAATC
TAAATCTCCTCCACCGAGTATGTCTTCAAAGCCCCAAGTGTTATAACCAAGGTTGGTAACGTGGT

CAAAATTATCCACATTGGCGTTTTTATAGGCAAAGAAAACATTTTCAAACTCTTCTGCTGAACCA
TTAACCGCCATAAAGGGTGCAAACAATTCATCCCCACTGAGGTTAAAGCTAAAAGGCACTGTTTC
TAAATCTGCTAGGCTAAGATTTGCATTGGTATCCGTTAGACGATTAAACTCGCTGAGAGCGATGG
TTTGATATTCGGGGTTATTTGGGCTATAGGTATTACCATCAGAGTCTATGACTGTACCATTTTCA
TCTAGGACACGGTAAAATCCAACGGTATTGTTAAAGGCGGCTTCACGGAAAATTGAACCAGAAAT
GGTATATTCAATTTCACTGGAATTATTCTGGGTGTCAACGAGGTCAATATTAGGATTTGGGTCAG
TAATGGGGATGGTATTATTTTCCGGTTGGAAACTGGTATAGTCAGTTACCGTTGATTGCAAAATC
GCCAAGGGTAATTGAGATTTGCTTAAACCATCATCTACCGCTTGATTCCATCGTTGGATTTCCTG
AGAAGAAGGATTGCGAGAGTAAAGGGTTTCACTGGTGACATTAACTATGGTTTCAACGGATGAG
TCTTCAAGGGATGCCCCAAAGTGGTTAACAATTTCTGCTTGACTTTCAGGAGAGCTTATAAAGCT
ATCGACAAAATCATCAACGTTGTCAAAATCGGGCATTTTCGCAGACCAATTGGCACTACCTGTGG
GATCACTGAGTCTTCCGAAATAGGCTTGATATAATAAACGGGTAAGGTTATATCGATCCATGGTG
TTATCAGGAATATTATTATCCCCAATACCTTCTAAATCAAATACTCCTACTTGACCAATAGTTTC
AATATTTAGGTTGAGTTGCTGAGTATTATCTAGTAAATTTGAACCGCTAGAAGGTGTACCATTTA
CTTCATTGCCTGTGGCAAAAAGAATTTTTGGATCTTGATTTTCTGGGGCTAATCCAGAGGTAAGA
TCGAAACTTTGTACTAATAAACCACCATTACCTAATGCTCCACCAAAAATCGGGGTAACGCTATG
ACCTAAACCTTCTGCTTCAGGTACATTGAGAGTGCTTAAGTCAACTAGGTTAAAGATACTGGGAT
TAAGATTGGTAACAATATCTTCTGTTCCCATGTCCATATTATGGTTATTGTGTTCACTATGACCC
CCATGACTTCCATGACCCCCATGACCAATGGTGCTACCGTCGGAAGTGGCGATAAAGTGTCCTGCT
CCTAATTGGAAGGTATAAACCCCCGTTTCTGTGGGAGAATTGGCAGTTAAAATCAGATTTTCTAG
GGCTATTTGTGCATATTGGTTAAAGCCACTGGTAAGGGTAATTTCTGAGGCTTGGGCAAGGGTTT
CATTTTCGATTAAAGCATCGGCTAATAAGGCTTGATCTGGTTGGTAGCCTCCTTCAAATTGTCCT
GTTTGTAATAAAAGTGCGATCGCAGCTCCATCTAAAATACTTAAATGAACTCCATTGGTAGTACG
ATAGGCAATAGCTAAATCCGCAAAATTATCTAAATTGTAATCGCCTGTACTCATAGTAACCTGTT
CAGGACGTAAACCCGCAACAGGATCAAGATTATCCCCTTCAATGTCAATGAATTCCCAAGGATCA
AATTCAAAAATTTCCGTCCAGTCTTCTCCTTGAGAGTCAGTATTCCAACCTGTTACGGTGAAGTC
GGTTAAATTAACAGAACCGTCGTTATTTACCTGAGTAAAACCTGCGGTAGTTATATCATTGCGGT
TATCTCCGCTCAAATCAGAAATTACTGCCCAAGGTGCTAAATTACTATTTTCATAGGGTTTAAAA
CTGCTGAGTAAGTTAGTTTGGTTTGTCAATAAACTAGCACCATCATAGACTCGGACTAAACCATC
TCCTTCGGTGGTAATTACGACTTCTTGGACAAAATCACTACTAACATCTCCTGTTTGTCCTCTTCT
AGGAGTTACATCAAGATTTCCGTCAAGGCTCAAAGTATAAGACTTAAAATCAGAAGCTCGATAAA
CTGTGACATTAATATTATTATCATCAGTGCGATCGAAGTTAAAATGTTCTGCAGGAATTAACCAA
CTATCATCGGTATTTTCAATCACTTGAATTGCCATCATCATCCCCGCATCTTCATGGGGCAGAAT
ATGACAGTGGAAAACATAAGTTCCTAAAAAGTCTTGGAATAGCATCCGAATAGTTTGATTATTCG
CTCCCCAAGTTGCTAAAGGCAGAGTATTATTAGGGTCGATCGCCTCTGGTAAAGTGGTAAAAGTA
CCCTGTAAAGGATCTTTTTGTAAAACCTCTCCAGAACTAGCATCATAGTAGTGATAGCCAGAACT
ATTGAGCATTGTCACATCTTCAAGGTTACGTTTTTCTGGTAACTCAGTATCACTATCCAACACTT
GATAATCATTGATATGGATGTGGAAAGGATGTCCAATATACTTAGTGGGTGCGTTGATACTCCAA
TTTTTAAGAGTCCATTCTTCGATCGTACCCAACTGAGCTGTAACCAAAGGACCAAAAGGAAAAAC
ATGATCATTAATCAGAAAAGGATTTCATAACCTAACCATGTACCCTCCTCATCCCTTGGCAAAG
CCGTAAAAGTTTGATATTCCTGTCCTGTTGACTCTTGATAGGCTTTTAAAGCCTCTTGAGTTGCC
GCATCTCTCTCTGTGGGATCTCGCCCTACTAATACATCTTTTGTCCAGTTAAACTCCCTTCCCCGT
GTAGTTTGCCACTGTTCATCAAATAAATTTACACTAGGTACTTTATTGGGATCATACTCTTCTGG

AGTTGTTGTCGGTAAAATATCTTGTACAGGAATAGTAGTATTAATCTGATTAATAAAATCTTCCT
GTTGAGCAGGGCTTAAATTTTCAGTACCTTCAGGAACAGTCAAAATAGCCAAAGGACCTGCCGCC
TGAGAATTTTCTGAACTTAATTCAGGATAACCACCCATATTCGTAACATTGTAGGTTAACCCATC
TTTTTCAAAAGAGTAAACCGAAGTTAATTCTGTAGTGCCACTCTCGAGATAAAATAAAACATCGA
CTCTTTTCCCCGGTGCTAGAGAAATAATATCTTCTCCTTTTGTGTAATCAGTCGGTGCAGAAGCCC
CTTGAGGAGTATATGAACCTAAAACCCCTTCCGCTCGGCGAATTTCAGGGTATTGATGACCATCT
TCTCCATAAATGAATAAGGGTATGGGTTCTAAGGTTGTATTACCATCATTGTCTGTAACTCTTTG
TTGCAAACTAACATTATAGTAGGCTTGATTACTTTGATTGCTTAAAGAAATACTTTGCCAACCCC
CCACCGTTTCTCGATTAGGTTGAAACTCTCCATTCACCGTCACCATAGTTAAACGGTTAACTATGC
CCCCTAAACCGTCATAGGAAGTTAATCTTAAACTGGGGTCTTCTTCTCCTTCAGTTTCATCTGCAC
CAAAATCAAGATCAATATTTTTGAGAATGGCTAAATTGCGAGGTATATCTTCCAAGCTAGGAATA
CGACTGAGAGGATCTCCAATTTGAATAAATCCCGATTGCCCCCCATAAACCTGAGCGTTAACAGA
AGGGTGATAATGGGGATGATACCAATAAGAACCAATACCATGATCATCAGGTATATCAATATTTG
TTGTCCAACTTTGTCCAGTGGTATAACGACTAACTACATTATCTCCAAAACCTCCGGGGGCAGTA
TGGGCGCCGTGAAAATGGAAATTAGTGGAAGTTGTCCCTCCCAAACCAGCACTACCACCATTGCC
ATAGGAACTATTACGCACTAGAGTAGCCGCTTGAATTTGTTCGGTGGTTAATCCGGGAATTGTGA
TTTCATTATCAAAATTAATGCGTAAATCTTCCCCGGGTTGCATAATTAATGTAGGTCCGGGATAG
CTAGTGGTAACTGTATCTCCATTGGGAAGACCATAGGTATATAAAAAGTTTGGCAACCAAAGAGG
GGGGGTTTCTCCTTGGGCAACATCTAAATTATCTAAAAGATTTTGAGCATCGAAAATAGAGCGGA
CGATTTCATCGTTATTATACGTACTCCATTTACCCACAGGATCATAGTTATCGTTGTCTTGGATT
GCTGTATAAAGTTCATCGGTGGTTTGTCCTTGGGCTGACCAAAATTCGTCCGTATTCGCCCCTTG
ATCAAATCCCGGTCCTGTATAGCCAATAAGTCTTAAGTTATAGATAGAATTATTACGAGATTTGC
CTGTCCAGTTTCTGACGATCGCATTTTTGTATTGAGTAAACAGAGAATTAGGACTATTAACAGCA
GTTAAACCATTTGCTCCCAATAAAAAATCATTAACAAATTGATCCACATTCGTAATTTGTACGGG
GTTACTGCTAATATCGAGATCAATTTCTAAATCTTGACGAGGTTGATTATTATTTTCAATAATGC
CCGTTTGAGGATCATAACTGAGACTAGAACTTAAAACAATGTTGGGATTACCTTCGGGAGTTGTA
GGGAATATAGCACCGAGGCGATAGAAAAAAGAATCTGACATGATCAACTATCCATTAAAATCGGT
AAATTTAAAATAATTAAAATTTTAATGATTTTTGGCGAAAAAAAGACCTCTTGTCTCAAAAATT
TTATTTTTTCTTTACAAACTGTCAATTTTAGATCTTTATTCTCAATATTGCTATAAAATCCTAAT
ATATTAGAAATAAAAAGAGAAGTTAATAGTCACAAAAAGAAAACACCAAAATACAGCGATAAAA
GGTTATATAGCAATACTAGCTAAGTTTTATTAACTACTGTTTGATAAAAGTAAAAAGCCTGAAGC
AAAAGGTAAAAGGGATAGTATCTTCAATGTTTTTTTCTGATAATTGATTATTTATAAATGATTA
AGGATTACTATAAGTTAACTTGATTATTAATTTTTAAATAATTTTTGGTTTATTTTTTTAGTGTG
GAACAAAAACAAATTGATAACTTTTTTTAAATTTATTTAAAGGTTTCTATTAATAATAATTAAT
ACTAATTTACCTTATATTTTTTATGGTAATTGTATATTTTTGTTACAGTCATTAATATTTGTTGC
GTCGGTTACTGGTAATTAATGCTATTTTGTATGGTTGAATGGAACTAATAGCAAGGAGATAGAG
TTAGTGGGTTTCGGTTTAAATGCCCGTTCGGCGGGTCTAAATTTATGTCATGAGAATGCGGAAGA
ATTTGCAATGACAATGCCTTCTGTGGATTTTGTTCAAGTGCATCCAGAGCATTTTCTACAGGACT
TAGGGGGTAATTATGTTCATGCTTTTGAAAAATTAAAGGAAAATTATCCTCTTACCTTTCATGGT
TTTGGTTTATCTATAGGTTCGATCGCACCTTTGCCAGAAAATTATCTAAAATTGGTTAAACGCTT
ACTAGCAGAAAATGAAGGAAGTTTTTTTTCAGAACATTTTTCATGGAGTTCTGTTTCTCATCACC
ATTTCCATGATTTAATACCCTTTCCTTTTACCCAAGAAGTTTTAGACTATTTTTGCGAAAGAGTT
GAACAAGTGCAAGAATATCTCGGTAGTGAGATTTTATTAGAAAATATTAGTAGTTATATGCGTT

TCAGCATTTCTGAAATGGACGAAATAGCCTTTATGAATGAAGTGTTTAAACGTACTGGAGCAAAA
ATGTTATTGGATCTTAACAATCTCTGGGCTAACGCTCAAAACTTTGAAGAAGATGCCTTAGAGAG
AATGATGGCAATTAAGCCTGAATATGTCCAAAGTTATCATTTAGCTGGGTGTACAGCTTGGGGAG
ATAATAATTATATTGACTATCATGGAGAAGCCGTTAGAGATGAAGTATGGCAACTATATCAAAA
AGCAATGGAGGTATTCGGAGCTAAACCGACTTTAATTGAATGGGAAAATAATATTCCTCCCATTA
GCCGAACCGTGGAAGAAGTCCATAAAGCTAAAGCTATTTTAACGAAAGCCTAGAATTCTATTCAA
GCTCAATTCGTGCCAATAATCGCCGATGAGGACAGGTTTCAGATATGACAATACGGCTAGTAAAT
TAGTTAAGAGGAGGAGAAAGGGGGATGTGGGGAGAGGAGAAAGCCAGTCATAAGTTCAGAGTTT
TTAATTCTTAATTTTTAATTCTTCATTTTTTCTTGCCCTACCATAACCAGAAAAATTTATCTTGA
ACTCAGATTAGTTTACTTTTTCGAGAAGATTGGAATTTCTAATTTTTGAAAATAATATGATACAT
CCTCAATTAGAAGAAATAATTAGTAAGTTAAAACAGTCTTTTATTAACTTATATAATACTAAAT
TAGACAAAATTATTTTATATGGATCTCAAGGGAGAAAAGATGCTCAAATAGATTCAGATATTGA
TATTTTAGTAGTTCTTAAAGACAACATTGATCCTTATAAAGAAATAGATAGAACTGGAGATATT
ATCTATCAAATAAGTTTAGATTATGATGTGGTTTTATCTCGTCATTTTATCTCTTCTGATAAATT
TAATTATAGTAATAATCCATTTTTACACAATGTAAAAAAAGAGGGAATTATACTGTGAAATTAG
AGGTTAATAAATTAATTAAAAAGGCTAAAGAGAGTTTAGTTGCCTCAGAAATTTTAACTCAAAA
CAAACTTTAGAGACTACATACATAATGACAAAAATAGAGACATGGGAAGATAAAGGGCAAGGTT
ATCCTCTACGTTGGTGGGTTTTGCCGATTGTTTGTTTACCAACTCTGGCGGTATTGCTAAATGGG
AACATTGTCTTTATTGCTTTACCTGCCATTGGTGATACTTTTCGTGACTTTCCCAATTTAGGGGA
ATGGGTATTAACTTCTTTTTCCTTAGTGGCGGCGGCTCTTTCCTTACCTGCTGGGGAATTGGGCG
ATCGCATCGGCTTAAGAAAGGTATTTAGCTGGGGTTTATGGTTATTTATCCTCGGTAGTGGTATT
TGTTTTTTTGCTTTTAAAGGCTGGATATTAATTTTGGGTAGGGTGATATGTGCTTGTGGTGCGGC
AATTCTTGCCCCCGTTGCCTTGGCTTGTTTACGGCGTATTTTTCCCCCAGAAGAACAGGCGATCGC
CTTTGGTTATTGGTCTGCTAGTGTCACCATTGGTACAGTAATTGGACCATTTTTAGGAGGATTTT
TAGAACATATTGCTAGTTGGCATTGGGTCTTTTTTGCCCCTGTAGTACCTGCTGCGATCGGACTT
TTAGGACTGCTTTTATTACCTTTGCTTCCCGGGAAACAAATCTCCACTAAGCCCGATTATATGGG
CATAACTACCATCGGTTTTGCCATCTTTTTCCTTCTCTTTTTCCTGATGGAAAGTCCGAATTTATC
CCCCAGTCTTAACATTTTGATTATCTGTCTTTTCTTGCTAACAGTGGTGATGTGGCGACAAAGTG
CCAATAAGCCATCAGCAATTATCCCTTTAAATATTATTACTCGCAATAGTTGGTATGTTCCTTCC
GTTTTACAGCTATTTATTCGAGCATTATTTGTATTTGTCATGACTTGTTTAACGGTTTATTTTCA
ACAAGGGAGAAATTTATCCGCTTTACAAACATCTATTCATATTTTGCCTTTAAGTATAGTTTCAG
GGGTTGTTTCTCTTGTCAGTGGCTATTTAGAAAAATCCTTCGGAGTCAAATATTTGATGATGGCA
ACTCTAACTTTAACCGCTTTAGGTTTTTGGTCTTTGCGAGATATTCCTCTATCCGGATTTGATGG
AGGTGAGTGGTTTGGCATAGTGGCTTTTGGTATCCTTTATGGCAACACCGCCCAACTTTCTCACC
AAGCCCTAGTTCATTTTCCTAAATCTCAGGCGATGAGGGGTTCAGCGATTAATACCTTAATCATT
AACTTAGGTTTGGCTTTAGGGGCGGCTATTTACAGTATTTTTGTCTCAATTGCTTTACCATTGAA
TTTAAAACAGCAGTCTAATTGGCAATTTCCCGTTGAGATACCTAGCAGTTTTCAAGCAATACTCA
ATCAGGTAGATCAAAATCTTCATACCTCAAGTCATAGTCAATCGTTACAGGCTTTACAAGAATCT
TTAGCCCACAGTTTTGGCTTTGGTTTTGCATTGTGTGCTATTTTCTGTTTAATCGCCATTGCCATT
ACCTACAGTATGCGTCCATTTTCATCGAATTAACTATTCACTATTCATTATTCACTAATTGCTAT
TGATGATTAAAAACAATCCTACCACTGCTAAATATGCAGTACAACGTCTAGCTGATTTAGGAATT
CGCCATGTTTTTGGCTTACCCGGAGATTATGCCTTTCCTATCGATGATGCGATCGAATCTAACCCT
AATTTAACATGGGTTGTCTGTAGTAATGAACTTAATGCCGCCTACAGTGCCGACGGTTATGCCCG

TGTTTTTGGGGCGTCTATCCTTAGCACCACCTATGGAGTGGGAGAATTAGGTGCAATGGCTGGTG
TCATGGGGGCTAAAGCAGAAAGATTACCTATTTTTCATCTGGTGGGGATGCCTTCCACTAGGTTA
ATGCGGACTCGCAAACCTTTACATCATACTTTCGGGGATGGAGAATTTAACCATTTTCATCAATT
ATCCTCCGCAACCGCTTGTGTTAGCACTATCTTAACCCCAGAAAATGCAATTACCGAAATGGAAA
GAGTAATTGCGATCGCACTTTCCCAACGACAACCAGCAATTATCGCCATAGCTGGGGATTACGCT
CAAATGCCAGTGATAGGCAATCCCATACAGGGAGTACCATTACATCAAGTTTCACCCCCAAAAAG
TAACCCTCAAGAGTTAGAGTGTGCCGTTAAAACTATCTTACAAAGATTAGAACAAGCTCAACAAC
CCGTTATTCTTCCCAGTTTTACCCTACTACGCTACGGATTACACAGAACAATTCACCCACCTTCTT
AAAGTTACTGGTATTCCCTACGCCACCACCAGAATTGATAAAGCCGTAATTTCTGAATCTCATCC
CTTGTATATGGGTATGTATCGAGGGGCAACTTCAGAATCCGCCCTCCGAGAATATGTAGAATCTG
CGGACTTAATTCTTGACATCGGCGGAGTCTTATTTGATGATGTTTCTACAGGTCATGGTTCAGCA
AGACTCGATCGAGAAAAGATTATTTCCATTCATCCCCATCATGTAGAAATTCTCTCTAATATTTG
TCATATTAATTCCCAATCCCATACTTATAGCCCTGTTTGGATAGGGGATGTCATTAGTAACCTTA
TAGAGAATATTAAACCCATTTCCTCCAAAAAATTTCCCCAACCTTCCCCTATTACCCTCGAAAAA
AATGCTTCTCAAGCTATTACTTGGCAAACCCTCCGAGCCACAATGCAAAATTTCCTGGAAGCGGA
AGATACTTTAATCTGTGAATCTGGTTTAAGCGTTGCCTTTTTATCATCCTTACTATTACCAAAAA
ATTGTAGCTTTCATAATCAAACCCTTTGGGCGGCTATTGGTTGGGCAACTCCTGCCACATTTGGG
GCGGCTTTGGCTAATCCCCAACGACGAGTTATCTTAATTACAGGCGATGGTGCTCATCAACTTAC
GGCAAATGAAATTGGCACTATGGGGCGTTATGGAGTTAAACCCATAATTATTGTCTTAAATAATG
GCATTTTTGGTATTGAAGAATTTTTAGCAGGAAATAAATCTAATATTTACAATGTTTTATCTCCT
TGGAATTATGCTCAGATACCTCATGCAATGGGTTGCGAAAACTGGTACTGTCAAAAAGTTACATC
TTTAGATGAATTAAACAATGCCCTTGAAAAAGCAAGACTTCACCATAATGGGGCTTATATAGAAG
TTATTTTAACCGAGAAAATTCCCCCCGCTTTATCTGAAACAATATACGCTCAACGCTATCAATTA
TTACCCATAGACACTTATCTGAGTTCGGGATAAATTTTCATAGGGAGTATTGAGAAAAGGAAAA
AAGTCAAGGGGCAAAGATGAATAGTGAATAGTGAATAATTAAAAACTCCGAACCCTCATTACTCA
TTATTCATTACTTATTGCTTTCTCCCAACACCTGCAACCTGAAACTTGAAACCTGCTCTTTCCCGA
TACTCCTACATTAAACTGAGGTAATATCTGAGTATAGAAAGCGATGAAGGAGAGGTAATGGCAA
AATTATTCGTAGCAAATACAATTACAGACTTAATCTTGGGTGATATAGATGAACATTTACCCATA
AATCGTATCCCTTTATCAGGAACTGTTTTAGGTTTAGGCACTACTACCGCAACAGATAACGGTGA
GTTAGTTTTTCTTCCTGATTTTGAGCAAAATAAAACTTCCATCTACAAATGTAACCCCACTGATG
GTAAAACTTATTCTTTATCCCCAACTTCAGAAGTAGATCATTTTCCTTATGTTTGCTTCACAAAT
GATATTAGCAAAGAAGGTATAGAAATCGATGTTTCTAACTTTAATATTACTAATTCTAGTCTGAC
GGAGATTGTGACTCAATCAATTAACCAATGCGGTGTCAAAGCTAATTATTATGGTGTATATATCG
AATCAACATGGTCATCGGTTGTTTTAACCATTGCATCGAAGTTATGTTTATGGCAGTGGGCGAGA
ATGAAAAATTTTGTCTCTGATGATATTTTTAGTGCAGAAGATAATATCTACACATCATTACAACA
TTTTTACTGTGCTGATCAAGAACACCCTGATAACCCAATGAAATACTTGGGTAAAGCGAATTCAT
GGCGCTTAATTGGAATGTATGCCAGTGAACCAAAAACAGGCTTAGTTACAGTACCACAACCTAAC
CAAAATTTACATATACATGGTATTTGTAAAGAAAAAAATTGGGGTGGCCACGTACACCATGAACA
TAAGATGAGCAAGTTACTCCATATTGATAGTTTAATTATCTATCCCCTTCAAGAAGTTGAAGTAA
TTGAATCGGATTTAACGATTAAGGATGCAGTATGGCTTGATGATAACCTTGTTTTTACTGTTGTT
AACCAAGGATTATTAGATGTGAGTAATCTTCAAATTCACATAGTTCCTAATAATCAATATAGCTC
ATACATTGGTTGTCGCATTCCTTGGTTAAGTGCCGGGAATTCTTTTCAAATCAATATTCCTAAAA
ATAAGATTAAATTAAAAACAGGAAAAAACATCATAAATATCATTGCAGACCCAGACAATTTGAT

TTTAGAAGCCAATGAAAATAACAATATAATGACCCTTGAGGTTTCTATTTCCCCTGTTTAATCTT
TGGGCAGGTTTCAGATAAAGACTATAACTTGATCTCAGTTCGGTTTAAGAATATCCGATAAGGTT
AGGTGTCAGGTTTCAGGTTGCAGGTTGCAGGTGTTAGGATGATGACCAGATGAGAAGAAAGGAT
GAGAGGAGGAAGTAGAGGCTTAGGGTTTGGGGTGTTAGGAGTTGAATATATTTTTAACGTTAGT
TCGGGTTAAGACAATTTTATCTTTCTTTCTAAGAAGCCATAAGGTTTCTGACTACAAATTGGGA
TGCTTTCAGCTTATCCAAAACTCAGGTTAACTTAATCAATTCTACGGCATCTTTACCATCGATCGC
ATCTAAATAAACTTTTACTTTTTCCTCTTTAGCGGTAGGTAGAGCTTTGCCGATGAAGTCAGGGT
GAATAGGTACTTGTCGATGACCTCGATCAACTAAAACTAACAATTTAATAATTTCTGGTCTGCCA
TACTCTGTAACAGCGTTTAAAGCGGCTCTAATGGTTCTTCCCTTATAAATCACATCATCCACCAA
AATTACGGTTTTACCCGTCAAATCAAAGGGTATTTCGGTCTTCTGAGGCGTTCTGGTTTTGATTT
GGTCTAAATCATCTCGAAAGAAAGTTATATCTAGCGCACCAACTGCCACTTTAACTTGTTCAATC
ATTTCTATCTGATTGGCTAATAGATAAGCCAAAGGAACACCCCTTGTATAAATACCCAAGAGGAC
AACTTGAGATAAGTCTGGGGCTTCCTCGACTATTTGTGAAGCTAAACGAATTAAAGTACGACGTA
TTTCGTCTGCTGAAAGTATTTCTCTTATTTGACGAGCCATTATTTGGATAGATTACATTTGAGTG
TATAGATTAAGACGTAAATAAGTGTTCGGGTTTCGGGGTTCGGAGTTAAACTTAACATATACTAT
AAATTGAATTGAAGAATCAATAATCGAGAATTAAAAATTAACCGTTCTTCATTTTCCATCCTCAC
TAATACCTCTACACCCAAAATAAGATCAACATTGACTGAGCTATGGATTCTCATTGTCATCTATC
GACTTGTTTTCCGTTTCTTCTACTGCTAGATTTTCATTTTCATTTAGTAATTGATTTTCCGTTTCC
CCTATTGACGAATTCTCATTTTTTTCACTGGATTGATTTTCTATTCCTTCCACCGAGACGAGAACT
ACAGTTATATTATCTGAACCTCCCCCGTCTTTTGCGTTTTGAATGAGAGTTTTAGCCGCCTGTTCT
AGGTTTTCTAGTTCTTGTAAAGATTTGTTAATCAAATCATCGGGTACTTCTTCGGTTAAGCCATC
ACTACAAAGCAAGAAGCGATCGCCATTTTGGACATCATCCATTTTTTGTATATCAATACCTTCGT
ATAAGTCTCTACGTCCTAAACACTGAGAAAGAACGTGTCTCCAAGGATGATATTTTGCATCTTCA
GGGGTAATTTCTCCAGCCCGAATCGCTTGAGAGATCCAAGTATGATCAAGGGTAATTTGATTTAA
CTCTCCATTACGCCAACGATAAAGGCGGGAATCTCCCACATGGGCTCGCCATGTTTCATCTTCTCT
AAACAGAACTAACACAAGAGTTGTTCCCATGTCTGCTCTTTCAGGATGAAGCACTTGATCTTCTA
AAATGCCTTCATTTGCCTGATAAATTGACTTTTCGATTAAATCATAAGAGTCGATGGGTGCATCC
CAATTGGTTTCTAGGTATTCTTTAATAACTTCTACGGCAATGCGACTAGCTTCTTCCCCTCCGGCA
TGACCTCCCATACCATCGGCAAGGATAAAAAAACGCCCTTCGGGGTCTGTATAGTGGTTATCTTG
GTTGTAGGGTCTAACTAACCCCGGATCAGTTAACCCAATAGATGTACATTTCATACGGTCAATTA
ATTGATACTAATATTTATAGTTTACGGTCTAATCTATCTAATTGTCTCAATAAACGGAACAAAGA
TACAGAGGGAAAAATACTTAAAACAAAAACTGCGATCGCAATTTGCCATAAGCCGTTAACAATTA
GTATCGTAGCAGATAAAATTAAAGCTCCCACAAATAGACTATAATTAGTAGCCATCTGCATCCCT
GCAATACGTCGCAAAAGGCGATCAGACTCTTGGGAACGAACTCTAATGCGTATATCTCCCCGTTC
TAATTTTTCGATGGCATCTTCTAAGCGTCGGGGTAAACCTAAAGCAGTATTGCTTACTTGCACTG
CCTGACGACTTAATTCATCAAACAATGACTTACCGCCATCATTACTAAATTCTGACATAATTTGT
AAAGCGAAAGGTTGTGCTACTTCCATAAAGTTAAATTCTGGATCTAATCCCTTGCCCACTCCCTC
TAAAGTAGAAAAAGCTCTCATCACAAAAGTAAAAGTAGCGGGAAAGCGGAAGGGTTGATCATAA
GCGATTTCAAATAAATCCTCGCTAATTTGATTGATGGATTGTTCCTCGAAAGGCTTATCCATGAA
GTTATCTAACATGAACTGAATTGACCTGCGCACAGGACCGGGATCATCTGTAACAGATAAAGCCT
CTAAATCCACCAACGCAGAAACGACGCGATCGGCATTTTTTTCGGTAATCCCGAATAACATCTCA
ATCAACTTTTCTTTGACATTGGTTTTAATATGCCCCATCATGCCGAAATCATAAAAGATTAAAGC
ACCATCGGGAGCTACAGCTAAATTACCCGGATGAGGATCAGCATGGAAGAAACCATCATTCAACA

ACTGCTGTAAATAGGCTTTTGCCCCTAATCTTGCCAAAATTTTGCGATCGAGTCCTGCCGCTTCAA
GAGCTTCATAATGGCTAATTTTAATCCCCGGCATATACTCCAAAGTTAGAACTCGAGGAGAAGTA
TAACGCCAATAAACTTTAGGAACATTAACCCAATTCACCCCCCGAAAATTACGGCGAAAAGTATC
TGCATTGCGCCCCTCATTCAAATAATCTGTTTCTAGCCAAAGAATACGACTACATTCTTCATAAA
TCCCTAACCAGTCTCTACCCTTACCCCAACGGGGATGATTTTGAAAATAGCGGGTAATTTGCTTA
AGAATCCCTAAATCAATAGTAAATAATTGGGGTAATCCGGGGCGTTGAATTTTGATTACCACTTC
TTCCCCTGATTTTAACTGTGCCTTATGCACCTGTCCCAAACTAGCCGCCGCCAGAGGAGTAGGATC
AACACTGAGAAATAAATCCTCTAAAGGCTTACCTAAATCTTTCTCAATAATCGATCGCACCTGTT
CAAAACTAAAGGCTGGAACTCGATCCTGTAACTTAGACAGCTCGTCCACATACTCGATGGGAAAA
ATATCTGCACGGGTAGAGAACAATTGACCCACCTTAATAAAAGTAGGACCTAACTCCAGTAAATT
TTCTCTAATCCATGCCGCTTGAATACGACGACGGGCGCTTCTTTTTTCCTCAGTATAACCATTAAC
ATAAGTCCATTTTTTACTGTTACGCCATAGTTTAAACAAAAGGATTAACACAAAAGTCCAAATAT
CAATACGACGACGCAAACGGGAATAACTACTGCGATTCCAACGATAAGTTTTTTTCCCTTTTTCT
AATTTAGGCTTCACATTAACAGAAGCAAAATCGGGGCTAGATTGTAAGTATTTTTCTGTATTAGA
GTAAGCTGAAGACACTGATTGTTATTTTATGAAAGTTTTACTAAAAAAATTTATTGACTTTATTT
TCAATCTTAAAAGAAACCAGAAAAAATTATATTTAGAACAAATCCAGAGACAAATATATTATTA
ATCCCTAAAACTTGCCCATCGAATATTTTTTCCTAAGAATCTCCATTATTTTGACGATAATTATT
TAATTCTGCCTTTAAACGAGCAATTTCCGCCCTTAGTTCATCTAATAACTCTTGTAAATCCACCTC
CGTATTAGCTTCTACTGTAATAGTGGTATCAGTATTACTAGATTGTTGTTTTGCCTTCTCTGCTC
TGGCTTTGACTTCTTGGGCAAACTCTTGTAATCTCTGCCTAGTCTCCGCATCAAACTTACCAATAT
CACTGAGAATATGAGTTAGACTATCTTCTGCTTTTTCGGTTAAAACCTCAGTTAAGGCTTTGCTG
AGATAATAAAAATCATTAATTGGGTTAGTCATTGGTAATGTTAATATTTCTTAATGGTTTTTAA
CAAATTATATCTGAGATTTTCAGATTCCGTTGAGATTATTTTGAACTATTCTCTCCTGAGTTGGA
TTTATCCGCCATTCTCTTTAACAGGTACGAGTGGGAAGTCCCTAGTTTCAGTGTAACGAACTAGG
GATGAAAACGAGTCCTAACGTGTTATAATAAAATCGGTCACTGACCCACCTAGCTGACTAAGAGG
CCGCCCTTGTGGCATAAAGTATGTACTCGGCAGAGACTAGGGAAGTAACAACCAGTAGCATAAAA
GGTCGTAAGACGCTGGTTCGACGCAGTAGAATTTTTGTTCTATGGGTCTGCGGAAGGCGGACTCC
TGCTCGGGTTCTAGCTAGACTAGATAAAGCCTAGAAATAGGTAGAGATGCTGAGTAAGACTCGAC
ACTACTTAGATTAGGTTCGGAGTAGTGTAAAGCATTGGCAGATGAGACGAGAAGCTCATTGTTCA
GCGTAGCGACAATGAGTACTTCACGATCAATGTATCTTTTTGTACCTGAGTTCGATATAAAATTG
TTGGGTGGTGGTAGGGAAAAAGGAAAACCTCCTCTGTGTCTTCCCTTAAAAGGGGTAGAAGGGGA
ACAGGGAACAGTATTTATACTTACATAACCTCAGTTCGGTTTAAGAATATCGAATAAGAGTAGGT
GTCATGTGTCAGGTTTCAGGTTTCAGGTGTTGGGGTGATGAGGAGATAAGGGGAGAGGGGGAAG
TAGGGGCGAAGGGCCATTCGCCCGTACAGGAGTTCGGAGTTTTTAATTCTTAATTCTTAATTTTT
AATTCTTAATTATCAACTATTCACTATTCACTAATTGCCTTGTCTTAATTACTAATTACTAATTT
TTAATTACCCCCTGTCCAAAGCAAAAACTATCAGTTAAACTGTTACATTTGTTTATGGAAAGTAC
GGTTAATTACGTCTAACTGCTGTTCACGGGTTAGTTTAATGAAGTTGACGGCATAACCTGATACA
CGAATGGTTAATTGAGGATATTGTTCAGGATGATCCATCGCATCAAGGAGAGTTTCTCGGTTAAA
TACGTTAACATTGATATGATGTCCTGTATCGTGGAAATAACCATCTAACATCCCTGCAAGGTTAT
TGATTCTCGCTTCAGGAGTTTTACCCAATGCGTCGGGAATGATAGAGAAAGTATAGGAAATACCG
TCTTGGGCATCTTCATAGGGTAACTTAGCAATGGAAGCCATAGATGCGATCGCACCTTTGGTATC
TCTGCCGTGCATGGGATTCGCACCGGGAGCAAATGGTTCACCTGCCTTACGTCCATCGGGAGTGCT
ACCTGTTTTTTTGCCATAGACTACATTAGAAGTAATAGTCAAAATGGATTGAGTGGGAGTGGCAT

TACGATAGGCTTTGTTACGGCGTACTTTGTTCATAAAATCACTAACTAAGTTTATCGCAATCTCA
TCGGAGCGATCGTCGTTGTTACCAAATTTGGGATAATCTCCCTCTACTTCATAATCTACCGCTAA
ACCTTGTTCATTGCGAATCACTTTGACCCGACTATATTTTATCGCCGATAAAGCATCCGCTACCAC
CGACAACCCAGCCATACCACAAGCCATTGTCCGATAAACGTCGCGATCGTGTAAAGCCATTTCGA
TGCGTTCATAGCTATACTTATCGTGCATATAATGAATCACATTGAGGGTGTTAACGTAGAGTTTA
GCTAACCATGTTACCAATAAATCAAACTTAGCACTTACTTCGTCATAATCTAGGTAATCCCCTGT
AATTGGTGCATAAGGAGGTGCAACTTGCATCCCAGTATTTTCATCTTTACCGCCATTGATAGCAT
AAAGTAAGGCTTTGGCTAGATTAACCCGTGCGCCGAAAAACTGCATCTGTTTACCGATTCTCATG
GCAGAAACGCAACAAGCAATAGCATAGTCATCCCCGTATTCAGGACGCATTAAATCATCATTTTC
GTATTGAATAGAACTAGAGTCAATGGACACTTTCGCACAATAACGTTTAAATGCGATGGGCAATT
TTTCTGACCATAAAACCGTTAAATTTGGTTCTGGGGCAGGACCTAAATTATACAATGTATGCAAG
AAACGGAAACTGCTTTTAGTAACTAAAGGACGACCATCTTCTCCCATACCGCCGATTACTTCCGT
AACCCAAACGGGATCTCCAGAAAAAAGCTGATTGTATTCTGGTGCACGTAGAAAACGAACCATAC
GCAATTTAATTACAAATTGATCGATCAACTCTTGGGCTTCTGCTTCGGTTATTAAACCTTTTGCC
AAATCTCTGACTATGTAAATATCAAGGAAAGTGGAAGTACGACCGAGAGACATCGCCGCCCCATT
TTGTTCTTTAATTGCCGCTAAATAGCCAAAATAAGTCCATTGGATAGCTTCTTGGGCGGTAGTAG
CGGGTTTCGAGATGTCAAAACCGTAGCTTTGTGCCATTTCCTTTAGTTCATTTAAGGCTTTAATC
TGCTCGAAAATTTCTTCTCTTAAACGAATGGTATCTTCATCCATGAAATCATATTCGAGGGAAAG
TAGTTGATTTTGTTTATCTTGAATGAGAAAATCAACACCGTATAAAGCTACTCGACGATAATCAC
CGATAATTCTACCTCTACCATAAGCATCAGGTAAACCAGTAATAATTCCTGATTTACGAGCTTTT
TTCATTTCGGTAGTGTAAGCAGAAAATACGCCATCATTATGAGTTTTACGATACTTAGTGAATAT
ATCACGAGTTTCAGGATCAATATCATAACCGTATGCTTTTAATGAACCTTCTACGACTCTGATAC
CGCCAAAGGGCATAATAGACCGTTTTAAGGGCTTATCGGTTTGTAAACCAACAATTTTTTCTAAA
TCTTTATTAACGTAACCTGCAGGATAGGCGGTGATGGTGGAGGGAATTTTGGTTTCTGTGTCTAA
AATACCTTTTTCCCGCTCTTCTTTCATCAATAGTTTTACTTCTGTCCAAAGGTTATTAGTGCAGG
AGGTAGCATCTGTTAAAAAAGATTCATCACCTGTGTAGGGAGTATAATTTTTCTGAATAAAATCT
CGAACATTAACCTCTTTTGTCCATTTACCTGTTTTGAATCCTTGCCAGGGTTGAGTTGAAAATTC
TTCTCTAGCGTGGTCTGTAGGTTTATCTGTAAGGTGAACCATACTTTTTTACCTCGCAAAATATT
TGGTTAATAATTTATTTTTCCTTACTTATTATTATAAACTAAATTGGCTTATTAACTAATAAAAA
AAATATTTTTTTATTTGCCAAAGATTATATTTATTTACCAAAAATAATGTTGAAATTAATTAGTC
TGTTATATTTTTTTACATATTATTTCTTTATAGTAAAAAATCTCTTGATTTCTTAGGAATAGTTG
CTAGATAAAAGTCTCCCTTGATTTTTGTTGTATATTAATCAATTCTTCTTAACCAAGAATAAAAAT
ATAATGTGTTTTCCCCTATACAATTTTCTAAGTTCGATTACAAATTAATAACGGCTAAGTAACTA
TATTTCTTTAATATTTTTTTTAGAATATTACTAAAACTTTATAAAAGTTAATTTTTTATCAATAT
TGTAAATTTTTATCAATATTTTCTCATTTTTTATCTTCTCCTTGATTAATGGATTTGCAGAGAAT
AAACAAGGGGCTTAAGCCCTGTCAATCAGCTAAGATGGAGTTAAATTAACCTCAGTTCTGTTTAA
GAATATCCCATAAGATTAGGTGTCAGGTGTCAGGTTTCAGGTGTTGGGGATAGGGGGATGAGGG
GATGGGGGGAGAGGTGGCAGGTATCAGGTGGCAGGTGTCAGGTTTAAGGGAGTAATGAGTAACC
AGTAACGAGTAATTATCCCTTTTTTGTCATGTTCCGAGATAAAATAATAGAAGAAAAAGAGAAA
TGGAACTTGGGAGGCTTAGATGATAATGGATGTAGCAGAGCAAGTTAGTAAACATTTGCCTCGAA
CTCCAGAACCCACTATACCTTTAGTGGATCAATATTGCAGTTACTATCAGGATTTATTTCCTGAT
GTAAGAAGTTATGAATATTTTAAGTTATTACATTTAGGAATAATCTCTAATCTTAAACGAAAAT
CATTACCAGAATTAGAAAAAGTACTAGGAGTATCATCACAATCCTTACACCATTTTCTCACATCA

TCACCTTGGTTATTATCGGAACTAGACCAAAGAAGATTAAATAAATTAGTAGAGATATTGCAGG
GGAAAAAAATCATTCTCATAGTAGATGAAACAGGAGATAGAAAAAAAGGTAATAAAACAGACTA
TACTGCTCGACAATATTTAGGAAATGTGGGGAAAATAGATCAAGGAATAGTTTCAGTTAATGTC
TATGGTGTTTATCAGAATATAACCTTTCCATTAATGACAAAAATATATAAACCAAAGTCAAGATT
AAAAGTAGGAGATAAATATAAAACAAAGACAGCAATAGCAGGAGAAATGGTAAGAGAATTAAAG
GAATTAGGATTAGAAATAGAAATAGTGTTAGCAGATAGCTTATATGGTGAAAGTAGTAGTTTTT
TGGAAGTATTAGAAGAGTTAAAACTGGAATATGTGGTATCGATTAGAAAGAATCATGGGGTGTG
GATGGGAGCAAATGAAAGAGTTAGAGCAAATAAATGGTGTAAATTTACTAGGACATTTAGGGAT
GGAAAAAGAGAAACAAGATACATAAGAGAAATAATTTATGGAAAAAAAAGGAGAATAACATACT
GGGAAATGACCACAGATACAGAAAATTTACCGAAAAATTCAACAAGTTTTGTGATGACAAATTG
TCAGGGAAATTTGAAGAAAAGATTAGGAGATTTGTATGGTCAAAGGACATGGGTAGAATATGGA
TTTAGACAATGTAAACAAGAATTAGGATGGAAAGATTATCGACTAACAAAAATAGAAAGTATTG
AGAAATGGTGGGGACTAATAAATAGTGTTTATACCATGGTAAGTTTACAAACAAAACCATTGAG
AGAATTACTATTTTCAGAAGAAGAAAAGAAAGAGGAAATAGAAGAAAAAGAGCCAATAAATAGT
TGGAAAACAAGTTTAAAGAAAATAATGATGATGATTGAGCCATTAATCTTATTTTGGTTAATAA
CACCATGGTTAAAAATGGTAAATTCATCAGGATTATTAGAAGGATTGAATAATTTATTATCGAT
CACTCATGATTCTAAATTATATTTTCCGACAGGATAATTGTTTCTATTTTTTGATAGTTGATTCG
GAGTTTGACAAAAGAGGGTTATCAACTATTCACCTTTGTCTTCCCCCTCGAGAGGGGGGATAAAG
GGGGGTTTACCCTTTTCGCCATCACTCATAGATAAAATTTATCTCGAACTCGGGTTATTAATAGT
CAAGTTTAAGAAAACCATTCTGAGCCGATTCCTCTTGCCATCATGGAAGCCATTAAGGGAATTAA
GGTAAATCCCACTAACTCAAAGGCAATAATTCCCTTTAATATTTTATATTTGTTGAGACTTATTT
CGGGGGCTTTATCTTCTAGTAAAGATTTAACCCAAAGAATAAAAGAGATGGTAGGGTATAAAGA
CACAGTACCAACAATGAAATAAAGAGCTATTTTTGCCCAAAATACTGGTTGGTGCATATAGTAAG
CGGTATCTTTCGCAAAATATAAAACTCTTAAGATTCCAGTGATTAAGATGACTACTGCGGCGGCA
CCATAGGCACTATCTGCCCAAACTATACGCCATGCTTCTTTTGTGGTTAATTTTTCTTTGAGGGTC
AAAAATTCGATCGCCAAGGATGCAAAAATAACTCCTATACTTAGATAATGAAAATAGGCTGTAA
TGGCACTAGCCCACATAATTAACTCCTGATTAATAATTTACGAGAATAACAGTATTAATTTTATC
CCAGTATCGTTATCCTTTTTTCTGTTTGTTAGGACTAGCTAATCTTTTCTCCACTCAGAATAAAA
ATAGGTGCGATCGCAGCAAAAACTTGACTTAATCCTCTTTTTTCTAATCTATTAATAGAAGATTG
AACTACCTCAATATTACAAGCTCTTAATTCGGCTAATCCTTCAGAAATTTGATAGAGGTTTTCCA
AATTACTTGCCGTTGCCACTAACCTTCCTCCTTCAGGTAAATAACCCCAACCAAGAAGTTAAAATG
TTTTTGATTGATTTGCCGCCACCAATACAGATGCGATCGGGTTTTTCTGGTAAAGTATCGAAACA
ATCTGGAGCATTTCCTTCTACCACTTCCACATTTTCCACCTCAAAACGCCGACAATTTTGCCGTAT
TAAACCTGCTACCTCTGGATCTCTTTCTATGGCGATAATTTTTGTATTTGGGCATAATAAGCCTA
TTTCAATGGAAATTGTCCCTGTACCTGCACCTATATCCCAAATAACTGATTTTTCTTCTAATCTT
AACCCGGATAAAATTAGTAACCTTACCTCTCTTTTTGTCATAGGAATACCCGGGAGGCGATCGAA
TAATTCATCAGGAATACCAGGAGTTTTATATTGCCACATATTGTTAAATAACCTCAGTTCAACAT
CATAATTGTAGATTTGGCGGAATAAATAAGTAATGAGTTGGGATAAGGGTTGATGAGGGGAGTA
TCCAGAAGCAATTCTTAATGATTACACTGTTTAAGAGTTCTATCTTTTAGACTTTTGTGTCCAAA
CACTAGAAATCAAAATAAAGCACATCAGAAAATAGATAGTTGTTACAACTCCATTCAAATATAGT
AAACGCACCGCCCAAATATCAGAAGTGATATTGCCTCCCATCCATAAACTACCATAAACTATCAG
CCAAATTAATCCCCACCTTAAAGATAAACGAGCGGTTTTTTTTCTTTCTGTTCTAAATTAGATT
TATAGTTTAAATTGACTAAAGACCAAATAGGTCCAAACAATGGCATTAGATAAAGAGATAGTTG

GAATTTGTCTAATTTATCTTGAGGATCTTCTTTCATAGATTAATTAAAATGATAGAATCTATAGG
GTTAAGGGAAAACGCCAAGATTAAAAACAAGGTTTAAACAGGAATATCTAAAATCTTTTCAATG
CCCTTTGCATTTCTCTTTGATCTTCCCTACGTTTAATGGTTTCTCTTTTATCATGAAGTTTTTTCC
CTTTACCTAGCCCTAAACTAACTTTAACCCATTCTCCTCTTAAATACATCTTTAACGGAACTAAA
GTTAAACCTTTTTCCTGTAATGCACCAATTAACTTATTAATTTCTTTTTTATGTAATAACAATTT
GCGATCGCGCTTTGGTTCATGATTAAAATATTTACCAGTGGTATCATAAGGAGAAATATGCACAT
TAGTTAACCAAGCCTCCCCATTACGAATTAAAGCATAACCATCTCTTAAATTGACTCTTCCTGCCC
TAATAGACTTAACTTCCGTACCCACTAATTGGACTCCTGCCTCATAAGTCTCTAAAATCTCATAT
AAATATCTTGCCTGACGATTATCACTAATTATCTTCACTTTGTCACTCATTTAATAACCTTCTAT
CACATCTTTTTATCGACAATCCTTTCATTCTAACAAAATTGGCAAAACCTTGAACCGATTGGAAT
ATTATGCTAAGATTAATTAAACTTTTCTGACTTTAATCTTCTAAATTCGTGGTTTTATAAGAGAA
ATATAAAGAAGTCAAACCTGACATCTTTTATCGAACTGAGGTAATGAAGAATTTTTTTCCTCTAG
TAAATTAATCTTGTTAAACAACTATAATCTAAATAGTCATGATAGCTTTTATAAATTTCAAAGCT
CGGTAATTTAACTTTAAAACTAGGGGGAATAATAAATTGATGAATAGGGTAATTTTTTCTTATA
TTAACAATATCATAATAAATTTTTTCCCATTTACTTAAGCACCTTAAAAATAATTGGAACTGGGA
ATATAAGTTTTCATACTTATCCACTAGAGCAATATACTGGAATATTTTAGCAATATTGATAAGTT
TAGTATCATCGTCTTCTTTGATCTTAATAACTTTATTCGCAATTTCAATTAAAAAATCTCCCTCT
GATTTTGCAAATAATAAAAATGGTTTAATATCTTCAATTCATTCCATTTTTTGTTTCTTCAATAAA
ATTATTAAGTCTCGTAAAATTATCCCAGTTAGTTAAATTGTTTATTCTGAGTAAATCGAGAAAAT
ATAAAAGATTTTCTACTTCAGATATAGAAATAAAATAAGCTAAATATTTGTCTATTATTTTTAT
TTCCTTTGTTTCTTTGAATTGGCATTTAAAACTATAGTGGAGTTTAATTAACTTTTCTTGAAAAA
TCAATTTATGATGATTTTCCTTGATTAGATCATATACGGAATCATAAAATGCGAGTAAAGATGGT
TGGTTATATTTCTTCTCAATTTTGGTGATAGATCTTAAGTTATCTAGGTTACTAAGAATAATCTT
AAAAAACTCTTTTCCAACTAGAAAATAATGCTTTTTGGTTTGACAATATTTTTGAAAATAGCCTT
CTAAACGAGCATAAATTAGAAAGTCTGAATGGGTAAATTTTTGATTAAATAATGCTTTTAATGT
AACACTAACGCTTTCTAGTTTTTCGAGATGATAGTCATGTAAAGAGATTATTTCTGGACAGATAT
GAGTACGAGAATATTGTTTAAAGAGGTAGTCAACAAGAGGTTTATCACCGACGAGAGGACGATT
CCACCAAGGCTTATCGGCTTTTCTTCCTATGGTTAATTCTATACTCATTTTTTTGTCCCCCATAGT
TAACTACAACACCTCCTTTACTTCCTATCATAATGTCTTTTTTTGCTGAATTAATCAGTATATTT
ATGGATTTTTACAAAATATTTATAAATAAATGTAACTTTGTCTAGTTATTTATTTTCTCTTTTTA
GAAATACTTAAATATTTTTATTTAGTTATTTATAGTTAAGTGATTGCAGATAGAAAAGGACATT
ATTTCAATTGCTTCAAGAAAACTAACACTATATGATAGTTTTGATGGTTTCTCGTGTCAGCTAGA
CCTAAAGAAAATTGATTCTATAATAGATTTAGTTGAATGATACTTGTCGTTGCGAGTTTCAAGGG
GAGCGTCAAACCGATCCACCCTTCTGAAACGATAGCAGAATCAGAATTGACTACTGAAAGTTGTT
GTAATTTGAGTTAGACATAATGAATAAACTAGAAATACAGAGTAAAATTTACCATTTTGATAAT
GATGATAACCTTTATTTTGTCACTGTAATTTTAGTTGATGAAGAACCCATCGCAGATTTTTCTTA
CTATGCTACATCTTTAACAGAATTAAAAAAAAGTATCGAACAAAAAGGAAATTATTTTATATTA
ACTTGTTGGTGTGGTATTCCTGACTGCGCTGGAATTGACCAAGGTATTCAGGTTATTCACTACCA
AAATGCAGTGAAATGGACTATTCATCAACCTAAACCTAATCGTACTTTTATTTTTCCTGCCAACG
ATTATAAAATAATAATTACTGAGGCAATAGAACAAATTAAACTCGATATAGCTAATTTATGGTT
TACACAGGTAGAGGAAAAGAATTCTAAATTAGAAATTGTACCAAGATGGGAAGATGATAAAGCG
TTAATTAAATTATTAAATATTAATCAATTCTTACCTTGACAGAATTGTTAACAAAAATTGACTAT
TATCATTTTTGTTCCTTATTAATCTTCATTAATCTTTTTCTGAAGATTTTTTTTAGTATAAGTAC

ATGGAGATATTATCTTCTTTTTTGTAAGTTACCAATTCAATTCTTTGAGAGCCTAAACTACTTAG
TTACGCATTTGCTAACAATAGCGGTTATCCTGCTCATAGCCGATGCTTAATACTTTCCAGTATCC
TTCTTCTATAATCTGCACTGCTAATAAATTAAATTCTTGATTTTATGGGCTAAGTTACCGTAAAC
AGCATTAATTATTTCAATGGGTTTAGAGACAGCTATAGCTAAATCCTCCCCCATTATCAACAATA
ATAGATTATTTATCTTTGCCCCTTGCCCTTTGCCTGTTGCCTACCCTAACTAATAATTTATACGAC
GAGAAGTTATAGAGGCACAATTTGCTTCAACACCTAATACCTATAAAACTTCACAAATACTTAAT
TCAACTAATTATCAAAGTTATCAATGATAGCTTGGGCAAATTCAGAACATTTTAACGGTGGTTCT
ACGGGAGGAGTCATCATTCTGGCTAAATCGTAGGTAACTTGACGAGATGCGATCGCACGACTGAT
ACCATTACGAATTAAATCGGCGGCTTCCTGCCATCCCATAAACTCTAGCATCATTACCCCAGAAA
GAATAACTGAACCCGGGTTGATGCGATCGAGTCCTGCGTGTTTCGGGGCTGTTCCATGGGTAGCT
TCAAAAATAGCACAGTTGTCACCAATATTAGCACCCGGTCCCATGCCTAAACCACCCACAATGGC
GGCGGCGGCATCAGAGAGATAATCCCCGTTTAAGTTCATGGTGGCTAAAATAGAATATTCATCAG
GGCGAGTTTGAATCTGTTGGAAGATACTATCAGCGATGCGATCGTTTACCATGATTTTATCTTGC
CATTGTCCATTACCATGGGTATCCCAAATCGTATTTAATACTGATTCTACTTCCTTTTTAATAGT
TTCTTTTTTCTCAGGGGTGAGAGAATCATAACCCGGTTCAATTTGACGGGCGTTATCTTCTATGG
AAATATCGGGATTAGCTTCTTTATTACTTAAAATCCAAGACTCCCTTTCAGTGACACATTCTGCA
CGAAATTCAGTAGTTGCTAACTCATAACCCCAATCACGAAAAGCCCCTTCTGTGTATTTCATGAT
ATTACCTTTGTGAACCAAAGTCACCATTTGCTTATGTTTTGGTAATCTAAGGGCATTTTGAATGG
CACGACGCACCAACCGTTGAGAACCTTTTTTGGAAATAGGCTTGATACCAATACCAGCATCAAGG
GGAATTTGTTTATTTTTAAACTCAGCAGTAGCAGGGATTAATTCTTCATTGAGGAGAGAAATTAG
CTTTGCACCGACTTCTGAACCTTGTTTCCATTCAATGCCAAGATAAATATCTTCTGTATTTTCCCG
ATAGACTATTACATCTAATTTTTCAGGACTTTTATGGGGAGATGGTGTACCTTCATAATAACGAC
AAGGACGCACACAAGCATAAAGATCGAAAATTTGTCTTAAAGCCACATTTAAAGAACGAATCCCG
CCACCCACAGGAGTGGTTAAAGGACCTTTTATCGCAATACCATATTCTCTAATAGCAGTTAAAGT
ATCTTCAGGGAGGTATTGATAAGTACCATATTTTTCACAAGCCTCATCCCCTGCATAAATTTTAA
ACCAATTTATTTTACGTTTGCCACCATAAGCCTTTTCTACTGCGGCATTAATCACTTTTTCAGTAG
CAGGCCAAATATCCACTCCTGTACCATCTCCTCTAATGAAGGGAATAATAGGATCATCAGGTACA
ATAGGTTGTCCATTATCAAATTTTATTTTACTTCCAACTTCAGGGGGAGTTAATTTTTCAAACAT
ATTCAATTAATTCACAGTCTATTTTTTTTACAACTATATAATGGTACGGCAAAACTATTCTTTAA
GGGGGTAAATGTACGAAGAAGTTAATGGATTTTTTTGATGAGTAGAGCTAAAAGAAAAACAAAT
ATATTTTGAGCGAAATCAAATACTATAACTATAGGAATACTAAATTAGTTGTAAAACTATATTC
TCTCTAACCCACTAATAAAGTGGAATTAAAGGAAAGTTATACACAAATTATTTAGGTCTGCGATA
TATCCAGAAATTATTGACTCCATAATGAAACCTTTTTTGATAATCAACCCTATGTTGGTTAAGCA
GGGTGTTTTGGTAGCTACCTTCGTTTTATATCCTTTATCTTGTTACGGTGGAGAAAAAGAACCAT
TTTTTCAATTTTCTGATAGAGAAGTTTTTCTAGTTTCTAGTCTTGATGATTCTTTGTCTTTGGAA
GAAGGAATAATTATTAGAAAATTCAATTTTATCGGTAATACTGCTTTTAGTAATCATCAATTAGC
GGAGGTTGTTAAACCTTTTACCCAGAGAAAAATTACTTTTACCGAACTTTTAGAGGTAGAAAAGA
TAATTACTAATTTATATACAGAAAATGGTTATATCAATTCTGGAGCGATTATTAATGCAGGACA
AAAGTTTAATCCTCGACAAGCAGTTATTACGGTGGAAATTATCGAGGGTAGTGTTTCTGAAATAA
AGGTTGAAGGTTTAAAAAGATTAAGAACGGACTATGTTAAAAATCGTCTGCAAATTGCCACGGC
AACTCCTTTTAATGTTAATAGATTATATGAAGCCTTACAGGTTTTACAACTCAATACTTTAATTG
CCAATATTTCTGCAGAATTGGTTGCGGGGGTAGTTCCTCAAGAAAGTATTTTATTAGTTAATGTT
ACTGAAAGTGATTCTTTTAGTTTTATGCCTATCTTTAATAATGGGCGTAGTTCGAGTGTGGGTAG

TTTTAGGAGAGGGGTGAGGATTAAAGAAGATAATTTATTTGGTTTTGGAGATGCGATCGATTTT
TCTTATTTTAATACCGATGGAAGCAATACTTTTAATGGTAGTTATAGTATTCCAGTGAATAGTTA
TAACGGAAAGATAGAATTTGCTGGGGGTTTTAATGATACTAGAATAATTCAAGAACCTTTTGAT
GTGTTAAATATTACAGGAAACTCCGAATATTATGAAATTAGTTTTTATCAGCCTGTGATGGAAAA
ACCGACCCAAGAATTTGGTTTAGGAGTGACTTTCAGTAAGGAATCTAGTCGTAGTTTTTTAGATG
GTGAACCATTTCCCTTTTCTTTAGGGGCTGATATTAAAGGTAATACCGAAGTGAGTGCCATTCGT
TTTTTTCAAAATTGGACTATCAGACAACCCCAAGATGTGTTTAACTTGAAGTCAGAATTTAGTTT
GGGTATAGAGGCTTTTGGGGCGACAATTAATGAAGAATTGCCCGATAGTCGTTTTTTGTCATGGC
GTAGTCAGTTGCAGTATGTCAGACAATTAGCACCAGATAGCTTATTTATTTTTCAAACAGATTTA
CAACTAGCTAATGATAATTTAGTCACCTTAGAGCAGTTTTATCTTGGAGGTTTATACAGTGTTAG
GGGTTATCCTCAAGATATTCGAGTGACGGATAATGGTTTATTGCTGACTACAGAGGCATGGTTTC
CCATTTTGCGAGTGAAAGATATTTGGCAAAATCAAGATGGTGTTTTACAAATTATTCCTTTTGTT
GATTTTGGGGTTGGCTGGAATGTGGGCAAAATTCCCAACCCTTCTCCTAATACCCTTATGGGAGT
TGGTTTAGGACTTCAATGGCAGATGGGAAATAATTTTAATGCTCGTATTGATTACGGAATTCCTC
TTATTCACATTGAGAGGTCAAAAAATTCTTTGAATGATGAGGGTATTTATTTTAATATAAACGCT
AATTTTTAAATTATTGTGATTTTGTTCTTTAAAGCCAGATTTTTGAAATAGTATCTTAAATAATC
CTTAATTTTCTCTTATTTATTAGTAATTATTCCCTAAATACATAATCGTTTAAAAAATTAGTTTT
TCAGGCAACAAAAAAAATATAATTGCTATAAGATAATAGTTATTAACCAATGTAAGTTTAATGG
TTTTCAATATCAACTATGAATGTTTATAAATCGATTAATCATAATTATGTCTTTTCTCGAATAAT
TTTAAGTACTTTATTAGGGTCAACTATTTTTTCTTTTGGTAACTTGAATGTAGAGGCTAAAAATA
GTCTTACGGTATCTAATGAAAATCAAGAACAAATTGACTATTTGAGCAAAAATTTAATCAGTCTT
GAAAATACAAATAATTCTATTCAATATCGTAAAGGGGGAAGTAATTCTGATATTCCCTACATCAT
CACTCCCCGTTACACTTTAATCTCTGATAACACTCCCACTCTACATTGGAATGAAGTTGAAGGGG
CAAGTAGTTATTTGGTGAAAATTCAAGGGGGAGATATTAACTGGCAACAGGAAGTTAAGGACAC
TACTGTTGTTTACTCGGGAGCAGAAATGCTCAAAAGTGGTATAGACTATTTTGTTTCTGTGGAAG
CTGATACGGGGGTATCTTCCTTGGATGATAGTGGTGCAAAGTTAGGTTTTCGCTTAATTACCTCT
GAACAATCAAATTCTTTACAAGATGCGATCGCGCCTATTCAAACATCAAATCTTAGTCAAGAGGA
AAAAGGAATTAAACTAGCTGAAAAATACCATCAATTAAATTTGAATGCAGATGCGATCGCAATT
TTAGAACAATTAAAAGCCGAAGGTAGTAAATCCGCTCAAGTGTATCGTCTTCTTGGTGATACCTA
TGCTAAAACGGGATTAAACTTACTAGCTGAAGAAAATTATCTTACCGCCATTGAACTAGAATCCG
CTAATAATAATTTGGAAAGTGTAACAGAAATCCAAGCCTCTCTTGTGGGAGTAAAAATAATGTT
GGGCAAATTAGAAGAAGCCGAGGAGTTAAATCAACAAGCCCAAGCAGGATATAGTAAATTGGGC
AATGTCGATAAAGGGCAAGAATTAAACCGCACTCTTACCGCTTTAACTCAAGGCAATAGCGAAAA
TCCTATCCGCACTCGTGGTAGTAGTCAACAGACTAATTCGGCTCAAAATCAAATCGATCAAGTTG
TCACCCCTAATTTTGGCAATGACTGGCCTTAAACTATCTAAGTTTTGCTCAAGTTAACAACTTAG
CTTATTATTACTAAGCACTTATTCAATGTATAAACACCGTCACTATCACTGGACTATATTTTCGG
GGAGATTATGGCTAAATTCCCTTGCTATTGTAGGATTAACGGGATTACTGACTTCCAATCAAATT
GATGCTCAAGTAATACCCGATCGCACTCTTGGTAAGGAAAGTTCCATTGTTACCCCGAATCTTAC
TATTAATGGGATTCCCAGTGAGCGAATAGACGGCGGTGCTGTTAGGAATACAAACTTATTTCATA
GTTTTCAAGAATTTAATATTGGTAACGGTAGAGGGGTTTACTTTAACAATCCTGCTGGTATAGAA
AATATTTTTACCAGAATAACAGGGCAAAATGTATCAAAAATATTAGGAACTTTGGGAGTATTAG
GTAACGGTAATCTTTTTTTGCTCAATCCTAACGGTATTATATTTGGTCCTAATGCCACACTGGAC
TTAAAAGGCTCATTTATCGGTAGTACGGCTGATAGTGTTGTGTTTGGAAATGGAGTGAAATTTAG

TGCCACAAATCCTGAAAATATACCCTTACTCACCATTGGCATTCCCATTGGATTGGAATTTCGCG
CTAACTCTGGAGAAATTTCCGTTCAAGGAGAAGGAAGTAACCTGTTTTTAGATTTTTCTAATGGT
TCTATAGTACCAGATCAAAGAAATGCGGGACTTGAAGTATCCACAGGCAAAACCCTAGGTTTATT
AGGCTCAAAAATAAATATTACAGGAGGTAACTTAACCGCAGACTCAGGTACGATCGAACTTTGGT
CAGTAATTGACGGTAATTTAAGGCTTACTTTTAATGATCAATCCTTAAGAGTTAATAGTAATGCT
GTTTCTCAATATGGAGACATTGAACTAATAAATAATGCCTCTGTTTACTTAAATGGAACAAGTTC
AGGGGGGATTAATGTCAATAGTCAAAATTTAACATTAAAAAATGGTTCTTCTTTTGTCACTTTAA
CTACGGGACAAACAAAAGGGGAAGATTTGAATTTTAAGATTGTCGATTCCGTTTTATTAACTGGT
TCATCTCCTAACCAATTACCGAGTGGCTTCTATACTCAAACTTTTGGTGCTGGTAGTGCCGGAAA
TTTAACCCTTGAAACGGGAAACCTAACCCTCGGAGGTGCTTTAATTTCTGCAAGTACTTTTGGCG
AGGGGAATGCAGGACAATTAAGAATATTAGCCAGAGATTCTATTAATTTAATTGATGATGGTTT
TGGTGTTACTTTTATCGTAGCTGACTCCGAATCATCGGGTAACGGTGGCAACGTTTTCATTGAAA
CAAAAAATTTAACTTTAGTAAATGGGGCTCAAATATCAGTAAGTGCTTTTGGTAGTGGAAACAG
TGGAAAACTGGACGTTAAAGCATCAGACACCATTCAAATTGTCGGTGAAGGACAAACATTTCCTT
CTGGATTATTTGCCCAAGCCATTCGCACTGGTAATGGAGGGGATTTAACTGTTAAAACAGGGCAT
TTGCTCATTCAAGATGGGGGTTTAATCTCCGTTGGCACTCGAGGCAGTGGTAAAGGTGGACAATT
AACCATTGATGCTTCTGAGTCTGTCGAATTAACTGGTACAAGAATAAGTGGTGCTGGTTCTCGTT
TAATTTCAGGTACGGTGGGAACTGGCAATAGTGGTAATATTATTGTTAATACCAAAGATTTAAG
AATTTTAAATGGTGCGGAAATTTCTGTTAGTAGTGTTTTTGATACATTTGTAGGATTGGGAACGC
CGGGTAACTTACAAATCAATGCTAATTCTATTTTTCTCAATAATCAGGGACAAATTAGAGCAGAG
AGTGGTTCAGGAGAAGGGGGTAATATCAACCTACAAGCGAAAGAAAGACTAGAATTGCGTAACA
ATAGTCAAATTTCTGCCACCGCAGGTACAGAAGGAGGAGGAGGAAACGGGGGTAATATTTTTATC
GATTCTCCTTTTATTATTGCTCCTCCCAATGGAGATAGTGATATTACCGCTAATGCTTTTGAAGG
GCAAGGGGGAAATATTATTGTTAATAGTCAGGGAATTTTTGGAATTGAGTTTAGAGAAAATCAA
ACCCCAAAAAGTGACATTACCGCTAGTTCTCAATTTGGACGCTCTGGAAATGTGGAAATTAGCAC
TCCTAATGTTAATCCTAGTCAAGGTTTAGTGGAATTGGTGAAAAATCCTGTTAATCCTGCTGATT
TAGTTGAAGTGAATGCTTGTAGTAAGGGGCAAGAAAGCGAATTTATTATTACAGGGCGTGGTGG
TTTGCCTCCTAATCCTGCATCAATTTTAAACGGTGATTTACTGTTGCCAGACTTTGGTATAGCAA
ATAATTCTCTAGCTCAACAAACTAAAGTTACTTCTGTTGCTTCTAAAACACCCGCTCAAGGATGG
CGAATGGTAGGAGAAAATGAGATGGTTTTAACGGCTAATACTTCTAGTAATAATTTAATTGAAA
AAGGAATTAACCATTATGAAAACGGTCAATTTCAAGAAGCTATTACAAAGTGGCAAGAAGCCTT
AAAAGAATTTGAAGAAGATAGTTTAACTCAGTCTATTATTTTTAATAACATTGCGATCGCACTTA
CACAATTAGGAGAATGGGAAAAAGCAGAGGAAACAGTTAAAACCAGTCAGGAAATCTTAAATAA
TATATCTAATAATAGTCAATTAAAACAGTCTATACAGGCTCAATTTTTTAATAACCTAGCCATAA
TAGAATTAGCCAGAGGTGATGGGTTACAAGCCCTAAAATACTGGCAATCTGCTACTCAAATTTAT
GAGCAAATAGGTGATCAAAAAGGAGTTATTCGCAGTTTAATTAATCAGAGTAAAGCCTTAAAAT
ATTTGGGTTTAAATCTCCAAGCGAAACAATTATTACAAGAAATTAATACAAATTTGGAACTAAT
TTCCGATCCTTTATTAAAGGTTGCTGGAGGAAAAAATTATGGGGATGTTTTGCGTTTGCTGGGTG
ATTTTTCTCAATCAGAACAGGTATTAGAAAAAAGTCTTGCGATCGCGCAGCGCCTCAGCTTTGCT
GAGAACGCACAGGAAAACTTATTACCTAATTATGTAAATCAAATTCAACTAAGTTTAGGAAATAC
TTATTTTGCCCAAAATAACATCACTAAAGCCCTTGAATATTATCAAGAAACAGATACTAATAGTC
CCTTTTTAAATTTAGAAATTCAAAGTCAACTCAATAAATTAAAACTTTTAATACAACGAAGAAG
ATTCTTTTCTTTGAAAAATTTACTTCCAGAAATTTATAATAAAATTAATAATTTACCTCCTAGTA

ATACCAGTATTTATGCACAAATTAACTTCGTTAATAGCCTTGATAACCTAGCTAAAATAACTTCT
GAATATCAAGATTTTGATAAAATTTTAATCTTAGAAAATGCTTTAAAACAAGCCCAAAAAATTG
GAGATAAAAGAGCAGAAGCCTATGTTTTAACATATCTAGGGGAGCAATATGAACAGCAAAAACA
ATGGACACAAGCAGAAAATATAACCAAATCTGCACTTATTATCACTGGACAAATTCAAGGAGATG
ACATAGCCTATCTAGCTCAATGGCAATTAGCAAGAATACTCAAAGCCAAAGGAGATAAACAAGGG
GCAATAACTGCTTATAATCTAGCTTTTGATACTCTAAAAGACTTACGCAACGATTTAGTAACCGT
TAACCGAGAAGTACAATTTTCTTTTCGGGAAAGTGTTGAACCTATTTACAGAGAGTTAGTCGCCT
TATTATTAGAGGGAGATATTTCCCAAAGCAATTTAATTCAAGCTAGAGAGATTATGGAGTCTTTA
CAATTAGCTGAATTGGATAATTTCTTTAAGGACACCTGCTCTACGACAAAACCAGTTCAAGTTGA
TCAAATTGATCCCACCACTGCGGTTATTTATCCTATCATATTGCGCGATCGCCTCGATGTCATTGT
GTCTTTACCGGGACAACCCCTGATTCATTACTCAACCCCTTTAAATCAAGATAAAGTAAGACAAA
TAGTCACCAATTTACAAAAAGCCTTATATGAAGACCCCCTTCAAACTGGACGTATTACAAACAAC
TATCAAGAAGACGCTCAAACCTTATATCAATTTCTAATTCAGCCCATAGAAACCAAACTGCAATC
CAATCAAATTAAAAATCTTACGTTTGTCTTAGATGGTATATTAAGAAATATTCCTATGGCAATTC
TCTACGATGGAGAAAAATATCTACTAGAAAAATATTCGATCGCACTTACTCCGGGCTTACAACTT
TTAGATCCTCAACCTTTTAAACGAACCCAATTAAGAGCTTTAACCGCAGGTTTAACCGAAGCCCG
TCAGAATTTTAGCGCCCTTCCCAACGTCAAAATAGAATTGACAGAAATTGAAAATGAAATAAATT
CTAAGGTTCTTCTCAATTCAAGAATTCACCAAGACTAATTTAGAAAAATTAATTCAAAATAATAAT
TTCCCTATCCTTCATTTCGCAACTCATGGTCAATTTAGCTCTAATATCGAAGACACATTTATCTTA
ACTTGGGATGAATCTATAAACACAAAAGAATTAGATCGTATTTTAAGCCGAGACTTTGCTTCCGA
CTCATCACCCTTAGAATTATTAGTTTTAAGTGCTTGTCAGACCGCAACAGGAGACGAAAGAGCGG
CATTAGGTTTAGCAGGAATTGCCGTTAGGGCAGGGGCTAGAAGCACTCTGGCAACCCTATGGTTA
GTAGATGATAGAGCAACCACCGAACTTATGGTTAAATTTTATCAGGAGTTAGCCCGAACAGAAGT
AACAAAAGCCGAAGCATTACGCCAAGCCCAATTGTCTTTATTAAAAGACGGTAAACATCAGCATC
CCTTCTTTTGGGCTCCATTTGTTTTAGTGGGTAATTGGTTGTAATAAAAAGGGCAAGGGGCAAAA
ACGAATAGTAAATAGTAAATAGTAAATAGTGAGTAGTTGATAATTACTCTTTACTCGTTACTCAT
TACTTTCTTCTGAAACCTGAAACCTGAAACCCTGTACGGGCGAATGGCCATTCGCCCCTACGTCTT
CCTCTCCCATCATCCCCCCAACAATCTAACACCCACCCTTATCCAAATGTTGTTAAGCCAAACTAA
GGTTTAGTTAGTTGGTAAGATACCCAGTTTAACAGCCACGCTTAAATCTTGATCATCTCGACGTA
AATTTAAAGTTAACTCTGAGCCAACTTTTCTGGATTCCACAATTTTTTGTACTTGAGTAGAATCT
TGAATTTTTTCTCCTTCTACTCCTTGGATAACATCCCCTGATTTTAAACCTGCTTGAGCCGCCGGA
GAGTTGGGCATAACATTAACAATTAAAACCCCTTCTTCATCAACTAAATTCGGTTTATTCATTTC
TTGTAATCGCTCTTTGGTTTGTTCATTGAGAGAAACCATACTAATACCGATATAAGGATGTTCTA
CTTTACCATCAGCAATTAAGGTTTGGGCAATTTGTGCCGCCCGATTGATAGGAATTGCAAATCCC
AATCCCTGAGCATTTTGAATAATGGCGGTGTTGATGCCAATTACTTCTCCGTTTGCATTTAAAAG
AGGACCTCCTGAATTACCGGGGTTAATAGCCGCATCTGTTTGAATAAAGTCTAAACGCTTATCTC
CCACGCCAATCTCAGAACTCGATCGCCCCGTAGCACTAATAATACCAGTAGTTACAGTATTATCC
AAACCCAAAGGATTACCAATTGCGATCGCCCATTCTCCGATTACCAAATCATCAGAATTACCTAT
TTCTAAAACGGGCAGATTTTCTGCGTTAATTTGAATCACGGCTAAATCAGTAAGAGGATCAGACC
CTAACACTTTACCTTCAAAAACTCGACCATCTTTTAGATTAACAGTTACCTCTGTCGCTCCATCAA
CTACGTGGGCATTAGTCAGAATTTTACCATCTTCACTGATAATAAAACCAGATCCTGTACCTCTT
TGAATTTGCTCATCAGGAATTTGAGGGACGCGATCGCCAAAAAATTGACGGAAAAAGGATCATT
AAACATCGGTGGAACATTAGTTGCAACCTTACGAGAAGCGTTAATTCTGACAACTCCAGGACCAA

CTTCCCGTACAACTTGAGACACGAAATTAAGGTTGTCATTATTGGGCAATGCACTGACGGTAGAA
TCATCGTTATAAGCTTTATCTTCTTTGAGAAGACTATTCTCGCCATCTCTTGTAAAGGCAAATAA
TTGAGGGGTATTAACTAAATAGTTAGTTCCCACTCCTATACCCGCACCTAACAATAGTAAAGATG
CACCTGTTGTTAATCTTTGAATAAATTTATTAACCATAACAGCTCAAACCTCTCTAAAAATAATA
CAAAATATAAAAATAATTAATTACTAGACTGAAAAGAAAATAAGATAAAAATAATTGCTACACA
AATAGATTAATCACACGAACAAAAAAATAAAAAAAGTCAGGGATTAAAGGTGCGAAGGAGCTAA
AGAGGAAAACCTATCTTTCTCCCTGACACTTAATACTATAAACAACCGATATGACTTTGATGTGA
ACCTAAGATGAAATTTTAGTGACATTTAGCCGCCGCATTAACTAACTCATTGGCTACTTTAATAG
AATAGGGTTTTATCAGCCACCAAATAAAAGGAGATAGCCACCCTCGGAGGGTAATAGAATAAGCA
ATATAACTACCGCATAAAGTGGACTCAATTTGATAAGTTATTCTTTGCTCAATACCCGGAATCGC
CAGTAAACGAATACTCAACAATTCTCTAGGGCGGACACTTTCTACAAAAGCACGAATAGGAAAAG
GAGTCAAACGGGTGACGACAGAAAAAATTAACCCCGGTTTAGGAATTAAGCCCTTAGGTAAATTT
GTTTGGGAAAAAAGAGGATGCCATGACATATCTGCTAAATTAATTAATTTTCCCCACACCATATC
CACTGGAACACTAGAAGCGACTCGATAGGTTTTATACAGGGAACATTTACATAATAATTTGCCAC
CTTCTGTCAATAATTTAAAAGAAAAATTTAAAATCATGGTCTTCACGGATTGACGAATACTTGGT
GGAAAAATCTTTTATATAGATCACACCATTATCTATGATAACCACTAATAAATAATGTGAACTAC
CCAACACCAAATTCTAAAATAATAGAGTGTTCTATATGTTGTACAATAATTCCATAAATTGGAAA
ATGACAAGGGTTTGCTGAGTTTGACCAAAGGAGTTTTAAAACCTTAGTTAGATTGAGAGTCTTGG
ATGTATATGTGTAAATATTTTAAGAATTATCAACATAATTTAATCTTAGAGGCAGTGAGCGAAA
AATAACGGTTTTTTGTAGAATAACAACATTGTCCTCCCTAGTAATTAGATTTCAAGAGAGAAAAC
GGTTGATAAGTTTTTTGATGATTCGCTTATAATAATCTGTGGCTATTATATTCTTTACTATGATA
TTAATAATATTTTTTTTGCACTAAATATGAAGCTATTAAGGTATTTTTCTCTCAAGCAATTAAGT
TTTTTTTTCTGGTTAATTTTATTATTTAATCCTCATATTAGTGTCGCTCAGGAAATAGAAAAACC
TAATCCTTTAACGACAAATTTTAGTAGTGATTTAATTCCTAGTATTGACAGAGAAATTACCCCAT
TTGAAAAAAAAAGAATTAGAAATAGAATTGATGAATTAAATAATTTAGCTGGTGCAGAATTATC
TCAAGGTAATGATGATGATGCTTTTTCTGCATGGTATGAGGCTATTAATTTAAGTAGATATTTGG
GCATAGAAGCTGAGATAAAAATGATTAATCAGGTGGGTAAAGTCGCTTGGGATAAAAGTAGAAA
TCAAGATATTAATTTTCTCACAGAACGATTAAATATAATTGAAAATACAGCCACAGAAAATAAA
GAATTAGATGAGGATTTTTTGGCTATTTTAATTGATGCCCATGAAGTTTTGCACAATATAGATCA
GTCTATTACTCTCAATCAAAGTAATTTAGAAATTGCCCGTAACAATAATGATACTGATCAAATTA
AGTCAAGTTTGGAAAAGTTAGGAAATCTTTATCTTGCCAAGTTCGACTATTACAGTGCTGAACCC
ATTTTTAAGGAACTTTTAGAAATTGCCCGTCAAAATGAGGACTATTTAGGAGAAGGTATTTATTT
ACGGAAGTTAGCTGAAATAAGTCAGGCTTTAGTTAATCCTGCTAATTCTGTTAAATATAAAGAGG
AATTAGTGGAAAATGGTTTACAAAATCAAAATTTAGCTTCTTTAGCTATGTTAAAAATTGCCATT
GGCGATGATTATAAAAGTTTAAATGACCCTCAATCTGCTAGTCAATCTTATCAAGAAGCCTTTAA
CACTGCTTGGAGTAATCAACAATATGCGATCGCAGGAGATGCTCTTAAGAAACTAGGAAAACTAT
ATCAAGAATATGAGCAATGGGATTCGGCACTGCAAATTTATCAAGAATTAATCAAGATAGAACA
ACAGTCTTATAATCTTTATGGTTTAATGAATACTTATGATTATATGGCAATAATTTATCAACAAA
AAGAAGAAGAAAATCAAGCACTTCAATACTGGCAAAAAGCCTTAGAAATTGCCCGTCATCTTAAT
TATAAAGAAGATTATTTTTTCAATAAAATTAGTCGTGTTAATGAGGAATAAGTTAGAGATTCGT
TTATTGGGGTATTGAGAAGAAACAAGTAACGAGTTGAGAGTTAGAAGCTACTAATTGTCAAATA
TTTAGTTTTGCCTTTTGCTTTTGCTTCCTCAAAACACTTTTCAGCACCCTTTAAATTGGTAAAAA
ATTCTTGATAATAGTTAATAATTGATTAGAAATAATCGGTTTGCTTAAATAATCATCCATACCTA

ATTGTAAACACTTTTCTCTTTCTCCTTTGACTGTTCCTGCGGTGAGAGCGATAATAGGAGTATGG
GTATTTGTCTCTTTTTCTTTTGCTCGAATCATGGAAGTTGCTTCATATCCGCTAAGTAGAGGCAT
TTGTAAATCCATTAAAATTAAATCGGGTTGGATTTCTATAAATTTTGCAACGGCTTCTTCCCCGT
TTTTGGCTTTAATAATTTTGGCTTGAGGTAATATTTTTGTAATCATGGCTTTGGCAAGAGTTAAG
TTAACTCGATTATCCTCTGCAATCAAAATTGTGATGTGAGAGTAACTTGGTTGTGAGATTGTATT
CGATTCTATGGGAATAACGGGGGTATTCGCTTTTATTTTTAGTTGTGAGAGAGCGGTAAAAAGTC
TATTTAAAGTAATAGGTTTGCTTTGCTGACTCTGAATTCCTAATTCTTTACAGGCTTGATGAATA
AAACTATCTTCTGGGGTACTATGGAGAAGAATAATGGGTAATTCTTCGGGAGAGATAAAGAGTT
TTTGTCTAATGTGGCGAATTAGTTCAATTCCATCCATGTCAGGCATTTCGTAGTCAACTATGGCG
GCTTGATATGTATTACTTTTTGTCAGTTTTTAATTGCGATCGCACTTTCGTTGACAATATCAGA
GTTAATATTTTTCAGAGCCAACATATCTTGAAGAATACAACTATTATTAAAATTATCATCAACAA
GTAAAACTTTGTTAATACTCTCTAAACCCTGATAATGGATTATTTCTCCCGCTTCGGTGATAAGA
TTAAGAGTAAAGAAGAAAGTGCTACCTTTACCTAATTCGCTGGTAAGATTGAGCTTACTACCCAT
TAAATTAAGTAGTTTGCTAGAAATAGTTAAGCCTAAACCTGTACCACCATATTTACGGGTTGTGG
ATTCATCTTCTTGAGTAAAAGCCTGAAAAATAATGTCTTGCTTTTGAGGAGAAATACCAATACCA
GTATCTCTAACGGAAAAAAGTATAGTGGATTTTTCGTCTTTAGAAGGTAGTAGTTTGATATTTAA
TTCAATCTCTCCTTTATTCGTGAATTTAGTGGCATTTCCTAGAAGATTAATTAAAATTTGTTTTA
GTCTAATCTCATCGGTATAAGCAAAACGGGGCAAATCAGGACTAACATTCAATAATAGTTCAATG
TTATGGTTATCTGTGATTTTGTAGCGAATTAAATCAGTGACTTGGGTTGCTAATTCCCATAAATC
AACTTTTTCATAACTAATTTCGAGTTTCCCTGCTTCAATTTTAGAAAAATCAAGAATATCATTGA
TTAACTCTAATAATAGATTTCCCGATTGGTGAACTAATTTTAAATATTTTTTTTGAGTAAAGTTA
AGGGAAGTTTGCAAAAGTAGTTCAGAAAAACCAATAACTCCGTTAAGAGGAGTGCGAATTTCAT
GACTCATATTCGCCAAAAATTCGGATTTTGCCTTACTTCCCGCTTCAGCTTTTTCTCTTGCTTGTT
GTAAATTTGCTTCTAATCGATTTCTATCAATGGCACTGGCTATACTATCAGCAAAACTAGATAAT
AAAGCAACTTCTCCTTCTGTCCATAATTTTTCTTGATGGCAACAACTAAAACCAATCATAGCAAT
GGCAATATTATTATTCATGATGGGAATATAAACTAATCCCTGAATATCCATTTTTAATAATTCTT
GTTTGAAGGGAATATTATCGTCTATGTGTTTGGTAAGAATTTGAAAGGTTTTACCGGCTAAAACA
TTCTCGGCGGCGGGGGAAAAGGCGGAGGAAGGAATATTCTGTAGGGAAGGATTTTTGATAGTCG
GTTGTTTGCCGTCACCATAGCATTCATATTCTTGGCTGAAAAATATTTCTGTTGCTTCATGGCGA
ATACTAATATAGTAAGACTTGTCGGCTTCTATTGCTTTAAGAATAATTGATAAGGATTTATAGAT
TGCTTGGTTAATATTTTTATTAGATAGCAACTCTTTTGTAGCTTGTGCGATCGCAACTAAGACTT
TTTCTTTCTGATATAAAATTTGATTGGTGTTTTTTAATTCATTTTCAAAGGTTTTTTCTGAGGTA
ATATCTCTGACAATTCCTTCTATTCTGTAAGGAATTCTTTTGTCATCATAAATATATCTAGCTTT
ATTGTTAATCCATTTAACTTGCCCTTGAGTGGTAATAATACGATATTGTGCTGAAAAACAACCAT
AATTTGACAAGTCTTGCCACATTTTCCCAAGCATGGGTTTATCTTCGGGATGAATAAATTTTTCC
CACAGAGAATTATCTGTTATCCATTCTTCATAAGGCAAACCATATATATCTTCTACACATCGACT
AACAAAAAGGGTTTTTAACTCTGGATAACTCATCGCCCATACTACTTCGCTTAAAGATTCTAAAA
TTGTCCGAATCTCATTTTTTGTTTCTCTTAATTCATCCTGAGCATTTTTGTAGTCTGTAATATCA
ACTTGTGTGCCAAACCAAAACCAATCACCATTTCTATCTTTTTGGGGCTGTGATTGAACCTGCAT
CCAGCGAGTTTTACCATTGACAACTACTCTCGCTATATGATTGAAAGATGACTTAGATGCGATCG
CTTTTTCACTTTTCTTGAGAAATAAATCTCGATCATCAGCATGAATTATGTTAATAGCACAAGAA
GCATCTTTTAAAACATCTTCCGCTTTCAAACCATTTAAAGCACACCAAACAGGGCTAACATAGGT
AAAACTGGAATCTTGGGCTAACTTATAAATGCCGATGGGAATAAAAGAAATTAACTGATTATAC

TCCCTTGTTTTTTCTTGTAATGCAATCTGACTTCGTTTTTGTTGATCAATATCTTGTATTGAACC
ATATATTCTCACACACTTACCCTCTTGAAACTCAGGTTGAATAATTGCCCTAACCCAAATCTCTTT
ACTCTTTGCTGTAACTAAAATTGCCTCTAAAAATCCCGTTTACCCTCATTAATGGCTTGATTAA
CCACTTTAATAACATAGTCTCTATTTTTCCCTTGCTTATAAAAATTAATACCTTCTTCTAAAGAA
GGTTGAGAGTCAAGGGGAATTTCATGAATTATTTCGTCATCTCAGTCCAATAATTTTCTCCAGT
CAACAGATTGACTTCCCAGCCCCCAACTCTGGCTAGTCGATTGGTTTCTGTGAGTAATTCTTTTGT
TTTAATTAATTCAAACTCTCTTGTCTTCTTTTCTGTTATATCCCTTGCCGTTGCATAAATTAAGCC
ATCTTGACGATAGGCATTCCAATGTAGATAAATATAGTTACCTTGAGAATGACGATAGCGGTTTT
CAAAATTGATTAATTTCCCCTGATTTATAAGTTGTTGATTTGCTTCAAGAGTCTTAGCAACATCA
TCGGGATGAATAAAATTAAGATAGGAAAAACTCTCTAAAACATCTAATGAGTAACCCAAAATAT
CTTCCCAAGCCTTACTCACTTTAACAAAATTACCTTGTAAGTCAGCAACACATAATAAATCTAAA
GATAGAGAAAAAAAGTTATCTAAATGAATTTTTTGCTTTCGAGTATCTCTTTCCCTATGAGGTTT
AAATTTATTTTCCGTCATTCTCTTTGCCCATATCAAAAAAATTGATCTTTATAAGTTCACATTGA
GTCTAAGATTAATAACTCAGTTTACCTGAATTCGATATAAAAGTATAACTTTTGTCTTTTCTTAT
CCTTATATTCTTAATCCTAGAACAAAGAACTAATTTTAGTATCTAATTCAGAAATACATCGAGAT
TTATAAAGACAATAATTCACTCCTAAATTAATTAAATATTTTTTTATTTTCTATTATTAATGGTA
ATAGTGTCATATAGAGATATATTCAAATTGAGAATATTTATGAATTGTTTGGATTATATTTAAT
TGTTTTTCTATGAAATGGAAAGAAAAATAAACTAAAATTGAATAAGGATTTTTTGAAAATAAAA
TCAAGCTGAAAATCTTTATTTTCTATTAATAAAAGATTCTATTTTTTGCGGTAAGTTAACTATTA
ACTGTGTCAATGTAATTCTTATTATCATTATTTTTTACAATTTTTTATTACTAGAATAGACTTTT
TCGGTAGCCGCTTTTACTTCTTGTAATCAAATGGTTTCCCCTTTTATTTCTATTTGCAAATTATG
GGTTAACTTAATTTTAATAATTTCTCTCCATAGTTGCGGTCACCAAAGGTAGGACTACGCCGATC
GCAACTTCATAACCAGTATGGATTAAAGTTTCTAAAGTATAACTAATGGACAACACTAAGAGATT
TGCGAATAAGAAACTCTATTTCTTCTTTTCCAAACTCTTTAGCTAGTTTTAGTATTTTTTTGTAT
AAAGATAAACGTCAATCATCAATTATCAATCAGGAGACTTAAGCCACCGCGTAAGCCACTTTCAA
AGCCCAGATAATTAAAGCGGCAATACTACCTAGAATAATGACCGCAGACACCGCCACAACTATGG
GTTTATCTGCCCCGTCAAATTTCATAATGCCACGATTTAAGTCAGACATAGGAAAATATTATTTT
TAATTTAAAAATTGCCTACCTTATACATTAACTTAAAACCAATCTCACGGTTCAAATATGAGAAA
AATGTCAATATTCTTAACTATCTTGGGGAAGTTAGGGGATGGGGGGATGAGGGAGATAAGGTTT
CAGGTTTCAGGTTTCAGGTGTTTAGGGGAATAATGAGTAATGAGTAACGAGTAATTATCAACTA
TTCACCATTCACTATTCACTATTTCCTGTCTTAACCAGAAAACCTAAATACACTCCTAATTTAGTT
AAGATTCAAATGTTCACAGGCTTTATCGGTGACAACCCTACCCCTTGGAGTTCGATTAAGAAAAC
CTATCTGTAATAAATAAGGTTCATATACTTCCTCAATGGTTTTTGAGTCTTCCCCCGTAGCGGCT
GCGATCGCATCTAGTCCAACAGGACCTCGGTTAAACTGTTCAATCATAGTAGTCAGAATTAATCT
ATCAGTCCAATCTAAGCCCAAACCATCCACATTATGTAAATCTAGAGCTTCACAAGCTAGAGTTT
GATTTATCGCCTTTTCTCCCTTCACTTGAACATAATCTCTTACTCTTCTTAGTAAACGGTTAGCTA
TACGGGGTGTACCTCTCGAACGACTAGCAATTGCGATCGCACCTTGTTCATCGATAGTCAAATTA
AGGATTTGAGCAGAGCGTAACACAATTTCTTTTAACTCATCAATATGGTAGAATTGTAGTCTTTG
GATTAAACCGAAGCGATCGCGCAAAGGAGAAGTTAAAGAACCGATTTTCGTTGTTGCCCCAATCA
GAGTAAAAGGAGCAAGGGGAATACTGCGAATTTTTGCTCCCTGCCCTTTCCCGATGGTAATATCT
AAACGTCCATCCTCCATCGCTGGATATAGCAACTCCTCTGTAACACGATTCAGCCGATGGATCTCA
TCAATAAACAATATATCTCCCTTTTTAAGACTCACCAGTAAGCCTGTAATATCCCTAGGACGTTC
AAGAGCAGGAGCTGCGGTAATCTTGCAATTTACCCCCATTTCCCCCGCTAAAATTAAAGACATGG

TAGTTTTGCCTAACCCCGGAGGACCATAAAGTAAAATATGATCCAAAGGTTCATTTCTTTGTTTT
GCCGCCTGAATGGCAATGGACATAACTTCCTTTAAGTCTTTTTGTCCGATATAATCAGCAAAGCG
GTGAGGACGAATTTTGTCATCACTTTCTAAGACATCCTCAACTATTTCCGTCGGTAAAAGAATCT
CTTTATCTTCATTCTGTGGTGTGTTAAAAGGTTTTTTCTTTAATTGTTTTTGTTTAGGTAGTTGA
TTATTCTTATCTGAAGATGATGATCTTTTTATAGCCATTGATGTTTTAATCTAAATTGTTGATGG
TTTCTCTGGACTTCTTCCCGAAGTCAAGCAACAGGCAACAGTAGTAAAATTAGTTTTAAACTGCA
CTAATTTTATCTTAATTCTTATTATCTTAATTCTTATCCCTCTTTTGTCAAACTCTGAATCAACG
ATCAAAAAATAGGAACAATTATTCCGTAGAAAAATATAATTTAGAACTATGAGCAATCGATAAT
AAATCATTTAATCCTTTTAATAATTCTAATGAATTTGCCATTTTTAACCATGGTATTATTAACCA
AAATAAGATTAATGGTTCAATCATCCTCACTATTTTCTTTCAACTTTTTTCCAAGTATTTATTTT
GGTTCTTCTACAGTGGTTATGATAAATGAATAAAAAATAACTTCCATAACCTTTATTGAATAGTG
TTTATAGTAAAATAAAAATTAAAAGTTTATAAATTATTATTTAATAATCCCCTATTGCCTATTGC
CTATTGCCTCTTGCCTCTTGCCTACCCTAACTAATAATTTACACGACGAGAAGTGATAGAGCCTT
TATTTTTTCTTTTTTACTTTCGGAAATAGTAATTTTTTTAAGGGTTAAAAGATTCTATTTGCTAA
CTTACTATGGTGTAAATACTATTTATCATCCTCCATAAATTAAGGATACTATCTACAATTCATTA
ATCTATCATTTACGGAAGTTGCGAATTATTGCTGAGTTAACGGAAGATTCTGATAAGTTGCTTAT
TCTCACAGTTTATTAGGATTTTAAATTAGCTAATTAATTAACGGAAAGTATGCAGGTCATTTTTT
TGATTCTGCATATTTTTTCTCACTTTTCCTGATATATAAATATAAAAAAAGACTTGGTCAATTAT
GGCAAAACTATTTCCCTCTTTAGCACAATTTCCTCATCTTCGTCAAAAACCAACGGAAGGAGAAT
TACATTTAATCAATTTTTTGATTAATAATTTAGATGATTCCTATGAGATCTATTTTCAACCTTTT
CTTAATGGCGATCGCCCTGATATTGTGTTGATGAGGAAAAATTCAGGAGTGATGATTATTGAAGT
TAAAGATTGGCACTTAAAAAATTATAGACTTAATCAGAAAAAACAATGGGAAGTCACTAAAAAT
GGCAATGATTGGTATGGTATAAAATCCCCCATAGATCAGGCTTTAGCTTATAAACAAAACCTCTA
TGATTTACATATTGAGAATTTATTAGAAAAAAATATCAAAGAACCAAAACATTGGGCGATCGTC
ACTTGTGCAGTTTATTTTCACTGTGAAACAGAAGAAAAAATTAACCACTTTTTAACAGCTAATTT
TTCACAAGATAAGGGATATTTAAACTTTTTAAGACACCAAGTAATATTAGGGCATAACTCTTTAA
CAAAACAAAATTTCAATAAGATCTTATATAAAAATTATTTGAGTCGTACATCAAAATATTTTGA
TGATCAACTATACAGCAGTTTTAAACGTTATTTACAACCTCCTTATCACACAAAAGAAGAAGGAA
AAGAATTAAATTACACATCTAAACAAAAACAATTAACTATTAGTAATGTAATTCAACAAAAAAT
TAAAGGTGCGGCAGGTTCAGGAAAAACTTGTGTCTTAGCAAAAAGGGCAGTAAATGCTTTAAAA
AGAATAGAAAAAGATGAAAAAAATCATATAAATCATGAACCAATTATTTTAATTCTCACTTTTA
ATATAACATTAATAAACTATATTCACGATCGCATTAGTGAAGTTAGAGAAGACTTTTATTGGGGT
CATTTTCATATTATCAATTACCATGATTTTATCACTCAAGAGTTAAATAATTTAGAAATAACAAT
GGATATTCCTGAAGATTTTGAAGACTGGAATAGTGAAATTCGCTCAGAATATTTAGAAACCAAA
TATTACAGTAATGTTGACTTGTTTCTCAACAATATAAATAATATTAAAAAATATCCTGTAATTTT
CATAGATGAAATTCAAGACTATAACAAAGAATGGGTTGATATTATCAAAAAATGTTATTTAGAG
GATAATGGAGAATATGTCGTTTTTGGAGATGAAAAACAAAATGTTTATGAAAAAGAAATGGAAA
AAGATAAGAAACCTTATACGGGAATAGGCGGAGCATGGAATCTACTAAAAACATCTTTTCGTTTA
TCATCAAATATTGCTGATTTAGCAAGTCAATTTCAAAATACTTTTTTTGCACAAAAATACGAAGT
AGAAAAAATAGAAATTAATAATAATCCCGTTTTAAATTTTAAAAATCCTATTGAATATCGATAC
TTACCTATATTTTCATTGGAAAAAATAATTAACATTATTGAAGATAAAGAAAAACAATATTGTA
TTCATCCAAATGATATTTGTGTTTTGGGTTCAAGAGTAGAATTTTTGAGACAAATTGATTATTCT
CTAAGAAAAAATCAACAGCGCAAAACAACTATTATGTCAGAATATGAGGAAATGTATTATTTGA

CTTTGATCGAATCTGTTGAAAAATATTTATTTCAACATCAACAAAGCATATTATTCCAAAAAAAT
GAGAAATTGGTTACTTTAAATCATAACCTAGAAAAATTATTGAATATCAGTGGGCTATTTAATA
AAAATATACATAAAATAGTTACAGCTTATTGTTTAGGAAGTCAATCTAAATTTTTAGGGATTAA
TAAAAAATTAATAGAATACTTAAAATTTAATAAAATTCAAGAAAATAATTACTGGATTTGGTTT
GAACAAGCTAGTATTTGTATAAAAAATTTGGGAAATAATCCTTTAATTAAAAATCGTTTTTATT
ACTATTTACAAAAGATTAGAAAATACAAGAAAAGACATTTTTGGATGAATACTGGTAATACTAA
ATTCTCAACAATCCATAGTTTTAAAGGTTGGGAAGTTGATACCTTATTTTTATTAATTGAAAAAG
AATCAACGGATGATCCTTTTATGACAGATGAATTGATTTATACAGCAATTACTCGTTGTCGCAAT
AAATTATTTATCATCGATTTAGATGGTACTCGTTATCGGAATTTTTTTGAGTCTTTTTTATCATC
AACTCCAGATTTATATATAAGAAAAATTAAATTCTGATTTTTGTGAAAGATCGTGCTTACAATAG
GTATAAATTGGCTTAAGTAGTCTTGAATATAGCCCAATTAACCTTAATAAAAAGCAGAGAAATA
AACCGAAAATATTAACTATTTTAGGGCATAGTGAAAGCAAAGCATAAACAATATACAAGTTATT
TATTATAAACTATTGGATAAATAATATTTTCTGTGATGGTTTGCATAATTTCCAAATTATTCCCT
GATATATGAGTAGCAGAAAAATCAGAGAGTAAAAATTATGGATACTTGGAAACAACTCAAATCC
ACTTATAGAAAAGCCTACCATCAAATTACCACTGAAAAACCCCATGAAGATGAACAAAAATATAA
AACCTTTGCTTCTTGCGTTGCTATACTATGTCATGATAACGTCAAATACTCTCTGCGCGATCGCCT
CTCCTATTTAAGTAAATTATTAGACTTATCAGGAGGACCTACAGTCAGAGAACACGCCTGTATTC
AGTCTGCCTATGCCATTGGTGATGCGGTAGAAATTAAACATCATGGAATGACAAGCTATGCTTTA
GACGAGAGATTTCCCCTGCCGGGTACATGGGATTATCAAAAAGCCTATAATTTCTCTATTCCCCA
TATCTTCGGCAAAATAATGCCTATCCACATAAAAGTGTGGTTAGAAGAAAATTGTTTTGATGACT
CTCCCGAAGACATCCTAGAAATTCAAACACTCTTAGAAACTTCATCCGATTACCAGTATTAAAAG
CACATAATTTTCTAATTTTTCACTGATATATAAGTGGAAGATAAAGTTATGAAAAAATCAACTA
TGGATAATATCAAAGAAATTAGAGACTATCTTTATAACGAATTGTGCAACCTTGAATCTAAAGAC
AAACATAATCGAGACTTAATGAGGAGAAAAATTAATCAACTTAATTTACTGTTAGAAATGTTAG
ACAATTTTGAAGAAGAATTAACCCGATACGAATTGATGAACAATACCCAACTTAAATTAAATCAT
ATTGAAACGCAGGAGTATCCCTTATAAAAAATATGAAGCAAATATTCAATTTATTTTACATGATT
ACTTTTATCTATAGTATAAGTTTTTCTGCTTATTTAGGTTGGTGGATTTTGCGTGATTGGAAGGC
TTTAGAGTATTCTCATAATGTCGAAAAAAATTATGATGTCTCTATACATCACCGAATCAATTTAG
TGGCAGAAGGCACTGGATTTTTATTATTAAACTGTCTTACTGCTATGAGTGTTATTGGCTTAAAT
ATTAATAATAGAAAAAAAGGTTAATTATCTTTTATATATATAATCAAAAAACATTATTATACTA
TTTTTAAATAGTATATTTTAGCTAAATGAAACAAATATTTAATGTATTTTATCTAATTGCTTTTA
TTTATAGTATCAGTCTTTCTGCTTATGTCGGTTGGTGGATTTTGCGTGATTGGAAGGCTTTAGAT
TATTCTCATAATGTTGAAAAAAATTATGACATCTCTATACATCACCGAATCAATTTAGCTGGAGA
AGCCCCTGTCTTTTTATTGTTAAATTGTCTTACTGCCATGAGTGTAATTGGGTTAAATATTAACA
ATCGTCAAGACTCTTAATCTATTTTTATCATTTTGTAGGGGTTTAATATTATTCAAACCATAAGA
ATTTTACTCTTTTCTTAACACCGAATATCTTACTCTCAATTTGCATAATTTTCCGATTCTTCCCTG
ATATATAAATAGGCAAACATCACCCATAACTAAACATAACTAAAATATGGAAATAACCTTAAAC
AAAGCTAATAAGCTACTTAATAAGTTTAAATCCGAACGCAAAAATTTACGAGCAGTGTTTAACCG
TGTGCGTTACTACCATGAAGACGAAAAAGTGGAAGCCATTACCTCCGTTGAAGTGAGTTTAGCTA
ACCTTACTCCCAGAGTAGCCGATGAAATCCAAAGAATGATCAATACTTATCGCAATCAACTGCAA
GAATGTTTGGATATTGAAGCGGATTTTTTCAAACTCAAAGAGGCTATTTTTGTTAATAATCAACG
CACGGGCATTTCTGAAAAGCTGAGTCAAATTGAGTTTTTAACCTCGAAAGTGGGCTTATATGATG
ATTTGATTAACGAGAAGAAACGCAATAAAAGGGTTACTTTAACCGTTGAAGATATTAACTATGA

GTTATTACGGGAAAACTTATCAGGGCAGGATGGTTCGGGAAAATTGTATATTCAAGTATTTGAT
GTTGACAGATTAGCTCAAGAGCGTCAAGAGGCAATTACTCAAATGAACCAAGCGGAGGATGAAAT
TGCTAGGATTAACAATAATACTACTATTAACATTGATTTACCCCTGCCAGTCGTCGCTTATTGG
GTTTAGATTAAATTTGCCAAAAATTTTTTCCAGAAATTTGCATAATTTTTAATTTCTCTCCTGAT
ATATAAATGGAGGTAAAATTCGTCTCTTGGCTACTCGGTTAGCCATTAAGTACATTTTATGTACA
TCCGAAGTGCATTGACCATAACAATAACATTACCTTAGACGTACCTAGCTGTCTGCCCTTAAACT
GTTGTTGGTTGTTGTCTTGGCGACAGTAATTTGTTTAGCTGATTTAAGTTCGTTGTTACCCTTAA
TGTGTTCAAAGTCAATGCACTATTAACAAGGGAGTAGGAGTATTTTGTTATTCTTACTCTCTTTC
AATTTGAATCCTCAGATTAAATCAATACCACCATATCCGCGATCGCAAAAATTATAATCATTTTG
TAATCAACGGTATTTACTAAATCATCTAACCTGTTTTTACTCGCTCTTCCCCTAATTTTCCTGTT
AAATTATTGATAAATATTTTTCTTAGTTCAAGTCAAGTAAGGGCGAGGTTTCATGCTTGGGCTAA
ATTTAATTTAGAATCCGCACAAAAGGAGTTTGAAGAAAAGTATCATCATAAGGTGAGATTTTCG
GGATTATCTATATAATAATTATTTATTTCAAATTTTAAGTAGTGGATTCCTAAACAAGCTAAAA
AATAACCGTCAAAACAAAAAATACTTTTAATATGTCTATTGTTTCACACATCCTAATCGGTTGCC
CAAGTAGCGGAAAATCAACTCTTGCTAATTATATAATTAAACAAAATTCTAATTATCAAATTATC
TCCACTGATAACATTAGACAACAACTTTTTGGAGATGAAAATATACAGGGTGATTGGCAGTTAAT
TGAAACGGAAATATTTAAACAAATTGACAGTTATATTCAAGCAGGAAAACCGATTATTTATGAT
GCCACTAACGCTAAAAGATGGTGGCGAATTTCTCTGTTGGAAAAGTTAACACAATATGACAATGT
TAACTGGATTGGTTGGTATTTAAAAACTCCTTTAAATGTTTGTTTGGAATGGAATCAAAAGCGAA
AACGTCAAGTACCAGATGATGTTATAACTAATTTATATCAATCCCTAAGAAATTTTCCACCTCTT
CCTGCGGAGGGTTTTTTAGCTGTTTACAACATACCTTTTAAAGAAGGTAAATTAGACGTTAATCA
ATTCAATGATAAAGTTTCAAAATTAAATCGAGTACAAGTCAATCGCACCAATCGCACTGCCAATA
GTAAAGTACAATTTCATCCTTATTCAAGATTACTAGACTTCGATCGCATCTTGCACTTAATTAGC
CTTATAATTAAATATCCTAATCTTGGCAACCTCTCAGAATCCAATCCTGAATTAATCAGAGAAAT
CTTTGGAGATGAAATTAAATTTAACAGTGAAATAGAAGAAATTTGTGGCTTTTTAACAAAATAT
TATGACAAAATTTATGCTCAACCAGAAGCCATTAAACAAGACTTAATCTGGCTAGAAACAATGGG
TATTATTAATCAACAAAATTATAAACCATTACCAGATATTGAAATAGTAAATATTGACGATTTA
GTAACCCATCCTTACTCAGATATTGAACCATTTACCAGACTAATAAAAACCATTCACTTTATCAT
TTATCATCCTCTAACTTATGAAGATAAAGAAGGATGTTTGCAAAGTTTAATTAAAGCCATGCAAC
AGGAAAAAGTAATTAATATTAATTGCGGTAATAGTATTCGTAAAGATATAGAAAAAGTTTTAAA
ACCCTTTGGCATTTTCCCAGATTTTACCATGAAAAAAGGCTATTTTATCGGCACAGGAATTTTAT
CTCAAACAGATTTAATCAAGGTATTTCGCTTATTAGAAGCCCAAGCTAATAGTTTATCAGATCCT
GTCGCTTTATCTGTCTATGAAACCTTTAAAAGTCGTTTAGGAAATGCTAAAATCGCTCACTCTGA
AAGTTACCCTGTCAGGGCAATTTATAATAAAAATATTGTTGATTTAAACAGTCTTCCTAATGATT
CTTTAGCCAAAAAAATTGATGTCTTAGAAGGTGCGATCGCCTCTGGAGAATTATTAGAATTAAAT
CGCTTTTTAGGCAGTGCCGAATTTTATAAAGATGACTCTCAATATAATCAAACAGAGGATAATAG
TTATTTTCTAATTTATCCATTACAAATAGTTTTTCATAATATTGGATGGTATTTAGGTTATGAAT
ATGCAGAAGGAGTAAATAAAGGATTATTTAAGTTTGAAAGATTAGATAGATTATTTTTATGTAG
AAAAACCCATCAATCAAGGGAAATGAAACAACAAATAAATGCCTTAAATAAATTACAAAAACTA
TATCAAGCTAGCGGAGGAATCTTCCTTGGAAATAACGTCAAATTGCAAAAACAATATCTCGATAC
TAAACAAAAAAGCAAAGCTGAAATAACTATGGAATTATGGTTTAATGATCATATTTTTAAGTTT
ATCAGTGAAGGTACAAATCGCTTTCCATCTCAACAAATGAAAATGTCAGCCCGTCTTCATAAAAA
TCATGAAACAAATCCTCTTTTTACCCTAAAAAAAAACAGAAGATAAAAACTTTCCTAATCGTTTTC

AAGTAAAATTACCGATATGGTCATTAGAAGACATTGATTTATTACGGTGGATTGTTGGTTTTGGT
GGACAAGTTAAGGTAATTAATCCCCCCGAATTAGTGAATAAAGTTAAACAAATAGGAGGGGCTA
TTTCCCTTTTATATTAAGGTTTAAATATCGAAATTTAACTATCCAGCAATTTGATAAATAATTGG
TTTATTTTTTTCTTTAGCAACTTCTAATCATGCTTTTTTAGCAATTAAAACTTCTTGTAATGCTT
CTTCTGGAGTTTCTCCATAAGCCGAACAAAATTTTAAATCAGGAATATCAGCAATATACCCTTGA
TCTTCTTCACTATAAAATATATTAATATGATAATCTTGCATAATTTATTCCTCGTTATCCAAAGT
TAAATTATATTTTTCAATCAATGCTAAAAATTGTTTGACTTGATAGGGAGAAACTTGTCCTTTTT
TGTTTTGAATATTAATAATTTCAGAGATGTCGTTATTTAGTTTTATTTTTATTCCCATAATATTT
CAATGGGATAAGCAGAAAATTTAGTATCTTTACTAACTAAAATTAATTCTTTTTCCATTGCTTGA
ACAATTAACATTCTGTCAAAAGGATCTCGATGATATAATGGTAAATTGCATAGTCTTTGAGTGTC
AGCAAACGTAATTGGTAATAAGAGAATGTCCGATAATTTTAGTTCATTTTCAATATGTTGATAAC
TTTTTTTAAGGGATAATTTCCCGATATTGATTTTAATGGCAATCTCCCAAAGGCTAATAATACTT
AAATAAACTTTTTCGGCGGAGTCAATTTTTTGTCTTAGATTAATTGGCAAATTAGGATCGTTTTC
TGACAACCAAATAAAAGCATGAGTATCTAATAAATAACCCTTCATCTCATATACTTCTCAAAATC
TTCTAAAGATTCATCAAAATCATCAGGTAATGGTAAAACAAAAGTACCTTCTAAAATACCTGAGC
GACGTTTTTGATTAGAAGTGTTTATATTTACATTATTTTCGCTATATTTTTCCTGAAGATACTCC
GCATAATGCAATAATTCTTCTTGCAAAGATATGGGCATTTTTCCCAAAGTTTCTAATATTTTAGT
TTCAATATTTATCATAGTATTTTATTTTTTAGTAAATATTTTTCCAGTTCAATATATTTAGAAAA
TAAAGTATCTATACTGGCTAATTGTGCTTTATATGTGTATCAAGGATAATCATTTTAGGCATTCC
CAGTCATCTTCATCAACAATAGGGCTAACAATATCCCCTAATGTTTTTCCTTTCCCTGCTATAAAT
TCAGGAGGTTGACGACGGCTAAAAACACTTTTATTCGATTCTTCAAGAATGGTTATAATGACGCA
GGCACAAGTAACTTTTGGTGTATCTTCCAACCATTTCACTTGTCCATTGTCATAGATAGCTTCAT
AGCTTTTTAACATACTTTTTAACTGATAATTTAGGGCTTTTTCCACATTGTAGCCAAAATCTCTT
TGTTAAAGAATTTTTAGAATCAGATTCTGTCATATAACAAATCTAAATTACATTTTCCTCATTCG
CATTAAATTATAAATAATTCTATAAATGTCAGGATAGGATAAACATAACCCTTGAAAACTAAAT
GTAAAAGTAAGTGAAAATAACCCCAAAAGACTTCTTGGATTCTGAACAACAAAAAACAATTAAA
TCAGGAAAAACGGACATGGAAATTATTAGTATGTAATCAACAAGTTAAACAATTAAAGTATAAA
GCTATAGCGTGTATATATTTTGTTATATATCTCCATAAATACAATCAAAATAGAATTAAACTTAT
GTTAAACATACTAAACATCCATATTAATGGTTTGCGTGATTTAATTAAATCCAAAATCTTGAGTA
ATAGCGTACATTTAAGTGCTACAATATCCAGATTACATTATTCCCATGTGTTGCATGATTGCAGA
TTATAAGCGATATGAATTTCTCGAAGATATAGAAAAAGAAAGTTTAGAAATGGTTAAGACTCAT
GCAATTAGAGACGTAGTAACCCTTCAGCCTAACTCTAAAAACATTGAAGAATTTTCACGCCAACA
ACAGATAAATAGCTCAAACTAAATAAAAAAGTTTGTAGTAACTATTTACTCTTTTTGGAGTTGA
AACCATCGACAAGGAATGAACGGCTAAACCCCTGAAATTTTGGCAGATACTCTCAATAACCAACT
TGCTTCAGTGTGGCTATTATTTTCCTCATTCGCATTGAGTGATAGATAATCTCGTAAATGTCGGG
TTATGATCAATCCTAACTCAGAGGATAAAATTATTTTTCAGCAACTTATTCAATCTTGGTAGTTA
TCAGAATATTCTTTTACTTCACTCGTAATTAACTATTATGTTTTAAGCACTTACTTCTGTCAGTA
AAGAAACTCTGTGTTAGTATTATTTGCAATAAAAAATTAGTTATTAAATTTTAAATGTCTCAAG
AATTAATAGAAAAAGCCAACATTCTCCTCCAACAAAATAATTTTCCAGAGGCAGGAAAAATATTC
CGTCAACTGTGGGAAGAAGACAATAATGCTTATTGTGCCAGTCGTTATCTTCATTGTTTAAGAAA
GGCTGGTTATACGTCACATGGTTTAAGTCAAGGAAAAAAAGCATCTTCTCAATTTCCTAATAATA
TCTATATTCAAAGAGAATTAGTTTGGATTTATTATGACATCATTAAAACTTCTACCCAAGAAAAT
AATTTACAAGATGCCCTCAATAATTTTGAGCAAATGCTAACACTAAATCCTGATTCATTACCCTT

AGAATTAGCTATATTTTTAATTATTGATTTAGCACAAGAAAAAAACCAATGGCAAATAATAATG
GATATTTGTAGTCAAGTTAATCCATCTCAAATTAGTAATGAATCTAAAATAATTAACGGTAATA
AAGTTAAGTCTAAAAGAGAGCGATATTATTTTAAATATATTAAAAGTTTAATTGAATTAAATCA
TTGGAATCAAGTTAAAAATATAGCTTATAATGCTATCTCTAATTTTCCTAAAGAAATTAATTTTA
AAAGATGGTATGCTTTAGCTTTAAGTAATAATGGAGATATTGAGCAAGGAATTTCAGAATTAGA
AAAAATCATCTTAAATGATAGACAAGAATGGTATTTATTTCAAGATATATCTGAACTTTATTGGC
AAAAAAATCAAGAAGAAACGGCTTTAAGATATGCTTGTAAAGCCTGTTTAGCTACTAAAGAACA
TAATTTAAAAGTAACTTTATATCAAAACATTGCTAAAATGTGTTTAGAAGTAAATCGGTTAGAA
TTAGCAGTAAAACATATTCAATTATCTAAGCTAGTCAGGCAACAAGAAGGATGGAAAATAAAAC
CTGAATTAGAGAAATTAGAAAATGAAATTAAACAAAAGTGTCAAGAAAATAATATAGAATTAAC
GGAGTATTCTTTTGATGAATTAGCTAAAAACTGCTTACAAATTTGGCGAAAAGAAAGTTATCGA
GGATTAGAAAGACATCAAGGAATAATTGAGTCAATCCCACCAGATAAATCCTTTGGTTGGATTCG
TGCATATAATGGAGATAGAATCTTTTTTATGCAAAAACAATTACCTCCATTTCTCAGAAAAGAAA
ATTTAAAAGTTAGTTTTATTTTAGAAGATAGTTGGGATCATAAAAAAAATCAATTATCCTCTAA
AGCTGTAGATATAAGAAAAGAATAATGAGATATTTTACTATGGTAACTATATCTAGTTAATCAT
GGCAAAATTTTCGTTTTGAGTTATGAAATTAACAAATTTTGAGCTATCTAAGCCAAAAAGAAAT
AAATCATCTTTTGCACGAGTCATAGCTACATATAATTGACGGATATTAATCAAAGAACCTTGTCT
ATTATTCACACAATCATCAAATTGTTCTAACCAAATAATCAAAACTATTTTAAACTCTAAACCTA
AAGAAGAAAGACAAGTTATTAATCTAATACCTTCCCTGTTATAGCTATAGTTAGATTTTGTTTTA
GAATCTTTTGTTACCCAATAAAAAGGAATGTGTTGTTGATTTAACTGACTCATTATATTTGATAA
GCTATTACCATTTTGTTGACGATATAAAATAGCAATATCTCTAGGATTAATTGAATCATTATCAA
TCAGTTTTTTTATAGTTTTAATAATTAGTTTTTCTTGGTTTTCTAAAGTATTAGCTAGATAAATT
ATAGGTTTATTCCCATGCCTTAAAGCTGATTCTGGTTTGATGATTGGAAATGTGATTTCTTCTGT
TTCTAGTACTTCTTTATTTTGTTGTATTTCTCTCAAAATTGACCATGCTGAATTTATAATCTCTA
CAGTGTTGCGATAGTTTTTATCTAGATCAAATTTTTTACTAATTGTACGATACCCTACGGCTTTA
ATTCCTAGTTCTTTCCAAGTAAAATTATCTCGCTTATAAACACCTTGACTACCATCGGCAACAAT
GATTAAATCGCCATTTTTGCTATCTTTTAAAGCCTTAACACAGCATCTAAACCAGCTCGGTACAA
AGGTATGAGCTTCATCAATTAAAATAGCATCCCATTTATCTTTATCTTCATATAAATCTAGTTTT
TCTAATACTAATTTTGCTGTATATTCGTCTCTATTACCTTCGACTTTTGCGGGTAACTTTCCTAA
AATATTTTTAGCCCATTGATCAAAATTATAAACTTCTATATTTTTATATTGAGGATTATTTACAT
CTTCATAGAGAATCGATTTTAAATAGGAAGCTAAGGAAATATTAAAGCAGAGAATTAAAACTTT
TGCCTGTGGATTTTGATTAATTAAAATTTTAGCACGGGCAACTAGAATTAATGTTTTTCCTGAAC
CTGCTACCCCTAAAAATCTTCTATGTCCATCACCAATTTTTTTAGCTAAATTTTCTTGTTTATAG
TCAAGAGTTATTGAAATTTGATCATCAGATAAAAGAGGAAATTTAGGAGGTACATGATTAATTT
TGGCGGTTTCTTTTTTGATGATAATTTCAGGATAAATTGCTCCTTTAATAGTATTAATTTGTTCA
TTAGTTAAAGTTGAGAATGGAAAATTAACAGTGAACATTGATTGTAAACGTTTAATTAATTTAT
CTATTGAAAAATCGTCTTTTTCCCAGTCCAATAATTCATTCCGATAAGCTACTTGAGGAGTAGGT
AAAACTGTAGTTAAGTTGATATTTTTCGCTTGATTTTCTGTGATATTGGACATAATCGCCCCCCA
ACCAATAGGAAAAGCTAATCTTCCTTGATATTTTCCACTCACATTGGTTAAAATCCTATATTTTT
GTAGTTGATTTAATAGCGAGTCTAAATAATCTTTTGCTTGTCTTAATGGGGATTTCTGATTTGAG
ATACTTTCATAATCTTCCCCTTCCGTTTCTATTTCAAATTTATCGGTTGTAGCTGAGATAATTTC
TTTAGGATACCAACCTTTTATTTCTACTACTAATAAACCAAAATTGGGTGCTAATACAATAAAAT
CTGGATGACGACTATTGATATTCAGTCGAGGTTCATACCAAATATAAAAATCATCTGGTAATTTG

TTTACTAATAAGGAAAAAAATCTTTTTTCGCCTTGACTAGTTTTTGGAGGTATGGAGGAAGGAAT
TGTTATCGCCATGACAAATTAACTATTTTATTCTTTCTTTATTACTTTATCAAACTATAAAACGA
TAGACTTCCAAATTTTTGACGACTCAACACATCAATTAATTTTGATAAAATCTCAGAAGAAATTA
AAATCACCCCCTTTTCTTTTGCCTCTCTAAAAACTTGCATAGTAGTAGATTCAGAGAAAGTCAAC
GCACTTACTATTACATTAGTATCAATAACAATTCGTTTAGTTTTCATCATTGAGAATTGATTGTA
GAATTTCTGGAGTTATTCCATTGGCGATCTCTTGTTGACTTGCTTCTTCCATAATGTCTAGCAAT
GGCTTTTGATTGATAGCCTTTTTTAGCATTACATTGAGAAACATACTAATTTTTTGCTGTTTTTC
AGGTTCGGTTTCTCGGTAAACCTTGGCAATTTTAGGATCAACTTGAATGGTAATTGTTTCCATTG
TTCCTTAACAGTTAATTAAATAGCATCTTTGACTGACAATTCTGAAAATAGCTGAAAAGCTGAAA
GTGTTAAGAATCAAACCATATGCTTAAATGAAAATGCCCAAAATATTTTTCCTAAATCATGAAAT
TTCATCTATCTAGCATAATTCAGATTTTCTTTGTCAGATTTAATAGATTATTTCGCTCTCATAAA
ATTGAAGCAGAATTAACTTGATAATCTTTGATGATATTAATAATTTGTTGACTACTAATATTAGC
CTGTTCTGATTTTGTATAAACAGTTAATAAAACTACTGTATTCTCTATTTTCACATAATAAATAA
AACGTTATCCGCAACTTTTTCTTTTTGAATACCACTATTTTTTACTCTTACTTTAAATACCTGAT
GATTAATACCAGATACTCTATCTCTCAGTAATTATTTTATGTTTTTACTTTTTAATAAGCGGGTA
ACGCGATTCGAACGCGCGACATTCACCTTGGCAAGGTGACGCTCTACCACTGAGCTATACCCGCAT
TTTTTTGTTTGCGACATTTAACATTGTGTCAAAAAAATAAAAAAATGTCAAGGTTTTTCTTAAAA
TTTTTCCCGTTCTCAAGGTAAACTCTTAGAAAAATGAAAATTAATCTTTCAACACATAGCCTACG
CCTCTAACAGTCTGAATAATTCTTTTTTCCTTATTCGATTCTAATTTCAGTCTCAGGTATCTCACA
TACACTTCAATAATATTAGAATCTCCGCCAAAATCATAACCCCAAACTTTTTCTAAAATTTGATC
ACGAGTTAATACTTGTCGAGGGTGGGAAATTAAATATTCCATTAAGTCATATTCCGTAGCGGTTA
AATCGATGATGCGATCGCCTCTTTTTACTTCACGGGTACGACGATTCAAGCTCAAATCAAGAAAG
TGTAGTTCGTCGGGGTCTTCTTCATTGCTTTTGCGTAAATGGACTCGTAACCTAGATAATAATTC
TTCAATACCAAAAGGTTTAACAATATAATCATCCGCCCCTGCATCTAAACCAGCCACACGATCAT
TGATTTCATCCCTTGCAGTTAAAAGGATAATAGGAATCATACAGCCTAAACTACGTAACTGACGA
CAAACATCAAAACCAGACATTTGGGGTAACATCCAGTCTAGCAAAATCAGGTCAGGATTAATTTT
TTGAGCAACCTTTAATCCTTCTTTTCCATCTGGTGCGATCGCGACTTCATAGCCTTCATATTGCAA
CTCTAACTCCAAAAACTTAGCTAATTTTTGCTCATCTTCTACCACTAAAATCTTACTCATTTTTCT
CTTTATCTTTCTCTATGGTTTATATCTTAACCTGAGTTCGTGATAAATTTTCATCGATGAGTCAT
GGCAAAAAGGGCAAAGGGCAAAGGGCAAGGGGCAAAGGTAAATGGTTGATAATTAAAAATTAAG
AATTAAAAACCCCGAACTGAGATTTTTTTATCGAACTAAGATAATAAAAAAGGAGGCGACAATG
CCTCCAGAAAAATGAGAAGTTATCAAATAGTTTGTTTAGACATCGTAATACAATGCAAACTCATA
AGGATGAGGACGAAGACGCATAGGGTTAATTTCATTATCTAACTTATACTCAATCCAATTATTGA
TAAAGTCTTCAGTAAATACCCCACCTTCTGTTAAGAAAGCATTATCCTGTTCCAAAGCCTCTAAA
GCCTGTTCTAAAGAGCCGGGAGTGGAAGGAATTTTGCTTAATTCCTCTGGGGAAAGATCATAAAT
ATCCACATCCAAAGGTTCACCCGGATCAATTTCATTTTTAATACCATCAATACCTGCACAAAGCA
TTGCGGCAAAGGCTAGGTAAGGATTGCTAGTAGCATCAGGACAACGGAATTCTAAACGTTTCGCT
TTAGGATTTGTACCAGATAAAGGAATACGTACAGAAGCCGAGCGATTACCTTGAGAGTAAGCTAA
GTTAACAGGAGCTTCAAAACCCGGAACTAAACGCTTATAAGAATTAGTGGAGGGATTACTAAAA
GCTAAAATTGCAGGAGCGTGTTTTAAAATACCACCAATATAGTGTAAAGCCATTTTACTTAAGTT
GGCATAACCATCACCCCAGAATAAAGGTTGACCATCTTTCCAAATAGATTGGTGAGTGTGCATCC
CTGAACCATTATCATTAAATAAAGGCTTAGGCATGAAGGTAACAGTTCTACCATACTTCTTGGCA
ACATTTTTAATCACATACTTATAAGTCATTAAATAGTCTGCCGCCTCTATCAAAGTGGCAAAACG

AAAACCTAATTCATTTTGTCCACCAGTAGCAACCTCATGATGATGCTTTTCAATGGGTACACCAC
ATTCAGCCATTGTTAAAAGCATTTCCGTACGCATATCCTGCATCGTGTCTGTGGGGGGTACGGGG
AAATAGCCTTGTTTGTAACCCGGTTTATAACCTAAATTTCCGCCTTCTTCTTCTTTTCCAGAATTC
CAACGTCCTTCTACACTGTCAACATAATAGTATCCCTTGTTTTCCGTTTGATCGAAACGAACATC
ATCAAACACAAAAAACTCCGCCTCAGGACCAAAATAAGCAGTATCTCCGATGCCACTTTGAGCTA
AATACTCTACAGCTTTTTGAGCGATACTACGAGGATCTCTGGAGTACCATTCCCCAGTACGAGGT
TCTTTGATACTACAAATCATGCTAAGGGTAGGTTCTTTGCAAAAAGGATCAATCCACGCAGTGGT
AGGATCAGGAACCATTGCCATATCAGATTCATTAATAGCTTTCCAACCACGAATACTAGAACCAT
CAAAAGGCACACCATCCACAAAAGAAGATTCGTCTATTTGATCGTAGTAAAAAGAGCAATGTTGC
CAAGTACCGGGTAAATCGATAAATTTGAGGTCGATAATTTTGATATTCTGATCTTGAATCATTTT
CAAGACTTCTTGGGGAGTTCCGGGCATTAAATTATTTCTCCTTTATTTCTTGTCGTTACTTTTAT
GTCTTACTCACAAGTCTTAATCCTAAGCACAGTCAAACCTAAGTTTTGTAGCTATTGATACATAT
ATATAAATATTTATTGTCTTTATGCTAATTTTGCATAACTGCTAGAAGACACAGCGAAGATGTAA
AGAAAAGTTATACTAGCACCATATAAACAAACAATTTTTATAATCTTTTTATATTTAACTACTCC
AATAAAGCATAAGATGACTGTTCAAATTAAGCTCAATGAACGCCATCATAAAGTATTACAGGCAA
CTATTAAGCATTACATCGCAACGGCTGAACCAGTGGGGTCAAAAACTTTAATGAAAGAGTATGAC
TTTAGCGTTAGTTCTGCGACAATTCGTAATGCGATGGGGAAATTAGAAAAGGCTGGTTTTTTATA
TCAACCTTATACTTCTGCTGGGCGTATCCCCTCTGATTCCGGCTATCGTGTTTACGTGGATCAACT
TTTAACGGAACAAAATACTGTTAAAATTTCCCTTGATCCTTTTCTTGATGCTACTTGGCTCAATT
TATCCGGGCGTTATGAAATTCTTTTTCAAAAAATAACAAAAATTCTTGCAGAAGTAAGTGGCTGT
ATTGCTTTAATTACCCTACCTCAAGTCTCAGGTAATATTATTCATCATCTTCAGTTAGTTCGAGT
TGCTGATAATAAAATTATGTTGGTGATCGTCATAGATAATTACCAAACAGAATCTTTCGTCTTAG
ATAGTAAGGAATTAACGTTTAATTCTCAAAAGTTACATCCTGAAGTTCTTGATAGTGAGCTAGAA
ATTTTATCGAATTTTTTAAATCATAAATTAAAAGGTAAATCTCTGAGGGAAATTAATCATTTAG
ATGGCAGTGAGTTGGATAATGATTTTAAACTTTACAAGGATTTTATTAATCATTTGCTCACAAAG
ATAAAGTCTAGTTATCATTTAGCTCAAAGTACTCCTATTTTATTGCAGGGATTCTCACAATTAGT
ACAACAACCTGAATTTTCCAGTATAGATCAGGTTAAAATTCTGTTAAATTTTTTAGAAACAGAGC
AAAAACAGCTTCTACCTCTTTTATTTAATATTAATGAAAATTTGGACGGAAAAAGAGTAAAAAT
TCATATTGGTTCAGAAAACCCATTGGAGTCGATGCGCTCTTGTAGCTTAATATCTGCTTATTATT
TTCAAGAAGATTATCCTGTGGGTAGTGTTGGCATTATTGGACCGACGAGAATGCCCTATGAACAG
GCGATCGCCCTTGTTGAATGTACGGCTAATTATCTATCTGAAAAAATGTAATTTACTTAAGTTCG
GGATAAATTTTCATCTATAAGTGATGGTGAAAAGGGCAAGGGGCAAAGTAAAAGGGCAAACCCC
CCTTTCTCCCCCCTCGATAGGGGGGAGAGCAAAGGTAAATAGTGAATAGTGAATAGTTGATAATT
ACTCGTTGCTCCTTACTCATTACTTTCTTCTAAAACCTGAAACCTGAAACCTTATCTCCCTCTCCC
CTCATCCCCCCATCTCCCAAATACCTACCCTTATCCGATATTTTTAAACTGAACTGAGAGTAAATA
TGTTTTGTTACTTAGATAATGTAAGATTTAGATTTGATTCATTATCCAAAGAGAAAATTCTTCCT
GACTCAATGGCTTAGAAAATAAATATCCTTGTCCATAATTACAGTTAAGTTTTTGTAAAATTTCT
AGTTGTCTTTCTGTTTCAATTCCTTCACAAACAACATTTAATTCGAGATTATGACAAAGGGTAAT
TATTCCTTGTAAAAATTTAATATTTTTAGGATGAGTATTTAATGGTTTAACAAATGCTTTATCAA
TTTTTATTTGATTTAAAGGAAAATTTCTCAATCTAGCTAGAGAAGAATAACCAGTACCAAAATCA
TCGATCGCAATTTTAACTCCTAGTTTTTTGATTTCTGATAAAATTTTTGTAATATTTTGAATTTG
ATGAGCAATGATTGATTCTGTAATTTCTATACGTAAATTATGAGGTGAAATTTGGGTTTTGTTTA
AAACATCTTTAATTTGTTGTAAGATATTTCCCTGCTCAAATTGTTGCTCACTAACATTGACATTT

ATACAAACATTTTCGGGAATTAAATTGGCTTCTTGCCACTGTTTCAATTGATTACAAGCCTTATT
TAACACTAATTTGCCAAGGCTAATCATCAGCCCACTTTGTTCGGCGATGGGCAAAAATTTATTAG
GATTTAAAAATCCTAATTCTGGATGTTGCCAACGCACTAAAGATTCAATACTGGTTATTTTTCCT
GTTTTTAAATCTATTATAGGTTGGTAATAAATAATTAAATTATCTTTTTCAATGGATTTTCTCAG
ATCATTTTGTAATTGTAAACGCCTGATAAAATCGTCATACATTTCTCTCCCGAAAACTTGATAAT
CTCCTTTGCCTTTATTTTTTACTTGATACATGGCTATGTCGGCATCTCTAAATAAATCCTCTGCAC
GAAGATAAGGAGAATTTGTAGTTTGATTATGACTTAAAACAATTCCAATACTACAGGTGGTAAA
AAATTCCTGATTATGAATATAAAAAGGTGAGCTTATCTCTACTTGAATTTTTTCTGCAACTGCGA
TCGCATCTTCAACTTTAGTGATAGAATCGAGGAGAATTGCAAATTCATCTCCCCCTAACCTTGCC
ACCATATGGGAAGAGGATAAACAACGGCGTAAACTATAGGCAATATCAATGAGAAATTTATCTCC
TGCTAAATGTCCAAGACTATCATTAATAGTTTTAAAATTATCTAAGTCAACCAATAATAAAGCAA
ATAGCTGATGAGGAGTATTCTTAGCTTCCTGCAACAAATTTTCTAAGGTTGCCATAAAATAAGCC
CTGTTGGGTAATTTCGTTAGAAAATCGGTATTCGCCGCTTCAATTAACTTAGTTTCCATAAAGCG
ACGTTTAGTAACGTCCTGAAAAATAGTCACGAAACATTGTTTTTCTCTTTCCCCAATAATAGTAG
TTGTTATTTCTAACCATTTAACCTCTCCTTTTTTCGTGATGATGCGTTTTTCAAGGGAAGCAGGA
TTAAGATTAGTAGAAAAGCAAATTTCTCGCAAATATTCTTGGGTGGATACCGTATCTTCTGGATG
AATAAAAAAATAGCCTGACTGTTGTCTTAATTCATTTTCTGAGTAATCCAATAATTTACACAAAG
TATTATTAACTATTTCTAGTCTGTAGTCAACATCATGGATAGCAATGCCCACAGCTACTTGGTTA
AAAATACCATTTAATAAGTCTTCTCGTTGCTTAATTTTCCCCGCCATTTCCCGTAAGGCTTTACTC
AACACCATTCTTTCATCTCGAAACACAGAAAACATGAAATTAGTGTCTGAAATGTCTATATCTGG
TTGATAATTACCTTCTCCCACGGCTTGGGCGTATTTTTGTAATTTTCCTAAACGGGTAGTTAAGA
AATGGGATAATCCGATAATGGAACAGAAAAAAATAGGATAGGATATGACAAAAAAAGATAAAAA
TCTTTCACGAATATCTTTTTGTATCTCATTTATTTCCTCTGTTTGCATATCTGCGCCTATTCCCGC
AATTATTTGTCCTGATGAATCTCTGATGGGTGCATAATAAGTTATCCAACTCCCCCATTTATCTT
TGTAAAAATTTCGATAAACTGTAGCCCCTTCTGCAAGAGTTTGTAAATGATATGATGTGGCGGGG
GCAGATTCTAAGAAATCAACGCTTTTTTGAGGGTTTTTTCCTCTCCATACATCTACTAAATAGAT
AATTTCGGTTTTATCTGTGGGGGATAATTGAGAGATATTGGGGTGAGTAAAAGTAAATAAAAAA
ATATTGGGGTGTAACTGGTTTATCTTTTCCATCCATTGTAGTTGACTTTTGTAGCGTGGATCGTC
TGAACTACCTTCTTCATTGGCTTTGCCTTCTTGGAATAGTTGTTTTAACTCTTCTGTATCAATCTG
TTGCGAAGATGCGATCGCAACTTGTTTCAAATTATTGGTCAAACGTTCTAAACTTCGCTTCATAC
TACTTCTATAAGACCACCAGTAAAGACACAGAAAAAAAAGAGTAAATACAACTGTGTAGCTAAT
TAGTATTTTTGTCCGCAGACTAATAAATTTTTTCATAGAGTTATGGACGAACTAGGAGCAACAGA
GACACTACAAGCGCAGAAAATAATTTGGCTTTAGTGATCACTGACACGATAAACTAGGAATGATT
TTTGGATAACAATATTAATTTAACTTAAATAAGCGAATACTCTCCTAGAAATAGGTATTTTATTT
AACAAATAATTTCTTTTATTTTCTTTTCCTGAAAATTAATCCCATTTTCTTTAAATTACTTGATA
ATTTATAGCTTACTAAGATTAAGAATTAACTGTTCAAGTATTTCTGGTAACAATTCTAAGGAACA
ATAGTTACACTTATATTGTGTGTTAACTGCGATCGCACCTACAAAATTATCATCCAAAGAAAAAT
TATTAATTTGCCATTGAGAGTAAGAGTTAGTACAGAAATTAATAGTATTTAATCCGCCGGATAAT
CCCTCTCCTATAGCTTTTAAATAGGCTTCTTTTTTTGTCCAAAATTGGTAAAAAGTTTTAACTTG
TTCTTCTTTTTTTAAATGCTGTAAATATTGATATTCTTCCTGTGCAAAAAATCTTTTTGCTATCC
TTAATAAATTATTATTATCTTCTAGTTCCTCTAAATCAACCCCTAAATTTAATAGCGAGAAACCA
TAAATTATATCTGTTTTACTATGGGATAAATTAAAATTTAAACCTATATTGTTTATGCTAAAATC
TAATACAGGCTTACCTTTTTTATTATAAGAAAAGAATATATCCTCTGGTTTTATTAATAAATAAT

TTGCTAAAATAACTCTTAAAATACCTTTATTTACTAAATAACTATTACATCTTTTTTGAGATTTA
AAATATAGAGCTTTATTCAGTTCATCATCAGATAAAAGACTTTTAAAAAAAGGAATAAAATCTA
AAAAATTACTAACATTACCATAAAAAAGATGAACATCATAGGGAAACAAAGAAAGGGGTAAAAT
AGAAATCTTTTGCCAAGATTGTAAATCCCAGAAAGTCATAAAAGCATCAAAACTTTAAGCGCATT
TAGGTATAATCAACAAACTAAAATTCCTCTATTACCTGAGTTCAGAGTTAAAGTAAAACGATATT
GATGAAAAAGGTAAATAAATTGATTTGTTATCTTCTATTTTTTTATTCACCAACAAATACTCAGA
AAATCTTTACCTTCAAACCTGTGGGTTGAACATTGCTCAACCCCTACAACACCGTCAAACCCATCC
AAATCGGATATTCTTATATCAAACCGAGGTTATATGATCCCCTCTAATTCTAACTCCTCAATCAT
TTGTAAACCACGAGGATCACCCAATTTTAGTAAAGCCATTTTCGCATCTTCCTTAACCCCTAAAT
CCTTATCCTCTACTAATGCCTCAATTAAAGCATCGATCGCAGTGGCATAAACAACATTGTAAGGT
AATTCACGACATAATTGTCCAATAGTCCAACCGCAATTACTACGCACTGCTGGAACTTCGTCTTG
TCTCAAGGCTTTAATTAACACTGGTAAAGCAGTAATGGTATCTTCATAATTCAACTTAGCAATAT
TTGCCAAACTACTAGCCGCCCATAAACGAACCGCACTAATATCAGTTTTAAGAGCATTTAACAAA
GGTTCAAAAGCTCGCTCGTCGCCATTATTACCCAAAGCCCAAACAATGCCTTTACGCACATAGCCA
TTCCAATCGTTTTTGAGAGTATTAATTAAAGGAACAACAGCACTTTCATCGGTATTTCGTCCAAG
GGCATAAGCAGAACTAACCCGAATTAAAGGACAACTATTATTTAAAAATTCGATTAAAATAGGA
ATTGCTCTGGGTTCGCTAATTTCGCAAAAAGCCCGAACAGCTTGGATTTGCTCCTGTTTATTATC
ACTGCGAATCAACTGTAACATTAATTCTGGATCAGGAGCAGGAGAAGACTCAACCTCAATGTCGT
CCAATGGATCGAGGTCGGAATCAAAATCAATAGCGGTTTGCTTTAAACTTAGATCATTCATATAA
ATTAATGATACATGATCAGCCCCAAGAGGTTAATAGTTTAGTATCAATTATGACGAAAAATTACC
AAGAAATAAGTACTTCATCAGAATACCAAAATGCGTTCCCCAATGAAACCCTCTCAGTAATTGCT
TTGGGGAGTAACTTAGGAGATAGTCAGAAAATTTTAAATGAAGCCATTTTCACCCTCAAAGAAAC
AGAAAAAAATACAATTAATTAATCATTCCCATTGGTATCAAACCAAGCCCGTAGGACCTCCTCAAC
CAGACTACTTAAATGGCTGTGCCATTATCAATACAACTCTTAGCCCTCATCAACTGCTCAACACTT
TGCTCGAAATAGAAAAAAAATTTGGCAGGGAAAGAAAAGAAAAATGGGGCGCTCGCACCCTTGA
CCTAGATTTAATCTTTTATGGAGACTTAATTTTAAATACTTCCACCCTAGAAATTCCTCATCCAA
GAATGAGAGAAAGAACCTTTGTCCTTGAACCCCTTGCAGAAATAGCCCCCCATTGGATTGATCCC
GTAACGGGTTTAAGGGTATCTGAATTACTAATCAACATCTCAGAAAATAAGACGTTAAATTTAG
AGGCTTAGAGTGTCACGGGGATGAGAGGAGAGGGGGAAATAAGTAATGAGTAACGAGTAATTAT
CAACTATTCACTATTTACCTTTGCCCTCCCCCCTCGAGAGGGGGGATAAAGGGGGGTTTGCCCTTT
TAACTTTGCTCCTTACTTTTTCAGCAATTCTAAATAGTTAAGATAAAATTAATATTAATTTTTGT
AACAAAATATTAATTAAATGTGACTGGATGAATAACCAGAAAAAACTAAAATAAATCGCAGAAG
AATAGGGATTATAAGGTTAAATAACTGAAGTAAAGAAGAATAATTCCGATAAAAATTGTTAATA
AAGACTCTTAATTTGATAAAGTAGATCACGATAAATCTAACTATACTTATTCTTTAGCCAAAGAG
ACTAAGTACCCATTAAGATTAAAAATTTAAAGAAGATTGACAAAGAGAGGTTTAACTATGAGAT
TTCGTAGCCTGTTCGTGGCTTTCCTCATCGTCTGCTTAGGTTTTTTGAATGCTTGTAGCGATAAT
GTAGCCAAAGCGTACGATCCTAATACAATTACTTATGATGAAATTTTAAATACTGGATTAGCAAA
TAAATGCCCCGAAATTTCTGAGTTTACTCGTGGTAGCATTGCTATTTCTCCTGATGAACCTTTAG
CTATTGTGGATATGTGCTTAGAACCACAAGAATACTTTGTAAAAGAAGAACCTGTAAATAAAAG
ACAGGAGGCTAAATTTGTTCAAGGTAAGCTCTTAACCAGAGATACCACCAGCTTAGAGCAAATCA
GAGGTACTTTAACCGTTGATGAGGAAGGGGTTTTAACCTTAACTGAATTAGACGGTATTGACTTC
CAAATCGCTACAGTTTTATTACCCGGAGGCGAACAAGTACCTTTCTTCTTCACCATCAAAAAAAT
GGTTGCTACTACCGAACCCGGTTTCACTGCGGTTAATACTTCTGCTGACTTTAGTGGTGAGTTTA

AAGTGCCTTCCTATCGTGGTGCTTCTTTCTTAGATCCTAAAGGACGTGGTTTAAGTAGTGGTTAT
GATAACGCTGTTGCTTTACCTGCTGGTGCGGACAAAGAAGATTATCTTCGCAGTAATTTAAAACA
GTATGAAACCAGAAATGGCGAAATTTCTTTACAAATCACAAAAGTAGATGGTGAAACTGGAGAA
ATCTCTGGAGTATTTGAAAGTGAACAACCTTCTTCTACTGATTTAGGTGCAGAAGAACCCGAAGA
AGTTAAAATTCGTGGTGTTTTCTATGCTCGTTTAGAACCCCAAATCTAGGTTATTAGTTCGGAGT
TCGGGGTTCGGGGTTCAGGGTTTTTTAAAGTAGGTAGGGGTTGAATATATTCAACCCGTACAAAG
TGGAGGATGAATCTTCATTCACCTGAAACCTGACACCTGAAACCTGACACCTATTTTATAAATTC
TACATTTTTCATTCTTCAATATAATGTCTTTATTTGATTGGTTTGATAATTTGCGTAAGTCTGAA
CCAGAGATAAAACCTCAGCAGGAGAGAGAAATTGCTGATGGTTTATGGACAAAATGTCCTAAGTG
TGGAGTTTTATCTTATACAAAAGATTTACAGGCTAATAATATGGTTTGCTTGGAATGCGAACATC
ATATGCCTGTTTATAGTGATGAAAGAATTAGTCAGTTAATTGACCACGATACATGGCAACCTTTA
AATCATCATATTAATCCCACAGATCCCCTCAATTTTCACGATCGCAAGGCTTATAGCGATCGCCTC
TTGGAATTAAAAACAAAAACTTCTCTTACCGATGCAGTACAAACTGGTGTTGGTTTAATTGATGG
TTTACCCATTGCTCTAGGGGTGATGGATTTTCGTTTCATGGGGGGAAGTATGGGGTCTGTTGTGG
GAGAAAGATTGACCCGCTTGATCGAGTACGCTACTGCGAACATGATGCCAGTGGTGATTATTTGT
GCTTCTGGTGGTGCAAGAATGCAGGAAGGAATGTTAAGTTTAATGCAGATGGCAAAAATTTCAG
GGGCGTTGCAAAGACATCAAGAAAAAAAACTTCTCTACATTCCCGTCTTAACTCATCCTACTACT
GGAGGGGTAACTGCTAGTTTTGCGATGTTGGGAGATTTGATTTTAGCTGAACCGAAGGCAACTAT
TGGTTTTGCGGGAAGAAGAGTCATTGAACAAACTTTGAGAGAAAAATTACCCGACGGTTTCCAAA
CTTCAGAGTATTTATTACAACATGGTTTTGTAGATGCGATCGTATCTCGTACTCAATTAAAGAAA
ACTCTTGCTCACTTGATTAGTTTACATCAGCCGTTTTATCCCATAGGAATGAAGAATTAAGAGGG
AGCATATCCCCGGCAAATGTGAATGAAGTTGGAGAATTTGGTTAAAGGGAAAGTAATATAGAGT
GAAAATATATTGAAAAAATAAACTCCGATGATTCAAAATAATTTAATACTTGCCACTGTTGATCA
AAATCTTAATAATGCGACTTGTTTACAAACTCTAAGATGAAATATTTAAAATTAATCTCTTTATT
TCTAGCTACAGTTTTAATTTTTTATAGTTGGAGTAAATGGAGTTATGGTCAACCTATAGTTGACA
ATCGAGGTGAAATCAGGGGGGTTTGGTTAACAAATATTGACAGTGAGATTCTTTTTTCTTCTCAA
AATACACAAAATGCGATCGCATCTCTCAAGGAACTAAATTTTAATAGTCTTTATCCTACCGTTTG
GAATTGGGGTTACACCCTTTATCCCAGTCAAGTGGCAGAAAAAGTTATTGGTAGTAAAATTGATC
CCCACGAAGGTTTGCAAAATAGAGATGTTTTAGCTGAAATTATTACAAAAGCTCATCAGAGTAAT
ATGAGGGTAATTCCTTGGTTTGAATTTGGTTTTATGGCTCCTGCTGATTCTGTTTTAGCCACGTC
TCATCCTCAATGGTTAACCAGAAGAAAAGATGATAGTGAAATTTGGATGGAAGGCAAAACCCAT
GAAAGAGTGTGGTTAAATCCCCTTCATCCCCAAGTACAAGAATTTATCACAGATTTGGTATTAGA
AATAGTTACTAACTATGATATTGATGGGATTCAATTTGATGATCATTTTGGCTACCCCTCTGAAT
TTGGTTACGATAGCTATACAATTGAACTATATCGTAGAGAACACCAAGGCAAATTACCTCCCTTA
GACTATCTTGACCCCGAATGGATTGAGTGGCGATCGCACAAAATTACTGAGTATATGGAAACCCT
GTTTCACAAAATAAAAGCAATCAAGCAAAATGTAATAGTTTCCGTTTCTCCTAATCCTCAAGAGT
TTTCCAAAGAAATGTTTTTACTAGATTGGGCAAAATGGGAAAGAAAAGGCTTTATTGAAGAATT
GATAGTACAAATTTATAGAGATAATATGGAGGCTTTTAACCGAGAATTAGCCCAAAAAGACTTA
CAATTAGCAAAAGATCATATTCCAACTGCGATCGGTATCTTAACAGGATTGAAAGGAAGACCAGT
TAATCTCAACTTAATTGAAGCACAAATTAGCACCACCAGAGAACAAAATTTTGCAGGAATCTCTT
TCTTTTTCTATGAAAGTCTTTGGAATATTGCCTCAGAAACTCCTCAAGAAAGGCAGGAATTCTGG
GCAAAAATCTTTAGTGATAAAGGAGTTTACTAATTGAACGTTAGCTCTACACCGCCTAGTGGAAA
TAAACCTTTACAATAATTAACAATACACATTTGAATCAATGAATTATCAACCGCAATTATGCAAT

CAATTTTTAATTTTGAACAATTTATTAACTACGAATTGCTAAAAAACATTTACGAAGGTTGGCAA
AAAATAAAAGCAGGGTTAAGTTTAGCCCACAAAAATTTTAGCACCGAAGTTCGTCAAAGAGTATT
TCCCTATCAACTACCCCTAGAAAAAGTTTCATCAGAAACATGGAAGGTTTTACAGGCTAGACTAG
ATCAAATTATTGAAGAAGATTGGCAGGATGCAAAAGAAGGAGTTTATCCCATTAACCTTCTATTT
GATGCCGATTGGGGAAAATTTTTACAAGCATATATCGGTATTTGGTTCGACTATCCTCAGACATG
GCAAAGAATCAAAAATAATAACTTTCAAGACTTACCCTCTAATATTGACTCCAATAAATATCCTC
AATACTACCGTCGCAATTTTCATTATCAAAGTGACGGCTATTTAAGTGAACATTCTGCTAACATC
TATGATTTACAAGTTGACATTCTCTTTAACGGTGTTACTGATGCTATGCGTCGCCGTATTCTCAA
ACCCTTAAAACAAGGCTTAAATACTTTAAATTCTAGTAGCAATCCGAAAATATTAGATGTTGCCT
GTGGAACTGGTAGAACGTTAAAATTCATTCGGGCAACTTTTCCCCATGCCAGTTTATACGGGATA
GATTTATCAGCCCCATACTTACGCAAAGCCAATGAATTATTATCCCAACTCCCTCAAGAATTGCCC
CAATTAGTAGAGGGTAATGGGGAAGAATTACCTTATCAAGATAACTATTTTCAAGGTGTCACCAG
TGTCTTTCTTTTTCATGAGTTACCTAATCCAGTGAGACAAAAGGTAATTAATGAATGCTACCGTG
TTTTGCAAACAGGTGGAATATGTATTTTTGCAGATTCGATGCAATTATCTGATTCTCCCGAATTA
GAAACGATGATGACTAATTTCCCCATTGCTTTTCATGAACCTTTTTACAATGATTATATTCGGGA
TGATTTAAAATCTCGTTTGAATGTAGCTGGTTTTAGAAATATTCGGGAAGAAGTTCACGGTTTTA
GTAAATATTGGATTGCGACAAAATAATTGATTCAAGTTATGAGACTGAAGCTGAGATATAATAC
CTGAATTTTAGTATTAAATGGCCTCAGTTCGGTTTAAGAATATCGGATAAGTTTAGGTGTCGGGT
GGTAGGGGTTGAATATACTCAATTCTTACGGTGTCAGGGAAGACGAGAGTTCGCTGTGCTTGTTC
CGCAAAGCGTTACGGGGTTAGTTAGGGGCGAATGGCCATTCGGCCGTACAGGAGTTTTTAATTCT
TAATTCTTCGTTTTTTCTTTGCCCTTTTTACTTTTTGCCTACCCTCCTGACACCATTTTTTTGTCA
GCCTCTATATGCTACCGACTTTTTCGAGTAAGATGTGAAAATGATAAAGCTAAAGTTAAATATTA
ATTAAGGATATACCTCATAATGGTTGACTCGATTATGTATCAAGAGGATGGTTATGTTGTGTTAG
AACCTAATCAGCCTGAACAATTATTAACAGAGATGGAATTATTACAAAAATTAGAACAAGTTAT
CGAAAGTTGCCAAGATATACCTTCAGACATTAATAGTATTGACTCTCTTTCGGAAAAAGCAAAGT
ATCTTTTAGAAAACTATTGCGAATTTAACATTGATGATGATAACTCCTTACAATGGTATGTGGTG
CGCTGGGAAAAAAGATAGATATAACAATCATGAGTTATTTTACTGATTTTAAAATTACTTTTGCT
CCTCTGTGTTTAACAGAAGTTTATCAATTAGCTGATAATCCTGCCAATGGTGCGATCGCACTCAT
GAGTGGTACAGTTCGTTGTCAAACAGAAGGAAAAAAAGTAGAATACTTGGAATATCAGGCTTAT
GAGCCGATGGCAGTGACAATATTTAAGCAGATAGGAGCAAATTTAGTTAACCAATATCCTGACAT
AAATAGTATTATCATTCATCATCGTGTTGGTAAGTTAGTCATAGGAGATATTAGTGTTTTAATCG
CCGTAGGATGCCCTCATCGTCAGGAGGCTTTTAAGGCTTGTCAATATGCCATTGATACTCTTAAA
CATAATGCACCTATTTGGAAAAAAGAACATTTTGCTGATGGTGCTAGTAGTTGGGTAAGTATTGG
AATGTGCGAAGCACTTATATAAACCTCAGTTCGGTTTAAGAGTATCCGATAAGGTTAGGTGTCAG
GTTGCAGGTTGCAGGTGTTAGGGGATGGGGGGATGGGGGGATGAGGTGATGAGGGGAAAGGGGA
AAACAAGTAATAAATAATAAATAAGTAGTAAGTATTCGTGGTTTTTCATTCTTAATTCTTAATT
TTTTATTCTTAATTCTTAACTATTTAACGTCAGTTCGGATTAAGGCAATTTTATCGTTATTTCTA
AGAAGCTATAAGGTTTTCTGACTACGAGAAAATAATGCTTTCAGCTTATCCAAAACTCAGGTTAA
TTAATCAATTATTAAGAAAAAAACGCTCCCCTCTACTGTGGGAGAGGGGTTGGGGGTGAGGGCAAG
ATAATTTAGCCCTGACTTTGAAAAAACCCTGCCTAAAAAGGGGATAAGGGGCAAAGGTAAAGAA
TGGGAATAAGAATTAAAAACTCCTAATCCCAAACTCTGATTCATCGACTGGTGACTAATTCATTG
GACAATTGACTAATGGCTTTAAAGGCAACATCCCATAATTTTTGGGGTTGTAACTCTGGTTGAAA
TAATGTCCATAGTCTTTTCACTTCTTCTGTATGAAGGCGGTCATCTTCTATTAGAGCGTTTAATA

GTTGACGGCGCAAGTATTCTCCTTCTTCTGAAACGAGAAACTGTAGTCCTAATCCTGCGGTGGGT
AAAAGGTTAAAGCGATGATCAGAACGTGCGATCGCAATCATATTTTCTAATCTTTCCCATTGAAA
CTTACCGTCTTTGAAAAGCACTTCTAGTAAACGTTTACGCATGGCGGGAGATTCTTCTGTTAAGA
GTCTTCGGGAAACGTAAGGGAAGGATACTTCAACAATACGGAAATCTCGATTTAAACTTAATGCT
AATCCTTCTTGAGTTACCAATGAACGGATAATCAGGGCAAATTTTGCTGGTACTCGGAAAGGATA
TTCATACATCAATTCAGAAAAATCATCGGTGATGGTTTTAAAGTTAAAATCCCCTACACTCTGAT
CTATGGCATTACCAAAAACCTTTTCTAAGGCTGGTACAATGGGCATAATATCTGTTTCGGGAGTT
AAAAACCCCAATTTGACAAAATCACGAGCTAAAGCCTCATAATCACGATTTATCAGTTGTACCAC
AGAAGATGCGATCGTTTCTTTTGCTTCTTCGCTTAATTGATCCATCATGCCAAAATCGATGTAAG
CCATTCTACCATCGGGCATAGCAAATAAGTTACCGGGATGGGGATCAGCATGGAAGAACCCATGT
TCTAATAACTGTTTTAAACCAGATGTAACTCCCACTCGAATAATATCATCTGTGTTCAGCCCTGC
GGCTTTAATCGTATCTAAATCGTTGAGTTTATAACCGTTAATCCATTCTAAAGTTAAAACATTTT
TGCTGGTGTAACGCCAATATATCGCAGGTACTTTCACCTCTTCATCATCACGGAAATTAGCGGCA
AATTTTTCTGCATTCCTACCTTCATTTAAGTAGTCAATTTCTTCAAATAATTTAGTCCCAAATTC
ATCAACAATTAACGCTAAATCATGACCTAAATTTAGGGGTAAAAAAGGACTAATCCAATGGGAA
GCCCAACGCATTAAATATAAATCTAGGGTAATTACAGGCATTAAATTAGGACGTTGTACCTTAAT
CGCCACTTCTTCTCCTGTATGCAGAAAGCCTCGATATACTTGACCTAAACTAGCGGCGGCTACTGG
CTTAGGAGATATTTCTCTGTAAGCGTCTTCTATTTTCAGGTCTAATTCTTTTTCAATAATACTGA
AAGCTAAATCATTATCAAAAGGCGGTAGTTGATCTTGTAATTTAATTAATTCTTCTAAAAAATCC
CTACGAATTAGATCAGGTCTGGTGGAAAGTGCTTGACCTACTTTAATAAATGTAGGTCCTAATTT
TGTGAGTATTTTTCTTATTTGTTCGGCTCTTTTTAGTTTGTTTTTTTCTTCTAAATTAAACCACT
TATCTAAAATTAATTGGATAATAAAAATACCAAAATAGGTAATAATTTTTAACCCTCTCCAAATT
GCTAACCAAGGACGACGATTGTAATATTGTGCGATCGCATCAGGAGAATATCTCAGTAAGGAGTC
ATTTTCAAGATTTTTGTCTGAAAAAGCGGATTTATTCACTTTTTGAATCAGTTTTAATATAAATA
ATTTTTAATAGTTAGGGTTTATTATAGCAGGGACTTTGTTCGAGTGAGTTAGGAGTTAGTTAGG
AGCGAATAACCATTCGCCCGTACAGAGTTTGGAGTTAAGAGTTATTAATTGTCAATTATTTAATA
ATCCCCTATTGACTATCCTAACTAATAATTTACAGACTCAAACTGATAGAGCCCCGATTTTTTGA
ATTTCCATTATAAATTGATGAGAGATAAAAGGAATTAAATTTCGCTGACGAGTTGCTTTTCCTTC
GGCGAAGATGACTAATAAATAGGAGGTTGAATTAGGTAGGGTAATATATGCACAATCATGACGT
ACTTGCGAAGTCCATCCAGCTTTAGACCAAATTTGAGCATTATTAGGTACACTTTCGCCTATAAA
ACCATTAATTTGATTTTCTTCGTCATTTATCCCTATCTTTTTTTTACTGGCATCTCTTTTTAGCAA
ATTTCGCATTTTTTCACAGGCTTTCTCTGTGACGACTAAATTATTCATGATGCTATAAAGTAATC
TAGCAACAGCATTGGTAGTTAACATATTTCGATTCTCATAGTTTTCACCCAAAAATATTCTTTCT
CTTCCATAAGCACCATCGCACCAAGTTTTCTGATTAACGTTGATAGAGGCAAATTCTTCCCAGTT
ATAACTCTGGAAATAGTCATTTATCATATTCCTTTGTTTTTGCCATTGAAGAAATTCTAATTCTG
GTAATTCATACCCTGACGTAGTTTGAGTTAGAGTATCCATAACTAGACTGGTGGCATCATTACTC
GATTCTACAATCATATCTCTGACAGCGCGATCGAACTCTGAGGAGGGTTGAATTTTTCCCTGATT
TAACCATTCTTCCACCGCCACTAAATAGAATAATTTGACAATACTTGCAGGATAAATTTTTTCGT
TTCCCCGATAGCTAAAACCCTGCAAAGGAGATTTCTCTTTATTCTCCATAACCACCGTCAAAGCA
ATGTGATTTTTTTTTAACGAAGGGAATTTTTGCCATGTCTTTTCTAAAATATTCTCTGTGGTTTG
AGAAAGATGATGATTAGTGACAAAAAAAGTCATTGGAAATCTCCAAAAATTGATAATAAAATTA
ATGTAACCAATTTTTCTCTTAACTAACCTCGATAATTGATTATTAGTTGTTAAAGTATAGATGTT
AAGTTTTATAACAATTGAAATCTTACTATGTTAGAACTATATCAATTTGAACTTTCTCAATTTTC

CGAAAAGGTTAGGCTTATCCTTGACTACAAGGGCTTAGAATACAAAAAAGTAGAAGTTACCCCCG
GTATTGGACAATTAGAAGTTTATAAAATTTCAGGACAAAGACAAGTTCCCGTATTAAAAGATGG
TGATACTGTTATTGCCGATTCAACGGAAATTGCTTTATATTTGGATCGCAAATACCCTGAAAAAC
CTTTAATTCCCACCGATGGAGTTGCTAGAGGTAAATGTTTATTGATGGAAGAATGGGCGGATGAA
TCCTTGGGTTTAAAAGGGCGTAAGGCTTTCTTTCATGCGTTAAATCATTATCCCAGTTTTCGCTC
TTCTTTTCTGCCCGAAAATACCCCTGATATTCTCAAGACTTTTGTTAATGCTTTCCCTTCAGAAAT
TATGGATGTTTTCAGTTTGGGAGTAGGTTTCGGAAAAGATGTTTTAAAAGAAGCTGAAAAGGGT
TTAAAGCAAGATTTAGAGGCTTTAACTTTGATTTTACATGACCAACCCTATCTAACCGGAGATAA
ACCAACTTTAGCCGATTTGACTGTGGCGGCTTTGACGACTATCATTAAGTTTCCTTCTGTGGCTT
ATTATGATTTGGATATTAATTTAGAAGGGAAGGGTATTCCGGGTTTAGCAGATAATAGCGCTTA
TGAGCCTTTCTTTACATGGCGCGATCGCATCTATGCTGATTTTAGACAACCCGTAGATAAATCTA
ACCGTAATAATTCTAGTGGAAATAGTGGAGATAGTAAACCAACCACCATTGAGATTGAGTAGTTT
AGTAATTATAATCATTCAAGAGAAAGGTTTATAGAGGCTTGGGGTATAGGGATGTTAGGGTGGT
AGGGGTTGAATATATTCAACCCTTACGAGAAGTTAATTAAACACTTTTGCCCTCTCCCCTCTCGA
GGGGAGATAAACGGGGTTTCTTTTAAAGGGAGACACAGAGGGGTTTGCCCCTTGCCCTTTTAATT
GTTAATTTTTAATTGTTAATTGGATGGAATTTGAAATTACTGGAAAAGATTTATTTAATTGGTA
TCAATCGGCAAAAAGAGATTGTTTAAATAACAATATTAATATTAGTGAACTTAATTTATTTATT
ATAGAATTAACATCTTTAAACAAACTTAATTTACAGTTAAATAATTATCAAAATCAAGAGTCAA
TAAACAGTAAATTTTCCTTAGATAAATTAGAAAAAATATGGAATTTGAGGATAACTAAACGTTG
CCCTATTCAATATCTAATTGGTGAGTGTCATTGGCGTAATTTCACATTAAAAGTAACTCCTGATG
TTTTGATTCCTCGTCCTGAAACAGAGTTAATTATAGACTTAGCTTCAGAAATTACATTTAATTAT
TCTCATTTAAAAACAGGTAATTTTCTTGATTTAGGAACAGGTAGCGGTGCGATCGCACTAGGATT
AGCCGAGGCGTTCCCAAATAGCTATATTTATGCAGTTGACAAGAGTAAATCTGCGTTAAAAATTG
CCCAAGAAAACGCCCTTAAATACGGTTTTGAGTCGAGAGTTAAATTTTGTCATGGTTCTTGGTTT
GACCCTATAAATGACCTCAAGAATAGCTTTAGCTTGATTGTTTCCAACCCCCCTTATATTCCCTCA
CAAATGGTATTAGAATTAGAGCCTGAAGTAGTAAATCATGAACCAAAAATTGCTTTAGATGGAG
GAGAAGACGGTTTAAAAGACATTCGTTATTTAATTGAAAATTCTCCCAATTTTTTAGCGAAAGAT
GGGTTTTTAATCTTAGAAATTATGGCAGGACAAGGTAAAATAGTTGAAAATCTTTTAAAAGAAA
ATGGCAGTTATAAAAATATTAAAATTGAGTATGATTTAGCAGGTTTAGATCGATTTGCGATCGG
TATAGCTACTACACCTTAAGTCACCCAAAGCTACGTTATGAGTGCATAGTCGCTTCCTCGGGAAA
AAATCATCAATTCTCCTCACGTTATGATATATTTTGAGGAGTCATTTTTGATGGTTAAATTGTAT
GAAAATTATCGACTCAAAAGGTCGTTTATTCGGCAAAATTAGTATTTTAGATTTAGGTGCGATCG
CAGTTATAGTTTTCGTTTTAATTGGTATTTTCATTGTACCAGGTCCAAAAGGTTCGATCGCACAA
GTGGGTAGTGGGGGAGATACTATAGAATTAAATTTATTGGTAAGGGGTTTAAGCGTCAAAAATC
CTCAAGAGTTTCTTAGCTCACTAGAAAATTCTTCCGTTAAAATTATCGTTAGAAATGAACCGGCA
GGAAGTTTAAATATAGAATCAGTGGAAGAATTGCCCAATTATGTGGTTGCAACTCAACCTGATGG
TAGCGTTAAATCTTTATTAGACCCTCGTCCCATTGTTGCTTTAAGTACAGATATGATTCTATACA
TGAATGGCAAAGGTTCAATGACTAATGATGGCGCAATTTTAGCTAACCAAAAAGTAAAAATTGG
TACAGTGTTAGAATTAGACGGCAGCAATTATAATTTTCGTGGCAGTGTTATTGATGTCAGAAAGA
AAGAATAACCGAAATTCAGAATTCCGATTTAAAATTTAGATTATAATAGCTTGGGCTTTGCCCAG
CTTTACCTACACTTACGGATTTAAAATAACGGCGTTGGTCAATTAAGCTATGATTTTTAATTTAG
AGCGGAAATGGGCAATAAGCAATGATACTTGAGTGTTGGACACTTGGAGCAATTCAATAATGCTT
TGCATCACAATTAATTAAGCAACCCCAAAATTACCATATCAACTGTTGTTTGCACAATGTTGTGA

TTGGGCAACGATAACCAACAATTTATCGATAGTATCTCTTAATTCTTTGCTTAATGCCGAGGAAT
TATTTACCATAATAGAAAAAGCTACATCATCATCTTTAGTTCTTAAGTAGCCCGATAAAGCAGAT
ACTCCTGTCAAAGTTCCAGTTTTTCCCCATAAGTTATTGTCGATTATATCTTCATTCTGAAAACG
GTTTTTTAGAGTGCCATTAACACCAGCTAAACTTAAAGAATTTCTGAAAGTTTTTGCATATTCAG
TTTTATCCATTAATTTTAATAAAGTTACAAGACTTTTGGGAGTTGCTAAATTTTGACGAGATAAA
CCAGAAGCATCCTTTAGAGAATAATCATTAGATAATAGCCCAACATTTTCTAATATCTTTGTCTG
GGAAATAAACTTTGTATCTTGGTTCGTTGCTAAATAACTTAATAAAATTTCAGCATATAAATTAT
TACTATTTTGATTAATTTCCGCAATTAAATTGTTACTATTAGGAGAATTAATTTCTAACAACAAA
TTAGCATCATTTAAATCTATATTTTTTTGATCGATAATTTCTGTTTTTTCTACTTGGATACCATT
ATTTTGCAGAATTGCTATTAAAGAATCTTGAAAATATTGCTCAGGATTGGGGATAGATAACCACC
AATTATCATCATGATTATTAACAGGGAGATTTCCCTCAATTCTTAACCTAGATTCTAAACCCATA
GGGTTTAAATTAATACTATATTCTTCATCTTCACTAACAGTTACTCCTTCATTGGTAATAAGCCA
TTGTTTTCCTGCTAATTTATCAGACCATTTTAATAATATTTGTTTACCGACTTGACTAGGGGTTA
AACTTAAAGTAACGGTATTTTCCTCCAAAATAAAACTATTAATAGGAACAGCATAGTAAAAATA
TAAATCTGAAAATTCCCAGCTATTATTAAAAGGATATTTAATAGGAGAATTATTCACTAAAATT
AAATTTTTAACATGATTAATATTTAACTGTTGCAGTTTTTTTGCTATATCCTCTAAATCAGACTT
CGTAATAGTGGGATCACCTTTTCCTATAATAATTAATTCATCTATAAATTCTTCTTTTCCCTTAA
TATAAATAGGAGTTTTGATAGTAAAATTTTCCCCAAAATTTAGTAAAAAAGAAGCAGTAGTGAA
TAATTTAACATTAGAAGCTGGAATAAAATATTTATTCTCATTTAACTTATATAAAGTCTGATTTT
GATTAAGTTTTTCGATTAAAATCCCCCAATTTTCTCTACTTCTTTCTGACTGATTAAGAATGCTA
TTAATAGCTGATTCTAAACCTTGAATACAAACCTGACTTTGATTATAATCAGATGATTCTTTTAT
CTCTGCTTTACTACTTAAACTATAAAAAAAGATATTATAAAAACCGATTAAGATACTCAATAATA
CTCTATAGAAAATATTTTTTTAATCATTGATAAAACCTATAAATAGAAAAATGCCAGAGAAAC
AATCCCTGACATTTAGCAATTATATTATTATCCGATAATTATGAGTAAAATGTAGCGCCAATAAA
AACCTTGTCTGCCACAATATCTCAATTTATTTATTTAAGATAATAAAGTTTTTCCGCTTCTAATT
GTTCAACTAAAGCAATTTGACCTTTTGCTTTTGCAACTTCTATGCGACGCTCTAAACTTTTAATT
AAGTTTGTTCTGTGCGCCTGACGAGCATTTTCAAGAGTTTTTTGACTAATCATTTTCTTTTCTCC
TATATATAGATTCAAATTAAAGATATAAAGAGAGAAATAGAGAATAAAATCAGATAAGCAAATT
TATATCCCTTCTCTCTTTTGCCTATGCTTATTGATATTTACTATTGAAGTCCTACTTGTTCAGATG
CTCTTTCTAACATAGTAGCTTGACGATTACTTTTACGTTGACGATTATCAACCATTAATAAACGT
GCTTGTTGACCAATAGAACTGATATTGTTAGTATTATTATTGAATTTAGCTTTCATAATTCGCCT
CCTCTAATTTTCGATGAGTTTAGATTCGAGGCGCGTTCCTTCAGAAGCCATTACTTCTTACTTCCG
TCTGACTATTAAGCAGATGAACGATTTATCCTTACTCTTTTAATATAACTAAAAAGTATCAAATT
TGACAACTTTCTTCTCTAACAAATTGTAAAGACTATAAGTATGATATTTTGGGAAAAATTGCTTT
ATTTTCAATTACTAAATGTAAGTTTAGTTCAATACAAAAATATCGGGTTTAAGGTGGTTTCAAGT
TTCAGGTGGCAGGTTTGACGTTGAGTATTTTATCAAAATTTATCAGTCAATCAAAGAATTTAAAG
AAAAAAATCAATAAACATAACTTTTTTGTAAATTCTGATGACTCTGGATTATAAAAACAAAACT
ATTGCCCGTTACTTCTTGTTTTTTCGCCTACCTTCACCTAAGAAATTTTTCCACAAAACCCAATTA
CTAATTCCTCATTTCTCAAGAAACAACACAACTATTCTTCTGTTTCTTCTTCTTCCTTAGTAGGAT
AAACGAAACCGTTTGCTTTACCAGTTAAAACAGATTTACCTAAAGATAAGGCTCTTTGAGCTTGT
AATTCTGCTTTTTTAAACCAATGAGCGCGACGTTGATTACGTTTAGATTTAGATGTTTTCTTCTT
AGGTACTGCCATAGGTTTTAAATAATATAATAAGTATTTTAGTTATTGACACAATTTTACTATTT
TAACCATAGTTGATAGATCGTGACAATAGTTTATTTGCCCGTATCAGTGGTTAATTGTAATATTT

CAGGGGAATCAAGGGTAGAAGTTGTTTTTTACCAGACAAACAAGATTTTATTTTTGCCAAAACT
TTTTCTTGATCAATCGGTTTTCCTAAAAACCCATTAGCACCGACTAATTTACTTCGGAGTCTGTC
AATCATACCATCGTTGCTAGTAAGGATAATGATAGGTACGTCTTTCAACGCAGAGACACGACGAA
TTTGGGAACAAAGTTCATAACCATTAATAATAGGCATGACTAAATCCAAAAAGATTAAATCGGG
TTTTCTTTTCAATATTTGCGGTAAGGCTTTTAGAGGTTCTCTAATAGAAAGATATTGATAACCTG
CATCAGTGATAATATGTTCCATTATTTCACAAATTTGTGAGCTATCATCAACGCAAACGATTAAT
TGTTGATAAGTGGTTTTTGTTATTTTATAATTCTGATTCGTGACACCAATTAGGGTAATAGCAAC
AGGTTGGTCTTCAATTTCGATGAGGGTAATTAAGTCTTGTTCAAAGTAGGGTTTAACCATACGG
TAATTTTAAAAATAGTTAGGTTTAATTTATCTGCTATTTCTGTTAAAGGATACTTCCCATTAAGT
AAAGAAACTAATTTTTTGTAGGTTTTTCTTTTCCTTTAAAATGTTGTTGCAATAAGACAGGATT
TTTAATAACAGGTGCATAATGGGGAATTAAATGGGTTAAACCGAATTTTTGCCAATGTTGCCATT
CGACTTCTAACTCCATCAATATTGATTCAATTTTTAGTAAACTAACAGAAGTTTTAGCCCAGAT
TCTTCCGGAAAATTGTCTTCAATGGTAATGACATCATATTTTAATTTTTCTATAAATTCAGCTTG
ATAAATATCAAATAAAGCCTGTTTAATTTTAATTTTTATCAGAGATAATGCTTGTTCTTTCGTAA
TTAAAAACCTTTGTAATAAAGAGACTATTAAATGATAGTTCCAACATTCAAATTCTTTGGATTTT
TGAATATCTAAAAGATGAGTATCTAAGTCAGGACAATTTTTTTCTAGCAACCTTTGCCATTCACG
ATAAGGATGATAACCGCCATCTGCCCAGACTAAACGACTCAAACAAAAATAAACACGCCATTCTT
TTCCGTCTGCTTTTTTGACATCAATTCTTCCTGTGAATTTTTTCCCTTTCAAAGATAGTAGTGCTT
TTTTAAATACTCCTGAACTACTCATTTCTTTCCTTTTTATGATATTAACTTTAGTTGGTAAATGA
CGAGATTAACAATGAGAGCTATTGTTAAGAAATGATGGTTTGATTGCTATTGCTAGTACGACAAT
TTTCTGCTTTTTAATATTCTATTACTAATGTGACTAATTAAATTTTTGTTTGAGTGTTATTGATT
TTATTAATACCTAGATTAAATATAACGTTAAAAGTTTATTAACATTTAGTGAGCTTTAAAATAT
TTATTAATTGCTGTGTAGAGTAGTTTACCTTTGCATTTTGATTGATTTTCAGTTATGATTAGCAT
TGATATTAAGATTCGTTAAGTTTGATGAAAACTTTTGGCTGTTTAATTACTAGAGAAAAAGCAT
TCTCACTTTCTTATTCTTACTCCATTGGTTAGAAGTTAAGGTTAAAAGCAAGGTGTAAAGGAATT
TTCAAAGAAAAAGTTTTTTTGATTTTTTCCCAATTCTGGAAACGGAGCAATAATTAATTTCTGCC
TTGGTTTTCTCTTCCTTTTTATAATTCCTAAATCTCTGTTTTCTGGGGCTGAAAAATATTTAACC
ACCTCTTTTGCTTTGCCTTTATTGTTAGCAACTATAAAGTGGTACAGTCCTCTATATATCATTTC
TATTGAGATTGAATCAAATGGCAATGATAATTCCTCTGCTACTGCGTCTCCTAAGTCTATTAATA
CTCCATAAAATAGCCAAGTTCCCCATATTTGTAACTTAATTCCATTGATTGAACCTGTCCATAAA
TAACTCAGACCTAATAATCTTTTTACTGTATTAAATGCCTCCTCTATTCGCCAACGTTTTCCATAT
AAGTTGACCACCACATGAGGGGGTAAAATTTCAGGACTTGTTACTGATGTCAAATAAGAATGCCA
CTTATTTTTTGACCTAATTTCAACCAGACGTAAGGTCACAATTGGCGTTTTCTTCCTTCCATCACC
TATATTTATTAGGCTGTCTCTCAAATCATGACTATTCGTAAATCGTTTTAATATTTCTATAGATG
CCCCTTTTTTTATTCTCGTAATAAAATCAATATTTCTTGTTATTAATTCTTGCCAAAATAGAAAA
TGATAAAATCCTCTATCCAACAATAATAGAGTCTGACTTTTTACTAAATTTAGAATATTTTCCTC
AAATTTAACCTCAGAAATACTGGGATTTTCTTCTACCCAAATTTCTACGGGTAATCTGGTTGCTA
AATCCATCACTACTGCCATTTTCCCCGCTAATTTTCCTGTAGGAATATCTTTTAAACTATCTAATT
TTCTGAACAATGCTTCCAATGTTGAACCATCAACAATCCAAATCTGCTTAAATTTTGTTAAAGAA
AAACAGACACTATCAGGGAGTGGACGATTCGTTCTTTTTTGCCATCTTTCCTTGAATTTTGGTAG
TAATTGTTTGAATACTTGTTCAAATAAAATGGAGGGAAAAGTTAAAAATCGGTCAGATAGTGCT
TTTTGACTTACTTTTGTCGGATCTACCCACAAAAAACCCTCCCGATTCAAAATACGAGTTAATTC
GTTGACACTGGGGACATTTCGCCATAAAAGAGTCAATATTGCTGCGGTCATTAATGATAAATTGA

GAACACGATTTCTCATTCCCAAATCACGAAACAGACTCTGTTGGGCAAAAATAGAAGGGGTTAAA
AGTTCAGACAAATGGGAAACAATAGCATCATTTTCACTCAAGGGATGATGTTTTTTCTGGGCGTG
GTCTCGGTTAATTTTCTTGGTCATTCAAAGTAAGTAAGAAAAAGTAAATAATCTTAAATAGTAG
CATTCATCAAGTTTTGAGCTGTTTATTAAGTTTTATTAGCAAAATATTGACTTTTTGCTCATTTA
TTTAACTTCTAGCTAATGTTCTTACTCTGATAGTCCCCCATAACTTAGATGTTTAGGGTAAGTAA
AGAGATAGTGAAAATATAGGATAATCTTTGACTTATATAAAAATATAAATCAACCTTAGTTCAG
CATAAAAAAGATAAGGGCAGGTTTTAGAGGTTGACTAATGTTCGATTTCGACCCTTATGATGAAA
GGTTTAGCGGTTGAATTTTTCAAACATTATTTCAAACATTAGATTTAGTCAATATCTTTAATTCC
TAATTGCTAATTGAAATAACCTTTACATTCCTGAAAATTTTATATCGAATTCAGGTTGGATTATT
AAATCCCTACTTTACGAATAGAGATTGAGCAAAATTATCCTTTACTACTCATGTTGAGATTGAGT
TTAACATGAAAAAATTTTTATTTAATTGTTAGTAATTTATAAAAAACAAAAACAATACAATGCA
AATAACTAATCAAATTCATTTCCGGAATCTCAGGGGAGATATTTTTGGAGGTTTAACTGCGGCGG
TGGTGGCTTTACCTATGGCATTGGCATTTGGTATCGCTTCTGGGGCCGGTGCATCTGCTGGTATG
TGGGGGGCAATCTTAATTGGCTTTTTTGCTTCTTTGTTTGGTGGTACTCCTAGTTTAATCTCAGA
GCCTACAGGTCCGATGACGGTTATTGTTACTGCTGTAATTGCTGATTTGATGGCGAGAAATCCTG
AAACGGGTTTAGCAATGGCATTTACCGTTATCATGATGGCTGGATGTTTTCAAATTTTGTTTGGG
GTGTTAAAACTCGGTAAATATATCACAATGCTACCTTATAACGTTATTTCTGGGTTTATGACGGG
CATTGGGGTGATTCTTATTTTTATTCAAATAGCACCTTTCCTAGGTCAAGAAACTCCTAAAGGTG
GCGTTTTTGGGGTGATCCAAAATTTCCCTGAATTATTAAGTAATATTAATCCTCCCGAATTAATT
TTGGGTATCATCACCCTTGTTATTTTGTTTTTATATCCTCGTAAATGGAAAAATGTTGTTCCGCC
TCAACTAACGGCTTTATTAATTGGTACGATTATTGCTACTCTATTTTTCAGTAGTTATGATATTC
GTACTATTTCCACTATCGGTGAAATTACCCCCGGATTGCCTAGTTGGCAAATGCCTACTTGGAAT
ATGGACAACTTACGCTTGATGTTTGTCAATGCCATTGTTTTAGCAACAGTAGGTTCGATCGACTG
TTTACTTACTTGTCTGGTTTGTGATAGTTTAACCCGTACAGAGCATAAATCTGATAAAGAATTGA
TCGGACAAGGAATTGCAAACTTAATTACTGGTTTATGTGGTGGTGTTGCTGGTTCAGGTGCTACT
ACTGCTAGTGTTGTTAGTATTCAAGCAGGGGGTAGAAGTGCTGTTGCAGGTATTGTTCGGGCTTT
AGTGTTATTAACCCTTTTTTGTCATGTTCCGAGATAAAATAATAGAAGAAAAAGAGAAATGGAA
CTTGGGAGGCTTAGATGATAATGGATGTAGCAGAGCAAGTTAGTAAACATTTGCCTCGAACTCCA
GAACCCACTATACCTTTAGTGGATCAATATTGCAGTTACTATCAGGATTTATTTCCTGATGTAAG
AAGTTATGAATATTTTAAGTTATTACATTTAGGAATAATCTCTAATCTTAAACGAAAATCATTAC
CAGAATTAGAAAAAGTACTAGGAGTATCATCACAATCCTTACACCATTTTCTCACATCATCACCT
TGGTTATTATCGGAACTAGACCAAAGAAGATTAAATAAATTAGTAGAGATATTGCAGGGGAAAA
AAATCATTCTCATAGTAGATGAAACAGGAGATAGAAAAAAAGGTAATAAAACAGACTATACTGC
TCGACAATATTTAGGAAATGTGGGGAAAATAGATCAAGGAATAGTTTCAGTTAATGTCTATGGT
GTTTATCAGAATATAACCTTTCCATTAATGACAAAAATATATAAACCAAAGTCAAGATTAAAAG
TAGGAGATAAATATAAAACAAAGACAGCAATAGCAGGAGAAATGGTAAGAGAATTAAAGGAATT
AGGATTAGAAATAGAAATAGTGTTAGCAGATAGCTTATATGGTGAAAGTAGTAGTTTTTTGGAA
GTATTAGAAGAGTTAAAACTGGAATATGTGGTATCGATTAGAAAGAATCATGGGGTGTGGATGG
GAGCAAATGAAAGAGTTAGAGCAAATAAATGGTGTAAATTTACTAGGACATTTAGGGATGGAAA
AAGAGAAACAAGATACATAAGAGAAATAATTTATGGAAAAAAAAGGAGAATAACATACTGGGAA
ATGACCACAGATACAGAAAATTTACCGAAAAATTCAACAAGTTTTGTGATGACAAATTGTCAGGG
AAATTTGAAGAAAAGATTAGGAGATTTGTATGGTCAAAGGACATGGGTAGAATATGGATTTAGA
CAATGTAAACAAGAATTAGGATGGAAAGATTATCGACTAACAAAAATAGAAAGTATTGAGAAAT

GGTGGGGACTAATAAATAGTGTTTATACCATGGTAAGTTTACAAACAAAACCATTGAGAGAATT
ACTATTTTCAGAAGAAGAAAAGAAAGAGGAAATAGAAGAAAAAGAGCCAATAAATAGTTGGAAA
ACAAGTTTAAAGAAAATAATGATGATGATTGAGCCATTAATCTTATTTTGGTTAATAACACCAT
GGTTAAAAATGGTAAATTCATCAGGATTATTAGAAGGATTGAATAATTTATTATCGATCACTCA
TGATTCTAAATTATATTTTCCGACAGGATAATTGTTTCTATTTTTTGATAGTTGATTCGGAGTTT
GACAAAAGAGGGTTAATTATTGTATTGTGGGCGGCACCTCTTACTTCTGGGATTCCTTTAGCAGT
ATTAGCCGGGATTGTGCTAAAAGTGGGGATTAATATTATTGACTGGAATTTCCTCCGAAGAGTTC
ACAAAATTTCTTGGAAAGCCGCAGGAATTGTTTATAGCGTGGTGTTATTAACTGTTTTTGTGGAT
TTAATGGTAGCGGTAGCAGTTGGGGTATTTATCGCTAATATTTTAACTATCGATCGCCTCTCAGA
ATTACAATCCAATGCGGTTAAAGCCATTACTGATGCTGATGATCAAATTGTCTTAAGTAATGAAG
AAAAACAGATTCTTGACTTAGCTAACGGACGTTTATTATTATTCCATCTCAGTGGACCAATGATT
TTTGGGGTAGCTAAAGCTATTTCCCGTGAACATAGTGCCATTGATAACTATGATGTGATTCTGGT
GGATTTGTCGGAAGTGCCAGTATTAGGGGTGACATCCTCCCTTGCCATTGAAAATGCAATCCAAG
AAGCGATCGATCTTGAACGAGAAGTAATCGTTGTTGGTGCTACTGGTAAAGTTAAGCGCCGCTTA
GAAAAATTGGGTATTGCAGGTTTAATTCCGGGACATCACTGGATGGGCGATCGCCTTACTGCATT
ACAAGAGGGTTTAGAAATTGTGCGAGAAAAACAAAGTCACAACTATGAAGCAATAAAAAATTAG
ATTTTTTGCAGAAGTCAGAGGACGAAAACAGTGATAAAATTTGGTTTTAAACGTTGCTGATTTTA
GCTTAATTTTTATTGTGCAAGAGATCTATAAAAAAAATTTTGATTCTAAATGATTACGGGTGTA
TAGTTGATACACCTTTTTTTTATCTCAGTTATTTCGCTAGAATATTCGATTTAGCAGGTTTAAGA
TTTTGGGGGCGAATAATCTTTCATCCTGTGGTTTGGTGGTTTAAGACTCTTGAAATTGTGATTAG
TCCATCAAGAATTGAAAATGAGAAAATCAATTCCTAAGAGATAAAATTTATATATAAAGGAGAA
TAAGCTCCTATCAATATCTCAATTATTAATAGTTAATGTCTATTGAAACAATTTACGGAAACTTA
CAAGGATTAAAACCTAGCCAACTCAAACAGTTACAAAAACTATATCATCAAAGAATAAGCGGCGA
TCGCATCTCTACCGTTGACCTCGCCGAAAGAATAGCCAACCTTAGCACAGAGTTAAAACAACCCT
TGAGCGTCTATCTAAATCGTCGTGGACAGGTGATTAGGGTAGGAGTCGGTACACCCCGTCAAACC
CAAATTCCCCCCCTAGAATTGCCTCGTTACGGTGAGTTAAGACTATCAGGGATTCGTTGTTTAAG
CACCTCATTAAAGCCTACTCCCCCCAGTGAAGCTAGTTTAACCGCAATGGTAAGACAAAGATTAG
ATGCTTTAGTTATATTAACCCTGACAGGTCAAGGAATAATACGTAAAGGAGGAGGAGCGAGAGG
ATTTGTGGATAAAGCCTATTTATGTCATTTACTACCCCTCAAAGATACCGATAATTATTGGGATG
TATCACATCCTCAATCCTTAGAAGATATAGTCGAACAAGATTTTCTCGATTTAGTTAATAACTTA
GAAGCAGAATTTAGCCGAGAATTTATTGCTCAAGAAGTTTCACCCCAACAAGAACGAGTTTTATT
AGTGGGCTTACACACAGGAGATAGAGAAGAACAAAAATTCCAAGATAATCTACAGGAATTAGCC
TTACTGGTAGATAGTGCCGGAGGAAAAGTATTAGCTACCATCGAGCAAAAACGCTCTCACCCCCA
TCCTCAAACCCTTGTTGGTGCAGGAAAGGTAGAAGAAATAGCCCTACAAGTACAAACATTAGGAG
CAAATTTAGTGGTATTTGATCGAGATTTATCCCCTGCTCAGGTACGCAATTTAGAGTTACAATTA
GGGGTCAGAGTGGTCGATCGCACCGAAGTAATCTTAGATATATTCGCCCAGAGGGCGCAGTCAAG
GGCAGGTAAATTACAAGTAGAATTAGCCCAATTAGAATATATGTTACCCAGACTAACAGGGAGA
GGACAAGCCATGTCTCGATTAGGTGGCGGTATCGGCACCAGAGGCCCCGGTGAAACAAAATTAGA
AACAGAAAGAAGGGCTATTCAAAAAAGAATCACCAGACTACAGCAAGAAGTAAACCAACTGCAA
TCCCATCGCTCAAGACTACGTCAACAAAGACAAGCCCATGAAATAACCAGCATTGCCATTGTCGG
TTATACAAATGCAGGAAAATCCACCTTAATTAACACCCTTACCAATGCCGATGTTTACACCGCAG
ATCAATTATTTGCTACATTAGACCCCACCACCAGAAGATTGACTATTACAGATCCTAACACATCT
GATACAACCACATTGTTAATGACCGATACTGTGGGTTTTATCCATGAATTGCCCCCTCCTCTTGT

GGATTCTTTTCGGGCAACCCTAGAAGAAGTAACCGAAGCGGATGCACTATTACACGTTGTGGACT
TGTCTCATCCCGCTTGGGAAAGTCATATTGAATCGGTTAAAGCAATTCTTGCTCAAATGCCTCTA
GCCCCTTCCGTAGAATTAATTGCCCTCAACAAAATAGATAAGGCAAAGAGTGAACATTTAACCAT
TGCCAAGGAAAAATATCCCCAAGCGGTTTTTATTTCTGCTCAACAACGTCATGGTTTAACTACAT
TGCGCGATCGACTTTTACAATTAGTTGCTAGTAATTAAAATAAGGCAAGAGGCAAAGGTAAATA
ATTAAGAATTAAAAACTCCGAATACTTATTACTTTCTTCTAAAACCTGCAACCTGAAACCTGACA
CCTGACACCTTATCCCCCTGCAATCTGAAACCTGACATCCCAACACCAGTTAGTGTTATGCTTTGG
ACATAACCACATAAGTATTTTGAGAAATAAATTTAAGCTGAGAATGACAAGTGAGCAGTACCAA
CAAAAATTAGTTGTCGATAAAGGAGGAATTAGAATTGATGTTTGGTTAGGAGATAATATCGACA
GTATATCTCGATCGCGCCTACAAAAACTCATTAGTGAAGGACAAGTTATCGTTAATCAGCAAGTA
TGTGAGAGTAAAAAAACTATTTTAAAAACAGGAGATGAAGTAATAGTTAGAATACCGCCTCCTG
TTTCTTCTCATCTTATCCCCGAAGCTATTCCCCTAGATATTCTTTATGAGGATGAATATTTACTCA
TTATCAATAAACCTGCTAACTTTGTGGTACATCCAGCACCGGGTCATAGTCAGGGAACTTTAGTA
AATGCTTTATTATCCCATTGTCCTGATTTAAAAGGTATTGGTGGTGTTGAACGTCCCGGCATAGT
TCATAGACTAGACAAAGATACAACAGGGGCGATGGTAGTGGCTAAAACAGAGCCAGTTTTACACC
ATTTACAGGGGCAAATTAAGGCAAAAACGGCAAAAAGAGAGTATTTAGGGGTTGTTTATGGTTC
TCCTTCGGTTTCAAGTGGTATCGTGGATTTACCCATCGGCAGACATCCATGCGATCGCAAAAAAA
TGTCAATTTTATCGGAGGATAAAGGAGGAAAACCGGCTGTTACCCATTGGCTATTAAAAGAAAG
AATCGGTAATTATAGTTTAATTCACTATGAATTAGAAACAGGCAGAACTCACCAAATTAGGGTAC
ATAGCAGTCATATAGGACATCCTATCATCGGAGATCCCTTATATTCTCATGGCAAATCCGTCGGA
GTTAATCTGACAGGACAAGCCCTACACGCTTTTCGTCTCACTTTAACTCATCCTATCACTAATAAT
ACCTTAGAAGCGATCGCACCTTTACCCCAAGAGTTCGAGAAATTATTACGCATTTTAAGACTAAA
AATTCATGGCTAGAGATGAGGGGAAGGGGGAAACAAGTAGTGAGTGGAGAAAAGGTGTCAGGTT
TCAGGTTTCGGATTGCAGGTGAGGAGGAGATGGGGAGATGAGGGGAAGGGGTGAGAGGGGGAAA
TGAGAGTTTGCTGTGCTTGTACACTCCTTTCAGTCGTGAACAGAGTTTTTAATTATTCACTATTC
ACTATTCACTTTTAAAACATTCTTAACAATTGAACCCCGATAAATTTTATAACCTGATAGTAGAG
AAGAAAATAATTCTTGAATGTAAATAGAAATAGCTACAAATCAATCTTTATGAGTCAGAATTTA
ATTACAGTAGCGTATATTGGTGCTAGTGCTTTGTTTATCCTTAGTCTAGCAGGGTTATCTAATCA
AGAAACTGCCCAGAAAGGTAATATATACGGAATTTCGGGAATGGCGATCGCATTTATTGCCACAG
CATTTAGTCCACAAGTAACAAACTATACCACGTTGATAGGGGCGATTATTCCTGCGGTAATTATT
GGGGCAATCTTAGCCAGTAGAGTGGCGATGACGGCAATGCCGGAGTTAGTAGCAATTCTGCATAG
TTTTGTCGGTTTAGCCGCCGTTTTAGTGGGAGTAGGTAATTACTTACAACCTGAATCAGGGTTAG
TGGGTGCAGAGGCCTTTATTCATCAAATAGAGATTTATGTGGGAGTATTTATCGGTGCGGTTACT
TTTACTGGTTCAATTGTCGCTTTTGGTAAATTAAGAGCAATCATTAGTTCAAAACCTTTGATGTT
ACCGGCTAGACATCTACTTAATATAGGAATGTTAAGCGCAACCATTTATTTGGGTTATGATTTTC
TTCACTCCGAATTTGGCTTACAACCTCTCCTCATTATGACGGGTATTGCTTGTTTGTTAGGAATCC
ATCTTGTTGCCGCTATTGGTGGGGCAGATATGCCAGTGGTTATCTCTATGTTAAATAGTTATTCT
GGATGGGCTGCGGCGGCCGCAGGTTTTATGCTCAATAATGATTTATTAATTATTACTGGTGCTTT
AGTGGGTAGTAGTGGGGCGATTTTAAGTTATATCATGTGTAAAGCGATGAATCGTTCTTTTATTA
GTGTTATTTTAGGAGGATTTGGCGAAGGAAGTTCTAACCAAGAGTAATTCTGCTCAAAAACAAGA
AGTGGGAGAAGCTGTTGCTACTAATGTGGAAGAAGTTGCTGATTTATTAGTTAATGCTAAAAAT
GTCATCATTACCCCCGGTTATGGCATGGCTGTTGCTCAAGCACAACACGGAGTTTCTGAAATTAC
TAAGTTATTGCGTGATCGCAAAATTAATGTTCGTTTTGGTATTCACCCCGTAGCAGGACGTTTAC

CCGGTCACATGAATGTATTGTTAGCAGAAGCAAATGTTCCCTATGACATTGTTTTAGAAATGGAC
GAAATTAACGAAGATTTTTCTGAGACTGATGTGGTTTTAGTGATTGGAGCAAATGATACTGTTA
ATCCTAGCGCTTTAGAAGACCCCAATAGTGCGATCGCAGGTATGCCTGTATTAGAAGTGTGGAAA
GCAGAAAACAGTATTGTGATGAAACGTAGTTTAGCCACTGGTTATGCAGGAGTGGATAATCCTTT
ATTTTATAAAGAAAATACAAAAATGCTTTTCGGTGATGCTAAAGCTAATGTTGACGCTTTATTAG
TAGCATTGAGAGAAAAAACCAGCAAGGGGAAAGCTAACAATAAAGTTTTAGTGACTGCATAGCC
TAAGACCTATTAATTTTACCTGAGTTCGATAAAAAATTGTCGATTCGGGTAAGCAATAGGGAACA
GAAAACAAGCAAAAGCATTAATAATTAACATAAAATACTTTTAAATTGATTTTAAATAGTTTTT
CCTTTAAATTAATTATGACTCTCTTGCTTCTCACCTTATCTGTCAACACCTTACAATACTCCGAAC
CCCGTTCACGAGCGAAGGGAGTGCAAGAGCCGAAGCTGAACTTTGAACCCTAACATCTCAATACC
CTGTCACCGACTTCTTTCTTACTTTTTGTCTAAGTTTTGGGGTTATTGAAAAAAAATTTTGTGAA
AAGTTGGTTTTTTTGTTGACATTTTTTAAAAGGTTCGTTATATTAATATATGTGCGTTAAACGAG
CAAAAACAAAGCAAGTCAAGCGTTACAAGTCCGAGCCCCCATCGTCTAGAGGCCTAGGACACCTC
CCTTTCACGGAGGCGACAGGGATTCGAATTCCCTTGGGGGTATATAATAAAGGAGGGTTAACAGC
CCTCTTTTTTTGTGCAAAAGATTATTATAGTTATCTGAATCTGAGTTCGGGATAAATTTTCATCT
ATGAGTGATAGTGAAAAGGGCAAGGGGCAAAGGTGAATAATTAAAAATTAAAAATTAAAAATTC
CGAACTACTAACTCAGGTGTTTTTATTTTTGTTTAGAGGATAATTCTCAAAAATGATTTAGGATT
GCTATATATAATTTAATTTGATCTAATCTAAGATTGTTTAACCAAGAAGAATGAAACTGCCTTCT
GTCAGAGACACCGCTAAAAAAGCACTTAATTTATTTCGAGTTGACGAAGAGGAAATAAAAAAAA
TATTAGCAGAAGTGCGATCGCAACTTCCCACTACTGAAGCTCTTTTGATTGGAAAACCACAAGCA
GGAAAAAGCTCTATTGTGAGGGGATTAACTGGGGTAGGAAAGGAAATTGTGGGGCAGGGGTTTC
GCCCTCATACTCAACATACCGAGCGTTATGCTTATCCTTCGGAAGATTTACCTCTATTAATTTTTA
CTGATACCGTTGGTTTAGGAGATATAAATAACAGTACAGAGGTCATAATTGAAGAATTAACGGC
TGAATTAGAGCGAGAAAGCAAAAAAGCCAGAATATTAATTCTTACGGTGAAAATTAACGATTTC
GCTACGGATAGCCTTAAGATGGTGGCGGAAAATCTCCAGAAAAAATATCCTCAGATTCCTTGCTT
ACTGGTAGTTACATCGTTACATGAACTTTACCCTAATTATGACACTAATCACCCTGATTATCCCCC
CACTTTTTCTGAAATAGATAGGGCATTTATAGAAATTAAAAATACTTTTCAAGGATTATTTGACC
AAGCTGTTTTAATTGATTTTACCCTAGAAGAAGATGAATATAATCCTGTTTTTTATGGTTTAGAA
GCCCTAAGAGATTCTTTAGCGGAGTTATTACCTTCTGCCGAAGCTCAAACAATTCACAAGTTATT
AGATCAAACAGTGGACTCTCAATTAGGAAGCATTTATAGAGATGTGGCTAGGCGTTATATCCTAG
CCTTTTCCATTATGGCTGGTACGGTTGCGGCAGTGCCTTTACCTTTTGCTACGATGCCCGTTTTAA
CTGCCTTAGAAGTGACGATGGTGGGGGTATTAGGTCAACTTTATGGACAGAAAATCAGCGCTTCT
CAAGCAGGTGGTATTTTAAGTGCGATCGCAGGAGGATTTTTAGCACAAGCCATAGGTAGAGAGTT
AATTAAGTTTGTACCGGGATTTGGTTCTGTGGTAGCCGCTTCTTGGGCTGGTGCTTATACATGGG
CTTTAGGGGAAGGGGCTTGTGTTTATTTTGGAGATTTAATGGGGGGCAAAAAACCAGATCCAAAT
CAAATTAAGGAAGTTATGAAAGATGCTTTTGATAATGCGAAAAAAAAGTTTAACGTAAATGGCA
AAAGAAGTCCCTAATCTCAGCATAGCGGATTAGCGGATGAATTGTGCCTCGATAAAAAAAATATC
CGTAGGATTCGACACAAAACCAAAATCATTACTTATAATAAATTTATTGGCAACCTAAGTTAGCA
ATAAACCTCAATGCACAGAGCGATAAAAGTCAGAATCTATCCCAACAAAACCCAAGCCAAGAAAT
TATCTCAGGTTATGGGTTGTTGTCGTTGGTGGTATAACTACGCTCTAAACTTGTGTATTGACACT
TATAAAGCTACAGGTAAGGCACTAAAGCAAGTAGCCCTTAATAAGTATCTCCCTAAGCTAAAAAA
AGAGGAAGATACGGCTTGGTTAGGAGATTGTTACTCACAATGTCTGCAATCAACGACTCTTAATC
TGACTAAGGCTTTCAAAAACTTCTTTGAAGGTAGAGCCAAATACCCCAGATACAAGTCATATCAC

GGAAAACAGTCTTGTCAATATCCCCAAAATGTCTCGATAGTTGATGGATGTCTCAAAATTCCGCA
ATTAGGCTTAGTAAAAGCAGTTATTCATCGGATATTTGAAGGTGAGATTAAGACTGTAACAGTA
AGCAAAACGCCCACTGGCAAATATTTTGCTAGTATCCTATTCGATACCCAACCAAGAATTACCATT
AGTAAATATGACAGGGAAAATTTGTGGTATAGACTTAGGAATTAAGGATTTTGCTATTGTTCAT
GATGGCAAAAAAACCAGTAAGTATGCTAACCCTAGACACATCAAAAAACATGAAAAAAACCTAGC
TCGGAAACAACAAAAGTTAGCTCGTAAGAAAAAAGGCTCTAAGAATAGAGAAAAAGCCAAAAAG
CTAGTCGCTAAAGTTCACGAACGCACAAGCAATACCCGTCAAGACTTCCTACACAAACTCTCCACC
AAAGTTGTGAATAATAATCAAGTGGTAGTCGTTGAACAACTGAATATCAAGGGTATGGTTCGTA
ACCACAAATTAGCAAAAGCAATAACTGATGTCGGTTGGGGAACATTTATCAACTTCCTAGACTAT
AAACTCAAGCAAAAAGGTGGGTTATTAGTGGAGATAGACAGATGGTTTCCTAGCTCTAAAACTTG
CTCTCATTGTCATTATCAAATGTCTGAAATGCCATTAGAGAGAAGAGAATGGACTTGTCCTAGTT
GTGATAGTCATCATGATAGGGATGAGAACGCAAGTAAAAATATTAGAGCAGAAGGTATCAGAAA
AATACAGACGGATGGAACAGCCGTCTCTGCATCTGGAGGATCGGTAAGACCAAAAGGCGGACGTA
AATCCGTTTTGAGGCACGAACCTGTGAAAGATGAAGCTCCCGACTCAGCGATAGCGGTCGGGTAG
TCGTTCACATAAGCGATATTCGATATTCAGGAAATAAAAGAAAAAAGGAAAATTTATTATTCTT
TATTATTCAAATTGTCTGCAAATACTGCTCTAAGTCTTCTACTAAACGAGTTAACTCTGCTTCGC
TAGTAAGTCTTATTTGAGGGCTTCTGAGGGTAACTAATACTTTAGCAGAAAAAGGACTAGGGTA
AATATTGGGATTGCAAAAAAGTTCTAAAAATACATCTCCTGTATATTGATATTCCATTGCTGTTT
TTGGGGTTGGTTTGCCTCCCGTTTTATTTTGCAAGGCGATTTCTTTAAGACTTTGCATTAAATGA
TTAATTTCTGTCTGTAAATTTTCCGCCGCACTAGAGGTAAAGTTAAAGGAAACTGAACCTTGAAT
TAAATTAAGTGTTAATTGACTCATACTAACGATAAATTAATGAATAGTGAATAGTGAATAGATT
TCAGTTAAAAATTGAGAGTTAAGAATTAGCCTTCGCCTCTCCCCCTCTCTCCTTATCACCCATCTC
CCCTCATCACCCTGAACCCCGAACCCTTAATTCAATTATCCTCGTAATCTTCAAAAAGTAATTCTT
CAAGAATCCAACTTACTTCTTCTTCTTGAGACTGTATTATATTGATTTTACCAAAACCATCTTTC
TCGCCGATGAATAAAATGGGGCTTAAAGGGGTGCAAATATTATATTTTTGTTCTTCTGAGTAAAA
ACTTGCTAAAAACTGTAATTCTTCGGGTTCTATTTCTGAGGTGTGTTCTTCGATTTCTAAGCTAA
TAATATTATTTTCTTCTAAAGGTGGTAATTCTCCACTAGCAGTTAAGATAAAACCTGTACGCTTC
AAATATAAATTTAATTCTCCTAAAACAGCTTTTGCATCATCAAAGATACCATCAAGTTCTTCTTT
CTCTTCTACCATAACGGTTTCACTATATTCATTATCTTCAGAATCTATTTCTTCCGTTAAAATGA
CAATGGCTAGATCAATAGGCATTAATAAAACGTATATTTTGCCATCATATTCAATTTCATTCTCA
ATATAACAATCGAGAGATCGCCCCTCATCGTCAAAGATGGTGACAATCTCAGTATCATCATATTG
CCCACTATCAGAGGAAAATCCGGGGGAGGACATAATTTTTAATTTAGCTAGAGTTTAGGTAAAAA
TAGTTTGTAAAATTCAGAATATCATGGTTTGAGATCAGAATGTTAAACTGATAGGGAAAATTAG
CGTGAAGAATAATAGTTTTTCTAATATTATTGATTATTTATTTACCCTTATATCCTTAATACGAA
TATTTTGAGTTAAAACAGTGGTGGATGAGACAACAAGTTCCTTTGCAATGACGGAAAATGCGGA
AGATGAATTAGCCCCTGTGGTAGCGGTTAAAAAAGGTAGTTTGCCAAAATTTTATTTGAACGTTT
TTAAGCGTCGTTGGTTAATTATCCTTACTTTTGCTGTTTCTGGTTTTGTTCCTGCATTTTTATGG
AGTAAAACAGATCCTGTTATTTACCCCGGTAGTTTTGAAATGTTAGTTGAACCTGTAACCTCCGC
AGAAAAACTAACTGAACCGACAACTTTAGCTCGAACGGGTGGTAATGTTAATGAAGAGTTACTTT
CTATTGATTATCCGACAATTTTACGAATTCTCAAGAGCAACAATATTCTTCAACAGATAGCAGAA
AAAGTTAATCAAAATAATCCTTTATATCCACCTGCTTACTTGATAAAAACATTTCAAAAAGATTT
AGTTGTAGAAAGAGCTCAACAAGGACAAAGTCGCTTTGATCAAACTAAGGTTATTTCCGTACGCT
ATCAACATGAAAATCCCGATTTAGTTATGTCAGTTTTAGAAACTGCGTCGGAGGAATATTTACGA

TATAGTCAAGAAGAACGAGAAAGAAACTTAGAATCTGGTGTCAGGTTTATTAATGAGCAATTAC
CCGAAATTCGTCAAAGAATTAATAAACTACAAGATAAACAAAAAGAGCTACAATCTCAATACGA
ATTAATCAGTCCTCAAGTCAAGGGAGATAGTTTATTTCAACGCAATACAGAATTCACCCAAGAAA
TTTTAAATGTTGAATCTCAGTTAAAAGAATTAAACATACTTGCTAATAACCTTCAAAAAGATTTA
GGTTTGACACCAGCAGAGTCTCTTAGTGCCTCTACTCTTAGTCAAGATCCAAAAAGACAACGGTT
GTTATTGGATTTACAAGCTATAGAAGCACAAATTGCTCAACAGTCAGCACAGTTAACAGAAAACC
ATCCCAATATATTAAATTTACAAGACCAAAAAGATAATATTACTAATTTATTAGAAAAAGAAAC
GACCAGAGTTTTACAGGAAAATAAAATAGATCCTAATGTTAACCCCAGAGTTTTTGCCTATCAAG
ATCCTAGTCGTTTAGCTTTAATTCAAAAGTTAATTGAAACACAAAATCAAATTGATACTCTTTCT
TCTCGTTATCAGTCTTTAAAAAATAGCCAAACTCAAACTGGTTCACAATTAGCAGTAATGCCGGG
CGTCATCAAAGAGTATAATGACCTCGATCGCCAAATTTCACTAGATACAACTATACTAAATCAAT
TAACTAATCAGAGGGAAACCCTAGGAGTGGAAATTGCCCAAAAACAGATTCCTTGGCAAATTCTG
TCCCCTCCACAAACAGCCATTGATGAATTTGGTAACTTGAAGGGATATTCTCCTGAGCCAGAAAA
AAAATTGGCACTTGGTACAGGTTTGGGTACGATTTTAGGGTTATTAGTTGCGATCGCGCTTGAGA
AGAGGAGAGATATAATTCATGAAACAAGTGATTTAGAGTATGCTTTTGGTTTACCAATTTTAGG
GGTAGTAGAATTAGAGGAAAAGAAAAAAGACCAAGTTACCCTTGAAGAAGAAAATTTTTTCCGT
GACTCAAAAAACGTTTATCAAAAGATAACTCCTGTACATATATCACTTTCTCAGGTTTATACAAA
TCTCCATTTTCAATTACCCAATAGTCATCAAAATCGCATCCTTATTACTTCTTTACATCCCACTGA
TGAACAGGCATATATTGCCGCTAATTTGGCGAAAACAGCAACGGAAATTGATCAAAAAGTATTAT
TGATTGAAGCAAATACTATTCAACCAGAAATACATGAATTTTTCAGTGACAACATTTGTTCTAAG
TTAAAAGATTTGTTATCAATTCCTGCTTTGGAGTTATCCATCTTAGATAAATTAGCAAAACAGGA
AAGTGTTACAATTCTCAGTGCTGAAGATTCTGACTCAGAAAAACCTTTACATTTAAGTTCAGAAG
TTGCTCAAAATTTAGTAGATCAAATTGCACAACATTATGCTTTGACTATTTATAATAGTTCTTTT
TTCCTTGAATCTTTTGATTTAAGTTTACTTGCTCAGAAAACAGAAGGTATTGTTATGGTTGTTAA
ATTAAGACAAACTTCTTTTTCTCAACTCAAAGATGCGATCGCACGTATTCAAACCTATGATCTCA
CTTTTTTGGGCTTTGTTGTCATTGAATAATAATTAACTTCAGTTCGGTTTAAGAATATCGGAGAA
GGATAGGTGTCAGGTGTTTGGGTAGAGGGATGGGGAGATGAGGGGATGAGGGGATGAGGGGAGA
GGGTGATAAGGTGTCGGGTTTCAGGTTTCAGGTTTCAGGTTTTAGAAGAAAGTAATGAGTAACG
AGTAATTATCAACTATTCACTATTCACTATTCACTATTCACTATTCACTTTTGCCCTTTTCGCTAT
CACTTATAGATGGAAATTTATCCCAAACTTAGAGTGTCTTAACCATTTGTTATCCTCAACTTGCT
ATATTGCCAAATCAAACCATTGGAATAAAAATCATGTTTGAAAAAATATTACTATCTAACCCCAG
TGCAGAGAATCCTAGCTCTTTACGTCCTCGGAAGGGAATTATTATTGGTTTAGGGCAAGTCGGAT
TAGCTTGTGCTTATTCGATGTTAATTCAAGATTGTTTTGATGAGTTAGTTTTACAAGATATTGCA
TCGGATAAACTTGAAGGAGAAGTAATGGATTTTTCCCATGGAATGCCTTTTATTCCACCCACAGA
CATCAAAGCTGGAACCATCGCTAATGAGGGAAGAAATGCGGATATTGTGATTATTACAGCAGGAG
TTGCTCAAAAAGAAGGGGAAAGTCGCCTGAGTTTGCTAGAAAGAAATATTGCCATTTACAAAAA
AATTCTTGCTGATGTTGTTAAATATTGTCCTGAAGCAATTATCTTAGTGGTAACAAACCCTGTGG
ACATTATGACCTATGCAACCCTTAAAATTACTGGGTTTCCTAGTTCCCGAATTATTGGTAGTGGT
ACTGTGCTTGATACTGCACGTTTTCGTACCCTATTAGCTAAAGAAATGGATATTGACCCTCGTAG
TGTCCATGCTTATATCATTGGTGAACATGGAGATAGTGAAGTTCCTGTATGGAGTACCGCTAATG
TTGGAGGTATGAAACTTGTGCCGAATACGTGGGCTGATTTACCTGAAGATGAAAAAGCTACTTTA
TCGGGAATTTTCCAAAAAGTTCAAAATGCGGCTTATGATATTATCAAATTAAAAGGCTATACTTC
TTACGCAATCGGTTTAGCTACTACAGATATTGTTAAGGCAATTCTAAATTCTCAGGAAAGAATTT

TAACGGTTAGTGGTTTAATGACGGGAATATATGGTATTGAAGATGTCTGTTTAAGTATTCCTAGA
GTTGTTAGTGAAAGAGGTATCATTAAGACTGTTAATTTAACTCTTTCTGAAGATGAGGAAAAGT
TATTACAACAATCGGCAAAAGTTTTAAAAGAAGTGTTTGATAAGATTAATTACTGAAAATGTAT
TTCATAAATACGAATGAATCTAAGTTATTTGTCCATAGAGGAACTTTTTTTAATTTTTGGCATAT
TAATATTAGCCAGTGTATTTGCTAGTAAAATAGCATCTCGTTTAGGAGTTCCTGCCTTACTTTTG
TTTTTGTTGATTGGTATTCTTTCTGGAAGTCAAGGTGTCGGTGGTATCGACTTTGAAAGTTATGG
TGTCAGTCAGTATGTTGCCGATTCGGCTTTGGTAATCATTCTTTTTTCAGGAGGGTTAGATACTA
AATGGCGACAAATACGTCCTATCATCAAAGAAGGTTTACTTCTTTCAACCTTAGGAGTTTGTATC
ACAGCCGTTTTGGTCGGTAGTTTTGCATGGCTTATTCTTGGTAGTTTTTCTAGTTTTAATTTGGG
GGTTAACGGTATTAGTTGGACGGAAGGTTTATTGTTAGGTGCGATCGTATCTTCTACAGATGCGG
CGGCTGTTTTTTCCGTATTAAAGTCCAGTAATATCAGATTAAAAAATAATTTACAACCTCTTTTG
GAACTAGAATCTGGTAGCAATGACCCCACAGCAATACTTTTAGCCACTTCTATCATCGGGGTTTT
AGCACAAGGAGTATTTAATCCTACTGTTATCATTTTTAGTTTATTACTACAAATACTAATAGGTT
CTTTTTTTGGCTACTACGGTGGTTTGTTAATGGTGTGGGTGATTAACCGTATTCAATTGGCTTCA
GATGGACTTTATCCTGTTTGTGCTTTTGGTTTATTACTTATGATTTTTAGTATCACGGCTTTTGC
TCGTGGTAATCCTTTTCTTGCCGTTTATATTATAGGTATTGTTTTTAGTAATAGTAATGTTTTGA
AAAAAGAATTAATTATTAGTTTTCATGATGGTTTATCGTGGTTGATGGAAATAACAATGTTTTT
AACTCTTGGTTTATTAGTCTTTCCTTCTGATTTACTTTCTAATATTAGTGTCGGAATTGCGATCG
CACTCTTTTTAATTTTAATTGCCCGTCCTATTAGTGTATTTTTGTGTCTTGCACCTTTTTCTAAAT
ATAAACAAGCGGATAAGTTATTTGTTTCGTGGGTTGGCTTAAGAGGGGCAGTACCGATTGTACTT
TCTATCATGCCTATTACTCAAGGTTTTGAGTACGCAGATCAAATTTTTAACCTTGTGTTTTTCTT
AGTAATTATCTCGGTTTTAATTCAAGGATTATCTCTTACACCGATGGCAAGATTTCTTAAAGTGA
TAGACTAGATTTCTTACAGAAATCAGGAAAGAGGGAATAGTGATGAAATTCACTGTAAAACGCCC
AAATTTTACTGAAAACTATCCCGTATAGTTATTCTTTTGGAAATTTTTTGAGCACAAAATAACAT
TGTTCAGCAATTTCCATACCTTTTTGCCAAATTATTAAATCTTTAAAGTCTTTTATCTCTGACAT
CTTGAAGTCTTTAATATCAAAAAATATTTTAACACCTTTGCCTTTTGCCCCTTGCCCTTTGCTCCT
TTAACTTTGCCCTTGAGCGCATAAATGTTATATCCAACTGGTATTTCTGCTGATAATATTGATGA
TTAATGAAAACTTAATTGAATAACTATGCTCAAAATCGAACTACCTCAAATTCCTTCCTCTGCAT
GGCAATGTGAAATCGGCAAAGAATGGGAAAAACCCTATACAGTACGCTACGCTAGCAATTTAGAT
GATGGAGCTAATCATGGTATGCCTTTGGGTGGTTTTGGTGGTGGTTGTGTTGGTAGAGGGGTAAA
TGGTGAATTTAATCTTTGGCACATTGATGGTGGTAATCATATTTATCAGTCTTTACCAGCTTGTC
AGTTTAGTGTTTTTGAGCAAGTAGAAGGAGAAACCCCCCAAGTATATGCCATGAGTACTAATTCC
CCTACTGATGGTAGTTTATCAAGTTGGGCATGGTATCCCCAAGGAAAAGGTACATATTCCGCACT
TTATCCTCGTAGTTGGTTTGACTATCAAGGGGTGTTTAAATCTCGTCTTATCTGTGAGCAGTTTT
CCCCTATTTGGGCAGGAAGTTACCAAGAATCTAGTTATCCTTTGGCTGTGTTTGAGTGGAGTGTG
CATAATCCTACTGATAAGGAAATAACCCTGAGTATTATGTTTACATGGCAAAATAGTGTGGGTTG
GTTTACCAATGCTATTAAGTCTCCCACTGTAAAAGTTAGAGATGATGGTAGTCCTGAGTATGAAT
ATCGACCAAAATGGGGAGATAGCACGGGTAATCTTAATCAGTGGATTCAGGATAATTTTAGGGT
AGGATGTTTGTTCGATCGCATCCGTATGGAAGAACAACCCCAAGAAGGGGAGGGGCAATGGGCAA
TTTCTAGCATCACAAACCCTAGTTTAGAAGTCTTTTATCATAGTCGTTGGAATCCCAAGGGAGAT
GGTGCAGATATATGGGATTATTTTGCCATGAATGGCTCTTTGCCAGATTATCAGGATGAGACACC
CGCCGAACCGGGAGAACAAATTGCGGGGGCAATGGCGATTAGATTTACAGTTAAACCCGGGAAAA
CAAAAAAAATCCCTTTTATCCTAGCGTGGGACTTTCCTGTAACTGAATTTGCTCAGGGAATTACT

TACTATCGCCGCTATACAGATTTTTTTGGTAGAAATGGGCGTAATGCTTGGGCAATTGTACGCAC
AGCATTGAAACACCATGATATGTGGCAAAATCGCATTAAAGAGTGGCAGAAACCAACCCTAGATC
GAGTTGATTTGCCAGAATGGCTAAAAATGGCTTTATTTAACGAATTATATTTGTTGAGTGAAGG
AGGAAGTTTATGGACTGCGGCAACGGAAAATGACCCTGTGGGACAATTTGGGGTTTTAGAATGTA
TTGATTATCGTTGGTATGAAAGTTTAGATGTCCGTTTATATGGTTCTTTTCCTCTCATTATGATG
TGGCCTCGTTTAGATAAATCAATTATGGAGGCTTTTAGTCGAGCAATTCCTACAGAAGATAAAAC
CCCTCGTATCATCGGTTATAATCAGGCACAGGCTATCAGGAAGGCAAAAGGAGCAACCCCCCACG
ATTTAGGCGCACCAAATGAGCATCCTTGGGAAAAAAGTAACTATACCAGTTATCAAGACTGTAAT
TTATGGAAAGATTTGGGCTGTGATTTTGTTTTGCTAGTTTATCGAGATTATTTATTTACTGGTGG
TTTAGATCAAGATTTCTTGTGGGAATGTTGGGATAGTGTGGTGGAAACTCTCCATTATGTGAAAA
CTTTTGATTTAGATGGTGATGGTATCCCTGAAAATTCTGGCGCTCCTGATCAAACTTTTGATGAT
TGGCGTTTACAGGGTATTAGTGCTTACTGTGGCGGTTTATGGATTGCAGGATTAGAAGCGGCTAT
CGCTATTGGCAATACATTGATTGCAAATCCCCCTATGAATCCTAGTTTACAACCTGATAACTACC
CCCAAGGAATTAAAGATGCGATCGCAGTTTTTCAACAATGGTTAACTCAAGCAAGAAGTCTTTAT
CATGAAACTCTTTGGAATGGGGAATATTACCGCCTCGACAGTGAAAGTGGTTCAGATGTGGTTAT
GGCTGATCAACTATGCGGACAGTTTTATGCTCAATTATTAGGATTACCCGATGTTGTAGAATCGA
AGTATGTAGAATCTACCCTCAGAAAAGTATATGATGCTTGTTTCTTAAAATTTCATGATGGTAAA
TTTGGTATTGCCAATGGAGTAAAACCCGACGGAAAACCTGTTAAAGAAAATGACACTCATCCTTT
AGAAGTGTGGACAGGTATTAATTATGGTATCGTTGCTTTTATGATTCTCAACGGCATGAAACAAG
AGGGGTTAAGAGTCGCAGAAACGGTGGTGAAACAAGTCTATGAAAATGGGCTACAATTTCGGAC
TCCTGAAGCGATTACAGCAGTAGGTACATTTCGAGCGAGTCATTATCTAAGGGCAATGGCAGTGT
GGGCTATTTACTATAGTCTGGAGAAAAAATAGCAATTTATGAAAAATAATTATCATTTTTAAGG
TCTTTGAGACTGTTTCTTGTTCTCTACTTTCTTGATACCACTTTTTCATCAACCTTTACTAAGCTA
AGACGCATTTAACTTGCATTTTTCCTTAGTTTTTGAAAATAACTCAAACCCTTATCTTTTCCCTT
TTCTTCTCCCCCCTCTCGAGGGCGGATAAAGGGCAGGGCTGTTTCAAAATAAAAAATTAGGTTAA
CTGAGATTAAGTAAGAGTAAGATATACAAGGATTTTTTTCTATTAGTTAAAACTTTATCAACAA
AAATCCCCTCATCTCCCTCTCTCCCGCTCTTTTTTCTTTCTCGAAAATGAAACAGCCCTGCGGATA
AAGGGGGGTTTCCCTCTTCCCTATCTTAACCAGACAACTTAAATGCACTCCTAGCTTAATCACTCT
TTTATTCCCTCAACAAGTCTGTGAAAAACCCACACAGATGCGATCGCTCTTTATTTTTTTTAAGT
AACGTTAAAATAGAACCTAGGTTAACTATAGTTAACAAAATGAACTGGATTAAACGCCTATAAT
TAGTTATCAGTATCCCATAAGGATCTAAATCTATGATTGATATAATTATTATTACAATTTTTGTC
CTTGCCTTTGGAGGTGTTGGTTTTGACATAGTCGAACTTCTCCCCTCCGAGATTCAAAATCAGGT
ATCGAACATCCAAGCCTTAAGATGGTTGGTGGCTGGTTTTGCATCCATTATCGGTTTAGCCATAG
GTTTAGTGGCACAAACTACCTACCGTCGTTTAGAACAAAAAATTCGTCAAACACCGATTGAAGTT
ATTATCACCAGAGCTATTGGTTTGGCAATGGGTTTGTTATTAGCAAACTTATTATTAGCACCTAT
TTTCTTATTGCCCGTCCCGAACAATTTTAGCTTTATAAAACCCATGATTGCGATTTTGGGTAGCG
TTATGTTTACTGTTTTAGGAGTATCTTTAGCCGACACTCACGGGCGTACTTTTTTACGTCTGATT
AATCCTAACAGTATTGAGTCGATGTTAGTTGCAGAAGGCACATTAACCCCCGTTGCCACAAAAAT
TTTAGATACCAGTTGTATCATTGACGGCAGAATACAACAATTATTAGCCACTGGTTTTATTGAAG
GTCAAATTTTAGTCCCTCAATTTATTCTCGCCGAATTGCAACAACTAGCCGATGCCACTAATGAC
CAAAAAAGAGTAAGAGGCAGAAGAGGCTTAGATATTCTCAATCAAATGCAGGAAGAATATCCTG
ATAAAATAGTCATTCATCCTGAAGAATATGAAGACGTTACAACCGTGGACGCTAAACTACTTCAT
TTAGCCCATGATATTAATGCAGTTTTAATCACCAATGATTTTAACTTGAGTAAAGTTGCAACTTT

GCAGAAAATTACCATACTCAATGTCAATGATTTAGCTCAGGCAGTACGCCCAATTTATCTACCCG
GGGATTATATAGATCTCAAAATTCTTAAACACGGAAAAGAACCCACTCAGGGAATTGGTTACTTA
GATGACGGTACAATGGTCGTAGTTGAAGAAGCAAATGGCCATGTAGGAGAAGAGCTAAGAGTTG
TCGTTACTTCGGCTTTACAAACTTCTGCCGGCAGAATGATTTTTGCTAAAACCTACGCTTCTGCTG
TTTCCTGATCAATCCTATTGGGATGAGTTTTTACTCTATCTCACATTTAATCTTATCTAGCTCAA
AAAAATGCCTAATTAACCTAAAACCTGATACCTGAAACCCGACACCTGACACCTAGTTAAAAGCG
TCTTTTAAAGCTGTACGAGCCGCTAAGTTGCTTCTTGTGGTTGCTAAAATGTCAGCTTCTCTTTG
TAGCCTTTCCCCTGTGTTATCTATTTCCAGCAGTGCTTGTTGTTCACTCGCTACCCCATAAAGGTT
ACTAGCAACCCAGTAGGATAATTCTATCGGTTGAGTAGGTAAGTTTTCAGGTAACTCTATTTTTT
GATCTGTTAATTTAGCTGAAAGTTTAACAACATCATGGAGTAATGTGGTTACTTCTTCGGCTTTT
GGTTGGAGATTTTCTTGGGGGGGTAGGTCTTCAAACCATTCCACTAAACCAACTCTATAAGGTTT
TTGTCTCACATATTCGAGTAGTCTAAACCTTTGTTGTCCTAGGGTAAGCACCTTCATGCGATCGT
CTGGTAATCTTTCAAAGTGAATTATCTCAGCACAACATCCCACTTTTGCTATTTCTCCACTAATA
GGGTCTATCATTAATACTCCAAAACGTCGATCATATTCAAGAATTGTATTCATCATCATGCGATA
ACGAAACTCAAAAATATGTAGGGGTAAAGGGCGCCCCGGAAATAGCACAACTTCAGGGAGGGGA
AATAATGGTAATTCTCTTACTGCAATGGAGGATGATGCCATAATTTTTATTTTTGTAGGTTGTTT
CTTATAATTTTACTAATTTTCTTCTTTTTCTTCTGTGGTTTCTCGTCCAAATTTCCCTAAAAATCG
ATAAATTTACCCAGTTTATTGGTTATGAATTTCAAATTAACCTAAGTTTAGAATAAATTTGTTTG
GTTATGGTAACGGATGAAAAATTAGGAATTAAGACAGGGCAAAGGTAAATAGTTGATAATTAAT
AGCTCCGAACTAGTCTCCCCCAACACCCTGACACCTGCAACCTGAAACCTACCCTTATCCGATATT
TTTGCATGAAACTGAGGTGTTAAAGTAATGAAAGCGTACATTAGAGACAATATTCTTTACATCAA
TAAAGAGGACATTCCTGAGTATCAAAAAGGGAAATCTGTCGTTAGAAATAATTATTTTTGGGCG
TTAAAATCTATCAGCGACTATGCACCTTTTAATGGTGATTGGGAATTTTGTTCTGAAATTTGGAT
TGCTTTAGGTAGGATGTTGTTATTTTTCTATAATTCTGGCTACTTGGGCGATCACGAAACCATGT
TAGAATTTGAGGACGATGTCTTAATCCCTGATGAATTAAGAGCAGTTTCAACAAAACTTTAAGAC
TTAAACCCCATGGCAACCTTTCTTCGTCCCTTAAGTTATAAATATCAATGGTTATACGATACAAT
TTCTCGTCTGGCGGCAATTCCTGTGGGGGGAGAAAAAAAATTTCGTGAATTAGCCTTATTAGATT
TACCAATAACCAAGGACAGCAAAATATTAGACCTTTGTTGTGGTGCAGGACAAACTACTAAATTT
TTAGTCAATTATTCCACTAATGTAACAGGTTTAGATATTTCTCCTCTTTCCTTAGAAAAAGCTAA
GAAAAATGTTCCTCAAGCAAAATATATTGAGGGTTTTGCTCAAAAAATGCCCTTACCTGATCAAT
ACTTTGATATTGTACATACCAGCGTAGCCTTACACGAAATGACGACAGATGAATTAGAGGCAATC
TTTGCAGAAGTTTATCGAGTTTTAAAGCCAGAAGGAATATTTACTTTTATTGATTTACATAAGCC
TCATAATCCCCTATTTATTCCGGGTATTTTTGCTTTTATGTGGTTATTTGAAACCGATACTGCAT
GGCAGTTATTGAAGACGGATTTATCGGTCAAATTAATTGATTCTGGATTTAGTATTTTAAAACAA
AAATTATATGCAGGAGGCAGTTTACAGGTTATTCAGGCAAAAAAGCCATTAATTTAAGAGTTGA
TTAAAAAAAAGTATTTTGATTGAAAAGTTTTATCATTCATTAATCACCATCAAAAAAGCCCCATG
ACTGGGGCTTAAAACTTCTTATTGGATTAATCTTAAGAATTTAGAGAGCGTTACCACGAGGTAAT
ACTTCTTCAGGGAAGACAAATCTTTCATGAGGTTGATCTTGAGGTGCCATCCATGCACGTAAACC
TTCATTTAATAAGATATTTTTGGTGTAGAAAGTTTCAAATTCAGGGTCTTCTGCCGCTCTTAACT
CCTGAGATACGAAGTCATAAGCACGGAGGTTGAAGCCTAAACCAACAACACCGATCGCACTCATC
CATAATCCTGTTACAGGAACAAATAACATGAAGAAGTGTAACCAACGTTTGTTAGAAAAAGCAA
TCCCGAAGATTTGAGACCAGAAACGGTTAGCTGTCACCATGGAATAGGTTTCTTCTGCCTGAGTA
GGTTCAAAAGCACGGAAGGTGTTTGCTTGTTCGCCGTCTTGGAATAAGGTGTTCTCCACTGTTGC

ACCGTGAATTGCACAAAGTAAAGCTCCTCCTAACACACCAGCAACTCCCATCATGTGGAAAGGAT
TGAGAGTCCAGTTGTGGAATCCTTGGAAGAACAAGATGAAACGGAAAATACCCGCAACACCGAAG
CTAGGTGCAAAGAACCAGCTAGATTGTCCCAAAGGATACATTAAGAATACGCTTACAAATACAGC
GATAGGAGCAGAAAATGCGATCGCATTGTAAGGACGAATACCCACAAGACGAGCAATTTCAAACT
GACGTAACATGAAGCCAATTAAACCAAAAGCTCCGTGTAATGCTACGAAAGGCCATAAACCGCCG
ATTTGACACCAACGGGTGAAATCTCCTTGAGCTTCAGGACCCCATAAAAACAAGAGGGAATGTCC
AAAAACATCCGCAGGAGAAGAAACAGCAACGGTTAAGAAGTTAGCACCCTCTAAATAGGAACTAG
CTAAACCGTGGGTGTACCAAGAAGTAACAAAAGTTGTACCAGTTAACCAACCACCCAAAGCTAAA
TAAGCACAAGGGAAAAGAAGGATACCAGACCAACCAACGAATACAAAGCGGTCTCTTTTAAGCCA
GTCATCAAGGACATCAAACCATCCTCTTTCCGGCGCACGTCCGACTGCAATGGTCATAAAATAAA
CTCCGAATGAATATGATTACAACAACGTTTTTTATTTCTCAAGTCTAAATTACTGTTAAAATCAG
TTTACAATGACTTACTGAGAAAAGTTTACTTTTCTTAACGTTATATTTATAGTACCATTCTATGG
GAAAATATCAAAATTTGACAACTCATTTGTTAAAAAAAGATTAAAAATATTAAAATCGGTTAAG
CTAAAATTAGAGCCTATTTATAAGATTACACTGCAATTATTAATATGATTACAATTGATTTATTA
GTGAGATATATTCCTGTTCCTTTATCTGTAGAAAAAAAAGAAGAAAGTGAAGCTCAAACCCTTTA
CAATCAGATCATCGAAGCGATGAAAAGTTCAGAAACTCAAATTGTAGAACTCACTTGCGATAAAC
AAACTGATAAAAAAATTGCTGTTATTAGTGATCAAATTACGGGAGTAGTAATTTCTCAGAAAGA
CGGTACAAGTAAAGGCAACAACGTACCCGGATTCTTTGCCGCCCTTGCCGAGAAGTAAGTTAAGT
ACATAGTTCAACGTTATACTTTTAGGGTTTTCGGGTGTTAGGGTATTAGGAAAATTTTTTGGTTC
TACATTCTAAATTTTTATTTTGAGTAAATCAGATATTGCGATCGCAGTTGAAAATCTCAGATTTT
CTTGGAGTCCACAAACATCGGTTTTAAACTCTTGTAGTCTTACTGTGCCAAAAGGTGAATTGTGG
ATGTTACTAGGAAATAACGGTTGTGGTAAATCTACTTTATTGAGATTGTTAGCGAATTTATTAAT
TGCCGATGAAGGTCATATTTATACTGATTCTCCTCTCGGCTTTGTCTTCCAAAATCCAGATCATC
AATTGGTAATGCCAACGGTGGCCGCAGATATTGCTTTTGGCTTAGTTTCTGAAAAATTGAGCTTA
TCTCAGATTAAAGTAAGGGTAAGAGAAGCCCTCTGTGCTGTTAATCTCCTCGAATTAGAGGATAG
ACCCATTTATGCCCTCAGTGGTGGACAAAAACAACGTATAGCCATTGCAGGTGCGATCGCACGTC
ATTGTCAGGTATTATTATTAGATGAACCTACAGCATTATTAGACCCAGATACTCAAATAGAGCTA
GTAGCTCTCGTACAAAAAATTGTCAAAGAAAGAAATATTACTGCCTTATGGGTGACACATCGTCT
CGAGGAATTAAATTATGCAGACGGCTATTTTTTATTAAAAGAAGGTAAAGTAGTATCACAAGGG
AATCCAAGGGAATTATTTAAGAATTAGTGGCAATTGTTACGACTTCAAGATTAAATTTTTGTTA
AAATCTACCATTATGACAGCACCAAAAATTAATCTTAGTAAAATATATATGCAAGGAAATGAAT
GCTACTATTTCTCAGGCTATCTTATTAGTGGATGGCTACAATGTCATCGGTTCATGGGCTTACCT
CCAGAGTATAAGAGACCAAAAAGGCCTTGAATACGCCAGAAACTCTTTAATTGAAAGTTTAATTA
ACTATTCTGGTTATAAAGAGTTAGAAACAAAAATAGTTTTTGATGCCCATTATCAAAAAACTGTT
AAAAATGAAGAAAAATACAGTAAAAGTCTTTCTGTTCACTACACATCATATTCAGAAACGGCAGA
TACTTACATAGAAAAATTTTGTGCTTCTTTTGTTCGTCAAAACCCAGAGAGTCGCACAAGGATAA
TAGTTGCAACATCAGATCAAGCTCAACGTCACACGGTAGTAGGTTATGGGGCAGAATGGATGTCT
GCTCAAAAACTAGCCAAAGAAGTTGATTTTACAAGTATAAGGCAGAAAAAAAACTTACGTTATC
GACCAAAGTCTCAAGGTCGTTTTCTGTTCAATTCCCTTGATAGTAAAACTCAACAGGCATTGGTG
AAGATGAGATTTGGAGGTGGATAAAGAAAAATTTAGCCAAAATCGTTGAATTATTCTATTAAGA
CTGTTATGATAGTATTTGGTCTTAAGCGGACGTGGCGGAATTGGTAGACGCGCTAGATTTAGGTT
CTAGTATCTCTGATGTGAGAGTTCGAGTCTCTCCGTCCGCATTCTCTCAACTTCTTTTCTCTTCAT
CGTTTCAGAAACTTTCTTTTATATTAATTAGTTTTTTTCAATTATTCTTCAATAGTTTGAAAAAA

CATTGAGATACTTGTGGCAAGTTACATAAAAATAAACGTAAGGTATCTACTTTTAATAAAAAAA
TAACGAAAAAACAACCTTTCAGAAAATAATAATACATTATAAAATATAAATATATCAGTGCTTA
TAGATTAAAAGTAGAGGAAAAGTAGAATAATACTTATAGAGCTATTTATAACCTTATATTTTAT
AAACATTTAGACAAAAGCTAAAAAGTAGAGGGTTAAGTAGATAAAAGTAGAGGATTTTTATTTA
TTACTGCTAAATCCGAAACAGGTTTCGTTTTTTTATTATTGGCAACACGTTGCCATTTTGAAGGT
TTCTATTCTGACAGGATTATATTCCCGTAAATTTAAAAGCGAAGGATTCTTCGTTTTTAATTCTT
CAAACCCGTTTTAGTTCTAGTTCAAACCTCATAGGATTGAAAATAATTCAAATCCATTAACTAGC
ATCCAAATAGTCGAGCAAACCTTTAAGGGTTGCGTATATGAAAAACAGTTTAAAACCCTTTCCTA
GAGTCTCACAGTTCATTATCAAACCTTTAACGATTGATCCTATGAGAACGCTCTATAATCACCTT
TTCAGTAAAGTTTTAGTGATTGTAAGTTGTCATCCAAACCTTTAAGGTTTGGATCTATGAAATCT
AACTTCGTAACCCTTGTATGGGAAAAATAACAAGGGCAAAATTAAGCAAATTATCTTAGGAATT
AATTAACGCTCTTAGTACCTATGCTGTGCAACTGTGCATTTTCTTGATTTTCTCTATAAATTCAT
TGTCTGAATAATAATCAAAAAATAAGTCAAAAAATAATGATTTATCGGTAGTAATAAATTCATA
AACCATTTTAGGTTTTAAATCAAATTCTAACTCTATCCATCGCTCCCAATCTGTAGGATTTAAGT
TAAGTTCGATTTTGGCTTTTAACCAAATTTGTCTCATTTGTTCTTCTTGATAATTAACCTCTAAA
GTATTGATTTTAATAGCTTGTTCATGTATAAAGTGTTTTTCTTTATCAGTAAAAACATCTATTGT
TGAATCACTATTTTCTAAGTTAACTAAGTTATTAGATTTGATATTTTCTCCATTATCAAGAGATT
TTTTAACTTTCTCAAAATTTTCAAAAGATTTATATTTAGAGATTATTTTTTTCTCTTCATCTTTG
AATCTTTTTTCAATATCTTTTTGTCTCTCAATACTATCAAAAATTAAGGCTTTACAATCTCTCAC
AGTCAAATCCGCCATCGTGGCGTTTTTCTTTTTCTCAGTTAATAATCTTTGTGCGTCATTAATTCC
AAAATTAAGTTTTTCTGAGTTATTTAGTTCGGGTTCAATCACATCCCACGATAAAGCAAACTGCA
TATATCTTTGAGCGGTTCTTTGAGGGATTTGAGTATTAGTTTCTAACCAATTTAGCCATGTTCCA
TGTTGACATTTACTTTTAGCTTCTATTAGCAATTCTCCGCAATTTTTAGCATAAATCAAGGCTTT
TGACTGATATTCTTTAGCTTTACCAAAGTTTTCTTCTGCTAATGCGTATTGTTCTTTAATCTGAA
CCGATAATGAGTCAATATTGGTTATCATTTGATTAATCCTATTTAATCTAACTACATATAGATTG
AAAAACCCCATTAGTAGAGAAATACTAATGGGGTTAAGTTTTATTATAGCTAATTATTAGAGAA
TTTCATTAAATCAAACATTCATTTTAAGACCACTAGAAGGCTTAATTTTCACACTACATTATTTT
AATGATTAATCATAAGCATAACTCAGTAAACATCGCAAATAAGTGTATATATTCTGAGAGTTAA
TCAAGACAATATTTTCAAGATTAAAACTTACAAAGTAAACATAATACTTAAAATAGCCTTGTTAC
AGGTTAAAAAGAGAGATAAATAAGGTAAAATAAAAATGAAAATAATTATTATATTAACCGTGTA
ACAGGTAACTATGGCTAATCATCATATAAAGTATCTTGGTGAGCAAACAAGATATAAGAGTAAA
TGGCAAAACACCCCTACAAAGTTAATCAGAATACCTGAACTATTTGAAGGTGAAATATTAGCTTA
TGCTCATCAATTAGATAATAGTAATAATTTTACTAATAACCATGTAACAGCTAAATTAAAAGAA
ATTAGTCTCAAGATTAATAATAAAGAAAAAGGTTATAAAACTAATTCAGCTAGTCAGTTAATTA
AAGATATTCAACAATTATTAGATGAGGTAAACTAATGCCAGAAAAACGAACTAATAAAGTAATA
AAGTTTACGGCACATCCAAATATAGATAAAGCATTGAGAGAATATTGCAAAGAAAATAATGTTT
TATTATCAGATGTATTAAAACTGATATTACTTGACTTTTTATATAATGCAAAAACCAGTTCACCA
TTAGTAAAAAGTTATATAAAAAGAATGTATAAAGAGGACTTAATGCCTAGCCCTCAAAGTCCTA
AACAATCTGATTTACATAAAAATTATGATTTGATGCAAATCATGATCAAGCACGTTTCAGATCCT
GAGATCTCCGAAATTGTAGAGAAAATTCTTGAGGATGATCCCACCAATGGTATGGACACAGTATA
ATAAAAAAAATATCTTTTAGGTGAATCCTAAAAGAAGCACTAAAAACACAAGAAGGTGTTTGCG
GTACACCCCAAAACCGTTTAATTAATTTTTTCAATCACTTAAATTATGACACATTCTAGCAGTTTT
TCTCTAACTGAGCTTTTTAGTTCAGATCGTTTTGCTGTATGCCGTGATAAAGAATACTATGAAAA

ATCATGGCAAAAAGGTAAATCTTATGACTATTGCCAACAACAGTTAAAAATCGGTAAACCTAATG
GCTATGGATTGTTAACAGGTGAATTTAATGGAGTAGGTATTGTTTCCATAGACTTTGATGGTGAT
AGTGCGGATCAAGTAGCTAAAGCCATTGGTTCATGGTTACTTAATCAAGATAGTATGTGTTGGTC
AAGTGGCAAAAAAGGACATTATCAGATTGCATATCAGATACCCACTGAACACTTAGCTCTATGGC
AACATATCAGCAAATATGACTTAACCAAATATAAAAACTATTCTGCTATCAATGGCGATCATCTT
GAGATACGCTATAAAGGTCATGCTAGTGTCTTACCCCCCTCTGTTCACCCTGAAACAGGTTATTA
CAAGTGGATTAATGAGATACCTCCTCGTTGTCTATCTTTTCAAGAGTCCTATGATTTACTTAATA
CCTGTTTAATTTTCATCAAGGATAATGATTTATCCCCCAAGACGAACTGAGAATGATTGAGGTA
GCTTTATCTCATATCCCTAGCGATGATTACCATACTTGGGTAACAGTGGGTATGGCATTATATAA
TCATGGAGTTGATTTTGATGTTTGGGATAGATGGAGTGCTACCAGTGCTAAATATAATGGCAAGG
GAATAGAGAAAAAATGGGATAGTTTTAAAAAGGGTTATAAAGTAGGAATAGGTACATTATTTTA
CCTTGCTAAAAACAACGGTTTTAATCAATCTCAGTGGATGAGAGAAAACCTCAAAAGAAATGTAA
CAATACTTTCTAAAACTTATGAAAAATCAGGGGATGATATTAATGAAAATAACGTCCCTTTTTTG
TTTGAACAATTAGTAAATAAGTTAGCACAACCTGAGATTAATGAGTCTCAAAGATTCTTAGAAA
TAGGTCAATTCTGTACACAATATAAAGTAGCCCCTAACTCACTTGTTACAGCGATTAAAACAAAA
ATAGAAAGAGAAAATAAAACCATTGAATTAGAGAATGTAAAATCTAATTTAGATGACTTAATTA
ATGTTCCTAATGAGCAATTAGATTTGAGTTATATTTTTGGGAATTATTTTGCAGAATATATAAA
AGAATCAGCTAAAGAAATTCCCACTAATCCCGATGCTATAGTCACGATATTTTTACCTGTTTTAG
CATCGGTTATTGGTACAAGATCAAGAATAATTGTTAATCCTAATACTCGTTATATTGTGCCTTTT
ATTATCCGTTCTATGATTGTGGCAAGAAGCGGTAATAAAAAATCACCTACGGCACGATTAGCGGT
TGATCCATTGCAAGACATCAATTTGAAATACTATCAGCAATATAAGAAAGAATTAGCAGATTAT
AATGAATTATCTGGTGATGAGAAAAAGACAACTCCCAAACCAGTGCAGAAAAGAACTATTACTCA
AGATAGTACCTTTGATGGACTCATTAAAATTCATGCAGAAAACCCTCAAGGCTTTTTATGTTATG
TGGACGAGTTAGCAGGATATTTCAAGAGAATGAATAAATTTTACAATGGTGATGATGTTCAGAG
AGATTTGGAACTATATGAGGGTAAACAGTTAATTAAAACCCGTGCTAGTGAAGAAAGTACATTT
TTCCTTGAAAGAACTGCTATCAGTGTCACGGGTACGATTCAAGAAGTGGCATTAAAGGAAATATT
AAACAATGAGTTGGATTTAACGGGTATTAGTGCTAGATGGATTATTTGGGCGGGTAAAATGCCTT
TGGGAAGGCTTACGGAAAGAAGTGAGGAAAATAACAATACATTTTCAGATGCTGTGAGTCAGGT
TATTGACATCTTACTAGATATGAATATCAACAGTGATTTGTTAATAGATGATACTGCTTATGAGA
TGTTTAGACAGTGGCAACACGGTGTGATGGATTGTTTGCAAGATTTATCCTTACCACAATTAGAG
AATAAACATAGCAAGATTGAGAGTGATGTAATTAAGTTTGCTGGTGTGTTGCATTATCTTTTTTC
TGTTATGGGTTCAGAATTGATTACAAATGAGATTGTGATTAACTGTGAGATTATGGCAAGGGCA
ATAACACTAGGCAATTATTATTTGAGACATTTTGCTTATATCGTCACTAAATGCCAAGAAGGTAT
GCTCAATAGCCAATTGCTGAAAATCCTTGAATTGTTAAAGAAAAAAGGTGAGATTACTAGCACGG
ATGTAAAAAGGTTTATTTGGGAGTTTAAGAGCGTTCCTAATGGTGATATTAACGAGTTGCTATT
ATCCCTAGTGGAATTAGGTAAAGCCCAACAAATACCTACTAAAAAGGGTATCAAAATTAAATTAA
TGTAACTTGCTTACAATGCGTCAATATAAAGTATTGAAATATAGTTACTCAAAATAAACCTAAAA
ACTAAAAGTAGATGAATCCTCTACTTTTTAAAAAGATGCTTTTAACTCTTTTATAACAAGAGTTT
TAAACATTTCTTTTTTTTTGACCTCTACTTTTACCTCTACTTTTTGGAATGCTTATATCATAAAG
GTTTAAGGCATTATTACAACGTAATCGTAGAGAAAGTAGAGGATTTTACACAAAATATGATCCTT
TTTCTTCATTTGAGCGTAAAAGTTTATGTTTACATTTCATTTTAACAACATATTTATACAATAAG
TATTTGAGATTTATATGTGACAATAATTAATCAGGATACTGTAGTTGATAAATCTGACAAAGAA
GTTTTATTAAATCTTCTTTTTTGTGATTAGTTGGCAAATCATCTAAGAGTTTTAATGCAGTTTCA

AAAGGGATCGGATTACGTTTAGCATTTTTGTATAAATCTATAGTTGTTTGACCTGAGAAATCTTG
ATAGTATTTGGCATTAACCGTTTCAGAATGTCCTAAAGACTTACAACGGACTGTGTCACTAATAC
CCGCTTGGATACCATTAGCATTACCTAATTTTCTTAAAGTGTGTGTTTGTGATACTTTATAGCCT
AAAAATTTAGTTGACCATTGTTCTATTCTTTGATTAGCATTTCTTGAGTAAATATTAAAACTTTT
ATGATAAAAGTTAGGTACATTCTTAATCTCTAATAAATCAATAATATTTGGATAGGTAGGGGGT
ATTAATGGCATAGCAATTCTAGCTCCTGTTTTTACTTTTTGATTACCCCATAAAGTGAAATTACC
AATATAAATTATATTATCTTTATTATCAGGATTATTTAATGCTTTAAATATGGTTTTATCCTTAC
TATTTTCTTGAGGATCATTTGCTGGAATATAGTCTATAGTTAAGTTTTTAATAGCTGATACCTCA
GCTATTCTTAAACCATAAACCAACTGAATAGAATAAACCCATAACCAAAATTTACGATTATTTTC
CCTTAATGAATGACTATCTTTAATAGATAATACAGCAACTCTTAATTTTAAAAAATCTTCAATAC
TAATTGATTGTTCTTCACGATTAAATGTATTTTTTTTCTTTGCATTAGTTTTAGTTTTAATTTG
CTTAATTTGTTATAAATTGTTTTTAGTTGATTGACTTCACATAGTTTAAGAAAAGCATTTAAACA
TTCTTGATATTTTTGTGTTTCTCTATCTGACTCATAGCTAATTAAAGAGTCATATAACAATTCAT
AAGATACTTGTTTCTCTTGATTATGTATTTTTTGATAATAATAACCATAAGCTCGTTTATAGCTT
TTATAGTCAGTTTCTTTACTACGTTGTAAGCCACGCCGATCATTTTGACTGAAAAACCATGATTC
AACTTTATTGATGGCATTAGCAACGGTTATAAGGTCATTAGTAGCGTTTTGATTTATTCCCTTGA
TGGTTTCATCATACCAAGCCCAAAACTCTATTTCTGACTCATAACCCTTTAATTGTTCATTAATT
TGGATTGCTTTTTCTAAGCAACGGTTTAATCCTAAAGTCGTAAAATTTTCTCCAGTGGCATGATC
AGATCTTTTTTTGTATTGTGTTTTGAATAAAAAATATAACTTATTACCACGTTTAAACAGTGATA
TACCTTTAGGCATTTGAGACTTGATATATTTAAAGTATTCAAGTATATCGTTATAACCTTGATAA
TCTTTTCCCATATTTTTCAACCAATTTTTCAACAAATCACTTAAAACCTTGATATTTAGTAAGTTA
GAGTTAATTTTAAATTGCCGATAATCATTGAAAAATAAACATTAATATAAATTAACTAGAGTTC
GAGTCTCTCCGTCCGCATTCTCTGAGTTTTTCATGAAAATTAAAACCTGTACAGTAACATATTA
TTTATTATCGGTAGCATATTAGTTGTATCTTAATAGATTAGATGCTTTTCGTAGCTCTGGTTTTT
TTATTACAATGTTAGATATTAACACTGGCTCTTCAAAGTGTGGTTATGATAAATTAATAGAACAT
AACTTCTATAACCTTTATTAAATAGTGTTTATAGTAAAATAAAAACTAAAAGTTGATACATTAT
TATTTAATAATTGCCTATTGCCTGTTGCCCACCCTAACCAATAATTGACAGACTCAAAGCAATAG
AGCTTTCGCACTCTTGTCTCTTCTCTCTTTTCTTTTTCCTATCTCCACTAAAATAGAAAAGAAATT
ATTTATTGTGAAGAAATATGACAGTTGCTCAGAGTAGTTCTATCTCTCAATTTTGGCAGGAGACT
TTTGCTCTAAACAAACGTTTATTCATACAGTTAAAACGTCGTCCTTCTACCCTTGTAGCGGGGGT
AATTCAGCCTTTTATGTGGTTAATTTTATTTGGGGCATTATTTAGGGGCGCGCCTCAAGGATTAT
TTGGCAATGATCTTAATTATGCTCAATTTTTAGCCCCCGGTATTATTGTTTTTACTGCTTTTTCT
GGAGCATTGAATGCTGGTTTACCTGTAATGTTCGATCGAGAATTTGGTTTTTTAAATCGTCTTTT
AGTTGCTCCTTTAACCTCTCGTTTTTCCATTGTCATGGCTTCGGTAATTTATATTATTACTCTTAG
TCTGATTCAAACCGCCGTAATTGTTATTGCTAGTGCTTTTATTGGTGCTGGTTTACCTAGTTTTG
CTGGACTAAGCGCGATCGCACTTATTGTCTTTTTAATCGTCTTAGGAGTTACGGGTTTAAGTCTA
GGATTTGCTTTTGCATTGCCGGGGCATATCGAATTAATTGCCGTCATTTTCGTAATTAATTTACC
CTTATTGTTTGCTAGTACTGCTTTAGCACCATTATCTTTCATGGCAAAATGGTTACAGATAGTTG
CAAGTCTCAACCCTTTAACCTATGCTATTGAACCCATACGCTATATTTATCAAAATAGTGATTGG
AGTCTTTCTAGTGTCGTGATGAATACTCCCTGGGTTGATTTTAGTTTAGTTACCGTATTATCTAT
ACTATTGACTTTTGACTTAGTTATTTTACTAACTATTAAACCCCTTTTAAGTCGCCGTTTTGCCT
AATTCCCTCGATTTTTCATCTTAAACTAAAGCATTTTTTTGTCTCTTTTTTCTAAATACCAACTTC
ACTAATTAATTTTTAAATTATCATTAAATTTCAGTGAGTAAGGGGTTTTACCCCTTATAAATACT

ACCCTTTTTTATCCTCATCATCGAGGTTATAATTATATTTATTATCTAAGTTCAAAAGTATAGAT
TAATGATTAAGCAAGGGGATTTGGTAGAAGTTGAAATCGATGATGTTAGTAGCGAAGGCAGTGG
AGTAGCAAGGGTTGATCAACAAGTAGTTTTTATCCCCAATACCGTAACAGGCGATCGCATCTCAA
GTAGAATAGTGAGAGTTAAAAAGAAATATGCTCAAGGGCAATTAGAAGAATTCAGAAAAAACTC
AGCTTATCGTATTCGCCCTCGTTGTATCGTTGCCGATAAATGTGGTGGTTGTCAGTGGCAACATA
TTAATTATTCATATCAATTACAACTTAAGCAAAACCAAGTAAAAGAAACCCTAACTCGCATCGGT
GGATTTGCTGATTTACAGGTAGAACCCATTATTTCAGGAGAAGACTTAAGTTATCGTAACAAAGT
TAACTACCCCCTCGGAGTTTCGCACACAGGACAAATTAAAGCAGGATATTATCGTCAGGGCAGTC
ATCAAATTGTTAATATAAATCAATGCCCTATTCAAGATCAACGTTTAAATCCTCTCCTTGCAGAA
ATAAAGCAGGATTTACAACAATTGCAAATACCCATTTATGATGAAAAAACTCATAAGGGTGCATT
GCGTCATCTCTGTTTTCGCATTGGCAAAAATACGGGAGAGATATTACTAACCCTTGTTAGTGCCG
AATTAAGCGATGATACCATGGATAAACAGGCAGAAAAATGGATGAAACGTTACCCTAATCTAGT
GGGAGTAACTCTTAACTATAATCCTCAAAAAACAAACGTCATTTTTGGGCAAAAAACAGAATTAC
TGGCAGGAAGACCTTATTTAATAGAGCAATTTGCCCGACTAAATTACCATTTAAGGGCAGAAACC
TTTTTTCAAGTTAATACGACTGTTGCCGAGGATTTATTGACCGCTATTTTAAATAAATTATCTCT
GACAGGAGAAGAAACAGTCCTTGATCTTTATTGTGGAGTTGGTACTTTTACACTACCCTTTGCTC
AAAAAGTGAAAAAAGCCATCGGTGTTGAGTCTTATTCTCTTTCTATTGAACAAGCCCAAAGAAAT
GCAGAAATTAATAACATCGACAATGTAGAATTTATCACTGAAACTACAGAAACATTTTTACCTAC
TTTAGAGATAAAGCCAGATCTAGTTATTCTTGATCCTCCCCGCAAGGGCTGTCAAACAGAAGTTA
TTGAATCTTTAAAACAGATCAAATCTAATTTCATCTTATACATTAGTTGTCATCCCGCCACCCTC
GCCAGAGATTTACAATTACTTTGTCAAGAAGGAGATTATCGAATTATGTTTGTGCAACCTGCTGA
CTTTTTCCCTCAAACTCCCCATGTTGAAACTGCTGTTATCTTGACAAGAATATCCTAAGAGAGAC
GCTAATGAGTATTTTAAGGGTAAAGGAAAATAGTTGATAATTAATAACTTCTAACTCCTGTACG
GGCGAATGGCCATTCGCCCCTAACTAACTCCGAACCCTGACACCTATCCTTCTCCGATATTCTTAA
ACCGAACTGAGGTTATCTATTATGAAGCCCAAGTTTTAGCACGATTAACCGCTTCTAGCCAGCGA
TCGCATCTTTGTTTTCGTTCATTTTCCGACATTTTTGGTTCAAAAACTTTAGTATCACTTTTAAT
CTGCTGAATTTGGTTTAAATTATCCCAGTAACCAACTCCTAAACCTGCAAGATAACCAACACCTT
TTGCTGTAAGCTCAATTTCTTGGGGTCTTTCCACAGGAATGCCGAGAATATCAGCTTGAAATTGC
AAAAGGAAATCACTTCTTGCCGCCCCACCATCAACAGTTAATAATTCCAAAGGTGTATGGGAGTC
TTCTTCTAAAGCCTTTACTACATCATAAACTTGAAAAGCAATACCCTCTAAAGTTGCCCTTGCAA
TGTGAGCTTTTGTTGTACCTCTGTCTATTCCAACGATTGTACCACGAGCATATTGATCCCAATAA
GGAGAACCTAAACCAGCTAAAGCAGGAACAAAATAAACATCTCCAGTATCAGGCACTGAATTAGC
TAGGGTATTTACTTCATCGGCATTATTGATGATACCTAAACCATCTCTTAACCATGTAATTGCCG
AACCACTAGCCAATATACTGCCTTCTAAACCGTAAGTAATTTCGTTATTAAAACGCCAACAAATA
GTTGACAAGAGGCTTTTAGCAGAAGGTACTAAAGTTTTACCTGTGGGAGAAATTAAAAAGCAAC
CCGTGCCGTATGTATTTTTGGCAATACCCTTATGAAAGCCTGTATGTCCAAAGGTTGCCGCTTGT
TGATCCCCTGCAACTCCTGAAATGGGTATTTGCTCTCCGAAAACTTCTGAAGAAGTGTATGCAAC
GATACCACTATTATCGCATAGTTGAGGTAACATAGATTCGGGAATATTGAATAAGTCTAATAAA
ACAACATCCCATTTTACCTTGTTGACATTGAGTAAAAGGGTGCGAGAAGCGTTACTTACATCGGT
AATATGTTTTTTGCCCCCCGTCAAATGCCAAATTAACCAAGAATCGATCGTACCGAAAGCTAGTT
TTCCTTGATTTGCAAGATTTCGAGCATTTTCTACATGGTCTAAAATCCATTCTACTTTACTAGCA
GAGAAATAAGCATCTAAAAGCAATCCCGAGCGATTTTTAACCTCTTTTTGTAATTCTCTTTCCTT
AAGATTGTCAATTTTTGCTGAAGTTCTTCTGTCTTGCCAAACAATTGCATTATAAATAGCTTTCC

CCGTCTCCTTATTCCACACAACCGTTGTTTCTCGTTGATTGGTGATGCCAATACCCTCTATTTGTT
TTTTCTCAATATTTCCTTGTTCAATGGCTTTTTCTACCACTGATAAAACAGATTGCCAAATTTCA
AGGGGATCGTGTTCTACTTCTCCGGGATTAGGATAGTATTGGGTAAACTCTTGCTGTGCCGAAGA
AATAACGTTACCATCTTTATTAAATATTAATGCTCTCGAACTGGTTGTTCCCTGATCGATTGCCA
AAATGTATTTATCCATTATAGTTAGCCTTAATTTTGTGTTCATTCTTTATGGTAACTATTAATGG
GGGTTGATAAAAAAGTGATGTCAGGGAAGTAGGTGTCAGGTTTCAGGTGTTAGGGGATGGGGGG
ATGGGGGGATGAGGTAATGAGGGGAAAGGGGGAAACAAGTAATAAATAAGTAGTAAGTATTTAG
TAAGTATTTGTGGTTTTTCATTTTTAATTCTTAATTCTTAACTATTTAACGTCAGTTCGGGTTAA
GGCAATTTTATTCTTATTTCTAAGAAACTATAAGGTTTTCTGACTACGAGAAAATAACGCTTTCA
GCTTATCCAAAACTCAGGTTAATTACTAATTACCTACCGCTTATATTTTCACATTGAACTAAAAC
TAAACATAAGGGGTAACATCTTCTTTACACTCATCAGCTAAAAAGGATAAAGAGCGAAATCTTAA
GCCTACTAACTGTTCATATAGAGGATTTAATTTACACATAGGGGGAATATGAACAATTTTTTTGC
CAAATAAAACAATATCACGTTCAAAAGGACATTGAGAAGGTACCATTTTACAAATAAAACGAGC
TAATCTAGGATCTTGAACGTTCATGTCATCCAACCAGTTTTTAACAGGTTTCAATAAGTCTGGAG
GGTGACTTTCTTCTTGACTTGTATCTTCTTCTTTCTCTGGATTCCATAAGGTGGCTTCTAAGGATT
TTAAGGCTTCAATATCTAAACCCAAAGCCTCCTGAAAAGCATGAACTGCATCCGCTTCTGATTGG
CTATATACCCCATTTGCGATCGCCATCATGACTGCGGTACGGAGGAAATTTTCTTTAGTATCGAG
ATCATCTCCTAAAGCCTCGGCTAATTCTTGAGAGGTAATAGGTTCTAAATCACCTAAATTTGTGT
TAGGTGCTAATTCATCTTGAGTTAAACTAGCAATAAATTCTTTCTCTTCAGGATCAAAATTGCCA
TCGGCATAGGCAACAGTTAGTAAACCCCGTAACCATGCAGAAATTTGTTTATCACTATATTGATT
TAATCTTTCGTTGTTTGTCATCGTTTTGAATAATATATTTGAATGATATTTATATTCAGCAATTG
AGCAATGGGCAATGGTAAATAGTGAATAGTTGATAATTAAGAATTAAAAACTCCGAACTCGTTT
CCCCTAACATCTTAAACCCTTACAACTAAGGCAATTTTAGGGATCAACCCATTTACCATCGGCTTT
GATTAAATTAATTAACTCTTTTACTCCCTCTGTCTCTGGTACTTTTTTGATTTCTTCTCTACCACG
ATACAGGGAAATATAGCCGGGTTGCTTACCTACATACCCATAGTCTGCGTCCGCCATTTCCCCCGG
TCCATTAACAATACAGCCCATTACAGCTATATCTAATCCTGTTAGGTGTTTGGTTGCTTCTCTAA
CCTGATGTAATACTTCTTCTAAGTTGAATAAAGTACGTCCGCAGGAAGGACAAGCCACATATTCC
ACCATTGTTTTCCGTAATCCTAAAGCCTGTAAAATGCTATAACAAACAGGAATTTCTTTTTCTGG
GGCTTCTGTTAAGGATACTCGGATGGTGTCGCCAATCCCTTCTGCTAACAAAGTACCAATTCCTG
CTGTGGATTTTATCCTGCCATATTCTCCATCTCCTGCTTCGGTAACTCCTAAATGTAGAGGATAA
TCCATTCCCAACTCATCCATTCTTTTTACCATGAGACGATATGCCGCCAACATTACAGGCACACGA
GAGGCTTTCATGGAAATGACTAGATTACGAAAATCTAAAGACTCACAAATTTTGATAAACTCTAG
GGCTGATTCTACCATCCCCTCTGGAGTGTCACCATAGGTAAAAAGCATTCTTTCTGATAAAGAGC
CATGATTAACGCCGATACGCATCGCTTTTCCTTGTTCTCTTAAGGAAACAACTAATGGTTCTAAT
GTTTCTTTGATTTTTTCCCCAATTTGGGCAAATTCTTCCGCAGAATAGGAAGTGCGATCGCTGCT
TGGTTTTTCAAATACGTATAACCCTGGATTTATTCTTACTTTATCAACGTGTTTAGCCACTTCTA
GGGCAATTTTCATTCCATTATGATGCACATCAGCAACTAAGGGAACAGGTTGATAAGTTTGTGCT
AATTTGGCTTTAATTTCCCCAACTGCTTTCGCATGGGCAAGACTTGGCACTGTCACCCTAACAATT
TCGCAACCAATTTCATGTAAACGACGGATAGCCGCAACGGAGGCATCAACATCGAGAGTATCTTC
GTTGATCATAGATTGTACTACAACAGAGTTGTTACCCCCAACAGTTACGTTTCCGACTTTGACAG
GGCGAGTTTTGCGTCTATGAATAGTAGTATCAAATAGGTTATTGTCTAAATCCCTTAGAGGTTTA
TCAATAGTTTGCATCGTCTTGCTTTTATGGCTATTGTTTATAATCAATTTTATTTTAAAGGACAA
TGACAGGGTTTTTAATAATAAACCTTTATTCTCTTGTTCTAAGGTAGATATTTTTTTTCAAGATG

TTAAGAATAATAACTTTATAATTATTAAATTTTCTTAAAGTATTTTTATGGAGCAAATCTTAAC
ATATTGACTAAAATCAGACAAATATTTACCATTTTCCATGGTTAGTATTTTTAATAAGTGATTCT
ACAGTATTTTTTAAGTAAGTGTCTTATATAGCAATGCTATTTGAGTTGTGTTAATTCTTTTGTTG
TAAAAAGCAAAAGGTAAGAGGCAAAAGGTAATGAAGATTATATATTCGGTGTTTTTTTCTAATA
ATTAATTTTTTATCAATGATTTATAAGTACTATATATCAAGAAAAAGATAAATATTCTTGATGG
TTAATTTAACGGATATATGGTTGAAAAAATTGAAAATAATTTAACATTTAACATCTTGATTTAT
CTATGGACAAAAAATAAATTACTAGATCTCATTTTTATTAATTTAGAGTTAATATATTAATTAA
GAGGCAGTCAGAAATTTGGAATTCAAGACTGAAATGATAATTATGTTGAAAAATAACTTGAACT
GTCATACAAATTCTTTAGGAATTTTTGTCATATTATCACTTATAAGTGTCAATAGTTTAATGGTT
GAGCCCAGTTTTGCCCAAACTTCTTCCTCACCCATAGAAGAAGATGTGTCTATTACTGGAGATTC
CTTACAGGGCTTGGACTCTAGCAGTCTTGCTGGAAATCAGTCCAATACTCCAAAATGGGATGTAA
CCGTTGAAAGGGAAAAGAAAAAACCCAGTTTTAGCACGGGAAATGAATTTTTTGATTCTTTATTA
CAAAAGCAATCTGAACCTCCCAAAACCTCTACAGAGGAAAAAATTAACACAAACAAGGGGGATGT
TCCAGTTAATCAAGGAGGTACAATTCCTTTATTTAATTTCTAACAGAGATTAGTTATTCGTGAAC
TACTCACATTCGCTACGCTGAAATGTGAGCTTCTTACCCAACAGATGGCGTTATAGTAGATTTCT
TTCGTCCTCTACTACCTCGTCTTACATCTGGGCGATAAGCTGAATCGCTAGTTCCTAACGACATTA
TTCGCAGTCCTTCATCTCTGATATTACATGAGGCGTTTACATCTCTATCGTGTTTTGTTTTACATC
TAGGACATTCCCACCGTCTAATATCTAAAGGTAAGCTCTTAACCACATTCAAGCAATGATTACAA
GTCTTAGAGCTAGGAAAAAATCTATTTATTTCAAGATAAACCTTACCTTCTTGTTCACACTTGTA
TTTTAGCATTGTCTGAAATTGTCCCCAACCTACCTGTTGTATTGATTTTGCTAGACAGTGATTTC
TTACCATCCCTTTGATGTTGAGATTTTCACATATTACAACTTGGTTTTCGTTTACTATCCTGCGT
GATAGCTTATGGTGAAAATCCTCACGAGTGCGAGATATTTTGTTATGAACCTTTGCTACTTTGAT
TCGGGCTTTGTTTCGGTTGTTTGAACCTTTTTGTTTACGAGATAAATCTTTCTGCTTATTCCTTA
GATTACGTTCATGACGAGCTAGCCATCTAGGGTTACTAAACTTAGAACCATCACTGGTTACACAG
AAATCTTTTATCCCCATATCCAACCCGATTGCTTTACCTTCTGTGTTTGGTTTTGGTTGTTCTTTT
CCGTCTTCAAATAAAATAGAAGCATAATACTGCTCTGCGTTATTTTTAGAGATAGTTACTGTCTT
AATTTCACCATCGATGGGACGATGGATTTGAGCATAAATCTCTCCTAACTTTGGAAAAATAATCA
TATCTCCTTTGATTTTGACATTTTGAGGATAGCTAAGAGACTGTTTACCATGCTTAGACTTGAAT
CGAGGAAAAGAAGCCCGTTTCTCAAAGAAGTTGATAAAAGAACGACTAAGATTGAGACATACTA
CCTGCAAACATTGGGAGTAAGTTTCTGTTAGCCATTCATATTCTTTTTTGAGGGAAGGTAATTGC
TTTTGGAGGTCATAACGAGATAAACCCTTTCCAGTCTGCTTATATGTTTCGTTAGTCAAAGCTAA
AAATCTATTCCAAAGCCATCTGCAAGAGCCAAAAGCCTTAGCTAACGACTCTTTCTGCCGATATG
TGGGGTATAATCTTACTTTGACAACCTTAAGCATACCAAGAAAACTTTTTACAATAAATTTATTG
TAACACAAAAATCTGGTTTTGTGTGGACTTGCTTTGCAAGGGTTTTCTTAGTTGGGCAAAATTCA
TCCGCTAATTCGCTTTGCTGAAATTAGGGACTTCTTTTGCCATTCACGTTAAAAAATTAAGCTCA
CTTAGCTATACTTCTGTTTAAGATGAGGTTTTTGGGTTGTATTAGGTAAGAAGCGAGAAATAATG
AGCTAAAGTGGGAAAAATTTATGGCTCTATCAGTTTGAGTATGTAAATTATTAGTTAGGTTAGG
CTGAAAGGCAAGAGGCAATAGGGGATTATTAAATAATAATTTATGAACTTTTAGTTTTCATCTT
AGCTATAAACACTATTCAATAAAGGTTATGGAAGTTATTTTCTACTAATTTATCATAACCACTGT
AGAAGAACCAAATTTATTTTCAATTTTTGCTGATTATTTAATGGCTAATTACTAAAGATTTCTGC
TTCTTTAAGCAGGGGAGCGTTTTTGTCTTTTTCTGAAAGAATTGGAGTTTCTTTTAAGTTCCAAT
TAAGGTTAATTTCTGGATCATTCCAACGAATTGATCTATCATGTTGTGAACTATAAAAATTATTG
GTTTTATACAAAACATCGGCAGTTTCAGAAAGCACTAAAAAGCCGTGAGCAAAACCTTCTGGAAT

CCAGAGTTGTTTTTGGTTTTCTGCACTCAGTTTGACAGCAACCCATTGTTTAAAAGTGGGTGATG
ATTTTCTTATATCAACTGCTACATCTAATATTTCTCCGTTAATCACTCTAACTAATTTGGCTTGT
TTTTGTTGTATTTGATAATGTAATCCTCTTAAGACATTTCGTTGAGAATGGGAGTGATTATCTTG
GACAAAGTTGGGATTTAAGCCTGTTTTTTCTTGAAAAGTTCGAGCGTTAAAACTTTCATAAAAAA
AACCTCTATCATCTTGAAAAACTTTAGGTTGAATGATAAAAACTTCGGGGATTTCAGTAGTGATA
ATTTCCATAATATATATAATTTGTCTAGTTTTTGAGAATAAAAAATACCTTGTATTGATTGCCCA
GATTTTTAATTAATAAAGACTTCTACTACTTTACTTAAACCAACGGAATTAGAGGCTTTAAATAA
GATGCGATCGCCTTCTTGTGTTATATTTTGTAAAGCGGAAATTAAATCTTTATGGTTGCTATAAA
TTTGAGAAGGAATACCATTTGCACCTTCAGCAATTTGTTTTGTTGCTTCCTCATCGGCTAAAATC
AAGACAAAATCAAGGTTTAACTCTTTTATCGTTTCTCCTACCTGACGATGTAATATAGGTGCATA
GTCTCCTAATTCTTTCATAGTCCCCAACACTGCTATTTTACGTTTTCCATCAGTTTGGGCTAAAAG
TTTTAAGGAAGCCAACATGGATTCATAACCCGCATTATAGGTTTCGTCTAACACCAGAATATTAT
TGGGCAGAGAATAACGTTTTGCTCTTCCTTTTGGTAAATTAACTTCTATACCTTGTTGTAAAAGA
GTTAAATCTAACCCTAAAACTTTGGCTGTAGCTATTGCTCCTAAATAGTTTAGGGCATTATGTTC
TCCAAGTAAAGGTAGTGGGTAGAGTTGATTATCTACTTTAAGGGTGGAGTCGTTGATTAATTCTC
CCCGAAAATTTCCCGATTTCAAACCATAGGTAATAGTTTCTCCTGTCCAAACTTTTGTTGCTGTT
GCTACCAATAAATCATTATCTCCGTTAATAATTGCTACTCCTGAATTATCCATATTTGCTAATAA
TTCACACTTTGCTTCGGCGATAGCTTCTCGTGAACCTAATAAACCAATATGAGCAGTTCCTACAT
TCGTAATTACACCAATATTTGGTTGGGCAATTTCAGTTAATAAAGCAATTTGCCCTTTCCCTCTC
ATCGCCATTTCTATGACGGCATAATTATCAGTTTCATCTATTTGTAAAAGAGTTTTTGGTACACC
AATTTCATTATTATAATTGGCTTCTGTTTTTAGGACTTTTCCTTGTGTTTTTAAAACAGCACTAA
TTAATTCTTTTGTAGTTGTTTTTCCTACTGAACCTGTTACCCCAATAACTGGTAATCCTAAATTT
TGTCGCCACCATTGAGCTATTTTTTGATAGGCTTCAAGAGTATTTTTTACCTGTAAAATGGGAAA
ATTTTTGTTAATTAAGTTACTATATTTTTCATTAACAATAGATGCGATCGCACCTTTTTCAAAAG
CATTATCAAGGAAATCATGACCATTAAAGTTTTCGCCCTCTAAAGCTAAAAAAATTTCTCCTCTT
TTTAAAGTACGAGTATCGGTACTAATTCCCAAACAATTTTCTGCTAAAATCTGAGAAGAAAAAGA
AGAACATTTAGCCTCAGTAATTTCACGGATAAAATCAACATTTGCAAAAGTTTTCATAAAAAGAA
AATATAAAAAATCAGTAATTTAAACGATATGAAAAATAAGCATTTATATTAGAATTTTAGTTAG
AGGTGAAAACATCCTTACGCAAAATTCTTCAACAATAATTAAACAAAAATATATCACATCATGAA
CTGCCGTGAATATCAACTCTTTATCAAAACGCCAATACAACAATATCATAGGAGATGAACAATAT
CTCTATGATCATTTGCTAGAGTGTGTTAAGACCAAATCACCTCAGCAGGTGTTGGATAGATTTAG
ACATTTATTCATTCAGGCAAGAAATTACGACGATCAGAAAGTATTAGAAGTTTTAGCCAAATTAA
TTGCCACCAAACGGGCAGAATTAAACTTCCCTTCCGTGTTAAATCGTTGTTGCCATATTCTCATC
AATATGTGGCAGTTGAACCCGAAAACTCAACCTTTTATCGTTGAGTTGGTTGAATTGTTTAACTA
TCTTCCTTCTGGAGTAGGTATTGGTAAATCTTATTCTAGTCGTATGGTGCAGTTGGTTAGACATT
TTTTAACCACTGATTACTATAAACAAATTCAACGTCTCGCAGTTATTATTGGCGAGAATCAAGGG
CGAAAAAATAACCTCATTCCCACAAAGCCGAAACAAGAAACAGTGGGAAACTTAATCACTCGTTA
TCCTTATTTGTATGATCATTGTTTATTAAGTGTTGATAGTAGTTCTGAACAACAACAAACTGTTT
ATCAAGTCAAAAATCAGTTAGAGAAAAAATTTGAAACGGAATTATCTCAATTTGTTACTTATCA
AATAAGGGTAGCACAGGCAGGTAGAGAAAAAATGGCACAACAGGAAAGAGAAAGAATTATTAAA
CCTGTTACTAATCCTACCCTATTAAGTGATCGTGATTTAGGAAGAAGTTTGAAACATTATGTGGG
ACCTGTGGAAAATGGCTATTCCTATCAGGATTTATCCCGTAGTTTTTTAACCCATACTAGTGATG
TGCGTAATTATCAATTATTCAAAGATGATTTATATGAGTACATAATACAATCCGTAGATGGCAAG

TATGGCAAACATAGCTTCAACAAAAGACTTTATGACAAAATGCAGGATTTATACCCCAAATATAA
TCAAACCAAACCCGATGAATTTCTAAAAATGCGTACTTATAGTCAGGTTTTTAATTATCTCATTG
TCGAAAGTCCCCAAAATCCAAATCATTTGGTATTTATGGATATGATTAGTAATATGGGAACAACA
AAAACCATTGGCTTAATGTTAAAGTTAGTCTTAGTTTGCAGTAAAGTTAAACCATACTTAGAAAA
AAGATTCTCCATCTTATTCAATCACTACGAGTCTTTCACCAGAGAAGGTGCAGGGTGGCTAGTTA
GAAGTTTGGAAAAAGTCCATGTAGCTTTTAGTGTTAATTTTGGTAATGTTGATATGTCTTTCTTG
AGAAGAATTTATGCCAAAAATTAACTTTCATGACAAATTATCCTCGGTGAGGTTATTTGCAAAAA
AAAATATGGCTCTCTTACTGTTCGTCGTGTAAATTATTGGTTAGGAAAAGGTGTCAGGTGGTAGG
TGGCAGGTTTGACGGTTAAAGAATAAAAATAATAAATATTAACGGCCCTATCACTTCTCGTCGTG
TAAATTATTAGTTAGGGTAGGCAAGAGGCAATAGGGGATTATTAAATAATAATTTATAAACTTT
TAGTTTTTGTTTTACTATAAATACTATTCAATAAAGGTTATGGAAGTCCTGTTTCTCTTAATTTA
TCATAACCACTATAGAAGAACCATATTAACTTATAACAATTACCTGAGTTTTGGATAAGCTGAAA
GCATTATTTTCTCCTAGTCAGAAAACCTTATAGCTTCTTCTTAGAAATAACGATAAAATTCCCTT
AACTCGAACTGACGTTAAATAGTTAAGAATTAAGAATTAAAAATTAAGAATTAAGAATGAAAAA
CCACGAATACCTGTACGGGCGAATGGCCATTCGCCCCTACTTCCCCCTTTCCCCTCATCACCTCAT
CCCCCCATCCACTACCACCTGTAACCTGCAACCTGAAACCTAACCTTATCAGATATTCTTAAACCC
AACTGAGGTTAGATAATTACTTAAATTTATCCATTCTTCAAGACTTAAATTTTCTGCACGAGCGT
GAGGATTGAGATTAAGTTTTTCTAAAGTGTCATTTAATTTTTCAGGAGAAATTAAAGATTTTAA
ATTATTTTTTAGCATCTTTCTTCTACTAGAAAAACCTAATTTAATTAAACTGTCTAAAAAAGCTG
GATTTTTAGCAGGATTAACTAGATTTCGAGGCGTTATTTTTACTACTACAGAATCAACTTTTGGC
GGAGGATAAAAGTCCCTAGCTGGAACGGTACAAACTATTTCACAGTCTGCTAAATATTGCACTTT
TAGGGTTAGCGCCCCATAAACTTTATTATTCGGCTCTGCTGTAAGTCTTTCCCCTACTTCTTTCTG
AATTAATAAAACGATCGCATCTAATTGTTTTTCTGCGGGTTTACTAATAGTGCCTAAAAGTTTTT
CGATAATAGGACCTGTGATATTATAAGGTATATTCGCAACCACTTTGTTGTAAGCTAAAAAATCA
GGAAAAGGTTGTAAAATTTCTGGTAAATTTAACTCTAAAAAATCTCCTTCTAACCAAGAGAAAAT
TATCTGTTTTTCCATAGTCATTTACTAATTTTTTACATAAATCCCGATCAATTTCCACTGCCAAT
AATTTGTCAACTAAGGGTAATAATCTTTTTGTTAAAACCCCTGTACCCGGTCCTATTTCTAAAAT
GCGATCGCCCTGAGTACCAGTTTGAGGATTACTTTTATTTAACTCAGCAGAAAAAATTATTGTAT
CTAAGGCTTTTTGACTTTTTAACCAGTGTTGTCCAAACTGTTTTCGGGGTCTAATGGTCATCACA
AACGCTTAAAGGTGCAATGTATAAGTATAACATTAACTTCATTTATCGAACAGAAAATGTAATCA
AAAAAGGGAAATTTGTTGCCCAATACTGATATGATGATAAAGACCCAATCATATTTAATAGTTAC
CAGTAATCCTCAAATGTCTATTAATCCTTCAATTCCCCTCAGTCAATCAATCCCTGTGGATAAAA
TTCAATATAATGAGCAAGGATTAGTACCAGCTATTACCCAAGATTATCTTGATGGTACAGTGTTA
ATGATGGCGTGGATGAATAAGGAATCTCTACGTAAAACCCTTGAGACAGGAGAAGCATGGTATTG
GAGTCGTTCTCGTCAAGAATTATGGCACAAAGGAGCTACTTCTGGGCATATTCAAAAAGTGAAAA
CAATCCGTTACGATTGTGATAGTGATGCCCTTTTACTTACCATTGAGCAGGTTGGTGATGTTGCC
TGTCATACAGGAGAAAGAAGTTGTTTTCATCAAATTGATCATTCCAAGTCTGCCCCATCTGCGGA
TACTTTAACAGAATTGTTTAAAATAATATGCGATCGCAAAGAACACCCCGTTGAAGGTTCTTACA
CCTGTAAATTGTACGAAGGGGGAGATAACAAAATTCTCAAAAAGATTGGCGAAGAAAGTGCTGA
GGTTGTTATGGCTTGTAAAGATAATGACCCCGATGAAATTGTCGCTGAAGTTGCTGATTTATTTT
ATCATACCCTTGTTGCTTTAGCTTACCATAAGGTTAACTTACGGGATGTTTACAAAAAATTGCAA
GAAAGAAGAAGATAATTTAGCTCAACGGACAAAAAAGTAAAAAGCAAGGAGTAAAAGGGAAAGA
ATTTTTTAGAATACTCTAATGACTATTTATTATTTTCCTAATATTTTTTGATTAATATTGACTGT

ACATTTGTATCTTGATAAAACTTAACTATAATTTTTCAAATGACAGATATAGATCTCTTAATGAT
GAATCGGTGTATCCAACTGGCTAAACAAGCAGAGGGAAAAACATCTCCTAATCCATTAGTGGGAG
CAGTCATTGTCAAAAATGGAGAGATTGTCGGAGAAGGGTATCATCCTCAAGCAGGATTACCCCAT
GCGGAAGTTTATGCCCTAAAACAAGCAGGAGAAAGGGCGAAGAATGCTACGGTTTATGTTAATTT
AGAACCTTGTAACCATTATGGTAAAACTCCCCCTTGCACTGAGGCTTTAATTGGGGCGGGAGTAA
AGAGAGTTGTAGTAGGAATGATTGACCCTGATGAGAGGGTTTCTGGTAGGGGAATAAAACGATT
AGCCGAATCAGGCATAGAAGTAAAAATTGGGGTAGCAGAAGATGCTTGTTTGCGTTTGAATGAG
GCTTTTATTCATCGTGTTAAAACAGGATTGCCTTTTGGAGTTTTTAAGTATGCTATGACATTAGA
TGGGAAAATAGCGACGGATACGGGGCATAGTCAATGGATTACAGGAAAGTCGGCACGACATTTTG
TTCATGAGTTGAGAAGTATATCATCAGCTGTAATTATTGGAGGTAATACCTTACGACGAGACAAT
CCTTTGCTTACTACTCACGGTTTAACGTCTCATAATCCTCTGCGGGTGGTGATAACTGCTTCTTTT
GATTTACCTACTTCTGCTCAATTATGGCAACAAGATACGGCTAAAACTGTTGTTTTTACAACTCC
TCAGCCTGATTCTTCTTTAAAAGATAGTTTACTGTCTCAGGGGGTGGAAATTGTGGAAATAGCGA
ATCTTAACACGACTATGGTTATGGAAAATTTAGCCAAAAGGGGATGTAATTCTGTTTTATGGGAA
TGTGGCGGAAATTTGGCAAAAGATGCGATCGCATCTGGTAATATTCAAAAAATATACTGTTTCAT
TGCTCCTAAAATTATCGGTGGAAAGGGTCTTTTTTCTCCCATTGGAGACATGAAAATAGATAACA
TGAATAATGCTTTGGCTCTAAAAAACACTTCTATTCGTCAAATAGATACTGATTTTTTAATTGAA
GGTTATTTGAATTTTAATTAATTTTAAAAATACAATATGGTTCTATCACTTCTCGTCGTGTAAAT
TATTAGTTAGGGTAGGCAAGAGGCAAGAGGCAATAGGGGATTATTAAATAATAATTTATAAACT
TTTAATTTTTATTTTACTATAAACACTATTCAATAAAGGTTATGGAAGTTATTTTTTATTAATTT
ATCATAACCACACTCTGAAGAACCTACAATATTACTTAAAAATACTAAAATTTCTCGAAAAATAG
TCTGATGAAACAAAAAATAAACAAAAGATATATTTATCAACAAATTTTTATAACCCTTAGCTTAT
CAATAATACCCCTAAACTCTGTTTGTGCAGAACCTGTTATTTATGATTTCACCGTTAACGTAACA
GAAGGCTCTCTCAAAGACAATACTTACGATGGCTTTATTATCTATGATGATGAAACAGTTACAGG
TGCGGAAGAAGAAATGATTACAGTTGAGGATGGTTTAAAAGTCTGTATGAATTTCTTTAATGAA
AACTATGACGAAACTAAAGACTCTGACTATCCCCAATACCCCCAATTGATTCTTAAAAACGGCAA
ACCTGAAAGTTTGGACTTTTGGCTAGAGCCTAATCCTCGTCGTCAATGGTGGAACAAAAATGGTT
GGTTAGTAACCGTTACACAACGTCCAGAAAATAGCAATTTGCCCGTTAATTGCCCAAATGGTGTT
ACACCATAAAAATATAATTATGGTGAGAGAGTAAGGAATTTGAACTGTTGCCTCTTTCCAGATGC
GAAATTGCTTATCCTCGGCGACACCACTTTTTCATCAATCTCTAATTATTAATCAACCTGAGTTT
TGGATAAGCTGAAAGCATTATCCTGTGGTCAGAAAACCTTATAGCTTCTTCTTAGAAATAATAAT
AAAATTGCCTTAACCCGAACTGAGGTTATACCCCAAAACCCCAAGCCCCTACTTCTCTCTCTCATC
CTCTCATCACTCTAACACCTGCAACCTGACACCTAGCCTTATCAGATATTCTTAAACCGAACTGAG
GTTAATTATTCATTTCATAGACTAAGTTAACTCAATTTGTGAGTCATCACCAATCATAAAACTTA
AGGCTTTAGGGCGTTTCGGCACAAAATGTATTTTTGCTCTTTGCCCAATTACACTATCAACTATT
CTTTGCTCAATACCTTTTACTTCCGCCGATTTTAAAATTACACTATGCTCAAGATCAATCTCACTT
AAGTGAACATTGTCAGCAATACTTGAATAAGGACCGATAAAACAGTTCTCCAGATGACAATTATC
TCCAATAATCACAGGACCTCTAATAGTTGAGTTAATAATTGTTGTGTTTTTTCCTATTTTAACTC
TGCCAATAATTTGACTATTATCGGTAATTTCATATTTATAATCTGCTGTTAAATTAGTATCCAAA
ATAATACGGTTAGCCTCTAATAAATCATCTTTTTTTCCCGTATCTAACCACCAATTTTTTAATTC
TTGAGCTGAAACAGGTTTTTTATTTTCAATTAAAGATTGAATGGCATCTGTTATTTCCAACTCTC
CCCTAGCAGAAGGTTTTAGATGATTAATCACTTCATGAATTGCAGAAGAGAAAAAATAAATGCCC
ACCAACGCTAAATTAGAGGGAGGATTTTTTGGCTTTTCTATTAATTCTAAAACCTCCCCTTTTTC

ATTAATTTTTGCTACCCCAAAAGCCGTAGGATTAGATACTTTTCTAAGTAAAATTAAAGCATCTA
ATTGTTTTTCCTTAAAATCTTGTAAATATAAATCTAAATCCTCTGCAATTAAGTTGTCTCCCAAG
TACATAACAAAAGGGGAATCCGTCAAAAAAGGCTGAGCGATTTTTACTGCATGGGCTAAACCGTC
AGGGCTATCTTGATGAATATAAGTTATTTTTGCTCCAAATCTGCTACCATCACCAGTTTTTTTCT
GTATTTCTTCCCCTGTTTCAGGGCTGATAATTATACCTATATCCGTAATTCCCGCTCTCACAATCG
CTTCGATACCATACCAAAGAATCGGTTTATTTGCCACGGGTACAAGTTGCTTTGCTCCCGTATAA
GTGAGGGGTCTTAAACGAGTCCCTTTTCCACCAGATAGAATTAATGCTTTCATGATAATACAAAA
ATTGGCGTTTCTGAGTTTTAGATATGATTTTTAGTTTAGGTGGGAAAGAGGCAAAGGAAAAGAG
GCAAAGGCAATTACAAAGTAATTAAAACTACTTACTACTTAACAGTTTCTCTTGTCCTAATACTT
TTATGCCTAACAGAGGTTGTTAGACCTCTTGCAAAATAAGAATTTAAGATAGAATTAGTGAAAT
TTAAAACCAATCTTTATCATTGTTCCCTGTTTCCTCACTTTTCCAAGAAGTCTATTTTACCGATA
AATATCTTTTGTGATTAATCAACGCTTTATAAATGAAAAAGAATAGCTACAATGGATATATGCCT
ATACATTGTCAATTAATATTAAGAAATATTAAGAAAAATGTTAAAGACTGTTGCAAAAACAGGG
AAAAATTTATCGGTGTGACCGTTTTAGCTTAAATTCCTAAACATAAGCCATTTATAATCAAATAA
GATATTTTTAACAACATTAATTTTTAGGAGATAAATCGTGACAATTAGGGTTGCAGTAGTTGGCG
GAGGTCCTGCAGGTTCTTCTACCGCAGAAGTGTTGGCGAAGGCTGGTATTGAAACTTATCTATTT
GAAAGAAAGTTAGACAACGCTAAACCTTGTGGAGGGGCAATTCCTCTTTGTATGGTTAGCGAATT
TGATTTACCCCCCGAAATTATTGATCGCCGTGTGCGTAAAATGAAGATGATTTCCCCTTCTAATA
TTGAGGTGAATATTGGACAAACCCTCAAAGATGATGAATACATCGGGATGTGTCGTCGGGAAGTT
TTAGATGGTTTTATGCGCGATCGCGCCGCTAAATTAGGGGCAAATTTAATTAATGGAACAGTATA
TCAATTAGATATTCCTTCTAACGACCGTGACCCTTATGTGCTTCATTATGCAGATCACTCCAATG
GTACAGCCCAAGGGGAAATGAAAACATTAAAAGTGGATTTAGTCGTTGGTGCGGATGGTGCTAA
TTCTCGTATTGCAAAAGCCATCGATGCTGGAGATTACAACTATGCGATCGCATTCCAAGAAAGAA
TTAGATTGCCTGAAGATAAAATGGCATACTATGAAGATCTTGCAGAAATGTATGTTGGTAATGA
TGTCTCTCCAGACTTTTATGCGTGGGTATTCCCTAAATATGACCACGTTGCCGTTGGTACGGGTA
CAATGAAAGTTAATAAAGCCATTATCAAGGATTTACAAGCAGGTATTCGTAAACGTGCCGCTAAA
CGCCTTGAAGGGGGAGAAATCATCAAAGTGGAAGCCCACCCCATTCCTGAACATCCTCGTCCCCGT
CGTGTAGTGGGTAGAGTTGCCCTTGTGGGTGATGCGGCTGGAACTGTTACTAAATCTTCTGGGGA
AGGTATTTACTTCGCCGCTAAATCTGGCAGAATGTGTGCAGAAATGATTGTGGAAGCTAGTAATA
ACGGTCAAAAAGTGCCTTCTGAAGCCGATCTCAAAACCTATATCAAACGTTGGGATAAAAAATAT
GGTGCTACCTACCTAGTATTAGACATTTTACAGAGAGTCTTTTACCGTAGTGATGCAACTCGTGA
GGCTTTTGTGGAAATGTGTGCTGACATTGATGTACAAAAAATTACCTTTGATAGCTATCTCTACA
AAACCGTTGTCCCCAGTAATCCATTGGTACAAATGAAAATTACTGCTAAAACCATTGGTAGCCTT
TTAAGAGGTAACGCCTTAGCACCTTAATTAAATCCCTGTTTCACGTCAAAGGGCAAGGGGCAAAG
GTGAATGGTGAATAGTTAATAATTAAAAACTCCTAACCCCCAACTAATATTACCCTAATATCCTA
GTACTAATGGATACTTTATTCGGAACTTGAGTTGATAAAGATTCTTTATAGGTTTTTAAGATGAG
CAATGGTTCATCTTATTTTTTTTGCAAATTTATTTCCCATTAGAAAAATCTTGGAATCCATCACA
AAACCCTTTATCATGGGATTAATGAATTATATTAGAATAGTTTTTAACTGATTTCAGGAAACTA
TCAACAAAAAATATATCAATAAATTTATTAGGAGAATAATCAATGACTGCTGAAAGTATGTTGT
TTAACGGAGCAATTCTTTGTTTTGTTGTTGTTTTAATTGGTTTAGCTTGGGGCTTTCTTTTACTA
AAACTCACAGGTGAAAGCGAGTAATAAAGTAATAAAAAAGAGAAAGGTGGGCATTGCCCACCCT
CAAATTAGATCAAAGAAAAATTAAACTATTTATTTGTTAGGCTGAGGAGTCATGCGCAAATAAG
GTTTAATTTCTGTAACTCCTTTGGGGAATTTTTGTTTCGCTTCTTCAGTGGGAATGGAGGGGACA

ACTACGCAATCTCCGCCATCTTGCCAGTTAGCTGGTGTTGCAACTTGATGGTTGTCAGTTAACTG
TAAAGAATCAATAACTCTTAAGATTTCATCAAAGTTACGCCCCGTACTAGCAGGATAGGTAATAG
TTAAGCGTAATTTTTTGTTAGGGTCGATGATAAAAACACTTCTCACAGTAAGATTATTGAGAGAG
TTCGGATGAATCATACCATATAAGTCAGCTACTTTACGGTCTTCATCGGCAAGAATAGGGTAGTT
AACTTGAGTGTTTTGAGTTTCATTAATATCTTGAATCCAACCTTGATGAGATTCAACACCATCAA
CGCTTAAGGCTAAAATTTTGACGTTGCGTTTATCAAATTCAGATTTTAAACTAGCTACAGTGCCT
AATTCTGTGGTGCAAACTGGTGTATAGTCAGCAGGATGGGAAAACAAGACGACCCAACTGTCTCC
AGCCCAATCATAAAAAGAAATTTCTCCTTGGCTAGATGCCTGGGTAAAATCAGGAACTTGATCTC
CTAACTGTAATGTCATAAATTTTATCTTTGTTTATATTTCAACTAAAATTTCTAGTTAAAATTGT
CTCATATTTTTCTGTATTCATTGTACCAATTATCATTTTTTCTGTTATTCTTTACAAATCTTTACT
TATTTTCTGGGTTAAAGGGTTATTTCACTGATAGAATCTTATAGCAACCGAGTCTAGTAATACTA
TCCATTTGGTAAATATGGGAGTATTTTCCCCTGAAGATGGGAATTTCTCTGGTTCAATAAAATCA
ATAAATATAAGATAATTATTAAAATAATCTCAATTCTTGGAGGTAAAATTTTGATGAAATTATG
GCGTAACTTTATTACTTTTATTGCTGTTACTTTTTTTGTTTGTGCTGGAGTTTTTCATGCACCTG
TACAGGCTTTAGATTTTAACACCGTACTACAGGCTAGTCCTCGCATGGTAGCGGCGGAAACAAAT
CGTCGCAATGCGGCGGATGATTTGTTAACCACTGAATTCGGTCAAAAAATCGACATCAATAACAG
TGATATTCGTGATTTTCGTGAGTTGAAAGGCTTTTATCCTAAGTTAGCTAGTATCATTATCCAGA
ATGCTCCCTATGAGCAAGTAGAGGATGTTTTAAATATCCCCGGCTTAAGTGAAAGCCAAAAGGAA
AGATTACAAGCTAATTTAGATAATTTCACTGCTAATCCTCAGTCTAGTGTTTTTAATGAAGGTGA
TGAGCGTTATAACGCTGGCGTCTATTAATTATTAACTCCATATATACGAAAAAGCGATCGCATCT
TTTTGATTTTTTAACTATCTCGGTGCGATCGCACTTTATTTTGTCTCAGCTCAGTTTAAAAATAT
CGGATAAGAACAGGTTTTCGATTTTAAGTGTACAGGTATTTTCGGTTAATTAATCATTAATTAAT
CCACGGTTTCATAATCTCCTGTAATAGTTTCATCCTTATCATAGTCAAAATCTAAATCACTATCA
TCCTCAGATATTTGACTTAAACCGCTTAAAATTTCATCCTCAATACTCTTAAAGTCGTCATCATC
CTCCAAACTTTCATATACAGTCGATTCTACCGAAGGAGAATCATTCCCCATAACAGTTGCGTCTT
CTGCAGAATTGTAAGCCTGAGCACCAATATTAACTACTTTATCTCGTAATTGATTAACCATAAGC
TCAACTTCTGAAACGGGAATATTTCCATTCTCCATCGCCTCAGTTGCCCTGCGTTCAAGGGTTTGT
ATTTCTCGTTTAAAATCTTCTCCCACCAGATTAGGAGTTTTTTCTACCATGAGCTGGTAATTGTA
CATCAAACTATCTAATTGTTTTTTGAACTCCACTAAATCTAGGCGACGACGATCCTGAGCTGCAT
ATTTCTGAGCGGCTTCTTTCATACTCTCAATTTCAGAAGAACTCAAAGCCCCCGTATTTGTAATA
AGAATACTTTTTTCTTGTCCTGTACCCTTATCTTTTGCCGAAACCTTTAAAATTCCATCCACATCA
ATATCAAAAGAAACCTCGATTTGGGGAACTCCCCTCGGTGCTGGTGGAATTCCAGTCAAGAGGAA
TTTTCCTAAACTTTTATTATCTTTTGCCATAGGGCGCTCGCCCTGTAAAACATGGACTTCTACCGA
AGTTTGACCATCCACTGCTGTTGAAAAAACCTGAGTCCGACTAGTGGGAATAGTTGTATTGCGGT
CAATGATTTTGGTACATACCTCTCCTAATGTCTCTAATCCCAAAGATAAAGGGGTTACATCCAAC
AATAAAAGATTTCTAACTTCTCCACCTAAAACACCACCTTGTACTGCCGCCCCCAACGCTACCGCC
TCATCAGGATTAACGGATCGATCTATCTATGGGAATTTGTTTTCCAAAATATTCTTCTATCTTAT
TGGAAATAGCAGGAATACGAGTTGAACCACCAACTAATACAATTCTGTTTACTTCTTCTTTCGTC
AATTCAGAATCTTTCAAAGCCTGTTCCATCGGCGGAATAATCATTTCTACCAAAGGAGCAATTAA
TTCTTCCAATTGGGGACGAGATAACTCTAATTCTAAATGTTTTGGTCCATTATCATCTGCCGTGA
TAAAAGGTAAATTGATCGATGTTTCCGATAAATTAGACAATTCTATTTTTGCTTTTTCGGCGGCT
TCTCTCAAACGTTGTAGAGCCATTTTATCCGTACTTAAATCAATTCCTTCCTGTTCTTTAAACCTT
TCAATCAACCATTTAACAATTATGGCATCAAAATCATCCCCTCCTAGCTGATTATTCCCAGAAGT

GGAAATAACCTCAAAGATACCATTACCTAACTGTAGTAAAGAAACATCAAACGTTCCCCCGCCTA
AGTCAAAAACTAAAACGTGTTGATCCTCATCTTGTTTATCTAAACCGTAAGCTAACGCCGCCGCC
GTGGGTTCATTAATAATTCTCATTACCTCCAAACCCGCAATTATCCCTGCATCTTTAGTTGCTTGT
CTTTGAGCATCACTAAAATAAGCTGGAACTGTAATCACCGCCTGAGTCACCGTTTCTCCCAAAAA
GGTTTCTGCATCAGTTTTTAGCTTTTGCAAAATCATCGAGGAAATTTCCTGAGGAGTATATTGCT
TCCCCTGAATATCGACGTCAACGGTGTTATCTTTTCCTTTGACGCATTTATAAGTCACTCGATTTC
TTTCTTCTTCCGTCTCTTCCCATCTACGACCAATAAAACGCTTGATACTATAGATAGTATTTTCAG
CATTAGTGACAGCTTGTCTTTTGGCTAATTGACCAACTAGGCGCTGACTACCCTTGCCAAAACCG
ACAATACTGGGAGTCGTTCTTGAACCTTCTTGGTTAGTGATGACGAGGGGTTTCCCCCTTCTAA
GACAGCAACGCAACTATTGGTTGTTCCTAGATCTATTCCTATTACTTTTCCCATAGTTAATAGTG
ATGGCGGTGTTACATTTGAGTTATTGTAATTTAATTATAGTCAGAACCTTTGATGATTTAAACAA
TTTCTTGACTATTCTTGACTATTTTCTGGGGTTTCCGATTCTGAATCGGTATTTTCAGATTCATT
TTCCCCCGGCATAGCTACTTTTACCATAGCGTAGCGCAATACTTGGTCATCAATCATATAACCTCT
CACCAACTGCTCTATGACAGTACCTTCAGGATATTCATTTGTGGGTTCTCTGAGCATTGCTTCAT
GGAAGTTTGGATCGAAAGGTTGATTTTCTGGACGCATTGCTGATACACCAACTTTTTTTAAACCT
TCGACTAAAATTTTATATACTCCCTGATAACTTTTATGAATAGTCATTTCTCCTTCATTGGCAGG
TTTAATTTGACTTCTAGCTCTCTCAAAGTTATCTACGGCTGGTAAAATTTCATTAATGATTTTTT
TCTTAACTTGAGATTCTAATTCTTCTTTTTCTCTACTGGTACGACGACGGAAATTATCAAAATCG
GCAGTCAGGCGCATAAACTGACTGGTAGCACTTTTTACCTGCTCTTCTTTTAGTTCTAGTTCTTGT
TTTAGAGATTCTATTTCTTTTTGTAAGGATTCAACAACACTATTTAATTCATTATTTTCAGTCTG
TGGTTCTGTGGTATCAGCTTTTGAAGGTTCAGCGTTTTCTTCATCGTTTACAACAGTTTCAGAAA
CTGGTTGCTGTTCAAAATCTGTGTCCATAACTTCTAATTCTTGGTTATTTATTTCTTCGGAAGGG
GAAGAGTTTGTTTCATGATCGGCAATATCTGTTTTTTTGTCTAATTCAGGATAGTTGTCCGCTTC
ACTCATCATAGCTTTAATATTGTCCTTCAATAAAATTTGTCAATAGTAATACTAATTATAAGCAT
ACTATTTTCATGATCCCAAAATCAAGATATTTATGGTTAAAAGTGGACGAAAATAGAAAATAAA
TTTTAGTAGATATACTCTTTTTGTGAAATCTTTTTAAAATGTTGATTATATATCATAAATATAGT
ATGGAAATAAATATCAAAAATTATTATCATAAATTTGTAATGTTTACTTTAGTGTTAGATATAT
TAACTACATTTTAGACCTAACATTATCTGAGTTTGATATAAAATTATCGGTTAAGGTTAGCAGAG
GGGAATAGGAAACAGGGAACAGAGTTTATAGTTGACATAAACTGGGGTTAAATTGATTTTAAAT
ACATTTTCCTAAATTTAATGATTTTTTCAGGAAAAATTTTGTCGATTACATAGATCTAAACTCCT
TATTTTTCAACACTTTTTTCATTAAAGTCAGATAGAAATATAATTGAAAAAGAATAAAGTCCAT
GTAGAAAATATTTAAGAAACGGAAAAGGTAGAAACACTCATTGAAAATTATTAGCGAAAAATCT
TCTTAAAAGGTTAATTAAAGTTATTGTTTTTATGAATTTTTGTCGTTTAAAGACGCTTTGTCTTA
TTTATTGAAGTTGACAAAATTTAGTTTTTGTTTACACCTATAATTTAATAAGAATTATGTTTGCT
TATAATATTGCTTAATTTATCAATTATTAGTTTCTTTATTTCTTATTTTCTGTCTAAACTTACGC
AAGTCCTTCATTACTTCTTCTGCATTTTGATAGCGATCGCTGAAGTGATAACGAGTCATTTTTTT
GATTATACTAGCAGTTGGGCGACTGACATATTCTTGACTATTCCAAATAATTTCCCCTGTTTTAT
CGTCTCTGGGTAGTTTGTTGGGAAAATTACCGGTTAGGCAGTGAATTGCTAACATTCCCGTGGCG
TAAATATCACTACCGAGACAAGGTTGTCCTGCTAATTGTTCGGGAGCGGCATACCCTTCATTGCC
AATGATAACGGTTTTCTGGTCGCTTTTACGAGATATTTGCTTAACCGCTCCAAAGTCAATCAAAA
CAATAGAATTATTGGTGTTGCGACGAATAATATTATCGGGTTTTATGTCTCTGTGAATTAAATTA
TATTCTTGAATAAAAGTCAGAATTGACATGATTTCTTCTATAAGAATTAAAACATCTTTTTCTCT
ATATTTTCCTTTTTCTCTTATTTCTCTCATAATGGTTTTTCCTTCAATATATTCTTGAATCAAGA

AAAACTCATTATTTTCTTCTATATATGCTAGTAGTTGAGGTATTTTTTCATGAGTACCAACTTTT
TCTAAAATTTTAGCTTCCGTTCTGAACAAACGTTGAACAATTTTTAAAAATTTTGGCTCTTTTGA
GGAGGGTGTTAACCTTTTAACGACACAGTAGGGTTTACCGGGGCGTTGTAAGTCTCTCGATAAAT
AGGTACTGCCGAAACTACCTTTCCCTAGGGCTTTGACAATTTCATAGCGATTAACAATTAAAGAG
CCTTGCTCATAGTTGATGATTGAAGGAGCATCGGTAATTTGCGATCGATTCTCCAATAACATTTG
TAGCATAGCGATGGATTTTTCTTGGTCTGCTACTTGTTTTTGAATGGTTAATTGTTCTTGTTTTG
CCTGATAAGCATTATAACTAACAAGTGCGATCGCACCTCCAAAAAAAGCAAATAGAGGAGAAATA
ATGGGAATCCATCCCCCCCAAAAAGAAATAATAACGGCACTACCAACAATAATCACAATGGCAAC
ACCTTGCCCTAAAATCATCACTAAAGGGCGAGAAAGAAACAGAATAGAACAACTACCTATCAAAC
CCCAAAAATAAATCCAGAGAATTTCTTGCCACTCTTGCCAACTCCAGATTAAAGGTTGCCCGTCA
ATGGCAGAAGTTAAAATTTGACTAGTCATATAACTATGAAGAGTTACCCCCGGCATTAACACCAT
ATCCTCACCTTGACGACTGAAGGGAGTATAAAAAGTATCTTTCAAACTAGGTGCAGAAACACCCA
TGAAAATAATTTTGTTAGTAATTAATTCGGGGCTAACATTCCCTTGCAAAACCTCATTTAAAGTT
ACCGTGGGAGTGGGCTTATCTCCTCGGCGATAGTCAATCATAATTTGATAACCATTCGCATCAAT
ATTTTGGTAAGCTCCAATAGTCTGATTAAGGGGTTTAAGGGTTGTTTTACCTAATTTTAATAAAC
CGTCTTCGGTGATTTCTGGCTCAATTCCCTCCGCCATTAAATAACTCAATGCCATTTGTAACCCCA
GAGAGTAAGGAGTGGGGCATCTACTTTCTTGAGGATCAACATAAAATAAATTACGCCTTACTACT
CCATCACCATCTATCACTATATCTGCAAAACCGACTAAGTCCATAGCAATAGTTGAAGGAGGAGA
CACCGCAGGTTGATTTTGCGATTCTAACTTACAAACCGCAGAAATATTGTTACTAACACTAAAAA
TATTTTTTAACTTTTCTGCTCCCGGTTCAACAGGCAAATCACGATATAAATCAATACCAATACCA
TAGGGATTATGTTGTAAAAGATTATTGAGGCTTTTAGCTAAAGTAGCGTCACTAATAGGCCATTT
TCCTTCTGCTTGAATATCGTCTTCCGTTATTAATACTGTCAGTATGCGATTATCTTCTTTTTCAGC
TAAACGTAATCTAATAAGTTTGTCATATGTTCCTAAATCAAAATATTCAAATCCTCCCCCTTGTT
TAAGAATTACTGTTAATGTACTAGCAATAACCGAAGCACTAATCACAGCAAAACTGGTTTTTCGC
CATGATTTTTGATTTAACCATTGAGAGAAGGATGTTGCCATGATGATAAAGGATTAATAATGAA
GAATGAAAAATTAATTAGCTTCTCCCCAATATCCCAATACCTAAAATACCCCAAACTCCGAAATC
CTAACTAAATAGTAGAATGTCCTAACGCCAATCCCATAACCAACATTCCTAAAACAAGGAAAGGT
TGAGCGCTGGCTTGATATTTAACATCATTTTTTAAGGGATCACGCAAAAAGTACATATCTTGGAA
GGTGATTTGGGGAATAACTAGGAGTAATAGTATAGTAGCGTAAAGATTTTGACCTAGATAAATT
AAATATCCCCCCATGGCAATCTGAAATACATCAATCATAATTACACAAATCCAAGCGGCAGTAGT
TACCCCAAACATAACAGGTAATGATTTTAAACCTAAAGTGCGATCGCCCTCCACGCTTTTGAAAT
CATTAACAACGGCAATACCTAAACCTGCCATACTGTAAACTAAAGTAAGAATAACAACAGTCCAG
TTTAACTCTCCGAATAAAGCATGACCTGCCCACCAAGGTAAAGCAATATAACTTGAACCTAAAGC
ATAATTACCCAGCCAACCATTTTGTTTTAATTTCAATGGCGGAGCAGAATAAATATAAGAAATAA
AAGAACCACCTATTGCTAAACAAGTAAGAATAGGAAATTCATGACCTGCCCACACATCCAAACCG
TAGGCAACCCCAATTCCTGCAAACAATAAAATTAAAATTTGAGCTACCACTTGGGGGACAGAAAT
AGCTCCAGAAGGGATAGGGCGATAAGGCTCGTTAATAGCGTCAATGTCACGATCATAAAAATCGT
TAATAGTTTGAGTATAACCAGCTAAAAGGGGTCCAGACATTAACATACAAGTTAAAGCCATCATA
AAATCCTCAAATCCCCACACATAACCCCCTGAAGAAGCCGCACCACAGACAACACCCCAAATCAAA
GGAATCCATGTGATCGGTTTCATTAACTGAAGCCTAATTTGCCAAATACTTTTTGCTTGAGAAGC
CCCTTTCATTCCTAGTAGTTGACGGGCTTTAGATCCTTTATCCTCTCGATCTAACTCTACTTTTGT
ATAGTCTTTATCTTCTGTATTAGTCATTACACTTAAATTTAGACAGCTAATGTTTCTGAATAATT
AACAAAATAGCATAACAGGCAGTTAACAATTTCGAATTGGGAATTAGTAATTAAGACAAGGCAA

TTAGTGAATAGTGAATAGTTGATAATTAAGAATTAAGAATTAAAAACTCTGAACACTCATTACT
TTCTTCTAAAACCTGAAACCTGAAACCTGACACCTGACACCTTATCTCCCTCTCCCCTCATCCCCC
CATTCCCCCAATATCCGCCACCTAAAACCTCTTTCCAAATTTTCCGAACCCTGAACCCCAAAACCT
TAACTTTCCAATTACCCAACGATACAAGTTTATTTAACTAACACTGGTTTTTTTGCCTCTTGATA
ATCATTAACTATTTTACGAAAAGCATCTCCGTCTATAGTTTCTTTTTCAATCAACAAATCAACTA
AAGAATCTATTAAAGGTCTATTTTCTCGGATAATAGCTTTTGCATTTTCATAGCATTGAGTAACC
ATAAAGCGAATTTGAGAGTCAATTTTTGCGGCTATTTCCTGAGAATATTCAGAGCGACTCATGGG
GTCATTTCCAAGGAAAACGGGTTGATCATTTTCTTCCAAAGCGAATAAACCTAAGTCGGACATCC
CAAATTTTGTCACCATCTGACGGGTTAAATAAGTTACTTTTTCAATGTCATTTCCCGCCCCTGTG
GTTACTTCATCATTACCGAAAATAACGTCTTCTGCGGCTCTACCTCCTAACAAGCCAGTGACACGG
GCAAATAATTGGGTTTTAGAAACTAATCCTTGGTCTTCTTCTGGGGTAAACCATGTTAAACCTTT
TGCTTGTCCCCGGGGTACTAAGGTAACTTTTTCAACAGGATCATGCCCTTTTGTTAATGTTCCAA
CGATCGCATGACCAATTTCATGATAAGCAATTAGTCTTTTACTTTTACTATCTACAAGGGGAGTG
CCTTCCATTCCCGCAATTACCCTGTCAATGGCTAAATCAATTTCCTCCATGGTAATCGCTTCTTTT
CTGCGTCTAGCGGTAAGAATTGCCGCCTCGTTGAGTAAGTTTGCTAAATCTGCTCCTGAAAACCC
GGGAGTGCGTTGAGAAATGGTTTTAAGAGAAACTTCGGGGTCAATTTTTTTATTGGCGGCATGAA
CTTCAAGGATGGCTAGTCTGCCTTCTAAATCAGGATAATCTACCATAACTTGGCGATCGAATCTT
CCGGGACGTAATAAGGCACTGTCTAGGACATCTGGGCGGTTAGTAGCGGCGATGACAATAATTCC
CGTATTTCCCTCAAAACCGTCCATTTCAGTTAAAAGCTGGTTAAGGGTTTGCTCTCTTTCATCAT
TACCACCACCGATACCACTACCTCTTTGTCTGCCCACTGCGTCAATTTCATCAATAAAAATTAGAC
AGGGAGCATTTTCTTTGGCTTTACGGAACAAATCACGGACACGGGAAGCCCCAACCCCCACAAAC
ATTTCTACAAACTCAGAGCCAGAAATACTGAAAAAGGGAACTTGTGCTTCACCTGCGATCGCTTT
TGCTAAAAGAGTTTTCCCTGTACCCGGAGGTCCTATTAATAATACACCCCTAGGAATTCTAGCCC
CGATAGCTGTGAATTTATCAGGACTTTTGAGAAACGTAACTACCTCTTGTAATTCTTCTTTCGCT
TCTTCTATTCCTGCCACATCTTTAAATTCAATACCCGTTGCTGATTCCATTTGAAACCTTGCCCGT
GACTTACCAAAATTCATTGCCCCGGCGGCACTACTAGCCGAGCGTTTGATTAACAAAAATAAGCC
AACAATGAGCATGAAAAGGATAGCTACTTGGATTGTATTAATAACTTGAGTGCTACTAGCAGAAC
CTGACTCAACCACAAAATCAATGCCTTTTTCTCTAATTTTGGCAATTAATTCTGGATTTTGATTG
AATAAATCAACTCTTTCTGGGGATTTTTGATCACCATTTTTTAAATAAACACTAGCTATATTCGT
AGTGGGATCAATAACTATTCTCTGTACTTCTCCTGTTTCAATACTGCGAAGTAAATCACTATAGC
TAAACTGTTGAGTTTTATTTTGTTGAGCAAAAGCAGAACTTCCCACCATGAAAGTTTGCATCAAG
ACTAAACTACTAGCAATTAAATTAACTATTTTATTTCTCTTACTTTTTTGACGACGCTCTAATTT
AAAAATACTCATTATTAATGATTCCTGAAAAATAATTATGAGAATAATGGCAAATGGTTCTTAA
TTTTTCATTCTACTTCCTATTCCCTGAAGAATGCCTTTGCCGATTAAACCAATACCCTTGCCGACT
ACTTCTGTAAGCAAATAAACCAGCAACTTGCCTAAGCTATCCCCTAGAGACTTGACTCCTTTAGC
GAGACTATCTCTTAATTCAATTAAAATAGTAACACCCCATGGTATTCCCTTGATGGTATTTAGTT
CTTGATAACGGGGATGAGTAATCTGACAACAGACTATTCCCTCATAGGTCAATCTTAACATTTCG
TATTGGTCTTCAAATATATTTTTTGGATTTTGCCAGTATTTTTCTCGGCGATATTTCCAAACTAA
ATTATTGCGAAACATTGCTACTTTACGGGAAGATTTTAATTCTGGTTGGAAAATATTATGTTTAA
GAATAGAGCTATCAGCAAAATTGTTAAGGATGAATTCTACAGTAAAGTTAGCGATATTTAAGAT
GGTATTTTGTAAAATAATTTCTTCTATTTCTAGGCTATTTTCATTTCCATAAGAGTAAGTAAAAT
TATCAATGGTAATTTCTTTTTTTTCTATTTCATATATAAGAAAGTCTTGAATAAAAGGAATTTTT
ATTAACTCATTGGTTGCTAATTTCTCTCCCTGATTAATAATTAAATTAACTAATTCTAAATTTTG

TTTGGATTCATTTTCTGTGGGTGAGGGTAAATATCTTTGTAAAAATTTGAGAGTTGAGTTTTGCC
ATAATTCTTTAATAAAGGTTTCAACTCTTTTTTCCAGTTCTTCCCTATTGAGTTGTAATTTCTCT
GTTTCTAATATTAATTCTTCCCATTCTTGTAAAATAATAATCAGTAATTCTTTTTGTTTACTATT
GATTAATATGTCTGTTGCTTGAATTTTTTTTGTATAATTTGTAAGGTTTTTCTGAATTTTTAACA
GGGTTAAATCATAGACATTTTCAGGTTGATTAACCAGAATAATAGCATTGGATTTTTCTGTTTTA
GCCTTATTTTTATCTTGCTTTTCGACTCTATTAGTATTGTCATCACCGTTTGAATTTGATATTTG
ATTAAAATTTAGTTGAAAATTTAATAAAGTGCGATCGCTCTCTGACAGAAAACGATTAATCAACC
ATTTTGCTAATTTTAATTCTCTTTTTCTACCATGCCAATAAATCACATCTGCGGGGGATAATTGA
TGAGTTTTTGTGTGATTATTAATTTCTTTTATTTGTCTCTCAATTTCTTTGATTCCTCCCTGACAT
TGAAGGGCTAACCAACTATTGAAATTATTTTGATTGCTTGATAAGCTCTGATTTTGAGTTAAATA
TTGTTCTCCCTTCGCACAAATTCGAATAATTTTAATTAGCTCATCAATTTCAATATTTTTATAAC
AATATCCTTTAACAATAGAAAGGGAAGTTAAATCATAAATTAATCCTCTCGGATTAACCATTAAT
AAGATATTTAACTCTGGATATTCTTTAACCAATAAAGATAAATCTTGGAAAAATTTTTTTACCCT
ATCTGGATACTTTTCCCAATCAAGAGCGACGGCAACTAAATCACAATTATAATTTTTGATCAGAG
AAAAAATATCTTTTAATTTTCCTGTAGCGATAATATTAATGTCTTGATAAATCTCATCTTTACAA
ATCTCTTTTAACCCTAAAGAAAAAATAGGATCTTCATCTAATAAAATGACATTGATTGAGGATTG
AGACTCATTAATAGACATCACATAAACGATTTATTTGAGTATGAGAGGGGGTTAGAAGTTTGGA
GTTCGGGGTTTGGAGTTCGGAAAGAGTTTTCAGGTTTCAGGTGTTTTTTATTATTTATTGTCAAC
TATAAACTATAAATATAAACTATTCACCATTATCCATTACCCTTCTCCCCTAAAAAGGGGAGACA
CAGAGGGGTTTCCCTGTTGTCTTGTCTTAATTACTAATTACTGATTATCTATGATGCTAAAGCAA
CTTCAATCATTTGCTGTAGTTCACCAGTTTGATACATTTCAATCATGATGTCACTACCGCCGATA
AATTCACCATTAACATATACTTGGGGTATGGTAGGCCAATTGGAATATTCTTTAATTCCCTGACG
AATATCATAATCAGATAATACATCAAATGTTTGAAAAGGAACACCGATAGTATTTAATATTTGG
ACGACATTATTAGAAAAACCACATTGGGGCATTAATTTTGTGCCTTTCATAAATACCACGATGCG
ATCGCTCTTAAGAATATCTTCAATACGTTGTTTTAATTCAGGAGTCATAAGTCTTCAATAAGATA
AAAAACAATAAATACAAAAAATTAAGAATTACAGGTTCAAAATTATCTCTGATATATTCAGGAT
TTAAGATTAACTCTTTAAACCAGACCAAGTAGAAGGGGTATAAGTTTTTAAAGCCAAAGCGTGA
ATCGCTTCTGATTGTAACTCCGCTTGAAGGGCGGAATAAACCAGTTGATGTTGTTTAACTTTGGT
TTTACCCTCAAATTGGGAGGAAACAACGATCGCTTCTAAGTGATCTCCTCCTCCCGTCAAATCCTT
AATTACTACCTCAGCGTCTGGAATTTCTTGCTGAATAGTTTTTTTAACCTGTTCCAAACTGACCA
TATTTTTTTTAGCTTGGTAATATTTTTTGATGCTTAAATAATAGTTATTTTTATCATATCTCAAT
ATGAGATTTACTTCAAAAGCAGTATTATAAAAAATGAAATAATGAGAAAAACAAGAGCAAAAAG
TAACTAAATTGACTTTAATAATATAAACATTGATTTTTACTTTTTTTGAGTTTAGTTTTTTAGT
ACTAACATAAATTTATTGGTATTAATAAATACTTTTACTATCTATTGTCCTTTATCTATTAACCA
AAATTTTTGTCAATAAATTTTTTTCCCCTGATCTTTTTTCTAATTAGAGTTTACTATTGGGAGTC
ATAAGCCCATCAAAATCAAGAGTTTTGAGCCATAACTAACTAAAAAAAGCCTCTAAAACCCAAAT
TTTCCGTAAAAATAAAGGATTTTTTGCCTATCTAGTCAAATATTTTTGATACAAGTCAGCCATTG
AAAAATAATAACTATGCTAAAGTCTTTGATTTCTAAAGACTATACTTTAAACCCGCCACCTGAAA
CCTGACACCTACCCTCGGCGACACAACTTTTTCATCAATCCCTAATTACTGATCAGGAGATTCAAT
GGGAGTTTGCACAAAACCGATTTCGTATAATTGTTGGTAAGATTTTTTACCTAATTCACTACTAG
GGTTTTGAGAACGAATAATTTGAATTAAAAGAGGTACAGCTAATTCAGGTTTATTTTGAGCCCTA
TGTACTAAAGCTAATTGATAAGTTGATTCATCTCTAAAACTAGCGGTTTTTAAAGCACCTGCCCT
TTGCTCTTCTGTGATGCGATTATCTATACCAGCAAAACTTTCTGCTAATTGTAGATAAAAATTGG

ACAATTGGTTAAAAATTCTTCTCGCTTGTTGTAATTTAGTCGCCGCCAAGTCATAATTTTCTTGA
CTAATCGCTTCTTCTGCTTCCTGCATTAATCTTTGTCCTCCTTCTAAGCTAAGTAAACTCTCCGTT
TGATTTATCGGGGTAACGGTATTTTCTTCGATAATTACTTCCTCCGTAACTGTTTCATTTTGAGC
TACTAAAGAAGAATTTATATTAGTTTGTGCGTAGCTAGGAAGTTGAAAAGCAGTAAGAGAGACA
ACTAATCCTAAACATTGACTAAATCTCATAGAAGATAACAGAGACAACATGAACCAATCCTGTTT
TAATTAAATGACTGAATTAAATTTTAAAGTATCTTAACACCTTATCAACCTTTTGCCCGATCGCT
TCAGGAAATCAATTTTTCCGCTAGGGATAAATTAAAGTATTTTTCCCCATAATTTTCACCCTGTA
TCACCCGAAATGGTAAGTCTTTTTCTCCCAGATGATTTAAAACTAATATTGCTTCATCAAAATTT
TGCTTTTCCGTATATTGATTTACAGTGACAACTGTTTTCAAACCTGCTTTAACGGCGGCAGTTAA
TCCTTGATTTGTATCTTCAATGGCAATACATTCATGGGGTGATAAATTAAGTTTTTCCAGTGCTA
ATAAGTAAATATCTGGTGCTGGTTTTTTTCTCTCTACCATATCCCCCGCCGCTATCACTTCAAACC
AACTAGCCATAGGATTTCCTAAACTGGTTTCTAATAGTGCCTCCACATTTGCTACAGAAGCCGTA
GATGCGATCGCAACTTTTACCCCCTGAGAATAAGCCTCTTGAATTAATCTTTTCACTCCCAAACGT
AAAGGAATAGAATTATTTTCCAGTAATTGACGATAATATCTACTTTTTAGTAAATGTAAATGGG
CAATAAACTCATCTAAAGACTGATTAATATTAAAAGAAGGCAAATAATTAGAAATATAATATCT
AATACGCTCCTTTCCGCCCCCAATCTCCAATAATTCTCCATATAAATCCACATCCCAATGCCATGA
CAAATTCTCTCGCTCAAAAGCCCGATTAAAAGCTATTCTATGACCATCTCTTTCCGTTTCTGCGAT
CGTACCATCTACATCAAATATAATTCCCTTCAGACTCATAAAGTTAATACCTCCCGTAAAAATCA
AACCCAATTATAACTTCAGTTCGAGATAACATAGATATAGTAAATATCCACAAAAAACTTAAACC
ATCATGAGTGAACCAGAAAACCTTTTACAAAAAGCATTTTATCTAGGAGTAGGTATTGCAGGATT
AGCCGTAGAAAAAGCTAACGATGCCCTACAGGAATTAAAAGAACAAGCCGATAAATTAGTCATTA
ATGGTGAGTTTCCCCACAGACTACAACAAATAGCCGATGAAATGGTGAATAAAGGGAAAATGAA
TACAGAAGAAGCCCGTAAATTTGTCGATGAAATTCTCCAACAACAAAATAATCAAAAAACCAGCA
AAGAAGAAAATAGCAACTCTTCAGAGCCTCGCATCATTGAAATTATCACTGAGGAAGAAGAAGAC
AAGGCATAAAGACTCAATTATGATATAATTTGAGAGATTTTAAACCCAATAGATTGACCAATGG
CAGAGACGCGCGCAAAAATAGCAGTGGATGCAATGGGCGGAGATAATGCCCCTCAAGAAATCGTA
GCTGGTGCAATTAGAGCTTCAGCAGAACTAGATGTCGATATACTTTTAGTCGGCGATCCTGAAGC
TATTCAGGCACAATTAGATCACCATGGTTGTACCTCGACCAATATTGAAATAGTAGCCGCCGATG
GAGTTGTCTCGATGGAAGAAGAGGCTCTTGTGGGGGTTCGTCGTAAGCCGAATGCTTCTATTAAT
GTGGCAATGGGGTTAGTAAGGGAAAATAGAGCCCAAGCAGTAGTTTCGGCTGGACATTCAGGAGC
CGCAATGGCCGCCGCTTTACTCGGACTCGGCAGACTCAAAGGAATCGATCGCCCCGCTATTGGTGC
TGTTTTTCCCACCATGTATGCCAATAAATCAGTAATTGTCCTCGATGTTGGAGCAAATGTGGACT
CTAAACCTAAATACTTAGAACAATTTGCCCTCATGGGAACAGTTTATAGTAAGTATGTATTGGGA
GTTGAAGATCCAAAAGTGGGCTTGATCAATATCGGTGAGGAATCAAGCAAAGGGAATGAATTAG
CAAAAGAAACTTATAAATTACTTTCTGAGAATCCGAAAATCCCCTTTATCGGTAATGCAGAAGGC
AGAGACGTTCTTTCAGGAGACTTTGATGTCATCGTTTGCGATGGCTTTGTGGGAAATGTCTTGTT
AAAATTTGCCGAAGCGGTAGGGGAGATTATGTTACAAATAATCAAAGAGGAATTACCCTATGGA
GTCAGAGGTAAAATTGGTACAGGTATTTTGAAACCAAATTTGCGTCGCATCAAGCAAAGAATTGA
TCACGCAGAGCATGGAGGAGCTTTACTATTTGGGGTGAATGGAGTTTGTATTATAAGTCACGGTA
GTTCTCAAGCTCCTTCTATCTTTAGTGCCATTCGTATCGCTAAAGAAGCGGTGGATAATCAAGTA
TTAGACCGTATCCGCAATTTTCAAGGAGAGAAATCCATTAGTAATGAGCAGTTAGGAATTACTAA
TTAACTATTGAATAGGTTTAGGTATCAGGTTTCAGGTGGCAAGTTTTTAGTGTAAATTGAGTACA
AATTAGAAAACGTCATTTTTACACTTTTTTTGTGATTTTATTCAGCAGACCCCAATTAAGAATTA

AGAAGAAAAACTATACCTTATACTTGTAAAATATTACATTTTTTGGCTTGGAATCAGAGTCTAAA
CATTAGGTAAAGTCTAGTGATGTTCGATGTAGCTTTTGTGAGGGCAAAACAAAATAAATAAAAA
TTCTTTTTCTACTAAATAAGTGCGTAACGTCAGTTAACGAATTAATCTTTATCAATCCGAACTAG
ACTAAAATAACTGATAAATGAATCGATATATTAAGTTTATTAAGTTTTATCCCTATTACTATGAA
TTCTACCAAAGAAAAAGTACAATCTATGTTGAGAAAATTACCAGATGATTGCACAATTGAAGAT
ATACAGTATCACTTATATGTACTGGGAAAAATTCGTCAAGGCTTACAAATAGCCGATACTCAAGG
AGTTTTACAACAAGCAGAAGTAGAAGGGTTGTTAAATAAATGGCTTATCGAGTAGTTTGGTCTCC
TAAAGCAGTAGAAGATGTAGAGGCGATCGCAGCTTATATTGCAAGGGATTCACCGTCTTATGCGG
CGGCAGTAGTACAAAAAGTAATTGAAATAACCGATGAATTAACCCAAGATGCCACGAAAGGCAG
ACTAATTCCAGAAATTGAGGAATCAAAAGTAATGGAACAATTTGCTTATAGCTATCGTTTATTGT
ATCGCATTGAAGGAGAAACAGTAACCGTAGCCGCCCTGATTCACGGCAAAAGACTACTTTACTTA
ACTGACTAGCCTATTTCAATGGGGAATGAGGAATGAAGAGATTACAAACTCTTCCAAACAAAAGT
TATCTGACGCTTATCATCCGTTAGTTTTATCAAAAAGCAAGAGGTTACTTTGAATAGTAGATTAC
TTCTGAAGCAAGAATTATTAGACAGTATTAGTAAAGTCGCTCAAACTCTCAATATTAATCCTCAA
TATCCGATTACTGATACCCTGATTTCCAGAGACGATAGCCTTAAAATAGAGAAAATAACACTTAA
TTTAGAAGCCCTTAATCCTTTTCCTAAACCATTAGTTTATGGCGTAAATTTATTAGATGGAATTT
GGCAATTAAATTATTCGACTGCAAGGGAAATAAGGTCTTTAAATAAATTACCTTTAGGTTTAAA
ATTGAGGCAAGTTTATCAAATAATTGATACCCAAAAAACATCTTTTTTTAACGTAGCTTTTGTGG
AACATTCCTCTGGTTTGGTTAAAGGTTATGTAAAAGTTACCGCTACTTTTTCCCCTCAAATTAAA
GATGGTGATTTGCTTCCCCAAGATACTATTAACGTTAATTTTGATAAGCGTTTTTTAGCTATTCA
AAAAATAGTTAATATAAAAACCCCCATTTTTGAACCTGTCAAAGTATTTAACGCCAGAAATCCTC
AAGGGCGAATACCTAGTTTAAAAGTCACTTATATTGATGAATCAATGCGTATTGGTAGAGGAGGC
GACGGCAGTCTATTTATACTTTCTAAAGTTAACAAAATTGCTGACTCATAAGTTTATTTTAAGGG
TAATGAGTAAAGGGCAAGGGGCAAAGGGCAAACCCCCCTTTATCCCCAAGGTTTTTTCAAAGTCT
GGGTAAAATTATCTTGCCCTCACCCCCAACCCCTCTCCCACAGTAGAGGGGAGCGTTTTTTCTTAA
TAATTGATTAATTAATACTTAAAAACTCCTGTATCTGTTCTGACGGGGAGACAATGAAACTGCAG
TGAGGGTAGGTTTTAGGTTTCAGGTTTCAGGTTTCAGGTTTTAAATACATGAATAGTGTTTTGTA
TTTACGAAATAAATAGCCAGACATATTATCTCAAAATGTTGATAATCCAATAGTTTTGCCTATTA
TTTGAACCATTGAGTAAATAGAGATAATGATAAATATTAGTGGAGACAAAACCTAACACCTACC
ACCTGCAACCTGCAACCTGACACCTGACACCTAACCTTATCAGATATTCTTAAACCGAACTGAGG
TTATTTGTTAGTTTCAAAACTTGACATTTTTGAGAATGAAAAAACCCTGCTTTATCCCCCCTCAA
GAGGGGCGAGGGCAAAGGTAAATGGTGAATAGTTGAGAATTAAGAATTAAAAACTCCGAACACC
TGTACGGGCGAATAGCCATTGCCCCTACTTCCCCCTCTCACCTCATCACCTCATCACCCTCTCCTCC
AAACTCATTTCCCCTCAACCCCCCAATACCCTATTCAATTTCTATCTGTTTGATATTTTGTCCTAA
AGTTTGTAACTCTTGCTTTATTTCTTCTTCATTTCCTACCACTAAAGTCACAATTTTGTCTGGGTG
TAAATATTCTTGAGCAACGTTCAAAACATCATCTATTTGAGTATTTTTTACCCCTTGTTGATAGT
CAAATATAAAGTTTTGGGGGTAATCATAATATTCATAAGTCATCATTCGAGATAGGGTTTGACTA
GGATTCTGAAATTTAAAGACAAAAGAGTTAAGTATAGAGTCTTTGGCATAGTTTAATTCCGCTTC
TGTAATGGGTTGAGTGCGTAATTTCTCGATTTCTTCTTTCATCGTTTTGATGAATTGAGTTGTTG
TATCAGTTTTTGTTTGGCCTCCTGCTACAAATAAACCGGGGTAGTCATAAGCACCTTGCCAAACA
CCATAGACAGAATAAGCTAATCCTTGGCGAGATCTGATTTCATTATGTAGTCTGCCTCCAAAGCC
ATTTAAAACCCCATTCATGACGCTAAGGGTAGGATAATTAGGATCATTTAATTTACCAGCTAAAT
GACCGAGTAAAATATTACTTTGAGTTAGTTGTGGTTGATCAGCAATAAATATTCCTTTGCTCTTT

TCTTGTTTTGAAGTGATTGTGTCAAATGTTGGTTTTACCTGTGAATTTCCTTGCCAGTTACCAAA
GTATTGGGCAATCTTTTCTTTTATTTGGGAAGAATCAAAGTCTCCGACTATACCCAAAATAATTC
CCTCTGGACGAACATATTTACTATGAAAATTTTTAATGTCTGATTGTTGTATATTGTCTAGGGTA
TTATACTCAATTGTTCTTGCATAGGGGTTATTTTCTCCATAAACAAGTTTTTTGAATTCCCTACT
GGCTATGTTTCCGGGGTCATCATTACGACGTGCGATCGCACCTTTTGCTTGAGTTTTCAATACTTC
TAGCTGTTGAGAGTCGAAGGCTGGAGATTGTATTACTTCGGCAAAAAGAGGAAAAATAGTGTCT
AAATCGTAACTGAGAGAGGAAAAACTAACACTACCCATCGTTTCATCAATAGAAGATTCGATCGC
AGCCGCTTTTTGCTCTAAAATGGAATTTAATTGCTCTGGAGAATAATTAGCCGTACCACCTAAAC
GAATTAAACTCCCCGTTAATTCAGCTAAACCGACTTGAGAAGGGGGATCAAACCTTGACCCTGTG
CGAATCACAGCATTACCACTGATTAAGGGTAAACGATGATCCTCTATCAGATAGATTACCATACC
ATTATCCAATTGATAACGTTCATATTCAGGCAACTTAATTTCCGCTAGAGGGGGAAATTGTAAAT
CCCGATAAGTAGAGGGAGTAACATCCGCAAAAGCAACATGATGGGTAGCAATGACAATAGCGATG
GTTATAAAAGTGAATCCCAACCATTGCCATATTTTGCCTAATTTTTTGATCATAATTTCTAATAA
TAATAGCTTTTAGTTAAACCAATAGCTAACACCTAAAACCACAAAAAACTACCTTAAATAAGTAT
AACTGCGTAAATTTTCCTCATAGTTTTTAATCAATCTTTGAGAATTATCTAAGTCAATTTGATTT
TTTGCCAAAGCATTTTCTGTCAAACAACGAAGTTTTTCCGTTAAATCCTCAGCAGAATATTCCAC
GTATTTTAACACCTCTGTTACAGTATCTCCTTTGACGACATATTTGATTTGATAAGCATCTTTTT
TACATTCAATATGCACCACATTTATATCCCCAAAAAGATTATGCAAATTCCCCATAATTTCTTGA
TAAGCCCCGACTAAAAACATACCTAAATAATAGGGTTTAAAATCATTACTTTCAGGGATTAAATT
ATCAATATTTTCATCATTTTTATTTGTCTGTAAAGAGTGTAATTCTAAAGATTTTTTTACATCTA
ATAAATCAATAAACTTATCTATTTTTCCATCGCTATCACAAGTTAAATCGGCTAAAATTACCTTT
TTATTTGGTTTTTCATTAAGTCGATGAATAGGTAAAACAGGAAATAATTGATCAATTGCCCAAGC
ATCAGGGGCGGATTGAAATACGGACAAATTAACATAATAGGTAGAAATCATTAAGTCAGCTAAA
TCATTTAAATCATCACTAACATAAGATTCTTTTTGGGTAATTTCATAAATTTTACGACAGCATCC
CCAATAAATTTGTTCAGCCTTTGCCCTTTCTTTTAAGGTTAAATAGCCAAAGTTAAATAAACTGA
TAGCTTCATCTTTAAACTGCACCGCATCATGGTACATTTCTTGATAGTTTTCTTCATCAATATTT
TCGTAAGTTTCCCATAAATTTCTAATAACTAAATGTTCTTTTTCTTCTCCAATAATCGGTAATTC
TTGAGGCGGATTATTACTATTTACTACGTTAAATATTAACACAGAATGATGAGATGCGATCGCTC
TTCCACTTTCACTAACCAAGAATTGGATGGGGAATATTTTTTTCATCACAGGCATCTTTTACCGCC
GCCACAATATCATTGGCATAGTTTTGCATATTGTAGTTTTTAGAGGCATAAAAATTGGTTTTTGA
ACCATCATAATCCACAGCTAAACCACCACCAACATCAAGATATTTCATCCCTGCCCCCATCGTTAC
CAACTGCACATAAATTTGACTAGCTTCTCTAATAGCATCTTTGATAACAGCAATGGAAGAAATTT
GCGAACCAATGTGAAAATGTAACAATTGTAAACACTCTAATTGATCAGTTTTTTCTAGTCTATTC
ACCACTGCAACAATTTCGGGAATAGTTAAACCAAATTTAGCTTTTTCCCCGGTGGAATCTCCCCA
ATGCCCAGAGCCTTTTGTTTGTAATTTTGCCCTTACTCCTAATTGAGGTTTTAAGTTAAATTTTT
TGCTTAGTTCTAAGATAATATAAAGCTCATTTATTTGCTCAATTACTATAATTAAATTATGTCCT
ACCTGTTGAGCTAAAAGGGCAGTTTCTAAATAATCTTCGTCTTTATAACCATTACAAATAAGTAA
AGTTTCTCCCATTTCATCTACTTCTAAAGTAGCCAAAGCAATCATTAATTCTGGTTTTGAACCCA
CTTCTAAGCCAAAATGATAAGGTTTTCCATACTCTACCAAAGCTTCTACCAATTGACGATGTTGG
TTACATTTAATTGGATAAACCCCTCGATAAACATTGTTATAATCATAGCGTGCGATCGCATCTTC
AATACAAGAATGTAAACGTTGTAAACGATCAGCTAAAATATCAGAAAAACGAATTAATAAAGGT
AACTGAATATCTCTTTTTTGTAAATTTTTAACTAATTCAAATAAATCAATTTTATAATCATTACC
TTGAGGAGAAACGGTAATGTTTCCTAACTCGTTTATGGCAAAATAGGGATGTCCCCAACCTTCAA

TTTGATATAAATTATTACTATCTTCAATAGTCCATAAATCGTTCATGTTTATTTCTACCAACTAT
GTCGTTATATAACTCTAATCAATATTAATTCCTTGTTGACGCAACTTTTCTTTTAATTCCTGTAA
CTCTTTTTCCGCCTTTTCTGCTCTTTTTTCTTGTTCTTTTTGTTCTAAAATAGCTTTTTGTGCGGA
CTCGTCTCCCGTTAAGAGTAGATTACCACTTTGATCACACCAACGCAACCATACCCCGTTAACATT
TTCAAATTCTCCCTCCCATACCGTTAACCCCAAATTAACTCCTTCTAACCAAGTATCAGAAGTTTC
CTGATAATGTATTCCCGTTAGTTTAAATATCTTTAAAACTTGATCACTTAGATATTTTTGTGGAT
CATAAACTCCATAATAACTCACCCTCATATCTTGATAAATTTTTAGTTTTTCTTCTAATTCTCCTC
CTACTTTATTAGAGACAATTTCGAGGGCAAATTCAGGAGGCTTACCAAATTCCCACACTAGATAA
CAACGATGATTTTTTTCCCAGAAATTCTCAGGAGTAGTAACATTTAAACTCAAGAAAAAATCTGG
AACAATGGGGGGTTTTCCAATACTATAATAAATACCAACATTGGCATCTGCTAAAAAAATCACTT
CTTTTCTGGCATGGTAAAGAATGCTAGTTAAAAATCTTTGTTGTTTTGCTGAACCAAAATTATCC
ACGGGGGTATCATCCTCTGTAATTAAATGACTAACATCAGGTTTGGGGATAAATTCAACTTCATT
ACCTGTTGGTATTTGTATTTGTGTTACCATTCTTATCTACTTCCTTAAACCTTTCTTTTCATTGTA
ACTTTAAATTTGCTCTTCTTTGCTGAAAACTTGTTATATTAAAACGAGTTAGTATGACCAACATC
AATGGAAATAAAAGGATATGTTAACTTTACCTCGCAAGAAATTTGCCATTGAAGAATATCATCA
AATAATCGCATCGGGAGTATTGAAAGAAGACTATTTAATTGAGTTAATTAATGGGGAAATTTTT
GAAATGTCACCAGTGGGATTTAGACACGCATCTTGCGTAAATAAATTAAATCAATTACTAAGTTT
AAAATTAGGAAATAAAACTATTATTAGCATTCAAAATCCCATTAAACTTAATGATAATTCAGAA
CCACAACCCGATATTGTTTTATTAAAACCTCGTCATGATTTTTATGCAAATCAGCATCCTACGGT
TGAAGATATTTTCTTATTAATTGAAGTGGCTGATAGTAGTATAGAATATGATCGCACTTTTAAA
ATTCCCATTTATGCAGAAAATAAAGTACAAGAAGTGTGGTTAGTTGATCTCAATCAAAACTTATT
AGAAGTATATCAAAATCCTCAGACAAATTATTATCAAAGTATCAAAAAACTTTCTTCTCAAGATT
CTATTACTTTAACTCAACCAGAAACTATCGCCGTAAAACTCGATCGCATTTTATTTTAGTTTATA
GTAATTTGATTGAATGAAAAATATTATATTGAAATCAGTCGAGATAGAAGAATATGTTAACTTT
ACCTCGGAAAAAATTTGCCCTTGAAGAATATCATCAAATAATAGCTTCAGGAGTATTGAAAGAAG
ACTATTTAATTGAGTTAATTAATGGGGAAATTTTTGAAATGTCACCAGTGGGATTTAGACACGCA
TCTTGTGTCAAAAAAAATTAATTATTTATTTGCGGAAAAATTAGGTTCAAAAGTGATCATTGGGG
TACAAGATCCGATTAAACTTAATGATAATTCAGAACCACAACCGGATATTGTTTTATTAAAACCT
CGTCATGATTTTTATGCAAATCAGCATCCTACCGTTGAAGATATTTTTTTATTAATTGAAGTGGC
TGATAGTAGTATAGAATATGATCGCACTTTTAAAATTCCCATTTATGCAGAAAATAAAGTACAA
GAAGTGTGGTTAGTTGATCTCAATCAAAACTTATTAGAAGTATATCAAAATCCTCAGACAAATTA
TTATCAAAGTATCAAAAAACTTTCTTCTCAAGATTCTATTACTTTAACTCAACCAGAAACTATCA
CCGTAAAACTCGATCGCATTTTATTTTAGTTTATGGTAATTTGCATAAAAATTATAGCTTTTATA
AAATTAAGTGATTATTGATTGTTAATAGTCAACTGAATCATCCCTTGTAAATGTTGTAAAACCGA
TTCTTTCCGTTTTTGTTTCGTTGAAGGAGAAACACTAGATTTCCAAAATAATAACTCCGTATTTA
TCAACTTCCAATAACGATGAGTTTCCCTTTGCCAAGATTGCCATTGAGGAAAAGCAGAAACATCT
ATATTATTCTCCTCAAAAGTCATTATCTCTGTTTGTAAATAATTTTGAATTTTTCGCCATTGAGA
AAATATTTGATCTATATCATCAGAATTACTCGTAAAAATCTTTTGGAATTCTACCAATAAATTTA
ACCAGTCAGATAATAAGGGAAAATGTGATTTAGGTAACATTTATACAAAATTGAGTTAGAATAA
AAAAATCTAATTACAATTAATATCAACTCTGAAAAAAAGTTTAAACAATCTTTAGTAGAAACAA
GATAAATCAGGATAAATTGATTCTCCCTCTGATTATCAGGATTAATGAAAATACCAGAGAGAGAT
GTCCTAATAGTTATCACTAAAGGCTTATTAACTAATTCAGATGAGAGTAAAACATCAAAGAAAG
TGAAAATATAGACTTCTTTGATACTTCTATAAATTTGCTTGAGAGAGAGTAATTTATTTAAGCAT

TTATAGAACAAAAATAGTTAATTATACCTAGAGTTTATCTAATCTTTATTTTACCCCAAATTATC
TGATTATGAGTCCTATTACACTAATACCTGAATCCACAGACAACAAAACTTATCAAGTCTATATT
CCTAATTGGCTAAATAACTGCTTAATTGGAGATCAAAACGATGAAAATAATAACCTAATATGTCG
AGCTTTTCAATTCGCCTATTCCTTACACGAAGGGCAAATGCGTAAATCTGGAGAGCCTTATATAG
CCCATCCCATAGCTGTTGCCGAGTTATTAAGAGACTTGGGAGGAGATAGCGAAATGATTGCGGCG
GGATTCTTACATGATGTGGTAGAAGATACCCCTACAACTCCCGAAGATATAGAAGTAATGTTTGG
TAAAAATGTGCGTCAGTTAGTAGAAGCAGTTACCAAATTATCTAAATTTAACTTTTCTAGCAAAA
CCGAGAGACAAGCGGAAAATTTTCGCCGAATGTTTATCGCTATGGCTCAAGATATACGAGTGATC
GTGGTAAAATTGGCTGATAGACTTCATAATATGCGCACCCTTGACTTTTTGCCCCCAGAAAAACA
ACGCCTCATTGCTGAAGAAACCAAAGAAATTTTTGCACCTTTAGCCAATCGCCTAGGTATTTGGC
ATTTTAAATGGGAATTAGAAGATTTATCTTTCAAGTATCTTCATCCCGAAGCCTACAACAAAATA
AAAAACTTAATTGCCGAAAGACGCACTGATAGAGAGGCAAGAATTGAAAACGTTATTGACACCA
TTAACCAACGTTTAGAAAACTTAGGCATTCATCCTGTAGAAGTGAAAGGAAGACCAAAGCATCTT
TATGGAATATATCATAAGATGATGCGTCAAAAAAAGGAATTTGAGCAAATTTATGACATCGCCG
CCGTGCGTATAATTGTTAATACCAATGATGAATGTTATCGTGCCTTAGCCGTGGTGCATGATGCT
TTTCGCCCTATTCCGGGTAGATTTAAAGACTATATTGGTTTACCTAAGCCCAACTTATACCAATC
GTTACATACTACTGTGGTGAATAGTAACGGGCGCCCCTTAGAAATTCAAATTCGCACCCTCGAGA
TGCACTATGTGGCTGAATATGGGATTGCTGCCCACTGGAAATATAAGGAAACGGGGCATTCTAAT
CATCGTCATCTTTCTAGTGATGATGAAAAATTCACTTGGTTAAGACAATTACTGGATTGGCAAAA
AGATTTAAAAGATGCCCAAGAATATGTTGATAACCTCAAGGAAAATTTATTCGATGATGATGTT
TATGCTTTTACTCCTAATGGGGATGTCATTGCTTTAGCCAGAGGTGCAACAGCAGTAGATTTTGC
TTATCGCATCCATAGCGAAGTAGGCAATCATATGAAAGGTGCAAGAATTAACGATAAATGGTCTC
CTTTAGATCAACCCCTGCAAAATGGTGATATAGTGGAGATTATTACGGCAAATAATAGCCATCCG
AGTCTTGATTGGTTGAATTTTGTTGTTACTCCCAGTGCGAAAAATCGTATTCGTCAATGGTACAA
GCGATCGCACCGTGATGAGAATATAGCCAGAGGAAGGGAGTTATTAGAGAAAGAATTGGGTAAA
AATGGTTTAGAATCTTTGCTGAAATCTGACACCATGCAGAAGGTAGCCCAAAAATGCAATTTTCA
AAAAGTAGATGACTTATTAGCGGCTTTGGGCTATGGAGAGTTAACCTGCACCCAAATTATTAACC
GTTTGCGAGAGGAAAATAAACAACAAACCACGGAAAAACCTCAAACCTTAGCAGAAGAAAGCAG
TGCAACCCTTACTAACACCCCTCAACAAAAAACTAATTATCGAGAAATTGATGGCAATAAATATC
CCATTGCGGGGATAGAGGGTTTGATGTATCACATTGCTGGATGTTGTAAACCCCTACCGGGAGAA
AATATTATTGGGGTTGTAACAAATACTCGTGGTATTTCCATTCATCATCATGACTGCCCTAATGT
TCATAATATCCCCGGTGAAAGACTAATTCCAGTGGGTTGGAATCCTCGCAAAACCAATCAACGGA
ATGCTACTTATCCCATTGATATAGTCATTGAAACTATTGACAGAGTCGGTATTTTACGAGATATT
TTAGGACGTTTAAGTGATCAAAATATTAATGTTAGTAATGCAGGAGTAAAAACTTATGTGGGTA
AACCTGCCTTAATTTCTTTAACTATTGATGTGCAGGATTATCATCAGTATGAATGTGTGATAGGT
AAAATTAAAGATATGAGCGATGTTTTAAATATTCGCCGTGTCAGTGAAATTGGCTAACTGTAACA
ATCGGAGTGAAAATCCCTACTTTTAGTGTGGCGAAGTAGGGAAAAATTAAATTAAACTTGACACT
TGAAACTAGACTAATGCTTTTTGCAGATAGGGTTTAGGATCATTGATAATTCATGATATGATGAT
AATGTGAGGGCTACTTATCCTATTAAAAAGTAAGTTATGGAGAAAGTTTTAATTTCATCAGGAA
CAAAACAAAAAATTACTGTGACAGTTGATAGTAATCTGATTGCTGATATTGATAACTTTACTAAT
AATCGCTCTGCCGCAGTGGAAGAAGCTCTTTTTTTATGGCGACAACAACAATTTGAGGCTAAATT
GCGTGAATTTTATTTGAATCGCTCTGGTAATGCTTTAAAAGATGATGAGAATTGGAGTGAAGTT
ACTCAAGATAATGCTATCGCAAATTGGGATGAATCAACGGAGAATAGTTAATGTCAGATTTTGAT

GTTTTTCCCCGTCAAGGAGAAATATATCTTTGTCGAGCCTCAAAACAATCTGGGGATACAAAAAA
ACGCCCCGTAGTGGTGGTATCTTTAAGATGTCAGAAATCAATATAGTTCAACCGTTTTAGTTGTG
CCTTTTTCTTCTTTTATCGAAGGAATTGAAAATAATCCTTGTCGAGTTTTAGTTAAAAAAGGTGA
AGAGGGTTTAATGGTTGATTCTGTAGCAATGGCGGATTTAATTACTAATATAACCTCAGTTCGGT
TTAAGAATATCCGATAAGGTTAGGTGTCAGGTTTCAGGTTGCAGGTTGCAGGTGTTAGGATGATG
ATCAGATGAGAAGAGAGAATGAGAGGAGGAAGTAAAGGCTTAGGGTTTGGGGTGGTAAGGGTTG
AATATATTTTTAACGTTAGTTCGGGTTAAGGCAATTTTATCGTTGTTTCTAAGAAGCTATAAGGT
TTTCTGACTACGAGTTGGGATGCTTTCGGCTTATCCAAAACTCAGGTTAATATAAAAAAGAATTA
TCTTGAATCCAGTGTTTATGGTGTCATTAGTCAATCTTATTTAACCAAAATTCAAAGGGCAATTT
CTTTGAGTATGGGTGTTTTTTTATAAAGCAAAATCAGCTTTTATACAGTAGTTATGATAAATTAA
CAGAAAATAATTAACAGGAACTTTGATAGCTATGGTTTTAATTATTTCAAAAATAAAATCGTTA
TAAATTAACATTTTTATTTAACCTGACACTTGACACCAGAAGCCTGACACCTTTCCTAAGCAACA
ATTTATATACTCAAAGTGAGAAAGCCAAAAAATGATTGATTTTTTAGCAACTTTTAATCAAATT
ATTGATAAGTCTTCTTCTAACCCTTCCACAAAAAATGTCTATGAGGCTTTAATTTCGGCGGAAAA
ATACGCTAATCAAAATAAGATTAAATATGATTTTTCTCAATTATTAGGTAGTTGGCAATTATGTT
TTATTACTGGGACAAAAAAAAGTCAACAACAAAGGGGAAACTTCTTAAAAAATGGCTTTTATTT
ACCAGCGATCGCATCTATTATAATTAATTATTCCATTCCTGAAAATCTCCAACAAAATAAAAATC
AAGGCATAATTGAAAATATAGTTAAATTCGGCTTAGTACAGTTTAATGTTAGTGGCGTTTGTAA
ATTTATTAATAACAAAAATATCTTAGCATTTGACTTTTTATACCTAAAAATGTTTATATTAGGAG
CAAAAGTATATGAAACGGAAATTAGAGGTGGTAAAGAAAATGAAACTACTTTTTGGGATGCCTC
TTTAAAAGACTTAGCTTTTTTTAGTTATTTTCTAGTGGATGAAAATTTTATTTGTGCCAGAGGTA
AGGGAGGAGGATTAGCTTTTTGGAAGAAAAAATAAGGCTATTTTTACGCTCATCTTGATAAATA
TTATTATCCGTTATCCACCCTAGGCGACAACTTTATATCGAACTCAGATAAATAATAAATAACCT
CAGTTCGGTTTAAGAATATCGGATAAGAGTAGGTGTCAGGTGTCAGGTTTCAGGTTGCAGGTGTT
GGAGAAAGTAATAAGTAACGAGTAATGAGTAATGAGTGTTCGGAGTTATCAATGATTAATTATT
CGCCCCAACACTATTTACCATTACCCTTTTTGCCATCACTCATCAGTGAAAATTTATCCCGAACTC
AGATTAAATAAAGTTACAAAAGTTTGATTTTATTCCCCTTCAGATTTTGAGCATTCTAGTTGGGT
TTATAATGGTTCTAGTTGCCTCTCATTAAGTCTTTATTATGAAAAAACTATTACTTACCTGTGCT
TTTATTGTCGGTGTTAGCCTTGCCTTACTTAATTTTCAAGGTTTAGCAGTTAAGGGTGAATTTGA
TTCTATTATTATTAATTTTCGTCAAGATATTCCTCAAACAGAGTTAGAATATAACCTGAGTGCGA
TCGCATCTCAACTGAAACAAGTACCGATTTTGAATAGTATTTTTTCCGAAGAAGAAAGAATTTAC
ATTGTAGAGGGAGATAAGAAAACCATCAAAAAAATCAGAAAATCTCCTTTTAAAAGTGATATTG
AATATGTAGAACCGAACTATATTTATCATAGTTTAGAAGTTCCCAATGATCCTGCCTATTCTCAA
CAGTGGAATTTGCGCAGTATCAATATTGAACAAGCATGGGAAGATAGTAAAGGAGAAGGTGTCA
CCATTGCGGTAATTGACACAGGAGTAAGTAAAGTACCCGACTTAGAAAATACCGAGTTTGTAGAA
GGATATAATTTTGTTGATAATACCATTAATGCTGACGATGATATAGGTCACGGAACGCACGTAGC
AGGTACAATCGCACAATCTACCAATAATAACTATGGTGTGGCAGGTATTGCTTACAAAGCAAAAA
TTATGCCCTTAAAAGTGTTAGGAGAGCATGGTGGTAGTATTGCAGACATAGCTGAAGCCATTAAA
TTTGCCGCCGATAAAGGAGCGGATGTAATTAATATGAGTTTAGGGGGAGGAGGAGATAGTCAAT
TAATGCAGTCTGCCATCAAATATGCCCACGATAAAGGAGTTGTCATCGTAGCGGCGGCGGGGAAT
GAAAATCGTAACTCTGCGGTATTTCCTGCTCGTTATCCTCAAGTTATTAGTGTAGCGGCTATTGA
TTCTGCGGGGCTTAAAGCTCCCTATTCTAACTTTGGGGCAGGAGTGGATATATCAGCCCCCGGAG
GTAGTGAAACAGGCAAAATTTTGCAAGAAACCATCAATCCAGAGAATAAACAACCCGAATTTATC

GGTTATCAGGGAACAAGTATGGCCGCCCCCCACGTGGCAGGAGTAGCCGCATTAGTAAAAGCTAC
AGGGATGGAAAACCCAGATGAAGTAAGAGAAGTTTTAATTCAATCAGCCCGTAGAGTAGAAAAA
GACCCCTTAAACCATTATGGAGCAGGGCAATTAGACGCAACTCAAGCGGTGAGAATGGCACTTAA
AGGAAAAATTACCCCGAAAGACTTTTTACGCTGGTTAAAAGAAAATGGCTATCTCAACCCTGTTT
TTTGGATTAATGGTGGCACTATCACCCTTTTACCGAAATTAGCAATGGTTCTAGGTTCATATTTA
CTAGCATGGTTTTTACGTAATTATGTCGTTTTCAACCTCAGTTTGAGTGCAGGATTAGTAACTGG
CAGTAGCGGTTTATTCTTCCTACAAGGATTATATTTATTTGATTTACCTCAATGGCCTTTTCGTT
TAATGGGTAGTTCTGTGCCAGAATTAGGTAATGTGGTTATGGGTACACCTAGTTTAAATCCCTTG
TTTGCTAGTGTTTTAATCCCCTTTATTCTGGTGGTTTTATTTCTTCAACATCCTCAACTTAAATG
GGGTGCGATCGGGTGTAGTTTAGGTGTGGCATCTTGTCTTGGGATTACTGCTTTTCTAAATCCTT
TTGTGTGGGGTTTAGGTTATGGGTTAATGTCTCAAATATTTCTCTTAATCAATGCTTTAATGTGT
GTTGGATTAGCTTATTTAGCCAGTAAAAGTGCTTCTTTCAATTAATCTGACCTCAGTTCGGTTTT
AGAATATTGGATAAAAGGTGAGGGGGTGATATCAAATCCGTTTAATTACCCACACAAAAAGATA
TTAATTAGGCCACCAATAAAAATTGGCTATTCCTTTAACTATTTCTTTTTCTTCCATAATTTTTT
TGCATCTACTAATAACTACCTCTTCTAACTCTTCTAAATTTTTAAATGTCCTATTAGCGATGGCC
TCATTTACTAATGGCCACAAACGTTCTGCCGGTTGTAATTCTGGAGAGTGAGAAGGCATCAGCAT
AACGTGGATTCCCTCTGGTATTTCCGTCGTTTCTTTAATCGGATGCCAACCAGCTTGATCCATTGC
TAAAATCACCCTTTTGTTTTTACCTATTTCATAGTGCTGTGCAAAATCTTTTAAGACTCGATTAA
ACAATTCAGTATTTACATATGGTAGTAGCCACCAGTATGTCTCTCCTGTATTTGGTTCCACAAAG
CCATATAACCACAACCATTGAAAACGCCAATTGACCGTAGCTATCGGTTGATAACCTTTTTCTAC
CCATACTCTTCTTAATACTGGTTTGAGTCCTAAACGATGCTCATCCTCAGCCCACACTTGAACGTC
CGCGTCGGGATATTCTTGTTGAATCTTTTTTTTTTTCTGCTAGTTGATTTTTCCATTCATTTTGTT
CAAGGCAGCTAGAATTTTGATGATTTGGACGAGGACATCGGAGACGATATTCTAATCCTTTGAGA
TATTCCCATCCTCTTTGAGGAGAAATAGTACGATCTAGAAGTTGAGTGAGCCATTTTGCTACTTT
GACACCATTCCAAAGTCCTTGATCATCGGGGGGTGATTGTAATCTTTGATATAGCAGAGCTAATT
GTTGATCGTCTAATAACGGTTTTCTACCCCGATTATGTTGTCTTTGATCCCCCAACATTTTTGCAC
CCGAACGATTATAACTACGAACAAGTTCGTAAATCCAACTTAAGCTATATCCTGTAACGTGGGAA
ACATAAGAGGTTCGTTTCCCTTGTGCTAATAACCAAATCACTTGGTAGTGAATTTTTTCAATCCC
TTGTTTGGCTTTTCGATACCCTTGTTCTAATTCTTGTAAACTTAAATGGGGTTCAATTTTGATTC
TTGAAGGCATTGGTCAAAATCTACGACAACAAATATACATCCTCTTGATTATACCATGTGCAATT
AAGCGGATTTGATATGAGAGGGGGAAGCGAATAGCCATTCGCCCGTACAGGTGTTCAGAGTTTTT
AATTCTTAATTCTTAATTCTTAATTCTCAACTATTCACTATTCACCATTTACCCTTGCCCTTTACC
CCTAATTGAAATCACATCCTAAGCTATCTCTAGTGGCAACCCCTGTGATAGAATTAACCCCAGAA
GCTCGATCGCACATTAAAGCTACTTGATAACGGTCAATTACTGCCCCTGTAAAGTCTGCACCCTCT
ATATTTGTATCATAGAAACGAGTACGAGTTGCGATCGCATCTGTAAAAATAGCGTTGCGTAAGTC
AGCAAAATCAAGGGTAACACGATCAATCAATGAGCCTGTAAAGTTGGTTTCCCGTAAATCAGCAC
CGAACAAAATTCCTTCTGTCATCACAGAATTAGTGAGATTAGATTGATAAAAAGAAGCATTGCGT
AGATTAGAAGCGGCAAAAACTGAACCTGTTAAGTCTCGGTGAGAAAAGTCTTCCCCTTCTAATTG
GGCATAGGTAAAATTAACCGCCGTTTGGGCGTTAACAGATTGGGGAGTAAGAAGAATAAAACTA
ATGGCTAATAACAGTAGAATAATTGCTTTAACGATGAGGGGAAAAAAGCGAGAAATAAAATTGA
CAATGGGAGAAGGAAGATTGAAAATTGAAAGACGAACTTGGTTAATTAATTCTTGATTAGCCAT
AAACAATTATTGCAAAAATTCTTGAGCTTCATGTTAATTGTAACCAGTATTAGTGAATTGCAAAG
GGAAATAAGCAGGGTTTCTCCTTCCCAAAAAATTGGGTTTGTGCCAACTATGGGCGCATTACATT

CCGGGCATGAAAGTTTAATTCAACGGGCAGTTACGGAAACAGATTTTGTGGTAGTGAGTATATTT
GTCAATCCGCTACAGTTTGGCAAAAATGAAGATTTAGATCAATATCCAAGACCATTAGAGCAAGA
TAAGGCAATATGTAAGCAGTTAGGAGTTAAGCTCTTATTTACTCCTTCTGTGACAGAAATTTATC
CTTCAGAAGAAGAAACCACCATCGTTAATCCTCCTACCGCTATGACTTCGGTTTTGTGTGGGAAA
TACCGTCAAGGGCATTTTCAGGGAGTGGCTACTATCGTCACTAAATTATTTAACTTAGTCCGTCC
AAATGTTGCCTATTTTGGGCAAAAAGACGCTCAACAAGTGGCTATTATCAAAAAAATGACAAAA
GACCTCAATATTCCTGTAAAGGTGCAATCTTGCCCCATTATTCGGGAAAAATCAGGGTTAGCTTT
GAGTTCTCGTAATCAGTATTTATCCTCTGAGGAAAAGGAAAAAGCTACTTTACTTTATCGCAGTT
TGAGCAAAGCACAACGAGCTTTTCAAGAGGGAGAAAGAGATTGTCAGAAATTGTTACAGATTAT
TAAGGAGGAGTTTAGATCCCCCCAAACCCCCCTTAATAAGGGGGGCTTAAATCTTAATGATTCTC
TTAATAAAAAAGGCTTAAATGATGTAAATATACAATTGCAATATGTAGAAATAGTTGAGCCTGA
AACTTTGCAACCCTTGACGGAAATAAGCGATTCTGCTTTAATTGCGATCGCAGCTTATATTGGTA
ATACTCGTTTGATAGATAATGTGATTTTAACGGTGACTAAACCGATTATAGCCCTTGATGGACCG
GCAGGGGCAGGAAAATCAACCGTTAGCCGTCGTTTAGCTCGTGAATTGGGTTATATTTATTTGGA
TACTGGAGCGATGTATCGGGCGATTACTTGGTTGGTGATGGAAAATAATATTAATGTTGAGGAT
GAAGAAAGTATTGCTTCTTTGGTGGCAAATGTGCAGTTAGAATTGTTACCTAGTGATAATTTACA
AATTCCTGTGACGGTAAAAATTAATGGGCAGGATATAACAAAAGAGATTCGCACCCCAAGAGTTA
CTAATCAGGTGTCAAAAGTTGCGGCACAATCTGCGGTAAGAGAAAAAATGGTAGCATTACAACAA
GAATACGGAGCAAAAGGGGGTATTGTGGCAGAAGGGCGAGATATTGGCACGAATGTTTTTCCCCG
TGCTCAATTAAAGATATTTTTAACCGCTACTCCCCAAGCAAGAGCGAAGAGACGTTTAATTGATT
TACAAAATCAAGGAGAAAGTAATATTGATTTAGATACTTTAATCAAGGAAATAGAGGAAAGAGA
TTACTTAGATAGTACAAGAGCTATTGCACCCTTGAAAAAAGCCGAAGATGCGATCGAGCTTATCA
CTGATAATTTAACCATAGATCAGGTCATTGAAAAAATCAAAAGCTATCTTATTTTAGTTTAGAGT
TAAGAAAGAGGTGACAGGTTGCAGGTTTCAGGTGTTAGGGGGATGAGGAGATGAGGGGAGGGGG
AGAAACAAGTAAGGAGTAATGAGTTCAGGGTTTTTAATTCTTAATTCTTAATTCTTAATTCTTAA
TTCTTAATTCTTAATTCTTAATTCTTAATTTTTAATTATCAACTATTAACTATTAACTATTAACT
ATTAACTATTAACTATTAACCTTTGCCCTTTCATAACCAAAAAATCAATACAACTGAGCTACTTA
ACTACACAATAGTTAACATCTACATAATAATTAACATTTAATCTTTTTTTGGGGTTTTTTGCTTC
TCTCAAATCATTTCTCCAAAGATGCGATCGCATTTTATTTTTCATTAAACCCTTACCAAATGGCA
ATTTAAGAGAATATTAAGCAATCATAAATATAAAAGTTTGCTTTTAAACATAATTACTATAAAT
TAATTGACAAAATACTAAAATTAATAAAATAAAGGCTTAAAAAAGAATTTAAGGCTAAAAAATT
AAATTAAAATCTAAATCAACAAAATAGAGTCCTATAGCTACCAAATATAAAACCATAGCACCTGT
TAATTTCCTAAAAATTAGTCAATAATGCAAAAAGAATAAATTTTAATCAGACATTAGTAAAATG
ACTTTAACCCTAGCAGTATATGGAAAAGGTGGTATCGGTAAATCTACCACAAGTTGCAATATTTC
CACCGCCCTCGCTAAAAGAGGTAAAAAAGTATTACAAATAGGATGCGATCCGAAACACGATAGCA
CTTTTACCCTCACGGGTTTTTTAATTCCCACTATTATCGACACCCTACAAGAAAAAGATTTTCACT
ATGAAGATATTTGGCCTGAAGATGTTATTTATAAAGGCTATGCAGGAGTTGATTGTGTTGAAGC
TGGTGGCCCTCCTGCGGGGGCTGGTTGCGGTGGCTATGTGGTAGGGGAAACGGTTAAATTACTCA
AGGAGTTAAATGCCTTTGATGAGTATGATGTGATTTTATTTGATGTTTTAGGAGATGTTGTTTGC
GGGGGATTTGCCGCCCCTCTCAACTATGCTGATTACTGTTTAATTGTTACTGATAATGGATTTGA
TGCTTTATTTGCCGCCAATCGCATCGCCGCCTCAGTCAGAGAAAAAGCACGCACCCATCCCCTTCG
TTTAGCAGGGTTAATTGGTAATCGCACCTCGAAACGGGATTTAATTGATAAATATGTATCCCATG
TGGCAATGCCTGTGTTGGAAGTTTTACCCTTAATTGAAGACATCCGAGTATCCAGAGTCAAGGGA

AAAACCTTATTTGAAATGGCAGAAAAAGACCCAACTTTAGATTATGTGTGTGACTTTTACTTAAA
TATTGCCGATCAAATTTTAGCATTACCAGAAGGAGTTGTCCCTAGTGAAGCCCACGACAGAGAAT
TATTCGGTTTACTTTCCGATTATTATTTGAATCCTCCTCAAGCCAAAGAAGAGAAAGAGGATGAA
TTAGAGTTAATGATAGTATAACTCAGTCAGAAGTGAGGGTTGGGCAATGTTCAACCTTTTATTGA
TCTAACTAAGGGAATTGTGGAAAATTTTGAGTTAAATAATTATTAATTCATCATAAAAGATTGA
GTTTGTGGTAATGGCTTCAGCCATTTTAGGTTCGCTCATTGAAAAATAACCTGTGAAGTTTTGAT
AAATCAATTACCAAAATTTAGTTAGGGATTTACTGTTTTTCCTTTAGATGATTTTAGACAAAAAA
TAAGGCAATTTAACAACACAATTTAACTCATTTATTCTCAGTAAATTAGAAAAAGGGCTAAAGCC
CTTACTACGAACAAAAAGAATTAGTTTATATCAACAATATAGAATTACAAAATTATGACTTTTAC
ACCCGAAAAAACTGCTACTCCTGAATTAAATTTTGAATGCGAAACCGGTAATTATCACACTTTTT
GCCCTATCAGTTGCGTGGCTTGGTTATATCAAAAAATTGAAGATAGTTTCTTCTTAGTTATTGGT
ACAAAAACCTGCGGTTATTTCCTTCAAAATGCGATGGGAGTAATGATTTTCGCTGAACCTCGTTA
CGCTATGGCTGAACTCGAAGAAGGAGATATTTCCGCCCAATTAAACGACTATAATGAATTAAAAC
GCCTCTGTGATCAAATAAAGCGCGATCGCAATCCAAGTGTAATTGTTTGGATTGGTACTTGTACC
ACAGAAATTATCAAGATGGATTTAGAAGGTATCGCCCCTCAGTTAGAACAAGAAATTGGTATCCC
TATTGTGGTAGCTCGTGCTAATGGTTTGGATTATGCCTTTACTCAAGGAGAAGATACGGTATTAG
CGGCCATGGCGAATCGTTGTCCGAAGGAAGCAGAGGTTAAACAGATAGAAGATAAGCAAGAAAG
AAATGCCATCCAGAAATTACTTAATTTTGGCAAAAAACAGGAAGAAACAGTTCATAGTGAGTAT
CACGAGCATCCCCCATTAGTCTTATTTGGTTCATTACCTGATCCTGTTGTTACTAATTTAACCCTA
GAGTTGAAGAAACAGGGAGTCAAAATCGATGGTTGGCTACCTGCGAAACGTTATACCGAGTTACC
CATAATTGATGAAGGTTATTATGTGGCAGGAGTAAATCCTTTCTTGAGTCGCACTGCTACCACTT
TAATGCGTCGCCGTAAGTGTAAGCTCATTGGTGCACCCTTCCCCATCGGGCCTGATGGCACTAGGG
CATGGATTGAGAAAATTTGCTCTGTATTAGGAGTTGAGCCTCAAGGTTTAGAAGAAAGAGAGGC
AAAAATCTGGGAAAATCTGCAAGATTATGTTGATTTAATTAAAGGTAAGTCAGTTTTCTTTATG
GGTGACAATTTACTCGAAATTTCTCTTGCTCGTTTCTTAATTCGTTGCGGTATGACTGTGCCTGA
AATTGGTATTCCTTATATGGATAAGCGCTATCAAAAAGCAGAATTAGAGTTTCTTGCCCAAACTT
GTCAGGAAATGGGGGTATCTCTACCTACTATTGTGGAAAAACCTGATAACTATAATCAATTACAA
AGAATCAAAAAAATTCAACCTGATTTGGTGATAACGGGCATGGCTCACGCTAATCCTTTAGAAGC
AAGAGGAATCAGCACAAAATGGTCTGTAGAGTTCACTTTTGCCCAAATTCATGGTTTTACTAATG
CCCGTGATATTTTAGAGTTAGTAACTCGTCCTTTAAGACGTAATAATAATTTGAAGGATTTAGGC
TGGAGTAAATTAGTTAAGGAAGAAGCAATTTTGTAATGGAAAAAAACTTAATTAACCTCAGTTC
GGTTTAAGAATATCCGATAAGGTTAGGTGTCAGGTTTCAGGTTGCAGGTTGCAGGTGGTAGGGGT
TTTTAGCAAGGGGCTTAAGCCCCTTGTTTAAGTCAGTTCGGGTTAAGGCAATTTTATCGTTATTT
CTAAGAAGCTATAAGGTTTTCTGACTACGAGTTGGGATGCTTTCAGCTTATCCAAAACTCAGGTT
AATTATTGGGAATTAGGAAAGGATAAAAAGGGCAAAGGAGGAATGAAAAATAGTTAACCACTAA
ATTCGATCGCACTTTGATAATATTGCATACCCCAAAAAATTAAATCAGGATCGATAATTAGTTTT
TGACTAAAATCACGATCAATATCTGCTAAATATCGAGATAATAATAAAGCGTCTCCCCCTGTCAA
AACGACTTTACTTTGGGGATATTTTTTGAACCAATCTTGAGCAAAATCTTTGATTCCCGCAACGA
TAGTATAAACGACACCACTTTGGATGGCACAGGGGGTATTTGTCGCCCAACGGGGAGTTATCTCC
CCCATAATTTGTACTTCAGGTAAAGCGGCTGTGTTAAAGAAAAGGGTTTGTAATTGTAATCTTAC
CCCCGGCATAATAGCCCCCCCTTGAAAGTTTTGTTTCTCATCTGCTCCCGAAAAGGTTAAAGCTGT
ACCACTATCAATTACTAGGCAGGGAAAACCATAATGACATCCTCCCCCCCATAAAGCTAAGATGC
GATCGCACCCCATACTATCATAAATATTGTGTAGGGGTATTTTTTGATAATTAATGATAATTGTT

TGAGGTAGGGAAAGATAAATTTTCGTAGCCGATGGCACAACCGAAGCCACATATAAAGACATTCG
ACTCTGAATATAGCCAAAAAGCTCCTCATCTAAAACCGCTTCTAATTCTTTGACAGTATCCGCCG
AAAGGGTATTCCATGTTTTTCTTAATTCTTTACCCTGAAAATAAGCCCAATGGAGTCGAGAATTA
CCAATCATCAAGCCTAACCAATTTTCCATTATGTCTTTTTATCTCACCCCCAAAATAATTCCTAAC
AGTAAAATAAAGCCAATCCAGACATTCTGACTAAAAATTTGACCATAATTAATCGAATCCTGATT
AATGCCCCTAATTTGGATATACTGCCCTACCCATAAAATAACTGCGATCGCCCATGCTACCCCAAA
AAAGAGGTTGAAACCATTAACCATCGCAATATAAGCAAATAAACCAGCCGTAAGGGCATAAAAT
AAGCCGACTGCATCCCCCACCAAATCATCAAAAAAGATGGCACTCGAATTGATTCCCACCTTGAG
ATCATCTTCTTTATCACTCATAGCATATACCGTATCAAACCCCATTGTCCATGTTACCGTTGCCCC
CCACAACAACCAGGTATTATTATTCAAATTCCCTGTCACCGCACTCCAACTAATTAATACAGCAA
ATCCCCACGCCAATGATAAAACCAATTGAGGCACGGGAAAAACCCTCTTTGCTAAAGGATAACAG
ACAATAAAAGGTACAGCCCCCACACAAAGCCAAAAACTGAGGGCATTAAGATAAAAAGCCAACAC
CGCCGCACAAGCTAAACTAATAAAAAAGACAACTATTCCCACCCTTACCGATAACGCACGGGAGG
CAAGAGGGCGAGTTTTAGTTCTTTCCACCTGATTATCAAGATTTCGATCCCATAAATCATTAATA
ACACAACCAGCCGCACTGGTAGCTAAACTGCCGAGAATAACAACCCCAACTAAAGGTAAAGGAGG
TTTTCCCTCTCCAGCTAAAAATAAAGCCCACAATGCAGGTATCATTAAGATAAGTCTTCCTGCGG
GTTTATCCCAACGCAACAATTTAACGATATTTTCCAGCAAAGATGATGATTTTAGTTCAGGGGAA
GAAACCATAATTTCAAGTAGATTCAAATTCTTTGTCTAAACCTTTTGTTATTGTATCTTAATCAG
TGAAAATAATAATTAAGACAAGGCAATGGACAAAGGTAAATAGTTAATAGTGAATAATTAATAA
TTAATAACTCCGAACACTCATTACTCATTACTCGTTACTTATTACTTTCTCCAACACCTGCAACCT
GAAACCTGAAACCTGACACCTTATCACCCTCACCCCCTCACCTCACCATGCCAAACCCTCCGATGC
TACCTGAAATAGACTGAATTATGATTTATTGCCAAGGAAATTAAGGATTGCATTAAAAACTCTTT
TATCTTCCAGCATCCACCGATGAATTAATACAGGGAAATTTACCTCTTTTCCTACTCCCATAACA
GAACTTTTAGGAGGCAAAATCATAGCATCAAAAGGAGTCCACATCACTAGGGTTTCAATTTTACT
TAATATATCTTTTACATCATTATTTAAGTCTTGTAAAAATTCACTATTTGGACGCATCTGTTGAA
TACCTTTTAGGGGTAGAAAATGAGCGTTATTTGTACCGTTATTAGGAGCAGAAATACTAACATAT
TTATTAACTCTATCAATACCGTCTAATCTCTGTAAATAGTATCTAGTAACTAATCCCCCCATGCT
AAAACCAATTAAGTTTATTTTTTCTTGCTCATGAAAATTATTGTTTATGAATTGTTTAAGTTGTT
GTGCTAAATCTCTTAAGTCGGCAATACCAATGCTAGGAATTAGGTCAATAGTATGGGGTTGATAA
CCGTATTTTTCCAAAATATTACCCATGGATTGAAATATTTTCCCTGTATCTGTAATACCGTGAAC
CATTAATACTTTTTTTTGATTCATTTTTATCTAATTGCCTCTTGCCTACCAAACACGAATAATCG
ACACGACGAGAAGTGATAGAGCCTAAATTACTGTTTAATAATTGTCTTTTCTCTTCCTAAACTAA
TAATTTACATAACAAGAAGCAAGAGATCAACAAATTTAATTTATGGTAAGAAAATTCCCTCATT
GGCAACAGATTGAATAAATTTTGTCGCCTCATCCACAGGTAAAGGATAACTGAAAAAATAACCCT
GCATAGTATCACATTGTAGATTTTTGAGTAGGTAGGCTTGTTCCTGAGTTTCTACCCCTTCCGCT
ACTACTTGTAGATTGAAGCCTTTTCCTAGAGTTATCACCGCCGAAATAATGGCTAAGTCGCTGGG
TTCATTTTTCAAATCTCTAACAAAACTTTGGTCTATTTTTATTTTTTGAAAAGGAAATTTTTTTA
AATAACCGACAGAAGAATAACCAGAGCCAAAATCGTCCATAGTAATCGAAATACCTATTTCTGTC
AATTCTTCTAAAATTTCTTGGGTTAATTCTGGATGAACAATAATACTTGTTTCTGTTATTTCTAG
CTCTAAATATTCTGGCTCTAATTTTGTTTCTTTAAGAATTTGTTTGAGTGATGTAACTAAGTTTG
GTTGACATTGCCTAGGGGATAAGTTAACTGCTATTTTAAGGGGAGTTAAACCTGATTGTTGCCAT
TTTTTATTTTGTTCACAAGCCGTAAATAAAACCCATTCTCCAATGCTATTAATAAATCCTGTTTC
TTCTGCAATAGGTATAAATTGGTCTGGTGATACCATTCCTAAATCGGGGTGTTGCCAACGAATTA

AGGCTTCCATTCCCGTTACTTTACCTGTGTTAATATTTATCTGCGGTTGATAATATAAGCAGAGT
TGGCTTTCTCTTATTGCTTGATAAAGGTTTGTTTCTAGGTGTAATAATTCTGTATTTCTTTTGTT
CATGGAGGGATTATAATACTGATATTGATTCCCCCACTATCTTTACTGCGATATAAGGCTATGT
CAGCATTTTTGAGTAAGTTTTCTGGGTTTTCACCGTCTTGAGGGTAAATAGCTATACCCATACTT
AAATGACAGTATAGTTGATATTCGGAAATAATTATCGGTTGTTTAAGGCTATTAAATATCCTTTT
AGCGACAATGGTTGCATCTTGAGGATTTTTGATTTCTGATAACAGTATGGTGAATTCATCCCCTC
CCCAACGGGCAATTACATCGGCACTCCTGAGACAGTTTTTAATTTTTATAGCTACTTCTTGCAAA
AAGCGATCGCCTACATCGTGACCTAAAGTATCATTAATATACTTGAAACGATCTAAATCAATAAA
TATTACAGCTAGTTTACTTTTATACCTTTGAGAACGTGCGATCGCCTTATATAGTTGTTCATTAA
ATAGATTTCTATTGCCTAGCTGTGTAAGTAAGTCATGACAGGCTTGATAACATAGCATCTCCTGT
GCCATTTTTCTTTCTGTAATATCTCGGACTGCATAGCAAATGTATTCTTTAGCACCATAGTAAAT
ACTACTAATATTGACTTCAACATCAATGAAGTGACGGTTGCGATGACGATGAATAGATTCTTGAA
CTAAATTAAGACGATTTTTTTGAACTTTACGGATTATACTATCATGTACTTCAGGATCAGTGGCT
AAAACATCATAAATTTTTAAGGTTAAAATTTCCTCGTTACTATAACCGATTAGACTACAATAGGC
TTGATTTGCTTCAATAATTTGTTTAGAAATAGGATCAACTAAAATTATTCCTTCAGATATTTGTC
TTACTACTGCTCGATATTTTTCTTCATTTTCTTGCAAATTTTTTTCTAAGGTATATCTATCTTCA
AAACAAAAAATATAGCTTTCAATAATATCTTGTTGCTGTTTATAATTAGCATACTGAGTATAGCA
TTTATTTTGATGTTTAATTTCTCTGATACAAAAATTATTGCCATTTGATTTCAAATCTTGATGTA
AATTATCCACAAAAGGATTAGGATAACTATTACGATTAAAATCGGGGAAAAAGTCTTTAAAAAA
TGAATTAAAGTGAATTATTTTTCCATCTTTATTTATATGAATGTAAGGGGCTTTAATATGGTTAT
TATTTTGATGTAAAGTAAATAAAACTTCCCTAATAAATTTTTCTTCTTGCTCTTGATTTTCTTCT
GTAATAGTAGTTTGTGAAGAAGATATTTTTGGGTATTTAGAATTTATGATAATGTTAGATGAAT
TAATTGATGATATATTCTCCTCTGAAATAGCTAAATAGCTACCACTTTCTTCATTAATAATTTCA
TAACAAATTTCAACTTCATTGCCCCCTATTTTTATGGAGTCTCCAGAACTCAATTCATACGACAA
GCATTTATTCCCATTAATAAATATACCATTAGAGCTACGATTTCCCTTTAAATCACCATCAATAA
TCCAAAAAAGAGTTTGTTGATTCTCTCCTTTGTATTTTACTGGTAGTATCGTACAATGAAATCGA
GATATTGTTGGATGATGAATAACAATTTTATTACTAGAATGACGCCCAATAGAGTATATTTCATT
GCTCAATACTATTCTTTGTCTTATATCCCCATAAGTAATATGTATTATATGTTTTAGTTCTACTG
GTTCATTCATTCCCAATAACCTCTAAAGTTATTTTATTGTTTAATTGAATTATTCTTTGATCTGA
AAAAATATTAACCTAAATTCGGTCTTTTAGTCATTAATAATTACTGTAAAATTTTCTAATTATTA
CCATTCTTTTTAAATTTATAACATTAAAGATTGTCAAACACAAAAAAAATCGCTATAATGTGTAA
GCGTATTTTATAAATGGGTGCGTAGCTCAGCCGGATAGAGCAACTGCCTTCTAAGCAGTCGGTCA
CAGGTTCGAATCCTGTCGCACCCGTCCATTTTTGTTCATTTTCCGTAAAGTCGATGAATGTTATT
ATCTACTGTGCTTATTTCATGTTGAAATCATTTATACTTGCTCTCGTCCGATATTCTTGAGGTGA
ATTAAGATTAAATAAGTGCGTGAAGCCAGTTAATATATATTCTGATAAACCATTATTTATCAATG
ATCAAGAATAATTATGCCTTTAATTAAAGTTCAAACATCTATCGCTAATCCTGATCAAGAAAAAA
TCAAAGATTTATTGAAGAATTTATCCAATAAATTAGCTAAACATGTAGGTAAACCAGAATCTTA
TGTAATGACAATATTTGAATCTGATGTACCCATGAGTTTCGCTGGTACTTTTGACCCTGTGTGCT
ATTTGGAAATTAAAAGTGTTGGTACAATGAGCTCTAGTCAGACTAAGACAATGAGCGAAGATTT
TTGCAACATTATCAATGATAATTTGGGTGTCTCTAAAAATCGTATATATATCGAATTTGCTGACG
CAAAAGGGTATTTATGGGGCTGGAATGGATCAACTTTTTAAGTTATCATTTCTTTGATTATTAAA
GCGGTAGCGGGGGCTAGTAAAACACCATTACGATAATGTCCCGTTGCTAAGATAATATTTTTGTA
GGGTTCTAATTTTTCAATTATAGGAGCAGATTTCCCTTCGGGACGTGGTCTTTTCCCTGTCCATG

ATAACATGATGGGAGCTTGGGCTAAACTGGGACAAAATGCGATCGCATCTGAGCGTAAATTTTGT
AATAATTGGTCTTTTGCAATGACTTCTCCCTTACTATCAGGAAATTCGACGGTTGCCCCTAACCA
AAACTCATTATTACCCATAGGGGCGATATGAATATCATTTCCCGTAATGACTGGATTAAAATCCT
CTTTGCTTTGCCATTGGTCATATTTTACTAACAATGCTTGTCCTAATACTGGTTTAATTTCGATG
GGTATGTGCAATGATTCTAATAGAGAAGAAGTACCTAAACCCGTTGCTATAATTACTATATCTGC
AGATAAAGATTGATTTTCTAAAATAACTTCAGTATGATTTTCCGAACGAATATTCAAACCCTTAA
CTTTCTCTCCGAAAATACATTTAACTCCTTTCATCGATGCACCTTTGACAAGGGCTTTAGTTAAA
AATGTAGGACTAACCTGCCAATCATCAGGGGAATAAACAGCCCCAACTATTGAAGAAGTGTTAAT
TTCTGGACAATGAAATTTTAATAAAGATTGATCCCAAATATCCAATTTATAGCCCTGAGAAGCCC
TAAATTGAGCTAATTTTGCGTATCTTTGAAGTTCTTCCTCTGTAAATAATAACTTGACAATACCC
TGCCGATTATGGGGAATTGATAAACCTGTGAAATTTTCTAATTCGGAAATCAAAGTTTTATATCG
CTTAAGACTTAACTCCCTTAACTTCCAGGCTCTGCCTTTCGTTTTAAGACTAATAATTGCCATTA
AAATTCCCAAAGCCGCCCCGGTTGCCCCCGTGCCTGGATTTTTTTCATCGATTAAGGTAATGTCAA
ATTCAGAATTATTACCTAGTTCATATGCGATCGCACTTCCTACAATACCACTACCGACAATAATA
ATTTTTTTCATTACTTTGATAATCTCATTCACAATAGGCGCAACTTTAATATATTGTAACCCCAG
TTATGACTCTGTCTCCATGGACTCAGTTATTCAGTAGGAATGTGTGATTTAGGAAGGTATCAGGT
TTCAGGCGGTAGGTGTTAGAGTGTTTAGGGGATGAGGTGATGAGGTGATGAGGGGAGAGGGCAA
TCATCGGAAATAGTGCTTTTCAAACAGATTTTTAGGTTAATTTAATTCTAGTTTTTATTTTGTAC
TAGGTCTATTATTGATATAGATGGATTTTTATAAGTAGAAATGAAGAAAAGAGTAACCCTTAGT
TTTCCCCGTACAGTGGTACAAATGCCCGTTACTTATCGATTGGCAAAAGAATTTAACATTGCGGC
AAATATTATTCGAGCGGCTGTTGCCCCAAATCAAGTCGGGAAACTGGTTTTAGAGTTATCGGGAG
ATATTGATCAATTAGAAGCTGCGATCGATTGGATGAAAAGCAATAACATAGGAGTATCCCTAACA
ACAGGAGAAATAGTTATTGATGAGGAAATTTGTGTTCACTGCGGTTTGTGTACAGGAGTATGTCC
CACAGAATCTTTAACTATTAACCCCAATAGTTATCAATTACAATTCAGGCAACAGACTTGTATTG
TTTGTGAACAATGTATTCCCACTTGCCCTGTACAAGCTATTACAAGCAATATGTAACACTTACTT
GGGGTTATAAGGAAAAATGTGAGTGGTAAAGGTAAGAGTCACCGAGTCACCAAATCAGTAAAAG
TGCTTATAAATTAAGGTATTTACCCTAATTTTTAGTTTTTAGGAGATATTCTCTATCATATTTTG
TTTCAACATAAGTCCTCAAAAAAATATTTAATTTTCAATTGAATTTTTAACAAAAAAACCTATGAA
ATATTCAACTATTTTTGGCACTGGAGCGTATTTTCTTATTTCATTCTCTTTAATTTCCTCTTTTTT
ACCAGATAAACTATTAGCGGAAACACAGATTAAGAATCAATTAGAGTTAACCAATGAAACACCA
GCATTTCAAGGCAAAAGGCTAACCGAAGGAGACTTATCCATTGAAATTGATTTCCAGCCCTATAA
TTTTGAAAATAATAACAATAATTTAAAATATAGACTTTTTTATCAAGGAAAAGAAAAATTAAAA
GCATCTAAAGAGACTTTTTTTTCTCAAGTTTATTTTCAACCCTCAGAAAAAGATAATCCCTATCC
TTACATTGTTATTGAAACCTACAGTGGTGGGGCTCATTGTTGTACGAGTTTTTATATTTATACAT
GGTTGGATGGAGAATTTGTTGAAACCAGTATCGAAAATTTAAACTCTGGCGGAGGAGTTTTTAA
AGACTTAAAAGGGGATAATTCTTTGCAATTTGTGACTAATGATAATAGTTTCTTTATGCTTTTA
GTTCTTATGCAGGGTCTTATCCTCCTAGTTTAATTTATGACGTAAAAAAAGGAGAACTAATAGAA
AATACCCGTAACTATCCTAATTATCTACGAGAAACCGCCGATAATATGCTAACAGCCCTACAAGA
AGGAATAGCAAATGATTATGAAGTCAATGGGGTTTTAGCTGGATATGTGGCTCAAAAAATTTTA
CTAGATGAGTATGAAGAAGGTTGGCAATTTATGTTAGAAAACTTCGATCGCAATGATACTACTGG
CTTAACTATTTATAATGCAGAATTTGAAGAAGTCGGACAACATCCCGATTTTCCCACCGCTTTAA
AAGCATTTCTTCAAGAAAGAGGTTATTTAAAAGGAAATTAAAGAGCGTCATAATTATCATTAAT
AAAAAAAATTGATGATTTTTTAATCGCTATTATTGATTATTAAACAATTGTAATAACTTAAATA

AATTAACTTTTGTTTCTGCTGTTGTTTCCCCCAAAAGTCCTGCAATTTCTTGGGCAACTTTCATCT
GATTATTAACAACATTATTTACTTGTTTAATCATTTGTTCTTGCATTTTTGTTAATTCTTTCTTT
TGTTTAATTTCAGACGTTAGATCTATCATTATGCAGAGAATCAAATTTTTTTCTTCTATAGGGTA
AATAAATTCTTTAACAAATATCTCTGGTTTAGGATATTCTTTAATTGAATTTTCTATATTTTTAT
TCTCTTTCCAAGCACTTTTTAAATGTTCTATATCACCGAGAATATTAACAGCTAATTGACCTTTG
ATTGCGCAACTTTCCAGTTTAAATAGTGTACAGAAAGCAGGGTTAACCAACTGAATTTCTAGGTC
AGAATTGACGGCAATTAGTGCATTGGGGTGATATTCCCACGCTAATTTATAGAGATAGTTATGAT
TATTTTCTAACATTATTTTAATCAGTGCTTTGAGGAAAATATTACTTTAAAGGGAGTATAATTTA
AGATTTAAACTTAATAAATAAGGAAAAAAATAGCTATAAATTAATTTTATTTAATCATGGGTAA
AATTTCATGGATAAATTTTTCTTCTAAGTTTTGAGGGCTAATATTAATAAATAAATGATCTTTAA
ATTTCATAACAATTCCTGAGCTACAAGTTTCTAAGCAAAAACAACCTTTTAATTCAACTATTTGA
TCTATGTCATACTCACTAATTAAGGCTTGTAGTTTTGGTAAAACTTCATAAACTCCTAACTGATG
ACAGGCTGAACCCATACATAAAAATAAGTTTTCTTTAAGCATTTTTCCTCTTTGAAAAGCTCGAA
CAAATGTTATTCCACATCAAGCTAAAAATCGCTTTATTATCCTCCTATTTAATAAATTTTCTAAG
GTCGAAATTCTTATTTTCCCCGTTTGAAAGACACTCTTTTTCTTGAGTAAGAATTAATAATATTC
TCTCACTATAGTCAACTGCTCCTTTTAATTGTTGTTGTAATCTATCTAATCCGCCATTGGCATATT
CTTCAAAAATTTTAATTCTGGCTTCTTCTTGATTCTCTTCATAGCTAGAAATAATATTTAAGTTG
TGGGTGCTAGTAATGCCTAACAGTTTAAATAAAGATTCATAACCACGGGAGTTAAAAACCTCTAT
ACTGATGGGGGAAGGTTCTTTTGCGATCGCACCTAAAGTCACAGAAAAATTATACCTTTTACCTA
AAGCGGCGGCGAAAGCCATTAAATCCGCATAAGTAGAAAATACTCCTTGGGGATTATCAGGAGAG
ATTACTAAATTTTGGACTAAATCAGCTTTATCCTGAGATATTCTAACTCTATTAGACATAATTTT
TATGAATTAAAAGGCAATAGGCAACAGTGTTTAATTAATTTCCCCCTCATCCCCCAGGGCTGTTT
CAAAGTAAAAAATTAGGTTAACTGAGATTAAGTAAGAGTAAGAGATACAAGGATTTTTTTCTAT
TAGTTAAAACTTTATGAACAAAAATCCCCCCATCTCCCTCTCCCCCTATCTCCCGCTCTTTTTTCT
TTCTCGAAAATGAAACAGCCCTGCCCTCATCCCCCTAACACCTGAAACCTGAAATCTGACACCTCT
TTCCTAACATCCCAAACAATAAATTAATTGCTATTTTTCTCCTCTTTTACTGGTTCTTTTTTACTA
ATGGAAGGAATTTCAGGGTCTGTTAAATCAATATAGATAATATCTTCTCGACGCACTTGGGCAGT
AATAACTTTAAGTTTTTCTAATACCATTAGTTGTTGTGGAAATTTTTTCGGGGTATAAGCACCTA
AGTATATTTTTCCTAAGTCCGTAAGTAGTACTAGGTTAGTGGGATTTTGCCAGTCAACGGTATTA
ATCTTGACAGTAGATTGAGTCAAAAACCCATATAATTCTTGCCAATAAGGAAGATAAGTCTGGGG
CGCTCCAATTATTTTTAATGTCGGTAGTTGCTCTGGATTTTCTTTTAAATTGTGGTACACTTCAT
TAGAAACAAATACTCCATCTTCATCTAAATATCCGATAGTTTGAACTGTTAATTTATTGGTTTTT
TCCTCTATTTCAGGAGCAAGTGCGATCGCAACTGGTTTTTTCTCTTCTAAAGAAATTTTTAAATG
AGGGGGGAATAACTCTTTTTGAATAACAACATTGTTAACTGGTGCTAAAGTTTGTAAATTTTTGC
CTAACTCTTGGGTGGATAGCTTCAATAAAGATTGAGGATACTGTAAAGGAATTAAACTACGCACT
TCATCATCACTAAGTAAACTATTACCTTCAATATCTATCTGCTGACTATTCTTAATTACCCAATT
AGGTAAAGTAATAAACCAAAACACCCCCCCTGTTAAAGATAAAATCATCAAAAAACGTACTACTG
CAATAAAATCTCGCCATCTACGCTCTTTTTTTCTTTCTTCTCGGCGACTTCTAATGGTAGTAGTGG
AAACAAAAGTTTGATTAGCCATTGATTTACCAATGATGGTTTCAACCACGATAATAAATAATTGA
CCCTATTCTAGTTAACAATGGACAATTCTTCACTATTTACTATTTACTATTTAACCTCAGTTCGA
TTTAAATATCCTATAAGGTCAGGTTGCAAGTGATAGGGGGATGAGGGGAGAGGGAGAAGTAGGG
GCGAATGGCCATTCGCCCGTACCGGAGTTCAGGGTTTTTAATTCTTAATTTTTAATTTTTAATTT
TTAATTATCAACTATTCACTATTTACCTTTGTCCATTGCCTGTTGCCTATTTCATAACGATTTAG

ACGGGTTCTAGCTTATCTGTTTTTATCAGTCTTAATATTTCCTTTATAATTAAATCTAGGTGAAG
TTATATTGAGAAATTAACAATAATTCTTTTACCTCATTAATTCTATATATTTCTAATTATTTACA
ATGATTTCTCGCCAACATTCTTCAGGTAATGTCATGGAAAATCCCATTTCTGCTATTTCATCTTC
ATCGACACAAAAATTATCTGACTTAGTAGGAAAAAATAGGTCAGAAGAAGTGACATTGTTGGCG
GCGGCGGTTCATAGCGCTCATGATTCTATTGTAATTACAGATACACAACTAGATTACCCAGGACC
AAGAATTGTATTTGTAAATAAGGCTTTCACGAAAATGACTGGGTATCAGGAGAGTGAAATTATA
GGCAAAACTCCTCGTATTTTACAAGGACCAAATACAGATCGTACAATTTTTAGAGAATTAAAAAA
TAATTTAAGAAAGGGAAAAGTCTTTTTTGGAGAAGCAATCAACTATCGTAAGGATGGCACAGAA
TTTTATAATCAATGGCATATTGAGCCTATTTATAACTCGGAAGGGAAATTAACTCACTATTTAGC
TATTCAAAGAGATGTAACTGAAAAAAAAGAAGCGGAGAAAAAATTAATCTATGATGCTTTTCAT
GATTCTTTAACGGGTTTATATAATCGAGGTTGGTTTCTCAATGAATTACATAAAAGTTTGATCAA
GGCAAATCAATGTCAAGATTCTATGTTTGCCCTTTTGTTTTTAGATTTGGATGGTTTTAAGTTAA
TTAATGATACTTTAGGGCATTCAGTGGGGGATGTTTTTTTACAAGAAGTAGCCCAAAGAATCAAA
AAAAGTATTCGCCCTCAAGATAAATTAGCTCGTTTGGGGGGAGATGAGTTTACAATTATCATTGA
GAACGTGAATGATTTAAGTATTGTTTCTAAAATTGCCGATCGCATCCAGTTTAACCTACAGAAAC
CTCTAATGTTAGGCTCTCAAGAGATATTTACGTCTTGCAGTATCGGAATTGCTTTAAGTAATTTG
GGTTATGAAACTCAAGAGGAAATGTTAAGAGATGCAGATTTAGCTATGTATCGGGCAAAATCTT
TGGGGAAGTCTCGCTCGGCAATTTTTAATAAAACCATGCACCAAGTGGCAGTTAAACGCTTAAAT
TTAGAAAATGATTTGCGTAAAGCCCTTGAAAAAAAACAATTTGAGCTTTTTTATCAACCAATAGT
TTCGGTTAGTGAACAAACCATAGTCGGCTTTGAGTCTTTATTGCGTTGGCATCATCCTGAAAAAG
GTGCAATTTCCCCCGCCGAGTTTATTCCTTTAGCCGAAGAAACGGGTCTGATTATTGATATTGGT
AAATGGGTACTAACAAAGGCTTGTGAGCAATCAAGAAAGTTTAATCAGTTAAATTCAAGTAATT
CTTTATTTATGAATATTAACTTGTCTCCCCGTCAATTTAAACAGTCTGATTTAGTTGCGACAGTG
GATGATATTCTACAAAAAACAAAATGCGATCGCCACTTAATTAAATTAGAAATTACCGAAAGTGC
TATTTTAGGAAATAATAATACTGCAGATTTAATGTTAAAAGAATTAAAGGAATTAGGTGTAAAA
TTGTGTATTGACGACTTTGGTACAGGTTATTCATCTCTCAGTCGCTTATATCAGTTTCCCATCGA
TATTTTGAAAGTTGATTCCTGTTTTATTCGTGCCATTGGAAAACATGAAAAAAAAGAAAAAATCC
TTGCAAGTATAGTGAATTTAGCTCATAACTTAGATATGGAAGTAGTGGCGGAGGGAGTAGAAAC
AGAATTTCAACTAAACAAAGTTAAACAGTATCAATGTGAATATGTGCAAGGTTACTTATTTGGTC
ATCCCCAGTCAAGTCAAGACATTGAAAAACTCATTAGAAGTTAGTAACTCTTAGCTCAGATAACT
TAATTCAAATAACTTATACTTAAAGATGGAAAGATTTTAATTATAATGGCTTTCTTTATCAATGA
AATATTCAAGACTGTTATGGGGGTTTTTCATCTTTTTTTGTCCACTAAAAATATCGGAAGTTTTA
GCACAAAATCCCAGTGAAACAGTTACCGTGGGAATTTATGAAAATCAGCCAAAGGTTTTTTTTAA
TGAGGATAAGAAACCTTCTGGTTTTTGGGTGGATATTATTAATGAAATTGGTAAGCAAGAAAAT
TGGTCTATTATTTATATTCCCTGTGAATGGAGTCAGTGTTTAAGTTTAGTGGAGCAGGGTAAATT
GGATTTGATGTTAGATGTTGCCTATTCTCCAGAGCGCGATCGCATCTTTGATTTTAACCATGAAG
TAGTTGTAGCTAGTTGGTCTCAGGTTTATGCTCGTAGAGGTCTTAAGTTAGAGACAATATTAGAT
TTAGACGGCAAAAAGGTAGGGGTTTTAAAAGATAGCATTCAACAAGAAGACATTAATCAATACA
TGAAATCCTTCGGTATTTCTCCGGAGCTGGTGGAAATAGATAGTTTTAATGAGATATTCATACTT
TTGGAAAAAGGGGATATTGATGCGGGAATTATTAATCATTTTTTTGGGAAAATTGCTCAAGCCA
AGTTTAATGTTGAAAAAACAAATATTTTATTAAATCCAGCCCGTTTACATTTTATCGTTGGAGAT
AAAGATCCGAAATCACTACTACCAAAATTGGATCTCCAATTACAAAAATTGATAAATAATGCCGA
TTCTGTTTATTATCAAGCGAAACAAAAATGGTTAGAACCAGAGTCTAAATTGAGTTGGTTAACCG

TTAAAAATGAAATAATAGATTCGGTTTTTATTTACCTTTTATTGGTTTATTTTTTCTTTTACTT
TGGAATTTTTCTCTCAAAAAAGAAGTTAATCACCGCATTGCTTTAGAAAAAAAGTTAAAGGATA
GTCAACAAAGTTATGCCAGTCTGGCTTCTTCTTTACCCGTGGGAATTTTTCGGACTAATAATCAA
TTTCAATGTATTTATGTTAATCATCATTATACAACCCTAACGGGTATTTCCTTTGAGGATGCGAT
CGCAGATAAATGGAAAAATGCCTTTCACCCCGATGATAAAGAATCCATTTTAAGATTATGGGAAA
AGTCCATCAAAGAGAAAACTATATTTGAGATGGAATGCCGTTTTCAGCATCCTGATGGCAAAGTT
GTTTGGGTTTATACTCAATCTTTACCAGAATATGACAGCGAAGGAAATTTAAAAGGTTATGTGGG
AACTTTAACGGATATTAGCGATCGCAAATTAGCAGAGTTGGCACTCAAAGAAAGTGAACAAAGA
TTCCGCAACATGGCGAAAAATGTACCCGGGGCGATTTTCCGTTATATTTTATATCCCGACTATAC
TGATAAAATTATTTATATGAGTGAAGGATGTTACGAACTTTGGGAAATTAAAGCCGAAGAAATT
ACCGATAGTATTAAAATTCTTTGGGATTTGGTTTACCCAGAAGACATACCAGCCATGCAGAAATC
TATACTCAAATCAGCACAAACATTACAACCTTGGTTTTGGCAATGGCGAATTACAACCCCTTCAG
GTAAGAAAAAAATGGTTAGAAGGCTTTGGCAGACCGACAAAACTAGATGATGATACGGTACTTTG
GGATAGTTTGATTATGGATGTTAGTAATCGTAAACAAGCAGAAATAAATTTAGCTAAAAGTGAA
GAACGTTTGCGCTTAGTCACAGAAAATATGAGTGACTTAATTTGTTTATTTAATGCCGAAAAAAA
ACTGATTTATGCAACTCCTTCTTGTGAGTCTTTATTGGGTTATACCGCAACGGAGTTAAAAGAAA
TAGATTTAGAAGCACTTTTTCACCCTGATGATCATAGCTATATTTATGATAATTGTTCTCTTTAT
TCCACCCATAGTGATGAAGATAGTCGAACTATTACCTATCGCATTCTTTCTAAATCAGGGGATTA
TATCTGGTTAGAAACCCTTAGCAAAAAAATCTATGATGACCAAGGAAATTTACTTTATATACAAA
CGACATCAAGGGATGTGAGCGATCGCATCTCTATGGAAGTACAATTAAAACATGATGCTCTCCAT
GATAAATTAACGGGATTACCTAATCGTAATTTATTAATCAAAAGACTTGATTTAGCCTTAAAAAG
AAATCGTCGTTACGCTCAATCTAACTTTGCCCTTTTATTTTTTGATCTCGACAACTTTAAATTAG
TGAATGATAGTTTAGGTCATTTAATTGGCGATGAATTATTATTAAAAATTGGGGCATTATTACA
AACTTTTATTCGAGATACAGATATAGCGGCCCGTTTAGGAGGAGATGAATTTGTCATTCTCCTTG
AAGATATAAAAGAAGTAGAACAAGCCGTAGTAGTTGCTAGAAGAATTTTAGACTCGATGCGATC
GCCGTTTATGGTTTCTAACCGACAGGTATTTACGAACAGTAGCATTGGAATTGTCATTGGTAATC
ATAAACATAAAACACCTCAAGATGTTCTCAGAGACGCAGATATAGCGATGTATAGGGCAAAACAC
CAAGGAAAAGGGAAATATGCCATTTTTGACCCCCATATGCACCAACAAACTGTCCTCAGACTAAA
TTTGGAAAATAATTTAAGAAAGGCGTTAGAGGAAAATCAATTTGTACTTTATTATCAACCAATT
ATTAATTTAGATAAACTAACTACCGAAGGATTTGAAGTTTTAATTAGATGGCAACATCCCCAAGA
AGGGATTGTATTTCCTTGCGATTTTATCAGTGTAATGGAGGAGATTGGTTTAATTAATGATTTGG
GAGAGTGGATTTTTAAAACTGCCTGTCAACAATTATCTCAATGGCAAAATCAGTTTGATTTACCC
TTAAAAATTAATATTAATCTTTCGGTGCAACAACTCACAGAATCTATTATTCCCTTACTCGACAA
AATTCTCGATATTTACCCCATTCAACAAAATACATTAGTGTTGGAAATTACCGAAAGTATGTTGA
TTAAAGATTTTGAAGACACGAACAATTTATTAAGCCAAATAAAACAAAGAAGCATTCAAATCAG
TATTGATGATTTTGGTACAGGATACTCTTGTTTAGGATATTTACATCAATTATCCGTGGACGCTT
TAAAAATAGATCGCAGTTTTATGAATTTTTCTTCTTCTAAAAACCACAATCAAGTAATTGTTAGC
TCTATTTTAGCTTTAGCTCAATCTTTAGGAATACGCGCGATCGCAGAAGGTATTGAAACGGAAGA
ACAAAGACAATGGTTAATTAGCCAAAAGTGTAAACTAGGACAAGGATATTTGTTTTCACCCCCCA
TAGCAAAAGACAAAGCGACAGACTGGCTAAATTTTCAAAGACTTAATTAACCTCAGTTCGGTTTA
AGAATATCGGATAAGGGTAGGTGTCAGGTTTCAGGTTGCAGGTGTTAGGGTGATGAGGGGATGA
GGAGAGAGGGAGAAACAAGTAATGAGTTAGTTAGGGGCGAATGGCCGTTCGCCCGTACAGGAGT
TAGGGGTTTTTAATTCTTAATTCTTAATTCTCAACTATTCACTATTCATCTTTGCCCCTTGCCCTT

TGACCTTTAATATCTTTCATCAATAATAATCCATTCATAACCATTGATAATTCCCTGAAAATAAT
CCGCCATTGTCTCAATAGGATAAGGTGTATTGCCATTTTGTCCAAACCATCGCTCATAAATATCG
ATCGCACTTGGAGTATCAGTAGTAACACCTTGCATAAATTTAACGATAGCCTTATTAACTAAACC
ACGCCATTGAGACTCATCTTCAGGAACAGTACAAGCATAAGACTCAATCATGTAGGGAAATTCAG
GGACAATTTCATAATCTTGACCGTTATCACTTTGTTTTTTCAAAGCCTGTAGTAAAATTCCATCT
CCTGCAAATCCGTCTAAATCGCCCTTCTGCAACATTGCCCATCCTTCCTCCTCACTTTTAACAGGA
ACAAGAGTTGCTGATGAAGCATATATTTTCATCGCTTTTTCATTAGTGGTATTAGGAATAACCCC
AACTTTTGCACCGGTTAGATTATCACTGTTAGCAAAACCACTGCCTCGGTTAACAATCATCTGAG
TGCCACTCGCAAAATAACTAACCGTAAAATCAACAAATTTTTCCCTTTCCCATGTTACGGTAGTC
GAACCACATTCAATATCAATAGAACCATCTTGGATTTGCTCAAAACGATTACTGGGATTGATTCT
CACAAATTCTAAGGTAACTGGTGTATTTAATTGCTTTTGAGCCTCTGCCTGTATTAACTCAAGAA
TATCAACAGAATACCCTACGGGTTTTCCCTGCTCATTACGAAAAGCAAAAGGGGGAGTATCATCT
CGATAACCAGCACGAATCACCCCTGTTTCCTTTATTCTTTCCAAGACAGTACCAGCCAAGACAGGG
CTTGAGCATAAGCCTAGACAAGTAAAAGCGACACCAATATTTAGTAATTTTTTCCACATGGACAG
GTTTTAAATAATTAAAGAATGAATAATTAATGATTAAAAGGCTATAAGCAACAGTTTTATTTAT
GAGACTTCTGGTAAAAATTACGAATTTACCACCGTAAAACAGGCATCTTGCCTATATTTCTGGAG
ATCTCTATTCTGCTGACATTTCGCCTCTTAATCTTATACCTAATTCCTAATTCCTCATTGTTGATA
ATTTCTAGTAGTTTCTCATTCTTTGAAAACGAGTTTGACCTAAATTATATCGGTTAGAAGAAGAT
TCTTCTCCGTCAATTCGGTCAATTTTTGTGGAATTGTTACTATTATCTTCAATTCTGTGATATTT
TCCTGATTTATCAATTTCTTTACGAGGCTCTGCTTCAGGGGCAACAATTTCTTGTATTGTGCCTT
GTTGTAATATTTCTTCCTCAGAAATCGGCTCTGTAGTGGATTCGATAGTATTTGGTTCAAGAATA
GACTCAATATTATTTTGATCACTATTTTGATTTTGTTCCTCTAACATATTGGGAACTACATCTTG
GGGAGCAGACGCAGAATTATAGCTAAAATAAACGTGACCTAATATTTTCCCCTCATAGGTTCTTT
TGGTAAAACGCCATCCGGGATTTAAAATAATTTTCATAGGAGATTCGCTGATTCCCTTTGCAGAG
CCAATTACCACGGGAGGTTGACCCGGACTACGACGATTTAAGCCCATTAACAATAAATTTCCGTC
TTTTTCCACTAGACTGAGAATATACTCTAAGCCATAATCTTGACTGTCATAACGAATAGAATAAC
CGTTAGCGTCCGTGCTACGGCGACAATGTCCACTAAAATCAAAGTTTAATAGTAATAGATCTACA
TTGACGGGATTTGCTCCTGTTTCATTCCAACATTGATTTTTACCCGGAATTTGTTCGATGACGAT
TAAATTATATTTATCTTCTCCGTAGGGTTGAGCTACCGCAATAAATTCTTCTTGAGGGACTTCTG
TTTCCTGAAAAGTAACTGATTTTGCGGAGTTGATAGGTAGAAAACTAGCTAAGGCTGTTAAAGCG
ATAATTGAAAGTTTCCGAGAGACTAGGGATTTAAACATATTTATTACCAATAGATAGATATTTT
GCTCACACTGAAAATATTGCCACAAGAAATAAAGATCAAGCAATAATCCTGACTAAAAAGGAAT
AAAGTAATTATCCTTTTCCTGATATGTTATCTGACTTGTTGTTTCTTAGTCATGTTCCTTCCATT
TTTATTTTTGTTTTTATCATTTTTATTACAAAAATTTCTTAATAGGGCTAAAGCATTTAGTTAGT
TTTTTAGCTCTCAACAAGTTGACTAATCAATATAATGCCCTAAGTTAATTTGCCCTTGGTTTGAC
GGAGGATATTGGAAAAAAGAAACTTCTCGTTGTATTTCACAGGGAAAAGGGGGAAATTTTATTA
ATAACTAAACAATAGAAAATAATTATTTATTTATATTATTTTGTGAACAAATGTTCAAGAATTA
AAATGTAATAAGAAAATTTATTTTTTTATATTTATTTAAAACTTAGATATAAGCCTAAAGGTCT
GAAATTATTATTAGACAATCAATTGATTCAGAGGTAATAGTTTTTTACTTAAAAATATTTTTTCA
AAATTATCCCCTATTTGGGTATTGAAAAATAAATAAATTCAAGTAATAATATACAAAATAAAGG
AAAATCTAATCTTAAAAATTTTGTGTGTGAGGAATTGAAAATGAATAATCGTTTTTTTTCTACTC
TCGTTTTAATGGGAGTAAGTGCGATCGCATTTTTTCCATCTCAAGTGAGAAGTAATCCTGTTAAT
AATGCAGATATTTTGCAACAGTTGGAAAACTATAATCAAGAAAACAGCAAAAAAAGCCAAAATC

AAGTCACTTCGGTTTCACAATTAAGAGATGTATCACCCACAGACTGGGCATTTGAAGCCTTAAGA
AGTTTGGTGGAGCGTTACGGTTGTATTGTCGGTTATCCCGATCGCACCTACCGAGGAAATAGAGC
TTTAAGTCGCTACGAATTTGCCGCCGGTTTAAATGCTTGTATGCAACAAATGGAAAGGTTAATTG
CTTCTAGTGAGTCAGTGTTAAGGGAAGATATAGAGAAATTAAAGCGTTTAATGAGCGAATTTGA
AGCAGAATTAGCGGCTTTAGGGGCAAAAGTTGACAGTTTAGAGGGTAGAGTAGCTTTCTTAGAG
GATCATCAATTTTCTACTACCACTAAACTAACAGGAGAAGTCGTAATTGGATTAGCTAGTATTTT
TAATGGGCAGAAAAATAACGGTAGCGAAAATATTGAACAAGTACCAGTTTTAGGGAATCGTACC
CGTTTAGAATTCAACACCAGCTTTACAGGAAGAGATTTACTTTATACTCGTTTAGCCACTGGTAA
CTTCCCGGATTTTGCTGATACGGCAAACACTCCTCAAGCAACCCTTGCCTTTGCTCAACCCGATGA
TAATGATGTAGCGGTGGAAGTTTTAAACTACAATTTCCCGATCAGTGACAATGTTACTTTATGGT
TAGAAGCCACTGGAGGTGCATTTGACGACTTTACCAATACCTTAAGTATTTTAGATGGAGATGGT
GGCAGTGGTGCGTTATCTGTCTTTGGTACTCGTAACTTTGCTTACTACGAAGGAGAAGGGTCTGG
TTTAGCCTTGGAGGGCAATTTTGGACAATTTGGTTGGAGTGTTGGATATTTAGCCTCTGACGCTT
CTTCTCCTCAAAATGGAGAGGGATTGTTCAACGGTGCTTATGGGCTTTTAGGACAAGTGGGCTAC
TATCCAAATGATAATTTCGGTATTGCTTTTGCTTATGGACATGGATATAACACAAGTGCGATCGC
AGCTAATAGCCGTAAATTTAGTGAAACCATTGCCCTTGCTAACGATGACATCAATACATCTCACA
ACACCTACTCTTTAACCATGTCATGGCAATTAGCCGAAAGTTTTGTTTTGGGTGCATGGGGTGGC
TACTCAAAAGTTTCAAACCTAAACTCTTTTGATAACGGTTTATACACTGTAGGGCGAGGCACATC
CGACTACTGGTATTGGGCGGCCACTCTTGGTTTTCCCGACTTATTTAAAGAGGGTAACTTAGGAG
GTATTATCGTTGGTATGCAACCTTGGCAGTCAGAGAGTAATATCAGAGTTAATGGAGAAAGACTG
AGAAATGATGATAATTCTTTCCATGTAGAGGCATTTTATCAATATGCTCTCAACGATAATATTAG
TATCACCCCCGGGGTTGTTGTTGTGACCAATCCTGCTAATAATACTAATAATGATCCTTTAGTTA
TTGGTACGATTAGAACTACATTCACTTTCTAAAAGGTTTTAGGGTGTTTAGGGGATGAGGAGATA
AGGGGATGAGGGGAGAGGGAGAAACAAGTAATGAGTTAGTTAGGGGCGAATGGCCATTCGCCCG
TACAGGAGTTAAGGGTTTTTAATTCTTAATTCTTAATTCTTAATTCTTAATTCTCAACTATTCAC
TATTCACTATTCATCTTTGCCCTTTACCCTTTTTTGATCTTTTATTGCTATAATTGTTTTTTTCGT
TGAGTGTGGCAATGGATAAGATCACCATCGTGTTAGTTGAAAGTCATGATCTAACGAGGATTGGA
TTAATTAACGCCCTTAAGGATTTTGCACATATAAAAGTTATTGGGGAAGCATCAAAAATTCAAGA
AGGGTTTAATCTTATTCAAGAAAAAAATCCCGATATTGTTATTATTGATTTAGCTTTATTCAGTA
CTGAAGAAATTGAATTTATCCGTCAGTTGAAAAAATTTCATTCCTCAGAAGAGAAGCCACTCAAA
CTTATAGTTTTAATGATAGAGAGTGATGAAGATTCTGTTTTACAAGCCTTTGCCCTCGGTGCAGA
CTCTTACTGTTTAAAAAACGTTTCCATCGATAATTTACAACAGGCGATACAAGTTACCCACGAAG
GAGATAGTTGGATTGAACCGGCGATCGCATCTATTATTTTAGAACAAGCAAAAAAAAGTTATCAG
AAAAAAACAGAAGATTTAAACACAAAAGATATAGAAAAAATAGAAATAGAACCATTATCTCCAG
AATCTCAAGAAATTATCGACAATTCATCCCTAACAGACCGAGAAATGGAAGTATTAGAGTTAATT
GTTGCAGGAAAAAACAATGCAGAAATAGCAGAAGACTTATATATTTCCATCGGCACTGTGAAAAC
CCATGTTCGTAGTATTCTTGCTAAACTTTGTGTGTGCGATCGTACTCAAGCCGCCGTCCATGCCTT
ACGCTCTGGTTTAGTAAATTAACCTGAGTTCGGTTTAAGAATATTGGATAAGGGCAGGTTTCAGG
TTGCAGGTGGCAGGTTTCAGATGAAATTAGTTCAGAGTTCAGGGTTACTTACTTTACTATTCACT
ATTGCCCTTCTCCCCTTAAAAGGGGAGAAACAGAGGGGTTTCCCTAATGCCCTTTGCCCTCTAAA
AAAACTATCTACCACCAACAGTGATAGAATCTACCTTAATATGAGGTTGCCCTACAGTGACATAA
ATACTCCCACTCACAGAACCGCAGAAACCTGCCGCTAAACCTAAATCTTGAGATGACATAGAGAT
TTTAGTCATAATTTCTTTCGCTTCACCAATCAGAGTAGCACCCTTTAATGGTTTGGTTATTTTGC

CATTTTCGATTAGATAGGCTTCATCTACACCGAAATTAAACTCTCCTGTGGGGCCAACACTGCCA
CCACCCATTTTTTTGCAGTAAATACCCTTATCCACCGAAGCAAAAACTTCATCAAGGGTATATTC
ACCGGGAGCAATATAAGTATTGCGCATACGAGAAGCGGCGGGAAGCTATAACTTTGTCTTCTAC
CACTCCCTGTACGAGGATGATTAGTACGAATTGAACCAGCACGATCTGCCAAAAAGTTCTTTAAA
ATGCCATTTTCAATTAAAAGAGTACGTTGAGGAGGCATTCCTTCATCATCCATATCAATACTACC
AAATGCTTGTTCTGTTAAGCCTTCATCCCATGCGGTTAAATTTTCATGGGCTATTTTCTCTCCTTT
TTTGTCCATGAAAGGAGTGCTTTTTCTTTCGATTTGAGTTGTTTCGAGAAGATGGCCACAGGCTT
CATGGAAAATTACTCCACCAAATTGATTTGCCATGATTACAGGATATTGACCAGATTCCACATAA
TCGGCATAAAGCATTTTTCCAGCGGATTCTGCAACTCCTTCCGCTACTGTGTCATAATTCCAGTTT
CTCAGAAAATCGGGGTTGCTGGTGTCTCCATCTCTTTTACCAATGGATGAGCGATGTTCTCCATC
TGCACACAATAAATTATAGCCCACGGATTGGGTTAAACGAATATCACGGGCAAAAGTGCCATCAC
TAGAAGCAACCAAAACTTCTTGCCAGTCACGAAAATAAGCGGCACGGCGGGATTGTACATGATTT
GCAATTTTGTTTAATTGATGATTGGCACTGAGGAGAATATCTCCCATCTCTTGCATATTGCTACA
GTTACCTAACCATATATCTTTATTTTTGGCGGTAGCGTAATCTCTAAATATTTCTAAGTTAATTT
CAGGAATAAAAGCTGTACCTTGGGGGATAGTTAAACCTAAAATGGAGAGGGCTTTTTCTAAGGCT
TGTTTTAAGCCATTAAAGGTTAAATCATTGGTACTTACATAACAGTCTTGTTTGCCCCGAAATAC
CCGAACCCCTGCCCCTGTCGAAAGTCTAGGGGTAATACTGGTAATAATATCATCTTCTGCCAAGC
AACTGATATAGTTAGCTTTTTCTAAGAAAAATTCAATAAAATCTGCACCAGATGCCCTGCCTATA
CCAAGAAGGGTTGAAAGGGGTTTTTCCCATGTTAAATCGAAGCGATCGCCTGTGGGATGATAAGA
TAAATTCGGGATTTCACGAGATATTAGTAAAGTTGGGGACATATTTTATAACTATTACTATAATT
ACTGTTGATTTTTTGCTTTATATCCTGTCTATTATAGTTACTAATTTTATGGACAAATATCCACT
CAATTAGCTTTATAGACTTTAACGTCTAATGGCTTAACTACAAAGCTCACTATCGAGTCCTTAAT
CCTAAAAGATTAAACTTCTCGACTACTTTTGGTGGTTTATTTCTAGCTAAAACTTTTACGTTGG
TAGCTTAATAGTTAAGGAAAAATTAACTGAATCCTTACCAACAACCCCATAGTTTTAAGGTGAAA
TATTTAATTTTTAAGGTTCATTTAATCTTATTTATAACATATTTGACCATAAATTCGGTAAAATA
ATTTAATACGTCAAGTTTTTGTTTCTTCCGAATGCCTTAGTTGAAAGAATTTAGGGTTGCTAATT
TTTAGATAACCTCAGTTCTGTTTCAGAATATCGGATAAGATTAGGTGTTAGGTTGCAGGTTTTAG
GGGAATGAGGGGATGAGGGGAAGTTAAGTAAACACTTTTGCCTCTTTCCTACCAAACATCAACAA
TTTACATACTCAAAGTAATAGAGCTATTGATAATAACGAAAAATATTTGGTTCTTCTACAGTGGT
TATAATAAATTAATAGAAAATAAATTTCATAACCTTTATTGAATAGTGTTTATAGTAAAACAAA
AACTAAAAGTTTATAAATTATTCTTTAATAATCCCCTATTGCCTATTGCCTCTTGCCTACCCTAA
CTAATAATTTACATACTCAAAGTGATAGAGCCAATATTTTACTTTTTTTTGCGATCTTCCCAACG
ATAAAGAAAAACCATAATAGCAATTACTGTTATTTGTACAAATAAATTATAAATGTTATCACTA
ACGTTTTTTCCTGCTAATAATTGAGCGAAAAAGATAAAAGCTCCGATCGCACCTGATGCTCCTAA
ACCTAAATAAATAAACTTTCTTAAACCTTTATAAGGGGCTTGGGCTTCTGCTTTTAAATAGGCGT
ATTTTTCAGAGCTTAATTTATTATTTTTCTGATTATCAGAATTATTATTCATAAAAATTATTTAA
AAATAAAATGTTGTTTAATCATATCAAAGAAAGAGCAATCCCAATAGTCATAATGACCAACAAT
GAGATCATCTTTCAATTTTAATTCACTTCTTCCTGACACAGAAATAAAAGGTTTCCACGGTAATG
GACTATTCCAAGACATTGTCCAACGGGTATTAATTTGATTTTCTATTTGATTTATTTCATGTAAT
TCTAAGTTTAAATTACTAAACCATTTTCTTAAAAAAGCAATCATTTCCTGATATTTTTTTAGTCC
ATAAAAATCATAAACAGGATCTTTAAAATGGACATTTTCGGCATAAATTGCATAGGTTTGATCTT
CAGGAAATTTTTGATAATCTTCTTTGATAATATCGATTAAATTCATTTTTTTATATTAATTTATA
CTAAATTAGAAAACTGTATTTGATAAATAGTCCAAATATCCACTAACCAGAGAAAAAAGGTAAA

AAAGAACAAAAAAATATACATCCAAGGTTGTGCTAAAGGACGAATATCTGTCGTATCAATGTTG
GTTTTATTCTGCACCTGTTGAACCATTTTGGCAAAACCGGCAATTCTCATGGGTAGTAAATAGGC
TTTATCTCTATTTTTTGAGACAAAATAATAGACTAATCCTCCTTGCCCTGTAGTACGCATTTTCA
GACTATCAATTTCTCTCCAAGATAAATGCCATCCCTTACGCCATAACCATTTTACCCATTGAGGA
TAAGTGACGGCTATACCCTCATTATCAACGATGACTCTTTCACTCAAAATAGCAAAAATTGCGAT
CGCACCTAAGCCAATGAGTAAGGAAAGTAACCAAATAGGGAAATTGAGAGAAGTTGTTTTGAGC
AGAAAAGGCAAAGGAATTGTTAAGGCGAAATAAAGATTAAGAAGGGTTATTTTGATTAAAGGAG
AAATAGGATAAATTGATGATTCCATAATAATTTTTTTATCGCATTTATTAAAATTATGAGGCTT
AAGCCCCTTTTTGCATCTTGCTTACCCTCACCTAGATTAAGTTATGCGGGGCCGTGAGTCTAATTT
ATCGACGGGAAATATCCATTAAAAACTGCTTACTATTTTTCACAAACTTTGATTTGGGCATATTA
CTATACCAACGAAATAAAACTTTTCCTAATAATTGAGGATCATGGCGAACATAAGCACTATCTCG
ATTCTCACTCATGACATTTGCTTTAACAATTCTTCGACCTAATTTTTCCACCTCTTCCCGATCCAA
ATATACAGGATGACAATGTTTTTCTGCATACTTTCTCAAAGCCTCTGGAGAAGGTTGTCTTCCCT
GCACTAAAACCGCATCAAATATTTTTTTCCCACACACTTTATCTATAGCTTTAATATGATCACTA
ACTGTATAACCATCGGTTTCCCCTTTTTGAGTCATTATATTACATACATAAATTTTAGGAGCAGA
GCTATTCAAAATAGCTTCTCTAATTTCAGGTACTAATAAATTAGGAATAATACTTGTGTATAAAC
TACCTGGCCCTAAAATGATATATTCTGCCTGTTGAATTGCTTTAACTACCGCAGGGGTAGCAGGT
GGATTAGTGGGATTACATCCTAGTTCAACTATTTTCCCTCCAGCATCAGGAATATTAGACTCTCC
ATAAATAACTCTGCCATCCTCTAATCTTGCCCACAAAGTGACATCTTCTAGGGTTGCCGGTAAAA
CTTTCCCCTGAATCGCTAACACCTTAGAACTTTTTGCGATCGCACCTTCTAAATCACCAGTAATAT
TAGTCATAGCGGTTAAAAATAAATTACCAAAACTATGACCATTTAATCCTTCCCCTGCGGTAAAA
CGATACTGGAATAACTCTGTCAATAATTTTTCTTCATCAGCCAATGCCGCCATACAATTGCGAAT
ATCCCCGGGAGGTAAAATACCCATTTCTCGTCTAAGCCTTCCCGATGAACCTCCATCATCAGCAAC
CGTTACAATAGCGGTAATATTAGCACTATATTCTTTTAAACCCCGTAATAAAGTTGATAAACCAG
TACCACCTCCAATAGCTACTATTTTTGTCCCCTTATTTAAACGACGATAATGCCAAAGCACATCG
ATTAATTCTTCATCTTTATCTGGTTTTAAAACCTCTGTAATTGAACCTAAAGTGCGAGTTTGCCC
CCACAATAATAAAAATAAACCCAGAAACAAAACTAAGGGGCCAGAAATATGGCTAGGAATATAA
TTAGTAATTTGATCTAAAGTATTAAAAATAAACTCAAAAATGCGAAAAACAGGAGTTAAGTTAC
TCCAAATAGCTAATCCTAGGGCAATCATAAAAGCCCCAATAATGCTCGTAAGTAGCCAACGCTTC
ACAAAAATTCCGGGGGAAAGCCATTTAAAAAGATGATTGACTTTTTTTCGGTGTCGGGGTTTTTT
TTCCATAATCTTTGAGGGATTTTCTGCAAAACAGGGATAGTAGTTTAATTAATCAAGATTATATC
TGAATTCTCAAAAACAAATCGGGGTAAATATTGAAGGTTTCAGGTGTTACGGTGTTTGGGGGAT
GAGGAGATAAAGGGCAGGGCTGTTTCAAAATAAAAAATTAGGTTAACTGAGATTAAGTAAAAGT
AAGATATACAAGGATTTTTTTCTATTAGTTAAAACTTTATGAACAAAAATCCCCTCATCTCCCTC
TCCCCCTATCTCCCGCTCTTTTTTCTTAATAATTGATTAATTAATACTTAAAAACTCCTGTATCTG
TTACTATTTGTTAGTTTCAAAAATTGACATTTTTGAGAATGAAAAAACCCTGCTTTTAAAGGGAG
ACACAGAGGGGTTTCTCTGTTCCCAATCTTCACCAGACAATTTTATATCGAACTCAGGTGATTTT
AGGCATTAAAACCACTATAATTGAAGGTTTAACTTGTTATTGACATCTAAAAACACTCGAGAAGT
TATTTATACTCATTGTTAAGCATAACTTGATCTATGTAGGTGGGCGACAAGCAATAGTTTTCTTG
TAGCAGAGCAATTATGACCTTAGATAGTACATAACTAATTGAGTGACTTTGATATAACAACCAAT
AAGGAGTATTGCGATTAGGAGAAGCCCACCACTTCATTAATCGCCTCCTTGAAACAAAATACATA
TTTTTTGATTAATACAACAAAATCGATACAGATACTAAACTTAATTAACATTTTTACGTGTTATT
TTTTTAAGGTCAAGATAAAATATAAATATGATAAAAAAGTATATTATAAAGCCTAAAAAAGTTC

TCAAAAATATTAGTAAAAATCAAATTATTATTTGTTCAATAACTATTCTGTATTTTGTTATATTG
ACAACCTTTGTCGTGACAGGAAAGCTAAAAGAATTAGATGAATATTTACTTCAAACTATTCATGA
AAACTTACCCGGTTGGTTTATTTATATTGCCAGAGCATCTTATTTTTTAGGAGAAGCTGAAGTAG
CTATATTTTTTGTTTTATTTACTTTAGGTGTTTTAGTTTGGAAAAAATATTGGGAAGAAGCTCAA
GTAATGGCTTTTTCTTGTCTCTTTATTTTAATTTTAATTGACAAAATTTTTAAACCTTTATTTGC
AATTTCTCGCCCTTTAGATCGTTTAGTGGAAAATGCTTTTGGCAACAGCTATCCTAGCGGTCATG
CGGCAGGAAATTTACTATTATATTTTCTTATTTGTTATTTTATTTCTCAGTCCTACCCTAAATTA
AAAGTATTTTTATACAGTTTAGCAGTCTTTTCACTAATTTTAATGGGGATAAGCAGTGCTTATTT
AAGAGTTCATTGGGTGAGTGATATTATTGCTTCTTATGCAGTTGGTTACATTATGTTTGTTTTTT
CAATTACTATCTTAAAAAGTTCTAAATAATCTAAGACAATATTGTTGGCTCTATTACTTCTCGTC
GTGTAAATTATTGGTTAGGATAAGCAATAGGCAATGGGCAATTTTTCTCCCTTAAAAGGGCAGAT
ACAGGGACAGGGCTGTTTCATTTTCGAGAAAGAAAAAAGAGCGGGAGATAGGGGGAGAGGGAGA
TGAGGAGATTTTTGTTCATAAAGTTTTAACTAATAGAAAAAAATCCTTGTATATCTTACTTTTAC
TTAATCTCAGTTAACCTAATTTTTTATTTTGAAACAGCCTTGCAAATACAGCAGGGTTTTTCAT
TCTCAAAAATGTTAATTTTTGAAACTAACAAATAGTAACAGATACAGGAGTTTTTAAGTATTAA
TTAATCAATTATTAAAAAAAACGCTCCCCTCTACTGTGGGAGAGGGGTTGGGGGTGAGGGCAAGA
TAATTTTGCCCTGACTTTGAAAAAACCCTGAGATACAGGGGGGGTTGCCTCTTTCTAAATTTGCT
ACCTAAATCAACAATTTACATAATCACATTAAACTTATCTATTTTTGAATTTTTGACTCTTATTA
CATTACATTCTTTCTAAAACAGAAATACCCAATAGTGATAAACCTAATTTAATAGTTTTTGCCGT
TAAATCTGCCAAAATTAAACGAGACAATTTAACTTCTTCTTCTGCCTGTAAAATAGGACACTGAT
CATAAAATTGATTGAATTTTTGACTTAATTCAAATAGATATAAACAAAGACGATTAGGTAATAA
ATCTTTCTCTACTTCCTTGATGATATAGTCTAACTGTAAAAGATGTTTTGCTAAGACTAATTCTG
TTTCTTCCGTAAGAAGAATATCCTGATTTGATTCTAATTTATCTAAGTCAATATTACCTTTTCTG
CTGATGCCTTGCACTCTTACATAAGCATATAAAAGGTAAGGTGCAGTATTTCCTTGCAGTGCTAA
CATTTTATCATAACTAAAACGATAATCAGTGGTGCGATTTTGACTTAAATCTGCATATTTTACAG
CACTTAATCCTACTATTTGACTAACCTCATTAATAAAATCTTCTTCTTCATTTCTTCCCTCGGTTG
TTAATCTATTTTCTAAGTCTTGTTTCGCTCTATTTACTGCTTCTTTTAATAAGTCTTTTAATTTA
ATAGTTTCACCTGAGCGAGTCTTGATTTTTTTGCCATCTTCTGCTAAAACTAAGCCAAAAGGTAC
ATGGACTAATTCCACATTGTCAGGCACAAAATTGGCTTTTTTTGCTACTTGGAAAACTTGAGCAA
AATGATTAGCTTGTCCAGCATCTGTTACATAAATTATTCTTTCTGCTTCATCTTCTTTAATACGA
TACTTTATTGCGGCTAAATCAGTAGTTGCATAGTTATAACCACCGTCGGATTTTTGTACAATTAG
GGGTAAAGGATTTCCTTCTTTATTCACAAAACCATCAAGAAAAACACATTTTGCTCCTTGATCTT
CTGTTAAGATATTCGCAGTCTCTAAATCTTTAATAACATCGGGTAAAAAAGGATTATAAAAAGA
TTCCCCTCTCTCTATTAGTTTAATATCTAATAGCCGATAAATAATTTCAAATTCTTTACGGGATT
GTTCACATAATAATTGCCATGCTTTGATACTTTCTGAATTTTTACTTTGCAGTTTTACTACTTCT
TGACGAGCAGTTTCTTGAAATTCTGTATCGTTATCAAATTTGATTTTTGCTTGTTTATAAAGAGT
AACTAAATCACCTATATCCACTGCATTGGCTGTAGTTAAAACTTCGGGTTTTTCTAGGCGTAAAT
AGGCAATTAACATTCCAAATTGTGTACCCCAATCACCCACATGATTAAGTCTTAAAACATCATGC
CCTCTGAATTCTAATACACGAGCTATACTATCTCCAATAATAGTAGATCTTAAATGCCCCACGTG
CATTTCTTTAGCAATATTTGGACTTGAAAAATCAACAATTACTTTTTGTTTTTCACTTACTTTTT
CAACCCCTAATCTCTCATCTTGTTGGTTTAATTTTATTTTTTCTGCAATATATTCTGGCTTAATA
GTAATATTAATAAAACCCGGTCCTGCAATTTCTAAGGGTAAGCAAAAATCTTGAGTTTGGGCATG
ATTAACAATATTTTCTGCGATCGCCCTTGGTTTTTGTTGTAAACTTTTAGCTAAAGGTAGGGCAA

TATTGCATTGATAATCACCGAATTTAGGATTACTAGCTGGAACAACTAATGGGTCAATATTAGCA
AATTCTTCTCCAAACGCATTCACTAAAGCGGTAGTAAAAATATTTCTTAATTTCTCTATCATGGA
ACAATCAATATAGCCTTAAACTAGGCAATTATAGCTTAAATAGACTTCAGTTCAATGCGAAAATA
TCAGTTAAACTAGAGTTCGATATAAATTTGTCGGTGAAGGTAGGCAACAGCGCCTCGGTAATAGG
CAATAAATAACCTCAGTTTAGTTTAAGAATATCGAGTCAGGTTTCAGGTTACAGGTGTTGGGGAG
ATGAGGGGATGAGGGGAGAGGTGGCAGGTATCAGGTATCAGGTATCAGGTGTCAGGTTTAAGGG
AGTAACGAGTAATTATCAACTATTCACTATTCACTATTCATTAAATTACCCTTTACCCTTTTACT
TTGCCCTTTGCCTCTTGCCTACCCTAACCAAAATTCCCATACTCAAAGTAATAGAGCCAAATTAGT
TTAGTCAATTCCCCACCATTTAATTGTTAATTCTATTTTTTCATCATTGGTTAATTTGATTAAAG
ATTTCTCCGTTTGATTATTAATAGAAAGTTTACTTATATCTAATAAATAACGGTTGTCAGTTTCA
GTTTTTGCCACAAGAGTATCAGTCTCATCTGCTAAGAGAATATTAGTTAAATAGTCTTCTTTACT
CTTAAACTTAAATGCCTGATTTCCCAAATCTCCTAAACGAATTTTACGCAAACTTTTAATGGGTA
ATAATTTACCGTATCCTAACGCAGAAATTAATAATAAATTGCTTTTACTATCAACTTTTTCACAG
CCAATGATTTTTTCCCCATATCTTAATTTAATACCGACATTTCCTTGCGCACTTTTACCCATAGTG
GCAACATTATCTTCCGTTATTCCTAGTCTTAAAATTCTTCCCCCATTAGTTGCGATCGCAGCTTCT
GAGTTTTCGCTTACTGGTAAGATAAACTGGAGGGAGTCTTTCGGTTTTAATTTAATCAGTTGAAA
GCCTCTATTACCTAAACCATCTAATTCACTGATACTAATTCGCTTCACATAACCTCCTTGAGTTAA
CATCAGTAAACCATTAGCTTTTGTATCCTCTAAACAAAGATAGGCAATGGGTTTCGCACCATTTT
GAGTGGCACTATTGGAAACCAGTCTATCAATGGTTTGTTTGGTGGGATAAGCAGGAATATCGGGC
AGAGAAAGAGGGTAAGCCTTCCCGCTATCGAAAAAGACAATCAATTTTTCTGCTTTGGGTTTAAC
GGTTTGTTGATAAATAACTAAATCAGTATTTTTCTTAACAATGGGTGTATCAGAAATAGTGGCAG
GTTTACGCCAATAAATCTTGCCCTTAGCAGTAATATGAATTAAAGCATCCTCTGGCATTTTTTGG
GAAAAATCGAGGGGGAGGGAAGGCAGTGATTCTTTTTTTGTCTGTTTCGTCGTAGTTTTAGAGGT
TTTTTCTTCTTTAATACTCTTTAAGTTTAAAATTTTAGTCTTTCTATTATCTGCAAATTTACGTT
TTAAACTCCGCAATTCTTTTTTTAAGGCTTTGAGTAATTCGTTTCTGTCTTCAAGTAACTGTTGT
AATTGGTTAATTCTCTCTTGCAGTTGCTTATACTCTTCTTCTAATTTCTGTTTCTCTAACCCTGTA
ATTCTCCTCAATGGCATAGCCAGAATAGAGTTAGCCTGTGCCTCAGAAATTTTTAAATCTGTTTG
TAATGTATTTTTTGCTGTTGTGCCATCGGCGGAATTACTAAGAATAGAAATAACTTTTTTAATAT
TTTTTAATGCTTTTAGCAATCCTTCTAATAAATGGATTCTTTCTTTTTTTTCCTCTAATTCATAT
TTATATTGTCTTGTTAAAGTCTGCTCTCTAAATTGTAAAAATTCATTTAACACCTCTTTTAAGGA
TAATTGACAAGGTTTATTGTTCACTAAACCAAGCATAATTATCCCGAAATTACTTTGTAATGCAG
TTTGTTTATAAAGTTGATGCAAAACTTGTGGCGGTTGGGCATCTTTTTTCAATTCTATCACAACT
CTAATACCAGTGCGATCGCTCTCATCTCGGATGTCAGCAATATCAGTAATTTTTCCCTGATTAAC
TAAATCTGCTACTTTTTCAATTCCACCCGGCCTTATTTACCTGAAAGGGCAATTCTGTGACAATGA
TGGCAGTTTTTTCCCGAATACGTTTCTTTTCTATCCTTAATTGTTCAATGGTAGCTACTCCCCGAA
TAGTTACAATACCTTTTCCTGTCAAATATGCGTCTTTAATACCATCTGGGTTAATAATCTCTCCT
CCTGTGGGAAAATCAGGACCAGGTATCAACTCTATTAGCTTATTATCATCTAAATCAGGGCGATC
AATCAAAGCAATTAAACCATCAACAATTTCTCCTAAATTATGGGGTGGAATATTGGTAGCCATAC
CCACAGCAATACCAGAGCATCCATTCAACAATAAAATCGGTAACTTAGCAGGAAGGACAACTGGC
TCTTGTTGAGAATTATCAAAGTTGCTGGTAAAATCAACGGTAGAATCGTTAACTTCTTCTAATAG
ACTCTCGTAGGAAATAGGAGCAAGACGAGTTTCAGTATAACGCATCGCCGCCGCAGGGTCATTGT
CCACACTGCCAAAATTGCCATGGCCTGCTAACAACGGATAACGACTAGAAAAATCTTGCACCAAG
CGCACCATTGCATCATACACCGCTTGATCGCCGTGAGGGTGGTATTTTCCTAACACATCCCCTACT

ACTCTAGCACATTTGCGGAAAGGGCGATCGGGGCTTAATCCTAATTCATACATAGCATACAGAAT
ACGTCTATGAACAGGTTTTAAACCATCTCTCACATCTGGTAACGCTCTCCCTACAATCACACTCAT
AGCATATTCCAAATAGGATCTCTCCATTTCTGTATGTAAAGCAGTAGGAATAATTTGACCTTGAT
TCAATAAATTTAACTGTTGTGCCATGAGTATTTTTGCTTAGTAAATGAAGTAAGGATGTTTATAG
GACTATTTTTAGAGATTAGCAAAGAATTTGAACGAAAGGAAAGATTAATTGAGAATTTAGTGTT
GGGGTTAATAGATTTTAAAAGAGCAAGAGGCAAAGATAAAGGTAAATAATTGATAATTAAGAAT
TAAAAAATCCGAACACTTATTAATCATTACTTGTTTCCCCATCACCTCATCACCTCATCACCTCA
TCACCTCATCACCTCATCACCCCTTCCCCTCTCATCCCATCCCTCTAAAACCCCAATCTCTCAATAC
CCCAAGATATTAACCGAGGTCAACGGAAGGAAAGTCAAAACTGAGAAAAAAACCTTGTATTGTA
AATAAATGTAACAAATTTTCAAAATAGAGAATTTGCAACCCTCAGAATCAATGAATTTAAGTCTT
GTAAGCCATTTATTAGCACATCTTTTTCGGATAAGATGTAAAAAAATATTACAAAAATCCTCATT
TTCATTTTTCATTGCCTATACTGACAATATGGGAACACATATCTAGTTGTTTATACTGAAACTTT
ATCTAAACTTTATATTGTTTGTTGTCTGTTTGTTGTCGTTAGAAATCTGAAGGAGCGAAAAGAAA
TGCACATATCAAATCAAAATACTTGGCACGATAGTTATTTGTTGTCTATGACAAAAAGTTTTCTG
GTTTGGAATTTAACCTTAACTGTTTGTTTTTTAGTTGTAGGCTTTCCGTTAGTGGTAGTATTGAT
GACGGTGGGGGTTTTAGCCGCAGTAGTTTTACAGTCCGTTTTCCCCGCTAGTGCGCTCGTACTTGT
GTCTAGTGGTATGCTAGGAGTAATCCTATCCTGTATTCTTTTCAGCTCTATTATTTTAACTCTCA
AAGGTGTTCATCCTAATGAAGTAAAATGGTTGGGGTGGTTACAAGATAATGAGAAAGTAACTCG
TAGAGCTTTATATGCTTCTTGCCCTTTAACTTGTAGTATTACTCAATATTAATTCCTTGAAAGAA
TAGGGAATGTACGATGTAGAATGTAGAAGAATGTTTGGTTAGATATATATTAATCATTGAGACG
ATAAAGGAATAAAGCCTTTACTTCTTCATTTTTCATTTTCAATTCTTCATTGTATATGACTAATT
CTTCTATGTGGCAATGGCGAGAGACGGAAAAAGGTAGATTTTTAACCTGTGATTTGCTAAGAAAT
TGGCAACACGGGTTTTTTACTTGTCATTTTCAGGGAGATTCCCCGAATATTCTCGTTGGTTATTT
GAATCCAAATGCTTCTGTTTATCGTCTTAAGCAAATTCATAGTGATATTCTCTTTTCTACTGCAA
CGTTAGATAGGCACTATTTATCTGGTGCTGATTTATTGGAGGGGGATGGTATTATCTCTAGTAAG
TCTTTAGATTCAGTTTGGTGTGCTAGTGCTGATTGTACACCTGTTCTTATTGCTGATAAAGGTAC
TGGGGGAGTAATGGCAATTCATTCAGGATGGCGTGGAACTTCCACTGCAATTGTACCAAAGGCGA
TCGCACTTTTACAATCTCAAGGGCAAAATTAGAAGATTTACTGTTTGCTTTAGGTCCGGCTATT
CATGGTAAGGTATATCAAGTCAATGAAGAAGTTGCCGTCGAGGTTTTAAAAACTATTTTCCCTTC
AGATACTCCCTCGGAATTAATCATTGAAAAAGCCTTACAAATAGAAAACCAACCAATTTTACCTG
ATGAGGAGGAAAATAAAGTTAGATTAAATGTGACTCAGGTGATTAGTTTACAAATTCAACAACA
AGGAATAAACTTAAATCAAATTGCGATCGCACCCTATTGTACTTATCAAACCCCAGAAGATTTCT
TTTCCTATCGTCGCACTAAAGAGAAAAAGATTCAATGGAGCGGTATTGTTTCTCATTAGGAGAGT
TTTAAGGCTCTATTACTTCTGGTGGTATAAATTATTAGTTTTATAGATAGGCAATAGGCAACAGG
GGATTCTTAAATAACCTCAGTTCGGTTTAAGAATATCTGATAAGGTTAGGTGTTAGGTGTCAGGT
TGCAGGTTGCAGGTGGTAGGGGTTTTTAGCAAGGGGCTTAAGCCCCTTGTTTAAGTCAGTTCGGA
TTAAGGCAATTTTATCGTTATTTCTAAGTTATTTCTAAGAAGCTATAAGGTTTTTTGACTACGAG
TTGGTCTGCTTTCAGCTTATCCAAAACTCAGGTTAAATAAGAAAAGATCAAATTTTAATTGTCGT
TATTCTGGAATGATTGATTTAGTGCAAGAAGTCTATTGAGCCATTGTAAATACTAATAGTGGACT
CATCTTGGCTTTATTTAAACTTAGCACCCCATCTTTAATGGTGTAATTATCCACTTGTTCTAAGG
TACGCAAAAAACTAATTTCTGTTTCCATATTTTCACAAGCCATCATGGTTGATGCCATTTGTTTT
AATGTCAAACGATTGCCTTCTTTTAATTCAAAACCGCCCATAAAATTATTACACCCTGCAAATCC
GTTGACTCTATTTTCTTCTGAGTTAAAAATGAGAAAAATGGGTTCTCTTTGATTTTCACTTTTTG

TGACAGGTTTACCCATCATTTCCGTTAATTGCCAACGAACATCGGTAAGGGAATCTTCAGTCATA
GATGCTTGTGCTACCTTGTTTAAAATGTATTTATCCGCTAAATCTCCTGAGATGCGATCGCCGTT
CATGTCTAATTGAATTAACCTATTTTCTCCTACAATAAACTGATTAAGAGCTTGTTCAATCCCTT
GCAAAGTGATCCGATTACCTGCTTCATTCCACTGAAATGTTCCTTTTGTCTCCCATGTTCTCTCAT
CAATTCCTAAATACTGTACTTGTTGAACAAAAGTTAAATCTTGATTCAAAATAATGGTAGTTTTA
ATTCCTTCACAACTAGCACATGGTAAAATTCCTTCATAAACACCATTCCAATCTAAAGAATTTTG
ACTGGTATCGCCAATGGCGGGAGTAACAGACTCCATAATATCCTCTGAATTTTGAGCTTTAAGAG
AATTTGACAAAAAATAGTTAGGAATTAACAAACTTAGTAAAACAGTTGGTATAAGAATTAACTT
GTATTTCATAATCGGTAATAATAATTTTTGACAGAACAAAGATAACTAGAATAAGGAATTGAGA
ATTGAAAGCTAAGAATCAAATCACTCTAAAACCTAATAACTCAATCACCCAATCACAGATGACGA
TTTTTAATTTATTTTTGTTCCCAACTTATTGTACTACTTCACCATCGGGGCGATCGAGTTCTGTTC
TAATGCCTAAATCAATAACTTCAATATCCCATTGACCTCGTTTAGCGGTTTCAAAAACGATATAA
CGACCATCAGGACTAATACGAGGATTTCTTAACCAACTGCGATAACCCTGAGTTACCAACTGAGT
TTGTTTTGTTGCTCGATCATAAATAAAAATATCTGGTCTGCCTTGTATGCTAGAAATATAAGCAA
TGTAACGTCCTGTACGACTTAAGCTAGGACTATCATTAATAGCATTACCAATGTTTAAACCGGGT
AAATCGACAAAATTTCTTAATTGTAAATCATAGAGTAAAATTTCCCTACGTCCATTGCGATTAGA
AACCATTGCCAACCAACGACCATCACCGCTAATGGCAGGTTGTTCATCATTATAGCGACTGTTAA
GAGACGTGGAAAAAGAAGAAACATTGCTGGAGTTACACCCATTCAAACTCACAGCAGAGAACACA
AGCAGTAACAGAAATAGTTGTTTCAAAACTAGACCAATTTAGTCAAATTAATAATCGAAATTAT
CAGGATTTGAGGGGCGATCGCCCTTGGGAGAATCATCATAGTCAGATTGTTCGGGTTCAATGGGT
TGATAGTCCACATATTCAGTGCCAACGCTAGAAACAATATCATCAGCAGACTTCGGACGACGTTT
TTTTGGTCTTGCACCAGTAGTAGGACTTACTCCTCTATCATTCCGAGGGCGAGAATAATCTTGAC
GGGGAGAAGAAGATTCATCATCCCAATGATCGGGGGTTTCTTGCCAATCTTCATAATTGGAACGA
CTGGGACGAGTTCGGCTAGTATTGCGACTGCGGGGATTAGGAGGCTCTTGATTATAACTGCTAGA
AGGACGACGGGGACGACGAGGGCGGGGATTTTCTTCTTCATACCCTGTTGTAGGGCGAGGGCTTC
TTACGGCTGTACCTTGCAAACGACGGCGGGGAATTTCCTCCTCCTCATAATAATCATCATAAGCA
TCTGTTTGATCCAACTCTGCACGATAAACACGACGATTAACAGGGCGATCATCTTCTACATAGGA
GTTAGCTCTCGACCCCCTCACTTTCTCAGAAGTAGCCCCTCTCAAACGAATACTCTCAACGGCAAA
AAAGATAGCAGAGCCAGAAAGTAACAGTTGACTAAATTGCAAAATGGGATCTAGCCGCCACCCTT
GAAAGAATAAGATAAAACCGCAAAGTAAGCCGACTGCGGCAAAAAAGATGTCATGATCCCTAGA
GACTTCAGGTCTAACAGAACGCATTAAATACAATCCAGCCCCTGCTAAAGCCAAAACGATACCCA
ATACACTGGCGGAGTTGAAACCAAAATTAACCATTGTTCAAACTAAAACTGCTATGTTTTCTTTT
ATCTTAACCAGATTATCTGATTCTGCACTGCCTATCTTTAACACCATGTCATAACTAGACATTAA
TTTTTAATCTTTCTCAAAAACTGTCAGGGGGTTTACCGTGTAATTAATTTATGGTTATGCCCCCT
GAAAAGAAATTGATAACAGACGTATCAAACTACTATATATTATTAACGGATTTTGTCCGCTTGGC
TAACGACAATTAAACCAATAGTAACGGGAATAACTACGATCGCAGCACCCCAAACCAAACTCCAA
AGAAAATTAGCTAAAGAAGCAGTCATAAATTTTTATACCTATTTATTTACTAAAAATGATCAAT
CTCAATTTTACAATAAATCCAGAGTTAAATTAGGGTAAACTATCCAAGGCAACTTTTCAAGGAAA
AAATTAAAAAGTCTTATGGTTAAATTGTTACCTGATAACTTACAAAAATTAAAAGAGCAAGTGG
CAATTGTTACGGGAGCTTCGAGAGGAATTGGAAAAGCAACCGCTATCGCCCTAGCGGAAGAAGGG
GCAAAAGTCGTGATTAATTATGCTAACAGTAGTCAATCTGCGGAAGAATTAGTTAAAGATATTA
TTGATGCTGGAGGAGATGCGATCGCAGTTCAAGGGGATGTTTCCCAACAGGAACAAGTAGAAGCC
ATGATCAAAACTACTACTGATAAATGGGGACGTATAGATATATTAGTCAACAATGCAGGAATTA

CCCGTGATACACTAATGTTGCGAATGAAGCTAGAAGACTGGCAAAAAGTAATCGATTTAAACCTT
ACAGGGGTCTTTTTATGTTGTAAAGCTGTTAGTAAAATCATGCTAAAACAAAGAAGTGGAAGAA
TTATCAATATATCTTCGGTAGCAGGACAAATGGGCAATCCCGGACAAGCTAACTATAGTGCGGCA
AAAGCAGGAGTTATCGGTTTTACCAAAACCCTCGCCAAAGAATTTGCTAGTCGGGGAGTCACCGT
TAATGCCGTAGCCCCCGGATTCATTGAAACAGATATGACAAGTGGATTAGAAGCCGAAGAAATTT
TAAAAGCTATTCCCCTTAATCGTTATGGTAAACCGGAAGAAATCGCCGGAATGATTCGCTTTTTA
GCCGCCGACACTTCTTCTTCTTACATTACAGGACAAGTATTTAACGTTGATGGTGGTATGGTTAT
GTAATAAATAGTTTAGTCTTAAGATAGGAAGATAGGAAATTTCTGGGTTAGGGTTTAGAAGTTA
GGAAAGAGGTTTCAGGTTTCAGGGTATTGGGGTGTTAGGGAAGCGTGTTAATTAAACACTTTTGC
CCTCCCCCCTCTCGAGGGGGGATAAAGGGGGGTTTGCCTTTTGCCTTTTGCCTTTTGCCTTTTGCC
CCTTTCACTTTGCCCCTGCTTACATTGCTTAAGTTTAACTCAACTATTTTTATTGCTATTTACATT
TCATGAAAGAAAAAGACTATCCGCAATTCCTATCACAATTAATTCACTCTTATAAAGATAATGTT
GATTTTTTGTCCATTCGCCTAGAATCTTCTCAGGGGAGTAACATTAAAATGCGTAACGGGCAAAT
TGAAAATATTAGCGAAGGCACTTCAATTGGAGGACAAGTTAGAGCTTGTCGTAAAGGAGGATGG
GGTTTTGCTACGTTTAATGATTTAACTCAGATAGCCGATTGCATCAGTGATGCCATTTCTGCCGC
TCAATTGATTGGTGATGACGAAACAGTCCTTGCAGAGGTTGAACCAGTGGTGACGAGTTGTAAAT
TACCCTTAACAGGCACAAATCCACGTCTGATCCACCTTTCTCAAAAAAAGGATTTAGTGGGAAGA
TGCGATCGCATTTTAAATAGTTATCATAAAGATATTACCAGTACCACTGTTAGATACAGTGATAT
TCAACAAAAAATAGTTTTAGCCACCTCCGAGGGCTCATTAATTGAGCAAACATGGATAGACAGCG
AAATGCGTTTTAGTGCCACCGCTAAACGAGGGGAATTAGTGCAAACAGGGAGAGAAACCACAGGC
ACAAGGAGAGGCTTTGAAGACTTACTCAACCTTGATAAACAGATAGAAAGTGCGGCGAAAAGGG
CGGTTAATGCCTTAGACTTGCCCACTGTCAAAGGAGGAACTTACACAGTGGTTATTGACCCCATA
TTAGCAGGATTATTTGTTCATGAAGCCTTTGGACATTTATCCGAAGCGGATATGGCTTATGAAAA
CCCCGACTTATTAGAAGTGATGACAATGGGGCGCAAATTCGGTGGTGAATTTTTACAAATTTTTG
ATGGGGCGCAACCAGAAGGACATCGAGGCAGTTATTATTATGATGATGAAGGTGTACCTGCCACC
ACCACTCAATTAATTAAAGATGGAGTATTAGTAGGGCGTTTACATTCCAGAGAAACGGCTGGTAA
ACTAAGAGAAAAACCCACTGGTAATGCCCGTTGTTTAAACTATCATTATCCTCCCATCGTGAGAA
TGACAAATACTTGGATAGGCAAAGGTAATACCCCTGTTAAAGATTTATTCAGCAATATCAAAGAA
GGAGTTTACGCCCGAAATTGGTTAGGGGGCATGACAAACGGGGAGATGTTTACCTTCACCGCAGG
AGAAGCATGGATGATTCGAGATGGTATCATAGCTGAACCTGTGAAAAACGTTACTTTATCAGGAA
ATGTCTTTAAAACCCTTGCCAATATAGAAGCGATCGGCGATGATTTTTATTGGGATGAATCGGGA
GGATGTGGAAAAGGTGGGCAAAATGGTTTAGCCGTTGGTTGTGGTAGCCCTAGTTTACGCATCAA
AGACGTAGTAATTGGAGGGGAAAATTGACAATTTACCATTGAGCGTTAACAATAGTTAGGTGTC
AAGAGTAGGGAGTGGGGAGTAGGGAGTAGGGGAGAGAGGAAATATTAGACATCTCTTGAAAATA
TCAATATAACCTCGTTCGTAGTGAGGGCTTTAACCCTTATTTCAATAACCATTGGATAAGTCCAT
TTTTTATATTATAACTCCGAACTCCGAACCCCGAACTCTGAACTCAAATCATTCTTCATTCTTAAT
TTTTAATTCATTATGTCCACTCGTGTAATAATAGCTCGTCATGGTCAAAGTAGTTACAACGCTCA
AAAAATGATACAAGGGCGTTGTGATGAATCGGTGATTACGGAAAAAGGAGAAAAACAAGCTCAA
TTATTGGGGGAAGCCCTCAAGGATGTTAAGTTAGGGGCGTTTTATAGCAGTCCATTACAACGAGC
TTATAAAACCGCTCAAATTGTTCAATCTCTCAATCAGTATAAACCTTCTATCACTGTGATGGAGA
AATTGAGAGAAATTAACTTGCCTGAGTGGGAAAAATGGAAAAAAGAAGATGTCAAAAGAGAATT
TCCTGAAGCCTATCGCACTTGGAAAGAAAAACCTGATGAGTTAAAGATGACTTTTGAGGGTGAAG
AGTTTTATCCTGTATTAGACTTGTATCAACAAGCACAAGAGTTTTGGCAAGAAATTATCCCGAAA

CACGATGGTGAAACCATTTTAATTACTGCCCATAATGGTATTAATCGTTGCTTAATTCTCACTGC
TTTGGGAATGTCTCCTGCTTACTATCACTGTTTGCAACAGTCTAATTGTTGTGTCAATGTCTTAA
ATTTTACAGGTAACTTCGGTGATTCAGTTCAGTTGGAATCTTTAAATCAAATTGGGCATTTAGGC
ACACCTTTACCAGATTTTCGCCCTGAGCAAAAACAAGGGTTAAGATTATTGTTGGTGCGTCATGG
AGAAACAGAGTGGAATAGAATGTCACGTTTTCAGGGTGTCAAAGATATTCCTCTCAATGATAACG
GCAGACAACAAGCAGAAAAAGCGGCTGAATTTCTCAAGGATGTGCAAATTGATTTTGCTGTCACT
AGCCCATTATCTCGCCCGAAAGAAACCGCAGAGATTATTCTTAAGCATCATCCCGATGTTAATTT
AACTACTAAAAAAGATTTAGAAGAAATTTCTCACGGTTTATGGGAAGGCAAATTAGAAACAGAA
ATTGAAGCAGAATTTCCCGGTTTATTGGCACAATGGAAAGCAAAACCTGAAACTGTACAAATGCC
TGAAGGAGAGAATTTACAGCAAGTTTGGGATAGAGCGATCGCATCTTGGCAAGAAATCGTCAAA
GAGAATATCGAAGAAGGCAAAATGAAAACAGGTTTAGTTGCCGCTCATGATGCTATAAATAAGG
TAATTATCTGTTATTTATTGGGGTTAGAATCTGCTAATTTTTGGAATATTAAACAAGGAAATGG
AGCAGTAACAGTTGTTGACTATCCTTACGGTTTGAATGGAAAACCAATTTTACAAGCAATTAATT
TGACGAATCATCTTAGTGGTGGTTTGTTTGATAAAACTGCGGCTGGTGCTTTATAAAATCCTTAT
AAGTTAGAAAAGAGGTGTAAGGATGTTGGGGTGTTATTAGGGAAGCGTGTTAATTAAACACTTT
TGCTCTCCTTCCTCGAAAGGGGGGTTTCTTTTAAGGGGAGACAAAGAGGGGTTTGCCTTTTGCCC
TTTTCTCCCTACTCATAATTAAAGGGGTATTGTCAAGAGTCGCCATAGAAAGTTAAAATAGATAT
TTTGATATTCTATGAAAAAACTTATCATTAAATAGTAGTCTAAGTAAAACAATTAAGGATAGTG
GCTAATTCAAAATCTGCTCTCAAGAGAATCCAAATTAACGAACGTAACCGCCTCCGTAACAAGGC
TTACAAATCTGCGGTTAAAACCCTCATGAAAAAATATTTTCAAGCGGTAGAAGCCTATGCAACTT
CTCCTAGTGATGAGTTAAAACAGGCAGTACAAACTTGTCAATCTGCGGCATACAGCAAAATTGAT
AAGGCAGTTAAAAGAGGTGTTTATCACAGAAATAACGGTGCAAGAAAAAAAGCTAGATTAGCTA
AAGCATTTTCTAAAATAAACCAAGCACAAGCCTCTTAAGTATTGGGGTATTAATTTTTACCGCTT
AAACACTCAATACGATAGCTAACAATAGTTAGCTAATTTTTTTCTCAAATAAAATAGGGAATCTA
ACCCTTGAAAAATTAGGAGTTATCAGAAATTTATCAGTATGAAAACGGAAATTAAGGATTAAGG
AATAATCGCAAAAGCTAATTTCTAATTCTTAGTTCCTAATTCCTAATTAAAATCCTGTACCATTA
GGCAAAAAAACACTAATGCAACTGATTGATACTCATGTACACATAAACTTTGATGTCTTTCAGGA
AGATTTGGATTTAGTTTCCAGTCGTTGGCAATCGGTTGGGGTAAATAAACTTATCCATTCCTGTG
TTGAACCCCAAGAGTTCGATCGCATCAAAGAATTATCAGAAAAGTTTCCTGAGTTGTACTGTAGT
GTGGGTTTACACCCCCTTTCTGCCAACAAGTGGGAAGGAGAAACTACCTACCAGCAATTATTAGA
ATTTGCCCAATCCAGCCCCAAAGTGGTGGCAATCGGAGAAATGGGCTTAGATTTTTACAAAGATA
ATCAACCAGAGTTACAAAAAGAAGTTTTCTGGAAACAGTTAGAAATTGCCTATCAATTAAATAA
ACCTGTCATTATTCACTGTAGAGATGCGGCTACCGCCCTACGGGATATTTTAGTAAGATTTAACC
AAGAACACGGTAAAATAACAGGAGTGATGCACTGTTGGGCAGGAAATCCAGAGGAAACTCAGTG
GTTTCTTGATTTAGGAATGTATGTTAGCTTTAGTGGGGTTGTAACTTTTAAAAATGCCAAAACCG
TCCATCAAAGTGCTTCTATTGTTCCTGATGATCGTCTTTTAATCGAAACGGACTGTCCTTTTTTA
GCTCCTACTCCCCATCGTGGTAAACGCAATGAACCTTCATATGTGCTTCATGTAGCGGAGAAACT
AGCCCAAATCCGAGGACAATCAGTAGAGGCGATCGCATCCCAAACAACCAAGAACGCCTGTAAAT
TATTTAACCTCTAAATTTAAAATAAAGCAAGGGTAAAGAAAATAAATAACCGAAACCCTGACCCC
AGAATCTAAATAGATCAATTCAATATCATTGACAACTAACCTATGAAAACCCAAGAACTGTTACC
AGATTTAATCGAAATTCAACGCTCTAGTTACAAATGGTTTTTAGAGCATGGCTTAATCGAAGAAC
TCAACAGTTTTACGCCTATTACTGATTATGCAGGAAAACTAGAATTACACTTTATTGGGGAGAAA
TATCGCCTCAAAGAACCAAAATACCACATCGAAGAAGCAAAAAAAAGAGATGCAACTTACTCAGT

ACAGGTATATGTACCAACATTGTTGTTAAACAAAGAAACAGGAGAAAGAAAAGAACAAGAAGTA
TTTATCGGTGATCTACCATTGATGACTGATAGAGGTACGTTTTTGATTAATGGTGCAGAAAGGGT
AATTGTCAATCAGATAGTGCGATCGCCGGGGGTCTATTTTAAATCAGAGGTGGACAAAAACGGAA
AAAGAACTTACTCCGCCTCCGTCATTCCTAATCGGGGTGCATGGCTAAAATTTGAAACGGATAAA
CATGGTGTCGTATGGGTCAGAATCGATAAAACCCGTAAAATCTCAGCCCAAGTATTGTTAAAAGC
TATGGGCTTAACCGATAGAGAAATTATGGATCGTTTACGCCATGCGGAATTTTACCATAAAACCC
TAGAGAAAGAGGGCAATCCCAGTACTGATGATGCTCTCATGGAACTTTACCGCAAATTACGCCCC
GGTGAACCTCCAACCGTAAGCGGTGGACAAGCCTTATTAGAAAGTCGCTTCTTTGATCCTAAACG
TTATGATTTAGGAAAAGTTGGACGCTACAAAATGAACAAAAAACTGCGTTTAACAGTCCCTTACA
ACGTAAGAGTTTTAACTATTGAAGACATTTTATCCGTTGTTGATTATCTCATTAATCTTGAGTTT
GATATTGGGCAAGTAGATGATATTGACCACCTCGGTAATAGACGAGTTAGAAGTGTAGGAGAAT
TATTACAAAATCAAGTCAGAGTCGGTTTATCCCGCCTAGAAAGAATTATCAGGGAAAGAATGACC
GTAGGTGATCCTAATACCTTAACTCCTGCGGCTTTAGTTAACCCTAAACCGTTAGTAGCGGCTAT
AAAAGAATTTTTTGGCTCATCTCAGTTATCCCAATTCATGGATCAAACCAATCCTTTAGCTGAAT
TAACCCATAAACGCCGTATTTCTGCTTTAGGACCTGGTGGTTTAAGCCGAGATAGAGCAGGTTTT
GCCGTGCGAGACATTCACCCTAGCCACTACGGACGTATCTGCCCTGTGGAAACTCCTGAAGGACCT
AACGCAGGTTTAATCGGTTCTTTGGCTACCTATGCCAGAATCAATGAGTACGGATTTATTACTAC
TCCTTACTATAAAGTTGAGAATGGAAAAGTTCGTTGGGACTTAACTCCTGAGTACTTAACTGCCG
ATGAAGAGGACGATAAAAGAGTTGCCCCGGGGGACATTTCCACCGACGAGGAAGGTAATATTTTA
GGGGAAACAGTACCCATTCGTTACCGTCAAGAATTTTCTACCACAACTCCTAATTCAAGTGGATTA
TGTAGCGGTGTCTCCTGTACAGATTGTTTCTGTGGCTACCTCTATGATTCCTTTCCTTGAGCATGA
TGACGCTAACCGTGCCCTGATGGGTTCTAACATGCAACGTCAGGCAGTGCCTTTATTACGGGCGG
AACGTCCTTTAGTGGGTACAGGTTTAGAAGCCCAAGCGGCAAGAGACTCTGGAATGGTAATTGTT
TCCCGTCATTATGGCAAAATTACTTATGTGGATGCAAAAACTATTAAGCTCAAAACCAAGGAAGG
AGAGGAGTTAGTTTACAATCTTCAAAAGTATGAGCGTTCTAACCAAGATACCTGTTTAAATCAAA
AGCCTTTGGTTGATGAAGGAGAGGAAGTAGTACCCGGACAAGTATTAGCCGATGGTTCTGCCACC
GAAGGAGGAGAGTTAGCTTTAGGACAGAATATCACCGTTGCTTATATGCCTTGGGAGGGTTATAA
CTACGAGGATGCGATCTTAATTAGTGAAAGATTGGTTCAAGAGGATATTTACACCACTATTCACG
TGGAAAAACACGAAATAGAAGCCCGTCAAACAAAATTAGGTCCTGAAGAAATTACCCGTGAGATT
CCTAATGTCGGTGAAGATGCTTTACTCAACCTCGATGAACAAGGGATTATCCGTGTGGGTGCTTG
GGTAGAATCAGGAGATATTTTAGTAGGTAAAGTAACCCCTAAAGGTGAATCAGATCAACCTCCAG
AAGAAAAACTGTTACGGGCAATTTTCGGGGAAAAAGCCAGAGATGTTCGTGATAATTCCTTACGT
TTACCTAACGGTGAAAAAGGAAGAGTTGTCTATGTGCGAGTGTTTACCCGTGAACAAGGTGATGA
GTTACCCCCCGGAGCGAATATGGTAGTCCGTGTCTATGTGGCTCAAAAACGCAAAATCCAAGTAG
GAGACAAAATGGCGGGTCGTCATGGTAACAAAGGGATTATTTCTCGCATCCTCCCCCGTGAAGAT
ATGCCCTATATGCCCGATGGAAGTCCTGTGGACATCGTTTTAAATCCTCTGGGTGTTCCTTCCCGT
ATGAACGTGGGACAGGTGTTTGAGTGTCTTCTAGGATGGGCTGGTGAGCATTTAGGCTATCGCTT
CAAGATGACTCCCTTTGATGAAATGTATGGAGAGGAAGCGTCTCGAAATACAGTCAATGGCTTGT
TAAAAGATGCCTCGAAGAAACCGGGTAAAGATTGGATTTTTGATGAAAATAACCCCGGTAAAAT
TCAACTTTATGATGGTCGTAGTGGTGAACCATTCGATCGCCCCGTGACAGTGGGTAAAGCCTATA
TGCTTAAATTAGTTCACTTGGTGGATGATAAGATTCACGCTCGTTCTACCGGACCTTATTCTCTG
GTGACACAACAACCTCTCGGTGGTAAAGCTCAACAGGGTGGACAAAGATTCGGAGAGATGGAGGT
ATGGGCCTTAGAAGCGTATGGTGCGGCTTATACTTTACAGGAATTATTAACAGTCAAATCCGACG

ATATGCAAGGACGTAATGAAGCCTTAAATGCGATCGTGAAAGGACAACCAATTCCTCATCCCGGT
ACACCTGAATCCTTTAAGGTACTCTTACGAGAATTACAATCTCTAGGTTTAGATGTTTCTGTCCA
CAAAGTGGTAGAAGGTAGCGAACACGTAGAAGTTGACTTGATGGAGGCTTCAGGGAGAGCGCCTA
AGCGTCCTACCTATAAGGAAGATTACAGTTTATCCAGTTTAGGTCAAGATGACGACGAGGAGTTG
TAATTTTAATCAGTAATTAGGAATTAGCAATTAGGAATTTAATCTTTAATTTTGTGGCAAGTTG
AGAAAGATAAAATCTATTTTCGACTTTTGCTTCTTGAATTTTGGTAAAAACTGTTAACCATCAAC
TAAACACCATGACTTCTAATCAGAAAGAGACTAAAAATAAAACTTTCTACAATAAAGTAATCGA
TAAAGGGGGCTTAAAAAAGCTAATCGCTAAAACTTTTACCGAACAAGGTTCTGCCCGTTGTGCTT
CTGTTTGTGACCAGTTAAAAACTTTGGGTTTCCGTTATGCGACTCAAGCGGCGGTTTCTATTAGT
GTGGAAGATTTGAAAATTCCTCCTATCAAAAAAGAAATGTTAGCAGAGGCTGAGTCCACCATCAA
AAATACTCTCAGTCGTTATGCTAATGGTGAAATTACTGAAGTTGAACGTTTCCAGAAGGTAATCG
ATACTTGGAATGATACTTCTGAATCTTTGAAAGATGAAGTAGTCAAAAATTTCCGTGAAACTGAT
CCTCTTAACTCTGTCTATATGATGGCGTTTTCTGGGGCGAGGGGAAATATCTCTCAGGTACGTCA
GTTGGTTGGTATGCGCGGCTTAATGGCTGATCCTCAAGGAGAAATCATTGACTTACCCATTAAAA
CTAATTTCCGTGAAGGTTTAACCGTTACTGAGTATATTATTTCTTCTTATGGCGCTCGTAAAGGT
TTAGTAGATACCGCTTTGCGTACCGCAGACTCTGGTTATTTAACCCGTCGTTTAGTGGATGTTTC
TCAGGATGTAATTATTCGGGAAAATGATTGTGGTACTCACCGAGGTATCGAAGTTACTCCCATGA
AAGACGGCGATCGCATTTTGATTCCTTTATCTGATCGTTTATTTGGAAGAGTATTAGCAGAAGAT
GTGATTGATCCTAGCACAGGGGAAGTTATTGCTACCCGTAATCAAGCCATTGATGATGATTTAGC
AAAAGCCATTGGCAAGGCGGTTGATAAAGTAAAAGTAAGATCTCCTTTAACCTGCGAAACCGCTC
GTTCGGTATGTCAAAAATGCTATGGATGGTCATTGGCTCACGGTGACTGGGTAAATATGGGAGAA
GCGATCGGGATTATTGCCGCTCAATCTATTGGTGAACCCGGTACTCAGTTAACCATGCGTACTTT
CCACACAGGAGGAGTATTTACTGGAGAAGTTGCTCGTCGTATTACTACTCCAACCTCTGGTACGG
TTAAATTCAGTAAGAAATTAAAAGTCCGTGAAACTCGTACTCGTCATGGAGATCAGAAATTAGTT
GTTGAGGTAACAGGTGATGTTATCGTTGGTGCTGAAAAAATTTCTGTACCCAAAGATAGTTTATT
AGCCGTCAGAGAAGGGGATGTAATTCAAGAGAATGACCTCATTGCTGAAGTAATGCCCCAAAAAA
CCAGATCCACAGAGAAAGTAACTAAAGACGTTGCTACAGACTTATCTGGAGAAGTGCATTTTGCA
GATATTGCCCCTGAAAGTAAAACCGATAAACAGGGTAATACTACTACAACCGCCTCTCGTAATGG
TTTGATTTGGGTGTTATCGGGACAGGTTTACAATTTACCCCCCGGTGCCGAACCTGCAGTGAAAA
ATGGTGACAAAGTTAAGCCCGGTTCTGTATTAGCGGAAACCAAGTTAAAAACTAAAAGTGGTGGT
GTTTGCCGATTTGAAGAAGGTAGCCGAGAAATCGAAATTATTACTGCTTCCGTTTCTTTGGATCA
GGCAGATATTTATCTCGAACATCATGGTAGTCAACAACAATACGTTATTCACACCCAAAAAGGCG
ATCGCTTCGCTTTAAAAGTAGTTCAGGGTACAAAAGTACAAAACAATCAGGTTGTTGCTGAATTA
ATTGATGATACCTATACCACTAACACTGGAGGGTTAATTCGCTATGCAGGGTTAGAAACAGGGCG
AGGTAACAAAAAACAAGGTTATGAGGTAATCAAAGAGGGAACTATTGTCTGGATTCCTGAAGAA
AGTCACGAAATTAATAAAGACATCTCCTTGTTATTAGTAGAAGATGGTCAATATGTAGAAGCAG
GAACAGAAATTGTTAAAGATATTTTCTGTCAGTCTAGTGGGGTGGTTGAGGTTGTTCAGAAAAA
TGATATTTTGAGAGAAATTATCGTTAAACCGGGTCAACTATATCTTGACTTAGATCCTGACTATT
TAGCTAATATCGACGAAGATAATATTATTCCCCCGGGTACTCAAATTGCTCCTAATAATATTACT
GAAGAAGAATACTTTGCCCAATTTGTTGATACCAATGAAGGGGTTGGTTTATTATTACGTCCTAT
CAGACAATTTGAAGTAATTAATACTACTCAGTCTCCTTCCCAAGAATCATTGAATACTCAAGGTT
CAGCTATTAACTTAAAACCCGTAATGCGTACTTTCTTCAAAGATGGAGAAAGAGTTAAAAGTGTT
GAAGGAGTACAGTTAGTTACCACTCAATTAGTACTAGAAACCCATGAGGGGATGAGTGCGGATAT

TGAGTTAGTTCCCCACGAAGCCGAAGAAGGTTGTTTCCGCTTACAAATTGTGGTATTAGAATCCG
TCCTTTTACGTCGTGAATTGGAAACAGAACCCAATATTATCACTCGTGTTTTAGTGGAGGATGGA
CAAGAAATTGCTCCGGGAGATGTAGTTGGTACAACTCAAATTCTCTGTCAAGAGGAAGGTATTAT
TCAAGGGGTAAGAGAAGGGGTTGAAGCAATCCGTCGTGTGTTAGTGGTCAGAAAATCTGACATG
ATTGATATACAAACTGAGTCTAAGCCTACTTGTAAGGCAGGAGATTTTGTCACTCAAGGGGATCT
ATTAGCGGAAAATATAACTTCTCCTGAATCAGCAAAAGTTCTGAAAGTGGAAGATAATCGAGTA
GTCTTACGTTATGCTCGTCCTTATCGTGTGTCTGCTGGTGCGATTTTACATATTGAGCAAGGAGA
TTTAGTTCAACGGGGTGATAATTTAGTATTACTCGTATTTGAACGGGCAAAAACAGGGGACATCG
TTCAAGGTTTACCAAGAATTGAAGAATTACTCGAAGCGCGTAAACCCAAAGAACCTGCCTTGTTG
GCACGTCGCCCCGGCTTCTGTCAAGTGGAATATCGTGATGATGAAGCGATCGATGTTAAGGTAAT
TGAGGATGATGGTACTATTACTGAGTATCCTTTGAGTCCTAACCAAAACATTATCGTGAATGACA
ATCAAAGAGTGGAAACGGGCGATCCTTTAACTGATGGTTTAGCTAGTCCTCATGAATTACTGGAA
ATCTTCTATGCCTACTATCAAGAGCAAGAGGATAATCAAGGAATGTTTGAATGTGCTTTAGCGGC
ATTAGAAAAGGCTCAACTATTTTTGGTTAATCAGGTTCAAGGGGTATATCAATCTCAAGGTATCG
ACATTTCTGATAAACATATTGAGGTGATTGTGCGTCAGATGACTTCTAAAGTAAGAGTTGATGAC
GGTGGTGATACTATCCGTTTACCTGGGGAGTTAGTGGAATTGCGTCAAATTGAAAAAGACAATGA
AACCATGTCTATCACTGGCGGCGCACCTGTACAATATACTCCTTTGATTATGGGTATCACTAAAT
CCAGCTTGAATACTGATAGCTTTATCTCAGCGGCAAGTTTCCAAGAAACAACAAGGGTACTTACA
GAAGCTGCGATCGAGGGTAAAAATGATTGGTTAAGAGGTTTGAAAGAAAACGTAATCATCGGGC
GTTTAATTCCTGCGGGTACTGGTTTCAATGCTTATGAACGTCAGGATTGGGAAGGAAACGACGTT
GTCAGAGAGGTAAATGGTAATGATTATGCTCTTATGGACGAAAGTAATCGCTTATCTTCTTCTCT
TACGGATGATGAAGATGTAATTATTGATGATCAGTCTGCTCGTACTTATTCTCCTGACAGTATGA
TTATTGATGATGATGAGTAATCATTGATTGACAAAGGGTTTAAACCCTTTGTTAGGAATTAGTTG
GTAATTTAGTTGGTAGTTCGGGCTTCTAGCCCGATTTATGTAGATTTTGTCTATTTATAAAATTT
TTTAGCTTATTCTTCTAAATCTTGAATGTCTTGTTTCCAACGAGATAGTTTTCTCTTTCTTTCCGT
AATTTTTTTCCCATTTAAAGGAGGCATTCCTGTCTCAAAACTATAAGGGAATCCTTTTTTATCCT
CAACAACTTTGAAAAATATAGTATTAGCATTTGGATAAATTAACTTACTTCTTAATAACCTTTCA
CAGTCATTTTGCCAACAAAATACGTGATCATGTACATTGTCTTTACTAATAGATAGACTTCTCAA
TTTTTTTAGAGAAATATCATTACTATTAAATAATAAATCTTCTAATTCACTGGTTTTAGCAAAAA
TTCTTGTTTCACTTCCTTTACCCCTGAAAAACTCCACAATAAGCCACTCATTAAAATCAAAAATG
CAAACTTCAGTAGCTTCGCTACCGTCATCTTTTAAAGTTATCATCTCTTCTATGTTCTCAACATTC
CCCACAATATCAAGCGTAGAAATAGGTAATAATATCCTGATTCTATCAAATCTATCGCTGTAATT
AGACCAAAATACTTTACGACTAATTAATTGCTTTGTTTCCCAGTCTGGTAATGATAATTTATCAG
TCATCACATTTATTAATTTTTCAAAATCTTTATAATTTAACGCACCAATCCACTGTTTTAAAGCA
TTTTTTGCCCTTACAGATAATTGATGCCACTGACTATGAGTACCAGTTAAACTATAATGAGTTTT
TAACCAAGTCACCAATTTAGGATAACTTCCTCCCACCATCGGCGGAATATTTAATAACAGATTTT
CTACCGCTATTAATTGAGATTTACCTGATAAGTTACTTGTAGTACATAATAGATAATTAATATAT
TTTTTATCTATATTTAATTTACTTATAGTTAAAGGAATCTTAACTTTTATTGTTTCAACTAAAGT
CAAATTGTTCTCCAAATTGAACTTATAGGGTAAATTATATTTTTGAAAAATTTCCTCAGGTTGCT
TCAAAAGTTGACAACAGAGTAAGCCCAATTCTTCATAAGAATAACTTAACAATAATTTAACAATT
TTAAAAGTATTTTTATCTCTATCACTTTCGGGAGTAAAAGTAATAAAACTATTGACTAATGAAGA
GGCAAAAATATTTTTGATTGGGCTCAAAATGATTTAAAATTAAACGCCAAAATAGTTTTAATTTCA
GAAAATTATCTCTTTGGGCTAATTTCCATATTTTTTGAGCAATTAAGGTACTTTTTTTCTGGTTA

TTTTTTTCCCACTTTGTCAAATTAAATAACAAATATATCCATTCTATAGAATTTATATTTTCTGG
ATGATTTTTTTCAATATCTTTGATAATCTCCTCAAGAGTGCGAGACTTTTTCTGAATATTATTAC
CCCTAGATTTGATGGTTTGTGCAACTTTTATCAACTCTTTAGGTTCTACATTATTCAAAGTTGGC
AAAATAAGGTTATTTGTAGAAAAAGCCATATTTACTCCTAATTATTGTTAACTTTTTGATTAAAT
ATTTCTTTTAAATCTTCTCTTTTAAATTGAAAACGAAACATTACTTTTCTATCTTCAGGAGAGTC
TATTTGTTGGTTAGAATCAATTTCTCCTCTACCCGCCGCTAATAATTTTCGTTTGAATTTATCTT
TATCAGGAAAGTCGATAGTATTAATATATTCTGTAACGGCTAATGCCCTTTGAAAACTTAATTCC
ATATTATCTTTTTCCTCTCCTTTTGAGCTAGTATGCCCTTCAATAATGATTCGAGAAACTTGATT
ATCAAATTCTTTATTGCTCAAAATAGTTCGACTATAAACAGGAATAAAACTATTTAAAAATTGTT
TTCCTTCTGGTTTTAAATTAGCTTTTCCCTCGTCAAATAAAATCTTATTATCTAAACTTAAATCT
CCATTCGGTTCAGGTTGGATATTATCTCCCACTCCTTTGATATTTTTAGTTGCTTCAATAATAGC
TAAAGGAATTTTTTTGATAATATTATTATATTCAACTAATTGGGCAGATACAGCAATAAATAAA
AGGGCAAAAAACATCAATAATCCTGACATTAAATCTCCCACAGATAACCATACACCAGAATCTTG
CTCTTCGATTAATTCTTCTTCAGTTTCATATTCTAAAAAGTTACTCATTTTTTCCTTTTAATTAT
TAGTAAAAGATATTATTCGTATTGTAAAAGTAGATTTAGAAAAGATTGAGGGTTTAAAATTATA
GTTTGATGAAAATGTTTTAAATTGAGTAAATGTTTATCTAAAAAAACTAAATAATTTGCTTTAC
TTTCATAAGAAGCTGATAGAAAAATATTATCTTTTGAGTCAATTTTATCTAAAAAGTTAGTTTG
ATAAATACCTTCTTTATAAAAAGCTAAGTCATCGAAAGCAGTTAACAAATTCAATGTTTTAGTTT
CTTTAATTTGATGTCTTATTAAAATAAGAGTAATTTCTTTCTCAATTTGCGGAGTGAGGATTAGA
GTAATTTTGTACTCTAACTAATATTTTATAATTAGATTAGGAGCAGATTGTAGATTAGAACTTCG
TAAGGCTGAAATAAATACTGAAGTATCTACAATAACTAAAGGTTTTTTCCTCATTTTTTAAGTAA
AAAATCTTGCCTAATTTCCTGAATAGCTTTATCTAACTTTTCCCATGCTTCTTGAGCATTTTTGG
GAGGATTTGACTTCACTCTTTTTTTTGCCTCCTCCATAATTTTTTCACTTTAATAGACTCTTCTTC
TGTTGGGGGAGGATAAACTCCAGATTGAGGAATAGAGTCTGGATAAATTGGTGTTTTCATAATT
TTATTGACTCCTAAGTTAAATTCAATCAATTTTAAATTACAATTTTGCTTAATTGCTGATTTATG
AGAGTTTCTAACAAATATTTTACATTTTAGTTATGTCGATTTTTTTTATTATCCATCAATTTTGG
AGGGCAATGCCCACCCTACAAATTATTGTTTAGATTGAGTTAATTCGTTAGCAGAAGCAACTAAA
TATTCAGCTACGTTAAATAATTCCGATGAGGTATTATTTAGTTTACTGCAAATTTCTGCCATTGC
TGAATCTGCTTTCTTGAAATTATTACTATAAGTCTCACTGGCTTTGCTTAAATAGTCACTGATTT
GTTGATTGGTTTTGGTTGATAATAAATCAAATTCTTGGGTTATTTCTCCTAAAATATTTTTAAAT
TCATTGGTGATAGTTTGAGAATTTTCTCTGAATTGATTCGCCATTTGTTGATAGGCAGTTTCAAC
TTTTTCCGCTTCATTGCCTAGAGTTTCTGCTAATTCTTGAAGTTGGGCATATCTTTCGCTGGAAT
TCATCCCTGTTGCGTTAACTAAGTTACTCACAATGCGAGTGGTTTCTTTGATATTTGTTAAACTT
TGATTGACTTCTTCTGCTAACTGTTTTCTCTTTTCACTTTCTTCTTTAAAGGTAGTTTGTAAGTT
GTTAACAACTTCTGCTAATCCTTCTTTTTGTCTGCCTAAAGTCTCATCTAAAAGTTGATTTTGTT
TATCAAAGAAATCTGTTAATGAAACTTGATATTCTAGGCGAAATCTCTCTAATTCAATTTGCACT
GTTTCACGGGTATTTTGCAACATTGAGTCAATATTTTGCAGGGTAGAAAGTAAATTTTCTTTCGC
CTGATTCATCAGTTGTGACGCTTTTTCCCCTACTTTGACAATGGTTTCTGTTTGCTGACTAAATGC
TTCATTGGTTTTCTGTAAAATGTCTTCGGTGGATTGTTTCACCCCTTCTAACATTTCTTTTTGTTG
GGTTGCCTGAGTTGTTAGTGCTTCCTGTAAGTCTTCTCTGATACCTCTAAAGGTATTAGCGGCTT
GATTGGCACTATTTTCAAAGGCTTGTCTTTGTGCCTCCATTCCTGCAATACTTTCCGCTACGGCAT
TATTTATTTCCGCAGCAACTTGCTGTAAAACCCCTTTGGTATCGTCACCAAATTGATTTAATATC
TGTTGCAGACTTTGGGCAAATTGCTGTAATTTAATGAGAGTATCTTGTTGAAATTCTTGTATGGT

TTGCACTGCACCTGCTAAACTTTCAGCAATACCCCCTAATTCGTCTTTTAATTCTTTGACAGCTAG
GGAGGCTTCTTCTGTAAGTTTAGCACTTTGATCGAGTCTTTGTACTACTGGTTCGATTAATTCAT
TACGTAATTCTCTGACTAATAATTGTACTGTGGATTGTTGCTCTATTTGTATCTGTTTTAAACTT
TTTATTTCTTCTTGAATGGGAGTAATTAATGGTTGCAAAACAGTAGCAAAAGATTCTGGTGTTAA
GGTTTGTAAATGATTAGTTTGGGCTTGAAGTTGTTGCACTAAAATACTATCATCAGATGCGATCG
CACTCTTTATCGCTAAGGCAATATTTTCAGGGGTTAAGTTAGCTAAACCCGTTAAATTATTCAAA
CTATGGGAAATTTCTTGAAAACCAGAAGTATCCAGACGAGATAGTAGTTTTTGCGATGATTCTAC
ATAGGCAATAGTAGATAATTCTTTTCTGACTTTATTCCTTTTATCTTGTCTAAATTTTCCACCTA
AAGCTAAAGTTATCATCATCAAACTAGCACTACCTAAACCAGCTAAAGAAGTAGAAAAAGCGGTT
TTCATTCCTTCTAATAGTTGATTGGAAGAAGCTAATAATGTTTGAGTATCGCCGATATTAGTGCC
GACTTTTTGCAAACCTAAAAAGATACCTAAAAAAGTGCCTAAAATACCTAATGCTGTTAACAAAG
TAGGTGCATAATAAACTGGACTTCGGGGAATTGGTTGAGTTAAAATACTGGGATAGGATGAAAA
AATATAATAATTATTATGATCTTTTTTAGGAATAATTTTATTAGGAGATATGTTTCCTTCTAAAT
GTTTTGTTAACCAATAGAATAAATCTTTATCTTCTTTGTGGCATTCTGCCTTAATAATATCTCCT
TTATTTTGCTTATCCTTCAAAAATTGAATCCCTTGATTTGTTAAGGAAAATTCTTTTTCCCTATA
GTAAAAAAAAATACCGAATACCTCGATAACAATAAGAATAATAGCGATGATGGAAATAAAAATT
ACTCCACCCCAATTTAAAAAGAAATCCATCATTATTGAACATACCTCTTAATCCTTGATTGTAAG
GAATAATATGTCAATTATTTTTGTGTGTCAAGTCTTTTGAATTACATTAGGTTAAATATTTAATG
ATGATTTACTGAAATGGCTGATTTCTTCTTCTGACAACATTTTCTTTGCTACTCTGATAGGAATA
TGGGCAACATCGGAGTAATTATTAATAGGAGAAACTAACATTAAGCCATATATACTTTCCTCTGA
TTCTATGTCTTTATCTTCTTTTAGATAAACATAATTAGGAGCAAAATTTTTTAACAGCTTAATTT
GTAATCTACTTTTTAAGTCTCTTAATCTTATGGCTAGAAAATGACTATTATCTTCCATTTCTTGG
CTTAATTTCCAAGCATGATTAATACAATGGATTTGCTTAATTAAATCCGTAAGAGAAGGTTTAAG
AAGGTCTTTATTCATTGATATTAATTTTATATGAGTTTTATTTTTCCGATACTTTAGCATAATTT
ATTAGGTTAATTATTCGCATAACTCTGCATTCCCCAAAAAATTAAATTGGTGGGCAATAATACCC
ACCTTACAATTTTTGAAGCTATCTCAACCTTAATTGAAGTCACTTTAGTTAATTAGAGTTAAGCT
AAATCAAAACGGTCAGCGTTCATTACTTTTGTCCAAGCAGTTACGAAGTCATGGACGAACTTCTC
TTTATTATCGTCTTGGGCATAAACTTCGGCATAGGAACGAAGAATTGAATTTGAGCCGAATACTA
GATCAACTCTTGTTGCTTTCCATTTAATTTGATCACTGGAGCGATCGCAGATTTCATACAAATTA
TTACCTACAGGTTTCCATTGGTAATTCATATCTGTGAGATTCACAAAGAAATCGTTGGTTAATAC
GCCTTCACGATCAGTTAAAACGCCGTGTTTAGTACCGCCATAATTAGTACCCAAAACACGCATTC
CACCAACCAAAACCGTCATCTCAGGGGCTGTTAATCCCATCAGTTGGGTGCGATCGAGCATTAAT
TCTTCTGGACTAACGGCATAATCTTTTTTAAGCCAGTTACGGTAGCCATCGTGTATGGGTTCTAG
GGGTACAAAGGATTCTACATCTGTCATTTCGTCTGTAGCATCACCACGACCGGGAGAAAAAGGAA
CGGTAATATCAAAACCTCCTGCTTTAGCGGCTTTCTCAATACCAACATTCCCCGCCAATACAATCA
CATCAGCAATACTTGCACCACTATCTTGAGCGATACTTTCGAGTATGGTCAATACTTTCGCTAAA
CGTACAGGCTCATTACCTTCCCAATCTTTCTGAGGACTTAGGCGAATACGTGCCCCGTTAGCCCCT
CCACGCTTGTCAGAACCCCGAAAAGTTCTGGCACTGTCCCAAGCGGTACATACCATTTCACGGATG
GTTAAACCACTCTCTGCAATCTTTTTTTTCACAGATTGAATATCATAGTTGCTATTTCCCGTAGG
AATAGGATCTTGCCAAATTAGGTCTTCTTGGGGTGCATCAGGACCAATGTAACGGGTTTTAGGTC
CCATATCTCGGTGAGTCAGTTTAAACCATGCACGAGCAAAGACATCGGCAAAATATTCCGGGTCA
CGATAAAATCGCTCGGAGATTTTCCGATATTCAGGATCCATTTTCATCGCCATATCAGCATCAGT
CATAACCAAATTACGGCGAATGGAGGGGTCTTCCACATCCACAGGCTTATCTTCTTCTTTGACGT

TGATGGGTTCCCACTGCCAAGCACCTGCTGGACTTTTTTTCAGTTCCCAGTCGTAATTAAGTAAT
AAGCGGAAATAGCCGTTGTCCCATTGAGTAGGATAAGTTGTCCATGCTCCTTCAATACCACTGGT
TACGGTATTGCGACCGATACCACGATGAGTTTTATTAATCCAACCTAAGCCCTGATCTTCTATGT
TAGAGGCTTCCGGTTCAGCACCCAGTAACCCTGCGTCACCGTTGCCGTGACATTTCCCCACCGTAT
GACCTCCCGCAGTAAGAGCCACTGTTTCCTCATCATTCATCGCCATACGAGCAAAGGTAACACGCA
CATCGTGGGCAGTTTTAAGGGGGTCTGGGTTGCCGTCCACTCCTTCAGGATTAACATAAATTAGC
CCCATCATCACGGCGGCTAAAGGATTTTCTAATTCTCGTTCACCAGAATAGCGACTTTGAGGATT
ATCGCTAGGCGCAAGCCATTCTTTTTCTGAACCCCAATAAATGTCTTTTTCTGGATGCCAAATGT
CTTCTCGTCCAAAGGCAAAACCAAAGGTTTTAAAGCCCATAGATTCATATGCGATCGTACCTGCA
TAGGTAATCAAATCAGCCCAACTGAGTTTGTTGCCATATTTTTTCTTAATAGGCCAAAGTAAACG
ACGAGCTTTATCTAAGTTGACATTATCAGGCCAAGAATTGAGAGGAGCAAAACGTTGATTACCAG
TACCAGCACCACCTCGACCATCAGCAATACGATAAGTTCCGGCGGCGTGCCAACTTAAACGAATC
ATCAAGCCACCATAATGCCCCCAATCGGCAGGCCACCAATCCTGACTATCTGTCATTAGTCTGTGT
AAATCTTCTTTGAGGGCGGCTACGTCAAGTTTTTTTACTTCTTCCCGATAGTTAAAATCCGCTCCC
AGGGGGTTGGTTTTGCTATCGTGCTGACTCAAAATATCGAGGTTGAGTGATTTCGGCCACCATTC
CATATTCGACATACTGTTTGTTGTTACACCACCGTGCATCACGGGGCATTTTCCACCAGTTGTTGT
CATATTATTTTTCTCTCTTGTGAGTTGATATAGGTTAATCTTAGCTAATGATTCTGAATAGATGC
GTGCTGCGTAGCAGATCCCTTCGGGATCGAACTTGGATGGTGGCAATTTTGGTGTTGCTGGTTTT
AGATATGATTTCTTGTTTAGGTAGGGAACAGGGAATCGGCAATGGGCAAAGGTGAATAGTGAAT
AGTTGATAGTTGATAATTAAGAATTAAGAATTAAAAACTCCGAACACTCGTACGGGCGAATGGCC
ATTCGTCCCTACTTCCCCCTCTCACCCCCTCACCTCATCACCTCATCTCCCTCTCCTCCGAACTCTG
AACCCTTATCCGATATTTTTAAACCGAACTGAGGTTACTTAAGCATATATCGAACTTAAGTAATA
TTTAAGATAAAATTAAAAAAAACTATTATTCACGACTACATCACTCAATGGCATCAATAATATCC
GTTAATCTACCCGAAAACTCTTACTCAATCCACATCAATGCTGGGGGATTAAATAATATTGGAGA
AAAAGCAAGTCATTTAGCTATTGGTAAAAAAATTCTCGTTGTTTCTAACCCAGAAATTTTTGACT
GCTATGGACAGATAACGATCGATTCCCTACAAAATCAAGGTTTTGAGGTAAATTATCATTTAATA
CCCGCAGGGGAAAATTACAAAAACCTAGAATCTATTGCCCAAATTTATGATACTGCTCTTAAATG
CCGATTAGAACGTAATTCCACTTTGATGGCATTAGGCGGAGGAGTGGTAGGAGATATGACAGGTT
TTGCAGCCGCCACTTGGTTAAGAGGAATTAATTTTGTCCAAATTCCCACTTCTCTTTTAGCGATG
GTAGATGCTTCTGTGGGGGGTAAAACAGGGGTTAATCACCCTCAAGGAAAAAATTTGATTGGAGC
TTTTTATCAGCCGAAATTAGTTTTAATCGATCCTTCTGTTTTGAAAACATTACCTGAAAGGGAGT
TTCGGGCAGGTATGGCAGAGGTGATTAAATATGGAGTGATTTGGGATAAAAACCTTTTTGAAGCC
CTAGAATCGGCTCAGAGTATTGATAGCTTAAATAATTTTGATGAAGCTCTCCTAAATGATATTTT
AACTCGTTCCTGTCAAGCTAAAGCAGATGTAGTCGGTCAAGATGAGAAAGAGTCTGGTATTAGAG
CGATTCTTAATTATGGTCATACTATTGGTCATGCCATCGAAAGTTTAACTAATTATGAGACTTTT
GTTCATGGAGAAGCGGTGGCAATTGGGATGGTAGCGGCGAGTAGAGTTGCTGTGAAAATGGGAT
TATGGACAGAAAATGAAGCCCATCGGCAAGATGAATTGATTGCTAAAACTGGTTTACCTACAACT
ATCCCCCATTATCTCAAGGTAGAAGAAATTATCGATCATCTACAAATGGATAAAAAAGTTAAAGA
CGGTAAAGTAAGGTTTATTCTTCCTGAAGCTATTGGAAAAGTACAAATATGCGATCGCATCTCCT
CTGAAATTTTATCGGAAACTCTCTCTGTTTTGATTGATTAAATTGGGCTGAATATTAAATATTAA
GAGATTAACCTGAGTTTTGGGCAAGCTGAAAGCATTATTTTCTCGTAGTCAGAAAACCTTATAGC
TTCTCAGAAATAACGATAAAATTGCCTTAATCTGAACTGAGGTTAGATATATTCACCCCTACCAC
CCCAAAACCCTAAGCCCCTACTTCCCCCTCTCACCCTCTCCCCTCATCACCGTAAGGCTTGAATAT

GTTAAACACCTGCAACCTACAACCTGACACCTAACCTTATCGGATATTCTTAAACCGAACTGAGG
TTAAGAGATTAAATAGATAAGGTGGGCATTGACCCACCACTTTCTAAATTATGATTGTATTAATT
TTTTGGTAAAAGAAAACGCCAACTATCAGGTTCAAAAGGCTTACCCTTCAGCATTCTATTCCAAT
ATAACCAAGGCAAAATATATTTTTTCACTAACCACATACTAGCTCTCTCTTGAGTGGGATCAAGA
GGAAAACTAGGAGTTGGTTGCTTACTATAGTCAAACTCTGCCATGATGGTTTTTCCATAACCCGT
AATTAAAGGACAACAAGCATAACCGCCATATTTTTCTTGGGGGATTTTTTGACTCATTAAAGCTA
AAATATTTGCCACAACTACTGGAGCTTCTTTCCTAATAGCCGCCGCAGTTTTTGAAGTGGGTAAA
GAAGAAGAATCACCTAAACCAAACACATTTTTATACTTAGTATGCTGACAGGTAAATTGATCAAC
ATCCACCCATCCTTGCTCATTCGCTAAAGGACTTTGTTTAATGAAATCATAAGTAGTCATCGGTG
GAGACACATGAATCATGTCATATTTAATCGTTACTGTTTCCCCTTCTCCCGTGGCAAAAATTGCT
TCTTTTGTATCTCCTTTTATCTCTACTAAATTATGTCCGTATTTAACATTAATGTTTTTGCGATC
GACAATTTTTTCCAATGGGGTACAATAAGCAGGAACACCAAACATTTTACCCGTTGCATTACCAT
AAACAATATTGGTTTTTTCCCTAACTCCTTGTCTAGTAAAGGCTTCTTCCGCTAAATACATAATT
TTCTGTGGAGCTCCCGGACATTTAATTGCCCCGGCAGGAAAGGTAAAAATAGCTGTACCCCCTTG
AAAATTCTTGATTGTTTCCCATGTATAAGGGGCATATTCCTTCATATAATTGGTTGTCACCCCAT
TTTTGCCCAAATTTTCTCTTAAGCCCTTAATTTTGTCCCAATCAATGGTTATACCCGGACAAACA
ACTAAATATTCATAGGAAAAAATATTCCCCGATTTAGTCGTAACTTGATTATTTTCAGGATCAAT
ATTTTCTGCAAAATCTTTAATCCATGTTACCCCTTGAGGAATCAAATTATCTTCATGTTTAACCG
TGTCTTCTAATTGAAATTCTCCTCCTCCTACTAATGTCCAAGCAGGTTGATAATAATGATTTTCA
GAAGGTTCAATAATAGCAATATTTAGTTTAGAATTTTTATTTTTTAACTGGGAAGCAACGGTAA
TTCCAGCCGCTCCCCCTCCAATAATTAATATCTGAAATTTTGTGTTTTTACTCATATTGATTATT
ATAATTTTTTAGATACTTAAAAATAAGTTTAGTTAAGTTTTACAAATTTTTCTAGTTTTTTAATA
TTTCTTTATTATGTCTATAAATTACTATTTGACAATATAATTAATTAATCTTTTTTTTTAGAGTT
TATTGCTTTATTTTACTGATTTTTTTGTTAAAAGTTTATTTTTATTTTGTTTTTCCTCTCAATTT
CTAGGATTCATTCATATTCTTATAAGTAGCTACCGCAGAAGGAGAAATACGATTAAGATAACGGA
AAATCCAATATTTTAAAACTGTATCGAGAATAACGGGAAAAGTAGCAATAAAAAGAAAATTAAA
ATCTCTATTTTCAGGTAAGCCAAAATGACGACTAATACTTTCTAAAATTACTTCCCAACCGTGGG
GGGAGTGAAATCCCACAAACATATCTGTAAAAAGAATAATCAGAAAAGCCTTTGCTGAGTCACTC
AAACCATAGATAGTTTCATCAATGAAAGACTTTAAAATTTGTAATTCTCTTTTACTAAAGTAGAT
AACAATACCAAAGGCAATTAAAGAAAATACATCAGCAAAAATATTTTTGATGGCGTTAGCACTTT
CCCGACGATATTCTTCTCCTAATTCTTGGGCTTTGGCCTTTATTTCATGTTCTTTTTCTTCAATGG
ATAACTCTGGAATTATACCTAACATCACTTTTAAATTTAGGTTTTCCTCAAAGTTTTTCAATTTCT
ATTAAGGCTTCTTCTTGTAAATCTTGATTAATAAAAACTATTTCTTCATGGCGACTAAAATAAGG
ATCGATAAATACTTTTCCCAATGCTATTTTGGCTACATTATGCACTAATAAAGGCACTATTATTA
ATAGTAATAAAAACTTAATAGATATGGCAGTTTTATATTTCGATTTTCTAAACTTATTAAGAAC
ATCCTGTTCACTTTCAGATGATTTTGGGTCTATTTCTTGCTTGATTCTATCAATAGTTCTTAAAA
ATGAGCGGGGCAAAACGCTAGTTTTATCTGAAACGGTTTCCAAACTAGATTCAAAGTTATTATCG
TTTTTATCCTGTTTTTGAATATTTTTTTGCTTTGCTAAGGAAGGGTTATTATTTATATTTTGGTT
TGGAGAGTTAGAATCTATTTTTACAGATGATGTTTCATAATAGTCAGCATTATATTTATCTAGTA
TTTGATCAATAAAATTTAATTTTTCTAGTATGATCTCTTGTTCATATTCAAAATTATAATCTCCA
GAGTGGTTATAGTTATAGTGACTATTTTTTGATTTAGATGATAAAAAAGTATGTATCGATTTAT
TTGTTTTAAATTGATTAATTTTTACTTTAATAACTCTCAAATTTGATTTTAATTCGCTCTGAAAA
TAAGCCCAAACACTTTCCCCATAGTCACAATTTTGAAATGATATTTTCTGACCTTTAAAATGCTC

GTCTTCTATTTTTTTGATCGTCAATGCTCTTTGATATGCTTCTTCTAGGGCATTTTCTAAACTAT
TACTAATCCAGCGTTTGGTAGAACGAAAAAGACGATTAAACATGGAATATAAGCCAAACTGATA
AGATTTAATTTTATATATTATCCTAGATCATTTCAGTTCGCTCTAAAATTACAGATTAAGGAAAG
AAAAAAGAAAAGTGATAGAACCTCTTGCAAAACTAAAATTAGAATTAATTTCTAAAAGGAAAAA
TCTGCTTTCCTTACTCTTTTCATCTGAATTCTTGACTCTCGCTTTGAATAGCTTAGGCTAAAATTT
TCTGTGAAAATAAGCAAAGACTTTTGATCATTTCTAACAAGGGGTTTAAACCCCCTGCTAATTTC
TAATGGAGTAATTATCCATTGACGCTCTATCAACTAGGCGTTAACCGCTAATTCAACTACTTTTT
GGAATGCTTCAGCGTCTTGAATAGCTAATTGGGCTAAGATTTTGCGATTTAAGCTGATATTCGCT
TTATCCAATTTATTGATTAGTTGACTATAGCTAATGCCATGTACTCTGGCAGCCGCATTAATTCT
GGTAATCCAGAGACGACGAAAATCTCTTTTTTTCTTGCGGCGATCGCGGTAAGCATTACGCAAAG
CCTTCATCACCTGTTGATTAGCCGTGCGGAAGAGCTTAGAATGAGAGCCACGAAAACCTTTAGCT
AACTTTAATATTTTCTTACGTCTTTTACGAGCGACGTTACCTCTTTTTACTCTAGTCATTTTTTTA
TCTTTACTATGGTTTTAATAATTTATTGACAAAATATAAAACAATACTTACCTTAATCTAATTCA
GCCTTATCTTTTAGAATAGGGCAACATTAAACGAACTTCTTTAACATCGTTCTCATGTACTAAGG
TAAGATTACTTAAACGACGACGTTTTTGTTCGGCAGTTTTATGTTCTAGTAAGTGATTTTTGTTC
GCTTTACGACGAAAGATTTTTTTGCCACTGCCAGTAACTCGGAAACGTTTAGCGGCTGATTTCTT
AGTTTTTACTTTAGGCATAACATTTTCTATTTTGACACAATTTACTATTATACGATTAAATTTAC
CTTACTAACTATAAAATTCTGAAAATAATTAGGGATTAAAGAAAAAGTTTTTTTAGGTTGCTTTC
TGTTATTTCTATTAGAATTTACCTTGAAAACTATAAATCTCTGATTTAAAAAGGATGTTCCGAAA
AATTTTGACAACTTTTCTCATGGCACTGATATTGTCAGTGGGAATTAAACCAGCTTTTGCTGAGA
CGGTAGTAGAGAGAATTTCCAGAACGGGTAATTTGGTGGTTGGTACTCCTTTTGGTTTAGTACCT
TATGCTTATAACAATCCTAACGAAGAATTAGACGGTTATTCTATTGATGTTGTTAAACAAATTCA
AAAACAACTAGAATTACGATTTGGTAAACAAATCCAACTTAGTTTTGTTGAAGTTAACAATATTT
CTGATGCCGTTTCCAAAATTTTAACAGGGGAAATTGATATTGCTTGTAATACCGTCTTTACTTGG
CAACGAGATCAATTTGTGGACTATACCCTAGGTTATACTCAATCCGATATTCGTTTATTAATTCC
TAAAGGCAAAACAATAGATAATTTCTCTGGGAAAAAAATCGGTATTCCGCCTTTAACTTTTGTGT
ATTCCGCCATTTCTCTGAATCATCCTGATGCAACTTTAGTGGAAGTTAAAACTACAGAGGAAGGT
TTACAAGCCTTAAAAGAGGGCAAGATTGATGCTTTAGCAGGGGACGCTATTATTTTGGACGGAGA
ATCTCAACAGGCACAAATGACGGCAGATTTTGAACTATTCCCGAAAGGTGTGCAAGGTTATGGTA
ACTATGGGGTTTCTTGTATCGTTCCTGAAAATAATTCTACTTTTCTTAATGTCGCTAATTATGCG
ATCGCACGTATGATGGAAGGTTACTTAGTGGGAAACGAAGAAATGACGACAATGGTTAACCGAT
GGCTAGGAGAAGATGGAGTTGTTACCATTGTGACAAAGGAACATTTAAAAACTTTCTTTCGTAAT
ACAATCAATAACTACGAACAAATTCCCTTTGATAATTAATCACACTATTCAAGATAAAAGTTAAA
TATTAACAAGGTTAACAGAATGATAAAAACAACGAATACAACTTGGATGGGTTTTTTATTAGCA
ATATCTACCGTTGCTTATTGTGGTTCATCCCATGCCACAACCGCAGACTCCATCAAAGAATCTTTA
AACCCCTTAGAAAATCGCATTAGTCGCATAGCAGAGGTGTTGAAAGAAAGAGAAAAACAATTAC
AACAAGAGGGACAATCTGACCACAACTTTACTTCTGACATCGATGATAACCTCATAGCAGGAGGT
TGGGGAAATGGAAGAGGAGGCAGTTGGGTTGACACCAATCGGGGCAGTTTTGTTAACAATCGCCG
AGGATGGGATAACTGGAGAGGCTGGGATGACTGGGGAAGATGGACAAATCGTTCTCCTTGGCGTA
ATGGTGGAGGTTTTCTCAATCGCCGTTAGTCAATGCTTAAACACTCTTTTAACAATTAACAATTA
GTAGTTAAAAAGGCTCTATTACTTTTAGTATGTGAATGAATTATTGGTTAGGGTAGGTAAGAGG
TAAACCCCTCTGTGTCTCCCCTTTTAAGGGGGGAAGGGCAAAAGGCATGAACTGTTAAAAAATAT
AAATTTTATCAATAATTAAAAACTTTTAAAAACTTAATAAATTCATTAAATATGTTATGTTAAC

TAGCCAAGAAAAAACCAATTATCTCCGTTCAAAAAATCAAGAATTTGGCCCAATTTCTTTAGTGG
TTATTCAGCCTAATTCATTTTGCAATTTAGATTGTGATTATTGTTATTTGCCCGATCGCAACTTA
AAAAATAAACTCTCACTAGATTTAATAGAACCTATTTTTAAAAGTATTTTTACTAGCCCTTTTTG
TCGAGATAGTTTTACTATATGTTGGCACGCAGGAGAACCATTAACAATGCCAATTTCTTTTTATA
AATCAGCTTTTGAAATAATTGAAAATACCAGTAAAAAATATAATCAAACTGAATTAAGTTTTAA
TTATTCTTATCAAACCAATGGTACTTTAATTACACAAGCATGGTGTGATTTTTGGCAACAATATC
CTGTTAGTATTGGGGTTAGTATTGATGGACCCGCTTTTTTACATGATGCTCACCGTAAGAATAGA
AAAGGAGGTAATTCCCATGAATTAACCATGAGAGGAATTAAATATTTACAAAAAAATAATATCC
CTTATAATACTATTTCCGTTATTACAAAGGACTCATTAGATTATCCTGATGAGATGTTTAATTTT
TTTGCGGAAAATGAAATTTATGACCTTGCTTTTAATATGGAAGAAACCGAGGGAGTTAATGATTC
TAGTTCATTAAATGGTAGTGAAGTTGAAAAAAAAATATCGTTATTTCATCGAAAGATTTTGGCAG
TTAATAACGGAAAGTAAATTACCCTTTTTTATCAGAGAATTTGAAACGTTAATTAGTTTAATTTA
TCATCAAAAAAGATTAAACCATACCGAAATGAATCATCCTTTTTCTATTATTAATGTAGATTATC
AAGGTAATTTTTCCACTTTTGATCCCGAATTATTATCAGTCAAAACTCCTGAATATGGTGATTTT
ATTTTTGGCAATATTTTAACTGATTCTTTAGAGTCAATTTGTGAAAAGGAAAAATTTAAGCGCAT
TTATCAGGAAATGAAAACAGGGGTTAAATTATGTGCTGAAAATTGTCATTATTTCGGTTTATGT
GGTGGCGGTGCAGGTAGTAATAAATATTGGGAAAATGGTACTTTTGCCTCTAGCGAAACTCAAAC
TTGTCGTTATCGTATTCAAATTTTAACTGATGTGGTTTTAGATGCGATCGAGCAATCTTTGGGAG
TCACTGCAAATAATCAATAATTCTTGAGGGTATTTTTGCGTGCTGCGAAGCAGATCCCTTCGGGA
TCGCGCAGTGCCATTTTGTGATCGAGAAAAAATCAAATCCTAATTCCTAATTTCTCGTTAAACAT
TATTCTTGAAATGTTTTATCCATTGCCCGTTTTCAAATCTTCGATTAAATCATGGAGATGATCAA
CATTAGCGTGATACACTTCTCCACAGAATTGGCAGGTAGCTTCTGCCCCTCCATCTTTTTCAATCA
TATCCGCTAATTCGTCAGTACCCAACATTTTTAAAGCACCTAAAACGCGATCGAAAGAGCATCCA
CAATCAAATCTTACCATCTGAATTTCAGGGAAAATATTTAAACCAAAATCCCCTAACAAATCCGT
AAAAATATCAGGTAAAGTTTTCCCTTGACGTAATAAAGGAGTAAAACCAGTTAACTGACTAACAC
GGGATTCCAAAGTTTCCACTAACTGTGGATCTTCTGCCGCCTTTGGTAATACCTGCAACAAAATA
CCCCCAGAAGCAGTAACTCCTTGTTTTCCCACAAATACTCCCACCAATAAAGCTGAAGGAGTTTG
TTCTGATGTACCTAAATAATGAGCAATATCTTCTCCTACCTCCCCTGAAATTAATTCAACAGTAC
TAGAGTAAGGATAACCATAACCCATATCCCTAACAACATAAAGATAACCTTCCTTGCCCACAGCA
CCGCCTACATCTAATTTTCCCTTATCATTGGGGGGTAATTCCACAAAAGGATTAGAAACATAGCC
TCTGACTGTACCATCTAATCTTGCATCAACTAATAAACCACCTAAAGGGCCATTTCCCTTAATTT
TAATATTTACCCTAGACCCTTCTTTTTTCATATTAGAAACTAAAAGCAAACCCGATGACATCGCT
CTTCCCAATGCCGCAGTAGCTACATAAGATAATTTGTGTCTATCCCTTGCCTCTTGTACTGCTTTA
GTGGTGATAACTCCCACTGCACGAATACCCCCATCGGCCGCTGTTGCTCTAATTAAATGATCTGCC
ATAATTTTCTTTACATTTCTCAATAATACCTTGATCTATCTTAAGATTAATTTGTCTAAAAGGCT
CATAGGGAGAAAGTAAATAACTTTAGCTCGGTTTAATAGGTAGGTGTCAGGTTTGACGGTTGCAG
GTGGTAAGGTGTTTAGGGGATGAGGTGATGAGATGATGAGGGGAAAGGGGGAAGTAGGGGCGAA
TGGCCATTCGCCCGTATAGGAGTTGGGGGTTTTTAATTCTTAACTGAGGTTTAAGTAGAGTTAAC
CATCTTATCTCAAACAGAAGTTCATTAATCATGAAGATATGAGATAATTAACTATTGTTAACAAA
ACTATTAAGCAATATATTATTTTTCGTGGATAAAAATCGCACTGTTTCCGACACTAAAAGAAGTT
TTTATCAACATCACCAACGCCCCATTAACTCTATCTATCGTCGTGTGGTAGAAGAATTAATGGTA
GAAATGCACCTCTTAGCAGTTAATGTTGATTTTAATCCCGATCCTATTTATTATCTGGGAGTGTA
TCAAAGTTTTCAACAATTCATGCAGGGTTATAAACCTGAGTCAGATAAAGAATCAATTTTCAATG

CTTTATGTCAATCCATTGAAAATAACCCTCAAGAATATATCAGCAAATCACAAACTTTACTAAAT
TTTGTCGAGGGGAAATCTGCTCAAGAAATTCTTGATTGGTTATTAAACCCTTCTGGAGAAGGAGA
TTTAGAAGTTGTTGCTTCTCATTGGCGCAGTAATTTAGAAAATCCTCGCTTTAAATATAGTCGTT
TATTTGCCATTGGTTTCTATACTCTAATAGAAAAAGGTGACGGTGAATTTATTAAAGATGAAAG
TAAATTTACTGATTTTATTCAGCCTTTAATTGACAAATTACAACTGCCAGTAGAAAAACTGAAAA
AAGATTTAGATTTATATCGTAGTAACTTAGAAAAAATGAATCAAATGCTAAGTGTAATGGCAGA
TGTTTTGGAAGCCGAAAGAAAGAAAAAACAAGCAGAGAGTAAATAGGAATATTCTAATTAGGAA
TTGGGAATTAGCAAATATTTCGTTAATATTAAATAACTTAAATACTCAGTTATAAGAAACTAAA
GTCAACTGAGAACAAGTTTTTAAAACTAAGTAAAAAGGTAATCATACTATTATTTCTATCTGTTA
TTTTTACTATAAAAAATAAGTTAATATCATATACTGACTGGTTATGTTAGACATCAAAAAAATT
AGAGAAAATCCCCAAGAAGTTTTAGATAAATTACATAAGCGTAGTCCCAATTATGAATTGCAATC
TATTCTTGATTTAGATAGTAAGCAAAGAGACATTCAAAATATTAGGACTGAATTACAAGCCCGTA
GTAATGAAATCGGTAAATTAATTGGACAGAAAATGAGGGAAGGGGCAAACCCTCAAAGTGAAGA
AATAATCACTCTCAAACAAGAGGGAAATGAAGTTAAAGCTAAATTAGCGGATTTAGAACCCCAA
GAAAGAGAGTTAAAGGCTAATATTGAGGCTTTATTGTTAGAATTACCTAATTTGCCCAGTGATAG
CACTCCTGTGGGCAAAGATGAAAGCCAAAATGTCGAGGTGAGAAGATGGGGAGATGAATTTATCC
CTGAAAATAAAGATATTATCCCTCATGGAGATATTGGGGAAAAATGGGGCATTATCAACTCAGA
AAGGGCAGTAAAAGTCGCTCAAAGTCGTTTTATTGCCTTAATTGGTGCTGGTGCGGCATTAGAAA
GGGCTTTAATTAATTTTATGTTGGATCAACAAATTGAAGCGGGTTATGTGGAAATTATGCCCCCT
GTGTTGGTGAATAGTGAAACTTTACAAGGTACAGGGCAGTTACCGAAGTTTGCGGAAGAAAGTT
TTAAGTGTGATAAAGATGATTTATGGTTAACTCCCACCGCAGAAGTGCCTGTAACTAATTTCTAT
CGTCAGGAAATTCTTGAGGCAGAAATGTTACCTATTTATCATTGTGCTTTTACTCCTTGTTTTAG
GCGAGAGGCTGGTAGTTATGGTCGAGATATGCGAGGATTAATCCGTTTACATCAATTTCATAAGG
TAGAATTGGTGAAAATTGTTCATCCTGATGTTAGTGTGGAAGAGCATGAAAAAATGGTCAAGGA
TGCGGAAAGAATTTTACAGGCTTTGAAACTGCCCTATCGAGTTTTAGAGTTATGTACAGGAGATA
TTGGTTTTAGTGCGGCGAAGTGTTATGACTTGGAGGTGTGGATGCCTTCAGCTAATTCTTATAGG
GAAATTTCTAGTTGCTCTAATTGTTTTGATTTTCAGGCGAGAAGGGCGGATATTCGCTTCAAGGA
AAAAGGTAAAAAGGGAACAGAGTATGTACATACTCTCAATGGTTCTGGTTTGGCAGTGGGGCGCA
CTATGGCGGCAATTTTAGAAAACTATCAACTGCCTGATGGTAATGTCCGCATTCCAGAGGTTTTG
CAACCTTATTTAAAGAGGGATTTGTTAATTGTTGAATAATTTGTCTTCAATTTGATAGAAATAAT
TATTTTTTAAGTGGTATGAAAAAATTAGTCGTTGCTACCAGTAATGCAGGCAAACTAAAGGAGA
TGGAAAAACATTTATCAGATTTTGGGGTGGAGTTGGCTCTCAAGCCCCCAGAATTTGATGTGGAA
GAAACGGGGGTTACTTTTGGAGAAAATGCTCGTTTGAAGGCTTCACAGACTGCTAAAACCCTCGG
AGAGTGGTCGATCGCAGATGATTCAGGACTAGAAGTAATGGCGTTAAATGGACAACCGGGTATTT
ATTCGGCTCGTTATGGTAAAACCGATCAAGAGCGTATTGCAAGGCTATTGAGAGAGTTAGCACCC
TTTGAAGATAGATCCGCTCAATTTGTATGTGCGATCGCAGTTTCTAATCCTCAAGGGGAAATCGT
CATTGAGACAGAGGGGGTTTGTTTGGGGCAAATATTGACAGAAATTCAAGGGGAAGGGGGTTTT
GGTTATGATCCCGTGTTTTACGTGCCTGAAGTGGGTCAAACCTTTGCTCAAATGCCTTCTGAGTT
GAAACAAAAATTAAGCCATCGTGGCAAAGCATTTGCTAATTTACAACCATTATTAAAATCTTTGA
TTGAATCAGAGGGAAAATAAAATGCAACCAGAAGTTATTGGCGTTGATTTAGGTGGTACTGCGA
TAAAATTAGGTCGCTTCACTCAAGATGGTAGTTGTTTGGAATCCGTTACCATTCCTACTCCCCAA
CCTGCCACTCCCAAAGCGGTAATAGATGCGATCGCCTCTATGGTTAAACAATTAAATAGCCAAAA
CCAAGCCATAGCCATAGGTGTAGGAACTCCCGGTCCTACTGATGCTCAAGGCAGAATCGCCCTTG

TTGCCATTAATTTAACAGGATGGGAAAATGTACCCCTAGCCGATGAATTAGAAGCAAAAACAGGC
TTAAAAACCACCATTGCCAATGATGCCAACTGTGCCGGATTAGGGGAAGCGTGGTTAGGTGCAGG
AAAAAATTATCATAACTCCATTCTCTTGACATTAGGTACAGGAGTTGGAGGAGCAATTATTATCA
ACGATAAACTATTTACAGGGCATTTAGGAGCGGCGGGAGAGCTAGGATTAATCACCCTTAACCCT
GATGGCCCCCCCTGCAATAGTGGTAATCGAGGTTCATTAGAGCAGTATGTTTCAGCTACAGCAAT
CATTCGTGAAACAGGAAAAAAACCCGGCAGAGTTAGCAACCCTTGCCGAACAAAATGACCCTACCG
CCTTGATGTTTTGGCACAATTATGGTAAACAATTAGGGGCAGGATTAGCCAGTTTAATTTATATC
CTTACTCCCGAAGTTGTTATTATTGGGGGAGGAGTTTCGGCTGGTGCAAAATACTTTCTCTCTTC
CACACAAAAAGAAATCGAAAAAAGAGTCTTACCCAGTTCTCGTATTAACCTAAAAATAACAGTTG
CTCAATTAGGAAATCAAGCGGGAATGGTGGGAGCGGCAAAATTAGCATGGGACAAAGGAAAGGG
CAATGGGTAAACCCCCTTTATCCTCCCATCTTCCAAAGACCTTAACACTATAGAAAATTTTATAT
CTAACTCAGGTCAATGAAATCCCTAGAAATAATAAAAACTTTACTTCCTACTCTGAAACTAGCAG
GGCAATATGCTTGTAATATTCAGTCTCAAATACAAACTCAACCTGATAAAGACTATGGCGAGAAC
TTTTATGCCACTGCTTTAACAGATGCTGATTTAACCATTCAAACTGCCATTGAATTAGCTTTGTT
AGCAAAATTTCCTGAGTTGAAATTTTTTGGGGAGGAATATGCAAAATCCTACAATACTAAATATT
TTCGAGGGACAAATTTAGAGGATGATGAGGAATTATTAGTTACTTTAGATCCCATTGATGGCACT
AGGGCTTATTTAGATAATTTATCTTGTTTTTCTATCATTTTAAACATCATTAAAAATAAACAATA
TGAGGCAGTATTTGTTTTACAACCTAAACGAAAACATTACTTTTATGCCTTAAAAGGAAAAGGTG
CTTTTATTGGTTCTATCGATGATGATTTAGAGTTAGCAAAGCCTTTAAAATTAAAATCTTTACAT
TCAAAAAAAATCTATTTAAGTTTTGCCTTAACCTCTTTAAAGACTAAATTAAACCCTATATTTGA
TACTTGGTGCAGTGCCACTGACTATAATCCTCCTGAAAATATACCAGATTATTTAGATTTAATTA
ACGGTGATTTAGCTGGTTTTATTATTGGTAAAGGTAATCTAATTGATAGTGCTAGTTTAGCTTTT
GTGGCAATGGAAGCAGGAGCGATCGCATCTCATTTTGATGGTAGTGATTTTCAACCTTTTAGAAA
CATTAATCAGATGAAAATAGATGGCTTAGTAATAGCTTTTAATCGGGAAATTCATCAACAAATA
TTGGCAATAGTTAACCAATAATCTAACAAATGATTTAAAATTGCAATCAATCTTAAATTACCAAC
AGAAATTATGATCAGATTTTTTATCACTTTACTGATTTGCTTATCTCTATTTATTTCCCCTGTAA
TTGCCGAAGAAAATATTAATATACCCACAGAGGCACAACTACAAGCAGGAGAAGAAATCGCCCGT
CAAGCCATTGAGGCAACGGAAAAAGGAGATTTTATTCTAGCAGAAAAGTTTTGGACTGAATTAA
TTAATGAATTCCCCTCTAACCCTGCTGTTTGGAGCAATAGAGGCAATGCAAGAGTTAGCCAAAAT
AAAATTGCTGAAGCCATCGGCGATTATAACGAGTCTATCAAATTAGCCCCTGATGCACCAGACCC
CTACTTAAATAGAGGCGTTGCTTATGAAGGTTTAGGAAAATATCAAGATGCGATCGCAGATTATC
AAAAAGTATTAGAATTAGATCCAGAAGATGCAATGGCATATAATAACCTCGGTAATGCAAATGC
AGGACTAGAAAACTGGGATGAAGCGGTAAAATACTATCATAAAGCCACGGAATTAGCCCCGAATT
TTGCCTTTGCCAGTGCCAATGAATCCCTTGCCCTTTATCAACTAGGCAGAAAACAGGAAGCCTTA
ACTAAAATGCGCAATATAGTACGTAAGTATCCAATGTTTCCTGATATGAGAGCGGCTTTAACTGC
GGTTTTATGGGAAGAAGGTAAACAAGGAGAAGCAGAGAGTAACTGGGTGGCAACAGTGGGAATG
GATAGCCGTTATAAAAATGTAGAGTGGCTTAAAGAAGTAAGACGTTGGCCTCCCAAAATGATAG
TTGCTTTGGAAAACTTCCTCACCCTTGACAATTAACAACGTTTAATAATTAGGGTTTGATGAGAG
TAGGTAATGGGTAATAGTCAAAAATACTTATCAATCAAAACTCTAACAAAATAATTTATTCTAA
GTTAAATTGATTATATGCCCTTGCCTAAGTTTATTTTATTCTGTCTCCACATTTAAGATCATTTT
TTTATTCAGTTTTTCTATGCGTAAATTAATGTTTTCAATTACATCATTATTATTGGCTTTATTTT
TATAAATTTCAAGATACCGAATTGCATCATTTATTTCATTTCTTCTTTCTTTTTCGTTAACTATT
ATTAAATAATCTTCTGGAGGAATATTGACAGATACTTCAGAATATGTCCTCAATACTGTGGGAAT

CCAAAGTGTACAACCTAAACCTAAAATTAAAAATGCCCTATATAAATCATTTTTACGATGGAAAA
CATAAGCAGATAATGACAAAGCTAAAATCATAAAAAAAACAATTAGTAACACACTAAAAGAGTA
ACTATCAAAATATGATTGTAAATTGCTGGTAATAGGAATCGTGTAAGTAATTCTACCAATACAA
ATACCCCCCAATAACCATGAAGCAGGATTTATATATTTTTTCCAATTAGGGAATTTATCTCCTAA
TGTCATAAAGAAAGAAATCAAACTTGCAATACCCGTAACTATAGTAAAAATATCAAACATATTTC
TCCTTATTATTGATCTTATTTTGAAGGGCAAAAGTCTCTAATCTATTTGTTTTAGCCTGAAAAAG
AAGGATGAGTATTAATATTGCTCATCCTAAATATAATTGCTAAACTTAACTAATTTAATTAATGT
TCTTTATCTATTGGAGGATGAGGATCATTACCTGGTGCAATAGTATCTTTACTGCGAATTTTTCC
GTCTCTTCCTTTTGTCGTTAATTCACCTCCCCCAGAATTTTGTAACATTTCTTTAGCTTTGCCAAC
AGCTTCTTTTTGAGTTGAATGTACACTAGAAGCCTTAGAAGCTGAATTTTTTTTGTTTACCCATT
CTCCGTTAGATTTTTGGTACACTGTGCGATCTTCTTTTTTTGACATAAGAATAACCTATTAATAA
TAAGTTTATACTAACAACTACATTTAAGAATGATATTAAAAATAAGTACATAATCAGTACGGAT
TACCGAACAAAAGAACTATCAATAAACAAAAATCAATTTTCCATCTCAGAAATAGCGATATTTAA
GTTTAATAATTGCTCTAAAATATCTCCATTTTCATCAAAATTATAAGCCTTCATTACTAATTTAT
CTAACTGTTGATGAAGTTGATATAACTTACTACTAGGCTCATGAAAAAAGGCATTATATAACTGA
GTAATTCCCCATGATTTTCGCTCCATTTGTTCAGTGCGATATTCATGTAATTCCGTCATTTTATCC
CTTATTTGTTGCACAATCTTATTACTCCCCTCGCCCTTTGGGAGAGGGGTTGGGGGTGAGGGAAA
CGGGAATGTTTCAAAACAAGTATTATGAGTATAACGAGTTGTTTGCCCTAAAGTTGATGATTGAG
CTTTAACCCAAAGACGATGTATTTTAGATGTTAAAATACCTAAAATATAAAAATCATCTGATGCA
ACAACAACAGTTAAATCACTTGGCAAAATACTGATACTAACAGGCAAAAAAATAAACCATTTAG
AGACTCTGGGAATAGCAAAATAATAAGGTAAATGTTTTATTGCCTCTCTCATTAACGGGCGTTTT
TCTCCAAATTTCCACCAATTTAATCTCGTAGTTTCTCTCCTATTTTTATCTCTTTCAGGCTTCACA
TTTTCCTTAACATATTGAAAAGGTTGTTGATAATCACAAGCTAATTCTAAATCTAAATCATTAAA
ATCAATTATCCATCGGCTAGGCTTACCATGAGGATTTTTTGCTAAATCTTTAGCGTCTAAAAACT
GTTTTAAAACCTTTTTATTTTTGCTATTATTGTTAATCCATTCCTTAACTTTTTCTTCACTAATA
ATAAAATCTTTTCCTGTTGGTTGCACTCCTTTAAAACAATAATTACTATTTATTTTTAATTTTTT
AGCCTGTGTAACATCTACAGACTTAGTTAAAGAAGAATTAATACTCTCGACTAATTTATCATCTA
AATAATATTCTTTTTGTTGTTCATAACACCAATTAACAATACTGACATAAACCGCCGCTTCTCCT
GACCATTTTTGACTAGAAAAAGCCTCATGAATATAACCTTTATTGGTTAAAATATAGTCCAAACT
AGCTTTTCTTGCGACTCCTTGACTAATGGTGTTTGTGGCTACTAAACCAATTCTTCCTTTTTCCTT
TATATTCTCGTGGGCTAATCGAAACCAATATGTGCAAAAATCGACTTTTCCTTTTACTTCTGCAA
ATTTTTTAAATACTTTTTCTGCATAATTATCTCCTAACTCTAAACGTATTCTTTGCCCTCCTAAA
AAGGGAGGATTACCGATAATAGCATCAGCTTTTACCCAGTCAGTAAATAAAGCATCACAACAAAT
AATATTGTTATCTAAACTATCTAAAGGTAAAGCAGGTTCAGTTAAATTAAATTTATCGATCGCAA
CTTTTCGAGCTATCATTAAAGTAACCTTTGCCAACTCCACAGCGAAGGGATTAACATCCATACCA
TAAAACTGATTAGGAGTAACAAAACTGAGATTAAACTGTTGATTATTGCCCTTAGTTTTACTTTT
TATTTTATCTAATAATAACTGCTCAATTCTTTTTAATTCTTGATAGGCAATATAGAGAAAATTTC
CACTACCACAAGCAGGATCTAAAACTTTATATTCCTGTAATTTAAGTTGTAAATTAGTTAAATCT
TTGATGGTATTTGCACTGTCAATTAAATCTTCCCAATACTTACTAATGGTGGGGCGCACAATTTT
CATAATATCCGCCTCAGAGGTGTAGTGGATACCATAACTGTGACGTTGTTGGGCGTTAACTGTGC
CTTCAAAAATATTACCAAAAATTGCAGGGCGTACTTTACTCCAATCTTGTCGGGCAACCGTTGCC
AATATTTCTAACTCAGGTTTGGTTAACTCGATGGCTTCCACTGTGGCAAATAAACCACCGTTAAA
ATATTCGACTCCTTGATATTTTCCTGCAGGAGTGACACCCCGATTATTCATTTCACGGAATAAAC

CCCCCAACACATCGTAACTACTAGCACCCTCTAAGCATTCTTCTACACAACGGATAAAGAGATTA
TGGGGTAACAATCCTCTATCTTCTGCAAACATGGCTAAGACGCATTGCAAGATGAAGCGTTGAAT
TTTGATAATACCCTCACCCCCAGCCCCTCTCCCACAGGAGAGGGGAGTAACTGTAGATTTATTTCC
TCCAACCTCTGTTAATAAGTGTTTATCCTCTCCAACTCCCCTTAATAAGCGATTTTGCAGAATTTG
GAATAACTCCCCTAGTCGTCGCCCCGCTTTTTCGGTAACATCAACTTGATTATTTTTAAAGATAG
ATTTGACATCCCCTCTCATCATGAAGTTAAAGGCGGATTGTCGGTGGATTAAATCCTCAATCTTA
ACTATATCAACAGGTTCATCTAATTGTAAATTAAAGTCAAAAATCCAAAACTCATCAAAATTGCA
CAGTATCACATAACGAGGGCGATTAGGTACTAACCTTGACCAATATTCAAAGGCTTGGGCATAAT
GTTTACTAAGGTCTTCCCCTCGCCTTTTCATCTCAATTAGTAGCTTGGGTTTCCAGACTAAATCGG
CAAATCCTGTATTTTTCTTTTTGCTACCCTTTTTAATAGCTTCTTCGTATTTTGCCCCTGCTTCTA
ACGCACCATCATAACCAAACCCTTGGAAAAATCTATCTAAAAAAATCTGAGCTTCTTTTCTTTCT
TGCCCTTTAATGTGTTGTTTACAAAAAGTAATAAATTTTTCAAGGTTTTCTGGTGAATTAGTTGT
CATGGAAAAAGCGAATAAAATTGATCTTAAACTATCAAAATTTTATCTTAGTTTTTATTTTCTAA
GAAGCCTAATAATGTCTTGGGGCATACATCATAATACTTGCCCCCAAAAGAATAATAACAACTCC
TAACCAATCATAAGCATCTGGGGGAATTTTATCTATTTTCCAGCCCCATAATAAAGAAAAGAAAA
CAAAAGCACCACCATAGGCACTGTAAACTCTACCGAAATTAGTGGGTTGAAAAGTGGGCAAAACG
CCATATAACATTAAGATTAACCAGCCAACAATAGCATACTTAAAACTTTTTCCTTCCCTCAACCA
CAACCAAACTAAATAACCTCCTCCGATTTCTGCTAATCCCGTAATTAGGAAAAATAACAATGATT
TAACTATATTCATTAAAAAAATTCTATTTAAGATTAAAGTATAATTCAGGGTATCATTTAATGA
TATATACTAAATAATTTGATAAAAAAAGACTTATTACAACGTCTTTATAAGATAACAATGATCG
CACTTTCAACAGTTAATTGGAGTAATTATTGGCAACAGGAATCAGAATCTTTCACTACTTTTTCA
GGAGAAAAAGAAGAAGTTTGGCGTGAAAAAGGTTTGTTAGGATATTCTGAGGAAAAATATATCC
CTTTACGTCGTGATTTATATTTAGAAATTTCTGATTATCATATGTATGAAGATGTGATGTTAACC
AGTCAATATGATCAGAAAAATGATGAGCATTTTGTAATTAATTTTGTGGTTTCTGGCAATGTTA
AAACTATTCATCATGGGGTAACAGATTATGTTTTAGAAACGGCAGGAAAAAACTATATTGAGTT
TTGGGAGTCTCGACAGGAAACGGAATATTGGCAAAAAGGCGATCGCATCTTAAAAGTTAGAGTCG
GAATTTCTTTGTCAACTTTAAGAGAAATGTGTGAAGATTCTTTAGACACTTTACCCTCAGAATTA
CAGTTATTAATTACGGGTAAAAATGTGCCTCCTTGTTATCGTCAGGGGGAAAATAATATTAATAT
GAATAGTGCTTTAAATCAGATTGTAAATTGTCCTTATCAGGGATTTACCAGAAACTTTTATTTAG
AAAGTAAGGCTTTAGAATTACTGGCTTTATGGTTAGGAGAAAACAACAAAATAGGATTTAATCA
AATTTCTTCTCTTAGTAGAAAAGATATTATTGCTTTGCAAGAAGTAAAAAATATTATGAAAAAA
AACTTACAAAATCCTCCTAGTTTAAAAGAATTATCTCGACAATTATGTATCAATGAATGTAAATT
AAAATCAGGTTTTAAAAAATTATTTAATACAACAATTTTTAATTATTTACACCGTCAAAGAATG
GAATATGCTCACAGTTTATTAAGAGATAAAAAAATAAAAATAACCGATGTAGCAATGATGTGTG
GTTATGCCAGTTTACCTTCTTTTTCTAAGGCTTTTAAAAAATATTACGGTATTAGTCCAAGATGC
GATCGAATTTGATAATCAAAATATATTTTGCACAACCCCTAAGTATAATAAATAAAGACATAAA
GTCCCTAATAGGAACGAGTACAAAGTTCAGAAAATGAGAATTAGTAAGAAGAAAAAAGCGATCG
AAATTTTAAATATACTCAAACAACTTTATCCTACCGCTACTTGTAGCCTGAACTATGATACTCCC
CTACAGTTATTAGTTGCGACAATTTTGTCAGCACAATGCACCGATGAGAGAGTAAATAAAGTTAC
ACCGGCTTTATTCAAACGTTTTCCTGATGCCCAAAGTTTTGCCCAAGCCGATAGAGAAGAAATAG
AGAATTTAATTCGCTCCACGGGTTTTTATCGCAACAAGGCTAAAAATATTCAGAATGCCTGTATC
AGAATTGTTAATGATTTTGATGGGAAAGTACCTCAAACTATGACAGAATTATTAACCCTTGCCGG
AGTTGCCCGTAAAACCGCTAATGTAGTGTTAGCTCATGCCTTCGGTATTATTGAAGGAGTAACGG

TAGATACCCATGTAAAAAGGCTTAGTAATCGTCTAGGTTTAACTAAACATTCTAACCCGATACAA
ATTGAAAAAGATTTGATGAAGTTATTACCCCAACCAGAGTGGGAAAATTTTTCTATTAGTATTAT
TTATCATGGTAGAGCCGTTTGTAATGCTCGTAAACCTCGTTGCCCAGAATGTACTTTAAGTCATT
TATGTCCTTCTTTTATCAGCTAAAAATCTTCTCCTTATTCTCTGAAAAAACTGTACACTAATCAT
CATAACCAAAGAACAGTTATTAACATAAATTATGGCTCTTGGCTATCTCGCCCTAGTATTACACG
CACATTTACCCTTCGTTCGTCATCCAGAAAGTGACTATGTATTAGAAGAAGAGTGGCTATATGAA
GCAATTACGGAAACTTATATTCCCATACTAAGAGTTTTTAGAGACTTAAAAAAAGATGGAGTAG
AATTCAAAATTACCATGAGTCTAACACCGACTCTTGTGTCTATGTTAAAAGATGAACTCTTACAA
CATCGTTATGATCAATATCTCCAAAAATTAGAGCAATTAATTCTAAAAGAAGTTATCAGCAATCA
AAATAATGGACACCTCAAGTATTTAGCTGAATATTATGAACAAGAATTTGCTCAGATTCGACAAA
TATGGAATGAGTCAGAAAGGGATTTAGTCTCAGCTTTTAAAACTTTTCAAGACAGCAATAATCTC
GACATTGTGACTTGTGGGGCTACTCATGGTTATTTACCATTAATGAAAATGTATCCCAATGCGGT
TAAAGCTCAAATTGAGGTAGCCTGTGACCATTATCATGACAATTTTGGACAGTTTCCCAAGGGGA
TATGGTTGCCTGAATGTGCTTACTATCAAGGCTTAGAGGATATTTTAGCCTCAGCAGGACTAAGA
TATTTTATCACTGATGGACATGGTTTAATTCATGGAAATCCTGTACCTCGTTTTGGTACTTATGC
CCCTATTTTTACCCCTAGAGGTGTTGCGGTATTCGGGCGGGATTCTGAGTCATCCCAACAGGTTT
GGTCTTCAGAAATCGGTTATCCGGGTGACCCTGTCTATCGAGAATTTTATAAAGATTTAGGATGG
GAAGCTGATTACGAATATATTAAACCTTATATTATGCCCAATGGTCAAAGAAAAAATGTAGGCA
TTAAATACTACAAAATTACCGATCGCACCTGTAGTCTTTCAGAAAAAGGTCTTTATGACCCATAT
TGGGCAAAAGAAAAAGCCGCAGAACACGCTGGAAATTTTTTGTTTAACCGAATTCAACAGGTGAA
GCACCTAGCAGGGGTGATGGGTAGAGAGCCAATTGTTGTTTCTCCCTATGATGCTGAATTATACG
GACATTGGTGGTATGAAGGTCCACAATTTCTTGATTTTTTATGTCGCAAAATATGGTTTGATCAA
GAAACCATTGTGATGACTACTTTGGGGGAGTATTTACAAAATCACCCCACCCAACAAGTGGCAAC
CCCCGCTCAATCTAGTTGGGGCTATAAGGGATACCATGAATTTTGGTTAAATGAAAGTAATAGTT
GGATTTATCCGCATCTTCACAAAGCCATTGAAAAAATGACTGAGTTAGCCCAACGCAATCCAGAG
AGCGAATTAGAATTAAGAGCATTAAACCAAGCAGGAAGAGAATTATTACTAGCACAATCATCGG
ATTGGGCATTTATTATGCACACAGGCACAATGGTAGATTATGCCGTCAAAAGGACAAAAAATCAT
TTATTGAGATTTAACCGACTTTACGAAGAGATCAAAAACGGGGATATTAACGGAGATTGGTTAT
TAAAGCTAGAAATAATGGATAATATTTTTCCCGATTTTGACTATCAGGTTTATCGCTAATTTTTC
TGATTTATTTGTTTCGTAATAATGTTTAATTGAAGCAGGTTAAGTGAAATTCAGAGTTCAGAGTT
TTTGATTCTTAATTCTTAAATTATCAACTATTTACCTTTGCCTTCCCCCCTCGAGAGGGGGGATA
AAGGAAGGGGGGTTTGCCCCTTGCCTTTTGCTCCAAAGTGACGAAATATCTAGGACTTAGGGAAA
TGATAGGATTGCTATATAATCTTTAGAAGTATTGACACTCCTTGACCTGAAGGTGCAAGGATTCT
TGGTTCACCAACAGCCCTTAAATTAACTTAATTGCTTAAATTAACCCAGAGGGGCTTATCTCCCC
AAGCGTATGACCCATAGAGCGAGTCGGTTCGGGTATGCCCTACCCTACCGAGAAAAACACTTAAT
CTTTGGTTTCTCTGTGCTTACTGCTTAGAGATTTTACCTTGAGAAGCATTCCTCTCAAGAACCCTA
TATCGCTAGTTTTCAAGGTTCAATGAATCTGCTAGATTCGAGGTTTTTTAAGAGGTTGATTACCT
TTCCTCTAAAAAAAATAATAACACACTGATGCACTAAAGGGCAGGAGACCTAAAACCCACAGATT
TATGGTAAAAATTAAAAATAAGATAAAACCAAATATTTACTCAGAACATAATGGATATAAAAGT
CAGCAATATTAACAAATTGGATTGGAGTGGTTCAGCTTTAGCATTAGGTTTTTTCGCTGACGACA
ACCTCAGTCTAACAGAAGAATTAAATCAGTTAGATCAAAAATTAGGAAATACCATCAGTGAATT
AATCGCTGAAACAGAATTTACAGGCAAAGAAGGAGTAAGTGCGATCGCACGTGTAGGGGGAGAT
AGTGCCATCAAAAAAATTATCATCATTGGTTTAGGAAAACCAGAAGATGTCAACCTAGATACTTT

CCGTCAGGCAGGAGGAAATATAGCCCGTATTGCCAAGCAAGGAAAAGTAGCAAAATTAGGTGTAG
CAATTAATTGTGATAATTATGAAGACGAAATTCTAGCTCAAGTTATCAGCGAAGGTATAATTCTC
GCTTCTCATCAAGACAATCGTTTTAAATCCGAGCCAGAAGAAAGTAAAATCAATCTCGATACCGT
TGAATTACTGGGATTATCCGCCAGTGAATCAGCAATCACTAAAGCCCAAAATATTGCTTCTGGGG
TAATTTTAGCCAGAGAATTAGTCAACTCTCCCGCCAACGACATCAATCCTGTTACCATGGCGGAA
ATGGTGCAAAATTTGGCCTCAGAGCATAATTTAGAATTAACAATTCTCGAACAAGCAGACTGCGA
AAAATTAGGCATGGGGGCATTTTTAGGAGTGGCTAAAGCCTCTGATTTACCCCCTAAATTTATTC
ACCTCGTCTATAAACCCCAAGGCACAGCAAAACGGAAACTAGCCATTGTCGGTAAAGGTTTAACC
TTTGACTCTGGTGGTTTAAACCTCAAAGTTAGTGGTAGTGGCATCGAAACCATGAAAATGGATAT
GGGCGGTGCGGCGGCTACTTTTGGGGCGGCAAAAGCGATCGCACTTTTAAAACCAGAAGTAGAAG
TACACTTTATCAGTGCAGTCACCGAAAATATGATTAGCGGTAAGGCTATGCACCCCGGAGACATC
TTAACCGCCTCCAATGGGAAAACCATCGAAGTTAACAACACCGATGCAGAAGGGCGTTTAACCCT
TGCAGATGCTCTTGTTTATGCCGACAAATTAGGTGCTGATGCGATGGTTGACTTAGCCACATTAA
CAGGGGCTTGTATCGTTGCTTTAGGTAACGATATAGCAGGATTATGGACAACGGAAGATAAATTA
GCCAACGAGTTAAAAATTGCCGCCGAAGCAGGAGGAGAAAAATTCTGGCAAATGCCCATGGAAAG
TAAATATTTTGAACAGATGAAATCTCCTATTGCCGACATGAAAAATACTGGTACTCGTGCAGGAG
GCTCAATCACCGCCGCTCTATTCTTGCAACAGTTCGTCAAAGACACTCCTTGGGCTCATTTAGATA
TAGCAGGACCAGTTTGGACAGATAAACCCAACGCTGTCAATAATGAAGGTGGTACAGGCTTTGCT
GTGCGTACCCTTGTCAATTGGGTTATCAATAATTAATTTTCATTTTATTAACTAGATTAGGGAAA
CTATCATGGGAGAATCAAAACGTAGAAAAGAAAACTTAGGGGATGATTACGGTAAAGAAGAAAA
ACTTTATCCTTGGCTACCCCTCACCAAAAAACAAGCCGAAGACGCTTATAAACTAACCACCCGTG
GAGCTTGGATTGGTATTGGACTCTTAGCTTTTACTTGGATTACCATTCGTTTTATCGGTCCGGGT
TTTGGTTGGTGGAGCGTTAATTAGGAGAGTTCGGGGTTAAGAATGGGGTGTTAAGGGGAAATAA
GTAATGATTAATGAGTGTTGGGATGAATAATAACTCCTAACTCATTGCTGGTTTCCCCCTCCTCC
CTCATCTCTCAAGACTTTAATAAAGAAATAAAGAAAAGTAGGCTATGAAATTGTTATTAAGGCT
AATTCAATTTCATTTTCCCCTGCTAGAGAAATTTTTGCCTCAATTCCGGGGCCAAAAAACTTCGT
AATTTTTTCCTGTTTTTCCTGCCAAGTTTCTAGCTTAATTAAGGGTGATTCAAAATACAAAATCA
AAGCATAATGATTATCGATTTTATCTTCTTGAATTTTTGTCAAAATGGGTCTTTCATCATCAGTA
GGGCTTAAACCTAAACGTTGTAAGGCTTCATCTAAATGGGCTTCTTGTCCGTAACGATAGCGAGT
AACATCTAATCTCAATTGATTTTGAGTGGCAGTAGCTTTTTCTTTTCTTAAAGCTAAAACTTCGG
GGGTAGTCTCTTTCAAGAAGGGAATCGGCTTTAATTCGGCGGCTTTTAATGCTAATCCTCCTAAC
AAGAGGGGAATACCATAAAAGAAACCGGCTAAATTAAGGGTAGAGTTTCCTGTGCCATAAGCAA
CAAAACCTATGACAGTGAGAATTGAACCAACCGTTAAACCAACTAATGCTAGGGGAATTTTTCGC
AACATAATCAATAAAAATATTTTCTATTATTTCGAGTAGATATAGATAATGTATCAAAATACGT
TCCTCTAGTTTAATCTCCATTCGAATCAAGGTTTGAAGCAAAGACGGAAACAACTACCCTGATTT
TTTTCACTTTCGACTACTATTTCTGCATCTAATTGTTGAGCTAAAAGTCTGACAAGGCGTAAACC
TAATGAGTTTGTTTGCTCAATGTTTAAATTATCAGGTAAGCCAATACCATTATCTTTGACTTCTA
ATTCTATGTCTTCTTCTTTGGCAATTAAGTTAATGGTGACTTCTCCTTCTCCTGTGGGAAAAGCG
TGTTTAAAAGCATTGGTTACTAATTCATTGATTAATAATCCTGCGGGGATAGCTGTTTCAATATT
TAGACGACATTCTTGTAAATAAGTAACGGGTTTTATACCTGTATGATAATGGTTATAGGAAAGG
AAAAGATTGGAAACGAGGCGATTGATATAGTCGGCAAAGTTAATTTGTGATAGGTCATCGGATT
GATATAATTGCTCATGGATAACTGCCATGGTTTGAATGCGGTTTTGGCTATCATCGAACAAACTC
CTTACTTGTTCATCTTCTATATAGCTAGATTGCAGATTTAATAAACCGGAAATGACATAAAGATT

ATTTTTAACTCGATGGTGAATTTCTTTGAGGAGAATTTCTTTTTCTTGTAAGGATTTAAACAGTT
TTTCTTCCGCTTCATGTCTTTCGGTGACATCGGTGGAAATACCATCAATGCTTACTGCTCTACCAT
ATTGGTCATACACAATGTGCGATCGCTCTCTTATCCAACGATGAAGCCCATTTGATAATTGAATT
TTATACTCAATTTCCCAATTCATCTCTTTTTGATTTTGTAAGACTCCGAGATGATAGTTTTCCAT
GATATTTTTCCATGTTGACTCTACTTCTTGATGATATTCGGGTATAACCATTTCTAAGAGAATCC
CCCGATTTGCTAATATGTCTGCAGGAGAATAACCGTATAGTATCTGACAAGCAGGGTTTATATAT
CTTAATTTTAGGTCTGGTAAAGACATTGACCAAACTACATCTTGCAGTGAGCTTAACACACTATT
TAAACGGGCTTCACTTTCCTGTAAGGATTCCTCTGCTTTTTTGCGATCGCTAATATCATTGATTA
CAGCAATAAAGTAAGATGGTTCAACGGCAACCCACACCACTGACATATTTAAGTTAATCCAGAGA
AAACAATCATGACTGTTACTATTACAACGAATACGCACTTCTCTCTCGAAAGTGGCTTGATTAGC
ATTTAAAAGATAGGATAAATCTGTGAGGGTGGCAGAAACATCATCGGGATGAATCAAAGAATGA
AAATCCACATCCACTAAATCGTTGCGATCGAATCCTAATAGTTGAATAAACTTATCATTAGCTAA
GACTAATTTACCCGGTAAACTAACTTGCACAATACCAACGGCGGCTTGATTAAAAATACTGCGGA
ATCTTTCCTCACTTAATTTCCATCTTAGTTGTGCGAGTCTAACTTCTTCGATGTCTTGCCCACAGG
CGATAATACCAATACCTTGCCCATGATTATCTGTAAAACGGTTAATATTCCATAATAATGTTCTT
TCACTACCATCGGCTGTTAGTATTTTTGTTTCTAAGTTTCTCTGTACTTGCCCTTGATTAAGGGT
TTTATTGAATATATCTTTTAAATCTTCTCGATTACTAGGGGCAACAAAAAGTAAGAAGTAATCTT
CCCCTAATACAGAGTCTCTTTGCCATCCAAAGATTTTTTCCGCTTCTTCATTCCATTCCACAATGC
GATACTCTTGATTTAAGACGACTAAAATTGTCCCCGCAGTTTGAACAAAAGTTCTTAATCTTGTG
GTGGTTTGTTCTAATTCTTCCGTGCGGTCCCTAACTCTAATTTCCAATTCTTCGTTGAGACTACGA
AGTTGAGACTCTGCTTGTTTGGCAAGAGTTATATCTCTAGCCACCGCATAAATTAAGCCCTCTTC
ATAGGGTAAACTTTTCCAAGCTAACCAACGATACTGCCCGTCTTTTGTTAGCCAACGATTTTCAA
AATTAACACTTTTAAAACCCCTACTTAATTTTTCTAATTCGGCTTTAGTAATATCTAAATCATCA
GGATGGATAAAAGATAGGATATTGCCTTTAATAATTTCTTTTTCTTCATAGCCTAATGTAGTTAA
AAAACTAGAATTAATCCGTTTAAAAGAACCATCAACTCCCGTAATACAAAACATATCTAAAGAAA
GATTAAAAAAGTAATCTAACTGCATTTGAGTTCTATTTTTATCAATAGCAGTAGCTAAAACATTA
GAAATTGTTTGCAAAAAATTAATTTCTTCTCTGGTAAATTTACGGGGTATTTTACTATAAACTCC
TATCACGCCAAAATAATTATCTTCTCCACCAATATTCACTGTCGCACCACTTAAAATTCCTGTATT
ATGTAAAAAAGGGGATGGACTAAAACGGGTTTCTACTAATAAATCATCCGTAATTACTGGTTGCA
TTACCTTCAGGGTATAGCCCGGCATCCAACGGGGAGATGAGCCTACTTGTGCGCTTCCAATCCAG
TCTGATGACCATCCTTTTCCTGCTTTGACTAAAAATGAGGCTTGATTAGCCTGTAATTCCAAAAT
ATCGCAATATTCTACTTTCAAGGTGTCGCTAACAATTTCTACGGTGTCATTAATCAGGGTTTGAA
TATCATTATTCAGTAAAGCCGTATTGCCTAAAGATGCGATCGCAGTTTGATATTTAGCAATTTCT
AGGGCTTTTTGAGCATATTCTGACTGTTGAATGGCAATAGCTACCTGAGATCCCAATTTTTGCAG
TAAGACGATTTCTTCTTCTAGCCAGTGTCGGGTTTGACGACAATTTTGAGCGGCTAGTAAGCCCC
ATAAACCATCATGATAGATAATTGGTACAACTAAATTCGCCCGTATTTGAATGCCTAAGAGCAAA
TCTAGGTGACAAGGTGATAAATTTCCCTCAAAAACGTCATCAACTGCCTGAATTCTACCATTGAG
ATAGGGTTGAATAAAATCCTTAGCAAAACAGTGATCTTGTACTACCCGCCCATGAATAGACATTA
CTCCTTCGACGACAGACTCCGCAATAATCACTCCATCCCCATCAGGATAAAGTTGATAAACAATG
ACGCGATCGCAATCTAAAAATTGTCTAACCTCATTAACAGTACTATGTAAAATAGTGTCTAATTG
TAGAGATTTACGAATACGATAACTAAGTTGAGAAAGAATTTTTTCCTGCTCAACCTGTTTTTGTT
CTATTTTAAAAGCAGTTTTGACAGTATCTATTTCCTGTTGTTTCCGTTTTAAATATTGAAATTGC
CAAGAAGGAGTTAGCACTTTTAATAACCGTTTAGGAGTTACTAAAAAAAGACGACTACAAACTCC

ACAAGAAAAATTTTTAACATCTTCTGATGGTAAATTATCAACTAAATCACAATTATAATTACTCG
ATTTCCCTTGAAAATTATTACTTACTAATAAGAGAGAATCATTGATAACTAAGTTACTAATAATA
CTTATTTTTTCGGTACTAATAAAACAAGATAAATCTTCAGACTCTAAATCTTTAACTTTTTTTAC
TTTCCAATCAGGTTCTAAAATAGTTTTTAAAATAGTACGATAGCTAAAAATTTCACACCTTGAAT
CCTTTTCTATAATAAGTCCATCATTAGACGAATCTTCATTTAAAATATTGATTATATTAATTAAT
GATTGATGAGGATGAACAATTTTTAAATTGGGCTTTATAACTTCATTAACAGAAATTTTTTGAT
AAATATATTCAGTATTAAAATATTGACATATACTATGAGGAGTAATAATTCCTTTTAAAAAACC
TTGCTCATTTAAACATCCATAAAGTTGATATTTATTTAGATAAAGATTATTCGCAATGGTAAAAA
CATCAGGAAGATTATCAATGGAAATATAAGGAAAAGATTGACAAAAATTTTTAGCCCTAAGAGA
ATAAAGATTAATATTCTCTGCCACAATTTTGATAATGATATGAGGAGAAATATAACCGAGAGGA
AATGAGTTATTAGCAACAATTAAAAAATCATGACCCATTAACAACTTTTTTAAGACATCAACAAA
GGAAGTCTCAGGAGAAATGACAAAAGGACAATAATCAATAATGATACTATTTATACGAGTTTCA
TGATTCATAAGCACAAGCATGAAGATTGTTATCAAGAGAATCTATCACTGCTTTTATATTTCTAG
TTTACATAATAATTTCTAAACTTTTAGCTTTTATTTTGCTATAAATATCATTCAATAAACGTTAT
AGAAGCCATCTTCCGCTAATTTATTATAACGACTGTGTGAGAGCCTAATTTATTATGACTCGTCA
AAAAAGAAATTAAATATAATTCTATTTAAGATTAATCAAGAATAATATATCTTTATACTGACCT
TTGCAAAAAGTATAATGATTGTCTTACAAAAATAATGTATTTTTGGGAAAATTATTCCTCTACAA
TTTTTTTTAGTTCTTGAATATTATTGCTACATAATACGGGAATATATTTTGTGATTTTTTCCAAA
GTCCAATCCCACCATTTCAATTCTAATAAAAAATTGATTATTTCATCATCAAAACGTTTTCTAAT
AATTTGAGCAGGATTACCGCCAACAATATGATAAGCAGGAATGTCTTTTGTTACCATCGAATTAG
TCGCTACAATGACACCATCCCCAATTTTCACACCGGGCATAATCGTTGTATTGTAACCTAGCCACA
CATCATTTCCAATGATAGTATCTCCTTTTGATGTTGCTTCCACTGACATTTTATCTTCCCATCCAT
TACCGAAAATAGCGAAAGGATAGGTGGTAAAATTATTTAAAGGATGATTTGCACCGTTCATAAT
AAATTTAACATTACTAGCAATAGCACAAAATTTACCGATAATTAATTTATCACCAATAAAATCAA
AGTGATATAATACATTTTTTTCAAAATTTAAAGGATTTTCAAAATCATCGTAGTAAGTAAAATC
ACCAACAATGATATTTGGATTTTTAACGATATTTTTTAAGAAACAAAGACGACTATAATTAGTT
AAAGGATATATTTGCTCTGGATTAGGATAAGTCATTTGACCTTGGTAAATTAGGCTATTATTTTT
AGTTTATATCACCAATAGGCAATGATAATAGTATTTTGAGTATTGATTTTTAAGAATAATTAAT
AATATTTAATATTCGTATTAAGCAACTTTATTGTTTTTAGAAAAAAGAGATTTTTAGGAATAAA
AAATTGTTTTAATGCCATAGATTTTCTATTTCTTGTAATTGGTTTTGCTCATTAAAATTTTCCCA
AATAATACCATTTACATCTATTTCTTGACTAAATGTTGTTGCTTCAGAATCTTCCCAGCCTAAAT
GTCTTAAACTTCCCTGTTTTACGCTTTTGTCTTGTCTTACAGAGAAATTAACAGTTTCTGTTTTCC
ACAAGGCATATTCATTCTCAATAATTATAATTGGTGATGCTGATAAGCGTTTACTATATTCTTCA
ATACTTTCTTTTAAATTATGGGTAGATAAAGCAATATGTATTCTCTTCATTGGGTGAATTTATCA
TGTTAATTAATGCCTAATTTTATATTGTAGAGTCACTTACATAACAATTTAAAGGGAATTGAGA
GAGAAAAAAGCTACAAAAATTAATAGTTTGTGACCCCAAAATAAGTTAAGTTGGAATTTTGATC
TGCATTTTAAAGATAAGAAAAGTTTATCTATGTCTCAACCAACTATTGAATCGATACTTAAAGAA
AAACGTACCTTTGCCCCTTCATCGCAATTTTCAGAAAGAGCTTGTATAAAAAGTTGGGAGCAATA
TCAAGAATTATATAATCGGGCGGCTCAAGACCCCATTAAATTCTGGGAAGAATTAGCAGAGCAAG
AACTAGATTGGTTTCAAAAGTGGGATCAGGTTCTCGATTGGACTAATCCTCCCTTTGCTAAGTGG
TTCGTGAATGGAAAAATAAATATTTCTTATAACTGCCTTGATCGCCATTTAACCACCCCAAGACG
CAATAAAGCCGCCATTATCTGGGAAGGAGAACCAGGTGACAGTCGTACTTTAACTTATGCTCAAC
TTCATCGGGAAGTGTGTCAATGCGCCAACGTGATGAAGCAATTAGGAGTGAAAAAAGGGGATGT

TGTTGCTATTTATATGCCCATGATTCCCGAAGCTGCGATCGCCATGTTAGCCTGTGCCAGAATTG
GGGCGGTGCATAGTGTGGTATTTGGGGGATTTAGTGCCGAAGCTCTCAGGGATAGAATTAACGCC
GCTCAAGCAAAATTAGTGGTAACTGCTGACGGTGGTTTCCGTAAAGATAAAGTAGTTGCTTTAAA
AGAGCAGGTTGACTTAGCCATTGCTGATAACCATACCCCTAGCGTAGAAAATGTGTTGGTGGTAG
AACGTACCAAAGAAAAAGTACCCATGGAAGCAGGTAGAGATCATTGGTGGCATGACTTACAAGCA
GGTGTTTCTGCCCATTGTGAACCCGAAATGATGGATAGTGAGGATATGCTATTCATTCTCTATAC
CAGTGGTAGCACGGGCAAACCGAAGGGGGTAGTTCACACCACAGGGGGGTATAACCTTTACTCTC
ACATGACTAGCAAATGGATTTTTGACCTCAAAGATGAGGATGTTTACTGGTGTACTGCTGATGTG
GGTTGGATTACAGGTCACAGTTATATCGTTTATGGACCTCTTTCCAATGGTGCTACTAGCGTCAT
GTATGAAGGTGTACCTCGTCCTTCTAACCTTGGTTGTTTCTGGGATATAATCGAGAAATATGGAG
TAAATATTTTCTATACCGCTCCCACTGCTATCCGTGCGTTTATCAAAATGGGAGAACATCACCCC
AACGCCCGTGACTTGTCTTCTTTAAGGCTTTTAGGTACAGTGGGCGAACCCATTAACCCAGAAGC
ATGGATGTGGTATCATAAGGTAATCGGGAAAGAAAAATGTCCCATTGTGGATACTTGGTGGCAA
ACGGAAACAGGGGGCGTTATGATTACTCCTCTCCCCGGTGCGACTCCTACTAAACCGGGTTCAGCT
ACCCATCCTTTCCCCGGTATCATTGCCGATGTGGTGGATTTAGATGGTAATCCTGTGGGTGATAA
TGAGGGCGGTTATTTGGTGATTAAGCATCCTTGGCCCGGTATGATGAGAACAGTATATGGAGATC
CCGATCGCTTCCGTAATACTTATTGGGAACATATCGCCCCGAAAGATGGACAGTATCTCTATTTT
GCTGGAGATGGTGCGAGACGGGATGAAGATGGTTATTTTTGGGTAATGGGTCGTGTTGACGATGT
AATCAATGTTTCTGGACACCGTTTAGGCACAATGGAAGTGGAATCAGCTTTAGTATCTCATCCTG
CGGTAGCAGAGGCGGCGGTTGTCGGCAAACCTGATGATATTAAAGGGGAGGAAATCTTTGCTTTT
ATTACCCTCGAAGGTGGTTTTACTCCCAGTGATGAGTTAGCAAATGAGCTTAAAGCCCATGTCGT
CCAAGAAATAGGTGCGATCGCACGTCCGGGAGAAATCAGATTTACGGATGGAATGCCAAAGACTC
GTTCAGGGAAAATTATGCGCAGATTATTGCGTAGTCTAGCCTCTGGGCAAGAGATTGCAGGGGAT
ACTTCTACTTTAGAAGATCGTGGCGTCTTAGATAAATTAAGAGAAGGGGCATAAACTATAGCAAT
CCTATTTGAGTTATGAAATTATCATAAAGGTAGGTGTCAGGTTTGACCCTTGCAGGTGTTGAGGT
ATTAGGGGATGAGGGGGAGGTTAATTAAACTCTTTTGCCTTTTCCCTTTTCCCTATCTAAACTAA
TAATTCAAATACTCTAAGTAATAAAGCCTAAATTCTTCCTTTAGGTTTGATAAAAGAAAATTCGG
AATTCCGGTTTTAAAATCCGACTTTTGCCAATATATTTAAAGGTTTATCTTGTTTAATTTTCTCT
AATTGTTCTTGATTTCTTACTAATAAAGCCCCAGCAAAACCTAATGAATTAACAGAAATTGATTC
GTATTTTTCTTGGCTTCTAGGAACTACTTTTATCCAGTCCTGAGTAATTAGTAAATTATAATCTT
TTGACGGTTGATTATCTTTTATTTCTATTCCTAAATCCCTGAGAATTTGTCGATAATATTCTGTT
GTTAGTTCGGCAATGTATTCTACACTTTTTGTTAAAACTTCAGATGAAAAATACATAATTCTGTG
TTCATAGGGAAAAGTATCTAAGGTAACTTTTTTATTAAATTCTTTTTTTTCTAAGAGTACTTTAT
TAATAGGTATATTGCTTATATTATTAGTAAAAATTTCAGGAATTAATTGTAAATGTTTATGAGG
TTGAGAAGCACCAGATAATTTACCACAATTATAAAAACCTAATCCCTTAATTTGTTTTAAAAGAG
TCCAAAGAGCATAAAAATCATGAAAGTTGAGAATGTCATTTTGAGACTCAAAATCACGGGTAAT
AATTAAGCAGTGATTATCAACAACATTATATTTGTTCAAAATTGCTAAATGAGTATCACTAATAT
TCCCTATAAATAAATTTTCTTCGTAGGGTAAAAAAGGATTAAAATTAGGGTCTTTTTTTTGTACT
TTATTTTGCTCTTTTTTCGCTTTGTCCTTACGATTTAGATTATCAACAACTCTAATAATAAAAGG
AATATTATTATCTTCAAAAATTTGGTAATGATTAGGAATAGAATGTAAAGCCTCTAATTGAATT
GCTTTTTCTGTTGCTAAGGTTATTTTTTGCCACCAATTATCCTTGATTGATTTCATTTTTATAGT
AAGTAGTTAAGAGTTAATAATTAGTCGGTATCAGATTGTAGGTAAAATTTTTAATCTTTTATTT
TAAATTATTTATTATTTCTACTTTATCTGGTGTTTGTAATGGTTGACAAGTTCTTGCGTTAGCAG

GGGGAATTGGCAAATTAAAGCGACTATGAAAGTCTTTTTGTACTTTATAACTTAATCTTGGTGAT
ACTTGTTTAACTATCTGTCTCAAGAGGCGATCGCCTGTATCATAAATTACTGACATTGGTAGTTT
GTAAATAAAAGGGGGAAAGGAGACAGTAACAAATAAATCTAAACACCAATTAATGCAGGTAATA
ATTTCAGGAGGGCTGATTTTATGACGATGATAGGCTTTTTCTATCCCTTTTGCCGCCGATGTGAT
GGGGATGGGATTAATCGTCATCTCTGAACGATAATTAACTTGATAACCAGAATGATTAAATTCGG
GATTAGAGATAGAATACATGGTGTAGTGATTTTCTATGGGAGGCTCTAAAATAACACTCATTTGC
GGTTCAACATTATAGCCAAATGCACCATAGTGTCCGATAATTAGAGTATAACCATTTTCACCATA
GGGAATTGCTTTCATCGGTTCAGCACAACGCACAAACCACCCTCGATGATCATTAAGATAGTTAC
AAACTGTTTCTGAGTCACTATACATTTCCATGATTCCTGTAAAGTGAGTTTGAAAGGTTATAGGT
TGATTTTCTCTAATTTCTTTATCTTGATTTTCTCTATCTTCTGGCATGGCGTTAATTTATCTTTCC
CTCATCATTGATTATGGGTAATGGGAGATTGGGTGGTGTTAAGATTTTGGGGAGAATGAAAAAT
AAAAAACCTTGTGGAATAAAGCATTGAGAGCAATAATTAACTCTCTTTGTACCGATAAAAACTAA
AACTGTTAAACTTAGAAAAAAAGATATAAAATCAGTTTATCTATTATTTTTATCAATACATTTA
AGGCTTCACTCTGAACTCTGTTTTCATCGATTCTAAATATTTTTCATAACCGATTTTATCTAATT
CAGTTTGTTTATCTTCTACCATTTGTCGCAAATTATTACTATATTTAAGCACTTTCTCTAGTAAA
TCGGGTTGTTGGGAGGCTAAAATCCTAATTGCCAATAAACCTGCATTTTCCGCATTTCCGATGGC
AACGGTAGCCACAGGAATTCCCCTAGGCATTTGCACAATGGAATATAAAGAGTCAACACCTTGTA
ACTGTCTCGTGGTTACTGGCACTCCTACCACTGGTAAAGGCGTAAGAGAGGCAACCATTCCGGGA
AGATGGGCGGCCCCCCCTGCCCCTGCGATAATTACTTTTATTCCTCTTTTATGGGCGTTTTGAGCA
TACTTTACCATTTTTTCCGGGGTACGATGGGCAGAAACGATCGCAACTTCATATTTAATGTTAAA
CTGTTCACAAACTGCGATCGCACCTTTCATGGTAGGTAAATCAGAATCACTACCCATAATAATGC
CAATTTCGGGATTAATTTCAGACATTTGTATTTGTTGAATAAAAGGGGTTTTAACTACAGTAATT
TTAAATCATTTTGAACTAAAGCATAGCCTTGAAATTATTAAAAATGAGTAACAAAACAAAAATC
TTATCTATTGGTATTCTTAGTTTTTTGCTATCAATTTTAACTGTATTGATTGTTCAAGCCCAGTC
ACAATTAACCATTGAGTCGAAAGTGAGTATTAATAGTATTGACTCGATAAAAGTAGGAATGAAT
CTGCCCGAAGCGGCAATGGCAACAAAGACAAAATTATATCTTGCCTATGCAGGAAGTGATAGTTG
TTACTACTTACAAACAGAAGGAGAATTAAAAGATGTATCATTCATGGTAACAAAAGATGAACAA
AAAAGTAAACAAAAATATATTACCAGTGATACCATTGCTAGAATTGATATAAATAACCCCAAAG
TCACTACTATCTCTGGAGCAAAAATTGGAGATAGTGAAACAAAAATTAAATCAATTTATGGAGA
TAAAATCAAAGTGTCTGCCCATCCATACAATCCCAAAGGACATTATCTCACTTTTATACCCCAAG
ATGAAGAAGATAAAAACTACCGACTAATTTTTGAAACTGACGGAAAAGTTGTAACCAGTTACAG
AGTTGGTAAATTACCAGAAGTGGAATATATTGAAAGATGTAGTTAAAAAACCTCTTTTCTAACC
AATAAATTTTATCCCGAACTGAGATGATATAATGTTCGCTGAGATTTCAAGTAAAGGAGAGAGT
ATGACAAAGGTAGCCGTATTAGGCATGGGGTTGATGGGGTATCCGATGATTTTAAGGTTAGCACA
AGCTCAAATTCCCGTCATAGCCTATAATCGCACTCAAGCTAAATTAGAGTCTCTTAAAGAAAAGA
ATATTCCCGTTACTAGCAATATTCATGAAGCGATCGCATTTGGAGATGTTATTATTTTAATGTTG
AGTGATATTGGTGCTATCCAAGAAGTTATTTTCGATTCTGGAGTTGACTTAGAGGGAAAAACTAT
TATTCAAATGGGCACCATTGCCCCCGGAGAAAGTAAAGGATTATATGTAACAATTCAAGAAAAAG
GGGGAGACTATCTCGAAGCCCCTGTTTTAGGTAGTATCCCTGAAGTTAAAAGCGGAAAACTTTTA
TTAATGGTAGGTGGTGATAAAAGCCTATTTAGGGCAGTGGAAGAACTTTTAACTCATTTTTCCCC
CAAACCTCTTTACATCGGTGAAATAGGCAGTGCGGCGGCTCTTAAACTGGCACTAAATCAGATGA
TAGCAGGGTTAACCAGTACCTTTGCTCTAAGTTTAAGTTATATTCAAAAACAAGGTGTTGAGGTA
GAAACCTTTATGGAAATTCTCCGTAACAGTGCTTTATATGCCCCCACGTTTGATAAAAAACTACA

AAGAATGTGTGATCATAATTTTAGTAATCCTAACTTTCCTACCAAGCACTTGCTTAAAGACATTA
ATTTATTTTTAGCCTCAGCACAAACTATTAATCTAAATACAACAACTTTAGAAGGAATAAAAGAA
ATAACTACCCTAGCAGTGGAAAATGGAGAAGCAGACAGCGACTATTCTGCAATTATTCAAGCTAT
TGATAATTAAAAGGTTTCAGGTTGCAGGTTGCAGGTGGTAGGGGTTAAATATATTCAACCCTTAC
GGTGTTTGAGGGATAAGGGGATGAGGGGAGAGGGAGAAGTACGGGCGAATGGCCATTCGCCCGT
ACAGGGTTTCAGGTTTTAGAAGAAAGTAATGAGTAACGAGTAATTATCAACTATTCACTATTAAC
TATTAACTATTCACTTTATGATAATTTTTGCTACCTTATCCCTAATTATTTGGATTTACTTAATA
ACAGCGAGGGGAAATTTTTGGTTATGTAATCAATTTTTAGAGTCACAAATATTAAAAGAAAAAC
TAAATGTAACAATAATCATACCTGCAAGAAACGAAGCAGAAAATATCTCTATTTGTTTAGAATCT
CTTTTAAATCAAGATTATCAAGGAGATTTTAAAATTATTTTAGTCAATGATCAAAGTGAAGATA
ATACTGCACAAATAGCAGAAAATTTAGCCAGAAAAAAAAATCAAACTCATCGACTAATTATTCTT
AATGGTAAACCATTACCCCAAGGATGGAGTGGTAAATTATGGGCAATGTCTCAGGGAGTTGAATG
GGCTAGACAAAATTTGTCAACGGATTATTTTTTATTTACTGATGCAGATATAAAACACTCGGTAG
ATAACTTAAGTCAGCTATTAGCAAAAGCGGAAAAAGATAACCTTGATTTAGTTTCTTTGATGGTT
AAATTACATTGTCAAAGTTTTTGGGAAAAGTTATTAATTCCTGCCTTTATTTTTTTCTTTCAAAA
ACTCTATCCTTTTCCTCTAATTAATAACCCTCGTAGTAAAGTATCTGGGGCGGCGGGGGGTTGCA
TCCTGATTAGAGAGAAAGCCCTAACTCGTATTGGTGGCATTGAAGCACTAAAACAAGCATTAATT
GATGATTGTACTCTAGCTAGTTTAGTGAAAAAAAGTTTACCCCCTAATCATGGTATTTGGTTGGG
TTTAAGTAACAATACTGTTAGTTTAAGAAAATACCCCACCTTAAACCCTATATGGAATATGGTAG
CACGGACGGCTTTTACTCAATTAAATTACTCGACTTTGTTGTTAATAGGTACTATTTTAGGAATG
TTTATCACTTATTTAATCAGTCCGATCGCATTTTTGTATAGTTTATATAGTCTCAATATACCTTT
ACTGATTATCTCTCTAATAACTTTATTATTGATGGCATTGAGCTACTATCCCACCGTAAAACTCT
ATCAGTTACCAATATTTTATTCCTTTTGCTTAAGTGCGATCGCATTTTTATATACTTTAATGACT
ATTGATTCTGCTTTTAGACATTGGCAAGGAAAAGGAGGGCAGTGGAAAGGTAGGGTTTATGGTA
ATTAAAATTATCAATATAACTTGAGCTTGGTAGAGAAAGACAATAGGCAATAGGGAATAGTGTT
GGGGTGAATAGTTGATAATTAAGAATTAAAAATTAAAAATTAAAAATTAAGAATTAAAAACTCT
GAACACTCACTACTTTCTTCTAAAACCTGAAACCTGACACCTGAAACCTGACACCTAACCTTATCG
GATATTCTTAAACCGAACTGAGGTTATCTTAATCCCCCAACGTCTTAACAACCGCCTTATTAATA
TACTCACTCAAAGTAACACCATCAACCCCCATTTCCGTATGATTTTGAGCGGCTAAAATTCCCCCT
TGCTGATGCAACCAACCACAAGTCGCAACTACCTTGGCAACGGGAAAAATGTTTAAATTAGTTTG
GGCGATAACTGCCCCTAAAAAACCACTTAATACATCTCCACTTCCTCCCCGTGCTAAGGCTGGTGT
GCCTAAATTGACAATCCAATTATTAATTCCCTTTTCGCTGACTAAAGTTTTTGCCCCTTTGAGTA
AAATAGTTGTATTAGTCATTTTAGAAGCCATTTTTAAGACTTCTAATGAATTTTCTGATAAATTG
AGTTGGGGAAATAGTCTTTTAAATTCCCCTATATGAGGAGTAAGAATGGTTGGAGATTTTCTTTG
TTTAATTGTCTGCCAATATCTCCCTTCCGCCAAAATGTTTAAACCATCTGCATCAATAATTAAAG
GCACTTCACTATTTAAAATTGGTGCTAATGTTTGCTCTTGATATGCACTAACTTCTCTACCTATT
CCCATACCAAATGCGATCGCATCATATTTGTTTAAATCCAGATTACGAAGAGAAATAATAGTTCC
TTCTTCTTTTACTGGAAGTCCAATTACTAAAGCCCAAGGAAAATGCGATATAAGGGTTTTTTTAA
GCCCATGAGGCACAGCAAAAGTAATCATTCCGCTCCCCCCACATTTTGCACCATAACCAGCTAATA
AAGCCGCTCCTGCGTACTGATTTGAACCGCAAATTAAAAGTAAATTTCCCTGTTTATATTTATGG
GTATTGACATGACGGGGTAAGGGTAAACAATTTTTGACATCTTCTTTAGTAATGATATGGGGATT
TGTCTCAGATACATATTGACAATGGAAACTATAATTAACATTTTCTGTCTGATTCTTTTCTCTTT
TTCTCTCCTCACTATTTGGGGGGTATAAAGGGGGGTTTCTCTCTTCCCTTTCATCATCATCATTTC

TGATTCCCTCACCCATAAGCAAAGATTCAGGAATACCTATATCAATCAAATGCAAACTCCCCGTA
TATTCCACCGCCGAAGAAAGAAATAAACCAACTTTCCACAAACCCAAACAGAGAGTATATACTGC
TTTTACTGCTATGCCTAAAACGTTACCTGTATCAGTGTGAATGCCAGAAGGGATGTCGATACTAA
CCACTGGTATGGATTGTTGATTTAACCAGATAATATCTTCCCTTAATTTGCCCTCAATATTACGA
GTTAAGCCAAAACCAAATAGACAATCCACAATAAAATCACAAGTGGATAAATCCTCAATAAATTC
GACAGAAGCAATACCCAAAAATTCAGCGTATTGTAAATGTTGTTTAGTCAAATCCTTTGATTTTT
CTCTCAAGGGAATATATAGAGATACCTGAAACCCTTGTAAAAATAATTCCCTTGCAATAACTAAA
CCATCACCACCATTATGACCACAACCAATAATTAAACCAACTTTTTGATAAAGATTTAGGGGATA
GAGTTGGGTAATTTTTTGACTAGCAAACACCGCCGCCTTTTCCATTAAACTAGCCACAGGCATCC
CCTCAGAGAATATTCTTTCCTCTATCTCCTGCATTTGTTTCGCTGTTACGGTAATAGATGTTTTT
ATCATCAACAATTAAATATTAGAAATAAATTAGGAATTAGTAGTTAAGACAAGGCAATTAGTGA
ATAGTGAATAGTTGATAATTAAAAATTAATAATTAAGAATGAAAAATCACGAATACTTATTACT
TATTTATTATTTGTTTCCCCCTCTCCCCTCATCCCCTCATCCCCCTATCCCCAACACCTGCAACCTG
AAACCCGACACCTTATCTCCCTCTCCCCTCATCATCTCATCACCTCATCTCCCTAACACCTAAAAC
CTGCAACCTGAAACCTGCAACCTCTAACTTAACTTACTACCCAATAGAAATAGACTAAACAATGT
GCCACTATTCCACAGACTATGTACCATAATAGAAGCTAAAAGACAACCAGAGCGAGTATAAACGA
TTCCTAAAATAATCCCCAATACTGCTAATGGTAAGGCTTCCGCTAAACTCAAGTGTGCGATCGCA
AATATAATACCTGAAACAACGATTGCACTACCCACTGACATATAACGAGTTAGAGAAGGAAGTAA
AAACCCCCTAAATATTATCTCTTCAAAAATAGGGGCGGCAATAGAGGCAGTAATGAAAAATATA
ATTAAAGCAAATTTATCCTGAGACTCTAAGGCTAACATTAACAAAGGATTACTTCCACCTTTCCC
TTGCCATATCTGTTGATTAAGTAAAGAAACAAAGAATACGAGAGGGATTGCTGTTAAATATCCTC
CTAAGCCCCACCAAAACCAATTTTTATTCGTTAATTTAAACCAATCTGTCGGTAGAGGGAAAAAA
GATTTAATAGATAAATATAAAACTGATAACCCTCCTCCGGCCATTAATAAGTAAGTAACTAAAAC
ATATAATGCTTTACCTTTTATGTCTAAATCAGTCGGATTAAAACCACTTACGCCAATGAGAATCG
GTAATAAAATCTGAGAAAGAAAGAAAAATCCAACGATTAAAACTTGCCCTATTATTTCCCAATCC
CAAGGAGAATCCCACGTTTTTCCCGCATTAGTTGCTAAAATTGATTCTTCTTTTTTCAAGGCTAA
TTGTACAATTAAGAAAATAATTAAACCAAAACCAATCAAGCCTCCGACAACAGGAATAACACTTA
GTAATAATAATCTTTTGATGGCTTTTTCTGCCAATTCTTGGGTTTTATTATTAATTTCTTCTAAT
TTGTTAAGATTATTTTCAATAGCATACAGCTTAGATAAAGTTATATTTTCAAACCATCCATTTAA
TTTTTTTTCGATGATTTCTTGTTTGTTTACACTAATATCACTATTGTTTATCCAAATTGAATTTA
ATATTTCTGCTGTGCTTTTATCGTTAATTTCCTGCCAATAAGCGATTGCTTTATCATTATTTTCT
TGATAAGCATAAATAATTCCTAGCTTAATTTTTAAGTCTTCAATAAACGCCTGATTTTCCTGTAT
TTCTTGCTTTAATTGCTTTCTAGGAGTTTCTGAAGCAATTGCTTTATCTTCTTGATTGGAAATCT
TTTCACTTCCTTTAACCGACTCTGCTTCAGTTAAGATGTTATTATACTGCTCCTGTGCGATTAAG
TAGGGATTATTGCCGAGAAGGTTTTGGGTAAAACTTTTTTGATTGTCTGAAGATGGTTGCCACTC
CGAGGCGTTAAGAATCAGATTAGTTTGATATAACTCCAAGCGTGACTGAATTTGAGTCTCTGTTA
AACTTTGAGTCAAGGCAAAAAAAACGCTGTATAGAGAAATAGCTGTTAATATGATAAGGAGCGA
TCGCTTCAAATTCATCGATTTTAGAGGGAAAAAAGAATAGTTATTTATAAATTCGGATATAGAT
ACAAAAAAATCATAGGGACTCGAAGACAAAATCAAGCCCCTAGACGTTAATAGTTGATTGTTGATG
GTTAATGGTTGATAGTTGGTAAATATTATAGTCAAGTTGGATCTTTTTGACAAGAACTTATATTT
CTACAATATCAAGAAAAACAATCTCAATTAATTAAAAAAGCTATTGATGATGTTTTAAATAATT
TGTTAATTCTTCACCATAATTCATCAGGGATATATAAAGTTTTTTTCATGATAATTATGTGTAAG
TATGATTAATATCATAATGGCTTATTTTTTATCTTTTCTTATATGAGAAAACTTAAATTCGGATT

TTTATGAAAGATCGCCCTTACAATGAATAATAGAGGATTTAAGGCTGATTTGATTATATGAGTA
AGCTAGTAGTGCTAAATTTAGGTGCGGGGAACTTAGATAATGGATTTCCTAATATCATCGCTCAA
TTAAGGGTGAACTACTACCCGACCGCTATCGCTGAGTCGGGAGCTTCATCTTTCACAGGTTCGTG
CCTCAAAACGGATTTACGTCCGCCTTTTGGTCTTACCGATCCTCCAGATGCAGAGACGGCTGTTCC
ATCCGTCTGTACTTTTCTGATACCTTCTGCTCTAATATTTTTACTTGCGTTCTCATCCCTATCATG
ATGACTATCACAACTAGGACAAGTCCATTCTCTTATCTCTAATGGCATTTCAGACATTTGATAAT
GACAATGAGAGCAAGTTTTAGAGCTAAGAAACCATCTGTCTATCTCCACTAATAACCCACCTTTT
TGCTTGAGTTTATAGTCTAGGAAGTTGATAAATGTTCCCCAACCGACATCAGTTATTGCTTTTGC
TAATTTGTGGTTACGAACCATACCCTTGATATTCAGTTGTTCAACGACTACCACTTGATTATTAT
TCACAACTTTGGTGGAGAGTTTGTGTAGGAAGTCTTGACGGGTATTGCTTGTGCGTTCGTGAACT
TTAGCGACTAGCTTTTTGGCTTTTTCTCTATTCTTAGAGCCTTTTTTCTTACGAGCTAACTTTTGT
TGTTTCCGAGCTAGGTTTTTTTCATGTTTTTTGATGTGTCTAGGGTTAGCATACTTACTGGTTTT
TTTGCCATCATGAACAATAGCAAAATCCTTAATTCCTAAGTCTATACCACAAATTTTCCCTGTCA
TATTTACTAATGGTAATTCTTGTTGGGTATCGAATAGGATACTAGCAAAATATTTGCCAGTGGGC
GTTTTGCTTACTGTTACAGTCTTAATCTCACCTTCAAATATCCGATGAATAACTGCTTTTACTAA
GCCTAATTGCGGAATTTTGAGACATCCATCAACTATCGAGACATTTTGGGGATATTGACAAGACT
GCTTTCCGTGATATGACTTGTATCTGGGGTATTTGGCTCTACCTTCAAAGAAGTTTTTGAAAGCC
TTAGTCAGATTAAGAGTCGTTGATTGCAGACATTGTGAGTAACAATCTCCTAACCAAGCCGTATC
TTCCTCTTTTTTTAGCTTAGGGAGATACTTATTAAGGGCTACTTGCTTTAGTGCCTTACCTGTAG
CTTTATAAGTGTCAATACACAAGTTTAGAGCGTAGTTATACCACCAACGACAACAACCCATAATC
TGAGATAATTTCTTGGCTTGGGTTTTGTTGGGATAGATTCTGACTTTTATCGCTCTGTGCATTGA
GGCTTATTGCTAACTTAGGTTGCCAATAAATTTATTATAAGTAATGATTTTGGTTTTGTGTCGAA
TCCTACGGATATTTTTTTTATCGAGGCACAATTCATCCGCTAATCCGCTATGCTGAGATTAGGGA
CTTCTTTTGCCATTTACGTTAAAAGACGGCAAAATAGAGCAATTTATTGGTAGTTTACCCCCAGC
ACCCGAATTGGCAACCCTTAACCAACAGTGGCAAACTCTTTATTATGCGTTACATCAGCGTTTTG
ATGTGGCTAGACGCAGACTTTTTGAAGTAGAAGAACAAGGAATTACTAATATCTCAACAGCAACA
TTTCAAGAGCTTTGTCAAGAAATTGAAACTAAATTTAATTCTTGGCTTGATTCTCCCTCATTTTT
AAAGATAGATCGCAAATTACGAACTAAATTAGACTCTAATGAAGAAATACAAATCATTATAGAA
ACGGGAGATTATACCCTACATCGTTTTCCGTGGCATTTATGGCATTTTTTAGAGGATTATCAACA
GGCAGAAATTGGCTTAAGTAACTTTGAATATAGCCCCATTAAACTTGAAAAAAAGCAGAGAAAT
AAACCGAGAATATTGACTATTTTAGGGCATAGTGAAGGCATAAATATTAAAGCAGATCAGGCAT
TAATTGCCCAAATAAACAATGCAGAAATAGTGTTTTTAGTCGAACCCAAATCAGCCGAATTTCAT
GAGAAATTATGGGATGAAAAAGGATGGGATATATTATTTTTTGCTGGTCATAGTCATACAGAAA
ATTATCAAGGAATTATCTTTATTAATCCTCAAGAAAGTTTAAACATTAAAGAATTAAAAACCGCC
ATTAATAAAGCTATCAAACAAGGATTACAATTAGCTATTTTTAACTCCTGTGATGGTATTGGATT
AGCAGAAGAATTAAGTAAATTAAATTTACCTCAAATCATTGTAATGCGCGAGGGTGTAGCCGATT
TAATTGCTCAAGAATTTCTGAAATATTTTTTATCCGCTTTACAACAAAATAAATCAGTTTTTTGC
TCATTAAGAGAGGCAAGAGAAAGATTACAGGTATGGGATAAAGATTTTTCCGGAGGCAGTTGGC
TACCGATGATTTGTCAAAACCCTTCTACTATTATTAAGTCAGAAAATCAGGTAAATTCTGGATTT
ATAATTAATAATTTATATGAGTCTATTCGAGAAAAGTTAGAGATTAATTTTCTAGGAAGATTTA
AGAAATCAAAAGAAATAAATGATAAAGTTGATAAGAATGATAATAATAAAGATAAGCTAATTGA
TACATCTGAAGATAAACAAGAAATTGATTCAAATAATGAAATCGATAATGAATTAAAAACTAAA
TCACCAGAAAATCCAAACAAACAGAATAAACCTAATAACACAAATCTTAATATCAAGATTGCTAC

TTTATTAATTATTTCTATTGCAACAATATTTGCTGTTCAGATTGGGAAAGCAAAACAAGAGTTTT
TCCTTTATTATTACTTATTAGCCGAACAAACAAAAAATTATTTATTAAATACAAAAACTGTTGCT
AATTTTTTTACCATAGAAACAGTAAATGATATTTTAAATCAAAAACCAGATTCATCAATTTACGA
TAAATTAGCTGTTATAAATGCTGAAAAAGGTGAACATAAAAAAGCATGGGATTGGTATAGTAAA
GCCATAGAATCTAATCCTAAAAATATTAACGCCTATATTGGTAGGGGATATGTCAATCTGTAGTT
TAGACTAAGAAAAAATTGCAAGACAATCAATAAGCTAACAAAAATTAGCTTACGGAGAAAATAA
ATTGAGTTAACTATTCCTTAAAAGTTAGGTTTTTCGCCTTTTTTTACTTTTGGATTGCTGTTTTT
TAATGGTGGGATAAACTTTAGCTTTGTTTCTTTTTCGCCCTTGAGGCCAGCCATGGGATTTTCCC
CGACTTTTAGGAGAAAGGGTAGGACTTCCAATCTCTATCAATAATGAAGTCATAGATAGAGCAAC
CCTTCCAGGAGTTAAATTCTGTTGTGGTTTTTGCCACGGCAAATGGTGTTCTTGGACTAAATCCT
TTGAGAGCCATAATTGCCAAGTGATATTGACGATTAAATTACTCCATCTTTGACACCTGTCAGGA
GTGCGAAAATTTGGCAAAGTCCAATGCAACCTCTGCTTGGCAAAACGATACCAGTGTTCTAATCC
AAACCTTCGCTTATATTGACTCCAAATATACTCCAAAGATAAAAATTCCTCTCCTACCCAGATTA
ACCAAAGTGGACGATGTAAACTCCCTGTTTTTTTCTCCTTTAATCTTTCAATTTTGAATAAAGTT
AATTGTTGTGTGGGGGCATTATAGAAATGAAGTTCCGTCCATTTACTGATGCGTATGAGTCCTAG
GGATGAATCTTCCATTTCCAATACTTTATTACTCTGAGGAAAATCATCACATTGATTTAACTTCC
ACTTTGCTCCATGTTTTTTCGGTCTTCCTCTACCGCTATAAGGGTCTGGCTTGCTATAAAGGCATA
AATTAGAACGTACTCGAATCAACTTACTTATCTCAATGTCCGCCGTCTGTTGGATAAATTTGCCA
TTGCCATATTCACAATCTAAGACCACTAATTTCCGTCTCTTTTCTGGCAATTCTCGACACACTTGT
TTGAGTTGCCATCGGGCTTTACTTAAAGGAGTTTCAAAGGATGTTATTCTTTCATGTCTTAAAGG
TAATGCCCAACTTCCTTTCTCTTTTAATGGTGGTAACCATGCTATGGTGCTATATCCTTGTCCTAC
TACTGATGAATTTAGGGATGAATGAGAATATTGATAACCTCTATCTTTCATCGTGGGACTATCTT
TACATTCCCACTGGGTATGATCTATGCCTAATAACGTATATTCTAAAGTGTCTATTTCTTGAGTG
TATCGTTTCATCAATTTGTTGCCATTGGGACGACTATCCTCTATCGCTTCATAGGTGCTACACCAT
TGCCTCTGGAAAAAAAGGCGATAAAGAAAATTCTGCCAAACTACGAGCATTTTCTCTGGTCATGAT
GCTGTCCATTAGCTCAAATGTGGCATCTTTACTTTTGACTAACATTTCATAGGTATCATGACGAA
AATTTTTTAGTCTGTTATATTTGTTCATGTAGAGAGATTTTAATTTGTGATTATTTTATTTTCTC
TCTATTTTTCTTTTTTGTCTTGTCCTTCCTCATTTTTCTCTACATTTAGTCTAAACTACAGGTCAA
TATAGAATTAAAAAACTATGATTTAGCGAATCAAGACTTAGATAAAGCTATTCAAATAAATCCT
AATTCTAGTAATGTTTATCATATAAAAGGGTTATTAGAAGATGATAAAAATAATTTATTAGATG
CGGTCAGCTACGCTGTGCCTTTGGCATCGCACTCTATGATCAAGCCATAGAATTAAACTCAGAAA
ATCCAGAGGAAATGGCAAGAATTTTTAATGATAGAGGGGTGGCATATAGGAAGCTAAGTTTGAC
AGCAAAAGGCGATAATATTAAAGAATATAATCAAAAAGCCTTAAGGAATTTTAACAAAGCAATT
TTATTAGATAATAATTATGGGTTAGCTTATTATAATCGAGGTTTATTATATGAGCAATTAGGCT
ATCTTGATAAAGCAAAATTAGACTTAGAAAAAGGGACAGGATTATTGAAAAAATAGAAGATAAA
ATTAACTATCAAAAATCTTGTGAAGAATTAAAATAAATAAATCAGTCTTTTAAAAATAAAAATC
TAATCTTTTCTTAACAATTCATTATGTAGAAAATAAGATAAAAAATAATTATTTTTATAATTAA
AATATATGCACACCTTTTAAATTTTTTGTGATAGTTTGCATAAAATACAAAGTACTCCCTGATAT
ATAACCCTCTTTTGTCATTTTCCGAGATAAAATAATAAGAACACCAATAGCTCCCACTGAAGTAA
TTAATTCTTTAAACCTGACACCTTTTTTCTTTTTTTAGGGATAATTTATTATATCCAAGGTAAGA
GAGGCATTTTTTGCACTTACTATTGTTTTCATTTACTAATTTATTTATACTTCTCAAGATATAGA
TTTAACCTAATAGGTAATAAATTATGTTAGAAGTGAATATCCCTACCATAAATAAACAAAAAAT
TCAAGATGAAATTATTGTAAAGATTGCATCTTTTTCGCAAAAAAATAAAACAAATAATCGCTAT

AATAATTATTGGGATTCTATAAATAAACAAATTGATTATTTACAAGAATTAATTGAAGTTGGTA
AACAGAGAGCAAAAGTTAGGGAGAATTTGCCTCAGAATTTTGATAAATTTCCCTTAATTTTAGTA
AAACCATTTATGAGCAAAAGTCTTAAGATCTTGCGATTTTTATTTAAGGATCAAAGAGAAGTTA
ATAATAATATTTTACAGGCTTTAGAAGAATCCGTAAAAATCAATAAAGTTTTGTTGGCAGAAAT
TCAAACTATGAATTCTAATTTTGAAGAAGATTTGCAGGTTTTAAGTTCCATTAATAGCAATTTAA
AGAATAAAAATAAATCTTTAGAAAAAACTATTAATCAACTATAAGAAAAACAGTTAATAATAAA
ATAGTTGTCAAATAAACCAATAATTGATAATATTTTTTATCTAGTAAAAAGATAAAATTTCTGG
TAAGATTTAGTGGTTTATTAATTTATAAATTTTAAATTCTTATTTTTACTGTACTTAGATTCAG
CACTGAGTGTGACATAAATCATGAAAATAATTGTTCTTTCAGTGTCAGCACCTTTTATTATAGGA
GGAGCTGAAATTCATGCAGATTCCCTGCTCAAAGCATTGAGAAAATTTGGTTATGAGGCGGAATT
GGTGACTATACCCTTTAAAAGTTACCCCAATCAACGCATCTTAGATACTATGTTAATGTTTCGTC
TTTTAGATATTACTGAATCTTGCGAACAAAAAATTGATTTAGTTATTCCTCTCAAGTTTCCTGCT
TATTTAACTCCTCATCCCAATAAAGTATTATGGTTACTCCATCAACATCGAGATGCTTATGATTT
ATGGGGTAAACCTGTATGTGGATTGGCTCAAAATCCTGATGGTCAACAAATAAGGGATACTATTA
TTAATGCTGATAATCGTGCCTTTGCCGAATGTAAGAAAATTTATGCTAATTCTCAGAATGTAGCT
AATAGACTCAAATACTATAATAATGTTGATTCTATTCCGTTGTATCACCCCCCCCAAAATGCGGA
AAAATTTTATAGTAAATCTGCTCAACGTTATTTCTTTTTTCCTAGCCGTCTTACCAAAATTAAAA
GACAGGAATTGATTTTAGAGGCAATGGCAAAAACTCATTATCCTGTTAAAGTTGTGTTTGCAGGG
GCGGCGGATGATGGAAAATATGACCGAGATTTACAAATCTTAGCGGAAAAATTAGGTATTGAGG
ATAGGGCTATTTTTGTGGGCAGAATCTCAGAGGAAGAAAAATTAACCTACTATAGCGAATGTTTA
GGAGTAATTTATCCTCCTTTTGATGAAGATTATGGCTATATTACCCTTGAGGGAATGTTATCTTC
TAAACCGATTATTAGTTGTCTTGATTCTGGTGGCCCTTTGGAATTTTTAGTTAATCAAGAAACGG
GTATTATTGTTGATTCTACCCCAGATGCGATCGCATCTGCAATGGATCAATTATGGGATAATCCT
AAACAGTCAATGATAATGGGAAAAAATGCTCGAAGCCATTATGAATCAATGAATATAAGTTGGA
TGAATGTGATTGAAAAGTTAACCAGTTAGTCAAAAAATAGAGAAGAAAAATAATGAAAATAAAT
TGGTTTTCCCCTTTACCTCCTGCTAACAGCGACATTACCAACTACACCATAAAGTTATTAACATTT
CTACAAGAAAAAGCTGAAATTACCTTTTGGACAAATCAAAGCCCATGGGATACTAAACTAAAAAC
AGAAGTAGCCGTCAAGGAATATAAAATTGAGCAAATATCGTGGTTAGAATTGAATCAAGGGGAT
GTCAATATCTATCATATTGGTAATAATGCCGAATTGTATGGGGATATATGGCAAATTAGTCGGCG
GTTTCCGGGGATAGTCATTCTTCATGATGAAAAGTTGCAAGATTTTTTTCTATATGCTTTCTTCTT
CTAAGAATGAGTATATTGAGTACATGAAACAATGTTATGGGGAAGAAGGAAATAAAAACGCCAG
ATTATTCCTTAATAATTATCATTCAAGGGAATTTATGGCAAAAAACTATCCCTTAACCCCTTTAG
CCCTAGAAAATGCCCTTGGAGTTATCACTTATAATTACAACAATTATTTAACACTCTCTGCACAA
AATCGCATTTTTGTGGGTTATATGCCTTCTTTGACTAATTTATTAATACCGATAACAATATAAA
CTCTAATCCTTTATCAGATGCAGATGCTCTTATTAATTTTGCTTCTCAAGTCTGTCAGTATCGTT
GTCAATCTAGCCTCGAGCGAATCGTTAATCGCATTGTACCAGAATTGACTTATCTTAACCCAAAA
ATTGAAACTTTAAATAATTTTGAAAACCTCGTTGATGCGATCGAATTTATTACCCTTCCTGTACA
TTCTAATAGTTAAAGATAATGATTTTTGTAACAAAATAAACAAAATAGATGCGATCGCTTCTTTT
AATAGTTAAGATGAAAGTGTAGCCATAAGAATCATTGATTTTTAATTGGGCAACGGGAAAACCT
AAAATTAACAGGGGGTAACTGTTATGCCATTAGAATACATAGATATGATTTCAATTATCGGTATT
ATTACCATTGCTAGTATTATTTTACTGCCCCTTTTGGCGTGGATTCTCACTTGGAATGACTCCCAT
TTGCATCCTTAACTCGAAGTTAAAAACCTTGTCATCGAAAAAAGAAAAAAAGCGAATCTGTGATA
ATGTAGATAGAGTTAATTAAATAGTGAGAACTAACCAAAATGCAAGTTAACGATTTGGGCTTTG

TAGCTACTATTTTATTTGTACTCGTGCCTACCGTTTTTCTCCTGATTTTATATATTCAAACGGGA
AAAAACGAAGCCTAATACATTTATTTATCTAAATCAAAAGATAATCTGTTTTATATATTATTATC
GTTTTCAAGGGTTAACGACTAGAAATCTTAAGGTCAGGTTCGATAGTATCTTACTTGATATTATC
TCTAGTTTTTAGCCCTTTAAATTTATGTTTGCAAAGATTTATTAAGACCACAGTTGCATTTAATA
ATATCTAATAATTCTTGAGGCTTATTTAAGATAAAATCTGGATTATATTTTTTTAAAACACTAGC
AGAATTAAAACCCCAAGTCACCGCCGCCACTTTGATATTGCTTTTTTTAGCAGCTTCTATATCAC
GGGTCTCATCACCAATATAAATCACTTCATCCTTTAGCAAATGGTGTTTATCAATGGCCTTATTA
ATTACTTTATTCTTACCAAAAAGAGTATTTGCAGAATAGACAAAATCGAAATAATTATCTAAGCT
ATTTTTCCTTAAAAACAACGAAACATTAGCCCCTAAATTAGATGTAATTATTCCTAAATAACATC
CTTTCTTCTTTAAATTACTTAAGGCTTCTTCCATACCTTCAAAAGGTTGTAAATAGGCAATATGT
TCATTTAATTGTCTTTTTACTCTTCTTAATAAAAATGGTATTTTATAGGCAGGAATAGGAGACTG
GATAATAACATCTTTAGAACTAAGATTACTCAATCTTACAATGTCACTTTCAGTAACGGGGTCAT
AACCAAATTCGTCTGCCAATTCATTCGCTATCTTTACTAAAGTAATGCGACTATCTGCGATCGTA
CCATCAAAATCAAAAATAAGCACTTTTAACGCCATGAAAGTTTCCCACATCCATATTCAAAATTT
AAAGTTTAAGGCTCGAAAATAAAAATAAACATATTTTGGGGAGTAATTAGTAATAAGTAGAGGA
GAGGTGTCAGGTTTCAGGTTTCAGGTGTCAGGTTTAGGGAGGTAATGAGTAATTATCAACTATTC
ACCATTGCCTATTGCCTATTGCCTATTGCCTTGTCTTAATTACTAATTACTATTGCCCGTCGCAAA
AATAACTAAAGACACTCTTGTTGAGAATAATCCCGTTACATTTATTGATTTTAACAGACTGAGTG
AATAATAATGGTAAGATGAAAACTGCGATTAATCAGCGTCAAAGTAAATTAACCATCGCAGTTA
GAGTTAAACATTATAGTTAATTAGTATGGCAAAAGTTCTCGTATCCGATCCCATTGATGAAGCAG
GGATTAAAATTCTTTCTCAAGTAGCACAAGTTGATGTTAAAACAGGATTATCCTCTTCAGAATTA
GCAGGTATTATTTCTGATTATGATGCTTTAATGATTCGTTCTGGTACTCAGGTTACGGAAGAAGT
CATCGAAGCCGCAACCCACCTTAAAATTATTGGACGGGCAGGGGTTGGCGTTGATAACGTAAATG
TACCTGCCGCAACTCGTAAAGGTATCGTTGTTGTCAACTCTCCTGAAGGAAATACCATTGCGGCG
GCCGAACACGCTTTAGCGATGATGTTATCTCTTTCTCGTCATATTCCTGATGCTAACCAATCTTTG
AAAGAAGGTAGATGGGATCGCAAAAAGTTTATGGGTTCTGAAGTTTACAAAAAAACCCTTGGTG
TTGTTGGTTTGGGAAAAATCGGTTCTCATGTAGCCACCGTCGCTAAAGCAATGGGGATGAAGATT
TTAGCTTATGATCCTTTTATTTCTCAAGAAAGAGCTAATCAATTAGGATGTACTTTAGTAGATTT
AGATTTAATTTTCACAGAATCTGATTATATCACCCTTCATGTCCCTAAAACCAAGGAAACAGCAA
ACCTCATCAATGCTGAATCTTTAGCTAAAATGAAACCCACTGCTAGAATTATTAACTGTTCCCGT
GGTGGTATTATCGATGAAGATGCCCTTTATGAAGCCTTGGCGAATGAGAAAATTGCAGGAGCGGC
TTTAGACGTTTTTGCTACTGAGCCTTTAGGGGAATCTTCTCTAAAATCTTTAGGTAGTAATGTTA
TTCTTACTCCCCATTTAGGTGCATCCACCGCCGAAGCACAAGTAAATGTTGCTATTGATGTTGCT
GAACAAATTAGGGATGTCTTACTTGGCCTTCCCGCTCGTAGTGCTGTTAACATCCCCGGTTTAAG
TCCTGATGTAATGGAAAAACTACGTCCTTATATGCGTTTAGCCGAAACTTTGGGCAATTTAGTCG
GACAATTAGCAGGAGATAGAGTCGAGAGTTTCAATGTAACTTTACAAGGGGATTTAGCTGATAA
TAACAGTCAACCTTTAGTCGTGGCGGCAATTAAGGGTTTACTTTCTAAAGCTCTTAGAGAAAGGG
TAAACTATGTTAATGCTGCGATCGAAGCTAAAGAAAGAGGCATCCACATCACAGAAACTAGAGAT
ACCACCGCTAAAGATTACAGTAACTCCATTTACCTCGAAGCCACAGGTACAAAAGGCACTCACTC
GGTGACAGGTGCGTTGTTGAATGATGGTGAAATTCGTATCATCAACCTAGATAACTACCCTGTTA
ACGTACCACCTAACAATTATATGTTATTTACCCTTCACAGAGATATGCCCGGTATTATCGGTAAA
ATAGGGTCTTTGTTGGGTAATTTCAATGTGAACATTGCGAGTATGCAAGTAGGACGTAAAATTGT
CCGTGGTGATGCGGTGATGGTATTAACCATTGATGATCCTTTACCTGAAGGTATTTTAGGGGAAA

TCTTAAAAGTACCCGGTGTCACTGATGCTTACACAGTAACTCTTTAATCGTAAATTTCCCGTTTC
TTTATAACTCCCCCTTATTAAAGGGGGCTAAGGGAAATCTCATTTTTAATTTTTAACTGATTGAT
TGAATTTATGACTGAAAATTGGTGGGAAATCCGAGTAAATTACATTCCCGAATTAGAAGATAGT
GTATTTTGGCGTTTACAAGAATTTGGTTGTCAGGGTACTGCTTTAATGAAAGAAGAGGACAATTG
GTTAATTAAAGGCTATGTCCCCACTATTAGTATTGATACCGTTGATTTAGCCGCTCTTGCTCTTT
CTCTCAAACAAGATTTTATTATGGCTGGACAGGAAAAGCCACCAGTTGTAACATGGCAATTAATT
AATAATGAGGACTGGGCAAGTAGTTGGAAGCAATATTGGCAACCGATGGATATTGGAGACCGTT
TTTCCATCTATCCTGCTTGGATTGAACCTCCTGAAAATTGCGATCGTATCATTCTTCGTTTAGATC
CCGGTTCTGCTTTTGGTACTGGTGTTCATGCGACAACTCAACTTTGTCTTGAATCTTTAGAAATG
CGCATAGATGAGGATGAAATTAATTTAAACATAGCAGATATTGGTTGTGGTTCTGGTATTCTTTC
CATTGGAGCAAGACTTTTAGGGGCGAAAGAAGTTACAGCAGTAGATACAGATCCTTTAGCAGTGA
AAGCAACAAAAGAAAATAGTTTACTCAATCAGGTGGATAATATCAAAGTCTTTCAGGGAAGCAT
TGAAGAAGTGAAATTGCAGGGGCAAAAATTTGATGGTATTGTTTGTAATATTTTGGCAGAAATT
ATTAAACCGATGATACCCGAAATGGCAGAGATTATAAAGCCCGATGGTTGGGTTATTTTAAGTGG
TATTTTAGTAGAACAATCTAACGAAATTGCTTCTATTTTAGAACAAAATGGATGGGCGATCGCAG
CCTTATGGAAAAGAGATAATTGGTGTTGTTTGAATGCGAGAAGGAGTTAAAAAATTGTCTTAAA
TTTTATCATATTATATAGAGGAATTAAATAGTTAATTTTTGTAATTAATCAAGAGAATTGAAAG
AATGTTTTACTATAATAAATACATCTAAAATTTATACTGGAGGTTGATCTATGTCTTCATCGACT
TTGAACAACAATCCGACAACTATCGGTGATTATGCCTATGGGGCAATTTCTAAGCATAGTCACAA
AATTTTTAAATATGAAAAAAAAATTTTTCAAGAAAATGATCCCGAAAATCTACATCAAATTCGG
GTGGCGATGAGACGCTTAAGAAGTTCTTTACTTAGCTTCGATCGAGTTTTAATTCTTCCATCTAT
AATATGCGATCGCACTGTAGGAAAAATTGCCCGTATTCTAGGACAGGAAAGAGATGCAGATATTA
TTAATATTAACTTAGAGACAAAATTTAAACCTAATTTACCAAGAGAAGAAGAAAAATTTTTATC
AAAACTAGAAGAAGAATTTAATCAAAAAAAAACATCTTCATTTTCCTAGTAGTAAATATATTTTA
AAAGGCAAAAAATATTTAAAATTAAAACAAGCCCTTTTAGATTGGCTAGACAGTCCGGAATTAA
ATAAAATTTCAGCTCTGAAAACAGAAAAATTATTACCAGATTTATTACTTCCACAAATTGCCTCT
TTTTCCCTACAATCAGCATGGTTAACAGGTACTGAATTAGAAAACAATCACGAAATAAAACTTCA
ATATAATCTAACAGATACAGAAATAAAAGAAATCCTTAATCAAGAAGGAAAATATTTACATCAA
CTAAGAAAACAAGCTAAAAAAGTACGTTATCAACTAGAATTGTTTACTGACTTTTACCCACCTAA
ATATCAAGAATATATTAGTTTTGTTAAACAGTTACAAACGGTTTTAGGAAATATTCAAGATAAC
TTATGCTTAATGAGTTATTTATCTGATTTAGTAGGAAAAAAATGGGAAAAAAATCTCCCACAACT
GTCATTATTAATCAAAGAAGATCAAGAAAGACAATGGCGAGAATGGCAAAAATTACAAAGTATT
TTTCTTTCTCCTAATTATTTGTCTCATTGGCGAGAAGTCATTATTAAATTTATTAATATTTAGCT
TTTCTACCAACAGAAAAATACAACATAATCTAATTAATTTAGATCCCTGTTTTCTTGCCTAATTT
TATGAGGAATTTCAACCATTATTTTTTGGACAATATCGTTAGGGTTATCATTTTCCTCAATACCA
ATAGTAATATCAGCTTGTTGATAAAGAGACTTTCTTTGTTCATATAAGCTGGTTAATTTTGTTTG
CAATTCTTCCCGCTTTAGTAAAGGGCGAGTGTCATCATCTTTTAATCTTTGTACCAAAACATTAA
TAGGAACATTTAACCACACTACCATACCATCTCTGAGATGACTCCAATTTTGTTGACGGAGGATA
ATTCCCCCTCCTGTAGCAATCACCGTATATAAGTAAGCAGAGACTTCTTGCAAAACATCACTTTC
TAACTGACGAAACTCATCTTCTCCATCTTCGGCAAAAATCTGATTAATACTTTTCTCACTGACAG
CGCTAATAATTGTATCCGTATCTAAAAAACGATATCCCAACTCTTTTGCTAATAACTCTCCTACA
GTAGTTTTACCTGATCCCATCATCCCAATTAAGTATATATTCAAACCCTTTAATAATTCTTTCAT
TTGATATTTCTGATCATTCTATAATTGCAATCACATAATATATCATTAACATCACCTCTGTTTAA

TAAGTGACGTTACGCAATTATTCATCTGTTTAGTTGTATTAGGTTTCAGGTTTCAGGTTTCAGGT
TTCAGGTTTGATTTTTTGAAAATTAATTCATTAATAACTTATATTGAAGACAATAAAATCTATTT
GTTAACTGTTTTCGTCAAAATCTATTAAATTTAACTCCGAATTTAGGTGTTTTTAAAGATTAACT
AATCAGTTATTTGGAGAAAACTTTCCCCTCGCCCTGTGGGAGAGGGGTTGGGGGTGAGGGAAAAA
CGTTTTGATACCGAATGCTTATTAAAGGGGATTTAGGGGAAACAAAAACTTCTTAGACAACTTCT
TAACTCCTAATTCCTAACTTAAGATAGTTATTATTTATTTCCGAGAAGAAAATAATTTACAATAA
AGCCGCACCTAAAAGTTGAGGTACTAATTGTAAAAGGAAAATAGCCAAAATGGGAGATAAATCT
AAACCCCCTAAAGGAGGTATTAAAGAACGGAAAATGTTAAGATAAGGATCTGTTACTGGGGCAA
GAAAAGAAATAATATTTGCCGCCCAATCTGCGGTTTGAAACCAACTCAACAAGATTCTAACAATA
ATTAAGGCTAAATATATTTGTACGAAATTAGCTACGGTGGTAACTATTAAATAAGAAATATTGT
TCATTATTACCGATATTGCTCCTGCTTTTCTACTCTATAATTATTCTAATCTTAACGGAATTTGC
TCTAGTTGGATTTTAATTTCTACGATTTAACCAAAAACTTTGCATTCCTACAGATTTTGCTCCCC
AATAATCTTCTTGTTTGCTATCTCCAATATACCAAGCCTCTTGAGGTTGGCAATCATGTTTTTCT
AAAGCCTTTAAAAATATTTGAGGGTGAGGTTTTGCTACTCCTGTTAAGGAAGAAATTGTAATTGT
TTGAAAATAGGTGGCCAAATTTAAATTCTTTAAAACGTCATAAATACGAGTATCAAAATTAGAA
ATAATTGCCAGTTGTATATCTTGATTTTGCCATTGTTTTAAACAGGATACTACTTCATCATAGAT
AAACCAAGGTTCGGCAGTGGTAAAGTAATCATATAATTGCGCAAAGAAGGCTTTAAAATCGGTA
AACTCTCCTAAGGCGTTTGCTTTGGCAAAAGTATCATAAGCTATCTTTTCCCACCATTGATATTC
TAATTGCTGAATTTCTTGTTTATTTTGCGTCTCAAAAGCTAAAGGAGGAGAAGACTTAAAACACT
CATAGAAATATTGGTTAATTTCCTGACAATTTCTAATTACACCGTATTGACTAGATATTTTAATA
TAAGCATCTCCCACACTATTTTTAACTCCAAAAATAGTACCGACGGCATCAAGAAATATCACTTT
GGGTTGATTCATAATCGATTTTAGTTGATGTCACTCCAGAAATTTATACACAAATAGAGATATTA
TCAGTCTATATCGTTAATGTGGAGATTTTACTGCTTAATTACTTATGAAATCTAGTTAGACTAGA
TAGCTTTTTTTTGTGGTGAATAAATAAACGGTCAATTTCTAACTTGAACAATTATGACTACTGAC
TCTTTTATTCACTATTTTTGTCAAAACTCATTAATTTTAACTCCTAACCCCTAATTCCTAATTTAT
GATCTCATTGACTACCATTTGAGGAGGTTAAATCTTTCCATGTCGATGGTGTCACGGTTGCGGTA
AATAGCGAGAATAATCGCTAAACCAACGGCCGCTTCCGCCGCCGCAATGGTAATTACGAAAATCG
TAAATATTTGACCTTTAATTTCTGCGGGATCTAAATAATTAGAAAATCCCATTAGGTTAAGATTA
ACAGCATTAAGTAAAAGTTCGATCGACATTAAAACTCGAACTGCATTACGACTAGTAATTAGTCC
ATAAATACCAATACAAAATAATGCTGCGGCTAAAAGTAAAAAATATTCTAATTGTATTTCCATCA
TTTTTGTTTAAACATTAATTATTGTCGTTATCATTACCTGTCGTAATTAATTCTCTAGGTTGTTC
AGGTAAAGTGAAGGATGTGGTATAAACTTCAGTGGTTTTTTGAGCTTCGGGAATTAAGTCTCTAC
GTGCTAAGATAATCGCCCCAACCATTGCCATTAATAATAAAACAGAGGCAATTTCAAAGGGTAAC
AAGTAATCACTGAAGAAATGTTTGCCAATAGCAACAATGGTATTTTCTACTACCGCAGGGGAGGT
TTCTGATAATTGCCATGGAGTAGCCAAAACCATAGTGCCTAGTAAGGCAAATAAGCCCACACAAA
CTAAAGCAGTAGATACTTGACGAATTAATCTACCTCTTACTTGTTGATAGACTTCAGTTTTGTTA
ACCAACATAATGGCAAATAGAATAAGAACATTTATCGCCCCAACATAGATAAGAATTTGAGCGGC
GGCAACAAAGTCGGCATTGAGAAGAATATAAAGCCCTGATATGCTTAAAAATACTCCCCCCAAAA
GAAAAGCAGAGTAAACAATATTTTCAAATAAAACAACTCCTAAAGCTCCGCCAATCATCAGGAGA
GATAAAATAACAAAAGCAACAAATTGGACACTTTGTGCGATTTCCACGATTTAATTTACCTTTTT
AATAAAATTTTTCACAAGTTATAGTATAAGTTTATTGTTCGATTAAATCTTCGGGGTGCTTTCCA
GCCCTTGGACTATTTTTAGGTAAGTCATGGGGAGATAAGACTCCTTTGGGAAGATAGCCTAATTC
TTTTAATGGAGTTACCATGGGATCTTGAGTGACTTTAGTGGGTAAACGTCCAAGAGCTACGTTAT

CATAATTTAATTCATGGCGATCGTAACTTGCTAATTCATATTCTTCTGTCATGGATAAGCAGTTG
GTGGGACAGTATTCCACACAGTTGCCACAAAAAATACAAACCCCAAAATCAATACTGTAGTGTTT
TAATTCTTTTTTCTTGACGGCTTTGTTAAATTCCCAGTCAACTACGGGTAAATTAATAGGACAAA
CTCGAACACACACCTCACAGGCGATACATTTATCAAATTCAAAGTGAATTCTACCTCTAAATCTT
TCAGACGGAATCAATTTTTCATAGGGATATTGTACTGTTACAGGTCTGCGTTTCATGTGGTCGAA
AGTAACGGCAAATCCTTGTCCAATATACTTAGCCGCTTCCACGCTTCCTTTGGCGTAATCTGTAAC
TTGTTTGAGAAATTTAAACATAGATCTTGATATGACTGCCAACAAATTTTATTTTATCTTATCAG
GAGAAGTCTATTAGTAGATAATTAAGAGGTATTGCTGATTTGAGGTATAAATAATTCAGATGCT
CGGTTTGAATTATGAAAGGGCAAACCCCCCTTTATCCCCCTCGAGAGGGGGGAGGGCAAAGATAA
TTAACAATTAATAAAAACACCTGAAACCTGCAACCTGCCACTTCTTTCCTAACCCCGAACTCCTAA
CCCACAATACTTTTTACATTTCGTAACTGAATATTGACAATTTATTGCAATAAAGAATTATACTT
AAGGTTAGATTTAAAAATAAATATTCATATTGTTAAGATAAATTATTAAAATCTTTAACAAAAA
CCAATATATCATCATCAATAAGTGTCTTTTAAGGGCAGTTTGTACATTTTGATTCTAAATTAGCA
ATCATAAATTGTTACACTTAGTAAAGAAAATTTAAGTATAATCATTATAAAAATCATGGGTCGT
ACAGGAGTATTATTATTAAATTTAGGTGGTCCAGAAAAACTAGAGGATGTACGTCCTTTTTTATA
TAATCTATTTTCTGATCCTGAAATCATCCGTTTGCCTTTTCCTTGGTTACAAAAACCTCTTGCTTG
GTTAATTTCTACTCTGCGCAGTAAGAAATCGGAGGAAAACTATTTAGAAATTGGTGGTGGTTCAC
CTTTGTTACAAATAACCGAGGCTCAAGCTCAGGCTTTACAGTCTAAGTTATCTGGGCAGGGGTCT
GATATTCAAGTATATGTGGGAATGCGTTATTGGCATCCTTTCACGGAGGAAGCGATCGCACGTAT
TAAGGAGGATGGCATTGAAAAGCTGGTTATTTTACCTTTATATCCCCAATTTTCCATTAGTACCA
GTGGCTCTAGTTTTAGGGTGTTGGAAGAAATGTGGAAAACAGATCCTCAATTACAAAAAATAGA
ATATACTTTAATTCCTTCTTGGTATAATCACCCTGATTATTTGTCAGCGATGGCAGATTTGATTA
AACAAGAGTTACAACAGTTTGAACAACCAGAGAAAGTATATATTTTCTTTAGCGCCCATGGTGTA
CCAAAAACTTATGTTACTGAAGCAGGTGATCCTTATCAAGTGGAAATTGAAGAATGTACTCAATT
GATCATGAAAACCTTGAATACAGGTAATCCTTATATTCTTGCTTATCAGAGCAAAGTTGGGCCTG
TGGAATGGTTGAAACCTTATACGGAGGATGCGTTGGAGGAGTTGGGGGCGAAAAATATCAAAGA
TTTATTAGTTGTGCCTATTAGCTTTGTTTCCGAGCATATTGAGACTTTACAAGAAATTGACTTAG
AGTATAGAGAAGTGGCGGAGGAAGTTGGTATCAGTAACTTTAAGCGAGTTCCTGCTTTAAATACT
CATCCGATGTTTATTGATGCTTTAACTAATTTAACTACTTCTGCGTTAGAAGAAACTCCTCTAAG
GTTTGATCAAGTTACCCATCCTAAGAAGAATATGAAGATGTATCCCCAAGAAAAATGGGAATGGG
GTTTAACTACCGTTGCTGAGGTTTGGAATGGGCGTTTAGCTATGGTGGGTTTTTTAGCACTTTTA
ATTGAGTTAATTAGTGGTCATGGTCCTTTACATTTAGTGGGCTTGTTATAATTTTTATAATAAGT
AATAAGTAATATGATTTTGTGAGTAATTAAGATACAATTTTAGACCAATATTATCCGTTAGGAG
TTATTAATTGAAAAGACGCAAATTCTTTAGTTTAACTCTTTTTTCCTTCATTTTTACTACTTTTT
TACCTGTTTTTCATAAAGTTTTTGCCCAAGAAACTGAAAATAATTTTTATAAATTAGGTTCTTTG
ACTGATTTGGAGGAAAATGGTTTTTTACTGAATGAAAATTTAGAGATAGGTGCTGTTTTAGTAA
TTTTAAAAGAAGATAATTCTCTGATTGCGATCGAGCCTACTTGTACCCACATGGGTTGTTTAGTA
GAGTGGAATAAGCAGGAAAATCTTTTTATTTGTCCCTGCCATAATTCTAAATTTCAAGAAGATGG
CAAGGTGGTGGAGGGTTTAGCCATTTCTGATTTGCCCATTTATCAAGTAAAGATAGAAAATAATA
CTGTTTTTGTTGCTAATCAGTAATTACTAACTGTTTTCTATTCGTTTTTCAATATCTTCAAAAGT
TTTTAATAATAATTTTTGGGCTTGTAATTTCCAACCATATTGTTGAATTTTAATAGCAATGGGAA
TTGCGATCGCAGGTGCAAGAAAATCTAAATATTGTTGAGAAGAAATACCCAATAATAAACCTAA
TCCACCGATACCCCAAAAAGGTAAGGCGAAAGATTGACGCTGACTTTCAATTTTTTTTGCCCAAA

ATCCTTGAGGCATTTTATTTAACAATTCTTGTTTAAGTTTTTGTTGTTCTTGAAAATTTAAAGCG
ATTCCTATTTTACGGCGTAATTTTTCTATTTTTCTCGCATCTGTACTATACTCAGTTAATTGATCC
TCTGCCATAGCGATCAGATTTTCTAAAGTTAAAACCATAAATATAGAGCCTAATGATTATTATTT
ATAATGTTTATAATTTTAGTTTTTTATCACAAAATTAGGCATAAAATCAATCAAAAATGACTCTA
TCACTTCTCGTCGTGTTAATTATTGGTTAGGGTAGGCTGAAAGGCAAGAGGCAAAAGGCAAAGGT
AAATAATAATTTATAAACTTTTAGCTTTTATTTTACTATGAACACTATTCAATCAAAGTTATAGA
AGTCCTGTTTCTCTTAATTTATCATAACTACACTCTGAAGAGCCAAGAAATTAATAATTAGTAAT
CGAAAATTATTATTAATGTTGAAAAAATTTATTTATTTGCTTATCGTCATTATCTCTATTCTGGT
TACAAATATCAATCCTATTTTCGCCCTTTCCTTAGAAGATAGAATTAATCAATATCCGTCATGGC
AACAGAAAATTTTATTACCAAAACCTGAAAAAGACTTAATTTTTCCTGATTGGTTTGAGGGAAA
ATGGGAAGTTACAAGTATTTTAAAGGAACAAATTGCCCCTTTAGCACCTAAGTTTCAAACACCGG
GTTTTGAGCAAAATAATGAATATATAGATAAAAAAATACAGTTTTCAGTTAAGTTTATTGAGAG
TAATATTCATCCTCAACAGGATAATTTTATACCGCAAAAAATTAATAAACAAACCATTATCATAG
TGGATCGTAAATTCAATAGTTTATCTATTGCACAAGCCTATTTAGGAATAGATAATGTGGAAAA
AGTTGTGATAAATCCTAATAATACAACGGAGCAAATTACTAAGTTTAGAGGAGAAAATGAACTA
ATTTCAAAGGTTATCGGTAGGCAACAAGAGAATATTTCTGAGAAAGATTTTATTACCAGTGAAG
TAACAAGGCAATTTTTTCGCCGTCCTAATAGTGTTTATTTAAACTTAGTAGAAACAACCACTAAA
TATCATTTAATTAACTCAAATTTGATTAAAGCAAAGCAATTTACTGCTATTTACTTATCACCTCA
AGACCCCGATTATTTTGTAGCTTTTGATCAACCTGTAGCCTTATATTACTATACACTAGAGTTGA
AAAAATAGGCTCTTTTGCTTCTTTGACTCAACGCCTTTAGGGGGAAGGTTTTTTCAAAATCAGGG
CAAAATTATCTTGCCCTCACCCCCAACCCCTCTCCCATAGTAGAGGGGAGCGTTTTTTCTTAATAA
TTGATTAATTAATACTTAAAAACTCCTGTATCTGTTACTATTTGTTAGTTTCAAAAATTGACATT
TTTGAGAATGAAAAAACCCTGCTTTAGGGGGAGGCAAGTCAGAATTTCCCTCTCTAAATCAAAAT
GATTGCTTTTTGCCTTCTTCAAATCGAACTGAGGTATGATCAACCATATAAGTACATAAACGGTA
AACTAAAAATAGAATTAGTCTCTAAACATTATGGATGCAATTACTTATACTCAGGCTCGAAAAAA
CTTTACCCATGTAATGAATGAAGTTTGCGAAAATCATACTCCGATTATTATTACTCGTCAATCTG
CAAAGCCTGTGGTAATGATGTCCTTTGAGGATTATAGCGCGATCGAAGAGACTTTATATTTATTA
CGGAGTCCAAAAAAATGCTCAGCGACTTTATAAAGCATTAGGAGAATTAAAACAAGGAAAATACG
AAAGTCATGAATTAATCGAAGAAGAATAGTAGTCATGAAAATTTCCTTTTTAGAAGACGGTTGG
CATGATTATTTATATTGGCAAAGTCAGGATAAAAAAAATCTTAAAAAGAATTAATCAAATCAGT
GAACTACTACCCGACCGCTATCGCTGAGTCGGGAGCTTCATCTTTCACAGGTTCGTGCCTCAAAAC
GGATTTACGTCCGCCTTTTGGTCTTACCGATCCTCCAGATGCAGAGACGGCTGTTCCATCCGTCTG
TACTTTTCTGATACCTTCTGCTCTAATATTTTTACTTGCGTTCTCATCCCTATCATGATGACTATC
ACAACTAGGACAAGTCCATTCTCTTATCTCTAATGGCATTTCAGACATTTGATAATGACAATGAG
AGCAAGTTTTAGAGCTAGGAAACCATCTGTCTATCTCCACTAATAACCCACCTTTTTGCTTGAGT
TTATAGTCTAGGAAGTTGATAAATGTTCCCCAACCGACATCAGTTATTGCTTTTGCTAATTTGTG
GTTACGAACCATACCCTTGATATTCAGTTGTTCAACGACTACCACTTGATTATTATTCACAACTT
TGGTGGAGAGTTTGTGTAGGAAGTCTTGACGGGTATTGCTTGTGCGTTCGTGAACTTTAGCGACT
AGCTTTTTGGCTTTTTCTCTATTCTTAGAGCCTTTTTTCTTACGAGCTAACTTTTGTTGTTTCCGA
GCTAGGTTTTTTTCATGTTTTTTGATGTGTCTAGGGTTAGCATACTTACTGGTTTTTTTGCCATC
ATGAACAATAGCAAAATCCTTAATTCCTAAGTCTATACCACAAATTTTCCCTGTCATATTTACTA
ATGGTAATTCTTGTTGGGTATCGAATAGGATACTAGCAAAATATTTGCCAGTGGGCGTTTTGCTT
ACTGTTACAGTCTTAATCTCACCTTCAAATATCCGATGAATAACTGCTTTTACTAAGCCTAATTG

CGGAATTTTGAGACATCCATCAACTATCGAGACATTTTGGGGATATTGACAAGACTGCTTTCCGT
GATATGACTTGTATCTGGGGTATTTGGCTCTACCTTCAAAGAAGTTTTTGAAAGCCTTAGTCAGA
TTAAGAGTCGTTGATTGCAGACATTGTGAGTAACAATCTCCTAACCAAGCCGTATCTTCCTCTTT
TTTTAGCTTAGGGAGATACTTATTAAGGGCTACTTGCTTTAGTGCCTTACCTGTAGCTTTATAAG
TGTCAATGCACAAGTTTAGAGCATAATTGTACCACCAACGACAACAACCCATAACCTGAGATAAT
TTCTTGGCTTGGGTTTTGTTGGGATAGATTCTGACTTTTATCGCTCTGTGCATTGAGGCTTATTG
CTAACTTAGGTTGCCAGTAAAATTATTATAAGTAATGATTTTGGTTTTGTGTCGAATCCTACGGA
TATTTTTTTATCGAGGCACAATTCATCCGCTAATCCGCTATGCTGAGATTAGGGACTTCTTTTGC
CATTTACGTTAAAGATATAAAAAGAAATCCATTTGATGGTATTGGCAAACCTGAGCCGTTAAAGT
TTGATTTTAGCGGATTATGGTCAAGACGTATTAATCAAGAGCATCGCTTAATTTATCAAGTCTTA
GACGATGAGGTTATTATTATTCAATGTCGTTATCATTATTAGTTAAGACTTACAATGAAAATGTA
TTGAAATATATTCTAAATCAATTTTTATGACACCTAAATTTGTCCTTGCTTCTGCTTCTCCTGCA
AGACTAAAGTTATTACGTATGATAGGTATTGAACCTATAGTTCATAAAAGTGACTATGATGAAT
CGTTGATTAATCTACCTCAACCTATAGATTTAGTGAATACTTTGGCAATGAAAAAAGCGGAGGTA
GTCGCCCAAAACTATCATGATTCTCTTATATTGGGCTGTGATTCTTTGCTTTTAGTGGATGGGGA
AATTTATGGTAAACCTGAAGATGAAGAAGTTGCGATCGCACGTTGGCAGTATATGCGTGGTAAA
ATGGGGAGGCTTTATACTGGTCATGGATTAATAGATACTATCAATAATAGTCAAATTGTTCAATG
TGGTGTTACAGATGTTTACTTTGCTGATGTCACCGATGAACAAATTAGAGCTTATGTAGCTACTG
GTGAGCCTTTAAAGTGTGCTGGTTGTTTTGCCTTGGAAGGAAAAGGGGGAGTTTTTATCGAAAAA
ATAGAAGGTTGTCACAGTAATGTTATTGGTTTGAGTCTTCCTTTGCTAAGGAAAATGTTGACAGA
ATTAAGTTATGAAATTAGTTATTTTTGGTAATTTTCTCTCACAATAGAGGCTTATATGGATAAAG
ATAGTCGTTTCGCTTTTTTAGTAATTGGTTTACCTTTTATCGGTTTAATTTATTGTTTAGGTATT
ATTGTTTTTTTTCTGGTGTCGCCCATCGCCCAAAATCACCCTTTAATAACAGGCATTATAGCCGCT
ACCGTTCCATTTTCGATCGCAGCTTTTATTTGGATTAAAGCATCAGCAAAGGCATATGGTAAAAA
ATCGAAAGGATAAAAGTAATGTGGATAATGAGTGTAAGTCGATTTGCACTTAAGTTGTCTAGTT
AAGGCAGGGAATGGGCAATAGATAAACTTTGGAGTTATTTTCAAGAACTAAGGGAGAATTTAAA
TAGAATGGTGATGGTAGGGAAAAGGGAAACCCCCCTTTATCCCCCCTCGAGAGGGGAGAGGGAAG
AGTTAAGACTTTGAGTCATTTTCACAAATTAATAAAAAATGTAAGTTAAATGCGTCTTAGCCTAG
TAAGTAATAAACAAAATAATCAGATGATGTGAGATGAAATTTGTTGACGAATATAGAGATTCAA
CGGTAGTAAAACAGTATGCTCAAAGAATTAGGGAAATTACGACTAAATCTTGGCAGATTATGGA
AATTTGTGGCGGTCAAACCCATTCTATTTTGAAATACGGCTTAGATCAATTATTACCTTCTGAAA
TCTCTTTAATACATGGTCCTGGATGTCCTGTTTGTGTCACCGATACTGAGAAAATAGACAAAGCA
GGTGCGATCGCATCTTTACCCAATGTGATATTATGTTCCTTTGGGGATATGTTAAGAGTTCCCGG
TAGTAAAGAAGATTTACTGAGTATTAAAGCAAAAGGGGCAGATATTCGCATCGTTTATTCTCCCC
TCGACTGCATCAAAATTGCCGAAGAAAACCCCCATAAAGAAGTAATATTTTTTGCTGTGGGATTT
GAAACCACCGCCCCCACTACCGCCATGACTGTATATCAAGCGGATAAAAAGCAATTAAAGAACTT
TTCCCTGTTAGTTTCTCATGTTTTAGTACCTCCTGCTATGGAAGCAATTTTATCCTCCACTCAATG
TCAAGTACAAGGTTTTTTAGCGGCGGGTCATGTTTGTACTATTATGGGTTATCATCAATATGAAA
CTATTTCTCAAAAATATCAAATACCTATTATCGTCACTGGCTTTGAACCTGCTGATATTTTACAA
GGAATTTATTTATGTGTTCAACAACTAGAAAAAAAGGAAAATAAAGTAGAAAATCAGTATAGTA
GATGTGTAAAAAAAGAAGGAAATAAAGAAGCTCAAAAACTGATTAATCAAATATTTGAAATCAC
AAATTATCAATGGCGTGGTATAGGGAATATAGATAAAAGTGGCTTTAAATTAAGAGAAAAATAT
GTCATGTTTGATGCAGAGACAAAATTTAATCATGTTTCCACTACCACATCAAACGCTGATAATAA

TTGTATTAGTGGTCAAATCTTACAAGGAATAAAAAAACCTCATCAGTGTCAAGCCTTTGGAAATA
AATGCACTCCTGAAAATCCGCTAGGCGCACCGATGGTATCTTCTGAAGGTGCTTGTGCCGCTTAT
TATCGTTATCACCTGAGTTTGATCTAAAATTAACATTAACTGTCTGGTGGTATTAGGCAACGAGC
AAAGGGCAAAAGTGAATAGATTAAAAAAGGGCAAAGTTAAAAGGGCAAGGGGCAAAGGTAAGAG
GCAAAAGGTAAGAGGCAAAAGGCAAGAGGCAAAGGTAAATAATTAATAATTAATAGTGAATTGT
TGATAATTAGTAACTCTTGACTCCTAACTCGTTTCTCCTCTCATCCCCTCAACATCCCAGGGCTGT
TTCATTTTCGAGAAAGAAAAAAGAGCGGGAGATAGGGGGAGAGGGAGATGAGGGGATTTTTGTT
CATAAAGTTTTAACTAATAGAAAAAAATCCTTGTATATCTTACTCTTACTTAATCTCAGTTAACC
TAATTTTTTATTTTGAAACAGCCCTGCCCCAACATCCCAATACCCCAATACTCGAATATTCTCCTA
ATACCTTCTCTCCTCATCTCTTAAAACTTGTATCATTTAAGCCAAGAAATAATCCAAGCTAAAAT
AATGGTGAAAATCAAAACTATTTCACTAATCTTTAAATAAACAGGAGTTTTTGTCCATTCATTAA
TCATAATTTCTCGATAATTTTCAGGCAATGATTTAATTTTATTTAGCCAATATTTAGGGTTTTCG
TCCTCTCTAAAATCGAGTTTAAAGATTGATAATAAAAAACTAAAACTGCCTATTTTTGCTATAGT
TAATACATGGAACGCTATAGCTAAAACAACAATAATCAGTGAAATAAGATGCAAATAGTACCAA
TAATGATTTAATTCTCCTTGAGGTAACCATTTTTCATCCATCATTTTCCCACTAAAAACGGCAAA
TACTAAAGCGAAAATACTAAAAGTATTAACTAGACGATGAAGGGAGTACCACCAAATAGGTTTA
TTAATTAATTTTAGTTTATCTAAACTATCTTTTTGAATTAATTTTTGGGCTTGTTTATGAATACT
ATAGATAATAAAAATAGGTAATATTAATAATACCCATAAGCCAAAAGTTCCATGTATCCCCTCTA
TTTCCTTCCAGTCAGGAAGAAAAGCAATTTTGCCCCAACGTCCATCATAAGTGTTATATGTCCAA
AAAGCAGTAATAACCGCTAAAATTACTGATAATCCTTGCAAATTATGCAATATTCTCAGCATTAG
TTTTTGATAAGGAGATTTAGAGTTACTTTTCATGTGTTGACTGCTAGATAAAGAAACACGAGAGC
ATTATTAATAAAATATAAAGCCAAATTCATTATCTATTACTTTGAACAAAATCAATCACTGCGTC
ACGAGTGGAAGGAATATCAGTCACTGACTGAATTTTATCAATATTCACTTCATAATGAACGGGTT
TCGCTTCCGTATTGATTTCAACTTCTCTATTAGTCATGTTTAACACTTCCTGTAACTCAATTACCC
CATTGCCGCAAAGATTAAATATTTCTTGAGAGTAGCCTTTTTTTATTATTTCCATCACTCTCATG
GCCGCTATATCGGTATTAATATACTGCAATTTGCTTTCTGGATGTAACCAAAGGCGATCGCCCCA
TAAAATATCAAAGATTGCATTTTTCTTTAAATTTTCACCCACAAATCCTCCCATGCGGAAAATTA
ACCAATCATGATGGCAATGTTGAACACATAATTCTGCTAAATATTTATGAAAACCGTAGGGGCTT
TGTTTGCTAATATCAATGACAACATCTTCTTTTGTTGACTCGTAACTTGAACAATCAGAATAGAC
ATCGCAGGAAGATAAAAATACATATTTATCACAGGGAAAATCAATTAGAGATTGTCGGAGCGAT
CGCACTGAAGCATCAAAATCAACTAAAGGCTCTTGAGTAGCTAAAAACTTTTTAGAGTTGCCATT
AGCATTAATTAAAATATCGGCTTTAGTACCAATATAGTCGTGATAATTATGTCTATTTACTATTA
AATGATCGATACTGTTTTTTTCACAAAAACGCTTATAAGCCGAACCGACAAAACCATTCCCACCT
ATAATAATTACTTTCATATCACTCTATATTGTCCCGTGCTTGTTCGACTCATCATTATATCTCAA
TGGGAAGGTTTAAAAGCAATCGCACCTTTCATTACCTAGTTTTACCGTGACACAATTTAAACTTT
TTGCCACTGTTGCACCAACAAGGTTCGTTTTTTTTGGGTTGAATGGGTGGTAAAATCTCTCCATC
AGTATAATACCATTTACCTTTTTCCTTGATAAAATTCGATCGCTCGTGCAGTTGAGCGACAGACT
TTCCTTCTTGATATACTGCGATAAACTCCACAATACCCGTTTCATCCTCAGATTTGCCTCTCTCGG
TGTTAAGTACGGTTAAACCAAGCCATATAAGACTGTTAGCAGTTGCTGTAATCATAGCTCTACTA
TTGGGTTTGCGGTATTTAGGATGTTCTGTGTTGAATAGGTAATCTATATTTTTGAGACAGTAAGC
CGAATAGCGCGATCGCATTAATTTTTCTGCGGTAGGTGCAGGTAATTGTCCTTGTAAATAGACAT
CACAACATTGATTTAACGGCTTTTGACTTCCACAGGGGCAAGGTACAGAAGAAAGACTATGATTA
GAAATAAACATGATTAAACGTCGATTAATAGCGAATTGAAAAATAGGTTTTTGTAAATGTTTTA

AATATATACTATTAGATTTGCTTATGCAAAAGTGTTGATCTAAAGTGCGATCGCATCCTATCTGA
AAATGTTATGGTACTATTCATTATTTTTTCTCCATAATTCCGTAAAGGACTCAACTCTTCCACGC
TGAATAACTTTTTTATATGATTTGAGAACTTCATTAGTTAACTCTTTAAGTTGTTTTGGAGATTT
ATAAATGGTTTTAAAATCATAATTTTCGCCCCCTTTATCTTCAAATTCACCATTTAAGTCAATAA
TCAAATCCCCTAAAATAGTGTGAAGACATTCAAAAAATTTTTTTCTTTACTAGGATGGAAAAGG
AAATCCTCTGGTTTTGCTCGAAAGTTTTTACCTATTTCATCTTCTTTAAAAACTTGTGAAACTAA
ATGAAGAAGTAAAAATCGAGTTATTTGATAACCTTGAAATAATTGAGGTTCTATTTTTGGCAAA
TCTTGTTGTATTTGTTGATAAAGTTTAAATAAAGAAACAATCCTACCACCTGTTACTTCTGGACG
TGAAAAAATTTTGGAATGAGAATCTTCAAATATTTTTTTAATTCCTTGTACTGCCCAAGGTTCTT
CTAAATCAAAAACTAATAATATACGACCAGCCAATTGATTATCAATTAACATTTTATCAGGAGGA
GATTTTTCTCCATTTTTTATTTGATAAAAATAGTCTGGATATTTTTCATTAATTTCTCGCTGAAG
TCTAACTTGAGTTTTTGTATTCGATCTAAAATCTCTTATTTTAATACCATTTTGATTATTACTAT
TATGAGTAATTTTTGTAATTAATTCTGAATCTGACTTAATTTCAATAATACGAGTTAAAATTCTT
AAGTCATCAGTTATAATGTCTCGTTTTGAGTATAAGCAAGAGACACTTTGACAACCATTGACTAC
TGCATAATCTTTAATTATTATTTTTCCTTGTATTTCAGTATTAAGTTCACCACAAATTATAGTTA
TTCCATTATGATAAAGTAGAAACTTTTTATGCTCATTTTCTTCCTCAATACTATGATAAATATCT
TTGTTGACTTTTGTTTTTCCTAAACTTTTTCTTAAATTCAAATCGAAAATTTTTTGATTATCTAA
ACCCTCCATTTTAACTATATCTGTCGCCTTAACTGCGGCTATCACAACCCTTGCAAAATTATCAAC
GTTATATTTAGAATAATCCCAACCAAAAACATCAAAAGCTAATTCTGAAGTTGCACGTATTGCTT
TATCACTATCAACGTACATTTCAGAAATTAAATTTTTATCCCATACTTCTAAAAGTATATTATCA
TCACCAGAAATAGTCTTAAGTAAATTATTTGCATTATCATCTTTATTAGTATTAGTAACAAAAAT
TCCCCGAACTTTATAATCAGGAGACTGTAAATATTCCTTACTTTCTAATAGTAAATTTTTGAGTT
GAATAATATTTTTCGTATTTGGTTCTCCTAGACTATCAATTAACCCATTTATGCTGTCTTCTGTA
ATTAATTGTTGTAAACTACCCAAAAATTCTTTAAGTTGAGTATCACCTAAAGTTTTAGATTCATT
TTGAACTATTTTAGATTGAAAAACATCTATTAATCCTTCATTATCATTGATATAAATACCGTCAA
TTCCTTTGTCGCCATGACCATCACAAACTATATTATCTACTTCAATTCTTTCTAATCTATAAATA
TTGACTAAAAACCAATGTAGAAAAGCTGTTGAATCTGTTCTACCTGATATTTTATAAGGTTGTAT
TAAATTAAGAAGGTTTGTGTAATTAATATCGATCATAAGCTATCGATTCTTTGTTAAAGTTTTTA
GCGTTGATACATCTCAAATTGATTTTTTGAAAATCCCTGTCACGACAATATTGTATAAATTTAAT
GAAACCATTTTTTAAAATAATGGATATTTTACTAATTTATTCACTCTTTATAATTAAACATACTT
ATTAGATTCTATCAATATAATCAACACTTTCTAATTCATCAGAAATTAATTCTTGCAAGTCCTTG
TCGCAATGAGAATTAAGATGAATTGTTGATGCTAATAATTCTGCTAAAATATCACCTTTTTGATT
ATTAGTTAAATTAGATAATTCCAGTTGATTAATTAAAGAAATTACCGTCTGACATTCCTCTTTTA
ACTCAATAATTAAATGATTTAAAGTATGGTTTTTTATATTAATTTTTTTTTCAGTTAGTTGCATT
ATTTTTTACCTAATCTTTTCAACGCTTGTTCAATTCCCTTTTCAAAGGCTTTGATTTCTTTTTGCC
TAGGTTGCGATAAAGTATATATTGCCCTAAAGCTAACTCAAAATCTTTCATAAGTTAAAGACTGA
TAAAACTTTTAATTTCAATAATAATTTTACGTTGTTTTTGTGTTTCTGAAATGAACTTTTCTACA
GCTAAATCGGCATAAAGTTCAACATCCTCATATTCAAGAGGATAAAGATCTGCCGTAATAATCCA
ACCATCTTTAATTAAAGCATTTTTAACCGCTTCATGGTAAATATCTTTTGTCATTCTCCTCGTTG
TAATTGGATTTAGGTGGGCATTGCCCACCCTACAATTATACTGGCATACCTAAAATGTCCTCGAT
TTTTGGCATTTGCTCAATTTCTGTGATTATATCTCTATCATCAATTCCCTCTTGAATGAGAATGT
TTGTTAACCCTTCCTCGAAATTATCATCTTCTATGATTATTTTATCGTCTATTAAACGAGCATGA
AATAAAATTTGATCTCGCCAAGATTCTTTGTGCCAACCTGTCATAATAATTAAAAAATGTCCTGT

ATCTCGATCGCACACAGAACTAATTTTCAAATTTTGTAATCGAGGTTGAATAGCTGATTGTTGTC
TTAAAACTTTAGTTAAAATGTCAGCATATGTTATTGATTTATCCATTGTAAAATATCCTCTTTTT
GTGAATTAAAAATAATTAAATTGATTTTATTTCGTTTAATAGCTAATTGAAAAATAGGTTTTTG
AAAATGTTTTAAATATACAGATTGACTAATTGCCAAAAACAATTTTCTTTTCGGTTCTTGCTCTT
CTAAAGCCCATTGATAAAATTGCATTTGTCCGATGGTTTTTCTAATTCACTAATCACAGAAGGA
GAATCAAAATCTTTAACTTCAACCGCAATTTTCTGATTATTTTTTTCTGCTGTAAAAAATTTTTC
AGCACCTAAATCAGCTTTTAGTAAGGTGTTTTCCAGAACTAAAATTAAAGGATCATCAGTAATTT
TCCAACCATCTTTTTCTAAAGCATGACGCAAGGATAAATGTAAACTGTCTCTTTTTGACATTATT
TTATATCAAAACATATCTTAAAAGGTGGGCAATGCCCACCCTACAATTATACTGGCATACCTAAA
ATGTCCTCAATTTTCGGCATATCTTCTAAAGCAATAACGCGCCCTTCATCCTCAAAGCCTTCAATC
TCATTGAAGTTTAAATAACGGTATAATTCACCCTTGAAAGGATCGATTTTCTCAGAGACGATCGC
GCTATATTCTTCCACAGTAGGAATTTTACCCAATAAAGCACAAACTGCCGCTAACTCAGCACTAC
CGAGATAAACCCTTGCACCTTTACCCATACGATTATTAAAGTTACGGGTAGAAGTAGAAAATACC
GTTGCACCATCTTCCACACGGGCTTGATTACCCATACATAAAGAACAACCCGGCATTTCAGTTCTC
GCCCCTGCCGCCGCAAATACACCGTAAACCCCTTCATTGCGTAACTGTTGCTCATCCATACGGGTA
GGAGGACAAATCCATAAACGCCCTTTCACTGTACCAGCACCCTCAAGGATTTTTGCCGCCGCACGG
TAATGTCCTATATTTGTCATACAAGAACCGATGAAAACTTCATCCACTTTATCACCAGCACATTC
GCTCATCAGTTTTACATTATCAGGGTCATTAGGAGCGGCAACAATCGGCTCTTTGATTTCACTTA
AATTGACGGTGATAATATCTGCATATTCAGCATTTTCATCCGCAGACATAAGAGAGGGATTCGCT
AACCATTCCTCCATCTTGGCAATACGGCGCAATAATGTACGTGCATCCTGATAACCCCGTGCGATC
ATATTCTTCATTAAACTAACGTTAGATCGCAAGTATTCGGCTACGGTTTCTTCACTGAGTTTAAT
TGTACTACCAGAACAAGAACGCTCCGCAGTAGCGTCAGTTAACTCGAAAGCCTGTTCCACTTTTA
AATCGGGTAAGCCTTCCATTTCCATGATACGCCCGTTAAAGACGTTGATTTTATTCTCCTTGCCA
ACGGTTAACTTACCTTCCTGCATGGCTACCCAAGGAATAGCGTTAACGATGTCTCGTAAAGTAAC
TCCGGGTTGCAATTCCCCTATGAATTTGACTAAAACCGACTCAGGCATATCTAAAGGCATCGCCC
CCAAAGCCGCCGCAAATGCCACCAAACCCGAACCTGCGGGGAAAGAGATACCTAAAGGGAAACGG
GTATGAGAATCACCTCCTGTGCCGACAGTATCAGGTAATAACATTCTATTTAACCATGAGTGAAT
GATACCATCTCCCGGGCGTAAGGCAACACCGCCACGAGTAGAGAAGAAATCGGGTAATTCTTTAT
GGGTTTTGATGTCAACGGGTTTGGGATAAGCGGCAGTATGGCAGAAGGTTTGTAAGGTTAAATCT
GCATTGAATCCTAAACAAGCTAATTCTTTTAATTCGTCTCTTGTCATAGGTCCAGTGGTATCTTG
AGAACCTACGGTAGTCATTATCGGTTCGCAGGAAGTGCCGGGGCGGATTCCTTCCACACCACAGG
CTTTTCCTACCATTTTTTGAGCTAAAGTAAACCCTTTGCCAGTGTCAGCAGGGGCTTGGGGGCGT
ACAAATAAGGTGCTAGGCTCTAATCCTAATGCTTGGCGAGTTTTATCGGTTAAGGCACGTCCAAT
TAGAAGGGGAATGCGTCCTCCTGCTCTCACTTCGTCTAAAATGGTTTCAGGCTTGAGGGTAAAAG
TAGTAATGGTTTCGCCGTTTTCGTCGGTGATTTTGCCTTCGTAGGGATAAATAGTGATGACATCC
CCTGTATTTAAGTTGGTGACATCGCACTCGATGGGTAATGCTCCAGAATCTTCGGCGGTGTTAAA
GAAGATAGGTGCAATCTTGCCTCCTAAGACATAACCGCCCGATTTCTTATTAGGTACAAAGGGGA
TTTCTTCTCCTAAATGCCACAAAACGGAATTGATTGCAGACTTACGAGAAGAACCTGTACCCACT
ACGTCACCGACATAAGCCACAGGGTGTCCTTTTTCTTTTAATTTAGCAATGGTTTCTAACCCTTC
AGGCATTCTCGATTCCAACATTGCTAAAGCGTGTAAAGGAATATCAGGGCGAGTGGTGGCATGGG
GAGCGGGGGATAGGTCATCGGTGTTGGTTTCTCCCGGCACTTTAAAAACTGTCACGGTGATGGAT
TCCGCTACTTTGGGCTTACTGATAAACCATGATGCTCCTGCCCATGCGTCAATTACTTGTTTGGCG
TAGGAATTGTTTTCGGATAATTCTAAGACATCGTTAAAGGCATCGAAGACTAAGAGGGTTTTACT

AAGGGCGTTGGCGGCTTCGGATGCTACGGTATTATCAGCAGATTTTAGTAAACTGACGAGGGATT
GCACGTTATAACCTCCCATCATTGTACCCAATAAGCTCACTGCTCCTTGTTTGGAGATAATGGGG
GATGTTAATTCTCCTTTTGCCACGGCGGTTAAAAATCCTGCTTTGACGTAGGATGCCTCGTCCACT
CCGGGAGGAATGCGATCGCGCAGTAACATCATTAATTCTTCTTTTAATTCCTCTGGGGGATTCTG
CAACATTTCGCACAGGTGTGATGTTTGTTCGGCGTTAAGGGGTAAGGGGGGAATGCCTAATTTGG
CTCTTTCTTCAACGTGTTGATGATATGTTTCTAACCAAGTCATAGTATTTATGCTTATATTTTTT
ACAATAATTTACTATTTTAAGCATATCAAGGTCATGAACAGGGGTTTTGTATCAACTCTATCAAG
GTTCGATCGATTTTGAACTATGATTAAAAAGAGTAAAATGTTTTTCAGTTTTAATTATGTCTTCA
TCTGGGGTTGTTTCTTTACAGAATCCAAAGCCTTTTCTTAAATGGGTAGGAGGAAAAACACAGTT
AATTAGTGAAATTGATCAATTAATTAATAAAATTGTTTCCAAAGAAAAAAAGAAATTTTGTTAT
ATCGAACCGTTTGTAGGAGGAGGAGCTATATTATTTCATATTTTAAATCAATTTGATAATATAGA
AAATATAGTTATTAATGATATTAATTATAATTTAGTTAATGCTTATCGAGAAATACAAAAAAAT
CATTTAAAATTAATAGATTTATTATTAGATATAGAAACACAATTTTATACCTTAAAAACTATTG
AAGAAAAGCAGAAATTTTATTTAGCCAAGAGACATATTTTTAATTCTATTCATGATTACTCTAGT
GAAAAGATAGCTTTATTATTATTTTTGAATAAAACTTGTTTTAATGGATTATATAGAGTTAATA
AAAAAGGCAAGTTTAATGTTCCTTTTGGAAAATATAAAAAGCCAAATATTTGTGATAAGGATAA
TATAATATCTGTTAATTATTATCTTCAAAAAGTAAAAATTTTACAAGGAGATTTCCAAGAAACT
TTAAATTATGCAGAAAAAAATACTATATTTTATTTAGATCCACCTTATAAACCTCTTAATTCAAC
GTCATCTTTTACATCTTATGCAGAAAAAATTTTTGATGATAATGAACAGATAAGATTAAAAGAA
TTTTGTGATGAAATTCATAGACAAGGAAATTATTTTATTCTTAGTAATTCTGATGTGAAAAATT
ATGATAGCTCCAACAACTTTTTTGATGAACTTTATGAACAATATAATATAAAAAGAGTAAAAGC
AAGAAGAAATATTAATTCAAAAGGAGATAAAAGAGGCGAAATTTTTGAACTTCTCATTACAAAT
TTTTAAGTTACTTTTACTATTAAAATAAATATTATGACTGAACAAGATATTACCTCAAAAACTAA
AGCATGGAAAGCTATTTTTGATACATATAATATCTATAATCATGATTTTTCCTCACCTTATTTTA
TAAGTGCAAAAAATATTAAATCGGCAACTAAGCATTTTGATAAAACCGCAGAAAAGGAGGTTAG
AATTATTTGTAAACAAGATACAAGAGAAAGTCGCCCAGATATATTTAAAGAATTAGGATTATTT
TTATTACCCGTTAAAAATGGCGAATATGCTATTTTAAAAGGAGAAGGTTATATCGATATACCCTT
AATTAAGGAAATTCCAAAAATTTATAAATCTAGCCTTAATTTTAAGTTAGATACCTCTTTGATAG
GAGATTCTGAAATGCAACATTTAGATTATGCTTATGCCTCTAGTTTAATTCGTCATTTTATGAAG
GATGATAGTTTAGTTTTAACAATTAGAGGGAGAAAATATACTCCTAAATTTGAAATGATAGTTA
ATAATAATAAGTTAACTATTGAAAGTGTACAAACAGAAGTTGATGCCGGATATGAAGGAGAAAA
GCAAGTAGTTTTAATTGAAGCAAAAAATGTTAAAACTGAGAATCTAATTATTAGACAACTTTAT
TTTCCTTTTCGCCAATGGCAATCACATACGAATAAAAAAGTCATAACCTTATTTTTTTCTAAGTC
TAATAATATTTATTCTGTTTGGAAATTTAAGTTTGAAAAAGTTGAAAATTATAATAGTATTATC
TTAACAGATTCAGCCAATTTTATTATTACATGATTAAGTTACAGAATAAAAATGTTTAATTTAAC
TTGAGTTTTGGATAAGCTGAAAGCATTATTTTATCGTAGTCGGAAAATCTTATAGCTTCTTAGAA
ATAACAATAAAATTACTTTAACCCGAACTGACGTTAAGTTAGAATGAATAAAAATATGGTAATG
AGATTTTAAATATGACGACAAAATCAGCAGAAATTAAAGAAATTATTTTTCAAACTCCTCCAGAA
TAAATAAATCAATTAATATGAAAAAATAGAAAAAAGACATCAAATTAAATTGCAAGAAATAAGA
CGAGAAAAGAAAAATGTAACCATTTTTAACCCATAGGGAGATTTCCACCAGTGAGCAGGAAAACA
ACCATCAGGGGCAGATAAACTAAAATCCCAATCATCATATTCAGAAAATCTGGGTTGTTTATTCT
CGTTTAACCATCCTACTTTGTCTGCAAACTTCGTTAAAACTTTGACATCTTGAGCCCTAATGGTA
TCAATATCACCCCCCGCCTCATAATAATTTTTTAATTGTACTCCAAAACTGAATTTCCCATTACTA

TACTTATACCACATTTGATCTATAACCTGTAGAGAGTTACACGGGTACTTACTAACATCTTCGGG
AGAGAGGGTTTCTCTTTGTTCTCCTGCTACTTCTAACATTATTCTAGTGGTTTCTATATCTGCTTC
TCGAAAATCCTTATTTTTTAAAAATTTTTGTAATGTACCGTATCTAGCAATATCTGGTAGTAAAA
TCAATTGTTCTTGTAATTCTTCAATCAGATTTGTTAGACGACTGATTTCAGTTTCCAATTTTTGT
ACACGGACTTCAATATCGTTCATAATGGAGAAATATTAACTGATTTTCTTTATAGGTTATCTTAT
TTCGTTAGAAATTAATCCTAATATTCTTGTATTGGTAACTCTTAATTGTTCATAAACGTGCCTCA
ATTCATAATCTTTAGTGCTACCAATTTTGACCACGAGAATTAATCCATTTGTTTGAGATGCTATT
ATTTTACCGTCAGAAAAACTTAATAAAGAAGGACTATCATAAATAATCAAATCAAACTGTTGAG
ACGCTTGTATTTGTTGCATCATCGTGGACATTGCTTGACTATACAATAGGCTACTTGGATCTGAC
AATACTTCTCCCACAGTGATAATAAAAAGGTTATCCCATTGACTGGGAGATTGAATCGCATTTTG
CCAAGGAATTTTGCCTGTAAGGATATGAGTTAGACCTTGATTATTGTCAACATTTACTAATATAT
GCAATTGAGGTAGTCGAAAATCTGTATCTATTAATAATACTTTTTGCCCCATACTAGTGGCTACC
TTGGCAAGGTTTAAGCTAATAGTTGATTTACCTTCTCTGGGAATGGAAGAACTAATAACAACAGA
TTTTGAGGGTAAATTGGGGGTTAATAAACTAATATTTGTGTAAAGATTACGGAAGGATTCTAAA
CTAGAAGATAGTGAAAGTTTGTCTTTTTCTAATGATTCTTTCTGAGGAGAATTAGAGGTAAAAA
AAGCGGGAATATTTTGTTTTTTAGTAGTTAAATTAATATTTTTATCAAAAGGTATTTCTGCTAAG
ATAGGCAGTTGAGTAACTGTTTTTAGTTTTTTAACATCGTAAATACTGCGATCGCATTTATCTAT
AATTGAAGCAACCACCGTACCAAAAGTTAAACCCCCGAATAAACCGAGTAATAGATTGTAAAGAG
GAATGGGATAAACAGGACTTTCTCCCACTTGGGGAGGAGAGATAATTTGCCAATTAAGATTATTT
TGAACATTATCTAATTCTAGTTTTTCTCTTGTTTCTAAGAAGCGCTTTAAACTTTCTGTCGAGGT
ATTTAATTCTCTTTGTAAATTATTATATTCACGAGTAACGGCGGGTAAACTGGCAATTCTTTGGT
TAAGGTTATCTAAGACTTTTTGTAAACTATCTTTTTTACTATTTAAAACTTTAATTTCGTCTCTT
TTTGCAATTAAATCTTGAGTTAATTTATCCCTCATTTTACTAGGAATTGCGATCGCTTCTTGTAG
ATTCTTTTGATTGTTATTTCTTAAAGAGTATCTAGCTTTCTCTTGCTCTATTAAATTAAGAATAT
TATTTTGTTTGTCTTTTAATGTAATAATTTGTGGACTTAAATCTGTAAAAACTGCCGACTGTTTC
GCCAATTCAATTTCAATTTCTTGTAACTGTTTTAATAAGTTTTGATACCTTGGTGATTCTGTTAA
ATAACTAAGTGCGATCGCATCTTCAGGAGATAATTTAAGATGTTGTTGAATACTATTATAAGCCG
ATTCTGCTTCTTGAATTTGTATTTGAGTGGCAAAGAATTCCTTTTCTGTAGCAACTAATTGTTGG
GTTAATTCCGTTGCTTGAATTTGTGGATCAATAAATTTATAATCGGTTCTTAATTTTTCTATTTT
TGTTTGTAAATTATTAATATTTTTTTCTAAAATAGGTATCTGACTACGAACAAATTTTAAGCCTT
CCCCAATATTTTGCTCTCCTGAATTCAGTCCATTTTCTAAATATTCAGAAGCAATAGTATCTAAC
ACAAAAGCTATTTTTTCAGGATCTTCATCTCGAAAACTAACCTCAATTAGTTTGGTTTTATCCAA
TTGTTTTATATCCAAATTCTCTAAATTTTGTAAATTAATATCCTCATAAATATCTGGATAACGAT
CAGAAATTTCCTTTAAAATAGGACTTAAAACACTAGCACTACCTAATATTTGAACCTCTGTACTA
TAGTCAATATTAGTATTTGTATTTCCTCCATCAAGCCCAGTTCCAAGTTGATTTTGCTGTGTTTG
GCTTTCCATGGGTTTTTCTACTAACAACATGAATTTACCCTGATAAATAGGAGATTGTTGAAAAG
TCCACCAAGTAACCCCAGTTGTGACTCCTAAGGTTGCACCCAAAATAAAAGCCCAACGCCTTTTA
AATAATTCCAATAAATAGGGAGTTTTACTGTTATCTTCTTCTTGAGATTGTTCTTTTTTCTCTAA
ATTATTTTCCAATGCTGAAAGAGAAGGGACAAAACCTTGATTTATTTCTCCTGAAGCAAAAGAAT
TTTTATCGTAATTATTAGACTTAGAAAAAGAAGAAATATTAGACTTTTCTGGTAACATTAATAA
CTCCTAAAATTAAAATAAATTTCAATATAAAAAACTCTTTACCCCCCACAATCATTTTACTGAAA
AATATTGAAAAATCCTAACAGTGAAAAACTCCTACCAATAGGACCTAAAACACTTTCTACACTAT
CTCCAAAAGCTGCGATCGCACTACGATTAATCACAATCACATCATTATTTTTGAGAATCGGATTT

GTTTGCTCATTCACCTCGGCCGCCAAATTGACAGGAATATTTCTTTTTGTAACAGTGCCATTAGG
ATTAACTCTAATCAACTCCACCGTACTACTATCTGCTCTTCTTTCATCAAAACCCCCTGCCGCTAA
AATCGCATTATTCAAAGAAGTATTAGGCTTTAAAACCGTTGAACCCGGATTTCGTACCGAACCAA
CCACATTCACCACAATCTCATCAGGAGCAAAATTAGCAGAAGCTAACCTTTGAGCCTCACTGGGT
TCAAGTTCCTTCGCCGTCGGAATAAAAATCGTATCTCCGTCACGCAATAAAACATCCCTATTTAT
ATCTCCCGATTCTAACATTTCCCACAAATTAACCTCTATTTTTTGCTGTTTAGCATCTCTAGTGGT
TCTCGAAATCACAATATTTCTAATATCCGCTAACTCCCGAATGCCACCACTTTGTTGTATCGCAGT
TGTAAGACGAGGAAGACCTTGTTCAATGGGTACAGTATAAGAACCCGGGCGGTTAACCTCCCCCA
CAATAGTAACATTGGGAGGTTCAACATATTTAATCCCAAAAGTTGCATCCGCTAATTGTCTATAC
TCATTAGGGTCAATGACATCTTTTTTAGGAACAATAATCACATCACCATCTTTTAATTTCACGTC
TTGGGCTAAATCTCCTTCTTGCAGTAGTTGCCAAAAATCTAGCACATAAAACACCTCTTTCCCTTT
TTCCCTTCTCTTGAGTTGAATTTGTCGAACATCCGCCGAAACCGTTAAACCCCCTGCAAGGGTAA
ATAAATCAGAAACCTTTGGTAAAGAAGGAGTATTAGAAGCTATTTGGGTTTGTCCTTCTTTTTGC
TGAATTTGAGTACCACCAGACAAATTATAGTTCCCCGGCACATTAACTTCTCCCACGATAGCAAC
TTCTAGGGGTCTAGGTTGTTGTAAGACAACTGTAATAGAAGGACGCTTTAAATAACGACTATATA
GACTTGCTAGTAAATCATTTACCTCCATAATCGTTTTTCCCCTCACATTAACAGTACCAATCAAG
GGAAAAGTTACAGTACCATCATTAAACACCTGTGCAACCCCTCCTTGATCAGGTAAACCAAAAAC
CGTTACTTGAATCACATCTCCCGTATCAAGAGTATAAGCCTTATTTTCTGGCAAAATAGACCGTT
GAGAAGTGGGTTTTAAAGGAGGTAAAGCATTATGTAAAAGACTATCGGAAGATGAAGAGGGGGT
TTGTGCTTGAACTGGTTTACTAATACTTATGACAAATAATGGAGAGACATTGAAAATAAAAATA
AAAGCTATTTTAGTTAGTGACATCTTGACCATATCAGCGATTACAAAGATTAATTTATTCCTAGA
TTAGTTTAAAGACATAGAAATTGTCTTATATTTAACCTCGGTTCGACATAAGAATGTTGGATAAA
GGCAGGTATAAGGTGTTTAATTATATTATTACTTGTCAACTACTTACCCAACACTATTCACCCTC
GCTTTTTACCCTTGTCTTTTTTCTGCCATAAACAGAAAATTTTATATCGAACTCAGATTATATTT
ATTATTTTTGAAAGTTATCTTAGGATAATGAGGAACAGTAAACAAAAAACATTGTAGCAATCAT
AACTTAATCATTACCAAAATGGCGAAAAAACAGAAATTCCCTCATCTTCTAGGTTCAAAGTGGAC
AGCCGCACAAAAGACTTGGGGGTGGAGGCATTTTCAAGTAATTAACCGCAAAAATGAAGATAAA
TGGGTATTTGCAGAAATGATGTCTTCTTGTGATAGCCAAGTCAGATTTTGGATTAACGCCAAACA
ATTAAAAGATCGTAATTTATGGACTCCGGGGTGGACTCCTTTAAAAAATTTGGAATAAAGAACA
ATTAGTGAATAGTGAATAGTTGATAATTAAAAACTCCTAATCCCTAACTAATTTATACCTAACAA
CTTAACTCATAAATAATTAACTCAAAAAAAACAACAGCCACCTCCTCGAACGCTACGGCTCAAGA
AAAGGTGGCTATGTGATATTCTCCTATATTATAACCAATAAAAGAATATTTTGTCAACAGTTTTT
ATGTTCTTTTTAAGAAGAATTAAGATAAATTTATACTAAAAAATTTTCAAACCTTCCTAATGCAA
TTAAAGGAAAATAATGACGATATAAATGATAGTTGATATAAAAATGTTGAGGGAATCCTGTACC
TGTAAATTCTTTTTCTTCCCATGTGCCTGTTTCAGTTTGAGAAGATAGTAAATAATTAATTCCTT
TATTAATACTATCCTGTTCAAATTTGCCCAAAGCCTTACCAGCATCAAGTAAACCAATTAATGCC
CATGCTGTTTGAGAGGCGGTGGAATTTCCTTTACCTTTCAATGCAGGATTTTTATAACTGTCGCA
GGTTTCACCCCAACCACCATCGGGATTTTGACAGTTAATTAACCAGTTAATACCTTTTTTCATGG
CTTCTTGACTGGTTTGAGGTGCAATTACAGCTAATGCGGATAATGCCCCAGATGTGCCATAGATA
TAGTTAACTCCCCATCTACCAAACCAACTACCATCATTTTCCTGTTGATGAAGTAAAAATGCGAT
CGCTTTTTGAACTTTACTAGAAGACATCGGCAAATCACAACTACCCACCATTTCTAATACTCTAG
CAGTAATATCCGCCGTCGCAGGATCAATCATCGCCTTTAAATCGGCATAGGGTAGTAAATTTAAC
CAATTAGCATCGTTGTTGATGTCAAAAGCGGCCCAACCATAATTATGACATTGCATGGTTTCAAT

CCAACGCAAACACCGCAAAATCGCTTCTTTTTTTAAATCTTCTCTAGGTAACTCTAGTTGGTGAA
GAGTCATAATTACCACAGAAGAATCATCCATATCAGGATAAAAACGGTTTTCATACTCAAACGCC
CAGCCCCCGGGTTTGCCGTGAGGATTTTTGACTACCCAATCACCATAATCTAAAATTTGCTTGTCT
AATAACCACTCTCCCCCTTTTATTAAAGCAGGATGATTAGGGGCAACTCCTGACTCCACTAAAGC
TCGTAAACACCATGCAGTATCCCACACAGGAGAAACACAGGCTTGTACTAAATAATCATCGTGGG
TTTCAAGGGCAAAATTTTCCACTGCTTCCAATCCCCGTGCTACCATGGGGTCATTGACGTGATAG
TTAAGAGTTTTGAAGGCAAACAGAGAGTTTAACATGGCAGGAATAATTCCCCCCCAATCTCCTGT
TGCTTCTTCCCTTTCAATAATCCATTTCTGTGCCGCCCTAATACTTTCTTCTTTGAAGGGTACAAA
ATTGATGTCTTCGGCAAATTTAAATAGGTTGTCTAATTGTACAAAAATATCTGTCCAATCTCCAC
TGCGGGGCAACTCATATTTAACATTTTCTTTGCCTTCGCTGTATAATTCGTCGAGGTTAATTTTG
GGCTCGATCTTGAAAACGGGTTTTTGATCAAATACGATTAATAAAGGTACAGTGCTACTTCTTGC
CCAACTAGACATTTCATAGATGGTAAAAATAGGATTTTGGGGTAATAACATAATCCAAGGAGGA
ATAGAAGGCACTCCTCGCCAATCATAGCAACCAATAATCGCTAGGTGAAATTTGGTAAAGATGCG
ACTTTTACTAATACCACCCTTACTTAAGATAAATCTTTTCGCTTTGGTTAGGGCTTCATCCTCTTG
GGCAACACCCAGTAATCTCAATGCCATATAGGCTTCTACTGATGTGCTTAAATCTCCTCCATCACC
GTAATATAGTTCCCAACCACCATGTTCTCTCTGCTGCGATCGCAAATAATGTTCTATTTTATGAA
GAGGTTTATGGGAGTCAATTCCCCAAATTTTATATAATAAGACTGCTTCTGCGGTGAGAGTGACA
TTAGATTCGAGATAGGCACACCAATGACCATCTTTTTCTTGTAGGGAAAATAAATAATCTTGAGC
TTGTTTGATTCCAGATTTAACTTTACTATGTTCTACTTTATTCGGATTAGTTAATTGGGCTGTCA
TAGATGGAAATTATAGCAATGAAACTTAATATTTTCTTTATAATAACCTGAGTTCGATACAATA
ATTTTTACCGCTCTTAGTTGTTGAGATGAACGAGTGGGAGTTTTAGATTAATTAGTGAGCTAATC
CCACCATAAGCTAACTTTTTGCTCAATTTTGAGTACATTTGCCAAAATTTGCAACCCAAAATCTG
GGTTATCCATGTCAACTCCTTCAAGTAATGCCCTTATTTCCTCATTAATTTCTTGACCCGATTCGG
ACATCATTGATAACATATCATCCATACAACCATAACCTTGATCGATGACATCAGGACAAAAATCA
TAAATTTCGATCGCTAATTCTTGTAAATTATCTGGTAATGTTTGAAAATATACCGTTACTTGATC
ATGCTCTACATCACTGATAGTAACACCAAATCGGCGATCCCAATCCTGAAGTTTATTGATAACAT
CTTTGGTAGTAACATCACAATTAATGCCATTAGTTTGATAATGTTTTATCACCTCTAAATTAATC
ATTTTTGGTTCTCTTAACTTATTGTTATCTAAATTAATTATCTAAATTATTGGTTAAGGTAGTTA
ACCGCCAGATAATACATCGTTAAATTTCTAATTGTCATTATGAACAAAAAAAAGAATTTTTGAGC
GTCCTAAACAACTTAGTGAGTGCTATAACAGAATTAAATCCAATATAGCCTTGGGGTTTTCCAAA
TAAATATCCTCTAATCTACTATCAGGATAAAGGAAAGAGTGAAAGTGAAAACTACCGATAGTTTC
ACCAATTATATAATGGTAATCTAAATCAACTAAATTACTTACCAACCAATAGCAAGGATAAACAT
AATTCGGTGCAAATATTTCCACAACTTTAGTATTAGGTTGACAAAAAACAAGGTTACTTAAACCA
CTACCATGAGGAGAAATAATTATTTTTGCCTGAGATAGTAATTCTGCTTGTTGTTTAACAGAAAA
TGACTCTAAATCAACTATTTCAAATTCATTTTTTTTCAATAATTTTATTAATTCTTGCTCATTAA
TAAGACGACGACTATGGGCATTATTTCTGGTTATATATAATTTTTTATGGGGTTTTTGCTCTTTG
TTGTATCCTAATAATTTACTTTTCAAATATTGACAACTCCAAGAAGGCATCCAAGCGATACAACC
GGGGAAGGAAGGAATAATTAATTCTTCAGCTTGAAGATATAAAGGAAATGAAAGGGGTAATATT
TTTTCTGGTGGTATATTAAATATTTGTAAAGTTTCTTTTTGAAAATTTGTTCTATAATCCACTAG
AATATAATCAATATCTTGCCAATTTATTCCCAATAATTCAACTAAATAGAGACGGGGCAAAATAT
CAAATAGCCAATGAAAATAAACGTTGTTTAATAAACCAGCTAATACTAAAACTTTACCTTTTATT
GTTGTTTTAGTCGGAAGAAAAGTGCTTTTTAAGAGAGAATGTTTACTAGGGTGACTATCAGGAT
GATTTGGACTTAAAGCAGGAGATTCAGGAGATAAATCACCAATTAAATAATTATTTTCTGTGAT

AATGGCACTACTTCCTTCTGTCTTTTTTAAATAAACTTTACCGTTAGGAATTTTGACAAGAAAAG
GTTTAGGTAGTTCTATTTTTTCAGGGAAGTAAAAACTGGGATGATTTTGTTTATGAATAGTTTTT
GGTGGTTTTAAGTATAAGTAGTTGTCTAACTCAAAAGGATAATAGTTTGTTTCTAATCGGTTATT
TTGTTTTGCCCAATCATAGGCTGTTTCATAATATTGATTGACGGGATTATTAATTTGATACTCAT
CTTCTTTTTTATTGTCTAAGTTAGAATTAATTTGTTGTGTAATTATTTGATATTGATGAGAGTTA
ATTACCTTCAGTTTCTCAAGATAAATATTAATATTATCGTACTGATTTTGGTAGAAAAGGCTAAC
AATTAAATTAGTATAAATGGTATCATTTTCTACTTCGTGTTCTAATGAAACTTGATAATAATAGA
TGGCTAATTTATATTTTCCTTGCTTAAATAATATATGACCGATATTCTGACTAATTTCTCCTAAT
GATTTATTCGTTTGTAAACTATCAATTAAGTCAATATTTAGTTTAATTACTTCTTCTTTATTTAC
TTTAATTACTATTTCTTGTTGATACCAGTTTTGGTAGTCATTAATAATTTTGGGTATTTGATTAA
TTAATATTTTCCATGTATCGACAACTAAATTTAATTTTTTTTCCATGAGATAAACATAACCCAAT
TGCCCATAGCTTTGAATGTGGTGAGGATTGATTTTTATTATTTCTTTTAAGATGTTTCTTGCGGA
GTTAAAATCATCAATTTGGAGGTAACAATCTGCTAAGGAGGAGAGAATTTGTAAATTTTGAGGC
TCTATTTTTTTACTTTTTTCATAGACTGCGATCGCATTTTTCCATTCTTTTATATCTTCCCATAAT
TTACCCAAATTTTCATAAGCAATAATACAGTTAGGTTGATTTTCTAGTAATTTTTTTAAGACTTC
TATTCCTAAATCAATATTACTTTGTTGAGCAAAAATGTGAGCTAAATCAATATAAGCACCGACGT
AATTGGGATTTAATTCTAATCCTTGATAATATAACTCTATTGCTAAATTAAAATTATGTTGACTA
TATTTAAGTTGGGCAATTTTAAAAATAGTTTCCGTAATACTATCTATTTCTAATTCGTCAATATT
TAATAACAATTCTAAGTAAATAGCTCCACTTAAATCATTTTCTTCTAAACAAAGATACGTTATTG
CTAAATAATTTTTGAGAAGATAATTTTCCTGATTTGTTTCTAATAAGTTTTCAATATATTTCTTA
GCTAAAAAATACTTTTCCTCGGTCAATAAATCCTCAATTATATAAACATTATTCTCACTTAACAT
CAGCAGTTGGGGATTGAAAAAAATTTAATTCAGCGTTAGTGAATTAGGCTATGATTTTTAGTTT
AGATCGGGAATGAGGAATAGGCAATAGATAATGGGCAAAGGCAATTAGTAATTAATTAACACCT
GCAACCTGAAACCTGAAACCTCTTTCATAACTCCAAACTCCGAACTAAATTTAATTACTATTGGC
TTTCTCCTTCAAAATCAAAAAATCCAGTATAAGCCTCCATACCATGTTCACCAATATCTAAACCT
AAGTATTCCTCTTCCTGAGAAACTCTTAAACCAAAAATGGACCTTAGCACAACCCAAAACATCGT
GCTAAAAATAATCATTGTAACTCCCACGCTAACAACCCCTAAAATCTGAGAACCAAGTAATGCAA
AAGTACCATTACTAAAAAAACCAGCAATACTTTCATCCCCTGTAATGTTCTCATCAGCGAATAAA
CCTACGGCAATAGTACCCCAAATACCGTTAACTAAGTGTACTGATACTGCACCGACGGGATCATC
AATTTTTAATTTATCCAACATATAGACAGAAAAAACCACCAAAATACCAGCTAATGCCCCAATAG
TAACGGCACTAGAATAAGATACCATTGAACATCCTGCAGTAATTGCCACTAATCCTGATAAAATA
CCGTTGATGCTTAGGGATAAATCGGGTTTTCCTGCTAATACCCAACTACTAATAGTGCCTGTTAA
TGCCCCAGAGGCGGCCGCTAAATTCGTCACCACCGCAATATAAGCTATATTTTCTCCTACCGCCAT
AGTTGAGCCGGGATTGAAGCCAAACCAACCAATCCAAAGAATAAAACATCCCAAAGTTGCTAAAC
TTAAATTATGACCGGGTAGGGGTACTATACCACCATCCGATAAATATTTTCCTAGTCTTGGTCCT
AAAATTATTGCTCCTGTTAAAGCACACCATCCCCCTACAGAATGCACTACTGTTGAACCCGCAAA
GTCCTGAAATCCAGCGTTTGCTAACCATCCACCTCCCCAAGTCCAATGTCCTATAACAGGATATGC
GATCGCAACTAAGAAAAAACTGAAAAATAAAAAATCAATGAATTTAACCCTCTCTGCCACCGCAC
CAGAGACGATTGTCGCCGCCGTACCAGCAAAAGCCACCTGAAATAAAAATTTTGCCGACAGAGGC
ACTCCAGCCCAACTCAAGGCGTTGTAAACCCCTTCATAAGCCTCTCCCGTGGCGGGGCTGTTATCT
GCTCCTTGTAAAAAAAATCCCTGAGTGCCAATAAAACTATTTCCATCAGCAAACATTAAACCGAA
TCCGATAGCCCAAAAAGCCAAAGTTGATACGGCAAAAACAATTAAGTTTTTGGAGAGTAAATTA
ACGGCGTTTTTACTGCGACAAAATCCAGTTTCCAACATCGCAAAACCCGCATTCATCGCAATAAC

CAAAATTGAAGCTATTACTACCCATAAAGTATCTAATCCTACTTTTAACTCTCCTGTAATTATTT
CTAAGGAAGTAGAACTATCTTGAGCATAAACCGCATAACTAGAACACAAGAGTAGAATAACACTT
AATAAAACACAACCTTGCCAACTAGAAGATTTCTGGATTTTGTTGATAATTAATTGAATTTTCCT
TTTTTGAGTTCTTGTAATTAACATTGCTTTAGATTCTGTTTTGACTCGCTTATTATAGGTTAAAT
GAGTTTTGATTATTAGCATCAAATTTTACAGAATTTTATAAGTTTTTTATTAGTTTTATTTTCAA
CTTATATTCAAAAATTATAAATAACTATTATAAATCATAATTAACTTTTGTTGAGGATTATATA
TTTCCACTGGTATTTGATATTTAGAGCTAATATATTGATAGTTTTTCTCTACCTGATTAATATGA
TTAATTATCTGTTTTTCTTGTTCATTATTATTCGTTAAATCACTCATTACCGAAAGAGAAGTAAT
TTTTTGGATATACTCATCTGTTAAGTAAACTTTAATATTACTATCACTAACAGTATAACTACATA
TTCTTTCTGAACTTTGACAAATTTCTCTTAATTTGGTTTTTATTTCCTCTCTTTTAACTGCTTTTT
TTAACGCACCTTGGGCGATTTCTAATTTTCCTTCAGCTTCGTTTTTTTGTTTTTCAATCTCATCTT
TTAACCAACTATCTGGGGAAATAGTGCCAGTTAAATTAATAACCTCTTGCCATAAATTAACTGCT
TTTGTCCATTGATTTTGTTTTTCTTGCTCTTGGGCAGATTTCATTTTCTCTTCTATATTTTTTTTC
AGATTTAATGCGTTTTCTTCAATGGTAATTTTCTTTTGAACTTCACTAATTTGCTGAAGATAATC
ATTAATAATTTCTTCTTTTTCCTGATATACTGCAGTTTGAGGTGGAATTTGTTCTATTTCTTGGA
TGGCAGATTTCCATTTTTTTTCTCTTTCTTTTAGGGCTTCTAAACTATTAGTAATATTATTTTCT
CCTTTGATTTGTGCTACTATTTTTTCGGCGTTTTCTCGTTTATTAATTGCTAAATTTTCTTTCTCT
ATTTGTCTATTTACAAAAGATAATTGTTGTTGATAGGTGTTAATTCTTAGGGTTTTAAATTCTTT
AAATTCTTCACTTTTTATCTTATTCAAAGGCGCGATCGCATCTTGCCAAAATTGCTTGACCCTTA
ACCATTCATCCAAAGAAAGAGGTAATTTTTCACTCATATTTTGAGCATTAGTAGCAACACTTTTA
GCATCTAAAAATAACTGTAAATCATTAATCTTTTCTTGGTAACTACTAATAAGCATTTGAGCTTG
ATCATGTTCCTTAGACCAAGATGGTATAGTTTTTAAAGTTTCGATATTTTGAACTAAATCAGTAA
TCAATTCTTTGATTTTACTATCCGTTAAATTATTTGTTTCTAACTGATTAAATAAATTATTTGTC
GCAACTTCTGTATCTGTAATCAAAGTACAAGTACCTAAAACACAGGGACGAGTGAAATAGTAAGT
AACACCAAAAATAGTTAAAGAAATAACTCCTAAAATAAAAATGATAAAACCTGCTGAAAAATTT
GCAGACTTATAAAAAGTATTTGAGGCTTTTCCATCTTCTATATTTTCTATCACATTAGATAAATC
TTGATTATTTTCAGATCCATTTACAGAAGAAAAATAAACTATATCTTGATTTTCAATATTAACAA
TTGAGTAATAATCAGTAATTTTATAATCAGCAATAATCTCTTTTATTAAATTAATAATTTTTGTT
AAAATATCTTCAGAATAACTATTAATAAAAATCAGCAATTGATCATTTTCTGGTAACTCAATTTG
GCACTCCAACTCAAGCTGAATTTCCTTCGCAACAACCGCTTTAATTTTTTCTTGGAAAGTTTCAG
GGTTTATTTTTAGACCCATTGCTGTTTTAATCATAGATAAAAAACCATTAACTTAACTACCTATT
AGGTGGATTATAAAATAAGCAATACAAAAGGCTAACCTCACAAGACAAAAATTAACCCTTATTCA
TTCAAAAAGTGTATCATTAAATTCAATTTTAATTTATGATCGTAATTAACGATTTATTAAATATC
AAAAACTTTACAGACTTTTTGCGTCAACTTCCCTAACTTTTTCTCTAATCTCTTCTGGTAAAAAA
TATGGCAGATAAAACATCTAACAGTAACTTACAGCCTTGGTGGAATCGCCCTCTTTGGGGAGATA
AAAGTATGCTAGAAAAATTAGAATCCATAATTCATAAACCTCATGATAGTATCCCGGAGGAAGTG
ATTGAGCATCATCAAAGAGTTTTCGGAGAGTTAAAAATACTGACACCTATAGCGAAAGCCCTAGA
TAGTAATGAATTTAATAATCCTGAATTTTTGGAATTTGTCCATATTTCTAAATTGTTTGCCTATG
AAATTGGAGAATATAAAGGTCTAAAAAATTATATTGCATTATTTAGAGTTGCCGTAGAAGCCAG
AAATAGTTTTTTGAAAATTGAACAAATAGAGTTATCCTATCGTAGTAGTAAACAACAAGAAATG
TATCGCTTTCTTCTCGGTTTACTTGAACAACAATTAAACTCTGAAGAATTCATTAAGAAATTAGA
ACAAAAACAGCAAGAAATATTACCAGAAATTCATAGTGAAGAAGGAAAAGATGCTATTAATGTA
TATACTGAAACTCTCAAAAAATTAGCCCGTCAAGATGAGTTAGGTATTAAATTAATGTACCTTTT

TAAAAAATATCAACTAGAGAATTTTTCCTTATTAAGAATAATATCGGAAATAGTCCAATACCTAT
TAGAAAGAAATTTGCTAGATTTTAATGATATAAAAATTTTGGTAAGAGCAAATCAAGATTTATT
TGACCAACTAGGGAAAGTCATTGAATTGCCTATTGATAAAACGAGGGAAGAAGATTATGCGAGG
ATGCTTCAATACATTGCTATGAAACAAAAATACCAAGATATATATATTCAATTTCTTCGCCTATT
AGAAGTGATGACTTCATGGAGTCATTTTTACCTAATTTTAAAAGAAATTCGTGAACATTATGACC
CCGATGAATTTGAAATACCTGAAGAGTTTAACACCCCTATCCCCGGAATCGAAATTTATAATAAG
TATCAAAGCGTAATTACGAAAAAATATAAATCTACCTAAGTTCGGGATAAATTTTCATCTATGAA
TGATGGAGAAAAGGGCAAACCCCCCCTTTATCCCCCCTCGAGAGGGGGGAGGGCAAAAGCGAATA
GTTGATAATTAAGAATTAAGAATTAAAAATTCCGAACACTTATTACTCATCATCCCTTAAATACC
GTAGGGGTTGAATATATTCAACCCCTACCACCTGACACCTACCCTTATCCGATATTTTTAGACCG
AACTGACTGAGGTTAAGTATTTTTGAGAAGGTAGGCAAGAGAGAATTAAGAATAAAAAAACTTG
AATTTCTAACTTTTTGGAGAATTAATTTTAGACAATTTTAAAAATTCTCTACAAAAGATTTTCTC
TAAGCAAGTCGGTTAGGAGAAGTAGCGGAGTTTATCAAGTTACTCAAATTTTGTCTAATTGCTGA
TTGTAATTTATCGCAATAAACTTCTTTACTTTGTTGGTATTGATTGGGTTCATCTACAAAAATAA
CACTATCAACGCCTTTCATTGTAAACATTTGAAAAAGTATTTGACTGACAGGAACTTGTTGGGCA
ACAATATCTGGACTATCAATTTTACTGTCGGGGAATTGTGCGGCGGATTGAACGCTAGGAAAAGC
ATAGATTACTTTCTTTTCTAACTCTGGATGTTGATCACTACCCAATATTGTTAATACCAAGTTGT
TGTCTAAAGTTTGTCTTAGATAATATTCTTGATGACCTAATTGTTGAGCAAAAGCATTAATAATA
GGGATTACCCCATTTTCCATAATAACCGCAGGGACACCATAATTAGGGGCTTCTTGGATCAAGTT
TTGTAGTTGCTGATCTAAGTTCATAATAGCTAATTTAATTCTTTAATTTCATTCTATAACGATTT
TTTAGTTAATTTAAACTAAGTTAAAAATTAGGGAGCAAGAAGATTTTTACCCATTCTTTTTTTAG
CATAGACATTCGCAACTTCAGGCATCCACTTTTGGAAATGTTCAAAAATTAATTCGGCTAAATTT
CTAATTTCTGGTTGTGCATCTTTTGGTAAACGCAAATCGCAGAAGTGTAGTAAACTACGGGCATT
AAAACTAACGACAAAATGTTGTCGGAGATTTTGAGTTAATAAATCCCTTGCGTGTTCAGGGGCGT
AGCCTTGTTGATTTACTTTTTCTGCATAATACAAGACTGCTTTTTGAGTAAATAAAATATCTTGA
TTACGGGCTGATGGAGAGTAAAAATATTTATTTCCTTCTCTGTCAAAATAATGGGCTACAGCACG
AAAATAAAAGACTTTTTCAATTAAATTTATGGCATCTTCTGGGGTTTCTTCTATTTGTTGAGCAA
GACGTAAAATTCTCTTACAAGTATATCTTTGAGACTGACAATCAAAAGATACACCGACTCTGTGG
GTTCTAGCCTGAACCATTACGGAATGGGGAAAATTGATAACATTAAAGGTGATAGATGGATGTTC
AAGAATACCCCAATGGGATTTATTCACGCAATTGCGGACGATTCTATCTCCCAATTCTGCTTCTG
AGTAATTAGCCAATTTAGACATCTCACTATAGACGAAGTCTTCGCTATAGTCTTGGTGCATAGCT
AGATAACAAAGTAAATTGGGGCGATCGCATTTACTTAATACTTCAACGGCAAATAAGGGATCTAA
CATTTCTAAATTATCGATCATCTCCATCTCCTTGTAAGGTTTGATTTACTTTTCTTGCTTGTAGTT
TTTTAATGTTTTTCTCGATTACTTGTTGAGGATCTAAACCTAACTCGTCGCATAATCTTGCCCAA
TACCACATCACATCCCCTAATTCTTTTTCTAATTTTTCTTTAGCAACATCTAAATTTTGATCCTTT
CTAATATATTTTTTTACTACTCCACTGACTTCCCCCGCCTCACTGGCTAAACCGAGGGTCAGATAT
TCCAAAAAAGTTTCTGGTGGATATATAGCAGTTTCTCTTGTTTTTAATGTATATTCGTTTAAATT
CATCTATGTAACTTATATTAAGCTAGTTTATATCTTAGTACAATTGTCAAGGTAGGACAAAGGCT
AAGAGGCAAAGGGCAAAGGTAAATAATTAAGAATGAAAAATTAAAAATCCCATTCACGACTGAA
AGGAGTGTACGAGCATAGCAAACTCTCATTACTTGTTTCCCCCTAAAACCCCAATACCTCCACACT
CTGACACCTGCAACCTGAAACTTGACACCTCTTTCCTAACTGCGAGGAAAACGACGACGTTGTAG
AAATTCAGGAATGTCTAATCCAGAAGATATACTTTGAGGATCAGTTTCAGGAGAGTTATTAGCGG
TTTCTTTATTTTCTGAGGTGGGAGAATCTAGGTTTGGCGGAGATGGGATGGTTTTAGCACGATCA

GGATTATTTTTTTTCTCTCCTGAAAAACCAGTGGCAATCACTGTTACTCTTACTTCTCCCTGCATT
TTCTCGTCAATCACAGCCCCAAAAATAATATTAGCATTGGGATCGACAATTTCATAAATAGTCTC
TGCGGCGGCGTTTACTTCGTGTAGGGTTAAATCCTTCCCACCAGTAATGTTTAAAACTACCCCTG
TTGCTCCTTCAATGGAAGACTCAATTAATGGGGAAGAAATAGCTGCGATCGCACCTTCTTTTGCC
CTTGATTTACCAGAGCCAATACCAATACCCATTAAAGCTGATCCAGCATCTGCCATAACCGCTCTG
ACATCGGCAAAATCAACGTTGACTAAGCCCGGGATGGTAATAATGTCAGAAATGCCTTGTACTCC
TTGACGCAAAGTATCATCAGCCATGCGGAAAGATTCTTGCAGGGGAGTTTCAGGAGGAATAACTG
CCAATAACTGATTATTCGGAATGACAATAAGAGTATCGACTTTACTTTCTAAAGCTCTAATGCCC
TCATCTGCCTGATTAGTTCGACGACGACCCTCAAAAGTAAAAGGACGAGTTACAACCCCCACCGT
TAAACAGCCCATATCCTTAGCAATTTCTGCCACAATTGGCGCCGCTCCTGTTCCTGTACCACCCCC
CATCCCAGCAGTAATAAATACTAAATCAGTATTTTCAAGGGCTTTTGCTATTTCATCCCTAGATT
CTTCTGCCGCCTTTTGACCAATGGCAGGATTACCACCAGCCCCTAAACCTCTGGTTAATTTTTGCC
CAATCTGTAAGCAATAAGTAGTCATGGATTGAGCTAAGGCTTGAGCGTCTGTATTTATTTGCCAG
AAATCAACCCCCACCACACTACTTTGGATCATCCTATTGACGGCATTACAACCGCCACCGCCAACT
CCAATTACTTTTATTTTTGCGATATTACTGGGAGTAATCGAATTAGAATTCATGTTTAAATCAGG
TGACTCATCTTTTGGTCGCATTTTTTTCCCTGCATTGGGGTTAGTGTGACTACCATTGGGGGACT
TTATTTTGGGAGTAGATTCTATAGATTGCTTGGCTCGGTTGAAATCAGGTTCGTTCATGGCTAAT
AAAATAAAGACGGCATTACCCTTTAGTATAAAATTAAAATAATAGAAGATAATCTAGTTGTTAT
TATTAATTTTAAGAATCTAGCCCTTTAATATTGCGTCAAAGGGTGACAGGAAAATTTTTTTCCTG
CGCAAAACTAATATAATTGCTTAGATAATTATACTCAGCAAGATTATAACTTAAGTCGTTCTCTT
TTTCAGAAAACTTTTAGGAATCTGGGTCTATGTAGTCAAATATATTTAACAACAAAATATGGGAC
TCACTCAACTGTCTGAGTTCAGAGTTCTATGTTCTGAGAGTTCGTTTTGGCTTTTACACTAATCT
CGGTCATGAACGAAGTTAAAATCAACAAATTTTGACAAAAACAGTAAATAAATTGATTTTCTTA
TTTTCAATATGAGTGACTAATGAGTCAATATTAAAAAAATAAAACCCGCCCTTATCCAATGATTT
GGCTAAATAACTAAGGTTTAATTATAAAAACTAGAAAAACTTTGAGTTAGAATTTTAAGCAAAT
TGCTTTATTCTAAGGCTTTATCATGGTATGAGTTATGAGCTTTGTTTTTGTTAATCGTTTTAGTT
ATATTCACCGATTTTTCAATGGGTTGAGGGACAAAAAGCATCTTTTTTTGTGGTCTTTGGTTTCC
TCGGGGATTTTATCTAGTTTGACGGTGATTAGTTTTGCTTCTTCAGCTTTTGCGGGTGATCCTTT
TCGCACAGTTAATCCTCGTAATATTAGCGAGGAAACGGAGTTGGCGTTTAAGGCTTTATTTGAAG
AGGGGAATTATGTAGAAGCGAAAGAGTATCTTGCTTTACCTAAAAAAAGCAGTTTTAATGATCCT
ATGTCGGCGGCTTTACGGGCTTCTTTGGCTTATACGGAACAGGATTGGAATAATTTGGATATTTA
TGCTCAAAAAACCTTGGATTATGCTAAAGCTATCCGCAATGACGATCCTTTACGTAGTAATCTGT
ATTTGGCGGTGGGCAATTTTTTAATGGGTGCTAGCGAATATCAAAGTAAGGGGGCGATCGCAGCT
ATTACTAGATTACAAGCTGTTTTCAACTATTTTGATCAGGCGGAAAGAATAGATTCTACTGATCC
AGAATTAAACTTAATTAAAGGTTATTTAAACTTATTTTTAGCGGTTAATTTACCTTTTTCTAGTC
CTCAACAGGCTATCGCTAATTTACAAACCTATGCCTCTCCTCAGTATTTAGTTAATCGTGGTATC
GCTCTTGCTTACAGAGATTTAGGAGATTATGATTTAGCTCTTACATTTGTTAACAAAGCATTAGA
AAGTACGCCGTTAAATCCAGAGTTATATTACTTAAAAGGGCAAATACTCAGAAACAAAGGACAA
ACAGACAATAATATTCTCGTTTTAGAAGAAGCGGTCAAAAATTTCGATATTGCGATCGCAAAAAT
TGATCAATTACCTTTAGAGGCAGTGCAAAAACCATTACAACGAGAAAAAAGAAAAACCCTAGAA
ACTATTAATCAATTAAAAATGGTGAGTCAGGGTAACTGAAATTGAAAGAAACTGAGGAAGGCAA
AAGTCAAAAGTATGATTTAGAGGGAGAAACAAATCATTAGTTAACCTCAGTTCGGTTTAAGAAT
ATCGGATAAGGTTAGGTGTCAGGTGTCGGGTGTCAGGTTGCAGGTAGTAGGTGTTAGGGTGATG

AGGAGATGGGGGGATGAGGGGAAAGGGGGAAACAAGTAATAAATAATAAATTAGTAATAAGTAT
TCGTGGTTTTTCATTCTTAATTCTTAATTCTTAATTCTTAAGGTTCTTCTACAGTGGTTATGATA
AATTAAGAAAAACAGGACTTCCATAACCTTTATTGAATAGTGTTTATAGTAAAATAAAAATTAA
AAGTTTATAAATTATTATTTAATAATCCCCTATTACCTATTGCCTCTTGCCTCTTGCCTACCCTAA
CTAATAATTTACACGACGAGAAGTGATAGAGCCATTCTTAATTCTTAATTAAAATCTCCTCTGGT
TAAGCGTATTTGAGCCAAAGCAAACACAACGGGTATAATTCTAGTATAGTTAGTTGCGATCGCTC
TCCATCCTAAATCAGCAAAAAAACAAGCCCATAAAGCAGGAGTAAGAAAAGCAAAAAAACTTCTC
ACCGCAGTATATCGAGCAGAAGCCATCACCATGCCCCTTTTTGCCATTTGTAAGCTGACATAACCT
TGTAATTGATTAGCAGTGGCTAGACTGCCTTTTATCAAAGTATTTTTAGTAACTTGATATGTAGC
GAAATGAATAGCAAATTGTTGGGCTATTTTTTGTAATAACCAAGGTTTAAAAAGAGAGTTAACA
GCAACTAAACCTCCTCCCCTAACTAAAATTTTGAGAGGATCATGTTGTAAACTGACAGGTAAGGG
CTGGGGTTGAGGATGATTTGCTAAAGAATTAATCACCTTTTGTCTCAAAGAATATTGCTCCCGAG
GAGACATTTTTTCCCATGCCTTCTGTAATAGGTGTAAAAAAATTTCTGACTCAAGCGCGATCGCA
TCCATGCTTCTACTATAAGATATTTGTAGATAACGACATACCCTAATTAAAATATCCTTATATCG
TAAAGAATGATACTGCCCCCGTAAAACCGTAAAACCATCTGCGGCTAAAAACCTAAATCGATTTT
CAATATAATCTAAAGATGTTTGCCAATCTAAGCTCTGAACATCATGGGGTGTAGGGGTTTGCCAG
TAGTCCAAAGGGTTGAAACGACGACAAAATAAAATTTGCGTAACTTGATGTATTTCTTCTTCGGA
GGCTAACTCTAATACTTCCCTTAACTCATCCACGACTTTAACCTATCTCACACGCATTTAATTATC
CACTTCATTATAAGCAGAAATTAGGAAATGGTTATCACTCTTAAGAATTAGGGAGCAACAATTAG
AGCGAAGGGAGGATGAGGTGATAGGGGGAAGTAGGGGCGAATGGCCATTCGCCCGTACAGGAGT
TAGGAGTTTTTAATTCTTAATTCTTAATTCTTAATTATCAACCATTGCCCCTTGCCCTTTCTCTTT
TTGAACTTGTACAGTAGAATTTTATACTATTAAAAATAAACATTTCTATGACTACTGTAAAAGTG
ACTTTCTTACCTGATAATGTTACTGTTGAAGCAAAAACAGGCGAACCATTGCTCGATGTTGCAAA
ACGTGGAAGTGTTTTTATTCCAACGGGATGTTTAATGGGATCTTGTCATGCTTGTGAAGTAGAAT
TAGATGATGGAACTCCTATTTGTGCTTGTATTAGTTCCGTTCCTAGTGGTATAGAAGAACTAACT
ATTAATCTTTATTCTGATCCTGTTTGGTAAATGAATTATAATCAGTCATCCTATAACAATTATTC
CTTCGCTGTATAATCAATAACCAATGAAATATCGCAAAGATAAAAGCCTTGATTGACAAAAAATT
TTCTTATATCTTACATTCTCCTCTCTCTTCATCTTCAAAATTTCTTTTACACATACCCAGTTTGAT
AATAGCTCTTTTTGGGGGGGTATTAATGGGGTTAACTGTTGCCCCTTTCAACTGCTATTTTCTAG
CTTATGTAGCTCTTATTCCCCTTATTTTTGCCATACATAAATCATTTTTTACCGCCACTTTTGCTG
GTTTTTTGTGGGGATTTGGTTATCATGGTACTGCTTTATTCTGGATTACGGGAATTCATCCCATG
ACTTGGATGGGTATTCCTTGGTTATATAGTTTATTAATAGCTTTAATCTGTTGGCTTTTTATCAC
TTTTTGGGGAGCAATTTTAGTTACTATTTGGTCTGGATTAATTAACTTTATCATCAAAATAGAAT
ATAGTAAATCACATTTTTATTCTTGGATTATCTTTATTTTAGGAATTAGTAGTTGGTGTATTTTA
GAAAAATGTTGGAGTCTTGGCTCTCTTTGGTGGTCATCTTTAGCTTATACTCAAAGTCCATATAA
TTTACCAATCTTACAGTTATTAACTATTTCTGGAACAACCACTCTTACCGCTTTTATTTTATTAA
TAAATTTCCTTATTTTTTATACTATTAAAACATATTTAATACCTGTAAATCAAGATAGAAAATAT
TTTAAATCAGTACTTTTTATTACATTATTTGTTTTTATATCTATCCATATTTATGGTTGGTTAAT
CTACAAAACACCTCTTCAAGATTCCCCTAATAAAGCGATTAAAGTCGGGATTATTCAGGGAAATA
TACCCAATGAAATTAAACTATATGACCCAGGTTTAAAAAAAGCATTAGTTAACTACCAAAAAGGT
TATGAAACCCTAGCACAAAAAAATGTTGATTTAATCATAACTCCTGAAACAGCTTTACCTTTTTT
TAATGAAGATATAGAAAAAAATATGAGTTTTTATAAAACAGTCAAAGAAAACAAAATACCCATT
ATTTTAGGTGCATTTAAAACTCTTAGTACTTACGATTATACTAATAGTTTATTCAGTATTGATAA

AAATGGAAATGTTATTAGCAGATACGATAAAATAAAATTAGTTCCCCTAGGAGAATATATTCCCT
TTCAACAAATTTTAGGAGATGTTATCCGTCGTTTATCCCCTCTTGATACTCAGTTAGTTGCAGGA
AATTATAATCAAAGTTTTATCATTACCCTACAAGACGAGAGCAAACAATTTAAATTGCCTGTTAT
AGTTGCTATTTGCTATGATTCTGCTTTTAGTGAAGTATTTAGACAACAAGCCTTGAAAGGAGGAG
AATTTGTCATTACTGCGGCTAACAATGCCCACTATAGCAATAGTATGCCTTTCCAACACCACGCTC
AAGATGTTATGCGTGCCATAGAAACAGGGAAATGGATGGCAAGGGCTACTAATACAGGATACTC
AGCAATAGTTAATCCTCGTGGTGATACTGTTTGGCTTTCTAATCTTAATGAATATCAAACTCACA
TAGATACTATTTATCGTCGTCATCAACAGACTTTTTACGCGCTTAGAGGAGACTGGCTTAATAAA
CTTTTTATCTTAATTATGGCAACTTATCTCTTTTTTAGAATAACTATCAACAGATTTATTCATTT
GCAGTAAAACGAATAATCTGAACTTTTGTTAATTTACTTTATCATGATATAATCAATTATTGATG
ATACGGGTAAAAGCTCAATAATAGGGACTAATAATAATCAACAATATACCCTATTTGTTTTCACA
TTATGAAATGTGTATTTAGAGAATCCTTTACTTTTAAACATCAATCAAAGCAGATAAGTATTCGT
TGAATAAAAATGATTAACAAGTCCTATTCTCAAGTTGAAGAAAAAAATAATACTCAATCTTTAT
CTGATTGCTTAATGGCACAAATGCCCCCTGCCGCTTTAGATTTGGTTGCTGATTTTTTTAAAGTC
TTATCAGAAACCAGTCGTTTAAATATTGTGTGTTGTTTAAGGACTGGTTCAAAAAATGTTACAGA
AATCATCGATAAAACAGGATTGGGACAAGCCAATGTATCTAAACACCTGAAAATCTTAGCACAAG
CGGGAATTGTGGCCCGGACTCAAAAAGGTATCAATGTATATTATCAAATTTCTAATCCCTTCGTC
TTTGAATTATGTGATTTAGTTTGTAACTCTTTGTCTATTCAAATACAGCAACAACACGAACAATT
AAGACAAATACAAAAAATGCAAAATAATGAAACAGTCAATTAAACATAGTTTTACAACAAAACT
GTATCTCTATCAGTAAAAATCATAAATATAATGCTGATAGATAAAAAAATCATATTTACTCCTAT
TTTATTCTCTTGAATTATTTAAGAGTACTTTCTTTAATTTTTTTTGCTCATTTATTTAAATAATA
AAAATCCATTATAAGGATTCAACTTTTATAAAGTTTGTGTAAAGAAAGTCATGAACAAAATAAA
TACTTAATAGATCTAAGTGTTATCACTTAAATAATAATTTAAAAAAGTAACCAAAATCAAGAAT
CTACTAAAATTGAGTGATAGAGAGGGTTTTTCATGGAAATGATTTCAGACAATAGAAATAAAAA
TAAATTAAATTAAGAAGTAATTTCTATGTCAAATTAGTTTTTTTTTGAGTATAATTTATCATTAC
AGATGGTAGAATAGATTACAGAAAAGTAAGTAAAAATAGATAACTCAAAGAATAATCATGAACG
AATTACTTAATAATAATGGGGAAGAACAAAATTTAACTCGTGATGAAAGAGTTAAGCAGTTAAA
AAATATGATTGACACTTTACATATTGCTGAAGATATTTCTCGTAATGGCTATTTAATTACAAGTT
CGGAATTAGCAGATTTAATGGATATTAACGCGAGTGCAGTCACCAGTAGAGGAGAAGAATGGTC
ATGGCGTAATTGGATTGTTTCTAGGGTTAGACGAGAGGGCAATCAAATTTTGTGGCAATTAGAA
AGGGTTGAAAAAGTTAACGAGTAAAAACGTTATTTATTTTTCCTCACAAAAAAATCAGAGATAA
TTATGGCTTAATTTTCCTTCTTTGCCGAAAATAATGGGTTAAAAACTCCATCGTTTATGGCAGAT
TTATGTTTTTTAGTAAAACATTACCATTCCTTATTGCTTATGATGAATTAATACTAATAAGTTAC
ATCAAGTCATTAAAGTGTTAAAACTATATCGCTATAGAATATATTCTACTGACGAGCAAAAGGTA
TTAGTTATCGCTGGTTTTTTAACTATAATTAAAAGTCATTTGTGTAGAAAATCCGGCAGTGAAAA
ACATAGCAACAAATCACAATTTAGCTAAGTCTATAAATAATTGTGGTTGGGAACAATTCTGCACT
ATGTTGAAATATAATGCAGAGTGAGAAGTTGACACTCCTTGATTTGAAGGTGCAAGGATTTTTG
ATTCAACTACATCCATTTTATTTTAATTGCTTAAATCAATCCAGAGGGGATTATGTCCCCAAGCG
TATGAACCATTTCAAAGTCGGTTCGAGTATGCCCCCTACCCTACCGCAAAGAATACTGAATTCTT
TGTTTCTCTGTGCTTATCAGCTTAGAGATTTTATCGTGAGAAGCCTTCCTCTTAATAACCCCATA
TCGCCAGTTTTCAAGATTCTGTGAATTGATTAGATTTGAGGTTTTTTAAGAGGTTGATTACCTTG
CCTCCAAAAAAATCATAACACACTAATGCTCTATATGAAAGGGCTTTAGACCCATAGGTTTTTGA
GAAAAAAACTTGCTAACTCTCGGACAATGTCAGCTTGACAACTCTCACCGTTAACCTTGCGGTGT

AACGGGGGATTCTTGGGCTGAATACTACCTCCACCAACAAGAGTTGATTTTGGTCTTACACATTC
ATGCTAGTCCGACACAAATATCGCTACTTGTGCCACTTAACGGAGCTACTCCCCAAGCGTGAATT
TCGATGTGTCCCACCGTATTTAATTGCTGTAACGCTCTGACTGTGTGGACTCTACCATTGACAGCT
AGAGATTTAGGGATTACGGAGTGTCTCTTGTTTCCTGCGTTCCCAAAGTCAACGACTTAATTTGA
TTATATCATAACGTTGGCGTTGTACGGGTCAACGGCCGTTGACCCCTAACATCTCACCATTAATA
ACATTGTTTGCCGTCACCTACGGTAACTCTACTCAAACTCGTTATTTAATGGGAGTCTTACGTCA
ACAAGATAACTAAAGTCAATTCTTTTCGTACTTTATAATTGTCTATAATTTAAGATACAACTGTT
CTGAAAATAATTCGCCTCTACATTCCTTAGTTTTATCTGAGAAAGGTTGCTTGTAACTTAACTTC
GGTTGGGCTTAAAAATATCCGATTAGGAGCAGGTGTCAGACTTTAATTAATTATTAATTTTTAAT
TGCCTATTGCCAATCCTCGGCGACACCACTTTTTCATCAGCCCCAGATAAAGATTGATGTTTTAGT
TTTGTTTCTTTTTATCCCCTAATTCAACTAATACAAGTAAAACTAAGGTTGTTTATCAAAAATGA
TGGTTGATGTTTGGGTAAATTTTAAGATATTATGAAAAGAAAATGAATAAAAAATGAAAAATCT
TTATGATAAGGTTTTCACAATCTCAAAATAAACTTTTAATATTTACTGTTTTACTGGGATTTGCT
TTGATAGGGTGTAATGGCTCGTCAGAAATAAACCAGAGCAGTAATCCAGAAAGTGAACAAATAA
ATAATACAGAGTTGAATAATGAGCAAGGAAAAAAGAAGGTTTTAACTAGCTTCACTATCTTAGC
TGACATGGCGAAAAATGTGGCTGGAGATAAATTAATTGTTGAATCAATAACCCGTGTTGGGGCTG
AAATTCACGGTTATGAGCCTACTCCTAGTGATTTAGTGAAAGCCGAATCAGCGGATTTAGTTCTT
TATAATGGCATGAATTTAGAACGCTGGATGGAACAATTTTTAGGAAATGTGCAAGATGTTCCTTC
TGTAGTATTAACTGAAGGTATTGAACCAATTCCGATTAAAGAAGGTGCTTATATCAATAAGCCAA
ATCCTCATGCTTGGATGTCTCCCCAAAATGCTTTAATTTATGTGGAGAATATTCGTCAGGCTTTT
GTGGAATTAGATCCTGAGAATGCTGACACTTATAATAGCAATGCAAAGATTTATAGCGATAAAA
TCAAAGAACTCGATCGCATCTTAGAGTCTGATTTAGCGAGGATTCCCGAAGATAAAAGATATTTA
GTCAGTTGCGAAGGGGCGTTTTCCTATTTAGCCAGAGATTACGACTTAAAAGAGATTTATCTATG
GCCTATCAATGCTGAACAACAATTCACCCCGAAACAGGTGCAATCGGTGATTGAAAAAGTTGAAG
CGAATAATATTCCTACTATTTTTTGTGAAACTACTGTCAATGATGAAGGACAAAAACAAGTGGCA
AAAACTACAGGGGCAAGATTTGGCGGAAATCTATATGTGGATTCTCTTTCAAGTGAAGAAGGTGC
AGTGCCGACTTATCTGGACTTATTAGAATACGATATTCGCACTATTACTAATGGTTTGTTAGCAG
GAGTGAACAATTAAAAATTTTTTCCTAATTGACGAATAAAAAATCAATGTCAACTAATAGTTAA
CAATACTCTCTGAAAACCAAAAATTGTCAACCAAAACATAACATAATTTTTACCCAAAAACCTCA
TTTATAAACTTTAAGGATAAAATCAATGATGAAAACCCCAACTCAACCCTTAAATATTAATTTCG
AGAATCTCAGCGTTACCTACAGCAATGGGAAATTAGCCCTTTATAATGCTTCTTCCAGTGTCAAA
CAGGGATCAATAACGGCTTTGGTTGGTCCTAATGGTGGAGGTAAATCGACTCTATTTAAGGCGAT
TATGGGTTTTTTAAAACCAACAGTGGGTAAAGTCAGTATTGGTAATTTATCCGTTTCTGAAGCCC
AAAAATCTCAATTAATGGCTTATGTGCCTCAAGCAGATGAAGTAGATTGGGATTTTCCCGTCAGC
GTTTTTGATGTGGTAATGATGGGGCGTTATGGTTATATGAATTTTCTACGTATTCCTAGTCAAAA
AGACCGTCGATTAGTAATGGAAAGTTTAGAAAGGGTGAATTTGGTCGATTTTCGTCATCGGCAAA
TAGGGGAATTATCAGGAGGACAAAAAAAACGAGCTTTTCTGGCAAGGGCTTTGGCACAAGAGGG
TAAAGTTATTCTTTTAGATGAGCCTTTTACGGGGGGTTGATGTAAAAACAGAGAAAAATATCATT
GACCTTTTAATACAATTGAGAGAAGAAGGACATACAATTTTAATTTCTACCCATGATTTAGCTTC
TATTGCTACATTTTGCGATCGCACTATCCTTTTAAATCGTACTATCTTAGCGGAAGGAGTCACGG
AGGAAACTTTTACGGAGGAAAATTTAACCATGACTTTTGGTGGTTTGCCCATGAATAGTGTTAAG
CAAATATTTAATTAAGGGATTAGAGAGTTCAAAGTTAGGAATGAGGTGTCAGGTTTTAGGTTTT
AGGTTTAGGGGAGCAATGAGAAAGAGGTTTCAGGTTTCAGGTGTCAGGTTGCAGGTGTCACAGGT

GATGAGGGGATGGGGGATGAGGGGAGAGGGGGAAGTAGGGGCGAATGGCCATTCGCCCGTACAG
GTGTTCGGAGTTTTTAATTCTTAATTCTTAATTTTTCCTTTGCCTCTTGCCTTTTGCCTTGTCTTA
ATTACTAATTTCTAATTAAAATGATTGTGTTTTCTAGTTTAGTCTCATGGTTACTTGAACCCTTA
CAGCATAGTTTTATGATGAAGGCTATTTGGGTAAGTGCCTTTGTGGGGATTGTTTGTGCGGTATT
GTCTTGTTATATAACCCTCAAAGGGTGGTCATTAATGGGAGATGCGGTTTCCCATGCAGTTGTGC
CGGGGGTAGTCGTAGCCTACGCTTTAAATATTCCTTTTGCCCTTGGTGCGTTTATTTTTGGTTTT
GGGGCAACGGTGGCCATTGGTTATGTGAAATCAAAAACTCGTCTTAAAGAAGATACCGTAATAGG
TGTAATTTTTACAGGATTTTTTGCCCTCGGCTTAGTTTTAGTTACCAAAATTCCTAGTAACATCG
ATTTATTTCATATTTTATTTGGTAATGTTTTGGGTATCTCCCCGGAAGATATTGTTCAAACTCTA
ATTGCAGGTGGTATTACTTTAGTTGTAATTTTAATACGTCGTAAAGATTTACTACTTTTTTGTTT
TGATCCCAATCATGCTAAGGCTATCGGCTTAAATACCAAAACTATCTACTATACATTACTATCTC
TTTTAGCTTTGACTATTGTAACGGCTTTACAAACGGCGGGAATTATTATGGTAGTAGCGATGTTA
GTAACCCCAGGTGCGATCGCATATTTACTTACAGATCGTTTTGATCAAATGTTAATCTTATCAAT
AGTTAGTAGTGTTCTATCTTGTGTTTTAGGCACTTATTTAAGTTATCATTTTGATGTTTCTACGG
GGGGAAGTATTGTCGTTTTAATGACCATAATTTTTATTTTAGCGATGATTTTTGCTCCTAAATAT
GGCATCATCAATCAAAATACCAAAATATATTCTGCTTAACTTGTTTACTGATACTTCAAATAATC
ATATAACCTATCTTCCGAGTTAAAAATAATGGATATTATCCAACTGAGGTCGAGAATAGAGTTTC
TTTTTTGATAGAATTTTTTTACACCAGTTATTCATTACTATCATGGGATAATGACAACTCATTTT
ATTAGTACGGAAATAGCCTTACCAGCGAATCCTCTTATCCTTAAACAGGAAATTGAACAAACATT
AAATCGGCAGGGTAAACCCTTAAGATGGGCAATTACCTCAGTAGATGAAATAGAAAAAAAAGTT
AAAGTAGAAGCAATAATAACTAGATAAGTGACGAACAATAAACCTTACTCCACAGTTTTAATTA
TTCCCACAGGAATCGGTGCTGATATAGGCGGTTATGCCGGGGATGCACTACCCATAGCAAGAGCC
ATAGCACAAGGATGCGATCGCCTCATTACCCACCCTAATGTTATGAATGGGGCAAATTTATATTG
GTCAACAGATAACATCTATTATGTAGAAGGATACGCCCTCGACCAATTTGCCAGTGCCAAATGGG
GATTGCAACCAGTCAGACAAAATAATATCGGTATCATTTTCGATCAAAGTATTGAAACAGAATTA
CTCTACCGACATATACAAACTATTGATGCTGTTAGGGCAACTTTAGGAATAGAAATCAACGAATA
TATTATTACCGATGAACCATTAGGAGTAGAGTTAAGAATTGCTGATAGCGGAGCAAGTTGGGGT
ACGATCAAAAACCCAGATAGTTTATTAAGAGCAGGAGAAAAACTGATTAAAGAAAAGAAAGTTA
ATGCCCTTGCCGTTATTGCTCGGTTTCCTGATGATAATGATAGCCAAGCCCTACAAAATTATCGT
TATGGAGAAGGTGTTGATGCTTTAGCAGGGGCAGAGGCAGTGATTTCTCACTTATTAGTAAGAGA
GTTTCAAATCCCTAGCGCTCACGCCCCGGCTTTATCCCCTTTACCCTTAGATATTAATATATCACC
AAAAGCGGCCGCTGAGGAATTAGGTTTTACATTTCTTCCTTGCGTGTTAGTTGGTTTAAGTCGAG
CCCCTCAATATACACTAACAAAAGACAACTATAGCTTTTGGGCAGAGGATGTAAATTGTTTAATT
GTTCCTGCAAATGCCTGTGGCGGAAGTGCAACCCTCAGCTTTAGTGGCTTGAATACTTTAATAAT
TGCTGTAAAAGAAAATAGCACGGTTTTAGATGTCACCCCAGAAAAATTAGGCTTAAATGTTGTCA
CCGTTAACTCTTATTTAGAAGCGATTGGGGTAATGGTTGCCCATCGTGGTGGCATTAGTTTACAA
GCGTTAAAACCGAAAATAAATTCTATTTTTACCAAAAAATCGTGGGTCTAAAGTTCCCTGCCGTT
TACGGCATTAGTGATATATAACATTTTTTTAGAGGAAAGGTAATCAACCTCTTTAAAAACCTCGA
ATCTAATCGATTTATCGAACGAGGAGCTGTCTCCTGAACTTGGTTCAGGAGCTTGTAAGCTCCGT
CTTGAAAATCAAAGTCATAGGGTTCTGTCGGGAAAAGCTCCGACAGGTAAAGTCTTTGAGCAGTA
AGTACAGAGAAACCAAGTATTTTAATTTCTCAGGTAGGGTAGGGCATACCCGAACCGCTCTTGAA
ATAGAGCATACGCTTGAGGAGAGAACCATCTCTGGACTAATTTAAGCAATTGGGTTAGTTTAAGT
GGACTCGATGAACCAAGAATCCTCGTGCATTCATGCCGAGGAGTGTCAACTTCTAGCTTGTATGG

CAATCCTATCCTGAATTCAGGATCAATTTTCATCTCTGAGTAATACTGGAAAGGGTAAAGGGCAA
AAGTGTTTAATTAATAACACTAACCCTAACACCCTAATCCCCTCATCACCCTATCACCCCATCTCC
TAAACACCCTGACACCTTAATTGATATTTTTAAACTGAACTAACCTCATATCTTACTGACAGGAA
AACGAGGCTGAGGATTGTAAAATAAAGCCCTAACAACAGCTTTTCTCAAATCGGGTGTTTCAGTT
GGTTTTTCTATCCAAAGAGTTTCATAATAGAAAAATGAGACACCTAATCTATATCTTCTTGCTTG
TCTAACCTTGTCTTCAATAAGAGTTATCGGGCTTGGATTATTTCTTAGTCCTGTTAGAATCGCAA
CCCCTGTAGGTATTTTTTTACGAGCATTTTGCACTTCAGGTACATTCAATTCCCGAACAAAACTA
TTCAAATCTGGGCGGTAAACTTGAATAATTAATTCATCCACAATACCTAAATTAACCCATCTTTT
CCAATCCTGTAAAAAGAGATTATAAGCAGTGGAGTAAGGATTAGGAGCAACAGAAAAAATAAAA
TTGGGATTTCTTTGTTTAAGAAGAGTATTTAATCTCACCATGAAATCAGTAATTTTATCAGCACG
CCATTTTACCCATTCTCTATCTTGAGGATTAGCAGGAGGGTTTTTTTCGTTTCTTGTTTATAAA
GATTTACGGTATAAGGATCATATCCAAAGTCTCGTGGTAGCGCCATATGATCATCAAATTGAATA
CCATCTACTCGATAGCGGGTACAAATTTCCAGAACTAAATCAGTTATAAACTGTTGCACTTGAGG
ATGAAAAGGGTTTAGCCAGACAACTTGCCCGGCCGCACTATTTCCTATCTTACTGCCATCTCGCCT
ATTGGTTAACCATCTAGGATGCTTAAGTGCTAACTCCGAAGAAGGAGGAGCCATAAAACCAAACT
CAAACCAAGGTACAACCAAAAGTCGATTTAAATGACCTTGATAAGTAATTTCTGCAATCATGTCT
TTATTATTACCCGCTTTAATGATAGGTATTCCCACTCTTTTTGCCACAGCACTATCATAAGAAAC
ATAACCAGAATTCCAGACAACAGGATAAATAGTATTAAAGTTAAGTTGAGCTAATTCTTGAATA
CTTGATTCCAATTTAGGAGTATCTAACCATATATCACTATCATTTTTCGTAATCCAGACTCCTCT
AATCTCTGCAAAGGATGTTTGACCCCATATTTTTGAAGGTTGAGGTAATGTTAGAAACATTAGTA
AGGAGATAATAAACAAAATTACTATTTTTTTCATTCTTCAAATATCATTCAATTTTCTAGGGAGT
TTTATTCTTGTTCAAAAAGTGATGTTATTTGTTTTTGATATTTTCCTGTATTTCAAATAATTTTC
ACCTTCACCATAATATCAAATGAAAATTTTTCTTATGATTTTTGCTGGTTGATTACATCAGTTTG
AGACAAAACTTCTTTGGGTTAATAGTGAATAGTGAATAGTTAATGGTGGATAGTTAATAGATTT
CAATGGAAAGTTAGAAATTAAAAATAAACAAACCATTCTTATCTAAGCTCGATGAAAAAAGTAT
TGAAAAATAAGGATTTGAGAGCTATTAATCGACAAGATTTTCCTTGAAAACATTATTAAATTTG
GGAAAAATCTGTTTAAAATTAATTTCATAGCAGTTTATATAACCTCAGTTCGGTTTAATAATATC
GGATAAGGGTAGGTGTCAGGGATGGGGGGATGAGGGGAAAGGGCAGGGCTGTTTCATACTAAAA
TCTTTGTTTAAGAGGGAGTAATAAGTAATGAGCAATGAGTAATGAGTTTTGATAATTTATTATG
ATTAATTGATTGTTTCAAGATTTTTTATTCTTACTTAAAATCTGCAAATCAAAAATCCCCCCATC
TCCCTCTCCCCTTATCTCCCGCTCTTTTTTCTTTCTCGAAAATGAAACAGCCCTGCTTTTAGTCAT
CACTCATAGATGAAAATTTATCCAGAGCTCAGGTAACTTTATATTATTGCTGAACAACAAAAGTA
AATAAAGGTTATTTTTCTGAGGTAACAATTATTTTTCTCGTTTTCTAAAATGGCTGTTTTTTCCT
TTTTTCAAAGTGTTAATACAGATTACCGAACAATTGAATGTACCCTCAGACACAGACTAACCCTA
ATCCTATGTTAAAATCAGCTATAAACATGGCATAGGGTGAGTAAAGACAATTATGTTTGAACGCT
TCACAGAAAAAGCGATAAAAGTGATTATGTTAGCCCAAGAAGAAGCTCGTCGCTTGGGTCATAAC
TTTGTTGGTACTGAGCAAATACTGCTCGGTTTGATTGGAGAAGGCACTGGAGTAGCGGCAAAGGT
GCTGAAATCAATGGGAGTTAATCTCAAAGACGCTCGGATTGAAGTAGAAAAAATTATTGGGCGT
GGTTCTGGTTTTGTAGCGGTAGAAATTCCATTCACTCCAAGAGCGAAAAGAGTCCTAGAATTATC
TTTGGAAGAAGCTCGTCAATTAGGTCATAATTACATCGGTACGGAACATTTGCTTCTCGGTTTAA
TTCGTGAAGGAGAAGGGGTTGCCGCTAGAGTTTTAGAAAATCTGGGAGTGGACTTAGGTAAGGT
TCGCACTCAAGTAATTCGGATGTTAGGTGAAACAGAAACCACTGCAGTAGGTGTGGGTGGTGGTA
GTCGCTCTAATAAAACTCCTACTTTGGATGAATTTGGCTCTAATTTGACGGTTTTGGCGGTGGAT

GGGAAATTAGATCCTGTGGTTGGTCGTCAAAAAGAGATTGAACGGGTAATCCAAATTTTGGGTCG
TCGCACTAAAAATAATCCTGTGTTAATTGGTGAACCCGGGGTTGGTAAAACTGCGATCGCAGAAG
GTTTAGCTCAACGTATAGCGAATAAAGATGTTCCTGATTTATTGGAAGAAAAAGAGTTGTCACC
CTTGATATTGGTTTATTGGTAGCTGGTACGAAATATCGTGGTGAGTTTGAGGAAAGACTCAAAA
AAATCATGGATGAAATCCGTCAAGCAGGAAATGTTATTCTTGTAATTGATGAGGTTCATACTTTA
ATTGGTGCTGGGGCGGCAGAAGGTGCGATCGATGCGGCGAATATCCTCAAACCTGCTTTAGCTAG
AGGTGAGTTACAGTGTATCGGTGCCACCACTCTTGATGAATATCGTAAGCATATTGAAAGGGATG
CGGCTTTAGCAAGACGTTTTCAGCCCGTCATGGTGGGTGAACCTAGCGTAGAAGAAACCATCGAA
ATTTTATTCGGGTTACGAGAAAAATATGAGCAACATCACAAGCTCAAAATTGCTGATGAGGCTTT
AGCGGCGGCGGCTAAATTGTCTGATCGTTACATCAGCGATCGCTATTTACCAGACAAAGCTATTG
ACCTTATTGATGAGGCTGGTTCAAGAGTACGCCTCTTAAATTCTCAACTTCCTCCCGAAGCAAAA
GAATTAGACCAAGAATTGAGACAAGTTCTTAAAGAAAAAGATGAAGCGGTGAGATCCCAAGATT
TCGATAAAGCAGGAGAATTGCGCGATCGAGAAATGGAGATTAAAGCCGAAATCAGATCTTTAGC
AGATCAGAAGAAAAAAAGTGCCGACATTAACGATAATCCCATTGTCGATGAAGAAGAAATAGCT
CATATCGTTGCTTCTTGGACTGGTGTACCCGTTCAAAAATTAACCGAATCTGAAGCTGATAAACT
CTTACACATGGAAGAAACTCTCCATCAAAGAATTATCGGTCAGGAAGACGCTGTAAAAGCTATTT
CTCGTGCTATTCGTCGTGCTAGAGTGGGCCTGAAAAATCCTAACCGTCCGATCGCATCTTTCATCT
TTTCTGGTCCCACTGGTGTAGGTAAAACCGAACTTACCAAAGCTCTTGCAACCTACTTCTTTGGCT
CAGAGGATGCAATGATTCGCTTAGATATGTCAGAATTCATGGAACGCCATACTGTTTCTAAATTA
ATTGGCTCTCCTCCCGGGTATGTCGGTTACAACGAAGGAGGACAGTTAACAGAAGCAGTTCGTCG
TCGTCCTTATACTGTAGTGCTTTTCGATGAAATTGAAAAAGCCCATCCTGATGTATTTAACCTCC
TACTACAAATTTTAGAAGATGGACGCTTGACCGATTCTAAAGGTCGTACCGTTGACTTTAAAAAC
ACCCTCTTAATCATGACCTCGAACATCGGTTCAAAAGTCATTGAAAAAGGTGGCGGTGGTTTAGG
ATTTGAATTGGAAGAAGATCAAAATGAATCTCAATACAATCGCATTCGTTCTCTAGTTAACGAAG
AATTGAAAAACTACTTCCGTCCAGAGTTTCTCAACCGCTTAGATGAAATAATCGTCTTCCGTCAA
CTCAATAAAGAAGAAGTAAAAGAAATTTCGGAAATCTTGTTAAAAGAAGTCTTTGCCCGTTTAA
CTGAGCAAGAAATTAGCTTACAAGTAACCGATAAGTTCAAAGAGCGTCTAATCGAAGAAGGATTT
AACCCTGCTTATGGAGCTAGACCTTTACGTCGTGCTATCATGAGACTACTTGAAGATATTTTAGC
AGAGGAAATTTTATCTAAACGTCTTCAAGAGGGTGACTCTGCTATTGTGGATATTGGCGAAGATG
GAAAAGTCATCATCAATGCCCAAAGAGAAAATCCTTTATTAGCAAAAGCTAACTAAATTTGGGTT
TAGGGTTCGGAGTTAGGGGTTAAAATAACTAGATAACCTGAGTTTGGGATAAATTTTCATCTATG
AGTGATAGCGAAAAGGGCAATGGGCAAGAGGCAAACCCCCCTTTATCCCCCCTCGAGAGGGGGAG
GGCAAAGGTAAATAGTTGAGTTCATAATTAAGAATTAAGAATTAAAAACTCCGAACACTTATTA
TTTATTACTTGTTTCCTCCTCTCCTCTCATCACCCTTTCTCTCTACTCATTACTCCTTACTGGTTA
CTCATTACTTTCTCTGCAACCCCCAATTATAAGAAATTATCTTTATTTTCTCTTTGTTGTAATAA
TTTGAGAGCTTTTTTAATATCCTGTGTTTTATTTTTATGGGCAATTAGAGTGGCATTTCCTTCCC
TAACAATAACTAAATCTTCTACCCCAATCGTCATGACAATTTCGTCAGACTGATTGTTATAAATA
ATATTATTTTCGCTATCTTGATTAAGAGAATCTCCCACCAGAATATTGTTATTTTCCTTTGCCAA
AAGTCTTTCTAAAGCATTCCAATCTCCTAAATCATCCCAACCAAATTCGGCCCAAATCATGTAAG
CTAAATCAGTTTTTTCCATTAAGGCATAGTCAATACTAATTTTTTCTAACTGTGTATAAGCTGAC
TTTCCTTTCTCCTGTAAAGGTTGTAGAATTTGCGGTGCGTGTTTTTGTAATTCTTCTAACACCAC
TTCTACGGGAAAAATAAACATTCCACTATTCCAACTATATTTTTGCGTTGCAATAAACTCTTTTG
CTGTCTTCTCGTCAGGTTTTTCAGTAAAGCGAGTTACTCGATAGAAGGAAATACCATCATTTTCT

CCTTGTTTCTCCCCTTGTTCAATATAACCATAACCGGTGGAAGGATAGTCTGGTTTAATTCCTAA
AGTGATGATACCTTGCTTTTCTTGAGCATATTTTATTCCAGTCTCAATGATTTTCTCAAATTGTT
CTTGATTCCCGATATAATGATCCGCAGGAAAAAAAGCTGTAATTGCATCTTCACCATAGCGACGC
TTTACTTCTAAACTAGCCCATGTAACGGCGGGGGCTGTATCTTTTGCTGTGGGTTCAACTAAAAT
ATTATCAGGTGGCAATGAGGGTAACTGCTGTTTCACCCCATCCGCTAATAACTCAGAGGTGATAA
CCAATAAATTTTGCCAACCTCCTGCTAGGTTTAATAAGCGATTTGCGGTGGTTTGCAATAAACTT
TCACCACTGTTATCCAAACACAGAAACTGTTTTGGACGTTGTAAACGACTTAAAGGCCAAAATCT
TTCTCCTTTTCCTCCTGCTAAAATAACAGGTATAAATTTGTTATTTCTCATAATTAAAAATGTTT
GCTGATTCTATATAAAATCTAAATCAAATGAAATTGGAAGGGTAAAAAATAAATAATAATTCTT
GCTTATAAATGTTCAATTATTTATCATACTTGTTCATAAGTCTAGCTATAAGCCCGAAATGCTCA
GAAAAATCGAATTCTTTACCAGATGTCTATTTCCCTCTTCCAGAAAAATTATAATTGAATTTGTC
AAGAAATTCTTTTTGACGAAGTTATTTAAGGTCACTATAGTATGGTATATGTATCAGTGATCTAA
ACCACAAAAACAACCTCAATTTTTGAACCCGAAAATAAATTGGCACGTCAATCCTATAATTCTAA
ATCTAAAATAAAAAAAACCTATTCCGTGAAAAAATCAGTTTCTCCTTTATTACAGGGCTTTATTT
GGGGAGGATGTTTTACCCTCACTGCGGCTGTTTCGGGTTTTGTGGGTATGACAGTGGCATTGAAA
AGCCCTTTGCCTGTAAATATAGAGCCTTTTACGGAAAAGTTACAGGCTTTGAAACGTTTTGGTTT
GTCAGCGTTATTTACGTCACCTCTTGAGCAGTCAACTAATATCTTAGTAATGGGTATTGATAGAG
TGCCTAATGCCGAGGGTATAGATAAATTTACCGGTCGTAGTGATACAATGTTACTGGTACGTTTA
GAACCTGAAACTCATACCTTAAAAATGCTATCTATTCCGAGGGATAGTCGTGTGCGTTTACCAAA
TGGGGCTTACACAAAGATTAATGGGGCAAATGCAAGAGGGGGAGTGCCTTTAGTTAAAGAGGTT
ATTCAAGATAATTTAAACGGTGTTACAGTAGATCATTATATCAGGGTGACTACTGACGCATTTAA
AAAGTTAGTGGATTTGGTTGGGGGTGTGGAGGTTTATGTGCCTGTAGATATGCAATACATCGATC
GCACTCAAGGATTGTATATTGATTTAAAACAAGGAAAACAAGTCTTAAATGGAGAAGAAGCAGA
ACAGTTTGCTCGTTTTCGTCAAGATAATTTAGGTGATATAGGTAGGGTACAACGACAACAAATAC
TGTTGAAAGCATTGGGTGACAAGATGCGATCGCCCCAAATGATATTTAAAACACCGCAAATAATT
GACTTATTGCAAGAAGAAATAGATACAGATTTAACATCTTCACAAATAATGACACTGGCAGGTTT
TGCCCTTGGTTTAGACAAAAAAGATATTAGAATGTTAATGTTACCCGGACGTCCAAGCTATCCTA
GAGAATATCAACTAAGTTATTGGCTAATTTCCGAGGAGAATAAGCAAAGCGTTATCGAAGAATA
CTTACAAATCGAAAATGCAACCCCATATAACCGCCCTTCTCCCAGCCGTATCCGTATAGCTATCAA
AAACGCCACTCCCAACCGACAATTAGATGACAAATTAGCGGAATTGTTAGGAGAAAACGGTTATC
GCAATGTTTACATTAGTCAAAAATCTACTATTCCCACCACTACCACTCAAATTATTGTCCAGAAA
GGGGATAATGAATCAGCCCAACAAATTCAAAGGGTGTTAAATTTTGGTGAATTAGAATATTCATC
TACAGGAGATATTGATTCCGACATTACCATTATCTTGGGTACAGACGCAGAAAAGTTATTAGAAG
AAAATAGTTTTATTAACTGACGTTACGTACTTGTTAACCTGAGTTTTGGATAAGCTGAAAGCATT
ATTTTCTCCTAGTCAGAAAACCTTATAGCTTCTTCTTAGAAATAACGATAAAATTGCCTTAATCC
GAACTGACGTTAAAAATATATTCAACTCCTACCTACCACCCCAAACCCTAAGCCCATACTTCCCTC
TCTCCCCTCATCTCCTCATCTCCCTAACACCTGTAACCTGCAACCTGCAACCTGACACCTAACCTT
ATCGGATATTCTTACTGAATAACAGAGGTTATATTGTGTGTAACTTCGGTTTTCTAGTAAAAAGC
ATCTAAATCGAGAGCTTTTGAGCCTCCTTCTAATAATTGAATAATCAGTTTTTGTAATTGATTCC
AAATTAAACCACCAATTAGAAGAGTTAAAGCCCCAGAAATGAGATAGGATATAACACGGTTATA
AGTAAAAATCTCTAAACCCGCCGCTAGAAAAATACAAATTCCGAGACAAACCCCAAAAAATGGTA
AAGTTAGACGAGACATCGCACTCATATCAGATGTTCTATTTTTTTGTCCCCGCTTCATCCAAATA
TTAACCTGTTGTTTTAATGTTGATTCAAAAGCTAAACCACAAGTAACACCAATAAAAAAACCAAA

AAACAATAACCCTAAAGGAGGCTCTGGAAAATAATACATAGATAAACTTTTAATTTTAATGACT
ATTGTAAATAATTTATTTGTTACAAAATATTAACACCATTCGACACCAAAGAATTAGGAATATTT
AACCTCAGTTCGGTTTAAGAATATCCGATAAGGTTAGGTGTCAGGTGTCAGGTTGCAGGTGGTAG
GGGTTGAAAATATTCAACCCTTACGAGAAACTAATTAATTACATTTGCCCTTTGCCCCTTGCTAT
TTTTTCAAAGGTTATTGTAAATCTCTCAGTATCAGTTTTTCTTTATCTTTAACTGTTGGTGGATT
TGGTTCGTTATCATAATTACGATATTTTACCCGCAAATCTTGTAAATCAATACTCATTGTTGAAA
ACAAAATTGGTTCTTTTTCTGTGTCAAAATTGGGAACATATAAATAACTAATGCGAGGAATATTG
AGATGGAATCTGATTAAATTTGCTGTTTCCATTCTGCCCACTGTTCTGCTGGCACACCCTTCATAT
AATCTCAGTAAGGTAGGTAAATTATCTAAATTATTAATATGTACTAAATAGCTTTTTTCAAAGA
GTTTACCAAATGTCATTTCCTCACAAATTTTTCTTACAATTTCCAAATTACCTAGGGTTATTAAC
ATTTCATCTGCTAAAAAACAAGCTCCTTGATAGCTACCAAATAAACTTTTTATATCTGCTTTTAG
TGTAGAAGATAGTTGTCTAATAGTAGGGCGTTTGCTAAAACGACTTAAGGCTAAATAAAGTAAT
ATATCTTGTCTTCTTTGATCTTCAATTTTATCCCATTCTTTTTGGTCTGTTACTTGTAAAATAAC
TTTAAATGCTTGTCGATAAGTTCTAAATTCTGCCTTAATTTTTTCTTCTTGAAGTAACTCCCCTT
TTTGAGGTAGTCTTCCTCTTTCACTATAAAAATCCATTAATGGTTTTAATAATTCTTCATAGTCA
GCGAATTTTTTGGCAGGGGATAAAATACGAGGAGATTTAACCCGTGAATGAAACCTTGAAAGAC
GAAAATTATTAGCTTTTTCTACATTTCTAAAAACTAAAAATACTCCTAAGCCAATGGGTATTGCT
TCTTCATTTAAAACTTGTTCAATGTAGTTTTTTAATTCTTCTTGTTCATAATATTTTTGAAAAGT
ATTTCTCTCGGTAACAATGCCATCTCCATAAATCATATAACCAGAAGTGCGATCGTCAATTAAAA
CTTGAGCAGAAACAATTAATATTTCTTTTGTTAATGACCATGCTTTGATTAATGCTTCTCGTCTT
TCTGTGACATTTTCAATCACATTTATTATATAACCTAAATTAACAATATCTGCTTTTTTGAAAGG
ATTATAAGGTTGATAATAAGGATCCCATCCATGACTGATAAAGCCTTTTTCTTTGATGATTTCAA
CATCTAAGCCATGACCGCAACCATAGTCAAAAAAACTATTTTTTTCTGCAAATAAACCTGCTTCT
AAGACTAATCTTACAGGACGAGAAAGTCTATTTCTTACCATAGCGGCACGGTGACGCTTAATTTT
TATAAATTCTGCTGGATTATCATTAAGATTACAAATTAAAAAGTGGTCGAAAAAACTAATAAAA
TTATCTTGTAATAATTGCTTCCATTGTTTTTGAGTTCCTATGTAGCGAGGATTGTCTAAAAGTCC
TAGTTTTTCTTCGAGATGGGTTAAATATGCAAATTTTTGATAGTTTGGATGGTTGGGAGTTAAA
AAAGTTTCTTTGCGGTGCAAAATAGGGGGATTATTACTCTGAGAATATTCTATATTTTTGGTTGT
TAAATCCTTTAAATTAACAACGATACTTTTGGCTAAAGGAGGATGAGGATTAGTTAAAAATTCA
GGATAAAATAAATAGGATATTTTTGGTTCACTCAAACTAAATTTAATGAGAGTGAAGGTGCTTA
TATTATTAATAGTTTTCCTCGCTTTTAATTCATATTCTTGAATAGATTTATCTAAAAAATCTAAA
GTAGAAATATGCACATAAAGATTCTGCGGAAGATGTTTTCCGATGGTACTTTGATGCAATAGTAA
GGAAATTTGATCAAAGTTATTCACCATGTTATTTTATCTATTACTAAAGAAAATATGATCCTGAT
TGTCTTAAATTTTAAGGTAAAATAAAAGATTAGTCCCAAATATGTTGTCAATTGTTAAGATATG
CCTTATGCCAGAACTATTAGTACTATAGTTGCGATCGCCCTTGCTTTGATAATGATTATATTGGG
GGGTTTATATTTTACCATCTGTTTTGGTGTGATAGTCTTTCTCGCCCAATTAGAGTATTTTCGTT
TAGTTAAAGCTAAAGGAATCGAACCCGCCGCAAAAACCACTCTAGTAGTTTCTCAACTATTATTA
ATCACTGCCACTTTTGTTCCTAATTTAACGGATGCAACCTTTGCCCTTGCAGGGGCGTTAATTTGT
TTTTATTTGCTCTTTCAACCGAAGTTAGCAACCATTGCGGATATATCAACTTCTATTTTAGGGCT
GTTTTATGCAGGATATTTACCTAGCTATTGGATACGTTTAAGAGTTAGTTTAGATCAAAATACTG
CTTATGCTCAATCTATTCCCAATATTCATAATTTACCTTTAGATGGTTATTTTCCCATACATCCTT
TTGATATTAGTAGTTTTCCTGATGCCCTTAAACTGACTTTTATGGCAATGGCTTGTATTTGGGCG
GCGGATATTGGTGCTTATTTAATCGGGAAGAATTTTGGTAAAACTAAATTATCTCATATTAGTCC

GAAAAAAACCGTTGAGGGTTCTCTGTCAGGAATTACAGGAAGTATTTTGGTAGGTTTAGTTGGTG
CATGGTTATTGCAGTGGGATGCGTGGATAATTACTGGTTCTTTTTTAGGTTTATTAATCGGAGTG
GTTAGTCTATTAGGAGATTTAATGGAGTCGATGATGAAAAGAGATGCCGGTGTGAAAGATTCTG
GGCAATTAATTCCCGGTCATGGTGGAATTCTCGATCGCACTGATAGTTATGTATTTACTGCACCA
TTAGTTTACTATTTTGTCACCCTACTTTTGCCATTATTTTCCCATAGTTTTTGAATAAAAAAAAG
GGGATTAAATTCCCCTTCTAAGATAAATATATGTTTAAATAACCTCAGTTCGGTTTAAGAATATC
GGATAAGGGTAGGTGTCAGGTTGCAGGTTTCAGGATTCGGGGTGTTGGGGAATGAGAGGAAGAG
AGGGGGAAACTGACTCTTAACTTTCAATTTCTAACTAAAATCTATTCACTATTCAAAAATAGTCT
TAAGAGCAAATTTAAGAGCCAATTACTGATTAGCCAAAGCTAAATCGGGATTTTCCTTTCTATTT
TCCAAAGCCGCATAAAGACGATTTAAAGCACTGACGTAAGCACGGGCAGAAGCAACAATAACATC
TGTATTCGCCGCATAACCTGAATAGGTTTTACCCTCATGTTTAAGACGAATAGTAACTTCTCCCA
TAGCATCGATACCAGCAGTAACGGATTTGACTGAATATTCCGTCAATTCATTAGGAATGTTTACC
ACTCGATTAATGGCTTTATAAACTGCATCCACAGGTCCAGTTCCGATGGAAGCGTCGCTCAACTC
CTTGCCGTCTGGGGTTCTTAGGGTTATTGTGGCTGTAGGAGAAGAATGATCACCACAGGAAACTT
GTACTAATTCCAAACGGAATAGTTCGGGGGGTTGTTGAATTTCGTCATTAACAATGGCTTCTAAA
TCCCAATCAGTGATTTCCCGTTTTTTGTCTGCCACTTCTTTAAAACGCAAGAAAGCTTTATTCAA
ATCATCTTCGGACAATTCAAAACCCAATTCCTGTAAACGGGTACGGAAAGCATTACGACCAGATA
GCTTACCTAATACAATTTGGTTAGTGGTTAAACCGATGGATTCTGCGTCCATAATCTCATAGGTG
AGACGGTTTTTCAAAACGCCATCTTGATGAATTCCCGACTCATGGGCAAAGGCATTCGCCCCCAC
AATAGCTTTGTTGGGTTGCACCATCATGCCAGTTAGGTTAGATACCAAGCGGGAGGTTTTATAAA
TTTGTTTTGTGTCAATGTTAGTTAAAGGTTCAGTAGAATCAGTTGGACGACCCAAGAAGGGGTTA
AAATACTGACGGCGCACATGAAGAGCCATCACTAATTCTTCCAATGCCGCATTTCCTGCTCTTTCA
CCGATGCCATTAATGGTACATTCTAATTGTCTTGCTCCGTTTTTCACTGCTTCAAGGAAATTAGC
AACGGCTAAACCTAAATCATTGTGTCCATGCACAGATATAATTGCTTGGTCAATATTCGGCACAT
TATCTTTAATTCCTTTGATTAATGCACCGTATTCGCTGGGAGTTGTGTAACCTACTGTATCGGGG
ATATTTACTGTTGTCGCCCCAGATGCGATCGCAGCTTCTAAAACTTGATAGAGAAATTCAGGATC
ACTTCTTCCTGCATCTTCAGGGGAAAATTCTACATCATCAGTAAATGTTTTGGCATAGGCTACCA
TTTCAGGCACAATTTGTAATACATCAGCTCTGGTTTTTTTCAACTTATACTCAAGGTGAATATCA
GAGGTAGCTAAAAAAGTATGGATTCTGCGTTTAAAAGCAGGTTTAAGAGCTTCTCCTGCACTTTT
AATATCTTGCTTAGTTGCCCTTGCTAAACCACAAATGGTGGGGCCATTTTCCGTGCCTACGGTTTC
TGCAATTCTTTGTACAGCGTTAAAATCCCCCGGACTAGCATGGGGAAATCCCGCCTCAATAACAT
CAACTCCTAATTTAGATAGGGCTTTGGCTATATTTAATTTTTCTTCTACATTTAAACTCGCCCCG
GGAGATTGTTCTCCATCTCTTAAAGTGGTATCAAATATAATAATTCTGTCTGGCTGATTGGTCAT
AATAAAATCAAAAATGCCCAGTTATAACATATCTCTCTATCATAAACAAAAAGAAACTCGATCGG
TAATGATTTCTCTTTCGGCTTAATCAGGCAGTAAAGGATAATGGCGAGGGTAAGGGAAAAGGTTT
TAATCTCAACGGCAAATCCCATATCATTAATTTATTAAAAAGGCGTTGTTGAATTAAGGTATGAT
TTTTACTTGAGATCTGGAATAGGCAATAGGCAATGGTAATATTTGAGTTTTAGATATTGGGTAT
AATTTAAGCACACTTAAGGCTTGAAACCATCTCAAATCCTATACTCTTATTATGCCAAATGGATA
TTAAATATTACCCCTGATATAAATTTTCGATCGCACTAACTAAATTTTCCTGTCTCATTAAAGAT
TCTCCAATTAAAACAGCATTTGCCCCTGCAGACTGAACAAAATCAAGGTCTTTTTTCTCGTATAA
ACCTGATTCACTAACAACGGTTATTTGTTTCTCCTTAATAATGTCTTGACGGGCTAATAAAATAT
TTTTTGTAGTGTTTAAATCCACTGAAAAATCTTGTAAATTACGATTATTAATTCCCATCAATTTT
ACCCCTTCAATATTTAAAATGCGATCGCACTCTTCTAAGGTATGAACCTCAATTAAGGCAGTCAT

TCCTAAACCATTAATAATCTTTAAAAAGTAATTAATATCAGCATCTTTAAGAATAGCGGCAATAA
GTAAAACTGCGTCTGCCCCCTGTAAACGAGCTAAATAAATTTGATAGGGATAGATAATAAATTCC
TTACACAAAAGGGGAATTTTGACCGCATTTCTTACAATACTTAAATTATCAAAACTCCCTTGAAA
AAACTCCTTATCCGTTAAAACAGACAAACAAGTTGCACCCGCTTTTTCATATTCTTTGGCAATGA
CTTCAGGATCAAAATCTTCCCGAATTAAACCCTTACTAGGAGAGGCTTTTTTCACTTCTGCAATT
AAGGCAGGTTGACGAGATGAATTTTTTAAGGCACTGAGAAAGTCAAGAGGTGCATCATTTAATT
GACTAACTTGTTTTCGTAACTCTAACAAAGATACCTTTTCTCGCATCCTTGCCACTTCTTCTTCTT
TTTGCCAAACAATTTTCTCTAAAATATTTTGAGGTTCACTCTCAGGTAATTTAACTTGATAACGT
AAGGTTTCGATAGCAACGGGAGGAGATGGTTGTTGACGACGAATTTTCATTAATATTCCAAAAAT
AAAATTACTTATTTAAGTACAGTTTGACAATTATAACAGTCTTCAATATAACCTCTGTTCGGGAT
AAAATCACCTGAGTTCGATATAAAATTGTCAGTTAGGGTAGGCAAGAGACAAACCCCCCTTTATC
CCCCCTCGAGAGGGGGGAGGGCAAAAAGCAATAGTATTTATAATTGATACAAACTACAGTTAAAT
TGATTTGAAATACATTTTTTCTCAAGAAAGTTAATTTTTTCGGAGAAAATTTCGCCAATTCTAAG
TCTCAACTCCTTATTTTTCAATGCCTTTTTCATCGAACTCAGTTAGAATCTATAGGTTAGAAGAA
AGTGTTAGATAATTGAGAGTTTTTTGATTATCAATTATTTACTATTCACTGTTTAATATTCACTA
TTCACTCTCATGTTAAATTTTAAATATTCTAGTGTCATTAATGCCCCCATCGACAAAGTATGGCA
TTTTCACGAGCGAGAAGATATTTTGCAAATATTAACGCCCCCTTGGCAACCAGTAAAAGTTATTC
GTAGGGAAGGGGGTTTAGAAGTGGGAGCAACATCAGAATTTGTCTTGATGTTTGGTTTTATTAA
TATTCCGTGGATTGCCAGACATAGTCAATACGAAAAATACAAACAATTTACTGATGAACAAATTA
AAGGTCCAATGGTATCTTGGACTCATTATCATCAATTTCAAACCGAAGGGGGTAATACTCGCCTT
ACCGATAAAATAGAGTATGAAATTCCGGGGGGATGGTTAAGCGAAATATGTTTAGGTTGGTGGG
TTAATTCTCGTCTTCACGATATGTTTGCCTATCGCCATCGAGTTACTAAACAACATTGTGAAACC
TAATTTTAGTTTTCTAGCAAAGATGCAATTTCACTATTAGCAAACAGTTTACGGGTATCTTCAAT
TAGCTTTTCTCCCCAAAGATTTTCAATTAAAAAGTCAATTTCACCATAATTGTTATCTAAACTTA
AAGCCTGTTGAGCTAGTTGTATCCCTTCTTGTTGTTTTCCTTGTGAGTACATAGCAACTGCGATC
GCTAAAGTAGGTTCAGCTTGTTGAGAATCAATTTCTAAAGCCTTTCGCCAAGAAGCTATTGCCTC
ATTCGTTTTTCCTTGTTCATACTCCACTAAACCAATATTATTGATGGCAGGCCAAAACTGTGACT
CTAACTTAATCGATTCCTGATAAGCAGTGATTGCCTCTCCATACTGATTCAGTTTGAGATAAGCA
TTACCTAAATCAAAATAAGCCTGTGGAACTTCATCACTAATTTTAAGACCAGCTTTTAACTCTTT
GACAGCAGATTCATATTCGGCTAACTGAAAATAAGCATTACCCAAAGAAAATAAAACCTCTGACT
CTTCTGGTGCTAGTTTTTTCGCTTCTAATAAGGCATTGACACCCTGCTCAATTTGTCCCGTCTGAA
TTTCTAATGTACCTAGAATAAACCATGTTTCATAATGATTAGGAGATAACTGCACTGCCAACCTT
GCACGAGAAGAAGCCAAATCAAATTCTCGAAAACGAATTAATTGCACAGCATCTTGTATTAGTTG
AAAACCATAATTATCCAAGTATTCACTATTTAATTCTGGTGTGTAGGGTAACAAAGTCTGTGCTT
TTACCACAGATGGGCAAACAGAAACCACACCAAGACTTAAGAGAATGGAAAAAATTTTAGAACG
AAACAACACTATCTTAAAATATTAATAATTAAATGGATTATGTATTTTAATCTTAAGCTATTGAC
CTTCAATTTGTCGGAATAATCATGAATTACCTAGGAAAAATTTAATAATCGCTTAAATTCTGATT
TAACGAATGTGTAACATTCACAGGCACTTAACTCTAAAAAAAGACGATTCAAAATCCGAATATGC
CCTCTACTGTAATGAATAATTCCATTTTCCTGAAACTTTTGAGCCACTTGCGTTACACTAGCACG
ACGCACCCCTAACATTTGTGCAATAAATTTCTGAGTTAAAGCTAATTCATTACTACCGACACAAT
CATGGGCATGAAGCAACCAACAAGCAAATTGATTATCAATATGATGATGACACTTACATACCACA
TTCTGAGAAACTTGAATGATTCTTGCCTGAGTATAAAGCAATAATATTTTTTGCAATGAACCACC
ACGCTTAAATTCTGCCTCAAATATCTTAACATCAAGTCTCATGGCACTATCTGGTATTTGCACAA

TAGCATCACCACTAGCAATATTACCGCCTAAAAACACAGATAAACCAACAAACCCCTCATTACCA
ATCAAAGCTATTTCCGTTGTTGTTTGATTTGATAAATTATGAACCAGAGAAATCATTGAATGCAG
AGGGAAATAAACATACTCGATTACTTTATGTTGTTGATAAATTACTTGTCCAGATAATAAAGAA
ACTTTTTCTAAGTGAGAAAATAGCATTTTATAATCATCTTGAGAAATCGAAGCTAGTATTTGATT
GTCAGAGATTTGATTTTTATTCACAGATAGTGCTAAGTTAAGATTCCGTTAATACTAACTTAGAT
ATGATTGCTAATTAACCTCAGTTCGGTTTAAGAATATCGGAGAAGGTCAGGTGTCAGGTTGCAGG
TTTCAGGTGGTAGGGGATGGGGGGATGGGGGGATGAGGTGATGAGGGGAAAGGGGGAAACAAGT
AATAAATAATAAATAAGTAGTAAGTATTCGTGGTTTTTCATTCTTAATTTTTAATTCTTAATTCT
TAACTATTTAACGTCAGTTCAAATTAAGGCAATTTTATCGTTATTTCTAAGAAGAAGCTATAAGG
TTTTCTGACTACGAGTTGGTCTGCTTTCAGCTTATCCAAAACTCAGGTTAATTAACAAAGATTAA
TTGTTTTCAAAAAAACTCAACAGTGATAAATAGAAAATAGTTGCTCACTATATTTTGCTTTATAC
ATAGCCATATCCGCACGGGATAATAAATCACATACTTTAGAACTTTTTATGTCCCCAATGACAAT
TCCGATGCTAACATTTACTGCTATTTCCATCTGATTAATTTTAAACGAAGTTGATAATACTTGTT
GGATTCGCATGGCACATTCTGTGGCCTCTTGTACGGAATGAATATGACTTAAAACAATCATAAAT
TCATCTCCACTTAATCTGGCAACATGATCAAAATTTCTGACACAAGTTATTAATCTTTTCGCAAA
AATAACTAACACTTCATCTCCTATTTGATGTCCAAAAGTATCATTAATGGATTTGAATTTGTTTA
TGTCTAAGTATAAAACAGCAAATTTGTTACTATGATTTTTTTCAGCTTCTATCAAAACATTATCT
AAAAACTCTAACAACGATAAACGATTTGGTAAATTTGTCAAACAATCATAGCGAGATTCATAAT
AAAATTGATCATGATTTTTTTGAATTTCTGTTATGTCATGACAAGATAAATCCCAACCGTTAATA
TTTCCTTTAGAGTCTAGTATTGGGGAGATTTTGATACTAACACTCAAAAATAAATTCTCCGGGAA
AGATAATCGTAAAGATAAAAAATTACTCTTACTTGAATTATTCAGATTACTTGTCTTTAAGTCTT
GAATAAATATTAAAAACTTTTCTAAACTATCAGTAGCGATAAAAGATGTAAAGTGCTTATTATT
TAGATCATCTACTGATAAATTAAAGAGTATATTTCCCTGATAATTAATATCCTTTATAATTAAAT
CGTTATCTAATAATAAACGAATATCGGGGGATTGTAAAAATAAATTTTTATAGTATTTATTTTCT
GACTCATTTTCCCATAACTGTCTTAGTATTAACTCATTTTCTTTTTGTAAATCCTCTAAATCGTT
AATAACAGTTAATTGATGGGGGTTGCCTAGTTCAATATCATAAGTGATTTCTTGGGCATTTTCAT
CTATTAAATCTTCGAGATAATCCTGAGAATGGTTCGTTGCTTCTGTGAAGTTTTCAAAAGGTCCA
AAATAATAAGTTACAGTGGGCTTAGTCGTCTCGTATCTAATCCACCAAGATAATTTTTGAGAATG
ATGTACTTTACTTTTTTTTTGAATAATTAGCTGATCATCAGAATTGGTAAAATCATTATATTCAG
AAACAATATCTTCAATATTACTACAAATTTTTTCATGGATTAAATCTTTTTCTTTCGCTAATTCA
ATATAAAAGAAATTATCATCTATAAATGGATGACATTGATCGATTAAAATATGATTATTGGGGA
TAGAATTAATTATTTTAGTGATAAATTTTTGACGAAAGTATGGTATTGACAACGCCTTTTGATAG
GAATGATTAACAGAATAATTTTTTAAGAAAATATCTATTTCATTTCTGACAATGGGCAAGGTTG
AATTAAAAATTTGATATGACATAATATTTTCAACCTCCAATAAAAGAAAAACTTAGCCAAATTT
AAGGTAAATAATAATTAAATATCCTTGCTTTTGTTAATCCTTAATTTGATTGGAAAATTACTACT
GTAGTTTGAAAAAACTAGGTATAAATTCTATATAAATTTGATTGAGAAATTGTCCAACATAAAG
CTCCCACAAAAAGTAAACAACATAATCCTTTTGCTTACTTAGTGATAATTATTCTTCCCAAAATT
TACCTTAAAAACCAGAAGATAAATATACACACACTCTCACAAAATTTAACCTTCTATTCTTGTTA
TATATCAATTCTTTTTGTGTGTCTGTACAATATCGTACCGATACCATCATTTATGATTTTGTATT
AATAGGGCAGAATGCAGAATTTTTATGGTTCTTCAGAGTGTGGTTGTGATAAATTAAGAGAAAC
AGGACTTCCATAGCCTTTATTGAATAGTGTTTATAGTAAAATAAAAACTAAAAGTTTATAAATT
ATTATTTAATAACCCCCTATTGCCTATTGCCTCTTGCCTTTCAGCCTACCCTAACTAATAATTTAC
ATACTCAAACTGATAGAGCCATTTTTATCCTCTGCGAGATCCTAATAAACGTATGAATTCATTTT

GAACAACCCGATAACATTCACAAGAAGCCTCTTCTAATTTTTTATGATCTAAAATCACTATTTTT
CCACGGTTATAGCGAATAATTCCTGCATCTTGTAGTGCATGGGCGGCGATTGTAACTCCCGCACG
GCGGACTCCTAGCATATTAGAAATAAACTCTTGAGTTAAATCTAATTCATCTTTTAGGACGCAAT
CATATACAGATAACAACCATCGAGCCAATCTAGCTTCTATTTTGTGTTGTCGATTACAGGCCGCA
CTTTGAGCTATTTGAGTTAGTCTTGCTTGGGTATGAAGCAATAAAATTTTTTGAAATATTCCTCC
TCTATCAAACTCTTGCTTAAGAATATCGGCTCTCAGTTTGAAACTGCTCCCCGCCATTTGAACAA
TTGCCCTACTTATAGTGTATTCACCACCTAAAAAAATAGGTACACCAATCATTCCTTCGTTACCA
ATTAAACCAATTTCTGTCGTTGAACCATCTTCCATAATTGACACCAAGGAAATCATTGCGGATAC
AGGGAAATAGGCATAATTAATAGCATCATAAGGTTCATGTAACACCGATCCTGAAACTAACATCA
CTTCCTGTAAATGGGGCAAAAGCCTTTGATATTCTGATAAGGGTAGGTTATTTAATAACTGATTT
TCAGAATTTATATTTTTCTTGCTCAAAGTATTATCTAAAAAACTTTATATTTACAATATTTTATA
AAATTTATTTTTTCGTCCGTACGCTAGCGTACATTAATATAATGATAATTTTACTTAATACACAG
TTCAAGCAAATAATAAATAACAATTAATATCTAGTGAGTTAGACCTGCAAAATGTTAGGATTAC
ATAGTGTATAGAGATAAAAGTGAAGTATAAGGAAATTTTGAATGGAAGAATATAATAAATCAAT
TTCTTTTTTTGAAGGAAGAGATATAAAAATACAGGTAGGATTATTAGCTCCTCAAGCTGGTGGTG
CGGTTTTAATTCAGGCTGGAGAAACAGCAGTATTCGTTACAGCAACTCGTAGTGCAGGAAGAGAA
GGTATTGATTTTTTACCCTTAACTGTGGATTACGAAGAAAGATTATATGCGGCTGGAAGAATTCC
GGGAGGATTTTTACGCAGAGAAGGCAAACCCCCTGAAAGGGCTATTTTAACAGGTCGTTTAATTG
ATCGCCCCTTACGTCCTTTATTTCCTTCTTGGTTAAGGGATGACATTCAAATCGTGGCGACTACTT
TGTCTATGGATGAGGAAGTACCCCCCGATGTATTGGCGGTGACAGGGGCATCAGTGGCAACTTTA
GTGGCTCAAATTCCTTTTGCTGGACCGATGGCGGCGGTGCGTGTGGGTTTATTAAATGATGAGTT
TATCATTAATCCTACTTTCCGTGAAATCGAAAAAGGTGATTTAGATTTAGTGGTGGCGGGTACAC
CTGATGGAGTCGTGATGGTGGAAGCTGGTGCGAATCAATTGCCTGAACAAGATGTCATAGAAGCG
ATCGAATTTGGTTATGAGGCAATCCAAGAGTTAATTAAAGCCCAACAAGACTTAATTCAGGAATT
AGGTATTAGTATTGTTGTGGAGGAAAAAGAAGAGGAAAATCAAGAGGTATTAAATTTTATTAAT
CAACAAGCCTCCGAAGAGATCAAAAAAGTTCTCTCTCAATTTGATTTAGATAAAAAACTGCGAGA
TGAAGCGTTAGATAAAATCCAATTAGAAAAAGTAGAAGAAACAATCAACGCTTTAGAGGATGAT
AACGAAATTAAAGTGGCGGTTACGGAAAACTCTAAATTAATACCAAGCCTCTTTAAGAAATTGAC
CAAAAAATTAATGCGTCAACAAATTATTGAGGAAGGGGTAAGGGTTGATGGAAGAAAATTAGAT
CAAGTGCGCCCCATTTCTTCTCGTGTACGTTTGTTACCTTCTAGAGTTCACGGTAGTGCTTTATTC
CAAAGAGGTTTAACCCAAGTGCTTTCCATTGCCACTTTAGGAACTGCTGGAGATGCTCAGGATTT
AGCGGATGATTTACACCCTGATATTGAAAAACGCTATTTACATCATTACAATTTCCCCCCTTACT
CTGTGGGTGAGACAAAGCCATTGCGATCGCCCGGAAGACGGGAAATCGGGCATGGTGCGTTAGCA
GAAAGAGCAATAACCCCTGTATTACCTCCTCAACAAGAATTTCCCTATGTGATTCGAGTCGTATC
TGAGGTGATGTCTTCCAACGGTTCAACCTCCATGGGTTCGGTATGCGGTTCTACCCTTGCGTTAA
TGGATGCAGGTGTACCTATTACTAAGCCTGTAAGTGGAGCGGCGATGGGCTTGATTAAAGAAGGA
GACGAAATTCGTATTCTCACCGATATTCAAGGTATTGAAGACTTTTTGGGGGATATGGATTTCAA
AGTAGCAGGTACAGATGATGGTATTACTGCGTTGCAAATGGATATGAAAATTACTGGTTTAAGT
ATCGAAGTGATTGCCAAAGCCATTAATCAGGCAAAAGATGCCCGTTTGCATATCCTCGGAGAAAT
GTTAAAAACCATTAGTGAACCTCGCAAGGAATTGTCTTCCTATGCACCTCGTTTAATGACCATGA
AAATTGATCCAGAAATGATTGGTTTAGTGATTGGCCCCGGTGGTAAAACCATTAAGGCGATTACC
GAGCAAACTGGTTCTAAAATAGATATTAGCGATGATGGTACTGTTACTATCTGTGCAACCCAAGC
AGAAAGGGCGCAACAAGCAAAACGTATCATTCAAACCATGACTCGCAAACTTAATGAGGGAGATG

TCTATTTAGGACGTGTTACTCGCATCATTGACATCGGTGCTTTTGTGGAAGTGTTGCCGGGTAAA
GAGGGCATGATTCACATTTCCCAATTAGCAGAGCATCGTGTGGGTAAAGTGGAAGATGAAGTCGC
TATTGGTGATGAAATTGTGGTCAAAATTCGTGGCTTTGATAATAAAGGGCGTTTGAATTTAACCC
GTTTAGGTATTCATCCTGAACAAGCTGCGATCGCACGTCAAGAATCCCAAATCTAAATTATTAGG
AGTTTAGAGTTAGGAATTAGGAGTTAGGGGTTAGGAGTTTTTTGAAATAATAAATAAAAGGATA
TGAGTGTTAGTCCTAAAACTGTGATGAAATGGACAAGTGCAGACTTGGAGTTATTACCAGAAAA
TGATTATCGATATGAAATTATTGATGGAGAATTATACGTGACCAGAGCGCCTCATTGGAAACATC
AAATCTGCATTACAAAAATAGCGACAGTTTTAGATATTTGGTCAAGTCAAAGTAAATTAGGAGA
AGCAATTATCAATCCAAGTATAATTTTCAGCGATGATAATAATGTTATTCCTGATCTAATTTGGA
TTAGTCATGAAAGATTAAAATCTTGTGTGGATGAGGCTGGACATTTTACCAGTGTGCCTGAGTTA
GTGATAGAAGTTTTATCTCAGAAATCCAATGATATAAAAAGAGATAAAGAGATAAAGTTAAAAT
TGTATTCTAATCGAGGAGTAAGAGAATATTGGATAGTAGATTGGCAATTAAAACAAGTAGAAGT
TTATCGTCGTGTTCAGAGTCAATTAAGATTAGTGATGACTCTAATGGAGGGAGATTCCATTAGTA
GTGCATTATTACCCGATTTTAGTTGTCAAATTAGTGAGTTTTTCTCAACCTTTAACTAAGTCTTG
ATTTTTATTATTTTGCAACTCTAAATAAATTAATAAAGCATTGACATCCGCAGGATTGACTCCCC
CAATACGGGATGCTTGTCCAACGGTTAAAGGTTTGACTTTCGTTAGTTTTTCCCTTGCTTCCATG
GATAGAGTTTCTATAGCCATATAGTCCAAATTTTCATCTAATTTCTTATTACTATGACGGGCAAT
TTGTTCGATCTGATTTTGTTGTCTTTTAATATATCCTGAGTATTTAATTTCAATTTCTGCCCCTTC
TTTTTCCGCTTGATTTAACTCTTCATTATGCAAATTATATCGGCTTAAATCTTCATAGTGAAAAC
CCGGACGACGTAACAAATCTGCAAGGGTAATTGAGCCTTTTATCTTCTGTTGGGTATCCTCCACT
ATTTTAATAGCTACCTCGTCTCTCTCTTTGATTCTCGTTTCATAGAGTCTTTCTTGTTCAGCGATG
ATATTCGCTTGTTTACGTTGATATAGTTCCCAACGGCGATCGCAGATTAAACCAATTTCTCGCCC
TAAAGGGGTCAATCTCTGATCCGCATTATCAGAGCGTAATGTGAGACGATATTCAGAACGAGATG
TTAACATTCGGTAAGGTTCACGCAATTCTTTAGTACAAAGGTCATCAATTAGAGTACCTAAGTAA
CTTTGTTCACGGGGAAAGATAATCATTTCTTTATTTTGGGCAAATCGGGCGGCGTTTATCCCTGC
CACAATGCCTTGCGCGGCGGCTTCTTCATAACCTGTTGTACCATTAATTTGCCCTGCGGAAAATA
ACCCCTCAATTTTTTTCGTCATCAGGGTAGGATAACATTGAGTGGCGGGTAAATAATCGTATTCC
ACCGCATAAGCAGGACGCAACATAACGCAGTTTTCTAGCCCAGGAAGGGTGTGTAACATGGCTAA
TTGGATGTTTTCGGGCAAACCAGTGGAAAACCCTTGAATATAAAGCTCTGGGATGTCTCTGCCTT
CAGGCTCAATGAAGATTTGATGGCTTTCTTTATCAGCAAAACGGACAATTTTATCCTCAATACTA
GGGCAATATCTCGGTCCTTTAGAATCGATAAAACCACCGTAAATGGGCGATAAATGGAGATTATC
TTTAATTAATTGATGGGTTTGGGCAGTAGTACGGGTTAAGTAACAGTTCATCTGTTCTTTTTCAA
TCCATACTTCAGGATCAAAACTAAACCAACGCACTTGTTCATCGGGGGGTTGTACTTCCATTTTG
CTATAGTCAACGGTGCGCTTGTCAACCCGGGCTGGTGTGCCTGTTTTCAGTCTGTCGGTTTCAAA
GCCTAAATCGTTGAGGGTTTCTGTTAAGCCTACGGCGGCAAATTCTCCTGCCCTTCCTGCATCCAT
AGATTTACCGCCAATCCAGATTTTCCCCCCCAAAAATGTACCTGTGGTAAGCACTACGGCTTTGG
TTTTAAAGCAAGTGCCGAAATAGGTTTGCACTCCGATTACTTCATCGTTTTTGCCTAATACTAAA
TCTGTCACCATGCCTTCTCTTAAGGACAAATTAGGCTCATTTTCGACAATGTTTTTCATCACGGCG
GCGTATTCTCTTTTATCGGTTTGCGCCCGTAATGCCCATACCGCAGGACCTCTGGAATTATTTAAA
ACTCTTTTCTGTAAATAGGTGCGATCGGCCATTTTGCCAATTTCGCCACCTAAAGCATCTACTTCA
TGGGTTAACTGAGATTTTGCAGGACCACCCACCGCAGGATTACAAGGTTGCCAAGCGATTTTATC
GAGATTGAGAGTTAACATCATGGTGCGACATCCGAGTCTTGCACTTGCTAAGGCGGCCTCACAAC
CAGAATGTCCTCCACCAATTACGATTACGTCAAATTCGTCTTGAAATTCTACGGGGGTTTTAATG

GTCATAATTTCTAGGAAATGTTTTTAATTCTGCAATATTTCAGACAAAGTATCTAAATTTCTATG
ATAGCGATTCATTGGGGTAAAAGGTAATTAACCTGAGTTCGAGATCAATTTTCATCTATGAGTGA
TGGTGAAAAGGGCAATTTAATGAATAGTGAATAGTGAATAGTCAATAGTTGATAATTACTCGTT
ACTCATTACTTTCTTCTAAAACCTGAAACCTGAAACCCGACACCTTATCTCCCCTTCCCCTCATCA
CCCCATCCCCTAAACACCCTGAAACCTGACACCCGAAACCTTCCCTACTGAGAATTAGATGTTAAA
GTATCTAGGAATTGAGAACAACTTCGGGTACTAATTTTTTATTTTCTTCCCGTTGTTTAAACTCT
TGAAGTTGAGCATCTATTTCCATGCGTACAATTTGACGATATTGGAGAAAATGCTCGTTATGGGC
GCATACAGTTAGATAATGTTCCCAGACGGGGGAGTTATTCTTGATAATACTAAAAAGATGATGCC
AGAATTTCCAGCGAGTATCTCTTTTGATTCCTTGTCGCCAAATTACTATGGCAAGGGCTTTTAAG
TCTATTAAGGAGGGTAATTTAAAAGGAGGTTTAGCTTTCGGCGCACCCAGTTTTAAAAAACAACG
ATAGGTGCGATCGAGGAATGTATGGGGATCATATAATTCCCAAAAGGCTTTAATATATTCTTGGG
CAATTTCATCTATGGGGCGAGTGGGGATAAAATTCATCAAAGTGCTTTGGTTAATATCCTGTTTG
CCGTCATGACGCAGTCTGCCTTCTTTTTCTAATCTTGTCCATAATGCGGTATTGGGTAAAGCCTGT
AACATCCCAAACATAGCCGTAGGAATAGCCGCTCTTTCTACAAATTCAATGATGCGATCGCCTGA
TCCTTGTTTTTCACCGTCAAAACCAATGATAAAACCTGCCATGGGTCTAATTCCTGCTCGAATAA
GAGTATCAATGGTTTCTTCAAGAGAATTACGGGTATTTTGAAATTTTTTGGTTAATTTTAAACTT
TCTTCATCAGGAGTTTCAATTCCTAAGAATACCGCATCAAAATAACACTCAACCATTAACTCCAT
TAATTCCTCATCTTGGGCTAAATCAACAGAAGCCTCTGTGTTAAAACGAAATGGATATTGATTTT
GTTGTTGCCATACTTTTAATTCTTTGAGTAATAATTTGACGTTGCGCTTATTTCCAATGAAATTA
TCATCCACCATAAAAATGCCTCTGCGCCATCCTAACTCATAGAGATAATCTAATTCTTTGAGCAG
TTGTTGTGGGGTTTTAGTGCGAGGTTTACGACCATAGAGTACAATAATATCACAAAATTCACATT
GAAAAGGGCAACCACGGGAAAATTGTACAGACATGGAATCATAGGCATCTAATTCCAATAAATCG
TAACGGGGGATGGGAGTTTCCGTGACACTTGGTTTTTCATCGGTGCGAAAAATGCCTTGAGTTTC
ACCTCTTTCTACGGCTTTCACAAATTCTGGTAAAGTAATTTCTCCTTCATCTAAAACGAGAAAAT
CTGCTCCTTCTATTTCTTCGGGTACAGATGTAGCATAAGGTCCACCCACAGCTACTAATTTCCCTC
TTTGTTTGGCTTCTGCTATTTGAGATAATAAATCCGTCTTTTGTACAATCATCCCTGAGAAAAGG
ACTATATCCGCCCATGCCCATTCATCCTCTGTAACAGCCCGAATATTGCGATCAACTAATTTCATT
TCCCATGTTTGAGGTAGAATTGCCGCAACGGTTATTAAACCAAGAGGGGGGAGTAAAACTTTACG
ATTGACTAATTCCAGACTTTTATCATAAGACCAAAATGTAGGTGGGAAAACGGGATATACCAATA
AAACGCGCACAATTTACTCCTTAAATTTTTTATATTATCTTGATTTCTCTTTCCCTCAGTTATAG
CTTACATCCGAGATTTTGTAGCTAAGTGAATTAAGAAGTATAAAGGGCAACAAGTAACGGGTAA
CGTTGTTTTATCAACTTCTTTCTAATATCTCATTGCCAAAATTTTAGTTCTTTAAGTATTTTTAC
TGATAATTTCATATGTAGATAATTACTGAGTTAGGCGTACAATTGTTCCTAAAAACAATATAGTA
ATTAACCAAGGAATAAGCCAAAGCAAAAATTCAATAGCTCTAAATTTAATAATTTTTCCTAGAGA
AATAGGATTTTGAAAAATTAAATTACGATAAATGAAATTAAGAGAAAACGAAAAAAAAAGAACT
AGAAATTTAACAAGAATATCATAAGATTCTGGGTAATGAAAAATAAAAATAGATAAAAAATTGTA
GGATAATTCCTATACAAAAAAAAGTTGCTAAAGGCAAATTTAAAAGCATAGGAATGAATACCAA
TTCAGGTTGAATAAAGACACAAATACCATAAATTAGTAGCCAAAAAATAAACCAAAGTAAGCTA
ATACCGATTTTTTTTAGAGTTACATACTTTTTATGGTTAGTTGATTGTAAATGTAATTGTTTAAA
TAAATGGTTGATATTATTCATTGAATAGGTTTTAAGAGAGTGAAATTAGATTTAGGTTTAGTAA
CGGCTTCAAAACAATTAAATGAATGGGTAGATTGTTCAGGAATATTATCAGATAAATTACAGTA
AATATTATCGTTATCAGAAACGAGATTTAAACTCTCTAAAATTTTATGATTAACTAATAAATAG
TTCGGAGATTTTATTAATTGTTTCAATTCTTCTTCATTAATGGCTGTAACTTGACGCTCACTATA

AAAGTTTAAGGATGGGCGCTCATAATCAAAATTAATATATACCTTTTTGTCCTTGGGTACAAATT
CATTGACCAAGTTTGCCACTGGTTTAACTGCAAAAGCCTCATTTAACTCCCACAACCAATGGTTT
GAGGAGAAAAACACCATCAGTGATACAAACATTCCCCAAAATAACACGGGAATAAATTGTTGGTC
TTTTTTTCTTAACAGTATAGTTACCGATAAAAAAGTAATAAAAAGTAGAATGAAAATTGTTAGT
AAATGTTGATCCTCTGGCTTCGTAATGGCAAAATAAATGAAGCCCCCCATAATAGCAACTGTAAG
CAAAGAGAAAAATTGTTTCCAAGCAGAAGGGTAAGAAATATAAGAGGGTAGAGATTTGACTTCT
CCTAAAGCCACTCCCACTGCTAAAGCAAGAGAAGGATATATGGGCATTACATACCAAGGAAGTTT
TGTACCCATTAAGGAAATAACAATAAGATAAATACTACTACTAATTAAAATAAATTTTGACCAA
CTCCAATTCAGATTTTCCCAAGCAAGTTTAAATCCCCACAATGCTAAAAAAATCCAAGGATAAGG
ATACTTTATTAATTCAACTAAATAATACCAAACCGCTCCTTGATTACCTTCTACAGCAGTAAAAA
CTCGATTCAAAGATTGTTCTTGAATAGCAGTATGAATAAAGGTTTCTCCATAATAGAGATACTGT
GCCGCATACCAAGCAACGACAGGTAAAACCCCTAAAATAATCCCACTCCAAAGATAAAAAGAAGT
AAGTAGTCTCGGTGTATCCCAAAGAAGAAATAAAAATGCGATCGCACCTAATAATACTCCCATCA
TCCATCCTTTAGTTAAACAAATTAATGCTAAACTAAGACCAACAGGAAAAGCCCACCTTAAATCT
CTTCTAGTTTTTAACAAACAAAGAAATAACAATAATTGAAAACATAAAGTTGCACCATCCAACAT
CGCCAAGCGTCCATGACGTATTACAGGCATCATGGTCAAATAAGCTAAAGATGAAAATAAAGCAT
AATAACGGGGCAAGAATAGCTCTCTGGCGAGTAAATATAGTAAAGGAACAGAACTAGCCGTCAA
AGTCGCTCCTATGATGCGTGTGGAAAATTCACTAACCCCTAAGAAACTATATACTACAGCCGTTA
AACTGTGAATGAGAGGAGGTTTATTGAGATAAGGTTCCTGCCAAATTGTTGGGAAAAGCCAGTGC
CAATGGGAAAAAGATGATTGATAAATCTCCTTTGCTACCTGAGCAAATGTACCTTCATCCCAATC
CCTTAAAGGTAATGCCCCTAAATTGAGAGAAAATAAAAAAATCGCAGCACAAAATAAAATGAGA
ACATAAAACCATTCCCACCAAAGTTCCTTTGATTTCAGTCTATAAAGGGGAATTTCCCATGTAAA
TATTTGACGGTTCATAGCATAGTAGATTGTGGGGAGATGATAGTTCTCTATTCATGCCAAAATTT
CCTCAATGGCTAAATGAGAGAACTTAAATAACTACTATAGCAAATAGTTCCAAAACGAAGAACTT
TAGTAATTAACAAAAGGGCTATGGGCAAAGGGTAAACCCCCCCTATTTAGCGGGGAGGCAAAGGT
AAATGGTCGATAATAAAGAATAAAAAACCCTGAACTCCGAACTTCCGAACCCTATTATGCTCCTC
TTAAGCCATAGGAGGACATTTTCATAATTTCACGGGTGGAAAAGCCGATATTAGCTAAAGCCTCT
CCATAACTAATCATAAAGTCTTCAATTAGCTCTTCTTTGGGCATTCCTAAAGTTTTTGCGTCTTT
TTCTACCTCATTTAACATTCTCCAAATAATAGGTAAATTTTGACGGTTAGCTTCTTCTAATTCAG
CTTTAGATGCCTCAAAATTGGCTTTTAACCATTCTTCACCAAAGTTAAGATGTAAGTATTCGTCT
TTTACAACACCTTCAGTAATTTTACGAGCAAAAGGATCAGCTACAGGGATATAAATATTATAAGC
TGCGATCGCAAATGCTTCAATAATTAAAGCCTGAATGAGTAAACAAGTGACAATTTTTCCTTCTT
CCTTAGCTTTTTGGAAATTACTGTGCAACTCGGCAAAATATTTTTGAGCAAAATCCATATCAGGA
GTGACACTCAAATTTCTACCACAGGATTGAAAACCCTTTTTGTGACGAAATTCCATTTTTGCCAA
ACTGGCTAATTCTTTTTCATGATCTGGAAGCATGGGTATCATGTCCATGTAATTATCATGGGCTT
CCTGTTCACCCTCGATTACAATGGCATTAATGCGACTATAAGCATCCTTATATACGTCACTGTTG
AAGTCTATGGTAGGTTCGACAAGTTGTTGCATAAATTGTTAAATCTCCTGTATAGTTTATGTGAG
CAAATCTAAACTCAACGACTTCAAAGGTCGTCTTTTATGAAGATAAGATAATAACTCATTTTAAT
CTAGTTGTTACCTATCTTATCGGTTTTTATTCCATATTGAGGTTTACCTCTGATTACTTTTACTG
ATACTTTATCCCTTCATTTATTAATAATTACTGGAAAATAGACGATCATTAATGGTTACAATAAG
TTATCGGGGTTAGGTTCGGGGTTCAGGTTATATTAACTATTCACTATTGACTATTTGCTAATTGC
AATAGTTCTTGTAATTTCTAATTCTGATGAATTTACCTTCTTTTCTTTGGTTGTGGAAAATCGCC
GCTTGGTCAATGGGATTAAGTATCACAGGATATTTACTTCTGGCATTTTCGGGAATATATTTGTG

GAAGAATCGTAAAGAAAAAAAACCTCGCCCTTCTTGGTTACGTCCTTTTCATTGGATTACAGGCA
CAATTTTAGTGATTCTTATCCTTATCCTTCTATCTATCGGTTTAATGGGAACTATAGGATATTAT
GGTAGTTTAGGACACTCTTGGCATTTACCAGCAGGTTTAACCGTTGTTACATTAATTTTATTATC
TGCATGGACTTCAACTCAGATACATCCTTCTCGCCCTTGGGCAAGAAAACTTCATGTTAATGTGA
ATATTGCTTTATTTTTTGCCTTATTAGCAGTGGGTTTGACTGGTTTATTTGTTGTTCAAAAATAT
CTACCGTGAAGTATTCGGTGTCAGGTGTCAGGTAAAATTTCCTAAATATTTGTCAGTGAATCAAA
AAATTAAAAATAAAAACGGCTTTATTACTTTGAGTATGTAAATTATTGGTGTTTTGTCGGCAAG
AGGCAAGAGGCAAAAGGCAAGAGCCATAAAAATTAATTTACAGCGCTTTTTTTATCAATAAACT
ACATTTTTTATCTCTCACAAAGTCGCAAAGGAGAATGCAAAGTGTGGTTTAACGAAATGAAAATT
GCTGTATTGACTTTTTTATCAATATCTTTTTGTTTTAACTCTAAATTCCTAACTTATTTTAAAAC
TAATTCTTCTGCCAATTTTTCTTTACCAAAAATCTGTGGGTCTAAGGTTCCCTGCCCTTTAGGGCA
TCAGTGTGTTATTATTTTCTTAGAGGAAAGGTAATCAACCTCTTTAAAAACCTCGAATCTAATCG
ATTCATTGAACCTTGAAAACCAAAGTCATAAGGTTCTGTTGGGAAAAGCTCCGACAGGTAAAGTC
TTTGAGCTGATAAGCACAGAAAACAAAGTATTCAAGTTTTCAGGTAGGGTAGGGCATACCCGAAC
CGACTCTGAAAAGGGTCATACGCTTGGGGACATAATCCCCTCTGAATTGATTTAAGCAATTAAGT
GAATTTAAGGGATGTGGTTGAACCAAGAATCCCCACGCCTTTAGGCTGAGGAGTGTCAAAAGGGC
ATGAAACTCCTAACTCCTAATTCCTAACTCATGTTAATTGCTAACTCCTTTTCCGAAGAATTTGG
GCGGTTTTGTCATAGTATAAACTTCTGTTTCTTTCTCAATACATTGACGGACATAACCCGGAGTT
TGTAACATTCCTAATAGGCTAATACCATCAAAAGAGCGAAAATCAGCAGTGGTTTTTTGTTCGAG
TAAAAGGTCAACTTTTTCAGGGTTTGGTCTAGTTTCAAATGTTGCCTCAGAGGCTATTGCAAACC
CCCAAGATGACCCATAAGTGCAAGTTGGGGCAAAATAAGAGTGAACGTTGCTAAAAACTGTTTTC
AGGGTATTTACTAAACGTCCATGAATATGTAAATTTGCAGGTGCGATCGGACCTGCTTGTATTAC
AACGATGCCATTTTCTCTCAAAATTTTCTTAAGACGAGCAAAATATTCTTGAGTAAATAATTGGA
AAGAAGGTCCTTCTTCAATGGGGTCAGACAAATCAGAAATAATTACATCCCATTTTGCTTCACTG
TTATCAATAACTTCAAAAGCATCACCAATCACTAAATCTAAACGAGGATCATCAAACGAACCTTG
GTGCATTTCTGGTAAATACTCTTTACAGGCTTCTACTACATCCCCATCAATATCTACCATTTTCGC
TTGTTTAACACTATGCCAACGGAAAACTTCTCTAGTTGTTGCACCTTCTCCGCCTCCTAGAATCAA
AACATTTTCAGGATTAGGATGATTTACCATAGCTGGATGTACTAATGCTTCATGGTATAAAAACT
CATCTCCCGTGCAAGATTGCCATTTACCATCCAATACCAAAGCCTTACCATAAGCACCACTTTCCA
CAATATACATTTCTTGATAAGGGGTTTTCTTATGTGCCAAAATACTGGTAATTCCGTGAGCATAA
ATATCGTAAGGGGTGATATACTCACTAATCCAAACATCAGCGTTGACGATTGATCCAGCCATAAT
TGCCGTTGTTCTCCTAATAAAATGCAAACTTATATTATAACAAATCTTTTCTTTGATAGTAACTA
TATTCAATATCAAATTCAGGTTGAAATTAACTCCCGTTTTATTGCTTTGCGATCGCGTAGCTCCA
TTTCGTGAGTATAAGTGAGGATAGTTTAAAGAGAAAGTTTTACTTGTGTAATTGCATTTTGTA
AAAGATTAATACCTTCTTCTACAGTATCAATGCGAGTACATTGATCTCTAAATTCCGCCGCCCCT
TTAAACCCCTGACAGTACCATGTAAGATGTTTTCGAGCTTGATAAATGCCCCTTTTACCCTTATA
TTCCCATAAACCAATTAAATGTTCTTTCGCACATTCTAAACGTTCTGATAGAGAAGGAATAGGTA
ACAATTGCCCCGTTTTAAAAAAATGATCAATTTGTCCGACTAAAAAAGGATAACCCAAAGTGCCA
CGAGAACACATGATGCCATCAGCATTAGTTATTTCTAAACAACGAATGGCATCCTCTACTGAAAA
AATATCACCATTGGCAATAACAGGAATGCTCAATTTCTCCTTTACCTGAGCGATTATATCCCATC
TAGCCTTACCAGTATAACCCTGCTCACGGGTACGTCCATGAATAGTAATCATTTGAGCTCCTGCT
ATTTCCATTTTTTCGGCAAAATCAAGGATATTAATTTCATCATCATTCCAACCAATACGAGTTTT
TACCGTAACAGGTACATTTACAGCTTTTTTTACTTCTTTAACAATCGCTTCGGCAATTTCTGGTT

GTCTGAGCAAACGAGAACCGCCCCCTTTTTTGGTAATCTTATTTACTGGACATCCCATATTTATA
TCAATGGTTTTTGCCCCTTCTTGAACTGCTTTTTGGGCGGCTTCTGCCATGAAATCAGGACGACA
ATCAAATAGTTGAATACTTATGGGTTGCTCATGGGGATCAATTTCCATTAATCTGGGTAATTGTT
GTAGGTGGTGAATTTCGGTCGCACTTACCATCTCTGTATAAGTCATTGACTCAGGGGCATAGCGT
CTTACTAATCGGCGAAATACTAAGTCTGTTACCCCTGAAAGGGGAGATTGTAAAACTCTACTATT
AACAACCACTGAACCTATTTTTAAAGGGGTTGATAATTTTTCTTTGAGTAAAGTATTTTTTATTT
CCATTTGAAGTCAGTGTCTAAATAATTAGGAGTTGATGAAAAAGTGGTATCACTACAGTTGCTTA
AGTTTGATATAAAATTGTTTGGTGAAGCTCAAGAAAAGATAAAAGCAATAATAATTGACAAAAA
ACTTATTAAAATTGATTTTAAAAGTTTTTAAGTATTACTTTACTAATGCGTGCGAGATCTCATTA
TATGTTAAAAGACTAGATTTTCAAAAAGTGTAGAGTCCCAACAGTCTCTTTAATTAGATTATCAT
TCGATGCTTTTTATTGTTACTGAGTATAATATAAAGAAATGTGTAAATTTTATCTGAGTTATTTC
AAAAATTATGCCTCCACGTTGGCCCAGAAAACCTGATCGTCAAAATGATCCTGAATTTCGTAAAT
TAGATGATCGCATGAATTTTGCTGTCCATACTGCACTCTTTATCGCTACTAATTCGGGTGTATGG
TTTTTTCACAATTTTAATCATCCTGAGTGGTCTTGGGCTAGTCAATTTAGTCTGATTTGGGGCGG
AATCTTAATTGTACATCTTCTCTATATAAGTGCGATCGCAGATTATTCTAACAGGAGCTAGTTGC
TAACAAGAGGAGGTGTCAGGTTTCAGGTGTTAGGGTAATGAGTAATGAGTGTTCGGAATTTTTA
ATTCTTAATTCCTAATTATCAACTATTCACCTTTGCCCTCCCCCTCTTGAGGGCGGATAAGGAGGG
GTTTGCCCTTTTAACTTTACCTTTTACCCGTTGCGTATTCTTAATTCCTCATTGAAACTTATTTTT
CGCTAATAGTTTCCCTTCGATATTTCCATTCTGTAGAACTACGTATTGCGATCCAAAGTAATAGC
ATAGCAATTAAACCTCCATTTTCAGGGGCATCCAAGGCAAATATAACTAAAATAGCAGATAAAAA
ATCTTCGATAAAAATAAATATGATTGGTATTTTTCCTAAACGGAAAAACCAGCCAACTTGAACTA
ATTTTAATACGAATGCAAATAAGCCTCCTATTAATCCTAAAAACCATAAAGGAGTTATTTGTACC
TCTAATAAACGAGCAACACCTACTGCCATTATTCCCCCCACAAAAGGACTAAAAATCAACTGAAT
AATTTGTATTATTCTTAATCCAATTAATTTTTTATTGCCTAATAGTTCAAATAATGACCAACTAC
TCAAAATGCCAATGACAACTTGCGGAGGAAATGACGAAAGAAAAGGTATATTATGCCAGAGTTG
ATCTATTCTAATTAAACCAATAATTAGTAGGGGTAAACCTATTCTCAATCCTCCTGCCGCTGATG
CAGACAAAACAGCTAATATTTGAATAAGAGAAATATTGTCAAAATGCACGAAAAAATATTATTA
AAAATAAAATATAGTTATTTAAAATCAAATTTTTGCTAGTTCATTATTGTTTAGACTTTGAATG
AAGTCATAAAGTTTGACTCAATTCAGAATTAAAAAAATATTTGTTGTATATACGATGTTACCAA
TAATAATTAATTTTTACTAAACAAAATTGCAATTGTAGCCTTTTCCTGATGATACTCTTTCATTG
AAAACTTTAGAGTTTGTCGTGATTTGAGATAAAATAAGAATAATAATAAGCAAAAATACTGGAT
TAAAAACACTTATGACTACTGGCAACACGTCAGAATTTCCAATTTTATCAGAAAGGGAATTAGAA
ATCCTTGAATTAGTGGCGACAGGACTAACTAATCATGATATTGCAGATAAACTAGAGATTAGTAA
AAGAACTGTTGATAATCATATTAGTAATATTCTTTCTAAAACCAAAACTGATAATCGTGTGGAGT
TAGTGCGTTGGGCTTTGCAGTGGGGAAAAATCTGTTTAGATGAAGTGAATTGTTGCACTCTCCCT
TCTGTTTCAAAAGATTAAATAACGACAAAAACGACAAAGATGTTAGATTTTGCCGATTTAATAA
TTTTAATGACGTTAGGGAATAAAAAAAGGCAAATTGCTGACATTTAGCATCTTGAAAAATTCTT
GACGTTGCTGATTTTAGTGATTTCTCATTTAGGTGTGAAATAGAGAATGGGTACTCTTGCGATCG
TGATAATATTTTGATGTCTCAATTTCTGGCATAATTTTAGTACATTTCATACTATGATTAAGCAA
CATCAAATTTTTTTTATTTAATTTATTTTATTTAATACGTTTCATATTGCACAAAAACCAGATTT
GAAAAATTTGATAACTCCTAAGGATTGAAAGCCAAGGATTCTGAGGAAAAATTGCTATATAGTT
TAAAAGATCGTTTACTTCATTTTCTTCGCTGTTATTTTGACACTCCTTGACCCGAAATTGAAAGT
ATTCTAGGTTCACTGACAGCCTTTAAATTAACTTAATTGCTTAAATTAACCCAGAGGGGCTTATC

TCCCCAAGGGTATGTATGACCCATAGAGCGAGATAGATTTCTGGTAAAACACAGTGGATAGCATC
AAAACGCAACAAATTCTCTTTGTATTGAGCTTATCTTTTTAACCTGAGTTTGGGATAAATTTTCA
TCTATAGAATGATGGAGAAAAGGGCAAAGGTGAATAGTTGATAATTAGGAATTAAAAATTAAAA
ATTAAAAATTCAGAACATTCATTACTCATTACTCTAACACCTGAAACCTGAAACCTAACCTTATT
AGATATTTTTAAACCGAACTGAGGTTTTTTATTAATACTCAATTTTTATCCGAAACGATAACCAA
ATCCTCTTACCGTTACTATATATTCAGGATTGCTAGGATCAAGCTCTAATTTTTCCCTTAGCCAAC
GAATATGGACATCAACGGTTTTTGTATCTCCTAGAAAATCAGCACCCCAGATATTTTCGATGAGT
TGTTCCCTATCCCAAACTCTTTTTGGATAAGTCATAAATAGTTCTAAAAGACGAAATTCTTTAGG
CGAAAGACTTACTTCTTCTCCTCTGACAGTTACCCTACACTCTGTTGTATATAAAGTTATATCTT
TAAATTGACGAATAGCACTGGAGATATTGTTATTGATGTTATTTCGGCGTAGTAAGGCACGACAA
CGAGCAATAAGTTCTTTCATACTGAAAGGTTTAGTTACATAGTCGTCTGCACCTATTTCTAAGCC
CAAGACTCTGTCTGTTTCAGCCGCTTTGGCACTCAAAATAAGGATAGGAATATTATTTCCTCGAT
AACGTAGTAAACGACAAATATCTAAGCCATTGACTTCTGGTAACATTAAATCAAGAATAATCAGA
TCTATTTTTTCTTTGGAGTTTTCTTCTCCAATACGCTCAAGAACGTTTAATGCTTCTCTGCCATTT
TCTGCTTGAATTACATTGTAACCCTCATCTGCAAGATTTAAGGCAATCATATTGCGAATCAACTG
CTCATCTTCAACCTACAAGAATATCATAGTTGACTGATGCTTTCTCATTAGCGCCAATTGGTGGTA
AATCCAGACACAACATAAAATTTCTTTATTTTTTGTTCCCTAGTAACATATATATCAAAAATATT
GAGTTTTGGTTATATTTTTTTCACTAGGGATGATATATTAGACTTCTTGCATAAATTAGGCAACA
GGGAATAGGCTACAAGGAACAGTGATAAAATGGGGTTTAAACTGCACTAATTTTATTTTAATTCT
TATTTTGCAAGAGGTCTATTGTTTATGACTCTACTATGGTAATTTCTTCTTCTCTTAAGTGAGCT
TTAAATTTTTTGCTAAATTGAACCAGATAAGGAAAATTAGCGCTAACAGGTCTTCCTTGCCACTC
GGTTACGATTTTAATCAATTCTCCTTCCATTCCTTTGATGTCAAAAGGTTCTTTTTTATGTTCAG
GATGATGATAAACTATCACAGATTCTTTAACTTTAACGCGATCGCCTATTTTCATAGTTAAGATT
AATTTTTAGATGAAATAAATTTAAAAATACGGATATACAGTCTTTTATTCTTGCATAGTTGGTCA
GTTGGTGCAAAGTTACCGTTAAATTAGTTTAAATTTTGCTCTAGTTGATCAAGATTTTATGGGGA
ATAAGGTTTGAGATAATTATTTTTGTCTATTTGCCAACGAATATTATCTTTAGATGGCTTTGTTA
TATCCGATGGTTGAGGGGTGTCTGTAGGAATCTCCGTAGTAATACGATGAAGGGGAACAACATAA
AAAACTAATTCAAATTCTCTTTGCAGACGTTGATTGAGAAAATCTTGTACTAGGCGTACTTGATT
AGGGGTGATTTCTTTGTCAGTTTCCAAGGTTACGTAAATGGTTGGTGTTTGAGTTGTCCATACAA
CCTGAATTTGAGTATCTTCCACTTCTGTACCAACGGTGATAGTTTCGTTGACGAGTTTTGCTCGA
ATTTCTCTTTCAATGCGTTGTTGCTGTATTAGGCGATAAAAGCTGGCACTAAGGGGAATAATTAA
TAATAAAGTTAACAAAGAAGTCCATCCCAATGCTTGACTAGGCTTAGTATAACCAGAGATGATAA
ACACAATCATACAAGCAAGAGTAATACCAAGTAAGTTCGTTGCATATAACAAAAAAGCTCCAAGT
GCATAACTATAATTATCCATAGTTAAAGAAATACCCACCACACATAAAGGAGGCATCAGAGCAAC
TGCGATCGCAGTTCCCGCTAAAGTATCACTAATACCTTTTCTAACCTTGCCAAAACCACTAATTGC
ACCAGCACTAATAGCAATACCTAAATCAATTAAATTAGGTTGAGTTCGAGCAGAAATTTCTGAGC
TAACTTCTTGAAGTCCCACTAATTGAGCAATAAAACTAGAGAGAAATAGTGAGACTAAGGTAGCC
CCCACAATTGATAAAAAAGCTCTCCGAAACAGCAATAATTCCCCCTCACAAGCGGAAAAAGCTAG
TCCTCTCAGAGGTAACATTAACGGTGCCACAATCATTGCTCCAATAATAACCGCACTACTGTTAG
CAATTAAACCAAAAGTGGCGATTAAGCAAGAAGAAACAGTACATAAAATAAAATCTTTGCTCCA
AGCGGAATCTTGAGATAATTGTAAATGAAGAGTCTTGATTTCTTCAGGATGAGAAGGAGGCATC
AAACGATAAGTTAGCATCCTCAACCAACGAAAAATAGCCTTAATTTTCTTTTTGTTACACCATTG
ACTATAGATTTGTTGAAAGCGAACAAAATTGATTTTATTCATTGCATTTGCACCTGAAACTTAAA

AGGAGATCTGATAAAATAATTATCTTTGTTGTTTGCGTATTTCTGTGATAATTCGAGATAAATTG
TAAAAAAATCTGAGTTCAGGGTCAACTTTTCTGGTTGTGTTGGTTAAAAAGCAAAGGATAAAGG
CAGGTAAAAATTAGCAGTTTAAGAATAAGTTTCTTCACTAACACCCTAATCTACTAAAACTAAAT
TCTTCGCTGAGTTCGATATAACCCACATTCTGCCCTAAAAAAATAATTGTTATTCTCAAATGAGA
AAAATAACCTTTTTTAGTGAAGATTGAAACTACTTATTTTTTAACATTTAATATTTATTTTTGCT
TACTTTTTTATTTCCACGATAAATTTACATTCACTTGAGCTTCTTTTTTCTTTTTATTTTATTCTA
CTTATTAATTCTTTATTTATCTTTGATAATCTTTTTAAGAATTATCACCATAGCTTATTGCCTAT
TCTTTATTAATATATTAGGTTTTAATTAAATCCCATAACAATTTTTCAATAGGTTTATGCTCAAA
AAAATGAGAAAAGTGAGGAAATTTTATGCTCACATTTACCTTTATTTATTTCCTTATTTTCAATC
TTTTCATAAACTCATCAGAGTGAGAAAAGTTAAACAGTAAAAACTGTTAGACCATAACTCGTATT
TAGTTAAATTCAGGTATTTTAATATCAAAATTCGATTAGTAATTCTATCTCAAGGGGATTTAATT
TATGAATGTTTTAAATACATTATTTTATCTTCAAATAGTTAGCTAAAATCTGAAAGGCTTTTCTA
GTAAATTCAAAATGATTCTTAAATAATTCTTAACCTATAATGAGAGCAAATCCTGAGAAGATAG
TAAAGTAATTGTTAAATTTATTTGGATTAAATTATAAATATGCTAGAACCAAGTGAAGTAGTAG
ATCTTTTTTTTAATCATTTACCAGTCAAAACTTTTCAACCCGGAGAGGTTATCTTTCGTCAAGGG
GAGAAAGGAAATTGTATGTTTGCTTTAATGTCTGGGGAAGTAGAATTAAGAAAAGATGATCAGG
TGGTGGAAACAATACATCAACATGATGTTTTTGGAGAAGGGGCTTTAGTCCAGCCTGAACATAAC
AGATGTACCACAGCGATCGCACTTACAGAATGCGAAATAGCGGAATTAAATAGAGAAAGATTTTT
ATTCTTATTACAAGAAACTCCCTTATTTTCTTTAGAAATTGTCAGGAGTCTTTCTAGCCGTTTGC
GCAAAGTAAAGCAGAAATTATTAAAATAAACCATGAAAGTCTTAGTTCTTAATGCTGGTTCTAG
TTCTCAAAAAAGTTGTTTATATGACTTAGATAGTCGGGGAAAAAAACCAAATTTTGTTTCTCCTA
TCTGGGAGGCAACTATTGATTGGACACTGAACTTAGGACATACTTTATTAAAGGTGAAAAGTAAT
GGTCAAGAATATTCTACCTATTTATCAGACCATAATCGCCTAGAAAGCATTAAGACTATGCTTAA
AACGATGGTTGAGGGTGAAACTAAGGTTTTAGAGCATTTATCCGATATAGATGTAGTTGGACAT
AGAGTGGTTCATGGAGGAACAAAATACTCTCAAGCAACCCTTATCAATTCTGAAGTTAAAGCCAC
CATTGAAAAATTAATTCCCCTTGCTCCTAATCATAACCCCGCCCATTTAGAAGGGATAGAGAGTG
TGGAAAATATTTTGGGTGACATACCCCAAGTAGCTGTATTTGATACTGCTTTTCATACAAATATG
CCTCTTTACGCTAAAATTTATCCTCTTAATCATGAATTCTATAAGCGGGGTATTCAAAGATATGG
TTTTCATGGTATTTCCCATGAATATGTTTCTCAACGTACTTCTGAGATTTTACAGCAACCTTTAA
AGAATTTGAATTTAGTCACCTGTCATTTAGGTAATGGTTGCTCAATTACAGCAGTAAAAAATGGG
CAAAGTGTTAATACAACTATGGGGTTTACTCCTTTAGAAGGGGTGATGATGGGTACTCGTTGCGG
TTCTATTGATCCTGCAATTGTAATTCATCTTATGACCGAATATGGCTATGATGGGGAGAAAATTA
ACCATATTTTGAATAAAGAATCTGGTTTATGGGGAATGTCCGAAATTTCTTCTGATTTAAGGACT
ATTTTAAAGGCTAAATCCGAAAATAATCCGAAAGCGATTTTGGCAATAGAAATGTACATTTTTCG
ATTACAAGAGGCGATCGCATCTATGTTACCATCATTGGGTAGTTTAGACGCTTTAGTTTTTACTG
CAGGAGTAGGGGAAAATTCTGCTTTTATCCGAGAAAAAGTTTGTCAAGGTTTGGGTTTTCTTGGT
TTGAAATTGGATTTAGAGAAAAATAGCCAATCCCTCTTCAATGAAAATATTGCTACTGCTGACTC
TAACTCTAAGATACTGATTATACCAACAAAAGAGGACTGGGCGATCGCTTCTCAGTGTTTTCAAT
TAATGGAATCGAAGAATTAAGAAAAGGGCAATTAGTGAATAGTGAATAGTTGATAATTAAGAAT
TAAGAATGAAGAATTAAAAACTCTGAACACTCATTACTTTCTTCTAAAACCTGAAACCTGACACC
TGACACCTGACACCTTATCTCCCTCTCCCCTCATCTCTCTTTCTCCCTAACGCTAATATCTACTCT
TCCTCTCCCCATGTAGTTTTAGGCTCTGCCGCCCTCAAGCCCTGATTGATTTGAGGATAAAAAAT
AATTTCCCCTTGATTGTTATCAGGTTTATTGACTGAGTAATTAGTTTCTCCTTTTCTCCTTGCAG

AAGTTTTTTCCTCACTGGTATCTTCTGCCACTATTTCATACAAAATTGCGAATTTTTCCCCTTGAT
TTGCCTTTCTTAAAATTCTTCCTAATCTTTGGATATATTCCCTTGTTGAACCAGTACCAGATAAA
ATTATTGCTATCTTAGCAGAAGGTACGTCCACCCCTTCGTTTAACACATGAGAAGCAACAATTAT
TTTATAATCTCCTTCTTTATATTTTTGGAGAATTTCATGTCTTTCTTTTACAGGAGTTTGATAGG
TAATTGCAGGAATTAAATATTCTTGAGAAATTTTATATACAGTATTATTGTCATTCGTAAAAAT
AAGAATTTTGTCAGGATAATGCTTTTCTATTAATTCAGTTAATAGTCTTAATTTACCTTGAGTTC
CCATAGAAATTTCTTTTGCTTCTCTATGGGCTAACATTGCTTGTCTGCCTTGGTTTGAACGACAA
CTAGCTTGAACAAAAAGTTGCCATCCTTGCAAACTGGACAGGGAAATATTATTTTGTTTTAAGAA
ATTATTTCTTGTTTTTATTGCTTGTTCATAACGTTGTTTTTCTTCTTTTCTTAGTTTTACTTTTA
AAGAAATAACCTGATGTTGAGCAAGAGTTTTTCCTGATAATTCTTCGGGGGTTTACGATAAATA
ACTGAGCCTATTAAACGGTTTAAATAATGGTGGCCGCCATCCTCTCTTTCTGGGGTTGCAGTTAA
ACCTAAGCGATAAGGTGCGATCGATTCTTCAGCTATAACTTGAAAAAAGTCTGTGGGTAAATGAT
GACACTCATCAAAAATTAACAGTCCGTAATGATTCCCTAAAGTTTTGGCGTGAATTGCGGCACTA
TGATAGGTTGCAATCAAAATGGGAGTGCGATCATGAGAACCTCCTCCTAATAAGCCTACTTCTAT
ATTACTAAAGGAAGCCTTTATTTCTGCATACCATTGGTGCATTAAGTCCAATGTTGGTACTACAA
TTAAAGTTGTTCTAGGAGTAGATGCGATCGCCATTTGAGCTAAAAAAGTTTTTCCAGCCGCCGTT
GGTAATACTACAACTCCTTGTCTCTGGGCTTGTTTCCATGCTTCTAAGGCTTCTTGTTGATGGGG
ATAAGGGGTTTTATTACCCTGTAATTTTATGTCTAAAGGATAAAATTCTTTTGCCGAGTCAATTA
GGTCAATATCTTCACTTTGTAAGGTTTCTATTAAGTAGCGATAGTCCAAAGCCTGAATACGGAAT
TTTTCGATGCGATCGTCCCAAGTAGCGTAATCAATCCATGCTTTACCTCTAGGTGGCGGATGTAG
GATTAATGTTCCTCTGTCATAGGTTAGTCTAGTTGTTCGACTCATAGAATAGTCATAAAGATCAA
AAAGCCAAGGTTTACCCATTGTGATCTACTAAAAATCAATTTAATCATTAAGTTAGTATTTTGTC
AAGATATACTTAAAGGATTATTTGATATAAAAAATATGATTTGGTTAGGAATTGATCCAGGGTT
AGCGATTATTGGTTGGGCAATTTTGTCAGGAGATGAAACTTCTTTACCAGAAATTATTGAGTATG
GAATTATTGAAACCAGCAAGAAGATGACGACGGGAGAGCGTTTAGCGGAAATAGAGATAGATTT
TGTTAGTATTATTAATGAGTTTCAACCAGAAGGAGTTGCCATTGAAATGCCTTTTTTTAATCGTC
AAATTAAAGCGGCTGGGGGAGTCTTACAGGCTTTAGGGGTTATTAATTTGGTTTGTTTTCGAGAC
GCTCATTTGACCCCTATTTTTCTTCATCAGTCTAGTTGGAAAGCTCATTTAGGCAATGGTAAGGC
AAAAAAAGCAGAAATTGCTTCTACTTTACAACAGTTATTTGATTTACCTGATTTACCTATTGATG
ATAGTGTTGATGCGATCGCGATCGCATATAGTGCTTGTTGTGGTTTAAGGAATATGATTGATTGA
GTATTTTAACGTCAGTAATGGAAATTTTACAGTTGAGAGAAAATGAGCCAATTTGAACAAGCAT
TAGACAAATCAAAACTAACTACTCCCATGTGGTTATTATGGATTTTATCCGCAGGATTAATCGCC
CTTGATGGCTTTGATTTTTTCATCATCGGCATTGCTTTACCTTTCCTCGAAAAAGATTTTTTACT
AACTCCTGCCCAAATTGGTTCGGTGGCAGTAGCGGCAATTTTTGGTTCACTAATTGGCTCACTAA
CTTTAGGGGCAATAACGGATAAAATTGGGCGGCAAAAAATGTTATTAATTGATGTCATAATTTT
TATCGTTTCTAGTGCAGGTACTGCTTTAGCTTGGGACGCTTTTTCTTTAATTTTCTTCCGCTTTTT
AGTCGGCATTGGCATTGGTGCTGATTATCCGATTAGTGTTGCCTATATTACGGAAAATATTCCTT
CTCGACTGCGGGGAAGAATGGTTGTCGGTGCATTCACTTTTCAGGCTGTGGGTGCTTTATTAGGT
GCTTTAACGGGTATTTTAGTCATTAAAGGATGTCTTTTTTTCTCTGATGATGCGACTATTTTTGC
TGTTCAATATGGTTGGCGAATTATGCTCGGAGTCGGTTTAATTTTAGCTATTTTAGTCGGCTTTT
TAAGGATGCAATTTTTATTAGAAAGTCCTCGTTATTTTATTGCTAAAGGGGATTATCAAGAAGCT
ACTCTTGCCGCCTCAGAATTATTAGATATAAATATAAAAATCACCCCTGAAACTGATCCTCAACC
TCAAGAAAACGACTTTAATTACAATACTTTATTCTCGAAAAAATATCTCAAAAATACTTTATTTG

CTTCTTTACCATGGTTTTTACAAGATATTGCCACTTATGGCATTGGCATTTTTACTCCTACGATT
ATCGCCGTTTTAGCTTTTACCCAAGAGGATAATTTTTTATTAAGTCAAAGTCAATCGGCACAAGG
TTCGGCTATTGTTGATTTATTCTTAATCATTGGTTTTTTAGCGGCTGTTTTGTTAGTAGATAAAA
TAGGCAGAATTAAACTACAAATTTTCGGCTTCATTGGTATGGCTTTAGGCTTATTTCTTTTAGCT
TTAGCAGGGGTCAGTAATTCTAATTTAATTTTGGTTTTTACAGGATTTTTTATCTTTAATTTTTC
TATGAATGCTGGTCCTAATTCCACGACTTTTCTACTTTCAGGAGAGGTTTTTCCTACTTCTATTCG
GGCTAGTGGTGCGGGATTGTCGGCTGCGATCGCAAAATCAGGGGCTGTTTTAGGCACTTTTTTAC
TACCGATTCTCAGGGTTAAAATAGGAGTTAATAATGTATTATATTTTTTAGTTGTTATTTGTTTA
TTAGCGGCAGTTTTAACTTATTTTTTAAGAATTGAAACCATGAATAAACCATTAGAAAATGTTTA
ATCTCCATCAAGTTGGGAAGCGATAGAAAGATGAAAAGTGTCTCGTCGTTCCTCTAACTTAAAAT
CAAAAATAACACGATCAATATCAGCAAAATATTTTAATTTTCTGTCAATAAAACCTTGTACAAAT
TTTTTAAAAGCCTGACTTTCTAAAGGGTCATTATCAATACCTAAATCTACTAAATAATTATTAAT
ATTATCTTTTCTTTCTTGTTCAGGTAATAGGGATAAGTTCCATGCTAATATTGCCACAGAAACTA
ACCTATGACCTTCTTCTAAATCACTTACGATTTCCATCTCTGGGGCGACAAAATCCCATATTACCT
CAGACATTTTCAATTCTCCTTTTTTGACTTTTCTCATAACTAAATCAGGATGGTTTTCCTTTATA
TCATGAGCAAAATTATCAAGAGAAGATGATTTTTTTCTACTTATTTAATCTTTTGTTTTTTTTC
TACAGGTTTTAGATTTTGTAAATCTTTAAATGCTTTAGATTGTTTTTTGCCCATGACGAAGATTG
TTTTTCTTTTTTATTCATTATAAACTCTAGTTTGAGTAGGGAATGTTTAACCCCGAACTCCTCGA
ACTCCGAACTTCTAATCCCTATTGGTGCATAGCTTTAGCCAATTCAGCCGCCACTTTAGGGCGAGA
GAATTCTGGAGGTGGCAATTCACCACGACGCAACATTTCTCTTACTTTTGTACCTGATAAGTGGA
TTCTTTCTTCTTTGGTAGCAGGGCTGGTTTTTGAGGTTGCCATTTGATTTGTGCGAGTACAATAG
AAGGCGTGTTCAAATTTAAGAGGGGTGATGCCTAATTCTTCTGGTTTAAATTCATCGAAAATATG
TTGAGCATCATAAGTACCATAGTAGTCGCCTACCCCTGCGTGATCTCTACCAACGATAAAATGAG
TACAACCGTAGTTTTTCCGAATCAAAGCATGGAAAATGGCTTCCCGAGGACCTGCGTAACGCATA
GCAGATGGGTTGATGGCAAGAATAACTCGGTTTTGGGGGAAGTAGTTGTCCACCATAATTTCATA
ACAACGCATTCTTACATCGGCAGGAATGTCATCGCTTTTAGTTGCACCAACGAGGGGATGAAGAA
ATAAACCGTCAACGATTTCTAAGGCACATTTGATGATGTATTCGTGGGCGCGGTGAATGGGGTTG
CGAGTTTGGAAGCCGACAACGGTTTTCCAGCCTCTTTCTACAAACATTTTACGGATTCGGCGGG
ATCAATTTGATATTTAGGGAAGTAGGGGTGATCGTCTCTTTGTAGTAGCCAGATAGGCCCTGCAA
GGTTGATTTCTCCTTGTTCGTAAACTACTTTAACTCCGGGATGTTTTTCCTCATCGGTGCGATACA
CGTTTACTGCTTCATGGGTTTTGTTATAGCGGTATTTCTGGGTTAATTCCAATACGCCAACAAAT
CTACCAGTATGATCATCTAAACGTACCCAACTACCTTCTTTTAAGGGTTCCGCTACTTCTGCACTA
ACAGAAAGGGTTACAGGTACAGCCCAAGGTAATCCGTTCATTAAACGCATTTCTTCTACGACTCT
TTCGTAGTCATCCTGTGCCATAAACCCGTTGAGAGGACTAAAACCACCAATAGCAATCATCACTA
AATCAGATAAGGCACGGTCATCTAATTCAATTCTAGGAAGGCTATCTGCTTGTGCCATAAATTCA
TCTCTTTCGGCAGGAGTTGCCACTCGATTGACTAAATGCCCTCCATGAGGCGCGATCGTCTCTATA
ATATTACTCATATAAATTTTTGTTAAGTTGATAAAGGATCAGGTTTTATAGTATCAGGCATTGAC
TCTTAGAAACAATCAGGTTGCCAAATTTAAGGTTTGGGAGATTGGGGAAATAACGATAAAATTG
CCTTAATCCGAACTGACGTTAAATAGTTAAGAATTAAGAATGAAAAATTAAGAATTAAGAATTA
AGAATGAAAAACCATGAATACTTATTTATTATTTATTACTTGTTTCCCCCTTTCCCCTCATCACCT
CATCACCTCATCCCCTACCACCTGCAACCTGCAACCCGACACCTGACACCTAACCTTATCGGATAT
TCTTAAACCGAACTAAGGTTATTTAATTCTTGATTGATTTTTTGTTTTTCTAAATTTTTGCCGAT
GATTACAAGGCGGGTTACTCGGTTATCTTCAGGTTTCCAAAGACGATCAAAAAATGTTTCGATGC

GATCGCCCACTCCCTGTAATACCATTCTCATGGGTTTATCGGGAACGTTAACAAATCCTTTGATG
CGGTAAATTTCAAATTTTGTGATTAGTCTATGTAATTCTTGGATTAAATCTTGTGGGTTGGAAGT
TTCGGGAATTACTAACTCAAGGGAATTTATATCATCATCGTGATCGTGGTCTTCTTCTGTATCAT
GATGGGAAGGGCGATTGTTTAAATCATCTTCCACGGCGGCGTTAAAACCGAGTAAAATATCAGGG
TTAATTTCTCCTTGATGACAGGGTATCACCTTTACTCCTTGACGTAATTCTTTTTCTAACCAAGAC
TTCACTTGAGTTAAATCCGTTTCGCTAATTAAATCAGTTTTGGTTAATAATACTAAATCGGCACA
AGCTAATTGATCCTCGAATAGTTCTTCTATGGGGGTTTCATGGTCTAAACTAGGATCATCCGCCC
TTTGAGCTTCTAAGGCTTCTAAATCTCCCACCAATGTACCATTAGCTAAGGCTTGGGCATCAACT
ACCGTCACCACACCGTCAACGGTGGCGTGGGTGCGAATTTCTTGCCATTTAAAGGCTTGTACGAG
GGGTTTTGGTAAAGCTAATCCGGATGTTTCAATAATGATAGAGTCGATCGCATCTTTCCTTGTTA
ATAACTCTTGCATGGTCGGATAAAATTCTTCTTGGACTGTGCAACAAAGACAGCCATTTGTTAAT
TCAATGATGTTATTTTCTTCTTCCTCGGTATCGCATACCTGACAACTTCGCAACAAATCTCCGTCA
ATGCCTACTTCTCCAAATTCATTGACTAAAACCGCAATTTTTCTTCCCTGATTATTTTGTAATAA
GTGTCGTAAAAGAGTAGTTTTTCCCGCACCTAAAAAGCCTGTGATTACCGTTACTGGAATTTTGT
GCATTTTTATGATTATTTATCTATTAATTTTTATATTTTCCCTTGTATATATTTTATTGAAAAAA
ATTTTTTATTCAACCTTTGTACAATAAAATACATACTTTACCTTTAATATCGTTACTAATAAATT
TATTTGAAATAGCTTAAAAAGAACATAAAAACTATTGACATTATTTTTCTAATTTGTTATAATT
TGAGCATAGGAATATAGCCACCTTTTTCTTGATCCGTAGCGATCAAGGGAGGGGTGGCTTATGTC
TTTTTTCCTCAATGCCATGACTTTTTCCTAATACCCAATGACGGCGGAAAAAGCGATATAAAGAA
GACTCTTCTAGGGATAAATAAATGGGTCTGCCATGGGGGCAGGTGTGGGGATTTTTCGTGATTTT
CCAATTATCAACGATGTTTTGCATCTGTTTAATGGTCAGTTTTTCACCGTTACGAATGGCACTTC
TACAGGCGATCGCAACTTGGGCAGAATTGAGATCTCCTCCATAACTTAATTCCATGATTGCCGAC
AGAGAATCATCTCTATTTAAAAGTATTTTAGGAATGGCTCTTAATGCCCATAAATCCTCCCCAAA
AGTTTCTATTTCCAAACCAATATTATTTAGTTGTTCTATTTGTTTAGGTAGTAATTTATCTAAAA
TAATGGGATTTTCTAAGGCTACTATTTCCCATTGATTTTGTAATTGTTCATAGATAATTCTCTCA
TGGGCGATGTGTTGTTCTATTAACCATATTCCTTGGGAATGTTCTGCAACAATATATGTATTTCT
TGCTTGAGCTATGACTTGTAAATCAATTAAACCGATATTTTCTTCTGGACTTTTTTCTCTTTCTT
TGATTTCCGGCGTTTCTAGGTGATAAATACTTTTATTCTCTGCTAATTTAAGAATATTTTCGACT
CTTTTATTCTCCCATTTTTGAGATATATTATTATCAGTTAATTTAAAAATTTCTGCAATAGTATC
TTTAATTTGTTCCTGCCAAAATTCAATATTATCTAAGAAGATTTCTGCTTTAGCAGGGTGTCGAT
TCCAGTCTATTTGTCGAGGAGATGTTTGTAAATGTAAAAAGGCGACGGGAAAACGATCCCTTTCT
AAGGTACGATGAAAACTGCTATAAATAGCTGATTCTAACTCAGGGCATTTGATTATTCTACCGTT
GATTCCTATCTTAACCCAATCTGGTCGAGGACGAGAGATGCGATCGGGTAATCCTAAAACTAATT
CAAGGTGAGAAATAGATTTTTCATAGGGAGTATCAATTGCTTTTTTTATAGTTTTTAAATCAGAA
TAATGAATAGTTTTTAATAATTGCGGTAAAATTTTATCCGCAGAATTACTACGACTAATGGAAA
AAATTAAGCGTTCATTCACTAATAATTGCCAAGTAATATGAGGATGACATAAAGCAAATTCTCCT
AACAAAATTTGAATTTTTTTCAACTGCTGTTTAAAAGGGGGATTTACCTGTCTTCTCACAGGCAT
ATTGCCAAATAAGTCGTTAACTCTTACTATTGTCCCCACGGCTGAAGGTTTGGGATAAATATGTT
GTAGTTGGGCATTTTCTGCCATAATTTCCCAACCGCAATCATTAGTAACCCGACTGCTAATATTA
ATCTGTGCTAACTGAGCGATACTATGTAAGGCTTCACCCCTAAATCCTAACGTGTGAATATTAAT
TAAATCATTAGTATTGTTTATTTTACTGGTGGCATGGGGTTGAATCGTCAGAGGTAAATCTTCTT
GGGTAATACCTTCTCCGTTGTCGGCTACTTGTAAATTCCATGTTTCTGGATAAATGGACACAAAA
ATGCGATCGGCTTTTGCATCTAAACTATTTTCTACTAACTCTCTGACAACGGCTACTAATGAATC

TATAACTTCTCCTGCGGCAATTAATTTAATTAAGTTGTCTGGTAATTTCCTGATAGCCATTTCAA
TTAGTGATTAGGGGTTGATGAAAAAGTGGTTTCATGAGGGTGGGCAATAGGCAATAGGCAACAG
TTTATAGTTTGATCATAAATAATAAAAACACCTGAAACCTGCTACCTACTTCTGACTCATTTACT
CGTTAGTGACACTGTAGTCAAATTCTATAGCTCGATTATGACACTCAATACAGGTATTGTGAGTA
ACTGGGGTTGTAAATTTTATATCAGGATGTAGAGCGAAAAAGTAACGAGACTGGGCAATAAATT
TTGGTCGAGGTTCATCTTTTAGCAGTTGCCTTGAGTATTGCTGTAAATATTGCCACATTAAAACT
TGGGTAAAACGGACAATGCCATCTATTCTTACACCATAATGATTACCCGGATTTTCCAAAATTGT
CTGCCATGTTTCCGAGGGTAAAACGGCGGGAGGAATTGGTATATGGCAAGTGGAACAATTTTCTA
AGTATAAGTCTTTACCAATAACAAAGTTACCTACTGCCTTATCAACAGATTTTGTTCCTGCAGTC
AAAGATTGATGATTCATCGCTAAGGATGCACCCCATCCAAAAATCGAACAGAAAAGGACTGTTAA
AGCTAATATTAGTGGTAAAGTTACTTTTTTTCTTTTTTTTGACCTAAAAAACATGATTCCCCTTA
AAATGAACAGTTGACAATTATCCCCTCCCCGTTTCGGTGGCATTATACGTTTGCCCTCGATATTAC
TCCGCGATCGCAACAGTATTGAGTAACCGAGCTACAATCACTTTTGCATTTCTGCCTCCTGCTGGA
ATCAAACGATGAGATAATACACTAGGTGCTAAAAATTTAATATCATCGGGAATAACATAATCTCT
CCCTTCCAAGAAAGCTAAAGCCTGAGATGCTTTTTGTAGAGCAATTGTTCCCCTAGGACTTACTC
CCAAAGCAATATCATCATCCTGACGAGAAGCATTGATTAAATTAACGATATACTTTTGAATCTCT
GGCGCAACCCTAACTGAAAATACCTCTTTTTGTAAATCTTTCACGTCTTGTAAACTCACACAGGG
TTCTAATTCTTCCACTAAAGTGGGATTTAACTGCTTTTCTAACATTTGCAACTCTTCCTCAAAGG
AGGGATAACCAAGGCTCAAAGAAATAGCAAATCTGTCCATTTGTGCTTCTGGTAAGGGAAAAGTT
CCCTGATACTCTACAGGGTTTTGTGTGGCAATAACAAAAAAAGGTATCGGTACAATCTTAGCAAC
TCCGTCAATGGTAATTTGTTTTTCTTCCATTACTTCTAATAATGCGGATTGAGTTCTCGGAGTAG
CCCTATTGATTTCATCTGCTAGTAAAACATTCGCAAAAGCAGGACCTGCTAAAAACTCAAATTCT
CGACTGTTGGGATTCCAAACATTTGTTCCTGTTACATCACTAGGCAATAAATCAGGAGTGCATTG
GATACGCTGAAACTTACCATTAATTGATTTTGCAAGAGATTTCGCTAAGAGGGTTTTACCCACTC
CGGGTACATCTTCTAGTAAAGCATGACCTCCACTTAATAAAGCAACTAAAACTAATTTAATGGCA
TCATCCTTCCCCACAATTGTACGGGCTAGATTTTCTTTGAGAATGGTAATTTTTTCCCTCATTAGC
AGTTATTTCCTTGTGTGATACTGAGTCCATATTTTATCAAATTTAGCCGAAGTGCGATATAGAAA
TATGACTCTCTAAGTAGGTTTCAGGCTTTAGGTTTTGGTTAATTTTTCTCAAAATTAATTTTGAA
CTATTAGTACCTTTGTACTTAAGAAGTCATCTAAGATCTATTAAACTTAACTTTTTTATTAGGCG
ACGGGCTTAAGCCCCTTGTTATCATTCAAACGTTTACTTGTTGCCTGTTGCTCACTCTTTCCCTGA
CGATTTGATTTAGATGTTTATATTTCCTAGCCCACTACTAAAATGCTTTAGAATGAGCATCGCTA
CCGTGTCGAGAAGTAGTTTGTACCATTAAAAAGTGCTGTAAGATAAAGATTAGTTAGACACAAG
AAATTAAAAAACTATCATGAGTAAATTACGCAACGTCCTCATTGGTGCTGGAATAGCGGCTGTTG
GTGCTGTGGGAGTAAAAAAAGCCGTTGATTATTTCCAAAATAAAAAACAGCAAGAAGCATTGCCC
GAAACCCCCGAAGATGCTCAAATTTCTAATCCAGAAGAAGAGGTTGCTTTTGCCGTTGTCGAAAC
TTCTTCCGTTCAAGATTTCCTTGATAAAAGTTTTGGTAAGTCGGGGCGCTACAGCCCTAATCGCC
CTCCCAAAGTATTTGAATATCAGGATAAACAGTATATGGTAATCTGGGCATACGATAACGAAAAA
TCTAAAAATCAAATGCTTGTTTTTCAATATACTGACAGTGGAAGACAAATGATTGCTAGTGTAGG
CTATACCGATTCTGCCACAGACTATAATTTAAAGATGGGAAATACTCCCTTTGCCGTAGAAATTA
ACGGAGCAAAATTACAATCTGGGGAAGGTGAAACGGAAGGCACAAGTGATGTTGATTTCGTCTTA
GCTTAGTTATTCTATTGGAGTTTTGATAAAAATTAGGATTTTTAAGGGGTAAAAAAGTCATTCT
GCCTGTATATATTTATGGCGATGTCTGTTGGGGGATTAGAAGAATTAGGAATTATGTTGATCGAG
AATTAGTGGTTAAATAACTTGTTAATATCATTAATTAGTGATCCACTATTAACTATTAATTATTC

ACTATTTATTATACAGATGGAGGTTATCAACTTTTATGGCAACTCAGGTTATTCAAAATCCGCAA
ACAAAAGCTAATAGTGAAAAAAAACATCATCCGGGTTATCGAGTTTTACTGCATAATGACAGTTT
CAATACGATGGAATATGTCGTAGAAACCCTGATGGAAACCGTTGGAATGAGTGAACCTCAAGCGG
TTAGTGTAATGATGGAAGCTCATACAAATGGTGTTGGTTTAGTAACAGTATGTGCTTTGGAACAC
GCTGAGTTTTATTGTGAAAGTTTAAAAAGTAAAGGTTTAACTAGCACGATCGAACCTGCGGAATA
ATGGTTTATTATTACTATTTATATTAATTATTAATTGTCCTCATTTTGCTTTTATCTCATATCAA
AAATTATCCTATAGTTGTAAGGTTAGGGCTATTTGTCTTAGCCTTACTATTTATTTGGTCTCCTT
TTTTACTCTCTACTTATTATTTTCTAGGAGCAAAAGATCCTAATTTAACTACTATTATAACCATG
GCAATTTTATTTGTCATTTTTCTATTTTTGGTTAAGTTTTGGGGTCAATTTGTGTATGATAAATC
GAATATTTTTAAAAGTTATGGTTTAGTTTTTTCTCGCACCAATTTTCTTTTTTTAATCAAAGGTT
TAATTATTGGTTTTATTTCTAATTGGCTATTATTTGGAGTTGAATTTTTATTGGGGTGGGTAAGT
TTTCAGTCTCCTTCTTTACCCTTGGTTAAACTCTTAACAGAAGGATTTATAAGTGCCATTGGTAT
TGGCTTGGCAGAAGAATTATTTTTTCGAGGCTTTTTATTATGGGAATTAGAAAGAGATTTTTCTC
TGGCTTTTTCTGCTAGTTTAAATGCTTTTATTTTTGCTTTTTTACATTTCATTAAACCCCTACTTG
AAGTAATTCGTACCAGTGTTACTTTTCCCGCTTTAGTTATTTTAGGATATATTTTAGTATTAGGG
AAGCGATCGCATCTTAACTTATTGGGTATTAGTATTGGCATTCATAGTGGCTTAGTGTGGGGATA
CTATATTGTAAATGTAGGACAAATGGTAAAATATAACAACTTAGTACCATCTTGGATAACAGGA
ATAGATAAAAATCCCATTGCAGGAGTTATGGGAATTAGTTTTTTATTGATATTATTATACAGTCT
AAAAATAGATAATAAATTGAGAAACAAAGTGAAAAATGATGTTAATATGAACTAATTTAAGAAA
AATATTAACTAAAAAAAAGTGTAATAGAGGTTAAGAATGTCCTTAATTAAAAAAAGTTTATTTG
TTGCCAGTGTAATTATTTCCCCTCTTATTTATTCACCGATACAAGTGGATGCACAAAATTCAAAC
ATGAGTTTTAGTCAATGGTGCGAACAACAATCATTATCTGCTGAAACAGAAAAAACCATTAATGC
AATCACTAAGGCTTTAAATGTAAAAAATTGTGCACAAGCCGCCGAAATCGCCCCTAGAGTATTCA
CCCTAACTATAGCAAATCAAAATATTTCCGACTTAAGACCACTCAAAAGTTTTATCAATCTTACA
GACTTAAATCTATACAATAACAATATTGTTGATATTTCCCCTCTACAATCGATGACTAATTTAAA
GAAAGTAGTTTTAGGTCATAATCCCATTCAAGATTTAACCCCTTTAAAATCATTAAATAACCTCG
AAAAATTATATATCAGCAATATTAATACTAATAATTTTGAAGCAATTAAAGATCTAAAAAAAAT
TAATACTTTATATATTAATCAGAATAAAATCAGTGATTTAAAACCACTAGAAAATTTAACTAAC
TTACAAGAATTATATGCTAGAGAAAATAAAATTAGCGATTTAAAACCCTTAGCAAATTTAACTA
ACTTACAAGAATTATCGTTAGATAATAATCAAATTTCTGATTTAACTCCTTTAGCAAAACTGAGC
AAACTAGAAGAATTATCTCTCAATAACAATCAAGTAACAGAAGCAGGAATCAAACCCCTGTGGGA
TTTAAAAAATTTAGAAAGAGTATATCTACATCAAAATCCAGTAGCACGACAATTTTGCCCCAACA
ACAGAGATAGTCTTTGTTTTATCGGTAATAGATAAATAAATTAATAAAGGTGAAAGGTAACAGG
TTTTAGGTGGAATTAAAATTGTTAATTTATAACCATTGTATTTTTATTTGTAGTTACCCCTTTAG
GGGTTTTAAACGTTTTTGAAATAAGGGCTTCAGCCCTTACTACTAACTGAACCTATTTTACGTTT
AATTAAGTGGAGCTACTTATTTATTTTCAATTCTTTGATTGACTAATCATAAATTTAAGGGTTTT
AATTCATCGCTACAAACAGAAATCCTACTAAATTAAAAGATTCTGGTAAAGCAATTAAGGCAATA
AACAAAATATTTGAACTGTGGTTGTACTTACCATCAGGTAAAACCACTTATTGTACACTTAATTA
TATAGTTATATAATGAAAATATTGAAGCTAAGAGTAATTATTAAATAAATTAAGCTCTGAACCC
ACAAAAATTATTATTCGGACTTATACAAAATTAAATAAATTAATGGAACATCAAACCTATTCTCA
TCAAATAGTAATAGTTGGTGGAGGTAGTGGCGGAATAACCACCGCCGCCCAATTGTTGCTCAAAG
ACAAAAATCTTGATATTGCAGTAATTGAACCTTCTGAAAAACATTGTTATCAACCGGGTTGGACT
TTGGCTAGTGGTGGTTTAATCGATCCGGATAAAATAATCCATGACAAAAAAGAAGTAATGCCTAA

ACAAGTTCATTGGATTAAAGAATTTGTAGCTAAATTTAATCCTGATAACAATACTCTCATTACCC
GTAAAGGCACAGTTGTTAAATATCAATATTTGATATTGTGTCCCGGATGTCAACTAGATTTACAT
TTAATTAAAGGTTTAAAAGAAACTTTAGGCAAAAATGGGGTAACAAGCAACTATGCAGGTTTAG
GGGGTTACAGTTGGGAGCTAATTGATAATTTCAAAGGGGGTACAGCCATTTTTACTTACCCTAAT
ACGCCTATCAAATGTGGAGGTGCGGCACAAAAAGTAATGTACATGGCTGATGCCACTTTCAAGAA
AAAAAGTGGTGTGGGTGTTAATAGTAAGGTGATGTTTTGTACAGCAGGTACTAGAATGTTTGGG
GTTAAAGAATATTCCGATGTCTTAGATGAAGTTGTGGAAAGACAGGGAATAGAAGTTAAATATC
AACATAACCTCAAAGAGATAAAAGCAGATACGAAAGAAGCTATTTTCGATGTAATTACAGCCAA
TGGTACAGAAGAAGTTATTATTAAATACGATATGCTTCATGTTGCACCGCCAATGAGTGCTCCAA
ATTTTATAAAAGAAAGCCCTTTTGCTCAAGAAAATAATGGTATTCACGGGGGCTGGGTTGATGTT
GATAAATATACCCTTCAACACTTACATTATCCCAATGTTTTCGGATTAGGAGATGCTTCTTCTTT
GCCCACTTCAAAAACTGCCGCCGCCGTGCGTGGGCAAGTGCCAATAGTAGTGGAAAATTTACTAT
CTCTTATGAAATCTCAACCTCTAAAAGCTAAGTACGATGGTTATACTTGTTGCCCAATTATTACC
AGTTATAATTCAGTGGTTTTAGCAGAATTTAATTATGAGGGTGAACCCATTTCTTCTTTTTTCAT
TGATTTAGCAAAAGAGCGTTATAGTATGTTTTTGGTCAAACGGTATCTTTTACCTTGGTTATACT
GGAATCGAATGCTGAAAGGGAAACCTTTTGAAACCAGTATCTTGAAAAAATTAGGTTTACTAAA
ATAACCTCAGTTCTGTTTAAGAATATCCGATAAGGTTAGGTGTCAGGTTTCAGGTTGCAGGAACA
GCAGGTGTTAGGATGATGACCAGATGATAAGAGAGGATGAGAGGAGGAAGTAGAGGCTTAGGGT
TTGGGGTGGTAGGGGTTGAATATATTTTTAACGTTAGTTCGGGTTAAGGCAATTTTATCGTTATT
TCTAAGAAGCTATAAGGTTTTCTGACTACGAGTTGGTCTGCTTTCAGCTTATCCAAAACTCAGGT
TAATTATGAAACCATTGCCTATTCCCTATTTCCTATTCCCTATTTACCTTTGCCCTATTCCCCACA
ACTTACTCTAATTGTCGAGCGGCAAGTCTAGCGAATAATCCATCTTCTTGGATCAGTTGATCAAA
ATTACCTACCTGCACCACTCGCCCTGCTTCTAAAACATAGATGCGATCGGCGTTACGGATGGTGCT
TAAACGGTGGGCTACCGCTACTCTAGTGGCGTTCATGCGATCGAGGCTTTTGGTGACAATGGATT
GAGTACGATTATCAAGGGCGCTTGTGGCCTCATCAAGGAAAATAATTTTTGGTTTTAAAACCAAG
GCACGGGCAATTAAAAGCCTTTGTCTTTGACCTCCAGAAAGGTTTGTTCCCCCCTCACTAATAACC
GTGTGCATCCCCATAGGCATTTGTTCAATATCTTCGGCAAAACCCGCCATTCTTGCCGCTTCCCAA
GCCTCATCAAGGGTAACTAAAGCCCCGGAAGTAATATTATCAAACAGAGAACCTGCACCGATACG
TCCATTTTGCAAAACAACCCCTAATTGTCGCCTGACGGCTTGAGGATTTAAACCTGCTAAATCTT
GTCCATCATAATAGATTGTTCCAGCTAAAGGGGTTTCAAAACCAAGCAACAAACGCATGATTGTT
GATTTACCACTACCGGAAGGACCTACCAGAGCAATGAATTCTCCGGGGTTTGCTTCAATGGTGAC
ATCATCTAAAATTAAGGGACCATCTTCACGGTAACGAAAAGTAACGTGATCTACTGTAAAACGTC
CTGTTAAACGCCCAGGGTCAGCACTGGTAACGTCTGCTTCGGGTTTCCCGTCTAAAATAGGTTTT
GCCCTTTCCCATAGGGGGACAATTTCTAAAACATCGGTAATAGTAACACTCAAATCGGTTACACC
ACTAAAAAAAGTGCCAAAAGCAGAGTTAAAAGCTAAGAAAGTACCTGCATTTAAGCCTCCCGGTA
TGCCTTTTGCGATCGCACCTTGAATAAACATAATGGCAAACCAAAATAATAATACCGAACTCATT
AAGGGTAAGACTTCTGTGACGACCGTAACACTATCATTAATTCTTTGAATACCAGCCATTAACTT
TGTCCGTTGACCGAATTTTTTTGCCCAAGCCGCAAAAGCCCTTTCTTCGGCGGCCGCTACCCTCAA
TTTTGAAACACCATTAATTAACTCGATACTTAGACCATTTAACTCCCCGTCTAACTCCTCTGTTTT
CCTTTGTCGGCGTACAAGCATGGTACTAGAAACAGTGGTAACAATTGCCGCTACAAGGGTTATGC
CCATTGCCACTAAAGCTAATTTGACACTGTAAATAAACATTAACCCCAAATTTAACAGGGCGAAA
ATACCACTTAAAAGAGTACGTTGAGTCGCCCCACTTAACTGAGAACGAATTTGCCCCACCGCCAA
CAAGCGCACCAATAAATCCCCTGTGGCATATTTACGGAAAAAAGCAGGACTGAGTTTAATTAAGC

GATCCCATACGGCGGGTTGCAAAACCCCATCAGCCGCACTCTCTACCCTTAAAGAAAAAATCCCTT
GGGCTAACTGAAAAGCCGCTTGTCCAAAAGCCGCCGCAAATAAAGCTAAACCGATTTGCCATAAC
AAACCTTGATCACTGTCAGGAATCGCATTATTAACTAAAAGGTTAGTCGCTTGAGGTGTTACCAT
CCCCATAAGAGTACCAACTGTACCCATAACACCAATGGCAATTACGTCTTTTTCATAACCCCGAA
TACCAAATTTAAACACTTCCAAAAAATGGGAAATGCCACTAGGCAGAGGGCGATAAAACATATAG
GCTTCCACCGACAAAGTTTCAGCAATCGCCTCATTAACAGGAGTTTTCGTTTTCGTAATTGGATC
AAACAAAACGTATTTATTACCCTGATGGGGTAGTAATGCCACAGGATTATTTTCTTCTAGGGTAT
AAGCTAAAAGAGGTCCGTTTTCTTGTTTCCACCATTCCCCCATTAATAACACCCGACGGGTACGA
AATTGAGAAGCCCGTGCGATCGCATCTAGGGGATCTTTAATCCGACTTAAATCTTCTGACTTAGC
AGGGGGAAGAACAGTAATTCCTTGAACTCTGCCGATAGCTCCTGCGGCAACTAATAAAGGCATTC
CCTCTTGGAAAAAGCCCACCTCTTCAGGGTGTAAAACTTCTTTTAATCCCCCCAAGGCACTTTCTG
TCACCTGAACATTGAGTTCCTGCCGTTGCTGAAACTGTTGAAAATTTTCCTCTTGTTCCTTGACA
ACAACTAAGCGAAGATAATGCAATAGATAACGATGAAATTGATTTAAACTAAGACTTAAATCTT
GACAATTTTCTAAATCAGAAGTGGGACAAGTATAAATTTCCGTCTGTTCTTTTGTATTTAACCAC
ATTTCAGGAGTCAGGGGAAAAACAGAGCAATTCGACTCAAGCATTATTTCCTCTAATCCCATCCA
ATGGAAACTTCCTGAGCGAACGTCAATCCAAGCTACCTGCCCTTTTCTAGCTTGAAGACTTTGCCC
TTCAATTAAAGAAATAAAATGACTACCCTCGGCTGTAACCAAACTTAAGGGAAATTCAGTGATTT
TTACATACTGTCGGGCTAACCACTCATCCCAATGATGTAACCATGTTTCTAATGGGGTAATCTGT
TTTAGATCTTGATTGGGGTCAGAATCAGAAGTTTTGTGGATCAAGGGTTCAAAAAAATCTTGAA
AAGAGAACTTTCTTAAAACCGTTTCCTCTAAACCCACCGCAATTATGCCATATTCATCCGAGAGA
AAATCAACTCCTAGTAAAATTTCTCCCTCTACGGCGGTGAATAAATAACGGCGAATATCTTGAGG
CTTATCATCTTCAATCAGGGTAGAAAATAATACTACTGAACCTGACTCCACTAACCACACGGTTT
GAGGGTCATTTAACAATAAAGGTTCGTTACCTTTAACTAAAATTTGTTCGGGTAATAAAGTTTGT
TCTGTCATAGCTTTAGATCAATACTCCTTGTTCTTGTTCTTCTTCTTCGGTTTCTTGGGAACTGGT
TAACTGGGCATAAATACCTCCTTGTTCCCTTAGTTCCTCATGAGTTCCCCGTTGTACTACTTTGCC
TCGATCTAAAACGATAATTTCATCACAATCTCGAATCGTACTTAATCGGTGAGCAACCACAACAC
AAGAACAGCCTCGACGGCGCAAATTGCGATCGATAATTAACTCTGTTTCTGCATCAAGGGCGCTG
GTGGCTTCATCCAAGACCAAAATTGAAGGATTTCGCACTAAAGCCCGGGCAATTTCTAGGCGTTG
CCTCTGTCCTCCACTCATATTCATACCCCCTTCAATTAACTCCCCTTCATAACCCCCCGGCATGGC
TAAAATCAAATCGTGAATTGCCGCATCTTCACAGGCTTGAATGATATATTCTTCAGGTACAGTAG
AATCCCAAAGAGTTAAGTTATCCCGTACTAAACCTGCGAAAAGGAAGATTTCCTGCTCTACCATT
GCCAAAGAGTTAGCTAAAACTGCTCTTGATAAACGAGTGCGAGGAATACCATCAAATAAAATTTC
CCCCTCCCAAGGCGCATAAAGTCCCGTTACTAATTTTGCGACGGTGGATTTACCTGAACCACTGCC
TCCTACTAAAGCTACTCTTTGCCCGGGTTTGATACTTAAGCTGAAATTGCTGATTAAAGGGGGTT
CTAAACGGCTATAACCAAAGCTAATATTGCGTAATTCAATATAACCTTTTAGTTTAAAATTATCT
TGGTTCACCGTTGCTAGTTGGGTATTGGTTTCTGTTTCTACCTGTTCCCCTCTTTCCACTTCAGTA
TCAATAGGATTGGCTAAAACATCATCTAAGCGTCCTAAATCTGCCTCTAATTCCTGTAAAGTGCT
ACCGAAATTAAGTAGGTTATTCACTGGAGATAAAAAACTTTGAGTTAAAGCCTGATAAGCAATC
AACATACCAATACTAAGATTGCCATTCATTACCCGAAAACCTCCTACTACTAAAATAGCGGTGGT
TGCAAGGGAGGTTAACAAACTAGGAATAGAAGATAAAACTTGAGAGGGTAAACTCAATTCCTGT
TGAGCATTAACCATTTTAGAATAGTAACCTGCAAATTTAGAAAATAGGTCTGACTCTAAACCTGC
GGCTTTCACTGTTTCAATGGTCATAATTCCCCCAATGGCAACTCCTGCCACTTTTCCGCTCTCTTG
GGCTAATCGGGCATTGGCATCTAATCTGGTACGGGCTAAATATCTCAGGGCGACGAAATTAATGG

CGGCAAAGAAAATAACCATTATTGTTAATACCCAATCATAAAGAAACATGATTAAGGCATAAAA
GACAATCATCACCGTATCAATGATCGTTGTGGCTAACTGTCCTGATAAAATATTAGCAACCTTTT
GATTAAGCTGCGATCGATTACTGATTTCTCCTGCGTATCTTTGGGCATAAAAACCAATGGGGAGG
CGCAAAGTGTGCCATAAAAATTGCCCTGACATAGAAATCGACAGTTTCAGCATCAATCTTCGCAA
ATAAGTCAACCTCAATCTTGCCAAAACTCCTATGAGGATAACGGCAAATAACATCACCAATAATA
AAGGTTTAAGCCAGTCTTGACGACCTTCAACTATCACCTCATCAATAAAAACTTGAGTAAATGCT
GGTACAGCTAATCGAGGAATCGTTAAAAATAGACCCGTTAAAAGACAAAATAAAATTGCGGCGT
GGGAATTTTGTAAACGAGCAGTTAAAGCAGGTATAATTCCTTGTTTTTTACCTCCTTTGGTAAAA
TCTGCCCCCGGTTCAAAAACTAAGACAACTCCTGTGAAACCTTGGTCAAATTCTTCTAAGGTAAT
GCTTCGAGGACCTGAAGCGGGATCATTGAGATAGACTTTATTTTTTACGAATCCTTCTACAACTA
AAAAGTGGTTAAAATTCCAAAAAACAATATAAGGGGGGAGGAAGTTTTTGCACTTCTTCTAAGGA
TTTTTTAAAACCCTTCGCATTTAAGCCGTATATTCTAGCCGCTTTTAAGACATTTGATGCTTTAC
TACCATCTCTGGATACCCCACACTCTCGCCTTAATTCGGGCAACGGTACAATACGACGGTGATAAC
CTAAAATAATCCCCAGAGCCGCCGCACCACACTCCACCGCTTCCATTTGAATAACTGTAGGAGTAC
GAACTCTTTTTGTTTGGGGAGAAGAAAACAGTTTTGATAAAAATGCAAAAGCCATAATTTATAA
CATCCAATCTTAAAAGTAAAAAAAGTAATCATCAGCCATTGTTATGACCTCAGTTCGATGCAAGA
ATATCGAGTTTGGGAAAGTATCAGGTTTCAGGCTTTTAATTAATTGCTAATTACTAAATTATTAA
TTGTTTATCGAACTCAGGTGTTGTGGTAATAAAATTAGTTAAATTAAATTCTAAAAATAGTTTT
TATAGACCTAATTATAAAATATAGGTTAAATTAAATTAGAGTGTTTTATTGTGAATTTTAGCTT
TTTTAAAGTATTTTAAAGCAAATTTTAATAAACTCCAGTCCAAGATCTAAATAAGGGAATAATG
TAGGAAATTGGTGCAACTTCTCCCACTTGAATACGAGTTGTGGTGGTAGTGCCGGGGGATAGTTC
TTCATTAGGACCTTTAGAAGAAGACCATTTATAACCGCTAAAAGTGTTAGGGTCTTTTTCCAATT
CAGCAAATACTTGTATGGGTGCTTTTTCTTGTCCTAATTGTTGGGCTAATTCTGGATTACCGATA
ATTGCAGTCATGTCTTGAGTAGTGACGGGAAAATCTGAAACATCGGTCACTTTGCCCAAAATTCC
TCCGTATCTTTCTCTTTTGACGAGACTAGGAGTAACTTGAACTGTCATTCCTTCTTTGACTTTTTG
TCCGTCTCTGTCGGCAAGATAGGTAACGCTAATCAAGGGGCTGTCTTTTTCATCGATTTCAATAG
AGCCAACACGAGTACCACTTTGAATTACTTGACCCGGTACTATCGCTAATTCTAAAATCTTGCCA
TGATGTTTACTGATAATTTTTCCTTGATCCGCTAATTGAAGTTCTAATTGGGCTATTTTACGCTT
CACTTCATCGACTTGATTTGATCTGTTAAAGGATGATTCTAGTTTTTGTTGATCTATACTGGCTA
TTTGACTGTTAACTTCGTCTATTTTATTTTGAATATCATCAAGACGATTGAGACTTTCCAGATAT
TGTCGTTGCATATCTGTTTTTTGTACTTCTATTTCTTGGAGCTTACTTTTAAGATTATTGATTTG
ATCTAAACCTTGCAGATACTCTCGTTCTATTTGGGCTTCTCTCACATCAAGTTGACTTAATTCTGC
TTCTAAGTTGGATAATGAGGAGATATTTTCAAATAATTGTTGTTCTGCACTTAAAAGGGCATCCC
CTGTAATTAGTTGCTTTTCGAGAAGTTTACGACGGCTTTCAATTCTTTCTTTGAGGGTAGGTAGT
AGGACATTAATTTGCCGAATACGCTCATTTAAACTTTTTCTTTGTTCTTCAAGGGCGGCAATACT
TCTTTTACGCACGAGAGGAACGTTTGTTTCGAGGTTCTCAAGACTTTTTTGTAGGTTGATACGGT
TTTTTTCTAATAGTGCAAAATTTTCTTCTCGTAAAACAGGTATGATAGATTCGCGATCGAGGGTT
TTTTCTAAGTTATCTTTTCTTTGTTCTAAAGTTCCTTTTTCTAATTCTCTTTTTAGTCCTTCTGCT
TCTGTTGTGTCCTGATTTTGGTTAAGTAAATCGGCTAATTTTTGTTTTTCTTGGGCTAATTGTTG
TCTTAAAGAAGATTGGTCAATAGTGCCTAATATCTCTCCCTCTTTGATCACATCACCTGATTTGA
CATTTAATGTGATAATTCTTCCTTGCCCTAGGGCTTGAACTTGAACCACACGGCGAGGTTGAACT
AAAATTCCTTGACCTGTTACCGTCAGGGGAATACGACCAAAAACGCTCCAAAGTCCTCCTACTAA
TACTAAAGAACTCATTGCGGTTAAAGGTACCCATGCTTTGGGGTTTATCACCTGCATTAATTGGT

CTAATCTTTCTGGGGAAGAAAGACGTTCGAGAGATTCTTGTCTGAATAGTTTACTCATTGCTATA
TGAATTTCCCTTATATACTAATATTTCGTTATCTGTTTGATGTTTCAACTAATGTGCTAATGTGC
CAATGGAAAATAAGGACATATCAAATTTTTGTCATCAAATATTAACTAACTTTTAATCTTCTTAG
CATCCAATCAAATCTTGCACCGCCGAGGGTCAGATTATCCATAACATCTAAGTCGATTTCATCTT
CATAAGTTGTATCTTGGGATAGTGTCTCTCCTTGGGAATAAGAAGTTTTGCCGAACCCCAGACGA
CTAATCATGCCTTGCACCCTACCTGACATTAGTTTTGCAATTACTCGATAAAAGCGTGATGCCAT
ACCGACGTCTTGCTGTAGTTTAAGAATCATTTGTTGTCTTGAGATGACCAAAACAGAACAGTTTT
CTAAGGTTTTAATTGTGCAGTAGGAGCGATGGGATTCTAAAACCGCAATTTCTCCTACTATTTCT
CCCCTTGATATGTGACCAATTTCTCGCTCTACAGGGGCGGAGGATGTATTGTCGTCACTTTCTAA
AAGAGCAAAAATGCTATTGAGACGATTTTTTTTCTGTTCACTCACAGAAACATTCATTACTCCTT
GAAGCAATATATAAATATTTTCTACTTGCTCCCCTGATTGAATTAAAACGGTATTAGCAGGGAAT
GTTTCTAATTTACCTTTTTGTAGAAACCAGTCCACATCTGAATCTCTTAGTTCACCAAAAATGAG
AGACACTTCTTGGAGGGGGGCTATTTGACCTAGTCTTTTTTTCAAATAGAGAGTGATTAGATGTT
GGAATCTTTCGAGTAACAGTATGGTCATTCCTCGATAAAATCGAGCGGCAAATCCGTAATCTTGT
TGTAATTTTTGATTTAGGGATTCACGGGGAATACTTAAAACCACAGAATCTGCGATCGCTCTTAT
ACTATTAGCGGCGGGATTCACTTCTAAAAAACTCATTTCCCCCAAAATTTCACCCTCAGAAAATT
TGCCAATTTCTTGTTCAAGACTTTGATTATCTTCTAAAGCGGCAAAAACTTCTCCTAGTCGTCCT
TTTTCATGACGGGTTAAAGTAGCAATAAATTCTCCCTGAATAATGATATAAACAAAATCAACTTG
TTTTGCCTGTTCAATTAAAAGGGTGTTAACAGCGACTCTTTGAGTTTGACCTGTGTTTTTGAGCC
ATTCAATATCGCTATTGCTTAGTTCCTGTAATAAAACATCTGTCATAATTCAATTTATTCTCCGC
AATCTTATAATTTGTTTTATTTTGGCGAAGTTGGTAATTTGAGATAATAAAAAATACTGATTAT
TATTAGGGATCTTGCTTTAAAATAACATCGACTAACTAGCTAAAATGGTACAAATATCTTGAGTA
TGCTTCAATTAAAATTATTACTCAACTCTGTTTGTCACCTTCATTAGTCATCTATTCTTTGGGTC
AAGATCTGAATATTACCTATTTCTCCTTGATTTTCTCCTTTTTTTCAGAGTCAATTATTCTTGTCC
AAAGTATAACCATATTTACTCAATCTTAAAGAATTTTTTTTGATAATTTATAGATTCTTGAACAA
GAGTCTCGAAATCATCGGAAGCCAATTCTTCTAATGTTAAAGTTGTTAAAGCCATTAGTAAGGAA
ATACTTTCTTGTTTTGTGGGAGTAGAGTTAAAGTATTTGTTATGAAATTGCTCTAAATTTTCTGC
GGCGGTTTTTACCAAATTCTGAAGTATTTTAAATGCTTGATCTGATAAAGGAGGGTTTCCTCGAT
AGTTTTCCAGTCTTCCTCCCTGACGATAATTTGGGAAGGATTCCAAACCAACAAAGTGGAGAAAC
CAATCAGCCCGAAAATACCCCAAAGCCGCCACAATTCCTTGGTAATCAGCAATAAGCTCATCTAG
TAAGTTATTGCCCATATAGTTAAGCACTCTACGGGTGAAATAATGGGTACACTCATGCTCTAAAC
GAATAGTGAGAGATAGTTTTTGCCACTGTTGTTCTGTTAAACCTAGTTGAGTGGCACTAACATTG
CTATAAAAGCCTTGACTAAGAATAATAAAACGGTCTTGATATAAGGATTTTTGGGGTATTAGTCT
TTTAAATTCTTGATTCCAATCTTCTTGGGTGCAATGGTTAGGATTATTTTTTTCCCATTCAAGAC
GATATTGTTTGATTCGATCCCAATTGTTATAACCCCCAATTATACAAGCTCCCATGGATGGGGGA
ATGGTTACAGGTTCATTTTTTTTGGTGATGGCTTGAATTAAAAGCTCAAAGTCTTGACGATTAGG
GGCAATGATAACAGGTATTGCTCCTGCAAGAGTATCTTTTATTTCCAGTTGTAAATTATCTGGGT
CTGTTAATTGTAATCCCCCCTCAAATCGGAAATTTTCTTCACATTCTCCCTTGCGCACTGCCCGAA
GATATTGTTCATTTTGACTCATGCCCTCTCGCACGGGAAATTTCAGTTGAACTAATGGAAAAGTT
AAGCTATTATTAGTACCAATTTTATCAGCTAAGGTTTTATAATTTTGCCAAATTTTTACGTGAGG
TTCTGATTTTAGTGGGAAAGAAATAAAATCATTGATATTACTATTTTGTTTGAACTTATTTTCGT
TATAAGTTAATAATTCTTCAATTTCGGATTCATTTAGTTTAAATTTGGTTAAGGTTTTATAACGT
GATTGTATTTTGTCCATTATACTTTTTAAACTTTTTTCCTACCATCATAAATGAAGATAATGAAA

AAATTTCTAATGATTTATATTTCTAAAAATAAAGTTTTTGGAGGTAGTGGTTTTAATCTTCTGAT
ATTAACTTTTGTTAACATAACCACTCTGTTTGTCGCTCAAAAATCTTTGTTAGCAAATGAACTTT
TGTTAAATATGGCAGACCAAAAAAAAGAAGAAATCACTCCTTTTATTGATGATCAGTATCAAACT
ATTGATACACAAGCATTTAATTTCTCTGAATATGAAAAATTATTCTTAAGTCAAAACCCTTCAGA
GGTTGAAAATAGTATTCCTCCAGACTTACGAGTCAACCCAAATCCTTTAGAGTTGCCAACAACTC
CCCAAGAAGTAGAAACCGTTACTATTCCTTTATCTTTAGAAGATGCGATCGCAGTTGCCAAGGGA
AATAGTCAACAGTTAAAAATAGCCAAAATTAACTTAGAGAAAAGTCAAGCCGTCTTAGACGAAG
CCAGAGCGGCACTATTTCCTACTCTTTCGGCGGCGGTTACTGCTTCTCGCCGAGAGGAGGCTTCTC
AAGGTTTATCCACCGAGGCGAGTGAACAAGACACGGAAAATCAAATAGCACAGTTAGAGTCTTCT
ATTCCCGCCATTGAAAATCAACTTGATCAGATTGATAGTATATTAGAACAATTGAACCCTGCTGA
TCCCACTGTCATACCAACCATCGTTTCTTTATTGATAGAAAGAAATAATGTTGAACAAAATCTTC
TGAGCGCTCAAAGTTCTTTAGAAAGCAGTAGGGAAGCCCTAAGAGACATCAAAAATTATGCGGGT
ACTTTCATTGATGGTACTGTGAGCTTAAACTATAATATTTACTCTCCCGGTCGTCAAGCAACTAT
CAACTCTGCCTCAGAACAAGTTTTAATCAACCAGTTAGAAGTTCAAAGAATTGAAAATGAACTGG
TTTTAAATGTCACCTTAGCTTACTATGACCTTCAACAAGCAGATCAAGAAGTAAAAATTAACCAA
AGTGACGTAACTAACCGACAGATTAGATTAGAAGGCTTACAGTTGCAACTAGAAGCCCAGTTAGC
CACCCGTGTGGATTTACTTAATGCCCAAGTTGAACTCGATAACTCCATACAAAACTTAAGAAATT
CCCAAACCGTTCAACAAACCTCACAACGTAATTTAGCTCGTTTATTAAATTTACCCCCCACTGTTA
CACCTTCCGCCTCAGATGCGATCGCAATGAGTGATCTTTGGGAATATTCCCTAGAAGAAAGCATC
ATCATGGCATACAAAAACCGAGTTGAACTACAACAAAAATTAGCCCAACGTAAAAGCTTTGAAGC
CCAAAGACAAATTGCCCTAGCACAAGTTAGACCAACTTTAGCCTTGTTTGCTAATTATCAAATGT
TACAATCTTATACCGATGACCCCAGAATCAATACCTTAGTAGATGGTAATTTTTCCACAGGATAT
GCTTTTGGCTTACAGTTAAACTGGAACTTTTTTGACGGTGGTGCGGCTAACGCAAGGGCAAGACA
AGCCGATGCAGACATTGCTAGAACCGATCAAGAATACTCTCAAGAAGCCGATTCCATACGCTTTC
AAGTAGAACGGTCATACTTACAATTACCCTCCCAAATCGAAAATATTGACACAGCAAGACAAGCG
GTCAATCGAGCCCAAGAAGCAGTCGAAGCCGCCCAAATTCGATTCAGTGCAGGTGTTACAACTCA
AACAGAAGTTTTAGACGCTCAAACTCGTTTAGTTCAAGCTCAAAATAATTTACTCAATGCCGTCT
TAGGTTATAACAGAGCTAAAGCAGAACTACAAAGAGCCGTTAAAAATTAAAAATAATTTATTAA
GTTATTTATTTTGTTGATTAAGATAAAAAAAGATTTTCAAGAGGATTTTTTTATGGCTAAAATA
ATTTCAATTCATTCCTACAGAGGTGGTACCGGAAAATCAAATCTCACTGCTAATATTGCCAGTAT
TGTCGCAAGTCAGGGAAATAGAGTCGGAATAGTAGATACTGATATTCAATCTCCGGGGGTTCATA
TTCTTTTCGGTGTTAATGAGAAAAAACTAGAATATACTCTTAATGATTTTCTTTGGGGTCAATGT
CCTATAGAAAAAGTCGCTTGTGATGTAACCCATGTTTTACAAGAAAATGCCCATAAAGATAGCAA
GATATATCTACTTCCTTCCAGTATGAAAATCAATGATATTTCTCGTATTCTCAGAGAAGGATTTG
ACATTGAGTTGTTATTTGATGGTTTTCAAAGCCTAGTGGACAAATTAAAACTAGACTATTTATTT
ATTGACACTCACCCCGGCTTAAACGAAGAAACTCTTTTATCTCTCACATTTTCCGATATTGTGTTT
GTGGTTTTGCGTCCCGATAAACAAGACTTTCAGGGAACTGCGGTAACGGTGGAAGTTGCACAAAA
ACTCAAAGTACCCCAAATATTTTTAATTATTAACAAGGCATTAGCGGCATATAACTTCATGAAAA
TGTCAGAACAAATAGAAAGTACCTATGGTCTTCCTGTAGTGGGAATTTTGCCAGAATCAAAAGAT
GTCATCAGACTTGCTAGTAATGGTATTTTTTCGTTGAAATATGAAGACCACCCCATTACAGAAAC
CATTAAGGAAATTGCCAAAACAGTAATGATTAAATAGAGGTTGACGAAAAACTGGTGTCACTGA
CTCAGATCTTGTAGCCAAAAATGGACTGGTGAAGGTAGAAAACAGGGGAAAGTAATAAAAATTG
ATAAAAAAATTATTAAAATTGATTTTAAAGGTTTCGTTTTCTAGTATTAGTGTACTGGTGCAAG

ATCTCAGACCGAGGGTAAGCAACGGAGAATAGCTAACGGACAACAGTTCAAAATATTTATAAATC
AAAGACTCGAACGGCAATAACTAATTTTTCATAAATTGTTATTTTGTATCAATTATGGCTCTATT
ACTTGTCGTCGTGTAAATTATTATTTTCGGTAGCGGATTTGGGAAACCCCTCTGTATCTCGCTTT
TTAAGGCAGGGCTATTTCATTCTAAAATCTTTGTTTAAGAGGGAGTGATGAGTAATGAATTAGG
GGTTTTTAATTATTAATTCTTAATTATCAACTATTTACCTTTGCCCCTTGCCCCTTGCCCTTTTTG
CCATCACTCATCGATAATTTATCCAGAACTCAAGTTAACTACTGTAGAACAGCCTCAATTATCTT
GATTCGGATTAGCTAGAAATAGTTACTAATTTTATGGATAAATATCCACTCAATTAGCTTTATAG
ACTTTAACGTCTAATGGCTTAACTACAAAGCTCACTATCGAGTCCTTAATCCTAAAAGATTAAAC
TTCTCGACTACTTTCTTTTGGTGGTTTTATTTCTAGCTAAAACTTTTACGTTGGTAGCCCAATAG
TTAAGGAAAAATTAACTGAATCCTTACCAACAACCCCATAGTTTTAAGGTGAAATATTTAATTTT
TAAGGTTTATTTAACCTTATTATAACATATTTGACCATAAATTCGGTGAAATAATTTAATACAGT
GTTTTTGGAGACAAAAAACTATTTTTTGCATTTTTTTCGGATGTTATTGTACTTAAAACTTTTAG
TTGAAAAGGGTTTTGATTTGTTCAGCTAATTCTAAGCAATGATTAATTAACTTAATATCATTTAT
TAGTGATAGCAATTTATTAACTTTCGAGGGAAAAGAAAGATTAAAAAAGAGGAAAAGTAATATT
AATCTACTTAAAATAGTGTTTAATTAATTGAAAAGAGTTATTAAAAACTTAATTTTTTTGCTGA
TAAAATTTTATTAATTAACAACTTCTATAAACTTATTTTGCTATATTTTCCTAGTTTAAACTGAA
TAAATATAGTTTAAATAATCGTATTTTTTGTTAACGCAATTTATATTTATAATCCATAAAACTAA
TTTTGATAATCCTAGAAAGATATTACTTTTTTCTTGAAACTTGAATTAAAATCGACAAATTGACT
CAAAAAAGCTCTTTTTTATCGGATATGAATTGGATATTAATTAAAATTTTTACTTTTTGGAGAAA
TATTTAAGAATAAATTAGAGAAAAGAAATTTGAATAAAAAATGAAATATAAGTAAGATGGACAA
AACGTAAAGAATCAAAAATAGTAAAAATAATGTTTGACAATTCTTTATTGAATCCGTCTTTTAA
GACAGTAAATATTCTCGCTCAGATTGAAAACCCCATAAACACATTCTCCTTAGATGGGGCATTGG
GAGAAAATCTGATTGTCATAGCAAAAGCCCTTTTAATTCTGGTAATAGGTTTGATTTTTGCCAGT
CTTTTTAAAGGGCAAGTAAAAAAAATGGTTACACAAAACTGAAATCGACAATAAAATTGCCGCTT
GGATTACTAATAGCGAAGACCCTTCTTCTTTACCGATTGAACAATGGATTGGAGAAATAGTCGGT
TGGATTATTAGTTTATTCGTTATTGTTGCTTTTCTTAACGCTTTAGATTTAGAAGTTGTTTCTCA
ACCTTTAAATGCCTTACTACAGGAAATAACTCAGTTTTTGCCTCGTATAGGGGGAGCCGTTTTAT
TGCTTGTATTGGCTTGGGCATTAGCCACAGTTATTAAACTTTTAGTGGTGAGTGCTTTAGGCAAA
CTAGAAGTAGATAAACGTTTGAATCAACAGATACAAGGGGAAAATTCAGAAACAGAAACTATTG
CTTTAAGCGAAACAATTGGTAACGCCCTTTACTGGTTCATTTATCTTTTATTTTTACCTGCTCTTT
TAGGTGTTTTAAGGTTAGAGGGAACTTTACGTCCTTTAGAAGAGTTAGTTAATGAAATTGTTGGC
ATTTTACCCAATGTTTTTGCGGCGATAATTATTGCGGCCATTGGTTGGGTAATTGCACAAATCAT
TAAAAAGGTTGTAACAAATCTGTTAATGGCTAGTGGTATAAATCAAATCGGAGAAAAATTCGGT
TTAGGCAGAAAGGAAAATAGTCAATCTTTGGCTCAAATAGTTGGTTCTGTGGTGTATGTATTAGT
CTTAATACCTATTGCCATAACTGCCCTTGATGCTTTGCAAATTGATGCCATTTCTACTCCTGCCAC
AGAAATGCTCAATCAGGTATTAAATATATTACCAAAACTTTTTGCTGCGATCGTAATTATTGGTT
TGGCTTATGTCGCAGGGCAATATTTATCTGAATTAATTACAAATCTTTTAACTACTGTCGGATTT
AATAATATTTTCATTTGGCTAGGAGTAACCCAAGAAAGTCCTCAAGCAGAAGAAACAGAAGAAG
CAGAGAATAATATCCAAACAACTACCCAGCAAACTCCTTCTCAAATTGTGGGGGTTATCGTCTTA
GTGGCAGTAATGTTAGTTGCATCTTTAACAGCAGTTGACATTTTGGAAATAGATGCCCTTAGAAG
CGTTGTGGCTTTTATTCTGGTTCTTTCAGGACAAATTTTAGCGGGGTTATTGGTTTTTTCCATTG
GCTTATACTTTGCCAATTTAGTATTCAGATTAATTGCTAACTCTGGCACTAATCAGTCGAATTTT
CTTGCCCAAACGGCTCGTATTGCTATTATTGTCATGGTTTCAGCTATGGCTTTAGAAAGAATCGG

TATTGCCCCAAATATTGTTAATCTCGCTTTTGGCTTATTAACTGGTGGTATTGCAGTTGCGATCG
CACTTGCTTTTGGTTTAGGAGGCAGAGAAGTGGCAGGAAAGGTTTTAGAGGATTGGGTGAATAA
TTTTAAAAAGTAGTAGTTTAGTAACCTCAGTTCGATTTAAGAATATCCGATAAGGTTAGGTGTCA
GGTGTCAGGTTGCAGGTGTTAGGGAGATGGGGAGATGAGGAGATGAGGGGAAGGGGTGAGAGGG
GGAAACAAGTAATGAGTTAGTTAGGGGCGAATGGCCATTCGCCCGTACAGGAGTTAAGGATTTTT
AATTCTTAATTCTTAATTCTCAACTATTCACTATTCGCTATATACCTTTGCCCCCTGCCCCTTGCC
CTTTTCCCCTTAATCATCCTTGATAGCCCTGTAAATTTTCAGGCTCAAAAACTTTTAAAATTCTA
GTAGCCTGATTTTTGGTCATTTGATCTCCTTCAACAATGATTAAATACTTACCCTGATTAAGACG
ATTACGATAAGGTAGAGCATCACCACTACCCTCTGCTAAACCGATACTTCCCCCCACAAAAAAAC
TACCCATAGCACCACCGATCGCACCTAAAAAACCACCTATAATATGGTTAAAAGGTTCACCAGCC
CAACCAAAGGTATCAATACTGGTAATGGCATCAAAAGTATAGCCACCAAAAAAACCAAACGGCAC
TAACCAATAAGCCATTCTAATGGCATTTTTACGCCCCTCATCTTCAGGTTTAATTAAACCAAACT
CATCGGCACTTTTATAACCTTTTCCTAAAATTGATATTTGTTTTGTGGGAATACCCGCTTTCTCA
AGCGCAGTGTAAGCATCTTCTGCTTGGATGCGATCGGACAATACAGCAACTAAGTAATTCATGTT
TTTAATTAATAATTAACAATTGAGAGGTGTCAGGTTTCAGATTTTATAAATGAATAGCCTGAAA
ACCCCTAGATTTTAATCAGGGGATGAAAGGCTTAAGGAGACTTGAGTCTCCCATTGTATCCTACT
CTTTTTGTGGTAATATTTTAATACAAGTTCAGTCAGCAATTAATGTAGTGCTATCCAATGAAGAA
TTATGGATGTCAGCAAAATCTGATTAATCCGAACCCTGATTTACTAGCAATACTAGAGTTTTTAT
GCGAGGAGTCAAATAAGCTCCATAATTGCGGAGTATATTTAGGAAGACAATTATGGTTTAAAGG
GAGGCGATACCTGAAAAAATTTGATCTCAATAAACTACTGAAAAGCAACAATCACTATCGGGTGT
TGTACTCACAGGTTGCTCAACAATTGTTGTTATCGGTAAAAGAATCGTTTAAATCTTACTATGAG
TTACAAAAACAATATCATAAAAAGACGATAACAGATAAACCCCGTCCTCCAAAGTATAGAAAGA
AAGGTGGTTTTTGTGGTCTAACCTATCCGAAACAAGCCCTTAAACTACGGGGTAATCAGATTCGT
TTGCCATTAGGGAAAACCGTTACTAGATGGTTTGGGCTGAGTGAGTTTTTTCTCCCTATGCCAAC
CAATTTATCTTTCTCTATCATCAAAGAACTTCGCATTGTCCCCCGTAATCGTGTTTTCTATGTAGA
ATATGTTTACGAAAAAGAAATAGTAATGCAAAAAAATGATCCTGAAAAGGTTTTGGCAATGGAT
CATGGTATAGGAAATGCCCTTAGTTGTGTTACTAATATAGGAAAATCAATCATTATCGATGGAAA
AAAAGCCAAAAGCCTAAACCAGTGGTATAACCAAGAAAGTTGCAAAACTAAAAGAAGGAAAGCCC
CAAGGGTTTTGGTCAGATGATTTGGCTCGTACTACCGAAATACGCAATCGTCAAATGCGTGATTT
CGTTAATAAATCTGCTAGATATATTGTGAATTTTTGTCGTCATCATCGCATAGGTATAATTGTCT
TTGGATGGAATCAGGGACAAAAGCAATCCGTAAATATCGGCAAGAAAAATAATCAATCTTTTGT
GCAAATCCCAATGGGTAAAATAAAAGACCGCATCCATCAATTGTGTGATGAACACGGTCTTAAGT
TTGTCGAATTACCAGAGGCTTACACTTCTAAATCTAGTTTCCTAGATAATGATTTGATACCTAAT
TTCGGTGAAAAACCCGAAGGGTATCAGTTTTCAGGAAAACGGATTAAACGAGGTCTTTACCGTTC
TAAACAAGGTTTTTTGGTTAATGGCGATGTAATGGCCGCCGCTAATATTCTCCGAAAATATGTAA
GCATACAGCCAGTGAATATCTCACTAGCCAAGGTGTCTAGGGCAGTTTTGACCCTGCCACAAAGG
ATAAACATATTTAATCGTGGTTTTAACCCGATTCAATATTCCTTTTTTGAATCCCCGTTACTTTA
GTACGGGTAGAGGTCAATTAATTCTCCTTACTCATGTACGAGCGAACGGCCGTTCGCCCCTACTC
ATTACTCCTTACTCAAAATTAACTTGCTCGTTTATAAGCCTCGTCTAATACCTCCGATAAAGTAG
GATGAGCATGGACATTAAAAGCTAATTCATGAACGGTTTGACGAGATGCGATCGCATTTGCCGCT
TCTTGAATTAAATCCGAGGCGTGAATACCAATAATATGGACTCCGAGTAATTCCCCTGTATCTTT
GCGGAAAATAATTTTAGCGATACCGTCTGTTTCTTTCTCTGCTAAGGCTTTGGAATTGCCCTTAA
AGTAGGTTCTAGCGGTGGCTACCTCAAAACCATCATTTTTGCCTAATTCCTTGGCTTGAGGCTCA

TTTAAACCCACATAGCTAATTTCGGGATGAGTAAAAGCGGCGGCGGAATACTACGATAATCAAT
GGTTTTGTCTCTACCGCACATATTTTCTACGGCAATGATACCCTGTCCTGATGCGGCATGGGCTA
ACATCATTTTACCATTAGCGTCACCGACTGCCCATAAATGAGGTACAACTTTACCATCTTTTAAC
ACTTGCATTTTATCGTTGACGGGAATAAAGCCCCTTTCGGTTTCAATGCCGAGATTTTCTAAACC
GAGGTTTTTGGTGGCAGGAATGCGCCCTGTGGCTACTAAACAAGCGTCAACCTCCAATACATCAA
CTATTTCTTTGGTTTTAGCGTCTGTTAATTCTATTTTTACTGGATTTCCGGGGGTAATTTTGGTG
GCAAATACGCCTGTGTAGGTTTCAATGTCACGGCTATTAATTAATACTCTTTCGGCAATTTTAGC
AATATCAGGATCAAAGGTAGGCATTAATTGATCCATGGCTTCAATCATGGTAATTTCTGAACCTA
AAGCAGTGTAAACATCAGCAAATTCTAAACCAATATAACCACTACCGATAATAGCAATCCACTGA
GGTAAAGACTCTAATTTTACCGCTTGATCGCTAGTATAAACGGTTTTTCCGTCCACTTCTACCCCT
CTAGGCACAAAAGGCACTGAACCCGGACACAACATAATATCTTTCGCTGTGATGACTTTCTCTGA
ACCATCATCCCCGATAACGATAACTTTTTGAGATTCAGCTACTTTACCCCAACCTCTGATAATATC
TACTTTCAAACGGGTTAAGCTATTAGTTAAATCTGATTGAATTTTACTAACTAAATCATTAGCAT
GAGATGCGATCGCACCTCTATCAAAACTAACACCACCGACAGTAACCCCCATAGACTGTAAATGT
TCAGTTTCTCTTAACTCTCTTACTTTTCCAGAAGCGGCTAAAAGGGCTTTAGAAGGGATACAGCC
ACGATTAACGCAAGTACCACCCATGTCTCCTGCTTCCACAATGGCAGTTTTCAAACCGCATTTAAC
AGCGTGTAAAGCGGCACCATGACCTCCGACACCAGCACCTATAATAATTAAATCATAATTAAAGG
ATTTACTCATAATACTTTATACTTTGTTTCTGAAATTGAGAAAAATAAACTATAACCCTTATAAT
ACCTTAGTTTGGGGATTAGGGGGATGAGAGTTCAGAGAGTTCGCTGCGCTCGTACACTCCTTTCA
GTCGTGAACGGAGTTCAGAGTTAATATAAAAAAGAAATTTCCCTCTAAAACCCTAATACCTGAAA
CCTGAAACCTCCCTTAACTTAACACATGAATAAGTGCGTAACGTCAGTTCTTAATTTTCAAAATA
GCTTGATTAAGTTCATTTATTTTCACTGGTTTACGAATATAATCATTCATTCCTGCAAATAAGCA
CATTTGTTGATCTTGATCTAAAGCATTGGCAGTTAAAGCGATAATAAAAGGTTGTTTGATGGACA
TTTCTCTAATTTTTTTTGTGGTGGTTAAACCATCCATTTCGGGCATTTGTACATCCATTAAGATG
ATGTCATAAGTCTTGTTTTCGATTTTGTTTAAGACTTCTAATCCGTTTTTGGCAATGTCTGCACT
ATATCCCAATTTTTTTAGAAAGGAAAGAGTCACTTTTTGGTTAACAAGATTATCTTCAGCTAAAA
GAATCTGGAGATTTGACGGTTTGATATTGTCTAAATTCTCAATAATACAAAAAGATTTTTTTGAT
GTTAAATAACATCTAAGAACTTCTTTTGCCCGAAATGTAAAATAAAATGTTGATCCCGGTTGTTT
TTGGCGATGATAAAACCAGTTTTCTGGGGGAAAACCACCGATATTACCGTTACTTTCAACCCCAAA
TTGTACCCCCCATTAGGTTGATCAAAGTTTTACTAATAGCTAACCCTAAACCTGTACCGCCATACT
TACGGGTAATGGAATTATCTGCTTGAGAAAATGGTTGAAATAAATAAGAGAGGCGATCGCGTTC
TATGCCAACTCCAGTATCTTCCACTCTGATCATTATTTCATATTCTTGCCATCCCTGTTGACTATA
AACATTTTTTATATCTGCATAAATCTTAATGCTACCAACGTGAGTAAACTTAATACTATTACCCA
TTAAATTAAGCAGTATTTGCCTGAAACGAATGCTATCACCCAATAAATTAGAGGGAATTTGTGGT
GCAATAAAACAAGAAATTCGGATATTATTTTTTTCTGCTTGGGGAGAAAGTAAATTAATAATAG
TTTTAATGATTTCCCGAAAAGCAAAAGGACGTTCTTCTAATTGTAAATTACCTGATTCAATTTTA
GAAAAATCAAGAATGTCATTAATGATAGTTAGCAAAGTTTCCCCACTATCGATAATAGTATGGAC
ATAGGTTTGTTGCTCTTCATTCAGATGGGTAAGAGATAATAGTTGTGCCATACCGATTACCCCAT
TCATTGGAGTGCGAATTTCATGGCTCATGTTAGCGAGAAACTCACTTTTAGCTTTTGCGACAATT
TCTGCTTTTTCTTTGGCTTTTGCTAAGTTTAACTGAGTTTTGAGGGCTTTATTATATTCGACTAA
AATTTTTTCCCTTTGTCGTCTATCTTCCCTTTGTTGTTTACTTAAATCCTTTAACTGTAAATTCTT
TGCTCTTAAGTTTCATTTGCTAAAGCTAAATCTTTTTCCGTTTGATTTTTTTCATTTAATACAG
CACTCAAAACCAAAGTTGTAATGGCAATACAAGCAATAAAAGATTGTAAAAAAAGTAAAGAACC

TCTAATATTATCTTGAGAAAAAGAAGTATTACCGTTGAGAGTTGTTACGCCTAAAGTAATAGCAA
TAATTAAAGTTAATAGTGTTCCACCTAATTCCTGAAACTTAAAAACAGTCCACACCAGTAAAGGT
ATAAGTAAATATTCAACATTGTAACCGATAGAAACATAATAAATGATAATGATAGTTAAAGCGA
CAATGATGACACTTTGCCAATAACAACGATTAACCTTCACCAAAAAACCTGAGAAATAATTATTC
CACGCAATAATTAAAGGGGTAAAAACCAAAATACCGAAGGCATCACTTAACCACCAAGTTAAAGC
TATATCAAAATATAATTCCCAAGGTGCTTTTCCTAATAGGCAAATGATTAAAGCACAGAGAAAAG
CTACAGGTAAGTGACTCAAGAAACAACCATAGGCAATAAACTTAATGGTATTTTCTATACGACAA
ATAAATTGATAGCTACCAACGGATAATTGTATTAAATACACAGCCAACAACTTACCTAAAGTGGA
AATTGCCGTGATAGTTAAAGTTAAAATTAGCTCAGGAATACCTAGCCAACTTTGATAAATCAATA
TTTCTGCCCCTAAAATGCCTATTAATAAAGCCCACCATAAAGAATCCCCCCAAATACAGATTAAA
CCTACTCCTACTCCTCCAGCAATCCAAATGGGAGTTGAACCTGTTTCTGGATAAGTAGTAAAGTA
ATCTGAAAGTATTGCTGTGACAAAATATGCGATCGCAACCGTGATATATTTCCAAACGGCGGGGA
GTGATTGCCGAGACAAAAATCTATTCTCTGATGATTCAATAGATGAAAAATTATCGGGGGAAGGC
ATTATTAAGGGTTTTGTGACAAGTAATTTAATTAAAAAGATTAAAAATATAAAGATACACCTTT
GCTGTCTCTATTTTAGACTAGATGTCAATTATGGCGAATAAACTTGTAATAATTTAAGTATTTTC
CGTATTATTCTCCGTAAAATTAACTAAGTAGCCTGAGTTCGGGATAAATTTTCATCTATGAATGA
TGGCGAAAAGAGCAATCATTTGAGTTCGGAGTTAAGATAAAAAAGAAATTTCCCGTAAGGGTTG
AATATATTCACCCCCAAACCCTAAGCCCCTACTTCTCCCTCTCACCCTCTCCCCTCATCACCTCATC
CCCCTAAAACCCGCAACCTGAAACCTGACACGTAACACCTAATCTTATTCGATATTCTTAAACAG
AACTGAGGCTATTTAATAATCGCCTGTTGCCTGTTGACTACCCTAACCAATAATTTACACGATGA
GAAGCAATAGAGCCTAATTTATGGATCTTCAACAATAGAATCTCCATTTATATCCACAGAGGAAG
AAGATTGATTAGGAATTGCTAAAACGATCGCATCTATGACCTTTCCGACACAGAGAATCTCTAAA
CCTAAATCGCTGGGGTAAGTTTGACCTTTAGGAACGATCGCTCTTTTGAAACCCAGTTTAGCCGC
TTCTTTAAGCCTTAATTCCATTTGTGAGACTAAGCGCACTTGCCCCCCTAAACCTATTTCACCAAT
TAAAACCGTACGGGGGTCAACGGTGCGATCGCGAAAACTAGCCACAATGGCAATAGCCATGCCTA
AATCGGCGGCGGGTTCTTCGACTCCTAATCCCCCTGCGGATGCTACATAAGCATCTAATTTGGAG
AGGGGAATTCCCACTCGTTTTTCTAAAACCGCTAAAATCTGTTGCAGTCGATTATAGTCAACCCC
TGTGGTAGAACGACGGGGAGAAGTATAACTGGTGGGACTTACTAAGGCTTGTAACTCAACAACTA
TACATCTTGTCCCCTCACAGGAAACGATTGTAGCCGTACCGGGGGCTAATTCATCTCTACTGCTTA
AAAACAACTCAGAAGGATTAGAAACTTCGTCTAAACCATGATCCACCATCTCAAAAATACCGATT
TCATGGGTTGCCCCAAAACGATTTTTCACAGATCTTAATAAACGATGGGAAGCGTAGCGATCGCC
CTCAAAATATAGTACAGTGTCCACTAAATGCTCTAACACCCTAGGACCTGCGATCGCACCTTCTTT
AGTTACATGACCGACGATAAATAAGGTAATTCCCTCTCTCTTTGCAACCTGCATTAAGGCAGAAG
TACATTCTCTTACTTGAGAGACAGAGCCGGGAGCAGAGTTGAGAGTGGCAAAATGAAGAGTTTGA
ATACTGTCAATAATCGCAACTTGAGGCTTGAGAGACTCCAATTCCCTTAAAATTTCTTCCAAATC
TGTTTCTGCTAAAACATAAAGATTCGGTTCACTATAGTCTTGATTAGTCTGATTCCCTTTGTGTC
CATTACCATTATGACTGTGATTAAGATCCTCCTCATCTTCTTCTATCACTCCTACCCCTAGACGAG
AAGCTCTTAATTTGACCTGTTGTCCCGATTCCTCGGCTGAAACGTAAAGAATGCGAGGAAAACGG
ATCGCTAACTGATTAGCAGTTTGTAACAATAGAGTTGATTTGCCAATACCGGGATCTCCCCCAAT
TAACACCAAAGAACCCGGAACAATTCCTCCTCCTAAGACTCGATCGAGTTCCTGATAGCCAGAAT
TCATGCGAGGTTGTTCTTCTTCTTTTATATCAGAAAACTTAACAGAAATTCTTGGTTGTGGGGAA
GTGACGTTTTTTTGGTGAGGACGAGCATTTGACTGCCAACCACCTCGGCTAAAGTTTCCGTTACT
ATTAAGGATGACTTCTTCCAAAGAACCGTAAGTATCGCAACTGGGACATTTTCCGAACCACTGAG

AAGATTCTGCACCGCAACAGTTACAGATATAAACAGTTCTGGTTTTTGCCATATTTTCTTTGCCC
TATTGCCAACAATTACCCCATAATGCTACCAAAAAAGTTCTTTCTACTATCAGGTTTTTACTAAT
TCTTAACTAAAATTTCATCTTCAAGAGATTTTAATTCACCAATTCACCTCAGTTCGGTTTAAGAA
TATCGAGTCAGGTGTCAGGTGTCAGGTTGCAGGTGTTGGGGAGATGAGGGGATGAGGGGAGAGG
GAGATAAGGTGTCGGGTTTCAGGTTTCAGGTTTCAGGTTTTAGAAGAAAGTAATGAGTAACGAG
TAACGAGTAATTATCAACTATTCACCTTTCTCTTCCCCCCTCGAGAGGGGGGATAAAGGGGGGTT
TGCCCTTTTCTCCATCATTCATAGATGAAAATTTATCCCAAACTCAGGTTAGCTTATCTATATAG
CGTTCCACTAACAGTATTGCAACTAGGTCATCAATGGGGCGAGGAGGAACTTTCAGGCTTTGAGG
AATTAATCTTCCTAAGCCTTTTGGAGGATACATTTGCCAGTATAGCCCTCTTGCTTCTAATGTGC
TGTTTTTTTCATCTACGAATTCAATAGCAGATGATAAATTTAGTTTATCTATTATTTTCTGTTTC
CACTCTTGAGAGGTGGTCAAATTCCCTATGATTAACTTATTGGGTTGATACTTAGACCACAATTG
TTGAATGGATGCGATCGCATCTTCACTTTTTATCACTTCATGCCATAAAATTTGCTTATCTATTG
TCACGATAGCCAAACCACATTTATCCCTACCCGGATCAAAACCTAAAAATATCATAAATGACAAA
CTCCGCAAAAATATAATAAACTTTTTACAAACTAGAGATAAAAATAGTATTACCCTCAAAAAGCT
AAAATATAAATAAAAAAGTAAACATCTGGAAAAAGGTAAAAAATCTGAATGTTAGAGCAAATTT
TACATCCTTCTATCGATTTCGGTATTGATACTGCTTTTATTTTAGTGATTTTGGTTGCGTTGGAA
GCTGTATTATCTGCGGATAATGCCATAGCTTTAGCCTCTATTGCTCAAGGCTTAAAAGACTCTAA
ACAACAACGATACGCCTTAAATGTTGGGTTACTAATGGCTTATGTTTTAAGAATAACCTTGATTA
TTACCGCCGCTTGGATTGTTAAATTTTGGCAGTTTGAATTATTAGGAGGATTATACTTACTCTGG
CTAACTTTTCGTTATTTCGTCAATTCAAATCAAGAAAATGGTGAAGACGATGAAAACTCAAAACT
GAATTTCAAAAACCTGTGGCAGGCAATACCGATTATTGCTTTTACTGATTTAGCTTTTTCCTTAG
ATAGTGTCACCACTGCCATTGCTGTTGCTGATGAAATCTGGTTAATTATCGCAGGAGGTACTATT
GGAGTTATTGCCCTACGTTTCTTAGCTGGTTTGTTTATCCGTTGGATTCAAATATTTACCCATTT
AGAAGACGCAGGATTTATTACTGTTGGTTTAGTAGGCCTCCGTCTTATTCTTAAAGTGGCTTATC
CTTCTTTAGTCCCTCCAGAATGGCTTATGATTGGTATTATTATTGGAATGTTTACTTGGGGATTC
TCGGAAAAGAACGATTTAGAAGTTAAAGATTCTCCTGAATCCTAAAAATATATTTGTAACATTTT
TGCAACAATAAAAAAGAGAAAGGAGCGGAGGAGAGGGGGAAACAAGTAATGAGTAATGAGTAAT
GAAAGTTAGGGGTTTTTTATTCTTAATTCTTAATTCTTAATTCAATAAGGGGATTGATGTCCATT
TCTTTCCGTAATATTGCCTTAGTGGCGATAACTGTTTTACTAATTCTTCTGCTGTGGCAGTTAAG
AAGTTTACTGGTTATATTAATGATTTCCGTTGTTTTGGCGGCTACCCTTGCTCCTATTGTTGATT
GCGGGGAAAAAATGCGTATTCCTCGCTGGTTAGGAGTTATTTTGGCTTATTTATCTATTATCTTA
ATTATTACAGGGGGAGGATTAGTAATTGGGCCTACTGTCATAGCTCAAATAGAGCGGTTATTACA
AAAACTTCCCGGATATTTGGACATTATTACAAATTTAACTCAGTCGTTAATTATTCGTTTTGGTA
TTACTGAGCCTCAAGCCTTAAATTTAATTGATCAGTGGCTTGATTTACAGGCTTTAGCATCTTGG
GCAGTGCGATCGAGTCAGAAGGTCATTTTAAGTTCTTTAGGGCTTACAAGAGGCATTGTCGGAGC
TATTTTTAATATCTTATTATCAATTATTCTTTCTGGTTATTTATTAGCTGGTTCAAAAAAACTAA
TTAAAGACTTTGTCAGTATTTTCCCTTCTCCTTGGGATGCGAAATTAGAAGCCCAATTCCCACCT
ATGAGTGATCGCATGGGAAAATATATTCAGGGTAGAATTTTAGTTTCTCTTATCCTTGGTGTTGC
CATTACTATTGGCTTAAAATTCATCGGAATCAGTGAATTTGCCCTTGGTTTAGGGGTTATTGCTG
GTTTTACTAACCTTATTCCTTTTTTTGGTCCTGTTATTGGCTCTATCCCCGCCTTAATAGTTGCGA
TCGCCCAAGGGGGGTTAACTTTTTGGTGGGTTCTACTCTTATTTGTTATTATTCAAAATGTAGAG
ACTTATGTACTAGATCCTTTATTAGTAGGTTCGAGTGTAGAAATTGAGCCTTTATACCAACTCTT
AGCAGTGTTAGGTGGGGTGCAAGTTTTGGGTATTATTGGAGCATTAATAGTTCCTCCTTGGGTAG

CAGGGGCAGGGGTGGTATTAGAAAATCTTTATCTCAAACCAAAACTGACGAAAGAAAATGCCAGT
AGTTAGTCTCTGATTGTTTAATCTCCTGATACTCAATACTATAAACAAATTGTCGAAAATCAAGT
TATCTTTAATGTTATAAGCCTCCATCGAAGCTAAACCCACTGACTTTTATACACCCATGATTATT
AGTTATTGTCTAAATCCTAATTGCTCCCAGCCGAAAAATCATCCTAAACTAAAACAATGTTATAC
CTGCGGTGCAAATCTGATTTTAAACAATCGTTATCGTGCCATTAAAGTTTTAGGAAAAGGAGGAT
TCGGTGCGACTTTTATTGGAGTGGATTTGACTGTAACTGAAAATCCTTTGTGTGTTATAAAACAA
TTACGCCCCAATGCAGATGACCCCCAAGCGGTTAAAACGGCAATTAATTTGTTTGAAAGAGAGGC
GAAAATTTTAGGTCAGATTAATCATCATCAAATACCAAAATTATTAGACTATTTTGAAGATCAA
GGACAATTTTATCTAATTCAAGAATTAGTTAATGGTCAAAATTTACAACGAGAAATAAAATATG
AAGGGGTTTATGGAGAGTTAGCTTTACGAAGATTTCTCCTAGAAATTACCCCTGTTTTAAAATAT
GTCCATTCCCAAAAAATCATTCATCGTGATATTAAACCTGCAAACATCTTACGTCGCAAAAAAGA
TGGGAAACTAATCTTAATTGATTTTGGTGCGGTGAAAGATGAAGTTAATACCAAATTGGCGAAA
ACCTTTGGGCAAACGGCATTAACTAAATTATCTGTGGGTACAATGGGTTACGCCCCCCCGAACA
ACTAGCCATGCGTCCAATATACTCTAGTGATATATATGCCTTGGGAGCAACGTGCCTTTATTTGT
TAACAGGGAAATCACCGAAAAATTTTCCCATCGACTCGGATAACGGTGAATTATTATGGCAACAG
GAAGTTTCCCTGAGTGACAAAATGGTTAAGATTTTAGAAAAAATGTTGGCTATTAATACTAGAG
ATCGTTACAAAAGTGTGGACGATGTGATTATTGCCCTTGACGTAGAATCCCTTGAAGGTAATATG
TTAAGTATTCCTACTCCTGTATCTAGCGTTGGAGCAAATATTTCTTCTTTGTCTGAAACGGAAAT
TACCGTTGGTGATATTAGTAAAACCATGTCTCCTGCCCAACAATTAAGACAGGCAATCCAAAAAC
GAAAAACAAAAAGCGATCGCCCCAAGAAAATGTTATTAAAATGGAATGAGGAAAGATTTTTTAA
TGCTCACAACAACGGCAAAAAAGACTTTAGTGACCAAGAGTTGCAAAACCTCAATCTACAAGGAA
GCAAATTAATTAAATGTATTTTTCGCTATTCTCAATTACAAGGGGTTAATTTTAAAGAGAGTAGT
TTATCTCAAGCCAATTTTTACTCTGCCGATTTGACGAATGCTAATTTTATTAATGCCAATTTGTT
TCAGGCTTATTTTTCCAAAAGCAACCTTGAAAACGTTGACTTTACAGGGGCAAATTTAGGCAGTG
CCGACTTCACTAATGCCAATGTGACTAACGCTAATTTTTCGGGGGCAAACTTAAAAAATGCCAAA
ATTAATCAGCAACAATTAAAATCTGCAAAGGTAAATCGAAATACAACGTTTCCTGATGGTAGTCG
TCTTTGGTGGAAGATTTTTTAAGTGAACGAATTTCCTCAAACTCTTAACTTCACTTGATTTCAAT
GTGAAGTTGTCTGCTGGTCGTAGGGGATTTGGGGAAACCCTTTTATTTTATTAGGCGATCGCCTT
TATTTTTCTTTTAAAACTTGAAAATGAAACAGCCCTGCTCCTATTTAGGGGGGATAAAGGGGGGT
TTACCCCTTGCCTTCTGCCCTTTGCCTTTATTAACTATGAATTCTCGGTTCAGGATGAAGGTTACT
TAAAGTATCACTTTCTAGTTGTGCCAATTCAGATAGGCGTAAATCAACAATGGTGCGATCGAAGT
ATTGAGTAGCCAATACCCAGTAAATGCCATAATGTCTAGCTTCAGAAGCCATTAACCCTCGGTAA
AACTTTGCTAACTCTAAATCAGGGCAATGTTGAGCAAGTAATCCTAATCTTTCATGCGATCGCGC
TTCAATTAAAGCAGAGACAAGCAGAGAATCTAAAAGCCTGTGAGGTTCTTGTTTTCTAATAGCCT
GTTTCAAAGTAGCACCATAAGGAGAAGGTTTTAAGGGAGCAAGAGGAATATTTTTTCTCTTTAAC
CATTGATTTACCTGCTCAAAATGTTCTAACTCCTCCTGTGCGATCGCTGTTAACTGATAAACTAA
ATCCTCATGGGCAGGATAGCGAAATAAAAAATTCACCGCCACCCCCGCCGCTTTGCGTTCACAAT
GAGAATGATCAAGTAAAATGAGATCAAGGTTATCCAAAGCCTGTTCTAACCATGCAGACTGAGTG
GGTTGACAAAGTAAGTTAATTTTAGTGAAACTAGACACGAATTTGTAAGCAATCAAAAAATAAC
CAGCCTACCCATAATAAAATAAATTTAGTTAGACTAGGATAAAAACTTATATATTTCTATTAGTA
AACATAAATATCTTAAATAAATAGAATGAGTAATAAAAGATTGGTTATCGGAGATGTTCATGGT
CACTACGAAGCATTATGTTCCTTGTTTAATCACATTGCTCCCACTTCAGAGGATCAAGTTTATTT
TTTAGGAGATTTAATTGATCGTGGTCCTGAAAGTTCTCAGGTGGTAGAATTTGTCATGAAAAATG

ACTACCATTGCATCCTTGGTAATCACGAAATTATGTTACTCGATGCGTTAGGAAACGGAAAACTT
AATCATCAAGCCTTCCAATCGTGGTTACAAAATGGAGGCAACGCCACTGTAATGAGTTATGGTCA
TAAAATGCCTTCGGCTGAACATATAGACTGGATGAAAAGTCTTCCCTTCTTCTTTGACTTAGGAG
ATTATTGGTTAGTTCATGCTGGAGTTGACCCCCGTTTAAATATTACTCAACAATCCCCAGATCAA
TTTTGTTGGATTCGCTCAGAATTTCACAGTAGTGAAAAGCCCTATTTTCAAGATAAAACAATCAT
CATTGGTCACACCATAACCTTTACCTTTAACGGACTAAAACCGGGTCAAATTGCCAAAGGAGAAG
GATGGCTCGGCATAGATACAGGAGTTTATCATCATGAGCAAGGATGGCTAAGTGCCTTAGATTTA
AACACGAATACTGTTTATCAGGTAGATAGTTTTGGCAAAAATTTCCGTTCTCTTCCTCTGTCTGA
AGTAACCCATAACGTTAACACTCGTAAATTGTTCGCCCGTCGTCAAAAGGTGTTGTTTTAAGTTT
TCAGAAGTTGAAGGGATGATTTCAGAGAATTGCCTCCGACTCTGACACTCCCTTCTGCGGTTCAA
ATCATTTTTTCTGAGCAAAATAAGCCGTTAAAACTTTCTGTACAATGGGACCTGCGGTTGAACCG
CCTCCACCTCCTGAATGCTCAACAAAGGCAACCACAACTATTTCAGGGTTCTCATAGGGAGCAAA
TGCTCCAAACCAAGCATGAGATTTTCCCGGTGGTGCTTCGGCTGTACCACTTTTTCCTGCTACTGC
CACTCCATCTACTGCCGCTTTACCCCCTGTACCGTTCGTAACGACGGCTCTTAAACCTTCTCTAAG
GGTTTTTAAGGTTTCAGGCTTTAAGTCTAGGGATTTACGTTTTTCAAGGTAGGAAGGATCTTGAC
GCAGGTGAGGTACTACAAGATTACCCCCATTGGCAGGAACAGCAAACATAACTGCTACTTGTAAG
GGTGTTGCGAGGGTAAAACCTTGTCCGATAGACATATTAACTGTATCGCCTACAGTCCATTCCCA
ATTAAATCTTTCCTTTTTCCACTGACTGTCAGCAATTAAACCGGGGGTATCTTCTTCTAATTCAA
TACCTGTCTTACTGCCAAAGCCATATTTTCTTGCCCACTCAATTAGGGTTTCTCCTCCCACTCCAT
TGCCAATTTGTCCAAAAAAAGTATTACTACTCCAAGCCATTGCCCTGACGAAACCCATGGGGCCA
AATCCAGATCGATTCCATTCTCCAAAGGCTGTGCCACCAACTCTTAAATAAGCATAGGTAGGAAG
AATTGTACTGGGTTTATATTTGCCTGATTCTAATCCCGCAGTGGCAGTTACGATTTTAAAGGTAG
AAGCGGGAGGAAATCCTCGTAAACTACGATTAATAAAAGGATTACCTTTTCCTTGTACTTCTTTC
CATAACTGAGTGGTGATTTTTCCAGAGAAAATATTGGGATCAAAACCGGGATAGCTAACCATTGC
TAAAACACTGCCATCACGGGGATCAAGGGCAACAACTGCTCCTTTGCGATCGCCTAATGCTTGTT
CTGCGGCTTTTTGTAAATCTAAATCAAGAGTTAAAGTGACATCATTTCCTGCTTTTGCTTCTTTC
AGCCCTAATTTTCCTAAAACTTTTCCTGCACCATCTCTTTCTAAAAGTATTCCTCCCCATTCTCCC
CTCAAATCGGACTCAAAAGCAGACTCAGCTCCCATTTTACCGATGACATCCCCTAAACGATAACCT
TGCTCTTTTCTTTGGGCGAGTTCTTCTGCGTTAATTTCTCTGGTATATCCTAATACATGAGAACC
ATTTTTCCCATGGGGATAATATCTGATGGTATCGGTATCGACTTCTACTTCTGAGAGTAAATTAC
GATTTTCTTCAATAGCCGTAATTTGTTGAGGGGTGAGGTTACGGGCAATACGAACTAAGGTAGGA
GAGTCGTAGTTTTCTTGTTCGAGGGTTTTTTGTAACTCTGTTTCAGGGATATTAAGAATTTGTGC
TAATACAGAGCGAATTTCCGCCCAATTTTCTTGTTTTCGTACAATAGGCCATAAATAAGCTGAGT
GGGATAAACGGGTAGCCCCTAATATTCTGCCTTTACGGTCATATAAATTACCTCTGACGGGGGGT
TTAGGGAGAATTTTAGTACGATTGTTTTCTGCTTTTTCCTGATATGTATTCCCCTCGACTAATTG
TATATATGCTAATCGAGATGCGATCGCACCAAAAAAGAAAAGACTAATAAGCAACATGATAAAT
ATAGGTTGGTCTTTTTGTCCCACATTCAACCAAATTTTTTGCCGTTGATTACGAGTATCAGAAAA
AGTAACTTGACGACTTTTTAAGGAAGATTTCTTAGACTTTTTGATAATTGGGGTTAGTTGCATAT
TATTTGAGATGGGGATGTTTATAAATCACAGGGTTAAGTCCAATAATTGCCTACGGCAAACCCTC
TTTAAAGAGGATAATTTATGACAATCATTTATTATATTAGAGGGCGTTGGAAAGAAATTAATTA
ACCTGAGTTCGGGATAAATTTTCATCTATGAATGATGGAGAAAAAAAGGCAAAAGGCAAGAAGC
AAGAGGCAAGAGGGGATTATAATAATTTATAAACTTTTAGTTTTTGTTTTACTATAAACACTAT
TCAATAAAGGTTATAGAAGTTATTTTCTGTTAATTTATCATAACCACACTCTGAAGAACCAACAT

TCTTATATAGCCGTTTATTCTTCATCTTCCCATAAGCAAGAATTTTGTCGGATACTTCGATAATC
TCCATTACCGATAATAAGATGATCTAAAATGGGAATGCCAATTATTTTTGCTGATTCCAATAATC
GCTTCGTTAAGTTCAAATCTTCGATGGAAGGCTCTAAATTTCCCGAAGGATGATTATGAGCAATA
ATTATATTTGTAGCAGACTGCCTAATCGCTTCTCGAAACAGCTCACGAGGATGAATTAAAGTTTC
TGTTGCAATACCAATCGTAATAACCTGAGTACTGATTAAACTGTTTTTGTGTCTAATAATAGCA
CGGCAAAACGCTCTTGAGATTGCCACATTAGTTCGTGACTGAAAGCCGCCGCCGCCGCATTTGGA
CTGTCAATAATCGCCCTTGCACAGGGGCGAAATTGAAATGCTCTTTTTCCTAATTCTACACCAGC
AAGAATGGTTGTGGCTTTAGCAGGACCTATGCCCTGAATCTCCATTAATTCTGTGGGAGAAATAG
TACGTAACATATCCAAAGGATCTTGTTTATTTTGACCCAGTTGATTCAAAATATATTGAGCTAAT
CCTACCGCCGATAGTTTGCCTTTTCCTTGCCCTGTACCTAATAAAATGGCAATTAACTCCGCCGAG
CTTAAATTCCTTGCTCCTAATTCTAATAATCTTTCTCTAGGGCGTTCACTTAAAGGTAAATCTGC
GATCCTCAGATTATAAACCATGTCATTGTTACAAAATGAAATCTTAATCTTTATTGTAACCTCGT
TTGTCGATTTAAGATCTGGTTTCAGAGTTTTTAATTATTCACTATTCACCTTTGCCCTCCCCCCTC
GAGAGGGGGGATAAAGGGGGGTTTGCCCTTTAATGACTTGTTAATTGCCACTGAATGCTTCCAAA
GCTGACTTAAGTGCGATCGCAGTGTGAGTCCAATGAGTTCCTCCCTGACAAAAAACAATGTAAGG
TTCACGTAAAGGACCGTCGGCAGAAAATTCAGAAGTACTACCGTCAATAAAAGTACCTCCTGCCA
TAACTAAATTTGTATCATATCCGGGCATGGGTGCAGGTACAGGGTCTAAATATGAACCGATGGGG
GAATTTTTTTGAATGGCACGGCAAAAAGCTACTAATTTATCAGGGCTACCTAGTTTAACTGCTTG
AATAATATCTCGACGGGGAGTAAAAGGTAAAGGGTTTACTTCATAGCCCAATTCAGAGAAAACAT
AAGCAATTAGGTGACTACTTTTCATTGCTTCGGCTACCATCTGAGGTGCAAGAAAAAACCCCTGA
AATAAAAGGCGATTTTGCTCAAATGTTGCCCCTCCACTGCTACCAATACCGGGGGCTGTTAAACG
ACAACAGGCTTTTTCTACTAAGTCTTCTCTTCCTGCGACATATCCTCCTGCGGTAACGATTGTACC
ACCCGGATTTTTAATTAATGACCCTGCCATCAAATCGACTCCCACGGCGGTGGGTTCACTTTTTTC
GATAAATTCTCCATAGCAATTATCCACAAAGCAGACAGTATCGGGATTTTGTTTTTTTACTAACT
GTACAATTCTGGCAATATCGGCAACAGAAAGACTTTGTCGCCAAGAATAACCACAAGAGCGTTGA
ATTAATACTAATTTGGTTTCTGGTTTGACGGCGGTGGCTAAACCCTCCCAATCAATGCCTCCTTC
ATCTGTTAGGGCTAATTCTCGATATTTTACCCCCCATTCTTGTAGTGTACCTTGTCCCTCGCCCCG
TACTCCAATTACTTCCTCTAGGGTATCGTAAGGATGTCCTGCGACTGCTAACATTTCGTCATTAG
GACGTAATACCCCATAGAGACAGGATGCGATCGCATGAGTTCCCGAAACAAATTGTATTCTCACG
GCGGCTTTTTCAGCCTCAAAAACTTGAGCGAAAATTTGGTCTAATACGTCTCTACCCAAATCATC
GTGACCATAACCGCTTACACTAGAAAAATGATGCACTCCCACTCGATGATCTCGAAATGCTGTTA
AGATTTTGTTTAAATGATGTTTTACCTGTTGATCAATTTCTGCAAAGGTAGGTAATAGTTTTTCT
TGGGCTGAATCAATTAATAAATCAATATTCATACTGGATGTAAATGTCGTTTAATATTTGTCGTT
GAATTCACGAATGGAAGCGTACTGCACCGATTGCATCTTATAATGAAGATAACTAAATATCTATA
ATTTATCAATTTAAGGGATAGGGATTCATCGTGTTTACAGCTACCACTTTACCTAACTCAAACTT
GCCTCAAGATTTATTTGGTGCGATCGCATCTTTGAAAAAAGAATTAAATGCCGTTATCCTCGCTC
ACTATTACCAAGAAGGAGATATTCAAGATATAGCGGACTATATAGGAGACTCTTTGGGATTATCT
CAACAAGCCGCTTCAACTTCTGCGGATGTTATTTTATTCGCAGGAGTCCACTTCATGGCAGAGAC
GGCAAAAATCCTCAACCCCGATAAATTAGTACTTTTACCAGACTTAGAAGCAGGTTGTTCCCTAG
CTGACAGTTGCCCCCCCGATAAATTTGCTCAATTCAAGGCACAACATCCAGACCATATCGTTATA
TCTTATATAAACTGTACCGCCGAAATTAAAGCATTAAGTGATATTATCTGCACCAGTTCCAATGC
CGTTAAAATTGTACAACAAATCCCCCCAGAACAACCAATAATCTTTGCACCCGATCGCAATTTAG
GTCGTTATGTAATGGAACAAACAGGGAGAGAAATGGTATTGTGGGATGGTAGTTGTATTGTTCA

TGAAACATTTTCTGAGAAAAAAATTGTTCAACTAAAAATAGAACATCCCCAAGCTCAAATCCTTG
CTCACCCTGAATGCGAGACTCCTGTTTTACGCCATGCAGATTATATCGGCTCCACAACAGCATTAT
TAAAATATGCCATGCAAAGTAATTCTTCCGAATTTATTATAGCCACTGAACCCGGAATTATTCAT
CAAATGCAAAAAGATGCCCCCCATAAATCTTTTATTCCTGCCCCCCCAGAAAATAATTGTAATTG
CAACGAATGCCCTTACATGAGATTAAATACCTTAGAAAAAGTTTATTTAGCTCTAAAAAATCGTA
CTCCTAATATTGAAATTCCCCCTAGCATTCAGCAAAAAGCCTTACAACCTATTCAGAGAATGTTG
GCTATGTCTTAATATTAAGTACTAAAGCAAAATAGATTGTATATTTTAAAATTAATGAAAATAA
CCCATAATCTTTGTTGCCTCTTGCATTTCATAAAGTAAATACATAACTAATTTTGCCTACCTACT
TAAGAGTGCGATCGAGCGTTTGTATGAATATTAAATTGCAACTTGTAGTACAAAAACGAAGAAA
TACATTTTATGTAGTTACGCATTTATACTGGGAATAAAAATTCGATTCTCTTTTCCCTAATTACT
GCAAGATGTCTATTGTTAAATTTGTTATAATCCAAGCAAATTTTTGATTTACTTATAAAATAATG
ATGTATTCAGAAATACTAAAACAAAAATTATTAACCACTTTTTTAGGTGAAAAACTATTAAAAA
TAAGAAATTTTTACGAATTATGGGAATGTTCTTGGAAAAACCCAGAAAATTTACCAACATTAGTT
AATGATCAAATATCATCCACAATTCTTACTAAACTTTGTGAAAAAGATAAAGTTTTTATCGATGT
AGGTTCTCACATTGGTTCAATTATTTCCGCAGTTAAATCTTACAATTCTCAAATAGAAATAATAG
GCATTGAAGCAATTCCCGAAAAAGTAATAAAATTAAGAAAAGCATTTCCCTTTGTGGAAATTTAT
GAATGTGCAGTAGGAGATCGTGAGGGAGAAGTTTGTTTTTATATTAATACCCTGAAATCTGGTTA
TAGTTCTTTAGGAAGAAAAAAAGATCCCAGAAATCAAGAAGAAATGAAAGAAATAAAAGTTCAG
TTAAAAACTTTAGATAATTTAATTCCTTCAGATAAAAAAGTTGGAGTCATTAAAATAGATACTG
AAGGAGCTGAATTGGGGGTGTTGAGGGGGGCGGTAAACTTAATATCAAGGAATAGACCTTTGAT
CATGTTTGAAAGTGGTCCCAATAACAATGATCTTGGCTACACAAAAGAAGATATGTGGTTATTTT
TTGAGAATATTAACTATCAGTTAATCATTCCTAATCGATTAGCTCATAATGGAGATTCTCTAACT
TTAGAAGGATTTATTGAATCCCATTTATATCCTAGAAGAACTACTAACTATTTTGCTATACCCAG
TGAAAAACGTGAGTTAGTTAAGAAATTAGCAAGAAATATTATGAATGTACAAGTTTAGTCACCT
CAGTTCGATATGAAATTTTATCATTTTGTTAAGTAAAATATCCTATAAAAACGGATTTACCCCCA
AGATTTATTTGGTGCGATCGCATCTTTGAAAAAAGAATTAAATGCCGTTATCCTCGCTCACTATT
ACCAAGAAGGAGATATTCAAGATATAGCGGACTACATAGGAGACTCTTTGGGATTATCTCAACAA
GCCGCTTCAACTTCTGCGGATGTTATTTTATTCGCAGGAGTCCACTTCATGGCAGAGACGGCAAA
AATCCTCAACCCCGATAAATTAGTACTTTTACCAGACTTAGAAGCAGGTTGTTCCCTAGCTGACA
GTTGCCCCCCCGATAAATTTGCTCAATTCAAGGCACAACATCCAGACCATATCGTTATATCTTAT
ATAAACTGTACCGCCGAAATTAAAGCATTAAGTGATATTATCTGCACCAGTTCCAATGCCGTTAA
AATTGTACAACAAATCCCCCCAGAACAACCAATAATCTTTGCACCCGATCGCAATTTAGGTCGTT
ATGTAATGGAACAAACAGGGAGAGAAATGGTATTGTGGGATGGTAGTTGTATTGTTCATGAAAC
ATTTTCTGAGAAAAAAATTGTTCAACTAAAAATAGAACATCCCCAAGCTCAAATCCTTGCTCACC
CTGAATGCGAGACTCCTGTTTTACGCCATGCAGATTATATCGGCTCCACAACAGCATTATTAAAA
TATGCCATGCAAAGTAATTCTTCCGAATTTATTATAGCCACTGAACCCGGAATTATTCATCAAAT
GCAAAAAGATGCCCCCCATAAATCTTTTATTCCTGCCCCCCCAGAAAATAATTGTAATTGCAACG
AATGCCCTTACATGAGATTAAATACCTTAGAAAAAGTTTATTTAGCTCTAAAAAATCGTACTCCT
AATATTGAAATTCCCCCTAGCATTCAGCAAAAAGCCTTACAACCTATTCAGAGAATGTTGGAAAT
GTCATAAAAGAGGATGTTATTGGAATTAGTAATTAACAATGAAGAAAAGGGCAAGGGGCAAGGG
GCAAAGGCAATTAATAAAAAATTAATAAAAGAATAGACTCCTTGTCATTCTTTACAAAACTTAA
TAGAATAGGAATCATACCACTAAGTATAAATTCTTATTATCATGTTTAGTGCAGAATTTCCTTGG
CTAACAGCCGTCATTGCCTTACCCCTTATCGGTGCTTTTGCAATTCCCCTCATTCCCGATAAGTAC

GGAAAAAATATTAGAAATTATAGTTTGACTATTGCTTTAATAAATTTTTGCTTAATCGTTTACGG
TTTTTGGCAAAATTATTCTATCTACGACACAAATTTTCAACTACAAGAATCTTATAGTTGGTTTC
CGCAAATTGGTTTAACTTGGAGTTTAGGGGTTGATGGTTTATCCATGCCCCTAATTGTTTTATCT
GGCTTAATCTCTACCCTTGCCATTCTCGCTTCTTGGCAAGTAGAAAAAAAATCGAAATTATATTT
CTTCTTATTGTTAGTTTTATATAGCGCTCAAATTGGTGTTTTTGCTGCTCAAGATTTATTATTAT
TCTTTATTATGTGGGAATTAGAGTTAGTTCCCGTTTATATTTTAATTTCTATCTGGGGTGGGAAA
AAACGTCTTTACGCCGCCACTAAATTTATTCTCTACACTGCTTTAGCTTCTATTTTTATCCTTGTG
GCAGGTTTAGCAATGGCATTTTATGGGGATAATTTCACCCTCAACATGGCTGAATTAGGCATGAA
AAATTATCCCATTGCTTTAGAAGTATTAGCTTATGTGGGATTTTTAATCGCTTTTGGTGTGAAGT
TACCTATTTTCCCTTTTCACACTTGGCTACCAGATGCCCACAGCGAAGCATCCGCTCCAGTTTCCA
TGATTTTGGCAGGGGTATTGTTAAAAATGGGTGGTTATGGTTTAATTCGTTTCAATTTGGAAATG
TTACCAGATGCCCATATTAAATTTGCTCCTTTATTAGTAATTTTAGGGGTAATTAACATCGTTTA
TGGTGCTTTTACTGCTTTTGCTCAAACTAATTTAAAACGTCGTCTTGCTTCTTCTTCCATCTCTCA
CATGGGTTTTGTCTTAGTAGGTATTGCCGCTTTTACCGATTTAGGCATGAATGGGGCAATGTTGC
AAATGTTATCTCATGGTTTGATTGCGGCGGCGTTATTTTTCCTCTCTGGGGTAACTTATGAGCGC
ACTCATACTTTAATGATGGATGAAATGGGCGGTATGGCAAAATCCATGCCAAAAACTTTTGCTTT
ATTTACTGCCGCCTCTATGGCTTCTTTGGCTTTACCCGGAATGAGTGGCTTTGTTAGTGAGTTAA
GTGTATTTTTAGGTTTTGCCCAAAGTGATGCTTATAGCTCAGTATTTAAGGTTGTGGTAACGGTA
TTAGCGGGGGTTGGTTTAATTTTAACTCCTATCTATTTACTGTCTATGTTAAGGGTTGTTTTTTA
CGGCGAAAATGTTACCGGTTTAAAGTTAGATGGTTTTCAACTTGATGCTAAACCCCGTGAAGTTT
TTATTACCGTTTGTTTAATTATACCTATAATTGCGATCGGACTTTATCCTAAATTGGCAACGGAA
AGTTATGATTTAAAAACTGTGCAAGTAGCCAGTAAAACAAGGGCTTCTTTAGAGGTAATTGTCAA
TCATTCTGTTGAGAATATTCCTGCTAATTTTACAATTGCAGAGATTAATAATTAACCTCACTTCC
GCTTAAAAATATCAGAAAGGGCAGGTGTCAGGGTATTGGCGATGGTGGAAAAGAGTGAGAGGGG
GAAGTAAGGGCTTAGGGTGGTAGGGGTTGAATATATTCAACCCTTACGGAAATTTCTTTTTTAT
CTTGTAGTTATTTAAATTAACTTTGAGACACTTTTAAAAATTTAATTCTTTGATAAACAAGAGCT
AGAGATTTTGATTTTGTTTCATTCTCTATTTAAAACGACTTTAACTCCAAACTCAAATTATTGTC
TTTTGCCCCTTTCATTTTCAACTTTGCCTGTTGCCTTTTCCCTATTCCCTTGTCTTAATTCCAGTT
ACTCGTTGCCCTTTTGATTTATTGATGTTGGGATTTAAGTTTTTCTTTTTCCCATGATAAAGTTT
GTTCTAACTCCTGTAATTCTTCTCGTCTTGTTTCCACTTCTAAGCGAATACGAGTTAAATCTTGAC
TCTGAGAAGTCAACTCTTGACGCCACTTTTCCAGCCTTGCCTCTTCTTCAGGTATGTTTATATTAC
TGCGAAAATTCCCTTCAATTAAATATTCTTTGAGTAAATTTAAAATCCAGTCTTTGGCTGATTGA
CAACGCAAAATTTGTCCTTGATTATTTAATTCTACTAATAATAATAATCCCTCTTTAAAATTATC
ATCATTGTCTAAAGGGATATATTGATTGTTGCAAAAAGTCCATTGTAATTTAGACTCCTCCACAG
CAAGAAGATTTAATAAAAGTTTTCCTGTGTCAGTATCTCTAATAACTTCGGCTAAATGCAACATC
TTTGGTAATCAACTTAGGGTTTAGGTTTTGGGGGAAATAGAGGCTTAGGGTGTTGGGGTTTTTG
GGGATTAGGGAAGACGAGTTCGAGGTTCGGGGTTTTTAATTATTAACTATTTACCTTTGCCCTCC
CCCCTCTTGAGGAGGTATAAAGGGGGGTTTGCCCTTTGCCCCTTGCCCTTTTAACTTTGTCTTTTT
CCCTTTACTCTAATTATTTTAGTAATTCTAGGCGTTTTTGTAAGATTTCTCCCTGAATTTTGCTT
TCTGCTAAGGCATTACGAGCATTTTCGATAATGTCATTGGGTGCTTTATTAACAAAATTAGGATT
GTTTAAACGCCCTTCTAAGGATTTAACTTCCCCTTCAATTTTAGAGAGTTTTTTCTCTAGTTTTG
CCGTTAGTTTCTCGATGTCGATTATACCTTCTAAAGGAATCAAAATTTGAATTGTACCTACTACA
GAGGCGATCGCTTTTGAAACTTCATCGGTTAAACTGTCAGTTATAGTTAAATTCTCGACTTTTGC

TAATTCTATGATATAGTTTTGAGTTTGTTGCAAAATCTGCCTTTCTTCAGGGTTTTCTGATTGTA
GAATGGCATTAACTTTTACTCCTGGTTTAATTTCTGCTTCCGCACGAAGGTTACGAATAGCGCGT
ATTGTCTCGAAAAGAAGACTAAAGCTAGTTTCCAATTCTTCATCGATGAGAGTGGTAAAGGTAGG
GGTAATAACTGAGGGAGTTTCTTCCGTTTCTATCTTAATTAAAGAGTCATTTTTTTGAATATTAC
CGAATATCTCTTCTTTAAAGTTGGCTAACCATTGCAAAGTTTCCTGTCTTTTCTCCACAAAGATG
AAATTTTGATAGATAAACCATCCCGTATAACCTAAACCAATTAGGCGTAAGAAAGGAGAAAGTA
AGGGAAAATCATGGATAGCGGTGGTAATAGAGGAAATCAATTGAATAACTACTAATACCAAAAA
TGCGATCGCAACTACGGTAAAAGGTTGTTGATATTTTCTCAGGAAACTGCTAATTTGTTCGGGTA
ATTGATCTAATAAACTTACTACTTGATGACCTAATTTAACGATAGTGCCTTCCTCGGTATTTTCA
AAACTGATAAGGGAAGGTTGAGTCATTTTTTGAATAGTAGTTAACTCCATCAATTCATCTACCAC
AGGTAAAGATTGCAGAGCTAGACTATCATCGCTATTTTGAGTTAAAGTGTGCCAAATTTCCTCGG
TGATGTGAGGCATGAAAGGATGTAACAATTTGAGGATACCTTCTAAAACATAAGCAAGGGTTTG
TTGTGCTACTTTACGGGAAGCAGAATCTTTATCTTGCCATAAACGAGTTTTCACTAACTCGATAT
ACCAGTCGCAAAAATCACCCCAAATAAACTCGTATAATCCCTTTGCCGCTTCCCCTAATCCGTAAT
TATCAAGATATGTTTCAGTTTCTTTGACAATTTGATTAAAACGTGATAGTATCCAGCGATCGCAT
CCTTCTAGTTGAGTTAAATTAGGATAGCCCAATTGGGCGGGGGTTTTACCATCTAAATTCATCAA
CACAAAACGTGCCGCATTCCATAACTTGTTGGCAAAATTACGGGAGGCTTCTACCGACTCGGATT
CATCGGTTTTACGGTTATATTGTAAACTAATATCCTGCCCCGCACCAGCTACTTCACGGATTAAA
GTGTATCTTAAGGCATCTGTACCGTATTTATTGCATAAAAGTAAAGGATCAATGCCATTATTGGC
GGATTTTGACATTTTTTTGCCATTTTCATCCCTTACCAAACCATGAATATAAACGTCCTTAAAAG
GCATCTTACCAGTAAAGTGACCTGACATCATCGTCATACGGGCAACCCAGAAAAAGATAATATCA
AATCCCGTCACCATAGTGGTATTGGGGAAGTAAGTATTAAAATCATCGGTATTGTTAGGCCATCC
CATGGTAGAAAAAGGCCACAACCCAGAAGAAAACCAAGTATCTAATACATCTGGATCTTGAGTTA
AAATAATATCCTCACCATATTTTTCAACAGCTTTGTCCCTCGCACTTTTTTCATCATGTGCTACAA
CAAAAGGAGTCTCATCGGTAATTTCCCCATTAGTTAAACTGGTAACATACCACGCAGGAATTTGA
TGTCCCCACCACAGTTGTCGAGAGATACACCAGTCTTGTAATTTAACTAACCAATCACGATAGAC
TTTTTCCCATCTTTCAGGCACAAAATGAGGGGATTGATTATCGTCTAATTCTGCTAAGGCTTTTT
TGGCTAAGGGTTCGATTTTCACAAACCATTGAGTTGACAGTAAAGGTTCAACAGGTACTTTACCT
CGATCGCTGTATGGTACACTATGACGATAATCTTCAATTTTGACTAAAAATCCTTCTTCCTCTAG
TCGTTTCACCACATTCTTACGAGCGACAAAGCGATCCTGTCCCGCAAATTCTCCTCCTTCTTCATT
AATTGACCCATCTTTATTTAAGATATTAATGAAAGGTAGATCATGACGTTTACCTATTTGAAAAT
CATTATAGTCATGGGCTGGTGTTACTTTAACACATCCTGTACCAAAAGCGCGATCGACAAATTCA
TCAGCAATAATGGGAATCTGACGATTAGTAATAGGCAAAGTAACGGTTTTACCAATTAAATCTTG
ATAACGCTCATCATAGGGATTTACTGCCACGGCGGTATCCCCTAACATCGTTTCAGGGCGAGTGG
TAGCAACCTCTACATATCCATCCCCTTCCGTTAAAGGATAACGAAAATGCCATAAATGTCCATCA
ACTTCCTTATTTTCTACCTCTAAATCAGACACCGCCGATTGAGATTCTGGACACCAATTCACCATA
TATTGACCACGATAAATCAATCCTTCTTCATACAGTTGCACAAAAGCAGTTTTTACCGCATCACA
TAAATTCTCATCGAGGGTGAATCTTTCCCTACTCCAATCAGCAGATAACCCCAATCGCTGTAACT
GATTAACAATAGTGCCTCCTGATTGATGGCGCCATTCCCAAGCCTTTTCTAAAAACTTCTCTCTAC
CTAAATCGTAACGGGTTTTACCTTCGGCTTTGAGTTGCTTTTCAATGATTGTCTGTACCGCAATA
CTAGCATGATCTGTACCGGGTAAACAAAGGGTATTTCTTCCTTTCATACGGTTATAACGCACAAG
GGTATCAATCAAGGAAGTATTAAAAGCATGACCCATGTGTAGAGTGCCTGTAACATTTGGAGGAG
GAATTACGATCGCATACGTTTCTCCTCCCTTATCGGGATTAGCTTTAAAGGTTTCTTTTTCTTGCC

AAAATTTTTGCCACTTTGCTTCTGTTATCTGAGGATCATATTGAGGAGGTAAGATGGTTTCGGGT
GTAGTCATAATAATAGAATTTTTTTAGTTAAATATAAATATTTATCTATTGTGATCTTATTTATT
TTCCCACAGATTAACTCTCAAAACTTACCAAATCAACATAAAAAAACCCACTCGATTGAACGAGC
GGGAAGATAATTTTTATTTTTTTATTTACACTTACTATTCGTTTAAAGGATATTAGAAAGTGAA
AGTGGTTCTTAATGTACCGATGATCTGATCATCATTTTGTCCTTGATTTGTGGAATTAACCCAAA
TTACACCGGGAGTCAATGAGATATTGTCAGTTAACTGATACTTGTAGAAAGCCTCTACGTGAACA
GGAAGAGTGGTACTAGAAACACGAACACGACGAGAGTTACCAGCCGCATCAGTGATGAAGTAAGT
AGGAGAAGCATTATAGGGTTGAACACCAGCAAAGATACCTAAAACACTTCCTTCTTTACCTAAAT
CAGGGAATGCAAAACCACCGCCATAGGTCCAGATGTCATCATTTCCACGACCAATTCTGGTTACG
TTGGAATAGTTGAAGAAGGCACTGAAGCTGATAGAGTCAGTGATACGCCATGCAAATTGGCTTCC
GTAAGAATTGACAATTTTATCTTTCTGATCAACCGTTGCTCCGGGGAAACGACTCTGAAAACGTT
CATTAAGAGCAGATGCACTTTGGTTAGCGAATGTTGTACCAACAATTCCTGCAGTACCGCCACCA
CCAAATAAAGCAGTATCAGCTTTTTGGAACGCATTAACATAGGTAAAACCTAAAGCAACAGTATC
ACTGAGGTTAAAGTTTAATTGGCTTAATAAAGCATAGTCACCATTGAAAATACCTTCACCGTCAG
CAGGAGAAGCGGCACTACTTGCACCACCAGCAAGATAACCTACGGTTAAAGTGCTAGGTCCGAGT
AAACCTTCTAAGAAACCTAATTGATAACTTAAACCGGCACCAGAACCACCACCAATACGATAGAT
GGGGTTTTCAGAAGCAAAGGTACTTAACGCACCATTACCACCGTCATAATCTTCAAAGAAAGGAT
TGAGAGTAGGAGTGTAGTCGCTGTGAATACCACCAAAAGCGGCTAAGTAGGCTTGAATTTGTGAA
TCTTTACCTAATTTGATAGGAGCATAGTATGCTAACCAGTCAACTAAAACATCATTTCCAGAACC
GGGATAGAGGTTGAAGGTTTGATTTGTAGTACCACTACTTCCAGAAGTTGGGACATTAGTAATAG
GTAAGCCACCTGTTCTGTAGCGGTTAGTACCTGTGGTATTAAATGCTCCAAAATTTCCAGCAGAT
AAACGGGTAACAAGGTTGTCTTCCCCTGTGAAACTGGTATTTAAAGCTAAACGAACACGATTACC
TAAAATAGCTTGGTTACCATCTTCAAAGGCTTGAGTTAAAGCAATGATTACTTCCCCTTGTAACT
TAGTAGTTGTAGAGAACTGATGATCTTCCAAGAATGCAACTCTGCCTTCTAAGTTATCAACTCTT
GCACCCAATGCCGCTAACTCAGCTTCAAACTCAGACATTAGGCGTTTTAGTTTTTCAATGTCTTCT
CTTAATACTGATTCGCTAGAAGCAATTAAACGTTCCATTTGTTGCATACAAGCGTTTAAACCAGC
TGCAAATTCATAACGACTTAATGCTCTGTTTCCTCTATAGGTACGATCTGGATAACCAACGATAC
AACCGTAGCGTTCAACAAGGCTTCTTAACGCTTCAAATGCCCAATCAGTAGGAGATACGTCTCTT
AACTGAGAAACACTGGTTACTTGATCTTGAGAGGTAACTCCGCCCTCTGCACCGTAACTATCTAA
TTGATTAATAATTTGAGTGTTACTATCGTTATTAGCTTGGGCTAAATTGATTTCTTCACCAGCTT
TAGCGGGAGAGTTTGCTAATAAACTAGCTCCTACGATGATAGGAGTAACTGTAATAGATTGCCAA
AAGTTTTTTTTCATTGTTGAGCTTCCTCACACCTTAATTTATTGGAAGTTAATAATCTGCTCTCA
AAGAGCTAGATAGATTTTTGTTTATTTCTGACTATCATATTAGCATGAAAAAATTTATTGTTCAC
TGAAATTTTAATCATTTTGTTAAGGTATCTTAACTTTTACTTAATATCATTTTATTTCCTATAGA
TTTCCCCGAATAGTAGGAAAAAGCATTTTTATCTCTATTATTTTTATGGTAATTAGAGTAAATGG
ATAGGAGAAAAAGTAAATATAACCAAAGAAAAACCCTACTAAATTAATTTATTCTTAAGTTAGA
GAAACATGACATAAGATACCAGCCTAAATTGCACATTCCTGCCGAACAACTGAGGTTGTGTCAAT
TACATTGATTCAAATGTAAGAAATACGGATTTTGCTGTAGTTAAAATCTTCCCTTGAAAAGAGTT
TTGATTCCTAGAGGCAAAACTTTAAGTATTTTCGATAATGTCCCATAAAGAAAGAGCCCCAAGAA
GTTAACTAGGCTGACCTACTAACTTGCTGGAACTCTCTCTTTAATTTTTTTATAAGGGCATTTAA
ATTATCAGACTTCTGACTGCTGGAAGGAATGGTTAAAAAAATGCTTATAACTCAATATTTGTTAG
TCAAATAGATGTATAAGTATATTTAAAAACTAAACAGCTCCAGCACACAGCCGAATCTGAGAACC
TAAAGACCCCACGAAATTACTACTACTATCTATCATGGGCATCACCTCCTGTTTCCCTTAACGGCT

TTAAAATTTTGTTTGATTTAATTGTAGATCATGATTCTTGGAAATGGCAAGAGTGTTTTTTTATT
TTACAGATTTTTAATTGAAATTCAATTCCTAACCCTTAATTCCTTAATACCACTGGAGATTTTAT
CTTCAACTAAAGATTGCTCAAGATTATGTTAGTTAATCTGTAATTAGCAATACTAAAAACTATTT
TAACGTCAGTTCGGTTTAAGAATATCCGATAAGGTTAGGTGTCAGGTTGCAGGTGTTAGGGGATG
GGGGGATGAGGTGATGAGGGGAAAGGGGGAAACAAGTAATAAATAATAAATAAGTAGTAAGTAT
TCGTGGCTTTTCATTCTTAATTCTTAATTTTTAATTCTTAACTATTTAACGTCAGTTCGGATTAA
GGCAATTTTATCGTTATTTCTAAGAAGCTATAAGGTTTTCTGACTACGAGAAAATAATGCTTTCA
GCCTATCCAAAACTCAGGTTATTTTAATTTGCCCGATCGCAATATACTAACGATAAGCCAAATAC
CGAGAAAAGTGGCGGCGGCAAAAAGAATATTACTGATAAAGATAAGTTGAGCAGTGGTTGCCCC
AGTGGAAATAATTGCGGCACCCATGGTCAGAGAACCAACAACTATGCTAAAAGATAATCTGTTAG
CAGAATCGTCTAAACTACGCCTTAAACCTTCTAATTCTCTAAGTTGAAGTTTCCATTGGAAAGTT
TCTGAACTTAGTCTATCTAAGATTACATCTATTTGACGGGGCGATCGCAAACTAATTGCTTTTAA
ATCTAAGACTGTCCTAAACAAAGTTTGGAAGGGAGTATCACCAATTAACTGACGACGGAATAAAT
CGGTAATTAAGGGCTTTATTTCTGCAAGAAGGTTAATTTCGGGGTTAAACTTACGAGCGACACCT
TCTAAATTAGCTAAACTCTTGGCATATAATCCCATATTGCCGGGGAGTTTGATTTTATTCCGACG
AGAAACGTCTAACACTTCGTAGAATACTTCACTGAAATTCAATTGAGATAAACTGAGATTATAGT
ATTTTCTCAACATACGACTATAATCATTTTCCAGTTGAGCTAAATTAGTTTTGTAGCTATTGCTT
TCTGATAACTCAAGAGTCAATTGGGCACATCTTTGAGCATCTATATCAACGATTGCTAAAAGCAT
TTCCGTCAATAGTTGCTGAGTACGAGGGTCAAGTCTGCCAATCATCCCACAGTCAATTATTCCTA
ATCTGCCATCACTCAAATAGAATATATTACCGGGATGAGGATCAGCATGAAAAAAACCATCTATA
AAAATTTGCTGAAAAAATGCTCTAAATAATAAGGTACTAATTTCTTGTCTTTTTTTGCTGTCAGG
TGCTATATCAGCGTCTAAGATTGGTTTACCGTCAAGCCACTCCATTAATAAAACTTTCTCAGTGG
TAAACTCCCAATATATTTGCGGAATTTCTAACTGTTTACTATCAAACCAATTACTATTGCTAAGG
TTCAATCTTAGTTTATCGGTAAATTGTGCTTCTTGGCGAAAATCTAACTCTGCTTGTACTGCTTT
CGTAAACTCATCAGCTAAAGTAACCACATCATAATCATTGCCAAAGTCTGTGAGTGCAACTATTT
CAGCAATACTTTTAATCAAATTTATATCTTGATTGACGATTCTTTCTATGTCTGGGCGTTGAACC
TTAATCGCAACTTCTTCACCAGTAGCAAGAGTTGCTTTATGTATCTGTGCGATCGAACCTGCGGC
GATAGGATTATGATTTATATCAGCAAAAATTTTATCTAGGGGTTGATTTAATTGCTCCATCAAAG
TTTTTTCAATGAGTCCCCAAGGCACTGGTGGTACTTGGGCTTGAAGGGCTGTCAGAGCCTCTATA
TATTGAGGAGGTAATAAATCTGGACGAGTACTTAATAGTTGTCCAAACTTGACATAAAAAGGACC
TAACTCTACTAAAATTTTACGTAGTACCTCCGGTGGTGGAAGTTGAGGGCGATCTGATTTTCCCC
CTGTCAAAACACTACGCATATAATCCCAACCATTACCTAAAACAATTTCTACAATTTCTCTTTGA
CGAGTACTGGTTTGAGTTAAGGAAAACATAATTGATGTATATTAGTCTTTGGATTTATAATTAA
TACTAATACTTTAAAGACAAACTTCTGAATTTTATTTTCGTGTTGACAGTAAATTAAATTAACTA
TTTCACCGTTTTTCTTAAACTAATTTATACTATTAAAAAACCTCCTATTGATTGATTCATAGCTC
CATGATGACCTCCACAGAACTAATCGTTAAAATTTCTTCTCAACTTTGGTTAAAACTTTACTCTT
TGTCAACTCACGTCCCTATTGACTTTGTTAAAAATTTCTTGGAGTTAATCAATCAAAAAGATTCT
CAAGATAAAATAAAATTATTCTTCCCTAATAAATATCAAAAAATTGCCCTCTTTTTTTCCGATAG
TAATATTTGCATATCCAGCTTTTTAGTAGAAAATAATGGGAAATGCGTTGTCAAAATAGTAGAT
ATTATTTCCGTTAATAATCTAGCTAAAAAAATTGAAAACTTTATTGATAATAATTCAGAATTTTC
TAAATATATAGAAATATTTTTTGATTCTAATCAGTTCAGTTACTCAGAGCAAATAATCGGTACCG
TTGACCCGAGAGAGCAGATAAACATAGAATATTTTTCTTTTAGCAAATATGATTTTTCCGCTAAA
TATATTTTTTCCTCAACTAAAGGAAGTAGTAATTTGGTTATTTTATCCCCTCAACCAAGAAGAAAT

AATTACTAAAAGTCCTCATTTTCCCGCTATTATTACGGGTCATAAAGGTACAGGAAAAACCACTA
CAGCCCTCTATTCTGCTCTAATAGAAGAGGGAAAGCTAGAAGAAGATTTACCAAAAAAAATACTG
TTTTTAACTAAAAGTAAATCAGATGCTCAAAAATATCAAAAGATTAGTCAAAAAATAGTTAGTG
ATAATAATATTAACTTTACTAATTATCTTCATTTAGGGAAAAATATTATAGAAAAATATCCCCTA
ATTTTTAACTATAAATTTCTATCCCAAAGACAGGTTACTTTTTATAAGTTTTATGAGGAGTTTTT
TAAGAGTCAACAAGTAATTTCCCTTAATCCAGAGTATTTATGGGAAGAAATCAGGATACTAATA
AAAGGCTCATTTAAAAGTATTAGGGGGGAAAATAATCTACTTAGTCTTAAAGAATACCTAGCTTT
AACAAATCAAAGTGTTTTTCCGTCAAATTCAGATTTTAATTTCATTTATAATTTAGCAACCTATT
ATCAAGAATGGCTAATTTCGCAAAACTATTGGGATGAATTAGATTTGACTCAATATTTACTGCGT
CAATTACCTAGCAATTATCAGGGAGATTTTGATGTTGTTTATATTGATGGAGTGGAAAATTTTTC
AGAATTACAAACACAATTTATATTAAAGTTACTAAAAATAAAATCCAATGATGATTATTTACCA
CAAATATTTTTTGTGGGGGATAACGATAATTATTTGAAAGTTAATAACCAAAGTTGGAATAGAT
TAAAAAAGATTTTAATTAATTGTTTTCACAAATTACCTCAGTGGGCAAAAATTAGAGAATTAAT
TGAAAATACTGAGTTAAATGGTAGCTTTTTATATAATAAAAATATAGTTAATCTTCAATCGAAA
ATAGCTAGCATAGCGGGGATTTCTCTCAATCATAAATCATGGATTCAATCCTTTAATAAACCTTT
AGTCATTTCAGAAATAAACTCGGAATTTATCATAAGTAAATCATCTCTCAATATTGATAGTGCGA
TTATTGTATTTAGCGAAGCAGAAAAAGCAAGATTACAAAATATTTTTTCTCAAGATAGTGAAAG
AATTATTAGCTTTAATGAGGTAGATAGCTTAGATTTTGAAGAAGTTTTAATTTGGAAGCCTTTTG
ATAGTAATAATGACATTTTTAATTCTCAGATTGGTAGCTTATATGATTATAATAATCCAACAGTA
TATAAGCAAGACTTAATATATTTATGTTCTAATTTAGGAAGAAAACAGGTATATTTTTATGATG
ATACTTTTGACAGTATCTGGAATGAGTCTGAGATTAATCAAACACTAGAAATCGGTTATGAAACT
GAATTAGAGTCAATGTTTAATAGAAAATATAGTTCGGATCAAGAAATACATATTGCTGATAAAT
ATTTAGAGTTAGGGAATAACAAAAGTTATCAAATAGCAGAGCAAATTTACAATCTTTATCAAGA
CGAATTTGGATTAAACAAAGTTAATGCTCTTTGGGAAGAGAAAAAGGGAAATTATGGTAAAGCA
GGGGATATTTGGAATAAAGTAGGTCTTTTTGATTATGCGATTAATTGCTGGAATGAAGTTGATC
ATAAATTATGGTTAGCTAAATGGTCTGTTTTGGATAATGAAGAATGGCAAAAAAGAGGATTATA
TTTTGAGAAAATTAATAATTATAAATTAGCAAAATTATGTTATGAAAAAGGTAATGATTTTGAA
GGAAAATTAAGATGTTTAGCACGGGATAATCAATGGGAATTAGCAGGTGATGAATGTTTGGAAA
GAAACCTATTAGAAAGAGCAAATGAATATTATGATTTAGCAGATAAATTTTATCGTCAATCAGA
GCAAATGAAGTTGGCGATAAAAATGTGGACAAAGTTAGGTCAATGGAAAAAAATTGCGTTAATT
TGGGAGGAGTTAAATCAATGGGAAAAAGCAGGTAATTGTTGGCAAAAAGATGGAGATATAGAAA
GGGCGGCAAATTGTTGGCAAAAAGCACAAAAATGGACGGAAGCACAAAAAATGTTGGGAAGAATT
AGGAAATTGGCGAGAGTTAGCTTTATCTTATGAACATGAAGAAAAATGGATTTCTGCGGCTCAA
ACATGGCTAAAAATTACGGAAATGGAAAAAGCCGCTTTATGTTATCAAAAAGGAAATCAATGGG
ATTTAGCCGAGAGTATCTGGCAGGAATTAGGATATTGGGGCTTTGTTGCGATCGCACTTCAACAA
CAAAATAAATGGGAAGAAGCGGCACAGGCTTGGAGTAAAACAAACCCAAATGAATTACAAGGAC
TATGTTATGAGCAATCAGAAAAAATGGGATCAAGCAGAAAAAGCGTGGTTAGAAGCCAAAAATTG
GTATAGAAGTATTCTAGCTGGTGAAAAACAAGGAAAATGGCAAGAAGCGGCTGAAAGTTGGGAA
AATTTGGGAGAATGGCAAAAAGCAGGAGAAGCATGGGAAAAAATTAATCAGTTGGAAAAAGCCG
CCCTTTGTTTTGAAGAAGGAGAATTATGGCATTTAGCTCAAAAACATTGGCAAACCTTGCAAAAA
AGCGATCGCCTCGCTGAAAACCTCGAAAAACAAGAAAAATGGCAAGAATCTGCCCAAGAGTGGGA
AAAATTGCAAGAATGGGAAAAAGCAGGAAGAGCATGGCAACAAATAGGAGAGAAGGAAAAAGCC
GCCCTTTGTTTTGAAAACGGGGAATTGTGGCACTTAGCAGAGGATTGTTGGCAGGAATTGGAAAA

TTGGGAAAAACTAGAGTATGTTTGTAAAAAACAAGGCTCATGGCAAAAAGCCGCTCTGGATTGGT
TAAAAGTTAATCAATTTGAAAAAGCCGCCCTTTGTTATGAACAATGCGACAATTGGCAAAAAGCC
GCTCAATACTGGGCTCGTGCGGCACAGCAAAACCCCTCGGGAGTGCAAAATTGGGAAAAAAGTGC
CAATGCTTATGAACAATTACAAGAATGGGAAAAAGCCGCTGAAAATTACCTTAAAGCAAGCAATC
ATGAGAAAGCAGGTTTATGTTATGAAAAATGCAAACATTGGGAAAAAGCCGAAGAATGTTGGCG
TAAATCATGGAAATGGGAAAAATTAGCCCTTGTTTGCGAACATCAACAAAAATGGGAAGAAGCG
GGAAAAGCATGGTTTTCCATTAAAGAAATAGAAAAAGCAGGTTTATGTTATGAAAAAGCCGAAA
ATTGGCAAAAAGCCGAGGATTGTTGGCGACAATTAAATAATTGGCAAAGGTTAGCAATGGTTTG
TGAGAATCAACAGCAATGGGAAGAAGCCGCACAACTGTGGCAATTTTTAAGTGAATGGCAAAAA
GCGGCTTTAGCCTGTTTAAAAATGGATGATTTGGAAACTGCGATAAAATACTATGAGAAGGGAG
GTTATACTCAGGAAGCAGAAATTTGCCGTCAAAAACTAAATAGTTAATAGTTATTAATTACTTCT
TCCTTCTTCCTACTTCTGACTTCTAGCCTCATTTTTAATTATTAATTTTTAATTCTTTAGATATGA
CATTAGAACAATTATTAATCAGTAAATTTCAAACATTATCACCCCACAGAAAGCAAGAATTGCTG
GATTTTGCGGAATTTTTAGCTCAAAAATCTGACTCTCAACCTCCATTCAAAAGTATTCGAGGTAT
GTGTGCTGATTTAAAATTAGAGCTTACTGAAGAAGATATACAGGAAGTTAGACGAGAAATGTGG
GAAAATTTTCCAAGAGAGGATTTATTATCATGACGGGAGTGGTTATTGATACTCATGTCTTGATT
TGGTATATTTTTGAACCCAATCGATTGTCTGAAACTGCCCTAACAACTATTGAAAAAGTCAATAT
AACTAATAGTCAAATATATATACCTAGTATTTCACTGATTGAGATTACATATTTAGTGGAAAAA
GGGCGTATTCCTCAAGAAGTTTTAACTAGAGTGAATCAAGCAATTGACAGTCCTAATGTTAATTT
ATTTCTTGTTCCACTAGACCGCCGTATTACTTTAAGTTTTGAGGAAATAGATAAATCGATTGTAC
CTGAAATGCCGGACAGAATTATTGCCGCCACCGCCTTATCTTTAGAGTTACCATTAATTACTCGT
GATTTAAAAATTCAAAACTTATCCAATATCCAAACAATTTGGTAATAGGTCAAAAATGTTTTAT
ATCTCAATTCCAACTCCGTATCGTTTGCGGAAGTGGACTATCCGAACTCTTATGCTAATGGTCAA
ATTGAGATAAAATTTAAAACTAGATAATCAGAATTATTCTTAATAAATTAATTAACTGACTAAT
GGATATTAAAAACGGCTTCGTTGGTACTATTGGCAACACTCCTTTAATCCGTCTTAATAGCTTCA
GCGAAGAAACAGGATGCGAAATTTTGGGCAAAGCTGAATTTTTGAACCCCGGCGGTTCAGTTAAA
GATAGAGCGGCATTATATATTATTCAAGAGGCAGAAAAACAAGGATTACTTAAGCCCGGAGGTA
CGGTGGTAGAAGGTACGGCGGGAAATACGGGTATCGGTTTGGCTCATATTTGCAATGCGAAGGGA
TATAAATGTGTAATTGTCATTCCTGAAACTCAATCTCAAGAAAAAATGGATTTGTTACGTACTTT
AGGGGCTGAGGTTAGGGCTGTTCCAGCTGTGCCTTATAAAGATCCCAATAATTATGTTAAGTTAT
CGGGTAGGATTGCAGAAGAAATGGATAACGCAATCTGGGCAAATCAATTTGATAATTTAGCTAA
TCGTCAAGCCCACTATGAAACAACAGGAAAAGAAATTTGGCAACAAACTGATGGTAAAGTTGATG
GTTGGGTTGCGGCAACAGGCACTGGAGGAACTTTTGCGGGGGTATCTTTGTTTCTAAAGGAAAAA
AATCCTAATATTCAGTGCGTTTTGGCTGATCCTATGGGTAGTGGTTTGTACAGTTACGTCAAAAC
AGGGGAAATAAAAATTGAGGGTAATTCTATTACTGAAGGTATTGGTAATAGTCGCATTACCGCT
AATATGGAAGGTGTACCCATTGATGATGCGGTACAAATCCACGACAATGAAGCCTTAAGAGTTAT
TTATCAGTTACTCTATAAAGAAGGTTTATTTATGGGCGGCTCTGTCGGTATAAATGTAGCAGGTG
CGATCGCACTTGCCAAACAACTAGGGCCCGGACATACCATTGTGACAGTTTTATGTGATGGTGGG
GCTCGTTATCAATCCCGTCTTTATAATCCTCAGTGGCTTAAGTCTAAGGGCTTAACTATTCCTCA
ATTCTAGGCAATTAATTTATTCTATTAAACTACAACATTATGCAATTTATTCGTCAACAAATTAT
TCCTTTTATTACGATCGCGATCGCACTTTTTGCCTTAGTGGCAGTCACAGCCCGTAGTTTTATTGC
TACAGATTTAGCAGAACCTGCCCCGATAGAAACGATTACGGGGAGTTCGGAGAGTTCGCTACGCT
CGTACACTCCTTTCAGTCGTGAACGGAGTTCGGAGTTCGGAGTTAAGATAAAAAAAGGGCAATGG

GCGATGGGCAAGAGGCAAGAGGCAAAAGGCAATAGGCAATGGTGAATAGTTGATAATTAAGAAT
TAAAAATTAAGAATTAAGAATTAAAAACTCCGAACACTTATTACTTATTTATTATTTATTACTGG
TTTCCCCCTCTCACCCCTTCCCCTTATCTACCCATCTCCTCCTCACCCGCCACCTGACACTTTTTCC
TAACCGATAATTTACACGACGAGCAGTAAGAGAGCCAAAGTTATTTTTCTATTTTTCAATCTGCT
TCATTAAAGTAGCCATTTCCAAAGCATTCATGGCATAATTCCATCCTAAATTACTTTTTATTCCC
GCTCTTTCTAAAGCCTGTTGCATTGTGTCTGTGGTGACAATTCCGAAAACAATCGGCACACCAGT
ATTTAAACCAACAGAAGCAATTCCCTTGGCCGCTTCATTGCAAACATAGTCAAAATGGGGGGTAT
CTCCTCTAATAACTGCCCCTAAACAGATAATGGCATCATAACGCCCTGTAAGAGCTAATTTTTTG
GCAACTAAAGACACCTCAAAACTACCCGGCACCCAAGCATAATCTACCTGATTTCCTTCTGGGTTT
ATATCCACTCCATGCCTTTTCAAACAATCTTGACAGCCAGATAATAACTTATTCGTAATTAAATC
ATTGAAACGTCCTATGACTAATGCAAAACGTAGATTTGATGTATCTTGAAAATTTCCTTCAAAAA
TTGCCATGTTTACCAGTAAATATTTTTGTCTAATTTGGTGAGCAAAAATACTCATTTTTTTGATT
GAATTTGGATTTTGCCCACCGAGTTAAATTTTGTCTTAATTCTTAATATTTCTTAACAAAAAGTG
AAATTAAACCACGAAAAAGTTTAAGACAGCAACAGCAACCACTAGGGCTAACCAGATGCCAGAAC
CTAAGTATAATAAGGGCTTAGACTGCTCCCAGTTTTGTGGGGAAGCATAAGCAACAGGCACATAA
ACGATCATTAAGAAAGAAAAAAGCACTAGCGCGGTTAATACTAATTGGAATATAACTCCCATTTT
CTTAATCTCCTAAAAATATATTTTTTTAACTAAACTTTATTAGATTAGTTTAGGCTAATTTGTGT
CATTCTGACTAATGAATCCTTTGACTGTTGATTGTTTTTATGAATAGTCAGAGAGAATCTACATT
ATAATAAAGTAATTTGTGTAATTTTACTATATCGTCTAATTTGTTATGACCGCTAATTCTCATTC
CTCTGCATCTAATAATCTCTCGTTTTCTATGGATGATTTTTCTCAGGCTTTAGAACAAGAACAGT
ATGACTATCATTTTAAGCAGGGAGATGTTGTCAGGGGGAAAGTGTTCGAGTATTCTTCCGACGGT
GTTTATGTAGATATTGGCGGTAAATCCCCCGGTTTTGTTCCTCAGACAGAGGTGGACTGGCGAGG
TTCTTCTAATGTTAAAGAAGTATTACCTTTAGAGCAGGAATTTGATTTTGTTATTATCAAGGAAC
AAGATGCAGAAGGACAAGTGAAATTATCTCGCCGTCAATTATTTATTGATCAAGCATGGGATAAT
TTAAAGGAGATTGAAGAAAAAGGTGCGATCGTGCAAATGTTGGTTACAGGAGTTAACCGTGGAG
GTGTTTTTGGTGAAGTACAAGGTTTAAAAGGTTTTATTCCTAAATCCCATTTAACTCAGAAAGAA
GATTTGGAGACTTTAATAGATCAAACTGTTGATACTAATATTCTACAATTAGACCAAAAAGAAA
ATAAGTTAGTATTAACCCAAAGAAATATTGCTCGTTCTTCCGCTCTATCTCAACTCAAGGAAAAC
GAAGTTGTTGCTGGTAAAATAGCTAAAATTCAACCCTATGGTGTTTTTGTCGATTTTGGTGGTGT
TAGTGGTTTACTGCACATCAAACAAATTAGCGAGGGTAAAGTAAACGCTTTAGAAAGTTTCTTTC
AAATAGGAGAAGAAGTGAAAGTTGTAATTATGGAAATTGATACTTTTAAAAATCGTATCTCCTT
ATCGACAAAAATATTAGAAACTTATCCCGGAGAGTTTTTAGAAAATAAAGAAGCAGTAATGGCA
AATGCGGAAGAAAGACTGGCAAATAAAAAAGCACAGAGTTAGTAGGTAGGGGTTGCAAAAAAAT
TAATTGGTGTCATGGGGGTAGGTTTCAGGTTTTTGGTTTTAGATATTTTTCACTGTTAATTGTTA
ATTACCTTTGCCCCTTGCCCTTTACCCTTTTAACTTTGCCCCTTGCCTTTTTTATTATACAAACTT
AAGATTTTCACTAACCTTAGTCTTGAATAAGCTGAAAGCATTATTTTCTAGTAGTCAGAAAACCT
TATAGCTTCTTAGAAATAACGATAAAATTGCCTTAATCCGAACTGACGTTAAATAGTTAAGAATT
AAAAATTAAGAATTAAGAATGAAAAACCACGAATACTTATTTATTATTTATTACTTGTTTCCCCC
TTTCCCCTCATCACCTCATCCCCCCATCTTCCCATCCCCTAGCACCTGCAACCTGCAACCTGACACC
TAACCTTATCGGATATTCTTAAATCGAACTGAGGTTAACTATGGTAAAACCATCCAAAATAAGCC
CACTAGGGTTAAGCTCATAATACCAATGAGATGATCAAAATCTTCTAGGATGCGAGGTAATTTGA
TAACCGCTTTTTTGATTAAGACAAAGTAAACTAACCAACCAATGAAGACTGTAATTAGGGCTTTA
ATAATATTACTTAGGGTGTAAGTAGATAGATAGAAACCGTTAGCAAAAATTAAACCAGCCAACA

GAAAAATAACTCCAAACCAAAAACCCCAATTTTGTTTTAGTATCTTGGAGGATGTATCGTGGGGT
AAAAAAATAAATTTGGCGAAGGAAATAGCTGTACCAACGGCGGCAATATTCATTAACATTACTTG
CCATGGATAAAGATTTTTAACCGTTAAAACTTTGGCACAATATCCAGCGAGTAAAGGTACACCGG
AAATGGATAAACTAGCGATGACTAAGGCTATCCATAAATAAGTATTAATATTAGTGTGTTTTAA
TTCTTTGATATTCCGTGTTGGCAATGAACCCGCCATTAAAAATAAGCTAGATTTTACTAAGCCGT
GAAAAAGGGCGTAAATTCCTGCAACGGGAGGAGAAGCCAAAACAAAACCTAATTGAGATACGGT
GTGAAAGGCTAACATTCTCTTGACATCGCTTTCAAAAATAGCGTAGATGACTCCTAGCAGGGCAG
TGGCTATAGCTACAATGCGTACAATATGATCCATCTCATCCACAATTAAAGCAAGGCGAATTAGG
GGATAAACTCCTGCTTTAACTACTACTCCTGATAACAATGCGGATACGGGGGTTTCAGCGGAGGC
ATGGGTGAGGGGCAACCATAATCCAGAGACAAAGACTCCTCCTTTAACTAATAATCCTAACAGAA
TAAGTGCGATCGCTTCTGGGGGGGAATTACTTAAACCGTTGAAACTAAAAGAATTATTAGCTTTA
TAGATTAAAATTGCTCCTATCAGGTAAAACAACATTGCTGTATTACTAACAAAGAGATACCGTAA
ACCGATCCAGATACTTTTGTCCGTGCGAGGATAAACTATTAAAAGAAAAGCGGCAATGCTAATAA
CCTCAATGGCAACATAAAAACTGATAAAGTCGTAGCCAATAAAACAAGCGTTAACACTACCGTAG
AGAATCATTAATTGGGTATAGAAAAAAGCCTTTCTCTCCTCATACCAACAATAAAATAAGACGGC
AATGGTAACAAGACTATTGGTAAGAATAAAATAGCCACTTAGCTTATCGATGAAAAGACTAACA
CCAAAATTGTCTAATAATTGTAAAGTTAAATCCTGCCCAGAAGTGAGTAACCAAAAAGGATACAA
AACAGAAATTGATAAAACTGCAAGGGAAAGTATGCGACTACATTTTGGCAATAAGTAAATAATA
AAACCTGCCAAAAAAGGTAAAGCTAACCAAATTAAAGTAATAGTATCCATTATTTTCCAATAGTA
TTTTCAATCTCGTCGGTTTCTAATGTAGGATTATCAACAGCTAATTTCATGACACCCACTAACAT
CAAAGCCTGAATGGAAAAACCAATTACAATGGCTGTTAAAATGACTGCTTGAGGCACTGGGTCCG
CATAATCAACGTTATCCTTAGCGGTGAGAATAGGGGTAAAAAAACCCTTCCGAGAAGCAATAAAG
ACATAATAAGCAATAACTCCACTACTCATTACATCCATGCCCATAATTTTCATAACTAGATTACG
CTTTAACATAATCCCTAAAAATCCACAGAGGATTGTCGCAAGAATAAATCCCTCTAAAATTATCA
TTGGCTTAATTGTCAGTTCTTTAAATAGCTAAAAATCAATAAATATAAAATTTTAAATAGAGTT
TAATCTATAATAATTGTTCTAATATAAAACATATTTTTACCGTTGACAACTGATTTATTCCTTCT
TTTATGGGTGAGGTGATAGAATTTTGATCATGATGAATATAAGTTTAACTTTCAGTAGTGCGATC
GCTCTTTTTACGGCGATGGTAATTTTAGCTTCAATACCCAGCATGAGCGTTTTAACCGTATCAAC
ACGAACCATTACTCACGGTTTAACTCATGGAATTTGGACGACTATGGGCATAGTTTCGGGAGATA
TTTTATTTCTAATTGTGGCGATTTATGGTTTATCAATTTTTGCGGGAATTAATATTTTTTTCAGT
TTAATTAAATATATTGGTGCTTTATATTTATTGTGGTTAGGCTTAAGTTTATGGAAAAAAACACC
GGTCAATCAAATCTTAGTTAAATCAAATGAAAAGTCTTCTTTTATAGCTAGTTTTCTCATGGGAT
TTGTGATCACAACTGCCGATTTAAAAGCGATTCTTTTTTATTTGAGTTTTTTACCTGCTTTTTTG
GATTTATCTAGTGTTTCTTTAATGGATACTTTTATTATCATTTTAATTACTATTATTGCTGTGGG
GGGAATCAAGTTTATTTATGCTTTTTTTAGTTATAAAACAGGCTCAGTTTTAAAGAAAAATAAT
AATTTTAAATATATTAATTATGTCGCTGGAACAATGATGATTGCGATCGCATTTTTCCTCTTATT
TAAAACCTGATACCTGCCTTGATATTATTAAACTGAACTGATTTAATTTAGCTTATAACGTGTAT
TTTCAAAAAATTAGTGCCTTTACTAATTCCCGTAGGAATACCCGCCATAATTAAGATGCGATCGC
CTTTTTTCGCTAAATTGCGTCTATGAATAACTGTTTCTACCTGATTGAGTAAATCTTCAAAGTTG
AGAGGCTCTTGATCAATTAAAATCGGTTTCACACCCCAAACAAGATTAAGACGATGATAAACTTT
ATCTTCTACCGTTAAAGCTAACACAGGAGTTTTAGAACGCTCTCCCGCCGCAATTATGGCACTAT
AGCCACTTTTCGTATAAGCAACAATGAATTTGAGAGACATAGTTTTGTCAATGGCATTTAAAGCC
TCGCTCAAAGCATGGGTTTCATCACTCAAAGAAGGAGGATGATTCGTAAAATTCAAATTAGGTTC

AACACTTAAAGCTATTTTCGCCATCATTTCCACCGCTTTAACAGGATATGAACCGACTGCCGACTC
TCCTGATAACATAATTGCATCCGTACCATCAATAATCGCATTGGCTACATCACTGGCTTCTGCAC
GGGTGGGACGAGGACTGCGTATCATACTATCTAACATTTGTGTAGCGGTAATTACCGGCTTAACT
TGTCGATTACATAAAGAAATAATTTGTTTTTGCAGTAAAGGAACTTTCTCAGGACTCATTTCCAC
TCCTAAATCTCCTCTTGCCACCATGATGCCATCACACTCATTAATAATTTCTTCGAGGTTATCAAC
CGCTTGGGGTTTTTCAATTTTGGCAATTACAGGGGTATCTTGTGCATTATTTTGAGCTAAAAATT
GTTTTAAAGTGATTATATCTTGCGCCCTACGAACAAAACTGAGGGAAACCCAATCAACTCCCTGA
GATAAGCCAAATTTTAGATCTTTTTTATCCTTTTCTGTCATAGAAGGTAAGCGCAGATTAAGACT
TGGTAAATTCACCCCCTTACGACTTTTAAGGATTCCCCCTTCTATTACTTCACAAATAACCATAGG
TTTCTCTATGGCAACAATTTTAAACTCTAACAAACCATCATCGAGAAGGATTTGTGTACCAATTT
CCGCTTCTTCTGCTAAGTAAGGATAGTCAATGGTAATGATATTTTCTCCTGTTTGCTCCCAATTT
TCGGGAATAAGAGTTAATTTATCTCCTTGCTTTATCTCTACTTGCCCTGAAGGTAATTGACCAAC
TCTAATCTTTGGTCCTTGTAAATCTTGTAATAAAGTAATAGGAGTATCTAATTCTTCTGATACCC
TTCTTAGTAAAGATACAATTTGAGCATGGTCTTCATAACTACCATGAGAAAAATTTAGTCTAGCC
ACACTCATTCCCGCCATTACCATTTGCTTAATAATTTCAGGAGAGGTACTCGCAGGTCCTATGGT
TGCGACAATTTTAGTATGGTGCTTCATTTTTTTCTTTATGTAATTTAGTCCTGTCAATATTTTCA
GGATAATTACTTTTTTTGAATTTGAGGTTAGGCGAAGGTTAACACTCTTATTTCATTGAAGGTTA
GGTGTCAGGTGGTAGGGATTGAATATATTCAACCCTTACGGTGTTAGGGTGTTGGGCTATCAGGA
GATTAGGGTGTTAGAGAGAAACTAATTCTTAAAGTGCTGAAGTTACTAATTACTAATTGTTTAT
TACCATTGCCCTTTTTGCCATCACTCATAGATAAAAATTTATCCCGAACTCAGGTTAGTTAGGTT
CTGCTGTTGGGAATCAAACACCTTATCAAGCAAAGGCTTAAAGCATAATTTAAACTGGAAAAAAT
AGTAAAATAGCCTTGTTTTGTCTCAAAAATCCTCTATTTCTTCCATTCTAATCAAATACTATTGA
TACAAATGATCGATTTGTGAAAAATAACTATTTGGCTAAAGTCTTTAATTTATAAGTATTCTGAG
CTGTTTCTTGTTCCTCGTCGCCTGTTACCTCCCCCTAACACCACTTTTTGATCAACCCCTAATTAA
TCATTGATTTCCTACTTTTTCTTTTCTCCCGGTTCTAATATCCTTTGAAACAAATATCCTGTACCT
CTAGCGGTTAAAATCAGTTCAGGATTGCTAGGGTCATCTTCTAATTTTGCTCTTAAACGGGATAC
GTGAACGTCCACAACTCTAGTATCGACATGACGCTCAGGTGTATATCCCCAAACTTCTTGTAAGA
TTTCTGAACGAGAAAAAGGTTCTCCTGATTTACTTACAAGCAGTTCTAGTAAGCTAAATTCCATT
CCTGTTAAGCGGATTCTTTGATCTCCTTTGTAAACCTGTCTTTTGTTGGTGTCAATTCTGATAGA
GTTAATGGAGATAACTCCCGAACTAGGTATTCCCGCAACTCCCGTTTTGTCAACTCTTCTTAACAC
AGAGCGGATTCTTGCTTCTAATTCTTTGGGAGAGAAAGGTTTTACCACATAATCATCTGCTCCTA
GCTCTAAACCTGTAATGCGATCTGCTACATCTCCTAAAGCAGTTAACATAATAATGGGTATATCA
GATTCTTTTCTTAGTTCTTGACAAACTCCATAGCCGTCTAATTTGGGCATCATTACGTCTAAAAC
CACTAAATCTGGTTGAGTCTCATGAAAAGTTGCGATCGCATCTTCTCCATCAGCGGCAGTGACAA
CATCATAACCAATCATCGAGAGACGAGTTTCTAAAATACGGCGAATACTTGCTTCATCGTCAACT
ACTAAAATTTTCTCTTTTTGAGTTTCCAATTTTTGCTCTCCTATACTAAAGATTTTTCTTATATA
TTTATTTGATCTAGTTTATAGGAAAAATGAGTCATGAGGAATCAGTAATCAGTAAATAATTCGG
GCTTACAGAAAAAGTATTTTGTCAGGTAGCTAAAGGAATAAAGAGCAAACCCCCCTTTATCTCCC
CTCGAAAGGGGGGAAGGCAAAACTTTTTAATTAACTTCCCGTAAGGGTTGAATATATTCAACCCC
TACCACCCCAATACCCTAACACCTGAAACCTGACACCTGCTACCTGAAACCTAACCCGATTTTGTA
TTCTTGGGCTGAACTGAGGTTTAATTAGTATTCCTCGTACTCTGGCATTTTCTGTTTTTGGGGAA
TATTTACTTTTTGGGGACGATAATCATCCTCAACATCATCATCCCACATATCATCTTCTAAGGAT
TCGTCATATTTAGAGCGTGTTTCCGATTTATCATAGTTATTGAGTTGATAACGAGGTGCAGGTTC

TCTTTCAACCCCACTTTCTCCCCAGTTGTCTTCTTCTATTTCTTCCTGATAAGCCATTGCTTCTTG
ACGACGGGCAGGAGGAGGAGTAATAACTTCTGCTTGTTCCCATTCATCTTCAGTCCAATTTTCCT
CTACCACAGCCGTGCGTTGAGGTTTCGGTTGGGCAACGGGGCGCATTGGTACTCCTGTTGGTAAC
TGATTTTCTGGGGGAGTGGTAGGAGCGTAATACATCTCGTCTTCGTCTTTTTCCCAAGGAGGACG
ACCAATTCCGAGTCTTTCTAATAGCCCTACTGTTATTTGTGTTACTCGTTCCTCTGCACCTTCAAA
AACGATAATACGATTAGGTCCACTACTAACAATTTCATCTACAGATATTTCATAGGTACTAATTA
ACTGTTCAGGTATTTGGGGAAGACCGATCGCAGCTATAATTAAAGAGTTTAATTGACCATTTTCT
TGATTAAATTGAAAATCTCTTACCTTACCTAAAGGTTCACCTGTTTCGGTGACAACTTCGGAATT
AATTAAAGCACTATAAAGGTCTATATCAACATCTTCAATGACATCCTCGTCTTCTACTAAAACCA
CGTCTCCCATTTGTGAAATGCGATCGAGATACATATATTTAGGAATACCAGACATGGCTAAACGA
TTATCTCTTAACCCTAGTGCAACTACTTCTCTGCGATCGACATCTACTAATACCTCCTTGACAACC
CCTAATCTTTTGCCTGTATTTCTAGTAATAACTTGAGTGTTTAAAAACTCATTCCTTAAGCTACT
TTGTGCATCTATCATAATAATTTAATATGTATCTAACAAATATACTTGACTTTAATTTGTTAATT
TAATTTAACTTAATCCTAGTCATTTGATAATAACTCATATTTATGATTTTTGATATTTCCCCCAT
AGCTTATCCTGTCCAGAAAAAAATTTCCCAATTTCAACCAAACTCAAAAATAGACCAATCATCTTA
CACCTAAGCTAGTTTGCATTTCTTATAGAAGAAAAACCTTTCTGCCTCCCCCAATGGGCTTTAAT
AAGTCCCTTACTAAAATTTATGATTTGGAAGTCTTTGGTTAAAGGCGGGTCAGAATTTCCCCCTA
AATCATACTTTTGCCTCTTGCCTTCTTCAATTGATTACAGTAAATAGCTATTGACAATCTGATTA
AAAAAATCTAAATACTTAGGATAATTACTGATGATATTTGAACCTGAATGATTATCTTGATATT
TATTTCTATTCAGCCTTTTAGTATTTTCATCATTAATTAATCCTCTAAATAATAGTCCAGAATAG
TTATTTTTCTTTTTTATGACAGGAGTATCAATTACACTTATTAACAAAGGTTGATTTTCTAACTT
GTAAACAAAGTTTAACTGTATCTTGTGTGGTGAAATACTATTTACTTGTATTCTACTCTCTTGCA
AACTTAAAAGATTATTGTTGATGAAATTTAAACCTGAGCATTTACCCTTTGGAAAAGTAATTAAT
CTACGAAAAACTATTTGAAACCGTTGAAACTGATAACTAATAATTTTAGCGATAAAGTTATTGAC
AATAATATCTAAAGAATTGGAATGAGCTTTTTTTTTATAACTGAAAGACACTTTTCCCATCTCAA
CATTAATATTTATGCCTTCATGAAAAGATAATTGGAAAGAATGAGGTTTACAAACTACAGGCTCT
TTTAATTGCCAATGGGGGGGGATAATTCCTTGTTGATATAAATTTACCAAACTTAATTGATTAGG
CTTGAGAGATTTCGCAAAAATTATAATAGTTATATCTTGATAATCATATATTTTTAAAGGCTTTT
GAGGTTCAAAGTTGTTCGGAAAAGGTATTTTCATCTGAAGTTAAGTATTGTAAAGTAAAATTAA
GAAGAGCATCACAGTTTATATTAATTGTAGGAAAATATTATGACTCTAGCAGTAGGTACTAAAG
CACCCAATTTTACAACCAAAGATGATCAAGGTAACACCGTTTCCTTATCCGATTTTGCTGGTAAA
GTTGTGGTTTTATACTTTTATCCTAAAGATGATACTCCCGGTTGCACTAAAGAAGCTCAAAGTTT
CCGTGATAACTATACCGAATATCAAGGCAAAGATATGGTAGTTTTGGGGGTGAGCAATGATGATG
AGGCTTCCCATAAAATGTTTAAAGAAAAGTATGGTTTACCTTTTCAACTTTTAGCTGATACTGAT
AGAAGTATTACTAAAGCCTATGATGTGGAAATGACTTATAATGGGCAGATTTATCCCCAAAGAGT
AACTTACATCATCGATGGAGAAGGAACAATTACCCATGTTGATGACAAAGTGCAAACTGCAACTC
ATGCTCAAGACATCTTGGCTATTTTAGGCTAATCAAACTGAGTTGGAGATAAATTTTTTGGGCAA
GGGGCAAGAGATAAACCCCTCTGTTTCTCCCCTTTTAAGGGGAGAAGGGCAAAAGAGTTTAATTA
ACTTCTCCCTAGTACCCTAACACCCCAATAACCCAATCTCCTAACCTCTCAAATTATTCCGCCGAT
TTTAAAACAGCTTCTGCGGCGGCTTCGGCTGTGCGTATTGCACCATCAAAAAAACCTGCCCACCTT
TCTGCGATTTCTGTTCCAGCCCAGTAAATACGGTTAAAAGGCTTATTGATTGATTGTCCAAATCT
TGTCCATACTCCCGGAGGCATGAAAGCGGCATAACCACCGCCTACCCATTGATTGGAAGGCCAGT
CTTGTATATCAAAGGTTGTGGGAGATAGGGCTTGTTGTCCTAAATATTTGGCTAAGTCTGATAAA

ACAACGGATTTTTGTTCAGATTTAGTTAATTTTATCCACCGATGATAGCGATCGCCCATGACAAA
AGTAGCTAAGACCCCTTTTCCACTGCGAGGGTCTGAACTATCGGCAAAAAGTTCAATCCATTCTG
TGTTTCCCATACCTACTCCGGCAAGTCCTTGAGAACGCCAAAATGGTGTTTCATAGGAGATTAAC
AATTTGGCTAATGTTCCCATGGGAAAGCGTTGAGTGAGTTGTTGCTTGATAGGTGGTAAGGGAGG
ATGATAGGTTATTTTTCCTGCTAAATAGGGAGGCATGGCTACAATGACGAAATTAGCTTGATAAA
TGCCTTTGAGGGTAGTTACTTCTACTCCATGATCATCTTGAGTGATTTTCACCACCGCTTCATTGA
GATGAATTTCGTTGTTTAATTCGGTGACAATTTTACTGGGTATTTGTCCTGCACCATCGTGGATA
AGTTCCGCTTCTGGATGTTCGGCTTGGGAGGCGGACTTGTTTCCCCATAAAACGTGTAATAGTGA
AACTTCATCGGGTTCAGCAGGTCCTAAAAACCCCACTGCACGGCACATATAGGCAAAATACCAAC
GACCGAAATCCGTTTGCGTATTTTCTTGAATCCACTGAGCAAAAGTTTGGCTGTCTAGTTTTTA
TTTTCTTCGTTAGCTTCTGGATATTGAGGGGATAAACTTTGGGAAAGCTCATCAAAGGTTTTCCA
TGCTTTCATTGCATCTTGCCATTCTTCCTCGGATACATTAGGGCGATCGCCTTCTGCAAAACCCTG
AAAAAAGCCATTAAATTCTTGCCGTTTGTCGTTGTAGATTAGCACTACTTTCCCTTGTAAAGGAG
AAGGAAAACGAGGAATATTATATTCGTCTAATAAAGCTAAAAAGCGGTCTTGAGTAGGACCTACC
CATTGACCACCTAAATCTATATACTGGTTGGGAGCAATATGTTTTCCTAACATACGCCCACCGAC
GTAGTCTTGGGCTTCAAAAACCAAGACATTTTTTCCAGCTCTTTGTAAGTTGCGTGCCGCAATTA
ATCCAGATAAACCAGCACCGACAATGATGCAATCATATCTATGCGCCATTCTTATTAAGATCCCA
TAAAGTTATTTATAGCCGAATTATAGTATCTATATAAAAAATATTGAAAAACCTTAATAAAGAT
TAAAGACTTAATTCCTGAGAGCGTAGATAGGCAGATTTAACGGCGGAAATAACGGCACTGCGAAA
ACCTTTTTCTTCTAGGGCGGCAACTCCCGCAATGGTTGTTCCTCCGGGGCTTGTGACTTGATCTTT
CAGGAGGGCAGGATGTAAGTTTTTTTGCTTAACCAGTTCTGCTGTACCCAAGACTGTTTGTACTG
CTAATTCAAGAGCGATCGCACGAGGTAATCCAGAAGCCACACCACCATCAGCTAAAGCCTCTATC
ATCAGGGCAACAAAAGCCGGACCAGAACCAGATAAACCTGTCACCGCATCCATTTGATACTCTGG
CACTTGAATCACAGAACCGATCGCTTCAAATAGAGATAATGCTAGACTTAATTGAGACTCAGAAA
CTTTGCCATTAGGTGCGATCGCACTCATTCCCTGTCCGACTAAAGCCGGAGTGTTAGGCATAACTC
TAATAATAGGAAGAGAAGCAAAAGCCCTTTCCAACTGAGAAAGAGAAGTACCAGCCAAAATCGA
TAAGATGAGTGGAATATGACGATGAGGATTTATAGTTAAACCTTCGACTACCCGTTCAAAAATTT
GCGGTTTAATCGCCAATAACAATACCTGAGAATCATCTAAAACCCTTTGATTATCAGTAGTGGTT
TCCACCTGATATTTTTCCTGCCACCATTGACGACGAGAGAGTGCAGGATCACTAATAATTATTTC
TGAAGCAATATATATATTTTGTGCGAGTAAGCGGGAAATAATCGCTTCAGCCATTACTCCACCGC
CAATTACTCCTAATTTAAAAGACACTATAGATTTTGCTATTTTAGGACGACTTTATTATAGATAA
TGATATGAGTAACCTAGCTTCTGTGAAAACTAGAATTATTGAGCCATAGAATCAGTTAACTCTGT
AGAGGAAGTTTCATTCCAAAGAGCATCAAAAGATTTAGTTAATCCTTCAGAGCCAATTTTTGTAT
TACCATTCTGATAAGATTTACCAGAGATTGTACTAACTTTGACATTTCTGGGAGTGAAAAGGAAA
ATACTTTCACCGATTCTCTCTTGATGACCATCAATAGCATAAGTGCCACCTGCGACAAAATCAAC
CGCTCTTTGAGCTTCTTCTGCTTCCATTACATTCAAGTTAAGAATAACAGATCTTTGCTCTTTCAA
AGTTTGAATTACTTGGGGAATTTCATCAAAAGAATGAGGCTCAATTACTACTACTTCATTCACAA
TATTATTTACTCCTGGCATTCCAATAACGTTGTTGGGTAAATTGCTCGAAAAATTGCCACTGTCA
AGATAAGAAGAAGAAACATCGCTAGATTTATGAGCAGAAGTTTCCCTATTATCTCTAGGGCGACG
ACGAGAAGAAGAAAGGGGAGGAGTAACTGGGGCTTCTGTTTGAGGTATAGCTACCTCATTCATCT
CAATATCAGAAGGGGGTAAATCTTGAGTAGTCATACTTTCAATTTCCTCCTCATAGTATTGGTAA
TCGCCTTGTCCTCCACCAATAATGTCTTTTAATTTGCCTAAAAAATTACTCACGGGTTTAATTCCT
TAACGACTCTAGTATCATAGTATAAACTTTGTTATTCTATACGATAATTTACCAAGAATTTTTGC

TTAAAATCCAATTTTGAATTCCATCGGCTAAAGTAAATGCTAACTGTTTTTGTGCAGAGCTGTTA
GTAATCCATTCAAACTCCTCTGGATTAATCATAAAACCTAATTCTAATAATACTGTTGGGGATTG
GTGAGGTCGAGTTAAAGCCAAGTTATTCCAAAAAATTCCTGCGGAATCTCTATCTAGCTGATTTA
TTAAATAATTGTGCAGATAAACGGCTAAATCCTGCGCTTGGGGATGATACCAAAATGTGCTAACT
CCCTTAGTTTTTTCGGCGTTACCTCCATCGGGTAGAGCGTTATAGTGGATGGATAAAGCTACGGT
CGGATTTACATTCCTAATCATTTTAGCGCGATCGCCCAGAGAAACAAAACTATCATCATTTCTCG
TTAAGTAAACCCTTGCCCCTCTTTTTTCTAACTCTTGGGCCACTAATTTAGAAACGATTAAGTTA
ACATCTTTTTCGGGATACCCTGTTGGTCCTAAAGCCCCAGATTCATTTCCACCATGACCCGGATCT
AGTAAAATACTAGCACCATTCAAACTCTGATTACTTTTCAGATTCGGGGGATGATTGATGGTTAA
AATTAAATTATTACCTTCATAACGCACATCATAACCCCACTGTTGAGGGGATTTAAAAGAAAAGA
TATAGTCAATTTGAGTAGGATTGACTTGATACCAGTCGAAACGCTTAATTATGGGATTATCATCA
AAACGAATAGTATCAGTTTGGGCAACAATATTATATAAACTTAAAGTTAAAGAATCATCATCTT
GCTTAATGCTAACAGGCACTGCACTTTCTAAGGGAAAAATTATTTCCGTTTGAGTCTCTTTGACT
TTAGAAGTGATACTGCGAATATAGCTGAGGGGAAGGGTATTTGTCGGTAAAACTCTGGTTTCTTC
TGCTTTAATCCAACCCCCATAATCGAGCCTTAGCCATTCTCCTTCTTTTCCTGTCACTTTTGCCCT
GACTCCTCTAGGTAAAGGTGTTAACCTTGAGTAATTAGTACCAGCACCAGTTCTAGCAATACCCT
GCTCTGCAATCACTTCCACTACTTGTAACTCTTGAGGATTAAGAATAGTTATGCTACCTTCTCCTT
TTCTTTCTGTGGTTTTACCTTGATAATCCATGACAAAAATCGGATTTAAGTTGGACACGGTATTA
CTAAATTGAACACATCCAGAAACTTTCTGCCAAGAATTGCCTACTGTGGAGATGGGGTTATTGTT
GTCAATCAAAACTGAGGCGTTACCGGGTAGATTAATGGTATTTAAGGCAGGAGTTAGGGGTAAA
GTATCCTGATTAAGTTTTACTGTGGTATTTGAGCCGAGGGGGGTAATGGCAGAGAAGCAGACATC
TTCATTGGGTAGTTTACTTATATCTACTTGGGGATTTAATAAATTTGCAGAAAAATTGTTAATTT
CTTCTAAGTCAGGCTGATTATCAACCCTTGTAATCACTCGATTTAATTCTTCATTGTCGTAACGA
ATTGTAATTTGATTTTTGCCCATTTTTAGAGGAATACTGGGGGCAAAATAGCCTAGAGAGGAGCG
ATTTATAGATTTACCATTAATTATGACATTACCAGAAGCAGGAGCAGAGCCAATAATAAAGATA
GATTCTGCTGTTGTTTGATGATTAACGGGGGGATAAACAATTTTTAAAGATTGAGATTGAGCAG
AGCTAGATTCTGCGATTAAATTCAAGCTAATGGTGATTAAAAAACTTTTCCAAAAAATATTGTTA
GACATGGAGATTAATTATAGTTAATAGTTTTAGTTCAGAGTTCAGAAAGAGGTGTTAGGTTTCA
GGTTGCAGGTTGCAGGTTGCAGGTTACAGGTTACAGGTGATAGGGAAGCGTGTTAATTAAACACT
TTTACCCTCCCCCCTCTTGAGGGGGGATAAAGGGGGGTTTGCCTCTTGCCCTTTTACATCTATTCA
CTAAATAATTTTAATTTGATGATAGGAGTATATCACCAGCTTTTTCTGGGGGTAAGGGATGGGAA
AATAAATAACCTTGTCCCCATATACAACCCATTTCTTGGATGATTTCTAGTTGTTTTTCTGTTTC
TACTCCTTCGATAATTACTTCTACCCCTAAGTCAAAGGCTAGAGATAGAATTGCTTTTATCATTT
TTGCTTTTTTTTGGTTTGTTTCTATATCTGTTACAAAACAGCGATCGAGTTTTAAGCCGTTAATT
GGTAATCTATGTAAATAGCTAAGACTGGAGTATCCTGTGCCAAAATCATCTATCCATAATTGTAT
ATTGCGATCGACGATCGCATCTAGTATATATTGGGTAGATTGTTCTTGAAAAATAGGAGAACTTT
CTGTTATTTCTAACTTTATATATTCAGGATTAACTTGGGTTTCTTTGAGAATATTATCAAGGGTG
GGTAAAAATTTTTCACTATTTAATTCTTGACTGGATAAGTTAATACTAATAATGGAATTGTTTT
ATGATTATCTGAAGAATTTAAAATATGATTATGCCATTTATATAACTGTTTACATCCCTCTTTTA
GTACCCATAAACCTATGGGAATAATTAAGCCTGTTTGTTCTGCTAAATTGATAAAGTGAAAAGGT
GTGACTATTCCTTTTTGAGGGTGATTCCATCTAATTAGACTTTCAAAGCCTTTTAATTGATTATT
AGTTAAATTAATTATAGGTTGATAATAAAGGATAAATTCTTCTGCTTCTAAGCCTTGTCTAATTT
CATTTTCTAATTGTAATTCTGCAAGGGCTTCGTTGTGCATATCTCCTTGGAAAACTTGATAATAG

GATCTTTTTTGGGATTTGGCTTTATATAAAGCTGTATCCGCATATTGTAAAATTTGAGGTGGGCT
AAGATAATTATATTGCTCTTTTTCTAAACTACTACTAAAAAAAATACCAATACTGATACCACAAA
ATAATTGATGTTTATTAATAACAAAAGGTGGTTTAAAGGCATTATTAATTTTCTCTGCTAAATTA
ATTATTTCTTTGATATTGTCAATATCATCCCAGAGAATAGTAAATTCATCTCCTCCTAACCTAGC
AACAATTTCTTTTTCTCCTAAACAATTACGCAGTCTCTCACCAATGGCAATTAGTAGTAAATCTC
CAACACCATGGCCTAAACTATCATTAACGGCTTTGAAGCGATCGCAGTCTAGGAATAAAATGCTA
AACTGATATTCATTGGTGGAATCTTGCTTGAGTTTATCTATACATTGACTTAGTTTTTGCATAAA
TAAACTACGATTGGGCAACTGAGTTAGTTGATCATAAAAGGCTTGACGATGGAGTTTTTCTTGTG
CTAGTTTGTATTTAGTTATATCTTCTACAGTGCCTTCATAGTAGAGCAAATCTCCTTTAACATTA
CGTACAGCTCTCGCATTTTCTGAAATCCAAATAGTTGTACCATCACTGCGATAAACTTCTGATTC
AAAGTTGCTAATAACTTCATTTTCTTCTAATAGTTGAACAAATTGCTTACGGCGGTGGGGTTGTA
CATAAAGCCTATTTTCTATATCGTTGAGATTTTTAATTAGTTGAATAGGAGTGTCATAGCCATAA
ATTTTGGCTAAGGCTTTATTGGCGGAAAGATAATAACCGTCAATGGTAGTTTGAAATATACCTTC
TACTGCATTCTCGAAAAAGTTACGATACCTGTCTTCTACTTGAGATAATAATTTTTCTGTGCAGG
GGCGATCGCCTATATCGGTTATAATACCTTCAATGCCTAATAGTTGCCCTGATTCGTCAAAAATA
CCTTTTCCTTTTTCCCATAACCACTTAACTTGATTATCTTTGGTAAAAATACGATACTCGATACT
ATAAATAACTTTCTTATTCAAACTGTTTTTGATGGTGTCTAAAACTCCCTGTAAGTCTAAAGGAT
GTGTAATTTTTACAAGATTGATTTTGTCATTTTCCATCAACTCATTTGGGGTATAGCCAGTTAAA
GACACGCAACCTTGACTAATATAGTCTTTTCCCCATTGAGGATTATAACTAATACGAAAGAAGAC
TCCTAAACTGGAATCAACTATATTGGCTAACTGTCTTTGACTTTGACGTAATTGGGTTTCAATTT
GCTTTTGTTTGAGAAAACTACCCAACTGGGAGGTAATTGTTTCCAAAAAATTAATTAAATCATTA
TTTATAGATAAGGATTTATGAGTAAAAAAGACGAAAATAGCAATAATTTCATCTCCTACCATCAC
GGGAATACCTAAAGCGGTTTTTAATCCAGACTGTTTGGCGTAGTTTTTCCTTCGAAAATGGGAAT
CTCGATGTATATTCTGCCACCATATTGCTTTTCTCTCTGCCCATACTTTCCCGGGTAAGCCTTCTT
CAAAGTGAAAAGAAAAGTCATAACTATGTTGCTGAAAATCTGCTAAGGCTAAAGGAAAATTATC
AAAATAGGGATTATCCTTGCGATACCATGCCACGGAATAGTTATAAACATTGCCCTCAAAATTGG
GTAGCCACACCTCGGCAAAATCCCATCGGGTAATCTGACATATTTCCTGTATCGTGTGCAATAAT
GCGGTTTCTAGGTTATTGGCAGTATTTATAACTTTTGTTAAATCAAAGAGAAAGGATTTTTCTTG
TTCATTTCTCCTTTTACCTGTAATTTCCTGAATTATACCGACTAAATATAATAATTCTCCTGTAG
GTGCATAGATTCCTTTTCCTTTTTCCCACACCCATTTTATATTGCCATTAGCGCTAACAATTCTAT
ATTCTAAATCAAAATGAGTACGAGATGCGATCGCGCTTTCAGTTTCTTCTCGAACAAAATTGCGA
TCGAGAGGATGAATTAAACTAGAATAAGATTTAGAGGTATTATTTAATAAGTATTGGTCTTCAT
AACCTGTTAAATCCCAACAATTTTTACTGATGTAATCCATTGTCCAATATTTATCATTACGACAT
TGATACACCACTACAGGTAAATTACTAATTAAAGAAGATAGCTGAGAAAGACGAGTGGCACAGTC
AATAATTGAATGGGTAGAGAGAGGGTGAAAAATAACAGCAATAACCTTTTCCGATTTCCAAGTA
ACTGTGCTAATAGATAAGTTATATAGCTCAAACTCGTAACTATAACAAGAATTTAACTCTCCTGA
AAAATAGTTATGATTTGTTAAAGGCTTTACTAAAAAACAACTTCCCTGTGAGAAGCCTAAAATTT
CTCGAATGGTATAGGGTTGAATATAGTCTTGCAAACGGGGCTTTGATTTAGAGGTAACAAACTTC
TCTTGAAAAAGATGATTTCCAGAAATTAGCTCAAAATTATCCCTGCGGAATAAAGCAATAGGTTT
ATCAATAGTTTCCCCATTGAGATTAATATTATCCAGATTTCCAGTCAAATATATAGATTCATAAA
GATTAGTCAAAGAATTGTAGCTTCTCTCTTGTTTTTCTTTATTTTTAGAATCCATAAATTAACCC
CATTAAAAATTTAGTAATGCTTTACTTACATACAATCCCGCCCCATTGGTAAGAAATTGAGCAAA
AGTGATTTTTGTCAATGATTTGTTTATAAAAATTAACAATCGCTCTAATCCTTTACTATTTATAA

CTTAGCGGATTTGATTACCCTAGACTAAATACTCAGAATTTGTTATTCTTATGATTTATGAAAAA
GTGCGATCTAAACCCAAAAATAGAGCCGTCTCACCTAAGTCAACAGAGAAAAGTCTTAAGAGACA
GAGTTAGCAAAGAATTGAGCTTGTAATTAATAATTGTAACTATATACTTGACAAATTGCACGCA
ACTTTAATCTCTAACTAATGACTACGTTTTAATCGCCTTAATTTTATGCCTTTTGAATTCCCAAA
AAAGTGATTATTGTATCGCATTTTTATTTTTTAATAATTTTAGCTCAAAATGATTCTCTAATTAG
CTGTAACAAGTAATGGTAACTAAAAATACCGTTTTACTGCTTATTTTTCAAAAATTTTATAAGTG
TCTTCCGGGTTATCTTAACTTTGGATATGATAAATTATCCCTAAAAAAAGATGTCAAGTTCTAGG
TTGAATTAAAATTGTTATAGTCATTTCAAATAAAAATGAAACAAAATCAAAGCCTGTAACTACT
GTTTATCAAAGACTTAAGTGGTTAAAAGTGTCTCAAAGTTAGTTAAAATAACGATAATTTATAA
CTCAAATTTTGTATCTGGCAAAAAATGAACTGCTCGAAGTAGAGAACAGGAAATAGAGATAAGA
ATTGACAAAAAAACTATTGAAATTTATTTTAAGGATTTATTTTTTTAGTATTATCGTACTAATGC
AAAAAAGATCTTAATCCCATAAAAATAAGCGGGTTAGCTATACTACATTGACAAAGTTGACAAA
AAATGCACAAAAATTGAGTTTTTTAGCCAAATTTTGGCTAAGCATTATATGTTTACATATTGAAA
TCCTTACAGTGAAAACCTTTCAAACCATTGCAAGAGTGCCTATTTCCCTCATCCAAGGATTTTTT
TAGTAAACTCTAATTAGGAATAAGAAATCAACCATTATTTAATTTTTCATTTTTCATTCTGCATT
GTTATAGATGTAGGCTAATATTAGTTTATATTTATAGAGATTTACTGTTCAACATTTAAAAATAC
TTGATTTTTATTACAGGTAGAAGAATAATGATTTTGATGATCGGTTGGTTTGAATGGGCTGAAT
ATTTAAGTATTGTATTAACTATCATAGGTTTATGTCTTAGCCTCACCATAGACAATCAGATTCTA
TTTATTATTGCTATTACCTTCACCCTAATTCTCAGTTTAATCAACCGTTTTCGCTCTGAAAATCGA
ACAAAAGCAAGAATTGCAGGGGCTTTGAGTTTACAAATGAGACGTTTTTCTGCGGAGGTTGCCGA
GGTTAAAGAAACTATTGATGAATATATTGAGAAACAGAAAACAGTTATAACCCCTCCTAAATTA
GCCAGTAATACCCCTAAGTCTGATAATCAAATTATAGCCTCCTTACAAGATGATCTTGACAGTAT
CAATAAATCTGTTACCAGCATAATTGAATATATTAAAGAACATAAATTAGAATTAAGATTAAAA
ACTTTAGAGCAGTTATATCAAACAAATCAAAACATTAATAAAAGTCAAAATTTTCCCAGTTCATC
TATAAAATTACCTGACAAAAATGCCTTAACTAAAACAGTTAATTATGATTTAGAAGCACCACCAA
AAATTGCTTGGAAATGTATTCATATAATCAATGCCCATCAGCAATCGGTTACAGATTTAAAAATC
ACAAAAGATAATAATTATTTATTAACCACTTCATGGGATCAATATTTACGTTTATGGTCTTTAGA
AACGGGAATTGAAATTGATGGAGTTGAAATTTCTGATCAAGGTGTCGTTGCTGTTTGTGTGAATA
ATCTTGATTATATAAACTATGGTATTGCTACAGGAGGCTTAGATCAAGATGTCAAAATTTGGTCA
TTGAAAGGAGATAAAAATAAATCTTGGCGTTTGTCATTAGAACATACTATGAGTCTTCATCAAGG
TTCAATTAATGGTTTAGCCATTGCTAATGAGCAGAAAATCCTTTTAAGTGGTAGTTCAGATCAAA
CTTTAAAACAATGGGATTTAGAGACAGGAAGACTTTTATCTAGTAGTTTTGACGAGAGTGGTGCT
ATTGGTGCAGTGGCAGTTTGTGAAAAACATGAATATGTTGCGGTGGGGGGAGGCGATGGAGTTA
TTAGTCTATGGCAGTTGGCAGGAGAAAATAAATTAGGGGCATTAATTGGCAATATGTCTTCCTTA
GATGCGATCTCAGTTAATAGTACAGGAGAATTAATAGCGGGAGGTTGTGCAGATGGTTCAATTA
AAATTTGGCGTTTACCCACAACAACATTTAGTCTTTACCTAGAAATAGAACCTTTTTTAGAGTTA
AAGGGACATCAAGGACAAGTGATGGATTTATGTTTTAGCCCTGATGATAAATTATTATACAGTGC
GGGTACAGATGGTTTAATCAAAATTTGGCACCCTAGTAGCTCATCAGAATTGGGACATTTAAAAA
TAAGTGATGACAATCGTATTTTTTCATTATCCTTAAGTAAAGATGGGACTATCTTAGCGGCTGGG
GGGGTTGATGGAACTGTCAAAATTTGGCAGCAGTCAACTTTATAAAAAAGGACAATGGGCAACA
GGCAAAGGTAAATCTTCAATGAAGATGAAATTGAATAAAATCAATCACCTTTTCTAATCCTTCTC
CTGTTTTTAAATTAGTGAAAATAAAAGGTTTATCACCACGCATTTTTTGAGAATCCCTTGTCATC
ACCTCTAAACTAGCACCAACATGAGGAGCTAAGTCAATTTTATTGATTACCAAGAGGTCAGATTT

TGTGATGCCAGGTCCCCCTTTACGGGGTATTTTATCCCCTGCGGACACATCTATAACATAAATGG
TCAAGTCCACTAACTCAGGGCTAAATGTCGCCGCTAGATTGTCTCCTCCGCTTTCCACAAAGACAA
TATCTAAATCCTTAAAAGTTGTTTCCAATTCCTCGATCGCCTCTATATTAATGGAAGCATCCTCCC
TAATAGCGGTATGAGGACATCCCCCCGTTTCTACTCCTTTAATTCTATCGGGAGTTAAGGACTGC
GATCGCACCAAAAATTGAGCATCTTCTTGGGTATAAATATCATTAGTAACTGCGGCGAGGGAGTA
TTTTTCTCGCATATTTTTGCATAAAGCGTCTAAAAGAGCGGTTTTGCCCGATCCAACAGGTCCTG
CGATGCCGATTCTAAATGTCATTGCTTAGTTTGTCTCTCAGTTGCTTGGTTAAAGTGGGGAGAGA
GGGAGGCGTGGGAAGCATGGGAAAGGGAGGAGAAATTAATTATTAAATAATTCCCTATTGCCTG
TTGCCCTCCCCCCTCGAGAGGGGGGATAAAGGGGGGTTTGCCTTTTGCCTACCTTCACTAATAATT
TACAGACTCAAAGTCAGAGAGTCGTTCTGTCTATACCATTCAATGGTGCGTTTTAAACCTTCTCT
GAGATTCATTTTAGCAGTAAAACCGAAGGTTTCTTTCGCTTTAGTGGTATCTAAACAACGGCGGG
GTTGCCCATTGGGTTTGTCGGTTTCCCAGATTAATTCACCGTCAAATTCCATCAATTCACAGATT
AATTCTGTTAAATCCTTAATGGAAATCTCAAAATTCGTACCGAGGTTAATGGGTTCGCTACTATC
GTACATTTGTGTACCCATAACAATGCCTCTCGCCGCATCATTGGAGTATAAAAACTCACGGGTAG
GACTTCCATCCCCCCAGACAGGGAGGGTGCGATCGCCTCTTTTTTGGGCTTCATGAACTTTACGAA
TTAAAGCAGGAATTACATGAGAGCTACGGGGATCGAAATTATCTTCAGGACCATATAAATTAACT
GGTAGAAGGTAAATGCCATTAAAACCGTACTGATTGCGGTAGGCTTCCAACTGTACTAAAAGAGC
TTTTTTAGCAATACCATAAGGGGCGTTGGTTTCCTCTGGGTAGCCATTCCAGATGTCTTCTTCTT
TAAAAGGTACAGGAGTAAACTTAGGATAAGCACAAATTGTACCGACACAGGTAAATTTTTCTACC
CCTGCTTGATAGGCGGCATGAATCAACTGCGCCCCCATCATCAAGTTATCATAGAACAATTCCGC
AGGTTTTTCTCGATTTAAACCAATACCACCCACGTGGGCGGCTAAATGGATGACAATATCTTGAT
TCGCAACGACTTTTTGACAATTTTCCCACACCGTTAAATCACAATCGCGCGATCGAGGTACAGTA
ATTTTATCGGGGTTTGCCCCTGCATTAATCAATTCCGCAACCACTTGTTTCCCTAGAAAACCAGCA
CCACCCGTGACAAGAATTTTTTTCGTACTTAAATCTAACATAACAATAAATAGTTAATAGGTTAA
TAAAATTGAGATGTATATATAGGAACAAAGCTAAGTGATGCTAAAACGGAGAATAATATCATCA
AAATCTCTATCTCCCCCTCCGTTTAAATCCTCAAAGCCAATGACATTAGCACCAAAATTGGTGAC
ATGATCAAGTTTGTCCGAATTTGCACCAGCAAAGGGAAAATAAATTTCTTCGCCCCCATTGCCAA
AATCTCCAAAATCTACTTTTAGAAATGGTGCATATAATTCACCGCCAAAGATGCTAAAAGCAAAA
CTTTTAGTTGTGAAATTTTCTGCGGCTAAAGTTGAGCCTGAAGTTGATAATCGGTTTGATTCAGA
TAAAGCAACCTCTGCGTAACCTGCTTCTGTTGGGTTAATTTCTGTACCCGTGAGAGGATCTAATA
CTGCCCCAGATTCATTTAAAATACGATAAAACCCCATTTCACTATTAAAAGCCGCTTCACGACTA
TAAGAACCAGAAACAGTAAAAGATTCCCCTTGAGTTAACTCCCTAGCATCAATGAAATTAATGGG
AGTGGGAGATGTTAGCTCATCTGTCAAATCGGTGACTAAATCCGTTGAGGCAGAAAATGAGGCAT
AAAAAACTTTACCATCAGGATCTAAATATTGATAAATTTCATAACCAGAGGCTAAATCTTTGACT
GCCACCCCCATGTTTTCCCCAATGGGTTGACCTGTATAGGGATTACGCATAACCGCTCCATCAACT
TCTACATAAGAAACATTGGAAACATGGTTAATATCATTATCAACATTGACCTGATGGGGTGTACT
CGGAACAGTAAAAGGACCTTCAGGATTACTGCTTGGATTATTACCGGGGCCGCCTGGATTGTAAA
CTACAGAATAATCCCCAGAGGAAGGAAGTTCAACAGTTTTGATTTGATTAATAGCTTCTTCATCC
CCAGAAAGAATAATATCGTAGTAATTGTCGTGATCTTCAATGTACGTATTATCATAAGTGGGTTG
TGCTAAAGATAAATCTGCTAACCCTTCCACTCGAATTGAACCAAATCCAGAAATTTCATAATCAA
CCCCTGTTTCTACAAGCTCAATTATATTACCTGAAGAATCTTCAGCTTTCACCAAGAAAAATTTT
TGATAATCCGTTGGCAAAATACTAGCAATACCATCGGGGGAAAATCCAGCACTGGTATAAATACG
AAGACGGAAAAGGGCTTGTTCTCCATACAAGTCAGAACCGCTATTATTTTGATTAGTAACCAGCC

AAAGGGGCGCACCTTCTCCTAAATCACTAAAACGATTTAACTTAGCCGTAACCATTTTAGGTCCA
TTACCTACAACATAAGGATTTTGGCTATCAACAGTTATACCTACTGCACTCATTAAACCATCTTC
ACCAATCATTTCTAGTGGGGTTGAGTCTAACACCGTAGAAACGGATACAGGATAAATTGCTCCCT
CTGTACCGGGAATTCCCCTATTGCCAAATTCTCCCGTAATTACTACTGTTTGCCGTTCATTATATT
CTGCATTGGGCAAAAAAGAAGCAATAACAGGTGTCACCACCACACCATCGTTACGAGTAATTCTA
AAGTCTGTAGGATTAACGGTTGTTGGTAACAAGGGAAAACTAAAAACAACGGGAATATTATCTA
ATTTTTCAATATCAACATCATAAGTTGCTTGAACTGTATCAGTGCTAATAGCAGAAGTATAGGCT
CGTTGATTCATGGTTGTATTGCCCCCCAAATCTTGCCAAGAAACATTATTCGCAAAGGCAATATC
CTGAGAGGCTTGATCTGTTCCCGTCATGCCGGGTATTCCCATATAGCCTTCAAATCCATAATTAGC
AGTAATAATTGTTCCTTCTTTACCAAAAAGATCTGCATTAAGCAAGATACTTGTATTAAGTGAAG
AACTTTTCCCAGATGTGTTTGACCTATCGTTGACGAGGTAGGCGATAAGTTGATCTGCTAGGGAG
GTATATTCAATTGTCATAAGCTATTATGCTCAAGTAATGTCAAGGAAATATAAATTTCGATCATC
GAAGGTAATGCTACACCGAACGCAATTTCAAGTTCCATAGTACCGTAGATCGAATAATCGATTTG
CTTATTTTTGGATACGGTTAATGGGAGAAGAGGAATTAAGTAATTTTTTATCTCCTTATCCTCTT
ATTTTTTTATATCTTTCTAGTTAACACTAGGTCGCATATTATGACGAATATAGGCTTGATCTTGG
ACTTTATCACCATTACTGATACCTAAAGCCTCTAAGTCTGCATCTACCATCAAATTAACTAACTG
GGGGAAGGTTAAAGATGGTTGCCAACCTAGTTTTTCCTTAGCTTTAGTGGGATCTCCAATTAATA
AGTCCACTTCTGCGGGGCGTAAGTAGCGCTCATCAAATTCCACATAATCTTCCCACTGGAGGTTA
ACGTGAGAAAAGGCTAATTCCAAAAATTCTTTCACAGAATGGGTTTCCCCCGTTGCTACCACATA
ATCATCTGGCTCATCTTGTTGTAACATTAACCACATAGCCTTAACGTAGTCTTTAGCATAACCCC
AATCTCTTTTTGAGTCTAAGTTACCTAAATACAATTTTTTCTGTTGCCCTGCCACAATACGTGCG
ATCGCCATTGTGATTTTACGAGTAACAAAGGTTTCACCTCGGCGGGGGCTTTCGTGGTTGAATAA
AATCCCATTGCAAGCGAAAATGTTATAGGATTCACGGTGATTTACTGTTTGCCAGTGAGCATAAA
CTTTTCCACAAGCATAAGGGCTACGGGGATAAAAAGGGGTAGTTTCCTTTTGGGGAATTTCCATT
ACCTTGCCGAACATTTCTGATGAACCAGCCTGATAATATCGCACATCAATACCTGTGCGCTGTCT
ATAGTCTTTAATGGCTTCTAATAGTCTCAATGTACCCATCGCCACACTATCAACGGTAAACTCAG
GAGCGTCAAAACTAACCCTTACATGAGATTGAGCTCCCAGATTATATACTTCTACAGGTTTCACT
TCTTCGAGAATACGTCTTAGGGTTGTACCATCGGTTAAATCTCCGTAGTGAAGGAAAAAACGAGC
ATCTTCTTGGTGGGGGTCTTGATAAATGTGATCAATTCTATCAGTATTGAAGGTGGAAGTTCGTC
TAATAATACCGTGAACCTCATAACCCTTTTCTAACAATAGTTCACTCAAATAAGAACCATCTTGT
CCTGTAACGCCGGTAATTAAAGCTATTTTGCGATCAGTCATAATAAAAATATATTCCGGATATAT
TAAAAAAAATGTTTCCTACTGTTTTGCTAAGTGTAACTTAAGTTGTATCTTTTTTATTAGGTAAT
TTTTAGACTTATTGAGGTCAAATTCAGAGAAAACTAGACAAGTAAAGTTAATATATTTGATTTT
TATCTGTGATAAATTAATGGTTTTCAATTGTTACCTAAAATCGACTCTTATCGTATATTCTTGGT
TCTAACTGAGGTTTTATTTAGGGCATTTGTCTAAATATCCATGATCCCTGATAATATATGTCAGT
GTTCAACAGGTTTTGTCATGACTTTTTCACTTCTTATCAGTTTTGGCTTGATTCTCTTTGCCTATT
TGTTGGGTTCTATTCCCACTGGTTATTTAGTGGCTTTGGTTTTAAAAGGTATTGATATTAGGGAA
TATGGCTCAGGTTCTACGGGAGCAACAAATGTACTAAGAACCGTAGGAAAAGGGGCGGCACTGAC
AGTATTATTAATCGATATGTTAAAAGGGATCTTGGCAGTAGCGAGTGTAAAATTATTTTTTATT
TATGGTGGTGGGGAAATATTGCCAATACAATGGCAGGATTGGCTGATTGCGATCGCATCTTTATT
AGCTGTTTTAGGTCATAGTAAATCTATTTGGTTAAAATTTTCTGGGGGTAAATCTGCCGCTATTA
GCTTGGGAATTTTACTCACTATGAATCCCTTTTTGGGATTAGGTACTTTAGGAGTATTTGTAATT
GTATTGTCTATCTCAAAAATTGTTTCTCTTAGTTCTATCAGCGCTGCGATCGCAGTTAATATATT

AATGTGGCTTTTACAGCCTACTGCCCCTTATATTGTTTTCGCTATCATTGCCGCTAGTTATGTTAT
TATCAGACATCGCAGTAACATCGATCGTATCTTATCAGGAAAAGAACCCCGTATCGGACAAACGG
CATAATTCAATCAGCAATCACAAAAAGGGCAAGGGGCAAAGGTGAATAGTGAATAGTTGATAAT
TAAAAATTAAAAATTAAAAACTCTAAACTCCGTTCACGACTGAAAGGAGTGTAAAAGCCTTCGGT
GCGAACTCTCGTCTTCCCTAACACCTGCAACCTGCAGCCTGACACCTAACCTTATCCGATATTCTT
AAACTGAACTGAGGTTATTTATTGACATTTTTTAGATAACTAATATCTGTATTTGTGCCAAAACT
ACTTAAAACTCTTGCGGCTTCGTCATAAGTAACCTCTTTTTGAGGTTGAAACAAAATAATATAGC
CAAAGGCTTTTCTCGTGTTGGCAGATTCACCATTTTGCCAATCTAAGTATAATGCAGACCAAGCC
CCCGGATCTATTTTACTTAAATCCTGAAATCCCCATGTTTCCTTTAAAGCATCTAAAGTAACATT
AGGCAACTTTTCCCGAAAATCTAAAGGCACTTTCCACGTGATCAAATTTTCCCTTGTTAATGGTT
TATCTGGATTAAAACTGGTAAATTCTCCTTGCTTTAATAGGGGGGAGGGTATTAAACCTGCCTCG
GCTAATCCTTGTATCACGGCAAAATCTGGATCTGAAACTGGTACATCGGTAAAAATCTGTTTACT
ATTGGGATTAGCTTCACGAATTAATTTACTGTTGACATCTTGGAAAAGAAGATTGTTAGTTTGCA
CTAACCAACGGGCATATTCTCGACGAGAAATAGTATTATAGGGGTTTATTACTTCTGAGTTGGAA
ACAATACCTAATCTTGCTAATTCCTGTAGAGGAGGGGTTAAATCTTCACTAACGGTATTTTCTGT
ATTTTCTGTGGGAGTATCTAAAGAGTTATTATTGGCACTTTCTGAAGCATTATAAGTCAGAGATA
ACTCTGTTTCACTACCTTGAGGAGTAAGGATAAGACTTAATGTTAGATTATCCCCTTGATGAGTT
GCATTAATGGAGTTTTCTTGGGATTTAGTTATTTGCCAGTTATTTGCTGATAACTCTTGCTGGTA
ATAATTTACTATTAAATTTAAGGGATCAGGCGATCGCCATTTAATAGTTTGTTGGTTAACATTAA
TTAGAGTAGATGGTTCGTAAATAGGAATAATTTGAGGAAAGTTAGACGGAAGATTAATCTTATC
ACTATTGTTTTCTGGATTATCGGCTAATGTCGAATTATCTGTCTGATTAGGATTATTAGCACCAT
CAGTGGTATTAGTTGTATTATCATTCAACTGAGAATCAGGAGCAAAACGAGATTCTAAAGCCTGA
TTACCGCTACATCCTGCCAAAATAATCAATAGAGGAAGAAAGTATAAAATTCGAGCCATTGCCTA
TTTATCTGAATTAACATTATCACTATTCTATAGTTTTCTCATCGTAAAGGTTTCTTCGTTAGGGT
TTTAGCCCCATTTCTGATGTCTAAATCAAAAATAATTGAAACAAATAGCAATTTATGAACAATCA
TTATTACTACTCAAGCCTTTGAGTTATCGTTATTTTAAACCGTTACCTATTGCTTAAACTTGGCG
ACACCACTTTACTTTTCTCTCAATCCTTATTTAGTTTGAACTATTTATTAAGTGTTAGATTAAAT
AAAAACTATTAAATTCATAACGAAAAAAACATAGTATTAATAATGATTACTTTACTGACGGATT
TTGGTTTACAAGATATTTATGTAGGAGTGATGAAAGGAATTATAAAAACAATTAATCCAGATAT
AGATATTATTGATTTAACTCACAATATTCCTCCTCAAAATATTATCGCCGCTAGTTTTGCTTTGG
CAAGTGCTGTGGATTTTTTCCCTGATGATACCATTCATTTAGCTATAGTTGATCCTACCGTTGGT
AGTGATAGAAAAATAGTTGCGATCGCATTTGAAAAGGGTTATATAATATGTCCTAATAATGGCA
TAATAACAGGAGTTTTAAACAAGTATCAACCACAACAAGTTTATGAATTAACAAATAGTAATTA
TTGGCTTAATCACAATCCTAGCAACACTTTTCATGGTAGAGATATATTTGCACCAATGACAGCTT
ACTTAAGTAAAGGTATCGGATTAGAAACTTTAGGTCATAATCTTAATCAAGAAGATTTAGTTAT
ATTAGATAATATTCATCCCATAATTAAAGAAAAGGAAATAATTGGTTCAATTCAATATATTGAT
ACTTACGGTAATTTAATTACTAATATCCCCTCTAAAATGTTAGAAAATAAATCTTGGTATGTACA
AGAAGGAGATAACAAAATTTTTCCTCAATTCACTTATAGCAACATTAAAAAGAAAGAATTACTT
ACCCTTATCGGTAGTCATGGATATGTAGAAATAGCCATTAATCAGGGTAATGCTAAAATTATTTT
AAATAAAGAATATGGTGATTTTATTACAGTAAAAATAGAGTAATTTCTTCGCAATGATAAATTA
TTTTGGCTCTATTACTTCTCGTCGTGATAAATTGTTCATTGAAAAAGGGAAAAGGAGAAACCCCT
CTGTGTCTCCCCTTAAAAGGGGAGAAAGGGAAAAGGGAAGAGAGGATTTTAAAACTTAAATTTT
ATAAATTTTTTATTTTTATTTAACCTGAGTTTTGGATAAGCTGAAAGCATTATCTCGTAGTCAGA

AAACCTTATAGCTTTTTAGAAATAAGAATAAAATTGCCTTAACCCGAACTGACGTTACACCCCAA
AACCCCAAGCCCCTACTTCCCCTCTCATTCTCTCATCACTTTAACACCTGCAACCTGAAACCTGAT
ACCTGACACCTATCCTTTTCCGATATTCTTAAACCGAACAGAGGTTATTTAACCTAACACCTGTA
ACCTGTAACTTTCTACCGACAATCAATTATGTTTTTGCTTAACACCAGTTATTAATTGCTACTAA
TCTAATTTCTTAAAAGTTTTCAAACTCCGCAGACGATTTATAGCACAAGCTAACTCGAAATGACG
AAACATGGTTAAATCCCCTTGAGGAAAATCTGTTAATATATCAAGCCCTTTCGTCTCTAAATCTT
TCATCAATAAATTGATAGCTTCGGAAAGGGATTTTTTTGTCCTAAATATTTTTTCTGTGTATAT
AATATAGAAAATGCGATCGCTCTTAGTTGACCATTCTCCACTAACTGAGAAACAGAAGCTAAATC
AATATTTTCTATCCCAAATTGGATTGTATCTAAATCTCTGACTTGAATTTTGACATCTCGCTTAC
CTCTTTTTGCGGAAATACTTTCAGGATTAATAAAACGGGGAGTAATCTTACCAAATTTGATACTA
TCGCCCCCCTCCCTAAATTGAGGAAAATCGAGTGCAATTTGTTTTGCCTTATCCGTTACATCTAA
AGGTTGAAAACTATCCATAGCAATGACAGTATTTGCTACTTCAAAATAATCCCCACTTCCCCCCA
TCACCAAAATCGTAGAGACACCATAATCTTCATATAATGCCTTAATTTTATCCACTAAAGGAGTA
ATTGGTTCTTTTTCTTTAGCAATTAACTTCTGCATTCTTCCATCTCGAATCATGAAATTAGTTGC
TGAAGTATCCTCGTCAATCAACAATAACTTACTGCCCACTTCTAAGGCTTCCATAATATTAGCAC
TTTGAGAAGTACTACCACTGGCATTTGGAGTATGAAAATTAACCGTTGATTTACCTTGAGGAAGA
TGATTAATAAAAGGAGAAATATCAACTCCTGTGATGCTACGCCCATCTTCAGCTCGAATTTTTAC
CCCTGTTGAATTTGTTATTACTAATTCTCTACCATCATCAGGAATATGATTATATATCCCCAATT
CGATGGCATGAAGTAAAGTTGATTTACCATGATAACCCCCCCCAACAATAAGAGTAATTCCTTTT
TTAATACCCATTCCCCTTACTTTACCTCGATTTGGACAGATAAATTCCACTTCCAGAGAAGGAGG
AGATTGAAATTTAATCACTTCTTTTCCTTGTAAGGGCAAAGGAGAAATCCCACTATCACGGGGTA
AAATGGCATCGTTTGCCACAAATGCCACTAAATCTTGATTCTCTAATTCTTGACGAATATAATTG
GCATCTTCAACGGTTTCTACATGGTATTTAACATCTTGATGATTAAGTACATCATATAACAGGCA
TTTGTTAACTATTTCGGGGATATTTTCACAAAGTAATTCTATGGCTTGAAATCCTAAAATTCTCC
TCCCATTAGCGGGTAGCCCCACCACTAAACGCACTTCTAACTCTTGTTTATTCAGTAAAACACTAC
TGCGTTGTATCACTTCTTGAGCAATGTCGGCAATGGCAATCAAACCACTTTTTCCACTACCTCGCC
TTTGACTAATTTGTTTAAGATGATCAATAATTTGGCGATGAATATAATCTGTTAAAGCAATATT
GCGAATAGAAGAATCAAATAGATTTTCAGGAAATTTAGCTATGGATTGATTTATATTTATTCTA
ATTTTACTAGGAGAAGCAAAAGGATCTCCTTGAACATGATCTATTATTAAGTTAAAGTTATCGAA
TTTATACTCACTTTTTATACTTTTATAAGCTGGATAACTTTTTCCATCTAAGTTTTTTAATAATT
CTTTTAATTGTAGATTATTTTTCATTGATAAAATAGCTTTCTTACTTTGGACGTGATAAATTATC
TCTAAAAAAAGGTGACTGGTTTTAGGTTGAATTAAAAAATTATAATCATTAAATTAAGACGCAT
TTAACTCGAATTTTTCCTTAGTTTTTGAAAACAACTCAAACCCTTATCTTTTCCCTTTTCCCCTCC
TCCCTCTCGAGGTAGGATACAGGGAGGTTTCACTCTTCCCTATCTTAACCAGACAACTTAAATGC
ACTCCTAACTTAAAAATTCATCCCAAACTTAGGTTTATTACTCAAAGGGTAGGGGCGGCGGCAAG
TAGAGTTTTTGTATAAGGATGTTGAGGATTGTTAAATATTTTCTCGGTGACATCTAGTTCAACTA
TTTTACCACCATTCATGACGGCAATGCGATCGCACAGAAAACGGGCTACGGATAAGTCATGGGTG
ATGAATAGATAAGTTAGCTTAAATTCCTGTTTCAGTTCTAACATCAATTCTAATACCTGCGCTTG
TACCGTTGCATCTAACATACTGACAGGCTCATCACAAATCACTAATTTCGGTTGTGTAATTAAGG
CACGGGCAATGGCAACTCTTTGTTGTTGACCCCCGGAAAGTTCTTTGGGATAACGGTTATAATAT
TCTTTGGGGTCTAATCCTACTCGTTGTAGCATAGTTTCAACCCGTTGACGATTTTCTAATTTATT
GCCTAGTTGATGAATGATTAAAGGTTCAATAATAGATTCCCCTATAGTCATCATAGGATTTAAAC
AGGCGTGGGGGTCTTGAAAAATCATTTGAATTTCCCTTCGCAAACCTCGTAACTGTGACCTAGAA

AGGGTTGTTAGGTCTTTTCCCTGCCATAACACCTGCCCATTGGTTGCCCGAATTAATTGCAAAAT
AGTACGGGATAGGGTACTTTTCCCGCAACCAGATTCCCCCACTAAACCGAGGATTTCTCCTTGCCA
AATGTTTAAATTGAGATCATCAACGGCTTTAATAAACTGACTTTTTTTCCCCAATAATTGCTCTA
TAAAGTTAACTGAAATAGTGTAATATTTTCGCAGATTTTTTAGTTCTAATAAAGGCTTATTTTCT
TCTTTTTTTTCTTCTTGATTCTGATTTTCTGTGTGATGAAGATGAAAAGCGGCTTTTAACAATGA
CTTGGTATATTCATGTTTGGGACTATATATAACATCTTTTACTGTTCCCATTTCTACCATTTTTCC
GCCATACATAACACCGATGCGATCGCAATATTTTGCTACCATAGCTAAATCATGGGAAATCAATA
ATAATCCCATATCTCTTTCTCTACATAATGCAGTTAATTCCCGTAGAATTTCTCCTGCAATAGTA
ACATCTAAACTGGTAGTAGGCTCATCGGCGATAATTACTTTAGGTTCTAACAATAATGCAAGTGC
GATCGCAACTCTTTGTCTCATACCACCACTAAACTCATGGGGATACTGATTAAATCTATCAGGGG
ATATTTTCACCGTAGCTAAAGCAGTGAGGGCTTTTTCTTTTGCTTGAGATGAAGATAAATGGGGT
TGATGAGCCTTGAGAGTTTCGATACAATGATCTCCAATAGTCATTAAAGGATCAAGCCTTGTCAT
AGGGTCTTGAAAAACTAAAGCAACAACTTCCCCTCGAAATCTGCGTAAAGCAGAAGCAGAATAAT
CATAAATAGACTTCCCTTGAAACCTTGCTTCTCCCTCTACCGTTGCCTGATTAGGTAAAAGACGC
ATCATCGCTCTGCCCAAAGTCGATTTACCACAACCAGATTCTCCCACTAACGCCATTATTTCCCCT
GTATTCAGTTGAAAAGAAACACCATCTACTGCCCAAGAGGGGTTACTATCATCTGCTCTGTGAAT
AGTAGAATAGGCAATACGTAAATTGTTAATGTCTAAAAGGCATTGACTCATAAAAGAAACAGGG
GATTAACAAACTGGATAAGTGCTAGTCAATTATTGATTACTAACTGCTGATTAGTCAATTTTTTC
ACTAAATCTAAATTATAAGCAAAAGGTCTTTTTATCGGTTGATAATCTTTGACATGAGTTTCTCC
ATGACGTAATACAGCATTAACTAACAAACAAGAATCATTACTCAAATTGATTGCACCATGAGGAA
TACCGGGGGGAATTGTAATCACCTGGGGATTATTTTCTGTTAAATGTAGATACTGATATTGACGA
TTTTGCAAAACCACTAAAACAAAATTACCCTTTACAACTAATAATTGATCCGTTTGAAAATGATG
GACAAATAAATCATCAATGGTGTGCGGTGGGATTTGCACTAACATGGTTTCATGGCTAGACTGGG
GAGTATAAAATAAAGCCATTCCTCCTTGAATTGATTGCAATTTTTGTTGTGAAACACTCTTGATT
TGTGCCATTTTGTTCACCTTTTCTTGCTCTTTAATTCTAAGATAATCAGAATATTTGTTAAAAAA
TGTTAAGTTAGTTACATAAATAAACTTTGAATATAACTAACAAAAGACAAATTATTATTAAAAG
ATATTGGGAAAAAATAGATCAGAAACTAATAAATATAATCATATTTTAATAAATATTGAAAAGT
AATTTTATCAAAAATAAAAAATAGAAATAGATATGAGTATAAGTGATATAGCTATTAATGAAAA
TATGACAAAAATATTGGGAGAAATAGTATTTTTAATGGGCAGTTGTGACAATTCCAGAAAATAC
CCTATTAGTTTTATTATTAACTACCTTTTACCATCAATACATCTAAATCAATATAGACTTTATAG
AACCGTAAAAGAAAACAAACCCATTGGTTTTGCTTGTTGGGCTTTTGTTAGTGATCAAGTAGAAG
AACAAATGATTAAAAATGATATTAACTTGACTATTGAAGAAAGAAAGTCAGGAGAAAATCTCTA
TATTCTCTACTTTATCGCCCCCTTTGGTCATGCAAAAAAAATTAGTCAAGACCTAAAAAATAATA
TTTTTCCTTATAGAATGATCAAGGGTTTAAGACTGACTAAAGATAGTAAAAAAATAGCCAAAAT
CGCCATTTATCATAACAGAAATAAAAGTTAGACAAAATCTTTCATCAGGCTATAATCTAAGGGTA
AATATCCTATGTGAAGGAAAAAATACTCCAAAATGGGCAAATTATATCAAGGCACGGAAAGCCAC
GATCGCATCGACATTAGAAATACTAACTTTAACGTAGTTTGGGCAAAATCTGGGGTTGATGTTGT
ACACGCCAGAGAAGATAATGACACCGTCTCAGGGGCAAATGATCAAGACATCATTCATGGTTATG
GCGGAGATGACTTTCTCATGGGTGAGGGAGGACGAGATTATATATATGCAAGTCAAGGAAATGA
CACTATTCACGGGGGTTACGCCAATTGGGTTCCGGGTGGAAAAGATGACCTAGCCGACTACATCA
GAGGTGACGAAGGAGATGATGCCCTAATTGGAGAAAGAGGTAATGATACCCTTTATGGTGGCTCA
GGTAATGACTATCTTGATGGTGGAGATGATAATGACGAGATTCGGGGCGGTTCAGGCAATGACAC
AATTTATGGCAGAAGGGGCAATAATAGCTTATCTGGAGAAGACGGAGATGACTATATCAGAGGA

GGAGTCGATCGAGATGTAATTGATGGTGGTAATGGTAATAATGAGATTTGGGGAGGTAATGGAC
AGGATACTATCAATGGTGGTGTAGGAAATGACAAAATTCACGGAGATGAGTTCATTGAAGTAGA
TGTCAGACTGCCTGTAAGTCAATGGAAATATTATTTATTAGGACAAAGGATGTAATTAATAGT
GGGGCTGGAAATGACACCATTGAAGGCATGGGACACGATGATGTAATTGATGGTGGAGAAGGTA
ACGATATTATTTATGGAGATTATATAAAGCCTATAGGAACATATGTAGAGGATATATATGTATA
TGGAAATGATTATATTAACGGAGGTTTAGGAGATGATGTCCTTTATGGCAATTGGGGAAATGAT
ACTCTTTTATCAGGAGAAGGTACAGATATTCTCTTTGGGGGAAAAAGAAACGATGTACTAAGGGT
GACAGGAGATCCCACCCAACAAGGAATCAATATATTATTTGGTGGTGAGGGAGATGATAGCATTA
TTGGAGGTGATCATTCTACCCCAGCAGAAAATCTCCTACTGCCCCCATTTCTGGAACAAGAGCTT
AAAAACTTAAAAGACTTTATCAAAAGTTTAGAAAATGCCAACAATTTTTTACCTACTATCGGTGA
AGTAATAGATGGTGGACGAGGATTTGACACCATTGAGGCAGGAGAAGGAATGGATATTATCATG
GCAGGATTACCCACATCTGCCAACACTCAAGACAAAGTGCAGTTGATTTACGGTAACTCCAAAGA
CTCGAAAATACCAGAGCCAGATGTATTTGTCATCACTACAGTAGAATTATCTCGACAAGAGCAGA
TTAATCAAGCCATGATTACAGACTCTCTAAATCTAGTTTTTAAAGGGTTGAAAATCTTAGCCACA
ATCGTTGATGTTTCACAAACCGTCGTACCTATCGGAAAACTACCCGGATTAGTTCTTGATATGGG
GGTTTATGGGGTAGAGCTATGGAAATTTATTGATAAATACACGGGAAAACCACCAGATGAACCG
AGTATTCTCGATAAAATGACGGTAATCCCGGATTTTGATCCCGGTTTAGATGTCTTGTATTTAGA
TAATGGCGGTGGAAAACTAAGATACACAGTAGAGTATAAAAGTCAGATTATAGGGAACAAAATT
TATCAAGGTCTTGTATTCAGTGATGCTCAAGGAAAGATACCACAACCGAGAGTGCTTTTACAGGG
TTTTACAAATCGTGACATGGAAAGAATTGTCAGAGATGAGAATCAAATTTTGCCAACTACTGGGG
AGTTTGTCGGTATTTCTTTCTTACCAAATCTCAATAGCCCCGATAATTCTGAACCCCCAAGACAAC
CGTCAGAACCGTTGAACTTAGGAAATTTTTTTACTCTGACAGAAGATAATGACTTTGTCCAAATT
GAAAATTCTAATCCCACTGAGATAATTACTTTAGCAGGAGATGACACAATTATAGTGGACACTCA
ACAAAAGGTTTTAGCTGGTATAGGAAATGATGAAATTTATGCAGGTGTAGCCAGAGGAGGCAAT
ACTTTATCTGGGGGACTAGGGGAAGACGCATTTTGGTTTTATGATGAGCATGGAGGAGAAGTAA
TCAATACGATCAAAAATTTACTAGGAGATGTTGATCAAGTTGATTGGGAAGTATGGCAAGAGAA
TGTTTACCAAGAATATATCAACACTTTTGGCGAGGGAAGATTAAATGTAGTTGTCGATTTTAATC
TTGAAGAGGATTTAATTGGTTTTACTGATTTTCTTATTCCTGTGGGAATAGATAATGTTAGCTGG
AGAAAACAGGGTAGTGATTCGGTTATTTCTTTATTTGAGAGAGAGATTATTCTTTTACAAAATAC
CCCCACGGAAAATTTAACTCCTGAGCATTTTGTCTTTGATAATTCTTTGTTTGAAAAAATTTCTG
ACGGGGATAATATTCTTAATACTTTTAGTATTTTAGATGAAGAAGAAAACTTAACTGATACTGA
TAATAATGGTTTTGATGATGAGACTATAAATATTAGACTAAGTAGTGAGGAGTTAATCAGTAAT
AGAAAGTTAATTAAGATTCAAGAGGAAGAAGAATTTTTATCTTCCACAGATACGAATATTATTG
TAGGCACTGATGATGATGATAGGATTATTGTCGATGGTAATCAACAGGTTTTTGCTCTTGGGAGA
AATGATCAAATCTATGCCAGTATATCTTCCGGAGGTAATATCTTATCTGGTGGTACAGGTAATGA
TGAATTTTGGTTTTATGATGACAGTGACGGGGATTTAGTTTTAAATATCGTTGAAAATGTGGTT
ACTGGTGGTGGTAGTCTCAATTTTGATGTTATCGAAGAGTATGGTAAAAGTTTTCAGGATGGAAT
TGTTAATACAATCACTGATTTCAATCCAGAAGAAGATGCGATCGCATTTGTGGATTTTCCTTTTC
CTTTAGGGCTAAATAGTTTTGAATTCAGACAAGAGGGAAATGATTTTATTATTTCTTTATTTGAA
CGAAATATTATTCTCTTGCAAAACATTCGAGAAGAAGACTTAACCACAGAGAATTTTATTTTTGA
TAATTCTTTGTTTGACCAAATTTTGACCAATACTCTTAATAACTACACTAATAAGCAAGTAATTG
TCGATGTTTCTTCTGCCGACACCGCCTTTCCAGAAGACGTTATTATCACTTCACCTCAGCCTGATA
CCTCAACAGTATTCATTGCAGGTAATCAAAATCATACCTTTGAGTCAGGCATTGCTCCAGACTTT

TTTGACCTGATTTCTGGTGGTACAAATATTATTCAGGGGATACTACCCCAATTAAATGAAGATAT
TATCGATGGATTCAGAGAAAATGATCAAATAACTATTCAAAATAGTCTTTTAAGTGCTGAACAA
ATAAAAGTATCTTTCGCTTTTGGTTCTGCTATTTTAGACATTGATGGTGATGGTGATGACATAGA
TTCCAGAATTACCATTGAAGGTGATTTCACTGACGCTCTATTCCTTGTCAATCCGAGAGAAGACA
ATACCGATATTACATTGAAAGTGCTGGATACTCCTATCTATCGTTTTCAAAATAAAGATGTACCG
GGGGCTTATATATTTGCAGGAGAAGAAGAAAGCCAAAGTATTCGAGATAGTTATACTAACTTCAC
AGAAGAAGGATTAGCTTTTAAGGTAGCTCACCAAGGAGATAGTTTAATCAGAGTTAATCGCTTCC
AAAATAAGGATATACCGGGCGCTTATCTATTTGCTAGTGAAGAAGAAAGTAAGAGTATTCGAGA
TAATCATACTAACTTTATAGAAGAAGGAATTGCTTTTTATGCGGCATCTGCGGGAAGTAGCACTG
GCTCTGGAATATATCGCTTTCAAAGTTTAGTTAATCCTGGTGCTTATATTTTTGTAGGGGAGCAA
GAAAGACAAAATATTATTCAAAATTACTCTGATGTTTTTGTAGAGGAAGGCATAGTTTTTGAAG
TGGTAGTTTAAAAAGTCTTTGGGATACCTCTGTGTATCCCCTTGAAAAGGAAGATGGGAAAACAG
TTAGATGGTCTATCTTAAATGTCAAAAGAGGACTCCTATCAGCATTTGACAATACAAAAAAATAC
ATTACAAATCCTTTAGTCCTGACGAAAGGAAGGAACTGGGAGATGAATTTTGACCAACAAAATA
AACCTTTAGTCCTTAGTATCAGTGGGTTTTGGTAATCGTTTACAGTAATCTTGGATAATCTCCGA
CATACTTACATTTTTAGACTTAGCGTAGTAAGCAAGTCTTGTTTTTTCGTCTTCGGTTAATCTTA
AGGTAAATTGGATTTTTCTGACCATTGACAATACATTAAATATGATGTGCGATTAACGATAACGT
AACCAGTAATGTGGGTGCTAGTGCGACAAATAATTACAGTACCTAGATAACTAAATAGTCAAAGT
GTAAGGTTGTTAACTCGAAAAACTCAGTTAACGTAAAACCTTTAGCCCTGACGATAGGAAGGGTC
TGAGAGAAATGACACCTAGATACTGGTTCCGTAGGGCATAGGGTAGCACAGATAAGAAACTGTGT
TTAAAATGCTTGTGGAGTCGGTCTGACGGGGGTTAAGAGCAATCTTGATCTAGTTAAGAGGCAAT
CTGAAACTTCAGCAAGAATCACCCATCACAATCTTTGATTTGATGGTGTGAGTGTCAACAAGTGT
AAATCTTGGGTGTACTAACAAGTAGGGCGATGTATCGCCCCACTAAAAAATTAAGATAAAGTCTC
AATTAATTTGTTTTTAGCAATAGTTAAAGCCCCTTCTAATTTACTGGTATCTTTACCCCCACCTT
GAGCAAAGTTAGGTTTTCCTCCGCCTTTTCCATCACAAGCAAGGGCGATTTCACGGATATATTCA
TTGGCGTTTAATCCTTTTTCTTGATAAATTTTATCGCTGAATACCCCTACAACGCTAACTTTATT
TTCTTCAGGAATAGAAGCTAAAATGACTGCTGAATTACCTAATTTTTCTTGCAATTTTTTCGCCG
CAGATTGTAATGATTTTGCATCCAAATTATCTAATTTAGCCACTAAAATGTTAAATTCTCCGATA
TTTTGGGCTTGATTAACTAGATTTTCAGACTTCAATAAAGCTAACTCTTGTTTAAGATTTTCTAC
TTCTTTTTGAGTTGCCTTTAATTCATCTTGTATATTGGCAAAACGTTCGGTAATTTCTTCGGGTT
TAGCTTTTAATTTATCCCCTAATTCTTTCACCACATTATCTCTTACTTTGAGATACTCTAAAACTG
ACGCACCTGCTACCGCTTCAATTCGTCTGATACCAGAAGCAACCCCCGTTTCGGAAATAATTTTA
AATAGACCAATTTCTGCTGTATTATTAACATGAGTGCCTCCGCACAATTCCATCGAAACGCTAGG
AATATCGATAACACGCACTTCTGAGCCATATTTTTCTCCAAACATTGCGATCGCACCTTTGGATT
TAGCTTCTTCTAAGGGCATTACGGAGATATAAGCAGTATGGGCTTCAGCAATCCAAGTGTTAATT
AAATCTTCAACCTGTTGTAACTGAGATTTAGTGAGAGCTTGAGGGCAATTAAAATCAAAACGTAA
CTTATCAAAGGATACTAAAGAACCAGCCTGAGAGATGTTTTCATCAACAACTTTTTTCAAAGCAG
ATTGTAATAAATGAGTAGCGGTATGATTGGTTCTGACACGGTTACGACAGGCTTTATCAACGGTA
GCACGTAGTTTTTGCCCGATGTTGATTGTACCTCTTTCCACTGTGCCATAGTGAACAAAAAAGCC
AGACTCTTTCTGCACATCCTCGATAGAAACTAACACATCGTCTCCTGCTAAATAGCCCTTATCGCC
AATTTGACCACCTGATTCAGCATAAAAAGGAGTTTTATTCAAGATTAACTGAACTTTACTTCCTG
ATTCTGCTTTTTCTACCGATTTACCCTCAACTAATAGCGCCTCAATCTGACTAATTAATTGATAT
TCGTGGTAGCCTAAAAATTCCGTAGGGTGGATATGCTCCGCTAACTTATCGATGCTACCCTGTAC

CGTTAAATCAATGGTTTCATGGGCAGATTGCGATCGCCCCCTTTGAGCTTCCATTTCCGCCTCAAA
ACCTTCAATATCAACGGTTAAACCCTCTTCTTCTGCAATTTCTTGGGTCAATTCGAGGGGAAAAC
CATAAGTATCATAAAGGGTAAAAGCATCCTCTCCTAAAATAACGGTTTTTTGGTCTTGTTTTAGT
TTAATAATCACTTCATTTAAGAGTTTTTCACCCCTTTCAAGGGTTGCCAAGAAAGCAGATTCTTC
TCGTTGCAACTCGGTTTTAATTACCTTTTCCCTTTCCCTAGTATTGCTATAAACCCCTTCCAAGAG
TTGTATCGCCTTTTCTGCCACTTCATTGATAAAATTACCATCAATACCAATTAAACGACCATGAC
GCACCACACGACGTATGAGACGACGTAAAACATAACCCCTTCCCGTATTAGAAGCAGAAACACCA
TCGGCAATCATTTGAACTACAGAACGAACATGATCCCCAATAACTTTTAAAGATACTTTTGTTTT
ATTCTCCGCCGTGCCATAGTCAATATCTGCTAACTTAGCCGCAGTTTCAATAATGGGGAAAATCA
AATCAGTTTCGTAATTATTCGGCACTTGTTGCAGAATTTGTGCCATTCTTTCTAACCCCATGCCCG
TATCAATATTTTTATTTTGCAAAGGTGTTAAATTACCTTCTGCATCTCGGTTATACTGCATAAAC
ACCAAGTTATAAAACTCGATGAAGCGGGAGTCATCTTCTAAATCGATGTTATCATCCCCCAATTC
GGGCTTAAAATCGTAGTATAACTCAGAACAAGGTCCACAAGGACCAGTAGCTCCAGATTTCCAAA
AATTATCTTCCTCTCCCATACGAATAATCCGTTTTTCTGGTATTCCTACCTCATCTCGCCAGATAG
CAAAAGCCTCGTCATCTTCTCTAAATACACTAACGACAATTCTTTCTGGAGGTAACTTAAACACT
TGGGTTGACAACTCCCATCCCCATGCGATCGCTTCTGACTTAAAATAATCACCAAAGCTAAAATT
CCCCAACATCTCGAAAAAAGTATGATGTCTGGCAGTGCGTCCCACATTTTCAATATCATTAGTGC
GGATACACTTTTGCGAAGTAGTAGCACGGGGATATTCAGGGGTTTTTTGCCCTAAGAAAATAGGC
TTAAAAGGAAGCATTCCTGCTATAGTAAGTAAAACGGTGGGATCTTCAGGTATTAATGAAGCACT
AGGAAGGATTTTGTGTTGTTTTTCGGCAAAAAAGTTTAAAAATTTCTCTCTGATTTCGTTTCCTG
TTAAAGAAGGAGGAATATTAGTCATAAAGTATTATCAATAATGGTCTATATCCTATTATTGCTG
ATTTTTGTTGCTATCATTAAGAAAGTGTTTCTAAGAGTCGTTGTTAGTGCGAAAGTATGGAATTT
GAGTATGATGAAAATAAAAGTAATATCAATAAAGAAAAACACGGCATTGATTTTGAAGAAGCCC
AGTTATTATGGTTAGACAATAATCGGATTGAGATAAAAGCAAATTCAACCAATGAATTTCGGAT
GATAATAATTGGTAAAATTAAGGAAAAATATTGGTCCGCAATAATAACATATCGCCAAACTAAA
ATTAGAATTATATCTGTTAGACGTTCGAGAAAACAAGAGGTAAATTTTTATGAAAGCTGAAGAA
TTAGATTTATTATTTGAAGAAGGAAAAGAAGATATTTTAGAATATTTTGATTTATCAACGGCAA
AGCGCCCTGCTTTGGAAAAGACAAAAATTGATTTGGATTTACCCCAATGGATGATTATTGCCTTA
GATAAAGAAGCAAAAAGATTAGGTATTGAGTCTCAATCTTTAATTAAAGTTTGGATTGCTCAAC
ATTTAGATTCCGTTAAAATCCGTTAAAATTATATGAACAATCCTAACGAAGATTTTACTCTAAAA
ATTAAGCCTCGTCCATCGGAAATAGTATCAATAAAAATTCCTTTAGATACCTTAGCAAACTTAGA
AATGATTGCCCAAAATCGGAATTTATCAGTAGAATCTTTAATTAAATTTTATATTGGAAAGAAT
TTAAGAGAAGATATTAGCCAAGAGTTTTCTGAAAAATTATTTAATTCTACCCTTAAGGTTTTATC
GAAATATATTTCTTCAGAATCTCAGCGAGAAAAAATTATTAATGAAATTAAATCTCAACTTTAA
TTGTTAATATCTCAGCTTAATGAAAAAGGTAGAAAACAAATGAGAAAACGAGATGTCAATTTTT
ATAAAAGCTGAAGAATTAGATTTATTATTTGATGAAAGAAAAGAAGATATTTTAGATTACTTTG
ATTTATTAACAGCAAAGCGCCCTGCTTTAGAAAAGATGAAATGAAACTTGATTTAGACTTACCCT
AATGGATGATTGTTGCCTTAGATAAAGAAGCAAAAAGATTAGGTATTGAGTCTCAATCTTTAAT
TAAAATTTGGATTGCTCAACATTTAGATTCTGTTGAAATTATATGAACAATCATAACGAAGATTT
TACTCTAAAAATTAAGCCTAATCCATCTGAAGTAGTATCAATAAAAATTTCTTTAGATACCTTAG
CAAACTTAGAAATGATTGCCCAAAATCAGAATTTATCAGTAAAATCTTTAATTAAATTTTATATT
GGAAAGAATTTAAGAGAAGACATTAGCCAAGAGTTTTCTGAAAAATTATTTAATTCTACCCTTA
AGGCTTTATCGAAATATATTTCTTCAGAATCTCAGCGAGAAAAAATTATTAATGAGATAAAGTC

GGAACTTCGATAATTAAAAACTAGCTATTACTATTCAACCAAACATAAATACTTTAGGAAAATGA
TTAAAGAAAAACCTCAAATTTGTACAATTGATTTGGATAAAAATATAATTCAAGCCATCGAAAA
TACTAATTTGCATTGCTATTCTGGAACACTAGGTAATCCAATTAAAGTTCCTAACTCATCTAATA
ATCAAACTACTATTTTAGATTTGCTTCATTTTGAGTTTCCTCCAAATCTTCAAGAATATGACATT
ATCATCATAGATTTAAAAGATAAAGAATCTATAGAGTATTGTGAACCAAATTTTGATGATTTTA
GTGGAAATAAAAGAAGTATTTTCTTAAGCAATTTTCCCGAAACCATTTTTGATCCTAGACCATTT
TCTTCTTATCATTTGAAATCAGAATTAAAAGAATTTTGTGATAAAGAAGTTTTAATAATTGTTT
TTTGTTCTTCAGATGTAGCAATTGATTATAGAATCCGAAAAATTAACTCTAATTATTCACTGATG
GATGAGATAAAAAATTATTCTTTATATGAGTTTTTACCATTTTTGCATAGATTTTCTAATGAACA
CTATCTATTAGGAAAACAAAAATTTGGAAAAAATATCAAACCATTTAATTTAGATTGTTCTTTG
ATCGATACCCTTTCTAAATTTATTGATAATTTTATCTATGAAATGATTTTTGATCATCCGAAAGA
ATGGGATCAAAATAATAAAAAAGAATCAAAAAGAAAGAGTTTTATTCCTTTGTTGTTAAACGAC
CAAAATGAAATTATAGGATTTTTAGATTTAACTGAAAATATTAGTATTTTAGCCTTACCACAATT
AAAAAATCAAGAGCAGAAAAAGAACCTTATTTTGGAACTTCTGAATAATAGTTTACCTGAATTA
TTTCCCAAAATTTTTCCCTATTCAACACAATTTGCTTGGTTAACATCTGAGTCATATTTTTTACC
TAATCATGCAAAAATATTGCAAAAGAAAAAAGAAATAGAGGAAGAATTTAAACTTAAATTAAAA
GAAATTGAACAAGAGATAGAAGTCAATCATAATAAATATAAGTTTCTTCATGATTTAATTACGG
AGACAGGTGAAAACTTAGTAAAATCGGTGGAATATTTTTTACAGTGGCTAGACTTTAAAAATAT
AACAAATATGGATGAAATTTCTACAAAAATTAAAGAAGAAGATTTACAAATTGAACTGGATAGC
GGTTTATTAGTTATTGAAGTTAAAGGAATTGCTGGAACATCTAAGGATAAAGAATGCTCTCAAA
TATATAAAATAAAAAATAGGAGAACTAAAGAGAGAAATAAATTTGATGTTTATGCTTTATATAT
TGTTAATCATCAAAGATTTTTACCTCCTCATGAAAGAAAAAAATCCACCTTTTTCTCAAGAGCAAA
TAAAAGATGCAGAGTCAGATGAAAGAGGATTATTAACTACTTATCAACTTTTTCAGTTGTATTTT
AAAATTGAGGAAGATTTTATCACGAAAGAAGATGCAAGAAAATATTTTTTAGATTATGGATTGA
TTGAATTTAAACCATCAAATTCTCATGAGTTGGGTTCTGCTATTGAAATTTACAATAACAATACA
GTTGCTATTTTAAACATTGAAAATATTACTATTAATCAAAACTCATCAATTATTATTTGTAATGA
TGATAAATGGTTTAAAGCTAAAGTCTTAGAGATACAATTAAATGGTCAAAAAGTTGATGAAGTT
TCTAATGGGGAAATTGGAATTAAATTAGATCAAAAAAATAACTAAGAAATCAATTTTATGGTTAC
AACAATAATGTAACTAAAATTATTTAACATATCTAAGAAGAAGGGATATTAGTAATCAAATATT
CTCAATAATTAGTCTATTACTAAAAAAATCTGATTATAATATCAAGAAATAATGTTGATGATACTC
AAAAACATTTAGAAAAACAAATTAATCAAAAAATAGCTAAATTAAAAAGAATAAAAGAGGATAA
ATAATTATGAATAAATTTGATTATACGATTACTATTCAATGGAGTGATGAAGATAATTGTTTTG
TGGTTTTTTTCCCTGAATTTAGTCAGAATGTTATGCAACCTGTTACTCACGGTGAAACCTATGAA
GAAGCCTTAAAAAATGGGCAAGAAGTTTTAGAGTTAATTATAGAAGAATATCAAGAAGATGGTA
AAATTTTACCTCAACCTAAAACCTTTGTTTTTGCTTAATTATTACCCCATATTTTCACCCATAAT
ATTCTTGAAAAACTTTATCTAATAATTCACCATTTAAGTTTAATATTTCATCAGTGAGACGAATA
ATTTCAATATTGGGCATCGCTTGGGGGCGTATTTCATTCACAATGTTTATCGCTTCTTGAATGTT
TTTAGTAAGATAAGTGAGAATAGTTAAAGCCATACCAGAAGAGCGACTTATTCCCTCATGACAAT
GAATTAAAAGTTTACCTTCACCTTTATAACTTTCTTGAAAATTATCAGCAAAATTTAGTACTTGA
TAAATTATATCCTTTGTCACAATTTCCAATCCTAACTCATCTTCTTCAGACCAAATATCATCAAA
TTCTAACTCTAAAACACTATGACGATTTTCCTCTTTTTTCTCTGTAATAGATAATGGAGCATAAA
TACGCCATGCAGGATTCATAATTGAAATAACTCCATCGGCTTCATCAATATAATCATTGACTTCA
CTCACTCCACAAATGATAATTTCCATATTACTTTTCTCCAATTTTAATGATAAGATTATTCACTT

CTTTTACTACTGTTATATATTTTATATTCAGAGTAAAATTCCCCTAAAAAGGGGATTAAAGGGGA
TTATTCAAGAACAAATTTAGCCAAACATACCGTAAGGGGCTTCTTCTAACCAAGTTTCTACTAAT
TTAATAACTTGTTTATTATTAATATCAATTTTCAGGTTATGATGATAATTAATATACTGAATTA
ATCCTGTTTGTAGGCTTTTTTTAATTATATTTAATTGATTTTCAATTTCTTTTTTATTCACTTGA
AAACGGATTGCAATTTCTTCTTTATCTAAACCGATTTTCTTTTCATTAATTAAATAATAATCGCC
TCCATAGGTTAACTTTAATAATTTTTTTTGAGCAGGACTTAAATTAACAAAAAGTTGTTGTAACT
TAGCATATAAAGGCTTTTTGTAGCAATTAGTTAACCAATTTTCAATTAATTTATTCACCGTATTG
AGTGGAGGAGTATTGAGATTGATAGCTTCTGCATCGTCATTATCTTTACTCAACTTACTTTCAAC
TACCTGAACAAAATTCTGGAAAATTGTTTCATTAAGTTTTCCTTTTTCTTTAGATATTTTATCCT
GACGACTGTCTAACAATTGGGCAATGGTAGTTTGTCTTAAACCTAAACCATAATTTAAAGTTAAT
AATCTTAATTTATGTTGATCATTTTCTTTTATCTTTTGTAGAATTTCAACCAATAATAGGTTTAG
TTCTTGTTTATCCAGAAAATTTGAGGAAGAATTACCTGTCATTTCTTTTATTTCTTCTTTTTCTC
TTTTGAATAAAATGTTTTCCAGACTTAAACTATTAATCGCATCTTCATTATTATCTATATCTGAT
TGTAAAGGAAGATTATAAGCATAAGCAGAAGGACGTTTTATTTTTTCATAAGCAACAAGAGAAT
TATGACATTTTCTCATAATTTTTTGAAAAATAGAAGTAGAAATATTGAGATAATTATTTTCACCT
CCAATACTAGAATCAATGTTGATTAGACTTTTATAAAGACCAACTATTTTAGTAATTTTTTCTAA
AGTTGGCTCAGAAATCATTTCCCCTTTTTTGATTTTTATTTCATTGCTATAAACTTCTTGAAAAC
ACTGCCAAAGTAACAAGTAAGAATTAATTTCTTTTTGACTATATCCTCTGTGATTTAAAGCACTC
TTTAAATTATTCTTAGTGGTATTTTTTAGCAAACCCCAATAACTATACTTAGCGACACCGTATTC
TTTATTAAGACTATCTTTCATAAGATTGTATAACCATCTTTGGCTATAAGTTTTAATATGAGAAT
AGTTAGGGTTAAATTTTCTAAATTCTTTTAATATTTTATGAGGATTACTGGTATAGTCTCTAAGA
ATGTTAAAGCCCTTAATATATTTCTCTGTTTCAGAAAGTTTACCTAAATAAACATTTTTTCTGGT
AAAAAAGTTTTTAATTACCCAATAGCAATTAAATTCGAGATAAGCCATTAAATGATTTTTTGCCC
AAGGATTATTTTCAATATGATTCAACCAATAAATAAACCACTGATTTTCATCCAAATCATCTAAA
TTAGAACTATGTTTATTAAAATTGAGAACTAATTTTACATCTTGTATCCATCTTAATTGAACGTA
ATTTTGTTTTTCTTCCCAAACTAAAAATTTAGTAAATTTATCAGTTAAATTGATAGGTGCAGAAA
TATAAGATATTGTCATTTTTTTCAGTGGCGATAAAGTTTATAATATCTTCATGAGCTACTTTTGT
TTCAGCTCGGAAATTAGTATTTAGTTCTAATTAATTCAGTTTTCCTTTCAGTCTGATATAATTAA
ATAAATTGTTTAGTATTTAGGTATTAGTTCGTTGCAGAAATTGAGGACAATATAAACAAATTGA
AAGGTTTTGATAAGCCATAAATACTCAATTAGCTTTCTGATAATTTCAAGTCTCCATTTATATAT
CAGGAGAGATTTTATTTTTTATGCAATTTGAACGATAATTAAAAAAATTTTCCACCGTAATCACA
ACAGTTAAGTTAGTTACTGATTACATTTTTATAATTTAATTGTTCTTGATAGAAATATCTACGGG
TTTGATGCGAACAAGGAAGATTAACTTATCAAGAGATATTTTGGCTTTGCCGTTGAGAGTAACAA
AGCCCTAAAAATTAATTGTTTTAAAGCACTATGACCTCAGTTTAGTTTAAGAATATCGGATAAGG
AGAAGTTTTAGGCTTTATTTTTCTAATACTTAATGAGTCAGTAACAAGATTGAAAAGAAAAATA
TCAATAAGTATTAAATTATTTCACTGAATTTATGATCAAATGTGTTATTGTTGGCTCAAATGAAC
CTTAAAATTGAATATTTGACCTTAAAACTATGAGGTTGTTGGTAAGGATTAAGTTAATTTTTCCT
TAACTATTAGGCTACCAACGTAAAAGTTTTAGCTAGAAATAAAACCACCAAAAGTAGTCGAGGAG
TTTAATCTTTTAAGATTAAGGACTCGATAGTGAGCTTTGTAGTTAAGCCATTAGACGTTAAAGTC
TATAAAGCTAATTGAGTGGATGTTTGTCCGTAAAATAATTAGTAACTATGAGCTTTCTGTAAATT
CCTATTACTTTTTCCTCCCCAACATAAAAAAAGATTTATGCAAAACTCAGGTCACTATAAACTGT
GAATAAATAAAACTGTGAATAAATGTTAAGAATCTCTGAGCCTCCTAAACAATTTGACGATCGAG
ATAATTCTCTAACCTAATATTAGACACCTGATACCTTTACGCCAAAGAAAATTTTATATCGAATT

TAGGCTTTGACTGGTGTTTTTATCGGATTAATCAAAATATGGACGCATTAAGAAGACTTGTAATA
ATATTATTGAATTTAAAGAATTAATAACATCCCATGGCGGTAGCTGTATAGGTTTTGCCCAAGGA
TTCTAACCCTCCTACAAATAAATTAACTTGATAGGGTTGAGCATCTGGGCGAGGGGTTGATTCTA
TTTTTAAGGGTTTATTTGTAACGATATTCACTGCTTCTTGATTAAAAAATTGATCAGCAGTTTGG
TCACTGTATTTAAGATATATTCTAATATCGAAAGGGCCTCCGTCATTGGAGCTAATTTCAACTAA
ATAACGACGGAAAGTGTTGTTATTACTAGGTACAGCCCAATCTGTGTTCCAGTTATTCCGAGTAA
TATTGATACCAAAAGGTGCGGGAATGGTGGGGGTGGAAACAGTTTTTGTCACAGTGCTACCTTGT
CCACCAACTAGAGGAATAGGGGTACAGTTTTCTGCAGAAACAGGTATTTGCAGTGCAAAAAAAAT
ACCCAAAAAGCTAACAATGCTCAAACTATTTATGTTAATCATTCTTTTAATGACCTCATTATTGC
CAAAATGGATTGGTTTCATCAGTGGTACAAATCATGTCTTGCTTTTGAAAAGCATCAATCCAACT
TTTCATTTGAGTTTTTGCTTGTTCTCCTTTTAACCAAGTAAGGGTTATCAAATTTATTTGATCTC
CGTCATTCTCACAAGAAAATTGGGGCATATTATCGGGAACACTTACTAACTCTTGAACACATTGC
CATGAGTCTTTTTCAATTACTTCTTGCCAGTTGTTAACTGACTTTACTTCTACTAATACTTCCTTA
TTATTTTGAGTGCAATACCATATTTCATCTTGACTAGAATCATTTGGTAAATCATCAATATTAGT
ATTCATTTGAGCAACTGAAGGGGTTGTTATCAAACTTCCGATGAGAAGAAATAAGCTCAAAAATT
TCTGATTCATAAAATTAAACTCTGATTTGTTGTTTACTGAAAAAATAGATTTTCCAAGTTAATTC
TAATTAAAATAATTCATAAAAACTCAATCTTCCAACTCCTAGAGGAAAAGCAGTGGGATTATTTC
CTGCAGGAAAAACAGTAACATCATACATATAAGTAGTGTCTTGACTAGGATTTCGCACCGCTTCT
AAACGAACAGTAAAAGTTGTACCGGGAGGCACGGGGGGATTAAAGAGAATATTTACTCCTGTTCC
TTCTGATAATTCTGAGGGTTGAATGGTTAATGTCTGACCTTGATTGTTTTGATCCCCAATGAATG
CGGAAGTGTTGTCAATATTGAGAGCTATCATTTCGGGGCTTTCTTGTTGTTGAATTGTTATTTGT
GCGATCGCTTCTATAGAATTACTGGGGAGAGTAAAGGTAAAGTAATAGTGTGCATCAGGTAAGT
TAACGGTGGAGTTAGGGGTTTTTACTCCAACAAAAACGGGAGGTTGACCTGTGAAAAAAGTTGCT
TGATTATCATTTTGACTATAAGCTGAAAAGGGAATAATGACAGTATTTGCACTGATAATTAAAG
GGGTAATTATTTTTAAAAATTTCATATTTTTATGAGAGAAATACTAAGTTTTAAAATTTTTATA
AACTTGTTGGTTTTTCAAAAAATAATTTAGCTAGTTTACCTTTACCTAAATAAGAATCAATTTCA
TAAAAAATATTGTGAATAATAAAAACTAGAGAAATCGACATTGTAACTGCCATAAAATGGAAAA
AATGACGGGTTTCAATAAATATACTTATTGCTCCTTGTATAGAATTGACTTGTCGATAAGTATGA
AATAAATCTTCTACCAAAGAGATAATGAATACTCCTCCTACATAGATTAGGCTCTTATACATTAC
TCGTATGTAACCCGGAGAGTTGGAATAGAGATTAATGAAAGGCATTGCATCCATAATTGCTACAA
CTTTAGCTGCCACTAATGCACTGATAATTGTTTTGGAAAAAACATAAACATCAATATTATACTCA
GCTAATAGAAATTTCATTGTAATCAATATATAGGCAAACCCTATTAAAAAAAATAGAGTTAATG
AGCCAACTTTTTTGAGTTCTTTTTTTATTTTTTCCTGCATAAAACTTGAATAAATTGATTAGATA
AAAACTTAATCTTAAACTATCCAAATTTTATCTCAGTTTTTATTTTGCAAGAGGTCTAATATAAA
ATTTTTATGAATACTTTTGAGGGTGATATATAAATCTATTTCTTGTTGTAATAATAATTTTTTTT
AATATAACTTGTGGTTAGATAAGAGTTTCACTATCTAAAACCGTTTCAATATTTTTATAGATGTT
AACAGCTTCTTCACGGGTATTGCCGATGCTTGTTAAGCCTAATTTGCCAAATTCTGATAATGCAC
CCATAAGATGAAAAATAGTACCTGTTTTAGTGCTACTGTCAAAGTGTAGTCGATGTTTTGTGACG
ATGTCCATTAAATCATTAGGTAATAAACCATGATAATTCGGTTTTTGTAAATTGTCTGATGCTAG
GTAATATTTTACTTTTTCTTCGGGACTATAAAACAATCCATCTTGATAATTATAAGTTCCATTTG
TTAAGAATTTTAAGGTCATAAAAGGGTGAGTTGTGCCACCTTTTCTTAAGTTTATTTCTATGGCA
TATAGTTGCCATTTATTATTTTCTTTAACTGCTAAAAAATCAACTCCAAATCTTTCCATTGCTCC
TTTTTTTGCCAATGCTTCTCCTACTTTTATACCTAATTCTTGCAGTCGCAAGCGATAGCCACTATG

AGCTGGAAAAGAACATCCTAGATAAATTTGTTGCTCATCTCCCCCTAAAATTTGGTCATGGGTGG
ATAATATTTCTACTTTTCCATTGGGTGTAATATAGCCCTGTACGCTAGGAGAAAATTTTTCTTCT
CCTTCAATAAATTCCTCTACAATTACTCCTAATTCAGGGATGCGATCGCAGTATGACTGCCATGT
TTCTTCTTCGCCTTGCACCTTAGCCTCATGAATAATTTGAGCGATTAAGTTTTCTGCTTTTACTAA
AGATATATCGAGAGAAAAAAGAGGGGATATACCACTAATATCTAAAACAGCATTACCCTCTCCAG
AAAAACCCTCATTAAGTTTAATTACTACTTTTTTTAAATGGGATTTTTTGATCAATAATTGTGCT
GTTTCTCTAATTAAATCATCAACAGTAAACACTAGCTGACTTCCTTCTGGATGAAGTAAATTACA
TTGACTAAAGATTTCTCTACTGCCACTTTTTGAACCCCAATAGCTTAATTCCGGGCTAGAAGCTA
GTAAAGGAATATCTAATTTGACGGATAATTCTTCCTCCAAAGGAGTAGAGTTAAAACAAACCATA
TAACTCTTATTAGGACGTAAAGAATTTTTAATTTTTGTTACCAAACGTGGTCTTTCTAGTATTTT
TTGTGTTAGTGGTTTAAGAGAACTGTCATAGGTTGTTAATAATAATAGTCTTTCTCTAGCATGGG
AAAAAGGAATACCCGGGAGAAGTTGTAAATAATAGTCAACAATAATGGGAGATAAGGGTTGGGC
TGTAATGTAAATGAGGCGAGTATGGGGATTTCTTAAACGAATCAGGGAAAATAAAAGTCTTTCT
TCATAATGTAAAAACCCCGCTACTTTTCTACCAACTCTTTGATCAATACTAAAAGAGGGAATTAC
TAATATATCTGTATCATCTTCATCTAAAATATCTCTATCTTGCCAACAATTACGTAGTTTATCTT
GCAATTCTTTAAATCTTTCTTTTTCTGATGAAGTAATTACCATGTTTCCCCCCTTTTTGCCTTTTT
TCTAATATATCTGAGTTCGGAGTTCACAGTTCTGAATTCGAGGTTATAGTTAACATATATTGACT
AATTTCTGGGCGTTGCTAATTTTAGATATGATTTTTCATTTAGGTGGGCAATAGGGAATAGGCAA
TAGTGATAATATCTTGACGTCCCAATTTTTGGCATAATTTCGGTACATTTCATACCATGATTAAG
CAACGCTAATTTTTGATTAGACTTCTTGCACAAGTCAGAGAATGGGGAAGAGAAAAGAGGGAAA
GGTTAAGCATTTTAGTCGTTTTTAAGACTTAATAAACACGGTAAGTTAAATGGGTCTTAGTTTAT
AATTGAATTTAGTTGTTTTAAATAACTCATATCCTCTTTTCGATTAGTTTGTATTTCCCATATAT
GAATATTTTGGGAAGGTAAATTACTTAAATTTTTCTCTAAATTTTGCCAATTTTGAATTAATTGA
TATTGAATATTATAAGTTTTGGCTAGTTGTTGAAAATTTATATTTTGTGGAGTAGCAAAGTAAT
TTTCAAAGACATTTTCATAGTTACTAATTGGTAGCATTTCAAAAATTCCACCACCATTGTTATTA
ATTAAAATAATAATTAAACAACCTTGAAAATGATTATTGATTAAAAAACCATTGGTATCATGTA
AAAGGGCTAAATCTCCCGTCAAAAGAATGGTTATTTTTTGTTTATAAGCTACACCTAAAGCAGTG
GATAAAGTGCCATCAATACCATTAGCACCTCTACTAAAATAAATGTCATATTTTTTATTATTTTT
TTGCCAGAAAAATTCAGCATATCTAACAGACATACTATTGGCGATAAAGATTGTAGTTTTTTCAG
GTAAATATTGAGATAGTAACCAAGCAATTTTCCCCTCAAATAATTTATTGTTATTTCCTATTTCT
TGATTGAGTTTAAGAGATATTTTATTGTCTATTTCTTGCCACTGATGTAGATATTTTAAATCATA
GGGTTGATTATTTGAATAAGTTTTTAAAAGACTAAATAAGTATTCATAGCTCACTCTTAAATGTT
GAGAGTTGCTATGTAAAGCATCAAAGTTATCATCAAATTCTGTGATAATATAAGTAGTAATTTT
ATGTTTATTTAACCATTTTCTTAACTCTTTACTGGTGGGATATTCTCCTATTAAAATAGCTATTT
CAGGAATAAGTTTTTCTCTATATTGTTCGTCTCTTAAAATCGTGTCATAAGTAGTTATTAAATGA
CTATTACAATGACTATAATTTCTCACGGGAGATAAAGCCTCTGCTAATACAGGAAACCTTAATAT
ATGGGCTAAAGATGCGATCGCATCACAGTATAATTCAGGTTGGGAAGGGGAATCAACTCCTGCAA
TGATGATACCTTTGGTCAAAGTTTGCCAATGTTTTAAATAGTTAATAAAGTCCTGATTAACACAA
GTAAATGAAGAGATAGATAGATGAAAATTAGAAAATAATTGTTTTTGTATGAAATCTTGTTTTT
CTTGATTAAAATTTAATTGCTCAATGGGAACTAATGGCTCGGTAAATGGTATATTAAAATGAACT
ACTCCCTTACTAGGAAATTGTGCTTTTTCCCATCCATAGATAATATTTTGTCTCAAATAAAATAA
GTCTTCTAATTCTGCAGAAGGTAAAGATAATTCTGTTTGCCAATTAGGATAACTACCATATAAAT
TAACTTGATTAATAGTTTGTCCTGCATGACAATTTCTTAGTTGAAAAGGTCGATCAGCCGTGAGA

ATAATTAAGGGTATATGACTATACTTAGCTTCAATAATTGCTGGATAAAAGTTCGCCCCTGCTGT
GCCAGAGGTGCAAATTAAAACCACTGGTTTATTTGTTCTTTTAGCTAAACCTAAAGCAAAAAAAG
AAGCTGATCTTTCATCTAAAATAGGAATACTTGTAATTGATTTATTTTCAGCAAAAGCAACTGTT
AGAGGAGTTGATCTTGAGCCGGGAGAAATGATTGCATATTCTACCCCTAATTTAGTAAAAGTTTC
AACAATTAAGCTACTCCAAAGGATATTTTTATTCCGAAAATCTAAAGACATTTAATAAGTAACAA
TTAATGATTATAAAGATTTTATTATCTCTAATTAATTTTTTGATATTTTTCACAAAACAGAAGCT
ATTATATAATGATTATGGACTATATATCAACTAAAATAATCGATTAATTTTTGATAATTAAATA
TTAACTATTCATGGAAATTATTGAGCTAGATTTTATTGATTTACTATGGGCTTGGGGACTTATTC
TCATGGCTATTAGTCTATCTATTTGGCAAAAGCTAGGCTTTGAAGGTCAATTTTTATTAGCGGCG
GGGCGATCGCTACTACAGTTGATGTTTTTAGGTTACATTCTTGAATTTATTTTTGTCATTGATAA
TCCTGTGGCGGTAATTGCGATAATTCTAATCATGATTACCATTGCGGCAATAGTTGCCCCGAAATC
GTATTAGTAAAAAAATCCCTCGTTTACTGCAAACAGTATGGTTAGGCTTATTCTTTAGTAGCTCT
TTTGTCGTTGTTTATAGCATTATATTTATCATTCAACCAGAAAGATGGTATAACCCTCAATATTT
AATTCCCCTAGTGGGTATGATTTTGGGAAATACCCTCAATGGTGCGTCTTTAGCAGGGGAAAGAT
TAGCCAATATGATCAAAAATAACCGCTTAGAAATTGAAACTCATTTGTGTTTAGGTGCAACGGGC
AAAAATGCGATCGCATCTTATCGTTTAGATGCCATAAAAGTGGGCTTAATCCCGATTATAAATTC
GATGATGGTAGTGGGGATGGTAAGTCTTCCGGGGATGTTTACTGGGCAGGTTTTAGCAGGAAATA
GCCCTTTATCGGCGGCATCCTATCAGATTTTGATTTTATTTATGATTGCCCTTGCTAATTTAATT
ACTATTTTAATCATCACCGAGGGAGTATATCGACATTTTTTTAATGCCCGTCAGCAATTAGTTAT
CGATTGATTAGGAATTAAGGGGAAAATAAGAGCTTAGGGTATTGAAGTATTAGGGAGAAACAAG
TTAGGGGTTAGAGGCTAGGAGTTAGTTAGGGGCGAATAGCCATTCGCCCGTACAGGAGTTAGAAG
TTATTAATTATCAACTATTCATCTTTGCCCTCTCCCTCTCGAGAGGGGATAAAGGGAGGTTTGCC
TCTTGCCTCTTACCCTTTGCCCATTCATTGCCAATTACTTACGAATTCTTAACCAACGCTTGATAC
AGCTTCTTTTGCCGCATATAAAACTTCTAGGGTAATTTCTCAAAACCTCTGTCTCTGGCAAACT
TTTCTGTATTACGTTTAACTTTACCTCGCACAAACCCCGGCACTTTATTTAACTCTTTTTGAGCTT
CTGTATCCCAGTTTAAATCAGAGTCGGCGGAGATTCCTTTGGTGATTACTTCTTTGGTATCATGA
CCACCAAATATTTCTAATAAATGATCTTCCATACCTAGGGTAAAGGAATTATAGACTAAATCGGC
GATCTGGTTTGTACCTTCATAACCCATAAAAGGTTTATAGCCAATGGGGAAGTTTTGAATATGAA
TAGGGGCAGAAATGACACCGCAAGGAATGTTTAAACGTTTGCCTACGTGACGCTCCATTTGTGTT
CCAAAAATTGCCGCCGGTTCAATTTTGGCGATCGCATCTGCTATTTCTCCATTATCATCACTAATT
AAGACTCGATCGCAATATTCACTTACTTGTTGAGTGAACCAATCTCCATCATACTTGCAGTAAGT
ACCGGCTAAAACCACATGGATACCCATTTCCCGAGCTAAAATTTTGGTCATTGCCACAGCGTGGG
TATTATCCCCAAAAACAACGGCTTTTTTCCCCGTCAAGTTTTGACAGTCGATAGAGCGAGAAAAC
CATGCCGCCTGAGAGACATAGAGGGTTTGATGTTCAATAAATTCTTCATAATCCACATTTGCCCC
TAAACCGTTGAGAATTTGCTGAATTTTACGCACACAACGGGCAGTTTCTACCACTCCCATGGGGG
TTATATCCACATAGGGCATTCCCCATTCCTCCTCCAAATAACGGGCACTCATTAATCCTAACTCTC
GATAAGGCACAAGGTTAAACCACGCAGAAGGAATCTTTTTCAAGTCATGTACCGAAGCACTATCA
GGTAAAATTAAATTAACTTCAATACCCAAATCTCTCATTAATCTCTGTAATTCCGTACAATCATG
ATTGTTGTGGAATCCTAAAGTGGAAATCCCGATAATATTTACTGAAGGGTGAGGGGTTTTTTCTG
TGACCACATCTCCTTTCTTCTGGGCTTTTTGCAAATAAAATTGTATAATTTGCTCTAAAGTGCGA
TCGCCTGCCTGTAATTCATTAACTCGATAATGGTTAACATCTGCCAATAAAACATCACTATTAGA
ATTTAACTGAGCCCGTTGCACAAAATTTTCTAAATCTTCCTGTAAAATACTTGAAGTGCAAGTGG
GAGTTAAGACAATTAAATCTGGTTTTTCTTCCTTGTCTTTACGAACAATATTATCAACAACCTTT

TCTTGCGAACCCCTTGCCAATACATTCCGATCAACTACACTAGCTGTAACAGGAGTAAAATCCCT
TTCCCTTTCCAACATCGATCGCATTACGTTAAAATAATCATCACCGAGGGGGGCGTGCATAATAG
CATGGACATTTTTAAAGGAACTAGCGATTCTTAGAGTACCGATGTGTGCTGGTCCGGCGTACATC
CAGTAAGCTAATTTCATAGGGTAATTTCTCCACTTGTTGATCTCTGTTTTCTAGTTTCCTTCTTTC
TTCTAAAATTAGAGATATTAAGTTACAAATATTTATAAAAAATAAATAAAATTAATAACTAATC
TAAGTTATGAGTTAGGAGTTCATATTTTTAGATTAAATTTAAATAATAGTCAAGATATATTGAC
AAAAAATAAGCTAAATTGACTATATTTTACTCAATAGTGAAGTTTTATGAATTTTTAACAAATA
ACCTTTATTTAATAAATGTATGAATAATTATGTTAAGAAAACTAGGTAAAATACAGGATTTATT
ATTGCTAATATTTAAAATTTCCCTACCTTTAAGCATTGTTTTATATGTGTTGAGAGGTTTGGGTT
TTCTAGGATTTGTGTCAGGGGGAGTTTATCTTTCTGCATTTTAATCTCTATTTTCTCCTTGTTA
GGTTTTTTTCTGGTAAAAACTTATATTTGAATCATCTAAAACCTCAAAGCCGCCTTTATCCGATA
TTCTAAAGCTATTGGAGTTTGGAATTTTTCTTAATTTAATGATTATTTCAGGGAAGATTTTTTTG
ATTAATTAGGTCTCAACTCTCTTATTTTTCAATATTTTTTTCATCGAACTTAGGTAAAACCGAAC
TGAGGTTAAATAATTAAAACTTAGATAACTCCTGATACCAACTTCTAATAATTTTAGGAGAAAT
ATGTTTTCCTCTTTCATCAATCAATTTTTGAGCATTTATTTTAACTTCCGCAACGGTTTTATAAT
TCGTTTTTACCATTTCCGTTAAAAATCTTAAAGCCCGAATATAAGGAATTTGATATTTTTCACAG
GCTTCCACCACCAACCCATCATCACTAGCACATAATAAAAAACGATGACTTGCAATAGCCAAAGT
TGATCGATCTGCCATACTTAATAAAGGAAATTCTACCCCAAAATCCATAAAACTCCTAAACATTT
CCTCAGTATCTAACGGTATGGGAATAAGATATTTTTGAGCAACTTCCCTTGTCTCTAATTCTAAC
TGATCTGAGTCTAAAACCTCTTGAGCAATATATAAGGGGGCGTAATGACTTTGAAGCCATTGCCA
ATCCCCCAAATAAACAAAATCAATTAGTGCTGTTGCGTCCAATATAATTCCCTCATACTGCTCTA
AAATGTCAACTTCAGGCGATAGCATAAACCTCCTGTTGCATTCTTGCCAATCTTAAAGCCTCAAT
GGTAATGTTTAAAAGTTCCGCCGCCTTTGATTCAGAAATTTTCTCGGCTTTTAATGCGTTCCAAA
TCAAAGTTTGATAACGTTGATTAACAGGAAAATTATTTTCTGCTAACTTTGGTTCAAGTTCAATT
TCTTTGGTAAGAGATTTATTATATAAACGTTTATAGTTAGCTCTTATTTTTTTGATAATTTCTCC
ATAACTATCTATTCCTAACTCATCAAGTCGTTTGATAATAGTGGCATAACTTACCCGAAAATGAG
CTTTAAGTTCCAGTAAATTAGTCATGTTTTGACAAGCTAAATCAAAAGCCGTTTGGGATACAAGT
AAATGACTAGCAAAATAATTAGCTACCTTTTCTCTTGCTTTTTCTTCCTCCCGACTTTTACTTATC
ATTAATTGGTTTTGATATTCGTCTCGATGAAAAATTAGATGTCCAATTTCATGGGCAAGGGTAAA
TAATTGTCTTTCAATACTAATATTATCGGTGTTAATTAGTACAAAAGCACCTTGACTATTGCTAC
AGGCACTTAACCCGAAAAAGTCTTTTTGCTCAATGGGTAGTCGTAAAACCTTTAAACCTAACTCC
TCTACCGCCTCAAATAGATTCGGAATGGGTGCATCACCAATACCTAAACGAAGACGAAAAGACTT
AGCTATTTGTTGAATACGACTTTCATTACCTTCTAGTACATTACAAGGGGTTGTTTCCGGAGCAT
AGGGAGGCAACCCAACGGCTACTTCAAGGGCAGTATAAGTGTTTAATAAATTCTCTACATAGCTT
ATAAATTGGGGTTTTTGAGTAAATTTACAATGACAGCGAAAACAAAAATTATTCGTTTGAGGTA
GTTGATCAATGGGACGTAATATATCATCAAGAGTTACCTTCAAAATTTTAGCTAAAACACTAAGA
GTTTTACTATCTGGTAAAGTTTTTGCTTTTTCATAATTATTAATGGTTTGTCGTGTAACCCCGGT
TTGTTCAGCTAAAGATTGTTGGGATATATGTAAGCCTTTACGATAACGAATTAAATTAGATGCGA
TCGTTTCTTTCATAAAGGTATTATTACGTTTATTATTTTTACATTTAGACTGAATAAAGTTAACT
AATTTGTAAAATATATTTTACATTATAACTAATTTTTGTAGATTAGCATTTAGCTTCATTTATCC
CCAAAACTAACCAAATTCTTTAATAAACCCTCCCTTTGTGCCTCTAAATCTAATATTTCCTTGCTT
ACCTCTTGTGAACTTCTCAAAGGTTGATACTGACAAAACGATTAAACTTATATTGTTTAATAAAA
TGAAAGATTTGAGTAAATTAAGAAAAAAGAACAGTTGGGAGGGATGAAAAAAAGAGAAACTCAT

GATATACTAGCAAAGTTCAGCAAATAAAAACTGCTGAGTTAAATATAATTATTATTGTGAAAGC
GATTCATTAAAAGGAAAGAATAGTAATTTTTATGAAAATCACCCAGGAAAAACTTCCTGCCAGTC
AAATCGGTTTAGAAATCGAGATTCCTGCAGAGACAGCTAAAAACACCTATGAAAAAGTAATTAA
AGAAATAGCAAAAACTGCTAATATACCCGGATTCCGTAAAGGAAAAGTACCTCGCCCGATTTTAC
TACAGAGATTAGGGGCAAATAGAATCAAAGCGGCTGTTTTAGATGAGTTAATACAAGATACCTTC
AAGAAAGCTATTGAACAAGAGTCCATCAATGCGATTGGTAACTATAAATTGCGTTCTGATTTTGA
GCAATTAGTTGAATCATTCCAAGTGGGAGAAGCCTTCACCTATCAAGCCGCCGTTGATGTGCCTC
CTGAAGTTAATTTAGGCCAATATCAAGGATTAACTATCAAAGCAGAAGAAGTAGTTTATAATCCC
GAAGATGTAGATAATTTAATTAAAGAGCGTCAAGAAAAACAAGCTACTTTAATTCCCGTGGAAA
ATCGCCCTGCACAAATGGGTGACTTAGTGGTCATTGATTTTGTCGGACGTAAACCGGGAGAGAAT
GAAGGAGAGGAAGGAGAGGTTATCCCCAATACAGAAGCAAGTGAATATCAGACGGAATTAAAAG
AAGGAAGATTTATACCTGGCTTCGTGGAAGGTATTGTAGGGATGAATTTGGATGAAACGAAAAA
ATTACCTCTAACTTTCCCAGAGGATTATCCTCAACAAGAATTAGCTGGAGAAAATGTTATCTTCA
CTATTACTCTTAAGGAAATCAAAGAGAAAGAATTACCTGAGTTGGATGATGACTTTGCCCAAGAG
GTAAGTGAATTTCAAACCATGGCAGAGTTGAGAGAATCCTTAGAAAAGCAGTATCAGGAAAAAG
CTAGTAACGAAACTAAGCAAAATATTCAGGCGGCAATTGTTGAGGAATTATTAAAACATACCACC
ATTGATTTACCTGAAACTCTGATTGAGGAAGAGGTACAAAATATTCTCATGCAAACAGCTAATCA
AATTCAAAGCTATGGCATGGATATTAATCAATTCTTCACTAGAGAGATGGTAGGGAGAATGAGA
GAAACTGCTAAACCTGAAGCGAATAAAAATCTTCACTCTACTATGATTATTGAAAAAGTTTCTGA
GCAAGAGAATATCTCTGTTAGTGAGGAAGAAATCAATAACAAATTTAATGAAATCAAGGAAAGT
CTTAAGGATAAAGACGTTGATGAGGAAAGACTCCTTAAATACGTTAAATCTGATTTAATAGCAG
AAAAAACCCTTGATTGGTTAGCAGAGAAAAATACTATTGAATTAGTGCCTCAAGGTACTTTAAAT
AAAGAGGAAAATGAGGCAGATTCTGAAGAATCTTCTGAAAGTTAATTAATCACTCAATTTAAAC
CTGAGTTTGATATGAGATTTTCTCGTTATAGTAGGTAGGAAAAGGCGGTTAATAATTAGCAATT
AGTCATTAATTAACCCGAGCTTGGGATAAATTTTTATCTATGAGTGAAGATGGAAAGGGCAAGG
GGTAAAGGAAAAATTAAGAATTAAGAATTAAAAACTCTGAACTCGTTTCTCCCTAAACCCCCAAT
TCTCCAACAACCTGACACCTGCCTAAACCTGATTTTTTTAGACATCGACCACATTTCTCAAAATA
AGGGAACCAAGCCCTAACTGCGAGCCTATATTAATATTTTCTACCAGATGTCTATTGAACTGAGA
TAATTTCAGAGTATTAAGTAAGATATTCGGGAGAAAATGATTGCTTGTTAGTGGTTATTTCTCCC
GAAACCTGAAACTGCACCATTAGGAAAATTCATCATTACTAATTTTTATGACGGAAAATCAAGCA
ACAGAAGTAAAATATGGTGAAAGGGCGATCGAAGAGGGTAAACTAATTACATTCCCTAATCCTCG
CCCGGGTAGAGTTTACAATATCAGTGTTACGTTACCTGAATTTACTTGTAAGTGTCCTTTTTCTG
GTTATCCAGATTTTGCCACTATCTATATTAATTACTGCCCTGATGAGAAGGTTGTAGAATTAAAA
GCGATTAAACTCTATATTAACAGCTATCGCGATCGCTATATTTCCCATGAAGAATCAATTAATCA
AATTTTAGATGATTTCGTGGCGGCGGCTGATCCTTTATCTATCACAATTAAGGGAGATTTCAATC
CTCGTGGTAATGTGCATACCGTAGTTGAAGTCAGTCACAAAAAGTAATTAGGAATTAGGAGTTAG
TAATTAGTAATTAAGGCAAGGCAATAGGCAATAATGAATAGTTTATAGTTTATAGTTGATAATA
AATAATAAAAACACCTGAAACCTGCATGCAACCTGACACCTAACACCTGACACCTCTTTAATTGC
TGATTCTGTGACCAATATCTCGACGATAATACATATTATCGAAATGTATTTTGGCTACTCCTCGG
TAGGTAGATGCGATCGCATCTTTGAAATCAGAGCCTCTACCGGTAACTCCCAAAACTCTACCGCC
GTCAGTAAGGATTTGTCCATTATTTAATTTCGTACCGGCATGGAAAACCATTACCCCTTCTTCTT
GGGCAGTGGGGATGCCAGAGATGACATCTCCACTGCGATAACTACTGGGGTATCCTCCCGCCGCC
ATAACCACACAAACGGCACTACCTTCATGCCATTCAAAAGGTGGTAATTCTGCCAGTCTTTTTTC

TGTACAAGCGAGTAAAATATCAATCAAAGGGGTTTTCAGCATCGATAAAACCGCTTGAGTTTCAG
GATCACCAAAACGACAATTAAACTCTAAAACTTTAGGCTCTCCCTCTGGGGTAATCATTAATCCA
GCATATAATACCCCCACATAGTCAATATTTTTCTCTTGCAATTGTTTTAATGTGGGTTCTAAAAC
CTCTTTAGTAATGCGTTGACTGAGGTTATCATCTAAAAGGGGAGTCGGTGCATAAGCTCCCATTC
CTCCAGTATTTGCTCCCGTGTCTCCCTCTCCGATTTGTTTATGGTCTTGAGCAGAGATAAGAGGGC
GAATTGTGTTACCATCGGTGAGAGCTAAAATGGACACTTCTTGTCCTGTTAAAAATTCTTCCACC
ACTACCTTAGCAAAATCCTGAGCAAAAAGTTCGTCGATCGCATCTAAGGCTTCTTTTTCCGTCAT
TGCTACAATTACCCCTTTTCCGGCGGCTAATCCATCGGCTTTTACCACAATGGGCGCCCCTTCTTT
TTTAATATATTCTTTAGCAGGAGACGCAGAGGTAAAGGTTGCCGACTTAGCAGTGGGTATTCCCC
CAGCCACCATTAAATCTTTTGCCCAAGATTTACTTGCCTCGATAATAGCACCATCTTGTTTTGGTC
CAAACACTTTCACTCCATGGGGTTGTAAATAATCCGCAATACCTAAAGATAAGGGTAATTCAGGA
CCTACTACTACTAACTCAATCCCTTTTTCCTGCACTAAATTTAAGATACTGGCAAAATCATCCACT
GCGATCGCCACATTTTCACATTTTTCTAATACAGCAGTACCTCCATTGCCCGGAGTACAGAAAAC
TTTTTCAACGGAATCAGATTGTAACAATTTCCATGCTAAAGCGTGTTCTCTCCCGCCATTCCCAAC
AACTAAAACTTTCACTTTACTTTTAATTATTTCTTCTATATCAATACAGTGTTTAATTTTACTGA
CTTTAACGGACAATAAGCAATAGTGTCAAAAAGTCTTTCTCATGAAGAATATAAATGGTTTTTTT
CTATATACTCTTGGATACTTTTAATTACAGTTGAATCATGATGATTCTCTCGATAATTACTAGAA
GAATAAGGGGGAATATGACAAGATGCGATCGCAAAGTGCCACTTAGTGAATGCTCCTGAGTTACT
ATGACGATTAATCTTTTCTATATCTTCCTGTGTTAAGGGATAATTTTCCCGAGGAATAACCAAAA
TTTTGACCTGAGACAAAAGTTTTTCGGCTTGATACCATTGAAAAATTTGATCAACTAAATCACTA
CCAATCACAATGGTAAAATTATTATCCTCTCCCCATCTCTGTTTTGCCTTTTCTAAAGTATGAAT
AGAGCGGCGATCGCTCACCTCTGGAGTATGATGAAGATGAGGATAATCTCTGTGTAAATCCCTAA
CCAACAAATCTAACATTTTTTGACGATGGACAAAGCCACTAGCATGACTCTTAAAAGGATTATCA
GAAGCATAAACCGATACTAAATCATAGTGATTAGCCAAATAAACTAAAATTTTTTGATGAGAAA
TGGTTGGAGGGTCTGCACTTGTACCAAAAATAGCTATTTCCATGCTCTTTATTCCATAACAGGGG
GATATTCCAAAGACACCGAACCTAAATAACCCTTACGAAAATCTTGTAATAATTGCCTCGCTACT
CTTTCCTTATCGTTTTTGTACTTCCTAGCCGCTAATTCATAAATAAACTCTTCTCCTGTCATCTCC
AAAGGATCTAAACCATAACGAGATTTTAGCACCTCATCAGAACCCAATTTAACTAATAAATCGAC
AAAATCAGCCGCTACCAACTGATTATCGTAAGCCGCCTCTCCAATATCTTCACAAATAGCTAATT
TTAAAGCATCCTCTTGATTTTCTAACTTCCAAGGAATAACTCCCGGTGCATCTAACAACTCTAAT
TGATCAGAAATACGAACCCATTGTAACTGCTTAGTAACTCCAGCTTTTCTGGCACTAGCCACAAT
TTTTCTCTGCAATAAGCGATTAATCAACGCAGATTTACCCACATTAGGAAAGCCGATTACCACGG
CTCGAACAGGACGAGGTAACATTCCACGACTACGACGACGTTCATTTACTTGTTTTTCCATCGAT
TGGGAAGCCTTTTTAACAGCTTTCACCCCTTTACCATCTTTAGCATTAGTAAAAAAAGGAATTTC
CCCTTGAGCGCCAAACCACTCTTGCCACTCTTTTCTTAAAGAGTCAGAAATCATATCTTCTCTATT
TAAAACTAAAAGACGAGGTTTATCTCCTATCCACTGCTTCAACTGAGGATGATGGGAAGCAAGAG
GAATTCTAGCATCTCGCACTTCTAAAACCACATCAACTCTTTTTAACTGTTCCTTGAGTTGTCTTT
CTGCCTTGCTGATGTGTCCGGGATACCATTGAATGATTGCCATGAGAGATATTTGCTTATTATCT
TTATTTTATTGAGATTGATAAGATATGAAGGGATTTTTAGGATTTTGCTAATGACCATTATACCG
TATCACCTGAGTTCGATGAAAAAAGGATTGAAGAATAAGGAGTTGAGATCAATAATAGACAAAA
CTTTCTTTTAAAAAATTAGTTCTATTTAGGAAAAATATATCAAAAATCGATTTTTGTGGGGAAA
TAGTCTATTGACTTTTCACAAATGATTTAGAATTGCGGTATATCAACAAACTTTTTAATCATGGA
AAAATTGCTTCTTCAGAGTGTGGTTATGATAAATTAAGAGGAACAGGACTTCCATAACCTTTATT

GAATAGTGTTTATAGTAAAACAAAAACTAAAAGTTTATAAATTATTATTTAATAATTCCCCATT
GCCTATTGCCTCTTGCCTTCCCCCCTCTCGAGGGGGGATAAAGGGGGGTTTGCCTTTCAGCCTACC
TTAACTAATAATTTACACGACGAGAAGTGATAGAGCCGAAAAATTTAATTATTGACACATCTTAG
TAATTTCAAATTAAGTCATGTATGAAAAAATTTATGGTGTAGTTCACGTGGGTAAAAACTTGTT
AATTGTAGGTTATAACAAGAAAGACAAATGGTCTTTTAGAGTTGTTACCCAAGAGGGAAAAATA
GTGAAAGAAACGACAGATTTTCTAACGGCTGAAAGTGCGGAAAAAGAAGGTTATATCTGGATAG
AAAAGAATCTCAGTTCAGTCTAATAATATCAGAGAAGAGCGTTGGAGTGTTAGGGGATTGGGGA
AGTAGGGGCTTGGAGTGAGGGCTAAATGGTTAATTATACCCTAACTTTGAAAAAACCCTGATTTA
TTAGAAAGGAATATCGTCATCATCTTCAGAAATTTCTATAGAAGGGCGATTAACTATTTTTGGAA
GTGCTAATTTTGTTTCTGTGAAAACAATGTCTCCCTTAAAACTTTTGACTACACTTTCTGCGGCA
TTTCTGGCTAATTGTGTATTGAATTCTTCATGATTAATTTCTACTGACGAGGACATTACTGGTTT
TGATGGTGGTTGGATTTTAGCTGTCTCAACGGGTTGATTAACTGGAGTTTGAGGAATAGAAGATT
TTGTTTCTGTGGGGGATGGATTTTCTATGGGTTTAGTGGGGGGTTGAGTAGGTTGGGGGGGAGA
ATTATCATTATTGTCTAATTTTTGATTTTTTAGGGCTGGATTTTCTATGAAAACAACATTCATCG
GTTTTTGGTAAACATGAGCAAATCCTGCTTCTATGTCTTGCTGTTGTCTTTTTCCTATGTCTAAT
AAAAATCTACTTGCTACTTCTAGGGTAACGGTTTGACCTTCTATTTGACGAATATGACAACTTTG
ACGCAAAAATGATTTAGTTGCAGTGGGTGTAACGGCTTCAATTACTTTTTCTTTTACATCTGTAT
ATGAGTCAAAATTGACAGGTGTTGAGGGTTGATAAATTTGAGGAGTTTGAGGAGTTGAGTTGTC
TTCAGGAGGGGGAGTAATTGCCTGTGATGTTTGTGTTTCTACAGTTGTATTTTCTCTTTTTTCTT
GAGTTACTCTTTTTTCTTGAGTTAAAGGTGCAGTAGCTGTTTGACTAGAATTATTTACCATTACT
GTGGTAAGGGCATTTGCTGAAGGCAATAAACTTAATAGGGTAATTTCTAGCCAAAGGCGAGGCTG
AGTAGTGTTTCTTATTTGTACTTCGCTATCTTTTAGTTTTTTTTGTCCTTGCAGAATTACTTTTA
AATCCCATTGATGGGCTTCTTGAATCAATTTTTGCCATGTGTCATCGGTTATGGCGACTAATTCT
TTTTTCTGAGGAGACGTTTTCGCAATTAGTAGGGTAAGATAAAAATTGGCTAAGTTTTGTAAGAC
GATTAATGGTTCTCTACCTCGATCGAGCAATTTACGACATTGGATGATTACTTCTTCAGGATTAT
CAGATGCGATCGCACTTATTAAAGCCAATAAATCTTGTTCTGAGACTGCACCCACTAAATCCCAT
ACCCTTTCGGGAGTAATAGTACCAGATAAAAGACTGAGTTGATCTAATAAAGTTTGAGCGTCACG
CATTCCCCCATTGGCTAATTGTGCTACTAAAGATAAACCTTCAGGAGTAATATCAATATTCTCTT
TTATGGCAATATCTTGTAAATGTTTAACCATTTCTATCAAAGGAATACGGCGATAATCAAACCTT
TGACAACGGGAAATAATAGTTGGTAATACTCTTTGAGGATCGGTGGTTGCAAGGACGAAAATTA
CCCTCGGAGAAGGTTCTTCCAATGTCTTTAAAAGGGCATTAAAAGCGGCCGTACTAAGCATATGA
CACTCATCAATCACATAAACCTTATAACGACACTTAACGGGGGCATATTGAGCTTTTTCGATGAT
TTCTCGAATATTATCAACTCCCGTATTACTAGCCGCATCAATTTCTACCACATCCAAAGCAGAGCC
ATTACTAATACTTTTGCAGACTTCACATTTACCACAGGGTTCTGCTGTAGGACGATCGCTTTCAA
TACAATTTAGGGATTTTGCTAAAATTCTGGCACTAGAAGTTTTACCGGTACCACGAGGACCACAA
AATAAATAAGCTGGTACTATTTTCTCATTTTTAATCGCATTAATTAAAGTTTGTGCGATCGCTTC
TTGTCCTGTCAATTGGGAAAAAGTTTGAGGTCTATATTTATGATGTAAAGGCTCGTAGTTCATGA
GTTGTTTCTTCAGATAGTTGTCTAGTTTGAGATATTAACGATGAAAATTATGAGTACTAAAAGTT
AACAATCTTACTTATTTTTTTGAGATTGACAATTAAAAGTTAACAATAGAGATATTGACAATTCA
AGACTATTGTAAACCGTTTCCTTCGTTAAATCAGAAGAATCAAACATCATAAACTAAAAGCCATA
GAAACATTCAACTAACATCTTGCAATAGTAGTCAGTGAGTGTCAGATTTGATTATTTTTTACTAT
TTTGAAAATTGAGGCAACAATTAAGATTTTTTTCAACTAATTCTTTTTTCTTAACCTAAAACCTA
AAACCTGCAACCTGAAACCTGAAATTATGCGTTTTTCATTATCTTTTTGATCATGTTATCTTTTA

CCATCATTTGTCTAAATACTTCAATTAAGTAATCTTGTGCCTGTTCCTTGCTCAGGGCTTTAACT
TGGTCTTTTAATACTTCTAATTTGAATTGTTGTTCTAGGGTGAGACTTGCTGATATATCCATTTT
CACTCCTCCGTTGATTTCGTTGTTATAAAGCTAACATATGGATATTAAAGCGTCAACTTTGTCAA
TCTTTCTGTTTATTTTGTTACTTTTCTTAATGATGTTTTTACATTTTTTTATATTTCAAGGAGAG
AAGGGCAACAGGCAATAGTGAATAGTGAATAGTGAATAGTGAATAGTTGATAATTACTCGTTAC
TCATTACTTTCTTCTAAAACCTGAAACCTTATCTTCCTCTCCCCTCATCACCTCATCCCCTAAACA
CCCTAACACCTGCCACCTGAAACCCTGTAGGGGCGAATGGCCATTTGCCCCTACTTCTCCCGCTCA
TTACTTATTTGTCTAACTGAAGTTAGTTGCTCCAAAAGTTTTTTCCGTTGGCAGACAATTTAAGG
GAGTTATTGTTTTCCTCGTGACGATGGATAATTTCTGTTTGAGGAACACCTGTTCTTATTCCAGC
TAAATCAAAAGCCTCTTTGACGCGCCAACGATATTCTCTACCGACATCCCATTGTTGTATAGGTT
TGGTTTTGATTAAGAGGCGAATCATAATTCCCTCATGGTTGATTTTATCGATACCGAGGATTTCG
GCGGAAGATAACATTTTTTCGTGCCAGTAAGAATCTCGATACATTTGATCGGCTACATTTTGTAA
AACATTGATGGTTTTTTTGATGTCTGCATCCCAGTTAATTTCTACGGTGAAGTTAACTCTTGACC
AATCTTTTGTAGCATTGATGACGGTGAAAATTTTGCCGTTGGGAATTACTTTTAAGTTACCATCT
AAATCTCTTAAGGAAGTGGTATAAAGGTTCATGCTTTCAACAAAGCCACCAAGTCCATTGATTTC
TACCACGTCACCGACTGCATAGCGGTCTGTAATTAGTATCAAAATACCGTTGAGCATATCTTCTA
AAAGATTACGAGAAAGAAAGGCAAATACTACCGCTACGGCACCAGCACCGGCAAGAACATTAGGA
CTAAGCCCCACAACGGATAAGGACATAAAAACTGCGATCGCGCCGAAGATAAAAGTTGTTGCGCC
TCTGAGGGCGGGGGAGTAGGTATTAACTCTTAAGTTAGCACGATTAGATTCTGGTTCAAGTTCTT
GTTTTTCTTTTGCCCAACGATTTAAAGCGTAGTCAATCCAAAAATCAACAAATTTATCGGTTAAA
ATCATCAAAATCCAAATCACTGGTAGCAATACCGCTTGGATTAAAAATAAATTAAATAGGAAACG
GGTTTCTCGATAAGTAAATGCGATCGAGCTTATGGTGAAAAGAACAATGGTAAATTGACTGAGA
AATAAAAGACTGGAAATCAATTCACTAATGTTAATTTGTTGACGAATAATATTTTGTTTTTTCAG
AACAAGGCTCGGTATTAATTTTGCGGAAAAAGAAATACCCTCAAAAAACATTTTCCTACCATTGT
CAAACATATTTTGAAAGGGATTATTTTGTGCTGAATCTCCTATTTCTCCTGATTCAGAAGTGTAA
TTTCTTTCTACGGGCTTGGCTTCAGGATCTACTAAAAGACTTTTTTGGATTTCTTTTAATTTATT
GCTGAGATTACGACGCCATTTATAAGCTAGTTTTGCGAAAAATTTAATGATCAGAATGCAAAGAA
GTGCGATCGCAACTGTGACCAAAGAAATTTGTATTCTCAACAAGGGTTGTTGACGATCCATTTCC
AATCCCCATAAAGCCTCACTTAATGAATTAATGACTATTCTTCGCCATCTTTCGGCTAAATCGTC
AATGGTTTGATTGTTGGCTCTGGCATCAACTCTGGTGACAGTAATTAAGGTTTGTTGAGTAATAT
TCAGCTCCGGTTTTGCCGCAATGAATATAACCTTTTGATTATTTTCAACTCCAATATCAACTACT
GGGGTAAGAGGATGAAGGGGTTTTCTTTTTGTCGAGAACCAAAATTGTAATTTCTGTTGAGGTA
ATTTACCTATTTCTCCAGAATCCGCCATAGACTCGAATATTTGGCGAAAAATATTTTGAATAGAC
CTTGCCCTTTGTTCTACTTCAAATGCAACCTCTTGAATTGGTCTATCAATATTTCGAGGAGAAGC
CACTATAATCATATCTTTATTAAATGCTGACAATAACCTCGTATTAATCCGAAGATTCCTATTGC
CATAGAAAAAAACATCACTACATAATGACCTACCACAAGCTCTAGCCCGATTTAAGTCCCATGCT
GGAGTTTCTGGTAACCCTGTATTTGTATTAAAACTAGGCATAAAAGGTATTTGACTAAGGGCTTG
AGGGCTAAAACCGACACAAAAAATAAATGAAAGGATAATGATAAGAGAATATTTATAAATTTTT
TTGATCATTTTCTTCTTGACATAAAGTTAATCACACCCCATTAATATAACAGTCTATTTTGGATT
AATGACTCATTGTCTTTACTCAACCTGAGTTCGATATAAAATTGTCGATTAGGGTAATCAACAGG
CAATAGTATTTATAATTGACATAAACTACGGTTAAATTGATTTTAAAAATTTAATCTTCTAGTGT
TAATGCACTGGTGTAAGGTCTTAAGTTTTAGCCAATAGCAAAAATAGTTATGCCACTGAAAGTAA
GAGTGAGCAGGATAATCACAGCACACCAACGAGCTAATTCTTTGCCACCGATGAATCGAGTTAAG

GCAACTTTAACCAGAGTATTCATAAATACAGCAATAAAAATACTTATTTTTGCCACGGATAAAGA
CATATTTTGATTGACGGCACGGGCTAGGGAAATACTAACTGCGTCCACATCAGCTAATCCTGATA
AGGCAGAGACTAAATAAACTCCTGTTTCTCCAAACCATTGTTCTCCTGCTTTTATTAACAAAGAT
AAAACAATTAAAATCAGAGCATATTGAACGGCTAAACCTAGTTCTACTGGATTAGATAAGGTTA
TGGGTACTTCTTGATTTTTTTCTTTGATTCCTCGCCAAATAAAGATTAAAGCAATTAAAATCGGT
GTCAAGCCTAAAATAATTAAAGGTATCGCCAATTGTTTCGCCAATAAGGAATTAATTACAGTTAT
CTGTAGCAATAAACGAGGTGCCATCGTTCCCGTAGCGAGTGCGATCGCAGTTGCCAGAATTTTAG
TTTGTCCTTGCCCCTGTTTTGCCATACGAGATAAAGCTATGGTGGTGGCGGTAGAAGAAGCAAAA
CCTCCCAAAAACCCGATTAATAAAACTCCTGATTTATCTTTCCAAAGACGAATAATAAAATAACC
TACATAAGAAATACTAACGATTAAAAGTACTAGAAGCCCAATAGCCCTCGGATTAATAGCATTCC
AAGGACCTAAATCCTGATTGGGGAGTAAAGGTAAGGTAACTAAAGCCAAAAGAAGAACTTTAAG
AGTAGCAATTAATTCTTCTTGTTTAAGCCAAATTAACATTTGCCGAATTTCTGACTTCAGTTTTA
GTAACCAACTGACAATTACTGCCACTGCAATAGATTCTAAGCTCAAATCTTTAACTACTAAAGCT
CCTAGTAAAAAGGTTATCAATAAAGCAAATTCAGTTGTTGTACCATAATCTTGGGATTTTTGAGC
CGTTAACACATAAGAGGTACTAACGAGAATGGCAAATCCTAAAAAAGTCACTCCTAAAATACCCC
ATCCCCATTCTTCTGCGAGTAACGCAGTTATGCCACCCAATAAACCCACTAAACCAAAGTTTCTTA
GTCCGTCACTACCTAATTCTTCAGGGCTTTCTCTGTTACTCCATCCCCTTTCTATACCAATAATTA
AGCCTAAAGTTAAGGCAATTGTTAGACGACTAATAATTACTAAATCCATTCGTTTGGAATATTAT
GATTAAAAGGGCAAGAGGCAAGAGGCAAGAGGCAAACCCCCCTTTATCCCCAGGGCTGTTTCATT
TTCGAGAAAGAAAAAAGAGCGGGAGATAGGGGGAGAGGGAGATGAGGGGATTTTTGTTCATAAA
GTTTTAACTAATAGAAAAAAATCCTTGTATATCTTACTCTTACTTAATCTCAGTTAACCTAATTT
TTTATTTTGAAACAGCCCTGCTCTTTATCCCCCCTTGTTTCTGTTTTATTTTTGAACTATCCCCAT
CCATTATGACATCTTTTACCCAGTGTAATTGGTTTCTATTCTCCAATGATTTCTTATTTTTTTCC
TACTTCTGTTGCTGTTTCTAAAATTATCTTAATTGTTTGTTTTTGATAATGAAGTGCATCAAAAC
TAAATGTTTTATTTTCTGTGGTCACTGCTTTTAATAAGTCTTGAATTTCTGGTAATTTGTTAACT
AAATTATTTAACATTTTTTCAACTTATAATAAATGCGATCTTTTGCCCTTTTCACCATCACTCAT
AGATGACAATTTATCCTGAACTCAGGTCAATTACTATTTAAAATTATTGTGGATACTGACTTCAC
AATTATCGCATTTACCACAACTAAAATCCCTACCTTGTGGAAATCCAAAAGCATCGAGGAGAAAA
CCCCAACGACATTTTTTTGTTCCCATATATTCTTCGGTTAATCTGACTAATTTTTTTTGTTTTTGA
ATTAAAAATTTGATATTTTCTTGAGGATTTTTGAGATTAATTTGATAGTTAAATGGGTCTAACC
AATATAATTGTTTACTAAGATTTAAAATTGAAAGATATACTTGTTTATCTCTGGATAAATTTTCT
ATATTCCCTTGAAGTGGCAATTTTTTAATTATTTTAAGTGCTTCTTGATATTGTTTAATTCGTCT
TTGGAGAAAGTATTTTTTTATATTTTTATCTTCGGGATTAAAAAAGCCCGTTTTTTCACTAATTA
AGGTTATTGCTTCTGCTAGTTTGCCGTCTCTGCCACCTCTACCTATTTCTTGAATATACTCAGAAA
GAAAGAGGGGGGCTTGATAGTGAAATATCCATCGCAGGTTAGGTTTATTAATACCCATGCCAAAA
GCAGATGTACACACCACAAATTTGATTTTTTCTTCTAACCAATCTTGTTCAATGTTTCTGCGAAT
ACTTGCTTCTAATCCGCCATGATATGCTTTATTTTGATAACCTAATTCATTTAGTAAGTGACTAA
TATTTTCACTATCTTTTCTGGTTCTGACATAAATTAAACCACTTTGTTTATTTTTATTTTTTATG
TGGTTAATTAATTGTTTTTTCCTACTTCTAGGAGTCCAAATAGTTTTAATTTTTATCCGTAAATT
ATCTCGATAAGGACTAATTAAAAATTTTTTAGGATTCGATAATTGCAAATATTCTTGAATAATTT
TTTGAGTATTATTATCGGCTGTGGCGGTGAAAGCGGCGATGGGTAAAATCATAGATTCTTTTTTC
ATAGCCATTTGAGAAGAATTTAGTTTAGATTTAATTAAACTAGAACGAATTGCACCTAAACGTA
GATATGTCGGGCGAAAACTATCCCCCCACTGACTAAGACAATGGGCTTCGTCTAAAATTAGAGCA

TTGATAATCAAGTTAGGATGAGAAATTATTTTCCAAATAGGCGGAGAAAGGAGAGTTTCTGGAG
AAAGATATAGCAACCTTAATTTCTGATTTTCTAATAAGTTTAAAGTAAAGGTGCGATCGCGCTTT
TTCATCTCACTATGTAAAATAGAAGCAGGTAAATTTTTCTGTCGCAACTCCAATACTTGATTTTC
CATTAAAGCTACAAGGGGCGAAATCACTATAGTTAAACCTTTTTGTAATAGAGCAGGTAACTGAA
AACAGAGAGACTTACCCCCACCTGTAGGCATTACTACTAAGCAATCTTTGCCTGATAAAATTGAG
TTAATAATTTCCTTTTGAGGATAACGAAAATTATCATATCCCCAATATTTTTGTAGATTATATTT
TAAGAATTGTAACGAATTATTCATAAATTTATGAAAGTCTAAATAGTGAAGGAAAGATTAGGAA
AAAGAAGGGATATTGCATTCAATTGGAGCAAAATTGATTACAATAAATTCTGTGTTATCTTATT
TTCAGTTATTTACGAAAAATAGTTACTATTTCTTCCTAGTTTCTTCTTGACTAACTATTATCAAT
AAAATTTAGGAAAAACTTAGTTAATATAGAAAAAAGATTTGAGGAACAAAATATGTTAAAAGCG
ATCGCATCTATAGTTAGTATTACTTCAATTATTTTACTTAATCCCACGAATATATCAGCACAAAC
TCAAGACTTTAATGCAAATAGTTATCAGAAAAATACCAACAATTCATTAATAGCCCAAACCATGC
CTCGCCTACGTTTTAAATTACCAGATAGGGGAGTGCCGGGGGCAAGAATTGGGGGGGCAACCAGA
AGTGGCGACAAATCAACAGTAGTAACCGCCATTATTCCTCCTGAAAAATTGGCATTAACCATTGA
CGACTCACCAACAATCTTTGTTTATCTGCCTAAGAATGATGCTTTTGAGGCTAACATCAAAGTGG
TAGAGGAAAATGGGAAAGAAATATACACTAAAAATTTCACCCCTCCCAATGAAGCAGGAGTTTTA
AGGGTCAAATTACCAGCTAACATTAACCTAGAAGAAAAAAAATTATATAAATGGGAGATTCAGT
TAATCTCAGAAGACGATAACCCATTAACAGCTTTTAAATTAAGAACAGTTGGTTGGTTAGAAAA
AGTTTCTTTACCTGAAGAAATGCAACAGGAAATAACAACAGATAAAGAATGGGATAGCTTAAAT
ACCTTAGCGGAAGCAGGTATTTGGTATGACACCCTAGAAGGATTAGCTTTATTACGTGCTGAAAA
TCCACAAGACAATCAAATTAAAAAAGAATGGGTTGAATTGTTGAATTCCGTCGGTTTAGATAGT
ATTGCTGAAATTGCTATCTTCCCTGAAGTGGTACAAATAAATTAGATATGAAAAAAGGGCAAAG
TTAAAAGGGCAAAGGGCAAAGGTAAATAGTGAATCACTTGTAACCTTCAACCCCTAACCCCGAAC
TCAGGTTATGATAATTGATAATGCTTTATCTTTGATTCAACCTTTGTAATTCAAAATTTTTTCTA
TTTTTCAATGCGCTTACCATTACCAAAACAGGAAAGAGAAAATCCCCTTTCTGAACATATAGGGG
AAGTCATAGAAACTTCAACCACAGAATTTTTAGCCCAATGCTTAGAGCCAGAAGAATTAAATTTT
CCCACTATGCCACCCTTTGGCAGTTGGGTTAAATCTCTTGATGAAGAATCTGGAAATAAAATATT
TGCTGTGGTGACAAATGTGACGACTGCTCCTGTGGATTCTGTACACAAAGCAAGAGCTTTAGGTT
TAACTTTAGCAGAATTAAGGGAACAGCAACCGCAAATTTTCGCTATGTTGAGAACCGAATTTAGA
GCAGTAATTATTGGTTTTGAAACTCCTGCTACTAATGACAATAATGGCTATAGTCCCAGTAATGG
TAATATATACCAATATTTGCCTCCCCGTCCTCCTCAAATTCATCAAGGGGTTTTTCGTTGTGTGCC
TGATGAGGTAATCAAGTTTACTGCTAATCCAGATTTCTTAAGGGTTTTATTACAAGTGCAAAATA
CTCCCGTTGAATCTCTCATTGCCGCTACCTTAAGAGAATCATACCGTTTGAGAAATGGCGATCGC
ACTTGGTTAGTTAATGCAGGTAGGACTCTTAGTGTCTTATTGAAAAATGATTATGATTGTTTAAA
GTATATTCTTAGTCAAGTTCATTGGTGAAATTAACAATTAGTAAGTAGTAATTAAGAATTAAGA
ATTAAGAATTAAGAATTAAGAATTTAAGAATGAAAAACCACGAATACTTACTACTTATTTATTA
TTTATTACGGCTCTTCTACAGTTGCTATGGTAAATTAACAAAAAATAATTGCTATAAGCTATGTA
AATTAAGAGATTAACGATAACAAAAAATTAATTGTTATAAATTAATATTTATTATTTAACCTGA
CACCTGCCACCTGCCGCCTGACACCTTTTCCTAACCAATAATTTATATACTCACAGTAAGAGAGCC
TTTATTACTTGTTTCCCCCTTTCCCCTCATCACCTCATCTCCCCATCCCCTAACACCTGCCACCTGA
CACCTGAAACCTACCCTTATCTAATATTTTTATGTCGAACTGAGATTAATTATTATTAACATAAA
GCGTTTGTGTGGGGTAAGCGAAAATAATTTTATGGTGATAAAATGCCTTTTTTATAGCAAAATTT
ACCTCATTTTGAGCCATACGAGAAATATATAATTCGTTGCTATGAACATAATAAATGACGTCAAA

ATTTAAGCTAAAATTACCAAATCCTGAAAAAAAGGCAATATCAAAGGTAACATTTTTTATCTCAA
TTAATGCTTCTTTTATTATATCGGGAATCGTCTCCAAAACTGCATTTTCTGTTTCATAAACTACC
CCTATTTTTAAAATTACTTGTCTGCGTTGCATTCTTTTAAAATTGCGTAATCTGGAGTTAGTTAA
ATCAGTATTTGCCAGAATTAATTGTTCTCCTGTTAGGGCTTTTATTCTTGTTGTTTTGATGCCAA
TATGTTCCACATAACCGATATAATCATCTATTGCTACTAGGTCTGAAATTTCAAAAGGGCGATCG
AATAAAATAGCAAAATAACTAAATAAATCTTGTAAAATACCTTGAGAAGCTAAAGCCAAAGCCA
CACCACCTATTCCCAAACCAGCCACGATTGCCCCTATATCAAAACCCAAATTACTGAGAATAAAA
ATAGTTGCTAATGTCCAAATTACCACCCTTAAAGCAGGAAATAAAGCATTAATTTTACGCTCAAT
TTCTTTATTAGAATCATGATATTTTTGACCATAATATTCAATTAAAATTTTTGAAATATCCACTA
ATAATCTTCCTACGCAAAAAGTTGTAGCAATAATTGAAAATGTTTTAACAGCACCGAGAATACTT
CTATGTAATTGAATTTCTTTAATGGTAAGGTAAAATAGACCAAAATAGGTAATGGGTAAAAAAT
GTTTTGCTAAAATATTTGTAATTTTATAAGCGAGTAAATTTTGATTATCTTTACTAATTTTTTTT
AATTGTCTAACTACAATATTTTTAGCTACCCTCAAGAAAAAATAACCTAAAACTAGCAGAAAAAT
AGAAATAATTATATCTTTCACTAAACTAGGTAGTTTTTCTGATAAATTAATAAAAAAATCTAAC
ATAAATAACTATTGATTAAAAATTTATTTTATAAAAACTATACAATCCTCAATGAGTCGGAATA
AATCAATTATTATAAAAAAGCGTTGCAGATATTATTGTTATGGCACTTATAAGAGTACCTATTCC
CTTTTGGATACCATTTTTTTTCACAAATGATTTAGTACTGCTATATAACTAATATATAATCTTTG
TATCATAGCACCGAATAGATATTTAAACTATTAATAATTATGAAAACCTTAAAATTTATACACTC
TATATTTTTCAAAAAAATAGCTAAATTACTAACTATATCTTTACTATTTATAAGTTTAAATTTAT
TAATATCTAGCTGTAACTCTATCAATAAAACAGACAATTCATCAAGAATTATACAATCAGTTTTA
AGCGAACCAAAAACTTTTAATGTGGTACTTTCTCAAGAATCACCCAATATTTTTGGTTTAACCTA
TGAGGGGTTAGTAAGAGAAAATCCTTTGACGGGTGAAATTGAACCAGCCCTTGCTAAAAGTTGGA
CTATTTCTGATGATAAATTAACTATTATTTTTACTTTAAAAGAGGATTTAAAATGGTCTGATGGA
CAACCATTAACAGTGGATGATGTCGTCTTTAGTTATAATCAAATTTACCTCAATGAAGAGATACC
AACAAATGTTAGGGATAGTTTACGCATTGGTCAAAGTCGGGCTTTTCCCACGGTAGAAAAATTAA
ATGAATCTCAAGTTAAATTCACAATTCCTGAACCATTCGCTCCGTTTTTAGATAGCACAGGATTA
GCAATTTTACCTAAACATATTTTAGAAGAAAAAGTTAACACTAAAGGCAGTGATGGAAAGCCAC
TTTTCTTATCTTTTTGGGGTGTGGACACTCCCCCTGAAGATTTGGTAGTTAATGGTGTTTATAAA
TTAAAAAACTATGTAACTTCTCAACGAATTATTTTCACAAAAAATCCCTTCTATTGGGAAAAAGA
TATTATGGGTAATCAACTACCTTATATAGAAGAAGTTGTATGGGAAATTGTTGAATCAACAGAC
ACTTCTTTATTACAATTTCGTTCAGGAAGTTTAGATTCTGTTGGCATTACACCCGAATATTTTTC
CCTTTTAAAGAAAGAAGAAAAGAGGGGTAATTTCACTGTTTTTAATGGTGGTCCTGCCTATGGTA
CAACTTTTATGGCGTTTAATCTCAATCAGGGATCGAGAAATGGTAAGCCTTTAGTAACCCCTTAT
AAATCAAGATGGTTTAATAATGTTAATTTTCGAAGAGCGATCGCGCATGGTATAAATAGAGAGA
GAATGATTAATAATATTTATCGGGGTTTAGGAGAAGCTCAAAACTCCCCTATTTCAGTGCAGTCA
CCTTTTTATGATCAAACTATTAAAGGATATGATTATGACCCAGAATTAGCCAAGCAAATACTTTT
AAAAGAAGGTTTTAAATATAGAGAAGATGGCAAACTTTTAGACTCAGAAAATAATGAAGTTAGG
TTTACTCTTTTAACCAATGCAGGAAATAAAATTAGAGAGTCTTTAGGTTCACAAATAAAGCAAGA
TTTAAGCCAAATTGGTATTACTGTGGACTTTACCCCGATCGCATTTAATGTTTTAGTGGATAAAT
TAAGTAATTCCTTAGATTGGGAAGCCCATATTATTGGTTTTACAGGCGGTAATGAGCCTAATGGT
GGTGCTAATTTATGGTTTCCAGATGGAAATTTACATCTTTTTAATCAAAAACCCCAACCCGGAAG
ACAACCAATTGAAGGTAGAGTTATCGCTGATTGGGAAGCCAAAATTGGGCAATTATATATAGAG
GGGGCAAGAGAATTAGACTTTGAAAAAAGAAAAGAAATTTATGATCAAACTCAGCAATTAGCCT

CTGAATATTTACCCTTAATTTATTTAGTTAATCCCTACTCTTTATCTGCTGTCAGAAATCGTATT
GAAGGAGTTGAATATTCTGCCCTAGGGGGTGCTTTTTGGAACATACAAAAACTAACCATTAGAGA
TTAAGAAAAGTTAAAAAGGCAAAGGTAAATAGTGAATAGTGAATAGTTGATAATTACTCGTTAC
TCATTACTCCCTTAAACCTGACACCTGCCACCTGATACCTGCCACCTCTTTCCGAACTCATTACTT
GTTTCCCCCTCTCCCCTCATTCCCCCATCCCCCCAACACCCTAACACCTAACCTTATCCGATATTCT
TAAACCGAACCGAGGTTATTTATGACCTAAAAATTTCATGGGAATGCCTCCTTCAGGGCTTAAAA
CGACTCCACGACGACGGGGTTTTACAGCTTTTTTTGTGACTAACTCCAAATCGTACTCTCGGAGT
ATTTTAGCCACTACTAAGCGCATCTCATAGGTAGCTAATGCCTCTCCCAGACAACGGCGCACTCCT
CCCCCAAAAGGTAAAAACTCATAGGGGGAATATTGTCGGTGTAAAAATCTTTCTGGTTGAAATTG
ATTGGGATTGGGATATAAGTCTTCTCGTTGATGGGTTAAATAAATGCACCCTGTCACTATTGTTC
CTTTTTTGACTGTTTGCCCTAATAGTTGCACATCTTCCGTTGCTACTCGGGGAAAGGTTAACATT
GCCACTGGATAGATTCGTAGTGTTTCATTACAAACGGCGGTAAGATAGGGTTGTTTATAAATTGT
CATACCGTCACTATCTGGAGGTAAGGTGTTTAATTCTTGCAGGAGTTTTTCTTTTACTTCTGGAT
AATAGTGAATCCAATACAATGCCCATGACATTGCGATCGCAGTAGTTTCATGTCCTGCAAACAGA
ATTAACATTAATTCGTCTCTTAATTCTTGGTTGGTTAATCCTTCTCCATTTTCGTCTTTCGCTTCT
AAAAGCAGGGATAACATATCTACTCTTTCTGAGTTTTTTTCTTTTCTTCTCTCTTCTATTTCACGG
TAAATTAAATCATCTAATCTTTTTTTGATTCTGAGAAAACTACCCCAAGGACTCCATTTTCCGAA
ATCTTTCCGTAGCCATTCATAAAATAGGAAACTAATATTAATTGGCTGGTTGAAGCGGTCTAAAA
TATCTTTGATTAATTGTGCCATTTCTAAGAAGCGATCGCCTTCTGTAAGTCCAAAAACCACCTTA
AGTATTACTTGTAATGAAACTTCCTGCATGGTTTCCCTAGTGATAAAAATATCATTAGTTTTAAA
CTTACTAAATAAATTATCAACTATTTCCTGCACTGAATTACCATAATAAATCATGCGATCGCCGT
GAAAGGGAGGCATCATCAATTTTCTTTCTCGTTTGTGTCTTTTTCCGTCCAAGGAAAGTAAAGAA
GAATAACCCACAATAGGAGTAAGTACCGTGTTTAACTCACCGTCAGCAAAAAAGGTTTGACGATC
ATTAGTTAATATTTGCTGTATAGCATCTGGATGATTAACAAATAAAATATTATTACCAAACCCAG
TAATTTGAGCAGTAAATAAATCTTGATACTCTTTGACTGCCTGACTCATATATTCAGGAGGATTC
AAAATCCACTTTACAGTCTGTAACCATACAGGAGTTGATAATTGATTGACGTAAACCATTTCTAT
ATTCTTCTTCTGTTTATTTTTTAGTCAATGCTTTAAATATAAAAAAGGAGGATATATAATCCTCC
TCTAAACTTAGATAAATATGCTATTGAAATTAACGATCAAGTTATTTAGTTTCGGAAAACTCTGC
ATCGATAACGTCATCTCCACCATCGTTGTTATCAGAATCGTTAGCACTTCCAGTAGCATCTGCCCC
GGGAGCCGCACCACCCGCTTGTTGATAAACATTACTACCGATGGTATAGAGAGTTTGTTGTAACT
CAGGCATTACGGTTTTGATTTTCTCGTCATCATCTTGACCTACTGCTTCTTTTAAGTCTTTGATTA
AGCCTTCTGCTTTGGCTTTATCATCCGCAGAAACTTTATCCCCTAATTCATTGAGTTGTTTTTCTG
CTTGATAGACTAAAGAGTCAGCTTGGTTTTTACGCTCAATTTTCTCCCGTCTTTCTTTATCCGCCG
CCGCATTGGCTTCCGCTTCCTTAACCATGCGATCGACTTCTGTGTCGGGTAGGGTGGAAGCTCCTG
TGATACTGATGGATTGTTCTTTACCAGTACCTTTGTCTTTTGCAGTTACGTTTAAGATACCGTTA
GCATCGATGTCGAAGGTAACTTCAATTTGAGGAATACCACGGGGTGCAGGAGGAATACCATCTAA
GCGGAAAGTTCCCAAACTCTTGTTATCTTTGGAAAACTCACGCTCACCTTGTAAAACATGGATTT
CTACATTGGTTTGACCATCAACGGCAGTAGAGAAGGTTTCTGATTTTTTGGTGGGAATAGTGGTG
TTTCTAGGGATGATTTTAGTCATCACACCGCCGAGGGTTTCTACCCCTAAAGATAAAGGAGTTAC
GTCTAATAAGAGAATATCTTTTACTTCCCCTGCCAATACGCCTCCTTGAATTGCCGCACCAACTGC
AACCACTTCGTCAGGGTTAACGGTTTGATTGGGTTCTTTTCCTAGAACTTTTTTAACGACTTCTT
TAACCGCAGGAATACGGGTAGAACCACCAACTAACACTACTTCATCAATATCGTTTGCACTTAAT
TTCGCATCTTTAAGAGCTTGTTCTACAGGAATAGCACAACGATCAATTAAATCAGAACATAACTC

CTCGAATTTAGCTCTAGTTAAGGTTAATTCTAAGTGTTTAGGTCCTTCTTGAGTAGCGGTGATAA
AGGGTAAGTTAATATCAGCCTGAGAAACGCTAGAGAGTTCTATTTTTGCTTTTTCTGCCGCTTCA
GTTAAACGTTGTAAAGCCTGTTTGTCTTTGCGTAGGTCAATACCTTCTTTTCCTTTAAAGTCTTC
GGCTAAGTAATCAACGATTTTTTTATCAAAGTCATCACCACCAAGGTGGGTATCACCAGAGGTTG
CTAATACTTCAAATACTCCATCACCAACTTCCAAAACAGATACGTCAAATGTACCACCACCCAAG
TCAAATACTAAAATGGTTTCATTACTTTTTTTATCTAAACCGTAAGCAAGAGATGCGGCGGTAGG
TTCGTTGATGATACGTTTTACTTCGATACCTGCAATTTTACCTGCGTCTTTGGTAGCTTGACGTT
GAGAGTCATTAAAGTAAGCCGGTACTGTAATAACAGCTTCTTTGACGGGTTCACCTAAATATTTA
CTAGCGTCTTCGATGAGTTTGCGCAATACTTGGGCGGAGATTTCTTCGGGAGCGAATTGTTTACT
CTGAGAGGGACAATCCAACTTCACGTTTCCATTTACGTTTAATACCTTGTAAGATACTTCAGTGG
TTTCGTTGGTAACTTCGTCGAATCTTCTACCAATGAAACGTTTTACTGAGTAGAAGGTGTTTTCA
GGGTTCATTACCCCTTGACGTTTAGCAATTTGACCTACGAGGCGATCGCCATTTTTGGCATAGGC
AACCACAGAGGGAGTGGTTCTAAAACCTTCAGCGTTAGCAATAACAACAGGCTTACCACCTTCCA
TTACTGCTACACAAGAGTTAGTCGTTCCTAAATCTATACCTACAACTTTTCCCATAAATAATAAC
TCCGTTTTACTATAAGTTAATTTCTATCTATAAAAGCAAACAACTAAGATTGTGAATAAAAAAT
AGATAAATTTACTCAATTGCTTATTTCTTTCTCTATATTGAGGGAAAGTGGCTATTTTCAGGTAG
TGTAGTTTTCCGTACCTATTTTCAGGGGTGTTAGCAAAGTCATGAGGTGAGGAGGAAGAATAGGG
ATTGAAGAATCACGAGGAAATATTTATACATTACATACAAATTACTTAGCCTAATATAACCTGAG
TTTTGGATAAGCTGAAAGCAGACCAACTCGTAGTCAGAAAACCTTATAGCTTCTTCTTAGAAATA
ACGATAAAATTGCCTTAATCCGAACTGACGTTAAATAGTTAAGAATTAAGAATGAAAAACCACG
AATACTTACTACTTAATTTATTATTTATTACTTGTTTCCCCCTTTCTCCTCATAACCTCATCCCCC
CATCCCCTAACACCTGCAACCTGACACCTGACACCTAACCTTGTCGGATATTCTTAAACCGAACTG
AAGTTAATATAGTCATCTATACTGAAAGAGAAGCAACTATTAGGATCATTTCAGCGAGAAAAGC
GTCTAAAAAGGAGAGAAAACTATATGAGTAAAAAAACTTTTTTGATATGTCAAAAGAAGAAAGA
CAAAAAACTTTCTGGGAAATGTCTGATGAGGATATTGATTTTTCCGATATACCAGAAATTAACCA
AGATTTTGTTAAAACTCTAAAAAGAATTGAAAATGATCATAAACCTCAGACAGATACCGTTAGA
ATTAAATCTTATCTACTCAATTGGTTTAAAAATAATGCACAAGAAAATAGTTATGAGGTTTTAA
TCAATAATGTTCTTGAAAACTATATTCGACATCAAACTGAGAGTTAATTATACAGAAGAGCAAA
AAGTCGATCGCATCTATATTTTTCATGCAAAATCTTCACCCTGACTCGGCAACTTATTACTCATT
ACTTATTTACTACGCTAAACCTAATACCTAATACCTTTCTAAACCGAATATTCTTGTCGAATAAC
TGAGGTTAACTGAACTATTACTTATTTACAAAACTCTTCCCCACGACGATATAATTCACGGGCAT
AATCAGTCCATGTACCTTGTCCCCAATAACGGAAACAACTGGTTTCCACTAACATGGTGTATAGT
AATGCCTCTTGATAGTCGTGTCTTTGAGTTACGCTTGGGTCTTCAGCAACAGCGCGATCGAATTT
TTGATGGAACATTGCACTTAATTTATTCATGGGTTCTAAAACATTTTCATAGCCTTTAACCCAAC
TCAAATCATTAGTCCATGAAGCCCCATCCATGTGGAATTGATGATCGTTTTCTTGCAGATGTGCG
ATCGCATTTTGGACAGATTCAGGGGAAATATCATCGCCAATAGCATTCCAGATTTTATGTTGTCC
AACGGGCTGACAAACGGGGAAATCTTCCTCTTTTACCCCCAAAGACTCTACCAATTCAATATATT
CTGTGCCGTTTAAACCAACAACCCCTTCCCCGCTATCTTTAATTTGATACCACACAGGATGATAAC
CTCCGGGAAACTCATTCATCATGACTCCACCGTTTTCACCGTCAGCGATTTGAGAAACACAAGAA
GGTACAGAAATATCACCAATATTTTGTCTGCCTCTACCCTTGGCTTCATAGAAAGGTTGCATTTG
TGCTACTAACTTAGTATCAGACCCTTGAGTTTTAATCAAAGCAACAATACTAACGCTTTCACCCC
TAGAATTTTTCGCCACCAAACGATTAGGTAAATATTTATCGTCATGATATAAACCGCCTCCTTCT
GGACGTTCTACAGAATGCTCCTGTACTAACAACCAACGATAACCACAATCTTTCAGGGCTTTAAT

ATATTCATAAGCAGTGTCAGGATGATTTGGTAAGTGCATTTCTGGAGGAGAGAAACCCTTGACTC
TTCTTAAAGCATCATCCCCGAAAATAGAAGCAAAATGATGTTGCCAAGCCAAAATTTGCAATTTA
AGATCGGGAATAGGAGTAGAAGGTGCAACAGCGTGAGACCACATTGTTCCTAACCATTCTACATA
ACGATTATAGTGAGGATCACAAGTAATTTTTTTCAGATTATCAATGATGTCATTGCGATTCATTT
GCTGAAATCCCCATAGCAAATTACCTGAGTAATCCAGCATAATTCTTGGACTGCAACCATTGCCC
ACTAATTCAGGGATAAAATCCCCCATGCGACTATAGCACCAAGCAAAAGTACCAGCGTTGTGATT
ATCTCCTTCTCCTTGATGCTCAAACATATATTGTAAGTTACAAATCAGACTGCCATCATGACCTG
CAGGAATAGTTGGCTGGTGCATATGTAAAGCACAGGCAAAAGCAGACTTAATTTGATGAGGATT
GATATTGGAATGGGGTAAAAAAATAGGATCTTGATGATTGACTATAGATAATATCCTTGCTTCT
GAACCGCAAATCGGGGGCATAAAAGTTGTTTGTGTAGGCATTTTTTCTCCTAATATTTTCTCTGA
AGATGTCTAGGTACTTTGAGCAATAATTCTTTATCTAATTACAACCAAAGTTCTTGATTTCACTT
TGATTTTACCTCAATTATTTCTCTTTATTTTTTCAAGTTTTGATAACTTTTTTGAATATTGTTAA
CTTTTTATTAAATTTTACAAAGATTTTCTAATTTATTCTTCTTGCCAAAGAGAAACCTAATCTTT
TATAATTGCAAAATAAGATTTAATTAATCATAATTGAGTTTCGTAATTTTCTCAAAATAAAGAT
TAAATTTTAACTAATTATTATCTTAATTAAACAAATTAAAAACAACCTCAATCAACGAATGGAA
ATAACAGTAAAATGGCGATCGCATCTTCAAAAAGCACTACATAAGAATCGCTCACAAGTTTTTAG
TAAATATATACAACTAGCAACCATTACAAAAGAAGGTTATCCCGCCAATAGAACCGTTGTGTTCC
GAGGTTTTTTAGAAAATAGTAACGTCATACAAATTATTACCGATACTAGAAGTGAAAAATATAG
CCATTTACAAACTAATCCCGTCGCAGAAATTTGTTGGTATTTTGCTAAAACTAGAGAGCAGTTTA
GAATTAGAGGAAAGATAGATTTAGTAATAGGAAATAATAATAATTTAACTTGGCAAAAAGCCCG
TCAAAACACATGGAATAATTTAAGTAAAAATGGAAAAGAACAATTTTATTGGGATACCCCGGGA
GAGGAAAAAATAGAGAAAGAAAAAGAAGAACAAATCCCTATCATTGATGAGAAAACCCCCCCCG
AAAATTTTTGTTTATTGTTATTAAATCCTGACAAAGTTGATCACTTAGAATTGAGAGGTAATCCT
CAAAATCGACATTTATATACCTTACAAGACAATAATCAATGGTCATATACTGCGGTTAATCCTTA
AAAAAAGTTGAGGGAAAAAGGAGTATTATGAGCGTAGTGAGCTCTCCGAACTCCGAACTCCGTTC
ACGACTGAAAGGAGTGTACGAGCGCTCGAGCGTTCTCTCCGAACTCAAATTATTGCCCTTTGATT
TAGTATAAATTCAGCAATAGTCAAATCTATAGGAGTTGTAACTTTTAAGTTGGTTTCTTCTCCTG
ATACTATGCGCACAGGAAAACCACATTTTTCTAATAAGGCGGCATCATCAGTAACTTGCCATCCT
AATTTTAAGCCGCGATCGTGACATTCTTTTAAGAGTTTAACATCAAAACCTTGAGGAGTTTGTGC
CGCCCAAAGATTATCTCTTGTGGGAGTATCGGTAATCAATAAATTCGAGTCAACTATTTTTATCG
TATCTTTTACAGGGATAGCGGCAATTAAACCCTGACATTCTTGTAAAACTTCGGCACAACGATTA
AATAAATCTGGAGTTGCTAAACATCTAGCACCATCATGAATTAGGACTTTTTCTGCTTTTTCTGG
CAATGCTTGTAAACCATTATAGACAGATTCCTGACGAGTATCTCCCCCCTTTATTAGTTTGATTG
GTTTACAGGGTTTAATTTGGTTAATAATTTCTTCAAATGTTGGAAAATCATAGGTTTGCCCCATA
ATACCTATCCATTCGATACTTTCCGCTTTGTCGGCAGAAATTAAAGTCCATGCAAGTAAAGGTTT
TCCTTTTAACTCTAATAATAGTTTATTACGAGAACTTCCCATTCTTTTGCCCATTCCCGCCGCCGG
AATTAATAAATACATTATTTTTATATTTTGTTTTTATTTTCTATTGAACAATAGTATATTTTATG
GGAATCTCAGATGTTCAGTTGTTCAGTTGATGGATTTTTAAAATCTTGTGATAATTAATGGTTGA
CAATAGCCACTACAAACTCATGATTGACAACTAAAAGTTGACGAACCAAACTATATTATTTTATT
TAGGATCGAACAAACAAAAACAATTAAGAGAAGCCAGAAACCTGCCACCTGAAACCTCTCTCTTG
CCGAACCACTGAGACTTATTGAGGAAGTGTAGCCGCTCCAAAAGTTGCATTGAATTGACGACACC
AAATGGCTACGGATTTATAATTTTCTAAATTTACCTCAGAAGGAATTTTATAACGTTGTTTACCC
TTCACTTTTTTGAGAGGTTCTAAAGTAACATAATCACCTTCTTTGATACTGTGTGTTGGTGCTTT

GGTTGTACCGATTACGTCTTTATTTTTGTGTAAAATGACGAAAAGATCGGGTCCAGAATCGGTTT
TAAAGTCTTCTCCAAATTCAATATATTTATTTTCACCTTCTTTTACCACAGATACACTACCACTG
GTGGGGTGTTCTCCAGAAACAAACATTGGCATTTTCATCGCTATTAATTGTCCAGAAGTATCAAT
GTGATTTGATAACCTTTGTGTTGGTTTAATCATGGCACAAACATCTGGTAACATCATCGCAACAA
TTGCAGGAACAACTGAAAAGGCAATAATCGCAGTGCGTTTCATTTTTCTTACTCCCTTATTATGA
AATTCTGCTTTTTCTGATTAAGAATATTTTTCCCCATGTTAATAGTAATCTTAGGAAATTTAACT
AACTACGAGAAAATAATCAATTCTTATGCCTTTAAAAAGTAAATGGATAAAATTACTCAGATTG
CTTCAATTTTATAGTGCCTAGATAAATAAATACTCAGATGCAAACTTCAATAGCTAACGCCATAA
TTTATTCTCTAACTCTCTAACCTGACGCTCAAGGCGATCGATTCTTCCCCTTAATTCATCTACTTC
CGACTGACGAGACACTCCTAAATCCTGTAATACATTGCGAATTTGTCTTTCCATTTGGGCTTGTA
AGTTACCTTGATCCGATTGAATTTGTTTCATGATGTCATCAACCATGTTTTTCGCTTGATCGGGA
TTGATTTTTCCTTCTTTAACCCATTGCTCACTTACCTCTTTGATTTTTTCGGCGGCTAGGGATGTA
GTACCAACACCTATCATTAATATTTGTTTTAACCAATCGTTATTTTCCATAATTAAACGAAATAT
TTATTAGTATCTACTTTTATCATAAGCCAGTCTTGATTTAAAAAGGTAAGGTATTTTCACCATCT
TAGTAACTGATGTTACGCAAAAAGATAATTAGTTGTTGTTTTGAGGTATCAGGTGTCAGGTATCA
GGTGTCAGGTTTAAAGTCAGAAAAATTCACCCTTTTTTATTGTGACTTAGTTTTTCGATTAAACT
ATGTAACATTCTGCCAATTTCCTCACATTTGTTTAAAGTAACTTTTAATTCTTGCTCTTTAAGAT
AGCCTAATCGTCCTACAATCATCAAATGAGTTTCTAATTCTTTGAGTGAGCTATTTGCCATAGAA
AGATGACGAATATAATCACCCAAATGATTTCTCCCTTTTCCTTCTGCGATGTTGGCAGGAATTGA
AACTGCGGCTCTTTGTATTTGACTACTTAAACCATAAATTTCTGTTTTGGGAAATTGAGACGTTA
ATTGATAACATATTACTACCAAATCCATTGATTTTTGCCAAACCGTTAAATCTCGATAACTTTTT
AGTTCAATCATTTTTATTATTTCCTGAAACCTGAAACCTCCCTTAACTTAACACATCAATAAGTG
CGTAACGTCAGTTAGTAATGAAAAGAATATCAAAATCAGGCGCAAATCTGACTATAATTAAATT
GCGTAACAGTTTCCCTTAGACTATTTTAAGTATTTTTAGTTATTGAGATAAAGATAACAATGATT
GCTACTGCAAACCTATCGGAAAATAATGACACACGAAGCCTTGCCGTGGTTGAAACCTATAAATT
AAGTAAGGTATATGTAACAGGTTTTTGGATGAATAAGAAAGTACCTTCTCTCAACGATTGTACTC
TCAAGGTTTATAAGGGGGAAACCTTCGGTTTATTAGGTCCTAATGGGGCGGGAAAAACAACTCTT
TTAAAAACTCTTTTAGGGATTATTCGACCAACTTCAGGTAAGGCATTATTGTTGGGTAAACCCCT
TGGAGATAGACAGGTAAAGCAAAGATTAGGTTATCTTCCAGAAAATGCTTACTATTACGATTTTC
TAACTGCGTGGGAATTTCTTAGTTTTATCGCCGATTTATTTAAAATTCCTAAATCGCAACAAAAG
CAGAAAATTTTACAATTATTGGATTTGGTAGGATTAGCCAAGCAAACAGCCCAGAAAAAACAAT
TAAGACAGTATTCTAAAGGAATGATGCAAAGGGTAGGTATGGCTCAAGCCTTAATTAATGATCCT
GAAATAGTCTTTTTTGATGAGCCAATGTCTGGTTTAGATCCTTTAGGACGTTATCAGGTTAGGCA
AATTATACTCTCTTTAAAAGAACAAGGTAAAACTATTTTTTTCAACTCCCATGTTTTATCTGATG
TGGAAAAAATATGCGATCGCATCGCCATCTTAGCAAGAGGGGAATTACTGAAAGTCGGTTCTTTA
GACGAAATTTTAGGCACGAAAAAGAGCTATCATGTTGTCCTTAAACAAGGAGAAAATAACAATG
ACTGGAATCAATGGTTAACTCACATCAAACAGGAAAATAATTTTATATCGGGAGAATTAACAGG
TAATCAAGAAGAGTTTATCCATTATGTTAAAAGTATCTGTGCAGAGTTAATCTCCATGAATCTAA
GCCGTGACTCCTTGGAGGAATATTTTATTCGCATCTTAGAAGAAAAAGGAATTACCTCTAGTCAG
TAAGAGAAATCTTTATTTTTCTTACTTGTTTTCTATCTGTTTAATTATTTCATCGCTCTTTTTAT
TGTTATATTTATTATTTTGTAAAACATCCTCATTAAAAGAACCCACGAGGACACTATTAATTGTA
GTTCTGGTGTTGTAACTCCATTGGTTTAAAGGGGAAGGCAAAAATTTGTCTCCGTAGGCGATGAA
GATAATAACATATACAGGGAAGAACTTGACTAATCGTATAGCCATTCCCCCTAGTAAACTGCCAA

AACCATTGAAAAATTTGAGATTTAGACCAATTTTCATAATCATTATCTTGGACTGATGAAACGAA
TTTATATGTCTTCACCTAAGATAACAAAAATTATAATTCACTTTTCTTTGTCTCGATATAATTCT
TAAATCTTTCTAAGTCTGCCTTCAAAGTAGATTCTACTACTTGCCCCAGAAAAAGATTATCCATT
AATTTGCCGATGATACCCGGTATGGCATAAGCCACCGTCAAACGAACAATACTATTAGAATGGCG
ATCATAAAAACGTACTGCCCCACGATTAGGCAAACCATCAACGGATTCCCATTGAATAATTTGAT
TTTTCTCTAATTTGACGATGCGAGATAACCAACTAAATTCAAACCCACCACTAGCTAATCGCCAA
CGAGACAAGTCAGGATCATCAGCTAAAATATGAACAGAATCAATCCATTTCATCCATTGGGGCAT
CTGTTCTAAATCAGACCATAAACTCCAAACCAAGTCAATAGGGGCGTTTACTTCTATTTGTACAC
TATGTTCGAGCCAATTTGTCATCGCAAATTTTGATCACTTAGTTTTACAGAATTACTCAATCATA
ACAATAACCAGTAATTGATCGGTAACTTAACCGAAAAAATCCGCTAGAATTATTAATTATCAGTT
AAAAATTAAGAATGGAGAATGAATAGTTAATCGTCCATAAAGGAATTGTTTAATTGCCCATAAC
TAATCATTAATTACTAATTAATTTTATCTGATCTGTTAATACCCTAACTGCCCGTCACTAATCGC
TCTTCTTGCTTTTCTCATACCCAAAATTTATTAATCATTATCTACTTTTACCTGAAAAGGCAGAA
AGGGAAAAGTTATATTTATTGAAATTTTAAAATAATCACTTTATTTCTTCTTTTTATCAACTCTA
ATATGAACTCAATTAAACTCGATTTTAATACTATTTTCCCTTATCAATTGGATGAATTTCAACAG
GAAGCTATTAGCTATTTAGACGAAAATAAGTCTGTTGTTGTCACTGCCCCTACGGGTTCAGGAAA
AACCATGATAGGAGAATATGCTATTTATCGAGCTTTAACTAATGGTAAAAGAGTTTTTTATACGA
CTCCTTTAAAAGCCCTTTCTAATCAAAAATTTAGAGATTTTCAGGATAAATTTGGACAGACTTGG
CTAGAAAATTTGGGAGTTTATGCCGAAATTGGTTTGATTACTGGGGACACCATTATTAATCCTAA
TGCACCAGTGGTTATTATGACCACGGAAATTTTTCGTAATATGCTCTATAGTACCCCTATTGGAG
AGGTGGGGACTTCTGTACAAGACGTACAAACAGTGGTTTTAGATGAGTGTCATTATATCAGTGAT
ATATCTAGGGGGACTGTGTGGGAAGAATCAATTATTTACTGCCCTTCTCATATTCAATTAGTAGC
ATTGTCAGCAACCATTGGTAATCCTGAAGATTTATGTCGATGGATAAATAGCGTGCGTAAAGCGA
ATTTACAACAGGGAAAAGTTAGTGAGTGTGTCTTAGTAAATTCGGATTTTCGTCCTGTACCTCTT
AAGTTTTATTTTAGTCATAGTAGGGGTTTATTTCCTCTTTTCAGTGAAAAAAATTCTCAAAATCT
TAAGTTAAATTCCCAATTAAAAAGGTTATTAGCTACCAATAAAAGAGGTAGATTTAGTCAAAGA
GATTGCCCTAGTATTAAAACTATTGTGCAACAATTAAGTAATAATAATATGCTCCCCGCTATCTA
TATAATTTTTAGTCGTAGAGGTTGTGATCAAGCAGTAGAATCTCTTAGTTATCTGAATTTAGTTA
CGGTAGAAGAAAGTAGAAAAATATTACTTTATTTACTTTATTTTCTCATGATAGAAAATTTAGA
GCTACAGGCTAAAATTATTGAATTTGCCAAAAAAGAGCATGAAATTGCTTATAACAAAATTATT
GACTTTATTGCTAATGAAAAAGAAGCAGGAGAGGCATTAGTTGATTATTTAATTGATAAGCCAA
TTTTTAAGGAGCGTTTACTAAGATTTTTAGCAGAACATAGTGAGGTAGCTAGAACTAATCAAAT
AGAACCTTTAACCAAGAGGGATTGCATCTCATCATGCTGGAATTTTACCTGCTTGGAAGGAGCTAG
TTGAAAGATTATTTGAATTGGGATTAGTTAAAATTGTTTTTGCTACAGCAACTTTGGCGGCGGGT
ATTAATATGCCTGCGAGAACAACGGTTATTTCTGCGTTGAAAAAACGTAGTGATGATGGACACCG
TTTGTTAACTCCTTCTGAGTTCCTACAAATCGCCGGGAGGGCGGGTAGAAGGGGCATGGATAAGG
TGGGGTATGTAGTGACAGTTCAAACTCCTTTTGAGGGAGCTTTAGTGGCTTCTAAATTGGCAAAA
GCAACTCCTGAACCCTTACGCAGTCAGTTTACTCCTAGTTATGGCATGGTTTTGAATTTATTGCA
GAAACATTCTATTGAGGAAGCAAAGGAGTTATTAGAGCTTAGTTTTGCGGAGTATTTGGCGGAA
ATTCAGTTATCCCCCCAAGAAGATGCGATCGCATCTTATACGACAGAAATAACTAGATTAGACAT
AGGTTTAGCGGAATTTAATAGTAAAGAGATTGCGGCTTATGAAAAATTAAAGGAAAGAAGAAAG
CAAGAAAAAAAAGTACTCAAATTATTTGAGAGAAATTGGTTACAACAAAAACAGAAATCGATTC
TTCCCCAATTAGAAGATTTAAAAATTGGTAGCATTCTTGATTTAAGTCGTCATCATAAGAAAAAT

GCCTATGAAACTCAAGGAGTTTTTGTTAACTATGTAGTTAATGGTAATCAAAGATTTTTGCTTTG
TTTAGGGAAAGATAATAACTGGTATCTCGCTTCTTTTACTGACGTGGTGGATATTAATAGTGGTA
GAATACCTGATTCTTTGATTGAAAAATTATATCTCCCAGAAACTCCCGAAATTAGAGCAGGGATG
ATTAATTCCGGTGATGAGCAATCTTTAACGGTTTCTCAACTAATTGCCGAATATGCCCAAGAAAG
TGTCGATAAAAGCGAAGAAATTATTTATCAAATTCAACGTATCGAAGAAGTAGAAGCTGCGATC
GCATCTCATCCTTTAGATAAAATAGATAACATCAATCAAATTGTCAAAAATAATCGTAAACGACA
ACAACTCAAACAAGAATTAAGCAGAATTCAAAGTCAATATCAACGCTATAAAGCAAATAGTTCCT
ATTATTGGCAAGAATTCCTAGCTTTAATCGAAATATTGAGAGAATTTGGAGCATTAGAAGGCTAT
CAACCCACACCTTTAGGAATCGCCGCCGCCACCATTAGGGGAGAAAACGAATTATGGCTAGGATT
AGCTTTAACATCCCATCGCTTAGACTATTTGACACCCCATCACCTTGCAGGAGTTATCACAGCTTT
AACCACAGAATCATTACGCTCTGATACATGGGTAAGTTATCAACCATCCCCGGAAGTATTAGACG
CTTTAGGAGTTCAAAAAATGGACGATGTAAGCTATAGTCCAGAATCAGAATTATGGGAAATCCGT
CGTAAATTATATCAGGCACAAACAAGACGAGATATTTCTATGCCCGTATGGTTAGAAAGAGATTT
AATCGGATTAGCCGAAGCATGGTGTTTAGGGGCTAGTTGGGAAGAAATTTGTAACAATACAACCC
TTGATGAAGGAGACATCGTTCGAGTTTTAAGGCGCACTGTGGATGTGTTAGTACAGATACCTCAA
GTGCCTAGCCTAGATTTTAGTTTAATTCAAACGGCGAAAGAAGCAGCAAAATCCATGAAACGTTT
TCCCATATAATTTTGAGCTCATTCGAGAGATTGGGGAAATGAGTTCGGGGTTAGGAGTTAGTTAG
GGGCGAATGGCCATTCGCCCGTACAGGAGTTAGGAGTTTTTATAGTAATTTCAAATAAGAAAGTT
ACATTTAACTGATAAATTCCGAATTAGGATGAGTTTGATGCCATCTTTGACGGTAAATAACCTCT
AAATCACTACCCGCTTTGCGAAAAATACGTACTTGGTTAAACCAGAATGCTTGAAATAAGCGGTA
GAAAACTACACTAATAATAGCCACAATTAACCCCATGGCAGTGGAAATTAAAGATTCGCCAATAC
CTAGTGTTACTCCAGTAGTGGAAGCTGTACCCAAATCACTGACAGAAATTGAACCTAGAGAGCGA
ATCAAGCCTAATACAGTACCTAATAATCCTAATAAAGGAGCAAGGGCAATAACACCTTCTAAAAT
TTTGTCCCCTTTACGCATTAAAGCCAATTCATCATCTGCCCCTGTTTCTAGGGCTAAATGAAATAC
GTCTGGATCAGGATTGTCCAACTGTAGGGGGGTATTTAAAAATTTGCCTAAAGGATGATTACGAT
AACGGGAGGCGATTTCCCCTGCTTTTCCCCAATTAGTCATTGCCGCATCCATAATGCTGTTAAGA
ATCAACTCTTCTTTAATTAAGACTTTTGACCAAAACCAAATACGCTCTATAATTGTTCCCAGAGC
TAAAATCGAGAGAAATAATAAAGGCCAGATGGCAACCCCACCTTTATCGATTAATTCAGCTAAAG
TCATTTATTTTTATTCCTTATATTAAAATCAATATATGTTTTTAATTTACCAATTATTAATTATC
TCCTAAAGAGTAATTAGTGAATAGTGAATAGTTGATACTAAAAAACTTCTGGCTTCTGACACTTG
AAAGTTGACACCTTTGTCTTAGCCTTTATGCTTCATACAGATTGATACGAGGAAGACGGTGTTTG
AAACTACAGAGAATTTCCCATGAAATTGTACCAATGGCGTTTGCCCAATCATCAGCACTTATGGC
TATTTCTCCTTCTTCGCCTAGTAATGTGACTATTTCCCCCACTTCTAAATCAGGAAATGCTGTAAC
ATCTATCATTAATTGATCCATGGTAATATTACCTATTTGAGGGGCTAGTTTACCTTGCACAATCA
CGTTCATTTTGTTGGACAATAGACGGGGAATTCCATCGGCGTAACCGATACCAACAACGGCAATT
TTCAGGGGGCGATCGCATCTAAAAGTATGTCCGTAGCTAACTCCTGTATGAGCGGGAATAGTTTT
AATTTGAGTAATTCGAGCTTTTACCTCTAAAACTGGTTTTAATGGCACTGTATGACGTAAATGGG
GGGCAGGATATAATCCATATAAGGCTAATCCAACTCTGACTAAATCATAATGTAAACTGCGATCG
CACAAAGTTCCTGCAGAATTGGCTAAATGTAGGCAGGGAATGGAAATATTTGCACTTTTGAGGGC
GAAAATGGCTTCTTCAAAACGAGATTGTTGTAGTTTCATCACCGTTGTGTCTTCTTCATCGGCGG
TGGCAAGATGAGAATAAACGCTTTTGAGTTGTAATTGAGGAAATTGCTGTACATATTTGACAAA
ATCTACCGCATTATGCCAATGAGTACCTAATCTTGACATTCCTGTATCTAATTTCAGATGCACTG
GTAAAACATGGTTTAAACTCTGCAAAGTATCAGCGAAAACCTGTGCTTGATGTGGACTGCATAAA

GTGGCTTCAAGATTATGTTTAACAATTTCTTTGACTTCATCGGCGGTGTTAATTGCCCCTAAAAT
CAAAATGGGGGCTTTTATTCCTGCTTGTCTTAACTCAATTCCTTCAAGCAAAGTTGCGATCGCAA
CTGCATCAACTCCAGCTTTGATAACTGTTTGAGCAACTTTAACAGCACCATGACCATAGGCATCA
GCTTTGATAACTGTCATGAGCTTTGTACCTGAATTTAGCCATTGTTTTAACACTTTCACATTGTG
GGTTAAGTTGTGATGATTAATGTTAACCCACGCCCGTTGTTGTTGTAGGGGAATCATTCGGTCAA
TCCTTGTTTCAAAGCAAATCTTACTAACTCAGTACGACTATTTGTACCTGTTTTACTAAACAAAC
GACTAACATATTTTTCCACATTACGGACACTGGTGTCTAAGGTTTTGGCGATTTCTTTATTCATT
AATCCCTGTGCCACTAAATCTAACACGCTTTGCTCTCTGGGAGTTAAATCAATTTTGATGGGAGA
GTCCACCGTTGTAAATTTTCCACTACCTTCTAAGCGATCTTTAATATCCTTAACATCTCTTAATAG
TTCATCCAACTGCACATTGCTAGATCCTTTATTATCTTTACTATTTCGTTGTTTTGCCAGTAAAT
TAGCCACAATGGCTTCTAATTCGTCAGGATCAAAAGGTTTTGGTAAATAAGCATCACATCCCGCC
GTATATCCTTCGATGCGATCACTTGTCATACCTTTTGCGGTCAAAAACACTACTGGTACATTACT
ATAACGACTATCTTGACGTAACTGAGCAAGAAACTGTAAGCCGTCAACTTCAGGCATCATAATAT
CAGAAATAATTAAATCAGGAATATCCTCTTGTATTTTGTCCCACGCTTCTTTTGCATTACTAGCT
ACTTTCACATCCCAATCATCATTACAACTTAAGTAGGCTTGTACAGACTCTCTTATTCCCGGCTCA
TCATCTACTAACAAAATTTTTTCCACTTCAGACATAAAATTCAACCCTAACCAAAGACAAACTAA
CAACTATAATAACCCGAGATCTACAATAACAAGTAAGGAAAGGCAAAAAGACTTCCGGTAAAAA
TTATGATTCTTGCAACGTATTTCGGACTTCTAGTCCGATTTCTATCAGATGTCTATTTGTCGGTT
AAGGTAGGCAATAAGAAAAACTCCTCTGTATCTCCCCTTCAAAAGGGAAGAAAGACAATAGTATT
TATAATTGATATCAACGTTAAATTGATTTCAAATACATTTTTAATCAATTTTTTTTACAATTCTC
CATTTACTACCTTTACCAGTCTATTTTTAGCTAGATGCAAGATCTGAATTGAATAAATGCACCTT
AGCTTTATTCCTAATTCCTAAATTCTAAACTAATTTAATCCTAACCAGCTTAATAAACCTTGTCC
ACTAAAATATTCAATAGCTAAAGTGATGATAAAACCAATCATTGCGGCTCTTCCGTTCAATTTTT
CAGCATAGTCATTAAAACCAAATTTAGGATCTTCCACATTAGGGGTTTTCGTTGGTTCAGGTTGA
TTCATGGTTTTATACTCCTTATCAGTACTTGTATGAAAAAATATTAACTTTTGTTAAAATCTTAC
CACAAAATCTTGACGAAAAAACTTATATAACCATTTTCACCTCAATTCAGTTTAAAAGTATCGGA
TAAGGTCAGGTTGTAGGAGTTGAAAGTTTTTCAACCCTTATGGTAGCAAGTTTTAGTTTTGATAT
TAGCAACAACTTTGTTTTTTATTAGTAGTCTTATTTTCTTGATAAAAATTTCCCTTTGTTATTTT
AGGATCACGTTTTATTTGACTATGTTCATTAAAACTTTTTGCCTTTTCTAAAGGGATTTCTTCCA
AAGGATTAATCCCTATTATAGATGAAGAATAAGGACTATTTTCTTTTGTCATAATTTGAAAAGTT
TTATCACAAACCGCCATTCTTTCTCCTCTATAAAAAGTATTTCCATCATCATCTTCAACTTTTTTC
CAAGGACCTTTATAAATAATTGCTTGATTTCTTTCTAAACATTCTCCTTCTTTACCTTTATAGGC
TCTTACGGTTAATGAGCGGAATTCAATACCATCAATTACTTGCCAAGGGGTATCTTGTCTTGTTA
GTATTTCGATGCCGTAAAATCCTGCTTCTGCAAACATTTCTAAAAATAAATCTTCTCTAAAAGCA
CCAGCAATACATCCACTCCATAATTCAGAATCATTAGTAATTTCTGATGGTAAATCTTCATCACA
AACTATATCTGAAATAATGGCTCTACCTCCTTTTTTTAACACTCGATATATTTCTGCAAACAGTT
GTTTTTTATCTGCTGTTTTGACTAAATTTAAAACACAGTTAGAAATCACAACGTCAATGGAATTA
TCTTTAATTAAAGTTTCTTCTTGACGGAGGCGATCGCACTCTGCTTCAAAGTTTCTTAAGTGTGA
TAAATTCTGAATAGGATGAAGGTTTAAATAAGATTCTACCTTACTTAAAGGCAACTGTAAATCTT
GAATTTTCCCTTTTACAAAAGCAGTATTATTATAACCAATTTTATTTGCTATTTCTAATTGATAT
TTTTGAGCTAAATTTAACATCTCATCATTAAAATCTACTCCGATCACCTGTCCATTTTTTCCTACT
TTCTGAGCTAGAATATAACAATTTTTTCCCGCTCCTGAACCCAAATCTAAGACAGTTTCTCCTAA
ATTAACATAACGAGTAGGATCGCCACATCCATAATCTTTTTCGATAATTTCTTCAGGAATAATAG

CTAAATAAGATTGATCATACTCCGTTGGACAGCATAAACTAGGTTGATGTTCTTTTGCTCCTAAC
TCATATCTTTCTAGTACAGAATTTTCTACATTATAATTCATAATTTATGGTAATTTTCTCAATAT
TTTTACTAACTTTAGTATAAACTTAAATATATCAAAAAAATTAAAAATGAGATTAATTTAAAAC
AATATATGATGCTAACAATTAATGCTAAAACTGATATATCATAATGATGTAAAATTAAAAATTA
TCAACAAATAACCCTCAGTGTCTTTTATGGATGAGATACCCGTTGCCCAAAGAAAAGTTAATACT
ACACCTCAAAATAAGCCTTTCCCATTTAGTATTTTCTTGGCTTTGGGAAGTGCAATCATAACTAT
GTCTCTTTTTTGGTTATTTTCTCAAACTCAATCATCTCAATCTCAGCAACAAGATACCCCGTCAGT
AACCGCCTCGGAAACAGTCTCAGACAATATCCCCACTACCGAAAACAATCCTCCTTCGCCAGATAC
GGTTGAAATACCAGAAAACATCTTAGGACATTTAGGCTACGAACAAGCCCCTATGAGTGAGTTAA
AATCCATTACAGCCGATGGTAGAATCAAGTTGAGAACAAAAGCCGCAGAGAAGTTTTTAGAAAT
GCAATCTGATGCGAGAAGAGATGGTATTACTCTGGTAGCTATTTCAGGATTCAGAACCATTGATG
ATCAAGAATATCTATTCTTTAATATCAAAGAACAAAGAAAACAGGAAACCTCAAAACGTGCGGA
GGTAAGTGCTCCCCCGGGTTATAGTGAACATCATACGGGTTATGCGGTGGATATTGGAGATGGAA
ATGCTCCAGCAACGAATCTAAATACTAATTTTGAACAAACAGCCGCTTTTCGTTGGTTAGAGGCT
AATGCGGCGAAATATAGCTTTGAGTTATCTTTTCCAGAAGACAATTTGCAAGGAATTAGTTATGA
GCCTTGGCATTGGCGCTTTGTGGGGGATACTCATAGCTTAGAAACTTTTTATAAAGCCCGTGAAT
TAAAGACTAATTTACAACCACAAATGTAAATCTTTCCAAAAATTTTCGCTCCAACCATAGTAATA
ATTGCTACATTAAATTTAGTCCAAACTATACTCACATAAAAAAATATTATGAGTCATTTAATTGC
GATCGCCTATGATGATATATTCAAGGCAGAAGAAGTAAGACTAACCCTCGCAAAACTGCAAAGAG
AGCATTTAATCGAATTAGAAGATGCGGCAGTAGTAGTTAAAAATGCAGAAGGAAAAGTCAAACT
CAAACAAGCCATTAATTTAACCGCCGCAGGGGCAACCAGTGGTGGTTTTTGGGGTTTATTAATTG
GAACATTATTCTTAGTACCTTTATTTGGTGCGGCAGTAGGCGCGGCAACAGGTGCGATCGGTGGT
GCGTTGAGTGACATTGGTGTGGATGATGAATTTATGCGCGAATTAGGGGAAACCTTACAACCTGA
TACCTCAGCATTATTTATCTTGATTAAAAAAGTGACTCCAGATAAGGTTTTGGATGAAGTAAGTA
AATATGGTGGCAAGATTCTTCGTACTTCTCTGAGTAAAGATGATGAAGAACAATTGCAAGAAGT
ATTAAATAATCGTGGGGTTTTACCCTTAGATGAATCCGCAATGTCGTAAACGATTCGAGGCAGCA
GGGCTGTTTCATTTTCGAGAAAGAAAAAAGAGCGGGATATAGGGGGAGAGGGAGACAGGGTTTT
TTCAAAGTCAGGGCAAAATTATCTTGCCCTCACCCCCAACCCCTCTCCCACAGTAGAGGGGAGCGT
TTTTTCTTAATAATTGATTAATTAATACTTAAAAACTCCTGTATCTGTTACTATTTGTTAGTTTC
AAAAATTGACATTTTTGAGAATGAAAAAACCCTGAGAGGGAAATGGGGGGATTTTTGTTCATAA
AGTTTTAACTAATAGAAAAAAATCCTTGTATCTCTTACTCTTACTTAATCTCAGTTAACCTAATT
TTTTACTTTGAAACAGCCCTGCCTCGAGAGGGGGGAGGGCAAAAGCCTTTAATTAACACTGACCC
TAACACCCCACCCCCCGAACTCCGAACATCCTACTTATTGACAAAATTACGGAGAATCGTTAATA
ATATACTTATTCCTCATTTCTCATTTCCGGGTGCACCCTGCACCCCCTCTTCATAAGCAGTCAGGG
TAGCCATGGACATCAATCAGCTTAAAACTGCCAGTTTATTACTGATTATGGGTTGTCGAAAATAG
GAATTAAATTAATTAACTATGGTAAGGTGTGCAGTTGACTGATAAGATAACAAACAATAGCAAG
ATTACTGATATAAGTAAAATTAGTTTCACATATAAGAGGAAAATATGACTCAAGATAGAGTAGT
TATTATCGGAGTTGCTGGTGACTCTGGTTGCGGTAAATCCACTTTTTTACGTCGTTTAGAAGATT
TATTCGGAAAAGAATTTATGACCGTTATCTGTCTTGACGACTACCATAGTTTAGATCGTAAAGGA
AGAAAAGAAGCAGGTGTTACTGCTCTTGATCCTAAAGCGAATAACTTTGATCTTATGGCAGAACA
AATTGCCGCTTTGAAAAATGGTCAAGCTATTGATAAACCTATTTATAATCATGAAACTGGAGAAA
TTGATCCTCCTGAGAGAATTGAACCTAACAAAGTAATTGTAATTGAAGGTTTACATCCTTTCTAT
GATGAGAGAGTCAGAAATTTGGTTGACTTCGGTGTATATTTAGACATCAGTGAAGAAGTTAAAA

TCCAATGGAAAATTCAGCGTGATATGGCTGAAAGAGGTCATACTTATGAAGATGTTTTGGCTTCT
ATTAACGCTAGAAAACCAGATTTTACTGCCTATATTGAACCTCAAAAACAATATGCAGACATCGT
TATTCAGGTGTTACCAACTCAATTGATTGAAGATAAAGAAGGTAAGATTTTACGAGTACGTTTA
ATCGAAAAAGAAGGTATTGAACACTTCGAGCCTACTTATTTATTCGATGAAGGTTCAACCATTGA
TTGGCGCCCCTGTGGACGTAAATTAACCTGTGCTTATCCCGGATTGAAAATGTATTATGGTCCTG
ATAACTACATGGGTAATGAAGTTTCTATTTTAGAGATTGATGGTCAATTTGATAATCTTGAAGA
AATTATTTATGTTGAAAGTCATTTAAGCAGAACGGGTACTAAGTACTACGGTGAAATGACAGAA
TTACTGTTAAAACATAAAGACTATCCCGGTTCTAACAATGGTACTGGTTTATTCCAAGTTTTAGT
TGGTTTGAAAATGCGTGAAACTTACGAAAAATTAACTGGTAGTGCTACTTTCTCCACAGAAGAAG
AAGTTGCTCAAGTATAATCAAACCTCAGTTCGGTTTAAGAATATCGGATAAGAGTAGGTGTCAGG
TGTCAGGTTTCAGGTTGCAGGTGTTGGAGAAAGTAATAAGTAACGAGTAATGAGTAATGAGTGT
TCGGAGTTATTAATGATTAATTATTCACCCCAACACTATTTACCATTACCCTTTTCGCCATCACTC
ATAGGTGAAAATTTATCCCGAACTCAGATTATTTATTTCGCCCTCACTCCCAACCCCTATCCCACG
CTTGAGGGGAACATTTTTTCCTAATAATTGATTAATTAATAGTTAAAAAATCCTGTATCTGTCAT
TATTTGATAGTTTCAGCAATTAAAATTTTTGAAAATGAAACAGCACTACCCTTTTTTATTAGACA
TCGACCACATTTCTCAAAATAAGGGAATCAAGCCATTACGGGCATAATGTATTATGCCCCTACGA
GCCTATATTAATATTTTCTACCAGATGTCTATTGATTACTGAGGGAATAGGTTTTTTCTTTCATA
CTTGCCACTACCAAAAATTGTTCAATCCCTACAACCTTCCCTTACCAGATATTTTTGCTGAACAAT
TGAGGTAAGATGCTTAAAATTTCATAATTGATTTAACTACTGTTAGAGCGGAATAACTAACTCCT
GCTGTGCCTTCTCCCGGATGGGTTGAGTCTCCTACTAACCAAAGGTTTTTGATAGGGGTGCGATT
AGCAAAGCCAAAGGGGCCAAAAGTATTGATTCTTTGACCTATTCCACCAACAATTCCTTTTTCTC
TAGCTGTATAATTGGCAAAAGTTACGGGAGTAGCACTTTCTTGATGAATAATGGTTTCGGGAGTA
AGATTAAAATATTCTGCTAAACGTGCGATCGCATCTTGAGTATAATTAAGTTTAAGGGCTTCGTA
TTCTGCTTGAGAAATATTCCACCAACGATTAACATCCGTAAAAGAAGAAGCGATAATAGTTGCTT
TGCCTTCGGGGGCTCTACCATCTCCGGGCTTACTAACAGAGACAAACAAAGAATTAATTTCTCCG
ATTTCCCTTTCATAATCATATAAAAACTGTAAATGAGGAGGACAATTAGAAGGGATGGCAGATTC
GTCAACCCCTAAATAAACCACAAAAGCACCGGATGCAGGAGGTAATTTTTTCACTCTTTGGGCGT
AACCACGGGAAATATTGTCTGTCATTGTTACTAAGTTATTCACGGTGACATTCCCGATTACATAA
TCTGCTTTTTCTTCTTTTATTTCCCCAGTCTTTAAATTTTTTACCCTTACTCCCACCGCTTTACCT
TTTTTTGTATGGATTTTGTCCACTTTGTGCCTCATTAAAAGTTTCCCGCCATATTTATTTAAACC
TTCTTCGAGGCGATCGCACAACACCTGCATACTACCCTGAAGATGGTATAATCCTTGAGGCTCTT
GAGAGACAGATAAGGCAGTGGCCGCATACAATAAAGCTGTTTCCTCTGTATCCACCTGAGAATAT
AACTTTAACTGCATATCCAGGAAAGTTTTTAACCTTTTATCATGGGCTAAACCCAACAATCTTAA
AGCATCAGCCACCGTCATAAAAGTAAAGGGTGCAGTGACAAAAGTATCTAATCTTATCGCTTGTA
ATAATTGACCGAAATCCCAGAGACTGCGAGGGGGTAAAATAGGATCACGGGCTTGAAAACGCCAA
CTAGCTTCAAATAAAATTCTCATTAACTCCCAAAATCTACCACTATCAGGAAAATGCCCTTCTCT
TTCCATCTTCCACTTTTCCTGATTTCTCCATACCATAATCGGTTGTTTCTCTTGGGGTAAAAAGAC
AGCACAGGCAGGATCACAATTTGTAGCGGGGGGTAAATCTACGCCCAACTCTTGAAACACTCGAT
GATGAATTCCTCCTACCTCTAAACCTGCTACTTGAGTCGCCCCCACATCAAAAATAAAACCTTTGC
GTTTAAAAGTAGAGGCACAACCTCCAGAAACAATGGCTTGATCATAAATAGTGACGTCATAACCA
TAACGAGCTAGTAATGCACCTGCGGTTAATCCTCCAATTCCTGCACCGACAACAATAACTTTCTCT
TTATGCCTCATATTTGAGTTTATTTGAATCTACTCTTGCTTAACAAATCTTAACATAAGTTAAAT
AACTTTTATTAATTATCAACTATCAACCATTCACCTTTGCCCCTTGCCTATTGGCACATAGAATA

ACCTCAGTTCGGTTTAAGAATATTGGATAAGGGTAGGTGTCAGGTTTCAGGTTGCAGGTTGCAGG
TTTAGGGGAGTAGTGAGTAATTATCAACTATCAACTATTCACCCCAACACCCCAACACCCCAACA
CTATTTACCTTTGCCCTTTGCCCCTTGCCCTTTTATTGCCTTTTGCCCTTTTATTGCCTTTTGCCT
TTTTTTCTCCATCATTCATAGATGAAAATTTATCCCGAACTCAGGTTAGAATAATGAGGACTTCT
CACCTGCATCCGTTAAAATGTTGATTGAGATTAATGGATAAGCAGGAGAATGAGTAGGATTAACC
CTGAAAAGTTTGACTGGGAGAGGATAGAAAAAGCCTTACAAGTAGAATCAGAATATGGCTATAA
AAATATTCAAGGAAAACTGTATCGTTTCAGTGAATTTCTTTGTTTAACTTTTGGTGAAACTCCCC
CCGTCACAAATCTTCTGGTTGCTTTTCGTTGGCAAGAAACCGCTAAAAAATTCGCTCGTTATTCT
CATCTCACTATCCTTGAGAGGGAAGATTTAATTAATCAAACCCTTAATTTGATTGGGGAGGTAAA
AGAGATGATTGTTAATAAACCTTACTATCATCCTGTGGCAGAAGAAGTAAAATCTCTGGCAGTGG
CTGAAGTCAAACCCCCTTCCAATACCTCTGTTGCCCCTGTTACTCTTAAGTTAGATGATCCTGTTA
CTTTACTCAAGGCAATTTCTCTTAGACAAGGAGAGTTGTTGAAAAAGTTGCAAATAGTCACTATT
CGAGATTTACTTTTCTATTATCCCAGAGATCATATCGATTATGGTCGCCAAGTGAATATTAAGGA
TTTAGTAGCAGGGGAAACTGTTACTATTGTGGGAAAAATCAAAAAATGTGACTGTTTCAGTAGT
CCCAAAAATAAGAAATTAACTATCTTTAGTCTCATTATCATTGATCGCACTGGAGAAGTCAAAAT
CAGTCGATTTTTTGCAGGAAATTTTTATAGTAATCGGGGATGGCAAGAAAAAATGAAGCGACAA
TTTCCCCGTAATGCTATCGTAGCGGCTTCTGGTTTGGTGAAACAGAATAAGTATGGTATCACCTT
AGAGAATCCAGAGTTTGAGGTGTTAAACTCTCAGGGAGGAAATATTAATTCCCTCAAAATTGGTC
GTTTACTGCCTGTATATCCCCTTACCGATGGTATTCCTGCCGATGTGATTCGCAATGGAGTGATG
GAGGCGATGGTGGCTTTACCGCAAATCAGTGATCCGTTACCTGTTATTTTAAAACAACAATATGG
TTTAATGGAATTACAAGAAGCGATCGCACGTATTCACTACCCCGAAGATCAAGATCAATTATCCC
ATGCAAGGCGCCGATTAATCTTTGATGAATTTTTCTATCTGCAACTCGGATTTTTACAACGCAGA
CAAGAACAAAAAGAAAGCCGTATAGCGGTGTCATTTACCCCTAATGGTAGGCTAATCGAGGAATT
TAACCAAATTTTGCCTTTCAGCCTCACTAACGCTCAAAAAAGGGTCATAGAAGAAATAATCCAAG
ACCTCCAGTCCCCTTCTCCCATGAATCGTTTAGTACAAGGAGATGTGGGTGCAGGAAAAACCATT
GTTGCGGTGTTTGCCATTTTAACTGCCTTACAATCAGGGTATCAAGGGGCATTAATGGCGCCAAC
GGAAGTATTAGCCGAGCAACATTACCGCAAAATAGTCGGTTGGTTTAATCTGCTTCATTTGCCCG
TAGAGTTGCTCACAGGGTCAACCAAAACCGCTAAACGTCGTCAGATTCATGGTCAACTCGAAAGC
GGTGAATTACCCCTTTTAATTGGTACTCATGCCCTGATTCAAGATCCTGTACAATTCCGTAATCT
GGGCTTAGTTGTCATCGATGAGCAACATCGCTTTGGGGTACAACAACGGGCAAAATTACTATCCA
AGGGGCGATCGCCTCACGTTTTGAGTATGACTGCAACACCCATTCCCCGTACCCTTGCCTTAACCC
TTCACGGTGACTTAGATGTGAGTCAAATTGATGAGTTACCCCCCGGCAGACAGCCCATTCAAACA
ACGGTTTTAACAGGCAAACAACGCCCCCAAGCCTACGAATTAATTAAGCGAGAAGTAGCACAAGG
AAGACAGGCTTATGTGATTTTCCCTTTAATTGAAGAATCGGAAAAATTAGATGTCAAAGCTGCGA
TCGCAGAATATGAGAAATTTAGCGAAAAAATCTTCCCCAATTTCAACGTTGGTTTATTACATGGA
AGAATGTCATCAGCAGACAAAGATAAGGCTTTAAACGCCTTTAGAGATAATGAAACCCAAATAA
TTGTTTCCACCACTGTGATAGAAGTCGGTGTCGATGTGCCTAACGCTACGGTAATGTTAATCGAA
AATGCGGAACGTTTTGGCTTATCCCAATTACATCAGTTAAGAGGAAGAGTGGGTAGAGGTAGCCA
TAAATCCTTCTGTTTGCTAGTGACGAGCAGTAAAACCCCTGACAGTATTCAACGCTTAAATGTCC
TCGAACAATCTCAAGATGGTTTCTTTATATCCGAAATGGATTTAAGATTAAGAGGACCGGGTGAA
GTATTAGGTAGTCGCCAATCAGGTTTACCAGATTTTGCCCTTGCTAGTTTAGTGGAAGATCAAGA
GGTGTTAGTATTAGCAAGGGAAGCGGCGGAAAAAATTTTATTACAGGATAAATATATGCAAGGG
GAGAATAGTCTTAAAGATGAGTTACAAAGACGTTATCAAAAATTAATAGGCAATGAAATTTTGA

CATAGAAATTTAATAAAGGGCAAAGTTAAAAGGGCAAAGGGCAAGGGGCAAAGGGCAAGGGGCA
AACCCCCCTTTATCCCCCCTCTCGAGGGGGGAGGGCAAAGGAGAATAGTTGATAATTAAAAACTC
CGAAAACTCATTACTTGTTTCACCGCCTCCCCTTATCTCCCCATCACCTCGTCTCCCTCCCCCCTGA
ACTTTAAGTTCTGAACCCCGAACTCCTAACTCGTAAATCATCTCCTCTGCCCCCTAGTAACTTCCA
AGACAGAAATTAGTTACCTATTAGTTCTAACTCTTTCACATTTTGTTACAATAACGTATGATCAG
TTTATAGTCTAATATCAACTCCTAAATGTCCATCATCAGAAATTTAGTGTCCAGAAAATGCTAAA
TTTTTAAAATCGATATTGTTGGACGTGAATAGAAATCGACTCAACTATCAACCATTAGGATCAGA
ATATAAAGAAGTTTGCTGATTCTTCAATTTCTCTAACCATTTTATAGAAACACTTATTATGAAAA
AAGACCTAACTAAATATAGAAATATAGGCATTTTTGCCCACGTTGATGCGGGTAAAACTACAACC
ACAGAGAGAATTCTTAAGTTAACTGGTAAAATCCATAAATTAGGGGAAGTACACGAAGGAGCTG
CTACCACAGACTTCATGGAACAGGAACAAGAAAGAGGGATTACGATTCAATCCGCCGCTACATCT
TGTTTTTGGAAAGATCACCAATTCAATATTATTGACACTCCCGGACACGTTGACTTTACCATCGA
AGTATATCGTTCTTTAAAGGTACTTGATGGTGGTATCGGTGTATTTTGTGGTTCTGGTGGGGTTG
AACCTCAGTCCGAAACAAACTGGCGTTATGCAAACGAATCTAAAGTTGCTCGTATTATTTATGTC
AACAAATTAGACCGTACTGGAGCAAATTTTTACAACGTGGTTAAGCAGGTAGAAGAGATTTTAGC
CGCTAAACCTTTGGTTATGGTATTACCCATCGGTATTGAAACTGAATTTAAAGGGGTTGTGGATT
TATTAACCCGTAAAGCATGGATTTGGGATGATTCTGGAGATCCAGAAAAATACGTCATTGAGGAT
GTTCCCGAAGACATGAAAGAAAGATGTAGAAATGTACCGTGAACAGTTGATTGAAACTGCGGTAG
AGCAGGATGACAATTTAATGGAAAAATATCTTGAGGGTGAGGAAATTTCCATTGATGAAATTAA
AAGTTGCATCCGTAAGGGTACAATGAATCTTTCTTTCTTCCCTACCTATTGCGGTTCTTCTTTCAA
AAACAAAGGAGTTCAATTAGTATTGGATGCTGTGGTTGACTATTTACCTGCTCCTAACGAAGTTC
CTCCTCAACCCATTGTTGACTTAGAAGGTAATGAAACTGGTGAGTTTGCTAAAGTTGATCCTGAT
GAATCTTTAAAAGCCTTAGCTTTCAAGATTATGGACGATCGCTATGGTGCTTTAACTTTCACCCG
TATTTATTCTGGGGTGTTAGAAAAAGGTATGACCGTCTTAAATACTGCTACTGGTAAAACCGAGC
GTATTGGTCGTTTGGTGGAAATGCACGCTAATAACCGTGAAGAAATCGATCGCGCTCAAGCAGGG
GACATCGTAGCTATCATCGGGATGAAAAATGTGCAAACTGGTCACACTTTATGTGATCCAGATCA
TCCTGCGACCCTTGAACCCATGGTATTTCCTGATCCTGTTATTTCTATCGCCATTAAGCCTAAACA
AAAAGGTGGTAACGAAAAAATGGGTCTTGCTCTTTCCAAAATGGTACAAGAAGATCCTTCTTTCT
ACGTTGAAACCGATCAAGAAAGCGGTGAAACCATTATTAAAGGGATGGGTGAATTACACCTTGAT
ATTAAAGTGGATATTCTCAAACGTACTCACGGCGTAGAAGTAGAAGTTGGTAAACCCCAAGTTGC
TTATCGTGAATCTATCACCAAAGTTGTTAACGATTCTTACACCCACAAGAAACAATCTGGTGGTG
CGGGTCAATTCGCTCGTATTGATTACACCATTGAACCAGGTGAACCAGGTAGTGGCTTCCAGTTT
GAATCTAAAGTAACTGGTGGTAATGTTCCTCGGGAATACTGGCCTGCGGTTAACAAAGGTTTTGA
GTCTAGTATCGATAAAGGTGTCCTCGCTGGTTTCCCCTGTGTGGATTTAAAAGTTACCTTAACTG
ATGGTGCTTTCCACCCCGTTGACTCAAGTGCGATCGCATACGAAATTGCCGCCAGAGCAGCTTATC
GTCAATCTTTACCTAAAGCAAGTCCTCAGTTGCTTGAACCCATTATGAATGTGGACGTATTTACC
CCTGAAGATCATGTAGGGGATGTTATTGGTGATCTTAACCGTCGTCGTGGCATGATTAAAAACCA
AAACACTACTCCCATGGGTGTCAGAATTAAAGCTGAAGCTCCTTTAAGTGAAATGTTTGGTTACA
TCGGTGACTTAAGAACTATGACTTCTGGCCGTGGTCAATTCTCCATGGAATTTTCTCACTATGCA
CCTTGTCCTAAAAATGTTGCCGAGGAGGTAATTAAAGAAGTTAAGGAAAGAGAATTAGCCGCCGC
TAAGTAAGGTTCGGGGTTCGGAGTTCGGGGTTCGGAGTTATGTTAGTCATGAGTAAGTGTCAAGT
GAATGAAGAATTAATTTTCTCCCGACTCCCCACTCCTTACTCCCCTACTTTGAAAACCGCCCGACT
CCCCACTCCCTTAGTTTGAAAACCTCCCCAGTGTATAAATGACAAAAATTGCCTATTTAGATTTA

GAATTTAAGGACAAAAAAAACGGGGATAATAGATATAAACAACCCACTGAACTGGCATATGCGT
GGTTTAGTAACAAAAAATATTTGAATTTTAAAACTTTTCATTTCGACTATAATCAAGACATAAA
ATTAGTTTTATCAGAGTTAAAGAAAGTTATTATAGACGCTACAAAAAAATATAATTTTGATACT
TTGATATTTTGGGATTATCGTCAGGATTTAAAAATATTAGAACAAGCGGGAATTGATCTTAAAG
TTTTTCAGATTGAGGATTTACAGAAAAATATTGCAGAAATAGTCAATAACAATCGCTTATCTCTA
GTTTATGCTGATAAATTATTTAATCTTAGTGATGAGTTGATTAAAGAAACTAATTATTTAAAAA
AGGAAAATCGTTTACCGTTAAAACTTCATACGGCGGTAGGGGATGCCACTCGTATTGCAATTATT
CATCGAGAATTTTATAAGCATAAAGAAAAGTATATTCATTTAATTCAAGAATATTTGAGTCATA
GTTATTCAGAAAATAACCCTGTTGAAAAACATTACAAAAAAGAAGGATCTAAAAAAAGTATTTT
ACAAAATATTCCTCTCAATTTAGAACAAATAAAAAGAATAAAAGAAGGGATTTTATTACTATTA
AGTTTAGATAAGCTAACGGAGGAAGAAAAGCAATTAGAAGAAGATTTTATTAAGAAAAATAAGA
ACTATCTATTGGTGAAAAAGCTAGAAGAAAATAACTATGATTTAGCTAATTTTACTACTGAATT
ACTAAAAATTAATCGTTTTTTGAATCTTAATTTTAATATATCTTTAAAAGATTTGTATGAAATA
GATTTTAGTCAATTGGTTAATAAATAAAATAATGTTTAACGTTTTTTTATAACATTTTCAAAAA
AATATGGATGAAAAAAATAAAGAATATAAGATAAAAGTTGCAGGAGAAATCATTAAACCGGGGC
AAGTTAAGCAAATCGAAATTCCTGTGGCAAGATTGCCTACTCAAACTTTAATTTGTCTGCCTGTA
ACTGTGATTAATGGTTTGGAAAAAGGGGCGAGATTATGGGTTAATGCGGCAATTCATGGGGACG
AAATTAATGGAGTGGAAATCATCCGTCAAGTGGCAAATCATGTCAATCCTCGGCTTTTAAAGGGT
ACTTTGATTACTGTACCCATTGTCAATGTATTTGGTTTTATCGAACAATCTCGCTATTTACCTGA
TCGCCGTGACTTAAATCGTTGCTTCCCGGGTTCAGAAACTGGCTCTTTAGCCTCTCGATTGGCGAA
TATTTTTATGAGGGAAGTGGTGGGTAAATGTACTCATGGTATTGATATTCATACGGCGGCGATTC
ATCGTATTAATTTACCGCAAATTAGGGCGAATTTAGATGATCCTGAGACGATGCGTATTGCGAAG
GCTTTTAATAGTCCGATTATGATTCATGCTACAACCCGTGACGGTTCATTGCGACAGGCGGCAAG
TTCTGAGGGTATTCCTACGTTACTTTACGAGGGAGGAGAGGCTTTAAGATTTGATCAAGATGCGA
TCGCAGTTGGGGTAAGAGGTATTTTTCGGGTGATGAAAACTTTAGGGATGTATGATAATCCTGTG
GAAAATGGAAAAGAAGAAACTTCAGTGAGGGTTAACCAATCAAAATGGTTACGGGCATCTCAAA
GTGGTATTTTTCAGTTGAGAACAAAATTAGGGGCGAAGGTAGAAAAAAAACAGTTGGTGGGTAC
GATTACGGATGCTTTTGGCAATAAAACCATTGCGGTGAAAAGTAATATTGAAGGTATTGTCATTT
CCCATGTGCAAAATCCATTGGTTAATCAGGGGGATGCTTTGATTCATATTGCGATGACTCCCATT
AACCCGAGTTAGGGTTTTGGGTTTATGAAAGAGGTGGCAGGTTTCAGGTTGCAGGTGTTTTTGTT
TTTTCTTATTTACCATTGCCCTTTAATCTCCACAACCCCAAATCATCTGTGTAGTGTGACTAAAAC
CTTAACTCCGTATTCTTGTTCAAAAATGGGCTTTTTATACTCTAAATTCCATAACTCTAAAGCAC
AGTCATCTCCAACAATGGTAGGATGCAAATGAAGATGTAATTGTTTATATTCTTCATCATATCTA
CATTCTAAGTCTTTAATTGCCCCTATTTGCCTTCGATCAAGTGCGATCGCAAGTCTTAATATCGC
ACTTAGTTGTTTAACCATTTTACGGTGATTATCCGATAAAGAAGCGTAACTTTCATGTTTACGTT
TTGGCTTACTCTTGCGATGATAACGAGCAATTTGAGCAATTAATTCAATTTCAATTTCACTAAAA
CCTAATAATTCAGCGTTGCGAATCAAATAATAAGAATGTTTATGATGGGCGGAATGGGATATAT
ATAAACCGCAATTATGTAAAATAGTGGCACTCCAGAGATATTCTCTTTCTAAGTCTCCCCAAGAG
TGCAGTTGACCCTTCATACTATCAAATATATTTAAAGCAAACTGTGCAATTCTTTCGGCGTGGGC
TAAATCTACTTGATATTTATGAGCGATTTTATAAATATTACGAGGTCGTACTTCTCCTTGATAAG
CCAGACGATTTTCAATATATCCATTTTGTAACATCCAGTCAACTATAACACCTTCTCTCAATGCTC
TCTCACATACAGTCAATGAATCCACTTCCAACATTTTCATTGCCTCTACTAAAATAGTTGTACCG
GCAATAATAATTTCCGCCCGACGCTCAGACATCCCTGTGATGGAAACTCTTTCGTTGTAATCCAT

TTTCGTTAATTTAACGAGGATTTCTTCTATATCCTTTAAAGGCATTTTATAACCATTCAGGGTTA
TGGGTTCAACGCTATTGCTATCAAGGGCATGAATCATGGCAAGGGTTTCTGCCGTGCCGGATGTC
CCCACTAGCATGGGTTTTTCTCCTGTATGAAGAGCATTTCTAAATTCTTCTACAGGCCGTTCTAAC
ATCCCTTGAATATAAGCACTAAGTCGAATAAAATCTTTACTGTTAATAGGATCACTATGGATAAA
ATCTTGGGTTAAACGTACAGCACCAACTTTTGTACTACTCAAAAATCTTGCTTCTTGGGCATCTC
CTAAAATAATTTCCGTTGAACCGCCACCAATATCAATTACAGCATGGACTTTTCCGCCAAAATCC
ATCCCAGACAAAACCCCTAAATAAATTCTTCTTGCTTCTTCTTGCCCAGAAATTAAATCGACAGT
AATCCCTAACTCATTTTCAACTCTTTGTAAAAATTCTCGTCCGTTGGGGGCTTCTCTTGTGGCACT
GGTGGCAACTGCTATCAACTGATTAACTTTTAAACTTTTGGCTAAATCCTTACAACGTCTTAAGG
CTTCTAATGCTCTATTCATAGCACTATCAGTTAAATTTCCCGTATCCAAATCGCGATCGCCCAATC
TTACGGTATCCTTTTCTTTAGCGATGATAGAAAAAGAAGGTATCTGAGCATTGATGTGTACTACT
ACCATATGAATAGAATTAGTACCAATATCGATCGCACCTAAAATACAATCTTGTAAGGCTTTTTT
CTGGACACTTTGATAAGATAACTGATTAGTATAAGTCATGAGTTTGAGTCAGAGAGATTCGATCG
TCCGATTACAATTAATTCCTGATTATCTTATTGTTCATGATCCGTTATTTATTGTCAATTAAATA
ATGAAAGAGACTTATTTTTTTATCGCTTTAGGCGCAATTTTAGGGGCATTAAGTAGATATTATTT
AACTTTATTCTGTCTCAATAAGTGGGGTAATAAATATAGTTATGGAACACTATTTGCTAATTTAT
CTGGGTCTTTTTTAATAGGTTTTATTTCCACTATTATTTTTTACAAAATTATTTCTTTAGAATTA
CAACAATTTATTTTAATCGGCTTTCTTGGAGCATACACAACTTTTTCTTCTTATATTCTTGATAC
CTCAAATTTATATCGAGAAAAGAGATTTATGGATGCAATTTTTTATGGGATAGGTACACCTCTTA
TCGGCTTTATTTGCATCGAATTAGGTATTATATCCGCCAAAATTTATAATAGTTTATGAGGTTAA
TGAGCGGGTTTATTTCAAGGTCAATCAGAAAATGTTGGGGTATTAGAAAATTAGGGAGAAAATA
ATTCTTAAATAAATTGTATGATAAAGCATAGTCGCTACCTTCTAAGCTCCGTAAATTGCTAATTA
CTAATTATAAATTAACCTTTTTTTAGTTATTTCAACTAATTTATTCTTCCTATAATTATGCAAAA
AATCTAACTTCTTGACTTAATAGATAAAGTAATTTGAGCTAATTTAATACCTATTTCAAGACAAA
ATAATCCCAGAATTGGACTGCCAATCCAATATAGCAAATCTCGAGATAAATTATTTAAGTTGACT
GCAGTATTTAATTCATAGGTAGAAAAAGTTGTATAAGTTCCTAAAAATCCTGTTATTAATAGTA
GAACTAATTCGGGATGTAAAGGATATTTTCTCGCCAGAATAGTAGATAAAAAACCCATGAAAAA
ACAGCCACTAATGTTTATCAAAAAAATGTCAATGGGGAATTGAGTTTTAAAATTTTGAAGTAAC
CACAAGCCTATATAATATCTACATAAAACTCCGGCGATCGCACCTAGAGAAATAATTATAGGAAT
GCGTATTTGTGGATTTTTAACCATACCATAATGACGATAAAGTTTTGTGGAAAGAAATTAATAA
AAATATAACACTTCATTTAACTAATATTAAATAACTTTTATTACATTAAAACCATAATATATAAT
CAAGCTGATTCTAAACAGATAGAAAACGCTAGAATTTAAAATTAGTGAATATATTTATTTATCG
TGAAACAAAATATAAAAAGTCATGAATTAAGAAATTTATACAAAATTAGCACTTTTTTCTTAAT
AATAATATTACTTCTAGGGCTAAATCGTTACTATAGTTTTTTTGCATCCTATGATATGGGAATTT
TTAATCAAATTTTTTGGAATAATTTACACGGAAATTTTTATCATAGCTCTCTTGCTTCCGCTATT
TCTACTCATGTCACCAGTGGCGAATCTTTACCCTTAGTTAATGACAGTTACTTGGGTCATCATTT
TAGCCCTAATTTATTACTATTTTTACCGCTATATTTTCTTTTTCCTTCCCCTGCCACTTTAGTAAT
CATACAATGTTTATTAATTACAGGGGCGGAATTGTCCTTTATTTTTTGGCACGACAACATATAG
ACCATATCATTTCTTTATTAATTGTAGTCAGTTTTTACAGCGCGATCGCAGTTTTTGTACCCACT
TTATCCAACTTTGATAATATATCTCAACTGCCCATATTAGTTTTCAGTCTTCTTTTCTGTTTGGA
AAAAAATCAATGGGGTTTAGTGGCTTTATTTTCCCTATTGCTTTTGGGTGTAAGACAAGAAGCAG
GATTAAATTTATTTGGAATAGGAATTTATTTAATTATCACTCGTCGCCATTGGATTCAAGGATTA
ATAATGGCTATTTTTTCCTTCATCTATATGATTTTTATTACTAATGTAATTATGCCCCAATTTAC

AGAGGATGTTGGAGAAAGATTCATGGTAGATAAATTCGGGCAATACTTACAAGATAATAACCCC
TCTACTTTAGGAGTAATTTGGGGTATGTTAACTCAACCTTTATTAGTATTAAAAGAATTAATTTC
TCCCTTTGATGATACCGTCGAATATTTGTTAGGACAATGGCTATCTTTAGGCTTAATACCAGCCC
TTTCTCCCGCTTCATGGATAATTACTTTTTCCCTCTCCTTACTCCTTTACTTGGTCAAGGAAAAT
CTATTTTATCCTTAAATATTCGTTATGCGATGATGATTACCCCGGACTATTTTACGGAACAATT
TTATGGTGGCGAGGACAATCTTTCGGTAATTTCTATCAAGATTTAAAAGCACTTAAACCGAGAAA
ATTAGGGGCAAGATTTAAAAAATTTTGGCTTAGTTGTATTGTTATCTCTTTACTATTAGGTTTTA
CCTCCAGTGCAACAGAATTAAGCCGTGCTTTTTATTTCATCATGCCAGATTCATTTCAACCCTTAG
TATATGTATCTTTACCCCGTCAATGGCAACATTCTGCTTCTATTTATCAGCTTTTAAAGCAAATC
CCTAAAGATGCCAGTGCGAGTGCAACTAATAATATTATTCCTCATCTCAGTGGTAGAGAGGCAAT
TATTCGCTTACCAATGATAGGGTTTTTAGACACAAACCACCAACCTCAAACCGTTGATTATATTA
TCGCTGATTTATGGGAATTAGAACGTTATAGCGTTGTTTTTACCAAAGAAAAAGGATGGTTAGA
AGCCATACAAGGGTTGATTAAAGAGGTAACAGAGAAAGAAGAATATAAGATTGTTGCTCAAAAA
GATAAGGTAATTTTGTTAAAAAAAACAAGCCAGTAGAAAAAGGGCAAGGGGCAAGGGGCAAAGG
TGAATAGTGAATATTGGATAGTTGATAATTACTCATTACTCCCCGAAACCTGAAACCTTATCTCC
CTAATACCCCAATACCCTGAGCTCCAATCATCCTTTTTAACAGCTTATTATTTGCCAATTCCTGCG
GTTAAAACCTCATTACCAGATTCTGTTACTAAAACATCATCCTCAATTCTGACACCAATACCTTT
CCAATAATCGGGAATTTCAGGTTGCCCTTCAGCGGGGGTGATGTAAGGAGAAATATAGATACCGG
GTTCAACAGTTACAATATGTCCTGCTTGAAATGTCGTCCAATTTTCCTTATCTTGTTTATAATTG
CCCACATCATGAACATCTAATCCTAACCAATGCCCCGTGCGGTGCATATAGAAAGGCTCGTATTT
TTTCTCCTCTATAATCTCTTTACTGTCTCCTTGCAACAGTTTCAACTCTTTTAAGCCTTCTACTAA
TACCTCCACTGCTTTATCATGGTATTGGTTATATGTATTACCCGGTTTCACCATCTCGATCGCAGC
TAATTGGGCTTCTAACACTAGCTGATAGATGTCTTTTTGGCGAGGGTCAAATTTGCCATCAACGG
GAAAAGTGCGGGTAATATCGCCATTATAATAGCCATAGGAACAACCAGCATCTATCAATAGCAAG
TCCCCGGCTTCCATTCTGCGATTATTTTCAATGTAGTGCAAAATACAAGCATTTGCCCCAGAGGC
AACAATAGAAGGATAAGCAACCCCATCTCCTCCTAAACTTCTGAAAGTGTGTTCAATTTCTGCTT
GAATTTCATACTCATATTTACCTGGTTGAGCAATTTCTTGGGCTTTTTTGTGGGCTTTGACAGAA
ATTTCCGTTGCCTTGCGAATCATCTCGATTTCTGCTGGACTTTTTATCATTCTTAACGGATGTAA
AACAAAATTACTATCCTCAATACCAGTAGGTCCTGTACCTCTTTTAGGGTAAGATTCTGTTAGTT
TTTGCCAATAATGAATAATACGGGCATTAAAGTTTAAGTCTCTACCTAAATGATAATAGATGCGA
TCGCCACTTACTAAATATTTAGGTAATATTTCGTCCAACTCTTCGATGGAATAAACTGCATCAGC
TCCATACAAATCCTTTGCCCCTTCCACTCCCACACGATAACCCGTCCAAATTTCCTTAGCTAAGTC
TTTCGGTTGAACAAAAAGGATAAATTGATGCTCACTATGATTAGGTGCTAATACTGCAACAGCAG
AAGATTCATTAAAACCAGTGAGATAATAAAAATCACTATCTTGACGATAAACATACTCTACATCA
TTGTGCATCACTGCATAGGGAGCGCTATTAAATACTGCCACCCCTTTACCGATTGCAGACATCAA
TTTTTGCCGACGTTGTTTATATTCCAAAGGAGAAATCATCTTGAGAAGTTTGCCATATACATTAT
TTTTAGAATTCTATCATTGACTATTACCAACCGAGTCCACAGAATTGAGTAATTATGCTTAAAAA
AGGTTTCAGGTTGCAGGTGTCAGGAGATGAGGGGATGAGGTGATGAGGAGAAAGGGGGAAATGA
GAATGAGTTAGGAGAGTTTGCTGCGTTCGGACATTCCTTTCAGTGGTGAACGGGGTTTTTAATTA
TCAACTATTTACCTTTGCCTTCCCCCCTCTTGAGGGGGGATAAAGGGGGGTTTACCCTTGCCCTTT
TACCGTGCGTGCATGAGTGCCTGAGATAGCAAAAAGTCTATAACATATGAATGATAAATTAAAGC
ATATTGAAAGCTAAAATCATCATAAAAAAAATACACAGTAATTAGAAAAAGAAAAATAAATTGC
TAAGATACTCAATTAAGGATCAGCTTAGAAACTATCAAGAGATAAAATCGTCTCTGTCAAAGTAA

GTTAAAATTTTTATTTCAATTAATTATGTTAGAACAGCAATCTCTTTTAAACAAGATCAAAAAAT
TAACCGTTATCGGTGCAACCGTTGCTAGTTGTTGGTTGACTCAAACTACTAGAATATTAGCTTTA
CCTCAAGAAGTAATTTTAGAAAAACTTAAGCCTATACCTGTATTTACCATCGCTGACTCTCAAGG
CGCACCATTAATCGCTTCCACAGAAGATAATAACAGAGTTGCAGGAGTTTTTATCAGTGAAAAAG
ATGCTAATTCCTTTGTTGAAAGATTAAAGCAAGATAATCCCGATTTAGGTAAACAAGTGCAGGTT
GTGCCAGTGTCTTTGGCAGAAGTTTATCAATTGTCGGAGAAAAATAGTCAACAACAAGATGGTGT
ACAATTTGCCTATGTGCCATCTTCTCAACAAATAGAACAAGCACAACAACTTAATAACCAATATC
AAGGTGGAGTGCCTTTGTTTGTGGCGAAAGCAGGAGAAGACCAAGGTTATTTAACTATTAAACA
AAATGATCAAGAAGTTATACCTTTCTTTTTTGAGAAGCAACAAGTACAACAATTAGTGGAAAAT
TTCAAAAAAGCACAACCTGATTTAGCTAATTCTGTACAAATAGAGGTTGTTATTTTAGAAGGAAT
GTTGGATGCTATGAAACAAGGGGATGACGAAATGTTGACGAGAATTGTTTTATGGCCTTCTAAAG
AGTCTCTTGAGTTTTTACGCTCTAATACCCCTCAATAGGAGTACTTTTTGAGTTAAAAAAAAAGA
GGGATAGGGGCTTGGGGTTTTAGGAAACTAGGGAGAGATTTCTTTTTGATTTCAACTCCTAACTC
TATCTTAAACTCAGGTCTTACATCTAGCCAAAAATAGACTGGTGAAGCTAAGGAATAGGGAAAA
ATAAACAATTAATAATAAGAATTGATAAAAAACTTATTAAAATTGATTTTAAGAATTTTGCTTT
ATAGTGTAAATGTACTGTCACAATATCTCAGTTAATCTAAGTTCGATATAAAATTGTCTGATGGT
GGTTGTTTGGTGGTGGTAGCGGATTTGGAGAAACCTCTCTGTGTCTCCCCTCAAAGGGGGAGAAG
GGGAACAGGGAAAAGTATTTACAATTGATACAAACTGTGGTTCAACTTATTTTAAAGACATTTTT
CTCCATTTAATGATTATTTCGGGGAAAATTTTGAGGATTTTTGATTCTTAAATTGACGATATTTT
GTCACTAACACAACGTGAGCGGTAAATTTATATATAGACCTAAAACCTTTACTATAAGTGTTATA
TATTTAAATCGCTAAGTATATAGTTAAAGTGTAGCCAATAATTAACTTTTAACAAGTGATTACCG
TTAAGTCCCTTAATTTATCACTACAAGCTAAAACAAATTTTTCAATTAGATATGACATTAGGTCA
AAGTTCATAGTATGATAGTAAAAAATAAAATTTGACGATCTGTAAAAATAAAAAAACACAATGA
AAAAATTAGGTTTATTCTTATCTTCCTTACTTTTGGCAGTGACATTCATGTTTGCTGCGATCGCT
CCTGCTCAAGCTGATACAGTAGAAGTAAAAATGGGTGCAGATAGCGGTATGTTGGCTTTCCAACC
TGCAAAAGTAACTATTAAAGCAGGGGACACTGTAAAGTGGGTAAACAATAAATTAGCTCCTCAT
AACGTAGTTTTTGATAGCTCTGTAAAAGATGCAGATAAATTAAGCCACAAAGGTTTAGCATTTAG
CCCCGGAGAATCCTTTGAAGTAACTTTTAACGAACCCGGTGAATACCCTTATTACTGTGAACCTC
ATCGTGGTGCTGGAATGAATGGTACTATTATTGTAGAATAATTAAAATAAAAATACTTACTTTT
CATCACAAGCATTGAAGACGGTTTTATACCGTCATTTTTTTTGTCTAAATAATTCCATTTATTTA
CCCAAATTAGTGGCTCTATTACTTCTCGTCGTGTAAATTATTAGTTAGGGTAGGCAAGAGGCAAG
AGGGGATTATTAAATAATAATTTATAAACTTTTAGTTTTTATCTTACTATAAACACTATTTAATA
AAAGTTATAAAAGTTGATGTATTATTTGCTTAAAAGATTAAGATTTTTCTGCAAAATATGGAAA
ATAGAAATTGGCAATTAATCGATACCATTGTCGAAATTAAGTCCCCTTGGGTTACTTTAATAAGC
GAAAGAATTAAAGATGATCAAGATAATATTTTAGATTATTGGAGAGTAGAAAAAGAAGATTCTA
TAATAGTTGTGACTAGATATAAAAATTCCTTAATCTTTCCTAAATCAACATTTCGTCCCGGAATT
AAGAAGGTGACTATTGATTTTGCAGGGGGGAGATTAAAAAAGAATAATAATTTAGAAAATACAG
CTTATAAAATTTTGCAAAGAGAATTAAATCTAACTCAAGATAAAGTTAAAATAATTACTCCAAT
TAATCATCAAGGATTTATTGTTAATAGTTCGTTTAGTAATCAAAAATTATGGGGTTTTTATTGTG
AATTAAAATCAGATATTAGTCTTGATAATCTCTTTATTGGTGCTATTTATAGTTTGAATAGCAAT
GATTTTAAACGCTTATTAAACGATTTACAGTGCCTTCAGTGTCGCAGTTTACTTTTAGAGTGCGT
TTCTCAAGGATTAGTTGCGACTTTTTAAAAATAAAGTATAGAATTTAACTGACAGATTTGTTTTT
TTAGTATCTATATTTCGCAAGACTGTAAACTTAACACTGCAATCATATTTTATATTATGACTACT

CAAAGTATCATTCCTAGCTACAATCATGATCTCAATTTTATTCGTGACGAAATTAGGGCTTTATT
AGAAAAAGGTTGTATTAGCCGTCATCAACCTTTATACGTGTTGGCTAACTATGTGCCGCCTAGAG
AATGGTTAAATATTGAAAGGCAATTGGAAGAAAAAGATTTCTTACTGCGCGATCGCATCTGTGAT
TTATTGACAGAGGAAAAATGGGACAATGATTAAGTTAAGTTTTACTGAAAAAGTGTTGTGATGA
AGATAAGCAATAGGCACAAAGCAAGAGTTTTAATGATTATTATTAACCTGAGTTCGGTTTAAAA
ATATCTGATAAGGTTAGGTATTAGGTTTTAGGTTTCAGGTTTCAGGTTTCAGGTTGCAGGTGTTA
GGAGATGGGGGGATGAGGTGATGAGGGGAAAGGGGGAAATGAGAATGAGTTAGGAGATTTCGCC
GCTCTCGAACATTCCTTTCAGTCGTGAACGGGGTTTTTAATTATCAACTATTTACCTTTGCCCTCC
CCCCTCTCGAGGGCAGGGCTGTTTCATTTTCGAGAAAGAAAAAAGAGCGGGAGATAGGGGGAGAG
GGAGATGGGGGGATTTTTGTTCATAAAGTTTTAACTAATAGAAAAAAATCCTTGTATCTCTTACT
CTTACTTAATCTCAGTTAACCTAATTTTTTACTTTGAAACAGCCCTGGGGATAAAGGGGGGTTTA
CTCCTTGCCCCTCGTCCTCAAATTAAACAGTTGATAGAATTAAGAGGACTTAATTTTTTCCAATT
CCTCTCTTAAAGCAATGACTTCTTGAGTAAGTTGTTGAATATTTTGATAGGGATTATTCTCCGCC
TTTGTTTCTACTGTTTCATCATAATTAGGATTATTATTGGTGTTTTCGCTCTTATTTTGAAAGAA
TTGCTCAATAATTTTTTTGGCTTCTTCTTCTGTTAGTGCGCCCTTCTCTTGCCATTCTTGAGATTT
TTTTTGTAATTCTGTTGTTAATTCTGAGACAATTTGATTGCGCTTGTTATTGTCTTGCAAAGTTT
CAATGGCTGAAGCCGTAGCACCCACTACGGTGCGAAAACCCTGTTGCACAAAATTGATTAAGTTA
TTATTCTCCATGATGACTAACTAATAAGTAATATTTTTAATTTAAGACATTGACCTTAATATAGC
CTAGCTTGAATAGTCCTACTTTTTTAGTTCATCACTGTTTCTTTCGATATATCCTTACTACTAATT
GCCCGTCGTTTTGGTTGTTGACTATCCTTAGACGTGGGAGAATCCACTGAATCATCAAGAGTAAT
TTTTATTTTAGGACCAGAACTAGCTAAACGAATCACCATTCCATGTTTAACTAATACTTTATTAA
TCTTTTTTCCGTTGATAAATGTACCGTTAGAACCTAAATTGATTAATGCCCAATCTTTCCCCCTCG
GACGAATTTCTAAATGATGTCGTGACACCACCGCACTATAAAGCACTACATCATTGTCATTAGCT
CGTCCAATTCTAATCACCGAGTTGGGATCAAAGTTCCATTTTTGAACTGCAACTGATTTGAGGGG
GTGTAATAGAGTTAAGGTAATCACCGACGTTCCATGGGAGTAAAGAGTGCAATTATTCTTAAGTC
AAATACTATTTTACTAAATAGTCTGACTAAATCTACTCTCATATTCTACATGATTTAGGAATTGA
TGAGTAATAATTAACCTCAGTTCGGTTTAAGAATATCGGATAAGGTTAGGTATTAGGTTTGACGG
TTACAGGTTGCAGGTGTTGGGAGATGGGGGGATGAGGTGATGAGGGGAGAGGTGGCAGGTATCA
GGTGGCAGGTGTCAGGTTTAAGGGAGTAATGAGTAACCAGTAACGAGTAATTATCAACTATTCAC
CCCAACACTATTCACTATTCACTCATAGATAAAAATTTATCCCAAACTCATACGAAATTAAGTTG
TAATTAATGAGGCTCGCCAAATATAATTACTGAGCGAGTAAGACTTTTGATAATTTCAGTAGTAA
CATCACTTACAGCTAGTCCTCCGGCAGTGCGTCGGCGAATGGATCTTAAAATTACGAGGTCAAAA
TTTTGTTTATGGGCGGTATGAATGATTGCTTCTGCGGGGTCTGAATAACGGAGAGTTTTGATTGT
AACAGATGTTTTTGGAGCTATTTGTTGTACTCCTAAGTGTAGGGCGTTTTTAAAATTTTCGATGT
TATTTTTGCTTAAGTGGCGATCATATACATGAAGTAAGGTGATAGTTCCTTGGTTACTTTCCGCT
AAAATACAGCCTAGTGCGATCGCATTTAGGGTTTTCAGATCAATTTTTTTGACGGGTACAAGAAT
TTTGCGTAGATGGGTAGGCTCGTCTAACATTTTGACAACGGCAACAGGGCAATGAGAAGACCAAA
AAACGTTATCTATTAAATTGCCAAATAAACGAGCTTGAATGGTGCTAGTGGGACTCCATCCCATG
ACAATAAGACTGCCTTTATTTTCTTTGGCGGTGCGACTAATTCCCTCGGCTAAGTCGTCATCTAT
GCGAATAATGGGCTTGACTGATACTGAAAATTGTTTACTATATTGCAATGCCCTATTAATTAACT
GATTACTGTTATGAAAGGCACAATCCAACTCAGGCTCATCCATGTGAACATGGGCTTTGACAATG
GATAGGGGGATAACTAAACCTGTTTCATTTCCTGCTAGAATACAAGCCATTTCTACTAAGGATTT
TTCTGTTTGAGGATTTGCGATCGAAACTATGACTGTGCGATACTGCTCATTGATGAAAGAATGTT

GATAGTTATTGGATAACTGTAGAGAGCGACTATTACTATTTTCTATTAAATTTGTATCGACTTCT
GATTGAAATAGATTACGGGCAAATTTGCTGGTTAAAATCGGTCCACTAACGGAAGTAATTAACAT
CATCACAATCACCCCATTAAATACAGAAGGAGAAAGCACCCCTGCATTAACACCTGCTAAGGTAG
CGGCTAAGGTGGCGGCTACTTGGGGGAGAGATAAAGACCACATAGTCAAACATTGATCCCAACTA
TAACCGAATAAGACTTTGGGGATTAAACTGGCGATAAATTTACTCAGAAATAGGGTTATGACGA
TGCCTAAAGTAAGCCATAAATCTTGGCTAAAAATAGTCGCAAAATCAGATATTTTCAGGATTAAA
CCAATAGAAACGAAAAAACAGGGAATAAATAGGGTGCTACCCACAAATTCAATTTTTTCCTTAAC
AGGACCATTTCCTACCACATCATTAACCGCTAAACCCGCTAAAAATGCACCGACAATTTGATCTA
CATTAATTAATTGAGCCCCCACTGAAGCCAAAAATACAGCTAATAGAACAAATAGAAATTGATTA
CTTTCTTCATCTCCTGTGCGGCGGAAATATTCTTTCCCAGCGCGATCGAATCCATACAAAACAATG
GCACAATAAATTGCTAAACTGGATAACTGTAAAATTAAACTAGCAAGGGTAAAATCTCCTCCATA
AATAGAAAGACAAATCGCTAAAACTAATAAAGCTCCAATATCTGTGAAAATAGTTGCCCCAATGG
TGATAACAACGGCTTCATTTCCCGCAACGCCCAAACGATTGACAATGGGATAGCCCAACAGAGTA
TGAGAGGCTAAAAGTGAGCCAATTAAAATAGAGGCATTCAAACCCATGTTAAAAGCTAATCCCAA
GGCTGTACCAAATACTAAAGGTACAAAAAAAGTACTAAACCCGAAAAAGAGCGATCGATTTTTGT
TCTTGCGAAAATCCTCTAAATCAATTTCTAAACCCGCCACAAACATCAAATAAATTTTCCCAATA
TCGGCTAAAAGACTCATGGATTCACTTTTATAATCCAATAAACCTAACCCATCACTGCCAAAAAC
TACCCCCGCAAAAAGTAATCCAACTAATCCGGGTAATTTGATACGCTCAAAAATAGGGGGAATAG
TAAGAATAACTAGAAGTAAAATAGTAAAAGTAGTTAGAGGACTATCGGGAATAGAGCTTAAGAA
TTCAGGCATTGAGTTAATATTTCGGCTTTTGTGCTGTTAATATTGTTACACTATGATGCTTAATT
AACCTAGTTTCAGCTCAAAATACTTACTTTTTAATTATACGACCCAAAGTCATAATTTATAGTTC
AAAAAAACTGTTGCCTATCCTCACCTAGATCAAGTTATGCCCAACCGTGAATATATTAGACATCG
ACCACATTTCTCAAAATAAGGGAACCAAGCCCTAACTACGAGCCTATATTAATATTTTCTACCAG
ATGTCTATTAATCATCAATCATTCACCTTTGCCCTCCCCCTCGAGAGGGGGGATAAAGAGGGGTT
TGCCCCTTGCCCTTTAATTATTATCCATTTCTGAATGCTGGTGCGATCGCATTGGGAGCATCTTCA
GTGACCGCTTTACAAAACTTAATAGTTAAGCAGTGATAACCAAGTAATAGTAGGATAGATGATG
AGTAAATACTTTAGTGCCTAAATATTCACCCTTGGTTAGTTAAAACCCTGAATTCGGCAATAATT
AAAGCATAATAAATCACCCTAATTGATTCGATGAGTAACCAAAATTCGATCATTACAGAGTTATT
ACAATTCTTTCCTGATCGGCGCTTGCAGATGTACTTTAAGTCATCTCTGACTGCCCTTTCCCACGC
TATGGAGGATCAAGTTTTAGCAGGAAATGAAGAGGCTTTAATCATTGCTAGTTTTCAAAAAGAG
AAGTTTTATCGTCAAGAAGCCCATCGTTACCTACGTATTGGGAAAAAATCTCGCCATGTTTATGT
CTTAGCCGCACCAGAGACGGAATTTAAACATAATTCTGATGTCTATGAAAAAATAGCTTTTGCTC
CTGATGATAGTTTGGCAATGGAGTGGCATTTAATTGTCATCAGTCCTAATTATGCTAATTGTTTG
ATTTGTCGGGAAAAACCTTACTCTAACTCGGAAAATTCCTCTGAAAGTTTAATGATGATGGATAA
TAGTCGTCGTTTTGAGGGTATTTGGACTTATGATCGAGATATTAGTGTTAAGGCGGCTGAACTTC
TTTTATATCGAATTGCTCAGTATCGCCCTGAGTTACAAGCCAAAATTCAAGATGCGATCGCATCC
TATTGTGCCGATTTTTGTGCCTTAGAAGAAACTAATAATATTATCAATCCTGATCCCTTTGTGCA
AAGGTTAGTCACCTATTTACAAGCAGGGCAGTACAAATTAATTAAAGCGAATCGTTTCCTGAGTG
CCAAGGAAAAAAAAGAGAGGTTATTAAATTCTGTTACAGGTGCAATACGCAGATCCCTTGATCCC
GAAGAAATTTTGCAAGTAGCTGTGGATAAGTTAGGTGAGGGTTTTGGGGTATGTCGTTGTTTAA
TATATCGCTGTCAGGAAAATGACTTAAATGCCCATATTAATCATGAGTTTCTCAACGAAAATATC
AATTCTGTTAAGGGGCAAGTATGGCCTTTAAGAAATAATCCTCTCTTTCAAGAAATTCTCTCTCT
GCGAGAGTCTATTAGTATTGATGATGTTACCATTGATCCTCGTATTCAAAAAGACCCTACTATCT

TATCCAAGTTAATTAAAAGCTGTTGTATTGTTTCTTGGTTAATTGTCCCCATTCTCTATCAGGGT
AAACTATTAGGGATGATGGAATTGCATCATTGTGACAGTAATCCCATTACTTGGAAACCTGAAGA
TATTGCCCTAGTCGATGCGATCGCAACTCAAGTAGGAGTAGCCCTAATTCAAGCCGAATCCTACG
CCCATTTAGAAGACTTAAACGAACAGCTAGAAGCCTTAGACAGAACCAGATCAAACCTTGTTGCT
ATCACAGGTCACGAATTACGTACCCCCCTTTCTACTATTCAAGTATGCTTGGAAAGTTTAGCCAA
TGAACCCGATATGTCTGAGGAGTTACGACAGGTAATGCTTACCACAGCTTTACATGATGCAGAAA
GAATGCGCAAATTAGTACAAGACTTTTTAACCCTTTCTCAACTAGAAAGTGGTAGAGTAGAATGG
AATCCTGAACCCTTATCCTTAGAAGAATGTATCGAATTATCCTTATGTCAAATTCGCTCTCGCAA
AGACAAAAATTCCCTGCCTAAAATTCGCATTAATATTAATCAAACCTTACCTCTAGTACAAGCCG
ATGGAGAGTGGTTAGTGGAAGTATTAAGCAAACTTTTGGATAATGCCTGTAAATTTACCAATGGC
AACGGTGAAATTGTGATTAATGTGGAGGAAAAAAATAATAATATCCTTAAAGTTACTATATCCG
ATAATGGTAGAGGTATAGAACCCGATCGCCTCGATAAAGTCTTCGATCGATTTTACCAAGAAGAA
GGAGCATTGAGAAGATCAGCCGGAGGTACAGGATTAGGATTAGCCATTTGTCGCCAAATTGTTAA
TAATTGGGGTGGGAAAATTTGGGCAGAATCTCAAGGAAAAAATCAAGGTAGTGAGTTTCATTTT
ACCATTCCTGTTTTTAATAATGATTCTATTTCTAGTTCAAAAAATAATTAATAAATTCTGCATTT
CAGAAGCAGTAAATAATGGAAAAACTGATTTTATTAACTACATTAATTTTGGCTTTTTTAGCTTA
TTATTATCAAACACAAATAACAATTAACTACCGCAGAAAAACTCTTAAACAAAGCTCATTTCCAG
AAGAATGGATTGATTTTTTATCTAAATATATTTATCTTTATCGAATTATTCCTCAAGAATTAAAG
CAAGAATTACATAGTCATATTAAAATATTTCTTCACGAAAAAGAATTTATTTCCTATGACAAACA
ACCAGTAAATATCGAAATAAAACTTGCGATCGCATCTCAGGCTTGTTTATTATTATTAGGAGATA
AAAGACAAAAAAGAAATTATTTTCCCTACTTAAAATATATATATATTTACCCTAATTTAATTGTC
CAAAACAAAGGAGAGCAGATGTCTGCAATCTTGTCAGGGCAATCTTCAGTAGGCAATAAATCAGG
TAAAGATGGAGTAGTTTCTCTCTCATGGCAGGAAGTCATCAAACAATCATCATCACCCCATCAGA
TTGAAAATGTTATCTTACACGAATTTGCCCATCAACTGGATCAAGAATTTGGTACAGCTACAGGA
GTGCCACGATTAAGCAACCTTCAAGAAACTCTTATTTGGGGGGAAATCTTAAAAAAAGAATATGA
AAACCACTATCAAGCCGTTCAAAAAGGTAGAATAACCATTATTGATCCCTACGGTGCAACTAACA
TGGCAGAGTTTTTCGCCGTCGCTACGGAAGCCTTTTTTCTCAAATCCAAATTATTAGCGGCTTAT
CATCCTTTACTTTATAATCAGTTAAGTAATTATTATGGAGTCAATCCCATTGAATGGAAACCCAA
TTAGTGAATAGTGTTGGGGTGAATAGTTGATAATTACTCATTACTCCCCTAAACCTGCCACCTGA
AACCTAACTAAGCGATTGCTATTCTTAATTTATGAACAATATTCCTATAGAAATTATTACTAGAA
TAATAGATCTAAAAAAGCAATTACAAAAGGCTAGTTATGCTTATTATGTATTAGATAATCCCATC
ATGGAAGACTCTGTTTATGATCAATTATACAGAGAATTATTGACCCTAGAAAATCAATATCCTAG
CTTAATAACCCCTGATAGTCCTACTCAAAGAGTGGGAGATAAATTAAGCAATCAATTCAGTTCAA
TACCCCATAATATTCCCCTTTATAGTCTTGATAATGCTTTTAATTTTGCGGAACTAAAAGAGTGG
GAAAATCGTTGGCAAAGACAATTAAATAATGTTCCAGAATTTACCTATGTTTGTGAATTAAAAA
TAGATGGATCTGCCATAGCTTTAACCTATGAAAATGGTATTTTAGTAAGAGGTTTAACAAGGGG
AGATGGAGTTACAGGAGAAGATATAACCAATAATTTACGAACTATTCGCTCTATTCCTTTACGCC
TACAAATAGATAATCCTCCTGAAGTTTTAGAGGTAAGAGGAGAGGCTTTTTTACCCTTAGATGAA
TTTACTAGAATTAATGAGGAGCGAGAGAAATTAGGAGAAAATTTATTTGCTAATCCTCGTAATG
CGACGGCAGGAACATTGAGACAATTAGATCCAAATATTGTTAGTGCTAGAAAGTTACAATTTTTC
GCTTATAATGCTCATATTTTACCTGAAAATAATAACCTAGATACTCAGGAGCAAGTATTAAAATA
TTTAACAGAAATCGGCTTTTTAGTTAATCCTAACTATCAAGTTTTTAATAACCTACAAGCTGTAA
AAAAATATCTTGATTATTGGGAAAAAAATCGTCACAATTTGCCATATATGACGGATGGAGTTGTC

ATTAAAATAAATTCTTTTTCTTTACAAAATCAACTAGGTTTTACTCAAAAATTTCCTCGATGGGC
GATCGCACTTAAGTATCCCGCCGAAGAAGTTCCGACAGTGGTAGAAAGAATAATTGTCAACGTCG
GACGGACGGGGGCAGTAACTCCCATGGCAATAATGAAGCCTGTACAGTTAGCAGGTACAACAGTT
CAAAGAGCTACTTTACATAATGGCGATCGAGTGCGAGAATTAGATATTAGAGTGGGAGATACTG
TGACTATTCGCAAAGCAGGAGAGATTATCCCTGAAGTAATAGAGGTTTTAACAGAATTAAGACCA
GAAAATACTCAACCCTTCCAAATGCCTACTCATTGCCCTGAGTGTAATTCACCCCTCGTCAAACCT
GAAGCCGAAGCGATACTTCGTTGTGTTAACACCTCTTGCCCCGCCATTCTTCGAGGTAATCTGATT
CATTGGGCTTCCAGAGATGCTATGGACATAAGAGGTTTAGGAGAAAAAATTATTATCTCTTTAAT
GGAATATCGATTAGTAAATTCGATCGCAGATTTATATAAATTAACTCCATTTCAATTGGTTGACT
TAGATAGAATGGGGGAAAAATTAGCACAAAAACTAATACAGGCTATTCAAGAGAGCAAAAAACA
ACCTTTTGCTAGGGTTTTATATGGTTTAGGAATCCGTTACGTAGGGCAAGTAAATGCAAAAATCT
TGGCAGAAAATTTTGAAAACATTGATAATTTAGCCAAGGCAACTCAAGAAGAATTAGAATCTGT
TTATGGCATTGGTGAAGAAATAGCAAATGCTGTCTTTTTATGGTTTAAAGTGCCAGAAAATCAAA
AATTAATTAGTGAACTACAAACTCTCAATTTTAGTTTAGCCATAGATACAAAAAACAAACAAACT
AATCAGCAATTGTCTAATAAAACCTTTGTTTTAACTGGAACATTACCAACCCTAAAAAGAGAAGA
AGCAAAACAAATTATTGAGTCTGCGGGGGGAAAAGTTACTAGCAGTATTAGTAAAAAAACTGAC
TTTGTAGTAGTAGGAGAAAAAGCAGGTTCTAAATTAGAAAAAGCTCAAAAACTAGGAATAAAAA
CCATAACAGAGGAGGATTTACTAGCTTTAGTTAAATAATCCTATTTTTCTATAACTACATTAGGA
TTTGGGTGGTTTTTAGGTATCCAATAGTAGGTATTAGATTCTATTTTTTCAACAATTAATAAGTT
AGCAAACTAAACTCTATTTATTATGTTGAGAATTTTGTAAAGACTCTATTTTTTCTAATAATTCT
TGAATCAACTTGCTTTGTTTTCTTAAAGTTAATTGAGTTTTAATTCTCGCTAGTAATTCTGGTTT
ACGAAAGGGCTTATTTACATAATCATTTGCCCCTAAACTATAAGCCTCTATGAGATGATTTTCTT
CAACACTAGCCGTAAGAAAAATTACTGGTATATCTTCATATTCAGGATTAGATTTAATACGGCGA
CAAACTTCTAAACCGCTAATATCTGGCATCATTAAATCCAATAAAATTAAGTCGGGATTTAAAAC
TTTTAATCTCTCTAACGCATCTTTTCCACTGAGGGCTAAACTGGTTTGGTAATGTGCCGATTCAA
GTATAATATTTAATAATTTTAAATTTTGTTTCAAATCATCAACAATTAGGATAACTGATTCTTCT
GGATTAACTAAATCTAAGTTACTCATATTTATGGTTTTGTCCTACCTTACATCAATAAAGTGCTT
TTAAAAATCCTCATCAATATTCATCTCTTGATTTAAAGTTAGTAGATGTTTTTCTATCATTTCCC
AATCTACTAATTTAGCACTTAAGTCCTTTTTTATTCTACCTTTATCCAACCAAATTACCCTTTGA
GCAAAATTTTTTACCCACTCCAAATCATGATTGATAACAATAATTGTCATCTTTTTTTCTTCATT
CCAAACTTGTAATTTTTCTCTCAACCAATGGGCATTACCCTTATCTAAGGCTGAAGTAGGTTCAT
CTAATAATAAAACCGAAGGTTGCATAATTATAGCACGTGCGATCGCAACTAATTGTTTTTGTCCC
AAAGAAAGCTCATTCTCTCTTTTTTCTAACCATGACTCAATGAAATTAAATTCCTGTAAACAATA
TATTAGTCTTGTTTGTATCTCTTTCTCCGATAAACTTTGAAGTTGTAAAGGATAACTCAAAGCAG
ATTTTACATTCATCCCCAATAACTTCGACTCTTGAGGAATTAACATTACCTGACGACGAAATTTT
AAAACATCCCAATGATGAACCAATTTTCCTCGAAAATATAAATCTCCTTTACTAGAAGTACATAA
ATGATTTAAAACTTTTAATAAAGTACTTTTTCCTGCACCATTACCGCCAATTAAAGCAATTTTTT
CCCCTTCATTAACATCAAAAGAAATATTATCTAACAAATGATTCTGATGAATTTAGTAAGCAAA
CTAATATTTTTCGCTTGTAAAATAGGAGAAATAGACATATATGCAAATTGAATCTAATTTATAT
TAATGGCTCTATCAGTTTGAGTATGTAAATTATTGATGTTTGGTAGGCAAGAGGCAATAGGGGA
TTATTAAATAATAATTTATAAACTTTTAGTTTTTCTCTTACTATAAACATTATTCAATAAAGGTT
ATGGAAGTCCTGTTTCTCTTAATTTATCATAACCACTGTAGAAGAACCATATTAATTATTTTATT
GTAAGCAAGAAATATTTAAATATTCAATATATCTGATATTTTTCTAGCCATTAATTTATCGCAAA

ATTATTAATAATTACTATATTTTAATAATCATAATTAAATCAATTTCCGTGCAATTTTTCAAACT
GATTTGTTTTTTCTGTTAAACCAAAATTATTCATTATAGTCACCACTCCCCAAGCGGTAAAAATG
CCAATCACTAAACCAATAGCTAAAAGTATTATAAACAATTTTTGAATTTCTTTTTTTTTCTGTGG
AGATATTTTTTCTACCTCATTATCTGCAGAAAAATCATTAACATTGCCAGAGTTTTGACCAGGAT
CAAAAAAGTTATTATTCATAATATTATTTCTCTTAATAGTAAATCTCTTGTTAATATAACATTCC
ACAAAAAAAATAGAGGCAATAAACTTAAGATTCGCCTCCTAAAATTTTTAACTATTTAGTTGAAAT
ATGCTTTTTAGATATAATTATTACTTTTACTTATTAATTATTACTTCCCAACGTTGACTCATGAC
AATTGAAGCCGATTCAAACCCTAAACCCAATCTCTGCCCGTCAAGAAATAATCGGTTAATCGAGC
TTCAGGTGAACCTTTTTCAGGGGTGTAGCAGTATTCCCAACGGGTGAGAGGAGGTAAAGACATTA
AAATCGACTCAGTGCGCCCATTGGTTTGTAAACCAAAAATTGTACCACGATCATAAACTAAGTTA
AACTCAACATAACGTCCTCGGCGGTATAATTGGAAGTTGCGCTCTCTTTCTCCGTATTCGGTATT
GCGACGACGCTCAATAATGGGTAAATATGCAGGTAAGAAAGCATCCCCACAACTTTGAGCAAAAG
CAAATAAATCTTCCCAATTACGAGACACTTTGCCTACTTTTTCGCTGTAAGCATTGGCTTCCCCAT
CAGCATTTGAACCAGCGTAGAGAATACCGCTATCATCCTGATAGTCAAAGAAAATACCACCAATT
CCTCTGGCTTCCTTACGGTGTTTTAAGTAGAAATATTCATCACACCAACGCTTAAAAGTAGGATA
ATATTCTTTATGATGGCGATCGCACGCATCTTTATGAACTTGATGGAAATGAATAGCATCTTCTT
CAAAAGCATAATAAGGAGTTAAATCTGCACCACCAGCAAACCACCAGATAGGACCTGCTTCAAAA
TAACGATAGTTGAGGTGAACCGTAGGAATATAAGGGTTTTTAGGATGCAACACCATAGAAGTACC
TGTAGCGTAAAATCCATGACCTGCTCCTTCAGGACGTTGCATTAAAATAGAAGGAGGTAAAGTAT
CACCCCAAACCTCAGAAAAATTAACACCACCTTGCTCAAAAATACCGCCATCACGAATAACTCTG
GTGCGGCCACCTCCACCTTCCTCTCTTTCCCATTTATCTTCACGGAAACGAGCTTTGCCGTCAATT
TCTTCTAATCCCTGACAAATTTTGTCTTGTAAATTCATTACAAAACGCTTTGCTTTCTCACGGGC
ATCGCTAGGGGGTAAAACTCTTTTTGTATCGTTGTTGATTAAAGGTTGGTTGACTAAACTGCTCA
TATTATTTTTGGACTGGTTAAAATATTCTTTTCATAACTATAAAGTGATGATACTAATGATGTCA
TCCTTGCTTAATAAAACTTCATATGAAAAAAAAGAAGAATTAAGAGGTATCAGGTTTCAGGTGT
CAGTTTTTATTTAATTCCTTTTATGTTTTCTCTGTTTTTCCCAGCTAATAGCCCCAATACCCCATT
CCTTACTCCAAATTCAAATTAAGTAATGTAAACATTAGTGCATAGATTTTTCTTTTGGGTTAAAA
TCATAAAAACAAAATATAACAATAAAGAGATTAAAGATGACTGCAACCGCAATTAAACCTAAAA
GTGATAAAAAGAAAGAATTAAAAGAAACTTTATTAACTCCTAGATTTTATACCACTGACTTTAA
AGCGATCGCAGAAATGGACATTTCCGCCCAAGAGAAAGAATTACGGGCGATGTTGCAGGAGATGA
AAAACGACTATAATCGCAATCACTTCGTTAGAGATGAAGAATTTGAACAAACATGGGATCATTTA
GATGAACAAACAAGATCCGCTTTTATCGATTTCCTTGAAAGATCCTGTACTTCCGAATTTTCTGG
GTTTATCCTCTTTAAAGAAATTTCTCGCCGTATCAAGGACAAAAACCCCTTACTGTCCGAAATTT
TTAACCTAATGGCAAGGGATGAAGCCCGTCATGCAGGATTTTTAAATAAAGCCATGAGTGATTTT
AATATTTCCCTCGACTTGGGATATTTGACCAAAAATAGAGTTTATACCTTTTTCAAGCCTGAATG
GGTGATTTATGCTGTATATTTATCTGAAAAAATCGGTTATTGGCGTTATATCTTAATGTATCGTC
ACTTAGAGAAAAATCCTCAATACAAGTTCTATCCCCTTTTCAAAAAATTTGAGCATTGGTGTCAA
GACGAAAGCCGTCACGGGGACATTTTTAACGCCTTATTACGCTCTCAATTTTCCATGTGGCAAGG
ATGGCAGGCAAAATTATGGTCAAAATTCTTCCTCTTATCCGTATTTGCAACCCACACCTTAACGG
TACATGAAAGAGAGGATTTCTACGAATCCGTTGGTTTAGATGCAAAAGAGTACGATCGCCAAGTC
ATCGAAAAGACTAACAACACTGCGGCAAAAGCCTTTCCCGTCATATTAGACACCAACCATCCCAA
GTTTTTCCCAGGTTTGGAAAAATGTTCTGAATATAACTTGCAGATGTCTAAAATTGATGAAAGCA
ATCAACCCAATTTCATCAAAGCATTGAGAAAACTTCCCTTACAAATTGCCATTTTCTTTAATTTA

CTCGGTTTATACTTTATAAAATCCATCAACGCAGAAGAATTAAGAGGTGCGGTTAATTAAAAAAG
ACATCTCCAAAAATTAGCATTTTTCCCGCTTAGTTCGGGCTTATAGCCCGAATTCGATCGAAGTC
TAAAGAGTTCGGAGTTAAAATAATCGATTCAAGCAAAAAGGCGATCGCAAAAAGATCTGTTATT
TAGAACCCATCTAAACTTAAGAACAATTTTTTGTGTCTTTGTAGTGAAAAATAGACACTCTAACC
AAAAAAAGAGTAATTATTATCCAAAAGGGGAGGGGGCGTAAAGCCCCCTGTTAGGAATGAAGTT
CGGAGTTTGGAGTCGCTAACTATTATAGCGAAAAAAAATATAGTTGGGAGCTTTTTTCAAATTTT
ATTGATAAAACCCCATCAAAGTTAGTTAATCATCAGCAAAAACATAACGATATAATTCAGAGCCA
TCGGGTTCAGGACTAGATTCCGCAAACTCTACAGCTTCTTCAATCAAAGCATTAATTTTTTTATC
GATCGCATCTAACTCTTCTCTGGTAGCAATTTTATTATCCGTGATATACTTACCAAATTGAATAA
TAGGATCTTTTGCGTTCCAAAATTCTTTTTCCGCCGGGTCTCTCAATTCATCGGGATCAGCCAAAG
AGTGACCACGAAAACGATAAGTTAAAGTTTCAATTAAAGTAGGTCCTTCTCCTGCCCTTGCTCGT
GCGATCGCCTTTTGTGCTACATCTCTAACAGCCAATAAATCCATTCCATCGACTTCATAACCCTCC
ATATTGAAAACACTAGCTTTCTTATAGATTTCAGTTTGAGAAGCCGCCCTTTCATGAGCCATACC
AATCGCCCATTTATTATTTTCCACCACAAAAAGAATTGGTAACTTCCACAATGCCGCCATATTGA
GACATTCAAAAAACTGCCCATTATTAGTAGTACCATCCCCAAAGAAACAAGCAGTCACCTGATCA
AAATCAGTCTCACCCAAAACCTTACGACGATACATACTTTGATAAGCCGCCCCTAAAGCCACGGG
GATACCCTCACCAATAAAAGCATAACCGCCCAATAACTTATGCTCGGCAGAAAACATGTGCATCG
AACCGCCACGCCCTTTACTACACCCAGTCGCTTTACCAAATAATTCCGCCATAACCTCCTTCGCAG
GTACACCACTACTTAAAGCATGGACATGATCCCTATAAGTACTGCAAACATAGTCTTCTCCCGGA
CGCATAGACTTAATGATACCGCTAGAAACCGCTTCTTGTCCATTGTAGAGGTGAACAAAACCGAA
CATTCTACCACGATAATACATTTCAGCACACTTATCCTCAAACATACGCCCTAAAACCATGTCCTC
ATAATACATGAGGCTTTCTTCTTTATTAATTTTAACTTTGGAGTTATCAAATTTAGGTATAGTTC
TTTCTTTTACCATAATATTTTATTTTAGTTGCCAATTAACAATTATTTCATTATCTATGATTAAT
CGGCAATTAGAATTAACGATTCAATAATTATTTATCTCCTAATATTTGACCTTGCTGATTAAATT
ACTTAAAGATGCTTGTGAAGTTCTTTTAATTATATATTTCCTTTAATTATATATGATGATGAATA
GAAAATATTTGTGGCAATATATCAATTTCCCTCTTATTACGAAAGCTATAATAAAACCTAAAGAA
GAGTGAGAATTAGCCATTTACTTTAGTGAATGGCAGAACGAACGTCGCAGAATTTATTCTGCCTA
TTTACCTTTTTGATTTTCTATGTATTTTTTGATTGTTTCTGTACTAGCATTACCTGCGGTTGAAC
AAAAATAACTAGGAGTCCATAAGGTAGGTAATTTTAATAATTCAGGAAATTCTTTTCTTAAATG
ATGAGATAATCTACCTTTCACTCTTTTGATGATTACAGATGGAGCGTAAGTAGGATTTGTTTCAA
TAAATAAGTGAATATGATCAGGCATAATTTCCAGTGCAATTAACCTCCAGTCATTTTCTGTACAC
AACTCAAATATTATTTCTTGGCAACGTTTGACTATCTTGCCTTTTAGTACTGCTTTTCTTCTTTTT
GGACACCAGACAAAATGATAATTTAACAAAGTTACTGAGTGAGGATTTCTGCGGTAATCTTCCAT
TATTTTGTTTATTTTGCTAATATATTCTACACAACTAATATACAGTAAGAAAATGTATGGAAGCC
AACAAGTTTTATTGAACCCAGATAAAGATACTAAGTCTGTACTAGAGTATATCTGCATTGAAGCG
AACAAACTCTATAACATCGGGCTTTATTATGCTAGACAAATATACTTTAAAAAGCATCGCTATAT
AACTAAGTTTGAGCTAGACAAGGAAATAAAAACTAACCTTCATTATCGGGCTTTACGCTCTTGCG
TAGCTCAACAAACATTGAGAAGTGTTTATGAGGGAATGTCAAGCTATAAAACTCTAAAAAGTAA
AGCTAACAAGGGTGATTTACACTTTAAACCTAAACTTCCGAATTATCGTAAAAAGGGTTTATATC
AAGCGGTTTACCCTGCTAGGTGGGTTAAGTTGGCAAAAAATAACCAACTAAAATTTACTCTAGGA
AAGCAAACAAAAGTATGGTTTGGAATAGATCATTTTTATTTGCAAATGCCTTCTAATCTCAATTA
TAAAGACATTAAGGAATACAGAATAATACCTCGAAATGGATGCTTTTATCTGGAATTTGTGTAT
GAGCAGAAAATAGAACCAATTTCTTTATGCCAAGACAATGTACTTGGTATTGATCATGGAATTAA

CAACTGGCTAACTTGTGTGTCTAATGTAGGTACTAGCTTTATAGTTGATGGAAAACAAGTTAAAT
CAATGAATAACTGGTATAACAAAAAAGTAGCAATACTGAAAGAGAATAGGACACAAGGTTTTTG
GTCAAAAAGGTTAATGAGAATAACCGAAAAGCGTAACCGTCAAATGAGAGACGCTGTTAATAAG
TCAGCTAGAATAGTTATCAATCATTGCTTACAAAATAATATAGGTACTATTGTTTTTGGTTGGAA
TCAAGGACAAAAGCAAGAAGCAAATATGGGAAAAAAGACAAATCAAAAGTTTGTAGTTATTCCA
ACAGCAAGATTAAAAGACAGAATTAAAGATCTGTCTGAGTTGTACGGTATTCAGTTCATAGAAC
AAGAAGAAAGCTATACGAGCAAGGCTTCTTTTTTAGATTTAGACTTCATCCCTACTCACGGTGAA
AAACCCGAAATTTGGAAGTCGAGTGGAAAAAGAGTAAAGCGTGGATTGTTCCGTACAGCTAACA
ACTTATATATCAATGCCGATTGCAACGGTAGTGCTAATATTATCCGCAAAAAAGTAACCGCAAAG
TCAAGTATTCTCAGTAATGTAAATCTTGATGGAGTGTCTATGGGTGCTTTGATACATCCCCAGAG
AATTAGGCTCTGGTCAGCTAAGACAAAGCGAAGCAATGTGGATTTATCCCATTGGTCTGCAGACG
CTTAGAATCCTCGGAATTTATTCAGTAGGAGTTATCAATTGTCATCGAAATTGATGGAGATACCC
ATTTTACAAATGACGGTATCGCTTATGATCTAACTAGAACTGCCATCTTAGAGAGTTATGGATTA
AAAGTAATTCGGTTTACTAATCATGATGTGATTCACTCTTTTGAAGGTGTTTGTCAAGCCATCGA
AAAAGCTCTTGAGATCCCCCCCCCCCCCCTTAATAAGGGGGCCTCAATACAGGCTTTTAGAAAAT
AGTTGAACTTAATAATTGACCCACCAAAAAATTCCAAGCAATAACTTCATCTTTACAAAAACTTT
ATATAGCAATTTAAGTAGTATTACGGTTATGTAAATCTCTTTTTTCGGGCAGTATGGTAATTAGA
GATCCTATGTATAAATAATCAATACAAAACCAGATTTATAAATAATATTTTATACCCATAACTTA
ACCTTAAACTGCTAGAGAAAAAAGAGGAAATTGCTATGCTAACCAACAATCAAAACAAAAATCA
AAAAAAACCCCTAGTTACTAATTTTATCCGTAATTTATTACCCTTCGGTGTCAGAAGCCAACAGG
GTTCAACTCTTCCAATGGCGATCGGTTTAGGTAGTGCCATGATGATTGTTGGTGCAGTGGCAGTT
ATGAAGGGAGGACAGGAGAATACAAATACTATATCTTCTGAACAAACTAAACAAGCGATGGCGG
CGGCAGAAGGCGGTATAACAAACATTCAGGCATTTTTGGCTGAGAATCCCGCTTTAGCAATGATT
GATTCGAGTGATTGGGAAAAAATTGTTGAAGAACCTACAACTAAGACAGAAGATATTGTGAAGA
AGGGTAATCTTGATTTAGAAAAAAAGATTAATGATAAAACCCCTACAAGTAGTAACAATAATAA
TGACGAGAATTCTCTTATATGCAAAGATTCTCCAGTAGGTGCAAATTCAGCAAAACCAACTTCAA
CTTCTTCTACAGTAAGCCCTTTGGGGGATGAATTTAGTGCAGATGAAATCAAAACAGGTTTGGAA
TCAGTTGTTGGAATAAAATTGGACTCTTCATCTTCAGATGCAAAGCCCGAATGGGTCTCAATGGG
AAATAACCAATACTATAAATTTATTGGATATACCAGAAACTCAAACAATGATGGTGGTCAAGTT
ATTATTCAAGGGAAAAAAGGTGATGCTGTTGCTCAATTAGTTGCTGATTTGAAAGGAACTTATCA
GGTAAATGGAGGCACTTCTTCTTCCAGTAGTTCTCCGACAATGGTTGATGGTTCTTCTGAAGGAG
CACCTGTACTATGGGTTACAGATAGTAAAAACAATAGCTTTGGTAACAATAATTTTGATGGTATC
GTAAAAATTTCTCAAGCCAGTTGTACATTTGAAGGTGAAAAACCAACACAATCTAAAAACTTTGA
ACAAACAACAAACAAGTTATCTTTACTTTTAACAAAAGAGGTAATGCCTTCTGTTCCAACTCCAT
TAGTAACTCCTAAAAAAATTGGTGCCATAAATTCTCCCAAAGGGAATTCAACTTTAACTTTTCCA
CTTGACGCAACTAATGATCCAAAAGACTCAGAGGGGCGTTATTACTATGAGGTTAGTAGCATAGA
TCTGAGCGGAAAAACAACTGTTAACATAGAAAAAAATGTTGTTTTTTATCTTACAGGAAATATG
ACAATGTCTGGAAATCCTGATTTTGTAACTAAAAACGGCTCAAAATTAGAAATATACGGTGGAA
ATGGAACAACGAATATAACCTTAAATGGAAAAGCAACTGCGGGGGCTAATATTTTTATTCATGCT
CCCAACGCCCACATTGGTGTAAATGGTGGAGGAAGTTCTAATCCAAACATTGCTGGTTCTATTTG
GGCAAAATCGTGGGGGCTTTCAGACTCTAACTCCGGTATATTGATAGGCAAACCAGGAACTTATG
GTGATTACATAGTAGGAGAAAATCGCCAAGTACCAGAAACCTCAACCCCTTCAAATCCGGTGACA
ACATATGATAACGTTGGACAAATGTCTCGTGTAACTAGACAAGCGGTTAAATAAGCCTAGATAAC

AAGAATAAAAAGTAAAATATTAATAATCAATCGCCTCTTCGGGGGCGTTTTTTATGGGATTGAG
AAATAAACAAGAAAAGATTAAGCTATGATTGAACTAACAATTAATCAATATTGTAGGTAGTTAT
GTGGGGTAAATTTCAACAGAGTGAACTTAGAATAGAAGTCTCCGCCGATGAAAAGAAATTAAAG
GATAGTCTTCTCAACATTGAACAATTAGAAAAATGGTTTTTTCCTCTTAAGTTTCAAGATAAGCG
TCCAAACCAACTAGAATCAGGGGATAAGTTTATCATTAAATTGGGATTAGTTGAAGTGAGAAAT
GAAGTAGAAATTATTAACAGTAATTGTATTCGCTTTTTATTAAGTGGAGGAATTGACGGCTATCA
AGAATGGTGTTGGGGTGATGGATGGATTCAATCTCGCTTAGAAGGTGTGTCAATACTGCCTTTGA
ATTTTGCCCAAACCATGAATTTATTGAGGTTAAGAACTTTTGTGGGTGCAGAAATTGGTGAGAGA
AAATAATAAATAATTTGCTAAGGAATATCGATTATTTTGAAATCATCAATTATGATCAAATATT
AAAACAGAGGGAAATCTGAAACTGCGAAAACAATGATTGAAAGAATTGGGCAATGGTTAAAATT
AATTCCAACAACCATTCAACCCTATGATCCTGAAAAGGATGATGTGAAAGGTAAGAATGGGGATT
CAACTGCTACTCATCCTGTGGTTAAAGATGGAGATTTAGAATTTTTGTTTTATCAGTTATTAGAG
GGGGTTGCTAACGGGTGGCAAGAGTCTCGTATTGAGCAATTTTTTGAGAAATTAGAGCCGAGAAT
TTCCCTTGAAACTTGGTTAGATTGGTTACAGCGTTACCGTAATCAGTTAATTAAGTCTTCTCATG
GTCATAGTCAATTGGCAGTAAGAATGATTCGTTTAGGTGAATTGACAGCAACATTACCTTTTATG
CGGGGAGTGGGAGATTTAGCTTATGAAATAGGTAAAGAGTTATTAAATCAGACAAAAAATAACA
ATTTTGTTAATTCGTTCAATTCCCATTATCAAGAAAATATAGATAAAAATATAGATAAAATTGA
AGATAAAACGCCTATAAATGAAGTAACCGAAAGTTTAACCGCAAAAATAAACTCTCCTACATCAT
TAGGGGAACTACTATCTTTATTACAAAAAGATGAAAATTTTGCTTCTGAAATTGCCCATCAGTTA
AACTTAAATACAGTTGATCCTAGTGTAATTATTGCTAAAATTGTCGAGCAAACAGAATCAGATTC
TTTACCTATTTTTTCTGCTTCTATTTCTGAAGAATTAGTAGAATATTTATTTAATTTAGGCTTAG
AAAAAGCATCGGCAGGAGATTTTGAAGAGGCTTTAATTTCATGGAATCAGGCTGTAACTCTTAAC
CCCGATTTTGCCCCCGCTTGGCATAACAAAGGTAGTGCTTTAGCTTACTTAAATCGTTTACCAGA
AGCAGTTCAAAGTTTCGAGCAGGCGATCGCACTTAATGTAAATGACCTCGATTCCTGGTACGATA
GAGGGAGTGCTTTATTTAGCATGGGACAATGGCAAGAAGCAATCATTAGTTGGGAAAGAGTAAT
TGGGATTCAACCTAATCATTATCAAGCGTGGTATCATCGAGGTCTAGCCCTAGAAAAACTAAAAA
AATTAGGGGATGCGTTGAATAGCTATGGAAAATGTTTAAGTATTAATCCTCTTTTTACTCCAGCT
CAGAAACGACAAGAAAAAATAAATTTCCTTCTATCCTCTAATTCAGAAAATTAAAACTTAGAACC
TGTTTGCAAAGCACTATTGCTGATTATTGATCCCCAAAAAATATATTGGAAAGTCCCCCTTATTA
AGGGTGATTTAGGGGGATCTAAAACTTTTCCAACAACTTCTCAATTATGGTCTTTTTTTGCTATA
TTTAACCTGAGTTTTGGATAAGCTGAAAGCAGACCAACTCGTAGTCAGAAAATCTTATAGCTTCT
TCTTAGAAATAACGATAAAATTGCCTTAATCCGAACTGACGTTAAATAGTTAAAAATTAAAAAT
TAAGAATTAAGAATGAAAAACCACGAATACTTACTACTTATTTATTATTTATTACTTGCTTCCCC
CTTTCCCCTCATCTCCTCATCACTCTAACACCTGCAACCTGCAACCTACAACCTGACACCTAACCT
TATCGGATATTCTTAAACCGAACTGAGGTTATATTTATGAATCGTTTTTAGTGAAAATATTGATA
GATTATCTGAGCTAAATTATCCCCAATTCCTGATACTTCTGCTAACTGTTTAACCGTTGCCTGAC
GAATATAATCCACCGAGTGAAAATGAGCTAAAAGCCTTTTTTGTCTCTCAAATCCCAACCCCGGT
ATCTCATCTAAAATTGATTTACGACTAGCTTTTTGTCTTTGTTGACGGTGAAATGTAACAGCAAA
GCGATGGGCTTCGTCTCTTAATCTTCTTAATAATTGTACCCCTGATTGTTCTTTATTCGTATCAA
GAGGTAAAGATTGATTGGGTAAAAATATTTCTTCCCTTTTTTTGGCTAAACTAATAACCTTAATT
TTCTCCATTAAATTCATTTCTGTTAAGATTTCACAAACCGATGAAAGTTGACCTTTTCCTCCGTC
AATCATCACTAAATCTGGATAATCTTCTGTGTGGGAAAAACGTCTTTTAATTACTTCTTGTAGAG
ATAAAAAGTCATCAGAGTGACCTATTTTGATTTCGGGATTTTTAATCTTGTAATGACGATAATAT

TGTTTTGCAGGTAAACCATCAATAAAAACAACCCTTGAAGCTACGGCATTTGAACCTTGCAAATG
GGATATATCATAACCTTCAATGCGGCGAGGCAGAGTGGGTAAATCCAATATTTTGGCTAAATCTT
CCATACCTTCTAAATTAGTTTGACTAAACTTTTGACTTCTTTCTAGCTCTATTTGAGCATTTCTA
GCCACCATAGAAATTAATTCCGCTTTTTGCTGTCTTTGAGGAGTAGTAATAATGACTTTTTTACC
CTTTTTTTCTCTTAGCCAACTAGCTAAAATTTCTTCTTCTTTAAGAGGATATTGAACTATTATTT
CCGTGGGAATTTCTGAGAAGTCAATATTTTGATAGTGTTGCTCTAAAACTCGTTGTAAAATTTCT
CCCTGTTCATCATTATGACCATTATCGGTAAAAAATCCTAATCTGCCAATTAAACGCCCTGCTCG
AATCTGAAACAGTTGAATACAAGTATAATTTTCATCCTGAGCAAGTGCGATCGCATCTCTGGAGA
TGGTATCATCAGGTAAAGCAACTTTTTGACTGGAAAATAATTGTTGTAAACTCAGAATTTGATCC
CTATATTGAGCCGCCTTTTCAAATTCTAGGGCTGATGAAGCGATTTCCATTTGAGTTTGTAATTG
TTTAATTAATTCATCAGTACGCCCCTGAAAAATAAAAGCAACCTTACTAATAATTTCCCGATATT
GTTCAGGAGTAATTAAATTTTGACAGACTCCGGGGCATTTTCCTAGATCATAATTCAAACAGGGG
CGATCTTTATATAAAGGCTTCGATCGCTGTCTTAAAGGAAAAGTACGTTTAATAATATCTAAAAT
TTCTCTTAACCTTCGACTATCCACATAAGGACCATAATAGCGATCGAGTTTATCAGTTAAACGAC
GCTTACGAGTAATAAAAATACGAGGATAATTTTCAGACCAAGTAATGCAGACATAGGGATATTT
TTTATCATCCTTAAGTAAAACATTAAAATGGGGTTGATGCTGTTTAATTAAATTTGCCTCTAAAG
CCAAAGCCTCCGCTTCCGTGTCGGTGACAATATATTCAATATCTCTCACCTGAGAAACCATTAAA
GCTATTCTATGGGTATGTTTAGCAGAAAGATTAAAATAAGATTTAACCCGTTTTTTGAGATTTTT
CGCCTTACCAATATAGAGAATATTTCCAGATTTATCTTTCATAAAATAAACCCCAGAATCATCAG
GCAAACTTTTTAATAAATCCTCTATTTTCATCCCATAACCATTCTATTTTTCATAAACAAGACAA
TTAAAAATGAAGAATTAGGAATGAAGAATTAGCTTCCCCCTCTCCCCCTCTCTCTAATACCTCAA
TCCCGAAAACTTCAAATTGCCTAATCATTCTTTTTATTATCAGACTTTGGACGAGTAAATTGAGA
AGCCGCAGGGAAATACTTCACAGGTTCATTTTGTAATGCTGTCACCATAAATTGTCGCCAAATGG
GGGCGGCGTATCCCCCTCCTGTCACCCCACTTCCTAAACTGCGATTGAAATCATCATTCCCAATCC
AAACAGCTGTTGCTAATTGAGGCACATAACCCACAAACCAAACATCCCTTTCCCCTGAAGTTGTA
CCAGTTTTACCCGCCGCAGGTCTGCCAATGTCAGCACTACGAGCCGTTCCCCCTTGTATTACTTGA
GTCAAAGTTGCAGAAAGAGAAGCCGTCGCCCATTCATTTAAAACTAATTTAGGTTTAGGGGTATT
ATCTAACAAAATATTTCCATCACTATCTGTGACTTGTACAATCATTGTCGTGTCTGATTGCCAAC
CATTATTAGCAAAAGTTGCATAAGATTTGGCCATTTCCATGGGTGTAACTCCCATAGGTCCTAAT
GGTAAGGATACAACAGGACTTAAGGGGCTTTCTATACCTAACGTGCGACATACTTCAATTACTTT
ATCTAAGCCTATTTCCTGCCCTAAAACCACTGCGGGAATATTTAAAGACTGACTGAGAGCTTGAA
TGATACTAACATCTCCTCCCCCGAAACTTCCACCATAGTTTTGAGGACGATAATAGCCACTTCCAT
CTCGATAACCTTTAGAATAGTTAGCAACTGATGAAGTAGGAGTGTATTTACCTGTGGCAAAGGCA
GTGTAAAAAACAAAGGGTTTAAAAGCAGAACCGGGTTGACGACGAGATTGTAACACCCGATTAA
ATTGGCTTTGTTCATAATCTACACCCCCCACCACTGCTTTAACAAAATGGGTTCGAGGGTCGATC
GCAACTAGGGCTATTTGATCCGCCTTAACTCCCCATGACACTAAATTACGATGGGATTTTTGGAC
GATTTCTTCTGCCTTAACCTGTAAATCATAATCAATAGTAGTTTGAACATGAAGTCCCCCCTTAG
TAATCATCTCTTGTCCAAAACGTTCTATTAATTCTTTGCGCACAGAATCAGTGACATAGGGAAGT
TTACTACTTTGCCATGCTTGAGGCTTACCTAAATAAAGGGGAGTTTTTTTAGCGGTTTCAGCCTC
TTCACTGGTAATCCAGCCCAAATCCACCATTCTCCCTAAAACTAAAGCCTGTCTTTGTTTAGCAAT
ATCATAATTAGCAAAAGGGCTATAAACTTCAGGGGCTTGAATAATTCCCGCCATCATTGCCGCCT
CTGCCAAATTTAGTTCTCTTGCAGACTTATTAAAGTAACTTTGAGCCGCCGTTTCTGCACCATAA
TTGTTATGCCCCCAATAAATATTGTTGAGGTACATTTCCAAAATATTATTCTTCTCAAAAACCTG

CTCTACTCTTACTGCTAAAACTGCTTCTGCTAATTTACGAGAAAAAGTCCTCTGATGGGACAGAA
AAACATTTTTAACTAACTGCATTGTTAAGGTTGATGCACCTTCTACCACCCCACCACTTTGAATA
TTAACCAGAATTGCCCTACCGATACTGGTAGGATTGATACCTTTATGATCATAAAAATTACTATC
TTCGATCGCCAATACTGCCATCTTTAAGTTAGGGGAAATATCCTGAAAATCTACTGTTGTGCGAT
GTTCCTCCCCGTGATATGTAGTTAGTAAACGTCCCTTAACATCATAAATGTAGCTAGTTTGAGAA
GGAACATAATTTTTTAAAACCCTTACATCTGGTAAGTTACGAAAGCTAACGGCTAAACCAACCAA
ACCACCTGCTATAGCTGATGTGGTCAATAATGTTAGACCTAAAAATGTCCCAGTGGTTACTTTTA
CCACTCCCATAAAAAAATTAGTAGGGGTTTTTGGATTGGGGACAGGATTATTAGTTTTACCTTTG
ATGCTAGTAGATGCCACAGTTTTTGACCAAAAAAATAATTTTATAAAATAGTTGCAAGAGTATT
ACACTAGCTTAGTTACATAACATTATAATTAAGCAGAAAAGGACTGCGCTTTATCCTAGCACAAA
CCTTTTATAATAAATCAAGATTCAAGAATTAAACTTGACATAACTAAGGTTTTTCTTTAGTTTAG
CTATAAAAAACGGATAATTAACCCATCAGGAGTTTTAAAAACATATGTTTGTACTTAATGGTTAT
GAGTATTTTTTAGGTTTTCTGATAGTTAGTAGTTTAGTACCTATTTTAGCTTTAACAGCGTCTAA
ATTACTACGTCCAAAAACAGGAGGACCTGAAAGACGCACTACCTATGAATCAGGGATGGAACCCA
TTGGGGGGGCATGGATTCAATTTAATATCCGTTACTATATGTTTGCCCTTGTGTTTGTAGTTTTT
GACGTAGAAACAGTCTTTTTATATCCTTGGGCAGTGGCATTTAACACATTGGGTTTATTAGCTTT
TGTGGAAGCCTTAATTTTTATCGCAATTTTAGTAGTTGCCCTCGTGTATGCTTGGCGTAAGGGTG
CTTTAGAATGGTCGTAAAGAGTAATTAGGAATTAGTAATTAAGACAAGGCAATAGGCAAACCCCC
CTTTATCCCCCCTCGAGAGGGGGGAGGGCAACAGTGTTTAATTAATTTCTCCCTTATCACCCTAAC
ACCTGAAACCTGCCACCTGAAACCTAAAACTTTTACCCTAACCCCCAACTCGGATCAAGTCCATTA
CAATAAAAATGATGCTATCTTAGTAAAGAAAAACTTATGAATACTTCTGCTACAGATTTAAAAA
ACTTAGAGAATCAGGCAAAGGAAAAAATCCTCAATCCTGCCGCCCGTAGTCAAGTAACTCAAGAT
TTATCAGAGAATATAATTTTAACCACCGTTGATGATTTACATAATTGGGCTAGATTATCAAGTTT
GTGGCCCCTGCTGTATGGAACCGCTTGTTGCTTTATTGAATTTGCCGCTCTAATTGGCTCTCGCTT
CGATTTTGATCGTTTTGGGCTTGTTCCTCGTTCTAGCCCCCGTCAAGCTGATTTAATCATCACCGC
AGGAACTATTACCATGAAGATGGCACCCGCTTTGGTGCGTTTATATGAGGAAATGCCTGAGCCTA
AATACGTTATCGCTATGGGTGCTTGTACTATTACAGGGGGTATGTTTAGCAGTGACTCTACTACA
GCAGTGCGTGGGGTTGATAAGTTAATTCCTGTTGATGTGTATATTCCGGGTTGCCCTCCCCGCCCT
GAAGCTATTTTTGATGCGATCGTAAAATTGAGAAAGAAAGTAGCAAATGAGTCAATTCAAGAGC
GTGGAACTGTTAGAAGTCAAATCAATAGGTATTATTCCACTACCCACAATATGAAAGCAGTTGAT
CCTATTCTGACAGGTAAGTATATCCGCAGTGAGTCCAGAAATAATCCCCCTGCTGAATTAGCGGC
GGCAATGGGAATGCCCGTAGATACAGCAAAATTAACAGAAAAAACCCAAAAGGAGGCTTAATCA
TCAATGACAGAAGCAAATAATAATAACAATCCAGAAACAGAAAATCAGCAAGAAAATAACGAGG
TGGGAGAAATTGTCCAAGCGGGGATAACATCTCAATGGTTGACAGAAAATGGTTTTGCTCATGAA
GTCCTTGAAAAAGATCATTCAGGAGTAGAATTGATTAAAGTTGACCCTGAGTTTCTTATTCCCAT
TGCGACTGCTTTACGTGCCTATGGATTTAATTATCTACAGTGTCAGGGTGCTTATGATTTAGGTC
CAGGAAAAGAATTAGTTAGTTTTTATCATTTACTTAAAGTGGAAGATAATATAACTAATCCTCA
AGAAGTGAGAATCAAGGTTTATTTGAATAGAGAAAATCCTCAAGTACCTTCTGTATATTGGATTT
GGAGAGGTGCAGATTGGCAAGAAAGAGAGTGCTATGATATGTATGGCATCATCTACGAAGGACA
TCCCAATTTAAAACGTATTTTAATGCCTGAAGATTGGGTGGGTTGGCCTCTAAGAAAAGATTATG
TATCTCCAGATTTCTATGAATTACAAGATGCTTATTAATTACTAGCCAATAATTAACTTAATAAT
TAAGGGGTATTGGGTTGGGGTGTTAGGGAGATAAGGTGTCAGGTGGTAGGGGTTGAGTATATTC
AACCCTTACGGTTTTAGGGAGAAATTTCTTTTTTATTTTAACTCCTTACTGCGTAAAGCTTTGCG

GAACGAGCGTACCGAATTCTCTGAACTCAAATGATTGCCCTTTTCGCCATCACTCATAGATGAAA
ATTTATCTCGCACTCAGGTTAAATAGGTATTAGCTCCTCAAAAATACCTATCAGTAACCCGCCGA
TTACTCTATAAAATTTCTCAATTTGTTTCAAACTTAATTGAATATAACTCTCAGTTGTCGAAAAA
GAAAATATAATCTACCATTGGAAAAGAGTATTGAATAAAAACAATGCTTTATTTGATAGCCATT
GAATTTTGTCGATGCTTGTTTTTGATGCTCAATTAAAAGTTAGGGAAAATATGAGTAGATAAAT
GAATAATACGATTACTTTAATTTGGTTAGCGTTACCTTTTGTTGCTGGTTTTCTCGTTTATTTAT
TGCCCAAATACTATAGTTATTTGAGTTTAGGGGGACTATTAATATCTATTTCTTATCCTGTTTGG
ATATTGACATCAGAAGTAGATTTTACTTTGCGGCTATTGGATAATTTTGGTGTTACGTTGTATGT
AGATCAACTTAGCGGTTATTTTATTCTTACTAATTGTCTTGTTACCATTGGGGTGTTAATTTATT
GTTTGTCGGAAGAAAGAAAGGCTTTTTTCTATACTCAATTAATGATTCTCTACGGTAGTGTTAAT
GCCTGTTTTATTGGCTATGACTTTATTAGTTTTTATGTTGCCATCGAGGTGATTAGTATTGCCGC
TTTTTTGCTTATGGTTTATCCCCGCAGTGATAAGAGCATTTGGATAGGATTACGATACCTTTTTG
TTAGCAATACTGCGATGTTATTTTATTTAATTGGGGCAATTTTAATTTATAAGGCGAATAATTCT
TTTAGTTTTGATGGTTTAATTAATTCTCCTCCAGAAGCGATCGCACTTATTCTGTTAGGATTATT
AGTAAAGGGAGGAGTGTTTGTATCTGGATTATGGCTTCCTCTTACTCATGCTTCAGTGGAGACTC
CATTGTCAGGATTACTTTCGGGCATCGTGGTTAAGGCGGGAATTTTTCCTTTTCTTCGTTTGGCA
TCTATCAGTGATGAGGTGGACTTTGTTATTAGAATTGTCGCCATTGCTACGGCTTATTTAGGAGT
TATACAGGCTATTTTTGAAAATGATACTAAAAGATTATTAGCTTTAAGTACTGTTTCTCAATTAG
GATTTATCATTGCCTCTCCTATGATTGCTGGTTTTTATGCCTTAACTCATGGTTTGGCTAAATGC
ACTCTTTTTTTAACTGTTGGTAATTTACCTAGTCGTGACATTAAACAATTGAATCAAGAAGGAGT
TAATTTTTATCTTTGGTTTGTGCTATTTTTAGGAAGCTCATCTATTATCGGTATTCCTTTATTAG
CTGGTTTTAGTGCCAAGGTAATGGTAATGAAAAATATTTTGAGTTGGCAAAATATACCTTTCAAT
TTAGCAGTTATCGGAACAGCGATCGCACTTTCTAAACTAATTTTTCTCCCCACTGCTAATACATCT
ATATCTGAAACAGGAGTGAAAAGAGGTTATTGGCTAGGAGTCAGTATTTTAATGGGAGGATTAA
TAGCGACTAATCTGATTTATCCTCAAACCTATACCATTGTCAATATTAGTAAAGCCCTAATCACA
TTTGCGATTGGAGCATTAATATATCTATTCATACTGAAAAAAATTACCCTAAAATTGCCCCGAAC
ACTAGAAAAATTTGATAACCTGATTGGTATCATGAGCTTAACTGTGATTGGCTTATTCTGGATGG
TTTTACCCATTGGCTAAAAAAAATTAAAGAATTATTAACTATTAACTATAACCATGAAACTAGCT
GAACCCGTTATCGATTATAAATATGATAATCAAGTACCACAACAATGGGTAGAGGTTTTAGTGGA
TTGTGTCTATGCAGAGGGATTATATACTTATCAAAATAAACCCGATATTCTTATTAAAGCAGGAG
ATATTGTTAGTGTTCCTTTTGGTAATACTATTGTTGGAGGGGTAGTAGTTAGGATTCTTTCTCAA
CAGCCTGATAATCTTGATTCTAGTCAGATTAAAGAAATAGATGATATTGTGAGTAGTGGTTTTTT
TCCTCCTCACTATTGGGAATTGTTAGAGCGCACTGCCAATTATTATTTAACGGATTTAATCAATG
TAATTCGGGTTGCTTTACCTCCAAAACTTTTGGGGCGATCGCAGCGTCGGGTTAAGTTATTATTC
AATAATATCCCTGAAAATCCTGAAGATTTCTGCACGGTGAAAGAATGGGAAGTAATTAAATTACT
CAAAAAAAGCAAACAAGGGGATTATAGCTATCGTTTTATCACCCAGAAGGTCAATAATAGCCGAA
AAGCAATTTCTTTGTTGGTAAAAAAACAATGGTTAGAAACCTATTTACAACCCCCTAGTAAGGCA
AAACCAAAATTTACAAAAGTTGTCACCTTTCTAGGAGAAAATAATAACTCAGAAAAATTGACTCC
TAAGCAAAAAGACGTTTTAAAAAACCTCAAAAATCATGATGGAGAATTAGCTTTACAGGAATTA
TTAAAAGTATGCGAATTGAATAGCGATTCTGTAGTGAAAACCTTGGAGAGAAAAGGATGTATTG
TTATTCAAGAAAGAGAAACCTTGCGTTTATTACAGGATTCTAACCCCACCGAAGATCAACCAAAA
TCATTAACTTCCCCTCAAAATCAAGCCTTAGAATTAATTAACAGTTTACATTCTTTTTCCACTGT
ATTATTACATGGTGTGACAGGTTCAGGTAAAACAGAAGTATATCTACAGGCGATCGCACCCATCT

TAGCACAGAAAAAATCTGCTCTTGTATTAGTCCCCGAAATTGGTTTAACTCCTCAATTAACAGAT
CGATTTCGGGCAAGATTCAAAGGACAAGTGTGCGTTTATCATAGTGCCTTGAGTGATGGAGAAAG
ATACGACACATGGCGACAAATGCTGACAGGTGAGCCTCAAGTGGTTATCGGCACAAGAAGCGCAG
TGTTTGCCCCTTTACCTAATATTGGTATTATTATCTTAGATGAAGAACACGATACCAGTTTTAAA
CAACATCAACCTATCCCCACCTATCATGCTGTTAAAGTAGCCCAATGGCGTGCAGAATTAGCAGA
TTGTCCCCTTATTCTAGGTTCAGCTACCCCCTCCCTTGAAACATGGCAGAAAGCCTTTTCTGTTGT
TCCTAAACCCCTTAAAATTAATAATATTGAGGATTTGTTAGTTAGCTCCTATCCCCATTATTATC
TTTCCTTGCCCGATCGCATCTTAGAAAGACCTCTCCCCCCAGTAGAGATAATCGATATGCGTCAAG
AGTTGCGTAAAGGTAACAGAAGTATTTTTAGCGATCGCCTCAAAGTAGAACTGCAAAACCTTAAA
CCAGAAGGAAAACAGGCGATTTTATTTATCTCTCGACGGGGTCACAGTACTTTTGTCTCTTGCCG
TAGCTGTGGTTACGTTATGGAATGCCCCCATTGTGATGTTTCCTTATCTTATCATTATAGCCATC
ATGGTGCAAGGGAATTATTACGTTGTCATTATTGCAATCATGCAGAGCTACACCCAAAGCAATGT
CCTCAGTGCTTTTCCCCTTATTTAAAATTTTTCGGCACGGGAACACAAAAAGTTGTATTGGAAAT
GGAAAAAGAGTTTCCTGACTTGAAGGTATTGCGATTTGATAGCGACACCACTAGCAGTAAAAATG
CTCACCGCCGTATCTTAACCGAATTTGCCCAAGGAGAAGCGGATATTTTAATCGGGACACAGATG
TTAACCAAAGGTATTGATTTAGCTAATGTTACCCTTGTGGGGGTAGTGTCTGCCGATGGTTTGTT
GTTTCATTCCGATTACAGAGCCTCAGAAAGAGCCTTTCAAACTCTCACCCAAGTGGCAGGAAGGG
CAGGCCGTGGGGAGGATAGAGGAAAAGTTATCATTCAAACCTACTCCCCCGAACATCCTGTTATC
CATGCAGTGCAAAATCATGATTATTTGGCTTTTTGTGACCGAGAATTAAAGGAAAGGGAGAGTTT
AAGCTATCCTCCTTATGGTCATTTGATTTTATTACGTTTTAGTGGCTTAGATGGGGATAAAGTTC
GAGATAGCGCAGATGCGATCGCTTCTTTATGTGCTAATTTATTACCAGATTATATGGAAATTTTA
GGACCTGCTCCTGCTAATATTATGAGGGTAGCAAGGCGTTATCGATGGCAAATTTTGTTAAAAAG
TGAGAAAGAATTTTCTTTAGAAATAAAACAAGAGCTAATCAATTTAAGACAATATTGTCATAAT
TCTGTTTCATTAGTTATCGATCCAGATCCCTTAAGAATTGAATAATTTGTCTTTATTGGTCAAGA
TTTTCTAAAATATCTTGTATAACCTGAGTTTTGGATAAGCTGAAAGCATTATTTTCTCGCAGTCA
GAAAACCTTATGGTTTCTTAGAAATAACGATAAAATTGCCTTAACCCGAACTAACGTTAAAAATA
TATTCAACCCCTAATATCTGCAACCTACAACCTGAAACCTGACACCTAACCTTATCGGATATTCTT
AAACCGAACTGAGGTTAATTAAAATATCTATCATTCCAGCAATTTCTTTCCAGTTCATATTAGAA
TATTTTTCCTTCACATAGGGCAAATTGTCAGTAACTTTTTTGGTTGCTTCTCCTATTCTTTCTAAT
CCGTGAATGACAGCGAAAATAGTTTTTTCATCTTCACAAAAATCATCAAAATTCAAACCATCAAT
AAATCGATTAATATAGTTAGCAATTCAAAAATAAGTTCTGTTAACTCTTTTTCTTTAAATCTTTT
AAGACTATATTGATTATTATTTATTGGCAAAAAAACTTTATCATTTTTTATGATGTCAGTTCATT
TACCCTAATAATTTTTTCGTTATGACTATTATTAAATCATTTCTTTTTTCCATAACTCTATGTTT
ATACTTACTTTCTTCACCCTTAGTGAAAAGTGCAGAATGGGAAGAAATTCAAGCTAGGGGTAAAA
TTATTGTTGGAGTTAAAGATAATTTACCACCACTAGGTTTTACTGACCAAAACGGTAATTTACAG
GGATTTGAAATTGATTTAGCTCATAAATTGGCAGAAGAATTATTAGGAGATAAACAAGCAATTA
AATTTGTACCTCTCCTCAATCAAGATAGGCTAAATGCTGTCATAGAGGATGATGTGGACATTGCG
ATCGCATCTATTACTTTTAATTCTTCCCGTCAAAGATTAGTGGATTTTAGCGACATTTACTATTT
TTCTGGAACAGGGATTATAGTCAAAAAAAATCAATTTTTGAGCAATATTAGGGATATTTCGGGA
AAAATAGGCATTTTAAACAATTCAAGAGCGATCGCAGAAATTCAATATCAATTTCCACAACTATC
ATTACAAGGAGTTTCTAGCTATCAAGAAGCCCTAGAATTAATGGAAAAAGGAGAAATACAGGGT
TTTGCAGGAGATGTAATGGTTTTAACGGGCATAACTCAACAAACATCAAACTATCAGTTATTACC
TCAAATTATTGGAGCATATCCCATTGCGATCGCACTTCCTAAAGGTAGACAATATCAATCACTAC

GAAACCAAGTTAATCAGGTAATGAGAAAATTAAGGCAAGAAGATTGGTTTAAGGAAAAAGCTAA
ATTTTGGGGCTTACCGTCAATTTGAAAAGGGCAAAGTAAAAAGGGCAAAGGTAAATAGTTGATA
ATTAATAACTCCTAACTCTGTACGGGCGAATGGCCATTCGCCCCTAACTCACTCATTACTTACTCA
TTGCCTAAGAGAAAATCCTGTAAAGAAGGTACTTTTAATCGTTGTTTTTGAGTGTGGGATTTATG
GGTTAAATCCATTAGTAATTGAGAGTAAATCCCCTCGATAATTGAAGGTGCATTCATCTTATTAT
GATCAATATTTTCTACTAATTGATTTACCACTCTGATAAATGGTGCGAGTCTGCCATCGTCAAAC
ACTTGCGGAAAGGCTAACCTTTCAGGAATTTTTATTTCTTCTAAGGGTTGATTGTCCCTAGAAGC
GTATAATTTAAAACCGTGTACATAATCCTTGAGGTTATCACTGCCTAAGACTAATGTGCCTTTTT
CTCCGTAAATTTCTAGCCAATGACCGCGCCCTTGATAGGTAACTGAGCTAAGATTAACCTGTACT
GGTGTGCCGTCATTCAATTCTAAGGTTATTAAACAGGTATCATCAGCAGTAACAGGCTTTAATTG
ATTGTTAGACAAAGGATCAGGGCGTTGGGGTATGGCACAACCTAAATAAGCAGAAATTGTCGCTA
TTTCCCCAAATAACCAGTGAATATAGTCAAAAGCATGAGATCCGATCGCACCTAATGCTCCCCCT
CCTCTTTCTTTAACGGAATACCAATTCCATGCACGGTTAGGATTGCCCCTTGATGCCACTAACCAA
TCGATTTTGATAAGTCTTGTTTTACCGACATAGTCATTATCTAAATATTCCTTGAGTAATTGCCA
TGCAGGAATAAAGCGAAATTCAAAGTCAGGGACGACAATTTTTTGCTTTTGTTTAGCTAAACGAT
AAATTTCCCTAGTTTCATAAACATTGAGATTAAGGGGTTTTTCGAGCAAAATATGCTTATCGTGG
TTAAGGGCAATTTTTGCCATGTCATAATGCAGAAACGGAGGGGTAGAAATACTGACTATATCTAC
TTTTGGTAAACGGCAAATATCCTCTAAATCATCCATTGCATGGGCAATATTATACTCAGAGGCGA
TGGCTTTTGCTTTATCTAAAACACGATGATGTATTGCAACCACCTCTGTGCGAGGGTGAAGTTGA
AAAGCAGGTAAATGAATTTGTTTACCAAAACCCGTGCCAACAATAGCAACGCCTAGTTTTTTTTC
ACTCATATCAATTAAACTCAAAATATATCAAAGTTCGTAGGGGCATAATATATATTATGCTCATA
TTGGCTTTAGCCATTAATTATTCCTTGTAAAGATAGAAACCCTAAAGGGTTTACTACAAACTAAT
TTTTATTAGACATCTGGTAGAAAATATTAATATAGGCTCGTAGTTAGGGCTTGGTTCCCTTATTT
TGAGAATTCTTGGAGATGTCTATTTACGTTAAACTACTCAGTGAGGGTTGAATATATTCAACCCC
TACATTACTTATTTCTCATTACTCTTTAAATATTAGAAAAACCAACCGTAAGAGTGTAAACCTTT
ACCCAATAAATTGACTCCTAAATAACAAATCCATACCACCAAAAAGCCTCCTGCGGCTAAAAGGG
CAGGTTTTCTTCCTTGCCAACCCTTGGTAATTCTGGCGTGTAAATAAGCGGCAAAGACTAACCAT
GTAATTAATGCCCATGTTTCTTTCGGATCCCAACTCCAATAAGACCCCCATGCTTCATTTGCCCAT
ACAGCCCCTGAAACAATACCGATGGTTAATAAAGGGAAACCTAAGCCGATAATACGATAACTGAT
ATTGTCAAGGGTATCCACTAAACTCAAACGCTCAGGAGAAAGTTGATTGACAATGGTTTTTTCCT
GGGTTAACACGGCTGTACCGCCTTGATTCTCAACTTCTGTGATTTTTTCTCCTTGATAGTTTAAT
TTTAAGGCTTTAACATTGCGGTAAGCTCCTGTACCGACAGAACTACCTCTTAATTGAATTTCTTT
GCCCTGAGTTAAAATCAAAAATGCGATCGCAACTAATGAACCTACCATCAAAGCAGAGTAACTAA
ACATCATGACACTAACGTGCATCATCAACCAATTAGATTTTAAAGCAGGAACTAAAGGTTCGCTA
TGTTGCATTTCTGGGGGGAGAGAAAGGGTAGCAAAAGCGGCGATACCCATAGCAGTAGGAGCACT
GACAACTCCCACTAAACGACTACCACTCATCGATTCTGCAATTAAATGCACTGTGGTGACACCCCA
TCCCAAAAAGAAAAGGGATTCATAAAGATTACTAATAGGGAAATAACCCCCTTCAATCCAACGCG
CCAACAGTAGAGAGGCGATAGATAAATTACCGATAATCATGCCCATTGTACCGAGTTTAGGCAAT
ATTGAAAAATTGGGAAAAGCTGTACCTACCCAATAAAATAACATGGTGATAAACAACACAGCGA
AAGAAACGTTATCTAAAATATTTTCTAAACTAACTAAATCCATAGGGTCAATATATTAAATTTAT
TTTCTATTATCCATGATGACCCACCTCAATTGCATCTACTCAAAAATTTACGTGGGGAAGAGGGA
GATGAGGTGATGAGGGGAAGGAATGAGAAGGGGAAATCGGAGCTTAGGGGTTAATAATTCAGAG
TTGTTAATGAACCTCAGTTCGGTTTAAGAATATCCGATAAGGTTAGGTGTCAGGTTTCAGGTTTC

AGGTTGCAGGGGTTGAATATATTTTTAACGTTAGTTTGGGTTAAGGCAATTTTATCTTTATTTCT
AAGAAGCTATAAGGTTTTCTGACTACGAGAAAATAATGCTTTCAGCTTATCCAAAACTCAGGTTA
ATTATTAATTATTCACTATTCACTATTCACTGATTACTCTTTAAGCAATACTGCCTCTTTTTCTT
GCTTCCTTGTAAGAAACCCCGACATTTTTGATTCCAGCTTTGATCATTTGTTCTTCTAATAAGGC
AAAAAAACGGGTGCGATCGCTTCCTTTGATTACAGCGAGATTATGGGCTTCAGCTAAAGCAACAG
GATAGCCAAAACCCTTATCAACTTGAGCAAGGGTAATACTTAGAGACTGATTGAGGAGAGATTCA
TTTTCCGCTACCCATTGGGGAAACTCAACCCTAGCAATTTCCGTGCCAACATGGAGATAACAGAA
GTAAATATGGTTTTCTTCTCCATATAAATCTAAAATACGAGAATTACTACGGAATATAGCACTTC
TTTCTCCTCGTTGTAAAAGGTGAGTCCAAAGATTAACATCTCTGAGGGGTTCAACTTTCTGACAG
GGTGTTTTATCCACTTCTTCCCCACAAAATGCCATACAATTAGGATTATCGTAGGGACATAAAGG
AAAACGAAGAAAGTTAATTGCCGATGTGCCACGGGAAGCACTAACATATCCCACGAAAGGAATTT
TTGTCTGTCGTAAACTTTCCCATGCTTTTAGGATAGGATTCAAAATTTCTTCTCTGGCCTCAACG
GGTAAAGTTTCTAAAAACCAGTAAACCAATGAGCCATCCATCATGGCAAGGTTAGGGGTATCAAA
ATGCGCTCCGGGAGGATTTACCCAAGTACAAGCCATTTCTGCTAATACCTCGGCTTCAGCTACAG
TGCGTCGATAACTCATCCATTCTTCGGTACGAATTCCCCATTTTCTGGATGCGTAGAGGTCTTCAG
TTTTATAGTATATTTCAGGAAGGGTATCAAGGAGAGGATGTAAATTTTGCCCATAATGCAACATA
ATTCGCCCAATATTGATCAAATAACAATAGGCAATTTCATGATGAGAGGGAGCAATTTGAGAACC
ATCTGCCGAAAAAACACTATGATTGTTCGGGGCAATGGGAATTTTTACTTTTTTAGTAATCGATT
CTTGAGGGGTAGCGGCAGTAAATAAGAGGCGATCGCTCCATTCTTTTTGAGCTTTAATAAAAGTA
TCTTGGTCTTTTTCAACTATTGCTAATAATTGTATTGCTTTTTCCAAACGCTGACTACTTTTTAG
GGCTTCTTTTTGGATTTCTTGCCCCATATCTGGCATTTGACGGGCTAATTTTGCTAAATCTAACA
CGGCTGTTTTTATCTGGGTATTTGGGAATGAGGAGATTTGGGAGATTGGGATATTGGGGAATTG
AGGTGTTAGGGTTCGGGGGTGTCAGGTTTCAGGTTGCAGGTGTTAGGGAAATGAGTAATGGGTA
ATGAGTGTTCGGAGTTATTCACTATTCACTATTTACCTTCGCCCTCGCCATTTTCTTAATTTCTAA
TAGATTTAAGCATTTCAACTAATGTATGATGTGCGTCTTTTGCATCAATTAGAGTATGATCAAAG
TTAATTCTTAATGGTTTTTCCTCGTTATTATCGATAATTAGCAACATACCTTCATTATCAATAGA
CAACATTTTCGCACTATTTAAATTTTCTAAATTACCATAATGTTTGGCATATAAGACTAAGGCAT
CTCCATGATCATCGTTCATATGTTTACAGATGCGATCGCTCACTGCGGTGGTAATAGTTTCACTC
ATGAGTTAATAAATATGATCCTATAAAATAGCTTTTTGACAAATTATAAGCTAACACCCAGTCAA
CTTGCCATTTTTATGGCTCTTCTACAAAATAAAAATATATTGATAAAGAAAAGGAAGCGATCTCA
ACTCAAAATCATAGCTTAATTCACCAACTCCTAATTTTTTACTCATGAAGTTTTTGGGCATTGAT
GACGTAAAAATTATTAAACACAGCAATAGTTTCAAATTGGTAAATATACAAATTAGTTGTATAA
AGACTTAGCAATAAATAATTATTTCTTTTCGTATTGGAAGCGATTAATTGGGGTATTTTATTTTT
ACCTGTATCGACTATCAAATTGCAAACCTGACTTTTGATCATCTCTTCCAAATTGGACGATTGAG
CAGGGCAAATATTTTCTTTAGCATACCGAACTAATTCTTCTGTGGCGAACTTTTCGTATTTAGTG
GAATTGGGGTTAGTAAGAGTCAAAATTATTCCCATAATTAAAAAAAAGCCCCCACCAACTGCTAA
CAAAACACGGGGAAACTTACGCTCTTCGAGAAATAATGGATTCATATTTAGATAGCTTTTTTTGG
ATAATTACTGGATCATTTTAATGAAATTATGTTATAATCAGAAATCGTAATGGCGAGCGTAGCCA
AGTGGTTAAGGCAGTGGATTGTGGTTCCACCATTCGTGGGTTCGAGTCCCATCGTTCGCCCTCCTA
AAAGTTTATGTTAATTTACTAACTCTTCACAAAACAGTTTACATCAATTTTTTTAGGTAAAGCTC
ACTAGAAATTCCTACATATAATTCTAAGATAATTGGATCTTTATTGTGATGTCACTCCGTTATTA
AATTGTCTTTATCTCAGATATTGCATTTAGCTAAAATGGACTGGTGACGGTAGAGAATAGAAGA
AATCCCTCTGTGTTTTCCATTTTAAGAGGAGAAGGAAAACAGGGAACAATAAATAATTGATGAA

AAACTTATTAAAATTGGTTTTAAGGATATTATTTTCTAATATAAAAGAACCAATGCAAGATTTC
AGTTTATCTTATACTCTTCGATTGAACTGGAGCTAACCATATCAGCAAAAAATCTGATTCATCAA
AAAATCTCAGAAATTGATATTATTATAAACAAAATGTAAATAAATATTAAGGTTTATTTTAATG
TTTCTATATTACGCTCTTGGTGTATTACTAATATCAGTAATCATAGGTGTTGTTATTTATCTAAT
AACTCCTCGGACTTTTGAAACTCCTGAAACTGTTGCAACTTCATATGATGAGTGGACAGAAGATG
GAATTTTAGAATTTTATTGGGGTGAGCATATTCATTTAGGTCACTATGGTTCTCCTCCAAGACGC
AAAGACTTTTTAGAGGCAAAAGCTGATTTTGTTCATGAAATGGTCAAATGGGGAGGATTAGATA
AATTACCCCGTGGTACTAAGGTTTTAGATGTCGGTTGTGGCATTGGTGGAAGTACCCGTATTTTG
GCAAAAGATTATGGTTTTGAAGCCACAGGCATTACCATTAGTCCAAAACAAGTACAAAGGGCAAC
GGAGCTAACTCCTGAGGGTGTTAGCGCTAAATTTCAGGTGGATAATGCTTTAGATCTTTCTTTCC
CTGACAATAGTTTTGATGTGGTATGGTCGATCGAAGCTGGTCCTCATATGCCAGATAAGAATAAA
TATGCTCAAGAGATGATGAGAGTTTTAAAACCCGGTGGTACTCTTGTGGTAGCCGACTGGAATCA
AAGAGACGATCGCAAAGTTCCTCTTAATTGGTGGGAAAGAATCGTAATGCGTCAATTATTGGATC
AATGGTCTCACCCTTCCTTTTCTAGTATTGAAGGCTTTTCCGAACAAATTGCTGAAACAGGTTTA
GTCGATGGAGATGTAGTCACAGCCGATTGGACTCAAGAAACTTTACCTTCTTGGTTAGAGTCTGT
TTGGCAAGGCATAGTTCGCCCCGAAGGTATTATCAAGTTTGGTTTATCTGGTTTTATCAAATCCT
TGAGAGAAGTTCCTACCATGATTTTAATGCGTGTGGGCTTTGGTAGTGGTCTTTGTCGCTTTGGG
ATGTTCAAAGCTGTTAAAGCTAAATCTGCTACTTCTACTAAAGAAGCTAATACTAATGAAGTGGT
TAATGTCTAATATCCATTAGGGGTTAGGAGTTAGTTAGGGGCAAATGGCTATTCGCCCGTACAGG
AGTTAGAAGTTATTAATTAATCTCGGTTCGGTTTAAGAATATCCCATAAGGTTAGGTGTCAGGTG
TCAGGTTGCAGGTTTTAGGGTGATGAGGAGATTAGGGGAGAGGGTGAGAGGGGGAAATAGGGGC
TTAGGGTTTTGGGGTGGTAGGGGTTGAATATATTTAACTTCAGTTGCCCTTCTCCCCTTAAAAGG
GGAGACACAGAGGGGTTTGCCCCTTGCCCATTGCCCTTTTAACCTTGCCCTTTTCTTAATTGTTAA
TTGCCATATTCACTCGATAATTTTTTTTTAATTCAAAATTAAGCATTTTGGACAAAGTTTCAGAG
AGAATTGTATTATCCAGACAAGAATTAACAAAACTGGTGTGATTTATGTTGACTCCTGTTAAATT
AGCTTGAGATAAGTTTGTATCGTCAAAAATTGCCCCGTCGAGGTTTGCCATTTGTAAATCGGCAT
TTTGTAAGTTTGCTCCTCGTAAATCTGCCCCTCTTAAGTCTGCACCAACCAATATTGCTTCTCTTA
GATTTGCATTCTGTAGATTGGCATTTTGTAAATTTGATCTACTTAGTCTTGCACCATATAAATTA
GCTTCTGTTAGGTTACTATTACTCAGATTTACTTCTTTCATAAATGAGCGGGTAAAATTAGCTTG
ATAAGCACTAGACTGTTTCATGTTTGCTTTACTCAATCTTGCTTCTTGAAAATTACTCCAGTTGA
GGTTACTGTTTCCGATAAAAGCCTCTCTCAAATTAACACGGTAAAGACTAGCTCCACATAAGTTA
ACTTCTGCTAAGTTTCCATTGCGTAAATTACTATGGCTAAGATTAGCGGCACTAAGTTGAGTTTT
AATAAGATGCGATCGCACCATGAAAGCTCCAGTGAGATTAGCTTTAGTCAAATCCGCTCCATTAA
AAACTGCTTCACTTAACTTGCTATACGTAAAATCAGCCCAGTTGAGTTGAGCATGAGAAAAGTTA
GATTTAGTCAAAAAACAACGACTAAAATCAGCTCCGATTAATTTCGCATATTGTAAATCCACCGC
CACTAACACCGATTCAGATAAAGTTGCTGATTGTAAATTAATCCCTTGAAACTGTCGAATACCCT
GCTCATAGTTTCTCAAAAATTGATTTATTTTCATCTCTATTAACCTGAAGAATTGATTTGACTAA
TGGGGAAGATTTTAAAATCTCACTTGAACGACCAAAATGCGAATTAATTGTATATCATTAAAAA
TGTCTTTTTAGCATTTTAATACTGAATAACCATTGATGGTTAGGGATTTGTAATTACTCTTAATA
AATATTTTAGATTCTTGAAAAAACCTTATTTTGTCTTACTTTTAGAAAAAGATAATAGCTACATT
AATGATATTTTCCTAGGGTATCGAGTTCATCCCTTTACTTTACTTACTTTTATAAATTAGTCCTC
TTGTAAAACTAAAATTAAAGTTAATTTACCATAGAGAAATAGTTAATTTTCTTCACTCTTGCCTT
TTGCCTTTCGTCTGACTACTCCAAGAAGTCTATCGATGGTGATAAGCGGAAAAATAAGGTTAACT

AAGATATAATTTAAGAAATGTAAATCTTAGCCCCATAATTCAATCAAAATATCGAAGAGTATTG
CAGTAATGGAACGAAAAAAAATAGTTGCGATTGTGACAGGAATAGTATCTGTTTTATTGGGAGT
TGTTTATTTAATTTTGGTAGAGTTATTGGACTCAAGAGGAGCGATGATTCCAGCCCCAATTAATG
ATGTTTCTTTACTTTTATCATGGTTATTCTCGTCTCATTTTTACCTATAATTTATCTGGTGTTACC
TGAATTCAGGATAAATATTCATCTCTGAGTGACGGGGAAAAAGGGAAGGGGCAAAGGCAATTAA
TAATTAGTAATCAATTAAAGCCTGAAACCTACAACCGAAAGTCTGTCTTTATCCAATATTCTTCA
ATCGAACTGAAGTGGTGTTCGATACAAAATGATTGGTTTGCTTCTTATTGTATTATTTTTCTTTG
TTATGACAACTATTCGATTAATGCACGCTAAATTACATCGAGTCAGGGTAACATCTGCAAATGTA
GATTATGTTGGTAGTATTTCTATAGATCCAGATTTGATGGAAAAAGTTGGTATTTTACCTTTAGA
AGAATTAGATATTGTTAATCTCAATAATGCTAAACGCTGGTCAACTTATGCCATTCCGGGGGAGA
GGGGAAGTAAGGAAATTTGTCCTAATGGCGGGGCGGCTTTGTTGTGTCAAGAAGGGGATATTTTA
ATTATATATGCCTATGAAGAGTGCGATCGCAGTGAGTTAATAAAAAAAGGACATAAAGCAAAAG
TATTAGTTGCCGATGAAAATAATAACATAGCGGATTTTTTTGAGCAAACCTTAACCCCCAATCAG
GATAAATTCCACTTTCAGGAGAGATGTTAGAGATAAATAAACAGTACGGAAAAAGTGGTTTTAT
TGCCATAGGAATCAAGGATTAAGAATTAAGAAGTCAGGGTGTTGGGGAGATAAGGTGATGAGGG
GAGGGGGGAGAGGGAGAAATGGGTAATGAGTAATGAATTATTATTCACCATTCACTAATTATCT
ATTGCCTGTTGCCTTTTCTTAATTCCCCAACCTTATCCATGAATCCTGCATCTAACAGATTTCAAC
GAAATTTAATAAATTATTCAACAAAAGTTTAACCATAAATTCAATAAAGCCATAATTTGGCGAT
CAAAAGAATCAGTTAACTGATGGCTCAATTGCCAACTATCCACTACGATACCTGAACAGAGTAAC
ATGAGGTAGAGAATGGAATATTTAAACATGGATTTAGCACAGTCTTTTTTTTCCGGTTGCTGTAG
TAGTTGCCATGCTTTATAGATGAAAGTTAGTCCCAAAATAAGAGCAAAAGAAAAATAAACAAAT
CCTGCACTATGTAAAGGATAGATAAAGATTAAACTAAAGGCAAAAGTAATCCAAGTATAAATCC
AAATTTGTTTAACCGTTGATTCAACGCCTTCCACCACAGGTAACATTGGCACACCAACTTCTGCA
TAGTCGTCACTAATCATCAAAGCCAAAGCCCAAAAATGAGGAGGAGTCCAAAGAAAAATTAATGT
AAATAATGCCCATGCGCTCCAACTTAAATCTCCTGTTACAGCCGCCCAACCCACAAGAGGAGGAA
TAGCTCCAGCCGCACCGCCAATAACGATATTTTGGCTAGTATGACGTTTTAACCAATGGGTATAA
ATAAGCATATAAAAGACAATCCCCGTCATAGCTAATAAGGCAGACAAAAGATTGACGGCAATCAC
TAAGATGCTAAAGGAAAGAATTGCTAGGAAAATTCCAAAAATAATTGCATGGTTAGATTGAACT
CTCCCCGAAGGAATAGGACGTTTTCTCGTGCGCTTCATGTCATAATCAATATCTTGATCATAAAC
GCAGTTGAAAGTTTGGGCAGAAGCGGCGGCTAATGTTCCTCCTAATAGGGTCATAATTAAGAGTA
AAGGATCAACTGTGCCTTTTCCTGCCATAAACATAGAGGCAGTAGTTGTTATTAAAAGTAAGGGA
ATAATACGAGGTTTTGTGAGGAGATAATAACTTTGAATAACTTGCCAAAAGTTCTCGTTACGAGG
TGAAATACTTGTTCCGATCATGTTTATTGTTTTTGCTGAATTATGTTTAAGGTTTTAGCCTTAAG
CTAATATTTGTTATCAGTTTTTTGATGTGATTAAGATTGATTGAAATAGACAGATTTTCAATTTA
ATTAGATGAGGGATCTAAATAAACTCTTTCTTCAAGGTTATGCTAATTTTTTATTTATGGAAAAA
CTAATTTAAAAGAGAAATGGTGGTATCTTCATCTCTGAGGGTATAAACAGTGAAAGTTATTAAC
ATCCCAAAAAGTGCCGCTCCCATGGTGTGGTGAGCTACGGTAAGAGGTTCTACTTGTAGATGAAG
GTAAAATGTAGCAATACCGAGTAATATTTGGCTTACTAAAATTACAGTGATATATTTAGTCAAA
TTTCGCAATTTTAGGCTTAAAGCAGGGGTTTTTCTCGCCAAAATAACTAAAGTGATAGTGGCGAT
GGTGGCAGGAAGCACTCCGATAATATGACTATTCATGACAAAACAAAGTTGATTCCCGGCAAAGC
ATTGATGTAAAGCCCATTGAGAGGCAACTAAACCCCCTAAAAGACATTGAATATATACGAAAATA
GATGCGATCGCCCCTATGAGAGTTAATTTACTTACATCCGTAGCTGTGCCTTGATATTCACTGAG
ACTGCTACCAATAGCGATTAATGTGCCAAAAAACAGTAAAGCAGTGCCTAGATGAGCAGTAACAA

TGTCAAAACGCAATAATTCTGTGACTGTCAAACCCCCCAAAATTCCTTGAAAGATGATTAAAAAT
AGAGCAAAACTAGAGGCATAGGGCAACCATTGAGGTAGTTTACGGCGATGCCACCAAGAAGCAAT
GACCAAAATAATGGTAGTAAAACCAATTAAAGCCGCATCCAGTCGGTGAAACCACTCTAAAAATA
CCTGTAAATTCATTTCCTGAGTCGGGACGATTTTACCGTAGCATAAAGGCCAATCAGGACAAGCT
AACCCCGCATTCATAACCCTCGTTGCCGCCCCAATTGCCATCAAAGCCAGAGTTGCGATCGCAATT
TTCCATATTAGCCACCTAATCCATCTAAGGGGATGAGAAGAAGAGAAGGAAAAGAAAGAATTGT
ATAAAGCTGAATCTGTCATATTAAAAAATGCCATTGAATTTCTAAGGTTGATCTTAGCTTAGGAT
CAAATTTCTGGTACTTTTGGTATTTTTAGAATCTTTCTTGATTATTTTTACAAAAGTTAACAAAA
CTCTTTTTTTCCTTAGCCATAATCTCTGATGAAGGGCAAAGGTAAATAGTTAAGAATTAATAATT
AAGAATTAAAAACCCTTAACACTCATTACTCGTTACTTATTACTTTCTCCCAACACCTGCAACCTG
CAACCTGCAACCTGAAACCTGACACCTTTGAACACTAATACCCCAACACCCTAAACCCCGTTAATT
ATGGCTTAAGTAACCGTTGTAAGGTACAATAAGCAAAATAAGATTTTATTGAGAAGGACAGATA
TTGTGGCTTTTAAGATCGGTTTATTAGGACTAGGTACTGTTGGCACGGGTACGGCAGACATTTTA
TTTAATCCCGATGGCAGACACCCCCTCCTTCAAAATATAACTATTAGTCAGGTAGGGGTAAAATC
ACTCGAAAAAAAACGCAACATTGATTTACCCTCAGAAATTCTGACCACAGATTTAGAAGCCATTG
TCACTAATCCCGAAATTGATATTGTTGTTGAGTTAATAGGTGGTTTAGAACCCGCCCGTAGTCTT
ATTTTAAAAGCGATCGCTCATGGTAAGCACGTTGTAACAGCGAATAAAGCAGTAATTGCCCGTTA
TGGTGGAGAAATTTTTGCGGCGGCTAATGAAGCGAAAGTTTATGTTTTGTCAGAAGCGGCCGTAG
GGGGAGGTATTCCCATTGTTGAACCTCTTAAACAGTCTTTAGGAGCGAACCGTTTAGAAAATATT
ATTGGGATTGTTAATGGCACTACTAACTATATCCTTACGGAAATGACCGAAAAAGGATCTGATTT
TGGACAGGTGTTAAAAGTTGCCCAAGATTTAGGCTATGCTGAAGCTGATCCCACTGCTGATGTAG
AAGGCTATGATGCGGCGGATAAAATTGCTATCTTAGCTTCTCTTGCTTTTGGTTGTTTAGTCAAA
CGGGAAGACGTTTATTGCGAGGGCATTACTAAAATTACTAGCAGTGATATTAATTATGCTGAAAA
ATTGGGCTTTGTCATCAAATTACTTGCCATTGCTCACAAAGTACAATCTACCACGGAAACTCAAC
TACAGCTAAGAGTTCATCCCACTTTTGTGCCGACATCCCATCCCCTAGCAAATGTCAATGGTGTTA
ATAATGCTATTTTAGTGGAGGGTTCACCCCTCGGACAAGTCATGTTTTATGGACCGGGTGCCGGT
TCAGGCCCTACCGCTAGTGCTGTAGTTGCCGATATTATGAACATTGTGGGGGTTCTCAATACTGA
TAAAACAGGAGATAATCTCGATTCTTTACTCAGTTGTTCCCCTCAAGGTCAGGGTCAAATTAGCC
CCATAGAGGAAGTTTTTAGCCGTTTTTATGCCCGTTTCCTCTGTGCAGACGTACCGGGGGTCATT
GGTCATCTAGGTACTGCTTTTGGCGAAAATAACGTCAGTTTAGAGTCTGTGGTTCAAATTGGATT
TCAGGGGGATTTAGCGGAAATTGTGGTTGTCACTCACCATGTTAGAGAAGGTAACTTTAGAGAGG
CGATCGCAGAAATTCGCAGTTTAAATGCGATTAAGAATATCCCCAGTATTATTCGAGTGCTGTAG
TTTGAATCAGGGGTTATGAAAGAGGTGTCAGGTTTCAGGTTGCAGGTGTCAGGTGTTAGGGTGAT
AAGAGGATAAGGGAGAAATTAATTAAACACTGTTGCCCTCCCCCCTCTCGAGGGGGGATAAAGGG
GGGTTTGCCTCTTGCCTCTTGCCTCTTGCCTCTTGCCTTTAGCCTTTTTGAAAGGAGATAAATACT
ATTTTTACACCACCTCTCGCTAAATTAATCGAACAATTACAAAAATTACCCGGCATTGGTCCTAA
AAATGCCCAGAGATTAGCATTACATATTATTAAACGTTCGGATAAAGATGCTGAAGCCCTAGCTC
AAGCTATTCTGGACGCAAAAAGAAGAGTAGGTTTATGTCAAAAATGTTTTCATTTATCGGCTGAT
CCTGTCTGTGAAATCTGTCGTAATCCTAGTCGAGATCAAAGTATTATCTGTGTTGTCGCTGACTC
TAAAGATGTAATCGCCTTGGAAAAAAACCAGAGAATATCAAGGTTTATATCATGTAATTGGGGGG
GTTATTTCCCCAATGGATGGTATCGGCCCTGAACAACTGCACATACAACCACTAATTCAAAGAGT
TAGTCAAGAAAAAATTGAGGAGGTGATTTTAGCTATTAATCCTAGTGTTGAGGGGGAAACGACT
ACTTTATATATTGGTCAATTAGTTAAACCTTTTACAAAAGTTACTCGTATTGCCTTTGGTTTGCC

CATGGGAGGCGATTTAGAATATGCAGATGAAATAACCCTTGCTAGGGCGTTAGAAGGGCGCAGAG
ATTTTGATTAAGATTGATCTATTTTTTAATATTCTTGCAAACAAAATAGAAATCAAAAATATAA
TGGAGATTATTTACTATTGATATACTTATCTGTTCCTTTTTCCTTTTTCCTGAATTCTGTAAAAA
TCCCTATTTTTCTACATGAGAAACAAAACGATGAGCAACAGTTTCTGGTTGAATAGCAGATAATA
CTACATGGTTAGAATCTGTTATGATTACGGCTCTGGTTCTTCTGCCATAGGTTGCATCGATTAAT
TGTCCTTTATCTTTTGCTTCATTGATGATACGTTTAATAGGGGCAGACTCAGGACTAACAATGGC
AATGACTCGATTAGCGGCCACTATGTTACCAAAACCGATATTTATTAATTGAATATCCATAAATT
TTACTGATAGTTGACAGTTTTAAATTTATCCTTTATCTTATCGCTATATTTTTAGAATAACAAGA
ATTTTCGTCAATATTCTCGGAAATTTGACATTGTTTTTATATTCCTTACGACTTTTTAATAATTA
CTAATTACTTACTCAAATGGATTTTTTGCGATCGCTTCCTATTGGTATATTTATAGAAAAGCCCA
TCTCATGGCTACATAAATTAGATCCTCGTGTTAAGTTAGCATGGTTGATGAGTGTAATTTTGACA
CCTTTGTTAGCAAATCCTTATTGGCGTATTTTTCTGGTCATTTTTTTGTTACTTTTGACAGTGTT
GGCAAAAATTCCGTGGCGTATTCTGAAAACACAGGTTAGTCTATTAGTCTTTTTTCTGGTTATTG
TATTTTGTGTCACCATATTTTCTGGGGATGGATTTAATCTTAATTACCAACCAACTTTGCCCCCA
AGCACATTACCAACCCCTCAACCCACGGATTATCGATATGTATTGCTAGAAATCGGTAATTTTAC
CATGACAAGAAGAAGTTTAGATTTAGCGATTCGAGTTACCACTTTAATTTTTACTTTAGTTTATA
GTAGTAATTTGTTTTTGTTAACTACAGCCCCTGAAAAAATTACCGCAGGAATTGAAGATTTATTA
TCCCCTTTAAGAAGATTTAATTTACCAATTACAGAAATTATTTTAACTTTAACTTTGGCATTAAG
ATTTATTCCTTTAGTGTTAGAAGAAGCCCAAAATTTAATTAGATCCATCAAAACAAGGGCAATTA
ATTGGAAAAAATTAGGTTTTAAAAAAAGTATGCAAATATGGTTAATTGTAGCAGAAAAATTATT
ACAAAATATCCTATTAAGAGCTGAACAAATTGCGATTGCGATCGAAGCTAGAGGTTTTACAAATC
CCCATGATCATTTAGTACAATGGCATGAATTAAAAATGATAAAAGCTGATTTTTTCGCTTTATTT
AGTTTAATAATATTTTGGTTTTTTCGGGTGATATATGGTGGTTAAATATTTTTCAATAAATCTTT
TATAACATTATAATTAATGGTTCTTCTACAGTGGTTATGATAAATTGACAGAAAATAACTTCCAT
AACCTTTATTGAATAGTGTTTATAGTAAAACAAAAACTAAAAGTTTATAAATTATTATTTAATA
ATCCCCTATTGCCTATTGCCTCTTGCCTCTTGCCTACCTTAACTGATAATTTACATACTCAAAGTG
ATAAAGCCGAATTAATTGATATAGAAAATATAGTTAACTTACTTCATTTTTACCTTTTACTTGAT
AGAATAATAAATCTAATTTACACCTAATGATTCTCCCACTTCTTCTATGTCTGTAATCAATTTTT
TCAATTTTTCTATAAATATTTCTATTTCTTTACTATTATCTGTAAACAAACTATCATGATCTTTT
AATTTTTCTAGTGCACTATCTGACTCAATTAAAGTTGTCAATTCTTCTCGAAAATTGTTGATTTT
ATCTTTAAATAAAAATCTAGCTTTTTCTATATTTTCCTCACTATTTTCTCTAAAAGAGCTTATTT
TTTGCTCAATATTATTAATACTCTTTTCTAATAATTCTTCTAAGTCTGTAGAATAATTAGCTAAT
TTTGGCTTTAAGTTATCTTCTAAATTACTGTTTATATTCTCTTGATAATCATTTATTTGGTTAGT
AAAATTTTCTAAATTATATTCAATTACTTCCTCTAAATAATTAACTTTTTCCGATAAAATTTCAA
TTTCTTGTTGTGTAGATTGCCAAGTAATCTCAATACTTTCATGATTAGCTTGTAAAGACTCCCAT
AATTCATTAAAAATAGTAATTCCTTCTTCTAAAATTTGTAAGTGATGAGTCTTCATTTTCTCTAA
TTGTTGCTCAAATTTATCAAAATAGCCATTGAGAGTTTGCATTGGTGAATTTAACTCTTTCTGCT
GATTAATCATGGTTTCATTCAGTTGTTTAATTTTATTAGATACTGCAACCATCTTTATCTGTAAT
AAACCTTGTTGAGTAATTATTATTTGAGGAAAATTAGATAGTTTTTTTTGCACTTCTATTAATTG
TCTAACGACTTCCTCTTGTTTGTTATTATTTTTTTCAATGACATTATTTAAGTTTCTCTAATAT
CGTTGAGAATTTCTGTCATTTTACTTAATTCTTCTGGTAAATTTTTTCTTACCTCACTTATATAT
TCTAAACTTGAAATTAACTTTTCATTAATTAACATTTTGATTTATCCTCTTTTAAAAATTTAGTA
CAAGTAAAAATTAAAATTAATTAGTAATAAAAAAAATAAATAATAAGTATTTTATAAAAGATAT

TAATTTAAATACTAAATATCAAAACTTTCTAATTCTGCATGGACTTTTTCCACTATTTCTTCTGT
ATCCATTAATTGATTACTTATATTTTCTATTTCTTGCAATGAAGGTTCAATATTGTCTATATTAG
TAATATTATCTTTAATCTGCTCTAAAACACTATTTAAGTTTTTTTCAACTGTTATCATCTCTTCT
TCTATTACTTCTTGATAATCATTATTTAGTAAATTTTCAATGGTTGTTAAATCAGAATCTATATC
TTGTTTAAATTCATTAAGCAAATCAATATTATTATCTTTAAAATCACTAAATTTATTTTGTAATT
CTTCTTCAAAATTATCTATATTCTCTTCAATATCTTGAGGGTATTTATCTTGAATATTTATCTGT
AATTTTTCCAACAATGTCTCTAGTTTATTTGTTTTATCAGTGCAGGAATCAGTAAAAGATGTAAA
AGTAATATTTGTATCTTCTATCATTGTCTCTAATGTGCTGTTAATATCTATAATGAATTCCTGAA
AAGGCGAAAAAGAATCATCCATTTTATTAATTACATTTTCCAAGTCACTGGTGATATTTCCATTC
TCATCTTCTCTATTTCCCCCAGTGAATGATATATTTTCCTCTAAAATTTTTGTCATTAAATCTTCT
AACTTTTGAGAAGCAGTATTTAATAACTTTTGATTTTCCTCTAACTCTTGTAAAGTATTTTTTTG
TTGTGTTTGCGAAGTTTCTCCTAACTCTTGAATTTGAGTTAACATATCTCCCAACAATTTTTCAA
AATCGACTAATTGGCTTAATGCTTCTTCTTCTTGAGTTTCCATTTCCTCATCATTTGTGGTTAAT
TTATTCATCAAATTAGTTAATAAATCATCTATTTGTTGGCTTGAATTTTCTAAATTTTCTGCTTT
CTGTTGGGATTCTTCTGTTAAACTATCAAATAATTCATTTATTTCCGCCATTTTTTGACCTATAT
CGGCTATTTTCTCTTGTAAAACCTCTATTTCTTCTTCTGTTTCTTGTATCCCTTCTTGTTTATCTC
CTTTTTCTGCCCCCCCTGACATCTTACCCGATGATTTTTCTTCCGCCGAAGATTTATTCTCTTCAG
ATTCTTTTTTTCCTTGTGAATCGTGGGGAGACTTTTTTTCTTCACCATCTTTTAAAGATGATTTT
TCACTGTCACCAAAAGAGTTTTCTTTTTCTCCCGAAGAATCATGGCTACTTTCTTCTACATCATCT
TTATTATCACCATTATTCGTTTTCGCTAATTCTCCCTCTAACTGGTTTAATTTTCCTTGTGTTTGT
TGTAATTCTTCTTCCGTTGGTAAGTCACCTTGTTGCATTTTTTTGGCTATCTTGCCAATATACTCG
CCTACCTCTAGCACGTTACTCCCCTGTTTTCCACCCAATTCTTCAGCTAAGGCGAGAAGACTTTGA
CTATTTTGCTGTAAATCCCCTGCTAAATTATTAATTTCTTGTGATTTTGCCTCCCCTTTTCCTTCC
AAGCCGAAACCGATTAAACTACCTAATACACCTAAACCACCGATACTAGAGGCAACTATTTTTTT
TGCTTTTTCTGATATTTCCATAATTTCTTTTGTTATGTGATAGATTTTATTATTTCTAAACTAGA
TTGATATAACTGTTGTGCGGTAGATAGTTTATTATTCATTTCCTCTAAGCTATCATTATTTTTTT
CGAGGGAAATCACTATTTTCTCTCCCGATTCTTCCGTTAAAGATTCTATTTTTTCTGATGTATTT
TGTAAGGTTTTTATTTCCTCAATAATATCTTGAAACTCTCCTTGTTTAATCTTCATATTTAGCCA
ACTATTGAGCTGTTGATTTTCTTCTTTACATACTTGTTTTAGTTCCTCTAAATTTTCTTCAACACT
ATTGAATAATTCGGGAATTAAATTTTTAATTTCTGCTATTTTTTCTTTAGATAAATTTTGTAAGC
TATCGCTTTTATTTGTTAAATCTTCTCGTTGTTCTCTAAATTGCGATCGCATCTTTTCACTAATA
TCTTGAAGGCTATTTAAATTGTTATTAGTTTCTTCTGAATAGTTTTTTAAATCATTATCTAAAGT
TTCTATTCTGTTTTGCCATTCATTAATATTATCTTTTAAACTATCAGTAAGGGTATCAGTTTTTT
GCTCAATATTATTAATATTTTCTTGAAAAGCTGAAGAATGTAATCTAATCTTTACCATTAAATCA
TCAGCAATATTATCAATAAATTTAACCTGCTCATTTAATTTTATCAAAGTAGCTTGAGAAACCCC
TGTTTGAGTCAGTAAATTTTCTTGTATTCTCATCATTTTTTCACTCAAACTTACGGTTGTATTTT
TAGTATTTTCTTGGAAATTAATAATACTTTGACGCAGAGAAGTAACACCATTATTAATCTTAGAA
AAAGAGCCAGAAGATAACTCCAATTTAGATTCCATTACTTCTAAACGCTCAGTCATTTCATCAAT
TAAATCATTTATTTTTTCCATTTTTAAATTCTTATTTAGTAGAGGAATAAAAGATAACAGTGAA
GCGAGAGAAAAAGACAAAGGGCAAAGGCAATTAAAAATTAGTAATTAACTAAAACCTAAAACCT
GCCACCTAAAACCTGAAACCCACCTCAATTAAATATTCTTATCGTTAAACTGAAGTTATAAAAAC
TCCCGACTCCTCTTTTTTCTAAAATCTTCCCAATAACTCTTGCACCTCCTCAAACACTCCCAAAGC
AGTATTTAAGCCAATATTAGTCGTATCCATTGCTGTCATTAGAGTATCCTTAGTGGTGTCAACAG

TGCCACCTAAATCCGTCACCAACTCACTCAAAGTCGTTAAAGCATCCTCAATATCTTCTGCCGTAG
AAAGTAAATCCTCAAACATACCCTCCGCATTGCCCGTAAATTCATCTAAAGCTGAATTAGCAGAT
TCTTGAGTATTATTAGCTTCTTCCTCAGCTTTTTCTCTCACACTAGCAGTAATTTCCTGTAATTGA
GTTGTTAAATTGCCTACCATGTCATTAATACGGGAAATAGCGTCTTGAGCGATTTCTTGTTCCAT
TTCTCCAGCTCTAACTTCGATTTCCTCCTCATTACTACTGAGGGCAGAATTAAAATCATTTTCCAA
CTTACCTTGAGACTCCGAGACAGTTTCCTCCAAATCTTGCTGCACTTCTTCTACCTTAGTTTGTAA
AGTTTCTAATGCTTCTTTAACCGAATTAAATACCTCCTCAAACTCAGAAGACTTAGTATCACTAT
CTTCTCCTAAAGCCGTTATCTCAGTTTTAGTATCTTCAACCACCTGATTGGCTTCATCCTGTGCAG
ACTGTAGATTCGTTTGTAAATCACCGATTCCCTGTCTTAAATTTTCTGTTTCCCCATTTAACTCAT
CTCTAGCATTAGAAATGCGCTCAAGTAAACCATCTGCACGAGTTGATAATTCATCCCAATTACTA
TCAAATTGCTGACTAACTTCCGCTAACCTTTCCCTTGCCTGATTTAAGTTTTCTTCAGCTTCATCC
GCACGATTTAACAATTCCTGTAACTGAGTTACGGTTTCGGCTATACTTTGTTCTAAATCTACCGC
CATAATTTTTACTCCTTAATTTGTTAATAATTGACTTTTATCAATGATTTGTAGTAAAGGCTAAA
GCCCTTATTTTGACTATTCTTATCGATGTCTAATAGAAATCTGGTAAAAACACAGGCAAGATGCT
TGTTTCACGGGGAAAATCGTAAATGTTTTGAAAATTTATTTCAAATCATCCGAAATCAATGCCAA
ATTTTTCCGCCGCAGATTTAACCCCATCAATTCCTGCCTCAACGGGCTCTTTAAATGCGTCTAAAT
CGTCAATTAAACCAGCTACAACCTCTCGAATACCAGAAGATTCACTTTCAGCATCAGTGACTTTT
TGCACCATATCTTCCACAGAGTCAGTCACCTTACTTACCGCAGAAGCAATCAATTCTTGTAATTG
AGCCTCTATCTCACTTTGAGCCTTATCCATCAAGCCACTGACACCCCCAGATATCGCCTCAGATGT
GGAACTTAAAACACTGGTAAAATTCTCACCAATAGAAGTAAACTCATCCGCCATTGTCTGAATTT
CACCACTAACGGTTTCTTGCCCTTGATTTACTTGCTCAGAAAAATTCTGCATTTCTTCATCCATAC
GACTACTAACATCTTCTAGTTTTTCTCTCAATGCCTCTATTGCTGACAAACTAGATTCAAAAGCA
CTTCTCAACTGCTCCTGACTAGCTTGTAAAGCCGTTTGAGACTCTTTCATAGCGTTTTGTGCCGTT
TCTTCCGTTGCCTCTGTAGATGCTTTTCCCTCCTCAAGATTAGTTTTCAACTGATTCATTTTTTCC
ATAGCTGTTTGTAACTCTTGCACCATCTCCTCTTGAGCCTGTGTCATAAAGTTACCCAACTCCGTA
GCACTACTCTCAAACTCATTAACGGCGGTTTCTGTTTCCGTTTGATTGTCTGCAATTTCCTGTTGC
ATTTGGGTTAATGCACCTTGCACTTCTCCTAATGTTTCTGCTACCTGTGCCTGTTTTTCTTGCACA
TTGGCAACTAATTCGTTAGCTTCGGCTTCCACCTCTTGCGCTAGAGTAATTAGATTTTCTAAACG
CTCAGGAAATTCCATAATTAGTTGAGATAGTAGATTGAGACTGTCACTTAATTCACTCATAATAT
ATTTTGTGTTGGTATTGGATTTTAGTAAAATTAGTAAAAATAGTTAAAACTTTTCCCTACCCTAA
TTAGTAAACTTAAACGGAAAATTAGCCTAAAGAGAAAAAGCGTCAAGCACAGCATTAATAGCTCC
GATCGCAATTTTCGCCGCCGCAATTTGTGGTAAGATAGGGCTTATACTAGCGGTAACGGAAGTGC
CAACGGTAGTCATAGAAATTTGTTCAACCATTTCACTAGCAAGAGACGACACCGCATCTTCCATA
GCTTGTTCAAAGGTTTGTGTTAATTCATCTTTAGTGGTTTGCCCCGCATATTCACTAGAATTTGT
CATGATTTCGTTGAGTCTTTCTTTTAACTCATCCGCCAAATTCATGTTTTCATCTTCAAAGGAGG
AGAGTAAATCATCAAAACTGCTAGTTAAATTTTCCAGTTGACTGGTTAACTCACTCAATTGCTCA
CCCGTAAGATTTCCACTAAAATCAGTTAAAAGAGAATCCATTTCTCCTTTATTAGTTTCTCCAAT
ATTAGTCGCGGTTTCCTCAAATTTAGCCTCTGTTTCTTCTTGAGTGCTTTCTAAATCACCTTGTAA
ATTACTGGTGTTGTCACTGAAACTGTTAAAAAACTCCTCAGCTTCGCTATTACTGTTAGCAAGGG
AATCTTCAAGATTACTAATCCCCTCAGCTAAATCAGCAAAAGTTTGGCTTAATTGTGCAAAAAAG
TTGTCTAACTCTCCTTGTTGATTATCAATTTCGGTTTCTACCTCTGTTTCTTTTTCCTCCACCGAT
TGACTAATTTCCTCAAGTTTTGCCTGAAGTTGAGTTAAGGCGGCTTGTAAGCCAGTTAACTCCGT
TATCATTGCTTCGGAGTCTTCGGTGAATTCCTTCTTAGAGTTTTCAAACTCACTACTAGCTGTTTC

TAAACTATTAGTAACTTCTTCTAAACAATTATCTAAGGATTGATTAGTTTCTTCTAATTGCTGAG
TAAACTCCGTCATCTGATTATTTGCTAAATCGAGGGCGGTGAGTAATTCTTCTAGCTTCTCCACC
GACTCATCAATATTCTGCTCCATTACTTGTGCATCTGCCATGATTTTCCCCTTTTGTGTGCTAATT
TGTTAATACTTGATAAGCCAAAATTTATTTAACTTAAATTATCTATTCATGTTTGAAGATAAATA
AAACTTTTGCTTGTCTTCACCAAATAAAATAAATTGAGATTACTAAGCCAAAACCGTTTGAATCG
CATCTAAAACGGGTTTAATATCCTCAATCAAATCAGTAACTTCGTCAACCTTATCAACTATGTCA
CCGATACCGCCATCGAGTAATTCTCCCGATTCTTCTCCTGCGTCTTTGAGAAAACCTAAAGCATCC
GTTAAACCTCCTGCGGATTCTGTTAAATCTCCCATTAATTCTTCTGTGAATTTAGAGGTTAGATT
ACTGATTAAGTCTCCTGCTTGAGATTCTAAATTTTGAGTCAGATTTTCTGCCCCTGATTTACTTT
GCTCTGCAACATCTTCAAATTCAGATTGTAAGCTCTCAAGTTTATTGCTTAACTCTTCAGCTAAA
TCGCTAAATTGTTGTAGGATATTTTCTTTTTGAGTTTGGAAACTTTCCGTAGCGGTATCTACTGC
ACCATTAAATTCTTCAATATTTCCTTTTACCGTTTCTTGATAGCTTCCCATTTGCTCCTCAGTTTG
TTGTATATTTTGGGAATATTGCTCAAATCCTGACTTATTCTCTTCAGTTTGAGAGTCTATTTCCT
CGGTGATTTCCTGTTTACGGGCTAGTAAATCTTGTAAACGGGTTTGAAAATCTGTCATTAGTTGA
GTAATTTGTTGATTAACCTCATTGGTTTTTCCGTCAAATTCTCCCATAATTCCTTTGGTATTTTCT
AATTCCGTTTCAAGGGTATTTTCAGCTTCTTCTAACTCACTAATCATTGCTTCCCATTTAGCTTGG
GCTTCTTCTCCTGCTTGAGATAAATTTTCCGCAATTTCTGTAGCTTTTGTAACTATTTCTTCTACT
TGAGTAAAAGATTTTTCCCCTTCTAAAATTAAAGCCTGTGCCCTTTCATCTACTTGCTCAATCAG
GGCATTAATTTCTTCTGTCGCCATTGTTTTTCCTCCATCAGAAGGTAGTTTTAATTAATATTGTT
TTTATTTTTGGTTAATAACAGTTACAATTAATAAGGTCATAACTCTACTGTTTCAATAATTTTTC
TCCACTTATAGTTGTCTTTACTCTTGAAATAAATATATTAATTTTAGGTTGGTTAATCGTTATAA
TTTATTGATACAGTAATATTTTAATTTTTTTTTCAGTTGTCGTATTAAACCACAGAAAAATTTAA
GATTTATATTAATTTTTATTGCACTTTTTTTGATTATTTTACTTTTTGTTAAGTTTTTTCTCTGT
TATGTTTTTTTCTCCTAAATTTCTTGAGCAAATTTTTCTTAAGTTAGTATTATTTTTCCTTCTTT
TTATAGAGGCTGGAGAAGCAAAAGGGTTAACAAACAAGGTGGTTGGGCAAAATAGTCTTGTGAG
GGAGATAAATAGTAGTGAGTCGCAGATAATAGCTAACAGTAAGAGAGAAAGGCTAAGGGTTGCA
ACAAAGTCTTTTCCTCCTTTTGCTTTCGAAGAAAATGGGCAGTTTGTCGGTTTCAGTATTGATTT
GTGGAAGGAAATCGCTAATGAGTTGGATGTCGATTTCAATATTTATGGGGAAAAAACTATTTCCG
ATTTGCTTAATTCTGTGACTGACGGTTATACAGATGTGGGAGTAGCAGGGATTACTATTACTTGG
GCAAGAGAAAAAGATATTGACTTTTCTCACTCTTTTTTTGAATCTGGTTTACAAATTCTTGTGCC
TATGGAAACTGTTTATCCTTGGCAGTTTTTCTTTGCTTTTATTTTTTCTCCCATTCTTTGGAGTAC
TGTTGCTATAATTGTTGTTGTAATTGCGATCGCATCTCATTTAATTTGGTGGTTAGAGAGGAAAA
ATAATCCACAGATGTTTCCTGAAAATTATAAAGATGGTATTTGGGAGGCGTTTTGGTGGGCAGTT
GTTACCGTTGTGACAGTGGGCTATGGAGATAAAACCCCAACGGGAAAAGCGGGGAGAATTATAGC
GACAATCTGGATGTTTACAGGCGTGTTGTTAATTTCCTATTTTACTGCCTCAGTAAGCTCAATTT
TAACAGTGCAGAGACTTGATAATAACATTACTACCATTCAAGATTTATATAACAAATCCGTTGGC
ACGGTGAAAGGTAGCACCGCCGCAGAATTTTTGGCTTCAACCAAAAGCTATGTTGTCTTATTTGA
TTCTATTGAGGACGCTTTCCTTGCCCTTGATGATGGTAAGATAAAAGCAATTGTCTATGATGCAC
CTGTTTTACGTTACTATTCTCGCAGTGATGGTGCAGGGAAAGTGAAAATTGTTGGCTCAGTTTTT
GAGAGACAAAGCTATGGAATTGCTTTAAAATCTAATAGCCCTTACCGAGAATCCATTAATCAAGC
TATTCTTAAACTAAAAGAAGATGGCACTTATGAGCGTATTTATCAACAATGGTTTGGTGCTGATT
AAAAAGTTGCTAACTAATATAAACTCAATTCTGCTTAAGAATATCCGATAAGGGCAGGTAACGGG
TTGCAGGTTTCGGGGTATTAGGGAGATGAGGGGATGAGGGGAGAGGGTGAGAGAAGGAAATTAA

CTCTTAACTCTCAATTTTTAACTGAAATTTATTCACTATTCACTATAAACTATTTACCCTTGCCCC
TTGCCCATTCCCTGTCTTAACGAGACAACCTAAATGCCAAACTAGCTTATTATAACTAATTCCTC
ATAATTTATCACGATGAAAGATAAGATAATTATTCATAGTAAATACCAAATGATTCAAACTCTT
GAGAATTTCAATGAGGAAAAGATTTATTTAGTTAAAAATATTGTAAATAATCAAAAATATCTAC
TTCAGTCATTGGATTTGTCATTGATTACCCCAGAGGAAATAAATCAGATAAGAGAGAAAATAGA
ATTAATTAAAAATATCGCCAAACAATCTGAGAATAATCAAAGATTTCCTTCTCTTGTTGATGTAT
TTGATGAGAAAAAAGCAATAGTTATTATTTATGAAAAATTTGAGGGCAACAATCTTCAAAACAT
TCTCAATAGCCGACAATTATGGACAGAATTAGAAGCTGTTAATTATCTTTACTATTTATTAGAGA
GTTTAGATTTAATTCATCGTGAAAACTTAGTTCATCAAATAATTAAACCACAAAATTTGACCATC
AATAATCAACAAATATTTATTAATAGTTATGGTAAATTAAGCAATTTAAAATCGTCTTTGATGG
AAACAGTAACCCTTGAAGATAAATTATATATCGCTCCTGAACAAATTAGAGGCAAAATTAAAAT
TAGTAGTGATATTTATGCTTTAGGTATGGTAATCATTAGCCTAGTTACGGGAAAAAATATTCTTA
GTTTAGAGGAAGATGACACAGGCAAAATAATTTGGAGTGAGGGAGAGACATTGACAGCAAATTT
TATTCAAATTATCGATAAAATGATTGCTTATCAAACACAAAATCGATATAGCAACTGTCAAGAAG
TTATGGGGGATATAGCTAAGTTTTTCCCTCAACGTGTTTCAGAAAAATCTCTTGATTATTCAACA
GAAAATATTGCTCATTATACCCCCACTGAAATTATAATTTCAACGGAGGAAGAGGAGTTTAGTCA
GGAAGAAAATTATAGTAACTCAAGTTATCTTAATCCCACAGAATATGTTAATTCAAATAATTCTA
ATCAAAAAGAAAAGGAAATTATAGTTAATGATACTGAGTTAATTAATGCTGAAGATGATATTAA
ATCTTTATTACCTTTAGCAGAAGAATATTTATCCGAAGAAAGCAAATCTTTTTCCGCCAGAATCG
ACAGTCAAAATCAAAAAGAAATTAGCAATGATAATACTGTCAGTAACTCATCATCTTTGCTACCT
TATTCTGAGCATAAATTCAATTTAATTCGTCAAATTAAGACTCCTAGAGGAATTTTAATTAGTTT
TATTATTTGCATTTTAATAATTTTCTCTTTTTCTTGGCTAAAAAGCTATTTATATCAAAAAAAGG
TAGAGAGTTTAATTACAGAAATTCAGAGCTATTATGAGCAAGAAAATTTTGATGAGTGTATTGC
ATTAATTAATTCAAATACAGTACAATCTTTACCTATTGCTAATAGTTTAACAGATGAATTTTTAG
GTAAATGTTGGTTGGGTTTAGCGGAAATTCAAGCAGTAGATGGTAATTTTGCTGAGGCGATTAA
TATAGCGATACAAATTAATCGTGATTCTTCTGATTATGAGAGGGCAAGACAATTTATTGATGATT
GGTCTGAACAACTTTTTTTAGAAGCTAAAATTCTTTGTCAAACTAATAGTGAATTGTCTTTAATA
GAGGAAAAATTAATGCCTATTCCTGAAAGCAGTAAATGGAAAAAAGATGCTTTAAATTTATTAG
ATCAATGCGAAAATAATACTAATAATAATCAAGTTATTCCTCTTTGTCCAGGTCCTTTATGTCCT
GAATAATCAATTAGTATTTTTGTTAGATTTAAGTTGCTCCCTCTATATTTCTTAAAAAAAAGTAG
GTATCAATATTTTCTTTTAAACGGTATAGTTGGGCAGGGCGTTTAACATCTTGACGGGTTTCCCC
TGTCGGTTCAAGAATACTCGCTCTTTCAATGCGACGACGAAAGGATTTGCTTTCTAAAGTGCGAT
CGAGTATTATTTCATATACAGTTTGTAAATCTCTAAGGGTAAATTTTTCTGGCATTAAATAGGCT
GGTAGGGAAGTATATAATACTTTATTTCGTAAACGTTCGATCGCTTTTTCCAGAATATAATTATG
ATCAAATGCTAGGGATTCTTTAATTTTGTTATTTTTTATGGGAGACCATTTAACTGCACTAACTG
AGGATGAGGTTATATCGTCGGAATTTGTCTTTAATTGGATATTTTCTGAGGGTAATAAAGCAAA
ATAAACCGTTGTGACAGACCATCCCCTAGGATCTCGTATTTTATTGCCAATAGTAAAACATTGCT
CTAAATAGGGCGTTTTTACTCCTGTTTTCTCCTCTAATTTTCTTTTGGCGGTATCTTCTAAAGTTT
GGTCATTTTCTAAATCGATAAACCCTCCTACTAATGTCCATTGATTTTTGAAAGGATATTCTCCT
CGCTTTACTAATAAAACCTGTAATGCCAAGTCAAAAACCGTGAAGATGACCGTATCGACAGAAGT
AGAAGGGCGATCGAATTTACTAGGGTCATAATTTTTAAAAAACTCTGTTTCTGAATTGTAGTTTT
GATTTTTATTCATTTATTCTCAGTAAAAATGACTCTCTTAATTGGGACATGATAAATTATCTCAA
AAAAAGGTGTCAGGTTTCAGGTGGCAGGTTTTAGGTTGAATTAAATATGTTTTATCGGACAAAA

TTATCTTGTATTTTTGTTGTAACATTTTTGACAAAAAAATAAATATTTAACCTTATTGTTGAATA
ATTATTAACATTAAATAATATTTTTTTCAATGATTTATTATCAATTAATATATATCTATAAATAC
CTATAAAATCTGACAAAACGTTCTCATTTACCAAGAGTGAAAACTGAATAAACGCTCATGTGAAC
AGACTAACAAAACAAAGCCAATAAATTTAGATATAATCTTCTATTGTGGTTGATTCAGAAAACT
ATGAAACGAATTAGTTACCCAATACTAAAATTATTCTTAATTTTAACGATTTTTAATTTAGTTGC
TTGTAACCAAGAAAAAATATCAACATCTACACAGGAAAACACAACAGAAATAAATCAGAATCAA
ACAGAAAATCGTCAAGAAAAATTAAATATAACCGTTACAATTCCTCCTCAGAAATACTTTGTTGA
AAAAATTGGCGGCGAGTTAGTCAATGTTAATGTGATGATAGGACCTGGATTAGAACCTCATACCT
ATGAGCCACAACCCCAACAGTTAACCAGTTTAGCCGAATCCAGTGCTTATGTTGCTATTGGTGTC
CCTTTTGAAGAAGTATGGTTGAATAAAATCACAACAGCCAACCCTCAAATTGTCATGATTAATTC
TGGAGAAGGCATTAATAAAATACCTTTAGTAGGTCACGATCATGATCACAGTCATGAAAATGAT
AATCATAATCATGAAGATGAACACAATGAAGATATTGAACATAATGAAAGTAATTATACATCAG
AAACGAGTAGTCCAGATCCCCATATTTGGTTATCTCCAAAATTAGCTAAAATTCAGGCGGAGAAT
ATTTATCAAGGTTTAGTCAAAATAAATCCAGAAAATCAGCAAATATATCAAGAAAATTTAACTA
AATTTCTAACAGAAATCGATAATATAGACCAACAAATAAAAGAAAAATTAGCACCAATTAATAA
TAGAAAATTTTTCATTTATCATCCAGCATGGGGATATTTTGCTCAAGAATATAACTTACAACAAA
TACCACTAGAATTTGAAGGACAAGAAGTCACTTCTCAGGAGTTAAGTAATTTAATCAAACAAGCA
AAAGCAGAAAAAATTAACACGGTATTTGCTCAACCTCAATTTAATAATAAAACTATGGAAATAT
TTGCTAAAGAAATTGGGGCAGAAATAGTTTTATTAGATGATTTGGCTCAAAATTGGTCTGAAAA
TATGTTATCTACTGCGGACAAATTAGCAAAAGCTATGACAAATAATACTAATTAGTGATGAATA
ATTTTGTCAAGAATAAATTAGATTTGAAAAAATAAAAGTGGTCATTTATATCTGATTTTTGATT
CATAAATATATGCTCAATGAATGTGTCATCAAATTAAAAAATATTCATGTTAGTTATGGTAAAA
ATCTAGTATTAGAAAATATTAATTTAGATGTAAAAGAAAAAGATTTTATTGGCGTAATTGGCCC
TAATGGTGGCGGAAAAACTACTTTATTAAAAACTTTATTAGGATTAATCAAACCCCAACAAGGGG
AAATTTCTATCATGAATTACCCTCTAAATAAAGGTAGAAAATATATTGGTTATGTTCCTCAAGCC
TTAGAGTTCGATCGCACCTTTCCTATTTTTGTGGAAGATGTAGTAAGAATGGGCAGACTATCGAC
AAAACATCTCTTTAAACCCTATAATCACCACGATGAAGAAATTGTTAACCATTGCCTTCAACAGG
TAGGAATGGCAAAATTGCGATCGCGTCCCATAGGGGAATTATCAGGAGGAGAAAGACAGAGAGT
ATATATAGCCCGTGCTTTAGCCACTAAACCGAGTATTTTACTATTAGATGAACCTACCGCTAACG
TTGACTCAAGAGTACAAAACAATATTTATGAGTTACTAAGAGAATTAAACGAATATATGACCAT
ATTACTAATATCCCATGATTTAGCGGCAATTTCTAGCTATGTAAAAACCATTGCTTGTTTAAATC
GTCGCCTACACTATCACCAAGATAAACTTATTACCCCAGAAATGATTCAAGCAACTTACCAATGC
CCGGTGGATTTAATTGCTCATGGTATTCCTCATCGAGTGTTACCAAAACATAAATAACCTGAGTT
AGGGATAAATTTTCATCTATGAGTGATGGCGAAAAGGGCAATTAGTGAATAGTGAATAATTAAA
AACTCCTGTAGGGGCGAATGGCCATTCGCCCCTACTTCCCCCTCTCCCCTCATCACCTCATCCCCT
AAACACCGTAAGGGTTGAATATATTCAACCCCTACCACCTGCAACCTGAAACCTGACACCTACCCT
TAACCGAGATTAATTTAAAACCAACCAATTATATTTCTGATAGCTAAAATAATTACCGTAATCAT
AAAGATTCTTTTTAACCATAAATTACTCATTTTTAAAGCCCATTTTGCCCCTAAAATAGCTCCAA
AAAACATAGTTATTCCTAAAATAATACCTAGCTGATAATCAACTAATCCTTGACTCATAAAAAATA
GCTGTAGCAATAAGAGAAGAAAAAATATTTAAAACCTTTGTAATTGCCACTGCCTCCACAAATGT
CATGTTTAAACAGCCAATAAAAGTAGCGGTTAAGGTAGTTACATAACCTCCACTAAAAAAACCAC
CATAAACTCCCAATAAGAAAGTAAGAAAATAGCCTATTTTTTCCATTAAAGGTGAAGATTTTTCC
TGTTTTTCTACTCCTTTATTTTTGTTAATTATAGAAAAAATTAACACGGCTATCATAAAGACAGA

AATCAGCAAAGGCATTGTTTCAGCAGGAATTATTAATACTAATAAAGCCCCTAAAATTGAACCAA
TAACCGTCAAAATTGAGAGTAAGGGTATATCTTGCCTTTGTTTAAGAATATCTCCCTTCAAAAAA
GGTATTGTCCCCCCGATACTCATAAAAATTAGTGCAAACATATTAGTTGCGATCGCATCAGTCGG
AGAAATTCCAAACTCTAACATTACAGGCACGGTAATGAGGGAATTACTACCCGTCACCACCCCAA
TAATGCTTGTGACAAGAAAAATAAAGACTAAAAAAAATAATTGAAATAAGGTCACAATTGATTA
AGATTAAGCCAAGATTTCTTCTAGTTGATGTTTTTGCCACAGAGATTTATATAAACCCTCTTGAG
ACAAAAGTTGCTCATGGCTACCCATTTGCACAATATTACCATCTTCCATAACTAAGATGCGATCG
GCATTTTGAGCCGCCGACATTTGATGGGTAATAAAAATAACAGTTTTATTTTTCTCCCGCTTAAG
ATTTTCTAAAATTTGCGTGGCCGTCTTATTATCGACACTAGACAGAGCATCATCTAAAATCAGAA
TGGGAGCATCTGTGTATAAAGCCCTAGCTAATGAGCTTCGTTGTCTTTGCCCACCAGATAAAGCA
ATTCCTCTTTCTCCCACCAAAGTATCATATTTTTGCGGAAAATTGACAATTTCAGGGTGAATTTG
GGCTTGTTTTGCCGCATATTCTACCTCTGTTATTTCACCCACAGGATCACTATAGGCGATATTATT
TTTAATAGTAGTGGAAAATAAGAAGCTATCTTGAGGCACATATGCGATCGCAGCTCTCAAATCTG
TTAAATTAATGGAGGTAATATCGATACCATCCAAAAATAACTGCCCTTGAGAAATATCAATTAAA
CGAGGAATAGCATTAGCTAAAGTAGATTTTCCCGAACCAATTAAACCTACAATTGCCACTGTTTC
ACCAGCATTAATTTGAAAATTCAAACTATTTAAAGCAGGTTGAAAAGCATTAGGATAAGTATAA
GTTAAATTAACAGCTTTGATATTTCCCTTGATATTTTCTATAGGCAAATTTACATTATCAACATC
ATTTTTAATTTTGGGTTCAACTTCCATAATTGCTTCAACCCTATCAATACTAACCTCACCCCGTTG
ATAAGTAGTAATGGTAAAACCAAGTAAAGCAGTAGGAAAAACTAATCTTTCTACATAAATAATT
AAAGCAATAAAATCCCCAACAGTAATTAAACCCTTTTCAATATCAGAAGTGCCAAACCACAGTAA
AACTAATAAACTAATATTGGCAATACCACCAATAATAGGAAACAGTAAATTGCGAGTTCTTGCTA
AACTTAAATTAGCCTTAAGCAGGCGTTGATTTTTGTGAGAAAAAGCCCTTCTTTCATTATCTTCT
TGAGCATAAATTTTAATCAGAGAAATACCGCTCATATCTTCCTGAATTAACTCACTAATATCCGA
TAATTTCTCTTGTACCTCAGCTTGATCAATTCTTAACTTATCACTAAATAACTGTACCGTAACTA
ACATTAAAGGATAAATAGCCAATGCCATTAAGCTCAATTTAACATTGATAAATAACATGGCAGGT
AAAGTCAAACCATAGGCAAAAATAGTATTAATTAAACTTAAAATTGCAAAACCAACTAAACGAC
GAATATTGTCCACATCGCTAGTAGCACGGTTAATTAAATCACCAGAAGTATTAGTATTAAAGTAA
GCCGGTTCTAATTTTAAAAGGTGTTCAAAAATTCTTTGTTTTAAATCAAACTCTACCTGTCTGCC
AATACCAAAAATAAGCACACGGGAAGCCATACGAATCGCCCACATTATACAGGCTAAAACAAACA
AAATGACCACATAACTAACAAGACGATTAAAGTCAAAATTAGCTTGTAAATCATCGAAAATATCC
CTAATCAACCAAGGAATATAAACACCAATAACATTGACAACAAATAAAGCGGAGATCCCCCAATA
TAAACTTTTTAAATGAGGTTTTAAGTATGATATTAACTTTGATAATCGATTAGCCTTAGCCATGC
TTTACTATCTTTATTTTTTCTTCATTTTTATATGATAAGCCAGTCTGCATTGAAGTTTCATCATA
GTGTTGGGATGAATGGATTTGAAAAGGGCAAAGGGCAAGGGACAAACCCCCCTTTATTCCCTCTC
GAGAGGGCAAAGGTAAATAGTTGATAATTAATAATTTCTAACTCTGTACGGGCGAATGGCCATTC
GCCCCTAACTAACTCCGTACCGCTTTGCGGAACGAGCGTTCTCTCGTTCCCCCCCATCCCCTAAAC
ACCCTGACACCCGAAACTGGACACCCCAATCCTCATCCAGAAGTATCGAGATCTTATGAAAGAAA
AAACAGTGGTAATATAACTTTATGATCAAAGACTTAAATGTCTGTTGGACTAAATTAAATCTTTT
CATATTCAAAATAAGTAAAAATTATGCCATTATTGACCACTGGAAAAGCATTTATTAAAGATTT
AGAAAAAGCTGGTTCTGTTGCGGTTTATGCTCCTTTAGAGGGCGGTTTTGAAGGACGTTATCAGA
GAAGATTAAGAAGCTATGGTTATCAAACTTTTAATCTTTCTGCTAGAGGTTTAGGAGATTTAAGT
GCTTATTTGATGAATGTTCACGGGGTGCGCCCTCCTCATTTAGGAAAGAAAAATATCGGACAAGA
GGGAGCGGTAGGACCTACTTATTTTGTACCACCTATTGCGGGTTATCAGTTAGAAAATCTATCTA

CCAAGGCTAAAGGTTTAGTTTTGTGGATTATTGAAGGTTTTGTGCTTTCTCGTCAAGAAAAACAG
TATCTAGTTGATTTAACGAAACAAGAACCAAAAATTAAAGTAGTATTGGAGCTAGGAGGCGATC
GCTTCTTCCGTTGGCAATCTTTATCCGAAGCGCTGTAAATATTTAGCAATTAGCAATTAGGAAAA
GACAAAAGGGCAAGGGGCAACGGAAATTAAAAATTAGTTTCCCTCTCTCTCCCCTTCGCCTCATC
ATCCTAACACCTGCAACCTGAAACCTGACACCTAAACTTATCGGATATTCTTAAACCGAACTGAG
GTTAATTACCTAAACTTCTCACTCAGGTGATTTTATTTTTCTGATTTAAAAAGGTAAAGTTTTGT
AACTTAATCACTCATTAACCTAGAAAATATTGTTATATTATTCATATAGAAATCAGATAAAGATT
TCTACTCGAAAGACAAAACAGCTCGTTGTAAACTTTGAATAAATTGAGGTAAAAAGGCTGAAAA
CAAGGTTAAATCTTTGTCTTTTGCAATCCGAAACAGCTTGTATTCTGTTCGAGATTTTCTGACCT
GTTAGTAATGAGAGCAAGGGAACTAATCGATGGTCAAAGTTAAATAATTGACTACTTAGTGCAA
CTCTGTATAGTGTTAATTTAAGGTAGAAAATATAGTCTAAAAATAAGGAGGTTAATTAAGCTGT
TTACGGTAAGTTACGTTGTCCATAAAATCAATAGCAGTAGCCAGTACCAGAGTCTTGAGTTTTCT
AAAATGTGGCTTTAAGGGAGTAAGATGATAAACTAGAACGTATAGTTGTTATCTCTCTGAATAT
TATCACTAAGGATAGAAGGCAAAAAAAGGACGGGTCAACTTTCGGGCTACTGTTGTTGTTATGAG
AGGTTTTTTATGATTACGGTTAAAGGTGAAATTCAATATCGTGCGATCGCATCTGGCACATGGGT
TTTAGTTAGCGACACAGGAGAAACCTATGAGTTGTACAAGCCTTCAGAAAATCTGCGTCAGGAAG
GATTAAAGGTAGAAATAAAAGGTAACATTAGAAGCGATGTTATGACCATTGCAATGGTGGGGCA
AATCCTTGAAGTGAAAGAATTTAGTATCTGTCGATAAGTCTGAAAATTAGGAATATCCCTTAAAG
TCAACATCCTAGCATCTTTGATCGTCCAAACATTTTTTCATCAAAGCCCAATTACCTTATAATTT
ATCAAGGATAATGACTTAAGAGGTTAAGATGAAAATCGGTGTCCCTAAAGAAATAAAAGATCGA
GAGTTTCGAGTAGGTTTAACTCCTGCTAGTGCAAAAGTTCTGATTGAACAGAATCATCAAGTATT
CATCGAAGATAATGCAGGTCTTGGTTCTGGTTTTACTAATCAAGATTATGAAAATATAGGGGCAA
ATATCGTTGATCAAAGCACTGCATGGAATCAAGAATTAATCATTAAAGTCAAAGAACCTCTAGCA
GAAGAATATCAATATATTAATCAGGGACAAATATTATTCACATATCTTCACTTAGCCGCCGATCG
CATCTTAACAGAATTTTTACTTAAAAGTGGCGTAACTGCGATCGCATATGAAACAGTACAATTAG
CTGATGGCAGACTACCTTTGTTAACACCCATGAGTATCATCGCTGGGCGACTCTCGGTGCAAATT
GGTGCGAGATATTTAGAAAAACAGCAGGGCGGTAAAGGAATTCTATTAGGGGGAATGCCGGGGG
TTACTCAAGGCAATGTTGTTATTCTAGGAGGAGGTATCGTTGGCACAGAAGCGGCGAAAATAGCG
GTAGGAATGGGGGCAAAAGTAACTATTTTAGATGTAAATATTGATCGTCTTTCTTACTTAGAAAC
TCTATTTGGTTCAAGAGTTGAACTACTTTACAGCAATTCCCAACACATAGCCCAAGTTGTGCCTC
ATGCTGATTTATTAATTGGCGCTGTATTAATTACAGGTAGAAAAGCACCTATTCTTGTTTCACGG
GATTTAGTTAAACAAATGTCCCCCGGCTCGGTTATTCTTGATGTCGCAGTGGATCAAGGTGGTTG
TATTGAAACTTTACGCACAACATCCCATAGTCAACCTAGTTATGAAGAAGAAGGAATAATTCATT
TAGGTATTCCCAATTTACCCGGTGCAGTACCTCGCACGGCAACTCAAGCCCTAAACAATAATACT
TTACCCTATATTGTTAAATTAGCAAATCAAGGAATTGGAGCTTTAAAACAAGATCCTAGTTTAGC
GCAAGGGTTAAATATACAATACCATCGTTTAGTACATCCTGCGGTACAACAAGTTTTTCCCGATT
TAGTCACCACTTAATCAAACTATACTTTAACTGGTCGGTTAAGGTAAAAAGGGCAAGGGACAATC
ATTTGAGTTAGGAGTTCAGGTTTCGGAGTTCGGAGTTAAAATTAACAGATTTCGAGAAAAAAAA
CTCATAAATCGATTTTCTCTTTTTAATCGTTTGAGTAACGAACCAACATTCAGAAAATCAAACCT
GAAACCTGACACCTATTTCCTAACCCTATCCTTAACACCAAACCGAAATTAATTAACTGGGGGAG
CGATATACTCTTTAATACCAACATAATCTAAGTAACGCTTAATAGCTTCTTCCGTATCCCCTGCA
AAGGCAACTTGTCCTTTATTTAACCAAATACTGCGATCGCACAAATCTTGAACATAGTCTAAACT
ATGAGAAACAAACAAAATAGTACTACTTTTTTCCCACAAATCTTGAATTCTTTGCCTTGACTTTT

GAGTAAAACGAGCATCCCCCACAGATAAAACCTCATCTAAAATGAGAATATCAGGTTCAACATCC
GTCGCTACAGAAAAACCTAAACGAGCTTTCATACCAGAAGATAAGGCTTTAACAGGTATCTCTGC
ATAGTCTTCTAACTCCGCAAACTCCAAAATTTCCCGAATACGCACCTTCATATCTTCTTTGCGATA
ACCTAGCAAAACTCCATAAAGAATGATATTGTCTTGCACCGACATTTCCATGTTAAAACCAGCTC
CTAACTCAATTAAAGGAGCAATTCTTCCCCTAACTCGAACACTACCTTCTGTGGGAGGTAAAATA
CCACAAATTACCTTTAAGAGAGTGGATTTCCCCGCACCATTAGAGCCAATTATCCCGACTTTTTCC
CCAGCACTAACGGAAAAGTCAACATCTTGCAAAACCTGTTTTTTAGCTGGTTTGATATGTTTCCC
TTGTAAAAAAGAAAAAATAGTTCTTTTCAAATCATAAGAAAATTCCTCTTGGGTTCTACGCCACA
AAGAAACTTGATCTAATCTAATAACTTCTTGAGTCATTTTTTATTTAATAAAATATTAATTTATT
TAAGTAATATCGAATAATTAAAATATCAATAAAATCAATTAGTTTTATAACAAATCCATAAAAG
CAGATTCAAATTTTCGGAAAAACAAAATTCCTAAAGTTAAAATAATAACTCCTGCTAAAATTCCC
TTAAACAATAACATAGCACTGGGTATATTTCCCATAAGGCTAATATCTCTTAAACTTTCAATGAT
TGACACTAAAGGGTTTAAACCAAGGATAGGTTGAATTCTGGGTGGTACGATTTCCGCAGGATAAA
AAACGGGACTGGTAATCCACATAACGAAAGTAGTAATTTCATAAAAGTAGCCTAAATCTCGAAAG
AAAACAAATAAAGCACTAACAAATAAACCTACTCCTGTACACACCAAAACTAAAGAAAAGAAGG
GAATTAATAATAAGAATATATAGAGAGGATTTTTGCTATTAACAAGAGTTACAATGGCTAGTAA
AGGAAAGGAACTAATCATAAATTGAAAAATATTCGCCGCAATCATTGAGAGAGGAAAAACGGGA
ATAGGTAATTTAATTTTATTGAGTAAATCTCCATTAACAACAATACTACTGAGTGCCTGTGTAGT
GGAAGAATTATAAAAGTTGATAACTAATAAACCTGTAAAAGCCGCTAAGATATAACTAATCAAG
GAGTCATTATAATAAGAAGCAAAAGTCGAACCGAAAATAGTAGTATATAATCCTGTCATAATAA
TAGGATTTAACAATGACCAATACACACCCAAATAAGAACCACGATAACGAGTATTAAGATTTTGT
GGTACTAAGACGGAAAGTAAATCCCAATAACGATGAAAATTTCGCCAAAACGCTGACTGCTCTTT
TTCTTTAATCTTTTTCTTTCCCGCTAAAATCATAAACAATAACAATACATCAATTGAGTAACTCC
CTTTGGAATAGGTCTATTGTATCCTAGAGTTACCTCCGCTCGATATGAAATTTTGGGGTGGTGGT
AAACAAAGGGCAAAGGTGAATAGTGAATAGTTGATAATTAAGAATTAAAAATAAAAAATCCTTA
ACTCTGAACTCCGTTCACGACGACTGTACGAGCAAAGCGAACTAACTCCCTAAACTCATTTCCCCA
TCCCTCCATCACCCCTTAAATCTATCTAAATCGGATATTCTTTATATTGAACTAAGATGAGTTCA
ATTATTTTAGTAAATTAAATTGTAGTAAAACAGATACATTTTCAGGGGTAAGAATTTTCTGTAA
TTCTTTGATTTCGTTTAATAAGCCTATTTCTTTTGAGCATTCCTCTTGTAATACTCTTATATAAG
ATTGGCGTTTTTCTTCAGATTCTGCTTCCGCTAGCATCTTGATAGCCATATTCATGTTATTAATT
GGATTGCTCATATTCACAATGAGTTTACTAATAATTTCGTCTTTAAGCTCCAGCATATTTTCATT
ATTTTTGATTTTTTTATCTTTTTCCTTAATTGTTTCGAAAATATCTTGGACTAATTTGCGATTTT
TCTCTAGTTTTGCTTCAAATTCTTGTTCTAATTCTTTTTTTCTTTGTAATCTCACGTCCACTGCTT
CTAGTAATTCATCAGGGGTAAAAGGCTTGGTTAAATAGTCATCAGCCCCCATTCTCATGCCCATA
CGAATATCTTTTTTATCAACTTTAGCTGTGACAAAGATGCAGGGAGTCATTAAAGTTTTCTCATC
TATTCTCAGTTTTTCTATAAATTCATAGCCGTCCATCTCTGGCATCATAATATCGCAGATAACTA
AATCAGGAGGATTTTCTCTGGCTACTTCCCAACCTAGTTTTCCGTTTTCTACTCCTGTGGCTTCAT
AATTATTCAATCTTAATATTTGTAGGATATTCTCTCTAATTTCCTTTTCATCTTCGATGACTAAA
ATTTTAACCATTAATCTTTAATTTATTTTTGTTAATAAAATTAATACGAACTAATTGATAATTCT
ATTTTCAGAGGTTTTCTATATTATTACATAAATTTTTTCATTGATTTTTAGATCTTTTCTTAATC
TTTCTTTAAACTTGAAGATTGTCAAAATAAAATATCTATTGTTTTTCTGAACATACTTACTTTTA
TCCGTTTATTCCCTTAGGTATTTGTTAATAATTTATAAATTCCCTGTGAACTGAATTAATTAAGT
TTATATTCAACTTATGTATTTCTACATAAACATTTATTAAAAAGAGTAAATAGAATGCTTAAGA

GTTTTAATTATTGTGTCTAATCTAATTATTTCTGATAGTATATAAATAGATATTTAAAAAATTA
TTCAATGTAAATATTAACTTTAATTAAGTTTTTTGGTTCTTTAAAATGAGAAATTTTAGCTGACC
TTTATTAGCCAAAGAATTTCTTTAATTGTTGTCTGTGTCAATTTTTTTGTTCTTTCTTGAACAAT
CTATTTATTATTATTCTTCATTTTCTATGACTTATCAGTTTTTGGAACTACAGTCTCTAATTATT
TCTTGTTTTTGTACTGTTTCCCCTGATGTGAGTATCGAGGATGCAATCAGAAAGTGGCACCGTGC
GATCGCATCTTTTCCGCCATTTAATCTATCTCAAGCTAATACTCATGATTACTTATTTGTCACTG
ATCAGGGAAAACCCGTAGGCGTAATTACTTACAAGACCCTATGTAAACTATGTATTAATCATGAT
GGTGAGGAAAAAATCTTGGTTAGGCAAGTGATGGAGATTCCAGAGCTAATTTGTGAGAAAGGAA
GAATTAAAGATGTTGAAGAAATTATTAATATTTTCAAGGAAAGTGATATTAATTGTTTGACTAT
TGTTAATCAAGAAGGTTATTTAGAGGGATTTATTAGTTGTGAAGATGTGGCAAAAGTTTGTTTT
CAATCTCGGCAAAAAGTAGGAGGCGAAAAACAAGAGGAGCTTTTAAAAGACACTGACAATGACC
TTGACAATTATCGTTCCTTAATGTCATCGGAAAAAGAAAAACAGTGGGAAATTGATTCTTTATAT
AGAAGGTTAGAAAGAGAAAAATTAGTTTATCAAATTACCTCACAAATTCACAACGCTATTGAAT
TAGAAGATACCCTCAATGTCGCCGTTAACCTCGTACGGGATTTTATGAAGTGCGATCGCATCTTA
ATTTATAAATTTGAAGGTCATTGGGGAGGACAAATTATTGCTGAATCAGTAGATAAAAAATTTA
CCAGTGCTTTAGGTAATTACGTAGAAGATAGCTGTTTTCAAGATCAAGTTTGTAACTTATATCAA
GGAAAAGACCCCGTTGTTGCCAATAACATTTATGAACAAGGTTACACCCCCTGTCACATTAAATT
ATTAGAACAATATCAAGTTAAAGCTAATTTGATTGTACCCCTAGAAGTATCAGGAGAACTGTGGG
GCTTACTTATTGGTCATCAATGTGAAACTTATCGAGAGTGGCAATTAGAAGATGTAGAGCTATTA
AAAGAAATTAGTTATCAAGTTGCCATCGCAATTTATCAAAGTGAAATTAATCAAAAATTACAAA
AAGAAATCTATGAACGTATAATTGCAGAAAAATTATTACAGCGACAAAAAAGGCAGTATCGAAA
CCTCATGGAAATTTTACCAGTGGGGATATTTCGCAATGATTTACGGGGAAATTGTATTTATGTCA
ACGAGCGGTATTGTCGAATCACAGGTTTAACTCCAGAATCCGCCAATGGTATTAGTTGGCAAAAA
ATTATTCATCCAGAAGATTGGGAAAAAGTGTTTCAGGCATGGGAAAATTTAGTTAATTATCATC
AACCTTGTGAGCTGGAGTGCCGTTTTTTACATCCCAATGGCGATATTGTTTGGGGTTATGTTCAA
TGTATCCTCGATCGAGATATTTCAGGAAAAATAATTGGTTTTGTCGGTAGCATAACAGATATAAC
AGATCGCAAAATAAATGAACGACAACTCAAAGAAAACAAAGCCAGATTTGAGAAAATAGCCACA
ACCTTACCGGGTATTTTATATCTTTCCCGTCAACAACAAGATGGCAGTATTGAGTTTGACTATAT
CAACAAAGAAGTTACCTCTATTTTTGAGTTAAGTGTAGAGGAAGTAATGCAAGATGGAAGTCTA
ATTTTTCAACAATTTCATCCTGAAGATATAGAAGAACATTATCAACGAGTAAAATATTGCTTAGA
AAATGATTCTTTATTTTTCCATGAGTGGAGGGTTATAACTCCGTCAGGCAAAATTAAATGGTTAC
AAGGACATTGTCGAGCAGAAAAACTAGCAGATGGGCAAATATATTGGGTTGGTTTAGCCATTGAC
ATTACCGAGAAAAAAGAGACTCAAGCAAAATTAGCCAAAACAGAATCCTTATTCCGTCAGGCAGA
AAAAATCGCCAAAATTGGTAATTGGGAATACCTTTATCGAGAAAATTATCTTTATTGGTCAGAAG
AAGTTTATCGCATTTTTGAAATAGAACCAAGGGAGTTTACCCCTCAGTATGACACTTTCCTTCGC
ACAGTTCACCCCGAAGATCGTCAAGGAGTCGATCGCGCTTTTAAGCTACATATAGAACATCGAGA
ACCCTATAATATTACCCATCGTCTTTTAATGGCAGATGGTAGAGCTAAATACGTTCGGCAACAAT
GTAAAACATCTTATGATTCCCTCGGTAATCCTCTTAAATCCGTGGGAACAGTCCAAGATATTACA
GAACAACAAATAGCAGAAAACATCATTAATAATATCATTCAAGGAACTTCCACCGCCGTTAGTGG
GAAAGAGTTTTTTGAAAACCTTGTTACTAATATTGCTTTATCTGTTAATCAACCCTGTGTAGTTA
TTACCAAACTTAAGGGGGAAATGCTTGAAAGTTTAGCTTTTTGTTATCAAGGAAAATTACAAGTC
GATGGTACTATTCCTCTCCAAAATACCCCCTGTGAATGTGCCATGAAGGAAGGATATTATTATTG
TACGGAAAAAGTAGCTCAAAAATTCCCTACGGATGCGTTTTTAGAAGCCATCAAAGCCGAGAGTT

ATGTGGGTATTCCCTTAATTAATCAAGGTGAGATTCTGGGAACAATTTGTGTTCTTAATGATAAG
CCAACACCCCATGAAACCATTGAAAAGATTAAAAGGATTTTGGAAGTATTTGCACCTCGTGCCGT
GGCGGAATTCATTCGGACGAAAGCGGAAGAAGAATTAAAAGAAATTAACTCAGTCTTAGAAGCA
AAAATCAAACAACGTACTCAACAATTAAAACAATCTCAGGCATTTATAAAAACTATTCTTGATAA
TATTCCCCTACCAATTTTATGGAAAAATACCCAATCACAATATTTAGGTTGTAATCCAGCATTCC
GGAAGTTTATGGGGTTATCTTTTTCTGAAGAAATTATTGGCAAAACTGACCATGATTTTAATTTT
AAACCCGAAGAAGTCCATCGACGTATAACCAATGATCGTTTTGTGATTGATTCTGGAGAATCCAT
ATTAGCTTTTGAGGAAACTATTACTATTCAAGGGGAAGAAAAATGGATTGAAAACTATAAAGTA
CCCTTAAAAAACTCTGAGAATAAAATAATTGGGGTTTTAACTATTTGTAAAGATATTAGTGAAC
AAAAAGCGATCGAATTTGCCTTAAGAACAAGTGAAGCTCGTTGGCAATTTGCCCTTGATGGTGCT
AGTCATGGAGTCTGGGATGTTAATTTAGTCACTAACGAAACCTATTATTCTCCTAAATTTATGGA
AATGCTTGGTTATGAACTTGGAGAATGGGGAAATAGTTTCGATGATTGGCAAAACCGTATTCATC
CGGATGATAAAGAGGTCACCCTTACTACAACACAAAAATATCTCAACGGTGAAGTATCTACTTAT
CAAACAGAACATCGTCTCCGTTGTCAAGATGGCAGTTATAAGTGGATTTTAGCAAGAGGTCAGTT
TGTCGAATGGGATGAGAATGGCAACCCTACCAGAATGATTGGCACAAATACAGATATAACCGATC
GCATCCTCATGGAGCAGGAATTGCTTAAGAGTAAAAATAACTTTCAACGTTTAGTAGAAGAAAT
AGGTAATAACTTTGTGATTTTTAGTCATACTTTAGAAAATGTTATTACTTACGTTGGCGGTGGCA
TAGAATCAATTTTTGGACTGACAAAAGAGGAGGTAATCAATAAATCTTGGGTAGAAGTGGTTAA
CTGGCATGAAAGCGGACTAGAAAGAGCCTTGGTATCCACATCGTTAATTCTTAATGCCGAAGATA
TAGTATTAGATGAATTTGATATGTCTTTTACCCATAAATCAGGGGAAGAGAAAATCATTCGTGTT
TGTCATCATGTGGTTAAAAATGATCAACAAGAAGCGATCGCCATCGAAGGAATTATCGAAGATAT
TACCCAACAAAAAAGAGCTTTGGATGCTTTACAAGAAAAAACCCAAGAACTCGATCGCTTTTTCT
CCCTAGCCCTAGATTTACTATGTATTGCCAATGTTGATGGTTATTTTCTCCGTCTTAATCCCCAGT
GGGAGAATATTCTCGGCTATAAAATAGAAGACCTAGAAGGCTCCAGATTTCTTGACTATGTTCAT
CCAGATGATATACAGAAAACCCTAGATGCGATCGAACATCTCAAATACAATGTAGAATTACCCTA
CTTTATTAATCGTTATCGTTGTGCTGATGGTAGCTATCGTTGGATTGAGTGGCGTAGTGCTTCTG
ATGGTAATTTATTCTATTGTGCGGCCAAAGATATAACCGAAAAACTGAAAACCGAAGCAGAAAA
ACAAACCCTCATTAATGCCTTAGAAAACAGTAATCATCTCCTGCAATGTATTAGTAATGCTCAAT
CTCAATTTATCACTGCTGAAAACCGTCTGACTATTTTTGAAGACCTATTGGCTAATTTATTGGAA
TTAACGGACAGTGAATATGGCTTTATCGGAGAAGTCTTATTTAGAGATGATGGTAGTGCGGAAA
TGAAGGAAACTTTTTTAAAAATAAAAGGAGTACCTTATATTAAAACCCATAGTATTACGAATAT
TGCTTGGAATGAAGAAACCCAGAAATTTTATGATGAAAATTATAAACGGGGTATGGAGTTTGAC
AATATGAATACCCTTTTTGGAGCAGTAATCATGACGGGTAAACCAGTGATTGCCAATAGCCCTAA
AACAGATCCTCGCAGTGGTGGCACTCCTAAGGGGCATCCTCCTTTAAATGCCTTTTTAGGATTAC
CTTTCTTTAACAAAAACAGATTACTTGGTGTGGTTGGCATTGCTAATCGTCTTGGTGGTTATGAT
CAATCTATTGTTGACTATTTACAACCATTTTTAGTGACTTGTAGTAACCTCATTGAAGGTTATAG
GCTCGATCGCGATCGCCGTAAAACAGAGGAAAAATTAGCCCTAAGTAATGAGGAATTAATCAGAG
CAACCCGTCTTAAAGATGAATTTCTAGCCAATATGAGCCATGAGTTAAGAACCCCTCTTAATGCT
ATTTTAGGTAATGCAGAAATTCTCACAGAACAAGTTTTTGGAGAACTCAATAACAGACAAATCAA
ATCTCTTAAGACGATTGAAAGCAGTAGTAATCATTTATTATCATTAATTAACGATATTTTAGATG
TTGCCAAAATAGAAGCAGGACAAATCACCCTAGAATATGTGCCCACCGATATTAAAAGTTTGAGT
CAATCCGCTTTAGTGTTTGTGCAACAACAAGCCCAGAAAAAAGGTATTCAGTTAGAACTTAGGTT
TGCTGATAATCTTCCTTACCTACCATTAGATCAAAGACGCATTCGCCAAGCTCTGATTAATCTAC

TTAATAATGCGGTTAAATTTACTCCACAGGCGGGGAAAGTCACATTAGAAATCAGTGTATTTGAA
GAAGAAGAGAGTCAGGAATTATTAATAATGGGGGAAGAGAATATTATCATGAAAACCTTTATTC
GTTTTGCGGTGATTGATACAGGTATTGGTATTGCCCCAGAAAATATAGAAAAGTTATTCAAGCCT
TTTATTCAGATAGATAGTGCTTTAAATCGCAAATATACAGGTACGGGCTTAGGTTTATCGCTAGT
GAAACAAATTGTTGAGCTTCATGGGGGAAGTGTAGGAGTGTCAAGCGAGTTAGGCAAAGGTAGT
TGTTTTTATTTTAATCTTCCTTCCAATTATTGTAATATCCCTCAATCTTCCATCAAAGAGCAAGA
AAAATTCCCCATCAAAGATAAGATGATAACACCTTCCGTGATTTTGTTCGTGGATGATGATGAAA
GTAATATTGCCACTATTTCTGATTATTTAGAAGCCAAAGGCTATAAATTACTAATTGCTAAAAAT
GGTTTACAAGCGTTACAAATAACTAAAACTCAACATCCAGACTTAATCATCATGGATATTCAAAT
GCCTGAAATGGACGGTTTAGAAGCGATCGCACTTATTCGTCAAGATGAAGATATTAAAGATATAC
CCATTATCGCCGCTACTGCCTTAACGATGGAAGGAGATGAACAAAGTTGCTTAGACGCAGGAGCA
AATGACTATATCAGCAAACCCTTACGACTCCGAGAATTGGTTAATAAAATCGAGACTTTATTACA
ATAAACCTCTGTTCGGCTTAAGAATATCGAACAAGTGTAGTGTTAGATAATAGGTCATAGGTTAC
AGGCGTTGAAAGTCAGAAGTCAGAAAAATTAATTTTCTTCCCACGCCAACATACATCCCATGCTT
TCCATGCTTCCTTTTCTTCCCACTTTCATCAACTCCGAACTCCGAACTCCGAACTTATTACTCGAT
CATGTCAAAAAAAATCCCCCTTTACCTTCTATTATCCCTACAATTTGCCATTCAAGTAATTGGTA
TCGTGGGGATAGTTGGCTATTTATCCTATCGTAGTAGTCGTGAAGCCGTTTATAATCTAGTAGAA
CATTTAATGGATGATACGGGCGATCGCATCGAGCAAGAATTAGATAATTATTTACAACAAGCCCA
ACAAATCAACCAATTAAACATTTCCGCCCTCAAATCAGGGGTGATTAATCTCGATAACTTAGATC
AACTACATCGTTACTTAATTCTTCAACATAAACAACTACCCAATATAACCAGTTTGCTTTTAGGC
ACAGAAAAAGGGGATTTTAGAACCATTCATCGAGTTCAAAAAGAAGAAATCAACAAAGGAATTT
CGATGATTAAACCCACAGATTTACCCCTAGAAGCAGGAATCTCGGAAATTACCCCATCAGGAAAC
AATTTTAACCTTTATACCGTTGATAAAAATGGAAACATAGATCGCTATTTAGACACCCTCCATAA
TTTTGATGTGAGAAAACGCCCCTGGTATCTTGATGCAGTAAAATCTAATAAGCAAGGATGGAGTC
AACCCTTTCAAATTGGCAAGACTAACATCTTAACCATTAACGCTTATGCCCCTTTTTCTAATTCTT
CCCAAGTCATAGAAGGAGTATTTTCGGTTCACTTATCCCTCAATCAATTAAATCAATTCTTAAAA
AAATTATCCGTTAGTCAACACGGAGAAATATTCATTCTCGAACGTAACGGCTTATTAATTGCCGA
CTCATATCACGATACTCCCTTTCATTATCAAATGGAGGAAATTCTCCAACCATCACGACAGGAAG
ATTTTAAGATCAAATTTGAGCGTATTTCTGCTTTTGATGATAAAAAGGGGATTCTTTCCCCCCTT
GCAGAAAAATTAAAGGCAAATAAGGTTAATTTAGATCACATTCAAGCCAAACAACAATTTGAGG
TTCAATTTCAGAATGAGAATTACTATTCTGCGGTTATTCCCTATCAAGACTCCTATGGCTTGGAT
TGGTTGATTGTCATTGCTATCCCCGAATCTCAATTTCTCGACACCATTAAAAATAACGCTAAGCG
TACCCTGATTTTATCCATTATCGCTTTAATAGTGGCAATTATCAGCAGTATTATTACTTCTCGCA
AAATAACTCACTCTTTATCAGGTTTAAGCCACATTGCCGATGATTTTAACCCCAAATCTGAACCC
CCACCATTGCAACTGTCTTATATTCAAGAAATTTCTAGCCTCTCAAATTCATTTCATCGAATGAC
AATCAATCTCAATGAAGCAGAAAAATTTAAACTTGACTATACAAGGAAATTAGAACAAGAGGTA
AAAGAAAAAACAGAAATTTTAATCGAAGCCCAAAAAATCGCCCATACTGGTAATTGGCAATATA
ATCTTATCACTCAAGAAGTAATTTGGTCTGAAGAACTATACAACATTTATGAAGCCGAAGATATG
ATGCCAGTACCTCACCCTGATCAAACTATTCAAAAAATACATCCAGATGACATTGAACGTTACCA
AAACTTAGTCTTAAAAGCAGTTGAAAAGGGTCAAAATTTTAATCTCGATTTAAAAATCATTACCA
CCAAAAATAATATTCGTTACATTCAAACTAAAGGAAAGCCCATATTTAATCAAGAAGGTAAAAT
TATTAAATACGTTGGAGTTGTCATAGATGTTACGGAACGGAAAAAAATCGAAACCGAATTATAT
CTAAAAAATCAAGAGTTAATGAGGGCAACTCGGCTCAAAGATGAGTTTTTAGCGAATATGAGCCA

TGAATTACGCACTCCCCTTAATGCTATTCTTGGTAACTCGGAAATTTTAAGTGAACAAATATTCG
GTACATTAAATGCTCGTCAATTAAAGGCGATCGCAACCATAGAAAATAGTGGGTCTCATCTGTTA
GACTTAATTAACGATATTTTAGATATTGCCAAAATCGAAGCAGGAGAAACAACCCTAGAACTTAA
TCCGACAGATGTTAAAAATTTATGTCAATCGGTTTTAACTTTTGTTCAACAACAAGCCTACAAAA
AAAATATTCAACTTTTCACAGATTTTCCGCCAGAATTACCTTATCTATTAATTGATCAAAGACGT
ATTCGTCAAGCCTTAATTAATCTCCTCAATAATGCCGTTAAATTCACTCCCGAGGGTGGCAAAAT
AACTTTACAAGTGAGCATAAGTGAAGATTTATCCCCCCTTAATCCCCCCTTAATAAGGGGGGAAG
AGAATAGCGTCATGAAAACCTTTATTCATTTTGCGGTGATTGATACGGGTATTGGTATATCCTCA
GAAAATATCAATAAATTATTTAAACCCTTTATTCAGATAGATAGTGCCTTAAACCGTAAATATAC
AGGCACAGGCTTAGGTTTATCGTTGGTGAAACAAATTATTGAACTTCATGGGGGAAATGTGGGA
GTATTAAGCGAATTAGGTAAAGGTAGTTGTTTTTATTTCGATCTTCCATTCAATTCTTACTGCAA
TATCCCTGAATCTTCCATCAAAGATCAAAACAACTTCCCGACAACAGATATTACTATCACTCCCTC
TGTTATTTTATTAGTCGATGATGAAGAAAGCAATATTGTCACTATTTGTGATTATTTGGAAGCCA
AAGGCTATCAACTGTTAATCGCTAAAAATGGTTTACAAGCCCTAGAAATAACTAAAATTCAACAC
CCTGACTTGATTATCATGGATATTCAAAATGCCCGAAATGGATGGCTTAGATGCGATCGCACTTAT
TCGTCAAGATGAAGATATTAAGGATATACCCATTATCGCCGCCACCGCCTTAACAATGGAAGGAG
ATGAACAAAGTTGCTTAGATGCAGGAGCGAATAACTATATGAGTAAACCCTTACGACTTCGAGAA
TTGGTTGATAAAATCGAGACTTTATTACAATTTGAACTCAGGTAGGTTGATGGATAAAGTGATT
TCAATTGCTTAACTGTTCAGTACGATTAGGGGCTTCTGTATTGCTTCCTGTAAAACTTTGGAAAA
GAGCAAAACCTGCAATCGCAGCTATAACAATACCGCCAACAATACCCGCCATTTTTAGTTTATTA
TTTGTGGGTTCGGGTTCAATGGTATCCTCCGTTGGGTCTTCTGAATGGGCATAACGATGGAATAA
GAAATCTAAAGCATAATTCCGTAATTCATAATATCTAGGATCTTCTGTGATTTGGGAACGATTAC
GGGGACGAGAAAAGGGAATATCAAGAATTTCACCAATTTTTGCACTAGGACCATTGGTCATCATC
ACGAGGCGATCGCACAGAAAGAGAGCTTCATCAATATCATGGGTAATCATCATTACCGTTAAATT
ATGTTCAGCACAAATTTGTAATAATTCCTCTTGTAACTCTTCCTTGGTAATGGCATCTAACGCCC
CAAAAGGTTCATCTAAAATCAAAACTTCAGGACGAATTGCCAAGGCACGGGCAATGGCAACCCTT
TGTTTCATACCCCCAGAGATTTGATAGGGTTTTTTCTGAGCGGCTTCAGTTAAACCAACCATCGC
TAAATGCTCTTTAACAATAGTGTCTTTTTCCTGTTTATTTTTGTCAGGATAAACCGCATTAACTG
CTAAATAAACATTTTCATAAACTGTTTTCCAAGGAAGCAGACAGTAGTTCTGAAATACCATCATG
CGATCGGGTCCTGGTTCAGCAATGACTTTATTTTTGAGAGTTACTTTACCAGTGGTGGGCTGATT
GAAACCTGAAACCATGTTAAGCATAGTTGATTTACCACACCCTGAATGTCCGATAACACAGATAA
ATTCTCCTTCCTTAACGGTTAGGTTGACTCCTTCCAAGACAGTGTAATTTCCGTTGGGAGTGGGA
TACACTTTGGAAATATCTTCAATGGTTAAAAATTCTGTTTTGGCTAAAGTTGTAGTTTTTTCCTC
GGTATTCATTAAACTTTGCATGGCGTTTTAGTAGTGAATAAATGTTAATTGGGAATGATTAAAA
GGGTAAAGGGCAAGGGGCAAGGGGCAAAGGTAAATAGTGAATAGTTGAGAATTAAGAATTAAGA
ATTAAAAACTCCGAATACCTGTACGGGTGAATGGCCATTCGCCCCTACTTCCCCCTCTCACCTCAT
CACCTTAACTCCTGACTCCTGCGTTAATTTCGATTTCCGATACTTCAAAATTACGGCGGATGGTG
AATCTTTCTAAATAACCGATGGGGTCATCTGGATTGAATACCATCTTGTCAAAGAGTTGGAAGGA
GGTGCGATCGCTCTCTAGGTCTGGAATGCCTAATTGACGACAGGCTTCCCCAAATAAATCAGGGC
GACGAACTCGATCAATTATTTCTACCCAGTTGCGGGGGAAAGGAGTATAACCCCATCGAGCTAAT
TGAGTTAAAATCCATAGGGCTTCTACTCTTCCCGGACAGTTGGCGCGATCGACATGAAATTGATT
GAATCTCAATAGAGGTTCAGTTTTTGTACCGTAATTGTAAGGATCAACAAAACCGGGGCGAATAT
ATTTTTGCTCAACTCCCACATATTCGGGACGAGCTAATATTTCCACAATTTCTTCTCGATTACGAC

GATCATCACAGTAGGCACAAGCCTCCATCAATGCTTTTATTAAAGCCAAGTGAGTTTGCGGATAT
GCTTGAGCCCAATCTTCTCTCACTCCTAATACTTTTTCTGGATGACTAGCCCAGATGTCTAAATCA
GTGGCAATGACATAGCCAACGTCTTGTAAAACTGCCTCAGAGTTCCAAGGTTCGCCGACGCAATA
GCCGTCAATATTACCAGCTTTTAGGTTTGCTATCATTTGTGGAGGAGGAATCACTGTTAAATTGA
CATCATTATCAGGATCGATTCCCCCTGACGCTAACCAATAACGTAACATCAGGTTGTGCATGGAA
GCAGGGTGTACCATCCCAAACGTGTGTACTGTATCTGGAGTTTGATGGATAATTTCTCTTAACCC
ATGAACATCGGTAACTCCCATTTCTTGAAATTTTTTCCCTAGGGTAATAGCGTTACCATTACGAC
TTAAAACAAGAGAAGTAATGATGGGAATAGATGGTTTACCTCCTGCTCCAACAGTCATACTGAGG
GGCATTCCCGCCACCATTTGCGCTCCATCAAGACTTTTTTCAGCGACTCCTCTAGCGATATGTTGC
CATGAATCCTCCCTAACGAGATTTACTTCTTCTAAGCCATATTCAGCAAAAATACCTTTCTCTTTG
GCAATAACTAAAGGCGCACAGTCGGTTAAAGGAATAAAACCGATATTGAGATTAATTTTTTCTAA
ACCATTACGGGCAATTACTTGAGGACTAGCAGTAACTTTGTTTTTGTTCGATCGCTTCTGTTGAT
TGAGGAAGTAAACGATTTCATTACGGAGACTGTAATAACTGGGATGATTTACCACCTCTAATCGT
TGTCTTGGGCGGGGGATTGGTACTTCGAGAATCTGCCCAATATGAGCCTCTGGCCCTGTAGTTAA
CATGACGATGCGATCGCTCAATAGTAAAGCCTCATCCACATCATGGGTTACCATGACACAGGTTA
CATGATTTTCCTGAACAATCTGCATTAAACTTTCTTGTAAATTCCCTCTAGTTAAGGCATCCAAC
GCCCCAAAAGGTTCATCTAACAGTAATACTTTCGGTTTTGTTGCCAAAGCCCGTGCGATCGCAAC
TCTTTGTTTCATCCCCCCAGAAAGTTGATTAGGACGCTTATTAGCGGCGTGTCTCAATCCGACAA
GGGCAATATTTTCTTCAATAATTGCCTTTCTTTCTCCTTCAGGATAATGACGTAAAACTCGATTA
ACCGCAAGGGCTATGTTTTCTCTGACCGTCAACCAAGGTAGTAAAGAGTAATTCTGAAATACCAC
CATGCGATCGGGACCCGGTTCAGTAACTTCTTTTCCTTCAAGAATAACCCCCCCATGAGTAGGATT
GAGTAAACCTGCGATCATATTTAAAAGGGTTGATTTACCACAACCAGAATGTCCCACAAGGGAGA
TAAATTCCCCTTTTTGAATGGAAAAGTCAATATTTCTTAAAGCAATATATTCACCACCATTAGCC
AGAGGGAATATTTTATCAACATGATCAACTTCAATAAAATTTGACATTTTTTTCAATAAACAATT
AATAATTAATAATTAGCAATTAAATAACTCCTAACCCCTAACCCTATACCGCTTTGCGGAACGAG
CGCAGCGAACTCTCCGAACTCTTATCATCTATTTTTGTTCACCCGGAACGACAATACTGGCAATA
AAACCAACAAATCTATCTAGTAATAAACCGACTACACCAACATAGATTAAAGCAATAATAATTTC
GCTCATTAAAGAACTGTTATAAGCATCCCAAATAAAGAAGCCAATACCTACGCCACCAATTAACA
TTTCTGCGGCAACAATTGCTAACCAAGATAAACCGATACCTATTCTTAAACCAGTAAAAATATAG
GGAACTGAAGCAGGAAATAAGATGTTAAAAAAGTATTCAGTTTTGGATAATTTTAATACCCTAG
AAACGTTGGTATAATCTTGAGGAATTTTTTGCACTCCCACGGTTGTGTTAATAATAATAGGCCAG
ATGGCAGTAATGAAAATAACGAAAATTGCCGATGGTTCTGCTTCTTGAAATGCCGCTAAAGCAAT
GGGTAGCCAAGCAAGAGGGGGAATAGTTCTTAATACTTGAAAAATGGGATCTACCGCATCATAAA
TAATTTTATTTGCCCCAATTAGAATACCTAAACCAATCCCAACTACAGCCGCTAAAGAAAATCCG
ATCGCAACTCTCGTTAAACTGGCAAAAATTTGAATTGCTAATCCTTTATCTGTACCTCCATTATC
AAAAAATGGGTCAATAATTAACGGATAAGTATCTTCAAAAACCTGAACAGGACTAGGTAAATTT
GAATTTTGACCTGTACAGACAATTTGCCAAATTACTAAAAATACGATGATAGCAACTGCAGGACG
AATTACTTTTTTAGAATACAAAAAGCTAAGGGGATTTTTTTTACGGAGTCTTAAATTAATACTAG
AATTAGCCATAGTTAAAAAAAGGAATGATTTGAATAAAAGATAAAATATAAGGTGATCATTTTTT
AGCTGAATTGTTGTGGCAAAGAGAAGTAAGTTAGACGCATTTTACTTATATTGTTTATTGGTTGA
TAGAAAATAAATAATACTTGTAATTATTGCCCCTTGCCTCTCGCCTCTTGCCTACCTTAACCGAG
AATTATTGATGTGAAGTTTATGTTAAGCCTTAATAGCTTTGATTTTGAGACTATCTAAATAGGCT
TGAGGATTTTCAGGATCAAATGTGATACCGTCAAAGAAGGTTTCAACCCCACGAGATGGGCTAGT

AGGTATTTCTGACTCAGGCACACCTAAAGCTGTTGCCGCTTCTCTCCATAAGTCTTCCCTATTGAC
TTCATCGACAATTTTCTTGGTGTCGGTAGAAGCAGGAATATAACCCCAACGGATATTTTCTGTTA
AAAACCATAAATCATGACTCTTGTAAGGATAAGAAGCGTTATCAGTCCAGTATTTTTGCATTAAT
TCCGTATCCTCAAAGGTTGGATCTCCATTACCATAGTTATATATACCCTTGGAGCGATCGATAAT
ATCCTCAAAAGGTACTTTAAACCATTCTCGTTTAGAGACAATCTGACACATTTCCTCTTTATTTT
CAGGCTTATCACACCACTGTTGAGCTTCCAACACTGCCATTAATAAGGCTTTAGCGGCTTTAGGA
TTAGCGTCAACCCAATCTTTGCGCATACCAAAGGCTTTTTCAGGATGGTCTTTCCAAAGTTGCCCT
GTGGTAATCGCCTGATAACCCACTCCCTGATTGACGGTTTGTAAGGGCCAAGGCTCACCCACACA
GAATGATTCCATAGCATTAACTTTCATGTTTGCTACCATTTGAGCAGGAGGCACAACAATAGTAG
AAAACTCTTTGCCCGGTTCTAATCCTCCGGCCGCTAACCAGTAACGAATCCAGCAGTCATGAGTTC
CACCGGGGAATGTCATCGCTACTTTTGGATCAGTTACTTTGGCAAATGCCTCTTTGAGAGGGGAT
GTATCTAAACCAACTTTAAGATCTTTATAGTTATTTCCCAACATGATACCTTGACCATTGACGTT
TAACCGTGCCAAAATATACATTGGGGTGGGTTTGCCATCGGTGACAGTTCCCATACTGATTAAGT
AAGGCATGGGGGTTAAAATATGGGCTCCATCTATACCGCCACTAGCCGAACCTAAAACTAAGTTA
TCACGGGTTGTTCCCCAAGAAGCCTGTTTAATTACTTCTACATCAGGCATACCGTATTTTGCATA
GAAACCCTTTTCTTTTGCGATAATTAAGGGGGCGGCATCGGTGAGGGCGATAAAGCCTAGTTTTG
CTCCAGTTACTTCGGGAGCATCTTCAGGAGAAATAACGGGGGCGTTTTCAGGAGTGGTATTTTCG
GTGGTGGTTTGATTTCCCCCTCCTTGGGCGCAACCGTGTAAAATTGCAGTAGTTAGGGCTGTTGC
CCCTGTGGTGAAGATAAATTTACGTCGGGATAGTTTGGACATGAACTATAAATTTCTAAGTAATT
TAAACATTTCTTAAGTTTGATGTACATAAATTTAAAACTTTATTTTTTCTTTATTTTGTATGTTT
AGTCACATTTTTTTGAAAATTTGCAGTAAATGAATTTTGTTTATTCTAAATCTGCATATAAACGA
AAAATAATGGTTAATCGCTAACAATTTTTATTTTTCCTTGGCAGGATAATTCTTCTTCATTAAGA
ATTAAATCAGTAATAGCAACATCATTTCCCAAATCAACTTCAAATTCTTTAATAATTACCATTTC
TGGGAATAAAATGCTGTTAATTTCAATGGGATTAAAAAGTAAAGTATGGTCATTTTTCAAGGTT
AAACTAGTGGTAATATTTATGTTTGATAATTCTTGTTTTTGATTTTTTATCTGTCCTTCTATAAT
TACTTTTTCTGTATATAAAGTAACATTTTGCCAATAAAAGCTATATTTTTCTAAAATTGATTCTG
GATTTTTTATTCCTTTATAATCCAATAGTAAAGCTAATAAATCAATTAATCCTTGGCTTAGTAGT
TTTGAAGCTAATGATTTTTGAATATCTTTTTTCTTTAAGTTAATTTCTCCTTCTGCAAAAATAGG
ATGTAATAACTTTAATGGTTTTCCTCTTAATATGCCTCCTAAATTAACTGCTATTTCTTCGGTTT
TTATTTGGGCATGATTAATACAAATTCCCTGATAGTTGGCTTTTTCTGCTGATAGAAAAACTTGC
TCTATTTTTCCTCTTAAAATTTGTCTATCTCCTGCATGAATTTCTATGTTTAATGTTTCGATTTCT
TGCAATTGCGATCGCACCCATAGTTTAATAGCAGGAGTAAGGATGTGAGAAATAATCTCTGATTT
ATGTTTTGCCATCAGTTTTTAAGATATAGTAATTAAATTTTTGGTTTGAGCTTTGAATTTTATCA
TTGATTAATTGATAATTGAAACTAGCAAAGTGAATCAATCACAGTAAACTAATTGTTGATGAAG
AATGGTCAACTATAAACCAAGAGATTGGGACTTTTGAGTCTGAAATAAGCCAATTATGGAAAGAG
TACAAAAAATTTTAAGGGAATGGGGTATTGCCTCTCGTCGAGAAGGGGAAAAAATGATTTTAGA
AGGGCGGGTAAAAGTTAACGGTATAACCGTTAAATTAGGTGATCTCGCTTCGGCTCAAAAGGATA
AAATAGAGGTAGATGGAAAGTTGTTAACCTCACAACGAAAACCATCTTTAATTTATTTATTACTC
AACAAACCAAAAGGATATATATGTACTTGCGATGATCCCCGTCAAAGAAAAACAGTTATGGATTT
GTTACCAGCAGAATTAAGAATAGGTTGCGGTATTCATCCTGTGGGAAGACTCGATCGCAATTCCA
GTGGGGCGTTAATATTAACTAATGATGGTGCATTAACTGTTCATTTAACTCATCCTCGGTATAAT
CATCCCAAAACCTATTCAGTGACACTTAGAGGAAAAATCCCCCATAGATTTATCAAGAAATGGGC
AGAAGGATTTATGTGGGAAGGGAAAAAAACTCTCCCAGCAGAGATTAATATTAATTACCAAGAT

GAGCAGAAAACTCAAATGCAAATCACCCTCAGAGAAGGAAGAAATAGACAAATAAGAAAAATTG
CCGAACTTTTTGGGTATCCTGTAATTAGTTTACACAGAAGTGCGATCGCATTTTTAAATGTTTCG
TCTCTTTCAACAGGAGAATATCGCTATCTAACCACAAAAGAAGTTAATTATCTCAAAGAATTACA
TTATCAGTAATTTGTATTATTTTACTGATTTAATCCGTCTAATTATCCTAATTAATTTAATTTTT
TATACTGTTTTATCTCTCTAGTAACAATTATGTTATTCCTTCGATTTTTTGCTTCTAAATCATCC
TCTGAAACCGTAGATTATGACGCTGTCAGAAAAACTATATTAGAAGAAATTGGTCAAACACTAA
AACAAAAAAAGACTGGAACTCGATTATACTGAAGAACTCGTCAGTAATCAAGTTCATATTCCTATA
ACTACTGTAAAAGCGATCGAAAATGCAGATTTTACCCATTTGCCTGAACCTATTTTTATCAAACA
AATTATCAACAAATATGCTAACTACTTAAAGATAAATGGAGAAGAATTAAGTAACTGTTTTCCCT
TAGAAAATAATAAAAAAGCACAAAAACAGTATCTCAAAAAATCATCTAACTTCAAATTTAATAT
CCAATTATCTCCGAAATATCTGTATTTAATTTACTTATTTTTAATCCTATTTTCTATTAGAAGTT
TGAGTAATATTTTGGAGTTTAGGGAATTTCAAGAAACTAAACTACCTCAAACAGAAATAGTAGA
AACAGATACCAGCAAACAAAATCAACAACCTCAAGCCATTCCTGCCGTAGAAAAAAAAGAAGAGT
CAGTTAAATCCGAGCCAGAAGAATTAAGTTTAAAACTCACCGTTAAACAAGATGCTTGGGTAAAA
ATAATTGTCGATGGTAAACCAGTTTATGAAGGAATTTTAAATCAAGGAACAGAGAAAGAATGGA
TAGCCAAAGAAAAATTAACTATTCGCACAGGTAACGCAGGAGGGGTAATGATTAGTTTAAACGA
GCAAAAAGCAAAAGAATTAGGTAAATTGGGACAAATTGAGGAGGTTACTTTTGAACTGCCAAGA
AGATCGTGAAATTTAGTAGAGGTTGATGAAAAACTGGTGTCAGGGAATGGGAAATGGTAAATAA
TTGATAATTAATAGACCTCTTACCAAATGAAAATTAAGATAAAATTAGTGCAGTTTGAAATGAA
TTTCACCACTGTTCCCTCTTCCCTTTTCCCTAACTTCTACAAGAAGTCTAATAATTGCGCGAGTAT
AAAACAGGAAACTACTATACAATAAATCTTAATTGAATATACGTAATTAACTATGACTACTCCAG
AACTTGAAATCAATCAGGAATTCAGCGATTTAGAAGATTTTACCCCCTCTAAAATCAGTCATAAA
GACGAAATTGAAACTGTTATTTACAGTTTAGAAGAAAACGATAGTGCCATGGTGCAAAAAACTG
ATGAGGGATATATCTGGAAATTTCAATATGGCACAGTAGAAGTATTTGTACAATTGACGGGAGA
AACAGAGGAAGATTTATTCAAAGTTTGGTCTGTTGTTTTACCTTTACCGGCAAAAAATGAAGCCG
AATTAATGCGTTTATTATTAGAAATGAATTGGGATGGAACTTTTGAAACAAATTTTGGTATTTT
TAATAATCAAATAGTGCTTTCCACTCAGAGAACAGTTGATGACCTTTCTGCTAGTGAAATATCCC
GTGCTATTACCCTAGTGGCATCTCTAGCGGATGAATATGACGAGGAATTACAATCTAAATACTTG
TAATATTTAGCGGACATTTTGCATCAATGGGACAATACTTAACCTCAGTTCGGTTTAAGAATATT
GGATAAGGGTAGGTGTCAGGTTTCAGGTTGCAGGTTGCAGGAACAGCAGGTGTCAGGTTTAGGGG
AGTAGTGAGTAGTGAGTAACGAGTAATTATCAAATATCAACTATTCACCCCAACACTATTTACCC
CAACACTATTTACCTTCGCCCATTGTCCTTTTAAATTTTCTCCTTGCCCTTTGCCTTTTTTTCCAT
CATTCATAGATGAAAATTTATCCCGAACTCAGGTTACTTAATAAACTTCTAGCTTTCTCAAACAA
AAGGCAAAGGTTGAGAGAATTGACTATTTTTTATAGAAATAGACCTCTCCATAAATTATTTTTCG
TTGGCTCGTAGTAAGGGCTTCAGCCCTTAAAATGTCTATTATTGGAGATGTCTAATGGATTTTAA
CTTTCATTTTGTATAAAGATCTAATGAGTAAAGCGGGTAAAATCAAATTAGTAGCCGTTGGGCAG
ACGGTGTTGCAGTCTGTGGAGGATAAAGGATACGGGGGCAAAGAGCAATCTTTGCCTAGTTCCTC
CCGAAGAAGCAGAAAATCCAAGTAGCGATATTTGGAAGCCCACGCCTGTATGCGTAGCATCAGCG
TGGGAGTGTCACATTATGGTGAAAACTAAATTCTCTTACCTTGCCCTAACTAATCAAAGATGTGT
CGAGTAGGTGCAGAATCTGATTCAATCCAAATCAAAAGCGGGACTGAACGGACTTGAACCGTTGC
TAACAGCGCTTAGGAGGCGCTTGCTCTATCCATCTGAGCTACAGCCCCAAATTTTAAGTTTTGTA
AAGAAGATAATTCTAGTTTAGCTAATTTATTGGGTCTTGAACAATTTTTTCTGGTAATTGGGGGA
GATTAGGTTTCCCGATTAGTTATTAATCAATTTTTACAATCAAAGATTATGATGAGTCGAATCAA

AATTTTATCTTGGTTAACTGTGATTTTTTTCTGTTGGACATTACTAGGTTGTAGTGATACGATCG
AAAGTGTTCAAGCCTTCAACAGTGAATCAAAGTCTCCGATTCCAAGGGTGGAAAATATCAGTGAA
GTTGCCCCCCCTAAATTAATTCAAGAATTAAATAAAAACTTTAGTCAGACTAAACCAGAGGTAAA
GATTCTTTCCCCTCAAATAGATGAAATCGTTAATTCTCAAGATGTCTCTGTTAGTTTACAGGTTA
AAGGGTTAGATATATTTAAGGATGAAGAATTAGGAATGGGACCTCATTTACATTTTTTCCTAGAT
GATAAACCTTATCAAGCTATTTATAGTGCCAGTGAGCCTATTATATTAGAAGGTTTAGCCCCCGG
TACTCATACTATTAGGGTTTTTGCTTCTCGTCCATGGCATGAAAGTTTTAAAAATCCCACAGCCT
ATGCTCAAACAACGTTTCATGTTTTTACTGCCACTGAGGACAATAATCCTTCTCAAGATGAGCCT
TTACTCACCTATAGCAGGCCTCAAGGCACTTATGGAGCAGAGCCGATTATGTTAGACTTTTATTT
AACTAATGCTCCCCTTCATGTCGTTGCCCAAGAAAATCCTGCGGATGATATTGCTGACTGGCGCA
TCAGAGTCACTATAAACGGAGAATCTTTTTTATTGGATACGTGGCAACCTATTTATCTGACGGGT
TTTAATAAAGGTCAAAACTGGGTTAAGTTGGAGTATATTGACGAAAATGGTAATGTGATTCAGA
ATGCCTTTAATAGTACAGTAAGGGCAATAAATTATGATCCTTCCCTTCAGGATACCTTGGCTAAA
TTAGTTACAGATAAAATTAGTTTAGATAAAGTAAAAGCAATTGCCCTAAAAGATTATCAAGGGT
TGCAAGAGGTGAAGACTCAAGAGAAGGAAGAAATTATTGAAGAAGAATTATCCGAAGAAGTAGA
GGAAGTGAAAGAAGAAAAATCGGAGATTGTGGAAGAAGAGTTAACTCCAGAAGTCATAGAGGAA
GAGTTAGTTGAAGATGTGACAGATATAGCTGAACCTTCGGTGAATGAATCTAAGCCAGAAAACA
GGAAGGTGGAACAAGATAAGGATATTTTACCGCAAGAAATTGAACCTACCGCAGATTTAACAGA
GGAGCAAAATGAGCAAGAAAACATAAGCGAGGAATTAGAAACAAAAGAAAATAAGCAGGAAGAT
GTGAATGATTCTGATAATCAATAATTAGTAAAATTTATTACTAACCTCGTAAAAAAGGCAAAAG
GCAAACCCCCCTTTATCTCCCCTCTCGAGGGGGGAGGGCAAAGGTAAATAATTGATAATTAATAA
CTTCTAACTTCTGTACGGGCGAATGGCCATTCGCTCCTAACTAACCCTGAACCCCTTACCGCTTGC
GGAACAAGCGCTTGATCGTTCTCTCGTCTTCCCTCACACCTACAACCTGAAACCTCTTTTCCAACC
CCTTACTTTTGTTTCATCACCATTGAAGGATAAATTATTTCAGACAAAGGTTGATGAGACAGACA
ATACCAATGACAGGTAAACAAATCTGGTTCTTGTGTACCAATAGATGCGATCGCAGGTGTGGCAG
GGAAAGGCCAAAGACGGTCAAAAATTATCTGTTGCTCTAATAAACGGAATTGCAATAAGTAAAC
AGAAGGAATAGCCTCAATAGAATCTAATAAATTTTCAGCCAAATGACAAAGTTTTCGGAAAATA
TCTGGGGATAAATGACAAGGTTGTTGATAGTCATTGGGAATCATTCCTTCACTAATGACTGAACC
ATAAATCAATTGTTCATGAGTAAAAATGATGGGCAACCATAAATCCCCTAAATATTGATTATCTT
GACTGGTGTGATAACCAAAATTCTTTTCTACCCATTGTCGTCTTTTTTCTAATTCACGACAAGCA
AAATAAATCTTCTGAGCGGGAAAAACAAACCAATCGGGTAATTTAATCGTCAAAGGACAATATA
CTAAGTTGGATGAAGGGGATAATAACTGTAGAGATTTTGACCACAAATCGGCACCACTAACAATC
TCAATTTCTGAATGGGGATTAGACTTTGTAACTCCTCTCGCTAAAGCTCTAGCCATCCTCTCCGCC
ACAGGGGCATTAGGACTAATTTTACCTCCTTCTACTTGAGCATTGACGATAATGATAACTTTTGA
CATTCTTTAACTGATGTAGTTCTATACTTTATTATCTCAGTAACGGTAACTTAATTCTTGATTTA
ACATGAACTTCTTAGTATTTATTTTTTCATCATTTTAACCTCGCCATTGATAATAACTTTTCAGG
GCATTGCAAATATCCTCTATTTTTACTGGCTTAGAAATATAATCATTCATTCCCACTGCTAAACA
TTTTTCTTTATCCCCTTCCATAGCATTTGCCGTCATAGCAATAATATAGGGTGATTGTTTTATTG
TATGTTTCTCTCGTATTTTTTGAGTAGCAGTTATTCCGTCTAAAATTGGCATTTGCACATCCATA
AAAATTATCTCATATTTTATCTCATTTAATCTATCTAATACTTCCTGCCCGTTGTTCACTAAATC
TATTTTATAACCTAATTTTGACAAAATTTTTACAGTTATTTTTTGAATGACAAGATTATCTTCTG
CTAACAAAATTTTTATATTCTTTTTAAATTGCTCAAGGTTAGAGGGCTGTAAATAAATATTTTCA
TCAGTTTTGAGAATCTCATTTTTTTCTGCTATAACTTCTAGGGTAATAGAAAAAATACTGCCCTG

ATTTAAGGTGCTTTTAACTTCGATTTTCCCCTTCATCATTTGAATCAATTTCTGACTGATGGCTA
ATCCTAAACCTGTACCTTGAGAATGTAAACGAGTTGACTCTACCTGCTCAAATGCTATGAAAATT
TTTTCTAATTTGTCTGAAGGAATACCTAAGCCAGTATCCTCAACAACAAAATTTAATTCAACTTT
ATTTTCTGAGAGTGGAATGGCAATAGTTTTAAAAATAATTTTTCCTTGAGGAGTAAATTTAACA
GCATTTCCTAAAATATTAATTAGTATTTGTCTTAATCTTTTTTCATCTATTTTGATAGTAACAGG
TAAAAAGTGACTGTGTTCAATAATAAAATCTAACTTTTTCTTTTGTGCCGCTACTTCAAACATTC
CTTCTAGTAACAGGAGAAAATCTTTAAAATTTACTACTTTTTCCTCCAAAAATAGTTTTCCTGCT
TCGATTTTTGATAAGTCTAAAATGTCATTAATTAATGTTAATAAATGTTCTCCTGATTCGTGAAT
TAGCCTTAAATCATGTCTTTGTTTTTCCGTTAAAGCTGTTATGTTATCAAATATTTGTGTTGTTC
CTAGAATAATATTTAAAGGTGTTCTTAATTCATGGCTCATATTGGTGAGAAAGTTACTTTTTGCT
AGGTTCGCAACCTCTGCTTCTCGCTTAGTTTTCTCTAATTCAATATTTTTTTCAATCAAATAATTC
CTAGATAATTTTAATTCTTGCAGGGTTTTATCTCTTTCGTGCAGTAAAGCAATAATGAGTAAATT
AACGCAAGCCATTACGGTAATAAAAATTTGTAAATCAACAAGGACTTGTTCTTTATTAGCACCAT
CTAAAATAGAGATATTAATAATGGTTTTATTTATACATAAAATTGAAATTAACGCATTAAAATG
GTTGGCAATTAAAGCCTTAAATCTAAAAGTACACCAGACCAAAGGAAGCAATAAAAAATATTTT
AAATGTAATGAATAATCAAAAGTAAAATAAACAAAAATAACAGTTATAGAAATAAAAAATATTA
ACTCTTTAGCATTATTAATATCATTATAAATCAAACTATTTAATTTTCTTTTAAAAGAAGTAAAA
TTTTTAATTTTATTAAAATATAGCCAGTCTTTCCAAGTAAAAAATAAAGGAGTTATGAGCAAAA
TTGCTGTAATGTTAGAAATCCACCAAGTAAACCACGAAAAAATAATTAAATCTGAACTAATTTTC
CCTTGTAATAACAAAATACTAATACCAAAAGTTGCGTTTAATATTGGTGCTAATAAAGCAGAAA
AAAAGATAAACTTTCCTACTCTTTGTAGATTTTGCAACGGGGATTCTTTACTATTAGAATGGCGA
AGATAGTAAGCAGAAACACCAGTACCAAACATTGTGCCTAATGCCATAATACTAATAGCGATAGT
ATAGGATATGACAGACCAGAAATTATTAAGATTAGAAAACGCCCAAAAATTAGCTAAAAATGAA
CCTAAAAAAACACTGGGTAAAACTTTATAACCCCATAAATAGACAAAAGCAACGGCAAAGCCATC
GGGAAACCAAACAGGGGTAACGGAATCAGGAGTCGATGCTAACCAACGACATAACAGGGAAATA
GCATAGTATAGTAGGGCAAAGATAAGTAAGACAAAAGGATTGCCTATTTTTCTAATCTCAAAACC
ATTGATGAGGTTTTTTGGTTTAAATGTCATTGTTGATTAGGCATGGTGCTTCAATGTTATATAAA
ACTTAATACCTGTGCTAAGGTGTGGGTAAGGTTGTTGATACTGTAATAATTTTCGCATCTCTGAC
GAGCTAGTTTGCCTCTTTGTTGTGCCTCTTCATAGTCACTGAAAAGAAGACTTATTTGTTGTGCA
ATTTCTGCGGGGGATTGAGGATTGATTAAGTAGCCTGTATCGCTCAAAATATGGGGAATTTCTCC
TACTTTCGTGGCGATTATTGGTTTTGCCATTGCCATTGCCTCAATCAATTCTAAGGGGCATTTAG
CAATATTTGCTGGAAGATTATGGGGAGTTATCATCATTATATGGGAAAGTGCGATCGCATTTTGA
ATTTGGTCAAAAGATTGAGGTTGTATAGTAATTATCCATTTCCCCCATTTTTCCTGTAGCTGTTG
ATAGTAATCTTTATTTTCTGGATTTCCTCCCACTAAAATTAATCTTAGCTGAGAATTATTTAATT
GATCTAAGGCTGTTAATATATCTTCTATGCCCTGCCCTGCTTTACAAGTGTTTGTTACCATCAAG
AGTTTATAGTTATTTAAGCCATATTTTTCTCTCAATTCCTCTCCATTGAAATTGTCAGGGTTAAA
TACTTGAGTATCTCTAACATTAGGTAAATAAGTGCCACCATAACGGTATTCGAGAAACTTACTAC
TAACCGTAATACCGCCCACACGATTCATTGCCTTTTCTAGCCATTTCAAATAAAGAGGATGCCCG
GGTTTTCTCAAATCACTATCTTGACTAAAAACATCATGGAAAATCTGCCCTTGATATTGCCAATC
ATCTCCGCCCCATTCTCCCATTTCCCAATCATCTAAATCAAGAATTAAAGGCTTTTTTCCTCGCCA
TGCTTTTATCAAACCCACTCCAAAACTACTTAATTGCGGTTTAATAACATATAGAATATCTCCGT
CAATTTCCCCACTCAAATCAAACATCGTTTTTACCAAAGAGGGTAAAGGTTTACCATGAAAATAA
GTGATAGGCAAACCATAGGGAGGAGAAGGATAAATTTCACCCCCAAAAAGGAAACCATAAATGT

TTACCTGATAACCTAATTTTTGTAACCCTTGAGAAATAGTATAACCCCTTGTTGTACCGTGAAGG
GATAAATCGGGTATTAAGACTGAGATTTTTTTTTCACTCATCGGTTTCTGGTTAAATAAAAAAGG
TATCAGAAGTCAGAAGTTAAGGTTTTAGAGAGAAACCTATTCTTAAGAGGCTGTGTGACGAAGC
GTCTCCGCCACCTTGTAAATGCCGTAAATAACTAATCACTAATTACTAATGACTAATTTTTCTCC
CTTCCATTCTAAACAATCACTCATTTTTTCTCCATCATGATAGGCGGCAAGTAATATTTCGCTGG
TTTTTCCCAATACTATATTACCTGTACGAGAAATTGCGATCGCACCTAAGTCTCTTTTATTTTCCC
AAGCCTCTTTCATGGATTTTTCCATTGCTTCGTTTAAAGACAAGCCATCAGTAACACGAATAACA
ATTTTTGCCGCTAAACACTCATCGATAATATCTTCTCCAATACCCGTGCAACTCACCCCTGCATAA
CTATTGGCATAATTACCCGCAGGCATAGCCGAATCGCTAACTCTTCCCACCTTTTCTAAACCTTTC
CCACCTGTCGAAGTACCCGCACTTATCCTACCATATTGATCTAATGCTACCACTCCAATAGTACCC
CTACGAGCATTCGGGCTAACTTCTGCTACCACTCCTGCCATTTTCCTTGTAAAATTATTTTTTCTT
TCTTCTATCCACTCTTGCAAACGTAAGTCGGTTAAAGGATCATAAATAGGTAACTGTAATTCTCT
GAGTAATTCTGCACTCCCGTAATCTGATAAAACTCTGTCTTCTTTATTTTGAAGGATATTTGCTA
AATTAATCGGATTTTTGACTTTAGAAACATTAATTACACCGCTAAATCTTTGCTTTTCTCCATCC
ATTAAAGCCGCACTCATTCTAATTTGTCCATCAGATTGTAATACAGAACCTGTACCAGCATTAAA
ACGAGGGTTATCCTCCAATAGCTGACAACCTTTGATAACTGCTTCTGTGGCGCTTTTTCCCTCTAA
CAACAAATTATAAACCTGTGTGACGATACCATAAAGAGATTCTCGTATTGCTGTTAATCCCCCCT
CACCCTTTAATGAACTTCCTGCTCCGCCATGTATAATAAGTTTTGGTTGCACTTTACTGGTCATG
ATTATTGTCCACTAATATTAAGTCAATCTCTATATTTTACAAATATTTACATTCTTCAGTAGTTT
ATAGTCATTTAGAGGATTTATTGTGATGCACTCTCTCTCAATTCCCACATGGATAGTTCATGTTT
CCAGTGTTATTGAATGGATAGCGGCAATTTGGTTTGTATGGCGTTATGGAGAAGTAAGTGGCGAT
CGCAGTTGGTATTGGTTAGCATATGGTATGTTACCAGCCCTAATTAGTGCTATGTGTGCTTGTAC
ATGGCATCTATTTGACAATGCAGAATCTTGGCAATGGTTAGTAACCATTCAGGCTTTAACGACTG
TTATCGGCAACATAACCCTTTGTATCGGTGCTTGGAAAATTTGGCAATCATCTATTACCACCTAA
GTTAGAGATAAAATTGTCGGTTAAGGTAGGGAAAAGGGGAAACCTCTCTATATCTTCCCTAAAAA
AGAGAGAAGGGAAACAGAAAAAAGTGCTTTTAATTGGCTTTTAACTGGGATAAACTGATTTTAA
ATAGATTTTTTGTGGCATCAGTTAATATTGAAACTATAAAACCGTTTCGGATTTAAGCATTTTTT
CATAACACCACCAGCCTAATAAAATAACAATAAAAGAGAATACAAATAAAAACCAAAAATGAAT
TTTAATATCACTAAATAATGCTCCTTTTCCCCATATTTGAATTAGAGCCTCATTCATATGAAAAA
TAGGATTAAACTGAGCTAATTGTTTCATTTTTTCAGGAAATAAAGAACTGGGGAAAAAAGTACC
TCCTAAAATTAATAAAGGCACTCCAAAAGTCGCTACAATAGAGTTAACATCCTCGGTTCTTTTTG
CTAATTGAGTACCAAAAATAAAACCTACTCCTACATAACTAATTATACTCAAAATAATGATAATT
AAGCCCAAGAAAAAAGAGCCTTCGATGGGTTTTCCTAACCAAGAAAAGTAATCCATAAATCAGAAT
AGCTTGACAAAATGCGATCGCACTATGAGCTAATAAAATGCCTAAAAAATAAGCAATTCCCATCA
GGGGTGAAATAAATAGACGAATGAGAGTTTTTTGTTCTCTTTCTCCGACAATAGTGGCAATAGTC
CCCCCTAAACAACTAAAAAATAAAGCCGCCCCCATCAAACTCGGAGGGGCAGATATTTTCATTGC
CAAACTCACATCAATTTTAGCTCTTTCCGCAATAATATAACTGTTTAAAACGAGAATTGTTAAAG
GAAAAACTGACCAAAAAATTAAATTTCTTTTTCTCTTTAATAATTCCTGTAAAATTCTTTGAAAA
ATAGCAATAGTTTGCTCATATAATTTCATATTAACTAATTAGAATCCCGTCTTCCTAAATCGTAA
ACAGCACAACAAATAACAGCTAAAATTCCGACACTAATTGACCAATTTCTCCCTAAACCTATCTC
CACTAACCAATTACATAAAAGTCCCAAAATAAGAAAAGGAATAACACTCATCAAAGAAGCTAAA
AAAGCATTTTGAGATTCTCTACCTTTCCGAGTCCGCTCAAATTCTGCTTCAGAAGTATAAAGGAA
ACGTTCAGCAAAATTAAACCAACGATATAAACCATCAATCACCATTTCTCTTAATTGAGCAAATC

CCAGATATAAGGCTAAACACCATAAAAAACTACCAGTAATAATAGCAATATTAATTTCCCAATGA
AAAAACCACATAATAGCAAATAATTAAATTTTAATTAGAATTTTTATTATATAAGAGAATTTTT
ATTTATATGAGATTAGGTAAAAAAATATTTAAAATCTTTGTTTTCTCAACTCATTAATAATTAA
TCTCAGTTCGGTTTAAGAATATCCGATAAGGTTAGGTGTCAGGTGTCAGGTGTCAGGTTGCAGGT
GTTAGGGGATGGGGGGATGAGGTGATGAGGGGAAAGGGAGAAAATAATGCTTTCAGCTTATCCA
AAACTCAGGTTAATTAGCAATTTAGTAATGGGGTGAATAGTTGATAATTAAAAACTCCTCATTCC
TAACTCTTTTCAATTCTCCAACACCCTATTTTTTATACATCATGCCGGGTAATTGGGTAACTAAT
CCTTCTAATTCTAACTGTAATAAAATGCCCGATACTTCCGCCGAAGTTAAATTCGATTGGTTGAC
GATAACATCAAAGGGAGTAGGTGAAGATGCGATCGCATCATATACTATACTCAAAGGCTCTGATA
AATTGGGTTTTGGGGTTTCGAGTAAAGTAGGAGGAGAAGAAGTTTCAGCAAAAAGGGATAATTG
TTGAGGAGAATTTAAGTCAGGAATCGCGCCTAAACTAGATAATAATTCTTTTTCAGTAATGATTA
CCTCCGCTCCATTATGAATTAAACGCAGACACCCCCTTGCTTGAATATTATCAGGAGTGTTTGGT
AAAGTATAAACATCTCGGTTAAATTCCGTGGCATAGTGGGCAGTAATCAACGCACCCGACTTTTC
CGGTGCTTCCATCACCAATATCGCACGACATAAACCCGCCACAATCCGATTTCGAGCGGGGAAAT
TACCCCTTTCAGGGAGACTTCCATATTCATATTCAGTTAAAATAAGTCCTTTTTCTGCAATTTCT
GCCATTAATTGTTTATTATTGCTAGGATACACCCGATCAAGTCCATTTCCTAAAACTGCGATGGT
TCTTCCTCCTCCATCTAGGCAACCACGATGAGCTATCGTATCTATTCCTTCTGCTAATCCAGAGAC
AATAGTAAAGCCATTTTGTGCTAATACCTTACTAATATTGTAAGTCCATCTTTTACCGTGTTCAG
TCGGCTTTCTCGTGCCAACAATGCCGATAAGAGGCGTTAAACCTTGATTTTCAGAAATATCGATT
TCTCCTTGATAATATAAAACGGGAGGAGGACTAGGAATTTCTAATAATAATTGTGGATATTTTCT
TTCAAAAGGAGTGATAAACTGAGGATTTTTCTCTTTATGTTTTATGTATAGTTGCTCTGGATTTA
TTTTTCCCTTTTCTTCTTTGATTTTATTAACTAATTTTTTTCCTATTCCTTCTATTTTTAACAATT
CTGATTCATTTATCTTCCACGCTAATTCAACACTGCCAAAATAATCATAAATTCTTTTTAAAGAA
ACCGCCCCAATACCTTTTATTTTTGACCAAGCTAACCAATATATTTTTTCCTTTTCAATGTTGTTC
ATTACAAATCAAAAATTGATAATTTGAGTATTTTCAACACTTTTTTCATACAAATGCCTAGCCTA
AATCAAAAACTTTCACACGACCTAAGTTCAACCTAAGAATATTGGTTTTAGGCTTTAGTTAATTA
CTAATTACTAATTGTTAATTGTTAATTGTTAATTAGAGATTGCTAATAATTTTCTGTACAATTTG
CATACCAGTCACCGCTTGCCAACCAAAAATTAATAACATACTCATATTTAAGCCAACATGGGTGA
ATCTAGCAAAATCATTACCTTTCTGCATATAAGGTACAAGAGAAACAGACACCGCCACCATTGCC
ATCATACCTAAACCCGCTAGTAAATGAGGACCTACAAATAATTTACCATTGTTAATGTAAGTGAC
AGCCATTCCCCCTACCGTGCCTAATATCACCAATGCCATAAAAATTGAACCGACTTTATGATGAC
GATTGGCAAAATTACCTTTGATTAATTCTTTTCTTTCTTCTTTACTAGCTTGACGGGTTTTTTTG
ACCTTAATGCCAAGATATAACGCATAAATTGCCATAGCAAATAACACCCACATCATAACGGGGTG
TCCAAATTGAGACCAAGTTTTCACTGCTTCGGGTATTTCAAAATTCATGGTTTTGAGAAAACATT
TAAAATAACTTCATATAATTTAACATAATTAGAGGTTGCGGAAAAAGTGGTGTCGCTGAGGGAA
AATTGACCATTTATTGATTAAGCATAAAAGAAACTATCTATTCTGACTGGATATCATAAATAAGT
AATAATTAAGTTATAATCTCTCTGAGTATAATTGATTAAGGTCAACTTTTTCAACATCGTAGCTA
CCAAGGATTTTTACGGTTTTTGTCACTTTTTCTAACTCTTTTAAGGCTTCTTGAATAGGTTTTCC
GTCGCTATCTCCTTGTAAATCAAGGAAAAAAAGATATTCTCCCAAACAGCGCTTGGTAGGGCGAG
ATTCTATCTTTGTCAAATTAATTTGTTTCTGGGCAAAAATTTCTAATGGTTTTACCAACACCCCC
GGAATATTGGCTTCAAAAGCAAAGCCAACCGACACATGATTTCCTCTTGTTTTTCCGATGCGATC
GACAACCCAAAAACGGGTACAATTATCAGGATAATCGTTAATATTTTTAGCTTTGATCGGTAAAT
CGTATAGTTGTGCCGCACGAGAAGAAGAAATAGCACCAGCGGTAGGCTCTTGATTTAAGTGGTGT

AAAGCCTCTGTCGTGGAATTAGTGGGAATTAAACGGGCTTGGGGAAGATTTTTTTCTAACCATTT
CTGACATTGCGCTAATGCTTGAGGATGAGAATATATTGTTTTTATCCCTTTTAAAGAACGACCAC
GAGAAAGCAAATTATGTATAATCGGGATAGTTAAACCTTTACGAATATATAAGCCGTCCAATTGC
CATAAAGTATCGAGAGTAATAGCGACAGTGCCTTCAATGGAATTTTCTATCGGCACTACTGCTAT
ATCTGCTTTTCCCTGTGCTAAGGCATGCAAAGTCTGTGAAATACTGGGTATAGGAATCAACTCAG
ATTCAATGTTATGAATTTCTTTGAGGAGTCGAGAATAGTAAAGTGTCGCTACTTCTGAGTATGTA
CCTTCTGGACCTAAATAGGCAATGATTTTTGTCATATTATCTTATCTCGTCGGAAATGATGCCCT
CTGCCGATATTTATCTTATCAAGGCAGAAGTAGGATGAATCAAAGTTTCTATTTTTTTATGATTT
CATCCCCCTTCCAGAGTTTATCCTAGTTTGTACCAGAAAATACCACTGCGGGAAACTTCTCTTGAG
CTTGAAACATGGTAGTTTTTCCAGCGTTTTCCTGCCAAAAATTAATGACTTCTGTTGCTTGGTTT
AATAATTCAATTTTCTCGGTTTCAGTAATCCAAGTTTTTGTGTCTAATTCATCTTTTAATTCTTG
CCAAGCATCGGTACGAGGCCAGAAATAGTAAGGAGTTAAAGGACTGATACCTTTACTAATAACAT
AATCTACAGCTAAGGCAACATTTTTATCTAACCAAACGACCTTAAGCACGAAACGACCTTCAATG
TTTGTTTCTAATTGATTTTCTATAGTGGTTTCCAATTAAACCTCCGAATAAAACTACTTAAATAC
AAATAATATTTATACAAACTAACATTATACCCTAAATACAGACTAATATCTTTAAAATCTCAAAA
GGAACAGGGCAAGGGGCAATAATTTGAGTTCGAAGAATTCGCTGCGCTCTTACACTCCTTTCAGT
CGTGAACGGAGTTCGGAGTTAATATAAAAAAGAAATTTCCCTCTAAAACCCTAATACCTGAAACC
TGAAACCTAGTACCCTTATCCAATATTTTTAAAACGAACTGAGGTTAAATTAACTCACGAGAGGT
GAGCTTTTCAATATTGGCTTGAGTTTGATATAACAAGTTATCACGACTATCTTTTACTTGGTCTA
TTTGGGGTAAAAGATTACGGGATTGCAGACTCATGTGTATTTGTAAATGAGCTACATAATCACTC
ACATCCTCTTTTTTGCGTTGATGAAAGGCAGATGACGGTAAATTCATGGGATGATAAAAATTTTA
TATATAGCCTGTTTGTTAGCTCAGAATTTATCATTTTTTTTCTGAACAGTCAAGATTATTCAGTG
GTATTAGAATTATTATTGCTAATTTTTAATATTTCTTAACGTAAATGGCAAAAGAAGTCCCTAAT
CTCAGCATAGCGGATTAGCGGATGAATTGTGCCTCGATAAAAAAATATCCGTAGGATTCGACACA
AAACCAAAATCATTACTTATAATAAATTTATTGGCAACCTAAGTTAGCAATAAACCTCAATGCAC
AGAGCGATAAAAGTCAGAATCTATCCCAACAAAACCCAAGCCAAGAAATTATCTCAGGTTATGGG
TTGTTGTCGTTGGTGGTATAACTACGCTCTAAACTTGTGTATTGACACTTATAAAGCTACAGGTA
AGGCACTAAAGCAAGTAGCCCTTAATAAGTATCTCCCTAAGCTAAAAAAAGAGGAAGATACGGCT
TGGTTAGGAGATTGTTACTCACAATGTCTGCAATCAACGACTCTTAATCTGACTAAGGCTTTCAA
AAACTTCTTTGAAGGTAGAGCCAAATACCCCAGATACAAGTCATATCACGGAAAACAGTCTTGTC
AATATCCCCAAAATGTCTCGATAGTTGATGGATGTCTCAAAATTCCGCAATTAGGCTTAGTAAAA
GCAGTTATTCATCGGATATTTGAAGGTGAGATTAAGACTGTAACAGTAAGCAAAACGCCCACTGG
CAAATATTTTGCTAGTATCCTATTCGATACCCAACAAGAATTACCATTAGTAAATATGACAGGGA
AAATTTGTGGTATAGACTTAGGAATTAAGGATTTTGCTATTGTTCATGATGGCAAAAAAACCAG
TAAGTATGCTAACCCTAGACACATCAAAAAACATGAAAAAAACTTAGCTCGGAAACAACAAAAG
TTAGCTCGTAAGAAAAAAGGCTCTAAGAATAGAGAAAAAGCCAAAAAGCTAGTCGCTAAAGTTC
ACGAACGCACAAGCAATACCCGTCAAGACTTCCTACACAAACTCTCCACCAAAGTTGTGAATAAT
AATCAAGTGGTAGTCGTTGAACAACTGAATATCAAGGGTATGGTTCGTAACCACAAATTAGCAAA
AGCAATAACTGATGTCGGTTGGGGAACATTTATCAACTTCCTAGACTATAAACTCAAGCAAAAAG
GTGGGTTATTAGTGGAGATAGACAGATGGTTTCCTAGCTCTAAAACTTGCTCTCATTGTCATTAT
CAAATGTCTGAAATGCCATTAGAGATAAGAGAATGGACTTGTCCTAGTTGTGATAGTCATCATGA
TAGGGATGAGAACGCAAGTAAAAATATTAGAGCAGAAGGTATCAGAAAAGTACAGACGGATGGA
ACAGCCGTCTCTGCATCTGGAGGATCGGTAAGACCAAAAGGCGGACGTAAATCCGTTTTGAGGCA

CGAACCTGTGAAAGATGAAGCTCCCGACTCAGCGATAGCGGTCGGGTAGTAGTTCACTAGCTTTT
GTGAGGATTTGTTTGGGGAAGCTAGTTATCAATGATCAATTTTTGTTCTAGTCGTTGAACTGTAA
TCACATCTGTTAAGTTATATTGCAAGGGGGTAATGGTAATATAGTTGTCTTTGTTTGCTTGAACA
TCGGTAGGTAAATCTGGAGGTAAGTAAATATGTTCAGGTTGTTCAATTTCTTCGACTACTTCCCC
TGCCAACCAATAATAACTTTTTCCTCTGGGGTCAAATCTTTGTTTAAAATTTTCTGTATAGCGTC
TGATACCCTGACGAGTGATTTTGACTCCTTTGATTTCTGGCTCGGAAACGGGGGGAATATTCACA
TTTAATAAGGTTGCTTCGGGTAATGGATTTTCAATTAACTGTTGGATTAACTTTAGAGCGTAATT
GGCGGCGGGTTGAAATTCCTTGATGGTAAAACTAGCTAAACTAAAGGCAATGCTTGTAATTCCTT
CCATTGTACCCTCCATGGCGGCGGACACTGTTCCTGAGTACAATATATCTGTTCCTAAGTTTGAGC
CTTGATTTATTCCTGAAAGAACAAAATCGGGTGGATTATCTTTTAAAATCGCACTTAACGCTAAT
TTTACACAGTCTGATGGTGTGCCAGAACACGACCATGCTGTTACTTTTTCGTGAAAAATTCCTTC
GATTATGTCTGCACGAATGGGTTGATGGAGCGTTAAACCATGTCCTGTAGCTGATCTTTCTCTGT
CTGGTGCTACAACTGTTACTTCATGATCACAGGATGCGATCGCATTTGCTAATGTTCTAATTCCC
TTGGCAAAAATACCATCATCATTACTAATTAATATACGCATTTTTTGAGCAATAAATTTAAGAGG
TTATAAAGTTAGATTGAGAAGTGAAATAAGAAAGAGATGATCAATAAAAATGATTAATTTTTAA
TTAGTTAACATCTTGCAAATTTCTGCCAAAAGGAAACAAAGCAATTGGGATTAACTTAAAATGTT
GTTGAGCAAAAGGAATGCCAATAATAGTAATAGCCAAAATTAAACCATGAACAAGATGAGAAAG
AGCAATTTCCCAACCAAATAAAATTACCCAAATAACATTAAAAATCATAGTCAAAAGATTACTAG
AATCTTCAGCAACGACATTAATTTTACCAAAAGGAGTTAAAGTAGCTATGCCAATTTTGATAGTT
TGTAAACCGAAAGGGATACCGATAATTGTTAAACAAAGACTTAAGCCACCAATAATATAGCCCAA
TCCACTCACAAAACCGCCAAAGATTAACCAAATAATATTGCCAAGTAGAGTCATAATAATTCTCT
AATTTTTATTTTGTAAGAGGTCTATTCCTGATTTTTGAATTTTATCACATCCCGATTTTTGTTTT
TATTTATAGTTGAGTCCATATTTGTTCATATTCTGCAAAAAACTGACTTTCGAGAGGCTCAATAA
ATTCACTTTTTGCTAAAACTTCGGGAGACATTAAGGGTATTTGACGATTAGTTTCCGAGAAATTT
GTTTCTACAGGAGAAATTCCACTACTAAATAAACTAATTTGCCGTGCAGATTTTGACTGCCAACA
AAAATCAATCCAATTATAAATTTTTTCCAACTCCGATGATTGATTGCGAGGTTTTACCCATATAT
CTGCCCATAGAGAAGTGCCAGAAAGAGGAATTACTGCTTTAATGTTATGGTATCTTTCTATTAAG
GGAATTATATCCGTTGACCATCCCACAGCTAACCATGTGTCGCCTAAAATCAGAGGTTGCAAATA
GTTACGAGAATCATAAAATTTGACTTGTTGATTTAGGGTTTTTAATTCCGGCAATAGGTTATTAA
TTTGCTTAAGATTCTTTGTATTGTATGAATAACCAAGTTTTTTTAACACTAAACCAATAACTTCC
CTCGGTTGATTGAGAAGGGAAAAACGGTGTTTTATTTCTGGTTTCCATAAGTCTTCCCAGTCTTG
GGGTTGCCATCCTAAATTTTGCAATTTATCTTCTCGGTAGGCAATCATAGTATATCCCCAACGGT
AAGGTGCACCCCAGATTTGACCTTGATTATCTAATTTGCCCTGATTATTTCTGATAACTAACTTT
TGGAATAGAGGAGATAATTTAGACCAATTTTCTAATATTTTTAAGTCTAGGGGATCTATTAATTC
ATTTTCAATGGCAAATGGTAGCCAATAGTTACCTAAAGTAGTTAAATCTTGTTTGTTGATTTCCC
TTTGATTATTGCCGATGATTGATAATTGGAGAGTGGATTTTGGGGAATTATTATTAAGGTTATT
AATTGTCTTTTCTTGTTGAGTCAATAGAGACTGATAAAGTTTTTCTAGGGTAGATTCTGGTTTTA
GTCTGACAATACCTAATTTTGCTGATTCTTGTTTGATATTTGTAATTAATTGGTTAGGGAGTGAA
CCTTCTAAAATGGCAATGGATAAATCAGAGGCACTTTCACAACCAGATAAAACGCTAGAGAGTGC
AACACTTCCACTACCTAATAAAAATAAGCGACGACTAAGCATAAAAATTTGATCTTGATTTCACC
GATAAGCGAATCTGCTTTCAACTTGGTTTTTATTGTGTTTTTTAAAGTATTTTTTTATGTTTTTA
TCTTTACAATAATTAACAAAATACCGCTGATAAATCTTAAATTAATCAGGAATACTGAGGATAAT
TTTATAACAGACTTTAGCATAGAAAGAAGAAAAGATAAACTATTTTCCTTAATTTTGCGAGATA

TTCTATTTATGACTGATAAACAACAAGATTTGAAGAAAATTCACAAGAGAATTGGCCAAATATA
TTTAATGGTGAAACAATTAGCCGAACACGTGGGATGGCAAGGAATAGATAATTCTCATATGAGT
TTAGACAATGATTTAACGAATCTAACTTCTCAACAAATGGATCTTTTAAATCAATCTTCTTCTGA
TTCTTTTCAACCCTCTACGTTTAATGGTAGAGAAGGTTTTTCTTCCGAACATCACCAGTTATTAA
ATAATCATAAAGATATTTTGGTGGATGATAATTCTTTACAAACTTCTTTTTCTAGTTCTTACTCT
TCTGTTTCTGATAATGTCAACATATCTTCTGAAGAGCAGGTTAATCGTTTAACTGCCCAATTAAC
AGCCGCCTATTACCGTATTGCTTGTTTAGAGGAACAATTATTGGCATTTCGTAATGATTTAGCTC
ATAGAGATGATAATTTTTATCGTTTACAGTAAGGTATTACCCAAATTAGACATAAAATTATGGG
TGGGGGTGAGGAAATTTTGTTAATTGATAGGTTTCAACTCTTTGTTTTTCAATAGTTTTTTCATC
AAATTCAGGTGATTATGAATCTCAAAGCCTTATTTTTCAATATTTCTTTTCTCGAATATCATAAC
TAGGGCTAAATGCCTGAAATTCGAAATTAGGTAGATTTAAAACTGAAGAATAATCTTTTCTATG
GCAGATAATAATTCTTCTTTTGTCATACCTAATAAATGAGATGCGATCGCACTTCCTCCTGCTCCC
ACCCCCTCTTTGACAAAACCTTGCTCATAAACTTGCAAACTGGGAAAACGGGAATTACTAAAATT
CAATTGTGTTGCACAAACAGACACATCCCCGATTGATTGAGCTAATCCGACGGTGTCTCCTGTGG
TGTCTTCGGCTACCCACCGAGTTGTACCAACGATAATATTGTCTAAACAGGTTTCAAGGTTATAG
TAACGATTTAGGGCTTTGATTAGGGCATAGATAGCTAACATTTGTGTGCCTCCTGCCAACATTAC
CCCCACTGTTGTACTAGCAGAAAGAGCGATCGCACTTACTACTATTTGCATCGGATCACCCACTGC
GGCGATAATTTCTAAAGGATCATTAAAATTATACTTACTATTTTTTAACCCTCGGCTGACAATCT
CCCATTTTTGTTGATGATTACATACAGGATGGCTACTATTAACTTTATCTTGAGCTTCTTCGATA
CCTAAAGCAGTTAACACCGCCAAAGCTGTTGTTGTACCTCCTACTACGCACTCACTCAGAATTAA
ATAGCTATCTTCTGTTTGTTGGGCAAGGATTTTTCCCCATTTTAATCCTTCTTGATAGAGATGTT
TGACAGTATCTAGGGGTAAGGCTTTTCCCGTGGAAACACAATTAGCACTTTTTCCTCCTAAGTCA
ATATTGGCAACGGAAGGAGATTGCAGTAACCCTGCATTGAATATAATTACAGGTAGATTAAATTT
TTCTACCACCGCACGACTGATGAAAGTGGGGGAGATTCCTACATCTAGGGGGGGTAAGGGAAAAA
TTGATTTTGTTTGAACACCTTTTACCAAAAATTCTGCATCAGCTAAAGCAGTATAGCGGCGAGAG
TCAACGGTTGCCCCTGCGGTGGAAATGCCTTCTATTAAAGCTGTATTTGTAAACCCTAATGTGCA
AGTGAAAAGAGGAAGACAATTAATATATTTGTTTAACCATCTCTTTCCCTGTTCTAATTGAGTAT
AGATTTTTATATCATTTTTAAAAAGCATCATTAAAAATTAGAAATTAGCAGTTAGTAATTAGCA
ATTAGAAAGGTTCTATCACTTCTCATCGTGTAAATTATTAGTTAGGGTAGGCAAAAGGCAAAAGG
CAAGAGGCAATAGGCAATAGGAGATTATTAAATAATAATTTATAAACTTTTATTTTTTATTTTA
CTATAAACACTATTCAATAAAGGTTATGGAAGTTATTTTTTATTAATTTATTATAACCACTGTAG
AAGAACCATTAGAAACAAAGACTATATTAATTAAAATTATGAGTAGATCAATAGTAACAGGATT
TGGCTTTTTTTCAGGAGTTTTTTATCCTTTTTTAGCTCTTAAAATTATTTGGCAAAATAAAACTT
TATGGCAATATTTAATTATTCCTATTTTGATCAATATTTTAGTCGGGATTATTTCTTATATTTTA
TTACTCAATCCTAGTTTAAATTTTTATGATAATTTTACTAATAATCTTATTCTCAGAGTTGATCA
ATTAATTGATAGTCTCCCTCAATGGCTAGAGTTTCTTACCTATATAATAAGTGCGATCGCATCTT
TTTTAAAAGGAATAATTATCATAATTTTATTGATTTTAGTTGGTTTTGTTATCTTACAATTTGGG
GGTATTTTGGGTTCTCCTTTTTATGGAAAGTTGTCGGAAAAGATAGAAATTTTGAAAAAAGGTA
ATTTAGATCTGATTGAAATTAATATTGTTAGAGAAATTTTTAGGGCTATTTTATTTGAATTAAA
GAAACTATTATTCATTATCGTTTTTAGTATTCCTTTATTTTTACTAAATTTTATTCCAGCTTTTG
GTAATTTAATCTCATTAATCGGCGGATTATCTATCACCGTAACGATTATTTGTTTAGATTTTTTT
GATGCTACCCTAGAAAGAAAAAGACTATCTTTTCGAGAAAAACTGAAATTTGTTTGGGGAGGTTT
TCCTCTTACTACTGGTTTTGGTTTGATTTGTTTAGCTTTAATAAGTATTCCTTTAGTTAACTTAA

TTGCTATTCCTATTTGTGTTTCAGCAGGTACTTTATTAATTTGCGATCATCATACACCACGTTAA
AATAACCTCAGTTCAGTTTAAGAATATCGGATAAGGGTAGGTGTCAGGGAAAACGAGTTAGTTA
GGGGCGGGCCATTCGCCCGTACAGGAGTTAGGAGTTTTTAATTATCAACTATTCACCCTTGTCCT
CCCCCCTCTTGAGGAGGGATAAAGGGGGGTTTGCCCTTTTAACTTTGCCTCTTGCCGACTTTCACC
GACAATTTTTATATTGAAATCAGGTGATTTATAGGATTTTTTTCATATAATTCCCCCATAATAAA
GCCGCCTGAGAGCTACTACTGTTGGTAGGAGAATTATCATCATTACCTAGCCAAACTCCCGTCAA
AATATTATTTTTTGGCACATAACCAATAAACCACAAATCAACCCCTTTATTCGTTGTACCAGTTT
TCCCTGCCTCCTGTTTGCCTAAATAAGCAGTTCTACCTGTGCCTGAAACTGTGACCTGTTGTAACA
TTCTATTCATGGTTTCGGCGGTAGTTTTTTTAATCGCCTGTTTTTGCTCATCTCCAGTTTCATTGA
AACGATAGACTTCTCTACAGGTAGTATGATTATCAAAATCTTCACAATCTCTACCATCCCGAATT
ACTTTAATAGCATGGGGTTTTGACCAAATTCCCTGATTAGCAAAAGCGGTATAAGCCCCCGTAAT
TTCTAAAAGGTTAACTTCACTCTGTCCTAATATTAGCCCCGGCACTGGATTTAAGGGAGATTCAA
CTCCTAACTTTTTTGCCATATTCACCACTGAAGATAAACCCACATCTTTCGCTACTCTCAAAGCTA
CAGAATTTTCTGATTGAGCTAGGGCTTGAGTCATGTTTGTCGCTCCCCCTGTTCTTTCACAGGCTT
TATATTGAAATCCTTGCCATAGCAATGGCGCGCAAGAATAGGTTTTAGAGGCACTTATACCGCTC
TCTAGGGCGGCTGAATAGGCAAATACCTTAAAAGTTGACCCCGGTTGTCTTTGGGCCATTGTTGC
TCGATTAAACTGGCTTTCGTTGAAGTCTTTTCCCCCCACTAAGGCAATTATTTCTCCATTTTTACT
ATTTAAAGATGCGATCGCACCTTGGGAAAAATTATGGGATTTACCGTTGCTATCAATATGATTTT
TGAGGCTTTCTTCGGCAATAGTTTGAATTTTGGGATTTAAACTGGTTTCAATGATAAAATCACCC
TCTTGGGTGAGGTCATCCCCTAATAATTCTCTCATTTCTTGGAAAACGTAACTATAATAATAAGG
GGCAATTACTTGAGATAAAGTCTGTCTAGCTTTAGGACTAATTTCAATGCGCGATCGCCTTGCTT
GGGATGCTTCTTCATTACTGATCATACCCAAATTCTCCATTCTGGCAATGACACGATTGCGTAAA
TTAACGGCAGTATCGTAGTCTTGCACGGGATTATAGGCATTAGGAGCGGGTAAAATTGCCACTAG
AGTAGCTGACTCGTTGATGTCTAATTCCCTAGCTGACTTATCAAAATAAAATTGTGCCGCATCTT
CAAAACCATATAAATTGATACCCAAATACACCCGATTCATATAGGTTTTGAGAATAGTGTCTTTG
CTGTAAATCGCTTCTAATTTGGTGGCTACAACCATTTCTCGCCATTTTCTTGCCAAATTATTTCC
CTACCCACTGCCGGATATAAACTTCGGGCTAATTGTTGAGTAATAGTACTTGCACCTTGTCTTCC
TCCTTCTTGATTTACCAATACTGCTCGAATAATACCAAGAGGATCAACTCCAAAATGCCAATAAT
AACGACTATCTTCAGAAGCAAGTAAGGCTTGGGGTAAGTAAGGAGAAAATTCTTTGAGGCTTTCT
AATTCTCGATGGGGGGATTCGATACGGGGGAAAGTAAAGTTTTACCATCTTCTGCATAAACTAC
CGTTGAACCTCCTGTATGATCAGGAATAGGATGAACTTGGTATTGGCTCCACTGTAAAACCATAA
AACCCAATATTAACAATAAGCCCACACTGGTGCTAAGTAAACCATAACGAGCAAATAACACCCAG
TTGGTAGGGGATTTATCGTAACGAATTTCGATCGCTTCTGCTAATTCTGGAGGTCCTAAAGTGAT
AAGATCATTATGGAAAAGATTAAGGGATTGATAACGCTTGCCTCCACAATATACCCCGTTAGTAG
AATTTAAGTCCTTAATAATAAATTGTAGAGGATTATTTTCATCTCTTTCAATCACGCAGTGAGTT
TGACTAATAATCGGACTGCGGATGACAATATCACAATTTTTACTACTACGTCCTATGATATAGCG
ATCTCCTAATAAAGGATATACTTGAGGTCTTTTTTCTTGACCATTTTTAACATAAATAGAGGGTA
CTTTTGCATTTGCCTTAAGCACGGGAAAATGTACTTGTGCCTGAATTGTTTGCCAAATCCCCGTA
AATGCTTGAGAAAAAGAATTGCTATTTGATTTGTTACCCATATATCTTTAATGAGGAATTATTAA
TTAGAAATTTGCAATTAGCTTTTATAAGTTAGTCACTATCGTTTTAAGAATAGTTAAATGGCTTA
AGTAAGTTCAAAAAATTTATGCCTCATTTTACCGAGACAATGACATTGGGACATGAATTATGAAG
TTAACTATTCACAAAATTAAGAAGCAATCTTAGTTTCCTTTTTAGTCAAACTTTCTTCTAACAAT
GAATTAATTAAGTAGCCCCACTTAATTAAACGTAAAATAGGTTCAGCTAGTAGTAAGGGCTGAAG

CCCTCGGTTGTTTCAAGAACGTTTGAACCCCCTAAAGGGGTAACTACGAACTTTCTTTTCTTTTA
TTGGATCTACTTAGATAAACTATTTATACTTATCTTCTCTTTCTGTGACAATGTTTCTAAACGTT
GGTATAAATTTTCAGGTATTTTTTGAAAAAAATTATCATTATTTTTTCTACCACTAAAAGGTTTA
GGAATAGGAAAACCAGTACTTTTTAGAGCATTAATTGTTTCTTGTAAAGCATCCAAACCGTTTTT
CATTGCTTCCTCTGGAGTTTCACCGTCAGAAATACATTCATTAAAATCAGGGAAAGAGATTAAAT
AGCCACCAGCTTCTTCTTTTGTTAATGGTTTTATTTCAAAGGGATATAACAATAAATTATTCATA
ACTCTAATTCTCTACAAAATTGATGAACTTTTTTATATTGAACTTTTTTATATAAATTGGCTTAA
TTGGACGATGTGCAGGTATTGATAATGTTTTTCCATCACTTCTAATAAAAACCACATGAAAGGTG
CTTTTTTGTCTCCATTCTATACCATAAGTTTGTGCTACAGTTTTCACATTTCCAATCCGCCAATCG
TGAGGATTGAGTTTCATTTTTGCTAAAATTCTATCTGCTTTACTCATTAATTAATATTTTGAGAA
TAGGTCGCTATCTTTGTTCAAGATACAATCAGTTTATCTATTTTTTCGTACATATCTCTCAATCT
CAGATCTTGCATCTAGCTAAAAATGGAATGATGAAGATAGGGAAAAGTTAACAGGTAAAGGTTT
GAGTTAGTTTTTAGAATTAATAAAAAAGGAAAGTTTAATGCGTTTTAGCTTAGAGATAAATATG
TTATAGAAAATATTAAAACTCCTAATAATAACCATAACAAAAAACAAAAACGAGTTAAAAATAA
AGCCTTTTTAATTACTTTTTCATCAATTTCCCTTTCAGGTTTTCCCAACAAGGGTTTAAACTTTTT
TACCCCCTGATAACTATTTTCACCTCCCAATTGCACCTTCAAAATAACAGCATAAATGCCTTCACT
CCAACCAGAATTAGGGCTAGGGTCTTGTATGGCATATTTTCGACATTCTTTAATGTTATAAATAG
GATTTAAAGAAATCAAGCCTAAAGTTAACACCGTTAAACGACAAGGCAACCATGTTATATAATCT
TCCCATTTTGCGCTAAACCAACCTATATCTTGATATGGTTCTCTTTTATAACCTATCATTGAGTCT
AAGGTGCTTAATGTTTTATATGCGATCGCACTTGGTACACAACCAACTATAGGAATAAAACTTCC
CAACAAAGCATAAAATAAGGGTGCAGTAACTCCATCAGTAGCATTTTCTGACACGGTTTCTAACA
CTGCCCGATAAATTTCTTCAGTGGATAAATTATCTGTATCCCTACCCACATAAAAACTGAGACGA
TGTCTAGCTTCGGATAAATTATTCTCTTTTAAAGCCGATAAAACATCTTCCGCCGCATCTGATAG
GCTTCTACCTGCAAAACAACTAGCTAATAAAATAACTTGAAAGACAAACCCTAACCAAAAATTAA
CTCTTGATAGTAAATAGATAATTAACCAAGTAATTACCCCACTACCCAAAACCAAAACTAAGCCC
AAAATAACTCCTGCTATTTTCCTAACGGTTTTATTCTCAGTTTGGTGAATGACGAAATTAACCGA
TTTTTCGATTATCCAACCTATCAACTGCACAGGATGATACCAATTTTTTGGATCGCCTATAAGAT
AGTCTAAAATAGAAGCCAGCCAAATAATGGTCATAAATTTAATGAGTAATTCAGATAAGGCAAA
GGGCAACATTGTTGAATTAATTTCTCCTAACATTCCAAAACCCTAACTTGTGACTCCTGATTTAT
TACTAATTATTTACTCCTATGCCAACTGCTTGAGATAAATTATCTAAATTGTTTTCTTCCAACTT
TCGGCTTAAACCTTGTAAAATATCGGGTACAATCCAAGGACCTTGATAAACCCAACCACTATAGA
ATTGTAGTAAAGTAGCCCCGGCAGTGATTTTTTCCCAAGCGGATTCGGTGTCAAAAATTCCTCCC
ACTCCGATAATGGGTAATTTTCCCTCTGTTTCTCGATAAATAAAGCGAATTACTTCGGTTGAACG
TTGTCTTAAGGGTAAACCACTGATTCCCCCTGCTTCTTCGGTGATTGGCTTTCCTGTTTCATATAG
TATTTTTGTATGTAATCCCTCTTTTTTGATGGTTGTATTAGTTGCAATTAATCCTGAAAGTTGAT
ATTGCTGAGATACCTTGATAATTGACCTGATTTGCTCATTTTCTAAGTCTGGCGCAATTTTGACT
AAAATTGGTTTTTCACCCTGATTATGGCTTTGTAAAGTATCTAAAATTGGCTCTAATTGTTCACC
CCCTTGAAGCGATCGCAATCCCGGCGTATTAGGAGAACTTACATTGATCACAAAATAGTCAGCAT
GAGCTTTTAAAACCTGAAAACTTTTTAAATAGTCTTCTTTTGCGTCATTTAACTCTGTTATCTTC
GATTTACACAGATTTATCCCAATGGGAATAGTTCTTTCTTGTTTTTGCCACGTTTCTTGTAATCT
AAGTGCGATCGCTTCTGCTCCTTGATTATTTGCCCCCATACGATTTAATAAAGCCTTATCCGCAGG
TAAACGAAACATTCGAGGCTTAGGATTACCCTCCTGACTATGGTAAGTAACAGCCCCCAACTCAG
CAAAACTAAAGCCAAAACTAGACCAAATTCCCGCCCCCTGAGCATTTTTATCAAATCCAGGAGCC

AAACCTAAAGGATGAGCAAAATTAAAATTCCAGAGAGACTGAGAAAGATGAGGATAATTAACGC
AAAAAGAATTTTCTAATTGTTGAATAATCCATCTCCCCCAAGTTTTATCTCGTTGAGACTGAATG
TTATGAAGAATATGAAGGGCTTGATTGTGAGCAGACTCTGGATTTCCCTTTAAACTAGCAAAAAA
AAGCGGATAAAATGGTTTAAAAAGATTAAGCATAATAATAATATTTTAAGGTTTTTTGTTAAAG
TTAATTTAACTTAATTAACTGTCAAGAAAAGGTTGAGATATGTTAAGCAATAGTTAAATAAATC
TAAAACGCAAACTGAATAAAAATATAAGTTGATCTTTAATGTAACTGGTTTTTAGCTCAAAAAG
CCTTGATCCCAAGTGGGTAATAATAGGGAAAGATAAAAACAAACGATAAATTTTAACTTAAAAA
GATGACACATAATAACCAGTGTACTTCAGCTTGTCGATACTGTCGATTCTACTCTCCAGAAGGGA
GACGGGGGGGAATGTGTTCTCAGCTAGGAGTTTCGGTGAAAGCTCACTGGAAATCATGCCATTTT
GCATCCCCCGTTTTTGATAATAATTGGCAACCTTTACCAGATATTGCCTTACTGGAAAAATCATT
TTCTTTAGGATGTGCAACTCCTCAAGTGAATTTTACTATTGAATCTAACAAGGTGACGGAAAGTC
ATGTTAATTCCACAGAAGAAATTGTTTCTAATTAGTTTAATTTTATTTACAGCAAGTTGAAATCT
AATTGATAACCATATTGAGAAAAAAAGGGAGTAAAAATAATTATTTTACAGTTATTTATCTGAT
AACACAATGCTGTAAATTTCGGTGGAACTTTTGGTATCATTTTTCCAGTATTTATTATCAAAAAC
GAGAGTATTTTAGTCTTCTGATTCCATGAATAAGATAGTCAACTATTCTCATCAACTTATTTGAT
TATTTATTCATTGTATTAACCCCTTGAAACAACATCACTAAACCAGCAATAACAATTACAAGGGC
AATAATTCCTAAAATTAAGTCTAATTGGCTATTATCTATCATCATCAATATAATATTTAAAATAT
TTGAAGGGTAAAAAGTTTTTTGCTTATTATTGAATAATTTTTTTTCCCTTTTCCCTGCTTAAACC
AATAATTTACATAATCATAATGAGAGAGTCATAATTAATTATGGAAGCCAAGGGTAATCACGGA
AATTAGGAGGACGTTTTTCCAAGAAAGCCTGTTTACCTTCTGCACCTTCTTCTGTCATATAATAA
AGTAAAGTGGCATTTCCTGCTAACTCCTGTAGTCCAGCTTGGCCATCACAGTCTGCATTAAAAGC
GGCTTTCAAGCAACGTATGGCGATAGGACTTTTTTCTAATATCTCCATTGCCCAGTTTATTCCTTC
CGTTTCCAATTCTTCCACAGAAACGACTGTATTTACTAAGCCCATATCTAAAGCCTGTTGGGCGT
TATATTGCCGACACAAAAACCAAATTTCACGGGCTTTTTTCTGTCCCACAATTCTCGCTAAATAA
CTCGCACCAAAGCCACCATCAAAACTACCAACTTTCGGCCCTGTTTGTCCAAAAATAGCATTATCT
GCGGCAATGGTAAGGTCACAAATTAAGTGTAAAACATGACCTCCACCAATGGCATAACCAGCAAC
TAAAGCAATGACCACTTTCGGAATACTACGAATCAACTTCTGTAAGTCTAAAACATTCAATCGGG
GTACTCCTGCATCGTCAACATATCCCCCTTTACCCCTAACAGATTGGTCCCCCCCAGCACAAAAGG
CATATTTCCCATCTGTATGAGGTCCTGCACCAGTTAGTAGTATGACACCGATTTTTTTATCTTCCC
GTGCATCACAAAAAGCATCGTATAGTTCAAATACAGTTTGTGGACGAAAAGCATTACGTTTATGA
GGTCGATTAATTACTATTTTGGCGATGCCATCCCATTTAAAGTAAAGTATGTCTTCGTATTCTTT
GACTGATTGCCAGTGGACATTCATTAGCTGTTAGTCTCTTGTTTTTTCTATGATTGACCGTGAAT
ATTATACTATAACCTTTGCTCCTTGCCCTTTGCCCATTCCTGAATACTGATAATTGGTGCATCTCA
GCATTGAGATAAACGCCATATCATAAATGATTTAGGATTGTTATATTGTAATAATAAGGAAAAT
TTGAGTCTAAAACTTTGGGGTTTAAAATGGCTGAAATCGATAATTTTTTACAGGATATAGAAAG
TAAATATCAACAAAAAAATAAACAAGTATCAAAGGTTAATCATAATCAGTTAGATAGAGAGCAA
AAAAAATCCAATAATTCTGATATTGATAGCTTTATTTCGGAAATAGAGTCACAAAAAAAGGTTA
CGGAAAATCAAGATATAACGGAGGATATAGTCTTAGAGATTGAGGGAAAATTTCAGCAAAAAAA
ATCTAGTAATTCCCTTAACTCAACATCGAATAAGAAAGATAATTTAATATCAGAAATAGAGTCTA
AGTTTAATCAAAAACAGTCTCAATCAAAAATAGATTCTGATGATATTATATCTGATATAGAAAA
ACAATATAAACAAAAACAATCTGCTCATAAATCGCAAAAAAAATCTGGAATTGCAGAGAATTAT
ATCAGTGAATTAGTTACTTCTTATCATCAAAAACAAAAAGAAATTAATCATAACAAAAAATCTG
AAAATTTAGAAGAATTAGACAACAAGAGTTAGAAAAACAAAGACAAGAAAAGTTAATCACTAG

AAAAGCAGAGGTATGGTTAAAAAATTTAGATCCTTATTCTGATGAAGGCTTTTGGTTTGAACAG
TTCGCTCTTTCTTATCCTACTAAATTAGAAGCTGCGATCGAATATTTAAAAGCACTGTCAAGTAG
TTAAAACTAATAATTATTCAGAAATATTTATGCTGACTGTGGGCGGAACATCCATTTCTTTAATT
GCCTTACTGCAAATTGTAATTGCTTTAATTTTAGTTATTATTATTGGCAAAATTTTCAAGAAAAT
ATTTAAGGAAAATATCCTTAATAAATTAAATATAGACTCTGCGAATAGAGAAACATTTGCTAAT
ATTATTAGTTATTTTTTTGGTGCAATATTATTTATTTTATTATTACAATATTTTGGAGTTAATTT
ATCCACATTAGCCGTTATTGGAGGCGGTTTAGGTTTAGGAATTGGTTTTGGTTTACAAGATATAA
CAAAAAATTTTATTAGTGGCTTAACTCTTTTATTTGAAAGACCAATTAAACCGGGAGATTTTATT
GAAATAGAAAATGTAAAAGGATATGTAACAGAAATTTCTACCCGTTCCACTGTGGTTGAAACTCA
GGATGGTTCAGAAATATTTATCCCTAATAGTTTTTTTATTAATCATCGTTTTACGAACTGGAGCT
ATAATAGCTTCACTGCTAGAATAATTATCCCCATCGCAGTTGCCTATCAATCAGATTTGGTATTA
GTTACAGAATTACTATTACAGTCAGCTTATCAAGAATCAAGGGTTGTGGCACATCCTTCCCCAGA
AGTTTTATTAGTTGGTTTTGGAGATAGTAGCCTTAATTTAGAGTTACGAGTATGGATAAATCCTA
TTGATCAAGAACCAGAAATAAGAAGTAATTTGAATTTTATTATTGAATATCTTTTTCGTCAATAT
AACATTACTATTCCCTTTCCTCAAAGAGATGTTTGGCTCAAAAATTATCCTGATGTTTTAGAGAA
TAAAAATATAAATATTAGTCAGGAAAAAAATAATCAAAATCATCATCATGAGTCAAAAAAATCG
ATTAAAGATATGTTGTGGGAAGTAGATTATTTTCAAGATTTTAATGAGATAGAAATACGAAAAT
TAATTGAGTCAGGATATAGAAAAAATTTAGAGAAAGAACAGATTTTATTCAAAGAAAATGAAAT
AGGAGATACTTTTTATATTATTCTTTCGGGTAAAGTTGAAGTATTTGTTGAAAAAATAAATAAG
CATTTAAACTATTTAAAGGCAGGTAGTTTTTTTGGGGAATTATCTTTGATGTTAGGAATGCCTAG
AACCGCTACAGTTAAAGCAGTAGAACCAACAATTCTTTTTTGTATTAATCAGGCTAATTTTAGAA
AATTGTTAATGAAAAATCCTGAACTTTATGACTTAATCGTTAATAAATTATCGGAAAGAAAAAA
TGAGTTAGTAGAAAGACAAAATCAGTTGAGAAGGATGGGATTAATTGATAAGGAGGAAGATGAT
AATAACCCATTAATTTGGGTAAGAAATAGATTGAAAAAATTGATTACTTAAACTGTTCTAATTCC
GCTACTAAACAATATATTTTTCCTTATTATAAAATTAATAGTTTTTAAGATTAAAATATTTTTTT
TTGTCCTGCTACCCATATTGCCTTACGTCTAAATCCCAGTTTTAACCATAATTCCGCCGATTTTAA
ATTACATCCTGCGTTTTGATATGCTTCTGCGGCTTCTTGCCATTTATGTTTGCCTTGGTTTTCACA
AGCATTGCCAATTCTTTCCCAGTTTTTAACTTGTCGCCAGCAGGATTCAGCTTTTTCCCATTTCTG
ACACCTATCGTAACATTCTCCCGCTTTATATAAATTGAGGATTTTTTCGTATATTTTTCCTGCTTC
TTCTAATTTTGACTGTTTTTCTAGGCATTTTGCTACTTCTTCCCATCTTTCCAATTTTCGCCAACA
CAATTCCGCATCCTCCCAATATTGTCCATTTTCATAGGCTTTTCCACTTTTTTCTATATTTCCTAT
TGTGAGCCATATTTGAGCTTCTTCCCAGAATTTTCTCTGATTACGAAGACATTTTGCTACTTTTT
CCCAGTCTTCTAAGTCTGTCCAGCATTTTTCGGCTTTCATCCAATTTTTATCATTTTCAAAACATA
TTGCCGCTTTCTTTTTTTCCTTTATTTCTAACCATTTTTCTCCTGCTTCATTCCACCTAAATTGAT
TTTCTAGGCATTGTGCAACTTTTTGCCATTGTTGTAAATTTTCCCAACATTTTTCGGCATCTTGCC
AATAACCACCATTTTCATAACAGATAGCGGCTTTTTCAACTTCATTTATTCGTAACCAAGCGGAG
GCGGATTTTCTCCATTCTTTTTCTTTTTGCCAAATTTCTGCCGATTCTAGCCATTTATCCTGTTTT
TCACACATCAAGGCAATCTTATGCCAATTACCCAATTCTCGCCAATAATATTCAGCGTCTTGCCA
ATAACCACCATTTTCATAACAGATAGCGGCTTTTTCTATCTCATTTATTTGTAACCAAGCAGAAG
CAGATTTTCGCCATTCTTTTTCTTTTTCCCAGATTTTTGCTGATTCTAGCCATTTATCCTGTTTTT
CACACATCAAAGCAACCTGATGCCAGTTACCTAACTCTCGCCAACATTTTTCTGCTTCTTCCCACT
CTTCACATTTTTCATGACATAAGGCTTTTTGTTTAAGGGGGTTGACTTTTGCCCATATTTTTGCC
GCTTCTGAGTATCTTTGTTCTAGTTCAAGTGCGATCGCAACTTGATGCCAAGATTCTGATTCCTG

CCATAACTCCATCGCTTTATCTAAATCATTAACCTGTTGATAACAGTAAGCCGCTTTATTTTTAT
CTTCTATTTTAAGCCAATTTCTAGCACTCGATCGCCATTTTCCTTGATTTTGACAGCAAATAGCCA
CTTTTTCCCAGTTTTCTAATTTTTGCCAACATTTCTCAGCTTTATCCCATGCCTCCACTTTTTCCC
AACACAACGCCGACAACTCAAATTCTTCTGCTTTTTGCCAACATTCCCCCGCTTTATGCCATTCTT
GCAAACTTTCCCAAAGAGGAGAGGCTTTTTCCATTCCTCTAATTTTGTCCACATTTGGGCTGAT
TTGTCTAATTGTTTATTTTCTTGATAATATTCGTTGGCTAATTCATAGTTATGAAAAGCATCTAT
TTCTCTCCCCATTTCCTTTAATTTATCTCCAGCTAATTCCCATTGATTTAACTTTTCTAAGCAATC
AATTTCCTTTTCTTTATTATTACTTTGCTGATAGCAATAAATAGCCATGGCAAAATCATTTTCTC
GATAGAAATAATCTCCTCTCAAATTCCATTCCTCTTCGGCTAAATATCCCCATTTTTTCTGCCATA
AATTATTATTTACTTCTGCCCAACAACGAAAAGCATTAACAAAATCATTAATTTTATCCCAATAT
TCCCCAGCATTTTCCCAATCTTCTTGCAACTCTGACAATAAGGCTTTGACTCTATTTATTTCTTTT
TCATCTTTTATTCTTTCATAAATTTGAATAGCAATTTTATAACTTTTTTCGTTACCTTCATTAAC
GTATTCTTCCGCCGCTTCTCTCATCTTATCTATGCTTTCATAAGACGCAAAAATAGTTTCTAATTC
ATCTAAATTACCTTGAGTTACTAAATTTTGAATTTCTGGTTTATTCCAAAAATCTATAATTTGTT
GATCATAAAAATATAACTTATTTCTTGCCCTTGTTATGCAAACGTATAAGCAGTTATATTTAAAA
TTATTTAGCTCCCTAATTGCTTGATTATTACGATTTTGCCAACTATCAAAACCAGTAAAAAAGTT
CCAAATTAAAACTTCATCAAATTCTAAACCTTTTACTTCTTGAATCGTTAAAATTCTTTCGCTAT
CTTGAGAAAAATACGAGGCTAATCTTTCTTTTTCATTTTCATCACTAACAATAATGGCATTTTTA
AGACCAAAAGAGGCTTTTTCTTGAAAAATTTGCTCAGGACTCTCTTTTAAAATAAAAGGTTTTAT
TTCTGATTCTAACCATGTTTCTTGTTTAATTAATTCTGATTGATTATTACTATTTTCTCGGATTA
TGTTGACAATAGCTGAACCTAAATTAACGATACCACTATAAGAACGAAAATTGATAGTTAAGTG
TTCAGGCTCAATTTGTTCATTAATTTGATTGATTTGATAATAATTTTGATAGGATTGATAAATAT
TGTTTTTTATTTTATTCCAACTAAAACCACTGGGGTTAATTGTTTGGGCTGGATCGCCTGTAAAA
AATATTTTTGGTAAGCTATAGCCATCATTATTTAAAATTAATAATCTTAGTAAAAATTTAACTTG
AATTTGAGTTAAATCTTTGAATTTCATCACAGTAAATCTCATGATACTCTCCTTTATAATTTTTCG
GTATTTGTTTTAATAAATAGTGAGTATAATCTAATTCATCCCAATATTCATTGGATTCTAACCAA
TTTTGATAATTAGTAACTAATTGATAAACTTCTTGATAATTTATATCTGGTGATAAAACACTTTG
CTTCGTTAATTTTTTATATTCCTCATAAGAAATAAGATGATTTTCTGAATTAATTGACTCTATTG
ATCCTTTTAATAAATGTCTAATTTCTTCCCATAATGAGACTGGATTTAACTCTTTTACCCTTGTT
GTTCTAGTCTGATAAAATTCATTTATAAAACGATATAAATTAACTTTATTTTCTCTGATAAATAG
GAATGGATTTCTAATTAAAAAATGGTCAAAATTGAGCAATAAAAACTGATAAAATTTTACCGCA
GATATAAATTCAAATTCATATCTTTTTTTGTGTTTTTTTTCTGGTAATAAATCAATGAGGTACAA
AATGACTGGATGAAGAATGAAACCTATTTTATGATCATCTATAAATTTTTTGCATAAATTATGAT
AATCTATATTAATGAAATTATCTAATTTTTCAGGATAAATACGCTTAATTATATTCTCTGCATAG
TTAGTTAATGAGCGATTATAAGTGACATATAAAACTTGGTAGGACGATTGATCATTTTTTTGATT
GTGGGCATTGTGAAGTGCTAAATAAAGTGCGATCGAAGTTTTTCCACTTCCTGCACTGCCTGTTA
ATAAAGTAGGTAAATTAGGTTGTTGCTTGATTATTTGATACTGTTGAGATGATAAAATTAAATC
AGGATCAGTTTGACTTTCTGGATTGATTGTAAAGGAGAAAAAATCACTTTCTTGATGAGTGAAA
TAACTATAATTAATACTTAAATTATTATCACGCCAAAGATGTTCTTGTACAAGATTTTCCTCTAA
TTCTTCATGGCTTAAATTTTCAATATCTTCTACGCTATATTCTCCGATACCATCTATAGGAAACT
TACCTCTATCCAAATCGTCATGGGGGATAAAATCAACGATTTTAATAACAGAATCATCCTGATTA
TCTTTTAATAAGGTTGCGATAATTCGATAGTTTAAATTATATCTAATACGAAAAATCTTTAAGCT
ACTATTAATTTTTAATTCCCTAATTTGACGGTAGGGAATACTATTATGAGTAATTCTTCTTACTT

TAAATAAAAAACGACTACGCTCTTTGGGGCTTAAAGCTCTAATCTTATTCCAAACTCGAACGGAA
AATAAAATTGGTATAGTCACGGGAAGTCAGAATTTGAAAATAATGAAGAATTAAGGGCTTATAG
AACCTCTTAAATCTTTACTAACTTAATTCAATAATGACATCCAGAAAATAAGTGAATTACAAAAA
GTAATAAAATTTAACAAATACATTGGAATAAAAAAATATAATCTGACACAGACATCTTACCCATC
ACGTATCAAAAATAAGAAATCTTGTTTCATAAACCACTCTTTGGTTACTGAGGAATTAATTTAAT
CAAACTTCTTAATGTCGAATTAACAGACTCTGAATCGGGAAAATACTTTTTCACGTCAGGATCAA
GGATAATTATGTCAGAATTATTTTCTGGAGGGAAATATTCTGTTTTTCTCGTACCGTCTTCATAG
TGAATAGTAACACTGTGTCCTTTTTGATACTGTTGATAATATTTACCTCTCACTGGGTTTTTGAG
TTCAGTTAAATCATATTCTTCTCTCAAATTATCTTCTAACTCACATTGTATTTTATTTTTATCCT
TCATAAAACCTCCTTTCTTTTGGTGTAACTAATCTTGCACTAATTAAACGAATTTTCTTACCACG
TTCGACAAAAGAAACAAACAAACATCTACCCTGACTAGACAAACCTATTATAATATAGCGATTTT
CTGTTTGAGAATGATCGGGATCGTCAAAATTGATTGATAATGGATCGTTAAATACTGAACTTGCT
TCCTCAAAAGAAACTCCATGTTTTCTCTTATTTGTTTTAGCCTTACTATCATTCCATTCAAACTCT
AAATCCAATTAACTTTCTCTCCTTTTTTGCTTAAAATTTCATGAATTTTTGTTGGGTTACGTTAA
CACTAACCCAACCTACAGAGTTATCAATAAACGTTAATAAAAAAGGGTTGAACAATGTTCAACCC
CTACGATATAAAAGGTGAAACAGGCATCTTGCCTGTAGGTTTTAAGGAATTACATCATTCCCATA
CCGGGCATTCCCATGCCACCCATGCCGGGCATTCCTCCGCCCATGCCACCCATATCGGGCATTGCA
GGTTCAGGGGCGGGTTTTTCTACCACTAAGGCTTCGGTAGTTAAGACTAATCCAGCAATGGATGC
GGCGTTTTGTAAGGAGGTACGAACTACTTTTGCAGGATCGATAATACCAGAAGCGATTAAGTCTT
CATATTGTCCTGTTAAAGCATTGTAACCGATATTAGCATCAGATTCTTGAACCTTGGCAACAACG
ATCGCACCTTCTACTCCTGCATTTTTAGCAATTTGAGCGAGAGGGGCTTGAATGGCTCTGAGGAC
AATGTCTGCACCGATTTTCTCTTCTTCGATGGTAAGACTATCTCTTAATTCGGCTACTTTGGGAG
ATAAGTGAATTAAACTTGCACCACCTCCGGGTACAATTCCCTCATCCACTGCGGCTTTGGTAGCGT
TAAGAGCGTCTTCGATGCGTAATTTTCTTTCTTTTAATTCAGTTTCGGTAGCCGCACCTACTTTG
ATGACTGCTACACCACCAGCTAATTTAGCGATACGCTCTTGTAATTTTTCGGTGTCGTATTCAGA
ATCAGTTTCTTCGAGTTGTTTACGGATTTGAGCAACTCTTTTTTCTATTTCGGCTTTATTTTCTCC
ATCGGCAACAATAATAGTGTTGTCCTTCTCGATGGTGATTTTACGAGCTCTACCTAAGTCATCTA
AATTGGTAGTATCAAGGCTTAAGCCAATTTCTTCAGAAATCAAACGTCCGCCAGTTAAGATAGCA
ATATCCTGTAACATGGCTTTACGGCGATCGCCAAAACTAGGTGCTTTGATAGCGGCTACATTTAA
GACACCACGGGCTTTATTAACTACGAGGGTAGCAAGGGCTTCTCCTTCAATGTCTTCGGCAACAA
TGAAAAGAGGTTTACCTGCACGGGCAACATTTTCTAATACAGGGACTAAATCAGCAATAGAGCTA
ATTTTTTTATCGGTAACTAAGACGAGGGGATTTTCAAATTCAACGATTTGTTTTTCTTGATCGGT
GACAAAATAAGGAGACATATAGCCTCTATCAATCTGCATTCCTTCCACTACTTCTAATTCGGTGG
CTAAGGATTTAGATTCTTCTACGGTAATTACGCCATCTTTAGTAACTTTATCCATAGCGGTAGAA
ATCATTTGCCCGATTTCTTCATCATTTCCTGCTGATACGGTAGCAACTTGGGCAATAGCGTCTCCT
TCTACGGGTTTAGCCATAGCGGAAATTTCTTTCACTAAAAGTGCGATCGCCTTATCAATACCACG
ACGTAACGCCACGGGGTTAGCACCAGCAGTAACATTTTTCAGGCCCTCTTTAATAATTGCTTGAG
CGATAACTGTAGCGGTGGTTGTGCCATCTCCTGCAATGTCTTTGGTTTTAGAAGCAACTTCGCGC
ACTAAACGAGCACCAGTATTTTCTAAGGGGCTTTCTAATTCGATTTCTTTAGCAACGGTAACACC
GTCTTTAACAATTTCAGGAGCACCGAATTTTTTTTCTAATAATACATTTCTACCTTTAGGTCCTA
AAGTCACTTTCACCGCATTAGCAAGGGCGTTAACTCCTTGTTCTAGGTGTCTTCTGGACTCTTCTT
TAAAAGATACAATTTTAGCCATAGATATTTCTGCTGTAAGACTATATTTATTTTTTTCTCATAGA
TAAATTTAGCACTCTTAGGTCATGACTGCTAATACGGTTATCAGCACCTTTTCTTTGATTAAGTA

ATGAGCAATTAGTATTAAGGTTTTAGGGGATTGGGGTGTTAGGGAAAAATGAATTAGGGGTTAG
GAGCGAATGGGCATTCGCCCGTACAAGAGTTTGAAGTTATTAATTATTCACTATTCATCTTTGCC
CTCCCCCCTCGAGAGGCAAGGTTTTTTCAAAGTCAGGGCAAAATTATCTTGCCCTCACCCCCAACC
CCTCTCCCACAGTAGAGGGGAGCGTTTTTTCTTAATAATTGATTAATTAATACTTAAAAACTCCT
GTATCTGTTACTATTTGTTAGTTTCAAAAATTGACATTTTTGAGAATGAAAAAACCCTGGGGGAT
AAAAGGGGGTTTGCCTTTTGCCCTTTTAACCTTGCCCATTGCCTTTTTTTATCTATTTACTGGTTA
TAGATGATGACTGAAAGTGTTAAGGCTACCCAAAACAAAAGAAACATTTTCTTCTCCACCATAAA
AAATGAGGGAGGCTTGGGTTTCACTCCAACGGCTGATAAAGTGGGGTTGTAAACCAAAAAGAAT
GATTAAAACAGCTAAAATCAGGGCAGGAATTCTCTCGCTCCACAAAACTCTGGGCAAATTGCTTA
ATTCGGGGGTTAAGCGTCCAAAAAAGACTCGATTCACCATTAACAAGAAATAAACTGCGGTTAAG
CCTGTGCCAATTAAACATAGTAGGGTGGGAATGGGAAAAATGGGGAAGCTACCTCTGAATACTAA
AAATTCGGAAATAAAGCCAACCATCCCCGGAATACCGCAACTAGCCATTACTGCTAATATCATTA
AACTACCCGTAACTGGTAAACCTTTTTCAGGATTGAGTAAGCCTCTCAGATAATTTACATCACGA
CTACCTGTTTTTTTATAGACAATACCCACCAAAATAAATAACATGGCACTAATTAAGCCATGACT
AACCATCTGTAAAATGGAAGCCTGTAAACTCAAGCGGGTAGTAGCAGATGCGGCAAGTAAAACAT
AAGCCATGTGGGCAATGGATGAGTAGGCAACTACTTTTTCATATCCTTTTGAGCGATCGCACAC
ATAGCACCATATAAAGCACTAATTCCCGCCAGAAAAGCCAAGTAAGGGGCAACCACGAGCCAACC
TTCAAGGAAAAAACCTACTCCGAAACGTAATAAGCCATAAGTGCCTAGTTTCAACAACACCCCTG
CTAACAAAACCGAAACTGGTGTACTAGCTTCAACGTGAGCATCAGGAAGCCAAGTATGGAAAGGA
AAAATGGGGATTTTAATAAATAAGCCGATTAAAAGGGGAATTAAGAGCAAAACTTGAGTGCCAA
CGGGTAAAGCATGGGACTTCAAAGGTTCAAAAGCAAAAGTTGGCTCTCCACTAAACCAAACTAAG
CCCAAAAAAGAAATTAAGACGAGGAATCCAGATAAAGCGGTGTAGAGTAAAAACTTCATTGCTG
CATAACCTCTTTTTGCTCCTCCCCAAACCGCAATGAGAAAATATAAAGGTACAATTTCTACTTCA
TAAAACAGGAAAAATAACAATAAATTTTCTGCTAAAAATGCTCCTGCAACTCCTCCTGTTAAAAG
TAAAATCATCCCATAATAAAAACGAGGTCTTTGAATATCTTTAGGAGAGATATAAATAGCAATT
AAAGTTAATAAACTGTTGAGAGAAATAAGGGGTAAAGATAAACCATCAACCCCTAAATCATAGC
CTAAGCCGAGCCAATTTAGCCAAGAAAAACTTTCAGTAAACTGAGTATCAAATAAGCTAATGTCA
AAATTACTCCCAATGATGATATTAATAATGAGAGTTGCGATCGCAATAATTAAAGCAATATTGCG
ATAAATGAGAGTATTTTTAAAGCTAGGTAAAAAACAAACAATTATTGCCCCAAAAATAGGCAAC
CATATCAAGGAACTAAGCATATTAAATAATTAATAGTTGATAGTTAATAATTTTGAGAAAAACT
TTTTAATTAAACATTTAGCAGATAATATCTATATAGTTTGTAATGAGGGCTTTAGCCCTTTTTT
GAGGGAATTAATAAAGAAATTTATTATTGATCAATTCCTCATTAAAATGATCCCCAATAGTTAAG
AATATTATTCCATTGTCCACTGAGGATAGACCACATTAATAATACCACTCCGACAATGATGGTTA
AGATATAGAACTGAGATTGACCAGATGTATTATATTTAAGGGTATTGCCTCCGAAAATAGTTACT
AAACTAATCAGGTTAACCACTCCATCAATAATGTAGCGATCGCACCATGTGGTAAACTTAGCTAA
ATTAGAGACAAAAGCCACAACTGTAAATTGATAAATCTTGTCTAAATAGAAATCATAGGCAAGT
AAATCTTGAATGACTCGCACGGATTTTTCCACAGGGCGATAAAAACCTTTTCTGAGGGGAATTAA
AAAGCCGATAACACAACCCAACAAACCAGAGGCGATAATTAAAGGTAAGGCATAACTAACCACCA
TAGCAGTATTGTTTAAAATAGGAGCATTAGGACTTAACCAGAGAGAATAATCAAAAGGTAGAAT
TGGAGCAAGTAAAGTAATAATAGTGAGGGAAACCATCGGAATTGCTAACTGCCAAGGAATTTCA
GGAGCTCGACGGGTTTTAGGTTGAGTTTTGCCCAAGAAAACAGCCCGAAACACACGGGTAAAATT
AAGGGCATTAATCAAATTAACTAATAAAAGAATCCCTAATAACCACCAAGAAACCTCTAAACTAC
CATTAAACCAGCGATGGAAAGTAATTAACATTCCCGAAGGCAATAAAGCAATGATTCCCGCACTG

CCCGTCATGTAAGCAAGGGTTGTACTCGGCATTTTAGACCAAATTCCCCCATTTCCGTAATATTT
TGGCAACTGGTAGTCAGAATAATTGAACCTGCACTCATAAATAGTAAAGCCTTGGCAACCCCATG
ACAGAATAAAATCAAAAAGGCAATATCCACATGACCTAATCCCACCGCAATAAAGACTAAACCTA
GATAAGCACTGGTAGAATGGGAAAAAGTCCTTTTAAGATCTATTTGTGCTAAAGCAATTAATGAC
CCTCCAATGGCAGAAATCGCACCAATTACCACCAAAGTATGAGAAGCCACAGGAGAAAGAGTGAA
TACTGGTTCTAATTTAATTAATACATAAGCACCAGCAGAAACTACGATCGAATTTCTCATAATAC
TAGCTGGATTAGGACCTTCCATCCCCTCATCTAACCAAAGATTTAAAGGGAATTGAGCGCATTTG
CCCGTAGGACCTGCAATTAGAGCTAAACCAATCAAAGTTGCCGTAAAAGTGGGAATAGAGGGGTT
TTCTGCCCATTCTTGTAACTGAGAAAAACTTAATCCTGCTCCATCACTAGATAAAGCAACAATAC
CCATTAATAACAAAATATCCCCAACTCTTTTGGTAAGAAAGGCATCTCTAGCCGCCGTAACTACA
AGGGGCTGTGCATACCAGAAACCTACTAATAGATAAGTAGAAAGGGTTAACAACTCTAACAAACC
ATAACTGAGTAAAAGGGAGTCACTTATAGCAATACCCCCTAAAGCCGCTTCAAAAAAGCCCATTA
AGCCAAAAAATCGGGCTAATGACCAATCTTTCTCCATATAGCCAATGGCAAATATTTGTGCCAGT
AAACTAATAATGGTGACTAACTCTAATGCACCACTACTGACAGGAGAAATTTCGATGGCTAAAGA
CAAATCTAATTCAGCAATACTAAACCAAGGAAAAACAATACTTTGAGAGGGAAAATTACCAATA
TTAAAAAAGATGATACTACCATGAAAAAATGATAGTAAGGTCATTAAAATATTCAGATAAGCCG
CCGGGCGAGGTCCTGTTTTGCGAATAAATCCTAATGCCCAAGGCAGACTAAAAATAGCCCCTAAC
AATCCATATAAGGGGATAAACCATATATGCTGAATAAGAAAATTAATCATGCTTATTGAATAGT
ATGTTTATGTATTAAAATAACTTTATCTACTTTTTTATCTACTTTTCTTGATGAATCGATTTTAC
AGCAATTTGTTAAGAATAAATAGACCTGAGTTCAGAGTTTGAAGTTTAGGTTGTGGGTTAAATT
TTGTTAGAAATGCAAATCAATTTTTCTATATTTAAAATCAATTTTTAACCCTAAATTATCAATTA
TAGCCTAGTAATGTTTTAAATAAGTGCAACGAAGATAAGGGATGTTGCGTTTAACAATAATGAT
ACAATTATTTATTAAGTTTTATTAACAATAACAAATAACTGAAGTATGGGAAATCAAATTGAGG
ATTTATTACAACCTTTTCAAAGATTTGGGATTAATTTGGGATTAAAAAGAATCAAAAATTTATT
AGCTTTACTAGATAATCCTCAAGAAAAAGTGCCAATAATTCATGTGGCTGGAACTAATGGTAAAG
GCTCTGTTTGTGCTTATTTATCATCAATTTTAACCGCTTCGGGATACAAAACAGGACGTTACACT
TCTCCTCATTTAGTTAACTGGACGGAAAGAATTTGTCTTAATGAAAAACCCATAGAAGAAGATAT
TTTAATCGCAATTTTAGAAAAAATAAGATCGGTTATTAATCCTAATGATCCTGAATGTCCCACTC
AATTTGAAGTAATTACGGCGGCGGCTTGGCTTTTTTTTGCTAATTCAGAGGTTGATGTAGCAGTG
ATGGAAGTGGGTTTAGGGGGAAGGCTTGATGCTACTAATGTGTGCGATCGCAATTTAGTTAGTAT
TATCACCTCTATTAGTCGAGAACATTGGCAAAGATTGGGGGATACCCTCGGTAAAATTGCCACGG
AGAAAGCTGGAGTTATTAAGGAAAAATGCCCTGTAGTGGTGGGAAGTCTTCCCGAAGAAGCCGTT
GGGGTAGTCAAAAATCGAGCGAAAGAATTAAACGCTCCCACAATTTGGGTAGAATCAGCCCAAAA
ATTATTCCCTAATCAAGCTGAATATGAAGGGGTTAACTACCCTTTAGCTTTAGGAGGAGATATAC
AATTACAAAACTCAGCAATTGCGATCGCAACTATCAAAATATTACAAGAAAAAGGTTGGCAAATC
CCCACATCTGCCATAGAAGAAGGCATGAAAAACACCCGTTGGCGAGGAAGAATTGAATGGGTGAA
ATGGCAAAAACGAGATTTACTGATTGACGGGGCGCATAATGTCGCTTCTGCTGAGGTTTTAAGGC
AATATGTGGATACCTTGCATAAACCCACTACTTGGGTCATGGGAATGTTAAACACAAAAGACCAC
GAAGGTATTTTTAAGCAATTATTACGTCCTGAAGATCAACTTCATTTAGTACCTGTACCAGATCA
TAGCACCGCCGAACCCGAAGAATTAGCAATATTAGCAAAAACCATTCAACCTCAATTAAAACAGG
TACAAACTTACGGCGATTTATTCACTGCCCTAGATTCTGTTACCAACAATCAGGAAACTGCAACC
ACATCTATTGTCCTTTGTGGTTCTTTATACCTACTAGGATACTTTCTTAGTAAGTTAGGGTAAGG
GCAAGAGGCAAAAGGCAGGAGGCGAAGATAAAATTGATAAGCGCATCGACTTTTAATAAGCAAA

ACTTATCTAAAACTAAGTAAACTTCTATTTATATTATATTGCTATAAATAAAAAATATTATTAG
ACTGATAATAGTTAAGTAAAGTTAATTAATCAGAAAAAAAAGATGTTGAACTTTAATTTGTTAA
TTTTGATGCGATCGCCTTTTAATTTTATCAAAGAAAGATTAGAAAATTTAGAAATTAGCACCAAA
GAAACCGCCGAAAAAATAGTTAACTTAATTCCGAATACCTGCCCTTTTGCCAGAGAAATTAGGCT
TTTTAATCGTTCAATCTTTAAAATTCCGCCTTTGTGTCAACTAAATCCTTTTTATGAAGATTTAA
TGATGTTACGTTTTAGAGCATTATCTTTTCTGTGTGAAATAGGAGAAGATATAACCCCTTATTGT
CAATAAAATTCCTTTTTTTCTTTTATCGATGTTTATTGTCGATTAATCGGTTTCAACTCCTTATT
TTTTAATACTTTTTTCCTTAAACTCAGGTTAATTAACTTTGTTAAAATTAGGCATTAAATGTATT
TTTTAATATTGAGCAAATAAGATAGTGACCAATAATAATAGTTATCAAGATTTATATAATTTAC
AAAGATTTGTTGATGCACAGGAAAAAGTATATAAAACTGTTATCGATGAATTAAAAAATGCAAA
AAAAAGATCCCATTGGATGTGGTTTATTTTTCCTCAAATTAATGGTTTAGGTAAAACTTCAACTT
CTATTTTCTATAGTATTAAAAGTATTCAAGAAGCAAAACAATATATCAATCATGATTTACTTGGG
AAAAGACTAAAAGAGTGTGTAACAATATTGCTCACTATAGAAGAAACCTCCTCAGAAAAAATCTT
TGGTTTTCCTGACTGTATGAAATTAAAATCTTCTCTGACTTTATTCTCGGAAATATCCGATAGTG
ATTCTATTTTTCATCAAGGGTTAAATAAATTTTTTCGAGGTAGTAAAGATGAGAAAACTTTGCA
AATATTATCTACATTAGAACGGTAAAAGATTAGATACATTAATTAATTTTGTGAGGGCTTCATTT
ATAAAATATAAGATAATACTAAATGAGTTGTAATCAATAAATTATTAGAAAAATAAGTTGCAGA
TATTGTCAATGTTCCCGTATTACCTGAGTTTGATATACGAAAAATGGTAAAAGTAAGCTGGGGAG
AGATGAGAAGCGTGGGAAGCATTAAAAGAAAGAGTTTAAAAATGAGTAAAAACAACCATTAAAA
TTAGTAAATGGTGGAGAAAAATCAATTTTAAATAGTTTTAAGTCAATATTAATTATTTTTAACT
CTAAAATTAGAAAGGCAAACTCATAACTCAGGTCATGTTCTATCACTTTTTTGGTGGTTTTTTTA
GACAAATGATTTGGTACTGCTATATAATAAAATAAACTCAAACCTATAAAATATAGGTCTAATC
AAAAAAGAAATAAAATACAGTTTATTAATATGATTAATTATAAAATAGTCCAAGAAGAATTAGG
AAATATTAAGCAAATTACGGAAGAAGAAAAAATTAAAAAACTCTCTCAAGATTACTATTATTTT
AGTCCAATACTGTCAGAAAAATTAGCTGATAAAACAGGAGATTTAGTCGTACAGCCAACTAGCGA
AGACGAAGTTCTGCACATTGCCAAAATTTGTGTCAAGCATCAAATTCCAATTACTATTAGAGGGG
CAGGTACAGGCAATTATGGTCAGTGTATCCCTCTCAATGGGGGAATTATACTAGACATGACAGAA
ATGAATAAAATTCAGTGGATAAAGTCTGGTATTGCCCGTGTTGAAGCGGGAGTAAAAATGAGTA
TTTTGGAAAGGGAAGCTAATAAGCAAGGATGGGAATTGAGAATGATTCCTTCTACCTATAAAAT
TGCTACTTTAGGGGGATTTATTGGCGGTGGTAGTGGTGGAATTGGTTCAATTAATTATGGTCAAT
TAAGAGATAGGGGAAATGTGCATAAAGTTAAAGTTGTTACTTTAGAAAAAGAACCAAGAATTAT
TGAATTAATAGGAGATGAAACTCAACAAATTAATCATGCTTACGGTACAAATGGTATTATTACG
GAAATAGAAATTCCTCTTGCTCCTGCCTATAATTGGTCAGATTTTATTGTAGTTTTTGATGATTT
TATGAACGCTGTAAAATTTGGTCAAGTCTTATCTGATAGTGATGGAATTATTAAAAAATTAGTT
AGTATTCACGCTTACCCTATTCCTAACTATTTTCTTGCTCTTAAACCTTATTTACCCCAAGGAAAA
AGTTGTGCTTTTTTAACTATTGCTGATAATTCTTTTTTACCTTTACAAGAATTAGTAAAAGAATT
TAGAGGGGAAATAACTTATAATCCTCAAGATAAACAAGAAAAACCTTTACAGCTTATTGAATTT
ACTTGGAATCACACCACATTTCATGCTAGAAATATTGACCCAAATTTGACATATTTACAAACTTT
CTTTTGGAATTTAGAGCAAGTAGAGAAAATGTACAAACACTTTGGGGATGAAGTGATGATTCAT
TTGGAATTTTTAAGGGTAGGAGGAAAAGCAATTCCTGCAGGATTACAGTTAGTTAAATTCACTTC
AGAAAAACGACTTAATGAGATTATTAAATATCATGAAGATAATGGAGCAAATATTGCCAATCCA
CACACTTATATTTTAGAAGATGGAGGCAGAAAAAAAGTAGAACCTGAACAACTTAAGTTTAAGC
GAATGGTTGATCCTTTTGGCTTAATGAATCAGGGTAAAATGAGAGCATGGAATCAATAACTAAG

TCAACTAAATTTTTAAGTTATTAATTATGATTTACTAAAAAAATTAACTATTTCTTGAGCTATAA
ATTCTGTCCCATTTTTGTCTAATTTATTATTAGTTAAAGGTGCGATCGCATCTTGTTTTAAGAAA
TCCCAATTGCCCTCAAAAAAATCAGCATAATTAACAATGCGATGATAGCCATAGTTACGTAAACC
ATCAAGGAGAATTTGAGCTTCTGCAAAACCATCACGAGTAATACTAATTACAGGCACATCAAGAC
GTAAACTTTCTGCAAAAGTGCTATAACCCGGTTTAGAAACAACTTTTCCGCATACAGGCATAAAA
TCCACAGGGCGTAAACTATGGCCATCAATTCTTAATAAATTAGGCAAATCAGGGGCATTTTTCGC
AAAAGTAATAAATTGCCAATCAGAGAAACTAGCAAGATTTTCGTAGGGAATTGCCTCTAATCCTA
ATCCTCCAAAAGTTAGTAAAACGGTTTTCTCCTTTGGCTTAATCAACTCAAACTTATCCCTTAAA
ACTTCACAACTATAACGAGGATTGCCCCCTGTTAAACCCACATCTTCAACCAAGGGAAAACTAGA
CATATCCTCTGCTAAAGGTAAACGAAATAAACGCTCAACCTTTTCATAACGACTTCTCAGCCAGT
GTGTAACTTCTTGAAAATCATCCCCCCAAGCCTGATAGATAAAATCCCAGCCGAAATTGCTCATC
ATCCAACAAGGAATACCCGCTTTTTGGGCAATAGTAGAAGCCATCGCTGGAATATCAGCTAGAAT
TAAACCAACCTTATTTAAACGAATAAAATCTACCTCTCCGGCTACAATCTCCGCTTCTTTTTGAC
AATACTCTTTCATACTTTCCAGAGTTGCCCCCAAATCCATGGTTAAACTATCGGATTGAATAACT
CCCACGTCAAACGCCCGTAAACGATGAATAAAATCACCCTCTATATAAGACTCTAACAACCAACG
GGGTGCAGTGGTAACGACTACCAGTAAAATATCGGGATTGAGCTTTTGTACTTGTGATGCTACCG
TAGCCATGCGCACTGCGTGACCAAAACCATGATTGGTTATTGCTAAATATAGAACAGGTCGAGAC
ATAATTAATAATTAAGAATTAAGCTAAAAAATATTCTAATTGATTTAAGACAAATTTATGTAAC
CTGAGTTCGATATAAAATTGTAAGTTAAGGTAGGCAAAAGGCAATGGGCAATAGTGATTGATGT
CCCAATTTTTGGCATAATTTCAGTACATTTCATACCATAATTAAGCAACAATTAAGCAACGCCTA
AAATATAATTAACTGTCTAAGACCTTTATATATTTTGATTAAAGTTTTTTATACTAAGTAAAGT
AATTAATTCTTGTCCAAATTATATTGAAAAAATATTAGATTTTATCATTATTTAAAAATTTTTT
ATTTGCGCATATGCCAACTCAAACTTTTTTTAATTTACCAGAAGCAAAGCAACAAATAATCATTG
AAAAATCTCTTGAGCAATTTGCTAATCACAGTTATGAAGCGGTATCTATATCTCGAATCGTGCAA
GAAGCAAGAATTGCGAAAGGTAGTTTTATCAATATTTTGAAGGGAAAGAAGATTTATATTTAT
ACTTAGTTGATATTGGAATTGCTAGACAAAAATTTTTTATTGAAAATGCTGATTTACCACCTATT
GAAAATGGTTTTTTTCCTTATTTAAGATCTCTATTAAAAACAAGTCTTGATTTTCAAATAAGTCA
TCCCCATTTTACACAAATTTTATTTAGATTACCTAATTATGGAGATATTCCTTTCCGTGACGAAG
TTTTTCACCGCACAAAAGCTGTTTCTATTGCCTTTATCAAAGAAAAAATTGAATATGCGATCGCA
CTTGGACAATTACCAAAGTATCTTAATCCTGATATGGTAGCTTTTATGATCGTAACCTTGGGTAA
TGAATTAAGACAATTTATCCCCAGACACTTAGGAATAAAAGTGGAAAAATTAGTCAAAGAAGAT
ACACCACTAGATGTTAATGCTATTAACCAAACCCTTGATGATTTCGTCAGCACCCTTGAAAAAGG
TATCGGTAAAGATTAAGAGGGATTGGGGTGATGAGGGGAGAGGTGGCAGGTATCAGGTTGCAGG
TGGCAGGTGTTGGAGAAAGTAATAAGTAACGAGTAATTATCAACTATTCACTATTCACTATTTAC
CTTTGCCCCTTGCCTACCAAACACCAATAATTCATATACTCAAAGTAATAGAGCCTTTAAAACTA
TTTGATTCAGCTAAGTAAGGCTTTAGTTTCAGACTCTCCAGCTTTATTCAACATTTTCTTGAGCT
GATAATTGAAACCGATAATTACAGCAACCTGAGTTAAGAAACTACCCAACAAGGGTAAAAATGA
AACCTCCCCTACTGCCACTGCGGGAAAAGGAGAAAATAGCCAAAAATTAGCCAATCCAGAACCAC
TTGTGATAATAAAACTCCACAATGGAGCAATAATTAAGAAACTAACTACACTCGTAGCAATGAGA
TTTCTTTTTCTGGTTTTTAACAGTAACAATAGTTGATAAATGGTGGCATAAATTACCATCATCAG
CGCCCCAAGAATTAATCCTGTGAGCATCAATACCTTATCTTCACCCCATGAAAAGATGACAATAG
CAGGGGTTACATATAAGTTCATCAATAAAATATTAAGTGCGATCGCACCTATAGCAGGACTTTTC
TCTCCTAATAATAAATCTTTAACTAAATGTCGATGGTTAGAATTTTCGTACCGAAAACGACTCCA

ATCAAGTAAAGTTTGTCGTTGAGGAGTTAAAGCAAGAGTTATCAATAAAAGATAAAAGAAATTA
ATAATCAGAAAAACGATTAATTTATCCTTAAAATCAGAACCTGAAAGACTGTTTTCCTGTAATGT
AAAACCAAGGTTGAAGACAATAAAACAAGCGGAAATTAAATAACTATGATATTTGGAAATCGCT
ACCTCATGGGGATTATGAAAACGACGTTTAACCCCCTGCCATAACCAGAAAGTCCAAACACCATA
ATTGGCGATAATTAAAGCAAAACCAGTCCAACTATTTTGCCACCAAAAATTACCATACCAATTAA
GATTACTTAATCCTTCTATAGATAAATAGCCAATGGTATTAGGAGAGAGAAAAGTTGCTTTGACT
AAATAAGCTAAAAACATTCCGGGATAAAAAAGTGCAAACCAATCAAAAGACGTTGCACTCCAACG
ATAAGCCTCTAAAGAAACCCCCATATTAGTTAAACAGAAAAAGAAAATAGCTAAACTACCTAGCC
ATGCCTGAAAACCACCTAAACCATTAGAGATTAAACTAAACAATAAACTAGCTGTATAAAAGAAT
AAACAACTAGCCCCCAACACTAGATAAAAACCGAAAATTAAAACAGGGGGAATATTTGCTTTCAA
ACCTGCGAAAAAATGTAGAGGAAGAGCTAAAGCACCAAAAAGATAAACTAAAATAGGTACACCA
AGCATTTTACCCCCCATAATCGTCATGGCAGATTGGGGAGTTAAACGAAGAAAATTAAGAGTATT
TCGAGTATTTTCTTTAGAAATATCAGTAATTAACATATAACTACCAACTACTAACAGAATAAAAA
TACCCATAATGCTCATGGTAGTAAATAAATCTAACCACCATAATTCTTTCAGAGCCATGATATTA
CCTAATAAATCTTCTTGACACACTCCTAAATTGGAATAAGAATCGGCGGGAGTGGAAGTGCAATA
ACGGTTACTGTATCGATGTTCTAACGTAGGTAATAAATTATTTAAGTAAAGATATAGTAATACT
TGACCAATAATTGACGCTAAAGAAACGATGACAACATTTTTGGGTTTTAACTTACCTTTTACTTC
CCGAAATAATTGGGGATTTCTTGCACCTATAAACTCGATAACATGGGGATAAAATTGCCTAAACA
TACTCCCTATCTCTCCTTATTTATTTACTCAATGAATTATTCTTTTCTCATAATAACGTTAGTTAT
GGTTGTTAAGTTCCTAAATTTAACGTCAAAGAGTGAAGGGAAAGGGGCAAGGGGCAAAGGAAAA
ATTAAGAATTAAGAATTAAGAATTAAAAACCTCTAACTCCTGTAGGGGCGAATGGCCATTCGCCC
CTACTTTCCCCTCATCTCCTCATCCCCCCATCCCCCTATCCCTGACACCTGCAACCTGCAACCTGAC
ACCTGATACCTACCACCTCTCCCCTCATTCCCTCATCACCCCAACACCCTAAACCTAAGCTCCAAG
TTAGACAAGAGGTCCTAAACCAACTTTAGGGGCATAAATAGCTCGATCGCCCAATTCATGCTCAA
TGCGCAATAATCTATTATATTTAGCCACTCTCTCACTACGGCACAAAGAACCTGTTTTAATCTGC
CCTGCACGGGTAGCAACAGCTAAATCAGCAATAGTAGTATCTTCTGTTTCTCCAGAGCGATGACT
AATTACAGAAGTGTATCCCTTACGGGTAGCTAAATCTATAGTGCGTAAAGTTTCGGTTAAAGTAC
CAATTTGATTTAATTTGATTAAAATGGAATTTGCAACTCCTAAATCGATACCTTTTTGCAAACGG
GTAGGATTAGTTACGAATAAGTCATCTCCCACTAATTGAATTTTTGAACCTAATTTTTCGGTCAA
AATTTTCCAGTTATCCCAATCTTCTTCTTGTAAACCGTCTTCAATGGAAATAATTGGATATTTAC
TTACTAATTCTTCTAAATATGCAATAAACTCACTGGGAGAATGGGCAGAACCATCATAGACATAT
TGTCCATCTTTGTAAAATTCACTTGCCGCTACATCCATCGCTAAAGCTACATCTGTGCCGGGTTTG
TAACCAGCAGATTCGATCGCAGCTACTAACAAATCGAGAGCTTCTTGGTTAGAACCCAAATTAGG
AGCATAACCGCCTTCGTCACCCACACCAGTTAGTAAGTTTTTATCTTTTAAGACTTTACTCAAAC
AACCAAAAATTTCAGCACCCCATCTTAAAGCCTCAGAAAAAGACTCTGCCCCAACAGGCATAATC
ATAAATTCCTGAAAGTCCACGTTATTATTGGCATGAGAACCACCATTTAAAACGTTCATCATTGG
CACAGGTAAAACATTAGCCAGAGGACCTCCTAAATAACGATATAAAGGAATGCCCAACTCAGCGG
CCGCCGCTTTTGCATTAGCCAAAGAAACCGCTAAGATTGCGTTTGCCCCTAAATTTTTCTTATTTG
TTGAACCATCTCTATCAATCATTGCTGTATCCACAGCAACTTGATCAAAAGCATCTAAACTTAAC
AGTACAGGGGTAATTTTTTCAATGATGTTATCTACTGCTTTTAAAACTCCTTTACCTTCGTAACG
ACGAGAATCCCCATCGCGCAATTCATGGGCTTCAATGTGCCAGTAGAAGCACCACTGGGAACTT
GAGCAATACCCACAGCACCACTTTCTAAACGCACTTCAGCTTCTACAGTAGGTTTTCCTCGAGAA
TCTAAAATTTCTCTAGCGGTAATATCTTCAATTAACATGGACTCTTGTTCTAACATAAATCAACT

CTATTTAATTTCTTGTCTATTGTTCAACAAAATCTAAACCAATAATACTCCTAGAACTGTCAAAA
ATAATGACAATTTGCTCATCAGCACGATTAAATTGCAAATCCATCACAACTATATCTGTACTATC
AATGCTTGAACCTAATCTCACTCGCATATCTTTAATACCTTTGAAATCGCCATTTTGAGCGATAA
TTTGATTCCACTCAGTTTCTATTTGTGTGGAAAAAATACTTTCCTTGAGGAAAGGATGAAGAAAC
CCTCTTGCTTCGCTGTAATTTCCTTCTGCTATATCTTCCACAAATTGTTGAGCGATAGTTTCAATA
TTTTCAATGTTAGGAAAGTCAACGCCCACAACTTCTTGGCTATCGTTAAAAATAACGATCCAATC
TTCGGTGACATTTTCAAATTCTAAGGTAACGAAAACTAAATCACTGCCGGGAGTTTCAATGACTT
GAGTCTGCTTTCTTCCTTTGAACTCGCCATTATTTGTATTAACTCTATTCCATAAACGCTCCATTC
TTTCTTGAGATAAATCATTTCTTAATTCTGGAGTTGCATAAGGAGTGATTTTTTCAAAATCCTGA
GCAAAAAAAAGTTCAATGATTGTTTCTGCTTTTTCTTTAAGGATTGCGGATGTATCTTGATTGTT
TGACTGTGCTGTTAGGGAAGGTAAATGTAACTTTTGAGCATAGCCAGAGGTTTGATAACTAAATC
CTGCCAACAAAGCGGATAAAGTGCTAACAGCGATAAAACGACTAATTTTCATGAAAATTAAATA
ACTAAATCTGAGTAGGTTAATACTAAAAATAATCGTTAGCCTAGTCTTTATTAGACTACTGCTTA
ATCTTATACCATTAGAATTACCATTTCTCTTTAAGTAGTTAGACAAAATGAGTTGTAAGTTTTGA
CTTTGTCTGCTTTTAATTTTTGTTTAGGTCGTGGGAATTATCTTGATCTATAGTCATTAATCATT
GATAATTGATCAAGAATCAGATCAAAGCATACATCGTAATTATATTGTCAACACAATGGTAAAA
ACCGATATTAAACCTAATATAGATAGCCCTTTTAACTTAAAAGTTTATTTACAAGAAAAGAAAG
AAATTGTTGAGAAGGCACTAGATGACTCTTTGCCCATAGAAAAGCCTGAGAAAATTTATGAGGCA
ATGCGCTATTCTTTGTTAGCTGGGGGAAAAAGATTACGCCCTATTTTGTGTTTAGCGGTAGCTGA
ATTGTTAGGACAAACTGAGGATGTGGCTATGCCTACGGCTTGTGCTTTAGAGATGATTCATACTA
TGTCATTGATTCATGACGATTTACCAGCGATGGATAATGACGATTATCGTCGGGGAAGACTGACA
AATCATAAAGTTTATGGTGAAGATATTGCAATTTTGGCAGGGGATGGTTTATTGGCTTATGCGTT
CGAGTTTGTGGCGATCAAAACATCTTGTATTCCATCGGAAAGGATTGTTAAAGTTTTGGCTATTT
TAGGTAAAGCAGTTGGAGCTAACGGTTTAGTGGGTGGGCAGGTTTTGGATTTAGAATGTGAGGG
AAAAGCGGATGTTACTGCAGATACTTTAAGTTTTATTCATCGTCGTAAAACTGGAGCTTTATTAG
AAGCCTGTGTTTCTTCTGGTGCTGTTTTGGCTGGTGCTTCTGAGGATGATTTAGCGAGATTGTCG
GTGTATGCCCAAAATATTGGTTTAGCTTTTCAAATTGTTGATGATATTCTTGATGTTACTGCTAC
TTCTGAGGAGTTGGGTAAGACTGCCGGAAAAGATATTTCTGCTCAAAAAGCTACTTATCCTCGTA
TCTGGGGTTTGGATACTTCTCGTCAAAAAGCAGAAGAGTTAATCAAAAATGCGATCGCACAGTTA
GACATATATGGTGACAAGGCTCAACCACTACGTGCCATTGCTGAGTATATTATTAACCGTAAAAA
TTAGGAAAAGGAAAAAAATGGAAGTATTAAGGGATATATTTTCTAATAAACTTTTAGTTATCCC
CTTATTAGCCTGTTTTTTGGCTCAAATCATCAAAGTTTCTGTGGATACTATTGCTAATCAGAAAT
TTAGTTTTCGCTATATCGTAAGTACAGGGGGAATGCCTAGCGCTCATTCTGCGTTAGTGGGTGCA
TTGGCTACAGGCACAGGACAAATTTTAGGTTGGTCATCTCCAGAATTTGCGATCGCAACTATTTT
TGCAGTAATCGTTATGTACGATGCGGCAGGAGTTAGACAAGCGGCGGGAAAACAAGCGAGAATTT
TAAATCAAATTATGGATGAATTTTTGCGTCATGAGGATTTTAACGAAGAAAGATTAAAAGAATT
ATTAGGGCATACTCCTTTCCAAGTTTTAGTCGGCTTGTTGTTAGGGATATTAAGTTCTTTATTTT
TACTTCCTTTATTCTCGAAATAAATCATATTAAACACAAATATCTAGGCTCTCTTACTGTGAGTA
TATAAATTATTGGTTAGGAAAAGGTGTCAGGCGGCAGGTGGCAGGTGTCAGGTTAAATAATAAA
TATTAATTTATAACAATTAATTTTTTGTTATCGTTAATCTCTTAATTTACATAGCTTATAGCAAT
TATTTTTTGTTAATTTACCATAGCAACTGTAGAAGAGCCAATATCTATATCAATTGTATTAAAAT
AAATAACAACAAATTTTATTTTAAATAAACTATTCTTAATTTATTTCCAATTTCAAACCAGGTAA
AAATTTCTGTAAATTTTTGAGAGATTTTTTTTGCCAAGGAATCTGTTCTGAACCCAAAATAATTA

ATTGTAATTTCTTTTTTTTTCTCAAACTAATTACGAACTTTGATAACATACTAATACCAGAACTG
TTTAAAAAATGTAGCTCTTTAAGGTTAATAGTCATATTTTCTGGGTCATTATCTGCTACTGATTC
TAGTAAATCTTTGATAGGAGAATATTCTTGAGGACCACTTAAACTTAATTCTCCTTTGAATATAA
CAGCCCAATTATCAGCGTTAAACTCTACTAAATAATCTTCTCCTTGGATTTTTTTATCTTGCATA
TTTTCTCTGTTTAATAAATATTTTTTTTATTACTATTAAATATCTTATAACAAAAAATGAGATTA
TAATTATTGATAATAATTAAGACGGAGAAATTTAAACTTCTATCTGCACCATTGTCGTTACTAGG
AAAAAACTATCATGGTTTTGAGAAGACATTTTTTCAAACTTCCAACCTATTTCTCCGTTGTAGTC
ATTGATAATGGTTAAAATACCCAAACCAGAACACTGATCTTCTTCTAAAGCGTTTTTTTCTAAGT
GTTCTATGTATAGGTTTTCTGGGTCATTATTTTGCAATTTATGAATAAAATTCTCTAATTTTTTC
TGATTATTGACAGTGACACTATTACTGGCAAAAACGACAGCAATTAAACTACTATTTTGTAGAAA
ATGAACTCCAAATTTTATTTGGGAATGGGCTTCTTCGTAATTATATTTCATCGCATTTTCTAATA
GTTCGTTAGCCACATAAGAAACTGCCGATTTACTCTCATGAATGCGTTTTTGCTCTTCTATTTGG
CTATCATCCAAAGGTAAAAAAGTAGTAAAATAATCTGCAATAAAATAAGCAGATAAGCGATTAT
TTCGCCAACGTTTTTTAAGAGGAACAGAAGTCGGCGTAAAGGTTAATTCTAGGGAATCTTGTTCT
GGAGGAAATTTATCAATAAAGTTACCAAAAGTTTGCATCCTATGACCCATTTTAGTTCTATATAT
TGCGTCTATTTTTATTATAATATTTTGCTTAACCCTCCGAACTCATCTCTCCCTCACCCTATGCAG
TTCTCCTTTTTATAATTGTTCTTATCTCAAACTTGGGCTATACATGAAGCTATTAATTTTGATAG
TCTTCAAATTTATTCTGTAAACTCTAAGCCATTATTTTTCACACAAATTAATAATTTAAAATGTA
GAATGTAAAAATAGCGGAAGTAGCGGGGAAATTTTTATCAGCAAAATCCAATTCTTACCCTTACT
GAAAGAATGTGACTAACTAGGCATTTTGTCCGCTTCTTCTCATTGAACAAAGACCAATTACATTA
TTCTCAATTCTCAGACTTATGAATCAGTCAGGTAACGAATCTAGCAGAAAAAAACACCCCCTTTT
AAAGTCGTTTCTCGCCTCTTTTGCCATTGGTGGGTTTTTAACCTCTTCTATTCCTCTACAAGCTCG
AGCGGAAATGGTGGATAGCCCTAAAGTAGTAGTGGATGAGGTGTGGCAAATTATTAATCATGAA
TTTGTCGATCGCAATTTTAATCGGATTGATTGGATTAAGAAAAGAGAAGAATTATTAGAAAAAA
ATTACAGTAGTAAAAAACAAGCCTATCGTGCCATTAACCAAGCCTTAAAAGAATTAGGTGATCCC
TATACTCGGTTTTTGCCCCCCGAACAATTTGAAACTCTTACCAGTCAAACATCAGGGGAAGTGTC
AGGGGTAGGTATTCGTATTGCTATTGATCCTCGTACTCAGGATTTATATATTATTGAAACTATTC
GTCAATCTCCTGCAGAAGAAGCCGGTTTACAAAGAGGCGATCGCATCGTCAGAATAGATGGAAAA
CCAACCGCTTTGATGGATTTAGATCAAGCCTCAGAAGCCCTGAAAGGAGAATTAGGAACTGATGT
AAACCTAGAAATAGCCAGAAGAGGAAAACCCGCCTTTAATGTAAGCGTCACCCGCGCCCAATTTG
AAGTTCCTTCCGTGGATTTTGCTATGAAAAGAGAAGGAGAATTGAATATTGGTTACATCAAACTA
GAAGAATTTAGTTCCCATGCCGCCGAACAAATGCAAAAAGCCATAAGAGAATTGAATCAACAAAA
AGCTCAAGGTTTTGTCTTAGACTTACGGGGTAATCCGGGGGGATTATTATTTGCCAGTGTGGATA
TTGCCAGAATGTGGATGTCTCAAGGAGAAATCGTGGATATAGTTGACCGTCAAGGGGGACATCAA
ACTTTTTCTGCGGATAATTCTGCCATTACTGATTTACCCTTAGTGGTTTTAGTCGATGGTGATTC
TGCTAGTGCTAGTGAAATTTTAGCAGGGGCATTAAAAGAAAATAGTCGAGCGACAATTGTGGGG
ACAAATACTTTTGGTAAGGGTACAGTTCAATCAGTTCATTCCCTTTCTGATGGTTCAGGGTTAGC
TGTGACAATTTCTCGTTATTACCCTCCTAGTGGCACAAATATCAACAAAAAAGGAATTGCACCCG
ATATTGTTCAAGCCTTAACCCGAGAAGAACAGTATTTATTAAGTCAAGACCCATCATTAATCGCC
ACTAAAGCTGATTCCCAATACTCAAAGGCGATCGCAATCTTACGTACAGTAGGCAAGTTAAATCA
TAACAATAACAGTTATGGCAGTATTTTAAAATAACCTCAGTTCGGTTTAAGAATATCGGATAAGG
TTAGGTTTCAGGTTTCAGGTTGCAGGTGTTAGGGGATGGGAGGATGGGGGGATGAGGTGATGAG
GGGAAAGGGGGAAGTAGGGGCGAATGGCCATTCGCCCCTACAGGTATTCGTGGTTTTTCATTCTT

AATTCTTAATTCTTAACTATTTAACGTCAGTTCGGGTTAAGGCAATTTTATTGTTATTTCTAAGA
AGCGATAAGGTTTTCCGACTACGAAAAAATAACGCTTTCAGCTTATCCAAAACTCAGGTTAAAAT
AGGCAAAGGGTAAAAGGCAGGGGAAAGAGGGCAAAGTAAAAAGGGCAAGGGGCAAAGGGCAAAG
GCAATTAAGCAATAATTAATAATTAGGAATTAATAATTTAAGAATAAGTTTTTCCCTAATACCCC
TATTCCCTAACTCCCCAAATTCCGAACTCCGAACTCCCTATATTATTATGAAAAAATATGCTAAA
CAGAAATATCACTTAACTCACGAGTAAGATGATCAAAAGGAGCATCCACTAAATTAACATCATCC
ATTCCCGCTTGAAATGCCATATCCTTAATCATTTCTTTCATGATTTGAATACCGCGCACAGTAGG
GCCAATAGGTACACCTAAAGAGTTATAGGTTTCCTTTAAACCCTGTAAAACACGCTCATCTAAAA
TAGCGTTATCTCCTGCCACTAAAGCATAACTAGCATAGCGAAGATAATAGTCCATATCTCTTAAA
CAAGCCGAATAACGACGAGTAGTATAAGCATTACCTCCAGCACGGATTAATTCGGGTACTTCCTC
AAATAATTGACGAGAAGCATTTTTGACGATGTCGGGAGAGTTACCGTTAATCATCGTAGCAAGTT
TAATTCTGGTAGTACCAGACTGAAAATAAGATTTTAAAGAATCTAGGGCATTACGATCTAAATA
GCGCCCCGTAACATCATAGTTTTTGATTAAACTGGTGACAGCATCAACTAACATTTTTTTCTCCT
AAACAAGATTTTATAAATTCAAGTTAAACAGTTAGTTTTGATCTTATATCTGATTATGTGATAA
AAGCCAATATGCTAAGGAATTACCTTTTTACAAATTAAGACTACTGTTAACTCATTCAAGGAAAG
AGTGGCTTCCTTTTACAATAATGATTATAAACAGCGAAACCATTATCTTATTTCTTTACCTCAAT
TGATAAGAAAAGTATAAAATCTTTACATCTTCCTGAGAGTTTTTGAAAAATTGCAGGGAAAAAG
ACAAGAGGCAAGGATAAATAGTTGATAATGAATAACCCCGTTCACGACTGAAAGGATTGTACGA
GCACAGCAAACACTCCTCTCCCCAAGCCCTTACTTCCCCTTCTAATTATTCTACTTAGACCTTGAG
ATATGCTAAGATTACTTAGTTGAAACTCTGAAATAATTATCGGAAAAATTCAATATCAATGACT
AAATTAAAACGCCCTAGCTACCCTCGCTTAGAAATACATCTCCTTAGAGAAGGAATTGTCGAATC
AATACATCAAGCTGAGGTTGTGGTTGCTGATGATCGAGGAAGAGTTCTTGCAGTTGCCGGGGATG
CGGAAACATTCTCTTTTATGCGTTCGGCAATGAAACCTTTTCAAGCTCTTGCTGTTACTAGTACA
GGAGTTATTGAGCGTTTTGATTTAGATGATCAGGATTTAGCTATTATTTGTAGTTCTCATCAGGG
TACTATTGAACAAGCTAGGCAGGTTTTTAATATACTTTGGCGTGCGGATATTGAGCCTAGTTTTT
TGAAATGTCCCATTCCTGCAGGAAAAAATAGTGCTTTACAACATAATTGCTCAGGAAAACACGCT
GGAATGTTAGCTACTTGTAAACAGAGAAATTGGAATTTAGATACCTACTACTATAAGTCTAATCC
GATTCAAGAGTTAATATTACATAAAATTGCTGAATTATTGTCTATTCCAGCCGATGAGATTATGA
GTGCGAGAGATGATTGTGGTGTGCCTACCTATGCTCTGGAATTGTCACAAATGGCAACTCTTTAC
GCTAAGTTAGCTAGTGGTAATAGTATTGATTTAGAACGAATTGTCAGAGCAATGACCAATCACCC
CACCATGGTAGCAGGAGAGGGTGCTTTTGACACTGAATTAATGAATTTAACCGATGGCAGATTGG
TTAGTAAGTCAGGGGCCGAAGGTATTCAATGTGTGGGAAATATTGGACAGAATATGGGATTAGC
AATTAAGGTTTTGGATGGTGCAAAAAGGGCAAAATATGCCACTGCAATTCATGTTTTAAAACAA
ATGGGGTGGATAACTCCTGCGGTGGCAGAAAATTTAGGAGAAATGTTCTTAAGAATTGATGATT
ATAGAAGGTTGGAAGTGGCAGGGGAATTAACTTTATTGTAAGAATATTGAATAATTAAGGCTCT
TCAGAGTGTGGTTATGATAAATTAATAAAAAATAACTCCTATAACCTTTATTGAATAGTGTTTAT
AGTAAAATAAAAACTAAAATTTTATAAATTATTATTTAATAATCGCTTCTTGCCTTTTGCCTCTT
GCCTTTCAGCCTACCCTAACCAATAATTTACATACTCAAAGTAATAGAGTCATAATTAATAATGA
TGGGGTAAATAGTTGATAATAAATAATTTGATAATAAATAATATCTCAACTTCTAACTCCGAAT
TCTGATTTCTCAATCCTCCTAATCTTCGACACCTGAAAGCTAAAACTGTCATGCAAATATACCTT
GATTCTTGTGCCACTACTGCACCTCATCCAGAGGTTGTTAAATTGGTGCAGGAAATAATGAAAGA
TAATTGGGGTAACCCCTCTAGCTTACACTTTTGGGGGGAAAGGTCAGCGATGGTTTTAGAAAAGG
CTCGTTTTTCCGTGGCTTCTTTGCTTAATGCTGATTCTGGAGATAATATTATTTTTACTTCTGGT

GGCACGGAGGCTGATAATTTAGCTATTTTTGGCACGGTTTATAATTACTCCCAACCACAACACAT
AATTATTTCTAGCGTAGAACATTCTGCGATCGCATCTCCTGTTACTATGTTGGAAAAGCAGGGAT
GGCAGGTAACAAGATTACCAGTGAATCGAGAAGGCAGAGTCAATCCAGATGATTTAAGGAAAAG
TTTACGGGATAATACTGTTTTAGTGTCTATTATTTATGGACAAAGTGAAGTCGGCACGATTCAGC
CCATTACGGAGTTAGCTGATATAACAAAAACCCACAGTAAGGCTTTATTTCATACTGATGCAGTG
CAGATAATGGGGCGCTATGAAGTGGATGTATCTGCTTTAAAAATTGATTTATTGTCTCTTTCTGG
ACATAAATTTTATGGTATGCAGGGAGCAGGAGCATTATATATTAGAGAGGGTATTGAGTTGCAA
CCTCTCATAGGCGGTGGAGGACAAGAATTAGGGCTACGTTCAGGTACACAAGCACTATGTGCGAT
CGCCGCTTTAGGGTTAGCATCCCAATTAGCACAAAAAGATTTAGAAAGTGAGAGTTTAAGGTTAA
GGGAATTAAGAGACTATTTCATTGACTTAGTAGAGCAAGAATGCCGTTATTTACAACTAACGGGA
GATAGATATTATCGTTTGCCCCATCATGCCAGTTTTATCATTAACCATCCTCATCGAGAAATTAC
AGGGCGAAAAATGGTGAGGGATTTAAACCTAGCTGGTATAGGTATCAGTGCGGGTTCTGCTTGTA
GTAGTGGTAAATCTTTACCATCCCCCACTCTTTTAGCAATGGGATACTCAGAAACTGAAGCCCTC
CGAGGAATTAGAATAAGTTGCGATCGCACTACCACTAAAGAAGATTTAGAATGGGTAGTAATGG
TAATCAATCAATTAATGAGTCGTTAGGGAAGATGAGTTAGGGGCGAATGGCCATTCGCCCCTACT
TCCCCCTTTCCCCTCATCACCTCATCCCCCATCCTCCCATCCCCTAACACCCGCAACCTGACACCTG
ACACCTAACCTTATTGGATATTCTAAAACTGAACTGAGGTTATTTATCTTTGCCCCTTGCTCTTT
TGCTTACTAAAATCTTTTTTCAGCAACCTCTACTTATAGAAATTTACCAATTCAGTTAATTTATC
CCATGGCTTTCCACTGGCAATTATATCGAGACATTTTTCAATTCCCTGAAGATGTTTTCCAAATG
GAATTAATCCTGCCACTTGCAACGCTAAAGAAGCATTTAAAGCCACGACTTCAGTCTGTGCTTTT
GTCCCTTTTCCTTGCAATACAGAGCTTAAAATAGTTGCATTCTCTTCCACGTTACCCCCTTTCAAA
GCCGCTAAAGACTCATGGTGCAAATTTAACTCTTGGGGATTAATCGTTGTTGTGGTAATTTCACC
TTCATTGAGCAATGCTATATCGGTAATGTCTCCTAAACCTGCCTCATCTAATTTTTCTCTACCGTG
TAAAACAACACCTCTATTTATCGATAGTAATTTTAAGGCTTCAGCCACAGGATTGATGAATTGAG
AATCATAAACTCCGATAACTTGCCCTGTGGGGTATAAAGGATTAACCAAAGGACCTAATAAATTA
AATATCGTCCTAATTTTTAACTCTTTTCGTATCGGTGCAACGGATTTCATCGCAGGATGCCATCC
GGGGGCAAATAGAAAAGTTATTCCAACTTCTGAGAGGGCTTCTTTTGCTATTTTGTCACTAACAT
TCAGTTTAATTCCTAAATATTCTAATACATCCGCAGATCCCACTTTACTGGAAGCAGAGCGATTA
CCATGTTTTGCTACCTTAACTCCTGCCGCAGATGCAACGAAAGCAACAGCCGTGGAAATATTAAA
AGTAGATGCACCATCCCCCCCTGTGCCACAAGTATCAATCACAGCCTCTGAATAATCAATTTTCTC
TGATTGTAGGGATTGATTGTGTAAAATTCTTGCCATACCTGCTAATTCTTCTGCACAGACACCTT
TTGCTTGAATAGCGGTTAAAATCGCCCCAGAAAGGGCAGGAGAGATTTCTTCTTTGAGCCATCCC
GTCATTAACTCTCCTGCCTGAGTTTCGGTGAGGGATTGTTTTTCTAATAGTTGTTTGAGTAAATC
AGACCAATCTTTATCCATTAATCCTTTAATCTTTAATATAATGCTTATTATCGCTATAATCCTAT
CAGAGAATAACCTAGGAAGCAGATAAATCATCAGAAAGCAGTAGGTAAAAGGCAATGGCTAACA
GTCAACAAGTAAGGTTTTGAATCTTTTTTAAGAATCAAATCAAAATTGTTAATTGATAATAATC
TCATCAATAATTGCATTTTTCTTGAGTAGTTCGGCAAAATAATTTTAAGGAATTCGGGGAAAAGC
AGAAAATTTTAAGTATTCCCTTAGATAAATGGATTTTAATTTCAATGTTACAAGAGTTCTAAAA
AATAGTTAAGAAGATGAATTATATATAAAGTATTATTTTTCACAACCAATTATCTACAAGTATT
ATCACAGTTATATATATATAAAGTTTTATTGCATTATTTCCCGAAAAATTGTAATCTATAATACCAT
AATCTGAATAGCAAAATATCATTATAATATATTTAAGGAATAAAATAACTATGGCTAGAAGAAA
AACTAAAGCAACAACAGGTTTAAGTAATCCTAAATCTAAAGTAAATTTGACTATAACTCCTGAAG
CTAAAAGTGCTTTAGATGTTATCACCAAAGAAATGGGGTTAACCAGATCTGCTTTATTTGAGGGA

CTTTTAAACGGTTCTATTTCTCTTTCTAGCCAAAACTCAGAAAAGAATATTGATATTAGTTTTAA
TGAAGATAAATCTGATGGGAAACTATTTCCCAAATCAATATTTTAGAAGGAGAAAACGTTGAT
AATCAAGAAGAAAAAAATGGACTCTATCTCTGAAGATTTAGAGGATTATAAACATAAAATTAGTG
TGTTAGAAAAAGAGATTAAAGCACAAGAAGAAAAAAATAATTCTCTTGAACAGGAGTTAGTTAA
AAAATCTTCTGAAATTCAAAGTTTAGGAGAACAAGTCAAAAATTTGAGTACAGTAGAAAAAGCA
GAAAATAATCACAATTTTGAAGCAAAAGTTACTGAGTTAAAGAAAATTATAGATGAGAAAAACA
GTGCGATCGCATCTTTAGAGGAAAAAATCAGTCACTTAGAACAGGAAGTTAAGCAGTCAAAAGA
ATCTGATGATTTATCTGTACAACAAGACCTTGTTGAGCAACTAAAATCTCAAATAGAATCTAAAA
ATAGACAAATCCATGATTTAGAACAGGCAAAAAATAACGCTATTTCTTCTACTAGAAACAGTTAT
GATCGTGGCATTCAAAATCACCTTACCCTAACTAACGAAAAACAAAAAACTATAATTGAACACTT
AGAAAAAAGAATTGCTGAATTAGAAAGTTTTGCATCCATTGGAGAACAATTCCTCAATAAATGG
CGTTCAAAAGCATATTAATGAAATGATATGAGTTCAGAGTTCGGAAAGAGGTGGCAGGTGGTAG
GGGTTGAATATGTTCAACCCTTATGGTATTTAGGGGATGGGGGATGAGGGGAGAGGGAGAAAC
AAGTAATGAGTTAGGAGTTAGTTAGGGGCGAATGGCCATTCGCCCGTACAGGAGTTAGGAGTTTT
TAATTCTTAATTGTCAACTATTTACCTTTGCCCTCCCCCCTCGAGAGGGGGGATAAAGGGGGGTT
TGCCTCTTGCCTCTTACCTCTTACCTACCTTAACCGACAATTTTATATCGAACTGAAGATAAGAA
TAATTTTTTGATCCATAGACGATCAAGCTATGTCACAATGAAGACGATATAAATAAATTGAACAA
TAACTAGCATGGATAGTTTGAAATTATACGAGTACGCATGGTTAGTACCCGTTTTACCCCTACTC
TCTGCAATGGTAGTGGGTCTGGGATTAATTTCTTTTAACCAGTTTACTAATAAATTACGACAAGT
TAACTCTATTTTTATAATCTCTACTACTGGAGTTTCTCTAGCTTTATCCATCGGACTTTTTTGGA
GTCAGTGGCAAGGTCATGAAGCCTTTACTAAAATGTTTGAATGGGCATCGGCAGGAGATTTCACC
CTCAGTATGGGTTATACCATTGACCACCTCAGCAGTTTGATGATGGTAATTGTCTGTACTGTTGC
CCTTCTGGTCATGATCTATACTGATGGTTATATGGCTCATGATGCTGGTTATGTGCGTTTTTATG
CCTATCTGAGCATCTTTGCTTCTTCCATGTTGGGCTTAGTTATTAGTCCTAATTTAGTACAAGTC
TATATTTTCTGGGAATTGGTGGGGATGGCTTCTTATCTCCTCATCGGTTTTTGGTACGATCGCAA
AGCGGCGGCGGATGCCTGTCAAAAGGCTTTTGTTACCAACCGTGTCGGTGATTTTGGCTTACTCC
TCGGTATGCTTGGTTTATATTGGGCAACTGGTAGCTTTGATTTTGGCGTAATGGGCGATCGCCTC
CAAGAATTAGTTATTTCAGGTAATATCGGCAGTTGGTTAGCGATTTTATTCGGTATTCTCGTCTT
TTTAGGACCTGTGGCTAAATCTGCACAATTTCCTCTCCATGTGTGGCTTCCTGATGCGATGGAAG
GTCCTACCCCCATTTCTGCGTTAATTCATGCGGCGACAATGGTTGCGGCTGGTGTATTTTTAATCG
CTAGAATGTACCCTGTATTTGAGCCGATTCCTGAAGTGATGACGGTGATTGCTTGGACAGGTTGC
TTTACTGCTTTCTTGGGTGCAACTATTGCCTTAACTCAAAATGACATCAAAAAAGGTTTAGCTTA
TTCCACCATCTCCCAGTTAGGTTATATGGTGATGGCTATGGGCATTGGTGGTTACAGTGCTGGTT
TATTTCACCTGATGACTCACGCTTATTTTAAAGCGATGCTATTCTTATGCTCTGGTTCAGTTATT
CATGGCATGGAAGAAGTAGTTGGCCATGAACCAGTTTTAGCTCAAGATATGCGCTTAATGGGAAA
TTTGCGTAAATATATGCCTATTACCTCCACCTGTTTTTAATTGGTACTTTGGCCATTTGTGGTA
TTCCTCCTTTTGCTGGATTCTGGTCTAAAGACGAGATTCTCGGTTTAGCCTTTGAAGCCAATCCTG
CTCTCTGGTTAGTGGGTTGGTTAACGGCTGGTATGACCGCATTTTATATGTTCAGAATGTACTTT
ATGACCTTTGAAGGAGACTTTAGGGGGAAAAATGAAAAAATTAAAGCTGATTTACTTCAAGCGG
CAGGAATGAGCGTTTCTGAAGGTGGTCATCACGGAGAAAAACCCCATGAATCCCCTGCAACTATG
ACTTTTGCTTTAGTCATGTTAGCAATTCCTTCTGTGTTTATTGGTTTGATTGGTTTTCCTTGGAA
TAACCGTTTTGAAGCCTTTATTTTCTCTCCCAATGAAGTGGAAGAAGTTGCTCATCATTTTGACT
TAACCGAGTTTTTAATCATGGGTGGTAATTCTGTCGGTATCGCTTTAATCGGTATCACCATCGCT

TCTTTAATGTATCTTCAAGGTAAAATTGACCCAAGTGCGATCGCATCTAAGTTTCCTAGTTTGTA
TAACTTATCCTTGAATAAATGGTACTTTGATGATATTTACGACAAAATCTTTGTCAAAGGCAGTC
GTCGTATTGCTCGTGCCATTATGGAGGTGGACTATAAAGTCGTTGACGGTGCAGTTAACTTAACA
GGCTTAGTAACCATCATTAGCGGTGAAGGCTTAAAATATTTAGAAAATGGTAGAGTACAATTCTA
CGCTCTCATTGTTTTTGGGGCTGTTTTAGGTTTTGTGGTTGCCTTTAGTGTAGTTGGTTAACCTC
TTGAAACTAAGTAATAAGTAATAAGTAACAAATCGAACTGAAATAGTGCTATGGTGAAAAAAAT
TATTGCCATTACAGGATTAGCCCTGATTAATTTGAGTGTGGCTTTCAGTGCTTCCGCTCGAAACA
TTGTAATTAAGTTTCCCTCTGGTAGTTATTGTGGATTTTATTCTGGAGAGATTGGCTTAAATGAT
ACTTTTAGTCTTAATTTGGGGAAAAATCAAACTTTAGTTATAACTAATCCTTACGGGCAGGAATA
TTCAGTTATCGCCCCTAATGGCAGAGTTTTACCGGTGGGAAATTATGTTTATCAAGACGTAAAGG
AATACTATACAGGCAATCAAGCAGGAGTTTTTAAAATTAGAGTGGATCATCTCTACGATACTCCT
AGTATTGAATTGCAAATTTGCGCTTATTAAATTTATCAATCATTTGATTTTAGGGGAATATATTA
TGATTCAAGTCTCAACTGAAAGCAGAATAGAACTATAATAAGTTCCTAAACATTGTTACTGCGAA
CCGATGAATATAGTTCCCCGTTTAATACCTTTTTATCAACCTGATATTAATACATGGTCATTGGA
AGCAAGATTGTTACGCTGGTTGACATTTCTCTGGATTGCTCTTGGTTTAATTATTCTTTTTTCTG
CTTCCTATGCAGTGGGGATTGAGGATAGTGGAAATGGTTGGTATTATTTTCAACGACAATTTTTT
TGGGCTGTTATTGGTTTAATTCTTTTTAAGATAATTATTCAGATACCAATTAGTTATATTTTAAA
GTGGTCAGGATGGGGATATGTTGCTTGTTTATTTATGATTTTTCTGACGGTTATCGGTTTGGGAC
AAAATATTAATGGAGCGGAGAGATGGTTAAGTTTAGGTCCTATTCAAATTCAACCTTCAGAATTG
CTTAAACCTTTTTTGATTTTACAGGGGGCAATAGTTTTTGGTGGTTGGAAAAAGTTAAATTGGAC
GGTTAAGGGTACATGGTTGGGAATTTTCGCTTTTACTTTAGCCTGTATTTTGAAGCAACCTAATT
TGAGTACAACGGCTTTGTGTGGTATGACGTTATGGTTAATGGCTTTAGCTGGTGGTGTGCCTTAT
CTTCAGTTAATGGTAACGGCGATGGGGGGAGTGTTGGTGGCGGGTTTAAGTGTGATGGTAAATCC
TTATCAGTTAAGACGTATTACTTCTTTTGTTGATCCTTGGGCAGATGCTAGGGGAGATGGTTATC
AGTTAGTGCAAAGTCTGTTGGCGATTGGTTCGGGGGGAGAGTATGGAGTTGGTTTTGGGATGTCT
CAACAAAAAATTGTTTTATTTACCTTTCCAATATACGGATTTTATTTTTGCAGTGTTTGCGGAAGA
GTTTGGATTTATTGGTTGTCTATTGTTAATTTTGCTATTAGTTAGTTTTACGACTATTTCTTTTT
TGGTGGCTATTCGCTGTCATCATCCGACTAAGCGTTTAATTGCTGTGGGGGTAATGATTATTTTA
ATTGGTCAGTCTTTAATTAATATAGGTGTTAATATAGGTGCGTTACCAACTACTGGTTTGCCTTT
TCCTTTTTTAAGTTATGGTGGTAGTTCAGTGTTAAGTAGTTTAATGTTGGTTGCTTTGTTAATTC
GAGTTGCTATTGAAACTAATATTACTGAAAAGACTTCTGTTTAGTTGGTGAGTATTTTAAAGGG
ATGATGGTACAACCTATGGAGGATTATAATTTCTTAAATAATCGCAATCAAGCCATTGAAAATTA
TATTCAACGGGTGACGGAATTGACTCAGTTAAAACAAACTATTCCTACCAATGAGGAGTTGCTGA
AAATTGCTTCAGAATTAGGTATTAGTGATGGGGAAATTGCTATTGCTCAAAAACAGTCTCAAGCT
CATTTTTTACGGGCTCAGGGTTATTATTCTCTCAATCATTGGGAAGATGCGATCGCAGAATTGGA
GGAGGCCTTGGCTTTTAATCCGTCTCATTTACCGATGTTACATTTATTAATTAACGCTTATATTG
GTAGATGGAAAGATAAGCATAACCGTCAGGATGAAATACAAGCAAGACTAAGAATTAAGCAATG
TTTAGAAATTCAACCAGATGATCAAGAATCTCTCAAATTATTAGCTAAATTAGATCAATCTATTC
GTAACTATCAATATCGATTTTGGAGTTTAGGGGCGATTACCGTCTTATTTTGTGGTTCGATAATT
GGATTTTTTATTTCTGATAATTTATCCTTAAATTTATTTAATAAAAATGATCAAATTTTGCAAAA
TTTACAACAGGAACTCAATTTAGAAATAGATACTTTAAGAAAAGAACAAACTTTGTTATATAAT
GAGTCTTTAGAACAATTAAGAAATCAACAAAGAATCAATAATGATTTAGAAAACAGAATCATAG
AATTACAAAATCAAATTAAAAATTTAGAGCAGAAAAACCAAGAATTACTCAATAAAATTAATCA

AAATCAATCATCAGAAAATCCATTTAACCAAATACCTGAATCAGAAATTAATGAATAAAAAAGG
ATTAGCTTTACACACTACAACGGGAGAATTAGGAGTTGCTTTTGTTGATAATCAAGGAAAATATT
ATTGTCAGTCGTGGGATTTAGGTAGAGATTTAGCCAATCAATTACAGGTAAAATTGCAGGAATTT
ATCGCTCCTTTATGTTTATGGTCAGATTTGGATTTTCTGGTAGTTGCGATCGGACCTGGAAGTTA
TACTAGCACACGAATAGGAGTAGTAACTGCTAAAACTCTAGCACAACAATTAAATATTCCTGTTT
ATGGTATCTCCACTTTAGAAACTTTGGCATGGAATCAGGCAATAAATAAGCCAGAAAATTCTCTT
TTAGCAATAGAAATGAGAGCTAATAATGAAGATGTTTATGGTGGAATTTATCAAAAAAAAAGTA
ATGATTTATTAATAAAAAATACTTGCTGATGAGCAGTTAAAAAAACAGAAATGGATGGAAATAGT
TAGTAAGTATCAAGTTAAATTTGAAGAAAAAATAGAGGTGATATTTGCTCCTGATAAGTTGGGA
TTTACTGCTAAAAATTTATTAGAAATAGCTGAAATTAAAATTAATTGTGAAGGGATAATAAATG
TTGATAGTTGGCAGACTTTACAGCCGTTTTATAACTAACTTAGGGGCTTGGGGTGTTTGGAAGCA
GGATTTTTTCATTCTCAAAAATGTCAATTTTTGAAACTAACAAATAACCTCAGTTCGGTTTAAGA
ATATCTGATAAGGTTAGGTGTTAGGTGTCAGGTTGCAGGTTGCAGGTGGTAGGGGTGAATATAT
TTAACGTCAGTTCGGATTAAGGCAATTTTATCGTTATTTCTAAGAAGCTATAAGGTTTTTTGACT
ACGAGTTGGTCTGCTTTCAGCTTATCCAAAACTCAGGTTAACAAATAGTAACAGATACAGGAGTT
TTTAAGTATTAATTAATCAATTATTAAGAAAAAACGCTCCCCTCCACTGTGGGAGAGGGGTTGGG
GGTGAGGGCAAGATAATTTTGCCCTGACTTTGAAAAAACCCTGTTTTGGGAGATAAGGTGTCAGG
TGTCAGGTTTCAGGTTTTAGAAGAAAGTAATGAGTAACGAGTAATTATCAACTATTCACTATTCA
CTATTCACTATTACTATTCCTTCTGAAATTTATCCATAACTCAGATTAAGAATTAAAATTCCAAA
ATTTCAGTTAATGCTATACTGATAACTTAAGCAACTAATTTGTTGTTTTATTCTACCTAATTAAA
CTATATATCATTAATTTCAGCATTTCTTCAGGAAGACTATGAATACTTCCCTCCAATCATCTAGT
AAAGAAGCAATCTTACAATATTTGCTGAAAGAAAATCAGGCTTCAGCACAAAATATAGCAGAAG
CTATGAATATTAGTACCCAAGCAACTCGCAGACACTTAAAAGATTTGCTAGAACAGGGTTTAATT
GAGTATGATTTAATTCGAGTGAAAACGGGCAGACCTCAATATTTGTATTCTCTGTCACGTCAAGG
GCGCGATCGCTTCCCTCAGAATTATGGAGAATTTGCCGTTTCTTTTTTGGATACTCTCACCGAAAC
AGTGGGAGAAAATGAAGTTAGTAGGGTATTACAAAAACAATGGGAGAAAAAAGCTGATAACTAT
CGTAAATTCATGACGGGTAAATCCTTACAAGAAAAGGTAACTCGGTTAGTAGAAATGAGAAAAG
AAGAAGGCTACATGGCGGAATTATTTATAATCGAAGAAAATCCCCCTCAATTTTTTATTTCTGAG
CATAATTGTGCTATTTCTGATGTAGCTGAATCTTATCCTCAAGTTTGTAGTCACGAGTTAGAAAT
GTTTAGTTCTCTGTTTCCTGAGTGTTTAGTAGAGCGCACAAACTGGATTCATCAGGGTGAACATC
GTTGCGGTTATTTAATCAAGGTTTGCTGAATACATCCATTCACTACGACACGAAATAAACCTGAA
TTCGAGAAAAGAGCGTTGCTGATAACTAGATATGATTTCTCATTTAGGTAGGGAATTAAGAATT
AGAAGTTATTAATTATCAACTATTTACCTTTGCCCTCCCCCCTATATTTAGAGCAGGGCTGTTTC
ATTTTCGAGAAAGAAAAAAGAGCGGGAGATAGGGCGAGAGGGAGATGGGGATATTTTTGTTCAT
AAAGTTTTAACTAATAGAAAAAAATCCTTGTATCTCTTACTCTTACTTAATCTCAGTTAACCTAA
TTTTTTACTTTGAAACAGCCCTGCCTATATTAGAGAGGATAAAGAGGGGTTTGCCCCTTGCCCTT
TTCGTCATCACTTATAGATGAAAATTTATCCTAAACTCAGGTTACTTATTACTTATTACTCTAAG
CTAACTAGAAATTTTAGAATCAATCAGCCTTGCTTAATTTTGGGGGACTTGGATTATCATTCCTT
ATCAAAATCAAACTGATGATAACACTGAACCTTGAGCAACTAAATAAAGGGCATTGTTTACTTTT
CCATAGGCGGCGGCGTGAGGGCGATCGCAATCATCACCATAACCATAGGTAATCAATCGATTTCG
ATTTAAACAGAGTTTGGTAAACTGGGGTGCGAGTAAATTAAATAACTCAAATCTTGACTTCAATT
CGGGAAAACGGCTTTGATAATTTAAAATTGCCTGTCGAACTTGACTCCAAAACTTCCATTCAGAA
TAGTTGGCATAGGTTTCTAATAAATCAGAAAGATAACGGTGATGACAGATAAATAAACCTGCAA

AAATAAATTGACATAATCCTTCTGGAGGTTCAGTTAATAGAATTTTGCGTAAATCATCGGTTAAG
TTTTCGAGTTCGGGCAAGGGATATTCACTAACATTCACATCATCGACAAAATCTTTCATGGCTAG
ACGGTGAGGAGTATGGTTTTTCAATACTAAAATTGTATTTTCTCCATGGGGTGAAAACACCACTC
CATAACGATAGAGGTAATGTAAAAGCGGTGGTAAAACAGTATTAAATAGGCGATCTAACCATTG
CTCTAAGGTTAAACCTGATTTTTCCACTAACTGAAGGATATAGGGAGAATTTTGATAGTCACAAT
GTAAGAGAGATGCGAGGGTGATGGCTTGTTCATCGTCTTCGAGGTAGCTTAAAACACTTTCCCGC
CATAGGCAACCTAACATTTCCTTGTATTGATAGGGTGCGCCAGAGAGATTTTGATAGTAGGGGTG
ATTGTAGTTTAAACTCGCAATTTCTCCGGGTAAAATGAGGCGACATTCTTGACTTAAAAATGAAT
CTCCATCACAGATAGATTTTACCCACTCAGTTACTTTTGGGGCGACTTGGGTTCGCTCGTTGGGT
AAGCCACGGTAAACTAAGGTGTTTAAAATACTTAGGGGGAGTTTTACGTGATGTTTTTGGCGATG
GCTAATATTGACAAAGGTACGAATGGATTGTTGTGGGAGATATTGATCTTGACTTTTGCCCAAAT
ATATGATTGATTTAAGGGCGATCGCCTCTGCAAATAAAGGAATCACAATATTTTTCCATTGCCAA
TCATGGACAGGAAGGAAATAGTAATCTTCTGGGTTTAGTTGTTCATTGAGCAAAATTTGCTTAAA
TGTTTGGGTTGTTTCGGGTTCTAATTCTTCTGTGATTAGCCGTTGATAATCTAATGATGGAATGG
CATTAAAAGCACTGTCTTTGCGTGCGATCGCAATCCATGTCATACAAATTGGCTCTTTACTCTCT
GGGGCATGGAGAAGATAGTCATCATAACCGAATCCGATACGCCCTTTATTAAAGGTAATCCAAGG
ATGTCCTTCCATTTCCCCTTCAAGGTGAGCATAGTCCAAAGCTAATAAATCCACTTTTTCCGCCGT
TTTATTCCGTTGAATATGGGCATCTGCTACCAAAGTATTACTCAATTCTTTAATTAGATGGGCGG
TGGTTTCTGCCGTCATGCCCACGGTTTCATGAATGTCAATGGCAAACTGAAATGGACTTGTAGCG
GTTTCCCACTCACCGTTTTCTCGACGATAAATAGATTCTGGCAAAACACGGTAACTATCAAAAAG
ACGTTTTTTTGCTTGAAATTTGTAACCAATACCTTCTGATAAGTTTAGTTGATAGGAAATCAAAG
AATCTCGTTGCTCAACGATTTCAGGGGAAATAATCTCCTCATAGAGAAATTCCGATAGCATTTTC
GCTAATAATTTTTGACTGACATTTTGCCAAATTACAGGGTTAAAAGCAGAGTTGAGAGTATTAA
ATTGCATGGTTTTTTCTCCTAATATTTGCAAGTAATTAAATTCAAAAAAAGATATGGATGTGAA
GGGAAATAATCCTATGAAAACCCTTCAGAAAGTTTGAGTCTAAAAATCGTCAATTTCGCCAAAGC
TAAATTCAAAATACATAAAGTGGCGGCGGCAATGAGGGGGAATGCCAAACCATAGCTATTCACCA
AACTAGAAGCGAGTAAAGGCGCACCTAGGTGTCCAATGTTAGCAAAGGAAGTAATCAAACCATAA
TTAAAATGTAGATGATTGGATTTACTATTTTCAAACAGTTTGAGTTCTAAAACCCCTTGGGTAAT
CGCCAGAAAGAAACCATAAATCACCCTTGCCATAACGAATATAGGCAATGATTGGCTAAAACCTT
GGAGGTATAGACTTCCAATTAATAGCATTATTCCCGTGGTATAAAGCATAGACAAACGGCTAGAT
GTGGCAAAACGGCGGATAAATGGCATAGATGCGATCGCCATTACACTGGGAATAAGAAATAGGA
AACTACTGGTGATTAAATCAACACCAAAGGGCTTAGGCTCTGTCACATAGGCAGTGAAATAAGGT
CTTACCAAATTATTCGCCAATTGAAAAGTGAGAACCACCAAACCAATAGCGATAATAAATCCCCA
CGGGTTTTTCCCTGAATCAACATTTTTAGCAATATTTTTTTGCTGATTTAACTTGGGAATATTCT
TAAGTAGCATAAAACATAAAGCGAATTGTAAAACATCACCCAAAGCCACCCCATAAAACAAGTGC
AAAGGATGCTGTAAATTGAGAACCCAAGCCCCAACCACCGTCGATGACACAATCGCCCCATGAAA
AACCGCCTGATAAACTCCCGTTAAAGAACTTCTCCTCTGCTCCCCCCCCAATTCAATAATCAACGG
ATAGAGCAAAAAGTAACTACTCTTAAAAATAAGTAATAGAACCGTAATAAACGTAAATTGATAA
ATATTTTGTGTTAACGCCATCATTGCCGTCATTACCGCCGTGCCTAATTGTCCAACAGATAACAG
TTTTCTCGCCTCAAACCGCTTCGCTAAAAACCCCCAAATGGGAGTAAAAAGCACCACTGTTAATC
GACAAATAAAGATATAGTATCCCGCATAACTTAAATCCTGAACCCCGAAAACCTTACTAAAAAAT
AAAGGGTAAAAAGGAGACAGCAACACCTCCGAAAACAAAGACAAAAATACACAGAAAAACAAAC
CGATAATCATTTTTTTGTGAAATAGTCTTCATCCAGCAACCCCAAACTCTTGGAAAACATTTTGAG

AGGAAACAGGATAAATTTCTTCTCCCAACAGGGTATTAATAATCACAGAACTTCGGTGAGCGCCT
AAGCCTAAATCTGGGGCACCAATGCCATGGGTATGTAGTTCGCCATTTTGAAGAAAAATAGAGTT
AGGAATATCTTGGGTTAACTGCAAACGATAATCAAAATTAACATCATAGCGATGTTGAGAATCCC
ACTGAATTAATGAATTTAATCCGTCCATAAATGGGGGAATGAAATGCTGATAACCTGTTGCCAAA
ATAATACAATCAGTATCATGGCTTACATAAGCATCCTCTTGACAGTGACGATAACTTAATCGATA
ACCCATCTCGGTTTGTTCAACTTCTTTGACTTCTAACAAAGGACGAAGACGCACATTGAGGGCTT
TTTGACCAATAGAACGCTCGTACAATAGATTGTAAATATCAGCAATTAAACTAAAGCTAATTCCT
TTGTAAAGAAGATTTTGCCTACTTCTGATCTGATCTCGTTTTTCCTGTGGTAAATGATAAAAATA
TTTGATGTAGTCAGGGGAAAAATGTTCTAGCCCTAACTTGGAATATTCCATGGGAAAGAAGCCCG
ATGAACGGGTATGCCATTCTAAATGGTAATGATACTTTTCTTGATCTTGCAAAAGCTGATAAAAC
ACTTCGGCGGCACTCTGCCCAGAGCCAATGACGGTGACAGATTTTGCAGTTTGATATTGATTTTG
TTTATGTAAAAAGTCTGCGGAATGGAAAATATTTTCCTGAGATTGAAAGTTTTTAAAACAAGGG
GGCATCGCAGGAGTTGTGCCAACCCCTAAGACTAAGTTTTTAGCTTTGTAACTGCTAGTTTCGGA
GGTTAAGACATTTTTTGATTGAACTATAAAATATCCCTCTTCATGCCATGTAATGGACTCCACTT
TTTCTGAAAAGCGACAACTTTTAAGCTGTTGTGAAACCCATTGACAATAATGATTGTATTCACGG
CGATAAATCTTAAATTCTTCCAGAAAATAAAAATTATAAAGGCGAGAATGTTCCCGTAAATAAT
TCAAAAAGGTGAAACGACTACATGGATCTGCCATAGTTACCAAATCCGCAAGAAATGGTACTTGA
ATGGTTGTTCCTTCCATCAGCAGTCCTTCATGCCATTGAAATTCTGTTTTTTGTTCCAGAAAAAT
GGTTTCTAGATGGGAAATGGGTTCTATGAGGGCGGCTAAACTCAAATTAAATGGGCCAATACCAA
CCCCTAAAAGATCGTAAATTTTTTCGGTCATTGTTGATTGTTTGTCTAAATTTTTAGTGTGTACA
AAACTTGCTTATTGAGAGGCTCTAAAATAATGCCTTTAGCTTTTACTACGAGCTAATCAAAATTT
GCTGATGGGAAAATATTTTGATCCCAAAGTTTTTCAAATTTGGTGCGATTTCCTAACACAAGGGC
TAAAGCCCTTACTACGAGCTAATCAAAATTTGCTGGTGAGAAAATATTTTGATCCCAGAGTTTTT
CAAATTTGGTGCGATCGCACTTCATCAATGCACCGGTTTTGTCGGGTAAATCAATTTCTTTTTGG
AACTCAAAGCCACATTTTTTAAAGATATGGATCATTTTCTGATTTCTGGCATCGGGTTCAGTCAT
AATTTTTTGAGTTTCTGAGGTCTGAAATTGAAACATAGTCATGGCACGTAATAAAGGTAACGCCA
AACCTTGCCCCAAAAATTGGGTATCCCCAATCAATAAATGAATGCCCTGATCAAATTTTTCTGCC
GGATAGTAATTAGCAATAATATCATCCATCGCCCAGTAGGACTCCCAATAACTCATGGGAACACC
ATTTAATGAACCGATATATAATGTTTGGTGAGGATCTGCGATCGCCTTAGTGAGATGAGCGTGCA
TTTTTTCTAAGGAAAAGTTCAACTTCCAAAAAGGAATCACATGGGGACAATTCATCCATTGATGA
ATCCGTTGTAAATCCTCCGTGAGAGTCACAGGGCGAAAAGAAATCTCTTTATCAATGGTGCGATC
GTAAATAGTAAAATTTTCCTTCCTCGTTAAAATTGATTTAGACATAAGCACCTCCTAAAACGGGA
TTTTTCTTTTCTACTAAGGGATTGTGAATATCAACATAAACAGATTGAGTAGCCACAGAGCCAAC
CAATTCGTCCATATCATGGAAACGAGTTAACAAATTAGCTTTACTGCGCAGTGTGGTTTGTTTTA
AATTCTCCAGTAATAATGAACCAGACAAATTGACATTTTCAAATTCTGTTAAATGTTTTCTTAGT
TCCGCAAAAAGTAACGACTCATCAATCAATCCCGCCACACCAAAAGCATTAATTAAACCAAAAAT
ATTATTAATGAAGAGATAGTACATTAATCGTTCATCAATCACCCCATCATCGCAGATTGTCTCGC
TTTTCTCGCTAATATTGGGCAAGATGCTATTAAGCAAATATTGAAAAGAGCGACGGTAATAATA
ACCTTGATTGTCTCGATAAAAAAATCGATGGGGATAACCTTCTTTCAACTCTAAAAGGCTATTTT
GTTGGTGAGCTTCTAGGGCAATGCCATAGGTAAAATAAAGCCAAAGAATAGGTTTTAAGGAAAT
CTGCAAATACTTTTGAAACCACTTTAAGCTAATTACCTCCGTGGAAACATTTTCTTTTTCGGCAA
GGGTACGAATAATAGTTCCTAAACGGGAACTTTGACCTGTAACAGAATCTTGACAGAGGGCTATT
AAACAGGTTGCATCGGTGGTGATGTTGGTAGGTTGAGCATTGCTATGGCTTTGATAATTTTCCGT

GTGAAAAGGATTAGTTCTCAAAATCGTGGCGAATCCAGATTCTAAGCCATCAAGGGGGATAGTAA
TATAAGCGGGATCTCGAACAATATCAAAATTAGGGAAATGTGCATACAAATCCTCCCCAATGGGA
CTAGAAAGGATGCGATTAACTTCTAAACCCCGCTCTAATTCCTTATAAAGATTCCCTCGAATGGA
GTTGGTAATTTTCACATTTAAGGAAAGTTTAAACATGAATTGTGATTGAGAATTATAAACGGTG
CGAATGGAGGAAGTGGGTTGATAGGGTTTGCCCTGTTGTCCTATGTCTTCTAATAAGCCTTCGTT
TAACAATTCCTGCACTTTGGGGGAATGTTTTAACCATTTTGCCTGCCATGGATGGACGGGAATCA
GGGCGTATTCATCTGGGTTACAATATTTATTTTTAAAAGATTCACTGATGTTTTGATCTGCCCGT
AGTTCGTCTTTGATCAATTCTGTGGCTGTTTTTTCGAGGGTTGAACCTTCGAGGATGATGGATGA
ATGGGCGCGGAAGTAGTGTAAAGAAAATGCTCCTTGGGTTTCTGGGGAATAGATGGGTAATTCTT
CCTCTAAAAATCCTTGACGACTTTTGGGGGTGGGGTGAAAATGATGCCCAAAAATCAAAGATTGT
TCGCTGTCAATAAAGTTTTGATGGAAGTCGTATAAACTATCAATGTCTGGCTGACGATGGTTTAA
AAACTGCTCGATATTTTGGCAACTTTGGATTACCCGTAAAATTAATTCGTCACAGTTTTCTGAGC
TATTACTATTTAGGCTTAATTCTTTGGAAATTAGTGCAGTTAAGGTTACGTAGTCTAGTTTTAAC
AGTTCACTGGTGTTTTGGGATTTGTAAAAAATGGGAAAGGTGAATAAGTGGCGATCGCACAATG
ACCAATAACGAACTCCTGCAAAAATGTCTAAATTTTGACGGGGTAACGGACAACGAAGGACGACT
TTTGCTTCGGTTAGTTCACTGATTTGCATTGCCAATGGCATATCGGGTGGTAATTTTTCATAGTC
TCTGGTTTCTCGCAGATAACAATTGAGAAAACTTTGAATGGTAGCTTGTTCAGCAATTTTTTTTG
AGTTCATGATTTATATCTGTTGTAATGTTTTACCTAGGGATTTGATTTCGATTAGTAGTTTTTGG
AGATCCTCCAAGGTTGTCAGAGGATTTAGGAGGGTAAATTTGAGATAGTTTCTGCCCTTGACTTG
GGTTTGAGCAATAATCCCTTTGCCTTCGAGCATCAATTTTTTTGGGATTTGTTCATTGATGCGAT
CAATTTCGGTTGCTGTTCCTTGACTGGGTTGGTAGCGAAATACAACGGCGTTGATGGTGGGTATA
TTCGCCAACTCCAATTCTGCATCTTCGGCAATAATAAGTGCGATCGCCCCTGCTAACTCAATGGTG
CTATCAATCATTTCCCCAAAGGTTTCCACCCCCAAGGTTTTCAAAGATAGCCATAACTTTAAGGC
ATCAAACCGTCGGGTAGTTTGAATAGATTTAGTTACTAAATCGGGGATACCCTGAGCTTCATTGC
TTTCTGGGTTGAGATAGTCAGCATGGAGCTTGATTAAGCCAAAATTAGCCTGATTTTTGAGCAAA
AACGCACCGCAACTAATGGGTTGATAAAAGAGTTTATGAAAGTCAACGGTGATGGAGTCGGCAAG
TTCAATGCCTTTGAGTTTATGACCATGATTTTGACTGAGTTTTAAGGCACCACCATAGGCGGCAT
CAACATGAAACCACAAACCATGATCTCGGGCAATTTTTGCCAATTCAGGTAGGGGATCGATACTG
CCGAAGTCCGTAGTTCCCGCTGTGGCAACGATGGCAATGGGTCGTAAATTATCCTGTTGTAAAGT
CCTTAGTTTTGCCGATAAAATCTCTGGTTTAAGCTGGAAATTTTCATCGGTTTCAATGGGAATAA
CCGCATTTTCCCCCAACCCCAGAATCGCCGCCCCCTGACGAATGGTAAAGTGAGCCACATCGGAAC
AAAAAATCCGAAATTGTTGGGCTTCTGGGGGTAATCCCTGCCGTTGAATGTGCCATTTTAAATGG
GTTTTGGCATAGTTATCCCTCGCCAATAGTAATCCCATCAAATTGGATTGAGTACCACCACTGGT
AAAAGTGCCGTCAGCAGAGTTGCTGTAACCAAACAAATCACAGAGCCATCGGGTTAATTGTTGCT
CCAAAATGGTTGCACTGGGGCTTTGATCCCAAGAATCCATCGATTGATTTAAACTGCCGATGATC
AGTTCAGCAGCGATCGCAGGAATCAGGGGAGGACAGTGGAGATGGGCAACACAGGCGGGGTGAGT
CACCATGGCGCCATGGCGGATGATTTCGGCTAATTCAGGCTCAATTTGTGAATAATCTTGCTGAG
TAAAGTTTTTTGGACAAAAATCCCGAAAACTCTCTTGCAATAAGGAAGGATTTAACCCAAAATAA
GGCTTATCTTCTTGCAAAATATTATTAACTATTAGTTGTTGGGTAAGGGCGATCGCCTCTTGATA
ACTTTCAATACTTTCTGTTTGAGCCGTCAGAAAATATCGGTCAAAAGGACGTGCCTGACTCGATG
ATGACATAGAAAAAATGGATAAATGAATAATGGGATGAGGTGAGAGAGGGAGCAGGGGAGCAGG
GGAAATGAGTTCGGGGTATTGTGATATTTGGGGATAAGGTGATGAGGGGAGGTTAATTAAACAC
TTTTACCCTCCCCCTCTCGAGATAAAGGGGGGTTTGCCCCTTGTCCTAAATAGGTTTACCTTTGCT

CCTAGCTCCTTTGAAATCTATTAACTATTGGTCAATTGCTAATTTTAATAACCTTTACACTGCTG
AAAATTTGATGTAAAAATCAGTTTACAACCTCTACCGCTTGGGCAAATATTTCACTGATGGCATC
AATTTGATCTGCCGTGATAATCAAAGGTGGGAGAAAACGAACTACAGTACCAAAACGTCCGCCCA
ACTCTAAAATTAAGCCCCGTTCCAGACAAGCCTTTTGAATTTTTCTTGCTAGTTCTGGATATGCA
GTCTTACCACCATGGGGATTAACAATCTCTACCCCCACCATCAATCCTCGTCCTCGTACATCACCA
ATACAAGGATGATTCCCTTGGATCTGTTGTAAATTCTGCATCAGGCGATCGCCCAAAACTTTTAC
AGAGTCAACCAGTTGATTTTCCATGATATACTTAAGTGTGGCAGTACCCGCTGCCATAGCCAACT
GATTACCTCGGAATGTACCAGCATGGGCGCCCGGTTTCCATAAATCGAGTTTCTCATCATACAGA
ACCACTGACAAAGGCAAAGAACCACCAATCGCCTTGGACAGTAACAACACATCCGGGGTAATCCC
CGACCTTTCAAAGGCATATAAATTTCCCGTGCGTCCCCAGCCGGTTTGAATTTCATCCACAATTA
AGGGAATATCCCGTTCGCTGGTAATTCGTCTCATCTCCTGCAACCAAGCATCTGGAGCAGGAATC
ACCCCCCCTTCACCTTGGACAACCTCCAAAATCATACCCGCAGGTGGCACAATTCCACTTTCTGGA
TCTTCCAATAAATACTCGATATAACGACTACTAATCTTATGTCCATTGTCACCACCCACCCCAAAG
GGGCAACGATAATCATAGGGATAAGGAAGAAAATGAACATCCGCCATCAACCCCGGTACTGCAGA
CTTGGGAGCAATATTCCCCGTTAAACTTAAAGCTCCGTGACTCATGCCATGATAACCTCCGGAAA
ACGAGAGGACACTCCGTCGCCCTGTGGCTGTTTTCACCAATTTAAGAGCCGCTTCCACCGCATCAG
CCCCCGTGGGACCACAAAATTGAATCTTTGCTCGTTTGGCAAAATTTTCTGGCAAACTGGCGAAT
AATTCTTCTACGAAACGATCCTTAATCGGTGTGGTCAAATCCAAAGTATGCAAGGGATAATTTCC
TGTCAATGTTTGTTGCATTGCTTCGATGACTGCTGGATGATTATGCCCTAGGGCAAGGGTTCCAG
CCCCTGCCAGACAGTCAAAATAGACATTTCCGTCCACATCCGTCACAAAAATTCCTTTTGCTTCGC
GAATCGCCATGGGAATTCGCCTTGGATAACTACGGGCATTGGATTCTCGGAGTTGTTGACGATCA
AGGTATGCCTGAGAATGATTCCCTGGCAGCGATCGCATTTGTACCTTTGTTGAATGAGGGGCTGA
TGATATCATTTGCCCTAAATCATTACAGAGTTGTTGCATCAGTTTTTTGTAATGAAGATTTTTAT
TGCGAATATCTACTAACAAGATATGGTTTCATATAATAGCTATAAATAGAAATAAATGTCAATA
AATTTTGAAAATAAGGTTAAACTTCTTTTTTCTGATAACAGCTAATAAATTGATGGGTTACTAGC
TCGGTGTCGTTGATTTTGATAAAATGGGATGTTGAATTAATTATTTATATTATCAGTGCGATCAA
CGTAGTCGCACCTTCGGTGACTGCGTCTAAGATAAAATTTCGCATAACGTATCGACAATTAATTC
TCTTTCAATCCTTATTATTGTTAACGCATTCAGTATCTAAACATCAATGAAGCATACTATCTCAG
TTTTGGTAGAAGATGAAGCAGGGGTTTTAACAAGAATTGCTGGTTTATTTGCCCGTAGGGGTTTT
AATATTGAAAGTTTGGCAGTAGGACCTGCTGAACAATCTGGTATTTCAAGAATAACAATGGTTGT
CCCCGGTGATGATGATACCATTGAGCAACTAACAAAACAATTACATAAATTAATTAATGTTATTA
AAGTTACTGATATTACTAAAATTCCTTGTGTCGAAAGAGAGTTAATGTTAGTAAAAGTCAGTGCT
AATAGTAGTAATCGTAGTGAAGTTTTACAAATTGTTCAGATTTTTAGAGCAAAGGTAGTAGATA
TTTCTGATGATAGTCTTATTTTAGAGGTAGTGGGTGATCCGGGTAAAATGGTGGCAATTATTTCT
ATGTTAAGTAAATTTGGTATTAAAGAAATAGCCCGAACAGGAAAAGTTGCCCTTGTCAGAGAGTC
TGGAGTAAATACCGAATATCTCAAGTCTTTACAAGTAAAAGTCTAATCAACCTCACTTCGGTTTA
AGTATATCGGATAAAGGTAGGTATCGGGTTTCAGGTTGCAGGTGTTGGGGATAGGGGGATGAGG
GGATGAGGGGAGAGGGAGAAACAAGTAATAAATAATAAATAAGTAATAAGTATTCGTGGTTTTT
CATTCTTAATTCTTAATTCTTAATTTTTAATTATCAACTATTCACTATTCACTATTTACCTTTGCC
CATTGCCCTTTTCTTAATTCTTAATTTTTTATACCTTGATAGGCAATAGTTAAAAATTGATCAGA
AGTTAGAAAACCGTCACCCCATTCTTGATCTAAATCTGCAAGGGGTTTTTCTGAGATAATGTCAT
CTAAAGTCATGTTTTGAGCTATTCTCTCTTTTACTCTTTCATTAACTGTTTTTAACATCTCTTGA
AATTTAATTAAAGAAGCGCGATCGCCTCTTTCTCCATGACCCGGTATAATAATAGTTTGCTCATT

AGACAACGCCAAAATTTGGTCAATAGCAGAAATCATACCATCAATAGATCCTCCAGCCTCTGTGT
CAATAAAAGGATAGAAGCCATTAAAAAATAAATCTCCTGTATGAATAATATTTTCTTTGGCAAA
ATGAATCACAATATCGCTATTTGTATGAGCAGGTGGTACAGCAAAAGCCTTGATATGATTACCGT
TAACGTGAAAATTACTCACATCATTAAAAGTAATTTTTGGTAGTGCCGAAGCAGGAGATGCTTTT
ACATCCATTCCTAAGACAGGATAAGAGTGGTCTTTTTGCATCTGTTTAAAAGTGTTATCGTGGGC
GATAATAACCGCTCCGTCATTAGCAAAGTTTTCATTACCTCCGACGTGGTCAAAATGATAATGAG
TATTGATTAAATAATTAACGGGTCTTCTATTGATGAGCTTAATTTCATCTTTGATTTTATCTGAT
AGATAAGCAAATTGACTGTCAATCATTAAAACTCCATCATCTCCAACGGAAAGTCCAATATTACC
GCCTTCTCCTGTTAGCATATAGATATTGTCTTTTAGGGGAATGATTCTAACATTGATTGCTTCTG
AATTTTGGCTAACTATTTGTCCATTATCTACTAATGGTTTTTCATGAGCGACAGTAGCTTTATTA
ATAATAAAAATACTAATAATACTCACAAAAAAAAATTAAACCCAGAAAACGAGATATTTTTTTCA
ACATAAAATTCCTCAACTACAAGTTCTTTTATATATTACTTTAACCTGAGTTTTGGATAAGCTGA
AAGCATTATTTTCTCGTAGTCAGAAAACCTTATAGCTTCTTAGAAATAACGATAAAATTACTTTA
ATCCGAACTGACGTTAAATATATTCACCCCTACCACCCCAAAACCCTAAGCCCCTACTTCCCCCTT
TCCCTTCATCACCTCATCCCCCCATCCCCTAACACTTAACCTTATTCTTTATTCTTAAACCGAACT
GAGGTGAAGTTGCAGAATACCCATGGGGGGTTACAGCATTGTAGAAAAATAAATATTCTTTCATT
ATTAAGGTTGTTTGGTAAAAATATGTGAAAACCCTAATAATTGTGAAAAATTGGTTTAATTTTA
GTTTTGTTAGCTTCATCGCTATTTTATTTCTATCTTCTTGCGGTAATAACTCTTCTAATACTACTG
TGAGCGAAAATGAAGATTCTGCGGGGGAAACTTTAACTCTTGTGGCCAATGGAGAGGATTTTATT
CGTCAGGGCTTTACAAGTAAAGATGGTTGGCGTGTCGATTTTAATCATGCTTACGTTACTCTTGA
TGAAGTCATTGCATATCAAACCGACCCTCCTTTTAATGCTGAAAGTGACGAAATGATTAACGCTT
CTGAGTCTATTATTTTAGTTGATGAGGCGATAACTATTGATTTGGCTCAAGGAGATGAAAATGCT
TCTCCCATCATAGTAGCAGAAGCGATCGCACCCACGGGAGTATATAATGCTATTTCGTGGAAATT
AATCAATGATGCCCAAAGCAATTCTAGTATTGTTCTTGATGGTATAGCAGTAAAAGATGGTGAAA
CTATTAATTTTGTTCTTAACTTACCCATACCCCTAGAATATCAATGTGGGGAATTTGTGGGAGAT
GAAAGAAAGGGGGTTTTAGAAGCGGGGAAAGATGCCCAATTAGAAACAACTTTCCATTTTGACCA
TATTTTTGGAGATGGCGAGGCAACCCCAGAAGATGAGATTAATGTAGGTGCGATCGGATTTCAAC
CCCTAGCACAACTTTCCAATAATGGAGAATTAAATATTGATTTAGCAACCATGAAAGCCCAACTA
TCCCCAGAAGATTATGAAAAACTAGAAAAAAATCTTCAATCTTTGGGTCATGTGGGAGAGGGGCA
TTGTCGCTTAGTTAGTTAACCTGAGTTTTGGATAAGCTGAAAGCATTATTTTCTCCTAGTCAGAA
AACCTTATAGCTTCTTCTTAGAAATAACGATAAAATTGCCTTAACCCGAACTAACGCTAAATATA
TTCAACCCCTACCGCCTGCAACCTGCAACCTGACACCTAACCTTGTTGGATATTCTTAAACCGAAC
TGAGGTTAGTTAGAGTCTTAGAGAGTATTTGAAAAGTTATCAAATATTCTTTTACTCCTCTCCTG
TGGGAGAGGGATTGGGGGTGAGGGTAATAAATAGTGAAACCCACTCTGAAATAGTTTCATCCTTA
TAATAAAGGGAATTTAAAGGACTTAAAACTCCCCAATATCCTAAGACCATAGCTGATAGTTATGA
TCACAAAACAAGGTCTAGTGAAACTTATCGTCCTAAAATTTGAATACGAACAGGGGCAACACCAC
TACTTTTCAAGCCAATTACCCTAGCCGCTCCAGCCGATAAATCGACAATACGACCACGAGTAAAA
GGACCTCTATCATTAATACGCACTACCACTGAGCGACCATTATTAAGATTAGTCACCTTAACTCT
TGTACCGAAAGGTAAAGAGCGATGAGCCGCAGTTAACGCATTTTGATTAAATCTTTCCCCACTAG
CAGTTTTTCGTCCATGAAAACCCGGTCCATACCAAGACGCAATCCCCTGTGCCGCCTTGCGTAAAT
TTTTGACCCCCGCAAATGTTGCTTTCTGAGTTTGTTTTTTCGCAACAACTTTAGGTTGCCCTTTAA
CTTCCTTTAAAGGAGTTGCATTACCCATTAAACGACGCAAACGATTAGTAGCCTGTAAAGCATCA
TGGGCTAAATCTTCAGTGCTATCTGGTAAGATAACGTATTCGTTAATTTTCACTAACTCGTTATT

AGCAAAAGTAATTACATAGTCCTCAGTTTTGGCATTCCAAGCAACGTTAATTTCTGATGCTTGAG
CAGAGTTAGAAGATAATTGTTCTAGTTTTTTTGCAAAATTGGTGGCGTTATTGATTGCTTTACCA
TCAGGAGTATCAAGAAATGTTAGTAGGGGAATATTGTTATAGTGAAGCGTCACAGCATCTTGATC
ATCCCACTGATGTTGATAGAGTTTGGTAACGAGAAATTCTTTAACTGGGGCGGTAGAGGTTTTCG
CTAGGACATTATGAATCGGAGAAGTGACTTCGCCATCTTGTGCTTGATAGCCATTAGCTCGAACA
GTTGGAGCGATTAAAACCCCACTGATACTTAATCCGATGATAGTTGATAAGGCTGTTTTTACAGC
TCTTTGACAATTTTTGTTATCCATAATGTTTCTTTAGTGAACAATGAAAGCCTTTAATATTGAGC
TTTTCTGTTAGTTTGCTCCTTGTGAATGACACTTTATTTATGTGAGTGAACTTAAATTAAGTTAA
CATAAGGATTTTTACTTATGGTTAATTATTTCTTATTTAAAGCATATTGTAATATTTAATATAGT
TATATAGGTGGATAAAATAAATTTTAATATGTATCATAGTTAGTTTATTTAATTTTAAAAAAGC
TCTCAATGTTTTAAATAACTAATCAATTAAACAGCTAAATACAAGTAAAAAAAATAGGATCTGT
AAAAATAGTAGTATTTTGAGTAAGAAAATAACTTAAGGATTTATGTTTCTTGTGGCTACATGAA
TGTTTAAAAAAATAGAAGAGGCTGATGAATTGATTTATAAAGGAAAAAAAGATTATTAAAGTGA
AATTATCTCATAAAACAAACTCTTTTAAAGGATAAATTTTGACCAAAATTAGACAATAATGGTA
TGATAATTTGGTCGAAAAATGATCAATAATTCCCGTGTCGATACGATAAGAAAATTTGATCAATT
TAAAGAGTAAAATTTCATCTATTGAAAAAATTTATCTAAAGGAGATTGCACAATGGATTATAAA
CAGGCAGGAGTTGACGTAGAAGCGGGAAGAGAATTTGTCAAAAAAATATCGGAAAATGTAGCTA
GTACCTATCGTAGAGAAGTTTTGGGCGGTTTAGGGGGGTTTGGTGGTTGCTTTGAAATCCCTGCC
GGTTATCAACAACCTGTCTTAATTTCAGGTACAGATGGTGTTGGTACAAAATTAAAAATTGCCCA
TCAATTGAATCGTCATGATACTGTGGGAATTGATTTGGTGGCGATGTGTGTTAATGACATTCTTA
CTTCTGGGGCTGAACCTTTATTCTTTCTGGATTATTTAGCTACGGGTAAATTAAATGGTAATCAA
TTAGCAGAAGTTATCAATGGCATTGTTATTGGCTGTAAAGAGAGCGGTTGTGCGTTATTGGGGGG
TGAAACAGCAGAAATGCCAGGATTTTATCAACAGGGTGAGTATGATATAGCTGGTTTTTGTGTTG
GTATTGTGGAAAAAAGTAGAATTATTGATGGTAGTGCTATTCAAGCAGGAAATGTTGCGATCGC
ACTTTCTAGTAGTGGTATTCACAGTAATGGTTTTTCTCTGGTACGTAAAATAATTGAATCTGAGG
GGTTAAGTTGGGATTATCAATGTGAAGAATTAGGAGAAAAAACCCTAGGAGAAATTTGTTTAAC
TCCCACCAAATTATATGTAAAACCTGTGTTGTCAGCATTAAAAACGGGGATTCCCATAAAAGGAA
TGGCACACATTACAGGAGGAGGCATCCCCGAAAACTTACCTCGTTGTTTGCCAACAGATAAATCC
ATAGAAGTAAAATTAGATAGTTGGACAATTCCCCCTATATTCCAATGGTTAAAGAAAAAAGGGA
ATGTGTCCTTAGAAGATATGCTTAATACTTTCAATATGGGAGTTGGCTTCGTGGTGATTGTGGAA
GCCCAAGAAGCTGATAAAACAATTCAACATTTTCAAAGTAGTGGTATTGATACTTTTTTAATTGG
TAAAGTAATTGAAGGGAAACAAACAATCATATTTAACTGACCATTAGTACCTATTTATATTTACC
AACCCTCTATTCATTACTCATTATCAAGAAGAAAATGTCTATTGAAACAGAAAATCAGGATTCTC
TCACAGAATCCCCGACACCTCCCCCATTAAAATTATTGAGTAGCAAAGAGCAAATTAACTTGATC
AAAAAAGGGGAAATAATAGAAGTTAATCTTCCCCCAGTTAATAAAAGTGAAGCTAATCAGTGGG
GCAGAATTATTGACGATTTTAAAATTAGATTGCAAAAAATAGACAAATCTTGGCAATTAGGTAC
AAAAACGGTCATTAAAAGTGGCGATCGCCTCTTAGATATAAGACAATTAAGTGAATTAGAAGGA
ATACTCGAAAACATCGGCTTAACCTTAGACATTATAATTACCCGTCGTCGTCAAACGGCTGTAGC
GGTAGCCAGTGCCGGTTATTCTGTGAAGCAAGAATCATCTTTAGTCACCTTTAGCGACACTAAAA
AAGAAAAAAAAGAAGAAAATCAAGCTCTCACAGAACCTTTATACTTAAAAACAACCATTCGCTCA
GGAGTAGAAATTTGTCATCCTAGCACGATTATTATTGCAGGGGATGTTAACCCCGGAGCAACAAT
TATTGCAGGGGGAGATGTCTTAATCTGGGGTAACCTAAGAGGAATTGCCCATGCTGGTGCTAATG
GTAATAAACAGGCATTAATTATGGCATTGAAAATGTTACCCACTCAATTACGTATTGCGGATTTA

GTTGCTCGCGCCCCCGATAATCTTCCTGATAACTATATCCCAGAAATTGCTTATATTAGTAATGA
AGGAATCAAAATCAACCCCGCAGAAAATTTTTATCGTTCCTATGAATTTATAGCAGACAAAAACT
ATTGGCAAATTAGATGATAACTTTTTTTGGGGATGAGGGGGTTTCCCCCTTATTTATACAGGGCT
GTTTCATACTAAAATCTTTGTTTAAGAGGGAGTAATAAGTAACGAGTAATGAGTAATGAGTTTT
GATAATTTATTATGATTAATTGATTGTTTCAAGATTTTTTATTCTTACTTAAAATCTGCAAGTCA
AAAATCCCCCCATCTCCCTCTCCCCTTATCTCCCGCTCTTTTTTTTTCTCGAAAATGAAACAGCCC
TGCCTTATTTATATCAACGAAAATTTTATAGCGGACTCAGGTATAATCACAATGAATCGCCGTTT
GTTAGTAATCAGTAATGGTCATGGAGAGGATGTAATTGCAGTCAAAATTATTCAAGAATTATGG
AAACTACAAAAAATTAATTTCTCCTCTGATTCTAGTCAAGAGTATTGCCCAATAAATATTACTGC
TTTGCCCATGGTAGGGGAAGGTTTTGCTTATCAAAAAATAGATATACCCCTATTAGGAAAAGTTC
AAAAAATGCCCTCGGGGGGGTTTATTTATATGGATGGTCGCCAGTTATGGAAAGATATTAAAAG
TGGCTTAGTGGGTTTAACCCTTCATCAATATCAACAAGTTAAACAGTGGAGCAGGGAAGGGGGTT
TAATTCTGGCGGTGGGGGATATTTTACCTTTACTTTGGGCAAACTTAAGTGGTGTTAATTATTTT
TTTGTCGGCACGGCAAAATCTGAATATTATTTAAGAAATGAAGAGGGGTGGTTAGAAGATGTTA
GTATTGTTGATCGCTTTTTGGGTTCGATTTATTATCCTTGGGAGCGTTGGTTAATGAGCAGTAAA
TCTTGTTTAGGTATTTTTCCCAGAGATAGTTTAACGGTTGAGTCTTTGCAAAATTACGGTATTCG
AGCTTATGATTATGGCAACCCGATGATGGATGATGTGGACATATCTTTTTCTCCTGTCCAAAATC
CAGATGATGAATATACCTTAAAAGTATTATTGTTACCCGGTTCAAGATTACCAGAAGCCCTCGAT
AACTGGCAATTAATCCTTTCTGCTGTGGATAGTTTATTAGAAATTCAAGATTTAGATTTAAGATT
TGTGGGTGCGATCGCATCTTCTCTAAACTTAAATGTCTTTGCCGAAATTGCCCAAAAATTTCAAT
GGAAACCGATTCAAAAAAAATCAGTTAACATACCCATTGAAGATCCTGATAAAATGATTCTTACT
AAGAATAAAGCTCAATTAATCTTGAGTCAAAATAGTTATGCTCAAAGTTTACAATATTGTGATA
TATCAATAGCGATGGCAGGTACAGCAACAGAACAGTTTGTCGGCTTAGGAAAACCTGCGATCGCA
TTTGCTGGTACTGGGGCGCAGTATAACCCAAAATTTGCGAAGAATCAAAGTCGCTTATTAGGAAT
ATCCTTACAACTGGTGGAAAATCCTCAACAAGTAAAAGATAAAGTGCAAGAATTACTTTCTAATC
CAGATTTATGGCAATCAATCTCAGTTAATGGTAAAAAAAGATTAGGAAGTGCAGGGGGTGCAAA
AAAAATCGCCCGTGAATTATTAAGCAAACTAACCAAAAGAAGTGAAGTATAGCAAAGGTAAATT
TCAAGATGGATGCCCAAGAATTGATAGAAAGATATAACCGAGGGGAAACAAACTTTGAAAAAGT
TGATCTTCAAAATCAAAAATTAACCAACATAGTACTTCACAGAATTAACTTAAAAGAAGCTAATT
TGAGTGGTGCTTGTTTTGAAGGATGTTTTCTTACTCAGGCAAATTTAAAAGAAGCTAATTTAGAA
AATGCCGTTATCCAAGGAGATTTAAGTGCCGTTAACTTGATTCAATCCCAATTAATTAAAGGAGA
TTTTCAAAATAGTGATCTTACAGACGCTAGTTTAAGAAATGCAAATCTGAGTCATAGTAACTTTA
GTCATAGTAAGTTAATTTGTGCATTATTACATGATGCTATTTTAAAAAACGTCAATTTGAGTCAT
GCAATTTTGATTAACAGTCTTTTAAGTCGAGCTAATTTGACAGATGCTAATTTACAGGGTGCTAA
CCTACAAGGGGCAAACTTAACTAATGCCATTATGATTAACACTAATTTAGAAAATGCCAATTTAT
CCCTTACTCAAATGAATTCTGTTAATGGTGTGGGTATCAAAGCATCCCATGCTAATTTCAGTCAT
GCTAATTTAAGCGGAGGGAATTTTGCTAACAGTATTTTTCATGGTGCTAATTTTTATCATAGTAT
GATGACTTGGTGTAGTCTCAAAATGGTGGATTTCTCCGAAGCCAATCTTTCTGAAGCAAATCTAT
TATGGTCTAATTTAACTCGTTCTAATCTAACTAAAGCTAATTTAGTTCATGCTAATATTAGTCAG
GCAAACTTCGATCGAGCTGACCTAACAGAAGCTGTGATGAAAGAATAGCTAGAGAGTGTTTTTAT
TCTTCCAACTAAATTACTGAGTTCAATATAAAATTGTCTGGTGGTGCTGGGGGGAGTAGAAGAGA
GAAAAGTCATAATAATTTGCAGAAAACTTATGTTTATTGATTTTCTTCTTGTTATTTTTTGTTAA
ATAATTGACGCTAAAGAATATCATGCTTGAAACCAGCCACCGGCAATTTGTCTAAGTCAGACAT

TCCTACTGAACAACTGAGGTATATTAGACATCTGGTAGAAAATATTAATATAGGCTCGTAGTTAG
GGCTTGGTTCCCTTATTTTGAGAATTGTTGGAGACGTTTATTAAATAGCTTGAGTGTTGTGGGGA
TTATTTTTGATAATCAAAATCGAAAAATAGTTTAACTTTAAATGGGGCAATAAAGTAAGATTAC
GATATACTTTTTCTTCTGGTTGAGTTGCTTTTTCTACTATATAAGAAGAGGATAATAAACCTTTT
TGTGCCAAAATTTGCCATACTTTTTCATAAACAGAAGCTACTTTCATTAAAACAATGACTTCCGC
CCAATCCAAAACTTTTTCTAACTCTTCTACCGAATAAAAAGCAGGTAAAACCACTAATTTTTCAT
CTTGCACAGTTAAAGGAATATCTAATACAGAAGCGGCAACCATTGGAGAAGATACCCCTGCAATT
CTTTCTATTCTGAGATTAGAGTCAATTTTTTTGATATATTCAGCTAAATAAGTAAAAGTGCTATA
AAAACTAATATCCCCTTCACAAGCAAAAGCCACATCTAAACCTTTAGCTAAAAATTCCCAAACCT
GTAATGCCACTTTCCGCCATGCCCTCTCTAAAATTTCTTGGGACAATGTATAGGGGAAAGTGAGA
GATAGTTTTTTTTGGTGGGGTTGCAAATAATTTTGAATGATAGTTTCTGCTTTGCCCAATTTTTG
ATTAATTCCAGCAGGAAAAGCAATTATAGGACTATTTTTGATGACTTTTAAGGCTTTAACGGTTA
TTAACTCTGGATCTCCTGTACCAACGCTGACACCATAAAGAGTGCCAATTCTTGAATTCATCAAT
ATTTATATGTCAATCTTATTTTTATTGTGCTAAATATATCATTTTATCTTACAGCGTCAGATTGA
CAACTCCCACCGTTAACCTTGCGGTGTAACGGGGGATTCTTCCTTCACAGAAAAATGCCTTACAA
AAGCCTTTCGTCCTGAGCGAATAGCGAAGGAACAGCCTTTGCTTAGTCTTACTCTTTCTCCAGAA
GCTGATGCGATATGCCCTACCGCACGGGGAAGCCTACAAGGTTTGGATGTAAAATCCAAACACAG
CCCCTTCAAAATTCGTAAACCTTCATCTCTGATATTTTTCGCCGCATTTACATCTCTGTCATGTAT
TGTTTCGCATTTAGGACATTGCCAACTACGAATATCCAAAGTTAGACTATCAACTCGATGTAAGC
AATGGTTACAGGTTTTGGAACTAGGAAAAAATCTATCTACCTCAATGTAAGTTTTTCCTTCCCAT
TCCGCTTTATATTTGAGCATGGTGCAGAATTGTCCCCAACCACAATCACTAATTGACTTAGCTAA
TTTGTGGTTTTTAACCATGTTTTTTACTGCTAGATTTTCTACTACTATGACTTGGTTTTCGTCCA
CTAATTTACGACTTAGTTTATGTAAAAAATCTTCTCTACATCTAGTTATTTTTTCATGTACTTTA
GCAACGGCTTTTCTTGCTTTATTACGGTTATTTGAGCCTTTCTGCTTCTTAGCTAAACGTTTCTGT
TTCTTAGCTAATTTTTGCTCATACTTACGGTAATGATTAAGATTACCATATTTACTACCATCACT
GGTGATACAAAAGTCTTTTACTCCTAAATCAATACCAATGGCTTTACCTTCTGAGTTTCGTTCTG
GTAAATCTTGCTCGTCATCATACAGACAAGAAGCAAAATACTTGCCCGATGAAGTCATCGATACC
GTAACCGATTTCAGTTTGCCTTCAGGTAAACGAGATACTTTACAGTAAACTTTTTTAAATTTAGG
TAAAGTCAAGTAATCACCGTCAAGTTTAATTCCTTGTGGGAATCGTACTGATTGTTTATTTCCTT
TCTTTTTGAATTTAGGAAATTTAGCCCATTTCTCAAAGAAGTTAAGAAAAGCACTACTTAAATCT
AAAGCCACCTGTTGTAAAGCCTGAGATGGAACTTCACTTAACCATTCCTTCTCTTTCTTTAATTG
AGGTAGTTTATTGATTATTTCATTACGGCTTAAACCTTTTCCTGTGGCTTTGTAAGTTTCGTTAG
TTAAATTAAGAGCAAAATTATAGAACCAACGACAGCAACCAAAAGTCTTAGCGAGTAGGACTTTT
TGCTCATCAGTAGGATAGATTCTATACTTGTATGCCTTTAACATTTAACTATTTAATCGCACTTT
TAATCTGATTTTAGCATAGATTGCCTATTTTGTTAAGAACTCCCTGTGGCTCGATTTTTTTGTTG
GTTCGTTTACACGAGGGGCTGTGGGTATTTTTTCGGAGAAATTCAACGGGGGTCTTTCACTCACA
CTTCAAGATAAAGACTCCTGTTATACTTGGGCATTGATGAAAAAGTGTTGTCGCTGAGGGCAGGA
AAAGAGCAATGGCAAGGAAAAATGAAGAAATATTTGATTTGTCAGCGAGGGTGTCAATGGAAAT
GAAAGTATTGGTAAGCACTATAAGCCATAGTAATAACTCCAGTAATAATTGATGATTCGTCTAAT
TCAAAGCGGGGATGATGCAGAGGATAATTTTTTTTATCTTCCATTCCAATACCCAAGCGAAACAT
TGTACCCGGTGCATAATCTAAATAAACAGCGAAATCTTCTGCTCCTAAAGATGGTTCCAAGATAT
TGATAACTTGTTCTTCTCCTAAAGCCTCCATTGCCGCTTTTTCCATTATTTTCGTTAAATTGAGGT
CATTCTGTACACTGGGGAGACGATGATTATATTTAATTTCACATTTTGCACCGTATATATTGCAA

ATACCTTCAACAATTCCTTTTATCCAACTAGGTAAATTTTCTCTAATTTCAGGATGGAGCGATCG
CACTGTTCCCACCATTTTTACTTGATCCGCTATGATATTAGGGGCACTCCCACCGTTAATTTGTCC
AATGGTTAAAACTAAAGGATGTAAAGGATTGTGGGTGCGACTGATGGCCTGTTGTAAAGCCGTG
ATTACCTGTGCCGAAATCCAAATGGCATCAATAGCTTGATGAGGTCTTGCACCATGACCTGCTTC
ACCTTGAATTATAATTTCTAAATCATCCATTGCCGAAGTTAAAGCACCATAGCGAATGCCCACAG
AACGAGCAGGAATAGTTGGAAAAACATGAACCCCAAAAATGGCATCCACATCCTTCATCGCTCCA
TCAGCGACCATCCAAAGAGCTCCCTGTGCAATTTCTTCCGCAGGTTGAAACAAAAAACGAATATT
ACCATCGATGCGATCGCACATTTGAGCTAAAATCATCGCCGTTCCTAATCCTACAGTGGTATGTA
CATCATGACCACAGGCGTGCATGACCCCCGGCACACGGGACACAAAATCAAGGGAAGTGGCCTCA
TGAATCGGCAAAGCATCCATATCCGTGCGAATAGCTAAAGTGCGATCTTGACTACCATTACCCTT
CAATTCTCCAATTACCCCAGTTTTGCCAATACCCTCTTGCACCGACAAACCACAGGAAGACAAAAC
CCCTGCCACATAAGCCGCCGTTTGATATTCTTGTCCACTTAATTCAGGATGAGCATGGAAATGAC
GACGGATTTCAATTAATCTAGGGGCAATTTCTTGAGCAATATCTTTAATTTTATCTAGCATTTAT
GGTTTGAGTAATGAGTAATAAGCCTCTTACGAGGCAGGGAACAGAGAACAGAGAACAGGGAACA
GAGAACAGTGTATAATTAAATTTTACTCTTAACTCCGAACTCCGTTCACGACTGAAAGGAGTGTA
CGAGCGCTCGAGCGTTCTCCCCGAACCCCCAACTCAAATCATTCTTCTTCTAAACTAACAGATTCA
TCAATGAAATCTTCTGGGATATTGTTCTCTTCTATCACCTCTTCCTGTTGTATAGGCTCAGGCTTT
AACATCAATACACCCAAAGGAGGTAAACATAAATCGAGAGAATGATCGTAGTTATGGAAAAACC
ACTCATCCGTCATTTTTCCTCCTAAATTACCCATGTTACTACCACCATAATCTCTTGAATCACTAT
TGAAAATTTCCCTATAAAAACCAGCACGAGGCACTCCAATACGATAATGACTATGGGGTTGGGGA
GTAAAATTACAGATAATCACAATAAAATCATTAATATCTGCTCCACGACGAATAAAAGAAACTAC
ACTATGTCTGTTATCGCTACAATCTATCCACTGAAAACCATCCCCCTCAAAATCCCTTTCATACAA
AGCAGGTTCGCTCTTATATAGATGATTCAAGTCAATAAAAAACTTTTGTAACTGTTTATGGCGAT
CGAACTGTAATAAATGCCACTCCAAATCAGTATTTATATTCCACTCGCTCCACTGTCCAAACTCCA
TACTCATAAACATGGTTTTTTTACCCGGATGAGCATACATATAACTAAATAAACTCCTTAAATTA
GCAAACTTTTGCCACTCATCCCCCGGCATTTTACCAATCATACTACTTTTGCCATGGACAACTTCA
TCATGAGAAAACGCCAACATAAAATTCTCATTTTGGTGATACCACATACTGAAAGTAACGTTATT
CTGATGGAATTGACGAAACCAAGGATCCATACTGAAATAGTCCAAAATGTCGTGCATCCAGCCCA
TATTCCACTTCATGTTAAAACCCAAACCCCCCATATAAGGAGGACGAGAAACCATTGCCCATGCA
GTTGACTCCTCCGCAATGGATAAAATACCGGGATACTGCTCAAAAATAGTCGTATTAACTTGCCT
AATGAACTCTACCGCCTCAATATTCTCTCTACCGCCAAATTCGTTGGGAATCCACTCTCCATCTTC
TCGATCATAATCCAAGTAAAGCATCGAAGCCACCGCATCCACCCTAATCCCATCAATATGATATT
TATCAAACCAGAAAAGGGCATTAGCAATCAAAAAATTACGGACTTCATTTCTACCATAATTAAAC
ACTAAAGTACCCCAACTCTTATGTTCTCCTTTTCTAGGGTCTTTATGTTCAAATAAATGTGTACC
ATCAAAATAGGCTAAACCATGAGCATCCTTAGGAAAATGCCCCGGCACCCAATCTAAAATAACTC
CAATCTCATTTTGATGACACTGATCAATAAAATACATTAAATCTTCAGGAGTACCATAACGAGAA
GTAGGGGCATAATATCCCGTTACCTGATAACCCCATGAACCATCATAAGGATGCTCTGCTAGAGG
TAATAATTCAATGTGAGTATAGCCCAATTTTTTAACGTAGGGAATTAATTTTTCTGCCAACTCAT
AATAGGTTAAGTAACGGGCAGAGGGTTTATATTCAGAAGCATGGACAATTTCAGCTCTTGCGCCA
TACTGTTTTAAAGGCTCTTCTTCTGATGTGTGCAACCATGACCCCAAGTGAACTTCATATACGGC
TACAGGTTGAGTTAAAGGATCGCTTTTACTACGTTTTTCTAACCATTCTTGATCTTGCCATGGAT
AGGTATCTAAATCACTGACCACTGAAGCGGTTTTTGGACGTGCTTCTTGAGAAAAAGCAAAGGGA
TCTGATTTAATATAGATATGACCCTCCCAATTTTTAATTTCATATTTGTAGTGAGTACCAAAATC

GAGATCAGGAATAAATAATTCCCAAATCATATCCTTTTTTTTACTCATTTGATGTTCTCTGCCAT
CCCATGAATTGAAATCCCCAATCACAGAGACATTACGGGCATTAGGCGCCCAAACGGCAAAATAT
ACTCCCTTCACTCCATCTAATTCAATGACATGAGCCCCTAATTTTTCATAGATGCGATAGTGGTT
TCCTTCTCCGAATAGGTAAATATCATAATCAGTGATGGGGGAGTCTTTGAATTTATAAGGATCAT
AAATTATCTTTTCTTGTTTACCTTCTTTTACTTTTAATTTGTAGGTATCTAATTTTTCGGTTTTG
ATTTCACATTCAAAAAAATGGGGATGATGTACCGATTTCATTGGATATTGTCGGCGACTATCTGG
TAACATCACTGATACACTATCAGCTTTTGGTAAATATGCACGGATTACCCATGTTTTTTGCCTTT
GCCTTTCTGAAATTAAGTGACAGCCTAATATCTCAAAGGGATTATGGTGCAGGTTGTTAACTATT
TGATTAACTTGCTCAATAGTAATTTTGCTAGGCATTTAATTCGAGATTCTTAACATTTTTTCTAT
ATGTTTTAATCATAATAGCTAGTTTGATCTTGAGGGTAAATCTGTTTTTCTACTATATTTTACTT
AACAATTAATTTTAACTATTGTTAACAATTTTTCAGCATTAAGCATTAAGGGAGGATAAGAGGT
ACATCAGGATGAATGTTAAATAATAAAAACATACTAATAAAGACGTCCGTAGAACATTTTTTGT
TGGCTAAAATTCATCACTCGTTAAAATAGCTTCATTAATAAAAAATCTCTGTATAATGATTTAAC
CGCTAAATCTAAAATTCTTGATAGTAGGATAAAGATGACCATTCTTGATGTTCGTGATTTACAAA
TAACTTTCACAGACGATGAAAAACAATCTCATGTTGCCGTAGATAAAGTTAATTTTAGTCTTAAA
AAAGGTGAAATTTTAGGCATAGTGGGGGAATCAGGTTCTGGAAAATCCGTGACATCATTAGGAA
TTATGGGCTTGTTACCTCCAACGGGAAAAATTAGCGGGGGGGAAATATTGTTTTCTGATAGTCAA
TCATCTAGTGTGGTAGATTTAGTTAGTTTATCCTCAAAACAAAAAAGAGACTATCGAGGCGGGAA
AATTGGTATGATCTTTCAAGAGCCAATGAGTTCCCTTAATCCAGTTTATAATATTGGTTTTCAAA
TAATTGAAGCTATTCGTCTTCATCAAGATGTTTCTGAAACTCAAGCAAAAAGAAAGGCTATTAGT
TTACTTCAAGAAGTTAAATTATTACCCACAGATCGAGATTTAGAACTGGAATATAGAGAAAATA
ATTCCTTAAAAAAACCCACAGATAAGGATATTCAACAATATATTAATAAGCGTAAACAGGCTATT
CTCCGAAAATTTCCCCACGAAATGAGTGGCGGACAACTACAAAGAGTAATGATTGCGATCGCAAT
TTCTTGTCAACCGATTATTTTAATTGCGGATGAACCCACCACAGCTTTAGATGTTACAGTACAAG
CGACAATATTAGAATTGCTAAAGGAATTGTGCCGTAGTCGAGAAATGGCATTAATCTTTATCACT
CATGACTTAGCCGTTGTCGCCAACATAGCTGATAGTTTAGCAGTAATGTATCAAGGTAAAATAGT
GGAGTATGGCACAGCTACAGATATTCTTTTAAATCCTCAACAACCTTATACCAAAGGCTTGTTAG
CCTGTCGCCCTCGCTTAGACGAAACTAGAAGCTACTTACCCACCGTCACCGATTTCTTAGAAAATA
AATTAGAAACTCAAGAAGAAAAATTCCCCGTTAAACTAAAAAAAATTCTTCTAACTCAACCTTTG
CTTACCGTAACAAATCTCAAAGTTGGTTTTCGGAAAAAAACCACATTTCCTTGGCAAAAAGATTA
TTTTTGGGCGGTTAATGACGTTAGTTTTCAACTTTTTCCCGGAGAAACATTAGGTTTAGTAGGAG
AGTCTGGCTGTGGTAAATCCACCCTCGCTCGTACTATATTAAGACTGATTCCCAATCACGAAGGA
AATATCGAGTTTTTAGGACAAAATTTAACTCAATTACCCTTAAAAAGTAAAAGTTTAAGACTTTT
AAGACGAGAAATGCAGATTGTCTTTCAAAATCCCTATAATTCTCTTAACCCTCGCCGAAATATTG
GTCAAACCATTCTCGAACCAATGATAATTCATCGCACTGGGGGCAATCAAAAAAAACGTAGGGAA
AGAGTGAGATATTTATTAGAAAGAGTTGGTTTGAATCCTGATTGGTTTAATCGTTATCCTCATGA
GTTATCTGGGGGGCAACGTCAGAGAGTTTGTATTGCAAGGGCATTGGCATTAAATCCCCAGTTAA
TCATTTGTGATGAGTCTGTTTCTGCCTTAGATGTTTCTATTCAAGCACAGGTACTAAATCTACTC
AAAGAATTACAAGCAGAATTTGGCTTAACCTACATTTTTATCTCCCATGACTTAAGTGTCGTTAA
ATTTATCAGCGATCGTATCATCGTCATGAATCGGGGTAAAATAGAAGAAATGTCCACCACAGATC
AAATTATTAATAATCCTCAAAGAGAATATACCCGTCAACTTATTGCTTCTATTCCTAAATTCCCC
AGTGTGGCGTAATCAAGTTATCTAGTTGAGATAAGACTTCCATTAAATAACAAGTTTGAGTAGAT
TTACCGTAGGCGTCAAGATGTTGAGGGGATGGAGGGATGAGGGGATGAAGAGAGAGGGAGAAAT

GAGTTAAGAGTTAGGAGTTTCGGATTTTTAATTCTTAATTATCAATTATTTACCTTTGTCTTCCC
CCTCTCTAGGGGGAATAAAGGGCAGGGCTGTTTCAAAGTAAAAAATTAGGTTAACTGAGATTAA
GTAAGAGTAAGAGATACAAGGATTTTTTTCTATTAGTTAAAACTTTATGAACAAAAATCCCCCCA
TCTCCCTCTCCCCCTATCTCCCGCTCTTTTTTCTTTCTCGAAAATGAAACAGCCCCGGGAATAAAG
GGGGGTTTGCCCTTTACCTATTTCCCTTTTCCTCATCACTCATAGATAAAAATTTACTCTGAACTC
GGATTATAACGATTAGAATGCGATCGCACCCTTGTATTCCGTTTTTTGAGCGTTGGCAGACTCAC
CACAGGTGCGAGGAGTAGGGAAAATTTTAGTAGGGTTAGCCAATCCTTTGGGGTTGAAACTACTT
CTAACATATTGCATCGTTTCTAAATCAGTTTCCGTAAACATATCACTCATATAGCATTTCTTGTC
GCTACCAATGCCATGTTCACCAGAAATACTACCACCAACACGGACACATAACTTAAGGATTTCCC
CCCCCAATTCTTCCACCGTTTCAAAAGCGCCCTCAACCGCTCCATTATAAAGGATTAAAGGATGTA
AATTGCCATCTCCTGCATGGAAAACATTAGCGATACGATAACCATATTTTTCGCTTAATTGATTG
ATTTCAGCTAATACTTGAGGTAACTTAGAACGAGGGACAACCCCATCCTGTACAAAATAATTGGG
GCTAATTTTACCCATAGCCGCAAAAGCCGCTTTTCTCCCTTTCCATAATTTTAAGCGAGTTTCTTG
ATCATTCGCAGAGGTTATACTACCTGCACCACACTGACGGCAAATTTCTGCAACCTTCTCTTTATA
GTTTTTCACCTCCACCTCTAAACCATCCAACTCCACTAATAAAATAGCCTCCGCATTGCGAGGATA
ACAGTCAGTCGCTACTACATCTTCCACCGCATTGATACTAAAATTATCCATCATCTCCATACCTGC
GGGGATGATACCAGCCTGAATAATACCAGCAACGGCTTTTCCTGCATCTTCCACACTTTGAAAAT
CTGCTAAAACAACACAAATAGACTCAGGAGTTTTGAGAATTTTTAAGGTAATTTCCGTGGCAATA
CCTAAGGTACCTTCTGAACCCACAAATAAGCCTGTTAAGTCAAATCCGGGAGTTTCTGGAACTTT
CCCGCCTATTTTAAGAATTTCTCCTTCCGCAGTTACTATTGTTAAACCTAAAACATGATTAGTTG
TTGTACCATATTTAAGACAATGAACCCCCCCCAGAATTTTCTGCCACATTACCACCAATAGAGCAT
ATAATCTGACTAGAAGGATCAGGAGCATAGTAAAAACCTGCACCACTAACCGCTTGTGTTACCCA
ATTGTTGATGATACCGGGTTGCACAATAATACGCTGATTATCATAATCAATGTCTAAAATTTGAC
GCATTCTTGCCGTAACGATTAAAACGCAATCTTCCACAGGTAAAGCTCCCCCTGATAAACCTGTA
CCTGCCCCTCTAGCTATCCAAGGAATTTCGTATTGGTTACAGATTTTGACAATCTCTGCTACTTCT
TCTGTGGTTTTGGGTAAAGTGACTAAGGCAGGGCGTTGACGATATTGAGGTAAACCATCGCATTC
ATAGGTAAGAAGTTCATCTTTTCGTTTGACAACTCCATTTTTACCTAAGACAGATTCTAGTTTTT
TAATGATGGGTTGCCACTTATTGTCAATGGAAGATAAAAACATAGCTTATTTATAAAGATGGGT
GAGAGGTTAAAAATTTGAATGAATACTTTTTAAGTTTCTATGATAACCTCAGTTTGGTTTAAGA
ATATAGGATAAGAGCAGGTTTACTTTATGAAAGAATTTAGGTATTGAGGGACTTATAAAGAGTG
GGCGATAGGATAAAATCGTATTTTAATTGACAATTTCTCTCTAAACATCTATATTGTCAACCAAA
AGTTGACATTGTCTTTATGTAGAGTTAGAAGCAATGGAGTTGAAAGTGTGAGAAATAGTGGTTT
TGAGAGGAAGCTAACTTTAAAATACTTCGGAAACAAATTTTTGTGAAAATGATCACATTAGATA
GTTTATGGATAACAAAAAATAGACAAAAACAAGTTAGAATCTGATTGTTGATAGTTAATTGATG
TCTATATTAAAGTTTTAAATTAATCTCGAATTCCTCATGAATAATTATCAAGTTCTTTCTCCTCA
ACATTTACCATTAATTTTGTTAACGTCTCTATGGCTTCTCACACCTCAGAAAGTTAGTGCGGGTG
CGATCGAAATTAGCATTGACCCTTATAATGATTCGGGATATGAGAGTATTCCTCTTGAAAGCAAT
CAAAATCCTAATGACAATACACACGCTTCTCCAAAACCTAACTTATCCGTTATCGATAAAGAAAA
TATATCTAGTGAAGGAATTTATTATGTAAAAGGAAACTCTCCTCCTCCTCCCCCGCAAATAGTTG
ACGGCAATCTCATCCAATCAAACCAGAGCTTACCTGCAATACCTCCTCTAAATGAGCCTTATAGCC
CCCAACAAACTGCTGATGTTAATCCTCGAAATATTACTCCTACTAACGAATTGTCTGTTTCTGTT
TCCCCTTTGAACGTTACACCTTCGACAGAAAATACTACCTCTAATAATGCTCCCACGACGAATAA
AATTGTGGTAGAAAACTCTAACAATAGTATCCGCAATATAAGCGTAAGTCCTCAAAACTCTAACT

CAAATTCTGTTTCAGTTAACAACAACTCAAATAACTCCTCTATGGGTAATTTAGGTAAAAGGAGA
AATCTGCGGGAAATTTTAGTTTTTTCTAATTCAACAAATTCTGAGAATATCAAAAATAATGGCAA
TAATATGCCTTCTGAATCCTTACAATCTTCTTCTAACAATAGTCAAACTGTATATAAAGTTTTAG
CTAAAACTAACGGTAATAACCAAGAAGTGGGAGTAAAATCTCTCTATCCTGAAGCCTTTAAAACT
AATTATCAAGGACAATCTTTACTACAAGTGGGGGTGTTTAGCACTCAGGAAAACGCTGAACAAGT
ATCCCAGTCTCTTGCAAATATAGGGGTTAAGGCTATTATTTTAAAATAACCTGAGTTTGGGATAA
ATTTTCATCTATGAGTGATGGAGAAAAGGGCAATTAATGAATAGTGAATAGTTGATAATTAAAA
ATTAAAAATTAAAAATTAAAAACTCCGAACTCATTCTCACTTCTCCCTCTCACCCCTTCCCCTCAT
CTCCCCATCTCCTCCTCCCCTCATCACCTCATCACCCCTTGTCTCCACTCCTTACTCCTTACTTTCT
CCTAAAACCTGAAACCTGCCACCTGATATCTTTTCCTAACCAATAATTTACACGACGAGGACTAA
GAGAGCCGCTTATTTCTCGGGAAAAACCGAAATATTTTAGTTTTCCCATAAACGTCTTCTTGCAC
TGCCTTTAAAGCGATCATGACTATCAGCTAATAATGCCGGTATATTGAGTTTTTCTGGGCATTTT
GGCAAACATTCACCGCATTCAGTACATTTATCTCCTTTTCTTCCCCAAAACCAGTGTCCTGCGTTT
TCAAACATTTGATAGCGATATTTTGCATATTCTGTCATTTCTAAGCCAACTCCTAAATTTCGTAA
TCTCAAAATTTCAGGAATATTTATATTTTCAGGGCAAGGAAGACATTGATAACATTGAGAACAA
TGATCTGTTTTTAGAGTTTGTTTAAGGGTATTTTCTATGTGGTTGAATGTGTCTATTTCCTTCTT
ATTTAATGGTTCATCTCTGTCGGCAACTGTGAGAGGATAAATTAATTCTTCTGGGTTACTAGCAC
CTAAACTTAAGGTTGAAATACGGCGATCGCACAGTAAAAAGCGATAAGTTAGAAGTAGAGGAGA
AAAAGGTTTACAAAGTTTTTCCAATTTCGGAGAAGGTTGATACAAAAGTCCTCCTTTGTCGGCGG
GAGAAATAATAAATACTCCTAAATCTAGTTTTTTTGCTAATTCTAATACTGAATAATTACGTTGA
AAAAAATAATAATAATGAATGTTAATAAATTCAAATAAATCAGTTAAAATAGCTTCTTTAATTA
GTTCTAAACTGCCATGACTAGAAAATCCAAAATGTTTTATTTTTCCTTCATTTTTTGCCTTAGTT
AATATTTCTAAAAAATCTGTTTTTTGTATTAAATCTAAGTGTGATGGTGTATTTATTCCGTGAAT
AGCAAGACAATCTATATAATCAGTTTGTAGATTTAAAAGAGACTCATCTAACCATTGACTAAAAG
TATTTTTATCTTCATGGGGGGTTAATTTAGTCGTAATAATAACTTTTTCACGGGGAATGTTTAAC
TCCTTTAAAGCACGACCCAAATAGGCTTCGCTGTTACCATATCCTCTTGCTAATTCTAAATGATT
AATACCCAACTCAAAGGCTTTTGCTACGGTTGACAATAACTGAGATTGACTGTAGCAACAACGCA
TTAAACCGAGGGAAAACACGGATATATTTAGTTCTGTTTTGCCAAAACGGCGATATTTCATTGTT
AATTACCTTTGCCGCAATGCCTCATTTAACTTTGCCTTTTCTTAATCTTCGCTGTTACTTTTTCGG
GCGCGACGGATTAATTCTTCAGGGCTTAAATTAGAGACAAAATCTCTGAATGCCTCTCTTTCTTC
CTCGTCTGCATCTCTATCAACGGGGATTGAAGCATCTAGTATTACTTCTTCCATCACCCAAATAG
GACAACCAGCTCGTAGGGCAAGGGCGATCGCATCACTGGGACGACAATCAATTTGCTGTTCTAAT
TCTCCGCCTTTGAGACATAAAAGAGCATAAAATGTATTATCTTCTAAAGCATTAATAATAATGCG
ATCGAGTCTTAAATTCCAAGCAGAAAGAATATTAACTATTAAATCATGAGTTAAAGGACGAGGA
GAAACCTGTTGTTCTAACGCCCCAATAATTGACCTTGCTTGATCCTGTCCGATATATATTGGCAA
TGCACGGCGTTCTGTGGCATCCTTTAAAAGAATGATCGGACTCCTACTAATAGCATCAAGTGCGA
TCGCCGCCACTTTCATTTCAATCATTGATTTATTCCTCGAAAAGAATTTTAGCAGGTCAATAATT
TCATTATAATTGCATTCTGTACCAAGTTTGACCAAAAATTTCAGATAGTGAATAATGAATAATTG
GGGGGTGTCAGGTTTTAGGAGAAAGTAATGAGTAGAGAGAAGGGGTGATGAGGTGATGAGGGGA
GGAGGAGATGGGGAGATGAGGGGAAGGGGTGAGAGGGGGAAGTGAGAATGAGTTCAGAGTTTTT
AATTTTTAATTATCAACTATTCACTATTCACTATTCATTAAATTGCCCTTTTCTCCATCACTCATA
GATGAACATTTATCCAGAATTCAAGTTACTTACTCTTATTCTACAAATTAAATAGAAACCAGCTT
ATGACATCAACAAAGACTAGCTTTATCATCACTAACGGGAGGCTAGTGGGTTATTCTGACTTACA

AACTCTAGTGATCGAGAATGGTAAAATAAAATCTATACAGCCCTCTCATTTCAAGACTAATTCAG
ATTTGGATTTAGAAGGAGATTATCTTTCTTTAGGGGGAGTTGATTTACAAATTAATGGCGGATT
AGGATTAGCTTTTCCTGATCTTTGTTTTGATGATATAGATAAATTGCATGATATTTGTGCTTATT
TGTGGTCAACGGGAGTAGATCAATTTTTACCCACTATTGTCACCACTTCTGTTGAAAAAATTCAT
CAATCTTTAAAAGTTATTCAAGAGTTTAAACGGGAAAAACCAAGAAAAAATGAAGCTGAAATTA
TAGGGGTTCATTTGGAAGGACCTTTTTTAAACTATGAGAAAAGAGGCGCTCACCCCCCAGAATAT
TTGTTATCTTTAACCCTCGACAACATCAAAATAGTTTTAGGGGCATATGCAGATGTAGTAAAAAT
AATAACTCTTGCTCCTGAATTAGATGAGAAAAAAGAAGTTATTCCTTACTTGCAGTCTTTGGGCA
TAGTGATAAGTTTAGGACATTCTCAAGCTACAGCAGAAGAGGCTATGATTGCTTTTCAACAAGGA
GCATCCATGGTTACTCATGCTTTTAATGCCATGCCGAATCTTCATCATCGACAGGCGGGTTTATT
GGGGGAGGCTATTGTTAATCCTCATGTTTATTGTGGTTTGATAGCAGACGGTAATCATGTTTGTC
CGACTATGCTTAAATTGATTTTACAAGCTAGTAATTATCATCAAGGGGTGTTTGTGGTTAGTGAT
GCCTTAGCCCCCATTGGCTTGGGCGACGGTGTTTATCCTTGGGATAGTCGTACTATTGAGGTCAA
AAATGGTACTGCTAGGCTTCCTGATGGTACTTTATCTGGTACTACTTTGCCTTTGTTTGTTGGGG
TTCAAAATTTGTATGAATGGGGTATTTGTAATATAGAAGATGCGATCGCACTTGTGACTGATGCA
CCAAGAAAAGCCTTAAAGATGCCTTGTTTAGAAGTTAGTTCTCCCGCTAATTTAATCCGTTGGCA
TGAGGATAAGCAAAATAAAAAACTCCATTGGCAAAGAATTAATAACCTGATTTGAATATAATTT
TTTAGTAATTGAGATTAAATATAATGAAGACATACAATTATCTTTGCCTATCATCAAAAAATGTT
ATTAACAAATAATCATAAAAATTTAATTTTAAATAATTGTACAAAACAAGATATTATCAATTAT
TTAAACAGTGCTTGGGAAACAGAAGATATTTTATTTAAAAGTATTCAAGACGATCGAACCTTTTA
TCTTAATCCTGATTCTCTGCGTAATCCTCTCATTTTTTATCTTGGTCATTCGGCGGTATTTTACAT
CAATAAATTGCTCATTGTTGGTTTATTAGACACTGGAATTAACCCTCTTTATGAAAAATTATATG
AAATGGGAGTTGATCCAGATACCCCAAATGAGTTACAAGATGCGATCGCATCTATTCAATGGCAT
GATGTAGAAAAATTATGGACATATCGCCATCAAGCCAGAAATCTATTGATTGACATAATTAAAA
AAACTCCCCTTGAGTTACCTATCACCCCTGAAAGTCGATGGTGGGCAATTATTATGGGGATAGAG
CATCAAAGGATACACATAGAAACATCTTCGATGTTAATACGACAATTACCCGTAGAATTAATCGC
AAAACCTTCATCTTGGTATTATGCCTCAAGCAAAGGAAAACCTCCTAAAAATGACATGATAACAG
TCAAAGGAGGAGTCGTGAACCTTGGCAACCCAGAAAAGGCTTATCTCTACGGTTGGGATGTAGAT
TTTGGCACAAGAGAAGTAATTGTCAATGATTTTGCTGTTAGTAAATACCCTATTACCAACGATGA
ATTTCTGCAATTTGTGAAGTCTGGAGGTTATCAAAATTCAAACTATTGGCAGGAAGAAGGATGGC
AATGGCGAGAAACAAATCAAGTTACCCATCCTAAATTTTGGATACTAAATCAAGATAGTTACCGT
TATCGTCTCATGTTTGATGAGATAGATTTACCCCTCGATTTTCCAGTAGAAGTCAACCATCATGA
AGCGATCGCATTTTGTAATTATTATAGTCAGAAAATAGGTCAAAAATGTACCATGATGAGTGAG
GCACAGTGGCACTTAGCCAGTGGGAAAAACCAAGATGACGAGAATAACTATAACCTCAACCTAAA
ATATATTTCCCCGACTCCTGTTGGTAGCATAGAAACAGCACAAAGTGATGAAGGAATTTACGATT
TAAGAGGAAATGTCTGGGAATGGTTAGCGGATATATTTACACCCCTAACAGATTTTCAACCTCAT
TATCTATATCAAGACTATTCTGCTCCATTCTTTGATAATCGTCATTATCTTTTAAAAGGTGGTTC
TTGGGCTACCAATGGTTATGAAGCCACCCCCTATTATCGTAATTGGTTTCGTCCTCATTTCTTCCA
ACACGCAGGGTTTAGAATATCAAACCAATTTTCCTAGTTATAGTAGCAGATTTGGAAAGAGGCAA
AGTTAAAAGGGCAAGGGGCAATGGTGAATAGTTGATAATTAACCTCAGTTTGGTTTAAGAATAT
CCGATAAGGTTAGGTGTTAGGTGTCAGGTTGCAGGAACAGCAGGTGGTAGGGGTTTTTAGCAAGG
GGCTTAAGCCCCTTGCTTAAGTCAGTTCGGGTTAAGGCAATTTTATAATTATTTCTAAGAAGCTA
TAAGGTTTTCTGACTATGAGTTGGGATGCTTTCAGCTTATCCAAAACTCAGGTTAATTAAGAATT

AGTTTCTCCCTAGCACCTCTTTTCTAACCCCTACTCAGGTGTCCTTATCCTATACTTTCAAGCCAA
ACTAAGATTAATTTAACTTTCTGTAATCAATTCTCTATCTTCTCTAATTCTTTGCTCAATATTTT
TCCCGCCAATGAAAGAGCGTAACATCCACGCCATTTCTTCGTGTTGTTCCATAATACCCGTTAAG
AAATCGGCTGTACCTTCATCATGAAATTTTTGAGAACATAAATCAATATGCTTACGAAGGTTGCG
AATTATATGTTCGTGGTCTATTACTAAACGCTGTACCATTTCTGTAGAGTCAGGTAAGTCGCTAG
GATGCTCTTTTAAAGTTGTATTCTCCAAAAATAGTTGCGCCGTACCAATGGGATATTCTCCTAAA
GCTCTAATTCTTTCCGCACAGTTATCTATATTATCTGAAATGATCTCGTATTGACCTTGCCAAAT
TTCATGGAGGGTTAAAAATTGAGGTCCAACTACATCCCAATGATATTTTTTAGTTTTGATTTGCA
ATAGATAGAAATCTGCTAAGTTTTGATTTAATAATTTAACAACTTCTTGAATTTCTGTTTTGCTT
AATCCTATATTAATAGTATTCATAAATTAATTTTTTCTTATTTGCACTTACTCTCTATCATGACA
ATTATTGAAATTATTTTCATCTCTCAAAAGTATTAAATATCTCTACCGAAAGAAGTATTTTATTA
CAGAAGAATAGATTAAAAATATCATCTTTTAAGATGAGTAATTTGAGGTTTATTCCTCTACCTAC
AAAAGTTGCCCTTACTCGCACACACTATGTAAATATAACTCTTTGAGTTTTTGATTTTTTCAAAT
TATTTTATTCTTACAAGCAAAACTAGCTAATTCAGTACGATTAGTTGAGCCTGTTTTTCTTAACA
AGTTACTCACATATTTTTCTACTGTTTTTGAGCTTAAATATAATTCCTCTCCTATTCTTAAGTTA
GAAAATCCTTTAATCAAGAGCCTTAACACTTGTCTTTCCCTATTTGTTAACGAAATATCTAATTT
TACATCGGACTTCGTTTGACTTAATTGAACATTATGACGAGTAAATAAATGCTTAATAATAGCAA
TTAATTCTGGTGGGTTAAAGGGTTTTTCTAAATAAATATCACATCCTGCCTGATAACCATTTACC
CTATCTTGAATACCGTCTTTATTGGTTAAAAAAATAACAGGTAATAATTCAAATTCTTTTATATC
TCTTAATTCTTTTACTAGCTCAAAACCATTTTTCAAAGGCATAATTATATCAGAGATTAATAAAT
GAGGATGATAAATTTTTGCTAAAGAAATCGCTTCCTGCCCATTACCCGCTAGAATTACTGAATAA
TTTTGTAATTCTAAACATTCTTTAAGTAAATTTCTAATAATTAAGTCATCATCAGCTACCAAAAT
TTGCCATGTCATAATTATCACAACTTTAAAGTATTTTTATGCGAGGCTATTGACCTTGTTTATAA
TTTCATTCTATATCCATTTTTATTTGTTTACGAATGTTTATTTAACAATAATTAAAATAATAATT
ATATTGTTTAAAATGTAACAATTTATGCTTATTTTTTTCTTTTATTACCATTCATTGCTATTTGA
TAAAGTTGCCAAAATGCTGAATAATTATTTACTGATAAATGACTATTAAGCTAAAATTAAAATT
TATGATAACGGATATAGAATCTCGACTTAAAACTCTTGCCCAAGAAGCCCAAGAGGCGATCGCAT
CTTCCGAGAGTTTATCACAACTAGAAGAATTAAGAGTTAGTTATTTAGGTAAAAAAGGTCAATT
ATCCCTCATTTTAAAAGAAATGGGTAAACTATCCGCCGAAGAAAGACCCCGTATCGGTGCGATCG
CAAATGAAATCAAAACTACTATTCAAGATAACTTAAGCAATAAACAACAAAATTTACAACAAGC
AGAAATCGAAAAGCAAATTTTAGCAGAAACCCTCGATGTCACCATGCCGGGGGTGAGCTATCCCT
TGGGTAAATCTCATCCCTTACAAAGTACTATTGATCGCATCTTAGATATATTTGTCGGCTTAGGA
TATACAGTTGCCGAAGGACCACAAATCGAGAGCGACTACTATAATTTTACCGCCTTAAATACCCC
CGAAGATCATCCAGCTAGAGACATGCAAGATACCTTCTATTTTGAAGATGGTAGCCTTCTGCGCA
CTCATACATCATCCGTACAAATTCGTTACATGGAAAAAAATGAGCCACCCATTAGGATTATCGCA
CCGGGTAGAGTTTACCGACGAGATACCGTTGATGCCACCCATTCCGCAGTATTCCACCAAATCGA
GATACTAGCCGTGGGGAAAGGCTTAACCTTCACCGACTTAAAAGGTACAATCAAAGAGTTTCTCA
GACAGATGTTTGGAGACGATTTACCCACTGTTTTCCGTGCCAGTTACTTCCCTTTCACCGAGCCTT
CAGCAGAAGTTGACGTACAATGGCAAGGTAAATGGTTAGAAGTACTCGGATGCGGTATGGTTGA
TCCAAATGTACTCAAAGCAGTAGGTTATGACCCAGAAATTTACACAGGATTCGCCGCAGGGTTAG
GAGTTGAGCGCTTTGCCATGATTTTACACAAAATCGATGATATTCGCCGTTTATATAATAGCGAT
CTCCGTTTTCTGCAACAATTCTAACATGAGTTCGGAGTTGGGGGTTCAGAGTTAAACTTAAAAGA
ATATTTAGCGAAAATTATATCACTTTTCCCTGTTCCCTCTTCCCTTTTTCCTCCACTTCAGCAAGA

AGTCTAATAAATTTATATCGAAGAGAGCTACTAATAGATTAGGATTAGATATAGTCGTTTTTGG
GGTTTATTTGTGTTTTTTAGCGTCGTAATTCCTACCTACAATCGTTTACCGATTTTACAAAAATG
TCTGAGGGCATTGGAAAACCAACAATTTGATACAGGCTTAATCTCTGATTATGAAATTGTCGTGG
TGGATGATGGCTCAACAGATGCGACTATCTCATGGTTAGACACAGAAAAAGCCAATTTACCCCAT
GTTAGACTATTTACCCAAAATCATCGAGGAGCAGGAGCGGCTCGTAATTTAGGAGTAGAAAATGC
CGTTGGTGAGTATATTATCTTTATTGACAGCGATTTAGTAGTGACAGAGAGCTTTTTACAAAGTC
ACGCTCAAGCCCTAATTACCGCCGAAAACAAATTACAATCTGACTTAATTTTTACCTATGGTGCA
GTTATCAATACTTGCAACTTTGACAATCCTACATCAGAACCTTATAAAATTACAGACTTCTCCGC
CGCTTATTTTGCGACAGGTAATGTAGCCATCAAAAAAAAGTGGTTAATAGAAGCGGGAATGTTTG
ATACCATGTTTAGTCAATATGGTTGGGAAGACTTAGAATTAGGCGTAAGACTGAAAAGGCTGGGC
TTAAAACTAATTAAATGCCCATCAGCAGTAGGTTATCATTGGCATCCTCCCTTCAATCTCGAACA
AATACCCCAATTAATTGAAAAAGAGATAGAAAGAGGCAAAATGGGGGTACTTTTTATCAGAAA
CATCCCACCTATGAAGTTAAAATGATGATTCAAATGACATGGTTGCATCGACTATTATGGGGCAT
TCTTTCCCTTGGCGGTAGCTTAAATGAAAAAACAATGAAACCCCTTTTGCAATGGCTAATTAATC
AGGGAAAACCCCAACTAGCCCTTGAAATAGCCCGAATCTTTCTTAATTGGTACAACGTGCAAGGA
GTCTATGATGCTTATAAGTTAAGTAAAAACACTTAAATTTTATTAAAAAAAATAAGTAAATAAG
ATTTATCCATATAATACTGTTAGACCAAAAATAAATAAATACTCATAAAAAAACTCTTATAAGA
AAACACGGAGGAAAAATTAATGCAAGTTTTATTCTTACTTATAGGTTTAGTTGTTGGTGCGGCAA
TATTGTGGTTTATTAACAAAGACAAGGTTGATGCTTCTGTTGTTGAAGCAGAAAGGCAGAAAGT
TTTAAACCAAGCCGAAGGAGAATTTGAAGCCCGTAAGCAAGAGTTAATTGCCTCTTTAACTCGAG
AATATGAAAACCAAGCTAATGAAAAAATTGCTGAAGTTGAAAGTAGTTACACTAACCAAATTCA
AGAATATCAAAATCAAATTAGTGAATTACAAACAAATATCACCTATTTAGAATCACAAACAGAG
CAGGTAACGGAATTACAACGCAGAATTAATGATTTAGAAAATGCAGAAACTCAAATTTCACAGT
TACAAGCTAAAATTGCCGAGTTAGAGGCAGGGAAAGGTGATTCTGATGATGTGGATGGGTTAAA
TACCAGAATAGCTGAATTAGAAACTCAATTACAATTACAGCAACAGCAATTTGAAGATTATCAAA
ATCAGCTACAACAGTCTTTTCAAAGCCAGATAGAAGATTTACAAAATAATCATCAAACAGAAATC
GAGGGCTACAGCATAAGAATTCAACAGCTCGAAAGACTAATTCCAGATTCTGACGCTATTATAGA
TTCTATTGCAGGTACGCAAAACGCTTCAGAGGAATTTTTACAAGAAGAAAACTCAGATATTAATA
ATCAAGAAGAATCCCTAATAGAAGATGAAACCCCTGATTTTGGTGCTTTTTCTGAAGAAGAATCT
TCTTTTGATGTGCCAGAAGAATCTAGTATTGCAACTGAAATCGCAACACCTTTTGATAGTGTAGA
GGAAGTTGAGGAAGCCTCTTTAACCTTTGATGAAGTAGAAGAAGGTCAAGATTTTGTATTTACCC
AAGATGATACTTCTGAAGAATCTTCCCTCGACTTTTCTGAAGAGGAAGCCAATATTGCAACAGAG
ATTTATCCCCCTCTTGATTCTGTTGAAGAAGATACTCCTCTTGATTTAGAAGAAGTGCAAGAAGA
AATTAGTAATGAAGTAGAGGAAGAATCGCCTTTTGACTTTGGTGCAGACGAAAGCGAAAATGAA
ACTCCCGATTTTGCTTTGGAAGAAATTAGTGAAGTAGAGGAAGATTCACCTTTTGATTTTGGTGC
AGATGAAAGCGAAAGTGAAACCCCTGACTTTGCTTTGGAAGAAATTAGTGAAGTAGAGGAAGAT
TCACCTTTTGATTTTGGTGCAGATGAAAGCGAGAGTGAAACCCCTGACTTTGCTTTGGAAGAAAT
TGATGAGGATGATTCTAGCTTAGATTTTGTGATGGAAGAGAATGAAACAGAAATAGATATGTTT
TCTTCTGAAATGGATATATCTAGTGATGATGATTTAGGGGATGATTTGAATTTAGATGCTTTTGG
AGAATCTCAAACAACTTCTAGTCAGGATGAATTAGACATTTTTTCTGATGATTCTGATTTTTCTT
CCACTGACGATGAATTGTTAGAAATTAGTGATTCCGATGATGAATTTAATTTTGATTATACTACT
GAATCTGCTAGTGCTTCTGCATCCATCGATAATGATGATCAAGATGATATTTTAGGAAACCTAGA
ATTGTCTGATAATGATGATAATGACTTAGGTTTAGATTTTGGCTTGGATGATGATTCTGATGAGG

AGGATTTTGACTTAGATAGTCTTTTAGAAGATTCAGATAAGAATTAAAAATTAATATCAAAGGT
GTTTAGGTGTGAGGTTCAATAAAAACTATTCTTTTATAATAAGAGGTAATAATTTTATTTGGTTC
TTCAGAGTGTGGTTATGATAAATTAAGAGAAACAGGACTTCCATAACCTTTATTGAATAGTGTTT
ATAGTAAAATAAAAACTAAAAGTTTATAAATTATTATTTAATAATCCCCTATTGCCTATTGCCTC
TTGCCTCTTGCCTACCCTAACTAATAATTTACACGACGAGAAGTGATAGAGCCTTTTATTTTTGT
TATCAGTAGTTAATAATAACAAAAAAATAAGGGGTAAAAACCCCTAACTCATTACTTGTTACTTA
TTACTAAAGTCAATTAGGCTTCATCCAAAGCGGCAATACCGGGTAAAACTTTACCTTCTAATAAC
TCTAAACTAGCACCACCACCAGTAGAGATATGACTCATTTTCTCAGCAACACCGACTTTTTCTACG
GCGGCAACGGAATCACCACCACCAATGATAGTAGTTGCTCCTTTTGCGGTAATTTCTGCTAAACT
ATGTGCGATCGCTTCTGTACCTTTAGCAAATTTTTCAAATTCAAATACTCCCATAGGTCCATTCC
AAACAACGGATTGACAGTCGGCAAGAGCAGTTTGGAAGGTTTTAACGGAATCAGGACCAATATCT
AAGCCCATCCAACCATCAGGAATGCTATTAATATCAACGGTTTGAGCGTTTGCATCAGGAGCAAA
ATTATCAGCTACAATTACGTCAGTAGGTAATAAAAATTCTACACCTCTTTCTTTTGCTTTTGCTT
CTAAAGCCTTAGCTAAATCTAATTTATCTTCTTCTACTAAAGACTTACCAACACTTAAACCACGG
GCTTTATAGAAGGTGAAAATCATACCACCGCCGATAATTAATTTATCGCATTTTTCTAATAAGGT
TTCGATAACACCGATTTTACTAGAAACTTTAGAACCACCGATAATAGCTACTAAAGGACGTTTGG
GAGATTCGATCGCACTTTGTAAGAATTTCAACTCTTTCTCAATTAAGAAACCAGCCACACTGGGG
GATAAATAGTGAGTAACCCCTTCAGTGGAAGCATGGGCGCGGTGAGCAGTACCAAAAGCATCATT
AACGTACAAATCAGCATTACTAGCTAATTTTTTCGCAAATTCAGGATCATTAGCTTCTTCTTCAT
TGTAAAAACGGAGGTTTTCTAATAAGACAACATCACCGTTAGCCATCCCATTAACTGCATTAGTC
ACATCATCACCAATACAGTCATCACACTTAACCACATTTTTACCCAATAACTCAGAAAGACGGAC
TGCCACAGGAGTTAAACGGAGACTATCCTTAACTTGACCTTTGGGACGACCCATGTGACTACACA
AAATTACTTTCGCACCTTTTGAGATTAAATCATTAATGGTAGGTAAAGCCGCTTTGATACGAGTA
TCATCGGTAATCGCACCGTTATCCATAGGCACGTTGAAATCCACGCGCATTAATACTCTTTTACCT
TCTAAATCAGATTTTGTTAAATTTGCAACACTTTTTTTAGCCATAGTGATATTTTGGAATATTTA
TCAGACAATAAAAATAGAAGAAGTAAAAAAAATTACTCTTTGCCGATTTTACCTGACAATGTCAC
TAGAGGTAAGTGCTATGTTCAAAAACATTCTATTTCCCATTGACCAAAGTAGAGAAGCCAGAGAA
GCAGTTGCCGTCGTAAGCAACATCGTTAAAACCTATGAAAGTAAACTATATCTACTATCAGTTGT
AGAAACAAATCAAGAAGAAGATGCTGTGATGAGCGACATGAAAACTGTTGCTCAATTACTCAAA
AATGCTCAAGATTTATTTTCCCAAGCTGAAATTATAGCGGAAGTAATAGAAAGAGAAGGAATGC
CTCCTTTTGTTATTTGTGATGTTGCTGATGAGATTGAGGCTAATTTAATTATTATGGGATGTCGT
GGCTTAGGTTTAACTCAAGAAGGAGTTGAAGATAGTGTCACTAATCGGGTGATTAATCTTGCCCC
CTGTCCTGTTTTAGTTGTACCGTGAATTAATTAGCAATTAGTAATAATTAACCAAAGCTCTGGAT
AAATTTCCTTGGCTATGGTAGCTGATTTGGGACACCCCTGTATATCTCCCCTTTTAAGGGGAGAA
GGGAAAAAGTCATTAAATTGAGAAAAAAACGTATTGAAAATCAATTTAACCGCAGTTGATGTCAA
TTATGAATACTGTACCCTTTTTTCTTTACAACAGACAATCCTATATCAAACTCAGTTAACTCCAT
CTCTAGGTAAAGACTTTTTGAGCAATCTCTAATTACTAATCACTAATTGCTAATTGCTAATTGTT
AATTCCTCATTAATTCTAACTAGGCTTTTTTAGGAGAAAGAAGCATTATCATATTGCGTCCCTCT
TTTTTGGGTCTTTGTTGAACTTCTGCCATTTCCCCTAAATCTTCTGCAAGACGCTCTAATAATTCC
TGTCCTAAGTGAGTGTGTTGAGCTTCTCTTCCTCTAAAATTGATTGTTGCTTTCACTTTGTCACC
AGCTTTAAGGAAGCGTTTTGCTTGATTGAGACGCACTTGATAATCATGCTCGTCGATTTTGTAAC
GCATTTTTACTTCCTTAACATCCGCAGTATGTTGCTTTTTGCGAATGGCTCTTGCTTTTTTCTCCT
GCTCAAATTTGAATTTGCCATAGTCCATGATGCGACACACAGGAGGATCAGCCGTTTCACTGACT

AAGACGAGGTCTAATTCTTTCTCTTCCGCTAGACGATTTGCGTCTCTGGTATCAAGAACACCAAG
TTGTTCTCCGTCTGTGTCAATTAATCTGACTTTAGGAAAACGAATATTTTCATTAATTTTGGGTA
AATCTCTTTGGTTATTTTTTTTGTTGCTATAGGTCACAGGCTTTGATTATTTTCTCCAATATGCT
TTCTATAAAATATTTAGTTGATTTGAATAAGTACGAAGGATTAGGTTTTTTTCCTAGTCAATATT
ATAATTGGTGATTGCTAATCTGAATTAGTTTTTCTCGATATTCGTCGGTGAAATACTTTTTCTAC
TGGTAATTTAGTCTTATTATTAAAAAAAATTAACAAGGTTAAAGGTTAATGGATTCAACGATAT
TTCAAGCCCTTTTCAATGGTTTAGCGGTGGGAAGTATTATTGCATTGGCGGCGGTGGGTTTAACC
CTAACTATGGGTATTTTACGTCTGTCTAATTTTGCTCATGGTGATTTTTTGACCGTTGGTGCTTA
TTTGACTTGGTGGATTAATCATTTTGGGTTGAATATTTGGTTGTCAATGGCGATCGCACTTTTGG
GGACAATACTTATTATGTTAGCAGGAGAACAACTATTATGGAAACCTTTGAGAAAACAAAAAGC
CAGTAGTACCAGTTTAATTATTGTATCCATCGGTTTAGCCTTGTTTCTGCGCAATGGTATTTTGT
TCATTTGGGGGGGGAGTAATCAAAACTATGATTTACCGTTGGTTGAAGCCCTCGATTTTGGTGGT
TTAAAAATTGCTTATTATCGTCTTATTGTCATTATTGTTACTATTTTGGCTATAGTGGGACTTCA
TTTGGTACTGCAAAAAACGAAAATTGGGAAAGGCATGAGGGCGGTGGCAGATAATATTGATTTG
GCGAGGGTATCAGGGATTAATGTTGAGCAGATAGTTTTATTCACTTGGGTTTTAACAGCAATACT
TACCGCCACGGGAGGGGGATTATATGGATTAATTACTGCAGTGCGTCCTAATATGGGATGGTTTT
TGATTTTACCTATTTTTGCGTCTGTGATTTTAGGGGGCATTGGTAATGTTTATGGTGCGATCGCC
GGTGGTTTGATTATTGGTGTGGCGCAAGAATTGAGTGTTTTTATCGTGGGTTCAGAATATAAACT
CGGTGTTGCTTTACTGATTATGATTGTAATTTTGCTAGTAAGACCTCAAGGCATTTTCGGTAAAT
AGTTGATAATTAATAGTGAATAGTGAATATTTAATAATTAATAACTTCTAACTCTGAACTCCGTT
TACGACTGAAGAGAATGTATGAGTGCTTGAGCGAACTTTCCGAACCTTAACACCTGAAACCTGAA
AACTGTCCCCATGGATGAGATGTTAAAATGAACCTGATTTGACAAAACTAATATATTTGGAGAAA
AACATTGGATCGTCAAACTACAGGCAATATTTCAACTTTAATAGGCGGAATTCTTGCCGCTTTAA
TTGTTTTTATTGGTTTTAATTCCTTTATTATTATCAACCCCGGACAAGCTGGTGTATTAAGTATT
CTGGGAAAAGCCCAAGATGGAGCATTATTAGAGGGTATCCACTTTAAACCCCCTTTAGTTTCCAA
CGTTGATGTTTATGATGTCACGGTACAGAAATTTGAAGTCCCTGCTCAAAGTTCGACAAAAGACT
TACAGGAATTAAGTGCTAGTTTTGCCATTAACTTCCGCCTTGATCCCATTCAAGTAGTTAATATT
AGAAGAACTCAGGGAACTTTGCAAAATATCGTCGCTAAAATTATCGCTCCTCAAACTCAGGAATC
TTTCAAAATTGCCGCCGCTAGGCGTACTGTTGAAGAAGCTATCACCCAACGTAATCAGTTAAAAG
ATGATTTTGATAATGCCTTAAATGAGCGTTTAGAAAAATATGGTATTATTGTTCTTGATACCAGT
GTTGTGGACTTAAACTTTTCCCCTGAATTTGCTAAGGCGGTAGAAGAAAAACAAATCGCTGAACA
AAAGTCACAAAGAGCGGTTTATGTAGCCAAAGAGGCTGAACAACAGGCACAAGCGGACATCAATC
GGGCAAAAGGTAAAGCCGAGGCTCAAAGACTCTTAGCAGAAACTTTAAAAGCTCAAGGAGGAGA
CCTAGTATTAAAGAAAGAAGCGATCGAAGCCTGGAAAGAAGGAGGGGCTCAAATGCCTAAAGTT
CTTGTTATGGGTGGAGATAGTAACGGTGGTGGAATTCCTTTTCTTTTTAATCTCAATGAACTGGA
AAGATAATTTCATGTTGATATAGCCATTAAAAAAGGGGTCAGTAATAGTTTTACTGACACCCTTC
TTAGTTATGGAGCTGAGCAGAGTCGAACTGCTGTCCAAACTAGCTATTTACTTTTCGCTCATTCA
CAGGTTTAGCTCATCTAATCCTCAGAGCGGGAACTGTCACTTATCCCTGACAGTAGGATTTTCTA
GTGTTTTCTTATCTCAAAGACAGACTAGAAACCATCTTTAAGAGCATCCGTTGGGGTTTGCCTAT
AATGCTTAACGGAGTCACACTATAAGCGCTCGAACCGGTTTTAGGTCTTAGGCAGCAACAGGAGC
TGCTTTACGAGCAAAAGGAACGATGTTGTTCGCAGTTACATTTTTTTTGAGCCTTTTATTTGCGA
GAGGAGACTCACTCTCGACCTGAATCACGGGTCAGCTTTCACTAGCCTGTCGAAGCCGTTACAGCC
CCTTGTCTGACCTGTTAATCTCTATCGTAGCTTGTTTATTTAAAAGTGTCAACTACAGGTAATTA

ACAATTAGTAATTGGAAATTAAGACAAGGCAATAGGGAATGCAGGGCTGTTTCATTTTCGAGAA
AGAAAAAAGAGCGGGAGATAAGGGGAGAGAGAGATGGGGGGATTTTTGACTTGCAGATTTTAAG
TAAGAATAAAAAATCTTGAAACAATCAATTAATCATAATAAATTATCAAAACTTATTACTCATT
ACTTCCATTACTCATTACTCCCCGAAACCTGAAACCTGCTACCTGACACCTGTAATCTTTTTCTGA
ACCCCACACTCGGGTGGACTGAGGTTATTTACTCAGAGAAAGTTCTCCTTTTCTGCCAAGAATGC
CTTGAGGACGCCAAATCATTAAAATCATAAGAATTAAACCAATTACCATGACTCTAAATGCACCA
ACTTGAGAGGCAGTAATAAAGGGCATACTACTAAATATAAATCGAGTTAAGGAGTCATATCCCCA
AAAAATCATTGCTCCTAATATAACTCCCGCATTATTTCCTGAACCACCCAAAACAACTATAATCC
AAGCATTAAATGTCATTAAAGGTTCAAAACTACTAGGATAAATGGTTGTTAATTGCCAACTGTA
AAATGCACCAGCTAAACCTGCGATCGCACCTCCTAACATAAAGGCTTGTAATTTATACCAAAAAA
CGTTTTTACCTAACGCCTTTGCAACTTCCTCATCTTCCCGAATAGCTTTCAACACCCGCCCCCAAG
GAGAATGAATAATAATATCTAAGCCATAGTAAATCACTGCCAAGGTTATCAATATCAGTAACATT
AACCCAGCTTTATAAGTATAGTCCATAAGTGCGATCGCACCGTTAATATAACAGATAAGAATAAA
AATAAAAGCTAGACTACCCCAAACATAAGTACTTAATAAACTGGGAGGTTGATAACTTTTTCCCT
GAATGTTTTTGGCTAAATTTAACCTTTGACGAACATTAATCCACAAACGCCAAACCACAAACACG
GCTAAGACTGTGAGAATGGCAACCATCAATAATTTTCCCAAGGGATTAGGCTTAAAAGTACCAAA
AGGCAGATTATAACCTTGAATACCAAAAGTTCCTTTGGTCAACCATTCTTCATTTTGAACAATCA
AACGGACTAATTCTCCAGCCCCAATAGTGACAATAGCCAAGTAGTCTTCTCTTAACTTAATACTA
CTAAAACCAATAATTAAGCCAAAAATAGAAGCCCCTAAAGCACCAATTATTACCGCTAAAAATAA
AGGTACTCCTTGTGACTCAAGCAAAACAGTCGTGTAAGCTCCAATAGTCATAAAGGCTACATGAC
CAAAATTAATTAACCCCGTAAAACCCCATTGTAAATTCAAACCAAGAGCAAAAAGACTATATAAA
CTCGCAGAAATTGTTAAAAAGACTAAATATTCAACCACTTTAACCTCATGTAAAAAAAATAAGTG
AGTCAGTTTGCATCTAAGTTTACTTGTGAGGGTAGTTTACAGGCAACAGACAATAAATAGTGAAT
AGTGAATAAATTTCAATTAAGAATTAAGAATTAAAAACTCATCACTTATTACTTTTCCCTAAAAC
CTGAAACCTGAAACCTGATACCTCATTACTCATTCATTACTTGTTCCTCCCTCTCCCCTCATCCCC
CTATCCCCTTATCACCCCTTCTGCTCAATCTTGTAATGTTTTTAGTAAACCCCCTCTAATTAAGCG
ACTAATTAACTGAGTTTTTTGTTCTTCCTTTAATGAATCGGGAAGCATTTGAGCGGTAAATGGTT
CTTTTTGTTTTGCTACAAACTCCATTGCCTCTCGATAATGTAATCCTCCATTGATACTATTTCCGG
GGAATTGAATACTGATGGAAAATCCTTTTTTAACTATACGGCATCTCATACCCTCTCTTTTTACT
ACTAATGTATCAGGTGCGATCGCATCTACCAAATTAACTTGCTGAAAATGCCCGTCAGGAATTGG
GGTAATCTTATGAATAAATTGATCATAAATTAATTCCATTGCCTCATCTATAGAACTTTTTTCCG
CAAAAGTTTGTAATAATTGATCGAATTGCTCCTTAATTTCTCCTAACATTTCGGGCTTATCTAAA
AAGCCAATAGGTAAAGCCTTACGTAAATCTTGATTTTTCAATGCCAACATTGTTAAAGCATTAAC
CATCAAATCAAGCCATTGAGTGGGATAAATACCTAAAGTTAAATGAAGGGAAAAACTTTCTTGA
GTAGCCGCATGATGCACAAAACCTCTGGGTAAATATAATAAATCCCCCGGTTGTAAACAAACTGT
ATGTAATAATTTTGGTAAACTATTTTCAGGAATAACCGGTTGAAAACTTCCCAACAAAGGCACAG
GTTGAAAACACTGATGCACTTGCCATTCTTTTGAGCCATCCACTTGTAAAACAAAGACATCATGA
GTATCATAATGGGGGGATAAACCCTTTGAGTCTTTTGGAGATAGATAAAGATTCGCTATTACCCC
ATGATTAAAAAAGTTTTGTAATTTTTGACAATAAATCGCCAAAGGTTTCCAAAAATTTTGTAAGC
CATTAACAACCAAAGTATGATCCTCATAATAGGCTTTATAAAGTTGATTAAGGTTAAGACTACCA
TCAGCTTTAACATATCTATCGGGGAGAAAATCCTGTTGATTTTTCACCACCCGTATCTCTGGGGG
TTTCAGTTTACTATATTGTAAAATTGAGTCTAAATCCTCTACCGAGAGTAAATCTTGATAGTAGT
TTTTTTGATTACGAGAAATAATTAAGGGTTTTTTTTCCCAATATTCATTGATAAATGTATTTAAA

TCAATGGGGGCAATTAGTTTAGCAAAATCAAAATCATCTATAGACATAATCTCAAAAAAAGAAT
AGTTTAATCTTTACGAAGAATATCAGGAAGCCAGAGTAAGCCAATAATTAACAAGCAAGAGATA
ACTAAAAAGATTGCATATTTAGTTCCGTCATCCATAAGTTTTTACCTCATTATAAATAAATAGCC
TGAAAACCCCTAGATTTTAATCAGGGGATGAAAGGCTTAAGGAGACTTGAGTCTCCCATTGTATC
CTACTCTTTTTTTGGTAATATTTTAATACAAGTTCAATCAGCAATTAATGTAGTGCTATCCAATG
AAGAATTATGGATGTCAGCAAAATCTGATCAATCCGAACCCTGATTCACTAGCAATACTAGAGTT
TTTATGCGAGGAGTCGAATAAGCTCCATAATTGCGGAGTATATCTAGGAAGGCAATTATGGTTTA
AAGGGAGGCGATACCTGAAAAAATTTGATCTCAATAAACTACTGAAAAATAATAATCACTATCG
GGTGTTGTACTCACAGGTTGCTCAACAATTGTTGTTATCGGTGACAGAATCTTTTAAATCTTACT
ATGAGTTGCAAAAACAATATCATAAAAAGACGATAATAGACAAACCCCGTCCTCCAAAGTATAGA
AAGAAAGGTGGTTTTTGTGGTCTAACCTATCCGAAACAAGCCCTTAAACTACGAGGCAATCAGAT
TCGTTTGCCATTAGGGAAAACCGTTACTAGATGGTTTGGGCTGAGTGAGTTCTTTCTTCCTATGC
CAACCAATTTATCTTTCTCTACCATCAAAGAACTTCGCATTGTCCCCCGTAATCGTGTTTTCTATG
TAGAATATGTTTACGAAAAAAAAATAGTAATGCAAAAAAATGATCCTGAAAAGGTTTTGGCAAT
GGATCATGGTATAGGAAATGCCCTTAGTTGTGTTACTAATATAGGAAAATCAATCATTATCGATG
GAAAAAAAGCCAAAAGCCTAAACCAGTGGTATAACAAGAAAGTTGCAAAACTAAAAGAAGGAAA
GCCCCAAGGGTTTTGGTCAGATGATTTGGCTCGTACTACAGAAATACGCAATCGTCAAATGCGTG
ATTTCGTTAATAAATCTGCTAGATATATTGTGAATTTTTGTCGTCATCATCGCATAGGTACAATT
GTCTTTGGATGGAATCAGGGACAAAAGCAATCCGCAAATATCGGCAAGAAAAATAATCAATCTTT
TGTGCAAATCCCAATGGGTAAAATAAAAGACCGCATCCATCAATTGTGTGATGAACACGGTCTTA
AGTTTATCGAATTACCAGAGGCTTACACTTCTAAGTCTAGTTTCCTAGATAATGATTTGATACCT
AATTTCGGTGAAAAACCCGAAGGGTATCAGTTTTCAGGAAAACGGATTAAACGAGGTCTTTACCG
TTCTAAACAAGGTTTTTTGGTTAATGGCGATGTAATGGCCGCCGCTAATATTCTCCGAAAATATG
TAAGCATACAGCCAGTGAATATCTCACTAGCCAAGGTGTCTAGGGCAGTTTTGACCCTGCCACAA
AGGATAAACATATTTAATCGTGGTTTTAAACCGATTCAATATTCCTTTTTTGAATCCCCGTTACT
TTAGTACGGAGAGAGGTCAATGGAATTCTACTCCCAAAGTAAAATTGATGGCAATAAATCTGGAT
GGGCAAACCTCAGTAATTGATAGCCAATACCGCTACTACCGTGATGGAAGCTAGGATTAAAAATC
AAAGGAGGTAAACCAGAAAATAATTGAAAATTACCTGCTGATTCTGCCCGACTCAGTAAAGAATT
AACCCCTTGATGGGAAAATTCTAATAATGAAGGTTGGTTTAATTTTTTAGAGGCAACTAACAGAG
TTTCTAAGCGCCCAAAATTACCCCAACAAAGGTTATCCACTTCCCCAAAACAATATTTTTGCGTT
GTTTCTAGGGCGTTATTTATATCCTCTTGAATCAAATTATCATTATAAATATGCGAAATACCTAA
ACGACTTAAAGCTATCCCAGAAGCTCCATTTGTCCAAGAAACGTTAAATACTGCATTTTCTGAAG
AATTATCAGCCCAATTTTTGTCTATAGGTGAAAAGAAATTTTGCTCTTGTTGAAGGGCTTGTTTT
CCTGCTTCCAAGAAACGTTTATCATCGGTAAGGGCAAATAGTCTGAAAAAACTATAACTAATACC
TGCTAAACCGTGAGCGAAACCTGTTTTGATATTTTTATCATCTGTTTGTTGATATTGAAGTAAAT
ATTCTCCGCACTCTTTCGCTATTTCCAAACTATGAGATTTTATCGATGCTGAGGCTAATTCTGTT
TTCGATAGTGCTAATAAACCTAAAATGGAACTGGCTAACCCGTCAATAATAGAAAAAGAAGACTT
TTTGATTAAATCAGGAGTTATTAACCCAGTTAAACTATGGGCATCTTCCAATAATATTGGTTCTT
GTAACAATTCGCTTATCTTAACTAAGGAATAAATTATTGAGCAAATTCCCCTTGTGCCACTAATA
CCCGTTTTTTTGACCAGTCTTTCTTGAATTTTAGGATATTTCGTTAGTTGTCGAATATTGTTTTG
TATAGTTTTGAGACTTCTATCGGTTAAATTTTTCCATCTCTCATCCTGTGTAATTTTATATAAGG
CAGAAAGAAAAAGAGCAATTCCTGAATTACCATCATATAAGCCCTGATTTAACCCCGGGAAACGG
AAACCATTAGTTTTTGCTCGATACTCTAAACTGAGCCATGTTATCCCTTCATCGGTTGACATCAA

AGAGTTTCTTTGGGCTTCTGAAGAAGTGGTTAGTGCGACCGCATTTTTTTCTAAATCTTCTGCAA
TTTTAACTACTTGCCTGAATAATAACTCTTGATTCAAAGAAGTAGAGTCAGATAAAGATAAATTC
TGTTTTTGAGAGTAGTCATCAGTAGAAATAAGAGAAATAGGTTGAGGCTGTTTAACTAATTGGG
CATAAAAAGCACTACGAATCATTTGTACTTGTTGCTCTAAATCCTTATGACTGAAATTTTCAAAT
CTAGTCATCAAAGATTCAAAACTGGGCTTTTCAAACATATCTGCTACCATTTCTCCCTTGCCCACA
TAAACCCCATTTATCGAAGTATTGGAGGTAAAACAGGGAATGTCTAAATCTTCCATTTCTCTTAA
TTCTGTTCTTAATATGGGTAATAACTTCTCTGGATTTTTCATCCCTAAAAAAGCACGACTTAAAA
CATCGAGGGCAATACTTCGCTTGATACCCGATTTAAGTAAACTAGGATGATAAGAATTAGATAAA
ATTGTGTTGTATGTGTTTGTGTTGCGAAATAAATAACGAGTATTTTGATTTTTTAACAATAAAA
AAGGGCTTTCTTCACTCAATAATAACTTTTTCTTTTTCAGCAAAAACTCATAAGTTTCTTTAAAT
CCTTGAACTATATCTTCTAAATAATCTTTAGGAGAAAGGATAGCATTATTCAGGACTGGTAAATT
ATTTCTCTGTGTAAAAACTACTTCCTTCAAGCCCAAATTCATGGCATCGGAATTTATATTGTTAA
TTACAATTTGTTGTATTTTCTTTTCTTCTTCTACTGCCAATCCACCAACGTTAACATTGACATCAT
CATTGAGAAAACTATATTCAATTGGTATCAAAAAAGTGCGAAATACAGACTCTTGAATTAATTTT
CCGACAGATGCAGTAGATGCGTGCTGTGCAGTAGATCCCTTCAGGATCGAACTTTCTGCTCCTTCT
GTTAAACTGATACCATTATCATCGTTATTTGCCTTAGTTCGTGGTTGTAATAATCCCTCCAAATC
AATCAACATGGGATTATCACCATGGGAAATTAAATTTTCAAAATGAAAATCAGAACCCTCTAAAG
TATAGGTAAGACATACCAACATTCCATAACGATAATAGAAATTTTTTGCCTCTTGCTCACTGTTA
CAAGGTAATGATGATAAAAACTCAATCCAACCATAATTATCACGATGGATAATTTTAATTGTTTT
TAAAGGTATAAGATTAGACTTTTGATTAAACCAGTTTAAAAATTGGTAAAAGTTTACATCAAGA
TGGATATTTCTAGGCTTATAAATTAACTTTAGTCCTGTGTCGAATTTTAATGCTATAACAGACCT
TCCTCTTTCATGAGAATCCGATAAACCAACCTGAATATCAATAACTTGTTTCAAAGGTTTATCCG
AAGAAAAAAGTCTTTCTATTTCTGGTAAATCATTATTTAACCTAGTAATAAATTCTTGATTAGCT
TCCACCCAATAATTAATTAATATAGCACTCAATTTTCCCAAAACGCTATACTTTTCAAAAAAAGA
AATCAGATTATCCGATAGTAAATTATCTAAAAATTGCTTATATTTATTGTTACTTTTACTTTCTT
TTATCTGAGTTAAAATAAACTCCTTCATCTCATTTCCTGAAGAACGAAAATTTTTAAATTCTTCC
AATAAAACAGATGTACAAAGATAGGCAAGTTGATTAATTAACTGTTGTTCTAAAACTACCAAAG
AATCATTATTTAACAAATCTAAATTAGATTTTGTTCTTTGACGTAATTTATCCCAAGCCACTTGA
ATAAAGGGAGTATAAATAGGCTCAAAAGGAATTTTAGTCGAATTAACAATATTCTCAGGTAATT
GCGATAATTCTAAATTTTCAGCTTTTTGTATAACTTTTTTTAGAGTAATCAACCATTCAGGTAAA
GGGGTGTCAGGAGAAAAATTTCCTTCAGCCAAAATCAATGAAATTTTATCTAATGTGAAGCCTTC
AAAAGCCAAGCGCTGTTTAAATTTTTCTTTATTGCCTTTTGCCACGATGTGACACCATAAAGTTA
AATTTTCTTCTACCTGAGATTGACAGGAATTTGAAATATTGGGAATCCAATGATAAGTTAACCTT
TCATTAATTGAACTAGCTTGATTTTGAATAAAGCTCAATGTTGTTTCTAAATGATGATTTTCCAG
TTTCATAGGTAGCAATATTAAAAAAATTTATTCCTTATTTTATTTAGTTCTTAACTATTTTTTAT
CCTTTTAAAAAAATAAATTTAATCAAAAAATTGTCTTTATATTTATATTTTTTAAGGTTATGAT
ATTGGTTGCCATAAAAATTTTACGTTAATTGTTATTTAGAAAAACTAATTAGTAAATCAATATA
TTTTGATAATAATAAACATTGAAAATATTTAATTTTTATGAAAATAACATAGTTATATATAAAA
CTTTATCGATAAAATCACTTGGTATAGAAACTATAAAAAACTAAAATGAGGTAATATTTTCATCT
TGAGTTCAGCTTAACTCATTAATAAAAGGAATGCCATATTTGTTCACAAGCTATGAATAAATTCC
TGAACTTTTATGAACTTTTATGTATAGACATTATAAGAAAAATTATATTACCTTAATGACAGTTA
CTTGAAGTCAACGAAAATCATAAAAATTTTCCAATTTATTTTTACAAGAGGTCAACTAATATGAG
CAAAGAAGCCGTAGAACAGTTTTTTGCAAAAATTCCTGAAGATGATAACCTGCAACAAAAATTAG

TATCTATTCTTCAAGCAGATATTGATCATCGTCAAGAAACTGCTAAATTAGCACAAGAATATGGA
TTTGATATTACAGCCGAAGAACTTTCGGAAGAAGTCAAAAAACGACAGGAAGAATTTGCACAGCG
CCAGGAAAGTGGAGAATTAAGTGAGGAAGAATTAGAAAGCGTTGCTGGTGGTTTATCTTTCCCCG
GTACTTTTAGCTTCCCCTCGCCTATACCAGGCACTTTTCCTTTTCCAACCCCACGTCCTGGACCTA
TTCCTTTCCCTAAACCAAAATGGTAAGAGTTTTATGATTCACGGGTGAATAAACAGACACAATAG
ATAGACAATTTTAGTTTCATTATATTTCCGTTGCTGTTTAGGTTTTTCCTTTTCAGTGACGGTTT
AATTTTGGAAATTTTTTGACCAGTAAAAATTCTAAAGTTGGCGTTGCTTATTTTGTATATGATTT
CTCATTTAGGTGGGGAATAAGCAATAGTGATAATATTTTGATGTCTCAATTTTTGGCATAATTTC
AGTGCATTTCATACCATGATTAACCCACACTCTAAAGTTTTATAAACTTTTATGAACTTTTGTGT
ATTTGTGGTTTCATAAAAATTATATTGCCTTAGCCTGTAATAGTTGTTTTTTCAAAACAATTAAT
TAACATCAATTTTCATTCTCATATGGAGTTTGACTAATAATGAGTAAAGAAGCAGTAGAGCAGTT
TTTCGCTAGAATTCCTGAAGATAATGAGCTACAACAAAAATTGGTTTCCATTCTTCAAGAAGACA
TTGACCATCGCCAAGAAACAGCTAAATTAGCTCAGGAATATGGGTTTGATGTCACGGCAGAGGAA
CTGTCGGAGGAAGTAAAACAACGGCAGGAAGAGTTTGCACAGAACCAAGAAAGCGAAGAGTTAA
GCGAAGAAGAATTAGAAAGTGTTGCTGGAGGCTCTATTCCTAACATCACCGTCGGTATTTTTCAA
CCTCTTACAATAAACACTTACAATATGCCGGGTTCTATTTCAAACCCTTTTCCGAAACCAAAATG
GTAAGACTTGAACAATTTAAGAGTGGGCAAAAGAGAATTTCAAATTTCTCATATCTGCGTTATTG
TTCAGTTATTTTTCTGTCAGTAGCAACTTTCCGTTTATATATTCAGTGAATAAAGAGGTTGAAAA
TTATGAGTAAAGAAGCTGTTATTAGTTTTTTAGATGCGGTTCCTCAAAATGAAGAATTACAACAA
AAATTAGTTGCCATTCTCGAATCTTCGGAAAATGATCGTGAAGATGCCGCTCAGTTAGCGAATGA
ATACGGTTATGATATTACCCCCGATGAACTCTGGGCAGAAATCCAAAAACGTCAACAGGAAACTA
AAGTCCGTCAAGAGGCTGGTGAATTAACGGATGAGGAATTGGAGGCAGTAGCTGGTGGTGAAAT
GATTGTGGCAACGATCGCATCAACTGTTGCTATGACTGTTTCTGTTGGTGGCAGTATCGCTGTGG
GAATACTAGCTCCTAAAATTAAATGGTAACAAAATACTGAACTTTTCTGAACTTTTCTCTATTGA
CTCCCATAGAAATAGTTTCTAGTATGTACCCATGGATTGCTAATAATATACATTAGTAATATTTG
CTTAACATTATATCGAGGAAGAATAATGAGTCAAGAATCTGCGGTACAGTTTTTGGAAGCTGTCC
CTGAAAATGAAGAACTACAGCAAAAATTAGTTGCTATTCTCGAATCTTCAGAAAATGATCGTGAA
GATGCCGCTCAATTGGCGAATGAATACGGTTATGATATTACCCCCGATGAACTCTGGGCAGAAAT
CCAAAAACGTCAACAGGATTTGGAAGCCCGTCAAGAGGCTGGAGAATTAACAGATGAAGAATTG
GAAGCCGTTGCAGGTGGTGAGTTCGTTGCTACTGCAGTAGTTGCCACCGTTGTTGGAGCGGCTGG
TCTTGGATGGACAATTGGTAGTGGAATCGCACCCAAAATAAAATGGTAAAGGTTTTTGACTTGCC
ATTTATCCAGATTAATAGGTTTTTCATCTTTTTTTGCCACCTATTTATATATTCAACAATCAAAT
GTTGATTTTCATTATTAACAGTTAAAGTTTTTAGGAGATTTAATCATGAGCATAGCCGCAGTAGA
GGCTTTCTTAGACAAAGTTCAAACTGATGAAGAGTTGCAGAAAGAACTTGCCACAGCCCTAGAAG
CTGAAAATGATCGTCAAGCAGTTACTGATTTAGCTAACTCTAAAAATTATGAATTTACTCCCGAT
GAACTTTGGGCAGAAATCCAAAAAAGACAAAATGATGCTTTAGCCCGTCAACAGTCTGGAGAATT
GAGCGATGAAGAATTAGAAGCCGTAGCTGGGGGAGAGTTTGTGGTGGGAAGCGTTCTTATCACCG
CCGCTAGTTTTGCCGCTTCGGTCACTGCTGGTGTTGCTATTCCTAATATTAAATGGTAGGAAAAA
GTACCGAACTTTTCAGAACTTTTATCTATTGACGATCGCCTCCCCAGTATTTATCATCGTTTTTAA
AGTTTTAATAAACTTACGTAAAAACCCTTAGGGGAGGTAAAAAAATGAGTCAAGAAGCCGTAGT
TCAATTCCTCGATGCAGTTCCTGAAAACGAGCAGTTACAACAAAGATTAGTAACAATTCTCGAAA
CTTCAGAAAATGACCGTGAAGATGCCGCCAAACTAGCTAACGAGTATGGTTATGACATTACACCA
GAGGAATTATGGGCTGAAATTCAAAAACGTCAAGAAGAAGCTGTCGAGCGTCAAGAATCTGGTG

AGTTAACCGATGAAGAATTAGAGGCTGTTGCTGGTGGTGAAGTAGTTGCAATTACTGTAGCTGCG
ATCGGTGCTGCTTCCACTATCTTCTCCGCATTAATTCCCAAAATTAAATGGTAGAAATACCTTTT
CATCATAGTTAAAAACTATAGACTTCCGGGGTATTTCGGTACTTTGGAAGTTTTATCTATTAATA
AGAATTAAATTTACCCAACAAATATTTATTGTTTCTCACAAAACTTTATTAAAGAGTTTTAAAAT
ACCTTTTCTGATATTATGGACAGTGCAATTTCCTTCGCCAACGGTTCTCAAATTCTTGCTCTCAAC
AGTTGCGATCGCCTCTGTTATGTAATTCAACCCTTTATTCAAGAAATACTCAATCAATTTGTCCA
ATTAAACAGCCTAGATCAAGCTATTGTCATCGGTGCAAAAAAGCATCATAAAATAGCAGAAAAAC
TTTTTGAGTTTGAATTAGATAATCCAGCAGAAACCTTTTCTCTTAAAGAACAATTTTTGTTTCCT
GAAAAAAGTCCGCTCCTGTCTTTGCAATACGGGAACACCCGCTATCCTTTATTCTTAGAAAAATA
TGGCTCAGAAATAATTTATGATCAATCCACCTTACCCGCAATTCATAAATTATTTTATCTGTGTG
GTCAATCCAACCTCTCCTATGAGCAAATCTGTGGGCAACTCGAACAACCCATGATCGACTTTTTA
GATAAATTAATCAATACAAAAGTAGTTCAAAATAAACCCAATCAACCAAAATCCCTTGAATTAAA
AACTCCGGGAGTTTTTCGCTTGCAACACGCCGCCCTACTTTACCGCACCAAAACCACAGGTATTTT
AGTAGATCCTCATCTTCACTCTAACTATGGCATTCCCAACCTGAAAAAAGATATTAGTCGTGCCA
TGTTGGGTGACAATGTTGATGCTATTTTGATCTCTCATCCCCACTATGATCATTGGCATTATCCC
ACTTTGATGATGTTTCCCCCAGAAATTCCCATCATTGTACCAAAAGTGCCAAGGGGATCAATTAT
GTGCGAAGACATGAAAGCCAGACTTGAAAGTTTAGGATTTACTAATGTTATGGCTGTTGATTGGT
ATGCAGAACCGATTTTAGTGGGGGATATAGAAATTAATGTATTACCCTTCTATGGCGAACAACCT
TTAGTACCGGAATTTAACCAACCAAAACACCCTGATTTGCGTAACTGGGGAAATACTTACTTAAT
TAACACGGAATACTATAAATCTTGGTTTTTAATCGATTCTGGCGATGACGATATGGGAACCATGG
CAGAAGTCGCAGAATATGTCAAAGAAAAATTTGGCACATTGGATCATGTCCTCAGCAATTTTCAA
CCCTTGTCTTATAACTCCATTGGTACTGATTTATCAGGGTGGGGAATAGATATTGTCGCCAATTT
GCTCAGTAATCCTCAAATTTTTTCTGTTACCAATAAAAAAGAAGGAGAACATATCGCCCTTTTAG
GACCTCAAGGAGTTGCTAAAATTTGTTCAATTGTTGGGGCAAAATATTGCTTACCTTATGCAGAC
AGTTGGGCTGAATTGGGAGAGTCAGGAATCCATGATGCTACTTTAATTAAAGACGTAAAAGAAG
AATTGCACAATTTAGATTGTCTTACTCAAGTTATCCCGTGGAAAATAGGGGATCAATTTGTAATT
AATGGCACAAGCAAAATAGAAGAAAACATTTTTATCAATCTTTAGATAATTAGTTAATATGAGT
AGAAGTAAAACTATTGCTAATTTTTTAAGTCCGAAAAAAGAGCCTAAATATTACGCTCAAAGATC
AGATTTATTCTCTATTGATTATCTTGAACAATTAGAAAAATCAATCCTATCATCTCCTTACTTAG
CAAAAAATCAACTAAGTGAAAATTTTGAACAAACTAGAGGTTTTTCCGTAGTTTTTAAAAGTAG
CAGTATTGACAAAGTAGAAACGGAATTTCCTTTTTTTAAAACTTACTTAAACACTGCCTTAAAAA
AGCCTTGTAATGCGTTCTATCTCAATGCCCTAATTTTAGACAACGGTAGTCGAGTTGATCCCCAT
ATTGATTGCAGTATTTCTTCCTATGAAATGGTTATGCTTGTTCCTCAATTAGTTAGTGTTTTGTA
TGTAAAAATACCCGATGATTTGCGAGGAGGAGAATTAATTTTAGAATCAGAAAATAAACAAGTA
GCAAAGATAAAACCCCAGAGAAATAATTTAATTTATTTTATTGGTAATTTACTTCATTCTGTCAA
TTCTGTGACTAGCTCTCAACCAAGAATAAGTTTAATTTGTGAGCAGTATAATTTACCGCCAGAAA
GATTAACAAAAATTCCAGATCTTGATATTAAATCAAGTCAAGAATAATATAGTTTTTTATTATT
TATTTTTTAAAATTATGATTAAAAACTCTTTAAATGATTATATTAATTTAATTAGACCAACTATT
TCTACTGATATTATTGATGAGAATAATTGGCAAAATATTAGTAAAGTTGCTCAATATCTTCCTAG
TGCTTTAACTACTTTTTTTGGCTTTGAATCTCGTTTAGGAACGAAAAAAGCTCATTGTGATTTTC
TTTTGTGTGCAGATGCTACAGAAGCAGGAAAAAAAGTATTAGGGGATAAAGAATATTCTATTCA
ATTATCGGAAAATTTGTTAATTCATCCTGTTTGGAAAAATGTTAATATTTTTGGTCAATTATGGA
ATGATAAGGGTTCGATTTTATCAGAGAAAATTAATAATATTTGGTTAGAATTTGATATTGATGA

GACACTAGATAATATCCCGATTCCCAGTTGTTTTTTTGCTCCTCAAGCTATTTATGCTAATCAAG
CGGATGAGGCGATTAAGTGGGTTTGTGATACGGCTTTAAATTTGTTAAGGGGAAAATCCATTAAT
CCTGAAATTCAGGCAAAATTATTAACTTGTTTACAATCATTGCCTTCTGGTGCTTATGTTTTTCA
AATAGGTTTAATGTTAGCAAGGGAAAGCGATTTTATTCGGGTTTGTATCCGTGATATTTCTCACA
CAAAAGTTATTGAATTTTTACAAAAAATTGGTTGGATTGGTTCTGTTAATGAATTAAAAAGTCT
TTTAAATGATTTAGCTCAATACTGCGATCGCATCGATTTAGATATAGATATAGGGAGCGAAATAG
CTCCTAAAATTGGTTTAGAATGTTATTTAGAAAGACAACCTTCTCTTAACCCTAAATGGCAATTA
TTTTTAGAATATTTATTAGAAAAAGGGTTAGTAATTCCAGAAAAAAAAGATGCTTTACTCAATT
ATACAGGATATATAAGAGAGAAAGATTACCCTGAATTATGGCCTAAAAATTTATCCAAATTATC
GAGTTTAATTGGCTCTCAATATCAAAGAATTTTCTTTAAAAGTTTGCATCATATAAAAGTAGTTT
ATCAGGAGAATAAATGTTTAGAAGCTAAAGCCTATTTAGCGGTAACGAATACTCTAATTGATCAG
CAAAGAATACAAAAATCAAAGGAATTTAAAAATAATAGCATTCAGATAAATAATTTTCTGTCCG
AACAAGAAAATAAACAATTATTAAACTTTATTATTCGTAATAAAAATCAATTTCAATCAGCTAC
TCTTCATGAAGATTATCAAAATTTAGGCAGGAAAGAAGAAAATTATCGTCTTTCTTCAGTATTAT
TTGATTTCCCTGAATGGGAAACTATAATGCGCGATCGCATCTCCTCCATTTTACCTGATGTGATA
GATAAACTAGGCATTCCGCCCTTTCCTGTTGCCCACATAGAAGCTCAAATTACAGCCCATAATGA
TCATAATTACTTCAAACTTCATAACGATAACGGCACACTAGAAAGCTCAGGACGAGTGCTTACAT
TTGTATATTACTTGTGTCAAGAACCACAACCATTTACAGGAGGTGAATTAAAAATTTACAACAGC
ACCAGCCCCGAAAATTTAAAACCAGATAGTATTAAAACTATTGAGCCTATTAATAATAGTATCGT
CTTTTTTCTTAGCCAATATATGCACGAAGTAAGGCCAGTAAATTGCCCTTCTCAAGATTTTGTGC
ACAGTCGTTTTACCGTCAATGGTTGGATTTGGAGAAAAAATTAACCATTATTACTTATTACTTAT
TACTTATTAACAATGCAAGTATCAGAAATTTTACAAACTCTCCCCCATTCCCTTGAATGGATGGT
ATTATTTAACATCAGCGCGATCGAACCTTTAACCGATCACAATACTATTAAGGCAATGTATCATC
TGCCCGAAGATGTGGATTTAAAGCCTTATTCTCACGTGGTTTTAACCAGCGAAGGTCGATTTTTA
GCATCTGGGGATAATCTTCAATTATTTGATCCTGTATCTGGAAAACGTTGGTCAAAAGAAAATAT
CAAGGATAATTTATATACCAGATTTTCGCCTCAGCTAAATTTATTCTCCGTTGATGAAGCCGATT
GCCTAGGATTAGGAGAACAAAACCCTTACTCTCCCGTTTTACTTCATGTTAAAATCGCAGAAGGT
TATGGACAAGCTCAAGCAATATTTGATCATCAACCCAATTTTGACCATTATCCTCTTTTAAAAGC
AGTAGGAGTTAAGTTTCTTTCAGGAGAGATAAAAAACTCTTACTACTTAGCAAAATTTCAAAACC
GTTTACCCATTCATATTCATGCAGGGATTTTATCTCACTTTAGTAGAACTGCACATTGTAACCTC
TTTTTCTTACAACATGGAAATATTGATCCACCTTTAGAAGAAGGGTTATGGAAAGCCTCAGAAGT
ACGCAGTAATTGGGGTAAAAATTACAACTTAACAATTTTAGCAAACCTTGTTAATCAGGTAGAGG
AAAAACCCTTAGCGATGGTGTGTCAACCTCCTCCACCGCAACCATTATTTGGCTATGGCGATTTA
GTGCCTTTAGGGTTTGTTTTAAGAGCCTTAAATCTTGCTACGGATGAAAATACAATTAACTCAAA
AGATAAATTAGAGAAATTTTTGCTTAGTAAACAAGAAGGCAAACTCTGGGCTTTTCATAGTCAA
AGATTAGTTACCGCAACGGATTCAGCTTTAGTTTTACAAGGTTTTAATTTACCTGAGTCGGTAGA
GGCATTGGAAGTTTTTGCAGATGGTAAGGGCGGTTACTATCCTCAACTTTGGTCTGAAGAAAAAC
AAGAGGGTAAGATGGTTTATGACGACAGTTGCGCCCATTGGTGTCAAGGTGATTATGCCACTACC
TGCATGGTAAGAAGTCTAAGAAAAAGGGCAGGATTAGAGAGTAAAACCCCTTTAGGTTATTTAT
TATCTGGTTTTGAGCATCGTAGTGGTTTATATTTTGCGAATCCTTATCTAGTGGATTGGTATTTA
GCACAAGCTATTACAGATGAAGAAGAAGGAGACATTTTGAGACAAAAATTAATAACAGAAATTC
TCGCTAGTATAAATGAGGATTATTCTTTTGGGTTGTATGATGTCGCTTTTTCTACGGCGTTGGCT
ATTTTAACTTTAACGGAGTTAGGGGTAAGAAGTAGAACAATTCGAGTAATGCAGTTACGTTTAT

TAGAGCTAATGGAAGCAAAAACAACTCTAACCATTCCCTTTTACTCTAGTTTAAAAATTGATTCA
GAAATTACTTCTCAAAAGGAGTTTTTCACCTTATTAATGGGTCAAAGTTTTACGAAAAATCCTTC
TGGTATCAATCAAAAACAAATTCGCAAAATAGGAGAGGAGTATCATGGTATTTCTCTTTATTTGG
ATACATACCGCTTAATTACTCATTCAACAATGGCTCTTGCGTTAGCTGAAAAATGTGATTTAGAA
GACGGTTATTTAGATTTAAGTCATTATCAAGACTATATTCATCCCCGTTATCAGTGTCAAAGTCA
TTGCGAGTATATTGCTAAGTTTGCTTTACCACCTTATTTATTGGAGGGGCAAAGTTAAAAGGGCA
AGGGGCAAAGGAGAGGTGAGTGAGGAGTTAGGAATTATTAATTATTCACTATTAACTATTAGAC
ATCTCCATTAATTGCTAAAACAAGGGCTGAAGCCCTTACTACGAGCCAATTAAAAATATTTTTTG
GAGAAGTCTATTAACTATTCACTATTCACTACCTTAAGTGACAATTTTATATCTAATTCAGGTTT
AATTCTATGAGAACAATTGCAATTTGCGGAAATAACGTTAAAAATATCATTGCTAGTCCTAGTTT
TGTGCAATCTTTGCAGATGAATGGGGTTACGGTTAATTGTGGTTTAATCCATACTCAAGCTATCA
AATTTGATCCTAGTCATCCTGAATATCAAGATCATGTTTTAATCAAAATTAAAGGTTTTTCTTGC
AACTATCGAGATAAAACTTTGATTTTACAAACTGCCACGGCGGGGGTTGATAATCGTTTTAATGC
TGTTGGCTCAGATTGGGTGGCGGAAGTGGTGGATTTTGGTTCTAATGTTACGGATTTACAAAAAG
GCGATCGCATCATTAATAATAATGCCTATCCTGATTCTGGGGTGAAAGGACTATTTGAAGGAGTT
CCCACTAATCACGGTTCAAAAGAGTATCGAGTGTTACATCGAGCTAAATTGATGAAAATTCCCCC
TCAAATGCCTGATGAAGTAGCCGCCGCTTTTAGTATTGGGGCGCAAACCACCTATAGTATGATTC
GCAGATTAACTCTTTCTCAAGGGGAAAACGTCTTAGTAACTGCCGCCAAATCCAATACATCTTTA
TTTGCGATTCAAGGTTTACAAAAACATAATGTTAATGTTTATGCTTCTACCACTTCTCGTAAATT
TGAACAAGAGTTATTAGATATGGGGGTAAAAAAAGTTGTGTTGATTGACCCTAATTCCCCTAAAT
TAATGAGTGAAGAAACTATTGCATCTTTAAAACGGGAAATTACTGGTTTTGATGGTATTATAGA
TCCTTTTTTTGATGTTCACTGCGATAAACTAATTCCCGCTCTCAATGTTTGGGGTAGATATATCA
CTTGTGGCTTATATAATCAATATTCTCATTTAACGGGAGAAGAATTTAATTCTATTGGTTTAACT
CCTACTAGTATGGTCAATATGATGATCAATAACATTCAGATTATGGGTAATTGTATCGGTTTAAC
TTCTGATTTAGAAGATGCGATCGCAGATTATGTTTCAGGAAAACTCAAAGTAAATATTGACTCAG
TATTCACAGGGAATCAAGTAGCAGAATTTTTTGAGCGCACCTATAACGCTAAAGACAGATTTGGC
AAAGTCGTTTACTGTTATGACTAAGTTAACTAAGCTACCAGTTATAATATTAGTTATAACAATAT
ATAAAATAAGACAATGTTCAGACTAAAAGTAACTACCGTTGGTAATTCTACAGGAGTTATCTTGC
CCAAAGAAGTATTAGCCAAAATGCACGTAGAAAAAGGCGATAATCTTTATCTGACTCCCACTCCT
AACGGATTTGAAATTACCCCCTATAATCCCGAATTTGAACAGGAAATGGAAACCGCACGAGAGAT
AATGAAACAATATCGTAACGCTTTACGAGAATTAGCCAAATGAGTATTCATTGGTTATCAATGCG
CTTAGTTATAGCCATTCATCAAGAACAAATTAGCGAACATGGAGGGGCAGATGGTTTAAGGGATA
AGGGTTTATTAGAGTCTGCCTTAATACGTCCACAAAATCAATATTACTATCATAAAATTACGAAT
ATCTCATCCTTAGCTTCTACTTATACCTTCAGCATAGTTAAAAATCATCCCTTTATTGATGGTAA
TAAAAGAACAGGTTTTATTAGTGGAGTAACCTTTTTAATACTCAATGGTTCTCACTTTACTGCTT
CTGAAGTGGAAGTAGTACATATCATCCAAACCTTAGCTAGTGGCAGAATTACCGAGGAAGAATTA
CAACAATGGTTCGTAAAGAAAAATAAGCAGATGAATAATTAAAGCATCATTTCATCCTCATTTCA
TATTCCCCTGTCACCATAGTATGGAAGATTAGGTAAAAATGAGGAAAAAGTTTATTATGACCCAG
TATAGTCTAAATAGGCGTAATTTTTTGCAATTGACGGGGGGTATTGCCACCAGCGTTGTTTTACA
TCAGATTCTACCCGCATCCGCAAAGAATATAAACTCCAACGGTTATTTAGAAGGGGATGTTATCG
ACTTAGTGATAGCAGAAACAAAAGTTAAGATAAGCGATCGCTCCTCTAGTGGTAAACTGGTCAAT
GGTAGCCTACCCTCTCCCACCATCAGACTTAAAGAAGGGCAAAACGTAACCATTAACGTCAAGAA
TAACTTAAATGAAGATACTTCTATTCATTGGCATGGTTTAATCTTACCAGCAAATATGGATGGAG

TGCCGGGAGTAAGTTTTCGGGGCATAAAACCAAAGGAAACCTTTACTTATCAATTCCCTGTTAAC
CAAAGTGGTACTTACTGGTATCATAGCCACAGCAATATGCAAGAACCATTAGGAATGTATGGTGC
TATTATTATTGACCCTCTTGAACCTGAACCTTATAAATGCGATCGAGACTATGTAATTATATTAT
CAGATTGGAGTTTTGAGAATCCCCACGCCATCCTCGCCAATTTGAAGAAAATGCCCACTTATTAT
AATTATCAAAGACGCACCATAGCTAATTTCGCCGAAGATTGGGAGTGGAAACAAATGCGAATGGA
TGCGGCGGATATTGCTGACGTGACGGGGGCGACTTATACTTATTTGATGAATGGTAAAGCCAGTA
ATGATAATTGGACTGGTATTTTTAACAAGGGTGAAAAAGTTAGACTCAGATTGATTAATGCTTC
GGCGATGACATTATTTGATGTTAGGATTCCTAATTTACCCATGACGGTTATACAAGCAGACGGGC
AGAATGTGCAACCCGTGATGGTAGATGAGATAAGGATAGGGGTAGCCGAAACCTATGATGTGAT
AGTTGAACCCGATACTCAAGAGGCTTATACTATATTTGCCGAAAGCCTCGATCGCAGTGGCTATG
TCAGAGGTACACTTGGGATAAAAGAAGGATTATCTGCCGAAATTCCTCCCCGTAGAGAGCGTCCT
TTACGTACAATGGATGATATGGGTATGGATCATAATGCTTATAGTGGTCATAATAATCAATCATC
TTCATCAAGTCAAGACCACAGTAGTCATAATTCCCCATCTTCTAATCATAACAATCATCAGAGTT
CATCAGTGGATCATAGTAATCATAATCAGCACAATGATCATGATATGTCGAATATGAGTCACGGT
GATCATAGTAATCATGATATGTCTAATATGTCGGATATGGATCATAGTACTCACGAGATAGGCGA
AAATGAAACGATAGATTTTTCTTGGCAACCGAGGGGAGAGGATAATAATAGCATCGGTAACGCCG
CAACCCCCATGATGGTAAAAAATCGTTTAAATGAAGCGGGGGTTGGCTTAGAAAATGTCAAACAT
CGAGTGTTAGTCTATACGGATTTAAAGAGTGTGAAACCCTTAAAAAACAGAAGAAAATTCGATA
GAGAAATAACCCTATATTTAACAGGAAATATGGAGCGTTATATGTGGTCATTTAATGGGAAAAA
ATATTCAGAAGAAAAAGAAATAGACTTTTATTATGGAGAAAGATTAAGGCTAACGTTTGTCAAT
GATACCATGATGGAACATCCTATTCATTTACACGGAATGTGGATGGAGTTAGTAAACGGTAACGG
CGATTATCAACCACGAAAACATACAATTATTATCAAACCTGCTGAGAAATTATCCACAGAAATAG
ATGTTTCTGCGAAGGGAAAATGGGCTTTTCATTGCCATTTAATGTATCACATGGATGTGGGAATG
TTTCGGACTATTAATGTTATTTCTTAAAAAATAATAGTATTAAAGCCTAAAATTTTTATAAAAA
AATTCATGTCTTTTATTAGGGTGAGCATTCTTTCTTTATGTCTCCTTATTTTACCTCTTTAGAGG
TAACTACAAACTTAATCAAAAAATTTAGATAATTAATTACATCAATGCTATATAAGAGTAAAGT
TATAGTGCGATCGAGTCTTATAACCTGTTTAATTTTAACTAATTTTATTCATCAAATTAAAGCCC
AAGAAATAAGGGAAACCATTATTAAAAAGCCATTAATTGTTAATAATTTTATCGATGAAAATAA
CTACCAAGAAAAACAAGAATTTTTTAAGCAATTTAAAGATGACCAATACTTAAATTCAAGAAAAA
AACTCTACTTTTGCTCAACATAATCATAACAACAATAATAATCAAGAAAATGATTTTGGTGATCC
CATCGACGATAATCAAATTTTTTTATAAAATCCTGTTTGATCAATTAGAATATCAAGTTAATGATA
GTCAAAATATTTTTAATTGGGATATTCAAGGATGGATAGGGGGAGACTATCAAAAATTATGGGT
AAAAACCGAAGGTGATGTTAGTTTTGATGATGGTAATGGAGAAGCAGAATTGCAACTTTTATAC
AGTAAAATGATTTCTCCTTATTTCGATTTTCAAACAGGTTTAAGATATGATCAACTATACGGAGA
TAAAGGAAATAGTCGAGGTTTTGCCGTCATTGGGATTGAAGGATTAGCACCTTACTTTGTAGAAA
TTGATACCGCTTTATTTATTAGTCATCAAGGAGATATTTCTGCAAGATTTAAAGCCGAAAAAGAA
TTATTACTCTCCCAAAGATTAGTTTTACAACCGAAAATAGAAACAAATTTAGCCATACAAAAAGT
AGAAGAATTTGGTATTGGTAGCGGTTTTAATAACTTAGAATTAGGTTTAAGATTACGCTACGAA
ATTAATCGTGAATTCGCCCCTTATATTGGTGTTAGTTGGAATAAACTATTTGGCGATACTGCTAA
ATTTGCCGAGGAAGAGGGGGAAAGTAGTGATGATATTAAATTCGTAACAGGAGTGAGATTAATA
TTTTAAATTAATCCATTAAAAACATCGTATAAGATACAAATCTCATTTTACTGAGTTCGATATAA
AATTGTCGATTAAGGTAGGTAACAGGAAATAGTAAACGAGTAAAGGTATGTACCATTGAGATAA
ACTCAGTTTAAATTGATTTTAAGTAAATTTTTCCCAATATTAGTGATTTTTTAGAGGACAATTTT

GTTGATTATAGGTATCAAATTCCTATTCTTCAAGACTTTTTTCATCGAACTCTAGTTCATTTGGT
TTTCTAGCTCAATCTACTCTTTATTTTAGTGTTGATGATGCCCGTCATGATCTTCTTCTTTATCTT
TTTCTTCTTTTTTATCATCATTATCTTTTGTAAATCTATTATTATTTATTTCATATGGAGACTTA
TATTTTTCTCGAAAATGAGAATATCCTTCGTGACTAAAAACAGGAGAAATACTAAGAGTAAAAT
TAAATATTAATCCTGTAAAAAATAACAAACTAATTAACAATTTACTCATGATTTGAAAATCTTTT
TACTAAATAATAATCGTTATTGTAACTATCAATTATGAAATCACTATGAAAAAGAATTTTTCTCT
ATCTTAATCAATCTGATCAATTATCTTATATCAAATCCGTTTAATTACCCACACAAAAAGATATT
AATTAGGCCACCAATAAAAATTGGCTATTCCTTTAACTATTTCTTTTTCTTCCATAATTTTTTTG
CATCTACTAATAACTACCTCTTCTAACTCTTCTAAATTTTTAAATGTCCTATTAGCGATGGCCTC
ATTTACTAATGGCCACAAACGTTCTGCCGGTTGTAATTCTGGAGAGTGAGAAGGCATCAGCATAA
CGTGGATTCCCTCTGGTATTTCCGTCGTTTCTTTAATCGGATGCCAACCAGCTTGATCCATTGCTA
AAATCACCCTTTTGTTTTTACCTATTTCATAGTGCTGTGCAAAATCTTTTAAGACTCGATTAAAC
AATTCAGTATTTACATATGGTAGTAGCCACCAGTATGTCTCTCCTGTATTTGGTTCCACAAAGCC
ATATAACCACAACCATTGAAAACGCCAATTGACCGTAGCTATCGGTTGATAACCTTTTTCTACCC
ATACTCTTCTTAATACTGGTTTGAGTCCTAAACGATGCTCATCCTCAGCCCACACTTGAACGTCCG
CGTCGGGATATTCTTGTTGAATCTTTTTTTTTTTCTGCTAGTTGATTTTTCCATTCATTTTGTTC
AAGGCAGCTAGAATTTTGATGATTTGGACGAGGACATCGGAGACGATATTCTAATCCTTTGAGAT
ATTCCCATCCTCTTTGAGGAGAAATAGTACGATCTAGAAGTTGAGTGAGCCATTTTGCTACTTTG
ACACCATTCCAAAGTCCTTGATCATCGGGGGGTGATTGTAATCTTTGATATAGCAGAGCTAATTG
TTGATCGTCTAATAACGGTTTTCTACCCCGATTATGTTGTCTTTGATCCCCCAACATTTTTGCACC
CGAACGATTATAACTACGAACAAGTTCGTAAATCCAACTTAAGCTATATCCTGTAACGTGGGAAA
CATAAGAGGTTCGTTTCCCTTGTGCTAATAACCAAATCACTTGGTAGTGAATTTTTTCAATCCCT
TGTTTGGCTTTTCGATACCCTTGTTCTAATTCTTGTAAACTTAAATGGGGTTCAATTTTGATTCT
TGAAGGCATTGGTCAAAATCTACGACAACAAATATACATCCTCTTGATTATACCATGTGCAATTA
AGCGGATTTGATATTAAGTGATCATCCGAACTTAATATAACCTGAGTTCGGGATAAATTTTTATC
TATGAGTGGTGGGAAAAAAAAGAAAATAGACAATCACGGTTTTGCTTTGATTGAACAAATAATT
CAACAAAAGCCCGATCTGATTTTTTCAGAGTTATGCCAATTGTTCAAAGAGAAAACTGGAGTAAA
AGTCAGTGAACCAACCATGTATCGATATGTCCAAAAATTAAATATTACCAGAAAAAAAAGTCTAT
CCGCCAGTGAGCAACAAAGAGATGATATAAAAAAAACGATACGAATATCGGAGGTGGCTCGATT
TTGTCGATATTAACAACTTAGTCTATGTAGGCGAGTCAGGGGTAAATTTAGGGATGACTCGTTTT
TATGGTTGAGGAAAAATAGGGGAAAGAGTGGTGATGGACTGTCCCAGAAACAAAGGTAAGAATA
TCTGTCAGGCAAAAAGCATTTAAAAGAAAGAATACACAATTGTCAATATTGTGGTTATACAGCAC
CAAGAGATGTTGTGAGTGCAGAGGTAATTAAAAATAGAGAATTAATAGCGGTAGGGCATACCGT
AAATCAAAATGATTACGGAGACGTACTGACGGGGATTAGTCAAGATTTGATTAGTCTAGTTAAG
TGTCTGTGAGGTAAGAATCGCTCGTTACAATCTTTGATTTAACGGGTGAGTGTCAAATCATAACT
ACTGTAATAGAATCAATATTAATTAACTTTTAGAGTCCAATCTTCTTCAATGGGTTCATAACCAA
TATCAATTTCCTCCTTTTCAATAATACTTCTCACTTTTTCAAAAACCTCTATTCCTTTAGGTCTAA
TTGCGGCAATAATATATTCTTGATCTCGTTTTTCCCTAACATCTCTAATTAACTGTAAAAAAGCT
GAATTAGAATTTTCCAGTTGATTTGTAGGAATAAATTCTTCACTAGGGTATATAACTACCCCATC
TTCTCTACATTCTATATAACGAGGCTGTTTTTGTTGATTAGCACCATCTTCCGCTTGGGCAATAA
TCTTAACTTCTGGGTTAGAATCTAAAGTCTGAGTACTGACTACAATAATTAATAAAATTAAACTG
CCAATAGTACAAGCTAAAACCGAGAGAAATGGAAATAATTCTAGTTCTATTTTTTTATGAGATTT
TCTAGTCCTAGACATGGTGATATTTTGAATATTTAAGGATAATTTAAAGTTCTTAATTTTTATA

ATTCTTCATTCTTAATTTTAGTTATCAATTTGCTCCACTAAACGAATAACCCTAGGTTTACTTAA
GTCTTGTAATGTAGGTTGAATATTTGTAATTTTGTCTTGAATATTGACTAATGTTTTTTCAATAT
CACCGACATTATGTAAAACTTCGACTATTTTTCTAGGTTAGATTGTAAAGATTTTAACTCAGCA
ATTCTCGCACTAGATGCTTCTAAAGAAGTAACTTTTTCTTCTAAACTGACGGCGGCTTTATTTAA
TTGTTGACTGATAGCTTGAGATGCTTCTACTAAACTTTGACTGTTAGTTTCATTATTTTTTTGGA
CTAAATCCACTAATTCTTTTATGTTAGTAATTATGGATTGATTTAAGTCATTTTGTTGTCCGAAG
GTTTGAATAAATTTGTTTCTATCTTCTAAAATAACGGCATTGACTTCTTTAATTGTATTAAGTAA
TTGTTGCTCTTGTTCATAAATTTTCTGAAACTGATTCACAAATTCTGTTACTAAATTTTTAGCGG
CTTCTTGAGCATAAATTTGAGCGGGAGAAATTAATTCTTCTGGAGTGGGAAGTGCTTCTTTTATA
GGTTTAATTAATTCTTCTTTTGTCGGCAGTTTTTTCTCAAGGGTTGTATTAATTGTATTAACAAT
AACTTCTGTATTGAGGTCATTTTTCTCGCCTGTTTGTTTCTCATTTAAACGAGGTAAAATATAAT
CGTTAATATAAATATCTGTTGCTAAAAGTAAGTTTGATTCCATTCTTTCAATTAAAACAAGGGGA
ATCATTACAACCACAGATAACAATAGTGCCAATAGGGTGGTATCAAAAGCCACCGCTAAACCACT
GGTAACAGTGCCGATGCCTTCTTTTATTTGGTCTATTTCGGCGGTATTTTCCAAAAAGCCAGTAA
AACCATTCACCGCACTACTGATGCCTAATACTGTACCAATAAAGCCCAATAGAGGGATAGCCCAG
ACTAAAATTCTTGGCACTGCGTAGGAAGATTCAGAGGCACTTAGATAAAATGATGAATCATCAAG
GGCAAAATCTGTAACGGTTTTTCTACTTCCTGAGTTGATATAGGCATTAATAACTCTCCTTAAGC
GATTAGTTATCATGGTGGAGGTGCGAGATAAGTCTTCTTGAAGATGGAGAAGTTGCACTGATTG
ATGATTTTCAAAGGAAAATTCTTCGGGGATTCCTAACTTTTTAAAAACTTTATTTTCTTTGCTAA
TTTTCAGATATTTATCAACGGTAATAGTAATAACTAAAAAAGCACAAAAAACGGTTAAAAATTG
AGTAAATCCTCTTTTTAATAACAGCATTCCCACATAAGTTTCTTGAAAGGGTAAAGCTAAAACAT
AAATTAATATTGTGAGGACTAAGGCGGCAATTATTGTATTAAGAAAATTAATGTCTAATTCTTG
ACGGTTATTATTTTTTTGACCCATGATTGCGAATATTAATAGTTTAGTTGAGAGTATATTTTACA
TTAGAATATTTCAAATAATAAATAAATTTTATTGATGAAGAGTGATTTTTTCTTAATGTTTAAA
GAAAGATTTGATTTGATTATAGATAGTAAATCAGAGATAATAATTTGGATAGGTAAATAGTAGC
GTAAAAGTAAGAGAATTGATAATGACAAAGAATGTTGGGGTAATTGGAGGTGGGCAATTAGCAT
GGATGATGGCTCTTGTTGCACCACAAGAAAATATTAAAATTTATATTCAAACTCCTCATGAGACT
GATTCGGCGGTGTGTTTAGGGGAAAGAACTATTTTTGCCCCAGTTGATGATGTGTCGGCAACAGC
AGAGTTAGCAAAATATTGTGATGTAATAACTTTTGAAAATGAGTTTGTGGATTTGGAGGGTTTA
CAGTCTTTGGTGACTGAAGGTGTGTGTTTTTATCCTCGTCTTACAAGTCTTTCCCCTTTATTAGA
TAAATTTGATCAAAGGTCATTTTTTCAATCACAGGGTTTTCCCGTGCCTCGTTTTTCTCTGTATG
AGTCGGATTCGGATTTTCAAAGTTTTTCTTTTCCTTTAGTGTTAAAAGCTCGTCGTCATGGTTAT
GATGGACAGGGAACTGTAATTGTCAAATCTCCTCTGGAATTACAATCAGCTTTGGAAAAATTTAA
ACAAACACCATTATTGATAGAAGAATTTGTCCCCTTTGAGAAAGAATTAGCGATAATGGCGGCAA
GAAGTGCCACTGGAGAGATTAAAGTTTATCCGGTAGCAGAAACCTATCAAGAAAATCAGGTTTGC
CGTTGGGTGATTGCCCCTGCTTTAATTAATCAGGATATTACAGAAAAAATAAGTGCGATCGCATC
TAAATTATTAGTAAGTTTAGACTATGTGGGAATATTAGGAATAGAATTATTTATGAAGAATAAT
GGAGAGGTGTTAATTAATGAAGTTGCCCCTCGCACCCACAATTCTGGACATTATACCCTAGATGC
CTGTGAAACTTCCCAATTTGCCATGCAACTACAGACTATCACGGGAAAAAAATTGGGCAATACTC
GATTAAAAAGCTCAGGTGCAGTCATGATTAACTTATTGGGCTATAAATCCGCTCAAAGTGAATAT
TTACCCCAAAGAAAAGAAATAGAACAACTAGGAGCATTTGTACACTGGTATGGTAAAACAGAAT
CTCGTCAAGGCAGAAAACTAGGTCATGTAACAGTATTATTAGATGAAAAATCCCCTGAAAAAATG
TATCACCAAGCCCAAATGATGATCGAAAAAGTAGAATCTATTTGGTATCCCCAGAGGTAATGAGA

GGATGGATTTAGGGTTCTGTGTTATTAATTATTTAACCTCAGTTCGGTTTAAGAATATCGGATAA
GGTTAGGTGTCAGGTGTTGGGAGAAAATAATAAGTGAAGAGAAGGGGAGGGGGGATAAAGGGCA
GGGCTGTTTCAAAATAAAAAATTAGGTTACCTGAGATTAAGTAAGAGTAAAATATACAAGGATT
TTTTTCTATTAGTTAAAACTTTATGAACAAAAATCCCCTCATCTCCCTCTCTTTTTTCTTTCTCGA
AAATGAAACAGCCCCGGGGATAAAGAGGGGTTTGCCCCTTGCCTCTTTCCTAATTCTTGATTTAG
CTGTCAGTTATAATAAGCTGTATGTAAAGAAATATTAAGTAATTTTACAAATATAACAATGCAA
CAGCTAGAAAAACCAATTCAAAAACCATATAGCTTAGAAGACTGGCGAAAAGGTTATGAATCTCA
AAAACAAGAATTAGAATATTGGATTGAAGATATAGAAGGAGAAATTCCCCAAGAATTATCAGGT
ACACTATATCGCAATGGACCTGGATTTTTAGATGTATATGGAACACCTTTACAACATCCCTTTGA
CGGTGATGGTATGATTACCGCTTTTACTTTTAATGAAGGTAAGTGTTTTTTTCGTAATCGATTCG
TTAAAACCAAAGAATTTTTAGAAGAACAAAAAGCTGGTAAAATGCTTTACCGAGGTGTATTTGG
TAGCCAAAAACCCGGAGGATGGTTAGCAAACTTATTTGACCTCAAAATTAAGAATATCGCCAATA
CCAATGTTATTCACTGGGGGGGTAAACTTCTTGCTTTGTGGGAAGCCGCCCATCCTTATCACCTT
AATCCTGAAAACCTTGAAACTGTCGGCTTAGATAACTTAGATGGCATTTTAGAGGCTGGAGAGGT
GTTTTCTGCTCATCCTAGAATTGATCCAGCTTCTAGTTTTAACAATGGAAAATCATCTTTAGTTA
ATTTTGGTGTTAAAACTGGACTTTCTAGCACCATTACTATCTATGAATTGGATGAAAAAGGCAAA
TTATTACAGAAATATAGTCATATCATTGACGGTTTTTCATTTATTCACGATTTTGTAATTACTCC
TAACTACTGTATCTTTTTCCAAAATCCTACTACTTATAATCCTTTTCCTTTCCTTTTCGGTTTGAG
AGGCGCAGGAGAATGCGTAAAATTTTTAGACAATCAACCCACCAAAATTATTCTTATCCCTCGTA
ATGCACCCCATAAAGATGTGATTACCTTGGAAACAGAGGCAGGATTTATTTTTCATCACGCCAAT
GCCTTTGAAGTAAATGAGCAAGAAATTGTCGTTGATTCTATCTGTTACGCTAAACTGAGTCAAAT
TGATCCAGATACTAGCTACAAAGAAGTAGATTTTGATAAACTAGCACCCGGTCAACTATGGCGTT
TTCAACTTAACTTACAAGATGGTAAAGTTACGAAAAAATTAATTGATCCTCGTTGTGTAGAATTT
CCCAGCATTCACCCCAATAAAGTAGGCAGAGATTATCGTTATCTGTTTATTGGTTCTGCTCATCA
TCCCACAAGTAATGCTCCTTTACAAGCCTTACTCAAATTAGATTTGCAAACTAACGAACAACAGT
TCTATTCTTTTGCTCCGAAAGGTTTTGCCGGTGAACCAGTTTTTGTGCCGAAAGCAAATCCTCAA
TCGGAAGATGATGGCTGGGTATTTAATCTTATCTATGATTCTAGTCGTCATAAATCTGATTTAGT
TATTTTTGATGGTCAAGATATTTCTTCTCCTGTGGCTACTTTGCACTTAAAACAACATATACCCT
ATGGACTTCATGGTAGTTGGAAAAGCAAAAGCTAGTTAATTGAGTTCGGGGTTCTGAGTTCGGGT
GTTAGGGGGAAGTTAACTAAACACTTTTGCCTTTTGCCCATTCCCTGTTGCCCTGTCTTAACCTGA
GTTTTGGATAAGCTGAAAGCAGACCAACTCGTAGTCAAAAAACCTTATAGCCTCTTAGAAATAAC
TTAGAAATAACGATAAAATTGCCTTAATCCGAACTGACTTAAACAAGGGGCTTAAGCCCCTTGCT
AAAAACCCCTACCACCTGCTGTTCCTGCAACCTGACACCTAACACCTAACACCTAACCTTATCAGA
TATTCTTAAACCGAACTGAGGTTGTCTTAATTTTTGCTGTCTATGGGAGAGGATTGATAAAGTAA
AAAAAATTGATTAAATATTCCTAAAGTTTGGCTTTTATATACAGGAGTTTTAACTTGATAAATA
CTAAAAATAAGATAGTTATTTCTGATGGTATTTTCTGATAAAAATTGTTCGATCGCACCTGAACT
TTGATCCGCAACAAAGTTACAAGTTAGACTTTTTAAACTACTTAATATATCCTCAGATTTATTAG
CATTACAAATTTGATCTTTTAATGAGCCTTGAGTATTTTCAGTAATGAATTCATTAAACTTTTTT
TCATTGGGATTAGTGAAATAGCTGTAAATAAAAAATACACCTAATATTAATAAAGAAATTCGAG
ATAATTTCATAATTTTTATACATAAAATAAACTACAAAAAAATTATTCTTCGGGATAAATATTA
GTAATCACTCTGGTACTATTATCTCCTTGGACAGTAATATTTTTATCTCTGAGATTACCCCATGC
TTTAATACCTTTTAAGTTTAAATCGGGATTTAATTGACTATAATAAACATTTCCTTCTGCATTAA
TTTCCTCATTATCCACATTCCAAAGCATTTTATCTGCATAAATTTCTGACTCCTTATCTTTTGTTT

CTCCGTAAATTCCTTCTTGTAAATAAACCTGATTTTCCTCCAAATTAAATTTAACAAAATTAGCA
GTTAAAGTAGTTCCTTTGTCTGGTTGTATCAACTTAGTTGGTTGATTAGATTCCATGATGCGATC
TCCATAATACCAAACAACACGATTTGTTGCCGCTTGCAGAGAAGGCTTAATAGATTGATACTCCA
CATTCCCACTTATGACGGCTACGCTATTATTCAAATCAACTTCTACTTGTTGGCTATTTAGTTTA
TCCGTGATAATTTTTTCATCAAAACGAATCATATTTAAAGGCTTATTACCAATCACTTTATCTTG
AGGGATTCGCCAATATAAATGTTCCGTAGTTAATTGTAAAGTTGGCTTATTTGTGGTTGCAAAAA
CATCTTTACTTAACTCTAAAACCTGATTTTTAGTATTATATATTCCTTCCTTTGCCGTCACCACTA
ATTTTTCATGACTAGCCTTAATTTTATCTTTTCCCGTTACAACCAAAAAATTATCCTCTGGTTTCC
AAACTACTTCTTCTCCTCTAAATTCAGCTTTATTCCGAGGATCAATAACTAAAATATTGCCCTCC
AAAAAAATTTCCTTACCTTCTTTTGTTACCTCTCCTCTATCGGCTTTCACTTTCAAAATAATCTCA
TTATTTTCAAATAAATTACCTACTATTTCTTCGATTTTTGCACTTTGATTATCTTGAGCATAAAT
TGCTTTTTCCGTACTAATTCTCCAAAGAGTATTACCATCAGCATTAGACTGTTCAATAGTAGAGT
TGTAAAGAACTAAACCTTGTTCAACTTCCCTATTCGCCTCATTATCATTAACTTCTTGAGGAGGT
TGACTATTTCCCTGACAACTAACTAAGAAAAAGCACAAGCAAAGAAAAGCAAAGGATTTTTTAAT
GAACATTATTTGTTTTCAATAAGCCTTAAAATTGTTAACTAAAAATAAATCAAAACTGAAAATT
TCATATCAAAGATTTATCCAGTTTTAGCTGTGCCTCCGACATCAAATCCTCTTGAAGATGATCCA
CAAAAAGAATGCCATTAAGATGATCTAATTCATGTTTGAATAATTCTAGCGATAAAATTATTATA
TTGTTTTCGATGCCGTTGACCATCACGGGTAAAATATTCTACCTCTATATCTCGATAACGGGCAA
CCATGCCTCTTTTTTGCCTAACACTTAAACATCCTTCTTCCTTTATCACTATCTCTTGAGAATGGC
TAACAATCAAGGGATTTATCATGGCAAACGGTGACATAAAAGGTGCGTCAGGATAACGAATATT
GGGATGAGAAGCCACGACAATGATTCTTTTTGATATTCCCACTTGAGGGGCCGCAATGCCAACAC
CGTGAGACTTGAGAGTTAAATCTAATAATTCATCGATGAGATTTTGTGTTTCTTGGCAGTGAATA
TCTTCAACTTCTTGTGCAATTTCCCTTAAAACTGGTTCACCTATTTGTAAAATTTCCTTGATCAT
AATCACATTTTGAAAACCATTATTTAACAACCAATTATCTGTCCTAGTATATCGTTTTCCCTAAT
CACATCAACTGAATGTATAAACCGCATTACCTGACTATTATTGGTATCGAGCAACACAAATTTTT
CCAAACTTCTTTTTATACTTCTCTTAATTATTTCTTAATTTTTTCTTAAATAACTGGTGTGAGGT
CTTCAATGTCGAAAAAAAATATCTATTGCCTAGCCACAGTTTTAAGTGTCTTAGCAAATCCCGCT
ATTGCCCAAGAAACTCTATACAGTTTCAATGAACCAGATTCGATGGCACAAATTAATTCCGTGTC
GCAATTGAGAGATGTTTCTCCTACTGATTGGGCATTTGAAGCCTTAAGAAGTTTGGTTGAGCGTT
ATGGTTGTATTGTTGGCTATCCCGATCGCACCTACCGAGGAAATAGAGCCTTAAGTAGATACGAG
TTTGCCGCAGGTTTAAACGCTTGTATGCAACAAATGGAAAGACTTATTGCCCAAAGTGAAAGCGT
GTTAAAAGAAGATATAGAAACCCTCAAACGCTTGATGGCAGAATTTGAAACAGAATTAGCCGCCC
TTGGTACAAGAGTTGACAACCTCGAAGGAAGAGTGGCATTTTTAGAAGATCATCAATTTTCTACT
ACCACCAAACTAGGAGGAGAAGTTGCTTTTACGCTTGCTCAAGCCTTTGGTGATAAAGTTACTAA
TCCAAATACTGGTATTAGCGAAGATTTAGACACTCAGGCAGTTTTAACTACTAGAGTAAGACTTC
AATTAGCCACCAGTTTTACAGGTAAAGATGTGTTATTCACTCGCTTAACGGCGGGTAATTTAGGA
GCATCTTTTGCGGGTCAAACCTTCACCGATGAAGGGCGTTTTGCTTATGATGGACAGTTAAACAA
TGACGTTACAATCGATCGTCTTCATTACTACTTTCCTATCAATGACAATCTCAGAATGTTTGTCA
TGGCAAGTTTAGGGGGACACCACTTCTACGCAGACACTTTTAACCCCGGTTTAGAAGCAGGAGGA
GGAGCAAACGGTGCATTGTCTCGATTTGCCGAAAGAAACCCCTTATATCGCCTTGGTTTAGGTGG
TCAAGGAGTAGGTTTCCGATACAAACTAGGCACTAACTTTGAACTATCCGCAGGTTACTTAGCAA
GAGGAGGAAATGACCCTTCCGAGGGGCGAGGTTTATTCAATGGTAATTACTCCGCTATGGGGCAA
TTAGTCTATAAACCCACTGACAGAATTAAATTTGGTTTTAGCTATTTAAACGGCTATGATCCTAA

CGGAGTGGGTTCCGCATTTTCCTTTGGAGGTACGGGTACAAGATTTGCGAACTTACAATTAACAG
GTTTAGGAGTTCCTAACCGACCAATTTCTAGCAATTCCTATGGCTTACAAGGTCAATTTGATATT
ACTCCTAATTTCAGTCTTCGGGCTTGGGGGGGTTATACTTCCGCCAAACTAATCGGCTTGGGTGA
AGGAGAAATTTGGAACTATGCCCTTGCTTTAGCCTTCCCAGATTTAGGAAAAGAAGGAAGCATGG
GGGCAATTATCGTTGGTGCTGAACCCTATTTAGGGGGTTTAGATGTACCTCAAAATCCTCGCTTA
ATCAACGATACTCCTTTACACGTGGAAGTTTCTTATAAATATCCTCTGACGGATAATATTACACT
AACTCCCGGTTTAATTGTTTTAGCGGCACCGAATCAAAATAGTGGCAGAAATGACCCTATTTATA
TAGGTACTATTAGAACAACCTTTACCTTTTAATTGCCCCTACTCGCTCCTTATACTAATAGCCTAG
ATGAGTTCAGGTATTGGGGAGTAATAAGCAATAAATAATAAGTAATGAGAAAGGGAGATGAGTT
AGGGATTGGGGGTTTTTAATTATTCACTATTCACTAATCATCTTTATCCTTTCCATTTTCCTAAG
TCCATAACTCAGGTTAATTAAAATTTTCCAGATTTTGGTAAGCATTAATCATCCCCTGAGCGCCT
TGATTATATCCTTCTCCTTCATTTAATTCAGCACTAACTTGAAATAACTGATTAGCTAACTCTAC
GCCGGGTAAATGCCTATCTTGAAGAGTTTCTTGTATTAAACGTAAGTCTTTTAATATATGCTTAA
TCATGAATCCGGGTTCAAAATCTTCCTCAATAATTTTTTTGCCTAGATTACTCAATGCCCAAGAA
CCAGCCGCTCCCGTGCTACAAACTTTTACAATCAAGTTGGGATCTATGTTTTGAACTTGGGCTAA
TTTCATCGCCTCGCATAAGGCTACCATATGCAAACTAGCTAAAACCTGATTACATAATTTTACCC
CTTGTCCACTGCCTACATCCCCACAGTGAGTAATATTTTTTCCCATAATCTCTAAATAGGGTAAA
CATTCTCTAAAGTCTTTTTCTTTTCCCCCTACCATAATCGTTAAAGTGCCTTGTTGAGCTCCTATA
TCCCCACCAGAAATAGGGGCATCTAAGAAACGAATTTGTTTTTTCTCTAATTGACTGGCAATGTT
TTTCACTGCCGTTGAGCCAATGGTGCTAAAGTCAACAACTAAGCTATTTGGAGGAGCAAAATTAA
TTACCCCTGACTCTGACAATAAAACTTCTTCCACATCTGGCACATCTCCTAAACAAGTAAAAATT
ATTTCGGCGGATTCTACCGCTTCGGCAATAGTATCAACCATTGTTAAAGATGATTCTTGTAATAG
AGGTTGTATATGAGGGCGATTTTTAGTACGATTCCACACTTTAATCTGACATGGGGCATCCGCTA
AATTTTTTGCCATTGGTGCCCCCATTACTCCTAATCCTAAAAATGCTAGTTGATGATTCATGATT
AACACTAACTAATCTTATTTTTGATGGCGTTGACTACAGTTTCTAATACATGAACCCTAGCATAA
TATTTATCATTCCCGGGTACAACTGTCCAAGGGGCAAGGGCTGTGCTAGTACGGGCGATCGCTTG
ATTTACTGCCACATCATACAAATCCCATTTTTCTCGGTTGCGCCAATCCTCTTCTGTTAGTTTATA
ACGTTTATAGGGGTCTTCTTTTCGATCTTGAAAACGGTTTAATTGTTCTTCTGGGCTGATATGTA
GCCAGAATTTTAAAACAATATAGTTTGATGTAGCTAATTGAGCTTCAAATTCATTAATTTCTCTG
TAGGCTCTCCGCCATTCTATCTCCGTGGCAAATCCTTCTATTCTTTCTACTAAGACTCTACCATAC
CAACTGCGATCGCATATACCAATTTTTCCTAAAGCTGGTAAACTACGCCAAAAACGCCAAAGATA
ATGATGTTTTTTTTCTTCATCGGTGGGGGCGGCAAAGGTGTGAACTTCATAACTACGGGGGTCTA
AAATATCGGTAACACGCTTAATCGCTCCTCCTTTCCCGGCGGCATCCCAACCTTCAAATAATAGTA
AAACCCCGATATTATGTTCATAAATATCTTTTTGTAATTGTCTGAGTTGCACTTGATATTTTTTT
AATTTAGTTTTATATTCGTCCTTTGGCAAGTGTAAAGTTAAATCAGTTTGCCCTAAATAATCAGG
CTCAGTGGGTAATAATTGACTTTGGGGGGCAGAAGATGGGGTTGCGGTGATAATTTCTCTTTTGG
CTAAACCTTCTCGAATAGTAGCAACTAATTGCGTTAAAACCTTCATTTTTGCCCAACGTTGACAA
TCTGCCTCCACTAAAACCCATGGTGCAAATCCAGTGCTAGTATAAATCAACATTTCCTCCGCTAA
ATGACTATATTCTTCGTAATTTTTTACTTGTTGCCAGTCTTCTTTGCGCACTCGCCACGATTCTAG
TTCGTCTTTTTCATATTTTTTAAGACGGTTTTTAAGTTCTTTTTTGCTAACATGAAGCCAATATT
TAGCAACTACCGCCCCATCTTCCACCAACTGTCTTTCAAAAGCATTAATATCCCTCATTATCAATG
GCACTCTTTCATCTTCTAGGCGCTTAAATAACCTGTCTTCTAACACGTGGGTGTACCAACTATGG
TAAAAAATACCAATACTCCCTACTGGGGGAAGTTTCTGCCAAAAACGCCAAAGAAAGGGGTATTG

TTTTTCCTGCTCTGAGGGTGAAAAGATAGGATGAACCGTAAAACCACGAGGATCCATATAGTTTG
TCATTTGTTTGACTAATTCCCCTTTTCCTGCGGCCGCCCACCCTTCTAAAACAACGATGATAGGCA
TTTTTTGTAACCAACAAATATTTTGGAGCGATCGCAACTCTCGCATTAATTCTTCTATTTTGTAG
CGGTAGGTATCTTTATCAAGAGAGTTACTTAAATCAAGGTTATCTAACATTTAAAAACTTTATTC
TACATTCTATTTTCAAATATATAGTTTATTTTGTTGATACTAATTCATTCTTAATTAATTCTTAA
CACAAACAAATCAAGGTTTCAGGTGTTGGGAAGAAAGTAAGCAGTCATGATTAGAGAGAAAGGG
TGATGAGGTGATGAGGTGATGAGGTGATGAGGTGATGGGGTGAGAGGGGGAAACAAGTAATGAG
TTTTTAATTATTAATTATTCACTATTCACTATTCACTATTCACTATTCTTAATTTTTAATTCCCG
TATCACTTACCCCTTGAATAAACTGTTTTTGACCGAAGATAAATAAGATAATGACGGGAATAGTA
GCAATTATTACCGCCGCCATGAGTAACGACCAATTACTGGTAAATTGTTCTTGAAAGGATGCTAA
GGCTAATTGTACTGTGGTTAAATTAGGGGTGTTAGTAAATACTAGAGGTTTAAATAAATCATTCC
ACTCCCCGATAAAGGTAAATAGGGAAAGAGTTATTATCGCCGGTTTTGCTAAAGGGAATAAAATC
TGCCAGATAATTTGTAAGGAGTTTGCACCATCTAGGGTTGCCGCTTCTTCTAATTCCACGGGAAT
AGAAATAAAATATTGACGCATCAAAAAAATACCGAAACCACTGGCAGCAGAAGGCAATATTAAC
GCCCAATAAGTATTGATTAAATGTCCCCATTTTAAGATTAAAAAAATCGGAATTACTAATACTTG
AAAAGGAATAATTAATGTAGATATAATAAGTAAAATAAGAAAGTTTTTTCCTCTAAATTTTAGT
CTAGCTAAAGCGTAACCTGCCAAGGTGGAAGTAATTACTTGAAAAAAAACAGATGCGATCGCAAC
CAATCCAGAGTTAGCAAAAGCTAATAAAAAATTGCCTTTTTCCCATGCCAACTGATAATATTGGA
AAGTAAATTTAGACTCAGTTCCTGTTGAAAAAAAAGAAGTATATAAAATAAAAATAAAGGGAGA
TAAAATAATAATTAATCCCCCAAATAATAAGATAAAACTAAAGATTTTAAATGTCGAATTAGTA
AACTTATCCATAAAAATTGATTCTCATGAGGGATAAGGTAATAAATGAGGATACAGAGAGTGGA
TTGGGGGAAGTAATCAGGAGTCAGGAGTTGGGGTGTTAGGAAGAAATTAACTGTTGCAATAGTT
TTTTGTCTAAATTGCTAATTGTAATTGAAATCTATTCACTAACTGCTTTTTACCCAAGGTTTTTT
CATTTTCAAAAACGTCTATTTTTGAAACTAATAAATAGTAACAGATACAGGAGTTTTTAAGTATT
AATTAATCAATTATTAAGAAAAAACGCTCCCCTCTACTGTGGGAGAGGGGGTGGGGGTGAGGGCC
AGATAATTTTGCCCTGACTTTGAAAAAACCCTGCTTTTTACCCCTATCAAAAAAATTAAACCTAA
TATCCAGTCACCTGATACTTAATACCTGAAACTTCCTAAAGTCGAAAATTTCTGTTAGGAAAAAG
TTAAAAGAGGTTTAAAGCCGTGCTTAACAGAGGCTTCGCCGATTTGTTCCATTTTTTCGACACTA
ATTTGGTTTCTTCCCCAAGAAAAATTAGTGTGCCATTTCTCAAACTCTAACAACATAGCCTCAGC
AAAACAAGCAAACATTTGTCTCCCGGGTATATCCATATTGACAATTTCCATAATTTTCCAGTCAA
TATTTAAGGAATGTTCTACAATGCCGCCATTTAATACCTTAATATCAGGATGATTAAATTTAGTA
CCCAAATTCTTCGGATAACCGCCATCAATTAATAAACAAGGTTTTTTCAAGGTTTCGTTATTAAT
TTCTACCCCTCTGGGCATACTGGCAACCCAAACCACTATATCAGCTAAAGGTAAGGCTTCTTCTA
AGCCCATGATTTTACCTCTACCTAAATCAGATTGTAAAAGCTCTAAACGCTCACGATTACGGGCA
ATCAAGAGTAAATCAGCAACATCAGTTTTTTGATTTAACCAACGACAAACAGCACTGCCAATATC
ACCCGTAGCACCGCATACAGCCACCGTTGCTTTACTCAAGTCAATACCCAATTGGGCGCTGGCTTG
TTCTACTTGACGACAAATAATATAAGCTGTATGAGTATTACCAGTGGTAAAGCGTTCAAACTCTA
ATTCTACGTTACGCACTTGCTTATTTTCCTTAAGATTGAATTCTTCAAAAATAATGGAAGAAAAA
CCACCGAGAGCAGTAATATTTATGTTGTGTTTTTGAGCTAATGCCATCGCATTTAAAATTTTCCT
GATTGCAGTTTTGATGCGACGACTGTTTAACATTTCTGGGAGGAAACAAGATTCGATATAACAGC
CTTCAATGGTTGCCCCTGTGACGCTTTTGACTTGAAACTCATCCACTAATATGGGAGGTGCAGAA
CACCAAAATTCAAATCCTTCATTGGCATATTCAGAGTAGCCCAGTTTGTGAGCTTCTTTTTGAGC
GTGTTCTAAGTTGGTTAAGTGACCGATGAGACCAAACATTTAATAATATTTTACTTTAATATAAT

GTTTTTTTTTGAAATACAGAAAGTCAATAAAAGCTAAGAATAAGTTAAGACTTTTTATCATATT
ACTAAAAAATGTAACAAAAAAAAAGTGGTGAGTGCTGATTTCTGATAGTGACAGGGCAAAACTTA
ATCAATGAATGCCTAATGATAAATAACTGTCATCTCCTAGCAGGTTATAATTACTAATTGCTAAT
TACTAATTGAATTATGTTGCCTAGAGAAGAATTATTAAAGAGAGTTGAAAATAAAGAAGATATT
GCCCGTGTGATTGATAAGGCTGAAAAGGCGGTTAAAACGTGGGAGTTAGTGGTGACAGATTTTCT
TTCTCCTCCTGTGTTGGCAGAGGTTAATGAAATTTTTGCTTCTCTGACAGAGGTAAAAATAGTTT
GTTGGGGGGGCTATCCTCAAGCGGAAAGGCAACGGGTGGGGATTCATCGAGAGGAGGTTTATGCT
GGTGTGGCCGAGATTCCTCTAATGGCTTTAGATGTCGCTGGTAATTTTCTTTTTGATACGGCTAC
TCATCGAGATTTTTTGGGAGCGATTTTAGGTACGGGTATTGTTAGAGATAAAATTGGTGATATT
ATTGTTTTGGGTGAAAGAGGGGCGCAGGTTATCGTTACTCCCGAAATAGGGGAGTTTCTGGAAAC
ATCTTTAACTCAAGTGCGTTCTGTACCAGTGAAAACTGGTAAGATAGATTTATCAGATTTAAAAA
TTCGCCCTCCGAAAAAGAAAGAAATGACTACGGTAGAGGCTTCTTTAAGGTTGGATGCGATCGCA
TCTTTTGGTTTTGGCATTTCCCGCAGTAAAATGGCCGATGCGATTAATAATGGAGATGTAAGGGT
TAATTGGAAAGAAATTACACAACCCAGTTATAATGTCAAAGATGGCGATTTAGTCTCTTTTCGTG
GTAAGGGTAGATTAGAAATAGGTGCGATCGCACTTACTAAGAAAGAACGTTATCGCATTAACTTA
ACAAGGTTTGTATAGGATTTATTTAGAGATATTTAATGAGTTTAATTACTATTTTTTTGACTTCT
TTTGGACTGGCAATGGATGCTTTTGCTGTTTCTGTGGGTAGCGGGATTAGCCTCAAAAAAGTAAC
TCTGAAAGATGCTATCATCATCGGTACGTTTTTTGGCGGATTTCAGTTTTTTATGCCCTTAGTTG
GCTGGTTTGCAGGGTTAAGTTTTCGGGAATTTATTGTTAGTTTTGATCATTGGATTGCCTTTGGT
TTATTAAGCATCATTGGTGGTAAAATGCTTTATGAGGCTTGGCAAGAAGAAGATGAAGAAGAAG
TAGGTACAGACTTCCGCAACTTATATGTTTTGTTAACTTTGGCCATTGCTACCAGCATAGATGCT
TTAGCTGTAGGTTTGGGTTTTTCTGTGATTAAAACTCCTATTATTACTGCCGCCGTCATTATTGG
TATTATCACCTTTTTGCTTTCTTTTTCAGGTGTATTTTTAGGAAGTAAATTCGGTCATTTATTTG
AGAAACAAGCGGAAATAATTGGTGGTTTTGTACTGATAGGCATTGGTTTTAAAATTTTATTAGA
ACATCTTTAGAAATTTATATTCATTTCAACATTTTTTGTTTTTTTAAACAGACTAAAAATATAGT
TAGCTCACTTTTTCTAACCATTTTTCAAAATCAGATATATTTTCAAAATCGAATAAATCCTCTGC
TAAAGATTCTAATTGAGGAATAGATAGCGATCGCACTTTCTGTTTTTGATTTTCAGTTAAACTAC
CGAAACGGCGATTTAATTGACGAATCAGCAAATTGGCTTCTCCTTCTTCTAAACCTTTTTGTATC
CCTTCTTCTAAACCTTTTTGTATGCCCTCTTCCAAACCTTTTTGTATCCCTTCTTCTAAACCTTTT
TGTATCCCTTCTTCTAAACCTTTTTGTATCCCTTCTTCTTTAGCCTTATCTTGCCAGTATTTGACT
AAGGTTTCGTAGGCATCTAATTTGGGCATAGTGGCAGTTTTTAAATCAAACATAGTCAATATATC
CTCTGTTTTTAATTGCGGTAATTTACTTTTTAGTATAGCTTCGATTAAGGTGAATTGTCTCTGGA
AGTCTTCTTCTGTTTTAGCACTACTTAACACAAGATTTCCTAAATGTTTTATCTCAGAATTTGGA
CTAACAATTAATCTGAGTAAAGCTAAATTAGGGCTTAATTTTTCTATATTTGATAATTCTTCTAA
GTATAATCTAGTAATATTATTATTCAATAATATTTGATAAGGAACTTCTGAACCTAAATCATTAT
TTTTGTTTTTTAGTATTAATAAACCTTTCCATGGTCTCTTGATTTCATATTGACTTAAATAGAGA
AATATTTCTGCGAAATATCGATGATAGAATTTTATATCTTTTTGCATTTGTGCTTCTAAAAAGAC
TAATGGAATATTTTTATCATCAGATAAAGGAGTTAAGAGTCCGTCTAAACGAAATTCTGTATCTT
TAACGACTGGTGCTGTATATTCAAATTGACAGTTACTGGGAATTTCAGGTAATAATTCGTTAATT
AAACTAGGTTGATTAATAAATATTTGGTAAAAAAGTTTATCAGTTTTCATTTTTTATCATTTTCT
TAAACCAATCCGCCGTCATTTTTATCCCTTCATCTACGGTATAAGGAGGTTGCCAATTAAGGGTT
TTTCTAATCTTACCACTATCCACCTGCAATGATCCTAAGAGGCGATCGCCCGCTTCTCCTTTTCCT
AGTAATTTTGTTCCTATTTTTATCAAACTAGAAGGTATAGGTAATAATAAGGGAGTTTTATTCAT

GGCTTTCCCAATGCGTCTTATTAACTCAGGGGTTGACAAATCTTCTCCGTCACTGACGATAAAAG
TCTGATTTTGAGCTTGAGGGGAGTCAATACAAGTGATAATAGCATCAACTAAATTGCCCACGTAA
ACAAGGCTACGAGAGTTATTTATGCCTCCTAACGGAAGGGGTAAACCCTTGGCAACTAATTTTAA
TAACCTTTCCATATTCCCCGGATTATTTGCGCCATAAACTAGAGTGGGGCGTAGAATGGTATAAG
TCATGGTGCTATTTTGACAAAGTTCTTCCAAGGCTTGTTCTGCTTTTAATTTGCTCTTACCGTAG
GGAGTGTCAGGGTTACAAAGAGAAGATTCGCTAATAATAGTATTGCTTAAAGTGGTAACAGCACC
AATCGAACTTAGGAAAATGAAGTGTTTTACCCCCACTTCAAGACATTGTTTCACCAGATTAATTG
TACCTTCAGCGTTGATGCGATCGAACTCTACTTCGGGATTAGTTACAGATTCTTGCAAAATGTGA
GCCCTTGCCGCTAGATGGATAACAACATCAATGTTTTCTAAGGCATTTTTCCAAGAAGTGCGATC
GTCTATTTCTCCAACAACAATAGTTTTATGATTAGAATTGGATGGATTACGAAGTGCAATGGTTA
TTGAATAGTTATTTTGTTCTAGTATGCAAAGAAGGTGATTGCCAATAAATCCCGTTGCACCAGTA
AGTAAAATAGAGGTCATATTTATTTCAAGTTTAAAACTTTTATATATTCTTTTAAGGCTCGATCG
AGCCTATATTGTTTTAAATAAAGTTGATGAGCATTTATTCCCATATTTTTCACTTTATTAGGAGA
ATTAGATAAGTCAATTAAAATATCTGCTAATTTATCTGCTTCACCACAAGGGCAATTAATACCAC
ATTCTTTTTCTGTAATTAATTTATCGATATATGAACCTGTTTCGGAAACAGAAATTATCGCCCTT
CCTGATGCCAAAATACCATAAAGTTTACAAGGAGCAACCACCGATTCTGCACCCTTAACTAAACT
AACTAAAGAAACATCACAGGCAGTTAAAGTTTGGGATAAAAGTTCTCTCGGTTGAAAAGGTAAT
AATAATAAGTTTTCTAATTTATACTGGTTTTTATATTGTTCTAATATTTTAAGTTTTGCACCATC
ACCGATAAATACAAATTGAATATTTGGGCTTTTTTTATTTAATATTTTAATGGTTTCAGCAATAG
TTTCAATATCGTGAAGACGACCAATATTTCCTGAGTAAAGTACCGTAAATTTATTATCTAACCCA
TGCTTAATAGCAAATTCATTCCTTTTTTTGTCTGATAACTCAATAGGTGCGATCGACCAATTTTC
AATAATGCAAAAATTATCTAAATCAAAATATTTAGACTCTAATAATTTTTTCATCGATTCACTTA
AAACAATGGTTTTTTCACTGTGATAGCAAATGAGATAAATCAAATAGTTAAAAACCTTAATAAG
CCAACTTTTTTCTTTTATAATCCCACTCAATATTGCTGACTCAGGGAAAACATCCTGTAATAAAA
AATAATATTTTCCTCCTTTTAAAAACTTGAAAATAAGTCCTAAAACTCCTGCATAAGGAGGATTA
GAGGCAATTAACAATGAATCTTTTTTCTCAACTTTAAATAGGACAAAAAACAAAGCCCCTAACAA
AAAAAATAACGAGCTAATTAATTTACCCAATATACTCTTACTTTTTTGAAAAGGATCGGGCGATC
GCAATATAGCAAAAGGTTTTGATTTTTCAGAATTACAAGGAGTACCTGTATAAACCTTAATTTCT
AAATCTTTAGTAAATAAGCCATCCGCTAAATCAGTTAATAATTGAGAAGTAGAGGCTTCACTAGG
GTAGAAATATTGACTAATTATAGTAAAATAAGACATAAATTTTATTAAGATATGTAGCCACTAC
CAATCTAAATCTTTTTCATAGTTAAAATCAACTAAAAAATCCCCTATTTTCTCTTTATAAATATT
TTTCAAATTTGGCGTAAAATGGTTTTTCCAATCCCCAGCAACTCCTTTTCTAAAAAAACTATTAC
TACTTTCATTTCCTTTATTCCGTCCGCCACTCAGGTTGCTAAAGGAATTAATTTCTACTATTTTTC
GAATTTTTTGAGGAGAATTATCCAAAGCACAATGTTTAGCTATTTTCGATAGTTCTATTTCTGGA
TTACTTAATAAATCCTCATAACGGATAATTAATCTTAAAGAATTCTCAGTGGTTTTTGTCCAAGA
TTCTATCCATTTAGCATATTCACACAAAAGATTTTCTGCAAAGAATTTTAACCCATCTTCTACTG
TCAGATTTTTATAAAGATGATATTGAGGATGCCATGGAGTTTGCTTAACATAAAAATAGTAACT
TACTGCGACATCTCTTAAATCACGGTAGATTACTACAAAAGGTATTTGACCATTTTCTAATAAAT
TCAAATTATGCTCTGAACCATGAACATGTAATTTTAAAACTACTAATTTATTTTTTAGTCTAATA
AAAGTATCTTCTGGTAAATCAAAGTCATGACTACCACCATGATTAATTTCATAAGCGTTGGCTTC
AGGTATCATTACTTCTTGATAACCCTGATAACAACAAATCATTTTCTTGAGCCATGAAGTACCAC
TTTTAGGTAAACCAGCTATAAAAATTAAATTTTGCGGGTATAAATTACTAAATTTATTAAATCCC
TCTTGAGCTTGTTTTAATTGAAACCAATAACGAGGTTTACTGACTATGTTTTTTTTTAAAAACAT

AGCTATATCTTGGGGTAGATAGTTTTTCAGTTTTTTTTCTAACAACTTATTCATCGGTATTAGTT
AAATTTTAGAATTTTATATTATCTGTATCACAAATTAGAGACATTAAGCGTTTTAAATGAACTTC
CCTGGTAAAATTTTCTTGGAAATGTTGAATTGCGTTTTCGCTTAGTTTTGATAGTCATCAGAAG
ATTTTATACCTTTCAAAATAGCATTAACAATGGCTTGAGGATCGTTATTTTTGATCAGATAACCA
TTATATCCATCCTTAACTTGTTCAGGTATTCCCCGATGATAAGTGCTTATAACGGGTGTGCCAAA
GGCAAGGGCTTCAATAATACTCAGAGGTTGTCCTTCCCATGGATACAGCGTTGGTAAAATAAAAA
AGTGAGCTTCTCTCAACATTTTTTCTTTTTTTTCTCCTGAGACTGTACCATGATAAACGATAGAA
TTTTCTAAACCTGCTTTTTTTATTTTTTCAAAAAACTCTTCTCGTTGCGTTTTTATATCTAGGGA
TTGATCATCAACTGCTGTCTTGTTAAACTCACCGCAAAAATCGCATATTAACTTAACATCATAAT
CTTGAATTAGTTTAATACAAGCATCAAGTAAAGCAAGATACCCTTTGGAAGGAATCAAATTGGAC
AGATATAACAAACGGAGATTTTGGTTCGTTGAGGGTAATGATTTTATTTGAGCTAACTTAGTATT
TAAATTTGTTGTTAAACCATTAGGTATCACTTTTAGTTTTTTATCAATGTCTGGAACAAAATAAA
ACTGATCTCTCAGTAATTCTCCCAAAACAATAATATGATCACTTTTTGCGAGAGTGTTGCAGATT
AATTTCTGGAATAGAGAAGATTGGTTAAGATAAAATTCTTTATAACCTCCCCCTTTGAGGTGTAG
GATAATTCTTTTTTTAAACAACCAAGCAAACCAGACGACCATCATATCTCGAATAAAACCCAACT
TTGAAGATGCGATCGTCATATAAACAACGTTTTGATAAGGGATTTTCTTCCATGCTTGTAAAATA
GCCTTTAGAGAATCTTTTGTTCTAACCCAAGAAAAAATACCAAACACTTCTGATTCTCGTGAGTC
ATATATATATACAATTTCTGGTTGATAACTAATAGATTTAAAGCCTTCGATCAGAGATTGTAAAC
CTAATGCGGCTCCAGTTGCTCTTGTCTGAGGATAGTAACCTATTAAAAGGATTTTTAGTTTCATT
GCAAGATAATGGATTTTAATTAAAAGAAAACATTTAGAATAATATAATTTTAAGAGCTGTTAAT
ATAATTATTATTAATTAAATATTAGTTTATTTAAGTTAAATTATTTTTTTATAACTCTCTTTTTA
TAAGTCTACTCTCCGCTACTAAAGTTGATTTTCGAATGTTATTGAATTCCGACCCTTCTTATTTT
TTCTAATCATAAATAAATACAATTTATAAGCAAAAGATAATATAAAAAAAAGAATTAGATTTGT
CAAAATGCCTACAATGCCGCTCGGAGACAATACAGAAACTCTACAAAATTCCGTGTAAAGAAGTA
TTGAGCCTATGGAAAAAGAATGAAACTTCATAAAATTATAAATCCAAGCGGGAATAACTCCGGA
AATAAAAAATAATAATATAAAACCCAAGATTCCAAAATTCATGTATCCGGCCCAGTTAGCTTCTG
GCCAAGAACTTGGAGGCAATTCGGACACTGGCATTGGTGGATTAATAATTCTACCTTCTGCCATA
CCTAAAAGATATCGCCCTGTGTCAACAGGGGGTTTGTCTGGAAAAATATTTCTGGGGATAGGGGC
TGTGACTAAAGATAAATAAGAACTACCTAACCAAAAATCATTTTCATCAAAATATTTTAAAATCA
CAACATCTCTTTCAACTCTTGAAAATCTTAATAAAAGATGAGTTTCGAAAGATTCAAAAGCATCG
GTCATTAATAAACCTACATTACCAGAGTATTTTTCTACTGCGTTAATTTGTCGAAATTGAACGGA
AATTAAAGAAATAATTATCATCAAAATAATGAGGAAAAGACGCTTTAAGGTAAATAATTTTCTA
AATCTTTCAATTCCATAATGTCTCAATATCATCACGGAAATAATAAAAGATAGTAATGGTCCTCT
TTGTCCTAAAGATAGCAAAACAAAACTATTGAGCAGGAAAATTAAAATGGAAGTGAAGTATTTA
TTCTTTGAAAAAGTAACATATAAACAAATAAGACTACCCATACTAATTGCAAAACTGTAAAAGG
ATTGAAAATATCCCAGGCCAGCCGCTCTTTCAACTCTTTGGTTTAGATTTAGCCATAAGTCTACT
AATCCTCCCATTCTCATTAGAAAATAGATATAGACACATATTCCAAACCCAATAAAAAAAAGACC
TATAGTAAATATATTTTTTATGTCACTTATTTTTTTTATTTTAAAAACTGTTATGCCTATAATTT
TATTAAATATTAAAAATAGACTTTTATTTTTGATACTATAACCACAAACTATTCCCAAATAAGTT
AGGATCGATGAGAAAACAGTCATAATATAAGCAAAAATCATGTAATCATAGCTTCTAAACTCACT
AGAAAATTTTAGAAAAATTATGTTTTCGATAGAATCTGTATTAATGGATAAGAAAAATAAAGGT
AAAGCTGATAATACTTCAAAATAAATAAATAGATTACCAGGGGAAAAAAAATCAGGTTTCTTTT
TGTAATTATTTTCGAAATAGTAAAAAAGACTAAGGCATAAAAAAATATGAGAGTGAGATTAATAA

TAAGAATATATTCATTAGATTAGTGTCATTTTCTTCTCGTTTTTTCAGTTATAGCAAGGGGGAGA
CAATCATAATTTTTAGGATTCAAAATAGTAGGGTAAATAAGGATATAAACTGGAAGTTTTAGAA
CTAATATATAATTTGACACTATGAGGAATCAAAAGTAGTTCATTGATACCTTTGAGAATAGAATC
AATGGTAAGTTCACCATAAAGTGGATCGATAGTTTTGTGAACTGAACCAGACTGAATATTTTCAG
GTATCGGAATGTTAAGAGATTGATAATAATAATCTTCGTAAAAGTATTTAGTATTATTTAAATC
AACTCCACACAACACGATTTGTTTGTATCCAAAGTTTAATCCAAGTAATATCGCTTGACTCAAGG
AAGCTCTTTTGAAGAATAATGTATTTTTAAAGTTCCTCAAAAGATTGAATTCATAAATTAATTTC
AAACTGTTTCTGAACACTGAAACATGATTTCCAGGAATAGAAATTTTATATGGTGCATAAGTATT
TTTCTTGATTTGTTCTGGAATTTGTTCCCAAGCAAACTGGGAAAGGGATGAATTTTGAAGTTCTT
TTTCAAGGATATTGATAATAAAAGGAGTATGTTGATAATTTTCACATTTTAGACTCATTAGCTTT
AAAAAAGTTTTTATTCTTTCTATATCTTTTCCAAATTCAAACATATAAAAAGTGGGTATAAAATC
ATGAATCATCCAAAAGTTTATGCCTAAAGAGTCATTCTTTTTTATTGTATTCCACTGAAAATCGG
TTAAATTGTTAATAGATTGACCACTACCTAAAATAAAAAGGGTTTCGCTAGATTTATATTCTTGA
TAGTTTAATTTTTTGTACTCCTTAATATTATTTAATTGAATGAACTTTTTAACTTTCTGTAACCT
TTTTAATTCAACAAAAACCTGTGTAATCGCTAATAGTTCGGGTGTTCGTTTTTCAAGTACAAGTT
TTATCTTTGATTTCATTATTGATCAATTTGCTTCATAACTTTATTTACATATATGGTTCTAATTT
TTCAAGTGCAATCCAAAAGCCTTCACCATCATTTTTATGTCCTTGCCAAATTTCAGGAATAAAAG
AAGCATAAGGTGCTGTTTTATTTAGATCTTCAGCAAAAGCTGGAAAATCTATTTCCCCTTCACCT
ATTTGTAATCCTTCACCATCTAAACCTTCAGAGTCAGCTATGTGTAAATGAGCCACAAAAGGTCC
GACTTGCTCAATAAACTCTTTGAATGACCAGTTATGATTATTACAAGCCAATTTAGAATGAGATA
TGTCTAAACAGACTCGATAGCTATGTTGAGAGCAAAATTGGGCAGTATCTTGGGGATCTACAAAA
AGATTATGATACCTTTGTCCACCAAAGTGCCAAGGAAAAGGAGGCATGGTTTGAGGTATGATTTC
CACACCTTCTGCATCTAACTGAGAAAGGCTGTCAATGAGTAATTCATAGAGTTTTACTCTTTCGC
TAAGACTCAGAGGGGCGTCAAGGGTAAAACCACCAACGTTAGTAACAATACAAGGACGATCGCAC
CTTGTAAAGTAGAGTTTTAGTTGTCGGGTAATATCGATAACTTTTTTCATTTCGTTGATCGACTT
TTGACGATAGGTAGTATCAGTAGAACAGAGATTAAGCAAATGATCTCCTGCAAATAATTCAGGG
GCATGAACCACTAAATCAAGATCATAGGGGTGATCAAAAAATTCGTGAAGATCTTGCTCTAAATC
TTTATAACTGAGATGAAATTCTAACAAATCAGGATTCGTTTTTGTCAAAAGATTTTTAAAGTCGT
GGTAACGAACAGGTAAACCCCAAGGGCGTTTAAATTTATAATTTCTCGGCTTGATTTGTTCTTGT
TCTAAATCGCTAGGATAGAAAAAATCCCCGGCTTGTAAATCTCTTTTTGCTGGTTTGCCGATGAG
TTCTTTTTTTCGATTGGGTTGTAATCCCTTACCCGGGCTTTTTACCTCAAGCATTGATTCAGTGAT
GATTTCTCCTAATTTTAGATCACAGTTTATTACTAAACTTTTAGCAAGGGTTTCCCTATTCATCA
ATTCCCCTTGACTGATTTTCCGTTCTCCCACTTCCCCTAAAGATTGTTCTACTTGACGAATACCTT
CTACCATCAAACGAAATTCATCAGGTAACAAGCTGACTTTATGATCATTGCCCTCCATAGTTTTA
TCGAGGGTAAAATGTTTTTCAACTACCTTTGCTCCTTTGGCAACAGCAGCGATCGCAACGTTAAT
ACCTCTTTCGTGTCCGGAATAACCTATCGGACAATCACCTATTTCTTTAAGTCGATCAAGATAGT
TAAGATTAACATCTTTAAAAGGAGCCGGATAAGTTGAATTACAGTGTAGTAATATATACATCGCT
CCCAATTCTTTGAGAAGTTTAACTGATTCGCTAATTTCTTGTTCCGTTGCCATACCTGTTGAACA
AATAAGGGGTTTTCCCGTTTTCGCCAAGGCTTTTAATAGGTCATGATTAGTTAGATCTACAAATA
AGAAAAAGAAAAAGTCTGATAGTATTCCTCGTTTAAGCAGTCAAAGTGTCTTTGATGATGAGTCG
ATTACTTTTAGTTTGGATAGCTATTGCTCTTTATACACTAATATAAGTCTATTAAGTTCAGATAC
TTCTATTCGTTCGATTGTGGTGGGTGCGGCAGAGGCTAGTGAAGGAAAATCAACTACATCTTTAT
TTTTAGCAAAGGCGGCGGCACTTTTAGGGCAAAAAGTTTTAATAGTTGATGCGGATATGCGGAAA

CCAAAAATTCATACTTATTTGGGGATTAAAAATAAAATTGGCTTGAGTAATTTAATCGCTGAAG
ATATACTTCCTGAAGATGTTATTCAGTCTTTTGATGATGGTCTTTCTGTTATTACTGCGGGTTCA
AAACCGCCTAATCCTACTCGCCTAATCGCTTCTCGTAAGTGGCAAACTTTGATGCAGAAATTTAA
GCAGGATTTCGATTTTGTAATTTATGATACTCCTCCTTTGATGGGTTTTTCTGATGGTAAAATAT
TGACTCCTCTTACAGATGGTTTAGTTTTTGTAGTCAGAATGGGTAAAACTCGTCGTCCTAATGTA
CAACAGGTAATTAATGATTTACAGGTTTCTAAGTTGACAGTTTTGGGAATGGTGGCTAATGGTGT
TAAAAATTATATGGGGGGTGCTTATTATGGTTATTACAATTACAAGAACTATTACAATGATGAG
CGTTAGGGGTATTTAAAAATTAAGGATAGAAATTTTTAGTTTTTGAGCATTTGTACGATCGCACT
TAATTTTTGTAAACTAATGAGGGCATTTTTGATTAATTTTATTGCTTTCCACGGTTCTTTTAAAA
ATGCGATCGCAACTTTCCTGTTATTCAATTTGCCATCAAAAGTAATAGCTGAATTTAAGTCAGGT
AAATCATCATCATAATTATCGCTAACAACCCTAATCACCGAAACCGACTGAAAATAATTTAAAAG
AGCAAAACTTTCCATGTCCACGATGGAAGCACTGGTTTTTTGAAACAGACTCTTTTTTTGACTAG
ATTGGTTAATTAGTGTATTGCTAGTTAATCCTTTTACCAAAGAACAATTTAACTTATCTCTCAAA
TACTTATTTAACTGTGAATCGCAATTTTTAGTGACTAAATTACCTTCATCTAAATAACAACAACT
TTCATAAATAACCACATCTCCCACTGAATAATTAGGACATAAACTTCCTCCCAAACCGATTAAAA
GAATAGATTTGTCTTTGGGAATTTGACTATTTTTTAAGAGATTATTCACTGGATTAATTCCTATG
GGAATAGGAAAAATTTTACTTTTTAAATCAGATGAAAACAAACTTTTTTGAAGGGCTTTTAACTC
TTCTCCATCACACACGAAAATATAATCAATCATGGCTCTCTTACTGCTCGTCATGTAAATTATTG
GTTAGGAAAAGGTGTCAGATGTCAGGTGGCAGGTTTGACGGTTAAATAATAAATATTAACTTAT
AACAATTAATTTTTTTTATTATTAATCTCTTGTTTTACACAGCTTATGGCAATTATTTTATATTA
AATTATCATAACTATTGCCAAAGAGTAAAAGACCTGATTTATTTTTCTCAACTCATATAGTAATC
CCTTGTACCTTTAGCGGACATAGCCATATTTAAACGATTTAAACCATGAATGTAAGCACAAGTGC
GGAAAGAAACCCCTAATTCTTGATGAATTTTCCAAACTGCTTCTGCTTCCTCAATCATCTTTTCTT
TTAATTTTTCTTGAACCTCTTTTAAAGTCCAATATAAGCCATTGCGATTTTGTACCCACTCCAAA
TAACTAACTGTAACTCCTCCTGCATTAATCAGAATATCGGGGAAAACATGAATACCTTTTGCTTC
TAGTATCTTATCCGCCTCGGAAGTAATCGGACCATTTGCCACTTCAAAAATAAATTTTGCCCTTA
CTCCAGAAGCATTTTCTACGGTTATTTGTTTTTCCAATGCCGCAGGAATCAACACATCCACATCA
AGGGTTAAAAGTTGTTCATTCGTAATGTTTTCATGCTCAACTATATTGCAAACGGTGTTATGACA
ATAGACTCCTTTTATGGAAAGATGCTTTCTTTTGTATTCTCTGATGCTAGAAATATCTAATCCGT
GAGGAGAATAAATCCCCCCCTGAGAATCGCTAACCGCAACCACTTTATAACCAGATTTAGCCAAT
AAATGGGCAATTTCCGCTCCTGCATTACCAAATCCTTGTACTGCAACCGTTGTTGTTTCAGGTTT
TTGATTAAATTGAGGTAAAACGCTGTTGATGACGTAAAAAGCCCCTGTAGCTGTGGCTGTATCCC
TTCCCTGAGAACCTCCCATGGTTAAAGGTTTTCCTGTCACTACCCCGGGGGAAATGCGACGGCGA
ATAATACTATATTGATCCATCATCCACCCCATAATCATCTCATTGGTGTAAACATCTGGGGCTAA
AATATCCACATCTGGCCCAATAAAATCTGCGATCGCCTCTATATAACCTCTGCTTAACCTTTCTAA
TTCAGCTTTAGATAATAATTTTGGATTAACGGTAATTCCTCCCTTCGCACCTCCAAAAGGTAAAT
TAAGTAAGGCACACTTAAACGTCATCCAAAAAGCCAGAGACTGTACTTCATCTAAACACACAGAA
GGATGATAACGAACACCTCCTTTTCCCGGGCCTCTTGTATCATCATAACGAACCCGATAACCCTGA
AAAATTTTTAAAGAACCATCATCCATTCTCACGGGAATAGAAACACTAAGACTAGCTTTAGGATA
CTTTAATCTTTCACTTGCGTCCTCAGAAATAGTGACATATTGTAAGGCTTTTTCCAATCTGAGAC
TCGCATCGGCAAATAGAGAGGACATAAATTTAGATCTACCAAATTTAACATGGATAAATCTATAA
TAACCCCAGTTCGTTGTAAATTCGGAGTAAATTTCCTTAGTTATGTTAAAGTAAAGGGCTTTGTT
TTATAATTAGATTACCTAGCTATTAAATAAATTTCCAATAAGTTTCAAGTACAATTAGAAGATGC

GATCGCATTCTAACTAAAATCGCTGTAGTCAAAGAGTTTGCCACTTCCGCCATGAAATTATGTAA
AAATCCCCCTCATTTAACCATTACTTGGCAAGAATGTAGCAATTTATAGCAACAATCCCTTTATC
ATTTAAAATCTATTCCTTTAGAAGATACAGATTATTTAGAAACCCAAAAACTATTAAGTAACTAT
ACAACTAATCTAGCAAAAATGAAAATCCAAGAGACAAAAGAAACAGAATCTTTAGAATACTTAA
ATCAAGCAGAAAAAGGAGTAGAAACACTACTAAAAAGTAACAATAATCAGCCTAATTATATCGC
TAGTCAACTACAAGCTATTACCAATGATTTGGAAAAAGTTCAACCTGAAACAACTGCTTATCAAA
AGTCCCAAGAATTACAAAAATTTGCAAAAGAAAGAAAAATTAAAACAATTTCAATAACTGATGT
TACTGTCAAAATACTGTAAACTAAAAATCTAGCATCAATTCAACCAATAAATTTATCTCGTTGAC
GTAAGATTCCCATTAAATAACGAGTCAACGGCCGTTGACCCCTACAACGCCAACGTTATGATATA
ATTGATTGTGTGGAGTTAACGTCCACCTACAGGGGGTCACCTCGAAAGTTGCGGTTAACCATGAA
TCGTCATGGTGTCTAGGTTTCCTAGATGGATGTACGACCCATTAAGGTAAGTACAAGTCAGCATA
ACAACCCTCGTAAGAGAGGGGGGAATTGCAAGTAATTGCAGTTCAGAATTTCCTATTACAATCTT
TGATTTAATGGTGAGAGTCGTCAAATATCAAATTATTCCCAACATGGAAAGCATCATAAACAATC
TTCTCAGTAAAGAAAACTTATTTGCTATATCCTTATTTCCCTATCTAGGCTTTTTATGGTTTCTC
ACTAAATCAGGAAAAGTACCCAAATTAGCTTTAATTGGTTTTTATCTTCTTCTCCTTTTTGTTGC
TATTACTATTCCTGCCGGGCTGTATGCCGAAAATCATTATCATACCACTCTCGCTAATGTTGACT
GGTTACACGGAGGGGCAGAATTTTTCTTAACTTTATCAAATATTTTCGTGGTTTTGGGATTCCGT
AAAATGGTAATTACCGCAGAAAATCAAAACAATACTCAGTAAAATTAGTGGCTCTCTTATTTCGA
GTATGTAAATTTGTGATAATAATTTGAAGCTCTTACTTTTTATTAAATTATCATAACTACTGTAA
AAGATCTGAAAAATCCCACTTATGAAGCTAAGAATAACTTATTAATTTACACTTTTTACTAACCC
TTAAAAAAGACTATTAATCTTATTTTATCATTGCCATATTCTGGATTTAAAAGTACCATCTAAAC
CCCTTTTGTCTCTTAAAAATTGCCAAATAAGAGTAAAGTATTCTTTAGCGGCACTGACGGAATCA
CCCATATAACGACACAATAACCCCTGTTGTAAAGCTGTTATTCCTATTCCTATATTTTCGTCATA
TTTTTCTTTTATCAAATGGCGTAAATTGGAAATATATTGATTAATATCTTCTGACCCTATATAAA
CTAAATTACCCACCACTGGATTTCCCCCTAAACCATTGGAAGCGTAAAATAAAGGACTATTTCCC
ATAAAATATTGCCTATCAATCCAAATCGGTTTGTTTTCTCGCCAAATCTCTAAATAATTTAACCA
ATTACCTTCACTAAAAATTTCCCCCCTTGCACTTCTACCGAAACGAATTATTTCCCATCCTAACCA
ACTAGCATTATCTTCTAAATCAACTCTCATTTTTTGCTCAAAATTAGCATTGTTAAAAACAATCA
TTTCTTGTGGCAAATATTCTAAATAAGCATTTTTTTCTAGCTTAATTTTTACTTCTTGAATAGCT
TTTTTTCCCTTAGAACCATATATTTTTGTTGCCGCAGGGGTAGTTATTAAAACCTGAGAACTATT
TGCTAGACAAATATTCTGCGAGAGAATATCATCTCCAGCTATTCCTCCAGCAGTATGTAAAATAA
CACTGTGACAAATGGATTTCCCTTCAGGATAAAAAGAATTTTGAAACTTAAAAGGTGCTTGACTA
AATACTGATTTTATTGTCGTTTCTTGTTTTTGATGTTCATATACCAGATTTATTATTCCTTGCCA
GTGTTTCATTAATTAATATCTATTTACTAAAAATTATTAAGAATTTTTTATACTAACAAAAATAC
CATTTATTCTATTCTAATTATTGTATTGTTCTTGCTCTCTTAGCTTAGTTGATAGCAATTATCCC
TTAAAAAATTGTTTGGAGTTCGGGGTAATATTTTTAATTATCAACTATTCACTTTTGCCCCTTAC
CCTCTTACCTTTTCCCATAAGACAATTTACACAACTCAAATAGAATTGTTATGTCCTCCATTGTG
AAAAAGGATTATTGGCTAGATAAGAGTTATAGTAACGCAAATCGTCAGTTACTTCCTCTCCTATC
CATTCAGGGAGAATAATATCTTGATTTTCCTCTGTCAATTCTACTTCTGCCATTGTCAAACCTTT
GTTCTCCCCTAAAAATTCATCTACTTCCCAAGTTAAATTACCTACAAGTATTTTATAGCGTATTT
TTTCAATAAAGGGGCGATCGCACAAAGTTTGTAACATTTCCTCAGCTTCTTCCACAGGAATAGAA
TATTCAAATTCTGATCTCGTCTGACCTTTTGTTTTACCCTTAAGAGTGAGATAACCCTGCTCACCA
ACAATCCGAATCCTCACAGTATTACCACTGATAGTATAAATATATCCCTGCCGATAAACCAAACC

ATTGTCAGGGGGAAACCACTTATCCAAGTTAACTAAAAATTTTCTCTCAATTTCGATACCCATTT
TCTTGCCAAGAAAAATAAATTAATGTTATACTAGCAAAGTATCAAAAATTAGGGCGGCGTAGCCA
AGTGGTAAGGCAGAGGTCTGCAAAACCTTTATCCCCCAGTTCGAATCTGGGCGTCGCCTCTTAAG
TAAGACTATTTTTATTATAGACTCTTTGGTTCTCACCTCTAAACCTTGAACTCCAAATCCTGAAT
CCTGAACCTCTAACTCTTTCTTTATTAATAGTTGTGTAGAAATGATAAGTAATCTAAGTTTGTTG
GGTTATATTGAAAAATAAACTTGGACTTATTGTAAAGGAGAAGGCGATTATGGAGATAATGATT
GAAGATTTGATGGAGCAGTTAGTAGAGTTAGGGGGGTCAGATATGCACATTCAGGCAGGAGCCCC
CGTCTATTTCCGTATTAGCGGTAAACTAACCCCCATTGGAGATGAACCCCTTAATGCTCAAGAGT
GCCAAAAATTAATTTTTAGTATGCTCAATAATACTCAACGTAAAACATTAGAGCAAGAATGGGA
GTTAGACTGTTCCTACGGGGTAAAAGGCTTGGCTCGATTTCGTGTAAATGTTTATAAGGAAAGGG
GTTGTTATGCCGCTTGTTTAAGGGCTTTATCTTCCAAAATTCCCAATTTTGATCAATTAGGCTTA
CCAGATATTGTCAAGGAGATGTCCGAACGCCCTAGGGGTATGATTTTAGTGACGGGGCAGACAGG
TTCTGGAAAAACTACTACTTTAGCGGCAATGTTGGACTTAATTAACCGCACTCGTGCAGAACACA
TCCTAACAGTAGAAGATCCGATCGAGTATGTTTTTCCCAATATTAACAGTCTATTTCACCAACGT
CAAAAAGGAGAAGATACTAAAAGTTTTGCCAATGCCTTAAAAGCGGCGTTAAGGGAAGATCCAG
ATATTATTCTAGTGGGTGAGATGCGCGACTTAGAAACCATTTCTTTGGCGATTACGGCGGCGGAA
ACTGGACACCTTGTCTTTGGTACTTTACATACTAGCTCTGCGGCGGGAACAATTGATCGTATTAT
TGATGTATTTCCTTCAGCAGAACAAGCCCAAATTAGAGCAATGTTATCTAACTCTTTATTGGCAG
TATTTGCTCAAACTTTGGCTAAAAAGAAAAATCCCAAACCGGGCGAATTTGGTAGGGCAATGGCT
CAAGAAATCATGATTGTGACACCTGCGATCGCAAACTTAATTAGAGAAGCAAAAGCCCCACAAAT
TTATTCTGCGATTCAAACAGGGGGTAAATTAGGAATGCAAACCATGGAACAGGCTTTAGCAAATT
TGGTGAAAACAGGTACAATTGCAGTGGAAGAAGCCCTTGCCAAGAGCAGTAAACCAGATGAATTG
AAACGCTTATTAGGTTCTTCTATGGATAGCGGGATGGTTACTCCTAGCAAACGTCGTTAATATTT
GTTTGAATGAAAGGAAGGATTAAATCATGGCTACTTTTATAGTTCAAGTTAAAGATAAAACAGG
AAATGTTTTAGAGGAAAGGGTTGTTGCTAATTCTCAAGATGAAGCTCGTCGTATTCTTCGCAAAC
GTTATGCGGCAGTGGGCAAAATTCGCAAAGCAGGAATGGACTTTGATTTAGCTTCTATTGAAGCG
GCATTAAGTAAGGTTTCTGTTAAAGATAAGGCTATTTTTTCCCGTCAATTTGCCGTATTGGTTAA
TGCGGGGGTTGCAATTGTTCGCTCTTTGTCAGTATTGGCAGAGCAATCAGGAAATCCTAAGTTTA
AGAAGGCTCTAACTACTATTGGAGAGGATGTTCAACAGGGGGTTAATTTATCTGAAGCAATGGCA
AAGCATCCCGACTGTTTTGATAGGCTATATGTGAGTATGGTAGAAGCGGGAGAAACAGGGGGGGT
TTTAGACGAAGTCATGAATCGTCTTGCTAAACTTCTGGAAGATGTTGCTCGTTTGCAAAACCAAA
TTAAATCAGCAATGGCTTATCCTGTCACTGTTGGTATTTTTGCGGTCATTGCTTTTTTGGGTATG
ACCATTTTCTTGATTCCTGTATTTGCTGGTATTTTCGACCAATTGGGTGCAGAATTGCCGGCTTT
GACTCAATTTATGGTTACTCTTAGTGATTTTTTGCGTAGCTGGAAAGCAATTATTCCCGTGGGTG
TGATTATTGGGGTTGTTTTTGCCTTCCGTCAATATTATAAAACTCCTGCTGGACGTTTACAAATA
GATACGATCGCACTTAAAGCTCCTATTTTTGGGGACTTAAATGAAAAAAGTGCTGTTGCTCGTTT
TTGTCGTATTTTTGGTACTTTAACCCGTTCTGGTGTGCCTATCTTACAATGTTTAGAAATATCTC
AAGAGACTATCCCTAATAAAGTAATTTCTAATGCCATTGGCGCCGCAAAAGATTCTATTTTAGAA
GGAGGTATGTTAAGTGTTGCGATCTCGGAAAGAAAGGTTTTTCCCTCTATGGCAATTCAAATGAT
GATGATTGGTGAAGAAACGGGGGAATTAGATGCAATGATGATGAAAGTCGCAGATTTTTATGAA
GATGAAGTAGAACAAGCAGTTAAGGCATTAACCAGCGTCATCGAGCCTTTAATGATGGTTTTAAT
TGCGGGTATGGTCGGTACAATTCTTTTATCGATGTATTTGCCAATGTTCTCTATTTTCGATCAAC
TAGGATAACAACAATTAAGAGTTAGTAATTAGAAATTAGTAATTGTTAGAACTACCTACACACTC

ATTATCTTATTATGGTATAATGGTAGTCTGTGTCTTGCAACCTGTTCGGATCAGGTAAGGGCATT
TATAAAGCAGAACTAAATAAAATCTCTGCTACCTGTACGGCAGTATATAAAGGAAATTAATATG
ACTTACGCAGTAATTGAAATCTGTGGAAAACAACTTAAAGTTGAACCAGGCAGATTTTACGATAT
AGACAGAATCAATCAAGAATCAGAAACCGAAGTATCCATCGATAAGGTACTTTTAGTTAGTCATG
ACAATGATATTCATGTAGGACAACCTTACGTGGAAAGTGCGACAGTAAGCGGTACAATCATTGAT
CATCGTCGGGGCAAAAAAGTTTTGGTCTACAAAATGAAGCCCAAAAAGAAAACTCGCAAAAAAA
GAGGTCATAGACAAGAATTAACACGCTTAATGATCGATTCTATTAGTATTGATGGCAATGTAATT
GCAGAAAAAACCTCTGAATCTAAATCTGTAGAAACAGAAGTTGCGATCGAGGCTTAACATAAAGC
ACAATAAAAATAATATTTAATTTGATTTAACCTTAAATTTTTGCTCAGGAGTATATAAAATGGC
ACATAAGAAAGGTACTGGTAGTACTAGAAACGGAAGAGATTCTAATTCTAAGCGTCTAGGTGTA
AAACGTTATAGCGGAGAAACAGTAACCGCAGGAAGTATCTTAGTTCGTCAACGTGGTACTAAAGT
TTATCCCGGTAATAATGTTGGTATTGGTAAAGATGATACTCTTTTTGCCTTAATTGAAGGAGTTG
TTAAATTTGAAAATAAAACTACCAGTCGTAAAAAAGTAAGCGTATATCCTGCTTAAATTTATGAC
TAGGTCAAAATTAATTAAAACTTAGTTTTAACTATTAATTAACAAGGGGCTTAAGCCCCTTATTT
TTTTGTCTGCTCGACTGATTACTTATTTAATTTCAAGTTAATTGTTAGCTAGGAAGATGTGATAA
AAAAGACGATTGCTATTATTCCATGTCCTGTAAAATATTGTGGAATATGTAATCAAAATGAAACT
AAAATATATGCTAGAACTAATTTCTATTTTTAGTGCCGCCGCATTGGGGGGAGTATTAGCATCCA
TACTTCGACAACCGGTCATTCTAGGCTATTTATTGGCAGGAATTACCATTGGACCTTTTGGATTA
GGTTTAATTCAAGAATATGAACAAGTGGAAGTGGTAGCAGAATTGGGTGTTACTTTCCTTTTATT
TGCTTTAGGGGTGGAATTTTCCTTTGAGGAGTTGAAGAAAGTTAAAGTTATTAGTCTAGGAGGA
GGGGGTTTACAAATCAGCTTGACTATTTTAACTACGGTAATTCTCTCCACAAGTTTGGGATGGGT
AAATTCTATTCCCGAAGGGGTATTGTTAGGACAAATGCTTTCTCTTTCCTCTACGGCAGTAGTCA
TCAAGGTATTGATGGATAATAATGAAAAGGATACCCCTCAAGGTCAGGTAATGCTGGGAATTCTT
ATAATTCAGGATTTAGCTTTAGGTTTAATGTTAGCGGTTTTACCTGCGTTAAATAAACCGGCGGA
AGAAATTGGTATTGCTATGATTATCGCTTTAGTCAAATTAATTCTCTTTGCTTTAGGGGCGATTA
TCGTGGGTAAATGGTTAGTTCCTCCTATTTTAAGATTATTAGCAAAAACTGAGAGTAAAGAACTT
TTCTTGTTAGGAGTTGTTACTATTTGCCTAGGTGTTGCTTTATTTACTGGTAAATTGGGACTTTC
CACCGAAATGGGTGCATTTGTTGCAGGTTTAATGATTTCAGAGATTGAATATGCTGATGAAACCC
TCTCAGATGTTGAACCGTTACGGGATATTTGTTCTATTGCTTTTTTCGCCTCTATCGGAGTGCTA
ATTAATCCTATCTTTCTTTGGGATAACTTACCAATTATTTTGGGGTTAGTAGCTTTAGTCGTATT
AGGAAAAGCCTTATTAATTACCCCCATTGTCCTTCTTTTCCGTTATCCTTTAAAAACGGCTCTTGT
TTCTGGTTTAGGATTAGCACAAATCGGGGAGTTTTCTTTTGTTCTTGCAGGAGAAGGCAACCAGT
TAGGAATTTTACCCGAATCCGTTTACTTAATGATATTAGGAACTACAGCAGTCACTTTAATTATT
ACTCCTTTTGTCTTTAAAGCCGTACCTCTATTCTTTAAATATGTAGAGTCTATTCCACAGCTAAA
AACCTTTTTTGCCCAATTTGATCAACCAGTAGAAATAGCAGAAAATTTACCATCTAAGGATCATA
TTATTATTTGTGGTTATGGACAAATAGGTAGCAACATTATATCCCTATTAAAAGAAAGAAATTA
TCCTATTTTAGTCATTGAACAATCAGAACAAAAAGTCAAACAATTGAGAAAAGCAGAAATACCTT
ATATTTATGGTAATGCGTCCAGCAATATTGTTTTAGAAAAAGCTAAAGTGAATGAAGCTAAAGG
AATGGCTGTAGCTTTGCCCGATGCTCTTAGTACTCGTCTTTGTATCAGACGTTCCCTCACTCTTAA
TCCAGAACTAGATTTGGTTGTCTGTGCCTATGAAGAAAAAGATATTGAGTTATTTTATCAAATAG
GAGCAAAAGAAGTTGTACAGCCAGAGTTTGAAGCTAGTATAGAATTATCCACCCATCTATTCAAT
GGTTTAGGATTCCCTCTCGAAGCTGTACAACAAAATATGCAAAAAATTCGCTCCTCTCACTATCA
AAAACTGAGATCAGATCAAGATAGTAATTTAATTTTCCGTCAACTAGAAGAAGCTACCCGCACCA

TGAGTAGTCAATGGTATCCTTTACCAGTAGATTCTCCCTTAGTGGGTAAAACCCTAGAAAAAAGC
TATATTCGCCGTTTAACTGGTGTAACTTTAATGGCTATTATTCGCTTTAGTGGAGAAAAGTTAGA
TTATCCTGAACCCGATACCGAAATCAAGTCTCAAGATAAATTATTAATTGTGGGAGATAAAGAAG
ATTTAGCGGCTTTTGATCGATTAGCAAAAGGAGAAATCAAAAACCTGCCCCAAGAAGACGATTCT
TGTGTTTGGATAAAAATCATTGAAAATGGACACTTAGTAGGTAAATCCCTGAAACAAGTAGAGG
AGTTATCAAAATTAGAAGTGTTCATACAAAGTATTCGCCGTGAGGGAAAATATATACGCTTCCCC
GACAGGGACACTATCATTGAAATTGGGGATCAAGTGTTGATGTTCGGTAAAATTGCCTCCTTAAC
ACAACTGAGTCAGAAATTAACTATACCTGTTGGAAATAGAGAATAGTAAATAGTTTTAGGCAAT
AGTTATGATGTTGTAGTAATTTCAATTAAGGTTTGAACAATAAAAAAAATTGCAGGGTATTCAT
CGACAACCAATCGGTAGTTTGAGATAAATACTACTTAACCTCAGTTCGGTTTAAGAATATTGGAT
AAGGGTAGGTGTCAGGTTTCAGGTTGCAGGTTGCAGGTGTTGAGAGAAAGTAATGAGTAACAAA
TAATGAGTGTTCGGGGTTTTTAATTCCTAATTCTTAATTTTTCCTTTGCCCTCCCCCCTCTCGAGG
GGGGATAAAGGGGGGTTCGCCTCTTGCCTCTTGCCTTTTGCCTTTTTTTCTCCATCATTCATAGAT
GAAAATTTATCCCGAACTCAGGTTACTTATTTATTAAACTGTTTCACTTTTATTTGGAGTTACTA
TATTTACTCCTAAGTTTAACTCTGGGTTATAGGAATAATCTGGTTTCCCCCCACGACAAAATATC
ATTATTTTTTATGGTATTCAGGGGGGGTTTCTTTTTTTAAGAAAGAAAATTAATCTCTGCCATTT
ATGGCAATTAATAAACGTAAGATAAATATGAATAAGTTGATGTAAGTAAGATACATAGAAAGGG
CGGCACATAAGTATTGCTCATCCCGATAAGCGCGAGGGAGAAGATAAAAATCAACTACAGACGCT
CCCGCAAAAAGCAACACTCCTAAACCAGAAATACCGACTTCCAACCAACTAGGAGTAAAAACACC
AAAAAATCCCAATACAACTTGCAACAGAATTACGACGAATAATCCCATAATACCAAGCTGTACTG
TTTTAGTTAGGGCTAATCCATCCTCTTCACCTAAGTTAGAACCAACATTTCGGGCAATCACAAAA
GTTACACCACATCCCAAGGCGGCAATGCCCACTCCTTGAAGTCCTACTCCTGCTGTACCTAAAGCA
ACAAAAACAATACCACTGAGGGTATATCCTGAAAGAAGACTATAGACAGCTAAAAGAGGCAAGG
CGGTGCCATTATCTGCCCTTTCTGCGATGCCTCTAACTACGAAAAATAAAACTAACTCGGCAATC
ATTGCCCCAAAAAAGGTAGGCATGAATATACCGGGGTTGGATTGAATTACTCCTAAACCGCCATA
AACACCTACTGCGGTTAAAACTAAACCTCCACCTAGATAAGGTAAGGCTTTATTGATAACATTAG
GTCCTACAAGGGCTTGACCTTGAGCCTTTTTGATTGCATTTCTAAAGTTACTGGTTGTTGCCATA
AATTCTCTCTATTTAATAATTCGTTAATTTTATATTTTTTCTTTCAAGTGGAGATAACCTTCTGT
AATAATTACACACTCTCCTTTTACCTTTATATCTTTTATTATGCCCTTTTGTTTAGTAGCGATCGC
CGTAATTTTACTATTTCTTCCGATTTCGATACCTTGTTCAATTAACCACTGCCAACTACCATCAGT
TTCCTGTTGAGATTGAGCTAAATAAGCCGCAAAAGCCGTTGCCGCCGAGCCTGTGGCGGGGTCTT
CTATTATATTCAAAGCAGGGGCAAACATTCTCACTCGCCAAAGATTTTTACTGACAGGGTAGCAA
GGGTAAACATGAGGGAAAAGAGTATTTCTTAGGGTTTTTTCCCAATGAGATAAATTTATTTTTGC
TTCTGATAACGCTTTCTGACTATTTAGAGGAATAATCATAAAAGGTAAACCGCAAGAAACAGCTT
GAGGACTAAAATCAGAATGTAAGTCTGTCTCAGATAATGATAAAACCTCTGCTAAGGCTTTTTTT
GAAGGAATATTATGGAAAAATTCGGGAGGATTAGGAGCAGTTAATTCTGATGATTTAACGATAC
CATTGTCCGTATAAATATTAACGGGTACAAGACCAACTTTTTCCTCAAGAATAATTTGTGTTGCA
GAAGATTTGAGAGTAACCATGCCCAAATGAGCTAATAGGAAAGCTGTACCAATGGTTGGATGTCC
AGCAAAGGGTATTTCTCCACCTGGGGTAAAGATTCTGACTTGATAGTCTGCCTGAGAATTAGAGG
CAGGAAAGACAAAAACTGTTTCTGAAAAATTAAACTCAACGGCTATTTTTCTCATTATGTCGGAA
GATAAACCTTCGCTATGGGGGAAAACCGCCAATTGATTACCCGCAAAAGGTTGATTTGTAAAAAC
GTCTAAGGTATAAAACTGATATTTCATTCAGAAATTTATGGTAATTAATAACAGGTCTGATTTTT
AAATTTTTTATGGCTAAAGCCATTACTACAAACTATAGAACTCTGAAAGTCAGTGGGGCAATACT

TACTATCTCAATTAGGATTTATTGATACTCATTCAACAACTTATTTAGCATAGCTATATGATTGA
ATGAAAAGAAGTAAATTCATTCGAGGGGGATATATTTCCATGGTGACATCAATCACGAATAAAA
CTACCAATCAAACTATAAGTTTAGAGGAATTTCTACAACTTCCAGAAACAAAACCCGTCCGAGAA
TATTTTGATAATAAAATTACACAAAAACCCATGCCCCAAGCAAAACATAGCCGTATTCAAGGTAA
ATTAACTAAAGTTATTAACGATGTAGTAGAAGAATCCAAAATAGCTTTAGCTTTTCCTGAATTAC
GATGTAATTTTAACGATAAATCCATCGTGCCAGACATTGTGGTTTTAACTTATAATCACATCCCT
AAAGATGAAAATGGCGAACTTACTAACACGATTTCTATTCTTCCTGATTGGATGATCGAAATTCT
TCTGACAATTCTGAAAATGGCTGAAAAGCTGAAAGTGTTAAGAATCAAACCGTATGCTTAAATG
AAAATGCCCAAAATATTTTTCCTAAATCATGAAATTTCATCTATCTAGCATAATTCAGATTTTCT
TTGTCAGATTTAATAGATTATTTCGCTCTCATAAAATTGAAGCATTTGAAGCTATTATGATTTTA
GCAATCAGTCATGCTTATGGCTGTTTTAATCCTAAACAATTGGCAGATTTCTTAGGAGTTAATCA
TCAAAAAATATATGCAGAAATTTCTTCATGGACATTATACAAGCTCCAAAAAGTTTTAAAATTAT
TGATGGTCAATGTGGCAGTAGAACAGTTACAAATTATTGAAAAAAAGAGTAATTCTACACAGTC
AAGGGCAAAGATAACACTTGCTGTAGATGATAGTGTTATTGACAGAGTAGGAAAGAGGTTACGC
TGTACATTTACTTGGTATAGTGGACGTTGGAAAAAAGTGGTAAATGGACAAAATATATTAGGAA
TCATATTAACAATCAATGGAAAAGCAATACCCATTGGATTAAAATATTGCTCCAAACAGGGAAGA
AAAAATACGGATAAACCAAGTATTTTAATTGCGATGTTAAAGGAAATTAAAGAAGAATGTCTCA
AAGAAAATATCAATATTAGTAAATATCCTATTACTCTCGATTCTTGGTATGTATCAAAACAATTA
AAAGAACAATTAGATGAACTAGGATTCACCAAAATTATTATGGCTGGGAAAAGTAGTTATGTAT
TTGAAGGAGAAGATTTTAAAGGAAAAGGAAGTGAATGGAAAAAAAGAGTGGATTATCAAGAAAA
TCATATCAAATCCGTTTAATTACCCACACAAAAAGATATTAATTAGGCCACCAATAAAAATTGGC
TATTCCTTTAACTATTTCTTTTTCTTCCATAATTTTTTTGCATCTACTAATAACTACCTCTTCTAA
CTCTTCTAAATTTTTAAATGTCCTATTAGCGATGGCCTCATTTACTAATGGCCACAAACGTTCTG
CCGGTTGTAATTCTGGAGAGTGAGAAGGCATCAGCATAACGTGGATTCCCTCTGGTATTTCCGTC
GTTTCTTTAATCGGATGCCAACCAGCTTGATCCATTGCTAAAATCACCCTTTTGTTTTTACCTATT
TCATAGTGCTGTGCAAAATCTTTTAAGACTCGATTAAACAATTCAGTATTTACATATGGTAGTAG
CCACCAGTATGTCTCTCCTGTATTTGGTTCCACAAAGCCATATAACCACAACCATTGAAAACGCCA
ATTGACCGTAGCTATCGGTTGATAACCTTTTTCTACCCATACTCTTCTTAATACTGGTTTGAGTCC
TAAACGATGCTCATCCTCAGCCCACACTTGAACGTCCGCGTCGGGATATTCTTGTTGAATCTTTTT
TTTTTTCTGCTAGTTGATTTTTCCATTCATTTTGTTCAAGGCAGCTAGAATTTTGATGATTTGGA
CGAGGACATCGGAGACGATATTCTAATCCTTTGAGATATTCCCATCCTCTTTGAGGAGAAATAGT
ACGATCTAGAAGTTGAGTGAGCCATTTTGCTACTTTGACACCATTCCAAAGTCCTTGATCATCGG
GGGGTGATTGTAATCTTTGATATAGCAGAGCTAATTGTTGATCGTCTAATAACGGTTTTCTACCC
CGATTATGTTGTCTTTGATCCCCCAACATTTTTGCACCCGAACGATTATAACTACGAACAAGTTC
GTAAATCCAACTTAAGCTATATCCTGTAACGTGGGAAACATAAGAGGTTCGTTTCCCTTGTGCTA
ATAACCAAATCACTTGGTAGTGAATTTTTTCAATCCCTTGTTTGGCTTTTCGATACCCTTGTTCT
AATTCTTGTAAACTTAAATGGGGTTCAATTTTGATTCTTGAAGGCATTGGTCAAAATCTACGACA
ACAAATATACATCCTCTTGATTATACCATGTGCAATTAAGCGGATTTGATATCAATGGGGAATAG
ATGTGCCTTGTGTGAGAAAAAAATTATCAAATCCGACTTTTGGGGAGTTAAATTTGTTATTTTTT
CAAAAAAGTAATAGTAGTTGTTATTTATTGATGGATTTAAGTAGTATATCGTTGAGAGGAGTAG
AAATATGGCGGATATGGTCTGCACACAATATTATTGAACAGTTTTGGAAAATGTTAAAATCGGT
ACTAAAAATTGCGGAGATGAAATTAAGGAAACAGGGAATTTATATAGGATTATTAATTAAAGTA
ATAATGTATTTAATACTGTTATCAATGCAGTTTATGCCTAGTTTAGGTCGTTTATCATTGACGCA

AATAATGAGAAAAATAGAATCAACAACTAAGCTACCTGATGTAATCAAAGAGCATTTTCAGCAC
GATTTTCTAGGGGTTTCAGCTATGGCATAGCTTTTCAGGGTTTTTGGAATTATCAGTTCTTTCTC
CTGATCAAAGTCAAACACTTATCATAAAAAAAATCTTACGTTGTCTCGAATCTGGTTGTCAATTA
GCATGGATAATTGATCCTGAAGAAAAAATTATCTTTGTCTATTCTCTACAAAAAGTGTCTTATTT
CGAATTAGATAGCGATGTTTTACCTATGCCTGATTTTATGGCTGATTTTAGCTTAACTTTAGGAG
ATATTTTTGGTTGGTTAAAGTTTTAAATGTATTTTGGAAGATAATAACTTGATTTAAGTGAAAA
TCGGCAACAGGCAACAGTTTTTTTACATCAAAATATAACCTTTACTATTATAATTGTTGATGATA
AACGGGATAAACATGATACAGCTACGCTATTATACACCCCGATAAAAAAGTAGGGTGAGTAACAC
CCACCCCATCAATTAAGATTTAATTAATAAGTCATCAGGCAAACGCTTTAAACTCAATCTCTCGT
CTTCTACACCTACAAAAATGGTGTCTCCGTCTTTAAATTCTCCTTTTAAAATAGATTTTGCGATC
GCAGTTTCTAAGTATTTTTGTACTGCACGTTTGAGAGGTCTAGCACCATAAACAGGATCATAGCC
AATTTCTGCAAGGAAGTCTAAAGCAGATTCAGATAAAGAGAGAGACAATTTTTGTTCTGCTAAAC
GAGTTTCTAAACGGGCAACTTGCAATTTAACGATATGACGTAGCTGGGATTTTTCCAAACTATGG
AAGATGATAATTTCATCAATACGGTTAAGGAATTCTGGACGGAAATTCGCTCTCATCGCATCCAT
TACTCGGCTACGCATCTGTTCATATTTACTATCATCCCCTGACACATCAAGGATAAATTGCGATCC
AATATTGGAGGTCATGATAATAATGGTATTAGAAAAATCCACAGTGCGTCCCTGAGAATCCGTCA
ATCTGCCATCATCAAGAATCTGCAACATGACGTTAAACACATCAGGATGGGCTTTTTCAATTTCA
TCAAATAATACCACCGAGTAAGGGCGACGGCGAATGGCTTCGGTTAATTGCCCTCCTTCTTCATA
ACCTACATACCCCGGAGGCGCTCCCATTAAACGGGAAACAGTGTGTTTCTCCATATATTCAGACA
TATCAATACGCACGATCGCCTCTTCAGTGTCAAAGAGAATACCAGCTAAAGCCTTCGCTAATTCC
GTTTTACCGACACCCGTAGGACCTAAAAAGATAAAACTAGCGGTAGGGCGATTAGGATCGCTTAA
ACCAGCACGAGAGCGTTGTATAGCCTCAGAAACAGCGATAACTGCTTCATCTTGCCCAATTACTC
GCTCATGAAGTTGAGATTCAAGGTGCAATAATTTTTCTTTTTCTGATTCAACTAATTTACTAATG
GGAATACCAGTCCATTTTGAGATAATTTCCGCAATGTCTGATTCTTCCACTTCTTCCCGCAAGAG
GGATTTACCTGTAGTTTGTTTTTCTGCTAGGGCAGTTTCTTTATCTTTGATTTGCTGTTGTAAAT
CGTTTAATTTGCCGTAACGTAACTCGGCGGCTTTATTATAATCATAATCTCTTTCCGCCTGTTGA
ATTTCCACATTAACTCTTTCTATCGATTCCCGTAAATTGCGAATTTCATCGATAATATCCTTTTC
AGACTGCCATTGAGCATTAAAAGCTGATTGTTGCTCTTTCAAATTTCCTAATTCTTGCTCTAGCT
TTGCTAATCTTTCTAAAGATGCAGGATCATCTTCTTTTTTCAATGATAATCTTTCCATCTCTAAT
TGGAGGATTTTGCGATCGACTTCGTCCAATTCTTCGGGTTTAGAAGTTATTTCCATTTTCAACTT
TGCCGCCGATTCATCCACTAAATCAATGGCTTTATCTGGCAAAAAGCGATCTGTAATATAGCGAT
TCGACAACATTGCCGCCGCTACGAGGGCATTGTCAGCAATTTTAACCCCATGATGGACTTCATAA
CGTTCTTTTAATCCCCTTAAAATAGAGATAGTATCAATGACATTAGGTTCGCCTACATATACCGA
TTGAAAACGTCTTTCTAGTGCCGCATCTTTCTCAATATATTTACGGTATTCATCGAGAGTTGTCG
CCCCGATACAACGTAATTCTCCCCTTGCTAACATGGGTTTAAGAGGTTTCCTGCATCCATCGCCC
CTTGAGTCGCCCCAGCACCGACAACGGTATGAATTTCATCGATAAAGAGAATGATATTACCCTCA
GATTCGGTAACTTCTTTCAATACGGCTTTGAGTCTTTCTTCAAATTCTCCTCGATATTTCGCCCCT
GCAATCAATGCACCCATGTCTAAGGCAATAAGAGTGCGATCGAGCAATGATTCAGGTACATCTCG
GTTTATAATACGTTGGGCTAATCCTTCTACAATTGCAGTTTTTCCTACCCCCGGCTCACCAATTAA
GACAGGGTTATTTTTCGTGCGACGGGACAGAATTTGAATAGTACGACGGACTTCATCATCTCTAC
CGATAACGGGATCTAACTTCCCTTGTCTTGCCAATTCTGTTAAATCTCGACCATATTTAGTCAAA
GATTCGTATTTTCCTTCAGGATTTTGATCGGTCACTTTCTGGCTTCCTCTAATTTCTTTAATGAT
AGTTTTTAGTTTATTTTCATCGAGATTAAATTCTCTTAATAAATTTTTCCCAAAACGGTCGTCTT

GGGCATAACCTAAGATAATATGTTCAATGGAAATGAAATCATCTTCAAATTCTTTGCGGTATTTT
TCTGCCCTATCTAAAAGAGTATCAAGGCTTTTTCCTAAATATACCGAGTCAGAAACGTTTTTAAC
CTTCGGTTGACGATTGATAAATTCATCGGTGCGATCGCGCAAACGAGTTAAACTAATATCAGCTT
TATTAAAGATACTTGTAGCTAATCCCTGTTGTTCTAAAAGAGATTTTAACAGATGCTCTGACTCA
ATTTGCTGGTGTTGACTTTGTTTGGCAATGTCGGGAGTTTTGACTATTGCCTCCCATGCTTTTTC
TGTAAATTGTTGTGGATTTGTTGGTTGCATTTTTTTATTTCCTAATAAGAATAATGATAAAAAT
AGCTTGTTTATTTAATAAGTAAAATTACAACAATTATATTATTCAGTTATCTAAACATGATTATA
AAACAATATAAATATTATCTTAGTTGGGAATACTCTACTAATTTAGTGGGGATTAAATCACCTAT
TGAGTTAGGGATTTTTGAAAAAATAGACATCATAAGAAATAAAGCATAAGCGGAAAATTAATTT
ATAATGACGTAACAGCTATCAATAAGAAAAAACCATGATTGCTCAACTAGAAAAAAAATACTAC
TCCGTAGAAGAATATTTTGAGTTAGAAGAAACTGCCGAATATAAAAGCGAATATCATGATGGAA
AGATTATCCCCATGACAGGAGGTACAACTAATCACAATAAAATAGCACTTAATTTTTGTTACAAT
TTTAAGTCTATTACTAAGGGCAAAAATTACGAAATTTATATTAATGATGTGCGTCTTTCTATTCC
TCACCAGAGACGTTATACCTACCCTGATATTATGATTATTAAAGATAAACCAATCTATGAAGGGG
ATAAACAAACAACTGTCACAAATCCCCTCATAATTATTGAAGTTTTATCAAATTCAACGGAAAAT
TATGATAAGGGAGAGAAATTTAAAAGTTATCGTTCTATTAAATCTTTTCAAGAATATATATTAA
TAGATCAGTATAGTTTTTCTGTGGAACAATTTATCAAAGAATCTGAAGGAGAATGGAAATTTAA
GGAGTATTTAGGAGAAAATCAGGTATTAAAATTAGGTAAAATTGATTTTGAACTATCTTTTTCT
GATATTTATGAAGGGGTTAATTTTGTTGAAAGTATTTAAAATTGTCGATAGCAACTGATGGAAA
TTATCTATCAAATTTTGTTAAAACTTTCTCAATTTCAAGTAAGAGTAGTTGGAGATCATCATGGT
AGTTTATTTATGGCATTGATAGAGATTTATATCCCCATAAATCTTACTTTAATATCTTCTAAAGA
TAAGATAGAAATTCCTGCTATTATTACATTTTTAATGTTAATTTCTGCAATCTTTTTTATTCTTT
TTTCTATTGTAGTTTCTAGCCCACAGTTTTGGATTGGTTTATTACAAAATCCAAGCAAGAATTGG
TTTAAAATTATTCTGATACAGCTATGGTTTGTGTGCTTATTATTTATATGGATTACTGCTTTAAA
ATACACAATCCAAAGATTTTTGTTAAATTCTAATTAGTATTTTGCTTTTAGTTAAATAATTGATT
TTTTATTATAAAGACTCATAGTTTTTTCAACACCATCTGTTAAACTAGATGCGATCGCATCATAT
ACTAACAACATTAAACTATTCATTTCCTTTCTTTCTTCGGCGGAAAACTTGCCTAAAACATGACC
AATAGTAGCGTCTTTATGACCAGATTTTCCAATACCAATGCGAAAACGAGGAAAATCCTGACCTC
CTACATGAGAAATAATCGATTTCATCCCATTATGCCCTCCTGCAGAACCAGATAAACGCAATCTA
ATTCTACCAAACTCCAAATCCATATCATCATAAACCACTAAAATAGATTCTCTAGTCAGCTTATA
CCAGTCTAAAACCGCCCTCACCGACTGCCCCGACTTATTCATATAAGTTAAAGGTTTAACTAGCTT
AATTTTCTGCCGATTGGGAGTAACAACTTCACAAAATAAAGCATTAAATTTCGTATTTTTTTGCA
AACTTCCCCCCCATTTATCCGCTAAAAAATCCACTACTTCAAAGCCGATATTATGCCTAGTTTGTT
CGTATTTTTTTTCAGGATTTCCTAAACCAATAATAACTTCGGGAATAACCAATTTTTTTTCATTC
ATGGTCTAAAATTTTAAACTATAATCTATGAATTATATGAATAATTTTCGTAAGCTAATCTACTG
ATTTTTTATCTTAAATCATTAATTATTTTATGGCTTTTCTACAGTAGTCATGATCAAATTTTAGA
AAAGTTTATAAAATATGAGATAAAATATGACAAAAACAAATTTTTTATGAATTAACAGTTTTTA
TTTCACCTGACACCTTTTCCTAAGCAATAATTTACATACTCAAAGTAAGAGAACCTATTTTATTT
CCCTATTTAATCATCAATCCAACTCGATCTCCAAGCGGATTCTGCTAAGGCAATTTGATACTGTT
GATATTTATTTTGGTACTCATCATCAAGGTTATTTAAACTTTGAAAAATTCTACTAATTTTTTGC
CGATTCTGATTTAATAAAGGATCATTAAATTCAATTTCATTAATTCTTAAACATAATGCCACAAC
AGGGGTAATATTCATATCCTCTGATTTATCATCTTTTTCAATCAAAAGACTTAACAGATTTGGAG
CACCACTAATTAAAGATGTATTTTCTTCCGCTTGTAGGGCAACTTCTAAAATTTTAGCAGGAATG

TTTTTAGGCAAGATTCCAATTTCCTGTAAATACTTATTTACCTGATGAGATAATTTTATCAAACA
TTCTTCGATCGCATCATCTACTTCCACCTGTAACTGAATAAGATTTTCAGGACAAATTTCTAATA
AATTATCATCTTTTAAGAGACTATCATTTTGATGATTACCTTCTAAAATATTATCTTTATCAAAA
GTTTCTGTTTCATTTTCTTTTGATTCAGTTAATAAAGTAACATCATTTTGATAAGGAACAGATGC
TAAGATACCACTCTTTAAATTGTGTAAAAAAATATTATCTATTATTTCTATCTCAGAAATTTTCC
CATATAAATCTTTAGGAAAAAATGAATGTAAATCTTTAATATGTTTCTGTAAATTAACTTTATCC
TGATAACTTAAAGCTAAAAAAGCGGAGGGATATTTTTGAGTGCAAACTTGATAAGTGGCTAAAA
TTAACTGACGTTTAACAACTGCAATTAACTGAGGAAAATAATCTTGATATAAACTTTGTAATTCT
GTCGCTAAATCCTTGATTTTAACATTTAAAATGCCCAAATCATTTCTTAATTGTTTAATTGATCT
AGTCATTATTTCATTTGCAATCTATTTCAATCAGAAATTAGGAATTAAGAGAAGGCAATTCGGAA
TTAATAATTAGTAATTAACAATCAGAAACGAAGAATGAAGAGTTAGTTTCCCCCTCTCCACTCTC
CACTCATCTCTCCTTCTCCCTAATCAAATTACTTATTACTCATTACTTATTAATCATTACTTCATA
CCAGCTTGAGCGGCAACTTCCTCAGCAAAATTAGTTTCTTCTTTCTCAATGCCCTCACCTAAGATA
AAGCGTTGGAAACGACGCACTTGGATATTTTCACCGATTTTCGCAATTTGCTGGGTGATTAATTC
CTCTACGGTAATACCTTGGTCTCTGATGTAAGGCTGAGAAAGCAAACACATTTCACCTAAACGCT
TATTAATTCTACCGTCAACAATTTTTTCACGGATATTTTCAGGCTTACTTGCTAAGTCTTCTCTA
CCCATTTCAATTTCTTTTTCCTTAGCAACTACATCTTCAGGAATGTCACTAATTTTAATATATTC
AACATTAGGACAAGCCGCAATTTGCATCGCAATGTTTTTCGCTAAGGCTTGGAAATCTTCACGAC
GGGCAACGAAGTCGGTTTCGCAGTTAACTTCTACTAAAACTCCGATTCTACCACCAGTGTGAATG
TAGCTTTCTACAATACCCTCAGCGGCAACTCTACCAGATTTTTTCTCCGCAGAAGTAATACCTTTT
TGGCGTAACCATTCCATGGCTTTAGCCATATCACCATCATTTTCTGCAAGGGCTTTTTTACAATCC
ATCATCCCTGCATTAGTCTTATCTCTTAATTCTTTTACCAGTTTTGCGCTTATTTCTGCCATAGTA
CTTTTTTGTATAAAAATTTAATAACTGACCATCAATAATAATGACATGATTTGCCCCAGATGAAA
ATTAGGAAAATCATTACTAGATGACTAGGTGGGCATTGCCCACCATTATTGGGGAAGATTGATAA
CTTTTAAGAAAAAATAGTTGAGGATAATTTAGAAGTGTACTCAGATTATAATATTATAACTACT
CTTCTTCGCCTTCAGAGTCGCTATCGTCTTCATAATCTTCATCGTCGTAATCTTCGTCATCATCAT
AGTCGTATTCAGCTTCCGCAGATACAGATTCCATAAACTCATCTAATTTACCTTCTGCGGCGGCTT
TACCATAACGCCCTTCATAAATCGCATCGGCTAATTTCCCGATAATTAATTTGATGGAGCGAATA
GCGTCATCGTTAGCAGGAATAGGGTAATCTACCAATTCAGGATTACAGTTAGTATCTAACATGGA
GATAACGCTTAAACCTAATTTTTCGCATTCTTTAACGGCATTATGCTCTCTACGAATGTCGATAA
TTACTACAATGTCTGGCACTCGACGCATATCTTTAATCCCGCCGAGGTATCTTTGTAACTTAGTT
AATTCACGACGAAGAACAGAGGCTTCTTTTTTAGGGCGTAAATTAATTGCACCGCTTTCTTCTAA
TTCTTCTAATTCTTTTAAACGTCCAACACGGGATTTAATGGTTTCCCAGTTAGTTAGCATACCAC
CTAACCAACGCTGATTGATATAAAAAGCTCCACAACGGGCGGCTTCTTGAGCGATGATTCCTGCG
GCTTGACGCTTTGTACCTACGAATAAAACTTTTTTGCCGTTTTCGGCGGCTTTTTTGAGATAAGC
GTAAGCCTCTTCTAATAATTGCGCGGTTTGCACTAAATCAATAATATGTACCCCATTTCTAGCAG
TGTAGATGTAGGGTGACATTTTTGGATTCCAACGGCGGGTTTGATGCCCAAAGTGGACACCTGAG
TCTAAAAGCTCTTTTAAGCTGACTACAGACATTATTTTCTCCTTTTTTCGGGTTAATCCTCCATCT
AAGGTTGTTTAATTTCTTTGGCTAGAAACACCCGATGCCTTAGATGTGCGAATTTTGACAACTTT
TCTAGTATATCATTGTCTTTTTTTTTTTTTCCAGATTTACCCCTTGTTTTATTTTCTTGATGGAAG
TTAAGATCTTTTTAGGGTCATCTCATTTAACAGTTAGTTATAGGGAAAATTGGCGATGTAATCAC
CAATAAGATTAAGCGTAATATCAAAAATGCGATCGGCGTAGCCGCACCTTCGGTGACTGCATGGC
TGTATTTTATATGAAAGCAAAGCACTACCTTACAATGACAACATCCGTAACACTGTTAATATTAG

ATAAATGTAGTTATTCTGTCCATAAAAAAATACCAAAAACAAAAATATTGACTTGCATAATTAT
CGTTTTTTAACTGGATGTACTAATTAAGAAACCTAATTTTTTTTAAGATTAAGAACATATAATGA
AAGATAATCGAAAGCAAATACACATAGGAGACGCTGTCAAAGTTAAAGGAAAACTTTATTTAGT
CTGGGATATAAAAGGAAATCATATTTTTGGTCGTCAATTTACATTTACAAAAACAGGACGTTTTC
AATATTATCAAGACCGTATTTTTTCCGTTAATATTTGCCAATTAGCCAATCAAAAAGAAAAGCAA
ATTATCAATGAACAAAGGAAACAGGCAAGTATTCAACAAAAATCCTATTCTCCCATTGGCGATAA
CTATACCCGTCATTTAATTCGTTGAAAAAATATGGATAACTTTAGCTATGAGAATAACAAGAAAT
AGTACTGTTAATAAATTCTCTACTTTTGTCTTTTTTCCTGATGAGAATAATCAATTTTTTTGCTG
GCAAACAGATTTATTATTAAAGCGAAATATAGAAAAAATTGTTGCTGAGAAGAAAAAGCAATCT
TGTGAGAAGATAGCCCGTTCTTTTTTATCTTCTATTTTAAATAAGCATGATAAAATCAAACAAAA
TCACCTTTCTGCTTACCTACAAGAAACTTGTTTATGGAGTGCGAGAATAATTTATGAGCGTCTAT
ACTTAACATTAAAATTACTCACTTTAGAAGAATGTTTTTCATCGGGGAATCTAGCATTAATTCAA
CCAGAAAAATTACTAAAAAATTATCAAGTTAAATATGGGACGAAAATCACAACTTATGCTCAAA
CAAGATTAAACACTATTATTAAAGATACTGTTTATCGTAATCGCCAATGGAAATTATTAAGTGAC
TGGGGTTTATTGACAAAAATAGGAATCAAACGAGCAGAAAGTATATTAACAATTCAAGGAGGTT
TAAAAGATAACAAATTGCAAGAATGTCTTTTAGCTTGGCAATGCTATCTTGATAATTATCAAACT
TGTGGCATAAGAAAAAAATAAAGTATTATCAGAGCCAAATTCGACACATTTACAGTTAATGGTTC
AACAATACAATTTACAAATTAAACAACTTAATCAAACACAAATTACTCCCGATGAATTAAAACAA
AAACTCAAATTTTGTATTGAAATGGCAAGATTAGCCACAAATCCAATTACTGTTATGTATGAAGA
AAATGACCACTCTCTAATAGAAGCAAATTCTGATAATTATATTGATGATTTAGCAATAGAAAAG
GAAAGTCAGGAAATTAAAAATATAATCATTAATAGTTTTCAAAACCAAGAAAGTATTAATCAAG
CAATTTTATATTTCATTTTTAGTTTAAATCTAACTCAAGGAGAAATTATTACAATAATCAATCAA
AGTTATCCTGATTTTATTAAACAACAATATCAGTTATCTCGCAAAATTAACAGTATAAAAAAATC
AATTATTGATGAAATTATAAAAACAAAAAATCTTCCTAAAAATCAAACGTCAGCGAAGGAAATC
AAACCTCTTATCTCTCTTTTAGATGCTTGTATTCAAGAGTATATAGAAGATGATATTTTTACATT
AATTGATCAATGTTATAAAAATCTTAATTCGGAACAGCAAAAATATTTAGAACAACAATACTTT
AAGGAAACAAAAAATAGCAATAAAAATAAAAATTCCTTTAATCATAATAATGAACTTATCATCG
ATAATGTTAGAAAAAAACTAGCAAATAAGTTAAATATTTTTATTCCTAAAATATCTATGATTGA
TAATAACATCAATATGCAAATTCAAAAATTTTGTGAAAATTATTTAATAGAATTAACTAATTA
TGAATATCATAAACTTTATTTCCGACTGCTACGCCAATGAATTAAAAATTGCCTTAGATGAAAAT
ATTAAACGTCAAACATGGCAGGAGGTTAAAAATTTAAGTACCGAAATTAATCGTCATTGTGGTT
ATATTAATCTTCTTTCTCGTAAATATTTTATTCAATGGCTAAATTTAGTTTGCAATATTAATATT
ACAGATACAAACAACATAATTGACTATATCACTATTTGGGAGTTTATTAATGGTAATGCGATCAA
TGTGAATAATAATCGCTTAGTTTTAATACCAACGGAAACCCAAGATAGAGTTAGTATTTCTATTC
CTCAAGAATGGTTAAAAATTCCTGAATGGGTTGGTAGTTATTATGTGATAGTGGGAGTTAACTT
AGAAGAAAATTATTTAGATTTTTGGGGTTATATTAACTACGATAATTTGCAAGAAAATGGGGAA
ACAGATAATTTAAACCATACAGTTAATTTACCCTATGAATGTTTGGAAACTGACTTAAATTTAAT
GGCTCTGGAGTACGAATATAGTTGGGAAAATATCCCTCAAGTTTCTCCATTAAAATGTTTATCAC
CTCAAATACAAAAAAAATTTAATTACAGAGTTGGAAAATAGTTTCTTTCCACGTCTTAGTCTTGAT
TTTAGTGATTGGTTGAGTTTAGTTAGTATTGCTGATGCCCGTTATAAATTATATTCCCATCGTCA
TAGTATAAGTTTAGGCGATTTATTGAAAAACAAAACAGAGAATATTTTGAGAAAAGGATGGCAA
AGTTTACAAGATTTTATCAAAGATAATTTTGAGACTGATTTCGATTTAAAGATGTCTCCTGCTTT
TCGCTCTATTTCACCCCAAGAGGGTTTAAATGTCTTAGTCAAAAATGATAATTATGGACAAATAT

TAGAAATTTTAAACACTATTCCTAATTGGCAAATAAGGGAAAATTTACAACAGGAATTTATTAG
GGTTTTAGTTGACCTAATTAATAATACTAATGACGAAGAAATTCTTTGGAGTAGTGTTTTCGCTT
TAGAGTCTTTACAAGCAAATCATTCTTTATGTTCTAAAGGATATGGAAAATTAATCAATTTCGAT
GAAGAAAATAAAAATTATTGTTTATTAATTAATATTTTACCCAGAAATTTTGGGCAAATTAGTA
TTTTTGTGAGAGTTTCCGTTTTAAGTAATGAGGATAATTTACCAGAAAGTTTATCTTTGAAAATA
TTAGATGAAAATGAAGATCTTTTTCAAGCAATTATTAGTGAAAAGGGCGATAATACTATTCAGT
ATAAATTTTGGGCAGATGAAGGAGAAACTTTTAAGATTCAAGTCAGTTGTAATAATAATTATAT
AGAAGATACATTCAAGGTATAAGTGTCATTAAAAATACGTTCGGAAACCCGTACAAAACAACCTA
TTGCTATCTCTAATGAATTTCGGTTAAATTAGCACTCGTAAGGTGAGAGCGCTAAAAACTAAGCT
CTGACCCTCAAAATAACTAATCTTTGAGAAATTGGAGTAAACAATCTATGGCAGCTATTTCCATC
AATGTATCCACTGTTAAGCCTTTGGGCGATCGCGTCTTTGTCAAAGTAAGTGAAGCAGAAGAAAA
AACTGCTGGTGGTATCTACTTACCCGACAACGCCAAAGAAAAACCCCAAGTAGGTGAAGTTGTTA
CCGTTGGGGAAGGTAAAGTAAATGATAAAGGTGAACGTACCCCTGTGGAAGTTAAAGTAGGCGA
TAAAGTACTTTACTCCAAATACGCTGGTACTGACATCAAACTCGGTGGCGATGACTATGTTTTAC
TCTCCGAAAAAGACATTCTCGCCGTAGTTTCCTAGTTTTATCGTAAATGAGGTTAAGGTACGAAT
CTCAGAATCAATCAAGTACCAACATTATTTATTTTCAAATATTACACAAAAATTAGTTAGAAACT
AAACAGAATCAATTATGGCAAAATCTATCATTTACAACGAAGAAGCCCGTCGCGCTCTCGAAAAA
GGCATTGACTTATTAGCTGAAGCTGTAGCTGTAACTCTTGGTCCTAAAGGACGTAACGTTGTTTT
AGAGAAAAAAATTTGGAGCACCTCAAATCGTTAACGACGGTGTAACCATTGCTAAAGAAATCGAAT
TAGAAGATCACATCGAAAATACTGGTGTTTCTTTAATTCGTCAAGCCGCTTCTAAAACCAACGAT
GTAGCTGGTGACGGTACTACCACTGCAACCGTATTAGCTCATGCTATTGTTAAAGAAGGTTTACG
TAACGTTGCCGCCGGTGCTAACCCCATCGCTTTAAAACGTGGTATCGACAAAGCTACTGAACATT
TAGTGGCTAAAATTGCTGAACACGCCCGTAAAGTAGAAGATAGCAAAGCTATTGCCCAAGTAGGT
GCAATCTCTGCGGGTAACGATACCGAAGTCGGTGAAATGATTGCTCAAGCAATGGACAAAGTTGG
ACAAGAAGGAGTTATCTCCCTCGAAGAAGGTAAGTCCATGGAAACTGAACTCGAAATCACCGAGG
GTATGCGTTTTGACAAAGGTTACATCTCTCCTTACTTCGTAACCGATACCGAGCGCATGGAGTGT
GTTTTAGATGATCCTTACATCTTAGTAACCGACAAAAAAATCACCTTAGTTCAAGACTTAGTACC
CGTTTTAGAACAAGTTGCTCGTCAAGGCAAACCTTTAATGATTATTGCTGAAGACATCGAAAAAG
AAGCGCTTGCAACTTTAGTTGTTAACCGTCTTCGTGGTGTCTTAAACGTAGCGGCAGTAAAAGCT
CCCGGATTTGGCGATCGCCGTAAACAAATGTTAGAAGATATTGCCATCTTAACTGGTGGACAATT
AATCAGCGAAGATGCAGGATTAAAAATTGATACTACCACCATTGATCAATTAGGTAAAGCTCGTC
GTATCAGCATCACCAAAGACAACACCACCATCGTTGCCGAAGGAAACGAAAAAGAAGTAAAAGCT
CGTTGTGAGCAAATCCGTCGTCAAATTGAAGAATCTGACTCTTCCTACGACAAAGAAAAATTACA
AGAGCGTCTCGCTAAATTAAGCGGTGGTGTGGCAGTAATCAAAGTTGGTGCGGCTACCGAAACCG
AAATGAAAGATCGTAAATTACGTTTAGAAGATGCTATCAATGCGACTAAAGCCGCAGTGGAAGA
AGGTATCGTCCCTGGTGGTGGTACAACCTTAGCACACTTAGCTCCTACTCTCGAAGAATGGGCAA
ATGCTAATTTAACCGCAGAACAATTAACTGGTGCTTTAATCGTAGCTCGTGCTTTAACCGCTCCT
TTAAAAAGAATTGCTGAAAACGCAGGTCAAAATGGTGCTGTTGTAGCTGAAAGAGTTAAAGAAA
AAGACTTTAACACTGGTTATGATGCCGCTAACAATGAGTATGTGGATATGTTCTCTGCTGGTATT
GTTGACCCTGCTAAAGTAACTCGCTCTGCTATTCAAAACGCAGCTTCGATCGCTGGTATGGTATT
AACTACCGAGTGTATCGTAGTTGACAAACCTGAAAAAGACAAACCTGCGGCTCCTGCTGGTGCTG
GTGACTTTGACTACTAAAATCTAACTTAATTAAATAAGTCTGAGGTGGGCATTGCCCACTTTTTT
TATGCTTAAAATGAAGTTAGATCATGATTAATTACTACCAGAGAGATGAGCTTAATTATTTATTT

TTTGAGACACGGAGAAACAACTGCCAGTACGACAGGGGGTTATTGTGGTGTTTTGGATTTAGATT
TAACTCCAGAAGGCTATCTTATGGCGGAAGACTTTGCTAAAGCCTATAAAAGCATTCCTTGGCAG
GGTATTTTTTCTAGTCCCCTCACAAGGACGATCGCAACTGCTACTCCTTTAGCTAAAACTATCGGT
ATGGGAATTCAACAACGGGATGGATTGAGGGAAATTGCTTACGGTAATTGGGAGGGAAAAACCC
CAGATGAGGTTAATCAACTCTATCACGACGAATATGTGCGTTGGTTAGCAGATCCGGGTTGGAAT
GCACCGACAGGGGGTGAAAAAGGAATTGATATTGCTCGTCGTAGCAATGAAGTGTTAGAAGAAA
TTGAACGTAATTACCCTACTGGTAATGTGTTAATTGTTTCTCATAAAGCTACTATTAGGATTATG
TTATGCTCTCTTTTAGGAATTGATATTGGACGTTATCGCGATCGCATCTCGATGCCTGTGGCTTC
TGTGACAACGGTGACTTTAACGGATCGAGGTCCTCGTTTAGATACTTTGTGCGATCGCACCCACT
TACGTCCAGAATTACACGCTAGAAAAGGAACTTAATCAGAGTTGAGAGTTTGAGCTTAGGAGATT
TAAGAAGATAGGTTTTTGACTATAGTACAGTTTATTAAACGAATGGAGTGAAGTGAACTGAATA
TCAAAAACTAAATCTAGCATTACAATAAAAGAAAAATAACTTTTATCATAGGTCGATTATGGCTT
ATAAACCTTTAACTTTATCAACTCCTAAACCCGTTTTGTCATGGGCTGGTCATGATTTAGCCAGT
CAAGAAATGCAAATGGCGAAAAATGTGGCTTCTTTACCTTTTGTTTTCAAGCACGTTGCATTAAT
GCCTGATGTACATTTAGGAAAAGGGGCTTTAGTGGGTTCTGTTATTGCCACGAAAGATGCGATTA
TACCTGCGGCGGTGGGAGTTGATATTGGTTGTGGAATGTGTGCGATTAAAACACCTTTTCAGGCA
AATCAATTGGAAGGAAAATTAAAAAAAATTCGTCAGGAAATAGAGGCAATTATCCCTGTAGGTT
TTAATGAAAATAAGGAAGTAGAAACAAAAGTAACTAATTGGCAAGGATGGAGAAATTTTAGTGA
TTTACATTTAGGGGTGCAAAGATTAGAAACTAAAGCCCTTAAACAAATGGGGTCTTTAGGAGGA
GGAAATCACTTTATTGAATTATGCTTAGATACCGATGAGCAAGTATGGTTAATGTTACATTCTGG
TTCTCGTCATATTGGCAATAAACTGGCAGAATGTCATATTCATACAGGGAAAGAGTTGGCTAAAT
TAGCAGGTTTAAAACTTCCTGATCCTGATTTAGCTTATTTTGTGGCGAATACTTCCGAATTTGAT
GCTTACTGGAGAGATTTACAATGGGCTCAAAACTATGCCCGTTTTAATCGGGATGTGATGATGAG
TCGCTTTAAAGGTATTGTCGAAAAATACTTAGCAGGAGGTAAACCTTGTAAACCTTTACTAACGG
TTAATTGTCATCACAACTATGCAGAAAAAGAAATTCATTTTGGTGAAGATGTTTATGTTACCCGC
AAAGGTGCTGTAAGGGCAACTCAAGAGGATTATGGTATCATACCTGGTTCAATGGGAGCAAAGTC
CTTTATTGTCAAAGGTAAGGGTAATCATGACAGTTTTTGTAGTTGTAGTCATGGGGCAGGAAGAA
AAATGTCTCGGGCGAAAGCAAAAAAAGAATTTGATGTTGCCGATTTAATTCAACAAACTGAAGG
TATTGAGTGCCGTAAGGATAGTGGGGTTATTGATGAAATTCCTAGTGCATACAAACCCATTGAAG
AAGTAATGAATCAGCAAACCGATTTAGTGGAAATCGTGGCTACCTTAAAGCAAGTTGTTTGTGTT
AAAGGATAACCAGAGTTCGGGGTGCGAGGTTAGTTAGGGGCTTAGGGTTTTGGGGTGGTAGGGG
TTGAATATATTTAACGTCAGTTGCCCTTCTCCTCTTTTAAGGGGAGACACAGAGGAGTTTGCCCT
CCCCCCTAAAGTAGGATAAAGGGGGTTTTGCTCTTTGCCCAGAGAGTTGTCAACTACTCATCCAT
GTCAAAATTATCATCATCGGAATCATCCCCTGAACTTCCGAAACTGACGTTATTAATCTCTAATT
CAGCTTTGACTTTTTCCTCAATGGCCTCGGCAATTTCGAGATTTTCCTCTAAGTATTTGACGGCAT
TATCTCGACCTTGAGCAATGTTATCACCTTCATAGCTGTACCATGCCCCTTTTCGGTTAACAATGT
TGGTTTTTTCGGCTAAGTCTAACAAACAACCAATACGAGAAATACCAGAACCAAAAATAATGTCA
AATTCAGCAATGCGAAAAGGTGGAGCAACTTTATTTTTAGCCACTTTTACTTTTGCCCGAATTCC
GTATTCCCCTTCGCTACCCTTTTTCAAGGTTTGAATACGACGAATATCTAAACGGACAGAAGCAT
AAAATTTTAAAGCTGTACCTCCTGTAGTTACTTCTGGACTGCCGTAGCTAATACCGATTTTTTGA
CGTAGTTGGTTTAAAAAGATGACTGTTGCTCCAGATTTACCAATATTTCCCGCAACTTTTCTTAA
TGCTTTACTCATCAAACGAGCTTGTAAGCCTACTTGTAAATCTCCCATTTCTCCTTCTATTTCTGC
ACGAGGCACAAGAGCGGCAACGGAGTCAACTACAACAATATCTACGGCGGAACTACGTACTAATT

GATCGACAATTTCTAAAGCAGATTCACCATTGTCAGGTTGAGCTACAAGTAAATTGTCAATGTCC
ACTCCTAAAGCGGCAGAGTAAGTAGGATCTAATGCGTGTTCTGCATCTACAAAGGCGGCGATGCC
ACCAGCTTTTTGAACTTCTGCGATCGCATGAAGGGCAAGGGTTGTTTTACCTGAGCTTTCAGGAC
CATAGATTTCAATAATACGACCTTTAGGCAAACCGCCACCGAGGGCGAGATCCAAGGTTAAAGCA
CCGCTAGAGATGGTTTCTACCTTCATTTTTGCGGCATCTCCAAGACGCATTATTGAGCCTTTGCCA
AAACTACGCTCAATCTGATTGAGGACGAGGTTTAATGCTTTTTCCTTATCGGGGTTTTGAGCCAT
TGTACTTGTAGCCATAGGTTTTTTCTGCTGTCTAACTCTCTCAATGATAATGTAATTTTTTTCTC
TATGTGACCTTTATTTCAGTCTCAATATTTTTGAATTTTATCTTATTTGGTACATAGGTATTGTC
CTTCTATCTTAGCTTATTTTTTCCCAAGCAAAAGATTTTTATGGTTCTTTTTTTGAGTTAGATGT
TATGGGGGGATGAGGGGATGAGGGGATGAGGGGAGAGGGTGAGAGGAGGAAATTAACTCTTAAC
TCTCAATTTTTAACTGAAATTTATTTACTATTCACTATTCACTATAAACTATTTACCTTTGCCCC
TTGCCCTTTGACCTTTTTTCTTTGTCCCTTAACTCAAAAGTTTTATATCGAACAGAGGTTAAGTT
ATTGACACTCACACCATCAAATCAAAGATTGTGATGGGTGATTCTTGCTTTTTTCAGATTGCCTC
TTAACTAGACAAAGATTACTCTATGCCCCCGTCAGACCGACTCCACAAGCATTTTAAACACAGTT
TCTTATCTGTGCTACCGTATGCCCTACGGAACCAGTATCTAGGTATCATTTTCTCTCAGACCCTTC
CTATCGTCAGGGCTAAAGGTTTTACGCCAACTGAGTTTTTCGAGTTGACAACCCCATATTTTGAC
TATTTAATTTTCTAGGTACTTAAGTTTTGTTGTTTTAACGCCCACATTACTGGTTACGTTATCAT
TAATTGTATCACTATTTATGGACACTGTCAATGACTAGAGAATATTCTTTAAGAGTAAGGTTAAC
CGATGATGAAAAATCAAGACTAGCTTATTATGCAAAGCGCAAAAATGTAAGTATGTCTGAGATT
ATTCAAGATTACTGTAAACGATTGCCAAAACCCCCTTCTGCTAAGGACTAAAGGTTTTATTCGTT
GCTCATTATTCATCTCCCGTTAAAACAATTAGATTTTTGAGTTTCTAACGGGAGTCCTCTACTGA
GACTAAAGATAGTTCTCGAATTTTGAGATTTAATTCTTTTAACCGAATCGGCTTACTTAAATACA
TATCTGCCCCTGCCTCTAAACATTTTTGCTCATCTCCTTTCATAGCTAAGGCAGTTAACACAATTA
TTTTACATTGATCTAAAAGGGGATTTTGGCGAATGATTTTAATTAATTCTATGCCCGAAATATTT
GGCATTTGAATGTCTGTAATGATTAAATCGGGATGGTGAGTATCGAGAATATTTAAGGCTTCTTC
TCCGCTATTGGCACATACTAAAGAAAATCCGTTGGCTTCTAAATAACTGGAAGTAGTGAGTAAAT
TAGGGTAATTATCATCTACTAATAAAATTGATATTTGTTGATTTTTTTCTTCTTGAGAGTTTTGT
GGGGACAAATCTTTTGATTTTTCTGGAGATACATCGCTATTTTGTTGATCTAGCTCGTAGGGAAA
ATCTACCATAAAACAACTACCAACTCCTAACTCACTGGTTAATTCTACTTTTCCGCCATGTAAATC
AAGAATCTGTTTCACTAAGACTAATCCTAAACCTGTACCCTCAAATTGACGATTCAATGAGGCGT
CTAATTGTACAAAAGACTGAAATAGTTTGCTGGTGTCTTCTTTTTTGATGCCAATACCTGTATCC
TCAATGGCAAAACGCAAATAGTTTTTCGTTATCTCTTTATCTTCTTCTGAAATTACTGACACTTT
AAGAGTAACTTTTCCCCCTTCTGGAGTGAATTTGACAGCGTTATTAAGTAAATTAATTAAAACTT
GACCTGTACGTCTTTCATCTGCCATCAAAGGAGTTAGATTTTCAGGAATCTGGCTAGTAATTTTG
ATATTTTTTTTCAAGGCTTGTTGTCTAAGGAAAGATAAGCTGTAGTAGCATATTTCTTTGACATT
GAGAGGTTTAATTTCTAGTTCTAGTCGATTAGATTCGATTTTGGCGAGATCGAGAATATCATTAA
TTAAGGAAAGTAAATGAGAACCTGCGGTTTCAATGGTTTTAAGTGCTTTACTTTGTCGAGGATTG
ATCTCTCCATATACCTGATCTTGCAAAATTTCAGTCATACCTAAAATAGCATTAAGAGGAGTTCG
CAATTCATGACTCATATTGGCTAAAAACTCACTTTTAGATCTATTGGCTATCTCCGCTTTTTCCGT
CGCTATTTGCAGTTGTTGGTTTTTTTGATGTAATTCCTCTTGAGCTTTTTTCTCTTCTGTTATGTC
GCTATTAATCCCCACCAGAGTAGCGGGATTTCCTTCCCTATCCCTTTGTATTTGTGCGATCGCCTT
TATCCAACGAATTTCACCATCAGGTTTAATAATGCGAAACTTAGCCACAAAATCCTTTTCTTCTT
TCACTACCAAAGCAAATTTATTTTCGATGAGGGAAACATCTTCGTGATACACACAGGCAATGAAA

TCAGCGTAAGTGAGGGAAGATTGTCGCTCACCCATGCCAAAAATTTCATACATTTGTTGATCCCA
GTGTAAACTCAAACTTTTAAGGTCTAGCTCCCAAATACCATAAGCTCCAGCTTTTAGAGCCACAC
GGAGGCGATGGGTTAAATCTTTTAATTGATTTTCTGCTTCCAGACGCTCTGTAATATCTTGAGCA
ATACCCAAAATTCCTATTAATCGACCTTTCAGATTTCGCATGGGAACTTTACTGGTTTCTAGCCA
AATATTCCTGTTATCTCTGAGAGAAACACGCTGTAATTTTAATAAAAAGCTATCTCCAGATCCGA
TCACTTGTAAATCTTCTTCTTGAATCTTTTGAACAGTAATTTCATCCCAGGGCAAATCCGCATCG
GTTTTACCAATTATCATTTCAGGATTATCAATACCCAAAATCTGTGCGGCCGCAGAATTCATTCC
CAGATAAACTAACTGATTATCTTTCCAAAAAACTCCAATGGGTAAAGTTTTTAAGATGGTGTCTA
GTAAATTTTTTGATTCTTGGAGAGCTTTTTCTGTATTAATTCTGTCCGTAATATCTGTGGACATT
CCACAAATACCATAGATTTCCCCTGCTAAATTGCGAAGGACAAACTTATCAGATAAAAAATATCG
TTTTTCACCATTGGATAAAACTGTTTCTTCAAATTGTTCAAACCTCCCAGATTCAATTAATTGTT
GATCATTAAATGTCAACTTACGGGCAATATCTAGCTCGAAAATTTCTTCATTGGTTTTGCCCACA
ATATCCTCTAAATTACGATTAAAAAGATCTAAAAAGGCTTGATTAACTAAAGTATGTTTTCCCTG
TAAATCTTTAACATAAATAACAGAAGGAGAAAAATTGAGAATTGCCTGTAATTTTGCTTCACTGT
CAAGAAGTCTTTGTTCTATGACCTTTCTTTGTGTAATATCTGTAAAATGCCCAATAATTTCTTTT
GGCTCACCCCTGTTATCTCGAACGAGGATTAACTCATCAGCAACCCAGATATAATGTCCATCTTT
ATGACGCAAACGATATTCATGGGTAATAAAGTTGGAAATGAATAACTGTTCAACATCAGCAAAA
ACTCTCTCTTTATCTTCAGGATGGAGTAATTCTTGCCAAAGAGTGGGATGATTAGTAAAGTCAAT
GGGTGTATATCCTAAGACTGGAAAAACTCTTTTACTGATAAAATTACATTTATAATCTTCATGGG
CTTGACTGCTATAAATCACCGTAGGACAAGAGTTTAAAAGATAAGCAAAACGTTTTTTCAAGGCA
ATAATTTCTTGTTCCGCTTGTTTTCGGGAGGTTATATCAAGGGCTGTTCCCAAAATTTGCAATAT
TTGTCCTTGTGAGTCTCTCTGAAATACTAAATCTCGACTATAAAACCATAACCAATTACCATTGG
CGTGTCGCATACGATATTCAAAATCAATAACATCCTCTGATTTACTGCGATGAAGTTTTGCGAGA
TGTTCTTCAAAACGACGAAAATCTTCTGGGTGCATTAACTGGGAAACAAAATTGCCACCCATTGT
TTTTATTTTTTCCGTTTCATAACCAAGACAAGTGAGAGCATAGTTGCTCATGTAAAGATGAGTTT
TGCTTGTCATGTCATATAAATAAACAATGCCGGGTATAGTTTCAATAAGGCTACGAAGCCATTTT
TCTTGTTCGAGGCGATCGAGTTCTGCTCGTTTGCGTTCACTAATATCAATAATGGTGCTACGACT
TTGTAAATAATTACCCCTATCATCGGTTATTATCTGGGTGCTAATTAAAACGGGCAAGATACTAC
CATCTTTAGCAACCATTTCTAATTCTGAATCCTTGATATATTTCAACCATCCTTTTTGTCGCATTA
GGGGAAATATATTTTCAAAAATAATGCGACTTTTTTCGCTCAGAAAAGAGATAAAAGGTTTTCCA
ATCATTTCCTCTCGACGATAACCAAGCCATGTTAATTCCGTATCATTAACTCGAATAAATTTTCC
TTCTTGATCAAGGGAATGATAACCACAAGGGGCGTTATTATAAAGATCCTTAATATCTGCGATAA
CCTTCGCCTTTTCTGCTTCCTGTCTTTTTCGTTCTGATATATCGGTAAAGTAGCCTACTACTTCCA
CAGGATTACCAAAATCATCCTTAACCAACACTAATTCATCTCTAACCCAAATATAATGACCATCT
CGGTGACGTAGGCGATATTCTCGTTTCAGGTATCCATGTTCAAATAAATCGGTAATTTCTGCGAA
GACTCTTGAAGCGTCGTGGGGATTAATATTTTTCTGTAAAAAATCTTTTTTCTCTAGTAAATATT
GAGGAGAATAGCCTAATAAATTTTCTACGCCCTTACTAACATAAGTATATTGATAGTTTTCTTCA
ATTTGTGAGCTATAAATTGTGGCGGGAGTGTTCTCCAATATAAAGTTAAGACGTTCTCTCAGTCT
TTGATTTTCTAATTCTATTTGTTTTTTTTGGGTAATATCTTTTGACACTCCTAATAAGCCGATAA
CTTGATCCTGTTTGTCTTTTACTACGGATAAACGTAATTCTACGTGAACGACATTACCATTTTTG
TGGCGACAGGCAAGTTCTAGCTGATAATTTCCCCTTTCCAGTAAGACGGGAATTATATCTTTTGT
TGGTGTTTCTGAGTCATAGTAGATAATACCCACTGGTTGACCGATAGCTTCTTGTTTACTGTAGC
CGTAAACTTCTTCGGCTCGATGATTCCAGTCGGTAATAATACCATTGAGATCTGTTGCAATTAAG

ATATTATCAGTATCTCGCAGAATATTCTCCTGAAAGTATTTCTGAGATATTTTGTTCTTGGATTT
AAACGGTTTTTGTTGGGATATTTGGGAAAAGGGCGATCGCTTCTTTTCTATAATTTGCTTAATTT
CTGTGGCTATTTGCTGAGGGGTTTGGGAAGGATTAAAAATAATTCCTCCTGCACTTTTAAGGATT
TGTAGTCCCCTTTTGCCGTCTTCGGTTTCGGATGATAGTTTTATGGCAAAGGTTTTAGTAGCTTG
TTCTTGGGCTAAGGAATCTAAAAGAATATCATTGGGAAAATTAAAAGGAAGTGCGATCGCTTCTT
CCGCATCAATTTTTTTTAAAAAACCATCTTTAACGACGTATTTATGATTATTGGACAATAGGTAT
ATGCCGCCCACTTCTATAGAGTTAGAGGTTGTTGGTTTGACTAAATGATAATTACTGTAGTCCAT
AAAAGAATCTAAATCATAACCATCACCATATGAGAATGAAGAAGCACCATGTTGAGTAATAATT
ATAGAGATATTTTGAGCCACAAATAAAGAAGATAAATTAGCTAATAACTCTTCCAAGTAGTTAT
AATTTTCAAAAGAAATATTTGGAATTACCAAATAAAATTGCTCCATCATTCTCTTCCAACTCCAT
GATTTTTTGATGGATTATTATTACCTTATTCTATAATGTATATTTATTGAATTGTAGAAAATTAA
GAAATAATAACAAAGAATTCTGTAAGTGCAATCTATAAAAATTAATTTTGGGAAAAACCTTATT
TTCTATTTCCTGAAATTTTGAGGATATGAAATTTTGAGGATAAATAATTGTACCTAATTAATTAG
GGGTTAGGAATTTAGATAAAAATATGACTCTCTCACTATGATCATATAAACTATTAGTTTAGGTA
GCGGATTTGAAAAAAGGGAACTTGCTATGCCTCTCGTCTTTTCAAGGGAGAAAGAGAAAAGAAA
ATTATGGCTCTATCACTTTGAGTATCTAAATAATTAGTTAGGGTAGGCAAGAGGCAATAGGCAAT
AGGGGGTTATTAAATAATAATTTATAAACTTTTAATTTTTATTTTACTATAAACACTATTCAATA
AAGGTTATGGAAGTTATTTTTTATTCATTTATCATAACCACTGTAGAAGAACCAAAATTATTAAA
TAATTTATAAACTTTTAATTTTTTTTGTCATAGATACTGTAAAAGAACCAAAAACATGGATAAA
AAAAGTATATAATTTGGGAAGTTAAGTTTTTCCTCTTTATTTTAATGAGAGTAGGCTAAGAAGT
ATTAATTTCAATTTTTATGGCTCTATCACTTCTCGTCGTGTAAATTATTGATGTTTAGTAAGCAA
GAGGCAATAGGCGACTATTAAATAATAATTTATAAACTTTTAGTTTTTATTTAACTATAAACACT
ATCTAATAAAGGTTATAGAAGTCCTGTTTCTGTTAATTTATCATAACCACACTCTGAAGAAGCAT
TTTTATTAATTCTTAAAATGATTCAACCGCTTATCTGTTACCTGCTACTCGTTACCTGTGTTGTTC
ATCAGCCTACCACCCCTTGACGACTTAGATACGTTCTAGCTTATTAGACCTCTTGCAAAATAGAA
ATTAAAATAAAATGTGGACATTTTCATAGTGAATCTTATCACTGTTCCCTGTTCCTTCTTCCCTG
ACTTCTGAAATGAAGTCTATTGATTTATCTGATTGCTTATCTTCACTGGTATCAAGTTTATGATT
TTAAAGACGGTTTCTCCTTTTATTTCCTTTTCCGAGGGTGTTAATTTACTTCAATCAAATCTTCA
GGAGCATAAACAGTGGTTACAAAATACTGCTGATGAAATGAGACATCAACAAGTGGGAGATACA
GTAACCTATGTAGTTAATCGTAATATTAATTTTACGAATATTTGTGAACAACATTGTAGTTTTTG
TGCTTTTCGGCGTGATCAAGGAGAAGAGGGGGCTTTTTGGCTAGAAATTGAGGATATTATTGCTA
AATCAACGTTAGCGGTAGAGAAGGGAGCAACGGAAATTTGTATGCAAGGGGGTTTAAATCCTTT
GGCAAAGGTTGATGGTAGCAGTTTGAAATACTATTTAAAACTCGTTGGTGCAATCAAAAAATCTT
TTCCTCAACTACATTTACACGCTTTTTCACCCCAAGAAATACAGTTTATCGCCCGTGAGGATGGT
TTAACTTATGATGATGTAATTGTTGCTTTACGTGATGGAGGAGTCAATTCGTTACCCGGTACGGC
GGCTGAAATTTTAGATGATACTGTGCGAAAAATTATTTGTCCTGAAAAAATAGATAGTCAGACA
TGGATGGATATTATCTCTAAAGCCCATCGTTGGGGATTATACACTACTAGCACAATGTTATGTGG
TCATATTGAAACTTATGAGCAGGAAATATTACATTTGACAAAAGTGCGATCGCTTCAAGAATTAG
CTTTAGAAAAAGGTTATCCTGCCAGAATTACAGAGTTTATTTTGTTGCCCTTTGTGGGGGAATAC
GCTCCTCAACCCCTAAGAAAAAGAGTCGGTAAAATTCAACCTGATTTAGAGGCCACTCTCAAATT
AACAGCCGTTGCCCGTTTATTCCTCGGTGATATTATTCCCAATCATCAGCCCAGTTGGGTTAAAT
TAGGTTTACATGGTGCAATAGAAGCTCTTTCATGGGGTTGTAATGATATTGGTGGTACTCTTATG
GAAGAGCATATTACCACTATGGCTGGTGCAAAAGGTGGTACTTGTTTGGAAGTTAAAGAAATAC

AAGATGCGATCGCATCTATTAATCGTCCTTTCCGTCAAAGAAATACCCTTTATACCTTAGTTTGA
AACGAGAATATCTAATTGAACCTAAGTTCGGAGTTAGGAAAGAAGTGTCAGGTTGTTGTAGGTG
ACGGGGGGATGAGGGGATAGGGGGAAGTAGGGGCGAATGGCCATTCGCCCGTACAGGAGCAATG
ATTGTTCAGAGTTTTTAATTCTTAATTTTTAATTTTTGATTATCAACTATTCACTATTCACTATT
CATTATTCATTATTTACCTTTGCCCCTTGCCCTTGGTGTGAGAAAAATTAGACTAACTCATTTTG
AATTGCCCAGCGAGATAATTCTGTGCGATTATGTAAACCTGTCTTGTTTAACATATTAGACACAT
GACTTTCTACAGTTCTTTGACTAACACCTAATTCTTGAGAGATTTCTTTATTAGACAAACCTCTA
GCCACCAGATTTACAACTCTAGTTTCTGTGGGGGTTAATTCTACACTTTTTGGTACTTGAATTCT
TAGAGCCGGGTCAATTTTTTTCTGACTGCTTCCCATTAAGCGCATTGTTTGATTTAGTGTTGACT
TTACTTGGGCGACTAATTCATCAGGCTCAAAAGGCTTCACCATATAAACATCTGCCCCCTGAGTT
AAACCTTTAATTCTGTCTTTACTTTGCCCTTTTGCCGAGAGGAAAATAACAGGTAACCATTCTAA
ACTCGGATTTTGTCTGATTTCTTGAACGAAGTTATAACCATCCATTTCAGGCATCATCACGTCAC
AAATGATTAAATCTGGAGTGTCTTGTTCAAGAATTTTTATTGCTTCTCTACCATGATTAGCTGTG
ATTACTTCATAACCATTAAATTCGAGATAGTCTTTGACTAATAGAATTAGGTTAGGATCATCATC
TATAAGGAGTAATTTTTTAGTTTCTGAGTCTTTCATGATTTTCGTAATCAGTTTTCTTCATTTTT
AGTATCTTCAAGACAAAGTTGTGATGTGTCTGAAAATGGCCAAGGATGTTTATGAAACACATATT
CTTTTACCATTTTAACCTGAGTTCGGGATAAATTTTCATCTATGAATGATGGAAAAAAGGGCAAG
GGGCAAAGGTAAATAGTGTTGGGGTGAATAGTTGATAATTACTCATTACTCCCTTAAACCTAACA
CCTGCAACCTGCAACCTGAAACCTGACATCTATCCTTATCCAATATTCTTAAACCGAATTGAGGT
TAACTATTTGTTATCCCTATTTCCTCTCATTCGTTTTCATGTTAGGATGTGCTAAGAAAATTAAA
AGCTGAACAATCAATAGCTTAATTCTTTAAGAACTGATTATGACTACAAAAATTGAGGGTAGTA
ACTGGAAAGTCTCATGGCAATTTTTAAGCCTGATTTTAGCGTTGACTTTCGGAGGCATCGGTTTC
GCCGCTACTTCTGTACTGTTGCGATTGCCCACTAATCCTAGTTGTAGTAAGGTTTCCCTTTTTTTT
AGTTCCGCTACTAATCGTATCTACTGTGCCCAATTGAAAGCGGAAAAAAATACTGTTGATGATTT
ATTAGATGCGATCGCACTTTTGCAGGTACTATCGGAAGATCATCCTTTAAGAAATGAAATAAATC
GTCACATTAGTGAATGGTCTAATGATATTTTGATTCTGGCACAAAGTAAATTTGATCAAGGAAAT
TTAGAAGAAGCGATCGCAATTGCCCGTAAAATACCCGAAGATGTGGCAAATCGCACCATGATAGA
GGAAAAAATCAGTGAATGGGGTGAAATTTGGCAAAGTGCGGAAAAAATCCAAGAAGATGTAGAA
GCAAAATTAAGAGAATCCCAGTGGAATCAGGCATTCTTAGAAGCCGTTAACTTGTTGAATGTAGA
TAACGAATACTGGCAAACCACCAAGTATCAAGAAATTGTCAAAACCATTAATCTTGCTAGAGAAG
AAAGCAAACAATTAGATGGTGCATATGTTGCCCTAAGACAAGGAGGAGTTGATAATCTGCTCAAA
ACCATTGAAATAGCATCCATCATTCCAGCAACTAGCTATTCCTACAATGAAGCAAATAAGTTAAT
CGAAGAAGCAGAAAATAAAATTGTGGTCATAGTAGAAGAATATTTAGGGGATAATGATTGGAAT
AATCTCAGTAACCTAGCCAGTCAAATTCCTGAAAAAAGTCGTCTTAAAAATCAGGCAAGAGATTG
GAATATTTTAGCCAGTGCAGGAAGAAATGCTAATCTTGGCACAGTTTCGTCTATGGAATTAGCCA
TTGCCGAAGCCGAACAAATTCCTAGCGATAGTAAGCTATATCTTCAAAGTCGAGAATTAATACAA
AATTGGCTTGCCCAAAAAGAAGATTTGGTTTATCTTGCTAGTGCCAGAGAAATAGCGAGATCTGG
AGAAATAGAAGATTTAAACGCTGCGATCGCCAAAGCAGAGTTAATTGGCTCAGAAAATCCCCTTT
ATCGAGAATCTAGGCGAGAAATAAATAAATGGCAAAGAGAAATACAAGTCATACAAGATCGCCC
TATTTTGATTCAAGCCAGACAAATAGCTGAGGGTGGCAATGTTCAATCATGGCAAAATGCTATCA
ATACAGCCACTCAAATCAAATCAAATCGAGCCTTATACAGTGAAGCTAGTGATCTAATTCGTCAA
TGGCGTCGTAATATAGAAAGAGTCGAAGATCGTCCCATACTAGATCAGGCGATCGCACTTGGTAG
CAACAACAACTATCAACAAGCCATAAATGTAGCCAGTCGTATAGGTAGAGGCAGAGCCTTATATT

CCGAGGCTCAAACCAATATTCGTAATTGGCGCCGAGAAATAAATGCCCAAGAAGACTTAGAAAAT
GCTTATCAAATAGCACAAAACAGCGATCCTCAATCCTTACTCCAAGCCATAAATATAGCCCGTAG
AATCCCCTCTAATAGTTCTGTGGGATACCAAAGTCGTCAGGCAGTTGAACAATGGTCAGAACAAC
TACTTAATAATGCGATGAGAATTGCTGAAACTTACTCCCTATCGGCTTTAGAAGATGCTATAAAC
ATCGCCCAAATGATACCGAGAGGCACTCCTGCTTATAATACAGCTCAACAACGAATCAAAACTTG
GAAAAGAAATTTATTTCCGGGTTTTAACGGTGAAAATCCCTCTAATATTGAAGAAGTTCCCTTAC
AAGAAACAAGTTACTAAGATTAATTTTTTTAACAATTAGAGTCATTATTGAGGGTTTTTTCCGT
GTAATATATACAATGATTAACCATCGGCGACTATTTATTAATAGCTAGAAATTATGAATATTTTA
ATCAAAAAACCGATTTTAGTTGCAGGCATTAGTCTTTCCTTTCTACTATGGATAGGACAAAAATT
GACTAATTATTGGGATGAAATCGGGAATGTCAGTATTTTTAGCTTAATTTGTCTTGGTGGTTTAT
CTTATTTTTTACAACAAATTGGTCGGAAAAAATCAGTTAATCAGCTTAATTTGTTAGATATTACC
ATCAATGATTTAACCGCAAAAATAGCATCTATCAAAAATCAAATAGAAGCCTTAAATTGTGAACT
AAATGAAATAGAAGAGAAGGATAATCAAGACGCAAAAATTAATATAAAAATCAACAATTTTCAG
CAACAATTAAATACTATCCAACAGTCATTTCCTCGGAATAATCTTAACATTGCCATTTTAAATTT
AAGTCAAAATACTAATTCTTTTGCTCTGTCTCAAGATATAGAGAAAAAAGTTAAAACTCTAGTTA
ATTTTCATGGAATAAATGATGTTTTAGAAATTTTTAATAACGCAAATAATTTAACTAACTTAGT
TTTGGAATATGATTTATTGTTATTAATTGTTAGTGAAGATTTAACCCAATCCCAAAAGGAAATA
ATTTCTTACTGTAAAAACCAACAGCAGAATTTAATTATAGTTTTTGATGATATTAATTACACTTT
ACAAGAAGAAAAGAAACTGATTTTGAATAGTCTTAAAAAAGCTCTTGATTCAGTTATAGAAGAA
AAATATATCATCCCCATATCCAGTAAAAAACAAACTATCAAGGTAAGAAGATATGAAGAAAATA
GTACCTATAAAGAATGGGAAGAAACTTTCAATGCAGATCATGAAAAACTTATCGAAACTCTCTTA
ATATTAACGGAAAAAGAATTAGATAAATTAACATTATCCACAACCTACAGACAAGCAATTAACCT
AGAAAAACAGATAAGAGAAGAATTAAACGCTATCAGAAAAAACAAAAGTTTGAAAGTGGTGGAA
AAATATCAAATGGTTGCGGCAACAGCTACGTTTGCAAATCCCGTTTCGAGCTTAGACTTACTAGC
AACTGCGGCTATTAATGCTCAAATGATAGTTGATTTAAGTAAAATTTATCAACATCCTCTATCTC
TCAATCAAGCACAACAAATATCTTTAACATTGGCTAAAGTAATGCTTAAGTTAGGCATTGTGGAA
ATTTCTAGTCAAACCATTAGTGCTATTTTGAAGACCAACATGATAACCTTTGTCGCAGGGGGATT
AATTCAAGGTATTAGTGCCGCCTATTTAACTCGTGTTTGTGCTTTGAGTTTAATTGAATATTATG
AAGTGGCGGACTTTACTGTTAATGACAACATAAATATTAGTAAATTAAAAGAGAAAATACAACT
AATTTTTGAGCAGAATAAAGAAAATAATTTCTTGAATCAATTTGTTAAAAAGACATCTTTATTA
CTAACATCAGTTCAGGTTAAGAGTATTGGATAAGGGCTAGTATCACCCCAGTTTTATTTATAATG
ATAAAAGGATAAGGGGCAAGGGGCAAACCCCCCTTTATCCCCCTTCTCGAGGGGGGAGGGCAAAG
GTAAATAGGTTATAGTTGATTATAAATAATAAAAAACACCTGACACCTGCAACCTGAAACCTGAA
ACCTATTTTATAGCTCCGAACTCCAAACACTGAAGCTATTTAAAATCCCCAGCGAAAAACTGTTG
CTCCCCATGTTAAACCTGCACCAAATCCAGAGGTAACGATGGTGTCCCCCGTTCTAATTTTTCCTT
TGCTAACGGCTTCATCTAAAGCTATGGGAATAGATGCGGCGGAGGTGTTGCCATATTCGCTCAGA
TTTGAGATTACTTTCCATTTGGGGATATTAAGTCTTTCAGCTACTGCATCGAGTATTCTTTGGTT
TGCCTGATGAAGAATCAACCAATCTATCTTTTCTGTGGTTAAGTCTGCTTTGTAAAGAGCTTTTT
CTATGACTTCGGGAACTTTATTGACGGCAAAACGATAAACTTCTCTTCCATTCATCGTAATGGGG
TTATATTTTCCCTGAGTAACTTGGTGATTATTACCTAATTTTTGAGTTTCTCCTGTAAAGGCAAG
ATTCAGACAACCGTTAAGTGTTCCGTTACTACACATTTCAAAGCCTAACAGGTTATCTTTTTCTT
CGCTACTTTGACAGACTACTGCCCCAGCACCATCACCAAATAAGACACAAGTAGTGCGATCGCGCC
AATCAACCCAACGAGATAAGCAATCAGCACCCACCACAAGGATATTCTGATAAACTCCATTGCGT

AAAAATTGGGAAGCAGTATTTAAAGCAAAAACAAATCCTGAACAAGCGGCCGTTAAATCAAAAG
CAACAGCACGGTAAGCCCCTAACATTCCTTGAACTTTACTAGCACTACCAAACAGATCATCTGGA
GTTGAAGTTGCTAATATAATCATATCAATGTCTTCGGAGGTTAAACCCGCATTGAGAATAGCTTT
TTCCCCGGCTTTCACTGCTAAATCACAAAGAGATTCAGAGGAACAAATATGGCGTTTTTGAATAC
CTGTACGAGTTTTAATCCAATCGTCAGAAGTATCAACTATTTTGCTTAAATCGTGATTATCTAGG
ATTTGTGGGCTAGTGGCTGAACCGCTACTGATGATCGTTACCCCTCGTTTTATTTGCTCCAATGA
TTTTCTCCTTTTTTGCGATGATGTTAATTATTAATTATCGATTATTATGGTTTTATATTAATTAT
TTAGACTATCATACCACCCTACTTTCACAACAGCGATAAAATGGTTTTAGATGGATTTTGATAAC
TAGGAAAAGTTAAAGCTAATGAAAATCGCTCCCTTACCTAACAACGAGTCTGAGCGAATTGAAGC
CCTATTAAGATACGATATTTTGGACACGGAATTTGAAAAAGCCTATGATGAGATTACTCAATTAG
CCGCCGCCATTTGTGGTACTCCTGTCGCTTTGATTAGTTTCATAGATGGCGATCGCCAGTGGTTCA
AATCAAAAAAAGGCATAGAGCCAAGAGAAACCCCCAGAAATATAGCCTTTTGCTCCCACGCCATT
CTCGATACAGAAATATTTATTGTTGAAGATGCCTCTCAAGATGAACGCTTTGCCGATAATCCCTT
AGTTTTAGAAGACCCTCCCCATATCCGTTTTTATGCAGGAGCGCCTTTAATTACACCAGACGGAT
TTGCATTGGGGACATTATGCGCCATTGATTCTCAACCACGTTGTTTAACAGAGGAACAATTAAAT
TCTCTACGAATACTCGCAAAACAAGTTGTTACCCAGTTAGAGTTAAGATTAGCAATGAAAACTCT
ACAGAATTACTCTCAAGAATTAAAAGAATTAAATGCTAGTAAAGATAAATTTTTTTCTATTATT
GCTCATGATTTAAAATCTCCTTTTAACACAATTTTAGGATTCGCTGAGGTTCTTAAAAATAATGC
CTTAGAACTTACAAAAGAGGAGATAGAAGAATTGGCAGGGGAAATATATAATTCTGGGAAAAAT
GCCTTTAAATTATTACAAAATCTTTTACACTGGTCTAGGTTAGAAACAGGGAAAATGCAGATTAG
CCCCGAAATATTAAATCTTAATAAATTAATTCAGGAAGTTTTATTAATTGTTACCCCAATGGCGA
AGGAAAAAAATATTCAAATTAACACTAATTTTTCAACTAATAATTTTACAGTGAAATGGGACTA
TAATATGTTAGTTTCAACTATCCAAAATCTGTTAACTAATGGTATTAAATTTACTCCTTCAGGAG
GGCAAATTACTATTGAATTGGAAGAAAAAGAATCTCAAATACAGATAAATATTATTGATAATGG
GGTAGGAATGACAGAAAAACAACTGGAAAATTTATTTAAAATTGATTGCTGTCAAACAACGGAA
GGAACATCAGGAGAAATAGGCACAGGACTAGGCTTATTATTATGTAAGGAGTTCGTCGAAAAAA
ATGGAGGTCAAATTTGGGTTGAATCTCAAATCAATCAGGGATCAACTTTTTCTTTTACTATTCCA
TTGAATTAACGCTCTTTTAAGAATTAATAAAAATTGAAATTAATGGGTCTTAGCTTAACTGGAA
AAATAATTTTGGTTCTTCTACAATGGTTATGATAAATTAATAGAAAAAAACTTCTATAACCTTTA
TTGAATAGTGTTTATAGTAAAATAAAAACTAAAAATTTATAAATTATTAAAGTTTATAAATTAT
TATTTAATAATCCCCTATTGCCTCTTGCCTGTTCAGGGTTTTTTCAAAGTCAGGGCAAAATTATC
TTGCCCTCACCCCCACCCCCTCTCCCACAGTAGAGGGCAGCGTTTTTTCTTAATAATTGATTAATT
AATACTTAAAAACTCCTGTATCTGTTCTGACGGGGAGACAATGAAACTGCAGTGAGGGTAGGTTT
TAGGTTTCAGGTTTCAGGTTGCAGGTTTTAAATACATGAACAGTATTTTGTATTTACGAAATAAA
TAGCCAGACATATTATCTCAAAATGTTGATAATCCAATAGTTTTGCCTATTATTTGAACCATTGA
GTAAATAGAGATAATGATAAATATTAGTGGAGACAAAACCTAACACCTAACACCTGCTACCTGCT
ACCTATAAGGTTTTTAGAGTTATTGTCCCCCCTTAAGGTATCTGTTACTATTTGTTAGTTTCAAA
AATTGATATTTTTGAGAATGAAAAAACCCTGTTTGCCTTTCAGCCTACCCTAACTAATAATTTAC
ATACTCAAACTGATAGAACAGTTTATTTTAATCTTTAAGGATTATTTGTAAGATTTTATCTAAAT
TATTATCGATCTTAATTGGTAGTAAATTTAAGGATTCTTGCTCTGACAATAAATGAGGAGAAAA
GGTTCTGATAGACCATTGAATATATCTATACAAATTATTTGTTTTTTGTTTTGATTTTGGTGTTT
CGTTCCATAGGCGAGTACCCGTTGGTCTTTCTTCTCTATCTTGAGATATTAACTCTTTATAGTTA
ATTTCTTCTTGGTGAGATTTTTTGATTTCCTCGATTAAAGCATCAACAAAATTATCTTGATTGGA

ATGAGTAATTATTATTTTTTTTACCGATGTATTTTCAATAATTTGTCTAAAAACTTGAGTTATAT
TAGATGTCCAAAATTGTTGATTTTTATGAGTTAGAACATATAATTTTTGAGTAAAAATATTTTT
GCCTTCTAAGTGATTTAAAATAAAAATAGCCTCATCAATACTATCAGAAATAATGACAATATCAC
TTTTATTATTAATTTCTAAACAAGAATAAACTGTGGATAAAAATTGATCTTTGTTTAAATATTT
TAATAAATTAGGATGTCTGTTTAAACTATCATCGTTAATTCTAATTTTGCCATCTAGTGCCATTA
CTTCACCATTATTTTTCACCCCAAGAGGATTAATTTCTATAATATCAAGATCATTTTGGATGAAT
AAATCATACATTTTTTCAATGATGGCGGTGACTGGTGCGATCGCTTTTTTTTTCAAATTCATTTT
CATAACTAAATGACGGGCATGGAATGGCAAAAACTCCTCTTCAATCACACAAGTTTGTAAATTAT
CTAAAAGAATTTCAACATTAATCCCTCCCTGAGAAGACCCCATTAAAACAGGTTTTTTAAGGACA
TAATCAAACATAATCCCCAAAAATATCTCGTTTTCCACATCATAGCGACTCTCTGCCAAAATTAC
CTTGGGATACTCTCCCTCTATGGATAAATTAAATAAAGATTGAGCAACGGCGATCGCATCTATGG
TATTCTCAACAAAGCGTACACCCCCAAACTTAGCTCTACCACTAACTAAAACTTGAGATTTTAAG
ACGATGGGGTAATGAATATGTAAATTTTTTAATTCACTAGGGTCAGCAATTGACTGAGAAGGTA
ATACAGGGATACCAACTTGGGAAAATAATTCTTTAGCTTGATATTCTAATAAGTCCATTAAAGAA
ATAATCCATAGAAAATAGTTTCATTTTAACCTGAGTAGTTGGGTTCGGCGTTAGGAAAAAGGTTT
CAGGTTTCAGGTGTTAGGTTTCGGGGAGTAATGAGTAATTATCAACTATTCACTAATTGCTTTGT
CTTAATTCCTAATTCCTAATTCCTAATTGAATCTAATATCCCAAATCTACAGCTATACGATGGGC
GCAACTGAAACCGGAAAACGCAACCGCATTCAAACCCTGCCCCGGAAATGTACTATCTCCCACAC
AATATAAATTTTTAATAGCAGTACGATTGAAAGGCATTGATAATAAACCTAAAAGACGACGGGC
AGGAATAGGCCCATAAGTACCGTTTATTCTTCCTAAGAAGCGTCTATGAGTGCGAGGAGTGCCAA
TTTCCATATAGTCTAACCCCGCATCCAAACCGGGAAAAATCTTTTCTAAACGTTCAATTAAACGC
CCTGCCGCTTCTTCTTTCTTGAATTCATATTGTTTCGGGGTTAATCCTTGCCAATAATCCATCGAA
CTGGGGGTAAAAGTATGAATGATATGGTAGCCTGATGGTGCTAAACTGCGATCGAGGAGAGTTG
GAATAGAAACAAAAATAGTTCCCTGTTCAGCTTCTAAGTTGTTCCAATCCTCTAAAATAATGTGA
TGACATTCAGGATTATTTTCTAACACTTTAGCTTCAACTCCCAAGTGTAAACTCAAAAAACTAGG
AGATTGACGATAATTCTGTTGCCACTTAACCTCTTTTTTCGGTTTTTTATTCTCTATCAACTTCTC
AAAAGTATCCCAACGGGTAGCATTAGATACAATTCTTTGAGCATAATATTCTTTTCCATTTGCCA
ATCTCACCCCAATGGCTTCATGGTTTTCCACAATGATTTCTGTAACTCTCGCCTGATATTTTATTT
CTCCCCCTAGCTTTTCTAAACCTTCTGCCAAAGTTTCCCCGATTTTGCCTACTCCACCTTTAGGAT
AATTAATTCCACCATAATGCCGATCAGAAAATACCATTCCCGCATTTATCATCGGAGTTAAGTCA
GCCGGTACAACAGACCAACAATAACACTCCATATCAATAAATTTTAATAACTCAGGATTTTTGAT
ATACTTGCGAGCAACATCACCGACATTTAAAGGTAAATATTTAACTAAACTTAAACAAGCAAGGG
GTTTTTGGAAGAAAACACGAGTTAAGTAGCGAATTTCCTCTAAGGAAAGCAATTCCATGGAGTTA
AGAGCGTTAAAAACAGCCCAACACTCATCATAAAATTTTCTAATTCCCTCTTTTTCGTCAGGAAA
ATAGTTATATAATTCCTCTAAAAATAAATCATAGTCCCGATGCACTTTCAAATCGAGATTATTGG
GTAAATGATAGTGTATTTGAACTGGGTCAGGGACAGTCTCCATTTTCATATTGACAGCATCTAAA
GCACGAGTTAACAAATTTGTTGTGCCTTCAGTGCCAAAACCAAATATCATTGAAGCACCAATATC
GAAGCGATAACCCTGACTCTCAAAATAACCTGCACTACCTCCCGGAATGAGATAACGTTCTAACA
CCAGAACTTTAACTCCTTTTGCGGCTAACTGAGTGGCTGTCACTAAACCACCTATACCCGAACCAA
TTACAATTACATCATACTGATTCATACTTTCTGTTCGATCTTAAAACTTTCCCTCTAATCTCCCTT
TTACCAGTTTAGCGGTTTATGGTCTCAAAAAAAGCCGTATTTATCCCTAAATTCTCACTTCTCAA
AAACAATATTAACCTTAGTTCGGTTTAAGAATATCGAATAAGGTTAGGTGTCAGGTTTCAGGTGG
TAGGAGTTGAATATATTCAAATCTTACGGGAAATTTCTTTTTTATCTTAACTCCGAACTTCTGTA

AGGGCGAATGGCCATTCGCCCCTAACTAACTCTGAACTCAAATGATTGCCCTTTTCGTCATCACTC
TATGGATAGATAAAAATTTATCCCAAACTCAGGTTATATTAACCTCAGTTGTTGAGCAAAATATT
ATTTTAGGGATGCTTCGAGTTTCAGGTGGTAGGTTTTGAGGAAATATTGGATGAACATTTATCAG
TCAATTGAACTAATTACCATCCTTTACCATCAAAAAAGTACAGTTTTTAACAATCAATACAAGGA
AACAAAAGATAAATCATGTAAAATAAGTTTTGTCTTAGATTTTAATAAATTTTAATCAATATAT
ACTATTTTAAATTGTTTATATATTCCTCAAAATTAGTTTGAATAAGTAATCATCAAATGTTAAAT
AAAATAATTTCTTGGCTAAATGATTTTTTCAAAGATAGAAATTTCATGAAGCTAATTCATATTAC
CGAAAATTTAGTCTCTAAGGTTCTTTCTATTGCTTTAATAATAGTTATCTTTGTTTCTATTTTTG
ATTTAATTTTAGTCCTATCTCAAGATTTATTTGTGCAAGAGCCTGTAGGTTTTTTCAATGTTACT
TTAATTGAAATTTTTGGCTTTTTTTTAAATGTGTTGATTGCCCTAGAATTATTAGAAAATATAAC
TGTTTATATAAGAAAGCACGTCGTGCAATTGGAGTTAGTATTAACAACAGCCTTAATTGCAGTGG
CAAGAAAAATTATTATTTTTGATACTAGCAAATATGATAAAGTCGATCTAATAGCTCTTGGTTTT
GCAACCCTCTGTTTGGCGTTAAGTTATGGAATAATTTGTTATATTCACAAAAGAAAAGTTTAAAT
TTCTTAGTTGTTTGGACTGAAAATAGCTGTAAATCTGAAACCTATTACCTTAAACTTGAAACCTA
CCCAAATCGAAAGGTATTGCTCAAGAACTGAGGTTAGCAATACATCATTGCCTTATAATTGAATT
TAGTCTATATGGGGATTTCAATGTGTTCAAAATATTCAGAACATTAGACAAATTTTATTGGCAAT
TAAATTTATATTGTTTAATATTTACTGGTTTCTAGGTAAACAAATGACCAGAAACTGAGTATTAA
ATTCAACTTTTACCCCTTAACTATATATATATTAATTAATAATTTGTAACCGATGAGTTTAACTT
TATATAATACCCTTACCAAGCAAGAAGAAGAATTTAAGCCTTCTAATCCAGAAAGAGTAACGATG
TATTGCTGTGGAATTACTGCTTATGATTATTGTCATTTAGGTCATGCTCGTACTTGTATCATTTG
GGATGTAGTGAGACGTTATTTGACTTGGAGAGGTTATAAAGTTAGATACATTCAAAACTTTACG
GATATTGATGACAAAATTCTCAATCGAGCAAAGGCAGAAAATACAACTATGGAAGAGGTGGCTG
AACGTTTTATTCAAGCCTATTTTGAGGATATGGAAAAATTGAATGTACAAAAAGCGGATTCTTAT
CCTCGTGCGACTCATACTCTTGATGGAATTAAAAGGTTAATTTGGGAATTGGAGGAGAAAGGTTT
TGCTTACGAGGCTCAAGGGGATGTTTATTATTCAGTAAGTAAGTTTCAAAGTTATGGTAAGTTGT
CTGGGAGAAACTTAGATGATTTACAGGCGGGGGCAAGTGGTAGAGTTAATATTCAAGATCAGAT
TAAGCAGGAGTCTTCTGATTTTGCCCTCTGGAAAAGTGCTAAACCTAATGAGCCTAGTTTCGAGT
CTCCTTGGGGTAAGGGGCGCCCGGGGTGGCATATTGAATGTTCGGCGATGGTTAGGGATAATTTA
GGTGAAACTATTGATATTCACGTGGGGGGAAGTGATTTAATTTTTCCTCACCATGAAAATGAGAT
AGCCCAATCAGAGGCAGTAACAGGTAAACCTTTGGCTCGTTATTGGTTACATAATGGCATGGTTA
AGGTGGAAGGGGAAAAAATGTCTAAATCTTTGGGTAATTTTATTACTATTAGGGATTTATTGGC
TAAATATGACCCTATGGCGGTGCGTTTATTTATTCTTCAGGCGAATTATCGTAAACCTCTTGATT
TTACTGATGGGGCGATGGAAGCGGCAACAAATGGTTGGCACACTTTAATGGAAGGTTTAAATTTT
GGCTATAAGTATGGCAAGGATTTAGATTGGATATTAGAAGGAGATATTCCAGAAATCCCTTCTGT
AAAGGATGATTTTTCTCAGGCGGTAGATCAAGATTTTAATTTTGCTTCGGGTTTAGCCGTCTTAT
TTGAATTAGCTAAGGAGTTACGCAAAGAGGGAAATTTATTAAGTCATGAAGGTAGAATAAGTCA
AAATTCAGAGGATTTGGAGCGTAAATGGCATACTTTAGTTAATTTGGCTCAAGTTCTGGGTTTAG
AAGCGAAGTTTGATCAATCTCTTTCTGTCTCTTCTCAAATTAGTGATCAAGAAATTGAAGATTTA
ATTTCTCAAAGAACTATAGCACGTCAAAATAAAAACTATCAAGAGAGCGATCGCATCCGAGATTT
ACTGAAAGAGAAAGGAGTTACCCTTGTAGATGTGGGCAAAGGGGAGACAAAATGGCATAGAGAT
TAATTTTGACAAAGGTATTGTCAATTCTTTAAGGATTAATCTACCCCGAAAATTTCATCTTTAAC
TTAGGTGAAGATAATAGTGAGAAATGAGGAATAAAAAAAGCTGATAGAAAAAACTGATAAAATT
GATTAATTAAAACTAAACAAGATTGAAGAGTAATAACCTAAATGTTTAATAACATACTCAAAAG

TTTAACAATGCGACTAATGACTTAATTTTATTTCGTTAATTTTTTTAATAAAACCATGCTTAAAA
AGCTAAAAACTATTGACATAGCCCTTATATTTCTTGTGATATTGGCTTATTTAGGTAATTATTTC
AAATTGCCTTTGTTTTTTGGGGTGGATTTTATTTTTGGTAGCATTTTTGTTTGGATAATTACTTA
TTTTTATGGTTTTCGGCAGGGAATTATTGCAGGTTTTATTAGTAGTATTGAAACCTATTTTTTAT
GGGGACATCCTTACGCCATCGTTATTTTTACCATAGAGGCTATTTTCGTTAATTATTTTTACCAT
CGTCGTTATAATAGAAATTTAGTCTTAATTAATATTATTTATTGGCTTTTTCTTGGGATTCCTCT
CATTGCTTTTTTTTATGGTTTTATTCTTGATGTTTCCTTGACAGGAACAATTTTAATTATTTTAA
AACAGTCAATAAATGGAATTTTTAATGCTTTAATCGCCACACTTATTTACAGTTATTCTATTTAT
TTACCAAGCAAATTTTTCCACATAAACAAAAAAAATCATAATCTATTTTTCAGGCAAAATTTATT
TAATTTACTTGTTAGTTTTGTTCTTATACCGACGTTAATTATTTCTGTTATTAATAGCTATAATT
CCATTAATGAAATTGAGAGCATCATCATATCTGATTTAAAAACTACCGCCAATCAATGGCGAGAT
AATTTGATTTATTGGCAAAATATTCATGTTAGAGCTGTAAGTAATTTAGCAAATAATTCATCTGT
TTTTAATGACTTGAATTTGCTATCGGAAAAACTCAATAATACCAGAGCAATAATTGATGATATAG
GTTTATTATATGTGAGCGATCGCAATGGAAATATTGTTGCATCTAGCCCTATAAAAAATCAATAC
GGAAAACCTCTTATTGGTTTAAATATAGTCGATACAGAAGAGTTTATTAAAGCAAAAAAACAAG
TAGATTTCTATTTTACTAACATTCACTATGACTCTGTTCTTTCAAATAATCATGTTTCTTTGGTT
TATCCGATTAAAAATCAAGCTAACCAATTTCAAGGAATAATATATGCTTCTATTTCAAATAAGAA
CATATCTGAAATTTTCATGAATCAAGACAGTATTTCTGAAATTTTAATTCTGGATGAAAATCAGA
AAATAGTTGCAAGTAGTACGGGAGAGATAAATAGTAAAAAAGTCAATCAAAATGAAGATAACTA
TGAAATTATCGATTTAGAAAATGGTTTTTCTCAATGGCTACCAATATATCCGGGTATGCCTATCA
TGACTAGATGGAGTTTATCATTTTATGTTCATGAAATCCCCATTAATAAAGAGACTCCTTGGAAA
ATATTAGTTAAAATATCCACTTCCCCTTATATAGAAAGGTTGAACATAATATACATAAAATCATT
AGGAATTTTATTTTTTATTACAATAATAGCCTTATTTATATCTGACAGTATTAGTAAAAAATTAG
TTAAGCCATTAGAAAAACTAGGTAAAATTTCCACAAATTTACCCGATAAAATATTTAACAATGA
AGATATTTTTTGGCAGGAAACAGATGTTGCAGAAATAGAAATATTGACAGAGAATTATAAGTTA
ATGGTTCGTGCTTTACAAGAAAAATTTGCTCAACTAAAAGATTCTGAACAAAATTTGACCAAAAA
AGTGACAGAAAGAACTAATGAATTAACCATAAAAACTCAACAATTAGAAGCAAAAATAGAAGAA
AAAAAGGCGATCGAATCTTTACTTAGAGAAAAAGATGAACGTTATGAATTAGCCGTTTCTGGTAC
AAACGACGGTATTTGGGATTGGAACTTAAACACTAATGAAGTATATTATTCCCCCGCTTGGATGC
GTATAATTGGTTATGAATCTGAACCACTACCCCCAATCTTAGAAACATGGCTCAAACGCATTCAT
GAAGATGATTTAGAAAAACATTTACATGATATTCACGCCTATTTGAATCAGGAAACAAACTTATA
TCAAAATACCCATCGCTTAAGGCATAAACAAGGGCATTATGTTTGGGTTTTAGCCAAAGGAAAAA
GAGATAATGATGCACAAGGAGAGGCTTATCGATTAGTCGGAACTATAACAGATATAAGTGATAA
AATCAGGGCAGAGCAAGAGTTACAGTTAGCAAAAGAACAAGCTGAGAAAGCTAATCAAGCTAAA
AGTGAATTTTTAGCCACTATGAGTCATGAAATTCGCACTCCTATGAATGCGGTAATCGGTATGAC
TGGTTTATTATTAGACACCCCTCTAAATAATGAGCAAAGAGAATTTGCAGAAATAATTCGCACCA
GTGCCGATAGTTTATTAACTATTATCAATGACATCTTAGATTTTTCCAAAATTGAATCAGGCAAA
TTGGAATTAGAATATCAACCTCTATCCATTATCAACGTTATCGAAGAATCACTAGACTTATTAGC
CCCAAAAGCAATTAATAAAGGTATAGAGTTAATTTATTTTATTGCCCCGGATATTCCTAAAACCA
TCATGGGGGATGTAACTCGTTTAAGACAAATATTAGTGAATTTATTAAATAATGCTGTCAAGTTT
ACCAACGAAGGAGAAATAGTTGTTTCTGTAGGGGTTAGTCGTCAATCCATTAAAGAAAATATCCT
TGAATACGAGTTAATTTTTTCTGTGAAAGATACGGGTATCGGTATTCCTAGCAGTCGTATGAATA
AATTATTTAAACCTTTTTCCCAAGTGGATGCTTCTACTACTCGACATTATGGCGGTACTGGATTA

GGACTAGCGATTTGTAATAGATTAGTTACAATGATGGGGGGACAAATATGGGTAGAAAGCAAAG
GAGTTATTGCCGGAGATTTTCCTTCAAGTTATCAGCCCAATTCTTCATTAAGTGATTTAGGATCT
ATATTTTCATTTACCATCAATACGAAAGTATCTAGTTGGTTAGTAAATCAAGAAAATCCCTCTTA
TTCCCATATTCTTCAGGATAAAGTAATTTTAATTGTTGATGACAATGAAATTAATCGTCAAGTGT
TGATGATTCAGTGTCACAATCTTGGCATGAAAACCATTATTACCGCTTCGGGTAGAGAGGCTTTA
TTAGCCCTAAAAAATGAGCAAAAACCTGATTTAGCGGTGTTAGATATGCAGATGCCGGGGATGGA
TGGTTTAACTTTAGCAAAACAAATTCGTTTACTGCCCCATTGTCAGAATTTGCCCTTGATTTTAC
TTAGTTCTATCGGTAGTGCAGAGATACAAAAGTCTTTACAAGAAGTGGATTGGGCGGCAACTCTC
ACGAAACCCATCAAACAATCTCTGTTAGAAGATATTTTTATTAACGTTTGTCAAGAAAATCGTAA
TTATCCCCGCTTATTTTCCTCTTCTTTTTCCTCTCCCTTTGAAGATATTGCTGGTATTGCCCCATT
AAAAATTCTTATAGCAGAGGACAATATTGTTAATCAAAAGGTAATTACTAACATTCTCAAACGTC
TAGGTTATCGGGCAGATGTGGTGGCTAATGGTTTGGAGGTAATGGAAACCTTGAATCGTCAATCC
TATGATTTAATCTTGATGGACGTGCAAATGCCAGAAATGGATGGTTTGACTGCTACTCGTCAGAT
TCGCACCCTGTGGAATTCTCCTAATAGTAATTTTCAGGGTATTCCCCCTTATATCATAGCAATGA
CGGCTAATGCCATGGAGGGCGATCGAGAAATTTGTCTAGCCGCAGGAATGGATGATTATTTATCT
AAACCCGTGAGAGTAGAAAAATTAATGGAAAAGCTGAAAAATTTACGAAAAACTTATCCTAATT
CAGAAATTAACCCTAAAATTGAAACAAAAAAGAATGATCAAAATCATATGGTGCAACTAGACAG
AGAAGCAATTAATGAACTAAAAGAAATGTTAGGAGAAGATGATTTTCCCATAGTCTTCGCTGAA
TTAGTACATACTTACTTAGAAGAAAGTCCTAGTTTGATTCAAGGCTTACTGAATGGATTGGAAAA
TCAAAATTACCAACAAATCAAAATTAATGCCCATAGTCTTAAATCTAGTAGTGCCAGTTTAGGGG
CAAAACATTTTTCCCAATTATGTAAGCAATTAGAAATGTCTGTGGTAGAAGAAAATTTTGAGTT
AATACCCCAACTAATCCCGACAATTGTCAAAGAATATCAGGAGGTAGAAAAATATATGAAAATA
GAGTTAAATAATCTGACTTAAATATCAAATTGTCGCTTAGGATGGACAATGGGCAAAGTAAAAA
GGGCAAAGGTTAAGAATTAAGAATTAAGAATAGCTCTTCTACAGTAGTTATGGCAAATTAATAA
TAAATAACCTCAGTTCGGTTTAAGAATATCCGATAAGGTTAGGTGTAAGGTTGCAGGTTGCAGGT
GTTGGGATGATGAGGAGATGAGGAGATGAGGGGAGATACAGAGGGGTTTCCCTTTCCAACTAAC
AATTTATCACGACGAGCAGTAAGAGAACCTTAATAATTAAAAACTCCGAACACTCATTATTCATT
ACTCATTTTTCTTCAACATCTTATTTGCGGAAATCCTAACAAAATCGAGCAAATTATGTCTTTTC
CACTATTTATGATTCTGACACAGTGGGTTAAGATATGGGAATCATTTTTAGATAAAAATATATA
GTATGGTCAATTTAAAAGGTAGAGATTTATTGAGTATTGCTGACTTAAATCAACAAGAAATTCG
AACCATCTTAGATTTAGCCGCCGATTTAAAAAGTGGAAAAAAACAGTTTCAAACCAATAAAACTT
TAGGACTATTATTTTATAAAGCATCCACTCGCACAAGGGTTTCTTTTAGTGTGGCAATGTATCAA
TTGGGGGGTAATGTTATTGACTTAAATCCCAGTCGCACTCAAGTGGGTAGAGGAGAACCTTTACA
AGATACTGCGAGAGTATTGGATCGTTATTTGGATATTTTAGCCATTCGCACTTTTGCTCAAGAAG
ATATACAAACTTTTGCTGACTATAGTGATATTCCTATTATTAATGCTCTGACAGATTTAGAACAT
CCCTGCCAAATTTTAGCGGATTTACAAACTATTGAGGAGTCTTTTGGTAGTTTAGAAGGAATAAC
AATGACTTATTTAGGAGATGGAAATAATGTTGCTCATTCTTTGCTTTTAGGGGGAGTTTTGATGG
GGATGAATGTCAGAATTGCGACTCCTGAAGGATATGAACCCAATAAAGACATTATTAAACAGGCT
CAAGCCTTGGCAAAAAAATCAGAGCAAGTAGTAATCACCCATAATGCAAAAGAAGCAGTGGAAA
ATACTCAAGTTTTATATACTGATGTCTGGGCTAGTATGGGACAAGAAGAAGAAAGCGCTACTAGA
GAGCCTATATTTCAGCCTTATCAAATTAATGAAGAATTATTAGGTATTGCGGATAAGTCGGCGAT
TGTTTTGCACTGTCTTCCTGCTTACCGAGGGAAAGAAATTACGGAAGAGGTGATGGAGGGTAATC
AATCTCGCATTTGGGATGAGGCAGAAAATAGAATGCACGCTCAAAAGGCATTAATGGCTTGTTTG

TTGGGATTGAGTGATTAAAGAGGTTTCAGGTGTCAGGTTTCGGGTGTCAGGTTGCAAGTTTGAAG
GTTTTAATTAATTATTAATTGCTAATTGAAATCTATTTACTATCGACGATTGAAGGTATAATATC
GGCTTGGAACTTTTAGAAAAATGTATCATAAATGACTAAAACAGCATGGGTATTCCCCGGGCAGG
GTTCACAAACTCAAGGCATGGGGATAGATTTAATTGAAGTGGCACAGGATAAATTTAAACAGGC
AGAGGAAATATTGGGATGGTCTATTATCCAGAGGTGTCAAGGAGATGAAGAAACTCTTTCTCGTA
CTCTTTATACCCAACCGTGTTTATATACCATAGAAGCAATTTTAGTCGATTTATTGAAGGATAAA
GGCAAAAATGCCGATTTTGTCGCAGGACATAGTTTAGGGGAATATGTGGCTCTTTATGCCGCCAA
TGTGTATGATTTTGCTACAGGTTTAAATCTCGTGAAGATGAGATCGCAGTTTATGGATCAAGCCC
AAGGAGGCAAAATGGTAGCTTTAATGAAATTTAACAGAGAAATCCTTGAGAAAACCATAGAAAA
TACAGAAAATGTCGTTTTAGCCAATGATAATAGCGATGGTCAGGTGGTTATTTCTGGAGAACCTA
ATGCCGTTGATGAAGTAGTAGAAGCAGTAAAAGCTAAATTAGCTGTGCCTCTTAAAGTTTCGGGG
GCGTTTCATTCTCCTTTAATGGCGACGGCCGCCGCAGAATTTGCCAAAGTTTTAGACGAAGCGGA
GTTTAAAGACGCTCAAATACCTGTTCTAGCAAATGTTGATCCCAATCCTAGTATTGATGGACAAG
AAATTAAAGAAAGATTAAAACAACAAATGACAGGGGGAGTGCGATGGCGTGAAATTATGGCACA
AATGCCCTCATTAGGTGTTCAAGAAGTGATAGAAATTGGACCGGGGAAGGTGTTAACGGGTTTAT
TTAAGCGTAGTGTCAAAGATGTAAGTTTAGTTAATATAAATGGCTTAGAAAATCTTTAAATGGG
TTTGACGGTATTGGGTGCTAGGTTTGAAGGTTTTATTTACTCGACCTATTGTTAATCGACCTATT
GTTAATTTATTCTTTAATTATCTTCCAAGTTGAGAAGTCATCAATAGAATGAAAGAGATAATCCC
ATATAACTATGTAATCAACAATTTTATTTTTTATGGATTTTCTTTCTGACACTCTCATTCTTTCT
CGAATGCAATTTGCATTCACTGCTATTTTTCATATGTTATGGCCTGTTTTAACTACAGGTATGGC
AATTTATTTAGTGATTGTCGAGGGTTTATGGTTAAAGACGAAAAACCCCGATTATTACCATCATG
CCCGTTTTTGGTCTAAGTTATATGTGTTAAACTTCGGTATTGGTGTAGCTTCAGGATCGCCTATG
GCGTTTCAGTTTGGCACTAATTGGGCGCCTTTTTCTGAGTCGGTGGGAGATTTTTTCGGTAGTGT
TTTAGGTTTTGAAGCAACCATGGCATTTATGCTCGAAGCTGGATTTTTAGGTATTATGCTATTTG
GTTGGCAAAGGGTTAACCCCACAATTCACTATATTGCTACTATCTGTGTGGCAGTTGGTGCAAAT
CTATCTACTTTCTGGATTTTAACGGCTAACTCTTGGTTACAAACTCCTGCTGGGGGTGATTTTGT
CGATGGCAAGTTCATTGTTAAAGATTATTTTGCGGCCATTGCTAATCCTTTTATGCTTAAGAGTG
TATCTCATATGTTTTTAGCTACCCTTGAAACTTCTCTTTTTGTCATCGGTGGTATCAGTGCATGG
TATATTCTCAAGAAGCGTCATCAGGCTTTTTTTGCTAAATCTTTTAAAATAGTATTAGCCGTTGC
GATCGCAATTACACCTTTACAAATATTTGTGGGTCATTTAAGTGCAGAACAAGTATATCATCATC
AACCTACCAAATTAGCGGCAATGGAGGCACAATGGGAAACAATTCCTGCGGGGGAAAAAGCCGAT
TGGAGTTTATTAGCTTTTCCCGATGAAAAAACCGAGAATAATAAATGGGAAATTACCATTCCCAA
TGCCTTTCCTTACATTCTTGAGTTTAAGTCCCGTTTGAGTGAACCTGTGTTAGGTTTAAAAGAAT
GGACTCCTGAAAATCGTCCTCGCATGGTGGGTTTAATCTATTATTCATTCCGTTTGATGAGTGGT
ATAGCCTTTGTCTTAGTAGGCGTAATGCTTGTGAGCGCCATTCAATGGTTAAGAGGCAAACTATC
AGAGGAAAATATTATTCAACAAAAATGGTTAATGATTTCTTGGATGTGGTGCGCACCTTTGGGGT
ATTTAGCCGTTGAGTCTGGTTGGATAGTGCGTTGCGTAGGAAGACAACCTTGGACTGTTTATGGT
GAAATTCGTACGGCAGATGCGGTTTCTAATTTACCCGCCGGGGATGTTTTAACTTCTTTAACTAT
TTTCATTTGTCTCTATACAGTGTTATTCTTTTCTGCTTTATATTTCGGTAGTCGTATCATTGCTA
AAGGACCTAAATTTGACTTACCTCTACCCGGAGATGATAAAGATTTACCAGTGGAAGTAGATTCT
GCTCAACATATTCCTGATAGTCGCCCTATTTAGTTAACCTCAGTTTGGTTTAAGAATATCGGGTA
AGGGTAGGTGTCAGGTTTCAGGTGTTGGGATATTGAGATATTAGGGGAAACGAGTTAGGAGTTA
ATTAAACACTTTTGCCTTTTGCCTTTTGCCTCTTGACTCTTACCTAGGGTGTTAGGGAGAAACTC

ACCCTTAAAGTGCATTGTTACTAATTACTAATTATTAATTAGCTTCAGTTATAAGGTGCGATCGC
ACCTTGTTACAGCTACATCGTAAAAAATCTGTAATCACTTAAAACAGTAAAAAAATGGAATCACT
TAATTATCTTTTACCATTGGTATGGTTTGCCATCCTTGCTTTATTTCTATTTATGTATGTGATGT
TGGATGGTTTTGATTTAGGGGTAGGGATTCTCTCTCTTACCTCTTCTTCTGAAGAAAGGCGTAGC
GTTTTAATGACAAGTTTAAGTAATGTCTGGGATGCTAATGCTACATGGTTAATCTTAATGGGGGG
AAGTCTTTTTGGTTCGTTTCCCCTCGCTTATAGTACTATTTTAAATGCTCTTTATATCCCGATTAT
GATTATGATTTTGGGGTTAGTGGTGCGTACGGTTTCTTTTGAATTTAGGGAAAATTCTCGTCGTA
AGTTATTCTGGAATTTGGCTTTTGGGATAGGTAGTTTTGTGGCAACTTTAGGGCAGGGTTTCGCC
CTTGCAGGTGTTATTGAGGGTATTCATGTGGATGCTAGTGGACATTTTATTGGTTCGACTTGGGA
TTGGTTTAACTGGCGTAGTGTGGTGGTTGCCTTAACTCTTATTCAGGGTTATGTTTTAATTGGTT
CTTGTTATTTGATTATGAAAACGACGGGAAGTCTTCAAGAAACTCATTATAAAACCGCTAAGATT
GCCGCTATTACTACTCTTATCGGTGCGATCGCAATTACTACTGTAACCCCTATATTCTCTATTTTT
GCTAGAAATAGGCTCTTTGATAAACCTTTTATTTATGTATTTAGCGCCATTCCCATTTTAGGAGT
AATTCTGGTGATTTTACTTTTAAGGAGTTTAGCTAAAAAAAGAGAAGTTACCCCATTTATTTACA
CAATTTTAATTTTCTTATTAACTTTTGTGGGATTAGCTTTAGTTGTATTTCCTTATATAATCCCT
CCAAGTGTAACTATTTTTGATGCAGCAGCATCTCCTAGCTCTTTAGTTTTTATGTTGACTTTTAT
CGGTTTTTTAATTCCGATTATGCTGTTTTATAACGTTTATAATTACTTTGTTTTTCGAGGCAAAA
TTACAGGTAATTAAAAGATATGGTAATCAGGTTTGAGTTTGGAAAGAGGTTTCAGGTTTCAGGT
TTCAGGTGGCAGGTGTTAGGAGTTAGGAGTTAGGAGTTTTTAATTTTTAATTCTTAATTCTTAAT
TTTTCCTTTGCCCATTGCCTATTGCCCCTTGCCCCTTGCCCTTTTTTAATTTTGATAAAATTCTCG
ATACCATTTAACAAACTCATCAATACCCTTTTCAATGGGAGTAGAAGGTTTAAAGCCAACGGATT
TATATAAGTCATCAACATCCGCATAAGTTATAGGCACATCTCCGGGTTGAATTGGCAAAAATTCT
TTCTCGGCGGTGACACCTAACGATTTTTCAATGGTGGTAATAAAATCAATGAGTTTGACGGGTTT
ATTATTACCAATGTTATACAGACGATAAGGTGCTTTACTATTAGGATTATCTGTTTGAGGTGTAG
GAATTTGCTTCATAACCCTAACTACTCCTTCAATAATGTCATCGATATAAGTAAAATCTCTTTGC
ATATCTCCATAGTTAAAAACTTTTATTTTTTTCCTTCTAAAATATTTTTAGTAAAAATAAAGTA
AGCCATATCTGGTCTTCCATAAGTACCATAAACCGTAAAGAATCTTAAACCTGTTGTGGGTATGT
TGTATAAATGACTATAGGTATGTGCCATTAACTCGTTGGCTTTTTTCGTGGCGGCGTATAAGCTG
ACAGGGTTATCAACATTATCTTGAGTGGAAAAAGGAATTTTTTTATTTGCCCCATAAACCGAACT
TGATGAAGCGTAAACTAGATGTTGAATGTTATGGTGGCGACAGGCTTCCAAGATGTTAACGAAGC
CTATTAGGTTACTATCAACGTAAGCGTGAGGATTTTCTAAAGAATATCTAACTCCTGCTTGGGCG
GCTAAATTGACAACAGTGGTAATTTTATTTTCCTTGAATAAGTTCATTATAGAGGTGCGATCGAC
CAAATCCAAGAGAAAGAACTGAAAATTATGATGTTGTTTTAAAATTTCTAAACGAGCTTGTTTT
AAAGATACGTCATAATAGTCATTCATATTATCAATACCGATAACTGAATAGCCATCATTCAATAA
ACGCTGACTTAGATGAAAGCCAATAAAACCGCTACTACCTGTAATTAAAATTGTTTCCATAATAT
TAATCAAATTTATTAATTTTTTAATAATAGCTGAATACCGTTATTTGAAGTAATTAAGATAATA
AGGTAGCCTGAATTTGATATAAAATCTTTAACATGGGTGAGTTTTAGCTATTTTTTTGAGAACAA
TTTACACTAACTATTTTATTTCATTAATCTTTATCAAATTAATGTTTTATATTTTTAAGTTACAG
TACTTTTCAAATTGAAAAAAATATTGAAAAAATAACTTATCAACATGATTAAATTCACTTACTATT
ACTATGCGTCTGGGATATTTTTTATTTTTCATTAGTGTATAAATATTATTAATCATTTAATCTTT
ATCTAACATAAAAATACTTGACTTATTCTTTTTAAAAATAAATTTATTGGACCCATAGACATCTT
ATAAAGATAAATTAATTATAAACATTAAAAACGATGTTCAAACTCTAATACTCCTCTTGTATCTT
GTTCAAAATCACTTGAACCTCTAAAAATAGTTTGTTCATTAAGACGATAACGAATACTATATCTA

GGAGATTGTTCATTAGTTAATATTTTCATAATAGAAATAGACAAATCATCAGTCAAATCTAAAGC
TAATTCCGCCCCTAAGCCAAGAGTAGAAATGCGGTTTTCTGGGTTTAATATTTGAGTAGGAAATA
ATCTTAATTCATCAAAACCAAATGCTTTTTGAATTTCTCCTTGCACACCACCTAAAAATGCCGCA
CTAGCTAAATTAACTAAATCAAGATTACCATTTGCTTCGGTTAAATTATTGAAAAAACCTCCTCC
TAAAAGGGCGATAATTTCTGCTTCATTTCTTTGAGGACTACTACTTAATACTATATTATTTTCTA
AATTATCACTCCATCCTTTTATTCCTGCTTTAATTTTAATTGTTTGAACTGTATCGATAGAAAAA
TTAGTTAAATCTCTAATCTCATTACTATTAGATGTATCCGCAAATTGATAGCGACTTGTCTCGGT
TACTGAGCTTTCCAACTGCAAATCAAGATAAGGGTTAAAACCATTTTCAGGAGTAAATTTCGCTG
TATTATTATAGTTATTAGCAAGACTTAATTGACTTGTAAATAAGTTTATATTACCGTCAGTTAAT
TTAATTATTCCTTGGGGGGATAAATTATTTAATTTGCCTTTTAAGTTTAGTTGTCCTTTAGCTCT
TAAATTTAAAATCGGAGGTTGTTCAATAATAATATTATCTTGTAAATTAATATATAAATCGCTG
ATATTAATGTTATTAATTAAACCTTGCTCAGTGATTATGTTCTCTGATTGATTTTTGCTTAACTC
AATCTGTCCATCATATAAATTAATTTCTCCTCCCAAATTAGGGCGAATAAAACTTCCAGATATAT
TTAACTCAGCAGAAGCATTTCCTCTGTATAACTCATCAATATTTAAACTTAAATCACTAACATTT
AAACTCAACAATTGATTATTCAATTGAGGGTCAATCAGAGGTAAACTACCATTCAAGCTGATGCT
TCCTCCACTAAATTCTCCCACTAAATTAGGAATGTTTAATTGGCTAAAATCAAATAAAACTTGAC
CATTAATATTTGAAATCGTTTGATTATCAATAGCTTTTCCTTGGACAACCCCATTGTTTAATGTT
ATGATACCTTCTGTATTAACTTCTTTAATAGAGTTTTTTTCTTGATTATATTTTCCTTTTATATT
CAAATCAATATTGCCGTCTCCTTCTAACCATTTCAACTGATTATTCGTAACTATATCGAGCAAAG
TAAAACCATCTTTATTTAAGTTTATATCAAGTTTAAAATCATTGTTGTTAGGTTTTGAGGAATTA
GGAAAAATTTGAAAAGGAAGACTTCCTATTAGTTTTAATGATTCATTATTATTAGTTAAATTAC
TACTAGCTAAAAAATCAATGCGAGAATTTCTTAAGCCGAAACTAGCGTTAGTTTGATCAATATTT
TTATTATTTATTTGTACATCAATAGCCTCAATATTTCCTTTTGCCAGAGGATTTTTTTCACTACC
ACTAATTGCGATCGCACTATTGACATTTCCTTGTATATTTAAAGTATCAGGTAAATTAGCTATAT
CAGCTATTTGAGAAAGAGGAAAATTAGATAAAGTCACCTCTCCAGATATTCTTTCAGGTTGAAAA
GTCCCAGTTAAAGATAAAATTGTTTCATCGCTTTTAATGATAACGGGGAGAAATGTGAGTAAATT
ATTATGATAACTACCCGTAACCTGTAAAAAATTGCCTTCGTAATCCCCCCATTGCCAACTATCCCC
ACGAAAATCAAAATCAGCTTTAACACCATCCTGAGATGAAAAACCTAAGTTTATAACACCATTAA
ATTTACCTGTTAATTCCTCTAAAGAAGGTAAGTTTTTATCTAAAATTTTCTCCCTTTTTAACTTC
AAATTATTCTCAACTTTTTTCAAAGTGTCTATTGTCTCTAAAAGAGAATCTTCCTCTGAATTAAT
GGCAAAAAGCGGGGGATTAGCAGATAAATTACTCTCTGAAGATGGAGAAGCAGATTTATCAGAA
ACAACCGAAATAGTATCATTTTTAGCCGAATATCGGTTATTACTATTACTATTCGCAGAATATAA
ATCTTTTGCAGATGCGTATTCCCTTGGCTGAAAACCTTTTGATATATCAGCAAACTCAAAAAATT
CCCAACTGGTAAGAAGATTTTGAATATCTCCATCTTCAATTTCTATACCTAATCTTAATTGTGGA
CTATTAGTCAAAGAAACTAATTTTCCCGTAAACTTATACTCGTTTTCCTGATGGTGTAAATTACC
ATTGAGAAATTCAATCACATCATTTTTAATAATGACTTCACTGGTGAGAATATCCCCGTGAAAAT
GATTTAAACGGGGTTTTGTAATGGTTATTTTTGATGCACTAACACTATAGTCATTTAGATTGAAA
CTTACCTCCCCAGAAAGAATACCCCCTACTTCTTTGATTTCTGTCGGTAGATAATTTTTCCAAGAT
TTTGTCACCTTACCTAAAGGTATCTCACTCATATCAACAACGAAATTTTCTGACTCCCTTAAACCT
CTTAATTGTGTGGTGTCTTTTTGCAGATAGATTTCTTGAGGTTTTAATTGACTGTCTAGGATTAC
GGCTAATTTATCTTCACTGTTACTATCATTTAAAGTTAAATCCAATCCCTGCTGTGGAGAAAAAG
ATAATTCACCTCGGAGAGGAGAAAAATTTAAACTGGCTACCTCAAATTTGTTAAGGGTAACATCT
CCTTTTAACTGAGGTTGAGAAAGATTCCCAATTAATTTACCTTGAAAATCAACATTACCCTGATA

ATTAAGTAAACTCAAAGAAGCAGGTAAAGATAGTTTTGACAAACTCATTTGTTGAGTAGTCACA
TCCAAATCAAAATCTTTTATGGTTTGAGTTTTGGCATCTAAATTAATTATTCCCCTTGCTTGAAA
ATTGTCACTAACTGCCTGATTAACTTGTAAGTATTCTCCATTCCAGCCAAAAGATACTGTCACTG
GTTGAGAAATTAAGCTAATTCCCTGAGCAAAAGATAAATTACCATCAACACTTATTTTATCTGGG
GTAATAGAATTCACATTCCCTTTTACCGTTAAATCTCCTTTGACTTGCCCATCCCAACTATTATTA
AGTTGAGAAAGGGAAAAACCCTCTGTAAAGGTGTTGGCAACAAAATTATTTTCTGCAACTTTGAT
TTGATTAACTTTAATTTTTCCTTGAGGTAAAAATATATCCCCTTGCCCTTCAATATTTATATTTT
CTTGATTATCTAAATCTCCCTTAACAATTAAACCTGCTGTTATATTACCCTTATCAATTCCAGGC
ACATTGAGAAATTGATTTTCTGGATTATTCGATAAAGATAAAAGTTGAGTAAGGGGAAAATTAT
TAACCTCCAAGGTGGTTTGCCAGTTATTATTCTTGAGATAGGTATTTTCTAATACAATCTGATTT
TCTCCTAGGTTAAAAGAAAAATCCCCTTCTAGCTTTAAGTTTTCTTGGTTTAGGTTTTGAGTATT
ACCACTTAAATTAAAATTCCCCTGAAGTATAGAATCTTGACATTTTAATTTTTCACAGTTAATGA
AGGATAAATCCCGTAAACTTCCTTGGGAAATTTCTATATTGGCTAACCATGAATCAGAGTTGAGT
TGTAGGTTTTTAACCCTTGCTTCTCCTTGAGGTAACGTTATATTAGAAGCACCTATTAATTGAAA
ATTAGAAAATTCTCGCCAATCCCCAGAAAAATTATAATTACCTGAAATTTTCCCCAGAGGAATTG
GTAATGTTTTTTGATAAAGATTGAGAAGTTTTTCACCATCAATTTCTTTTCCTTGCCAATCAATA
CTAAATTTGCTGTTAGCTTTTTGTAAGTTGATAAAACCGCTACCTTTTATTTCACCTCCTATTTT
CGGCAATATGGTGACATCTTCTATATTTAACTGAGAGTCAACAATAGCTAAATTAGCGGTTATTT
TATCAAGAGGAATTTTGGCAACTTTAATTTCTTCATCTGCATTGGCAATTTTTACTTTAACTATC
GGATTATAGACATTACCTGTTAAGCTAACATTACCTTTAATTTTTCCCTCTGTTTCTAAATTAAA
TGGGGTTAACTTCAAAGAGGAAGCTACATCGTCAACAGTTAAAGAATTAGTAATATTGGCATTA
ATATTTAAGTTTCTATTTTTATCGATAATGCCTGTAACTTGAGTGGGAATTAAGCCTAAATTAGT
TGTAATGTTATCTAGTTTAATTTCTCCATCTGTTGATAGGTTTATATCTCCATTGCTGTTAACTA
AGGGATAAGGTAATTGTGAAAAAGAAACATCTACTTGATTAACGTTAATTTTACCTTCAATACTA
TCAAGGGTTTCATTTTGAAATTCAAGGGTAAGATTGCTCTGTAGTTTACCTGAGTTTATTTGTAA
GGGTAATCTAACTAAATTGAGTTGATTGAAGGTATCTGTCAAGATATTTTCGGCGTTAATATTTA
ACACCCATTGGTTATCTGAGGGAATTCTCCATCCAGTTAGGTTGACATTTCCCCCTGCAACAACAT
TTCCTTCTACATTGAATAAATTTTTATTTTCTTCAAAGATGAAATTGGCTAGGTTGATATTTAAT
TGAGAGTATTTTTCTGGGTTGAGATTATTTTTGACGAATAAGTTACCACTATTTAGTTGTATATC
ATTAACATTGATACTTAATCCCCAAGGTAAGCTACTTTTTTGGTTACTAATATTTAGTCTTAACC
AACTATTATTTTTATCTTGTTGTAGGTAAATTTCTCCTTGATTAAGAGTGATGTCTAAATCTAAT
TTTTTTGATAGTAGTTTTAGGGGGTTGAAATGGACTATAATTTCTTCTACGATGGCAGAATCATT
TTCCGTGGCAGTAGAGGGAATAATTGTTTTCCCGATGGTTAAATGATTAAAGGAAATCGTATTTA
TTTTACCTAGTTCAACGGGACGAGAAAGGTAATTACTAATAGATTTTTCTAGTAAGGGATTTAGT
TTTTCTGTGGTGAAATGCCATCCGTAGCTTAATCCTCCTCCTACAGATAATAAAGTTAGGTAGGT
AAACCAGCGCCAAAGGTTTTTTTGATTCTGAGTGGCTTCTGCGGTTACTTTATTTTTCTTAGAGT
CTGATAGGGAATAGTCGGAGGAGTCAATCATCTCTTTTGCGGATAATGGTCATGTTTTTGTCTCT
AGCTTATCCTAATTATTCTAAGTTAAGAATTAAATGATCGTCCCTTCAAATAATTAATTTTTTTC
TAGTTATCCATCAATGATTTCAGATTAGCTAAAGCTGACCAACGTTTATCGTTTTTATTGTTATC
TGTGGATGTTTTGCCGGGGGGTTGACAATCATTGCTACATAAACTGCGTAAGGGAATGGCTAAAG
ATAGTTGTTGATAAATCCATTCTTCGATAATTAATTCTCCATCGGGGGGTAAACTTTCCCATAAA
TCTTCCCCTGTAATTTCTCTTTCGGGGGGATAATATTGATCTAATTCGGCTTCATATTTAAGATA
AATAATTTCTGAGGTATCGACTTCTAAACGATAATTAAAAGTCTGTAAACATCGATCGCACGTTA

ATGTAAGGATAGTATCTGCTTGTAATTGTAAGTCTAAGAAGTTGCCCCCATGACGTACTTGAAAT
ATTCCTTTTACGGGGGTAAGAGTGGTTAAGCCTGAGATGCGATCGCACAACTTGATTTCCATGGT
TTGCTCAGGCATTTTCAGCAGTTGTGGAATATAAATAGTTTCCATCAGTGAATAATGAATAGATT
TCAAAAGGGTAAAAGGCAAAAGGCAAGAGGCAAGGGGCAAAGGAAAAATTAAGAATTAAGAATT
AAGAATTAAAAACTCCGAACACCTGTACGGGTGAATAGCCATTAGCCCCTACTTCCCCCCATCCCC
CTATCCCTGACACCTGCAACCTGCAACCTGACACCTAATCTTATCTATCGTCTTAATTTTACGATC
AAACGGCGATCGGGTTCATTTCCTTCGCTTTCTGTGGCTAAATCTCCTGATTTTTCCAAAAAGCT
ATGGACTTGTCTTCTTTCTACCGATGATAAATAGCGTATTTCCTCTGGCATTCCCGTAATACGAA
CTCTATCGGCGGCCTTTTGTGCGATCGCCATTAGTTCAGCTTGACGGCGAATACGATAACCATTT
AACTCCACTGTGAAAAAACGTTCTTCCCCATCTAATACTCCTATATTTAACAAAACATTAGCTAA
ATACTGTAAAGCATCAATGGTTTCGCCTTTTTTTCCAATCAAATTTTCAATGACGGTGGGAGTTA
ATTTAGTTTCATCAATGGTTAGCCAACAGGAAATTGCTCTTTCTTCTTGTTCAACTCTACCAATG
GTAACACTGGCAGGAACACCCATTAAAGTTAGTAATGTTTCTAACCATTTTTTACCCCTTTGGAT
TTGTTCATCCATAATTTATCAATTAATTCCTACTACACTACTAAGAAGTTTTTTCTTTCTTTTTC
AGATTTTTTTTCTCAAAAGGAAGATTATCTCTGGTTTTATTGGCTTTTTCTCTTTCTGCCACAAT
TTTTTGCAAGTTCTCTGGCAAAGGTTCTCTCATGAGAATTAAAGTTTGAATCGTTTGGAAAACGT
TAGCAATAACGATATACATTAAAACCCCTGCAGGAAGGGGGAAAAATAAAAACATCCCAGAGAA
AATAACCGGTGTAATTTTATTCACCGTTTGTTGCTGTTCATTTCCGCCAGTATTTTGTCCGCCACC
AGATAATTCTTGGTTAAGATAAATACTGAAACCGAAAAAGATAACCATTCCGAGAATATCCCAGT
TAATCGTACCATCATCGCCGACAACTCCTACACGTCCGAGGGCTTTAATAAACAAAAACCCTTTA
TCGGCGGCAATACCGGGGATAGTTATTTGAATGGTTGCATCTCCAGTGGAAAGGGCTTGAATCTC
ACCATCTTCAGTCACCATGACTCTTTCTTGCCCTTTGGTAACTTCCCAACGGGGTTGTAAATGACT
GTCTGAATATTCGCTAACGATAGATTGTAATGATTTGCCTTCTGTGGTTTGCAATTCAATTTGTT
CTTTTTCCCCTACCACTAACTTGTTTCCAGCCGGTAGAATGGCAGAAATACGATTATGTACCCCGT
CATTGATGAACACGTTTTGAGCTTTTGTGGCAAAGGGTTGAGGTTGAATACGCTCGATTTGTTCT
TGGGGAAAGATTTGCACATCAACGGTATAGTTAATATCCGCAAATGGTGAACCTCTGAGGGTAGC
AAACAAAGCGAATAGGATGGGCATTTGTAACAGCAAGGGTAAACACCCCGCTAGAGGATTGCCTA
ATTCCTGCATTAATTTCCCCATTTCTTCTTGTTGTTTTTGGGGATTATCTTTATATTTTTTCTGAA
TCTCTGCCTGTCTTTCTTTCATCAAAGGCTGAGTGATACGCATTTTTCTCATATTGCGAATTTGTC
CTGCACTCAGAGGATAAACCGCAAACCTAACGACTAATGTTAGGGCAATAATTCCGAATCCATAA
CTAGGCACAATCCCATAGAAGAAGTCAAGGATTGGCAACATGATGTTTGTGGATATAAAACCTAT
ACCAAAATCCATTTTGATCTCGTTTTTTCTACTTTTACTTTATTTAATTTGATTGTCAGCAATTA
TTAACAAATACTCTAACGCTTTAAAATAATAGTTAATTATTGAATGTGTTTTGGGAAAAACATA
GTCTATTTTAAGTTATTGACGACATCACTTTGGATCTATTTTTTTCCAGAATGTCCCAGAATGTA
AATGATAAGTAAAAATTGCTGATAACTAATCTAATGGCTAAAGATGCAAAGCTCTATATCATTA
ATCCAAAGAAAAAGTAATGGTCAATGAATCTTATTTTGCGATCGCATCTATGGGTTTACCAGTTT
TATCTGCCGCTTTTTCGGCTATATAGTCATAAATTTCCCGAAAACGAGGTAATGCTCGCATTTCC
AAACGACTTTTATCTTTTAAAGTAACCACAATGTCACCCCAAAAACCTAAACCACGAGGCACTTT
TACCACTCGGACAATTTCCGAATAAATAATATCGGTGCGATCGCGTCCCATCCAACCACCAGTAA
CAGAGATACGACGGTCAGTAATACGATAACGCAACCAAATAGAACGTACGATCGCTCCCACCGTT
AAAGGTAAGCAAATAACAGTAAATGCCAATAGAATATTGATAATTAGATCCCCCACATGAGGACC
TCCTTCATAAAATACGTCTTCTCTAATACCCATTGATTATATTCGCCTTAATTAACAACTTCTCTA
ATTCTCTCAAAAAATGTTCATAATTGCATTCTTTAACTTCAGGTTTTACCACAATCACAATAAAC

CAAGGGGATGAAATCTGTGGCAACAAAGAGCGAAAAGCAACTCGAATATGCCTTTTGATTTGATT
ACGAACCACCGCCTTCTTACTCACTTTACGGCTAACACTAATTCCAATTTGGGTATAAACAGAAA
CAGACTCATCCACCCGTAACCCTCGGACAATAAAATAACGACTAGAGCGGCGAATACCCTGTTGA
TAAACAGATTGGAAATTTAAACGCCGTTTCAGTCGATGCTTTGATGGTAGTCCCACCTATATTTC
ACAGTAAATTTAATGATTAGTAATATCAAACTTAAAATGCTAAACTTAGCTTCCTTATTTAACAG
TAATTTTATACTGCTAATCTTTTTCTACCCTTATTACGACGAGCTTGAATTACTTTTCTACCATT
TTTGGTTCTCATACGGGCGCGGAAACCAGAGGTTCTTTTCTGCTTACGTCTAGTACCATTTAGTG
TATATTTACTCATAGTTTTTAAATATTTAGCTTATTTATTATAAAAAACAGCGTTTACTATTATA
AGTCACACTGGCTAACCCCGTCCAGATTTGCACTTTAATACAATTTTAGTTCAAAATGTCAAGGG
GTTCGGAATGTTGAGGGGATGAGGTGATGGGGGGAGAGGTGGCAGGTGTCAGGTTTAAGTGAGT
AATGAGTAACGAGTAACGAGTAATTATCAACTATTCACTATTCACTATTCACTATTAATTTGCAA
ATTACCCATCATGCCTAAGTCTTCATGATCTAAAATATGGCAATGATAAACAGTTTTACCTGTAA
AATCGTCAAACCGAATGCGAATCTTTACCCTTTCTCCTCGTTTGACTAACACCGTATCGCGCCAAG
CTAATAAATTTTCGGGAATATCATTACGGCTAATCACTTGAAATGAGTTAACGTGAATATGAAA
AGGGTGATCCATCATACCAGTGTTGATCAATTCCCATTCTTCTACGGTATTTAATTTTACTTCCG
TATCGATACGTTGATGGTTATAGGATTTGCCATTAATTAAGAATGCCATACCGTTAGCCATGCCA
TGATTAAGGGTAAAACGTCTTACCTGACTAGGTGAGGGCAAAGCCGTAATAGTATTTAACTGATT
TGGCAATGAGACAGATTGATTATTTTGATTAGACTGATTAAGGTTAATAGTCGCCAAAATTTGCG
AGGAGTTGTTATTTCTTCTTCCCATCATGCCACCCCCCATCATACCGTTACCCATCATACCCATTC
CCCCACGATTATAGGGTAAATTAATCAGTTTAAGAGATGATTGCTTATTCTCTCCTTTGATCATA
ATTTCCGCCCTTTGCCCCGGAGTTAATAGTAATCTTGTCATTTCTTGGGGAGATGCGATCGCACCT
CCATCGGTAGCAATTAAATAAAAGGGGTTATCATCGAGAGATAAGTTATAAAAACGAGAGGTAG
AAGCGTTTAAAATCCTTAATCTTAACCATTCTCCTTTATTAAGGCTAATTTGAGGCTTAATTTCT
CCATTGACTGTAATGACATCCCCTTCCCTACCCATCATTAGTGACATCATTCGAGAATCAGTTAAC
CTGCCGTTGTTGTCTAAGGGGAAATCTTGTAAAACGATAAATTCTTCTTTTGCTGAATTTATTTC
GGGAATATCGTCTAATTCTCCTTTAATAATAAATAATCCAGCTAATCCTCCTGATAACTGTTGTG
CTGTGTATCCGTGTAAATGAGGATGATACCAAGCCGTCATACTGGGGTGATTGTCGGGAATGGTA
AATTCATAGTTAAAGGTTTGATTTGGTTGGATTTCTAAAAAGGCATTATCTGCGTTACCTGTGGG
GGAAATATGTAAGCCGTGATAGTGTATATTTGTGGGTTGAGCTAGTTTATTCGTAAATTTAATTT
TAACTTTATCTCCTGCTTTTGCTTCTAAACGAGGAGCTGGAATTTGCCCATTATAAGTAAGTAAA
TAAGCAGTTTTTCCATCTATATTTACAGGAGTTTCTTGAGCGATTAATTCTACTTCTAATAAACC
ATTTTTACTTTTATATATTTGTGGTTTTAATTCATTAGCCCATAATGTATCTTGATTAGAATTTT
GCTGAAGGTTTTTAATTATTATTGCTGTGGTAGCAAATAGGGTTAAGTTTAGTAATTGACGACGA
TTAATTTTCATTTTATGATATTATGAATAATGGTATCGGCATTGTAAAAAAATAATTTTATCAG
AACTTATTTGATAGACTAATCTGTGTTCCTGAGTTATTCTTCTTGACCATGTTGAACCGGGTAAA
TATTTAAGATGTTCTGGTTTACCAATACCTTCAGTAGGATTTAATATAGTAGCTTCGATTAAATT
TAAAATTCTCTCACTCACTTTTTTATCTTTGCGAAACCACCAGCGTAAGTCATCCCTAAATTGACT
ATCAAAAATAACGGCCTTATTGCTCAATTTTCAATTCCTCACAAAGTTCAGATACGCTCTGTGAC
TCGATTTTAACACCTTCTAGTTCATTTGCACGCTCCATTGCCGCTAATAATTTTCTCGCATTAGCC
GGAGATTTAAGTAGATATAGGGTTTCCATCATACTAGACAGTTCTTGAGCTGACAACATTGCTAC
ATCTTCTTTCCCTGTTCTATTGACAATTACAATACCACGATCCTCGATGATTTCATCTAATATTG
AGGCTAGATTCTGGCGTAAATTGGTATAATTTGTTTGTTTGGTTAACATGACAATAAGTACATAA
ACTTGTACTTTATAATTCTATCTTAAAATTTTCTGGTTGTGCTACGGAAAAGGAAAAAGAGAAA

AGCAATAAGAATTTACGAAAAACTTATGCTTACAGTAGAAAAACTATCTTTAGTAATTTTTATAC
TGATTAAATACTGTAATATTTCCCTTATTATTGAAACTTAAGACAGAGAAGGGTTGTTTTATTTT
ATCAGATTCCATACCGGGGCTACCAATGGGCATCCCTGCAACAGCTAATCCTTTAAGATTACTGG
GTTTTTGGCTTAGAAAACGCTTAATATCATTGGCGGGAATATGCCCTTCCATCACATAACCGTTA
ATAATAGCAGTATGGCAAGATGCTAATTCAGGGGGTAAATTGTTTTTCCTCTTAATCGCCTCTAT
ATCATCGGTTTTTATGTCTGTCACCTCAAAGCCGTGTTTTTTCATGTGAGAAATCCATTGTCCAC
AACATCCACAAGATGGGCTACTATATACGGTAATCTTCTTACTGCCGTTATAGTTAACGGTGGAT
TTATCCCAAACACTGGCAGTTATAGCCTTATTTTCGTGATTTTCGTGGTTTTCATGGGCATAACT
AGGTATATTGAAGGAAAAAGCAATACTGCTAATAATAGTCATCGTTGCCATAGCTGTTAATTTTT
TTATTAATCTTGCTTTTGTCATGACTGTTTCTCTCTTTTTATCTTTACCTTTGATTCTGTCAAAAG
AATATGAAATTAGGATGAAATGATTTTCGGTAAATAAACGGTAAAAATACTGCCTTGATTTTCT
TGACTTTCTAAAATTATTCTACCACCATGATTAAGCGCGATCGCATTTGCAATAGCTAAACCTAA
TCCGCAACTGCCTTTCTCTCTATTTCTTGCTTTATTGATTCGATAAAAACGATTAAAAATTAATT
TTTGTTCTTCTTTATTAATACCAATTCCTGTATCAATTACTTTTATAATGACTTCTTTTTTTGTT
GTTTCTAAAAATATAGTAACTTGACCATTTTTTTTATTATAATTAATAGCATTAATAGTTAAATT
TGCTAATAATCTATATATTTGTTCTTCATTACCGAGAATAAAAACCTTACTTTCTTTTATTTCTT
TTGTTAAATTAACCTGATTCTTTAATGCTAAATAAGATAATTCTTCGGTTAAATCATTGATTAAA
TCAGCTAAATTGATTTTATTTTTTTTGATTTTATCCGTATTTTCTAAGCCTGAATCAAGGCGAGT
GAGGATTAATAAATCATTGACTAAATTAATCAGTCTATCATTTTGCCGATGAATAATTTTAAAAG
TTTCTTGACTGTCTTCTAAGGTTAAATTGTGACTTAAAAAAACACTATCAATAGTTGCTTTTATC
GCTGATAATGGGGTACGCAATTCATGGGCAACGTCAGAACCAAATTGTTGCATTTGTTGGTAAGA
TTGAGCTAAGGGTTGTATTGCTTTTTCTGCTAAATACCAACTGGCAATGATCACCAAAATAATCA
GTAAAGGTAAGCCTAATAATAACAACCATTTTATATTATTTACATATCGGTCAAAATCTTCTAAA
CTTCTGCCTATTTGCAAATAACCCCATTCTTTATTTTCTTTAGTGTGTAAAAGTAAGGATATTTG
ACGATAATTATTATTTTGATTATCTTGAATACTTGTGAATTCTTCTAAGGAATAATTAAGGGATA
AATTATCAATTTTAACTCCTGCATTACCTAGCAAATTACCTGATAAATCATAAAACTTTAGATAG
TATTTCCCCTGCTGAATTAGTCCTTTTATGTAACGATTTTCGTAAGATTGATTTCCACATTTTTC
ATCAATCAAGCACAAATCTAAGATGATTTCTTTGACATTTTTTTCTAGCTTATTAGGTTGAATCA
ATAAAGGTTCAAGGGTATCATGAAAGGTTCCAGCAACGGTCTTTAATTCTTGATTAATAGTTATA
TAATGAGCGTGTGCGATCGCTTCATAAACAAAAAAAGCACCTAGAGAAATAATGCCAGAAAAAA
CACTAGCATACCAACAAGTTAAAGATATTTAGTTTGATTAAATAACCTATTTATTTTCACTCAC
CATCCAATTTTAAACGATAACCTAAACCATAAACTGTTTCAATTAAATCACCATAACCATATTGA
TTAAGTTTCTTTCTTAGCAGACGAATTTGAGCGGCTACCACATTACTAATAGTATCAGATCCTAT
TTCCCAAAGTTGATCTAATATTTGATCTCTAGTTAAAATTTGTTGGGGATGACGCATAAAAAATT
CTAAGAGGTGAAATTCTTTTTTTGTCAAGGGAATGATCTTAATTTTATCTGTTTTTAATGTGATT
TGAATAGTGTTAGTTTGATAATCTAAAATCAAATTTTTGACTCTTATTTGTGATGGTTTAATCTC
CTGTGATCTTCTCAATAATGCCCTAATTCTCGCAAACAATTCTAACATATCAAAGGGTTTAATTA
AATAGTCATCTGCTCCTGCATCTAACCCTTGAATTTTATTTTCCATTGCATCTTTTGCCGTTAACA
TTAACACTGGTAAAGGGTTATTTTTTTTCCTAATTATCTTTAATAATTCAATGCCTGATAATTCA
GGTAACAACCAGTCAAAAACGCCTAGATTGTAGCTAATATTAGAATTTTCTAATAAATCTAATCC
TTCTTGTCCTGATTCTACCCAATCTACCACATAATTTTGTCCTAGAAGCGATCGCTTTATTGCTAT
TCCTAAATCTTTTTCATCTTCAATCAATAATATTCTCATATACCCCATATTAAGCTGTTTGCATCT
AAGTTTAATGGTTAGGGTAGTGAAAAGTTAAGCGTCTGAGTAATAACAAGGGGAGGCTATCCCCT

CACCCAATAGATATGTTGACGACTCTTACTATTAAATCAAAGATTGTAATGGGAGATTCTGAATT
ACAATTACTTGTAATTCCCTCCCCCTCTAATGTTTCCTGTTTCCTAACTTCACCAGTCCATTTTTG
GCTAGATACAAAATCTGAGTCTTATCTTACCATTTAACCTTTGTTGTTAGTAGAATTGTTTGACC
TGCAGTAAAAGCAGTTACCGCAAAAATACTCATTGCTGTTGCCACAAACTCTCCACCAGCGACAG
CCTCTAACTCTTCATCACTTAACTCTCCTGCCTCTTGTCGCTGAGAAAAATCTGCTTGGCGTTTTT
GAATTTCTGCCCAAAGTTCATCCGCAGTAATTTCATAACCCGATTCACTAGCGAATGTTGCAACA
TCTTCCCGATCATTTTCTGATGATTCGATTATATTCATTAATCTTTCTTGTAACTGTTCGTTTTGT
GACACAGCCTCAAAAAACTCAATCACGGCTTCTTGACTCATAGAAAAAATCTCCTAATTTATGTG
TTAAAAAATCGATAAACCATGAAACCAATGACACAAAAAAATAAATCTGTCAATTTAAAGATTA
TTAACAAATATCATTAAAAACGTTAAATAATTATCAATAATCTTATTTTTTATATGGCTAAATAC
TTGAAAATATATAATTTGTCTTTTTAAAAACCATATATTTATTTTAATTTAACAGGCTATTTTTT
ATTGTATTATTTAATTTGGAGCAAGTAGTAAATAGTCTTATCTTAAACGCTAAGACATAAAAAA
CCAAAAGCTATTAAGCTATACAAACAAGTTGACAAAGGTATATATGCAAAGTTTATAAACAGAT
TATGCCAACTTTAAAAACAGTTTACTCTAAATTATTTTTTCTTTTTCTCCCTAGATTTATAGTTT
AATTAGATGATAGATAGAATTATAAATCCTTAAGATTATAAGCTGATAAAAATAAATATTTTTT
TAATGTTTACTTAATTTTTATTTATAGATTAAATTGATTTATTTCTAGTTTTCAAAAACAGACTT
ACTACTTAAATCAACTAATACCCAATCTAAAAAATCCACGGGGGCGGTAAATTCTATTTCTGCTT
CGGGGGCTGGACTTTTATTAAGATCGATCGCAACTATGTTACCAATAGGAATATCAGGGGGGAAT
ATGCTACTTAAATTAGAAGTTGTCACCACGTCTCCCACTTTGACATCAGATACCTTTTCATAAAA
TTCCATCAAACCAATAGAAGTAGATTGCCCTTTAATGAATCCTTGATAACCCGTGCGAGGAAGGG
AAGCCCCAACACGACTATTGTAGTCACTGACTAATAATACCCGACTGGTATTACTTGTGACTTCA
CTAATTTTCCCCACTAATCCTCCCACACTCATCACCGCTTGATTTCTTTTCACACCATCATTACTA
CCCACATCAATAGTAATAATTTGCCACCAAGCATCTGGGCTTCTGCCAATAACTCTAGCGGGAAT
TAGATTTGCAGAGTATTTTTCCTGATAATTAACAATCTGTTTTAACTTTTGATTCTCACTTTCTA
AAGTTTCAATTTTATTACTTAATTCCTGTATAGTTCTTTGCTCATAAAGTGCTTGACGATCAAAA
CTTGGAGATGATAACCAAGAGGGTGACAAACGATATAACAATTCATTGAATAAAAAACCCTGAT
GACGATATATAAACCAAGTGAGGATTAAACTAATTAACCCCCAAATGACTTGTCTTCCGTATTTT
TGCCACCAACGATAAAAGAATTTCAAAATTGCAACCTATCATCCGCATTTATTCTTGATTAATTA
TTATCAATCCTTGACTATTTATTGTCCATAGCATTTTAAATCTTTTTATCTCGACGACGGGGAAT
GTCATAGCGGAAAAAATCGTAGGCTAAAATGGTTTCGGGAAAAATTGCCCTTGCTTCTGAGAGTA
AATCTTTCATTTGAATGGGATTACCCGGAGCATAACGAGGACTAAAATGAGTCATGATTAATTTT
TTCACCTGTGCCGCTAATGCTACTTGTGCCGCCATCGTGGTGGTAGAGTGCATCTTTTCAAATGCC
ATCGATGCGTCTTGATGGGCAAAGGTAGCCTCATGAATTAAAACATCTGCATCTTCCGCAAGTGC
GATCGCATTTTCACAAAAAACTGTATCTGTACAGTAAACAAATTTCCTTCCTATTTCTGTAGGAC
CGCAAAAGTCAACTCCTGAAAAGGTTTTACCGTCTTCGAGAGTGATGGTTTCTCCTGCTTTTAAT
TTGCCATAAAGAGGCCCAGAAGGAATACCTGCTTTTTTCGCTTTTTCTACATCAAAAATTCCTGC
TTTATTTTTTTCTGAAACCCGATAACCATAGGCTGTAACTCGATGTTTCAATAGTTCACAAGTAA
CGGTAAATTCTTCATCTTCATAGACTAAGCCCGGTTCAACGGTTTTTATGGTGACACCATAGGGG
AAATAAGTATAAGAATACTTCATACAGGATTTTAAATAAGGCTCAATCCCCGGAGGCCCAAAAAG
TTCAATATCTTTTGCATCCGCCCCCAAACCACAACTAGCCAACAAACCCATCAAACCAAAAATAT
GATCCCCGTGCATATGGGTGATAAAAATTTTTTTCAGTTGAGATGTTCTTAAATCACTACGCAAA
AGTTGATGTTGAGTACCTTCGCCACAATCAAATAACCAAATTTCTCCCCTTTGAGTTAGTCTTAG
GGCAACGCTAGAGACGTTTCTGGATTTAGTTGGTACACCTGAACTTGTACCTAAAAAAGTTACTT

CCACTATGATTGACAATGGTTGGTTTTGCTGATTCAGACCCAATTATAACTTAGTTCGGAATAAA
ATTTAATGGGTTTGCAAAAGTGCGATCGCATCTTCTAGGCAAACAGGAGGAGAGAATAAATATCC
TTGTCCGAAATTACAACCCAAATTAAGCAAAATATCTAATTGTTTTTCATTTTCAATACCTTCAG
CAACAGTCGTCATATTTAAACCGTGAGATAAATCAATTAAAGCACGACAAATTGTTTGATCTTTA
CAATTACTATCTAAGTTTTGTACAAAACATTTATCAATTTTTACCGTATCAAATTGGAAGTTTTT
AAGATAGGCTAAATTGGCATAACCAGTACCAAAATCATCTATAACTAAAGGTAAACCAAAATCTC
GACAAAATTCTACTAAAAAAAGTGCGTCTTCTCCTTCAAATAAACTTCTCTCTAAAATCTCCAGT
TTAATTTGACTAGGCTTGACCATTGATTGGTTAATTTTTTCTTTTAAAATTGGGAGGAAATTGCC
ATCAGAAATTTGTTTTTCAGCTACATTAATACTTAGAAAAATATCTTTACCTGTTATAGTTTTTA
TCTTTTTAATTGTGTCTAATGCCTGATCTAAAATCCATTCTCCAATGGGAATAATTAGAGAAGTT
GATTCGGCTAAAGGAATAAAAAGATCTGGGGCAATATGGCCTCTCTTATGGCAAAACCAGCGTAA
TAATACTTCAAACCCTGCAATTTGATTATTTTTTAAATTAATAATTGGTTGATAAAATAAAGTTA
ATTCTCCTCTTTTAAAACCATTGCGTAAATCACTTTCTAAACGTATTAAATCAATTGCCTGACGT
ATTTTTGATTGGTATTCTTGTTTTTTTTGTTCTATATCTTCTGTTGAAATATCTATTTTACTGTT
TCTTAATCTTCCCGTTTCTAAACGAAAATACTTCATTACCACCATTAACAATAACTTTAGAATGG
GGTCAGCATCATCAATTCTACTTCTTACCTGTTCACCCGTTACAACCGTTAAAACTACATCGGTTT
TTGCATAAGCAGAAGCAGAGCGAGGGCTTCCATCAACTAATGCTAATTCTCCAAACATTTCCCCT
TCTTTTAAAGTATTTAAAACTGTAGGACAATCATTAACTATCGTGGTAATCTCTACTAAACCTTT
TTCGATAATGTAAGCAATATTACCTTTATCTCCTTCTCGAAAAATACATTCCCCCGCTAGAAATC
GACGATTTTGTAATTTCTGTTCTGTATTACTATGGAGATTCATTGTTAATAGTTAATAGATTATT
TTTTGTTCTTCTTTAATTTAACATTGATTTTGTATCAAGATAATTTTTTTAGGTTTTGAAAATAA
AATTTACCCACTATAAATACAAATAGTAAATAAATAACTAAAGGGATAATATATACTATATCGT
GATAACCAAAAACTATTTTTATCAAAATAAAGGGAAATTTATCTTCTGGTAAAATTTCATGACTA
GACCATAATATTGAACCCAATAAACAAGAATTTTGCTCGAATAAACACCAATTTGCTGTAGTGTC
TAATTCATTGATAAATAAAAGACTTTCATCTAAGTTATATAAGCTATCAATGATTAATCCTCCTG
TAACCAAAATTAAAAATATTCCTGCAATTCTAAAAAAGACATGATTAGTTAATCTAGTTTGTAA
ATAAACAAAAGCAAGGGTCATCAAGACTGTTAAAAATAGAGATGCTATTACTGCCAAATTAACA
TAAGTAGAATTATTCTCAGGCATAACATTTAAAAGCAAAAAAATATTTATTCCTTTGATTAAAA
CTAAAAATAAAGCCAAAGAAAAAATAGTTAATCGTAAATTTTTTCCCATTAAAAGCTCATTTGTG
CTACCTAAATTTTGCCAAAAATTTGCCTGTTGAGAAAACCACAAGATAAACCAAATAAGTAAAGA
AATAGCGATAAAGACAAAAATATTAAGAATCAAGGGAGTGAAAATCCAAGCATTATTACTGGTT
GAATCAATATTATTAATTCCTTGAGAAATGACTGAGCCAACCAAAATAGAAGCCACTATCCCCCC
AGAAATTCCCAAATAAATTTGACGAAAATATCCTGAATGTTCTATTCTCTGAAAACACGCAAAAA
TAATGGCTAAAAGTAAAGAGATAGGAAAAATATTTCTCAGAATAATCACAAAAACAGGTAAAAA
AATATTCCAATACATAATATAACCCTAGTTCTATTTAAGAATATTGAATAAAGGTAGGTGTCAGG
TTTTAGGTGGCAGGTTTCAGGTGTCAGGGTGATGAGGGGAGAGGGTGAAGTAGGGGCGAATGGC
CATTCGCCCCTACAGGGTTTCAGGTTTTAGAAGAAAGTAATGAGTAACGAGTAATTATCAACTAT
TCACTATTCACTATTCACTATTTACCTTTGCCCTTTTTTATCTTAATTCCGAACTCAGAGTTAAGT
AGTATATTCAGCGTTGATTTTGACATAATCGTAGCTGAGATCACAACCCCATGCTTTACCGCTAC
TGCTACCATCACCCACGGAAACGGAAATTAAGACGGTATCATTCTTTAGATATTCTCCTGCGGCC
GCTTGTTTGAGATAATTACTAGCAGAAGTGCGATCGAAGTTTAAAGGTTGTCCTTTTTCCATTAA
GAGAAAATCCCCTAATTTAATCATTAAATCTTCTTGCTTAAATGGTACACTTGCTCTACCTGCGG
CGGCGGCGATTCTTCCCCAATTGGGGTCTCGTCCAAAAATAGCCGATTTGACAAGGGATGAACCA

ACAATGGTTTTAGCAATTTGACGGGCAGATGCTTCCGTAGATGCCCCTGTTACTTCTACTTCTAT
CAAACAAGTTGCTCCTTCTCCATCTCGTGCGATCGCTTTTGCTAAATACTGACATACTTCAGTGAG
CATGGCTTCTAGTTTTTGTCCATTTTCATCGAGACTGGTAATGGCAGGGGTGCGAGATTGACCAT
TGGCTAGGGCGATTAAACTATCATTAGTGCTAGTGTCACCATCAACGGTGATTTGATTAAAACTT
TTATCCGCCGCCCGTTTTAACATCTGTTGCCATAACTGGGTAGAAACTAACCCATCACAGGTAAT
AAATCCTAACATCGTTGCCATATTAGGATGAATCATTCCTGAACCTTTGGCAATACCTCCAATGC
GCACAGGGCGCTCATCTATCATCGTTTCTAAGGCAATGGATTTGGTTACTAAATCGGTGGTAACA
ATGGCTTTAGCGGTGGCTTCTCCTCCATCTTCAGATAATTCACTAACTAATTGAGGAATGGCGTT
TAACATGGCATCCATTTTGATTCTTTGCCCAATTACCCCCGTAGAAGCGAGTAAGATACTATTAG
CATCTATATTTAATTCCTTAGCTAAAGCCGAGGCACTATCTAAAGCATCTTGCCAACCTTGTTCTC
CTGTAGCCGCATTTGCTTGTCCTGCATTACACAAAATTGCTCTGGCACTAGCTTTGTCTTGAAGTC
TTTGGCGACAATAGTCCACACAAGCCGCTCTTACTTCTGAAGTCGTGAATACTCCTGATGCGATC
GCCTCTGTTTCTGACCAAATCAAGGCTAAATCAGGGGCATTAGAGGGTTTTAACCCTGCCGTAAT
TCCAGAAGATTTGAACCCTTTAGGAGCGGTTATACCCCCTTCAATCACTTTCCAATCTGCCATAA
GTTAATTGTTATCGTTGATTATTGAGAATTTTAGATAATTCTACATCGTTTTTTCTAATAATTAA
TTTTTTACAAATGATTTAGGAATGCTATCTTAAAGAATTAAACTCTGATTAAAATTAGGAGAAT
ACACTGATGAAAACTCAAGATAAATTAACCGCATTAGAAGATTATTTAAACTTAAATTATCCCAT
TACTTTTCATCCCGAAAATGAAGGAGGATATACTGTTATAATTCAAGATTTAGAAGGATGTATTT
CTGAGGGAAAGACTGTAGAAGAAGCGATGAAAAATATATTATCTGCTAAACAACCATGGTTAGA
AACTGCATGGAAACATGGTGATTTTATTCCTTTGCCTTCTCATTTTATTCTTGATAATTAGACTT
GCTTAATTTAATGAGAACCGAAATCTAAAAACTAAAATGCTTTCTCCAACAATTCGTTTTTTGAC
TAAAGTTGTTTTTCCCTTATTTTTGATTACCTTTATCGGATTTATCTTTGCTATACCTAGCATTA
GTCAGGAAGCAGAATCATCTTTACCCCCTTTTAAAACTTATCCTTTGCCGCAAACTTTACAAAAT
TGGCACTCAGACAACCAAGAAAACTATTTTTCCCAACTGGATACTCACCCGGCAGGAGCTTTGAT
TTGGACTGATTTTCCCGTCAAAGTTTACATTCAATCTCCTGCCGAAGATGATTTATCACCTTCGGC
CTTGCAATCATTTCAACAGTGGCAAAAAGCGGTAAAAGATGCGATCGCACCTTGGCAAGAATATA
TAGAAATAAGGCAGATAGACAATGCAGACATTGCCGACATAGTTATAACTCGTCAACCTCCTGCC
ATTAAAGCCGAAATCAACCCCGAAACAGGATTATATGATTTACCCCGTAATCGAGCGGCCACAAC
AAGCGTTAGGTTTTATCTAACAGAGGATAATCCTCCCTTATTAAAACACAAAATGGCGATCGCAG
TTAATCCGAATCAAATTTTTGAGTATTTAGTCAGTAATATTAGTCACGAAATGGGACACGCCCTA
GGTATTTGGGGACATAGCGACAATCCACAGGATATAATGTATTATGCTCATACCAGAGAAATCCC
CCAAATTTCACCCAGAGATATAAACACCCTTAAAAAAGTTTACCAACAACCAACCCGTTTAGGGA
ATTATTTAACCTCAGTTCGGTTTAAGAATATCGGAAAAGGTTAGGTGTCAGGTGTCAGGTGTCAG
GTTGCAGGTGTTAGGCTGTTGGGGTGATGAGGTGATGAGGTGATGAGGGGAGAGGGAGAAGTAG
GGGCGAATGGCCATTCGCCCGTACAGGGTTTCAGGTTTCAGGTTTTAGAAGAAAGTAATGAGTAA
CGAGTAACGAGTAATTATCAACTATTCACCCCAACACTATTCACCTTTGCTCCTTATTTTGAGCG
AGTAATATCAATAGTAATTGAACCTTCAAAGTCGGGAATAGGGGGATTTGGCTTAGATAGTTTT
ACCCTAACTTGATTTACCTTTTCCATCTCCAATAAATCTTGAGCAATAACCTCGGCTAGTTTTTCT
ACCAACGCAAACTTAGCGGTTTTGATAATATGCTTAACAATGGTGATGGCTTGACGATAATCTAA
TGTATCTTTTATGTCATCGCTTTTTGCAGAAGGTTGTAAATCCACCCATAGAGTTAAATCTACCT
CAAACCATTGTCCTAAACTTCTTTCTTCGGGAAATAACCCTGTATAACCATAACTGCGAATACCT
TTAACTTCGATCGCATCCATACTTTATCTAAATCATAAAAAATGTGTCCTATTATACTTTAGCAC
CGAAAATCACCTAATTTATCTTAATTAAGAGGTTAAATCAAAAGATGGTATTAAAACTAATAAA

TCAGTGATCCCAAGCTCAAGATTTAGTTGTGATAATAAAGTACTAACTTGTCCTCGATGATGGGT
TTGATGATTGAAAAAATGTAGAATAATATAAGAAAATTTTTTATTAAAATGTTCTCCTTTCATA
TTATTGAAACTGAAACTATTTTCTAAGTCTTCCTCTGTAATTTCTAAAATAAAGTCCTTGATTAT
TTGATCAATTTTCTCCCGCTCTAACGTTAAATCCTCAATGCGAGCGTATATTATTTGATTTAGGG
AATGAGGTCTTTTAATACTTCTGATATAGTCAAGACTAGAATAATTTTTAGGATGGTCAGAAAA
TCTTTTTAGCCAAATAATATCAGCTACTAAAATATGATTTAAAGTGCCAATAATAGAAAGAAAA
AAAGCTCCTAAATTTTGATGTAGTTTTTCATGAGGTATTTGCTTACATATGCTGTAAATTTTTTC
ATTCATCCAACTGTTATATTCAGCCATTAATAAATAGTTTTGTTTTTGATTCATGATAATTATTT
ATATTTGAATATCTAAGATATAATTTTAATTTGATTTATTATAAACCATCATATTCACTTTTTGT
AATTCTTCTTTAATCAATAACTCAGAAGATTTAATAACAATGATTTTTTCATCTTCTTTTATATT
TTTAATTGGGACATATATTCTTCTATAATTAATTCTAAAACTTTGTCTCACCAATTATGTTTTCA
TTAAATTTAGATAAAATTGAAATATAAGAATTTAAATATTTGTCGAAAATATTAAGAATTGATT
AAAAAATATTTCACAAACAAAACACTCTCGTTATCGTTTACCAGTTAATTTTTGTACTAATCTTT
CCTCTCCTTCTCCACTCATTACTCATTTTCCGTCTTCCCTTTTCCCTACCCTAACCAGTAAACTTT
AAGATGTACTCCTAGCTTACTTAAAAATTAAGAAAGCCACAATTAATAATCTCGATGAAAGAATT
ACAATCTAGTCTTGTTATATCTTGCCAAGCTCCTAGCAATTCGCCTTTACACAATCCCGATATTAT
TGCTAATATAGCCTTAGCTTGTGTAAATCAGGGAGCAAAAGGATTACGTATTGATAGCCCCAATC
ACATCAAAGCAGTACGAAAATTATTACCTGATATTCCCATTATCGGTTTATGGAAACAAATGGGA
TTAGATTCTGATGTCTATATTACTCCTCGTTTAGAAGATGCGATCGCAGTTGCGGAGGCAGGGGC
TGATATAATTGCTATCGATGCTACCCAAAGAAAACGCCCTAATGGGGAAACACTGGAAGAAATAA
TTTCCCATATCCAACAAAATTTGCATAAGTTAGTAATGGCGGATATTGACACCAAAGAAAATGCG
ATTATCGCCAGAGATTTGGGAGTGGATTTTATTGGTACAACTCTTTATGGATATACCCAAGACAC
AAGACAATTTACTCCTCCTAATTTTGATTTAATAGAAGAATTAGTCAGGGAAATTAATACACCTA
TTATTTGTGAGGGGGGAATTAAAACCCCAGAAGAAGCTAAAAAAGCTCTTGATAAGGGCTGTTTT
TGTGTCGTTGTTGGTACCGCAATTACAGGTATTGATTTATTAGCTCAAAATTTTGTTAAAGCCCT
GTAGTAGCTTTTTATTTCCGTTAGACTTTCACTAAATGCGATCGCAATATAAATAATTAGTTTAA
CTTAATTGAATTTCTCCTGATTCGGCTAACCGCCAATTATTATTATTTTCTTGAACAACTGCAGG
TTTAATAATATGAATTTCATTACCCTTAAAACCTTGAGACATATTTCCTTTACAAATAAATAATT
GAGATTTATTAATATTCTGTAAAGTAGGCACATTAAACTTAGTATCTGGGTCTAAAAATAGATA
AAATTGACCACTATTATTATTAATAATGTAAAATTCTCCTTGACGATTATTTTCTTCTAATTCAA
CAATACCTTCCCATGTACCTGCTAAGATTTTGTTTAAGGTTTCCTTCGTCATGCTTACTCTAATGG
CATTTTGAACTAAATTATGGGGATTTTCTTGATAAGATCGAGCTAAACGTAGATATTCAGGTTCA
GGTTCAGACTTAGTTTGTTCTTCTAATTCAGGCTTTAATTCATTATTTTCATCAGTGATAATCTC
ATTTTGCGGTTGTGTCTGATTACTACTATATTGCTTTTCAAGAGGATTAACTTTTAATAAAATAT
TATTTATTTGAGATGTCAGATTTTCTATTTCTTTTTGCAAAGAATCATTCTTATTTTTTAAATTT
TTAATCTGAGCATCTTGCTTAGTAACTTGTTTACTTTGATTCTGATTTCTCTCGGATAATTCTTC
TATTTTATTTTCTGTTTTCTCAATATGATTATATAATTTGTGATTTTCTTTTTCTATTTTTTTGT
TTAGCAAGTAAATCCCAATAAATGCTCCAATTAATCCTAAGGCAATAATAAGATTAACAATCCAA
AGAATTAAAAAACTACTATTATCTTCTTGAGATGAATTATTTTGTCCTGAAGTTTCCTCTGAATT
ATTACCGAAACGAGGTAGCTTTTCTACGGTTTTTATTTCTTTATCAATCGCTTCTATTTGAGTAT
TAATAAATCCTGATAATTTGCCATAGGTTTTATTATTAAAATTGCCACAATTAGCATCATTCTTA
GACAAAAAAAGCCCTAGAGGTATTTGTATGCTTTCACAAATTTCTGTTTTATCTAATCGTCCATC
TTCTAATAATTTGGTAAATGCTTCTACACTTAATCCCTTTTCCTTTTCTGTTTGTAGTGGTGATA

ATAAATTATTTAATTCTCTTTTTTCTTCTTCTGATAATTGATTTTTTAAATTGTTAACACTGGCG
ACTAATTTATCATAGTTTTCTTTGATTTTAGTATCTATTCCTTCAATATCTGTAGTAGGTGGGTC
TTTTTTACTTTCTTCCTCCAATTTTTTTAATAGGAATTTATTATCATTTTCAAAATTATCATAAC
TGTTTTCTATATCTGTTAGTAAGCCTTGAAAATTAGGAGATGAGGAGATATTTTTAGATTCATTC
TGAGCAAAAACGGGGTAGCTAAAATTAGTAATAAGACTAGGAATAAGAGAGGATAAAATTATTG
ATTTAAGGATTTGATTTTTCATGGTTCACCTCTTGATTATAATAATTAAAATTGTAAGACTCCTT
TTTCTGTTAACTGCCATTGGTTGTTAATTTCCATTACTTTAGCAGTTTTTAATACTCGAAAATTT
TTTGATACATTATCTTGATAATTATTTAATTCAAAAATAGTTTTAATTGCTTTTAAATTGGTGTT
TATTTTCAGGTTTAAGTCTGGTAGCAAACAATATCCTCCACTTTTTAATTCCACTATCCAATAGT
CACTTTGGTGAGACTCTTGAAAAACTATTTGATTAGCTTTGTTAAGATAAATGTCTTCTAAGGTT
TTTTTTGTAGCATTTACTTTATAAGCATATTGTTCTAAAAATTCGGGATTATTATAATATTGTTC
GAGAATATATTGTTCATATTGATTAATTTCTATATTGGGTATTTTTTCTAAATTAGGAGATTTTT
CTGGGTAGTTTTCTTTATTTTCGATAAATTCTGTTTCAATAGTATAGGAAGAATTGTCAAATTGA
TTGTCTTGATTGCTGATATTATTTATCGGTAATTTCTTCAATGATTCTTGGATAATTTCTCTAAT
CTCTGCTTTTACTTCGGAAATTATTTCTTGTTTTAATTCCTCTTTGATGTTTGTTGATAAAGGCT
GTTCTGGCGATAAGTTTTCTAGTTTTCTTTCTATGTCTGAAAGTCTTAATTTTATCTCAAATAAT
TCTTGTGTAAAGTTTGGTGATTCTTCCATTTTTTTTCTTAACTCATTTCTCTCTTTACGGTATTGT
CTAATTTTACTAAAAGCTATTCTTGCCCATTTTTCTGCTGTTTTAATTAGTTTCTCTGGTGATTG
TTTTTTATAGTTTAAGTAAATTCCTAAATCCCAATAACATACTGTACATTTTTCCTGATTAATTT
GATATTGTGTGCCGCAGATGGGACATTTATTTTCGTGTTTAGAAAACATGGTTACATAGTAATTT
TTATAATTTTGAATTAGGAAAATTTTGTTTGCCTTTGCCCCTAAACTTATCTTTAATTTTTTTAA
TCTAAAATTTGTCTAAATCGGATATTTAGGGATAACTTAATTTTTAAGTTTCTGTTCATAAGTTG
ATTTTTTCAATTCCTTTAATCTCCCTTTCCCTTGAATAAAGTATTTTGATCCCCCCTAAACCCCCC
TTAATAAGGGGGGAATTTAATGATGGGATTGTTGGTTAGTCTCGGTATGCGTCTGCCCATCTTCT
GCCTAAGACGTGGAGTAAGGATTTTAAGCCCATGATAAAGGGGGTTTTTTCCCGTATCATTTTGG
CATCTCCTTTGATGTCCAGGAGTTCGTTTTCTAGGTGATATTTGACTTCTTTCCAGAGTTGGGCG
TTTTTGTCTTCGCTTTCTTCTGAGTTTTCGCCGTTGGATTCGTTGGGTTTTTGCGGTTTGTAATCG
GATTTGGGTAAGGGTTGGATGGTTTGAATTTTGAGAGACTGTAATCCTGCATGAAATTCATAGA
GGAATTTTTCGAGGTGGGTTAATTCGGGGATGTCGAATTTGGTGATTGTACCTTCAAGGTGAAGT
CTCGATTGCCAGTCTTTTTCTTCTTTGAATATTTGATCGTCGTCTTGGTATTCAACGTAATATTT
TTCCCCTGCGATCGGATCTATTTCGGTATTGTCTGTAAGCCCTGTTAATTCAGCACCATTGAGAAC
TAAACCACAAGCTACCTCATGTTTTGGTCTTTCACTCAGAACGGTTTGTTCTTGGGTATCGTTAA
ATTCTGAGGCTTTACTTAGGATATAACTTAAGAGTTTATTAAAGTCAGCGTTTCTGTCGTATTTT
CCTGTAGGGGATAACCAGTGGAAGAAACGAGAGGCATTACCGCCAACATAAACGGGGGTTATTTG
GGCTTGGCTATATTTACCTTCTTGATGAAGGGTTTTGAGAATTAAGCCAACATAGTAGTACAGTC
CTGCAACGCCGATCGCAGTTATGGTTATTAGCCTTTGGAAATAGGGATCTTCGTTTTTGTAAACT
CGTTTATTATTGAGCCATTCTTGGGCTTTGTGACGAAGACAAACATCTATTTTTGTCATGATAGA
CATTTCGCTCGTGCTTCCCCAATTACCACTGATAAATTCCTGCATCAATTTTTCAGCATAGCGAGG
ATTTTGTGCTAAAACTTCGCTGAAAATGTTTCTTCCTGCCAATAAAACAGAACATTGATAAACAA
TGCGACGATTTTCCCAGATGGATATATCGGAAGTACCGCCACCAATATCAATACAAGTAGTACGC
ACAAGGGGATGTCTTTCTTTATCGTTGAAATATTGGGCTAAAGCGACACTTTCGGTGCAAAAACG
TTGGTTATCGTCTATTTGGGGAGTGGAATAGTCAATGCCTGTTTTCGGTTGTAACTCGGTGGCGA
TGTCTTGCCAGTTACGATGGAAGGTCATTTTATTAATGTAGGAAAAGGCACTGGGATAAGAAACA

CACCATTGAATAGCGTCCATCCCTTCAGAAGCTGCGATCGCGCTTATGTGCAGGGCAAGATGACG
TAAAAATGCCTTAGAATCTTTGATGTTAGACCATTTCAAATCTAAGCGAATCCATTGTTTATTGG
GGTCAAATTCCGCAGGATCAGGAATATATATACGCCCGTCTAAAATTGGTTGCAATTCATCAACA
TTGCGATCGCTATTTTTGGTGGTTAAAACCGTACATAAGGGCAAAGGTTCTTCCGATGGTAAAAA
TGTATCAGGGATAAAATTTTCAATGAGGGCAGGTAAACGAGTACCGAGAGGACTTTCAGTCACAT
TAAGTTGTAAATCCCTGTTGATGTCTAATAATGAATCTCCTGTATTATCGCCTCTAACATAAACA
TTAGTAAAAGAAGTTCCAAAGTCAACCCCCACTCGCCATGTGCCTCTGGAATTTATTTCGGGTAC
AGTCGGCAGTAAAATTAAACCTATATCATGGCCATAGGAATTTTTACAAATCACATGGGTGGGCA
TTTCCTCTAGGGTAGAGAGTTGGTAAGCACCCGTACCGTTAGGATCTTTGAAACTGCGTGGTTGT
TGGGTAGGATTGGGGAAATAGACTTGGAAAGTTTCGTCTCCATAGTCGCCATCGTAATAAAAAGT
ATAATAATTTTTCCAGTTTTTCGCACGGAAATTAGGCCATACCGTTAATACTGGCACATATTTAA
TGGCGTTTTCTTCCTTGAGGATATAATCTTTTTGCAGACGGAGACTGTTAAATTGCCTCTGATTA
CCCTCTCCGATGCCCGATAAGGGGATAGTTAAGGTTAAGCGAATCCCCTGCCCGTTAACATTCAC
AAAAGGCTCTAATTCGATGTTTTTGAGTAAATCTTCAGGAGTAAAATAGTCTAATAAAATTTCAT
TAAGAGGTAACAGAGGAGAGATTTTATTTCCGTTAAAAGTAATCGGTAATTGAGACTTAGGTGC
GATCGCACCGGGGAAAGCCCCTTCTAAATCTACAAAGGCTAATTCCTCTAAAAATAAATCTTGAG
ATGCGATCCAGTTCACATCATCCCAAACTAAAGTACCATTTTTGAGATCATTAATATTGAGAGAA
GCGAGGGTTTTGTCACGATGTATCCAGATATTTTGCGGTGCAACTCCCCAATATTCAGAAATGTT
TTGATCAATTATGATTAAAGGTTTTTGAGCGGTTTTAACGGCACTGGGAATCACTCTAACGTTAG
AAGGTTTCGGAGGAATTTTTAGGGGTTGATTGAGTAAAATTAACGCCCCTCGGTTGATGGGTTCA
CCAAAATAAGTCGGGTTACTGCTTAATTGTAAGGGGTTTAAGCCTTGTTCATCTAATTGTAAATC
GTTGCGGAATTGGGTAATTAGACGAATCATGCTATTTAAAGCAGTGGGCGAACCTTGATAGTTAA
CTATTTGTGCCTGTAAGTTCTCTAACCAACGCCATAATAACTCTTTTGATTCTTGGTTTAAATAA
GGCTCAGGACTAACTAACATTCCATTTTGATACCATCGTAATCCATGCCAGTTACCTTCCTCTGAT
GGGCATACAAGAGTGCTAGGGCTACTCATACCAACGGGTTTACCCTTCCACAAAAATACATAAAT
TTCTTCCCAAGGGTTTTTATTATCTCTGGTGTAAAGGGCGTTAACGGGTTCGGGCATCAGTTGAT
ACAAAGCATCCCCAAAGGGTTGATAACGATATTGGGATAAGTCTAAAAACTGAGCTTTTAAATCA
AATCCCTCTACCTCCGCCAGTGCGATCGCAGCTATCATTCCCCGCCATTGATCCACCATTACCTTA
TGAATGGGGTGTTTGGTATCATAAAGAGCCATTTCTGTAATCAGAGGTCTAGCCCACATCGTAGG
AACAGAGCTAACGCTTTTCATCGCCCCCGGTGCTTGATAATCAAGGCTATCGGCAACATCACGAA
ACGCCACATCGCCTTTACTATCCCATTTACCGGGTTGTCCTCTGGTGATGGTAGTATCTTCTTTTA
GTTTTGATAAAAGTAAATCAGCCATAGTTGTTAGTTAATAATTTCTGAATAGTTGGGTTAGGGA
GTTTGGAAAGAGGTGTCAGGTGTCAGGTTTCAGGTGTCGGGTTTTAGAAGAAAGTAATGAGTAA
CGAGTAACGAGTAATTATCAACTATTCACTTTTGTCTTCCCCCCTCAAGAGGGGGGAAAAAGGGG
GGTTTGCCCTTTTCTCCATCACTCATAGATGAAAATTCATCCCGAATCTAGGTTAATTCTTCATTC
TTCATTTTTAATTTTTAATTTTTCATTTTTAATTCATTCACACTTTGCAACAGACATAAACCGCT
TTTGCTAATCCTGTCACTCCCTGATTCGGTTCGCCAAAATCACGGGTATCTAATTCCCCTATTTTA
TTTTTGAGGGTGATAGCGTTATCATCTTGCAATAGTTCTTTGGAGCGAGAATCATCCCACAATAA
ATCTGAGAATTTTTCCCCTTTCAAACTCTTAGGATTACCGAAATATTCTCTTGCTTTGAACAGTT
GTACCCTTTCGTTATCGCAGTAGTGAATATCTTTTAACCATCGCAAAAAGTCCTCACACCAAGCG
GTAACGCTTTTAATGGCATCCTGTTGATCAGATTCACTCAAATCTGGTAAATGTGGTCGAGTTCT
TCTGCCAAACATTCCCACTCCTGAAAAGAATTTATCGAACCATGCTAAACCGTTTAATTCTTTAA
TACCAGCAGTTTTCGCTTTCTGTAATTGATCTTCAAAATCCGCTAACCAGATAAAGGCAAAACGG

GCGGCGTTGACTAGCTTGGCTTTCACTTCTTCTACTTGGGGTATATCATCCCACTGTAAAATCCCC
GCTTCATTGCGACTCATAACGTGTACCCCTTCGGGGGGATTATCACAGAAAAAATGACGGGCGGC
TAAACCTGCGTAAAATTCCACAAAGTGAGGGCGATTAACTTGGGTGTCTTTTCCCACGCTAAAAT
CCCCTAGTTTTTCCATACTCTGATTACCGATGAGATATTTAGTGTCAAATTGGTTAGCTTGTTCT
TTGTAATATCTCAAAGCGGCTTCGGTGTTGAGTAAAAAGAGATCGGAACTGGCGTAAACTTGTTC
ATTTGTATTACTATTTTTAGGAAAATTAAAATAGGGTAAGCCAAAAATACAACCGATTTTCACCC
TATCTCTTACCCCGTCTTTTTCTAGTTTATTGGCAATTAATCGTCCAATGGTGGGTAAGCCTGAT
GCGCCTGTACCGCCAAAAATTGATCCACAGAGTAAGACTTTGGTTTGAGAACCGCCCCCCGCATT
ATTTTCGATTTCGTTGATCAAACTGCGCCAAGGCTCTTGTTCAAGGCTTTCGAGGTTAATTTGGG
TCATAATACTAGATCCGATCGCAGGTCTGCCCCTAAAGCCCACTTCTAGGGATACTTGTCTTTCGT
CTTCGGTGTAAAGTACATCGAATAGGTTGCCCAATGGGGGAGAAGTCTGTTTTAAGGTTTCGTAG
GAGAAAAATGACCCTAGTTTGCGATCGGCGTTGTTGTTGGTGAAAGGTGACCAAACTCCATAAGA
TTCAAGGTTTGTGGTCATCCATGCACATTCTTGATGTTTCCCTTTAAGTAGTTGAAAACATCTTT
GATAAATACTAACCGTACGCTGCGATCGCTGTAGATTACCGTTAGTTTCATCTGCATCTATATAT
AAAACTTTTAACATATTGGTAGGAATTAAACCCACAGAAGCCAAATGTGCGATCGCTTCTATACA
TTTCGCCCCCGTGCCACCAATGCCGATAACATAAAAACTCATAAAATTTTCCTCAATATGAACAT
CAATTTCTATCGATTAAATCGTTTACGAGAATAGCAAAAGGAACTGTTTGGCGTCGTAATAATCC
GGGGGTACTCGTAACAGTGGGCAACCAATATAATAGGAAAAGAGAGTCAAACAAGAATAAACCC
GTCATAGAAACTACTGTTTCTTGAATTAACTCTCCATTCTCATCTTGATTATTAAAGTACATCGC
CAAAAATACTACTACCGTCAAAACAAATAAACCAATATACCAAAGTAGTTTAAAGGAACTGGCAC
TTCCCCCCTGATTCTTCAGTAAAATGATGTACCATAAAACCGTAAATAGTACAGAAAAGCCAAAC
ACAATCCAAGCGGCGACATAATATTGTTGAATCCAATCACGGATAGGAATACTTTTAATTACACC
AAAAAATCTTTGCTCATAGAGAAAGGGTTGCAACCACATCATCACAAAGAAACCCACCAAAGCAA
AGACAATAATCCTAATAATTTCTCCGACTTTCATCGATTAACAAACCATTAATTAAGTGAATGTA
AATATAAAAACTCATTAGGTGTATTTTACACTCATTCAATCTATAAGACTTATTAAGTTTTCTTT
CATATCTAGGAAAAAATTTACAGTAATTCAAAAACTTGATCATAATTTTTTCCAAATTCAAATCG
GATTGCTATGACTTATACTTAAAATTGACATCGATAATAGAACAAAGAATTCATGGGCAACCTGC
GTGTAAAAACTATCAAATATCTTATCCCCCTGACTCTATCTCTATCTTTTCTCATTGGTTGTCAA
AAAAAACCTGTAGAAGAAATATCCTGTGCAATGCCAGAATGGGCGAGTGAAAATTCTAACTATGT
TAGCAACCCCACACTAGACATAGCAGTTCACGTTGACGGTAGTGATTCCATGTTAGGTTATGTCA
CGATTCCCAATAGCAACTATACCAGAACCATTGAAGTTTTATCGAATAGTATTATCGGTACTAGC
AATGTAAATGTAACATATCATCGCATCGGGGACGAAAAAAATATCACCAGAGATGATTTCCGTAA
AATTGCGAAGTCAACCACTTTCTATGATAGCAATGACGGTACTTATAAACCCGTATCTAGTCCTA
TTCAATCCGCTATTACTCCTCCCCCACAGGGTAAAGAAAAATTAACGGTTATTGTTACAGATTTA
GAAGGGGATGACGCTGGTAGAATTGCAGAAGCCTTAAGTAAGAACTATCTCAATGAAGGAAGAA
AAAACTACACCATTGGAGTCTGGGCAGTAAAAAGTCAGTTTAATGGTACAGTCTTTAATCCCAAT
ACAGGCAGTGCTAAATTTATTTATAATACCCAAGGTAAATCTTCCGAAGCCTATCGACCTTTTTA
TGTTTTGTTCATAGGAGAATATGATAAAATAAACGATTCTTTTAATGAGATAAAAAAACTAGAT
TCTCAACTTCAAAGCTACAGTGAAATGTTTATTTTTCCTAGTCAAAATATTCTCAAGGAAGCAGT
CAATTTAGGGAATTTTTCAACCATAGAACGCAATTCTGTTTTGCCAGAAACAGGAGATTTACAAA
GAATTTATAGCCTTAATGATGATAATGTGGTAGTGATTCCTAAAGATCAAAATCAAGATACTTA
TGACTTAATAGAAGTGGTTAACCCCACTGAAATGCAACCTAAAATTAACTACTCTGTTGCTTTTC
CCCAGTTAGCTACGAATGATAATAATAATTATTCTTTATTTATTAATGAAAATAATTTGATCACT

CAAACTAAAGTCTTTACCTATGGTAAAAACAATCAGAATATAGAAGAACCTGAATTAGAAGAAA
AAGAAGAAGAAACTAACGCAGAATCAACAGAAGCTAAAGAAGAATCCCAAAAATCAACGGCTAA
TAATCAAGAAAATAATACTCCTTTAGAAAATACTCAAGAAAAACAAAACTTTATCGAAAACTCT
AGTAACACTTTGCGTCAAGCCTTAATAATTGATAATCTGAAACTGGATGAACAAAAACAAAATTT
AGAGTTTGTTACTAATCTCAATCTTAATAATTTATCTAATCCACAAATTTATTTATTTGAAGTTG
ATTTAATATTAGATGATGTAACCAGTTTAGACTGGTGGAATGATTGGAGTGTAAATGCCGGAGA
ATTAGGCGATGGCTCAAAAACTCAGAATTTATCTATTTTTATGAATAAGTTAAAAAGTTTAAGTC
TGGAAACACTAGAAAATGAAAATGGTGATGCAGTAATTGGACGTTTTTGTATTGGAATTCAAAA
AAATTAAAATTAGATTAGTTCGGGGTTCGGAGTTTTTTAATTCTTCATTTTAACTGAAGTAAATA
AGATTATGACTAATTCAGAATCAGACATTCAAGAAATTATTAAATATGTACAAAAATTAGAAAA
AAGAGTAAAAAAACTTGAATCAAAAGATGCAATTATCTTAAAATCTGTTCCCAAGGATATACAA
CCAAAACCGTTAAAAATTTCCCCAGAAAAATTAATAGATATTTATAATGATGTTCCTTCTATTTT
AGAAGAGTATGTAGTGGAAGTTTCTATCACCGCACAAAGTTGGCGATCGCACTCTGAGGATAAGA
CAATAGTAGAAAAGAGTATTGGCGGTAATTACTGGATAATTTTATTAGAAAATTTAGATGAGAA
AAAATATTATTTGTTACCCAATCAGAGCAAAAAATTAAGAATAAGTAGATTAAGAAGTCTGAAC
AATATGTTTAAAATACAAGGAGAGAAAGACACAAATAATGATAACTATATAATAATTAAACCAG
CCCTATTAAATATGATTCCCAGTGGAAAAGAATGGGAAATAATCGAAAAAGGAATACTTTATTT
AGGCAAAACATCCCCCACTCAAAAGTTAACCACAGAATTAGAAAAGTTAACAGAAAATCAAGAA
ACAATACCTTCAAGTCTTCAAGGCTTATTAAACGTTTTAGAAAAACTAAATAAAAATAATAATG
AGTTAAAAATAAAACTAAATTCTTTAGAAAAACGTATTCAAAAACTAGAACCTGAAGAAATAAA
ATGGGTAGAATTATATCATCTTGAACCAGACAAATTTGCTACTTTGGGTGGAGGAAATACTAAAT
TAAAACTCACTGAAACAACTGTTAATAAACTTCTACAAAGCTCTACTAAAGATATTTATTTAGAG
GCTGATTCTCTCGGTCAATATTTATTAAAGCAAGGAGAAAAAGGATTATATTTATTTCCTGATCC
TTTAGGAATATTTGATAAAAATAGTTTAATTTTAACCCGTTTAGCGAATTTATTTATAACTCATG
GAGAAATTCCGATCGCATCTTTAGGGAAAGATATAAAAGTAATAAAACCTGCAAAATTGAAAAA
AATGAATAATAATCAATGGAGCTTATTAGAATCAGGAGAAATTAATTTTTAGTTTTAGTAAAGC
AATTCAAATATTTTAAAAGATTTTTTCATGAAAACAGCTAGTACATTAAAACTAATACTCTTGCC
TTCTTTTCCTAAAACTCTGATCAAAGTAAAAATTCTGCAAAAACCGTCTTAAGTTTTTATAAAAT
AAATATGTTAACTATAAATAATCTTAAATTTATGACTACTCAAACGATTGATTTAACCGAGATGA
TTTTAACGAAAATAAAAGATTTACCCTTAGAGCAACAACAAGAAATTTTAGATTTTACTGAATTT
ATTGCTCATAAATATACCAAAAATTCAGAGGAAAAAAATAAATCGAATGGAAAAAGAGTTGCAG
GTTTATTTAAGGATAAAGGTTGGATAAGTGATGATTTTAATGATCCTTTACCACCTGAAATATTA
GGGGATATTATATGAAAATATTGCTAGATACTCATGCCTTTATTTGGTGGAATTTAACTCCTGAA
AAATTATCTTTAAATGGATTAAACTTAATAGAAGATAAAAAAAATATTATCTACTTGAGTATTG
CTAGTGTGTGGGAAATGCAGATTAAAATATCTATCAATAAACTACATTTTGATAATCCATTGTCT
CAAATAATTACTAAGCAGAGAAATATTAATAATTTTCAAATTTTGTCCATAGAATTAGAGCATA
TTTGGGAGTTAAAAAATTTGCCTTTACATCATAAAGATCCTTTTGATAGAATACTCATTGCTCAA
GCTATCATAGAAAAAATCCCAATTCTCACCATCGATTCTATCTTCCAGAATTATTCTATTAATAC
TATTTGGTAAATAATAATTAGCTCAATATAGGAGCTTTTTAAAACAAATTTTTTAGGGAAGATT
AGGATTTTTATTCGTCATTTCCTCATAAACTAATTTCGACACTTCAGGATAAAAGGTTTTATCTT
CGTAAAAAGCAGGATCATCCCCTAAAATAAAGAAAATATGATACTATTGTTAATAAAAAGACAT
CCTTTAAAGGACTTCTGCTTATAATGTGCAGTTTTTTCCTGACTTCTCTTTTGGTAATTCTTAAT
TCTTAATTAAAAATAGATTTACGAGAAAAAGGTTGATTATTGATTTTCAAATTCTGCAATAATT

TGTTCAATAACTTTGGTTGAAATATCAGAAAAATCTGACTTAGAATAAATTGTCACTAGAATAA
TTTTACTGCTAGTTTTAAGATAGTAAATAACTCTATAACCTGCACTTTTACCCTTTTGAATATTA
CTATTTTTAAGACGAACTTTAAAAACCTGATAATTAGAACCCGAAATATGTTCCCCCAAAATTTC
TCCTAGTTTCAGACACTCAATTAAGGGCTCTAAATCTTTACGAATAGAGCGATAACGTTTAGCTA
ATTGAGTCAAATTTTTTTTAAAGTATTGTGTTAATAAAATTTCGACGGGTAACGAATTATTCGAC
ATCAATTCCCTCCCACATCTGGGATAAAGGAATTGTTTGCCCCGTGAATGCTTCATTAAGCCCTT
GTTTAATCCCTTCTATCACTATTTCATTGGGAGTATCATCAGGATCTAAATTAGTTTTTTGCATT
AAATATTTTAATATTTCTTCTTGTTCCGATAAAGAAAGTTGATCAATTAAATTCAATATTTGATT
TTTTGTAATTGTTAAAGTTACCATAAATTTTTTCTTTTTTTATTAATTTCTCAAAGTAATCTTTA
AATTGATTTTAGCATTTCCTCATAAACTAATTTCGACACTTCAGGATAAATATGTTAACTATAAA
TAATCTTAAACCTATGACTACTCAAACGATTGATTTAACCGAGATGATTTTAACGAAAATAAAAG
ATTTACCCTTAGAGCAACAACTGAAAAAAATAAATCGAATGGAAAAAGAGTTGCAGGTTTATTT
AAGGATAAAGGTTGGATAAGTGATGATTTTAATGATCCCTTACCACCCGAAATATTAGGGGATAT
TATATGAAAATATTGTTAGATACTCATGTTTTTATTTAGTGGAATTTAATTCCTGAAAAATTATC
TTTAATATGTACTAAAGTTATATTTTTAAAAATTTATTCCTAGTCTCAAGCACATTCTAAATAAT
TAGCATTTACCCATCCAGTTCTGCCATTCAAAGAGCGATTAGATCCTTCAATTACTCTTATATAA
TACCAAGGAAAATCCCCTCGAATATATTGTACAACATCATCATTATTTAACCCAGCAAGTGACTC
TCCCCCCGGACTTTTACGCACAGCTAATTGTCCAGTTTTGATGTTAGTAACTAAACAACTAGGAT
TTTTTTCAACATGAACAGTGTTTTCAAAATGGATTTGACTATGATGTATATAGCCATTTCTACCA
CAAAAATCAGTTACATACCAACCATTTGCAGAACCATAAGTATTAATAGTAATAGAAGTAACGGC
ATTTACGCTACAAATAATTTCCCCATTAGGTGTTGTTCTAATATTGGAAGGGGGATCAAAAACGA
TAGCACTTATATTTTGACTGCCACTATCAAAAGAACCTGTAGTGTTACTAATTTTCTCTTTATTA
CGATCTTCTTCTTTAGCTTTTTGATAAAGCCTTTCCGCCTCCAAGCGTTGTTGCTCCACTTCCTGT
TTTAGAGCCTCTAATTCTTCTCTTTCTTTATTAAGATTTTCTTCTGATAATTGTGATTTTATTCG
CTCTTTTTCTTGCTCAATTTCAGCTATTTTTTGTTGAGTTTGATTGATATTTTGAATAATAAAAA
ACCCGACCAAAGAACCTAATAAAGCTAATATTAAAATTAATAAAATAATCAGAATATTCGATGA
AGATTTATCATTCTGTTTCGGATAACTATTACTTGAAGTATTAATTTCTGGCTCTGGATATATTG
GCTGACTTACAGGAGGTTGAACTACAGGAATAGGATCAGTATTTTTTTCGATATTAATGTTAGAA
GTCTTGACATTATCACCTTTCGGAGGAACAAATGCAACGGTTTGAAACTGACTTAAATATAAATC
CTCTTGCATCGTTTCGGCAGTGAGATAACGATTAGAAATTTCCATTTTTATCGCCTTTTCTAACA
CTTTTGCTAGTGTAGGATGCAAATTCGCCACTAAATTTTGCCATTCTAACTCCCCCGTTAAATTG
TTAGAAGGAAGCTCAATGGGATATTTTCCCGTTAAACTATATATCATGGTTAACCCTAGTGCAAA
TAAATCTGTACTAAAAACAGTTCTTCCCATACTTTGCTCAGGGGCGATAAATCCTCTTGTACCAA
TGACGACAGAGGAAACCGTTGAACCTGATGAAGTGTTGAAACTCCCCATTGTTTCCTTCACCGCG
CCAAAATTGATCAACACAGGAAGCCCGTCAGAGGCTCGGATAATGATGTTTTCAGGCTTTATATC
TCTGTGAATGATATTTTTGCTGTGAACATATTTGAGGGTATCGAGAAGGGATGTTAAGATTGTT
AAACAACGATCAGAAGATAGAATCCCTGATTGACTGAGGTTTTTTCCTTCTATGTATTCTTGTAC
TAAATAATATTTATCATCTTGACAGAAGTAGGCATAAAGGGTGGGAATTTGCGGACAGTTTTGTC
CCAATTCTTCTAATACTTGGGCTTCTTTGAAAAATAGTTTTTCGATGATTTCTGAGCTAGTATTA
TGATTAACAGATTTAAGGGCTTTTACTACTACTTTTCGTTGAGTTGGGCTGTGTAAATCCACTGC
TAAAAAGGTTTCTCCAAATCCCCCAATACCTAGAGGATTAAGTATTTGATAGCGATCGCACAAGA
TTTCATTCATAATGATGCAATCCCCTGATTAACTGTGAGGGATAAAAATTATGTTTATATGCTTA
TAATAGTGCATATTATTTCTTTAACTGTTTTAACTTCTCCCTTCCGAATTTTCAAGACCGATTAA

TTCTGGTTTGACTTTCTCCAAAAAACTAATTAACATTACTGTTAAAATTCCTTCAATTAAGGCTT
GAACAGCATAACCAATTAGAGAAGTAAAAATAGCTGTTTTTTCTGCGTCAATATCTAAATCAGGG
GCAATATTATTAATAGTAATAAAGACAAAAATTAAGGCAGATAAAAAAACACTTAGCCCCCCTCC
CAAAAAGGCTAAAGTGGACTTGCCGAAGGGAGTATAAAAAAAAGATGTGGTGCGAAAAGACCAT
ATATAATGAGCTAACAAAGCAGGAACACCCATAATAATAGCGTTAATTCCCAGAGTAGATAATCC
CCCATGTTGAAAAAACATCCCTTGAAAAAATAAGCCAATTAAAATACCGGGGAAAGCATAATAAC
CCAAAACTAAACCCATCACTCCATTTAAAACGAGATGGATACTGAAAGGAGGAATGGGAATATGA
ATTAAAGAAGCAACAAAGAAAGCCGCAGTTAGTAAAGATGCTTTAGGGATTTCTTCTTGATAGTT
TGTCTGTTTATGAATTTGTTTTAAAGAAAACCAAGTTACACCTCCTGTAATCGCATATCCGGAGA
TACTGACAGCAGGAGGTAATAATCCATCAGGTATGTGCATAAAATAACTAGAGAAAAATTAGAG
ATAAGAAATTATAGATGAGATGAGAGAAATAATAATTGTTTCAATCATTAATAATTAAGGCTCT
ATCACTTTGAGTATGTAAGTTATTCGTTAGGGTAGGCAAGAGGCAAGAGGCAATAGGCAACAGG
GGATTATTAATTAAATAATAATTTATAAACTTTTAGTTTTTGTTTTACTATAAACACTATTCAAT
AAAGGTTATGGAAGTTATTTTCTGTTAATTTATCATAACCACACTCTGAAGAACCATAATTAATA
CTAATTACTAACTTTTGATTCCTGCTTTTTGCGAGAAAAAAATAAAGCTGTACCCACAAAACCCC
AACTACCTGTTAGTGCCATTAAAATTTTTTGGGGGGTTGATAATTCTCCTGTGATGGACGAACTA
GCACGGGGGCTTTGCCCTAAATTAGTGTTAGGATTTTCTGATATTTCTTCTTGAATTTGATGGTC
ATTTTCCGCCTGTAATGGCTCAATAGGAATATTAACTACACTACCATGACCTGCTGATCTTACTT
TTATCGTCCAATTACCGTTAACATTCGGTTCAGGGACAAAAGTAAATTTACCATCACTATCTGTA
ACTCCTGTTTGCCAAGCCTGAGTCGGATTATTAGGAGCATAAACCACTACTTGAGCATTGCTCAT
TGGTTTTCCACTATCATATTTAGCTTGAATTGCGATCGCTTCTGTAGCTTGATAGTCGATCGCCA
CCCCATGAGCAGAAGTTTTTGGCTGATGGATTACACTGGCTAATACTAACCCCAATCCCAAACTG
GATAATGTTCTTAATTTCATTTCCTTTTTTCTCACGAAGTGCAGAATTCTTATTTCATAGTCGAA
CAACTTTTCTTTTTTTTCAATGCCCACTCAGGGTATTTTTTCATTAAAATTAGGGGATTAGGGAG
ATGAGGTGTCAGGTTTCAGGGAAGATGAGAGTTCGCACTGAAGGCTCTTACACTTCCTTCTGTCG
TGAACGGGATTTTTAATTCTTAATTATCAACTATTCACTATGAGGGTAAGCCATTTGGCGTAAGT
CAATACAATCGTCAAATTCTGTTGCGGTTAAATAACCTAACTCTAAGGTTGCCTCTTTTAAGGTG
ATATTTTTTTCATATGCTAAATGGGCAATTTTTGAGGCTTTGTCATAACCAATTTTAGGAGTTAA
TGCAGTGACTAACATCAAGGATTGATTAACTAATTCTTCGATACGTTGACGATTGGGCTTCATTT
CCTTGACGGTAAAGTCAGTAAAATTGTTACAACTGTCACTAAGTATCTTGATTGATTGCAAAATG
TTATAAATCATCATAGGCTTATAAACATTCATATCTAACATCCCACTCGCTCCGGCAAAACTAAC
GGCGGTATCATAGCCCATAACTTGTACTGCAACCATGGCTAAAGCCTCACACTGAGTAGGATTGA
CTTTTCCCGGCATAATTGATGAACCCGGTTCGTTAGCGGGTAATTCTAACTCATTTAATCCTGCA
CGAGGGCCACAGGCAAGAAAGCGAATATCGTTAGCAATTTTGTATAGTGAACAGGCTAGGGTTTT
TAGTGTACCGCTCAAAGATACGATCGCATCATGAGAACCCATGACAGTAAATTTGTTCGGAGCAC
TAACAAAAGGTAAACCAGTGATATTGGCAATATGATGAGCCGATTTTGTGGCAAAGCCGACTGGG
GCGTTAATACCAGTACCTAAAGCAGTACCTCCTAAAGCTAATTGATAGACATGGGGTAAGGCTTG
GTTAATACGGGTAAGGTTATCATCCAACATCCCCACATAACCTGAAAATTCTTGTCCTAGGCTCA
AGGGTACTGCATCTTGAAGATGAGTTCTACCAATTTTTATTATATCTTTCCATGCTTGTGCTTTT
TCGGCTAATGCGTCTCTTAATTTTTTGACCTTGGGTATTAATCTTTCTTGAATTGCGATCGCACT
GGCTACATTCATAGCGGTGGGAAAAGTATCATTAGATGATTGAGACATATTGACATCATCATTGG
GATGAATAGGATTTTTACTGCCCAACTCACCCCCCACAATTTGAATGGCGCGATTGGAAATGACT
TCATTAACATTCATGTTAGTTTGTGTACCGCTTCCCGTCATCCATACATATAAGGGGAAATGTTC

ATCTAACTTGCCTTGCAAAATTTCATCGGCGACTTGAATAATTAATTTCGCTTTTTCTGAAGGTA
ATTTCCCTAATTCTTCGTTAGCCATAGCCGAGGCTTTTTTGACAATGGCAAACGCCCGAATAACC
TCTATGGGCATCATATCTTCCCCAATAGAAAAATGATGGAGAGAGCGTTGAGTTTGAGCACCCCA
GAGGCGATCGCTTTTTACTTCTATTTCACCGATACTATCTTTTTCGATACGATTCATGGGTTTAC
TAATTAGTGTATATTTTATAATTTATAGAAAATTAATCTATTTTTGGCATAAGTGCGATCGTCAT
GAACCATCAATTATAAATAAACTTAAAAGTAAAATGCTAAATATAGTTAAATGGGGTTAAAAAA
GATAAAACTGATCGCATCCTCCCATTCCCAAAAGAGCGATCGTCCCTTCATTCCCAAACAATTCAT
CGTTACAAAAATTTATCTTAAGGGCGATCAACCTTCTCAAAAGATAGGGTAACGTAACTATATAG
TAAAAAACATAAACTATCCCTCATATTTAAGAAAAGGTTTAGCAAAAACAATGGTCACAATAAA
TAAACTTCTCTTTGTCGGTGATACAGTTCTTAACTCCTCCTTCGTCATTGGAAAATCATTAAGTG
ACTTATGTACAGCACATCATCTTAGAGTTTTTAACTTAGAAGGGACATTTTCAGAAGCAAAAAGA
CCTTTATTTAAAGCTGGACCACATTTATTGTTAAATCCTCAATTTTTAGAGCCAATCGCTCAACA
TTTTAACGTTGCTGTTTTAGCGAATAACCACAGTATGGATTTTGGCATAGAAGGATTAGAAACAA
CACAAAAACTTTGTAAAAATCATCAAGTAATTACCATTGGAGCAGGAATAAATAAACAAAAAGC
GTTTACTCCTTTTGATATTGATAATATTAGAATTATTGCCATAGCTGAAAATGAATTTGGTTCTG
CACAAACAAATAAGGCAGGGATAGCCACAACCGATAAACCGTTAGAAATATATCACCTTATTAAA
GAAGGAAAAGAAGAAAATAAATTTGTAGTAATAGTAAGTCATGGAGGTACAGAAGTTATCCCCA
TTCCTCCGCCTTATTTGCGTGACAGATATAAATTGTGGGTTGAATATGGTGCTGATTTGATTATT
GGTAGTCATCCCCATAGTGTTCAAGGACATGAAATTTATAAAGATAAATATATATTTTACTCATT
AGGAAACTTTGCTTTTAAAAATGAAAATCATAAAAAATATCTCAATACTAATTGGTCAATTGCT
GTTAGCGTAGATATTGATTCTCAACATCCTCAAGTTATTCCTTTAACTACTGAGAACAATTGTAT
AGATATTAGTGATCGTTCTGATCTGAAGAAAGAATATCAAAGGTTATGTGATTTAATCCAATCTC
CTGAATATTTGGAAATGTATAATAATATTGCTTCTGAATTGTATCCTCTTTGGTATAAGCGACTA
GGTGTAGCAAATCAACAAGATGCGGCTCTGTTATTACATTATTTACGTTGTGATGCTCATAGAAA
TCTTATTGAAAATGCTTTATCTCAAATTATTGGAGAAATTGACCTGAAGGAATATCAAACTAGAG
AAAATTATGATGAATTAAATGCAAGAAATAAAATACTCAATGAATGTTATCAAAAAATGCAAAT
GTTGCCAGAAGAAAAAGAATATCTAACGAATATTTTACAAAAAAGTAAAACTTATTTAGAAATA
GGCTCTGGCTACAGCACATTATGGTTTTCACAATTTGTTGATAAAATAGTTTCCGTTGAAACTAG
AAAACAGTGGTTTGATAGTATATCTGCAACGATCAAGCAAAGTAATATTTCCACCATAGAAATTT
ATCATTTTCCACCCGAACCTTGTGCTTATGACGAACAAGGTAGAGAAAAATGGTGTTATAGAAGA
AATTATCATCCAAAAACAGATTATGGTTTATATGAAGAATTTACAGGCTATTTAGCAGGAATAG
AAAATTTACTTAATCGATATAAGTTTGATGTAATTTTGGTTGACGGAAATGTTCGACAGCTAGTT
ATTGAAATGTTAATTAGAAGAAATTATCAAGGGGTTATATTATTACATGATGTTATTCCCGAAA
GAAATTACTTAAATCAACCTATCTTTGATTTGAAAGGTTTAGCTAAAATCAATCAAGTAGGTAGT
TTAGTTAGATTAGCTGTAATAAAAAACAATTCAAGTCAGCAACTTACTATTTCTGAAATGAACAC
TTCAATTAATTACAAACAACAAAGAATATTTGAAACAGATTGGTTAGCATCCCAACCAGTTTTTT
ACAATGAGAAAACAGGAAAGATTAGCCACAACGTTAATGATGTAATTGATTTTGCTAATCTTGA
ATTTCATCCCGAAGGTTTTAACAATTATCTTGATTTTGGCTACTCCATACTAGAACAAACTCCGA
TAAAAGACGTTAAATTTTTAAGACATTCATCCAGATTAACAGTTGGCGATGATGGTAATATTAA
TGTTGAATATTTAGAAGACATTGTAGAAAAATGGATTGGTAAAACTTCCTATGAAGATGATGTG
TTGCATTTACTCCATACAGCGATTCGTAATTGGGAAAAATCTGTAGAAGGAGAGATTATTCTTCC
TACCAGTGGAGGCTACGACTCTCGGATATTAACTTATTTTATTGAAGATAAATCAAGAATTAGAT
CGTTTACCTATGGTTTATCACCAAATCAATCAGAATCTTTTGAAGTAGTCCATGCTCAAAAACTA

TCCGAAATCTTTGGAACAAGATGGGAGCAAATTCCACTAGGTTATTTTCACCGATATTTTGATGA
CTGGGATAAATTATTTGGTATCTCAACCCATGCCCATGGAATGTATCATATCGAATTTTATAATC
AAATAATGCCAAAAATAGCAGGGGGGAATCCTTTTCTAAGTGGAATTATTGGTGATGCTTGGGCA
GGAAGTGTTAACATTGACGATTTATATTCTCCAAAAGATTTACAAAAGTTGGGTTATACCCATGG
CTTAAACAGTAATTCAGAGTACAGCAATTTTAAAGGTAAAAATTCAATAATTGAATACTTTTAT
GAAAAAAATGTGGAATCCTTGAAGATTCCTCGTTATCGTGTCATTCAAGCAATGAGATTTAAGAT
AATATTACTATCTTATCTTTTCACTGTACCACAACAATTCGGATTTAAAGTTTGGTCACCTTTTC
TAGATTCCGAAATTGCTTTATCAATGCTAACTTTGCCACCATCAAGACGAAAAAAATAGAATTTGG
CAAAGAGAGCTTTTCCAAAGAAACGGCTTGGATTTAGAGTCAATGAATTTACAAAGAAGCACTCA
GAATAATCTAAATTACCAAGCAATGTGTAAGATGCCAGTTAAACCACTGGATGTTAAGCTTTTAG
CCGAAGTTGTTAATCCTCAATATGTTCAATCTATCAACTACTATCTTCAGGAAAAACATTTAGGG
GCTTATTGTGCTTATTTAACTTTAAAACCTATCGAAAATGCTATCAAAAAAAGACAAGAGTTAAA
TATGAGTCAATCTTCAGCATCTATAAGTTATCAACAAAACATAAGTAAACCTTTGATTCAAATTA
ATTCTTTTCGTGTTCAGCAGTGGAAATTAACTACCCCCGTAGCCTTTATCATCTTCAATCGCCCTG
ATACTACCCAAAAAGTATTCAATGCTATTCGTGAAGCTAAACCTCCTAAATTATTAGTTATCGCC
GATGGTGCAAGAATTGACAAAGAAGGAGAAGCAGAATTATGTAAACAAACAAGAGCAATTATTG
ATCAAGTTGATTGGGATTGTGAGGTATTAGTTAACTATTCCGATGTTAATTTAGGATTAAGAAA
ACGGGTTAGTAGTGGTTTAGATTGGGTATTTGAGCAAGTAGAAGAAGCGATTATTTTAGAGGAT
GACTGTTTACCCCATCCCACCTTTTTCCGCTACTGTCAGGAGTTATTAGAGAAATACCGAGATGA
TGAAAGAATCATGATGATTTCGGGGGATAACTTCCAATTTGGACGCAAAAGAAGCGAATATAGT
TATTATTTCTCTCATTATGGTCATTGTTGGGGTTGGGCTAGTTGGCGCGGGGCATGGACAAAATA
TGATGATTCAATGCAACTATGGCAGGAGTTAAGAGATAATAAATGGTTAAAAGATGTGTTAGGA
AATGATCAAGCCGTAGCTTATTGGTCAAGAATATTTCAATCAGTTTATGATGGTTTTAGCAGTTG
GGCTTATATTTGGCAATATACCTTGTGGCTGAATAGTGGCTTGACGATTTTACCCAATGTTAACT
TAATTTCTAATATTGGTTTTGGTTCAGGCACTCATACAACTGATGTGAATAGTCCTTTAGCTAAT
ATGAAAATAGAAGCTATCAATTTTCCTTTGCGACATCCACCTTTTATTATCAGAAATACTCAAGC
AGATGAATTTACTCGTTTAAATCATTTTGGAGGTAACTTACAACAATCAAAATCAACAAATACAA
ACAATATTACGATGATTACAAAACAAGCAATTAATTACTTAAATCAAAATCAAGAAATACAGGC
ATTAAATTTATTTGAGGGTATATTGCAGAACAATTCAGAAAATATGGGGTCATATTATGGAAAA
GCCTTATCACTAGCAAGATTAGGTTCTTTAAATACTGCCGTAGAAACCCTTGAAAAATTGTTAAC
TATTCAACCTCAACACAAAAAAGCAAGGTGTTTGTTAGAAGAAATACGCCCAAATAACCTTGATT
TACTGTTAGAAAAATCTGTCCAAGCCATACAGGATAATCAGTTACAAAAAGCCTTTAATTTGCTG
AATACTTGTAAATCAATAAGAGAAAGAAAAAGAGGTTTAGATTTACTCAGAGCAGATTGTTTTC
TAAAAATGAAGCAACCAATCGCCGCCTTACAATCAGCTTACGAAGAATTAAGATATTTTCCTGAT
AACGAAAAAGCTCAAAAATTAGTTGAAGAATTAAAAGAACAATATGGTAAATTTCTCACCAGTG
TAATTGATGATCCAGAATTTCAAGAATTATTCCAACAAATAAGACCTTATACAATGGTCAGTGAG
TATCGTCTTTATTCTTTATTTTCTCTTACCAAAAAAATATGTCAGGAAAATATACAAGGTAATTT
TATCGAATGTGGCGTTGCTGGTGGTGGTTCAACGGCACTAATGGCAAGTATTATTAAACGTTATA
GTCAAATCCCTAGAAAAATTTATGCTTGTGACTCTTTTGAAGGAATGCCCGAACCTTCAGAAAAA
GACAAATCAAGGGGCGTTTTAGCCGACTTAACAGGGTGGGGAAGTGGAACTTGTGCAGCACCCGA
AGCTAGTGTAATTGAAATTTGCCAAAAATTAGATGTTTTGGACTTTGTAGAAACTGTTAAAGGT
TATTTTGAAAACACATTACCCGAAATAAAAGATCGAGTAGGTAATATTGCATTTATGCACATGGA
TGCGGATTGGTATGAATCAACTAAAACAATTCTTAAAAATTTATATGATCAAGTCGTAAGTGAT

GGATTTATTCAGGTAGATGATTATGGACATTGGGATGGATGTCGTGAGGCATTACACGAATTTGA
AACAGAGAGAAATCTTAAATTTAACTTGAATCAAATTGATTATAGTGGGGTTTGGTTTTCTATGC
CTAAATTGGTTAATGCTAGAAATGTATGATCAAGTGGTGCGTAACGAGTCTGGGGGTGACATGA
AACGCTATCTCGCCCAAAATCCTTTACCTAACCCAGAATTTATTATGGAAAGGTTTAATGTAGAA
GGAGAAATGTTAATCAAAGCCTTTCAAGGAAGAAACTTCCCTAGTAAATCCCTAGCAGATTTAAA
CCCCACCCAAATCGGACAATTTCGCCAAGGAGGAGAAGTGCATCAATGGATGTATGACAGATACT
CCCTTAAGGTTTTATTAGAAAAAGTCGGCTTTGTAGATGTGAAAGTTTGTCAAGCCAATGAATCA
AGAATTGATGGTTTTAATTATTTAGATGTTTTACCCGATGGTAGGGTGCGCAAAAGTGACTCATT
ATTTATGGAAGGAATTAAGCCTTAAAATTTAACCCAACTATGAACAATCTTGAGGATAATGACAT
TGAAAAGCTAATTAAAGCCATTAAGTTAATCCAAACTCAAGTAAAATGGAAATCAGCAAATAAA
GCTGAAATTCATTTAGCTAAAAGAATTAAACTAGGGCATTTAAAGAATAGTTCATCACTGGATG
ATTATGAAAAAATAATTCAAATAATTATCTTTAATCCTGAATCAGAAATATATATCTTTCGAGA
TGATGATTCTTTTTATCCCAGTATTACTAATCAAATTAATAATCAATTATGGCTAGTTATGTTTA
GTCTTGACGGAATTATGGAAACGGCTTTTCCTCCTAGTAATCCTGAAAAATATCTGAAGAATAAT
CCCTTTGTTTATCTTGGCAAATTAAAGGAGTTAGTTTAAATGAATCAACTATTAAATCAATATCA
AATAAGTCTTCAGTTTCTTGATGTTAGTGGAGCGGAATATTTAGATTTATTAAGTATCAGAGACG
AATTAGCCATTTCAACTTTGACAGAAGAAGATCAAAAGATTATGGAAGAATCTGATCAAATTTT
AATTAAAAATAGTGTTTTAATCTATCAAGAGTTATCTCGTTTTATTAATTTAACTAAATATAGAC
AAACAAATAAAATTAATCCTCAACAATGGTGGTGGTATTTAGATGTTGTATCTTATTTACCAGCT
AAGTTGTTGACAGTAAAACAAAAAACTTATCCAGAAATTGCTTAAATAAAAAAATAATACTTTC
CTTACCTATATGATTAAAATTTTACATATTATTGATTTTCTCTGTCTTGGTGGTGCGGCGCGCTC
CATGATCGCCATTTCTAAATATTCCTCCCGTATCGATAACAAACTTCATCATCAAGTTATTTCCCT
AAAAAAAGCCCATCCTAGCGCCATAGACTTAGCACAACAGGCTGGAATGGAGTATTTAGAATATC
AAGACGAAAAAGAAAGAAATGAGATTATCGCCTCGGCGGATATAGTGCATATCCAATATTGGAA
TAACCCCCAGATGTTTTCCTTTCTTCATTCAGAATTACCCCCCACAAGATTAATTATTTGGTTTCA
CGTTTCAGGAGATAATGCCCCCCAAGTTATTACCCGTGATTTAATTAATTATGCTGATTTTGCCA
TCCCCTGTAATCCCCATTCCTATGAATTGCCTGTTATCAAACAACTCAAACCAGAAATTCGCCACA
AAAAAGTAGGCATGGTATATGATGCGGCAGATTTTGAACGAGTGGAAAACGTCCAACCTCAACCC
CATGAAGGTTTTAACGTTGGCTATATGGGATCATTATCCTTTAGTAAAATGCACCCCAACTATAT
CTCCATGAGTGCTAAAGCTAACATTCCAGAGGTTAAATTTATTCTTTGTGGTGGGGGGAATATTG
AATTATTAAAACAACAAGCCTCAGAATTAAACGTTCTTGATAAATTCGATTTTCGAGGATTTGTA
GAAGACATCGCCTCAGAAATTGCCAAATTCGACGTTTATGGTTATCCTCTGTGTGAAGATACCTA
CGCCGCCGCCGAATTAAATCTACAAGAAGTCATGTATGCGGGAATCCCTCCCATTGTGCTTCCTCA
TGGGGGTGTCAAAAAGCTAATCATTAATAACTATACAGGGTTAATGGTTGATAGCGAATTAGAA
TATAGTCAAGCCTTAGAGTATTTATATCATAATCCCCAAGAAAGATTAAGATTAGGGAAAAATG
CCAAAGAATACGCCAGTCAAATATTCGGGGCAGAAAATGCCGCTAAACAATTAAACCCTATTTAT
CACAAACTACTGGAAACACCTAAGCGAAGCCGACAATGGAGTATTCCTGTGGATGGAAACCTACT
AGATGAACCTATCTCCTTAATTGATGTTGTGGACATTCCCACTCAATTTTACGGTGCAAAAACAT
TTATCGAATCTTTGGGAGATACAGCACCTCAATTTGTTAGCAATTTAACCTCTAATGATACTCAA
GAATTATTTAAAACTGACGAAATAATAGCTAAATCATCAACTCTTTTAGGTATGGGGGAAGGGG
GAATCATTCAATATTTGAACCATTTTCCAGAAGATGGTTATTTGAGATTTTGGACAGCTTTAATT
CTTGAAAAGGAAGGAAAAATAAAAGATGCTTTTGGACAATTCATTGTCGCTATCAATAAAGGAT
GTAATCATTGGCGAGTTTATTGGTATCTAGCTCAAGTGGCAGATTATTTAGAAGAAATAAATGTT

ATGGAACAAGCCTTAAATAATCTTGAACAAAATGTGCTTAATTTTAAACCAGTTCAACAATTAAG
AGAAAAATTAAATCAATTATTATTAGCTGAAACAGAATTACTCAATATCAAATTCACGGAAATA
AACTATTTAATCTTCCCCAATTGGCAAAGCGATGAAGAAACCTTAACCGAAGAATTATATAACCT
CATTTTCTCCCTAGCTAAAAACTCCCCCGTCTCTGACTCCCCCCTCAGTAAAGAGAGTTGGGAGGG
ATCAACCCTCGTCATTGACACTACAGGTATAACGGAAGAAGATGCTAATTTATTCTTATCAGGTA
TTGCCATGAATCTGATGATGGAGGAAGAATTAGACTTAGAAAACACTTTAGACTTTGCCCTAATT
AATAACCTAAACGATCGCACTTGGAATAAATTACTCCCGAAAATCACAGCGAAAATAACCCTTGA
AAACGAAAATCAAGACATTGTTAATCAACTTAATCTTGAGGATTTAGTTACTATTGAGGGTAAT
GGTAATAACTATGCTATCTTTCCTGATTGGAAAGCAGATCAAGAAGAATTAGCTTTAGAAATTGG
TGAGGTATTAATGAACCTTTCTGATCAAGAAAACTGCACTTTATTAGTCAATCTCAATAATGTCG
ATCAAGAAGAAGTAGGCTTATTTTTCTCAGAAATAGCCATGAATTTAATGTTAACAGAAGATAT
AGAATTATCGAATAATTTACAGATTAATTTTGTCAATTTTACTGCTCACCAGTGGCAAAGGTTAG
GAGGATTAATCAAGGAAAAAATGACCATTAATTATGAGGATTTACCCGATAATTAACCCACTAA
ATAATGAATTTTATGACTGCAATATAAATTTGTAAAATTATGTAAATAGCTTAACGATGAATCA
AAAATTAATAGAATTATTACAAAATCAGTATGATAATTTGCCTTATCCCCAAATTCCTTTAGAAC
AATCTCCCCAAGAATATTATGACACTCTTTTTATTCATGATTTGACTACCCCATATTATCTATAC
CAACAAAAAATTATTGATACGAAAGATAAACTTATCTTAGATGCAGGATGTGGCACTGGATGGA
CTTCTCTATTCCTAGCCTATGCCAATCCGGGGGCAAAAATTATCGGTGTTGATTTATCTCCTAAA
TCCATAGAAGTCGCCCAAGCAAGATTTAGACATCATGGTTTTACTAATGCTGAATTTTATGCCTT
ATCTCTCGACAACATAAAACAATTAAATTATGTTTTTGACTATATTAATTGTGATGAAGTTATTT
ATCTTTTACCTAAGCCAGAAAATATCCTTAAATTATTTCAATCTGTATTAAAAGAACAAGGAATT
ATCAGAGTTAACTTTCATAGTTATTATCAAAGATCTAACTTTTTCCGAATGCAACAACTGTTCCA
GAATATTGGTTTACTTAATGATGACTATGGCGATTTAGAAGTGCAATTGGTGCAAGAAACCATG
AGTAACTTAAAAGAATCCGTACAGGCAAAACAAGTATGGCAAAGTCAATGTTCAAAATTTTTGA
GCCAACCCAAAAAAATGAAAGAAATTATTATGGTTAATTATCTTTTGCAGGGAGATAAAGGTTA
TACTGTGCCACAGGTTTTTAATATGTTAAAATCTTCGAGTCTCAACTTTTTAAGTATGGTGATGT
GGCGAAAATGGAATATCAGAGACTTATTTAAAAAACCCCATGATTTACCGGAAATATGGGAAAT
GGGATTAGAGGATATATCTTTAGAAGATGAATTGTCTTTATATGAATTAATTAATCCCGTACACC
GTTTAATCGATTTTTGGTGTGTTAATAAATCTACTCCCTCGATGATTTCTCCCTTAATCACTTGG
CATAAAGAAGATTGGCAAAAGGCGAAAATTGCACTACATCCTCGCTTTAAATCACCTAAAATCAA
AGCAGATTTCGTCAGGACAATTGAAAAAAAAGAGTCATTTCTTTTTAACCGCTATATTGCTTTCC
CCGCACTAGGAAATGTCGAAATAGAAAGTTCCATCGCCAGTTGTTTTCTGCTATTATGGGAACAA
GAAAAAGTGAGTTTTTCTGAATTAGTTAAACGTTGGCTAACTATTCAACCCTATGATTTACTCTC
CCTTCAATCCAAATCAGAATTATTAGCAATAAAAGAGTTAATTAATGAAATAGTTAAACTAGAG
AGTTTTTTATATATATTAGTAGAAAAATAGCTGAGTCTAAATTGTCTAAATTTTATTACAATACT
TGTACAGATTTAGTTTGTTTATCATCCAAAAATTCAATTGTATTTACTGAATTTATCATTGAGTT
TATATTCGGATTAATCAATATTTCGTGAGAATGTACAAACTCATAAATTTCAAAATCAATATGAG
CATATTTTTCAATTAAATTAATGGTTGAAGACCTTTTAATAACATCTTTGCTATGTGAATTAGGA
TTTTTTCTTTGATGGGGTAATTTAATACCGTCTTTGATTAAGCCCTACATCTTCATCAAGATAAA
AAGTTGTTCTAAATGGCAAAAATCAGCAATGAATAGATAATAATCATGAAAAATAGTATGAAAT
GATTTTCTATTTGATGGATTAATATAAAAGTTTAATGAATCATTGATAGGATTACTCAAATGGG
GCATTTCATCGTAAAATTTAATCAGAAAATCATCAAAATCATATTTATCCTTCGGTTGCTTATCT
TGATCGCATAAAAAACCCCTCCAAAAAAAATTATTCTCCCATTGATGATTCCACAAAGATACTAA

CCTCTCAACTGGATGACGGACAATAGAAAAAAAATAACCGTCCAAAAGAATATCATTTAAAAGA
AAAGGTCTGATATTGATTAATTCGATAGAATGAAATTGAATGAATTCTGATTTGTGAAATAAGG
CATTCAATTGGTGATAGCTATATAAGTTTAAAATAGTACTATTTAACGATAAGTAATTGGTATA
ACTATTTTGAAAAATAGAAAACAAAGAAGTGCCACCAGTTTTTGGGATATGATTAAATACTAAT
TTCATAATAAGGTCAAAAGCTATTTTTATAATGACAATAGTAGATAAAAAAGTAGATAAAAAGG
AAACAGAAGCGACAAAAATAAAATTTAGTTACTAGAATTAACATATATATCCCACATTTGTTGA
TAAGCCTTTTCCATTTCTCTAGCAAATTGTTTTGCATTCCAAAGAGGGGATGTTTTTTTCGATTG
TCTTAACTTCCAACTCACTTCCTTTCTCAAATTTTCATCTGTGCCAAATTTTATTCCCCATTCGAT
ATATTTTTCATTACTCCATGCAATGCCTTCGGTAATACCTGCATTCATCATATAAGTATAACTAT
TGCGAGCCGCAAATTGCTCCCCCACTCTCGTCACTACTGGAATATCTAGCCAAAGTGCTTCTAAGG
TAGTTGTTGAACCATTATAGGGGTAAGTATCAAGAATTACATCAGCAATTTGTAGATTTGCTCGG
TGAGTTTCAATAACCATATCAAAGGGAATAAATCTTAATTTATCTATGGGTAGATTTTCTAAGTT
AGCAACACTTATTACTAATTCTTTTAATGCACTTTCATCCTTTCTTATTTTAATGAATAGATAAC
TATTGGGTACTTGTTTGATTATTTCCAGTTGAGACTTCAGAATATCAGGATTAAGTTTGGCAGGA
TTTTGTAAGTTCATAAAAACTACATCATCTTTTTTTATGCCAAAATCTTCTCTTCTTAAAGTAGG
GGTAGCGATATTAAAACCATCAATGGCTAAATAAGTATTCGGTAAGCGCCAAATTTTCTCCCGAT
AATATTGTTCCGCATTGTCAGGCAATACATAGGGATCTGCAATATAATAATCGATCGAGGGTAAA
CCAGAAGCATCTAAACCTAACCAAGATACTTGTATCGGTGCAGGTTTATTAACCATCACAGATGC
TGTTAAATTTAAAGTCACACTGTCCACTTCTATTAGAATGTCAATTTGATCATCATATATTTTAT
TGGTGATTCGGTTGACATCTCTTTTCCCATCATAAAACTGTTTGATTTTTTCATTTTGAAACCAT
TTTTGGGTAATATTATCCTGATGTCCTTGTACTGAATAGATATTCACATCGAATAAATCTGGATT
ATGATAATTAATTAACCAGCGACAGAGAAAGCCCACAGAATGAGTCCTAAAAGTATGTCCGATAT
AACCGAGCCTTAGTTTTCTAGTTTCCTTTTCTTGAGCAAAAATCCTAGTTTGGTTCTCTTTATTG
GCTTGATAAAAAATTTTTGAGATCCCATTAAATAAGTGTCGGTTATGGGCTAGATCATCTTTTAA
GTAAAGAGGACAAATTAAACCAACGATAAACGCATTTTTAGCCAAGGGATCTTTTATTTCATAAT
TTTCCCTGATCATTGTTTCCCAATATTGAATTTGTTGCTTTCCTGTTTCGGTTGCTAAATCCCATT
TACCAATAGTTAAATAGCAATTGAAAAGAAAATTATTGCCTAAAATTTTTCCTTCGAGGGAATCA
CTTTTTTCTTTGATTATTTCTGCTACTTCTAAGGCTTTATCATAATTTTGAATTTTCATATAAAG
AAGATATAATTCCCGATATGTATGCCAATTATCAGGAATAAATTTCGTATAAATACATAAAAGA
TCAATAGCATAAGCAACATATAAGTTATCTATTCCCATAACATGAGCAATGGTTTTGATTTTATT
AGCTACTTCATTATTAGTGTCAGTGGCAATTAAACTAGCTTCGGCTAAATCAATACTCGCTTTAG
TTGGTAATTTGAGAACTTCATCTAAAACTTTTATTAATCTATTGATTTCTATTTCAGGAATCTTA
TCTTGGGTTAGGAGTTTTAAAATATTCCAATCATAAATAAAGTCCACCTGAAAAGTTTTTAGGAT
AACAGACAATCTTAAAAGTTCTAATAAATTCCCTATGTTCTCAGGGTTAATTTCTTTTAAGGCAG
AGCGAATTACCCAAACCAATTCATATTCTTGTTTTTCTTCGATTCTATCGGCTTCGAGAATAATA
ATTTGGCTTAATTCTTCGAGCAAAGACTGGTGCTTATCTTCCTCCGTTTCAGTAAAGGGTATAAA
CCAAGTAGTTTGAGCTTCTGCTCCCTGTTTATTTAATAAATAAGCTAATCCTAAATACCAATAGT
TAGACAAACACTCTGGATCCTTTTCCACAAAATTCTCATAAAGATTAACGACTTCTTGATAGTTG
TCATTGGCTAAAAATTGTTTTGCTGAAGTTTGCCAATCATTATTATTCATCTTAATAATTTTACC
AATATTTAATTTTATAAGCTCTTATCGTCATTGCAAATAAGAATAAAACAGTTTGATGATATTAT
CAATCCTCAAATATCAGGATTAGTGAGCAATGCCCACCCTAATTTTATTTATCTTCAAACTTAAC
AGTAAATCCTGTGGGTGTGGGAATAATTTTAGCACCAAGGGCTTCGATTTGTTGTTGAGCAAATT
CTCTGACTACTTTTTCTGGAGTATAAACATAAATACTTAACCATGCACTGGCTCGAGCTTTAGTA

CTATCGGGATTAGTTGCCAAAAAATCTGCTATTTCTCTGGCTCTAATACCCGTATTAAAACTATT
GCTTGTATGATGATATTCTGACCATTGTGCGGCTTTTTCAAAGGATTTTTTTGCCAAAGAAGGAC
TATCGATAAAGAGTAACTCTGTAATACCCTTATAAAACCATACTTGATAGGTAGAATCCTGTTGT
GGAGACATTTTGGCAAGTCCTTGATTCATTAAAGTAACACTGACTTGAGGCATCCCACTAAAAAG
AGTTGTAGCAGGTTCGAGGGTAAAATAAGCCCCCGTAAATCGAGGATCATTGTCAACTATTTCTT
GAAAGTACAACGGGTTGAGAGTATATCCTGTATGATTTCTGGCTTGGGTATCCCCGAAATACTGA
ATAAACTGTAAATACAACCAATCTGCCACCAAATTATTAAAACCAAGTTTGGGAATTTTTTTAAT
TAACTCTAATTGAACCCTAGAGGTATCTTCTAACCTTTTCAATTCTTCTTGACTCAGATCTTTTTC
TCTAAGGTTAATTTCTCTTGTTTGTAGGGAAATAATACCAGTAATGGTAAGTAATAAAATTAAG
AGAAGAATGGTATAAGACCTAAATAGTTTTTCCATTTTTTGATTCTGAAGAAGAAGATATTATC
TGAGGATTTAATAAAACACAAGAGTTTGCTGTAAAAAATTGCTTTGTGCCTAGCAATAATTATCT
TGTTTAACTCAATTATTTAACTTTTATTTCTTCGTTAAAATATATTATCTCAAAATCAATAAAAA
AATTAGTTAATTCTTTCCGCATCCGCCGCACACGTACCTGTGTTTGTAGGTGCAGTGGTTTGAGC
CTGTACATTAATAGAACGACATAAATAAATAGAATAGTCAGCACTAATAGGATCAAAATAAACA
GAAGAGGCATAAGCTCTTAAACCGAATTCTAAAGGATTATTGTTCACTGCTTCTATGGTTACATT
ATCGGTAACAGGAATAATATCATAACCACTCACATATATTTGATCGACTTTTATTCCTAAAGCAT
CCAAAATTTCTGAAGAGGTGTAACCACCACAGCAAAACCTTCTTTGTTCCCAATGAAAGGCAAGT
TGGCTACGATTAATATTGCCTAAAGTATTTTTCACTTCTGCTTCTCTGGCTTTTCCAGTTTGATT
GACAAATGTAGGAATAGCGATTGACGCCAATATTCCCGCAATAATCATCACCACCATTAACTCAA
TCAGGGTAAAACCTTGATGTGAAAAGAAAACTCCATGATAAAAATTTAAATTTTTCCAGAGTTTT
TGTTTTTCCTTTTTATTCCTGCACATCATAATTATTAGTTCCTTAGAAAAAGCTCGAAATACATT
AACCTTCAACAGAAAGAGAATTATAGTAGGTATATTTATTTAATATGTAAATTTTATCAAAAAC
CAATGATTTTTAAATTCCTCAACACACTCTTCACTAGGTTTAGAGGGCAAAGCTTACCGAAACTG
CATATCTCATTATTAGTATTTAGATTCCACAGCAGTCCCACAGTAGTAATATGTTTCAATTTTCA
ACTCATCAATATTTTTTAAAACAAAAGTCGGAGTGAGCCTTAACTTCCCCGACTAAACGTTTTTA
TATTTAGGCAACAGCCTAAGAATACATTATTTACACCTGAAATCAATCATATCGTGCAAATTATC
TGATAATACCAGCACTTACCCCATCACAGCCCGCAGAGTCCGCCGTAGCGGGTGCACCAATGGCAT
CAGTAATTTCATTAGATTGACAAATTGCTTGAGCGAAAGTACCCTCTCCATGAATAATCAGACCA
GAAAACGCACGAGTACCATCCTTCGCGAATGCTGTATTCTGACTAGCATTTGTAGCAGAATCAAC
AGATCCTGCAATCGCCATTGTTGTATCGGTTAAATACTCAGTAGGCAACTTAATACCTAAGAAGT
TAATATCATCTGCAAAGGTTTGACGTTCAAAGTGATAAGCCTGTTGAGAGCGGTTGACAGTACCC
ACGGCGTTTTTCAATTCGGTTTCTCTAGCTTTACCTACTTGTCCTAATAAGTTAGGGAGGGCAAC
GGCGGCGAGGACACCGATAATAATTACTACGACTAAGAGTTCAATTAAGGTAAAACCTTCGTTAT
TGGCTTTTTTGTTGCGGAGGTAAGCTAAATATTTTAAAGTTACTTCGGGTTTCATATTTTAATGC
TCCTGTGAGGGATATTTTAAATAGTTTGTTTTTTCTCTTATTCATACATTACCCAGATTCGATCG
AAAACCTATCACCTGATCAAAAAATTTTTTTCTTTTTTCTGATTCCCCCCTTATTAAGGGGGGCT
AGGGGGGATCTTTCATTTGGGGGAGTTTCGATCGAGTGGGGTAATGGTTGGAGTCATAAACCGCT
TTGCAATGAATTGACAAAGCTAATAGCTAAAGTCGGCTAAAGCGACTAAAAAAAATCAATTTTA
TCCCAACGAGTGAAAAAACTTTGCTTAATAAGTGAGGGATTGGAGGTCGGGGGAGGCACGATCGA
GTTGATCCCCCCTAACCCCCCTTCGTAAGGGGGGAATTTGAATTATGGGTGCGATCGCTTCTTAG
AGCGTGAGATTTGTGGTAAAATCCGAAGCAAAATGAGTTAAAAATCATGGAATGATGAATGAGC
AACTTTACAATACTGACTATACTCTATTGCTGAAATTCTCGAGCGTGAAGTCAAGCAATAAATAT
ACCATATTTGATTTTCGGAGGTGCGTGCGACGTAGCTGTGCCTTTGGCATCCCATCTTTTTGAAT

TTATAAGATAGATTTTAAAGCTGAAGATTGAATACTATCATAGTCTATTTTTAGCATTAACTCTT
CGGGATTATATTTACCTTCAAAGGCTTCAAGAGATGCTTTTCTAATCGCTGTATCAAAGGCATCT
GCAAGTATTTCCTTTAATTCTTCCTCTGTGGCATAATAACCCCCTGATTTACGCCTTTTATTAATT
TTTTTAATTTCTTTGATGGAGTTATAAATAGAAAAATCCCAAGAGCGAGTTGTTCTTTTTTCCGC
CTGTTGTTTAATTAAATGTAGCAATAAAATTACGGCATAACTAAAAATTTTGTTAAGTTTATCTT
CCTTTGACATTTCTTCTAATTCAGCAACCAATTCCAAAGCGCGATCGTAGTTTTTTTCTTCTATGT
ATTGACGCAGTGCTAATAACTCTTCCATTTTCTATGCTGATTGGATACTCTAATCAAGACTCTTT
CTTGCTCATGATCATAGCATTATTGTCCAAATCATGAAGAGACTTTTCCTCATACTCCTCCAAAA
CTTGATCCTCAACACTCGGGGTATTTGTGTCTTAGGAGGTGCGTGCTGGGTAACAAGTCCCCTCG
CGATCGCACTCTATGTCTTTGGCATCGCATCTATAACAGACGAAAAGAGATGTTAAAATTATAAA
CAAAAATGATTTAATCATTATGGCGACAGTAATTAATGTTGAATCCCTCAACCAGTTAACTAGCG
AAGCATTTTATCAGTTATGCCTTGCTAATCCTGATATACCGATGGAAAAAAGTCCTCAAGGAGAA
TTAATCATTATGTCCCCTGTTGGTGGTGAAAGTGGTAATCGGGAAGCTAATTTAATCGCTGATTT
AATTTTCTGGAATCGTCAATATAAGTTAGGACAGGTTTTTAGTTCCTCCACTATCTTCAAATTGC
CTAATGGGGGCGATCGCTCTCCTGATGTGGCGTGGGTATCACAGTCAAAATGGGATAGTTTAACC
GCCGAAGAAAAAAAGAAATTTCCCCCTATTTGTCCCGATTTTGTGATGGAATTAAGATCTGAAAG
TGACAGATTAAAGCCTCTACAAGAGAAAATGGAAGAATACTTGAATAGTGGCTTAAAGTTAGGT
TGGTTAATTAACCCTCAAGATAGAGAGGTAGAAATCTATCATCATCAAAAACCCAGAACCGTTGT
CAAAATGCCCTGCGTATTATCTGGGGAAGAAATATTGCCTAATTTTGCCTTAGAAGTTGATTTTA
GTTGAAAGATTTGGCTAAAAAAAAATACTTGCATGAGTTTCCCTACCCCTTCTTAACAAAGAATT
ATAACCATCAAGAAGATAGGTATATTCTCCACTTGGAAACCATCTTAAAACTCTCTAAATATTTT
TAAAAAGATCAAGACTTTATCTATAATGATTGATAATAATTGAAAACAAAAACCTCGATCGAGC
TTGATTAACATTCCTATTATGAACTACGCTAGTTTCTAACGCTAAGGATAATTAGGGCGGTTTCA
CTCCTAAGAAAACAGAGTAAATATATGAGTATTTTTGAAAACTTTACCAATTTTTTAGAATCTAA
ATTAGATGAATTTTTACAAAGTAATCCTGAACTAAATTTGACAATTATTGCTCAAGAAATCAAGC
AAGAAAAAGAAGATACTATTGGGTTAATTAATAAGTTAGAATCAAGAAGAAAACAACTAGAATT
AGATATTTTAAAATTAGTTCAAGATATAAAAATATGGTGTGAAAGAATAGAAAAAGCGGAAAAA
GCGAATAGAAATGATTTAGCTAGAGAAGCAGAAGAAAGAAAAAATACATTACTTTCTCAAGGAA
ATTTTGCTTGGCAGGAAATGGAAAAAGTAAAAAATCAGATACTTGAAAATAATAGTAAACTGAT
CAATCTTGAGAATAAAGAAAGGGAAATTAATTTAAAGGTTAAAGAGTTAGAAAAAGCTAAACAG
TGGACACAATCTAACTCCAATACCTCCAATTATAACTCTTACGGGTCTTATAGTAACTCTGGATT
TAATAACAGTAATGATGATTTAGAGAAAAAATTTAGAGATTGGGAAGTATCTGTGGAATTGGAG
GAATTGAAAAAAAGATGAGTTAGGGATATACTCGGGAAGTCTATCCTGAAATGTCGTCACAGG
ACTCCCGTTAAATTTCACGAACAAAAGAAAAGCCCACGGAAATTAACGGGAGATGAATGACGACC
AGCAAAGAAAAAGGAACGAAAGTGACATCTACACACTTGACAAAATCCTGTAGATAGTTTAAGG
TTGATAGTAAGGTAATTGATTAAAATCATGCTAAACCTTTCCTTCGAATACAAACTTAGTTTAAC
AAAAATCCAGATCGAGCAAACAGAGCATATCCTTGAGGTGTGTCGTTCGGTTTGGAATTTTGCGT
TAAGGGAGAGGATGGATTGGTCAAATTCCCGAAAATCAAGGAGCAACGCCTGTTCAATTCAACAG
GAGTATATAATACCTGCGCCTGCTCCCTACCCCAATTACCATATTCAAGCTAAGGCACTAACTAA
AGCTAGAAAGGAAAACCCTAGACTAGCTTCTGTAAATGCACAAGTACAGCAACAAGTCTTAAGGA
CTCTGGAAAGAGCTTTTTTAGACCGTGAACGTAAGGGATTAGGGTATCCTCGTTTCAAAAACCAT
AGTCGTCTGCGGTCATTCGTTTTTCCTCAAATGCTTAAAGATTGCATTAAAGGAAATCGAATTAA
GTTACCCCAATTAGGTTGGGTTAAATTTAGACAATCTCGCCCCATTCCCGATGGTTTCTTGGTAA

AACAAGCTCGGATAGTGAGAAAAGCGACAGGGTATTTTGTGATACTGTCATTACAGTCAGATGTG
GGCATTCCCCAGCCAATCCCTCATGGGCATCCTAAAGGTTTGGACATCGGCTTTATCTGCGCGGTG
GTGACATCAGACAATGAATCCATCCCTAGACCTAGGTTCTTAAATAAGTATTCACGGGAGTTGAA
AAGACTACAAAGAATGCTGAAAAAGAAACAGAAAGGATCTTGTGGATGGAAAAAGTTACAAAAA
CGTATCGCTCGTTTACATTTTAAGACTACTTCCCATCGTAAAGATTACCATTTCAAACTCTCCCAC
CACTTGGTAAAGGATGCAGGGATGATATTTGTCGAGGATATAAATTTCCGAATGTGGCAAAGGTC
ATTCTTGTCAAAATCTAGTGCCGATTTTGGTTTTGGACAGTTTATTGACATTTTGGAATGGGTTT
GTTTCCGAACAGATACTTATTTGTTCAAAGTAGATCACCGTTACACAAGTCAAGAGTGTCCTGAA
TGTGGTGCGAGAACAGGGAAGAAAGACTTAAAAGAACGAGTACACCAATGTCCTGAGTGTGGTT
ACATTACAGGGCGAGATCATGCTTCTGCTTTAGTAATTAGGAATCGTGGTTTAGATTTAGTGGCG
GTCGGGCAGCCCGTCCAAGAAAAAGCTAGTGGAGATGGTCGCAGCGGGGGCGAGTTTATTCGTCT
AGCCACGAATCTGTGAAGCTAGAATCCCCCGACATAATCTTTGATTTGTCGGTGGGAGTGTCAAT
TACAAAATCTCCCTAATGCCTCAAATATCTAAACACCCCAATATTTTTGCTAACTGACATTGAAA
CAGCCCTGAGTTACTTATGGATTTTAAGGGGTTAGGGATAAGTGAGTAATGATTAAAAAAACTT
AAAGAAATATCAGAGAATGATTAATCCTTTGCTACAGATAAAAAAATTTGACTTATCCTTAAAA
AAGAAAAAGTTTTATTATTCAGCAGTTTAGGATTTAATCGTTGTGAATATACCAGGTAATATTA
TCACTTTGATCGCAGGTATTCTGCTTACTTTGATCAGTTTGTGGTATGGTCAAAATCACGGCTTG
ATGCCCGTTGAAGCCTCGGAAGGGGCGGCACAAGTGGATGGTTTATTTAACTTGATGATGACTAT
AGCTACGGGCTTATTCTTAATCGTAGAAGGAGTTTTAATTTATTCCATCATTAGATTTAGGCGAA
AAAAAGGCGATCGCACTGATGGACCTCCTATTGAGGGAAATGTACCCTTAGAGATAGTTTGGACT
GCTATTCCCACAGTAATCGTGTTCATTTTAGCGGTTTATAGCTTTGAAGTTTATAACAATTTAGG
TGGTTTAGATCCCGAAACTTCCAGAGATTTTCCCCAAGAAGAAATGCAGATGGCAGAGAATGGAG
CAAAATTAGTCGCTTTTAATCCCCATCAAGGACATTTATCTCTTGGTATCGGTAGCAGTAAAGCG
GATTTGGAAGTAGATGTCAACGGTATTCAATACGCATGGATTTTTACTTATCCTGATACTGGGGT
AGTTACGGGAGAACTTCATGTACCTGTGAATAAACAGGTTAAATTAAACATGAAAGCTGGAGAT
GTGATTCACGCTTTCTGGGTACCTCAATTAAGATTAAAACAAGACGTTTTACCCGGTAGGGATTC
AGAGTTATCTTTTAAAGCCAATAAAATTGGGGAATATCCCATCATTTGTGCTGAATTATGCGGTC
CTTATCACGGCGGGATGAAAACGGTGCTTCATGTAGATAGTGAAGAAGACTATCAAGCATGGGTG
CAAGAAAATACCATCGCATCTTTAGAAGACAAAGAGGAAACAATGGCTTTATTGTCTCAGCCCAT
GACAGAAGAATCTCGCCTGGAGATGCACTCTCATCATTTAGGAGTTACTCCTGAGACGTTGGCAC
AACTCCGCTAGGTTTTGGGGTGTGGGAAGTAGGGAGTAGGGAGAGAGGGAGGCACGGGAAGCAT
GGGAGGCGTGGGGAGAAATCTTCATTCGCCTAACACCCGACACCTGACACCTGATACCTGACACC
TGATACCTCTCTCTCGTCAATAATATTTTCATTCATCTATCCAATTTATGAGCAATACAACAACA
TCTAATCATAAACAGAGGAAGTTAATAGATTACTTTACCTTTAACACAGATCATAAGGTTATCGG
TATTCAATATCTCGTTACTTCCTTTCTCTTTTACTTTATCGGTGGCGCATTCGCCGAAGTGGTGCG
CACGGAGTTAGCTACTCCTGATCCTGATTTTATCTCTCCTGAGTTATACAATCAAATGTTTACCCT
TCACGGTACGATCATGATTTTCCTCTGGATTGTACCTGCAGGGGCGGCTTTTGCTAATTATCTCA
TTCCTTTGATGATTGGGGCAGAAGATATGGCTTTCCCTCGTTTAAATGCCGTTGCTTTTTGGTTA
ATACCTCCGGGGGGAGTATTATTAATCAGTAGCTTTTTGGTGGGTGCGCCTCAATCGGGTTGGAC
TTCTTACCCTCCTTTGAGTTTAATGACTGGTAAATGGGGAGAAGAGTTTTGGATTTTGAGTATCC
TGTTATTGGGTACTTCTTCCATCTTAGGGGGTATTAATTTTGCTACTACCATCCTGAAAATGCGC
ATCAAAGACATGGATTTACACAGTATGCCTCTATTCTGTTGGTCTATGTTGGCAACTTCTGCTTT
AATTCTTTTATCTACTCCCGTTTTAGCGGGGGCGTTAATTCTTCTTTCTTTTGACTTAATTGCAG

GTACAAATTTCTTCAATCCCGCAGGAGGAGGCGATCCTGTAGTCTATCAGCATCTTTTCTGGTTC
TACTCTCACCCTGCGGTTTATATCATGATTTTACCCTTCTTTGGGGTAGTTTCTGAGGTTATTCCC
GTGCATTCCCGTAAGCCTATTTTTGGCTATCGTGCGATCGCATATTCTAGTTTAGCCATTAGCTTC
CTCGGTTTAATCGTCTGGGCTCATCATATGTTTACCAGTGGTACTCCCGGCTGGTTAAGAATGTT
CTTTATGGCAACAACTATGCTGATTGCCGTGCCTACGGGGATTAAAATTTTCAGTTGGTGTGCCA
CCATGTGGGGCGGGAAAATCGAATTAAACACTCCCATGTTATTTGCAGTGGGTTTTGTCTCTTCC
TTCCTAATCGGCGGTTTAACGGGGGTAATGGTGGCTTCTGTACCCTTTGATATTCATGTTCATGA
TACTTATTTTGTAGTAGGACATTTTCATTATGTATTGTTTGGAGGATCGGCTTTAGCTTTATTTG
CAGGGTTTTATCACTGGTTTCCCAAGATTACGGGTAAAAACTATCATGAGGGTTTAGGAAAATTA
CATTTTATCCTCACTTTCTTAGGATTAAATATTACCTTTATGCCCATGCACCAATTAGGCTTAAT
GGGGATGAATCGCCGTATTGCTCTTTATGATGTAGAATTTCAACCTTTAAACTTACTTAGCACCG
CAGGGGCTTACACTTTAGCTGTTTCCACAATTCCTTTTTTGATTAATATGGCTTGGAGTTTATCG
AAAGGTACACAAGCAGAGCGTAATCCTTGGAAGGCTTTAACCCTTGAATGGCAAACTGCTTCACC
TCCAATTATTGAAAACTTTGAAGAAGAACCAATTTTATGGAGTGGTCCTTATGACTACGGTATTG
ACAGCGAATCAGATTCAGAAGATGAAACCGTCAAGGATATGTTAGCGGAAGTCAGTGCGAAATG
AGTTCGGGATATTGAGGGGTTAGGGAGATAGAAAAGTCGTCAATTTATCGGCTTTTCATAAAAA
CTCCCTCATTTCTGCAAGAAATTTAAGAAAAATTCACAAACTAACTAAGTTACTATGCAAAGTTC
AGCAATAGAAAATCAAGTTACCGTAGGTTATGAAGCCGAATCAGGGCATCATCATGGCCACCCTG
ATTATAGAATGTTCGGGTTAGTCTTGTTTTTAGTGGCAGAAAGTATGATCTTTTTTGGGCTTTTT
AGTGCCTATATGATTTACTATGCTACTTTACCTGAATGGCCTATGGATGGTATTGAGTTAGAATT
GGGTTTACCTGCCATTAATACCATTATTCTTGTGTCGAGTAGTTTTGTTATGCACAAAGGGCAAT
CCGCTATTAGAAATAATGATGTCAAGGGTTTACAGTTTTGGTTTGCCCTCACCGCAATCATGGGC
ATAATCTTTTTAGTTGGTCAAGGTTATGAATATATGCACACTGGTTTCGGTTTGACTGACAACCT
TTTTGCTAGCTGTTTTTATGTCTTGACTGGTTTTCACGGTTTACACGTTACCGCCGGTTTACTTTT
CACTTTGGCGGTTTTGGTGCGATCGCGCTCTGAGGGTCATTATACTGCTAGTAAGCATTTTGGTG
TAGAAGCCTCTGAATTGTATTGGCATTTTGTGGATGTAATTTGGATTATCCTTTTTGTTTTAGTA
TATTTATTACCGTTGGTATAAGATAACCTGAGTTCGGGATAAATTTTTATCTATGAGTGATGGGG
AAAAGGGCAAGAAGCAAAGGTCAAAGGGCAAACCCCCCTTTATCCTCCCTCTCGAGGGGGGAGGG
CAACGGTAAATAGTGTTGGGGTGAATAATAACCCCTAACTCCTGTAGGGGCGAATGGCCATTCGC
CCCTACTTCCTCTTCTCTCCTCATCCCCATCCCCTAAACACCCTGACACCTGCAACCTGCAACCTGA
AACCTAACCTTATCTGATATTCTTAAACAGAACTGAGTTTAATATAAAAGTTGTGGGGTTTTACC
CACCGTATGCGATTATATTTGGGAAAGCAAATTCAACATTTATTGAGTGATATAATCAGCTAAAA
TAATTTTCTCCCGATGGGCTTTTATGAGAGTTAAATCCTGTTTACTGACTTGTTCTAACATATAG
CAAATATGGTCAGGAGTTAATAAAGAGCCATCATTGACAGCGCTACCTAGATATTTTAATTTAAC
GGTATTTGTTGCTTGATCATCATTTTCTACTAAAGAAATATCATCAAGATTAATCCTTAAATTTA
CCATTTTTTTCTTGCCCTTTTTGTCTGTTTTTGACATTAAAATTTCTGAAGAATTCTTCACATCTT
CAATCCAATTTTGCCATTGAGATAGGCTATATTTATTATCACTGGAAAGAGTAATTAAATATTCG
GCTTTGTTCAATAACTGGGTAGCTTTAGGAGATTTAACGGGTAATTCTTGCACCGCTAGGATGGG
TAATTCTAGGGGTAATTGAGCAATTAATTTCTGCTTAAATTCCTCTATATCTATGGGGGTATGAT
TTTCAAAATCCACTATCTCCCCATTACTCGTCATACCCAATGATAAAGCAAGGGCAATGGAAATT
TTTGGTCCGGGATGAAATCCCCCTGTATAGGATATGGGAATACCTGCCCTTCTTACAGCCCTATC
AAATAGTCTAACTAAATCCAAATGACTAACTAAAGCCATATCCCCCTGCTTACCAAACCATACCC
GAATTTTTTGGGCTTTCTCTTGATTGGGTTGAAAATCCCCTAAATAGTCGGGAATAGGAGGAGGT

GTTACCACGATATTATGACCAAAATCAGGACTACACACACCGCAACTACTACAACCATCAAAGGC
ACAATCAGGCACAGTGGCTTCCTGTAACGCCCTTTCTAAGTCTTCTTTGAGCCATTGTTTCTCAAT
ACCAGTATCTAAATGATCCCAGGGTAGGGGTGCATCGTAAACCCCCCCTCTTACCCCACCCACAGG
AGGAGATTCCCCCCGCTCTTTGGGAGAGGGGTTAGGGGTGAGGGTATTCCCCTCGCCCTCTGGGA
GAGGGGTTAGGGGTGAGGGTAAACCGTCAAAAATATTCCATTCACCATTTTGCACCTGACGATAT
TTCCAACTCAAACCTGCTTCTTCAATGGCTTGATTCCATGCACCATAAGCCTTATCGATGCTTTCC
CACCATGAATCCATACCAGCTCCCAATTCCCATGCACGACGTACTACAGGAGCAAGAGTGCGATC
GCCTCTCCCCACAAAATCTTCAATGGCAGAAATACGCACATCAGTGTAATTGACTTTAACTCCAC
GCATTCCGGTGAATTCTTGCTTTAAAAGGATTTGTTTGCGTATAAATTCGGAAGTAGAAACGGAG
TGCCATTGGAAAGGAGTATGAGGTTTAGGGGTAAAGTTGGAAATGGTAATGTTAAAATTGAGAC
GCTTACTGCTGAGATGACGACATTCTTGACGCAACCATCTGACGGTTTCTGCTATACCAATCACA
TCCGCATCGGTTTCTCCCGGTAAGCCAATCATGAAATAGAGTTTTACCTGATTCCAGCCTTCCCTC
ACTGCGGTTTGAATACCCCGCAATAATTCCTCATTGGTTAAACCTTTATTGATTATATCGCGCAT
TCTCTGTGTACCTGCTTCCGGGGCAAAGGTTAAGCCCGTTTTCCGAGTACCACCGATAATATTAG
CAATATTTTCATCAAAGCGATCGACTCTTTGGGAGGGAAGGGATAAGGAGATGTTTTCCTCATGG
AGACGGTTTTTTATTTCGATGCCTACCGCAGGTAATGAGAGATAATCAGAGCAACTGAGAGATAA
TAGACTAAATTCGTTAAATCCTGTCTGTTTCATCCCTTCTACGATGCTATCTACCACCTCATCAGG
CTCAACATCCCTTGCAGGGCGAGTTAACATCCCCGGTTGACAAAAACGACATCCTCTGGTACAACC
CCTTCTAATTTCTACTGTGAGGCGATCGTGAACTGTTTGCACATAGGGAACTAAACCGATAGAAT
AGGCTGGGATAGGTGTGGCAACTCGTCTTAATACTCTCTCAGGCACATCATCACGATTGGGCTTA
ACACTACCATCGGCTTGTAAATCGTAGAATTGAGGCACATATACCCCGGGTATCTGAGCTAAATC
TAGTAGTAGCTCTGTTCTAGTTAACCTGTCTCGTTTGCCTTCTTCAATAATTAAGCCGATTTCAG
GTAATAATTCTTCCCCGTCACCCAATGCCACGAAGTCGAAAAAGTCGGCATAAGGCTCAGGATTA
GAGGTTGCAGTTTGCCCTCCTGCGAATATTAAGGGGTAATCGGTATCTTTTCTTTCTTGCCAAGT
TAAGGGAATATGAGCTAAACTAAGCATTTCTAAGATATTAGTCGCTCCTAATTCATAACTTAAGC
TAAACCCTAAAATATCGTATTCTTTAACATCTCTTTTTGTTTCTAAGGCAAATAAGGGTATATTT
CTTTCCCTCATCTTACTGGCTAAATCGGGGGCAGGTAAATAGGCTCGATCGCACATTTGTTGCGG
TTGTGCGTTAATAATATTATAAAGGATGATATGCCCTAAATTGGATGCTCCTACTTCATAAACTT
CAGGATAAGTCAATACCCATCTAACTTTTGCGGTGTCAAAGTCTTTATGTTTTGCCCCCAATTCG
TTACCTAAATAACGGGCGGGTTTAAATATTTCTGGTGTAATTAGTGTATCAGATGCGATCGACAT
TATTTTCTCAATAAAAATTACTTTAAACCTTAATTATAAGCTGTCCTGTTTGAAGATAAATAAAA
TCAGAAAATATAAGCTAATCTATGGAAAAGTTCATTAAGATTCACACAAAGAATTAGAAAATAA
TTATGAATAATTTAAAAATAGTTAATTTATATATTTATCCTATTAAGTCTTGTCAAGGAATCGA
AGTTAAATCAGCACAAGTTACAGCAAAAGGTTTATGCTTAATCAATAATTCTTGTAATTGTACCG
TAGGCGATCGCACTTTTATGTTAGTTAATGAACAAGGTAAATTTTTAACTCAGAGAGAATATCCT
CAGTTAGCAACTATAAAAGTAGATATTAGTGATAATAACTTGATTTTAAGTAGTGAAAATAATG
ACATTTCGCCCTTTGAGTTGACTATTTTAGAAGAAAATATTTCCAGAAAAGTTACTGTTTGGCGA
GATGAAACCATAGGTATAGATCAAGGAGAAGAAGTAGCAAAATGGTTTAAAAATGCTTTAAAAT
TAAATATCAATTGTTACTTAGTAAAACAATCTCCTCAGTATATTCGTCCTATTAATAGTAAATAT
AGTTTAAAAGAAAATCAACCTGTAAGTTTTGCAGACGGGTTTCCTTTTTTATTAACAATACTGC
CTCTTTAGCTGAACTAAACCATCAATTAAAAGTAAAATATCCTCAAGATAACTTGCAAATTCCCA
TGAAAAATTTTCGCCCTAATATTGTTATTTATACGGATACTCCCTTCATTGAAGATACGTGGGAA
GAAATTGAAATTAATTTGATTAAATTTAAGTTAGTTAAACCTTGCAGTCGTTGCATAATCATTAC

TACACATCAAAAAACGGGAGCAAGAAACCCTTATAAAGAGCCATTATTAACTCTTGGTAATTTTC
GAAAAACTCAAGATGGAATTATCTTTGGTCAAAATATGATAGCCTTGTCAGAGGGTGTTATTCAC
GTTTCATCGCATCAAACTATAAAACACGAAAAATAAATAGATATTTAAGATTCTTTTACAGTAAT
TATGGCAAACTATGAAAAATAACTTATATAAATCATGCTATACAGGAGTTATGGTAAAACAAAA
GTTAAAAGTTTATTAACCCTTTTTTGTCATGTTCCGAGATAAAATAATAGAAGAAAAAGAGAAA
TGGAACTTGGGAGGCTTAGATGATAATGGATGTAGCAGAGCAAGTTAGTAAACATTTGCCTCGAA
CTCCAGAACCCACTATACCTTTAGTGGATCAATATTGCAGTTACTATCAGGATTTATTTCCTGAT
GTAAGAAGTTATGAATATTTTAAGTTATTACATTTAGGAATAATCTCTAATCTTAAACGAAAAT
CATTACCAGAATTAGAAAAAGTACTAGGAGTATCATCACAATCCTTACACCATTTTCTCACATCA
TCACCTTGGTTATTATCGGAACTAGACCAAAGAAGATTAAATAAATTAGTAGAGATATTGCAGG
GGAAAAAAATCATTCTCATAGTAGATGAAACAGGAGATAGAAAAAAAGGTAATAAAACAGACTA
TACTGCTCGACAATATTTAGGAAATGTGGGGAAAATAGATCAAGGAATAGTTTCAGTTAATGTC
TATGGTGTTTATCAGAATATAACCTTTCCATTAATGACAAAAATATATAAACCAAAGTCAAGATT
AAAAGTAGGAGATAAATATAAAACAAAGACAGCAATAGCAGGAGAAATGGTAAGAGAATTAAAG
GAATTAGGATTAGAAATAGAAATAGTGTTAGCAGATAGCTTATATGGTGAAAGTAGTAGTTTTT
TGGAAGTATTAGAAGAGTTAAAACTGGAATATGTGGTATCGATTAGAAAGAATCATGGGGTGTG
GATGGGAGCAAATGAAAGAGTTAGAGCAAATAAATGGTGTAAATTTACTAGGACATTTAGGGAT
GGAAAAAGAGAAACAAGATACATAAGAGAAATAATTTATGGAAAAAAAAGGAGAATAACATACT
GGGAAATGACCACAGATACAGAAAATTTACCGAAAAATTCAACAAGTTTTGTGATGACAAATTG
TCAGGGAAATTTGAAGAAAAGATTAGGAGATTTGTATGGTCAAAGGACATGGGTAGAATATGGA
TTTAGACAATGTAAACAAGAATTAGGATGGAAAGATTATCGACTAACAAAAATAGAAAGTATTG
AGAAATGGTGGGGACTAATAAATAGTGTTTATACCATGGTAAGTTTACAAACAAAACCATTGAG
AGAATTACTATTTTCAGAAGAAGAAAAGAAAGAGGAAATAGAAGAAAAAGAGCCAATAAATAGT
TGGAAAACAAGTTTAAAGAAAATAATGATGATGATTGAGCCATTAATCTTATTTTGGTTAATAA
CACCATGGTTAAAAATGGTAAATTCATCAGGATTATTAGAAGGATTGAATAATTTATTATCGAT
CACTCATGATTCTAAATTATATTTTCCGACAGGATAATTGTTTCTATTTTTTGATAGTTGATTCG
GAGTTTGACAAAAGAGGGTTAATTATTATTAAATAATTCCCTTTTCCCCTTTTCCTCTTTTAGGG
GGAGATACAGAGGGGTTTCCTACTACCTAAACCAATAATTTACATAGTCACGGTTACATAGTCAC
GGTGAGAAAGCCATATTTAATTACTCTAAAAATAGATTCAAAAAGATGTGTTGCCCATGCCTAAA
CGAGTTTTTATTCCCGTGCCACAATAATTTGAATAATTCATTAAAAGATTAACTACATTAATCAA
TAAAGTGTCAACTTTTAAAGGTATTTTGAGTTTAACTTCTCCGATAAAACCTGTCTTTTTTTGCT
GACCAATATATTGTTGGCGACTATATAGTTTATAATAATTAAAATAGATGTTTTCTGATAAATA
TTGTGTTAATTCATCTCCTCCTAAATAAATATTACAAAAATAATTCCATTTATCTAACCAGCTAC
GAAACATTAAACTAGGAATCGGTAAAGGTAAATTAATTTTTCCTTGTGCAAAAGCAGTAGGAGT
CAAAAAATTAATAGTATATTCTCTTATACTATGAGGATCACTTGCTACTAATTTACTATATATTT
CTTCATAAGAAGTAATTTTATTTTCTCTATTAATTACATCTAATTGAATGCCTAAAAAGTCTAAG
GAAGAATTGAATTCTAAGTTTTGGATAGCATTGCTGGCATTAAATTCTAATCCTGACAATGAAAG
ATAATATTTTTCATCAGGAAATAAAATATAAAAGTTTCCTTCTTTTTTACAATTACCAATTATGC
CAGAAAAAGTAATTTCAGGGATAGGATTATTTATAAAATCTAAGGACATTTTTTGATACAAATT
TTTAATTAAAAAAACTGTATAACTACAAGGCAAAAGAGTTATTTCATTTACTGTTAATGTCCATG
TTGACTTAATTAGCATTGATAATTTTCACTGAATTATTTTATTATGTTTTGCTTTCATCTCCAGC
CCTTTTTCTACAGGGTTAGGGAAGTTTTTAACTATCAACTATTCATTATTCACTAATTGCCTTTA
CTCTTGACTACCTTAATCAAAACACTTTTTAAATATTTCCTAATTAATAATATTTAACTTTGTCC

AACCCGGCACATAACGAATGTGACTATCTCGATTAAGAATGGTGCGGCGGGATTTTGGGGCTTGA
AAATTAGGAGCTTTTAACCCACAAGTGTCTCTTATTTGCTCTCTTAAATCGTCATTTATCAACCA
GTTAATGGTAGTGCCTCCCATACCTGTGCCCCACCCAAGTCTTAACTGATAAGGGCATTCTTCCTC
TTCATAAAAATCTCTAATTGACTCAAAATCTAAATTTTTTCCTTTATCTTTATTATCATTAATAT
TATTCCAATAATCATATTCCCCATCCCATTGAATTTGAGTGAATTCTTGACAGATATTAACTAAT
TCTTCAATACTATTAAAGGGTAATTTCATACCTTGATTATGGTTAAACCAAGATAGCATTTCTAA
ATCTAAAGTAATGGTAAATTCACAGCGTACATTAAAAATCATTTCTGTGTATAAAGAGGCTTTAT
ATTTAGCATTATAATCATGAAAATGACTAGAAACAATTACTTCAGTAACAACGGGAATATTATA
AGTTTTTCGTTTTCCTGTTTTTTTATTGGGTACAGTTTGGGCTAACAAGGGGGTAGAATCACTAA
TATCTAAAGCTCTTAAAAAGTCAGTATAAGGACCTATTCTTGGTTGATATTTTTTATCTTTATAT
CCTAGGCTAAAATCTTCGAATAAATCACTCATAAATAGTCTATCATCAGCAAATTTTTGTCGCCG
TTTAAATTCTTTTTTTCCTCTATCATTTGCCAAATTTTGTCTTAATTGTGCTTCTATTTGACTAA
TTTCTTTTGGGGTGTTATATTTTTCTCCATGTTTAAGTAGAAAAAATGCGATCGCACTTCTCATT
GCTCCTTTTATGCTACTACCGGGTATATAAGGCTCACCAAAACCATTACGAATCATGGGGCGTAA
GTCAGAGATTTTACCATTTACCCATTTACGACTGATACCAATAGCAGGAAAAACTTGATTATCGT
AACTATCTGTTAAATTCTGCCAGTTTTCGTCAATACTGTCTAAAAGATATTTAATATCTTCTTTA
TCCTCAATTTTTTGAATATATTGCTGTAACTTGCCCCTCTCCAATAAAACCCGTGCTAAGGCTTCC
TGATTAGGCAAATAAACCTTACCACCAGCTTGTATATATTCATAAGGATTAAGTTGAGATACAGC
ACCTCCAATTCTCAAAATAGGGCTAGTTAAACGTATCTTAGCAGTTTGATAATTCTTGGTGAAGG
TGAGAGGTTGTAAAGTTGTGGTATTCATGATTATTCAGAGATAGAAGTTAATTGGGCTTGAATG
GGCAAAGAAAGACTAATACCACTGCGATAAACTTTATGAACTTTTTGAAATCGCTAGGGGTGAC
ATCTGCTAATTCCCCTTGAGGGATAGTGGAAAATACTGAACCTTCTGTAAACATTCGTACCATCT
TCCGTCTTAGTTGTCGCCCTGAAAAAGGTGAGCCTATCCATCCTCCTCTTTCTTGAATTTCATAGT
AGCTTTTATCGATAATTTCTGAAGAAATAGAAGAATTCCAAAATAGACTAATTAAACTGTATAA
ATCTCCCTGATTAAAATTAATAACATCTTGCCATTGGGAAGGCAATTCTAACCATTCGGGGGTAA
ATCTTCCCGCACCACTGGATTTTTCTCCACCGATGCCCTCTTCTGCCAAAAGGTGTAAAGCGGCGC
ATATTTTTATTTCTAATTCTTTTGTATTTTCAGCAAAATTAATCAGAAAATATAATCCAGAATAT
TCCTTTTCATCTTGGTAATTGTACTGTACAAAACCCGTGTGATAAAAATTGGTGCTACTATCATT
TCTATCGATTGCTACTTTGGGATTTTTCTTAATTTCAAAATTATTTTGATAATCAAATACCCCTT
GTTGTGCTAAGTAATTATTACTATTATTTTTTCCTTGAGTTTTACTTGTTAATTCTTCTATATCA
TCTTGAGTAAAATCTGCTTTTTGATACCATTTTTGCCAGATTGTCAAGGGTAAATGTTTAAGTTT
TTTGTAGGTTTTAGTAAAAGCTAAATCGTCTTTAATGGGATAGTTTTTAGGAAATAATAAAGGT
TTGGGTAGATAGTAAGTAAATTTATCTTGATTACGATGATAAATAAAAGTAGAACTATGGCGAA
ATAAATGAGGATTTTGATTAATTTGGTTCAATAAATCTTCAACTTCTTTTTTGCCAAATAAACGA
GCGTAGGAGGTAATTAAAGCACTAAATAAAGTATCCGATTTGACTCTTTCTTGGGTTTCTTCTAA
ACCTATACCAACTTCGCCGAAATGAGCTAAATTTTTAGTAAAATTAAGATGAATTAGTTTCCAAT
TAGACATGATTTATTTGTAAAATTTTTCTCTCAAGCAGGAAAAATATTTAAGTTGAGTTACTTTT
CTATTTATTTGCACTTTTGTCAGATTAAAAACCTAATGATATAGGTATTTTTTGCGTAATTTTGT
AAAAAACTTCATAGCAAAAAGGCTCAATAAATTAAGCTACTACAAGCTAATTTTGTTTCGGATTC
AACAATCCTTTTACATCTTGAAATCTCTCTAATAAATTATCTGTATTATCAATATTATCCAGAAG
AAATTTATCATCTTGTTTCTTTGTAATAGCTTGACGATAAAAGTCAAGAGTGCGATATTTTATGT
TAAAGTTAGTAAAGTTTATTTTGCCATATCCCCTTGAACCATGCCCCCCTAGGGCATCATCTTCCA
GAATTTTAAGTGCGATCGCAATATTCTCCAAGTCTTTTTCTACCTGAGTTTCATCCTCTACCGTAT

AAACCATTTCAAACTCAAATACAGAACCTGCGGGAATACGCTCCACTTGACGGGGATTGGCGGCG
GAAGTGACTCGATCAATGCCATTTTCAAATTTCCACTCGGTCATATATAAGCCCGTGTCAATATT
TTGCAGACGTTGAGCCGAATCGGCTTTTAAATGAGCATCTCGGACAATTAATCTAGCACTGTAGT
TGCGCCCCTTGATTTTGGCATACTCTTTCCCTTCAATGGTTTTTGTCTGTTTATAATCGAGTTGTT
GACTATCGGCAACAGTCTTTTCTATCCAACAATCTTTGCCCGTTGAACCAAAAACCCGACTTAAT
TCACAGTGTTTTGCGCCTTCAAACTTTACTAAATTACCTTGCACATCACTATAACCATCTTCTGTA
TCATCACTTTCGTAGCGATATGTACCACTACCACCGGAGCGATTTAGGGGTTTATTTAGCATTCT
CTCTAAAAGTGAGCGAAATTTACCTTTCATGGATGAACCGGGTAAATATGGTTGTAGGGTTATCG
GATCTCGAATAACAGATTTATCTAAGCCTCCTATGTCAAGATTTTCAGCACCACCACCAATATGT
AAACCAGTTTTAACAAGAATGTCACTTTTAATGATAATTTTACCTAATAAATTTCTTTGGGGCTT
GTCGATAACTTGTGCCATGATTTATTTTCCTCCTGCGGCTTTATGATAGGCAATAATAGATTCGA
TGAATTGAACTAAACGCTCGAAATCATCTAATGATTGAACTTTATCGATGACTTGTGCCATAACC
TCACTCAAAGGTTTAGCGGCATCCACTCTAGCGGCGGCGTAGGCTAATTTGGGTTTTAGTAAGAC
GATTTCGGGTTCAACTAATCGAAATAATTGGGCTTTATCCTCATTTTCTCCTGCTTGAGTTAGTT
TAACTTTAAGACGATTTATTGCGTCTAAAAACTTACGAATTTGGTTGGTTTCTAACCGTTGATTT
TTTAAATATCTACCAAATTCTTCCGAATGTTTAACTAAATCTCGGATGGGATATTCTTTAAATGT
TTCTGTGTTTTTTAAACTATTTAAAATTGTCTGAGTGCTTGTCATATTTGTCTGTTTTTGACCCT
CACTTTTATTCTGACTATTATTATTTTTGTTAGAATTTTGCGTTTTTTGAGTTATGTTACCTCGA
TTTGGTGATTGGTTTAACCTTGGTTTATCCATTATTTATTTTTTTATCAAGGACCAATATATTTC
TAAATTTAATCCAATAGTAAAAACAATCGAATCGTTAATTCAATTATCTGGTATTGGTATTTTTT
TATATATTTTTTTATTAATTAATTTGGACATTATTTTCCCCCTTTTTATGATGAACGATTTAATA
ATTCTAACCAAATTGAGATCGCACGGTAATAACGGGAATTATAAGGGCTTTTTAAAGAAGTTTTA
ATAGGTTCAAATTGAGGATGATTTATAACCTCATTAGGTAATCTAGTCAAGGTATAAGCTATTTT
TGGTAAGTGTAAAAAATATTTTAAATCATCTATTTCCTCTTTATAAATAAGACCTGTTTTTCTTT
TTTGTGCTTCTTTAATTTTTTGTTCTTGTATTTTAGCCGTTGCCAATAAATTCTGAATAAAATTA
CGAGAATAACTATTATTTATTTTCTGTAAACTATCCACAATAGGTTTAACACCAAACAAACTTAA
ATGAACATCACCCCCTAAATATTCTCTTAAATTATCTAATCTTTCTAACTGATTAACATTAATAT
CTTTTTTCCCTAGCCATTCTTCCCACTTAAAAACTTCCCCAAATAAACCCAAACTATCACGGCCAT
TTCCCTTAGCTTTTTCTTCCGCTTCTCCTGATTCATTTGCTGATTGATAAAGAGGATATTTCGCCC
CAGCAAAACTAATTCCCGCCGATAAAGTAACGCTAGGATTATAACCAGTATATTTCCGAAAAGAT
TGATAAATTTCAAAAGCAAAATCTATAACTTCATTCCAAACGCCACTGATAAATAAATCATCTCC
CCCAGCGTAGATAAATAACAAATTATAATTTTTTTCTGTTGCTAAAGTATTAAGATAAACTTTAA
AAAAATAGGTCATTTGACGGGATAAACTAGCCAAACGAGGTAAATTTAATTTCTCACCTAACCCT
TGAGCAAAAATCTTGCCTAAGTTATCAACATCCATGCGTAAATAAGCAACTCGCCCAATTTTTCC
TGAATTTTCTGCGATTTTCACCATTTCGCCCGCAGACATGAAACCCGATTCATTTTCTAATTCTGT
TTTTTTGCCGTAATTACCTAGTAAAATAGGCTTAATTTTTTCATTTTTATAATTACTTAAATCCC
AATTGTTAATTAAAAAAATTGTCTCTGCATTAACGAGGGATAAAGCCTTATTAATATTTTCATAG
AGATAGTATTCATAACCGGGTAAAACAATTTTATTTACCCTTTCAGTGCGATCAAAACTTTTATT
CTTTGCTCTTTTTGCTCGAACAATAGCCTTAATTTTAAATAGACGACTACCTAAATCAAACATGG
TGCGACACACCCAACAAGCAGAGGAAGAATCAGCATCATGAGGATTTAAAGGCTTCAACTTGAGT
TGGTCATCTCGATGGCAAACTTGACAAGATTCATAACTAGGTTGGGGTGTTAAAAAATCTCTAAT
TTGATTACTAAATTTTTGCTGTTTTTGTTTTCCTAATTTTTTGGGAATTTTTTGCCAATATTCAG
ACAATTTATCATTGCTTAAACTATCCACTGGTAAAGTTACATAGTCTAAAGCAAGAAACATTTTT

CCTTGATAGCTTTTACTAAACCAATCATTAAAACGAGCTTTAATAATATTAGTTATTCGTTTATT
TTCTTCATGATTATTATTGGTTAGAATATACAAATTTCCTCCTCCTGCATAAATAACATTAGTAA
GAGGTAAATTCAATTCTGTTAATAATTGTTGAGTGATTTCCGTTGTCACTAATTCAAGGAAAAAA
CTTCTAGCTCGTAAAGATTTTAAAGCACCATCAGCAGAAATTGTATAGATAAATTTTTGAATTCC
TGATAAATCTCCTCCAATCAAACCAATACTATCGGCGGAGGGATTATTTTTAGACTAACGGCTA
TCATGACGATAGATTTCACAAAATCACTATAAGCAATGTCATTGTCTCCATAACTAACATAAGAG
CCTATTTTTTCTAAACAGAGATTCAATAAAGACAAATTATTTAAATCTTCCTCATTCAAGTTACC
AATTATTTCTTTTACTTGTTTTTTAAAATTGCTAATGTCGGGAATTTCTAATGCGGGTAATTCTA
AAATAGGGGATTCGTTTTTAATGATAGAAGGCGGTAAATAATGAACATTTTCTTGTCGCTTATAA
CCTTGAAGATGAATGGAGTCAAAAAGTAAATTCAAGGGTTTAAATTCTGATTCATTTTGCCATGA
CATCACTTCTTTTGCCCGAATAATTGCTTGATGTTGCATTAATGGCAGTGAGGGAAAATCCAACT
CCAACCACTGGGCTAGTTTCTTTATTCCTTCTTGCAAAACAGAAAGGGCGATTGAATGACTTGTT
ACTTCTTTATTTTCCATTAATTCTTGTTGTGTGTAAAAAAAAATAGGATGTGATTTTTAAGAAGT
TATTTAATGTTGGCTATCATCTTCAGGGTTAAGAAAACCAAGAATACTACTTGAATATTAAAATA
GTTGGATTAAATTTCAGTATAAATGTAATATTTCTTAAAAGAAACTGAATTAAATGTAAGAATA
GGGCTTTCTAAGAGGCTTCTAGTTTTTTTGGGAATAAGGGAAAGGGAGAAGGCAGGATGAGGTG
ATGAAGGGAGGGAGAGAAAAAGGTTATTGTTAAGGTTAAGGAGAGCATATGCACTCCCTATTGA
TTATTTTTTTGGTAAGTTGGATGCAAATACTTTGAGGTTAAACCATAGTTTCGGGTTATATTCTG
GTAGCCATGATTCTTGAGAGGCTAACCATTGGGGAAACCAAGGGCTAAATAAAAAGTTTTTGAAC
GCATCAATAGTAAAGTTAAAATAAGAGCCTAACCATTTAAAAATATCTTTATTTCCTGCCATTTG
CCAAATCCAAAGAAGAAGGGGGGGATTCTGACTAGCCGCTTTTAGTGCTAATCTGTTGAAAGTTA
GCCAATCTGTGCGATCCTTAATAAAAGTATCTGCTGTTTCAGGCTCCGAGGCAAGTAAACCAAAG
AATGTATTTAACATAGAGTTAATTCTTTGAGGTGGCAAGGTTTTATGGGTAGGCACCATCATCCC
TTTGGAAAACATCCAAGTAACGGCTATATTGCTTTGATATGCCTGAATTTGATTCAATGATTCTC
CATCAAGCAGGTTATGCTTTAAAGCTATATCTAAAAGATTGGTTAGTTTGCCTAAATTTCGCACT
AATGAGCCAAATCCTGTAAAAATCAAAGGAGACTGCACTGATGCTGCATCCCCAATACATAAAAT
GCGATCGCATCCTATTTTTCGATCATCTTTAGAAACGGTGAAGCGCCCCGGAATGTAACCGAAAG
TAGGTTTTTTCCAAGTTAATTTATCCAGATCACAACGACGATATTCTGGTAAAATCGAGAAGAAA
TCCTCATACATTTCTAGTAATGAGCCGGGGTTATCAGGATGGACTTGATGATAGTGGAATAAATA
AATTGTTATCTCCTTACCTTCGGCGGGGAATAATTCCCAAATCAACTGTCTTCCTCTTGAAATAT
CCCCATGAGAAAATAAAACATCCCCATAGTCAAAATCCCACACTTCAGGCTCAAATCCTTCAACA
ATAGCACCAACAGTAGGGCAAACACTATCAAAAGTCTTATCTCCTGCCAATTGCCAAGCAATAGG
GGAAGCAGTGCCCATGGCATCAATTAATAATCTTCCCGTGGCTTCTTTTTCCTCTCCTGTAGGGG
AATAAGTCGTATTTACTGTGACTAAATCTTTGCCAATAGTGACTCGATTAAACTCCGTCTCTTCC
CAAATATCCGCCCCATATTGCTTTAATTTTTGTCCACATAAAGTTAACAACCTGCTGGTATCAAT
GGCAATATTTAAAACCTTGGGAGTATGCAAAACTTTAGCTTTTAAATTACTAGGATTATGGGCAT
CAAAAAACTTACTAAAACCGTCTTTATATTCAGCCGCAATAATTGATTCAAATTCTTTTTTTGTA
AATAAACCATTATCGATTAAAACTTGAAACTCTTCTCGTGAAATATTCCACTCTCGGTTCATTCT
GCCAAATTTTAAGCGTTCAATTAATAAAACATGGTAGCCTAGTTTCGCCATTAAAGCGGCATGAA
TTGCACCTAAAGCACCTCCTACATAGATTAAATCATAATCTGCTTTACCTGTCGTTTTATCTTTA
AAGATAACTTGTTTAGGAGTTAAATTAATATTTTTATCTTTATTTTTAACACTTTCGCGCCAATT
TTTTTCCCACCAATAAGCCCGTTTTAAATCATATTCTCCTTGGGGCATTTTTTGAAAATATTTAA
CAGTTTTAGGGTAGTAAGATTCAAGAGCTGAAAAGATGTTATTTTGATTTAAGTCAATGTCGGG

TAAGGAGGGATAATAAGGAGGAAATTGAGCCTTAATTGCTTTTTCAAATTCTTTTTTGATTTTAT
TTTCTGCTAATACTTGTTTTTCTCCCCAACGAAAGATTTTTAAATATGTAGTTCTCTGTAATCCC
CAAACAAAAATTGATAAGTGATGATTGTCAGCTAATTCTAAGCGGATACCATCAGAAGTGTTAAC
TTTTTTACCGACACTGGGTTGCCAAGTATTATGCAACCACTGACAAACTATATTAGGATTGGGTT
GAGAGACTTCTATATATAAAATTTCTGTAACTTTCATTGCTAAGAGATATTTTAATTAATATTTA
TCAAGATTTTATGATTATTAATTAATTATCTCTAATTGATGGTGTTTTTTGCTATTCGTTAACTA
CTCCTCAAATTTTTGGGTTGTGTTAGGCACAGTGAAAAGAGAAAAGTTGGTGTGGGAAAAGGCA
AAGGTTAAAAATTGAAATAAATGCGTTTTAGCTTACTTCCATCATTTAATTTTTTTCCACACGAT
TCAAATAACTGTCATAGGTATTTTGTAATAACATTGCCACTGTCATGGGTCCCACGCCTCCGGGT
ACAGGAGTAATATAACTTGCTACTTCTTTAACTTGCTCATAGGCAACATCACCCACTAAATATCC
TTTTCCGTCTTCTGTTTCTAAGCGATTAATTCCCACATCAATTACTACCGCAGAGGGTTTAACCAT
ATCGGCGGTAATCATTTCTGGCTTTCCAACGGCGGCAATTAAAATATCGGCTTCACGGGTGATGG
CGGCTAGATTTTCTGTGCGAGAATGGGCAATAGTAACCGTAGCGTTTTTTTCTAATAGCATCAAA
GCAAGAGGTTTACCAACTAATATGCTTCTACCAACCACAACGGCTTTTTTTCCTTCTAAATCAAT
ATTATTATCCGCTAAGATTTCCATAACTCCTGCGGGAGTGCAACTTCTTAAACCTTTTTCTCCTCT
TACTAATTTCCCTAAATTCTCTGGATGTAAGCCGTCTGCATCTTTTTCTGGGGCGATTTTATGAA
GTAAGCCAACCGCATCAAATTTTTCTGGTAAGGGTAATTGTACCAATATACCATCTACATTGCGA
TCGCAGTTTAATTCTTCAATGACATTTTCTAATTCTTCTTGAGACACCGTATCAGGAAAGTGTTT
GCCGAAAGATTCCATACCTATTTTCTGACAGGCTTTTTCCTTGTTTCTCACATAAACTGCACTAGC
AGGATTATCTCCCACCATCAAAACAGCTAATCCGGGGCGTCTATTTTTTATTGCTACTTGTTTTT
CAATGTCAGCTTGTAAACGGGATTGAATTTTTTTTGCTAAGTTTTTACCGTCTAAAATTTGAGCT
ACCATATCGTGATCAATATTATATTTACTAAATCAAATATACTTGAAAATATATAGTCATTCTAC
TTTTTTATTTCACCTGACACCTTTTCCCTTTTTCAACTAGCAATTTATCACGACGAGAAGTAATA
GAGCCAGTTTTTAAAAGTGTCTCAAAGTTAATTGAAATAACTATAAAATTTGGTCTTTTATTATT
GAAAAATCCTCTGTTGCTTATCCAAACTAACTAATAGCGATGTCTATTAATTGATTAATATCTTT
AATAGTATAAATTTTTGTTGTCAATTTTTGTTCTAATTCTTCAATCGTCATATCATCCAAGAATT
TTTTTTCATCATGCTTCAGCATTAAAGAAGGTAAAAGAATCTGTTTATTTATGTTCTCTTTTTCT
AAATGATATAGTAAATCTTGACCAGTTAAAAGACCTGTGACTGTAATTTCTTGTCCCCAATAAAT
ACTATTAAATGCTCTTAATTCTACTGTTAAATTTTCTACTTGATTAAGTCTTTTTACTAATGGTT
GAAAAGCTGTTTCTACTGCATTTCCCACTATCCAAATAAACTCTTTTTTTTGTTCAATTTTACTA
GGTAATTTAGCCTCAGCAATCATTCCAAATTCTTTAATAAATTGACGAATTGAGCCTACACCGTT
GCCAATTTGAGGATAATCTTCATAATGAGATTCTGGTGGCAATTCTTCTTGGGCAATTAAAAACC
ATTCATCGGCTAACCATGCAAAAGTTGAACCTGATTTTTCTCTAAATTTCTCTTGTAATTGTTGC
ACTTGTTTAATTACTTCTCTTGCTTTTTCCCTACTAACAGGGATTAATTCATCTTTATCTGGGCG
AAATTTTGTTAAGCCAACAGGCACAACTGCTGTACTAATTACCGCAGGAATATCTCCATTATGGA
AAGAAAATAAATCCTGTAATGTTGTCGTTAAATGTTCACCATCATTTATATTAGGACAAACTACT
ACTTGAGCGTGAATCTGCAATCTTTTTTCTTCAAACCAAGCCAGTTGTTTTTTAATTTCTCCTGCC
CGTTGATTTTTTAATAAATGACTCCGAATTTCTGGTTCAGTAGCATGAACTGATACATAAAGAGG
GGATAAGCGCATTTGCTCAATTCTTGACCATTCTTTGGCAGTTAAATTAGTTAATGTTAAATAAC
TACCATAAAGAAAACTAAGGCGATAATCATCATCTTTGAGGTAAAGGGTTTCTCTTTTACCGGGG
GGTTGTTGATCAATAAAACAAAAAGGACATTTATTATTACATTGAATTAAACCATCAAAAAGGG
CTGTTTCAAACTCTAATCCTAAGTCTTCATCATAGTCTTTTTCTATCTCAATAAGGTGATTTTTT
CCCCATTTATCCACTACTTCTATTTCTAAATATTCATCGGCACACAAAAACTGATAATCAATTAA

ATCTCTAGGTTTTATACCATTAATACTAACTATTTTATCTCCTATTTCAAAACCAATTTCAGATG
CGATCGAATCTGGTATTATATGACTGATAGATGCTGGTTTCATTCCCTTTAATTCTGTAGATAAA
AAAGCTGTACTGATAATATTTTATCTTTAAATTAAACCTTTTGCTCAAACAAATATTACTGTTGA
GGGTATTGAGCTAGTAAGGATTTATTGTTAGTTTTGAAGGGTTATGCTAAATTAAGTGAGGGAT
AAAATTTTAATTATCATATTTTCAACAGAGTAAATTTTCAGTTTTACTGCTCAAAAATAATGCTC
ATGGTTACATATTCATCTTTATTTGCTTCTTATTGGTGAAGACTATCTAATTATTTAGAATATAC
ATAATAAAACAGGGGCATAATAGGATTATGCCCCAAGCAATTAGCAATTAGTAATTAACTTTAA
TTATAAATTATAATGCAAAAAAAAAAAAAGGGTCAAATGTCCTCAAAAATAGTTATTCGTGGCGC
AAGGCAACATAATCTTAAAAATATTGATTTAGAATTACCCCGCAATAAGTTAATCGTATTCACTG
GGGTATCTGGTAGTGGCAAATCATCCCTTGCTTTTGATACCATTTTTGCAGAAGGACAACGCAGA
TATGTTGAGTCACTCAGTGCCTATGCCCGTCAATTTTTAGGACAATTAGATAAACCCGATGTGGA
TAGTATTGAAGGCTTATCCCCTGCTATTTCCATTGATCAAAAATCCACATCCCATAATCCTCGCTC
AACAGTAGGCACAGTTACGGAAATTTATGACTATCTACGGTTACTCTATGGTAAAGCAGGAGTAC
CTCATTGTCCCCATTGTGGTCATTCTATCGCTCCTGAAACTATTGACCAAATGTGCGATCGCATCT
TAAGTTTACCTGAGAAGACAAAGATACATCTATTAGCCCCTGTTGTCAGAGGAAAGAAAGGGAAT
CATCACCAATTATTATCTAGTTTACGCTCTCAGGGCTTCGTGAGGGTGAAAATCAATGGAGAAAT
TAGGGAATTAGGAGATAATATCGAGTTAAAGCCCCAATCAAAGCATAATATCAGTGTTGTGGTT
GACCGTCTCGTAGTTAAGGCTAGTATTCAAGAAAGACTCGCTGATTCTCTTTCTACTTGTTTGAA
GTTAGCGGAAGGAATTGCCATAGTCGAGATTTTGGACAAAGGAAATGCCCAGAATACCATTGATA
ATTTCAATATAGCAGAAAATGATCCCTCCCAGTCCCCTTTAATAAGGGGGGAGTTTTCGGAGGGT
GACTTAATAAGAGGAGAATTTCCTGATGAGATTGTTTTTTCAGAAAACTTTGCTTGTCCTACTCA
TGGAGCGGTAATGGAGGAGTTATCTCCCCGTCTTTTTTCCTTTAATTCTCCTTATGGTGCTTGTA
GTCATTGTCATGGTTTGGGTAGTTTAAGGCAGTTTTCTCCTGATTTAGTCATACCAGATCCCAAT
GCTCCTGTCAAAGATGCCATTGCACCTTGGGCGGAAAAAGATAATCCCTATTATTCGTCTCTATT
GGAATACTTAGCAAAGGAATATAAGTTTAAACTGACTCAATCTTGGTCAAGTTTAACAGAAAAG
CAACAGCATATCATTCTCTATGGTGATCAAGAAATTTTACAGGTACAATATGGCTACTATCGGGG
AGTTATTCCCATGCTCGAAAAAACCTATCAGGAAACCAACTCAGATTTAATTAAACAAAAATTAG
AGCAGTATTTAGAGTATCAAACCTGTGAAGTGTGTCACGGTAAAAGACTTAAACCCGAAGCCCTT
GCCGTTAAATTAGGACAATATTCCATCTATGATTTAACCAGTGTTTCCATCGCCGAAGCCCTACA
ACGTATCGACAAGATTCAACTCACTCCCAAACAAGCTCAAATCGGCGAATTAGCCCTCAAAGAAA
TCAAAGACCGTTTAAAATTTCTCCTTGACGTAGGTTTGGATTACCTAAGTCTCGATCGCACTGCC
ATGACTTTATCAGGAGGAGAAGCCCAACGCATCCGTTTAGCCACTCAAATTGGTTCAGGCTTAAC
AGGAGTATTGTATGTATTAGATGAACCTAGTATCGGTTTGCATCAACGGGACAATGATCGCCTCT
TAGCTACCCTGAAAAAATTAAGAGATCTTCAGAATACACTTATAATTGTAGAACATGATGAGGA
TACTATCAAGAGTGCAGACCACATCGTCGATATTGGACCAAAAGCAGGTATTCACGGAGGAGATA
TTGTCTGTCAAGGCAATTTAAAACAACTACTTAACAGTAAAGAATCCCTAACTGGGGCGTATTTA
TCAGGCAGAAGAAAGATTGAAATTCCCTCCACTCGCAGAGAAGGTAACGGTAAATCTCTTATTTT
GAAAAATTGTCATCGTAACAATCTCAAAAATATTGATGTAGAGATACCTTTAGGTAAATTTGTCT
GTGTCACGGGGGTATCTGGTAGTGGTAAATCTACCCTCGTCAATGAGTTACTCTATCCCTCTTTG
CAACATCATCTCAGTCGTCGTGTGCCTTTTCCTAAACAGTTAGACTCCTTAGAAGGCATCGAAGC
AGTGGATAAAGTCATTGTAATCGATCAATCTCCCATTGGACGCACACCTCGATCGAATCCTGCTA
CTTATACAGGAGTTTTTGATGTGATTCGGGAATTATTTGCTCAAACCATCGAAGCCAAAGCAAGG
GGATATAAAGCAGGAAGATTCTCTTTTAATGTCAAAGGTGGTAGGTGTGAAGCCTGCGGTGGGCA

AGGAGTTAACGTTATCTCTATGAATTTCTTACCTGATGTTTATGTCCAATGTGATGTGTGTAAAG
GTGCAAGATATAACCGTGAAACTCTCCAAGTTAAGTATAAGGGAAATTCGATCGCCGATGTCTTA
AATATGACCGTAGAGGAGGCCTTAAAAGTATTTGAGAATATTCCCCGCGCAGTAAATCGTTTACA
AACCCTTGTGGATGTAGGCTTAACTTATGTCAAATTAGGGCAGTCTGCACCCACTTTATCAGGGG
GGGAAGCCCAACGGGTAAAATTGGCAACGGAATTATCTCGCCGCGCTACAGGTAAAACCATTTAT
TTGATCGATGAGCCGACTACTGGTTTATCATTTTATGATGTTCACCACTTATTAAATGTCTTGCA
AAAATTGGCAGACAAAGGTAATTCTATCATTGTTATTGAGCATAATTTAGATGTGATTAAATGT
GCGGATTGGATTATTGATTTAGGCTTGGAAGGGGGAGATAAAGGAGGGGAAATAGTCGCTATTG
GGACACCTGAAGATATTGCAAAATCGAAAAAATCCTACCTCGCAAAATACCTCAAAAAATTATTA
AACTAAAACCATTTCTCATTTATTTAAAGTATATTTATGATCAAAACAGAAAAAATACTGATA
ATTAGAATATAATTAATTAATTTTTTGTGTCCAATCTTCTATTAACAAATCTTGAATTTTCTCAA
AATCACGACTATTTCTGGTAAGTAAAATAGCTCTGTTTACTATGACAATAGAAGCAATTTTTAGA
TCCATAGTTCCTATTTTAGGATATTTTTTTCTGAGTATTTGAAATTCTTTTCCAGCTTTTTCATC
AAAATCAATAATAGGAATTTGACAGTAGTTTATTAACTGTTTTTTTATTCTTTTATAAATATTTA
TTTGTTCATCAATATTATTAGATTTATTAATTAAACTTAACCACCCCTGTAGTTGTTCTTGATAA
GTAATAATAGTTACAGATATTTCTTGAGGATTAATATTTTTTAATCTGAGTTTCAAAAATTCGGC
ATCTTTGCCATTCCGTTCTAAAATACTAAGATGATCAGTATCTAAAATATACATTAAAAGATTAA
CTAAAATTTATTGATAAAAAAGGTTAAAATTTGCAAGATTCTGAAAATAAATTAAACATTGGAA
TCACGATAATATTGTCCTAATTTCATCGCTTCATCATAAAGAAAATCATCTTTAAATGTACCACC
AATATTTTCCCACCACAATGCCGAAGATGAATTTGGTTGTACGGTGTCTTTAAATGGTTGTTCTT
CCTGTTTTAAATATTCATTTACTCTCAAGATAATTAAGCTACAATAACTTCTTAATTGAATAATT
AATTCATGCTTTTCTGTGGATTCTTGAAAATATTCTTTCAAATCATAAGTAGTATCCAACAAAGT
TTTCTGAGCATTTTTAGAGAATTGTATCAATTCTGATCTTGAAAATATATAACCAAATACATCAA
TAGTTTCAGCAATTAATTGATACCCTTTAATTACTTTTTGTGAGTCTATTTCTTCATCTCCAGTT
TTCTCCTCATTAACCATAGAAATACCCAAAAGCAATTTAGTATATCCCATCAAAGAGTTTGTCAT
ATCATCAACTATATATCCCAATTCAATGGCTTCTACTATATGTTTGTAACGTCCCCACAAATCAA
ACCAAGATTTAGTTAAAGTATCCAATGATTCTGCTTTTTGTCGATAGGTAATCGAATTATCACAA
CATTTATCTAAAATAATCTCTGTTTCTTCTATGAGATTTTGAAAAAGTTTTTTATCTGAATTAGC
AAAAATATTATTCAAATATTTTGTGAGAGTTTCAAGATTGTTTAAAAGATAATAATGACAACTG
TTATTAACATTTTCTGTTGATACTGTAATGTTTTCCATGATTTATTAATTTTTATTATACAAAAC
TAAAAAAAGTTATAAGATAATTAATAATTATGAGTCAAAAACTTAGTACTTATTACTCTGTCCA
AAATAAAAAAAATAAAATGAAACAGTTCTACCGTGTCTCCTGAGTCAAGTAAGTTTTAATTATTTC
CTAATAACCACATAACATTAAAGCCAACACTAACATTATTATCCTTTTGGCGAATAACATTACTG
ATGTTTGTACTTCCGGCGGCATTACTTCCATAAACCCCAATACCAAAATTAGTTTTCGGAGGCAT
ATAACGAGCTCCTGCCGCCCAAACTGTGGGATCATTACTTACCATGGGAGTAACTTCACCAATGA
ACTGTAAACCGGGAAAAGGTTGAAAATTGATCCCTAAACCAGCACCTAAAATACGATTATCTCCA
AATAATCCTGCTTTGGGATTGAAGAAAAGGGCGATGTTATCGTTAGCGGAATAAGTGAAAGCGG
CTTCAGCAGAAAATGAGCCTACGCCATCTCCTGCATCGGATGATTCACCAAATGCCCCTCTAATAC
CAAAAGAAAAGGGGTCTCCATTAGCTTGATTTAAGAAGTTTAATTTGGTAGCCGCTCCTAATTTG
AAGGAGTTACCTATGGGTTCATCAGTATCTGCAATTTGTTGTCCTGTAAATTCTAGTTGGGCGTT
GTTGCTTAAACCATAACCTAATTGAAAATTATAGTTGGGAGATAGTCCTGTGTCTAGGGAAAAAG
CACCCTGACGAATAGTATTAGGGGTTGTTAAGATAAAACCGTCAAAGAGTAATTGTTTATCTCGA
TTGGTTAGGGGTGTCATCGGTGTATCTCCAAAACTAGGGGCATAATTGGCGAAAATGTCGGGGGT

ATAGCGCACATTTAACCCAACTCCCACATTGCCACCATCGGGGGGAAAAGCTAATAAACGAGTGG
CGGGGGTGCTTCCTAATCTATTGGTGATGGATAAATCTACTCCTACTGCGGGGCTATGTAAATAG
TTTAATCCTGCACTCCAAACTACGCTTTTACCAATATCTCCTCTGGTGTTGACGCTGTTACCACCG
GGACCTAAGGGTACGTTAACATCGGCAAAGAAGCCAAATTGTTCGGCAAATTTAAAACTTATGCC
TGTACCGATATTGAAAAATGTACCGTAAAAGTCTCCGCCATTGTTGACAGTGTCGGGAAAAACGC
TTAATCCCCCGACAACGTGCCATTGATAAACATCATCAAAGCTATAAGTAAAAGGTACTTGAATT
GTTCCTGCGAGGGTGTTATCTTGGGAGGAGCGATCGCCTCCATTAAATAAACCATTTTCTGAAGT
TACTTTTAACCATTCTAAAGAACCAATTACGGCAACATCATAATTATCTTTATCAATAAATTTAT
ATTTGATTCGAGGAGCAAGGGAGAAAAAACCAAAATCTGTTTGCCGTCCATTAAAACGGGTACTT
AAAGTGTCATCAAAGAAAGATAAAGCTAATCCTATTTGTAAATCTTTATTAATTCCATAGTCAAT
AGAGCCATTGTAAACCTGTAAGCCAGTTCCAGCACCTTCTGAACCTGTACTAAAAATATTCGCTC
CATAAGTGGTATAAAAAGCCCCTTGAGGCAGAGTATTAGCGGTTTCCACCGTCATAGGGTCAAAA
GGGGTAAGTTGTAAAGGAGTTAGGGTATATTCAATGGTGGTATTATTTTCGTCTAGGATGTCTTG
ATTTTGTGCGGTTTCTGAGTATGATTCTGAATCGGATAGAAGTAACTGTGCATTTTGACTATCTT
GTAAACTGTTTGCGGTAGTCGGGTTTTTTACTGTGATTGATTCTTCCATTTCCCCCGCATTTACG
GGAATGTACAAACTACTAATAATTAGAGTTAAACAATTATATAGAGCAATATTTTTTATTTTCA
TGGGGGTTATAAAATTCTTTAAAATAATGGCTTAATTATAATAAGTGATGGTAATTCCGAAAAA
AGTCTTTTGCTTAATACTGAAATGCAATAGTAGTTGATTTGTTATGAAATAATTTAGCCCGTTGG
GGGGGTTGGGAAAAAAATGAGTTCGGAGTTATCAATTATTCATCTTTGCCCTTTGCCTCTTGCCTT
TTTAGCTTTGACCTTTTGAGGGGGTAAATTCTGCGAGAATCTCATTTTGGCTACTGTGTTCAATC
ATTATCTCTAAAATATAATCTTTTACCTGTTGTGTATGTTCTAACATAGCAAAGTGTCCGCAGTT
ATCTATCTCATAAACATTTTTTTGATTTTGTTGAAACAGTTGATGAAAACTGGCTAGATGCTTTA
CATATTGTACTTCCATTACCTTATCTTGTTTTCCTGCGATGAAATACACTGGTTGAGATAAACGG
GATACTATTTGAGGTAAGTAGTGTACTTGTTCTTCTGTGGTGGATTCTAATAAAGATGCGATCGC
AGCTTGTTCATTAGCGCTGAGAAAATCTTTTAATCTTTGTTTGCCCCATTGTCTGCCTAAAGGAC
GTTTCACCATCATACGGGAAAAGATGAAATCAAAAAAAGGTACATTGACAAACCAAGAAGGACG
AAATTTAACTAAATTTTCCCCTGCCTTACGAAAACGCTCAAATTCTTCTTGTAAATAAATCCCTC
CTCCTGCGTTAACACAAAACACTCCTGTAACTTTTTCTCTACATATATCCGCAGTCCATAGGGCAA
CACTGCCCCCCAAGGAATGCCCCACTAACCACGCTTTTTCAATGGCTAATTCTTGTAATAAAATCT
TTAAATCTTGAGCATAACCAGATAAATCAAACTCACTCTTATTAACAGAAGTGTTTTCATCCCAT
TGAGACAAACCGAATCCCTTTAAATCATAAGTTAAACAGGAATATTCGTGTTTTATTTGTTCAAT
TAACGGTAGCCAATAATGATGACTTAATAACCAGCCATGAATAAAAACTAATACAGGCTTAGTTA
AATCTTGCTGAGTTTTAATGAATTGATAATGATGATTAATTCCACGAATCTTTATTTTTTCCATG
TTTTGACTATATTTTTTAGAGGATTTTTCTTCATTTTAGCTCTATTAAACCTCCGTTTGATGCAA
AATATCGGATAAGGGAAGGTTGCAGGTGTTAGGGTGATGAGGAGATGAGGGGATGGGAGGAGAG
GTGGCAGGTGTCAGGTGGCAGGTGTCAGGTTTAAGGGAGTAATGAGTAACCAGTAACGAGTAAT
TATCAACTATTCACCCCAACACCCCAACACTATTTACCTTTACCTGTTGATTATGGAAGAGAGAT
AAACCCTTGTCCAACATAGATATTAAAAGTTTCTGTGATTTCTCCAGACCTTTTTAAGGATATTT
GGGTGATTTTATCCCATTTATTAAAAAATTGATTATATTTTGTGCCATTACTTTGATTTTCTGCA
AATCTTTTTGATGTAATATAAATACCATTTTTGCCGATTAATTCTTTTTTTGTCGGATGCCAATA
GTCAAAACCTCGACTATCATTACTAAAGCAGGTTACAGGAATGGTTGTTAAAGGTGCGATCGCCA
TTCCGATATAACCACCTAAATAGTATTGATTGGTAAAGATAAAGTCTGCTTCTTTTAAGGCTTGA
TTAAATTGAGGAGAGTTTGCCAATAGTTTTCTTAATTGTACAATATCGATTAATTCTGTAGAAGG

GTCATTTTGAGGGGAAATGATACCGTTGAAAAAAGTGTTTTGATTCGGTTTTTGTAGTAATCCGA
TTTGAAAATGCAATAGTCCAACGATGAAAATAGTGATAGTAAATAGAAAAGATAAATAAAACCA
ACGATAAATGAATTTACGCCTATATTTATACCATTTTGAAGTGTAGTCACCCAAAATTAAAGTTA
ATCCCCAAAAACCAGGCATAGACCATGTTGGTAATATTTGAGTTACCGCTCCCAAAACAGTAAAA
CCAACGGTTAAAGGTAAAGATACCCAGAGAATTAGTCGATAGGGATAATGACAGGATTTAACTA
ACTCTTGATAACTACTGTTTCCACTAACCCACCATAAAGGAAAACCAAAACTAGGAAATAGATAA
CCAATAGTAGCTAAGGCAGTAACTACTATATTAAGTAGATTAATAGAGAAAGGTGTCGGGTTTTC
TGACTGGAAGCGCCCAGATAGTTGAAAGGCAAAAGAAGCCCAATTATGTTGCCAATTCCAGTATA
AAAGGGGAAACAGGGTAAGAATAAAACAAATAATTCCCAGTATTAGCCAACCTGAAGTAAAAAC
TCGCCGATAGGGGGAATTAAAAAGGCAAAATCCGACTAATCCTAAACCAAGAATAAAGCCGTGGT
ATTTACTCAAACAAGCTAAACCAATACAAATGCAGATAATAACTAAACGAAAACTGGGTTGATA
TTTTTCTTGGGGGGAATAGAAAAAAAATTCTTCACTGGCATAGTATAAAGTAAGAGTCCAAAAA
AAAATTAAGGGAACATCAGGTAAAGTTAAAACTCCGATCGCAACTGTAAAAATGGGAATTAAAG
AGGCTATAATCAGGGAAAACAATGCACTTTGAAAACCAAATAACTTTTTTCCTGTTACATATAAC
AAATAAAAACTAACTGTAGAAATAATTAAAGTGCCAATTCTAATGGTAAACTGATTAACTTCTCC
TGTTAGCCATACACCTATACCCGTGGTTAAAGCCACAAAAATAGGATGATCAAAATAACTCCAAT
CTAAATTTTTACTATAAAGAAAATAGTATGCTTCATCATATCCCGGAAACAAAAATAAAGCGATA
ATGATTCTGATAATTAATCCTAGTACCAGAATAAAAACGACTGGATAAGATGAAAAAAGTAGTT
TTGTTAATTTGATCATTGATTCTAGTTATGAATCTCTAACTAAATGAAGTTAATATATAGGGTGA
AAAATTCTTGGGTTATCAATTTATTATTTTAGGTTGAATGCGTCCTTTTAAATTCTCTATTTTAG
ATTCTTATTTATTTCAGGAGTTAATTCCTCCTTTTATTTTTAGTGTTGCTATTTTTTCTACATTG
GGAGTTGCGATCGCAACTTTATCTGATTTAAGCTATAAAATAGTCAATGCAAACTTACCCTTTAT
TTATGCGTTACAAATTTTCTTTTTAAAGATACCTGAATATGTAGCTTATGCGCTACCTATTTCTG
TATTGTTAACCACATTAATGACTTATAGTCGTCTTAGTAAAGATAGCGAATTAATTGCTTTTTAT
AACTGTGGTTTAAGTCTTTACCGATTGATAACACCCGCTTTAATTTTGAGTTTAATAGTGACAGG
ATTAACCTTTATTTTTAATGAATTAATTGTACCCAATGCTAATTATAAAGCAACAACAATTTTAG
TAGAACAAATCAACGAACAAAGGAAATTTCTATTAAGACAAGATATTTTTTATCCTGAATATGT
AAATGTAAAAGAAAAAAATGGTAAAAGTAATAAATACTTAAAAACCTTATTTTATGCCCAAGAA
TTTGATGGGGAAAATATGCAGTCTTTGACAATACTAGATACAGAAAAAAAAGGACTAAATAAAG
TAATTATTTCTGAAAAGGGTACTTGGAATAATCAGGATAAAGTATGGGATTTGTTTAACGGATT
TATCTATGACATCACCAAAAATCAGATAAAAGCTGAGGGTAAATTTTTCGAAAAAACACAAATT
TCTTTACCAAAAACTCCCCTAGAATTAGCGTCAAAAAGTAGAGATCCTTACGAAATGAACATCAT
TCAATCATTAGAATATATCAAACTATTAAGACTATTAGGGGATGATAAAACAGTATTAATGTTT
CAAGTTCGCACCGCTCAAAAAATATCTTTTCCTTTTGTTTGTGTAATTTTTGGTTTGGTGGGTTC
GTCTTTAGGGAGTCGCCCAAATAATGCCAGTAAAGCAACAAGTTTTGGCTTATGTGTCGGTATAG
TTTTCTTTTATTATTTAGGTAGCTTTATGATTAGTAGCTTAGGATTAATTGGGATTATTTCTCCT
TTGATGGCGGCATGGATTCCGAATTTTATTGGCTTATTAATAGGAGGTTTTTTATTAGTAAAAGA
AAATGGTTAAAGAGTTAAGAATTAAAAATTAAGGCTCTTCTACAGCAGGGTTTTTTCAAAGTAA
GGGCAAAATTATCTTGCCCTCACCCCCAACCCCTCTCCCACAGTAGAGGGGAGCGTTTTTTCTTAA
TAATTGATTAATTAATACTTAAAAACTCCTGTATCTGTTACTATTTGTTAACCTGAGTTTTGGAT
AAGCTGAAAGCATTATTTTCTCGTAGTCAAAAAACCTTATAGCTTCTTAGAAATAACGATAAAAT
TGCCTTAATCCGAACTGACTTAAACAAGGGGCTTAAGCCCCTTGCTAAAAACCCCTACCACCCGCA
ACCTGCAACCTGACACCTAACACCTAACCTTATCAGATATTCTTAAACCGAACTGAGGTTATTTG

TTAGTTTCAAAAATTGACATTTTTGAGAATGAAAAAACCCTGCTTCTACAGTAGTTATGACAAAT
TAATAATAAATGGTATCTCAAACTTTTATTTAAAGAGGGTTTCAGTTAAACAAAAATAAAAAAT
TGATATAATTTAAGTCTTAAAATCCCCTCTTTCCTCTTCCCTATTCCCTATTCCCTATTCCCTTTT
TCAACTAACAATTTATCACGACGAGAAGTAATAGAGCCAGAATATTTAATCCGAGTTTGATATAA
AATTCTCGACGGTGTCAAGTTTATTTCAAGTCGTAATTAACAATTAACTAAAACCTGATACTTGA
AACCTCCCTAATTGATTCTTAATCTTCAATTCTCAATTTTTATAGATCAAGCGATTAAACTTTCT
GCATAAAGATGATGTTGATTTATCTCAATCTCTGTTGAATTATGCAATTCTATTACTTTGCTTGA
AGGTAAATGAATTTTTTCAACATGAGGAGTAAAACCTAACCATTGCTTGGATGTTATCGCAAAAT
CATCGCTGTTACCACTCACACAAAAACGGGTAGGTAACAATAAACCATGATTACGTAAGTTCATA
AGTTCTAGTTCTTTTTCTGCCGCTCTTACAACATAATCTGCAGGATCAATAAATCGTACCGAAGA
GGGGAGAATTTCCGATAAAACTCCTTTAAGGTGAGGATAATGGGTGCATCCATAAATTAAAGTA
TCGATATTCGCTTCTATAAGAGGCTTGAGATACTGTTGAGCCACTTTTTTGGTGTAAGGATCATA
AATGCGATTTTGTTCAATCAGAGGCACAAATTCGGGACATCCCATTTGCCATACCTGTGTATTAG
GATTAATTTCCGCGATCGCATCTCTATAGGCATTACTTTTAGCTGTTGCCACCGTGGAGATAACG
CCGACTCTTTTTCCTGCCTTAACAGCACCTTTTGCTCCGGGATGAATTAAACCCAGAATAGGGAA
ATCATACTCAGACTGTACTGTTTCCAATGCTAAAGCAGAACTGGTGTTACAAGCCATTATCACCA
TTTTGACACCTTCAGACTGCATCCAATCGAGAATTTGACGCACAAATAAAATAATTTCTTCCTTA
GTACGAGTACCATAGGGAAGACGTAAGGTATCGGCAAAATAGAGAATTGATTCTTGAGGTAAAT
AACGATAAAGTTTTCTGAGAACAGTTAAACCGCCCACTCCACTATCAAACACACCAATTCTATTA
CCCAAAGAATTTTTCATATAACGTTCAATATTGCAATCAAAATTATCTTAATTTAAGCAAAACTA
CTCAGAAAAGTAAATAGACAATTAGGAATTAACCAATAGCTAAATGTGAGTTCGGAATTCAGGG
TTAGGAGTTAAACTTAACATAGATTGATGAACAAGCTCAATAAATTGATTTTCAAGAATTTTCTG
CTCGAGTTAATCATTTAATAAAAGCAAAATTTTATTTATGATAGTTCTCAAAGCCTTATTTTTTTT
CAACATTTCTTTCATCAAACTCACATTAGTTAAAAGCCCTAAAACTTCCTAGGCTAAGACGCATT
TAAGTTACCATTTTTAATAAGTTTTAAATAAGTTCTAAAAATGACTCAAAGGCTTAACTTTTCTT
TCTTCTCTGATAACTGATACCGAATCAAAGACGGTTAGTTTTAATATACTCAATAATTCCAGCGG
CGATCGCTTCTGCCATTTGTCTTTGAAAAGTAGGATTAGTTAACTTGGCAATATCTTCTCTACCC
GTCAAAAAACCAGCTTCTACCAACACCGCAGGCATATTAGAAGTTCTTAACACATAAAACCTTGC
TTGTCGAACCTTGCGATCATTTACATCAACTCTTCGTAAAACATTACGATGAATAGTATCCGCTA
AAGCCTTACCATTTTGAAAGTAATAGGTTTCATAACCACTCACTTGAGGCTTATTTGCTCCAGCA
GAATTAGCATGAATACTCACAAACAAATCAGCATTAATACGATTAGCCATTTCTGTACGTCCTTG
GAGACTGACAAAATAGTCACTATCTCTTGTCATTCGTACCTGTATGCCCTGCTGTTCTAAAATAC
GAGCTAATTGTTGGGAAATACTTAAAACAACGTGTTTTTCCTGTACTCCTCCAATACCGATCGCA
CCCGGATCTTTTCCGCCATGGCCTGGATCAATCATAACTACTATTTTACCATTAGTAACAGGTCG
ATTAGGTAAGGGAGCAGAATTAGCAGAAGGTAAAACAGGGCGAGAATTGTCCCTCGGAGTGTAA
TCAAAAGGACTTACACTATTAGGAGCAGGAGTAACGGGAATATTTGTAACAGCTTGAGAATTACT
ACTGGAAGAATTTAAACGATTGGGAGTAACAGGGGAAGAGGATGAACGACTAACATAACCACTT
AAGGGTAGTGTCACCACTCGCTCATTTGATTGACTAGGACTATAAATTCTAGTTCCCAAAGCAGG
TTCAATTAAAAGAACCACAGTCTTATCATCAGGTTGCCAAATGCGTAAACGAGAAATGGGGCTTC
CTGTGGGCAGAGAGGGGGATTTAAAACTAGGGGCTAAATCCGTGTTTTCCATGCGTAATTGGTAA
ACCTGATTTGCTGTTGCCCAACTTCCCTTCGCTTGAATACGTTGATTTCCTCTTACTACCAATTGG
TTATTATTAATGTCAATGGATTCTATTAACGTTTTTTGAGAATTATTAATGCTACCACGGGCGGG
AGAAACATTAGATTGTGCCACAACAGACTTATTATTATTTGATAAATCTATTACCCGACTAATTC

CCCCCTGAGGCCATAAAACAAACCCCCCCATACGACTAAAACTTCCCTGCCAGTCTGGACTATCAG
GATGAACATTCAAGGTTAATAATGCAGAATTACGTCTTTGAGTAATTTCTATATTTTCTACACCA
TATTTATTAACAGCCCAAGATTTGAGTAAATTAGAAGGAATATTAATATTACTAATTTCAAAAT
TAATTTTTTTTCTGTCTCGACTGCGATCGACTTTTATTTGATTACGATGATCCCCATCAATACCCA
CAATTAAGCCACTAGAAGTAACTTGTATCTCATTGCCGCCACTAGCGGTAGTCTCATTAGTATTA
TTACTATTATTGTCACTACTGTTGTTATTAGCACTAAGACTGCGAGTATTGCTAGAACTATTAGA
ATTACTAGGATTACTGGAAGAATTATATGGATCAGAAGGAGAAGAAGGAGCGGGAAAATTACCC
CGTTGAGGTTGAGGTAAATCCACAGACCATTGAGTGGGGGATAAGCCTTTAATTTTTATCTGTTG
GGGATCTAAAATATATCCGGGAGCTAATTCAATAACTAATCTCGTGGTACGAGAATCAAACTGAC
CAATTCTTAAACTGGTAATCAGACCACCGTAAGTTTCATTAATAGTTGGTCTTCCCAAAGTAGTT
CCGGGTAAGTCAATTACTAAACGAGTAGGATTAGCAATTAATTGTGCCGTAGGTTGTACCCCTTG
ATCTGTGGTAAAAAGTAATCTATTTTGATTTGTTTCATAGCGCCAAAATAACAATCTACCCGCAT
AAGCCGGGGTTGTTACCAACAAAAAAGTTAGACAACTTAATATAAAAGCATAAAATTTAATCAT
AGTAATTCTCCGCTATAAATCTTAAATCCCCTTCATAGTAATGTTAATTTTTTATAAACATATTA
TTACTTAATGACAGAGACATGAAAAAATTGTTTCTTAGGTGAAAAGTCGAAAGTTTTAGCAATT
AGCTTTTTAATAACCTCAGTTCAACACGAGAATATCGGATAAGGGGAGGTTTAGAGTTAAATACC
CTCGAATTTGTTATTAATCAGTCAAAGAATTGAAAATAATAAAATCAATTCCTGATATTTTTTCC
TCAAAATATATAAATTTTAACTCCGTTCACCACTGAAAGGAGTTATAAGAGTCGCAAGCAAACTT
TACGAACTCAAATTATCTGTTGCCAATAGTTTTACTGAAAGCAGGACGTTGAGAGATTGTGTCAA
TATAATTGCTAATAGCAGGATAATCCCTCAAGTTTAATTTCAACAAAATTTGAGTGTAAGCCAAC
ATAGAACCAACTGCGATGTCGGCAACCGTTAATTTGTCTCCTAAGAGAAAAGAATGGGATTGTAA
GATTTTTTCTAAAGGAGGAAGTAGTTTCGATATTTCCCTTTCCCGATTCGCTTCTATAAATAAAC
CTGTAGAGAGGGTGGAATTAGCAAATAAAATCCATTGATTAAAAATACTTCTTGTTTCTAAAGA
GTCAATTTCCTGACCATATTTTTCTGCTAAGTATAGCAATATTGCTCCCGATTCCCAAAGAATAA
AATCTCCGTCTATAATTACGGGAACTTTCCCCATGGGGTTTAGTTTTAAAAATTCTGGGGATAAA
TGTTCTTTATTTGCCATGTCGAGGAGAATAAACTCATAATCTGCTCCTATTTCCTCAAGATACCA
TTGAATAATAGAAGCACGACTGCGACTTCCTCCGTAAAGTTTTATTTTCCTATCCATTACTATTT
CTTGCTAATTATTATTTACTGTTAATGTTTCCGTCGTTATGAAAGTATTTTTACTCATTTTCGGA
GATGAATACTCTCTATTATGACGTTTTGCATGGTAATTTTGAAAAACATTCCCTGCTTTTACACA
TATAGCAGAAAAACAGAAACAATTAAGGCAAGAATCAAAAATAACTTTTATGCCAAGACATTGT
TTCACCACTCACACCATGAATAAATTTATAAAATTAATACTTTAAAGTCTTTTGTGAGTATTAGG
ATGTTGTTTTACATTATCATCATCGGAAACAATATTGGTATTATTCAGCATTCTTTTTCTTTCCG
TTGAATAGTTTAAGTCGGTTTTACAATCACCTAAGTTGATTAACTCTTTTGCACTTGGCTTGTTT
TGGTAGGTGCGGGTAGCGGCAAATGTGCGATTGATAAAGTCTTCGCAAAATAAACTTTCCGAAGG
AAATAGACTTGGTTTAATCAGTTTATCTTCTTTGATTATTTCACCAATCGTAAGTGCGATCGCTT
TTTCGTAGGAGGTAGGAATTCCCTTAAATAATGCTTCTAGGGCGGCTGAGGGGATAGGTTCGATG
ATTTTAACCTCTTTGGTTTCTCCTTCTTCTCTTAGAAAGTAGGTGGCTAATCCGAAAACGCAATA
ATCTTGAGTTGCAATTCCTTGAGTGATTACTTGATTTGTCATAGTTAATGTCAATAGATGTTTTT
TTTATCTTAATACCTGACTGAATTAAGGGTTATTATAATTGAACGTTACGAAGAATAAAATTGCT
CTGGATTTAGTGAAGTGATTGAGCTTAGATTTAAGATAGTAGTCACAATTTTTCTCTTTATTTAA
AATTAATTTATTGTAATTTTTTGTCACTGTCTCTTAATTTATTTTTTACTCTAAACCTATACTCT
TAAACCCCAATGGTGAATCCCTAAGCCCTAACGTCAAATTTTGCCCCTAGAACTGGGAGAGAAAA
TGACATGACTTAGAATAGAACTGCTATATAATTTATGTCTGATTATTCTAATATAAAATTACTTA

TAATCGAATTGCTAAAAGTAATCTTACCTTTATATAAAAATGTATTCCAAAAAACGTTCTTCTCT
GTTATTGACTACCTATTTACTATTGAGTTGTGTAACCCCCTCTCAAGCTCAAGATGTTGAAACTA
ATACTTCTCCTCAAAATCCTTCTTCTGCTCAAGTTCAATCAACAGACAAATTTAATTCTAGTGGT
AATCCCCTTCTTTTTCCCACTAAAACTCAGGAGGTAGAAATTGAAGAACAACAGCCTGTAACTTT
AAATCAAGCCATTGAAATTGCGTTGAATAATAATAAGGACTTGGAAGTGGCAAGATTACAATTG
GATAGATCCAGAGATAGTTTAAATGCGTCCTTAGCAACTCAATTACCCACTCTTAATGGTCAATT
AGAGTTTAACCAAAGAGGAGATGATACTCCCGGCGAAGATGACTTCATTATGCGCGATCGCAGCC
TTTTATTAGGACAAATCGAGTTAAGTTATAATGTGTATGATGGTGGTCGTCGTAATGCAAACATT
GCTCGTTCTAGCAGGGAAGTTTATCTTAATCAGTTAGAAGTTGAGCGTATTTCTGAGGATGTTAG
GTTTCGGGCGACAAATGCTTATTATGAATTGCAAAATGCTGATGCTCAAGTTGCCATTGCCCAAG
CGGCCATCGAAGACTTTTCCCAAACTTTGAGAGATGCCCAGTTATTAGAACAAGCTGGATTAGGA
ACTCGTTTTGATGTATTACAAGCAGAAGTAGATTTAGCCAATGCGAACCAAGCATTAACTAGGGC
GATCGCAGAACAGAGAAATGCTCGTCGAAATTTAGCACAAGTGTTAAGTGTGGGTCAAAATGTGG
AATTAACCGCCGCCGATGAGATTCAAGAAAATGGGGAGTGGCCTTATGATTTAGAACAAAGTATT
GTTATGGCTTATAAAAATAGAGCGGAATTACAACAACTATTAGTACAAAGAGAGATTAATGAAC
AAGACAGAGAGATAGCCCTTGCCGCCACTCGCCCCACTGTCAATTTATTTGCTAACTATAACTTT
ACTAACACTTTTACAGATACTTTTGACAGTACAGGTTATGCTAATGGTTACAATGTTGGGGCAAG
ACTTAACTGGGAGATTTTTGACGGTGGAGAAGCAAGTGCAAGGGCAGATGTAGAAACCAGAAAC
ATTGAAATTGATGAAACTAATTTTGCCAAGCAGAGGGATGAAATTCGCCTGAATATCGAAACTGC
CTATAATAATATGATTGCTAACAAAGAAAATATTTCTACCACAGAAACTGCTGTTACTACGGCGG
TGGAAAGTTTACGTTTAGCAAGATTACGTTTTCAAGCGGGAGTTGGTACACAAACGGATGTTATT
AATGCCCAAAGAAGTTTAACCGAAGCTAGGGGAAATTTCCTCCAAGCTATTATTGGTTATAATCA
ATCTTTGAATGAATTACAAAGAGCAGTCAGCAATTTACCTGACAATAAACTTTTTGAGGTGAGAT
AGTCAGAATTAGGAAAAGGGCAAACCCCCCTTTATCCCCCCTCTCGAGGGGGGAAGGCAAAGGTA
AATAGTTTATAATTAAATCCTCAACACTCAGAACCCTTAAACCCATTACTGTCAAATACAGATAA
GTCAAGATGAGAAAATTCTTGGAAAAAGACAGTTAATCGAGAGGCGATTTTGTTTACTCCCCCTT
GGACATTGGTTTCAATATTAAGGGGGATAACAGGATCGTGTAATGATAATCTTAGTAAAAACCA
TCCTTGTTCTTCTGGGGATGAACAACTAACTCTAATGCCTTCATAGTTAGGAGTGACAATTTCCC
AGTCGGCTTGATTTTCTACATAAAACCCCAGTTTATCAATGACATTATTACCGTAGTTTTGGAAA
TCTTCCACATTGATTTTTAGTCTAAATTCTTCGCTTTCTATGGGTTCAATGAGATTTGAAATTAA
ATCTGTTAAATTTTGTCCTTTTAAGCGTAATTTTGCCAATTCAATCAATAATTTCGTTACTAAAT
AAGCTCCATCATCAAGAAAATAGTTTTCTTTTAAGGCGGCGTGGCCTGAAGTTTCGATCGCAACC
CATGATTCTTCGCCTACTTTATTTAACCTCAAGGATTCATTAATTACGTTTTTATAACCTCTTTT
AAAGCGATGATGTTTTCCACCTAAATCTTCTATGAATTTTGTTAAACCATCGGAGGTAATAGAGT
CGGTGACAATAGTTGAGCGTGGATGTTCTTTCAATATAATTGAGCTAATTAAGGCAATTAAACGA
TTACGATTCAATTCCTTTCCCTGACTATCTACGGCGGCGGAGCGATCAACATCTGTGTCAAAAAT
AATACCAAAGTCGGCTTGATTATCGATTACTGCTTGGGCAATAGAAGCCATTGCCTCTTTATTTT
CGGGGTTAGGAATATGATTAGAAAAATTTCCATCAGGTTCTAAAAATTGACTTCCAGTTATATCC
GCCCCTAATGGTTTCAATACTTTTTCGGCGTAAAAACCTCCTGCACCATTTCCCGCATCGACAATA
ATTTTTAAGCCTTTTAAGGGTTGCTCAAAGTGTTGATGGTGGTTAACGTGCGATCGAATTTTCTC
AACGAATAAGTTACTATAAACGGAAATAAAATCGTGATTTTCTACCTTTCCTTCCTCATTAGTTT
CCGAAAAAACATTTTTTTCTGCGATCGCCAAAATATCAGCTATATTCTGCTTTTCTAAACCACCA
TCAGGGGTAAAAAATTTTAATCCATTACGATTAAAAGGCAAATGACTAGCAGTGAGCATAATTG

CACCATCACAATTAAAACCCTCAGTAATGCAACTCATAAACATAGCAGGGGTTGAACATAAATCA
AAATTAAGAACTTTTGCCCCTAAGCTAGTAATACCTAGTGTCACTGCTTGAGCTAAAACCTCCCC
TGATAAACGACTATCCCTACCAATAGAAATAACTAATTGATTTAATTTCTTCTCTGTTTTTTGTT
GCAACCATTGAGTAAATGCTTTTCCTAAGGTCTTGACTACTTCTGGAGTTAAATTAACTTCTTCC
CCTTCAATTCCCTCTAAAGCTACCCCTCTTATATCTGAACCGTTTTGTAATTTTTGCCAGTTAACC
ATAATAAAATCCTCTGATAGATGATTAATCCTTTGTTGATAATCTGAGACTTATTTAACTTATAG
CAATTCTCTTTATGGTGAGCGACACCAATCATTCGTATCGAGGGATAAATTTCAATAGACATCTG
GTAGAAAATATTAATATAGGCTCGTAGTTAGGGCTTTAGCCCCCTCTGGTGGCAGGGTTTTTTCA
TTCTCAAAAATGTCAATTTTTGAAACTAACAAATAGTAACAGATACAGGAGTTTTTAAGTATTA
ATTAATCAATTATTAAGAAAAAACGCTCCCCTCTACTGTGGGAGAGGGGGTGGGCAAAAGTGTTT
AATTAACCTCCCGTAAGGGTTGAATATATTCAACCCCTACCACCCCAATACCCTAACACCTGAAAC
CCGACACTTTAAACAGATGCCTTTTCTTACACAAAAAATAATTCCAGTTGAAGTAATGATTAAAT
AAAACCACATATCAACTGAAACTTATTGACTAATATAAACTTTTTAATTTACTATAAATTTTGCA
AAAATTTGATTGATAAAGAAATGAATTAAATCGGTAGAAAAATTTTCCACTTAGGATGACCAAA
AATTAAAAACGATCTAAATTGGCACAACAAATGACGTTTTAAAAAACTTAATAAGGTTAGTAAT
AATCTGTTTCTGGTTGCATTTGAGTCGCCATTAGTTCAAAATTAGAAGGGTCTCGCAGAAAACGA
TATGCCTCTTCTTCTAAACGGTGGATGAGGTAGCCTTCGATAACATTAGTTTGACGGAGACAAGC
TAAATACCCCTCCATATAAATACGTAGTTCTTCATAACGACTACCTCTATGCCATAAATCCACCA
TTCCATCAGTTAATTTTTGATAGTATCGGATGGCAAAAGGATCTGTTAACATTGTTCTTTGGGTT
GAATTTTTATTTTTGGCAAATTGAAGTTTTAAACTTCTCTTAATTTCATTGTAACTTAATTACCG
GGGAATCCCACATACCAGAAATATGTCACCATTGGAATAACTGTTGCTAAAACTAAAAGAACTAT
TGTCCAAATAGTGGCACTACGATCATCTGTTTCTTCCGCCGTGGTAAAAGTCCCTTCTATGCTGAT
TTCTTCCACTTGAGGAGCTCCGGGGTCATCTTGTCCAGACAAAACAGCTACAAGGCGATCGCCTGC
GGAAATTAAAGCCTGATTATACTGTAAGTCTTTAAGGGAATAAGGCAAGGTTTCTTTTACTAAAC
TTTCGCTAATTTCTGGGGTTAATAAATTGCTCACTCCATCTCCAGATGCGATCGCACCTTTATTAC
TAAGAGTATCAACCACTACCAAAGTTTGATTTTTTGCTGACTCTGGAGTAGGATACCATGTCTGA
AAAAGCTCAGTCGCTAAAGAGTCGATAGTTTGACCATAATCAAGACGATTAATTACAACCATTCG
TACTTCATTACCAGTATCCTTAGCCAAATCTTGTAACATAGAGGTTAATTTGCCCTCCGTAGCGC
GACTAATCGCATCAGCATCGTCATAAACCCAAACATCCTCCCCCGCATTAATTACAGGTAAATCA
TATACCCCTGTGGCAAAAGCAGTATTGATTCCTACATTAAAAAGCAATAGAAGGGAAAGACAAA
AATAGGCAAATATATATATTTAATTTTATTCATAAAATAATTGCTCCCAATTTGAAAATAATGATT
TAATTATCTCATGTTATAACCGCTCATTAAGAAATTTTTAGGGTTTTTTTAGATCATTTCACCCT
TGGGGATATAAATACTTATTTCATCTATTTCAGGAAAAATTGAACCAGTTAACTGCATCTCTAAT
AGCTTTGTCTATATCAGTTTGAGGTAAACCTAATTCTTTTACAGCCTTCTGAGAATTGTAAAACA
TATACTGACTAGACATTTTTACCGCTTCCACAGCTACGGAGGGATTTTTCCCTAATTTACTCAAA
ACATACTCATCTAAATAGGCAACAGCTAAAGGAAACCAAACAGGAAATTTAACTTTTGGGGCAGA
TTTATTCGTTATTCTGGCTAATTTATCAAGAAATTCTGCTAAAGTTAAGTTTTGATTTCCTAAAA
TATATCGCTCCCCAGTTTTACCTTTTTCCAAAGCTAAAATATGTCCTTTTGCTACATCTTGAACAT
CAATAAAATTTAAACCGGTATTTACAAACCCCGGCATTTTTCCCCTTAAAAATCTCACAATAATT
TCTCCCGTGGGAGTTGGTTTTAAATCATATGCACCAATAGGAGTACTCGGATTTACTATGACAAT
ATCTTGTCCCGATTTGATGGCTAAATGGGCTTCTTGCTCCGCATAATATTTGGATTTTTTATAAT
TACCAATTAAATTTTCTACAGGAGATTGATAATTTTCATCAGCTAAAACGCCATCTTTTCTGACC
CCAATTGCCGCCACTGAACTGGTGTAAATAGTACGTTTTATATTTGCTTTTCGAGCGGCGTGAAA

AACATTTTTTGTCCCTAAAACATTAGTTTGATATAACAATTCTTTATCTTTTGACCAAAGAGAAT
AGAGAGCGGCAACATGAAACAAATAATCACAACCCATCATTTTATCATAGAGATTTTCATCATTT
AAACTCCCCTCTACTAATTCAATATCTAAATTTTTAACGTTCGTTAAATCTGCATTTTTTCTAAC
TAAAGCTCTTACCTGATAATTTTCTTGTAATAATAATCTTACCAAATTAGCCCCAACAAAACCTG
TTGCACCAGTAACAAAAACTCTTCCTTTCATAATAATTAAAATACATAAAATTAAATAAATTTAC
AAAAAAATAACCTCTTATAGAGAGGTTTTAAATATTATTATTCAGATTAAACTTCTTATGATTA
AACATATTTAATTCAAAAACTGTCACCCTCAAAAAAATTGTCTAAAGTAGCAATATTATTATTTT
TGATATTATTTTTCGGTTGATCATTATGGTTCATTAATAACCAGTAAAAACCACCAAATATTAAT
ACGCTAATCATGCCAAAAACTAAAGAGCCATCCCCTTCATGTAAATAATCAAAAGCCTCATTGTT
ACCCTTTGAAACGAGAATAGCCATTAATCCTACTCTAATGGCATTTACAAAAAATGCAAGAATAA
TCGCACCTAAAATAATTGACAAGTTATATTTTTTCTTAATAGGAAACATCTCCACCGCCAAAACA
GACAAACCTAACATATAACAGATTAAATCAACTCCTGCACAAGCAGTATTAACTTCAACACTGCC
AGTAGGTAAATGAATATATACCCCTTGTAAGTTAACAGAAAATCCAAGATAAGATAGTAAAAAA
GCAGTGGTCGAGGCGGTAAATATAGCTATGTTAACTATTCCTTGATCTATCAGCCCAGTTAAAAC
AAAAGATGGAATGCCTAAGAAAAAAAGGATAATTAACTCAGATTTGAACTGTTTTAATCCTTGA
AAGCCAGAGGCTATCAAAGCACAACCTAGTCCCACAAAAAAGGGTAAAACTCGTTGTATATCCGC
ATGTTTAGTCGCTTCTACTCCAATTGTACTTTTGGAAAGAACAAATAATAGAACTATTATCCCTA
AAATCAAAGCAGGAATAGTGGTGTGTAAATTAATGTTTTTTCGTCGCTCCCAAATCATCGAACCA
ACTCCGAGAAAAAACAATGCACTTAAGCCAAACTGAGCAGAATCCTCCGCTTTCCACAACAGAAA
TAAGTTTATTATTGCAAATGCGATCGGAATAATCCCCAGAATCAATCTAGGGATACAAATTTTTT
TGTTCATAATAAGATTTTTATTCATAAGCTATTGATATGATTATCAATATAAATCATATTTCTAC
TCAGTTTAAAGAGATAATAATTTATGATACAAAAGCAAACTATTCTGTTAATAACTTCTACGATC
CAACCATCCTTAAATACTTATTTATTAAAAATAACAGATCCTGAAGAAAGATTAAAAGATTATG
AAAAAGCATTTTATTTTTATTGTAGTCTTATAAAAAGCTGTATTGTTTCTGCAATCATTTATTGT
GATAACTCAGGTTACGATTTAAGCTCTCTTAAAAAAATAGCTGAAGAATTAGAAGTATTATCCAC
TGTTGAATTTATTAGTTATAAATCTAGTATAGATACCATTAATAATGGTCGATTTTATTTAGAA
ATTAATTTGGTTAAATATTGTCTTGACAACTTTTTATTTATAAAGCATAGTCCTCTAGCTACAAT
ATATAAGGTGACAGGTAGGTACATTATAGCAAATATAAAAAAAATTATTTCGCCTTGGAAAAAT
AATAATTTTGACTGGTTTTGTAATTGTCGCAATTATCCTTACAAGTGGACAGATTTTTATCTTGT
GGGTTTTACAGTACAGAGTTTTAAAAAGATAATTTCAGAAACAATGTCATTATATGAAGGGAAA
AAAGATGGAGAAATTATATTAAGAGAATATTTAGATTCGTTAAAAAATTCAGATTTAAAAATTG
TTAAAAGATTTCCAGTTACTCCTCTAATTAAAGGAAGAAGGGGGTACGATGGGGGATACTATGGG
CAGGGAAAAGATTATATCAAGTATATATTCCGTTCAATCACTAATTATGTTTTACCTTTTATCTG
GTTATAGTAAATAGTTTCTTAATTTTTGTTTATTACACTTTGATAAATTTCTAAAATGCGATCGA
TCTTTTTTTCCCAATCAAATTGATTAATTACTTTGTTATAACCCATTTCTCCCATAGATTTTCTTA
AATCGGGATTAAGAATTAATTTCTCCATGGCTTGGGTTAAATTTTGTATAAAAGACGCTTCTGAA
TCAGGATCGACTAAAATACCACAGGTTTCATCCAAATAATCCGCAGGTCCTCCCCAATTAGTCGC
AATAACGGGAATTTTCATTGCCATTGCTTCTAAAACGACAGCTCCACCACATTCATAAAGACTGG
GCAACACCATCATATCCGCACTTTTTAAATGTTGGGCGCAATCTTTTTGCGATAATTGTCCTTGA
AAGGCAATTTTACTGGAAACACAATTATCATTGTTATCAATAATTAATCCTAAGGATTGAGCTTG
TTTCTCTAAAACTGTTCTTTGTTTACCTTCCCCAATAATTTCCAAGGTTATTTTATATTCCGAAA
GTATAGCAATTAAATCTTTGAAAGCAACTAAAAGTAAGTCAACTCCTTTCCATGAAACTAATCTT
CCTAAATAAACAATTTTTATCTCTTTTAAATTTTTTTTTGTAGCGTGAAAGTTTGTCTCAATTTC

TTGTTGCCAAATAGATAAATCAACACCATTTTCTACTAATTCAATAATATATTTATTTTGCAGAC
ATTGGGGTAAAGCATGTTTGCTTCTTTTATTGGCAACTAATAGTAAATCTGCTTTAATTTTACCC
GGAATTAAGATATTGATAAAATTGGAAAAATTACGCCCCAATTCTAGGAATAAATGAGTCCAAG
GTGACTCTCTATCTTTAAAGCCTTTAGGGTAGTTCATCCCCCCATTCATCGGACCGATGATAATG
GGTACTCCCATATCGTATATCATAGATGGTTCTTTTGGAGAAACGGAATAGGTTGATGAATAAG
ATCAATTTGATACTCTTTGATTAAAAACTTTGCCAATTTTTTTGTGCTAAATTGAGTTAGCATTC
TTAATAAAAAACCAAAAGTAAAATAAGAAATTCTTGAAGGGAAAGGTTTACCCAAAAAGAATAC
TAATTTGTGTAAAAATGAATCTTTTATAAAAAAAAATTCTCTCTAAATCATGGGCAAAAATTTGA
TTTAATTCTGTTTTCGTCCTTTCATGAACAATCATCCATACATCTATTTTTCTTTGTCTTAAAACT
CTAAAATAGTGTAACGGCAAAGCGGCTTCACCTCCAAATTTCAGGGAGGCGTGTTCTGATATAAT
CAAAACTTTCATACTATTCAAAAATTTCTAACATTATTATTCTTCTAATTCTACAAATATTATTC
AATAATAGGTGTTAAAATTAAATATTTATCTCTAATAATTCCTTAAATTGTTGAAACGGTAAAG
CACGACTAAATAAGTAGCCTTGATAATAATCGCAGTTATTTTTATGCAAAAATGAGAGTTCTGGT
TCTGTTTCTACTCCTTCTGCAATAATTTTTAAGTTTAATTCATGAGCCATTTCTATTAAAGATTT
TGTGATAGTGGCATTTTTTGGATTACTTTCAATTTTATTGATAAAGATGCGATCGAGTTTTAAAA
TATCAAAATATAAATTCTGTAAATATCCTAAAGAAGAGTAACCTGTTCCAAAATCATCGATCGCA
ATTTTTAAACCTATACTATGTAATTTTTTTAATTTACGTGAAGCCATTTGATGATTATTAACCAA
GAGACTTTCACTAATTTCTATTTGTAATAAAGAAGGATTAAAATAATTATCTGCCAGAATCTTTA
ATATTTTTTGTTCTAAATAATCTTGATCAAACTGCCTTTTTGAAAGATTAATAGATAATCGCAAA
TCAGATTTAAAATTTTTATGTAGATAATTAAATTCTTCACAGGCAGTATTTAAAATCCAATTATT
AATAGTTTCAATAATACCTATTTCTTCCGCTAAAGGAATAAAAATTTCAGGGGAAATATATTTAT
CTTCAGGATTATTCCATCTCAATAAAGCCTCAGCACCAAAAATTTTATTAGTTCTCATTTCGACT
AAAGGTTGATAATAAATTTCTAATTCCCTATTTTCTAAAGCATTGTGTAATTTTGCCTCTAAACT
TACCTGTTTTGCCGTAAAACTATGAAAAGCAGGGGAGTAAATTTTATATTGATTTTGTTCTAAAT
CTTTAGCCGCTTCTTTTGCTTTATACAAAAGTTGATCTATATTTTCTCCATGTATAGGATATAAA
CTAATTCCTATTTTAAAATCTATATATATTTCCTGTTGTTTATAAATAAATGGTGGAGTTATTTT
TTCTAACAAATTAGTTGCTAAATCAATTGCTATTTGTTTTGTTTTGATTGGAGGAATAATTAAAG
CAAAATCATCTCCATCTAATCTTGTTAATATATAGTCCTCATTTAAGTTAGCCTTTAATCTTTTT
ACCAAAGCCTTAAATAACATATTAGCAAGGTCTTGTCCCAACTCATCCCGAATTAAATAAAAACG
ACCAAAATTCAAATATAATAGAGGAATAAATTGAGATTTATTATCAATATCCAAGTCTGCTTGA
GAATTTAATTGTTGATAAAATAACTCTAAAATACCATTAAAGTTTTCTACCAAAGATAATTGAT
TTGGTAATTGAGTTAACTCATCATATTTAGAGATAAAATTAAGTCTTTCCTGCTGAGATGTTAAC
TGATGAGTAATGTGACTTATTTGATTATATTTTTGCCATGCAATCCTAATATTTGTAACTAAATC
TTCAGTTTTATAAGGCTTAACAATATAGCCATAGGGAGAAGTTTCCATAGCTCTATTTAACGTTT
TCTCATCAGCATAAGCCGTTAAATAAATAATCGGAACACACATAATTTGATTGATTTTTCTTGCC
AAATCTATGCCATCAACATCTCCCTTAATTTTTATATCCATTAAAATTAAATCCGGTTGATTTTC
CTTGATTTTCGGTAATACTTTATCATGACTATTTTCCTTTCCTATTACCTGATAACCTAAATTTT
CTAAATCTAACTTAAGACTCTCGGCGGCAATAATTTCATCTTCTATGATGAAAACTTTAAGGGGA
GTATTCATGGTTTAGTTTTTTATATTTATTAATACATCAATGAGAATTTAAAAATCTTTATTTCA
ATGAAAATTAAATTAAAGTTACAAATTTATTATATTTATATTTGATTATTTATTTTTTTATTACC
CTTAATAACATAATTTAACTATATGTTAAGATTCATTCTAAATTTCTGTAGTTGCCTCTAACATC
TTATTCTCTAACAATATAATAAATTAGTTTGTCTATTTAATGTTCAAAATAATAACATTTAAAGT
GATTCTTTACAAAACTTTTCAATTAATTTATATCTAGCTAACGTGATTAAAACTAGAAAAATCCT

CAATACCTTTAGAAATTAAGAGTCTTTCCCTTTTTCTCCTCGCAATTTCTCTCATATTGACGGTTT
TATCTGTTTCATCAACAATATCACCTGTCAAAACTTCTAATACATCTTCTAATGTTACCACCCCAG
AAACGCCACCATATTCATCAATTACTACCATTAAATGTTGTCGTAATTCCTGAAAATTTTTCAAT
AAAAGATCCGCCCTAATAGTTTCAGGTACAAAGTTCACATTTTTGGCTAAATTTGCGATTTTTTC
TGACCCCTTACCTTTAATGACTGCGGTTAATAATTCATTCTTAAATGCTATTCCTAAAACATCAT
CAATGGAATCTTGAACGACTAAAATTCGACTGTGTTCAGACTTAATAATTGTATCTTGACATTCA
CTTAAAGTTAGATCACCTTTTAAGTAGGTAATAATAATTCTTGGAGTCATCAAATCGTACGCAGA
TAAATCATTAAGATGAAAAACTCTGTTAATCATTTCTGCTTCATCCGCCTCAATTACTCCCTCGT
TGCTACCAATATTCGTTAATAACTTAATTTCCATTTCATTGGTTGTCGGCAAAACTTTTCCCTTG
GTTAAAGGCTCTGTTATTTTTTCCATCAACCACACCAAAGGATTTAAAATAAAGGCTAAAAACTT
AGTAGGAAAAGCCAACCATAAAGCAATATTATCAGCATATCTTTGTCCTAGAGTTTTGGGAATAA
TTTCACCAAATATAATAATTAAAAAAGTCAAAATGGCAGAAAAAACTCCTAACCATTTACTACCT
AAAGCCTCTGTGGTGAAAGTACCAATTATGATACTACCCACAATATTAAAAATATTATTGAGGAT
AACGATCGCAGCTATAGGGCGACTCATTTTCTTTTTAATTTGTAACAAAACCAAGGCAGAAGACT
TTTTTGACTGAGCCCACTGTTTTACTTTTATATCAGAAACAGATAGAAGCACAGTTTCAGTTAAA
GAACAAAAAGCAGAACCAGCTAAAACAATAATGACGGAAATAAATAAGTTGAACATTATAAAGG
AGTTAAGAGTTAGGATCAAATGTTATTAATCATGGTTAAAGGGAGAAGTAGAGAGTAGAAATTT
CTAAAATATTGATAAAAATTTCCCCAAAACTCAATCTCCTAATCCCTCAATAAATATTTATTGTA
CCCCTACAGAACGCCCTCCAGAACCAAAAGAAATAACTCTATCTTCTGTGAAAGGTATCCAGCCG
ACTTCATAACCCATACTTCTTGCTTTTTGTCTTGCTACACGAAATGCCTCAAAACTGTCGGGACGT
ACTAAGAAAGCAACGTAGTTTATTTGTGGATCTAATTGTTTTAAAATAGTACCATAGTCAGAATC
TGTCGCCATTAACTCTTTCTTGTTTTCACCAGAGGAAGTCGCTACTGGCTGATAAATAATTGATC
CTTGTTCACTAATGGTAACTTGATAATCTTGATTACTGTAGTAAAAATTTTTTAATTTTTGTTCC
CTTGAATTAACACAAGCCTGATATTCTTGAATTTCTTGAAGATAGAAACTATAGAGATAATCGGG
AATATCATTGGGTATGTTTGGATCAGAACAACGAGGTAAAGTGGTAACAACTTCATCTATTTCTT
GACTAATTTGAGGATCGCTGACATAAAATATCTTGTTATCTCTCACTTCAAAAAAATTAGGTATT
TTGTTTGTTTCTGAACGTAAAGGAGTGCGAATAATCGTACCTGCTTCTACTGCCAACAAACTCAC
AAATAAACCAATAAACATTAATACCCCTACGGTGTTAGTTAGAATGTCTAAGAAAGAATCTAAA
TTTTGACTGGGGTTACTTATTTTTCTGGCTCTTCTGTTTCTCATTGTTATATAAACTGATAATCT
ATATTAGAAATTTTACTATTAGGGCAAGGAAGATATAAGGGCTTACCTGAGTTCGACATGAAACT
GTCGGTTAAGCTAGGGAATAGGGAAAAATCCTTCTGTATCGACTCTTTTAAGGAGAGAAAGGGA
ACAGGTGACAGACAATAGTCTTTATAATTGACATATACTATAATTAGATTGATTTTAAATACATT
TTTTCTCAATTTAATGATTTTTTCGGGAAAATTTTGCTCATTCCATGTCTTAAATCCTTATTTTT
CAATACTTTTTTTATCAAACTTAGGTAACTGCAATTGGGCATAAGAATATTAAATGTAAGTAAGT
TGTAGAGATTGAGCATTGTTCAAACCTTATGGTAACAGGTTTCAGGTTAAACATTGGGAAACAAG
ACTTTATGAGAATTTTGCGATCTCTCCCCTAAATAATGGTATTTTTACTCACATAATGATTTTTT
TGCATTTTTGAGAAATACTTATGACTGTTACTGTTATCCCTCAATCCATAGAAGAAGCTATTATT
CAGGCAAAATCAGCGTTACAAATTGCCCTTGAAAGTGGTTGTACTCGCATTACTATTGATTTAGT
CATTCCTGAAATTGCCTTAAAAGCTCAATATTTAGCCCAAGAGTTTGCGGATTTTTTCTCTGAAT
ATGGTGCAGGGCTGAAATTATTTTTCCCTGATACAGGGGCGGCGGCTTTGGCAAGGAGAGATTGG
GGAGAGACGGAATTTCAAGTAACAGATATTGGTAGTAGTCGTAGTCCTATTGAAAGAAAAATTA
GCGATACAGATCAAGTTTTTCTGGTAATTTCTCCTTCAGCAGTGGAAGTTAATTTAGTGGAAAAG
TTATGTAACATAGCAGGCGATCGCCCTGTTATTTTATTAATTCCTCAATTAGAGGATGTTTCCAT

TGTGGGTATTGGTTATGCGGCTAGACAGTTAAGGGAAAGATTTATTAGTATTCTCGAATCTGCTT
ATTATTTCCGCCCTTTAGAATCGGCAGTGGTGTTAAGAAGCTATCCTCAATTATGGCAGGTATGG
CAACAAAATGAAGAAAATGAAGACTATGAGTTAATCGCAGAAGTTCCTCAAAAGCCTTTAGGGG
AAAACCTCGATCGCATTTTGCAAGGTCAAACGGAGGATGATTCTGATAATAACAATAACACACCT
CCTCCCTCTGCTTCTGGCTTGTTTGCTTCTCTCAAAAGTTTCCTCAAAGCCCTAAATAATTAGAGA
TAGGGGCTTACTTGAGTATCTTATTTCTTATTCTCGTTTAAAAATAGTGCGGCATTGAGTAAGGC
TTGGGCTTTTAACATGGTTTCTGCAAATTCTAATTCGGGGGAAGATAAATAAGTAATTGCTCCGC
CAACACCGATAGCAGTTTTTTCTGGGGTTATAATAGCAGTACGAATAACTATGTTTAAGTCCATC
GTGCCGTTAAGGGCTAAAAATCCGATACAACCTGAATAAATGCCCCTTGCTTCCGTCTCTAAGTC
GTCAATAATTTCTAAAGTGCGTTTCTTTGGTGCTCCAGTCATTGAACCACCGGGGAAACAAGCCT
TAATACAATCTGTGGTTTTGATTCCTTCCTTGATTTTTCCTTTGACTGTGGACACCATTTGATGT
ACTGTACTATAAGATTCGATCGCCATTAACTTAGGAACAGTAACACTGCCCACCTGACAAACTTT
TCCTAAATCATTGCGTAATAAATCGACAATCATAAGGTTTTCTGCTTGTTCTTTTTCACTACACT
TTAATTGATTTTTTAGCTGTTCATCTTCATCTTTAGTTTTCCCTCTTTTTACTGTGCCTTTGATGG
GCTTTGATTCTAAATTTCCCTGTTTATCCAGATGCAAAAAACGCTCAGGAGAACAAGAAACAATA
CTTAAATCATTAAATCTAAAAAAACAACTATAAGGAGCTGAATTTTGAGTTCTTAAATAAGAAT
AAAAATCTAAAGAGTTATCAATATTAGGCAAATAAAGATGGTTTGTAAGACAAATCTCATAACT
TTCTCCTAATCTTATTTTTTGTAAAGATGATTCTATATTATCTAAATACTCCTTTTTATTTCTAC
TTAAATAAATCGACAAGCCACTACATTTATCATGATATAAATTGTCATTATTAGCTTTGAATATA
TCATTTAGATGCTCTTGCTTAGAATATTTGCCTGTATTTGAATTATCTTTTATACTAGAAACTTT
TGAATTAACATCCCATAAAATGTCTGCTTTTTTAGGAGAGTTTTTTAATTGAAGATTAGAAGATT
TTTCCCATGATTGATTATCATTATACTGATGTAGTAAAAATAGTTTTTCCGCTAAATTTTTAAAC
CAGTTATTAGCTTTTTGTTCAGAATTTTCTTTTCCTAAATAAACTAAATAAATATGCTTGTCTAA
ATGATCAAATACAATCATCCTATCTGCAAAAATAAATTGAGCATCAGGATAAGAAGATATATGT
TTATTTTTATACCCACATAAGGCTTTTAATTCATAACCGAAATAACCAACAAATCCCCCATTAAA
GTTAAAGGGTAAATCCAAGTTATAATTATTATATTGGTCTAAATATTGTTGTAAAAAATCAAAA
ATATTATTCTTAATTATCTTTTTTTCTTCTTTTTTAAACTGAATTATAGTATTGGTATTAACATC
ATAACTAATAAAAAAACTATTTTCCCCTTCACAATCTCCCATATAAGAAAATCGAGATAAACCCT
CTGCTATCATACTACTGTCTAACCAAAAACTAGGATTGGCATCTCGATATAAATGCTCAAAAACA
ATTTCGGAGTCAACATAAAAATCTAATTTTTGACTAAATAACTTGAATTTATCCTGTTTTTTATT
ATCTATACTCTTATTTAAAATTATTTTGTCTGCTACATTTTGAAAGCTATTTAATTTGATATTTT
TTATATCTTTATTATGAGATAAATAATATTTTTCTGAAAGAATATTAAAGTTAGTTAATATTTG
CTCACCATATTCAGAGCAAATGGATTCAGGGTGAAATTGCACACCCCAAAAAGGTTTTAATTTAT
GTTTTATGCCCATAATTAAATTATCTTCAGTATGGGCTATTATTTCTAAATTAGAAGGAAAATTG
GCAGGATTATTCGCAATTAATAGAGAGTGATAGCGAACCACAGAAAAAGGAGAAGGAATACCAT
CAAAAATACCATTACTATTATGGTAAACTCTGCTAATTCTACCGTGATAAGGCTCTGGTGCTTTA
GTTATTTGAGCATCATAATAATATCCTAATCCTTGATGTCCCAAACAAATGCCCAAAATTGGAAT
ATTTGAGTTTAATAAAACATCTTGACAAACACCAAAATCTTTTCTATTTTTTGGATTACCTGGCC
CCGGAGAAATAATTATATTATCAAAATTATTGTCTTCAATTTCTTGCCAAGATAATTGATTATTA
GTTATAACAATAGGTTTTTCTTGATTTATTTCTGCAACTAATTGATATATATTGTAAGTAAAAGA
ATCATAATTATCAATAATTAAAGTTCTCATAAAAGTTGTTACAAAACAGTGCTTTAAAGCGTTAA
TTATTTTACTCTTGCCCTCAACCTATCTCCCAGAAGACAGGGAAATTTTTTCTAACAATTCCTTAT
TTAACTTATTTAAGAATATTATATATAGCGTTTATTAAAACTAAGTAAACAAAAGTTTGTAGTT

GCCCCTTTAGGGGTTTCAACAGTTCCGGAAATAAAGGGCTAAAGCCCTTACTACAAACCTTTTAC
CGTTTTATGAGGTACATTTTTTTATACTATTATTACTTATTTTCCGATAATGAGAACTACTATAT
ATTTAACAAGGATTATGAAAAGGGAAAAACAGCCAAGGAATAAAAATAAATAATAACCAGATTA
TTTCTAAATAATTATTGTTAACCAAGATCTTCTTTTTCTGCCATTAAATTATCAATTCTGACTTGT
AAACGATTATCGTAAAATGTGCATTCTAGGTTGTGATTAAATTCAGAAGGTAGCGTCATTATAGA
TGATGTAACTTGTATCAAAGATTCTGCTATTAAAAGATAATCAACTTTTTTCGCCGCAATTTGAT
CCGCTCTTAATTCCCTCAATAATATTAAATTATCCCAAAGTTGTTCATTATTAGGTAAACAAAAA
CCTAATTTTTTAAGATAAAATAGCCAGAAAAATAAAAAAGGATCTCTACATTTCTTATGGGCTGA
TTCATGGGCTATAACTGCTAATAAGTGTTCTTGAGAAAGTAGTTTTATTAACCCTTGACTTAACA
CTAATTGGGAGTTCCAAAAACCTATCTGCGCGGCATAAGGAAAATCTGTTTCTAGGAGTTTAAAC
TTAAAATTATTAATTTTTTGCACTGAACACTGTTGCAAAATTGACCAAGTTTTGAGATGATCTAA
GGTATTTTTAATAAGAGTGAATAAAGCATAAATTAAGAAACTAATAGATAAGTAATAGCTCAAT
TTACTAGCTTTTATTCCCCACATTTCTCCTTGATAACCCATACAAATAATGGCAATACAGGTCAT
CAATAAAATTAAAGTAGGAAAGGCAAATAAAAATAAAGATAAGCCCCATTTTTGCTGATATTTT
TTACTAATAAATTGGCTTAATATTCTCAGACCATAGGCTAATAATAAACTGATAAAAATTAATA
ATAAGTGCATTTTATTGTTTCTCTCCTCTTTGTTTTCTTACTTCTGCTAAACGAGTGGCGATCGCT
TCTATTTGTTCTATACTTGCAGTATCTAAGCTATCAGCGAAAGAAGCAACTACTTCTGGGTTACT
AATAGCTAAAAAGTTATGTAATTGTTCATAGGATTGCACTGCTTGGGCTTGTTCACGAGAAATGA
GAGGATACCAGAAAAACGCTCTACCCTTCTTTTTGTGCGTTAACCAGCCTTTTTTGGTTAGTCTT
TGCAACACTGTCATCACTGAAGCATAGGCTAATTCTCGGTCTGGATCTGCTAAAATGCGATCGTG
TATATCCTTAGCACTAGCACAGCCTAAATCCCAGATAATTTCTAAGATTTCTTGTTCTAAGAATC
CTAAAGATAATTTTTGCGGTCGATATTTTGGTAAAAAAGACATTTGATTCAATGAATAATTAAT
TTAGATTCAGAGCGATAAAAGAGGTTTCAGGTTTCAGGTTTCAGGTGTTAGGGAAGACGAGTTA
GGAGTTCAGAATTACAATAATAAATACCATTGCCTGTTGCCTGTTCCCTATTGCCTACCTTAACC
GACAATTTTATATCGAACTTAGGTTCTATTTAGGGTTAACAAAATTGGCAAAACTCAACTCATTA
ATACTGTTCCATTGTAAAATACCCTTTCTAAATTCTAACCATGATTGGTAAATTTCTTTAAAAGT
AGGGTTTTGTTGAGCATTACTTTCATACAATTCAAAGGATATTTTTTCGGCTTGTTTAAGAATAT
CTTGAGGAAAAGGAACTAATTTTGTGCCTCCTGTAATGAGGGATTTTAAGGCTTGTTGATTGACA
AAATCGTAGTGAGCTAACATGGTAATGTTGCTTTCTCTGGCACAGACTTCCATCATATTTTGATA
CTCTGTGGGTAATTTTTCCCAAGCAGATTTATTTATTAAAATTTCCAAAGAAGGACCTGGTTCCC
ACCATCCGGGGTAATAATAGTATTGAGCAACCTTGTTTAAACCTAATTTTTCATCATCATAGGGG
CCCACCCATTCTGCCGCATCGATCGCTCCTCTGTCCAGAGCTAAAAAAACTTCCCCTCCCGGAATA
TTTTGAACTGTTACCCCTAATTTACCCATTACCTCCCCACCTAAACCGGGAATACGCATTTTTAAA
CCTTTTAAATCCGCCAGAGATTTTACTTCTCGCTTAAACCATCCCCCCATTTGCGCCCCCGTATTT
CCTGCAGGAAAGTTGATAATATTAAAATCACTATAAACTTTTTGCATGGCTTCTAAACCGCCACC
ATAGTACCACCAAGCGTTATGTTGCCCCGCCAATAATCCAAAAGGGATACCCGTGCCAAAAGCGA
GAGCCGAGTTTTTACCCACATAGTAATAACTAGCAGTGTGTCCACATTCAACAGTAGCATTTTGC
ACTGTATCTAATACCTCTAATCCCGGTACAATTTCCCCCGCCGCATAAGGAGTAATTTGAAATTT
ACCGCTACTCATCGCCTTGAGGCGATCGCACAAACCCACTACAGATCCATAAATAGTATCTAAAG
ATTTAGGCCAACTCGTCACCATACGCCATTTTATTTCAGGTAAACTATTCGCCGTAACCGAAGAT
TTATTTTGACTTTGACAAGAAACTAAGGTTGCTGAAGTTACCGCACTAATTCCCGCATTAGTTAA
AAATTGTCTTCTTTGCATATATTTGATTAAATAATTATTTGATTGTCCTTAAAAAATATCATCTT
AAAGAAATTAATTAATCATAAAATCATAAAATCATAAAATAAAAAATCATCATGATAACGATAC

GTTGTAATCAACATTTATTTCGCAACATAGAAGCGATTATTTTTGACAAAGATGGTACTTTGGCA
GATTCAGAAAAATTTTTACGGGAATTAGCACATAAAAGAGCTCGTTTGATTGATGCAAATATACC
CGGGACTTATGAACCTCTCTTAATGGCTTTTGGAGTAGAACAAAACTATCTTAATCCCACAGGTT
TAATGGCAGTAGGTAGTAACAAAGAAAACCAAATTGTAGCGGCGGGATATATTGCAGAAACGGG
AAAAAGTTGGTTTGAAGCCTTAGAAATAGCCTCCGAATGTTTTGAAAATGCAGAAAAAGCTTTTC
CCACCAGAGGTTTAACTTCACCTTTATTTGCAGGAAGTTTAGAAGTCTTAGAAATGTTATCAAAA
AAAGGCTTAAAAATAGCTATCTTGTCCGCCGATACCACTCAAGGAGTCAAAGATTTTGTCGAAAA
TCACCAACTTTCCCCCTACATAGATTTTATCATGGGTGTTGATGGTGGTCTTTCCAAACCAGACCC
TCGCCTCTACTTACAAACTTGTCAGCAGTTAGGAGTCAATCCCCTAAATACTCTCATGGTAGGAG
ATTCTCAAGGAGACATTGCCATGGCAAAAAATGCCCAAGCAGGAGGCGTTATAGGTATCTGTTGG
AAATATCCCCACGCAACCCACTTAAATACTGCTGATGTCGTAATTAGTGATTTAGCACAAATCAA
AATTGCCTAACTTCCACTTATTACTCATTATCCATTTACTCGGCTTCGGGTGAACTCCATATCTGT
AACCTGCAACCTACAACCTGCAACCTTTTTTCTAAACCCCAACTTCTAATTACTCCCCTCATATAG
AGAGGCTTGTGATTGTAACAGTTTACTCACTTGTTCAAAAACTCCCCCTGTTACCCCAGTCTTGTT
AATTGCTTGTCTTTCAGGATAAAATAAGGGGCGATCGAGATATAGTGCAATAGCCTGCTCAACCT
GTTCTAAAATTAAGCCGTGTAATTCTTCTTTAGCTTTTTTTCTTTGAAACTCCGCCCCTTCTTCGG
TAGTAGCATAGAGAGGAGGTAAACGGTTTAAAGCATAGGCAGAAATATCTCCCACATCTAACAAT
GTATCGCTAGTAGTTTCAATTTCTGCTACTCGGCTAATGACTTCGCTTACCACAAGCTCTTCCATA
ACATTAATGAATTGTTTTTTAGGAGTGGCAGTAATTTCTCCTGTTAATAACGCCCCCATCAACTT
ATCTAAAGCCTTGTATTCTTCGGCAGAAAGTTCTCCAGAAGCATCACATAATCTACCTACTTCCG
CTTCCATGGAAGGAGTGAGATAGCCCGTTTCTAATGCCTGTTCGACAATTTTTTCAATATCCATG
AAACTTTGACAATTATTAAGATACCGATAATATTCTAATACTATTTGCTCAAGGAGATAGAATTA
GATGAAATATTTATCAGAAATTACTGTTAGTTTAGTATGATTTATACAGTAGAGAAACATTTAG
AAACTAAATGAGCAGTTGGAAAAAGGGAAAAAAACTTAATAACGGTCAATATATAGTTGAGTCC
ATTTTACTTCGAGGTGGCTCTAGTCCTTGTTATCGGGCGAGAGATGTTAAACGAGATATGTTAGT
CACTCTTAAAACTACGTATGTCACCAAAATTATGGAAGCTGAGAGAGGAGACTTAGCAGAAAAGT
TAATCAAACAAGCTCAACAGGTTGCCCAAAGGTGTCAAAGTCCTTGCTTAATTAAGTTATATCCT
CATGTGTTTCTCGAAGATGATCTCGCTTATATGGTGATGGATTATCCTGAAGGTAATGATCTTGC
TAACTATATTGATGGTAATGGCAAATTATCCCCAGAAGAAGCTCTGAAAATAATTACAAAGGTTG
CCTCGGCGGTTAATATTCTTCATCAAAATAAATGTCTTCATCTCGATTTAAAACCTCAAAATATC
ATTATTGAAGATAAAACTCAAAATCCGATTATTGTTGACTATGGTTGGGCAGTAAAATTGTTTGC
TTTAGCGGCAAGAAAGACTCCGATTTCTCCCAACGACTGTTTTTCCCCTCCAGAAAAAATCAAGG
ATTCTGGAAAGCTAGGGGTTTATAGTGATATTTATTCTTTGGCGGCAATTCTCTACGTTTTAACT
ACTGCTCAACTACCTTTAAATGCAAATTTAAGAAGTTTTCAAGATTTAGTACCCCCTATTCAATA
TAATAACCAGATGAGCGATCGCTTTTCTGATGCAATTTTAAAGGGTATGGCATTGGATATAAAAC
AACGCCCTCAATGTTTAAAGGATTGGTTAGATTTATTTAAACAAGCTCAAACCCAAGAAGAATCT
TCAAGCCAAGAAAAACAAAAAATAATCGAACAAACAGAGTTTATTGCGCCTCCTCCAGAGGAAAC
GGTAGTACAAAGGTCAACTCAGATTATTACTCCCCCCACTATTACAAAACCAAAATATAATTATC
CAAATATTGAAGAATTTAGCTTTGAAACTGTTACCCTCACAGAAGAAAAAAAATTATTTGGATTT
GTTTCTAAGATTGAAAAAAATTTAACGACAAAAAAAGGACAATATTTCGTTGAATATTTAGGAC
AAGGGGTAAATTTAGAAATGATATTTATTCCCTCTGGTCAGTTTATGATGGGTTCAAGTAGTGAT
GAGCGTCATCGTGAAAAAGATGAAAGTCCTCAACATAAGGTTAATCTAAATTCTTTTTATATCAG
CAAATATCCCGTTACTCAATTACAGTGGAAAACGGTTTCTTACTTTCCAAAAGTAACTCGCAATT

TGAAGCACAAACCTGCTCTCTTTAGAGGTGATGCTTTGCCAGTGGAAAGGGTTTCTTGGTTTGAT
GTACAAGAATTTTGTAAGCGATTGAGTAAATATACGGGCAGAAATTACCGTTTACCCACTGAGTC
GGAATGGGAATATGCTTGTCGTGGGAATACTAACACTGCTTTTTCTTTTGGGGATGTAATTACCT
CCGAAGTGGCAAATTTCGATCGCCCCAATAAGGATAATAAGAATAAAAACATTAAATACGAAAA
GAAAACAACCCCTGTGGACAATTTTTATCCCAATCCTTTCGGCTTATATGATGTTCATGGCAATG
TCTGGGAATGGTGCGAAGACAATTATGTAAGTAGCTATACCCAAAAACCAAAAGATGGTACGGCG
CATTTAACTTCTATGGTTAATCAAAATCGAGTTGTGAGGGGAGGATCATGGTCACTATCTTCGGA
ATATTGTCGTAGTGCAAAAAGAAATAGTTATGCTCCAGATTCTTCTTATAATTTTATCGGTTTTC
GGGTGGTGTGCGTCTTAGATTGATTTAACTGAGGTTCAAGGGAATATTATGGCTCTTTTACAATG
GTTATGATAAATTAATGGGGGATAACTTCCATAACTTTTCTTTAATAGTGTTTATAGTAAAATA
GAAACTAAAAGTTTATAAATTATTATTTAATAATTGTCTCTTGCCCTTCTCCCCTTAAAAGGGGA
GACACAGAGGGGTTTGCCTATCCTAACCAATAATTTACAGACTCAAAGTAATAGAGCTATTGTTT
TTTCAACCAAAATATCAGAAAGGAAGTAATATAATTGCTAATTACTCATTAATATAAATACTCA
ATAGGTGCGATCGCATGAATATTTTTGATAAATTAGAACAGGATATAAGTAATCAAATTCAGAG
TTTACAAGCTAAACAGCAAGAATTAATTGAAAAGAAAAAATACTTAGCGACAACCAAAGAAAAA
ATACAAAATTTTCTTACCTCTGCTCAAGAAATTCAGCAAATAGTAGGAGGTGAACCAGAATTGCT
TAAGTCTATCCAAAAAGAATTAGGAAAAATTTTTTCCTCTCATAATAATCCACAACCAAAACTAC
CGATAAAAAAAGAAAATAGTACTACTCCTAACCTAGAATCTCCTCCAGAAAATAAACCTGTTGAT
TCTGCTCTAAAAAGTGCAGATAATTCCGATGATACTGAAGAGGGAGAAGCCATCAAAATTTTTAA
CTTTGTCGATGCCCAAGGGAAAAATACCTCATTTTAAATCTTTGGTTGTAAGGGCTTTCTGATTC
CTGAAAGCAAACTTTAAATTAGAGATAATAGAGAAAACTATGTTGAAGGAGTCAGGAGTTATAA
TTCTATAGAAGGAATTGACTAATGATTGATGTTGGCGATATTCGGACAAATATTATCTTTAACAT
TTTCAAAATTTTTAATTTCTTGCCAACTAGATAAAATACCATTTAACTCGTCTTTCAACATCTTC
AATTCATTGATTTTTGACTCTATTTCTTCTAATTTATCTGTTAGTAATTGTTTTGCTACACTACA
GGGAATTTGTTGTTGATCATATACAACTAATATTTGTTTTATTTCTTTTAATGTTAATCCCAAAG
ATTGCGATCGACGTATAAAATTTAACCTTTGAAAAACCGCAGGAGAAAATAAACGATAGCCATTG
TTATCCCTCGAAAGATGAGGAGATAGTAACCCTAAATCAGCATAATAACGAACAGTTTTCACTGG
TAAATTAATTTGTTTGGCAACATTCCCAATAGTAAAAGAATTAAGCATTTTTTCTGTGATTTATC
ATTCTTATTTCATTACACAACTTTAGCTTTAAGGTAATAAAAACCATTCGGATGAAATTTGAATA
ATCAGAAACGATAATCTCGTTTTCGTTTTAAAGGAGGCAAAGGAGTTAAACTACTCCTACTGGGT
GGTAAGTATCGTCTAGTTCGAGACACGGTAGTTATTTTTTGAGGTTTTGTTGTTTGTAACCAACG
CATAAATAAGCCTGTAGTAGCTATTAACAGACCTAACGATAAAAGAGTCCAACGTTCCTCAAATC
CCCCTAAAATAGAATCCACAAAGCCGAATATAATAATAAAAGCAGAGATAGGTTCTTTGCGATAG
ATTTTTTTAATTGAATGAGAAAGTGACTTTTTCATAGTTTTAATTTAACATCTTTACTTTAATTT
TACTCATAATTTAAAACTTTATTTTATAGAAACAAAAGTTCATCTAATCTGTTATATTGACAATT
TAATTAACCTCAGTTCGGTTTAAGAATATCTGATAAGGTTAGGTGTCAGGTGTCAGGTTGCAGGT
TGCAGGTGGTAGGGGATGGGGGGATGAGGTGATGAGGGGAAAAGGGGAAACAAGTAATAAATAA
TAAATAATAAATAATAAATAAGTAGTAAGTATTTGTGATTTTTAATTCTTAATTCTTAATTCTT
AATTCTTAATTTTTAATTCTTAACTATTTAACTTCAGTTTGGATTAAGGCAATTTTATTGTTATT
TCTAAGAAGCTATAAGGTTTTCTGACTACGAGAAAATAATGCTTTCAGCTTATCTAAAACTCTGG
TTAATTAGCAATTAACAATTAAAAAGCAATGAGCAATGAGCAAAGGAAGAATAAAGAATGAAGA
ATAATTAACCCTTAATTTTAATTTTATCCTCACAATTTCTAACTTCTGACCCTTTATTTCTACCCA
TTACTCATTACTTATTTCGTTCCCATATATTGAATATGTAAAATTAGACATCAATTACGGTAATC

CAATCCTTATAAATTGGATCTCTATTCTCGGTAATTTCAGTTAGTTTTTCTTTCAATTTCATGGT
TATTTTTTTATCCGCAGGAAGATGATAATTTTCCACTCTTTTTACGGGAGTAATCTTTGCCGCAG
TACCACATAAAAATACTTCATCAGCAATAAATAATTCTGTTTTATCAATTTCTCTTTCTTCTACT
TCAATTTCTAAATCTTTAGCAATAGTAACTATACTATCCCTAGTTATACCTTCTAAGATATCTTG
ATCTACATTAGGAGTGATTAATTTATTATTTCTAACAATAAAAATATTCATTCCCGTGGCTTCGC
AAACTTTTCCTTGAGAATTCATTAAAATTGCTTCATCAAAACCACTATCTACTGCCTCAGTTTTT
GCTAAAGCAGAAGTAATATAAGCACCACTAATTTTGCCCCGTAAAGGTAAACTACGGTCTTCCTG
ACGATACCATGAACTGATTCGGCAACTAATACCATCAGGGGATAAATAATCTCCCATTTCTAAAC
CGTAAACGAAAAAGTTTTTTTCAATATTGTGTAATCTAGGGGCAATACCTAAATCTGAAGTGTAA
ACAAAAGGGCGAATATAGAAGGGTTTACTTGGTTTATTTTTACTAACAAAATCCTTGATAACGGT
CTTTATCTTATCCGCAGATAAATTATAGTGCAAGTATTTAGCACTGTTGCTTAAACGACGACAAT
GACGGTCTAAGCGAAATAATAACACTTGATGAGGATTTTGAGGATTAATTGTACCTCTCAAACCA
CCAAAAGCACCTGTACCATAGTGTAAGCCGTGAGTAGCGATAGAAATTTTTGCATCAGCAAAAGG
AACAAACTGACCTTCAAAATAGGCTATTGGTAAAAAATCGGGCATGATTAACTATAAAAATATT
TATTGATTCGATCGAAAATACAGATCCTTCAATAATATCATTAAATTCTATAATTTATCTGTTGA
TAACTCTTCAGTTTTTTTATCCATACTTTAAATCATCATATTTATTTGGTTATTTCTCACTTATC
AGTTAAGATAATCAAGGAAATATATAGTAAATTGTTATGTAAGTCTCTGCAAAAAAATAGGTAT
CTCATAACATCTTCCTGTTATTCACGCAAAAGAATCGAACACTTATTAGGAGTGATAAAATCAAT
GGAAAATAATCAATTAAAATACCATCAATTGAAAAACAAACTCATTGGAGAAATTTTGCAAGAA
GCAGGGTTTGTCAATTCTGACCAAGTTGCAGTTGCATTGATGGAACAAAGTTTTTATACTCATTT
AAAGTTGGGAGAAATTCTTGCACTTCATGGGTGGGTGAATCAAGAATTAGTAGATTTTTTCGCAG
AAAGAATACAATTGCCTCCAAAAAAAGAAGCAAAAAAAATAGGGGAATACTTACTAGAAGCAGG
TTTATTGACAGAAAAAGATATTCAGCAAATTTTAAAAGAACAGCACAAATCTGGGGTTAAATTT
GGCTCTCTGGCTGTCTTAAGAGGTTGTATTAAACAAGAAACTCTCAATTTTTTCCTGAAATATTT
TACAAATGAAGGTTTAAACGATAACCACTTACAATATAAAATTCGCACCCTTTCAAAGCAAAGTA
AGTTTACAAATTCTCATAAAAATACAACGCATAATGATATGGAAAAAGAAAAATCTTTTGTAAC
CTCATCCAACAATCACCGAGATAAACAAAGTTTAAACTCTAAACATACAAAAAATATAATTGACA
ATCAAGAATTAGTGGAAGATGATTTAGAATCAATGACTTGGCTGAAAACCTCTTATTAAAATAG
GGAAAGTATGTATCCGAGAATAATAGTATCAGATTCTGAATGAAGCGTTGATAAATCAGGATAC
TCAGTCTTATTATTGGGGTGTTGTGATATACTCAATTTTGGTTTTCCCTCAAATATGGAAAGTCC
CGTGATTGTTTGACTATATTTATAAAGTCATAGGAAGTTTTTTTTCGAGAACAGTTAATTAATTT
GAGGGAGAAAAATCAAAACACTAAACTACGTCTGTTTAAGCTGTTACTAACTCTTCAAACATATT
AGTGGTGTTTTGAGAAGCAAAGACTTCGGGGAAACCTCTGTCAATTTCTCTCATAGTGGCTTGTT
TAGCTTCTAATGCTGTTTGTAGAACTTTCATGGCTTCTTTGGGTTGACCTTGACCACAGAAGAAT
AAGTCTGCCGCAAAATAACCGTACTCGGGCCAGGTATGAATAGCAATATGAGATTCTGCTAAAGT
AACAGTCGCTGTAACCCCGTGGGGACTAAATTGGTGTACACATATATCTATTAAAGTGGCTTTTC
CAGCTTCAATTGCATCTAACAAGCCTTGACGGATTCTTTCAGGACTATTTAAAAGATCTGCGGGT
GCTTGCCAAGCATCAACGACTAAATGTGTACCCACTTTGTTCATTCTTCTCAACTCACTCCATACT
GTTTTTGCTAATAGGGACAATTGATTTTAAGGATTCTTTGCTATTACTTCTCCATTTTCTAATAT
ATGTTGTTTTGTTTTTTGCTAGATATATTAGGTTAAGATAATGTAAAGAAAAAATCACTCAATT
ACCTAGCATAGACAATATAAGTAAAAAGTGTCAAGAGTTTTGAGTAATTGATTACTACTTAATT
TTACAACAAAGTCGATAGGTGGCTTTCAATGATTGGTCTTAGTTACTTGAGTTCAATATTAAGAT
TGTCAATTAAGTTTAGACAACAGAGAACACCCGGGCTTATAAAGTGAATCCGCAGGAAGCACAAA

TAGCCCTTCGGCAACAGTTAATAGTATTCATAATTGGCATAAACGGGGATTAAACTGATTTTAAA
GATATTTTTCCTAAATTCAAGGATTTTTTCGGAGAAAATTTTGTCAATTTTATAAATCTCAACTT
CTTATTTTTCAATACTTTTTTCATAAAACTCAGTTAGTTTATTTTTGTGCGATCGCATCGTGCAT
TATCTCCCATTGAAGCAGGTTTAAGAAAGATCAAGAGGCAAAGAGTAAGGATATGAAGATTTTA
TATTGATAGAAAAATTGTTTACATAACTTTACTAAATTTCAGTAAAAATGAATATAAATTAAAA
ATAAGTGTTTTTTAGATAACAAAAGTGCTAACCTATAAAATATGAGAGAAAGAAGAGGGGGTTT
AGAAAAATTAAGTAAAGACTGGAGTGAGAAAACGGTTTTATTTAGAGTATGATAAAATGACAAG
GAAAGGATTATTTTCTCTTGTTTAAATTCTCAAGATTCTTATGCTTATTTATTTTATATAAGTGT
CTCTTTTCCTTGAAATAAAAAGATAAAAGTGGCTAATTTTGAGAAAAGCTAACAACGCTTTGGTT
AACTAAAAATCAAAAGTGAGATTACTGATCGCTTAAGAAATGGAGTATTGATTATGAAATTATT
AATCCAAGGAAACAATATCGAAGTTACTGAATCTATCCATGATTATGTTGAGGAAAAATTAGAA
AAAGCAGTCAAACATTTTCAAAATTTAGCGACAAAAGTTGATGTTCATTTATCCGTCGCTCGTAA
TGCTCGTATCAGTAATAAGCACAAAGCAGAAGTCACGGTATATGCCAATGGGACAATAATTCGAG
CCCAAGAACACAGTGAAAATTTATATGCAAGTATTGATTTAGTTTCTGACAAAATCACTCGCCAA
TTGCGCAAGTATAAAGAACGTAATCTTGCTAAGAAAACTCATACTGCTGAGAAAACATCGGACGC
AGTAGAAGAGAAAACTGTAGGAACTGATTTAATTGGCGATCGCGAACCCGAATTACCTTCAGAGG
TGGTGCGGATGAAATATTTTGCCATGACACCTATGACTATTGATCAAGCCTTGGAACAATTACAA
TTAGTAGATCATGATTTTTATATGTTCCGCAATGAAGAAACAGGAGAAATAAACGTCATCTATCA
ACGTAATCATGGTGGTTATGGCGTGATTCAACCTCGCAACCTAAATTAGAGGTTAAATAGCCTTG
TCAAGATATTGTACTTTCTTGTCCAATAGGGTTAGAAATTAGGAGCTAGGATCAACTTTTCTTGC
CCTCACCCCCGAAACTCACCCCCCGCCCCTCTTCTTTTCGGAGAGGGTAGCCCCACAGGAGAGGCG
AGACTTTTTTTCTAATAATTGATTAATATAATAATTAGAACTTTTATACACTACAACGAAGCTGT
TGCCCGTTGCCTACCCTTGCCCAAATCAAGTTATGCGATTGCTGAGTATAACCGAACCTAGTTTA
ACCAACCTGCACTGAGAACTACTGTTAAACTGATAAAAGTGAGGCTAAGAATCCAAGTTACACGG
TTAAGAGTAGCTTCTGCACTTTTTGTACTACTAAATAATTGAGCTTGTCCGCCAATTCCACCTAA
ACCGTCACCTTTGGGGGAATGTAATAAAACGAGAAGAATTAATAAAAATGCTGATACAAGCCAA
ATAATTTGCACTAGTTGATATACTGTCATAATTTATCTTAGTTTTTGATGGACTTTAATTATTAT
AGTGAGATCTCGTCGATGAAAATACTTTGCTTCTAGCGGGTGAACCAACGAAAATAGTTAGCAGT
GGTGAATTCTTTTATGGGGAAAAGCATATAAGTTAAAGGATTAATGGTCACAATTATATCTCTA
AAGTCCCGCACCGCTAAATTCACAATTTGTTGTGCTACCCATTCTGCGGACATTATACCAATGGG
ATTAAGGTTACTTTTAAAAGGGCCAAGAATTAGCTTTCTAATGATGCAGGGGGCATTAACTCTTT
TCATGGTTACTAATTCTCCCATAGTACGTTTACTCATTTCGTAGAGTGGGCTAAAAGCTGGGTTA
ACTTCTGCTTCAGATGTGTTGACCCAAATTTCTTTTGTGGCTTTATCTTGATTAGTTTTAACGGT
ATCTAAAAATAATTCCATTAGTCGCCACCCTGAAAATGTATTAACTTCATAGGATTTTTCTATGG
CTTCTGCACTTTTTTCTCCATGAACATTAATGCCATGATTGATGATTAAAATATCTGTTTTTGTC
AAAAGTTGGTTTAATTCTGATTCTTTACCAATTTGCCAAGTAACAGTTTTGATGGGAATTTGTTG
ATTTTTTACACTAATAGATAGTTCTTTTTCTTGAGAAGTTAATGCGGTAATTTTTGCACCTTTCA
AATATAGCTGTTTAATTAACGCCTGTCCTAATGTACCATTTGCTCCAGTGACGATTACTTTTTTT
CCTTTCAATGATAAAGCAGTACCCATGATGCGATCGATAACGGTAATAGTACCACAAAAATAGGC
ATTTTGATCATCGAAATGGTGGCGCCAATGATAGGGGCGGTTAACAAATAGATTGGCTGGAATAT
TAGTAAAATCTCCTTGGCGGTGAGTTACATCTGTAATAGCATCAGCATAAGGAACTCCCAAACCA
CGGGCGATCGCACCTGCGGTAAATGTTAATGTATAAAAAGACCCTGTCCAAACTAACCATTGATA
ATCTGGATTAATCCATTGTGTTTGAATAACAGTAAAAGCATAGATAAAACTAAACGCCAACATTA

CTAGGGATTCTGGTAAATCGTTATACCAGTTTGCCTGACGATAAATAGCTTCACTATTAACGGAT
AAATCAGGTTTAAAAATGCGATGATGCCATGAATGAAGACGATATAGAGGTTGCCACACATGGGC
GAGAAAATGATATACGTCTCTAACTAATTCTACCCAAATTACAGAGCAAAGTGCGATCACCAAAG
GTGCGGCTATGGCGAGCGCTGACGAGATAACTATTTCCAATACTTCAGTATGACCCATTGAATTA
TAAATATTTTAATTTTTATAAATTGAAAATTATAAACAAAAATTAGAAACCTAACGCCTAAAAC
CTATTATTTTTAATCTCTAGGCTCAAATTTTAAAGCCGCAGAGTTCATACAATAGCGTTTTCCCG
TTGGCTCAGGGCCATCATTGAAAACATGACCTAAATGAGCATCACAAGCATTACATAAAACTTCA
GTACGTTTCATGAAAAGACTAAAATCATTTTTGTAAGCAACACTATCCTCTCTAATTGGTTGCCA
AAAACTAGGCCATCCTGTACCAGAATTGTATTTAGTATCAGAGACAAATAACTCATTTCCACAAC
AAACACACTTATAAACGCCCTTACCTTTAAAGTCGTGATATTCTCCCGTAAAAGCTCTTTCTGTG
CCATGTTTTCTGGTAACTTCAAATTGTTCAGGAGTAAGAATTTCTTTCCACTCCTGTTCTGTTTT
TTTTATTTTCTCTACCATAATTTTAAGAGTTGATTTAATGTTAAGATTTGCAAAAAAGACCATCT
TTATTTTAAGACATGGAGCAGTAAAGATAATTCTCTTTGAGAGAAAAAGTCTTTAAGTAAGAGA
TTAAGAGAAATGAGTGAGGATAAAGAATAAACATCGACGGAATTTGGGCTTCTAGCCCGAAATA
CAGCAAAAATCATAATTTTTACAATCACAATTTCTACCAGAAGTCTAATGATGAGTTTTTGATAT
TTTTATCTCTGAGAGTTTATGGAAAAGCCTTTGCTAGTTAATTTATCTATGTTGTTGGCAAAACC
CACAGGAATAGCCAATTATGTCATCAATATTTTACCCTATTTTGATTCTTTAGAATATATCAGTT
TAGTTGCTAAACAATATTATCAAGAAAATATATACAAAAATCCCTATTTTGTCAGTGAAAAATA
TAGTCCAGATTATGGTAGTAAGGGTCATTTTTTGAGATTATTATGGACTCAATTAAAAGTACCAA
ATATTTATCAGCAATCAGGTAGTAATTTACTTTTTTCTCCTGTGCCAGAAATGCCGTTATTTTCT
AAAGTTAAATCAGTGATAATGGTACATGACTTAATTCCATTAAGGTTTCCAAAAAAAAATTCTCC
TCTTACTCCTTATTTCCGCTATTATATTCCCCAAGTTTGTCAACAAGCAGAGCATATTATTTGCA
ATTCTAAAGCTACAGCCGATGATATTATTAACTTTTTTGGGATTAATGCAGATAAAATTACTCCT
ATTTATTTAGGTTATAATCCTGAGACTTTTAAGGTTATTGATAAAATCAAACCTATAAATAAAA
AGCCTTTTTTTTTGTATTTAGGCAGACATGATCCCCATAAAAATGTCGAAAGAATTGTATCTGCT
TTTGCTCAATTTAAACATCATCAAGAGTATGAGTTGTGGTTGGCAGGTCCTACGGATTCTCGTTA
TACACCTTTACTCAAACAACAAACAAAAGAGTTAAATATTACTCATCTAGTTAAGTTTTTAGATT
ATGTAAAAAGAGAAGATTTACCTATTATTCTTAATCAAGCTCAAGCCTTAATTTTTCCCACTCTA
TGGGAGGGTTTTGGTTTTCCTGTATTAGAAGCGATCGCATCTGGTACTCCTGTAATTACATCTAA
TATATCTTCTTTGCCTGAAGTGGTAGGGGATGCAGGAATTTTAGTAAATCCGTTGGAAGTAAAAG
AAATCGCTTCAGCTATGAATTTAATGGCAGAAGATGATAAATTAAGACAACAATTAAGGGAATT
AGGCTTAAAACAAGCACAGAAGTTTAGTTGGCAGAAAACTGCTCAAGCTACTATCGAACTTTTAT
CTAATGTCAGATAATCAATTATGGGCAAATAAGAATTAGTAATTAAGACAAAGCAATCATTTGA
GTTCAGAGAGTTCGCTGCGCTCGTTCCGCAAAGCGTTACAGAGTTAGGGGTTCGGAGTTAAGATA
AGAGTAATAATTAACAATTAAAAACACCTAATATCTGCAACTGAAACCTGACACCTAAAACCTTG
ACTTCAATTATTATTTATCACTTATTAATTATTTAACAATAATGAAAGCCTTATTTTTAGATAGA
GATGGAGTTGTCATCGACTATATACCTTATTTGAGTAAGCCTGAACAAGTAAAAATACCTGAAAA
TGCACCATTTGCATTGAAACAATGGCAAAATCAAGGGTATCGCTTAGTTATCGTCACAAATCAAT
CGGGAATTAGTCGAGGTTATTTTACCTTAGAAGAAGTTAAGGCAATACATGAACGATTAATAGC
AGAATATGCTAATTTTGGCGTTTATTTTGCAGATATTTTAATTTGCCCTCATCAACCTGCGGATA
AATGTCTTTGTCGTAAACCTTCTCCTTTTCTCATTAAAACCTATGCAGAAAATCATGGTATCGAT
TTAAGCCAGTCTTACTTCATTGGTGATGCCCCTAGTGATATTGAATGTGCCATCAATGCGAAATG
TCAACCCGTTTTACTTTTGACAGGAAGAGGTAAGGAAACTTTAACTCATTTATCTCAGTATTCTC

TTTCTATTCCTGTTTTTGAGCAAATGAAAGATACTATTTTGTTATTAAAGACGTTTGCAGAAAAA
TGAACTTTTTTGATTCCTTCCCGTCTTTTTCTCTAACTACATTCACTTAAAAGTTATCTGATTATT
TATATGTTAAAAACTAACACTTTATTTACTTTTTCTAGTCTTTTGCTCTTGACTTTTTCTGGTTT
TTCTGTTAACAAAGAAGCTATCGCGCAATCTTCTAATAAATTTAACCGTCAACAGTGTATTCAAA
ATTTAGTCAAGGATGGTTTAAAAAGCGAAGATGCCTCCGTATGGTGTAATTATAACGAACAATGC
TTAGTGCGATCGCAAAAAGAAGGATTACACGCCGAAGCGGCAAAAAGTGTGTGTGACTGTACCAT
TAAAACTTTTAGAAATAAATATACCACACAAAAATTTAGGGAAATAACCAAAAAATCAGAAACC
GATAAAAAAAATAGCCAATCAATTGAGGGAGGTTGGAGAAATTTGTTTTGAAGAAATATTATTTG
AATAATTAACAAATATTTTCTAAGATGGAGAGATAAACTGTAAGTATTGATCTTGAATTGAAAA
TACTTATTTTCATTTAGCTTATTAGAGAAACCTGCACAATAATTTTTATTCCGTGTTGGTAGTTC
ACAGATATTCTTTCCATAAAATTCATGAATAACGCAGATTATTATGATCAATGTCAGGAAATTCT
CACAGATGCCACCAGCAGACTGGGTTTTCCTCTAGGAGATGATAAAATCTGCGAAATCTCTAGCT
TAATTACCGAAGCGATGGGGGGAAATTATCGTTGTTTTCACAATTTTGAACATTTGTTAATGTTT
AAGGATCAAGAAGACCCCATCATTACTATAGCAGGTTTATTTCATGATCTAGTTTATATACAGGT
CGATCGCAAAATTCCCTTCAATTTAACTGTTTATTTAGCTCCTTTGATTCAAGAAAGAATAGACG
GTTTATATATAAAATCTCGTCAAAATAAAAAAGATAAATATTTAGAAATTATTTTAAAAATATT
TGGTTTAAATATCGATGATAACTTATCAAATTTTCGAGGGCATAATGAGTTTTTAAGTGCTTTAT
GTACTGCTCAAATTTTAGATAATTCTTTACCCTTAACTGTTATAGTTCGTATCGTAACAATTATT
GAATTAACTATTCCTTTTCGACAACTAGAAAATAATATCTCAATATCTCAACAGTTAGAAAAACG
TTTATCTAAAGTCAATCAACAATTTCAATTAGGTTTAAAAAATGATGAAATTATCTTAACAATT
ATTCAAGGAGTTAAATTAGCTAATTTAGACGTCAGTGGTTTTGCTTCAACCAACGTTAAAGATTT
TATCAATAATACTTGGTTACTTTTACCAGAAACTAATCATATTTTAAATGACCAATATCATTATT
TAATTAAAGATTGTTGTATTGCCCTGATCAAAACCACAAAATTTATTCAATGTTTATTTCCAGAA
AATATTTTCCATTGTTATCATGATTATCCTAGTTCTGATGCTTATGAGTCATTAATTAATAGAAG
TAAATATAACTTAAATATTGCTCAATATTATTTTGCATATCACATAATTGGTTTAAGTATTTTAC
AAGCATTTATTTCTCGCTTTACTTTCTCTTTACCATTATCTTTTTTCTTTCCTCATAAGTCTAATT
CATCTAAAAATAATTCATCTTTTATTGACTATATTATCCCTGTTAACAAGAAAAATCTTATCCCT
AATTCTGTTGAAGATATAGTAAGTAATTTAATTAGTGCAGAGTTTCAACCTTCTTTTTTTCCTTA
TAGTGATTTAGGCAATTTTGTTATTCTTATCTTCAATTATTTAACACTAAAAGATAGCCTAATTT
TTATCGATAAATGTTTGTTATTTTTTGAAGAAGAAATATGCCAAGATGATTTTTTAAATTTATTT
CCTTCACCTTTAATAAAAATAATAGAAGATGCGATCGCACAGCACCTTGCTGAGGTAAGATCGCA
CTTTGTTTTATAAAGTAAATAACAATAAATTAAGCCCCTACTTCCCCCTTTCCCCTCATCACCTCA
TCCCCCCATCCCCTAACACCCCAAAACCCTAAGCCCCTATCCGCCGATACTGCTGATTACTTAAAA
CTGGATGCGATCGCGATTATTCCCTGATTCTACATTTTTTTCAATAAATTCGATAATTTTATCAG
CGATGTCAATATTAGTAGCCTTTTCTATTCCTTCTAAACCGGGAGAAGAATTAACTTCCATTACC
ACTGGTCCATGATTTGATCGTAGTAAATCAACCCCTGCAATTCTTAACCCCATTGCCTTAGCAGA
ACGAATGGCAGTGCTTCTTTCTTCAGGAGTGAGCTTAATTTTTTCCGCTTTTCCCCCTCGGTGAAG
ATTCGAGCGAAATTCCCCTTCCGCACCTTGCCTCTTCATGGCCGCTACCACTTTATTACCAATAAC
AAAGCAACGAATATCTGCTCCTCCTGCTTCTCTGATAAACTCCTGTACCAATATATTTGCATTCA
AACCCCTAAAAGCCTGAATTACCGACTTTGCCGCTTGATAAGTCTCCGCTAATACAACTCCTATAC
CCTGAGTGCCTTCTAATAGCTTAATCACTAACGGAGCGCCGCCCACAGTCTCGATTAAACCATCAA
TATCTTCCGTATCATGGGCAAATCCCGTAACAGGTAAACCAATGCCTTCCCTCGCTAAAATCTGT
ACACACCTTAACTTATCGCGCGATCGAGATATAGCCTGAGATTCATTGGCACTAAATACCCCCAT

TACCTCAAACTGACGCACCACCGCACAACCATAGAAAGTACGAGAAGCTCCGATGCGTGGTATGA
TTGCATCAAAATTTTCTAACTCCTTGCCACCGTAAACTACCGAAGGTTTATGGGAAGTAATGTTC
ATATAGCATCTCAGATAGTTAACTACCTTTATTTCATGTCCCCTTTTTTCTCCTGCATCTCTCAAT
CTTCTGGTGGAATAAAGGCTACTATCTTGAGATAAAATAGCGATTTTCATGGAATCTTCTCAAAT
TAATTTTTCATCTTCCTTATTGTACATTTACAATTGATTTAAAGAAATTGTGAAAATCTCGTTAG
TAAGTGCTATAATGTGTTAAAGTAACCAGAGGCGACAATATTCTATTCACCGTTGCCCAGTATTG
CAATCAAGTTTAACAATTCACTTATGAATCCTGTCATAAATCGCTTAATTATTTATGTGTATTGA
ATCGTCAAAATTAGTAAGCTGTTTCAGGAATAGTAAATAATGTGTCTGAAAATAGATAAAAAGT
AATGGTTGCGATAAAATCCAAGCAATCATCATCGCTTTCTTTGTACTTAGATTGAGTATGTATAA
ATTTTCAATCCATACTTGTCTTGTACTAGACATAATTACTGAGTAAGTTTAATTGTTAGCGTAAA
CTGTCATTATTAGAAAATTTAAGAAATATATGCCTAGAAAGCAAGTAATTCACCCAATGGTGAA
GTTGCAACAAAAAGTGTCTTCTTTAGTAAATTCACAAATTATAAGCCCTGATGATAGTATCGGCA
AAATAGCCCTGCTTTTCGGCAGTGATTGGGCTTTTTTGAAATCAGAATTAATATCTTTTGGTTTT
TCAATGCAAGACCCTGTTCGTGATTTATTAGTAGTACAGAAATGGGATGATTAAATAAGTAATT
AATTAGTAATTAGTAATTAAAACCTGAAACCTTTCCAATGCTATTTATTTTTACAGACTTAGATG
GTACATTACTCAATCAAAATGACTATCGTTATGATGATGCCTTACCAATTTTAGAGATATTAAAA
AATAAAAATATTCCCGTCATCCCCGTCACCAGTAAAACCAAAGCAGAAGTTGAGAGTTTGTGTGC
AGAAATAGGCTTAAATGATCCTTTTATTACGGAAAATGGTAGCGGTATTTTCATTCCAAAAGATG
ATTTTCGTTTTGATACAACACAAGCATTAACTACAGAATCGTATAATTATATAAGTTTAGGTTTT
GAGTATCATAACGCTAGATGTATTTTATGGGAAATATCTCAGAAGTTAAAAACTGATTTAGTCGG
GTTTGGAGATTTATCTCCCGAAGACATTGTTAATTTGACTGGTTTATCTTTAAATGAGGCGATTT
TAGCCAAAACAAGAAATTTACAGAGCCTTTTCTTACCCCTAAGCATATTAATCCAAGTTTATTA
CAAAATATTGCCCAAGAGTATCAGGCTACAATAGTAGTGGGCGATCGCTTTTCCCATTTAATTAG
TGCAAAAGCAGGAAAAGGAAACGCAGTGAAATGGCTACTCAATAATTACCATAACCTTGAGGAA
AAAATAACAACTATAGGCTTGGGAAATAGTCCCAATGATATTAGTTTATTAGAAAGCGTAGATA
TTCCTATTATCATCCCAAATCCTGATGGAGTACACAAAGGACTAAAAAATAAAAATTGGCAAGTT
GCCCCCCATAGTGGCAGTAAAGGATGGAGTCAAGTTATTACAGAAATAATTTTAAGCCATAAAAT
ATAATAGCTTTTCTCGATTTTTTCGTTGATTAATAGATGTTTGAAACATTCAACCCCTATCACCT
GACACCTAACCTTGTCCGATATTCTTAAACCGAACTGAGGTTAAGATAAGACAGTGCAAAATCTA
AGTTATGATTAAATACTGCATAAAAAGTATGAAGAAAAGTTAAAAATGAAAGTTTTAATTACTG
GTGGTGCAGGTTATATCGGGTCAGTTTTAACCCCGACACTTTTAAAGCGAGGTTACGAGGTAACT
GTTGTTGATAACTTTATGTTTCATCAAAATAGCCTTGCTGATTGTTGCCAATACGAAACTTTTAA
TGTTGTCAGAGGCGATTGTCGAGATGAGTCTTTAATGAAAGATTTAGTCAAAGATGCAGACGTA
ATCATTCCCCTAGCGGCTTTAGTGGGCGCGCCTCTGTGTAATCGGGATAAAATCGCCACTGAAAC
CACAAACAGAGATGCGATTCAAATGTTGTGTCGTCTAGCCAGTAAAGAACAAAGATTTTTAGTCC
CAATTACCAATAGTGGTTATGGTGTCGGTGAAAAGGGTAAATTTTGTACAGAAGAAAGTCCTCTA
CGTCCAATTTCTACCTACGGAGTGACTAAAGTTGAGGCAGAAAAAGCCATCTTAGAAAGAGAGAA
TAGTATTAGTTTCCGTCTTGCTACTGTCTTTGGTATGTCTCCTAGAATGCGTATTGATTTGTTAG
TGAATGATTTTGTCTATCGTGCTGTCACCGATCGCACTGTTGTAGTATTTGAGGGGCATTTCAAA
CGCAATTACATTCATATTCGTGATGTGGTGAATGTCTTTTTACACGGATTAGATAATTTTGAAGC
GATGAAAGGAAAACCTTATAATGTGGGCTTAGAAGACGCTAATCTTTCTAAATTGGAACTATGTG
CCGAAATACAGAAGCAAATTCCCAATTTTGTTTATATGGAAGCCCCTATCGGCGAAGACCCAGAT
AAAAGAGATTATATTGTTTCTAATGCTCGTATTTTAAGCACTGGTTTTGAACCCCAATGGCCTTT

AAGTCGTGGTATTAAAGAGTTAATCAAAGGTTATACGATGATTCGTAATACCATTTATTCTAATG
TTTGATAAATTCTTGCACTCAGTTTTCAGGCAAAAATATGGCGCAAGGGTAGGTGTCAGGTTTTA
GGTGTCAGGTGTTGGGGATAGGGGGATGAGGGGAGAGGGAGACAAGGTGTCAGGTTTCAGGTTT
CAGGTTGCAGGTGTTGGGAGAAAGTGATAAGTAACGAATAATGAGTAATGAGTGTTCGGGGTTT
TTAATTATTCACTATTCACTATTTACCTTTGCCCTTTGCTTCTTGCACCTTCAGATCAAGGAGTGT
CAAGGTGTTTGGCTTACCTTTTCCTCTGAATTAATTGTTGGAACGAATTTTTCCGCAAACATTTT
GATTACAATATAGACAATTGGCACAACAAATAAACTCAAAAAAGTTGCCACTAGCATTCCTCCAA
ATACAACTGTACCTAATGATTGACGACTGGCAGAACCGGCCCCAGTAGCGATCGCAAGAGGTAAA
ATACCTAAAAGAGTGGAAAATGCGGTCATTAAAATGGGTCTGAGTCGAGCTTGAGATGCTTCGAT
CGCAGCTTTGACAATGGGTAAACCCTCATTGCGTAACTGATTAGCAAATTCGACAATCAAAATCG
AGTTTTTACTAGCTAAACCAATCAACATCACTAGACCAATTTGACTGTAAACATCAAAGGAAAAT
CCCCTAGACATTTGCGCTAACAATGCCCCTAAGATAGCCAACGGTACAGCTAAAAGAATGATTAG
AGGATCAATATAGTTCTCATATAGTGCCGCTAAAACTAGGAAAACAAATAATAACCCTAAACCAA
AGATAATCGGTGCTTGTCCTCCTGAAACAATTTCTTCCAAAGATTGCCCAGACCATTGATAGCCA
AAGCCCCTTGGTAGAATTTGAGCAGATAACGTCTCCATAGCTGCGATCGCATCTCCTGAACTTGC
CCCCGGACTAGCAGAACCACTAACCTCGATCGAACGAAATAAATTGTAGTGATTGATGACTTGAG
GACTGGTGCTAGGAGTAATAGTTGCCAAATTAGATAAAGGAATCATTGTATCATTTGCACTACGA
ACATATAACTCTTTAATGTCATCAGGATGAGTGCGGAACTTACCATCTGCTTGTACATAAACTCT
ATAAGTCCTGTTTTGTAAGGAAAAATCATTAACATAACTAGAGCCAAAAGCAGTTTGCAAAGTCC
GGAAAACTTCATCAATATTGACATTGAGAGCTTGAGCTTTATTGCGGTTAATATCAACTACTAAT
TCGGGGCTATTGGCTGTAAAGGTCGTAAAAACCCTTGTTAAACCTTCTGTTTGGTTAGCCTGTCC
CAATAAACTACCCAAAACCTCCATCATCGAATTGATGTCCCCATCACTACGCAAATCTTGTAACT
GATAGGTAAAACCGCCAAAACTACCTAAACCCCTAATGGCTGGAGGATTTAAGGGAATAATTCTC
GCCTCTGGAATCATGGCAAACTTACCCCATAAACGACCAATTACTGCCTCTGCCGATTGTTCTGGG
CTTCTTCTTTCTGCCCAAGGTTTAAGAGGAGTAAAAATCACCCCAGAGTTGGAACTATTACCACT
AAATGCAAAACCTCCTACCCCGAAGGTTGCTTGAACTTCAGGTTCTTCTAAAATAGTTTTTTCGA
TCTTCTCCATTACATCGCTAGTATATTGTAGCGATACCCCTTCAGGTGCTTGAATAATAGTGATA
AAATAACCCTGATCTTCTTCTGGTAAGAAACTTTGAGGTATTGTAATATAAAGCCATCCTGTACC
GGCTAAAAGTAACACAAATACAGCAATAACGACGGTTCGCATCCTTACTAAACCGACTAAACTAC
GACGATAAGACTTAGTTACATTATCTAAAAAGCCATTGAAAATATCGCAAGGAACTTGTAACCAT
GCTGGTAATTTTTGATGATGTCTTAGTAATAATGCACATAAGGAGGGAGTTAAAGTTAAAGCCA
AAAAAGTAGAAATGGCAATAGAAAATGCGATCGTTAAAGCAAACTGGCGATATAAAGCTCCCGT
TGTACCCGGAAAGAAAGCCACAGGTACAAACACCGCCATTAACACCAAAGAAGTAGCTATTACCG
CCCCTGTCAACTGTTTCATCCCTAACTGAGCCGCTTTAAAAGGACTCATATCCTTATCTTCAATAT
AGCGGTCAATCTGCTCTACAACTACGATCGCATCATCTACTACCATCCCCGTTGCTAAAGTCAAAC
CAAATAAAGTTAAACTGTTGATAGAAAAATCAAATACCTTTACAAAAGCAAAAGTACCAATTAA
AGAAAGAGGAATTGTCACCGCAGGAATCAACGTAGTTCGCCAGTTCTGCAAAAACACCAAAATAA
TCAAAATTACTAAAAAAACCGCCATAAATAAGGTATTAACTACTTCTCGTAGCGATTCCTGAACG
TAAAGGGTTGTATCAAAGGCAATCTCATATTTTAATCCTGGGGGAAAAGTCTTAGATAACTCCTC
TATTTGTGTTTTTACCTGCTTTGCCACCTCTAAAGCATTAGAACCTGTTAACTGATAAATCCCTA
ATCCCACCGCTGATTGCCGACGATAACGCAAGAAAGAAGAATAATCCTCCGCCCCTAACTCAACCC
TACCCACGTCTTTAAACCTTACTAAAGCACCATTAGCATCAGTTTTTAACAATAAATTTTCAAAC
TCCTGTGCCGAAGTTAATTGACTAACCGCCCTCAAATCAATCTGAAACTCCTGCCCATCTGGTACA

GGTTCAGCACCTATTTTTCCCGCTCCAATTTGATTATTTTGAGCCGAAACCGCTTGAATTACATCT
GCTGTTGTTAAACCACGACTAGACAACTTTTCTGGATCTAACCATAGGCGCATCGCATAACGTCT
TTCTCCAAAAATAGTTACATCTGCTACCCCGTTAATCCGTTTTAATGGATCAACTAAATACAAAT
CCGCATAATTACTCATAAAAAGGTTATCATAGCGGTTATCTTCGCTATAAAGTCCAATCCCTAAA
AGAATATTATTAGATTGCTTAGTAACACTAACTCCATTTCGTTGCACACTTTCTGGCAATTGGGC
TTCCGCCACCGAAACTCTATTCTGTACATCTACAGCCGCCAAATCCTGATTCACATCAGAAGCAAA
GGTTGCTGTTATACTGCTTGTACCGCTATTGGAACTACTAGAGGTAATATATCTCAATCCCGGAG
TCCCATTAATTTGACGCTCTAAAACGGTAGTAACGGCATTTTCTACCACCTCAGCACTTGCCCCAT
CATAATTAGCTGTTACTTGTATCTGTACAGGGCTAATTTCGGGAAATTGAGCCAAAGGCAAGTTA
AAAAGGGAGATTAAACCCACAAAAAGAATAATTAATGCTGTTACGGATGAAAAAACAGGGCGTT
TAATAAAAAAATCAATCATCTCATTATCCTGTCTAGTTTACATCTAAAATTACTAATGAATATCT
AGTACCTATTTGTTGCTTATTTCCTCTCTGAAGCTCAACACTTAATATTAATTTTTATTTCAAAA
TATGGTAAAAATCGCAAGATAAAAACATAATAAAAGATATGAATTGGGTTTGTTTTTGATTTTA
GTTTTCAGAAGTAAGAACCTGAATTAATTACTAGAATTAATAGGTTTAGTCTCATTTTCAGGCAT
TTCCTGTATGGGACTACCTTCTTGTACTTTCAAAATACCTGCACTAATAATTTTTTCGCCTGCCTG
TAAACCTTCTAAAACCTCATAGGAGTTGCTGTGAATTTCTCCTATTTTAATTGGTCTTTGTTTTG
CGATTAATCCTTCCCCTTCAGGATTTTGTTCCGCTACAAACACGAAACCTTGACCACCAATGCGAA
AAATAGCACTAGAAGGCACTAAAATTCCCTGACTACTATCCCAAACAATTCGGGCGGGAATAAAT
TGGCGATTTAATAATTTGTTTTGAACATCATTGCTAAAGAAGGCTTTTGCCAAAACTAATTGGGA
ATCAGAGGTCACATTCGGAGAAATAAAATCAATTTTTCCCACCGCTATTACTTCTTCTCGGGAAT
TTAAAACCTCTACAGGTAAACCCAATTGAAGCTCAGGAGCTTTTTCCAACGGTACGGAAATATTC
AATTCTAAAAGATCATTTTGGGTGATACTGGTCAACCTAGTATCTGTTTCGATAAAATCACCGAC
TTTAACGGGTATATTACTAATTTTACCTGCGATCGGAGCTTTGATCATTGTTTTATCTAATTCCA
CCTTTAATCTTCTCACATTGGCTTGTGCTTCCTGCACAGAAGCTCTGGCTTGATCAATATCCTCCT
GCCTTGCCCCATTTTGTAACAACAGCAAATTTTGTTCGGCTTCTTCAACTTCTGCTTGTAATGCTC
TCACGTCAGACTGTGTACCTTCTTGTAATTGATTCAAACGTCTTTGAGCCTGTTCTAAATTTGCA
CTAGCAGTTTTTGCTTCCGTAACAAAAGTGTCAAATTGGTCACTAGAAATTGCCCCTTCGTCTCT
CAAATTACGATAACGATTTAATCTTTGCTGTGCTAACTCATTTTGAGCTTGAGCAGAGTCTAATT
GAGCTTGTGCTTGGGCTATTTCTTCTTGAGAAGAACCTCTTTTTGCATTGGCTAATCTAATTTTA
GCCCTATTGAGTCTAGCCTGAGCAGAAGCAATTTCTTCAGGACGACTTCCCGCTTCCAGTTCTGCT
AATTGAGCTTGACGAGAGGCTAAAGTTGCTTGAGCTGAATCTAGTTGTGCTTGTAATGAGTCACT
TTCTAAAGCAAACAAAACTTCTCCCCTATTCACATTTTGCCCTTCTCGAACCAGAATATCCACCAA
TCTTCCTTCGATTTCTGATTGTAAATTCACTGTTTGACTAGCTTCTAACGTACCAAGTACATCAG
TGCTTGTTTTTATCTGTTCTGTTTCCAAGGTGCTAAGGGTAACTGGTACAGGAGGCATGGTGTTA
CTGACTCCTGCCGAAGTTGCAACCTGACGATTTTGCCAAAAAAACCAACTTACAGTTCCTGCACCG
CCAATGAATAGAATACTTCCCCAGACCAGCCATTTTTTCCCAGACTTTAAGGAGGAAGAATTTTT
TCTATTCTGGTTAGGCTTTGTTTCTTCTTCTTTTACTTGTTCTAATTGTGTATCAGCCACGGGATT
ATTTTTTGTCACGAGTTCATCATTTCATTATAATCTGCATTTTGAATTTATTTTTACTTGAAGTA
TCCATAGATAATGCTCAATGATTCATCGATACCTTGACAAAAATCTTAACTTTTATTTTTAATAC
TAACTTTTTGTCAGTGTTATACTTAAGTGTTATTACTAATAAAAAATATTAAACTCTTTTAGTTT
TTGATTAAACACAATATCTTATTACTTAGCATCAATGACTCAATTTAATCAAGAAAATATAAATC
CAACCTACTCTGATTATCCAGAAGATAATAGTGACTATCAGGCTAAATTGACGCAAAAAGTCGAT
AAACTAGGAAATTTTAGCTTAAACATGCTAAGATCTTTAGTTCTATGGCGTTTAATTGGCTACGG

TTTTTTAGTTTTATTTGCTTTAGATGTAGTGGCAATTGTTGTTCCTCCCAACTTTTTAAATCCTC
AGTGGGAATTTCAAATTTTAGGAGAAATCGTAGAACGAGTTGCAATTCCCTTTATTGCTCTCTTA
TTAATTTTTTTGGGGGTAATTACCTAAGAAAAGGATGGGAGTATTTAGTATTGACTAGTCTGTC
ATGGTTATCCCTAATTGTAGGTGTTTTATTTATTTTGGCTGTGCCTTTAGGAGTTATTAATACTA
TTCGTATTGATACTCAAGCAACTACCCGATTAACAGAACAAACTAATCAAAGATTGGAAATTTTG
AAACAGGTAGAAACAAAACTGAAAGATGTCAACAGTCGAGAAGAAATGCAAGTATTAATAAGTC
AACTAAGTAACAATAACGCTCCAACCATTGAAAATGATCAACAATTAACTGAAGCGAAACAAAG
ATTAGGGGAATTTATCACAAGCTCTCGAAATCAATTGAGTACTCAAACTGATTTGGCAAATAATC
AACGAAGAAAGTCTCTGATTAAACAATCTGTGAAATGGAATTTAGGAGCATTGATTTCAGGCATT
TTGTTTGTTATGACTTGGCAAATGACTAAATGGGCTCGACAAAAAGTTGAATAAATCGATTAAT
AACTCTACGACAAAATCATAATAAATTAACAAAAAGGCATTAAAAATAATGCCTCTTTAACTCTG
AGTATGTAAATTATTGGTTTAGATAGCGAATTTGGAAAGAGGGAAACCCCTTTGTATTTATCCTT
AAAAGGGCATAAAAGGAAAAGTAAATTATTTAATAATGATTGATAAACTTTTCACTTTACCCAG
TTTTTTTATTCATGATTTGCCAACATTCGTCAATTTTAACCAAAGCTGAAGAAGTGAAGTTGTTA
ACACCTAATCCCAATCCTAATATCTAACTAATAGAGGTGTATTTTACACCTTTTTAACTGTTCTC
ATTAAGCAGAAAGAGTACGACGCTTGAATACCATTTCATAAGCCTCGATAATATCTCCCTCTTGC
CATTGGCTAAATTTATTAACAGCAATACCGCATTCAAAACCAGACGCAACTTCTTTAACATCATC
TTTGACACGGCGTAAAGAATCTAAATTACCATTATAAATTTCTGTTCCGTTGCGTAACACTCGAA
TAAAGCGATTACGGACAACTTTGCCTGACAAGACGTAACATCCAGCAACAGCTCCTTTGCCAACG
GAGAAAACGGCTCTTACTTCAGCTTTTCCTAGACTTTCTTCCACTTCTTCTGGATCTAGTAAGCCT
TCCATTGCTCCTTCTATTTCATCTAATAATTTATAGATGACGTTATATTCTCGGATATCTACTCCT
TCTTGTTCGGCGGCTTGACGTGCACCGGGGGCAAGAGTTGTATTGAATCCAATAACTACAGCTCC
AGAGGCAGCGGCTAAATCTACATCCGTTTCTGTTACTTCTCCTGCACCTGATAATAAGACTCTGA
TTTGTACTTCATTTTGCGGTAGTTGTTTTAATGAGCCTAAAATCGCTTCCACAGAACCTTGTACA
TCGGCTTTAATAATTAAATTCAGTTCTTTGAGTTCTCCTTGTTGTGCCTGTGCTGATAAGCTACT
CAAACTTACTCGACGAGATGACATTGCTTGTTGTAAGCGGTTATCCCGTTTGGCGGATGCTCTAC
TTTCTGCGATCGCTCTTGCGTCTTTTTCATTGGAATAAACTTCAAATTCATCTCCCGCTTCTGGAA
GTTCACTTAAACCCAGAATTTCAACAGCAAAGGAGGGACTTGCAACTTCAACTTTTGCTCCTCGA
TCATCGATCATCGCCCGAATTTTACCCATGACAGAACCCGCAACAATCACATCACCAACTCTTAAT
GTTCCATTTTGAACTAATAATGTTGCCACAGGACCCCTTGCTCGGTCTAAATGGGCTTCAATTAC
AGTACCCTTGGCGTTGCGATCTGGGTTTGCCAACAATTCTTCGACCTCAGACACCAAGACAATCA
TCTCTAAAAGAGCATCTAAGTTATCACCAGTTAAAGCACTCACAGGAACCATTACGGTATCGCCT
CCCCAGTCCTCTGGAACTAATTCTAATTCAGCTAATTCCTGTTTGACGCGATCGGGATTTGCTTCT
GGCTTATCAATTTTGTTAATAGCCACAACAATGGGGACTTTTGCCGCACGAGCATGACTAATGGC
TTCTTTTGTTTGAGGACGTACCCCATCATCTGCCGCCACCACTAACACAGCAATATCAGTAACTTT
TGTTCCTCTTGCCCTCATTGCCGTGAAGGCCTCGTGACCCGGAGTATCTAAGAATACAACCTGTTG
AGTTTCCCCTTCATGTTCCACATCCACATGATAAGCACCGATATGTTGAGTAATACCTCCTGCTTC
TCCTTGAACTACTTTAGTATTTCTTATCGAATCAAGTAGGGTTGTTTTACCATGATCCACGTGTC
CCATAATTGTTACAACAGGAGGACGTTTTTGCAATTTATCTAAATCTGCCTCCGTCAACATATCT
GTTTTAGCCGCACTGGCTTTTTCTTCTTCTGTAATTACTTCCACTCCCAACTCTTTAGCTACCATT
TCCGCAATTTCTAAATCAACGGTTTCAGTAATATTAACGGCAATTCCTTTGAAGAAGAGAAGTTT
AATAACTTCAGTATCAGGAGTAACTAATAGATCCGCTAATTCTCGAACTGTAGGAGCACTTTTAA
GGACGATAAATTCAGGTTTCTCTTGTTTTTCCTGTTTTCCTTTTCTTTCCGATTTAGTTTCTGTTT

TATCTTTTTTAACTGGTTGTTTTTTAGGCGCACGAGTCTTTTCTGTGGCTTTTGTTGCTTCTTTTT
TAGGTTTAGGAGGACGAGCTAAAGCTAAATCGATAACTGTAGAATCTTCTTGGTCATCATCAAAA
TAATCTTCTAAATCATCGTCATCATCAATGACTAAAGAACGACGCTTCTTGACTTTCTTTGCGTC
TTTTTTGCCCGATTTATCAGAATCATCGTCATCATCATCCCAAGAAGAGTTTTCCGTGGTTGATT
TTTTCTGTTTTGGCTTTCTGATTTTTGGTTTTTCCAACAACATTGGTTCTGAATCTAAATCATCT
TCATCAGTACTTTTTGCTTTGGTTTCCAAATCATCATCGTCTAAATCTGAGTCAATGATTGCAGG
GGGGCGTTGGGGGGGTTTTTGTAGTTTTGGTTTCGGTGCTAATTTGAGTTTATCTAAATCTGCTG
AATTTTTTGCATCCTTAATAACAGGTTTTGCTGGTTTAGGAAGAGAGTCTTTTTCTTCTGTTGTT
AATTTTGCACTGCCTATACTTTCCTTATCATTACGACGGGGTAAATTTTGATTATTTTTAGTGCG
GCGAGAATTATTTTTACTCTTATTATTATTCTTGCTTTTTGCCCTTTTTTCTTCTTCTGAAGTGG
ACACAGCTACTTCACTACGACTGACTACTTTTTTCTCATCGCCTTCTTTACTATTACTATTTGCTT
TATTAATAGTGCTTGGCTTTTTCAATTCGGGGGCGAAAGGACTTTTTAACTCTGGTTTTTCGACT
AAAGAGGGACGAGCTGGAGGTTGTTTAAGACTTTTATCCATACTTTGAACGTTGTTTGGGGAACC
ATTATCATTTTCCTGTACCATTTCCGAATGATTAGGAGCTTTTTTATAGTCCTTTGAAGGCTGAG
GAGGGCGAGGAGGACTTAACAATACAGGGGAGTTAGCGTTATTTTTTTCCTGTTCTGACTGTTTA
TCATTCTTATACACAGCCAATATTTCCTGTTTTTTATTAGGCTTTTTAATACTGGAAGATGAGGT
TTTTGACTTGTCAGGGGTGTTTTTGTTTACTTGATTTTGTGCCGATGTCATGGAGACTTTTTTTA
CTGCTTCTTTAATGTCTTGTGCTTGGGATTCAGTGATGGTACTACTATGATTTTTTACCTCAATG
GATAACTGGGAACATATTTCTAATACGTCCTTGTTGTCTAATGCAAGCTCTTTTGATAAATCGTA
TATTCTTACTTTTCCGTTTTTCATCCACAAATTGCCTTAAGTTTACTATATTTTGCTTTTTTTGAG
TGACTTTAAGAAAGTTTTAAGATGTTTAATATAACAGTGTTTAATCAGATGAAATTATTTATAT
TCATAAAAAATGGTCTGTTGTTGAACATCTACAATATTATGACTATTTTGATTTACAAATAATCA
AGAATCTATTAAGAAAAATTAAGTCATAATTATCCTGATTCTTATTTTACAGTGTTTTTCGTTTC
TTTGAGTTTTTGCCACAATTGTTGATAGATGTCTGAGGGAATGGACGTTTTAAGGGCTTTTGCTA
GACGTTTTTTTTCTGCGATCGCAACACAATCGACATTTTTGCAAATGTAAGCGGATCTTCCCATT
CCTTGATCAACGGTAATAATTTGTTGGGCATTATTTTTCACCACCCTAAAAAAATTACTTTTATG
ATCAATTTTCTGACAACTAATACAACGGCGGTAATTTTTGATTTTATTCGACTTCCTCATTCAAA
TAGTCAGATTTCTTTTTGATGTCAATTTTCCATCCGGTGAGACGTGCCGCTAAACGAACATTCTG
ACCATCCTTACCAATGGCAAGACTAAAGTGATTATCAGGAACGACAACTAAAGACTGCTTTTCAT
CGGGATCAATCAGTTCCACGATGTCTATTTTGGCGGGACTCAAAGCGTTTGCTATGTAAGTAGCA
GGATCTGGAGACCAACGAATAACATCTATTTTTTCTCCCTTAAGTTCATTCACTACTGCCTGAAT
ACGAGATCCCCTTGCACCAATACAAGCCCCTACAGGATCAACATCTCTTTCTAGGGTATCTACAGC
TATTTTTGTTCTTGCGGTAATATCTGGTTTACTGGGATTAGCCTCCCTTGAAATAGCAACAATGC
GAATAATTCCTTCCTCAATTTCTGGCACTTCATTGGCAAATAAATAAACGACTAAACCAGCATCC
GCCCGAGATACGATTAACTGGGCTCCTCGATGAACTCCCTCTTTTACCCGTTTTAAATAAACCTTG
AAAGTTGCATTGGCACGATAGTTATCACTGCTTAGTTGTTCCCCTCTGGGCAATTCGGCTTCAAC
TTCTGGTCTTCCATAGGCACTTTGCACCGTCATAATTACAGACTGTCCCTCAAAGCGTAATACCTT
AGCAGAAAGAACCGTTCCTTCTAACTCCGTAAACTCTGCTTGAATAATCGCTCTTTGTTGCTCTT
TTAGTTTTTGTTGCAATACCTGCTTTGTTTGCATCGCCGCCATGCGACCAAAATCTTTCTGTTCAG
GAGTAACATCAACTAAAACTTCATTGCCTAACTGTACTTCTTCATTAAACTCTTGAACTTCAGCT
AAAGCAATTTGATGATCAGGATCTTCCACAGAATCGGTCACCATTTTCAGTGCTAAAACTCTAAA
GCCTTCCTCTTCTAAATCCAATTCTACTCGGAAATTATCAAAATATTCAGGAGGTAACATACTGT
GTGCTTTTTCTCCTAATTTAGAGCGACGAAAACGCTCATAGCCTTTAAATAATGCTTCTCTAAGA

GCTTCTTCTACAGAAGTTCTAGGTAAATTGTGGGTTTGACTAATTTCACTAATTAACGCTTGTAA
ACCTGATAATTTAACGATGCTCATAAATTTTTGACAATAAAGTAATATATTTGTATATTTGATGA
TGTAGCTGGGAAAAGTTAACTGGGATTATGTAATTGGACTGATTGAATAATGTCTCTAGGAATT
TTTGTAATTTTACCTTTACAGTTTACATAGACAAATTGATCATCTCTGCCTTGTAATGTGCCTTC
CCATCGGTTTTTTTTCTTGAATATCGTATTGGTTTCGACAATGACGGGAAAACCTTTGAAACTGA
TAAATTCTCGATCGCTCTCTAAGTTATTACCAATTCCAGGACTAGAAATTTCCAAAACATAAGTA
GAGGGAAGAGTATTTTCCACATCAAGAATTTCTTCTAGTTGACGACTCATTTTTTCACAATCATC
TAAACTGGTATCTCCGCCAACACTTTTTATATCTATTCTCAACACAGGTGGGTTTTTGTTTGTTT
GAAAAACTATATTTGCGATTTCAAGATTCAATTTTTGGGCAAGAGGTTCGGCTAGTTCTAAAATA
GAAGGTATTAGAGGATGAGTCATAGAATGATTTTTCTCAGACAAATTTTTCAGTTACATAGTCAA
AAGACAATAAAAAAAGCGGGATAATCCCACTTCGATCATGGTTTTTTACCCTTCTCAAAAACTTT
GACCATTCTCAGGGTAAATCGAAAGAGGCACAATCAACTTCTGATAACAAATCAAAGCAGTAGAT
AGTCTTAGAGTACCCACTTTAACCCCAGAAAGAAATTCAGAGAAACAGACTTATATATTAATTTC
TATTAGATAAAGTGTTTTAATTCTGATCTCTTCAGTCAAAGTAAATGCTTACATTCCCTAAGTTT
ACTAAGCATTTTTTACAGTAGAGCTTTTTCCTGAAAAGTATTATAACATGAAAAAAATTTTTTTC
CCTCTTGAATTTGTTTGACGAAGCACCGAGAAAAAATAGTCAAAGTGTAGGGAATCATTGATAAT
GATTAGTGTTCAAAGAAAAGTTTTGTAATTTTATGTATCAAAAAGAAAAAGAAATTGCTATTTC
GGCTGTTTTACAAGCCTCTGAACTATGTCAAAAAGTTAGACAAGATATTCCCCCTGCTTTAGAAA
AACAAGACAAAAGTCCCGTTACTGTGGCTGATTTTGGTTCTCAGGCAATTATTTGTAAAGCCTTA
AAAGATATTTTTCCTGATACTCCCATTGTAGGGGAAGAAGATGCTACAGAGTTAAGACAACCCGA
ACAGAAAAACACTTTGACAAAAATAACAGAATATGTGAAGCAAATTATTGATAACGCTTCGGAA
AATCAAGTTTTAGACTGGATTGACTACGGTAATGGCAAAGTTAGTCGTCGTTTTTGGACTTTAGA
CCCCATTGACGGTACAAAAGGTTTTTTAAGACAAGATCAATATGCGATCGCACTTGCTTTAATTG
AAGATGGAGAGGTTAAATTAGGGGTATTAGGATGTCCCGCTTTGAACATTAATCAAAATGAACA
AGGTTGTATCTTTGTAGCTGTGCGAGGAGAGGGAAGTTATCGAATGCCCTTAAATGGCGGAGAAA
TGACTAAATTACAAGTCGTTAGTAATGATGATGTACAACGTTTCCGTTTCGTAGAAAGTGTTGAG
GCTAGTCATGGAGATCAAGAAAGACAAAATGCGATCGCACAAGCCGTAGGAATTACAAGTCAATC
AGTAAGAGTTGATTCTCAAGCTAAATATGGTATTGTTGCCAGTGGAGAAGCCGCTCTTTACCTAC
GCTTACCCTCCCCCAAATATCCTGATTACAGAGAAAATATCTGGGATCACGCCGCAGGTGCGATC
GTTGTCGAGGAAGCAGGAGGCAAAGTTACAGATATGTATGGCAATCCTTTAGACTTTTTTACTGC
CACTAAAATGATGGAAAATAGAGGGGTAGTGGTTAGCAATAGCAAAATTCATGAAAAAGTTTTA
GAAGCTCTTAATCAATAATCTTAAGATAATATTTTTCTTGCAAAAGATTTTTTCATCTCATAAAA
ATTAAGATTACTTTCACTTTGAATGAGGAACAGGAATAACTAATCTGATTAAAAATTAGGCGTT
AATAAGCTAAAATTTTTCATTCTGAAATTGCAAAATAAGGATATGTTCCTTGTCGATTGATAAAT
CGGTATTACGATGGTCAATTCCTGCAAACTCATTCTCACAAACGTAATCTATATACTTTACATTA
AACACAAATTTAATATCTAATTGCTAAATGTGAAATGACCATTGCCAGAACTATTTGTCTCGGAT
TTATTGCTGTCATCTCTGTTGGAACAATTCTTCTCATGATGCCTTTTGCCGTTGAAACAGGGAAT
TGGGGAAGTTTTATTACTGCTTTGTTTACATCTACCTCTGCGGTTTGTGTAACAGGTTTAATAGT
CGTCGATACAGGCACATATTTTTCTTTTTGGGGGGAACTAATAATTCTTTGTCTAATCCAAATAG
GCGGACTAGGCTACATGACAACCAGTACTTTTTTAATTCTCCTTATCGGTAAAAAATTTGATTTT
CGCCAAAAAAATCGCCATTAGTGAATCTTTTGATCGACCCTTTTTACAAGGGAGTCGAAATTTAGT
TATATCGATTTTTGTTACCACCTTTATATTAGAAATAATTGCCACTATAGTCCTTTACTCTATTT
TTTCTCAAGATTATGGTTGGAAACAAGGTTTATGGCTAGCGATATTTCATTCTGTTAGTGCTTGG

AATAATGCCGGATTTAGCCTTTTTCCCGACAGTTTAATCCAATATCAAAGTTCAATACCGATAAA
TATAGTAATTACAACTTTAATTATTCTTGGCGGTATTGGCTACCAAGTTATTATTGAGGTTTTTC
TATGGTTTCTTGACTTTTTCAAACAAAAAGTACATCCTCATTATGAATTTTCTCTTAATTTTAAA
GTTGTCACACGAACAACTTTGATTCTTCTGATTTTGGGTACAGTTGGATTTTTTATCACTGAACT
TCATAATAATAATACCCTTGCTGAACTGCCTTTTAAAGACAAAATAATTACAGCTTGGTTTCAAT
CAGTAACAACAAGAACAGCAGGATTCAATTCTATCGACATTGGTGCTATGACAAAGGCAGGACTA
TTTATCACTATCGGCTTAATGTTTATCGGTGCATCTCCTAGTGGTACAGGAGGTGGAATAAAAAC
AACGACCTTAAGTATTCTTTATAACAGTACTAAAGCGGTTTTAAGAGGTCAAGAACAAGTAGTAA
TGCACAAAAGAGAAGTACCTGTTTCTTTAATTCTTAAAGCTATGGCAGTTGTATTTGGTTCAGGA
GTAACAGTTGTTTGTATCACCTTTTTTATTTCCTTACTTCATTCTGATTTTGAATTTCAAAGTAT
ATTTTTTGAAGTCGTTTCTGCTTTTGCTACCGTCGGTTTATCCACTGGAATTACTTCTAGTTTATC
GGTTTTAGCGAAACTAGCCATTGTTTTTACCATGTACTTAGGACGAGTTGGAGTATTATTATTTA
TGGCAGCCATTGTGGGAGATCCTCAACCGAGTCGAATCGAATATCCAGAAGAAAATTTATTAGTT
GGTTAGAAAGTCATGACGGAAAACAAACAAGCTCAACCTGATAATTCTCAAAGATTTAGAACCTT
TTTTAACTTGGATTTAGGTTCTTTAAAATTTTTAAGCAATCTAAGAAAAGAAACTCGTCAGTTTG
CTGTTATCGGTTTGGGAAGATTTGGCAGAGCAGTATGCGAAACACTATACAACATGGGATATGAC
GTATTGGGGGCTGATGTAGAAGAAAAATTAGTGGCTCAAGCATTAACCGATAAAATCGCCTCAAG
TGCCATTCGTTTGGATTGTACCGAAGCTAGTGCTTTAAAAGAAGCTGGAATTTTTGAATTAGATA
CTGTTATAGTTGCGATCGGAAACTACTTAGAAGAGAGTATCATAACAACTTTAAATCTCAAAGAA
GCGGGAGTGAAATATATAGTAGCTAAAGCATCTTCAGAAACTCACGGAAAACTCCTCAAAAAAGT
TGGAGCCGATTTAGTTGTGTATCCTGAGTACGAAGCAGGATGTGAATTAGCTTATACCCTTACTA
AGCCCGGAATATTAGATCGTTTTGAATTAGACCCCGACAACAGTATTGTAGAAATAGCTATTCCC
GAAGAATTTGACGGGAAAACTCTAGCAGAAATGAAACTTAGAAGCAGATATGGACTCAATGTAT
TAGCTGTTGGGAATGATGATAAATTTATTATTAATCCACCACCACAATTTGTATTAACGAAAGGC
TTATCAATGGTAGTAATCGGTTCAAACAAAGATATAAACCGTCTCTAAACAATCATCTAACTTAG
GGTGAGCAATGCTCACCTTTTTTTTTGCTTAGATTCTTAACAACAATGGACATTAAATAAAACAT
TTATTTAGAAAAATTGTAATTCTGAATGAAACAGAAGAATCTGGAACACATCCAAAGCATCATTC
TGAGAAATAATACCTCAACCTGAGTAGTGAATAATAACCCTGTTAACGACTGAAAAGAGTGTACG
AGCGTAGCCAACTCTCTGAACCCTAACACCTATCCTTATCCCCCTTATCCGATATTCTTAAACCGC
AATCTTGCTCATAACTTTTTCTCCAAAAACACTCATGGATGTTCCTAGGTCAAAAAAACAAGAAA
AATCTTAAAAACCTTAAACAGTTGCGATATATAGGTTTTAGCGAATAAGGAACAAGAAAAAAGA
AAAAAATGTAGAAAAAAAGTTGCCATTTTAAAAAAGGTTAGTTATATTAGTATTTGTGCGATTG
AGCCACACGCTCAACTCTGACACACCACGAACCTCGATAATTCAATATTTTTCTAGCCATCATAAC
CAAATACAAATCCTTGTTCAATTCATTTAATTGAAAAACAAAAAACAGGAATCAGGTAGTGAAA
GCAAGTAATTGTAAGTAATAACCACTATGGAGAGTTTGATCCTGGCTCAGGATGAACGCTGGCGG
TATGCCTAACACATGCAAGTCGAACGGGCTCTTCGGAGCTAGTGGCGGACGGGTGAGGAACGCGT
GAGAACCTGCCTCAAGGTCGGGGACAACAGTTGGAAACGACTGCTAATACCGGATGAGCCGAATA
GGTAAAAGATTTATCGCCTAGAGAGGGGCTCGCGTCTGATTAGCTAGATGGTGAGGTAAAGGCTT
ACCATGGCGACGATCAGTAGCTGGTCTGAGAGGATGAGCAGCCACACTGGGACTGAGACACGGCC
CAGACTCCTACGGGAGGCAGCAGTGGGGAATTTTCCGCAATGGGCGAAAGCCTGACGGAGCAATA
CCGCGTGAGGGAGGAAGGCTCTTGGGTTGTAAACCTCAAAACTTAGGGAAGAAAAAAATGACGG
TACCTAATGTAAGCATCGGCTAACTCCGTGCCAGCAGCCGCGGTAATACGGAGGATGCAAGCGTT
ATCCGGAATCATTGGGCGTAAAGAGTCCGTAGGTGGCACTTCAAGTCTGCTTTCAAAGACCGAAG

CTCAACTTCGGAAAGGGAGTGGAAACTGAAGAGCTAGAGTATAGTAGGGGTAGAGGGAATTCCT
AGTGTAGCGGTGAAATGCGTAGAGATTAGGAAGAACACCAGTGGCGAAGGCGCTCTACTGGGCAT
ATACTGACACTGAGGGACGAAAGCTAGGGGAGCGAAAGGGATTAGATACCCCTGTAGTCCTAGCG
GTAAACGATGGATACTAGGCGTAGTGCTGTTAGAAGGACTGTGCCGAAGCTAACGCGTTAAGTAT
CCCGCCTGGGGAGTACGCACGCAAGTGTGAAACTCAAAGGAATTGACGGGGACCCGCACAAGCGG
TGGAGTATGTGGTTTAATTCGATGCAACGCGAAGAACCTTACCAAGGCTTGACATCCTGCGAATC
TTGGAGAAATCTGAGAGTGCCTAAGGGAACGCAGAGACAGGTGGTGCATGGCTGTCGTCAGCTCG
TGTCGTGAGATGTTGGGTTAAGTCCCGCAACGAGCGCAACCCTCGTCCTTAGTTGCCAGCATTAA
GTTGGGGACTCTAGGGAGACCGCCGGGGAGAACTCGGAGGAAGGTGGGGATGACGTCAAGTCAGC
ATGCCCCTTACGTCTTGGGCTACACACGTACTACAATGGTTGGGACAAAGGGGAGCGAAACCGCG
AGGTGGAGCGAATCTCATCAAACCCAGCCACAGTTCAGATTGCAGGCTGAAACTCGCCTGCATGA
AGGAGGAATCGCTAGTAATCGCAGGTCAGCATACTGCGGTGAATCCGTTCCCGGGTCTTGTACAC
ACCGCCCGTCACACCATGGAAGTTGGTCACGCCCGAAGTCGTTATTCTAACCCAAGTGGAAGGAG
ACGCCGAAGGTGGGACTAGTGACTGGGGTGAAGTCGTAACAAGGTAGCCGTACCGGAAGGTGTGG
CTGGATCACCTCCTTTAAGGGAGACTTCGAGAAGAAGTCAGCAAGGAAATGGGGAGGGCTAGAAA
AATAAAGAAAGAGGTGTTGGGGCTATTAGCTCAGGTGGTTAGAGCGCACCCCTGATAAGGGTGAG
GTCCCTGGTTCAAGTCCAGGATGGCCCATTGGGGGTATAGCTCAGTTGGTAGAGCGCCTGCTTTG
CAAGCAGGATGTCAGCGGTTCGAGTCCGCTTACCTCCAGAAGAACGAGTTTAGCACGGTGAGGTG
TCACTACTGCTGAACAAAAAGTTCAGTGAGAACCTTGAAAACTGCATAGAAAAATAGGGAAGAT
TAAGGTCAAGAAAAGAAGGGCCGATGGTGGATACCTAGGCACACAGAGGCGAAGAAGGACGTAG
CAACCTGCGAAAAGCCTCGGGGAGTCGGAAGCAGACGTAGAGCCGAGGATGTCCGAATGGGGCAA
CCCTAAAAACACACCTAAAAGAGGTGATGAGCGAACCTGGTGAACTGAAACATCTTAGTAGCCAG
AGGAAGAGAAAACAAAAGTGATTCCCTTAGTAGCGGCGAGCGAAGCGGGAAAAGCCTAAACCAT
TGTCTAAGGAGAATGGGGTAGTGGGACACCAAAACATGACAGAGAAGGTTAAATGAACTAGCTG
AAAACTAGACCAAAGAAGGTGAAAGTCCTGTAATTGAAAACCGACGAAGCATAGGTGAATCCCG
AGTAGTCAGGGATAAGTGGAATCCCTGGTGAATCAGCCACGACCACGTGGTAAGGCTAAATACTA
CTGTGTGACCGATAGAGAAAAGTACCGCGAGGGAAAGGTGAAAAGAACCCCGGGAGGGGAGTGA
AAAAGAACATGAAACCATCGGCTTACAAGCAATGGGAGTCCCATTAGAAGGGATGACCGTGTGCC
TGTTGAAGAATGAGCCGGCGACTTACAGGTCGTGGCAGGTTAAGACGAAAAGTCGAAGCCAAAGG
GAAACCGAGTCCGAAAAGGGCGAGAAGTCACGGTTTGTAGACCCGAACCTTGGTGATCTAACCAT
GTCCAGGATGAAGCTACCGTAACAGGTAGTGGAGGTCCGAACCGACCAATGTTGAAAAATTGGCG
GAGGAGGTGTGGTTAGGGGTGAAATGCCAATCGAACCAAGAGCTAGCTGGTTCTCCCCGAAATGT
GTTGAGGCGCAGCGGTGTGAAAGGTCATCGGGGGTAAAGCACTGTTTCGTTGCGGGCTGGGAGAC
CGGTACCAAGATGAGACAAACTCAGAATACCGATGAATGGTAGCACTAGTGAGACGGTGGGGGAT
AAGCTTCATCGTCAAGAGGGAAACAGCCCAGACCATCAGCTAAGGTCCCCAAGTACTAACTAAGT
GAGAAAGGAGGTGGGAGTGCTTAGACAACCAGGAGGTTTGCCTAGAAGCAGCAATCCTTGAAAG
AGTGCGTAATAGCTCACTGGTCAAGCGCTCCTGCGCCGAAAATGAACGGGGCTAAGTTAGTCACC
GAAGCTATGGACTACATTGTAGTGGTAGGGGAGCGTTCTATTAAGGGTGAAGCATTAGCGGCAAG
CAGATGTGGACGAGATAGAAGTGAGAATGTCGGCTTAAGTAGCGAAAATTAGGGTGAGAATCCC
TAACCCCGAAAGCCCAAGGGTTTCTCCAGAAGGTTCGTCCGTGGAGAGTTAGCCCGGACCTAAGG
CGAAGCGAATAGCGTAGTCGATGGCAAGCAGGTAAATATTCCTGCGTGGCTTAATTGGAGTTGCT
ACAGGGACCCATGAAAACTAAGCACACCCTGAGTGGATTGGGAGCGAGTAATCGTGAGTGTGGAA
AGATAGTGGCAAGAAAAGCTGTAGTAATGTTGAGAATTAAGTCCCGGTACCCGAAACCGACACAG

GTGGGCACGTAGAGAATACGAAGGGGAGCGAGGTAACTCTCTCCAAGGAACTCGGCAAAATGACC
CCGTAACTTAGGGAGAAGGGGTGCCACCGAGAGGTGGTCGCAGTGAAGAGTCCCAGGCGACTGTT
TAGCAAAAACATAGGTCTCTGCAAACTCGAAAGAGGAGGTATAGGGGCTGACGCCTGCCCAGTGC
CGGAAGGTTAAAGAAGTTGGTCAGCGAAAGTGAAGCTAGCGACTGAAGCCCCGGTGAACGGCGGC
CGTAACTATAACGGTCCTAAGGTAGCGAAATTCCTTGTCGGGTAAGTTCCGACCCGCACGAAAGG
CGTAACGATCTGGGAGCTGTCTCGGAGAGAGGCTCGGCGAAATAGGATTGTCTGTGAAGATACGG
ACTACTTACACTTGGACAGAAAGACCCTATGAAGCTTTACTATAGCTTGGAATTGTGTTCGGGCT
GGCAGTGCGCAGGATAGGTGGGAGGCGTAGAAGTATTCCTTGTGGGGGATACAGAGCCAACGGTG
AGATACCACTCTCTGCTAGCTAGAATTCTAACTCGAATGAGGACAGTTTCAGGTGGGTAGTTTGA
CTGGGGCGGTCGCCTCCAAAATGGTAACGGAGGCGTACAAAGGTTACCTCAGACTGGTTGGAAAT
CAGTCGAAGAGTGCAAAGGCAGAAGGTAGCTTGACTGCGAGACCAACAAGTCGAGCAGGGTGGAA
ACACGGTCTTAGTGATCCGACGGTGCTGTGTGGAAGGGCCGTCGCTCAACGGATAAAAGTTACTC
TAGGGATAACAGGCTGATCTCCCCCAAGAGTTCACATCGACGGGGAGGTTTGGCACCTCGATGTC
GGCTCATCGCAACCTGGGGCGGAAGTACGTCCCAAGGGTTGGGCTGTTCGCCCATTAAAGCGGTA
CGTGAGCTGGGTTCAGAACGTCGTGAGACAGTTCGGTCCATATCCGGTGTAAGCGTGAGAACATT
GAGAGGAGCCTTCCTTAGTACGAGAGGACCGGGAAGGACGCACCGCTGGTGTACCTGTTATCGTG
CCAACGGTAAACGCAGGGTAGCCAAGTGCGGAGAGGATAACCGCTGAAAGCATCTAAGTGGGAAG
CCCACCTCAAGATGAGTGTTCTGGTGTAAGGTCACGGGAAGAAGACCCGTTAATAGGCACAAGGT
GGAAGTGCAGTAATGTATGAAGCCGAAGTGTACTAATAGACCGTCATCTTGACCTAAAGAACTAT
GAAATTCTATGCAGTCTTGAAGGTTATTCCTTCGAGAGTATAGCTGGTGTGACTAGCACTATGGA
ACCACTACGAACCATCCCGAACTCGTGGGTGAAACATAGTAGCGGCGACGATACTGCTTGGGAGA
CCGAGTGGGACAATAGTTCCATGCCAGCTTTAACATTCTTCCTTTACAATTCTATCTTTCATCGA
AACATTAAAGAATCACCTCACATTGTGTAGGTGGTTTTTTTATTTATGTCTCTTAATTGTTCATC
ATAAGCAATTCCTAGGGAAATAAATTTGTTATGTTGTCTAATGGTTGATAACGACTGACTAATTC
AAATAAATTGTGGTCAGATAATCAAATTATTTTAACTCGATATGAATTCTCCTTTTTGTGAATCG
GCTTTTTCTGAAACTATTAGACCAATTATTGCTACTTCTGTTGATGGTCTTATTTTTATTAATAA
TCATTTATTGGCGATCGATTCTCGTAATGGTTATTTATTATTAATTAATCCGAAGAATAGTGTCA
CGAAAATTGTCAATAATTCTTCTTGGCAAGATTTTATTGGTGCAAAGGGACTCTGTATTTCTGAG
AATAAGCTATGGTTTACAAGTAGGGAGTATATCTATTATTGTTCCATTGAATGGGAAAAGGATA
ATCTTGTTATTACTTCTAAGCCTTTTTGTGCTTTTTCTCTTTCTTATCCTGCTAATGGAATTGCTG
TTTGGGAAAATACTATTTATATTACAACCCAGAAAACAGGACATATTCTAGTTTATTCTTTGAAT
GGAACCGCAATCACTAAATTTTATGCTCCTGGTATTGGTGCGGAAAATATTACCGTGAAAGGAGA
AGAAATTTGGTTATGTGACTCCATTGAACAAACTGTATATTGCTTGGACAGAGCTACAGGAGATG
TAAAATACAGTATTTTAACTCCTTTTGAATCTCCTACGGCTTTGGCTTTTTGTTATGATGAAAAA
ACTGGAAAGGATATTTTATATGTGGCTTATAGTGATCAAGAGCCTTATATCCGTGATAATCCCAA
TGCAGAGCCGAATCATGAATTATTATATCGCGATCGCACTTTTATTCATCCTTTACATTTCTATT
TTGATGAAGAAAATAAATATACTCTTTCTAATGGTTTCTTATTGGAAATGACTTATGTAGAGGA
AATTTCTCCGTTAGATCATTTAGAACTTAAAAATTTAGAATGGCGTATTGCTCTACCCACAGAGA
GTGCGAGACAAAAAATAAAAAGTATCGAAGCGGTGGGTTTGCCATATATAGAAGAGGATTATCA
AGGTCAAAAAGTGGCATTATTTAAGTTCCCAGAATTTAATACTGATGAACGCTATGTTTTTGGTT
GGCGAGCGATATTAGAGGTTTGGAGTATTAAGTATCAGTTAAAGCCTCGTGATTGCGAGGCATTA
CCCCCGTTGTCGCCTGAATACGAAAGTAAATATTTGGTTGATGATGATAATTTGGCAATGAATAC
AGATGTGATTTTAAGGGCGGCGGAAGATGCTAGGGGTTCAGAACGAATCTATTGCGAAAAATG

TATAATATTCGTAATTATGTTTATGATCATTTAACTTACGGTATTAAACCTCATATTGATACCCC
TGATATCGTTTTAAAAAGGGGCGTTGGCTCTTGCGGTGAATATTTGGGTTTATTACTGGCTTTAT
CCAGATTAAACGGTATTGCCTGTCGCACGGTTGGTCGTTATAAATGTCCATTAAAAGTACTTCAT
TTTGGGATACCTTTAGTGCCAGATTTTAATCATGTATGGATGGAGTTTTATTTGCCTAGTATTGG
TTGGTTGCCGATGGAGTCTAACCCAGATGATTTAGAAGATTCTAATATTTATCCAACTCGATTCT
TTATGGGTTTATCTTGGTACCATGTGGAAATGGCAAAAGATGTACCTTTTGAAACCTTAAAAAGT
GAAGGGGAGTTGATTAATAAGGAAACAACTTCTATTGGTCAATTAGCAATTAATCATGTTTCTTT
TACCATTATAGAAGAATTAAAGCCTACTGGAAGTGAAGAAGGTTGATCACAATAGCTCAAGTTG
AAGCTCATTAATTGATAGAATTGTCAATATCGCTATTTAGTTATATAATGATAGTTAGTTGATTA
AGATTTGTAAATAAAATAACGGTCAACAGTAATCATTTATTTTGTCATAGCATTTCGATTCAATA
AAAAATCAATTGGGGTTGAAAAATGATGTTAAACAATGTAGGAAGAAGCGATCGCTTTTTTCGT
TTAATTTTTGCTTCCATTCTAGGCTATTTAGGCTTATTCATCTATGATGGAACTGCTTTGGGTCT
TGGTTTAGCGACTGCATCGGTATTGTTAACAATTAGTGGTGTATTTGGTTTTTGCTTTCTTTACA
CTGTATTGGGCATTAATACGAACAACGCCCGTGAAAGTTCTAAATACTAATATCAAATACTAAAC
TTTATCTGATTCTTAGAAAAATTAACCGAGATATACGTGGTTTCTATCTTGATTTTTCGCAAAAT
AGACTTCATAAACAACGGAATATAAACAAACAATTAGGAGATATGTAGATTATGGAACAGAGTT
TTCCTTATGATGAAAGTGCATCAATGTCGAGACGAAATTTACTAAATTTTATCACAGGAGCAGCG
ATCGCAGTTACGGCTGGTGGAGTATTATATCCTGTTGGAAAATTTTTTATTCCCCCTTCTGATGT
GGGAGAAGATGGCTCGATTTTGGCAAAAGATGTTAATGGTAATTTAATTCCTGCTTCTCAAATTT
TAGCAGAACCGATCGGAACAAGAGCCTTAGTAGCGGGGTTAGCAGGAGAGCCAACTTACCTAACT
ATTAAAGATGATGGAACATTACATCCTTGGGGAATTGTCGATAATTGTACTCATTTAGGTTGTAC
TTTTCCATGGAATGGTAATGAAAATCAATTTCAGTGTCCTTGTCATGGATCAAGGTACGATGAGG
AAGGAAGAGTTGTTCGAGGTCCTGCCCCTTTACCGTTGAAATTAGCGCATATAAATGTTGATGGT
GATTATATCAGGATTTCTCCTTGGACGGAAAATGATCCTCGCACAGGGGAAAAACCTTGGTGGGT
TTAAATCTTTGAAAAATTAGGAATTAGGAATTTATCTGAATCAGCAATTCTTTCTTGCTAAAGAA
TTGAGATAACTTGGCATTGGTTGAACAGAGACAATAGCTATTTTTCATTTTCTATGAATGTAGAA
GTAGTTAATAACTCCATCGGATTCATTTGTAAATTGTAGGATACCCAAGCAAAACCACTACTAAA
CAAGATAAAAGTTGCCACTAAAGTACCCGTTAAAAGTTTTACTTTATGTCTTAAAGATTCAATTT
CTAAACGATTTTTTCTAATAGTTGGCAACAATTCTGTTTCTATTATTTCAGGTTCAATAGCGGGA
ATTTGTTTGTCGGGAAACTCAACAAGACTATATAACCAATCTGTTATTAATTGAGGACATTTGGG
TTGTAAATAGAGTTTTACTCCTAAAGATTTGGCTAAAGGGCGAGGAAAACGAGTTAGATATTTG
AGAGTTGCGTTGATTCCCCACAGAGGACAATATTTATTAATTAAGTTGATAGTTGCAATATCATA
GAGACAGCCAACAATTCCTTTTGCTGTAGCTTCTTCTCTACGGAAAAGTTGATCAAGAAGTATTA
ATATTTCTCTTAGTTTTTTATCTTCTAAATTTTTTTCTTCTTGAGTTTTCGTTTTAGACATGATT
TTTATTTAGCTTAAATTTAATGGTGTTTTAAATCTAATTAAACTGATTTAATCTATATAATTTTG
TTTTTAATTGTTATATAAAATGTTCTTTTACTGTATTTATTTAGCAATAAATATTAATGTCTTTG
TAAAGAATATTTATTTAAAATAGATTTGTTTTGTTTTGATGATTACGATAGTTTTTGAATAACA
TTACTAAAAAATTTTATTTATCTTTAATCTTATAATTTATTCTTATAAAATTCAATATCTATTTG
ATTAAGATCAATCTAATTCAAAATACTTATAGAGAATTGACAATAATGGGTATTTTTTGTAGCG
AGATAAAATAATCAGTAATAACTTTTAATTTTTATTTTCATATTTTGGATAAAAGTAAATAACA
CTAAATCATTATGGATAAATGCGTCTTAGCTTCTGTCGGTGTATCATAGATTAGGGCAAAAGCAG
TCAATATCCTAATAGTGAAAAAATAAATATGTTAGAGTCTTTTTCTAATCAACTTTATTATCTAC
AACAGTTTGCCAATGAATTAGTTAATAATCAATTACATCAAGTCTCTATTTTTAGTTTTTTGATT

ATTTTTGTGACTGGTTTAATCACCAGTTTAACTCCTTGTATGTTATCAATGCTTCCTCTAACTAT
TGCTTATATTGGCGGTTATGAAACTAAAGGAAGATTATCTTCTTTTTTGCAATCAGCCTATTTTG
CTTTAGGATTAGCCACAACTCTTGCTTTGCTGGGAGTTTTCGCCGCTTTATTAGGTAGGGTTTAT
GGGCAAATTGGTACTGGTTTACCAATATTTGTAAGTGCGATCGCAATTTTAATGGGTTTAAATTT
ATTAGAAATAGTACCTTTAAAATTTCCTAATTGGGATGCTAGTAATTGGGTAAAAGATAACTTTC
CTAATAGTCTTAAATCTTATCTTCTAGGACTAACTTTTGGCTTAATTGCTTCTCCTTGCAGTACTC
CAGTTTTAATTACACTATTAGCTTTTGTTGCTAGTAGCGAAAATATATTTTTGAGTTCTTTATTA
CTGGTAAGTTATGCTATTGGATATGTTTTTCCTCTGATATTAGCAGGTACATTTACAGGCACATT
AAAAAGTTTATTAAATTTACGAGTTATTACCCAGTGGATTAATCCTATTAGTGGGGGGATATTAC
TAATTTTTGGTATTTATTCTTTGGGTTCTTATTTAATTGCTTAAAACTTTACTTTCTTTTTAGCT
CTTAAGCTCGTTATTAATAAAAATAGTGAAATTAAAATTAAAATCAAGCTAAAACTTAATCAAG
TCAAATATTCTTATGATTCACCGAAATAATTTTAAGTTATGAAAATCAAACAATTATGGGAAACT
CTAACTTTTTTTGAAGTTATTCCTTTTTTTAACTGTTTTACAAATTTTTTCAATATTTCTCAGCA
CAAAACTTCACCTCTAATTTCAACCATGAATTTTACTCTAATTTGTTATGATCATAGTCCTTTAG
CTCAAGAAACCCTCAATAATCTTCAACAACAAAATATACCTCTGCGTATTCTTAACACATCTTCG
TTTAATCCTAACCCTTCCGAAAAAACTGAAATTATTAAAATTAACCTTCTTCAAGACAGTTTATC
ACCCCATTTACTAGAAAGAGTCGATCGCATTTTTTGTTTTCCCCAACCCGGTATTAATAATTTAA
TCAATCTGATTAATCAACAGCTTTTTCCCGGTGAAATTATTTTATGTGACTTACGCTATGGCAAT
GAAAATCAGAGATATATTTGGGGGGCAGTAGATGATGTGGTAATGGGAGGAGTAAGTAGTAGTA
ACTTATTTTTTAAGGAAAATAAAGCCATCTTTACTGGCAACGTCTCGACAGATAACAACGGCGGT
TTTGTGTCTGTAAGAAGCAAAAATTTTAATCCCCCTTGGGATTTATCGGCTTATGAGGGTATCCG
ATTGAGAGTACAAGGAGATGGAAACAGATACAAATTTATTACCCGTTGTGAGGGAAAATGGGAC
GGTATCAGTTATTGTTATTCCTTTAACACGATTGCAAATGTGTGGATGACTATTGATATACCTTT
CAAAGATTTAATACCTGTATTTCGGGCTAAAACCGTCAATGCAGATCAATTTGATCAAACCAGAG
TTTACTCTTTACAGCTAATGTTAAGTAAATTTGAATATGATGGTGCATATAATCCCACTTTTCGG
GCAGGTGTTTTCAGCTTGGAGATAGAGTCCATCAAAGCCTATGGTAAGCCAAATTCACCGCAACT
AATTTTGATGGATAATCAAGCTGACGGGGAGACAATGAAACTGCAGTGAGGGTAGGTTTTAGGT
TTCAGGTTTCGGGTTGCGGGTTTTAAATACATGAACAGTATTTTGTATTTACGAAATAAATAGCC
AGACATATTATCTCAAAATGTTGATAATCCAATAGTTTTGCCTATTATTTGAACCATTGAGTAAA
TAGAGATAATGATAAATATTAGTGGAGACAAAACCTAACACCTAACACCTGCTACCTGCTACCTA
TAAAGTTTTTAGAGTTATTGTCCCCCCTTAAGATAAAAATTTATCCTGAACTCAGGTTATTTATT
ATCCCTCATTAATTTAACAAATTGTTCAAACAAATAATCGGCATCATGAGGACCTGGACTTGCTT
CAGGATGATATTGTACTGAGAAAAATGGTAGGTTTTTGTGTTTTAAACCCGCTACAGTGCGATCA
TTAAGATTGAGATGGGTTATTTCTACGTCCGCCCCTAAAGATTCTTCTGTCACTGCAAAACCATG
ATTTTGGCTAGTAATTTCTACTTTCTGCTTTAATCCTGCGGGTTGGTTTAAACCTCTATGTCCGA
ATTTTAGTTTAAAGGTTTGTGCTCCTAGAGACAAACCAAGAATTTGATGCCCCATACAAATGCCG
AAAGTGGGCTTTTTCGCCTCTAAAAGGGCTTTTGCTGTGGCAATACCTTCGGCTACCGCAGAAGG
GTCTCCTGGTCCATTAGAGAGGAATATACCATCGGGATTATACTTGAGGATTTCTTCAGGGGGGG
TATTAGCAGGAACAACAATTACTTTACAACCATAACTCGCTAGACGGCGGAGGATATTACGTTTG
ATACCAAAGTCAATGGCAACTACTGTATATTTTTCTTTGTCCGCTAAACTTGCTTTAGGACTAAA
TTCCCATTCAGAATTCGTGCTTTCATGCCATTCGTAAACTTCTTTGGTAGTTACTTCTTTAACAA
GGTTTAAACCTGCCATAGAAGGTACTGTACGCAGTTGAATTAATAATTGTTCTGGGTCTAGGGTT
TCTGTGGATATAGCCCCATTCATCGCACCAGAGATGCGTAAACGGCGAGTTAGGTCACGGGTGTC

GATGCCGTAGATAGCAATAATATTATGTTGCTTCAGATAATCTGGAAGGGATTGAGTAGAGCGCC
AATTACTGGGGCGATCGCATATATTACGGGCAATAACTCCTTTAATGTGGGGTTGATGGGATTCA
TCATCTTCTGGATTAATTCCTGTATTTCCTAATTCAGGATAAGTAAAAGTAACAATTTGTCCGCA
GTAACTAGGGTCTGTCATCACTTCTTGATAACCTGTCATTCCCGTGTTGAAAACGACTTCTCCAA
AAGTTGTACCTGTAGCGCCGCAGGAGTAACCCTGATAGGTAGTGCCGTCTGCTAAAACTAACAAT
GCTGGTTTAAAACTAAAAATAGACATAAGCAGTGGTGATGAATTTTATATTTTACTTAACAATTC
TCTTGGTTGATTTATTTATTCTCATCCTCATCAACATACACTAATTTAACGTTTCTATGCTACTG
TTATTTGAGCTATTCTTGACAAACACAACTAATATCAGTCACATTAATAATGGCAATAGTTTGTT
TGAGTTAGACGCTTAATTAATTGGGGTAGGGTTGAGATTATGAGTCCAAGAAAATTATCCGATG
GCGATCGCCAAGAAATATTATCTTTATATCGCAATACCAACGAGACAACCTCAACATTAGCGGTG
CGTTTTGGGGTCAGCAGTTCTACGGTGAGTCGTTTTCTCAAAAATAGTCTCACGGAAACAGAATA
CGAAGATTTAATTCAGGAAAAACGTTTAGCACGTACGGCAAAAAACCCAGAGGAGGAAATGGAG
GAAAAAATAATTGATCACTCCTCCAAAGATTCGAAGGAAAATGATAATGATATTGATGAATATT
CTAGTGTCATAAAAGAAGATCAAGAAAAGGCACAATTAAATTTAGATTTTGAGGAGGAAAACGA
GAATTTATCTCTATCTTCAGAAACACAAGAATTAGAGCCAATCTCTCTTCATCAAGACAACACTT
CCGTAACTCCAAAAATACAACTTAAGGAAGAAGATACAAGAGCTAATAATGTGATGTTAGATGT
ACAACCAGAAGAAACAGAGATTAACGAAGAGGAAGAAGAAGTTCTAGAGTCTGTCCAAGTTTTG
GCGGCAATGTATGGAGAGGATATGGAAGATGAAATAGAGGACATAGAAGAAGACGAAGAAGATT
TCGAGGATGAAGAAATAGAAAAAAAACTAATTGTTTCTAATCCTATGGCTGAGAGTCAGTTACA
AGTACTACCCCTAGAAAATGCGACTTTCCCGAAAGTTTGTTATCTTGTGATCGATCGTTATGCAG
AATTGGTGACTAAGCCTCTCAAAGACTTTGGACATCTGGGCAAAATACCGGGATCAGAAATTTTA
CAATTAACCCTACCTATTTTTGATAATCATAAGGTGGCTAAACGATTTTGCGATCGCAAAGGAAA
AGTCATCAAAGTGCCTGATGGTAAGATGTTACAAAAAACTTCTATTTACCTTAAAGCAAAAGGTA
TTAGTCGTATTCTGATTGATGGCAAAATTTATTCTCTTAATTGAGAAATAGGGCAAGAGGCAAAC
CCCTCTGTGTCTCCTCTTAAAAGAAACCCCCCTTTATCCCCCCTCGAGAGGGGGGAGGGCAAAGT
GTTGAATAAATACTAACTCGAACACCCCCATACCCCAATTCCCTGACACCTGCAACCAGAACTCCG
AACTCCTATCCTCATTATTTGTTTCTCCCCTCCAACTAATTATTTTTTTGCCACCTGTTGGGAAAG
TATTTCTAAGGCTTTACGATATTGAGCGTCTTCTGCCGTACCGATTTTATCACGGTCTTGGATGA
GTACCTCTTTTTCTGCATCTGTCATTTCATAAACAACATCAGGAACGATTCCTTCTTTATTGATG
TCTCTGCCCTTTGGAGTTAAATATTTAGCAATTGTTACCGCTAATCCTGAACCATCCCCTAAGCCT
CTAACCGATTGTACTAAACCTTTCCCGAAGGTTTGTGTTCCCACTACTGTGGCTCTGTCATAATCT
TGTAAAGCACCAGAGAGGATTTCACTGGCACTAGCAGAACCGCCATCGACAAGGACAACGATAGG
TTTATTAGTTAAAACACTACCATTAGCATTATGAGCATCTACTTGTCCAACTCTGTCAACGGTAG
AGACTATTTGACCTTTATCGATAAACATTCTTGCAATTTCGACACTAGAGTATAACAAACCACCA
GGATTTGATCGCAGATCTAGGATATAACCATTCACATTTTCTTTTTCCCCAAGTTCGATCGCATC
TTTCATTTCCTGGACAGCATTACCACTGAATTGAACTAAACGAATATAGCCAATTTTTCCCAAAT
TAGGGGTATTTTCGATTCTTGCCCTGACAGGATGAATTTCAATTTTGGCTCTGGTGAGATTATAA
TCTCTTATGTTTCCTTCCCTTTCGATGGTCAACTTTACCTGAGTGCCGGGTTTTCCACGGATTAGA
GAAACAACATCATTTAAGTCCATCCCTTCGGTACTTTTACCATCAACTTTGCGAATAATATCTTT
AGAAATGATACCAGCCTCAGCCGCAGGAGTATCTTCAATGGGGGAAATAACGACGATATTATTTG
TTTCTTCTTCTTTCGTGATTTGGATTCCTACTCCTGTCAACTCTCCTGAAGTATCAATTTGCATAC
TTTTAAACTCTTCAGGATTCATAAAACGAGTATAAGGGTCATTGAGCCTTTTCAGCATTTCCTTG
ACTGCTTCATAGGCTTCTTCCTTATCGCTATATTCTTTGTCGAGATACTCTTGTCTAATTCCTCGC

CAATCTTCTCCATTAAAAGTGGCATCTACATATTGTCTATTAATTACTTGCCAAACTTCATCAAC
AATTTCCTTGGGACTATCTTCAAAAAATGCTTGACTTTGGGATACTCTTAAACCAATACCTGTCA
TGGCAACACTAGAAACAGCTAAGGCTGTTGTACCCAAAATAAGCCTACGTTTAGTAATTTTCATA
TTCAATTAACTCGTCTCAAAACTCTATATTAGGATGTGGGAATATCCAGTTAATTTTAACTAGAG
GATGGTTTTTCTTTATTTAGATGCTAACACAATAGACCTTGAGGAATTTGTAATTAGCTTAACCC
GACTATCTATTCTTTTTTACAATTGTGTATAAAGATCAATAAAATCTTGACGGGGTTTAAATTGT
TCCACTTCCCTGATTTTGGCGTACAGCTCCTTCTCCTCCTCGCTCACTTCAGGGGGGATAACAATA
TTAATAATAACTAATTGGTCTCCTCTTTCTCCTCGTATATTAGGATAACCTTTATTGGCTAATTT
TAATCTTTGTCCTGATTTTACCCCGGCGGGGACATTCATTTTCACTAAACCATCAATGGTAGGAA
CTTCGATCGCACCTCCAACTATTGCTTCAGAAGGAGTCAAAGGAACTTCACAGGAAATATCAGAA
CCTTGTAAAGTGAAAAAGGGATGAGGTTCAATTAAAATCTTTAAATACAAATCCCCGCCTCTGAT
ACCTTGACCTTTCAAGCGTATTTTTTGCCCATGATACATACATGGTGGCATATCCACTTCAAGCG
ATCGCCCATCTTCCAATCTAATTCTTTCTCTTCCCCCCTCATAGGCTTTAGTCAGAGGTAAAGTCA
GTTTAGCCTCAATGTCTTTGGGTTGGGTGCGAGGAGAGGGGGGTGGACTTGATGGTTTTAGTAGTC
CCCGGACGAAAATCACCGATATTAGGAGGATTGGGGCGATAAGTAGAATTTTTATTACCAGAGGA
AGCACCATTAGAGGATCGAACATTATCTTGCCAAAAACCGCCAATATTAGCATTAGGAAAAGGAA
AGCCATTACTAGGGCGTTTTTTCGTTTGACTACCAACAAATCTACGTTTGCCACCCAGAAATTGA
CTATCATACTGTTGACGTTTTGTGTAATCAGAAAGGATGTCGTAGGCTTCATTAATTTGCTTGAA
CATTTCCTCAGAATTTTGATCTCCCGGATTAACATCGGGGTGATAGCGTCTTGCTAAGGTGCGAA
AGGCTTTTTTAACGTCTTCAGGAGAGGCATCACTAGAAATGCCCAATACTTGGTAATAGTTAATC
ACTGGTGTCCGTAAATTTTGCATAGTCGATACTTAATCAATCCTAACCAGAAAAAGCAAGATTTT
GTATTTAACCAAGTTTAGCGTAGATTTGATGTTTCAATTAACCTTAGTTGTGGATTTCTCTAATG
AATAAAAATATTGTTAAATAAAAATGGAACAAAAGTAATACTAAGCTAGGGAATAATAGACTTT
TTGTAGAAGTCAGACAACAGGCAAAAGTAAAAAAATTGACTCCAACCCCATAGAAATTGATTTTA
ATTTTGCATTTGTGCCTAAATCATTTTTATGCCACTTGAGATTGTAAAAACTTAATAAGACCTTG
TTTCTCGATCAATCCCACAACAAAACCATTTTCTTTAACTACTGTTAATTCTAAATCCTGCTGTT
GTTCTAAAAGTTTAACCACTTCTAATAAAGATAAAGTATCCACAACGGTTTTAACTTCTTCTTTT
GATTTCATTACTTGATGGACAAATACTTCATTCCATTGAGAAGTTGCGATCGCTCTTATATCATC
TAAATGTAAGACTCCTGTTAAAATACCATCTTCATCGGTAACGAGAAACTTACGCCATGGTTGTT
TACCAATTACGTATTCGTTAACAAATTGTCTAATAGTTAAATTACCTTGAATAACAGGACTTTCA
GGGGAAATCGCATCTTTAGCGTAGTAATGATTTAATTTATCCTCCAATTGGGCAGACTGTGCCGC
CGTTCCCGCATTTTGTAGTAAAAACCAACCAATTAAGACAGTCCAAAAATTACCAAAAGGAATTA
TTCCTAATGTTCCCATTGCCCCTATAGTAATAGCTGTCCAACCAAAAAACTGTCCAAAACGCCCCG
CAAAAATTACTCCTTTCTGGGGATTACCTGTGATTTTCCACACAATGGCTTTTAATACGTTACCA
CCATCCAAGGGTAACCCCGGAATCATATTAAATAATGCCAAGACAAGGTTGATGGTTGCTAAAAG
AGATACTATTAGTTGTAGTGCGATCGGTAAAGTAAAATTCGTTTCTATGATGAGGAAAATCGCA
AAAAGGATTAAACTAACCACAGGACCTGCGATCGCCACAAAAAACGCTTGTTCAGGGGTTTAGA
CTCTTTTTCAAGGGCGGCTAGACCCCCAAAAATAAACAATGTAATTGATTTTACTTCTATTCCTT
GACTAATCGCTACAAAACTATGTCCCAATTCATGGGCTAAAACGGAAGAAAATAGCAGTAAAGAA
GCGACTAATCCCAATAAAAAAGGAACTAAACCGGGTAAATTTGTTTGATAGGCTAAATCTGTACC
AAAAGTTAAAGTCATCAACCCCAAAATCAGAAACCATGAGGGATTGATATAAAAAGGAATGCCG
AATAAACTACCGACTCTGATGTTACCATTCATAAAAAAATAATTCTCCTTGTTTTCAGAGTGATT
AATTATTAATTACTAATAACTAGGAAAAAATGTAAATAATTATTACTAAAAAATCTTATTTTTA

ATTCACTTTCTATTATAAATCAACTGAAAAAACTGTAATATTTTATTCTTTCTGGTTTTCCGTCA
TTTTTAGTTCGGTTATTCTATATATTTATTTTCTGATTGGTTAGTTATGTCAAGAATCAAGATAC
TTGTTTTTTTATAACCAGTGACGTAAATATAATAAAATATGTCACTAAAACGCTACTTTAATCAC
TTTTTTAAAGTGGGAATGTCAGTCTTCTACTTATAGAATAAAACTAAAACACGGCAATAGTTCTT
TTAACAAGGGTAATTTTAAGTATTTTTATTGATAAAAATGGTAGTAGCAACAAAAAAAATATTA
CAAATCACAGACACTCATCTTGACGAAAAATCCACAACTCATGGGCATAACGTTTATGATAAACT
TGCCATAGTTTTTGAGCAAACTATTGAAAAAAAAGATTTTGACTTAATTATTTTTTCTGGAGATA
TTAGCGATAAGGGTAGTTTAAATTCTTATGAGTGGTTAGCAAAAAAAACAGAAAACTATAACAG
TAAAATTATTTGGATGGCAGGTAATCATGATAGTGTCGATAACATGAAACAGGTTTTTAATTTG
TCTTTTCTGGTTGATATTGAAGAAAATAAACTTAACTATCAATTAAATTTGTCAGGAATAAATA
TTTTATGTTTGGATACTAATGATAAATTACTTTCTGATAATCAAATTGATTGGTTGATTGAATCC
ACAGATGACAATACAATTATTTTTATTCATCATCCTCCTATTACTTGTGCTTCTCCATTTATGGA
TGACAACCACTCATTGGCAAACTGGAAAATTATCAAAGAAAAACTCTCAACTATATCAAACAAAA
CTTTTAGATTTTTTTGTGGACATTATCACCAAGAAAGTTATATTTCCTATCAAAATATACATCTT
TTTCTCACTCCATCAACAATGTTTCAAATTGACAGACATAAACCCCATTTTCAAATTGCTAATAC
TTTGTCAGGCTATCGAATAATCGAAATATTTGAGGATAAGTCTTTTAAAACCTATTGTGATTATA
TTTTTATTACTTAAATTAGATTTTTTAAGTAAATTAAGGAAAAGAAAAAGCAAAGAGAGAATAG
TTGAGAAGAAAAACTCCTGATTTGGTTACTTCTACGGATTACTAACTCAGTTAAACTTGATACCT
GACTTTATCAGATATTCTTAAGCCAAACTGAGGTTAAATAACTTTATTTTTCGGTTCTTCAGAGT
GTGGTTATGATAAATTAAGAAAAACAGGACTTCCATAACCTTCATTGAATAGTGTTTATAGTAA
AATAAAAACCAAAAGTTTATAAATTATTATTTAGTAATCCCCTATTGCCTATTGCCTCTTGCCTA
CCCTAACTAATAATTTACATACTCAAACTGATAGAGCCTATTTTTCTTCTAATACAAAGTATCCT
TGAGAGTTTTTATTGTAACTAATACTATATTTTTCGGGAGGGAATTTAATGATAGTTTTAGCATT
TTGAGAGGAAATTTTTATCAATAAATGAGCATTTTTGTTAACTCCTAATATTTCTCCTTTTTGCT
GATTAATTAGTGCTGTTTCTTTATGATTGTAAAGTAAGCTATTATATGTTTTTACGACAAAATCA
ATACCATAATTTTTATAAAAGTAATAACCATTTATTATTGCTTTAATAGCCAAAAAACCTATTTT
TTCTATACTATCAATATCAGGATTTTGACCGACACACAAATAATCATTTAAACTGATTCCATTGG
CAGGAGAATTATTTTGATAATTAATGCCAATTCCGATAACTGCTTTTTTGATTATCGATTTCTCT
ATTTTTGTTTCCCATAAAATACCCCCTAATTTTCTGCCTTCCAAAATCAAGTCGTTTAACCATTTA
ATGGCAACAGGTATTTTTATATTATTAAACTCAGATGCGATCGCGTTTGCACTGAATAGGGTAAG
ATGGTGTAAACAATCAACAGGCAAGTTTAACTCTAAACCTAAACTTAAGTATAAACCACCGAGAT
GCGATCGCCAAATATGACCTCTTTGACCTTTTCCTGCGGTTTGTTGCCTTGCTATAACCACGAATG
GAAAGGGAATATTATTCTGCTTTTCCAGTAACCATGCTTGGTCATTAGTAGAAGTTAAGGTATCG
TATAAATAACAATCTATTTGATTGAGCTTAAAAAGATTATAAGCAAAAGTTTCTGGTAAATTAA
AAACTATTTTATTCATATTGTAGTGATATGTTAGAAGAACAAGATAACGTGCAAGAAAATTTTA
TTGATGTAGAAAAAGTGAACCTTACTCCTAATAAAATAAAGCTAATTTATTTAGGTATTTTAGCA
TTAGGAATTAAACTAGAATCAATGGTTATTCCTATTAGTAAATCGGAGCTTGATTTAGTGGTTGA
ATATCTAAGTAAAGTATTGCAAAAAAACGAAGAATTAATCAGAAGAGCTTGTTCTTTGCTTGAA
CAAATAGAAAATTCTGAGCAGAATAATTATTATGGAATAGTCAAAGAATATCTCGATAACTTCT
TTGGTTTATCAGAATCAGAAGAAACTCTGTCCCTAAATTTAACTCAAGAACAAAAACTATCTTTA
GCCCTTAAAGTTTTGACAGATTTACTATTTTATAGTAGTAGATCAGGACAACGTTATCTACACAA
ACAATTACAATGTTTATAGGATAAAAAAAGTAGATAAATTGATTTTATTTTTATCGAAAGTTAA
TAAAAAAATGTAAAACAGTAATTAACTTCAATCAATAATAAATAACCAATAATAAATAACACTC

TGGCTTATTGTTTATGATCATAATATCATTTTTAATCAATCAATCCCTAATTATTGACTCTTTTT
GAAAGATATAAATTAGCAATTGATAAAACTAAAATAAAAACAAATAAAACTAATCCCATCGTAC
AAGCATAACTAATGTCTAACTCCTTAAATGCTTTTTCATAGAGATAATAAACTACTGTTTTAGAG
GCGTTGCGTGGTCCTCCCTGAGTGAGAAGATAAACTTCTTCAAAAACTTTAGTTGCACTTAATGC
TGAAATTACCGCCACCAAAAAAAATATAAGGACTCATCAAAGGGATTGTAATATCAAGATGTTTAC
GCCAACCATCTGAACCGTCAATTGCTCCTGCTTCATACAATTCGGGAGAAATACTTTGTAATCCT
GCTAAATAGATAACCATGTAGTATCCCAATCCTTTCCAAACAGTGACAATCATTAAGCTCCAGAT
TGCCCAATTAGGGCTAGTTAACCAAGGAATACCGTCATCTAAGCCTATGCTACGCAAAAATTGAT
TAAAAAGACCATTTGACCAATAAAGAGCCTTCCAAGCCAATCCTGCCACTACCATAGAAACCACT
ACAGGTGTGTAGTATGCTCCTCGAAACCAGGTAATTGCTTTTAATTTCTGATTGACAAGAATCGC
TAACAAGAGAGAGAGAATAACTAAAGGAGGTACAACACCAAGTAAATAGATAAGGGTATTAATT
AAAGTCTGTCGAAATACCTTATCTTCGCTCAACTGTCGAAAGTTTTCCCAACCAATCCATTCAGG
CGGTTGAGTTAAATCATATTCGTAACGGGTAAAACTCAGAGAAAAAGCCTGTATTGCGGGGAAA
AAGACAATAATCCCTAAAACTATTAAAGCAGGAAGCAGAAATAAATAAGGATTAAGGGTTTTTA
ACCAACGGGAAGAAGTCATATTTTTTTGAGTTCAAAGTTTAGAAAGAGGTTTCGGGTTGCAAGTG
TCAGGATGTTGGGGGATTGAGGTGTTGAGGGTTCGGAGTTTTTAATTTTTAATTCTTAATTATCA
ACTATTGACTATTCATCTTTGCCCCTTGCCCTTTGCCCTTTTTTTATCTAATCACTAATTGCTGAA
AACTCTAATTAAAGTACCTTTTGGCGGTAAGTCTTGAGGTTTGATGGTAGTTTGATTTCCTTGAA
TGATTTTGATACCCATATCTTCTTCTTGCTTGGCTAGTTCCAAAAATTCATCTACCCTTGTTAAGT
CGCATTTTCCGGGTTCAGCCGCATCGGTTAAATATTGGGTGGGAAAGACAACTTTGGGATTTAAA
GCCTTGATAGCTGCGATCGCTTCTTGGGCATTATAGGCTTTAGCACTTCCCCCGACAGGCACAAAT
AAGACATCAGGAGAACCCATGAGAATTCTTTGTTCAATATCAATGGGAGAGGCAATTCCTCCTAA
ATGGAGAATTTTTAACCCCCCCTGAGTCCATTTCCATGCCACATTATCACCAAATCTTCTACCTCC
TTCTCTGTCATGAAACGTTTTGATACCTTGAAATTCGATCGCTTTTACTTGATATACCCCCGGTTC
AACCATTAATTGAGGGTTATTAGGTAAACCTGCCGCCGAACCTTCGTCTAACAAGCGACTGCTAA
TCATCACAACTTCGACTTCTGGATTTGGTTTGGGGTATCCTGCGGTACAACCCAAAGGCGTAAAA
GGATTAACCAAAATTTTCGCTTGAGGATTGCTAAAAACAAAAGCACTATGACCTAACCATTGAAT
TGTAACCCCCGTATTATTTTGAGCAGAGCTAGAGGGGGAATACTTGAGAGCTAAAAAAGTAGTTA
ACAAACCACAGCTTGTGAAGCGAATCCAATCACGGCGTTTCATACTCATTAATCTAAATCTTTCA
GGAAATTGCCTAATAATTTTAACCCAGAATCGGTAAGGATGCTCTCAGGATGAAATTGAACTCCT
TGAATATGCTTAAATTCTTGATGACGAATTCCCATCACAATTCCGTCTTCTGTCCATGCAGTTAT
TTCTAAAATAGGGGGAATAGTCTCTTTTTCCACAATTAAACTATGATAACGGGTAGCAGTCAAAG
GGTTAGGTAAATCTTGAAAAACCCCGATGTTGCTGTGTTTAATTTCCGATGTCTTCCCGTGCATT
AACACTGGTGCAGATACCACTTTTCCTCCAAAAACTTGTCCGATGGTCTGATGTCCTAAACAAAC
CCCTAAAATGGGATATTCTTGACCTAATTTTTCGACTAATTCTAAACAAATACCAGCAGAATCAG
GAGTTCCCGGACCGGGAGAAATTACGATCGCATCTGGCTTTAAAGCGGAAATTTCCGTTAAAGAA
ATTTGATCATTGCGAACAACCCTAATATCCTGTGCTACCTTATAATCCGTAGCTAACTCCCCTAGA
TACTGTACTAAATTGTAAGTAAAACTATCGTAATTATCGATAACCAGAATCAACCTGTTTTCTCC
TCTATATAATTTTTAGCTTAAGATTGAGATTATTAAGTCTATCCCATGAGGTAACAAAATAAAAC
CTGCCACCAATAGAGAAGCCAGAGAACCAATTAAAACGGCTCCTGCCGCACAATCTTTGGCAATT
TTTGCTAATTCATGATAAGTCTGTTTGACGGTAAGATCAACTACAGACTCCAAAGCTGTATTCAA
CAATTCTAAAACCATTACCATAGCACAAGTAAGGGAAATTAAAGCCATTTCGACAGTAGTTGCAT
GAAGATAAAATCCTAAAGCTAAAGCCACGCTAGTCATTACCGTATGAATCCGAAAATTACGTTGA

GTGGTAAAGGCATATTTCACTCCCTGCCAAGCATATCTAAAACTCAACCCCAAATTAGTTGCTAC
TTTCCAAGCGAGATAACGTTTTTCTGACTTGTAATGATAAGTTTTTTCTGAAGAGAAGTTATGAT
GAACTGGTTGTTGTGCCAAAAATTGCGGGGAATCACTTTTAGAAGTAATTGGCTTAGAAGTAATT
GGCTGATCATTGCTGATTAGTTGAAATTTAGGCATATTATTCACTTTTTATTAAATTTCTTTTAT
ATTAGATTCTTCAGTATAAGCCGATTATTTAATAATTTATGTTCCCACAGTGAAATTTTAGCTAC
ATTTTTCTTGATATTGTATTCTTTCTACTTATTATTATTATCATTTAGATAAATAAAAGCTATAT
TTAGTTATAGTTGAGGTAATGATTGAGATTATTTTTAACTTCAGTTCGGTTTAAAAATATCGAAT
AAGGTTAGGTGTTAGGTGTCAGGTTGCAGGTTGCAGGTGTTTGAGTGATGAGGTGATGAGGGGA
GAGGGGGAAGTGGGGGCGAATGGCCATTCGCCCATACAGGGGTTCGGAGTTCGGAGTTTTTAATT
CTTAATTTTTCCTTTGCCCCTTGCCCCTTGCCCTTTTACTTTGCACATTGCCCTTTTCGCTATCAC
TCATCGATGACAATTTATGCTTAACTCAGGTTATTTTTAAGTAACAGAAACCACAGTATTTGAAT
TAAGAAAGATAAATAATAATTTAATATTACGAAAAAATAGTTTACATTTAATAATTGATCATCA
TAGAGAGTAGGAGGTTATATGCGTGCTGTTTTAATGGCTGGAGGTTCTGGGACAAGGTTACGTCC
ATTGACTTGTAACCTACCTAAGCCGATGGTTCCAGTTTTAAACAGACCCATTGCAGAACACATCA
TTAATTTGCTTAAGCGTCATGGTATTCATGAAATTATTGCTACTCTCCATTATCTCCCCGATGTG
ATGCGAGATTATTTTCAAGATGGTCGAGATTTTGGCGTTACCCTCAACTATGCTGTTGAAGAAGA
ACAACCTTTAGGTACTGCTGGTTGTGTGAAAAACATTGAAGAAATGCTCGATGATACTTTTTTGG
TCATTAGTGGTGACAGTATTGCTGATTTTAATTTACAGGATGCGATCGCATTTCACCGAGCCAAA
AAATCAAAAGCAACTTTAATTTTGACCAGAGTACCAAATCCCCTTGAGTTTGGGGTTGTTATTAC
CGATCAGGAAAACAGAATCCAAAGATTTTTAGAAAAACCCTCCGCCAGTGAAATTTTTTCTGATA
CCGTCAATACTGGTACTTATATATTAGAACCCGAAGTCTTAAAATATCTGCCCCAAGATGAAGAA
TGTGATTTTTCCAAAGATTTGTTCCCTCAACTGTTGGAAATGGAAGAACCGATGTATGGTTATAT
TGCTGATGCTTATTGGTGCGATGTGGGACATTTAGAAGCCTATCGAGAGGCACATTATTCAGCAC
TAGAAAAAGAAGTGGAATTACAATTTCCCTATCGAGAAATAGACCAAGGAGTGTGGATTGGCGA
AAATACCTATATTCATCCCACTGCCATTGTGGAAAGTCCTGTCTTAATTGGTAGTAACTGTCGTA
TCGGTGAAAGAGCAAAGATTCTTGCTGGTACAATTATTGGTGATAATGTGACCATTGGTGACGAT
GCAGAACTTCATCGACCGATTATTTGGAATGGCGTTATTGTGGGCGAAGAATCTTCTTTATCAGC
TTGTATTATAGCACGAGGGACAAGGATAGATCGGCGATCGCAAATTTTAGAAGGAGCAGTAATCG
GTTCTCTGTGTAATATTGGCGAAGAATCCCAGATTAACATCGGTGTCAGAGTTTGGCCTAACAAA
AGAATTGAGCCGGGGGCAATGCTTAATATTAATCTGATTTGGGGAAATATAGCCCATAGTAACCT
CTTTGGACAAAGGGGGGTAACAGGACTCGCAAACATTGACATTACCCCAGAATTTGCCGTTAAAT
TGGGAGCGGCTTATGGTTCAACCTTAAAATCAGGTAGTAGTGTTGTTGTCTCCAGAGATCAACGC
ACCGTATCAAGAATGGTGAGTCGTTCTTTAATTGCAGGTTTAATGTCTGTAGGCATAGATGTACA
GAATTTAGAAGCGGCGGCAATTCCCATTTCTCGAACCATGACTCCCAAGTTAGGAGTAGCGGGAG
GAATTCATGTGAGGTTGCATCCCGATCGCCATGACTATATGTTAATTGAATTTTTTGATACCAAT
GGAATTAATATTTCCAAAGCCAAAGAAAAGAAAATTGAAGGAGCATATTTTAAGGAAGACTTAA
GAAGAGTACAAGCCATTGATATTGGTGATATGTCATATCCTGCCCAGGTAGTGGATACATACCGC
AAAACCTTTGAAACTCAACTTAATTTAGAGGCTATTCGTAATAGTGACACAAAAATTGTCATTGA
TTATGTTTATTCAGTTTCCGGGGCAATCTTACCGCAACTATTGGCAAAATTTGGTTGTGATGCCG
TTGTTTTGAATGCTAGTCTTCGTCAAAGAGCAATTTCTAACATTGAAAGGGAAAATTTGCTTTCT
CAATTAGGTCATGTGGTAGAGGCTCTAAAAGCAAACTTTGGAGTCCAAGTATCAGCTAATGGTGA
ATTATTAATTTTAGTAGATGAATCAGGTATTCAAATTCGAGGGGAAGAATTAACAGCTCTGATG
GTAAATACAATTCTCACGGCACATCCTAGAGGCACTGTTGTTGTCCCCATGAATACATCCTCTGC

GGTGGAACAAATTGCCCGTCGTCACGATGGTAAAGTTATTCGCACAAAAGTCAATCCCAGTGCTT
TAATGGAAACCAGTCAAAATACTCCCAATGTGGTTTTAGGAGGAAGTGGAGACATGGGGTTTATT
TTTCCGAAGTTGCACCCCGGTTTTGATGCGATGTTTACCATTGCCAAATTAATAGAAATGCTAAC
CGTTCAAGAGCGCTCTTTAACACAGGTAAAAAGTGAATTACCGATGGTTTGTCATAAATCCATTA
CTCTTCGTTGTCCTTCTAAAACGAAAGGCTCACTAATGCGTTATCTCATTGAAACCGAATCCCCCG
AAAAGTTGGAACTAATTGATGGAGTCAAGATCAAAGAAAATCATACTGATAATTGGGTTTTAAT
TTTACCTGATGGTGGCGAACCCTTAGTACATGTTTATGCTAATAGTGATAGTAGAGAATGGGTGG
ACGAACATTTGCAGGTATATAGAGATAAAGTCCAAAGTTTTATTATTCAACAACATAGCCTTTAA
CTTTTAGTAAGGGGCAAAGGAAAAATTAAGAATTAAGAATTAAGAATTAAAAATTAAAAACTCT
GAACACCTTTACGGGAGAATGGCCATTCTCCCCTACATCCCTCTCTCCCCTCATCTCCTCATCCCCC
TACCACCTGCAACCTGCAACCTGACACCTAACCTTATCCGATATTCTTAAACCGAACTGAGGTTAA
ATAGTGTTGCGGTGAATAGTTAATAACTTCTAACTCCGAATTCTGACCCCCAACATCTCTAATCT
ACCATCCCCAGCTACCAGCAACACCCTTGAAAGGACCGACAATATTAGAGGTAATCCAACCACCA
TAGAATCCTCCGGGTTGAGGAGTGACTAATTCCCCATTAACGTAACACTCAGACATTAATCCTGC
ATAAAAGGCGACATAATGTGCGATCGCATCAAATTCTGGAGTAGGATTAGGATAGTACCAAGCGG
CTTTATAAATTAATTTATCATCGACACAAACATCATAGTATTTCGCACTTCCTTTCCATTCACAA
AAAGATTGTCCCTCACGGGGAATCAAATATTGTATTTGTATATCTTGAGGAGGTAAATAGTAAAC
AGGTGGATGGGAAGTTTCTAAAACACGATAACTAGAAGTTGTATCGGCGATTTTAACCCCATTTA
AAATAATTTCTATTCTACCTGAAAATGCTTCTAAACGAGGGGGACGAGGATAATCCCATACAGAT
TCTTGCCCTTCCTTTGGTTCAATTTTTTGGACTTTATTAAACATATATCAAATTAATTAGGGGTT
GATGAAAAAGTGGTGTTGCAAAGCTAGACAACAGTCAAAAACACTTATAAATCAAAAACTGTAG
TACAATTTTACAAACGTCTAGCTAATTACTTCGATGATTGCTCATCATGGGACTCTCCTGCCATA
AAAGCCTTCTCAAAATTCATTCTCTGCTCTGCATTACGTAAAAAATAACCGCTCATCATTGCCGA
AGCGAGAAGTCTGCCTAAATTTTCCCTACTAGTACTTACCATCACCCCAAAATGTTCAGGAGGTA
AACTGCCTAACATTCCTACAATATTACGTTCCATAACTTGGAAAACTTCTGATGATTGAGGTTTA
GATAATTGAGCAATGGTTTCGGGAGTTAGGGATTGGATATATTCTAAAAAATTATTGTCTCCTTC
TCCTGTTTGAAAAAATTTATTGCCATTACTTTGATAGTTCACTGTTTTTACTCCTAAAAATAATC
TATATGTTTTTTTATAAAGATTTAATATTTACAATTTACCAATTAGATTATTGTTCGTGATTAGG
TTAAACCGAACTTAAAGAATAGGTTTTTCCTGAAAATTATTGATTATAAATTTAAGTTTATTTG
ATTTCTTGTTTCATTCTTTCTAAAGTCATGTGCATTTGTTGAAACATTTGATCAGGAGTAGTACC
AAATTGACTTAACTGAGTCTTTAATTGTTCAACGGTCATTTTTGCCATAAAATCTTCAGAGAGTT
CAAAACGTTTCATAAAAATACGATAACGCTCCATCATATTTTCCATTTTTTCAATAAAAATTTTC
TTTCCTTCCCTGTCAAATTTTCCGAACTCACCGCCTAATTTCATCAGTGATTGATAATCCTCGAAA
AGTTGCTTTGCCTCTTGTTGAACGACTTCAGAATCAAAAAATCCCATGTTAACCTCTTTATCTCA
GTAAAATATAGATGTTTGTTTTTCTACAAATTTTTATAAAAGTTTCGTATATTCTAATTATAGAA
TTATCGATATTGTTTTGAAACATACGGATTTCCGTAACCAAAAAATTATGGTTTCTCAGCTTTTT
AATTTTTATCAACAACCTAAGAAGAAAATTTTTTCTGTTGTTTTGGCTTTAATTGGTACTGTAAC
CCCTTTAGTGGGACTACATAAACTTTATCTTGGACAACCATTATGGGGTTTAATTTACTTTATCC
TCAGTTGGGAAAGTCCTATGGCTAGAATTGCCTGTGCGATCGATGCTGTTTTATACTTAGCACAA
GATTCAAATTATTTTTATTCTCGCTTTAATCCTGAGTTACCGTTAACTGAAAATAATTCAATAGA
TGCAAAACAAATTACGGAAGTGGCACAAGCCATTAGAGAATTAGAAAAATTACGTCAAGATGGG
TTGATTTCCGAAAACGAGTTTGAGCAAAAACGCCGTAAATTAATTGCGTGAAGTACTTACATTTG
TTTCGCTGAAATATGAGCTTTTCGTCTCATCTACCAACGCTTTAGCTTTACAATACTCCGAACTA

AATCTAAGTATTGTCGAGTCTTATTCCGCATCTGTGCCTATTTCTAGGCTTTATCTAGTCTAAGC
TAGAACCAGAGCCGGAAACCGCCTTCTGCAGTCCCATAGAACAAAAAAAATTCTACACCTTCTCA
ACCATGTTCATAAAAACTTGTTTAGAGGATCAGTGGGTTGATTGCTTCCTCGATCTGCTAGTAGT
TCGCAACCCTTTTAGACAGTCGGGCGGGTTAATAACCAACTTCGTTATAACACGTTAGGATTCGT
TTTCATCCATTTCGTTACACTGAAACTAGGGACTTCCCACTCGTACCCGTTAAATTAAAGATAGC
AATATTTTTGGTGAAGCCAAGTTTTATTTATGATTAAAATATTGAGGTTTTTTATTTTTGGTTTT
TTTCGTTGGTTAATCATTGTCTCGACTATATTCATATTTTCTTCTATCGCTTTAGTTACTTATAT
TTACCAAATAGAGCCTCAATGGATTCAAATAAAAAATCTTGATATAACCCTGCCTAATTTAGAGG
AAAATTTTGAGGGGTGGAGAATTTTACAAGTTAGTGATATTCACATTAACCAATGGATGACAGA
AGAAAGGTTAACCAATATAGTTAATTTAATTAATGAACAACAGCCCGACATAATTGTTTTAACGG
GGGATTTTTTTTCTCAATCGGGTAGCTATTCCACTAGATTAGATAGATTAGAAGAAAGAATTATT
CCTAAAATTCCCTACGCAGAAGAACATCCTAGCTTGATTAAAAGATTTTTAAATAAGGCGGGTTT
TCCTATTCAAGATACTCCCCGAAGAAGATATTTTGATGATGATCAAATAACTTTAATTAAAAGTT
TAAGTAAACTTAAGCCCCATTACAAGAGTTTTGCTATATTAGGAAATCATGACTATGCCACAAGC
CATAAGTTAGTCAGAGAAGGTTTAAAAGATAGCGATATTATTACTTTAAGAAATAGAGTTTATA
CCATTGAAAATAACAGTAGTTATCTGAATATTATCGGTGTTGATGATGTTTTATATGGTAAAGA
TGATTTAGATAGTGTGTTAACACAAATACCTGACAAGGGTGCAAATATTTTATTGGTTCATGAAC
CAGATTTTGCGGTTTCAGCGTGGAAAACCCATCGTTTCCAATTGCAATTATCCGGACATTCTCAC
GGTGGACAAATTAAAATTCCTTTTAGAAAACCTCCTTTTCTTCCTCCTTATGGACAACAATTTCC
TGAAGGATTATATCGATTTAGCGATTTGCTACTCTATACTAATCGAGGTGTTGGGATGGCTTACC
CTTATATTCGCTTTAATTGTCGTCCAGAAATTACTATTTTTACATTACACTCTGCAAAAGATAAT
TAACCTAAGGTCTGGATAAATTTTTATCTCTAAGTTATGGTGAAAAGGGCTATTAGTGAATAGTT
GATAATTAAGAATTAAGAATTAAGAATTAAAAATTAAAAATTAAAAACTCTGAACACTCATTAC
TTTCTTCTAAAACCTGAAACCGTCAAACCTGAAACCTGACACCTCTTTCATAACTCCTGTACGGGC
GAATGGCCATTCGCCCCTACTTCCCTCTCCCCTCATCACCGTAAGGGTTAAATATATTCAACCCCT
ACCACCTGTAACCTGCAACCTGACACCTAACCTTAACAGTATCCAATGGGTGTCTTTTACCTTTTT
TCTTGCGAAAGTCTTGAATTTCTGGTAATTTGTTAACTAAATTATTTAACATTTTTTTAACCTAT
AATAAATGTGATTTTTTGCCCTTTGCTCCCTGTAGAAGAGCCATAAAAATTTATTTTAAAACTAT
GAAAAATATATTAGTTTGTACAGATGGATCAATTATTGCTAAAAATATTTACCATTATGGTGCTT
GGTTTGCAAAGAGATTAAATGCCCAAATAAATGTTTTATCGGTTACAGATATTCGTTCTCAAAAA
GTAGTTTCTACTGGCAATTTTAGCGGAGCAATTGGGTTAGGGGCTTCTGAAGATTTATTAGAAAA
ATTAGTGGAATTAGAACATCATAAAGCTCAATTAAATCATGAAAAATCTAAGTTAATTTTAAAA
ACTGCAAAAGAAATCTTACAAGCAGAAGGGGTAATAAATGTTAATTTAATTTCAAAAAAAGGTT
TTTTAGTTGATACTTTACATGAGTTTGAAGGTAATAATGATTTAATTGTGTTAGGTAAAAGGGG
AGAAAATGCAGAGTTTGCATCAAAGCATTTAGGGGGAAATGTGGAGAGGATTATTCGTAGTAGT
CATAAACCTTGTTTAGTTGTTCCCCTTCAGTTTAAGCCCATTGAGCGGTTATTATTTGCCTATGA
TGACAGTGCTAGTTGTCAAAAACTATTACAATTTATTACTTCTTCTCCTATTTTTCAAGGTTTAG
AGTTGCATTTAGTAACCGTTACAAAAGATAAAAAGGATCAAAAAGCCCATGTTAATCTCATGAA
GGGACAGGAATTGGCTACTTTAGCAGGTTTTAATCCAATTTGTTTACTCATAGAAGGACATCCTG
AAGAGGCGATCGCATCTTATATTTCTGAAAATGATATTAGTATGTTATTGATGGGAGCTTATGGT
CATAATCGTATTCGCCAATTAGTAATTGGTAGCACTACTATTCAGATTCTACGCTCTGCTCAAAT
TCCTGTATTCGTTTTTCGTTAACTGAATTTTATAATAACTGCGTACATCAACCCCTAACTCTTAA
CTCACCTTTTGATTTATAATAACTAAGAAAAATAAAGCTATATGAAAGTTTCATCTTTTAATAAA

AATCTTAATTTAATCGTGGTTCTCTGTTTAATCACGGTAATAGGTTTATCAGGATGTCAACAGTT
AAATGCTACTTCAGGGGTCGATAATAACACTCAAGAAACCGCTACCTCTGCGGTAGATAATAGCA
ATTCAGAAGACAATCTTTCTTCAGGGGAAACTAATTCCAATGGGAAGGAAAGTAACAATCAAGA
TAACATTAGTCTAAATAATAACTCTATGAATTTACCTCGTTTAGAAGGAAACGCAACAGTAGTAA
TGAAAATTAACGGACAATCCGTTACCATTGAACTTGACGGCAATAATGCCCCCATTACCGCAGGA
AATTTCGTCGATTTAGTACAACAAGGCGTTTATAACGGTTCTATGTTTCACCGTGTTATCAAAGA
ACCTCAACCCTTTGTAGCACAGGGTGGAGATCCTCAAAGTAAAGATCCCAGCGTTCCTGTAACTG
CTTTAGGTACTGGTAGTTATGTTGACCCCACTACAAAAACTTCTCGTTATATTCCCCTTGAAATT
CGTCCTGAATATGATGAAACTAAAGAAGGAGTTACCCCTCCCGAAATTATTTATGGTCAAACCAT
TACTACCGCGCCTGAGTTAAAACACGACTATGGTGTTATTGCGATGGCACGTTCTCAGATGCCTG
ATTCTGCTTCTGCTCAATTCTATTTTACTTTAGCTGAATTACCTTTCCTTGACGGTAATTATGCCG
TTTTCGGTAGAGTAACAGAAGGTATGGATGTAGTTACAAATATTAACCAAGGCGATCGCATTGA
ATCTGCTGAAGTAATTTCTGGAGCGGAGAATTTGAAGAAATAGGAGTTCGGGGTTCGGAGTTAT
GAAAGAGGTTTCAGGTGTCAGGTTTAGGGGAGTAATAAGTAACGAGTAATTATCAACTATTCAC
TATTCACTATTTACCTTTGCCCCTTGCCCTTTAAAAATCTATTCCCTTTTCTACATTTTTATCTCT
AGTTTTGAAAGTAGTTGTTACGGGCGTTAGCCTTTTTACCTGTTTAGGAAATACTCAAGATACTT
GGCAGAAAATCCTTAATGATGTTTCTGGGATAAAAATTCAACAACCTTTTTCTTTTCTCCCTCCT
CTACCTTTGGGGATGATTGAAAAACAACCAGTGTCTATCCATGATTTAACTAAAATCCTTATACA
GCAATTATGTCAGGATGCAAAATTAACTCTCCCTCAAGATAATATTGGGGTGATTGTGGGTTCAA
GTCGGGGTTGTCAAAGTCAATGGGAACATTTTTTATCTTCTTTTGCTCCTGATTTTTCCTCTTTGC
CTTGGTTGCATACTTTGCCCTCTCAACCTGCTTTAATGATTGCTCAATATCTGCAAACTCAAGGTT
TTGTTTCTGCTCCTGCTAACGCCTGTGCTACGGGCATAGTTGCGATCGCTCTTGGATATGAATTA
ATTAAACAAAATAGATGCTCACAAGTTGTAGTCGGAGGAGTAGAAACCGCCATTACTCCTCTAAC
CATCACGGGATTTAATCAGATGAAAGCTCTTTCTCAAGAGGGTTGTTTCCCTTTTGCCGTGAACA
GAAGCGGTTTAGTTTTAGGAGAAGGAGGAGGATTATTATTATTAGAAACGGAGGAATCTGCCCG
TTTAAGAAATGCCAAAATTTACGGAGAAATTCTTGGTTGGAGTATGAATTGTGATGCTTTGGCGA
TGACTACTCCTGAGTCAGATGGTGAAACTGCCATTCGCTGTGTTAAAGATTGTCTCTATAATTCT
GGTTTATGCCCCCAAGAAATTGATTATATTCATGCTCATGGTACAGGGACAATTTTAAATGATCA
ACGAGAAGCACTGATTATTAAAAAACTATTTCCCCACTCTCCCTATGTTAGTTCTACGAAAGGAT
TTACAGGACACACATTAGGGGCTGGAGGTGCGATCGCATCTGCCCTCAACTTTTTAGCTTTAGAG
AAACAAACTCTCTTACCAAATACGATGAATCTAACCCTTGATTTTGGGCTTAATTTTGTGAAAAA
ATCCCATCACCATCCTTTAAAAAAGGTACTTTGTTTTAGTTTCGGTTTTGGAGGTCAAAATGTTG
CGATTGCTATGCAGAATATTACATAACCTGAGTTTGGGGTAAATTTTCATCTATAAGTGATGGCG
AAAAGGGCAAACCCCCCTTTTTCCCCCCTCGAGAGGGGGGAAAACAAAGGTGAATAGTTGATAAT
TAAAAACTCCTAACCCATTACTTGTTTCCCCCTCATCCCCTAAACACCCCGACACCTGCAACCCAA
AATCTAGTCCAATCTAAAATTACTACGTCGAACTGAGGTTAAATATTGTTAAAATGAATCGGTAT
TTTATCTAAATTTGAGAGAATATAAATATGTTTGGTTTAGGTTGGGCTGAAGTAATTATTATTT
TGATCGTGGCGGTTTTAATTTTTGGTCCAAAAAAAATACCAGAGTTAGGTAGTTCTTTAGGAAAA
ACCCTGAGAGGATTTAAGGATGAAGTAATAAGAGATGATGATAACGATAATAATGATAAGGAAT
CAGATAATTAACAATTGCCTCATCAACATAGTCGAGTTAATATATAATTTTCTAAATAGCAATGG
AACTTATCCCCATAACAAAGATTAAAATTCTGTTATAAGCTATAGAAAGATAATAATATCAAAG
TTAAAGGAATTAAAAACTATGAGCAATATTCAAGACCAAATTCAAGAAGAATTAAAACACGCTA
GGGAAGTGTGCGATAGCCCTGATAGTAACTCTGGAGAGTGTGCGGCGGCATGGGATGCAGTGGAA

GAATTACAAGCTGAGGCTTCCCACCAAAAAACTAAAGAACCCAAGAAAAACTCTTTAGAACAATA
TTGTTCCGAAAATCCTGAAGCGGCTGAATGTCGTCTTTATGATGACTAATTGTCCTAGTTTCAAT
AATTCATTTACTATAGGGTGGGTTTTCCCCACCTATTTTTGTTTATTGGTGTGATTTTATGGAAG
AAATTTTATTAAAAGCTCTAGTTTGGACTAATTATAAGTTATTTTTATTGATTTGCTTAGTTTTA
CCTCTTATTCTTTCTGTTTGGAGCTTAAAAACTCAAATTCCCTCGATTCAACGCTTACTACTTATT
TACTGGCGGGTGGCTAGTCTTTTAGCCATCGCAATTTATCTGTTTATTCCTGTGTGGCAAATTGG
TTATTTAGCATGGTTTGCTGGACATATTTTAATTGTCATTTGTCTTTGGTTTTGGGCTGATATTA
ATGATGAAATACGAGATTTACCTAAAAGTAGTTTACGTTTGGCTCTCACCTCTTGGCGTTGGGCG
GTAAGTTTATATGGCATAATAAGTGCGATCGCATTTATTCCTTTTTTACGTTGTACATTTGTTCC
TTCTGCCAGTGCAGATACTATGTGTAGATTGTGGCTAGAAGCTCCTTGGCATTATAAATCGTGGT
TTCATCCTAATTCTACCACTGGATTTTTAGGCTTTTTGGGAATGAGTGGATTAATTATTTATGTT
ATTTATTTTGTTTATTTTCTCACTTTTCGTTTAGTCAAACAGGGGAGAATCGCCCTTGAGCAATA
ATAAATAAACCTCAGTTCGGTTTAAGAATATTGGATAAGGGTAGGTGTCAGGTTTCAGGTTGCAG
GTTGCAGGTGTTGAGAGAAAATAATGAGTAACAAATAATGAGTGTTCAGGGTTTTTAATTCCTA
ATTCTTAATTTTTCCTTTGCCTCTTGCCTCTTGCCTCTTGCCTTTTTTTCTCCATCATTCATAGAT
GAAAATTTATCCCGAACTCAGGTTAAATAAAGGATAAATAAGGGTTAAAATGGGAAAATGGGGG
ATTGGGGGGATGTGGAAATTTAGGAAAAGCTAATTCTTAATAAGAAGAGGTATTGGTAAAAATA
TTTGCGGTGTTTTTTGTTATTCTGAGAAAATCAAAATATAAACAGACAACAAACTAGCCTAAAAA
AAACAAACATTAACTTATCCAAACAATATGGTAGAGAGATACTTGCAAACTGATAATCAATGGG
AATTGGATATATATAATATCCCTATCTATTCAGTAACAGATGCTTCACGATATCTTAATATTCCT
CTGCCCACTATACGCACTTGGTTAAATGGACGTAAATATAAGACAAGAAAAGGTGAGCAAAATTT
CCTACCCTTAATACAACGCCCATCAGAAGAAATGTCGCAACTTTCTTTTACTAACTTAGTTGAAG
CTCACATTTTAAGAGTCATTCGTACTATTCATAATATTTCTTTAGATAAAGTGAGAATCGCCCTT
GATTATATTAGTAAGAGATTTGATACGGATCATCCTCTTGCTACTAAGCAATTTAGTACCGATGG
AGTTGACTTATTTTTAGAACAAGTAAGAGACTTAGTTAATGTTAGTCGCTCTGGACAATTAGCGA
TGAAAGAAATTTTACTGCAATTATTAACCCGTGTAGAATGGGATGAAAACAACTTAGCCACTAGA
CTCTACCCTGAATTAGATAATACTAATGATGATAAAATTTTAATCATTGACCCTCGTATCTCTTT
TGGAAAACCTGTAATCAAGGGTACGGGCGTACCCACAAAAGCGATCGCTCAACTATATGACGCAG
GGGATTCAATAGAGGATATAGCGGAAAACTATGACTGTAAAGTTATAGATATTAAACAGGCAAT
TTTATTTGAATCATATATAAAAGTCGCATAAGTTTTAATATAGCTCAAATAAACAATTATTCTCC
GTTGTTAATAGGCGAAAGTTTTAGATATTTTTTACTTTGAATAATTATGTTTTTGCTCAATCAAA
AATAGAAAATAAATTCATAAAAAATGCCAAGATACCTGAAAATAAATCCTAAGTACAATAAAAA
TTAAATGAGTAAACATCACAAATTTAAACACACTTATTTTATTGACAGAGCATTAGGTAAATCAG
TGGCTAAGGCATTACAAAACTTAGGGGTTAAGGTTGAACTTCATAAAAATCATTTTCCCTCAAAC
TCCCCCGATATAGAATGGCTACCAATTGTTAGTAAAAAAGGTTGGGTTGTTTTAACTAAAGATAC
AAATATCGGTAGAAATATTTTGGAGATTAAAGCTATGGCTTATTTTAAAGCAAAAGTTTTTACCC
TTGTTTCTGCTGATTTAAATAATGCTAAAATGATAGAGATTTTTGAACAAACTATAGAAAAAAT
AGATAATTTTGCCACTACTAACACTGCCCCTTTTATCGCTAAAATTTATAAGAATTCTACTATAA
AATTATGGAAAAATCATACTAAACTAAATAAAAATTTAAAACAAAAATAATTGTCTTTTTTACT
TTAAATTCGATCAATTTTGGCTTAACCAAGATGTTTAGTTTTCAGTATGAGAAGTTTTTAAAGCGT
TGCTTAATAATCATCTGAAATGTACTGAAATTATGCTAAAAATTGAGACATAGACATCAGAATA
TTATCACTATTGCCTAGTGCATGGTTTTCTATTCTCCACTTAAATGAGGAATCTTGTTTTAAATC
AGCAATAACGTTTTTAATTATTAATTACTAATTGCCCTTTTGACTTTTCTTAATTTCTAAAATTT

CTTATTGAAAATATCTCTCTAGGGATTCCAACCAATAGTCACCATTAATATTATAGTTATTCCTG
ATTTTTCTGGTATTGAGAACAGAATAGGCAGGGCGCTGGGCAGGAGTTGGGTATTCTGCGGTGGA
AATGGGTATAATTTCTTTAACTTTCAGGGGATAATTTAATTTTCGGGCTTGACGAAAAATATTGA
CAGCAAAATCGTACCAGCTACAAACTCCTAAATTTGTAAAATGATATATGTCTTGACGGTATTTT
TCTCCCTTATTTTGTTCAATTATTTTTTTGATTATTATGGCTATATCTTCAGCATAAGTGGGGCA
ACCAATTTGATCTATTACTACTTTTAAAATATCTCTTTCTTCTCCCACTCTTAACATAGTTTTTA
AAAAATTTCCCTTACCATATTTTCCGTACACCCACGCAGTTCTTAAAATTATATAATTATCACTA
TTTTCTTTTATATTTTCTTCTCCTTGCAATTTACTTTTGCCATAAACACCTAAAGGATTAGTATT
ATCAGACTCTAAATAAGGAATATTTGATTTGCCATCAAAAACATAATCTGTGGAAATATGAATA
AATTGTGCATTAATTTTATTACTTTCTTCTGCGATAATTTGAGGTGCAATCGCATTTATTTGTTG
AGCTAATTGGGGTTCAGATTCTGCTTTATCAACGGCAGTATAAGCGGCGGCATTGACGATAAATT
GAGGTTTACAATCTCTGATACAATTTCTAACATCCTTTTGTGAGGTTAAGTCAACAATACTTCGA
TTAATCAGGGTTAAATCTCCTATTTTGGATAAATGAGTGCTTAATTCAGTGCCTACTTGTCCGTT
ACTTCCAAATAATAATATTTTTGTTGTCATTGTTATAAATAAGTTAAATTAAGGTTTTACGGTTT
GATTTTAATCGCAAATGAAGCTAAAAGATAATTCTGATAACGCTCAAAAATATGGTAAACAAAG
GGATTGGTTTTGGCATGGTTGGCGTATCCGTTATAGTTTTTATCCTGCCAATATGTCAGGAAAAA
ATCAAATACCAATTCTCCTTTTACATGGTTTTGGGGCTTCTTTAAAGCATTGGCGATATAATATA
CCTGTTTTAAGGCAAAATCATTCGGTTTATGCCATTGATTTACTAGGTTTTGGTAATTCAGAAAA
AGCCTATGCAGAGTATGGTATTCCTTTTTGGTCCGAATTAGTGAAGGATTTTTGGGACAATTTTA
TTAATCAACCTTGTATTATTATTGGTAATTCTATTGGTTCTTTAATCGCTTTAAATGCGGTAGCT
AATTACCCTAAAATAGCACGGGGATTAGTCATGATTAGTTTACCTGATATTTATGGTCGTAGAGA
AGTTATTCCCCCTTTTTTATATCCTATTCTTCAAAAAATAGAGAATTTAGTGGCATTTCCTTTGT
TAATTCGTTTAATTTTTTATCTTGTTCGCCAACGGGGTATTATTACTCGTAGTCTTAAATTAGCC
TATGTTGATCATAAAAATGTGAATGATGAATTAATTGATATTATTATGACACCTCCCCAAGACAA
AGGGGCGGCAAGGGCTTTAATTGCTTTAACTCGTTATGTGAATGATTTTAATGTGTCGGCAAAAA
CTCTTTTATCTCAGGTTCATATCCCTATATTACTACTTTGGGGAAAATGCGATCGCCTCATTCCTC
CTATAATGGCAGAAAAATTAGCTCAGATTAATCCCCAAATCACTTTAAAATTATTAGATAATTTA
GGTCATTGTCTTCATGATGAAAACCCTGATTTATTCCACCAGTTGTTTTTTGAATGGGAGAAATT
AATTATTAACCTTAGTTCGGTTTAAGAATATCGAATAAGAGTAGGTGTCAGGTTGTCAGGGTGTT
TAGGGGATGAGGGGATTAGGGGAAAGGGAGATAAGGTGTCAGGTTTCAGGTTTCAGGTTGCAGG
TGTTGGAGAAAGTAATAAGTAACGAGTAATGAGTAATGAGTGTTCGGAGTTATTAATTATTCAC
TATTCACTATTCACTATTTATTTTTGTCTCTTGCCTTTCAGCCTACCCGAACCAAAATTCCCATAC
TCAAAGTAATAGAGCTTTTTTAATTCTTAATTTTTTAATTAAATTCTTTCTCATGTCAACATTAA
AACATTCTCAAGACGAAAAATTTTTTTTGTTAAATTGGCTTAAAGGCAGTTTAATCGGTAATTTA
GTCGGCTTACTATCCCCATGGCGATCGAGTAGTTATCTTCTCGGTTATAGTGATATTATTACGGC
TATTTTTGTTTGTTTAGTTATTATCAGTTCACCTTTTACCAGTAATACCCTAGTGGGAATTATTC
TTTTGGCAGGAGGTGGATTTTGGGTTTTATTAACGGTTTCTGATATTGACAAAAATCAAATTACT
CCCATCCATCTTTGCATTTTTGCTTATTGGTTAATTTCTGTGGCGGCTACTGCTTTTTCTCCGTTG
AAAATGGCCGCTTTATCAGGATTAGTTAAATTTACTCTCTATTTACTTCTGTTTGCTTTGGCTAG
TCGAGTTTTTGCTAAAACAAAGATGCGATCGCTGATCATGGGTACATATTTATTGGTATCCCTGA
TTGTAAGTGCTTATGGTGTAAGACAACAATTTATCGGTGTTAAACCTCTAGCTACTTGGACTGAT
CCCACTTCTCCTTTAGCTGATACTACAAGAGTTTATAGTTTTTTGGGTAATCCTAATTTGTTGGC
TGGTTATTTAATTCCTGCGGTAGCTTTTTCCGTAGTGGCTTTAATTGTTTGGAAAACTAAACCAC

AAAAAATTTTGGCAGGATTTGCTTTAGTCATCAATGTTGCTTGTGTTTATTTTACAGGTAGCAGA
GGGGGATGGTTAGCATTAATTGGCACAGGTATTGTCTTTCTCCTTGGCTTTAAGTTTTGGTGGAA
TGAGTATTTGTCACCCTTTTGGCGTAAATGGTTAATCCCAACAGTAATTGCCTCTTTTCTGCTTT
TGGTTGGCGTTGCTATGATTTTTGTTGAACCCTTGAGAATCCGAATTTTTAGTCTTTTTTCTTGG
CGAGGGGATAGTAGTAATAATTTTCGTATCAATGTTTGGATTTCTGCTTTACAAATGTTACAAGA
TTATCCTTTAATTGGGATTGGTCCAGGGAATGAAGTTTTTAATCAAATTTATCCCCTTTATATGC
AAACTAAATTCACCGCCTTAAGTGCCTATTCTATTTTTCTCGAAATAGCCCTAGAAACTGGTTTA
ATTGGTTTATTCTCTTTTCTAGCCGTCATTGTGACGACGTTTAAGAGGGGGATGGTATTAATCAA
AAAAATGAAACCTAGTAATGATTTACAAGCAATCTGGATTTTAGGTGCGATCGCATCTCTGGCAG
GTTTAGCAACTCAGGGTTTATTTGATACAGTATGGTATCGTCCTCAAATCAATACCTTGTGGTGG
CTATGTGTAGCGATAGTAGCGGCGGTTTATTCATTAAAATCGGAAACCGATAATCATGTTAGTTA
ATTTATCGAATCAAATTGGTTCAATTTTACTAATTACCGTATATATAGGAGGTTTGTTATTAATC
GCAGAAATTCTCAATCGTCTTCACAAAACAGATAGCGAATTAACTCGCAAAATAGTTCATATCGG
GACAGGTAATGTTATTCTCTTGGCATGGTGGTTAAATATTACCTCAGATGTGATTTTATTAGCTG
TTATTACTGCTAGTATAGTTGCGATCGCATCTTACTACTTACCAATTCTTCCGAGTGTCAATAGT
GTGGGCAGACATAGCCTTGGCACATTATTTTATGCTATTAGTATCGGTATTTTAACTGCTCTGTT
TTGGCATGAAGGAGAAAAACAATTTACCGCCATTGGTATTCTTATCATGAGTTATGGAGACGGTA
TGGCCGCATTAATTGGACAAAAATGGGGTAAACATAAATATCAACTATTGGGCAATAAAAAAAG
TTGGGAAGGTTCATTAACCATGACATTAGTGAGTATCTTAGTAGTTATTTCAATCTTCGGGATAG
TTGCTACAATACAGACAAAACTATTCATTATCGCCTTACTCATTGGAATCTTTGCTACAATTTTA
GAAACTTTTTCCCCTTTTGGCATTGATAATTTAACCGTACCCGTTATTTGTGGAACTTTAGCCTA
TTATTTACAAAATATTTTCACTATTAACTAATTAAATAACTTTTAAACTTATATCCCTTAACTAC
CGATTATTACTCATCATTCAAAAATTTTGACTTTTGCTTCTTATTCTTAATTGTTAATTTTTAAT
TTCTAATTTCTCACCATGTTTAATATAGCCGTAAAACAAGAAGAATATTTAATATATATTCTCAA
AGATATAGAAAATGACTCTCGTTTAGAAATTGTGCCAGAAAAAGGAGGAATCGTCACCAATTGG
AAAATACAAGGTCAAAATATACTGTATTTAGATCAAGAAAGATTTAAAAATCCCAGTTTAAGTG
TCAGAGGTGGTATTCCCGTTTTATTTCCTATCTGTGGTAATTTACCTGAAAATATCTTTTATTTT
GAAGACAAAAAATATATCTTAAAACAACATGGTTTCGCTAGGGATTTACCTTGGAAAGTAATAG
GACAATCTCACAGTAAATCAGCTAAGATTATTTTAAGTTTAAAAAGTAATCAAGAAACCTTAGA
AGTTTATCCCTTTGAATTTGAATTAATATTTAGTTATGAATTGTTAGGAAAAAAACTTATTATCG
AACAATCCTACGAAAATAAATCAAACAAAAAAATGCCCTTTTCTACAGGGTTTCATCCTTATTTT
TACTGTGGTAATAAAGACAAATTAAAATTGAATATTCCTGCTACTGAATACCAAAATAAAACAG
GAGATAAAACATTTAGTTTTGATGGTAATTTAGACTACAACAGTCCAGAAATTGACATCGCTTTT
ACAAAACTTCAAGGAAATAAAGCCTCATTTACAGATGAAAAAAGAGATTTAACTGTTAAAGTAA
AATACGATGATTTCTTTTCAACTTTAGTATTTTGGACATTGGAGGGTAAAGAGTATATCTGCCTT
GAACCTTGGAGTTCACCACGCAACAGTATTAATACAAAAGAACAACTAAAATACCTACAACCTCA
AGAAATCTTCCACACTCAAATTGAAATAGAAGTGAGTTAACACTCTCCTGTCACCTAATCAAAAG
ATTATGGTGAGGGATTCTAACTTCTATCAACCATTCACGAATAGACATCTCCAACAATTCTCAAA
ATAAGGGAACCAAGCCCTAACTACGAGCCTATATTCATATTTCCTACCAGATGTCTAATGAGAGC
TTAATTTGCTAAAGGCAAAAGTACTGATGTAAGATCTCAGTATGTATCAAAGTTTTGAATTAAT
AAACACTTCACCTGCCACCTGAAATCTAAAACCTATCTACCAAAATTTGATATTCTTATGCTGAA
CAGAGGTTAGCTAAAAAGTTTCTCCCACTTGTAAATAAACTGACTGATTTTGTTTACTAACTACC
GCCTGTTTTTCATTAACTGCCATTAAAACCCATCCTGTTGCACCAATTTCCTTTCCAAGGGATACT

TTTTCTGTTATGTTGTTTACTTTGAATAAAGCAACACTTTTACTATCAGGTAATTGAACTAGCCC
AATTAAAGTATAATTTCCTTGGGTTTTTTCTTCTGTGCTTTCTGGAACGGAATTTAAAGCTATTT
GCTCAGGTATATTAGAGTTATCTTTAGAAAGAGAAGCAATTACAGGTACTTTGTTTACCTTAGGA
ATAGATGAAGAAGAGGTAAGATTCGGTTTTGTATTATTATTCGATAGGTTATTTGTAGTGATTG
ATGAATTAGCAACTGGTTGGGGCGGTTGAGGAATTGGAAGGGGTTGTAAATCGGGGGGAGGTAA
CGGAATATTATTTACCGTTGATTGGTTACTGGGCAGAGGTGGTATTTCTAATGTCGATGTAATGT
TATTACCTGTGGATAGTATTTGATTTTCAATATCTTCTTGATTTGGAGAAATATTTTCGTCATTG
AGGAGATTTTCTTCTGTATTTTGGTTATTGATAGTATTTGTTTCTATGAGGTTGCTTTCTGATAT
GATTTGCTTATTCGGGTTAACAGTTTGAGATTGAGAATTAACTGTTTGTTGCCGATTTTTTAACC
ACAAGCCCAGTATCAAAATTAAATACAAACTAGCAAGAATAAAAAGTAGTTTATCAAGATTATT
TTTCCATCCTATTTTTGTTTTTATCTTATCCATATTGACTAAAATACCTCTAATCACCAAAACTC
ACTTTTATAGTTTAGTTTAAAAAATAACTTTCATTTATTAATTCTTGTTTATTTTAGTTGCCTGA
CATGACAAAATTACGTCAACTTATCCCCATTTTATTTGTTATTATTCTACTCTCTATATCCGTAG
CCGCGATCGCATCTGAGCTAAAAAAATATAGTATTAATAGTGTTTGGCAATACCTACAAAATATT
TCTCAACAAAGAAAAATGTTAGCCCTATTAGTAACCGGATTTGGTTATGCCTTAATGACAGGATA
TGACATTTTAGGATTTATTCATATAAAACAAAAACTATCCGTTTTGAAAATAGCCTTTACTGCTT
TTGTCAGTTATGCAGTAGGTAATACTGTGGGTTTTACAGCTTTCTCAGGCACAGCAATTCGTTAC
CGCTATTACGGCTTATGGGGAGTAAATAAAATCAAAATAGGAGAATTAATTGTTTTCACTCACCT
AACATTTTGGCTAGGCTTACTAAGTATTAGTGGAGTCGTCTCTATTTTAGATCCATTAACCCTTC
CCAGTGCCATTAAACTACCCTTTGCATCCATTCATCCCCTAGGATTTATATTCTTATCCTTAGTGT
TAATCTATTTCATCATCAGTGTCACTATTAAACATTCCGTTACCATTGCAGGAGAGGAAATCACC
TTTCCCAAACCGATCATTTCCCTTGGTACAATTATTGTTGCTGGTTTAGATTGGGGATTAGCCGC
CTTAGTGCTTTACCTATTATTACCTCCCAATCTTTCTATGACATATATCGGTTTTTTTGGTATCTA
TATTGTTGCTTTAACTGCTGGTTTAATTAGCACTGTGCCGGGAGGATTAGGAGTATTTGAAACGG
TGATGCTATATTTAAGACCAGAATCGATAAGCGCACCTCAAATGTTAGGAGGCTTAATTGCTTAC
AGATTTGTTTACTACCTATTGCCTCTAATTTTTGCCGTAGTTTTAATTTTGCTCGAAGCATGGAA
AAAAAGACACCCTAAGTAACTGACATTAGGCAAAATAATTTGGGGTTGCTTAATTATGACATGA
AATATTATTAACTCCTGCAACAAGTCTAATCATAATTTTTCTTTCTTATGGATTCAGAATATCTC
ATAGAAGGCAACTCTGAATCTACAAATTTTTGATTCTCGCCAACACCCGTTACACCATTAAAATT
TCCCTGTCGAAGCATCATAAATAAAAAGAAATCCCCCACTAAACCAAAAATCACACTACTTAAAA
AAACTGCGATCGTACATATAATTACGGTAAATAAACCTAACGGTGCAATAATTAAAATAATCCAA
GTAATCACCCCATTGAGAATCGCCACAAAAACGTTGCGAAAGCGAACTATCGTCCTAACTAAAGC
AAAATTCATAAAAATTCTCCTCAACAATAAAAAGTATTACTAACATAATAAGAGAATCATTGTA
AATATGTTGTATTCTTAACGACAATAAAAGATAAAGAATGACTGCTTTATATTTAACCGAACCCG
GTACAAAAGTTCACTATAAAAATCAAACCTTTACCATTAAAAAAAATCAAACCTATACTTGTCGG
CTAAGTGAACTAGAATTATTAGTCATATTACCCGGGGTGCAATTAACCGATGCCGTTATTTCCGC
TTTATTAGACCAAGGTATCGAAGCAATTTTTTTAAGGCAAGATGGACAATTTAGAGGAAGATTA
CAAGCTAGTTTTGCCTCAAATCCTCTTATTCGTTTAGCACAATATCGCACTGTTGAAACATCTTT
TGGTTTAGCTTTTGCCCAAAAATTCGCCTACGGTAAAATCCGCAATCAACGAGCATTATTACAAA
TAAAAAACCGTGCCACAAAGGGAAAAATAGCTCAATTATCAGAATCCATAGATACAATCAATGTA
TATCAATTGCAACTCAAAAACATTGATAAACCCAGAACAAGAGATGAATTAATGGGCATAGAAG
GAATTTCTGCCCGAAGTTATTATCAAGCCTTACAACATTTTTTCCCACCTCATTGGCAATTTAAC
GGCAGAAACCGTCGCCCTCCAAAAGATCCCATCAATGCCTTACTCAGTTGGGGTTATGGAGTCTT

ATTAGCTCGTATTTTTGCAGTTTGCGTAAAAGCTGGATTAGATCCCTATTTAGGTTTTTTTCATT
CTATTCAACCTTACCGCCCAAACCTAGTCCTAGATTTAATGGAGGAATTTAGACCGATTTTAGTT
GATTATGCAGTCATTTCCCTCATTCAATCTCAAATGTTGGATATAGCTGATTTTCAACCTTCCTCT
GATGGACAAGGAATCTGGTTAGGTGTCACCGCCAAAAAGTTGTTGTTGCGAGAATTAGAAGAAA
GAATGAACCAGTATTTATTATATCCCCCTCAAAACCGCAAACTAAAACTCAATCAAATTTTTCTC
GAACAAGCCCGAATGTTAGCACGATGTTTATTAGAATCCAGCTTAGATTATGAACCCTTTTTTAT
TAAATGATTAAACTTTGGCTCATTTGTTACGACGTAAAAGACGATAAAAGACGTACTCAATTGGC
AAAATTATTAGAACAACATTGTGAGAGAGTGCAGTATTCAGTTTTTGAATGTCCTTTAGATGTA
AAAAAATTAGAGCATCTTCTCTCTACTCGATGGTTAAAAGTTCTAAATTTACAGGAAGATAGCCT
TAGAATTTATCCCCTAGATGCTTCCGCAAAACAAAAAATCAAAATTTATGGACTGAAGGATAATC
CTCCCTACGAGTCTCCTGACTATCTCATCTTGTAAATAAGTGTTAAGCTGACTTGTCAATCTGTCG
CAGTTAGTAAATAATAGTTAAAAAGAGTTTTAGCAAAAGAAATTTAGATAAGTTCATTTTTTTG
TTAGGATTCATTTTTATACTCAGAGTATTTTCAGTTTTTGCTCAAAAAAACCTTTTTCTACCTTG
ACAAAAAAATAGTGTTAAGAAATGTTAAGCGTTATTCTGTAGCTTCTCGCATACATTTTGTACAA
GCAAGATGTAGTAAAGGTTTCCAAAGGTAGGGTTAATTTCCTCATTTACCCCGTAAGGGGACGGA
AACTCCTGAGAAAGATTTGCATACAATGACAGCTAATGGTTAATTTCCTCATTTACCCCGTAAGG
GGACGGAAACTTGGGTCAGTATTTAGCTTTGGGGCAGGAACTCAAAGCACCGCGTTAATTTCCTC
ATTTACCCCGTAAGGGGACGGAAACCATATGACACTAGGGCATTTACCTGAAGTTAAGCCTTTTC
AATCCAAGTTAATTTCCTCATTTACCCCGTAAGGGGACGGAAACAATGTTCTTTTGCTATTTCTA
ACATTTCGCTAGGATCAAAGCCTAGTTAATTTCCTCATTTACCCCGTAAGGGGACGGAAACCATT
CTGTTAACTATAGCATTATTAACAATAGCAGATTTACCGTGTTAATTTCCTCATTTACCCCGTAA
GGGGACGGAAACCTATATCTTCTTTACGCATAACGCAACCCCTCGCTTAAGCAAATGTTAATTTC
CTCATTTACCCCGTAAGGGGACGGAAACGCTTTTGATAAGTTCTACCGATGTTGAAGATGTTAAA
TAAGATATAACTGAAATTTCAGATTAGTGTATTAATACTAAAAAAGAAAATTTTAAAATCGATT
TTAATAAAGTTTTCATCAATTCTTATTACTGTTTTCTGTTCTCCACCTTCAACAGTCCATTTTTGG
CTAGATGAAAATCTGACCAAGTATAAAGAAGAGTCACTCATAAGCCGGATTCTGTTCCTTTAAAT
AAATTAAAGGGGTAGTTATCTATCTGGGATGAATATTACTAATCACCTCAAGCGGTACACCAAAA
GCGGAATCGGGAAAAGACCAACCATAATTCCTTCGACCTTGCTCCCAACCGGGGTTTACCGAGCC
AGTACCTCTCGATACTGCTGGTGGGCTCTTACCCTCACCTTTGCACCCTTACCTCTAATCGGAGGC
GGTATTTTTCTGTGGCACTATCCTCACGCTCACGCGCACTGGGCGTTACCCAGCAGGTTTGGTCTT
TCGGGAGTCCGGACTTTCCTCAAACTAAATCTTAAACCTCACAGGAAAAGTTTAGTTCGCAACTA
CCTCCATGACTCTTCTTGCTATTATTTTAATGATTTTTTCTCTTTAAGTGATTTTAATGACTATC
AGAACAGCGATCGCATCTACCATAAACTGTTACCTCAAAGCGATCAACTTTCAAACCCTGACGTA
AGTTACTGATATTGATACCGGTTAAACTTTCCCAAGGAATATCCTCTATTTCCCCGCAACACTCAC
ATAAAAAATGATGATGGGGATCAACATTAGCATCATAACGACAAACCCCCTCCTCTAATAGAACT
TCTTTAACTAAACCGACATTTTGAAGAGTCTGTAAAGAAAGATATACCGTTGCCTGAGAAAGAGT
TGGCACTTCTTGATTTAAATCCGTGAGAATCTGTTCAGCAGTAGGATGATCACACCTATGAAGTA
AATTGGAATAAACCGCAAATCTTTGGGGTGTTACCCTCAACCCTTTTGATTTCAATGTTTTGATA
ATATTATCTTTGCTTATAACCATTTTTAAATTGCTTTGAAATCTCGAAAATATACTTATTCATAC
TATTTTTCTCTATACCTTCTAAGGTAACAAAAATAAAATGATTATTGCCATTGACATATTCTTAA
ATAAGAGTTATTCTTAAATAAGAGCAGTAAAAAAGGCTCAAAATATTGAAAACATTTAGATTGG
AGAAATTAACCCATGGCAGTACTTGAAAGAGTTCCTGACGTAGTATTTAAAACCCGTGTCCGTGA
CGAATCTATTCAACCTAATCCTTACCGTTGGGAAGACAAAACCACTCAAGATATTTTCGGTGGTA

AAAAAGTAGTAGTATTCTCTTTACCCGGTGCATTTACTCCTACTTGTTCTTCCAATCACCTTCCCC
GTTACGAAGAATTATACGACGAATTCAAGGCTTTAGGGGTAGATGAAATTGTTTGTGTATCCGTC
AATGATGCCTTTGTTATGTTCAAATGGGGTAGAGAAATCGGAGCAAAAAATGTTTTCTTACTTCC
CGATGGTAATGGTGAATTCACCCGTAAAATGGGGATGTTAGTTGATAAATCTAATCTCGGTTTTG
GTTTAAGATCTTGGCGTTATTCTATGTTTGTTAACGATTGCAAGATCGAAAAAATCTTCGTTGAG
CCTAATTTCTCTGATAACTGTCCTACCGATCCTTTTGAAGTTTCTGACGCTGATACCATGTTAGCC
TACCTCAAAGGAGAGCAACCTAAAGGAGTTTCTGAGCCTCGTAAAGAGTTTGTAGGTTAATACCT
GTAGATTTTTCTATACAAGTGTTTTGGCGGCATAGTTTTTATTATGCCGTTTCTTTTTCCTATCA
TAATAAGCTATACCCCAGAAGGGCATTAATTTAGGTAGGTTTCAGGTTGCAGGTGGTAGGAGTTG
AATATATTCAACCCTTGCGGTGATAAGGTGATGAGGGGAGAGGGTGAGAGGGTGAGAGGGAGAA
ATTTCTTTTTGATTTTAACTCCGAATTCAAATTATGCCCTTTTCGTCATCATTCATGGATAAAAA
TTTATCCTGAACTCAGGTTAATCGTAGTATTTATCTCAAAATACCGACTTCTTGGCGATTACCAC
TCTATTCTCCCTTGGGGAGTAACTTTTTTAAGGGGTGTAAGTAAGGATAGAGATGCTTATATCAA
ACTAGGACGAATGTATAATACCAAAATCAATTAAATTGGTAGAGGCCGTATCGGTTCTCAAGAAT
CTCCCACGTTTATCCCGTAGGTTTTAGTGATGAAAGTTGTCAAAAATTATTCACTACAAAAAATA
GATGTTGTACTAGGCTAGATAATATTAGATTTTGATTATTATGTAGTTATACAGTAATTTAGTT
ACAGGAGACATAGCATATGAGATTATCCAGCAAAAATTTAGATACTCGTTTAGATACGGTTTATG
ATACCATTGTGGTTGGTGGTGGCATGGGAGGTTTATCTGCGGCTATCTACTTAGCTAGGTATGGT
TTAAAATGTCTAGTCATCGAAAAGGGTAAAGGGCGCTCAATGTGGATGCAGGATTTACGTAATTA
TGTAGGTCTTGACCCAGATACCCCCGGTAGAGATATTATTAATCATGGCACGAAACAAGCTGTTG
ATTGGGGTGCAGATCATTTAAGGGGATATGTGGAAGATGTCACAGACGAGGGGGATACTTTTGCT
GTTAAGGTTAAGGTGGGCAAGAAAGACAGTATCTATCCTGTTTTCCGCAGTAAGTATTTAATTGC
CGCTTCAGGGGTGATTGATGTTTTACCCGAATTAGATGATATGCAAAATGTTTATGATTATGCTG
GATACACTCTCCATGTGTGCATGATTTGTGATGGTTTTGATATGTGGGATCAAAAAGCCGTTTTA
ATTGTTGGTAAAGAGTCCCAGATAAATGCGGCATTTGTACTCAATTGGTTTACTCCTTATATTAC
TGTTTTAACTCATGGCTTATGCACTGTTAGCGATGAAATGAAACAGAAATTGGCGGAGCATGGTT
ATCCCCTCTATGAAAGTGCGATCGCACGTTTTGTGGGAGAAGATCATAAAATGCAAGGGGTAGAA
TTAGTTGATGGAACTTTTATTGAGGCAACCACAGGTTTAATTAATATGGGTTCAATTTATCATAA
CCACTACCTCAAAGGTATAGAAGGCTTAGAGTGGGATGGAGAAAATCTTGTCACCAATGAGATGG
CACAAACAAGCCACGATCGCATCTTTGCGTTAGGAGACTTGAAAAAAGGCTTAAATCAAGTGTCT
GTTGCCGTAGCAGATGGAACTTTAGCCGCTACTCAAATTTGGCGTAATATTCGCCGTCAATCTCC
TCCGAGAAAATGGGAGAATAATTTATCTACCACAATCCAATAAATTTTATCTCAAACTGAGATAA
CGGAGAAAACAATGATCTATGATACAAGTAACAGTTTTAAAATATCTCGGAGTAGTAAGCAATC
ATGGATGTTAAAATGCTAATGGTCATCTTAACTGGCTTATTTATTGTATCGGCTTTATTTTTCGG
TACTAGAAACGGTTACTATGACACCGATTACTATGATGGTAACGGTTCGGCTCATTAAAGTAGTA
GTTTTTTTATGATTAAGCGGCAAGAAAAATTTTCCGCACCTGAAATTAGCTTTTCTTGTTATCCC
TTTGGTGTTGGGCATTACACCGAGGGGGTTGCCTTACAACTGACTCTGGGTAATTATAGACTATT
ACTTGATTGTGGTGTTGAAGATATTAGCTTATTAGTTAATCAAGATCAACCTCCTTTTGATTGGG
TGTTTTGTACCCATGCCCATCAAGATCATGCTCGTGGCTTACTTGCTCTACATAAGTGTTACCCAA
ATTTGCCCATTTATACCAGTGATGTCACGGGAAAGTTATTACCTCTCAATTGGTTGGATAAAAAG
GATTCTGCTTCCATTTCTTTTCCTCAAGTGTTACCATGGCGATCTCCTTTTCATTTACAATCAGAT
TTAACGGTAGAATTGTATCCTGCGGGTCACTTACCCGGTGCATCGGCTTTCTTGTTTCAATATCA
TCACCGAGATAGGGTTTATAAAATTTTCTATACAGGAGATTTTTGTCTTTCCAATTTACAGTTGG

TAGAGGGGTTATCTCTGGATAGTTTACGGGGTTTGAATCCTGATATACTAATTCTTGAAGGAAGT
TATGGCACAGCTAGACATCCTCATCGTCGTCATCAGGAAAAACAGTTGATGGCAAGAATAAAAAC
TGCGATCGAATCTCAGGTTAATGTTATTATGCCTGTACCTACCTTTGGTTTGGGACAAGAATTAT
TGAAATTATTGCGATCGCATCATGAATTTACGGGAAGAAACCTTGATATATGGGTAGATGGAGAC
TTAGCCTCAGCTTGTGACTTATATTTAGATTTAATTAGCTATTTCCCCGAAAATGTTCAGAATTT
TGCTAAACATCAACCTCTTTTTTGGGACGATAAAATAAAACCCCGCATGAGACGAGTAACAAAAA
ATACTGCCCGTCATCAATTAATTGTTGAACCAAGTATCGTTTTAACAGATAAAATAAGTCATTTA
CAGGAATATTGTCAAGATAATGGACAAAAATGGCTGATATTAATTTCAGAACAGTCAAAAATCA
AGCAAACTGATGAATTTAAGTCACTAACTCAAAAATATGATCCAGAAAGAATCATTATCGAAGAC
TATCTTTTAGCAGAACATAGCGACGGCAGAAATACAACTCAACTCATTCATAACTTACGCCCCCA
ACATATTATATTTGTTCATGGTTTACCCAATTACCTAGCAGATTTAACCAGTTTAGAGGAATTAC
AAAATCGCTATCAACTCCATTCTCCTGCCGTTGGGGTAAAAGTCGATTTACCCATTGGCGCCCAA
TTCATGCAACCAAAAATCACCACTCAAAATAACTATGAAGGGGAATTGAACGAAACAGGGGCTTA
TGTTACTATTACCTTACCAGAACAAATTAATAAAGATCCTCGATGGCAAAATTTCGCCGACACGG
GCTTAATTGAAGCAAGATGGCAAGGGGAAGAATTAGTTTTAAGGGGATTATCTCAACGGGAATT
GCTACAGCAAAATAGTCTCTTAAGAAGGCAACTCAATCCAGAATCCTGCGGTAACTGTCGTCATT
TTGATAATCAACATTGCCATAATCAAGAATCTCCTCTTAATGGCTTTACTGTGCCTCATGATGGT
TATTGTCCTCTGTTTGAAGTTGATAATATTTAGATTGACCTATAAGAAAACTGTAGCCCTTCGCC
CTTTTTCCGTTGCTTACCCTCACCTAGATAAAGTTATGCGGGGGCAGTGAGTGTATATTAAAATG
ATATTGTCAAAAAAATTAATCATTTGCCATCATGACTACACCTATTATCGCAGAAAAAATAAGAG
CGATCGCATCTAAAAAAGAAGTATTAGAACAACTTTTAGAAAAGCCAGATTTAGGCAATCTTCGT
CTTGATGTTAATCAGGCTTTGGAAGAATTAGAAGAATTAATTGAAGAATTAAATAAAACTTTTC
CTTCTTAATTTTATAGTTATTAATAATTAGCTATCAGTTTTACCAAAAAATTCTCGATGGTAAAA
CTAAATTCCTTCTTGAAATCAATTATTCATAATCGGAAGTATAAAATATGGCAATTCTCTTATGG
TAAGGCACATTACTTATCCGTATTGAATGATAGGTAATAGACAACGATGCACTGGTATTAGAAAA
ATCGTGTCTCATAAGTTTAGTGAAAGCTATATAACCGAAAAACTAAATATTGAATTCTGACATCG
TATTTCTATACATCATCATTATCACTATTTCATAACCTTAATAGAAGCACTATAGTTCATTTTTG
TGGTAGGTTAATTAAAGAATTTTATAATATTACTTATTCAATAATGGCAATGAATAATTACTCA
CTATTAATTAGTTATATAGTAGCTTTAATTAAGACTGTAACAATAATATAATTAAATAAATATT
GATTTCTCTAACAACGTTAAACTATATGCCCCTTCAGGAACATCTTTAGATGAAATTATCCGTGA
TTATTACACAAATTAAATGTAACTTTCTTATTTGAAATTACTATAACTTACGCAAAAAATAAAAC
AACTTAATAATAACTTAGATACCAGAATAATCAACCACATCAAAACTAAAGAAAGTTTAAAAAT
TAGCCCAAAAGATTTAACCATTAATTATAATGATTTTATTAAACTAAATAACTTATTCAGTAATC
TTGATAAAGAGAGAAAAGATATATTTTAAAATTAAGAAGAATTCGTAAATATTCATCAAGAAAG
TCCTCTGATATTATCAGGAATAAAAAATACTGGTAAAACAACTATTCTATTAATTAAAGCTATTT
TAAATGCTAAAAGTAAAGAAAATAATAGTCAAAAAACTATTTTGGTTAGCGATAGTCTAATAAG
TAAATATCAAATTATTAACCGCTATAAACAATTAACAGGAGAAGCCTTAAACAAAAATAATTAC
CTAGAGATATTATCTCTTACAGAATTATTGAAAAAACTATTAACAGAAACATATCAAGGAAGCG
ATAACCAGTTTGTCAGAAGTAAACAAATTAATTTTCATCTCTTCAAAGAAAAATTTAGCACCCAG
TATAATCTTGAAGAAATAGACTCAAAGCATTTATGGCAAGAAATATATTATCTCCTCAAAGGAA
ATGCCACTTATTCCATCAATGAAAAAGGATTAATTGCCTTAGAAGACTACCAACAACAAATTCAA
AACTATAGTTTTCTCCCTCTAAATGTCAATCACCAAAAAATATATAAACTGGCAGAAAAATATCA
ACAATGGTTAAAAGAAAATAACTATTGGGATGACAACGACTTAAGCAGATATTTATTAACTCACC

TACATGAAGATAATATCGGTAAATATTATATCTATGGCGATAACATTCATCATTGGAGTGAAAT
TAGTCTTAAATTAATTTGCCAATTAACCACCGCAGAAAAAACTTTTGCTTTAACTTAGTTATTTT
CAACATCTACAAAATAACCAAGAAGGAGAAAAACTTTTATCCGAATATCTTTATTAAGTTAAACT
TCAATGGGAATATTCTGATTATCTAGCTTTGTAAAGAAATATTAACTTAACTTGTCAATCTGTCG
CAGTTAGGAAATAATGGTTAGAAAGAGTTTTGGCAATAAAAAATTAGATAAGTTCCGTTTTTTG
TTAGGATTCACTCTTATACTTACGGTATTATTCATTTTTACTCAAAAAAGCCCTTTTTTACCTTG
ATAAAGAAATAGTGTTAAGAAGTGTAAAGCATAATTTTGTATTTTTTCGCATACACTTTGTATA
AGCAAGATGCAGTAAAAATTTCCAAAGGTAGGGTTGATTTCCACATTTACCCCGTAAGGGGACGG
AAACTTAATAATATAACAATCTGTCATGACAGATGTCAAGGGGAGGTTGATTTCCACATTTACCC
CGTAAGGGGACGGAAACTCGTTGTCGGGATAGGGCTAAATCTCCCGCCATGGGATTGAAAGTTGA
TTTCCACATTTACCCCGTAAGGGGACGGAAACTGGTAAGACCCACCGCCCCCTAATATATGGGGC
ATTATAAATAATGTTGATTTCCACATTTACCCCGTAAGGGGACGGAAACTGTACATCTGGAGATG
TTGCATATGCCTCAAAATTATGCGTTGATTTCCACATTTACCCCGTAAGGGGACGGAAACAGCAT
TGAAGATTGCGAGTATGAAAATTGAAGATGTTTTCGAAGTTGATTTCCACATTTACCCCGTAAGG
GGACGGAAACTCATAACTTCTTTAGTTTTATCGCAAATAATAATCATATATGTTGATTTCCACAT
TTACCCCGTAAGGGGACGGAAACTCTACCCACTCCCCTGCGTCTCCACGGTAGAACATATTGAACG
GCGTTGATTTCCACATTTACCCCGTAAGGGGACGGAAACGTCATTACATACTTTTCCTCTGTTGT
AACTCCTTTTCCCGTTGATTTCCACATTTACCCCGTAAGGGGACGGAAACAGTAAACCTCGACAA
AAGAGAAATTAAAGAGATGGCACGCTCTTGTTGATTTCCACATTTACCCCGTAAGGGGACGGAAA
CTGATACAGGCACATGGGCACATAGATTAATAGCAATAGCCTGTTGATTTCCACATTTACCCCGT
AAGGGGACGGAAACTTTCTCTCACTTCATCCTCGGTAAATCCCGAATTTTCCATTGTTGATTTCC
ACATTTACCCCGTAAGGGGACGGAAACAGGTGTTTTTTATCTTGAGGAGTTCCTGCGTAATTAGT
TGATTTCCACATTTACCCCGTAAGGGGACGGAAACTTGAGCATCACCAGTTTTAGCACTTAATAA
TCTTTCACCAAGTTGATTTCCACATTTACCCCGTAAGGGGACGGAAACTGGCTTGACCAATACTT
TTCAACAAGATCAAACCATTCCTGTGTTGATTTCCACATTTACCCCGTAAGGGGACGGAAACACT
AACCCAGATTCATTTATTTGATTATCAGACCCAGGTAGTAAGTTGATTTCCACATTTACCCCGTA
AGGGGACGGAAACCTGTTTATAAATCCCCATCCTGATTGGGTGATTTGTTTTCCATGTTGATTTC
CACATTTACCCCGTAAGGGGACGGAAACTTGGAGTAATTGAAGGTTAAGACTACGTCCTAAGTCC
TGAGTTGATTTCCACATTTACCCCGTAAGGGGACGGAAACGTGGGTGTATAACCCACGGGGGTTT
CTTTTTAACTAGCATTGTTTTATTCTCCCGCGTTGATTTCCACATTTACCCCGTAAGGGGACGGA
AACATTCATAGAATTTGATTCGGAGAGAATAAGAAGATTTGAAGTTGATTTCCACATTTACCCCG
TAAGGGGACGGAAACGTTATGTAGTAGGGTATGATGGGCGAATTTGCCTGCAATCTTAACATCCA
GTGCGTATCAATCCTGCTACGGGCTATGGTATCGTACTCCTAATAAGCGGGTCGGTATGCATCTT
AGTCATTACCGTTCTAGGTTGCCCCCCCCAATCAGTAGAACCAATCAATGAGTAGAAAATGTTAC
CTTCTGAAACTGTTAGTATTTGCCAACTTTCTGCCAACGATCTACCACTCATGGAGGGTTTATTA
ACTGTCTTTGGCGAGGCTTTTGATGAAGTTGATACCTATAGTTCATCACGTCCAAGCAGCGCTTA
TCTCAAGCAGTTGTTAGGCAGCGATTATTTCATTGTGCTTGCAGCGTTAAAAAATGGAGCGGTCG
TTGGAGGTATCGCCGCTTACGAACTCCGAAAGTTTGAGCAGGAGCGAAGTGAAATCTATATTTAC
GATCTGGCTGTTGCAGCAGCACATCGACGCAAAGGCATCGCTACCGCATTAATCCAGGAATTAAA
AAAGATCGCCTCTGTGCGAGCAGCCTACGTTATTTTCGTACAAGCAGATATCGGAGACGATCCGG
CGATCGCTCTCTATACAAAGCTTGGCACACGAGAAGATGTGCTTCATTTCGACATTGCTGTTGAG
TGAGATCGGCGATGTCGATGTATAACAATTCAAATGCGGCTTTTGTGCGACTCAGCAGCGTCGCT
TCTTTTTTGCTCAATTAGTACAGATAACGCTACTCTCAATGCAGCGCACAGAGTAGCGACAGGAG

TGGCATTCAGGGTTGATTTCCACATTTACCTTACCCCGTAAGGGGACGGAAACTCCACAATTTTC
GAAAAAAAAGAATTTGCGATCGCATCTTTAGGGTTATTTCCTCATTGACTCAGTAGGGGGATAAA
GACTGATTTTGAGAAAAAGTTAAGAGAGAATTAACAAATAAAACTACAATGATGGTGATAGATA
TTTAAGTTGACGCAATTTAGTTGTAATCAGTGACACAAACTCAAAAGTATATTCACAAAACGATA
TTAAGTAATGGTATTACCCTAGTGGTAACGGAAAACCCCACCACGGATATTATTGCAGGGAAAAT
TTTTTGTCGTCAGGCGGGTAGTTTATGGGAAGCAAAACATCAGGCTGGATTATTTCATCTTTTAG
CTAGTGTCATGGCTAAGGGTACTCGGAATTTATCTTCTTTGGAAATTGCGGAGAAGGTAGAGTCT
ATTGGAGCGGCACTAGGCACGGATACTTCTAGTGATTATTTTTTAGTCAGTATGAAGACTATTAC
AGAGGATTTTCCTGAAATTATGGCTTTAGCTTCCGAAATTCTCCGTTTTCCCTCTTTTCCTCTGGA
TGAGATTGCACTAGAAAAGAATATTACAATACAAAATATTTTGTCTCAAAAAGAACAACCTTTT
AATGTGGCTTTTTCCCAATTACGTCAGATGATTTATGGGCAACATCCCTATGGTTTTTCTCTTTT
GGGAACAGAAGAAACGGTGGCAAATTTGATTCAGGAGGATTTAAGATTTTATCATCAACAACAT
TTTAGACCAGATCGTTTAGTTATTAGTTTAGCGGGAAATATTGACTTAAATAGTGCGGTGGAGGT
TGTGGAAAAAGTCTTTGGTGATTGGTTTCCCCCCGAAGATGCCCTTATCAAGATTGATTCCCTTG
AGCAAAGAGAGTTATTATCACCCAAGGCTCGATCTCACCATACTTCCCAAGACACTCAACAGTCT
ATTATTATGATGGGCTATCTTGTGCCAGAAATGACACACCCCGATTATCCCGTGTTAAAGTTATT
GAGTACTTATTTAGGAAATGGGCTTTCTAGCCGTTTGTTTGTGGAATTAAGAGAAAAAAGAGGT
TTAGCTTACGATGTTTCGGCTTTTTATCCTACTAGGGTTGATAAGTCTCAATTTGTAGTCTATAT
GGGTACTGCCCCTCACAATGCAGACATTGCCCAAGAAGGTTTATACAATGAAATAAAAAGGTTAA
GAGAAAATCCCCTCACCACAGAAGAAATCCAAACGGCAAAAAATAAATTATTAGGGCAATACGCT
TTAAGTAAACAAACCAATGGAGAGTTTGCTCAAGTTTTTGGTTGGTACGAAACTTTAGGCTTAGG
CATTGAATATGACAATATTTTCCCTCAAAAAATTAGTAGTGTAACCATTGAAGATATACAAAGAG
TCGCTCAAGAATATTTACAAGATCAATTTTTGTGTGTTTCTAAAGTAGGTCCGAAATAATTAGGA
GATAGGGGGGTTAGGGTATTGGGAAGAAATTAACTATTGATTATTTATTAGTAATTTACTATTT
ATAAATGAAATATATTTTTGCCGACTATCATCCGCTTCATTTTATAACAACTTCACCAGAAAAAA
TCGAACAATTAGAATATTTAAAGAAGATAACTCAAGGATTTTTATGGCTACCAAAAAAGATACA
TAATGATAATTTATCCGCTAATTTTTATGACTCTTCTTTAAGGGAACATTTAGTAAATATTTGGT
CTAATAGTAAATGTATTGTTTTTTGTTTAACATTGGGTGCTGTTACTCGTTTAATTGCTCCTTTA
TTAACTAACAAAAAAACTGATCCGGCTATTATTGTAATTGATCCAGCTTTTGAATATATTATTTG
TTTAACGGGAGAACATCAACACCAAGGCGATAAATTAACAGAAATATTGGCAGAATTATTATCTG
CAAAAGCAATTATAACCAGTGCTTCTTCTAATCTTAATTTACCTGCGATCGATTCTTTTGGTTAT
ACTTTTGGGTGGAAAAAAGGAGAAGGAGATTGGACAAAAGTTAGTGCAAATATTACTCAGAATA
AACCTACTTTAATTAAACAAGAATCGGGATTAAATTGGTGGTGTCAAAATCTGCCTATAAATCAC
CCTTTTATATTTGGTGAAGGGATAAAAGATAATAGTAAGGAAGGGGAAAAGAGAGAAGAGGACT
TTTTGAAACAGTCAGATGATGACTTTGCTAAGTCTATTTTTACGGAAGCAGAAAAACAGGTTCAA
GCAATGGTTTATATTGGAGTCAACAAAACTCCTAGGGTTGATATTCCTATCGTCAGTTGGCATCC
GAGAGGGCTATGGGTTGGCATTGGGTGCGAAAGAGATACTTCCCCCATACTCATTGAAAATGCTG
TTAATTCCGTTTTAGCTCAATATAATTTAGAAAAAAATGCGATCGCATCTTTAGCTACTATTGAT
CTTAAAGCTGATGAAGTAGGTATTTTAGAGTTAGCCCAAAAATGGCAATTACCGTTAAAAACCTT
TACATCTGAGGAGTTAAATTTAATTGATGTGCCTAATCCTTCTAGTATTGTTGAGCATGAAGTAG
GAACAAAAAGTGTGGCGGAGGCTTCTGCATTAAAAGCGGGTTCTTGGTCAAATTTACCTGAAAAT
AATCTTTTTCCTACCCTCATCGCCCCGAAACAAATTTTTAAACAGGAGGACTTAAAAGGTGCGGT
AACAGTAGCCATTGCCCTTTCTAATTTAGAGTATAATCCTCGTCAGGGTAAATTATATCTCATTG

GTACAGGACCGGGTGCGATCGAATATCTCACGACGGCGGCAAAAACAGCCCTCAGAGATGCTGAA
ATTATTATTGGCTATGGTTTATATATTGATTTAATCAAACCTCTGTTGCACCCCTCACAAATTAT
TGAAACTTCAAATATTACTCAAGAAAAGCAAAGAGCAAAGAGAGCGATCGCACTAGCTCAATGGG
GGTTGAAGGTAGCGGTAATTTCTTCAGGAGATTGCGGAATTTACGGCATGGCGGGGTTAGTTTTA
GAAATTTTAGCAAATCAAAACTGGGATGGAAAACAGCCCCCTATACAGGTCTTTTCTGGCATAAC
TGCAATGCAGAGCGTGGCGGCAAAAATTGGATCGCCCTTGATGCACGACTTTTGTGCCATTAGTT
TAAGTGACTTACTTACTCCTTGGGATGTCATCGAAAAACGCCTTCACGCCGCCGCTACAGGAGAT
TTTATCACCGCCATTTATAATCCTCGCTCTCAAACCCGTCAACAGCAAATTATCACTGCCCAAAAT
ATATTTTTACAATATCGCTCAGGGAATACCCCAGTTGCGATCGCACGTTCAGTAACAAGAGAAGA
TGAAAGTATAATTCTGACAACCCTAGAGGAAATGTTAAACCATCCCATTGATATGGTAACTACAG
TCTTAATCGGGAATAGTAGTACAAAAAGATACCATGACTTATTGATTACTCCCAGAGGATATTTA
GGGCAATGTTAAAAAAAGGGCAAGGGGTAAGGGGCAAAGGAAAAATTAAGAATTAAAAACTCCG
AACTCTGAACTCCGAACCCCTGTATGGGCGAATGGCCATTCGCCCCTACTTCCCCCTCTCCCCTCA
TCACCTCATCACCCAAACACCTGCAACCTGAAACCTGAAACCTAACCCCTCTTTCTGATAAAATCT
TAATGAAATTTAAAAATATGTAAACAAAATTAATAATCAATGAAAATATTAGTAATTGGAGCAA
CAGGGACTTTAGGCAGACAAATAGTCCGTCATGCTATAGATCAAGATTATTCTGTGCGTTGTTTA
GTGAGAAATCGTGGCAAAGCAGGATTTTTAAAGGAATGGGGGGCAGAATTAGTTAAAGGGGATA
TATGTGAATTTAAGAGCATTGAGTCGGCATTAGAAGGGGTTGATGCGGTAATTGATGCGGCAACG
GCAAGAGCGACGGATTCTTTAACTATCAGACAAGTTGATTGGGAAGGAAAAGTAAATTTAATTC
AAGCCTGTGCCAAAGCAAATATTAAACGATATATATTTTTCTCTTTGCTAAACGCTGAGAAATTT
GAAGATGTACCATTAATGAATATTAAGCATTGTACAGAGTTATTTTTACAAGAATCTGGATTAG
ACTACACTATTTTTAAAATAGGTGGCTTTATGCAGGGTTTAATAGGTCAATATGGTATTCCTATT
CTTGATAATCAACCTGTGTGGGTAAGCGGAGAAAATACTCCCATTGCTTATATGAATACTCAAGA
CATGGCAAAATTTGTTATTAAAGCCTTAGAAATTCCTGAAACAGTCAAAAAAACTTATCCATTAG
TAGGTAGTCGTGCTTGGACTGGAGATGAAATAATTCAATTATGTGAACGCTTATCCGGAAAAACC
GCTAAAATTTCCCGTATTCCCCTTGGCTTACTTCGTTTTTTACGGGGTTTTACCCGTTGGTTTAAG
TGGACAAGAAATGCCTCTGATAGATTGGCTTTTGCTGAGGTTTTAGCCAGTGGTATTGCCTTAGA
TGCTCCTATGGATGAAGTTTATGAAACCCTTAAAATTGACCCTCAAGAAATTACTACTTTGGAGG
CTTATTTACAAGAATACTTCGATCGCATCATGAAGAAAATTCGAGAAATCGATTATGAGAAAAGC
AAAATGAAAAAACGGAAGAAAAATAGCTTTTTCAAATAATGTCTTGATTTAACTTTATTTAGGG
TATTAGGGAATGGGAAATAGTTGATACAATTGGAGTTGTTATAATGAACACAACCCACAATAGT
CAATTATCTATCTTAACAATAGAAACCATATGTAATTGTTAATATTTATTATAATTATTAATTAT
TAATTCCTCATTGCTAGTTAACTATGACTCTTACTTATAAAAAAACTATCGCCGATGAAGTTCGT
CAAGACTTCCCTATTCTCCATCAAAAAATTCACGATAAACCCTTAATTTACTTTGACAATGCCGC
AACATCTCAAAAACCGAGAGCGGTAATAGAAGCCTTAAGACACTACTATGAGTATGATAATGCTA
ATGTCCATCGTGGAGCACACACTTTAAGTGCAAGGGCAACGGATGCCTATGAAGGTGCAAGGGAT
AAGATAGCTAAGTTTATTAATGCTAAGAGTCGCAATGAAATTGTCTATACCCGGAATGCCAGTGA
AGCAATTAATTTAGTTGCCTATAGTTGGGGTTTAGCACATCTCAAAGCCGGAGATGAGATTATTT
TATCTGTAATGGAACACCATAGTAATATTGTTCCTTGGCAAATTATCGCTCAAAAAACAGGGGCA
ATTCTCAAGTTTGTGGAGTTATCCCCTTCTCAAGAATTTGATTTTAATCATTATCAGTCACTACT
TTCGGAAAAAACTAAATTGGTTTCCCTTGTTCATGTTTCCAACACACTAGGTTGTATTAATCCCG
TAACTGGGATTATTACCTCCGCTAGAGAAAAAGGGGCAAAAGTACTTATTGATGCTTGTCAGAGT
TTACCCCATTTACCCATTGACGTTCAAGGAATGGACTGTGATTGGTTGGTTGGCTCTGGACATAA

AATGTGTGCTACTACTGGTATTGGGTTTTTATACGGCAAAGAGGAATTATTAGAAGCAATGCCCC
CCTTTCTCGGTGGTGGCGAAATGATTGCAGATGTGTTTTTAGATCATTTTACCTGTGGTGAATTA
CCCCATAAATTTGAAGCTGGAACTCCCGCTATTGGAGAAGCGATCGCATTTGGTACTGCTATTGA
TTACTTAAGCAATATTGGCATGGAAAATATCCATCAATACGAAGAAGAATTAACAGCTTATTTAT
TTCAACAATTAAATGAAATTCCTAACCTCACCATTTACGGTAATCAACCCACCACAGAAGGTAAA
GGGAGAGCTGCTTTAGCCGCATTCAATGTCAAAGATATTCACGGTAGTGATTTATCAACACTGTT
GGATCATGAAGGTATTGCAATCCGTTCAGGTCATCATTGTACCCAACCTTTACACCGTTATTTAA
ACATTTCTGGCAGTGCAAGGGCAAGTCTTTACTTTTATAACACCTTTGCGGAAATCGACGCTTTT
ATCGTTGCTTTAAAAGAAACTATTGATTTCTTCACCCAAATGAATTAGTAAGAAAAATGTTAAA
ACAACTCCCCTCGCCCTGTGGGAGAGGGGTTAGGGCTAAGAGTAAAAGGCTTTGAAGAGGAAATA
TTTTTTCATGGGAAAGGGAGACAGAGTCTCCCCTAATTGACAATTGACAATTGACAGTTGACAAT
TTATTAGTTGTCTTTCTGGGTTAGATCATAGCATATCTGACGGAAGTTTTCACCTCTTTGCTCAA
AGTTCTGATACTGGTCAAAACTAGCACAGGCAGGGGATAGTAAAACAACTTTTGCTTTTAAATCA
GAGGCTAATTCCCTTGCTCTTTTAACTGCATTTTCCATCGTCTCTACTATTTCATATTGTCCGAAG
CCAGAAGCCTCAAACCGCTCTGCAAAGAAAGAAGCGGCTTCTCCGATGAGTAAAACAGAAACACA
TTTTTCCTTAATCGTATTTATCCATGCAGTATCATTTCCTTCTTTTGCTTCTCCTCCTGCAATCAA
AATCGTGGGGGAAGCCACAGAATCTAGCCCTACTTGAGCCGCATCATAATTGGTAGCTTTGCTAT
CGTTTATATAAGCTACTCCATCAATATTGGTAATTAATTCGAGGCGGTGAGGCACTCCTGTAAAT
GACGAAATAGTATCTGCGATCGCTTTTTTGCTAACACCGGCTAATCTAGCGGCGGCAGTAGCCAT
TAATAAATTTGTAAATTATGTTTTCCCACCATCTTAAACAAAGACACAGGGGCAATTAACTCCT
TAAAAGCCACAATCCATGAATCTTCGAGATAAACACCCTTTTCTATATCCCCTAATAAATATTCT
TTTCCTCTTACACTGGTCCAATAAACATTTTTCCAATCTAAATCTAAACCGAAATTATGAAGATA
AGGATCATCCCCATTGAGAATTTTTAGGTGGGATCTTTGTAATAAACTTGCCTTAATACGATGGT
AGTTTTCTAAAGTTTTATGTCTGGCTAAATGGTCTGGAGTAAAGGTTGTCCAAATACCAATTCTA
GGAGATAAATCCATAGAAGATTCTATTTGATAACTACTAATTTCAGCTACCACCCATTCAAGGGG
ATTATCTAAAGACTCTAATGCCAAATCACAAGCGGCATAGCCAATATTTCCACAAGCAGAGGTTT
TTATACCCCCCATTTTTAACATCGCTTCTACTAAAGCTGTTGTGGTAGTTTTTCCATTTGTCCCAG
TAATACCAATCCAAGGAGTTTTTGATAAATATTGCCATGCTAACTCCATTTCACCAATGGTTTTT
ATACCTTTTCTCCTCGCTTCCTCTAAAATGGGTATATCCCAAGGCACTCCGGGGCTAACGACAATT
AAATCAGGATTTTGAACATTATTTAAAGGTAAATTTTCTCCTAATTTAACAACTATATTTTCTTT
TGCTAAATCATTTTTTATTATTTCTAAAGATGGCGAATTAGAACTATCATAAATTGTTACTTCTT
CATGATTTTTTGTAAGAATTTTAGCTGCCGCAATACCTGACTTTCCCAGTCCAATAATATAAACT
TTACCCATTTATTTATTTTGTTGATAATGAATCTATTAATAGGGGATCGCATCTTGATTGTAACT
GTTTTTCTATTTTTTTCTCTTTTTTTGCCAAGTTTCCTTTGCTTCTTTAAAAAGTTTTTTCAGTCT
TAAATCTAAGGAGAATCCCCTACGAGTCTGAATAATTATCGTATCACAGAGGCGATCGTGAAAAG
CCTGATTATATTCATCATCAGAAAAAAATCGTTAAGCCATCCACAATAATAGGGGTTAATAATAAA
ACCATTAGCAAAGAGTCTTTAAAGTTAATTTTTAAACCAATCATCGCAGTAAAAGCGATTAGGGC
GATGACTCCTTCTCTTTTTGACATTGACACCATAGAAGGTTGTCTTTTTCTGCGTATATCTGCTA
TTTTCAAATCCATTGCCCAACGTCCTAAACTTTGCCCCTTATTAATTTGTACAATTATCACTCTGA
ATATAAACCAAAGAAGTACAAATATTACAAGCTCAAGGAAAATATTAGTCACAAGGGAAGAAAA
AATCCAGACTATAATAAAATCAATCAGGAAAGCATAAGCTCTACGTTCAAAGGGTGCTTTGGGAA
CTCTACGAAATTGATTTTCTTCGCTATACATGGGTAGTTAAATAAATAATGGGCTTTATTAAAAG
ATTGGGGGATTAGGGGGAAACAAGTAATGAGTTAGGGGTTAGGAGTTAGTTAGGGGCGAATGGC

CATTCGCCCGTACAGGAGTTAAAAGTTAGAAGTTGAAGTTATTAATTATCAACTATTTACCTTTG
TCCTCTTCCCCTATTTAGGGGGGATAAAGGGGAGTTTACCCCTTGCCCTTTCTTTGCCTCTTACCC
TTTAATTGCTAATTTCTAGTTTAAATTGATCTGAGCTAATGAAATTCAATTATTTTTTCTTAATT
TTATTTAAAAAAGAATTACCCGCATCCACTAGGAAACGTTTGCGAATAGCTTCCCTTCCTAAAAG
CATCCGAAAGCCCATAACATCTCGATTTGTTAGGGTTAATTCAATATCCCAATTATAATCTCCCA
ACTGAACTGAGGTAATAATTACAGGTCTAACAGTGGCATGACCTCCAGAACTCGTGACCTTACGA
TATTCTAATAAGGGAGCTATACAACTAACTGTCGTATGAGTTTCCCTTTGAATGGGATGTACTTG
AAATTTTATCAAGGTGCGTCCTGTACCGCCGATCCCTTCGGGATCGCGCTCTTGATAGGGGTGTA
TGTGATAAGCGTGTAGTGCTGATGTTCTTGCACCAGTATCAATTTTCGCTTTGATTTTTTCTACT
CCTAATTCAGGTAGTGATAAGTATTCTCTCCAACCAATTAAAGGTAAGTTTTTATGGTGGGAATG
ATCATTCATAATCAATAGTCAATAAATGTCTGTTCAAAATATTCAAGGGAAACAAGTAATGAGA
AAAAGGTTTCAGGTGTTAGGTTTCGGGTTTCAGGTGTTTTTAATTTTTAATTACCCTTGTCCCTA
GCCCCTAGCCCCTTGATTACTAATTCCTAATTCTTCTAATAGTCTTTTTTCTCTATACAATTTCTG
ATCTCATTAAGATCTATATATCTATCTGTTGCGTTACGCAACTCTCTAGCAATCATACCATCAGT
AGAAACCACCGTTATATGAGTATTTTTAGAGCGTAAAAGTTCGATCGCTCTTTCAAAATCACCAT
CACCACTGAATAAAACAACACGATCGTACTGATCTACAGTATTGAACATATCCACCACGATTTCA
ATGTCTAAATTCGCTTTTTGGGAAAATCTACCTGAACTATCGTCATAGTATTCCTTAAGAATTTT
TGTTCTTACGGTATAACCCAAGCTAATTAAAGCATCTCGAAAACCCCTTTGATCTTGAGAGTCTT
TTAAGCCTGTGTACCAAAAAGCATTGATTAACATGAAACCAGACTCTTCCGAAAAATAGTCAATA
ACTTTACGAGGATCAAAAAACCAACCATTTTTTTGTTGTGCATAAAACATATTATTACCATCAAC
AAAAATTGAAATTCTATCTCTTAAGCGATGAGGATTTACATGGCTATTATGAGTATGATTTTCAT
AGTAATTATGATTTATTTCCGTACTATTAGTAATAATATTAGTAGAATTTACTTGGTAATTAGG
GTTATATTCTGGACGGGGAATTAGTTGAGGTTTAGGGTGTTCTTTTTCTGTAAGATTTTCCTCTT
CTAAATCCGTTGTATCCTCAAAATCTAGGTGATTTTCTATGTATTTTCCTTCTAGGTTTAAATTA
TCATTATTTATGTCAAAGTTGTGCATATAAATAACATCTATTTAATAATATAAAAATAAGAATA
TAGTTATAAATATTTTCATATAAGTGACATGATATACAAGCCTGAGAATTTCAGACAAGGTATA
AAACAATTTTTTCAATTAATAAAAATATTTAGTTTTTATGGCAAAAACATCACAAATGTTAGTA
GCAAAATATACAGTTTACCGAATAAGTTAAATCAAAATAATTTGATAATAATTTTTAGGAATAA
TAGAAACATTCAATAATAATATAAGTTTTAAAATATAATAATTTTTCCTATAGTTTAAGTAATT
TAACTTAAATAAAATAATTAGCGAAAGTGTTCAGGATAGGAACACCAATCACTCCATCCTCCTGC
ATACAATTTGCTTTGAGAAATGTTAATTTTAAATAGAGCTAGTAGATTGACACAAGCAGTAACA
CCAGAACCGCAATACAAGATTATTTCTGGATATTCAAGATAAGAAGACCACAGAGAGGTTAATTC
TTCCTCAGATTTAAGAAAGCTATCATCTGTTGTCATTTCTTGCCAAAAAATATTTTTAGCACCGG
GAATACTACCTGCCATCGGATCTATGGGTTCATGTTCTCCTCGATAGCGATCGCCTGATCTAGCAT
CGATAATAATAGTAGAGTCAAAATTAAGACGAGATTCCACATAATCTTTATCTACCAGCCAATCA
GAACGAATTTCAGGTAAAAATGAGCCTCTAACTCTATCAGGAATTTTATTACTAACTGGATAACC
TAGTTTTTGCCACTCCTGCCATCCTCCATCAAGAATAAAGATGCGATCGTGACCTAAATAACGTA
AAAGCCACCATAAACGAGCAGAAAAAGCTAAACGAAGATCATCATAGGCAATGACCGTAGTTTCC
CCTTTAATAATGCCAAATTCCTCTAATTTCGAGGTAAAAGCATCTATTTTGGGCAAAGGATGACG
GCCACCATGACTCTGAACAACGTCTGACAAATCTTGGTTTAAATCCAGATAATAAGCATTTTCTA
TATGGCTTTTTTGATATTCTTGATAACCCCATTGGCTATCAGCAAGACGAAAACGACAATCGATG
ATCTTGAGATCTGACCGTCCGAGGTTGTCTTTGAGCCATTGTGCCGAAACAGTTACCTGAGTGGA
ACCCATGGAATCTAGTAATAGTTAATAAGATAAAGTTCAATTTAGTGTTAATAAAAAATTATAA

GATAAGAATAAATATATAAGTATAAGTTTAAGTTTAACCTTAGTTAAGGACTAATGGCTAATAT
ATACACATACTAATGAATTTTAACAGTTTTGACACTGATGACTTTTGAACCTCTGAACAGACAAA
ATTTGTAGGATGTAGGTGATATAAATAATTAACCTCAGTTCTGAGTCCTAACTTATCTCAAAGAT
ATTGAATCAACTATAATGAAAGTGAAATAAACAAAATCTGTTAGGGAAAAACAATGAAGAAGTT
ATCTAATTTACTATGGTTTTCGGGACTTCTAGGGCTTGTTACACTCGGTTTAGGTGCAACTATGA
GCGAAAGTTTGGCTCAAATCAGAGGAGAAGATTCCGATCGCTTCTTTCGTCAAGGCAACGAGATG
ATGGAAGAACAAATCAGAGAAATACAAAGAGAAAGCGTTAGACAACAACAGGAAGAAAAAGCCC
AAATGGGTGAAATGGATGATATGGATATGGATGATATGGATATGGATATGGAAAATCCTGAAAA
TATGGACGGGTTTGAAGAGGATAGAGATGAAAAAGAAACAGAACTAGAAACAGAGCTAGAAATG
AAACAACCTTCAGGTGTGGAATTTAAAGAAGTAGAGGAAAATCCAGAAGGTTTAGATGATAATG
TGCCTGAATCTCCAGAGAATGAAATAGAAGTACAAATGTAAAATTTATGAATTTTTCCATTGAG
ATAGTTTAACCCGAACTCGATGAAAAATGAAAAAAGTACTGAAAAATAAGGAGTTAAGACTCAT
TAATTAACCAAGATTTTCCTTTAAACAATTATTAAATTGAGAGAAAATGTATTTAAAATCAATTT
AACCGCAGTTTATATCAATAATAAACACTTTTCCTTTTTCTCTTTGCCCCATTTATATCGAATAG
AATTATATCGAACTCAGGTAGGTTAGTAACGAATCCTTGGATCAATCAGAGCATTAACAATATCA
ATAATAATACTGGCAAAAACAACGATGATTCCAAAAAAAACCATAATTCCTTGTACTGTGGGGTA
ATCCCTCAAAGAAATAGCCTCATATAAACGATTGCCCAGCCCGGGCCAAGAAAAGGTAACTTCGG
TTAAAACAGCTCCCCCTAATAGAGCCGCAAAAGTTAAACCCATAACGGTAATAACGGGAATTAAG
GCATTTTTAAAGGCGTGGGAAAAAAGTATTTTTCTTTCTTGGATTCCTCTCGCCCTTGCCGCTTCC
ACGTATTCAGCTTGTAGAGTTTTTTTCAGATTCACTCGCACTATACGCTCAAAAATACCACTCAA
TAAGATTCCCAGAGTAAAACAGGGTAAAACTAAGTAATGAATAGAAGTGCTAAATTGATGACCA
TTAAAAGACAATAAACTATCAATGGTATATAAGCCAGTAATTTTGTTCGGAGTGTCTAAACCGAT
AGGGAAACGAGTACCTAAAGGAAACCATCTTAACTGCACCGCAAAAACTAATTGCATAATCATCC
CTACCCAAAATAAAGGCAGAGAATAAGTAATAATTCCGAATAATCTACCTCCTAAATCCCAAATA
GTATTGGGACGTAATGAAGAAATAATCCCTACAGTCATGCCAATTGCGATCGCAACTAAAATTGC
AAAAAAAGCTAATTCCACCGTAGCAGGAAAATATTGTTGGATAACCTGCCAAACACTTTCTCCCC
GACTTGTGAGAGAAGAGCCTAAATCAAAGCTCAATACCTGCCCTAAATATTTGAAATACTGTACC
CATAAAGGGGCATTTAAACCCAATTCTTCTCTTAGTGCCTCCTTTGCCGCTTCAGGGGCTCTAGTA
CCTAGAATAGCATCAGTGGGATCACCGGGTGTAGCTCTCAATAACAAAAAAACAATTGTTACAAT
CGTCCATAACATTAAAGGAGCTAACAGCAGACGAGCGATAATATAATACTGTAAAGATGAAGAG
CGAGACATAGATTTGAGTAAAGATTACAGTGGGCTAATTATAATCTATTTTTGATCATTAATACC
CAAATTTTTGATACTTTTACGCAAATTTTGCCATAGTTCTTTAATTTGGCGATATGCTTCTTGGG
GGGCTATTTTGCCATTAGTTTCTAAATTGCAAATATAACTTACTTTTTGAGCAAATTCTTGTAAA
TTAGAATTAAACATTAAATTCTCAGGCGTAAAATCACCTCGATAACTACTTTGGGGAAATAAAAA
CTTTTCCTTATCTCTTTGGTTTTCATCCATGACAATATTTTTTCTTTATTTTAAATTAACAATAA
ACTGATAATTCCGTTTCATTTTATCTTAATATTAACTATTTCGAGATTTTAGGTTAGGAAGTAAT
CAAAAGGAGAAAATTAGTAAAGCATAGATATTTTTTTTGTTGATTCTTGAAGCTAACACCTCAAA
CCTGAAACCTTGTTCTAACTAACCTGAGTTTTGGATAAGCTGAAAGCAGACCAACTCGTAGTCAG
AAAACCTTATAGCTTCTTAAAAATAACGATAAAATTGCCTTAATCCGAACTGACTTTAAATAGTT
AAGAATTAAGAATGAAAAACCACGAATACTTACTACTTATTTATTATTTATTACTTGTTTACCCC
TTTCCCCTCATCCCCCCATCCTCCCATTCCCTAACAACTGCAACCTGACACCTGACACCTAACCTT
ATCGGATATTCTTAACTCGAACTGAGGTTAATAAAATTAATGATAAACCTGCCCATCAGGCATAA
TTGCCCCTTGTAAATTAACTCCTGTTAGGTTAGCACTACTCAAATCAGCGCCAATTAAATTTGCA

TTAGTTAAATCTGCGGAGGTTAAATTAGTACGAGCAAAAAACACTTCCATCAAAGAAGCATTACG
CATAACCGCACCGTATAAATTGGCACGAGTTAAATCACCTCGATTGAGACGAGCAAAACTTAAGT
TTGCCTGAAGAAGATTCGCACGGGTAAATTTCACATCGGGCATTGTGGCACTTTCTAAATTTGCC
ATACTTAAATCTGCATCTACTAATTTAGTCCGTTCTAGTTTAGCATTAGAAAGATTAGCTCCGAT
TAACTTTGCCCCCATAAATTCTGCTTCTGTTAAAAAAGCTCCTTTTAAGTTGGCTTGAGTCAAGT
CTGCATTAGTTAAATCAGCTCCCCTTAAGTTAACTTCGACTAAATTAGCTCCGACTAAACTTGCG
TTTCTTAAATCAACCCCACGAAGATCTGAGTTTTTTAGGTCTGCTTTGTCTAAGTTTACCCCTCTT
AGGTTTGTTGGTTCAGAACGTTTTTCTCTCCTCATATTTACCGATCTTAAACAAGCCCCTTTAAG
ATTTGCATTGCTTAAATTTGCATAGCGTAAGTCCACTTCAAATAAGTTAGCGTCTAGTAAGTTAG
CTAAAGTTAATTCTATTCCTCTTAAATCTGCTTTTTGTAGAATAGCCCCATGCAGTTCCGCCCGAT
GTAAATTTGCTCCCATTAAGTTAGCTTGATTGAGGTTTGTACCGCTAAAATTAGCTTCCACTAAA
TTAGCCCTACTTAAATCGGCACGATTAAGATAGGCAAAGGTAAAATTCGTCTTTTGTAAATTTGC
CTCTCGGAGAATAATACCAATCAAATCACTATTTGTAAGGTCTATTCCTGCTAACATTGCACCCG
TAAAGTTATGATCTCCTGAAGTGTAACGCTCAATAAATTGGTTAATTTCCATAATTTTCCTAGAA
ATAATATTTCGTTATTTTTTATTGCTGATTTCCGATTTATTTTTTTTGGATTGAGTTTTATCGGC
TGATTTATCTATTTTTTGTAGAAGTGTTTAATACATTCTACTAAGGCGGTGGCGGCTTGGATGTT
TTCTTCTACTTCTAAACCTTCAATATGGGCGTTGGCTACTTGCAATAAATGATGTTCTACCAATT
TTAAATCTTTACCATACTGTTGAAAGAGTTTAATTAGCAAGTCATCGAGGTTACAGGTGCGAACA
ATTGACCAGTGTTGCTCATAGGGGTCAATTTGATAAACAGTATAGAATAAAACTATTTTAGCCCT
GAGAGGATTAGTGTATTGCATTATGTCTTGTTTGAGATCAAACAGACTATCAACTTTTAACCAGG
GCAAAACTTGTCCTTCAGGGATGGCACTAGGTTCAACATCTGTACCTTGTTTGATGGATAAACTT
TCTTCTTCTTTGGTGGGATTTGGGCTTTCTTCCACTGACTCGATCACAGAGTGGCGTTGCTCGATC
GCAATTTGAGTTTCTTTGGAAATATCAGAAGACTGATTGGGTTTTTTGGCTCTAATCAATTTCGT
ATCTTCATCTTCATTCCCTTGCTCTCTTATCTCTGCAGGTTCATGTTTATAGAGATAAGTTAACTC
TCCAATGATAGTATCTGCGATAAAAGAATAAAAATTCTGACGGTTGATACTCGCAACAATTTTAT
CAAGGGCTTTTCTTAGTCTTTTGGCATTGGGATACAACTCATGTAATTCTAAAACTAATTCCCTT
AAATCATACATTTCAATAACGTTAATGTCATTTTCCCAATATTGCTTACAAGCGGCAAAGATTAA
CTTTTTAATTCGGGGGGATTCTTCATGATATTCTATATTCTCACAGATGCGATCGATTAACTCTG
GTTCTAGCTCATTCCCTTGATATTCCGATGCGTTAGACACTGTTTGAGCATCTTCTGTATATAAC
TTTGACATTCTTTTATACACATACTGAGCAATTTTTACATAAACTCCTTTGCGATTAAGAGTATT
AACCGCTTGATAAAGTAATGCCTTAAGGTCTTCTATGGTAGGATTACTCTCATATAATACTTCTA
GCAACGATTCAAAAGAATAGTTATTGACGATTGAAAAATCTGACTCCCAGTATCTTTGACTTACA
TAAAAAACTAATTTTTTTGCCCTTTTTGCTTCTGGTAAACGACTTAAATCATCGGCAATACTCGC
AATTATGTCTGACGATGCGATCATAGAACTTCGTCTGGGATAATTTATTTTAACACTTTGAAATT
ATAACTAATACCGTTAATCCTAGCACAGTATGCTTTGATTATAACTTCAGTTGTTCAATAATAAT
ATCATTTCAGAAATTAAGAAGATGGGTTTCAGGTCGTAGGGGTTGAATATATTCAACCCTTAAGG
TGTTGAGGAGATGAGAGTAGAGGGTGAGAGGGGGAAATGAGTAATGAGTTAGGAATTAGTTAGG
AGCGAATGGCCATTCGCTCGTACAGGAGTTAGAAGTTATTAGTTACTAGTTACTATTTATTAATT
ATTCACTACCAACTATTTACTCTAATTTTTGCCAGTGAGAAAAAGTGGCGGCTTGACCACAATAT
TCACCGTTACTGTCAAATAAGTTCCAAAGGGTAATATCTTGTATTAAATATCTTTTGCCTGTACT
AGAGACTCTTACGCCTCCATAGTTGGTGATATAGCCTTTTAAGTTGGCTTCTTTGAGTAGTGCTT
CTCTTTCCTCTCTTGATATGGGTTCTGTGGTATTGCGAGAGGGAGTTTGTATTAATTGTTGCCAG
TTCATTTCCCATAAATCTAGGGCTTGTTGATTTCCATAATTGTAAATGGGATCTGGTTTATTATT

GTGGGATAAAACCACGAAATCAGCATTATATAAGTTTTTGGAGTCTGTTAATAAGTCCTCATTTC
TTTCTATTAATTCTTTATTTAAAAGACGTTTATAACTATTTAAAAGTATTTGTGTCCAAGATATT
ATTTCTGGTTTTTCCCAAATTTGATTATCCATTATTTACATTTGTTTTTAGAAAAAAAGTTAATT
TCAGTTAGTAATTAAAAATAAAAAAAATGGCTCTATCACTTCTCGTCGTGTAAATTATTAGTTAG
GGTAGGCAAGAGGCAAGAGGCAAGAGGGGATTATTAAATAATAATTTATAAACTTTCAGTTTTT
GTTTTACTATAAACACTATTCAATAAAGGTTATGGAAGTCCTGTTTCTCTTAATTTATCATAACC
ACACTCTGAAGAACCAAAAAACTTATTACTTACCCATTCCCCATTGACTACACCAAACCTGAACCC
CTAATGAATAAGATATGATTGTGCTGAACTTAGGTTATTATATTATCGATCGCCGATAGTTAGAA
AATCATTTGCTCCGAAAATAGCTTCATAATGACGATAAAATTTCATTTCCAAAATATTATAAAAT
GGGTCTTGTAAAAAAAAGGTTTGATGTTCTGTAATTTCACCCTTAAATCTGAGTTTTGGTTGTTG
GTATAATTGTAAATATTTTTCCTGACATCTATTTAGCAAATCACTCCAATGGGATTCTTCTGTAA
AAATAATACCAAAATGACGGGGATAAATACTTGAGGGCGGTACTAAAGTTTCTTTGGTAGTATG
TCCTACTAATTGATGTCCATAAAAGTTGAAAATAACAGCGTGTTGATTTTCTCTGCCAATATCAC
AACCTAAAATATCACCATAAAATTTTTTTGCGGTGTCAATATCATTGATGGGTATAGCTAGATGA
AAAAGATATTTATTCATATTTTTTGGTAGTTTTACATAACATTAAAAGCTGTTCCTTCTAGGATA
AAATTAGAGTGGTCTTGCAATATGGTATTTTTTTCGGTTTCACCCACAAATAAGTAAGTACCTAA
AAGGTCAGAGTTTTGAAAACGATAAATGGAATTACCTTGATTGCTACTAGGGGGAATAACATGA
AAAGCAGTGCCTTCGAGAATAAAATTAGGATAGTTGTCTAATATATTATTTTTTTCTTCTTCCCC
TACAAATAAATATGTCCCTGCAATGTTTTGATTCCGAAAGCGATAAATAGTTAATAAATCTTCAT
CGGCGGTTTCACTCACATTAAAAGCCAACCCTTCTAATTGAAATTGAGGATAATTACGCCCGATA
TTTCTCACTTCTTCCTCTCCAGCATACAAATATGTTCCGGGGCGATCGAGGTTTTGCAGACGATAT
ATAGGTGTGTCCATAAACATAGAAAGATCATCAGTACTTTGATTTGTTTCGCTTTCATTGCCTAC
GTTGTTTTCCTCTACGGGGTTTTCCGTTTCATTATTTTCGGTGGGGGAAGCAAGAATTCCCGTGG
CTAAATTAGGATCGTCTGTGGCTAAATCGAGTTTAATATTTACATCACTAATGGTTACTTTACTC
TTCACAATATCATCAGCAGGGTCTTGATCTAAGTTCCCAATAAAACTAATCTCATAAGTGCCAGA
GGGTAAAAATAAAGAATAACCTCCAGTGGAAAAATTAACAGTCTCGATGCGATCGCCTGTATCTA
AATTTTCTGCGAGAATACGAACATTAGCAATGCCCTCCCCAAGACTATAAAAGTTATCGTCAATT
ATCTCATCGGTGTAAATTACCCCCGTTAAAAAAGCACCATCCTTCTTACTGTAGGCAAAATTTTG
AGTAGTCATCACCGCATTATAATCTCTACCATTAAGAGAAAAAATGCCTTCTAAAGAAGCAATGC
CTACTTCTTGAAACTCATCTCTCATTAAACTGACACGATGACCTCTATTGGGATAATTTTCATCC
ACAAAAAGGTTGGTATAGTTATCAATTACAAACTCATTAAAATCTAAATCTCCGGTTGTCCCCTT
CCATGCAATATTTTCTCCACTCCCCCAAGGGGCAATAAATTCATATCCTGCTCTTGCCATACGTTG
TTGCGAAGTAGTGTTATCTATACCCGTATGACTAAAAATATCATTAGCTAACATCCAACTACTAT
GATTATAAGCCGAGTCGATAAGATAATAATTAAACGCTAAGGGTTGTTTAGGAGCTAAGGTAAT
TAGACTATTCGGAGATACCCCCTCATTCAACTGTCCATTTAAGAGGCGATCGGCTTCTTGTTGAG
GATTTGCCCTTGCTACATTGATTAACTCTAACATTAACTGATCAATAGCACTGGGGTCTCTGTTC
TCCATTTTCGTGATAGTTTAAGTTATATTTTATTTTACATTTTAATATAAGTTGATTTTTCCTGA
AATATTAATTACAATATTAGGGGTAAAAACCATTAAGGGGCTATAACTCAGTTGGTAGAGTGTC
ACAATGGCATTGTGAAAGTCAGCGGTTCGAATCCGCTTAGCTCCATTGAAAGCCTTAGCAGATTA
AGTTTGATTTTTTAATCTGGATTGTGGTTTATCTTATATGTGAACTACTACCCGACCGCTATCGC
TGAGTCGGGAGCTTCATCTTTCACAGGTTCGTGCCTCAAAACGGATTTACCTCCGCCTTTTGGTCT
TACCGATCCTCCAGATGCAGAGACGGCTGTTCCATCCGTCTGTACTTTTCTGATACCTTCTGCTCT
AATATTTTTACTTGCGTTCTCATCCCTATCATGATGACTATCACAACTAGGACAAGTCCATTCTC

TTCTCTCTAATGGCATTTCAGACATTTGATAATGACAATGAGAGCAAGTTTTAGAGCTAGGAAAC
CATCTGTCTATCTCCACTAATAACCCACCTTTTTGCTTGAGTTTATAGTCTAGGAAGTTGATAAA
TGTTCCCCAACCGACATCAGTTATTGCTTTTGCTAATTTGTGGTTACGAACCATACCCTTGATAT
TCAGTTGTTCAACGACTACCACTTGATTATTATTCACAACTTTGGTGGAGAGTTTGTGTAGGAAG
TCTTGACGGGTATTGCTTGTGCGTTCGTGAACTTTAGCGACTAGCTTTTTGGCTTTTTCTCTATTC
TTAGAGCCTTTTTTCTTACGAGCTAACTTTTGTTGTTTCCGAGCTAAGTTTTTTTCATGTTTTTT
GATGTGTCTAGGGTTAGCATACTTACTGGTTTTTTTGCCATCATGAACAATAGCAAAATCCTTAA
TTCCTAAGTCTATACCACAAATTTTCCCTGTCATATTTACTAATGGTAATTCTTGTTGGGTATCG
AATAGGATACTAGCAAAATATTTGCCAGTGGGCGTTTTGCTTACTGTTACAGTCTTAATCTCACC
TTCAAATATCCGATGAATAACTGCTTTTACTAAGCCTAATTGCGGAATTTTGAGACATCCATCAA
CTATCGAGACATTTTGGGGATATTGACAAGACTGTTTTCCGTGATATGACTTGTATCTGGGGTAT
TTGGCTCTACCTTCAAAGAAGTTTTTGAAAGCCTTAGTCAGATTAAGAGTCGTTGATTGCAGACA
TTGTGAGTAACAATCTCCTAACCAAGCCGTATCTTCCTCTTTTTTTAGCTTAGGGAGATACTTAT
TAAGGGCTACTTGCTTTAGTGCCTTACCTGTAGCTTTATAAGTGTCAATACACAAGTTTAGAGCG
TAGTTATACCACCAACGACAACAACCCATAACCTGAGATAATTTCTTGGCTTGGGTTTTGTTGGG
ATAGATTCTGACTTTTATCGCCCTGTGCATTGAGGTTTATTGCTAACTTAGGTTGCCAATAAATT
TATTATAAGTAATGATTTTGGTTTTGTGTCGAATCCTACGGATATTTTTTTATCGAGGCACAATT
CATCCGCTAATCCGCTATGCTGAGATTAGGGACTTCTTTTGCCATTTACGTTAAATTTAACGGTT
TTGTGACTAGGATAGGATACTTTCCATCACCCAATCTCTTATTTCCTCGTAACCATTGAGAAACT
TGTGGATTGATAATCACTTTTACCCTGTCAAGTTGTCCAAATTGTCGCGCTTTATGGTAGTGGAA
ATTCTCTTGTAAAGCAGTTTCCAATTTTCGGATAATTTCTTCTTCTTCTTCCTGTGCTTGTTCGAG
TAAAAGACAGTAATGAGGAGGATTTCCCGAAACGGGTACTAAACAACAAAAACCCCATTTTAAA
AAATTATTCAAACATTCTCTGACAAATTCAATATTTAATTTTTCTCCCACTAAATCACTAATCTG
CTCACCCCTGCCGACAAAATTCAAACAGGGAGTATTAAGATACCAATGGGAAACCTGAATGCGAT
CGCCTATTCGATAACGAGACAAGCCCCCCAACTGAGAAATCACTAACTCATAGGTTTTTCCCACTT
CCAATTCTCTTAAATTACAAATCTCCCCTTCCGAAGTTAAAAATTCAAAAACTACTTGATTTAGT
AAAGGTATAAATCCCTTAACGGGAATTAAAGGAATAGTCATCGGTGCTTCTGTTGCCAATAAACC
TTTTCCCTGCAAAAAAACTTTAGGAAAATATTGTTTGAGAATTTCCGCCGAGTCTGCGGCATAGT
TACAATCCCAACAGGAAATCAATTTTAATTCAGACCATAATTCAGACCAATTAATTTCACTTTCT
CCTAGTAAACTCAAACGTCTTGAACTTATCTTATTGACTAAACAAGATTGTAATTGTCGATTGTT
TGTCTGAATAAAGTCTAATTGAGTGGTTAAAAAACTAGGACTCCAAAGAGAAAAAGTTTCTAAA
TCAGGGGCTTGAAGTAATGCACAAGCTAAACTCCTACGAAAATCCTCCACTGTGGAAAAATGCCC
TTTTATTCGCAATAAAAACTTCCCTAATAACCATTTTAGTAAAGGATTAAGATACTTAGAATCAT
CAATTCCTTGCCCAGTTGTACCAATTTCAGGGGAAATGCAAAGATAGGTTTTACCACTGGAAAAA
GCCGGACCATTACTAATTAAATCATAAGCCCAAATACAGAACATATTACTGAAACTAGCTAGGAG
TGAAGTAGTATAAGGAATCCATTTTTTTGATCCTGTACTTCCGGAGGTACATTCCCAAAAATGTA
TTTTTTCTGGTGTTATAATCGCAGAATTTGGTTGTTGACGTTGTTGTGCAATCCAAGGTTTTAGA
GAGTCATAATCTACAATAGGAATGTTTCCCCAATCTTCTAAATTTTTAAGTTTTAATTTAGAACC
ATAGGCGGTTTTAGTTAATTTTTGAATAACTTTATTTTTGACTTTATTTTGTGCCTTTTGAGAAT
CTTGCAAATCTAGGTGAAATTTTTGATAAAAGGGGAAAAATACGGCACTTAAACCCTTGATAAA
AAGTTGGGACATTTAGATTTTAATTTTCTTTTACTTGAGTAGAAAGAGAGTTATCATTTTCTGTT
TTGTTAGCTTTTAATTTAACTCGGGGATAAAGTTTTTCTCCATAAGGTACAAATGCTTTCTCTTC
AATCTCTACCCCGGGGGCTAGTCCCACATCTCCACTTATAAAAGAATAGTTTCCAACTTTTATTTT

TTTTGTAAAAACTATCAAGTTATCTTTTTTTGGTTTAACATTGTGTGAACAAAATACGGTGCGAT
GACCAATAATAACCCCATCACCGACTTCAATCAAGGGGCGATCGAGAATAGACACAGTCGGAACC
CAATAAACATTTTTGCCTATTTTTGCTCCCCATAAGCGTAACCAAAGAGAAAATAATCCGGGTAC
TAAGTGTAAAATTTTTTCTAAGAAAGAGAAACTATTATAAATAATTTGAATTTGCTGAGTCCCCC
ACCAAGGATTATATTCTTTGCCTTTCAAATAGCTAGTACCTTCAAGATTAGGATAAAAATATTGG
TGAATACGATAAACTAATAAAGGAATAATATACAAACAAATAAATAGTGTAAATAAAGGCAGAA
AACTTGGGTTAATTATTAACCAAAATAAATTACTGAAAACTAAAAGTAAAATAAGGGTTGGAAA
AACATATAGTATTTTGCTAAGTATTGTCATGACCTGAGTTCGAGATAAATTTTTATAGAGTGATG
ACGAAAAGAGCAAAGGGTAAACCCCCCTTTATCCCCAGAGTTTTTTCAAATTCAGGGCAAAATTA
TCTTGCCCTCACCCCTAACCCCTCTCCCACAGTAAAGGGGAGCGTTTTTCTTAATAATTGATTAA
TTAATACTTAAAAACTCCTGTATCTGTTACTATTTGTTAACCTGAGTTTTGGATAAGCTGAAAGC
AGACCAACTCGTAGTCAAAAAACCTTATAGCTTCTTAGAAATAACGATAAAATTGCCTTAATCCG
AACTGACGTTAAATATATTCACCCCTACCACCTGCTGTTCCTGCAACCTGACACCTGACACCTAAC
CTTATCAGATATTCTTAAACCGAACTGAGGTTATTTGTTAGTTTCAAAAATTGACATTTTTGAGA
ATGAAAAAACCCGGCTCGAGACGGGGGAGGGCAAAGGTAAATAGTTGATAATTAAGAATTAAAA
ACCCCGTTCACGACTCCCCCTAAACACCCTGACACCTAAAACTTGACACCTCATCCTCCCAACGCC
TTGATACCTGATACCTGACATCTCTTTCCGAACTCTCAATCTTCTTTATTAAAGGGAGTAAAGGG
AAAAAATAGCTTTTGAACTATATTGATATTTAAAGTAATTCCCATATTTTCATCGTGGTAGTTGG
GTTTGATATAAGTGCCATGAAAAATATCCCAAAGTTTTAAGTTAGCGGCAAAATTTCCCAGTTTA
TCTCCACAGTGGTGATGGGCGTGATCTTGGGGTAAGATAAGCCATGGATTCAAGATTCGGTGAAT
TATATTATTTTCAGGCAAATTCCACGAACTATGTCGCCATAAATCTAACAAACAAGTCAACCAAGA
AGCCGAAGGCGTATCCCCGATAATCACTCAATAAATATAACATTAAACTATTTAGCCAGATATAA
GGAAGGAACAAACTTGACCATAAAGTGTTACGAGAAGTCCCTAACATATCCATTTGAGTTACGCT
GTGATGAACTGCATGAACAGAAAATAGTATTTCATGAAACAATAAGCGGTGAGTCCAATAATAA
ATATAGTCAACAATAACAAAGCTAATAATAAAACCTAACCAAGGTTCAATGATTAGACATCCTTG
GCTCTGTGGTAAGATTTTTTGATATAAATGTGTTGCTATTCCTATTTGAAGTAAGGGAATAATTG
CTCCCTGAAAACATAAACCGATGCCATCTAAAAACCAGTCATGTAGAGACTTTGTAATTAATAAC
TTTCTTTGTTTGCTTTGTAATAGGGTAGTTGCAAAAAATAGCCAAAATATAATTATAGGAAACA
TCGGTTAAACCCAAAAGGTAACTTCAATGGTTTTATCTGGAAGGGTTCGGGGATCTTTCCCCTGT
GCGATCGCCTTCCTAATTGATTCTACAAAAACATGAATGGTATCTGCTTGAGTGTGGGCGAATTT
CTCGCCATATTTTTCGATTACTTCTTGCTGAATTTGCAAAATCTTCACATCTTGTCCAATAATTCG
TTTCGCAGTGTACCAAACAAAAGGTGTTGCTAAATAAGACCAAATTCCATAGGAAAAAGTGGCAT
CGGTATAGACTAAGGTGGAGGTTTCCGTTTCAGGTACGGATTGACTTGTTATAAATAGGTGACGG
CGAGGAGCAAATTGATATTCTACGGAAGTAATATTAGGCATAAAAAAGCGATCGCTGTGATAAA
TCTTATGGTGACTAGGGTTTAAAAACCGTGTCCACCAGCCCAAATTAGTATCCTCATTGTGATAG
TTAGCGATCACAGTTCCTTGAGTGCGGGTGACAGTCATTTCTAATTTTTGTTGGTAGGATTTTCT
AAAAATTCCCGGATGTACAGAAACTGTGTGGGGAATATCAATAAAATTTTCAGCACAATTAGTA
ACATTATTATTAAAACGGTGAACCACCCGTACATAATGCCAACCGGGTTGTTGATAGTATGGCAT
AGAAAAAGGGGAAAACTGTGGCTTGATTTCTTCTGTTAAGCAGACATAGATAAAACCATCCTGCT
CACAGGTATGATATTGTTTAGCTTTTAAACTTGCCCTTGGTTTAAAATTTTCTCCTTCAGAGGGA
ATAGAAACAACTCCTCCACTTTCATCATAAACCCAACCATGATAAGGACATTGTAAATACCCTTG
TTGGACTTTACCACAAGATAAACGACTATTGCGGTGAGTACAGCGATCTTGTAACGCAACTGGTT
TACCATTCTTTGTTCTAAAAATTGCCAACCATTCCCCCAAAACTTGACGACTTAAAACTTGATTC

GATTTTAATTGTTTACTTAGAGCGACTACATACCAAAAATCTTCAAACTTCATAAGTTTTAATGG
TTATATCTTGAGTTAAATCCAGTTGACAAGCTAAACGGACTCTGGGGTTTTCAGGGGCTAAAATT
TGTAATACTTCTTTTTCTTCTTCCTTTGGAGGAGGAATATTCCCGATAATTTCGACTAAACAAGT
ACCACATAAACCTGTACGACAACCAAATAAAATAGGGGAATTTTGGGCAGTTAAATGGTCTGCTA
ATGATTGGTGAGCAGGAAGTTCAAGGGGGGGAAACTCATTATTGGTGAAAGATATTTTACAAAG
CATTGGGTTAATAAATAAAAGGGATTTAAGTTTTTATTGACGACTATTTTGTAGTTGGGTAAGCC
TAAAAAATTTTGCTAGGGCTTGTTTTTGAGTTTTTAAATATTTCTCGAACGAGTTGGAAGCCATT
ATTTTCATTGTTTTATTTTGGGCAGAAATAAAGTCTAAAGGTTCTAAATATAATTGATAGGATG
AAATTGCTTCGTCATGGGTTTGAAAACTTCTGTGTAAACCTTCTGTTTCTTTCGTAAAGCAATCC
TTGAGCATGGCTTTGGCCTCTTCCATAGAAAATCCAAATAGAGGCGATCGCATTATTTTTAAGAT
CGCACCATAAAGAGCAGGCTCGTACCAAAAAATACCATTGATGGCAACGGAAACATGATAATGAT
CTTTTTGACAACCCCTGATACCTAAATTTGTTACATAGCGTTCAAAATATGTGGGGGGTTTTAGG
CATTTACTAACATCATGAGACAAAATAGTAGAAGTGTTAAAGTGAAAACTCTCATCAAGAAAAT
GATGATAGGATATTTGAGCGGGGATAGGAGATTTTTCTTGGTTTGGATGTTGGAGATGAAAATT
ACTTAGCCGATGTTGAACTAATTTACCGTTTAAAGTGCGAATACCCCGAACAGTTAAATATTGAC
AGGCTAAAAAAGTATTTCCTGCGGAGAGTAAGCCGAAACAGTGTAATTGTAAACTTTTCCACCAA
TTTTTAAAACTATTGGTATCAGCAAAAATCATGGTTTCTCGAAAGGGCGATCGCATCGAAAAACT
AAATATTCTTTCACCAAAAAGTTTGTTTTCAACTTCATCTATTACCCTACGAAAAGCATTAATGT
GAGAGCGTTCTTGACGGGTTTCTAAGTCTAAAGTATCACAAATTAACCGAAAACCCTCTATAGAA
TAAAGGCCAGTTGCACTTGTTTGATTAAAAAAAATGGTAGCAATTTCGGCAGAAATAATTTGAG
AATAATAAGCCACCCAATAAAGTTGATTTAAAATTATTTTTTGAGACTCACTAGCTTGTTCCCAA
AAAGGTGTACCATAGAGCAGAGAAAATTCAGGAGGATTCCAATAACAGTTTTTGCACTCTTCATA
GTTAAAATTAGCCGCCAATTGATCTAAATCTGGACTATGATCTGTTTGTTTTGCCCTTTTATAAT
TTAACTGTAACTGGCGATAAGTTTTAGATTCTGGTGTCAAAGAAGGGCGATTTATTTCATTAGTG
GAACACTGTAAAGATTTCATAACATCATCTTTTATTTTTTTTATTTTTCCAAAATTATCTAAGTA
GATCCAATTAAATAAAAGAAAGTTCGTAGTTACCCCTTTAGGGGTTTCAAACGTTCTTAAAACAA
CGGAGGGCTTCAGCCCTTACTACTAGCTGAACCTATTTTACGTTTAATTAAGTGGGGCTACTTAA
TACACTGTGAGGGACTATAAGGATGAAAAGAACCTTGTTTTTCTAAAGATTCGATGATTGAATCA
GGCACAGTTCCCACTAGAAGCGATCGCAATAAATCTAACTTTTTACTACTATGCTTTTCCGTTTCC
AATTCAGTTAAAATTTCCACTTTATCATTTGCCGATAAATAACCTAAAACCCTTTCAATATTGAT
TAATAATCTCTGGGGTCCAACTTGTACCATATGCAGAAAATAAGTTAACGCAGAATAAATATTCC
CTAAACTTTCCTGACAATAATCAGTAGAATTTTTAACAAGAGTCACTCCCAAAGCATGATGACGA
GCTTCCGCATCCAAAAATCCCTGCAATACTTCCCCTAAGTGGGGATTCAAACAATACTTTGCCAA
AGAGCGATAATGACCCATACCCCAGCCCTCTAAAACCACTTGAACCAAGACCATTAATAACAACT
TATCATCTGATTCTATTGCTTTTGCCATATAACTTAAAAAAGTGTCCCCAGAAAAAACCGGTACT
TCTTTTAAAAAAGGAGTAATAGTTGCTAAATGAGTAGCCTCGTCCGCAGAAAATAAGCTGTATAA
AAGTCGTTCTTCGTGGGTTTGAGCCAGAATCACCATTTTCGCCATATAACCAACCCCCGCCTGTTC
CACCCAATAAATTTCCTCTAGTAAATCCTTGTTGGCAAATTGAAGAATTTGTTGTTGCTGTTGAG
GAGTTGCTGAGCAGAAATATTTAACCTGATTTAAGCCGAAATAATTGGCATCCCAAAATAAAGAT
TGAGGTTGACAAGAAGTACAGATATTAGCCCTTTTTAAAGCTGATGTAAGCAAACGTTGGGTTTT
AGATTCTAAATCGACGTGATTTAATGTAAAACCGCTAATTTTGTTAGAAATTTCCGACATTTTAT
TCACTAATTAGACTAAGGATTATTTGTTAAATGAGAATCAATCCCCTCGGTAATCTGTTCCCAAC
TTCCCCACTGTAGAACCGTTTGATGTTCAACATGATCAATAAATTCCATAATAGAATGATCAGCC

CAAACGGGGGTTTTTAAATTAAACTTAATAGTTTTAAATATTTGTGTATCACCCTTGGCAAAATA
TTCGCCCACTAAAATTGTGATCCAAAGTATAAGTTTATTAAATACTGCTCCAAAAAATCCTGAAC
GACGTTTTGTCATTGATACCATTGCACCTTCAGTTTTACCATTTTCTAACATTCGTAACGCAAAC
ATAATATAAAAATGCAAAAAATCTGGTCCAAGAGTTACAGTTCCTACAGTGCCATACCAATAACA
CATACTATAAGTGCCTTGTTCCTTATAAAAAGGTTGAATTAAATTAATTAACCAAGAATCTTTGC
CTCCTTTGGTAGTGTTGCTAAAAGTAATAACACTAGGACTAATAATTTCCGATTTAAACTTAATT
TCTACGGGAAAATTATGTACTGTGTTAAAGTGGTGGGCATCAATGGCATTAATCATAACCACATT
GGGATGACATTCTTTGACAAAGATTTTACTTATACAAGTAGAAACTTCTTCCTTTTCTAATTCAG
GAGCAAAAGGAATCGGTAGCTTCACTTCATTTCCAGTCCATAACCAAATCAAACCATAAGCCTCT
TGTACGGGCCAAGTATTGACTTTCAGGGGTAATGTTTTGACCAAGCAAGGAATTTCTACACAACT
ACCATCCGCACTAAATTTCCAATTATGAAAAAAACAGCGCAAACTTTCTCCTTCTACTTTACCTTC
GGCTAAATGAGCTCCCATGTGTGGACAATAAGCATCCATGGCAACAACATTTCCTGTATAGGTAC
GATAAACGGCTAAATCTCGTCCCAACAAGGATACCGCTTTAACCTTATTTTTTGGTAAATCTTTC
GAGGGTAAAAGCCAATACCAACCTTCTATAAAGCGTTGAGGATGATTAAATACTCGCTTATTTTT
TGTTTGTTGTATCATCAGAATTTTGTTTATTAATGGAGAAAGGTATATGTATGGAATCAGAACTT
CGCCAAATCCTTAATCCTGCTTTTGTTAAATGAGTTTCAGAGACTTCTGCTAAACAGACTAATTC
ATCTCCTTCATTATGGCTAGGGTTTTTTCTGAGAAAAAAAGCGATATGGGAATTTTTTAATCTTT
CATCGGGGATTTCTTGCAAATGAGATTTTAAAATTTGAGGTTGTGGAAAGCGAACAATTCCAGTG
GATACATCGTAGTATTCACCAAATTTCTCTTGAGCTAAATTGTGCATAAATTCGGCAATTTCAGG
GGGTGTATTTTGTGCATAACAAGGATAAAATGTTTTCCAAAAAATACTTAAAAAGCGATAAGTT
CTAAACCCTGAAGAAATTAAAAGCCAATATAATTTTTCTTTTCTTTCTTGATGAATAGCTTTGAC
TGAAGCAACCCAAGCCCTTGCCAACGCTGGACTTGACCACATGGAGGGATCAGTAATTGTGTCTC
CTGAATAAACTATTCTTACGGGTTTGTTGTGAAATTTTGCATCATAAAAATTTATTGTTGTAAAT
CCTTTTAAAACTAGATTTTCTTTTAATAAAATAACCCAGTCTTTACCACTTAAATCTTCTTCAAA
TTGTTGACGATTTACTCCTTCAAAATGAGTTTTAAATAAAGAAAACATCTCGTCTTTTTCTGAAT
GCAGTTCTTCCACCGGTAAAAGTATTTTCTCAATAGACATCTTTAATTTTGAACCGATAAATATT
TATTTTGGTTGCATACTCAAAAAGTAGAGCAGTTTACTATATTTTTTTAATAAACTTTAAGAAAA
TTAAAATTTGCCAAGTTGTCAGAGATATTTTAGAGATATTAAAGACCAAATCAAACAAGTTAGA
AATTGCATTGACCCAATGTGAAAATGCAAAAATGTCAGATTTTGTTTTTGTTAATGATAAATATT
AAAAAATCACTGGTTCTTCGTTAAAATGGGAACTAAATAGAATTTATTTAAAATATTGAGAGCG
GTAAATTAGCTAATTGGTGGTGTTAGGAATTTAACAGTTAAGGTGATGACAGCTAGGAGTAATC
CACTAATAACAGTAATGAAGATACCCCTGTTGGTAAATTCAAGGTTATCAATTCTTTTATTGGTA
TCATCCAACCTTTTTCCTAATCCGTTAACATCGCTTTTAATGGTAGCCACATCTACTTTTATATCG
TTTAAATCTTTAGATATGCTCTCGAATTTAGAGGTAATTAAATCCTTTAACTCTTTTAAGTCATT
ATCCGTTAATGCACTCATGTTAAAATCCTCAATAGATAAAAGAACTGAGAGGATAGAAGTATTCG
CCTTACCCTTATTCCTTGCTTTTATTTTACACTTGTCAACTAGCCTTATCCTGACAGATTCTATAA
ACTACATTAAAACTTAATTCCTTGCCTTGTTTAGTGGGAATACTTTGGGCATTGAGACAATCAAC
TATTTTCTGGTAGCTTTTCCCAGAATATATGATGTATTTTAACAATCTTTGTAACTAAAAATTTT
TATCTTATCCACTGAAGACAAAAACATCATTTACAATATTGATATTATTGCTAACAATTTAGAGC
TATAACTTAATATTCTCCTGATCACCATGACTCAAACTCCTATTTCTTCTGCTTCTAAAGAAAAA
CTAAAAACAGCCTTAGTCAGTCCAGATTATTTCAATCCCTTTGGAAATAAACTAATTCAAGCAGG
TTACATTGATAAGGAAGATTTAAAAAGGGCGTTGGTACAAGTGAGAAATACTAAACAGCCTTTA
GTAAAAATCTTGGAAAATATCACCCACAAGGCTTTGTCTCCTGAGTTAGTGCGACAGTACAAACA

ACATCAACTTTTTGAATTAAAAATTCTTTATGGGGTTGATTGTGTTGACCCAGAGTCAGAAGAAA
TAGCAGCGGATCAAATGACCGATTTAATCGAGTCTATTATGGGAGTTCAGGTTTGTCGTCGTCAT
AAAGTTTTACCCTTGCGCCGCATTGAGGGTGAACCGCCTGTAATTGTTGTTGCTATGGTTAATCC
TGATGATTTACAGGCGTTGGATGATGTCAAAAAAATATTACGAGCCAAAGGCCTTGACTTACAAA
GAATTGTTATTACTGAAGAAGATTTTGATAAGTTAATTAATAGGTTTTATGAAGAAGATAAGCA
AAGACAAAAAAGAGAAGAAGAGTTAAAAGAAAAGCAAAAATTAGATCAGTTAGGGGATGTTAGT
GATATTGTTGATGATTTGGATCATGATTTAGATGAAGCTGAAGACGAAAATCCAGATGGTGATT
TGACTTCTAATGATGCTAATCAAGCCCCTATTATCAAGTTGGTTAATAAAATTCTCATCAAGGCT
TTACAAGAAGGGGCATCAGATATTCATGTTGAACCCCAAGAAACCAGAATGAGGGTGCGTTTGCG
CAAAGATGGGGTTTTAAGGGAATATTTTACTCTTCCTAAACATATTACAACTGCGGTGGTAGCTC
GTTTCAAAATCATGGCTGAGTTGGATATTGCAGAACGCCGTTTACCTCAAGATGGGAAAATTCGT
CGGGTGTTTCAGGGAAGAAATGTGGATTTTCGAGTTAACTGTTTGCCCAGTCGTTACGGTGAAAA
GGTCTGTTTAAGGATATTAGATAATTCGGCTACTCAATTAGGTTTAGACTTTTTAATTTCTGATG
AGGAAACATTGGCGAAAGTTAGAGATATTGCCAGTCGTCCTTTTGGTTTAATTCTGGTTACAGGT
CCTACGGGTTCGGGTAAATCCACAACTCTTTATTCTGTTTTGGCGGAGAGAAATGATCCGGGAGT
GAACATAAGTACGGCGGAAGACCCTATTGAGTATGCTTTAGAAGGAATCACTCAGGTGCAAGTAA
TCCGAGAAAAAGGTATGGATTTCGCTTCAATTCTTCGGGCTTTTCTCAGACAAGACCCTGATATT
ATTCTGGTGGGGGAAACACGGGACGCTGAAACCGCAAAAACTGCGATCGAAGCCGCTTTGACTGG
ACACTTGGTTTTAACCACTCTACATACTAATGATGCGGCAGGTGCGATCGCACGTTTGGACGAAA
TGGGGGTTGAACCCTTTATGATTTCTGGTGCATTGTTAGGGGTTTTGGCACAAAGATTAATGCGA
AGAGTTTGTAGTGAGTGTAAAATTGCTTATGTTCCTACTAAAGAAGAATTATCCCGTTTTGGTTT
ATCCGTATCTGGAGAAGAATCTGTTACCTTTTATAAAGCGAATACTTTGTCAGGAGAAGCCCTAC
AACAAGCCCAAGCGAATGGCACGGTTTGCCAAAAATGTGGTGGCGGTGGTTATAAAGGGCGTGTG
GGTGTTTATGAGTTTATGGTTATGAGTGAAGAGTTGCAAAAACTCATTAACCGAGGAGCAACCAC
TGACATGATTAAAGAAAAGGCTGTTGAAGAAGGAATGGTAACGATTTTAGCCTATAGTTTAAAT
CTAGTTCGTCAAGGTCATACAACCTTAGAAGAAGTTGAAAGGGTAACATTTACTGATTCTGGTTT
GGAAGCAGAATTGAAGGCTAAACGAAAATCATCTTTAACCTGTAGTGGCTGTGGAGCAGAATTAC
AACAGGAATGGTTAGATTGTCCTTATTGTATGACTCCTCGTTTCAGTGAGTAGATTTTACAGAGC
AATAAAATTAGGTTGCAGGTTGCAGGTGTCAAGGTTTTGGAGAGATGAGGGGAGATAGGGAAAT
CGAGTATTATTGGGGTATTAGGGGATTAGGGTGTTAGGGGGAAACCAGAGAACGCTCGAGCGCTC
GTTCCGCAAAGCGGTACGGAGTTAGTTAGGGGCGAATAGCCATTCGTCCGTACAGGAGTTAGGAG
TTATTAATTATCAAATATTTACCATTGCCCTTTTAACTTTATCTCTTGCCTCTCTCTTGCCTATTA
TCCCTTGCCCATCCTAACCAGATATTCTTATATCGAGCTGAGGTGATTTGAAATAATCATGCTAT
AATTCATTAACTTGAAGAATTAGTGGGACTTTTTGTGTTTAAATTTATAGTCTAATTGTTCTTAA
ATTAAAAATAAAAATATGTTTATCTTTCCCTAGAAAAAATGCCTCAAATTTTAGTTATTGATGAT
GATCCAACGATACAAATTTTGTTAAAAAGAGCTTTTTCTTCTAGTGGTTATAGTGTTCAAATAGC
TGGAGATGGACAAAAAGGGATTGAATTAGCCGAAAAAATTAAACCCGCTTTAATTATTTGTGAT
TGGTTAATGCCTAAACTATCAGGTTTAGAAGTATGTCAATTTGTCAAAAAAAATCCCCTATTAAA
TACTACTTTTTTTGTCTTGATGACTTCTCGTGTTTCTGTGGAAGATAGAATTCAAGGATTAGATG
TAGGGGCTGATGATTTTATTTGTAAACCAATAGATTTACAAGAGTTAAAAGCAAGGGTAAGAGC
TGGTTTAAGATTACATCAATTAAGTAAAGATTTAGAAATTCAAAAACAACTTTTAGAAACAGAA
TTGAATGAAGCCGCCGAATATGTTTCTTCTTTATTACCAGATTTTTTAGATGATAAAAATATCTT
AATTAATTATATGTTTATTCCTTCTTTGCATTTAGGAGGAGATATTTTCGATTACTTTTTTTTAG

ATGAGCAACATTTAGTTTTTTATTTATTAGATATTTCAGGACATGGATTAAAAGCGGCATTACCT
TCTATTTCTATTCTAAATTTACTGCGTTCTCGTCGCTTAGGAATATTAAATTATTATTCTCCTTG
TGAAGTTTTATATCACCTCAATAATAATTTTCAAATGTCAGAAAAAAATGATAAATATTTTACT
ATTTGGTATGGGGTTTTAAATGTTAAAACTGGTAACTTAACGTATTGCAGTGCAGGTCATCCTCC
TTGTGTTTTATTGTTAGAAAATAATCGAGAAAAAATGAACCAAATCTGTTTAAAAACTCTTGGT
TTGCCTATCGGAATGTTTCCTAATATTGCTTACCAAAATGCTTCGATTAATGTTGGTTCTTCGGC
AAATTTGTACTTGTTTAGTGATGGTATTTATGAAGTAAAAAATGACCAAGGAGATATATTAGGG
TTAGATAATTTTGTTGAATTGTTATGTCAATTTAATAATGAGTTAAATCCTATTTTGAATTTTTT
AAAAACTTATCACAACTCATCTAATTTTTCAGATGATTTATCTATTATTCATTTGAAATATTCTC
AACAAGTTTTACCTAGTTAATCTTAATTCGGCTTAAGAATATTGAATAAGTGTAGGTAGGCTAAG
ACGTATTTAACTTATATTTTTTTGTTCTTCTACAGTGGTTATGATGAATTAAGAGAAACAGGACT
TCCATAACCTTTATTAAATAGTGTTTATAGTAAAATAAAAACTAAAAGTTTATAAATTATTATT
TAATAATCCCCTATTGCCTATTGCCTCTTGCCTTTCAGCCTACCCTAACTAATAATTTACATACTC
AAACTGATAGAGCCTATTTTTTTTAATTCTTGAAAATAACAACTTCAACGCTTACCTATTGTAAG
GGTGGACTTTCCCTATTCCCTTTCTTAACCAAACAACTTAAATGTAAACTAGCTTATCAAACATC
TGGTGGCAGGTTTGAATAAAAATTAAGGGCAGTATTTCCATATATTTTTGTCTGCAATAAATCAA
GGTTTTGTTTTTGTTTAATGATTGATTTTTTAGGGTCATATTCTACTGCTATTTGTCCATTTTTT
GCTAATAATTCGTATTCTAAGATTAAATCTAAAACAGTATTATATATATCTAATTCGTAGGGAGG
ATCGAAGTAAATACAATCAAATTTTTTTCCTTGTAAATTTTTTAGTCTTTTTAAAACATCGCCCC
TTAAAACTCTAAAATTTTGATCATTATTAGCTACTTTTTGCCAGTTTTCTCGGATAATTTGACAA
GCAGAAGAAGATTTTTCGATTCCAACAACTTCTTTTACTCCTCGACATAGAGCTTCCGCCCCCATT
GTACCATTTCCTGCACACAAGTCTAGCCAAGTTGCATCTAAGATGCGATCGCACCAAATATTAAA
TAATGCCTCTCTCACTTTTGCGGTGGTAGGGCGAGTATCCCTTCCTGACAAGGTTTTTATTTCTCT
GTTACCATAAATACGCATCTTTATATCTATCAAATATTCCTTAACCCTTATAACTCATAACTAAC
AAAGCGTTAATGATAGCGGCGGCTACGGTAGAGCCTCCTTTATTACCCTTGATTTGTATTTGAGG
AATGCCTAACCGAGATAGATATTCCTTTGATTCTAAAACATTTACAAATCCCACTGGCACGCCAA
TAACAATCGAAGGTTGAATTTTTTGTTGCTCAATCTTTTTACATAAAGCTATTAAGGCAGTTGGA
GCATTCCCAATTACATAAATAGCATTGGGATAATCAAGGCAACATTTCAATAAACCCGTTTCCGT
GAGAGTATGACCAGATTTGGGGATTTTAGCTTGTTTTACGGCTACAATAACTTGATTTTGGTAAG
TTTTCCCCACCATATTTTTAATACCTTCTTTTACCATACTCACATCGGTGACAATGGGAGTTTTAT
TTTGTAAGGCGAGTATGGCGGTATCAATGGCGTTGTTACTACAATAAAATAAATTTAGGTAGTCA
AAATCAGCGGTGGTATGAATAATACGGCGGACAATATTATACTCCTTTGCAGACAAATCATGATT
GCCAATTGTGTTATCAATAATAGCAAAACTTTTTTCAATAATAGGATTACTTAATTCTGTCAAAA
TAATCTTTAGTTACCTACTTTTATACTAGCCCAATTTTGTTCTCTAATAAAATTAGTCATATCAG
TAAATTTTCTTAATTCCCACTGGCTTAATTCAAAAGTAGGAAGATTTTTTGATACTAATTGCTCG
TTTGTTTTTGTGATCACTAATTCTACTTGTTCTCGGTCAATATTATCTTCTACTTTATCAAAATA
ACCTAGGGTAATATGTCCAACAAAATCATATTGTTGCTCAATTCCTAGTTTAATAATTTGCTCGT
TTTGATAAATCAATTGTCTCAGTTTGATGATAGGATTATAACTATCTTCCGTTGGTGCTAAACAA
ACTGCGATCGCACGTGGAAATACGGATAAGCCTAAAACTTCTAATTCTAAGGTGTCAATTTCGGG
ATTTAATTGAGCATATTCCTTAAAAATTTTATCAATTTCTGATATTAATAAATTATCAAAATCTG
GATTTTCTTTCACCGCATTAGTATAGCTAGTATCCCAAATTAAATCTGCTATAGTTAAATGGAAA
CTTTGTGGCGGTACAGGTAAAAATAAATTTTCTGGTAATTCCTGTGTAATTTGAGTTTGAATTTC
TTGTAAATAACTATAAAATTTTTTGTTATTAGTTTCTTCCTTCTCAGGAGGAGTAATAACGCTAA

AACCGGGAAAATCTATGGCTTTACCATCGACAAACTTTGGTGATGATTGAATGTTTTTAACTTGT
TGCTTGTAGTTGCTAGGGAGAGTCATTTGCACAACTCTGTTAATATAATCTTGATAATTGCCATC
CATAATTTTTATATTTCTCTTATCGAAAAATTAACAATCTTATTTTTATTGAAATATTTTATATA
AATTATATTATAAAATTAGTTGGTTTTTTCTGTCAATAAATTGATATATAAATCAGGTTTGAAC
AAGTCAAAAAAGTCACTAATAAAAATAATAATCAACTATTTAAAAATGAATCAAAGATATTTTG
TAGCTTTTAAATAAACAGAAATTCAAACTTATAATTAATAATTCATTTGGGTTCTTCTACAATGG
TTATGATAAATTAAAGGCAAGAGGCAAACCCTACTTTAGCCCCAAGGTTTTTTCATTCTCAAAAA
TGTCAATTTTTGAAACTAACAAATAACCTCAGTTCGGTTTAAGAATTTCCGATAAGGTTAGGTGT
CAGGTGTCAGGTTGCAGGAACAGCAGGTGGTAGGGGTTTTAGCAAGGGGCTTAAGCCCCTTGTT
TAAGTCAGTTCGGATTAAGGCAATTTTATCGTTATTTCTAAGAAGCTATAAGGTTTTTTGACTAC
GAGTTGGTCTGCTTTCAGCTTATCCAAAACTCAGGTTAACAAATAGTAACAGATACAGGAGTTTT
TAAGTATTAATTAATCAATTATTAAGAAAAAACGCTCCCCTCTACTGTGGGAGAGGGGTTGGGGG
TGAGGGCAAGATAATTTTGCCCTGACATTGAAAAAACCCTGCTTTAAAAGGAGAGATAAAGAGG
GGTTTCCCAAATCGGCTACCCTAAATTGATAATTTACACAACAAGAGGCAAGAGAGCCAATATTT
TCATAACTTAGTCATAGGTAAACCTTTCAATAATTGTTTGATGTTGGGGAAAAATTTTAAGTAAG
TCTTCTTTTTTGCCAAGGGTAAAATCATTAAAATCAAAACCCGGAGCAACAGTGCAACCAATTAG
ACTATATGAATTAGGTTGACTCACCGCCGCCGCAAACCAACTATTTTGAGGAATTACTAATTGCA
AAACTTCATTTTCTTCGGGATTATTTCCCAGTTTTATTAGTTGATAGTCTCCTTGTGAGTTGATA
ATATGAACATCTAAACTTGAGCCAGAATAAAAGTGAAAAATCTCATCACTCTTGAGAAGATGAA
AAGCTGAAAACTCGTCATTAAGGAGAAGATAATAAATTGCAGTACTAGCATTTCTTGCACCGTTA
TATTTAGCCAAAAAATTGTCATTATTAACCATATCTAAACAACGATAATTTTCTCGATAGTATCC
TCCTTCTGGGTGTTTTTGAAGGTCTAGTTTGTCGATCCAATATTGAGCATTTTTGGTCATAATAC
AACAAGTCCAATCATTATTTGACAATAATTTAATCAATTAAAAGTTTAGTGTTAGGAGTAAAAG
AATTATCAATTTTTGATGATAAATTTTTAATTCATTAAAAACCTCTTATCTTTTTCTTTTTGGTG
GTTGACGGGGTTAATTTTCCTGTTTCAATCCAATGAATATGTTTTTGAATTAAATCAGACAATTT
ATAATTTTCAACAATAGTTTGCCTAGCTTTTTTTCTAATTTTAGCCATTAAATCCTGATTGAATA
AAGCATATTCTACTTTATCTGCGATTTTTTCAGGTTCAAAAAAAGGTACTAACAAGCCATTATAA
TTATCTACAATTATTTCTTTAACAGGTTGAGTATCAGAAGCAATTACCACGCAACCCGTTGCCAT
TGCCTCTAACATTGACCATGATAATACAAAAGGACGAGTTAAATAAATGTGGGCAGAAGAGGCTT
GTAAAACCTTAAGATATTCACTGTAGGGAAGTAAACTAGTAAACGAGACTTTGGTTAAGTCAAG
AGGGAATTTTGCTAACATCAAATCTTGATAGTTTTGCCCCTCTGGTAGCTGTTTTCCATAAGCGA
CTCTATTTTGTCCAACAATAACAAAATGAGACTGAGGACGACGTTTTTGTAAAATATAAATAGTT
TCTATTAATTGGGGAAATCCTCGATAAGGCTCCATGCCTCTGGCTACGTAAGTTATTATTTCCGC
TTTATCACTCAAGTCTAAGTTAAGGCTAGGAATAACTAACTTAGTGTCAGCATTCGGTTGAAAAT
AGTCTGTGTCAACACCGTCATGTAAAACAGTTATTTTGTTATGATATTCCCTTGGAAATTGTTGT
TTTTGCCATTGAGTGGGAGATAAGCCTCGATCGCAGCTATATAAATCGATTAAAATGGGAGTATT
CTTCACCCTAATTCTAGCTTGATCGTCAGCAGTTAAAGGTTCATTGGGGTCAAAATCAGCATCTG
TGCCAATGGCGTGGTAAAACCATTCAAAATAAGCCAAAAATAAGGCACGGGGAAAAATATCCTTC
ACAAATAAAGTTGAACCCCAACCAGAATGCCCATATATAATATCAGGGTAAAATCCTTTTTGTTT
TAAATTAATTAAAGCACGATAAACAGCTTGACCTTGTAAAACAGCATTTTCAATGGGGCGTACAT
AATGATGAGTTTCTGGCTTTGCCTCCCTAGAAGGTTGATAAATAATTTTTGTGATACCTTCTATT
GCCCCCTCCTTTTTCTTCGTGAGAAAAAAAACATCATAGAGAGGATTTTTACCTAAAAGTTGCCC
CAAATGACGAAATTGAGCAGGAAAATTCGGATGAATAAAAAGTATTGTTTTTCTTCTTTCTGACA

TCTATTCTGTTTGATAAGTTAATTTATCTGAAAATTTAAGTTTTTCCAAGAAAATATGAGTACAA
ATACTCATGACAAAAACAGTGAAATAGATAAAGATCTATTCTATAATTAAAAAATTAGTTATCT
TAGAGTAATAATTGTTGCAATTGTTAAAATATTATATATTTGCAATTTTTATTAAAAATATTTA
ATCTCAAAATATGTCTTTCTACTATGATGAATTTAAAGAGAAAGAATGTAATAGATACAAGAAA
GTTTGTTTGAGATCAGAGACGTTAATTTGAGTGTTTAAGCATACTAGAGGTTGTTATCATGGAGT
TAATTATTTGTTTTTTAGCTGTTGTTTCCGCTTTCACTGCCTTAATTATTAGTGTAGTCGGATTA
GTAAATGCACCCACCCCTATTCTGTTAGCTATTTTAGTGGCGGGGATGATGTTAACACAAAAATC
GATTGATAAATTTACTGAACCTACAGTTAATCTTACAGAAGAAAAAGAAAAAAATAGAATTGAA
AAGGTAGAGTTGAATTCTTCTAATTCTAATAATGAGAAAGAAAAAACTATTCAATCAATGGTTT
ATCGAGGTTCTAATTATAGCCGAAATTCTGCTTTAGATAAGTTATTTAGCTTTAAAAATAAGGGT
GTAACTCAATATCGTGGAGCAAAAACTAATCACGACGGGAAGGAAATTGTCTGAGTTTATTAGAT
AGAGTTTGTAAAAAAAGTCAGAAGAAACGTTAAAGTAATCAATGGGCAAAGGGTAAAGGTTAAT
AGTAAATTAAATAATAACTCCGAATTCATTTCTCCCTAAAACCTGACACCTGACACCCGACACCT
CAAAACAAAAACTAATTATCTTTTTGCATAACATCAGTTAACAATTGATTCTATGGTTTTGCATA
ACTTTAGTTATTAATTGCTTCAATTTTGCCCAAAAATTCCTGAGTTAATTTATAAAAAGCCTTTG
AACCGTTACAATTAGGAGAGGCTAGAGTGACGGGAATAAATAAGTCCACAGCTTTGGCAACATTG
ACATCCATAGGAATATCCGATGCAAAGATTTTGTCTATGGTAAAGTCTTGTTTAACTCTTCTTTT
TACTTGCTCATAGTATTTATCGTAACGTCCAAATAAACCTGATTCGGATAAGATAAAAACAACAC
CCAGTAGTTGTAATTTAAGGGGATTATCTTCTTGATGATTTTCTTTTAATCTTTTAATTCTTCTT
TCTAGTAGTTGTATCCCTACTAATGATAATGGTTCTGGTCTTGCTGGTAAAATATAAAAATCACT
AGCTGCGATCGCACTTCTTGTTAATAAATTATATCCCGGGGCGCAGTCCATAATGATAAAATCAT
AGTCATTTATGACAGGTTGAAGAATATTATGGATTAGGTTACGTTCAAAATTATTCCATACCCAC
TCAAAATCATTACGTTTATTTTCTTTTACTGCCTGTTGATGCAACATTTCTGAGACTAAATACTC
ATCATATAATTCAATATCTCCGGGCAATAAACTCAATTGGGGTAAATCGCAGATAGAAGGAGTGA
TAATATCGTGAATACTCAACTTAGCATAAGGATTTGGTTGTACAACTAAATCAATCAAATAACTA
AGAGTTTTACGTTTTTTACGCAATTTACCAAACTCATGGGGGGGTAATAAACTGAGTGTGGCACT
AATTTGAGCATCTAAATCTAATACTAATACCTTCTTGCCAAAACCTTTTGCTAAACAAGTGGCTA
AGTTGACGGTGAGAGTGGTTTTACCAACCCCACCCTTCATATTTACTGTACTGATAATATATCCC
ATAATTTTGAATAATAATCACCTTAAACTTTTTGCAAACTATCTTGTAACCATTGAATATATGCC
ATAGAAGAATTAACTATCGGTAAAGCAATAATTTCTGGCACATCATAGGAATGTAAATCTTGAA
TTCTAGCTGACAAAGTCGAAAAAAGTTGCTCATCAGTTTTGATTACTAATTGCCATTCAGCATCT
TCACAAATTTCATTTTGCCAACGATAAACAGAAGTCACAGGATAAAAATTAACACATCCTGCCAA
CTTTTCTTCCACTAATACACGGGCAATTTTTTTCGCTTCGATTTCATTACCTGTAGTCACAAATAT
GACGATATGATTTTTTCGTTGCTCCATAATATAAGCTAGTTTGCTGAATAATTAGAATATTTAGG
GTAGGGAAGAGGGAAAAGTAACAATAATTTATTCTAAAACTATTAAAATTGATTTTTTTTATCT
TGCTAGTATTAGTATCTTGGAAAAAGATTTCAGTTTAATAATATTTCCAGAAATCGGGGTTTTAT
GCCCAAAATACATACAGCAAAAATCATAACTTTTTTCAGAAGTCTAATGATTGATTCTTCATAAA
CTAAAGATTGGCCCTTGCATCTTTCACCGCCAAAATAAAAGTAATCAAAGTTAAGGGGATACTTA
CAGCCAAAATGACAGTAGTGCTAGTTGTCCCTAGCTGAGGTACACCAGAACTTAACTCAAATACA
CAACCAACAGATGCGATCGCGGCTATACAAGAAAGCAGTAAAAGTACACCGCTTTTAGGAGTCAT
ATACATAAAAAATAGTTACCAGTTAATTGCATAAGATACATAACTGTTTTTAACACAAGAAAGG
ACAAATAAACAATTTAGACTATGTTAAATATGGCATTGCTAATTTTCGATACGATTTCTCATTTA
GGTAGGGTGGGGGGAATGAGTAATAGGCAATAGTAATAATAAATATTTTGATGTCTCAATTTTT

GGCATAATTTCAGTAGATTTCGTACCATGATTAAACAACGCCTTAAATATTAATTACGGTATAAT
CTGAGTTTGTGATTAAAATTTAAAGTTAAAATTTATTTTATGAGTAATACTTATACTGTAAAAA
TTCATAATGCAGGTCAATCTTATACAATACAAGTATCTGAAGACCAAAAGATATTAGATGTAGCA
CAACAACAAAATATTGAGCTTCCCTCCTCTTGTAACGCTGGAGTTTGTACTACTTGTGCGGCAAA
GATAATTACTGGAAAAGTTGAACAAGGTGAAGGAATGGGGTTATCTCCTGAATTGCAAGGGGAA
GGATATGCTTTATTATGTGTGTCTTATCCCCGTTCTGATTTAGAATTAGAAACTGGTAAAGAAGA
CGAAGTTTATGATCGTCAATTTGGACAAGGATAGAAAAAGGCAAAGGCAATTAGTGAATAGTGA
ATAGTGAATAGTGAATAATTAAAAACTCCTAACTCTCATTACTCATTAATTGTTTCTCCCCAATC
GCCCAACACCCTAAGCCTTGATAAGTGGGAAATGGATTTGATAAAAGTCTAATAATATTTGTCGG
TGAAAAGGGGCGAAAAAACGCATCTGCTTGGCTATTTTTGAGTAACATTGACCTTGAAGGATAGT
TTCTGGAGGTGCAAGGGTAAAATCCCATCCTTCCTTCAGAGTTAATTGTTTTATTTCAACTTTAA
GAGGGGCGTAATAAACATGGCGGATAATATTTCCTTCTTGGTGACAAATAAATTTATGAAAATCC
GTGATTTGATAGTCTATCTCCTCCTGAATTTCTCTGATTAAAGCCGATTCTGGGGTTTCTCCTGTC
TCTAAATGCCCTCCAAATAATGCCCAGCAACCCGGATGTATAATGGTGGGAATATTATCTCTTAA
TTGTAATAAAAATTTGTTTTCTCGATACAAAATTGCGATCGCAACTTCTTTTACTGGTGAAATCA
TAAAACAACCTTAGTTCAATATAAGAATATCAGATTTATATAGGTTTCAGGTTTCAGGTTTCAGG
TGTCAGGGTTTAATTAATTACTAATTACTAATTACTAATTGTTAATTACCCCTTGCCAAAGCTAC
TAACATAATCAGAAATTTTATATAGAAATCAGGTAATAATAGAGAAAACCTGCTAAACCTCCAA
TTAAGACTAGCCACACAGAATTAATCTGATAACGAAAAATAACAATAGTAGTAATAATTACTAG
GAAGAGGGTAAATAAATCCGTTACAGAATTAGTCATCAAAATATAAGTTACCCCTGCCATTAAAC
CTAAAGAGGCGGCATTTACCCCGTCTAAAAAATTACCCGCTTGAACAGATTGACGGATTTTTTCC
ACCCAAGGATTGACTAATAAAACTAAGATAAAAGAAGGTAGAAAAATACCGATAGTTGCCATGA
[truncated: 381,248 more chars]
