# Supplementary material for: Traces of a Primitive RNA Ring in Current Genomes
Source: Biology (Basel). 2025 May 12;14(5):538. doi: 10.3390/biology14050538 (PMC12109556; doi:10.3390/biology14050538)
Supplement: Supplementary file 1 [file Supplementary material Biology S2.pdf › biology-3598710-supplementary/Supplementary material Biology S2.pdf]

>NZ_CP020120.1 Methanococcus maripaludis strain OS7mut1 chromosome, complete genome

---> Sequence

ATTTTTTTGATAGTTTAAAATATCGCTTAATCTAAATATATCAATTAATATGCCTAAAATAAAA
AGTGACTAACAGCGTCACATTGTTCCCACCAGATGGCAGTACTTAATGAATCGCAGGAGGGCTTA
ATTTCCGAGATCGGGATGGGATCGGATGTAACTCCTCCGCTATGACCGTTATGTCAAAGTTTAAA
TTAAAAGTGCATCTATTGCCCCCGTATCTTTAAGTGTCCAGATGCCAATTTATCTCTTGAACTAT
TAGTACTAGCGGGCTGAACATCTCGGAAAACCTTGATGCGTACACCCCTAGCCTATCAAACCTGT
CTTCTACAGATGTTCTCGTTCCCGAAGGAAACGGCTGTCTATTTTCAGGATGAATTTCGAGCTTA
GATGCTTTCAGCTCTTATCTCTTGGCGCGTGGCTGCTCGGCAATGCCTTGTCAGACAACCGATCGA
CTAGAGGCGCCGACGGCCCGTTCCTCTCGTACTAGAGCCACCTTTCCCTCAGACAGCCAACACATC
CAGCAGATAGCAACCAACCTGTCTCACGACGGTCTAAACCCAGCTCATGATCCCCTTTAATGGGCG
AACAGCCCCACCCTTGGGTCCTGCTGCAGACCCAGGATGGGAAAAACCGACATCGAGGTAGCAAG
CCGCGGGGTCGATATGGGCTCTTGCCCGCGACAACTCTGTTATCCCCGGGGTAGCTTTTTTGTCAT
CCCTGGCCCCCATCAGTGAGGCACAGGGGTTCGTTAGGCCCGGCTTTCGCCTCTTCGTTGCGTTTT
GTTGGCAACAAAGTCAGGCTGGCTTTTGCCCTTACACTCAACAGCGGAGTCCTGACCCGCTTGAGC
CAACCTTTGGGCCCTCCTGATGCATTTTTAGGAGGGTACCGCCCCAGCCAAACTGCCCACCTACCG
GTGTCCCCTAAAAGGGTAAGGAACATGGTCATGAGAGGGTGGTGTCCCAGGGTCGCCTCCACCTT
TCCTGGCGAAAAGGTTTCGATGGCTCCCACCTACACTGTACACTCACAACCATGCCCCAACGACAG
GCTGCAGTAAAGCTCCACGGGGTCTTCGCTTCCCACTGGAAGTCTCCGGCCTTTGCGCCGGAATGG
TAGGTTCACCGGATTCTAGTCGGGGACAGTGGAGGTCTCGTTACGCCATTCATGCAGGTCGGAAC
TTGCCCGACAAGGAATTTCGCTACCTTAAGAGGGTTATAGTTACCCCCGCCGTTTACTGGCGCTTC
GCCCGGTTGGAACCGGGGTTCACGTACCAGCACTGGGCAGGCGTCGGCCTTAGTACACACCTTTTC
AGGCTCGCTAAGACCTATGTTTGTATTAAACAGTCGGACCTCCCTGGTCACTGCGACCAGCGATCC
CATATATAAACAAGATCACTGGCACTCCTTCTCCCGAAGTTACGGAGCCAATTTGCCGACTTCCCT
CGACTAGATTCATCCGCTACGCCTTAGGATACTCACCTAGGGGCACCAGTGTCGGTTCTGGGTACG
GACATCTAAAATCCTTGTTAGTTCCCTTTTCACGGGCTCCAGAGATCGGCCGAACCCTCCTAACGG
AGAGCTCATCACTCTTTCATCCGGTTCTCGTTATTACAACTCTCCCCGGACTTATAAGCTTGAATG
CCCAGACAAGAACACTCAGCCTACCCAGAAGCGTCGGAAACTAACCTAGTGTTGCCACGCGTATT
TAGATGGCACAGGAATATTAACCTGTTTCCCTTTCCTCTCAAAGAAGTTAACTCGAGAGTTAGGA
CCGGCTTACCCACAGCTGAAAAACATTGCTGTGGAACCCTTGCCCCTTCGGCGGTGGGGATTCTCA
CCCCACTTATGCTGTTACTACTACCGGGATCTTCATTTCTACGAGGTCCACTCGACTTCACAGCCG
AACTTCTGCCCTCGCAGAACGCCCCCCTACAGGATCACCTTTTGGTGCCCTTGGGTCTCGGCGGCC
GATTTAGCCCCGTCCATTTTCGGGGCCCTTGATCTCGACAGGTGAGCTGTTACGCACTCGTTAAAG
GATGGCTGCTTCTAAGCCAACCTCCCTGCTGTCTTAGACCAAGGACGCCCTTCGTGTTCACACTTA
ATCGGCACTTAGGGGCCTTAACCCAAGTCTCGGCTATTCCCGTCTCGGACATACAGCTTACCCGTA
TGCCCTCACTCGGAGCATGCGACGACGACAAGTTCGGAGTTTGACAAAACAGCGAGGAATTTCTT
CCCCTAACCACCTTATCAGTGCTCTACCTTGCCGACTATCTAAACTCCGGCTGACCTGAGAGTCAC
TTCGGAGGGAACCAGCTATCTCCGGGCTCGATTGGCCTTTCACCCCTAGACCGAGGTCAGAAGAG

TGCTTCGAACGGCAACAACTCTGCAGGCCTCCATCCCTCAGTTGAGGGACTTCACCTTGCCCCGGC
CTAGATCGCCCGGTTTCGGGTCATATTGCTGTGACTCCGGGCCCTTAAGACCCCGTCCCTCAAGAT
TTTGCGGACTTGTTGGTTTCCCTACGCCTACGATCGTGAAGATCTTAAGCTCGCCACAACAATAT
ACTCCCCGGCCCGTTTTTCGAAACGGACGACACAACCCTGGCTCTATACTTCGTACAGCCGTCTTG
CGACGGTTTCCTTCAGTATAGTTGCCTTTGGGGCCGTGTCATACCATACCTATCTGGTTTCAGGCT
CTTTTCACACCCGATTAAAGGTGCTTTTCAGCTTTCCCTCGCGGTACTAGTACACTATCGGTCTTG
GAACGTATTTAGAGTTGGAAGTTGATGCCTCCCAAATTCCCGCGCGATATCCAACGCACGGTACT
CAGGGACACTTCAAACCTGAATCGATTACGCTTACGGGACTTTCACCCTCTATAGTGTGACGTTCC
AGTCAACTTCAGCTTTTCTCAACAGGCGAAAAGAAGGCCCTATAACACCACATCTCCCCGAAGGG
ATTCAGTTTGCTCTGATCCGCGTTCAATCGCCTCTAATAACGGAATCTCTATTGATTTCTCTTCCT
GCGGGTACTAAGATGCTTCAATCCCCCGCGTTCCCAATCCTTAACGGATTACAAAGTAGGAAGTC
CCATTCGGAAATCTCAGGTTCAATGACTGTATGCGTCTCGCCTGAGCTTATCGCAGCTTACCACGT
CCTTCATCGGCGTTCCAAGCCAAGCCATTCCCCAGATAGGGTAATAGATAAATCTAGGCATCTGG
ATAACACCAGATACGGAGGCTTATGCACGACCTTCACTAAAAATCCGGTCCTCGGTCCATAATAC
TCGGTATTGTGGACTGAGCAACTAACAGCCTACAATCTTTTAGTGCCTAGTAGCAACCATTGTTC
TTTTTTTAGGAGGTGATCCAGCCGCAGGTTCCCCTACGGCTACCTTGTTACGACTTCACCCCCCTC
GCTGAGCCCAAGTTCGACCCTAGCCAAAGGCCAAGGCCTCACTTCAACCCAACTCGGGTGGTGTGA
CGGGCGGTGTGTGCAAGGAGCAGGGACACATTCACCGCAGTATTATGAACTGCGATTACTACGGA
TTCCAGCTTCACGTGGGCGAGTTACAGCCCACGATCCGAACTACGACTAGGTTTAGGAGATTTGC
GTCCCTTTTCAGGGTTGCGGCCCATTGTCCTAGCCATTGTAGCCCGCGTGTAGCCCAGGAGATTCG
GGGCATGCGGACCTATCGTTGCCCGCTCCTTCCTCTGACTTAGCGCCAGCGGTCCCCTATGAGTGC
CTTTCTCCCGGAGGAAAAAGTAGCAACATAGGGCACGGGTCTCGCTCGTTACCTGACTTAACAGG
ACGCCTCGCGGTACGAGCTGACGATGGCCATGCACCACCTCTCAGCGCTTCAGGCAAGGTCGTCAA
CCTGGCCGTCATCATGCTGTCGCTCCTGGTGAGATGCCCGGCGTTGAATCCAATTAAACCGCAGGC
TCCACCCGTTGTGGTGCTCCCCCGCCAATTCCTTTAAGTTTCAGTCTTGCGACCGTACTCCCCAGG
CGGCGAACTTAACGGCTTCCCTTCGGCACTAGATGGGCACGGGGCCCACCTAACACCTAGTTCGCA
AAGTTTACGGCCAGGACTACCCGGGTATCTAATCCGGTTCGCGCCCCTGGCTTTCGTCCCTCACCG
TCGGACCCGTTCCAGACAAGTGCCTTCGCCATAGGTGGTCCTTCAAGGATCAACACATTTCACCGC
TACCCCTGAAGTACCCTTGTCCTCTCCCGGTCCCAAGTCCAGCAGTATCCCTGCCAGTCCTGCGGT
TAAGCCGCAGGATTTAAACAAGGACTTACTGGACCGGCTACGGACGCTTTAGGCCCAATAAAAGT
GGCCACCACTCGGGCCGTCGGTGTTACCGCGGCTGCTGGCACCGAACTTGCCCAGCCCTTATTCCT
CAAGCTGTTTACACTTGATAAAAGCCCATGCTGTGCATGAGCACTTGGGATCCCCCCGTCGCACTT
TCGTGCATTGCGGAGGTTTCGCGCCTGCTGCGCCCCGTAGGGCCTGGGGCCGTGTCTCAGTCCCCA
TCTCCGGGCTTCCTCTCTCAAGGCCCGTACCGATCGTAGGCTTGGTAGGCCATTACCCCACCAACT
ACCTAATCGAGCGCAGTCCAATCCTCAGGCGCATATGCTTTCGAAAAAGAATCGTTCCAGACTGC
TTTTTCTATGGGGTATTATCCTCAGTTTCCCAAGGTTATCCCCCACCTGAGGGTATGTTAACCACG
TGTTAATGAGCCGTCCGCCATGGCCGAAACCATAGACTCGCATGGCTTAGTCGAATCCCAATAGC
AGTGGCCTCCGGCGGGATCAACCGGAATTAGTAGCGTAAAAGAATTTTACGCCACCCTCTCGTGG
AGTACGTCATTTTACATTAAAGGAAATGACATTTGGTTGCTATCTAGGTTTCACCTAGATTGTAG
GCTTACATCGCACCTGAGTATTTCAATCAGATGTTCACTTCAATTGCATAATATGCAAGTGATAT
GGATAGTGTAATGTTTTTAGGTTTGAAACGATAGTATATATTGTACACTATGAAATATTAGAAT
TGAGGTGATTCTATGACTCTTGACCGCAATGGTAAACTCATAGAAATCGGTTCCTATGTAAGATA
TATTAACACAGGAACTCATGGAACCGTCAAAGCAATTGAACCAAAAAATGATGAAGAATGGGTT

CTTTTAGAAAATGACATATATTACAGACCCGAACTTCTTGAACTTGTTGAAAGAAGAAAAATCGA
AAAAAAAGAATCGGAAGAAGAATTAATCGAAAGAATAACTAATGAAGATATTGATCTTGAAGCC
GCTGAAAACGGATGCGGATGTTCAGGGGCAGGTTAATTAGTATAATTTAAGTATCAAGGTGCATT
TATGGACAGTCTTTTGGAATTAAACCGGGTAAATACAGCAAGAGAAATAATAAGAAAAAAAATA
CAAAATACTGGAAGAACCAGTATTTATGATTTAACGGGTCTTTGTGGCGGATTTGAAATCTGTGA
AGAAAACCTAAAATTAATAGAAACATATGTTGGGCCAGCAATATTTTCAGAAAAACTAAATAAT
GCTGGTTTAAGCCATTTATCAGGAAATTCCAAAATTCATAATGCAGTATGTTTTAACAGGACTTC
TTCGGCAATATTGTCTACAATAATGACCCTATCAAAGTCTTTCGAAAAATTGGTGCATTATGTTC
CTGAAAAACCTGCACACCCCTCAATTCCTAGGAGTTGCAAAATATTTGGAATGGAATACTTTGAA
AGTGATTCTTTAGAAGAAATTCTCTCAAAAATTGATGAAAATTCATTTACTGCAATAACTGGAGC
TACCATGGATCACAAGGTAGTTTCTGAAGAAATTGCATGCAAAATAATAGATTATGCAAAATCT
AAAAATTCTCCGGTATTTTTTGATGATGCGTCTGGAGCAAGGTTGAGAAAATTGTATAAAGAATC
ACCAGCTCTTGAAATGGGTGCAGATTTAGTTGTAACCAGCATGGACAAATTAATGGACGGCCCAA
GAGCCGGCCTTCTTGCAGGGGACAAAAACTTAGTTGACAAAATATATTCTGAAGGTTTAAAATTT
GGACTTGAAGCGCAAGCCCCAATAATGGCCGCAGTTGTAACTGCTCTTGAAAGGTTTGATCTAAA
TAATTTAAAAGATGCATTTGAACGTGCAGAAAAAGTTGATTTGTCAGTATTTGAAGCTGAAAAA
ATTGAGTATAAAAAAACTCCGACAGGATTTATAATAAAAAATTCATCAGAAGAAAAATTAATTG
AAACTGCACTAAAATTGCTTGAAAATTACGGAATAGTTACAATCACTGCGGCAGGTATGCCCGGT
GCAAGTAAAAACATAAGAATAGATTTCTGTTCAAAAGATGCAGAACGAATTTCTGATGAATACG
TAATAAATGCGGTTTTAAATTCTTTAAAATAATATAATAAAAAAATAAAATAACTTTTTTTAAA
TTTTTTAAATTAATTCTTTTAAGTCTTCGGGCCTATGGAGTTCTCCACCAATTTTTCCACAACCG
ACTACTTCGCATTTTGCGTATTTCATAACTTCACCAGAAATCTGTCCTAAGTTCATTTCTGGAAC
TATAATTTTTGAGGCTTTTAATCCTGCTATCAATTTATCTGGAAATGGAAATACGGTTTTTAACC
TGATATATCCAACATCTTGACCTTGTTCCCGTAAAGATTCAACAGTGTGTTTTACAGTTCTTGAA
GGGGTTCCGTAGCAGATAAACATTGTTTCGCTTTCTAAATACTTGCCTTCGTAAAGTACAATATC
ATCTTTATTTTCCAAAATTTTATTGCAGATTCTTCTAACCAGTTTATCGTGTGTTTCTGCTGAAA
CGTCAGGGTAGCCATTTTCACCATGTGTCAGACCAGTTACGTGGATATTATATCCTTCACCAAAT
GTTGGCATTGGTGGAACTTCCTTGTCAAACGGATATGGTTTTTCTTCTGGCTTTTCACGGTTTAA
TATTTCAATGTCATCGTGTAAAACTACTTTTTCTCTCATGTGTCCTAAAATCTCATCAGCCATAA
CAAAAACCGGTATTCTGTATTTTTCTGCATAATTAAAAGCAAGTATTGTAAAATCATACATTTCC
TGAACTGATGAAGGACAGAGTGCTATCGGTTCATAGTCTCCGTGACTTCCCCATCTTACCTGCAT
CATGTCTCCTTGAGCGGCTGCTGTTGGCTGTCCTGTTGAAGGGCCACCTCTTTGGACATTTACTAA
AACGCAAGGTGTTTCAGTCATGTAAGCGTAGCCGATATTTTCAAGCATTAAACTCATTCCAGGGC
CACTTGTTGCAGTCATTGATTTTGTACCTGCCCAGCTTGCACCGATTATTGATGCGATACTTGCA
ATTTCATCTTCCATTTGCGAATAAAACCCGCCAATTTTTGGAAGTCTTTTTGCCATTCCTTCTGCA
ATCTCAGTTGAAGGAGTGATTGGATATCCTCCGAAAAACTTACATCCTGCCGTTATTGCACCTTC
AACGCATGCCATGTTTCCCTGCATGAAGTTAACTTTTGTCATGAAATCACCGAAAAATCCGTAAA
TTATTGAAAAATCCATTAAATATTTTCGCTATTTATATCTCCAATAACATAATTATATAACTTAT
CTATGTCTTTATCGATTCCATACTTTTTAAAGTAACGAAGCAGATTTTCAAGATCTCTTTTTAGG
TAATATTCTTCGTCATATTCTACTTCTTCAAATGGTAATTTATCTAATTTTCCTTTTTTCCCAACT
TCGATTGCCTGAGGCCAGTCAATAATTACAAAGTCGCCTTCTGAATCAATCAATATATTAAATTC
ACTTAAATCGCCGTGAATAATTCCTAATTTGTATGTTTTTTTAACTTCTGCCATCAACTTCCAGA
AAAATTCTTCAGGATCCACGTTTATTTCAAGAAGATTAACACGCTTTAATTCTTCCCCGTGAAGT

TTTCCCATTATTATTGCGTGCCTGTTCTGTTCGATTGGTTCAGGAACTTTTACAACTGGAAAAAG
CATGTTTAAAACTTCAAATTCTCTTGCAGCGGTGAGCCTTGAAGCGTAAAGCCAGCTGATATGCC
TTTTATTTGCAAGGTATTCTCGGTATCTTTTTCCCATTGAAAAGCAGGTCCTTCCAAGCTGGTGG
AATTTCAAAACAGCTTCACGGTGATTCGTAAGCAGAACGTGATAAACGTCGCCTTCTTTTCCAAC
CCCAACTTTTCCACCAATTCCTGCAATTAATTCCTTTTTTATAAATGCATTCATCGCTAATGCATC
GTATCCCCAGTGAGAAAGCCTGTATCCGTAATTAGACTTGTTGGTAAACTTAAATTTATTCAATC
TTCCAAGCCTGAAAACTATTTCTTTTTCATGAAATCCCGTTTTCCGCCTTAAATCTTGAATAGGT
ACCCATTCATGATTTTTCATTGAAATTTCAATTATTTTCAACAATTTCCAGTCATTATCTTCCAT
TTCCGCTATTGAATTTAAAACTTTTTCGATTTTTTCACGCTTTTATTTTTTAATACTATTTTTCT
ATGGTGCTATAAAATTTTAATTATTGTCTTGTTTTGAAATTTTTTCCAGTAGTTGTTCTCTTAAA
CTGTCTGCACATTTTATTTTTTCAGAATTATCTATATTTTCAACAATATCCCCAATTCCAAAGAT
TTGTTCAAATTTCCCGTTTGAAATCTTCCAAATTTCTGAAATATCTGATAAAATCGTATCTATTG
TTTTTTCAGTCGTTTTTTCGCATATCAAATCTAAGATTATTCCATATATTTTCAGTATTTCGGTT
AAATACTCGTTTAAACTTTCATTTTCTTCATTTGTTTTAGAAATTAATTTTACAAAATTTTTCAG
TGTTTCTGAAGTTTGAATTGTGATTTCTGATAAAAACTCTGAATTTTCATTTGTTTTTATAAATT
CAATTGCAGGTTTTGAAAAATTATTCAGTGCATCTAATATCAAAGTTCCAGCAGCTTCATTTCTT
AATATTTCCCTTGAGTTTAATTTATCCAAAATTAGTTCAAAAATCTTTAAAATTTCAGAAAACAT
CATAATTTTGAACGTTACCTGTTTTGAAGTTATTTTTTCGTTGTGTTTAGGTTTTGAAAGTATAA
ATTTTATAATATTCCCAAGTTTTTCAAACACAATATGGATTAACTCTTTTTCGTCTGATTTGTCG
ATCTTTAATTCGTATTCTTGGGCATACAAAAGTCCGATTTTTTTAATTCCCCTTAGCGCCCTTAA
AGATAAATCATAATTTCTATTTAGTTCAAATTTCGAAATTTTTTCAAGTGAAATTATTGCTTCCC
GCGAAATGGAAAATGAAGTCCCGTATGCAGTTAATCCCAAATTTTTAAGGTTGTCTATGTAGCTT
TCAAAAAATTTTTTATTTTTAAATTTACCTTTTTTCGCTAAAACTGAGTAATTTGCACCAAGAAG
GATTATCCCGTTTCTTGCAGTCATGTAGTGATCAGATTTTAAACTTTTTCCAATAATATAGTAAA
CTGTTCTCAGTGTGTTTTTTGGAATTTTCCTTGCAGATTTGTAGTCTTTGTTTTCTGAATCCAGT
GAACGGTAATTTTTTATTTCGTCTTTTGAAATGGAATTTATTAAAGCATCTATAACATTTTCGGG
CTTTAACCCATTAATCGTGTTCTTCATGTATGGTAAAAGTATCAAAAAGTCAAAATAGACGAAAA
AGAACAAAATACCAATTAAAATATGATATGATATCTCGTATCCGCCAATAAATACCGGAATTCTT
GCTGTGGGCATAAACCCGAGTATCAAAACTTCGTAACTCATAGAAACGATGTACGCTATTATAAA
TCCCCAAAACGTCTTGTTAAGTAAAAGATGCGTAATTCTCGTTGAATACTGCTGGCTTACCATCT
GGATTGCAACAATTGAAATCGATGCAACAATTCCAACAATCGCTCCTTGTGTTTGTGGCAGCGTG
CTTAAAATATACCTTAAATTTTCATCAAATCCCGTTCCCCATGCAAAATAAAATAGGGAATAATA
AGCGAGTACTGCAGAAATTGCTGCAATTATCAGTACTTGGAACCATTCTTTTGATAAAAAATAAT
CCATAACATAAGCGCAGTTTTTACAAATCTTCGAGAGCATCGTGACCACACAGTTTCTTTTAAGT
AAATATGTTAATTAATTATAAATAAAAATATATAGATTAAATACATATCAATTCAAGAATCAAT
TCGGCATATTTGGTTCAAAATATAGGCATGGTGAATATATGAAATTTGGATGGATAAAAACAAC
AGGAAATGACCTTGAAGAGAGAATGGAATCGGTAAAAGATGCTCTTGAGAGTTCAATTCCAGGA
ATCATTACAGAAAAGGAAGAAATAAGTTCTGTAAGAGAACTTGGAAATATAAAAATTGTTTCTG
ATAATCTCGATGCAGATGTCGTTTTAATTAATAAAGGGGAAGATTTAGAGATTTTAAAATCTGC
AAAACTCTCTGGAAAAGAAACCGGTGTTTACGTTGTAATAAACACTAAAGAAGATGAAGTATAC
GCTACAGACGTAAGCAGACTTAATTTTGTCGATTACGTTGTTTTAGAAGGAAGCGACTGGACAAT
TATTCCTCTTGAAAACATCATTGCAGATTTATTCAGCGAAGAAATAAAAATCGTTTCAGTTGTAA
CAAACGTAAAAGATGCGGAAGCTGCATACGAAATTTTAGAAAAAGGAGTTGACGGCGTAGTTTT

AATTCCAAAAGATATTAACGAAGTAAAAGATTTCTCAAAATTAATTGAAAGAATGAACTCTGAA
AGTGTAAAACTCGACTACGCAACAGTTACAAAAATTGAACCTGTTGGAAGTGGCGATCGAGTCTG
TATTGACACATGTTCCATGATGGAAATGGGAGAAGGAATGTTAATTGGATCCTACTCACGAGGGA
TGTTTTTAGTTCATTCAGAAACTGTTGAAAACCCGTATGTCGCTACACGACCATTTAGGGTAAAT
GCCGGACCTGTTCATGCCTATATCTTGTGCCCTGAAAACAAAACTAAATATTTAAGCGATTTAAA
AGCAGGCGATAAAGTTTTAGTTGTTAATAAAAACGGTGAAACGAGAGAAGCTATTATCGGACGC
GTGAAAATCGAAAAAAGACCATTATTCCTTGTTGAAGCAGAATATAACGGGGAAAATTTAAGAA
CAATTCTCCAAAACGCAGAAACTATCCGATTAGTTGGAGAAGATGGAAAACCAGTTTCAGTTGTT
GATTTAAAAGTTGGAACCAAAGTATTGATAAAACCAGATGAAAACGCGCGGCACTTTGGAATGG
CGATTAAAGAAACAATTATAGAAAAATAATTTTTATTTTTACTTTTTTTAATTTAAAAAAATAA
TAAACAAGAAAATGATTGTTATCGATTTTTAAAGTTTTATAAATCTTTTAAGGTAAATTTTGTA
AGCTACGTGTGACATTATAGCCAAAAATGCCGCATATGCTCCCAAAGTTACCATTATTATTTGTT
CCATATTTTCACCGTATCTAACTGTCCATTTGAGGACTATTTAGGTACTTCTTTTGCAGATATAA
ACCTATTTGTGATAATTTTCGTTTTTTATAATATTATTGGAAAAATCTGCCCAATTAAAAATGAA
TAAAAAAGCCCAAAATTACCTAAATTATTAAATAAATGAAAAATCATAACCGTTATAATTATGA
TTAAGAGTTATAGAGGGCCCATGATGCAGATCAAAATTGGAGAAATTGAGTCTAAGCTGAATAA
AATTATCGAAGAAGAAGGTGCAGCTTACTTCGTACTTATAGACCCTGATGAAAAGAACTATCGGG
AAATTGCAAATCACGTAAAAGATTATGCTGATGCAATAATTATCGGAGGAAGTATCGGAATAAT
AAACCTCGATGAAGTTACAAAAGACATTAAGGAAATCACGGGACTTCCAATAATATTATTCCCTG
GAAACGTTGATGGCGTCACAAAAGAAGCAGATGCAGTATTTTTTATGAGCCTCATGAACTCGAAA
AACACGTACTGGAACATGACTGCACCAACTCTTGGCGCCCTTACAATCAAAAAATACGGACTTGA
AACACTACCTATGGCATACCTTGGAATTGAACCAATAAGTAAAACTGCCGTTGGATTTGTCGGGG
AAGTAAACGAAATTCCGCAGAAAAAACCAGAAATTGCAGGACTTTACAGTCTTTCAGCGTCCTAC
TTTGGAATGAGGTGGGCTTATCTTGAAGCAGGAAGTGGTGCAGAATACCCAGTAAGCAATGAAA
TGGTTGGAATTTCCAAAAAATTAAGTGGAATAAATATTATTGTCGGCGGAGGAATAAGAACCCCC
GAAGTAGCTTACGAAAAAGTCATGAGCGGTGCAGACGTAATTGTAACTGGAACCCTGACTGAAAA
AGACCCGCAGGCTGTTGTAGAAATGAAAAAAGCAATTAAAAAGGCAGGACTTGATAAATTAAAA
ATGCTGTCTAAAAAATAAAATAAAATAAAATAAAATAAAATAAAAATTAATTTATTTCGATTTT
TCCCTGATTCCATTCAATCATGTGTTGTTCTCTTTTTGATAAATCCTGTATCGGAATAATTCCCG
GCGTAGGCTTGATACCCATTCTTTTCTGGAAATCCGTCTGTTCTTGGAACGTACCGCTGTTTATCA
TCCTTACCCCGTGATAATTATCATACCCGTTGATGTGGATGTGCCCAGTGTGGAAAATATCAGGT
TCAGTATGTATTGCAAGATAATCAACGTGTTCTGGAGCTATCGGGCATCTTCCCCCATAAGTTGG
GCACAAATGCCTTCTTTTTAAAAGTTCAGTCATGATTGATGGAGGGTTTGTATACTGGGCTGATG
AAATCTGGCCTATAATATCATCAAAACTTCTGCCGTGGTACATCAATAAATCAAGGCCGTGCATA
TTTACAACACCTGGGTTTCCAACAAATGTTACATTTTCTTTTGGAAACAGGCTTGTTATCGATTC
ATCAAATGTTGGCTGCGGTTCCGCAGGTCTTAACGCATCGTGGTTACCCGGACAAATTATAACTT
TTATATGTTCTGGAACCTGCTCTAAATAAGCTGCAACTTCAGCATACTGTGAAATTATATCTACG
TCATATAAATCGTACTCTTGGCCAGGATAAATTCCAACACCATCCACTAAATCCCCGGCAATTGA
GATGTATTTTAATCTGCTTACTATTTTTTCTTCTAAACCGTTATTTACTTCGCCATTTAAGAACC
GGATAAATTTTGCGAAGACTTTATTTAAAAATTCACGACTTCCTACGTGAACATCTGACAAAAAT
GCAGTATATATCTTTTCATCGGTTGTTTTTGTGGGTTTTGGAGTAATATCTGGCCTAAATGCCCG
ATCTACAAACATCAAATCTCCTTTATCATTTATAGTTCCTTCAAAACCAATAACTTCATCTAAAA
GAACGTCTGATGGAAGATCTCCTTTTTCCATTTTATCTTTCATCAAAAGTACCCTAAAAGTTCCG

TTTTCATCTTCTAATTCAACCATTTTATGTCCGTTTTTGGTGGAATTCACATCTGACACGATCCCT
GCAATAAAAACTTCATTTTCTTTTTTTCGCCTAAAAAGCTGGTTTAAAGGGTATGCTTTCTTTTG
ATTTTTTAATTCAATTATTTTTCTTAAAGTACTGCATCTATCCTTAAAATATTTTACAAAGTCAT
CGAGTGTTCCTTCGCACGTTGATTTTCCGGTAACGTCATATTCGTCGTAAACGTGTATCTGTGCA
TCAATATCTTTTGCATAGTAATTTATCTTGTTATTAACACTTTCTCGGATAGACCTTACTTCTAA
AATACGTTCTTTTCGCTCTTCCTCTAATTTTTTATGTATTTCATCAAGTTCAGGTTCCTCTTCTTC
AATATCTTCGAAATCTAAATCGTAAACTTTATTTTCCCGTTTATGTTCCATAATTTCTTCATTTT
CAGGTTTAACTTCACTAACTGGCTCAAAATCATCTTTTTCTATAATTTTTTCTTCGGATGCCACT
TCGTTTGATGTGTAATATGCTAAAAAATCGAATTTTCCGTATTCTTCAAAAATATTTTTTAAATT
ATTTCCCAAAAAAATGTTTAAAAAGTGCGAATCGAGGAGTATAAAATCGTCCTTTTTGCTTTTAA
ATTCTTTTATTTTATTAAATAAATCTGAAAAATCATTGCTGTCGAGATTTTGAAGCCTCGAATAG
CTTTCAGGAGCTAAAAGTACCTGAATGTTCAAAAAGTCGTTTATCATAATTTCACGGTTATTCTG
ATTTTTTTACATGTCTGTTAATGTTGTGGCATAAGTAATCGAGAGGATGAATTATATCTTTCCTT
GAACAGGTTGGATCAGGCCTGCAGTAATATCCAAGACCTTTTCCACCATCATCACGCCAGTTTTT
GCAGTACTCGCAGTGAGTTATGTGGTTTGCAACTTTGTACCCAATATTATGACTTATGTAGTATT
TAGTTTTTTCAACATCGAAATCTCCAACAGATTTAAAAATATTTATTATTTCATCTAAAAAGCGT
TCAATTTGCTCTTCTGTTGCGATATCCTGTGCACTTCTATTTGTAATAATTCCTTCTTCGATCGGC
CTTAAATTAGGATCAAATTGGGACACAAATCTATAAACAACAAAACTACGCCTTGCATAGTGCGA
AGGAGATCCCCCTGACAAAATATCGTTTAAAATTTCCCTCATGCATGGGGGATGCCAGTCTTCAG
GAATTTCTCCAGAGTATTCTTTAGTACTTAATTTAATTCCACTTTCAATGTTATATTCAATATTA
ATTCCGTCAAATGCTTCTTCTGATTTTTTAACAAGTAAATCCTTTATTTCTTTTGGCATTTCTGC
ATTTCGAACCATATCTACGTAATTTTTAATTTTTAACCTTATTTTTTCGAAATTATAAATTGACT
GCCTTTTTTTACTCAATAAAACTACATTTCCTTCAAGAATCATTCTTTCAAGGTGTAAATCGTTA
TTTCTTGAATTTGCCGCTACATCGATAAAATCCCAAATATTTACCCGTGAATCGCCTTCTTCAAGC
AGTACTTTTTCTGCATATTCTTCTGCAAGCTTTTTAATATATATGTTGTGATTTTTGGAGTTTGC
TGCAAGTATAAGTGTTAAATAATCTATTTTTTCTGAAAGTAGTCCGCTGTCTCCAGATTTTTCCC
GAAGCGCATATGCACTTAATCTTTCCTGATAATCCCTTAACGCCTTCAAATATTTTTCTTCGTTG
GTTTCTTCCGATTGTTTCGAGTTCAGATCTTCTTTTTCGTCATCGTTTTCTGAAACTTCAGGGTTT
TTATTCCAAAATAATAGTTTTAGTCTATTATTAACTAAAGCAATAGTTAAACTATCATTTATTTC
GTCAATCAAATTCAAATCTTTGATACTATCCATATTGTCTAAAAACACTAAAATCACAAATAATA
TTTTATAAGTTATTCGTTCATTTTGTAGAGAAAAACTGCAACTTCACCAATTGCACCGACATTAT
TTTCTAATATATGCATTCCAGTTCCTTTTTCAAGTTCCTTTTTTGAAAAGCCTGATGAATTTCCT
GAAAAAATTACCAAATCTCTTTTTCCAATTGATTTGAAGTCAATTTTCTCTTCTGCAATTTTACT
GTCTGCTATTAATATTACTCTTTCAGGGTTTAAAATCTCTACTGCATCATCGATATCTTCGAGAA
ACATTAGATTTTTATTCAGTTTTATAGCCATTTTCTGAGCGATTGGAATTCCATTCTGAGCTGCT
GAACCAGTTGCCCTTGTAAATATTACGGAGTCAATTCCCATTCCAAAAACTGTTTTTGTAAATTC
TTCTATCTGTTTTGAACTAAAAGTGTTGTGAAGTGCGATAAACATGAAATTCACCTTTTTTATTC
TTTATTTTTAATTTTGAAATTTTTATTTATTAAAATATTTTTTAACTTCTTTTGCAATTGCTAAT
GATAATGAAGGAGGAACGCTTTCTCCAACCTGATTGTACTGGGCGGTAATTCCCCCTGCAAAAAT
ATGGGTATCCGGATAACTCATGAGCCTTGCTTGCTCTCGAACTGTTAAAAGTCTATCTTCCTCAG
GATGAATAAATCTCCTTTTTCCCATGACAGTTTCAGCGTTTCCTTCCATTGAAAGCCTTATGTAG
TTGTCTAAAACTCCGCCACAGGCTTTAAACTTAACTGCTGAATCACCGACATTTATTTTATGAAT
ACTTTTCGCAATTTTTTCTGGAAGCGGATATTTTTCATTATTCAATGCATTTCTGTCAATATTTA

ATAAAGCGGTTTTTACATCCACATTATCAGTTTTTTCAAGATTTAGAGGGATATTTGAAATAATT
ACCCGTTTTCTAAAGGAAGGATTTCCGTAATTTTCAGAATCAAAAACATGAAAGTACAATTCGTC
GTACCCTGCTCTTGAAAACTCTTTTTCGATATTTTCCCTAGTTTCATAGTTTAAAATTCCGGGAA
CATTTTCCATTACGAAAACTTCTGGAGAAATATCTCCAACAATTCGAATATAATCTAAAACCAGT
CTTCCAACAGGATCTCGATAAAGCCTGTTAAATGGACTCCTTTCACGCTTTGGATTTGCACCTGT
GTATCCTTCACAGGGCGGTCCACCTATAATCAAATCGATTTTTGAAGAGGACGTATCTTTAATTT
TATTCGTTATGTATTTTGAATTTATTTCTCGTATATCCTCATTAATTACAACAGGATTTAAATTT
TCATAATTATTTGATTTTTTAAATTCTTCAATATCATCCTGAACTAAAAAATCTTCGAGTTTAAA
ATAGACATCTTTTTCTAAAATATCGTTTAATTTTTTTTCAAAAACCAATCCATTGTAATTTAAAG
CATACGAATTTGCCGCATTTTCATCCAATTCAATTGCCAAAAGTGGTTTAAATCCCATTTCGACA
AATCCTCTTGAAAAACCGCCGCATCCACAGAATAAATCTATAAAATTCATTTTCCCACGTAAAAA
TAAGTGCTGATAACCATATAATTGTGATTACCTATAAAAATACATGGTGCTTAAATGAATCGTTC
AAACCACACAAACAATTCAAACCTTGGAAAAATTTTAGAAGGGTTAGAAATTAAAGAACTCCTT
AATTCTTCAAAAAATATTGCGGTTATTGGAATTTCAAAAAATAGTGAAAACCCGAGTTACTTCGT
TTCAAAATATTTGATAGAATCGGGATTTAATGTTTATTTTGTAAATCCAAACTATTCTGGTGAAA
AAATACTAAATCATGTTGTATATTCAAATATTTCTGAAATAAAAGAAAGAATAGACATTGTATC
GATATTTGTAAATCCTTCAAGGACCAGTGCTATTGCCAAAGATGCTGTTAAATTAGGATTTAAGG
CTTTTTGGTTTCAGCTTGAAACTTTGAATAAAGAAACTGCGGATTATGTTTTAAAAAATGGTTTT
GATGTCGTTTTGGAAAAGTGCATCATGACTACAAATCAAAAATTTTTATAGTTTAAGGAAATAG
TTAGTTTAATTTAAAAAAGGTGACTTTGATGAACATTTTAAGAAGGGGAAGACTTGGAAGCAAC
GTCAAGGAAGACGTGATGAAATTCACTACAAGCCTTGAATTTGATAAAGAAATCTTTGAAAGCG
ATATTTTATGCGACATTGCGCACACGACGATGCTCGTTGAACAGAACGTGATTTCAGAAGAGAAC
GGTAAAAAAATAATTGCAGAACTTAAAAAAATAGCGAAAAGGGGCATGGAAAATCTTGATCTTG
ATCCATCTCTTGACGACATTCACATGGTAATTGAAAGTGAATTAATAAAGGAACTTGGTGAAGAT
GTTGCTGGAAGAATGCATACTGGAAGAAGCCGAAATGACGAAGTTGCAACTGATTTAAGGCTTTC
TTTGAGGAAAAAAGTTCTTGAAATAATTGGCCATTTGATTACAATGGAAAAAAATATGCTCACG
GTTTCAGATGCACATAAAGAAACATTAACCGTTGGATACACACATTTACAGCAGGCACAGCCTGT
AACTTTTGGACATCAAATTTTAAGCCATGTTTCAGCAATTGAAAGAGATATTTCAAGGTTTTTTG
ATACATACAGCAGGATTAATATCTCCCCGTTAGGATGCGGTGCAATGGCTACAACGGGATTTAAT
TTGAATAGAAAAAGAACAAAAGATCTTCTTGGATTTTACGGAATAATTGAAAATTCAATGGATG
GCGTGTCTTCAAGAGATTTCATCGTTGAAACAATGGCAAATATCTCAATGCTTGGAACAAACCTC
TCAAAAATATGTGAAGAGCTCGTCGTATTCTCAAGTGCAGAATTCAACACCATTGAAATTGCAAA
TGAATACACATCCACCTCTTCGATTATGCCGCAGAAAAAAAACCCTGACGTTGCTGAAATTACAA
GGGCAAAACTTTCAACGCTAAATGGAGAGCTTGTAACAGTTTTAACGATTATGAAAGCGCTTCCA
AACACATACAATAGAGATTTGCAAGAAATCAGCCCGCATCTTTGGAAGAGTGTCTACACCCTTAT
TGATAGTATTCAAATGGTTGACGGAATGATATCAACTGTTAAGGTAAATAAAGAAAGAATGAAA
GAAAATGCAGAGAAAAACTATTCTACCGCTACAGAACTTGCAGATACACTTGTAAGAGAATGTGG
AATTGCATTTAGAATGGCTCATGGGATTGTTGGAGAATTAGTAAAACGATCTATTGAAGAAAAA
GTTGAAATTAAAGAAATAATTTCAGAGGTTCTTGAAAAGAATAACCTTTCATTAAGCCAGGAAA
AAATTGATACTGCACTCGATCCTTTTGAAAACGTGAAATTAAGAAATGTAATCGGCGGACCTGCA
CCAGAAGAAGTAGAAAGAGCAATATCTTCATTTAATGAAAAAATTTCAGCACATAAAGACAAAT
TAGATGAAAAAATTTCTGAAATTAAAACTGCTGAAAAAAATCTCTTAGAATAAAAAATTAAAAA
AATTATAAAATATTAAAAACCCTTTTTAAAATTATAGTTGCGTAGCTTCCTTTCTTTAAACTAAA

TTCTAAATCTATTTTGTACTTTTTTGAGTTTAATTCGTCAGATTTAAACTTTCCAGTATTGACAT
CTCCAGGAATTGAAATAATCGGTCTTTTTGTGTATTTTAATTTTCCAAAATCTAACTTTTCAAAA
TCTGAAATTTTAATCCGTTCTTTTTTCAAAATTGCCTTTATTATTCTTTTTTCTGAATCAGAGTA
TTCTACGTCTGAAACTATCGTTGGAAAATCCATTTCTTTTAATTTACAAAACAATTCTTCTTCAA
TATTTTCGTAAAACAAAAATGTCCCGCAGGAATAATCGACATATATTCTATTTTCTTTTGGAATA
ACTTCTTTTAAAAACTCTTTTAAGCATTCATTCCAAAGGTAACTTTGATATGCAGAAATGAAAAG
CTCTTTTAACCGATTATCTACGTATTTAAACGCTTTTTTAAAATCATTTTCGTAGGTTAATGACT
TTACCATATTTCTAAACATTTTACTTCTTATCTGCTTCTTATCGATATATTCTAAGCATTCATCCC
AGTTACCCCAATTTTTAGCAATAAATCGTTTTAAATCCTTTAACTGCTTTTTCTCACTTTTCGTA
TAGCTTGTTAAAAGTATTTTAACGACTTCTTCATAATTGCCTTTCAAAATCTCCTTCGCTATAAA
TTTACCGTTAAATACACTTCCAAATCTCTGATCATCGTAGTAATTTGGCGCTCCAGAACTTAAAT
TTCGTATATTGTCTGCAATCTTTAAAAATTCGTCTTTATCGACAGCCCTTGCTATGATTTCAAAC
CGGTTTCCATACAATCTTCCAATTTTTAAGGGTTTTTCAATGGTTCCAACGTATTCAAGCTTTAA
ATTATCTTCGTCAAGGGATAATTTACCGTATTCTTTTGGAATGGAAACATATTGAGTTGTGATTG
CGTGCCTGTCCTTCAAACCGCAGTAACCAATTTCTTTTGTAGGAACTTCAAAGTTTTTTGAAAGA
TACGATATAGCTTTTAAATTCTCTATTCCGTTTTTTTGAAGCGTATATAACGAATAATTTCCATT
ATCATTTAATTCATAATCTAAAATCTCATTTACGATGAAATCTTCGTGTTTTTGTCTAAATTTCA
TGATGCACCTAATTTAAATCGTGTTCATCTGATCTATTTCTTTTAATATATCATAATCATTTCTT
TTTACAGCGATTTCTCGAATTTTTTCGATTATTTTTAAATTTTTACCACATAATTCCCGATATTC
CTTACAATATTCTAAAATATCTTGGTAATGTCCTTCGATCAAACTTTTATCGTATTTTGATAATT
CATATGCTAAAAGTCCTTTTAAAGTGGATTCTTTTGAGAATAAAAATACTGGGTGAAGCCAGAGC
ACCAGTTTTTCCTCCTCTTTTTCCATTATATTTTCAATTTTTTCTAAATCCATGAATACGTCAATA
TAAACCAGAACTTCAGAAGAAATTTTTGAATTTTCCGAAATTTTCTGAACTATTGATTCCACGTT
CACGTCAAACTCAGAAATCTTAATATCCAATTCTTCGTCGCTCAATTCATTCCAAAGCAGTATTC
TTTCCCTGTGTTCGTATGAAGCCGTTGAAAATGTCTTTAAAATAACTTCAAATGTATTGTCTACT
GAAAAACTGTATTCGTCCGCTTTTCTCGAAGAAAGGTGTATTATCATTACACCATCATCAATGTG
TTTTAAATTTACTTTTCTTAAGACATATTTTCTATCCGTTGCTTCAAAACTAGGCAATTCATCGC
AATCCATTAAATTTTTTATAGAATGGCGTTTACCATTTTCATCCCAAAGATTAAGTCCTTTTAAT
TCAACGTCAAGACTTTCCGCTAATAAAGGTCTCTTGAATATTCGTCAATATTTTTTACGATATC
TCGTAATATCTTATTTATAAGTTGTTTAGACTCCATTTTAGTCACCAAATTTATCCGCCATATCT
TAAATACCATCCGCGATCCATTATATTTTTAATTAATTTTCCATTGTATTTCCCAATTCCAAGAA
GTTCCTTATTTTCATTAAATACTGCAACTTTTCCGTCGCCTTTTAATTCAATAATTGAGGAATCG
AAAACATCTCTTGAATAAAGAAATAATCCTTCTCCTTTTTCATTAATAACTGCATAATTTTTTTC
AATATCTTTTGAAACCAGCGTTATACCTTCAAGGGATGGTAAAAACTTTTCTTTTCCCGAGATTT
CTTCTAGATCTCCAATATGTATTCCTGCACTGTAAATATCCTTGTGATTTTTTAGGGATTTCATC
ACTTGTGAACTTACATGGACTACGTTTTTTGACTTGCCCCTTAATACTAAAAAATTATTAAAGTC
AAGTTCTTCAGCTTTTTTACCAATATACCTTGAAATGGCCGCTTTTATAGCGCTAATTTCTGATT
CTTCGAGCTTTCTGTGTTTCATAATATCCCGCGTAGATTAGATCATTGTTTCTATTTAGATCGTG
TAACTATATATATCAAATTATAAAAAAGTCGGATTAAATTAGTTATGTTAAATTAAGTTAAATT
ATGAATATATTATATTATTTTTGAGATTTCCTGATTGTGGTTTTAACGTAGCTTTCTATTTTTTT
ATCCAGCTGTTTTGAAAGACTGTTTATAAGTTCGAGGTTTTTTTCAGAGATATCTATTTTTTCGG
GTACATATTTAGTATTTTTTAGGGTTTTTTCACCAGTTAATACATCCAGATCAGAAAAAGTACTA
TATAATTCCCTTCCTGTTGACGCGATCTCGTTTTCAAACAGTTTTTCCTTCTTTTTTACCATAAATT

GAAAAAAGTGGGAACTTTGTAATTACGTTTGAACCTGCCATTACGAGCGGCCCGATATTTGTAAG
TTTATCAACCCATGTTCCGGTTATTATCTTCATTTTTGGGAAATTTAACCTCACAGTTGAAACCC
AGTTCATATATTCGAGGGTCGTTACCGAAGTTTTTCCCTGAAAAACGGTTTCATCTTGCGGATTT
AGGGAATAAAATGTAATCCTATCAAGATTTAGTTCTTCGATTAAGTTTAAGAGTAAATCTATATC
TTTTTCAGTTTCTCCCATTCCTAAAATTATCGTAATTCCAGTTTTAAGCCCCATGTCTTTTGCTTT
CTTTAACATTTCTTTTGTATTTGATAAAGGCTTTCCTGGACACAATTCTTCGTGCAATTTTGGAT
TTACAGTTTCTACTGCACCAACAACTCCTTCAATATTGTTTAAATTTAAATTTTCAAAATCAATT
ATTCCAACATTTAAGTACTGTTTTGCCTGCTGGATGTAGGAAACCATCTCGATAGTATCGTTTAA
TTCTTCAGTTTTGTACCCATATCCTCCAGAAATAAATTCAAGCTTCCAACCTATTCTTTTCATTAG
TATACTTTCTGCAAGAATCGATTCAGGCTTTCTTCTTGCTTTTAATGGATCTTTTATTTTTTCTT
TTTGTGTGGACATGAAGCAGAATTTACACGGGTCATCTAAATTACAATACCAGCCTAAAAAAAGA
GCTCTTTCTAAAGTAGTAATATTTCCGTGGTGTTTTTCCGTTAATTTAAATGCCTTAATTGAATT
TTCCAAAATCTCCGCAGAATTCATAATATCTCCTGATAAATTTAAAATACTTGAGTAAAAGATAA
TATTATACCACAATTGTTAGGGGTTGTTTTCGTGAACAAATTATCTGAATCTGATTTAAGTGTGA
CCTTGAATATAAACTATAGAGGTAAACAGATCACGAAAACTCAAGTCGAAATTTTAAAATCAGTT
TCTGAAACCAGATCCCAGAACAGAACCGCTGAAATTCTGGGCATTCCCCCATCAACAGTCAATAT
TCAGATAAAAAGGCTTGAAAATAAGCTAGATTTAAAATTAACATATTCTTCTCCTGCCGGAACCA
TGTTATCCCCTGATGCAGAACGTATCGTATCATATTATAATGCATATCTTGAAAGAACGTCAAAA
GAAAAATTTATTGCATGCGGATTTATAAGTGGAGAAGTGGGACGTATTCTTTTTGATGATGTGCT
CGTTTCATCATTTTCTAATGTTTTGAGGCTTCACAGGTCAAATTTAACCAGTGTCGTTGGAATTG
ATGATCCTTACTGGAGCTACAGGCTTGGAGATCCTGTTCCTGTTGCATTCGACCATTTTGTAATG
GTTTCGAAAGGCGATTTTAACTTTAAAAATCTTCTCGGGGTTAACTATTCTGCACACAGGATTGT
TTGGAAAACCCTTAAAAATGAAAAAATAGATTTTAAGGTATCAAAAGTTGTAAAAAAATCCATTT
TATGCGATAGATCTTTTAGAAGAGGGCTACAGTATGTTTTTAAATACGTGTCTGTTAAGATACGT
TAAAAAAGACTATTTAATCGAAACTCCAGACTATTATGATAAAACAAAACATACTATAAACTTT
ATTTTAAATAAATCCATCCCTGAAGATGAATTTGAAGAAGTATTGTATAAAAAAGAAAAAGAAA
TAAAATTGGCGGGTTTTGATCCGCTTTTGTAAATTTGAGTTGTCCAATATTATTTTTAAACTTAT
TTGATAATTATTTTTGAATTTATTTTTCCAACAATTGGCGAAACAAAGTATGCGGTGGGAAACGC
TATACAGTAGCCGATTAAAAATACCTTTGGCCAAAATGCTAAAACATCTAAACTAAATCCTCGAT
TTACCATTGTCATTACTAAAGACATTACTAATGCCATTGAGCCAGACATCAATAATGCAAAGGTT
ATTCTTTCGTATTTTTTGGGGATAATCATGTAATTCACCTAATTTTAAATTAAAGTAACAATTAA
AAAATAATAAAAACAATAAAGATTCAAAGTATAAAAAGTTAAAATAAAATCGTATTTACTGGGG
AATATTTCCGCCAGGAACCATTACGGTTAATTTTACCTGTTTTACGCCTTTTTGTGACGTAAGTT
TATCAGTTAATTCTTTAATTTCTCCTGCATCCCCCTTAACGAGAACTACTTCCATACAGTGGTCGT
GATCGAGGTGCATGTGGATTGTTGCTACAATTAACTTTTCGTAATCGTGCTGTATGTTTGTTAAC
TTCTCCATTACGTCAGTGGAGTGGTGGTCATAAATAATACTAATTGTTCCAGCCCGTTCCCCTTC
AAGACTGTGAATCCACTTATGCTTTATTAAATAATCCCTAATAGAGTCTCTTATAGCCTCACTTC
TGCTTGCATAGCCCCTTTCTTCGATAATCTCGTCGAATTCTGCAAGTAAATTTGTAGGTAACGAG
ATACTTATCCTATCCATATCCACCATTGTATGCCTCCCAGAGTTAATAACTGAAATCTACATGTA
ATAGATAGTAACTTCGTAATATTTAATAATATCTTAAGAGTCCTATTTAAATATTATGTAATAA
TAAGGTGAAGAATTTGAGTGTTGTTATCGGATACTACGGAAAAAATGGAGCTGTTATTGCAGGC
GATAAAAGAAATTTACTATTCAACGGAATTGAAAGTAACAGGGCAAAGCTTGAAGAAGTTCTTT
ATTCTGGAGAAATTAAAACTGATGAAGAATTGTTTAAAAAAGCCTCTGAATTTGATGTTACAGT

CCATATTAATGATACGCGAGAAAAAGTTAAGAGTTTAGGAAATTTATTATCTGGAGAAGTAGTA
TCAATTGGAAAAGACTCCAAAAGAAGAAGAATGTATTTAACAAAAGAAAAATGCGCAATAATTG
ATATTGAAAATGACCGGATAACCAACAAATCAGTAAAAACCGGGTCGGGAATTGTTGTATTTGGT
AACCGATACATAAAACATTTTGTTGAATCAGAAATCAAAAAACACGTTCAAAAACTTTTAAAGA
TGAGTGCCCGCGAGATTAGAGATTTATTTGAAAAAATCCTAAAAAATATTGAAAATGCAACATT
AAGTGATACTTTCGAATATTATGTTGTTGAAACAGGAGAACCCGAGTTTGAAAAAGCAGTTAAC
AAGGATTTGGATGACTTATTTAATTACAGACATGATTTATCCATAAAAATGGCTGAAATGCAGA
TATTAACAATGATTGCTGAAAAAATAGTAAAAATAGGGGATGTCGGAGTAATTAAAAATGGAAC
TTTAGTACTATATGACGAATTCCTTGCAATTAATAAGATATGTCCTGAACCAGAAATTTACTCGG
AAATTGAAATCACGGGCGAATTTATTGAAGGAGATATTATAACAATCGATAATGAATCTTTAAA
AGTAAAAAGAACAGGCAGTCCAGTAGCAGTTCAAAAAATAATCTGTAAAAAATAATAAATTAAT
TGAATTTAATTTTTTTCCCGATTAAATCAGCAGTTATCCCTGAAGCTTTCACTTCAAACATTCTA
TTTGATTTTACTTTTGAATTGTAAGTTTTCCAAGGAATTAAATTTGTTGCAACGTCAACAATTTT
ATCATCTTTTAAAAAAATCACATCTATAGGATATTTCATAAAAAATGTATGAATATGAAGTCTTC
TATTCCCATATTTAAAGAATAATCCTTCATTTTCTGAAATATCCCTAAACATGAGCCCAAATGCC
CGTTTTATAAAATTATCTGCAATATTTACAGAATAAGTTTTACCGTTTATTTCAAGGGTTTTTGG
ATTCATTTTTTAGTTCCTCTCTTGTATGGCACTGGAACATACTGTACAGATGGCCTCTGATTTAC
AGGCTGCCTGTAAAGGTCGAATAACTCATAAACGATATTTGGCAGGTCTGTGTTTACTTCAATTC
CGAGATCTTTTAATTTATTAATTGTTAAAGGATAGTCGTGCGTCCACTTTCCCGTAGAAAGAGTT
TCTGAGAGGTATTTTGCTTTTTCTTCGCCCATTTTGTCTTTCAATATATCATAGACAAATTCTTT
AACCTGAACTATCGCTTTTCTTGCAATATCCCCCAATATTAAAGTTTCATCGTCCAATTCATCCGT
ATATTTTGTATTTATTGCATTTATAATTGAAGCTGCAGGGTACTGTCCAATCTGAGGATCTACAG
GCCCCATTACTGCATTTTTATCAATTATAATTTCATCTGCTGCAAGAGCTATCAAGCTTCCACCGC
TCATTGCATAGTGGGGAATTATAACTGTCGTTTTTGCCTTGTGTTCCTTTAGAGCGATTGCAATC
TGTTCACTTGCAAGTACGAGCCCACCAGGAGTATGAAGTATTAAATCTATCGGCATATCTTCGGG
AGTCATTCTAATTGCCCTTAAAATTTCCTCACTATCTTCTATTGTAATATATTTATAAAGAGGAA
TTCCAAAAAGTGCCAGCTGCTCCTGCCTGTGTATCATGACAATTGCACGGGTTTTTCTAACAGCT
TCGAACTGCTTTATAAATTTGTACCTTTGTAAAAGCCGATATCTAAAAAGCACCTGCGGATAGAA
AAATAAAAACGCAAAAAAATATCCAGATAATTGATGAAGGATCCATTTTGATAGCCCCCGTGAAA
ATTGTCTTTTTGATCCAGTAAAGTCCTTAGATAACCTAAATATCTTATCATATATATATTCCTTT
TATATTAGATTAACATACTTACTTAACCTTAAATAATATCTTAGTCTAATCAGATTATTCATATC
AAATTATCATATAATCAAGAGGTGCACTGATGAGCAATATCAATATCAGTAACCTGAAACTCAAA
AAAACTCTTCAAAAACCACCCGTAGTTCCAGAAGAAGCCATGTGTTTAAAATACGTGATTTTGGA
ACCCGTTGGTTTTCCGATAAATGTTAATGGCGAGACTTTAAAGGTTAGTGTAGAAAACCACGCAC
TATTTAACTCATACGCTCGAGAACAGTGGGAAAACGAGATTGTAAAGGAAGGCGCATATTTATTT
GACAGCACGATTATCCCGGAATACGCATTTAAAGTTGTTTCCCTCTACCCAAAAGAGGGGGGAAT
TATAACAAAAGACACTTTATTTAAATTGGAAAATAATTATAAACCGCAAAATTCTCCAAAATTT
AAAGAAACCACTTTTGAAGAAGTCGTCGGACAGCTTGATGCAAAGAAAAAGTGTAAAATTGTAA
TAAAATATCTTGAAAATCCTGAATTATTCGGAGAATGGGCCCCAAAAAACATCTTATTTTATGGA
CCGCCTGGAACTGGAAAAACAATGTTAGCAAGAGCACTTGCAACAGAGACGGATGTACCGCTTTA
CTTAATAAAGGCTACTGAACTTATTGGAGACCATGTTGGAGATGGCTCAAAACAGATTGAAAGTC
TTTACGAAAATGCATCAGAAAATACGCCCTCAATAATATTTATCGATGAACTCGATGCAATTGCA
CTTAGTAGGCAGTTTCAGTCATTGAGGGGAGATGTATCTGAAGTAGTAAATGCTCTTCTTACAGA

ACTCGATGGAATAAAAAATAATCTGGGAGTTGTAACAATTGCAGCGACAAACAACCCAGAAATGC
TTGATAATGCAATTAGAAGTAGATTTGAAGAAGAAATTGAATTTAAAATGCCTGATGATAACGA
AAGATTGAAAATTCTCGAACTCTATGCAGAAAAAATGCCGATAGCAGTAAATGCAGACCTGAAA
AAATATGTTGAGAAAACAAAAGGCATGAATGGAAGAACGATTAAAGAAAAATTCCTAAAACCTG
CACTCCATAAAGCAATTTTAGAAGATAGAGAATCGATTACTCAAAAAGATCTCGATGAAATAATC
AAAAAAATCGTTAAAAATTCTACTCCATTAAATTTATACGGATAATCTATATATTTATACGGTAC
TATACCGGTATGTTGTAGTATATAGATAGTGTAAAAAAGGCGATATCTTGACGGGGTCTAAACCT
TATAGATTAGAAGTTACATTTCCTGCGGAACAGGAAAATGAAATTAGGCAAATGGCAAAAGAAT
GTGGATTCAGAAACGTTGCAGAATATGCAAGAGTTTCTATGAAATTTTTTTATACAATGAAAGA
CGTTATGCGTTCAGTAGTTGATTTTGACTCAAAATTAGATGTTGTTTTGGAAAATACAAAATCTA
TCAATAGTTCTTTTGAAGACCATAGAAAAATTGTGGTTGAAACTGCTGAAAAAGTAAATAGACT
TTATCAGCAAGAAGAACTTGAAAGTATTTTAAAAGAGATTATTCGTATTTTACAGGCTAATTATC
CAAAAGGATATAATGTTCATGAGCTTGTTGAGTTAATCGGTGCAGATACGGATAATAAGAAAAA
TGATTTATTTTGGTATGTTTTAAATGAAAATAAGTTTTTAAATGTTATGTGAGTGTTGATAAC
GAGTTTGTGTCATTGAGAGAGAGACCTTTATGGTCCGGGTATGAACTAGATACATTTGGTGTTAG
TCATGAGGGTAAATAAAGAGGAAAATTTAAATCCTGGTTCAAAAGAAGTTTTTCAAAAATTTAT
GAATTATAATTCACACCATAGTGAAAGTTACACAAAAACTCTTAGATATTCAATTTTAAATTTTA
TGGAATTTGTAAAAAAAGATTCATTTTTTGATGTTGATGTATCGGATTTGATAGACTTTTACAAT
AATAAAAAATGTTCAAAAAATACAAAACTGACTTATTTAACTTGTTTAAATGTTTTTTATACAT
ATTGTATAGATAATAACTATATTTCAAATAATCCGGTTTTAAAATACCGTAAATCTTTGAAAAA
ATCAAAAAAAGAAAGAGTTTATTTGACTGAAAAGCAGCAAGAGATGGTATACTCAAGACTTGCA
GATATTGAGTATAGAATACTGTATGTATTTTTGGTTCAGACTGGACTTAGAAGAAGTGAACTTA
TAAATTTAAAATTTATTGATGTTGAACTTGATGATAGAATAATTCATGTACATGGTGGAAAGGG
GGATAAATACCGTTTTGTTCCGATTTCAGATGAATTGTATATTTCATTAAAAAGATGGAATTATG
AGAGAGCTTTGAGATATCCTTTAGATGATACTTATTTTATAACCCGACGGGGTAAAAGGTATAAT
GAGAGGACTTTGGATTCTTTTATACATTATATAGATAGAGCAGTGCCTAATTTAAATGTGAGGGT
TACTCCACACATTTTAAGACATACATTTGCGACTAACTGTATAAATAAAAAAATGGATTTAAAAA
CGCTTTCTTTGATAATGGGGCATGAAGATATTGGAACGACTTCGATTTATCTTCATAAAAATAAA
GAACAGATTAAAAAAGACTTTTTAGAAGCTTTAAGATAAAAATTAGTTTAAGTCTTGTTTTTCT
TTTATTGCTTTTGACATTCTGTTATAATAATCTTCATATTCGTAAAAGTTATTTCCTTTTTTTAT
TTCATCTAATGACATTTGTGCAGCAGTATATTTTTTTAACATATAATAACACTCTGCTTGGATAC
AGTATGCCATATATGTGGGTTTTGCTTCGATAGATTTTTTAGTTTCTTGAATTGCCATTTCAAAG
TTGTTTCTGCTTGCATATACCATTGCCATTTGGTAGTGATATATGCTTACTTGATTTTTGTTGTT
GTAATCAGTTATTTCATATTGTGGTGGGTTGTAATAAGCATTTAAATCGATGTATTCAAATGGGG
GTAAATCTTCTGATTTTTCGTAATTAATAGTACTTTCTGATGTTTGTATACTTGTTTTAGTTGTT
TCTTTGACAGTATCTTGTTGATTTATTTGATTATCAATTATCAAAATACTGGCAACTGTTGCAAC
TCCCCCTAAAACCATTAAAAAAATAATTGCAAATAAAATAACTATTATTTTAGTAATTTTCAAGT
TTTCCCCCTCACTTGATATTTATTCTTTATTTTTAGAATGTCCGTTGTAGATATTTGTGTTTCCA
GAACCGTTAATGTTGATATTTACAACTATTTTTTCTTCAAGTTTTTCTCCTTGTTTGTAGAAATC
CCTTGCTTCTATACCATAGATTGTTATTTTGTAACAAAATTTATCCATTTTTCCCTTCCAGTCTA
ATATTTCAATTGTTATAAGGCCTGCATCTGCTAAATCTTTTAACACTAAACTTGCTGTACTTCTA
TGAACACCTAATTCGTCAGCAATTTCTTGTAATCCCAATTCTCCTTCTTTATACAATAAATTAAG
GATTTCTTTAACGTGTTTTTTACCAAGTAGTTTTATAATATTCATGATTAGCAGTCCCTTTTAAG

TTTTTGTAATTTATTTTTCACTGTTGGGAAAAATTCGTATGTTGAACTTTTTATTTTTATTATTA
TTTTAATCATTGTAACATTTTATTCTCTGTTGAATAAAACCGAAACAGTTAAATAGTTATATATA
TAGAATAGGTACTGTTGGTAAAAGTCCAACAGTGAATAAGTTTTAAACGGAGAATTTTTACTAA
TTTGAAAAGAGGTGAACTTATCAAAGAAACTTATGAAATTGACGTTCCCGAAGGTTGTGAAGTA
ATAATACTTCAAGATAAACCTGTACCCCCCTTTGGAGATTCTAAACATATTATTATCCCCAAGAA
TTTAAAAATCGTTCGGGCTGACGTTATTTTGTGTGTAGAAGCTGAAAACAGATAATCCATTTGAC
AAGGTGAACATCGTGGTTAGATGATTAGACGTGTTGACATATCGTTCATTCCCCAAACTTATCTT
GTTATGGGGACTGGAGAAAGGGAACATTTGACAGTTTTTAAAAGGGATTTTGAAATGAATCCTT
TGGTTTCAGATTTGAAAAGTTATAGAAGATATGTTCTCAGGAAGAACGGAATTGATTATTTAGC
ATACATTGATGCAGAATTTAACACGATGGATGAAATAGAGCTTAAAAAAGAGTTTGATGGCGTA
TATGCCAGATTTGTAAAAAGAGGGTCAATACAAGACCAGTTTAAAAGAGTTGATGTACATGGTT
GAAGAAAACACCAAAAAAAATGAACAAGAAAACAAAACCGTTGTAAAAACAGAAAAAAAAGGCT
TAAAAGCCGTTAGTTTAGCAGATATACCAAAAATGCAAAAAATCGAAATTGGAAATACAACAGT
TACATTTAATGCAGGAACCGAAGGAATTCCTTCATTTACTCCAGATGGTCAAGCTATTATGAATG
GTAGATACCCTAAAAATGAGTATGTTCCATCAGTAAAATCGCTTGGAAACGTAAAAATTAAAGT
TACTAAAGGATTTGGTAGTGATAATGTAGAACTTTCATACACGGTTATGAAAGCGATTATTACAC
AGGTCGAAAATAATCCAGTAACTAAAATGATGGTTGACATTTACGCAACCGCAGAAGTTGTATCA
CAGTGGAGAACTGGATTTGAAACTGGAACAATGGACAAAGATACTATTTTAATGCTCGCAGAAA
GAAACGGAATCCCTGAGGATCACGTAAGAGCTCTCGTGTTCGACGAAGATGTTGGATTTAAGACG
ACTGCTGAGGACAAGTTTGTTGTAGAAGCTTAATTTTGGGGATTGTTATGAGGATACCTAAACTT
TCAAAGCCTGAGCAGAAACGAGTGGACAAGGAAAGTGTTAGAAAAGCAAAAGCTCTAGAAAAAA
TCAGAGAAATTCACGGAGATTTACCAACATTGGAATTTCCAAGAATTTCTCTTGATAGAGCTGTT
TTTTCCATTTTTTCAAAAGATATTTTTAGACACATCTATCGAGGAAAGGATAGTTATATTGACAG
ATATGATCAAGCCCGAATCAATCGAATGGTTGAGGATATTAAAACTAATGGTTATCAAAAATTG
GACTGTGATCTTCCAGAAAGAAAAGATATGAAATACAGGGGCGATGTTTTCAACGATTATAACCA
GTTTAAACAAAATATGATTGATCATAAGGGTCATGGAAACGGTTTACACTTTAAACTCTGTGAAT
TTGAGGATGAGGTTATATCCTGGATTACTATTTCTATGAGAGAACCCCACATGGCGGTTGCATAT
GTTAACTTTCAAAAATACTACCGTTATTTAAACAGTATTCCAAAACCCAAGTTTGGAAACTTTGA
TACAAACTTTTTACCGCTTGAATGTGATAATAAAGAGAAAATCTTTGATGATTTCGCTAAATTGT
ATCTTAGATGGTGTCAGGAAGTCGAAATAGTTTATAGAAAACTTATTGATGATTTGTTTGGTTAC
GAGCTTGACCCTGAAAAAACCGAAGTTATTCCTGCAAGTATTGAGATTCCTTGTGAATATCTAAA
TAGAGATATTGCGGAATACTCTTGGCTTTCAGATATTGCGCAATGTAAATCAGTTGTAAAGATGT
CTGACCTTACAAGAACTATGTATCTTAATAAATCTTTGAAATCAAGACGTTTTCAGATGAAACTT
TATCAAAAAGCATTTGGACTTGCAAGAATGGAGTTAACAGTTCATAAAGATTGGGCTTCTCACTT
CTTTTGTGAAACTGATCCAGCAGTTTTGAAAGATAAAATAAAAATGTGTATTGATGCAGTTTTA
GGAGAATACAAGCTTTCATTGTACGAAATTAAACCAGTAAAGATGACTTATGATATTTTTTTAA
GTCAACTTGCAGCAGATCTGAAATGGGATCGGGATATGCTTTTGGCAATAATTGAATCTAAAAAT
TCAGAATTTACTTTTAAAAATGATAATCAAGGACTTAGAAAAAAGCTTAAAAATAAGGATTTAA
TCGAAAAAGATCACCGTGGAGTGTGGGTTAAGTCTGATTATGCAAAATGGCTTTCAGGTACTCTT
GGAAGATACTTTATCTGTCCGCATTGCCGAACTTATATGTTAAAAAGTGAAAAATCACTTGAATA
CGTTTGTACGACTTGTGGATTTTCTAAAAAAAATGATTTAAGGGGTTATTATGGGAGATAAAAAA
GAACCTTTTAAAATTACAAATACAAGACTATTCCAGATTGTGGACCAGTTAAAAAGGGAAGGGA
ATCTTCCAAGAGATTTTATTGCAATTATTGATAATACTGGAGCCATGAAAGTTGCAGGACGTGCA

ACTCAAGATCAGGTTGAAAATTTAGTTTACACAGTACAAATCGAAGAAGATGGATTTAAAGTTT
AAAAATGGCGGTAAAATGCCTGAAATTAGAACTAGAGAAGAATTCTTGAAAGTTATGGATGAAA
ATCGACTTTGGGGCGGTTGGATGTTAGCCCTTGAAGATGGCTCCATGAGTGGTGCTGAAAGTTAT
CAGGAACAGAAAGACACCGCTGAAACATATAAAGAAAGATTTCCTGATTGGGCAGAAGAAATAA
TTAAAGAAGCTGAGCTGGTGAAAAATACAGAAGAAAAATCGGTTTAGACGAAAGTTAAAGAGGG
AAATTCAATGGAATCGAGCGAAAAACTTGAAGAATACAAGAGAATTACCGATGAACAACAAAAA
CTCCAGTTTGAGTTAATAAAAGAAGGTGTTCCTGTTGAAGTTTTAAATCGGATTTTTAATGATGC
ACGTTCTAAAAATTCGCATATTCCATATAGGTAGGTGAAAAATGGCTAAAAAACATTTTAAAAA
TAAAAAAACTCGAAAAAGAAATTTTTCTAAAGAAAATGCAGAGTTACAATCAGTTATCAAGCAA
TTGAAAGAAAAAGGCGAAATTAACAAGAATGTCCTTGCTGTTGTGAGTTCTACTGGTAAATGGCT
TTGTATTCATGCTAATAATGAGGAAAAAACTGTAATTTATCATAATTGTATGATAATGAGCGAT
AGATTTAGAGTTGAGGGGTGAAAATGAAAATTGTTCGGAAATTTGCACCAACTTCAAAAAATAC
GTTTTCCATAGGTCCGGTGAAAAAATTAATTAAAAGATATCAAAATCACCAGCTGGTGAGTATTG
ATCCATTTGCAAATACTTCTAAATTAGCAAAAATTACCAATGACTTGGACCCTGAAATGCCAACA
GATTATCATTTGGATGCTTTAGATTTTTTGAAAAGGTTTGAAGCGAATTCTGTTGACCTGGTATT
ATATGATCCTCCGTTTACTCCGAGACAAGTTGCAGACTGTTATAAAAAATTTGACCGGACCGTAA
ACAAAGAGACTACTCAAATAAGCTATTGGTGGAACCAAAAAAGAGAGCTTTCGAGAATTGTAAA
ACCTGGTGGAATTGTCATAGGTTGTGGTTATAATTCAAATGGATGTGGCAAAAAGTATGGATTT
TCCTTAAAAGAAATTTTACTCGTTGCACATGGCAGTAATCACAATGATACAATTGTGACAGTAGA
TCAAAAAGTTCAATCTAAAATTTATGAGCTGGTGAATTATGATATTAACTCTATCAAAAGCGTTT
AAAGACGTTCCAAAAAAAGAAAAGCTAAATTAAAAACGACTGTAAAAGCTGTGGAGTTGGTGGT
TTAATCCCTGGAGGGTAGCCGATATCGACTACATCTTCAGTAATTTAGGTGGTAAAAAAATGCAG
CATTTCTCAGGTTGTGAAGCGGTTATTATGATGTTGTTGTATCCTGAACTTGAAGGATTTTCTAT
TATAATATCTGCCCCGATAGCTGAAATATTAACTTTTTATTCGTATGCAGGAGTTAAAAAGGCTT
TAAAAAGTCTTTATGAAAAAGGCTGTTTATATCGCTTTGAAAAAGGGGGTTATTATTCTATAACT
AAGCTTGGAAAGAGCGAACTTCTTAAAGTTAAGGAAGATTCCGACTATGACATGATGTATAAAC
AACTTCAATATTTCCAAAAGACTGGTGCATATTTGCCATATTTTGATCTTGAACGTAGGCTGAAA
GAAAGAGGTATAACTGAGGTGGAATTTTGGCGGGTTATGGAATAAGACCTTTTCCTGGTACGTGC
TGTCCTGAATGTACGTGTAATAATGGGAAAGATACTTGTGATGGCTGCGGTCATAAGCATTCTGA
ATGTCATAGGAAAGAGTACACTAAAACTGGTGAAAAAATCGTAATTGAAAAAGAAGTTGAGTTT
GATTTATTAAAAGAATTCTGCATTAAAAAAATGGGTAAAGAACGATTTGAAAAAAGATTTAATG
ATCTTTTAGGGGGTCCTGATGATTATTTTCCTTTCTATAATAATCCCAATAAGGTAATTTTAATA
ATCGCTGATGAACTCGAATTAGTAGACATTAATGAAGATTTTGAAGGTTTTGAATGGATTGGAG
AACATTATTGCAAAAAAGGGTGAAAAAAGTGAATAATTTTAAAACAGATCTTTTTTGTTATCTA
GTTTTTTTATTATTCATTTATTGGTATAAATTACTACCATTTGAAGCTGTAACCGTTATGACTCT
TTCGGGAGTTTATTTCTATCGAAAATATGGGTTACCTGTCCTTATAGTGTATTGCTGTACTTTTG
ATATGTTTTATATTATCATTAGGGAGCTATTAACTGTTGGATTTACTGGAGATATAATTCACGTG
TTTGTATTGGGCAGTGTTGGTTGCATAATGCTGTTCTTTGTTTCGACATTTAGTGAAAAATTAGT
AAGTGGATTTGATTAAAACGAGGTGAAAAGCTTGGGAAAAAAGAATAAAAGAATTTACTTAACT
CAATCAGATTTTAATAAGCAATACAATAAGATTTTACTTAAAATTGGTTTAATTTTATTTGTTTT
GTCGTTATTTAGGGTAATCCCCTCAGATGTATTCGTAATTATGCTGTTAGTACTTATTTTAGAAT
TTAAACTTATTTCTATTTATCAATTTAAAAAATATTGTAAAAAAAACAAGTTGCCTTTTGATTTT
GTTTCGATACGATATTGTGATGGAACTGGAGAAGCAATGCGTTTTAAGACCCCTGCTGAGTATGA

GGAGTTTATAATTGATTTATTGGCTAAATGCAATGAGCATGACGTAGAATTAGAAAGATTTGAT
AAAATTAGTATGTTTGTGGAGTTGGAATTATGCGAATGATGGAAGTTAAATCGGAAGTTGATTT
TAAATTTACAGAAAGTCAAGAAAAGCTTTTTGCTTTGTTGTTTGTATCAGGTCTAGTTGGTATGA
TAATAGGGTATGTTATTTCTTTTGAAATAGAAGTTATTGGTTTCTTTTTAATGGTAGCTACTTTA
TTATTAGTATTTTTAATTAAATTTCAAATGAATAGAATGTATGATAAATATATTAGTTCTTTGCC
ATATGCGAATAAAATGATTCATATGTTGTTTGTTAATGGTGATTTAAAGGAAGAATGTTATCTT
ATAATAGATTCTCTTGAAGCTGAAAACAGATATTTTAGAAAAATTTATTCCATGATGGAACAGG
GTTATTTACCTGTTGAAGTTGAACTCATTGATGATTGGGTTGAATAATCATGATTGCCCTTTCTT
CTTTTCAAAGTCCTTCGGTGGAAATAGGTATTCACTCTGATCTTTCAGGTGTGGATAAGAAAAAA
TTGGATCTTGCATCCAAAAGAGCATCATATTTAGTTTAGTTTACAACATTCAGGAAAAACTTCC
AGAAATATGTGATAACCTAAATCAAATTTATGACGAAAACGACTCTCAAATAATATCTTTAGGA
GTTCAAACAAGGAAAAAAAGACATTTAGCTTTAAAAATTGAAAAAAACAGTTTTCAAATTGTTT
CACGAGTAAAAGGGGATTGTAATATTTTGTTAAGTGATGACTCCTGGAGTGATATTAAATGAAA
AAAGTCATGCTTTTATTTTTACTTTTCATGATTCCTACAATTTACGCAAATGATTATGATGTATT
TGATTCTATGACCGAAGAAAACATGAATCCGTCGATGGATGCTTGGGATGGTATAGAATTGGTTT
CATTTTTTTATAATATCTCAGATGATTCTTTAACTTTTGAATTCTCGAATCAAAAAGATAGTACT
GTATCATTCAAAGGTTATGATGCTTACACAAAACAGATTTACTATATAGATTTACTTCCAAATCA
AATTACAAAATATAGAGTTGAAGATCCTGGACTTAAAGTTGGAAATATTTACTTTGCTTTCTGGA
ATAACAATGGATTTATTATTGTAAAAGAAGCTGCGACTCCTTTAACTCTACCAAACAAGATAGTT
TTAATTTCATACTCTGCATTAGGTTGGTTTACTGTTAAAATGTGGGCTTATCTGATGATTTGTAC
AGGTCTTGCAATGTATGTTACAAAAACTTACTTTATAGAAAAAATGGTTTATGAGAGAAAAAGA
CAGGTTCTTAAATATATTGGATTATCTTTTGGGATTCTTGCAGCATGGATTTTAGCTGTGAAATA
TGGACTACCTTTTGGATTGTATGAAGTTTTAACGATTGGAAAATGGAAATTTGTTTTAAAACTGC
CGTTCTTAGATTTTTCAAACTGGAACTTTAGATATTTACTTTCATATGGTAAATGGCTCTACTTT
GTAGCGTTATGGGTTGGATTTTGGGCAGGATTTAAAATATTCAAACCCGAACCCTTTAATCAATA
TGCAATAGCTCTCCATAAACTTGAAGCTGACGGCATTAACAAGAAATTAATTAATAAAAGGGAA
TTTTTAAGCCTTCCAATGATTCAAAAGTTCGGTAAGAAGTTTGTAAGATATCAAAATAAAGTTTA
TGAACTTAGATTACCTCTTTGCCTTCCTGCAAAAGTTAGTTATGGTGGAGATTATACTGAGGATC
TTTACGAAGTTATTGATTATACAATAATAGAACCTAAAAAAGAATACAATGAAACTACGTTTTG
GAATAGAATATTATCACATTTGGAGAGATTAAAAGAGAAATTGTGTCCAGAACCTGGAATTATA
GTAGTTGAATTATCAAAAATAATGAATGCTGATGCAGTAGATGCGTTCCACTTTACTTGGAGTTT
AAATGAATTGATGGATAAAAACCATGAATTGAATTATGAAACTATGGATTTAAAGAGAGTGAGA
GGAAACGAAGTTATTGCTGTTTCAAGAGTTGTAGTGGAGAAATTTACTTCTGTAATGGATGGGTT
CCAGCAGTTTTCAGATTATATAATATATTGTATTCAGAAGATGAAAAAAGGAGACAATCCTTTCT
TTGATGAAATTATTGAAAGATTAAGGCTTGAAAGTGCTGAAGGTGGTGAAGATGGAGAAGGAGG
ACAGACTGGAGATAGTAACCCAACTAACGAATCCAAATAATATAACAACGTGTGAACCGTTATTT
AATTCAGTAATTCCGAAGAAACATAAAAAAGGGTTTCAAAGGGGTAGAACTGAAAAAACAGTAC
TTAGAAACCCTGAGAAAAGGGGCTTTTTGTATAAATTAATGGATATGGAAGAACATAAAAAAGC
TCAAGAGATTGAAGAAAATAAGAGGTTAGAAGAGAATGAATAATTATTTTTCATTTATTGATTT
AATATTGATGAATCGAAAAAAAGTTGAACATTCTGGTATTCTTGTTTGGGGATACCGAGGAATG
GGTAAAACTACCTGTCTTAGAGGTATCTATCACCAAATAGAAGAATATAATAAGTTAAATCCTAG
AAATTCAATTTCTTCTATTTTTGCAACAGATATAGACCAACTTGATATCTGCGATTGTCTTTTTT
TAGATGATTTTGGTACAAAATTCAATAAAAGGGATTTTTCAACTACTGAAAATAAGGAATTTGT

AAAATTACTTCAGGAAGTTAGAACTAGTATTCCAGTTATTGTCGTTTCAAGTCCAAGTTATTTTA
TGCTTGACAAAGATCTTAGAAACTTTTTCACCCCTGCACAAATTCTCTCAAAATCGAGTGAAAGA
TTTTTATTAAATGTATCAGGTGCTATGTTTTCAGTTCCAAAACCTAAAGATGAACTTTTAAAAGA
GTTTAGGGATAAGGAACTTGAAGAAAGGAAAGAAAGATGTAAAAAATCTATTGAAAAAGTTAAA
ACTGCCCATGCCAAGAAAAATGGAATTAAATCATAATTACTCTTTTTTAGATATTTAGATATTCA
GACATTCACGATATACAGATATTCGGGATATTTAGACGTTTTAGTTATTTTTATACCCTCTAAAA
GTTACATATTAATTATGAACTTATGAGGTGGCTCTCGTGTGGATTTAAAGTTGAACATGGTTTCA
GTTTTTGCATTTATCGGTGCTTTTCTGTTGCCTTCGTTATATTTACAGAATAAAATTGGAACTAT
CTTTATTGATTTGTTTCCGTTTCTTTCTCCACTGACTCCTCAAGGGGTCTGCGTTGTTTTGGCATT
GCTGCTTTTAATTGTTGCAGTAATTTTAGCTTATGGTAATCCTATGGGTCAGATTTGGGTAAAAG
ACAAATCAAAGACTCCAAAGACTAAGAAAACTAATTTTAACAATTCTAAAAATAAAACCACCATG
AACAAAAAAAGGAACTTCTAAAAAACCTAAAAAGAGGTGAAATTATCGCACAACCTTGGAGAAAT
TGGTATCCAAGAAGAAAAACCATGTCATCAGAAGCCAATAGTCTTGCAATGACTCCGTCGTTGTT
GAAAAATGTTATTTTCGGTTTCATGATTCTGGCTGCTTTTGTAGGGTATTCTGGAGACATATTTG
GAACTACCATTCCATACATTGGACTTGTAATTGACTTTATAGTTCAAATTGGTGCAAGTTTGGCT
TATGGTATTCCTGGATTGATCTTGTTCTTTTTATCAAAAAGAAATAGAGATGCGGATTACTTCGC
TTTGATTTGGATAATTATCGGCATTTTGTATGCTGTTACTGAAAATACACTAACTGGAATTGCAG
GATTAATAACTCCGGTTGTTTCAAAACTGCCAACGCTTGTTACTGGCTTGATTGTACTTGCTTTG
GATATTTATTTTGCGATTCAATTATACAGAGAAGATTAGAGGTGAATTAATTTGGCAAGAATGA
ATGAAAAATCAGTAAATATACCAACAGGTTCTCTTTCATATTTTGAAATAGTGGGTCTGTTGTTT
GTTTTAGGTGGATCCCTCACCGTTGTTTGTGGGGTCGAATGGTTCATTAACTCAATTTTTGGAGC
ATATATTTGTACAGGGATAGGTCTTGCGATAGTGTCTCTTAGTGGAAAGGACAATTTAAGACGTG
TTAAACGTACTAGCTACTGGGAAGGCGTTGGAATTGGTTTAATTGGAGTAATTAATTTACTTTTT
GGATACTTCCAGTTAGAAGCGTTACAGACTTTAACTTATCAAATTTTAGCTATGGGATTGATTTT
CATAATTACAATTGCAGTAGCTTATAGGGATTAAGAGGTGAGTGATTTGGATTTAAAAAAATCA
GTAAAAGGATTTGGTCTTTTGTTCATGCTCTTGATTTCATTAATTTCGAGCGTATGTGCAACAGA
TGTTACGTTTAATGTCGGTATGGATGGCGTTACAGTTACAGAAGGAGAAACGGATTTAGGAATA
ATTAATACTACTGGAACAATCAATTTTACAGATGAGGAACACAATCTTACATTTTCCGCTGTAAA
TTACGATTCATATAACTTAACAGTTAATCCTACAAGTGCAACGGAATATAATGTTACACTAAATG
AAACTACCTATACAATTGTCATAAATTCAGAGTTAGACACTTTTGAAATTTATGATGAAGAAGG
AACATTAGTTGGAACTTTTGACGATGTGTCAAGAATTCTTGAACTTACAGCTGGAACTTACAATT
ATACAGCTGTTAAAACAGGATACGAAAATGAAAGTTTAGATTTCATTGTTCCTGATACAGAAGA
ATTAAACGTTTCAATGATTTTATTACCTCAAAATACAACGGATGATACTGCCGATAATGCAACAG
ATGAAGAAATTCCAGTAACTATTATTGTAATATCAGATGCAGATTCAAAACTTGAACAGTTAAA
TGATTTCTTCCATGATAACGTAGCCGTAATGTGTGTGATAGGGCTTGTAGTTATTTACGGAGTCG
GTAGAAGTATGAAAAAAACACAACATAAACCGGGAAATAGAAATTAAGGAGGTAAGATAATGAA
GAAATCTTATCTTCTTTTTTTATTATTGCTGCTTTCAGTAGTTGGTTCTGTAAATGCAACTGCTG
TTACAATTCCTGAAACGTGTACGGTTACAAGCAATTATGTAGTATTAAATGAAGAATTAGATACT
AATGTGACAAAAGCGTTACTTCTCACATATTCGTGGAATGATTATACTTCCGGTTCAATAATTCC
GACTATTTCAGGTGTTGATGGAAATATAATTTATTTTTCTGATGTGGATAGCTTAGCTCGTTTTT
GTTCTATTATTTCAGGAACTAGTCCAAGTTTAGCGTTTGATGGGAATTATAAAATTTTTAAATCA
GGTGCAGAAACTTACGCTACGTGTATTTTAGTAAATTCGCCTACTCCATCGGCAAGTGGAACGTA
TCTAAATAATATTACTATGAATCTTGAAAGTACACAATTAATGTACACTTATGGATATTACTTGA

CCACATTAACTGGAAACACCGGAACTGTGTTGTACAATTCATATCAGATATGGACTCAAAATTCA
GGTGGGATATGTGATTTTTCATCACTTACTGCTAATCAAGAAGGAATATTTACAGTTTCTCAAAA
GTGGTCGGGTAGCTCTGTAACAATGGTAGTATCAGTATTGGTTTTTGATTATGTAGATTATACTA
CTTCAATTCCAGATGGTTCAAATGCGGTTGTTTCTGAATTGTTAAGTCCTCCTACACTATATGAT
TTAACAATTAATACTAATGTGGACGGATTAACCGTTTATAATGGAGAAACTGTTTTGGGTTCTGC
TGATGATGGAACGGTATTTCCTTTAATTGCTGGAACTTATGATTTAACGTTTGAAAAGGACGGTT
ACTGGAATGAAACAGAAACAGTTATTATTACTGATTCTGGGGTTTCTGTTGATTTGGAAATGTTT
CCTGATTCAGAATTGTATTTGATTTCTTCAAATGTTACAACTCAAAATATTGTAACAAATATGGA
TGTTGTAATGACCTTACATATTGAGCCAAAAGTTGAATCTCAAAATACAATGATTTCATTCTCAA
AAGAGATAGTTTCAATAAAAGAAGGAACGGAAGAACTTACGAAAGATGGAGATAACTATATTAT
CGGGGATTTTTATGAACCGATTGATTTAACAGTTACTTTTAATTCTGGAACTTTAACCGGAAACC
GATACATTGAAACTACTGTATTTGGTACTGCATACGTTGGAACTCAGAGTACTGACTTTTCAACA
ATAAAATCAATTACATATGCTGTAAATTCGCTTCCAATTATTGTCTATATGCCTGATTGGGAAAG
CGGAGTAAACGAACTTAGAATTACAGAACAGGAAGGAAACACTTTAATTTTAAATGTTGAGGTG
TTAGATAGTTCTGATGATTCAGTGTTTACTCAAAATGTGGTTTTCAATCCTTATGGAGTTGAAAC
ACTTGATATTAATTTAACGGAAGGTATTTACTATTTGCATCTGTACTGTACTGACTTTGATACAA
GTATTCCTTTTACAGTTTCTGCAGCAAGTACAACAGATGATGATTCAGATATTACTGGAGTAATT
GTTACAGGTGATGATTCATTAGTGGATCTTTTGTATGATTATTGGTATGTTCCAGTAGCTTTAGG
AATTGCAATTGGAGTTTACTTTGTATTTTCCAAGAAAAAAGGTAAAAAACCTTCAAAATTAAAA
GGTGGTAAAAATGGTAAAAAATAAATTTGCCCCTTTTTTGATCCTTTTACTTCTTTGTGTAAGTC
CTTCTCATGCTGCATGGACTGATTATATTCCATTATATGGCACATTTACTGGAGCGGTTGATACT
GCTACTGGGTTAGTAGATTCATTTTCTGGAATGATGGATTCTATAACTGATGCAATGGATTCTAT
TTCTTCATTTTTTAATCCTGATGATACCACAATAATTGAAACTGGTGACATAGAAAATCAGACTG
CACTTGGTATTGAAGATGGGGAATATTTTTCAGCAGATGAAGTAAATTCTGTTGGAATGGCTTCA
TTGGATAATATTTCAAAAACGATTAATTCAGAATATACTTGTTTGTTGAATACCTCAGTTACAGG
TACTGGTCTTGGAAAGATTGATATCGGTCTTAGGGCAAGGGATAAATTATATGGATACTCGGCTT
TTTCCGTTGCTATGTCGATATATAAACCTGAAACTTACGCATATCAAGATAATTTCCATTTAACA
AGTTATTCTTTATGGGTTGAAGATATTGAAAATCCAGGTGTTAAACTTTGGACATATTCTGCTTC
AAGTAGTGAAGTTGCAACTCCCGGGGGGTCTGCAATTACAGATGCTACAATTTTAAAAGCTCCTG
ATGATAAATATTCAGTCGTAAAATCCTGTTTAACTTCAAATTCTAATTTAATGGAGTTAGATGGA
ATTATAACTTCACAAAATAAACAGTTTGAAATTCACGGAAACGTTGTAGGGTACATTGAAAATT
GGCAGGAAGTTGCGGTAACTACTTCAAATCCTGATGGTTCTACAACTACAACTTGGAAATGGGTA
AGGAAAGACAATATTCCGGTTGATTTAGATATTGTAACTACGTCAATGTGGCAGCATGAAAACAA
TGGAGCTTATTCACTTGATGGTGCTACTGGAACTTTACCTGTAAGTGATTGGTATGAATATGGTG
GTCTTTGGGTTACATATGCATCATTAAATCAAGGAAGTACTTCAAATTTACTCGCTCAGTTTTGG
TCTAGCCCGGTTCACATTTTAGATTCAAGCTCCAGTTACAAATTTTTTGCATTGTATAATGCAGA
GTATAACGCTGTAACTGATTTAGATATGGATTTATCAAATGTGGATACAAGGATAGTTACTTTAG
CCGTATCTAATGATTCAAATTTTGAAGTTGCAGGGTCAGATGAATTCAGTAATTTGAATATTGAT
TCAACATATGCAATTTCTGGAAACTTACCTTTCATGTCAAGTAGTGATAACGTAGCTTCTTGGAA
AGTTTATGCTATTACTTATGGTGAAATTACAAGAGATGACGGTCAAAAATTACCAGTTTGGACTG
TTGTAAGACCTTGGATTGCCGTAAACGATAACGTTCAAATATTAAATTTAGATACTGCGATTGAA
CTTGAAGACCTTTATTTAAGTGGTGATATCAATTCATCTGCACTTGAAGAAGTAAAAACTACTGC
AATTTCTGAAATAGAAACTATGCTTGATTCAATAGAACAAGATTTAGGCTATGCAGAAAGAAAT

GATAATACTGAAATGATTAATGCACTTAATAAAGCAAAAACATGTCTTAATAATGCTAAAAGTG
TAATTGAATCTTTATCTTCAACAGATACAGAAGATGAAAAGACTGCAAAAATCAATAAATACAA
GCTTTATCTTGAAGCTGCTGAAATGTACTTAAAAGCATCTGCTTGTTATAAAACAGGTCTTGATA
GTGATGGAGAGTACTATGAAGACATGGCAGATGAGTTAGTATCAAAGGAATCAATAGTAATTAC
TTCTGATGGAATCTTTGATGTGTTAAATGATATTCCAATCCTTGGAACTTTGGTTTCATATATTC
CTGGAGGATGGTGGACTGTATTAATCTGTGGAGTTTTAGCTGGTGGATACGTTTACCTTGAAAAT
GACAAGAAAAAAGGATTTGGAAGAAAAAGAAAAAGATTCTAATTAATTAACTTTTTTTAATTTT
TTACTATAAGAAAAACTGTTATTTCTCTTTTTAAATTATTAAATGTGTATTTTTTAAATATCTTG
TAACATATTGTATTTTGTTAAATTTTGGGGGAAAATATGAAAAAACTAATTTTATTGATTTCAA
TTTTAACTTTATTTTCAGGATGTGTTACAGTAAATGTTAATGTTCCAGATAATGAGCTTACCGAA
AAAGTTGTTGATACAGTTGATAGTTCAAATGATATTGAAACTAAACCGGTTCAGAATAGTTTGG
AAGAGAATACTATTCAAACGTCTACAAAAAATGAAAATTATCCTTGGAATGCTCCAATATTTGTA
AATAGATATGACTTTTGCCTTTATGCATGTGATTTTAAAAATGTATCTACTTATGAGTTAAGATT
TAATGATCAGGATATTTACCAGTATTCAGAAAATGAACTTTATACTATTGATAAAGATCCCGGAG
ATTTGACAGATGTTACTTTGATTCATCAAGATGGTACTAGAAAGCATTTTGATATTTATTTTAAA
CTAAGTACAGATTATGATACTTATGTTAGTTATGCTGAGAAATTTTTACAAGGTGGAGATTATA
GGAATGCAGATTACTGTTTAATGAAAATTGAAAAGTTTAATGCAACTTGTGATTTGTATGCTACT
AGAGCTTACTGTTTAATGAATCTTGGAGAGTATGAAGTTGCAAGAGATTATGCTGAAATTTCAA
AAACTGACCCTAACTGTGTTACTGTGTCTAATCAGATTATAGATACTATTGATTTAATTGAAAAA
TATGATTAATTTACTTTATTTCTTATATTATTGGTTATTTATTGTTATTTAGTATTTTTATTTGT
TATTATTCTTTATTTATTATTTAACCTTTAATTATTTGTTATTTTTGTTATTATTCTTTATTTAT
TCTTTTTTATTTAATTTAATTGTTGTATTATCGTTAGTTTATGTTTAGTCTTTACAGTTAGTTAG
TTGTTAGTTTGTTATTAATTCAGTTCCTGGAACTTGTATATGGGTTTCCGCACCCGAAGGGGCAT
AAATCAATAAGCGCTAAGCTCTAAAATCGCCATAGGCGACATATACCCAAAGGGTATATAAGCCA
TGGGCTTGTCGTTTGAAGTTCCTAAATTGCGTGGACTACCGTCGAAAAAATGCACTTTCTAGAAA
TAAAATCATATAAGCAGTTTTTCAGGTAGTTCTGAGCAGGTCGCGACCTGCGAAAGCAATTGAAA
CATTGCTTTGATTGTTTAAAATTAAGCTTGATTATATGAACTGCGCAGCAGTTCTTAACTCCGAT
AAAATCAATAAGTCCAAAGACTCATTTTATGGGCGAAGCCCATTACTAGTGTGGTAGCCATACAC
CCGGAGGGTGTAGGGCGTACCTATATGATCCGCCCGTTAGGGCTATTAATAGATAGTCAACATGT
ACAGCACTTTTTTTGGTTTTTTTGAAAAGAAGTCCAAAACATAACTTGTTGTTTTTTACTGTTTT
TTTGTCAAAGCTTGTTTGGTTAGTCTTTATTTGTTTTTTACTGCTCTAAAAAAATAAGCTGTTTT
AGGCGTTTATTTTGGAATCTAAGAATATTAACTTTCTTCGATGTAAAAAAAATAAATTATTGGT
ATATGACATACTACCGGTAATATACCTGTAAAAACTTATATATATACGGATAAATTCTTTTTTAA
ATTTTTAAAAAATAAATTGTAAAAAAATAGTGAATAAATATTTAATTTTTAAATTTAACTGCTG
TTCCGTAGTAAGTGTATTCTACCATTTCTTCGCCAGTTTCTTCTGATTTGTAAGAAACTGAATCT
AATTTACACTTATTATTCCGTCTGCACCATCTTCTTTTCCTTTTCTAAATAATCCGTATAAAGCT
TTCTTTTTAGCTTCCACTTGATCTTTTGTACCTGCTTTAGCAGCTAAAGATCCCATGATAAATCCA
GTATAACTTTCAATTTCTTTTCCTTCAATTCTATCGCTTGTTGTTACGATCATAAAAACCACCAT
GTTTTTTTAAAATAACTATGGAAAGAAGATATATATTAATTTCTAAATAATTTCATTATTTAAA
GAAATCTTCAATTTTAACCTGCTTTCTCTTGTCGTTTACAAATAAACTGTCAATTCCTGACTTTA
TAATTTCAAGTCTTTGTTTAATATAGTCGCTTGCATCATACTTTTCAGCCATTGTCTGGGAAACA
TTCATATACTTTTCAACAGCCCCTTTTGAAACTGTCAAAATCAGTCTGCTTCCACATTTTCTGCAA
ACTCCTTTTAACGGCATCCTTCGGTATTTTGCACCGCATTTACATCGGACACCCTGTCTTGAAAAA

GCTCTTAAATTTCCGATTAAATCAGGTACGAAATGTGATTGAATAACCTTTTCAGCTACGTCTCT
TTCATCAGTTGCCCTGATTTTTTTTGCTACGGCTAACTGTGCTGAAGTTTTTCCATCATCGAACC
TAATGTTTTATACGCACAAATTGGGGGCCCTTCATCGATTTTTGATGTTTCATGAGTATAGCCAA
TTCCTTCGTACTGGCTGTCTTTATCAAGCCTGTCTTCAATAGTTTCCATCATTTTTTTAATTTCTT
TTGGAGCGATTCCTTCCAGAGATTTTTCATAAAATTCAAGCGGGTATTCCCACATCGAATCCATG
TTGTGAACCTCACCATCTACTTCCTTAGGGTCCAGAATAGTTGTAAGAACCAGTGGTGCATCCAT
CTGGCCGCCCCTTTTATCAGGCAAAAATCTTTTTGAAAAGTTCATAAATGCATCCAAAAGGAGGA
AAAATGCATCTTCATCCCCATCACAATTTCTCCTTTTTGAAGCGTGGAAATAAGGATGTGCATAG
CCTACGTTTGCATTGCTGTATCCAATAATTCTTCCAACCATTCCTGCAGAAGTGTGTGGTGCCATT
CCGATTATTAAATGGCCTACAAGGTCTTCTCTTGTATTCACGTTGTAAAATCTATCTTTTTTGTA
GTATTTTTCCAAAAGATCGTCTATAAATCCTGAAACATTTAAAAAGTAATCCATACAGGATTCAG
GAACTATTACATCCTGAACTTTTAATTCTAATACCTGATCTTCATTTTCAAGGGGATTTCCGTGA
ATATCTTTTAAATATCCTAACGATTTTAATTTTTCGATAGGTACGTGTATTTCTACAGGTTTAAA
ATGCGTTACAGGAACATCTGTACAGTCAAACCGGGTAGTTCCGTCTTTAAATACATATATGTTAT
TTTTTGCCCGCAATATTGCTTTTTCCAAGGGTTCAATTATTTTATCCTTTGAGGTCATACCTTTA
ATACATTTTACGTCGCCGGGCTTGTTTATTTTTAAATTTTCAAGAGTTTTATACCAGTAATCCTT
TATTGGAAGCTTTATTCTTGAAGGGCCTGTTGGTTCAACAGAATTTCCACAGAACGGACATGTCC
TGTATAAACTTACCTTTCCACAACTACATTTTACATTTGCAATTTCGATTTCGTCAGTTTTTCCTT
CTTCTACGGCCTTATTAATAAGTCTTACAAGAGCTCCCGCATTTCCAATAGGAAAAAGTCCATTT
ACAGGCGGTTTCATCTTTCTAGCAGCTGCTTTTTCAGGCCTACCCATTCTTGCGCCAACGTAAATA
TAGGCGTTTCTTCGGATTTCAAAAGGTGCGATCGTATTTATAAAGTGCATGTTATTTTTTGTATT
TTGAAGTTTTTCATCAATATTATCTATCATATCCCTTTTTTCATCGAAATCATAACCTAAAGAGT
ATAAAAATGGATAATATTCAAAAATTTCTACTTTTTCTTCCATTACGGTATGAGGACATCCAATA
AGTTCAAGATACCTTTTCGCATTTAAAATTTCCTCATCTTCAGGATCGTAATTTAGTTCCCATGA
ATCATTGCTTGGATTGTATTTTCCACCAATAACCCAACTTCGAAGGGTAGAAATATTCTCTTTAG
AAATATCGTGCCAAAAATATGTGTATTTTGGATGCAGTGGCGTTTTTGTAATTTTAGCATAATTT
ACTGCTTCGTTTTGCCCTGGATTTTTAATAAATTCTTCCGTGTATTCCAAATTTTGTGATTTTAA
GATTTTTTCATACCATTCCGTGCACCAACTGCTTGGAAGTACCGTATGGTTGTTTTCTAAGAAAT
CCCCGTAATTAACTAAAATATCGCCTAAAAATAGAATTTCTTGAACTTCATCGGTGTATTGTTTT
GCTTTTTCAACAGTGTCTATTTTAAGTACGCTTCCGTCATTTAATTTAACTATCGGACCTTCGAT
ACTGTCAACAGGAACTACACATGTTGCCTTTCCAGGCCTTTCAGTTTTTAACTGTGTTCCTACAGC
CATGAAGTCATCAACTAAATACATTATTGCAGGATGAAAACCATCTGTTGCAAAACCAGTATTTC
TGCTTCTTCCATATCTAAGTCTGAAACCGCCTTTTTTGGAAGGGTGTGAAAATACAGGCCTTCCT
GCAATAACCTCCCCAATAAACTTAGTTACAGCTTCTACTTCATAATCTTCATACTGGGATAAGTC
TTCTTCATCTTCGTAGTTATATTCATCGTTTTCTTCTTCCAGTTCTTCGATTACTTCTTCTTTTTT
GCTTTTTAATTCTTTTAACCAGTTCCATCCTTCGATATTTAATTTATCAACGTGTCTTAAAATTT
TAGGAGCTTTTAAAAGAACACCTTCAACCAGTGCTAAAAGAGCACCCCCTCGAATCTGGTTTGTT
TCCACTCTTGGTAAATCCCGGTGTCCACTAACTTCAACGTCGTCAGTGGCTTCCCCAGTAATTTCT
ACAGAGATATTTCTAACTGCAACCCGTATCTCTTCAGCTTTTGGCTGGTATTGGAATGTAGTAAC
CTCAGATTGATACAAATCAACTTCTTCACCGTACCGTTCAACTTCATCTTCTGTTGGCTTAAATCT
ATCTAATCCCATGTTTTTTCTAACATAATCCCCAACAAGTACTGCAAGTGCCTGTGCAGTTCCCCC
TGCACTTCTAATCGGCCCTGCAAAATAAATTGCAAGATACTCACTGTTATCGCTGTTTTTCTTAA
TTTTAACATACGCAATTCCTTCTAGGGGTGCTGCAACAATTCCCTCAGTAATAACGGCCAATGCA

GTTCTTACAGCCTGTTCAGCTCCAACTTCTCGCCCAAACTCGCCAAATTTTCCTTCGACTATCTCT
TTCGCAATTTCAAGCGCTGCAGGTTCTTTTCCAAATTCAGATACTAGTTCCCTAATACGCTCTGCA
ACATTTTTTGGTCCAACGATACCTTCAACCCTGTCTGCCATATCTTTTGCAAGCGGATTTCAACA
TGATCCGTTACGTCGTACCCGTTCTTTCTACACTCTTCTGCAAGATTATAAAGGTTGGATACATC
GTCTAAAATATTTTTAAAATATTCAGTCATTCCTTTTGAAGCTGAAACATGGAGCATGATTATCA
CGATTAAAGTTGGATTAAACGTAGGTTAAAGAGTTATGTCTTAAAAGAATATATAGTTTTAACT
TGGTTATGGGGGTACCAATTATAAAATAATTGTTTGTATAGCCTAGGTTAAAAAATTATTTTGA
AAATGTTTAAAATTTTGTAAATTTTTTTGAAATGGCATCGGGGCTATAGCCTGCCTTCTGTAATT
TCGGTGACATTCAGCTTAGCGCATATGCTTGCACCCGTGTAAAATAAGGTCGTTTCATCGATTTT
CTAAGAATCCTCAATTTTGAAAATATCATAGTCATATTTCTTAGAACCGTGTCGTTTCTATACCC
TATTCATTCCTTCTCAGGAAATCTACACGATTGGGTGGGCAGAGCTTCCTCCCAGTGGATAGTCA
CCAGCCCCCTTGCCACGAGTTTGAGTATATACTACCCGAAAACTCGAAAAATGAAATGAATAAAA
AAAAGTGACTAACAGCGTCACATTGTTCCCACCAGATGGCAGTACTTAATGAATCGCAGGAGGGC
TTAATTTCCGAGATCGGGATGGGATCGGATGTAACTCCTCCGCTATGACCGTTATGTCAAAGTTT
AAATTAAAAGTGCATCTATTGCCCCCGTATCTTTAAGTGTCCAGATGCCAATTTATCTCTTGAAC
TATTAGTACTAGCGGGCTGAACATCTCGGAAAACCTTGATGCGTACACCCCTAGCCTATCAAACC
TGTCTTCTACAGATGTTCTCGTTCCCGAAGGAAACGGCTGTCTATTTTCAGGATGAATTTCGAGC
TTAGATGCTTTCAGCTCTTATCTCTTGGCGCGTGGCTGCTCGGCAATGCCTTGTCAGACAACCGAT
CGACTAGAGGCGCCGACGGCCCGTTCCTCTCGTACTAGAGCCACCTTTCCCTCAGACAGCCAACAC
ATCCAGCAGATAGCAACCAACCTGTCTCACGACGGTCTAAACCCAGCTCATGATCCCCTTTAATGG
GCGAACAGCCCCACCCTTGGGTCCTGCTGCAGACCCAGGATGGGAAAAACCGACATCGAGGTAGC
AAGCCGCGGGGTCGATATGGGCTCTTGCCCGCGACAACTCTGTTATCCCCGGGGTAGCTTTTTTGT
CATCCCTGGCCCCCATCAGTGAGGCACAGGGGTTCGTTAGGCCCGGCTTTCGCCTCTTCGTTGCGT
TTTGTTGGCAACAAAGTCAGGCTGGCTTTTGCCCTTACACTCAACAGCGGAGTCCTGACCCGCTTG
AGCCAACCTTTGGGCCCTCCTGATGCATTTTTAGGAGGGTACCGCCCCAGCCAAACTGCCCACCTA
CCGGTGTCCCCTAAAAGGGTAAGGAACATGGTCATGAGAGGGTGGTGTCCCAGGGTCGCCTCCAC
CTTTCCTGGCGAAAAGGTTTCGATGGCTCCCACCTACACTGTACACTCACAACCATGCCCCAACGA
CAGGCTGCAGTAAAGCTCCACGGGGTCTTCGCTTCCCACTGGAAGTCTCCGGCCTTTGCGCCGGAA
TGGTAGGTTCACCGGATTCTAGTCGGGGACAGTGGAGGTCTCGTTACGCCATTCATGCAGGTCGG
AACTTGCCCGACAAGGAATTTCGCTACCTTAAGAGGGTTATAGTTACCCCCGCCGTTTACTGGCG
CTTCGCCCGGTTGGAACCGGGGTTCACGTACCAGCACTGGGCAGGCGTCGGCCTTAGTACACACCT
TTTCAGGCTCGCTAAGACCTATGTTTGTATTAAACAGTCGGACCTCCCTGGTCACTGCGACCAGCG
ATCCCATATATAAACAAGATCACTGGCACTCCTTCTCCCGAAGTTACGGAGCCAATTTGCCGACT
TCCCTCGACTAGATTCATCCGCTACGCCTTAGGATACTCACCTAGGGGCACCAGTGTCGGTTCTGG
GTACGGACATCTAAAATCCTTGTTAGTTCCCTTTTCACGGGCTCCAGAGATCGGCCGAACCCTCCT
AACGGAGAGCTCATCACTCTTTCATCCGGTTCTCGTTATTACAACTCTCCCCGGACTTATAAGCTT
GAATGCCCAGACAAGAACACTCAGCCTACCCAGAAGCGTCGGAAACTAACCTAGTGTTGCCACGC
GTATTTAGATGGCACAGGAATATTAACCTGTTTCCCTTTCCTCTCAAAGAAGTTAACTCGAGAGT
TAGGACCGGCTTACCCACAGCTGACAAACATTGCTGTGGAACCCTTGCCCCTTCGGCGGTGGGGAT
TCTCACCCCACTTATGCTGTTACTACTACCGGGATCTTCATTTCTACGAGGTCCACTCGACTTCAC
AGCCGAACTTCTGCCCTCGCAGAACGCCCCCCTACAGGATCACCTTTCGGTGCCCTTGGGTCTCGG
CGGCCGATTTAGCCCCGTCCATTTTCGGGGCCCTTGATCTCGACAGGTGAGCTGTTACGCACTCGT
TAAAGGATGGCTGCTTCTAAGCCAACCTCCCTGCTGTCTTAGACCAAGGACGCCCTTCGTGTTCAC

ACTTAATCGGCACTTAGGGGCCTTAACCCAAGTCTCGGCTATTCCCGTCTCGGACATACAGCTTAC
CCGTATGCCCTCACTCGGAGCATGCGACGACGACAAGTTCGGAGTTTGACAAAACAGCGAGGAAT
TTCTTCCCCTAACCACCTTATCAGTGCTCTACCTTGCCGACTATCTAAACTCCGGCTGACCTGAGA
GTCACTTCGGAGGGAACCAGCTATCTCCGGGCTCGATTGGCCTTTCACCCCTAGACCGAGGTCAGA
AGAGTGCTTCGAACGGCAACAACTCTGCAGGCCTCCATCCCTCAGTTGAGGGACTTCACCTTGCCC
CGGCCTAGATCGCCCGGTTTCGGGTCATATTGCTGTGACTCCGGGCCCTTAAGACCCCGTCCCTCA
AGATTTTGCGGACTTGTTGGTTTCCCTACGCCTACGATCGTGAAGATCTTAAGCTCGCCACAACA
ATATACTCCCCGGCCCGTTTTTCGAAACGGACGACACAACCCTGGCTCTATACTTCGTACAGCCGC
CTTGCGACGGTTTCCTTCAGAATAGTTGCCTTTGGGGCCGTGTCATACCATACCTATCTGGTTTCA
GGCTCTTTTCACACCCGATTAAAGGTGCTTTTCAGCTTTCCCTCGCGGTACTAGTACACTATCGGT
CTTGGAACGTATTTAGAGTTGGAAGTTGATGCCTCCCAAATTCCCGCGCGATATCCAACGCACGG
TACTCAGGAACACTTCAAACCTGAATCGATTACGCTTACGGGACTTTCACCCTCTATTGTGTGAC
GTTCCAGTCAACTTCAGCTTTTCTCAACAGGCGAAAAGAAGGTCCTACAACACCACATCTCCCCGA
AGGGATTCAGTTTGCTCTGATCCGCGTTCAATCGCCTCTAATAACGGAATCTCTATTGATTTCTC
TTCCTGCGGGTACTAAGATGCTTCAATCCCCCGCGTTCCCAATCCTTAACGGATTACAAAGTAGG
AAGTCCCATTCGGAAATCTCAGGTTCAATGACTGTATGCGTCTCGCCTGAGCTTATCGCAGCTTA
CCACGTCCTTCATCGGCGTTCCAAGCCAAGCCATTCCCCAGATAGGGTAATAGATAAATCTAGGC
ATCTGGATAACACCAGATACGGAGGCTTATGCACGACCTTCACTAAAAATCCGGTCCTCGGTCCA
TAATACTCGGTATTGTGGACTGCTAATAAAAGTGGACCCGGCGGGATTTGAACCCACGGCCTCCG
CCTTGCAAGGGCGGCGCTCTCCCAACTGAGCTACGGGCCCATTCGTTAAGCCTACAATCTTTTAGT
GCCTAGTAGCAACCATTGTTCTTTTTTTAGGAGGTGATCCAGCCGCAGGTTCCCCTACGGCTACCT
TGTTACGACTTCACCCCCCTCGCTGAGCCCAAGTTCGACCCTAGCCAAAGGCCAAGGCCTCACTTC
AACCCAACTCGGGTGGTGTGACGGGCGGTGTGTGCAAGGAGCAGGGACACATTCACCGCAGTATT
ATGAACTGCGATTACTACGGATTCCAGCTTCACGTGGGCGAGTTACAGCCCACGATCCGAACTAC
GACTAGGTTTAGGAGATTTGCGTCCCTTTTCAGGGTTGCGGCCCATTGTCCTAGCCATTGTAGCCC
GCGTGTAGCCCAGGAGATTCGGGGCATGCGGACCTATCGTTGCCCGCTCCTTCCTCTGACTTAGCG
CCAGCGGTCCCCTATGAGTGCCTTTCTCCCGGAGGAAAAAGTAGCAACATAGGGCACGGGTCTCG
CTCGTTACCTGACTTAACAGGACGCCTCGCGGTACGAGCTGACGATGGCCATGCACCACCTCTCAG
CGCTTCAGGCAAGGTCGTCAACCTGGCCGTCATCATGCTGTCGCTCCTGGTGAGATGCCCGGCGTT
GAATCCAATTAAACCGCAGGCTCCACCCGTTGTGGTGCTCCCCCGCCAATTCCTTTAAGTTTCAGT
CTTGCGACCGTACTCCCCAGGCGGCGAACTTAACGGCTTCCCTTCGGCACTAGATGGGCACGGGGC
CCACCTAACACCTAGTTCGCAAAGTTTACGGCCAGGACTACCCGGGTATCTAATCCGGTTCGCGCC
CCTGGCTTTCGTCCCTCACCGTCGGACCCGTTCCAGACAAGTGCCTTCGCCATAGGTGGTCCTTCA
AGGATCAACACATTTCACCGCTACCCCTGAAGTACCCTTGTCCTCTCCCGGTCCCAAGTCCAGCAG
TATCCCTGCCAGTCCTGCGGTTAAGCCGCAGGATTTAAACAAGGACTTACTGGACCGGCTACGGA
CGCTTTAGGCCCAATAAAAGTGGCCACCACTCGGGCCGTCGGTGTTACCGCGGCTGCTGGCACCGA
ACTTGCCCAGCCCTTATTCCTCAAGCTGTTTACACTTGATAAAAGCCCATGCTGTGCATGAGCACT
TGGGATCCCCCCGTCGCACTTTCGTGCATTGCGGAGGTTTCGCGCCTGCTGCGCCCCGTAGGGCCT
GGGGCCGTGTCTCAGTCCCCATCTCCGGGCTTCCTCTCTCAAGGCCCGTACCGATCGTAGGCTTGG
TGGGCCATTACCCCACCAACTACCTAATCGAGCGCAGTCCAATCCTCAGGCGCATATGCTTTCGAA
AAAGAATCGTTCCAGACTGCTTTTTCTATGGGGTATTATCCTCAGTTTCCCAAGGTTATCCCCCAC
CTGAGGGTATGTTAACCACGTGTTAATGAGCCGTCCGCCATGGCCGAAACCATAGACTCGCATGG
CTTAGTCGAATCCCAATAGCAGTGGCCTCCGGCGGGATCAACCGGAATTAGTAGCGTAAAAGAAT

TTTACGCCACCCTCTCGTGGAGTACGTCATTTTACATTAAAGGAAATGACATTTGGTTGCTATCT
AGGTTTCACCTAGATTGTAGGCTTACATCGCACCTGAGTATTTCAATCAGATGTTCACTTCAATT
GCATAATATGTCAATGGTTCGGATAATGTAATGTTTTTAGGTTTGAAACGATAGTATATAAAAA
AAATTAATTTAAGAATAATTAAACCATACTTTCAAGAACATCCAACTGTTCGCCAGTCCCAAAAG
CATATATTTGATCATTATATTCTAAAATAATCTCAGGACCTGGATTTACAAGCAAATCATTCTCT
TTTTTAATTCCGATAATTGTTACCCTACTATTATATCGTATTTGCGATTCTGTTAGTGTTTTTCC
ACGAATTTTTGAATTTTTCCCAATTAGTATTTTCCTAATTTCCAAATCTTCATCATATTCATATT
TTGCAATAGACATAAAAGTTGAAACAAAATCCAATATCCCTGGTTTCATTGCAAGTTCTGCAACC
CTAAGACCCCCAATCATATAAGGGGAAACAACCTTATCAGCACCTGCAATTAATAATTTATCCAT
TGCTACCTGCTCGTCTGCTTTTGAAACCACATATAATTTTGGATTCAGTCTTTTTGAAGAAAGTG
TAATATATACATTGTCTGAATCTGTTGAAACTGTTGAAATAAGTGCTTTTGCACGGTCTATCTGC
GCATCTATTAAATATTCGTCGTGTGTGGCATCCCCGACAATATAATTAAAATTTGGATCTTTTTC
AAATTCTGCTTCTAAAATTTCTGAATTATTATCAATTGCAACATAATCAAGACCGCTTTCCCTTA
ATCGATCTATTACAACCCTACCAATCCTACCATAACCGCAGACAATGTAGTGATTTTCGAGCATT
TCTATTCTTTTTTTCATTTTGATCCGCCTGTTGTATCTTTTAAAATAACCGCCAATAAACGACTCT
GCAAACGCGCCCAGCGTATAAAGTCCAGTACTTACCCCAAAAACAATAAACAAAATTGTCAAAAG
TTGGCCGAGTTCTGAAGTTGGGTAAAAATCGCCATAACCAACTGTTGAAATTGTGGCTACCGAAA
AATAAATTGCTTGTAAAAGGGTCCAGCCCTCGATATACATGAACATTAACGAAAACGAAATTATA
ACAACAAATAATACAAAAATTCCGATCTTAATTTTTTCCATTGATTCCATAACATACCCGAAACT
AATTATAATCCCATATATATCTTATAAAATATATATTTCATTCTAAAGGGGAACGTTATGGAAAT
TGGATTAATTGATATTAAAGGAACTCTGCCTTGTTTTGAAAATTTTGGAAACCTCCCAACAAAAA
TTATTCATGAAAATAATATTTCCAAGATAAAAGACCTTGAAATGCTCATAATACCTGGGGGAAGT
ATTGTTGAGAGCGGATCTTTTACTGATGAATTTAAAAAACAGATTTTAGATTTTGATAATTATA
TTGTTGGAATATGTAGTGGATTTCAAATACTCTCTGAAAAAATTGATATTGGGAGAAAAAGCCC
GATTCCAATAATAAAAGAAGGACTTGGTCTTTTAAATGTCGAATTTTCACCACTTATTTGTACTG
ATTTAGTAAAATTCAACTTTGAAAATAATAATTTATTCGGAGAAAAAAATCAATTAGGACACGG
TTTTCACTGCCACACCTACGGAAACATTGAAATTACTAATAATTCAACTGAAAAATGGACAATTT
CAGAAATTGAAAAATTAAACTACAAAATAGTTGGAAAACAGAATATTTTGTCTGGCGTTTTTAA
AAATAAAGTATATGGAACGATGGTTCATAATTTACTCGATAATGATCTCGTTGTTGAAAATTTTT
TAAAAAATATGAAAATTAAAGACGATGAAAGGGAAGAAATTTTTAAGAAAAATTCGATTTTAAA
GGAAAAACTAAAAAACAGATCCAAAATCTCTGAAAACTTCAAAAAAATTGAATCTAATGACAAA
AAAGGAATAATTTTACTTGCAACAGGTTCTGAAAGTGGGAAAACATTTCTTTCAACAAGTATCTG
CTCAAAATTAAAAGGAAAAACATTTGTCTCAAAAATAGGTCCTGATGTAAGAGATATTGTACCTT
CATTATATCTTTTAAGAGAACCTATGACGAAATTTAACAGTGTTAAAATTTCCGATAGAGGCTGG
TCTGAACCGTCCGAATTTTTAGAATTCGTTAAAAATTCCAAATTTAATAATTATATTATAGAAGG
CGTCATGGGTGCATTTACTGGAGCCTTAAATAAGAAAAATTACAGCGGATCAGAAGTTTCGAAAA
TTTTAGGTTTTCCAGTATATATTATATCATCCTGCAGTAAAACCGGAATTGAAGGTTCTTTTGTT
GAATGTATGGCATACCATTCATTGCTAAAAGAAATGGGTGTTAATGTTGCTGGAATTATATTAA
ACAGAGTATATAATACAGAATTACTTGAAAAAGTTAAAAAAATTGCAGATGACTTAAATGTTGA
TTTAATCGGTGTGAATAAATTAAATGTAAATGTTGATAATAAAAGAGGGCTAATTCCTGAAGTT
GAAATCGATTACGAAGAATTTTGTAACGCCACGATGAAACTTGATTTTGAAATAGATATTCCAAA
AATTAATCCTGAAAAAATTAATGAAGATTCTAAAACTTTCGAAGAATATTTGAAAATATGGTCT
GAAAAACTAATAAATATAAATTAAATCCTGTTTTTAAAATTTTAAAAAAGAAAATATGTTATTT

TTTTAATTAAACTATTATTTCGCCGTTGTTTATTTTTTTTGCAGTGTCGTATGCATCATATATAC
CAAATATCCAAAAAAGCGGATAAGTAATGAAGCCTATTACCAAAAATATTAAAAAGATGTTTAT
AATCTGAACAACGATAAATACAAGCCCTTTTCCAATTTGCCCATTATATATTTGGCCTAAACCAA
CGAATAAAAATGATAAAACTGCAGCAACTCCTGGATTTTTTTCTGCTTTAGGCACCATTACGCGC
ACCCCGCATTTAGGACATATCTCTGCATTTTTATTTATTTCTTCCCCGCATTCTGAACAGAATTTT
GTTTCCATATAAACACCCACTTAAAAATAATTAAAAGTCTTTTGATAGGTTATTTGTATTACTTA
ATATATTTTTCCCATAATGTAAGCATATACTAAATAAAAGTAAAAATCCACAAATATCGCAATAA
TTAATCCCAAAATTGGAATTACTGATGCTATAAATATAACAATTGCACTGGTAACAATTACTATT
AAATATAATATACAGTAATCTGAAAAATTATCTTTTATTTTTTCAAATATTTCGGAAAACCTAAA
TGCTGCTCCAAAATCATCGTATGCGATGTAATTTGCATAAGCCATTGGAATAATAAATCCAATAA
TTAGCGTAACTAAAAATGTAATTAGCCCCACAATACCTAAACCAACGATACCCCCTACAATTGCT
TCGTCACTTCCAGTAAACATTGTTAGTATAGATAAACCGCCGAATACAGCCCAAATGATTGATGG
AATTAACATATATATGAACATAATAATAAATGCAACTAGCCCTTTTACAAATTTACCGCCAAATT
CAGACCATTCTGGAAGTTCTTCTTTTGAATCAATTATATTTTTAAGAAGTTCTAGCGCATAGCCA
AATGAAATAAAGTTTACTATTGGAATTATAGATAATATCGCACCAATAACAACTTTTTTGATCCA
ATCTGGATCGTCCGTCGGGAACTTAAATGCCCGTTCAAAATCCATAGTATCACCCCATATGTTAT
AATATTAAATTTTCGAATATATAATCCTTACTTTTTGTTAATAATTATATAAAATAGTTTTTTGA
AGATTTGAAGCATTTTTAAGAAATTTTTTAATTTTAAATTGATAAATTTTAACTTTTGGATACCT
CATTAAAACATTTCATAATTATTATATAGATCTGAAAACATATGTGAAAGGTACCAGAAAATTT
GTGAAAATAATGGATGATAAACAGATTCAAAATATTCGACCACGACTTACAGAATGCTTAAAAA
CGCTAAATTTTCAAGATATTATCTCTGAAAAAAAGAAAGTAGCTGTTGATATAGATAATATCTA
TAAACACGGGGTAATTGACTTTATAGAGTTTTTAGAAGAATTTCCAAAAGAAGGAATAGAACTC
CTTGAAGAGTGCTACAGGGATGCATATTATTCAATAAAAACGGAAAAGCCCGATATTGTAATTAC
TGTAAAAAATATACCTGAAGAATTTAATAAATCTGAAAAAAAGCAAAATTACACTATCGAAGAT
ATTAAAAGCGGCACTCTTGGAAAATTAATTGAATTTGAAGGAATAATTGTTATCGCGACAAAAA
TAAAATCTGCCTTAAAAAAGGCAACTTACGTATGCACACAATGTGGCGAAAAAAAAATACAAGA
AGTTGAAAATCCATTTGAAGCTTATTTTGAACCACCATGTCCAAAATGTGCTCAAAATATGACTT
TAATTGAGGACGAATCTGAATATATTGATTTTCAAGAAATTAAAGTTCAACAGCCCCTTGATTCT
ATGGGTGACCCTGAAGAGCCACCAAAATATATTACTGTATTTTTAGAACACAGTCCTGGAATTTA
TTGTGGGCGTGTGAAAGTTACAGGAATTCCAATAAAAAACCAGAAAAACAAAAATATCCCGATT
TATGATATTTATGTTAAAGGAATTAATTGTGAAGTTTTAGAAGATAAAATTGAAGCAAATTTAA
ATGAAGAAGATATCAAAAATATCGAAAAAGTTGGAAAAAATCCAAAAGTAATAGATATATTATC
TGAAAGGATGATTCCTGAAATTAAAGGTTATGAAATTATCAAAAAAGCGATATTTTTACAGCAA
GTTAAAGGGGTTAAAAAGGGAAACAAAAGAGCTGACAGCCACCTGCTTTTAATTACAGATCCAGG
TATTGGAAAATCAGTTATGCTTAGAAAAATTGCTGAAATTCCTGGAAATTTGTACGGGTCAGCCA
CTACTGCATCAGGTGTTGGTTTAACTGCCGCGGTTATTCGTGAAAAAACCGAAATTGGAGACGAT
ACATGGGTTATCAAACCAGGTCTTTTGGTTAAAGCGAATAAAGGAACTGCATGTATTGATGAGCT
TACAGTTAATCGGGACCTTCAAACTTTTGTTCTTGAAGCTATGGAATCTCAGACAATACACGTTA
GTAAAGGTGGAATCAATGCAAAATTGCCATCAGAATGTGCAATTATTGCAGCATGTAACCCAAGA
TGGGGTAGATTTGATCCAAATGTCTCGATTCCTGAACAGATAAATATTCCTGCACCTATGCTGAG
CAGGTTTGATTTAATATTCCCGATAAAAGATGAAGCAGATCGATCAAAAGATAAAGACATTGCAC
AGCACATTATCAATGTTCATAGGGCATATTTAAGTAAAGAAGTTTCGAAAAATATGAAACTCGA
CCATATTATAGTCGATGATGTCTTGATTGATAGGGATTTTGTAATAAAATATATAGAATATGCA

AAAACTAAATCACCGATAATTTCAGAAAGTGCTGAAAAGCTTCTCACTGAATTTTATTTAAACAT
GAGAAAAGGTTCAGTTCAAATTACTGCAAGACAGCTTGAAGCGGCAATCCGTATTGCAGAAGCGC
ATGCAAAAGCAAAATTAAAAGATGTTGTTGACGAAGAAGACGCAAATGAAGCCATTTCAATAAT
TACTGAATCTTTAAAGGAAACTGCATATGATCCTGAAACCGGACAATTTGATGTCGATAAAATTT
CAGGAGTTGGAAAAAAAGATAGAAGTAAGATGATGGGAGTATATGATTTAATAAAAGCACTTGC
TGAAAAAAGTGATAACGATCTTGTAATAATTGATGAAATCGTCGAAGCTGGAAAATCAAAAGGA
ATTGATGAAGAACATATTTTACTTTCAATTAAAAAGCTTAAGAGCAATGGGGACATTGATGAAC
CTAAGACTGGAAAATTCAGGATAATTTAAAAATTTGTGAAAATCATGGAAATTATTTACAATAA
ACCCGAAATTTATGCAACAAATGATATCTGGATTCAGCCTGTTAGCAGGAGCTCTGTTTTAGAAT
CAGATGTTGTTGGAAATTGTAAAATAATTAATGATATTCCAATTTTAAGAGTATTAGGAAGTCC
GTCTTTAAAAAAGACTGTTTTAAATATTTTACATGTTTTAAAAGATAAAATGACCTTTTTTAAA
AATAATTTCGAAATTAAAGGAATGGATAATATTTTCAAATATTTAAACGATGTCTATTTTGATA
AATCTGTTAATAATTATTATAGGGCCAAAAAAGCATGGTACCGTGTAATTGGGGATTTATTCGAA
GATATTAATAATTGGAATTTTAAAAAAGTAATGGGTAAATTAATAACTATTTTGAAAGTTTTAA
ACCCAATTTTAGTATTTGACCTTCACGATGTTTCAAAATGGAATCATTTGAAAACATTTCAGAGA
TTTATTCAATTTTTGAAAAATGAGGAAATAAGTATTGTTTTAAGATGTCCTTTTGAAGCTTATTT
GTATTTAAAAAATAATTTTGAAAATTATAAAACAAACAGTGTTGCTGCAGTAATATATTATGCA
AAAAATAAAGGCCATTTAATATCTGAAAAAGTTTCAGAAGAACTTTTGAAAATAAGCTGCGGAA
ATTTAAGAATAATTGATTTAATACTTGGTAATTCAAAAAGAGACCTTAAAAATATGCGAGATTT
AAAAATAAACTGGAAAAAAATAATTCCAGAAGTTCTGCCTAAAAAATACAAAAATATTGTTCAA
AAAATATTCCTGTTAAAAAAATTCAATATCAAAGAAATATCGGACATGTTAGAATACAGTTCTTC
GACAGTTTATAATTATCTAAATGAACTTGTTGGATTGAATATTATCAAAAAAAGAAAGGTTAAC
AAAAATATCCTTTTTAAATTGGATGTTGATAAAAAAGCAGCTTTTGATATTATTCAATCGAATA
TTGATTTTAAAGATATATTTTTTGAAATTAATGATTTTAAAAATATAAACTTGAAAAAGCGTAC
GGAAATTTTCAATTGGATTTTTAATTTTTGGATAGGTTTGGATCCAGTTTTTATAAGCTATTTAAA
CAAGGTTTTAAAATGTTAATTATTTTGTTTTTTATTATTGGATAGATAAGGCTATTTTAATTTTT
AATAGATTACTACACTATTTTTTTATTTATCAGTTGATTCCTTGATTTTTTAGAGTATAACATTA
TTTTTAAAATCTTTTTTTACTTGATTTTGATTTTTTGTTTTTCGGTGTTTCGAGTATGATGTATA
TATGGTGTGTTTCTTTTTGATTTTTTTGTTTAGAAAAATTTGATTATAATTTTATTTTACTTTTT
TAACCTATATATGTATAATAATAAAATATAATTAATATAATAATATAATAATAAATAAAAAAAG
TTATTTTATAAAAATAAAGTTAAATAATATAATAAATACTAAAAAACATGGTTTTAACAGATAT
ATATTATAAAAAAGCATTATATAATTATTATATATAATATAACCCTTAAAAACATGGTTAAATA
TATAAAAAAACCAGAAATCTCAAAAAACAAATTCAAAACAAAAACATGTTATATACATGAAACT
CGAAAACCCGAAAAGATAATCTAAAAAATATACTTTTCTAAATCCATAAAATAATACATAAACA
GTATAAAAAAATGAAAAATATTAATTACGTTTTTTAGAAGATGCTTTTTTTAAGTACAAAACAT
GTTCACAATCATTACTCTGGCAGGCTACACACCAGTAATTGTTTTCATTAGTTTTGAATATTTCA
AAAGAACTATCATTATTATTTAAAATAAAAGTAGTTCCTCCAAAACCGTTGTCTATTTTTTTAAT
ATCATAATCTTTTAATGTATTTTCAACAGAAAGGGTGGTTTGGAATTCGTCTTTTTTCAAGCTGT
GTTTTAAATTCTTCTTTTCTTCAGTTTTTTTGTCTTCAATTGTTACTGCGTTATATATTTTCAAG
TAATTTTCAGGATTTATCGAATAGTATACTTTATGACTGATTCTTTCTCTTTTAACAAGATTTAA
CGAAGTACATAATTTTAAAAATTGTGCAGGAAGTCCTTTTGATATATTCAATTCTTTTGAAAGAT
CTTCACAACACCCTGGCTCTTTTAAAAGTTTAGTTAATATTAATTTCTCTCTTTTGTATTTACT
AGTTTTTTTAAAATAGCTTCCATAAAATCCCCAATTTAAAATAACTTATAAATTTATTTGTATTA

TATTAATTTATTAATTAAATATGGTTTAAATTTAGTAATACATTTATATTCACAATATAATATA
TTATTGTTTTAATTTACTCAATATATTATTTAAAAAATTAATATTATTACGATATTTCATCAGTT
TAATCATGATTTAAACTCTTTTTATTAATTATCTTGATTTTTTATATTTCATTTGCAATTTAATT
AATAATTATCTGTTTTAATATATATTACTTATTATATTCACAATTTATTTTATTTTTAAACTTAT
ATTCACAATTAATATTTATAAAAATACCTTATTTTTCATAATTCAAATTAATTTTATATTTATTG
TGATATATTTATATAATATACTGATTTTATTAAAATGATTTTTAATTAAAAATAAGGATTTAAA
TTAATTAAATACTTAAAAAGATATTATATTCACAATTTATTTTATTTTTAAACTTATATTCACAA
TATTATTATTTTAGATGTTTTAAACTTTCGGTTTTTCGAAACTTATAAAAAAGATAATTCATTAT
ATATTTTAAGACACTTTTCGGACTATTTTCATACTGGAGGGATATGGCTTATGTTAGACCCAATT
GAATTTATTCATAATGCATCATCGATATCGAAAAAATCAATGAACAGGTTAAAATTAAAAACGA
ACCCATTTTCAGAAAAACCAATTAGGGGTAACACCAAATTTTTTGTTGGAAGAACTTCTGAACTT
TCTGAAATTGCGGACATTTTGGGTGCAGCACAGTATGGTAGTGTTGCAAATGCTGCAATAGTTGG
TACGAAAGGAATTGGAAAAAGTTCGATACTAAATATATTGTATTACGCTTCAAAAAGACACAAT
CATTGGGTTGTTGAATTAGAAGCTTCACAAATCACTGCAAGGCAGTTTTTAATACAATTAATGTA
TGGAATCATCAAAGATAATCTGTTTTCAGTAGATGGGACTCTTTCATCGGATTATATGAAACATT
CTCAAAAAATAATTGAGATTTACAAAAGATTGAGCAATTACAGCGATAAAACTCCTGTTCACTAC
CCTCGTGAAAAAATTGAAAGAGATTTAAAATATCTGTTAAACGAAGTAAAGGAAGAAAACAAAC
TCTGCGTTATATTAATTGATGAAGCAGACCAGTTTGCAAAAAGAAGCTGTCTTGGACTTTTACAG
TTTTTCCACTCGTTTTTATATGAAGATGATATTTTAACGTTTTTAGCAGGCCCTCCAACAATGGT
TGAAGATTTGACAAAAATATCCCCTGCAATAAGAGATAGGATCCCAAAAATCATAAACATGCCCC
CTCTTACAAAAGAAGAAGCATATGATCTTATAAAAAGAAGACTCGAAGATTCACAAATAACTGG
TGCGAAAGGATATCAACCTTTTTCAGAACAGAGCATTGAGAAAATAATTGAGGAATGTGACGGA
ATTCCAAGAAGAATTATAATGACATGTTCTGAAGCAGTTTCTCTCGGACTTAAAAATGGTGTAGA
TGAAATAGATGATGAAATTGCAAGAAATGCAATGAAAAATCTTGGGATAAGTGTTGGACACCAG
ATTTTAAACCACCTAACACCTGCCCAGTCTAAAATAATAAAAGCAATGGCTGAACTTGGTGGAAG
CTCTACGGTAACTGAACTTTCAGGAATTTTAAACAATTCTACTGGAACAATTGGAACACACCTTT
CAGATATTTATGAAATGGGGTATATTTATAAAGAAAGAGATGGATATAATGTATACTACACTTT
ATCCAAGGAATTAAAGGACGTTTTGATAACAAAAGAAGATATTTCGTAAATTATTTCAGGTGTT
TTTTGATGCAATGCAATGACGTTCAGGCAACAGAACCTGATATTAAGGTTTCATTAACCAGGGTT
GGCGTAACAAACCTAAAAAAACTTGTAAAATTAAAAAGAAAAAATAAAAGAGACATCGTTCTTC
TCCCTACCTTCGAAGTTTTTGTGGATCTTCCAAGTTCGCAAAAAGGAATTCATATGTCAAGAAGC
CCCGAAGTAATCGAAGAAGTAGTTGAAAACATACTTTTAGAAAAGGAAATTTATGGTGTTGAAG
ATTTATCTGTTGAAATAGTTATGAAACTCTTCGAAAAACACGAATATGCGACAAGAGCCGAAGTA
ATGCTTTACAGCGATTACATGATGGAAGAAAAATCCCCTGTAACAAAAAAAGATTCCCAGGAGAT
TGGAAAGATAATTGCAAGAGCATACGGTGTAAAAGACAGCAATGGAAAAATAGATGTAAAAAAG
ATGGTTGGTGCAGAAGTTGTTGGAATTACAGCATGCCCTTGTGCTCAAAACATGTTAAAAGAAAA
TGCAGTTGTTAGTTTAACTGAAAAAGGATTTTCAACTGAAGAAATTGAAAAAATTCTTGATTCA
GTTACCATTGCAACACACAACCAAAGAGGAATTGGAACGGTTATGATAGAAGTTCCAAATGGATA
TACTGTTGGAATTTCAAAAATAATCAAAATAATTAAAGATTCAATGAGTGGGGAAGTTTACGAA
CTTTTGAAGAGAAGTGACGAAGCTTTTGTTGTAGAGGCAGCTCACAAAAATCCAAAATTTGTTGA
AGACTGTGCAAGAGAGATGATAAAAAGAGTTGTGGATGTTTTTGATTATCTTCCTGAAGATACA
CAAGTTCTTGTAAGGCAGGTAAATAAGGAGAGTATTCACAGACACGATGCTTTTGCAGAGCGAAA
TTCTACAATAAGGGAATTAAGGGACGAACTTAAAACTTTAACAAATTAATTTTAATTTATTTTTT

AGTGGTTTTATGAAAGCTCTCGTTGTTGGGGCATGTACGCGCCCTGTTGTAAATTCTTTAAAAAA
ATTAAATTTTGAAGTTTATTCAGTTTCCCACTATAACCCTGTGGATTTAAATGCAGATCATAAAA
AATATACAATTAATGATAAATTTCACGGACATTTGGCAGATAAATATTCTGAAAAAGAATTATT
AAATCTTTCAAAAGAATATGTGGATTTGGTTGATTATATTTTTGTATGTTCTGGAATATTTGAG
AGTAAAACTTCAAAAACTCCAAACTGGGACGTTGTTGGAAACTCACCAAAAAGAATTAAAAATA
TAAGTGATAAATATAAAACTGTAAAGAAATTGGAAAATTTAGGCTTTAATGTGCCGGTAACATA
CCTCGTAAATAATAAATACCAGTTTGAAAAATGCTTATCGGAGTTAAAGTCTGTAGTGGTAAAAC
CTATTTCAGGTAGCGGCGGAATTGGTGTAACTAATATTGATTTTCAAAATACGGATAATTTGGAT
ATAAACTATCCTGTACTGGTTCAGGAATGTATCAAATCTGAATCATACAGTGCTTCATTTATCGG
TTCAAATTTTTTATGTTTTAACAAGCAATTGATAAATAACAATATTTATGTAGGAAACATATCCC
CGTATGTTTTAAACTTGAAAAACGAAACAATTCAGGAATTTGAAGAAATAATAGCTTCGCTGGA
TCTTTTAGGGATGAATGGAATTGATTTCATGCTAAAAGATGGAATTCCATATATTATCGAAGTA
AATCCTAGGATTTTAGGAACTTATGAAACAATAGAATTATCTTCAAGATACAATCTTTCAAAAGC
AATTCTCGAAAATAAACCCGTAAAACCAAAAGACTGTTTTTTAAAAAAATAGTTTTTTCAGACA
AAGGTTCATCATATTCTATCGATAAAAATCCATTTTTAAAGGATATCCCACAAAAAGGAACCTAT
ATCGAAGAGGGAGAACCCATTGTTACAATAATCGGAAGAAACATGGCTGATGTAAGAGAAATGG
AGTGCGGTATTTTAAGAGGTGTATGAAACATGAATTTATCAAAAGATGAGATATTTACAATGCT
CGAGAACCCCCTGATTCAACAAGTACTTTTCGAAATAATGGATGAGGACGTTGTTGGTTTTGATG
TTTTGTCAGTACTTATCAATACTAATGAAGTAACTGATGATGAAATTTCAAGACAGTTGGATGTA
AAATTAAACAATATTAGAAGGATACTTTACAAATTATATGAAGCAAGGCTTGTAGACTACAATA
GGGAAAAAGATGAAGAGACAAACTGGTATACTTACACCTGGAAACCATCTCTTGAAAAAGTTCCT
GCATTAGTTGCTAAAAAGATGAAAAACGTACTCAATGGTTTAAAAGAACAGCTAAATGTGGAAG
AAAATAACATGTTTTTTTTCTGTTCTGACTGTGAAATTAAATTTACATTTGAAGATGCGATGGAT
AGTGGATTTAGATGCCCCCAATGTGGCGAGATGATGTATGAATACGATAATAAAAAGGACATTTC
ACTATTAAAAGAACAGATTAAATATTTAGAGGACGAATTTAATAAAAACCCTCTTTTTTCTACTT
ATTAAGGTTGCATCTATGAATATTGTTTTAAATGATCTTCTAAACGACGCTACTTTATATGAAAA
ATTTTCTAATTTTAAAAAAATGCAAGAAATACCCAATTTTAATGAATATTTAACTTTTTAACAG
AACGTTGTGAAAAAAACGTGGTTCTACACAGCATTGCGGTAGCTGATTATGTTTATGAGTTTGGA
ATCAAATTATTAAATAAAAAATATGATTTTGATTTAGATACGGCTGTTTTAGGTGCTATTTTACA
CGACATTGGGCGAAGCAAGAGCCACAATATCGATCACGGGATAATTGGTTCTGAAATATTATTAA
AAAATAATTTTAATGAAAAATACGCTAAAATTGCAGAACGGCATATCGGTGCAGGAATTTCTAT
AAAAGAAACAGAAGATTTGAATTTGCCTAAAAAAGATTATATTCCAAAAACATTGGAAGAAAAA
GTAATTGCAAATGCGGATAATTTAGTTTCAGGACATATTCGGGTTGATATTGGTTTTGTAATTGA
TAAATTTAAAAAAAGAACAAATAATGAAGTTTCAAATAAAGTTTACGCACTTTATTTAGAAATA
AATAAGTTAATTGGTAATTAACTAAAATTAATTAAAACAATTGATTTTTATGCAAATAACAGTC
CGGTAAGTCTCAATACTGCATAAAATGTGCCGATTGCAAAAGTCATACATACCGCACACGTAATT
ATCATTCTATTTACTTTTAAACCAAATAATGGAACAGCAAAGAAAAGACCAAACAATCCAGATCC
AAACTGAATAGTTTCAATTGCTTTTGATATCTGTGAAGCTTCAACAGAAGGCATTGTTGGTGCTA
ACTGAGATATTACTGTTAAATAACCCCCAATTAAACCAGCAGTTGCGGGGATTACACCACAAAGA
ATAATAAGACCAACTGCAAGACCGTTTGCAGTGACACTTAGAAGTTGAGATTTTAAATTCTGAAG
ATCTTCAAGGGTTTTTGCGAGTTTTTTTAATGTATCTGCAAGTTCCCCCCCGTATTTTCTATTTTC
AATAATCAATCTTCCAACTTGCCTAAAGATCTTTGAGTCAAGTGAATTTGAAACAAGAATCATTG
CATCCTCAAAACTCAATTTTCTTTCAGTCATTAATACGACAACTTTTGAAAATACAACATCTACT

TCAGGAATTCCACTTCTTATAACTTCATTAATAGCTTCAACAACAGAACGTCCGGAATCAAGTGA
TAAAACCATAACATAAAGGGCTTTTGGTATATTTTTGTCGAATTTATCCATCTTTGATTCATACA
TTATAGCAGGTAGTGAAAGAACGGATCCTAAATACACGAAGGTTAGGATTAAAGTGGTTTTTAA
TGTAAAACCTAAGAATATTTTTAACAATACTGGTAAAATCGTTATAACCAATAAAATACCGATA
AATACCCTTTCATCAAGTTTTTTACCTGTCTTTTTTAATATCTTAATATTTCGTACAATTAAAAC
ATGATATACATGATCTAGGTATTTTTTGGCATCCATAATTTCACTCAATCTTCATTTTAATAAAG
ATGGTAAATATAATTGTTGACATTAACCCTATTTTGCCCATCACGAGGTCAATAGCATTGATAAT
TCCGTCAAATCCTTGCTGAGCACCCAATGCTGACTGTATCATTCCTGAAAATGGGATAATCAAAC
CAATACCAAATATAAGATTTCCCAAATTTGATATTTGGAATTTTGAGCTATCTATTTTTGAGATA
GATTCTCTAACTATTTCATTATATAAATTTTCAAGTAAAAGCTGCGTTCCACCTTTATTCGCTGC
AAGAATCAGTTGGTCATAAAGCTGTCTCATCAGAAATATTTTTGTTCTTATTTTGGCCCTTTCGA
GTGCTTCTACGAAATTATAACCGCCCTGATTAATGTCTTTTATGATACTTCTAAATTCAAAAGAA
GTAATTCCATATTCAGGGTTATTTGCAATATTTTTCAAAGCTTCCTGAACAGAAGCGCCAGAATT
TAACATCGAAATAAGGTGTAATATACTCATTAACACCTGAATTTTAATTTCCCCTCTAAACAATG
TTAATTTCAGTTTTGGATAAAATATACTACCAACAACTATAATAACTGCTAAAAGTACCCCATTA
GAAACGCCAGAAAATACATCGCCGGTCAAAACACCGTAAATTATGAATAAAACAACCGTGGTGAT
TGCTGCAATCATCATCTTTAAGAAATAAACTCGCTCATCTACAATACCTGCATACTGGTAATCCC
TCTTAGATGGTAAAAATGAGGTGTTTGTAACCCAGTATGAAAAAGATCTGGAGTATCCGCCAAGT
GCTCCAAATTCTTTTTTTTCAAAAAGTAAATAATCTAAGTCAATACTTACTTCGGGAGTTTCATC
GATATATGGTTCGTAAAACTCTAAAATCTCATCTTTATGTATATCTTCAGTTTTTCTTTCGAAGA
TTTTTTTTAAATATTCCGATCTTCCAACCCTAGATATTTTTCTTTTTTTCGGAGTTTTAACTCCTT
TTAATGTATTGGCGAGTTTATCAAAAGTACCCTGTTTTTTTTCAGTTTTTCTTTTCATCTAAACC
CCAATTATTCCAGAATGTTTTTCAAGACATTTTCTGGATTTTCCTGATATTGCTTAAGTACATCC
CCTACTTTTCTAATATCATTAATATTGTTGTTCACCATATATTTTAGTACTTTCTTTCTATTGAT
TCTATCGTCCATTAATTCATCTCTTGTGATACCTGCAATCTGGCACACGTCTTCTTCCCACATACA
AATTCCTGTTTTTACAACACTGTCGCTTATTCCATCGTATTTAAAGAGCTCAGTTTTTGTAATAT
TTTCCCCACTTCCCCCGATTTCAACAACACCGAGGATCCTTCTAACAGTTTTTTTATTACGTTTAA
TACGCTGCTGGTTTATGATAAAATCAACTGAAGACATCATAACTTTTGGAACGTTCATAGGTGGG
TTTATTAACCTAATGAGTGCTTCATCTGCGCTGTTTGCGTGCAGAGTTCCGGAACATCCGTCATG
CCCGGTGTTCATAGCAACAAGCAACGAATGGGCTTCACTACCCCTAACTTCCCCTACAAATATCCT
GTCAGGCCTCATCCTTAAGGAGTTCTTGATCAAATCATCCATCGTGATTTCATAACCCTGAATAC
CAGGTCTTCCAGGTCTTGTAATCATTTTTATCAGGTGAGTTAGTGGAACCTGTAACTCTGGAGTA
TCTTCAATAGTTACAAGTCTATCGGTATACATCGAAAACATTGAAACAACGTTTAAAGTTGTAGT
TTTACCCGAACCAGTTCCACCTACAATCAAAGTGTTTGCAGGTTTTGCACCAAAGTATCCTTCAA
CAGCCTGCCAGAGGAATGCCGCAAGTTCTAAATCAAAAGTTCCAAAATTAATTAAATCAACAATA
GTTAATGGATCTTCACTAAACTTACGAATGGTAATTGTGTTTCCACCCAATGTAACGTCACTCAT
TGTAGCGTTAACCCTGCTTCCATCTGGTAAAAATGCATCAAGCATTGGAGTTCTTGAATCAATAG
TCCTGCCTGCAAGATAAGCTATACTTTCAACAACCCTCGTTGCTTCGTGTCTATCAAGCCTAATAT
TTGTTTCACACATCTGGTATTTTCTGTGAAATACGAAAGAAGGTGATTCAACACCATTTACCATT
ACTTCTTCTAATTTGCTATCATTTAATGGAATTTCTAAAAGCCCTAATTTTCCAATTATTAAGTA
AAAATAGTGTGATAAATGGAGTATTTCAATATCTCTTAAATGTAAATTATTTTTTTCCGAATAA
TTTTTAAGGTATCCTTGGATCTGTCCTAATTTTTGAAGGGTAGATTCTGAAAGTTCCGCTTTTAT
TGTTTTAATATCCAAATCTGAAAATTTTGCAAGAGCTGTATTCATAAGAGTGATTTCTGGAATCT

TGTATAGTGTGACACCATTTTCTTTCTCAATAATTACATCAAAATCAATGTCATCAACTTTTACA
AAGTATTTATCGAGTATTGAGCTTGTAGAATCTCTTTCAAATTCTTCTACTGGCTGTTTTGCCCG
TTCATTCACTGAAAAACCCACTTTTATTTCGGGTTTTTTGACTTTTTCAGAAGTATCAAGTATAT
TGGGGGTTTCAGATACTTTTTCAGATACATTATTTTTTTAGCGACAGATGGATTGGAATCTCTG
CTTTGGATTCTGTCAAAAAGCCCCATATTATCACCTTATAATGTTACATCGATATCTAAGTGTTC
TGTTAGCTCTTTGTACCTATTTCTTATTGTAACTTCAGTAACTCCTGCAACATCTGCAACTTCTCT
CTGAGTTCTTCTAGTTCCTTGTAAAACACTTGCAATATAAATTGCAGCAGCAGCAACTCCAGTAG
GTCCCCTACCACTCGTAAGACCGCGTTCTCCTGCTTTTTGCAAGATTGATATTGCTTTTGACTCTA
CTTCTCCAGGGAGTTTTAATTCTGAAGCGAATCTTGGAACATAATCCACAGGATTTGTTGGAGCA
AGCCTGATGTTTAATTCTCTTGAAATAAATCTATATGTTCTTCCAATTTCTTTTCTATCTACTCT
TGAAACTTCTGCAATTTCATCAAGAGTTCTTGGAACTTTACATCGTCTGCATGCAGCGTAGAGTG
CAGCAGCAGCAACACCCTCAATACTTCTTCCTCTAATTAATCCTTTTTCTACAGCACCCCTGTAAA
GAACTGCAGCGTTTTCCCTGACGTTTCTTGGAAGACCCAATTTTGAAGCTATCCTGTCAAGTTCG
GAAAGTGCAAAGGCTAAGTTTCTTTCAGATGCATCAGAAACCCTGATTCTTCTCTGCCATTTTCT
TAATCTATAAAGTTGAGCTCTTTTATCTGCAGAAATATCTTTTCCGTAGCTGTCTTTATTTCTCC
AGTCGATAACTGTTGATAAACCTTTATCGTGGATTGTATAAGTCATTGGAGCTCCAACCCTACTT
CTTTTTACACGCTGTTCGTGATCAAAGGCTCTCCATTCAGGACCCACGTCAAATAAATTCTGCTGT
AAAACACATCCACACATTTCACAAACGATTTCAGCCCTTTCATAATCCTTAATAATACTTTTACT
ACCGCAGACAGGACAGATTAATTCTTCTTCCTTTTCTAAAATAACATTCTTATTTGAGTAGTCTT
CAGGCTTTGCGATAGCAATTTTAATCTTTCTTTCAGGCTTTTTAGTATCTTCTTTTGTAACAGAT
TCGACTTTCATGATAACACCGTCTTAAAGTTATCTTCTTCCTTTTTTACGGGGTTTTGATTGTTT
TTTTGATTGTTCATTCATAACGAAAACATTATCCGATTCCAAGGTTTTTTCAGCGTCGTCTTCAC
TATAAGGAATTATTTTTACATACGGTTCTTCAACAGGTCCAAATACATCGTAGATTTTTCCAATC
TTTTTAACTCTATCTTTAAGTTTTATTCCAACAGGCGAATTAAAACGAGGCTGTTTTTTAACACG
CCCAATTATTTTTCCTTTTGGAGTTCTATGCAGTATTTTTATCTTTTCCAACGAGATTCCCCCATA
TCGAATGATTATGGTATATCGAGAGTTATGAATATTTAACAATAAAATCGATTCATATATAGTT
TTCGCACGATAGTATTTATATATTTCGAAAAGTATATATATGATTTACTAAATAGAATCGCAATT
CTAAGAACAAAACGGAACTAAAAACAGTGTATGTAATTAAATCATCGATAGTTTTATATACTGT
GTGCAAAAACCTTAGCGTTTTAGTAATGGGAATCATAAACCACCCAAAAGTATTTAAGAAATAA
AATTAATTTAGTTGGGAATAAAATCTGTTTTTTACATCTTAATTACTGCCTAAATTAAAAAAACA
GGTAGAATTTAAATTTATTAAAAATCTAAAAATTAAGCTTAAATTTAAATTACTATGGCCATTT
TTTTTATTCTTTCATCTGGAAAAGCTTTTTATCATTTACCAGCCCTTGTTTTTATAACTGTAATT
TTTCGGACTTTAGATAATAATATATAGGTGGTTATTCGTGAATAATAATAGCATTGAATATAGA
AAGTTGGAACACCTTATTGTGTGCGACCACTGTGACGTGGAGTATAAAAAAGGGACCCTTCTTGA
GGACGTTGAATTAATTCACAGCGGTATATCAAACTGCGATTTAGACGATATCGATACATCTATCG
AAATTTTTGGAAAAAAATTAAATGCTCCGTTAATTGTAGCAGCAATAACTGGTGGACACCCTAAA
GCAAAGGAAGTCAATAAAAATATTGCTATTGCTGTTGAGGAACTGAATTTAGGTATGGGTGTAG
GTTCACAAAGGGCGGCTATATCAAAAAGCTACCTTGAAGATACCTATTCTGTTGTAAGGGATCAC
ACTTCCTCATTAATTATCGGAAACCTTGGAGCAGTTAACTTTGTGGAAGATTCATGGGATGAAGA
AATTATTTCAAAGTCTGTAGAAATGATTGATGCAGATGCCATGGCGATTCATTTCAATCCACTAC
AGGAAGCTATACAGCCTGAAGGCGATGTAAACTTCAAAGGACTTAATATTTTAAAAGAAATAAT
TTCAAATTACAATAAAATTCACGGAAAAATTCCATTTATCGCAAAGCAGGTTGGCGAAGGATTTT
CAAAAAAAGATGCCATTTTTTTAAAAGAAATTGGCTTTGATGCGATAGATGTCGGCGGAAGCGGC

GGAACTTCATGGGCTGCTGTAGAGCTTTACAGGATAAAAGATGAAGAGCAGAAGAATTTTTCAA
ACCAGTATTTTAACTGGGGAATTCCAACTGCCGCATCAATTCTGGAAGTAAATTCAGCCTTTTCA
GGACCGATAATTGCAACAGGCGGAATCAGGACTGGAATCGATATTGCAAAATCAATTTCAATTGG
TGCAAACTGCTGTGGAACTGCTTTACCAATTTTAAAAGCAGCATTAAAGTCTTCAGAAGCTGTTA
CAACTGTTCTAGAGAGAATGATTAAAGAGTTAAAAACTACAATGTTTTTGACAGGCTGTAATAA
TATAAACGAACTAAAATCTGCCAGATATATTTTAAAAGGCGATTTAAAGAATTGGAAGGATCAG
ATTTAATAATTTAAATATTATCCATCGGAATTTTTTCAATTTATTTATTCATGTATTCAAAGCAA
AGGATGTGATAATTTGCAGTTAGAAGTAGTCGCAATAGGTGGATACGAAGAAGTCGGAAGGAAC
ATGACTGCCGTAAATATCGACGGCGAGATCATAATTTTCGATATGGGTGTAAGACTCGACAGGAT
AATGATTCACGAGGATACGGACATCTCAAAAATGCACAGTTTAAATTTAATTGAGATGGGCGTAA
TTCCAGATGATACGGTAATGAAAAATATCGAAGGAGAGGTTAAGGCGATTGTTTTAACGCACGG
GCACCTCGACCACATCGGAGCAATTACAAAACTTGCACACAGGTACAACGCTCCAATTATCGGAA
CTCCATACACTTTAGAACTCGTAAAAAGAGAAATTTTAAGCGAAAAGAAATTTGATGTTAGAAA
TCCATTAATTACTTTAGAAAACGGAAATAAATTAGATTTAACAGCAAATATTACTTTAGAATTT
GTAAAAATTACACACAGTATTCCTGATGCTGCAATGGCAGTACTTCACACACTTTATGGTGCAGT
AGTTTATGGAAACGACTTTAAATTTGATAATTTCCCTGTAGTTGGCGAAAAACCAGATTACAAAG
CGTTAAAAAGAATTGGAAAACAGGGCGTAATTGCAATGGTTTCCGAAAGTACAAGAGTTGATTT
TGAAGGAAAAACAGCATCAGAAGGAGTTGCTGCAAACCTTTTGAAAAACGACCTTTTAGGAACTG
ACAATGAAGACAATGCAGTAGTAGTAACTACATTTTCATCACACATTGCAAGAATTAAATCCATT
ACTGATATTGCTTCAAAAATGGGTAGAATTCCAGTACTTGTTGGAAGATCCATGTCAAAATACTG
TGGAATTGCACCAAGATATCGGTTTAGTAAAATTCCCAAAAGAAACAAAAATTTGCTGGGACCCTT
CATCAATCGATAAAACATTCCACCAGATAATGAAAGACGGTAAAGAAAACTACCTTATGGTTGTT
ACAGGACACCAAGGAGAAGAAGGTGCGGTTTTATCCAGAATGGCAACAAATAAAACTCCATTTAG
GTTTGAAAAATACGATCAGGTTGTATTTTCAGCAGATGTTATTCCAAACCCAATGAATGCGGCAC
AAAGATATTTATTGGAAGCAAGATTAAATTTAGCAGGAGTTAGGCTATTTAAAGGAGCTCACGT
ATCTGGACACGCGGCAAAAGAAGATCACAGGGACATATTAAGATGGTTACAACCAGAACACTTGA
TACCTGCTCACGGTGATTTTAATTTAACTTCAGCATATGCTAAATTGGCAGAAGAAGAAGGATTC
AGACTCGGTGAAGATGTACACTTACTCAGAAACGGTCAAAGCTTGAAATTTGAAAGAGTAATTTA
ATTGAGGTGCTAAAATGGTGTTTGATAGGGAAATATTGTCAAAAATCGATTCTGAACTCAAAAA
ATACATGGAAAAAGATACTAAACTTTACGGTGCATCAAAACACCTATTACTTGCAGGCGGTAAAA
GAGTAAGACCTTATCTTTCAATTTTAACATATCTCTTAAAGAAAGATGACCTTACTGAAATAATG
GCACCAGCACTTTCTGTTGAATTGATTCATAATTATACATTAGTTCATGACGATATAATGGATAA
TGATGACCAGAGACGGGGAATCCCTACAGTTCACACGGTTTATGGTGAACCAATCGCAATTTTGG
CTGGAGATTTGTTATATGCAAAGGCTTTCGAAGCTCTTTCAAATATTGAAGATAGTAACAAGGCT
CACGAAGTTTTAAAGGTTTTATCTAAGGCATGTGTCGAAGTCTGCGAAGGACAGACTGAAGATAT
GGAATTTGAAGAAAGATTCCCGACTTTAGATGAATACCTTGAAATGATTTCCAAAAAAACAGGT
GCTTTAATTGTAGCTCCTGTAGAAATTGGAGCAGTTATGGCAGACTGCACTCCTGAAGAAAGAGA
AGCACTCTGTAATTATGCAAAAAGAATTGGAATGACTTTCCAGATTCAAGACGATGTTTTAGATT
TAATTGGGGATCAAAAAACAATCGGAAAGCCTGTGGGTAGTGACATTGTTGAAGGTAAAAAGAC
AATGATGGTAATTCACGCAATGGAAAACCTTTCTGAAGATAAAAAAGAAAGATTATTGCAGATT
TTAGGAAATCAAGATGCAAATCAACAAGAAGTTGCAGAAGCAATTGAACTTTTAGCAGATTCTA
TTAATTATGCAAAAGATACAATAAAAAACGCAACAGAAGAAGCTAAAGATTACTTAAAAATATT
CGATGCAGAAAAAAGAAAAAATCTCGAAAACATTGCAGATTTTATCATTGAAAGAATTCACTAA

TTACATTTAATTAGCTATTTTAACTTTTTTTAAAATTTAAAGGTGTTTTAATGAAAATTCCTAAA
ATTTACGTTGAAGAAGAATTAAACGACGGCGATCGTGTCGCAATCGAAAAAGATGGAAATGCAA
TAATTTTTTTAGAAAAAGATGAAGAATATTCTGGAAACGGCAAGCTTCTTTATCAGGTAATATA
TGATGATTTAGCAAAATACATGAGTTTAGACACTTTAAAAAAAGATGTTTTAATACAGTATCCT
GATAAACATACATTTACATATCTTAAAGCAGGAACTAAACTTATTTCGGTACCTGCTGAGGGCTA
CAAAGTCTATCCAATAATGGATTTTGGTTTCAGGGTTTTGAAAGGATACAGGCTTGCAACACTTG
AAAGTAAAAAGGGGGATTTAAGGTACGTTAATTCTCCAGTAAGTGGAACGGTCATATTTATGAA
CGAAATTCCGTCAGAAAGAGCAAATTATGTATTTTACATGCTCGAAGAATAAATTTTCATTCTAA
ATTTTTGGAGTACTGGTATCCCCACGAACCAGTAATCTGTCCTTTTTTTGCCAGTTTTCCTAAAA
TATCTAAGGTTTCTTTTTCAAGTAATTCTGGGGTGCTGTTTTCAAAGTAAGTTCCACAAAGGGGC
ATAAAGTAGTGTGCATGGACTTTTCCATTCTTTTTTATAATGTATTTCATCAGATCAATACTTTC
TTTTCTTTGATCTTTTGTTTCGTCTGGAAAACCAAATATAAAATCAACTTTTGGAATTAAATCAC
ATTCTTTAGACGTTTCAACAGCATTTATAACATCTGAAACCGTGTGACCGCGTCTTATATGTTTT
AAAACTTCATCGCTTCCGCTCTGCGCGCCAAAATGAATGTATCTATTGTCGCAATATTTATTTAC
TAAATCGAGAGTTTCAAAGTTTACAAATTCCGGCCTTACTTCTGATGGAAAAGTTCCAAAAAAGA
GCCTTTTTTTGATTTCAAATAGACTTTTCATCAGTTTTTCAAGTTTTTCAATATTGGGTTTTGTG
CCTGTTTTTGAGCCATATGAAAACGCGTTTGGGGTTACAAACCGGATATCGCCCATAGTTTTTAC
AATTTTTACGATACTTTCAATACTTCTATGCCGTATATTTTTTCCAAATATTTGTGGAGTTTGGC
AGAATCTACAGTTATACGGGCATCCTCTTGTAATTTCTATTGGTGCAAGAGGCCAGATTTTTTCA
TAATCGTCAAAATTTTCAATAGGCTTTCCTTTAATTATTTTTTCAGGGGTTTTTCCAGATTTTAT
GGAATTTATAAGATTTGGCAGTGAAATTTCTCCTTCGCCAACGATAACATGATCAAAGCCTAGCT
TTAATGTATCTTCTGGACATCCTGATGGATGAGCTCCTCCCGCAATTAATATAACATCTTTTTTT
AGTTTTTTTAATTCGTTTATTTCGCTTAAAACTTCTTTTTCCTGCATACTCATAAATGAATATAT
TAAAATATCTAAAGAATCCAGATTTTCAATTATTTCGCCAAATTCTTTGAATAGTGTCCCGCCTA
TCAGGGGGTATAATTTTGAGACACTGTACTTGTTTTTTGAAGTCATTCTAAATCCAATCATAACC
TTAAATAATTATGATTGGTATATAAAATTGACAGTAGAATTGGAGGTGCTTTTTTGAAAAAAAG
ACCAACTCTATATATCAAGATACACTACCCTGGAAGTATCTTGGAAGGAGAATACGACTACGAAG
CGGTAGTAAAACTTAAAAATAAATATATTAAATATTTAGAAACTGGAAAAGGAACAGGCACACT
GTTATTCAACGAAGACAAGAAAAACTTTGCAGTGGTTAATTTTCAACAAGTTTGTGCAATAGAA
GTCGATAAAACCCTAATGTTCATGGATGAATTTGAGCGAGAATGGGATAAAGTTTATAAAACAA
GGTAATTTTAATTTTTAATAATGATTGGATGTATTGGTGATATAATGGCGAAGATTGCAAAAAA
TGCGGTTGTTATTGGCGATGTTGAACTCGGTGAAAACGTAAATATCTGGTATGGGGCAGTATTAA
GGGCAGATATTAATAAAATAAAAATAGATGATGATTCAAACGTGCAGGATAACTGTACTGTGCA
CTGTTCAAAAGATTATCCTGTTTTTATTGGAAAAGGCGTTTCAGTAGGGCATGGTGCAGTAATTC
ACGGGTGTACCATTGAAGACAACGTACTCGTTGGAATGAATTCAACTGTACTAAACGGCGCAAAA
ATTGGTAAAAACTCAATAATTGGCGCAAATGCACTTGTATCACAAAATAAAGAAATTCCACCAAA
TAGCATGGTTTTAGGAGTTCCTGGAAAAGTTGTAAGAACTTTAACGGATGAAGAAATAAATTCT
ATAAAAGAGAATGCAAAAAACTATCTTGAACTATCAAAAAACTTATAATGTTTAAAAATCAATG
AGACTATTATTGTAAATCAATAGGGTGAAAAAATGGTCAACTTAGGATTTGTTATCGCAGAATT
CAACAGGGATTTAACATTCATGATGGAAAAAATGGCAGAAGAACATGCTGAATTTTTAGGTGCA
AATGTTTCCCACAAAATAATGGTTCCAGGATCATTTGACATGCCTCTTGCAATCAAAACACTTCT
TCAAAAAGACGACATCGATGCAGTTGTTACAATTGGATGCGTTATCGAAGGAGATACTGAACACG
ATGAAATTGTAGTTCAAAACGCGGCAAGAAAAATTGCAGACTTATCTTTAGACTTTGGAAAACCA

GTTGCACTTGGAATTGCAGGCCCTGGAATGACAAGAATGCAGGCTGAAGATAGAATTGATTACGG
TAAAAACGCAGTAGAAGCTGCTGTTAAAATGGTAAAAAGACTTAAAGAAATTCAATAATTTAAA
ATTAAATTTATAAATCTTTTTTAGTGATTTTATGAATGTTTTAAAAGAAGTATATGCTACGATCG
AAAAAAGAATCGAAGAAAAACCCGAAGGCTCTTACGTTGCTAAATTAACAACGGATGACAAAAA
AACTGCAGTAAATAAAATCTGCGAAAAAGTCGGTGAAGAAGCTGCAGAAGTTATTATTGCAGCA
AAAGACAACGATAAAGCAGAAATAATTTACGAAAGCGCGGATTTAATTTTTCATACAATGGTCCT
TCTTGCAAAATCAGGAATTACCTATGAAGAATTAAGTGAAGAATTCAAGAAAAGAATGAAATAA
CTTATTTTAAATACTTTTTTAGAATTTTGGAAAGATTGTACCTTCAATTAGATTTAAAATATCTG
TTCTTGTAAGGATTCCAATTGCGATTTCTTCATTATCCAGTACAATTAACCGTCCTACATTATGT
TTTTCCATTAAAATTAATGCATCATATATTTTCTCGTTTTTTGAGATTGTGTGTATCGTTTCAGC
CATTAATTTTGTAACATTTTCATTTTCAAGGCCTCTTGAAACTGCATCTGCGACATCATGTAGGC
TCAATACTCCAAGAAGTTTTTTTCCATCCATTACAGGAATTCCACTGATATTTGCATCTGAAAGT
AGTTTTGCGGCATCTCGAATCGTTTTTTCAGGTGTTATGTATATCAATTCTTCTTTTATTCCAAC
ATCCCCAACAGTGATTCCCGGAACGCTTGCAACACTTATTACGTCCATTAAAAGGATATGGTTTA
TATCATCTCTTCCAACGACCCTACCCAAAATAACGATTTTGTTGTGGTGCGTTGGCCCAACTTTTA
CAACATCCCCTTCTGAAAATTTCCTGGTGTCCCCATTAACGTAAATTTTTGAAGAACATGATTTT
TCTCTTGTAACTGTGTCAAATACGATTTTTATGACACTAACTCCTTCAACGAGGTCGTCTCTTTT
GTAAATTGGAACTGTAATTTCTTCTTCTGTTTCAAGCCCTAATGAACGGTAGGTTAAACTCGTTG
GAATGTATCCTCCTTTTGGTCCGGGAACTCCATCAACAAGATTTAGTGCTCTTAAAGCCTGCATC
TGGTTTCTAATCGTTCCAGGGTTTCTGCTTAATTTGACTGCAATTTCTGTTCCCTTTACAGCTTTA
TTTTTTTCCCTATAAATTGAAATTAATTCTTGAAGTATTTCTTTCTGAACTATTGTAAGTTCCAT
AGTTACACCGTTAAAAATGATTATAAATAGTGATTATAAATAGTGACATATATATTATGGTTTT
TAATTGGTATAATAAATTTATGATTTAATTTAAGTAGAAAATCTGCAAAAAAGAGCTTAAATAT
ATTTAAAAATCTAAAGATACTGTTTTACAAGGAATGTTCTTTTCTAAGTATTTTAAAAATGATTT
TAAGCCGTAAACTTCGGTTCCGTAGTGAGTTCCATCGATAACTGTTATCCCTAGTTCTTCTGCAA
TGATTTTTGAGTGGTGAGTTAAATCTCCAGAAATGTAAACATCTGCAAAATTTGATACATATTTA
ATAGAATCTTGCGAAAGCCCGTATCCTGAAAGAACTGCCACTTTTAACTGTTTTTTATCATATAT
GCTTTTAGGAACGACGGTTTGTGGAACTGCGAATATATTTTCTTTCGTAATTTTTAAAATATCTT
CAAAAGTTCCATCAAATGTTCCAACTCTTCCAAGACCATCTTCATATAAATTTTTAACATCTTTT
AAATTATATAATTCAGCCAAACAGTCATTAAGTCCGTTTTTACAAATATCTAGGTTTGTATGCGC
GCTGTATAGCGGAATATCTTTTTTAGTTAATATTTTGAGTTTTTTGTAAATTGGTCCTGTAAAAT
TTCTTATTGGATCTTTTAAAATCGGGTGGTGGGTAAACAAAAAGTCCACGTTTTCAGATTCTGCT
TTTTTTATAACTTCTAAGGACGGGTCAAGTGCGATTCCTAATGTTTCAACTTCCTTTTCAAGGTT
TAATCCAACCTGAAGGCCGATATTGTCCCGAGGTACGGCAAGTTCTTTTGGAGCAAATTTTTCAA
TTATTTTTATGATTTCAAAAGCTTTCATTTTTTCCACCCTGTAATCACCTGTAATTCTTCAAATG
AAAATTTTAAAGACCCAGTTTGTTTCGCAACAAATCCTAATTTTTCCATTTTTTCAATGGTTTTA
TTTCCATCGGTAAGTGATGATTGAAGAATCTGAATAATTCCATCTTTTTTTAAATAGTTGGCTAC
TTCATCTAAAAACTTATCCAAAACTTCTCTTCCGTCTTTTCCGCCATCAAAAGCGTAATTTAAAT
ATTTTTCTAATTTTTCCTCGTCAGAAGTTGGAAGGTATGGTGCATTGAATAAAATTACGTCAAAT
TTTTCATCAATATTTTTGAAGAGGTCGCCTGTTTTAAAGGAAAGTTTTTTGGAATCAATTTCGTT
TAATTCTGAATTGATTTTTGCACACTCTACTGCATAGGGGTTGATATCAATTCCAATAACTTTTA
AAGCACCCTGTTTTACGGCATTTATTGCCTGAATTCCGCTTCCAGTTCCAACATCTAAAACTGACT
TATTTTTTACATCGACTAAATTTTCAATTAAAAGTTCGCTGTCTTCCGCAGGAACGTATACTTTA

GGGTGTGTTTTTATTTTTATCCCGTTTATTTCAATTATTTCTATCATAATAATCTATTTTTTACA
TTTTCCAGATTTCATCGCCGATAAAATGAATGGTTTTGATTGCTTTTCCTTTTTTTTCTTCAACC
ATTGTTTTTCCATCCATTAGTGCCTCTCCAACAGATAATGGCTTTTTGTGAGTTACGTCAACTAC
AAAAACAATGTCCCCTTCTTTTATATCTTCGTCAGCTCCGACGATTCCGGGAGCCATAATGTCTGC
ACCGTTTGCGATGAATTTTACGGCACCCATGTCTACAATAACCCTGTTGTTTTCAATATCCATTTC
TAAAAGGAATTTGAGTGTCGGAAATATTCTCCCGCTGTTTACCATTGCAAGAGGAGTTCCGTCTA
AAAGGATTATTTCGCCTTCTTCGGTTACAGCTTTTTCAACAACTGCTTTTTTTGGAATTATTTTT
TCAACGTCAAAAATATTTCCAAGTTCTTCTTTTAATTCCTTTAATTCTTTCTTTTTTAACATGTA
TCTTCTTTTTATTTCCATATTTTCACAGTTAAATATTTTTTAAGATTTAAAAACAGTTTAAAATA
GTGTTTTAAAGAATAAGTAAGTTTAGGTTAAAAAACGTAAATTGGGGAAATCTATTTTATTTAT
TCTATAATTTCGTAACATGTGTTTCTCTGTTTTGGAATTCTTCCAACTGCTAAAACCATGTCTTT
TAATTTTTCTTCGCTCATGTACGTTCCGTAGCTTCCGCCAGCAGCTTTTGAAATGCTTTCTTCCAT
TAAAGTTCCACCGATATCGTTTGCACCACAATTTAATGAAATCTGGCTTAATTTTGTTCCAAGTT
TTACCCACGAGGTTTGTATGTTTTTGATATAATCTTTAAAGAAAATTCTTGAAATTGCGTAAACT
TTTAAATCAAGCATTCCAGAAGCTCCGCTTTTTACTCGTTCCATGTGATAAAGGGGTGCGTTTTC
ATGTAAAAAGGTTAATGGCACAAATTCGGTAAAACCGCCAGTTTCTTTTTGAATTTTTCTTAAAA
TTGATAAATGTTCTGCAAGATGCTTATTTTCTTCGATATGGCCGTACATCATGGTGCAAGTTGTA
TTAATTCCAGTTTTATGTGCAGTTGATACAACATCAATCCATTCAGAAGTTTTTAATTTTGTAGG
GCAGATTTCAGACCTTACTTCGTCATCCAGTATTTCAGCAGCAGTTCCAGGCATGCTGTTTAATCC
ATTTTCTTTTAAAATTTTTAAAGCTTCTTTTACACTTAATCCTGCATTTTCTGCAGCGGATTTAA
CTTCCATTGGGGAAAATGCATGTATATCAATTCCCCCATAGGTTTTGGTGATTTCTTTGACTTTT
TTTAGGATTTCAGCTTGAAAATAGGTATCAATTTCTTTTAAAAGGCCGCCTTGAATACAAACTTC
AGTAGCACCCGTTTTTCTTGCTTCAAGTGCTTTTTTAGCAACTTGTTCAGGACTTAAAAAAAACG
CGTTTGGATCGCCGTGTTCTGCTTTAAATGCACAGAATTTACAGGTTCCGCTACAGACATTTGTA
AAGTTAATATTTGCATTAACAACATAAGTAACCGTATCCCCTGCAATATCCTTTCTAAGAGAATC
TGCAAGTTTCAATATATCAAAAAAGTTTTCAGTATCTTCAAATAATTCAACGCAGTCTTTTTTTG
AGATTTCTTTTTCTTTAAAAGACATCAAATCCATCAAATCACCAATCGATCTATAAATATCTTCA
AGACTATTTTTTAATATTTAAATATTTCGATGATATTAACTGCTAATTCGGGACTAAATCATTAA
AAAATTAAAAATTAGGATTTATTCAATTATTTCATAAAGTGTAGTTCTTTCTTTTGGAATTCTTC
CAATGCTTATAATTGCATTTCTCATTAAATCTACGCTTGCATCTTCGTAGATACTTCCTGCAGCTT
TTGAAACTTTATCTTCTACTAAAGTTCCACCAAGATCGTTTGCACCGGATTTTAAAATTAACTGA
CTCAATTTCGTTCCAATTTTTACTCTTGGTGCCTGAATATTTTTGATAGATTCTTTAAATAAAAT
CCTGGCTATTGCATAGAGTTTTAACTCGTAAAGTCCAGATGCACCGTCTGTAACTCTTCCGGATT
TGTAAAGGGGTGTATTTTCATGTAAAAATGACATTGGAATAAATTCTGTAATACCGCCGGTTTCT
TCCTGGATTTCTTTTAAGATTGATAAATGATCTACAATATGTTTGTATTCTTCAACGTGACCGTA
GATTATTGTAGAAGTTGTTTTAATTCCGATTTTGTGTGCAGTTTTCATGATATCTATCCATTTTT
TAGTAGACATTTTCAAAGGACAGATGTCTGATCTTACTTCATCATCGAGTATTTCAGCAGCAGTT
CCGGGAATTGTTCTTAAACCTGCTTCATTTAACATTTTTAAGGCTTCTTTTATACTAAGTCCTGC
ATTTTCGGCGCCATTTAATATTTCCTGTGGGGAGTATGCGTGGGTATATATTCCGCCGAGTTTCG
AAGTTGCGCTGTTTACTTTATTTATTGTTTCAACTTGGAAGTGTGTATCAACATCAGGATGAATT
CCACCGTGAATTGTAACTTCTGTAAGTCCTGATTTTTTAGCAATAACTGCTTTTTTAGCAATCTC
ATCCGAATCCAATTTGAAAGCTTCGGGGCTGTTTTTTGAAACGTTGAATGCACAGAATTTACATC
CCATGGTACAGACATTTGTGGTTTCGATATTTGTGTTTTTTACAAAAGTAACCGTATCCCCGACA

ATATCTTTTCTAATGGAATCTGCAAGTTTTAAGATATCAAAAAAGTTTTCTGTATCTTCAAATAA
TTCAACGCAGTCTTTTTTTGAGATTTCTCTTTCTTTAAAGGACATTAAATCCATCAAATCACCGG
ATAATTTCTAAGTTCCACTTGGGCTTTTTCGTATATAAAATTGAGTATAGCTAATTAATACGTTA
ATTGGGGTAATACGTTTATTAATCGGTCAAAATTTCGCTGTAGCTCCTTTAATATATATAATTTT
CTATTTATTGAGAATAGAAAGTACATTATATTGCTGATTAGTAATTATACTTGTTATATTAAAA
CTCTAGAAATAGGAAGGTGGCAGAATGAAATTTGGTATCGAATTTGTTCCAAACGAACCAATAAC
CAAACTAGCATACTACGTTAAATTAGCAGAAGACAACGGATTTGAATTCTGCTGGATTACAGACC
ACTACAACAACAGAAACGTATACATGACCTTAGGAAACCTTGCAGCAGCAACAAACAAGATCAAG
TTAGGTCCTGGTGTTACAAACCCGTACGTAAGAAGCCCAGCAATCGCAGCATCCGCAATGGCTAC
CGTAGATGAATTATCAGGCGGTAGAGCTACATTTGGTATCGGCCCTGGTGACAAAGCAACATTTG
ATGCATTAGGTATTGAATGGACAAAACCAGTAGGAACCATCAAAAAAGCAATCGCAGACTTCAAA
GAATTAACCGCTGGAAAAAGAATGGCAGAAGGTGCACAACTTGCAATTAAACCTTCAGCAAAAAT
CCCAATTTACATGGGAGCACAAGGCCCTAAAATGTTAGAAACCGCAGGAGAAATTGCTGACGGTG
TTTTAATTAACGCTTCAAACCCAAAAGACTTCGAAGCAGCAGTTCCTTTAATTAAAAAAGGAGCA
GAAGCAGCAGGAAGAAGCATGTCAGAAATTGACGTTGCAGCATACGCATGCATGTCAGTAGACAA
AAAAGCAGACAAAGCTAAACAAGCAGCAATCCCAGTTGTAGCATTCATCGCTGCTGGTTCACCTC
CTATGATCTTAGAAAGACATGGAATCGACCCTGCTAAAGTTGATGCAATTAGAGCAGGATTGAAA
GAAGGAAACTTCGGTGCAGCATTTGGTAATGTTGACGAAAACATGTTAGAAGGATTTGCTTTATA
CGGTACACCTGAAGAAGTTGTAGAAAAAGTAAAAGCATTAGAAAAAATGGGTGTAACCCAAGTT
GTTGCTGGATCCCCAATCGGACCAAACAAAGACACCAGCATCAAATTAATTGGAAAAATCATTAA
AGAATTCCAATAATTAAATAACTTTTTTAAATTTTTTAAATTTCAAAAAAAAAGAGTTTTATTT
TAGTTTTAAAGGTTTATTTGGCCTTTGATTTTTCAATGTATTTTTCAATCATTTTAGCTTCGATT
TTTGAAGTTATTCCTAGTCTTTCTAAAACAATTTCTGTAAGAAGTCTTTTTAAAATTTCTTTTCC
TTCTTTTGTATCATCAATTGTTCCCAATTTTTTTGTAGTTCCTGGAGACTTTAATTTTGTTAATG
CTCCTGAAATATCTTCTTCTTTAACTCTTCCCTTTTTCTTTTCGTATATTTCGTCTGCAATTGCCC
TGAGTTTTTCCATGTCAAACGGCAAAAAATCTTTTATTTTTATAGAGAGTGTTTCTTCGATTGAT
GCTAAAACTCTAGCAATTTCTTCGTTGGATGTATTTTCATCAATTTTGATTTTTTTAGAAACGTA
CCATTTTCCTTTTTTAGCGTTAAACTGTATCCGTTCTTCAGACATAAAATCACCTGAATAAAACA
AGTTAATATAATCGTAAAATTATATTCTGTTTAAATTATATAAAATAGTTTCAATTGAAATCAA
AACAATTTAAAAAGAGTAAAATAATTAGTTCATTTCAAGAGTTTTTTTCAAAATCGGGCTTTGA
ATTTCTTCTACAGAAATTTCTGAAATTTCTACAACTTTGATATTTGCTCTTTTTACAGCATGTTT
GCTGCCAATTTCTGAATACATAGTTTCTAAAGCATCATTTTCTTTTACAACGTTGTATTCTTTTG
TAAATACCATTGGTTCATCTTTTCCAACGATTTCTCCTTTTATTCTTACGATTTTTGCCATATTAT
CACCAAAATAATATTAATCTATTAAATCTAGAGCTTCTTCAATTTTTAAGATTTCAGGACCTGAC
GTGTCGCCTCCGACAACAGCTCCTTTGGTGTTTGCGAGAATGCACGCGCCAACTGAAGTTACGCCC
CTATTTGCAGTGCCTCTTTCAACATAGTCCACTCTTAAAATGTCTTGAATCCATTCTAATTCTAA
TTCATCAGTTAGTGGGTGAACCAGGCATCCTTTATCGGTTGCAACTCCATTTGAACCAACTGTTG
GGAGTTCAGCGTAGTTTCCTACTCCGGATTCTACGTCTAGTACATCCTCAATAGTTTTTCTAAAT
CTGGATAATTCTTCCGAAATTATGCATCCTTTATTATTAGTTAATATTAAGTTACCAAATGCCGT
GTTTTTAGCTTTAAGTCTTTCTAAATTAACGTCCAGACTGTTTTCTTTTAAAAACATTTTAATTA
ATTCAAGTTCTTTTTCAGTCGTTATGTTAGGTACTAAAAGTCCGTTGGAGTTTCCAACACATAAG
GAGCCTATTAAAGAGCTGTTTGAAATGTTTAATTGGGTTGCAGGAATGTCGAGTACGTTTTTCAT
ACGTTCCATCGTTTTTTGATCAACTGAAAGAGGAAAAAACCCATAATCTTCAGTTGCGAGTGAAT

GTACTCCTAGTGTGGACACTCCAGAAAAATATGTTTTCATTATCATAGTACCACAGTGTTTCTAA
AGAAAGAAGCAGGTAGATTATTCTACTAATGTTGCTTTAACAACATCTCCTTCTTTTACAACTTT
AACTCTAACTTTAGCAGGGATTTTGTTTAAGCTTCTTTCCCATACTTTTTCGTTTATTGTATTGT
CAAGTTTTACAATATCTGATTTCATGTGTTTCTTCAAGAACTCCCTAATTGCCCTTATAGCTCTT
GGAGCTCTTTTTGTTGTAGGGCTTTTGTTTGTTACGTCTCTCAAAGGGATTGTATAGATTCTTTC
TTCTTCCATATTATGCACCTCCGCATGGATTAAAAAATGTATAAATATTAAATCATATAGAATAT
GAAAAAACAATATTTAAATATTTTGGTGAGCGGGTAACATGGAAGACTACACAAAAGCAGAAAT
TTTAATCGAAGCCCTGCCTTACATCTGTAAATTTCACGATCAGAAGATTTTAATTAAGTACGGCG
GCCACGCTATGGTAAATGAGCAGGCAAAGAACTGGATTGCAAAAGACCTCGTGCTTTTGAAATAT
GTTGGCATAAACCCGATAGTAGTACACGGCGGCGGCCCTGAAATCAATCGTGCGATGGAAAAAAT
GGGAAAAACCCCTGAATTTATTCACGGGCTTCGTGTAACTGATGAAGAAACACTTGATATTGTTA
AAATGGTTCTTATTGGTAAAATAAATGGTGACATTGTTTCAAAATTAGAACGATACGGCGGAAA
ATCTGTCGGTCTTTCTGGAAAATCAGGTCAATTAATTAAAGCAAAGAAAAAAATCCAGTATTTAA
TGAAAGACAGCCAAAAAATCGAAGTAGATCTCGGAATGGTCGGGGAAGTCGAACACGTCGATACA
AAATTAATAGACATACTGGTTGAAAAAAGGTACATTCCGGTAATTTCACCAATAGGTGTCGATCA
CCAGGGAAATGACCTGAATTTAAACGCAGACATTGCTGCAGGAGATATTGCAGGTGCAATGGATG
CAGAAAAACTTATAATGGTTACCGATGTCGATGGAATAATGGACGACGTAAATGACCCATCTACC
CTTCACAGGAGGTTAACTATTCCTCAAATTGAAGATATGATCGAAAAAGGGCTTATTACTGGTGG
TATGATTCCAAAAATCGAAGCATGTGTCAATGCACTTGACAAAGGAGTTCAGAGTGTACACATAG
TAAATGGAAAAACTCCTCACGCAGTTCTTCTTGAAATTTTCACGGAAGATGGTGTGGGTACCATG
GTTGTAAGAGAATAACTTTCTTTTTTTAATTTTTCAAACTCTTTAAATTCTTCTTTTATCTACAA
AATATATCTATTTATTGGTCTATTTGGATTAAAAAAGATTTAAATTTTTAGATTTAAATTATCT
TTATATAGGAGATAAGTCAATGTAGTATTATCGGAAAAGGAATTCCGGTTCCTTCCGAGACGCAC
CAACCAAATCACTATATCGCACCATTAACCTTAAAATAGCCAAAAACCATTAAAAAACCAAATAA
CCGACATTACCTTATTAAAAAATAAGGTAATATACCCCTATTTACAAAATATTAAATGACCTATA
AAAATCGAAATTTCATACGCGTGAATCATGATTACCACTTTATCAATCTTAAAAAAATATCAAAG
ACACGAAGGGGTGTTTAAATGAAAAAAATTGAAGCTATAATCAGAATAGAAAGATTGGATATCG
TAAAAGGCATTCTTTCTGAGAATGGCTTTTTAAGTTTGACAATTTCTGAAGTAAAAGGTAGAGG
AGTTCAGGGTGGAATAACTGAACGATACAGAGGAACAGAATACGTAGTTGACCTTCTTCCGAAAG
TTAAACTTGAAATGGTCGTTGATGATAAGGATATTGACGAAATAATTACAATAATTCGTGAAAA
TGCAGAAACTGGAAAACCCGGAGACGGAAAAATCTTTATTATTCCAGTAGAAGACGCAGTAAGA
ATTAGAACAGGCGAATCAGGCATAAAAGCTATTTAAATTAAAAATAAAATCAGGTGAAAATTAT
GGCAACGGCAGATTTATTTGCTGACCCCACAAACATCGTAACAGGGCTCAGTACATTGGCAACTT
CTGGAGATGTAATGTTCTTAGTATTGATGGGAGTACTTGTGTTTGCAATGCAGTTAGGATTTGCA
CTTCTTGAAGGCGGTCAGGTCAGAAAGAAAAATGTTAACAACGTAATGATGAAAAACATGGCAG
ACTGGTTAATTGGAGCAGTTTCATGGCTTTTCATAGGCGGTGTTCTTTCAGTTTCACTTGTTCCT
GCAGACTTCTTCAGCTGGTGGGGACAAATATTTACAACAAACTTTGCAAATAATGGAATAGACTT
AGCAAACTGGTTCTTTGGACTGGTATTCGCAGCAACTGCAGCTACAATCGTATCAGGTGGTGTTG
CAGAAAGGATTAAATTTAAATCATACTTATTAATGTCAATAATCATAACTGCGATACTCTACCCG
CTGTTTGTGTACCTTGGACCTTGGGGTTCAGGAATGATTCCATTCAACGATTACGCTGGAAGTTT
CATTGTACACGGTCTAGGTGGTTTCTTAGCACTTGGAGCAATTGCAGCTCTTGGACCTAGGATTG
GAAAATTCGTTAACAAAAAAGCAATTTCAATTCCTGGACACAATATCCCAATGTCAGTATTCGGG
GCATTTGTTCTCGCAATCGGATGGTACGGATTTAACGTAGGAAGTTCATTTGCAGTTGCAGATAT

TTCTGGATTAGTATGCGCAACAACAACACTTGCAATGGCAGGTGGAGGTATTGGGGCACTCCTCG
CTTCAAAAGATGATGTATTATTTACAGCAAACGGGCTTGTTGCAGGTTTAGTTGCAATCTGTTCG
GGAACGGATGTTGTAAGCCCAATAGGGGCATTAATTATCGGGCTTGTTGCAGGAATTCAGGTACC
AATCGTCTACAAAATCGTTGAAAAAATGGGATTGGATGACGTATGTGGTGTAATTCCAGTACACG
GTACAGCAGGTATTTTAGGAGGTATCTTAACAGGTGTCTTTGGAATGGCTGCTCTTGGTGGTGCT
GGTGGAGTAAGTTTGGTAAACCAGCTTATTGCAGCAGGATTAACAGTAGTATACGGTACAGGATT
AGGATTCGGTCTTGGAAAATTAGTCGGTGCATTTACTGGTGGAATTAGAGTTACTGAGGACGAAG
AAAAAATGGGACTCGACCTTGCAGAACACAAATTGCCAGCTTACCCTGACGAATAATTAATTTTT
CCTTTTTTTCATTGAAAAATAGATACTATTATATATTTTTCAGAATATATTTTTTCAAAATGTTG
AATATATTTACTATTTTGGCGGTGTTAGTATGAGAAAAATAGAAGCGATATTCAGACCTGAAAG
ACTTTTAGCAGTTAAAAATGCTCTTTTTGAAGAAGGATTTATCAGTTTAACGCTTACAGAAGTAA
AAGGAAGAGGAGTTCAGGGCGGAATTGTTGAAAGATACCGTGGAAACGAGTATTTAGTAGATTT
ACTTCCAAAAGTTAAAATTGAGATTGTTTCTAATGACGGCGATGTTGAAAAAATAGTTAAACTT
ATTTCCGAAAACGCAGTTACTGGAAAACCTGGAGACGGAAAAATCTTCGTAATTCCTGTTGAAGA
TGTAATTAGAATCAGAACAAATGAAACAGGAAAAGACGCTATTTAAATTCTAAAAATATTTTTT
TATTCTTTTTTTCAAATATTCAAATTTATCTATTTTGATAGGAATATTTATATCAGTTTTATTTT
TAAGTAAGAATAAGAATTCCGGTTCCTATATCACACATTTTTCAAGGTATGGGTTTTCCTTAAAT
TTCGATTTTAATCCCAAATTTCATTTTAGTTGGTTTAGTTAAATGGTGCAATAATGAAGAAACTA
GAAGCAATAATAAGAATGGAAAGAGTAGGACTCGTTAAAAATGCTCTTTACAAAAGCGGGCATG
CTAGTTTAACGTTAACAGAAGTAAAAGGAGAGGAGTTCAGGGCGGAATTGTTGAAAGATATCGC
GGAACAGAACATGTGGTAGACCTACTTCCAAAAGTCAAAATAGAGCTCGTAGTCAATGAAAAAG
ACGTAGACTCTGTAATAAAAATAATTGCAGAAAATGCATATACCGGAAAACCGGGAGATGGAAA
AATCTTCGTAATTCCGGTCGAAGACGTATGTCGTGTAAGAACTGGTGAAATGGGTCCAGATGCTA
TATAATATTATTAAATTTTAATGGTGCGTAAATATGGTGACGGCAGATTTATTCAATAACCCTAC
AAATATTATGGATGCGTTAAATACGCTTGCAAACTCAAGCGATGTGATGTTTTTAATTTTTACTG
GTGCTTTTATTTTTATAATGCACCTTGGATTTGCAATGCTTGAAGGCGGTCAGGTTCGAGAAAAG
AACGTGAACAACGCAATGATGAAGAACATGGGCGACTGGATCATTGGATGTATTTCCTGGCTTTT
AGTTGGTTCAGTAATTGCTAGAACATTAAACCCTGCAGATTTCTTTATTTGGTGGGGAAAAATTG
CAAGTGCAACACCTTTCCTTACAAATAATGGATTGGAACTTGCAAACTGGTTTTTAGGACTTGTA
TTTGCAGCAACTGCAGCAACTATCGTTGCAGGGGGAATTGCAGAGAGAATCAAGTTTAAATCCTA
TATTTTAATTTCAATCGTTATAACGGCTTTGTTGTACCCATTCTTTAGTTATCTTGGTCCATGGG
GTGCAGGAGTAATTCCATTCCACGACTATGCGGGAAGTTTGATGGTTCACGGTGTTGGCGGATTT
TTGGCACTTGGTTTAATTGCAGCAATCGGTCCAAGATTTGGCAGATTTGTAAATAAAAAAGCAAT
AACAATTCCTGGACACAACATCCCAATGTCGATTTTAGGTGCATACATTCTTGCATTTGGATGGT
ATGGATTTAACGTTGGAAGTTCACTTGCATTGAGTGATATTTCAGGACTTGTATGTGCAACAACA
ACACTTGCAATGGCTGGGGGAGGTCTTGGAGGATGTTTATTCTCTAAAAACGATGTGTTATATAC
TGCAAACGGACTTTTGGCAGGAACTGTTGCAATTTGTGCAGGAACGGATATTGTAAGCCCGCTTG
GAGCTTTAATAATTGGATTTATTGCAGGATCCCAGGTCCCATTAATATTTAAAATTGTTGAAAAA
TTAGGATTGGACGATGTATGCGGTGTAATTCCAGTACACGCAACATCTGGGGCTCTCGGTGCAAT
TTTGGCAGGGATATTTGGAATGACTGCATTTGGTGGTGTTGGTGGTGTCAGTCTTACCGAACAGA
TAATCGCAACATTAATTTGTGCAGTTTATGGTACCGGTGCAGGATTTATTCTCGGAAAACTTGTT
GGGGCAGTTACTGGCGGTTTAAGAGTTAAAGAATCTGAAGAAAAAGCAGGACTTGATATTTCAA
TCCACAAACTCCCGGCATACCCTAAAGAAAACTAAATATTAACTTTTACTTTTTTTAGAAATTTA

TTTTTATATTTTAAAATCAAATTATTTTTTATGAAAACATTCAATGAACAGTGTTACGACCTTAT
AATGCAGATTCCAAAAGGAAAAGTCACCACGTACAAAATAATAGCCGAGGCTCTAAATACTAAAG
CTTATCGGGCTGTAGGAAATGCGATGAAAAATAATCCAAAAATTTTAACAGTGCCGTGCCATAGG
GTTGTAAATTCTAACGGTTACGTTGGCGGATATGTCAACGGGGTTGAAAAAAAGATAGAAATTT
TAAAAAACGAGGGGGTGGTTATTTCTGGGGGTAAAATAACCGATTTAAAGAAATATTTATTCGT
GTTTTAGGTCTATTATCGTTGGATCTTTATTGGGTCATTTAACTGTCGACAATAATGATTTTAAA
TGTTTTCAGCAAATATTTTTTTGAGGGGTTTAATATTCATTGTGGGGTATTGTCCACACAGTTTA
CTATATTTTCATAATATATAAAGTTGCTTAATGGGGAATTTAATAAATATTATTTTTTAAAATC
AAAAAAGCCTAATTTTAATACTTTTAATTAAAGATTTATAAGTATAATTTTGTCCAGGGTGGTTT
CATGGTCTTTGGAATATTTAAAAAGAAAAAAAAGGCAGTCGAGGACTATTTAAACGATAAAAAT
GTACTTGAAATTACATTAAATGATTCTAAGGATTGCATTCTTGATTTTACTTACAATTTTATATG
GCAGAGTAATTTTGACATTAAAAGTCAAGTATTCAATGGAATATCTTATGAAAAATTAGTAAAC
CATATAAAAAAATCCTGAAAATGTAGTATATATTAAAGGAGATGTGGGACACAGGTTTTGCAGTT
CAATGGGTGCCGATTTAAAGTATTTTGGAGGATCGGGCGGTAAAATTCAAACCGGAACTGTTATT
GTTGATGGAAACATTGACACGAGATTTGGGATAAGTATGGTTTCTGGAACTGTTTATCTAAATGA
AAAATTTAGCTTAAAAGAGCCAATTGGAAATATAATTGAAGTTGAAAGTGATATTTCAGGATAT
AAAAAATATATTTCAATCACTGAATTAATGGAATCTAATTCACAAGAAAAAGTTTTAAAACCAA
ACATTTTTAAGAACAACGAATTAATTTTAAATGATAATGTAATTCGGGACACTATTGGTGCCAGA
CTTGAAAATGGATCAAAAAATTACCGTAAACGGAAATGTAGATTTGAGCACGGGAATTTTGATGA
AGAATGGAACTGTTTTGGTTAATGGCAATGCTGGAAAAAAATACGGGATCAGTGTTAAGGGGTGG
AACTGTAATTATAAATGGAAACACAGGGGATTTCACTGCATCTGACATGATATCTGGAACAATAA
TTATCGATGGAAATGCAGGTAAATTTTTAGGTTCGAAAAAGAAAAACGGGGTAATTTATGCGAA
GGATGGTTCGGCAATTCCTCCTTTAAAAAAACAGTCGTTAAATTCAAAAGATAAAGAACTACTTT
CTAAAAATGGATTTTTTCGGGGAGTTTGTAAAATTTTAAAATATATTTGAATGTATAAATATATT
TTCCCAAAATAAAAAACGAGCTTCAGGCGGGGTTTGAACCCGCGGCCTCCTGCTTACCAAGCAGG
CGCTCTACCAGGCTGAGCCACTGAAGCACACTTAAACCTACAATTTGCATATCAACTAACTCAAA
AACAATATATAAACTTTTTGGTATTGTTCATATTTTTTATTAATTTTTTTATAAAATTAAATCCA
ATTTTTTGAAATTATTTAATTCATTAATAACGTCTATTTTTAGAATTAATCCGAATATCTCTATT
TAATCAATATTTCAAACGCTTTTAAAAGAATGAATTTTACCGAAAACTTTATATACCGTAATTCC
AATCTAATTAGTGCAACTATTAAAGAAGCCCAGTAGTGTAGTGGCCAATCATCCGTGCCTTTGGA
GCATGGGACCGCGGTTCGAATCCGCGCTGGGCTACCATTTTTATTTTAGGCAAATTAATTTATAA
ATTTAGATATACCATTTTAATTTTTTAAAAAAATAAAAATTATTCTAAAATTTCAAAGTCGATA
CCGTGCCCAAAGAGGAACAATAAATAGCTTGCAGGAATTTCTGTTGAATTATGGTCAAAAGTATC
TCCTTTTAGTGTTAACGGATCAAGTTCCTGTTTTATTTTTACATTGTACATTTTTTTATGGAATT
TGGAAAATACGCCATGATTTAATGGATCGAACTGGTTATTTTTCAATGAATTATAAGTTATTTCT
TTTACCATAAGTCCAGTGTACCCGTGAAGTGATCCAATGGTTCTAAGAAGCCGGATATCATCATC
CATAACTTTTTCGTCGAGTTCAAGCCTGCTTTTTACTTTATCGATCGTTTCGTATATGGTTCGCCT
TGTAGTGTCTTTCCTATTATCGATTTCAGTTTTCCAGTCTGAAGTTTTTTCGAACTTTTTTGTTG
CAATCTTGTTTTTATTAAATTCTCTTACCAGTATATTTTTCCACCGACTCCCCATATTCCTTAAAT
TCAGATTTTTTCCCAAAATATATTCAATAAAGTATCTTCTTTCGTTTTTTCCCCATTTTTCTATTC
TTTTAAGCAATTCGGGGTCTTCTGGTTTTAAGTATATGTGGTAGCCTCGGTTTCCAGTAAATACT
ATTTTTAAATCTTTTTCGTCGAGTCCAAAATCAGGAAACAAAAAGTCCTCAATTAAAATTAAAAC
CTGATTTCTAGCTTCACCCAAACATTCTTCGCATATCCATGAATCATCATGAGTACACTTTTCTGT

TTTATGCGTATCAATATCAAATGCAACTTCTCTTTTAAACAAACCTTTTTTTGCAGCGCCCCCAAC
AATATCCGGGTATTCCATGTATGAAAGTGATTTATATAGGTGAAATGGGGATTCTTTTAAAACCC
AAGCTAAATAGTCTCTTTCTGTATTAAAAGACATATTTCTATTGTCTACTTTTTTTAACATCCCG
TAGCCAATTTCCCTATGACCTAAATCATCAGGAATTTCAAGCCATTTTCGAACAAGTATGGCATA
TTCAAAGTATTGTTTATATAGGCTCGAAACTTCTTTAAAAACCTTATCTGTGGCAGGATTGTCGT
TCATAATTTCACATAACGCTTAATTAACCAAATTAACTTTAAATAATATTGTGATTATCTATAAT
TAAACTTATCTTTTAAAAATGATTTTTTATTTTTATTTAAATATGGTGTGAAAATGATTTTGGG
AGTAGATATTGGCGGAGCAAATACAAAAATAACAGAACTTCACGAAAACGGTGAATTTAAGGTT
CACCACCTTTATTTTCCAATGTGGAAGAATAACGATAAATTAGCAGAAGTTTTAAAGACTTATTC
AAAAGATGTATCGCACGTAGCACTTGTTACGACCGCAGAACTTGCAGATTCTTATGAAACAAAAA
AAGAAGGCGTAGATAATATCTTAAATGCCGCAGAATCTGCCTTTGGTAGCAATATATCCGTTTTT
GATAGTGATGGAAACTTCATTTCTCTTGAGAGCGCAAAAACAAATTATATGAAAGTTTCTGCATC
AAACTGGTGTGGAACTGCAAAATGGGTTTCAAAAAATATCGAAGAAAACTGTATTTTAGTAGAT
ATGGGTTCAACAACAACCGATATTATCCCTATCGTGGATGGAAACGTGGTTGCAGAAAAAAACAGA
CCTTGAAAGACTTATGAATCATGAATTGTTGTATGTTGGAACGCTTAGAACCCCGATTTCACATC
TTGGAAACACGATATTGTTTAAAGGCGTTAATACAAACGTTTCTTCAGAATATTTTGCAATAACT
GCGGATATTTCGGTAGTTTTGGAAAAAGTAACAACTGAAGAATACACATGTGATACACCTGACGG
TAAAGGAACCGATAAAAGAAGCAGTTTAGTTAGAATTTCAAAAGTTTTATGCTCAGATTTGGAT
CAGATTTCAGAAATTGATGCAGAAAATATCGCAAAAACTTATTATGAATTATGGAAAGAGTTAA
TTTTAGAAAACGTGGAAAAAGTGGCAGAAAAACACGATCTTAAAAAAGTTGTAATAACTGGACT
TGGAGAAAATATTTTAAAAGATGCATTAGGGGATTTTGAAGTAATTTCTGTTGCAGAAAGGTAT
GGAAAAGACGTTTCCCTTGCAACACCTAGTTTTGCAGTTGCAGAACTTCTTAAAAATGAACTTTA
ATAACTCAAATTTTATTTTCACATTTTTTTACAATAAAGTTTATAACTATTTAGATAATATATTG
AATAAATTTTAGATTACAATTTATTTACATCAAAAATAGAACGGAAAAATAAAACACAGGTGAT
AATCATGCAAGAAAAAATTGCAGTTTTGGCATATTCTGGAGGGCTTGACACGAGCTGCTGTTTAA
AATTACTCGAAGACAAATACGACTACAAAGTAATTTCAGTAGCAGTCGATGTAGGTCAGCCTGAA
GACGATTTAAAAGAACCAGAAGAAAAAGCAAAAAAATTCGGTGTATTAAAACACTACACAATCG
ATGCAAAAGAAGAATTTGCAAAAGATTACATTTTCAGAGCTATTAAAGCAAATGCACTTTACGA
AGGATACCCATTATCAACAGCTCTTGCAAGACCTTTAATTGCTATAAAAATTGCAGAACTTGCAC
AAGAAGTTGGAGCAAGCGCAATTTCACACGGATGCACTGGAAAAGGAAACGACCAGTTCAGATTT
GAGTCAGTAATGAGAGCAAAAACACCAGAAATTGAAATTGTAGCACCAATAAGGGATTTAAACC
TTACAAGAACTGAAGAAATAGCATATGCAAAAGAAAAAGGAATTCCAGTTCCAGTAGACTTAGA
AAAACCATTCAGTATCGATGAAAACTTATGGGGAAGAAGTATTGAGGGCGGAATTTTAGAAGAC
CCAATGACTGAAACTCCAAAAGAATGTTTTGCATGGACTGTTGACCCAACAGATGCACAGGACAA
AGAAGAATACGTAGAAATTGACTTTGAAGAAGGGGTTCCTGTAGCAATCAACGGGGATGCATTG
GAAGCAGTTTCATTAATAAGAAAAGCAAACGAAATTGCAGGAAGAAACGGTGTTGGAAGAGTAG
ATATTGTAGAAGATAGAGTTTTAGGCTTAAAATCAAGAGAAAACTATGAATGCCCTGGTGCAAT
GCTTTTAATAACTGCACACAAAGCTCTCGAACAGCTCGTTTTAACAAGAGAAGAACTCGTATTCA
AAGAAATGGTTGATTCAAAATACGCAGATTTAGTATACAAAGGACTCTGGCACGAACCATTAAG
ACTCGATTTAGATGCATTCATTGATAAAACGCAAACAAGAATGAACGGAAAAGTAGTTTTAAAA
CTCTACAAAGGTTCAATGAGAATCGCTGGAAGAGAAAGCAAAGATGCAATCTACAACGAAGAAA
TGGTTTCATTCGAAAACAAAGAAATGGACCAGAGAGAAATTGTCGGAATGGTTAAATTCCACGG
TCTCCAAGCAGCAATCTATGAAGGATTAACAAGAAAATAATTACTTTTTTAATTATTTTTTTTAA

TATTTCTCATTTTAAAAATTTATTTTAAATTTCCACGTGATATTATGAACATTTTTACTTCAAAA
GGTACCATAAAGTATGAAAAAGAAAAAAATAATAAAATTAAGTTCGGAAATGTTTCCTGACGACC
TCTGCGAGCAGTGTGGGCGGTGCTGTATAATCCACGTTTTTAATTCAACCGAATGCGGTGAACCC
GAAGTTGTATACTGCAATCACCTTGATACTGAAACAAAACGCTGTAAAATTTACAAAAACAGATT
TAAAAAAGAGAAAAAGTGTCTTTCAATGCTCGAAGCTATAATGGTTTCCGCACTTCCAAAAGACT
GCCCTTATGTTAAAAATTATGAATCTTATGAAGAACCGTGGTTTTACGACTGTTTACGGTCAAAA
TCAAAAGATTAATGATAAAATATCGTAAAATATATGAATTAATATCGTAGTGGATGTTGATTTT
CTCTCTTTTATCATTCCAAGGATGTAGCTTATTATAAATACATCTAAAAGTACCATTGATGACAG
ATACTGGGCATGAAGGCTTGCAAAAAGTAAACTTGTATAAATGTTTCCAAATCTCGGCTGTAACG
CTCCTCTAAACATCAATTCTTCACTTATCCCAACTACTATTGATAAAAATACTATTTTTAAAGGG
GTAGATGCTGACGTTTTTATGGTTTTTACTACTTCATTGACATTTGAATTTTCAACAACAGCCTG
TTCTGCAATATGTTCTGAAATTGCCGGATCCATTGACTGGAAGACATATATAAAAAAGTCAAAAA
GTCCCCATATAAATTTATCAACCATTAATAGAAGGATTACTCCAAAAATCGCGTATTTTAAGGAT
GGAATTTTTATATCGAGCCTTTCAATAGACTGTTTTAAGTTTTTTCTCGTTCCAAAGCCTATTCC
AACAAAAGCATAGGCTAAAATTAAAGTTGAAATGCAGCAATATCTAATATTTCTAAAAAGTCAG
AAAGGATAATTATAAGACCTGCTCCCGATGCAAAAGCGATTATAGGATTTAATAAAATGTATGT
AGAAAGCGAAACTGAAATCTTATGAATTGAATTGTCAAACGAAAGATCCATTTTTAAAAGTTTT
GTAAATTTTTTAAAAAAGCCCTGATTTCTAATCAGTAAAACGCTCAAAAAAGAAGCAGCATAAAC
AGAAAGTCCAAAAACATAATTTACATCAGACATAAAATATATCGAAAAGAAAAGCGCTGATAAG
TTGAAAACAAAAATAATGTCAAGAGTACGTTTTTCAATAAGTTCCAGATTAAATGCACGAATTA
AATTGATCCAGTATATAACTAGACTAATAAAAAAATAAAAGAATCGAATGAGTGATTCGATCAT
CTTTATCACTGCTTAACTACTGGGGTTTCCGATAAAAGCCCACAGGTATTAAGGGAAGACGTGAA
ACACTTATACTCTTTCTGCGAAAGCGAATTTTTCTTTTACAGCGGTAACTTTAACGTTAATAACG
CTACCTTTTTCTGCGTTAGGTACGAAAACAACGAATCCATCGATTCTAGCGATTCCGTCTCCACCT
TTACCCATGTCTTCAATTGTTACTTCATATTCTTTTCCTGCTTCAACAGGAACATTTTTCATTGCG
GGTTTACCGAAAGCCATATTTATTCACCTAGTATTATATACATCTCTTATATTGATGCAATATTA
CACTGCTTTTGATAGTATATATAAGTATCTGAGTTCATTATTTGAGAACTTAACATTAAATACGC
TTTGAATTTTAAAAACGATTTATCGGTGGTTAAATGATTAGAGAAGTGTTTTCCTCGATTATGGG
GGAGGGGAAGTTTATTGGTAAAAGATTTATATTTGTGAGATTTAAAGAATGCCCCCTTGACTGTA
TATATTGTGACGAGCCAAATGCTCCGGGGGGAACTGCAAGAGTCGAAGAAATTTCCGGATCAGGC
GAATTTATGGAATATCTAGAAATTGAACATGAACTTATCGAAATTATTGAAAAATTGAGGACGC
CTGACCTTTTTGCAGTTTCTTTTACTGGGGGAGAACCTTTGGTATATTCCAATAAAATAAAAGAA
TATTCTGAAATTTTAAAACATAAGGGATATAAGACGTTTTTAGAGAGCAACGGAATGTTTCCAG
AACGATTGGGTAGCTATGACTATGCTTCAATTGATATAAAGTTGCCAGAACACTTTGAAAATAAA
GACGACGATTTTTGGTATGGATTATATAAAAAAGAACTTGAAACGATTGAAAGACTCTACCTGGC
AGGAACCGATGTTTATGCAAAAATTGTCGTGTTTGAAGAAACTTCTGAAGAATTAATTGAAAGA
ATCGCGATAGATTTATCAAAAATTGGGAATATAACGCTCTGCATTCAGCCAGTATCACCAACTGA
TAAAATAAAAACAACTACTTCGAAAAAAAAGATATTTGAATTAATGGCCATTTGTGGCAAGTAT
GTGGATGTAATGTGCACGCCACAAATCCACAAATGGATGGGAATGTTATAATATCTGTTTGCAAA
TTTAATAATCTTTTAATTTTTCTTTTAAAGAACTAATTCCCGTTCTATCGATTGTATCGCCAAAT
CTTTCATGGGAATCTGCATTTTCTTTGAAATAATCCATAATTTCAGAAACTATTTTTTCAAGATT
TGGGGCATCGTATATTCCGATATTTTCGCCAATTTTATATTTTTTACCGAATTTTCCGCCAACGG
ATATCAATACGCCATCTTTTAAAGAGATACATTTTCTTTTGCACGATTCCCCGCATTTTCCGCAGT

TAACGCACTTTTCATAATTTACAACTGCTTTTTTATCAACGATTGTGATTGCATCGATGCTACAG
GATTTTAAGCACAGTGTGCATCCATTACAGTTTTCTTCATTTATTTTTGGTATTCTCTGTCCAAT
AATTCCTACATCGTTTAACTGCGCTTTAGCACAGTTATTTGGACATCCTGAAATTCCTATCTTAA
ATTTATGCGGTAATTTTTTTCCAAAAAACAATGAATCAAGTTCGTTACAAATCTTTTGACTGTCA
ATAAGTCCGTGAGTGCAAACAGTTCCCTTACAAGCGACAAGGGGCCTTACAGTGTTTCCAGTTCC
ACCCGATATCAGGCTTGAATTTTTAAGTTCTTCTTTTATTTTAGGTATATCTTCGTAGTTTATCC
AAGGAATTTCCACACAAAGCCTGACGGTGAAACTAATATAATTTCTACCGTATTTTTTTGATATT
TCTGACAATTTGTCAATTTTTTCTGAATCCATGCATCCTGCATCGGTTTTTATTCTTAATGCAAA
ATGTTTTCCATCGTTTTGAAGGATAAAACCTTGCATTTTCAGATTAGCTATTTCATTTTTGTTCA
TATTACCATCTAAATTAGTATTTGGGTTTAATAAACTGAATATTGTTTTATAATCTAAAACGGCA
TTTTATAAATCTATCTGATAAAAATATTTCATGAATTTCTGAGAATATATGTCATTCTTTGGCAT
CTATCAAAATCTAGCGAAATATAAAAATAATATAAAAGAGTATTATCTGGTAACGTGGTGATAT
CATGAAAAAAGAACTCATACTTAAGGCTTTAAAATTAAGGGATATGGGTTTTCCAAGTGGGGAT
ATTGCAGAAGAACTAAACATATCAGTTAAAACGGCATTATATCTTACACTAAATGGTGAAGAACT
TTTAAAGGCTGAAGAATGTCCAAAAGAGGATTCTGAAAAATTGGATATCTTTTTAGAATGGGAC
AACGTTCGGGCTTCATCAAGAAGGCTTAGAAATATTTCAAAAATAATCTGTGATATGCTTTCAGA
TGTTGAATTTGACGGTGTTGTCGGAATTTCTTCAGGCGGGGTTCCTCTTGCAACGCTGATTTCGG
ATGAACTTGATAAAAATTTCTCTATATATGTTCCAAAAAAACACATCCATACTGAAAAAGAGAA
AACAACAGGATTTATCGGTCAAAACATGTCCAGCATTGTTGGAAAGGACGTTATTATTGTTGATG
ATGTAATGACATCAGGAAATTCTGTAAAAGAAACAATAAAATACTTAAAAGGCATTGCTAACCC
TAAAAAAGTATTCGTGGTAATGGATAAAAGTGGAATCGATGAAATCGAAGGCGTTAGTATAGAA
CACCTGTTTAGAACTGGTGTTGTTGATATTAAAAAATAATTTTTTATATTTTTTAAAATAATTGA
ATAAATAGTTGGATTAACCTATAAAAAATAATTTTTCAGAGTATTTTGGCTCTTTTATATTCAAT
AATGTATATAAATGACTTTTTTCATAATAGGATAACCTAAAGTTGGAGGCTTATCATGTGCGGAA
TTATCGGATTTATGAGTAGAACTGGAAGACTTATCAGTGGAGACAGGATTGCTACAGCCTTGAAT
TGTTTAAAGGAAAGAGGAAATGGTGAAGGTTCAGGTTATGTAGGATATGGAATCTACCCAGACT
ATAAGGACGACTATGCAATACACGTTTTTTTAGATGCAACCAAAGATTACGATAAAATTAGACAC
GAAGTCGAAGAAGTTCTTGTAAAATACGGATACATCTTAAAAGATGAAGAAATTCCAACAGAAG
AAGGAATTATCAAAAAAGAACAGGTTTCGTGGAGATTTTTCTACAGGTTTGATGAAAAATATAA
ATCAGTCGAAGAAGACATGATGGTGGATATCGTAATGGATATCAACGCTAAAATCGACGGAGCAT
TTGTATTTTCAAGCGGTAAAAATATGGGCGTATTTAAAGCTGCTGCATGGCCTTTAGAAGTTGCA
GAATACTTTAAAATTGACCAATACAAAGGTTTCCAGTGGCTTTCACATGCAAGATACCCTACAAA
CACGAAAGGATGGTGGGGAGGTGCGCACCCATTCAACCTGTTAGGATGGTCCGTAGTTCACAACG
GAGAAATTACAAGCTACGGTGCAAACAAAAGATACGTTGAAAGTTTCGGATACAAATGTAAACT
TTTAACCGACACGGAAGTTGTTGCATATCTCTTCGATTTACTCATCAGGAAACACAAAATCCCTG
TAGAATATGCAATGAGTGCATTAGCTCCAAGATTCTGGGACGAAATCGAGAGCATGTCTGAGGAA
GATAAAAAAATACACGAAGCAATCCGTATGACCTATGGCGGTGCAACAATGAACGGTCCTTTCGG
AATTGTTGTTGGAACACAAGAAGGAATGGCTTTCATGAACGGCGAAGTCGAAGAAGGAAATACT
ATGGTTGGCTTAACCGATAGAATTAAATTAAGACCATTAGTTGCGGCAGAAAAAGACGACTTGTT
ATTTGTATCAAGTGAAGAATCAGCAATTAGGAAAATATGCCCTGAATTGGATCGAGTATGGATG
CCAGATGCAGGAAGAATGGTTATTGCAAAATTAGAGAAAAAAATTTAATTTAAGTTAATAATTA
GGTGAGAAAAGATGATACCTTCAACAGTTCCGCCAAAATACAAAGTTTTTGTGGATCCAGAAAGA
TGCATGCTTTGTGAACGATGTACTACTGAATGTTCATGGGGCGTTTACAGAAGACAGGGAAATAA

AATTTTAACATACCCTAACAGATGTGGGGCATGTTTAAGATGTGTTTCACTGTGCCCTAGAGATG
CTATTACAGTTACATTAAATCAATCATGCGGCAGGGAACACCCTGTATGGACACCTGAAGTAAAA
CAGGATGTTGTAACACAGGCGAAATCAGGATGTATTCTATTAAGTGGTATGGGTAATGCAAAAG
AGTACCCAATATACTTCGATAAAATTGTTCTTGATGCATGTCAGGTAACGAACCCTTCAATAGAT
CCATTAAGAGAACCAATGGAACTCAGGACCTATGTTGGTAAAAAGCCAGAAAAATTAGAATTTG
ACTATGTTGAAGAAGAAATCGACGGTAAAAAGGTCAAAAAAGCAAAATTAAAAACAAAAATTGC
TCCAAATCTTAAGTTAGATACTCCAATAATGATCGGACACATGTCTTACGGTGCACTTTCGTTAA
ATTCCCACAAAGCAATGGCAAAAGCTGTAAAAGAATGTGGAACGTTCATGGGAACCGGTGAGGGT
GGACTTCACAGGGATTTGTACGGATATTCCGATAATGTAATTACCCAGGTTGCAAGCGGTAGATT
TGGTGTAAATAGCGAATATTTAAACAAAGGAGCTGCAATTGAGATTAAAATCGGTCAGGGTGCA
AAACCTGGAATCGGAGGACACTTACCTGGAGAAAAAGTGTCAGCAGAAGTTTCAATGACAAGAA
TGATTCCACAAGGGTCAGATGCAATTTCACCAGCACCTCACCACGATATTTACTCAATTGAAGAT
CTTGCACAGCTTATCAGAAGTTTGAAAGAAGCTACAAGATGGAAAATGCCAGTATTTGTTAAAAT
TTCGGCAGTACACAATGTATCAGCAATTGCAAACGGTATTGCAACATCAGATGCAGATGCAGTAG
TTATTGATGGATTTAAGGGTGGAACTGGCGCTGCACCTAAAGTATTCAGGGATAATGTTGGTATT
CCAATCGAAGTTGCAATTGCAGCAGTAGATGATAGATTAAGAGAGCAAGGAAACAGGCATAAAA
TAAGCATCATTGCAAGTGGAGGTATTAGAAACTCCGCAGATGTATTCAAATCAATTGCACTTGGT
GCTGATGCAGTATACATTGGAACTGCCGCAATGGTTGCAATGGGCTGTACCGTATGTGGAAGATG
CTACACAGGACAGTGTGCATGGGGTATTGCAACCCAGAAACCTGAACTCGTAAAAAGATTGGAAG
TAGATGACGCTGCAAGAAGAGTAGCTAATTTAATACATGCATGGACTCATGAAATTCAGGAATTA
CTTGGAGCTGCAGGAATTAACTCCATAGAAAGTCTCAGGGGAAACAGAGACAGGTTAAGGGGCGT
AGGTCTCAGCGAAATTGAATTAAATACATTAGGAATAAAACAGGCAGGAATGTAATGGCCAGTC
TAGGTGAAAGAATGAAAGAACTTAAAATTGACGCCAAGGACATGGACTACAGGGAATTAAACGA
GAAAATCCACGAAGTTTTGGACGAAAACAAAGACTTGGAAAAATTAGTACTTGAAAATGTTTTA
GGCCAGAGATTTATTGGAAATGGAGTTTCAAGAAAAGATTTAACGATAATTGTAAACGGAGTTC
CTGGTGGGGACCTCGGAATGTTTATGAAAGGCCCTAAAGTAGTTGTAAATGGAAATGCTGACCAT
GCTCCTGGAAATACAATGGATGAAGGATTTGTCGTAATTCACGGAAGTAGTGGGGATGTAACCGG
CCACTCGATGAGAGGTGGAAAAGTTTACGTTCAAGGAAATGTTGGATACAGAAGCGGAATCCACA
TGAAAGAATACAAATACAAATTCCCTGTTTTAGTTATCGGTGGAGCTGCAAAAGACTTCCTTGGG
GAATACATGGCTGGAGGAATTATTTTAAACCTCAACATGGACCAAGAAGATGGCGAAAATATCG
AAGGAAGGATGATTGCAACAGGAATTCACGGCGGAGTAATTTACGTCCGAGGAATTGTAAAAAG
TAGTCAACTTGGAATCGCAGCAGATATTAAGGAATTTACAGAAGAAGATAGGGATAAAATAAGA
CCACATATTGAGGAATTCTGTAAGGAGTTTGGATTAAGTGATGAAATTAAGGAAAAGTTAATTA
ATTCAGACTATACAAAAATAGCTCCAATATCAAAGAGACCATTTGGAAAATTATATACTCCTGAT
TTAAGGTAAATAAGGTAAGTAAGTATGCCTTTGGATCCAACCCAAGCCTTTTTAAAAGGCTGGAT
GCCAAATATATCTCTTCATGAAGTTAAGAAGTATTCTATTGGGGATCATTGAGCGAAGCGAGATG
AGCTCTATGAAAACCACTGGGTTTTCATTTAATCCAACACCTCTCGACGAAACCAAAGGTTTTCG
TGAGTCTCTACAAAAATTCGAAGAATTTTTGTGAGTCAGCAAATCTTCGATTTGCTTCGACTTAA
CGAAAACCTTTCAGGTTTAGTTGAATAATCGTAGATTATTCAATTTCCCCGCAACTATGTTGCGG
TGGATCGTAAGTCAGCGAATCCTTTGGATTCGCTTCGACGATAAATTGAAGATTTGTCTCGATTT
TTAAAGGGGTTGGAAATAAAGGTGCTGGATTTAATTATCTTTTTTAAATGAATAGAAGGTGGAA
TTATGAAGTCATACTTGAATTTAAAGGAAGAAGTTTGGGATAAGAACATCTGTTCTGGATGCGG
AGCTTGTGTTGCAGTCTGTCCTACAGAAAATATTTATTTCAAAGAACAAAGCCCTGTAAAATTTG

TATGTGATGAGTGTGCATGTATAATTGCTCCTACCCAAAATGGAGAACACCCGATATCTGCGGAA
TTCTGTAAAGTTACCCTTTATGATGTCCCATGTGGGGCCTGTTATGATGCATGTCCAAGAACCTC
TGAAGGAAAATTGTCTCAAATTGAAAATGGGCTGGACAATATAATAAAGGCAGTAAAGGCCAAG
TCCAAGCTTAATACAAAGGGAGTCCAAAGCGGTGGAGCAGTTACTGCGATTCTGGCGAATGCATT
TGATGAAGGGATTATAGACGGAGCTCTTGTAATGATGGAGGATAAATGGACAATGAAACCTGAG
TCCTATTTGGCAACTTCAAAAGAAGATGTTTTAAAATCTGCAGGAAGTAGATACAACTGGAATGC
TCCTATATTGGAGGCTTTAAAAGAGGCTGTAATGGATAAAAAATTGAATAAGTTGGCAATTGTG
GGAACACCTTGTGTTATTAACGCAGTTTATCAAATGATGTCATCTGATAACGACCTTGTAATTCC
ATTTAGAAATGCCATAAGGTTTAAAATAAGTCTTTTCTGCTTTGAAACATTCGATTACTCTAAGA
TGACTGAAAAGCTTAAATCAATAAATGTAGATCCGTGGGAAGTACGTAAAATGGATATTGATAA
AGGTAAACTAATCGTCGAGCTCTTAGATGGGACAATCCATAGCTTCCGATTAAATGAAGTAGAAG
ATGTCGTAAGGTCTGGATGCAATGTATGTACAGATTTCACAGGTATAACTGCTGATATATCAGTT
GGAAATGTTGGATCTCCTGAAGGATATTCAACAGTGCTTATAAGAAACAAGTGGGGCAAAGGCCT
CTTTGATAGGGCAGTTATAAATGGATATTTAGAAGCTGAAGAAGGAGTTGACATAAAACCAATT
GAAAGTTTAGCTGAAAAAAAGGCTAGACGAAAAGATGGTTCTGAAATTTAATATATTTTATAAC
TTTTATTTTTGTTCAGTCATAATTGGGCCTGAACTTTCTGTTTTACATAGTCAATACCGAGTTCT
TTTATAAAATCGTAAATTCTCCCTTTTCCAAATTTTGAATACATTTCAATTAAAATATCGACAAC
TTCTAAAACTTCATCCTCTGTTTTAACATCTATTAGATGCTTAGCAGCTTTTGGAAACTTCCCGCC
TTTACCGCCTATATTTATGGATATTGCCTTATCTACCACATTTATGGCATCGCATAATTTAAGAC
AGTCTCCCTTTCCAATACAGTTTTCTTTTATAGCTTTATTTTTCCAATCTATTGCATTTATTTCAC
AAAAATTTTCACATTTTCCACATTCTATGCACTTTTCTTTGTTGATTTCTAATATATATCTGAAG
TATATCCCAATATCGCAAAATTGATGATGAGTACAGTTGTTTGGACATCCGCTAACATTTATTTT
TAATTTTTTTGGGACCCAAATACCTTCAAATTCATCATGTAGCTTTCTAGCCAGGCTTACAGAAT
CACCAATTGAGCTATAACATTCGGATCCAATACAGCATACGATTTGTCTAATCCTTGTTCCGGTG
GACCCTATTTTAATACCTTCTTTTTCAAGGAGCTCTTCAACTTTTTCAAGATTTTCATATTTTAT
TTTAAACTCTAAACCCTGCCTTGTAGTTAAATGAACCTTTCCAAAACCATATTTCTTAGCAATTT
TTCCGATTGCAATCAGCTGTTCACAACTTAGATAACCTGGCTTTAGGGATAATCTTACCATAAGT
CCGTCTTTTTTAGGAATAACTCCCATCTTATAATTGTTCCTACATGCCATTTTTTCACTTTAAAA
ATTATTGCATTTATATTCTATTACAGGACGTGTGCAAAACTCATGCACACAATAAATAGTATTAT
ATACTATCATGCTCAAGTATAATGTGTGAATAAATTAATCACAGGTGGTATTGATGTACGAATGG
AAATTGAATGATATTGTGGATAATGGGATATGTGCTAAATGTGGTACATGTACAGTAGTATGTC
CTAATGGTATATTAACTTTTGAGGATAGGCCAAAATTAACTGAAGAATGTCTAAGAAAAGGAAA
TGGGATGTGCTTTGAGGTCTGTCCAAGAGTTTCATCAGGAAAGTATCAAATAAAAATCAGAGAA
AAATTTAAAGAAGAATACTACTATGGAAAAGGAGACGTAGAAGGGCAGGACGGGGGAGTTGTAA
CAACATTCTTAAAGTATCTCTTAAAAAACAAAAAGATAGACGGTGCTATAGTAGTTGGAGATGA
ATGCTGGAAACCAGTTTCATTAATCGTTCAAAATGAAGAGGATTTAATGAACACTACAAAATCAA
AATACACTGTATCAACACTTGAAGCTTTAAAAACTGCTGGAGAAATGGGCTTAGAAAAAGTCGCA
GTTGTTGGATTACCATGTCAAATCAACGGTCTTAGAAAACTTCAGTACTTCCAGTACTTGGCAAA
GCACGATGGAGAACTAGGTAAAAACGGAAAACCAGTTAAACTTCCAAAAATAGAATACCTAATC
GGATTGTTATGTACTGAGAAGTTTGAATACGACGAATTAAAAGAAACCCTTGCAAAATACAACA
TAAACATGGACGATGTAGAGAAGTTTGACATCAAAAAAGGAAAACTACTCGTCTATGTTAATGG
AGAAGAACACAAAATACCTTTAAAAGAGATTGAACTAAGTGCAGGCTGTAAAATGTGTAGAGAC
TTTGACGCTGAAATGGCTGATGTGTCAGTCGGATGCGTTGGAAGTCCAGACGGCTATTCAACAGT

TATAATAAGAACAGAAAAAGGGGAAGAGATTAAAAACGCCATAGAGTTAAAAGAAGGCGTAAAT
TTAGAAGCTATTGAAAAATTGAGAGACTTAAAACTCAATAGATTCAAGAAAGAAGTTGAAAGAA
GAAAAGCAGAAGATGAAAAAGTATCATTCTACTGGACCGCTGACTATGGTGGAGTAGGTAAAAG
AGCAGATGGGACCTACTTTATAAGAATTAGAGCAAAACCTGCTGGATGGTATAGTATTGATGAA
GCCAGAGAAATACTGGAAATAGCTGAAAAATACGATGGTAAAATAAAAATGACAAACAGAGGTG
CATTTGAAATCCATGGTATAAGTGGATTTGATGTTGAAGCTATGGTTTTAGAGCTCATGGAAAA
AGGATTCATAACTGGTTCAGAAGGGCCACTGGTTAGGGCAACACTGGCATGTCCTGGAGAAGGAA
ACTGTGGAAGTGGATTAATAAATACCACCGAACTATGCAAAATACTTGAAGATAACTTCAAAGA
ACATCCTGCACCATATAAATTCAAAATTGCAATAAGTGGATGTCCAAATAAATGTGTTAGACCAC
AAATCCATGATATAGGTATAGCTGGAGTTAAGTTCCCAGTAGTTAATGAAGAAAACTGTAACGGC
TGTGGAAGATGTGCAGAAGTTTGTAAGATTGAGGCAATAGATATTAGGGGAGAAACATCATATA
CAAACTACAATGTATGTATTGGATGTGGAAAATGTATAAAGGCATGTCCAAATGAAGGTAGGGA
TGTTAAAGAAGAAGGATTTATGGTGTATGTTGGAGGTAAGACCGGAAGGGAGGTAATAGAAGGA
GTAAGCATGAAATTAATGAGTGTTGAAGAGATACTCAATTTAATAGATAAGGTATTGATAGTAT
ACCACAAATACGCTAAAAAACCACAAAGAGAAAGATTGGCCGCAGTTATGGCAAGGATTGGAAA
AGGAAAGTTCTTAGAAGAAGTTAAAGAACTTATGGAACAAAATTAAATATTTATTCATATTTTT
TAATTTAAACTACTGTTTTTTTAACCATTCTTTTACACCCATATACCAATCGTCCAAAAGTTTTT
CCATGATATCCTTAACTTTTTCAAATTCTACAGGTCGGTATATGTATTTATATCCAAAAGTCTCT
GTTACTTTTTCTCTCTCTACCAATCCACAGTTTATTAAGCTTCTTAAACCTTTCTGCACCGTTGTT
CTGTCTCGTCCAATTTTTTTGGCTATTTCATTTACTGGAGCTTCCTCAAGATTTAGGAGCTCAAA
GTATATTCTAATCTCTATTTCTTGCAGGCCCAAAATACATCTCATTAAATCCTCTATTGTGAATT
TCTGCATTCTAGCTACTATTAAGTTATTCATTAAATCCCTCCAATATTTTACTAAAATATTATTT
TATAGGGTGGATAAAATTACAAAAAACTGTTTCATTCTATTGGAAACCTTAAAAGTGCTCCCACG
CCTCCCAACGCCTTTAACTGCTTTCCGGCATCATGTTCGGTAGATATAATTACAACTTTTCCGCCT
ATATTTTCAACTGAGTTCATTAGGTCTTCAACAGCCCTTCTATTTTTTCTTATAAACTCGTCTGT
AACTAAAAGTGTATCAATTGCAGAATAGTTCATTGCATTTTTTACACTTTCAATTCCATACGCTG
CAAGACCTTTTTTAGATATTTCTTCGAGTAATTTTTCCACTACTTGCGTTTCTTTGCTGATTCTTG
CTTCGCCGTAAATTTTATCAATAATTCCTGATTTTAAAATTTCAGAAAGCCCTGATTTTCCAGTA
TGGTTTGCAGATTCTACAACTACATTTGGAGCTAAACTTTTATATTTTTCAGAAATGTAATTTTG
AATATTGTTTCTTCCAAATCCGGGGCCTGCAAAGAGTATTTTTCCTTCAAATTCCGAAATCGAAT
TGATAATATCCGAATAATAGCTAAATTTAGCCTGTTTATTTTGTTTGTAGTCCAATTTTTTTGAT
ACTCCCGATTTAATGCTTGCAAGTTCCTTAATTCCAAATTCCCGAACTAAAAAGATGTCAGCTTC
GCTATCATCCATAATAACGACTACTACTTTTGGCTTTTTAGAAGAGTCTTCTGCTTCTTTTAATC
TTTCAATATCCCATTTTTTCCAATTTTTTTGAACAGACACCTGCAAAAGTGGTTCAATATCAATT
GTATGGTATGATCCAATTGGAATATCGTCAGGTGCATGTATAATTTTTCCACTGACTCTAAGCCT
GTTGAAATCTTCATGAAAATTAATTTTTTCAGCTTTTATCCCTAAAAAAACTCGTCTTTTAGTCC
CTCTGTCGGCACGTAGTTTGTCTCCTTTATCTTCCGTTCTTCGCTCGGTTACCGCGTAAATTGCAT
TGTATGGCTGTATTATATGTGACAAATGCCATAAATCATCTAAATTTCTGGAATTAATTTTATT
ATGTTTCTTTCAGGTATTTCTTGAATGATCTTCATTATATCTCTTTTTTGGGCATTTTCTCGTTT
TCTTCACTTTCTTCGTAATCGTGAACGAATATATCTCCGTTTTGAAGTATGGTTACAACAAATGG
TTCTACTACCATTCTGTTTTTGATGTACCTTTGAGCATTCTGGTTTACAAACTCCATGATTTGAA
GTGAAGAGTAGTTTACATGTATTTCTTCCTGATCGGACTTTGAAAAAATAATTTTTGGGATACTA
AATACGTCAGTTCCCTTATCTATTTTAATCTTAGAAGGTGCGATTATATCGTTATAGATACTCAA

TGTATTCCGCGCTTTTTCAATGTTTTCGCGAGCTCTTTTCTCGATCATGCTGATGTTTGCCCTACT
TGTCCCCATAATATTGGCAATTTCATCTTGAGTGTGGCCTCTTTTCCTGAGGTTTAGCACATTTA
TCTGAATTTCTGTCAAAAACGATTCCATAGTTCCACTTTTCATAAGTAATTATCATATATTATCT
TAAAATTGGTATATAAAATAAATGATTTAAATGTTTTTTTAGTTTGTGAAATGATTTATTCTTT
AAAAATTCAAAAAAAGTTTTTAGAAATGAAAAATTATCTTCCAAGTTCAGAATGCGCTTTTGCA
AGATGTCCTGCAGCAAGGGCACCAATAAGTGATAATTCACCGGCTAAAACAGCAGCACCTGCAAT
TTCTGCAAATTTAACTGCTTTTTCAGCACCTGCACATCCAAGTATTTCGAGACATTCCCTCTGAGT
TTCAACTCTAGTTCCGCCACCGATTGTTGCAACAGGTAAATCTGGAAGCGTAACTGAGAAATAAA
GGCCATCTCCAGTACATTCTGCAGTCGTAATTCCCATACTTCCTTCAACAATATGCGCTTCGTCCT
GCCCTGTTGCTAAGAATAATGCACCAACAATATTTGCGTACTGTGCGTTAAATCCTAATGATGAA
CTTATTGCTGAACCAATCAAATTTTTGTAAGTGTTTACCTGTTCAATTGCTTTTGAAGTGGTTTT
TAGATATTTTTCAACGTATTCTTCTTTTAAAAATACTTCTGCAACAACACTTTTTCCCCTTCCTTC
AATAAGGTTTATTGCAGCAGGTTTTTTATCAACGCACACATTACCGCTTAATGCAACAGTATCTA
TAATTATTCCTTCTTTTTTAAGTTCTGATTCGATAAATGAACAGGCTTTTTCGGTTGCAATTGTT
ACCATGTTCATTCCCATTGCATCCCCGGTTTTAAATGTAAACCTTGGGTACACGTTTCTTCCAACT
ATCAAAATCGGACTTATCTGAATTAGTTTACCATGCCTTGTTGTAGATTCTGCAATTTCTTTTAT
CTTTGCGAAGTTGTCTAATATCCACTCTTTTAATTTTAAAGCATCAACTACAGAGTTCGTTTTAA
TTATTGGCGCACGGGTCATCTTGTCGTCGATCACGCGTACTGTTGCACCACCACATTTTGTGATA
ACACTGCACCCTCGATTAATTGATGCAACGAGAGCACCCTCCGTAGTTGCGAGAGGCACGTTAAA
CTCACCTTGTGCATATTTTCCATTGATTGAAATTGGGCCTGCAAACCCGAGCGGAATCTGGACTG
CACCAATCATATTTTCAATGTTTTTCTGCATTGCAAGTTTTTCATCAAGAGTGTATTCCTGAATA
TGTCTTGTTTCAACGTTTGATAGTTTTTCAACGTATTTTCGCCTTGCAATAACTGCATTTCTTTCT
CCAAATTTTGAATCAAGCTGGTATACTTTTATTTCCTTTTTAAGAAGTTTTTCAACGATTTCATC
GATATTAATGTTATTTTCCATTTTTCCACCATGCCGAGTTCATAAGGTCTTCTTTTGAAGTATTA
AGTGAGTAGGACACAAAATCATGGGTTATTCTTTTTGCAAACGCTTTAGAGAAGTTTTCCATAAT
TTCTTCAGGATTTTTGGTTTTAGAAAGTTTAATTGCTTTTTCAAGCTCTTTTACCCGAATTTCTT
CAATATAATTATTAAAATCTTTTAAAACTTCTTCAACATTGATTTTTTCAATCTGTTTCATAAGA
ACGTCATATTCTTCATCAATAATTTTTTCAACGGCAGGAACTTCCTCAATTCGTTTTTGAAGATT
TTTATCAGATATATTTCTTAAATCGTCAATGGTATATAGTTTTATATTTTCAAATTCCCTTACCG
CATCGTCAACGTCTCTAGGGTTTGCTATATCAATTATAATCTTGTTTCCAACGTCAATTAAATCT
TCTTTTTTTAAGATGTAGTGGGGTGAAGATGTAGCACAGATTATAACATCAATAAAGTTAATTGC
TTCTTTTAATTTGTCAAAATGAACTGCCATTCCTTTTAATTCTTTTGCAAGGGTTTCAGCACGTT
CGTAAGTTCTATTTGCAACAATAACGGCTTTTATATGTTTTTCCATAAGGGCTTTTGCAACCAAA
GTTCCAATTTCTCCCGCACCGATTAGTAATACATTTTTGTTTGCAAGTCCGAAGTTTTTTTCCGCA
AGTTCAACTGCCGCACTTCCAATTGAAACACTGCCTTCGTTAATTTTGGTTTCAGTTCTTGCTCTC
TGTCCAACATGTATTGCTTTTAAAAAAACGCTGTCTAAATAACGGCTTGTTTTTTTAAGTTCTCG
AGCTTTTTGAAAACTTGTTTTAATCTGGCCCAAAATCTGGTTTTCTCCAAGTATCATCGAGTCCA
TTCCACAAGACACTTTTAAAAAATGAAACGTTGCATTTTTTCCTTTTAAAATGTCAAATCCTGAA
AAATCGCTCAAAATACAATCTACATCAGAATTTACCTCAGTATCAAAGTAAGCTTCGATCCTGTT
GCAGGTTTGGACTACAACACACGCGTCATATTTTTTATAAAATTCATCCTCGTCAATTCTCATCT
TTTCAAGAACTGGTATCGGGTATTTTTTGTAATCTGCTCTAACAACCAACATAGATTCACCTATG
TTTTTCAATAAGTTTTAAAAGTTCTTTTTTAAATTTTTCGTCACTAAATACCGTTTCAAGAACTT
TTTTTCTTTCGTTCTGATCTTCAATCTCACTTTTTAAAAAATCACGAAGTTTATTTTGGAAATCA

AGTTCAAATTCAGAATTTTTTAAATACTCTTCAACATTCTTTCGAATTTCTCTTGCAATAATCGG
ACTTTTTCCGCCAGTATATACTGCAAATGAGACACCATCTACCTCACAGCATGCAGGAACTATCA
AATTGGATTCTTTTTCAAAAGTAGATGAGTTTATAAATTTTTTTAGGCTTTTTGCAACCGAAACA
ATCCGTTTATTGTTTTTTTCGTCAATTGCAGTTATTATAATATCGTGATTTTTTATTATTGAATA
TAGTTCTTCATCACTTAAATTAGTTACATCGGATTCAAAAAATATCGCATTTTTGTATTTTGAAA
TATCATTTTTATCAATACTTTTGGAATAAACAGTAAGTTTTGCACGACTCTCTAAAAATTTTTCA
AATCTTCTTTTTCCAACGCTTCCAAATCCAAAAATCAAAACATTAAATCCACCCAAGTCTAAAAA
AACAGGAATCATATTTTCACAAGCAATTTATCGATTCAACAATGAATTATATATCTCATAAGTTT
TTTTTGAAGATAATCCCCAAGAAAAGTTTTTTGAAGTATCCAAACTTTTTTCTCCAAGGGACTTT
CTTAACATTTTATCATTAACAAGCATATTTATTTTATCTTCAAGAGCTTTTGTATTTTTTGGAGG
GACGATGTATCCATTTATTCCATCTTTGATCAATTCAGGAATTCCGCCAACACTAGTTCCAATAA
CTGCTTTTTTACATGCAAACGCTTCTATAATCGTCATTCCAAGCCCTTCTGAAATTGAAGGAAGC
ACCAAAAAACTGCAATTTTTTAAATATTCTGAAACTTCGCTTTGCGTTTTTGGACCGAGCAATTC
AACGTTTTTGATATCGTTTACTTTTATAAAATCCAGTATTTTATTATAAAGTGGGCCACCACCAA
TGATTTTAAAATTAAAATCGATATTTTTGATACTTTCTAAAAAAATAAAAGATTCCTTTTTGTTCA
ACTAATGATCCAACAAATAATCCGTAATTTTTGTCGCTATCAGATGGATTAAATAATTCTTCATC
AAATCCGTTGTATATAACGTTTGAATCAATATTAAAATTTGATTTTAAATTATTTTTTAAAAAT
TCACTAACACAGATTATTTTTTCGGAATTATAAACTGCATAGTTAAAGAAGTATTTTCCAAGACT
GCTTTTTGATAAATTCAATACATCACTTCCATGAAGTGTCAAAACTGTTGGAATACTGCATTTTT
TTCCCAAAATTGCCCCTAAAAACCCCTGAGGTGCTGCATAGTGGCTGTGAATTAAATCAATTTTT
TCGTCTTTAATTATTTTTTTTCCGAGTTTATAACCGTTTAAAATGTAGCTCGGGCCCCTTAATTT
TGAAAAATAAGGAACTTTGTGGATTGTAACGTTTTCAAAAGAATTTTTAACGGTTTCATCAAAA
TTGTAGTTTAGAATATGAAATTCAAAATCGTCTAGTTTGTTTAAATTTTTTATTAAATTTTCAAC
ATGTATCGAAACTCCTCCAATAAATGGATAATATATGGAGGGAAGTAATATTTTCAAAATAACA
CCAAAAAAAGAGATTAGTGGGTAGTTTTCCACTTTACAAGGACATAGTGTTTTTGAGGCTCTTTT
AATTCCTCTTCTATTCTTTTTGCTAACGGATCAACTATATTTTTTCTAACCCTGTCTTCAAAATCC
TTAAAGCTTTCAAATTTCTTTATTTCCCGTTCTTCAATTACTTTCCACATTGAAGTTTTACCAAT
TCCCGGAAGCAGTTGTAATGTATGCATCCTGTTTGTAATTGGCCCGCAGTTGTTTAAAAATTCGA
CAAACCTTTCTTCCTGAGCTTTAACTGCTTCTTTTATAACGTGAAGAAGTTCCGTTTTTGCAGTA
GGAGTTAGTTGATCGTATTTTAACATTTTTGATACAAACTCTACTTTTTCCCGTTTACCTTTCCC
GATATAGAGTTTTTCTGCAAGATCTGCAACCTGATTATCCTTAAAAGTTAATTCCATCATTACAA
ATTGTTTTTCACCAAAAGCTTGTGCAATTGGTTTTTTTTGATGAAGTGGCCTAGTATCTTCAGGG
TAACCATATTCTAAGAAGTCTAAAACGTATGCGTAATCTTCAAACTCTCGTTTATTTTGTTCATG
CCCCTCTCTCATACTCACACCTATCAATGTTGAAAATTAAAATAACATTACTTAATATCGAGTTA
ATTCAATAAAAAACTAATTGGTTTAATAATAAAATGGTAGAAAAGCGTTATTTGAATTTACTAA
CTACTTCTAAAATTTCGTCCTTGTTTTCAGGGACGTCAACTCTGTAGAATATGATTTTTAAGTCT
TCTTCTGTTTCAGGAAGTAAGTCAATAATTTTAACTGCCATTTTTTCAGTTAAACCTAAATTTAC
GAGCTGTTCAAACATAGCGTCAGCATCGTCTTTACTTATTGTTGAAAATTTTCTTAAATAGTCGA
GTGAACAACCGTGTTCATAAGATAATTCGTCAAAATCTGCTCTTTCATCCATAATTTCGGTTGCA
TGAGCAATTGTTGTGTATTTTTCGGAAATAATTTCTTTTCCAATCATAATCTCCCTCAAAAACAG
ATTAGTCTAAAAAAAAGATATCTAAAATAATTATAATTTAGATTCTCTTAAGTGTTGCGGTCTA
GCAATAATATCTTTGTATTTTCCGCCATCTTTAACTCTAACTAAGAATGAACTTCCTCTTTGAGC
AACAACAACTCCAGTTTTACCGTGGAATTTTGGGTGAGGCATTCCTTTCTGTACTGAAGGGTCGA

GTACTACGTGAACAAGGTCTCCTTCCGTGTAGCATTTTAAAGCTCTTGTTAAAGGAGCCATTCCT
TTCTGTCTTGGGTGTTTCTGAAGTTTATATCTTGTTTTGCTTCTAAATCCTTCACTTTTTTGCAT
AGTTATTCCTCCTAATGTGTTTCATGAACATAATTGGCATAGTGTCTATCCTTTTTTTCACTACC
AATATATAACCATTTCTATTTTAGATATTTTCTGCATCTTCATAGTCGTGAACATCCAGAACATC
TAAAAATTTACAGATACATTTTTTATTTAACAATTCCGAAACGGAAGGGGTTGTTCTTCCTTCAT
CACTGTTAATAAGCTCTTTGATGTATAATCCGCCATCGCAGAATATTTTAAGTTCAAAGTTATAA
TCATCAATCATATGCGGCCAAGCTTTATATACTTTCCGTACACGCACGAGGTCTGCCCTTCGATGC
GAGACTCTATCGGGCGTTCTTTGATCAATCGTCAAATCTTCGAGTTTTTTTACTAATTCGGCTAC
TTCTTCATTAGATACTTTTTCTTCGCACTCAACGTGGGCAAGGTATGTTTTTTTATGCGGTTCGT
TTTTGAAGAAATGAACGTCCTTTTTAACCCCGTATTCAACATTTATTATTTCAACGTTTCCAGAT
TCATTAACTTTTGAAGCAAGCTCTTCTAAATTAACTTTCTAATCTTTGGCTCTTTTATTTCGAT
TACAAAAGGTCTTCCATTTCCAAGCATTCTAACGTCGATGTCTTCTCTTCCCGCACCGTGAAGTGC
TTCTGAACTTCCTTTCATAACATCCATGAATGGTTCTGCAATAATTTCTTCAACTGAGGTCATGT
ACTGTTTTCCGGTAAAGTTACATTTTTCACAGCCTTTTCCTTTACAAGATCTGCAGTGCCAGTGG
CTTTGTGGAATTCCTCTAACGAGCTTTTTATACCGCCCTTTGATAAATACTGGATTTACCTGAAG
TGTAACTTTCTGGTTGTATGGGTTTACCATTACAACAATGTCTGGATTTTCCTTGTCAACTCGCC
TTTTTACAAGAGGTACTACAACTTTTCCAAGTTCTCTTCCAAATTCCTGTCGTATGCTTTCTATA
AATGGGGTTTCAATCTCTTTTTCAAGTTCTTTAAACCTTTTTGGAAGTTTTGTACCGATTAAAAA
ACTGTCAAACTCGTATTCAGAAAGTGCGTTAACTACATTTTCAGCAACTTTTTCAATATTTTCGG
GTTCAAAAATTCCTTTACACCAGGGGCAGAGTTCGTCACACTCTTCGTTTTTTTCGTATTCAGCTT
CATTTTTGAATTTTGTTTCGTATTTTTCAATATCGTCAATTAAATCAAGTTTTATTCCTGAAATC
CCTGTTTTATAAAGACAATACATCAATTCTTTAATTTCTTCTAATTTTTCAGGGTCTACTGGTTT
TAATTCTGGATTTTCAGAAGCTTCTTTCATCTCTTCAAGGGCAAGTCTTAAGTTTTCTTCGAGTT
CGATTGCTTTTGCAAGTTTTAAAGCATGCCCCCTCTCAAAATTACTCGACCTAATAAGTTTTCCG
TAAAGTCTTCCAAAACATCTGTCGCACAATGGGTATTTTTTTAAAACAGAATAATCTATATTCAT
ATAATCTCACCAGGTGATTTCATGATAATAAATGCGGATCTACATATTCACTCAAAATATTCTAT
GGGAACTTCTAAGTATATGGATATCGAGCATATATTAAAATATGGTCCAGTAAAAGGTCTCGATT
TAATTGCGACAGGAGACTGTTTACACTCAAAATGGCTCGAAGAAATCGAAAAAAATGTCAATAA
ACCGCTTCTTCTTTCAACAGAGGTCGAGGATAGAAATCGGGTTCATCATTTGATATATTTACCAC
ATACCTCTCAAGCGCATGATTTAAGAGATAAATTTTCAAAATATTCAAAAAATATCGATGCTGAT
GGAAGACCAAGAGTTTCATTAAATGGAATGGAAATTATCGATGCAGTAAGGGACATTGGCGGAC
TTATCGGACCAGCTCATGCATTTACCCCCTGGACAAGTATTTACAAATCATTTACTTCAATTTAT
AATTGTTATGCGAGAAAACCAGATTTTATCGAGTTAGGACTCTCGGCCGATACAGACATGGCAGA
CATGGTTGAAGAACTTCGAGATCTGCCTTTTTTAAGTAATTCTGATGCCCACTCATTTTATTCTC
ATCGGCTTGGACGAGAATTTAATCAGATGGATGTAGATGAACTTGGTGGACTTGAAACAAACTTC
GAAGAAGTAAAAAAAGTTTTAAAACACAATAAAATAACTGCAAACTATGGACTCGATCCAAATC
TTGGAAAATACCATTTAACTGCATGTACAAGATGCTACACCCGGTTTACGTTAGAGGATGCCACA
GAAAACAACTTTAAATGTCCTGAATGTAAAGGAACGATAAAAAGAGGGGTTCATGATAAAACTC
TGGAACTTTCAAAGGATAAAAAAGTAATTCACCCAGATTTCAGGCCAAAATATCACAAAATAATT
CCGCTTGCAGAAATAATCTCCCTTGCAATTGGAAAAAATATTGGAACAAAGTCGGTAGATACGAT
TTGGGAGCAGTACATTGAAAAGTACGGAAATGAAATCGATGTTTTGATAAATGAAGATATATCT
GAACTCAAAAAAATAAATGAAAAAGTTGGGCATATAATAGAACTTTTCAGAACCGGTAAAATAT
ACATTTACCCCGGAGGAGGCGGAGAATACGGCCATATTTCAAAGGTTCCAGTAAGTGTTAAGTGG

TTCAAACCAAAATTAACCCTCGATTCATGGGTAAAATAATAAAAAAGTAGAAATTAAAATTTAA
TTTAAATATTCTTTTAAAACTTTTGAAAGCCTTTTAATTCCTTCAACTATTTTTTCGTCAGGCAT
ATTTGAGTAATTTAACCTGAAAGAATTTTCATGACCTCCGTTTGGGAAGAAAGATCCACCTGGAA
CTATCGCAACGTTATTTTTAATGCATTCATCTAAAAATTCACGCGCATTATACTTTTCAGGAAGT
TCAACCCATGTAAAAAGCCCGCCTTCTGGATTTGTATATTTAAGCCCCTCTGGGAATTCTTCTGCA
ATTGTTTTAATCATTAAATCCCGTCTGCCTTTGTATAATTTTTTTAATCTTGATACTCTTTCATC
AAAATCGTAATTTTCAAGCATTTTTGAAATAATTCTCTGAGAAAATGACGAGGTGTGCAAATCTG
CGCTCTGTTTTATAAAGATGTATTTTTCAAGGATATCTTCAGATGCAGCAATCCATCCAACCCTA
AGCCCTGGGCAGAATGTTTTTGAAAATGTTCCTAAATGAATTATGAGGCCCTTTTTATCCATTGA
TTTTAATGAAGGCAAAATTTCTCCATCAAATCTTAATTCCCCGTAAGGATTATCTTCGACTACCG
GAATTTCATATTTTTCAATTATTTTCATGAATTTTTCCCTTCTTTCAAGAGTCCACGTTTTTCCA
GTTGGATTTTGAAAATCAGAAATTGCATAAATGAGTTTTACATTTTTGGTAGTTTTTAAAATTT
TTTCAAGTTCTTCTGGAATCATTCCAAAGTCGTCAGTTGGAACTTCTTTAAACTGCGGTTCGTAT
GATTTAAATGCATTAATTGCTCCAAGGTAGGTTGGACTTTCACATAAAACCACATCATTTTCATC
TAAGAATACTTTTCCAGAGAAATCTAATCCTTGCTGGGATCCACAGACGATTAAGATATTATTTT
CTGTAAGCTCTGTTTTTAGTACTTTATTCATCCGTTCTGAAATCTGCTGCCTGAGTGGTGGGTAT
CCTTCCGTTATATCGTACTGGAGAGATTCAGGACCCATTTCATCGAGAACAATTCCGGAAATTTC
TTTTATTTCTTTTACGGGAAAGAGTTCTGGTGCAGGAAGTCCGCCTGCAAAAGATATTATTTCAG
GTTTTTGAGTAACTTTTAATATCTCCCTTATTTCAGAAGATTTAATGTTTTCCATCCGTTTTGAG
TAGCTTAATGCCATACGCATCCCTTAAATTTTCGATAAAGATATATTTTGGTAGTTCATAACATA
CAAATATACAGTATATTGGGTATATTGTAGTATATCTAATCTATATATACATTATAGTGCACGAG
GAAAAGTCATGAAGGATCTTAATAAAGCAATTTCTAAAAATCTGAAAAAAATCCGTAAAAGAAA
AGACCTAAGTTTAGATGCGTTATCCAATCTAACTGGGGTAAGTAAGAGCATGCTCGGTCAAATCG
AGCGAGGAGAAGTAAACCCTACAATTTCAACAATTTTAAAGATTTCAAATGGCTTAAAAGTTTCA
TTCACCTCACTTTTAAAGAACGAAAAACCCGAGATTGATCAGATACCATTTGAAGAGTTAAATCC
TGTTTTCGAAGGTGACGGCCACAAACTCTATCCTGTAATTCCTTTTGAGGATGATAAACGATTTG
AAACATACATTGTCGAGATAAAGCCTGGACACAAGTATTATTCTGAAGGCCACGTGAAAGGAACC
GAAGAAATCATAACAGTCTTTGAAGGTGAACTGGCACTTTCAATAGGGGAAATTGATTATTTCCT
TAAAAAAAATGATACAATCAGGTTTAATGCCAATAAATCGCATGTTTATGAAAATAGGGGTTTA
GAACTTACAAAATTAAATCTTATCCTTCATTACGAGTAATTTAAAAATAGATTAAAAGGTTAAA
ATAAATTATTGGCAGCATCCACAGGTTTTTTCCTGTTTAATGCATTCTACAGGGCATACGTCTGA
ACAGTCGCCACAGTCGTTACATTTAGCACAGTCAATAACTGCTTTACCATCAGCGTTTTCTGAGA
TAGCTTGTACAGGGCATACTGGAACACATGCTCCACATGCGATACATTCGTCTTCGTTTACTTTA
TATACCATAATTACACCTCAATTAGTAATTTTTTCAGGTTTATTCTCATTAAGCATTACATATTT
TTATAGGTTTGTAAATGTTTCTAATTGCCCCATTGTATTTATACCTCAAGGGAGATTTTTGTTAT
AAACCATGAAGTGAAAAGTCATGAAAGAAGAAATGAAAGGAATTTACGTTCTATGGATCACAAT
TTTTGTGACAATGCTCGGTATTGGGCTAATTTCGCCACTTCTCGCGATATTTGCAAAATCATACG
GGTTATCAAATTTCCAGATAGGACTAATTTTTGGATCTTTTGCACTAGTTAGGACGATATTTCAG
CTTCCTGCGGGAACCCTATCAGATTCTTACGGAAAAAAAGTATTTTTGGTAGGGGGAACTCTTCT
TTATGGTGTAACAACTTTATTTTATGGTTTTGCATCTGGATTTGCAAGTATATTGATAGTTCGGA
CATTCACAGGAGTTTTTTCTGCATTTGTAAATCCTGTCGCAGGTTCATATATCGCACAAACCTCT
CCAAAATCAAAACTTGGAGAATACATGGGGTTATTTAATTCTGCGATATCTTTAGGATTTGGGGT
TGGACCGCTACTTGGAGGATTACTTGCAGATATGTATGGATTTATGGTTCCTTTTTATGTAGGCG

GGCTTTTAGGTATAATTGCATCAATAATAGCATATTTTAAACTTGAAGATACTCCAAAAACCCAA
GAAAAACAAAAATTATCTTGGAAAAATTTATTTTCACTGGAATTTTTAAAAATGAGGAATTTTA
GCGCGGCATTTATAATAAATATTGCACTAACAATTTCTAGAGGCGCAATTTTAGCGTATCTTGCA
ATTTACGCTTATGATTATGGAGTTTCTGCCTTTAAAATTGGAGTAATGCTTGCAGCAATGAATCT
GGTTCTTGCAGTAACTCAGAAAATGTTTGGAAGAATTTTTGATGAAAAAGGAAACAGAATAATT
TTACACGGAATAATTATTGGAAATTTGGGAATGTTTATCCTTGCAATGTCCACGTCATTTACTAC
AATGTTTATTTCGTTAATTATCTCATCAATAGGGGCTTCAATGAGTACCCCGGGCATTAATTCGA
TCGCAATACGAGATATTCCTGATAAAAGACAGGGCGAAGCAATGGGCCTTTACACTGCAAGTATA
AATGTTGGAATATTTTTTGGAGCAGTTTTACTCGGCTACATTTCCGATATCGTTGGAATTTCTAA
CATGTATAAAATAGGGGCAGTATTTTCATTTTTAATAAGTTACATTGCATATATCGCGATAAAAA
AATAAAAAAAGAATAAATAATGATTTAATTTTCTACTTCTTCTGTAAATGCCCATTTCAATAGTG
GCGGTGTTGAAATAATCGATATTGTAATGAATATCATTGTTGCAGCAATGAATTTTCCGACATTG
GATTCAGAAATTACTCCTGCATGTATTGCAACCATAAGGTTGATCAATGCAATTTCTGCTCGAGG
AATCGATCCAATCCCCATTTGAAGCGATTTTTTCATATTCCAACCCATGATTCTCGCACCAAATCC
TCGTCCAACGACTTTACTTACAATGGCAATTGCAGTCAGTACCAAAGCAAGGTAGAGTGCATCAA
AATCTCCAAAAATTGTTAAATTTAATAATGCCCCGGTATATACGAAGAATAATGGGATTAATAT
ACTATAACCTATTGTTTTAACGTCTTCAATTAAGTTTTTACCTTCGGCAGTTCTTGAAAGTACTA
AACCTGCCATAAATGAGCCTTCAATAGCGGCCTCAAACCAGTTTTCTGCTAAAAACGAGAATAAA
AACATGACTGCTATTGCAAGAGACAATATTCCTTTTTCTACATGGAGTTTTTCTGAAAACGAAAT
AAACTTATTTACAAGTTTCATGGCAAGGAATCCTGTAATTATAAAGAAAATTGCGAATTTTCCTA
AAAGTCCAAGGATGCTTCCAGTACCAACTGCAAATATTATCAGCATTATGCCTAAAAAATCGTCC
ATAATGCTGGCACTTAGCGATGCTGCACCAACTTCGCTTTTTAAAACACCCATGTCCATCATTACC
CTTACAGTAATTCCAATACTCGTTGCAGTTAATACCACTCCTCCTGCAAACGATTCAGCGGCACTG
TAACCCATATACTGCAGTGCAAGATAACCCATTACAAGGGGAACTAAAACACCAAAAACAGTAGA
AACTGTAGCAATTTTGCCAGTTTTCTTTAAAACCGACAGGTCAGTATCAAGTCCGCCTAAAAATA
ACAAAAATATGATACCCAAACGTGATAAAAAATCAAAAATTTCATTATTCTGAATTGTGAGATG
GTCAGGACTCACTAATCCAAAATATACTAGATTTCCAAGGATCATTCCCATAATCATTTCACCGA
GAACTCCCGGAAATCCTAATTTATTCATCAGGTGGTCACCAAGTTTACCAAAGATTATCGCAATT
CCAATGGTAAACAACACCCAGCTAATATCCATCAAAACCCCTCTAATTATTCTTGAGCCAAACAA
AGACTTAAAAATTTGTCAATTAATATTTTTAAACTTATTTCTCCAATTAACCTTTCTTCGTCATT
TAAAACTGCCAATATCTGCACATTGTACCTTTTCATCTTCAACAGTGCATCGATGACTGTAGAAT
TATGATTGATCGTGAGCGCATTTTTTTCCATGACTTCTTTTGCAGTTTTCGCACCGCCAATAACA
GATTTCAATGCAGAACGTGTACTGCCTATGTACAGGTTATGCTTGTCTGGAGGAAGTAAAAAGTC
AATTACACTCAAATATTTGATAACTCCAAGTAAACTGCCTTCTTCTTTACAATTTATAACCCAAA
CGTGGTGTCGTGATCTCAAAAGTTCCATAACCTTAATTATTGGGTCATTATAGCAAACAACAGGC
ATCATGTCTACAGGGGGTATTACATGTTTTAATTTAATGTGGTGATACTTTCCAAGTGCATTTTC
AAGATTTTCCATATGTTCACCAAAGAGTTTAGAGCCGAATTATATATCTTTTTGAACATATAAAT
ATTAAACAATTTTTTTAACAGGGGGTGGTGGGTACATGCCTTTAAATGAAATTATTCCTGTTATT
GAAAACCTGATTTCAAGAGGTTATATTAAAAAACAAAGTGTAATCGACGCACTTTTAAGTGTTCC
AAGGCACAAATTTATTTCAAAGAGCATGGAACGTTATGCGTATGTTGATGGCCCTTTAGAGATAG
GATACGGGCAGACTATTTCTGCGATACACATGGTTGGAATAATGTGCGAAGAACTTGATTTAGAT
GAAGGCCAGAATGTACTTGAGGTTGGAACTGGTTCAGGCTATCATGCAGCAGTAGTTTCAGAAAT
CGTTGGCGAATCTGGAAAAGTTACAACTATCGAGAGAATACCGGAACTTTTTGAAAATTCAAAGA

AAACACTCTCAGAACTTGGATACAATAACGTTGAAGTGGTTTTGGGAGATGGTACAAAAGGATA
TTTAGAAAATGCTCCATATGATAGGATATATGTTACTGCATCGGGACCAGAAGTTCCAAAAGCTC
TATTTGAACAGTTAAATGATGGCGGAATTTTACTTGCACCTGTTGGGGCCCATTTTCAAACGTTA
ATGAGATACACGAAAATAAACGGTAGTATATCTGAAGAAAAACTTTTAGAGGTTGCATTCGTTCC
ATTAATTGGTGAGAATGGCTTTTAATATTAATTATCTTTTTTAAATTAAAATATTAAAAAATCA
ACATACTTTTTTATATTTGAAAACAAAATTAATTTTGAAACGCGTAGTATATGGAACGTATCTAA
AATGGAGAGATAACATGAAAAAATTAGGAACAGACCTTTTAAAAAGAGGATTTGCGAAAATGGT
AAAACACGGGGTTGTAATGGATGTTACAAACGTGGAACAGGCATTGATTGCAGAAGAAGCAGGT
GCTACTGCTGTAATGGCTTTAGAAAGAGTTCCTGCTGACATTAGGGTACAGGGCGGCGTTGCAAG
AATGTCTGACCCAGAAATGATTTTAGAGATTAAAGACGCAGTATCAATTCCAGTAATGGCAAAAG
CAAGAATTGGACACTATGTAGAAGCTCAAGTGTTAGAATCAATCGGTGTTGACATGGTTGACGAG
AGTGAAGTTTTAACTCCTGCTGATGAAGTAAATCACATCGACAAACGAGCATTTACAGCACCATT
TGTTTGCGGTGCAAGAAACTTGGGCGAAGCCTTGAGAAGGATCGATGAAGGAGCAGCAATGATTA
GGACCAAAGGGGAAGCTGGAACTGGAAACGTTGTTGAAGCTGTAAAACACATGAGAGCAGTTAA
CGAAGGAATTGCAAGAGTTATCGGATACAAAGAAATGGGCCTTGAAGCAGAATTAATTCAAATG
GCAAGAAATGAATTGAAAGTTCCAATGGAGCTTATTTCAGAAGTTGCAGAACTTAAAAGACTTCC
TGTTGTAAACTTTGCAGCTGGTGGAATTGCAACACCTGCAGATGCAGCTTTAATGATGCAGATGG
GATGTGACGGAGTATTTGTGGGCTCTGGAATCTTTAAATCAGGAAATCCTGCAGTAAGGGCAAAA
GCAATCGTTGAAGCAACATATAACTTCGACAAACCAGAAGTAATAGCAGAAGTAAGCAAAAATT
TAGGAGAAGCAATGGTTGGAATTAATATCGATGAAATTCCAGAAGAAATGCTTCTTGCAAAAAG
AGGAATTTAATTTTGCTTAAACTTACCTAAATTTTTATTTTAATTTGGATTTTTATAATATCTTT
TTAAAAAATAAAACACGATAATTATTTGAAAAATAATTTACCAAGAAGAACTACCGAAAGCTTT
ATATATAATGATACTATTATTGAGTATGCGAACAAATTGCCTCGGTGGCTCAGCCTGGTAGAGCG
CCTGACTTGTAATCAGGTGGTCGGGGGTTCGAATCCCCCCCGGGGCTTGAGTTACCTTTATAAAT
AGGTAAGTTCAAATAATAATTGTCAAATGGGCCTGTGGGGTAGCCTGGTCATCCTTTGGGATTTG
GGATCCTGAAACCCCAGTTCGAATCTGGGCAGGCCCACCACTTATATTCCCGCGATAGTTCAGACT
GGTAGAACGGCGGACTGTAGATCCGCATGTCGCTGGTTCAAATCCGGCTCGCGGGACTCACATTA
ATTCTGGGCCCGTAGCTTAGTCTGGTAGAGCGCCTGACTTTTAATCAGGCGGTCGAGGGTTCGAA
TCCCTTCGGGCCCGCTCAAAAATTTATCAGAGTTAATAACTCTGAAGGGTCTGACATAACGGTCA
TAGCGGAGGAGTTACATCCGATCCCATCCCGATCTCGGAAATTAAGCCCTCCTGCGATTCATTAA
GTACTGCCATCTGGTGGGAACAATGTGACGCTGTTAGTCACTTTTTATTTCACATGCTCTGGTGG
TGTAGCTCGGCCTATCATACAGGACTGTCACTCCTGTGACTCGGGTTCAAATCCCGGCCAGAGCGC
CTTATTTCTATTTTCAATACAAAACGCAATCTGGGCCCGTAGCTTAGTCTGGTAGAGCGCCTGAC
TTTTAATCAGGCGGTCGAGGGTTCGAATCCCTTCGGGCCCGCTCAAAATTTACTTTCACATGCTCT
GGTGGTGTAGCTCGGCCTATCATACAGGACTGTCACTCCTGTGACTCGGGTTCAAATCCCGGCCAG
AGCGCTTTTTTAATAATAATTATTATAATCGATATATTTGGATTTATTGTGAAAACATGTCTCAC
GAAGGCGAAGAGTTTAACAAAATTTTTTCAAAAGGAAAATTTTTAAAAAATATTTTTTTCAACC
TGTTCAGATCATACAAAAATCCTGAAATTATTGGAAAAACCACAAATCCAGAACTTGATGACTGC
ATATGTTGCGGTTTATGCGTTGATGTATGTCCAACAAATGCGATATCTGTTTTTAAATTTCGAGA
TATAATCTGTGAACACTGCGGTGCTTGTTTGAATGTATGTCCTGTTGATGCAATTGAAAAAAATC
GATTTTCAGTGGATCCTGAAAAATGTATAAAATGCGGAGTATGTTCTATATTTTGTACAATTCCG
ATTTTAAAAAATGAAATTCCTGTTTTAAACGTTCCTGAAGTTACAACGGAATGCAATAACTGTAA
ACTATGTATTCAAAAATGCCCTCAAAAAGCAATTTATTTTGAAGATGGCAAAATTTCAATAAGTT

CAAAATGTAATTTGTGTCTTAAGTGTGTTGAATTCTGTCCTCTTAATGCATTAAAATCTCCAAAA
GAGTCATTAAAGTCTAAAATTATTAAAGTAGATCTAAATTCCTGTATTTTTTGTAAAGACTGCGA
AGAAATATGTCCAGTTAAAAAATGATTACGAAATCAGACGATTTTAATCCACTATTAACATTTAT
ATATTATTTTCTCAGATATGAATAACATTCATTCGGGGGAGGAATTTTTTTGAAATACGGAATGT
TATTTTCAGGAATACTAATGTTTAGTATCGGACTAAATGTTTTTGGCACGTATCTAGAGAGCATA
TATTTATTTCCGCTTTATCTTGATAGTATCGGAACGATATTTAGTGCAATAATATTGGGCCCGCA
TATTGGGGCTCTTGTGGGGCTTATTACAAATTTGATAACAGGATGTTTACTATCTGTGGAAAACT
TTCTTTTTTCCGTAGTAAATGTTTTAATCGGGCTTACAACCGGGATAATGCTGTATAAAAATCAA
ATTAATATTAAAAACATATTCTTTGCTGCGATAGCTGTCTCGATAGTCTCTTCAATTATGGGGAA
CCTTCTTTCGATATATTTCTTTGGCGGAATTACTGAAGGAGGGGTAGGCCACATTACCCAGACTT
TAACCGCTTTTGGAGAAAGTATTCCAACAGCAGCTTTTGCAGCGGGGCTATATACTAATTTTGCA
GACAAGATCATATCATTTACTGCTGCATTTTTTATGATATATGTTTTGCAGGACAAACTTTCCAT
TTATGACTTAAAACTTGGGAATTAATCTTTTATTTTTTTATTGGGTTTATTAACTATTAATAATT
TTAATGACATTATTATATACTTTATATTTCAGAAGTACTACCATTAAATTGACGGTGGTATCATG
GATAAAAAAGTACTTTATATGACAACAATCCCTGTTGGAATCGCAATAAATGCAATAGGGGGCCA
AATTGCAACTTTTTTAAAATTGCCTATATTTTTAGATAGTATTGGTACTGTTTTAAGTGGATTTT
TGTTAGGTCCAGTTGGAGGCGCTTTGGTGGGTTTCTTTACAAACGTTCTTCTTGGTTTTATACTC
GACCCGTCATATATCCCATTTTCAATAGTAAATATTGTAATTGGGCTAGTTTCTGGATATGTTGC
AGTTAAACACGGAATAACTCTTAAAAATTCAATAATTGTTGGGTTAGTGCTTGCAATAATTGCAC
CAATGGTTGGAACCCCAATTGCAGTTTATTTATACGGAGGGCTTGTTGGTGGAGGCGTTGACCTT
TTAACAGCGGTATTTTTACATTCGGGTCAGGATATTTTTTCATCAGCGTTTTTGGCCAGAATACC
TGCAAATTTAGTGGATAAACTGCTTTCATGTATTTTGGTATACTACATCATAAAACCGTTCCCAA
AAGACATACTTTCAGAATTAGGCGTAAAAGTAAATTAGGTAAAAAATATGAAATACCTTAAAAT
TAAGATTTATCTAATTTTTACCCTTTTTTTACTTGTTTTAGTAATTTTTAATCCATTTTATGGAA
TTTTAGCGTCCATTGTGGTTGTTTTACTTACCAAAAGATTTGAAGTATTTTCAAAAAGATGGATT
TTGTTTTCCCTGTATCTGGTTGTTTTTTATTATTTTATAATGGGCCAGGATGGGCTTAACAATGC
ATACAGGCTGCTTGCATATATTTTTACAGTGCAGTGGTTTATAAATTCAGTTTCAATTGAAAAGT
TAGTTGAATTTATTTCAAGTTACAATCGTGATTTGGGAATTGGGATTTGGATGACCTTTTCAACA
CTTGAGGTTGCTAAAAAGGAGTTTGAAACTACGAAAAATGCCCAACTTTCAAGGGGGCTAAATAA
AAAAGGATTAATTAATAAATATCGGTCTTACTATGCAATAATCTCGCCATTAATTGTAAAATTAT
ACATTTCAGCAATTAACCGGGCAAGATCGCTTCTTTCGAAGTGTTACGACTGAATTATTTTTTAC
ATATTTTTATATTTTATATCTTTTTTAGATATTAGAAATTATAATTAGTATATATTTTATTTTAA
GACCTAATGATATCATTAAATAAGATAATCACTCTAACTAATGTATTTTCGGAGGTGTAATATTG
AAAAATTTAAAAGGCATACTCGCAATTTTCTGCGTTATGGCAGTACTCGTATTTGCAGGCTGCAC
TGAACAGCAAGCTTCTAATGTTTCACAAGAAGAACAGATTGTAACGGTATATGTTGCTTATGGCA
GTCTTGACTTCATTGCAGATGAATTTGAAAAAGATACAGGCATAAAAATGGAATACGTGTCACTA
TCATCGGGTGAAGCTCTTTCGAGGCTAAAAGCTGAACAGAGCAATCCTTCAGCAGACGTCTGGCT
TGGTGGAGGAATCGATGCATTTATAAGTGCACAGCAGGATGGGCTACTTGAAGCCTACAAATCCC
CAAATGCAAAAGATATCGATCCATTGTTTGTTGAAGAAGACGGCTACTGGACAGGAGTTTCTCTT
GTTACAATAGGCATCTTAGAAAACACGGATTTGTTATCTGAAAAAGGATTACCTCAACCACAAAC
GTGGGATGATTTAATAACTGATGATTACAAAGGAGAATTAATTGCATCCAACCCGACAGTTTCGG
GAACTGCATACTTTACGATATGTGGAATTCTTCAATGTATGGTGAAGATGAAGGCTGGAATTAC
CTTGATAAATTCTATCAAAACACTCCATTCTTAACAAAACGTGGAAGTGGGCCTGGAAATTTAGT

AACTGCTGGTGAATACGCTTATGGTGTAGCGCCTGACCCACACACGACATTAATTGCAGATTCAG
AATTACCTGTAAAATCTGTGTTTTTAAATAAAGTTATCTGGTGGCCATCCCCAGTTTCGATAGTA
AGCGGTGCAAAACACCCAGAAAATGCGAAAATATTTGTTGACTGGTGTCTTTCAAAAGAAGGTCA
GGAAGTACTTATGCAGGCAAGCCCTAGGGTGCCTGTAAGGGGCGACATTGAGGTTATCGAAGGCG
TTCCAAACCCTAAAGATCTTGATTTCCTCGACATGGATTTCATGTACTGGGGAGAAAACAGGGAC
AGGATTTTAGATGAATGGGAATCTAGATACAGAGAAATTTCAACTACAGAATAATTTTTTTACT
TATTTTTTTTCAGGTGCAGTAAATGAAAGAAGTATTTAATAACGAAATATTAAAAAAAATTTAT
CCAAAAATCCTTGAAAATGGTTTTTATATTCTTGCAAACGTTTTTTTATTGATATTCATAATGTA
TCCTGTATTTACAGTATTTTTACAATCATTTTACGGTTCAAACGGTTTTACGCTTGATGCTTATT
CTAAATTTATCTCAGATTCGTACTATTTTGGCATTTTAAAAAACAGCATTGTTGTAGCATGCTTT
TCAACGATAATTTCGATATTTTTGGGTTTTATTTTTTCAATTATCATTTTTAAAACCGATTTTAA
ATTTAAAAATTTTTTCAAAATTGCAGTTTTTCTACCAATAATTACTCCAGGGTTTATTTCATCGC
TTGCATATGTTTTCCTGTTTGGAAGGCACGGAGCAGTTACTTACGGCTTACTGGGGCTTGAACCA
AATATATATGGCTGGAAAAGTGTTGTAATAATGCAGTCTATCGATTACACGACAACTGCATTTTT
TATAATTTCGGCAGTACTTCTTGGAATTTCTGGAGAATTTGAGGATGCCGCAAGGAATTTAGGTT
CAAATGAATTTCAGGTTTTTAAAAAAGTAACATTTCCGTTATTAATTCCTGGAATCTTGAGTGCA
GGCCTTTTGATATTCATGCAGTCTATGGCTGATTTTGGAACGCCGATAATTGTTGGTGGAAATTT
CAACACGCTTGCAACGGCATCTTATTTTGAAATAATTGGAAGGTATAACACCCAAATGGCATCAA
CACTTAGTGTAATCCTTCTGTTCCCATCAATGGCCTTATTTTATCTTTATTCAAAATATCATAAA
GGCTACGAGCTAAAAAAATCTAAACTAACAAAATATTCATTAAGTAATATACAAAAAGTAATTC
TTGGACTTCCTGCCGTATTTTTCTCAATAATTGCATATCTTTTGTTTTTTGCAGTTTTTGTGGCTT
CTTTTACGAAAAGTTTTGGTCACAACTACGCTTTAACTTTAGATTATTTTTATGAAGCAATAAGC
GCAGGACAGCTTGCAATAAAAAAACACACTTATTTATGCCCTGTCTTCAAGTATTTTGGTGGCTTT
TTTAGGGGTTGCTTATTCTTATATTGTATATCGGGGAAAATTTTTTGGTCGAAAACTGATGGATT
TAGTGATAACACTTCCATTTGCAATACCTGGAACGTTTATGGGCTTGGGATACCTTTTAGCATTT
AATAATTATCCGTTATTGCTAACAGGAACATCTTCAATCATAATTTTAAACTGCATGGTTCGAAA
ACTCCCGTTCTCATTTAAAACTGGAAATTCAGTACTTTCCCAAGTAGAAGAGTCTGTTGAAGAAG
CATCTCTTAATCTTGGTGCAAGCCGATTAAAAACATTTTATAATGTTGTACTGCCCTTACTGAAG
CCTGCAATAATATTCAGCATGATATATACTTTTATTGCAACTATAAAGTCACTGGGTTCGATAAT
ATTTTTAATGACGGCAAATACAAAAGTACTATCAGCAATGGTCTTTGAATCAACGATAAACAGGC
AGCTTGGTGTCGGGGCATGCTACTCGATGTTCATGGTTATTTTGTCGATAATTGGGATATTGGGA
ATATTGAGATTGAAAGGGGATAAAAAATGGATTTAAGACTCGAAAACATTTCAAAAAACTTCGA
AGAACATGGGGTTTTAACAGATATTGATTTTGAATGCGGCGAAGAACTTGTGTCACTTGTTGGTT
CCAGCGGTTGTGGGAAAACGACTTGTCTCAGGATTATTGCAGGATTTGAAAAGCCTGACGCGGGA
AAAATTTTTTTAAATGGTGAAGATATAACGAACGTTCCACCAAACAAACGAAATATTGGATTAG
TATTTCAAAATTACGCATTATTTCCCCATTTGAACGTTTTTGAAAATATTGCATACGGTTTGAGG
TTAAAAAAAATTTAGTAAATTGGATATTTCTGAAAAGGTAAAAAAATTCGATAGACCTCGTCAATC
TCGGAGGGTATGAAAAGTACCCGATTAGTGAATTAAGTGGTGGAATGCAGCAGAGGGTTGCAAT
TGCAAGGGCAATAGTGATAGAACCAAAGGCAATGCTTTTGGACGAGCCTTTAAGTAATTTGGATG
CAAAATTAAGAATAAAAATGAGAAAAGAGCTTAAAGAACTGCAGAGAAATTTTGAAATTCCAAC
GATTTATGTAACGCACGACCAAGAAGAAGCATTGGCCATTTCAGACAAGGTTGCCGTAATGAATA
AGGGAAAAATAGAACAGTTTGGAGACCCTTATTCTATTTATTCTAACCCAAAAACCGAGTTCGTT
GCAAAATTTATTGGAATTATCAATGAACTCCCCTCATCAATATTAAATAAATTGGGTATTGAATC

CGGCGATAAAAAGTATTTTGTAAGGCCTGAATCAATAGAAATCGGTAAGGGCAATTTTAAGGGA
AAATTGGTGGATTATGAATTTTTAGGAAGTATTGTAAGGTATGTTATAAAATATGGGGATTCTT
TATTTACTATTCAGTCGAATAAATTGGTAGACTTTAAACTCGGGACTGAAATTTTGTTTGAATTT
GATAAAAACAGTATAATTTCAATTTAATTATTAACTTATTTTTAAAATTTTTAGGTAGCTGTCA
ATTATTTTTTGATACTTTTCATTTAACTGAAAATCAGAAACTGTGTAAAATGCGGTTTTTTTCCA
ATTTTTACAGGTATTTTCTTCTATATTTAATTTTTTTAAAAATTTACATTCTCCTTTAAAAATAT
AACTTTTAAAAGAGTCTATTTTTACATCAAATCTATTTTCCAAAAAACTGTCAAAATAATTGTAG
ATTTCGGCATTTTTTTCAATTGATATATTATTTTTAAGTCTTATTTCGTATTTCCAGATATTATC
CAAGTGTATGTGAAGTTTATAACCTTTTAAAAATGATTTTTCAAAAAAAGTTCTATTTTCAAATA
AATCTTCCGGATCTGATTCATAAAAATGAGTAATTTTTCTTTCAATACCTGAAAAATAGCCAATA
TCTGGAACACAAGCTCCAAAATAAAAATTATTCATATCAAAATTCTCATTTACGTTTAAACGTTC
TTTTTCAAGAAATATTTTTGATAAAACAAGATGGGAGTATAAATTGGGCATATTTTACCTATTTC
AAATGTTCAATGGAATTATTTTCTGGAATATAATTTAAAAGTTTATTTCCAATTCTTGTAGTGTA
AGGTCTTGTTTTGTTTGTATCGTTTAGTATTAAATTCATCAAATCTTTCGATTTAATATTTATAT
ATCCTTTTGAACACTCTTTTTCGTTAAAATATTCAACACGATCCTTTATAATGCCATTTTTAAAT
ATTGCAAGCGGAACGTCTTCTCCCGAGTGTATTAATGTTCCAACAGATGGCGTAGAATGATCGGC
AGTAACAATAATCAGTTCATCATTTAATTCATTTATTTTTTGAAGGCATTTATCTATTTTTTCGA
TAACTTCGACTTTATTAATCGGATTTTTTGTATGTCCTGCTTCATCAGGCTCTTTGGTGTGGATA
TGCACAAAATCATAATTTTTTAATTCAATTCCTTTTAAAAATGCTTTTTGAAAATCGCCTTCAGG
ATAATAATCCATTTTCAGCAATTTTGATAAACCTTTAAAAACGCTGCTTTTTGCAATAATTGCAG
CATTCATGCCCCATTTATCATGGAAAGAATCTATTTCCCTATATCTTCCAGCCCACTTTGTAAGCA
GGAAATTTGCAAGTTGGAGATTTTTTTCCTTCCTTTTAATATTTACGGGATGATTTTCAAGGATT
TCACTGCATTTTAGAAGATATTTATTTAATACTTCGGCAGTTTTCACAGCATTTTCAATTTCTAT
TCGTTCTGAAATATTTTTAAGCGGTTTTACCTTTAAAACGTGTCTTTCCCGATAAAAAGGGTCGC
TATCAGATATTTTATCCGATATTTCGTAATTTGAAGAACTTATTTCTAAAATACAGTGTGTATCA
AAAGAGTAGTGTAGATTAAATTCATAATCTCCAATTTTTTTTGGAAGGGAATTTATAAGCTGTTT
TATTTCTTCAATTGATATATTTTTAGTACGCCTATCTTTTACAAAAAAGCCATTATTTCTTTTTA
CATATCCAAAAGTGGCCCTTAGATAGATAGAATTATCTTTAAGTTTAATATCTTCGCCCAAAGCT
TCGATAACTCCTCTTCCAGGAAATTCCGATAGTTTGTAACCCCAGAGCAGAAAATGAGCAACTTC
AGTTCCAAGTGGGACTCCTTTTTTATACGGCGTCATCATTCCTGTGACGCTTTTTTTCGCAAAAGC
ATCTAAGTTTGGAGTTTTTGCATATTCAAGTGGAGTTTTATCTTTCAAAACCTCAGAAGGCCTAT
CTCCAAGTCCATCCAATAATAAAAGTAAGGTTCTCATGGTATTACATTAAAGGAACTTTTTCACG
GTTTCAATATAATTAAAGTTGTTTTTATACCCGTTTTTCAATATTTCAATGGATGACGGATATAT
AAATTCATTTAAGATTTTTACGATTTTTTCTCTTTCAAAAATTACTTTTCCGTCCAGATCCCTGT
AATTTCCACTAAAAATGCATGAATATTGGTTTTTTCCCTGTTCCCATGGTTCACCAATAATTTCT
TTTATTTCTGACCCTTTTTCAATATCTTTTACTGGAAGACAGAGTTTTACGTCAAATTCATCGCA
TATTTTGTAGTATGTTTCAAGAGTTTCCTTGAACTGGTCGATTTTAAAATCTTTGTCGTGGAGTA
TTCTCTCCCCGCTAATTATTTTTTTTCCAAGGTGTTTTGCAAGCGGAATTCTTATCATTCTTAAAT
ATGCATGGCACCCAATGCAGGGCGTGTAATATCCATATTTTTGAATTGTGTGTGCAATAAATCTC
CCATTGAGTGCATTCCAGAGTTTTGGTTCAAAAATAAAATGAAGTGGCAGTAAAAATTTATTTTC
ATGCGTTTCTTTTATTTTTTTATTTATTACATTCCAGTTTCGGTAGAAAATTTCTTTTTCACCGT
AATCTGTACCCGTAAATGTTAAAACTGGCAAAACAGCATCAATATCTTCATCTTCAAAAGCTTTT
ATAATTGCTGCAGCGCTGTCTCTTCCGGAAAACTCCCCGACGGCAATTTTTTTTAAATCAATTCCG

TTTTCTTCGATAATTTTCAATGAATCTTTAGATAAAGTTAACTCTTTAAATGATTTTTTAAACTC
GATATCCAAAATAACACCTAAATAACTTAAAGATACGTTTTATTTTATTTATGAAATTCAAGGTA
TTTATTTAACATGTTTTTAGTGCTGATGATTTCTTCAACATCCATAAGTTCGATAAGCCAAAAAT
CGCAGCCGACATCCAAAAGGCCATCTAAAACGGGTTTTAAGTTGGTCAAATCTTTTGGAGCAACG
TGCCCTATTTTGTCGCATTCTTTTTCGTAAGTATGCACATTGTAAATTTTATCTGAAAAAAGGTC
GATACAGTTCATTCTGTTTTCATAAGGGGTGTGTCCTATATCAAAAGTTATTTTACACCCTGTTT
CTTCTGCAACGTTTATTATGTTCTCAGGTTTTGAAGAAAATCCATTTCTTAAGTTTTCAATACAA
AGAGTAATGTTTTTTTCTTTTGAATATTCAGTTAATTCCGAAACATTTTCTAAAAGAGTATTGTA
TTCGGGTTCTTTCCCGTTATGAAGGTGAATAGTTAAATAATCTGCATTATCTAGCTTATTAACTA
TATTTTTAAGTGTTTCAAGTCCTTTTTTATTATTCGGAGTTCCGATATCTACGATAGGTGCATGG
AATCCATTAAGTCCTTTTTTGAAGTTTCATCAATGTTTGAGCAAAAATGAAGGCCCCAATCCGT
GTATTCCAGTCCTTTTTCTGGTTTTGAAAATCGCATGCAAGTTCCTATCATAAAAGCACCGATAC
AGATTATACCAATCAAGATATATAAAATTGTCATTAGAATATCTAATAGATATGGGTTAGCAGT
AAAATATATGTTTTTGAAAGATTGATATTTTATATGTAGAAACCAAATTTGGGGGTTACATAAC
ATTTAAACGAAAAAGAGGGGAAATATATGAATGCAATATTTGAAAGAAGGAGTATTCGACACTA
CACTTCTGAAGATGTTTCAGAAGAAATAGTCGACGATCTTTTAAAAGCAGCAATGTCTGCACCGA
GTGCATGCGACCAGAGGCCATGGGATTTTGTAGTAATTCGTGATAAAAACACACTTTCAGGAATT
TCTAAAATAAACAGGCACGCAAGGATGTTAAACGAAGCACCTGTTTCAATAGTTGCATGCGGTAA
CATGGATCGGGTAAATGGATCATGTGGGCAGTTCTGGGTTCAGGACTGTGCTGCTGCAACTGAAA
ATATTTTAATAGAAGCTCAGGATCGGGGAATTGGTGCAGTATGGCTTGGATTTTATCCTGTAGAT
GAAAGAGTCGAAAAAAAATGAGAAAAGTTCTGCATGCGCCTGAAAATGTAGTTCCTTTTTCAGTTG
TGGCGTTAGGAAATCCTGCGGAAAAGCCAGTTCCTGTAGAAAAAATTCGATAGAGAAAGAATACAC
TATGAAAAATGGCCGACAGGATATCTTTAAAAATCTTTTTTAAAACTTTTAAATTTTTAAACTTA
TGGGGGTGGTTATTTTAAAGATAATCATTACTTTTTTATCCCATCAAGTTTTAATTTATACAGCA
AAATTTTCGCATTATTCTAAAAGGTGGTTTTAATGAAAACAGTTTCGATAATAGGCGGAACGGG
GTATACGGGCTCAGAACTTTTAAGACTCCTTTCAAACCACGACGAAGTTGAAGTTGTGAATGTAA
CATCAAGAAAGGAAGCTGGAAAAAAATCTCACTGATTATCACCCTCAGGTCAGAAATTTAAGTAAC
TATAACGATTTAAAATTCCAAAATATTGCTCCAGAAGATATCGATTCTGATATCGTGTTCTGTGC
AACACCACACGGTGCGTCAATGGCAATTGTCCCGACACTGCACGAAAAAGGAATAAATGTAATCG
ATTTAAGCGGGGATTACAGGTTTGAAGATATTGATATGTATGAATCATGGTATGGATTGAAACA
CTCTGGAAAAAATAGATTCTGCAGTTTACGGCCTTCCTGAACTTCACCGGGAAAAAATAAAAAAGT
CAAAAACAATTGCAAATCCGGGATGCTACCCAACAGGAGCAATTCTTTCAATGGCGCCATTGGTT
GCAAATGATCTCGTTGAAGACAGGATAATTTTTGATTCAAAATCGGGAGTAAGTGGTGCAGGTGT
CGAAGCAAGTCAAACTACCCATTTTGCAAATGTAAATGAAAATATCGGACCCTACAAAATAACGA
AACACAGACACAGTCCCGAAATTGGAAAAGAGCTTCAATATCTTGCAAACAAAAATTTGAAAGTT
TCATTTACTCCTCACCTTCTGCCAGTTACACGGGGAATATTAACTACTGCGCATTCATTCCTTAAA
GAGGATATAAGTCCAATAGATGTTATCGAAATTTACGAAGAATTCTACAAAGACGAATTTTTCA
TAAGAATTTTCGAAGAAGGAATGCCTTCATTAACGGGAGTTCGAGGAACCAACTTCTGCGACATT
GGCGGATTTGAAATTGACCAATATGGCAGAATTGTTGTAGTTTCAGCAATAGACAATTTAGTAA
AAGGAGCAAGTGGTCAAGCAATCCAGAACATGAATATTATCATGGGATTCGATGAAAAAGAGGG
TTTAGGCGTTGGCGGGCTTAAGCCTTAACTTAAATTTTAAAAATATTTTATTTATTTTCTTATTA
CATTAAATCTTCCGGCAGAGGAATGTTTTGGAAGCACATCTACACCGAGGCTTTTTAAATAAGCA
TATGTTTTCGTATCAGTTCCGTGAATGTAGATTTCTCCCAAATCTTCAACAGTGTTTACATCTAT

TGACGTATAGAAGGAATGGCATTTTATAACTTTTAAATCGTTTTTTTCGGCTTCTTCAATATGTT
TTAAAAAACTGAATCCTTCAAACTGTGTTTTCATGCAATTTTTTGGATTTAATAAAAGTAAATTC
GTACCTCCGCCCCTTGATGGGCAGATTACTATTGAATTTTTAACGGATTTACTAATAATTGCTTT
TAAATTTACTTCTTTTAAAAGCGGAACATCTGCAGGAGTTATTAAAATAGACTCCTCTTTTATAA
AATTTAAAGAAAAGTTTATAGCTTCGTTTAATCCTTTAACATTGCTATCTTCCCTAATGGGAATT
ATTCCAAGATTTTTTGAAAATTCTAAAATTTCTTCATCCTTACTTACAACGTAGCAGGCATCGGC
ACATTCTTTCACGGTTTCGTAAGTATCCAATAGAATGTTTTTTATAAGCTCTATTCGTTCACCAG
AACTTAAAAAATCTTTTAATCTTGATTTTACGTTTGATAGGGGGGATACGGGTATTAGTGCAGCT
AACATCTTTTACCACAACGTTTTAAATAATGATTAGTTTTGGGTGTTTAAATTATATAAATAACA
TTGCATTTGAAAATGCGCTTAAATCGCCAGCTTCTCCGTGGCCAGGATATATTTTTAAAATTCCT
TTTTTATAAGCTATTTTTTCAAGTAAATTTATCGATTCTCGTAACTCGATTAGGCTTCCCGTTGG
CAGATCGGATCTTCCAACGCCGTATGAAAACAGGGTGTCTCCAGTAATTAGTGAATCTTTGTATA
TTATGCTTATTCCACCCGCAGTGTGTCCTGGGGTATGTATTATCTCAATTCCTATTTCTTTTAAGT
CAGAATCAGCTTCTTTTATTGTAAAAATTTCTTTTGGTGGAACCAGATCCATTCCAAAGAGTTTA
CTTACTGTAACATTACTATCTTCTGCAAGGTGGGGTAACTCTAGTTCATGTATTAAAACAGGGGC
ATTAAATTCGTCCTGAAACAGATAATCGCTTCCAGCATGGTCAAAATGGCAGTGTGTGTTAATAA
TGTAATCAATTTTATAAGTGTATTTTAAAATATCTTTTTTCAAAAATTCAAAAGTTCCCGGAGTT
CCAGGATCTATCAAAATAACTTTTTTATCGACTATTAAATATGAATTTGAGTCATAGCCTCTTCC
ATTCAATTTATAAATCAAATTATCACCTCTTTATCTCAAATGAATGCAGTCTCCAACGTTAAAGA
CTTCAACAATTTTTCCAGTATCAATATGAATTCCATCGTAAGAAATATCGACAACTTTTCCAGTA
ACTGTTTTTTCATGCATTATTAAGTTTACATCATTAAACAAAGTTTTCGAGCATTTTTTGTAACT
TTCAAGGAGTAATTCATCGTCAATTTTTAGATTTTTGACAAATATATCAATAAATTTCTTTAAAA
TTAAATCAACGTTAAATTCAACATTTTCAATTTCTTTTAATGAGGTAGCGATGTTTTTAATTTCA
GGATCAATTTCATTATTGATATTTAACCCTATTCCAAGTACTAAATAACCTTTGCTCACATTTAG
TTCTGTTAATATTCCGCATATCTTTTTATTTTCAAATAAAACATCGTTTGGCCATTTTATCTCAA
ATTTTTGATCTGAAAAAGATTTTAGTGTTTCTATAACACAAAGAGACCCTAAAAAATTTATTTTT
TCAAGTTTTCCAATATCTTCGATACCAATTAGGACTGAAAAATAAATTCCGCCTTTTTTAGAAAA
CCATGACCTATCTAATCGACCTTTTCCGCGATTTTGGGTTTCTGAAACCACTATTTTATTTTTTTT
GCCCAATTTTCCAAGATTATACGCGTAAGTATTTGTAGAATCGATATTTTTGTGAGAAATTATTT
CAAAATCCAATTTTAACCACACTAGTAATTTTCAAATACATTTAAGAAAAAATTCGTCAGCTTCAA
CTTCTCTAACGATTTCAGGAAATACATAAATATTTTCCTTTAAATCTTTTCTAAGCCTTCCAACA
ACCAGTATAACGTCTCCTTTTTTGAAAGTTTCAAGCTTTTTGGCCATTCCTTTAAATCCGGAAAC
TCTGATATCGCCCGTTAGATCTTCAACTGTCACGGAACAAAATTCTTTTGCCGTATTAGTATATT
TTGAAACAATTATGCCGTATATTGCAACTCTTGAAACTTGTTTTGAAGTTTTAGTTACAATGTAG
TTTGATTCCCACTGGCCTTCTGTTTCAACGAATTCTCCAGAGGTTATTTCATTTATCGGTATCTTT
TTTGCAGGAATTCTCATGAATTTACACCGTTAAATCTCGTAATTTTTTATTTTTTCCATAAATTT
TCCAAGTAATTTTGGAGAAGATTCGCTAATATCTACAATGGCTTCTGAAAATATGATTAGTCCTT
TTGGAGTGATTTTTAACGTTCCAAAGGCCAAAATCCTTGAACCCGTAGTTAATTTAACAGATTTA
TCTTTGGAAATAATTATTTTTCCAGAAGCATCTTCAATTTCTAATTTATCGTAATTTTCATCATT
TTCAAGTCCAATAACGGTTCCAATTATCGCAATTTTATTTTCAATTAGCGACTGATCAATTTCCC
CAATTTTTTGAAGGTTATACATGATTTATCCCTTTAATTTCTTGTTAAATTCTTCCATTATATCT
ATTTTATTCCAATTATTGCACCTGAAAGCTGTATCTTTTAAATATTCATCGTATATTGTAAATCC
TGAAGTTAATATGTCCAATCCAAGTAATTTATTGTTTAAAACGTCCAATATCTCTTCATCATCGA

TAATAATTTTTTTAAGTTCTTCAAGGTCGAGATAATTTGTATTGTTATTTAAAGTAACCCGAATG
GTTCCAGAACCGTCATCAAGGGTGCACGTGCATAAAAAAAGCCATTTTAAATTTTCATCTTCTTC
ATCGACTTTATTTCCGCACTTACAGACTGCATAACCTTTTTTCAAGGTCATTTTTTTATTGCATT
TGTCACAAACTGGAAAATATGGTTCTTTTGAATGGATCGCAACGATTAAACCCCTTAATTCTGCA
CCGATTCCTTCTTTTAATTCGGAAATAGATTTTCTTGAAAAATCGTTCCGCTGAGCACTGGATTT
TTTTAAAAGTTCATCTATTGTTGGTAAATCTGACTCAATTTTTGAGATTTTGCCGGATTTTGAGA
GAGCTAAATGAATTCTATCTTTATATTTTTTTGGAGCATGTGCATTTTCAATTAATATTGCATCT
CCTTCGTTTAATTCATTTAATAAACTTATTTGTTCAGACCACAATGATAAAAGCCCTCTAGCAGA
TCCATCATTTACAACAACGTTTACAACAGGGTTTTGAGATACAATTTTCTCTTCAAAAATCTTTT
CAATTCTAACGATCGAGTTTATTTCGATCATTTTTTCGATTATATCGCTTAATTTTATCGTATCT
ATGCTGTCAATGTTTTTTTTATTAGAATTATTTTCCAATTTTTCAGAATTTTCCAGTATATCATC
AAGACAGGGGTCTTTTTGGAGCATTTACTTCACCAATTACTTTCAAAATTATATTTGCAAGTTTT
AATTTTATTTCAAAACAATCAATCAATGTTAAAATTTAAAATAAGCCACGATTAAACTTATTAT
ATATTATAACAAAATTTATTTATATTTTTTATCTATAAACTGTAAAATAACCTTATTAACACGCA
TGGTGAAACAATGGTTGGTACTCCTCTTTTTTCAAATGCTAAGAAAATTTTACTTCTCGGAAGCG
GGGAACTCGGGAAAGAAGTAATAATAGAAGCTCAAAGGTTTGGTGTAGAATGTATTGCAGTTGA
TTCTTATGAAAACGCGCCAGCAATGCAGGTAGCACATAGATTCCACGTAATTGATATGAAGGATG
CTGGGGCTTTAAGGGCAGTAATCGAAAAAGAAAAGCCTGACTTAATAGTTCCTGAAATTGAAGCG
ATAAACACAGATACTCTAAAAGAAATGGAAAGCGAAGGATACCACGTGGTTCCAACTGCAAATGC
GACAAAACTTACGATGGACAGAGAAGGAATCAGGAGACTTGCATTTGAAAAATTAGGATTAAGA
ACCGCAAAATACGAATTTGCAGAAAATTTGGAAGAATTAAAAGAAGCAGTAAAAAGAATTGGAA
TTCCATGTATTATAAAACCAATCATGTCATCATCTGGGAAAGGACAGAGCACGATTAAATCTGAA
AGCGATATCGAAACAGCGTGGAACTATGCAAAAAGCGCTGCAAGAGGAATTGGAACAAAGGTAA
TCGTTGAAGAATTTATCAAGTTTGATTATGAAATTACGCTATTAACTGCAAGAACTGCAGAAGGA
ACCCGGTTTTGTGAGCCAATAGGCCATATACAGGTTGATGGGGATTATCATGAAAGCTGGCAGCC
TCATCCAATGTGTGCACCAACAAAAGCAAAAGCTCAAGCAATGGCAAAGAAGATAACTGATGAAC
TTGGAGGATACGGTATTTTTGGAGTAGAACTCTTTGTTTTAGACGACGAAGTTATATTCAGTGAA
GTATCTCCAAGACCTCACGATACAGGAATGGTTACAATGGTAACCCAGAAAATGAGTGAGTTTGA
AATTCACGCAAGGGCAATTTTAGGGCTTCCAGTAAACGTTGATATAGTTTCTCCTGGTGCAAGCC
ACGTTATAAAGTCAGAAATTCTCAAATGGGCTCCAGAATATGAAATTCATGAAGCTTCGAAAGTA
AAAGATACAAAAATAAGATTATTTGGAAAACCTATTGCAAAAGTTGGACGTAGAATGGGTGTCG
CCCTTGCAGTCTCAGATGACGTAATAAAAGCAAGAGAAAATGCAGAAAAAGTAGCACACCTTGTA
AAAATTAAATAATTTTTTTATTTTTTTTATTTTCAGTTGTTATGCGAGTTTTATAACGTAAAACA
CTAAAAGTACCCAGATTGCTATTGCAAAATAACCTTCTTTTTTATGAAATGTATAATTTAATGGA
TAAAGTGTTCTGCACCCCATCGGAGTCATACAGTCACCGACAAGATGTGATAAATAACCAATAGC
GATTGGAATTATGTAAAATTCTAACCCCTGAAATGTCGTAAAAGACGTTGAAATCATATTGTACA
AAACCCATGCAAAAACTGAAATTGCGACACTTTTCCCAAGAAGTTTGTTATTTGTAACCATTATT
AAAGCAATCAGTGAGGCCATTATTGGAGAAATCACTGATAATTTAAACGTTAAATAACCTAAAA
TAATTGACATAACACCTAAACTTAAAAAAGTGTGCGTAATCCCCCTGTGTTCTGCATAATACGGG
GTAATATAAATCACACCGCTCAAAATAGCGGCTATAAAAAAACCGGTGTTGAAGTATTCTGGTCG
AAATAGGTATGCTAAAATAGAAACCAAAATAAGGCCACCCGATATGTAGAGTCCTCTCTGAACTA
TTTCTGACTTTACGTCGTGATCTAAGTCAGGATAGAGTGCCCCTGCAACCAGTAAAAAAATCTGT
TCTGGGGAAGAAATAAATGGGAGCCCCATTAATATTCCAAGAGTAATATGTCCTTTCCAGTTCAT

TTTAACCACCAAAAGAAGCGCCTGCCTCTTTTTGGCAGGTTTAAAGCAATGATACAGCATCAAAT
TTATTTTTTATTCTTAAAACGATTTCGGGAATCATTGTAGTAATTACGGATTTTTCAGCACTTTC
AAGAGCCTTTTTATCGTTTCCATTACAAGCATATGCATTTATTCCATTATCATTTAAGATTTTGA
CGGTTGCATGTTCAACCCATGGGTCAACATCGCTTACGTATAGTTCTTCAGGTTTAAGTGCATCT
GCAATACCCCATGAAATATATCTTCCACCAAGAACACCGACTTTATCGCAATTTAACTCTTCTTC
AAGAGAATCGAGTGTTTTTCCAAAGTATGGCGCATTGTACATCTTGCTGTTGGTAATTGAAACCC
AGTGGGCATCACCTATAAAGTAATTTCCAATCATTGAAGGATATACAAGATGATTTTCTTTGTGG
TTTAAAAATCTACTGCTCCTTGCAAGCACTTTTAAAAGTTCCCTGTTTGTGATACAGATGTTAAT
TTTTTCAGCTTCTTCCTTCATTATTTTTAAAACTCTTGGAAGTCCAAAGTTATTTCGTCTTGCTTC
TTTAATATTATATCCGCACATGTATCCGTGGTGGTTACCGCTGAATCCAGATAGTATTACGTTTA
ATCCGCTTCTAAGTCCTACTCTGCATTCATCTTCATACGCACCGTTTGTTGCAACAACTCCCCCTT
TAACAAGTATTCTTGAAACAGCAACAGATTTTGCAAAATCTTTAAGTCTGTCTTTGGACCTGTTA
AATGGGCCCCCTTCAATTACGAGTACGTCAACATCCAAATCGTTGCAGGCTTTTAATCCAGTTAT
TAAATCTTCGTAACCGTCACCTATGTGGAATATTCCTTCAACTCCCCTGCCAAATTTTTGAGCGGC
TTTTGCAACGTCTTCCATTTCTGAAATATCTGCACCGTGGTCTCCACCGCCCTGTTCTTCAACAAC
GTTTACACAGACCGTTGCTGATTTTTTTATCCATTCTCCAAACTCATTAGAATACTCTCTTTCTTT
TGTAATGAGTCTTTTGTGAATTCTGTCTCTTGGACAACCTTTGTATGGCGGGCCGTTGTAGTAGC
AATCGCCCATACAGTGGGATATTTCTTTTGGAAATCTCATTGGACCAAATCTTCCAAAATGGTCT
AGGTCCAGTGGAACTTCAACGTTATTATAAACTTCGTTCATAACTTCGATTGGTGTTAATCCTTC
TTTTTCTGCAATATCTGCAAGAGCATATCCGCAAAGGTGTATTGGAAAACCCATATGATCTGTAA
AAACACAGTTTTCGATGAATTGTGGCAGTGTTAACGATGAAGAGCAGGGTCCAACCCCAATTTCT
AATAAATCACAACCTAATGGGAATCTTCGAAAAATACTTCCCAATTTTTGTGTATCTTCAAAACT
TAAATCATCAACAGTATCTACAATTTCAATAATATCCGATTCTGTCAATTCTTGACTGTTAATTT
TATTAATAACAAGTTCACGAAGTTCCATTGCAGAATCTAAATCATTTAATGAATTTTTTATGAGT
TCTTTCATAATTTCACCTTATTTGCAGATATAGCAGTTAATTTAGTTTTGGGGTTAATCCAAGTT
CTTTTATCATGCATATAACATCTTTAAGGTTCCCGGTTTTTGGATTTCCAACTCCTTTAATGTTC
ATTGGGTGGTTGTCTGGAATTACTGTAGCAATATTACTTGCACCGCCTAAAAGTGCAAATTGAGC
TAATTCTGGGCCCATTGTGGGGGTTGGCGAGGTAATTCTAATATCTGGAAACAGGAGCCTTACAA
TAGCGATAGTTTTTGCCTGTTCAATCGCCGAGCATTTCGGGCAGGTTTCCATCGGCGTTTCTTTGT
ACGGGTTAAAACCCATTATTGGAATTTCTTCAACATCCAGTTCTTTTAAAAAGAAGAGGTGTTCA
ACCCTATCTTCGTAAGTTTCCCCAATACCTATCAAAAGACCGGAAGATAGTTCGACCCCGTGTTT
TTTAACCATTTTACAAACATTTATTCTATCTTCTAAATTTTCACCGGGTTTTACATTGTTAAACA
CGGTTTTATTCGTAGTTTCAAGATTACAGCATATTGTATCTATGTTATATTTCTTAAGTTCTAAA
ATACATTCTTCAGTTAAGTCTGCTCCCGCATTTACCAAAACTTCTAAATTGGATGCTGATTTAAC
TGCTTTTAGGGCCCTTATTACTTCTTTTCCATTATAGCCGTGTCCTGAGGAACAGCTGACTCTTTT
AATTCCTGAATTTTCAATTGCAACTGAAGATGATTTTATCTGGCCGTCATCAGATCTAAATGGTT
TTACATAGCCTTCGGAACTCGTTCCTGCTGCAAAACCGCAATATTTGCATTTTGGGGTTACAGAA
CAGATATTGGTTACATGGACTGTTGATGTGATTTCAATTTCGGTTTTAAAAACGTCTCTAACTTC
AGAGGCTATTGAAAAAAGTTCTAAATAGTCTTTCCAGTTCTTAATATTAAATAATTCAATTGCAT
CCGATTTTGTAATTAAGCCCTGTTTTACATCTATTTTTCCATCTTTGAGGTCCTGAAAATTGCCTC
TTATTTTTGAAAATATCATTAAACCACCGGATTGTAAATTACGCTGAAAATTAAAAAAGAAATA
TGTTTTAGTAATACTTAAGGTTTTATTGAACAGCCGCATTCGCATTTGTTTTCGTTTGCGTGTTT
TTCAAGAACTTTTAAGGATGCAGGAAGAATATCTGCTAAAGGACCAAAGCACATGCTGTCTGCAG

TACCGGTTAATGCTTTAGGGTTTAATTTGTCTTCCATGTTTTTAATGCCTTCGTTTCTCATTAAA
TCTAAGATCTGTGAGATTGCCTCATCAGCCATCATTTGAGCGAAGTCTGCAGGAGCACCTAAGAT
CTGGGTTACTGCGTCTCTGTAAGCTAAGATAGCAGCGTAAACTGGTGCAGTAACTGCTGAACCCA
TGTCACATACTGGGCTGATTAAGTTTGCAGGCATTTTGAATGCTTCCCCTCTTGCAGTTTTTGCC
ATACAGTAGAATTCTTCTACTTTTTCTGCATCAGCTAAACCTTCTGAAAGGAATGCTTGACCTTT
CATTTCTGGAACTGCTCCTGGGTGGTATGCAATAATGTTTAAATCGTCTCTTCCTAAGTCTTTGA
AGATTTTTGCGAATTTTGGTGTTGGAATTGTACAAGCGTGTGTTACAATTGCACCTTTTTTAATT
TCGCTTGCGAATTTTTCGATGATTGCTGGCTGGCTGCCGCCTTTTGGTAACCACGTAATTACAAT
GTCAGCATCTTTAACTGCTTCAACATCACTTGCAGTTACTTTTAAACCTACTGTTTCAGGGTGTA
CAAAGTGTATACATCCTTTAGGTGGTTTTGGTGTTTCTTTAGCTTTAGCCTTTACAGCTTCTCTG
ATTTCAGGCATTACTTTTTCCGCATCTCCTGCTAAATGTGCTTCCATAACTGTTTTGTAGTCAAA
TTGGTCGAGTACTACCATTCCAGGTTCTTCAGCAAAGCAAGGGTCTGAAACAACAACTTCTGTAA
TTTCAGGGATTAAGTGTAACAATTCTGCACCCATTGTGATTGTTGAGTGGGTCATTGCAATTTCT
GGGATTCCTGCTTCTTTTGCAACTTGCGAAGCTCTTGAAAAGTTTGTGATTCCTGATGCAGCGTG
CGTTCTGTAGCATCCTGCACCTAATATTGCCACTTTCATATCATACACCTCAGTATTTACTATAGA
TTAATCTATGTAATATAAAAGCTATTAAGGGTTAATACCTATATAAATATTAGTCATTAGATTA
TATAATGTAATGAGTTAAATATAAATATGTTAATAATATTTTTCACGTATTTCATATTATCGTA
TTTGTTATTAATATTTTATACATACATAAAAATTATTTTTTTAGCAATAAATACTATAATGTGCT
ATGCAAAATAACTGATGCTATTAAAAAATCAAATATCAGTATAACAATTTTTGAAAAATCGAAA
AATAAATTAAAAAAAGGTATTTTTAGAGAAATGCAACCGCATTAATTGGAGATCCGTCAATTTT
TTCGATATTTAGTGGGAATCCAAAGAATTTGAAAGTTTTGTTTTCCAAAATTTCAAGGTTTTTCA
TATTTTCAATTATTTTAATTTTTGAAGATAAAAGTTTTTCATGGACAGAAAAGTTTCCAATTGAA
CAGCAGTCTATTCCAACAGCTTTTAAATTCATAGAAATTAGTTTTTCAATGTGATTTTCAATGTC
GCACTCTTTTTCAAAGTATTCAGGATCTTCCCAGTATTTTGAAAAATCAGTTTTTATGAGCAAAA
TTTCTAAATTTTTAGGTGCATTTTTAAAAAAATCATTCATATTTTTAAAAGAAATACAGTACCCT
TTTCCAAAAACATTTTCAAATTTTTCATTTGTAAGTCCAACATGCATCGGGTAATCAATATGTGT
TCCAGTATGAACGCCCATGTTTAATTCTGAAACCGAGTATTTACCATTTTTTTAGTTTTTACAA
TAAATTCTGGGTCATTTGGATATACAAAATTCTTAATTTCATGAGACAAGTTTATTATTCTTTCT
TCCACCATATTTTCTCCACATAGAAATTATTTATTTAATATTAGTTATAATTGGATACATTATAA
ATTTGGGCTTTGGTGATAATTTGAGGTACGACTTTCACACTCACACGGTATTTAGTGATGGAGAA
CTTATTCCTTCAGAACTCATACGAAGGGCGTCTGTATTGGAGCATAGGGCAGTAGGAATAACTGA
TCATGCAGATGCGAGTAATTACAAGGATTTAATCGAAAAAAATACTTTAGCAAAAGAAGAATTA
TCAAACTACATGGATTATATTGAGATAATTGTAGGCGTTGAATTGACACATATTCCCCCAAAATC
AATTCCAAAAATGGCTAAAAAATGTAAAAACGAGGGTGCAGAAATTGTTGTAGTTCACGGGGAA
ACTCCAGTTGAGCCTGTGGCTGAAAAAACAAATTATTATGCATCAATTTCAGAAGATGTTGATAT
TTTAGCACACCCTGGACTAATAGACATTGAAACTGCACAAAATGCTCTTGAAAATAATATATTTT
TTGAGATAACCTCCAGAAAAGGCCACTCACTTACAAACGGCCACGTGGTATCTGTTGCAAGGGAA
TTAAAAATTCCAATGGTGATAAATACTGATACACACGCTCCAAGTGATTTAATAACTTACGAATT
TGCAAGAAAAGTGGGACTTGGTGCAGGAATGTCTTTTAGGGAACTTGAAAATTCATTAACAACA
AATCCCTTAGAAATTTTAAAAAGAATATAATTTACCGGTGTTTTTATGAGCAATATTTCAGAAAT
TGTTGTTGAATTATTTAAAGAAGCCTCAATTTATCTTCCAGACGACGTTAAGTCTAAATTAAAAC
ACGCTGAAGAAGTAGAGTGTGGTGCATGTAAGGATACGCTACAGGCAATTGAATTAAACAATAA
AATTGCGGAAACTAAACAAATCCCCCTCTGTCAGGATACAGGAGTTCCAGTTATTTTTTTAAAAA

TTGGAACGGGGATTTCAAGTGAAAGAATAATTGAAATAATCAAAGAAATTGAAAGTGGCGTAAA
ACGGGCAACCGCAGAAGTGCCATTGCGGCCAAACGTTGTAAACCCAATTACTCGGGAAAATTTAG
GCACGAATACTGGAATGGGTGCACCGTTTATAAATTTTGAATTTAGTGAAAATTTGAATAATGA
AATCGAAATATCTGTGTTTCCAAAAGGAGCAGGAAGCGAAAACATGAGCGCTTTAAAGATGCTG
ACTCCTTCTGAAGGGTTAATGGGAATAAAAAAATTTATCCTTGAAACAGTTGCAAATGCAGGCGG
TAAACCGTGTCCTCCAATAGTGTTGGGTATAGGAATAGGTGGAACTGCAGATATTTCATTGAAAC
TTGCTAAAAAAGCTCTTTTAAGAGATATTGGAAACAGGAATCCTGAAAAAACAATTGAAAATCT
GGAAAAAGAGTTGCTGGATAGTATAAATCAGCTTGGAATTGGTACAATGGGACTCGGCGGTAAG
ATAACTGCTTTAGATGTATTTATCGAGGTAAATGGATGCCACACTGCATCCCTTCCGGTTGGAAT
TTGTATCCAGTGCTGGGCTGATAGAAAAGCGAAAAGAAAAATAAAATTATAAAAGTTTGTAAAC
TTTTATATCACTACAAAAAATCTCTTTTTTTAAATAATCAAACGATCTGATACAAAGGTTTTTTA
AAAAGTGCAGCGATTTTATAAACAATTAACTCAAAGAAATTAAAAAAATCTTACCGGTAGATTA
CCAATGGATGAGCAGGATATTTTAAACGAATTACGAGAATACAGGAATCAGGATTTGAAATACG
AAGAAGGCTATATTTTAGGATCAATGTGCACAAAACCCCACCCGATAGCTAGAAAAATTTCAGAA
ATGTTTTTTGAGACAAATTTGGGAGATCCAGGGTTATTCAAAGGTACTTCAAAGCTAGAAAAAG
AAGTAGTTTCAATGATTGGCGGTATTTTACACAATAAAAATGCTTTTGGTTACATTATTTCGGGA
GGAACTGAAGCAAACCTTACGGCAATGAGGGCATTTAAAAATATCTCAAAATCTAAGGGTAAACC
TCAAAATATCATAATTCCTGAAACTGCGCACTTTTCTTTTGACAAAGCAAAAGATATGATGGATT
TAAATGTTGTACGGCCTCCTTTAACGAAATATTTTACAATGGATGTAAAATTTATCCAAGATTAT
ATCGAAGATTCTAAAAACGAAGTATCTGGAATTGTAGGGATTGCAGGATGTACAGAACTTGGAT
CAATTGATAATATCTGCGAATTATCAAAAATTGCGGTTGAAAATGATATTTTGCTTCACGTGGAT
GCTGCATTTGGTGGTTTTGTAATTCCATTTTTAGACGATAAATACAAATTAAAAGGATATAATTA
CGATTTTGATTTTTCACTAAATGGGGTTTCTTCAATTACTATTGATCCGCACAAAATGGGGCTTG
CTCCAATTTCTGCGGGAGGAATTCTGTTTAAAGATAATATGTTTAAAAAATATTTGGATGTGGAT
GCCCCATACTTAACTGAAAAACAGCAGGCAACGATAATTGGAACCCGATCTGGCGTAGGTGTTGC
TTCAACTTGGGGAATTATGAAATTACTTGGAATTGAAGGATACAAAAAACTCGTAAACGAATCA
ATGGAAAAAACAACATATTTAGTGAAAAAAGCGAGAGAATATGGGTTTGAAACTGCAATAGATC
CTGTAATGAACATTGTTGCATTAAAAGATGAAGATAAACATGAAACATGCATGAAATTGCGAGA
AGAAAACTGGTATGTTTCAGTTTGCAGGTGTGTTGATGCTTTAAGGATTGTTGTAATGCCGCATC
TTGAAATTGAACATATCGACGGATTTTTAGAAAGCCTTTCAAATACAAAAAAATATTAAGAATT
AAAATCTTATCTGCATTAAAAGTATTTTTGATGGTGGTTTAGTCATGAAAATTTATCTCGATGGA
AACTTCGTAGAGAAAGAAGATGCGAAAGTATCCGTATATGACCACGGGTTGCTTTATGGTGACGG
GGTTTTTGAAGGAATTAGGGCATATGATGGAGTAGTATTTAAATTAGAAGAACATATTGAACGT
TTATACGATTCAGCAGATTCTATCGCTATGAAAATCCCTGCGTCAAAAGTAGAAATGGAAGAAAT
CGTTATCGAAACTTTAAAATCAAACAACTTAAAAGACGCATACATAAGACTTGTAGTTACAAGGG
GAGTTGGGGATTTAGGCCTTGATCCAAGAAAATGTCCAAAAGCAACGATTTTCTGTATTGCAGAG
AATATGAAACCACTTCTTGGAGAAGATGGAATAAAGGTTATAACCTCTTCAATAAGAAGACTGCC
TGTGGATGTTTTAAATCCTGCTGTAAAATCTTTAAATTATTTAAACAGTATTTTAGCAAAAATTC
AGGCCAATTATGCGGGAGTAGACGAAGCTTTTTTACTCGACAGCGAAGGATACGTCGCAGAAGGA
ACTGGTGACAATGTATTTGTTATTAAAAATGGTAAAATAAAAACACCTCCTCTTTCATCAAGTGT
TTTAAAAGGAATTACAAGAGACACTGTTGTAGATCTTGCAAAAGAAGCAGGTTATACAATTATA
GAAGAAAAAATGACGTTACACGATTTATTTGTTGCAGATGAGATATTTATCACGGGAACCGCGGC
AGAATTAATCCATGTTGTTGAAATTGATGGAAGAATAATTAATAACGGAAAATTAGGCCCTGTT

ACAAAAGATTTAAGCGATAGATTCAAAGAAATAAGGTCTAAAATCGGTAAAAAATATATCGAGT
AATATTTAATTTTTTTAAATTATTTTAAAAACTATTTTTAAACTTTTTTCAAAGAAATAAAAAA
TAAAAAAGAGAATTATCTTTCAGCGTTTATCGGATAGTTTGGAGCTTCGTTCGTGATTAAAACAT
CGTGAGGGTGGCTTTCTTTCTGTCCACTTTGAGTAATAATTACAAATCTTGCCTTTTCATGCATT
TCAGGAAGGTTCTGTGCACCACAGTATCCCATGGATGATCTTAAACCACCAATTAACTGGTATAC
GATGTCGCTTGTAGGTCCTTTATATGGAACAGCACCTTCGATTCCTTCAGGAACGAGTTTTGAGT
GTTTCATATGTCCACTCTGGAAGTATCTATCTGCAACGTTTCCAGAACTTCCACACATTGCACCAA
GGGAACCCATTCCTCTGTATTGTTTGTATTTTCTTCCGTTAATGGTCATTAATGATCCTGGAGCT
TCTTCTGTACCTGCCAAAAGACTTCCAACCATTACAGCATTTGCACCAGCTGCAATTGCCTTTGCA
ATGTCACCGCTGTATTTTATACCGCCGTCTGCGATTACTGGAACGCCGTACTTTTTAGCAACTTCT
GCAACTTCTGCAACAGCAGTTAATTGGGGAACTCCAACTCCTGCAACAACTCTTGTTGTACATAT
TGAACCGGGTCCAATTCCAACTTTAAGTGCGTCTGCACCTGCTTTAATTAAATCTTCTGCAGCTTC
GCCGGTTGCAATGTTTCCAACGAATAATTTTGTTTTGGTTCCTTTTAATATTTCTTTTAATTTTT
TGACGTTTTCTACAACCTGCATGTTGTGTGCGTGTGCACAATCAATTGCAACTGCATCAACGCCT
GCTAAAACTAATGCTTCAGCTCTTTCAAAGTCGCTTGGACCGCAAGCTGCTGCAACAACTAGTTT
TCCATCACTATCTCTTGCTGCGTCAGGGTATTTTTTTCTTTTTAAGATATCTCTTAATGTAACCAT
TCCGAGAAGTTCTTTCGTGTTTTTATCCAAAATAGGAAGTCTTTCGATTTTGTTTTCATAAAGTC
TGTTTAAAATTTCTTCATAAGGTGTATCTTCATGTACGTGAAGTACTTCTTTTGTCATAACTGTT
TCTACAGCCACGTTTTTATCTGGAACGAATTTTAAGTCTCTTGTGGTGAGAATCCCGACAAGCGT
CTTGTTTTCAGAAACTACTGGAAGACCGCTCACGTTGTATTCGTACATTATCCTTTCAGCATCTA
AAACACTGCTGGAAGGTTCAACTGTTACGACATCTCTAATTACTAGATTTTCTGCCATTTTAACA
GCCTTAACGTGTTTCAACTGTTCTTCAACAGTCATGTTTCTGTGGATTACTGCAATTCCCCCTCTT
CTTGCAAGGGCAATTGCTAAATCTTTTTCAGAAACTGTGTCCATTGCCGCAGATATAATCGGGAT
ATTAAGCTTTAAACCTGCAATATCGACCGTAACATCCGTAGTTTTTGGATCAACAAAGGATTTGT
TTGGAACTAATAATACATCGTCAAAAGTGTAGGCTCTTTGAGCACTAACTATTTTATCTAAAAAC
AAAATAACCACCATTACCTTTTTTTGTATATTATTTTAATTAATATATAAATCTGGTGGTAACGT
GGATGAAATCAGTATGGCTTCAAAAGAGCTTGCCAAGGGGGCAAAAATGCTTGGAAAACACTGCA
GTTCTTGCGGGTTTCCAATGTTCGAAAAAGACGGTTTGGAATACTGCCCAAACTGCAGTACTTCT
GAAATTGAAATCAAAGGCGATTCAAAAGAATCAGAAAAACAATTAAAAAAAATAAACGAAAATA
AAATTTCAAAAAATGATGATAAATTAAGGATAATAAACAGAAAAATCGAGTATTTATTTTCTAA
ATTAGATTCAGAAGAGGAAATTGGCAGAATTCACGAAATAACTTCATCAATTAAAATACTTCTA
AAAATAAAAAAATATCTGTAAATTTGGAAAGAAAAAACGCTTTCCCAAAAAATAGGTCTTATCC
AAGATGCACAATATTTACAATGGTTTGGTATCAATTGGTGGCATATAGCTAATAATTAGATTCCC
CAATTTCCCTATATTAGTATTAGTATTTATGATTTGTGCTATTAGATTTTCTAAATCAAAAGTTT
GAAGTCTCGTTTTATTAATTTCAAAGTTCCATGATTAATGATACATTTATATTTTATAATTTGAT
AGTGGTATTCAATCAAGATAACAAGTGAGAATATGATTCAAAAATGCAGAGTATGCGGAAAAGA
ATACGATGTTGATGCCATAATTTACAACTGCGAATGTGGCGGATTACTCGAAATTAAATACGATT
TTGAAAGTATCAAAGAAAAAGTTTCAAAAGAATCCTTAAGGGAAAGAGAACTTGGTGTATGGAG
ATATTTAGACTACTTACCAGTTAAAGATCCTGCAAAAATTGTGAGCCTTCACGAAGGGGGAACTC
CACTTTACAAATGTGAAAATCTCGCAAAAAAATTAGGAATGAAAGAGTTATATGTTAAAAACGA
AGGTGCAAACCCAACAGGAAGTTTCAAAGATAGAGGGATGACTGTTGGAGTTACAAGAGCAAAC
GAACTTGGTGTTGAAGTAGTAGGCTGTGCTTCAACCGGAAACACATCTGCATCACTTGCAGCATA
CTCTGCAAGAGCAGGTAAAAAATGTATTGTTCTTCTTCCTGGAGGAAAAGTAGCTCTTGGAAAAC

TCGCTCAGGCAATGTTTTACGGTGCAAAAGTTATCCAGATCAACGGAAACTTTGACGAAGCTCTT
GTAATGGTTAAAAACCTTGCATTAGAAAACAAACTCTACTTATTAAACTCTGTAAACCCATTCAG
ACTTGAAGGTCAGAAAACAATTGGTTTTGAAATCTGCGACCAGTTAGACTTTGAAGTTCCAGACA
TGGTTATTTTACCGGTTGGAAACGCTGGAAACATCAGCGCAATCTGGAAAGGATTTAAAGAATTT
AAAGAAACAGAAATGATTGACAGATTACCAAAAATGACTGGAATCCAAGCAGAAGGTGCACAGC
CAATTGTAAAAGCGATAAAAGAAGGACTCGACGAAATAGTTCCTGACGAAAATCCTGAAACAATC
GCAACTGCAATCAGAATCGGAAACCCTGTAAACGCTGCAAAAGCACTTGATGCAATCAAATCTTC
AGGAGGGCTTGCAGAAAGCGTAAACGATGAAGAAATTATCGAAGCTCAGAAACTCCTTGCACAAA
CAGAAGGAATTTTCGTAGAACCTGCTTCAGCTTCATCAATCGCAGGTCTTATCAAATTGATAAAC
ATGGGATTAATTGACAAAGATCAAAAAATCGTATGTATTACAACTGGTAACGGATTAAAAGATC
CAGATGCTGCAATCAAAGCAAGTAAAATGCCTACAGAAATTGAATGCGACATGGAAGTACTCAGA
AAAGCAATAGAAGAATAATTAAATCTGGTGGAATTATGAAAAAGACTGTCGAAGGCAAAGCATG
GGTTTTTGGAGATAACATCGATACTGATGCAATATTACCTGCAAGATACCTTGTATACACTACCG
AAGAACAGCTTGCAAAATATGCAATGACTGGAACCGACCCAGAATTCCCTGAAAAAGCTACAGTT
GGCGATATCATCGTTGGCGGTAAGAATTTCGGTTGCGGAAGTTCAAGAGAACACGCTCCAATTGG
TTTGAAAGGTTTAGGTATTTCAATGGTTATTGCAGAGAGTTTTGCAAGAATTTTTTACAGAAATT
CCATCAATATCGGATTCCCATTGTTAGAATGCAAAGATATTTCAAAACACGTAAAAGAAGGCGAC
GTTTTGAGAGTTGACCTTGATAAAGGTACCGTTAAAAACGTTACAACTGGCGTTGAACTAACTGG
TCAAAATCTCCCTGATTTTATGATGGAAATCTTAAACAATGGCGGATTAATGCCGCATTTGAAGA
AAAAGATTTCAAAAGCTTAAATTTTTTTATTTAGTTTTTATTTTATTATTTTTTACATTATTTTT
CGGTGATTTTTATGTCAGATCCAAAAAATGTAGTTTTTAAGGAAAGTGAGGATTTAAACGGTGT
TTTTATCGAGGGGCCTGATTTTGATGAAACTATCGATTTAAAAGACGTTTTAACAGATTATTATG
AAAAAATAGGTTTTCAGGCAACACACCTTGGAAAAGCCGTTAAAATTTGGAAACAAATCGAAGA
AGTTAGAAAAAATGAAGAAGTAGTTGTTTTCATGGGCTATACTTCAAACATGGTGTCATCAGGTC
TTAGGGAAATTATATCCTACCTTGTAAAACACAAAAAAGTCGATGTACTGGTTACTACTGCAGGT
GGAATTGAAGAAGATTTTATAAAATGTATAAAACCGTTTGTTCTTGGAGAATGGGACATGGCAG
GATCAGATCTTCGAGAAAAAGGAATAAACAGGATTGGAAATGTATATGTTCCAAATGATAGGTA
CATCGAATTTGAGACTTACATGACTAAATTTTTTGAAACGCTTTTGAAAAAACAGCAGGATGAAA
ATAAAATAATAAGTGCAAGTGAATTTTGTTTTGAACTTGGAAGATTTATGGATGAAAATTTAGG
GCAAGAAAAAGAAAAATCCATTATTTACTGGGCCTATGTGAATAAAATACCAATATTCTGCCCCG
CAATTACTGATGGTTCAATTGGAGATATGCTCTACTTCTTTAAAAAGAATGAAAAAGGAACCGAT
TTAAAAATAGATGTTGCAAGCGATATTGTAAAACTAAATGACATGGCAATTGATGCAAATAAAA
CAGCATGCATCGTTTTAGGTGGATCTCTTCCAAAACACAGCATAATAAATGCAAATCTCTTCAGG
GAAGGAACAGATTATGCGATATACATTACAACAGCAGTTCCATGGGATGGGTCATTAAGCGGTGC
CCCTCCAGAAGAAGGTGTTTCATGGGGTAAAATTCAAGAAAAAGCAGATTTCGTCGAGATCTGGG
CTGACGCAACGATTGTATTTCCAATGCTCGTTTATGGAGTATTTAAATAACAATCACAAAATACA
ATTTTTAAATTTTTTAAAATTTTTTGAAAAAAGCTCTTTTAAAGGATATAAAGAAAAGATATTT
CTTGATATCTCTCTATTGTTGGGACAAATATTTTGGGTGATCTTTAAAAGTGGAATATGAATGTC
ACTTTCTAAGATGTGCAACTATCGCACATTACACTATGTAACTCTCGTAATATATATAAATTACT
ATATCAACTGAGGTAACCCGTATATAAAGGACTATTTTACAGAATATCAAAAATTAATATTTATC
GATATAGGTATTATTTAACAGGTTTTAAAGAATATTTTTCGTTTTTAAGAGTTTTAAGTACAAG
ATTGATAATTTAAGAAACTTTTTAACGAGTAATAAATATATATTAATAATTTTTAATTACATAA
AATCGATAAGTAATTATATGTAGTAACTATATATGTGTGGGTGTGGCCCCACAAGACCACAATAT

TCACAATGGAACATTCAAATTTTTGAGACTAAATCTTCATTTTCTCAAAAAATATATTCAAACTC
ATTTTTAACTTTGAATTTGTGACTTAACGGCTTTTAAATTTTTAATTTAGCCCGTATTTCCCATT
CTTAGTTAAAAATAAGTATAAAATTAAAAGGTGGAATAATGACCGAATTTAAGGTTGTTCATAC
CATATGCCCCTACTGTGGAACGGGTTGCGGTATTGACCTCGTAGTTAAGGACGGTAAAGTAGTAG
ATTCCCACCCTTTCAAAAGACACCCTGTAAACGAGGGTAAAGTCTGTATAAAAGGTAATTACTGC
TATGAGTTTGTTCATAGCGAAGACAGGCTGACAAAACCACTCATCAAAAAGAATGGGGAGTTTAT
TGAAGCCACATGGGATGAAGCATTAGATCTTATCGCAGGAAAATTAAAACAATATTCTCCTGATG
AAGTAGCATTCTTTTCATGTGCGAGAGGCACAAATGAAGAGAGCTATGCTTTGCAGAAATTTGCA
AGAACTGTACTCAAAACGAACAACGTAGATCACTGTGCGAGGATATGACACGCTCCAACAGTTGT
AGGTCTCGGAGAATGTTTTGGTTCAGGTGCAATGACCAATTCAATCACTGATTTAGCACAAGCAG
ATGTATTGCTTATCTACGGTTCCAATACTTTTGAAGCTCACCCTTTAATAGCAAGAAGTATTGTA
AAAGCCAAAGAAAATGGAACTAAAATAATTGCAATCGATCCAAGAACAACACACACTGCAAAAA
TGGCAGATTTACATTTAAAGCTTATTCCTGGATCAAATATTGATTTGATAAATACTATCACGAAT
ATCATCATCCAAGAAGGAATGGCTGATGAAGAATTTATTAAAAACAGAACCGAAGGTTACGATG
AACTAAAAGATGTAGTTTCAAAGTATACTCTCGAAAAAACAGCAGAACTTTCAGGAATACCTGCA
GAAACAATTCTTGAAGCAGCAAGAATGTATGGAAGTGCAGAAAACGCATCTATAATGTATTGTT
TAGGTGTTACTGAATATACATTTGGTGTGGACAACGTTAAATCATGCTGTAACCTTGCAATGGTT
ACTGGAAACCTTGGAAGACCCGGAACTGGTGTAAACCCATTAAGAGGTCAGAATAACGTTCAAGG
TGCATGTGACATGGGTGCACTTCCAAACGTATTCCCAGGATACCAGAAAGTTGGAGAAGCTTATG
AAAGACTCGAAGACTTATGGGAAACAGCAGATTTAAACAGAGAAATTGGATTGACAAGTCCCGA
AGTTTTACACAAGGCTGGAGAACAGGTAAAATTCCTTCACATTGTTGGGGAAGACCCAATGGTTG
CTGATGCAGATATAAACCACGTTGAAAAAGCTTTGAAAAGTTTAGATTTCTTTGTAGTTCAAGAT
ATCTTCTTAACTGAAACTGCAAAACTTGCAGATGTTGTACTTCCTGCAGCTTGCTGGGCTGAAAA
AGACGGAACATTCACAAATTCTGAAAGAAGAGTACAGAGAATCAGAAAAGCAGTAGACGCACCT
GGAGAAGCACTTCCAGACTGGCTTATTGTTCGAAAACTTGCTGAAAAAATGGGTGCTGGCGAAAA
ACTCAAGTTCGAAAGTGCATCAGAAATATTCGATGAAATGGCAAAAGTTATTCCACAATATGCAG
GAATGTCTTTTGAAAGACTCGGCATTGATGGACTTCAGTGGCCATGTAAAACTCCAGAAGACCCT
GGAACTCCAATACTCCACAAAGAAAAATTCTTAAGGCCAAACGGACTCGGTAAATTTACTCCAGT
TGAACATAAAGATGCTGATGAGCTTATTGATGAAGAATACCCACTCATACTTACTACGGGAAGAA
TAATCTTCCACTACAACTCTGGAACAATGACCAGAAGGTGTGACAGTATCACAAATGAGATTGAT
GAAAACTTCATTGAAATAAATACTGAAGATGCCAAAGAATTGGGAATAAAGCCTGGAGAAAAAG
TTAGAGTTTCCTCAAGAAGGGGAACTGTAAATGCAAATGCCCGAGTAACTGAAAACGTCATAAAA
GGAGTAGTTTACATGTCATTCCACTTTTTAGAGGAAGCAACAAACAAACTAACAAATTCCGCGTA
TGACCCAGTATCAAAAACCGCAGAACTCAAAATATGTGCCGTTAAAGTTGAAAAAATTTAATAAT
TATTTTTGAGGTGATTTAAATGAGCTATTATTTAGTTCAGTCAACGGACGAAGATATTTTAAAGC
ATGCGGAATGTGGTGGGGCAGTTACTGCATTTTTCAAGTACCTTCTTGATAAAAAACTGGTTGAT
GGAGTTTTAGCACTTAAAAAAGGAGAAGACGTTTATGACGGACTTCCTTATTTGGTTAACGACTC
AAAAGAACTGGTAGAAACTTGCGGTTCACTTCACTGCGCTCCAACAATGTTTGGAAACATGATAA
GCAAGCACTTGAAAGATATGAATCTTGCAGTATCTGTAAAGCCATGTGATGCAATGGCGATAGTA
GAGCTTGAAAAAAGACATCAAATCGATAAAGATAAATTATATACAATTGGATTAAATTGTGGTG
GAACCGTACCTCCACAAACTGCAAAAAAGATGATTGAACTCTTTTACGATGTAGATCCAGAAGAT
GTAATCAAAGAAGAAATCGATAAAGGAAAATTCATAATTGAATTAAAAGATGGAAGTGAAAAAT
CCGTCAAAATCGATGAACTCGAAGAAGAAGGATACGGAAGAAGAACAAACTGTCAAAGATGCGA

ATTAAAAGTTCCAAGAAATTCAGATCTTGCCTGCGGAAACTGGGGTACTGAAAAAGGATGGACTT
TTGTAGAAGTTGGTTCCGAAAAAGGAGAAGAATTACTTAAAAACGCACAAAAAGAAGGATACAT
AAATGTTAAAGCACCTTCTGAAAAAGCGCTTGAAATCAGGGGAAAAATCGAGAAATCAATGATT
AATCTCGGTAAAAAATTCCAAAAAGAACAATTGGACGAAAAATATCCTTCACCTGAAAAATGGG
ACGAATACTGGAGCAGATGTATAAAATGCTACGGTTGTAGGGATGTATGTCCAATATGTTTCTGT
AAAGAATGTGCTTTAGGTGAGGACTATCTTGATAAAGGAACAATACCTCCTGATCCAATAATGTT
CCAGGGAATCAGGCTTTCACACATGAGTTTCAGCTGTATAAACTGCGGACAGTGTGAAGATGTAT
GTCCTGTAGAAATTCCACTAGCAAAAATATATCACAGAGCGCAACTGAAAATCAGGGAAACTACT
GGATTTGTGCCAGGAATTGACGACAGTATGCCGTTTTTATATAAATAATGATAGTTAAAATTAT
ACTTCTTTTTCTTTTTTTAGAATAAATCAATTAGAATTTAAAAGATAGCCAATGGCTTGACCGAA
AGAAATTCCGCCATCCCCGTTTGGAATTCTTTCATGAATTAATGGTTTTAAAGACTCTTTTTTAA
TATTTTCAACGATTCTTTCAGAAATTATTTGTTATATGACACTCCGCCAGTTACTCCGATATAT
TCAATACCCTGTCTTTTTGCAGTTTCAATTGCAATTCTTGATAATCCATCTGCAATTGCAATATG
AATGAAATATGCTATATTTTCAATTTTTTCATTGTTATTTAGCATTTCCAATGATTTAACAACTA
AAGATGTAGTATCGAGTATGTTGTTTTCAATTTTAATGGACTCTTCTGCTAATTTTTCAATTTCT
AATTTATTTTCACTATATTCATTTGCAAGCGCTTCAAGTCTTATTGATGGTTCTCCATCGTATGT
TCTTTCAAAACAAAGAGAAACAAGTGCAGAAATTGAGTCAAGAATTCTTCCAGTTGATGTAGTTT
TTGAAACATTGATATTTTTTGTAAGCTGGAACAATATTAATTTCAGTTCTTTTTCTGAAAAGTAG
TTGTATCCCTTAATAATTTCAGAAACATTTAAATTAGTTTTATTTAATATTGACGCAAGCATTCT
TAACGGATATTTTGATGCAAGATCCGCTCCTGGCTGAATCTGTTCTTCCAAGTGTCCAGCTCTTTC
AATTTTTCCATTTTTAAATAAAAATACTTCTCCACCCCAGATATTTCCATCATTCCCGTATCCAAG
ACCGTCGATTGCAATTGTGACGTTATTTTCAAAAATATCTGAATCACCCATTAAAGAATAACAGT
GGCTTTCATGGTGCTGCACCTGAGTTACAGGAATTTTGTACTTTTCACCGAGTTCTTTTGCAAAA
AGTGTTGAATTAAACGAAGGATGCAAATCGCAAACTATTGCATCGATTTTGTTTGTATTCGTAAT
TTTTATCAAATTTTCGACAGCTTCCTTTAAATAATTAAAAGTTTCGTATTTTCCAGTGTTTCCAA
TATACTGTGTTAAATAAAACTTGTTATTTTTAACCAAGCAAGCGACCGAGTTTAATTCTGGGCCA
AGTGCAAGAATGTTTTTTGTACTGTTTTTAATTTTTTCATAATTTACTTCAATTGGTTCTGGAGC
AAAACCTCTTGAACGCCTTAAAAGTTCCATTTTTCCATTTATTTCTTTTAAAACGCTGTCATCAC
ATCGATTTACAATTTTTCGATTGTGGAGTAAAAAATAATCTGCAATATTTTCCAATTTTTCTAAA
ATCTGTCTATTATCTATTGACATTGGAAGTCCGGGCAGGTTTGCAGAAGTCATTATGTATGCAAT
CTGGTTAGTATTTTCAAAAAGAAGGTAGTGTAACCCGGAATAAGGAAGCATTACTCCAATCGTGT
TCAGGTTTGAAACGTGTTCTGAAAAGTATTTTTCGTAAAGTTCATTTTTTTTCAAGGCAACAATC
GGTTTTTTTGGAGATTTTATTGCATTAAGTTCATTTTCATCTCGTTTTGAAAATAAATCAAGATA
TTCTTCCCGACTCATAACTGCAAAAGCCTGAGTTGGCCTATTAAGTCTTTTTCGAAGTTCTAAAA
TAGTTTCATCAGAATTAATTGAACAAACAAGGTGGGTTCCCCCAATTCCTTTAATTGCAAGAATT
TTCCCTTCTTCAAGGAATTTAACGGCATCGCATATGTCATTACTTATTATTTTTCCAAAATTGTC
AGTTATAAAAATTTCTGGACCACAAATATCACAGCAGGTAGCTTGAGCGTGGAATCTTCTATCTT
CTGGATTTTTATATTCTTCAATACAGTTTTCGCACAATGGAAAATCATTCATTGACGTGTTTTCT
CTATCATAAGGCAGTTTTTCAATTACTGTGAACCTTGGGCCGCAGTTTGTGCATGCGGTAAATGC
GTAATCTGATCGTCTGTCCTTTTTATCCATTATTTCTTTTAAACATTCATCACATATCGAAATAT
CTGGTGGAATCGTTCCTTCTTCCTCGATTTTGGAAGTATCACTTAATTTTATTGTAAAATCGCCA
TAAATTTCGTTTAAATTTTCATTTTCATCTATATCTTCAATTGAAATGTTATCGATTTTTGATAG
TGGCGGTTTTTCGAGTTTCAAATCTGATAAAAAAGCATCAATATTATTTAATTTCCCAGAAACAA

CTATTTCAACGTAATTTCCCATATTTTTGACGTAGCCGTTCAGGTCATTTTTTTTAGCAATTCTA
TATACAAATGGCCTAAAACCAACGCCCTGAACGATGCCTTTGACATCTATTCGTTTTGCAGTCAT
TGAAACTTCACCAAAAAATCGATAATTAAAAAAAAGAAATATTTTATTTTTTATTTAAGAATCG
TCTGTAAAGACATTTAGGGTATGAAGAGCATCCTAAAAATTCTCCTTTTTTAGTTTTGATAAGTC
TTAACTTTTCTCCACACCAAGGACAGGTATTCTCATCACTATGTTTTTTAACTCTTCAGCAAGG
GAGTATCCTGAAGTACAGAGTCTCTCACCATTGTATCTAACAACACTGTTTACTGTGTCAACGTA
GATAAGGTAGTTTTCGAAAACTTCCGTATTTTCAAGGTCTATTGTTAAATCACAGTCTTCTTCAA
TGAAACCTTTAGTTAATTCCATCTGGGTTTTCAAAGGAAGTAACCAGTAGCCCTGACCTGAATAG
TTGAATAATTTACCTTTATATTTTAATCTGCCGATTAACCAGTTGTAATCTCTGTCAGCAATATA
CTGTGCACTTTCTTCAACGGATTCAAAGACATTTCTATCTTTCATTCTTTCTAAAACTTCGATAG
TATGTGCATCTCCTCTCCTGTACTCTTCAAGGAGCATACTTTTGAAAGTGTTTATAAATTCAGTT
TTTCCTATTTTTATTCCAAGTAATAGGCCGTTAAATCCATTTGCAACAACGTATTTTTCTTTTTC
AACAATTGTGTCCTTACAGAATCTGAAGTAAACTCTGGCGTTTATGTATTTATTGCCATTTTCTG
AAGTTCCTTTTTTTAAAACGAAGTTTGTCCTGTAATTTTCAGTAAGCCCGTAGTAAAAGACGGGT
TTTTTACCATTTTTAAGTCCTACAATTGTAATAAGATTTTCAGGAACCATCCAGTTCTGAGTCCA
CGTTTTTCTACCCATTTCTGTTACATTAAAGTGAATGTTACTTGTAAGGGTCTCAAATAATTCGT
TTGACATATTTTCACCGAATAAGTTATGTTTTAAGTTTAAATAAACGGTTTTGGAAGTTTTTCAT
TTTCACATTCTACAACCATAACGTATGGAATGCTCTTTTTTAAGAAGTAAGCTACATTTTCACCG
ATTTCATCTTTATTTTGAATGTAAACATTTTCAATTCCGTATGCATCCGCAATTTTATTGAAATC
GGGCGTATTGATTTTGCCAAACATGTTTAAATGATTGTTTTTCATCACTACAATAAGTATTTTTA
AGTTGTTTTCAGCGACAGTCCCAAGTTCTTGGATATTCATCTGAAAACCGCCGTCTCCACTGATG
GAAACGACTTCTCGGTTTATATTGTAATCATTGCATCCAAACTTGACACCAATTGAAGCGGGAAG
TCCAAAGCCCATGGTGCCCATTGAATGAGAAGAAATGATATTTCGCGGAGCCACACATTTTTTTA
ATAAAGAAACGAAAACAGTGTGATTTCCAGCATCCGTAGTTATTATTGTATCTTTAGGTAAGTTT
TCCAATATTTCATGGATTTTAGTAGGATAGTCGCCGAATTTAAATTCGTTTCGGCTTAAATTTTG
TCCGGACGGCTTAAAGTGGCCGGAAGAAACGTTTTCATTAAATGTATTTACAATAGTCTTAATGT
CGTTGGTACTATTGATACCTAAATTAAGGGGAATTACATTATATAAAATTTTATCCCTTATGCTT
TCTGGAAGTGTATTGTATGAAAGACTTGCACCTATTGAAAATACTGCATCGCTTTTTAAAATGAG
GTCATTTGACTTTAAACTTCCCCTTCTTCCAACCATTGCATATGCAGTTTCAAAATTCTCATCAAC
AACCCCTCTTGCAGGATATGTTGTTAATATTGGAATTTGAAGCTTTTTTAAAATTTCACCAATTT
TAATCATTTCAGAATACTTTAAATTCCCATATATGCCCTGTCCTAGAAGTAAAACTGGATTTTTA
GCATTTTTGTGCTTTTTTATCGTTGAAACAGCATTTTCAAGTGATTCAGTATTTTTATTTTTCAA
ATTTATTTTTGAATCAATAACATCTTGATTGTAGATATCTGCAGGAATATTTATAGAAACCGGCT
TTTTAAGGTTATAGCTCTCATTATATGAATTTATTAAATATTTGGAGTCAGGATTCTCAACAAAG
TATCCTTTTTCAAAATTCAAAAAAATCCATTGGAATTTCTTGAAAATAATTTTTTCCAATGTAATT
TGTAGAACATCTGCCGGTAATTGCAAGAACCGAAGAAGAATCTTTAAAAGCAGTAGCAATTCCCG
TAGTTAAATTTGTAGCGCCCGGTCCAGCCGTTGCAAGGCAAATTCCAACTTCATTTGTAATTCTA
AAATATCCATCAGCTGCGTGAGCTGAAGCCTGTTCATGTTTTACATCGATACAGTTTATTTTTGA
ATTTTCATGAAGTGTCCTGTAAATTGGAAGAATCTGCTCTCCAGGATATGCGAAAACAGATTTAA
CACCTTTATTTTCAAAAAAATCGATCATAAAATCCAAATATTTCATAAAATCCCCATTTTAAACT
CAATGGATTATTTCTTCAAATTCAATACATTCTTTCTTCATTATCTGTTTTATAGTTTCCACGTA
TTTTTCAAAATTCCCTAATTTTTCGAAATTATCAAGTTCTCTTTCAAGTAATTCTCCAACATCGT
CATCTTTTTGATTTTTAATCAGAAATACAACAGAACCGCTCAAAATACTTGGATTTTTCAAAATA

TCGTCTCTATCCCCATCAGTAATTCCAATTATTTTAACTCCGAATCTTGAAAGAATATCTCCACA
AATTGTTGTTGTATCGTCACCAACAGTCACAACAGCGCATATTTTTTTGTTTTGTATGGTTTCTA
AAACATCTTCTCCGGCATGGTTTACAAATAAAACTTCACCAGATTCTGAATTTAGATTATATTTG
GCAATTTTTTGGTTTTTTGAAGGGTGGCGCCTCAAAAGTCCGGTTTTTACAACTACTTTTTCAAG
GTCGATATTTCCTAACTTTTCAACACCATGCCACTTGACAGTTCCGCCAATAATATCAACAATAT
TTCCGTTTTCAGAAACTATTATAACGTCGTTTTGGCTACTTCGTCCAATTATGATTCCGTTTAGC
ATAATGGACTCGTTAATATCTACACCATTTATTTTTCTAAATGATTTACTTCCTTCAACCCAGAA
ATTTAATCCTTTACTGATACATTCTTCAACGTTTAAATTGAGCTTATCTGAAATTTCAGATACAA
TATTCCCTAACTTTTCATCATTTTTTGTAGTATTCCACAAGATTATCGTTCCATCAGATTCTCCCG
GTCTTTCAATCTGAATTACCGGTTTTTAACGTCTGAACGTTCAACAACAATTTTTCCAAAAGTG
TGGCCAGTGATTTTTGATTTTCCGTAGTTTAAAAGAATTAAAATGTCATTATTTTCAAGTTTTTT
AAGCGATTTTGATGGAACCAGCTTTTCTGAAATATCGATGATATCTTCAAGAGAATTGTCAATTA
CTGCAACTCTTCCAATGGTTCCCCCTAGTTTAATCTGTAAATTGATATTTTCATCTAAATTTTTC
AAAATAGCAAAAATTTTTTCTGCAAATCCTGAATCAATGATTTCAGGCCCGTGTACAACAATTCC
TACGTTCAAATTATCACAAAAATAAAAAATATTAATTTATTCAACGAAATTTTTCAAAATTCCGA
TATTTTCAATTTCAATTTCGACAATATCTCCTTTTTCAAGCTTTCCAATTCCCGGCGGAGTTCCTG
TAGAAATAATGTCATCAGGGTAAAGGGTCATTATGGATGAAACAAATTCAACAAGTTCTTCGAC
ACTAAATATCATGTTATTGGTGTTTGAATCTTGTTTTATTTTTCCATTAACTCGCAACTGTATTT
TTAAGTTATTTGGATCTAGGTTGGATACTATTTTTGGGCCAATCGGGGAAAAAGTATCAAAAGA
TTTAGCTCTTGTCCACTGCCCATCTTTTTCCTGGAGGTCTCTTGCAGTAACGTCGTTTAAAATTGT
AAACCCTTTTATGTAATTTTTAGCATCTTCTTTTTTTACAGATTTACACGTTTTTTCAATTACGA
TTGCAAGTTCAGCTTCGTAATCTACTCTTTCAGATGCTTTTGGTATTTTGATTGCTTCTTCGTTA
TAAATTATAGAAGAAGTTGGCTTTAAAAATATAACTGGCTCTTTTGGTAACTTCATTTTAAGTTC
TTTTGCATGATCTTTATAATTCAATCCTGCACATACTATTTTTGAAGGTTTTATTTCCAAAATTT
CACCCTAAAACTTATTCTAAAATGTTTACTTTTCCGTCAACAACTTCAATTTTTACAATAGTTTT
TACTTTGTATCCTGTTTTTTCTTCAACAATGTTTTGCCCGTTTCCTCTATCGATTATACACAAAAC
ATCTTTGATATCTGCACCAGCTCTTTTCAACGCGTTTATAATTGCAACAAGGGTTCCGCCTGTTG
ATATAACGTCATCGAGGATTACAACTTTGTCCCCTTTATTTATACCGTTTAGGTAGAGTTCTCCT
TTACTGTATCCAGTTTCTTGATGAACCGGAATTTCCCCTTCGAGCAAGTACTCTCTTTTTCTCATT
ATAACATATGGAATATCCGTTGCAATCGATACCGCAGTTACAATTGGGATTCCCATGGCTTCTGC
AGTCACTATTTTATCAACATCCGTATCTATCATCTTGATAACTCTTGTTGAAACATCTCTCAATA
ATTCTGGTTCAACAAGGGGGACTCCATCAGATATCGGATGTATAAAGTAGTGATATTCTCCTCTT
TTAATAATGGGGCATGTTTTCAATGATTCTTCCAATAATTTGCTCATATAATTCACCAATTTAAA
TTTAATTTATATTTATAATTTATTTTTTAGCTGTTTCTTTTTGGGCATCATAAATATTTTTAAAT
TCCGATAACATAACTTCAACAGAATATGCGGCTCTCGTTATACCCATACTTCTAGGATCAGAATC
TGTTCCAACAACAAAGAAAGTATCATCGATTTTTGGGTAAACGAAAAACTGTCCAATGGAACAAG
AAGCCTGTTCTGGAACTGTTTCTTGAACATAGAGCATGTCGACTTTGTCAGTATTTAAATTTAAT
TTACTTTCAATAGTTTTTAAAGCATCTTCTTTTTTCGAATTTACAAAAGAAAAGCCCATTAAGTG
TTTTCCTTTTGGGGCAAGACTTTCATCGTAGTTTGAAACCGTAGATGCCCAGCAGGGGTTATCTA
TCCAGATTTCTGAGCCCAAATATGGGAAATAATTTTTTTCATCCCCGATCCAAACAGTTGTCGAT
TTTGTAAATTTGATGTTGCTTAATTTTTCTTTGTTTTTTTGAATTTTATTTATTTTAGCTACTTT
TGGTAACGTAATTGCAGGTGCGGAGTAAACAATAATGTCTGACGTGTATTCTCCTTCAGAAGTTT
TTACGGCGTATTTTTTACCGTTTTTTGTTATTTTTTTAACTTCTTCGTTTTTTATTTGAGTATTT

CGTAATGAATAGGTAATACAATTACAAATGGACTGTACTCCCCCAACCGGGTAGCCTTGTGTTGA
ATATCTGTGCTCAGTCATGAATTTATTGATGTATTTTTTTGACCCGTTTTTTATGGAACTTATAA
ATCCATCGTTTAAGATGTTAATAATCCTTTCAGTTCCAAACTTCTTTACAAGCGTTGATTTAAGT
GGATTTATCTTCATATCCTTGTAGATAAATGGTAAAATGTCATCTTCTGGAATGTATCCTGCACC
TGTAAAAAATCTCCAAAGAGGCGTATGTTCAAGATTTTCTCCACTTAAAAAGTATGCAATCGTGT
TGAAAAAATCTACAGAGGTACTGCTAAGGCCCATTTCTTTTACAAAGTCATATACTGACGCATCT
TTTGTAAAACCCATGGTCATTAAATCCATAATCTTTGAAGTAATCAAAACTTTATCTTTATTTGG
AATTACCGGAGTAGTCATCCATTCATGCATCGAAACAGGTATTCTATGCAATTTATCCGTTCTAA
TGTAGTAATCCCCGTAATTTCTGAAATTTGGGAGGTATCTTGAATATTTATTTAAAAGTCGGATT
AAAGGCCCGTTATCAAGCATGGTAATTGCATGGACTCCAATGTCTACAGAATACCCATCAATTTT
TTCACTTCTACACAAACCGCCTACATAACTTTCTTTTTCAAATACTATTACATTATGCCCTTCTTT
TTCAAGCGCAAGTGCTGAAAGAAGTCCCGATGTTCCGCCACCAACTACGATTATATCCATGGTTT
CACTCGCATCTATTCAGTAATTTTCTGTTTTTTTATTCTTTCTTTAATCATTTTTCCAATCTGCAT
TATTTGGGCGTTTTCCAATGGATTTGGGGTGGTCCCAGTATTATTTTGGTATATACTTTCTGCAA
GTTCCATATATATTTTACTTATTTCTTCTTCTGGAGCATATTCTATTGCTGTCTGTCCATCAATTT
CAGCTTCATTGATTATAGGGGAATTTGGAACTTTTCCAATAATGTTTGCATTTAGCTGGTTTGCA
AATTCACTCACAATATCAAATGCGTCCATTGAACCCCTTACGTTGTAAATTATACCTCCAAGTGT
GCTTCCTCCACGTTTTACAAACTGGCTTATTCCACTGCATATATTGTTTGCAGCATAGAGCGCCAT
GTAATCTGAAGATGTTACAACGTAAATCTGGTCAGCAAGGCCCATTCTAAGCGGCATTGCAAATC
CTCCGCACACAACATCGCCTAAGACATCATAAAGTACTACATCAACACCGAGATCTTCAAAAACG
TTCATTTTTTTGAGGAGGTCTATTACAACAATCACACCTCTTCCCGCACACCCGTATCCGGGTTTT
GGGCCGCCTGCTTCAACGCAGTAAATCCCGTTAAATCCTTCATAAATTATATCTTCTTTTTTTAG
TAAATTTTCACGAATTATTGTTTCAATTCCAAGTTTATCGATTCCCTTTTCCCTCAAAACATCTA
AAACAGTAGGAATATCTTCTCCACTTCTTAAATTAGATGTACAATCGTGTTTTGGGTCGCATCCA
ACAACAATAACCTTTTTACCACTTTTGGATAGCGCAGCTGCTAAATTACAAACAGTAGTGGATTT
TCCAATGCCTCCTTTCCCGTAAAATGCAATCTGTTTCAAAATATCACCGTTTAACATGAATATGA
AGGATTCTCGTAAAATTAATAAAACAATATAGTACATATAACCGGAATAACTAATATACTTTAT
TATTAATAAACTATTGATTGAAAAATCACGATAATGCTGTTTTTATTTAACAACCCTTATTTTTA
AAAAACTGATAATTAACTGAATCTAAAAATAATTCCTATTTTATTCAGCAATATTTCGTTACTTG
TCGGATTTAATAATTCCTCTGTAGAAATTTATATATACTTTGTCGTATCTATAATGATTAATAAT
GACAAATAAATTTGAGGGATGATAATGAAAACTGGAGACAAATACATAATGCTTGAAAAATATT
CAGAATTACAGCAAAAAGCTTTGGATGACCCTGAAAAATTCTGGGGAGAGCAGGCTAAATGTTTA
GAATGGGATAAAACCTGGGATAAAGCACTGGACTGGAATCCTCCTTATGCAAAATGGTTTAAAGG
CGGCAGTTTGAATGCAAGCTACAACTGTGTTGATAGGCACATAAAAGGAAACAGGAGAAACAAA
GCAGCAATAATTTGCGAAAAAGAAGACGGCAGTTGCGGAATACTAACATATTACGAGTTATATCG
GGAAGTAAATAAATTTGCAAACGTTCTAGAGAATTTGGGCGTTGAAAAAGGCGATGTTGTCACA
ATCTACATGCCAATGATGACAGAGGCAATTGTTGCAATGCTCGCATGTTCGAGAATTGGGGCAAT
TCACAACGTTGTATTTTCCGGATTTTCTTCAGATGCACTTGCTGATCGGATAAACGATTCAAAAT
CAAAAATATTGGTGACTACGAACCTTTTATACAGGCGTGGAAAAGAGATTGACCTTAAAAAAAT
ATTGGATAAAGCTTTACTGAATTGTAATTCAATAAAACACGTGGTTTATGCAAAAAGGGGAATT
GAAGACCTCAATTTAGTTTCAGGAAAAGATTATTACTGGGATGAATTGATGGGCGGTGCTAAAA
GTTACGTAGAACCTGTTCCCGTAGAATCAGAGCATCCTCTATTTATTCTATATACGAGCGGTACA
ACAGGAAGTCCAAAAGGTGTAGTTCACTCAACAGGCGGATATCTAACTTATGCTACAAAAACCAT

GGATTGGACATGGGGATTAAATGAAATGGACGTATTTTGGTGTACTGCAGACATTGGATGGATT
ACAGGACACACTTACGTAGTATATGGGCCGCTTTCACTGGGTGCTACAATAGTACTCTACGAGGG
TGCAATAGACTACCCTGAACCCGACAGATTGTGGGGCATTGTAGAAAATCACGGAGTTACAATTC
TATACACTGCACCAACCGCGATAAGAATGCTGATGATGTACGGTGAAAAATGGGTAAATACTCAC
GATTTATCGACATTGAGGCTTTTAGGAAGCGTTGGGGAGCCGATAAATCCAAGAGCTTGGAAATG
GTACTATAAAGTAATTGGAAAAGAAAAATGCCCGATCTGCGACTGCTACTGGCAAACTGAAACTG
GTGGGCATATTATATATCCCCCAATTGGAATCCAGTCAGTTCCGTTAAAACCAGGTTCTGCAACA
TTCCCTGGAATTGGAATTGATGTTGATGTTGTAGACAATGAAGGAAACCCCGTTGACGCAAATGT
AAAAGGAAATCTAATTATAAAACGACCATGGCCAGGAATGCTTGCAGGGCTCTGGAACAACGATG
AAAGATACCGTGCTGCATACTGGGATAGGTTCAAAAACAAATTTTCAACATCTGACTATGCAATA
AAAGACCAGGATGGATACATTTGGGTACTGGGACGATCTGACGAAGTATTGAATGTTTCAGGACA
CAGGATTGGAACCGCAGAACTTGAACACGAACTTGTATCAAATAAAATGGTTGCAGAGTCTGCCG
TAGTTGGAAAACCCGATGATGTGAAAGGAGAAGTTCCTGTTGCATTTGTTATATTAAACGAAAA
ATACCGTGAACTTCCATCAAATGAAGTAAAGGCTGAACTTATCAAACATATTCGAGATACTGTCG
GCCCAATTGGAACTCCTGCAATGATATTCTTTGTAAACAAACTTCCAAAAACAAGAAGTGGAAAA
ATAATGAGAAGAATATTGAAAAAACTTATAATGGGTGAAGAGATAGGCGATGTTACCACATTGG
AAGACAACACAAGTTTAGAAGAAGTTAAAAAAGAGCTTGAAGATTTCAAGTATGATTAAATAAA
TTTTTTCTTTTTTTAAGTTTAATTTTTTCTGTAAACGTGAATATATCTTGTAAGGCTTTTATGAA
CTCTCATTTCGTAGAATTCCACTAAATTTAAATCAAGTTCCATTCTGTTTGGTGCTGCAAATACT
AAATAATCATTATCTTTTAAAACATCTACAATTTTTTCAAATATTTCTAACATGTCCCCTTTTTT
AGATGTGGAAATTCCATATGGTGGATCAGTTACAATTCCATCTATCTTTTCAATTTTCAGGTCTT
CCAAATATTTTGAGACGTTTTTAGCATCGCTATGCTTTATTGAAATGACTTGTTTTGATAAATCA
TACGTGTTTAAGTTTAGTAGTGCACCATTTACCATCTGTTCATCGATGTCACTACCAATTAATTT
GCATCCTAAAAATCCGGCTTCAATTAAGAATCCTCCTGTTCCGCAGAATGGATCTAAAACAATTT
CCCCTTCTTTAACTCTTGATAGATTTACAAGGCATCTTGCAAGTTTTGGCATTATGCATCCTGGA
TGAAAATATGCTCTTAAGTGGGGCCTGTTATTTTGGAAGTATTCAACATCTCGTTTTTCAATAAC
TATCCCTGTATACATTGTATCGTTTGAAATAACGACTTTAACCGTTTTTGAAGGGTTTTTTAAAC
TCACCTGTGCAGAAGTTTTTTTCTTTATAATTCCCCCTAGTTTTCTTTCGATTTCCATCGAATTAA
ACGGGCTTTCTTTTTCCAATTTTAAAACACGAACTGCAAAAGTTTCATCTCTTTCAACTGGAAAA
TCGAGTGTTTCAGAACTTTTAAAAAAATTTTCGAGGGTTTCATCTACATTCTGGTTAATTATTGG
GGATTCTGAAAATATAACGTGTCCTTCGTTTGTGTATCCTCCCCTTTTAACCATTTTTTTTATAA
TTTCTTCTGAGATTGAACTTTCCAATATTACGTAATTTTTAAAGCAGTTTAAATTACCTCCAAGA
CTAAAAATATCGATTAAAGCATTTAATTCTGCGATGGGTAGTTTTTCGTGTTCTGCACTTAACAA
AAAACCTGCTTTTGTGTTTTTTAAATTCAACATATCACCAGTAGGTTTAAATTTCAAAACATCAT
AGGTGTTATATTCATATATATCTTAGTGATTTTAATGAAACTTTTCATATATCATGAAAAACAGT
GCGACCCAAAAAGGTGTACTGCACTAAAAATGGGAAGACTGGGTTACGCAAAGATAATTACAAA
TCCAAACAAGGTCCCGAGGAATTCACTTCTACTAAATCCGTATTCTGAAAAGACAGTTTCCGTTG
AAGATAGGGAAATTATCGAAAAATATGGAATTTTGGGACTGGACTGTTCGTGGAAACAGGCGGA
AATTGTATTTAAAAAAACAAACGCCAAGAATCAAAGAGTTTTACCCTTCCTAATTGCAGGAAATC
CTGTAAATTACGGAAAACCCTGTAAATTAACGACTTTGGAAGCAATAGTGGCTACCCTCTATATA
GCAAACTATAAAAATGAGGCCGTCTCTTTATTGAATGGTTTCAAATGGGGAAACACTTTTATAGA
TGTTAATAAAGAATTACTTGAACAGTATTGTGGAAAAACATCTGATGAAATCATTAGGATTCAG
AACGAAATTTTAAATAACATGCGAAACAGGTGAAATAATGGCATTCGAAGAAGCAATCAAAAGA

GTCTTCATGAAAAAGATTTGCATGAAATGTAACTCAAGAAACTCATGGAAAGCTACAAAATGTA
GAAAATGTGGATACACAAATTTAAGGCCTAAAGCAAAAGAAGCTAGGGCATAATCCATTACTTTT
CTTTTTTGGTTTAAATTTATATTCTATTTTGTAAAATCGCTTTTTTAACGAATTATTTTTCAAGT
TTAACGAATTATTCTGTTCTGATTCAAATGAATAGTAATAAATATCACACAGAATATAGGAATA
AAAGTTCCTGATAGAATGAAACATTATCTATTCAGGTAATTTGTCGGAGACTAAACTTTTAACTC
GGGAAAAACTCCCAGAAGTTCTTTAAAAACTACCAATCAAACAAGTTAGTAGTTATTCAAAAACT
GATAGCCTAGTTTTATATGCATTCTCATTTTACACGATTTTTCAAAAAGTTTTTCTAATGTCGAT
TCGACGAGGTGAATTTATCGAAATTTAGATAAAAGAGGTGATTTTATGAAAAAAGCACTGATTT
TACTCGTGCTCGCCGTATTTTTAATTGGCTTTACAAAAATCGCGTACGTGGACGAAGAATCTGCT
GACGGAATAAACAGCCAAAAAGCGATAATTACACTTATAATTCTTGGAACTGCGGCTGTGTTATT
TTTCACAGAAGCGCTTCCACTGCCTGTTACCGCAATGTTAGTTCCGGTGGCTTTGAGTTTTCCAGG
GATAGATATTTTATCGAGTTCTCGGGCCTTTGCCGATTTTGGAAATAAATGGGTGGTCCTGTTTA
TGGCTGCATTCATACTTGGTGAAGCAGTATTTAGGACGGGCTTTGCAGATAAAATCGGGCAACTT
ACCGTGAAAGCAGCAGGAAAGAGCCAGTTAAAATTAATGCTTCTTGTAATGCTTGCAATCGGTAC
GATGTCTGCATTTTTATCAAACACAGGAACAACAGCAGCATTTATTCCTATTGTAATGGGTATAT
GCGTTTCTGCAAGTTTAAAACCTGGAAAATTGTTAATTCCTATGGCTTATGCTGCATCGCTTGGT
GGAACGTTAACTTTAATTGGAACTCCTCCAAACGGGCTTGTAAACGATGCACTTGAAAAATCAGG
GATTGCTCCATTTGGGTTTTTCGAGTTCGCAAAAATTGGAATTCTTATATTTATCGTAGGGATTC
TTTACTATGGAACAATTGGGCACAAATTACTGCCTGAATCTAAGGCAAAAGCAAAAGATTTTGTG
TCAGATACTTTGAAATACCGAACTAACAAGATGTGGATTGCAACACTCGTGTTTACATTCGTTGT
ATTAATGACAGCAACTGGAACGATCCCGCTTGTAACTGCATTCATGCTTGGTGCGTGTATGGTTG
TAATGACTGGATGTATCACGATGCAGGAAGCTTTTAACAGTATTTCGTGGACAACAATCTTTTTG
TTTGCAGGAATGCTTCCACTTGGAAGTGCAATGGCGGCAACTGGTGCAGCTACAATGATTGCAAA
CACGGTTATTACGTATGTTCACAGCCCATTGGCACTTTTAGCCGCAGCATACATACTGACTGCAT
TAATTACGGAAGTAATGTCAAACACTGCAACAGCTGCCTTGGTAATGCCTCTTGGAATTGCTCTT
GCAGATGCGTTTGATGTTAGTGCAAAACCGATATTGATGGCAATTGCAGTTGCAGCATCTTCATG
TTTCCTAACTCCAATTGCAACGCCTCCAAACATGATCATACTTGGTCCTGGAGGTTATTCATTTA
AAGATTACGCAAAATCAGGATGGCCACTTGAAATACTTTGTGCAGTAGTTGCAATTGCAGTTATT
CCAATGATATGGCCATTTTAATTCTATTTTTTTATTGATTTTATTGATTTTATTGATTTTATTGA
TTTTATTGATTTTATTGATAATACTAATTTTAAAAAATATGATTTTCAGTATGATTTTTTTTTAA
TTCGTAATAATTATATACTATAAATGTGAATTATTATTGTGGTTGCAACCATTAAGCCATCAAAT
AGCCACATTATGTACAAACATGACGTTACCCCAAATATCACTACTTATCTCTCTTAGTCACCACTC
CAAACTAATTGGGAAAATTTTCTATTTTAAAAATTTTTTAAAAAAATTAAATCAAAAACGGTTT
AGATTAATACGCTTAGCGCATAAACCAAAAGGATTATCGCTGCAAAAAGCAATATCAATTTTATC
GTTGTAAATACCATTTTAAACATGATTTTTAAAATTAAAAAGACAACAATTGCGGCAATAATTG
CAGTTATTATCCAGCTAAATCCAAACATATTCCCACCTTAAAAAAGTTTTAAGTTATTTTATGAT
TTTTTCTGCAATGTTTTTAAACACTTCGTCGCTTATTTCAGTACCGTCTTCTCTAGTTTCTTTAGT
TTTCTTTACAATTTCCCATAATTCGTCTCTTGAAGCTTCAAGTCCGAGTTCCTGTAGTCTGTGTGC
GACAGCCCTACAACCGGAATGTTTTCCAAGGATAATTTTTCTTTTCTGACCGATTTTTTCAGGCA
AAAATGGTTCATACGTCAAAGGATTTTCTAAAACTGCATCGACGTGTATTCCGCTTTCGTGGTAG
AATACCATTTCTCCAACAAGCGGTTTATTTTCAGGGATTTTGATTTTTGAATAGTTTTCAACGAG
TTTAGATATTTCAGTTAGTATTTCAGTATTTAATCCCATATCGTGGTCGTAAAGTACTTTTAATG
CCATTGCAATTTCTTCAATTGCAGCATTTCCTGCTCTTTCACCTATACCATTAACTGTGGTAGATA

CAGCTTTTGCACCACCGATTAAACCGTATATTGAGTTTATAACTGCAAATCCAAAGTCATTGTGG
CAGTGTACTCCAATATGTGCCTTTTTTAGGTGTTTGCTTAGTTCAGAACATATGTGGTACATTGC
CTGTGGTGTTGCACATCCAAGAGTATCTGCAATATGGACCCTATCAGCGCCGTGTTCTTCTGCAT
TTTTATGAATTCTAATTATGTCTTCAAGAGGGGTTCTCGTTGCATCTTCTGCGGAGAATGCTACA
AAAAGGCCGTGATCTTTTGCATATTCTACGGCTTTCATACCCATTTCTTCGACTTCATCAAGATTC
TTATGTAATTTATATTTCAAATGCATTGGAGAAGTTGCAATAAATGTAATTATCCCGTCAACGTC
ACATTCGATGGCTTTATCAATATCTTCTTTTAAAACTCTTGATAAGGCTAAAATCTGCGCATTTA
GGCCTTCACCAGTAATTGATTTGATGGCTTTTCTTTCATTTTCAGAAACAATTGGAAAACCTGCT
TCAATCTGTTTAATTTTAAGTTCATCCAGTTTCTTAGCAATTTCAAGTTTTTGGTCTCCTGCAAA
ACAAACTCCTGGGGTCTGTTCACCATCTCTCAATGTTGTATCATACAAATAACAGTCTTTTAAAT
CTAATTTTGGGTTGTACGGAGATACAGCTTTCCAGTCCATACTCATTCCTCCATTTATATCATAA
ATAAACAAATTTCATATAATTTTACTAATTTGAATTAACTTCAAGGATGTATATATTTTCGCCCT
GTTTAACGCTTTTTAAAATTTCTGCGTTTTTAACTACTTTCCCAACGATATTTGTACCCTCAAAG
GTTTCCCCTGTTGGCCCATATTTATCATGTGATGAGGTCCTGACACCCATATATCCTTTATATCTT
TTAGCCATATTTGTAACAGCAATTATTTCAGAATCAACAGATCCTTTTGGAGTATTTTCAGGTAA
TAATCCTTTTGAATAATCCCAATTTCTTTCAAACATTGATATATCGTCGTGTTTGAAATATATTT
TTAACGTGCCTATTCTTTTTGTTGTAAGGCCTGTAGTTTTTCTAAAGTACCACGCGGTAGTTGGA
GCTTCTTTATCAAATATTTCAACATATAGTAATTTTTCAGGTTCTATTCCTTTTGTAGTAACTTC
TTTCGATTTTAATATGTCCATAGTATATTCAGGCACCTGTTCAACGATTACTGCATCGTCATCTG
TATTTCCTTCCATTTTCAATGTGATTCCATGTTTTTCAAACAGAAGTTTTGCATCTTTTTGCGTT
TTTCCAATTAAATTTAATCTTTCAGGACTCGTTTTTACAGTAATTTGTCCCGAATTTGAAAAATC
AACGATTTCAATTCCATCAGTTACTTTACCAACTACAGTATGGGATAAGGATGATGCTCTATTCT
CTTGATAGATATAAACTTTTCCAACACCATTTCCGACATTTCTAACCGTGATTGTTCCCCTATCTC
GGTTTTCAGGATTTTCTTCATCAATTAATAATGTTTGAAGCCTGCAATCTGCAACGTATGTATTT
GTATGTTCTGAAATTTCCAACGTACCGTTTTCAAGTATTGCAAGGGCATGTTCTGTACTTTTTGA
AGGTCCATTAAGCTCTGCTTTACAGTATGTGTATATTTTCCAGTCGTCCTGAATTTCTTTATCTA
AATCAGTAGTTACTTCATAATCAATGTTTTCTTTTGATTCTCGCATTTTTTCAATGGATATAATC
TGGTCATCGGCAGTTAATGCTTTTAAAACTCTTTTTCCACCAATTAATATGCCTATTTGTGGATT
TTCAAGACCATATAATTCCGTTACATCTCTTTGAACAAATATTAAATTTCCTTCACTCGTATCAA
GCCCTGAAACGCTTAAAACAACATCCCACTTTTTAAAATTCTGTTTTAAACTACTTACTGGGAGA
TTTATCTCGATAGATCCAAAAGATACATCAGAAATACTTTTCCATATCAGGGATTTTTTTTCAAA
AGATTTGTAGTTTTTATTCCAGAAATCGACACTTTCGTTTTCTTCAGTTATTCCAACTACAAAAG
AACCCTTGGTCGTTTTTATCATGTATTTTTTGGTTTCTTCCTTAATTACTGTGGCAGTTCCCTTTA
CAATTACAATATTTGACCCAGGGATATAATATTCGTCTTTTATTACATCTTTTAACGTTTTTCCA
GATTTTGGTTTATCATTTACTAATACATTCATATAACCAGCCCCTTTGGGTTCATAACTAGATCT
CAGATGTGTTGGCAAAATTTTTCCGAAAATATATTTATACTTAAATAACAATAGTATGTTGCTAA
CTGTACATTATATATATGATATTTTAAATAACTTAGCGGACAGATTAATTTATTGATTATCTTAA
TGATTTTACTGTTTTAATTGAAACTTTAACTTATGGTATCAAATTATACGCATACATATCAAGGA
GAGAGATTATGGCCGAAAATACTGAAGTAAAAATATCATCCAAAGCTAAAAAGATATTAAGGTC
CCAATCGCACGAAATCGAACCTGTAGTTTGGGTTGGAAAGGAAGGGATCGAAAAAACCATAGAA
GAAATTAAAAGGCAGATAAAAGATAAAAGCCTTATAAAAATCAAAGTGCGAAAAAGTGCACTTG
AAAATGGCGATAAAGCAGAAATGGCCGAAAAAATATCTAAAGAAACTGGTGCAGAAGTTATCAG
CGTAGTTGGAAATGTTATTACTATTTTCAAACCAAAAGAAGGCTGGAAAAAGTATTCTACAAAG

AAAACAAAGAAAGAAAAGTATGTTGAAGAATTTGAAGAAATGCGGTCTAAAAAAGCACTCCTTA
AGAGATAATACGTTATTTTAATAACGGATGATATTTTAACCTTTTTTTCATTTTTTACTCATTTT
TATATTACTGGGAATCAGGGAAAGTAATATATATGGGGTGCAATTATTATAATAAGATTGCTCG
GTCTTAAGGATTTTTAAATATTATTTTTAATGAATCCGTTAATTGTCCAGATTATCCGAATATGA
GTTACAAAAGGATGAACTGAACGTTATTATTAGAGGTGTTTACATGGTAACCGTTTACGATGTAC
CAGCAAACGATTTAATCGCAAAATTAGCTGAAAAATTGAAAGAAATGGGCGTAGAAGAGCCTGA
ATGGACAAACTTCGTTAAAACAGGAGCTCACAAAGAGAGAAGACCTGATGACGGAGATTGGTGG
TACGTAAGATGTGCTGCAATATTAAGAAAAGTATACATCAACGGCCCTGTTGGTGTTGAAAGATT
AAGAAGCGTTTACGGTGGAAGGAAGAACCGAGGATGCGCTCCTGAAAAATTCGTTAAAGGTAGT
GGAAACATTATCAGAACAGCTTTCCAAGCTTTAGAAGCTAAAGAATTACTTACTAAAGCTGAAAA
AGGTGGAAGAATCATTGCACCAAAAGGCCAATCTTTAGTAGACAACACTGCAAAAGAAGTAGCTG
ATTCAGTAGCACAATAAGTAATTTTATTGATGAGGGGGTTTGATGAACCCAGAAGAAATAAGGC
AAAGAAGATTGCAGGAAATGCAAGCAAAAGCTCAAGCCCAAGGGGCAGCAAATGACCCTGAAGCA
CAGAGACAGATGCAAGAGCAGCAAATGCAGTACGAAATGCAAAAACAAAAAATTCTCAGGCAGA
TTCTTTCCGAAGAAGCAAGGTCGAGATTAGCAAGGATTAAACTTGCGAAACCTCAATTTGCAGAA
CAGGTTGAAATGCAATTAATACAACTCGCACAGGCCGGAAAACTTCCAGTTCCGCTTACTGACGA
GTATTTTAAAGGCCTTCTCGATAAAATATACGAGATGAACAGACCTGCAAAGAAAGAAATCACTA
TAATGAGAAAATAAATGATTCACATGGCTAAAGCTCACGTTTTATTCAGCGGTGGAAAAGATAG
TTCCCTTTCTGCCATAATTTTGCATAATCTGGGCTATGACATTCAGTTAGTAACCGTTAATTTCG
GAGTTTTGGATTCATACCGTCATGCAGAAGAAACTGCCAAAATAATTGGCTATCCTCATAAAGTC
GAATCAATGGACAGGGAATTAATTGAAAAATCTGTTGACATGATCTTAAAGGATGGATATCCTG
GAAACGGGATTCAGTTTGTACACAAGCAAGTTCTCGAAATAATTTCCGAGAAATACGATGTAATT
GCAGATGGTACGAGAAGAGACGACCGCGTTCCAAGACTTGATCACTCTGAGATTCAGAGTCTTGA
AATGAGGAAAAATATTCAGTACGTGACTCCTTTAATGGGATTTGGTCACAAAACTTTAAGAAATC
TAGTTAACAATTATTTCATAATTTCTGAAAAAGAAAGTGAAGAACTTTTGAAATCAGACTATGA
AACCGAAATAAGGGAATTAATTCGGCAAAGAGGAATCGATCCGCTTGAATACTTCCCAAAACACA
TTCAATCAAGAGTTATTGGTTTAAAAAGATAAGTTCAGAGGTGAAAAGATGGCAGGCAACAAAC
CTTTGGGTAAAAAAATTAGACTTGCAAAAGCTTTAAAACAAAACAGAAGAGTTCCTATGTTTGCT
ATTGCAAGAACAAAAGGAAGCGTTAAACAACACCCAAAAATGAGACACTGGAGAAGAAAAAACC
TTAAAAAATAATTAAAATATCTTCTTTTTTTAATCAAGTTCAAAATTAAAATTTAAAAATTCAA
AAAAATTAAAAAAGTAATTTAATTCACGTTTCTTAAGTCTTTTACACGTTTTGCTTTACCTTCAA
ACCTTTCAAGGGTTCTAGGTTGTACTATATTTATTTTAGAGCTTAATCCAAGCACAGTTTTTAAT
TTATGGTCTACTTTTTTACTAATCTGTTCAAGGCAGCTGAATTTTTCGAGGAATTTTCCATCTGC
AAGTTCTATATTAATCGTCATCTGGTCTAGGTGTCCGTTTTTGGTAACGATTATTTCATAATGCG
GTCCGATTTCTTCGATTCCTTCTAAAACACTTTCAATTTGTGTTGGGAATACATTTACTCCTCGA
ATAATTAGCATATCGTCGCTTCTTCCCCTTATTTTTAGCATTCTCGCGGTAGTTCTTCCGCATTTA
CATTTTTCGTAAGTGAGGCTTGTCATATCTTTTGTTCGATATCTAAGAACAGGAAGGGCTTCTTT
TGTAAGTGTGGTAATTACAAGTTCTCCAATTTCTCCTTCAGGGAGTACTTCGCCGGTTTTTGGAT
CGATTATTTCTGCAATGAAGTGGTCTTCAGAAATATGCATTCCGCACTGGTATTCACAATCTCCA
GAAACTCCTGGACCCATTAATTCACTCATACCGTAGTTTTGGGTTGCAAGTATGCCCCATTTTGA
TTCAATTTCTGCTCTTGCAGATTCAGAGCATGCTTCTCCACCAAAAAGACCTATTCTTACTTTCAA
GTCTTTTTTAGGGTCAATTCCCATTTTTTCTGCAACTTCTGCGATATAAAGGGCGTATGAAGGAG
TACATATTAAAACGGTTGTTTCAAAGTCTTTCATGAGCATAATCTGCTTTTCCGTGTTTCCGCTT

GACATTGGAATGACAGATGCCCCAACATTTTCAAGACCATAATGTAACCCGAATCCACCGGTAAA
AAGTCCGTAACCAAATGCGATTTGAGCTGTATCTTCATCAGTTACACCTGCAGCCGTTACAACTC
TAGAAATCAGTTCTGCCCAAGTTCTGATATCTTTTTTTGTGTAGCCTACAACGGTTGGCTTTCCA
GTAGTTCCTGATGAAGCATGAATTCTCACAACTTCTTTTTAGGAACTGCAAACATTCCAAATGG
ATAATTTGCCCTGAAATCATCTTTAACTGTAAAAGGGATTTTTTAAGGTCATCTAACGATTTTA
TATCTTCAGGTTTTAATCCAACGCTGTCAAATTTTTCTTTGTACAATGGTACATTTTCATAAGCT
CTTTTTACAGATTCTTTTAATCTTTCAAGCTGTATTTTTTCAATTTCTTTTCTGTCCCATGTTTCT
TCTTTAGACCATATCAATTTACTCTCCTCCACCGTTTTTATTCCATTTAGCCGTAATTTATTAAG
TTTTCGAAATTAAACGTTGATTAAACATTTAAATTTAAAAATAATTCTTCATATAAGATTCTTAA
TATAAAATCCAAGTTTAATTATATATCAGTTTCTAAATTAAAATGAAGAAATGCTTCCAGATTTT
TTATCGAAAATTCCAACGGATTTTTGAGTGTTTTTTGATGTACAGAATATTAAATCTTCAAAATA
ACCTAATTCTTTTAAATTTTTTCCAGAATTTGATTCAAATATCTTTCTTACCCAGTCTGATTTTG
TCAACATTCTTGCAGGGATCACTTCTTCAAATTTTTCATCAGGAATTTTTTCATCGTATTCTTTG
AAAATATAATCAGCAATAATTCCTGCCCCAATAAAGTCTTCAATTGCAAAATTTCCTTTTCTGTG
TACTGGAACCAGCATTATTCCTTTATTATTTTCTTTTGCGAGTTTGTAAGCTTTTTTTGCAACAC
TTTGTGCGTTTGTGATTGAACCGATTAAAATATGGTCTGAAACGATATTTTCAAGAACTCTTGTT
CCATTTGTCGTGGTTAAAACAATATTTCCCCCATTTTCAAAATGTTCTTTTATTAAATTTTTATT
TACTGTTAATTCAACAGGGGAATTTCCAAAATTAAATCCTTCAATTTTTTTGGCGTTTCTTTCAC
CTATTTTTATAGAATTTTCGATATTCTCTGCTTTTTCGATACTGCCTGTTATATATATCTTATCG
CACAAGTCAAGTAGTGTACAAATGGTTGCAGAAGCCCTTAAAACATCGATTACAATAACGCAGTA
ATCACTAAAATCAACTGTTTTCTGGAGTTCACTGCTTCCAAAAAAATCAAAAGAAATTGAAATTT
TCATGAATTTCACCGTGATAAAATGAATTTTACTGATATAGGGATTTTTGATTATTTAGGCATGA
TTTTGTTAATACTAATTGTTTTAGCAGCAATCTTGTTTGTAACTGCAATTGGTCTTGGTTATTAC
CTTATAAATAAAAATAAAATCTTGTTTCCTAAACTATCATTGTATATTACAAATAACTTCTACTC
CATTCTATTAAGAATTTTTCTGATAATTGGAACCGAAGATACATTTTATAAAGTTGCAAGTGATT
TTTATAACAAGTACTATTGCGAAGACTTTAAAAAAGCCAAAAATAAGGTTTTAATCCTTCCACAC
TGTTTAAGGGATCTAAAATGTCCCGGAAAGCTGGGTCCCGATGGTGTAGAATGTGTATTTTGTGG
AAAATGCCCGATTGGAAGGGTAATAAAAGTTGCTGAAGAAAACGGGTATAAAACTTACGTAGTT
CCCGGTTCAACATTTTTAAAACGGGTATTAAAAGATAAAAAACCGGACGGCGTTTTTGCAGTAGC
GTGCCATAACGACATGTTTCACGGAATGACGTTTCTTTCTCGAAAAAATGTACCTGTTCAAGGAC
AGCTTTTAATGAAAGATGGATGTATTTCAACACTTGTGGATGTCGAAGAATTAATTAGCCGACTT
AAAAATGAACCCTGTTAATTAAAAACCTATAAAAACGTGTAGTTACTAACTATCTTAGATTAGCG
ATAAGGTGATATTTTGGTATTATCTAAAATAAAGGAATCATTGAAAGGAATTACCTCTAAAAAA
CTTGAAAAAGAAAATGGAACCAAGTACATCATGTTTGGTGGAAAAGGGGGAGTTGGAAAAACTA
CAATGAGTGCCGCAACAGGTATTTACTGTGCAGAACAAGGCCTGAAAACAGTTGTAGTTTCAACC
GACCCTGCTCACTCATTAAAAGACAGCTTTGAACAGTCATTTGGTCACGAACCAACAAAAGTAAA
CGGCATGGAAAACCTCTATGTTGTAGAAATCGACCCTGAAGTTGCAATGTCAGAATACAAAGAAA
AATTAAAATCACAAATGGACGAAAACCCAATGATGGGTGGAATGCTCGAAGACCAGCTTGAAAT
GGCTTCATTATCTCCAGGAACCGACGAAAGCGCAGCATTTGATGTATTTTTAAAATACATGGACA
ACAATGAATTTGATGTAGTTGTATTTGATACTGCACCAACAGGACACACCTTAAGATTCTTAGGC
CTTCCAGAACTCATGGACAAATACATGACCAAAATGATCAAGTTCAAAAAACAGATGAGCGGTTT
TATGAACATGATGAAAAAAGTCATGCCATTTGGCGGTAAAGGCGAAGATGTAGATTACGATAAA
GCCTTAGAAGAAATGGAAGAAATGAAAGCAAGAATTACAAAAGCAAGAGGAATCTTGGCAAACC

CTGAAAGAACAGCATTCAGACTCGTTGTAATTCCTGAAGAAATGAGTATTTTGGAAAGTGAAAG
AGCAATGGAATCTTTGAACAAGTTTAAAATACCTGTTGATTCAGTTGTTGTAAACCAGATTATTC
CTGAAGATGTAGAATGTGACTTCTGTAGGGCAAGAAGAAGCTTACAGGAAAAAAGACTTGAACT
TGTAAAAGAAAAATTCGGCGACAAAGTTATTGCAAATCTTGAATTATTAAGAACCGAGGCAAAA
GGACTCGATGTTTTAAAAGATATTGCACACAAACTCTACGGAGAAGCTAAATCCGAAGAAGAAA
AAGAAGTAATTGTAGCTTAATTAAATAAAATACTTTTTTTATTTTTTTTAAAATTAAAAGATTA
AATTAAAGTCCGTTAGCTCTATCGAGGACTATATCTATAATTCTATCATCTGCACCCATCGGTTT
TCTGTACACTAATTCAACACCTTCGGGAATTTCGACAGTTTCTGTGTCATGATGGTGGTGATGGT
GTTCGTGGTCATGATGGTGGTGATGTCCTCCTTCATCGTCTCCTTCGTAAATTCCAAGTATTTTT
GGGATATCTCTTTCAGTGTGGTTTCCTGGAGCTAAAAATACTGGTGTTACAATTACTTTTTAGC
ACCTGCATCAATTACCTTTTTTATCGTTTGGGGAATTGTAGGTTCGTTGAATTCCATCATACCAA
CTTCTACAATGTCGTAAATATTTCTTGCTTTTATTTTTTCAGCAACTTCCATAACAACGTTTTTT
GAGTGGGGGAGTCTGCTACCGTGGCCTACCAAAACCAATGCTTCCATAATCTGCACCTGTAATAT
TTTTTAATTCATTTTTAATATTTATGACTATTTTATATTACAGCCATATATAATTTACGTTTTAA
ATCGGAATTATTCAATAACCGCCTTTTCAAACGTGTACATGGCTAAAACTACGAGAAAAACGCTC
AAAACAATTAAAATTGAAACATCGAGCATTATTGGAAATCTTGAAAAACCTGCTAAAAAATATC
TTGCAGAATCAACTGCATATGTTAAAGGATTTATATATGCCAGAATTTTCATCCAAGTTGGCATT
GCATCTATCGGGTAAAATGCACCGCTTAAAAATATTAGGGGCTGCATTAAAAGTCCAAATATTAA
GTGAAAACCTTCTTGGCTGTTTATTTTTAATCCTAACGATATTCCAACGCTTGCAAATGCTACCG
CAAGAATGAAACTTGTAAAAATTACCGGAATCATTCCGAAGGGGTTTAAAGATACTAAAAAAAG
GCTCAGTACCATTATCAAAACGCCCTGAATGAGTGCAGTTATGGAGTCCCCCATAATTCTGCCTA
AAATTGCTTCGCTTCTTGACGTTGGGGCTACCAAAGTCTCTTTTAAAAATCCAAACTGCTTATCC
CAAATAACAGTAACACCGCTGATGAAGCTTCCGCTAAATACTGTCATTGAAACGACTCCTGGAAT
TAAATATGTCAGGTAGTCAACTCCTCCAAATATTTCTCTTGCGGCTGGAAAATCAAATGCCCTGC
TCCAGCCTATTCCAAAGAAAACTAGCCAGACGAGCGGATTTAGTAATGATCCAAGTACTCTGGAT
TTTGCACGGGTAAATCTTCTAAGCTGCCTGTAAAACATGGCTGAAAATGCGTCCATAATAATCAC
TTACTTAAATTTTACAGTTTCTGGAGTTAATGTAGTCTTAAACTGTCTGATTGTGTATATTCCAA
GTAGATATGCAATAGAAACTGCGATAACGTGTCCTAAAATCATTCCAAACCAGACACCCGTTAAT
CCCTGATTAAATATTATTCCAAATGTATACGCCATCGGTATCTGGAGGATTATCGTTCTTAGTAA
GGAGATAAATAATGAATTGTTTCCTTTTGAAACGCCCTGAAACATGGCCGCAGTAAGCATTCCAA
ATGGGAGTGTTGGGTAGAATAAGAACATATACTTTAAAAATACTAGTAAACCCTCCAAAATATG
CACGGAATTTTCAGAATATGTAAATAGATAAGTAATCTGATTTTCAAATATCAATATTAGCGCTG
CAATTATTGTTTCAACAAGAATTCCCATTTTTAAAGCATATTTGTAAGAAATTTCTAATTTTTCC
GGGTTTGCAGATCCGTATGCTGCTCCTGTTACTACTGTAACCCCTGTTGCAAGCCCTACGAGGGGG
ATTGTTCCCATAGATACAATTCTCCAGCCTGTTGAAAATACTGCAATTCCATCGTTTCCACCAGCT
TTTGCAACAATTGCAGTCAATAAAAACATTGAAAATGCCATAGTGAGGTGTGAAATAGAAGCAG
GAAGTCCTATCGAGAATATTTCTTTTATGATGTTAATATCCAGCTTGAATTTTTCAAACGAAATT
TTAAGAAAAGTATCTTTTTTAATAAAAAGCCAGTAAACGAATAAAATGCCCGTAATGGTCATAG
AAAGAAGCGTGGCCCATGCGGCACCTGCTACACCCATATTTAATACATATATAAAAATCGGGTCA
AGCACGATATTCAAAACTGAACCAAGAATTATCGCATACATTGCTCTTTTAGTATCTCCCTCCCCC
CTTAAAATCGCATTTGCAATATTGGTAAATAGTAGTACAACAGCCCCACCAAATAAAATCGTGGA
ATATTCAACTGCCATCGTAACAGTAATTCCTGATGCCCCTATAAGTGAGAAAATTGTCCCTAAAA
ACGGAATAACTCCTGTTAATAAAATTCCAATGATTACCCCTAGTACAATCGAGTGAACTGCAATA

TTATCTGCTGCATCCTTATTGTTTTGTCCAATTCTTCTTGAAATTGCGGAGCTCCCTCCAATTCCA
ACACCATTTGAAATCGCCATTATTGCGAACATGAATGGAAAGAACAGTCCAATTGCCGCAAGAGC
GTCTGAGCCGAGTCCTGCAACCCATATTGCATCGATTACGTTGTACAATGACTGAAAAGACATTG
CAATAACCATCGGCAGCGAGATTTTTACAATGGCTTTTTTCGGGTCTCCGAGCAGATTTTGAACT
CCCTTTGTCGTTTTCATTTAGAATCTCCTCCTTTTTCCCATTGGAAGCAAAGATTTTTGATTTTTA
TCTTTTTCAGACCTTATTTCCCGTCCCGTTAATTTTATAAATACATCATTTAAATTCGGCTTTTT
ATAGCTTATTTCCTGAATTTTTAAGCCGTTTTCGTGTGCAAATTCGAATATTTTTGGAATTTCTA
AATCAGCATTTTCAGTATAGTAAATTACTTTTCCGTTTTCTTTAAGTTCGCAATTTTCAAAAGGC
AGATTTTTTGGAATTTCTTCGAATTTTACCGAGATGCAGTCATTTCCAACGATTTTTTTAAGTTC
GTTTACAGTTCCTTCAGCAATGATTTTCCCGTTATCAATTATCGCAACTTTATCTGCCAAAAGTT
CTGCTTCTTCCATGTAGTGGGTGGTTAAAAATATCGTCATATTATTTTTTGCCTGCATCTTTTTA
ATGTATTCCCAGATGTGCGACCTAGTTTGAGGGTCAAGGCCGATTGTTGGTTCATCTAAAAATAA
GATTTCGGGTTCGTGAATCAAAGATCTTGCAATTTCAAGTCTTCGAACCATTCCTCCACTAAAAT
TTCTTACAATTTTATCTTTAAATTCCAGTAGTTCTACAAAGTCTAGCAATTCATCAATTCTATTT
TTTAAAATTTCTCCTTTGTATCCATATATTTTTCCGTGGATGTAGAGATTTTCATAAGCAGTTAA
CTGGTTATCAAGGGTAGAATCCTGAAAAACAATTCCTATGGTTTTTCTAACTTTTTTAGGGTCTT
TAGATGCATCAAAACCTGAAATTACTGCACTGCCCGACGTTGCTTTAAGAAGAGTTGTAAGTATA
TTTATTGTGGTACTTTTCCCGGCCCCATTTGGTCCTAAAAATGCAAAAATTTCTCCTTTATTAAC
ACTAAACGAGACGTTATTTACTGCCACATTTGTATCAAATTTTTTTACCAGATTTTTTACCTCAA
TTGCATGCATGTTAATCATCCCCATATTCGATCTGATAGCGAATATCTTTTGCACATTTTTGCAT
GATTTCCCCAATTTTCTTTCTTTTTTCATCGTCCATGTCGATAAATTCTTCAAACGCTAAATGCAA
TGCTTTTTTAAGTTCAATTCCGCCAAAGTTATGGAAATGATAAAGTGCTTCGGATTTTTTCATGC
AGTACCCTACTTTAGCCTGGTTTTTGTCTAAAAATGAGTGGCCGTCTTTAGTTAATTTATAAATT
TTTTTACCTCTATCTTCCGTTTTCTCAACTTCAATAAGTCCTGCAGTTTTTAAATTAGACAATAT
TGGATAAACCATGCTTGGACTAACTGAAATTCCCGTAATTTCCTTAATATTTGAAATCAAAAGGT
AACCATGGAGGTTTTCTTTTTCAAGAAATTTTAATATCAATATTTTTATGAGTTCTTTTACTTTT
TTACCATGCTTCATTATATCACTAAATATATCTTAAGATATATTTTAAAACACACGATATAAAAA
GTTAAGTACTGTCGAAACATTTCTATTTATAACGTTGATAATTACAATCGGTGAAGAAAAAATG
GAACTTACTTCAACAGCAGTACTGTTGTTTTTTTTAATATTTTGGACAGTTCTGTATATTATAAA
TCACAATAAAGATAAATTAGGAATTAATCTGGATTTAAAAACTTACATGGGTATTTTTGGAATA
TTGAGAACGCAAGTAGGAATGAATATAATAAAAAAACTTGGAAAATATAAATTATGGCAAAAAC
TTGCATATCTATCGATTCCTGTATGTGTTGTAATAAGCATCTACACGTTTTACGCATTTATTCTG
TCAACTGTCGGGCTTTTTGACGGAACTGTTGAAAAAGAAGCTGCCAAACCAATTATATTTTTATT
TGGAAGTACAATTCCGTGGATTCCTGGAATTTTGGCAATAATTATTGGAGTTACTATTCACGAAC
TTTCACACGGGATTGTTGCAGCATCTTTTGGGCAAAAAATAAAGAGTTCCGGGCTTTTATTAGCT
TTGGGAATTCCGATGGGTGCATTTGTGGAGCTTGGGGACGAATTTAAAGACTCAAAACCTAAAAT
TAGGGGTGCAATTGCGGCAGCAGGCCCTATCTCAAACGTATTAGTATTTTTCCTAGTACTTTTTG
CAATGCCTTATTTTACGGGGATGAATTCAAAATTAACGATAACGGATGTTTTGGAAGATACTCCT
GCTTACGGAATAATTTTTGAGGGCGATGTAATCTATTCGATAAATGGAAAAATGGTTAATTCTTT
AAATGACTTTTACGATGCAGTGGGCGATATACAACCTGAACAATCAGTAGAACTTGTTGTTTTAA
GAAATAACGAAATAAATTCGTATTACATAACGACATCAGAAGAAGGAAAAATGGGAATTGTTTC
AGAACCTTCAAAAACGGTAATGTATATTTTACAAACACTCTACTGGACTTCACTATTAAACATGC
TTTTAGGATTCTTCAATTTACTTCCAGCAGCCCCTCTGGATGGCTACCACATCTGGATGGCTCTGC

CTGATGCAATAAGAGACTTTAGAAAGAATAACTGGTTTGTTTCAAAAATTGCAAATCTTGTTGG
ATGGGTAATTAGTGAAAGGAATTTAAACTCGATAAGTATATTTGTATGGCTGGCAATACTAGCG
TCAATGATTTATTCATTTATTTAAACGTTAATTTTCAGGTGGATGATGATAACAATTGCTTGTGC
AGAAGGGGGGTCAACAATATACTCCCTAAAAAAAGCTATTGAAGATTTGGGCGAAAAATGTAAT
ATTTTATTACTTTCTAGCGATAATTTGTTAGTTGATACGGATTTCAATATAGAAACTGATTTGAT
ACACTCAAGATGCGGTATCGGGGATTATTTAGATAGGCTGACCCTTTTTTCATGGCAGGTTTTAA
AAAATCTGGAATCTGAAGGCCACTATTTCATAAATCCACTCGAAACAATTTATAATTCATCGGAT
AAATTTAAAACAACGAAAATTCTTAGTAAAAACGGGTTAAAAACGCCAAAAACTGCACTTATTA
GGGATTATGCGGATGCAAAACATTTTTTAGATACTAAAAATATGAACTACCCAGTTATATTGAA
AAATTCATTTTCAAAATGCGGAATGAAAGTACAAAAAGCAAGTTCTGACGATGAATTAAAAAAA
CTTTCCAAAAATTCGATATGGGAAAGCAAACTAATTCAAGAATACGTTGATTTTAAAAATGGGG
ACACTTATAAGGATATGAGAATACTCGTAATTGATGGGGAAGTTGTTGGCGGGTATAGGCGAGT
AAGTAATAACTTCATAACGAACCTATATGTTGGCGGTCAAATCGAGCCTTTAAATGTATCCTCAG
AACTTGAAGAAATCGCTTTAAAGTGTTCTGAGTGCATGAATGGATATATTATGGGAATTGACATC
TTACCAAAAGATGGAGAGTACTACGTTGTTGAGGTAAACACTGCACCCGGTACAAAAGGATTTAG
GTCCCTTGGAATTGATGTTGATAAAAGAATTGCAGAATGCCTTGTAAAATACAAAAAAAGCTAA
AAGATGATAAAAGTGTTTGAATCTCTTTTAATTTCTTTTTCATTCGTTTTACTTTCCTTAATATT
AATTTTTAAGGAAAAACTCGGGATCGGAAAAGAAATATTTATTGCAGAAATCATGGCACTAATC
CAGCTTGTTGTTCTTGGTTACGTGATTGGAATAGTATTTAACCTTGGAATTTATTATGCCAGTAT
TATGATACTGTTCATGGTGTCAGTTTCAGCATTTATGGTTAAAAGAAATGTAACCAAAGAAAAA
AATAGAAAAATAGTATGCGCTGCATTTTTATCAACGATTACAATAACAATAATTTCTCTGATCAT
TTTAACAGTATCAAAAACGGTATTATTCGAAGTACGCTATTTAATCCCTTTGATGGGTATGGTTA
TTGGTAATTCTACAAATGCAATGTCTATCGGGTTAGAGAGATTCTTAAATGATTTAAAATCTGAA
AAAGACGTACTCTGGGGCTATCTTGCACTAGGGGCAACCGAAAAACAGGCGGTGAATACGTTTGT
TAAAAAAGCAGTCCGTGCGGCATTAACGCCCCATTTAAACTCCACAAAAGCAGTAGGGCTCATAT
TTATTCCAGGAGCAATGGTTGGAATGCTTCTTGCAGGCGTGGACCCTTTAGAAGCTGCAAAGGTT
CAGATAACCATAATGTGGATGATAATGGCATCTAACATATTTTCTGTAACGATTGCATGCTACCT
ACTCTACAAGGAATTTATTCACCAGATTTCTTCTTAAATTTAACGAACTTTTCTAAACAATATTA
CGAAGAATACTGAAGAAACGGCCGCTAAAATGACTGCAACGAGCCTGCATTCGTAAGGCGTTGTT
GGAAGTGCGAAAATAACTATTGCAGTTACTATCAACGCAATAATACTTTTGTTAACGCTTTTAAA
TACAAAAAAAACAAACATTGAAACTATTCCGCCTATAAACATCGGGTAAAACATCCCGCAAAGGT
CGTAAACTTGATTAAATAATAAATCAGATAAGCTATTCACTATTGTAAAAAGATCTATGTTCAA
AACTATCCCCCCAGATAATTCCCAATAGAATATTAATCTAATAATTTAAAAATGTAACTAAAACA
ACTTTCTGGTGACTAAATGTTAATTGTTGATATTAACCACGGTGCTTTAGATTTGGCATTAGAAT
ATGAAAAATCAGGGAAAGTTCCAGTAATTTGGGATATTTACGGTAAATTGGAACGAGACAAAAA
TTTTAAAGAAGAAAATAAAAATATTTTATCAAAATTTAAAATAATTTCAAAAAAAGAAACACCT
GAGTTTTCAAATTATAATGAAGTTATTGCACCAGTTCACTGTCCAATAGACGTTAAATTTAAAAC
TTTTCACGAAGCAGTTTCTGAAATTATATCAAAAAAGTACCCGAAAACCGTTGAAAAAATGATAA
CTGTAACGGGTGTTAAGGGAAAAACTACAACTACTGAACTTTTAAAACATATTTTATCCGAAAAA
TATAGCGTTTTCTGCCATAACAGTAGCGATGGTTCAATTACGCCAGTAACTGTTTTAAATATATT
AAATAAACTGAATGAAGAAAAAAAACTTCAAATTTACGATTTTTTGATATTTGAAATTTCTTTA
GGAATAGTTTCTTCAAATTACTCTATATTAACAAATATTTTGGAAAACTACTCAATTGCAGGCGG
TAGAAGAGAAGCCTCTATCAAGATAGATTCTCTAAAAAATTCAAAAATCGCATTTGTGAACGAA

GATCTTAAAAACAGATTTAAAATATTCCACAAAAATTTAAAAATAGTTAAAAATGCAAAAATAA
TTTCAAAATACCCTTTAAAATTCAATTATTTGAAAAATGATTTCGAATTCAACGAATCTGTTTTT
GGAAGCCACTTTATCGAAAATTCAGTTTTTGCAATAGAACTATGTTCTTATTTTATGGGCTTTGA
GGAAATAAAAAACAGTTTAAAAAGTTTTGAGATTAAAAACCGAATGAATGTAGAAAAGAAAAAC
AATTACGCAATTGTTAAAAATATTAATCCGGGACTCGACTTAAAAGCAATAGATTACGCAATTTT
TGATTTTTTAAACTTATTTGAAACCGGAACAATAATTATTGGCGGGGATTTGGGATGCACCTGTG
AAGAAATAGATATTAAAAAGCTTGCAGACCTGATAAAAAAATATGATAGAGATGGCGTTAAGTT
CCTGCTTTCTGGAAGTGTCGGAAAGAATTTAAAAGAGATTATTAATTTAGAATTCGTAGATATT
AACAATTATGCTGTTGAAGAAAATACGCTTGTGATATACCGTTCAAAATTGTGTTAATTATCAAG
TTGGGCGGCAGTATAATCGTATCTATATGTTCCGCGTACTGGTTCGACTTTTTTGTCAACCAAGAC
CACAAAATATTAATTTTTGAAGTTCGGAATGGAATTCTGTATCGTTTGGGCTTTTTTTAACAAGT
CCTGCAAGTTTTGTAACTCCCTGAACGGTTCCCTGTTCATTTAATACGTTTAAAATATTATTGTC
AATAATTTCATCGCTAATGAAGTCAAATAAAAATTTATTCTTATCTTTTACAATTTCAGAAGATT
TTTTTGCAGTTTCGGGTTCAGAATAAACCATTACACTCCAAGATTTCTTGTAAGCATCTTTTAAA
CTTTCAGCAAGGGGCGAAATTTCATCAAGTCTCTGTTTTTTATCCCCCATTATGACAGTAATTCG
GTGTTCTGCCATTTTAGGGGGTTTTGGAATATCTAAAAAAATTCTCGATCCAAAAAAGTCACTCA
TCTCGTTTTCGATGATACCCAGTTCATTCTCAGATAAATTTATCAAATTCCATCTTTCTTTTGGG
GATAATTCATTGTATCGCTGGACTGAAATACTTTTAAATAAGTCTCGTGCATCTATTTTCCTCAT
AATCTCATTTGAGGAACCTTCAGACCTTCTTAATGTACATATTAAATCAATATCATCCATTAAAG
ATAAATCTCTTAATTTAAAGACGTCGTCTTTAATTGCGTCTATTGTTGCATTTTTAATCATGGTT
TCCGAGATTCTTGAGGTGGGGTGCATATAAACAGTTGGATACATCTGATATCTTGCAGTAAGGAG
TGATTCAACAGTAGTTCTTCCTTTTTCGATAATTCCAAGTTTATTTGTATCTTCAATAACCACAA
TACTTCTGATAAGTCTGGGGATATCTATTGAACCGTACGCAACTCCGGTGTGGTGACTGTCCCTT
ACTAAATAGTCCATTCTGTCAGCATCGACATCTCCGTGTATTAAAGATCCTTCCAGGCCTTTTGA
AGAATACACATCTAAAACTTCTTTTGAAGTGTAATTCTCAAAATTCATTCTTTTTATTTTTTCTT
TCGTAAATTCTTCATGGTCATATCCTGCAACTTCTAAAGTATGTGAAAAAGGAGGGTGACCGATA
TCGTGGAGTAATGCAACGATTTTAGTTAAATTTTTGTCAATATTTTCTAAATTTTTGGCAATTTC
GCCTGCAACGTGCATTGTACCTATAGAATGCTCAAAACGGGTATGATTTGCACTGGGGTATACCA
AGCAAGTAAGTCCGGTTTGCTTGATATTTCTCAATCTCTGAATTTCGGGCATATCAACTATTGAT
ATCTCTTCTTCGTTAAGTTTGATATCTTTATGGATTGGATCCCGTATTATTTTGAGTTTTGACAT
GCTAAAAAAATATCTTTAAACAAATATATAAATTTATATGGATATATTGGGAGATTAGTTAATTT
ATTGTTTAATTTTGGAGTTAGCATAAGTATAATATACTAAATTTGTATATTACGTTATTACTAAT
TCAGGTGGCAAAAATGGAATTTAAAAATAACTCTTATTTTGTAGATAATGTCTCAGAAAGCATT
TCAATTTTATCATTACTCAAAGAATACGAAGAAAGATTATCTGGATTTGAAAAAGATTCTTTCAA
AGTAAAAGAACCATATGTTTACGTAAAATTTTGTTTATATACAACATTACTTTTTAGAATACTTG
AAAAAGAAATTTCAAAAATAAATCTTTCAGAAGACGAAGAAAAAACAGTAAATATTCTTAAAAA
ATATAAATATCGTGATTTCGAGGCTCCTTACGAAGAAAATTATATTAAATTTACAGTATGGAAA
AACGAATCTGGAACATTAGTTTACCAGTTATGTGATTTACGAGAGAATGAGTCATCAAGTGAAA
ATTGGAATAAGATTTATTCAGTTTATATGATACACCCAAAATACTTTAAACACATTAAAAAAAT
TGTTTTAAAACTAATAAATGAAAATTAAAATGATATTAAATCATTTTTTTATTTATCCGTGGTA
TCCGGTATTTTCAGGGCCTTTTTCAATCTCGGATACGCCTGCGTTTTGACCGTATTCTTTTGCGAG
GTCTTTGTATTCATCCATATCATCATCTTTTACAGAAGGTGCAATTCTTTTAAACGCACCGTCAA
AGTGTCTCGATTCGACATGTTCTGAATTTATATTTTCCCTTAATGCAATCATTGCAGCTTCTCTA

CAAACTGCTTCTATATCTGCACCAGTATATCCTTTAGTTTCTTTTGCAAGTTTTTCAAGGTTCAC
GTCTTTTCCAATAGGCATTCCTTTCGTGTGAACTTTAAATATCTCAAGTCTTGCTGTTTCATTAG
GAATAGGAACTAATACAATTCTATCAAGTCTTCCGGGCCTTAAAAGTGCCTGATCTAATATGTCT
GGCCTGTTTGTTGCAGCAATTATCACTACATCTTTAGGTTCTTCAAGACCATCAAGTTCCGTTAA
AAGCTGATTTACAACTTTTTCAGTAACGCCACTGCTTCCAAAGTCCATTCCTCTTTTTGGAGCGAC
TGAATCGATTTCATCAAAGAAAATAACTGTTGGAGCAGCCTGTCTTGCTTTTCTAAATATTTCAC
GGATTGCTTTTTCGCTTTCCCCGACCCATTTGCTGAATATTTCTGGGCCTTTGACGCTGATGAAAT
TTGCTTCACTTTCATTTGCAACAGCTTTTGCAAGCAATGTTTTTCCAGTTCCTGGAGGACCAAAG
AGTAAAACTCCTTTTGGGGGCCTTATTCCCATTCTTTCAAACATTTCTTTATTTTTAATTGGCCA
TTCAACAGCTTCTTTTAAATCCTGTTTAATGTCTTCAAGCCCTCCAACATCACTCCATTTGATATT
TGGAACTTCTACAAGTACTTCTCTCAATGCAGAAGGTTCTACTTCTTTCAAACCGCCCACAAAGT
CTTCTTTAGTTACTTTAATTTTATCCAGTACTTCTCGAGGAATTTCTTCTTTTTCAAGGTCGATG
TCAGGCAGGAATCTTCTTAAAGTTTTCATTGCAGCTTCTTTTGAAAGAGCAGCTAAATCAGCACC
AGCAAAACCGTGTGTTTTGTCTGCAAGTTCTTTTACCATTGATTGATTTAATTTTACTTTTACTT
TGTCTTCCACTTCGCCGTCTTTTAATATTTTTTCGATTTCTTCTTCTGATGACGCTTTTTCGACTA
ATTTGACAAGGCTTTCGATTTTGCTCCTATCATATTCTCCAACAAGTTCATTTAATATTGAAATT
ACATCAGATTTTTCATAATCTGGTTGAAGTGGCATATTTCTTGTATGTATCTGCAAAATCTCGTT
TCTACCGTGTCTATCGGGAATTCCAATCGTAATTTCCCTGTCAAGTCTTCCAGGTCTTCTCAAAGC
CATATCGATTGAATCAGGTCTGTTTGTAGCTGCGAGAATTACGAGCTGACCTCTACTTTCAAGGC
CATCCATCAATGTTAAAAGCTGGGCAACCATTCTTCTTTCAACTTCGCCGCTTGCTTCGTCCCTTT
TAGGAGCAACTGCATCGATTTCATCGATAAATATAATTGAAGGCGAATTTTCTTCAGCTTCTTCA
AATATTTTCCTTAAATTTTCTTCGGTTTCACCGACATATTTACTCATTATTTCAGGGCCGTTTAT
CGTGTAGAAGTTAGCTCCGGCTTCATTTGCAACAGCTTTTGCAAGCAGAGTTTTTCCAGTTCCAG
GAGGTCCTGCAAGTAATACTCCTTTTGGCGGTTCAATACCAAGTTTATCAAATAATTCAGGGTAT
CTCATTGGAAGTTCTACCATTTCCCTGATTTTTTTGACTTCTTCTTTTAAGCCCCCGATATCTTCG
TAAGTTACGTTTGGAACTTTGGTTTCCTTTAATTCTGTAACTGGTTCGGTTTTTAATTCAATTTG
TGTATATTCGTTAATTTTCACTGGTCCTTTTGGAGTAGTTCCAGTTACAATGAATGGAAATGCGG
TTCCTAAAACCCCAATTACAACTTTAGAACCTTTATCTACGACCTGTTCTACAAGTCTGCTTCCAA
CATAACTTTCAAATCCTGTACTGAATCTTACAGCCTGCATTGGTGCGAGTGTTATTTTTTTAGCT
TCTTTTACTTCAACGACGGTAATTTTTACTTTATCCCCAATACCGGCTTTAGTATTCTGTCTTAA
AATACCGTCCATTCTTATGATACCTTTTCCCTGATCTTCGAGGTATCCTCTCCAGACGGTTGCGTA
AGTTTTTTCTTTTCCTGCAATTTCTATGGCATCTCCAGCTTTTAAAGAGAGTTTTTCCATCGTTA
GCGGATCAATTCTTACAATTCCCTTGCCTACATCTCCCTGGTAGGCTTCTGCGACCATTAAATCTA
CCATAAATCCACCTCACAAAGTATAATGCAATAAACATTTAAATTTAATATTTGACTTAGTATTC
ATTTGAAGTTCTATATATATCTTTCTGTTATCAGGTATTACTTAACCGTATTTATTTTCCTCCAA
CAGGGTCGTGAATGTGGTATCGGTAGTATCTTGTAAATCCACAGTGTCTGCACCGAACATATATC
CCATTTTCAGTGATTAATATGTCTTGCGGACCAAAATGGCCACATCTAGCACAGTCAATGTCTTT
TTGAAGTACCCAGTTATCGTATTTTCTTTTAACGCCTACTTCTTCTTTTAAACTATTTTCATCGTC
AATATCTGCTTTTTTAATAACATAATATCTTGTAGCATTGCAATTACTGCATTTGACTAACGCTT
GATTTGGATAAATTTCAATAATTTGATCTGCAATTTTTCCGCAACTGTAGCATTTTGTAGTGTGG
TTTAATTTCCAGTTTTCCATCAAATCCCCCTTTTAGTTATAAATTTAAAATATGTGGGCGGAATT
ATTTATAAATTTCTGAAAAAAGTTAAAATTTCAAATTAATGTATCGAAAAATGATTAAAATGAG
TATTGGTATAAAAAATGGGTTTTTAATTATTTATTGAAATAGTCAACCGCATTTTTGAATATTTG

AAGGTTGCTTTCAATAAATTCTGGAATAATATCGCCATTTCGTTTAGCAATTTCTTTTTTAAGTT
GATATTCTGGTTCACTTATTGAATAAAGTGACCTTTCAGGATGAGGCATTAAACCAAATATTCTA
CCGGTTTTATCACATATTCCTGCGATATCTTGATATGCGCCATTTGGATTTAATGGGTACTCTCC
ATTAGCAGGATTTCCTTCAGAATCGCAGTAACTAAATACAATCTGCTTATTTTCTTTTAATTCAT
GGTAGGTTTTTTCATCGCAGTAGAATCTACCTTCACCATGGGCAATCGGAACGTGTGTTACGTTG
ATTCCTTTTGTAAATACACAAATGGACTCGTTTTCTTTAATGTGCACCCATCTGCATTCGTATCG
GTTGTTTGTGTTTGCTTCAAGTGCGCTAATTCTTTCCCCGTAATCTGTGGAAGGAAGTGCAAATA
ATCCCAAATTGGTCATTACCTGAAAGCCATTACATATTCCTAAAATCAATTTTCCAGAATTAATA
AATTCTTTTAAATCGTCAAATAAGTTGTTTTTAATTTTATTTGCAAATGCGTTACCTGATCCAGT
ATCGTCCCCGTATGAAAACCCTCCTGGAAACATGATTATTTCATAATCTGCCATTTTCTTTTTTCC
GGCGATTAAATCGTTAATGTGGACAATGTCCGTTTCTGCACCAGCTTTTTGGAATGCGTGGGCGG
TTTCTGTTTCACAATTTATGCCGTATCCAGACATTACAAGTACTTTTGGAACCACTTTTATCACC
TAGAAATCCTCAAATCTACTTCTGTACACTTCGTCCATGTTTTCAAGGCTTGAAGTTATCACAAT
ATCTTTATTATTCTTAATTACGAAGTCTTTTTCGGTAACTTTACCTATTTTTCCTATTTTGAATT
CGTTAATTAAATTTTCGAATTCTTTTTCATTTTCAGGAGCAATAGTTACTATGAATCTTGACTGT
GTTTCCGAATACATGATTTTTTCAAAAGAAATGTTTTCATTTGGAGCTTTTGATAAATCAGCTTC
TAATCCAAGGCAACCTGAAATCGCCATTTTTGCAAGTGTAATTACAAGTCCTCCAGCAGCAACTG
ATGCGCAAGAGCTAAATATTCCTTTCTGGCTTAATTCGTACATTTTTTCATACAATTTTCTAGCC
GATTTTGCATCGACTTTTGGAGATTTTCCGCCAACTTCGCCAAATGCATGGTAGAATTCACTTCC
ACCGCATTCGTCTTTAGTTTCTCCGATAACGTAAATTAAATCGTCTTTCAATTTTAAATCCATTG
AATTTGTATGTTCTATATTATCAATTACACCAAGAGCTGACATCATCAGGGTTGGATGCACTGAA
ATTTTGATTGGATTGTTATTTTCGTCAAATCCTTTGAAATCGTTAAACATACTATCTTTTCCTGA
AATCAATGGAGTAAGATATGTTTTCGCGTAATCGTAGCATGATTTTGCAGCATCTTTTAATTGGT
CCAATCTTTCAGGTTCATCTGAGCTGCACCAGCAGAAATTGTCCATTATCGCGATGTGGTTTATA
TTTCCACCAACTGCAACACAGTTTCTAATTGCAGTATCCATGGCACATGCAGCCATGTCGTAGCA
GCTAATTTCGCTTAATCTTGGGAACAGAGACTGGCTCATTACAAGCCCTTTTTGACTACTCAAAA
GAGGCCTTATAACTGAAGCAGTTCCATTTACTCTTCCTTTTCCCTGAAGTGGTTTTATTACTGAA
TTATTCTGTACTTCGTGGTCGTACTGTTTTGAAATGTACTCAAAGCTACAGTTGTTATGGTTTCC
AAGTATTTTTTCAAAAGTTTCCAACTGGTTTTCAACAGTAATTTCTGGTTCTTCAGTCCTGTAGG
AATATGGTTTTGTTATTAATTTTCTTTTTGGAAGTCCGTTGTGTAAAAAGTCCATATCTAAATCC
ATTATGGTCTTTCCGTTGTAGTTTACAACTGCTCTATCGGTACTGTTAAATTTACCAATTACAAC
AGCTTCAACGCCCCTTATTTTCATTAAATCCATGAATTCTTCCAATTTATCGTCTGGAACGGATA
GTGTAACTCTTTCTTGTGATTCACTTATCCAGATTTCCCAAGGTTCAAGTCCAGGGTATTTTAAA
GGAACTTCTTCAAGTTCTACAACGAAACCGCCGCATTCTTCTGCCATTTCTGCAACTGAACAGGA
AATTCCTCCTGCGCCGTTGTCGGTAATACTGTTGTAGAGTCCTTTATCTCTTGCTTCCTTGATCAC
TGCATCAGAGAGTTTTTTCTGGGTAATTGGGTCTCCAATCTGAACTGCGGCTGCAGGGCTTCCTT
CATCCATTGCTTCTGATGAGAAAGTTGCACCGTGAATTCCATCTTTTCCAACTCTTCCACCGATAA
TTACAATGTTATCTCCATTTTCAGCCGCTTTTTCATGGCTTAATCTTCCGTCTGGAAGTTTTCTTG
GAATTAAACCGATAGTTCCAACGAATACGAGAGGTTTTCCTTTGTAAGTATCATCAAAGTAGACA
AATCCGTTAGGTGTAGGAATTCCAGACTGGTTTCCACCAACATTTACACCATGAACGATTCCATC
GAGTATTCTTCTTGGTAGAAGTGATGGGTTTGTTTTATTTTGTCCCCTGTATATTATTTCTGTAT
CTTCTGGTCTTCCAACACAGAATCCGTATTTATTTGCAACAGGAAGTGCTCCAAGACCAAATCCT
ACGGTATCCCGGTTAACTCCAACGATTCCCGTAATCGCACCACCAAATGGGTCGAGTGCTGAAGG

GCTGTTGTGGGTTTCTGCTTTGTCAGTTACCATCCATTCATCGTCAAAGATGATTCCACCGGAGT
TATCAGAAAATACTGAAATACAGAAGTCTTTATCACCGAGTTCTTCCCTTATTTCATTTGTAGCA
TTTCTTATGTACTTTTTAAAGATTCCTTCTTCGATATCGTCGATTTGGGATGCAAAAATAGTGTG
TTTACAGTGTTCAGACCATGTTTGAGCGAGTGCTTCTAATTCGATATCTGTTGGATTTCTACCTT
CTTTTTGGTAGTATTCTTTTATTGCGGTAATGTAATCAAGATCGAGTGCAAGAGGACCTCTTCTT
TCGCCAGTTTCTGGGTCTAATATTCCTTTTTTACCAATTTCGATTAATTCATCGTCGCTGACATTT
AGGCTGATTTCAATAACCTTTGGTTCAGACGTTAAATTTACTTTTGGGATAATGTAGTCCATTCC
GCAGTCTTTAACGTAATTATCATAGGTTTTTATGTGATATCTCTCGATTAAAATGTTGATAAGTT
CTTCTGCAATTTTTTCAACGTCGTGTTTCGCTAAATTTCCGCTTAAATATACTACTTTTGATGAA
AATACCTTGCTGAGCCCAATATCGATTTTTAAAAGATCGTTAATGATTTCAGAGGTCGTGTTTGC
AACGTTATCAGTAACTCCTGGTAAAAATCCAAGTTCAAGTGCCCAGTCGAATTTATTGTTTGACG
GAAGATTTATTCTTGGTATTTCGGTTACAGGATTGTGGAGTGAATCTGCAATTTTTTCAACATCC
GCAATTGAAAAATCGTAGTTTATCGTGTAGACCTTGCTTACATCTACTGATACATCGTATCCAAG
TTCGAATAGCTTTTTTTCGATTACCTTTGCTTCAGAACTTTTTTCTTTTGTATCTATCTCAATTCT
GTATATTTTTTCGTTGTTTTCAGCATTAATCGTTTGCATGAATACATCCCTCGTAGTAAATTAAG
GATAATAAGAATAATCTAATGTATAAATTTTTTCTGTCGATAACAAGAATAGAATATAATAAAA
CTAGATGAATTCAAGGTTTAAATAAATAACATAAATCCTGAATTTTTAAAATCTTTTAAAATTA
AGAATTAGGAAAAAAGTAATTTATTTTCTTGCAGCTCCTGCATCTTCAAAGCCCTGTCTTAAACC
TTTACCTGCACTTTTTTGCAAAAATTCCGGTTTAAAAAGCATGTAGTACACATTTTCAGCATGTT
CTTCAGCTCTTCTTTTTGCAAGCCAGTCTAATTCTTTTCCATTCACTGCTTCGTCTTCGTGAACAA
AAACTTCAATGATGTGTTTATTGGTCATCAACTGGGCCATCATCAAACCTTGAGAAGCTTCATGG
GCGCAGACTTTGTCTTTGTCTTTTCCGCCAGGCATTCCAAGTGCCATTGTAATTTCACAACCCTGT
TCTTCCATCAATTTTTTACATGCAACAGGAAGATCTTTCATTCCTGGAACCGTGTACCTTATTAT
TTTGATATTGGAAGTCATTTCGTTTAGTTTTTTAATTGCAGCAGATGCCATGTCGACCCGTGCAA
ATGTTGTATCTGCAATACCTACTTTGACTGTCATGATTATCCCCATACAAATTGAATCTACAAAG
TTATAGCTTATTTAAACTATAAAAAACATGTCACAAACCCAAATTAAAATCAATTTTAAAAAAG
TAAACTTAAAAAAATTAATAATTTAAAAAAACTCAAAAAAGCTTATTTTTTAGCCTTTTTAGTT
CTTCTACCTTTTTTGGCGTTATTTTCCCGTTCCTCAATTTTTGAAATTGCATCGTGGTAAAATTCT
TTATATTCGCCTGCTTTACTAAAAAGTTCTCTAATTTTAGTTTTATTCTTTTTAGCCTGCACAGT
ATGCTTCATTTTAAAATGCATGGCTCCAGATCTTATATTTGATGGTAAAATAAATTCCATATTCC
CCCTCTGGTTTTAGTTAACAGTAATAAATTTATTTTTAGGGGTATTTATCTATATCCTTGAATTA
ATAAATACGCTAAGTATATCAAAGAAAATCGACGAAAAAAAGCAATTGATGGATAAATTTTTAA
AAAAGAAGATAAATTCGAGTGAATTCAAAAAATAAATTATTCTTTTGAGAATTCTTTGAGGTAG
TATTCAATTAATTCTTGACCATTGATTCTAACAAGGTCGTTTTCTTCAGCGTACTTTTTAACCAG
ATCTTTTGACATTGCTTTTCCATTATCGCCCATCATTTCACAAATGGTCGTAATAGGGGTGAGTC
CAGCCATTTCTGCAAGTGCAACGGTCATTTCTGTGTGGCCTTGTCTATTTTTAACGAGTCCATCTG
TAGCTCTTAAAAATGCAACGTGTCCAGGGCATCTGAATTCTTTTCCAAAGTCTTTAAATCTTTCT
TCGCTGCAGAGTTCTGTTATTTTTTTAACAGTTAATGCTCTGTCGTTATCTGTAATTCCCGTAAA
TGTTGTTCTGTGGTTTGCATAAAGTGCAAATGTTGATTTTTCATCATAAGGAATATCGTTTGGAT
ATAATTCTTTTAAAACAGGGTATTTTTCAGACGCAACATCTAAAATGTCAACCATAAATGGAATA
CCCAATTTATCACAGAAATCAGGGTGAAGACAGGTGCAGATCAATCCTCCTGCATTTTTCCTCAT
TTCTCTGATATATTCTGGAGTAATGAATTCAGATGCAACGACCATGTCGGTTTCTCCTTCTCGAT
CATCGCTATCGTAAAGTAATACCATTTTTCCGCTTTTTAGGGCATCAATTGCTTTTTCAACGTTA

CTGATTTTTTCATCTCCCTGTATTTTTACTTCTGAGTTTCTCGCATTTTTTGAATCTGTTGTCACA
TTATCACACTATTATTTTTAAGTTTTTACCAATCTGTTTTTAAGACTATATATCTGTTAGGATAC
GGATTTAGGATTTAAAAATAAAAATAAAAAAAGAGCAGAATTAACTTAATTATTTTAAATTACG
ATTTTTACAACATCTGAATTATTTAATGATAAAAATTTTCGAAGTTGAATGGGTGCAATAATTTC
TAAGGTTTTTTTAGAATGATCGGTCTTTTTTGGGGCAACAACTGCACAATTTATAAAATTGCCGT
GTTTATTGAATAGTTTTATTAAAAGTACCTTTCCACCAAAGTATTTTTTCCCCTCGATTTCAAAT
CCGTCGAATTCTATTGGATTAAATTCATCTAAATTGAAATTATGCTTTAATTTTACGTTCAATGT
TCCTTCAAACGGAGTAAATCCTGTTAATTCTTCAAATTTATTTTTGTAAGGGGTTAATCCAACAA
AAAACTTACCTTCCCCAAGTCCGCTTACAACCTGCCCAAAAATTTCCAAAAGTGTCACCGTCACTA
TATATAATTTGAAACCGTTAGTATTTATTCAAAAATATTATCTAACAATTTTATTTTATCGCATA
AATATTTGGCACATTCGGGTGATTATTTGATTGGAATTATCGGCGGAACAGGAATTTCTTCAATA
TTAAACAAGGGCGAAGAAAGAATTATTGATACAAAATACGGAAAATCTAAAGTTTTAATTGACA
AAGAAAGTGATGTTGTATTACTCTTTAGACACGGGGCTGAACACAATACCCCTCCGCACAAAATA
AACTATCGTGCAAATATCTGCGCTTTAAAAACGCTCGGTGTTGAAAGAATATTGGCATTAAGTTC
AGTTGGATCCCTTCGCGAAGATGTAGTTCCAGGAGACTTTTTAATTCCAAACGACTTTTTAGAAT
TTACAAAAGCAAGAAAAAGCACGTTTTATGACGGTGAAGATGGAAAAGTTGTTCACGTTGATGT
AACTGAACCGTACTGTTCAGAATTAAAAGAAGTTACAAAAGAAATTCTCAAAAAAAGAGATTAC
AAGTTTAACGAAGGAGTTTACGTATGTACTGAAGGCCCAAGATTTGAAACAAAAACAGAAATTC
AAATTTATAAAAATTGGGGCCACGTTGTTGGAATGACGGCGTACCCCGAAGTAGTTTTGGCAAGG
GAAATGGAAATGTGCTACACTTCAATCTGCAATGTTTCAAATTATGCGGCAGGAATTTCTAAAAA
TGTTTTAACGGTTGATGAAGTTTTAGAAACCCTAAAAGAAATGGAAGAAAAGATACTAAATGTT
GTTGACGACTTTATTAATTATGAATTTAAGGAAAGAACATGCTTTTGTAAATCCGCGCTTGAAAA
CGCGGTAATGTAAAAAAAGGGATATTTATGAAAACTTTCGAGATTTCAGAAAGTAATCTCAAAA
ATTGTGAAAAAGAGATAGAAACCGCAAAGAAAATGATATTGGGAGGAAAAATTATTCTTTGTGG
GACTGATACTCTTTACGGCCTTTCTACAAATGCATTAGACAAAAATGCAATTGAAAAAATCTATT
CAATAAAAGAAAGGGATCCAAAAAAGCCAATTTCGATAAGTCTTGGCGAAAAAAAACAGATCGA
AAACTACGTTTATGTGGACGAAACCGCAAAAAAAATAATCGAAAAATTTATGCCTGGCCCAATTA
CAGTTATTTTGAAGAAAAAAGATATTATTCCAGATATTTTAGGAAAAGATGATGTAGGGGTTAG
AATTCCCGATAACGACGTTATAAGAAATATTGCAGTTGTTCCACTAACGACAACAAGTGCAAATA
TCAGTGGAAAAACTGCTCCAACGTCGCTTGAAGAAGTAGACCAAATAATAAAAGAAAAAGTTGA
TTTAATAGTTGATACCGGCCCCTGCAAATACAAAGTGCAGTCCACAATTGTAAAAGTGATTGACG
GAAAAATAGAATTGGTAAGGGAAGGCAAAATTCCATTTGAAGAGATTTTAAAAGCAGTAAATGA
ATAATTTTTTATCTATTTTTGTAAATTTTAAAATTTTAATAAAAGTTAAAAAAATTAAAAAATC
AATTATATTATCTTTTTCCAAGTTCTTCGACCATTAAAAGGGCGCAGTAGTCTCCGCAGACACTG
CAAGCTTTTTCATCTTTTGAAGGGATTTCTTCTCTCATTTTTCTTGGTTTATCGCTGTCAAGTGCA
ATTTCAAACTGTCTTTCCCAGTCGTGTTTTATTCTTGCATCAGCCATTTCTTTTTCAAGTTTCCAG
GCAACAGGATGTCCTTTTGCAACGTCTGCAGCCTGTGCAGCAATTTTTGATGCAATCAATCCTTC
TTTTACGTCATCTTCTTTCATGAGCCTTACATGTTCAGCAGGAGTTACGTAGCACAAGAAATTTG
CTCCGCTGACTGCTGCAAGGGTTCCCCCAATTGCTGCGGTGATGTGGTCGTATCCTGGTGCTAAAT
CGGTTACAATAGGGCCTAAAACGTAGAATGGCGCATTTTTACAGAGCGTTTTTTGGATTTTCATG
TTTGCTTCAATGTTGTTGTATGGAACGTGGCCGGGTCCTTCAACCATTACCTGAACTCCTTTTTCT
CTACATTTATCAACAAGTTCCCCTAAAGTAATTAATTCTTGAATTTGAGCTCTATCTGTGTTGTC
CTGTAAACATCCTGGTCTCATTCCATCTCCAAGGCTCAAAGTTACGTCATGTTCTTTTAAAAGTT

CAAGCAAGTAATCAAATTCTTTGTAAAGCGGGTTTTCCTTGTCGTGGTACATGATGTACGCGGTT
AAAAATGCACCGCCCCTGCTTACGACACCCATTTTTCTTGGATCGTTATTTAAAGCGCTGACAGTC
TGTTTTGTAATTCCACAGTGAAGGGTCATGAAATCAACACCTTCTTTTGCCTGCCTTTCAATTAC
GTTAAATATCAAATCTTCATCCATATCGATGACTCTTCCGTACTTTTCTTTTGCATCGACACCGAC
TTCGTAGATTGGAACAGTTCCGATTGGAAGATTCGTGTTTTTAATAATCTCTTTTCTAATTTCTG
GAAGATTTCCGCCGGTACTTAGATCCATAATCGCGTCAGCACCGTATTTATTTGAAATTTCAACT
TTTTTAAGTTCGCAAGCGATATCAATAAAATCTGGTGAGGTTCCAAGATTTACATTAACTTTGGT
TCTTAAATTATCGCCAATTCCAACTGGTTTCGTGTTTCTGTTTACGTTTTTGGAATTACGACGT
ATCCTTTCGCAATCAGGTTTTTGAGTGTTTCAACATCCATTCCTTCTTCTTCTGCTACGAACTTCA
TCTCTTCGGTAGTAATTCCGGATTTTGCGTTAGTCATCTGGGTCATAATATCACTTTTATAACTT
TAATTAAATAGTATACAAGCATCTGTATTAGTATGTAGTGATTATTTAAATAAATATTCGAAGT
GGTATTATGTGGGATTTAGAGACCGATAGGGTCTTAAACGAGATAAAAACAAGAAATGCGAGAA
AAGTACTATTGCAAGCACCGGAAGGGCTTAAACGAGCTGTTGAAAAGGAAATAGAATTTTTAAA
AACGAAAGTTGATGCAGAACTCATGATATGGGGCGAAACATGTTTTGGCGCGTGTGATTTGTGCG
ATGAAGAAGTTAAAATTTTAGGAACAGATTTAATTATCCATTACGGGCACGAAGAGCTTTCATAT
GTCCATTCAGAAATTCCAGTAGTATTCGTTCATGCTTATTTTAAGGAAAACGAGTATTTTTTAAA
AGATGTAGAAATTATTCTCGAAAAAATGGAAAATCCAACCGTTACAACAACAATTCAATTTAAA
AAAGCGCTTTCAAAGTTTAATCCAGTTGTAATTTTGGGATGCAGGGCTCCAGTTGAAAACGCACA
AAACGTACTTTTTGTTGGAACTGGAAGGTTTCACCCTTTAATGATGGCATATAAACTTAAAAAAA
CCGTTAAAATTTATAATCCTATTACGAGAGAGATTTCTGAAATAAGCGACCAAGAGATAAAAAA
GCTGATAAAATTAAGAATCGGGCGAGTTTCAAAGTTATTATTAAATCCTCCTAAAAAAATTGGTG
TAGTTTTATCGACAAAAAAAGGACAGTGCAGATTAAAGTCGTTTGAAAACGTAATTGAACTTTT
AAAGAAAAATAACATTGAATATATTCCAATAATTTTAAATAATCTATCTCAGAGCTATTTATTTT
ACAAAGTCGATGCGTACGTGATCTGCGCATGTCCAAGGATAGTAATGGATGACTACCAAAATTAC
GAAAGCACGTTAATTACTCCAGAAGAACTCAGAATGTATCTTTCAAATGATTTTGAATACAAATT
CGATGAAATTTTGGAAAATAATTTCTACTAAATTTTTTAATTTTAAATTCACCGATAAACGAAA
AGTATATATATTAGATCAGTATAGGTATAGATGCGAAGTAATGTGCTCATTGGAGGGATTGCCA
AGCCTGGTCAAAGGCGTCGGACTTAAGATCCGATCCGGTAGCGGTTCGAGGGTTCAAATCCCTTT
CCCTCCACTTTTTGAAATAACGTGCTCTTTTTTGGTTTCTTTCAAGACCGTGGGTTTTTAACAAG
ATTTAGCTTTTTTGCAGTATTTTACATCTAAAGGACCAGGAATTGTTCCTCAAACTATATCGGAT
TTTTACCAATGGCACCGATTTTGATGGTTTTTCACTAGTCCGATCTATCGATTTTCAAAAATCCT
GAGTCCGATCTATCGAGTCTATTTTTAAGAATTTAAAAACGGTTTTTACAGACTTATACGCTATT
TAACCGACAAATTATTTATATTTCAAACAAAAGACCTTTCATGTAGTATATTACTATCAATAACT
ACGTGAGGGGTTTTGAATGATTAGAATATGTCCAAAGTGTTTGAAAGCCGTGGTCCATTATGAAA
AAGAATATTTGACTGGAAAAGTCAGGCCAGTTTTTGTTGATACTGAAAACAAGTGCTGCGGGTAC
CTGCAGACCGAGTCGGTATTTAATAATGCAAAATGTTCGAAGTTGCAGATCTTGAAAACTCAAGC
TGGAAAAATATCAAAACAGCTCAACTTATCTGAAGAGTTGAAATATGAATTCTTTGAAAATCTG
AAACGCTTTAAAAGAACTCATAATGAGTGGAAAGATTATCGAGTACTTGAAGAAGCATTAAACT
ATACTCTATCGACATTAAACATAAAGCTTCAGAACGAACAGGTAGTTTTGAAAAAGAAACTTAA
AATATAACCATATTACTTTTTTAAAAAGATTAAAAGAGATATAGAACAATTCAAAAATCCCCCG
AAAAATTGAACTGTTCACGAAGTATGGAACGAATTTAACAAATATGAAACTCTCCATTTCCCAAG
GTATTTCCCACCTTTGGGCAAGTATAATTTTTAAAAATAGCATTACTTATACTTTTAGAGTAATC
AAAACCAATATAACGGGTTAAAAAAGAAAAAAAAGGGAGACGTACCTTATTAAAATATATATCC

TAAAAGATAAGCTAAAAATATCAAATAAATCAAAATATAAGAAATACCATCAATTCTATCTATT
TTTGAGAAGTAATCTGTTCTAAAGTTTTTAAAGTTCAACATCTTCTTTATTTTTTCCCGGTTCAT
TGTAAAAATTATTAAAAGTGCCATTATTGTATTTACCATCAATTCTACGTTGTATTTTGGAATAT
CCACAATCATTGCAGAAAATGATAAGGCAATACAGGTGTTTGCAATATTACTTCCAATTACATTT
CCAAGAACAATACTTCCAAGTTTTCTTTTTGCAGCTGCTACTGAAGATGCAAGTTCTGGAAGAGA
AGTTCCAAATGCAACAAGTGAAAATCCAATAATACTTTCGGGAACATTAAAATATTCTGCAATTC
CTTTTGCGCCCTCGATAAATAAGTCACTTCCCAAAATTACGGCCAATAATCCGACTACAATAAAT
ATCGAAGCTTTTTTAGTGGTAAGATCTGAAATAATTTCTTCATCGTCAGATTCACTCGTTTTAAT
CGTATATATTACATAAGTAATCATGAAAAGAAATAATATCAGTCCATCAAACCTTGAAAAACCGT
TGTATCCTAAAATAACCAGTAAAATTGTTGCAAGAAGGTAAATATTTCCATTTTTAATAATTGCT
CGTTTTTTAATTATTAATGGATACATCAATACGCTAATTCCCAAGATAATTCCGATATTTGCAAT
GCAACTTCCAACGGCATTTCCAACAGCAAGCCCTGGAGATCCCGAAAGTGCAGCACCGCTACTTGT
TACAATTTCTGGAAGAGAAGTTCCAAATGCAACAATCGTTGCACCGATTACAAAATTTGAAACAT
TGAAGTGTTTTGCAACCCGAGTACTTCCTTCAATAAACCAGTCACTCCCGTATGCAAGTAAAATT
CCCCCAAATATCATGTATGATATCAAGTACCCTAACATAATCCCCCATTTTACAAATCTCTTAAT
AAAATTTTGATTTATCAATATATAACTTAAACGATTGATTCAAAAGAAGTTTAAAATAGTGGCT
TTATATGCAAACCTAGTTTAAAATTTGAAAAATTTTTCAATTTAATTTATGACGGGTACCCATTT
TTTTATTTTTTAAATAAAATTTTAATATTGAATTTAATTATTCAATTTCTTTTTTCAGCCAGTTT
TCAAAGGAATTTAACTGTTCTTTCGTATGGAAATAGTGTTCACCTGATTCTACGATTTCTAAATT
ACAACTAAACTGTTTAACGAAATTATTTACTGTATCATATTCACATAATTCGTCTTTAGATCCGT
AAAGAATAGATGTCTTAAAATTCCAAGATTTAATTGGATGTTCATTTACATAGCAAAAGTAATCC
CAATATAGTTTCTGACCAATTGGGGTTTCTATCTCTTTTTCTTTTTCTAATCGTTCAGGGGTTAC
ATCAAACCAGTTCATCATATTTTGAATAATCCGTTTCATGTCAACAACGGGTGATAAAAATAAAC
TTTTTTCTAAATTTTCATCAGGATATGCCAAAAGACTAAAATATGCGCCCATACTGCAGGCAAAC
AAACTTATCTTATTCCAGTGGTTTTTAGCATATTTCATTATTTTTTCAAGATCATTTACACAAAT
GTCCACCTTACAGGGAGTTTTATCGTTTTTTCGATCCCCGTGTTCAGGTAAATCAAAACTCAAAA
CTTGATATCCCTTTTTAACAGCAATTTCTGACAGCATCTGAATTACAGTATCATCCTTACAGGAC
ATATTTCCGTGAACTGCAATAAATACACGGTTTTGTTCTTTTCCCCACAAAATAGCAGGAATTCC
ATCGATTGTAATGTTACTTTTAATCATTTTTACCACGATACGCGTATTAATTTCAATTATAACTA
TGTTTTAATTCCAAATAAAATTTCAAAACATTATTATTTTAATGAATTATTTTTACAAAATATTG
ATTTTTAACCTTTTAGTATTATCTAATATAATATTAAGTTCCAACAAAAAAATAAATTTTTATGC
TATATTTTAATAATTTATCTATTATTAGGGGTTATTTTTTAATAATCCCGATTAAAACACTTTTA
ATTTGGAAATAGTTATATATTACCGGATATATAATTAAATTGGGGTTAATTTACCATTACTTAGT
AAAAACTATTTTTAAAAATTCCGAAAAACTTTGGAGGGGGTCTTCATGAAAAAGTTTTTAGTTG
GATTTATTTTATTTTCCATGGTCTTTTCAGTAATCCCTGGAGCTGTGGCGATAGATTACGATATT
ATTGTAGGACAGAATGGAAAAGTAGGAACTTTGGCATTGAATGCAGTTGGAGATACTTTGTATT
TTAATTATGCTATAAATTCACCTTGGGAGCTTTGTGAAGTACATTTAACTGCATTAAATGAAGTT
CCTGAAGCGGATAAAGAGAACATCTGGCATACTAAAGGATATATTACAAAAACAGGAAATCCGA
AAGTTGGACAGTTTGAAATCAATGAAGAGTTTGATCCCTATTTAACGGAGTATTCTGGTTCGCTT
AACATTGGTGAATTGGGTAATTCAAACTTAACAATTGCAGCGCATGCCTTAGTTTGTAGACAAGG
TGATGAAATAATGGATTATGGCTACGAGCCAACTGATTATCCTTGGGCAATAAATTACAGTAATT
ATATTAACGGTTCGCCTGATTCAACTGAATCATGGAGATACGATCCACTACTATGTCTTGAAGAA
AGTGATAGTTCAAATCAGGGTACAAAGACATTTACCAGTCTCGGACATGGGGGATCAATAGAATT

GTATTTTGATGGCAGTATTTGTGGAGATGGAGATAATTCCACACCCGATTTGGAAATTTATGAAG
TAACTTATAATCAGAACACGAATTGGGTTGAATCTATTGGTATTGAAGCATATTATAATGGAAA
TTACTACTTGGTAGGAAATGCTACGAATAATTTGGGTAGCACCACCGGCGTAACGAATATGTTTA
TAAACTGCATTTATGATCTTCCTAGTGGTGTATGTATCGAAAAAATAAGAATAAATGATACATT
GTCAACGCACGACTATGACATTGATGCAGTAAAAGCGAACTACAAATGCGTTCCGATATATAATT
GCATCTGTGAAAGCGCATGGGCTAACGACACCTGTTTTGGAGGGGAAACGTTCCCTGGAAATAAC
TGGGCTACGTATGTTATATATCCAGAATGCGATTAAATTCTTTTTTTAATTTTTTAAACAATAAA
CAACAGAATTTACACTTTTTAAATTCAATTTAAAAATCAGAAAAAAAGAAAATATTTTTTTAAT
TAAGTTATTCAACAACTAATGGCCCATTTGCGGTCCATTCGACTATGATTGGCCCTTCTGGCAAT
TCATCTAAAGTTTTAAAGTATTCTAAAGCAGCTTGTGTTCCAAGTCTGTCAGATCCTGCAATATA
TACAATAGTGTAACTTTTAACGACGTTTCCCGAGTTGTCTTGAACTTTTAATACTTGAATAACCC
CTTTATTTTCTCCAGGGTAGTCGTTTGAAATTGGCATTTCAAACTGGTCGTTATATTCTCTTGCA
AAGCCATTTGCTAAAGGTCCACCAACAATTACTGCATTTCCAGAAATTGTGAATCCTTCTGCGTT
CTGTACTCTTTCCCTTAATTGGTTTGCGTAATTTTCATCGATTTCGTTTCCATATATTACTGTTGC
ACTTGAAACGAAGTTTTTAATAACTTTTGAGCTTATTTCATCAGATATATCTGAGTCATAGCTTC
TTCCACCGCCACCAGATGCGGGGGATGAAGTTGTTGTTGGCGTTACCGGTGCTTCTGTAATTATA
TAATTTTTAGATTTACTATCTAAAGCAAATATGTCTGCAATGAGCACCCTTTTCTCCGATTCCAA
AGGTGGATAGAGTGTTTCAAGAACTTCGTAATTTTTAGAAGTGCTAACTAATGGGTATGCATCAT
AAAGCGTACTATTGCCTTCACCATATTCATAAGTTATTTCATAAATATTACTATAAACTCCTTCA
TCTTCGATTTCTGTTCCTGTGTACCCTTCGTAGTAGTTTCCAAGTCTGTAGGTGTATTCTTTTCCA
TTGTAATTGTAAACTAATTTTGGTGAAGACATTATGAAATCATCTTCGTCTTCTGGCCCAAATAA
TAATTGACCTTCATGAATTGTATTTAGATAGAAATATTGTTCCACTGCACCTGAATTTAAGAAAT
ATTCGTTATCTTCAAATTCACAGTTTGTAATTGTTACATTTTTAACATTACTTTCTATAAATATT
CCGGTTGACCAGTTTTTAAGTTTTAAGTTTTTAATTGTAGTGTTTTCTATCAGGTATTCGTACGC
ACCAACATATATCCCAGTGCCTACAGAATTATTACATATTATCTGATGGTTTTGACCATCTAAAA
CCACGTTGCTTGTATTAATTCGTATTCCGCCGTAATTTACAAGCATGTCCTCTGAAAGAACGTAT
GTTCCAGATTCATTTATTGTCATATTGTATTCTATATCTCTTGCATCTGCTGAAACTAACATACT
CAAAGCAATTATTGCTAAAATTAAGAATTTTTTCATTTTTTATCACCGCCTTACCTAGGAATTAA
TGGAACTATTCCATATGGTTTATCAAATATTCCTAAAACGGATGTTCCAAGTTCTTCACCAACGT
AGTAGTTTCCAAGTCTTCCTTCGTAAACCACTCCATCGTATTTGTACGTTAAGACCGGTGAATGG
AAGTAATTTGTAAATGTTGAATCATAGTTGAAGTTTGCCCAGGTGTTACTTTCAAAGTTGTTTAG
ATAAATCCAGTTATATTCTCCATTAAGATTTAAACCCCATGAATTTTCGATAAATTCATTGTAGG
TGATCGTACAGTTACCAATTGCAGATTCGCCTTGAGTATTGATTCCAATCATTAAATTGTTAAAT
TTACAACCAGTAATGGTTCCATTTTGCATAGATATAATATTTATTCCCCATCCGTTGTCTATTCC
AGTAAAATTACAGGATGAAATGACAATATTTTCGCCATCAGCAACTATTCCTGCATTAGTTACCA
AAAATACACAAGATGAAATTGTTACATTATCAGCATTTGCTAAATTTAATCCATTATCACAATTT
AATCCAAGATTTTTAATCGTAATATTTTCATACGATCCCATGAATGTACTTACTCCTGAGGTGTT
CATGTAAAATCCATTTCCGTCAATTGTGACGTTGTCAGAATCGATCGCAATTCCCATACTGGAGT
TTATATTTTCTTCTAAAACGTAAATTCCCGTTTTAGTTATTATGTATAATCCGGTAGCAAAATCA
GCTTGCGTTAAGTGGATTTTTCCATCGTCATATTCAATTTTACCAAAAATGCTGTTTGTATCTTT
ATCCCAAGGTCCTGCAAGCGGTTTTGAATCGTTCGTATTTATATTTATTACATATGGAATATTCC
AAGTACCATTTTCAATCACTGCATCTTCTTCATCGTAAAGGTACCAGTAGTTACCAAGAATTCCC
GTGTAGTTTTTACCTGCGTAGGTGTAATTTACTTCATTTTTAGAAGTAAAATAATTGATGGGCCT

TGATTATCAACTGAACCGTTTATATTGTTCAAATAAATTCTATTCCAGTATGATGCAGAAATGCA
TTTAACTGCAATGTCATTGGAATTAATGTCCGATCCATTTATTTCTATATTTTGCCCATTAACAT
AAAGCGTATTATTTTGAGCAGTTATCGTACAATCAATTACATTTTGGTACGTGCCACCAACTGTT
AAAGCATTGTACGTGGTTGCCCACATATTACTTCTTAGTATATTATTATCTGAACCTGAAAGGCT
TACTAATTCGTGATTTCCTGTTAATGTACAGTTTGAAACATTGTTGTCTGAACCTGAAAGGGTCA
AAGCTTCATATTGTGCGTAGATAGTACAATCAATAATATTATTGCCCGTATATGACACTTCTAAT
CCGGAATATTGGTCTGCTCTTATAGTAGATCCCATTACGGTACAATTGTCTGAATTTAAGTCAAT
TGTATTGGTGGCTGCATTGACAGTGGAATTTATAATTGATGTATTGTCTGAATTCCCGAGATATA
CCCCATAATCATCAGGTGTAGTTACATTACATCCTGAAATTAGGTTATTTTCAGAATCAGTAAGT
GTAAGTGTATATCCATCTATTGAGTAGATATTACATCCTGAAATTAGGTTATTTTTTGATCCAAC
TAACTGCATTCCTTGGCTAGTTGAATTGATAGTATTTGCAGAAATCACATTATTTTCCGAACTAC
TGCCAATATAAATTCCAGTGAAATTTGATGTTATATTATTTTCAGTAATTGTTGAATTTATAACA
TTACCCATAAGGTAGATGCCACGAGTGTCACTGTTGTATTCCGTGGAAGTAATCTTATTTCCAAT
TATTCCACAATCTTCCGCGTTAAAGATGGAAATGCCCCTATTTTTGGAGGTAATTTCTGAATCCT
CGATTACGGTATTATTTACATTTTCAATATGAATACATTCGACTGAAGACCAGTCAGACTTCAAA
TTTTTAACAGTTACATTTGTAGAATTTATTATGTAAACTGCATGATTCAAACCCGTACTGGTTGC
ATTAATCCAATTATCGTTACAATTTAAAACAACGTTTTCGGTGTCTTTGATCATAATTACGATCT
TATGGTCGAGATATGAAGTATTTACAAAATCTGCAGTTACGTTATACTCACCAGATTCATTGATG
AATACAAACACATCATAAGTTCCAATATGGGTCGTATTTTCAAAATCAGTTTCATCGATTGTATA
ATCTGGAACTGCGCTAACGCTTCCTACTACAAAAAGCAGTAGTAAAAATAATAAACATTTTTTTA
TCAAGTGTTCACCCCGAACATTTTAATAAGTGTTAAAAAAATATGTCGAATACTCGAGAGTTATG
ATTTTTTTAATTTATATTTTTAACGTCCATTTTAAGAAAAATATACAAATGGGGAACATTTTAA
AAAATTTAGAAAATCTAAAAAGATATAAAATCGGACTTTTTTATCAAATATTACATTAAATAAT
AAAAAGAGAATTAAAAATCAGAAAAAAAGAAAATTTTATTTTACGTTAAATGAAATTACAGGAT
CATTTAACGCATCTTCTGGTGTTTTCGTAGGATGGGAGCTAAATTTCTTACCATCCGGAAGCTTT
AAAATTGCATAGTATTCTCCAGTTTTTAAATACTTTTCAATCGTTATTTTTGCAGGTTTTTCGCC
TTCTTTAAAGTAAACACGATATTTTTTTATCAGCTCATATGTATCGTATTTTGAAAGATCAACCA
TATTTACCACCCCCTGATTTTCATTATACTTTATTTTCCCAAAGGTATTTATTTTTTTCAGTTTTT
TAAAAATTAACCCTAATAATACGTTAATATCATTAAAACGGAATAATAAATCCTTTAAATTGGG
TAATTATAATTTTTAAAAGTTAAATTTTAGTTTAAAAGAGTTATAAATGTTTCATCGAGAGGTT
ATAAACTTCGATATACTTTTGAAATTTAATTAAAGTTTGATCCATTAAATATATACCTCGGTTTT
AAATAAATAATAATTATTAACGGGGGTGATATTTTTGAGTAATAAAAAAATTATTACGAGCTTT
CTTTTATTATTAGTTATTTTAAGTTCAATATCTGCATACGGCAATCAAAATAAAGGTTATGGGCA
AAATCAAGGCGGCCAGGTTTACCAATTATATGATGATACGCAGGTAAATCACCAGCAAGAAATTG
GTAGTTATCCTGTTGAAGAATTACGTCAAGAAGAAATTGACGGATTATTGTTGATGAGAGAAGA
AGAAAAATTGGCAAGAGATGTTTATTTGGAATTATATGATATTTGGGGCCAGCAAATTTTCTTA
AATATCGCAAATAGTGAAAGTACTCACACCAATGCAGTAAAGTTACTCTTAGACAAATATAATTT
GACAGACCCTGTTACTAATGATACAAGAGGAGCATTTACAAATCAGGATATGGCAGAACTTTACA
ACGTATTGGTCGAAAAAGGATCAGTTTCACTGCTAGATGCTTTAACTGTTGGTGCAACAGTTGAA
GATTTAGATATTAATGATTTAAAAAAGTTAAATGGAATATCAGATAATCAGGATATCACTGCAG
TATATAATAACCTTGAAAAAGGTTCTAGAAACCATTTAAGGTCATTTACAAGAGTTATCGAACAA
AATGGTGGAATTTACGAGCCTCAGTACATTAGCGAATCGGAGTATAATGAAATAATATCAAGCG
AAATGGAAACGGGTACGGGATATTCTGCAAATCCTGCAGTACAGACTGAACAATCTAACCAGTAT

AATGCCAGCAGCTCAAACAATGAGTCTAAAAATGTATTTGAACGGATGTGGCAGGGTTTCATGAA
CTGGTTTCGTTAATTCTAATTAAATTTTAATAACCCTTTTTTTTAAATTCAAATATTTTTTATTT
ATTTTTTAATTTTTAAAATCATTAAGCTTTAATTTTTAATTTTTTTAAATAGGAAGATATAACA
GAAATACATTACATTATTTAATAAAAGTTAAGTAATATATATTATATTTAGAACAAATGTCTAA
AAAATGGAGGGGGTGTGACTACATGTCTGATTTGATAGATAAAGAACTTTTAAACTATCTTTAT
GGCCTGAAAACTCGAGAAAAAACTTTTAAAGTTGATATAAATGAAGTTTTTGAGGAATTTAATG
TCGAGAAAAAAGAAAGGTTAATGGAAAGTATTGAAACGTTATCCCTTGAAAAAATGATTTATAT
TCAGGGAAATAGTATTGAAGGATTTAAAATGGGAATATCTGACAATGACCCAATATTTCTTATA
ATAACTCGAAAAGGAATCAGTAACATTGAAACAAATGAAAAAATGTCGCACGATAAATTAAGTG
AACTTTTTAAATATTAAATACTGATTTAAATTTCAAAAATCTTTTTTAAACGATTTAAAATTTCA
TAACTTTTTATTATTTTTTAATCATCAAATATGCAGTCCACCTTAAAATTTCAAAAGTTAATTAA
AATTAGTTATAAAAATATTTAAATAGTAATTTTATGTAATTAACATGTCAAAGAATAAAAGGTG
TACTCATGAGGACATATTTGGTGTTATTTTCAATACTTCTGGTTTTTTTGGCCGGATGCATATCC
ACAGAATCTAAAGTTGAGGATACAACAACAGCGGTTACCTTTGTTTCGGTTACCGATTTTACGGG
TGAAACTTCTACAATTAATTCACCGGTTGAGAGAGTAGTTTCTAATTATGGCCTGATACCGCCGA
TAATTTACCTTTTTGGTGAAGGGGATAAAATTGTTGCAGGAAGAGGCGTTATGGGGGCTGATGA
ATTTATTGATGCAGTTGACCCAGAATTCAAAACCCGGTCAAAAGATATTAAAAGCGACAACGTTG
AAGAAATTAAAAGTTTAAATCCGGATGTTATTTTTGCGCCATCTTGGGGTTCACAACAGGGACAG
TACCCACAATTAAAGAGTTTGGGAATTCCTGTTGTTTTTATCGACATAGAAACAGCTTCTAATTA
TTATAAAACTATTGAAATGATGGGCGAAGTATTTCAAAAAGAAGCTTATGCACAGGAATTAATC
GATTATTACAATCAATCTACAAACGATGTTTTAAAAAGAACAGCATCAATCAGTGAAAATGAAA
AACCAAGGGTTTTATTTTTAGTTTACGACCAGAGAAAGAATTCATACTGGACTCCTGGTGGAGAA
TTCTATCAAAATAATTTAATTACAATGGCAGGTGGAAAAACTGTTTCCAGCGATTTACCAACCGG
AAAAGCACAAATTAACGTGGAACAAGCTGCAAGCTGGAATCCAGATGTTATAATCACAAGTGCTC
ACACGATGGAGTATCCAAGCACGAAAGTAAAAGAAGATGTATTAACTGATGAGGGTTGGAAAGA
AATTAATGCAGTTAAATCTGGAAATGTGTACGCAATGCCGTTTGATGGTGAATCCTGGGATACAC
TTGGTCCAAAATATGTTCTCGGGCTATACTGGACTGCAAAAACACTTCACCCTGATATGTATACT
GATGTTGATATCTTAAATCTTACATCATCAGAATATGAACGGTTTTTCAATGTAAGTATTGACGA
AGTTACGCTCGTTGGTGATAATATTGAATAAAAATTATGTTTTTCCTTTTTTGTTTGCAGGCGTG
TTTTTATTTTCCCTGTTTGTTGGAAGATACATGTTTGATCCTTCTTTAATTTTTACAAATTCACT
TGCAAACAATATTTTTTTTGATATTCGGCTTCCAAGGGCGATTGCAGTATCACTTGCAGGGGCAT
CTTTAGCGCTTGCGGGAGTTTCTTTTCAAAATATTTTTAAAAATTACCTTGCAGGCCCAAATATT
TTGGGGGTTACAAGCGGGTCCGCATTTGGTGCATCTATTGCAATTTTATTTTTTGCGTACAATCC
GTATCTTGTACAGTTCTCCGCGTTTATATTTGGTGTTATTGCAGTATATATTGCATATAAACTCA
GCAGTCTTTTAAAAAGTGGAATTGTTGGGCTTATTCTTTCTGGAATGGCGATTTCTGCATTTTTT
TCTGCGATGATCGGCCTTATAAAATACGTTGCAGACCCTTATGAAAAACTGCCAACGATAGTTTT
CTGGCTTCTTGGAAGTTTTGTAGGCCTTCGATGGGTGGATTTGGGAATATCAGTTATACCCATGT
TAATCGGGATTATCGGGCTTGTAATGTTAAAATGGGTTTTTAACATCCTTGCAACAGGGGATGAA
AATGCAAAAAGTTTAGGGGTGGATTCAAAAAAACTGAAAAATATAACGATATTTCTTGCAACAC
TTGCTGCATCTGCATCGACTTCGCTTGCAGGGATGATTCAGTGGGTCGGCGTTGTAAGTCCACAT
ATTGCAAGGCTTTTGGTAGGGGTAGATAACAGGAAACTTGTTCCTGCATCTGCTTTTGTAGGTGC
TACACTTCTCCTACTCTGCGACACGCTTGCAAGAACTTTAACGCCTTCTGAAATTCCAATAAGTAT
TATAACTTCGTTTATTGGTGCACCAATTTTGATTATTATTTTATCAAAAAGAGGGGTGAAATAAT

GTTGAGAATTGAAAATATAAATTTTAATTACGGGAAAAACAAAATATTGAATGATTGTAGTTTT
AAACTTGACAGTGGAATTGGATGTATACTTGGGCCAAATGGTGCGGGAAAATCCACGATTTTAAA
ATGTATTTCTGGAATTGTTCCTGTTAAATCTGGAAGTATCACAATTGACGGGGAAAAAACTACCC
AAATGAAGTATAAAGATAGATCAAGGTTAATTTCATATTCGCCGCAGGAATTTTCGATAAATTT
TCCATACACGGTATTTGAAGTTGTTTTGATGGGAAGAAATCCACACGTTAATTTTTTAACGGGCC
CTTCCAAAAAAGACGAACAAAAAGTAACTGAAATACTAAACTTACTAAAAATCAGCCATTTGAA
AGATAAAGAATTCATATCGCTTAGTGGCGGTCAGAAAAGGCTTGTAATGATTGCAACTGCACTGA
TTCAGGATGCAAAATTGATGATTTTTGATGAGCCGACATCTTTTCTTGATTATAAAAATCAGTAC
CTTGTACTTTCAGTAATCGAAAAAATATCAAAAGAAATGAATAAAACCGTTTTACTGTCGCTTCA
CGATCCAAATCTGGCGTTTTTGTTCTGCGATAACCTATTTTTAATGAAAAATGGAAAAATTCTCT
CTGAAATTGCGGATAAAAACTCAGTATGCCCTGAAAATTTTCAGGAGCTTTACGGGATAAATACT
AAATTTGCAGATATATTCGGGCAAAAAGTTGTTCTTCCGGATAAAGACTATTTAAAAACTATGAT
TTAGTGATTATAATAAATTATTGAGGTGTTATAATGATAGTGGACGATTTAGCTGATAAAACAC
TTGAAATTTTAAAAAATGGAGATAAATTAACCTTAAAAGAATATTCAATAGCAATACCTTATGC
TTATGTAATTGTTGAGGGTGAAAAGGGGGTTAAAATGGGAGTTTCGATGGTTCTCGATTCAAAG
TGCCATTACCGACGAGATTTTGAGGAAACTTCTCTTGAATCCATTATAGAATGTGCTAAAGGCTA
CAATCTGACTGAAAGAACACTTGCAATGGCTGCGATAAATGCAGTTTCTCAGTATTATATTGATT
TTACGGAGTCAAAGAACGAAGATGTTTTGGATTTGATTGTATCTGACAAAGATATTAAAAAAAT
AGGGTTTGTTGGGAATATTGCACCAGTTGTAAATGAATTACGTGAAATTGATGGCAAAAATTAT
GAAATATATGTATTTGAGAGAAATTCTTCGTCTTGCGGTGCAGAATTACTTGATGATACGTTTGA
ATATCGTTTACTTCCAGAAATGGACGCGGTAATAATAACTGGAACAACCCTTGTAAATGATACGA
TAGATGTTGTGCTGGAGCGCTCAAAAAATGCAAAATTAAAAATTTTAACAGGGCCAACTGCTCAA
ATAAACCCCGATTTCCTGAAAAATTCTGGAATTACGCACACTGCATCAACAAGGGTTATAAATAT
CGATAAAACAGTCACAGATTTAAAACACGGTTCTTCTTTTGGAATATTTAGCAGAAATTCTTCGA
AATATTCCATGAAAGTTAAGGATAATAATGAATAGATTAATCGGTGAAAAAAATGAATGATGTA
TTATCAAAAGTAACGGACAAAGAATATTTAAAACAGATTGAAGACGTTATCAAATTTCACGGGC
ATTTAAGTGGCGGTGCATTTATCGGGATACAGATGTACAATATTGCAAAAAAAGTTCTTGATATA
AAGGACACAGATCGAGTTTACGCAAATTGCGAAACTAGAAACTGCATGCCTGATGCATTTCAGGT
ACTTGGAAACTGTACAACTGGAAACAAACGTTTGAGCATAAAAGACTACGATATTATGGCCGTAA
CTGTAAACCAAGATAATTCCTGAAAATGAGGTTGGAACGGGGGTTAGGATAGTATTTGATCATAA
AAAAGCTGAAAAATACCCTGCACTTTACAACTGGTTTATGAATATAAAAAAAGTGCCGCATGAAG
AAGTAATTTCTGATTTAATCGATGCAGGGGATAGTATTTATTCATGGAAATCTGTTGAAATTCAT
GTTTCTGAAAAAAAGCCAAAATTTTCTGCAGTATGCGGGCTTTGTGGTGAATCTTTTATTAAAAA
AGGCACTGATGATGAAAATATCTGCCCAAATTGTGCAGAAAAATCTGCTGAATAATTTTTGAGTA
ATGCTGGTGTATTTTTATGAAAACTGCTGATGAAGTTCAAAATATTTTAAATGGATTTACATGTA
ATCGTGAAGTTTCTGTTGAAATTTATGAATCATACGGGAGAGTGCTTTTTGATGACGTTTATTCT
AAGGTTGATGTCCCCCTTTGTGATAAATCAACAGTTGACGGCTACGCAATAACTAATGAAGGACT
TAATAATGATTTTTTAGTAATTTCGGATGAAATTCACGCTGGAGACCCTGCAGACATGCTGATAA
ACCCTGGAAAATGTATATGGGTTGCGACAGGTTCAAATATTCCAAAAAATACGGATAGGATAATT
CCTTTTGAAAACACGATAAATTCAGAAAACACGATAAGAATAGTCAAAATGACAAAAAATTCAA
ATATTTTTAAAAAAGGAACAGATATTCCAAAAAATACAAAAGTTCTCGATAAAAATACCTTGAT
TGATGAAAAAGCGATTGGAATTTTGGCATCAATTGGAATGGATAAAATAAATGTTTATGATACT
CCAAAAGTTGCGATACTTTCGGTTGGAAGTGAACTTTCAGAAATTAACGAAGTAAATTCAAAAAC

ACTAACTGCGATTGTTAGAAAGGCAGGTTGTGAGCCTGTTTTTTTAGGGGTTGTTGAAGACAGTA
TTGAAAAAATAACCTCTGCAATAAAAAACGCTGTTTTACAGTATGATATTGTTGTAACTTCGGGT
GGAACATCTAAAGGTAAAAAAGACTACATGTTTGAAATTATATCATCTATTGGAGATATTTTAT
TTCATAAAGTAAAATCAATTCCCGGAATGCCAATATTTGCTGGAAAAGTTAGTGAAAAACCCATA
ATATGTCTTCCTGGAAATGTTGCATCGTGTCTTGTATGCGGTTATGCGTATCTTTTACCAATGTT
TTTAAACGCGGCTCATAAAACGTTTAAGCGAGAACTGGTAACTGCCGACCTTTCAGAAGAATTTA
ATTCAGAACTTGGAATAAAACACTATTTTCCAGTTAAATTAAAGGACGGGATTGCTTACCCTGCA
TTTCGGGGTTCAAACATGATTACTTCAATTGCGAATGCGGACGGTCTTGTTGATGTTAGTGAAAA
TGAGGTAAAAGTCAGAAAAAAATGTTAAAATCTGGCTGATTTAATATTATTATACATATTTGATT
AAAATATCAAAAATACATTTTATTAAAAAGATTAAAAAATAAAAAAAGTATTGGGTCAAATTTT
TAATTTTAAACACAAAGGCCAATCTGGTTTAAAATTAAGGAGTGGTGGTTACTTTAAAACAGGG
TGTCGAACTGTTTTTTTAAGTAAAACTATTTAGGGAGTGTCATTTAACATGAAATGTTAATGATA
AGTAGTTACTTTTTTAGATAAATTAATCACATCCAATTAATAGATATTGTATACAACCCTAATAT
AGTTTTCTATTTAATTTATTATGTCGGGTATATATTATCTATTTTTACATATCTTTAGAAAAGAG
TTTGATGCGATTTTTTAAAATAATAAAAAAATTGATTGAATTTTATATTAAACAATTTAGCCTA
TATTTTGTATAACATAAAATGGTGTGGTAGTTTGAATTTAATCGAATTTATGTTGTGGAGTTTA
TTTACCAAATTAGCCAATAGTAATTTTTATAAATTACTTAATTTAATTAAGTTAATTAACTCCAA
TTAATACTACTTATCTCGTATTTTTATAATTATCTATTTATTGTAATGAGTATATATCTATATAT
TTAAATATTTTAAAAATATATCAATATATATCGATATATATCGGTATATCGAAAAGTATAAATA
TATTTTATAATATACCTAACTCCTGTAAAAAGGTATTTTAAAATTCAAAAGCCCATGTTTTCCAA
TTTAAAAATAAGAATATGGGTAAATCACGTTTTATGTGATTTTTTGGTGAAAAAAATGTTTGGCT
GGTTAGATTATGGTGCGAGATATATTGTAGAAAATATCCTGAATATGAGTATGGATACTGCAAT
TGGCTCATCCGTTCATTTTTTCATATACGACAGCTTAAAAATTGTTATACTGCTGTCTATTATGA
TATTTTCGATTTCATACATTAGAAGTTATTTTCCACCTGAAAAAACAAAAAAAATACTTGAAAGG
TACTCTGGAGTTTCTGGAAATATCATGGCATCTCTACTTGGGACTGTTACGCCATTTTGTTCATG
CTCTTCAGTCCCAATATTTATTGGATTTATTGAAGCAGGAGTTCCATTAGGAGTTACTCTTTCAT
TCTTGATTACTTCTCCGATAGTAAACGAAGCTGCGTTTGCAGTGCTTTTAGCATCATTTGGATGG
CAGATAGCAATGATTTACGTGGTTTCAGGAGTACTTATCGGGGTAGTTGGTGGACTTATAATTGG
CAAACTTGGAATGGAAAATCAGGTTGAAGAGTATGTATACAGCATAAGAAGCAGGGCTCGAAAA
ATAAAAGAATTAACGCAAAAAGAGCGGTTAAAATTTGCATACGACAGCACTAGGGATATTGTAA
AGAGAGTCTGGCTCTATATCCTGATAGGTATAGGAATTGGTGCAATAATACATGGATATGCGCCA
GAAGAAGTTCTTGCAAAATATGCAGGTCCTGAAAATCCATTGGCAGTAATTTTTGCAACAATTAT
TGCAGTGCCCCTTTATTCAAATGCTCTTGGAACAATTCCTATCGCAGAAGCTTTAATTGGAAAAG
GGGTTGGAATTGGAACTGCACTTGCATTTATGATGGCGACAACTGCGTTATCATTCCCAGAAGCA
GTTTTACTTAGAAATGTAATAAAACCAAAATTAATCGCGGTATTCTTTGGAATTACGAGCATTGC
AATTGTAATTACAGGATATTTATTTAATATTATCCTTTAATTTGATATTTTGACTAAAACACACA
TTTAGAGGTAAAATCATGAAAATAACAATATTAGGAATGGGATGTAAAAAATGCGTTGAAGTTT
ATGACAACGTGAAAAAAGCAATAGAAGAACTCAAAATAGATGCAGAAGTATTAAAAATTACAGA
CGCTGCAAAAATTGCAGAATATGTAATGTCAACTCCAGGAGTTGTAGTTGATGATGAAGTAATCT
TTGAAGGAAAAGTACCAAGTGTTGAAGAAATTAAAAATGAATTATCTAAACTTTAATATTGGAA
TATACTAATATGTCTGATATAGAATAATTGAGATTTAAAAAGACATATAGGTAGATCGCCCATG
TTTAAAGACAAAAATATTGTGGTAGGTATCTGTGCATGCAGTCCTGCAATAAAAACTCTGGATTT
GATTGGACAATTGCGCGAAAGGGGTGCAGAAGTCGATGTTATCATGACACCGAATTCTGTCAACT

TTGTGTCACCCTTGATGGTTCAAAGGGAAGCTAAAAGACCGGTTCAAATCGAAGCTTTTGAACTT
CCAAAAATGTATGATCCAAATCATAAATCATTATCACAAAAGGCAGATTTGCTGATTTTAGCACC
GGTTTCAGCTAATACATTAGGGAAAGCTGCAAATGGTATAGCAGATAACTTGTTGGCAACAACAA
TCATGTCTACAAAAGCTCCTATCATTGCTGCAATGCACATAAACCCAATGATGTACAGCAATCCT
GCCGTTCAGAGAAACATAAAACAATTAAAAGAAGACGGATTTATCTTTGTTGACAATGGAAATG
AAGAAACCCCTAGCAAGTTCCCTGAAATTTCCCAGATCATGTCAACAGTTGAACAAGCGCTAAAT
CAAAAATAAGTTTCTATATAAATATACTACTTTTTTATTTTTAAACGATTAATATTGGCAAAA
TCGGACAATGCGGTTAATAAAAGATACAATTTTAAAAAATTAGTTAAATTACGTGAAATTTTAA
CATTAATAACATAATAATTCAATAAAAAAAGAAATTAAATGGTTTTTGGGTTAATATTTTTTAA
GAATATTTCAGGGGCATAATATTTAATTAATCCATTATGGGGGGGTTAAATTAATTAAACGCCGG
ATACTAATGATTAAATTAAGTATGTATTGTAATATATTGTTTATGTTACATTTAATATAATTTT
CCATTTGATTTTGATTATTACCCATTTTTGCAAGTGTATCATTCAATTTCATTCCCTTAAGGACC
ATTAAATAACGTAAAATCTGTTTACAAATTTTATTTACAAATTTAAATATTTTAATAGCTCCATG
CGAAATTATATATACCGTAAATCTATACTTTCCCATAACCCGGAATAGTACTTCCGGTTATAATA
TATAAAGGGGAGAAAATATGGCCAAAAAGTCATCAGGAATAATCACAGGACTTATAACCGCAAT
TTTATTATCTGCAATGTTACTCTTTGCAGGATGCGTTTCTGATTCGTCATCAGCAGAAGAAACTA
CAACGATAACAGTTTCGGCAGCATCTAGTTTAACTGATGCAATGACCGATATTGCAGCAGCATTT
GAAGAAGATAATCCAAATATTAATGTTGAATTAAATTTTGCAGCATCTGGATCGCTTCGACAGCA
GATTGAAGGGGGAGCTCCTGTAGATGTATTTGCTTCAGCTTCTAAAAAACACGTAGACATACTTG
AAGATGAAAATTTAACAAGTGCCGGTTCTAGAACGAATTTTGCATCAAACAGTTTAGTATTAATC
GTTCCAGCTGGAAATCCATCGAACATTAGTTCAGTTGAAAATTTAACAAGTGATGCAGTAACAAA
AGTTTCAATTGGAAATCCTGAAACTGCACCTGTTGGAAAATATGCTAAAGAATCACTCACAGACT
CTGGTTTATGGGATGAACTCGAAGAAAAAATGGTTTACGGAGAGAATGTAAGACAAGTTTTAAC
TTACCTTGAAACTGGGGATGTTGATGCAGGATTTGTATACATGACAGATGCAAAAATTGCAAAA
GAAAATTCAATCGAGATTATAACAACAGTACCAATTGTTACAGAAATTATTTATCCAGTATGTAT
CATAGATTCTTCTGAGAATAAAGAAGAAGCCCAAGTTTTCTTAGACTACCTAACAAGTGAAACCG
GAAAACAAATCTTGACAGATTATGGCTTTACAGCAGAAAATTAGTGATACTTCATGGATTCAATA
GTATTTCCTTTAGTACTTACATTAAAAATTTCTTTTATATCTACATTTTTTGTAGTTATTTTTGG
AGTTATTCTTTCATACATTCTCGCTAGAAAAAGCTTTTACGGGCGAGATATTCTGGAGAATATAG
TAACTCTTCCAATGGTTCTTCCACCGACCGTTTTGGGTTATTTGTTAGCATTACAGCTTGGAAAA
AACGGATTTATAGGAAGTTTAATATACAAATTTACAGGCTCAGGAATTTTATTCACCTGGCAGGC
GGCAGTAATTGCTGCGTTTATCGTATCATTTCCGTTAATGGTTAAAACAACGACTGCTGCAATCT
CTGCAGTTGATAGAGAACTTGAATATGTATCTTATACTTTAGGAAAAACAGAATTACAGACGATT
TATAAGATAACCCTTCCACTATCAAAAAAAGGAATAGTTGCAGGCGCAATTCTTAGTTTTGCACG
AGCAGTTGGTGAGTTTGGTGCGACATTAATGGTTGCAGGAAATCTTCCTGGGAAAACAAATACAA
TGTCGCTTTCGATTTACCAGGCATTTCAAACCGGAAACTATGATTTAGCAAATGTATTGGTAGTA
TTGTTAATTATAATGTCATTTTTGACGATATTTCTTACTGGAAGATTTGTTGAAAAATGGAAGTT
TTAAGTGGTATTTATGATTTTGGAAATAGATATTGAAAAAAACTACTACGAATCGAAAAAAATTA
AAAAAGAATAAAACTCCTTCGTTTAATTTAAAATCTAAATTCAGTATAGATAGCGGTTCGATAA
TAGTTTTATTTGGTAGGTCGGGTTCTGGAAAAACAAGCACGTTGGGCTGTATTGCCGGCCTTTTA
GATCCTGATAACGGAAGGATTATTGTAAACAATGAAATTTATTACGATTCCGATAAAAACATAA
ATATGTCTCCACCAAGAACGAAATTTAGGTTATGTTTTCCAAAATTATGCTTTATTTCCCCATTTA
ACAGTCAAGGAAAATATTGAATACGGAATTAAACATTTAAGCGAAGAAGAAACTGAAAGAAGGG

TATCTGACCTTTTAACATTAACTCAGATCGAAGGACTTGAGAGCCGATATCCTGATCAGATTTCG
GGCGGTCAAAAACAGCGTGTAGCTCTTGCAAGAGCACTTGCACCAAGTCCAAAAATATTGCTACT
CGATGAACCGTTTTCTGCACTGGACATGATCTCAAGAATGCGTTTAAGGGAAAGTCTGAGGAGTA
TTCAAAAAGAATTAAAAATTCCCGTAGTTTTCGTGACGCACAGTTTTGAAGAAGCTTTTGTAATG
GCTGAAAAAGTTGTAACATTTCATAATGGAAGAATTCAGCAGATCGGAACTCCAGAAGAGATAT
TTTATAATCCAATAAATGCAAGTGTAGCAGAACTCGTTGGATACATAAATATTTTTTCAAATGCG
GAAATAGATTACAACGAAAATAGCCCCGATTTTTCAATATTGGACTATCAGGACATGAAAATGTC
GATAAAAAAATTCAATAAATCTATGAATGAAGTTTCCTTTGGAATTAGGCCTGAAAATATAAAA
TTAATAGATATAAACAGCGAAAAAACAGAAAACGTTTTTGAATGCATAATAAAAGATGTAGTTA
ATGAAGGTTCTACAAGCCTGGTTTACCTTGAAGTAATCCAAAATGGCTTAAGACTCATGTCAAAA
ACTCAAAATGACAAATTCAAAAAAATTAAATTACGCTGTTGGAGAAAAATGTAAAATTGAACTTT
TAAAAGACAAAATAACAATTTTAATGACGCAAAGTGTGAAAAAAATCGATTTAACTTAAAATTT
TAATCTAACTTTTATTTTTAATTTTTTAAAAAGAAGTTAATGCCTAATGCTATATATATCACAAA
AACAAAGTACTATTTGACTGAGTAGTCAATCTTAAAAATATGCCAATTATTTATCACAAAAAATC
TCGGAAATAGGGATAATTATGCAGTACCGCGAAATTAAAAAAAATGGCGACAAAATCTCAGCAC
TCGGATTTGGTGCAATGAGGCTTCCAACCAAAAACGGATCGATCGATAAAGAAAGAGCAAAAAA
ACAGATTTACTACGCGATAGATAACGGTGTAAACTTTATTGATACGGCATATCCATACCACGGCG
GTGCTAGTGAGTCTTTTTTAGGAGAAATTCTTTCAGAGAATTACAGAAATAAAATAAAACTTTC
AACAAAACTTCCGCAGTGGCAGGTTAAAAAATATGAAGATATGGAAATGTATCTTGATATCCAGC
TTAAGAAGTTAAAAACTGATTTTATCGATTACTATTTTATCCACAGCCTTTCAAAAGACAGCTGG
GCAAAAATGAAAGATCTTGGAGTTTTAAAATTCTTAGATGAAGCTAAAAAAAGCGGAAAAAATAA
AATATGCATGTTTTTCTTACCACGACAATACAGAAACTTTTAAAGAAATAATCGATGCTTATGAC
TGGGATGCATGTTTGATTCAGTATAATTATTTAGATGAACAAAATCAGGCAGGAACTGAAGGTT
TAAAATATGCTGCATCAAAAGGAATCGGCGTATTTATCATGGAGCCTTTAAGAGGTGGAAATCTT
TCAAAAAAAGTTCCAAAAGAAGCAGAAGAAATTTTTAACAGTTACGAAGTTAAAAGAACGCCTG
CTGACTGGGCACTGCGATGGGTTTTAAATCATTTGGAAGTCACATGTGTTCTTTCTGGAATGAAT
GAAGAGGCTCATGTAAAAGAAAATTTAAAAGTTGCAAGTGAAACTAATCCAGATTCTCTTTCAA
ATGAAGAATTGGAAATTTATGATAATGTAAAAGAAGTTTATCGAAAATTAATGAAAATAAACTG
TACTGGTTGCGGATACTGTATGCCATGTCCATTTGGAGTAGATATTCCTGCATGCTTTTCAATGT
ACAACGAAAAAAGTATTTTTAACGATAAAAAGACTGGATTTTTGTATCTTGCAAGATTAGGTGG
AGTAATGGGCGGTGAAGAGTCTCACGCAGGCCTTTGTGTAAATTGCGGTAAATGTCTTAAGGCAT
GTCCGCAGAATCTTGATATTCTGGAACTGTTGAAAGATGTTTCCAAAGAACTTGGTGGAAAAGGA
TTTAATTTAAAAATAAAACTAGCTAAAATAGCAATGTTGATTTATAGTTCTTTTAATTCGTTAA
AACAAAAAATATCAAAAAATTAAATTATTTTTATTTTATTTATTTTGGGTAATATATTATCATG
TGCATTTTTGTCATTTCTGAACCGTTATTTTCGTATTTGTGCGGTACATCTGCCTTAAAACGAAT
TGAATTTCCTTTTGGAATGGTGTAAGTATTTTCATCTATTGATAGCGTTAATTCGCCTTCATAAA
CGGTTATTGTTTCTTCTGAGTTTTTAATGTGGCCTTCTGAACTCATTACCATTCCAGAATCGGCT
TCAACATGATATATTTCAAAAGGTCTGCTGTCTTCAAAAGTAAATAATGGATAAGATCTCCATTT
TTGAGTGTCGTCGTAAATTGGTGACACTTTTTTAAGATCCACTATTTTTACATCTGTTTCCTTTT
CACTTAAAAGTGATGTAAATGATATTTTCAATCCATTCGCAATTTTTAAAATAGTGGTTATTGTT
GGATTTACTTCTTCGCGCTCAATCTGTCCGAGCATTCCTTTACTCACCCCAGTAAGCTTTGAAACC
GCGTCAAGGCTTAATCCGTTTTCATTTCTTATTTTTTTTAAGTTTTTAGAAATTACGTGGCTAGG
ATTTTCCATGAATACTACCTTTATTTTTTGTATGGTATATCATACGTATCGTATAGCATAATATA

TTTATACTACATAATATCATACAAATCGTGTAACATACTATACAAAGTTGTTCTCTGATGTATTA
TTGTAGTGGTGTCATGAATGAAAAAACTTTTGAAAAAATATGAATTATTTCTGTTAATTATTCC
GGCAGTTTTTTTCGGGGCCGGAGCATTTATAACTGGAAAAATCGGAATTTTGGAACTTTCATGGG
ACGAATTAACATTTTTAAGATTTTTAATAGCTTCTGCAATAATTTTACCCTTAGTTTTAAAAACT
GAACATAAAAATTTGAAGTTAAAGAAAAATGATGCATATATCGTAATTTTGGCAGGATTATTTG
GTATGTTTGGATACCACGCACTGTTTTTCATGTCGCTTGAATATATCACTGCAATAAATTCTGCA
CTGTTGATGGCAACAACTCCAATGGTAACTTCGATAATTGCTGCAGTAGTTTTAGGCGAATATTT
TGGAATTAAACGGATATTTGCGGTATTTATTGCATTTTTTGGAGTATTACTTACAATTACAAACG
GAAATCTGGAAGTTCTTAAAAACCTGTCATTTAACATCGGGGACGTTATAATGTTTTCTGCAGTT
TTATGCATGGCGTTATACGCGGTACTCAGTAAAAAAGTCGCTCAAAAATATTCTCCATCGGTAAT
CTTAACATACGGGTATATTTTAACAGTCGTACTTTTGATACCTTATATGATTTTAAAAAACCCGA
TAAATGTGATATTAAATGCATCCCTTGAAACATGGCTTTCAATAATATACATGGCAGTATTTGCA
TCGGTTTTAGCACAACTGTTACAGCAAATATCTTTAAAATATCATGGGGCTAGTAAAACAATGTT
GTTTTACCAGTTTGTTCCAATAATTGTAATTGGGCTTTCAGCAGTTTTTTTAAAAGAGCCATTCA
CAAAAATAACTGCAATAAGTACTTTATTTATAATGGGCGGAGTATACATAAATTCAACCATAAA
AAGAGACTAGTATCTTTTATTTTTAACTTTTTTATCTACTTTAAATTTCTAAAAAAATTTATTTC
ATAAATTCTATAATTTTATTTAATCAATAAAGGCGATTTTATGAACTCATTTTTTGAAACTTATC
GGGACTTAAAATATCTTTTAAATCGAAATTATAGAAAAAAATCCGCTTTAGATTTTGTTGCAAAC
CACTACGGCTTAAATGCTTTCGAAAGAAACTTTTTTGGGCGGTGTGTATTTTCTGACGATCATTT
AGAAGTTGTAAATAAAAAAATAACAGATTTAACAAAAGATTTTAAAACAAAATCTATTGCAATT
GATGGCTTTAATGTTTTAATAACTTTAGAATCTTTAATCGAAGGAGTAGCGATATTTTGTGAAGA
TAATGTAATTCGGGATTTAAAATATCAAAAAGGATATAAATTAACTGAAAAATCTGAAGAAAAA
ATTGAAATTATTTTAAAAACTATGTTGAAGTTAAATCTGAATAATTTTGATGTATATTTTGATG
AACCGACTAGTAAAAGTGGCGAAATTTCAAAAATAACAAGGAATCTAATGGAAAAATATTCAAT
TTCTGGAGAAGTTATTTTATCAAAAAAAGTCGATTTTGAGCTTAAACATTACGAATTTGTCGCAA
CATCTGATTTTCATATAATTAAAAATGTAACTGGATTTTTAGATATTCCAAAAATTGTTTCAAAA
AATTATAATTTGGATGTTAAAAATTTCTTAGAAATTATCAAAAAAGGAAAATTAGAATTTTAGT
TTAGAGTTTATCTTTAATGTATGGCACAACTTCGTTTATCGGGATTCTGAACTGTTCTCTTGAAT
CTCTTTCTCTGATTGTAACTGATCTGTCTTTTAAGGTTTCTCCATCGATAGTTATGCAGAATGGA
GTTCCTACCTCGTCCATTCTCATGTATCTTCTACCAATTGCACCGCTGTCGTCGTATTCTGCAATT
AAACCGTTTTCTCTTAATTTATTTTTCAAATCCATTGCAATTTCAGGCATTCCTTCTTTGTTAAC
AAGTGGGAAAACACCTACCTTTATTGGAGAAACTGCATTTGAAAAGCCCATTACGGTTCTATCTT
CCTCTTCTTTGAAAGCATGCTCCATAACGCAGTATGTAATCCTGTCGATACCATATGAAGGCTCT
ATCACGTGAGGGATTATTTTTTCCCCTTTAACGATCTTTTTAACTTTTTTAGCAGTTAAATATTC
TTTTAATATTTCGAAGTCTCTTTTGCATGTTTTTAAAACATATTTTCCTTCTGTTTCAAGAGCTT
CTACCAATTCTTCCATTTTTTTGTCGTCCAATTCTTTTAAAGACTCTTCCAAAACTTTTGCATCGC
CTTTAAATGTTCTTCCGACTAATTTGTAGTTCAATTCAATTTCGTAAGCTTCAACTTCTTTATCT
TCATCGAGTTCTACAAATACTGATAAATCATCGCCACTATTTTTCATGTGTGCTAATAAATCGTA
GTTTGTCCTATCCGCAACACCTACACACTCAATCCAGCCGTATCTGTCAGTATACAATTCTGCATC
CCAACAATCAGCAGCATAGTGTGCCATTTCGTTTGGAAGGTGCTGTCTAAACCTTAATTTCGATT
CATCGATTCCAATTTCCATCAAGAATTTTTTTGTAACTGCAATATAATATGCAATTGCTTCGTGT
GCGATTATTCCGTTTTTAACCGCATCAGAAAGGCTCATTCTGACAATCTTTTCTTCAGCGCTTAA
TGATTCGTCTTCCTGATTTTTACCTGAAAGTAATGGTAAAACCATATCTTTTACGCTTTCAAATT

TCTCAAAATTCTCTTTTTTCCTTGAATCAATGAAAAATTCACCTTCAGCTTGTGTAAATTCTCTT
AACCTGATAACTCCTTGTCTAGGGGAAATTTCGTTTCTGTATGCTTTTCCAATTTGAACTGCACC
ATATGGAAGTTTGTTTCTAAAGAACTGGCTTATTCTTTTAAACTGTATAAAGATACCTTGAGCAG
TTTCAGGCCTCATAAATGCAGCTCTTTTTCCACCAGGGCCGATTGAAGTTGCAAACATCAAATTA
AAAGTGCTAACTTCTTTAAATTCTCCACCACATTTTGGACATCGAATGTTATTTTTTTCAATCAT
TTCTTGAAGTTCCTGTAAAGTTTTTCCTTCAGTATCTACATCGATATTTTCTTCGATAATGTGGT
CAGCTCTGAAAGACTCAAGACAGTCTTTACATTCAACGAGAGGATCTGTAAAGTTTTCAACGTGT
CCGGATGCTTTTAAAACTTCGTAAGGAGTTACTGTCGGGCTGTCAATTTCGTAAAATCCTTCATT
TACGATATAGTGTTTTCTCCACGTGTTTATAACATTGTTTTTTAAGATTGCACCAAGTGGTCCGT
AGTCAAAAAAATCCTGCGATACCGCCGTATATTTCAAAAGAATTCCATAAGAATCCTCTTCTTTTA
GTTAAATCAATTATTTTATCGTATTTATCCATAAAATCACCTTTTAATAAAGTATCGTTTTTTCG
TTGCTGATTCTATCGAGTAAAAATATTAACAGTGAAGATGCGATTAAATCTGCCGTAGTTCCGGG
ATTTAACTTATTACCTTCTTTTGATAAAAATTTATCGAATTCCAAAACCGATTCTTCATTGAAGT
TATTTAAAACTTCTTTAGCTTTTTCTGAAACCATTTTAGAGACATCGATACCTTTTTTCCTTGCA
ATCAACGTGTCCGGATAGTTTGAGAGTAGATTGATAAATGTTTTGGTAACTGCAATATTGATATT
GTTGTATTTTTCGTAGTATTCTTTTATTAAATTATAGCCTTTGTAGGATATCTTAAAATTTTCGG
TCCATTCATTTGAAATACTGTCCCAAGTGGATGAAATTTTAAAAACATCATAAAGTGTAAGATTT
TTTTCAATAAGTTCGTCTTTAGCATCCTTACTTTTTAAATCAGGTCCGTTCTCAGGAGCGTTAAC
AAAAGCAAGTGCAATTTCAATAGCCCCGTACACGTCAATTGCATCCTGAACGGTCGTGTTTTTAA
TGATTTTTTCCGTTTCTTTTTTTAGTTGCGATTCGCTGAATTTATCCAAATTTGATGCGGCAATT
GCTATTGGCATGTGAAGCATTATTATTCCAAGGTTTGCATTTGTTGGAGACCATTTTTTGGATTC
AATGACTCCTTTTTTAATAAAGTTTCCGATATTGTTCTTTTCAAGGCAGGCTTGATGAATTACAT
CTCCAAATGCAATTCCTGAGCTTATAAAATGGTGATATTTTATATCGTCGTAGTCTCTATTTTTA
TGGACGTTTCCAGGTTTAAAGCTTCCAACTTCAAAGCAACAGGCGATTTGAGAAGCTTTCATTAT
GTCAAATGCTTTCATTTTTTCACTAGTTACAAGTTTTCCAAATAATTCTTAAGTTCATAAAATGC
TCTTTTTCTGTGCGATATTTCGCTTTTTTCTTCTGTCGTCATTTCTGCAAATGTTTTAGTTTTCCC
TTCTGGAATAAAAATGCTGTCGTAGGCAAAACCAAACCCTCCGTCTTTTATTTCTGTAGAAATGG
TTCCTTTTACAATTCCAGTAAAGAGTTTGATGTTGTCGCCATCATAGTAGCCAATAACTGTTTTA
AAGTACGCATTTCTGTTAGTTTCGTTTTCTAAAAGTTTTAAAATCCCTTCATTCCCAATACTTTC
CTGAACGAACCTTGAATAAGTTCCCGGAAAATCGTTTAGTTTTTCGACGAAAAATCCGCTGTCTT
CAACTATAACGGGCCTGTTAAATTTTTCAAAAACTTCTAAAACCCCAAACGCAGAAACTTCTTCG
AGTCTTCCCTGAACTTCCGCGTAAGGAATTTCAATCTGTTCGATTTCGCAATTAGCTTCTTTTAA
TATAATTTTTGCTTCATCAACCTTATTTTGATTCCCTGTTGCAAAATAAATTTTCATAAAATCAC
CAGGATGCAATCTGCCTAATTTTCGATACTATCATTTAACTCGGGCTTGCACATATTTATAGCCT
TACAAAAAATAATTTTATTATAAACTATCCGGGGGGATAATAATGGCCAAACCAGAAACTGGGG
AATTAAATAGATTTCAATTAAACCTTCTTTCAGCATTTGAAGGAAGTGTGTTATCGTTAGATGAA
ATTGAAAAATTGTACATCGAACACAAATTGAAACATTATGGTGAAGATAGGGAATCCGTAAAAG
AATTAATATTCGATTTAGAAAAAGGATTTTTTAAATTAAATGATGAAGTTATCAAAGAATACGT
TGAGATTTTGTACAATGTATATAACAGCCCATGTAAAGAATCTATTGATAGGTATGAAATGATT
TTAGAAGACATTGGTTTTAAAAAGGTTGAAAAATATCTGGAAGAGTTGGAAAAGTCAGCATATA
TCTCAAAAATGGGTGATGGATACAATATAACTGAAAAAGGTTCAGAAATGTTAAAACATAAAAA
ATAATATTTTTTTATTTTATTAAATCTTATTTTAAAATTCTAACTGCATTTAAGACGGAAAGAAG
GGCAACTCCAACATCTGCAAATACTGCCTGCCACATCGTGGTTTCTCCAAATATTCCAAGGCTAA

TAAATGCTATTTTTACTATTAAAATAACAAAAATGTTCTGGAATGCAATATTCTGTGTTTTTTTC
GAAATTTTAATCGCAGAAATCAATTTTGAGGGTTTATCGTTCATTATAACGACATCCGCAGTTTC
AATTGCAGCATCACTTCCAAGGGTTCCCATCGAAATTCCAACATCTGCTCTTGCAATTACCGGTGC
ATCGTTAATTCCTTCACCAACAAATGCAATCGTTTCTTTTTTAGATTTATTAGCTTCAATCTCTT
CGATTATTTTGACTTTATCTTCAGGAAGGAGATCTGAATAATATTCGTCAAGATTAAGTTCAGAT
GCAATTTTTTCAGCAATATCTTTTTTATCCCCCGTAAGCATTGAAACTTTTTTAATTCCTAGTTT
TTTAAGTTCCAGAACGGTTTCCTTTGAATCTTTTTTTAATTTGTCGGAAATTAAGATATATCCTG
CATAAACTCCATCAACTGCAAAATGTACCGCAGTTTCGTAAACATCCAATTTTTCAAAATTTATG
TTTTTTTCTTCCATCAGCTTCTCGTTTCCCGCAATTATCTCGCTACCATTTATTCTTGAGATTATT
CCTTTCCCTAAAACTTCACTAAATTCTTCAAAATCATCAAGGGAAGTTTTACATGAAATTGTTCC
AAAGTCCATAATAGTTTTTGCAATCGGGTGGTTTGAGTTACATTCTACCATTTTAGCAATTTCAA
GAAGTTTTTTTCCGCTAAATTCGTTTTTTGAAACGACTTTTGTAACTTTAAACTTTCCTTCGGTC
AAAGTTCCGGTTTTGTCAAATGAAACGTAGGTGGTTTTGCTTAAAACATCGATATAATTGGAACC
TTTTACTAAAATTCCTTCTTTTGCAAGTCTTCCAATTCCTGCAAAGTATCCTAATGGAATTGACA
GCACAAGAGCGCAGGGGCACGAAATAACCAATAAAATCAGAGCTTTGTAAAACCAAGGAACAAA
TGGTTCGTTAAATATTATTGGCGGAACAACTGCAAGTAAAACTGCAATAAATACAATAATTGGA
GTATATACTTTTGCAAACTTTGTTATAAATTTCTCAGTTTTGGTCTTGTTGATACTTGCAGATTC
AACGAGGTTTAATATTTTAGAAACTGCTGAATCACTGAAATTTTTAGTAGTTTGAACCGTTAAAA
GCCCGCTTAGATTAATCATTCCTGATAGGACTTCTTCACCACGATTTATCGATTTTGGAGTACTT
TCTCCAGTTAAAGCAGAAGTATCTAAACTTGATTTTCCATTTAAAACTATTCCATCAATTGGAAC
TTTTTCTCCAGGTTTTATAATGATAATTTGACCAATCTGTACATTTTCAGGCTTTACTTTTATCG
TTTCCCCGTTTTCAAGAACATTTGCATATTCTGCTTTTATCGAAACGAGTGATTTTATCGAATTT
CTGGATCTTGTTACTGCAATGTTCTGGAAAAATTCTCCAATGCTGTAGAATAACATTACTGCAAC
TCCTTCAGGATATTCCCCAATTAAAAATGCGCCGATAGTTGCAATACTCATTAAAAAATTTTCAT
CAAAGAAATTGAGTCTTTTAATATTTTGAAATGTTTTTTTAAGCACTTTTTGTCCGACTAATATG
TAGCTTACAATAAAAAATAACAGCTGAATACTTAATGAATATCCAAAATAACTGCTAAAAATTCC
CAAAATAAAAAAAACCGAAGAAATAACTATTTTTTTTAATTCTTTATAATCGATTTCGTTTGTTA
TTTTTATTTCAGACTGTTTTGGAATTACAATTACTCCCGGCTCAATAGATGTTACTATTTTGGTA
ATTTTTTCAAGATCGATCTCTTTTTCAGTCAATATTAACTCACTTGTAGCCATATTGATAACTGC
AGAAGGGTAGCCCTGTTCTTTTAATAATTTTTCAATTTTTGAAGCGCATCCTGCACACCAAAGCC
CTTTAAGAATATATTTTTGCATAATTATTTCCTCAATTCAACAATTTCTTCTAAAAAATCCCTAA
TTTTTTCATCGTAAAGTTCGTAGTATACAAATTTACCTTCTTTTCTGAATTTAACGAGTTTTCTA
TCGCGAAGTATTCTTAACTGGTGCGAAATTGCTGAAATGGATGTATCAGCAATATTTGAAAGTTC
ACACGAACATAATTCACTTTCTTTCAATGCTAAAAGTATTTTTAACCTATTTGGATCTCCAAACG
AATTCATAATGTTTGAAATGTTGACAATATCCTCATTTGAGGGTAATTTTTCCATTATATCCTTA
ATTTTTTCGTTACGAACGGAATATTCACTGCAAAGATCAGACAAACTATCACCTATATATGAACA
ATTGTTCAAATGTTATATATAATTTTTTATATTCGTATATTGAAAATTAACAATTACAACTTTAT
AAAAAAACTAAAAAACTCGATCCAAGCGATAGTTATGAAAACAAAACAGCACAAAATTTTTTGG
ATTTCTGCATTAGTAGCGTCTATTTTAATGATTATTTAAGTATAATAAATTCGTTTTTTGGAAAA
ATAGCGTTTGTTCCAATTCAGGTATTCTTAATAACGGTGGTAATTTCTGGAATTTTAAGCGACAT
GGAAAAAAGCTCTAGACTTGAAAAATTAAACATACTAATAGGCACATTTTTCAGTGAAACCGGA
ACTAAATCTTTAAAATATTTCTCGAAAATCGATTCCCTCATTGACGAAATTGGAAGTAAATTAAA
AGTAACTGATTCGTGGTCTGACGAAGATTTTAAAAATGCGCTAAATTATGCTAAAGATAGGGAT

TATACATTAAATGCTTCAAAAGAAGATATTATAGAAATCTATGAATTTTTATCAAAAAATAAAG
AGTTTTTAATGAGGCTTTTAGAAAATCCAAATTTAATGGAGCACGAACACTTCACAGAATTACTT
AGAGCAGTTTTTCACCTTTTAGAGGAACTTGAAAGCCGTGAAAATTTGCATGAAAGTTCAGATAA
TGATATAATGCATTTAAACGGGGATATGGTTCGTTCATACAGATTAATAACAATAGAATGGGTA
AATTACATGAAATATTTGAAAAATAATTATCCTTATCTGTTTTCTCTTGCAATGAGGAGAAATCC
ATTTGATAAATCTGCTAAAACATCCTTAAAATAGTATTCATTCTTCTTTCAATTTTTACTTCTAT
TTTCTTTTTCATTGAAAAAGTGTCCCTCGACATTAACACTAACTTTCATCCTTGGAAAATACCAT
GTCGGTCAGATAACCCTATGTCGAGAGTAAATGTACGAATTTTTGCTGTATTGTGTTCACAGTAA
GGATCCTACCAACTCTTACAACTCGCCCAAAAATTCGATCAAAGTTTTGTTACTCCAATTTCGAA
CCCGCTATCACAGCGAGAGTATTCACAAGCTCCATGTAGGTCGGTTAATTGAAGATGTTATTAAT
AAGTATCATTATGTATTTATAGTCTTTCCCGGCTTAAATAATATAAAAATCATTAATTTTTTCAT
TTTTGGTGATATAGATGATGTACGTTGTAGGCCACAAAAATCCTGATTCAGATAGTATCTGTTCT
GCAATAGCACTTGCATATTTCTTAGATGCATTTCCTGCAAGACTTGGAGAACTAAATCCCGAATC
ACAGTTCATTTTAAAGAGATTTGGATTAATGGAACCAGAATTAATTAAAACTGCAGAAGGAAAA
GAATTATTTTTAGTAGATCACGCAGAAAAATCACAAAACCTCGATGATTTTGACAAAGGAAAAT
TAATCGGAATTATTGACCACCACAAAATCGGTATTTCAACAACTGAGCCAATTATTTACCTCTCA
AAGCCAGTTGGATCAACTGCAAGCGTAATTTCAGAACTCTACTTTAGGGGAATACTCGACATTAT
TGGCGGAAAAAACAAGGAATTAAAAGCAGATATTGCAGGGGTTTTACTTTCAGCAATCCTTTCAG
ATACATTACTCTTTAAATCGCCTACAGCAACACCACTTGATAAGGAACTCGCAGAAAAATTAGCA
AAAATTGCAGAAATTTCAGATATTACTGCATACGGAATGGAAATGCTGACTGCAAAATCAACTGT
TGGTAAAATGACTTCAGAAGAAATTTTACACATCGATTATAAACCATTTGACATGAGCGGTAAA
AAGGTAGGTATCGGCCAAGCCGAAGTAATCAACATGTCCGAAGTTGCATCTAAAAAAGAAGCAAT
TCAAAATTTAATCGACGAAATGATTGAAAAAGAAGGATATGACATGGTATTGTTCCTTTTAACA
GACATCATGAAAGAAGGAAGTGAAGTTTTAGTTGCAGGAAACAAATCCGCTTTTGAAACTGCATT
TGAACTTAAATTGGATGGAAAAAGTGTATTTATTGATGGACTAATGTCAAGAAAGAAACAGGTA
GTTCCTCCGCTTGAAAAATACTATTCAAATTAATTATTTTAAATATTTTAAAATTTTATACATTT
TTTACTCTTTTCTCAAAAATTAGATAAATATAAATAAAAATGCTATAAAATAGGAATTATATTG
TTGAATTTTAACTATTTTTTCAAATGTTATCAAAGTTTTTGAGGTTAATTATGTGGCACTATTTG
TTTTTGGCGGTTTATGTTGCTATGATTATAGGAATAGCTACTGTAACGAAAAAAAGAATCAAAG
GAGTTTCTGACTTTTTACTAGGGGGGCGAAACGTAAACCCTTGGATGTCTGCGTTTTCGTACGGT
ACTGCTTATTTTTCAGCGGTTATTTTTATCGGATTTGCCGGAAAAATGGGTTGGAGTTTTGGAAT
CCCGACAATGTGGATAGTTGTTGGAAATACGATTATTGGAAGTTTTTTGGCATGGCAGGTTCTAG
GTAAAAAAACAAGAGAAATGACGCAACGATTGGGTGCTTCAACAATGCCGAGTTTCTTAGAAAA
AAGGTATAAAAGCAAGAATTTGGAATCATTAACTGCGTTTATCATCTTTTTCTTCTTAGTTCCTT
ATTCTGCATCTATTTATAAAGGTTTAAATTTCTTATTTGAAGGATTTGGAATATCTCCAATGAAT
GCGTATATTTTAATGGCGGCATTAACTGCGATATATTTATATTTCGGAGGATTTATTGCAGCAAC
ACTCGCAGACTTTGTTCAGGGCTGTATCATGATTTTAGGGGTACTTTTAATGGTATTCTTTTTAG
GCAGCAACCCTGAAGTTGGCGGGTTTTCTGGAATGCTGTCGAATTTAAGTTCAATAGATCCAATG
CTGGTAACCCCGATAAATGAAGCAAGCTTTATTCCAATAATTGGGCTTGCGTTGCTCACAAGTAT
TGGTAGCTGGGGTCTTCCTCAAATGATACATAAATTCTACACGATAAAAAGTGAAAAAGCAGCAG
TTTCTGCAAAATGGGTTTCAACAGCATTTGCGTTTATCATTACTTTTGGTGCAGCATATATTGGA
ACTGTAAGTAGACTTTTCTATCCCGATGCTGCTTCAAAAGCTGCAGCAGGTTTAACGAGCCCTGA
TATGATAGTTCCAAAAATAATGGAAATTGCACTTCCTGACTGGGTTGCAGCATTAATTCTCGTAC

TTGTAATTTCAGCTTCAATGTCTACTTTAGCATCATTAGTACTTGCTTCAAGTTCTGTTGTTTCA
ATAGACTTTGTAAAAGGAAGATTAAAGACTGATTTATCGGATAGAAACACGATGATACTGATGA
GATTGTTGTGCGTTTTATTTGTTGTATTATCGTTCATACTTGCAATAGTTCCAAACAGCTATGTT
CTATCATTAATGGCTCTTTCATGGGGTTTGGTATCAGGATGTCTTTTGGCCCCGTACTTTTTAGG
ATTATACTGGAAAAGAACTACAAAAGAAGGAGTATGGGCAGGAATTACCTCTGCTCTCGCAATAA
TGTTTGGAGGAGCATACTACTTTGGAATAAACAGCGTTTATATGCCGGCAGTTGGTTCTTTAGCA
ATGATTGCACCAATATTCGTTGTAATGGCGGTAAGTCTTATCACAAAACCATTTGAACGTGAACA
CATTGACTACATATTCAATAAAAAATAATTTTTTTACTAATTTTTATTCCATTTCGTAATTTTTA
GAGACTTTTGAATGATTTTTCCCAAATTTTTTAAGTAATTTTAACTGCCAACTCAAAAACGTATT
CAAAATTTGTATATATTATTATCAAGATATTTGGTTGAGTATTACCAAATAGGATTTTTACATCG
CGGTGTGATTTTCCAATGGAGTTGAAGTTATGATATCAGAAAATTTGAGTATCGTTTTGGTATTC
ATACTCTATTTGCTCGTGGTAATGGGCGTAGGTATGTATTTTTACAGGCGAAACGAAACGATAAG
CGATTATGTGCTTGGTGGCAGAAAATTAAATAGTTGGGTTGCAGCGTTAAGTGCGCAAGCTTCAG
ATATGAGCGGTTGGCTTCTTATGGGGCTTCCTGGTGTTGCATATCTTTCAGGAATGAGTGAAATA
TGGATTGGAGTTGGTCTTGCAATAGGAACTTACCTAAACTGGAAATTCGTTGCAGAAAGGCTTAG
AAGATACACAGAAATTGCAAAAGATTCTATTACAATACCCGTTTACTTAGAAAATAGGTTTAGA
GATCAGTCTAAATTGTTAAGAATTGTTTCAGCGTTTTTTATTATGCTATTCTTCTTATTGTACAC
GTCTTCAGGATTAGTTGCAGGCGGAAAGTTGTTCAATCTTGTATTTGGGGTAGATTATACTCTAG
CAGTTACAATCGGTGCTTTAGTAATTATTGGTTATACATTCCTCGGCAGTTTCCTTGCAGTTAGC
TGGACTGACTTTATACAAGGTTCCCTCATGTTTATTGCGATATTTTTAATTCCTATCATGGGGAT
TTCACAGGTGGGTGGAATTGATGCTACAATGAATGCATGGAATTCAATAAGTCCAGATTACCTAA
ATCCATTTACCAACCTAGATGGGGAATCTTTAGGTATAATGGGACTTGCATCAGCTCTTGCATGG
GGTCTAGGATACTTTGGAATGCCCCACATCCTTGTAAGGTTTATGGCAATCAAATCAGCTGATAA
AGTTCCAAAAGCAAGAAAAATTGCAACTACATGGGTTGTAATCAGCCTTTTTATGGCAGTACTTG
TTGGAATGGTCGGTGCAGTGGCTCTTGGAGCACCACTCGATGATCCAGAACATGTATTCATGGCA
ATGGCACAAGGGTTATTCCCAAGTCTTATTGCAGGGTTATTTTTGGCAGGTGTTTTAGCAGCTAT
CATGAGTACTGCAGATTCACAGCTTTTAGTGACCGCTTCAGCAATTACTGAAGATATTTATGCAT
TATTAAATAAAAATGCAAGTCAAAAAGAGCTTTTATGGATAAGCAGGTTTGCGGTAATTGTTGT
GGCAGCAATAGCTTATTACTTTGCAATAGTTCCTGGAAGCAGCGTTATGGCTCTTGTTTCATACG
CATGGGCAGGATTTGGTGGCGCATTTGGTCCAGTAATATTGCTTTCACTGTACTGGAAGAGAATG
ACAAGAAATGGTGCTCTTGCAGGACTGTTATCTGGTGGATTTATGGTAATTCTCTGGAAAAACTT
GAGCGGTGGAATTTTCGACCTTTACGAAATTGTTCCGGCATTTTTGCTTGCATCAGTAATGATTA
CGGTTGTAAGTTTAATGGATAAAGAACCGTCATTAGAAATTCAAGAAGAGTTTGACAGGGCAGT
TTCTGAAATGAAATAGAAAATCTAAGAAAAAACTAAGAAATAAATATGATAAGTACCAATCATA
TTTAAAATAATTTTTTTATTTTGGGGTACTATGCATTATTCAATGATTAAACCTGTTTTCAAAGA
AGAAGAGTTATTAATAGACAAAGGGTCGCTAAAAACAAAGCGAAAGTTTGCTTTTTTACTAGAT
ATTAATGACCGAGTACTTATTAACCGTAATTTTTATGTAAATGATGAAGTAGATGTAGTTTTAG
ATTATACATATACGAATAGCAAAAGACCAAAAGAAAAGATTAAATCTTATGTTTTATCCGATAT
ATCAAAAGAATAGGTGTAATTTATGAAATTAGACGCAAAAGTTTCAATTTTTCATGCAATATTT
GGCGCAGCATTTGGATACCTTACAAACTACGTGTACATGTTTGGTTTAGGAATGTTTAGCGGTGT
TGCATCATTTGTATTCATGTTAATCACATTAGTTATTACTGGAAATTTAGCTTCAATGATATTTG
GAAGAGAAAGTATGAACCAGAAAGAATGGATGGGAAGTGGAGTGGTTCCATTTTTCTTCATCTG
GTTGGTATTCTGGATAATGACATACAACGGAGTCTTTTACTAAGGTGGTTTTGTGGAATTGAACA

TCAAAATGGATAGATCAAAAGAACTATTTGAAGAATCAAAAAAATACCTTGTTGGGGGAGTTAA
TAGCCCGGTAAGGTTATTTAAGCCATTTCCATTTTTTGTAAAATCTGCAAAAGACTGCTTTTTGT
ACGACGAAGACGGAAACGAATTTATTGATTACTGTTTAGCATACGGGCCAATGGTTCTCGGCCAT
GCAAATGAAAATATCCTAAACGCTGTAAAATCTCAAATGGATTTAGGAACTGCTTACGGGGTTCC
AAGCGAAAAAGAAATTACTCTCGCAAAAGAAGTAATAAATAGAATTCCTTGCGCAGAAATGGTT
AGATTTGTAAATTCTGGAACAGAAGCTACCATGGGTGCAATAAGGCTTGCAAGGGGAGTTACTGG
AAGAAATAAAATTATCAAGTTTGAAGGTGCATTCCACGGTGCTCACGACTACGTGCTTGTAAAAA
CAGGTAGTGGTGCTTTAACTCACGGTGCTCCAAATTCACCCGGAATTCCTGAAGATACTACAAAA
AACACGCTTTTAATTCCATTTAATGATGAAGATGCAGTAAAAAAAGTAATTTCTGAAAACAAAG
ATGAAATTGCATGTATCATCCTTGAACCTGTAATGGGAAATGTTGGCTGTATTCCTCCAAAAGAT
GGATATTTACAATTTTTAAGAGAAATTACCGAAGAAAATGGAATACTTTTAATATTTGATGAAG
TTATTACTGGTTTCAGACTTTCAAAAGGTGGAGCTCAGGAATATTATGGAATAAAATCAGATCTT
GCAACTGTTGGTAAAATTTTGGGCGGAGGATTTCCAATTGGTGCAATAACTGGTAAAAAAGAAT
ATATGGAACAGTTTTCACCAAATGGCCAGATTTATCAGGCAGGTACATTTAACGGAAACCCGATT
TCAGTTACCGCAGGAATTGAAACACTTAAAAATCTTGATGATAAATTCTACAAAGAAACAACTAA
AAAAGCAGGCATTTTATCAAATTGTTTGAGAGAAACTGCTGAAAAATATAATATTCCTGCAAAA
GTTTACAATGTAGCGTCAATATTCCAAGTTTACTTTAATGATAAAGAAATTGTAACTTATGAAGA
TGCAAAATCAAGCGACACTGAAAAATTCATGAAATATTTCTACACGCTTTTAGAAAACGGTGTAT
TTGTAGCTCCATCCCAATTTGAATGCTGTTTTACATCGATAAAACACAATGATGAAGTTTTAGAA
AAAACAATGAATGCAATCGATATTGCAATGAAAAAATTGTAAATTTTCTTCTTTTTTAATTTTTA
AAAATAGCAAATAATTAAAATAAATTAAATGATTTCAGATTTTTCAAGAATTTCTCGTGCTTTT
TCTTTCGAAATTCCGTTTCTTAAAATTGTATGCCTGTTTCTAAGTGCATGTGCAGAAGAAAGTGC
TTCAATTACAATTTCTTCATCGAACCCGAGCTGTTTTGCAGTAACTGGTGCATCAACTACTTTTA
ATGAAGTTTTAAGCATTTCAAAAGTTTCAACTTCAAGTTTTCCTTCTTCAAGGTAAATTTGGGCA
GTTGCAAGTGTTCCAACTCCACACTGTTCTCCGTGTAGCGAATTTGTATCGATACTATATTTTTC
TTTCATTGTGTCAAGTGCATGCGAAAACAGATGTTCGCTTCCTGAAGCAGGTCTTGACGAATGAG
CAATTGCAATTGAAATTCCGCTTCCAATGAGTGCTTTTACGAGTTTTTTTGGATATTCTTCTAAA
TTTGAATTTAAAACATAATCCATAAGTTCAATAGCAATAGTTTTAGAAAAAATACCTGAGCTGTC
ACTGTATTTTTCAGATTTTTCAATTTTTTCCAAGTTCCCAGTCTAAAACCGCGGTGATGTTTGAAA
CAATGTCGCCCATACCTGCTGAAAGAAGTTTTTTTGGAGATTTTTTTATTATATCAATATCTGCA
ATTATTGCAATTGGAGCTTCAGTCATGTATGAAGGCTGTGTTAATGAAACTATCGGGGATGCAAT
CCCGTCGTTTGATGCAGTTGTTGGAACACTCAAGAAAGGCTTTTTTGATTTGTGTGCAATCAATT
TTCCTATGTCTATCGGTCTTCCGCCACCTATTCCTATGATGGAATCGTAATCATTTGCATAATTTT
TTATATTTTCTAAATCCGAAGTTTCAATTTCGTCGTAGTAAATAAAATCAAAATCTTTATTATAT
TTTTGAGTGTTTTTGCCAGTAATTACGAGGGGATTTTTTAAATTAAGATTATCAAGGATTTCTTG
TATCCTAAAAGATGCTTTTTCCTTAATTATCGTATATCTTGGAATTACAATCATTTTTTCACTAT
TTCACTATTCAACGATGTATTTCGTTGGATCAGTTATTCCTGCTTCTTTAAATGCTCTTTTTCTTC
TCATACAGCTTTCGCAAGTTCCACAATGCAGATAATCTTTACCATTATCTTTGTAGCATGAATAG
CTGTATTTTAATACTTCTAAATCAAGTTTTAATTCGAGTTCTTTTCCTAATTTTGCAATTTCAGG
TTTATTTAATTCATATAATGGTGCAACCATCTTAACTTTATTTAATGTACCGTATTCAAGGCTTT
TATTAAATCTTTCAATGAATTCAGGAGTGTTGTCAGGAAATGTTACTCCTTCTTCTTTATTTAAC
CCTGAAAATATTTTTTCTGCACCTATTCCTTCTGCAAACCCTGATGCAATAGAAAACATTATCAT
GTTTCTTGCAGGAACCCAAACTGCCTTCATGGTTTTTTCAGATTTTTCAAAATCATCGAGGTCTT

CTTTTTCAGGTTCTGGAATGTTTCCGGTATTTAATCCGCTTTCACTGAATTCTTTTACGAAATTA
ATATCAATTACGTGATGATCAGCATTTAAAATATCTGATATTTTCTTAGCAGATTTTATTTCTTG
ATTTAGCGCCATCTGCCCATAGTTAAATGTAACAGTACCGATATCATAGTTTTTAGATTTTGCAA
ACATTGATGTTACTGCAGAATCAAGTCCCCCACTAAGTACACAGATAGCTTTCATCAAAACACCT
TTATTATTTTAAATTAGTCTGGAATTATATTTTCAAAGGTATTTAAAATTTTATAAATTAAATG
ATAAAACTTAAAAAAAGAACAAAATGAAATTAAAATAAAAAATATTTAAAAAAGATTAAATTCT
TCTTCCAGGAAGTGTACTCTTTCTATCGAGTAGTTCCTGCTGTTTTGCTTTGTTCCAGTTTGAAAC
GTTCTGTAAGTAACCTGTAATTCTTGAAAATTTCTCTATATGGTCACTTCCGCAACCTGTACAGT
TGTCTTTTAAACCGCTCATTGCAAGACTGCATTTTTCACAGATGCTCAAGTTTTTCGTGTATGTC
CAAAAACCAATATCTGTTTTTGTCATTTTTTTAGTAATGTCCATTAAAACTTCAGGGTCTGCATA
AGCTTCTGCATTCCAGAAATGGCCTATGTGTCCACCGTTACAAAGTTCATGGAATTTTTCTTCAA
TACCTACTTTTTCACCCAATGTAACGTCCGCATTTACTCTAACGTGCGAAGAATTCGTATAGTAT
ATACTGCTTAAATCATTCAAGTTTCCGTTTACCACTGATTTTGTTTCTTCTGGGTAGTATTTGTG
GTCAAATTTCGCAAATCTTCCAGCAGTGCTCTCAGCAGGAGTTTGTGTTACAGTCCATCTAAGTC
CAGTTTCTTTTTTCAAATCATCAGCGTATTTTCTAATGTGTGAAATTACTTTTACACCAAAGGCA
AGTGCTTCTGGTGACTCGTGAAGTTCGCTTCCAACGTGGTATTTTAAAAGTTCGTTTAAACCAAC
GAAACCGAAAGTTTTCGTGGTGCTATCATACCGGTAATACTGTTCTCCATCGAATTCCTGTGTTA
AGAATGGTAAAACATTGTCGTGGTAGAGTCTTCTTTTAGAAATTTCATGTTTTATCTCAAGCGCT
TGTTTTGCAATAGCTAATTTTTCGTCAAGTATCTCGAATAATCTTCCGTCATCACCGTCAGCTTC
GTAAGCAATTCTTGGAAGGTTTATTGTATACCACTGCATGTTTCCAGTTCGAAGTGAACTTTCTT
CAATGGAATTACCCCATTCACCGCTTAATCTGGTTCTGCAACCCATTGCATTGGTGTTTCCATGCT
GGTAATCGGCAAACATATTTATAAAGTACGGTAGTCCCCATTTAGCGCTTAATTCATGTATTTTT
ACGAGTAAATCTTTATTTTCATCTTTGAATGCATTTTCTCTTAATTTGAAAATGACATTAGGGAA
TAAGAATGGTTTTCCGTATGCATCCCCATCCATTAAAACATCTACAAGCGCTTCTGCAATCATTT
TAACTTCGTAATCGTATTCTTCGTAAGCTCCAACAACTTGACCTGCTTTTATTGCATCTTTGCCTT
TTAGGTATTCTGGAACTTCCAGTTCAAGGTTAATTGAACTGAATACGACCTGTCCTCCCCTTGCA
GCATATATCTGGTTCATTTCATAGATAAATAACTGCATGAGCTGTCTTACTTCGCTGTATTCAAG
TCCTCTCATGTATGGGGCAATCCAGATGTTAAATTCATCTATTGACTGTCCTCCAGACATTTCAC
ACTGTGCTGCACTCAAAACTTTTGCCGCATGCTGTAATGCAACATCCGGGTGTTTTGCAGGTTTT
GAAACACTTGTATGCCTTCCAGTACCGTCTACTTTTAAACCGTATTTAAAGAAGTTTCTAAGGTC
GTGCTGACAGCAAACAGTTCTAACTGCCGCATATTCTAAATCGTGTAAGTGAATGTCCCCTCTCA
TGTGGGAATCTGCTATATATTTTGGGAATACATTTAAAAGTGCAAACTGTTTCATGGTTTCGTCA
GCAACCCATTTATGAATACTTTCAGGGTTGTGCATGAGGTTTGCATTGTCTTTTGATCCTCTTTC
GATTAATTTTACGATATCGTAAACAGGAATACCAAGTCTCGTGTGTTTGTGCCTGAATTCTTCTA
ACCCGTGTTCAATAAGTTTTGTATTAACAATTTCTCTTATCATTGGGGCAGTAAGGTACGTTACA
TTTAAATTTTTAACTTCTGACTCTACTTCTGTAGCTATTCTTTCTGCAGTTTTTAAATCAGCGCCT
GCTTCCTGAATTAGGGCTTTTACAATCTTTTCTTTATTAAATGGTTCAAATTCTTTTTCAGATGT
CCTGATTTTCAGACAGTTTTCAGACATGTATTTTTTAGAGTAACTTTTGCCCGTTTTTTTGTCGT
ATTCTTTTAATGTTGTGTAAAGGATTGATTTTAAGTCATCTGTAGACATTCCATTGTAGACTTTT
CCGTAAATTTCAGACAATATTTCATCTATATTTTCGTAATCAACTCCTGAATTTAAAAGTGATTT
TACGAGTTTGTTAATGTTGAAGGTTTCTTCATTACCGCTTCTTTTAACAATATTTATTTTGAAAT
CGACGTATTTCGAATTAGAGATCATTGAACCACCTAAAAGAGAATAAAAAATAAATAATTCAAT
AAATGTTAATATCGGGAGAATGACCCCGATTTAAATCCAGATATTACCGGGGTATTTATAACTAG

AATTATATTAATCAATATCATAGGATAAGTAAGATTAATATATATCATTATCGATACGAAGTTC
GAGCTTTAATTATATAAATAAGCTTAAAAACGGATATTTATGGATGTTTACTGGTGAAGCAGAT
GAAAATAATTAGTGAAGGCGAGACAAAATTAATGGTTCCAGAAGAATCAACTTTATCAAAGAAA
GATACTGTATTTTATAATCCGGTAATGGAAACAAATCGGGATATTTCTGTTTCAGTTGTTCAATC
ATTTTTAGACGATTTCAAAAGAGATGAATTTTTAATGTGCGATCCTCTCGGGGGCAGTGGTGCAA
GAGGGATCAGGTACGCAAAAGAATTGAAATTTAACGGGGATCTGAAAGTTTCGATTGGAGATAT
TAATCCAAGCGCCGTAAAAATGATAAAAGAGAATTTAAAATTAAATGAACTTGAAAACGTCGAA
GTATTTCACGAAGATGCAAATGTACTTCTTTCAAAAAACTTTAAAGTATTTAATGTAGTTGATTT
AGATCCATTTGGTTCCCCGGTTCCTTATTTGGACAGTGGAATCAGGGCAAGTCTCACAAAAGGGG
GACTTTTATGTATGACTGCAACAGATACAGCGGTATTATGCGGAGCATACCGAAAAACATGCATT
AGAAAATACAATGCGGTGCCTTTAAAAGGCGATAAGGAACTTGCAGTACGTTTAATGATTGGAT
ATGCTGTAAAAATGGCTTCAAAATATGATATTGGATTAAAACCGATATTTTCACATGTAACGGAT
CACTATGCAAGAACTTTCATGGTTACAGAACGGGGAGCCGGAAAAGCAGATTCTGCAATCGAAAA
TATAGGATATATCCGGCAGGATAGTGAGCAAAAATCATTTAAGACATTTGAAGAAGGGTCTGAA
AAAGGATACGCAGGTCCATTTTATTTAGGTGACATTTCAGATAAAAATATCGTTCAAAATGCATT
AAATACTGCTAAAACTAGAAATTATTCAAAAAGAGCAGTAAATATTTTAGAACTGATTTCAAGA
GAATCAGAAATCAATCAAGTGGGCTGTTTTGATATTCACGAGCTTTGCAGTTTTATAAAAAAGCT
GGTTCCGCCGGTAAACGATATCATGGAAAACTTAAAAGAAAATGGATTTAAAGTTACAAGGGTT
CATTACAATCCTTATGGTTTAAAAACAGATGCAGAACTTTCAGATCTGGTCGTTTTAATATCTGA
ATACCATTCAAAAAAATATTAATTTATTTTTATGTTATATCTATTTTTAAAATTATTAGTTGTGC
GAAAGAATTTTTCCAAAGAAGTTTTCGTTCTTTCATTTTTAGGATTGCTAAATACCTCTGAGGG
ATTTCCGTCCTCTAAAATCTGGCCATCGTCAATAAAAATGAGCCTGTCGCCGACTTCTTTTGCAA
AACCCATTTCGTGAGTTACAACAACCATCGTCATTCCTTCATATGCAAGCTGTTTCATTACATCG
AGTACTTCTTTTACGAGTTCAGGGTCAAGTGCAGAAGTTGGTTCATCAAATAACATTGCATCCGG
TTTCATTGCAAGAGCACGTGCAATTGCAACTCTTTGCTGCTGTCCGCCTGAAAGTTGTATCGGAT
ATTGATTTTCTTTTCCTTCTAAGCCCACTTTTTTTAAGAGTTCTCTTGCTAACTCTTCTGCTTCTT
TTTTTGAAACGTTCAAAACTTTTCTCGGAGCAAAAGTAATGTTATCTAAAACGGTTAAATGAGGG
AATAAATTAAATTGCTGGAATACCATACCGATTTTTTGCCTAATCTTATTGATATTTACTTTTTT
ATCGGTTATATCGTCCCCTTCAAAGAAAATATGTCCTTCAGTAGTTTTTTCAAGTCCATTAATAC
ATCTGAGAAGTGTAGATTTTCCGCTACCACTTGGACCGACAATTACTAAGACTTCTCCTTTAGCC
ACTTTTAAATCTACTCCTTTTAAAACATGAGCCTCTTTGAATTTTTTGTGGATATTAACCATTTC
TATCAATTTTCCACCTGTTTTCAAAGTTAAAAGTAAAATAAATTAATTATTCGTCTTTTATTTCT
AATTTTTCCTTTAAAAATTCTTTTACTTTATCTTTTCCTAATTTTTTACGCATTTCATAAAGTTT
TAAAGGGGTAATTATTTTATAAATTTGTGAATCATCAATTCCAAACTCTCTTGATCTTGCTTGAC
ATATTCTAATTCCTATATCATTAATTGCAGCATCTACATTGTCTGCATGATGAATTAGCCATGCT
TCAATTGAATCGGGACTTACGGGTCCGTGGTCTCCGTGGTGGCTTGCTACAACCTTTATAACTTC
GAGTGGAAAATCTCTTCTGTAAAGTTCTGCAATTCCAAGTGTTAAATGATCAAGGTGAAAATCTG
AAAGGTGGTCGAATTTTCCATCTTTTAACTGGTAATTTTGCGGTTTCATAAGGTCGTGGAGTATT
CCTCCAGAAATTAGTAAATCACGGTTTAGTTCAATACCATATGTTTCTTCCAAAGCTTCTGCCAT
TTTTACAGCCATTTTTGTAACCGCAATTGTATGTTCGAGGAGTCCTCCGGGGTATTTATGATGCC
GTTTAACGCTCGCAGGGGAGTTCTCAATTGTTATTCCAGTACTTTCAATTTCAGGATGTTTTGGT
AGGGGATTATTTATATATTCAATAACTTTTTTTCTTAATTTTTCGTCCTTAATTTCCTCTGCAAG
AGCGATTAGATCTTTCATTAATTCACCTCGGGCATATATAAAACAATAAATAGATTAAATTACTA

TATATTTCATTCGATAAATGAAAATGGTGCATATATGATAGATTTAGCAAAAGACCACTTAAAA
AAAGTTCTATCCCTCTGCGGAGCAAATAGGGATTGCGAATATTATCCATGTCACTATGAAAATCA
ATCGTGCCTTTGGTGCTACTGCCCATTTTACCCATGTGAAGATGAAAACTTGGGCGAATTTGTAA
AAAGAAAAGACGGATCTTTAATCTGGAGCTGTATGAAATGCAACTGGATTCATAAGCCAGAAAT
TGCATCCGAAGTTTTAAAAGAGATTACAGAACTTACTAAAGATAAGAAAATAAATGATTCGATC
GAATTTATTGATAATCATGAAATTTTAATGAATATAAAAAGGCGAGTTGAAGAAAAACTTGGAA
AGGATAATTCAGTTTAAGGGCGGAAAAAACCATGTTTGGATTTGATAATTTAATTTTAGGAGTT
TATTTATTCAATTTTTTACTGATACTGACTGCAACATATACTGATATCAAAGAGCGAATAATTCC
CCATTTTGTAATTATATTGATGTTAATTGTTAATTTACCAATCGGGTATTATTTTTTTGGTTTTG
ATGCAATAACTTCATTTTTTGCAACGTTGATTTTATGTTTAATTCTTGGGGTCGGCATGGGTGGT
GGAGATGTTAAGATGTTTACGGCATTATCTCCACTTTTTGCAGCAGAAACTATATATTTCGTTCC
GAAGGATATTTTAATATTAATCGGTTTAAGTGCACTGTTTGCGGCAATTTTCCCAATGACAAAAA
TATTAAAAAATTACTGGAAAGATATTATTCCATCTTCCGCATATCTTGCAATGCTTATTGGAATA
ATAGTTTCGATTACAGAAATCTATTCTATTGGAAATACCAAGACAATACTATGGATTTACATAAT
TTTATCGATATTTATTTCCCGAAAAATTCCAAAATATCGAATTATATCTAATAAACTTGGATATA
TTACGCCTATTTACTTGGTTGGATTTTATTTACTCAATCCTGCGTATTTTGTATCCGAAAATGTT
CTTATTTCGTTTTTTGTGTATATTGGACAGCTTTCCTTAATTTCGCTCGTGATATATGCATTAAC
GGGTGCTGAAATATCTTCAAAAAAACAAATCAGCGATTTAAAAGAAGGGGACATAATTCGGGAT
ATTGTTACAATAAAAGAAAATGGCGAAGTAACTGTTGAAAATGCAAATATTTTGAAGCGTTTCA
AGCACATGATTTATGGAGAAATGGGTAAACTCGAAGGAAAATCGTTAATGACTGATGGAGAAGG
TCTTTCTAATGAAAATCTGGAATTATTAAATAAACTGCAAAATGAAGGGAAAATCGGCGGGGAA
TTAAATGTTTTAACTACGTATCCGTTTGTTCCATTTGTGCTTGTTGCTTACTGTGTAATTACCCT
ACTAAATATGGGCTTTATAAATTATCTGGTGAGATAACATGTTTAAACGTTCTAATCGGGGTCAG
ATATCTTTTGAATTTTCAATAATCGTTCTTTCGATACTTTTGATATCCACAATAACTATTACGTA
TTTTTTAACTTCTTCATTTGACGAAGGAACTAGATCACTTGATAAAATTGATTTGGGTGCAAAAA
CTGCGGTTTCACTCGTAAATTCCGGATATAATGGAACACAAGCAGAGGGAACACTTATTTATGCA
GGGATGACCTGGGATAATGCAGGTAATAATTATGAAAATATAACGGTGTATATTTCAAATACAA
CACCAGTTCCGGTTAATACCCGGGTTTTTGTGAAAAATTATACTATCGAATCTCAAGGTATTGAT
ACAACAAAATATGATTTTAACATAGCCTGGTCAGGTTAATAATTTTTTTAAATTTTAAAATAGA
TATTTTGAAGACAATAGAAAATATATTTAAAAGATCCTGACAATATATTTAATATCCATATTTA
TTATTTCATTGTTATAATTTCAGGAAGGAGGGAGTTTATGCCTGCTGAAGAGTATAGTGGTAAAT
TGAAGAGTTTAAAATCTAAATCAAAAAAATCTTCTAGGATACCTCCGAAACAGCTTAGAATGGCA
CTAGTTATTGTGGTTTCGGTTATTGTGATTTTTGAGGTTTACAATATTTATTTCACTGCAAAAAA
CAGGGAGCTTGAAGAGTTAGAAAATAACCGGGCGGTCGCAATTGATACAATTGATAAATTATTCT
TTGAGTATCCAAATGATCCTCAGAAAATCGCGTACGTGATTAAACTACAGCAAAGTCAAGATGAG
GAAAATCGCGTACGTATTTTGGATGACGCCCAAAAATATTTGGAAATAAAGCAGTATAAGACTCT
GGCCATAAATCAGATTAAGGATATGTATGGGGAGTATTATGGGAGCAGTATAAAGGCAAATGAA
CTCGAAAGAAAAATAAACAGTGCAAATTCAACTACCGAAGTTGATGAGCTGTTCAAACAGTCAAA
CGTGGAAGAAGAAGTTAAAGAAATTCTTGAAAAATCGCTTGAAAAAACAATATCGTCTGGAGAT
TCATACTTTTATGTTGAAATGTCTGGTCAAGAATATTTCATGACAAAAGACGACGTTTTAGAAAT
TACTGATTCAATGGGCATAATGCAACTCAAAGAATTTAGGGTAACTCCAGTATCTAACTTAAATA
AACTTACAGTTGTTGTATCTTCAAGACAGTGTGGTAAAATACCACTTTCTGGAGATTCAATATTG
GTATACAATAAAAATACTCCTGAAGCACCTATTGCTTATGCAGTAATTGATTCATCATACGTGAT

TCTCCCAGATATAGGATATAGTGAAGAAAGATCCGTATCAAATATTGTAAACGACGAAGGAAGT
GAATCAACTGTTGAAAGTACTTCATCAATTTCATACACACTTAACAACGTGCCCGGAGTTCTTCA
TGCAACTGCTGCTGATAAATTAGATATCGGAACAGTTATCGATAAATTTGGAAGCTACGGTGAAA
GACTTAATAAAATTGAAGACAACACTCAAATATTTGATGAAAATGTAAATTACTTATTAATAGT
TGCAGTTCCAACCGAAAAAGTTTCAAACTTGGTTGCAGTAAACAGTGAGGACCTATTCATTGTAA
AAGCAGCAGGTGGTAACTGATGAAAATGCTTAATATAAAATATTTAACTATTTTTTTATTTTTA
ATATCAATAATTCCAAATGCACATTCTGAAACTACTGTTGCTACTATGGATACTTACTGGAGTTT
AACTGGGGAAATTACAAACGAATATCCTTATTCCGTATTTATAACGATTCCTGAAGAAATTGAGT
ATATCGAGAAAGATTTAAAATCTTCAAAAAATTTCATGGAATTAACAAATGGTGAGAGTGACGA
TGACGAAGACGAAGAGTCTACCGATCCAGTTACATTTGATATCACTCCAAAATCAGAATTAAATG
GGCAGGAAGGATTTTGGATCCCACCTTATACGACTGCAAATATAAAGATGTATTCTACAAGGGTT
GACGTACTAACTACAAAAGAAGATGACGAAGAAGAAGATGACGAAGAAGTAGTTCGAACAATGG
AGATATCTGGCCCTGCAGCATATCCAAAAACAAAACTCATTGATTTAAGTGAACTAATTCCTGCA
TCTAAAAAAGATAAAATAGAGCTCGATAATTTTAAACTTTATGTAAAAGGCTCAATAGTCAAAA
ATGATACCGATGTGCTGAGTATTTTGATACCTGCACCAATTGTATTAAAGGATTACAGCACTTGT
GATAAATTTATCAGCAAATATTCAGTAAATACCTGGGTAGATTCATATTCCGACTGGGTAACTGA
TCACAACGAATTAAGCAGTTATGGATCAACAATGCTCGATAACACACTTGTTCCTGTGTTTGACT
TAGGTCCTGGGTTTTACAAAACATTTGATGTCCCTGCACTCGTATATACTACTTCATCAAATCAA
GAATTAAGTTTTTCATACAAAATGTATTGGTATGCTGAAGAAGAAGACACAAAAGACATCTGGA
TGCTTTAAACTACCTATTTTTAATTTTTGAGGAATGTTAAAATGTCAAAAGGACAGGTTTCAGTA
GAATTTATCGTATTATTTTTAGCACTTCTTGTAGCGGTTGTTGTATCTACAATGACTCCAGGAAT
TTTTGGATTGAATAAATCTGTAGAGTTATCTTCTGCAAGTTTAGCTCACGCTGCACTTTCAAAAG
TTAAAAGCAGTGTTGAATTGCTTTCAGTATCTGATCCGGGTTCATCAAAGCTGGTTTATGTTAAA
TCCCCAGTTTCAAAATGGGGGATTAATAATAGGTCAATTTCGATATCTGGGGACGGATATAATAT
TTCAACAAATACGACTGTAACTTTAAAAAATAAGCTTGGAAGCACTTCATTTACCCATGAAACTA
ATTCATTGAAGATATTAACAGTTAATTTAACAAGGATGGATGAGTATGTTCAAATTGACATCAA
CTAAAAAAGGACAGGTTTCTTTTGATTTTATACTCGCAATGCTTTTCCTATTATTAATTTTTGCA
TTTACTGGTCAAAATGTATTAAACATGGCAAAATCATTCAAAGAGTCCGAAACTGTTGAAAGGGG
GCATGCAATACTTGATAATTTCGAAAATTATGCAATCACTGCGTATTCAAAAGATGTTACCATAA
ATGCAACTTTTAAGCCTGTAGGTAACTTAAATTATACAATTATGATTTCAAACAAGACAATTGGT
GTAAATTCGACTACAAATATACTATTTAGTCCCGATCCCGATAATAATGGAGTTGTTAATATATC
CAGTAGTAATATTAACAATTCAGTAAATTCAATCCCTTTAAACACTGTAAATATATCTTTTGGGG
ATTTTTATGTTTCAAAAACATTACAAATATCTATTCAATAATCTTTTTTTCGTATTTTCTGTTTT
TTTATTAATTTCGTCAGTATATTCATATTCTGATTATTATAAGGTAAATGTGGATGAAAAATCTA
AAACTGAAACATTTAATATCGATAAAGTAGAGAATTATACCTATAAACTTAGTTATGTTCACTAT
GGAAATCTTCAAGAAGGAATGTATGTGAAGATTTTTGTAAATGGCAACAATATCCATGAATATT
CAAAAGATCTTTCAAATACTGGTTCAGGCGCATATAAAAAATCAGAAAACAAAACAGATATCAC
AAATTATTTGGTAAACGGGAGTAATGAACTCAAAATAGAATCAAATATCTGGAAAACAGAAAAT
TCGAGCCCCTATTATGTTTTAGAAAATTTTGAGATCACCGAACATGAAGTTTCGACAATAAAACT
TCCAATATCCCAAAATGTAAATTTTTTCGTATTTATTTTATGTTTGACATGTTTGATGAGAAGAA
AGGGATAAAATGACTTTAAAATCAAAATTAACTTCCAAAAAAGGATACATCTTTACTTATGAAG
CTGTAGCAGTTGTAATTTTATTTGTAGCTGTGTTTTACATGGGTTATTTTACATTTACCCACGTA
AATCTTACTAATCAGGAACAAAAACGAGATTTGGAAAATTTTGAAAAAGCAAATTTAATTTCAG

ACATGCTTTTCAAAATGCATGAATTTCCGTCAAATTCGTATGTTCCCGATTATTTAAGGTTTTTG
GATTCTGTAGCTAAAAGATATTCCGGTTTAGAAACAATTCCTGGTACTTTTGATCCATATTCTAT
GAATCAGGAATTTATATACAATGAATCGTGGTATTATGAAATAAATATTACAAATCCTGGAACT
CAGGATCTTACAGATTTTCAAGTGCTTGTAACATTAAATCCTTCAAATTTTAATTTTGATTATTC
CGAGGATGGTTCAGGGCTCAGTTTCTGGCAGGGAAACACTCAACTTCATTACTGGATAGAAACGT
GGGAGTATAACGATGAAGCAAGAGTCTGGGTGAAAGTTGACAGCCTTCCTGACGAGACGACAACA
CAGATATTGTTAAAAAGGAACCAGGGCGGGTCATATTCAAGTGACGGAGATGACACGTTTGTATT
TTTTGACGATTTTGAAAGAACCTCCTTTGGGGGCGACAAATGGAATGTTGTGTCGGGTAGTTGGG
AAATTATTGCTGATTCGGAACTTTCATATTATAATGGGGGTTTAGGAACTAATCATGTTGCACAC
CTTGAAGCAAGCGACACTGAAGATAGGAGGGCAGTTTCAAAATATTCCCTAAATCTAAATGACGG
AGATATTTATATTTTAGAAGGGGATTCTAAGGGCCATACTGGCGTATCGGGCGGTAGTGCAGATA
GCCCCGATACAATGTTGGGATTTTATTCTAATGGGGGAGGAACTACTTATTATAATACCTTTGAT
GGGTTTTATCAGGTTTTTGGGATATGTACGGCAGGTGATTCAAATGAAAAATTGTCAAGCATATT
TACCAAAGATAATGTATGGTATGTTAAAAAATTAATTCTTGAACAAAAAAGCGATTATTCTAAT
AGAATAGTCAAATCTTCAATATGGGAATTTTCAAATGGGTATAGGGGCGATCCAAATCCAAATA
ACAATGTAAATTCAATGTTTGATGTAAGTGCTGAAGCAAGAATTTGGACTTTTTGGGGTTGGGT
AAATTATATAAATCCTCTGGATTGTGACCACATATTAATCGGAACTGCACAGGGTAGTCACAGCG
AAGAATACTGGTTTGACAATATTAAGGTTAGAAAATATTCTCCAAATATTTTGGTTGACGTTAAC
GAAAATTTAGAGTATAATGCAGGTCAAAATGCCGTAGATGTTTCTGAATACCACTTTGATGGAAT
GCCTTCTACACTTTCATCAAATGTAAATTTGGTTGATGTAAATTATAGTTATACAATAGTTCCCG
ATACATATGTAATGACGAGGAATTTATATGTTCCAGTAAAAACATGGAGATATTCAAATAGTAT
ATCAGAAACTGAAAACATCAGTTCAGGAGAAATTCTATATTTTGCTGCAAGAAGGCCTTCAACCA
TATCCAATATAACTGCAAAAGCAGACGCTACAACCACAGCTTTGTTTATGGTAAATGGTGTACCT
TACGAACTTTCACTGGGTACTGGTGACAAACTAACTGACTTTGAAAAAGTTATTTCAACGCATGC
GTGGGAGTATTATGAACCAAATGAAGTAAAACTTTTGAGTACGGACCCCGTTACAAATGTAGATT
TAACTTTAAATTACGATGAGTCGTCTACAATATATGTTTTAAAATTAAAGCAGTATAATGTGTCA
TGTATTCTTAGTATGAACAATTAAGGTGTTTTAAATGATATTTGACATGGATTTGGTCATTGCAA
TAGTCATACTTACAATTGGAATGGCGTTTTTTACGACATCGCTGGTGGAACATTCCGGAGCATAT
CAGGATGTTATAAAATCAGATATATTGCATGATAAGGTATCTTATGATTTAAAAGCAATTATTA
CTGACGGGACTGCTGAAGCAGCCTTGATGTTAATAAATAATAATGAAGATTCCACTGCAGATGCC
CTAATAAAGAGCAGGTTACATGTTAAAAATTACGATTTTATTGTCGGAAATGAAACAATAGTTA
GTAATGGAAATAAAAATGACCAAAACATGGTTATCGTGTCTTCCGTAGTAATTATGAACCGATCT
GAAGGATGGTATGGGGTAGCATGGAATGAGGGGGGCAGTATTACTATAACTGGTCCTGAAACTTC
GGAATTACAAGCTTATCAAAGCGTTTCAGGAGATTATAAAAAAGCAATTTATTACTTTAATAAC
AGTAAACCCGTCGAAGTTTCTTTAAAAGTATATGGTAACTAAGGTGGTAATTTATGTATCTTTCT
CAAAGCACTACAACTCTTGTATTATTATTGTTGCTGACTGCCACTTTAACGTATAATACTTTGGA
TTTGCAAAAAAATCAAGTTTTAACTGAAATGGAAGCGTCTTCAATAGACTTGAAATCTTCTTCTT
TAGAACACATAATCGAAAGCTCTCTTCCAACAGTGTTTAACAGGGTATTAAATGATGCAGAGCTG
GAAGTAATCGACAATTATAATAACAACCCTTCGGGAGACCCTTTTTTTGAGTCTACTAATTATAC
TTTAGCGTATTTAAAAAATGGGACGGAAGATATAATAGTGAAGGATTATCTTGAAAATGTAAGC
GAAGAATATTCTAATTTAGGATATACTATCGATTATAATTTTAGCATGACAAATATTACGATGG
TTGACGGGTTCACCTTTAAATTGGATTATGATTTGTATTATAAAATCCAAGGAAACAAAGGCATT
ATCAAAGAAGAGAATATTTCTTCATACCAATATTCCACTGTAAAAACGGTTCTGGATGCTTATCA

TCATGTAAAACCTACATATGTCGGAGTAATAAACGTATCTAATCCTAATGCAGAAACACTGTATG
ATTTTCAGGTAAAAGTGGTTTTTAACGACTCTAATTTTGATTATTCAATAGAACCTACTGGAGAA
GGTCTTAGATTTTATGACGAAAATGGAATTATATCCCTTATTGGGTTGAAGTATGGGATTATAG
TGACGATGACAATGATCGAACATCAGTGTTGTGGTTAAAAGTTCCAATATTGGATGCGAATACAA
ACACTTCAATATCTGTATTATCTACATATCCAAAAATTTCAGAAAGCAATGGTAACCGGGTATTT
GAAATGTTTGATGATTTTGAGGATAGTAATTCAATCTATTCAAAATGGAATGTTTACCAAGGAA
GCTGGACGTATTCTACGACTTCAAATATATTTTTAAGTGCCACGTATAATACTAAAATGGTTCAG
TGCTTATCTGCAGAACCTGTATCGAGAATGATCTCAACAGATAATATTACCTTGGATAGTTATAC
TATAGAAGTTGATGCAAGGGGTACTTCATCTAATACAGATACTCCGAATACCATGTTGGGGTATT
TTGCAGACCCACAATATTTAGCAGAGACTACGACACATCCTGATGCTTTTTATACCTTTGAACTT
GGTGGTAGGAGAGATTATGGTACTAGAACATCGTTAGAAACTTATAGTGGGTATCAATTGTATT
GGGATAATGGTGATTATGGCACATATGCTGATCTTGCAATTGTTAATCAGTATCCTGTGGATTAC
TACAATGTTCACCGGGCAGACCTTGATTATTCTTCCCAAAACCCCTCTGAAATTTGGTATAGATT
AAAACTAAATGTTATTGAAGATACTGTTTATGGAATGTACTTCACTCAATTAGATTACATAAAT
AATAACGAACCTGACTGGATGATAACTGAAACTATCAGTAACCCTTATGGAAGTTATTTTGAACT
CGGTACATCTTTTGGAGATCATTCATCATATACTCAAAATATCTATTTCGATAATTTCCGTGTTA
GGAAATATGCTTCAATAGAACCAACAACAAATATTTACTCTTTATCAAAAACAGTAGGAATAAAC
TATATAACCCCTCCAAGAGCAGAAGGTACCAGGTATGTTTTGGGAAGTGATTACAATTTATACTA
TGAAGAAGCGGATAATTACCCATCAATAATCGACATGCTTGCAGGAGAAGATTTTAAATCTTGGG
AGTACGGTTATGGATTAAAATTAAAAGGATATCAATAAAAATAAAATTTATTCAATTTTTTCTA
AATTTCCTTCAATAAATTCTAAAATACAAACGTCAACATCGTTCTTTTCACAGAAATAGTTTTCC
ATTTTTCCATTAAATTTTTCATGTTTGTTTACAATAACTGCATTTGGTTTTTCTTTTAACATTTC
TTCAAGAGTTAATAGTGTTTTTGTATCAACAACTTCCTTTTCTTCGATTTTAAATATTAAAAAAT
ATTTTGCAGAATCAGCGTCTGAAATTGTGTCCATATCCATTGGAACTGCAACTTTCATTGTTTTC
ACCTTTAAATTTTTTCAACATACATTAGCGGATAATCAAGTTCTTTAATGTATTTTAATCTTAAA
AGAGCACGGTTTTTCTCTGTTTTTACAGTTAGTGATACATCTAGCATTTCACCAGTTAATGAAAC
ATATTTTCCATCTTCGATTATTCCAACAATGGATACAGAAACATCTTCAATAAATGGCTGGTTTT
TCAAAGATTCAGACATTGCAATTTCTAAACTTTTTTTAGTTTTTTTGCTAACAGGTGTTCCAACA
AATTGGTGGAAAAGTGCTCCAAGTGAGATACCTCCTTCAAAAACGGCTCTTTCCCGATCACTTAA
ATTTTTAAAGTATGAATCAAAAATTTCATTTTTTTCAAGGCTTTTTTCAGACATTTTCTACACCA
CTTAATTCTTCTTCAAGAGACTTAATGCAGACAGAAAGAACAGAATCAACTGCTTCTTCTTTAGT
TTTTTCTTTGTTCCAAGGGGCATTTAAGCCAGCAGCTTCAGCATGCCCTCCACCTTGCCCATCTAA
AATTTTTGCAACTTTTTCCATAAGGGCCCCCATGTGGATTTTTTTTGAAATGGCTTTTCTTGAGC
GCACACTTATTCTAATTTCTCTATCCCTTTTTCTTGCAGCAACAACGAATGCAATGTCTGCTCCAA
TACTTACAATTGTTTTTGCACAGGAAGCTTCATGAGAACTTGCATGGGATATTGCAATAGTGTAG
CTATCAAGTTCCATTAATTCCATCCTGCTACAAGCTTTCAAATGTGCAATTCTTTTGCTTGGATC
ACTTTCCTGGCTTAAAAGATAAAGTACTTTTTGAAAAGATACGTCTTTCATCAACCACGAGATTA
TATTAAAGGTTGATTCTTTTGCAATTTTCAAATGTTTTGTATCATACAAAATACCGCATAAAAGC
CCTATTCGGATATTTTTTGGAGGGTAAATCTTCATTTCTCTGAATAAATTTGCTACAATTTCGCA
AGTTGAAGTTGAATTTTCGTCAACAATATAAAATTTGCAGAAATTTATAAGATCGGTTTTTTTGT
GGTGATCTATTAAAATAACATCTGAATTAGTTAGTTCTTCTGAATTTACAGTTACCTGGTTTATT
GAAGAAGTATCGACTAAAAAAACAGTTTTTGGTAATTTTGGATATTCTATAATTTCAATTTTTTC
GTTTAATTCCTTTAGTATTGACCGTGAAAGTTTACTTACAGAATCTGCAGATATTTTTAAATTTT

TTTCTTTTGATTTTTTCTTAGAAAAGCTGTTTGCAAGCTGTTTTAAACCGATTGCAGCACCAACT
GCATCAGGATCTGCGTTATGGTGACATAGAAATATAATATCATCATTTTTTAGATAATCTTTCAA
TATTTCAATTTTTTTTTTCATAAACAGACCTTAAAAAAGAGTATAATAAAAAATTAATTTATTG
GCCCTGACCCATTACTTTTTGGAGGCTTTCTTGTAATTCAGTGTATCTTTTCTGCATTTTTTCTTC
TTGTTTTTCAAAGGTAGTTACTCTTAACTGGAGTGTTTCTATCTTTTCACTTAATTCTTCTTTTA
CTTCAGCCTTGTTTCTTTTAACGAGGATTCCACCAGCCATCTTAAATACTTCTTCGGAATCAGATT
TTTCCATTTCTTCAATAGCTTTTTCCATTTCCTTTAATTGTGTTTCAAACTGCTGTTTTTGGTACA
TTATCATTTGTAACTGCTGCTGGAGTTGTTGGAATTGCATTAATTGATTTTGAACGTTTGCAGGT
AATTCCATGATTTCAATCCTCTTTTAGGTTTAATTTATTTTTAAACTAATTTTATTTACCAATTA
TTATTCATCTTCTGTTAGTTTATATATACTTTCTGCAACTTTTATCCAACGAATATATGAATAAA
TAGACGCTTTTAAGATACTCAATTCGTCAGAAATGCACGAAATAATAATTGAATTTTCAAAAAGG
TCCATTTTGCATTTTGATCGAACTTGCGATGCGACATGTTCCGGATAAACAGATTCATAAATAAT
TCTGGCTTTTTTTGGAGTTTCAAAAGTCAATTTTAAGGTAAATTTGGGATATGTCATAATAACAC
TATTCAATATCGAGTATTTTTACAGTTTTTATTTTCAAACTCGGACCCATATTTTCTGAGCCTTT
GTGAACTTCAATGTAAAATGTGGACTCATCAACCGTTTTAAAGGTAATTACAACTTCAGAATCCT
CTTCCTTATAATTACTCAATTTTTTGAATAAGTATTCAAAGAAGAATTCTTTTAAATGTTCAGTA
TTTTTATCAAACTTTGAACCCAGTTTTCCAGAATTTTTAAATGCTTTTCCTGAAACTTCCCGTTG
AAGTTTTAAGGATAAGTTCATAGAAAGAATTTTATTATTTTCAAAATTAAATATTTTGAGCCTT
CCAGGGTTTCCTTTTAGTTCTTCTACAATTATCATATCGTCCTTATTTTCGATTAGCTCGGAGAG
AGGAATTTTTCCCCTGTTTAATATTTTAAAATTAAAAACCCTCGCTAAATCGTTTACAAGGCTTC
TGGTTCTTTGAGAAGGTTTTCTGGAAGTCGTAATTATCATTATCTTGCCTGAACTTTTTTAACTA
TTTTAGGTCTTAATTTTATCAAAACTCTATTACTACAGTGAGGGCATTTAGCTTTTAATCCTATT
TCATCAAGAGTTACGATTTTTCCGCAGTTGGAACATCTGTACTCTGCCATAATATCACCTAAAAA
AGCTTAAAATGAAAAATAAATGGTTTAAAAATAATTAAATTAGATTTCTCTACTTTTACTTTCG
ATAACTCTTCTGATTGCTTTTGTAACAACTTTACCAGCACCAGTTTCAGGAGTGTATGCTCCACC
AGCGATTTTTGCACCGCATTTTTCACATACCCAGATAGAGGTTCCTGCTCTTTTTAATTTAGGGA
ATGCACAAACAGGGCATTTGTAATCTTTGTTTTGTTTGATTTCAACGTCTCTTAATCTTACTCTG
ATTTTTCTACCGTATCTTGATCCAAATCTTCCTGCTGAACCAATTTTTTTAGTGTGGCTAAATTC
TACCATTATTTCACCTGTTTCTATCCGTAAATTGATTAGTCATATCTTTTAAGTACTTTAGTTTG
AACAGTTCCTTTTGTAAGTTTGTTCAAATGCATGTAAAATTCGTTTTCAATACCACTCGGAATTT
CGATTACAAGAACAAGCGAACCATCGCCCAACCATTCTTCCTGTTTAGTTGCGCCATATTCATGA
AGTGTGTGGTAGGCATTTGCAGCGAATTCTCCAAGAATTTTGACTGCAACATCCCTTTTTTCAAA
CTTAATGGGTAAAAGTTTCCTCAATTCCTTAATAATTTTAGGAATTTGTTCTTCAGCGCTTTTGT
AAATATCAACACTGAGTCTAGCTTCTTCCATAGCGTTTTCAATCCTTTTAGGCGGGTGAGGTGTA
TCAGTCTGGGGGTTTATCGTGTTTTTTGAAATTATCGACACGATTTGCTTTCTTTTTAGTTCTTG
TATTTCTTTTCGCTGGTTAGCTGTTAATTGAACAGTTCCTTTTTTTAGAATCTGTTCTGCAATTT
CCAAAGGATTTAACGTTTCAAAAATCTTCAAAAGTACATCTTCTGGAACTTTTTCTCCTTTTCCA
GAGTCCTTGTAAACCGTTTCAGCGGCCAAGATTTCTGAGATGCCTATTGGCTGTCCTTCTTTAAA
TTTAGCTGCCAAGTATGGATCCACGAGTATTTCGAATTTTTCCCCATGTGATTGAAGCCTTGCTA
TAACAGCATTGTCCAATGACACCATATCTATCCCTTCTTTTATTTATATACCTTTCCCCGCATTAA
ATAAAACTATAATTTGGGTTTTTTCAGGCATTTATTCTCAATTTATCTTAATTTAACTTATTCAC
TATCTTCTTCGCCTTTATTTTTTTCAGCTTCCTCTTCTTCAGCCTTAACTTTTTCGTAAACTGCTT
TTACGATTTTTTCAATTTCATCAACACTTATTTTTTCAACTAATTTTCCAGAATCTTTTATTATG

GCCATATCAATATTTTCAGGCTTTAATTCTTCAGTAGTTTTACTTAAAGCGTAAAGTGCTAATTC
CATTCCTTCGTTAACAGTCATATTTTCGTCGTATTTTGATTCAAGAACTTCCATTGCGATAGGCC
TTCCAGAACCAATTGCTGTTGCTTTGTATTCTATTAAGGCACCACTTGGATCAGTTTCGAACAGT
CTTGCACTGTGTCTGTCGATTCCAGTAATTAAAAGTGCCAAACCAAATGGTCTTGCACCACCGTG
TTGAGTGTATGCCTGTTTAATGTCACAGATTTTTTTGCAAGTGCTTCAACAGTGATTGCTTCAC
CATAAGAAACCCTGTTCATCTGAGCTTCGATTCTTGCTCTATCAATTAAAACTCTTGCATCAGCA
ACTAAACCTGATGTAGCAGCTACAATATGGTCGTCGATTTGAAATATTTTTTCAATTGATGAAAC
GTCAATTAATTTGCTGGTAATTCTCCTATCGACTGCGAGAACCACTCCATCTTTACACTTAATTCC
TACGGCAGTAGTTCCTCGTCTAACTGCTTCTCTAGCATATTCAACCTGGTAAAGTCTTCCTTCAGG
GCTGAATATCGTTATTGCTCTATCGTATCCTGATGCTGGAACCATTTGTTGCATATAATTGCCTC
CAAAATTTAAATTAATAAGTATGAATTGTAAATTAACGGATATAATAACTACTTTTCGCTGAAT
TTTTATATATATCTATCTTTTTATCCTCGAAACTCACTTTCGCTATATATTAATATATTAGAAAA
TCTAATATATTCATGTACAGATAGGTGATAGTATGATCCCTGTAACAATTTCTGAAGAAGCAATG
GTGTTTATAAACGAAAAAATAAGCGATACGGGTTCAAAAGACTTGATCGTGTTTTTCGAAGGAT
TTGGTTGAGGAGGTCCTAAATTCGGGATTGACACAGCAAGTAAAATCCTTGAAACAGATGAAAA
AATTTACGATGAAGATTTTAGAATCTTTATCGACAAAATGGCAAGACAGGTATTAAACGAAGTA
TATATTATCGTTAAAAAATCCGTTTTCAGCGGAAAATACCTGGCAGTAAAAGGAGCAGGTGGATG
CTGTTAATTTAAGAACCCTAAATAGGGATTAAAGACAGCATACCTTATTTTTTTCTATTTTTTA
TTGTATTATTTTGTTATAATTAATGCATCTCCAACGAATCCGCCTTGGCCTTCTTCTTTGATAAA
TGCAGGGTTGATGTCGATTTCACTTATTTCATCGTAAGTTTCCATTAAAACGCCAACTCTAACGA
TCAATTCTTTTAAGAATTCGATATCACTTCTATTTTCGCCCCTAACGCCTTCTAAGATTTTATAT
GATTTTAGGGAGTGAAGTATTTCTCCAGCGTATTCTTTTGTTATCGGGGTAATTCCAAAGGAAAC
ATCCTTTAAAACTTCTATGAATATTCCACCAAGCCCGGTCATAACAACAGGTCCAAATACTGGAT
CTCTTTTAGCACCAATAATGATTTCTTTTCCGGTAACAAACTGTTCGATCAAAACACCATCGATA
ATTCCATCGATTTTCCTATCGAACAAATATTTTTCCCCATTTTTCATTATCGTTTCAAATGCTTCA
GACGCATCACTTGGATCGATTATAACACAACCTGCATCTGATTTGTGTAATATATCTGCGGAAAC
AACTTTCATTACTACTTTTCCTAAATTTTCAGCGTATTCTTTAGCTTCTTCTTTAGTTGTAGCAAC
AAATTTTTCAGGAATTAAAAATCCGTTTAATTTTAAGAATTCTTTTGAATTATTTTCGTTTGGAT
TACTGAGTAATTCCTCAATTTTTTCTTTATTATTTATTTTAACGTTTGAAATTTCATTTCTAACG
TCATTTAAATATTCTGGACTGTCATATTTTCCTTTAAGCCTGCTTTGTCTGTATAAACAAGCTAA
TGCATGAACTGCAAGATCTGGGCAGATAAATGATGGAATTCCTTTTTCTTGTAAGTAACTTCTTG
CTTCTAATACGGAAGTTCCTCCAACAAATGAAGCAACAATTGGAATTTTATCTTTTTTACATATC
ATGTAGTTCTTAAGTTTTACAAGTTCTCTTGCAACACCAAGGGCATCAGTCATTCCTTGAGGGGT
TAAAATTGCGAGTAATCCATCTACGTTTGGATCATTTTCAACCAATTCAAATGCGTGTTTGTATC
TATTAACATCTGCATCTCCGATTAAATCCAGTGGATTTGAGATACCTGAAGTGTCGGGTAAATAT
TTTTTCAATTCAGAAACGGTTTCATCTGAGAATTTTGCAAGTTCTAATCCATATTTAGCAACAGA
ATCTGCTGCTAAAACTCCAAATCCTCCTGCGTTTGTTACAACAGCAAGTTTTTTGGATCTCATTG
GTGGCTGTACTGAAAATATCTTTAATAAATTTACGAGGTCTTCAAAGCTTTCTACGTTAAATACA
CGGCTTTTTTTGAATGCTGCATCGTATACTGCATTGTTTCCTGCAAGGCTTCCTGTGTGTGAGGA
AGCAGCTTTTGCACCTTCTTCGGATTTTCCACTTTTTAAAACAATTACTGGTTTTTTCCTTGAGA
TTTTTTTAGCAGCACTAATGAATTTTTCGTCCTTTAAGCCTTCGATATAAAGTGCTACAACTTTT
GTGTGCGGGTCATTTTCAAGGTAATTTAATAATTCATACTCTTGAACATCGATTTTGTTACCCAT
ACTCACGATTTTTGAAAATCCGAGGTTATAATAATTTGCAATATCAAGAAGTGCAGTCATAATTG

CCCCACTTTGTGATATAAACGCAATATTTCCCATTTTTGAAAATTCTTTACTGAATGACGCGTTT
AAATGGTTGTGAAGATTTATCACACCAAGACAGTTTGGACCGATTGTTCTAATTCCATACTGTTT
AATAATTGCTTCTAATCGTTCTTCTAATTCGTGGTTGTTGGTTTCCGAAAATCCTGCTGTAATAA
TTATTGCAGCTTTTACTCCTTTTCTTCCACACTCGTCCATTGCATCTACTGCATATTCTGCAGGAA
TCGATATAATTACAAGGTCTGGAGTTTCAGGAATATCTGAAACAGAACCGTAGCATTTAATTCCA
TATACTTCGTTGTATTTTTTGTTAACAGGGTATACTTTTCCACCGTGTTGTTTAAAATTCAACAA
GTTTTTCATTACGGACTGTCCTACTTTTCCTTCTATTTCTGATGCACCAATTACTGCAACGGATTT
TGGGTTGAATATTCCTTCAAGCGTGTTCATATAAATCCCTCGGTTTGTTATATTCTGATAAACAT
AATCATAATTATAATTAATAATAATTAACAATAGGTAATTACAGTTTAATTTAATATATGTATT
TTTCGGATTGAATGATTGTGATAAATTCAGTTTTCAGGTGTTGAAGGGTATGAAAGACATCAAA
TGCGTTCAGAAAACAGGAAATATTTTAGAATTTGAACAGCTAAGAGAAAAACTCGGGATTATTG
ATAAAAAGGACGTATCTTCTTTAGAAATAAAGGTGATTGACGAAAATTCAGAACAGTACGGAAT
ATCAAAAGAAAAATTAATGGAAAATGCTGGAAGGGCAGTTTACGAAGAAATTGTTAATTTGAAC
TTTGATTTTTCAAAAGCGTATGTATTGTGCGGAACAGGAAATAATGGGGGAGATGGGTTTGTTA
TTGCAAGACATATATCGAATAATTTTGCTACATTTGTCGTATTAGTTGGAAACGAAAGAAAAAT
AAGAACTGCTGAAACTCAAAAAAACTTTAATATCCTCAAGAATATGGAAGCTTTTGGTAATTTAG
AATATATAAATATTTTAGAAAATGAAAATGCAATAGATTTAATAAAAACGCTTGAAAATGAACT
AAAAAATGAAAATGTATTGGTAATTGATGCAATGCTTGGGACAGGATTTACTGGAAATTTAAAA
AATCCTTATTCAACAATAGTTGATTATTTGAATGATTTAAAGAGCAAATACTACGAAAATTTGA
AAATAATCAGTGTGGATATTCAAACTGGAAATCTGAAATCTGATCTGGAAGTTATACTCCACAAA
AGAAAAGTTGAAAACAAAAATAAAAATTTTGTTGTTAAAAATATTGGAATTCCAAAATATATTG
AAAACATGGTTGGAAAGGGCGATTTTTATTTATTGAATAAAAGAAAACTAAATTCTCATAAAGG
GCAGAATGGAAAAGTTTTGATTATTGGTGGATCAAAAGAATACCACGGCGCACCTGTTTTTTCAG
GTCTTGCAGCTTCTAAATTTGCAGATATTGTGACAGTTGCTTCAGTTTCAAATGTTATAGATACT
GTTAGAAATTATCCTGAATTAATGCCCCATGAATTAAATGGGGAATATATCAGTCAAAAAAATA
CCGATGAGCTTTTAGATCTGTCTAAAAATTATGACTGCACGGTTTTAGGTAGCGGACTTTCATTA
AATTCCGATACAAAAGAGTTTGTAAATTCGTACCTAAATGAAACAAATGGAAAAGTAGTTATTG
ATGCAGATGCAATAAAGTTGATTGATTATGGTAATTTTGAATTTAAAAATAATTTTATATTCAC
TCCGCACAAAAAGGAATTTGGATACATGAAGGATTATGTTAAAAAATATGCTAAAAACCAAGGT
TTTAATTCAACAGTGGTATTAAAAGGAAGTATCGATATAGTGTTTGATCCAGATAATATTAAAA
TGAACATTACTGGAAATCAGGGAATGACTGTAGGGGGAACTGGAGATATATTATGCGGAATTAT
CGGTGCAATTTATGCATGTAATGACGCATTTCCTTCAGCCTGCTGTGGAACATATATAAACGGAT
ATTGCGGAGATTTACTCGAAAAAGAGTACGGATATTATTACAATTCAACGGATATTATTAAAAT
ATTGCCAAAAGCACTTAATGAACTTTTATATTAATTAATTTATTGAGGGAACTCATGGAAAATG
ATGTATTTAAGGCAGATATGCACATACACACCAAATATTCGGGAATGATGAAATACATGGGTTTA
AAATTTCCTGATTCAGTGGAAGAACCATTAAAAGTCATGAAATGTGCAAAAGCAAATAATATGG
ATGTTATTGCAGTTACTGATCACAACTCAATAAAAGGTGCTCTTGAGGTTAAAAAACTTGAAAAA
GAGTTTGGAATTGAGGTTATCGTTGGTAGCGAAATAATGTCTAGTGAAGGGGAAATTCTCGGTCT
TTTTTTAAACGAAGACATTCCAGCAAGGCTATCCCCGGAAGAAACTATCGAAAGAATCCATGAAC
AGGGCGGACTTGCAGTATGCCCTCACCCTTACAGTCCGATTTGTGAGGCAATAGGGGATAAAGTA
TTTGAACTTGATTTTGATGGTGTTGAAGTTTACAACGCATTCCACAGGGATGGAATTGTAAATAA
TATTGCACTTGATAAAGTTTTAAAAAACTACCACAAATCTCCTTTTGCATTTTTAGGAAATAGTG
ATGCGCATTTAGCAAGAATGATTGGAAATGGAAACACTAAATTTGAAGGAAACTCTGCAGACGA

TTTATTTAATGCAATAAAACACAGGAAAACAACTTACGAAGGAACGCCAACTCCACTTTCAGATA
TAATTTTGTGGAGCTACAACGTGATTTACGCTTCAGAAAAAGCACTTTTAAAATCAATGGTCAAA
AAAGACGATAATATCGTAAATTACAATGGTTCAAAATTTAAAAAAGGCATTGCCGCATTTGGCG
GGTTAATGTACATTGGAACACCTCTACCGCTTGTAGCAGGGGTTTTAGGGAATATATATCTTAAA
AAGAAAGCAAAACAGAAATTAAAAGAAGTAATTCAAGAAGTTATTTAATTTTTAAGTTTTTTTA
TTTTTTATTTGTTAAGTAATTAAATTAATTCAACGAAGTTTTCAATCATTTTTAAACCGTTTTTA
CCACTTTTTTCAGGATGGAACTGGGTAGCATAAACGTTTTTTTTATTCAAAATGCACGGGAATTC
GTAACCGTAATTAGTAGTTCCTGAAATTACGTCTTTTTTTGAAGGATTCACGTGATATGAATGTA
CAAAGTAGAAATATTCATTATTTGCTATTCCTTCAAAAAGAGGAATATCTTGAACCTGATTTACA
GTATTCCAACCCATGTGGGGGATTTTTTCAGAATGTTTGAATTTTATAACGTCCCCTTTTATCAC
GCCAAGGCCTGGAGTTTCTGGACATTCTTCACTTTTTTCAAGTAACAACTGCATACCTAAACAAA
TTCCTAAAAATGGAACCTTTTGAACGCATTTATTAATTATTTCATTTAAAGAGCAGTCTCCTGTT
TTTTGGGAAATATTTTTCATTGAATCTCCAAAATTCCCAACACCTGGAAGAACTAGCTTGTCAGC
ACTTAAAATAGTTTCAGGGTCACTTGTAACAACGATGTTTTTGTGTATAATTCAAGTGCCTTTT
CGATACTCCTCAAGTTGCCTGCATTATAATCAATTATTGCAATCACGATTATCCCGTTTAATATA
ATTCTTCTTCAAGTTCTTTTAATGTTTTAGATATTTTATCAAATGCTATGTATGCATCTTCGATT
ACTTTTAATCCGGTAATTACAACTTTTCCACTACCAAATATTAATACAACAACTTTAGGTTCACT
CAATCTGTAAACTAATCCTGGGAACTGTTCTGGTTCGTATTCTGTACATTCTAATGTGGATATGT
CATCTAAATTAGGTTCCATTCCAAGTTCGGTTGTAGCAACCATGTTTTGTACTTTTACTTCAGGA
TTGTCAATAAGGTCCATTCCAGCTTCTTTTAATTCTTTAACGATTTTGTTTATTGCAATTACTGC
ATCTTCCTTACATCGTGCACCGGTGCAATTTAATTTACCACTTCTAAATATCAAAAGTGCAACTT
TAGGTTCGCTCAATCTGCAAACAAGTCCCGGAAACTGTTCTGGTTCGTATTCTGCGTTATCCAAT
ATATCTGCGGCATATTCTAAGTCAATATCAGTTCCTATCTGTGTTGATACAACTACGTTTACAAC
TTTGATTTCAGGTTCCATCATTTTCACCAGTTTATATATCTGCTATAAATTTCTATTTATATAGT
ATATAAACATTTATACTTCTGATTATATATTTAAATATACAAATATATATGCCCAAAATAAAAA
GAGAGTATTCGGAAAAGTAATTATCCGAATAAAGCACCAAGACCTGCAGCAGCAGCTGCTCCTGT
GTCTTCTTTTTTCTCTTCTTTCTTTTCTTCAACTGGTGCAGCAGCTGCAGCTGCAGGAGCTGCAGC
AGCTACTGGTGCGATTGCAGCTTTTTCGATAGCTTCTGCAATGTCAACACCTTCTAAAGCAGCAA
CCAAAGCTTTAACTCTAGCTTCGTTTGCTTCAACGCCACCAGCTACTAATATAGCTTTAACAGCGT
CTTCTGTGATTTCTTTACCTGCTGAATTTAATAATAATGCTGCGTATATGTATTCCATAAAGTAC
ACCTCCAAGATGTATGAACCTTTTTTTGAAATTATCCGTTTATGTATATATTTTTATGGGTATTC
GCATTATAAGTTATTTTAGAAGAGCATTCCAAGACCTGCAGGAGCTGCAGCTTCTTCTTTTTTCT
CTTCTTTCTTTTCTTCAACTGGAGCTTCTTCAACTGGTGCAGCTGCGCCTGATGAAATTTTTCCTT
TTAAGTCTTCGTCTAAAGCGTCATCGCCAGCAAGACCTGCAACAGCAATCATTTGAGCGTAAGCT
TTTCCAAGGATTGCATCAGCGGTTTTATCTGTTACGAATGCACTTTCAACGGATACAGCTTTTGC
ATCGTTGAATGCTTTTTGAACGATTGTTTCAACTGTAGCACTTGTTGGGATAACAGCGTTAACTG
ACAAGTTGAATGCGCTTGTATAAGCTTTTTGGATTTTTCCGACGAATTCTTCTTCATCGATTTTT
AAATCACTTTCAGTGTAAATAACACCATCTTCGTAAACTCCGAGTACATTTAAACCAACTGTAAC
TGGTTTAATGTCTAATGCAGAGAGTACAACTGCAAGTTTCTGTGAAACAACTTCGCCTTCTTTTA
CAACGATTTTGTCTTCTTTGATTCCGATTTTACCCTTATCAATAGCAGCAGGGATTCCAACTGCT
TTCAATTCACTTAAGAATGGTCCTGGTGGCATTCCTGTTGATCCAGCTTTAACTTCAATATCGCA
TGGAGCAATAGCGCCTCCTTTAACAGGTGCAGGGCTTTTACTTTCATCAAGTGTTTTGTATAATT
TGAAAGGGTTCATTTCGGTAGCAATGATTGCAGCCCCTTTGTCCATGTAATCTACTAATTTAGCA

AATTCTGGATTTCCGGTTTCTTCAGCAACTTCTTCAATTGCCCTTTTCATGAGTGTGTTTCTTGAC
ATTTTTAATGTCATTTGATCTCTGATTTTGTCCCTGATTTCTTGGAGTTGTACCGCAGGAACTTC
CATCATGTCAATTAATGCAATAATGTTTGAACTTTTAAGTAATTCTTTTAATGCATTAACTTCTT
CAATTTTCCAAGGGGCAATCTTATGTTCTGATTTAGCTTCGATCATATTTTCAACCCCTCTATTTC
TCAATAGGAGCAGCTGCGCCCATTGTAAGTTTTGTAAATGCATTTTTAACGTGGTAGAGTCCTTT
TTCGTATTTTTTAGACACTACGTTTAAAATTGCTTCGGCATTTGCAGCAAGTTCATCGTCGCTCA
TTGATTCTGTTCCGATGTATACCTGGAATAATGCTTTATCCCTGGTGTTTATTGCAACGGTTTTT
TGGAATCTAGCAACTAATGGAGCTAAGTTTGCATTTGCAGGCACTGGTTGTGGCATTTTACCTCT
AGGACCTAGAACCGGACCTAATGATTTACCGACCAATGGCATCATATCAGCTTGAGCGATAAAGA
AGCCGTGTGCGTTAGCAATTTTTTTAGCTGTTTTTTTGTTTTTACCTAATTCTTCTAATTCTTCCT
GTCTTATTACAGTGAGGCCCATATCTTCTGCTTGAGCAGCCAAGTCACCTTTCGCAATAACCGCA
ATCGCTACGTCTTTACCTCTTCCACTTGGTAAAACGATTTGCTCTTTCAACCTGTTTTCAGGTCTT
GAAAGATCCAACTCTTTTAAGTTGACGATGAGATCAACGGATTGCGTGAAGTTTCGCGGCTTAGC
AAGAGTTCGAGCCTCCTTCACTGCGTTCAGTATTTTTTCACTGTCCATGTTGATGCCTCCTGTAAT
TATTTAATCGCGCCAATGCGATCTAAACATGGGGCAGACATACTATCACTTACTATTATATATAA
GTTACTGAGTTAAAAGAGGCCACTTTTTTAGAATTCAAGTTAAAATAAGGTTTTACAAGTTAAA
AAAATAAATTATGCCGATATAAAATATCAATTATAAATCTTCAAGGGTTTTGTTACAAAGATCG
ACGATATCTTTCAAAGCTTCTTTTAAAACTGTTTCAGCATCTGTTTTTTCATCGGTTTTTAACAT
TATCGTTGGATTTGAAATGTATCTTCCAGTGTCAGGATCTAAAACCGGGTGTTCTACGCCATATG
ATGCAAGAATAACGCCATCTTTTGATAACAATATTTCTTTTACTAAATTGCTTAAAGAGTGGTCG
TCATTTACTAATTCTAATTCGATGAAGTTTTTGTTTTTTTCAATGATTTTAACGTAGTTCATTTT
TTCACGCTCAAAAGTTCTTTTCTTGTTAGATTTATATCTTTATTGAGAACAATTTTTTCGTCTTC
TTCTCTAAATAATTCTTTTTCGAGTCCTTCTTCGATTAATTCATCGATATCTCGTCCTGTTATATA
TACTGCAAGAGTTAATACATTTTTAACAGGTTTTGAGCCAATATTATAAAAGTTGAGTTCAGAAT
AAATGTCAAGAATGTCTTTTAAGATGCGGTTAATTTCTTCAATTTTTCTTTCACACTTGATTACG
TTTTCATCTTTTAAATATTTAAAATTAATTTTTAATGCAGAAAGGGTATCCAAAACAAGGTCTTT
GTTCACAGGATATTTTAAATCATTTAATTTGTACTCGAATAATCCTTTTGTGTCTGCATTGTATT
TATTATGGACTGTATCTATAATAGTAAGTATATTTTTATGGTCTTCTACGAGTTTGTACTTTTCA
TCGCCAAATATCTTTATTCTTGCACTGTTATATGCCCTATTTTCCGCATAATTTGCTTTTGCGTCA
AATGAATAATCAATGCCTAATTTAGATATCCTTTCGCAGATTTCAATAATTTCTTCTTCAGTTCC
TTTTATTGATATTATTCTTTTCATAATTCTCACCAGATAAATCCAAAAATCGTCTCTTCAAAAAT
AGATATAAAACTTCAAAAAGGTTAAGCCGTGTCGTATATAAAACTATGTAGTTATACATTACGTC
TCAAAAATATAAAACTGTTGGCAGAATAAAAATAGTTTTTTTAACAATTGGCGACTACACAACA
AAACTGTTATATAGATTATTGCCCATATGGGTAATAGTCCTGCAAAACTTCTATCTGATTTTTAT
GTTTGATAAAAACATTAAATATTTGCAGGATAATTCTATTCATCAAATTTCACTCTGCCGATTAA
AAAAGAGAAAGTAAGGCGATTATTAAAAAATTTATTTATTCTGCTGTTTTTGTTGCTGCTGTTGC
TGAGCCTGTTTTTGTTGCTGCTGTAGTTTCTTTTGTTGCTGCTGTTTTTTAGTGTCTGGCATATG
TATCACCCCTTTTTGTACAATATATATTTTAATTCTGATATATTTAAAATTTATCAGATTAATGG
AGTCATTTTTTTAATTAAACGTATAATTGTAAATTATAATCAATAATATCTATTTTTAATCCGTA
AATCGTTTCACGGCTGAAAATCAATTTATAAATTTAGCGTCAGTAAAACTCTAAAAATTTATTTA
ACTGGTTTTGAAGAGAATTATATCTATAAATGGGGGGGATAGTAATGGTAAATGTTTCATATTC
GAGCGTAAATGAAATGCTAGACCACTGGAAATTTAAAAAAAGCATGCGAAACAGAATCAAAAAA
AGCTTTAATTTAGAAAGACTTTTCAAAACACATGAAAAATATCGGGATTTGGAAAGCACGGTTT

ATAATAAAATAAAAAAACGAGAAGAAAAGGAAAAGAAGTCAAAAAAGTAGATTAATTATTCTAA
AATTACTCTTTTTTAGTGAAAAACTTAAATGTTCAGACTTTTTTGCTGTTTTATTAAATCAGATT
TTTGTTCAATTTTATAAAAAAATTAGAAAAGTATAAATCGGTAAGTTTAGACATTAGTATAAAC
ACAATTACTTTTGTAAAAGAATTATTATAATAATGCAAATTACGAATAAACGTTTTTATTGTTA
ATATTTGTAAAATCAGGCGAAAACATGTTAGAATCTATATTAAAAAACACTTCAGAAATAGTAT
CCATCGAAGAATTAACTGAACTCTTAAAAAAAGAAGAAAAAATTGCATATATCGGATTTGAACCC
AGTGGAAGAATTCACATGGGCCATTACTTGCAGATAAGAAAGATGATCGATCTTCAAAAGGCAGG
ATTTAAAATAGTAATTTTACTAGCGGATTTACACGCTTACTTAAACCAAAAAGGAACAATGGATG
AAGTAAGGGCTCTCGGTGAAGAAAACAGAAAAATATTTGAAGCAATGGGACTTGTTGCAGATTA
CGTTTATGGAAGCGAGTTTCAGTTAAAAGACGAGTATACAATAGATGTTTACAAACTCGCACTTT
CAACCACGTTAAACCGGGCAAGAAGGAGTATGGAAGTAATTGCCAGGGAAGACGAAAACCCGAA
AGTTGCAAGTGTCGTATATCCACTAATGCAGGTGAACGACATAAAACACTTAAATGCTGATGTTG
CAGTTGGCGGAATGGAACAGAGGAAAATTCACATGCTTTCAAGAGAAATTTTACCAAGCATGGGA
TACAAAGCGCCAGTATGTATACACAATCCTGTTTTAACTGGGCTTGATGGTGAAGGTAAGATGTC
TTCGTCAAAAGGAAATTTCATTGCAGTAGATGACGATGAAGCTACAATAAAGAAAAAAATGAAA
AATGCGTTCTGCCCTATGAAAGAAGTTTCAGGAAACCCCGTTTTAGAAATTGCTAAATACTATTT
AAAATACCCTGCAACTGTAAAAAGGCCAGAAAAATTCGGTGGAGATTTAATTTTAGAATCTTACG
AAGCTTTAGAAAATGCATTTGTTGAAGGACTTCACCCAATGGATGTTAAAAACCTCGTTTCAGAA
CAATTAATTGAAATTTTAAGACCTATCCGCGAAAAAATGAATAAATAAGGATAAATAGTGAAAT
TATGGCGTTAGATCAATTATATTTTGGAAATACCCTGTATTCATACATGATATTTTTAATATTCA
TATTTTTCGGGGTATTCTTTGGAAAAGTAATGTACATATTTTTAAATAAGTACGTAAGAGTTCTT
GCAGGAAAAACGAAGACTAAATTAGATGACGTAGTACTTGATGCAGTAGAAATTCCATTAATAA
TTGTGGTTTTCGTTTTATTTTTCAAGTATGGATTAAATAATTTAGTTTTACCAGATAATTTAGCT
TTGTGGCTCAATGAATCTTTAACTGTGGCAATAACCTTTGCAGGAGTTTTATTTTTACTTAAAAT
TGTGGATGATATAATTGTAAATTATGTTGTCCCAATCGTAGAAAAGTCAGAAAATACGCTCGACG
ACCAGCTTGTCCCATTAATGAGAAAACTGGTCAAATTTTTGATTTTAATTGCAGGGCTCCTGCTT
ATTTTATCAAATGTCGGTTACAACATTTCAGCACTTCTTGCAGGACTTGGTATCGGTGGTTTGGC
AGTTGCGCTTGCTGCAAAGGACACGATTGAAAATTTAATTGCAGGATTTATTATAATTGTTGATA
GACCTTTTAAATTGGGCGACTGGATAAAATGGGGCGGAAAAGAGGGAATTGTAGAAGAGGTAGG
AATTAGGAGTACCCGTGTTCGTTCATTTGGGGACACATTAATTACAGTTCCAAACGTAAATATCG
TTCAAACTGAAATTGAAAACTTTTCAGAGCGAAGAAAAAGACAGGTAAAAGCAACAATTGGTTT
AACTTACGATACGCCAGTTGAAAAAGTAAAACGTGCAAAAGAAATAATAGAAAACGTTTTAAAT
GACCATCATGGCGTTGTAGATCCAATAAGGGTATCTTTTGTTGAATTTGGGAGTTTTTCACTTGA
TTTAAGAGTTGAGTACTTTGTAAGGGATTTCGGGTTTGATTTCTTCTTAAATACTAAGGATGAAA
TAAATATCAAAATAAAAGAAGAATTTGAGAAAGAAAACATTGAATTTGCATTCCCTACCCAGAC
AGTATACTACAAAAAAGAATAATTAAATTTTAAATTAGATTTTATTTTTATATTTTTTAAGGGA
AAATTATGGATAAGACGTGGGCGTTAATTTTTGCAATTATTGTTGGATTTGTAGCATCACTTCAG
CCGATAATAAATTCAAAACTAGGGGACCATATCGGCTCTAAAAATGCAGTATTTGTAAATTTCAT
AATCGGACTCGTTTTAATTGGTCTAATAATTTTAATTAGTGATCCGTCATCAATTAAACAGCTTT
CAAAAGTACCAGCTATAAATCCAGTCTATCTTTTAGGGGGAATTATGGGCGTAGTTATTGTTCTG
CTTTCATTAATTGTAGTACCTGAACTTGGAGCACTTTCTGCATTTTCAATCTTTGTCGGGGTTCA
ATTGATAAGCGGTGCGTTGATTGATCATTTTGGAATATTTGGAATCCAAAAATCACCCATAACGC
TTCTTAAACTATTTGGGATTTCATTACTGTTGTTGGGGATGAGAATTCTGATTAAATAACATAAA

AAAAGAATAAAAAAGTTTTAAAAATAATTAAATTATCCAAAGACCATTAATATTCCATCAAGGG
CCATCTGGATAGATATTGCAGTAAGTACAAGACCCATCATCTTTGTAAGTACTTTAAATCCTATT
TTACCGAGCATTTTTTCTAAATCTTTTGAAAATTTGAGCACGAAGTATGATATAATTATACAAAG
TGATATTGCAATTATCACGGAAAGTTTTCCATCAAAATCACTTGTCTGTGCCATCATAGCAATAA
CTGCACTGATTGAACCCGGTCCCGTTAGTAGTGGAACAGTTAAAGGGACTATTGCGATTTCATCC
ATATCTTCAATTTCTTCGCTGTGACTTTTTAAATGGGATTCAAGTTTTGGGCTTTTTTCTACCTT
GGAAGTGTTTCCTTGAATCATATCAAGTGCAATTAAGAAGAGTAAAACTCCACCTGTAATTTTGA
TTGCAGGAATTGAAATTCCAAAAAATCCGATGATTTGATTTCCAAGAAGTGCAAACATCATTAGA
AGTGCGAATGCGGCAAGCATGGATTTACGTATGATTTTTATTTTCTCAACTTTGGAGTACTGGCT
AGTAAGGACTAAATAAGTTGGAATTACTCCAAAGGGGTTCAATATGGAAAATAATGATGTAAAT
GCCAGTATTATAAATTGTACGTCCATATTTTCAACTTTTTTTCATTTATAGCAGGTTATTTTTTC
TTTTTATTCCATCATAAAGATCGATAGCATTTCCGTTTAAAAAGGCAGTCATTGTATAAATTACT
CCAGGCGGCAGAATATTTGTTGGTGATCCCCTTTTAACAAAGTAAACGTTTTCAAATCCTAAAAG
GCCCCCATAAATCATACCTGCAATTTTTTGCAAGTCTTTCACATTTTCAAGAGTGATTACCACGA
CAGGGTCATCAGTAGATACTCCAGAATATCCAATTCCTGGAATTTTTGTAGGGACGTCCGAAACG
CTTGACGAAGAATCGTATCCAAATTTTTTTCCAATTTCTTCAACAAAGAGGGCTGCTTCATTTAC
AAAAGATTCTCCGCAGTATGTATCGAATCCAATAATAATTGAATCTCCATATTCGGAAGGTGTTT
TAGAAAATGCATATGATACACCTTCTCCAGCAGTTTTTGGATCATTAGAAACCCCTTCTAAATCT
TCAAAACCTTCAGCGTGTTTGTAAAGTAATTTAAACACGATGTTGTTAACTTCTTCAATTCTGTC
CTCTGAAACAAAAGATGAAATTACAATGTCGTCACCAGTTACGTTTGTAATTGTGGATTTTACTC
CAATTTTAGACATTTCTAACTGGAGTTCTCGCTCTATTTTTTCATTAACAACTTCGCTAGTTTCG
ACATCATTTGAAGATATGTCGACACTTATTGAGGAGATGTACTTCACACTATCACTCTTTTCAGG
TTTTCGATAAAACGGTTTTACAGAGTCCAAGTCTCATCAAACTGTTATTCAGGGATTTTTCCCAG
CTATTTTTTAAGTTTCTAATAACATAACTATCCAAATCAATGTCAATATTAGTTTTGACGTCAAC
CAGTATTAAACCTTCGGCAGGGGATGGAGGATAGCCTTTTTTGTAGTCCTCTGATAAAATTAGTT
CGATAAATTTTTCAGGATTCTCAATTTTATGTTTTCCAATATCTGAGAGAAGCCTTATGATTCTT
CTAACCATGTTCCAGAGAAAACTCTCTCCAAATATATCAATTGTGATGAAATAATCATTTGATGA
TATTTTAATGTCGTAAAGTGTCCTGACGGGATCTTTTACTTTTGTTTTATCCCTTTTTGATAAAT
TCTGGAAGTAATGTGTTCCGATTAGTTTTTCAGATGCCTTTTTCATCAGTTCGACATCATGATTT
TTATTTGGTAAAGTATACTGGTAGTGCCTGTGTTCAATTTCTGGAAAAATTTCAATCTCTTTACA
TCCCAAAACCCATATTCCATCCCATTTAAGTTTAGACTGAACTCTTGGGAGCACAGGTTTTTTAT
CCATTTCGTAAACTACGTAATTTCCAAGTGCAGCTACTCCCCGGTCAGTTCTTCCCCCGTGGTAAA
GTGGAACTCTATCCTCGTTATCTAAATATCCACAGTCTAAAAGTGCATTTGAAATTTTGTCACAA
ACGGTTTCTCCGTGGGGCTGTGTTTGAAAACTTAATTTTCCATCGTATGCAATCTTAAATATGTA
CATCTTATCACAATTATATTTCCGGATTTCTTGGAATGTCCTTTTCGATATATTTTACAATTATA
TTAATTTATCGAAATTAGGAATTTCAAAATAATTATCAGTAATATTTATTCAAATCTATTTTTTA
ATTGTTGCATTCAATTTTGGAGGGAATAGAATTTAATTTTTTTGTTTTGAAAATTCAGTTTTTTT
AAAAATATTTAAATTTTAACATAAACATAGGTTAGTTTAACACCAATATCTTGTGATACAATGA
CAAAAGTTTTAGTAATCGACCCAAAAATTGCAGGGATGTCTGGAGATATGTTTGTTTCATCCTTG
CTTGAACTTACAAATTCTTACGATTTAATGGATGAAGTAATAGCGGAATTAGAAAAATTAAACA
GTTGCAACTCATTTAACGCAGTATTAATGGATGAAAAAGTAAATGGAATTTCTGCAAAACAGCTA
AAAATTGAAATCGACGAAGAAAAAATAAAAAATCCAGATGAATTAAAGGAAATCATCATAAATA
CTTCTAAAAATCTAATGTCCGAAAAAGGAGTTAAAATCTGTGAAAATATTATAAATGATTTAAT

TGAAGCAGAAGAAAAACTTCACGGGGATCATTTTCATTTACACGAGATATCTTCACTAGATACAG
TTTTTGACATAGTTTTACCCGTATTGATTTTAGAAAAAAATGGATTTTTAGATGGAAAAATTTA
TTCAACGCCTCCTGCACTTGGAAATGGAAGAATATCAATGGATCACGGGATTATTCCAAGTCCTG
CACCTTCAACACTCGAAATTTTATGCAAACACGAAATAAAATGTTCAAAAGTAGATTCTAATGTT
GAATTATTAACCCCGACAGGAGCTGCAATACTTTCAAACGTCACAGATGAATTTATTGATTCATA
TCCTGAAATAGTTCTTTTAAAAACTGCATACGGGGCCGGAACCAAAAGAATGGAACACGTTCCAA
ATGTTTTAAGGCTTGTCGAAGGAAAAACCGATGAAAAAATAATTGAAAAAACAGTAATTTTGGA
AACTAATATTGATGATATTTCTTCAGAAGTCTTGGCATACGCTGTTGAACGGCTTTTAAATGAAG
GAGCGCCTGACGTATTTATAACGCCAGTGTTTGGTAAAAAAAATCGGCCATCGAGTATGATTTCG
GTAATATGTCCATATCACGTTCATGAAAAATTTGCAAAAATTTTAATTGAGGAAACTGGAACTTT
GGGGGTTAGAATTAACCATTACGATAAAATAAGGGCTAAAAGAAGAATGGAAAAATTAACGCTT
GATATAAATGGTGTTAATTTTGGATTTGATGTGAAAATATCTGATTTAAATGGGGAAATTGTGA
ATATAAAACCAGAATTTGAAGATTTGAAAAAAAATTGCAAAAAATCTAAATATTCCGTTAAGAGA
TGTTTTAAGACATGCAAATAATAAAATAAATGAAAAATATGGTTTTTAAAGTATAATATTACCA
TACTTTTAAATCAATTCCTTCAATTAAAACCCTATTTCCTTCAACTTCAACAACAACTCCGCTGTG
AACTTTCTGCATCATACAGTGGGTTGGGATAAATCTAACAGTTTTTCCAACTTCTGGAGTTTTAC
CGTTATAAACTCCTTCAAATGCAGCAATCAGTGCAATTCCAACTTCAATATAATCTATTTTTTCT
GAATTAAACCTGTTGCAAGGGCCTTCCAGATGTACTACATACATTCCGTGGGCGTCATCAATTAT
TTCCGCAAAATGTGGAATTGGGCATCCAAGAGGCCGGTATTTGATATGATCTCCCTTTTTTATTG
GATTTTTTGAAAGGATTATTAAAGTTTCCCTACAAGAATGCTCTTCTGGAAGCGGATCGAGTAAA
AAGTCAGCTTCGTATCCATCTAAAAGTCCAACAAGTCCAGTTGTGGAGTTACATGTTTTTTTAAT
TTCAAGAGGTTTCAAAGTTGATGCAATTTCTTTTATTTTTTCAATATTTCCTTTAAATTCTTCTT
TAAATAACAAAGGGCAGTTTTCAGGGTTTTCTCCTTCAAGAATTTTTTCTGCGAAGAGATCACAC
CTTTTGAACCCACATGCCTTGCAGTTGTATCCTGGAAGCAGTTTTACAATTTCTTTAAGGTTTTC
CATGTTATTCACCGACATATTTGGTAAAACCATCGATTCTTCTTAAAACGCCCCTGTGGTGTTTT
TTAGAAACTTTTGTTTCCCCAACACACAAGGTGCAGACGCAAAGTGGGGCGTTATGTCTTAATTC
TTCATTTTCAAGGTCCACGATATCTTTTGCTTCCATTATTTCTTCAGAGATTTCTGCACAGCCCTG
TCCGCTAAGTCCATTTACATCGTATATTGTACATTTTGGATTCATTTCGAGAACTCGTTCTCTAA
ATACTTCCCTTTCAGCTTGGGATATGATGTCTCCTTTAGTTATTGCAACAACATCGGCACTCGTT
AAAAAAGGTCCAACTTTTCTAGGGGTATTTGGGCCGGATGTTGCATCAATTACACAAATTCCAAG
GCTGTTTTCGGTGTATGGCGCACACCTGTGGCAAAGACCTGCGGTTTCGATAATTAATGTATCAA
CGCCTTCTTTTTCAGCCCATTCTGCCATTTCTTCTAAATTGTAAATTGCAAAGTGATCAGGACAC
ATATCTTTACTTAATCCGACAAGTGTGGGAATTTTAAGTTTTCCGTATCGGGTATCGTCATCAGT
ATATAAACAGTCGATTTTAACAACTGCTGGCTTATTTCCTTTGTTTAAAAGCTGTTTTATTGTGT
GGGTCATAACAGAAGTTTTTCCTGCACCAGGGGTTCCTGCAACAATTATCATTTTCAATATTTCA
CGTCCAATTTAATTAATTACCTGAAACTGCTGGTGTTTCTATGTCTTTTAAGTTTAATTTTTTGC
CATGTCTTACTTTTTCTCTCATAGAGAGCATCCAGCTATCAATTTCGCTCAATCTTTGTGATACTT
CTTTTTCTTCTTCCGTAGTTTCGATGATTTCGGTCATTCCCCCGTTTCGCATAACAACTCTTCTAG
TGGTCATTAACGCGAGAACCGGATCATGCGTAATTACCATGACAATTTTTCCGCATCCTGCAAGG
AGTTCGAGTGCTTCATGCTTTTTAATTCCTGCATTTTCAATTTCATCAATTAAAACAATCGGTGA
ATCGCTGATAACTGCAACATCTGCAACCATCAGGCTTCTCGACTGTCCGCCACTGAGTATTGTTA
AGTTGTAGTCTTTTTCAATCGGCTCTCCTGTGAGATTGTTTGCTAATTTGATTACTTTATCGACA
ATTCCTTCAGAATTCATTCCTCTGCTCTTTGCATGCATAATTAAAAATTCTTCAACAGTCATATC

TGCTAAAAAATTCATATTTTGTGAAAGCTGGGCAATTCTTCTTTTTCTAGGGTCCCTTCTCATTT
CAGTATCTGGAACGTTTCCGTTTACGAGTATTTTTCTATTTGATGGAGTATCTCCCTGAGCGAGC
TGTTCAATATCGCTTATTAAGTTAGATTTTCCACTTCCAGTTGGTCCAACTACTCCGAAAATTTC
TCCTCGTTTTACAACTAGTTCTTTTACTTTTTCAGATTCTCCAAACTTGTTGAATCCGCCCATAAT
TTTAATTTCTTCGATATTCATAAGTTACACCTTTTTAACTTAAATTTTTCCGAAAGTGTCCCCTC
TTAATCCTCTTCTCAGAGTTTCCAGTGAAATTACTTCTTCAAAGGATATATTTCCAAGATTTACG
TCATTCCCAAAACGTAATATAAATTCGACCTGCTGATTTTTTTGAGGTGCTTCAAGTATTATTTT
ATCCATATCAACATTTTCAGCAAGTATTTCAAGTTCTTCCTTTTTCACGTTCCCTTTTTCATCAAA
AAGCCCTATTGATTTTCCGCTTTCTCTACCTTCAATAATTACAAAGTCTGCACCGGATTCAAGGTC
TTTTTTTACGAGTTCTATTCTTTCGTGGATTTCAAGTTCGCCATCTAAAACGATACTTTTTTTAC
CAACTTCTGAAAGAACGATAAAACCTGCTTTTTTTGCCTGTTTTATTGCATAATCTTTATCTTCT
GGTTTTAATTCCATTGAACCATCAGATATTTCAACACATTCAAATCCGAGGTCTTTACATTCTTT
TAAGTATTCTTCAAAAAGGTTTTTTGAGAAACATACTTCAAAGAGTGTTCCACCAGGGTATGTTT
TTATGCCGTATTTTTTGTAATTTTCAATTTTTTCCTTAACCAGTTCTCTGGACTGAACAGCGCTT
GTGCCCCATCCAAATTTTGCAAAAGTGATATATTTTCCACATACTTTTAAATAATCGTTAATAAA
GTCAGGGGACAGCCCTTTATCAATTATCATGGTATTTTCAGTACCCTTTTCCAAGTTTAAAAAAG
AAAATGCGTTCATATTTCCACGTCCATATCTTATAAGAAATAGAATTGGTAAAATTTTTATTATA
ATATCATTTTAAAATATTATATGATTTATCAATTATATATTTTTTCATTTGTACACGGAATTTAA
TTTACAATAAGAAATATCGTGCATTAAAACAATATCGCAACCAATAAAAAATAAAATGACTAAT
TATCAGTAGTCTAAATTTGGAGTTGATCGCATGAATATTACACGAATGCATGGTGCTGGCGGAAC
CGTAATGCAAAAGTTAATTAAGGAAACAATACTCGGTAACTTAGAAAATACAAAAATTGAGGGC
GGAATTGGTCTTGAATCTCTTGATGATGCTTCAACTATACCTATTGGGGACAAAGAAATAGTTTT
TACTGTTGACGGACACACTGTGCATCCAATATTTTTCCCAGGCGGCGATATCGGGAGAATTTCAG
TATGTGGAACAGTAAACGATCTTTCAGTAATGGGAGCAAAACCTGTTGCACTTTCTCTTTCGATA
GTCCTTCCAGAAGGATTTGATATTGAAAAACTTGATAAAATAATGAAGTCAATAAATGAGGCGT
GCAAAGAAGCAGGTGTTGCAATAATTACAGGGGATACAAAAGTTTCAAATGTTGATGATATAAT
CATTTCATCTGCAGGTATAGGAATTGTTGATTCTGGCAAAGCTGTAAGGGATTGCGGAATGAAAG
AAGGGGATGTAATAATCGTGAGCGGAAATATTGGAGAACACGGCCTTACAATACTTCTTTCAAGA
GAAGGATTTGAATTTGAATCAGAATTAAAATCAGATGTAGCTCCAGTAAACGGGCTTGTTCAGA
GTGTTTTAAATGCAGGATTTGAAATTAATGCAATGAAAGATCCGACAAGAGGCGGTCTTGCAGAT
TCTTTAAATGAAATGGCAGAAAAAAGTGATTTGGGAATATTAATTCATGAAGACAAACTCCCGA
TAAGTGATGAAGTTCAGGCTATTGGTGAAGCTCTTGGAATTGACCCATTAACTGTCGCAAATGAA
GGAAAAGTTGTAATGTCTGTAAAAGCAGAAGATTCAGAAAAAATACTTGAGATTTTAAAGGCAC
ATCCTCTTGGAAAAAACGCCAAAATTATTGGAAATGTGACTTCTGAACACAAAGGCGTTTTAATG
GAAACAATTGTTGGAAAAAGAATCGTTGACACGCCAATGGGCGACCCAATACCAAGAGTCTGCTA
AAAATTTTTGAAATATCCAAAAATCCAAAACTTACTTTTTTTATATTTAAAATTCGGCTTTTTTC
AAACATCTAAATGATTATTCTTTTATCACACGATTAATTCTATATTTTTGGTTTAATCTATTTTA
TGGGGGAAATTAATCCTTTATTTAAACAATAAGCTATAATAATAGCGTTTTAGAAAGTTATAAT
TGTAAGAGTTATATTGAATTTGGGGCATTCAATATACGATTTCGAAGAGGGGGTTTTAATGAAA
ATTTTCAAGGTATTTCTGGTAATGGTAATATTGGGGGCCATTTCAGGAGTATGTGCGGAAGATAC
TACAATATATGTAAATAATACTCACTACTGGTACCAAAGTGGTGCATTTTTCGAATCAAATAATT
CTATTCAGGATGCAGTAGAAAATGTGCCCGAAAATGGATTAGTTGAGATAACTGCTGATTTAAA
AGTTTCAAATGGCATTGAGGTTAATAAAAGCAATATTATAATTGATTTAAAAGGAAATAAGCTT

TCAGGAAATTATGAAGGAAGAGGAATATCCCTATCCGGAAATAACGTAAGTTTAAAAAATGGGT
TAGTTTCGAATTTCGATTACGGAATAGTTCTTGAAAATGCAGAAAACTGTAAAATTTCAAACAAT
GAAGTGTTTGGAAATACCTATGACGGAATTTACATACTCAACTCAAAAAATAACGAAATTTCTGA
AAATTTAGTTTATGAAAATGGGGTCATCGGTATCGTTACTTCAGGAATATTTTTAGATGGGTCTG
AATTTAACAATATTACCAAAAATACGGTAAATAATAACATTTACAATGGAATCGAGTTATTAAA
TTCAAAAAATAACCTTATTTCGGGAAATAACGTTTTTGAAAACGAAGATAACGGAGTTTTTGTTT
GGAATTCAAAAAATAATTCGATAATATTTAATGAACTCTATCAAAATGAGAATAACGGGATTTT
AACAAGAGAATCTGAGTTCAATACTATTGAAAGCAATAATATCTTTGAAAATGACGATTCTGGA
ATTTATTCATGGAAATCTTTTGAAAATACCATATCTAAGAATAAAATTTCTAAAAATTCCGAGGG
AATAATCTTGTGGAATTCTGATTTAAACGTGCTTTTTAAAAATAAATTATTAAATAATGTAAAA
TCAGGAATTTCTATTGAATTTGGTACGAAATATAATACCATTTATGATAATTTATTTAATAATTC
TTTAAACGTGCGTTTTGAAGATTCGGGTAAAAATTACTGGAATGCTCCCGTTATAAATGAAAGTA
ATATCCTTGAAGGGGAATTTACCGCAGGAAATGTTTGGTACACTCCTGAAGGTACAGGATTCAGT
CAAAAAGCAATGAATCAAGATTCTAATGGGGATACACTTTCGGAAACGTATTATGAACTTAATTC
TGAGAATATTGACAATATCCCATTAGCACCTGATAGCGTACCTCCTGTCGTAAGCATTGTTTCCC
CTAGAGAAAATGCTTTCTTTGGTGAAAATGAAACAATATTAGTTGACGTTTCAGTAACCGACAAC
TCAGAAATTCACTCTGTAATGCTCGAGGTGGATAATAGTTACAAAGAAATAATGGTGCAAAACG
GAACTACCTATTCCAAATCTTTAGATAATTTGGACTATGGGGCACACACGATTAAAATATATGCA
GAAGATGCTGTTGGAAATGTAAATTTCTTCGAATCAAGGGTATTTTCAATATCTACTCCAGATTC
AACTCCTCCGGAAGTTAGAATTATTTCCCCAGTTTCAAAATCTTATGCTGAGGGCTCTGAAGTTT
CGATAAAAGTTCAAATCACTGACGAATCCGACATATATTCAGCTTATGCAAAGATCGACGATTAC
ACTGTCGATTTAATTGAAAGTAACGGGTATTACATAAATATTCTTGATAATTTGGAATGGGGAG
CACATACTTTATGGATAATTGTAACAGATGCTGCTGGAAACTCAAATTATAACGAAAAAGTCACT
TTCGACGTAAATGAAGGGGATATCACTCCTCCTGAAGTTGAAATAATAGAACCTGAAATCGGGGA
TTCTTTTGAAGAAAATGTCTCGATTAAGGTTTCAGCAACAGATGATTCGGGAATAGCTTCTGTAA
AAGTAATGCTTGATGAAAAAGTAAGTTTTACGCTGACTAAAAATTCAGAGTACTATACTGGAAT
ATTAAATGATCTTTCGTACGGGATACACACGTTGAGGGTTTATGCAGAGGATAATGAGGAAAAC
ATAAATTCTGATGAAGTTATTCAGTTTGAAATAGAAGTTCCAGATTATTCATATACAAAACCAAT
AACTGATGATATCAAAGAACCTGCAGAAACTTCAAATACTGAAAACAAAACAACAGATACTCCAT
TAAACAATGAAACTGGTGAAATTACGCCGAATGAACCAGAAAATGAATCAAATGGGGAAATAGA
AAACAATACGGATGAATCACAAAATAGCGAAACACAAAATAATACAGATGCAGATGATGGGGGC
AATATTTCAGATAATCCTTCAGAAAATGATTCTCCTTTAGAACCAGAAGATTCAGGTAGTGCCGA
CTCAGGAGATACCTCTGACACTGAACCTGACAATAACGATGAAGGTTCTGAAGACTCAGGAGATA
TTAATTCAGAAACGGGAGATGCTACTGGAGAAGAACCTACAGAAACTTCTCCAGATGATGAAACA
CAAAGTAACGATGAAAACCTGTGAATCAGAAACTTCAGATGATGAACAATCAGAAACTCCTTCGGA
AACCGAGGATACTACTGGAGATGAATCTTCAGAAACTTCTCCAGACGATAATTCGGGAGGGGAAT
CAGAAAGTAATTCCGAAACAGAAGATTCAGAAACTTCAGACGACGAATCATCGGATAGTTCTTCC
GAATCAGAAAGTAATTCAGATACTTCTTCAGAAGACAATTCAGACGGTGAAACCAGCGATAATTC
ACCTGAAGAATCAGGAGATTCAGTAGCTTAAAAATTTAATAAATTAAAAAAAGAATTAAATTAT
TTTTTCTATAATATCTATATTTTCTTTAGCATTTTCATTTTTAGGTTCAATTTCAAGGGCTTTTT
TATAACAGGAAATTGAAGATTCATAGTCTTTTAACCCAACATATGAATTTCCTTTTAAGATCCAA
ATTTGTACATTTTTTGGATCAATCTGAATTGCCAAATCAAAATTTGATATTGCAGCACTATAATT
TTTCATTTTATCAAAACAAATTCCTTTATTTATCAAAACATCTGCATTTGATGGATTTATTTCCA

ATGCTTCATCGTAATATTTTAAAGCATCTTCAAAGTTTTCAGTATCTCGATAAGCATCTGCTTTG
TAATATGGCGGATATTCGCTTTTTGGATTGATAGTTTCTGCAGTATTAAAACATTCTATTGCACT
TTCGTAGTTCTGCAGTTTTAAATATGAATTTCCTTTTCCAAGCAGTGCAGTTTCCATTTTTTCAT
CAATTAATAGAGCTTTATTATATGAATCAATGGATTTTTCATATTGTCCAAGTTTAAATTGTGCT
TCCCCTTTATTGTACCAAACGTCTGAATTTTTCGCATTAATTGAGATTGATTTATCAAAACATTC
AATAGCTTCTGAATAACTTTGCATATTAGTTAATGTAATTCCTTTATTTTTCCAGGCTTCTGAAT
TTTCAGAATTGATATTTAACGATTTATCGTACATTTCAAGGGCTTTTTGGTAATTGCCGGTTTTT
GAAAGTACTGTCCCTAATCCATTAAATGCTTCTGAATTTTCAGAATCAATTAATAGAGCTTTATT
AAAACATTCTCTAGATTCGCCATATATTCCCAGTTTTAAAAGCGAATTTCCTTTATTTACCCAAA
TTTCAGCAGATTTTGGATTTTCTAAAAGTGCGAGGTTATATTCAGATATTGAATCGACGTAATTG
CCTTGATTGTACAATTCAAGACCTTTATCGTTATAAGATTTTGATTTTTGATCTCCAATACACCC
TGCCATTAAAAGTACTAAAATTACGGTCAAAAAAACGACGATTTTTCGCATGCCTGTTCACCACT
CCGCGGATTTGAGCGTTAATTGGGCTTTTATATATTAATGCCAAAATAAATAGTTTGGGTATTTT
ATTTTTAAAAAAGCTATTAAAAAAATAGATTTCTGGAAAATACAAACGCATTATAAAAATCTGG
GTACAAAATAAAATGTTCCAATTGCGCTTCCAAGGTTTGCAAGTGCTGCAACTAACAAAACTCTC
ATCGCCTGATTTTCCATCAATTCTTTAAAATTTTCGGTATTCATCATTTTTCCAATATCGTTAAA
TGAAACGTCCCTGAATTTAAGTTCTACTAACCCTGCAACATATCCTGCACCAACCACAGGAATTA
ATGAGGTAACTGGTGCTGCTAAGAATGCTGCAATAATTGTATGAATCTTTCCACGTGCTAAAATG
ACGCCCAAAGATGAAAGACAGCCGTTTATAAGCACCCATTCCATTGTAAGGGTTTTTAATGCTTC
AAGATTTGAAGATGCCGTGTAAAATCCGTATAATATTATTAATAAAATTGCAATAGAAATTATT
TTACCTAAAATATTTGACTTTTTTTTTATTTCCATTAAAGATTTTAAGTCAATATCTTCCCCTAT
TTCCATTTTTTTCAGGTAGTTTTTAATTCCTGGGACGTGGCCTGCGCCAACTACTGCAACTATAT
TTTCTTTTCCTTTGCTTGCTTCAAAGAGATTTTTTGCCATATATCTATCTCTTTCATCAACGAGTG
TTTCATAAATTGACGGGGAAATGTCTTTCAAAAGTAAAATTAAGTCATCGGCATTTGAAACCATG
TCATTAACTGCTTTTTTATCTAATTCCAAGTTTTGTTCGGTTAAAAGACCCATTAAAAAGTCAAA
TTTTTCTTTAAGTGTCATTTTATTGACTGTTCTTGATAAAGTAACATTAATTGGTCTATCAATTA
GTGAAATTGGTTTTCCGTGTTGTATCGCAAGGTCGGTGGCTTTTTTCATTTCACTTCCAGGTTTT
ATCCCAAATTGTTCTCCAATATTTTTCTGGAAGTTTGCTAAGATGGAGTGCACTATGTAAATTCC
TACTTTCCCCTGTTTTATGACTGATTTTAAATCCACGTTATTTTTTTTATTTTGAAACATGGCTA
CAAATCGGTCTTTATCTAATTCAATAGCTACTATTTCTGGATCCGTTTCGATAATGGCGTTTTCT
ACTTCCTTAACACTATCATCAGATACGTGGGCCGTTCCTACCAATCTGATGTTGCATTCTGTAACG
CCATTGTTAACCCTTATATACATTTTACCACCAATATATCTAGTAGCTATATCAATCATGGATTA
TATATATTTGTTGATAAATAATCTGGTGGAAGAATGATTTTTGTTAAAGATGTAATGAAAAGGC
CAATAACACTGAATAAAGACGATAATATCGAAAAAGCAATATCAACATTCAGGGAAAATAAAAT
AAGTGGTGCGCCTGTTGTTGAAGGCGAAAAACTGGTCGGAATCTTATCCGAAAGTGATATTATTA
AGGCGTTAACTTCACATGACGATAGGTTTAGTTTAGTGCTTCCATCTCCTTTTGATTTAATTGAA
TTACCGTTAAAAACTGCGATAAAAATTGAAGAATTTAAGGGAGACATGGAAAACGCACTTAAAA
CAGAAGTTTTTGAAGCAATGACTGAAAAAGTAGTCAGTGTATCTTCAGAAACCCCGATAACGAAT
GCAGCAGAAATAATGGTAAAAAATAAGATAAAAAGACTACCTGTAGTTGAAGGCGAGAAATTGG
TTGGAATTGTAACTAGGGGCGACTTGATAGAGGCAATGATTTAAAGGTGAAGTTTAATGAAGTT
GAATTCTCCTTCCAGAATACATATGGGATTAATTGATTTAAATGGAGAACTTGGAAGGGTTGATG
GTGGAGTGGGAGTTGCTCTGGATTACCCTAATTTTCAAATTGAAGGAAAAGAAAGTAGCGAGAT
AGAAATAGATTTTAGAATTGAAATTGATGAGAAAGATAAAGAAAATTTGGAATCAAGAATTAAA

AATGCGGCAAAAAATGTTTTAGATATTATTGGAGAAAGTGGAATAAGTTTAAAAGTAAATGACG
CGATTTTATCCCATTCTGGACTTGGAAGTGGAACTCAAGTGTCGCTTTCTACTGGAAAAATTACT
TCATTAGTTTATGGCATAGACTTAAACGCAGAAACACTGGCAAAAATTACGGGAAGGGGCGGAAC
TTCAGGAATTGGTGTGGCAGCTTTTGAAACTGGTGGATTTATTGTTGATGGGGGCCATACTTTTG
GTGAAGGAAAAGATAAAATTGATTTTAGGCCTTCTTCAGCATCAAAGGATGTGAAACCCTCTCCG
GTACTATTCCGACACGATTTTGACTGGGATATTGTTTTAACAATTCCAAAAGGAGAACAGGTCTG
TGGGGATAGAGAGGTTGACATATTTAGAAAATTCTGTCCTGTTCCAACTGAAGACGTTCAAAAAA
TATGCAGGTTAGTTTTGATGAAAATGATGCCTGCAGTTATCGAAAAAGACTTTGATTCATTTGGA
AAAGTTGTAAATGAACTTCAAAATTTAGGATTTAAACGTGCAGAAGTTGGACTTCAAAAAGAAA
GTTTAAAAGGATTATTATCAAAGCTTCAAGAAGTTTCATACAGTGGAATTTCAAGTTTTGGCCCA
ACAATTTATTCTTTAGGGGATAAAGATACAATAACTGAAATTTCCAATGAATTTTTCGATAAATT
TGGAATTGAAGGCGAGATTATATCTACAAAAGCAAATAATTCAGGATATGAAATAATAAAATAG
TTAAAACATCTGTGGAAAAAAATGGACAAAAATATAAAAGAAATTATAAAAAACATGGATTTGAA
ATTTAGAAATATCGACGGAAAGAAGCTGTTGCTCGCAATTTCGACAGATAAACAAAAAAATGTT
TTAATGACTGCATTTATGAGCGAAGAATCTTTGGAAAAATCAATTGAAACAGGGTATATGCACT
ACTATTCCACAAGCAGGGATAAAATCTGGAAAAAGGGCGAAGAAAGCAAAAACGTCCAAAAAAT
AATAGATGTATATCGGGATTGTGACGGAGATGCACTTCTTTTCACGGTTGAACAGACCGGATGGG
CCTGTCACGAAGGATACATGTCGTGTTTTCACAACAAGATTGATTTAAATACTGGAGATTCTACC
GTTATTGGGACTAAATTAGATTAATATTTAATTTAACTTATTTTTTAAATTTTAAATATTTTTTA
TCGTTTTTATGGTGAGATTATGTCTATCGAAGAGATTAATCAAAATTTAAATGAAAATAAAAAG
TTTAAGGAATGTAAAATTACTGTAGACACCTGTGTGGTTATTGATGGTAGGATTACGGAATTAA
TACTTGACGGCAGAATTGAAAAATGTAAAATCATCGTTCCAGAAGCAGTGATTTCAGAATTAGA
AGCGCAAGCAAATAAAGGACGGGAAATTGGCTATTTTGGAATTGAAGAATTAAAAAAACTTGTA
ACTGTTGCAAAAGAAAATGAGATATTAATCGAGTATTATGGAGAACGACCTTCAACTGGTGAAG
TTTCGCTTGCAAGAGCAGGGGAAATAGATGCAATGATTCGAAAAGTTGCAAAAGATACAGATTC
GATACTTTTTACAAGTGACAAAATTCAGTACAATCTTGCAATAGCTCAAAATATCAGCGCACATT
TTTTGAAAATTCATGAAGAAAAGGTCGATTTAAAGTTAATCGAATATTTTGACGAAATTACAAG
TTCAGTTCACCTAAAAGAAAACTGTTTACCTTACGTTAAAAAAGGAAGACCTGGAAAAGTTAAAT
TAATTCCTTTTGCCGATGAAATAATGGATAGATCCGAAATAAAAGTAATAATTGATAATATTTT
AAAATATACTGAGCAAAATCACGGACTTGTAGAAATTGAACGAAGGGGAGCAACTGTAATTCAG
CTTGGAAATTTAAGGATTTCTATTGCAAGACCTCCATTTTCGGAAGCTTTAGAAGTTACCGCGGT
TAGACCAATTACAAAAATATCTTTGGAAGACTACAACCTTTCTGAAGACTTAAAAGTACGATTGG
ATAGTGCAGAAGGTATTTTTGTTTCAGGAGCTCCTGGAAGCGGTAAATCTACATTCGTTTCCGCA
CTTGCTGAAAGATACAAAAATATGGATAAAATCGTAAAAACAATGGAAAGTCCACGTGATTTGC
AAGTTGATGAGGAAATTACGCAGTACGCACCGCTTGAAGGAAGCATGGAAAAAACTTGCGATATT
TTACTTTTGGTAAGGCCTGACTACACGATATATGACGAAGTTCGTAAAACAAAGGATTTTGAAAT
CTTTGCAGACATGAGGATGGCTGGAGTTGGAATGGTTGGAGTAGTTCACGCTTCAAAAGCAATAG
ATTCAATTCAAAGGCTTATTGGAAGAGTTGAACTTGGAATAATTCCACAAATTGTTGATACTGTA
GTTTTTATTGAAAATGGTAAGATTGGAAAAGTCTATGAAGTGGAATTTAATGTCAAGGTCCCTC
ACGGAATGAAAGAAGCAGACCTTGCAAGGCCAGTAATTGAAGTAGTAGACTTTTTGACAAAAAC
TCCCGAATACGAAATATATACTTACGGGGAGCAAGTCGTAGTAATGCCGATAAAAGAAACTATTG
AATCAAGAGGGGCTGTTTTAGCATACGCGGAAGAAAAAATTGCAGAAGTTTTGAAAAAACACCT
TCCAAAAAAATCAAGACCTGATATTAGGGTTGTAAACAAAGATACTGTTGAAGTAAAAGTTTTA

GAAAAGTTCATTCCTGCAATAATTGGAAAAGGCGGAAAAGAAATCTCAAAACTCGAAGAAATAA
CTGGTTTAAGAATAAGTGTCAGGGAAAAAGATGAATTCGTAGAAAATGAAAAAGATTTTGAAAC
TTACGAATTTATAAACGAATATGAATCAACAAAACTGAGTCAAACTGGAAAACACGTTATTGTT
GAACTTGGAGAAGATTATATCGGTGCAAATATTAAGATTTACATTGATGGGGAGTACGCTTGCAC
TGCAACAGTCAGTTCAAACGGTACGGTAAATATCAGTAAAAAAACTGCAATCGGAAAAGATTTA
GCAAATGCAATGAAAAAAGGAAAAGATATTTACGTAGAATTTTAATTTAGTTTTTAAAAAGTGA
AGAAACTTCTAAATCATCTGAAAATATTTTTTCTTTCTGATTTTCTCTAAATTCAGCTAATTTTT
TTTCAATTCTTTCATCAGAAATGGAGAGCATTTCAGCAGCTAAAATTGCGGCATTTTCCCCCCTA
TCGATTCCAACGCAAGCAGCAGGAATTCCAGGGGGCATCTGAACAGCGCTTAAAAGTGCATCGAG
TCCATCCAGTTTACTTTCAACAGGAACTGCAATAACTGGTTTTGTAGTCATTGCTGCAACAACTC
CTGGTAAATGCGCTGCAAGTCCTGCAATTGCGATAAATACTTTTGATTTTGAATTTTTAACAATT
TCTTCGACTAATTCAGGGGTTCTGTGTGCTGAAGCTACTCTTACTTCATACTCAATTTCAAATTC
TTTTAAAATAGAAACTGCCTTTTCTGCAATTTTTACATCGCTTTTGCTTCCCATAATTATGGTTA
TCATTTAATCACCGTTTTATTTTTAATGAACTCAATAAAATTAATCAGTGGTTTTTACATATGAT
GTTTTTATCTTTTATTTCAATATTTCTAATTTTAAAGTTTAGTCTTTATGAACAATTAGTATACC
TTAATTACAAACAAAATAAATAATACTTTAAGAAAATTAAAATTAAAAATCTGGGGGATAATAT
GGTGTGTGAAAACGAAGTGGAAAGAACATTTGTGGCACTAAAACCGGATACTGTTGAAAGAAAG
CTTGTTGGACGGGTAATCCAAAGATTTGAAGATAGAGGTTTTGAAATCGTTGAAATGAGAATGT
TAACCCTTACAATGGAAATGGCGGAAGAATATTATGGTGAACACAAAGGAAAAGAGTTTTACGA
AAGATTGATAAAGTTCATGACTTCGGGTAGAATAGTTGCAATGGTTATTAAAGGAGAGCGCGCA
ATTTCAACCGTTAGGAAAATGCTTGGAAATACGTGTCCGTGCGATGCAGAACCTGGAACTATTCG
CGGAGATTTTGGACTATACACTCCCGCAAATATAATTCATGCATCAGATAGCTTAGAAAGTGCAG
AACGTGAAATAGATTTATTTTTTGGAAGATAATTTTTAAATAACCTCATTTGGCCATTTTTTATT
TTCAAGTTTGTTAGTAAACTATATAATATTTAGATTACTAACTTCTGTTTCGGATTTTTATCAAC
TTTATAATTTGTACTTGGATGAAACACAGATTTTAGAAATTAATATTAACTGGTGATTTTTATG
GCATTAAGATGTCCGATTGTGAGTGTTCTCGGTCACGTTGATCACGGAAAAACTTCCCTTTTAGA
TAAAATTAGAAGAACAAGGGTTACTCAGAGAGAGGCTGGGGGAATCACTCAACATATCGGTGCA
AGTGAAATACCGATAAATACCATAAAAAAAGTTTCAAAGGACCTTTTAGGATTATTTAAGGCAG
ATTTATCTATCCCCGGAATTTTGGTTATCGATACGCCAGGACACGAAGCATTTACTTCCCTTAGA
AAAAGAGGGGGTGCTTTAGCAGACATTGCAATACTTGTTGTGGATATAAATGAAGGATTTAAGC
CCCAAACAATTGAAGCAATAAATATTTTGAAACAGTGCAAAACACCTTTCGTAGTTGCTGCAAAT
AAAGTGGACAGGATTCCAGGTTGGAATTCAAGCGAAGGTCCATTTATTTTAAACTTCAACGAAAA
AAACCAGCACCCAAATGCAATGACTGAGTTTGAAATAAGGCTCTATGAAAATGTTATAAAACACT
TAAATGAACTTGGATTTGATGCAGATTTGTTTTCAAGAGTTAAAGATACTACAAAAACGATAAA
TGTAGTTCCAGTATCTGCAATGACTGGCGAAGGAGTTCCAGATTTACTTGTTATAATTTCAGGAC
TGGCACAGAGATTTTTAGAACAAAAACTGGCTTTAAACGTTGAAGGTTATGCAAAAGGCACAGTT
TTAGAATTAAAAGAAGAAAAAGGTCTTGGAAAAACAATTGATGCAATAATTTACGATGGAATTG
CAAAAACTGGCGATTTTTTAGTTGTTGGAAATCCTGACGGCGTTCTTGTAACAAAAATCAAGGCA
CTTTTAAAACCAAAAGAACTCGACGAAATGAGGGATCCAAAAGATAAATTCAAACCTTCAAAACA
GATTTCTGCGGCAACAGGTGTTAAAATATCTGCACCAGATTTGGATAGTGTTATTGCAGGAAGTC
CTTTAAGAATAGTTCCAAAAAATCAGGTTGAAGCTGCAAAAGAAGAAGTTCTTGAAGAAGTTGA
AGAATTTACAATTCTAACGGATGACGAAGGAATAATCATTAAAGCAGATACTATGGGTTCTTTGG
AAGCTCTTGCAAATGAACTTCGAAAAGTAAATGCTAAAATCAAAAAAGCAGAAGTTGGGGACAT

CTCTAAAAAAGATGTTATCGAAGCTTCATCATACGCTTCAACAAACCCTTTAAACGGGCTTATAA
TTTCCTTTAACACAAAAGTGCTGGCAGATGCAAAAGCAGAAATTGAAAAATCAGACGTTAAATTG
CTCGAAGGAAAAATTATCTATAAATTAGTCGAAGAACACGAAGAATGGACAAAAGAAATGGAAG
AATTAATGAAATCTGATGAAATAAACAGATTGACAAAACCAGCAATGATTAAAATTCTTCCAAA
CTGTATCTTTAGACAGAAAGAGCCTGCAGTATGTGGTGTTGAAGTATTATATGGTACTTTAAAAA
TTGGAAGTCCTATAATGTCTGAAGATGGAAAAAAACTCGGATATGTTAAGGAAATGAGGGACAA
CCAGCAGGAAAATATAAAAGAAGCAAAAGTTGGTATGCAGGTTCCTGTTTCAATCGATGGAAAT
ATAGTTCTTGGTAGAAATGCAAAAGAAAACGATATACTCTATGTCGAAGTGCCAGAACCCGAAGC
AAGGAAATTACACCACGAATTCAAGGACGAATTGAGGGGCGATGAAAAAGAAGCTCTTTCAAGA
TACATGGAATTGAAACAGAAGATCGAAAATAATATATTTTGGGGAATGTAAGCCATTAAACCGC
ATTTATACAAATGACGAAACATCAACAATATATACTCATTACGTTAAATGTAGTTATGGACTACT
ATTCGGAATTTTCAAATTTAACTTAACTTAATAATTTTCTCTTTCGGAGGCATAACATGATTTTA
GTCATAGGCTATGGTTCTTTAGGGCGTAAAGTTGTGAATAACGCTAAAAATATTGATAAAGTAA
CCGTAATAGATAAAAATGAAGCTGTTTTTGAATCTCTTGAGAATGGGGATTTCAATTATGTAATT
GGTGATGCTTCCGAATTAGATGTTTTAGAAAGAGCTAAGGTAAAAGAGGCAGATACACTTTTAG
TTCTCACAAACGATTACGAATTAAACAGGAAAATTGTAGAAATAACTTCAGAACTAAATTCAAA
AGCCTATATCATTGCAAGGGGCATAATAAAATACCCTGAATTATACAACGGTTTAGATATAAATA
AAATTATATATCCTCTTGAAAGTGCTGCAAAAGATTCTGTAAACGAAATAGAAAAATCAAAATT
GAGGAGAAAATTAGCTGAATTAAAAGAAGTTGCAAATAAGGCTAAAAAGTCATTTAATGAACAT
TACTCCGAAAAAGAAGATGAAACACAGGAAAACCACAAAGCTCCTTTTTTAATATTGATGCACAG
AAACCCCGACCCAGATGCAATGGCAAGTGCAATGGCGTTGAAAACTATTTTTGATAAATGGGGAG
TTAATTCTGAGATTGCTTATGGCGGAAAAATAGGCTATGATGAAAATAAAGCCATGGTAAACTT
ATTAAGTATAAAATTAAACCAGATTGATGAAATAAATTTATCTCGATACTGTTCTATTGCAGTAG
TTGATTCATCTAGTGCTAAAACTCTTCCAATTGATATTGAAGGTTCAAAATTGGCTGTAATAATC
GATCATCACAATGACAGCGATATTGTTGCAAAATACATGGATATAATGCCTGAAATCGGTGCAAC
AGCTACTATTTTAACCAATTATTTGTTGGGGCTTGATATAACGCCGAATAGGGATTTGGCAACTG
CATTATACTATGCGATAACTTCTGATACAAACTATTTCAAGAGAAAAACGTCTAAAAAAGATTTC
GAAGCTGCAAGCTATTTACAGGGTTTAATGGATCCAAAAGTTCTGGAAATGATTGAAAATCCAGA
TATGGATACAGAAACCATGGAAATCCTTGGAAAAGCGATAATGAATAGAAAAATCATAAAAGGA
AATCTTGCACTTTCATATGTTGGAACATTGAAAAATAGGGATGCCCTGCCAAGAGCAGCAGAGTT
TTTGCTTAAAATGGAAGGAATAAGCACGACTTATATTTTTGGAATTGCTGAAAATGAAATCCAT
ATAAGTTCAAGGACAAAAGATTTGAGAGTAGATGTTGGAAATATCATGAAAACCGCGTTTGGTG
GGGGAGGTCATCAATCTTCCGCAGCAGCGAGTGTTGAACTTGGTATCTTCCAATCTGTTTCAGAT
AAACAGTCTTTGAGAAAACTCGTGGAAGAAGCAATTCAAGCAAAAAATATTTGAAACCATGGGTA
TCGAAGAAGAAGAACCTGCTGGACAGGATTAGTTTGATATAATGCTAAAAGAACTTATAAACAA
GTTATTGTGGCATCCTGATTATAATCCCGAAGATTATATTATAAATTATCTACACAGGGGCGCAG
AAAACGATGAAAAATCAGTTCCTCTGAAAAAATATCGTTATTGAAGACTCTTTTCTTGTTTTTGAC
GAAACACACATTCCCTTTCACAGAATTTTGGAGATTGTGAATACAAAGACTGGGGAAATAATTTA
CAAAAAAAGGTAGGGATCATGGTATACGCAGAAGAGTACATGACAAAAAAAGTTCATTCTATAA
CCCCTGATGCTACGGTTTCAGACATAATAAAACTTGTAAAGGAAACTACCCACGATACATTTCCT
GTTGTGGTAAATTCAAAAGTTAAGGGTATTGTTTCAGTTCACGACCTTATTGGTAAGGATGAATT
GGATGAAGTAAGTGAATTCATGACTCCAAGAGATGACATGATCGTTACAAAGCCCCACACTAAAA
TAATGGATGTTGGAAGGATAATGTTTAGAACTGGATTTTCAAAACTTCCAATAGTTGATGAAAA

CAATAATATCCTTGGAATTATTACAAATACTGATGTAATAAGGTCGCAAATTGAAAAAACAACTC
CAAAAAAGCTCAAAAAAATTGTAAATTCGTACATAAATTTAGGTTACGAAGTAACAACAAAACG
AGAAACTATCCAGATTGATGATTTAATCCCTACTCAGGCCAACGTTTACGAAGATGAACTTTATG
GAAGGGCTTATGAATTAAAAAGAGGGCTTGCTGAACCGATAATTGTTATAAAAACAAATGTAAA
CGAAAAATATATATTGGTTGACGGCCACCACAGGGCGGTAGCAGCATGCCTTTCTAATATAAAAG
AACTCGAAGCACATGTTTTGGAGATAAATACGGATAAAACGATGGGAATTGAAAAAACTGCTGA
AAAACAGGGTTTGAAAAGTTTGAAAGATATTAAAATACTCGACGAAGAAAAAATGAACTGCAGC
AGTGCATATAAACTCAGGGCAAATATCCTTGAGCTTTAACTTCTAATTATTAAGTATTTAAGTGG
ATGTTTGCAGCATGCAGAAAACAGGCCGATTTTTGGAGATTGATTTTTTTAGGGGACTTGCAATA
ATATTGATGGTTATTTCAAATTTTGTAACTGATTTAAAGTATTTTTTAGGATACTCTGAATATCT
TTTATTTTGGAGTTTCTTTGCTCTTTTTGTAGCATCCAGTTTTGTATTTATCTCAGGAGTTTCTT
TTAATGTCAGTTTTTCAAAAAAAGTTGCTTCTAAAAATTTAACTTACAAATCTTACTTTAATCGT
TTTTTAAAACTTTCCGTAATTGCAGTAGCTATAACCGCGGTTACTGCTTATTTTTTAACTTCTGG
AACGATATATTTTGGAATTATGCACTTTTTGGCAATATCTGGTATTTTGGGAATTATATTTTACC
GTTTTAAAAAATTAAATCTGTTTTTTTCAGTTATTTTTTTGTTTTTGGGAGTTTTATTTAGCGAG
ATTGTTTTAGATACCTATTGGTTACTTCCTTTTGGAATACTGCCCACTAACTTTTACACGCTTGA
TTATTTCCCAATTTTTCCGTGGTTTGGAGTATATTTACTTGGAATGAGCTTTTCAGAAAAATATT
ACAAAAATGGGGTTTCAAACAATTCATTTGAAATTTTTAAAACAGGATTTTTTAATAAAATATG
TATTTTGGGGCAAAATACGCTAATAATTTATATAATCCACCAGCCAGTTTTAGTTGGACTTTTAA
TTTTAAAATACGGAATATTTTCAAAAATAGCACCGTAAAAAATAAAAAAAGGAATATTGTATTT
ATCTGAATTTGCTGTATTTGTAAAAGATTCTGCAAGTTCCTTCATCAGAAACCATACAGCTTCCA
ACTGGATTCATCGGATCACAAGTTTTTCCAAAGAGTTTACAGTCATGAGGAAGTTTTTCTCCCCT
TAAAATTTCACTGCAAATACAGGCTTTGTTTATTGTTTCTTTAATGTCTGGAACATCAATATTTT
CATGAACATCGTATTTTTTGAATTCTTCTCTTAATTTCATACCGCCGTCTTTAATGACTGGAAAG
CCTCTCCATGCAACATCAGCGGGTTCAAAAACTTTATTCATAATTTTTTGAGCAATCACGTTTCC
TTCTTCTCTTACTCCTCTCGTGTACTCATTTTCAACTTTTGCGTCACCATTTACAAGCTGTTTTAA
AATCATCACGATTGAAGCCATTACATCAATTGGTTCAAAGCCAGCAACAACCATTGGTGCATGGT
ACGTCTTACAAGGGTCGTAGTATGGTTTTAAACCTGTAATCGTTGAAACGTGGCCAGGACATATA
AATCCATCTAGGTTAAGTGAAGTTCCACCGCCGAGTAAAAATCCCATTACTGGTGGGGTCTGCCT
GTGTGAATTTAATATGTAGAAATTTTCAGGTTTATTTAGTAGTTCTGCGGCAGTTGTTGGAGCAG
TAGTTTCAAATCCGATTGCAACAAATACTACCTTTTCATCCTGTTTTTTTGCAAGTTTTACAGCA
TCTCCAATTCCGTAAACGATTTTTACATCTGCTCCTTCCGATTGAAGTTGCATTAATGATTTTTT
ACTTCCTGGGACCCTATACATATCTCCTAATGTTGCAATAGTATATCCCTGTTCTGCAAGATACA
TTGCTTTATCGATTTCTTTTTGTGTTGTAACACACACAGGGCATCCGGGTCCTGGAACTACCGTA
ATATTATCAGGTAAAACGTCTCTTATTCCATATTTGCAGATTGTATGTTCATGTGACCCACAAAC
GTGCATTATTTTTACTTCGTCCAGTTTTTCAGAGAGTTTTTGGATTGTCTGAATAGATTTCTTAA
TTAATTCTTTATTATTGATGTTCATGATCTTTGCATCCATAATTTCCCTCGTCTGATTAAGATAC
CAAAATTATGACAGAATACTATATAAAAATAGCATAGTGAATTTTTGTAAAAAAATCTAAATAA
TCTTTTATTGGCAATAAAAATTAATTAAAATATTCTAATTTATAAATTATTGGTTTTGAGCAGCC
CTAATTCAGATTCAATTTATCAATATATTTATATCATGTTATCAAATTATAGTTATTCTAAACAT
ATTCGAACTATTTATGAGGTGTTTATCATGTTTCCTGGCGGAGGAAAGTTCAATCCAAGAATGAT
GAAACAAATGCAGAAAATGATGAAAGATTTCGGAATGGATGCCGAAGATTTAAAAGCTGTCAAA
GTTACAATCGAACTTGAAGACAAACTCTTAGTTTTTGAAAAACCAAAAGTTCAGGTAATGGACAT

GCTTGGAAATAAAACTTATTCGATTACTGGAAAAGCTAAAAAAGTAGCAAAAGAAGAAGAAAAA
ATCGAAGATGTCGAAGTAAAAGTTGAAGTAACTGAAGAAGACATCGACATGGTTTCAAGCCAGT
GCGGAGTTTCAAAAGAAGAAGCTAAAAAAGCATTAGAAGAAGTTAATGGGGATTTAGCTGAGGC
AATTTTAAAACTCGGAAACTAATTTTTAATTTTTTTAATTATTGAATATTTTATTTATTTTTATT
TTTAATTTTTTTCTAAGGCCGGTGAGAGAATGGATATCGAAAGGTACGTAAGAAAGTGTCTGAA
AAAGGGCATTTCAGAGTCTAAAATTATTGAAGACGGTTTTAAAAAAGTGATTGAAGTTAAAGAC
ACTGATGAAGGATGGGCAAGAAGATTTGTCGAAGCAGTGGTCGAAGAAGTAAAAACAACTGAAA
AATACCGTGAATCAGAAGATAAAAATTTAAAATATATACTTGACTGCCCAGAATCCGGAATTACA
ATGGGAAAAATGGGTGTTGGAAGCCGAGGAGAAGGCGATTTTTTTGTTCACCGCGAAATTGCGAG
AATTATTGAAAGTACAAATCAGGATACTGAAGTTGACAGTACCGAACAGGACGATGCAGGAGTT
GTACGTGCAAATGCAGAACACGTTGTAGTTGCAGTTGACGGAACCCACTCAAGATTAAGTGATTT
TCCATTTATTGCAGGATTTCACGTTGCAAGAGCAGCTTTAAGGGACGTTTATGTAATGGGTGCGG
ATGCAGTTGCAGTTATAAGTGACGTTCACCTTGCAGATGATGGAGACGTTTCAAAAATATTTGAT
TACACTGCTGGAATTTGTGCAGTTTCTGAAGCTATTGATGTACCGCTTGTTGCAGGAAGTACCTT
GAGAGTTGGCGGGGACATGGTTTTAGGAGACAGGATGGTCAGCGCTGTTGGTGCAATCGGAACAA
TTGAAAAAGGAGAACCAACTGCAAGAAAAAGAGCAGAAGTTGGAGATGTAATTTTAATGACCGA
AGGAAGCGGTGGAGGAACAATTTCTACTACTGCGCTCTATTACGGAATGTTTGATGTGATAAATG
AAACGATAAACGTTGATTTTTTAAAATCATGCAAAAATTTGATTGAAAACGATCTTTTAAAACA
CATTCACGTAATGACTGATGTTACAAATGGTGGATTAAGGGGAGATGCATTCGAGATCACAAATA
CTGCAAAAGTTTCGCTTGAGATATACAAAGAAAAAGTTAATGAACTCGTAAACCCGAAAGTCTTG
GAAATGCTTCAAAAATTAAATATCGATCCACTTGGAGTTTCAATTGACAGCCTTTTGATAATTGT
TCCAAAAGATAAAGCTGGAGAAATAATGGAAAAAACCGGTGCTAAACAGATTGGGGTCGTAAAA
GAAGGAGAAGTTAGCTATTTAATTGACGAAAATGGCGAAAAAGAACTTTTAATTCCAAAATTCA
GGGAAGCTGCATACACACCAGTTAAAAAAGTAGTTGGTGAGCAAAAACCTGACAACTTTGAAGA
AATGAAAGAAAAAGTTAGAAAAACATGCGACATGGCAATAGAAAAGAAAGATTTTGTAGTAAAA
GAAATGGTTAAGAATTAATCAGTCTTAATATTAAGTTCTTTTATCAATTTTCCTATTTTTGACGG
ATCTACAATATTTATGTCTACATTTTTATTTTTTATAGAAATTTCAACTGTTTTCTGGTTTTCAA
CAGAAAGCTGAATTCCAACACCTTTTTGTTCAAGCGTATCAATAAGTCCGAGTATCATTTCCATA
TCCATAATATCACCTTTTAAGATTTTCTCTCGTTGTATATATTTAAAGCTTCTGAAACCGTTTTT
CCGTCAATTGTTATTGCATAAATTGCATTGCACATTGCAACGGCATCAGATAACGATTTTTGATG
GATATTTCGACCTGTCGCATTTCCTGACGCTCCGCTTATGTGAATCTGTTCGTAAAGTTGTTTTA
AAAATGATTCAATATCAGTACTTGATCCTCCTGCACATACAACTTTAGTTTTTCCGGCAGCCATA
ACAGCTTCTTTAAATGATTCTGCAGGATTTTCCGATTTAGGGTAGTTTACTTTTACAAAATCTGC
ACCCAAGCATGATGCAACACCAGTAGCTCCAGCAATCAGTTTAGGGTCTTTTTCATTTTGTACTG
CTTTTCCTCTTGGATAAATCCAGAGAATTACAACAAGTCCTCCTTTATGTGCTTCATAAATTATT
TTCGAAGCTTCAGAAAGCATCTCTGATTCGTATTCACTTCCAAGATATATTGTATATCCAATTCC
AACGATATTAACTCCTGAATTTTTCTTAAATTCCAATATGTCGTTAATATCTAATAATGCAAGAC
TTAAAGGGTCTTTTTGGTCCACATCTACAAGATTTGTTTTTGAATTTAATTTTACAATGTAATTT
ATATCTTTATAATCTCGACAGTATCTTGATAACAATCCAAACTGAACTGCAAAACCGCCTATTTT
TGAATTTTTAGCAATTTTAAACAGGTGTTCTGGATCTGCATCGTCTTTTGAAATATTTTCCCCGT
AAAAATCGTTATTCAAGTGTTCGATTTTTTGATCGCCTGCAAAAAGCATTAAATTGCCAGTATTT
CTTGTAAGTTTCAGGTAATTTTGAACGTATTCTCCATGCATTGATTCTGGAGTTTCCATCGGAAT
TAATACATCTTTAAAGGTTATTTTTTTAACCACTATCGTCCCCTCTTTAGTTTTTAACTAACTAT

TTTCAATAATACTTCTCTATTTTTTGATTTAAAATAGTTTAGATTTAATTAACGGCGATGAAAAC
TAATATATAACATACAAGTATAATTGTATGTGCAAATTCAAAAGCCATATCGGGGGATATCATG
TTGCGTGGGGGCGTAAATGATTGTAAAATCAAATGGGAATTTTTGATCGGAAATAGTATCGATT
CAAGTCCAATTTTAGCTAAAAACGGTACCATATATCTTGGTTCAAGCAATAAAAATCTTTATGCA
ATAAATCCTGATGGATCCGTAAAGTGGTTTTTCAAATCTGGCGAAATAATTGAATGTAAGCCATC
GATTGGAAAAGATGGAACAATTTATTTTGGTTCAGATAAAGTCTACGCAATAAACCCAGATGGA
ACTGAAAAGTGGAGATTTGATACAAAAAAAGCAATCGTATCAGACTTTACAATTTTTGAGGATA
TATTATACGTAACTTCGCTTGATGGGCACTTATACGCAATAAACCCAGATGGAACTGAAAAATGG
CGCTTCAAAACCAAAAAGGCGATATATGCAACACCAATAGTTTCAGAAGACGGAACAATTTATGT
TGGGTCAAATGATAATTATTTATACGCAATAAACCCCGACGGAACTGAAAAATGGCGCTTTAAAA
CTAATGATTCGATAACATCTGCAGCTTCGATTGGAAAAGATGGAACAATTTATTTTGGTTCAGAT
AAAGTCTATGCAATAAACCCCGACGGAACTGAAAAATGGCGCTTCAAAACGGAAAACGTATTGA
GTCATCTCCAGTAATTGGAAACACTGATACAATCTATTTTGGATCATACGACGGACATTTATACG
CAATAAACCCTGACGGAACTGAAAAATGGAATTTTGAGACAGGAAGCTGGATAATAGCCACTCCT
GCAATAGATGAAAACGGAACAATCTATTTTGGAACGAGAAACGGGAAATTCTACGCACTATTTA
ATTAATATTTCTTTAATACCTTTTTTATTACGTTCAAAAGAGTGAATTCTTAAGTAGCATTTTTA
TATCTATTTTTACTTATTGTTTATTGAGCGATAAACAATATTCAGGATTATTTTGGTGGTATTAT
GAAAAAAGTAAAGGTATGTTCTCCGGGAACTTCTGCAAATCTCGGTCCAGGATACGATATTTTTG
GGCTTGCTCTTTCAATCCTTATGATATTGTAGAGGTTGAAAAAACAGAAAATGGTATCACTATA
TCTGTTGAAGGGGAAAAAGCAGAAGAAATACCTACAAACGTAGATGAAAATACTGCAGGCGTAG
TTGCTAAAAAGATGATCGAAGATTTTAAAATCGAATCTGGAATTCATATTCATATAATTAAAGG
AATAAAGCCAGGAAGTGGTCTTGGAAGCAGTTCTGCCTCATGTGCAGGAATTGCTTTTGCGTTAA
ATGAACTTTTTGAACTAAAATTATCCAAATTAGAGCTAGTTAAATATTCTTCACTTGGGGAAGCA
GTTGCAGCAGGTGCTCCTCACGCAGATAACGTAGCTCCAGCAATTTTTGGTGGATTTACTCTTAC
AACAAGTTATGAACCTTTAGAGGTACTTCATATTCCAATAGATCTTGAAGTTTTGGTTGCACTTC
CAAATATTCAGGTAAGTACAAAAACCGCAAGAGAAATACTTCCAAAAGAAATCCCGATAAAAGA
TATGGTAAACAATGTTGGAAAAGCTGCAGGAATGGTTTATGCATTATATAACAACGATCTAGATC
TTTTTGGAAGATACATGTCAAAAGACTGCGTTGTTGAGCCATGCAGGGCCAATTTAATTGATGGA
TATGCGGAAGTTAAAGAAAAAGTAAAAGACATGGTCTATGGTATAACGATTAGTGGTTCAGGAC
CTGCAATTATTACAATACCTAAAAAAGAACACGTAATTGATATCGAAAATATATTTAAGGAAGT
CTGGAACTGCCCAGTTTACTACACGAAAGTGGGGCCCGGATGTTACGTTGAAGAAATTGAATAAT
TTAAATTAAAAAATTTTTTTATTTTTTAGTTATTCTATAATTTCTAAGATCCTGTTAATTTCTTC
GTTTTTAAATATTCTTATTTTATTTCCTTCTTTTACATTTTCAATCGAATTATTATACTCGTAAA
TCTCGTAATTTGAATCATCCATTGCTGTTATCGTATCAGGCGTAATTGAAATAACTGTTGCAGTT
TCACAATCTTCTCCTTTTTTAATTAATTTTGCAGATTTATCGATTTCGTTCCAGGAAAATGCAAT
TTTATCCCCAAGTTCTACAGGTTCCATATATACTCTCGATTCATTTAATGAAGAAACGACATAGA
TTTTTTCGTTATGTTCTAAAATATCTCCAATTTTAAATTCTGGAATCCTAACTGCAACTGTTATT
CGATACATGTCTTTTCCAGTATCTTTATCTACTCCAACGAGTTTTGCAGTTTCAATCGATTTTCCG
CCGTATTTTGCTTTTATTGCTGATGCAATGTTTCTTATCGCGCCCATTGAACCCATTTGGTAATCA
AGTCCTTCTTTTTGAGGGATATATTTTGAGATAAATGCCATCCTGTCTTTATTAAGTCGTTTTCC
AGCTTCATCCCTAACAAAGTCGTCCAGTTCTATCCTTTCTTTATCATTTAAAAACCGGTTCATTGC
TCTAACCTGAAGTGTTCCTTCATAGTAGTTTGACATACATCTTGAACATCTTGGGCACTGGACCA
TATCCAGATAAACTGTAATGTGTCTTTCTTCTTCTCTATCGTTATCTTCACCAACAAGCCTTCCTT

TTGCAAAAACAACAGTGGGAATTTCTACACGGGACCTTTTTCCCCCTGGAAGTTGGCGTGGCTCT
TGTGGCATTATTTCTACATCGATGCTCTTATTGGCCTTTTTTAAATTATCTTTAACTCCAAAATA
AGCAATTTCTTCCATTATTTCGTATGTTTCCCTTTTTTGAGGGTCTTGCCAAAGTTTTCGTTTGT
ATGATCCACACATGTGGCATACTTCGACGTGGACCTCGTTATCGACTTCAATAAGTGGATTCATG
TGGGTGTAGCAGATTTTGCAGAGTCCATCAAGAAGTTCGCCTTCATGACCGCATCGGTAGCAGAA
TCCTTTCATAATATCACCAAAAATGTAAAATTTATTAATTAAGTAAAAAAAGAAGAAAAAGAGT
ACTTATAAAGTCTTAACTGGTTTCATTGCACCACAAGCGGTACATTTCAAGAAGTGTATTCTTCC
TTCTTTTACGATTTTTGTGTCCGGTTTTCCACATTCAGGACAGAGTACGTATTCGTTTACGTAGT
TTTGGATTTTTTCATTTACCATTCTCCATCCAAATTTACCCTGCAAGATTAATCTGACACCTTCCA
TGTCTCCTGCGGTACCGAGTTCTTTCATTACGTATTTTGCAAATTCTTGTGGATCTCTTTCCATGA
ATTTTGCAATATCTTTGAAGTTTTTAATGATTGTCCTGTTTCCTTCTACAAAACTGTCTGCAGAT
GGAATTTCAAACCTTACGTCTTTGAATACTTCTTCTGGAAGTTGTTCTCGCGCTCTTGTTAATAA
ACTGCTGTAATCGAAGTAGTCGACCATGTTTTCACCAAATTTAAATTAGAAACTAATAGTAGTAA
AAAAGTGTACAAGTTCTAAACTTGTTTTCTAGTCCATTGTTTATATACTTGCCGTTAAATTAATA
TAGTTGTCACGATACATGATGTTAAGACTAGGTTATACTAGTAGTTTAGTGACGGGTGAATTCCC
GTAGGGCATGTAGCATTAAGCTGAACCCATAAACAGAATCGACGAAACAAGTGATACCATGAGTA
TGTACAACTACGTGAAAGAAGCATGGAAAGTACCTGCTAACTCATACGTTAAAGAGTTACAATGG
GCTAGAATGCAAGACTGGAGAAAAGAACCTTCAGTATTAAGAATTGAAAGACCTACAAGAATTG
ATAGGGCTAGAAACTTAGGATACAAAGCAAAACAAGGAATTGTTGTTGTAAGAGTTAGTGTTAG
AAGAGGAGGTTTAAGAAAACCAAGACCAAAACACTCCAAAAAACCTGCAACATTAGGTATCAACA
AAATTACAATGGCAAAATCCATTCAAAGAATTGCTGAAGAAAGAGCAGCTAAAAGATACCCTAA
CATGGAAGTATTAAACTCCTACTGGGTAGGACAAGATGGAAAACAAAAATGGTATGAAATAATC
TTAGTTGACCCATGCCACCCATCAATCAAAAACGACAAATCCTACAGCTGGCTCTCCACCGGAAAC
CACAAAGGAAGAGCAAACAGAGGTTTAACCTCAGCTGGTAAAAAAGGAAGAGGATTAATGTACA
AAGGAAAAGGTGCTGAAAAAGCAAGACCTGGTGTCAGAGCTAACGGCAAAAAAACGAAATAATT
AAATTTCCTTTATTTTAATTCTTTTAATTAACTTTTTTAGTTTTTATTTTACTCGAATTTACTGA
TTTTTATACGAAAATTACTTTTTACTTTTTTAAAAACCTGTATTTTAAAACAGCACGTACTTTTG
ATATTTTATTTTAGAAATAATAGTTAAAATTTATAGGATACCTATTAATTAATTTAATATATGG
TATATGCTTTTTTTTAAATTATTATGAATTTTGTGGTCCCCTATCCACAAAGTAGTCAATTTTAT
AGTTCTGTTGTGGATTTAACAGTAGATATCGTTTCAATTTTCGAAATGTATTTATAAAATAGAAT
ATTATTTAAAATTGGTCCTGTTTTAGTTATTAAGGTATCTATTGTTATGAAATCCTTGATACGGT
TAAATCCCCAATCATCACTAAAACAGGACCGCTTTTTTTATCAGGTCTATTAATATTTTTTTAGA
TTATTTGCGTTTAATAAATTTTAAAATAGATCATAATTGAAATTTGATTAAAAAGTAGTAATCT
ACCTAAATTTTTTAAAAAAAATGGTTTTTTTTATTAACAAAAATATATTTTGATACAAAAAATA
AGTTAAAAGTTTTGAATAAGGAATATGGGATCTAGTATATCTATTGTATACCTATTTATTCCTTT
AATTCTATCCTCGAGTTAGTTAAAATCCCCATAATAACAACTCATAGGAACCGAAGTTCCGTTTT
ATCCTATTATTTGACTCAGTACTAAAGTTTACGATCAAAACGGCATATGACGTTATATTTGGGAC
AGCATGGATTTTAAGTCAATATAACGTTTTGGGGCATTTTCGCATGAAATAGTGTTTAACGTGAA
TGGTTAGTGTATAATAACTTTTTTTTAATTTCCAAGAATAATAATTAAATAGTCATTAAAAACA
TAATTTAATATGTCGGGGATTTACGTGTAATTCAAATATTTTGAATTTTTTTAGGTGGGGGATTA
GCATGAATCAAGATGAAAAACAATTTAAAAAAATTCTTTTAGATAGTCTTTTCATATCCTGTATA
ATTATAGCGATTCCGGCGATTTTGTGTTTATTTGGGGCAGGGCATTGTACTTTGGGCCTATTTTT
TGGAGCAATACTCACTTTTTTAGTTGGAATAATGGTGCTATTTAGAAAAGATGAAATCATTTCAC

TTATTCTTGGGGGGGAGGCGTAGGTATCACATTAAAAAGTTAAAAAGTGAAGATTAAATTAATT
TTTAAAAAAGTATTTTTCGCATAAATTCTTTATTTTGTAAATTTTTAGATTAAATGTTATTAAA
AACTCTTAAAGTTGCGAAACTGTTGATTTCGGGGTTTTTTGATTATTTTTTGCAATATTTAGTTT
TTTATTAAATTATTTCTGTTCTGGTAATTTTAACACCTGCGGTATATTTTTAATATAATTCGTTT
ATATATTAATGGTAACTGTCGACTTATCCGGATTTAACCTATAAAGGTGTTTATGAAAATGGTAA
ATAACATCACAATATCAACGATTGCAAACGCAACCGAAGACGAAGAAAAAGTTCTCGATTCAATA
GTTTTCTTTTTACCTGAAATAATTGAAGAAGACGATTTAGAAACCGAGACTGTAGAAACAGAAG
GAAATTTTGGAAATCCTATTGAAATCCACACCGTTAAAATTTCTGGAAAAAAAGCAAAATTGGT
ATTTAATTATATTATTGATTTGATAAAAAGCGACGAAAGAAATGTAAACCGATTAAAAAAGGAA
CTTGATAGCAGGATCGAGAAAAATAAACTTTATTTAAGATTCGATAAACAGAAAGCATATCTTG
AAGAATGCAAACTTTTTGATGGGGACGATACCGTAAGAATCGTGGTAAATTTCAAAATTTTTGTT
CCAAGCGGAAAAGAAGAAAAAGTAAAAGAAATGGTTTTGGAAAGGCTTGATAAAAGAGGTGTTT
TTTAATGCACGAACTCTCATATGCAACGTCAGTACTAAACGCGATTCTTGATGCGGTAAAACAGC
AGGAAGAACTCGGTAGAAAAATAATAAAAGTAAATGATATAAATCTTGAAATTGGGGACCTCAC
ATTAATAAGTCTGGATCAATTGCAGTTTGTTTTTGAGGTTATTTCAGAAGATACGGCCTGTAAAG
GTGCAGAATTAAAAGCAGAAATGGTAAAACCAAAAATTTTCTGCAATGACTGTGAGTTCGAGGG
AAATTTAGATACTAAAGATGAACTTGAAGTAGTATGTCCAAAATGCGAATCAAGAAACATTAAA
TTGAAAGGCGGGAAAGAATTTAACATAGTAAATGCGACGATTGAATTTGACGACGAAGAATAAT
TCTTTTTTATACTTATTCAATTAGTTTTTATTTTAACGTTTATATAGTATCTTTTAAATAATGTT
ATTTGTTGAAATTTGGATGATTTCAAATGCAAAGATTGATATTCTGTTACTTCAAAGTGATGAA
ATGATTTTAATATATAACCAAATAATTAACCTGCAAAAACCCGAAGTTCCCTTGTTTTGGTGTAC
AAAATGAAAAAAGAAATTAAATTAAAAGAACTTTCGAATAATATCGTAAAATCACTTCTGGAAA
ATCCTGACAAGCCAGTTCATGAAATAATGGACGAACTTGATATTTATTCAATGAATCTCGACGAT
GAAGATTTAGATCTGGCAAAAAGTCTGGTCTGTGAATTATTAAGTAGGATTGACGTGGTTTCGAT
GAATCTCGACGATGAGGATATTGCACACACGATGTCGATGATTTCATTGAGGTTTCACATGCACG
ATAAGTACAATAAATTTGAAATAATGGATGTACCGCACCTTCAGGCACGAATAAAAGATGCTGCA
AAATACATAAAATATGTTAAAAAAAGTAATAAAGCTTAATTTTTTTATTTTTTATTTTTGTTC
AACGAATGCGGATTTTTTAGCACCGCAAATTGGGCATGCCCAATCATCAGGTAATTCTGAGAATT
TTTTTTCTTCTTTTTCTTCATCATATACATATCCGCATATTGTGCATTTCCAAACTGACATAATG
ATTACCTCCCGTTCAATAGGTTTAATTTGGAATTAATAATTTATATAATTTTACGAATATTTTAG
AGTTCTGTTAATTTAAGTAAATATCTAAAATTTTCATCAAGTTTATTCTCGCAAGTCTTAAAATC
AAAATTTCTTCCAAGAATTGGCCATATTATTGAATCTATATGTAATGGCGGAATATTTGTGGTTT
TAGAAACTTCATTCCAAAATTTTATCGGGTTTTCATCAGTAAATTTAAGCGTGTACTTTTCAATT
CTCGAATCTTTTGGAATTTCGATATTCATTGGATATGGAAAAAAATTCGTTAAATACAATTCTTGA
AGCATATCCAAACATTTTAACTGCAAAAACAACTGTTTTTGAATTTTTTTTAGAATTTAGGTATT
TTGAAAGATTTTCAAGTAATTTTCCCATATCTGAATAATAAGTCTTAAAATCCAAAAGATTTAAG
TTTTCAAGAAATGGAATAATCCTTTCAATTCTTTTTATCTTTACATTTAAAAGTCGCTTATTTCC
TTTCGAATTTTCAAGGAAATTAACGTAACTTTCCCCTAAATTTTCATTATTTGCATCATGTTTTG
ACCAGTATTTTGAAAATTCTTCCCACCAGAGTTCTCCTGTAGTACATAACTGGTAGCTTACAATC
GAATTTAAAATTACGAGTTTTATAAACATTTCATCGTTTTTCATGGAGTTTTTTAAGTTTTCAAG
AACGAAATATTGTGTGTCAATTTTTTCTTCCATGTATTTAGCAAAATTTACATCAAAATGTCCAA
ATATTTCTAAAAGTTCATTTATTTTTTCCAAGTTTCTCATAATTTTCACAAAATTTTTTAATAGT
TAATTAAATAGTTGAGTTTTATCAGCTTCTTTTTAAATTTATTATTTCTTCAATTTTTTCTAGAT

TTGGTTCTCTTTTCCAGATCGGGGTTTTTCTGAAATGTATGGCGGAACAACTTCTTCAAATACT
GGTTTTTCACAGGTATAAAATATTCCAAGCGGATATTTTTCAGTTTCAAGGGATTTTTCAAATGC
TTTTTTTCTATTCGTTATGTCGTGGTCTTCTAAGAAATAAGTATTTTCTTTGTACCATTGAAGAG
TATTTAATTTGTTAAACGAGGGACATGGATGGAAAATATCTACTATCGATAAACCCTTATGATTT
ATTGCTTTAATTATTATTTCTTTGGTTTTTTCAAGGTTTCCCGAAAATGTACGGGCAACAAAGGA
TGCATTTAAAGATATTGCAAGTGCTATTGGATTTAGCGGTTCTTCAAATACTCCCCAAGGTTGAG
TTGGGGTTTTTGTAGAAATAAGCGTTGTTGGGGAAGCCTGTCCTTTTGTAAGGCCATATATCTGG
TTATTATGTATTAAAACCGTGATATTTGGGTTCCTCCTTATCGCGTGAATGAGGTGATTTCCGCC
TTCCGCATACATGTCACCATCACCGCCTTCTGCAATCACTGTAAGATTTGGATTTGTAGCTTTTAC
GGCAGTTGCAGCAGGTATCGCCCTTCCATGAAGCGTGTGGAATCCATGTACTTTTATGTAGTGGG
GCATCTTTCCAGCCTGTCCAATTCCTGAAATAATTGCAACATTTTCAGGTTTTAAATTTAATTCA
GTCAGGGCATTTGAAAGCGAAGCTTTTATTGCAAAGTTACCACACCCGGGGCACCACGAAATATC
TAGTTTTTCGGTTCTTTGAAATAAACTGTTTTCCATAAATTTCACTCCCTCAAAACTTTTTCAAT
TTTTTCGATAATTTCTTCGACTGCAAAAACCCTTCCATCGTATTTTAAAATACTATCATTCATTT
CAATTCCAAATTCCCGTTTTAAAAGCCTTCCAAGCTGTCCGGTATAATTTAATTCAATGTTTATT
ACTTTTTCAGCATTTTTCAGATATTTTTCAGCAGAATCTGAGATAGGATAAACCTGGCTGAAGTG
TAAAAATGCAGTATCTTTTAGATTTAATTTTTCAAGTGCATCTTTTATCGGGTAAAATGTTGAGC
CCCATGAAACAATCAGGTTTTTGTAAGTTTCATTTCCAATGAATTCGGGTTCAATTGCTTCTTTT
TTAATTAAATCTAACTTTTTATTCCTTTTTTCAGTCATTTTGTTTATTAAATCGATATCTTCTGT
AATATCTCCAAATTCATCATGTTCGTTTCCACATGAAAGTACAATTCCTTCTCCAAATCCAGGAA
TTCCTCGTTTTGAAACGATATTTTCATTAAATTCATATCTTTTATAATCTAAGCTGGTTTTTACA
ACGTGATTTTCTTTTTTTAAGCTTTCAAGTTCATTATCTGACAGATTGTAATACGTGTCCGCCAA
ATATTCGTCAGATAATACAAAAACAGGAACTTGATATTTATCTGCCATGTTAAATGCAATCTGAC
TTATTTTATGGGCTTCTTCAATTTTTCCCGGCGCATATATAATTTTAGGAAATTCTCCATGCCCT
GAATAAAGTGCTAATTCTAAATCTCCCTGTGCAGTTCTTGTTGGAAGTCCAGTTGAGGGCCCCGG
TCTTTGTGCAAGATATATCACAACTGGAGTTTCAGTCATTCCAGAAAGGCTTACAGCTTCACACA
TTAACGAAAATCCGCCCCCTGATGTAGTAACAAGGCTTCTAGCTCCTGCATAACCTGCACCAATG
GCCATATTTATTGCAGAAATTTCATCTTCAACTTGTTCTACAATAATTCCGAGGTCTTTTGAATG
GGTTGCAAGAAACGTGGAAACCCCTGTTGATGGAGTCATCGGGTAAAATGATACAAAATTACAGC
CTGCTTTTGCAGCCCCAAGTGCAACAGCTTCTGTACCGCTTAAAATAATTTCATTATTAATTTTT
TCATTTTTTTCGATATTTAAGCTTAAATCTAAATCTTTTAATATTTTTTTTCCAAGTTCGTATCC
TTTTTCTGCAGCTTCTATATTTTTATCTACGATTTCTTTTCCTTTTTTAGAGAATTTATCTTCGA
GATATTTATTAAAAAGCTCTAAATCGCAATCAAAAATTCCAAGTATTATTCCTGAACCAACTGTA
TTTGAAAATAAAACGTTTCCAATGTTTTTACTTATTTCTTGGATTGGAATATCAATAATATTTTT
ATTATTCATAAATTCTTCGTCAATATTTTCTTTTTCCCCCAGTATAATCGTTTCATCAGAAATCC
TGTCTTTGAGGTGGTTTAAAACACCTTTTTTAAATGTTACTAACAAATCTAATCTTTTTAAAAAA
GAACGCTGTTTTTCCGAGGATATTACAATTTCAGTAGTGTTTATTCCGCCCCTAACTCTTGACAT
GTATTCTTTTGTAGCAAATACATTGTATCCTGATAATTTCAATATTTTTGTAAGGGATTCTTCAA
TTGTTTGTATCCCCTGTCCTGCAGCGCCGCCGAGGACTATCGAAACTTCTTTTTCAAGATTCATAT
TTTCCCTCCACCAAATACACATATAAATTTGAATTTGAATATATTTAACATTGCTAGATTCCGTG
ATAACATGAATGACGACATATATGATGAAATGGTTCGAGAAAGGGGACGCGAATATTTTTTAAA
AAACATGGTAAAATACTGTGTAAAACGAGGGAATACCCTTCATGGAACGGTTTATGGTAGTGAT
AAGTATATAACAAAGGTGGATTTAAAAACAAAAACTGGAATTTGTACTTGTCCTTATCAATACA

ACTGTAAACACGCATATGCTCTTTTAGAGTCATATAAATCTGGAAAATATGTCGATGGGGATGAA
TTATTTTTAAATTTTTCAAAACTTGATAAATTGGAAATTTTAAAAATTTTTGAAAATATGATTG
AAAAACACAATTTATGGGATGAATTTATAACTGAAGATAAAACCCTCCTTGATACTGCAAAAAAC
ATGTTAGAACTTACAAAAATAGAAAAGAAAAATGTATTTACATTTACTTCGTTTTTAAGAAATC
AATTTTTAAAAAATGCCAAAAATGAAGAACTTCTTTTGATAATTCCTGATGTAATAAAATACAT
TCAGGAGCGAAAAAAACTTGAAGAAATATTATTTTTAATAGTTGATGAGCTTTTTGAGCGGGGA
AAAACTAATAAAGATATTTTAAAGAAATTAATCGAACTTTCAAGAAAATATCGTGAATTGTGGA
TGGTAAAAGATAACATCCTTGATTATGAGTACTTTGAACTCTTGGAATATTAATTTTCAGAATAT
TTTAAGCTTTATTAGAATAATTAATAAATATATTTATATAATCTGCCTAAAAATATAAATTGTA
GCAGATTAGCGCTTTGATAAAAAATAAATCAATGTTTTAAAAGATATTAGTATATCTAATATAT
ATCGGGAGGTTTTATTATGGAACTTGACGTTACAGGAACAGTATGCCCTATGCCTGTTTTAAAAA
CAAAAAAAGCATTAGATTCATTAACTTCTGGAGATGAATTAACCGTTACTGGAGATTACAAGCCA
GCTCTTCAAAATATCGTAAGATTCGTGGAAGATAAAGGTCATGAAGTACTATCCACTGAAGAAAG
TTCAGATGGATTTAAAATAGTTATCAAAAAATAAGGTGAAATTTTGAAATTTACAGTTATAATC
GGAGATGCACCTTACGGAAAAGAAAGAGCTTACTCTGCTTTAAGATTTGCACTAACTGCCCTTTT
AGAGGGAATTGACGTTAATATATTTTTACTCGAAGATGGGGTTTATGTTGGTAAAAAAGACCAG
AACCCCGCAGGAGTTCCTAAATATTCAGAACTTTTAGAAAACGCAATCGAAGCAGGAGCAGTAGT
AAAAGCATGCGGTCCATGTGGAAAAGCAAGAGGAATTTCTGACGAAGAACTTCTTGACGGAATTG
CTTTTGGAACAATGCACGATTTAATAGCTTTCGTAAAAGAAGCAGATAAAACAATTAACTTTTAA
TTTCTCTATTTTTTGATTTTACTTTTTTTAATAATTTTACTCGAATTTTTAAGTCATTACTTTTA
GGTTAATGTTTTTATATTATTTTTTGAAACTTAACTTATTCTAAAAACGAGTGAAAATATGTTTA
ATGATGATAAAGAATTTACTCTAAAAAAACTCCAAGAAGCACTTGATAATGGCTTTGTGGACCTT
GAAGTTATGTATTTGGTAGATAAAATAAATGAATTTAAAGATTACTACACTACAAGCAGCTGTA
TAGGAAGATGCGGAATAATTGAGTTTCCAAAGGATAAAAATCCAAAAATAAACTCAAGATGGCT
TGGAAAATGGCACCATTATGCCAATTACGATGAAATTGATGAAGCACTTTTAAAAAAATCTGAA
AATTTCGAAAAAATTTCATTTGTGTTAAATTCTCCAATTTTGCACGTTGCATCAAGAAATGCGGA
CACCAGTAAAAAGCTTTTAGAACTTGCATACCACAACGGTCTAAAAGCTTCTTCAATAAAATCGA
TTTCAAGTAAAAGATACATCGTAGAAATTATGACGACCGCTAAAATGGATGCTCCAATTGCATAC
GATGGAAAAATGGTGGTTAATCGCGAATATTTGGATATATTGCTTGATGAAGGGAATTTAAAAT
TAAAACATGCTAGAAAATCATTAAAAAGATTTTATGAAAAGTTGAATGAACTCCAAGATTGATT
AAAAAATATTAATACTTTGAGATACATATGTTCTTAAATAGGTGGTGAGAGCATGCTTGATTTG
AAAGGCACCAGTTCTAAGGACATATTCCCGCTGGCTTATTCTATACACATATTGGTTAATAAATC
GCCAGTGACAATGCGTTCCAGGGATAAACCGGGAGTTAGGATTGAAAAAGGAAAATTATTAGAC
GATAATTACGAAGGATATGTCTTAAAGCAGGTTTTGGACTACGGGGAAATTTTAAGGGTAACTCC
TATTTGTGGCCCATATGCAGGAGTTCCTGTTGTAGTTGTTCCAGTAAAAGAAGAAGATGAAATTA
GTGGTGCAGTAGGTATTGTAGATTTAACTGCAGGAATATTTGAAGAAATTCATTCAATAACTCGA
AGAAAAGAACTGGCGAAATTTTTGCCTGACGAAGCTTTTCCAAGATAACTATCAATTCTGGTGAT
ATATTGAAAAAAGTATTCATATTCCCATCAAATAGCCTTATTTTGGCAGATCTGGTGGAGAGATT
TGGTCACAAGCCATTAATGATCAATAATGCAATCGGGGATAAAGTAAGAAACCTCGAAATTGACA
CGCCACCATTAAATATAACTGATGAAGACCCTAAAAAAGGTCTTAAATATGCGGCTATCGAGGTT
CCATCGGGTGTTCGGGGTAGAATGTCTTTAATTGGCCCTTTGATTGATGAAGCAGAAGCGGCAAT
AGTCATGGTTCATGCGCCAATCGGATTTGGATGCGTAGGATGCGAAAGGACAAATGAACTTACTA
AATACCTCATTAGGAGAAAGGAAATGCCCGTTTTAAATATTGAATATCCTGAAAATGATGAAGA

TGCAAAAGTAGTTGTAAAAAAGATTGCACTATTTTTGGAAAGTTTAGAAAAATAGGGATTTTTA
TGAAGAAAACAGCTTTTATGTTATTATTGGTTTTTTCATTGGTTTTGAGTTCAGTATCTGGGGAG
ATAATAAATTTATCAGAAAATACTGACCTTTTTTCTGAAATGAAAACAATTGCAATGGCACATTC
CAAAGATCTCGATAAAATTCCATTTGCAACAAATATTGCAAAAAATGAAAGAATAAATCTAAAT
GTAAAAACCAATGAAAGCGATTCATTTGTGTCTGTGATTGTTGAAGATGGAATTATTACAAGTTT
TGAAAAAGGAAGTTTAGAAAATGCAACAATGAATATGTACGCGGATGAAAGTACAGTTCGGGAA
ATTTTGGACTCTGATGATCCGGTAAGTGCTGCCCAAAGTGCTTTAAAGGCAGATAAAATAAGGAT
GGAAGGCGTGGGAATTGTAAATTCCATAAAAGTCTCAGTTGTAAATGTATTAATGTCCTTCTTTT
AAATATATTATTTTTTTAATCATTATTCTTTTTGTGAACTGTGTCCCACTTTCTGCAAAAGTATA
TATATTTTTAAAAACTAGGTTTCTCAGAAATAATACTTACCTACGTGATATATATGAGTTTAATA
AAAAAACTAATGTCAAAACACGCTAAGCTAGAGTCTTACTATTTATGGATGATATTTTTTGTGAT
CGCTGTTTTTTTATTGGCCTTTGGAGTTTACAGGCTTCCAGACTTATTGTAATGTAAATTCTTCA
AAACAAAATTTTAAAATAAAAAATAAAAAATTAATCCCTGTTGTCAAAGACCCTTCTACTCTTTT
TTTCACTTCTTGGAAGTTCTCCAACTTTTAAACATTCAACATCGGGTGAAATTCCGATGTATGTT
TTAAATGCTTTTATGATTGCAAGTTTTGTTTCTTCAAAATCTTTTTCTGAAACATCAACTTCTAC
CTTAAATAGCATAGAATCTCTACCTTCGAATCTGCTGAGGACAATCTGGTATTCGCTACTTACAC
CATTTAATTTATGTATTAAATCTTCAATCTGTCCCGGATATATATTTACTCCTTTTATTTTGATT
CTATCATCAGCCCTTCCAAGAATTCTATCGATTCTAGGATATTCACTCCCGCATTTACATTTTTCA
GGAATAATTCTCGTTAAATCTCTTGTCCTGTACCTTATAAGTGGGGCTCCTTCTTTTGTAAGTGT
TGTAATTACAAGTTCTCCAAGTTCTCCATCAGGTAAATTTTCACCCGTAACTGGATCAATGATTT
CAAATAATAAATGATCAGACCAGTAGTGCATTCCTTCGTGGTATTCGCAGTCGAGTGCGATTCCT
GGCCCATATATTTCAGTTAATCCGTAAATATCAAAGCTTTTTATTCCAAGTTCGCTTTCGATCCT
ATTTCTCATCTTTTCGCTCCATCTCTCAGAGCCGATTACACCTATTTTTAAGTGAATTTTATCATG
AATCCCTTTTTTTCGTATTTCTTCTGCGAGTAATAATGCATAAGATGAAGTAGATGTTAAAACAG
TAGATTTTAAATCCACCATCATTTGAAGCTGTTTTTCAGTATTTCCCGGACCCATTGGTATTGCC
ATTGCACCCAAACGCTCGGCACCAAGTTGAAATCCTATCCCTGCTGTCCATAATCCATATCCTGGA
GTTATCTGTATTCTATCTTTATTTGTAACGCCTGCCATTTGGTAGCATCTCATCATCATTTCTGAC
CATGTATCTACATCTTTTTGAGTGTAGGGTATGATTACAGGGCTTCCAGTTGTTCCGGATGACGA
GTGTATCCTGACGATTTTTTCATCAGGAACTGCTTGAAGACCTAATGGGTATGCACTTCTTAATT
CTTCTTTTTCAGTGAACGGAATTTTTTTAAAATCTTCAAGCGAATTAATTTCCTCAATAGTTATG
CCTGCTTTTGAAAATTTGTTTTTGTAAAATTCACTACCCTCTGAAACGTGTTGGAGTAATTTTTT
AACCAATGCATACGTTTCCAACTGTTTATTATTCATTATTGCCCTCCAGCATAATTGTTTCTGCG
GATAATTTGGCAGCATTTAAATTGATGGCCCTATATTTTTCAGGAATTTCTGATTCGATAGTTTT
TAAAAAGTCATTATATTCTAAAGGCAGAAATCCAGTATAAAATGCAGCACTGAGGACTGAAACA
TTCATTGATTTTGGATTTCCTGCGATTTTAGCAATTTTTGAGAAATCGATACAAATTGTTGATTT
AACGTATTTATTAATAAAATTTATAATTTGTTCTGAATCATATTTTTCATTTGAAGATCTCGCAC
TTGGAAGAATCATTTTGCAATTTGTGATTATTTTACCATTTTTTTTAAGAAAATGTAAATTTCGT
GCAACTTCGGATAATTCAAGGCCGATTATCAAGTCCGCACCGCCTTCTGGGATTAATGAAGATAT
TATGGATTCACCAAACTGTAAATGGCTTATTACAGATCCTTCTCGCTGTGACATTCCAAGTGTTT
CTGCATTATTTACATGAAAACCTGAATTCATTGCAGTAATTGCAAGGAGACGTGATGCTAAAACG
ACACCCTGTCCCCCAACTCCACAAATTATTACACTGAAGTTCATTGTTTGGTCACCTCAATTACTG
CATCCGCAGGACAAACGGCTTTACAAAGTCCGCAACCCGTACAGTTATCCATAATTTCTGGAATT
TCATCATTCATGGTTATTGCAGGACATCCAAGTTTTTCAACGCAGATTTTACAGTTTATACATTT

ATTTTTATCAATAACACATTTTTTATCTGATTTAACAATTGAAACACAATTTCCTCTAAAAACAA
CTGCAGAAGGACCATTATATTCAATAGCTTCTTTTGAAATTTCCTTACATGCAGAAAAATCATCT
GCAGAAACTGTTTTTACAAATTCAACTCCGCAACTTTTCAAAATTCCTTCAATGTCGATTAATTT
TGTAGGATTCCCAAGCGCAGTTTTTCCAGTTCCCGGATGTGGTTGGTGGCCAGTCATCGCAGTAG
TCCTGTTATCTAATACTGCAATTGTAACGTCTGCATTATTGTAAACTGCATTAATTACTGCAGGA
ATTCCTGAATGGAAAAATGTAGAATCCCCAATAAATGCTACATTCTTTGAATTTTTAGTAGTTCT
CGAAATTCCTCCAGCAATGCTAATTCCTGCACCCATGCAAAGACATGTATCGATTAATTCTAAAG
GTGGGGCATTTCCTAAAGTGTAGCATCCAATATCGCCTGAAAATATGGTTTGAATGTTATATTTT
TTTAATTCTTTTGAAACTTCTTTAAATGCGTAAAATACTGTCCTGTGCATGCATCCAGCACATAA
ATTTGGAGGCCTTACTGGAAGTGAAATTATATCAGTTTTATCGATTAATTTTTCGGAAATTTCGA
GTTTCTGAATTTTTAAACTATCCAATACTTTGTTAATTCCAATTTTTACAATATCTACGCTGTAT
TCTCCACAGCAGGGAAAAATATTATTTTTCTTTCCATAAACCGGCTTTAAAATACCATGTTTTCC
AAGTAATTGGAGTAACTGGTCTTCCAAATATGAATCGAGTTCTTCAACTACGAGTATTAAATCAA
CTTCGTTTGTAAATTCAATTACTTTGTTTTCGGGGAATGGATGAGGGGCCCCAATTTTTAAGACT
GAGAATAATTCTTTGTTATTTGAAATAGCTTCGTTTACGTAGTGGTATGACGCTCCTGAAGTTAT
TATCCCAATTTTTCCAGTTCCGGTTTTAAAATTAAATTTTGAATCCGAAAATTCATTAGATAGTG
TTTTTTGTAATTTTTCTAAAATTGGATGTCTTTCTGCAGTTAATTTTGGAAATATTGCCCATCTG
GAACTTTTTGAGAATCCTTCTATATTTTCATCTGAAGGTTTTAATTCTTCGAGCACTACATCATC
GCACCTGTGTGAAACTCTCGTTGTAGTTCGAAGAATTACTGGGGTTTCATAACTTTTTGAAATTT
CAAAAGCATATTTGGTCATTTCGTAGGCTTCCTGTGAGTTTGATGGATCGAGTACTGGAATATTT
GAGAATAATCCAAAAGATCTTGTATCTTGTTCAGTTTGGGATGAATGGGGTCCTGGATCATCGGT
TACGAGGAGAACTAACGGCCCTTTTATTCCAAGATATGTTAAACTCATTAATGGGTCGGATGCAA
CATTTAATCCGACCTGTTTCATAGTTACAATGGAACTTGCACCTGAAAATGAGGCTCCAATTGCA
GTTTCGAGTGCAACTTTTTCATTTGTAGACCATTCAACGTAATAATTATATTTTTTCGCATTTTT
AATCAGTGTTTCCATAACTTCCGTTGAAGGAGTTCCAGGGTACCCTGCTCCAAACTGTAATCCTG
CAGCAATCGCCCCGTACGCAATCGCTTCATTGCCCATTAAAAAATGTTTATTACTCAAAAAATTC
CCTCTAATAGATTTTAAAATTACTAATTTTTCCTTTAAAAGTTGTGACTTTAAAAATTTAACCAG
TATATAATATAAATATTTCGATATCGTGTCAGTGGTGTCGTAAAATAGATTAAACTGATTAATA
ATAAAAAAAGAAGTTAAAACTGTTTTATTTTTAAAATAAAGTAATCAGTAATATGTTATTTATT
TAACATCTCGGATTTTAGATAGATATATAAAACTTAAATCAATAGGTATATTAGATTTTTTTGA
AAATACTGTTTTTCGAAATTTGGCAAAACATAAATATTTTCTAATGACAATATAGAACTAAGGT
AGATTTGATTTATCCGAAACATGATGAAGGTGGAAAAGTATGGAAGAAAAAGTAACCTTAAGTG
TTATTAAGGCAGATGTTGGAGGGCTTTGTGGACACACATTAGCTCCAGAAGAATTATTAGACGCT
GCAGAGTATGTTCTCGAAAATGCGCTCGATGAAACATTAATTGATTATTATGTTACAAACTGTGG
TGACGATATCGACATGATCATGACCCACGATAAGGGAACAGACAACGAAGAAGTACACAAACTTG
CATGGGACGCTCTTGAAGCAGCAACAGAAGTTGCAAAAGAGTTAAAATTATACGGTGCAGGACAA
GACTTACTTTCAAACTCATTTTCCGGAAACGTTAAAGGACTCGGTCCCGGTTGTGCAGAAATGGA
ATTTGTTGAAAGACCAAGTGAACCAGTTGTAGTTTTCTGCTGTGACAAAACAGACCCTGCAGCAT
TCAATTTACCATTCTATAAAATGTTTGCAGACCCATTCAGCACTGCAGGATTGGTATACGACCCT
TCAATGACCTCAGGATTCAGATACGAAATTTTAGATGTTTTTGCACACAAAAGAGTATTTTTAGA
TGCTCCTGAAGAAATGTACTCATTACTTGCATTAATTGGAAACACTGAAAAATACGCTGTAAAAA
GAGTTTACAGAAGAAAAGACAACGAAATTGCAGCAGTATGTTCATCAGAAAAATTAAACATGAT
TGCTGGAGAATACGTTGGAAAAGACGATCCAGTAGCAATTGTAAGAGCTCAAAGTGGATTCCCTG

CAGTAGGGGAAGTTTTAGAAGGGTTTGCAAACCCACACCTCGTTGCAGGATGGATGAGAGGATGC
CACTTTGGACCTATCATGCCAGTTGCTGAAGAAGATGCAATGCCTGCAAGATTTGACGGTCCTGC
AAGAGTTATGGCTTTAGGATTCCAGTTATCAAAAGGAAGATTAGTTGGACCAAACGACCTCTTCG
CAGACAAATCATTCGATAAAGCAAGAGAAAAAGCACTCGACATGGCTGAAATGATGAGATCAAT
GGGTCCATTCATGCCACACAGATTACCTGAATCAATGATGGAATACACATCAGTTCCAAAAGTTT
TAGCCCAGTTAAAAGACAGATTCCTCGAAATGGACGAAGGATGCGAATGCAAACTCGATGAAATA
AGAGAAAGAGGAGACATGGAATAATTTTTCCATGAATTACTATTTTTTAATCATTTTTTAAAA
TTGAAATGTAAAATCTAAAATTTAAAAAAAAGTATTTTAGTTATTTTTTAATTATTTTAAAACT
GCTCCTTCGTCAGCAGATGAGACAAGTTTTGAGTATCTCGATAAGTAGCCTTTTTTGATTTTAGG
CTCAAATTCTTCCAGTTTTGAGAGTCTTTCTTTAATGACTGATTCATCCAAATCAACATTTATTT
CTTTTTCAATCATGTCGATTTTGATGATATCCCCGTTTTCAATTGCAGCAATTACTCCGCCAGCTG
CAGCTTCTGGAGAAACGTGTCCGATACATGGGCCTCTACTTCCACCACTGAATCTTCCATCAGTAA
TCAATGCAACGCTGTCATCAAGACCCATTCCACAGATTGCTGAAGTTGGGGAGAGCATCTCTCTC
ATTCCCGGTCCACCTGATGGTCCTTCGTATCTGATTACTATAACGTCCCCTTCCACAATTTTTCCA
CCAAGTATCGCAGAAATTGCTTCATCTTCTGAATTGTAAACTTTTGCAGGCCCATCGTGTTTGTA
CATTTTTGGATTTACTGCACCGATTTTTACAACGCAACCGTTTGGCGCAAGATTTCCTTTTAAAA
CCCTTAAACCAGAAGTTTCGTGAACCGGAGCTTCCACTTTTCTTATAACGTCGTAATTTATGTAT
TTAACAGATTCTGCAATTTCTAATATGCTTCTTCCATCAACCGTTTTTGTATCTCTAATTTTTTCT
TTTAAAACGTTCAATACCGCAGGAATACCTCCAGCATTGTGTAAATCAATCATGTAGTGTTCTCC
GCCAGGTTTGATCGATGCAATGTGTGGAACTTCATCGCTTAACCTGTCAAAGTCATCGAGAGTTA
TGAATTTATTTTCAATTTCATTTGCAATTGCAGGAATGTGTAATGTTGTGTTTGTTGATCCACCA
AGTGCAAGGTCAACTAAAATAGCGTTTTCAAAAGCTTCTTTTGTTAATATGTCTGTTGGTTTTAA
GTCTTCTTTTACCATATCAACAATTTTTGAGCCACTTTTTTTAGCAAGCCTAACTTTTTGGGCATC
AACTGCATGTGTTGTTGCACACATTGGAAGAGAGAGTCCCAACGCTTCTGTAAGGCAGGCCATAC
TGTTTGCAGTGTAAAGCCCTGCACAACTTCCAGCACCTGAACATGCACAGTCTTCAATGCACTTT
AACTCTTCTTCAGTAATTTTTCCAACTTGGTATTCTCCAACACCTTCAAAAAGGCTGATAAGTTC
GTATTTTTTACCTTGGAATTCTCCTGGAAGCATTGGCCCCCCAGTAACTACAATAAAGGGAATGT
TTAGTCGTATGGCACCCATTATCATTCCAGGAACGATTTTATCACACGTAGGAATTAAAACAAGA
CCATCAAATCCATGTGCTCTTGCCATTGATTCAACAGCGTCTGCAATAATTTCTCTTGAAGGTAA
AGAGTATTTCATACCTTCGTGGCCCATTGCAATACCGTCGCAAATTCCAATGGTATTAAATTCAA
ATGGTGTTCCACCGTTTGCATAAACACCATGTTTAGCCGCTTCTGATAATGTTCTTAAGTGAATG
TGGCCGGGAACAACTTCTGTAAAGCTGTTTACAATTCCAATAAATGGTTTTTCCATGTCTTCGTC
TGTATATCCGCAAGCCTTTAAAAGAGCTCGGTTTGGAGTTCTTATAACTCCCTTTTTGACGTTAT
CACTTATCATATCTTACCACCATTAACGATTAAGCTAATATTCGAGCAAATTAAATAAAAAAGTA
TCGATGTATAAATTATCTATTTATCTAATAAATTTCCGTAATCAAATAATATATCTTTTCTTTTT
AAATCGTCTAAACTATATATTCCAGTATGGTAGTAATCCGACCTTATTTCTTTAGAATATAAATT
TATTGGTAATTCTGGTTGGGTAATTAATTCAAAGCATATTCTGTCCATTTTATGTTCAAAACAGG
CTTCATAACTTGTATGGTTTGGATCTGGAAAGTTTTCTTTAAATTTTTCATTATATTCTGGTTTA
AAATAATTAATCAATGCAGCTTCAGTATAGTTTATTCTCTGATTAAGTGGTATGGCGTTTCCAGC
CTTACGATTTTCTACCCACTTTTCATAATCAAGCTGTTTAGACCTATTATTTAAAAGAATGCACT
GATATGGTTCATCAAAAGACATTAATAATAACCAAATTTCTTTATTCGGATCATTTATTAATATT
TCCGAATAAATTTTTTGTAGGGTAGAATGAGAAAGTAGTCTTTTGTCTACGTTTCTAGAGCCATC
TTTTCCGAAAGACTGTCCAATATATAAAACCTCAAAATCAAGTTTTTCATCTGCAAATGTGGTGT

ATAGCAATGTGCTCGAAAAATCAAGAGTTCCTTGGTTTTGATGGGTTGCAGTACCCATATTGTAG
GGATATTCTGAATTTATTTCAATTTTTGCATCTGATTCTAGTTTTAATTCTTCAATCGAATAACT
GTCTTTATTCCAGATTTTAAAATAAAGATGTACTATATTTTCAATAAAGTTGCACTTTTCCGGAA
TTATCTGCACTTTTGGACAAGAACAAACAAAATAAATATGAAAATCATTTTTTTCAAAGAATCCA
TTTTCTGATAATTCGTTTAACGAAGGCTGGTTTAATAACAAACACTCTTTATTTTTCATAATCAG
TGCGTTTTCACAGAATTCCCCCAAATTCCCACCAAAAAATAAATTTATTTCAATTTTTTAATAAT
TGCTAACGATAAATAATCTTTAAACGGTTCAAAATCAATTTCATCGATTTTTCCTAACGCAGTTT
TTTGATTTTTTGATGTAACCCGGTTTACGACCCCAACTAGATAGTTATCCTTTTTATCTTTTAAA
TTTTTCAATTTTTCTTCTAAATCGTTAGTTTTCATCACAATTAACGTATCGAATTCTTCTAAATA
TCTTTCTAAATCTTTTCCTTGAGGTAAAATTGCTAATTTTTCATCCCCTTCAACTAGGGGGATAT
TTAAAACGCCTGCAGCGGCAAATGGTGATGAAATTCCGTTTATAATTTCTACATCAATATTATTT
TCATTGAATATGTGCCAGACGTATGAAAAAGTACTGTATAGCGTTGCATCTCCAATCGTTACAAC
TGCAACAGTTCCATTTTCTTCCATTACTTTTTCTGCGGCATTATTCCAGTGTTTTTGTAAAAATTC
AACATCTTTACTCATTGGAAACAAAAGTTCTACCACTTTTTTGTTTTCTGGAATTATAGTTTTTA
TAATGTCATATGCAACAGAAGCTTTTCCTTCCTTTGAAATTGGAACAAATATGGTATCTGCATTT
TGAATTACATTTACCGCTTTTAAAGTTAATAAATCTGTATCTCCTGGACCAACACCTATTCCATA
TATTTTATCGACCATATTTTCACCAGTTAATTAGTTAATAAAGTATTTTAGCTCCGTCATTACTA
GGTTTTGTAATTGTGGTTTCCCCATGTTTTTTGAGTGCATTTTCAACGTTTTTTTCATCTTCAGG
GTAGCAGAGAGCAACATAAGAAGGACCGGTTCCTGAAAGCCCCGCAGTAATTGCACCAGCTTCTA
AAGCATCAACAGAAATGTTTGATGGAAAATTCAACGCAGAAGAATAAAGTAATCCATTTAAAAA
CAGTGCTTTATAGTAATCTCCATTCATACACTTTTCAAAAGCCATATCAACGTAATCTTTAATCA
ATTTCATTCTGTTTACATCAACATTCTTTTTAAATTCAGGCATTAATACAATAACTTTTATATCT
TCTTTAAATTTATCTTTTTTCAGTATTTTTCTTTCCATATTATTGCATACGGTAATTCCGCCAAA
GTAAGATGCAGTAGCATCATCATATGCTCCGGTAACCGTTAAATTTTCTTCAAAAGATGATTTTA
TTGCAAGATCTAAAACCAAATCCGGATCTACTTTTTCACCAAGTGCGCCGATTGTTGCCAAAACT
GCCGCATTTGAAGCTGCACTACTACTGCTTAATCCTGATTTTATCGGAATATCGCCAATTGTTGA
AATTTTTGCAGAATAATCAACTTCAAAGTGATCAAATACGTTTTTAACACACCTTTCTACTAGAC
TGGGTTTTAATGTAGGGTTATCTATTGAAATTCCGTTTACAATTTTTTTCCCGTTATCTATTAAC
TCAACATCAGCTTTTATTTTCAAATCAACACCGAAAGCAGAGCCAAAACCTGTTGCAATTGCATT
AATTATTGTTCCCGACCCTAAAGAAACTGCAGAACATCGCATAATGTCACCAAAAGAATTAAATG
AGTAGTTGAACTTTTTAAATTTAATAAGTGATACTAACATATAAAATCAATGTAGTAGTATAAT
ATTATAGTTAAAAATAGTATGTTTGAATGATTACAGTGGATATCTATGAAGTATGAAGTCGTTA
TAACTTCGGGCAATTCTAATCAGGCACCAATTGGAGCTTTTTTTAAAGGTGATTTCGTAACTTTA
CATTTGTATGGTGGTTCACACACTTTTGAAAATTTACAAAAAGATGGGTTTTATATTCTAAATAC
TTGCAGTCCTTATTTAATTGCAAAATCAGTTCTCGATGATGCGGGGGATTACGAATATTTGGATA
AAGAAGGAATAAAGATTCCATATTTAAAGGATTCTTACAAAATAGAATTAATCGAAATCAAAAG
CCGAAAAATAGTGGAAACAAAAAATGAATTTGGAAGTTCTAAACTCATGATTGTTGAAGGAAAA
CCTATTTTTGAAAAAATATTAAATTGTAATACTTCTGCTTATAACCGTGCAGATGGGGCCGTTGT
TGAAATGGCAGTTTTATATTCCCGGAAAAATATGATTTCAAAAGAAGAAATGAATGAAGAAATG
AATAGATTAATGAAAATTATTAAAAAAGTCGGCAGTAAAAATCATATCGAACTTGCAAAAAAGT
TAGGTGTTTAAATGGAAGAGTGGGTTGAAAAATACAGGCCAAAGTCATTAAATGATGTTGCAGG
GCACAATAAAACTAAACAAGCGCTTATTGAATGGATTGAATCTATCATAGGTGGCCAAAATCAAA
AACCAATACTCTTGGCAGGGCCTCCTGGATCGGGTAAAACCACATTAGCTTACGCGATTGCAAAC

GATTATGCTTTTGATGTAATCGAACTTAATGCAAGTGATAAGCGAAATAAGGATGTAATTTCAC
AAGTTGTGGGAACTGCGGCAACTTCAAAATCTCTTACTGGAAGAAGGACTTTAATCGTTTTAGAC
GAGGTTGACGGGCTTTCAGGAAATGATGACCGAGGAGGAGTAGCTGAAATAATAAAAGTTTTAA
AAACAGCAGAAAACCCCGTAATTTTAACTGCGAACGATGTATACAAACCTGCTTTAATGACGCTT
AGAAATTCTGTAAATCTGATAAATGTCGGTTCCGTTCACACAAACTCGATTCCGCCGGTTTTAAG
AAGAATTGCACTAAAGGAAGGTTTTGAAATTGATGAAAAAATAATTAAAATGATTGCAAGTCAT
TCTGGCGGAGATTTAAGGGCTGCAATAAATGATTTACAATCACTTGCAACGGGCGGCTCTATTGA
AATCGAAGATGCAAAAGAACTCCCTGATAGGGACAGTGAAAAGAGCATTTTCGATGCAATGAGA
ATAATAATGAAAACAACCCACTACGACATTGCGACAAGTGCTACACGGGACGTAAAAGAAGATAT
TGGAACTATTGAAGAGTGGATTTCTGAAAATTTGCCAAAAGAATATTTAAAATACAAGGATCTT
GCAGAAGGGTATGATTACCTTTCAAAATCAGATGTATTTTTAGGAAGAGTCTACCGAAGACAGTA
TTTTGGACTTTGGAGATATGCTTCAGCTTTAATGACTGCAGGGACTGCTTTAGCAAAAGAAGAAA
AGTATCGAGGATTTACCAGATATGGGCCACCTGCCATATTTACAAAACTCAGCAGGACTAAGGGC
AGCAGACAGAAAATGAAAGATATTTTGAAAAAAATAGCTTTAAAAACCCATACTTCAACAAAAA
GAGCAAGAAACACCGTGGATTACTTGACTGTAATTTTTGAATCAAATGCGGAAGTTTCTGCAGAA
CTTGTGGAATATTACGAACTTACAAAGGATGAAATAGAGTTTTTAACTAACAAAACAATTACAA
AAAAGATACTTTCAGTAATTGCGGGTAAAAAACCAAAAGTTAAAAAAGAAACTCCAAAGAAAAA
GGAAAAACCAAAAGAAGTAATGCCAATTATTCCAAAACGCCCGAGAATTTCGGAACCTCCAAAAG
AACCTTTAAAAGAAGTAATCGAAGAAACCCTCGAAAAATCTGTTGAAAAAGCAGATACAAAAGA
AGAAAAGAAAAAAGATCCAAAGAAACAGGCGACTTTGGACAGTTTCTTTTAAAAATTTAAGTTA
ATTCTTTTTTAGTATTTTAAAGTCAATTACAAATACGTATTCTCTCGGAGAATATGATTTTACGA
TTCTTTTGTCCAAAATTTCACATTCACACTTTGATTTGAAAAGTTCAATTCCCTCATCAAAATCT
GCACCTACTGTGTAGTAGTGGATTGTTCCACCCTCTTCAACGATTTCAAGGGCTTTATCTACAAA
TTCATGAGCGTATTTTGGTAAATTCATTATAACTCGTGTTCCTTTCACATCTACGTTTCTTACAT
CCTCTAAAATTGGGACTATTTTATTTTCAAGACTGTTTAACACGATATTTTGTTTTAAAAGTTCA
ATTCCATCCGGGTTTATATCAATCGAATATATTTTTTCTGCATTTTTACAAGCAATTGAATATGG
GCCAACTCCGCAGAACATGTCAACTACAATGTCATCAAAGGTTACAAGATCCATTATTCTTTTTC
TCTCCCATCCAAGCCTTGGTGAGAAGTAAACTTTAGCAACATCAACTAAAAGCCTGTATCCGTTT
TCTTTGTATAACGTAAGTGTTTTTTCTTCACCTGCGAGGTGTTCAAGATCCCTAACTCTGAAATC
TCCCTTCACATCGCTTTCCCGTCTAAAAACTGCTTTTACAGAAGGAATTAATTTTAAAGCATTTT
CTCCAATTTTTTTCCTAATTTCTGGAGCAATTTCTTCTGAAATCTGTAAAATCACAATATCTCCA
ACAATATCGTATGCATGAGCTATTGAATTTCCTTCAACTTCATTTTTGAAATTATTTAAAAGATA
ATCTTTGAAGCTGGTCTTTTTTTCAACATCAATCTTTGAAATTTTAGTTTCATCGATTTCTGAAA
TTTCAACTCGATATTTTCATCTTCAAAAATTTTTTTATCAAAATTACTGGAAGTTAAGGGGATG
TATAAAAATTCCCCTTCTTTTTTTAATTTGTAATTTTTACTGAGCAAATGATTGTCAAGCATGAT
TTTTCGTGTTTTTTCGCCCAGTTTGAAATTAACTTTGATGCAGAATACCAATATTTCACCAATAT
TTAGAAATTATATATGTTTTTGTTAATTGATCGTATTTACTGTGAAATAGCCTTTTTAATTTTAT
AATAATGATATTCAACAGTTTTTAATGGGATTTGTGTAATTTCGGAAATTTCTTTTGGGGTTTTT
TCTATATATTTTTCCACAATTTCTTTTTTACTTGTGGGTCGTCCGGTAACAGGTTTAATTGATTT
TACCTCAATTCCAATTTCTTTTAAAGCTCCAAGTACTTTTTTATTAGTCCTTTTGAATTTAGATT
CGGGTAAAATAATTTTTTTAACCTCACAGCTCTCCAGAATTTCAATTATAATGTTTTTAGAGAGT
TCTGTGTTTATATATATTGCATCCTCTTCAGCGTCTAAATTCTCTATTTTTTCGACAAATTCTTCA
AAAGAATGCCCTCTTAAGTATTTCATAAAATCACGTGTAAATTCAGGTTTGATATTTGTGTTAAA

ATATGTAAAAAAGCAGATTTAAATAAGTTATTTAATTAAACCTAGCGCTTTATTGGTTTTTTCAA
TTGATTTTAAGTTGTCGCTTTCCATTTCGAGCATTGCTCGGATACAGTCCACGTTTTCAGGGATA
ACATCGCTTTCCTGGTGTATTGCCTGCATCATGTATACTTCATTGTCTACAACGTTTACGCTTTCT
TCCCATACTGCAATTTCATTTAGATCGTATCTGCTTCTTCCAAGGTCTCTTGCATATTCAATAATT
TTTGCAGTTGAATCAAATCCTTCTGAAGCTTTTACGGTTAATATTCTTGGTGTTTTTGCAAGTGC
ATCTATTATTTCATCTCTATTCGTTCCAGAAGTTTCTACCATTATTGAGTGCATGTGCATCAAGG
TTGTTGGAACGATAACTGCTGAAGTCATGATTTTTCCATCGAGTTCGGGAATAACTGAAACAACG
TCTGGTCCGTGGTGTGATGGCACAGTTACTGGATTTGGAACGATTGCATTCACTGGTCCTGTTTT
TATGTCGTTAGGGTCAGCAGCTCTTCTTATTAAGACTATTCTTGCTTTTAAAATGTCTGCAACTG
AATTAATTGCGTACATTGATCTACAAAGTCCTGTCGTGTTGCACGATACGAGCCTGATGTAGTCT
TTTCCGTAGCATCTGTCGTAACTCCATAGTGAGTTAAATGAGTCTTGTGCGTCGCCTGCTTTTTCT
CCACCTTGGATTATTGCTTTTACGCCGTGTTTTTTGTATACGTTTTCGAGATTTGCTTTTCCAATT
TTTTTAGGGGCTCCGTCAACTACAATATCTGCATCTTCAATAATGTCGTCAAGTGTTCCTTCAAC
AGGAATTCCTGCTTCTTCAAAAAGATGTTTTCTTTCAGGAATTGCTGCAAATAATTTGTATCCTT
TTTCAACAGCCATTTTAGCTTCAAAGTCAGGTTTCGTTTTTGTAACTCCAATAACTTTCATATCG
TCCTGTTTAGCTACTGCATCGGCAACCCTTTTACCGATAGAGCCGTAACCGTTTATCAATACATTT
GCCATAAAATCACCAGTTTATATCTATTTTACCCCTATTTAGAAAGTATTTATTTTTAAAAAGTT
CTAACTTAGATATAAATATTATCTATATTAATTTATATATCGTTTTTGAAAATCGGTTTTGAATT
TCACAAATATCTATTAATAATCTATTAATAAAATGTCTACAATTCATTTTGTACGAATGTTAATA
AATTTTACGATTATTTGGAGGATTAAGATGAGGCATTTAGTTACAACTGCATTAGCATATACAAA
TGGGCCCCTTCATCTTGGGCACGCGAGAAGTACTTACATTCCTGCAGACATCTTTACACGATATTT
AAAACTAAAAGGCGAAGAAGTAATCCATATTGGGGGAACAGATAACCACGGAGTTCCCATTACAC
TAACTGCCGAAAAAGAAGGCGTAAAACCTATTGATATTGTTGATAGGTATCACAATGCAATAAA
AGCAGACCTTGACAGTTTAAACATTTGTTTTGATAATTTTGGAAGGACACACAGTGATATACACA
TTGAAACTGCTCAAGAATTCTATTCAAAATTAAAAGAAAATGGATACATTTACGAAAAAGAAAT
TGAACAGTTTTACTGTGAAAAATGCGACATGTACCTTGCAGATAGATACGTTGAAGGAATCTGTC
CATTTTGTGAAGGCGAAGCAAGAGGGGATCATTGTGAGGTTTGCGGAAGGCACCTTGAACCAACA
GAACTTGTAAATCCTTACTGTATACACTGTAATTCAAAACCAGAAATCAAAAGAACTACCCACTA
TTTCTTTAAATTAAGTGCAATGCAAGATGCTTTAAAAGAATACATTGAAAATTCTCCAGAAATGC
CTGAACACGTAAAAAATATGGCTTTAAGATGGATTGAAGAACTTCACGACTGGGACGTTTCAAGA
AACATAAAATGGGGTGTTCCAATCCCAGGATGCGACGATCAGGTGATGTATGTCTGGATTGAAGC
TCCAATTGGGTATGTATCTTTTACAAAACAGCTTGGAGATATCTGGAAAAGCTACTGGCTTGAAA
ATACCGATGAATCAAAAATTTCACACTTTATTGGAAAGGATATCACTGTACACCATGCGGTATTT
TGGCCGGGAATTTTAAAAGGAATTGGCGGATACAAAATGCCAAACGCGGTTGTTAGCGGCGGTTA
CTTAACTCTTGAAAACAAAAAAATGAGTACAAGTAAAAACTGGGTTGTATGGGTTAAAGATTTT
GTTGAAAACTTTAGTTCTGACTATTTAAGATATTTCTTCATGATAAATGCTCCATTAAATCGAGA
CACCGACTTTTCATGGGACGATTTCCAAAAAAGGATTAATACTGAATTAATTGATATTATCGGAA
ACTTTACACACAGGACTCTCGTATTTACGGAACGTAAATTTGGTAGTACTCCGATTGTGGACTTA
AACCAGTTAAAAGACGAAGATAAAAAGTTAATTTCAAAATGTGAAGATACAGAAAAACTTGTTG
ATTCATTAATTAGGGAATACAACTTTAAAGATGCATTAATGGAAATTATCCATTTGGCAAAAGA
AGGAAACGGTTACTTCCAGGGAATGGCGCCATGGGCAATTAAAGATGAAGAAAGATTAAAAGAA
GTAATGTACACATGTTCTGTTGTTTTAAAGTACATAATTTACCTTTTAAGTTCATTCATGCCTGA
AAAAACGGCTCTTCTTTTAGAATACATGAACGAAGAACTCGATTTAAAAGTTAGGGGAAATCCTC

TTAAAAAAACCGAAAGTTATATTTACCAAGGTAAGCGATGAAGATATTTCAAGAATGAAAGAAAA
CCTCATGAAAGCTACGAAAAAGGCAGAAACAAAAACAGACGAAAAGTCTAAAAAAGTAAAATCT
GGTGAAAAAATGGATATGATTGATATTGATTACTTTGGAAACATTGATTTAAGAGTTGGACAGA
TTTTAGAAGTTGAAGAAGTTCCAAGATCAAAAAAACTCTACAAAATTATCGCTGATTTGGGCGAT
GAAAAAAGACAAATTGTTTCTGGATTAAAAGGAGCATATGAAGCAGAAGAACTTGTTGGAAAAA
AAGTTATCATCATCTGCAACTTAAAACCTGCAAAATTATGTGGCGTCGAGTCACAAGGAATGCTT
TTAGCAGCAGAGGATGACAGTATTGTAAGTTTATTAGCACTTGATAGAGATCTTCCAGTTGGAAG
CAAAATCCACTAATATTCTTTAAAATTATATTTTTTGATTAAATTTCTTTTTTTATAAAATTTTT
TAAAAAAGTTTTTATTTTAAGTTAGATATAAACTGATTAGAGTTAATTTATTAATTTTAATAAT
AATCTGCGGGAGGGACATCATGCTATTCTTAAATGCAAAATTTATAGACCTTGATCTGGGCGAAA
ATGCGGTAATTGTTAACGAAGAAGATTTAAAAGGAACTTCTTACTACCCGCAGGATAGGGTATTG
ATAGAGTCACACGCAGGTTCTGTCATTGGAAATATTTATTCTACGAAAACAATGGTCCAAAAAGG
CGAAGTTGGAATGCTTGTCAGTGAACTTGCAGAAATTTCAATTTCAGAAGGGGAAGAAGTAAAA
TTAAGGCATGCAGAAAAGCCTGAATCGATTCCGTTTATAAAAAAGAAAATGGATGGTCAGGTTT
TAAATCCGCATGAAATAAGAACGATAATTGATGAAATCGTATCTAAAAAACTCTCAAACATTGA
ATTATCCGCATTTGTTTCATCTACGTATATTAATGGAATGAATATGGATGAAATAGGCGAGATGA
CCAAGAGAATTGCAGAAACGGGAGATATGATTTCTTGGGAGAAAAATCTTGTTGTCGATATTCAC
AGTATCGGGGGAGTTCCTGGAAACAAATATGCACTCCTTTCGATTCCAATACTTGCAGCAGCGGG
AATCACAATTCCAAAAACCTCATCAAGGGCGATAACTTCGCCTGCAGGAACTGCCGATGTAATGG
AAGTGCTCACAAATGTTGAATTAAAGGAAGAAGAAATAAAAAGAATAGTAAAAACCACAAATGG
ATGTCTTGTATGGGGCGGAGGAGTAAATTTAGCACCTGCTGACGATATTATCATCAATGTGGAAA
GACCAGTTTCAATAGACCCTCAGCCACAGCTTCTTGCAAGTGTTATGGCAAAAAAGATTGCAACA
GGAATTAAATATACTGTAATTGATATTCCTGTTGGAAAAGGAGTAAAAATTAAAAATGAAGCAG
AAGGGGCAAAATTAGCGAGGAAATTTATTGAACTTGGGGAATTGCTCAACATTAAAGTAGAGTG
TGTCTTAACTTATGGTGGACAGCCTCTTGGAAGGGCAATTGGGCCAGCACTCGAAGCAAAAGAAG
CAATTGAAGCACTTCAAGATCCCAAAAATGCTCCAAAAAGTTTAATTGAAAAAGCCTTATCTCTT
GCAGGAATTCTTCTTGAACTTGGGGGAGCTGCACAGATTGGCGAAGGGCAGAACTTAGCATGGGA
AATTTTAGAGTCTGGTAGGGCACTTGAAAAATTCAACCAGATCATAATTGAACAGGGCGGAACTC
CAAAAAAACCTGAAGAAATAGAACTTGGAGACTATATTGAAGAAATAATTGCTCCAATAGATGG
CTACGTTACAGATATAAATAATACTGGAATTACAAACGTAGTTAAAGAGGCTGGAGCTCCAAGGG
ATAAAAAAGCAGGACTTTTACTAAACTCAAAGATTGGAAACAAAGTTAAAAAAGGAGATGTGTT
GTATACCATCTATTCTGGCTCTGAAGAAAGGCTTGTATCAGCAGTAAATCTTGCGAGAAGAGTTT
ATCCTGTAAAAGTTGAAGGAATGCTTATTGAAAGAATAAGTAAATTCTAACTTTTAATTTTTTA
ATATAGGTAGAATTAAATAGAATTATTCATAGTTATTTTTTACAAATGTTTACTGATTTAGTCTT
ATTTTATCTAGATTTAACTGCGGTGACTTAATGAATGGAATGCTTTTAATCCGAGAATTTATTTA
CCGGTACTATATTTATCCGATAGATACAAAACAGGGCTATAATTTAATTCAGGAAATCACTTACG
GGATTTTACTCTTTGTAATGGTTTACACGTTTTACAGGATGTGTTTAAAACTTAAAATCGCAATT
GACCGCAGATTTGCAGAAGTTACTGTATTTTATGTAATTTTAATAACTTTGATGAGGGCGCTAGT
TGATGCAGGGCTTTTTCCAAGGCTGTATTACACAGTTACGCCTGGAATTGTAGTTGCAGTTGGTA
TTTACTACATGATTTCGATAATTATTTCAGGAATTCTTTTAAAAAAGAGATATTATTTACTTTCA
ATAGCAATGGCAGTTGTTCCAATTCTTTACATGCTTTTCGAGTTTTCGAGTAGGATAACTCACCC
CGAAGCAATAGTGTATGTGTCTGGAATACTGGTTTTAAGTTATTATTTATTAATCTACATCGTTG
AAAAACTGAAAAAGGCAAAAATTGAGCGGATAGATAAATACGCGATATTTTCACAGCTTGTAGA

CGCATCTGCAACATCTGTTGGAATTGGTGTATACCATTATTGGGAACAGCACCCGGTTCCAAGGT
TTTTTATGGATTATTTCGGACCTTACAGCATAATACCTTTAAAAATGCTTGTAGTGCTTTTGGTT
TTAGATATATTTAACAAGGAAGTTAAAGACGATAATTTAAGAAATATATTAAAAATAACAGTAA
TGGCTTTAGGGCTTGCACCAGGTCTTAGAAACCTATTTAGGACAGTTATGGGAGTTTAAATTAGG
TGAGAATAATGGCTGAACCAGAATACAACGAAGAAATCAATATGGAAGTTACAGAAGAAAAGAT
GAGAGAATACATGGATAACGAAGTAGACGAAGGGGACTACATAGAAGTTTACTTTGGAAGGTGC
CATGTTGAAGGAACCATTGATTCAAAAGAAGGAACTCATTATCGAGTTGATACGGATAATAAAA
CATTTGGACTGATGGAATTTGATATTGAGAATATTTCAAGGGATGTTTTAGAAGTAGCGCACATT
CCTGAAAACAGCAATAAAAAAATCATACTTTCAGTTCTGTAAGTGATAACATGTTTATGGGAGCA
GTAACCAAGGATGGAAAAGACATCGCAGACCGATCAAGAGAAATAGTAAAAACAAAAATAAGCG
AAGTTTTGGGAAAAAAATGTTGTATTATACTCTAACGACGAAATGGGAATAATTGAAAGGGTAGT
TCATGCAACTGCAGATCCAGAGTATTCAAAACTCGTTAACTTTGATAATAAACCTATTGAAAATG
GTTTAAATGCAATAAATGATAAAAGTCCGATAATTGCAGATATTTCGATGGTAAAAGCAGGAAT
ACGCTATGAAGACGTATTATGCACAATTTCTGAAAAGAAAGTCTTTGAAGTTGCAAAAAAAGAG
CAAGTTACAAGGGCAGTTGCATCAATTCGGGCTTCAAAAGAGTTAATCGATGGAGGCATTGTTGT
TATTGGTAATGCGCCAACTGCACTTCTTGAAGTCATAAGATTAAATAAAGAAGAAGGAATAACTC
CAAAATTAGTTATCGGAGCTCCGGTTGGTTTTGTAAAAGCAGCAGAATCAAAAGAATTATTAAG
AACTACAGATATCCCATCAATTTCTACAATGGGTCCAAAAGGTGGAACTCCTGTTGCAGTTTCAG
CAATTAATGGAATTATTGCACTTAGTAAAAATGAAAGAATTTAAATTATTCCATTATTCTATCGT
AAATTGAATCAAGGTCAACGTTTATTGGGTCTTTTAAAACATAAACTGGGTAACCTTCGTTTTCA
TATCTTGGGATGATGTGGAAGTGAACGTGCGGGACTTCTTGGCCTGAAACCGGTTTATTATTATT
TAAAAGGTTGTAGCCATCCATTTCTAATTTTTCAAGTTTTTTTACAATTTTGTGGATAACTTCCA
TCATTTCGCATGCAAGTTCTTTTGGAAGTTCGTCAAATGTTTCAAAATGCTCTTTTGGAATTATT
AAAGTATGGCCAACTGCTCTTGGAAATGCATCCATAAATGCTAAAAATTTGTCGTCTTCGTAAAT
TATTCTTGCAGGAATATCTCCTTTAACAATATCGCAAAAAATACACATGATTAAAATCACCTAAG
TTTATTTCGAATAACCGATAAGGTTTAAATGGTATATAAAAATGATATTTTTAATTTTAAAATA
TCGAAATTTAATAACTTCAAAAAATTTTAAAAATAGCCGTTACTAATTTTTACGGGGTGTTGGAC
CGCAATACTTTATATACTGGGTTAGACATAATTATTTTTCGAAATTACAGTTTTTACCATATTGA
TGCAGCTATTGGGTGGATTATTTAAGCATGCTGTAAATAAATCTGAATGATTGTTTTAATATTAA
TAATATTCATGGTATATTTAAGAATTTTAATGAAAAAAAGGTTAATACAAAAATTATCTGATTT
TTCCCGTATTATAATATAATAATGAAGTTAAGGTTAGGCTAGGTATCGTAAGATTTAATAATCTT
TTGTGTTTATGAATGTATGGTGAATTTAATGGACATGTTATTGAAGATAATTTATTCGGCAGCA
ATTACGGGACTACTTGCGGCACTAATTTATAAAAAGAAGTATCTCGACAAATGGGGGATATTCGG
ATCTTCGGTAATGGCATTTACAATATTGTTTTTGGCAGATTTAAAATGGCTACTGCTCCTTATCT
CGTTTTTAGTTCTGGGCAGTCTCGTAAGTAAAATGGGTTATGGTTTTAAAAAGACAATAAAAAT
GGCTGAATCAAGACGTTCTTTGAAAAATGTTCTTGCAAATGGGCTTATGGCAATATTATTTGTTT
TAGCGTATTCTTCAGGATTTATTACCGAAGAAATAGCCCTTGTAGGATACGTTGGAGCAATTGCC
GCTGCAAATTCTGATACTTTTTCCTCAGAACTTGGAATGCTTTCAAGAGAAACTCCAAGACTCAT
ATCAAATTTTAAAACCGTTAAAACTGGAACTGATGGTGGAATAACTGTATGCGGTACATTTGCAG
GGTTACTTGGATCTTTTTTGATAGGTTTACTTGCATACGCCCTTTTTAACGATATATTGATGTTT
TGGACTGCAACAATTTCGGGAATGATTGGAAACTTTGCCGACAGTTTTCTTGGTGCATTTTTCGA
GCGAAAAGGAATATTAAATAATGAACACGTAAATTTCATGGCAACTTTGAGTGGCGGAACGTTT
GCAGTTTTATTTTACCAGTTTGTAATTTAAAATAAAGCTGGCTTTTCTGTTTTTCGGGTGAGGTA

ATAATTAACTTATTTTTTAACTTTTTCAAAGATTAATATTTTTAACGATAATTAATATTTAATCA
GAATCTATAATAATTATAAAATTGAATTGGAATAGGAAATTAAAGTGAAAACTTGTGATAACGT
GAAAACAGTAGTTATATTGGTTAATCCAAAATATGGTGGGAATTTAGGGGCTATTGCAAGAAAT
ATGATGAATTTTGATGTTGAAGAATTAAGGATAGTTGGAACGCCTGAAATTTTAGATGATGAAG
CATATATTCGAGCAGTTCATGCGAAAAATATCCTAAACAATGCTAAATTTTATTCAAAACTTGAA
GACGCAATTTCAGATATTGATTTTACCGTGGCAACAACAGGGGCAGTTTGCGGTGATAGAAACGT
AAACCGGGTTCCAATTACTCCTCGAGAACTTGCTAAAAAACATTTGGGTATATGTGGAACATTGG
GAATAGTTTTTGGGCGGGAAGATGATGGTCTAAAAAATGAAGATGTAGAACTCTGTGATATTTT
AGTTTCTGTTCCAACTTCAAACAAATACCCAATATTGAATCTTTCTCACGCCGTTTCAGTAATAT
TGTACGAAATTTACTTTGAATCATTTTCAGAAGATATTCCTTACGATTTAAACATGAAAAACGCA
TCAGTTGATGAAAAAAATGCACTTCTTAGATTTTTCAACGAATTTGTAGATCGTTCAAAAGATGT
GCCAGACTATAGAAAAGACATATGCAAGACAATTTTTAAAAGAATTGTTGGCAGGGCGTTTATTT
CGGGAAAAGAAGCAAATTCCTTAATATGTGTTTTTAAAGATAAATGCCCGAAATAATATGGAAA
GTTTTTTAAACCGTGCAATGAATAATAATATTATCAATAGTATGGCGGAAAAGGTGAGGGGAAT
GAGTATATCTGGTGCACAGGGAAAGTCTTTGGTTTTGAAGTATGAAAACAACGAAATCATTTTTA
ATTTAAGCAATGAGGAAGAAGGTCTGTTAGATACTGTTTCGATGGATTTAGAAACTTCTGCACTG
TTTGTAACACTTTTAAATCATGGTGTAGTTTCTGCTGAAGAAATAAACCGTAAATTCAAAAAAGA
AGGTATAAAACTTCAAAAGATACAGGATGTTGGAACGTGTTTTGCAAATGAAAGTAGGGTATCA
ATTGCAATACTCCCAGCAGACGAAAAAAGAACTGCCTCGATATTGATAGGCATTCACAAGGAAGA
TGAACACTCATCAATAATAATAAAACCAAAAAGAGCAGCATACCTAAGCATTTCCATAACGAAAA
TGCTGATAAATACAATGGAATAATTTCAGACGTTATTTAATGATTAAAAAAAGTAAATAAATGC
ATTATATTACATTTCAGAATCCAACTTTTTTTGAATACTGTCTTCTGCAACATCTTTGATATCAA
GTGCGATAGGGCACAATTTTTCATAATCGAGTGCGTAATTTTTACCTGCCGAAGTTAAAAGATGG
GGATTTATAAATAAACATTTTCTATTAATTTTCAATGCAATAAACGGAATCCCGTCAAAAGTTTT
GGAAAATTCCAATAATTTTTCAACATCTTCATTTGGAATGTAATACTTGTCTTTTGAGGTTGACT
TACACTCAATTATAATTGGTTTTTTTCCTTTTTTTCCTGCAACTAAATCAACGCCGTGACTTCCAG
CACTTCTAATAACTGCAAATCCATGGCTTTCAAGTTTTTTCTTTAATTCTCGTTCAAAAGTAGAT
CCTTTCTGATATTTATGCGCCATAATTTCCCAAAAATATTTAGTTTTCTTTTAAAACTTCGTCTG
CAATTTTTTCAAAAGCATCGACTTTTAATTTTTTATTTTTCAAATTTTTAAATTCTTCTTCGATT
TTTTCTTCACTAATTTCTTCGTTTTCTACTGCATCAAGGGCCTTTGAAATCCTATCAAGTATTTTC
TGTTCTGCTTCAAGCTGGTGAAAACCTTCTCTTGGGCCCCCTTTTCTAAATGCCCTGCATCGTCTC
TGGTCCCATATTGGAAATCCCCTATCTTTACAAAAAATCTTATTTTTTTCAAGATCCCTTACTAA
TTTAACTACTTCATCGACAACGCCATCTTCGCCTTCGATAAATGCCCCAAAACACGTTTCTTTTAC
ATTAATATCATATTCAAGTGAGTTTAAATATCTAAAAAGCCTTGAAGGCGATGTTTTAGAATCTT
CTGCAAGAACTATGACTTTAGATTTCATGATTAACACCATAAGTCTTAATTTGGGAATTATTGAA
TAAGTTTTGAATACCATATTTCATAATTTTTTTCTACATATTCTCTTATGTCTTTTGGGTTTAAA
CCATTTGAGTAGCAGTAAGTTGCAATTCCTCCTGCTCCAACACCTTCCTTAACTGAACCATTACAG
TATGCTTTTAAGCCGTCAATTTTTGAATTTTCATATCCAAATTTTGATGCATACAACGGAATATC
TCCAATTTGTTCAATAATATCTTTTAAATCTGCATTTTTATCATTTAATACAAATTCAGTGGTAC
TTATCCCTAAAAGTCCACTTTTCAGTATTTCGGGACTAATTTCTTTAATTGCTGCTAAAACAGAT
GCCATCTGTGTTCCTCCTGCAAGTAATACGGGTTTACCTTTCGATACTGCTGATATGGCCATACCT
GCTACAACTGGCATCATTTTATCCCCAACTGCATTTAAAACATCAAAAACATCTTTTGATTTAGA
ATTTTTAAGTCCGCTTTCGACAACACTTAATTTCAAGTCTTTCGGGTTTTCGACTGAACCTGAGC

TTATCTTATCTCTCGCATCGTAGCCTAAACCTAGAAGAACACCAAGTGCAGTTGTAGTTCCTCCT
GGAACGCTTTCACCAATCACTAAACTATCATAAATATTGTTATTTCCAATTATTTTTCCTAAATT
TAACAGTTCTTTTGAATTATCCATTCCAAGACCTTTTTGAATATCTCCTGTTGGTCTTTGATCTA
CTGAAATGTATGGGATTTTTGGTTTTATGTAGCTTCCAGCATCAACGGTCATTACAGGAATTTTT
AAAATGTCGACTGCCGCTCTTGTAATTGTTGCAGGGCTTGGGCATCCTGTAGCATCTATTGGCGG
GTGGTTTAGTGATAGACTTTCCCCGGTAGTTACAAGTTCCATATCTGCTGCAGGCGTGTAATCTA
TGACACTTTTATTGACTCCAGATATCCCCACGTATTTTGTAGTTTCTATTGATGATATAACGCAT
GAAAATAATCCATTGGTTCCTTTAAGTTTATCTAAAAATCCGTTTTCGTTAATTGAGATAATTGG
CATGATTTCTCCTTTAAAATTTATTTAATATATTTACTATCTCATACTTTACTTTGTTTTCTATT
TTTTTTCTGTGTTAAATTATTATGGGAGTTTTTAGAAACTGTTAACACGGTGATTTTATGGAATA
TAATTTATTATTAAAAATAAAAGACGAATCAATGGAAAAAGAAGTACTGGATGAACTCCTCAAC
TTTTTGGATAAAAAATATGCAGGAAAAGTTGTTGCAGAGCCCTACCGCCGCATAAATTTAACAGT
GGACATTTTGATTAAATATAATTTTGGAATAGTTCTTATAAAACGAAAAAATGAGCCTTATAAG
GATTATTGGGCAGTACCTGGCGGTTTTGTTGAATATGGTGAACGGGTTGAAGAAGCTGCAAAAAG
GGAAGCTAAAGAAGAAACTGGTCTGAATATTGATAATTTAACGCTTATTGGAGTTTACAGTGAT
CCAAACCGTGATTCAAGGGGCCATACGGTAACGGTTGCATTTTTGGCTGATGGAATCGGAACTTT
AAAAAGTGGAAGTGATGCAAAAGATGCAAGAATATTTGATTTGGATGAATTAAATGGGGTTGAT
TTTGCATTTGATCACAAAAAAATTGATAGATGATTCAATTCATTATATATTTGATTAGCCAGTATA
TTTTGTATGAAGTATCTAAATCTACGAATTAGATTAAGGGAAACATTTATATACTAGAATACCCT
TCCTATACTATGCTCTTGAAACACTAAATGGAGACGGGGGCCCGTGGCCTAGTCTGGATACGGCA
CCGGCCTTCTAAGCCGGGGATCGGGGGTTCGAATCCCTCCGGGTCCGCCATTACTACTTTTCAAGA
TTTATGCAAATGCTCCAGTGGTGTAGTCCGGCCAATCATCCGGCCCTTTCGAGGCCGGGACTCGGG
TTCAAATCCCGGCTGGAGCACTTTTTTTAATATTGGTTAAATTAATTTATACATTTAAATTAATA
AGTTATATATAAATCCTTTTCGACAAAAGGCGTATTTTTTTTCGATGATATCCTTAAAAACATAT
AAATATCTAATTTAATATAACCAAAAGAGAATCTAATCAAAACAAGAGGAAACTCGTTTCTATC
TTTTTTACTAATATTATGGTATTAAGCGAGGGATAACCATGGTAAAAATAACCGATACCTCCTTT
AGGGATGCGCATCAATCTTTAATGGCAACAAGGTTAAGAACCGAAGACATGTTACCGATTGCAGA
GAAAATGGACGAAGTAGGATTTTATTCGATGGAAGTATGGGGCGGTGCTACTTTTGATTCATGTA
TTAGGTATTTAAATGAGGACCCATGGGAAAGATTAAGGGAATTAAAAGGAAAAATTCAAAACAC
GCCCTTACAAATGCTTTTAAGGGGACAAAACTTAGTAGGATACAGGCACTACCCTGATGATGTTG
TAGACAAGTTTATTCAAAAATCTATCGAGAATGGGATAGATATTGTAAGAATATTCGATGCTCT
AAACGATATTAGAAATGTTGAATATTCTATAAAAGCTGTTAAAAAATACGGTGCTGAAGTTCAG
GGTGCTATATCATATACTACAAGTCCAGTTCACACGACCGAACAGTTTGTGGAGCTTGCAAAACA
GTTTGAAGAGTTGGGTTGTGATTCTTTATGTATTAAGGACATGGCAGGACTTTTAAGGCCCTTTG
ACGCTAAAGAACTTGTTGGAATGTTAAAAGAAGAATTATCAATTCCAATCGATTTACACAGCCAC
TGTACTGCAGGAATTGCTCCATTAGCATATATGTCTTCTGTAGAAGCAGGAATTGATATATTAAA
TTGTGCAATGTCCCCTCTTTCAATGGGTACATCTCAGCCACCTGTAGAAAGTATTGTGGCAGCAT
TAAAAGGCACAAAATACGATACAAACCTGGATCTTGTATTATTAACAGAAATTAGGGACTATTTT
GACGAGATTAGAAACAAATACAAATGCCTGATAAATCCAATCTCTGAAAGAGTTGACGCTAGAAT
TTTAAGATATCAGGTTCCAGGAGGAATGCTTTCAAACCTAGTATCTCAATTAAAAGAACAGGGTG
CACTCGATAAATTTGAGGAAGTTTTAAACGAAATTCCAAACGTGAGAAAAGATCTCGGATACCCT
CCATTAGTAACTCCAACGTCCCAGATTGTTGGAACTCAGGCTGTAATGAATGTATTGACTGATGA
AAGATACAAAATCATCACAAATGAAGTATCTAACTACTTAAAAGGATACTATGGAAAATCACCT

GCACCAATCAGCAAAGATCTCGTTAAACGGGCACTTCAAGATGGTGAAAAGCAGATTACATGCAG
ACCTGCAGATTTACTCGAACCGGAATACGAATTAATGGAAAAAGAAGCTTTCGCAAAAGGAATAG
TTTCAAAAGAAGAAGATATTTTAACTTACGCTTTATACCCACAAGTTGCAGTCAAATTCTTGAGA
GGGGAATTAAAAGCAGAAACAATACCTCAGGAAAAGGACGTTTCAAAATTCATGGAAATTCCAA
ACGAGTACATTGTTGAAGTTGATGGGGATTCTTTCAACGTAAAAGTGGAACCCGTATACAGCTCA
GGAATTAAAAAAGAAGAGAAAAAAGAAATAATAACTGCAGAAACTGAGGGTGCAGTAACTTCAC
CATTTAGAGGTATTGTTATAAAAATAAAAGTTAGTACGGGCGCGGAAGTTAAACAAGGCGATCA
GATTTTAGTTTTAGAAGCTATGAAGATGGAAAACCCTGTAGAATGTCCTGTAGATGGAACTGTTG
AAAAAATAATCGTTAAAGAAGGTCAATCTGTAAACGTGGGCGACATATTGATGATTATAAAATA
AATTAAGCACTTAATTAATGATACAGCAATTCCAGAAGGTAATATTATGTTTAAAAAAGTATTG
ATAGCCAATAGGGGAGAAATTGCAGTCAGGGTAATTCGAGCATGTAAAGAACTTGGAATTAAGA
CTGTTGCAGTTTTTTCTGAAGCTGACGAACACTCACTCTATAGAAATATTGCAGATGAATGTTAT
CCAATAGGAGAACCTCCTGCGTCAAAAAGCTACCTTAACATGGAAAGGATCTTAAAAGTGGCTGA
AAAAGCAGGTGTGGATGCAGTTCACCCAGGATATGGTTTTTTATCGGAAAATGTAAAATTTGCAG
ATGAATGTCACAAAAGAGGCATTGAATTTATCGGCCCCCCAACAAACGCAATTGATGTTATGGGC
AGTAAAATAAATGCTAAGAAAGCAATGAAAACAGCAGGAGTTCCTGTACTGCCTGGAAGGGAAG
AAGCAATCGAAAGCGAAGAAGAAGCAATCGAAGTTGCAGATGAAATTGGATACCCTGTAATCAT
AAAAGCATCTGCAGGTGGTGGCGGTATCGGAATGAACGTGGTTTACAATAAAGAGGAACTCGTTG
ATACGATACAGTCTACAAAATCCATTGCACAGAGTGCTTTTGGTGATTCAACAGTATTTATTGAA
AAATACCTCGAAAAACCAAGACACATTGAAATACAGGTGGTTGCAGATAAATTTGGAAATGTGA
TTCACCTTGGTGATAGGGAGTGTTCAATTCAAAGAAGGCACCAAAAATTAATTGAAGAGTCCCCC
TCCCCAATCATGACTGAAGAATTAAGGGAAAGAATGGGAAATGCAGCTGTAAAAGCTGCTAAGGC
CATAAATTACCACAGCGTTGGGACGGTTGAATTTTTATACAGCAAAGGCGAATTTTATTTCCTTG
AAATGAATACAAGAGTTCAGGTAGAACACCCAATAACCGAAGTTGTTACGGGTGTTGATATCGTA
AAAGAACAGATAAAAATTGCAGCTGGAAAAAAATTATCCTACAAACAAGATGAAATTACATTCA
GAGGACATGCAATAGAATGTAGGGTAAATGCAGAAGATGCAATAAATGATTTTGTTCCAGCTCCT
GGAAAAATAAAATATTACAGGTCTCCTGGAGGCCCAGGTGTAAGGCTTGATAGTGGTGTATTTGG
GGGAGCTGAAATTCCACCTTATTATGATTCCATGGTTGCAAAGATAATTACGTACGGTCTTACAA
GAGAAGAAGCAATTGAAAGGATGAAAAGGGCACTTTCCGAATATATGGTTCTAGGAATAATTAC
AAACATACCTTTCCACAGGGCGGTAATTGAGGAAGACAACTTTTTAAAAGGAAACCTTTCAACGC
ACTACGTTGAAGATAATCTATGCTCATTTAGAAAAGCAATGGTAAATTATGCTTTAGAAGCAAA
AGATAGGGAAAAAAATATTCAGTGAAAAAGTATTTCACGGTAATAAAAAGGTCATTGCAATTGCA
GGCGGTTTAAATGCTTACATAACAAGCCTTTCAAATAAAAATAAAGATAAATACGATAAAAATT
AAGTGTTTGATTATACTTACTTTAAATATATTAAATCATCCACTTAATTTTATGAATAATAGAAA
AATATAAATACTAAATAAATTATCTTTTTATAGTGATTAAGTGAGTTCGTGGTCTAGTTGGCTAT
GATACCGCCCTGACACGGCGGTGATCGGGAGTTCGAATCTCCCCGGACTCACCATTTTTATTTTAG
AAAATAATATTCATAAATTTAAATAGATACATATACACAAACTTTATTTAAATATTGAAAAACC
TTTTTTTAATATCAAACTTAGGACTTGTGAGAATATGGGTTTTTCAGAAATTTACGGTGTTGCAT
TTCTTTCAATAATTTTGTTAATTTCAATAACGGTAATTTATGGAACTGTTGATTCGAATTTAAAC
AATATTTTAAGTGCAAATGACGACCATGCTTACAACTTGTTAAAAAAATCTAAGGAAAATCTTAC
GGTTCAGTACGAAGGAATTGATTCTGATTCTGGAATTTTAAATATAACTGTAGTAAATAATGGA
AATATATTGGAAGATGCTTCAAAATGGACGGTTATTTTTGAAGGGGACGTGGTAAACAACCCAGT
AATTGAAAAAACATATATTGAACCTCTTTCAAGAACTCAAATATATATTGAAACAATTTACAATT

CAACAACAATCGATGATAAAAGAGTAGTAGTTTCGGGAGAATTTGGAACAACCTTTTTAAAAAC
AATCGATGTTAATTAATTATTTTTTAGAAACAAGCTCTGCATCTTTTATATTGTAGCTTCCAGAA
ATTCCATCCCAAATGATTTCATAATCTAAGACTTCTATTTTTTTTTCAAAAAACGGGGCATCAAA
TACAAACGTTTCTGCTTCTCCGCCTTCAAAAGCTTTATTAATTTGGTATTTTTCCATTATCTTTA
ATAATTCTTCAATATTTGTTTCATCAATTCTTTTTCCAAGCCATTTTTTGTCCAGCCCGTAAGCTG
CAACACTAACCATCCTAAAATCAAAGAATTTTGAAGTATCTCGTAAAATCAATTCCTGATCTTTG
TGCCAGAGTGGTGCAAATGATTTTATTCCAATTTCTTCACAGATGTGGTCGATTCTTGCTCTTTG
GTATTCACTTGCAAGAGCTCCACTTACAATTCCATCGATATCAAGTTTTTCAAGGTTTTTTTGTA
AATCAAGAATTTCTTTTTCTTTTTCGCCTTTAGTAATTACCTTTATCATTTCAATTCCTGTACTTT
CTGATACGAGGTCTGTCAATTCAACGTTTGGAATGTGAAACATGTAGCTTTCTTTATTTTTTGAG
GCCACGTTTACCAAATAATTAACATCCCAGCCCTGATTTAGGGCCCAAAATAGTGCATAGGCAGA
ATCTTTTCCACCAGAATAAAGCGATGCAATCTTCATAAATTTCACTTAAAATCTTTTAAAATAAA
AAAGTAAAGAATAGTTTAAATAATTATTCGAGGCTGAGGTCAAATAGTCTCCATTCTCCATTACT
ATCTCGGCCATTTTTAAAGTATACTCTTGGTTCTTCAATGTTATCGGGAATATCTTCAAATAACC
AGTAACCTTCCACTGAACCCAAAGCAGGGATTTTGGAAACATTATTCAAAGAACCGCCAGAGGTA
AATGGGTATTCTTTAGTTCCAGAATACACCATAACTTCTGAAGGTGTGAAATCTTTTGGCTGTCC
GTATTTATTTTCTATTTTAAGGTCTACACGGAATATTTTTTTGGATTTTTCTCTAGGTATTGTGT
AGAATCCGCTTCTCAATGCAGTTATTTTGATATCATCGAGTTCGTATGAATCCCCGCTTTCAACT
GCATTTTCAAGGAATTCCTGTTCAGCAATGTTTAAAATTTCTTCTTCAGTGTAATATGGGATTTG
AATTGAGTTTGTAATTTTATTTATAACTTTACCTGTTTTTGAAGTAAATACTACTTCAGCATACC
CTGATTCAGTCATTCCTTTTTCAATATCATCTAATGAAACTATAAGCGTGTATTTACTGTTAGTT
CCTGCTTTTGATGGGTCAACAACGTATTCTTTTTCAAAGATTTTGTTATCATAATCATCATATAT
ATTTAATTTTGCAGTACCTGTTGCAGTTACAAGTTTATTTTCCGCATCGTTAAAAATTAACGTGA
ATTTCAGTGAATCGTCAGTTTTTGCAGCGTAAAATGTGCCGATATATTCTGCATCATCAGAAACA
ATATTTTCATCACCAGAATCAACTTCATCGAGTGCTTGATCTCCACTATCCGTTTCTGTGCATCCA
CATAGTAACAGAAGAGATATCATGCAAAAAACTCCAATAACAATTTTTTTAACCATAATCTATCC
CCTTTTAAAATTTACAGTTTAGACTATTGAATTTAAATTATTTTAAATTTTTGAATTGTTACGAA
TCATTAAAAAAAGAGTTATCAAATACTAAAAAAATAAAACAATAAAAAAAGGTAAAATTTTAAT
TTACCGGTAAAAATCCATCGTTTTTCACGATTTCCTGACCTTTTTCACTTAAAACGTATTCGATG
AATTTTTTAACATTTTCGTCAGGTTCTCCTTTTGTAAGCAAGTTTAAAGGCCCGTATATTGTATA
AATCCCTTCTTTAACATTTTCTTGAGTAGGTTCTACTCCGTTAAATTGTACTGTATTGATAGACT
CATCAACGTACCCTATTGAAACGTACCCTATTCCATTTGCATCTGAGGAAACTTTTGATTTCATT
TCTCCATTTGATGCGACCATTAGTGCTTTTTTAGAAATATTGCTCTGTAAAAGTCCTTCTTTCCA
GAAAGTATCTCTTGTACCGCTGTCTTCTTCTCTGGTGTATACATTTATAGTGCTGTCACTTCCACC
AACACTTTTCCAGTTTGTAATTTCGCCAGAATAAATTTTTTGTACCTGTTGAGTAGTTAAATCCG
AAATATTATTTTCATTGCTTGTAATTATTGCAATCCCATCAAGACCAATTTTATAAACCGTAATA
TCATTATAAAGTTCTTTTTCTTCGGATTTTAAGTTTCTTCCTGCCATTCCAATTGTTAAATGGCCC
TCACCAGTTTCTTTAACTCCAAAACCAGAACCGCCACCTGTAACTTCTATCTGGACATTACTATTT
TCTAACATGTATTCTTTTGAAATATCTTCGATTATTGGTATTAATGCAGTTCCACCTGCCATTTT
AATTGTAACTGGCTCTGAAACTTCGCCAGAATTATCCATACATCCGCTGAAACAGGATATTGAAA
TTAACAATACCAAAAACAGCGCTATTTTTTGAAAGTTCAAAATACCACCTGAAAAATTAAGAAA
AAAATAATTTTTAAATCATTAAATCATTTCAACAAATGCTTCAATTCTTGTTTTTAACTGTTCTT
TATCGCTTTCTGAGTAGTCAGTTTCTACTCTAATGATTGGGATTCCTGCTTTTTTGAGTTCTTTT

TCAATTAAAGCACCTTCCACGTTAAATGTGTGGCAGTACTGGAGAGTGTAGTATACAACACCGTC
AGCGTTTTGTTCTTTTACGAGTTCTTTAATTCTTTCAATTCTTTCTTCGTTGCTGAATTTGCATGC
ACAAGGGATTTTAAAGTACCTGTCAGCTAATTTTTCAATTGTGTATCCTTCAACGTAGTTTTCAT
ACTGTCTTGTTCCAGTACAGCTTTCTTCTCCAACAACTACTGCACCTGTATCTTCAATAAGTTCAG
GGATTTTCGTGTTTCCTGCAACCATTGGGCAACCTGCAATTAAAATTCTTTTTCCGGAATATCCT
TCGCCTTTTTTAACTCTTTCTTCAAGTTCTTCAACTAAATCTTCAAGAACTGATTCGATAGTGTT
TATATCTAACAGGTAGGAAAGTTGGAACAATTTTAAAGAATCTTTTCCGTTTATTGGGGATGGAT
TGTTTGCTCTTAATTCGTAAATTTTATAAAAAAGCTTTCTTACGTTATTTACTTTATCAATAGCT
TCTTTTAGTTTTTCTTCGGTTATTTTGTTTCCTGTTTCTTCTTCAATTAATTTTTTCAACTCTTCA
ACTTCAGCAAGCCACAGTTTTGCAGATCTGTCGTCTCTAAAGTGTGGTAACTGCATTATGTGAAG
TTTGGTCATTTCAGCGAGGTGTTCAAACATTTTTTTCTTTCCATCGCACGTAGTTTCTCCAATTA
TCACATCAGAGGCTTCAATGTACGGGCAAGCTTTGTCTTTTTTAAATCCAAATGATGATTTTATC
AAAGGACAGATATTTCTTGGTAAAATTTCTTCTGCTGATGGTATTGTACTTTCTTTTCCACCGCA
AAGCCCTACAGGAACTGCATTAGCTGCTAAAATTAATTCTGTTGGAACAAATGTACAAAATAATC
CAAATACTTTTCTTCCTTCATCTTTTTGGCCGTATAATTCATTTTTTTCTGCCTGCGAATGCTTCGC
TAATTTTTTTTATGGATTTCAATTCTTCCATTATTTTCACCATTTTTAATTGAAAGTTAGTTATT
AGATTTATTAATGGTAGGGCATTTAGCTAAGGTTTACCTTACGTATAAGCTTTTATTTATAAAAT
ATAAAAGTATCTATTAAATTAGTTAATGACAAATATATAATTAGAAAGATTAAAAATAACGATA
GTACCTCGATAAAAACTTAAAAAACGAAAAAATCCAAAAAATAATTCAAAAAAATTAAAATTTT
AAAATTTAGTAAACTGTTTTGCCTTTAGAACATATGCATTGCACAGATACCCTACCCAAGGAATA
ATTGCAACCAAGAAATAATTCTTTTGAAGATAAACTACAGTTAATAATTCCATTTTTAATAATTCC
AAAATTGTTTGCTGAACAAACCAGTTCTATATTTGGATCCATAAATAATAGCCGGTAATATGCTA
AAATTATCAAAATAGATCCAAAAACAAAAGAAACAGATTTTCCAAGTCCTATTTTAGTTACAAA
AATCCATAAAACAAGCTGACTTATAATTAAGATTAGAAATAATTCCCCATTCATAGATATTCCCC
CAAATATTTGAATGCACAATATTAATTTTTTAAAATCGCTTATATATAATTAAGCTAATCAAAA
AAAGAACAATGAAAGTTAAGGTTTTTTAGTTACAGGGAATATTGGGAGCACTGTTTTTTGATAT
GTTTCATTTAAAACTTCTGCAAATCCGGTATAGATTGCAGAAGATCCGCAGATTATTCCTAACAG
TCCTGCAACACGAGTTACTGTTAAATTTCCAGTAATATCTCCTGTTGCAAGTAATAAGAATAATA
TTGTAAGGGTTCCAAATACAAATTGAAGGGCCCTGTTTGTTTTGAATGTTCCAAAAAACATTCCT
AATGTGAATAATCCCCATAAACCTAAATAGAATGCCATTTCAATGGGTTCTGGTGCATTTGCAAG
TCCTAATTTTGGTAAAGTTAAAATTACAACAAGACTGAGCCAGAATAGCCCATATGATGTAAATG
CTAATGTTCCAAACGTATTTCCTTTTTTCCATTCCATTATTCCGACGATTACCTGTGCTAATCCTC
CGTAACACATTCCCATTGCAAGAATCATCGAGCTAAGTTCAAATAACCCAATATTGTGTAAATTT
AACAACACGGTCGTCATTCCAAAACCCATGAGTCCAAGTGGGGCAGGATTTGCACTTGTATCAAA
TATTGTCGTGTACTTTTCTTGCATGGTTTTACCTCAATCTATCATGATTAATCATTATAGATTCT
GGTATATATTCATATGGTTATTAAAAATGATAAACATGCAATCTGAAAAATAAAAATATACTAA
AAAATTAAAAAACAATGAAAGTTAAGGTTTTTCAAAAGCCATAATACAGGCTCCAACTGCGCAA
ACAATTTGCGGTTCTTCTGGAATTAACAGTGTTTTTCCAAGGTGTTTTTCAAGTACTTGAATTAA
AATTTTGTTTTTAGCAACCCCTCCACTAAATACAATATTTTCAAGCTTCATTCTTTTAACCATTG
GAACGATCCTGTTTGATATACTGTCATATATTCCCATTATAATTTCTTCTTTGGGTATTTTTTTA
GAAAGGAGGGATATTATTTCACTTTCCGCAAAAACAGCACACATGGAGGATATTTTTGCATAATT
GGGGGATGAATATTCATTTAATTCTTTATCTAAATTTAAAATATCTATACATTTTTCCAAAAACT
TTCCAGTTCCAGCAGCACACTTGTCTGATAGAATAAAATCGATGACCTGCCCATCTTTTAGTTTT

AAAACTTTGCTGTCCTGCCCCCCAATATCAATTAATCCATCCGCATCTTTAAAAAAATAGTTTGC
ACCTTTTCCAAGGGCAATTACTTCTGGAACAACCTTATCAGCAAAAGATATTTTGTGCCTTCCAT
ACCCTGTTGCAACAGTTTTTTAACATTTCTTCCTTTCTTAAATTCTTCAACCATTTTTAAAACAT
CACTTTCTTCGATTACTACACCCAAATCTTTTATTTTATAATCTATTATCTCATCATTTTCCATTA
AAACAAATTTTGTTGTAGTCGAACCGATATCAATACTTAAAATCATTTTTACACCCAAGTAATAA
TTATAATACTTCGCAAATCTTTGAATATTATTTTCCATCATTTTTTAAGTTTAATACTTATTTAA
AATTATTATAATTATAAATTTATCAAGTATATTATATGTTACTGGTATACTGGCATTAAAAATA
TATAACTGCAATGCTTTGAGTGTATTTATCATTTAAAACACGTGGAAAAAACATGATTACAGAA
AACATATTTTTTATAATGAATATCATTGGACTTTTAGCATTTGCAGTAGTTGGTGCATTAAAAGG
AATTAAAAAAGGCTTAGATCTACTTGGAATAATTGTTCTTGGAATTATGACTGCACTTGGGGGTG
GAATTACAAGAGATTTACTTGTAAATACAATTCCTTATGCATTAAGATCTCCAAACGATATGGGT
GTTGCATTAATTGGAGTATGGTGCGCAATAGTAATTTTTAAGGTTTTTAATGAGGATGTAAGCA
ACAAATACATCATACAAATTCCTGATGCAGTGGGCCTTTCTGCGTTTACAACAACTGGTGCAATG
ATTGCATATAACTTTGATGTGTCATTTTTTGGAATAATCATTCTTGCAACATTAACAGGTGTTGG
TGGTGGAATAATAAGCGACATATTGTTACAAAAAACACCTTCTGCATTAACCGAAGACTTTTACG
CAAGCTGTTCAATAATTGGAGCAATTGCATTTTATCTGGCAATTCTATCGAATTTAAGTCTTGAG
ATGAGTGCAGTTATCTGCAGTATCGTTGTTTTAATGATTAGAATTGTTGCAATTCTTTGTAAATG
GAGCCTTCCAAAATTTTACAGCGAACAAAAATAGAATCTATAAAACATCTAAAATTTTTTTAAAA
ATCAAAAAAAGAGAAATGGTTTGGTAAAATATTATTTAATTTAATGTAAATTTATTCTGGTTTT
TCCATTAATTTACATTTGTTTAAGATTTGTCCAGCGCCTGCGTGAGGTTTTCTTGAAACAGCGTC
AGTTGCCTTTTCTAATTCTCTAGCTTCATCAACTAATTTTGCAGCGTATCTTATTGCTTCGTGTGC
AGATGCAACGATTGGGATGTATTTTTCCATTTCTTTGGTCATGAAGCATCCTTTAACATCGATGT
CAGCTACTTTTTCAGCCATTACAAATGCAGCCATAGCTTTTGCTTTTGCGTATGGGTTTGCAAAG
TCCATTGCTTCAACTGCTTTTTGTTCGGTGATTACGATTTTTGGTAATGGTATTTCTTTTCCTTCT
TCAACTGCAAATACCATATCATCAATAGCGTTTTGTACAACTCTTAATGCACCGGTTCCAGCTAA
AACTCTGATTACATCAGCATTGAACATTGCCATTTCAACAGGATCTAAGAACTGTCTTCTAGCAC
CGATCATAGGGTCGCATTTAATTAAAACGTATCCGAGTCCTTCTTCAGCCATAGCGTCTTTGTCT
TTGATACCTGGTGCGTCACCAATGATAACTGCAGGGATTCCGCCAGCGCTTAAGATTTCTCTAGC
TTTTTTTGGACCTGGTGCAGCAGGGTTTGGACCAATGTATACGATGAAGTCTGGTTTTATTTCTT
CAACCATTTTTTTAGCAACTTCTTCTACTTGTTCAGGGCCCATTTTAGCTCCGCTACCTAAAACTC
TAACGTCAATGTCGTTTCTGTCTGCTCTTTCATCTAAACAAAGATCAACTACAGGGGACATTCCT
ATGTTTCCGCATTTTAAAATACCTATTTTTACAACCATTATATCACCGAAAGATATTATTTCCTT
TCCGTATTACCTTTGAATTCAGGGGCATATATTAATTGCTATTTGAAATCTTTATTACCCATATT
AATAAGTGTAATTACAGAATTATGGTGGTAAAAAATAAAGGTATAAATAATGCCAAATACAGAT
ATAGGAGCGTAAAAGAGATTTAATTTATTCAAACGGAGGGGAATTTTATGGACGACTTCGAAAA
AATAATCAATTCATTAAGTGACAAAGAAGTAATACTTCGCGTGGACTTAAAACAGAATATAAAA
GTTATTAACTTTAAAATTGGTTCGATGGATATAAATGGGGAACTTGGAATCAGTTTCAGAAGCA
TAAACCCTGAAAAAAAAGCAGAAGAAATTCCTGAAAACATCATAGAAGTTCCCGAAGAAGTAAG
TGAAGCTGCAGAAAAAGTCGAAGAAAAATTGAAAATTTAATGTCAATTTTAAATTAAAAAAATA
AAATTATTATATAATTAAAATAATTTTTTTGGTTTTTTCTTTTTATCATAAAACTTTTTACCTTT
TTTAAGTCTATTTTTTACCATTTTTCGATAAAGCGGGCTTCCGCAGACTTCGCATTCATCATCAA
GGTAATTTATCGGATACATCTTTTTACAGCCTTTGCAGATTTTTTTCCAGACGAAATCATCTTTT
GAACCAGCTGAAACGATATTTTCGTAAACAATTTTTAATTTTTTTGAAACATTCTGAAGGCCGTA

ATCGTCAGTATACAAAATTCCGCCAAGGTCGATTGCAAGCGCAAGTATTTCGATATCGTTGTTTG
AAATGGTGTCGCCAGTTTCTATTGAAGTTTTGACTACTTTTTCAATCGTTTCGGGTTTTGGATCG
AGTATTTTTAATTTTCCGTATTCAATTGCCAATTTTACAATATCCTCTTTTGAAACGACTTCTGA
AACAATTCCGTTTGTAGTGTAGTGCTCTCCTTCTTCAATTGATGGATTGTATCCATGTATAAATG
CTGAAGCATCGAGTATTTTTATCATAAAATCACTTTTTAACTAATCAAATATAGTTATATTATAT
GGATACTTACTATTTTTACATATTTAAAAATCTCCGGTGTAAATGTGACGAGAAATGCAAATAAA
AATAATACGGAATGTGAAAAGTGTTGGATTTTTGATTTAAATCAATTTAGGATGATTACCGATT
CAATATTAGACGAAAATACACTTGTTCTTGAAATAAACCAAAATTACGAAGTAAGCGTTTTGATG
GATTACGACGTATCACTTGAAAACGGAATTTTAACTTTCAAAGATTTTGTAAATGATAATTCTAA
ATCTGCAACAGTGCTTACAGGTTCATTAAAAACAGGAATATTGAATAAAATGTCTCAGAGCATTT
CAGAAGGACTTTTAAATAAAACTACAAACCGGAGAACTTACTATATCACAGAACCAACGCCTTTA
ATGGGGCATACTGCATTTGGGCTGATTGATAGGGGAACAAACGTAATTCAGGTAAGAGGACTTA
GTGGCTGTAATATAAACTGTCCATTCTGTTCTGTTGATGAAGGAATTCACTCAAAATCTAGAAAA
AACGACTATTATGTTGATATGGATTATTTAGTTTCGGAATACGAAAAAATCGCAGAGTTTAAAG
GATATACCAAGCTTGAAGCACACCTCGACGGACAGGGGGAGCCTTCATTATATTATCCACTTCCA
GACTTAGTCCAAAATTTAAACGAAATAAATTCGAAAAACAATGGAATAGTTTCAATCCAGACAA
ACGGAGTTCATTTAACCGAAAAATTGATCGATGATTTAGAAGTTGCCGGACTTCATAGAATTAAT
CTTTCAATCAATGCAATGGATGAAAAGTTCTCAAAAGGGCTTTCAGGAAACAAAAATTACGATAT
TGAACGAATTATGGAAATTGCAGAGTATATAAAAAATTCAAAAATTCACCTGTTGATTGCACCGC
TTTTACTTCCAAATTATAATGATGAAGAATTTAAAAGAGTACTCGATTTTGCAGTTGAATTGGA
ACAAAAAACTCCACAAACTACGATAAACCCGATAACAAACAAGAAAAATCCAATTGTCGGCCCTC
AGTTGTGTTTAACTTACCAATTTGGAAGAAAAATTCCAAAAATGAGAGTTTGGGATTTTCCAAA
GTTCTACAATCTTTTGGATGTTTATGAAAAAGAATATTCCAAAAAAGGAATAAACGTGGATTTA
GAAGTTCCATTACACGGATTTTTTGGAAGTCACAGCAGGAAAAGACTGCCATGCCCATTTAAATT
AAATGAAACGATTTCAGTAACTGTTTTAATGGATGGACGGGTTAAGGGTGAAGTTATCGGTGCA
TCAAAAAACAGGGTAATTCAGATAATCGATTGTAAAACAGATGTTAATAAATTGAATGGAAAAC
GGGTGAACGTAAAAGTTTTACGTGTAAAAGACAACGTGATTGTTGGTTCACTGGTAAAATAATT
AACTTTTTAAATTTTTTAATATATTGGAAAATAAAGCAACTCTCAGTAACCCTGTATTCATCATT
TCTTTAATCTGCAACTAATACCACAAATTAAAGTCAGCAGGGATGACAATCATCATCGAGTGTTT
TGCATATTATTTTAATATTCCTTTTATTCAATTGTATTAAAATGTATTTGCCATATTTTGAAATT
TCAAAAAAACCCTTCTATTTTTGGAATATTTTCGTAAAAAATCACAATCTTTTAAATTATCTAGA
AAACATTTTTTTTAACATTCTAATTTTAGTTTAAAAAAGCCAGTCGTTTTCGAGGGGCATATAAC
TTTCAGGTTCCCCAACCACAATTTAAGAATATGCCCCACACGACCACAATTATTCACATGGAGTG
TCCCTAAAGACATACTTAGATATAGTATCTATATATTTAATGTTTTCTAATAACAAAACCAATAC
ACAATGTTTAAATTACCATAAATTACCGTCAAGGTGACTTTTAATCTTATCTGACGTTGAATTAT
AGATTCTATTTATTTTGGCCTTAAATATATCTTTTTTAATATACTCCCTGCTTTCAACGGTAAAA
TTAATATGCATAGTTTTAAGGTAGTTTATAAATTCGTTAAATTCTGCTTCCGAATCAAAATAAAG
TTCAAAACATTCTTTTGCAACTGATTCTTTTGAAATTTCATTTATGGTTTCAACCATGGGGAGTA
AAATTGATTCTCCAAGCTCATGTGCTCTTTTTTTTCTTTCTTCTTCGCTTTCGCATTTTTCAAAAG
ACTGATAACCTTCCCTTACAAGTCGGCTGAACATAAAATCAATTCCAAGATCGTAAACGTAACTA
AATGCGATTGAAAGGTCGCCACACTGTGTATTGTGAGTAGTATATTTTATTGGATTTTTTATCAT
TTCAGGGTCTTTTAAAACTACGCCTTCTCGATGATTTTCGTTTAAATCGTCTAAAATATCCCATA
ATTCATCAATGTCTCTACTATCGATGATTTTTATCGGTTTTACGTAAGGAATATTATATTCATCC

AAAATTTTTTCTTTTTCATATGTCGAAAGAGCAAGGTTAGTTTCTTTATTTCTTACATCAAAAAT
ATAAAAACCGAGGTTTTCATAAGTCTTGTCTGCTTCAGGATAGTAATGATTCACGTAAGGATTAT
TTAATCCGACCATTTCACCACAGAGCATTAATTCTGGAAACTCGTCTAAAAATTTTGTATTAAGA
TATTTTTTTACTTTTTTAGTCGTAAATGGACAGATTTTTCCTCCGCGAGTAATTGCAATGATTTC
ATCGTGCATTTTCACGATTCGTATGTTGTATCCATCTAATTTTTCTTCTACAGCAATTTTGCCGTT
AAAATATTTTTTAACAGATGATTCAAGCACCATCGCACGCCTTATTTTTGGATAACCAATAATAA
AATCTAAATTATCATTTAAAAAAAGAATTGTTCCTCTTTCAATATGTTTCAACTTTTTTTTAAAA
CAAAGGTATTTTTCCCCGTTATACTCATATTTAGTGATTAATTTTCTTTTAAATCCGCTTTTCAG
ACTATCCTCATCAATATTTAATTTTTTAGATGCATTTATCATAAATTCTTCAAAATTTAAAATAT
CTTCTGAAAAATAAGTTTTGTCCATAAAAATCAGATTAGTTTTTAATTCTTTTTGAAAGTTTAC
TGCATACAAGCATGTTGCTTGGGTGTGACCTTTTGCAGTAAAAACCATTTTTATCGGCTTTTTTT
AGAACTTTTAAAAATTCTTTTGGATTATCTAAATCAAAATCTTCAACATTCAAATATGCATTGCC
TACTTCCCATGGGAAATGCGCATCACTTCCAACACCCATCAAGAGGTTGTTAGTTTCTGCAAAAG
AGTATGCTTCATCATTTGGTCTTTGCTCGACACACCGGCTGTTATATACTTCGACTATATCCACAT
TTTTTATAAATTCTGGATTTTCAAGTTCCCCATATTTACAAAGAGACCTTCTTCTTCTCCAGTCA
AATGGGTGGGGCAGATAAATAAGCCCGCCCTGTTCTTTTACCTGGTCGACAGCTTCGAATATGTT
TTTTTCCTGAATTTCTTCAGTTAAAAAGAGTCCACAAAATTCCCCCTTTTCCGTCGAAATCTCTTC
TCCCGGTATCCCAAAGTCAACTTTAGTCATGGCATTGTGATCAGTAATTATCGGCAAAATATGTT
TTCTACTGCACATCTTTTTTAACATTTCCAAAAAGTTCATGGAGCATCTGGACGTGTTTGTGTGT
ACGTGCATGTCAATTTTTATCATCAAGTCACCAGAAGTTTAAAGTGATAAATTACTCGAATTTTT
CCGTTATTTTCTGTATATTTTTGTAGATATTACTTATGAATGTTTCGGAGAATTTATATTTTTTG
TTCTCGAGTAAGATATTTCCACCGACTATTACGGTGTCAACATCGTTTCCAAACGCAGAATATAC
AATATTTGATTTTATATTATTAAATGGTCTTAAATGTGCCGAATCAAGGTCAATCAGAATAATAT
CGTTATTTTCTATTCCAATACTTTTACTATTAATTCCCATTTTTAAAACCGTATCAATACCAGCA
ACTGTTGGATCCCACCTGTGTGCTTTATGTAACAGGGAAGTCATTTTCATTTCTTTAAAGATATC
AAGATTATTATTGCTTGCAGGTCCATCAGTTCCAACTGAAATATCTGCGCCATATTCTAAAAGTT
CAAATAGGGGCATTACTCCCCCACTTGCGAGTTTCATATTGCTTGTTGGGCAGTGCAGAACTTTA
GCATTATTTTTTGCAAGCTTTTTAGCTTCGTTTTTTGTAATCCATACGCAGTGTGCAGCATGCAA
TTTTTCATTCAAAACTCCGATTTTTTCAAGGTATTCTACGGGGCGCATTCCAATTTTATTTTCAA
GTTCAACTACTTCGTACCTTGTTTCAGAGACGTGTGTGTGCATATTTACATTGTAATCGTTTGAA
ATTTCACTGCATTTTTGATAGGTTTCTTCTGAACACGTGTATGGTGCGTGAGGTGCAATCCCTAC
TTTTATATTTTTTTCACCAGCATTATTTTTAACAAAAGATTCAGCAGAAGTTAACAGTTTATCTA
TACTTTGTTCTTCAGGAGTTCCAAAATCTATAATTGGATAAGATACTTGCGCATTCAGTCCAATT
TCTTTTGTAGCTTTCAATATTTCTTCAGAAAAAAAGTACAGTTCATTAAAAGATGTAACTCCAAA
ACGAAGCATTTCAAGGCATCCTAAAAGTGAGCCGTAGTAAACATCATTTTTATTTAATTTCGCTT
CATTTGGCCATATCTTTTGATTTAACCAGTCTTGGAGAATCATATCATCAGCAATTCCCCTTAAA
AGAGTCATCGGAATGTGGGTGTGTGCGTTATAAAGCCCAGGCATTGCACATTTCTTTTCTCCAGA
AATGATTTTTAAGTCGTTTTGATTTAAATTTAATTTTTCAAGGAGGTTTTTTCCAGTAGTTAGTT
TTATTTCTGAATTTTCAACTTTTTCAATTAAAATATCGCAATTTTCATGAACATTTAAATTATTA
TCAATATAATATGACACATTCTTTATCAATATCATAATTTTCACTCCAATCTGACGTAAACGTAT
GATTCATAACATTTTCCATTTAAATCATAGCAAATAGTTTTATTTTTTGAGTAATATGTCCCTTC
ATCTTCATAATCGTCAAAAATTTCTAATTCAGTGGAATTTATATTAAATAGTATTATCCCACTTA
CAGATGAGTTTTCTTTGGGCTTAATTCCATAGTATCCTATTTTTTCATCAAAAAATTTTTCAAAA

TTATATATTTTTCCAAGATTTTTTTCAGGAACTCTATTTATTAATTCGAACAATATTTCATCCTT
CATCAATTCGCCATACGCAAAAATATTAAAATAGTCCATAAAATCACTTTAAAAAATGGTGCAAC
GTGTACTGCTTTTTAGAGTCTTTTAATTCGTCCTTAGTAATTCCTAAAGCTTCCATTAATCGTAT
AACTGGGGGTAAAATCTGATTTTCGATATAATAATTTGTATCGTAATTTTTTGCATTTTCCAGTA
TTTCCGCCCGCTCACTAATCGATTTATTTCCGCTTGTTATGATGTAGCTGATAACATCACCAACAT
TTACCCGATTTCCGGACTTCAATATTTTTTTTGCAACTTCAACATGTGGGGCTGTGGTTTTATAG
TCTTCGATTCTTTTTGTAAGCTGAGTATGAATCAATAAATCTTCATTATTTACTTTTCCATCCTT
TAATTCTTTAATGGTATCTTGAATCACTTTTTTTGCATTTTCAACGCTACCTTCTTTTAAAAGTG
CGTTCAATACGTTTTTTTGCGTATTTTTTGAAACATTAGACCAATCCCGCCTTACTACTTCAAGG
CCTTTTACCGTAATTTTTTCATTTTCGTCAATAAGCGCGTATTTCTTTTTGGTTACAAAAATTCC
CCTTTTGAAATACCCTTCAAATTCAAGTTCCATTTCTCCTGGAAGTGTGTTGTTTATGGTTTTTA
AAAATTCACTGGTTTTTTCTAAAAGCTCATCTTTTGACAGCTTTTCTTTATCGTCGGCCCATTTA
GAATAAAATCCATCCGTATCTGCATAAATTACTTTAAATCCAAAATTTTCAGCTTCTTCTATCGT
TTTTTGAATGTACTGTCTTCCAAGATGCGTTGTAATTTCTGCGCAATCTCTCGAATACCACCTTGC
CATTGGAAATGCAAGGTATCCATAGTGTGAATTTGCAAGAACTTTGATAGATCTTTGTTCGTAGT
CTAAAATCTGGTATTCTTCGTCAAATTCTCCTTTTTCAGCTTTTTCCCTTAAAACTTTTTTGACTT
TTTTTCGCCTATTTATTAAATCCATTAAGGTTTTTGGGATAATTCCAATACTTTTTTTACAGAAT
TTGTGGCCCAATATTTCTTCATTTTCACCATTTTTTTCATCACTGCAGCAAGTACAGTCTATTGTT
TCAGGGCTTAAGTTGTGGCTCATTATAATTGAGGGATAAAGGCTTAAAAAGTCCATCGAAACGAT
ATCTTCATGAATTCCCTTTAATGGCTCTTTTACGTATCCACCTTCATATGAACTTCGGATTCTTTT
TTGATATTCTTTTCCTGACGGCCTGTTTGGGGCTATAACTCCCATTTTGTAAGAATTTTTTAAAA
GTAAATATTCTACCATCTGGCTACTGCTCATACGGTTTATATCATAAAGGGACTGATTTACAACT
CGAGAAAACATTATTTCAAGAGGTAAAAACTGTTCTCCGATTCTTTGAGTATAATATGCATCCTG
ATGTGAATATTCTACAAGTGTTTCATCTAAATTATCCCACATTTTTGGAATATTTTCATGACCAA
CATCTACTTTTTTCACGTCAAAAAGAGCTTCGGTTACATTTTCGAGTCGGTATTTTGTTAAATTT
AATAACCGTCTTGCAATCGGGTATAAATCGATATGAACTCTTCCAGGGATGTAGCTTTTAGAATT
CATTCCGCCTTTCGTGATTTTTATTTTTTCATCGTTTTTCCCAAGTTTTAGTTCAAGCCCGAAACT
TTCAGCTCTTTTTTTAAGGTATGGAAAATCAAAATTGTCCCCATTATATGTGTAAATTATATCAT
AATCTTTTAAAATCTCGATAATTCGCTTTATAAGTTCTTTTTCATCTTTTACATACTCTAATTTT
TCGTGGTCGAATTTTTTGGTGGAAACTACGGTGTTAAAATCTTTTGAACTAAAACTTGCCATTAT
TATTGGGTCTTTTTTTGGATTCGGCTCTTTATTACAGTAAACTTCAATATCAAAAGAAACTGTTT
TAAAATCTGGAATTTTATTATCTATTTTTTTGTTATTATCAAAATCCCATGTGGACGTAGGAATA
ACACTATTATCTATTAAATAACGTCTTACAAATGGAATATCATACTCATAGAGCCTTTCAAATTC
CATCAATATTTCTCGAACTGTTTTTACGTTATTTGGGTATTTTACAGTAACTTTTGAGAGCTCTT
TTTCCTGATAATTTTCATCAAAAACTATTTTTTTAACAGTTTCGATGTTTTCAATCATTTTGCTG
ATTTCACTATCTATTTTATGTTTTTTTTCGTAATCTTTTAAAAATTCGAGGATTTGTTCTTTATC
CGTTGAATTTACGTAAAAATACGGTTTGAAATCTTTTTCTTTTATGATACTATTTATCAAATATA
GGTATATGCACAAGTCATCAGAATTGTAGTCTAAATCAATCAGGCTTTCCATTAAAACACCGCAG
AAATTTAGGTTATGAGTTTTATGATCAAAAACTATTTTTTAGATTTTAAAAAATATTAGTTAAA
TTTATATGTTCATTAAAGTAATATTTGAGAGTTACAGTTTTTAGTATATTTTTAATTAAATTCGG
AAAAATACGGTTTTATTTTAGTTATTGATGGTGACATGATGAAAATAACTGGTAAGGTGCACTT
GTTTGGGGATGACATCGACACAGATGCGATAATTCCCGGAGCTTATTTAAAAACGACTGATGAAT
ACGAGCTTGCATCGCACTGTATGGCAGGAATTGACGAAAATTTTCCAAAAATGGTCAAAGATGGT

GACTTTTTAGTTGCAGGTGAAAACTTTGGATGCGGAAGTTCAAGGGAACAGGCCCCAATTGCCAT
AAAATACTGTGGAATCAAGGCAATAATTGTTGAGAGTTTTGCAAGGATATTTTACAGAAACTGC
ATAAATTTGGGAGTATTTCCAATTGAATGCAAGGGAATATCAAAACACGTCAAAGATGGGGATG
TAATAGAATTAGATCTCGAAGAAAAAAAAGTTATCTTAAAAGATACTGTTCTTGACTGCAATCTT
CCGACAGGAACTGCAAAAGATATAATGGATGAAGGCGGGCTTATAAATTACGCAAAGAAACAAA
AAAATTAAAATTTAAACGATTTAACAAATTTGATACAAATTAATTATAGTAATCAGATAGTAAA
TTTATATTTTGGTGATTTTAATGTCGCGACTGGCGATTTTAGATTATGATAGATGTCAACCAAGA
CGATGTTCGATGGAATGCATGAAGTACTGTCCTGGAGTCAGGATGGAAGAAGAAACAATAGTAA
TGGATGAAAATTTAGGAAAACCAATAATTTCTGAGGAACTTTGTAGCGGATGCGGTATCTGTACA
AAAAGATGCCCATTTGGAGCGATAAGAATTATCGGGCTTCCTGAAGAGTTAACGGATGACAGGAT
TGTTCATTCATACGGTCAAAACCGATTTAGATTGTATGGATTAATTACTCCAAGAGATGGGGTAA
CTGGGCTTTTGGGTCCAAATGGTGTTGGAAAATCAACAATAATTAAAGCGTTAAGCGGAGAAAT
GGTCTTAAATTTAAACGATTTAACAGAAACTCCTGACATGAAAAAAGTTTTAGACTATTTTTCAG
GAACAGAACTTCAAAATTACTTTGAAAAATTGAAAAACAATGGGATAAAACCAATTCACAAACC
CCAGTACGTTGATGTTTTACCAAAAGTTGTAAACGGAAAAGTTGGAGAACTTCTCAAAAAAGTAG
ATGAAAAAGGAGAATTTGAAAAAATTACAAATGCTCTTGAACTTTCGAATCTGTTGGATAGAAC
TTTTGACCAGCTTTCAGGCGGGGAACTTCAAAGAGTTGCAATTGCAGCCGCATGCCTTAGGGAAG
GTGACATTTATTATTTCGATGAGCCTACGTCCTGGTTAGATGTAAAACAGAGATTTAGTGCAGCA
AAAGTAATAAGAGAAGTTGCTGAGGGTAAAAAAGTTGTTGCAGTAGAGCACGATTTGATTGTTT
TAGACTACCTTTCAGATTACATTCATATAATGTATGGTATTCCTTCAGCTTATGGGGTTGTAACC
CACCCGAGAGGAACGAGAGTTGGAATAAATACGTATCTTGATGGTTTCTTAAAAGAAGAAAACA
TAAGATTTAGAAAAAGCCCGATAGTGTTTGAAAAAAGACCTCCACAAGACAGTACTAATCGGCCT
TTACTTTTAGATTATACTGATATTTCAAAGAAACTTGGAGATTTCAGTTTAAATGTTAATGGCGG
CCAAATCTATCAGGGTGAAGTTGTAGGTATTTTAGGTCCAAATGGTATTGGAAAGACCACATTTG
TAAAAGCACTTGCAGGTGTAATTTCGCCAGATTCTGGAGAAGTTACGGGGGACGTTAAAGTTTCA
TATAAACCGCAATACATTTCATCAGACTTTGAAGGAACAGTTGAAGATTTATTAATGTCAATTAC
TGCAATTCACACTTCATACTACAAATCAGAGATAATAAAACCACTTGCGCTTGAAAATATTCTTG
ATTCGTCAGTAAAAGACCTCTCAGGCGGGGAACTTCAAAGAGTTTCAATTGCAGCATGCTTAAGT
CAAGATGCAGATCTTTATTTAATAGATGAACCTTCCGCATTTTTGGATGTTGAGCAGAGATTAAC
TACATCAAGAGTAATTCGAAGGATGGCTGATGAAAAAGAAGCAGCAATGTTTGTAGTTGACCAC
GATATCTTGTTCCAGGATTATATCTCAGATAGATTTATTGTATTTAGCGGTATTGCAGGATCAAA
CGGAACTGGTTCAGAGCCATTACAAAAACGAGCTGGTGCGAACAAATTTTTAAAAGAAATGGGA
ATTACTTTTAGAAGAGACCCTGACACAGGAAGGCCGAGAGTTAACAAGGAAGGAAGTCAAAGAG
ACGTTTACCAAAAAGAAATAGGCGAATATTATTACCTCGACGAATAAATGACTTACTTTTATTTT
TAATAAAATTTTTTTACAAATATGCGAAGTAAATTATGTCAATAGTACTATAAATTCGTAGATA
TTTGGATGTATGGATATTTTTTAAAATTGGTCTAAAAAACGTTTAACAAATGTAATTTTGTTAT
ATTTTTAATTGTTAAATTATATGGAATTTTAAAATTTTTCATTATTAAAAAATTCTTTAAAAAA
GTTGCAAATATTTGCAACAGTTTATTTTTTAAAATAATTACTTTTTTGGATTTATTTTTAAAATT
TAACCCCTATTAAATAGATTAATCCCCTGAAACTATTATTGCAACCGTTTCAAATGGTAAGGTAT
ATATAGTGATGGCGACAAGGCTATTCTTGTAAAAAAAGTAACACAACAAAGGTGAATTACTATG
GCTATGAGCATGAAAAAAATAGGTGCTATTGCTGTAGGCGGTGCAATGGTTGCATCCGCTTTAGC
AACAGGCGCTTTCGCTGCTGAAAAAGTTGGAGATGTAGATGCTTTCGCTGCTGATGTAGTTGCAG
ATGGAAACGCAAACGTAGATATTGTAGTAGGTTCAAACGCTGCTGCTTTAGACGTTGTTTCAGCT

GCTAACATCGCTGCAAAAATCGGATCATTAATGTTCAAAGAAGGAACAGTTGAAGACGGTAGTGC
ACAAATCACTGTAAGTGCAAGTGCAGAATCCGATGAAATTAAAGATGTATACACTGATGCTACTG
TATCCGTTGTTAAAAAAGCACTCGTAATTGCTGCTTCAGATAACGATTACACAAGTAAAACTTCA
GGTATTTTATCATTGATAGATGGTACCGCCCCAGCAAAAGAATTATTTGGAATTACAACTGCAGG
TGCAGTAGCTGATTTAGAAGACTTACAAACCTTAGCATTTATATCAGATGCTGACCCTGCAGACT
GGTACACAGGAGACTTCGAAGCAACAGAAGTACTTGTAGCTACAATTAACTCAGATTTAGCTACA
CTCGGTACTGCTGGTGCAGATATAGAAGTTTTAGAAGATGAAGTAATCTATGCATCATTAGCATT
CAAGGAAGATACATACGGTGCTGCTGCAGGTGACTACGCTGCAATTGCTCCAGGTATGAGAATGC
CACTTCTTGGTGAAGAAATGGTTCTCGTTAAACTCGATACCGACGATGACCTCGTTATTGTAGGT
AAGGAAGCATACGATGGAGTTATTAAACAAGGCGAATCATACGACATTGGAAACGGATACACCG
TTAAAATAAACAATGTTTTAAAACAAACAGTAGGCTCTTACAAAGTAGATGTTCAAGTATTAAA
AGATGGAAAAGTTGTAGCTTCAAAATTCGACACTGTAGGTCTTGGAGATCAACTTAAATTAGTTT
ACAAAGATGTGGGTATTGTTGTAAACGATGCATGGACAGATGTTGGAGATAACTACGGTTTCGCA
GAACTTGTAATTGCAGATAATGTAGTTGAAATGGAACTTGGAGAAGAATTCATCCCTGACTACGA
AGCATACACTGTAGAATTTGACGGTACAAGTGCATTAGCATTAAGTGACAAAGCTTCAGCTACTG
CTCCTGCAAAACTTGTAGGTATTGCTTTAAAATATGTTGGAGACGACGTTGACAAAATTTCAAAA
GATGAAACATTCACAATCGCAGACTACGCAAAATTAGATTTCGACGAAGACAGCGGTAAAATGAT
TGTTAAATTCTTAATGGACGAATCAAAAGATGTAACTGTAAACATCGGCGAAAAAGTAAGTGTT
TTAAACGCTGAAATTAAATTAGCTGAAATCGATGCAGAAGCTAAAGAAGCTGTTGTAATGACTGC
ACCTATTGCAGTATTAGACTCAGAAGCTTCATTAGATGCTGCTGACAAAGGATTAATCTTAGTTG
GTGGACCAGTTGTAAACGAATTAACAGCTGAACTCGCTGACGCTGGATTAGTTGCAATTGACAAC
GAATCACCTGCAACCTTAGCAGTAGCTGCTGGCGCTGCAAACGGTAACGATGTATTAGTTGTAGC
TGGTGGAGACAGAGCTGCAACAGAAGAAGCTGCTGAAGCTTTAATCGACATGTTATAATTCAAAC
TTTTTAAAAAAGTTTGATCAAAATTGTGGGTCTTCTGACCCACTTTTTCTTTTTTAGAAAATAAT
ATTATTATATTTAAATTTATAGTTTTATTTTTAGAAAATTAATAAATAATTGAAAAAAGAGAAT
AATATCATAAAATATTTTGCGTGATAAAGCTATGTTATTTAGTGCAGTGATCTACGAGGAAGAT
GGAATATATGTTGCAGAGTGTCCTGAAGTTGGAACTGTAAGTCAGGGAGATACAATAGAAAGTG
CAATATCTAATTTAAAAGAAGCAACTGAACTTTATTTGGAAGAATTTTCCGTAGAATCTAAAGTA
AAACCTATTTTAACAACATTTGAAATTAATTCAAATTGTGATGGCCTTGCCAAAATTTAGGGACG
TGTCTGGAAAAGATGTACTAAAAATTTTATGCAATAAATTTGGTTTTGAAGTAACGGGTCGAAA
GGGAAGTCACGTGAGATTATCTAAAAATAGTCCTGAAGGAAAAGTTGGAACGGTTGTGCCTATGC
ATCAGCCTTTAAAAATAGGAACTTTAAAAGGAATTTTAAAATTGGCAAAAATAGATTTTGAAGA
ATTTGAAAAATATATTTAGTTCACATTTCACTTTTTTATAACTACCCAATTCAAAGATTTGCAGG
TACCACAAATTGAAAATTTGTGAGTGAATGTTGATCAAAACTTTTTTGATGAATTAAAAAATGG
TAGCGAGGCGAACGTTTCAAACGAAGGGGCATGCTGCCCCCTTCACCCCTGCATTTTAACTTTTTA
AAAAATGGTAGCGAGGAGAAGATTTGAACTTCTGATCTCCGGGTTATGAGCCCGGCGGGATAGGC
CTGGCTACCCTACCTCGCTTCAACAATATTAGCTTTTCAGATACTTTTTGATCAACCTTTTTTTAA
AAGGTTGCTGGGTACCCTACCTCGCTTCAAACAATTAGCTTTTGATCGACCTTTTTTTAAAAGGT
CGCCTGAGTGCTCTAACTCATTTCAACAATATTAGCTTTTGACTAACACTTTGCTAACACATAAA
CATATTAACCGTAGTATTTAAACTTATGGGTTAAATATTTAAAAAAATTTTTTTAAAAATGCTT
TTTTATTAGAAAAATGCGGTGTATGCATAAACTGCCAAGATAACGAGCATCGCAACAAAGAATAT
TTGCTGGCTTCTCATCATTTTCTTTCTTTTCTGAACAAATTCTTCTTTACATTTTTGTGAACAGA
ATGATTCTTCTGGAGGGATAGATAATCCGCAATTTACACAGTGTTTGTGTTTTTCCATTTTTAAT

CACCAACGTGTAACATTTAGTTTTTGAAGTTTTAAATAAATACCTAAAAATCTTTCTTTTCCCAT
CATAGGCAGAAAGCAAAGCTTTCTGATATTTTGCAAAATTTAATTTTGCAAAAAAGGCTTTTCTT
TCTTTAGAAAGAAAAGGGTTTAGGGAGTTATAGTAGTTCCGTTATAATTTCCCGTAACAGATGCT
AAAATTTCTTCAGGAATTCCTTTTACAACTATAGTTTTTAATTTTGCCCTATCAATAATTTTTGA
TGCTAATGGATCAACGACACCAGATGACCCTGCACTCATTGAAGCTGAACCTGTGATTTCCAAAA
GTTCTTTTGTATTTATTTTATCAAGTTTTACTGCATCTTTATTGCATCTTGGGTCTTTATTGTAT
ACTCCATCAACATTTGTTGCAATAACGAGTAAATCTGCATCAATAAATTCTGCGAGAGTTGCTGA
AACTGCATCTGTTGTGTGTGCAGGATGAGTTCCACCCATTACGAGTATTTTATCCATATTTAAAA
TAAGTTCAGCATCTTTGAAATTTTCAGGAACCTGTTTTACTGCATCTTTTCCAAGTGCTGAAATT
AACAGCATGCTGTTCATTCGTGTTGCAAGGATTCCGATTTCATCACAAAAAGATTCATTTGCAAA
TTCTCGCGCAACTGATATGTATTGCCTTGCAGTGTTTCCTCCGCCAACAACGACAGAAACTCTATG
GCCCATTTCTTTTAATTTTTTAAAAGCTTTAGCATAATTTTGAATGTTTTCAGTCGACGCGCCTT
CTTTTGGCATCAAGACTGATCCTCCAAGTGCAAAAACTACATCCATGAAGTAACCTCAGTTATAT
TTTGTAAGTTTTCAAAAAACAATAACTCTAAATTATCCTAAATAGTATAATCCTTTATTATATAA
AATTTTAGCTGAAAAATTCTGAAAAAAGAAAATAAAAAAGTAACAATAAGATAAGTGATAACTG
TAATATTGAATCACAAAATCAAAGATTTTGATATCTGATAATTAACTTATCAGTAGTTTTAAACG
TGTAACTTCCCAATTAAGGGTAATTTACATTTTTAAAGCCATTTTAATGTCTTCAACTTTAACAG
TTTTTCTTCCAGCATGTTTTGATAAATCTGCTGCTTCTTTAGCAATATCCATTGCGATTTCTTCTA
ATGCCTCAACAAACATTTTTGCTGCATCTTCACTTACTCTTTCAGCACCTGCTTTTTTTAATATTC
TTACTACTGGTGCAACTGGTAGTTCTGCCATAAAATCACCTCAATCTTTTACAGTTTTAATTTTG
GAAAACTAAATATATAAATCTTTCGGAAAAAACCACAAATGACAAGCCTATACCTATATGAAATC
ATGACTAAACCCAAAACCACAATGTAATTTATATTAGCTTACCTATATTATTATTAAGAATCCAA
AAATCGCTAAATTAAATTAGAGTTTGGGTGGTATCATCAATAATAAAAATATTCTTGAATCATG
GACTGTATTTTTATTTGTCTTTCTTATTGTGTGGACACATGTCAATGTTGTGGTCTCAGATAGCA
TGGTTCCAGTAATGACGAGAGGCGATTTTGTAGTCGTGGAAAATTCTTTATTTGAGTTTGACCCA
GAAAGCGTCTCAGTAGGCGATATAATAGTTTATAGTGCCCACTGGACCAATGCTGGAGAAAATAA
AATAATAAACCAGGACTTATCGATAAACGGGCGAAATTTAAATTATTACGAGTTAAATTCCCGAG
AAAGTATGATTTATAACCTGCCAATAATTTCGAGTTATTCGCCAACAACGCCTGTAATTCACAGG
GTAATTGATACGTGGACTGATTCTGAGGGAAATAAATATTACATTACAAAAGGAGATAATAACC
CGACGTATGATCCAGAATTAATACGTGCAGAACAAGTTAGGCAGAGAGTAGTTGAATTAAATGA
CGAACCATTTATAATTCCATATCTGGGACACATTTCAATAATTCTAAAAGAGAATTTGATTATAT
TTGTGATATTGCTTGTAATATGGATGTATTATGATTACAGAAAAGAAAAAGTTGCAAAAAATAA
TTAAAAACTAAATAAAACCAAAAAATATTAATTTTTTAAAGTAATTCTTTAAAATGTCTCAAGA
GCGATGGTGCTTTTGCAACTATTTTAATTGCGAGCTTTCCAACATCTACTTCTTCTAAGTTTTCA
GGAATTGAGTTTGCAATTGCATTTAATTCATCATCACTTAATTTTTGAAGTATATTTTTGTATTT
TAAGTCGTTCATTAAAAGTTTGTAGTACTTTTCTTTCCATTTTTTTTCGTAAAGATCTAAAAACT
CTTCTGAATAGTTTTCTGCTTTGATACTCTGTGCAGCAACATTTCCAGCCATCAAACCACAATCCA
TTGAAAGACCTATTCCTCCACCAGTAAGTGGACTTACATGTCCTGCAGCGTCGCCTACAACCATG
ATGTTATCAGCAACTGTTTTTTTAATTGGGCCGCCTACTGGGTCACCACCAACTTTGAATTCAAC
TGGAACTGCATTATCAAGCCTTCCTTCAACTAAAGGATGGGTTAAAAATTCATCTAAGTAATCGA
TTGCTCTTTTTTTACTATCAATGACACCGATACCTACGTTTACAGTATCTCCTTTAGGGAATAAC
CAGATGTATCCTTTAGGGCATATGTCTCCAAAGTAAAATTCCATCATTTCGTTGTTCAATAATTT
TACATTGGTCATTTCATATTCTGCACATGAACAGATTTCTTTAGGGTTTTTCTTGGATTTTAGCC

CAGCATATTCTGCAATGTTACTTTCAACACCGTCTGCAGCAATTACGATTTTTGATTTAACAATG
TATTCGTCTCCAAGGTAGTTTACAAATACGTTGTATCCATCATCGGTTTTTTCAATTTTCGTAAC
TCTACTTTTAACTGCAATCTGTGTGCCAGCTTGACCGCTTCTTATTGCAAGGAATTTGTCAAATA
CTTTTCTTTCAACAACATAGCCGTCCGTTTTTCCGCCTTTTACGACTATTTTTTTACCATTTGGAG
CTATTAAGTACCCGCCCCTAATGTATGATTTAACAAATGAGGGATCAGGATTAATTCCAAAATCT
TCGAGTCTTGGAACCGCCTCAGCACATCTAACTGGTGTTCCAATTTCTTGGGATTTTTCAATTAA
AAGTGTTTTTGCCCCATTTTTAGATGCGTTGTATGAAGCCATGCTACCCGCAGGTCCTGCACCAAC
CACAACTACATCATAGCTATCGTTTAAAGCCTTCATAATTATTCCTCCAACGCATTTACAGGACA
TACAACCGTACACAATTTACAGTTGTTACATTTTTCTTCCTCAATGTATAATTTGTTTTCAATTA
ATTCTATTGCCATACTTTTACATACTCCAACGCATGCACCACAGTAGCCACATTTACTGTAATTCA
CTTTCATCGTATCACTTTTACCATTAGAATTATTATACAAATTAAAATCATTGTATAAATACTAT
CCTCATTAGATTGTTGTATTAATCCTATTTAAAACCTTATTTGTGAATTCCTTCTGTACTTTGGA
ATCATGGCCATTACAACTGAATCAATACATAATTGTTCCCTTAGTATATTGTTATCGGTAAGAAC
TCCAATAATTCCATTTTTCTGACCGATATTTTTACCCTGTAGGTTTCTTCTGCAATTTTGCTGCA
TTCATAACCTTCGTTTATCATATCAGAAATATCTTTCGGAATTTGGAAAGCCTGAGACGTTCCAA
CACTGTAATCCAATCCATTATACACAACACAAATGTGGATATCTAATTTTTTTCTCCAATTTCT
ACAATTCCTGCTTCAATTCCAATTCCATAAACACAATTTACCTTTTCAAAAGCACCCTTTGCCCGG
TTATATGCTCCTTTAAAAGTTTCTTCAAATCCTTGAGGCTGGTCCGATACCCCACTTTTTGTATCT
ACTGAAGATACAAGCATGGTTCCAACAGTTTTTTCAATTGCTTTTTCTACAGCAGAAACTTTAAC
TGGATTTGTACTTCCAACAGAAATAACTCTTAATTTGTGCCCTGCAGGGCCTCCACGAAGTTTTT
GTGCTTTTTTTAAACATTCTGGATCTTCACAAAGTAAACTTCCAAATTGATATGTTTTTGCAGGC
TTACCGCAAATATCGCAAAATCTGGATTCAAACATTAACTCACCAAAAAATAATTACATTAATTA
AAAAAATGAAAATAATGGTTAAAAAAGATTATGTTTTAATTTGATAGAACTTTTTTTGCAGCAG
CAACTCCGCCTTCTGCATTGTAGCCTAATTCTTTAAATGCAAGTTCAATTGCAGCTAAGGTTCCT
AAAACCTGGTATTCTTTAGCTGAACCCATGTGTCCAACTCTGAATATTTTTCCAGCTAAGTGTGA
CTGTCCGCCAGCAACTCTTATGTTGTATTTTTCTGCAAGGAGTCCTCTGAATTTTTTGTCGTCAAT
TCCTTCAGGGTATTTTGCTGAGGTAACCGTAACTGATCTTGCTCTTTCTTTTGCAAATAATTCAA
GTCCCATAGCTTCTAAACCTGCTCTTGTAGCTCTAGCAAGTAAATCGTGTCTTTTAAATCTGTTT
TCAAGCCCTTCTTCAAGTACCATTTCAAGAGCTTCATTCATTGCGTAAGTTAATGAAACAGATGG
TGTGTAAGGGGTTTCTTTTTTAGCATCCCATGATTTTTTGTATGCGTTTAAATCTAAATAGAATG
ATTTAGTTTCCGTTTTTGAAACAACATCCCATGCTTTTTCACCCACGGTAATTGCAGCAAGTCCCG
GTGGTGCAGCAATACATTTTTGTGAACCGGTAACACAGATATCGATGTTGAATTTGTCTACATTT
ACGTAGTCTCCACCAAGTGATGAGATCGTATCTACAATGTATATTGCATCGAACTCTTTTACAAC
TTTTCCTAAGTCTTCGATTGGGTTTTTAGCACCTGTTGATGTTTCGTTGTGAACAACAGTTACTG
CTTTTATTCCCTCGTTTTCTTCGAGTAATTCTCTTAATTTTTTAGGATCTGCCAAATCTCCCCATT
CAGGTTCGTATTTTATTGTGTCTGCCTTGTAAACTGATGAAATTTTGTAGAATCTTTCTCCAAAG
TTTCCGTTCGTGATGTTTATTACTTTGTCGCCTTTATCCAAGGTATTTGAAATAGCCATATCCAT
TACAGCAGTTCCTGAACCGGTTATGATATATGTATCATTTTCAGTCTGGAATACTTTTTTCATCA
TGTCTACGGTATCGCCGGTTAAATCTCCAAAGTCTGAAGTCCTGTGGCCAATAATTGGTAACGCC
ATGGTGTTTAAAACCCTGCTTGGAACCATTGTAGGGCCCGGAATCATTAACAGTTTTTCAGTATC
CATCTGTTTCATTAAATCCCTCTATTTAACTTATTTTTTACTAAATTCTATAATATTGATGGAAT
TTGATATATATTTCTATTATTTATATATTGTCATGAATTTTATATTTATTTAGCCAGTTTTTTTA
CTCGTTCTACTCTTTTTTTAATTAAATCAAGTGTTCCAATGTCACGTCTGTAGTAAACTTCGCCTT

TAAAGTTTTCAATTGCTTTTTGGGCGATTTTTTCAGCTTCTTCGATATTTTCTGAAATTCCAACA
ACAGCGGCACTTCTTGAACCAGTAATGTATAATTTTCCATTTTCTTCGTTAATTGAAGCGTAAAA
CAATATTGCATTCGCATCTTCAATTGCTTTTTCGTCAACTGCTAATTCTTTGTTTCTTACAGGGTC
TATCGGGTAACCGTTTGGAACAACATATTTACAAACAGTTGCTTTGTTTTCAAATTCAATGTTTA
TTTTATCAAGTTTTCCTTCTGCAATTGCAAAACAAACATTTAAAAAGTCAGTTTTTAATATCGGG
AGCAAGTTCATAGCTTCAGGATCTCCAAATCTTGCGTTATATTCAATAATCTTTGGACCGTCTGC
AGTAAGCATGAATTGACCGTATAAAAATCCTTTGTAAGGGCCAACTTCTAAATTAATCGAATTAA
CAGTTTTTTCCATTATTTTTTCTGCTTTATCAAGCATTTCTTCTGATAAAAATGGCAATCCGTGG
TTTGGACAGGAGTATGAACCCATACCTCCGGTTATTGGGCCTTCATCGTTGTTGTACGCGTGAGG
GTGGTCCTGAACAGCGGGCATAAATACAATATTTTCGCCATCTACAAACCCGTGAAGTGTAAATT
CAACACCAACAAGTTTTTCTTCGATAATTATTTTTCCGCCACCAATACTTTTATCGAATACTTCTT
TAGCGTAAATTTTAGCTTCTTCATTGTCTTTTAACTGTTCACCAACGACTTTCACACCTTTACCGC
CAGTAAGTCCTGCAGGTTTTACAACTACATCTTTTCCTAACGAAGTCATTTCATCGATAAATCCT
TCAAGTTCAGTTCCGTAATTGTTAAATGCCGCATATTTCAAAGAACCGTCGATTTCATATTTTTT
AAATAAGTTTCTCATGAATTCTTTGCTTGTTTCAATTTGTGCCGGAAGTTTTTTTGGACCAACTG
TTAAAATACCCATTTCTTCGAGTAAATCAGAAGCTCCAACTCCAAGTGGGGACTCGGGACCAATT
ACTGCAATTTCAGGTTTAATTTTTTCCGCAAACTCTTTTATTGCAGGAACGTCGGTTTCGCTGTT
AAATGATACCTCTTCAGAGAGTGCATATATCCCAGGATTTTTATTTTTCATTAACGTATAAAGCT
CCACGAGTTCGTTTTTTTTAAGGGCCATGGCAATTGCATGCTCTCTAGCTCCCCCGCCAATAAGTA
AAACTTTCATACAAACCACCATTTCGATTATTTAGTTAAAAAAACATTTAAAAATATATATTGAT
GTTAGTATTTTTTAATTTTTGAAAATGGAAATAATTTAATCATCTTTTGTTAATTCATTATAATA
AATAAAATTATCCATGATTAAAGAGTCGGGTACAATAAATAACTTTAAAAAAGTTTTTAAAAAA
TAGGATTTAAACTGATTTAAATTTCAGAAACTTCATCTAAATCGATTTCTTGATATTCTTTGTCT
TTTGTGTTTAATATTGCAACGGTTGGTTTTCCAGTTAAATATCCGCAGCACTCTCCTGGGTTAAT
TACTAAAACACCGTCGATTTCTTCGAACTGTCTTTCGTGAGTGTGTCCGTGAATTACAACATCGT
ACTCTTTTGACTGTATTACTGCTTCGAGAATTGGAGCATGAGTTCCGTGAAGAATGAAAAATTTA
TGCCCATCTGTTTCAAAACTTACATAATTTTCAAGTTCATTATCAGGATCAATTTCTTTTAACCA
TTCCTTTAACAGGGTTTTTTCACCGTCGTTATTTCCAAAAACTCCAACGATTTTTGAGTTTAAGT
TTTCAAACTCTTTGATTACAAACAATGATACAAAATCACCACAGTGAACAACCATATCAACTTTT
TCGTCGTTAAAAACAGCGATAGCTTTTCTTATATTTGGTAGATAGTCATGAGTGTCTGACATTAT
ACCGATTTTCATAGAAATCACCAATATTAGCAAGGGTAGTTAAACAATAAAATAATTATAAAAT
AGAAAATCAGATATTTAAAGCTTTAATTACTGGAACCATGGCTTCGACGGTATAATTTTTTTCAG
GCATTAACGAATCAAATCCAAAAGAATCTACATTTTTCTTTGTTATCGGCCCAATTGATACGATA
TACTGCTTTTTTAAAATTTCTAAAAACTCAGGGCTGCAGTGTTTAAAGAAATTTTTCGCAGTAAG
ACCACTTGTAAATGTTAGAATTACTTTTTTATCTTCGCTGTTTTCAGAAATAACCTGTTTTTCAA
GTTCCTTTAATTTTTCAGGTATATTTGCTGGCTCTTCAGAACTGTAAACAAAAACAAGGTCTGCA
TTTAATCCTTCTTTTAATACATCCCTAGTTGAAGGAGTTGTTGGTACAAGCACCTTTTCTCTATT
TTTAACCTGTTTTTGAAGTGCTTCAAGTAAGTTTTCTGCAGTATATTGTTTTGGAATAACGTCTA
CAGCTTTTCCAAATGTTCTTTCAAATTCTTTTGCAGTTTCCGTACCAATTGCACCTATTTTTTTAG
TTAAAACTTTTTTAGCATCCTCTAAATTTAAATTCCTAAAAAAACCTTCAACTCCCCGAGGACTT
GTAAATACTATCCAAGAATAGTTTTCAAGGTTTATTTGGGCTTTTTTAAAAACCAATTCAAGTGT
TGGAAGAATAACTGTTTCAAAACCGTTTTTTTCAAGCAATTTTGAAAAATCCTTACCTTTCTCGG
CCGGGCGCGTGATTACAACTTTCATGGGATCTCCATTTAATTAGATTTTTTTCATATTCAAATCT

CTTATGTATGCCCTTAATTTTTCAATATTTTCTTCAAAATATTCCATGTCTTCTAAATCCCTTAA
AATTTCTTCGCCCATGTAAATTCCCGCAGAAACTTTGTCTGCGAGGAGTCTGCTGTTTGGAACGT
CGTATTTTTTATATAATAATTCTAAATCTTTCTCCATATCTTCAAGTTCTTCTTTAAAGACTTTT
TCCATGGAATTTACAATAGTTTTGTCATCAACGGTAATTAGATTGCCAGTTCCTTTCATAATATC
ATCTTTTAGGTTTTAAGAATCATTTTAATTTTATTTTTTTAATTATAAATAACTTTTTTTATTAA
ATTATAAATAAGGAATGCTAACGGATTTTTTAAACAAAAATAACCTTTTTCCAATTGAATTAATC
ATGGGAAAACTATATATTTCTTAAACCAATATCATTGATGTAGTTTTAGCGGCATTAATCTTTT
TTAAACTTTAATAATTTGGAAATTTACCAGAGGTGAGACTTTGTTAAGAAACATGGACGATTCAT
TTGATATATATGAAATAAAAGCAAGACAAGTACTTGATTCACGGGGAAACCCAACTGTGGAAGCT
GAAGTGTTAACTGCAGGTGGTGGATACGGCCATACAATTGTTCCAAGTGGTGCTTCAACAGGAAC
TTTCGAAGCAGTTGAATTAAGAGATAAAAGCGAAAAATATGGCGGAAAAAGCGTATTAAATGCA
GTTTCAAACGTAAATGATATTATTGCACAGGAATTAATCGGTGAAGATGCAAGAAATCAGAGAT
TAATTGATCAAATAATGTTAAACTTGGATGGAACTGAAAATAAAGGAAATTTGGGCGCAAATGC
AATTTTAGCAGTTTCACTTGCAGTTGCAAAAGCAGCGGCAGACACTGCATCACTTCCACTTTATA
AATACATCGGTGGCTCTAACGCATATGTTATGCCTGCCCCAATGATGAACGTGTTAAACGGTGGC
CAGCACGCAGGAAATGCACTTGATTTTCAGGAATTCATGATAATGCCTGTTGGTGCAGATTCATT
TGCAGAAGCTGTAAGAATGTGTTCTGAAACATACCAATCTTTGAAAAAAGTAGTTGCTGAAAAA
TACGGAAAAGATGCTGTAAATGTAGGGGATGAAGGTGGATTTGCACCTCCTGTAAAAACAATCG
ACGAAGCTCTTGCAGTACTTTTAGAAGGGGTTAAAAAAGCAGGTTATGAAGACGAAATCGTATT
TACAATCGATTCTGCCGCAAGTGAGTTTTACGATGAAAAATCCAGGTCATACATGATTGCAGGAG
AAAAAGTTTCAACCGATAAATTAATCGATATTTACAAAGAAATGGTTGCACAGTACCCAATTGTT
TCAATTGAGGATCCATTATTTGAAGAAGATTTCGAAGGATTTAAAACTGCAACAAAAGAGTTAA
AAGGAATCCAGATTGTCGGTGACGATTTGTTTGTTACAAACACGAAAAGACTCAAAAAAGGAAT
TGAAATGGGTGCTGCAAACTCACTTCTTTTGAAAGTAAATCAGATTGGAACCCTTTCAGAATCAA
TCGATGCTGCAAACATGGCATTTAGAAATGGATACAGTTTGGTTGTTTCACACAGAAGTGGAGAA
TCAGAGGACTCAACAATTGCAGATTTAGCCGTTGCACTAAATGCAGGCCAGATAAAAACCGGTGC
TCCTGCAAGAGGGGAAAGAACTGCAAAATACAACCAGTTAATGAGAATCGAAGAAGAACTCCAG
ATTTCAAAATATGCTGGAAAAGATTTTAAAATTCCATTTTAATTATTTTAATTCTATTTTTTAAA
TTTTTTTTATTCAATTTTATTTAAAACATGTATTTTTCAAAGATTTTTTAAATTTTAATTTAAAG
TTATATATTATAATTTATGAATATAACTGAGCAAAGAATAAAAACAAAAAATACATATTGTTTT
AAAATCATTATCAATAATGTCAATTATTGCCGGATTGGTGGTTTGTAATGGAGATTAATCACGAT
TATAGAGTAAAACTTTTTGATGAATTAGGTTTTGAAAGAAAACAATGCACTGAGTGTAACCAAT
GGTTTTGGACACTTGATAAAGATAGGACTACTTGTGGAGACAGTCCTTGTGATGAGTATTCATTT
ATTGGAAACCCGATCACCAGTAAAAAATACACCTACAACGAAATGGTTAAAGAATTTACGAACTT
TTTCGACGAAAAGGGACACACCCCTGTAAAAAGATCTCCCGTAGTTGCAAAACGATGGAGAGATG
ATATTCTTTTAACTATTGCATCGATTGCAGTATTTCAACCATGGGTTACAAGTGGGCTTGTAAAA
CCTGTAAAAAATCCGCTAGTAATTGCACAGCCATGTATCAGGCTTAACGATATTGATAATGTTGG
AAGAACTGGAAGACACTTAACATGCTTTACAATGGGTGCACACCACGCATTCAATTCAAAGGACG
ACTACAAGTACTGGACAGATAAAACCGTTGAATATTGTTTTGAATTAATGCAAAGACTCGGAATC
GATGGAAAAACAATTACATTTATCGAAAGCTGGTGGGAAGGTGGAGGAAACGCTGGACCTTGCT
ACGAAGTTATAACGCACGGTGTTGAACTTGCAACACTTGTTTTTATGCAGTACAAAAAAATTGGA
AACGATTACGAAGAAATCCCTTTAAAAATTGTTGATACTGGATACGGAATTGAAAGATTTGCAT
GGGCCTCACAAGGAACTCCAACAGTTTACGAATCTTTATTTTCAGAAATAATTGAAAAATTAAAA

GAAGATGCGGGAATTCCAGAAGTCGATGAAAAAATAATGGCTGAGAGTGCAACTCTCGCTGGACT
TATGGATATTGAAAATGTCGGTGATTTAAGAGTTTTAAGGCAAAAAGTTGCTGAAAAAATCGGA
ATGGATGTAGATGAGCTTGATAAATTAATAAGCCCTCTTGAATACATCTATGCTATTGCTGACCA
CACAAGATGTCTTTCTTTCATGTTTGGAGATGGAATTGTACCTTCAAACGTAAAAGAAGGATACC
TTGCAAGATTGGTACTCAGAAAGACATTAAGATACATGGAAAAAATCGGAATCTCGATGTCTAT
AAAAGATATCATTTCAATGCAGCTTGAAAACATGAAGGAAATTTATCCTGAATTAAGCGCAATG
AAAGAATACATCATGGATGTTTTAGATGCTGAAGAGAAAAAATACATTCAAACGGTAAACCGTG
GAAGAGGAATCGTTGAAAGAATGGCTGCTTCAAAATCTGAAATCACGCTTGACGATTTAATCGAA
TTATATGACAGTAACGGGCTTCCTCCTGAAATTGTAAAAGATGTAGTTGATGAATTAAACAAAA
AAGGAAAGAAAACTATTGCAATTACTGTTCCTGACAATTTCTACACGATTGTTGCAGAAAGACAC
GAGGAAGAAAAACCTGAGGAAGTAGTTTCAACGAAAAAAGAACTTCCTGAACTTGAAGTTTCAA
AAACTGAACTTTTATTCTTCAAACACCCAACTCAAGTCGAATTTGAAGCTAAAGTCTTGAAAATC
GTTGAAAAATATGTTGTACTTGATAAAACTTTATTCTATGCTGAAGGCGGAGGTCAAAAGTACG
ACATTGGTCAATTAAATGAACTTGAAGTTGTAGATGTTCAGAAAAAGAATGGAATTGTATTCCA
CAAGGTTTCAGACATTTCAAAATTCAAAGAAGGCGACACAGTAAAAGGTGCAGTAAACTGGGAC
AACAGGCTTAAATTAATGAGAAACCACACTGCAACGCACGTAATAAATGCAGCGGCAACAAGGGT
TCTTGGAAAACACGTATGGCAAACCGGTTCAAACGTTGATACTGAAAAAGGAAGATTGGATATCA
CTCACTACGAGAGAATTTCAAGAGAACAGGTAAAAGAAATTGAAAGAATTGCAAATGAAATTGT
TCTTTCAAAAATGCCTGTAAATTCCACTTTTATGGATAGAAACGATGCAGAACAAAAATACGGCT
TTACAATCTACCAGGGTGGTGTAGTTCCTGGAGATACTTTAAGGATAATCGAAATCGAAGGAACT
GACGTTGAAGCGTGTGGTGGAACCCACTGTAGTAACACTTCAGAATGTGGATACATTAAAGTCTT
GAAAACCGAAAGAATTCAAGACGGTGTCGAAAGACTTGAATATTCAACAGGAATGGGTTCAGTT
TCAGAAATTGCGAGCCTTGAAGATACTTTGATCGATTCAGCTGAAATTTTGGGAATTCCAAATGA
CCAGCTTCCAAAAACGGTTAAAAGATTCTTCGAAGAATGGAAAGAACAGAAAAAAACGATTGAA
GAACTCCAAAAAAAAAGTCGGTGAACTCGTAAAATACGAACTTGCAGACAAATTTGAGAATGTCGG
AAATTACGAAGTTTTAGTTGAGCAGGTCAGCGGAACTCCAAACGAATTAATGTCAATTGCAGATA
ATCTTGCAGTCGGTAACAAACTCATAGTTTTAATGAATGAAAACGACTATCTCTTATGTAAAAGG
GGAGAAAACGTTGAATTAAGTATGAAAGACTTAATCAGAAATATTGGAAAAGGCGGTGGAAAAG
ATAACCTCGCACAAGGAAAATATTCTGAAAACAAGGAACAAATTATGGAAAAAATAATTCAGAT
TTTAAATAAATAATTTTATTTCTTTTCTGTTTTTGATAAATATTAGGTGCTAAAATGTTCATACC
CTCTCATATTACAGGTTTTTTCAAGATAACTAGACACGAAAATTTACTAAAAACCGGATCAACTG
GTGCAGGAATTACACTAAACAGGGGAGTATCCACAAAATTGGTTGAAGGGCCTGGAAACGTTTAT
TTTAACGATGAAAAAATAGATCTGTGTCCATCAAAAGATGTTATCCATTATTTGGGACTTAAAAA
CGAATTAAAACACGATATAACATATATTTCCGATTTTCCGCTCGGATGCGGGATGGGAACATCAG
GATGCTGTGCACTTGGTGCTGCATATGAGCTGAGTTCAACATACAACTTTAAGAACATGCAAAAT
GATATTGATAGAAGTAATTTATATAACAAAAATGAATCAGATATATCGGAACTGGTGAAAATTG
CTCACAAGGCAGAAGTACGCTGTAATACGGGGCTTGGAGATGTTATCGCACAGCACACAAAGGGT
TTTGTTATTCGAAAGAGTCCAGGATTTCCAATTGGTGTAGAAAGCGTTGACATTAAAAACATGGA
TGATTACAGTGTTTTGGTAGATATATTTGGCAAAAAAGAAACTGATACTGTAATTAACGATCCAT
CTTGGATAGAAAAAATAAACAATACTTCAGACGAATTACTTGGAAAGTTGTTGAAAAAACCAAC
ACTTGAAAATTTCATGGAGTTATCTTACTACTTTGCAAAAAATACCGGGCTTGCATCTGAAGAAA
TAATCGAAATATGTGATGATTTAAGATTTACTGCTGGAGCGTCCCAGGCAATGCTTGGAAATGCG
ATATTCTGCATCTGTAAAGACGAAGAATTAAATGACGCTGTTTCAATTTTATCCAATCCAGTAGT

TTGTAAAATTTACAAATAAGGTGTAAACATGATGTCTACAGTAAAAGTTCTTTTATTGATGGCAC
TTTTAACAGGAATGATTTATGGAATCTGTTATATGCTTGGATTCCCCCAATATTTGCAATACTT
TTAGCACTAATTCCTAATTTAATAAGCTATTTTTATAGCGACAAAATAGTTCTGAGTAGTTATGG
TGCAAAAATAGTTGATGAAAACGAAGCGCCAAATTTACATAGAATCGTTGAATCAATAGCAAAC
AGGGCAAATATTCAAAAACCAAAAGTTGCAATAATTAATACTGATACTCCAAATGCATTTGCAAC
AGGGAGAAGCCCTAAAAATGGTGTAGTTGCAGTTACTACGGGAATCTTACAACTTTTAAATGAGC
AGGAACTTGAAGGAGTTTTAGCTCACGAAATTGGACACATAAAGCACAGGGATATATTAATTGG
AACAATAGTTGCAACAATGGCTGGAGCAATAATGTACATTGCAAATTTCTTACAGTGGGGAATGC
TTTTTGGATATGGAAGAGATGATGATGGAAATCCTTTGCAGATTGTTGCATCCTTACTTTTCATA
ATATTGGCTCCAATTGCCGCGATGGTTGTTCAGTTTGCAATTTCAAGGCAGAATGAATACAATGC
TGATGAAAACGGGGCAAAATATTCAAATCCCATTTACCTTGCAAATGCACTTACAAAACTTGAAA
AAGGGGTAAAATACCATCCGTTAAGAAATGGAAGCCCTGCAACTGCGCACATGTTTATTGTAAAT
CCTTTTAAAGCAGGAAACGTTGCAAGGTTATTTTCAACACACCCTTCAACAGAAGAAAGGGTAAA
AAGATTAATGGAAATGGCAAGTAATCCTAAATATTTAAGATAAGGCGATTTAATGCAGCTTGGA
CTTACTGTAATTGCGGAAGACCGTATGGGAATACTTTATAGATTAACAGGGATAATTTCGGAATT
AAATGCAAACATAGTGTACACTCAGCAGTTCATTGTTGGAGAAAATACTGGTTTAATTTATATGG
AACTCGATGGAGTCGAGGACGAAGAAAATTTAGCAGGAAAGCTTGAAAAATTAGAATTTGTAAA
AAAAGTTGAAACTCACAAAACCATGAAGAAAATATTTGGTAAGAGAGTTTTGATATTTGGAGGG
GGTGCTCAGGTAGCTCAAGTTGCAATGGGCGCAATAAGCGAAGCTGATAGGCACAATATCCGCGG
GGAGCGAATAAGTGTCGATACAATGGCAGTTGTTGGAGAAGAAAATCTTGCAGATGCAATTTTG
GCAATAAAAACACTTCCAAGGGCTGGAGTTTTGGTTTTGGCAGGATCATTGATGGGTGGAGCGAT
TACAAAAGCTGTTGAAGAAGTAAAAAAGGATACAAACATTCCAGTAATATGCTTAAACATGTTT
GGAAGCCTTCCAAAGCTTGCAGATTTAATAGTGACCGATCCATTACAGGCAGGCGTTATTGCGGT
AATGACTGTTGCAGATACTGCTAAATTTGACATAACTAAGGTTCGCGGAAGAATACTTTAATTTT
ATATTATTTTTTTTATTTTTTTGGTGATTTTATGATAAAAACTGTTGTTGGTAGCTACCCGGTAG
TTAAAGGAAAACCTGATTCTATGCCTGATAAAATTAAGAAATTTTTTGGAAAATATGATAAATA
CGAATATTCGATAAAAAAAGCGTTAGATGATCAATTAAACGTTGGAATTGATATTTTAAGTGAC
GGCCAGGTCAGGGGGGATATGGTTGAGATATTTGTATCAAATATGTATGGCTTTGAAGGCAGGCG
AGTTGTCAACAGGGTTGAATTTGTAAAACCAATCACTTTAAACGATATAAAATATTCACTGAAAT
ATATTTCAAAAAAAGACCCTGAAAAAGGAGTAAAAGGAATAATTACGGGAGCATGCACGTTAGC
TTCATCCATTCGTGTTGAAAATTATTATTCCGATAACAAAGATGAAAATTTAATTTACGATTTAG
CAGGGGCTTTAAACAAAGAAGCTCTTTCGATTCAAAATCATGTAAAAATGATACAGTTTGATGAG
CCAATTCTTTCAACTGGGTTATACGATCTTGAAGTTGCAAAAAAAGCAATGGATATTATTACTTC
TGGAATAAATGTTCCTGTTGCAATGCACGTCTGCGGAGATGTTTCAAAAATATTTTACAAATTAA
ATGAATTCAATGTCGATATTTTGGACCACGAATTTGCATCATGCAGAAAAAAATCTTGAAATATTA
AATGAAATCACAAAAAAGGTTGGTTTTGGGTGTATAAACACTAAACTTAAATCGGTAGATTCTG
TCGACGAAGTAAAAGCATTAATAAGTGAAGGAATAGAAATATTAAAGAATAATTCTAAATTTGA
AGGTTCAATAAATGATTCCGTTATAATTGATCCGGACTGCGGTATGAGACTGCTTCCAGTGGATG
TTGCATACTCTAAATTAAATAACATGGTTACTGCAGCAAATGAGATTGAAAAGGATTTAATTTA
AACTTTTAGGTAAGATACAATGTCAAAGCCAATTTTAAATAATTATAAACTGTATTTATTAGTCG
CCCTAATGTTCTGTTCTATAAGTGCAGTTGATTCGTACCGTTTTGACGAGTATTCTGTGATATAT
TCAATAGATCAAAACGATAATTTTTATGAAGAAATAGATTTGAAAATTTACAACAACAATTCTG
ATGATTTATACGAAATTAGTTATACAATCCCTCAGGAAACTTCAAATTTAACATTTAATTCTTCA

AAAGAAATAGATTCTTATTCGATAACTACAGATGATGCAGGGGTAACTGAAGTTAGTGTTAGGT
TAACTGAGCCAATTGGTCCTTGGCAGACTGGGGATTTGCTGGTTAGTTTTACTGGAGAAGTTTCT
GATATCGGCGGAAAAAAACAGGTATATATTATAGTTCCTGCATTTGATTCAACTTTTAAATTATC
AGTACAGCTTCCAGAAGGAGCAGCCATAGTTTCGCCTGTAAAAGATGTTTTGAGTATAAGTCCTA
GGGATTATACTGTTGGGTCAAATGGTAAAAGTATCTTTGTAGAATGGGAAAAAGAATTAACCTC
AGAAGAAAAATATTTTGAAGTTACTATAAATTATGTGCTAACAGGCGTAATTACTCCTTCAACTA
CTGGCGAAAATAACGAAGATTTGTACCGCGCAATAAGCATTGTTTTAGGAGTACTCGTTGTTGCC
TTAATATTTTTCATATATCGGAATTATAATAAAGTAAAAATGATAAATGAACTTCAAAATGAGA
TAAATGGATTAAACAAAGGCCTTGAAATATCGCATCTTAATTTACAGAATAAAAATAAGGAACT
TCAAAGATTAGAAGAATTAAACGAACACGTTTTAAAAGAATTGGATTTATCAAAAAATACTTTA
AGAGATTATGAATTAAAAATTAATGAATTAAATGACGAAATATTGCAGTGTAGGGCTCAGGTTG
GAAACCATACTAAATTAAAAGAAGAATTAAATTCGAAAGATGCCGTGATTAAAAGTTTAAATGA
TTACAAAAAATTATCCGAAGAACTTAAATTAAAAATTAATGAATTAACTGGACTCATCAGTGAA
AAAGAGAAATGTATTATTAAATTAAAAGAAAAAATCGGAGATTACGAGTCCCATAAAAGTGAAA
CATTAATGAATATATTAACGGATGACGAAAAACAGCTTATTGAATTAATTAGGGAACACGGATC
AATTTCCCAAAAAGAAATCGTTGATATAACTGGACTTACCAAACCAAAAGTTTCAAGAATGATTT
CCGATCTTGAGCAGAGAGGAATGGTTAGAAAAGTAAAAATTGGAAGAATTAATAAGATTGCTCT
TTCAGATGACTTAGATGGATCGATTTAAAAATCCCCCATTTAAACCTTTTTTTTACAATATTTTT
TTACAATAGTAAACTATATGGAGTTAAATGTACTATTTTAAATCATAAATTTCAATGTTTGGAT
TAAGGATTTATTAATATAGTATAATTGTTAAATTGAAATCGAACAAGTATGGTGTGGATGTTTT
AAGTTACATATTGAAAATATCAGAAAATCCGATTTTTAATTTTTACCCCTTTTTAAGAATTACAG
TGTTGAATTAGGTTATTTTCGAAACCAGGGTATTTTATAACTATTTGGGCTTATTTAGGTGAATA
CTATGAAAATACTTATTTCTATATGTGGTGAAGGATTTGGACACACTACGCGCTGTATTGCAATT
GGAAATGAACTTTCAAAAAAACACGAAGTCAAATATGCAGCATACGGCAAAAGTAAGGATTTTG
TTGAAATGTCTGGAGGGGATGTTTTTGAAACATATCCTGAAATAAGGCTTTCTGGGAAAAAATGG
TAAGTTTGATGTTAAAAAGAGCATATTTAACAGTGATTATCACCCTGTAAGAGCGATAAAAAAA
GAAATGGATATAATAAAAGATTATGATCCAGATATGGTAATTTCAGACTGTAAATATAGCTCAG
TTGTAGCATCAAAGGTTTTAAAAAAGCCTTATTATATAATAACGAATCAAAACTGCACTAAAACG
GATAAAAAGGAAAAATACATTGTATATCCAGTAATGGAAATATTAAACGTTATAAATAAATCTG
CAGAAAAAGTCATAATTCCAGATTTTCCACTGCCTTATACGGTGTGTGAATATAACTTAAAACTG
CTAGAAAATCTTTCATTTATAGGCCCCTTAATTCGTTATGATGTAGTTCCTGAAGAAATTAGTGA
TGAAGGCTATATTTTATGTGTGATAGGTGGATTTGAATATCGATATAAAATATTGGAGCTTTTA
AACAAGATTTCTGCTAAAAAAGGATTAAAAGTAAAGATGGTATGCGGAAGTTACGATGTTGCAG
AAAAACTTGAAAAAATAAAATCTAAAAATGTTGAAGTAATTCCATTAACTACAAACATGGATGA
ACTTTTAAAAAAAATGTTCATTTGTGGTATGTCATGGTGGACACTCGACACTGATGGAAGCTTCTT
GTTTTGGAAAACCGGTAATAACTATTCCTGATTTGGATCATCCAGAACAAGAAAACAATGCGAAA
AAAATATCTGACATGGAATGCGGAATTGCACTTTCTTACAATACTATCGATAGCAACTTGGAAGA
ATCGATTGAAAAAAATGTTTTCAACTACAAAATATTACGAAAATTCCAAAAAAATTGATGAATTTG
TACCTTAAAAACAGTGGAACTGATAAGGTAATGGATATTGTAGAAAATGGACTTTAAATTTAAA
AAGTGAAAATATGAAGATAACTATTCTTTCAGGCGGAACAGGAACTCCAAAATTAATACAGGGT
TTTAAAGAAATTCTTCCAAACGAAGATATTTCAGTTATTGTAAACACTGGTGAAGATACCTATAT
TGGCGACATTTACCTGTCCCCCGATATTGATACTGTTTTGTACACGTTTTCTAATCTTATAAACG
ACGAAACCTGGTATGGGCTAAAAGGGGATACATTCTTCTGCCATGAACAGCTTAAAAATTTTGGA

TTTGACGAAGTTTTGAGGATTGGAGATAAAGATCGGGCATTAAAAATGCATAAAACATCTCTTTT
GAAAAAAGGAATACCTATGTCTGAAATCGTAGATATCGAGAGAAAATCGTTATCAATAAACTCA
AAAATTTATCCAATGTCCAATGAAAAAATTGAATCCAAAGTTTTAATCGAAGAAAACAATGAAA
AAATCCTTCTGAAGTTTCACGATTTTTGGGTGTCAAGACGCGGAAATGCGAAAGTTTTAGATATA
TTCTACGAAAACTCAAATTATGCAAAAGCGGCAGACGGAGTTTTAAAAGCAATTCAGAAAAGCG
ATTTTGTGATTATTGGTCCATCAAACCCGATAACCTCAATTGGGCCAATTTTAAGCATTTCCGAA
ATAAAAAATGCATTAAAAGAAAAGCTTGTTTTTGCAGTTTCGCCAATAGTTGGTGAAAATCCCGT
AAGTGGGCCTGCTGGAACTCTAATGCATGCAAAAGGCTACCCTGTGAATGCAGTTGGAGTTTATG
AATATTATAAAGATATTGTAGATGTTTTAGTTTTAGACAATAATGATATTAACAAAAAGAAAGA
AATAAATTGTGAAGTTTTATATGCTAATACGATAATGAAGACTATTGATGATAAAATAACTCTC
GCAAGAAATATTTTCGACTATTATAAATCCAGATAAGGATATTAGTAATATTATTGAGTAATAT
ATTTATTAAGTGTTATATTAAAGTCAAATTATTGCTTCGTAAACTGAAAAGTTAGCGGATTAAT
AAAGGAGATAATAATGATTCAAATAACCGTTGTACAAATTGATAATTACGGTCCATGGACGGTT
ACCCCCAATCCAAGAAGAGAAAGTGACTTACAGGCACTTCAATCAAGACTTTACTGCGATATGAA
CTTGCAATTTGGAGCACACAAAGGTCTTGCATTTTACACAAGATTTGACAATATTATTGCAATTA
CAAATGGGATTGATTTAGAAACCCACAAAAGAATTCAAAATAGTGTAAAAAATAGGTATCCATT
TACTGTCAGCATGGCGGTAGCTTCTGCAGAAACTGCATACGAAGCTCAAAAACTTGCAACAAAAA
CAATTCAGGAATACGGAAGTGCACAAGACGATGTTAGAAAAGAAGTCTTAGATGTAGCTAACGA
ATTTGTATCAAATGGCTACGTGCAACTTGCACACGTAGATATAAACGATATAACGGGAAAATTAA
CCGATCTTGAAACGGCATACGATACTTATCTTTCAGTGCAAAAAACAAAGCTTAAATTGATGGAA
GAACTTAAAAAATACGATTCATTGGGCTTTTTCATTGGTGGGGACAACTTCATGTGTCCATGTAA
TGGAATGAACGAAAAAGATTTCCTCTGCATGTTTGAAGACATAAAAGATTCCTGCGGTATTGAAT
TAAAAGCAGGTATTGGAATTGGAAAAACTGCAGAAGATGCATCAAACCTTGCAGATATCGGCTTA
GAAGTTATTCGGGAAGGAAAAACTGATTCTCAGGTTTATACCTTGAAACAGGAGATTGATGAAC
AAAAAAATGTACCTTACAACTACATGTGCCCAATTTAAACTAATTCTTTTTTTATTCGGGATTTT
ATGAAAAAATTAGATAAAATTTACACGATTCCAAAAAAAATAGTCCTTGCTCCAATGGCAGGGA
TTACTGATGGCGATTTTTGTTTAAAATATAATGATACTTTTGGAATTGTATGCCTTGGTGCATTC
AATTTAGATTTTGACACAAATTTGGCAAGCAAAAAAATTCAAAACCGCGGAAGAAAAGAATTTA
TTTATGAGTTAAGTGAGATTGATCAAAAAATAAGTTCTGAAATTGAAAAAGCGAAAAAAATCAA
TTCTTTAGTTTCAGTAAATATCCGTTTTAACGATTTTTTTAAAGCAAAATCTCACATAGTCAAGA
TTTCAAAAAATGCAGATATTTTAGAATTAAACTGCCACTGCAGGCAGCCAGAAATAACTGATCTG
AATCTCGGGCAGAATTTATTAAAATTCGAAAATGAATCCAAATTAATTAATTTTTTAGAAAATA
TTCAAAAATTAAATTTAGAAATTCCTATTTTTTTAAAATTACGGGCTAATTTTTTAAAAGCCGAA
GAATTAATAAAATCGCTTGACAAGGTTCGAGGATATTTTGATGGAATACATATCGACTGTTTTAA
TCCTGGAAAAAAATCATGCGGATTTAGAATACTTGAAAAAAAATTAGGGAAAATTTCCCTGAAAAA
ATAATAATTGGCAACAATTCGGTTAATTCTGTTGAAAGTGCAAAAGAAATGCTTGAATATGCCG
ATTTTGTATCTGTAGCACGATGTGTACTGTCAAATAATATCGAATGGATTAATAAAATGTAACTA
ATGTAGGGATTTTAGCATAGTTTTGAGATCGCCCTTAAAATATCTCCATCGGAAAGTATTCCAAC
AAGTTTATTTTCGCTGTTAATTACCGGTAACTGGTTTATAATTTCCCTTCCATCTCCGTAAAGAT
CCATCTTTTTCAAAGCATCAACTGCAGATTCGTCCTGTTTGATCGTTACAACTTTTTTTGTCATA
ACATCTGCTATTGTGGTTTCTAAAGTGTATTCATCAATAATTAAGTTATATCCTATGTCTGTAGT
CGTTACAATACCGATTGTTTCGTTGTTTTGATTTACAACAGGCAGACAACTTATTTTATTTTTTA
AAAGGGCTTCAAATGCTTCAACCACACCTGACTCAGGGCTAACTGAAACCACGTCACTGCTCATG

ATATTTTTAACAATTATTTCTTTGATTTTTTCCATTTTATTCTCCTGTTGTCTTTAATAATCCCA
AAAGGTATTTAAATTAAATACTCAATTGTATATTAGATTTTAAATTTATTAAATAAACTATCGA
TTTCAAATAGAAAGCTTTAAATACTAATATTTTTTTAGACAAAAAATCAGCGGTTGAATGATAT
ATGAAGCGGGCAAATCTGTCTCATCATGATAAGGTAGCCAATGGAATCCGACTTTTTGGAAATAC
GCTTATAGATGAGTTAAATTACGATACATTTATATGGACTAAAGAAATACTTGGCCCAGACGACG
TAAAAATTTGGAAAAAAACAATTGAAGAAGAAATTAAGAAAGGAAATAATATTTACAACATGGC
AAGACTTTGTAGGGTTGGGGAAAACGATTATTTAAAAGAATTAGCATCAAAATATAATGTAAAG
TACTACGATTCATCTGACCCAAAAAAATACGATGAATTGCGAGTTTTTGCTAAAACAGGGCTTGA
AGGGATTAATACAAAAGTAATGACGATCATGGGAACAAGCAGGCAGTCTGGAAAATTTACAACC
TGCATGATTTTAAAAAAAGAACTTGAAAAGAGAATTAATATTGGAATTGTCGGTACAGAACCGC
AGTCTTTAATTTGCGGTATTGATGAAATGGTGGTTCCTCAGACAATAGAAACCTGCCACTGTGCA
TCAACAATATTTGGAGCAGTAAAAAAACTTGATACTAAAAACAGGGATTTAATTATTGTTTCAA
GTCAAACGGGAATATTTGCAGACACTTTAGAAGTTGGAACTGGAAGGGGCGGCGGTTTAATTAGC
ATTGCAGTTTTATTTGGTTCAAAACCTGATTTTACAATTCTGGCATCCGACACTTTAGATGCGGA
GATTATAAAGAAGAATATTTCTGCAATAGAACTTTTAAGCGGAAAACCTGTAATTGCAGTTACGC
TAAATTCAAGGCGCATGGACGAGGAAAAATTGGAAGAATCAATTGAAAAAATATCAAAAGATCT
CAACTTGCCCGTTGTAGATGTGTTAAAAAGAATAAATCTTGATAGATTAGTGGATGAAATTATT
TTGAAATTGAAAAATTAAATAAAACTAAAAAAATAAAATTTAAAACTATTTTTTTAGGTTTTCA
ACAAATTTATTTGAAATCGCCATGATTCCTAAAATTGGCGGGGCCCCTGGTGCTTTTGAAATTAC
CGATGCGTCACAAACGTATAATCCTTCAATTTCAGTTTCAAGGTTTTTATCAACGACTTTTCCAA
CTGCACACGTTCCTCCAGGATGCGATCCTCTTGGAACTGTTGAATATATATTTTCAACTCCTAAA
TTATAGATGTATTTTGAAGCTTTAGAAACGCCTTCGCACAAATTCCGCGTATCATTCATCGACAT
TTCTTTTTTTACACTATTTTCAACACTTCTCCGGTATTTTCATCCTTAATTTTTACCATTAAACT
AAATATGTCATTTTTATTTACATTTTCAAATTCTGAAATTCTTTTATGGAGTAATTCTGAGTAGT
GAGTTGCAAGTATGAACTCATCGTATTTTTTGTTTATCGCCATTGAAACTTCTGATTTTAAGTTT
GAACCTTTTAAAATTCCTCCGACAGTTACAAAAGTATCAATGTATAAATTCTTACCAATTTCTTC
ATTATTTGATATTGTTTTTAAAATTCTTGGTGAATTTATTGCCCCAGCAGATAAAATTACTTGTT
TTGATCGGTAAATCTTAATTTCTCCGCTTTTTTTATCGAGTGCAACTATTGAAAAAATGCCGTTC
TGTTTAGCTAATTTCATTACAATCGTTTCTGTTACGATATCGTCGCTGTATTTTCTCAAAAAATC
ACATGGTATCCATTTAGCATCACAGGGGTCATGTGCACAGAGACCGCAGTTTTTACAGAGTTCAA
AGTTAATAAATTTTGGAGTTTTTGAAAAACCTGATTCTAAAAGTTTTTTTGAATTTTTTCCTAAG
TAATCATCAGGAACGTCTGAAATTGATAACTCTTTTTCAATTTTTTCATATACTGAATCTTTTTT
TGAAATATTGAACTCTTTTAAATCAATTCGAACTGCATTTGCAACAGAATAAACGCCTGTTCCAC
CAAATGAATTTGCATAGATTACTTCAACATTTTCCCCTTCAGATACATAGAAAGGATCACTTCCA
CTTTCAATCAGTCTTATATTATGGTTTGGAAACGCTTCTTTTAACTCTTTAAATACCGTACTTCC
CCCAACACCAGAACCTATCACAATAATGTCTTCCATGGTTTTACCTCAAAACTTACCGATAATTA
TAAATTATAATATAAAATACTCTTTTGATAGATTTTTTAAGATTCATAAAACATATTAACTTTTC
GGTAAATATTATTTTATAAGAGAAATATGGTGACACCATGATTATTATTCCTGGCCTAAATTCAC
AAAATCTGGCCAAAGAAGTTGCAAAACTAACAAATTCGGAACTTGCAAGAGTGGAATCAAAGCA
ATTTCCTGATGGCGAAATATATGTTAGAGTTCACAATGAACTAAAAGGAAAAGATGCAGTTATA
ATTCAGACCCAAAACACGCAGAATGATTCAATTGTTGAAGCAATTTTAATGTGCGAAGCTTTAAG
GGACGAAGGAGTAAAAAGCATAACTTTAGTTGCACCATACCTTGCATACGCAAGACAGGATAAAA
AATTCAATGCTGGAGAGCCAATCAGCATAAAAGCGCTTGCAAAAGTATATTCAAGTATTTGTGAT

AGAATCATCACGATAAACCCACACGAAACACATATCAAAGAGTTTTTGATATACCATTTATTTA
TGGGGATGCAATACCAAAATTAGCAGAACATGCAGGGGTAAAACTTGTAAAACCACTTGTACTGT
CCCCCGACAAAGGAGCAGTTGCGCTTGCAAAAAGAGCAGCTGAAGAGTTAAACTGTGAATGCGAC
TATTTGGAAAAAACAAGAATTTCTCCAACAGAAATTAAAATTGCGCCAAAAAATCTTGATGTATC
TGGAAAAGACGTATTAATTGTTGATGATATCATTTCAACAGGCGGAACAATGGCAACAGCAATTT
CAATGTTAATTGAACAAGGAGCAAGAACAGTTATTGCGGCATGCGTTCACCCGGTTTTAATTGGG
GATGCATTAAACAAACTCCACCTCGGAGGAGCTTCTGAAATTGTTGGAACAAACACGTACCCTTC
AGAAGTTTCAAAAGTATGTGTATCAACAATAATTGCAGACATTTTAAATGGAGAATAATTAAAA
TAAAATAATTTTAACTATTTTTTAATTAAAATTAGAAAAAAGGAAAAGATATACTAAATTTCAA
TTATTTGTCGTGTTCCCAGTAAAGTGCATCGAGAAGTATCGCTACAGGGTATTCCATGTTTATTG
AAAGTTCTGATGCATCTTTTATTAATTTATACGCTTCATCAGGAGTTTTTGGACTGAAATATGGA
ATTTCAACTGTTTCAAGCAATTTTTTGGTCCACTGGCCCATAGGAATTTGTGCAGAAATTTTTTC
CTTTAAATCCCCCCGATGACTTATTATGATTAAAATGGGGATTTTATAAACTTTAACTAACGAAC
CTATCGCATTTATGGAGTTTCCAAGGCCAGAATTCTGCATCAAAAGCGCAGTTTTTCTGCCCCCA
AGATAAGCTCCAGCACAAACACCAATTCCCTCCTCTTCACGAGTTACAGGAATGTGCTGAATATC
TTTGTCATCATTTAGGTAATTTAAAACAGTTTTTAAATTTGCACAAGGAACACTCGTTACAAAAT
CAACCCCTGAATCTAGTATTGCTTTATAAACAGCTTCACTTGCATTCATAACTCCACCATCCGGCT
TTATTTTGGTGATAAAATTGAAAATTTACATTGAAGGATATGGCTGCACTTTAAACACTGCAGAC
ACCCAAATAATAAAAAATTCAGTTAATGAATTTGAAGATTTTTGAATTGACCGATAATGTGGACG
ATTCTGACATAATTGTCATAAATACCTGTATTGTCAGGCAGGAAACCGAACACAGAATGATTTCA
CGAATAGAATATTTCAAATCATTGGATAAAAAAGTTGTCGTAGCAGGATGCATGGCAAAAGCGC
TGTCCAAAAAAAATTGAAAATCTTGCAGATGTTTTGATAATGCCAAGAGAAGCTCAGCATTCTGGA
AACATCTTGAAAGATAAACTTTTAAAAGATTGTTCTGAAAAAAACAATGAATCAACTCAAAATT
TAAATTTTGAAGACAAATTAAACGAAAAAATCAAAAAAGTAAGTTCGCAAGGTTTAATTACTGC
ACTACCGATATGTGAAGGATGCCTTGGAAGCTGTACTTACTGCATAGTAAAACGTGCAAGGGGAA
ATTTGGCGTCATACGATCGAGATTTAATTGTTAAAAAGGCAGAAGAACTTGTAAAATCAGGTAC
AAAGTGCTTGCTTGTAACAGCACAGGATACGGCATGTTATGGACTTGATAATAACGACAGCCTTC
CAAATTTAATAAACGATATTTCAGAAATTCCTGAAAAATTTGCAATGAGAATTGGAATGATGCA
TGCAAAATTTGCAGAACCAATACTTGACGAATTAATTGAATCTTTTAAGTCCGAAAAAGTGGTAA
AATTCCTTCACCTTCCAATTCAAAGTGGGGATGACCAGGTTTTAAAAGATATGAACAGAAATTAC
ACGGTTGATGAATATATTTCTGTATTAAATGAGTTCAAGTCAAAAATTAAGAATTTAAACTTTA
CAACTGATGTAATCGTTGGATTTCCAACTGAAACAGAAGAAGCTTTTGAAAACACTCTTGAAATT
GTAAAAAAGATAAAACCTGACTTTACACACGCTGCAAAATATTCTCAGAGAAAATACACAAAAGC
AGCAATTTTAAAGCAAGTCGATACAAAAATAAGGAAGGAAAGATCTGAAATATTAAATGAATTA
AGGCGAGAGTTAAGCTATGAAAATAATAAAAGACATATTGGTGAAACTTTCGAAATACTTGTAA
CCAAGGATAATATGGGCGTTACTGACAACTGTAAAAACGTGATTTTCGAAGAACCTGCAAAAATC
GGTGAGTTTAAGCGAGTTAAAATAACTGGTGCAAAGACTTTCGGACTTTCGGGTAAACTGATAAA
TTAATCTCTTTTTTTTTCGATTTTATTTAGTATATTTTTTACATTTTTAATTCCTTTTTCAACCT
TAAATCGGTGTACCTACACAAAGACCCACATAAAAACAATATATAGTTAGGATTTGACAGAATAA
TTGGGGGGATATAAAGGTGTCACTAATGAAATTGGGTACTAAAAAAATTGGAACAAAACTTTTA
ATATTTTCAATAATAACAGTCATAATTCCAATTGCGATCCTCGGCATTGTTTCATATAATACTAT
TACACAGGATATGAAGGAACACTCTGCGGATAAATTGGAATCAAGCAATGAGATGGCAAATACC
ATCATGGACAGTAGATTTACCGAACTTTGTACTTTGGCAGAATATGCCTCATACACTCAAGGTGT

AAAAACCAGTATCAAAACTGGAAATGTAGATGACCTTGAATTAAGGGCTGAAACGCTTAAAAGT
GCTTCTAACGCTGATTTGGTAATATTTACGGGTAATGGGGGAAGTACAATTGCTTCTTCATCTGG
TGAAGGTATAACCTTAGATTCAACAATTTTAAAAGTTTCAAAATCCTCCGAGAAATCAGGATTCG
AAAAAATACCTGAACAAACTGCTTTAAAATTTAGTGACGCAGACGTTACAGGCACTGATTCGGCA
CTATCAATTGTGGTGGCTTATCCTATCTATGATGATGCAGGAACACTTGGATATGTTGCATTCAT
CGATGTTTTAAATAATGATCATACTTTGGTTGATAGTATAAAAGCTTCAACTGGTGACGAATCTA
CGCTATTTTTAGAAAATTACAGAATATCCACGAGTGTAGTTTCAGGAAATAGCCGAGCTGTGGGA
ACCACTGCATCAGATGTAGTATATAACACAGTTGTAAAAGAAAACACTGAATTTATGGGTAGTGC
AAATGTTGTTGGACAAAGTTTCCTTACAAAATATGTACCTCTGAAAGATTCAAATGGTGCTACAA
TAGGCATGGTCTTTGTTGGAACTCCAGAAGCTCAATTTACACAATTAGTCAACAATGCAAGAAAT
AATACAATAATTATTGCATTATTTGGACTGTTAATTGCTGTGGTTATTTCTTTAGTTTCTTCAAG
AAGTATTACCAAGCCCATCAATCAATTAGAAGACGGGGTACATAAATTTGGTAGCGGAGATTATG
ATCACACAATAAATGTGAAAACTGGCGATGAATTAGAAGAACTAGCAAATTCTTTCAATAAAAT
GGCCAAAGACATTAAAGAACTGCACTCAATGCTGGATGCGGATAAATTATCCCTTGCAAAAACAT
TAAAAGAAATATTTGATATTATGGATCATCTTGCAGAAGGGGACTTTTCAGTAAGGGCTGATGA
AAACAGGCACAAGAATAAACTTCAAAAAACAATCAACCATGCAATTGAAAATGTTTCAAAAATG
ATGGAAAATTTGAAGTCAGAAATCACTGTATTAAGCGGTGAACTTGAAGATGTTAGTGAAGGAC
TGAAAAGAGCTAAAGAAACTTCAGAACAGGTTACTGATGCTGCAAATCAGGTTGCAACTGCTGCA
GCTGATCAATCTGCAAAATTACAGGATACTTCAGATGAACTTGAAAAAACTGCGAAAGCTGCAGA
CATGGTATATAATGATGCAGAAAAGAGCGTTGATTCAGCAATCGAAGTAAAAGATAATTCAGAA
ACCGGTGTTAAAAAGGTTGAAAATGCAATAGATACCATGCAGAAAATTACAAACGTAATTGACG
AACTTGGAAAATCTATTCAGGAACTTGGGGAAGAATCCAAAAAAATCAACGAAGTTACAGTTTT
AATTAAAGATGTTGCAGAACAAACTGGACTTTTAGCACTAAATGCATCAATAGAAGCTGCTCGTG
CAGGAGATGCCGGAAAAGGCTTTGCAGTCGTTGCAAGTGAAATTAAATCTTTAGCAGAAGAAATT
AAGAAATCCGTTGAAGATATCAATAGAACAATCAATGGAATTAACAAAAAAGTTGAAACAACAA
TTGATTTAGGCCTTGCAGGAAGAGATGAAGTTGATAGGGGAGTTGTTGCAATTGATGAAGTTAA
CAGCGCATTTATGAAAATAAAAGAAAGCGTTGAAACATCCGCAAAAATGATTGCACAAATTAAA
GATAATGCTAAAAATGCATCGAACAATACTCAAGAAGCACTTAGAAACGTGCAAGATATCGCATC
AATTTCCGAAGAGTTTGCAGCAACAGCAGAAGAGCTAACCGCATCCTCAGAAGAACAAAATAGGG
CTGTTGAAGAAATAGATGCTTCAGCCGAGAGAATGATGGAAATATCAAGAAAATTAGCAGAAGA
TGCAGATGAATTTAAGACTAAATAATTACTCTTTTTAATATTTTTAATTATTTCAAAAAAGTTTT
AAAAAAGTTTAAAAATTTAAATTATCTAAAGATAGGCTGCACTGAGCACTTCATTGGAAGTGGA
AGCGGTCTTTTTGGTGCGCCAATTGTTTCAGAACTTATTTTTCAATCAATTCTTTAACAGACAA
TGATTCTTTTTTAGGTTTTTTCAGTTCTGATTCTTCCCTAACTGTTACGGTTAATTTACCGCTTTC
CATTTCGCTGTCTCCAATTACAACAACGTAGTTTACCCAGTCAGTTCCAGCGTTTCTAATTTTTTT
ACCCATGCTTTCTTCTCTATCGTCGAAGTCAGCTCTGATTCCTGATGCTCTTAATTTATTAACAAC
TTCAAGAGCGTATTCTTCATGTTTATCCCCTACAGGAATAACTCTTGCTTGAATAGGGGTTAACC
ATGTTGGAAGTGCAGGTGGTTTATTTTCTAAGGTACTTAAGTAAGCATTTTCTAAAAGACCGCAT
AAAACTCTTTCAACACTTCCTGTTGGTGAACAGTGAAGGATGATTGGGTGAACTTTTTTGTCGCC
ATCGTGAACAACGATTCCAAATCTTTCAGCACTTTCAACGTCGATTTGAACAGTTGGGTTTTCAA
TTGGTCTTCCAAAGCTGTCAACAACTGCCATATCGACTTTTCCAACCCAGTAGTGTTTTCTTCCGG
ATAAGATTTCTAAAATTGCATCTTTTCCATATTTTTGTTTGTATTCTTTTGCCATTGCAAAGAAC
CATTCTTTATTTTCGTCAAAGAAGTCTTGTGTGAATCTAAATATTATTGCGTAAGGAGTTTTAAA

GTCATCTCCAGTTTTTAAACCCATCCAAAGCTGGTCTTCAAATGCTTCCATTGCCTGTTTCATATC
CATACATACGGTATGCATATCCGGCATTGTAAATGCTCTTAATCTTTTTAAACCAACTAATTCTC
CTCTCTGTTCGTACCTGAAGCTGTAGGTTGAGAGTTCGTATAATCTTAAAGGCAGGTGTTTTGGT
AAAAGATACATGTCTTTTTTCATCATGAACTGGCCAAAGCAAGCTGCAAATCGAAGCATTAAATC
TTTTCCGCCCTGTGTAAATCTGTACTGTCTTTCTCCAAATTTATCCGCGTGTTCCCTTATCGCGTT
ATTTTGAAGGTCGTACATTACAGGTGTTTCAACAGGCATTCCTCCACGTTCTACAACGAGGTTAT
AAACGTAGTCTGAGAGTAAATCTCTTATTAATTTTCCTTTTGGATACCATCTAAAATGTCCTGCA
TCTGAACTTGGTTCATAATCGCATATTTCCTTTTCTTTAATGTATTTTACGTGAGGAGGTTCTCC
TCTTTCGGTTGCTTCTTCTTTAATTTCGAGTTCGTGTTTTAAAAGAGCTAAAAGTCTTTTATCGT
CCATTTTGGAGATAATTTCATCGTTAACTTCTGTAAGATTTTGACTTTCTCCATCGATTATGTAG
AATTTTGAAACAACTTTTTCTTTTTTAACTTCTTCTTTTCTGTCAGTTGTTATTTTTCTTGAGAG
TTCGCTTAACGGGTGTCCTTTACAGCTTATTTTAAATGATTTGTACCATCCAAATGGTGCTCTCA
ATACTTCGTAGTTATTTGAAAGTTCGCTTTCAATTTCTGCTAAAATTTCTTTTGCAGTAGCCGGA
GACCCTAAATCACTTGATAAATGTGCATAAGGGTAAACTACAACGTTTTTCACTTTTAAATTATC
TGCAGTTTTAATTATTTCTTCAACAGCGTTTTTAACGACAGCGTTTGGATCAGATTCATCATCTT
TTTCAACAGCGATAAAAACGGTTAAACATTCGTCCATTTTTCCGTTTAAAACATCAGTTTTTTCT
GCAACCTTGGTTTTTTCCTTGGCTTCAAATTCCAAATAATCGGAATGTATTAAAAGTGTTTTCAT
TTTTTCCCTCTATTTAGTAAGGATCTAGTTGTACTGGTAATTTACATAAAATTTATATGTTTAAT
TGGGATACACAATATAAAAAATTAATTACTGTGTTTTACGGTGTTTATTTGTATTGATCTTTTTT
TTAAAAACTAATTATTTTACGTTTTATTAACTGGTGGCCATCATGGATAATTCTAAAGACTTAAT
ATGTATTGGATTTGAGGGAACTGCTGAAAAATCAGGTGTTGGAATAATTACTTCAAAGGGTGAA
GTTTTGTTTAACAAAACAATAATTTATACTCCGCCTGTACAGGGAATCCACCCAAGAGAAGCCGC
AGATCACCACGCGGAAACTTTTGTAAAACTTTTAAAAGAAGCATTAAACGAAGTTCCGCTTGAAA
AAATTGATCTTGTTTCATTTTCACTAGGTCCTGGACTTGGTCCAAGTTTGAGGGTTACTGCAACA
ACTGCAAGAGCACTTTCTCTTTCAATAAATAAACCTATAATCGGGGTAAATCACTGTATTGGTCA
CGTGGAAATTGGAAAATTAACAACTGATGCAGTGGACCCGCTTACATTATATGTAAGTGGTGGAA
ATACTCAAGTTTTGGCATACACTGGAAAAAAATACCGGGTAATTGGTGAAACTCTCGATATTGCG
ATAGGAAACTGTCTCGACCAGTTTGCAAGGCACTGTAATTTACCGCATCCGGGTGGGGTATACGT
TGAGAAATTTGCAAAAGATGGAAATAAGTTTATAAAACTTCCGTACACCGTTAAAGGAATGGAT
CTTTCTCTTTCAGGGTTATTAACGTCTGCAATGAAAAAATACGATTCAAACGAAAGAATTGAAGA
TGTATGCTACTCATTGCAGGAAACTTCATTTTCAATGCTCACAGAAATCACCGAAAGAGCGCTTG
CACACACAAATAAAGCCGAAGTAATGCTTGTTGGCGGAGTTGCTGCAAATAATCGGTTAAAAGAA
ATGCTTAAGGTAATGTGTGAAGAGCAGAATGTTGATTTTTACGTTCCAGAAAAACAGTTTTGTGG
CGATAACGGTGCAATGATTGCATGGCTTGGAATATTGCAGTATTTAAATGGAAAAAGAATGGAT
TTGAATGATACAAAACCGATTTCAAACTATCGAAGTGACATGGTTGAAGTTAACTGGATTCATGA
CGAAAGCAAAAACTTGAATGATGGAAATATTAAATCTCGAAAAATTCCAAAACATTTAATCGGA
AAAGGTGCTGAAGCCGATATATCAAAAGGAAGGTATTTAGAATTTGAATCTATTACAAAAGAGC
GAGTTAAAAAAGGATACCGTATTTTAAAACTGGATGAATTGATAAGGACAAGAAGAACAGTAAA
AGAAGCAAGATTTTTGGCAGCAGTTAAAGAATGTGGAATTCATGCTCCAAGCATTTATGACATTG
ATAAAGAGAATAAAAAAATTACAATGAGCTACATTCACGGAAAAATTGCAAAAGACGAAATTGA
AGAAGGAAATATCGAATTTTGTAAAAGTTTGGGGGAAACTATTGGAAAAATGCATTGTAATGGG
ATTGTGCACAATGATTTAACAACTTCAAACTTTATAATTTCAAAAAATCCGTATATAATAGATTT
TGGGCTTGGAAAATACAGCGATTTAATTGAAGACAAAGCAATTGATTTGATAGTCTTAAAAAAA

TCGATAATGAGCATCCACTATGATAAATTTGATTTGGTCTGGAATAAAATAATCGAAGGATACA
AAACGTATGAACTGGCTGAATCGGTTTTGGAATGCATAAAAGAGGTTGAAAAACGGGCAAGATA
TCTTTAATTTTTTTATTTTATGTCGGGGACTAAACTTAATTATATATAATTTAATGTCTAAATAT
ATTATCATGATACTTTGGAGGTAGAGTATCATGAAAAGACTAAATCATTCAAGATGCCGGATTA
ACGACTGTGCTTACGTGGTTGCAATAAGTCTGGGTCTGAATATTCTAGGATCCCAGGCTGTTACA
TACCTTCAACTGCCTGCATTTTTAGATAGTACTGGAACTATTTTGGCAGCTGTGTTATTGGGACC
CTTATTCGGCAGTTTAGTTGGTCTTTTAACAAATTTAATCGAAGGATTTTTTATCTATTCTGGAC
TGTACTATTTTGCAATAGTCAATATTTTGATCGGGCTTGTTACCGGAGTTATTTTTAAAAAATAC
CCGTTCAATGTGAAATATGTAGTTATAACAACAATAATTCTTGCGTTAATTGGAACGATTGTCGG
AAATATAATTGCATACTATTTGTTTGGTGGAATTACGGGTTCACCAATCGATGACGTAACCAAGAT
ATCTTGTAAGTAGTGGAATGAGCGTTTACAATGCAGTATTCGTTAGCGGATTTATCATAAATCTA
TTTGATAAAATAATTTCATTTGTTTTAGTATTCGTAATAATTGGATTTTTCCAAAAATACGTTGA
TAAATGCTATTTAAATATCAAATTTGATGATAAAACCAACTAATTCTTCCATTTACCTAAATTTT
TAGCTTTTTTAGCTTTTTTAACGTTATTTATTAAATCGTAATTTTACATTTTTAGAATATTTTGA
TTTTGAATCTTTTTTTATTTTTTAGATATTCATATATAGCTTGAAATTTAATGTAAAATAGATTT
TTAGCATACGTAAATTTTCTATTTTTTAAGATGTATTTACAGTAAGGTGAATATTGTGCTAGTTA
TTCCTGCAGTTGATATGAAAAATAAAAAATGTGTACAGCTTATCCAGGGAAATCCTGATAAGAA
ACACGTTGAATTAGATAATCCCCCTGAAATTGCAAAAAAATGGGTAAATGAAGGTGCAGAAATG
CTTCACCTTGTGGACCTTGATGGGGCACTTGACGGAAAACGTGTAAACGATGAATTTATCGAAGA
AATAATTAAAACATCCGGAGTTCCTGTTCAAATTGGTGGCGGAATTCGATCAATCGAAGATGCAG
AATACCTCGTTGAAAAAGGGGCTAAAAAAGTAATTATTGGAACTATTGCTGTAGAAAATCCAGA
AATAATAAAAGAACTTTCTAAACGTATCGACAGTGAAAAAATAATGGTTTCACTTGATGCAAAA
GACGGCAAAGTTGTAATAAAAGGCTGGAAAGAAAAAACAAAATACACGCCTGTTCAAATTGGAA
AAATACTCGAAGAAATGGGCGCTGGAAGTATTTTATTTACAAATGTAGATAGTGAAGGTCTTTT
AAAGGGAATAAATATCGAACCAACAAAAGAACTTGTCGATAACTTGAAAATACCCATTGTAGCA
TCAGGCGGAGTTACAACAATCGATGATCTTTTAAAATTAAAAGAAATCGGGGTATACGGGGTAG
TAGTGGGTTCTGCAATTTATAAAAATTTGATAAATTTAAAAGAAGCAATTGACGCAGTAAATAA
AAGTAATTAATAATGATTTTGGTGGTTTGATGGATAAAATTGGTAAAATAACAGCGGTAGGCCA
TATTGCACTTGATTATATATTCAATGTGGATAAATTTCCAGAATTAAACACTTCAATGCAGATTC
CAACTGCTAAAAAATACTATGGCGGTGCTGCATGCAATGTTGCAGCAGAAATTGCAAATTTAGGG
TTAAACTCAGAAATACTTTCATGTGTTGGAACTGACTTTAAAGCTTCAGGATATGGGGAATACTT
AGAAAAATTGGGCGTTTCAACCAAAAGCGTGTTTGTTTCAGAGGAAGAAGAAACTCCAAAGGCA
TGGATCTTTACTGATCCTCAAAACAACCAGATAACGTACTTTTTATGGGGTGCTGCAAAACACTA
TCCTGAAATCGAAGTTCCAGAATTTGATTCAGAAATTGTACACCTTGCAACTGGAGACCCCAATT
ACAATTTAAAATGTGCACAAAAAGCAAGTTCAAAAGGAATTCTCGTATCATTTGATCCGGGACAG
GATTTAACTTTATATTCTAAAGAAAACATGGAAAACATCATCGAAAACGTTGATTTTTTATTCAT
GAACAACCACGAATTCCAGAGAACCCTTGATTTATTGAATATTTCTGAAAAAGAGCTGATTAGTC
GAGTTAAAGTTTTAATTGTAACTTACGGAAAACAGGGAAGCATCATATATTCAGAAGATGAAGC
AATCAAAGTTCCTGCGATATTAACCCAAGCAAAAGATCCAACTGGTGCGGGAGACAGCTACAGGG
CTGGATTTTTAACTGCGTATTTAAAAGGGCACGATTTAAAGAACTGTGGACTTGCAGGTTCATGT
GTAGCTTCTTTTGTTGTGGAACAGGTAGGATGTCAAACCAACCTTCCATCATGGGACATGGTTAT
TGAACGACTTAACGAAAATAACCTTCTATAATTTTTTCTTTTTTTAGTTTTTATTGCCTATTTTT
CCAAAAGCTATTTTTTTAATGGCGATTTATTCAAATCTAAATGACAATTTATATATAATTTAAAT

TTTAAAAATTAACGTTTGTATTACAAAAAATGGTGGTGATCGTATGGCAAGAGAACAGTGGGCC
TCTAAAATGGGTTTCATTTTAGCGGCAGTTGGTTCTGCAGTTGGTCTTGGAAATATATGGCGTTT
TCCATATATGGCATATGAAAATGGTGGTGGAGCGTTTTTAATTCCTTATTTTGTTGCTTTATTGT
TTGTTGGTATAATGGTAATGGTTTTAGAGCTTGCTTTGGGGCATTCAACAAAAGGTTCAGCACCT
TTGGCTTTAAGAAGGCTTGGAAAGAACTTTGAATGGATAGGCTGGCTTGCAATAGCTACTGCATT
TATAATTACGACGTACTATACTGCAATTATTGCATGGGCATTAGTATACCTTGTTAAATTATTTA
TGGTCGGATTCCCAACTGATTTCGGCGGATTTTTCTTTAGTGATATTTTAGCACTTTCTTCAGGT
CACGGGGACCTTGGAGGATTTAACTTGCCAATATTGGTAGGATTGGCTCTGATATGGGGCGTAAA
TTACATTGTTGTAAATTCAGGAGTTAGAAAAGGTCTTGAAAAAGCAAATGAAATTTTAATGTCT
GTATTATTTTTCCTGATTCTCGCACTTGTTGTAAGGAGCATTACCTTACCTGGCGGTCTTATGGG
TATTGAATACTATTTAACGCCTAATTTTGCGGTGTTACTAACACCTAAAGTTTGGATTGATGCAT
TTTCCCAGATATTCTTTACATTGAGTCTTGGATTTGGTATCATGGTAGCATATTCAAGCTACTTA
CCTAAAAAATCAGACTTAACTGCAAGTGCATTTACAATTTCATTAATGAACTGTGGATTTTCATT
CTTGGCAGGATTTGCGGTATTTGGAACACTCGGATACATGGCAATGACACAAGGTGTGCCTATTT
CAGAAGTTGTAACACAAAGTATCGGTTTAGCATTCGTTGCATTCCCTCAAGCACTTTCATTAATG
CCTGGGGGAATTATACTTGCTGCAATATTCTTTATCGCACTATTTGTTGCAGGAATTTCCTCATC
AGTTTCACTTGTAGAATCAACTGCATCAGCGGTAATTGATAAGTTTAATCTTCCAAGACACAAAG
CAGCTTCAGCAGTTATTGCAACTAGCTTCTTTGCAAGTTTAATTTATGCAACAAACGCAGGTCTT
TACTGGCTGGATTCAGTAGACCACTACATCAACTGGTTTACAATTCCGCTTGTTGCAGTACTTGA
AATAATCGTTTCAATATGGATATTTAAAGGAAGCAAACTCGAAAAATACATCGACAACCTTTCAG
AATACAATTTAGGATCTTTCTGGAAATTCTTTGCAGGAATATTCTCTCCACTGTTCTTGATATAC
ATGATATTGAATGGAGCTTGGACAGAATTAACTTTGGGCTACGAAGGCTACGCACCAATATTTTT
ATTATTGAGCCTTGGAATTCCAGTATTTGGTCTTGTGGTTTCATTAATTATGCCAATGATTCCAT
GGATTGATAAAGGAAGAGAAATCGAAGAATGGGACGAGTTTGTAAAAAAGGATGAAGAATAAGG
AGGTAATAAGATGAGTACCGGTGCCATAATAATGTTCTTATTTGGAGCAATCGTTTTATGGGGAG
GTTCAGCATATTTCCTCCATAAAACGCTAAAAAATAATAAGGAATAATACAGGAACCAAAATTTT
TACTTTTTTTTACTTTTTTATTTTTAAAATAAATTAAAAAAATTAGGGTTTATAATCCTTTAAAA
AAAGATAATTAACAATGCAAATTTTCAATAGTGCTTGTTTTTTATATGTTTTATAATATATGAG
TTAGTAATTTTGATTAATCATTATCCTGTTATTGGCACGGTGGTTTAATGTTATTTAGGAAGCTT
TCAACGATAGAAAGGGTATATAATATTGGAATGGAACACGATTCAATTAATTATGTATTTAATT
ACATAACTAACTTTATCAAAGAATCAGAACGAGACCTTGTAAAACTTTACGATTTAAAAGTGCTT
GATAAAATTGTGATAAATGACATAATGACTAAAGATTTAGTCGTTCTTGATGAAGAAGAATCAA
TCGATGAACTTGGGAAACTTATAAAACAATACCGGCACATGGGCTACCCTGTATTTAATTCAAAC
AAAGAACTTGTTGGAATTGTTACATTTGATGATTTAAGGACAAAAAAATCATCAATGTCTAGGT
TAAAAAAATTAAAAATAAAAGACATAATGACAAAAAAAGATGAAATTGTTTCAATTTCGCCCTA
TTCCTCAGCTTCAGAAGCTCAAAAAATAATGGTCGAGTACGATATTGGAAGAGTATTGGTTGTCG
ATGATGGAAAACTTAAAGGAATCGTCACCAAGGGAGATATTGTAAGAACCTCTGAAATTTACAA
CCCTGAGCCTAAAATAAACAAATGCAGCCACATTACAAACATCTACTTTTACGGGGACAATCCTA
AAAAAGTTGAAGAACTTGCAGAAGAGATAATTGTGTTAATGGGTGCAAGAGGCTACATTATCAG
TGAAAAAGAAGACTTCATCGAAATTTCAGACAGGGAATGGGAGCCAATTGCAAAGATAATGAAA
AGGAATGGTAAAATTGACCACATTTCAATAGACACGAAACTTATAAACATTCTCGAATTAAATAT
TATAAAAGAAGTATTGAAATTAATTGAAAAACCTAAATTCGAAGAAGTAATAATAACTGATCAC
ATTACCCTCGTAGATGAAAAATCAGCAATTCAAAGGATAATTACCGATGTAACCATGTTTGAAAA

GAGCATGAAGACTTTTGGAACTATTACGGTCAGGATTGACTTCTAACCTTTAAGGTGGAAAAGAT
GTACCAGATTATTAGATATGAAGGGGGAGTCTATAAAAATAATATTTTGAAGGAATGGATTGAA
GACGTTGGTGGATTTATAATTCAAGAGCACGTAATGCAGCTTGATGTCTACATGACAATTGCAAT
TCCCCAAAATGAAATTGAAAACTTTAAGGAAGAAGCAAAAAAATACAAAGGAAAAATAGTAGAA
ACCCCACTTGCAGGAATTGAGATTGCAATCGTATCTCCAAGCCTTTCAAGACACCACCTGCCCCAC
ATTGCATGCGATGTTTCAGAATATGTAAGAAAATTCGGTGCAAAACCAAACATGATTGGACTTGC
ACACGGTGCTGGAAAAAGCATCAGTGAAATTCGAGAAAAGGAAAAAAGGCTGATTCAGGAACAC
GACATCGCAATTTATGTTATGGGAAACTTTGAATCCTGCATTTTAGATAAAACCCACCTTTTTAA
AGTCGATATTCCGCTCGTTGTAACTGGTGGCCCTGAAACTCTCGATATACCATATACTTATGTTG
GAAACCTTGGAAGGCGAGCGCAGAGACTTAGAAAAGGGGAAGAAATAAGGGCATTACGGCAGAT
GATTGATGAAGTCACTAAAAAAATAAACGATAAAAGAATGGAACTCTCATACGACCCTCCAATTA
TTCCACCTGTAGTTTTAAAAGATGAAATCGAAAAAAGAATAGATGAAGTTCGTGGAATTCTTGC
ACCGATGCCAATCGTGACACAGTTGGATGGTTTGAGAATTAAAATGGATTATGACAGAAACCACA
AAGAAATCGAAAATGTTAAAATCGGTAAGTATCTTTTAAAAGATATTGCATATGTTACAAGGTC
TGAGATGAAAAACTACATTCTGATCAAGTTAAAATCAACATCAGAACTTAAAACGGATGAAAAT
AAAGCTTAATTTAAAATCATCAAGAAAATAGGGAAAAATATGGAAATTGACAGAACTATTGAAA
ACGAAACTGAAATCGAAAACGAAGAGTCAGAACAGATAATCGAAGTTCCACTACCTCCTGGACTT
CCGCAATCCGTTATTGGAAGGCTTACATGCGTCTGCGATATTGGATACGAAATAAAAAAAGACGA
AATGATGGACAAAGAGTACCCGATAATTAAGGGTACACAAGAGCAGATAGACTACGTAAAAGAC
TACATCTTTTTGTTTACTGAATTAAAACTTGCTTTGAGGGAAATTTCAAGGCTTGCAAGACGTTT
CAAAACAGACGTTAAACTATTCACCGAAGACGATGAATTGCAGTACGTACTCGGATTTGCAGTAC
AGGATGTATCTGGAAGAGACAGATTTGAAGTATTAATGGAAAAGCCTGAAGGAGAAGGCGAAAA
AATAGTAATTCTTGAAAGAGAATTTTACGTATACCTTTAAACTGTTTTAAAATTTTTTTATTCT
CAAACATTTAAAACAAACTTTTTTAAGCGGTCTTTTCTTTTTTAAATTTTAATAACCATTCTCAT
CTTAAATTTTAAATTAATCTTTTTAAATTTAGTTGAAAAAAGGAAGAAAGGTTTATTTTTAATA
GTTATTTTGTGAGTTCATTTTTTTAGTCTTCAATTTTACCCATATAAAAAGGAATTTCTAAATCT
ACATTTGAGGGCATTTTTTTACTTTCCTCAATGCAAACTGCACAAGAAAGATATATTAAAGCATT
TCGAACTAGTTTTAACATTTTTAAAGTTTTTAAAGTAAATTCTTTCAAAGAAATATCTTTTGCAA
TATTTTCAGGTTCATTGAATAACTTATCTGATTTAGGATCGTAATCAAACATATGGATTCTAGTA
TGTTTATGTTCTAAAAAATTTCTTAGATCATCTAAGTTTTGAGCATCGGGTTCTAAACATTCAAT
ATATTCATCATTATTTCTTAAATTATTGGCATATATATCTCTACTTAACCAATATAATCCCAAAA
GCGCCTTATTATCAGTTTTTCTAATAAACTCTTTGAATTTTCTATGATTATTTTTGTTATACCAC
ACAGTTTTAAAGTATATTGTTTTGGGATTAACATTTAATTCTGCAAATTCTTCTGCAAAATAAAG
ATATAAAAAAATAGGCAATTTTGTCTAAAATCGAATAAACATTTTTATATGCAATTTTTATTTTTT
CGATATTCATTGAATACATGCTATAATCCATAGTATCGAGTAAATTTCGTTCTTTATCTGAAAAG
TGATTTTTTTCAAAAGTTGTACCTTCATAATACAGATATCGGGCAGTAATAAATTCTTGCTTTAA
TTGGTTATATATGCCCCAAAAATAAGGTTTAGAATATTCTATTCCATTTATTTCTTTAAATCGCA
TGTTGGGTAGTGAAAGGACATCGCTACAAGCTATTGGTTTTTTAAAAAGGTCATTTAGTGGATTT
ATAAACAAACAATTTTCTAAACACCACTTTCGATATTTAATTTCTTTTTTAGAGCCCCCAAAATA
TTTTTTGATATTTAAATATTTCAAATTTTTATTTAACTTTTCAGAAGGAATTTCTGATTCGATAT
CCTTCTTATACTTTTCAAATACTGGTTTCGCATGTTTTTCAGTGGATTCGTTAGTTAAACTATCT
TTCAAATATTGGTAACTTTCTTTTAAAAACAAAAATTGATGTCCGTTATCATAAAGTATAGATGC
ATAATAGTATAATGTCATTCCTTTGGCACCTAGAGCCATGCCAAAAGAATTATTTTCTTTCAAAA

GTCTTTCATAATAGTCTAATGATTCTACATACCTTCCCAGATCACACATCAAATTTCCTAAATTT
GTCAATGCCCTATTTTTTAATTCTTCATCGGGATGATTAAGATTATCGTGAATTAAAAGTGCTTT
TCGAAGGTAAATTATCTCTTTTTCATTGTATTCTTTGAAATCCCAAGATGTACTATCTTTTTCAT
TTTTTAAGTCATTTATAATAGTTAATCTCTTTAAAGTTTCATATGTGGACCCCAAAATATAATAA
ATATACATTGATTGGTAATCTTCAAGCTTTTTTTCAAGAAATATTTCATTTTCTTTAATAACCTT
ATCAATACCCTCAATTTTCCTTAAATCATTGGATAAATCGGATAACCTTGCAATATTTGGTAAAA
ATTCATTAAATTCAAGATCCGAATAATCCAAATTAAAAAGTTCATTATATTTTTTTAGATTATTC
TCAAAATCCTCGTTAACCATAGTTACCCCTCGATAAAAATTCAATTAAAATAATTATTCTTCGAT
AAAATATTAATCTTCCTTTTTTAGTTTTAACATTAAATCTACGATAAAATTATAAAATTATAAAT
ACGATTTTTAATAAATGGATATCCATTTGAATTTTAACAATTGAGGGGAAATTATGTTCAATAA
ACTTAAATCAGCAGTTTCATCAACATTCTCAAAACTTGCAGATGCATTAAAACCAAAATCAAGAT
CTGAAAAAATTGATGAAATTAGGGAAGAAATTAAAAAAGATCCAACGTCATTTGAAAAGGGAAC
CGCTTTTGAATTATGTTGTTTGGATATTTTTCCGGAAAAAGACTTTGATTTACTTGAGATGACAC
ATAATCCGGATTCTGCAAATGGTAGATTTGTTAAAAGCGATTTAAACCCAGATTTACAGTTTAAA
GATAAAAAAACAGGTAATAATTTTAGTGTTGAATGCAAATACAGAAGCAGTTTATTCAAAGGGG
CTTTTAGCTGGGCTAAAAATAAAGCTCAAGCTGATAGATACAGAGAATATGAAAAAGAAAATGA
AATTCCGGTATTTATTGCAATGGGTTTAGGCGGAACCCCTGAAAAACCAGAACAAATTTTTTTAA
TGCCATTAAAAGAAATAAAATATAATTCACTTTTCCCAAGCGTTTTAAAAGACTACGAAATAAA
AGAAGCTAAAGATGTATTTAAAATTTTAAAATAACTCGGTTTTTTCTTTTCTAAATTTTAAATA
ATATATCGACCCATAACATTTAACAAACCAGATACGGTGTTAAAATGGGACTCGAAGGAATATTT
GTTATCGCTTTAACGATAATTGTTTTACTCGGACTTTTGAAATTTATCACGAACAAATAATGTCT
TTTAAATTCTAATTTTTATAAACATAGATTATTTACATGACTTTTTTTAAAGATTTAATCAAGAA
CGCTTGAAGGGCAGGTGGAAAAATGCAAGAAGTTCACAGGTGGCACAACGACCTTACCGGCAAAA
AACTCGTAGAAAATTTAAAGAAAAACCTTTTTGATGCGTATTATTTTGAAGATGAAAGCGAAGT
TTCTAAATATATTATGAATTACGTTGGAACTGGAACTAAAATTGGTTTTGGAGGTTCAGTTACGG
TACAATCATTAAATATTGCAGAGCAGGCAAAAGAAAAAGGTGCGGTAATTTTGGATCACAATGA
TGCAAGCCTTACGCAGGAAGAAAAAATGGATATCATGAGACAGCAGTTACGTCTGATATATTTAT
TTCAAGTACAAATGCAATTACGGCTTCAGGGGAACTTGTAAATATCGATGGAGTTGGAAATAGG
GTTTCTGCGATTGTGTTTGGTCCGAAAAAAGTAATAATCGTTGTCGGATTAAATAAATTATGCAA
AGATGTTGAAACTGCATTTGAAAGAATCAAAATGCAAGCTGCTCCAAAAAACATGAAAAGATTG
GGATTTTTAAACCCCTGCATAAAAACGGGATACTGTGTAAATTGCGATGCGGAAACAAGAGCTTG
CAGAATCTATTCAGTAATTAAAAGAAGACCAATGCTTACAGATATGACTGTGATTGTTGTAGGA
AAGAGCCTTGGATTTTAAATTTGGTGAAAGCATGAATCAAGTTTTAGAAACAATAAAAAACAGG
AGAAGTGTTAGAAAGTTTAAAAAAGAGCAGATAACCGACAGTGAACTTGAAGCAATACTCGAAG
CTGCAATTTATGCGCCAACTGCACTAAATGAACAGCCCTGGCAATTTACAGTAATTCAGAACAAG
GAATTACTCGATGAAATAAATACTGTATCTAAAAAAACCCTTGCAGAAAATCCAGATTCAATGAA
AAATCTGCCTGAAAGTCTGATAAATGCAATGAGTAATCCGAAATATAATATACTTTACGAAGCTC
CAACTTTGATAGTTGTTTCTGGAAATAAAAACGCACATGCAGCGATTGTTGATTGTTCTGCGGCA
ATTCAGAACATGCTTTTGGCAGCAGAAAGCATGAATATAGGTTCAGTCTGGCTTGGACTTGTAAG
GCCGTACTTTACAACTGAAAATGTTAAAAAATTAAATATTCCAGAAAACTTTGAACCATTCTACG
GGGTTGCATTCGGTTATAAATTGATAGAAAAATCAAAGCCGGCGCCTGAACGAAAAAAAGACGT
TGTAAATTATATACAATAACTTTCTTTTTTATTTTGATTTTTTGAAAATTTAATTTAGAAAAATA
TTTTAAAAAATTTAAAAAAAGTTTATTCTAAAATTCCCATTTTTGCAAGGAGTGCACTTAACTGT

ATCCTTTCGTTTGAACCTTCAACGATTCTAAAGTCGCATTCTCCAATTGCTTCGACTAAATGTACT
TTTTCTTTTTCAGAAATATCTAAATTTGGAACTTCCCTAAATACCTGAATTAATATATCTTCGCC
ACTCATTCCCCAGTCGATCATTAAATTGTAGAGCTGTTCTCTTGCTTCAACGAATTTTCCATTTA
ATGCAAGTTGAGTCATTTTTTTGATTTCATCAGGTCTTGCTTTTGAAGCAACTTTATAAACTATT
TCTTCGGTTATTTCATCAGAAACTGCAGCTGCGGTTTGTAAAACGTTAATTGCTTTTCTCATGTC
TCCTTCTGAAACATATATTATTGCATCGATTCCTCCCTTTTCAAGGTTTAAATTTTCTTTTTCAG
AAATATCTTTTAAATTTTCAACTAAATCTTCGGTTTTTAACGGTGAAAATCTAAAGATTGCACAT
CTTGACTGAATTGGGGGAATAATTTTACTTGGGTAGTTACAGCTTAAAATAAACCTGCAGATATC
GGAATATTTTTCCATGGTTCTTCTAAGCGCGTTTTGAGCGTCTGAGGTTAAAGCATCACTTTCAT
CTAAGAATATTACTTTAAATGGTGCATCCCCAATAGGTTTTGTTCTTGCAAAGTCTTTTACTTTG
GTTCTGATTACATCAATTCCTCTTTCATCAGAGCTGTTTAATTCGAGAAAATTTTCTCTCCAAGT
TTCGCCGTATAAATCTTTTGCAAGAGCGAGTGCTGCAGTAGTTTTTCCAACTCCTGGGGATCCAC
TAAAGAGCAAGTGGGGCATTGACTTTTTTTCAACGTAATTTGTAAGTCTTTTGATAATTTCATGA
TGCCCAACAACTTCGGAAAGTGTTTCTGGTCTGTATTTTTCAACCCATGGTTTTTGCATTATTTT
CCCTCGTAAAAATGGTAACAATAATTTCAATTAAAATATTTCCATGATTTTATTAATATTTGACT
ATTTAAAAAAGAAAATTTTTAGAATTTCATAAAACTCAAAATTTCTCCAAATCTTGATTTTACAA
TATTTTTCTTACCCAAAACCTTTGCATGGGCATCGAGTTCGATTATTCCATAATTGTAGACTTCT
TTTATTGGATGATAAAGCCTTGGGCCGTCACTTATACCAACGGTTATTACCTTTGGATTAATTTC
TTTTAAATTTTCAATTAATAATGCATGAGGAACTCCTGACACAACAACAATATCTGGATTAATTT
CTTTTAGGCAATCCAATGCTTTTTCACCAGTTATCGGGTATTCGTCAAGTCCGCCCGTGATGTAG
TCTATTTTAAATCCGCATTTACTAAATTCTTCTAAAATATTTTTAGCGTCAGCCCTTACTTTATC
AAAACCGATATTTTCATCGAGATTTGCAATATTTATGCAGTTAAAATGTTTATTAAGTTCTAAAA
TCGGGTCTGAAAATAAATACGCAGTTTCTTTTTTCGCATTCAAAACACAGGCAATTTTTACATCT
TTGCCCTGTTTTATTTTTGCATCGGTTTCTTTTAAAATATCTATAACCTTTTCAAGGTCGTCTTCA
TATGTTGGTTTTAAATACCGATTTTTAGCCATTCCTCGCTTTTTTTCAACGTCAGTTGCACAAAC
AAGCATTTTTTTCTGCCTTTCGTATTCGGAATAGTCAATTAATCCTGCATTTAGCGCGCCATCCA
TTGCCCTTATTGCACCAACCGTGTTATCAACAAGTCCGCTGTGGATGTCTACTGGAATTATTGTA
TCATCAAAGTCATCGATGAGGCTTTCAAGGTCTTCTCCAATTATCATGCTTGCACAGGTTCCAAC
GATCCCAATTTTGTATTTTTCGCTTTTTGGAGTTTCTTTTTTTAAATATTCCAAAACTTCCGCTA
TTACATCTCTTAATTTATCCATTGCACCAAAAATAAAATCATTTTCACCCATTGCACTGGTAAAA
ACTCGTATTCCATCAATTTCAAGAAGTCTTGCGGTTCTAAAACAGCATCCACTTGGCCCGTGGAG
AATAATTGCATCAACGCCGATATCCCTTAACTGGTACATTGCAGCCGCAATTGGGGAAGGTCTTG
GATGTAAAATCATGATATCACTTTAAAGTGTGGTTTTTATTACTAATTTTGATATTCTAGGATTT
ATTCTCAAATTTTAAAAAATTTTGGGGTCAAAATATTTGATGAGTTATAAATAAAAAGATATTA
ATTCGTAATATAAATTTGCACTACAAATAATATATAGAATATGAGGATACAACTATAAATATAA
ATAATAGCTCTTTTAAAATAGAGAGAAATGAAAACCCAAAATATCTCAAAAATAGCAGGTTGTA
TTTAGAGGAGGATATTTTTGACCAAGTTAAGGAGAATGGTTCTGGATGTTTTAAAAACACACGA
GCCGAAATTAACCGATTTAGCAGTTAAACTATGTTCTGTAGATGGTATTGATGGAGTAAACGTAA
CAGTTTACGAAATAGACAAATCTACCGAAAATATTAAAATCACAATTGAAGGATTCAATCTTGAC
TACGATGCAATAAAAGCAATTATTGAATCCATGAACGGAGTAATTCACAGTATTGATGAGGTTGC
AGCAGGTAAAAAGATCATCGACGAAGTAAAAACTCCACAAGATAGATACTAAGTTCTTTTTTAAT
TTTTTTATTTTATTACCAGTTAATTTAATTTCAGGGGATTTAATGCTTGAAGGAATTTTTGATAT
AAATCATATTTTTGATGAAGAAGGGATAAAAACGTTAAAACGATTTGGATGGGACGGTAGCGTT

GCAGTTCAAAATCATAATGAATATTCTGAAGAAAAAATCAATGCCGCAGTTGAATACGGGGAAA
ATTGCGAATTTAAAGTGTATTCTGGAGTAAAAATTTCAACAAAAAATCAAAATGAAATGGAAAA
AGCGGTTAAAAAATATAGAAACAAAGTTGATATTTTACTTGTTGAAGGAGGGGATGTTAAAATA
AACAGGAGAGTTCTTGAAATGAACGATGTTGATATTTTATCAACTCCTGAATTAAATAGGATGG
ATAATGGGCTTGACCACATCCTTGCAAGACTCGGCAGTACAAACCGAGTTGCGGTTGAATTAAAT
TTTGGAAATCTTTTAAAAAGCAAAAATTATGATGGATCAAAAATTCTTTGGGCATTTCAAAGGA
ACCTAAAGCTTTCTAAAAAATACGACACTCCGGTTGTAATTTCAAGTGGTGCAAACGATATTTAC
GGAATAAAAGCACCTGGGGATTTAAGGGGATTTTTAAATACAATCGCTGATTCCTTATATTCTAA
AAAAATAATTGAGACAACGTCAAAAATCATTGATTACAGACTTTATTTGAAAAATAAAAATGTT
TTAATGCCTGGACTTGAAATTGTCGAAGAATAATCCTTTCTTTTTTAATACTTTTTCTGATTTTT
AAAAATTTAAAAAATAGTATTGTTATTTTAAAAATTCAGTTAAATTTATTTTATCTCCTATTTTT
GGAGTAAATACACTATTTTCATTGAATCCTTGTGAAACGGTCCACTCTTTAAATATTTTTACAAC
TTCTTCTTCACCGTGCTGGATTACGAGTACTTCGGGGTTTGCTTTCTTTACAATTTCCCTTAATTC
GTCCCTTTCACCATGTGCAGAAAATTGGTATGATGCAATTTCTAATTTTGGTTCAATATCAAATT
CTCCAATTTCAATTTTTCCGGTTTCAAGCAACTTTCTTCCAGCAGTTTCTTCAACCTGATATCCCG
TAAATATTAATGAATTTTTTGGATCTTCCATAAATTCGTGGATATATGGAATTATGGGCCCTCCT
TCAAGCATTCCTGCTGTACTTACGATTATTCCGCCATTTTGTTTAATATCTTTTATTATTTTTGGC
CTATCTTTTGATTCAACTTCGATTACATTTAAAAATGCAGCTTTCAAATCGTCAGGGTGATTCAA
AAATTTAGGATATTGTAACATTATCCTTGTAATTTTTCGCCCCATGCCATCAAAGTATACTGGAA
CTCCAAAATCATGTTTATTTAATATCATTAAGATTTCCTGACTCCTATCAACTGCAAATACAGGG
ACTAGTGGAATTCCTCCTCTTTCAAGAGTTTCTTTTATTTTATTTATGAAATTTTTTTCCGCATC
TTCCCTATTTTCCTGATTTTTATCCCCATAGGTTGATTCAACAATTAAACAGGCGATATCTTCTT
TTGTGTATGACAGGTCCGCTCCTTTTACAAGGTCTGTATCGCTTACTTTGGTGTCTCCAGTATAA
ACTACTCGTTTTCCGCCGTAATTTAGTGATATTGTTGAACTTCCAGGAATGTGTCCTGCATTAAA
AAATTCAAATTCGAAATCCTTAAAATTTTTTGGATGATTGTACCTAGCTTTTCTAAACAGATTTA
GTGATTTATTAACGTCTGCTTTATCATAAGGAATATCCTGCCCTTCTGCACCAGCAATTTTAATA
GAATCTCTTAATAATTCTTTTGATATTGCTTTTGTAACCTCTGTAGCATAAATTGCAGGAACCAT
ACCTTTTCTGATAAGTAAGGGGATTCCGCCAGTGTGGTCGAGGTGTGCGTGCGACGCAAAGACAC
AATCTGCGTTTAAATTATCTAAAATCGGGTATTCTGATTCTTCAGGGGATAATTTTACCCCGCAG
TCAAATAAAACTGTACTTTTTTCGGTTTTGAGTTCTACGCAACTTCTTCCAACTTCAGAAGCACC
GCCTCTAAAATAAAGTTCCATTCTACCGCCTCCCAAAATGAATACTGTCAATTATGTTTTATAAG
GTATATAAAGGGGTAAAAACAAAATTAGAATAGAAATGTATTAAAATTATCGTTGTAGATAATT
CAAACTTAAAAATTATGTGTTGTTTTAAATTTAAAATTAATAACTATCAAATGGGTGATTTAAT
GATTGATTCCCACGTACACGCCGATACAAGACCTTACGAAGATTTTGAATTGATGGCAACTGTTT
TTGACTGTGTTATAACGCATGCACACGATCCTTTAAAAATGAGGTCGTTTGATGTCGCACTTGAC
TATTTTGACAAAATATTGATCAATGAAATTGGAAAAGCACGAAAAAACGGTTTAAATATGAAAG
CATGTATTGGAATTCACCCAAGAGCAATTCCTCCAAAAATAGATTACGATGTTTTGAAAGAATAT
TTGAAAAAAGAAAATGTCGTTGGTGTTGGGGAAATCGGGCTTGAAAAATGCAGCAAAGAGGAAA
TTGAAGTTTTTGAAAAACAAATAACCATTGCAAGAGAAATGAACCTTCCAGCAGTAGTTCACACT
CCAAGAACGAACAAAGAAGCAGTTACAAAAAATATTATCGAAGTCCTCGATACTATGGATTTACC
AATCGATTTTAAGAAAAAAATAGTAATAGAACACGTTACAAAAGAAACTGTGCCAATTATCTGG
GAAAATGACTATAAACTTGGAATTACTGTTCAGCCTCAAAAATTAACGCCAAACGATGCTGTTGA
AATACTTGGGGAGTATTGTGAAAAAAGATTTTTATTAAATAGTGACAGTTCTTCGGCACCATCAG

ATATATTTGGAGTTCCAAAAACTGTTTTAAAATTGAAAATGGCAGGATTTGATGAAAAGGTAAT
TACTGCAGTTTCACACAACAATGCAGCAGATTTATTTAAAATTAACTAATTAAACGAGGGGAAAG
ATATGGATGAAAAAAAACTTGTGATATACTACTCACTTTTTGAAAATACTGAATATGTAGCTAA
TCTTATTTCAGAAAATACTGGAGCAGATTTATTAAAAATTGAAACTGTAACAGAAATTCCAAAA
AAAGGATTAACAATGTTTTTAGAACTTGGAAGATTTTTAATGTTTAGAAAAATGCCTGAAATAA
AAGAGATATCTGTAAATTTGGACGAATACGACACTATATTTATTGGAACTCCGGTATGGGCGGGA
AATATGGCCGCGCCTTTAAAGACCTTTTTTTCAAAATACAAATTCGATGGGAAAAAGTTGCGGT
ATTTTGTACTGCGGGAAAAACTATTGGACACACGCTTGAAAACATGAAAAAAGAACTCATTGACA
ATGAACTCATTGGTGGAACCGTATTTTTAGATGTTTTAAACAACAAGGCAGAAACTGAAAATTAC
GTAAAAGAATGGTTAACTACAATGTACAGGTCAAAAGAAGATTAAATCTTAAAAATTAAAAATA
AATTATTTTAATATTTTTTTTAGGTTTTTGAAGCATAGTAAATCATGTATATTGCAGATAGTCCA
ACAAGAATATATAGAATTCTTGCAACTGACGGGAAACTTCCAAATACTACCTGAATAAGGTCTAT
ATTAAAAGCTCCGACAAGTCCCCAGTTAAGTCCCCCAATTATTACCAAAACAATAGATACCCAAT
CTAACGCATCCTTTCTTGAGTGGGTTCGTGAGACAATACTGGGTCTTTTTTCAATATGATGTTCA
CCTTCCATAATACCCCCTCGATTTATGCATGGCTTTTTCGGCCTCAATATATTCTATTTTCAAAAG
GGTTAATATACGTTCACATGATTTTACCGTTTTTAGACCGTTAATTTACAAAAAAACTGTTTTAA
ATCGATTTTTTAAATTACATAAATTTATATATTCTAATGTTGTAGGAATCATGATACTTCGATTA
TATTGTGGGGGGAGTGTCGGGCCTTTAAAACAGCAATGAAGGTGAGTTTATTCCAAAAAAAGGG
TTTTTTTTGAATAAAATGTCTCATACAATAATAAAAGACGGAATAGACTGTGTTATAAGCAAAG
ATTATTTTTACAAAACTGAAAGTTACTACAAACTGATATCAAAAGAGATATCTGGAAAAAATAA
GGTTTTTCCAAGTAGCAACGATGTACTGGATGCATATATTGTTCCAGTAGCACTTAAACGGGCAG
AACTTGCAGGAATTCCTATTTTACAGTGGGAAATATCTTCAGGTGACGTTCCAAATCCTTCGATT
GTTTATTCAGTAAATTATTTTTCAGATCCTTCAACGTATTATATCGTTAAAAATCGAGAAGAATC
AAGAAAAATATCAAAACACGTAACAAACTGCGGAAAATATCCCCTGTGCTACCAAAAAGTTCCTG
AAAATTTTGAAATGGTCAATTTTTCGATATTTTTTGGCGAAGTATGCACAGAAAATGAAAATAT
AAAACAAATAGCAAATAAAATCTATCAAACCTTTAAAATTCCGCTAATTAATGCTTTATTTGTAA
TTATGGACGATAAAATATATCTTTCATCACTATCTCCTGTAAACGTATCAAAACTTTCAAAAGAA
GAAAAAGCAATGATTGAAAAGACCTTGTTGGTGGATTAAATGGGAAAATTTGGAGTGTTTGTTG
ATAGACAAACCCTTAGCAGTTCAAATCAACTCCAATCTTTAATCAGATTCCGAGATATTGCCGAA
TCTATGGGCCACGAATTTTATTTTTTATTCCCAAACGAAATACACAAAATAAAAACATTGAATGG
TCTTTTTATACGATCCAAAACAGATCCAATGAATATAAGCTACGTAGCATCAAGAATCGCAGAAT
TAAACGGCGTAAAAGTAATAGATGATTCAAAATCCATTCAAATATGTGCCGACAAGATAAACAT
GTATTTACACATGATGAAAAATAATGTGCCATTGCCAAAAACCAAATTTTTAAGAAAATCTGAA
AATAACATTGAAAATTTAAAATCCATTGTCGAAGAAATTGGACTACCTTTAATTCTAAAAGAGCC
ATCAACCTCTTTTTCGATGAGGGTTGAAAAGGTAAACTCATTAGAAGAATTTTCAAAGGTAGCTT
CCAGATTTTTGAGGCTTTCAGATGTATTGGTTGCACAAGAATTTATCGAAACCAGATTTGACTGG
AGAGTTGGAATTTTAAATGGTGAAGTAATTTATGCCTGTAAAATATCTGATTCCAAAAGAAACAT
TCAAGATACAGGACTGTGTAGATGGGCACAGGGTATACTGTATGGTTGAAAGCGTAATGTTTGAC
GAAGTTCCAAAAGAAGTTGTAAACACAGCTTTAAAAGCTGCAAATGCAGTTGGAACCGGTCTTTA
TGGTGTTGATTTAAAAGAAAAAGACGGAAGTATTTACGTAATCGAGGTAAATGATAACCCATCT
CTTGAAACAACGGAAGACGAATTTTACCCTGAAGCGTATTCTAATATTGTATCCTATATTATGGA
TGACTCAAGGCAGAAATATCCTGGCGGAAAAATGAAATTTTCAGAAGTAATCGATCCCGATGGTG
TAGCAAATGATAGGAACTGAACACGAATATTCCATCAACAATCCCGATTTAAAAGCAATGCCAGT

TTCCGATTTAATAATCGAAGAAATTTCTGGAAAAATACAGAATGAATTTTCTTTTGAGACAGTTG
GCATTTCAAAGGAATTACAAAAACACGTAATAGAATTTAAACCAATTAGTCCTTCAGAAAGTATT
TCAAATCTTGAAAATCAGATTTTTACAGGTTTAAAAAGTTTTTACGAAAGGACTAATAATAAAT
ACAAATTGATGGGGCTTGGAATGCACCCGACTTTAAATTTATCAGAAACTTCATTTTGGAACCAC
GATGAAAGGGAAGTTTTCGATACTTATGATCGAGTATTTAACTTAAGACAGCATGGATGGATGA
ATATTCAAGCTTTACAGGTAAATGTGGAATATTCAACTGAAGAAGACCTCGTTAGAAAGCACAAC
AAGCTTCGAACGCTTTTACCATATCTTGTAGCAGTAACTGCATCATCCCCTATAGTCGAAGGAAA
TTTAACTGGATATATGGATAACCGTCTTTTATATTACAAAAAAAATCAGAAAGAAATTCCAGAA
ATCTGCGGAAATTTGATACCTGAAAAACTCGAAAAATCTTCTGATTATGAAAAACTACTTGAAAA
AATGTACTTAGAACTTAGAAAAAAAGATGCAGAAGTTTTATGTGAAGAATGGGTAAATTCAAGG
GCTTTAATTGTAAGGCGTGAAAGAAAATGTGTTGAAATAAAAATAATGGATGAACAGGAATCTA
TTTATGCAGATATGGCTGTTTCAGCATTTATAAACTGTCTTTTAAGAGTCAATGATTTGAAAGTA
GCAGAAAACGACGAAACAATACTTGAATTAACCGATATTGCAATAAAATCAGGAACAAAGAAAT
TAAAAGCAGAATTAGAATCACTCTATAAAAAAGCAGTTTCTGTAGCAACAGAAGAAGAAAAATC
TTATTTAATCCATATTCAAAACAGGATTCAAAACGGAAATCTTTCAGAAGTTATTCTTAAAAAAT
ATAATTCTGAAAAAGACATTTCAAAAATATTAAATGAAGCAGAATCCTGTTTAAGAGAAAATAA
AACTATGTTTTAACTTTTATTTTCACTTTTTAATTTATCATTTAAGTTTTTTGCAGGATTTTTAA
ATGAACATGGAACATTGCATAAACATTTTGCACAAACGTCTGCAACTCCATATTTTTCATAAACT
TTTTGATTTGTTAAACATACTTTATAACATTCCTTTCGATCAAATTCGTTTATTTTTAAAGCTTC
TTTTTCACATTTTTCAATGCAAAGTTTGCAAGAACCATTATACTTATACAAACAGTATTCATAAT
CTGGTTTTTCACTAGGTTTTAATTTTGCAGATGTAATTAAACTTCCAAATCTACCGTTACAGCCT
TTTTCAGTAATTAACATATTGTTGATTCCAAATGTTCCAAGACCTGCAATTTTTGCAACGTGCCT
GTGCGACCAGTAGCTTATCAGTTTTTCTTTGTCAAAATTATGAGTTGGCGGAATTTTTGAGCAAG
TATGATTCATTTCACCTAATTTTTCAGAGAGATAGTCGTTTAAATCTTCTATCATTTTATTTGTT
TCAACGTAAGAAATTGCCCAAGTCTCAGATGCAAGTCCCGGGTGTACATTTCCAAGGTTAATTTC
CTTTTCAAACGGCAAAAAGTAGCATATTACACTTTCTGATTCACTTAAAACTTCTTTTGGAGTTA
AATGTTTTTCTGCAATTACTTGCTTTAATTCTAAAAATTCAGGATCACAAGAATCCGCAAATCCG
ACAATTGGAGTCAGCCACTTAGTTTTCGTGTCGTGTTCTTTTGAATAATTTTTTACAAATTCAAT
AATAAGATTTTCAATTAAATTTTCCATAATTTCCACCGATTTATAATCATAATTATCTGATTCTT
TAGTTTTTTATATGTCTTATTATAAAATATATTCAAAAATATCGGGAGATTTCAGATGCTAAAA
ACTAAACTTTGGGATATTGAATTTAAAAATCCAGTATTTCTTGCAGCAGGCGTCATGGGCGAGAC
TGGAAGCGCACTAAAAAGGATGGCAAAAAATGGTGCAGGGGCAGTATGTACCAAATCTGTTGGA
ATTGAAAAAAAACCTGGACACAACAATCCAACAATGGTTGAGGTTGAAGGGGGCTTTTTAAATGC
AATGGGACTTCCAAATCCGGGTGCAGATGAATATGTTGGAGAAATTGAACGAATAAAAGATGAA
ATGAAAAGAATGAATGTTAAAATTATCGGTTCAATTTACGGTAAAAACGATTCTGAATTTCAAA
AAGCTGCTGAAATTATTGGAAATTACGTGGATGTTTTGGAGTTAAACATTTCATGCCCCCATGCA
GGTGGAGGATATGGTTCATCAATCGGTCAGGACCCATATCTATGTAAAAACGTGGTTTCAGCAGT
AAAAGACGTTTCAGACATTCCTGTTATTGCAAAATTAACTCCAAATGTAACTGACATCAAAGAAA
TTGCAAATGCAGTTGTAAATGCTGGAGCTGATGGAATTGTTGCAATAAACACGCTTGGTCCGGGA
ATGGTAATTGATATCGAGTCAGGAGTTCCAATCCTTGGAAATCGGGTTGGTGGAATGAGCGGAAA
AGCAGTAAAACCAATTGCAGTTAAAAATGTTTACGATATATGTTCAGCAGTAGATGTGCCAGTAA
TTGGCGTTGGTGGAATTACTACCGGGGCTGATGCAATAGAATTTATGATGGCAGGAGCATCAGCA
GTTCAAGTTGGAACTGGAGTTTACTATCGAGGATACGACATATTCCAAAAAATAAATAACGAAA

TTGAAGAATATCTCCTGAAAAAAGATTTAAAAATGTCAGATATCATTGGAATTGCTCAGAAATA
ATTCTTAATTTTTATCCGATTTTAAGAATTATAAAAACTGTGCGTAGAATTTATATACCAAAAAC
GAGAATATATATAGTGAAACTGTTTTATTATTTTAAAATATTTCGTTATCTCAACTGTTTTAAAA
GTTTAATCTGCTACTGAGGTGGTTATTTGTACAAAATTTTAAAAATTTCTGATACCGTGAGAATT
CCCCCTAAAATGTTTGGAAACCCGTTAAGCGAAAACGTAAAAAGGGTTTTAATGGAAAAATACGA
AGGAATTCTTGATAAGGACATCGGATTTATCCTTTCAATCGTTGATGTGGATAACGTAAGCGACG
GAAGCATAATTTACGGAGACGGTGCAGCATACCACGAAACAGAATTTAATGTGCTTACATACGTT
CCAGAACTTCAGGAAGTAGTTGACGGAGAAATCGTCGATGTTGTTGAGTTCGGTGCTTTTGTAAG
ACTTGGCCCTCTTGACGGTTTAATCCACATTTCACAAATTATGGATGATTTCGTAAGCTTTGACC
CAAAAAGAGAAGCAATTATTGGAAAAGAAACAGGCAAAGTTTTAGAAAAAGGCGACAAAGTAAG
GGCTAGAATTGTTGCAGTAAGTTTAAGAGAAGAGAAGAAAAGAGGCAGTAAAATTGCTTTAACA
ATGAGACAGAACGGTCTTGGAAGACTTGAATGGCTCGAAGATGAAAACGAAACACTAAGTGAAG
AAAATTAATTTTTTCACCATTATTTCTATTTAATTTTAATTTGAGGTGAATTTAAATGAATGCAT
GTTTAAGATGCAAATACATCACAAAAGAAGATACCTGCCCGATTTGTGGATTTGATACTTCAGAT
AACGTTAGAGGTTTATTAATCGTACTCGATCCTCACAACTCAGAAGTAGCAAAAAAAGCAGGAAT
TGATATAAAAGGAAAATATGCGCTGAGCGTTAAATAACTTAAATTTGATTTTTGTTATAGTGAC
CTTTTATGTATTTATTAAACGACAAAGTAGCTCACGAATTGAAAAAACCTTTCGGAAAAGTATAT
AAAGAACTTCCCAGTATAGAAGGAAAGGTCGTTTCTATAGGTGATGTTACTACAAAACACCTTTT
ATCGAATGGTATAATACCAGACTTATCAATTTTAGACTTTAAAACTAAAAGAAATATTCCTGTTG
ACATCCCTCACAAATTTAAAACAATTTTTGAGGTTGAAAATCCTCAAGGGTGCATTTCTGATGAA
GCCATAGAAAGGATTAAATACCTTTCCACCATACATGATAGAGATATGGCTTTAATCATTAAGGG
TGAAGAAGACTTACTTACAATACCTGTAATCAAGTACTTCCCCGAGGATACCTCAGTCATTTACG
GACAGCCTGATGAAGGCATGGTACTGTTAAAAATCACAGACGAACTGAAACAGAAAATTGAAAA
ACTTCTAAAAGAAATGGAAGAAAGGTGAATGGATATGGATATAAGCATTATCTCAGACAGAAAT
AACCCATTATTACAAAGAAGGGAAATAAAATTCACAGTATCATTTGATGCAGCTACCCCTTCAAT
CAAAGATGTAAAAATGAAACTTGTTGCAGTTTTAAACGCAAACAAACAAGTTCTCGTAGTTGACA
CTTTAGATCAGATTTTTGGTAAATTGGAAGCAGAAGGTTACGCAAAAATCTACAACGATGAAAA
AGCAATGGCAACAATCGAAACAAAATCCGTTTTAGAAAAAAACAAAATTGAAGAAGAAGCAGAA
GCTGAAGTAGCGGAGGAATAATATGGCAGCTAAAAAAGGTGCAAAAACATCAACAAAAAAATCA
GATTACTACAAAGTTGAAGGAAACACTGTAGAAAGATTGAAAAAAGTATGCCCAAAATGCGGTG
CAGGCGTATTCATGGCTGAACACTTAAACAGATTCGCATGTGGAAAATGTGGATACATGGAATAC
AAAAAGAATGAAAAAACAGAAAGCGAAGAATAATTCTTTTAATTACTTAAAACTCTTTTAACGT
TTAATATTTACTTCTTTTTTAATATTACTTTTAATTTAAATCATTTTAGTGAAATTATACTTTTA
ATTAATCTAAAAGCCCGAAAACATCTGCAATTTTACCGACATTTTTTATCGGAACATATACTTTT
GTTTCAGGATATTTTTGAAATTTTCCAAGAATGCAAAGAAAATCCATTGAAAATTCCAAACTTTC
TTCAGGATATTTGTGGTTGAAATACTCCAAAATCCAGTTTGGTGGTTTTTTAAGGTCTATTGCGT
CTTCATACATTGCCCAATGGCATATTACGTTAATTAGTTCCATTGTCCCCCCCTTTCTATATTAAC
GGTACTAATTAGCATTTATTAGTTTTGATAAAATAGATACTATTTCACTATTTAGATTGTAATTT
AACCTGTATGATATAATTTTTAAAAAGCTATAATTATAATTTACTATTCGTCAAAAAAGCATAA
TTTGGATTAAACTTAAATTAATCGTTGATACGACGACTTTTAAAAAATAAAATTTTATTTATTA
TTGGTTTTTAAGGTATCATCTATGCATTCTTTTAAGGCGGATTTAATATCTTGAGTTTTAGTGTG
ATATAGTTTTACTCTGTCTTTTAATGTTTCAATTCTTTTTTCACCCATCGTGTTGTATATAATTA
CATCGGGGTTTTCTAAAAGCAATTTTTCAGCAGGGGATCCTCCACCGTTGCCGTCACAGGGTGTG

TTTTGAACCCTTAAAATGTTTACTACTTGATAATTTTCGTCAAGTTCAAAAATTAAGAATAACGC
AGTTTTTCCAAAATGTTCACAGATTTTGTTTTCAAATACGGGTACTGCTATTTTCAAGTTATCAC
TCTGATTTTTATTATCTAAAAAAATCTTTTTTCTTTTAAGTTTCTGTGTTTCGTATTTCTCATTA
TTCATATATTTAAATATATTTTTACAAGTATTTATGCAGAATTACTGATTGTGTAAAATTGAAA
TAAGTCATCATCTTTTAAAATTTAATTTATATATCATATTTATTTAAAAAATTTAAAAAAAAAG
AATAATTTAAAATTAGTTTAATTTTGTTAAATTTCCGCTTAAAACTTCTTTTAATACTGCATCTA
CATCTTCTAAAGACGTTTGATATAGTTCAACAACGCTTGCAAGTGCATTTACGCTTCTTTGACCC
ATGTTTACGAAAACTACTGCATTTACGCCTTCGGAAAGAAGTGTCTGAACTGTACTTCCAGGACC
CTGTCCTCCGTGACCTTGTTCAGTTTCGTGAGCAGCCCCGTGGCATGGGGAGTTTCCAACTTTTTT
CGAGCCAATTATTTCATTATTTTCGTTTACTTCAAAAATCATAAAAAATGGAGTTTTTCCAAAGT
GTGAACAAACTGTTTTTTCATCAAGTATTGGGATTGCTATTTTCAAATTATCGCCTTATTTTTTT
ATTAAGTTAAATAATTAAAATCTCCCTCTTCCCATTCCCATTCTGCCACCTCGGCCCGCACCAAAA
AAGTTTCTTCTTCCGCGAGCATCGATAACATCTTCTTCAGAGATTCCTTTTGATAAATTAGTTAC
TTCTTCATCAGATATATCATTACCTGTTTCTCTTTTAATGATATTTTTGATTTCTTCGTTAGTTA
ATCCCATTCTAAAGAACCTTCTAATTCCAAGTCCTACGTATTCAGTAGTACCTAATAATTTTCTG
CAGATGCAGTTTCTGTGTAGTCTATAATCCATGTGGTTCACCCCATTTCATAAAATATGTTTATA
ATAATTATTTTCGCACATATGTGCGTATTTCATTATATTGAAAAACAACTATATAATACTACCTT
TTTTAAAATTACAGGTTTTTTAGATTAATATCGGTTAGATAAGGGTTATAAAAAAAGTAATTAT
TTGAGTTTGTCGTAAAAATCATCCCAAATTCGTTTAATTTCATTACCTGCAGGGCAGTCGTAATT
TACAATAGGACATTCGTTTTGAATTGCTTTATTTACGGTATTATCAAACGGAACTTTTCCCAATA
TTTCAAAGCCTGATTTTTTACAGTAATCGATTATTTCAGAAGTTTTTTCTTCGTTAATGTCGTAT
TTATTGATTATTACATAGCTTTGAATATCAAAAAAATTGCGTAAGTTCTAAAACACGGCTCAAATC
ATCGAGTCCCGATGTTGTTGGTTCAGTTACGACTACTGCGTAATCACAACCTGTAAGCGATGCAA
TTACAACGCATCCTATTCCTGGAGATCCATCAATTAATAAATTTTCAAAACCCTGATTTTCAAGG
GAATTTTTCCTAACTTCGGTTACAACTTTTCCCGCACCTTCTGCCCCGATATTTAGCTTTGCGTAA
GATAAATATCCATCTTTGATTTTGCATGAATAAATGTGTCCTGTAACATTATCAACCATTTTTAC
AGCGTCAGCAGGACATACATATTCGCATAAACCGCAGCCTTCACATTTTAACGGATTTACTTTTA
TTTCAGGCGTTATTGCACCAAACCTACATTGTAAGCATTTTTCACATTTAATACATTTTTCAGTA
TCAATACTTGCAGTCTGGGATCCAATATACTCTTTAGTATTGATTATTTCATTTTCAAACATTAA
ATGTAAATTTGGAGCTTCAACGTCGCAGTCTGCGATATTTCTTTTATCCATCAATTCTGAAAATG
AAGATGAAATGGTAGTTTTTCCAGTTCCGCCTTTTCCACTTATTATGACGATCTGTTTCATAATA
TCAACCTCTTTTCAAGTTCCGAGGCAATATTTTGCAAGTTTTCTGTAAATTCGGCATCCGAATTT
ATCAGGATGTCTCCTTTTGAATATTTATTTGCAATATTTCTTGAAAATGGAATTTTTCCCAGAAT
ATCGATTTTTTCATTTTCGCAGTAATTTTCAATGATAAAGTCAAATTCATCAGATTTATTGATCA
AAACACCAAATGGGATATTTAGGTATCTCAAAACTTCAACCGCAATTTTTAAGTCGTGAAGTCCA
AATTTGGTTGGTTCTGTGACAAGAATTGAATAATCGCTATCTTCAACAGTGTTTATTACACTGCA
TGAACTTCCTGGAGGTGCATCGAGTATATTTATTGAATCAACATCCAAAAATTTTTTCAAGGCAG
ATATGACTGGAACTCCTGAGGGTTCACCAATATTTAAAACTCCCCGAAACGCGTTTAAATTCGGA
ATATCTTTAGATTTTCCGGATTCTATTTTTCCAATTTCCCGTAAAATTTCTGAAATGGCCTGAAC
AGGGCATGCAATGTAGCAGAGCCCACAGTCGTGGCATAATTTTTCAAAAACTGTTGAATTGTTTG
GAGTTATTGAAATAGCGTTAAATTTACATAATTTACTGCATTCACCGCAGTTTGTACATAATTCT
TTATCGATTTTTGGAACTTTTTTAAAGACTTTATTTTCAATTTCAATGTCAGGTTTTAAAAAAAT
AAATCCGTTTGGCTCTTCCACGTCAAAATCAAAATAACTAACATTTTCATGATTTTCCGAGAGAA

GAATGCTTAAATTTGTAGATATTGTGGTCTTTCCAGTTCCGCCTTTTCCACTCAATATTGAAATA
TTCATAATTTTTCCTTACATACCTTGATGTGGCTTTCCAGCTTGTGTTATTTCACTTAATTCATTT
TTTTCGAATAATTCAATGTTTTCAAGAACCGTTGATCCAGTTCCTTTGTAAACTTTAATATTTTC
GCCATCTAAAACCTGTTTTGCGTTTGGCCCCATATTTCCGCTGATTACTACATCAATATTTTTTGA
AACTACTTCTTGTGCAGAGCCAATACCTGCACCATGTGCCGAATCGCCATTTGTATTTTCAATCGC
GTCGAATTCTTTTGAATCTGTATCATAAATTATAAAATAAGGGCATCTACCAAATCTTTGTTCGA
AAGTGCTTTCTAACGTATTTCCGGTACTTGTAATACAGAGTTTCATTAAAATCTACCTCTTTTTA
TTTCTGTAAGAATGTGTGGCAGAATTCTATAAATACTTATCCCCATATGTGCGATATTAAGAAAT
TCTATTTTTACAAGTATTCTCAAAAATGCTGTCTAAAATACAGTAATAAAATATATTTGTGTTTT
AAAAATTTTAAAAAAGGAATTAGCGATATTTAAGCAAAATATAAAAGTAAAATAAATAAAACTG
CAACTGCGATTATCAATAGAGTTATTCCTAATAAGAAGTATGATCTAAAGTCATCGTTTTCATGG
ATTTGTTTATGTAATCCTGCAATTATTAATAAAAATCCAGACGTTGGGAGTATTAATAACATGGA
ATCCCAATTTTTTGTTAATTCAAGATATGCGGTTATTACTATAAACATTACTCCAAACGAAATAG
TTATTTTTTGTAGATCTCTTAACATCTGTCCATCAGATTTCATAAAAAATCACCTTCCCCCTCTAA
GGTAATATTTTTAAATCTGTTATCGGTTGTTTTTTAGTCCTAAAATACTTAAAGTTCTTACAATA
TCTGTTTTAGAAATTATTCCAAGGATATTTCCATTATCAACTACGTAAACCCTGTCAGTATTTGC
CATACCTTTAATTAATTCATTTAATGCAGCCTCTGGTGATACCATCACAGGTGGCGACATTATCT
CTTTTACTTTAGCTGTTTTTGCAGTTGTAGATGATATTTCATTCATAGTAATGGTTCCAATGATA
TTTCCATTTTCAAGCACAGGATAGCCTAAAAACCTGTTTTCAAGCATTAAATTTAGTACATCATC
GACAGTATCTTCTGAATTTACGTAAACTATATTTTTAGACATAATATCCTTTGCAGGCACATCTT
GAAATAGCGTATCTGCAACAAGTGCCCGGTATTCTTGAGATGCTCCAAAGTAGATAAATATTGCA
ATTAAAACCAAAATTACATTGAAGTTTATCAGTCCAAAAATTAACAAAGCTAGTGCAAAATACT
GCCCAACTGTTGATGCAAGTTTTGTTGCTTTTATATAACTCATGTTGGTTAATTTTGAAAGCAGT
GCCCTAAATACCCTCCCTCCATCCATTGGAAATGCTGGAAGCAGATTAAAAATCCCAAGAAGTAT
ATTCAATCCACCAACAGTTTGAAAAAGTGGATATTCTGCCAAGGTATAATCGGTTACTGTTGAAA
TTCCAAGTAATACCGTTCCAAGTGCTAAACTTACCAGTGGGCCTGCAATTGCGATTTTAAACTCC
CCTTCTTTTGAGATTTCGCTCATCATGGCCATTCCGCCAATTGGCAAAAGCAGTATTTTTTCTATT
TTTACCCCGTATTTTTTTGCAACATATGAGTGCCCTAGTTCGTGTAAAACTACGGATGTAAATAA
GAACATGTATAAAATTAGCCCTTCAATGCCCCAAAAGTAAAAAACAACGATTAACAGTAATATAA
ACGTAATATGGAGTTCAATGGGGATTCCCATTACACGCGCTATTTTAAAAGATTGCAAAAATTTC
CCCTCCGCATCTGATGTATTATATTATAGCCATTTACTTATTAATACTGTTTTGATGAGTTAATT
ATATGGGATAATTTAAAATATTTAAAAAATAGCCGATGTAGTTTATTAAAAAAATTAATCTTTT
CTTTTAGAATATTCTATAATTTTTAGGAGTATTTTTTCGGCTTCTTTTAAATTGTCGTTTGAAAT
ATTCATGTCAAGTTCACAGAAAAGTGTTTCTATATACTCAGTATATCTTTCTTTTTCAATTAAAA
GCTCTTTATAGTCGTAAATCATCTTTCTAAATTTTTCCTGTTTTATTTTTGCCATATCGTCTTTG
ATATTGTCCCAAATGGTTGATTTTTTCAGGTCGTATTCTTCGGCAACTTTTCGAATGCTTTTTCC
ATTGATAATTTCTTCAATGATTTTTTTCTTTATATCTCCTTCAACTTTTTTGGGCCTCCCAACTTG
ATCAGTAATTGATACATCGACCCCAAAAATTAAAAGACGATTTAATGCGCCCTTATTTTTCTTTT
CCATGTATTCGTATATCGATTTTGGCATTGTAACTTCGTCAACACCTTCTTTTATTAACTTTTCCA
CATCTTCTTTTGATAAATATTTTGAAGTATGGTATGTTTTGTATTTTTCAGACATTTGTTATCAT
CTTTAAATTAATCCGTTTAAATTTTGTTTTATCGGTAAAAGAGTAGTTTTTTTTCTTATTAACCA
GTAACTAGTATTATTTAAAAAAGTATTTAAAAATGCAGTATTAATTTGACCATTTCTGTTTTAAT
TCTTCAATGTATCCTTCTGTTTCTAGTTCGAGTAATACTCTGTCCATTTCATGCAATAATTCTTT

ATTGTTTTTATTTACTGCAATTCCCGAGTAAAGAACTTCCAGGGTATCAATTAGTTCTAACTGGT
CCATGTAGCTTGAAACTTTAAAATTATCCATTTCAGATATGGTTGCATCAATTTTTCCATTATTT
AAATCGTTATACAGTTCTATTGGGGTTTCATATTCAATTATTTCAAAATCCATACTTTTTTGAAG
ACTTTTTGCCTTAGTTTCGTGTATTGTATCATCAACAACGCCAATTTTTTTTCCCATTTAAATCA
GTGATTGTTTGAATGCCACTTTCTTTTCTAACCAGTACTACCAGCTCAGTATGGGAATATGCCCT
TGTAAAACTCACTATTTTTTCTCTTTCAGGAGATATTGAAATGTCCGATATTGCACAGTCAATTT
CATTATTTGCTACAGCATTTAAAAGAGTGGAAAACATGTATTCTTTAAATTCAACATTTAAATTC
ATTCTTTTTGCAATTTCTGAAACTAATTCTACTTCAAATCCTATTAATTCCCCATTTTGCTCATA
AACCGCAGGTGGTGCATGTTTGAATACCCCTACAGATAATGTTCCTGTTTTAATTGTTATTTGTT
CAGACGGAAGTGAAATACATGCCGAAATTGCTAAAATCAGTACGATTGCCATCAAAAAACATATC
TTTTTCATAGTTTTACTCACAACCTCTGTTTGAATTTATACTAAGTTTAAACTTTATGGTAAAAA
TACTTATTTATTCCATAAAATTGTCCGAAAAACATTTAAAATCTGGTTAAAGGTCGTACCAGTCC
CCAATTTTCACAAGTTCTTTTTTAATGTCGTCAACAGTAATTTTCAGATAGTGCATTGTGGTGTT
TATTGATTTATGCCGTAAATATTTACTCACCTTTTCAAGCGGAACTCCGTCATTTAACAGGTGTA
GGGCTCTGCTTCTTCTAAATGTGTGCGTTGAAAATTCTTTATGAATTTTAGCATCTTTAGTATAC
TTTTTAACGAGTTCCCATGCGTATGTCCGGTTTTTAAACTTAAAAAGTCGGTCATCATCTTTTAA
ATTGTTATCAACGATATGTCCGAAAATATCTTCATTTAACCACTCTGGAATAACGCAGATTTGGA
AGTTTTCAGTTTTAGTGTCCAGTAATTTACAAATGCCTTCGTTTAAATTTATGTTTTTTACTTCA
ATTGTTAAGAGTTCTGAGACCCTGAGTGCCATTCCATAGAGCATTTTAAAGAACAGTTTGTGTTC
TTTTGAGTCTACCGTATCGATAAATAGTTTTAACTCGTCTTTTGTAAGCCAGTCTTTGCAATAGT
GCCGTGTCATATTACCAACATAAGTTTAAGTTCTTAAATTTTCTTAATTGTTCTTTTAATATTGT
TTTCATTCTTTATATATTTATTTAGCCTGTTTTAACCGTTTTCGTTTATATGTATTATTTTATAT
ATTTAAATATTTTTACATATTGCTTTATTTACTTATTTATCTAAATATTAAAATATTTATTTATA
TAACTAAATAGATTCTATAAATGGGGTATTTTGTCCGGATATTTTAAACTAACAATTTACTTAAA
TTGTTAGTTGTTAAAAAATTACATTAAAAATCAAAAAAGAGCTTTTTAAAGCGGCAATCTAGGA
ATTATACGGTAAATTTAACGAAAAAAAGTTTTATCCTAATTTTAATTTTTAAAAAATAGATATT
ACTTTTATAAAATTTTTAAAAAATAGATATTACTTTTATAAAATTTTTAAAAAAGATGATATAC
GCCATATTTAGGTTAAAAAACAGTTTAAAATTGGATTTTGATGTATAAATTATAAAGTTCAATA
TTGTATTATTTTTTTATGTATGTTTAATAAAATATTATTACAAATTAGTAGAATTTTTCATAAA
GGCGATATTTTCCTAGCATAACCTATATATTGCCCTATAGAGAGAATATAGTTTGGAGTAGTTAT
CTAGTTAAACGTTATGTAACAACATTACAAAATGAGTAACTTGAAAATTTTGGGGTTAATCTAA
AGGAATCGGATAAACACGGTGTTATAGTATTTTTGGGGGATAAATATATTGAATTATATAATTT
TTGGTTAGCCACATTTCGGGGGGCTTGTGGCTGATCATTTATCTATTATTTTTGAGACTTTTTTT
GACATTAAATAAATTGTTTTAATTTCTATTTTTATTAGTTATAGTTCATTTCAAGGCATAACTTG
ATATATGAGTATACAGTATTACTACTAGTTGGCGATGGATTGAATGTAAATCCTTTTGATCGGAA
AGACTAATAGTCAAAGCCCAAATTTCTCCATGTCGTATGATTTACGAGGGTTTGACTACAACCTC
GTGAATCATAATTTGGGAGTTATTTTACTCTTTTTAATTAATTATGATTTTTTCAAAAGATTATT
CAGAATTTATATTCGCATTCATTTTTGACGTAAAATTCAAAAAAGAGGATATACTATGGAAAGT
TTTAAAAATTTAGGTCTATCTGACGAGATATTAGAAGCATTAGAAAAGAAAGGCTTCACAAACCC
AACCCCTATTCAGGAACAAGCAATTCCTATTTTAATTGAAGGAAAAAGAGATATTGTCGGCCAAG
CTCAAACAGGTACTGGAAAAACCGCAGCATTTGGTATTCCTATTTTAGAAACAATTGATGAACAC
TCAAGGAACACGCAAGCACTTATTTTAGCACCTACAAGAGAACTCGCAATACAAGTTGCAGAAGA
AATCGATTCAATCAAAGGTTCAAAAAGATTGAATGTTTTCCCAGTATATGGTGGTCAATCAATTG

ACAGACAGATAAGAGAATTAAGAAGAGGAGTTCAGATTGTTGTTGGAACTCCTGGAAGAATACT
TGATCACATCTCAAGAAGAACAATAAAACTTGAAAACGTTTCATATGTAGTTTTAGATGAAGCTG
ATGAAATGTTAAACATGGGATTCATTGACGATGTTGAAGAAATCTTAAAATCAGTAAACACTGA
CAAAAGAATGCTTTTATTCTCAGCAACACTTCCTGACAGCATCATGAAACTTGCTAAAAATTACA
TGAGAGAATACGACATCATCAAAGTTAAAAGACAGCAGCTTACAACTACTTTAACTGATCAATCA
TTCTATGAAATTCACTCAAGAGATAAATTTGAATTACTTTCAAGAATCATTGACACGGAAAAAGA
ATTTTACGGTTTAATCTTCTGTAAAACAAAAGCAGACGTTGACGAAGTTGCAAACAGATTGAATG
AGAAAGGGTATGCTGCAGAAGGTCTTCACGGGGACATGACCCAAGCTCAAAGAGAAAAAACACTC
GACAAATTTAAAGGAAAGAAAATTAACGTTCTCGTAGCAACTGATGTTGCAGCAAGAGGAATTG
ATATCAACGATTTAACACACGTTGTAAACTTTGATATTCCACAAAATCCTGAATCATACGTCCAC
AGAATTGGAAGAACAGGCCGAGCTGGAAAACAAGGTTATGCAATTACATTTGTTGAACCTTCAGA
ATTTAGGAAGTTTAAATACATCCAAAAAATTGCAAAAACTGAAATTAAAAGAGAAGAAGTTCCT
GATGTTAAAGATATAATCAGCGCTAAAAAAATAAAAATCATTTCCGGAATAAAAGAAGTTTTAG
AATCTGGAAAATACGCTGACTGCGAAAAAATGGCTTCTGATTTATTAGAAGATGCAGATCCTCAA
GAAGTTCTTTCAGCAGTTTTAAAATATTCTTTAAAAGATGAATTAAGTGAATCAAACTACAAAA
AAATTGGAAGAGGTTCACAAAGATCTGAAAGATCTGGAAGAAATGACAGCAGAGGAAGAAGATC
TTTTGCACCTGGTGAAAACGTAAGACTCTTTGTAGCTCTTGGTAAACTTGATAAAATGAACCCTA
AAAAATTAGTGGATCACATTTCAAGAAAATCCGATGTTAAAGGAAGAGACATCGACGATGTAAA
AGTATTTGAGAAATTTTCCTTTGTAACGGTATCTTCAAGCGATGCAGAAATAATTCTTGATTCAT
TCAAGAATGAAAGAAGAGGAAGAAGATCAATAATCGAAGTTGCAAGCGGCAACTAATTAATCTC
CTTTTTTACTTAAAATTATTTTAAATTCTTTTTTTAAATTTTTAACAGAATCTTTTAGAAATTTT
ATATTTTTCGACTGGAATTATTTAAAAATATAAATTTTAATATTTCTTAATCATTATCACAGTCG
TCACAATCCTGGTGATGATTATGACAGCATTCAGTGTCGTAATTTGAAGAATCCGCCCTATTTGA
ATTACCTTCTTTAATAGTTTGAATCTCTTTTTTAATCTTTCGAATAAATAGCGTTGTAATTGTCG
CTATCACTAAACAAGAGCCTGCTAAAATTGGTAGTAACGATACCATAATTTTACCCCCTATCTAT
TTTCATCATAATAAAATTAATATTTATGTCGGGTCTATTTTAATTTAATTATATTTCTCGAAAAA
GCGGTTATGGTGGTTTCTAAAATTTCGAAATTAAAATATACTTTTTATGACATATCTTTATTAAA
TCGCACGGAGGCGGTATTTTTGGATGCTTACGATTTTGATATCAACCTTCAAAAAGTATTTTATA
GTTATTTTAAGGATATTAAAAGCTCCAATTGGAAAAAATGGTTAGACATCTCTTCAGTCGAAGAA
TACGAAGATGCAATTTTTAAAAATTCTGGGCTTTCAAATGATATTATAACGGATACATATTCAAA
AAAAACTGATCCAAAAGAGTTTTCATTAAATCCTAAAACTTTTTTAAAAGAAAATCTCGAAAAA
GATGATATAAACAATTTATTTTCAAAAGACACTGCAATCGTAACCCATACATCTGGAACTTCTGG
AGGGGACATTACAAAATTAAAGTGGTTTTTAATTTCAGATGATTTTTTAAAGCGAGTCTGGGTTC
CAGGAATGCAGGCAATATTTGAATCAAGCGGATTATCAAAAAACAAATCAGCCGTGATTTTTGTT
CCAAGTAGAATTAAAGTTGATGGTTTATCAAAAGTGGATGATTTAAACCTTATTCGCCTTTACAC
GTCGGAATTTTCTCAACGAGTTGTTTTGTCACACATCCGCCCTTCTTCGTATTTAATCGATGAGT
ACAAAAATGCGCTTAATTTACTTACAATTTCAAAAATACTGACTCTTGACGATATTTCTGTAATT
TCTGCACCTGCAGCAACGATTATTAAATGGGCAAATATTGATAAATTAAGAACGGACTTGAAAA
ATCAATAAAATCAATTGATTGGGAAACAATAAAAGAAGAAAACAGAGAAGCTTTCGAATTAAAA
AAATTGTATGATCGAATTGGACTTAAAAATTTTGCAGAAGAAATCCAAAACAGGCTTTCCGAAGC
ACTTAAAGATTCTACGCTGATATTTAGCATAACGTCTCTTTCAAAAGATGAATGGGCGAAGATCA
GGAAGTTTATGGGCTGGAAAGTTGGCGAAGAAAAGTTTACAAACCTCTACGTTGGAAGCGAAATC
GGCCCATTTGCAGGGGGAATAAATAAAGGAGATAATTACAGAATGTATACATTTCCATTAACAT

ATCCTGTAATTGAAAGAAAGGGTAAAAAGGAATTTATCTCTAAAACTAAAGATAAATATGGAGT
TTTGCTTGTTTCAAGGCCTGGTTCAAATCCTTTAATAAATATCGATACAGGGGACGTAATAACTA
TCTTAAATCAGGAAGGGATTCCAAAAATTTCTGGTGAAATTTTAAGATCTGGATTTAAATTAAA
ATTAGAAATGAAACTCGAGGGTTTTCCGGAAAATACGGAATTTTTTGCAGGAGATTATTTTGAT
ATTGGTGGAATTGAGGTATTCAACCCTCGAAAGATGGTTTCGTGTATGGGGCAGAGATTTGGATT
AGAAACGGATAACCCTGTACTTTTAGTTTCAAGTGACTCTTACAAAATGGTTTTACCGCTTGAAA
ATAATAAAAACGTGCTTATTGATAAAATAAAAGAAGAAATGCCAAATTGTTCGATAGGGGAAGA
TATAGAAAAAGCTTTAAAAAAATGATAAAATTTCGCTTGAAATGGGAAATACAAATGTTAAAACT
GCAATTGACCATACTGAACTTTTAGAAAAAGTTAAAATGGGCGATCTTCCAAAAGGGGTTCTTAA
AAAATGGCCGTTTTATATATTAAATGTTCCAGAAAATTCAGGAATTTAAAAAAATCATTTTTTT
AAATTTTGGACATGGAAAAGTATTTATAGAATAATCACCCTTATTAAGTTGTTCGTGCGGAGATG
GCCCAGTCTGGTACGGCGCGGGACTGCTAATCCCGTTACTCTCTGAGTAACCCGAGTTCAAATCTC
GGTCTCCGCGTTTAACTTTTTTCTGAAAATATTAACTGATTTTGATTACATTTTACGCGTATTTT
TTCGGTTTAACGACAGAATTAAATCCCGCACTATAAGTTAGTCCCCTGTCTTTACAAAATACGGG
ATTAAATTTTTAAAAAATCAAAGACTTTTTTCTAAAGATTGACATTTTTGAATACTGGTTTTTAA
AACTCGTGAGGTTTTCAAACATTTGAGATCCTAACGAAAAAAAGTTAAAGTTTATTTATTCATGT
TAAATATTTTTCCAACATCTTCTCTAAAATCGCCCGATTTATATATTAATTTTCCTTCAGATGCA
GGATTATCATAACTTAGATCTGTTCTACATACAACTTTCATAAATCTGGGCCATGTTGGAAAAGT
TGCATTATAGAAATCTTCCCCCCTAAATGATTGGAAATATTTGAAGTCCTTATCAACATTTATGA
ATTTTTTGAATTTATCTGGGGAAAATACGTTCTGTAACTCATTTAAAACTAATCTTATATCTTGT
TTTGAGGGTTGGGCACTTTTTAACTTTAAATCTTCATAAGCAAGATTAATTTTTTCATTTCGATC
TCTCGATTCAATTAGTCCAACTGCAACTTTCAGCTGATTTTGGAAATAAACGGGTATTTTAGTTT
TTATATCATTATACACGGCCAGATCTACGAAAAAATCTAAGTCCTTAAGTTCTATATTTTTATAC
TCTGTTTTAAAAATATCTAAATTTTTAAGCCCTGCCTGCATCCAATAACCAATATCTGCATTGGA
TGGTTCTCTAGAAACACTTCTCCTAACTATTTTATCCAAAATTCCCTTTGTTTCCAAATCATATTC
TACTTCAAGCCAGTATAATGTATCTTCAGATAATTCCTTATAATTAATTTCAAACCCGCCATCTA
AACGCCTGATTGCTTTATGGGTTATATCTTCAATTGGTGCACTTATCGTCATTAATGTAACTTTT
CTTGCATTTCCTTTTAATATTTTAAATCCCGAAATTAAGATATTTTTTTTGATTTTAAAGAATAT
AGTATGTTTTTGGGCATTTTCAGGGCATAATATACTCAGTTTTTGACCTTCCATTATAAAACATT
CTTTAAAATCTTTGGATAATGATTGGGCCATCCCAACAATTGATTTAACGACTGGTTCAACAACT
AATTTAGTGGATGCCCCTAAAATAATGCTTTCAGCATTCATTCAACCACCCCATTTTCAAAAGTA
AATAACTGATAAAGCGGAATCTTGACTCGATCACTATTTGAAATAATGTTCAAAGGTATAGATA
ATTCAGTTGCAGGGTAATCATTATTTGCAAAATCAGATACTGAAACTTTTAATTCTAAGTTTTCA
AGCTCCTTAGATATATCTAAGATTGCAGTTGCTCCTATCAAAAGTATTTTTTTATCGGTGGTATT
TTTTAAAAACTCTAAAAATCCACTTAATTCAGTTAGTATATTCATACTCATCATTTGGGCGGAAT
AATGAGCTTCTGTAAAATCATAAGCAAATTTTTCATCTTCTTCAAATATTTTTTTAAATTTATCA
ATTAAGTTTGCAACTTGGTCTAAAAAATCTTTGTTCAACTCCATGGTTCCGTCGTTTTCAGTCAT
GGATTTTTTAAATTTATAATACTCTAAAAGAACCTCTTTATGGGGTTCGTAGGTATTCATTAAAG
TTTCGAGTCGTTTATACAATATTTCGTTATAATTTTTTATATATTCATCAATATTTTCAGGAAGT
GCTTCTTTAATATATTCTGATTCTCTAAGTGCTAAGCCTAACACCATAGTTCCTTTTCCTGAATT
TTTTATGGATATGTGGTCAGAAATATTTGTATGTGGCCCACTTTTATTGATTGTTATTGAGTTAG
GTATTTGTTTAAATGTAATTTCCGGTCTAAAAAGATATATTTTTTTAGTTTTAACGAGTTCATTG
CCATCTATATCCATTTTAAAATATATTTCTTTAATTTCTTCCTTTGTATTCAATTTTTCAGATTT

AAAAACAAAACCAACGTATCCATTATCCTCAAAACCGTAAATATCCAATGAATTTTCAGCATAAA
CAAATCCGTTTTCTTTTACGTTGTACATTTCAGAAACAACAATACCTTCAGGAATAATGACTTTA
ATTCTTGCAGGACTATATCTATCCCATGTTACATGACCCGGAAATTCAGCCCCTTGTAAACAGTC
TTTTGGGATATACAATGATTTTAAAAATCTATTTTCATCCAAAATGCCCCCTCCAAAAGTGTATA
GGTAATATCTAATATTTTATTATTTAAAATAATTACGAAATCATTCAACAATTCCATTACTTTAA
ATATTTTTAAACAATTTGTTGTACCGGTTCCCGTATTGAAATATTATTTTAATCAAAACCTCGTA
ATAAAATTTGTAACCAAAGTGTAACCAAGATACCCAAAATACGTTAAATACCGAATGAAAAAAG
GCACTATTACGACAGCAAAATTTTAAAACACAAATCACAAACACGCTATATCCATACTACGTTAA
ACAAAACACCAATATACTATACGTATTTTTCATAATTTTTTTACAAATATTTTTCTTTTACTCAC
TCTTACTAAAAATTTTGGTTACAAAGTTACACCAAAAAAACAAGGAAAAATATTAAGTTAAAAG
TAGATTAACCGCCTAAATGTGCCGGAAAATGTAACCGTTACATCAGGTTACAAAGTTACAAATTT
TTGACTCCAAAATAATACTAAGTATTACAAATTTGTAAAATACATACAAAATATGAAAAATAGA
AATGATTTCTAAAAAAGAAATGTAACTTTGTAACTTTTTAGTAATAGTTACAAAGTAACATGGT
TTATAGGGTTTATTTTGTAATCAAAGGATAATTAGCAATGTAACTTTGTAACCATGAAAATTTG
ATTTTAACTTAAACAAATATTTGAAAAAATAAAAAAAAGATCAGAGAGAAAAATCCCCTGGAAA
AGTATTGAAAAATTGGTTACAAAATTAAAAAATATTTCAAAAAAGTTATTCCAGTTTTTCCTGG
ATTGGATTTTCTATGAATTTATACATGTATTCTTTTGGTTTCACAACTATGTTTCCAACCATATG
TTCAAGTAACTCAATAGATTTTTCTGCAGGAATTCCATGAGTTAATTCTAATCTTAATGTAATAT
CTGATTCATGTGCTGCTTCTTTTTCTTCAAGACATTCTTTCAATGTATCAACTATTAATTCACCA
ATTCTCTGATCATCAGGATGTTCAGTAAATTCTTCAGTTATTGGAATCCAAACAGCTTTTTGTGG
TTTGTGTGATCCAGTGATTCTTGTAGCTTTTACTTCGAATCCATGAGCATTAAACTCAGCAAGTG
TTTTAATTCCATTTTCAGCAAGTTCAACAAAGTTAAGCCCTTTTGCAGTTAATAATATATTGTAA
TTATCAATCTTGTCCTTTTTAATGTAAATTCCATATTTTTCAAGCTGTTTTACTCCTAAAAAATA
TTCAGCTCCTTCGGGTTCTGCAATGTAATCTAAAATGTTATGTTTTACACCTGCTTCAACCCATTC
TGATAATTTTAAAACTTCTTTGATTATTGTTTTCTTGATCATATCTTTTGACGTGTAAATATTGT
TATTTCCGTAAACTACCTTTTTAGATGGTTCATATTCAAGTTCAAACTCTTTAAAAATAAATTTT
GCAATCTCTCTTCCAATCATTATTACATTATCGTTTGCATTTTCCCTTGATGGATCAAACTTTAA
AGTGTCGATATGTTCTTTTATTTTTTCAATATTTCTGTAAATATAAGCTCCTAATTTTGGCATTT
CATCTAATAATTCATTTCCAATCTCTTCATTATTGATTAATTCATAATCATATGCACTTAACACA
ATAAATCTGTCAACCAATGCAGGATCAAATTCTTTAAAATGATTTGTAGACATTATAAACGGCCT
TCTTAAAAGAAACGTGTAATCGTCTTTTTCCATGTCTTTATTTCCCCTTGTAATCACGGCTTCTTT
CCCAGTTCCCATCTGTTTTAACATATCAACGAGTCTTTCTGGAAATTCTTTTAATTCATCAAACC
ACATTGGAAGATTAACATACTGTTCTTTTGCTATTTTTGAAATGCTACCTTTTAAATCATCATTT
GAATACGTGTTTGTAGTATTCAGAAGTAATCTGTTGAATACGATGTTCATTCTCGCAGTTTTTCC
AATTCCCTGTTTTCCAATGATTAAAAGATATGAAAATTTGTCAAGTTTTTTAGTGTTTAAGAGAT
CATACCTGAAGAGTGATGCAAGTGAATATTCAAAAATTATTTTTGTAAAATCTTCTTTTCCAAGA
TAGTTCTTTAACAGCTCCAATATTGATCTAATTTCTTCTTTTTTGTATTTCATTGAAAGCATTTT
TTTAAAATCTTTTGCTCTTAAACTTTCTCTTTGTGATTCCCAGCCGTTTAATATTATTTTATCCGT
TCCGTAGTATCCAAAGGCAATTGTACGTTTAATAACCTTGTTTTTATCGTCATATCTCTTTAAAA
TGTAGTCAAATATTTCGCTGTATTCTTCCAGGTATTTTATGTAATTGAAATTTCCTGAAGGTTTG
ACTGCACCAATAAAATCATCAAAATAATTTATTTCATCTGTAAGATCCACTTCTTTATCTTTTAT
CAATAGTTTTTTCTGTGTTTCAAATTCTCTTCCATTAAAATATGTTACATTCTCAATTTTTGGCC
TGGTGTATGCTGCAATAGGTTTTAAATCTTTTACAAATCCTGCTTCATCTTCTATTTTATAATTT

ATCGCATGGAGGTTTTTATATCGCTAAATACTAAATTCCCGTCTTTACATAATTCTCTAAATAT
GGATTTTTCACTTATTTCAACACGTCCTGAAAGGCCCTTGAATTCAACTTTCACGCCAGGTATTT
TTAGGGTTCTGTAAATTTTATCATATATTGTTGTAAGATCACTTCCTTCAGGGTAAAGTTCCAGG
ATCTCATCATTATCCAGTTCATATTTTTGTGAAAATTCATCTAAATCAAAGACAAATACATTGTT
TTCAGCTTTCTTTTCAGCAGCATACTTTTCGGAAAACTGCCATACTTTTTCTAAAAGTTCAATTC
GTTTTACCCTTCTTTCTTCCAGTTCCTGTTTTGCTTGGATCACAATTTCTTTTTTCTGAATTTCGC
TTAAATTTCCCATTCCCCCACCCTATAAAATCTTTTTAATTTGTTTTAACATGTTTTCCTGTCTTT
CCTTAGAATGGGAATAGTAAAGAGTAGTTTCTAAGGATTTATGTCCCAAATATTCTTTTACAATG
TCGATTGGCATTCCTTTATCCAGGAGATCAACTGCTCGACCATGTCTTAAAGAATGGATTACTAC
TCTTTTATTTTCTGGAATTCTCCCCTGGCTTTTTAGTTCTTTTACTGCATTTCTGAAAACATCGCT
GATACGGTTTCTCTGTAATGGTTCGCCCCGGTACGTTTGAAATATATTATCTTCGGGTTCCTTTC
TGATATTTCCTTTTAAACAGTAATTAACAAGTTCTAATGTCTTATCTGAACATGTAACAATTCTT
TCTTCTTTTCCTTTGGTGTCACGCAGTCTAAATTCCCCTTTATCAAAATCAGAATCTTTATATTTT
AATTTCAAAGCTTCCGAAATCCTTGCTCCGGTATCCCATAAAAACCGGATTATCATTACATCCCT
GATTTTTGTAGTGCTTCTTGTTTCCAAGATTTCCTGGAAAATCATGTTTAATATTTCAGTGGATA
ATGAGTCGTAGTGTTTTATTTCAAAGCCTGAGAATCGTTTCCTGTCTTCACTTTCATCTTTAAAG
TCATTGAAGTTTTTAAGTCTCATAAGCTTGTAAAACACTTTTAAATGATTGTAATACCTGTTTTG
TGTATTTTTGGAAAGTTTTCTTTCTTTTTCAAGATAATTAAAGAATTTTATAAAATCTGCATTAG
TCATGTCTTCAGGTTCTTTTTTGAGCCTGTGCTCTACAAAGTTCAAAAACACTTTTAATCGATTA
ACATCCCCTTGAATTGTGCTTTTTTTAATCCCGTCGAATTCTCTTTCTTCAATAAACTTGTCAACC
CATTTTTTAGATTTTGGAGTTTCTTCGACCTTCTCTTTTCCAGGTGTAAATAGTATCATGTTTTT
TAGATTTTGCATAAAAATTCCCCGTTAGCTAATTTAAGAGTGTAGTGTTAGTTATTTGTTTAAAT
TGTAAAAAATTACAGAATTTTACTTTTAAACCACGTCCATGACCTGTTGTAATAACTTAAAGCTG
TCTGAATCCATTTTTCCCAATACTTTAGGGGCTTTTGGATCACAGATTGATAATCTATCTTTTTT
CCGGTTCGTGTTTTCCATCATTGCTAAAAAATCCCCATTTCTAAAAACTTTAACAATCAGCATTT
TAACGCCGTTTTCTTCAATTAAATTTCCTTCTTTGATTTCAACAAGTCCTTTTCTCACCATTCACC
ACCTCATAGTTTGATTCTGGATTAATTGGATAGATTAACTGTCCGCCGTCTCCATCTTCCATTAC
TTTTAAAATGGTATCACAGAGACTGCAAGTCACTTCATAAGTTCCGCCAAAGGCCCATATTTCCA
GTTCAGATCCGTCATTTTCAATCAGTTCACCACATTCTGGACAAACTATCATTCTACCACTTTCAA
GTCCGTATCTTTGAACATTATCGTGTTTTTACATCCTGGAATGTGTAATCCTATACGATATTCTT
CGCCTTCAGGATATTCTTCTGCAGGGTTCACTTCAACGACCACGTACTTTCCAGAAAGTTCTCGCC
ATATCCCACTGTAAAATATAGCTTCAGAATAATTTCTTGTAATAATTTCAACGGAATTTCCAACT
TTAATATCCTTTTTAATCCCTATTTCCTCTCCGTATTCTTCAAATTCTTCTTTTGTTACTTTTATT
AATTTTAAAGTCCCTTTAAGTTCCATTATTTCATGAAGAAATCCTAATCCGCATATTCCTGCATC
TTCGTTCCAATTCTCAACTATTTTTTTCAGACTTTTTTCAGATTTTTTTACAGTTATTGCTGGAG
AAAATCCGCAGATAATTTCACCAGTATCATCGTTAACAAGCAGATAATTTTCTTCTAATTTCATT
TTAAACCCCTCATTTCTGTATAATTTCTTCGATGGTTTTTCCAGTTCCTAATTCAAACACTTCATA
ACTACCCATTTTGAACAACGGGCATTCTTCTTTTTTCAAGTCTTCGAGACTGTTGTAAATAATCA
ATTCATCATCAAAATCCATTAATGGAATAGGAAACCCATTTGAAGGATTTTTCAAGATTACTATA
TACTGAGCCATTTTAATCCACCACGGTTATTCCGTATTTTTCAAAAATTTCTTTTAATTCGCTTT
TAAATCCATCAAAATCTTTTCCTTCAACTTTGAACTCCTGTATAACAATGCCGGATATTTCTGGA
GTCCCTTCTTCAAAATTTTCAATTTTTGTCACATCAATCATGCTTTCACTTCAGGTAATGAATAG
CCGTATATTTCAAACTCGTATTTGATGTTATTTTTATCAAAGAATTCCTTAACTTCTTTTAAAAT

TTCTTCATTAGTTGCACAGTTCCTTATTTTATCATTAATCTCTTTATGCTGTTCTAATGTTAGGG
GATCCATCAAATATGTGCTGCAATTAGTTTCATCTTCAAATTTCTCACGCAATGCAGAAAATGGT
AAACATTTAATACCCTCGTCAAATGCCATCAATTCAATCCAATCTTCTTCTTTTACAAGATCAAA
GAAATTAACAGTAAACTTTACGATCTTTGCACCACAGGTCTTAAAATCATGTTCACACGGGCAAT
CTTCGCAGTTTTCACAGTATTGGCAGTCAGGACTTACTTTTTGTTTCCAGTTTGTATCGCTCATGC
TTTCACTCCAATTACCTGTTTAAGCAGTTTTAACTCGTTGTTTCCCATATTCGAAATAACTTCAT
ATTTTTTTACATCGTAAACCGAAAATCTTTCAGGTTTTAAATCAACGTCATTGAGTGCGATAAAA
TCCCCGTTTCTGAATTTTTTAAGGATCAAATAAAGCACTCCTAAATCACTTTTTAAAAAATCTCC
TTCTTTCATGCTTTCGCCCCCATTAAGCAGTTAAGTAATCACATCGAACAGTTTTGAAAGTTCTT
TCCCTTTTTCTGTTCGTTTTCATATCAATTTTCCTTTTAAATTTCAGAAAGTCAGGACGGCTTAA
AACTTTTAAATTGCATTCTTCATCTTTTACAAGTTCAGAAAGTCTGATTAACTCTTTTTTACCGT
TTGCAACTCCGTAAGATTCGGGAATTGTTCCCCTTTCGATAATGTAATGGAATTTCATTTTCCCA
CCTCAAAATTTTAAAATTAAATTTTTTTCAACTCTTTTGCAAGATCACTGTCTGGTTCAACCACT
GTTACGATCAGGGTTTTTCCAATGTACTCTCTTGGAAGTGTGGGATACATTGTTGCAGCGTTCCC
GTGTGCTACTGCGTAACATACAAAGGAAGGCAAAATCTCCCGCTCTCTTTTCCAGCCTTTTTTAG
TTATTTGAATAGTGATCACCTTTTAAATTCGGTTTAGATTTTTGAAAATTTATGATTGTTATCAT
TTGGTTTACTACTATCATTTATGATAACAATACTACTGTATAATTATATGAGTATATAAATGTTA
TCATTTATGATACCGCTATCATTACTGATGAGTGCTATTTAAAAAGTAATCTTTAGATAATTAAA
TAGTAAAAAATAGCGCGGTATAATCATGTTTCTTAAAGTATTATCTGAGAAATACTCAAAAGAG
ATTTTATTATTGTTAAATGATAACGAAGAACTTTATTTTAACCAAATTCAAAAAGAAATTGATG
TATATAAGAGTAATTTAAGTAGAACTCTCAATAAGCTTTTAGAAGCAGGATTAATTGATAAACG
AGAAGAAATGCAATCTCCTAAAAGAAAAGTCGCCAAAAGCTATTATAAATTAACGGATTTGGGT
AGGCGTTCTTTAAAATTTTATGAATTGGAAAATGAGCTTTTAAAAATAAAAAACAATGAAGAAA
AAAATGTTAATATCCATATTGAAAATGTGGGTGAAAATAGTTTGAATAACAGTTTTAATTTTAG
TTCAAAAAAGTAAACGCCTCTGTTTATGTGGTTGTCAACAGAGACTGTGTTTGTAAATCTAATAA
GAACACGTAATTTTAATATTTATGTTTTTTGATATTTAGCTTGTTGTAACGGAAATTGTTCCTTC
TTCGTCATATTCTGTATCAAATGTGAATGTGACGTATTTAGAAGCTCCTTCATCTAAATAAATGC
TCTGTTTTTCTGAAAACGTTCTGCCTTTATCGGAATTGAAAACTGCTTTTACGTTTACATTTCCT
TCATACCCATTATTTTTTAAAAGAACTGTTACTTTATCGACTGAATTTAACCCATCAAGTGCTTT
TTTAGAAGTTACTGATACCACTTCAGGATCAGGCCCCATAACTCCAGCGCATCCGCAAGTGATTA
TTAAACATGATATTGCAATTAGTACCAATATTTTAGAAATAACCTTCATTTCATCCCCCGTTTAA
TTTAGTAATTTGAATGACAATTCTTTTATATTTCTCATATTTATAGTATAATTATACAATATCAA
TTAACGGGGAGGTTTTAAATGTTTAAAAAACTATTATTTGTTCTTTTAATTTCTGTTTCACTATT
ATTTTCAGGATGTATTGATAATTCCGCGACAACGGAATCTAATGGAGTAGCTAGTTCAACAACTT
CACAGGACTCATCAGTTGAATCAAGCATGATTATTTGGGGAACTCCTAACGATTTTGACTATTAT
AAACTACCTGTAAACCTTCATAAATCAGATCTTGAGGGTGCAGATAGGATCATTCTAAAATTAAC
TGATGGATCAAAAATTTATTATCTCGAAAATTACACTGGCGAAAAAACAGAAGTCGAAGAATTT
AACTCAGATGTTATTGACACAATTTATATTTATTTCTCTGAAACTGAAATGTGGACTATTCCAAA
CAATGATAATAAAAATATTCCAATATTACCTTTGAATGTTAATGAAAACGACATTGACGAGGTTT
ACAACTATTATCTTGAAGAATCTTATTCTTACTACAATGAAAAAAATTACAAGCAGTCTGACAAA
TATGCAAGGGCTGCTACTTATTATAAACCAAATGTTGAATCATACACATTAAGAGCAGACTGCAT
GAGGAAATTAGGCGATTATGAAGCTGAATCATATTTCCAAAATAAAGCAGATCACTCAACTTTAT
CTTTCGCAGAATTTGAAAAAAATGTACACTAAAAATATGAATATTCCTGATGATGCAAAAGATTAC

GAAGATTTAGGAGATAAATACCAAGATTCGAACCCTGAAAAAGCAATTCAGTATTATGAACAAT
CTCTTGATATATTTGACGGCAATGCATGTGTTTGGCATAAGCTCTCAAGATGTTACTATGACGTA
GGCGATGTTGAAGCATGTACAAATGCAAATGAAATGATTTACAAACTTGATCCTAACTTTTATAC
TGATTTCCCTCTTATAAGGGGATAAATTCTATTTAAATTTTTGAAAAGGTGGTAAAATGGATTTT
AATCTTAATAATATCGTTATCAGTGTAAAAGAAAATGTTATGGAAATTATTGGTCCTTACAAGTC
TGAGACAATTGCTCTAGACCATATTTCTTCAGTAGAAATTTCTAAATCTGGTTCCTATGTCATGG
GTATTGCAGTTGTTATCTTCCTCGCGGGGCTTGTTGCTGGTTTTTTCAATGTATATGTGGGTATA
ACTCTTGTTTTAGCAGGTTTAGTCTTAGGGGCTGTTGATTATTTTATGTATGTATTAATTATTTT
TCATGTAAATGGCCGTGGAAAATTCCCTTTAATGGTGCAGAAACGTAAAGCTAATAACTTATACA
ATTACGTTTCCGAACTCCTGAACACTAGGAAATAATTGGATAAAAAATAATCCTTTTTTTAAATT
TCTTATAGTAAAATTGCAGGGCCCTCTAACATTCGCCCTGGCTTTTTGTAGCCCAAAAATAAACG
GTTTATTTATGGATTAAAAAACAGAATAACGTTTTTCTTATAGTAAAATTTAAAGTAATTTAAT
CATTTGGACAAGTTCTCCATTGTTTGGGGTCTGGACTTTTTGAAGATACCTAAATCCATTTCTTT
CGTAAAATTCAATTAATTTTTCAGATTCTTTACACTCTATAATTATGAGTTTTCCGCCCACGATT
CCATTAGCTTCATATAACTGGTCTAAACAATCTTCTAAAAGCGTTTTTCCAGACATTACATCTTT
GGACGTTCTAAATTTATCATTTATGGCAAATTGTCCAATTAGGTATCCTGAAGTATTTTTCTTAT
CCTTGTCTCCAGTCATTTTTTTAATGGCTGTTTTCGAAACTTCATCACCAAAGTCTATAACTTTT
AGTGCGATTGAAAAATAGCCTAAAAATTGAAATGTATCGTTATAATTGCCGTATTTTATGTATGT
TCTGCTCCTGCTTTTCAGCTCATTAGGTATTGCAGTATCATATAAAAAATCCACAAGATCATGAT
CTTGAGAACATTCAAAAAGAGAAAATATTTTTGATTTAACTGTTTCAGGATTGTTTTTAAAGCCA
TTTAAAATAGTTACAAGTGAGGTAATAGTCGTTTGAATACTTCTTCACCTTTCTTAAGTTCTTCG
TGCATGTCTTTTTTTGGAACGTAACTTTCAGTCTGTTTATCTAATTCTTCACAGATTATTTCCGC
GGCTTCTTTACTTAATACAAATTCCCTTGAAAATGAGGACGTAGCCATAATTATCACCTGCATTT
CGTTGCAGTTACTACTTAGTAGTTTTTAGTAAGTAAATAATAATGGTTGATTTTACTATTTATAG
CTGTGGATTGGATAAATAAGCCATAACTATCTTTTTTTCAAATAATTATAAAGAGTAAATTTACT
TATTCCAGTAGCTTTTAAAATCTGTTTAACTTCGTATTCTTTTGAATCATACATTTTTAAAGCTA
ATTCAACCTGGTTTATATCTGCTTTAGGTCTTCCACCTTGGCGGCCTCGTGATCGAGCTGCTTTTA
ATCCTGATTTAACTCTTTCAGAAATTAAATCTCTTTCAAATTCTGCCAACCCTGCAAAAATAGTA
AAAAGTAGTTTTCCGTGTGCTGATGAAGTGTCAAGCCACGATTCTTTTAAACTCTTTACAGAAAC
GTTCATTTTTTCGCACTTATCCATTATTTCAACAAGATCTCTCGTACTCCTTGAAAGCCTTGTTAA
TTCACTAACAATCACCATGTCTCCAGGACGTAAAACTTCAAACATTCTATCAAATTCAGGTCTAT
TCTTTTTAGTTCCAGAAATCTTTTCTAAAAATATCTTTTCACAACCTGCTTTCTTCAACTCATCAA
TCTGTCTTTCTAATTTCTGATCTTTCGTTGACACTCTTGCATAGCCTATTATCATGTTATCACGTT
TGAGGTTTGAAAACTTAAAAAAAGTAGAAGTATTCTTACTTTGATTTTCTTACGGGTTTTATAAC
TCGAATATCTAAAAAAAATTTACAAATTTTAACAGTTCAAAAAAAAGTTAGAAAAACATTTGTTT
TTCTTACTCGTAAATATCTAGATTATTGTCCAGATTCTTAAAAATATATCTTCATAAATCCATGC
AAACCATGATGCAATCCAATATACACTGGTTCCTTCTAACATGGGCAATAATGTAGAACTAACTG
CCCCCAATAATATAATTCCGAAAACAACGGTAATTATAACACATACTGGATTCTCTTCTCTTTGC
GGCATTTTTTCACCTATACTCAGAGATTACCTTCTAATGTTTCTTTTAAAAACATTATATTCTGT
CCTACTTTTTTTGAAGTTCTTTTAACTTCTTTTTGATATGAAACAGAATGAATTTCTTTTAAATC
TTCTAAAATATCTATAATTATCTTGTCAACTACCTTTTCAAGGTGTACTGACTTTTTTCTTCCTG
AACTTTTTGGAGATTCTTCAGGATCTTCATCAAAATTTCTTCAGGTCTTGCCTTGGATCTTCCA
GTTGGAATGTCAAAAATAGGTTTAGAATCTGAAGAATTTTCTTCATTTTCTTCAGGAGTAGGAAC

TACAAAATCTCCTTCAATTTCTTTTTCAGGAGTTTTAGGATCTGGAACTTCAGACAAAAAATCAC
CTTCACATCATTTCATTTTTCATCTTGGAAATAGCATTATCAAATGAAAATTCTCCACCTTCAAA
GCTTTTTTCTTTTGTGCTCTTCCTGCTGCTTTCTTTTTCACTTCCTTTGTACATTTTCATAGTAGG
ATTGATGTATAACGCAGCATCGACAAGGTTTGGAATATCTTCAGATAATAGATTTTCAACCATTA
CTTCATATGCTTCAGCACGTTTCCTTTCAACAATCCAAGATACTCTCTGTCTTGGATCATCAGCTT
CGAGAGGAACTGCATATTTTACAAAATTTGCGTGTTTATCATCCCTAAAATGATCCACAAAAAAC
ATTATTATGTCTTCGGAAATGTCAACACAGGACTTAGGGGCATAAACAACTGCATAATGTCTTTT
ATAATTAAAAAGAGTTGATTTGATAGTTATTATTAAATCTTCAAGCCAGTCTGTGGCTTTATTTT
TATATAATATTTTATAAGTTCCTTCATCAGGAATTACTGCATGTGGATAACCGAATAATTTCGCA
CCGGATCCATTATGCCAGATTAAAAGTTTTTGTCTGGATAAATAGGGAGCAAGTGAATCGTATGC
TTCCCTTTCTGCATTATATTCTTCTTTTTTCTTTTTCACTTTTTTAATAACGTTGAAAGTTCCTGG
TTTTTCATCTTCAGTTTCATCAATATTTTCTTCAGTATTTTCAGATCTTTCACTTCTGGGATCTGG
ATCTGATTTTTTCTTTTTAATTTTCAAATTATCTAAAATACCCAAAATCTCACCTACAAGCTCTG
TGGTTGCCTGTATTCAATATGTTCTGTGAAATCTTTATTATATGAAAAATAGCCCCTAAATCGTA
GTTTTCTTTTCATACCTTCCCATTCTGAAAGACTTCCTGGTTCATCAAAATCAACAACATCTTTA
AAATCAGGAATTAATTTTTCAAGGGTTTTTGTATCAGTATTTGACGCTAATTTCCCCATTATTCC
GATATGGCTTGAGCCAATCAAGATATCTTTTGGGAGGTTATCCGTGTACTGTGTCGCACAAATTA
AATTGATACAAAGCGATCTTCCTTCTCTTAAAATCTTGTCAATCGCCCATTTTCCATTAGAGTCT
TTCATGAAGTTCTGAACTTCGTCAACTACAAAAAACACAGGAATTCTGTATTTTATTGCAACATT
TATCGCATGTTGAACCACTAATTTAAAAAGTAAACATTCTTCATAAGAGTCAAGCTCTGAGTTCT
GTGAAAAATCAAGAATATTTATCTTTTTATTGGTAATCTGTTTTAAAAAAGAGTCTTTTGTTTCA
TCAGTAAACAGTATATCGGTTTTTAATCGATTCATCGTTTCATCAATTCTTAAATATGTTTGAGA
AGGTGCAGAATTTGGTTTTTCGTAACTTTCAGCAATCGATAAAACTTCTTTTGGATCCCGGATTT
TTGAGATATCCCAAATATTTTTTAAAACAGCAAAGTATCTGGTCTGTTCACCTAAATTATTCATA
TCTGCAATTATGTCAAATGGAATGTCGGGATAAGCAAGTTTTGAACTGATTACTTCCGTTAACGG
CCCATTATCAAATGCAATATTTTCTATATCTCTTCGAGGTCTGAAAACAAACCGCCTTGCAGGAT
ATGCTGCAGGGCTTAACCCATGTCTTCCAAGAAGTGTTTTGTATTCATCGATCCCTGCTTTTGTG
TATTTTCCAAGGATATTTGGAAAACCAATGGCAAAATCACCCATTAAACCATAACGAAGTTTTGT
GTCATTTTCAAGTTCATTAACATAATGTCCAATGTAATTCCAGACTCCATCGCCATATTTACGAT
AAATATATTTTTCAGTGCTTTTTTTAGATTCTTCATGGTAAATTTCAATTACTTTTTTAGGTTTT
GAAGTGTCAACTTTCCTTTCAATCACAATAATTATGGGTGGAATTCCTGCATCTGAATAACTTGA
ATAAATTTCTTCTATCATTCCACGCATTATCGTGGTTTTTCCGTAACCTGACTTTCCTAAAAGAG
TAATATGGTTTGCTTCATCATTTTTAAGGTTTAAACTTCTTTTTGTTCTTTCTCCGTGTTTTTTC
GCTGAATCTGTTCTTCCTATACCTAAATTTATCATCACCACATTGGATCACCTTTACCTTAAATT
ACTAAAAAACCAGGGTTTGGACACCAGTGTCACTACCGTCACCAGTGTCACCGCACAGATAAAAA
AAACTAAAAAAATGGTTACCATACATAAATTAAATTCCAAAATCGAACTGTTTTTTCTTCCATCT
GAGTACTAAAAATAACATTAAACATATTACACCACTGAATAAAACACCAAATTTAAAACTATTT
ACAGTTTTTACGGCATTATTATAAGCTCCTGCATTGAAGTCCTTAGTATCATTACTTTGTTTATT
GATCAGGTCTGTGATCATGTCTTGATATCCTTCATTTTGAGTTTCTAAAATTTCCTTTTCAGCTT
CTAACAGTTTAATCTTTGAATTGAGTGTTGTAATGTTTTCTTTTAATTCAACGTTCTCAGCTTTA
GCCACGATATAACTTGAAAGCTGCTTATCTTTTTTACTGAGTTCTTCCTGTAGTGCAGTAATGTT
TTGTTTCAATTCATGGATTAGTGCATTCTGGCCTGAAACCATATTTCCATCATAAACAATTATCG
TGGAATAATTGAAATCATCATAATCTGTCAAAACATAAACTGTCTCATTGTCGCTGTATGCAAAA

ACAGATGGGAGCAGACAAAGGAACATAACTAAAAACAGTTTTTCTGCATTGGTTATAACCCTAA
GAAGCTAGTCAGTGCAGAAATGTTTACTGTTAAATATCCAATGTATTCCAAAGCTCCAGCAAGTA
AAAGACTTACAGAAAATGCTACATAACATCCTTTTGAGAATTCATCCATTAAAATCACCTTTTAA
AATAATCCTTCCATATCAACATAACCCTTTTCACGATTCACAGCAATGTAACCCATGAATCGACC
AGTTGAGCCAATGTCACCAAAATAGAATTTAAATTTCCAGTATTTGTCAGACTCTTCAATTGATT
TGGGTTCAACCCTAATCATCAGACCCTTTGACTCTAATGAAGTGCCATATTCTTTGTCAACGTAA
TTCTGTGCAAGATAATAAGCTTCAACCCCGCCGATTTCATACATCTTTGGTGGTTTTGACAATTC
TGTCTTTGAACCGTCTTTATAATACACGGTTTTATTTGAAATAATGTATCCTGATGAAGGATAAC
CAAATTCTAATAATTTTAATTGAATAATATCTTCATCTGTTTTGGATGAGTTTGATATTTGAAAA
ACAACCAAAAAAATAACTAAAAAAGTGAAGAAAATTATTTTCTTCATTTTGATCACCCTGTGATT
CCAGGGATTGATGGTATTAATGAAATATTGGCCCCGAGTGTTGCAAATGTAGCTCCAAGCCAGGC
AAAGAGTAATAATTCATCGTCTCCAGTTTCAGGGGCTAAAATATACACTGCACATATTCCTGCAG
CAACCAATGCAGGTGCTGCAATTGAACCTAATGATAAACTAATTGTTCCAAGTATTCCGTAAAGA
ACCGCAACAAAGGCGATTACTCCAATAACAACTGATTTTAACAGGTGGTTTGCGGTATTTTGGGG
AATTAATTTGTTATAAATGCCAGAAACATCTGCAAATTTGTCGAGAGCTTCAATCAAAAGAATTA
CTGCAAAAGGGATGGAAGAAAGTGATGTTGCAATTTCAGAAATCGTAACAACTTGAGTTCCAGTC
CACCAGAGTAAAATTCCAATCACAATCGAAGCACCAAAGGCTTTTGGAAGCCAGACATCAAACGA
AAAATTGGCCATTAACTATCACCTTACTCAGCAGCAACTGTGATATTAATCCATTCAGCACCTTC
AGCAGGGAGTCTTGATGTGTTTTCATCACATACTATTTCTAAATAGTAATAGAGATCTGTTTCAG
TACTGTTTGTAGTAAATTCAAAAGTTCCAGGAAGATCCAAAGCTGTTGAATTTACCGTATTTTCA
AAAGTTGTGTTATCATATGAAGTGAATAATAAAACATTAGCGGTTGTATTCTCTTCTGACGCTGA
AAGCGCGAATAAAACTACACTTGATGTACTGTTAAAGTCAGGTACTGCTGCAAACGTCAAAGGAT
CAACAGTTTCTTCAGTACTGGTTTCAACAGTTATTTCAACGGTTTCATAAGCTCCTGCATCGCTA
GGTAACCTGTAAGTTTTGCCGCTGTATGAAACGACTGCATAGTAGTAAAGGTTTTCTTCTCCTTC
TTCAGGTGTTACTTCAATATCTAACGGGAATGATACATCTTCAAAGGTATTTGTTTTTGTAAATG
TCAATTCATCATATGAAACATACAGTTCAATCGAATCTGCAGTTATATTTTCTTTTGAAGCATTA
AATGTAATTAAAACATCCCCAGTTTCATTGTCAAAAGTTGCATCTGAAGAGAATGTTGTTGAAGT
TGGAATAATAATTCCTCCAGATTCTCCTTGATTTAGATAATAATAGGAAGCCCCTGCAACGAGGA
TCACTGCAATTGCAAGGCCAACCCATAAATTATTATTTTTAGCCATTATTTTCACCGTTTAGATT
TTTTACCTGTATTTTTCTTCATTTCACTTCTACTCTTCTGAATCACGTTTCCCTTGCCGTCAACAT
AAACAAAAGACCCTTTTTTTCGATTTACCTTACCAATAATTTCTGCCATTCAATACCTCCGATTA
AAGAATTAACCTGTTCAAGGATTCCCGAAACATCAAAGTTTGAAAGATCAAGAAATCCAAAATA
ACTCATTAAAAAAACAACTGCGGTGGCAAGGATTATTGCCTTAATATTCTTAATCAAAACAGCTA
CAAGCATAATTAACGTAATTTGAATCCAGTAGGATTGAATTATTGGAATTATTGATTCAAAATTC
ACATAAAATCACCACCACTTTTATTTTTTAGCTGAATAAGCAACTATTGCACCGACGCATGCCCC
AAGCATAATCCTTGAACATTCAAGAAAGATCTGGGAAACGTCCTGGTAAAAATACATTAAAATA
AGTACAATTAAAACAAAAGAAGCGATCAAATCGCCTAAATTCCATTTATCAGAACCTATTTTCCA
GACACATATCAAGACGATTAATGTTGGAACCATCCATGAAAGAAGACCTGCTAATCCAAAGCCCT
GACCTATGACTTCAACTTGTGGTTTTAAAACCATGGATAAGACAAAAGACAGGATAACTACAATT
AATTGATTAATCATAATTTACACCTACATAATAAAGCACCTTAAAGCCCAAAGCCTGTGAAGCTT
GGAATTATTGATAAATAAGAACCCCATGTTGCAATTGATGCCCCCACAAACAGGTACAAAAGAAA
TTTTTCTTTTCCAGTGTTTGGAGCAAGGAGATACAATGCAAAGATTGCTGCAACCACAACACTTG
AAGCACCTGCTGAACCAATTGAAAGGGTGATTGAACCCGACAACGCCCAGAGGGTTACAAAGAAC

ATGCAAACAATCGTTATAATCGTTAAACCGATAGCCACATACCCAGAATAGTTTTTAAAAAGGGG
AGTGTAGACTACTTTGTTCATGTCCACAATTGTATCAAGAAGTTCAGCCATTAAATAAAATACAA
AAGGAACTGCTGCAAGGCCCCGAACAACGTCAGAACCCATAACAGAATTATTCATTACATACCAC
ATCAATGCAGCAAAACCAACACCACAACCAACCATTTTCAAGCTAAAAATAAGCCAGGGCGGCAA
TGATTTAGAGCCAGAGCTATTTGAATCAGAAGCCAAGAAAATCACCTAAAAAAAGTTTTAAAGA
ATTATAATATTTTAGTTACAATTATTGCACCAGATACCAAGACTCCGAGTACAACAATAATTAAA
ACAGTGTCTGGAACGTCACCAATGCCAAAACCAGACTTAAACTGAGTCCACCATTCAGAAACAGT
GGATCCTGCAGTAAACCATAAACTGGGTTCGTATTCAGCTGCCAAACTTTCAAGCTTTTCAGCAT
TACTTGCGAGTGCATCTCCTTGATCATCAAGTCCTAAATTGTATTTTGCTGCCGCTTCTTTCCAGT
AGTCGCCAGCTGGCTCATAAACGTCCCCTGCAAGATAAGTGTAAACTTTTACTTCTTCAGGATCA
TCAGTATTTTTAGCCTTTTCGTAGTACGCAGCTGCTTTTTCATAGTCTTCTAAAGCGTTTGAAAT
CGCATTTTTAGCTTTATCGTTATCTTCATATTTTCCAGATTCTGATTCTGCAACATACTGCTTTTC
TTTTAAGCTTTCTTCAGACATTTGGATTATTTCAGTGATTCTTTGAGAATCACTATCTGAAATTG
AACCATCACTCATTAATTCAAGGATTTCTTCTTTGAATTCCTCATTTAACGAATATTTCACGTTG
TCAATTACGGAAATACGTGGTTTTATAAGACACCAGACTGGAAGTGCTGAGTCAATTCCATCGTC
AACATCTGCAACAACTAAAAAGTAGGTTTCAAAAGCTTCAGTACTGCTGTCTTTTAGATAATTAA
GCGAAGTTGTAACCCCATTATGAACTGACATATCGTGGAAGTTTCCAGGAGTTTCTGAAGCAATT
GTTGCTACTCCATCATCCCTTAATTTTAGGACTAAAGCCCTATAATCATCCTCAATAGTTACTGA
AACAGGTTCTAAATTAGCAGGCTGTCCAACAAGAACAAATCGGTACTGTGCTGATGAATTATAGC
AGTGTACGGGACTTGCCCAAACTCGACCAATCACGTTAGAAGTAGCCCCATTTGAATATGAAGAG
TAAGCAATTGGTATCACTTCGTTTACGTATGATGTAGGAAGAGAAGCGTCAGCTCCATCAACATA
GTAAGCACCGTTGTGGATTCTATCCCATGCTTCAAGAGTTGTGAGTTTTGTATCTAATGTAATTG
TTTTATCGTATTCTTCCCAGGTTTTCCATACATAACGCCCAGTTTCATCATCTAACTTGTAGCCAG
ATTCTTTTTTGTATAATTTAACATATCCATCGATTTCAACGACAAGTTCAAACTGTTTAACTTCA
GCAGTTATAAGCTCCTGGAGTATTTCCCTGTCAGGAGCATTTGTAATTTGGGATTTTGCCTTTCC
AATGTATGGATCTGGTGCTTTCATTGTGAAATTTATAATCAGTTCTTCAATTGCCTCAGGATTAT
CGTCATTATTATCCTGAAGTTTTATGTTATCCGTATAACTTTTTGGATTCCATTTAACGCCATCA
GAATCTACAAGATAAATATTCACGTTTTCAATCATCACATAGGAACTTTCTGAACCCGTTAACGC
TTCATCAAAGTCCAACTGAACTGGAAACGCGGAAAATCCATATATTTGTCAGGCCCTTTCATTG
AAACATCAACATCTGATAACGTTCCTGTAACCGTAAACTTGTATATCCCAATTCCTGATTCTAAT
TTATCTTTAATTTCTGCAATATCCTTTGCTGCAGATTCTTCTGAATATTGCCTGTATCCTGCAAG
GAGGTCGTATTTGTATGTTCCATTAATGTATTCGTCATCAGACACATTAGTCTTTGCAGCTCCTG
TGACTGCCGCAGATTCTTCTACATCTTTTCCTGTGGCCCACCGGTATGCGGAATCTACTGCAATTG
CCCCAATAACCCCAACTCCTGCAGCCACAAGTACTGGAGCTCCGGCAGTTACTGCAACTGCACCAA
GTGCAACACCGCCAAGACCGAGAGCTCCAACATCATCCCATCCTGCATTTAATTCATGTCCTAAAA
CAGAAAATGCATGAGCTGAAGGCAAAGTTGCAATTAAAAAAATGCAAAAAATAGAAAATAGTTT
TGAATTTATCATTTTTTCAGCCTCAATGCTGCAAATGTTCCTGCAATTAACAGAACTGCAGCAGC
TAAAAGAATTAGTGTAGTGTTACCCCCCGGAATTGAAGCCAGGAAAAGTGAACTACTGGATATTG
TTAAAACAACGTTTCCAGAATAGATCGCATCGGTTTTATCAGGATCAAATATTTCTAATGAAAGT
TGGTAATTTTCAAGTTCAAGCTCTTCAGGAACTTCAAAAGAAATGTCAACTGTTTTGTCAACCGT
TTCAGGGGCTATTGAAAACGTTTTAGCAATTGATTCGTTATAAAACGGACAAGTTAACTCAGCAG
TGTAATATTTTACATTTGAAGTAGGATTTGAAATCTTAAACTGTACTGTTGCAACATTCCCCGGA
TTTACCGTGATTTCATCAGTAATCAAAGTTACTGGTTCAATAATATCAATTGAATAATATGTTTT

TACAGTTCCTGCTTTTGCATTTAATTCAAGTGTGTAATCTCCTGCATCATCAATTGGAACTTCAA
ACGTGTAATCACAGTATTCTAAAAGATTAGTGCTTGAACTCCACAGTTCAACGTTTTCAGAATCG
TACAGAACAATTAAAACTGAATAAGCAGTTCCAGACTGATCAGTTAATGTAATATCATTTATTCC
AATCCCAAAAGTTTCAGGCATTTCTAGTAAAAATGGAAGTTCCAAAACTTCATAATTAATCGTTT
CAAGGTTATTGTAAGCTGTACCTTCGATATCTGTTGCAGATAATTCGACAGTAAATGTTTTTTCA
CCCCAAGACGAAGGAGTTTTAAAATCAATCTCGATATTTTGTGTACTAGATACATCCCCTAAAAT
GTAATATCCATCAGCTGATCTTGGAAGCTCCTGGGTTAGATAATAAACTTTGTTTACATCAACTC
CAGATATTCTCAATTTTGAAGCATAAGCATCTTTTACAGGCGTTATATCCATTGAAAGAGTTCCA
ATAGAATTAGGGTACAAATTTCCAGAAAACTCTTTTGAAACTTGAAATATTGAACTTTTTGGGGA
AAGTGAAATATATACCTCTTGCGTTTCAATTTTTGCGGAATATGTTTCTAAAATATCCCAGTAGC
CCTCTTTAATAAATTTTAAATTATGTGGGCCTGCGGGGAGCTGTAATTCAATATATCCCCCGTTA
TCAGTCGTCCCCAAAAGTTCGGATCCTTCATAGATTTGAGTATCGGGTAAAGCGTAGATATTTAA
AATCCCCATGATTTGAGAATAGCCCTTGTCTTCAAATCCAACGAGCGAACTGGTAACGGGATTTT
GGACGTAGATATAAGATAATGTACCTCTATCCCATGCACCGCACCACTGGTACAAAGATAAAACG
CCCGATACGGTATTTACATCAAAATAACCGTAGGTATTTTGATAAGATCCAGAGGATAACGTATT
TAATCCAGTTCCGTCTTCAGATAGGGTATATCCTCCCCCATACCATGACTTAGATAATAAATAAA
TCTTTGTGGTATTGGACTCTATTGGAAGCGTCCGAGCACTTGCACCTTGAACATAAGTATATAAT
ACTTGCCCATTCGGGATCATTACACAAGACGGAGTGATAGCGTAATTATTATAATCATAGGGCAT
CAAAAACCGTGCAAGGTCTTTTTCAGAATTGAAATAGATTTTATTATTTTCAATTCCCGTTACGA
CTGGAGTATATTGTGAATAAGTTACTGCCGTAATTGCATCTTGAATTGTTGTGTCATTATAATTA
TCTGCAATTAAGACGTAATAGTCCGTAGGCGTAACCACGGACGGAACGACAATATTGGCATTAAC
TACATTAAATGAAAATACCAAAAGCAGTAAAAAAAGAAAATAACGTTTTATCAAATTAACCACC
CACCAAAACAGTGTTTAATGCACTCTGCAAAATAACATAATCCTTAAGATGGTTTTTATCTCGTT
TTAACTTGATAATTGAATTAAAAAAGTCGTTTTTTTGAATTTCAGATAAATTCAATTGTTTTGCA
ATTTTTCCTGCATTCATTTTCAAACTCTGAATGTTTGAACACGTTGCCTGATCTTTTACAAAACC
AGTCTGTAAAACACTGCAGTTCTTGTTACTCGTGTGAACATTGACAGCTTTGACTTCTGATTTAT
GATAATCCTTTTCAAAATGATCTACAACGTGTAGGCAGTTAGGACAAATTTTTATCAAAAATAGC
ACCTCAAAAAATAAGATAAAGCATTAGCTAACAGGTAATGTTTCAATCACAGATATTAATCTCCT
GAAACATGAAAAAAATACACTACATGATTTAATGTGGTAAAAAGCAATATATAGTGTTTATTAA
TTTAATACAGAATCAAAAAACCCGTATATTTGGACATCACAATTACAAGTCCTCTGAATGCAGAT
GATCTAAAATCAATAGATACCAAACGGAACATAGGGCTCTTTTAAAACAGAATATTTAAGCAGTT
ATTTTAACAAAAGAACGCGTTTTAGAATGAAAAAATAAAGCAAATTTAATCTAGGTCTGCTAAT
CCCGTTACTCTCTGAGTAACCCGAGTTCAAATCTCGGTCTCCGCGTCTTCGTTTTTAAGACGTTTA
AAGCTCTTTTATTCAGTATTTTACAAGACCCGTTATAACTTTATAGTCTATTTTGATGTATCTCA
ATTTTACGCTAAAAGCCCCCATTTGAACAGTCAAAAGTCACGGGACTCGGATCCCGTTAACAGTA
ACTGACTTCAAATTATCGTTTTTCGGAGTCCCGTGTGTAGTTTTCGGAGTCCCGTGTACATATAT
CAAAATTTTAGGACTAAAATTTTATTCCCTACGAATAATTTTACTAATTTAAGCGAATTATTCGG
GTAAAACGCTGTTTTACAGATTTAACCAATTTTAAATTTAAAATAACAAGTTAAAAAGTACTAT
TTTTAGCATTCTTCGAACATGGCCTCTTTAAAAATTTATTTTTATGGAATCGAAAGTCACGGGGT
CTTTTGATAATTTCAAGATTTTAAAACGTAATTTCTGGAAAAAATCAGTTTATTTTTATTTTATT
TTCGAATTATCGGACAGGTAATCAGTTTCAATCTCTTAGTGATTAAAAATTTAAGTTTTAACGGT
TAATCCAACAAAATTACGAAAATGTTATGTAATTTATTTACTTTTTAAATTTAAACCTAAATTTG
AAAAGGTATTTATGTGTAAATACATATATTCCCATGTAAATATAAAACTTGAAAGGGATAATAT

GAATGTTAAGAGAACTTACAGCATTGATGAAACAGTAGTTAAAAAATTTTCAGAATATTGTGAT
GAAAGGGGACTTAACATGAGTAAACAGATTGAAACTTTCATGAAATATGTTGTCGAAGGACCAG
AAGTTAGGCCAGAATACCTTGAAAAACTAGAAGAAATTCGAAAAGGAGAGTTTATTCCTGTTAA
GGATTTTGCTAAACACTATGGGTTAAAATAATGACTTATGACCTACTAATCTCTAAAAAAGCTGA
TAAAAAACTAAAAAAACATTCCCGTGATAAAAAAATTCATTGAAGCCGTAAATAAAAAAGTAAG
CGAAATTTTGGAAAATCCAGAACACTACAAGCCACTACGTGGAGATATGCATGGATCAAGGCGAG
CCCATGTTCAGTCATCTTATGTCATAGTTTACGAAATTGATGGAAATATCGTAAGATTACTGGAT
ATTGAACATCATGACAAGGTTTACTTAATTTTATCAATATTTGAATTATTAATTAAAAATATTT
AAAAATTTTAAATTTTTGCTTTTTAAAATCAAACTATTTTTAATATATTTTCGAATAATCAGGTA
ATTATCGATTTTAATTTACGTATCCGTTAAAAATTAAGTTTTAACAGTTGATCTGACAAAATGTA
AAAATGTTTGTCTTTTTTAAAAAAATTAAATACAAAAGGCAGCTTGATGTTGAAAATTTTTGAG
AGTTTAATTTTTAGAAATATAAAATTTTGTTTAAGATAAAAAAGAGTATATTAAAAAATTAATT
ATTCGATTTTATTGAAATAAGTTAAGCTAGAATCTCCTCCGGAATATAATTCCGGATGGATCATT
CTTGCAAAGTCTTCCATGTAATTTGCAGGATCTTTTGCTTCATCTAGATAATAAGATGTAACAGG
AATGTATAATTTGCCGTTAGAAAGTGATTCAAAACTTGCAAGATTTGGATCGTACTGCAATAAAT
CATCTCTAGATTCTATTCCGTAATATTCCATTCCCATATAAATAACAACGTCTGCACTTGCAGCTC
TTTCGTAGAATGTTTCTGCATCAATAATAAGACTGCTAGATCCAGGAATGTCGTTAAATATATTT
TCTCCACCAAATTCGGTAATCATTTTATTGTTATAGTTTTGAGAACCATAAAGATAGTAAGTTAA
TCCCCCGTAATCTACAGGCATAACATATACCACTGTTGGATAGTCATCTACCGTAGAAGCAGTCC
TAATTAATTCATTTCGTTTATGCCAGGCATCTTGGAAATACTGTTCAATTGCATCTGAATTTGAA
TCGCCCCAGAATGCAGCAACGAATTTAGCCCATTCGATTCTTCCAAGGTATGTTTTTTCATCGTA
AGTATAAAATCGGGCTACATCTATTCCAAGTTCAGTTTCCAATTTATTTTCTGCAACATCATGAT
CATCCCAGCTACCTAAAAATACCACATCAGGATTTAAAATAGCGATTTTATCGTAATCTATATCT
GTAATGCCACCCACAGTAATTGTTTCATTATTTAAATAGCGTGAATAGATTTCTTCCGAATTCGT
TGTATCCCAGTTTCCAATTCCATCAATCATGTCATATACGTCAGCATTATTCAAAACATCTGCAG
TTGAAAGCACTGGTGAATATTGGGTACATGCAAGACTGTTTATGGGGGCAGTTAAAACAGTTGCT
CCTTCAATTTGGGGATCGGTTGCTCCCTCTTTTAATAAAACAAATTCATTACCTACTGAATCTTT
GACATAACAGTAGCCTTCATTCCAGTGAGGATCCATTTCAAAATTTTCTGCAAATACTACTGGAT
CCCTAAAAGTATCGTAATTTTTAAACAAATAAACTACATCAACAACGTTTACCGCATTGTCGCAG
TTTAAATCTCCAACATCTAATCCGACATTTCTGTGTTTAAAAAGATAAACTACGTCTGCAATACT
GATGCTGCCGTCTCCATTGACGTCCCCAATAGCTTGGGCACAAGCGGATGGCATAAATGCAATCA
TAGCAAGCATTAATAGTGCTATTACTTTTTTCAAATTACCACCTTCGTAATAATAATATTAAAAA
TATAGTATCAAATAGTATGAGTAATCAAGTACTATTTAAAAGTTGTCAAGTTATCTTTAGAAAG
ATATGTGTTATTAAAAAAATGGTATAAAAATTAATAAAATGAAAAAAATAATTCTTCGTTAATAT
GGTTCTAAATGTCCTTTAAACATTGAATATTTCCATCATAGTTGATGACTGTTTTACCCAATTTT
TGAGCTTGTTCCAAGAGATTTAAATTTAAAATATTCATGTCGCCAACAGGGTAGTTCGTGTTTAT
GAATACATCTGCGGATTCTAGTTCCTTAAATGCTTCATCCAATTTTTCATTTGAAATTTTACAGA
ATGCTTCTTCGCAAATAATATCTAATTCCATAGATTCTGCTACTGCATAGTCAATGTCGTTTTTT
TGCAAGATTCCAACAGTAATGTCATACCCATTTTTTACCAAATATCTTAATACTCCGGAACCACT
TCCCCCTCCACAGTTTACAAATACTTTTTTATTTCCATTTTTTTTATCGTTAAATTCATTTTTTAA
CTCAAAATATCCAATTAAATTACTATAATTGGCGTTTTTCAAATCATAAAGCGAATTAACTGTTT
TTTTATCCATTATCTCTTCAGGGTACCCATAAGCGATTATTTTATGGTCTTTAATGAGTGCCATT
TTATCCGCTATTCTTAAACCCAATTCTATATCGTGCAAAGTTACAATTATCGCAAGATTTTTTTC

AGTGGCCAGTTTTCTTAAAAGAAGAGTTAACTCAATTTTATGTTTTGCATCCAAAAAACTGGTCG
GTTCATCCAGTATCAGTACATTTGGCTCTTGTGCAATTGCTCTTGCAATCATAATTTTTTGTTTT
TCACCATCACTCATCTCAAAGAAATTTTTTGAAAGAAGATGTTCAGCATTTACAGATATGGCCGC
ATTTGTAACAATTAATTTATCTTCATCAGATAATCTGCCAAAAAGATCAGTATATGGATGCCTTC
CAATTGCAATTATATCAAAACCAGTCATATTTCCAGGATTAACCCGATCAGTTAATACAACTGCC
ATTTCTTTTGCAAGTTCTTTTGGCTTTAATTCATGAATTTTTTCAGCGTTTAAATAAACTACGCC
TGTTTTTGGCTTTAAATATGTTGCAATAGTTTTTAGAAGTGTTGATTTGCCAACCCCGTTTGGAC
CGATTATACATAATATTTCGCCCTCTTTTATTTCGAGATTTGCATTTTCAACAACCACATAATCG
TTATATCCAACAGTTAAGTTTCTGTTTTTAGCAATATATCACAACGAGTTATTGTTTTAGGTAT
TCGAATCAAATTTTTACGATTTAAAATATTATTTTTATTTTTAAAAGACTTTAAATAATAAGAT
ATTATATTTTTTATTACTTTATATTAACTATGGTGGTTCAGTGAACATTTTTGATTTTTCAATAT
TAGGCAATTCTCTATTAAGTGCAGTGCTGTATACAATAAAACTATCCTCAGTGGTTCTTCTTACA
ATGTTTATCATGAACTGCCTGATAAATACCGGAATAATGAAAAAAGTAAGTAATTTTTTATATCC
TTTTACAAAACATCTAAAAATGAATCAGCTTTCATTATATTCTATACTTTCATGTTTTTTAGCC
CTACTGTGGGCTATTCGATACTTGCTGAAGGATATAACGATAAAAAAGTCAACGAAAAAGAACTT
ATTGGAAGCTCCCTTGCAAATTCATTTCCTTCAATATTTTCACATATATTTACGTTTTTTATACC
AATTGCAATACCGCTACTTGGAATGACTGGCGTTATATATATTTTAATCCGCTCAGGGGTTGCAA
TGGTGAAAACAATTATCGGCCTATTTTATTTAGCGCTAGTCTCGAAAAATACGGAAGAATATGAA
CCAAAAAACGAAGAACAAAAAAATATCAAAAAAAGCATGAAAAAATCGTTTGAGAGTACTTTAA
GGGTTTCAAAACGAATCCTTCCCATAATGTTTATTACAATGTTCATTGTCATCTATTTCTCAAAT
TTGGGAGTTTTTGAATTCATAAAACTGATTATAAATCCAGTTACAGGATATTTAAATCTTGATCC
GAGTGTTGGAATTTTAATTCTAACAGATTTAATCAATGTTCAGGCCGCAATGGTAATGGGAAGTG
GACTTTTGGAAAACGGGGTTTTGGGTTCAAAAGACGTGATAATTGGGTTAATCTTTGCAAATGTA
TTATCACTTTCAACAAGATATGCAAAACACTCTCTTCCATTACATGTTTCATTATTTGGGCCAAA
ATTAGGAACTAAAATCGTCATGATAAATGCAGGTATTACATTTGCAATAGATGTAGTCATAATCG
GGGCATTTTTATTGCTTGGCTGATTCCAAAATTAATTTAATATTTTTTAAATATGGTAATTGCA
ATAATACTAAAAAAACTATAATTTTTCTAAAAGTAGTAAAAATTCAGTATTAATTATATATTTT
CCATTTCAGCATTCGTAATTTCTGAGATTTTGTCCAAATAGGATATAGATGGATCGATTACCCCT
TTATTCCGGATATCATATATTTTAAATGAAATTTTCCTTTTTTGAATGATTTTTGAAAGTTCTTC
GATTTCGTATTTTATGTCTTTTTTAATGGGAACATTCGCTTTTCCATCACTTATCAGTATAAATT
CAACTGAAGATTTTTTGTTTTTATTCCTGAACTGTTTTGAAATGTTTAATGCAACTTTTAATGCT
GAAGAAAGTGGAGTTTTACCTCCTGTTTTTATAGAGTCCAACTGAGCCTTAATTAAATTATATTT
TCTTGTAAATGGGGATAATACTATAGCTTCACGCCCCCTAAAAGATATCACTGAGATATATTCTC
TTTTTACGTAGGAATCATCAGCGTAACGGTCCACAATTCCCTTGGCAATCCCGATTCTTTTGGAA
ATTCCCATACTTCCACTCGAATCTAGGAGAATCACTGAAAGTTTAGGAACCCTGTTTTTTCGAAT
TTTTACCTTGAAATCACTTTGTAAAAGTTTGTATGGTTTCTGATTTAGAATTGCACTGTTTATCG
TGGAAATTATGTCTATATCTTCGTAGTGTTCAGGTTTTGATACTGTTGTATATGATATTGGGTAT
CCTCTTTTAGATCCTATTTCAGATACCCTTACTTTTTTAGATCCACCATGATTTTCAAAACACGTA
TTTTCTATTTCAGAATTTTTGGAAATTATTTTTTTATCAAACTTTTCAGTTTTGAAATTTAATTC
TTTAAAGTTGTGGTTTTTTTCTTCGAAATCCGTTTTATTTCCATCATTGTTATCTTTTTCATCTA
ATGGTGGTGATGGGGGACTAGTTGGCGGATCTGTTTCCGTTTTTTTTTAGATTCGTCTTCCATTT
CTTCGTTATTTTCATTTTCCATGGACTGTGATGGCGGATTACTGTTTGGATTAGATAATCTGTGA
ATGAGTGCAAGATCCATCGCTGTTTTTAAATCCTCCAAACTAACTTCCATACGTCCATCAAGTGC

TGCAATGGTTTTCGCAGTTTTTATCGTGGTTATTTCTGCACGATGCGTTTTTATTCCTAAATGAA
TTATGGAATCAATTAACATTTTTAATAGTTCTTCTGAAATTGTTACGTCATTCAGAATTTTTTTC
GCAGTTTTTATCGATTCTGTTAAATTTTCTTCGATTGACGCATACGTTTCATAAAATGAAAATGG
GTCTTTTTGGAATTTTTCGACTCTTTTTAAAACTTCAAGCCGATCTTCTGCTTCAAAGAGGGATT
CTATTTCCACGTAAAGTCCGAATCTATCAAGTAACGGGGGTCTTAACTCACCTTCTTCTGGATTC
ATACTTCCAATTAGCACAAATCTTGAAGGGTGTTTAAAGGAAATTCCCTCTCTTTCAATCGTATT
CCAACCCATTGCTGCAGAATCGAGCAATATATCAATAACATAATCATCGAGTAAATTCACTTCAT
CAATATAAAGTATATTTCGATTTGCGTTGGCCAAAATTCCGGGTTGAAGAACTCTTTTTCCTTCA
TTAAGTGCTTTTTTGATATCTATCGTTCCAACAAGCCTATCAATAGTTACACTTAATGGTAAATC
AACTACTTTCATAGTTTTTTCAAGTTTTTTAAACTCATTGGCCGAATTTGCATTTTCGTAACAGT
TATCGCACATTTCAAGAGGGTTTTCAGGATTGCAATTAAATGGGCATTCCAAAACAGCTTCAAAA
TTTGGTAAAACATGTGAAAGTGACCTTACAAGTGTCGATTTTCCAGCACCTTTATCGCCAGTTAA
TAGCACTCCACCAATTGACGGATCAACTGCACAACAAAGTAGTGCTTTTTTTGCTTTTTCTTGAC
CGACAATTGCTCCAAAAGGATATACTACTTTTCTACTCATTTTACCACCTTAGATTTGTTCCAAA
GACTTTCCAATCCAGATAACGATTCTTTTTTTGCATCTTCGTTGAATGAACTCGTTATTATTTGT
CCACCTTGTATTTCTGATTCTCCATCCATTAATTCTTCTAAATCCCCTTCTAATTCAGAATATGCT
TCTTTAAGTTTTTCCAGCTTTTCTTCATCTGCATCCCATAATCCCCGTGCTGAAGCCTCTATTAAT
CGCCTTGCAATTTCTTCACAGGCGTAAATATTATTTTCTTTAAACCATTCCCTCATTTCATCATCT
AAAATGTATTGTTCAGTTATTTCATCGAAAACCCAATCATTAACCATTTTCGTAGTTGCAGACCA
ACCATAAATATGCTGAATCTTTTTTGAAAATTCATTTGCGCCCCTGTAACCGTGGACTTTCATTT
CGTTAACCCATGCAGGATTTAATATTTTAGACCTTGCTACACGTTCCAGTTCTTTTTCAATGGTT
CTTACTTCTGCGTTTGAAATATCTTTTGTATCCACAATTACAATGTCTATATCAGAATTACCTTT
TAACGTAGAAACCGCAGTATACATCCCGCCCTGATACGCATACTTTGAACAGCAATTTGTGATAT
CATGTTCATCGCTCATGTGGTCCATAGTTACAATATCTACAGTTTTTAAACCTGAAATTAATGCT
TCTGGACTGTATTTGCCGTAAGATTTTGCAGTGTATCTGTAAGAACCCCACTGTAGCCATACTTT
ACTTAAATCCTCATCTGAACTCCATGCTGATGCATCTACTGCTAAATTTACTCCTGAACCATAGGC
ACCAGGTGCATTTGCCCAAACTCGAGATTTTGCTTCGTCTAACAAATCTTCGGTTAATTGTTCTT
TGAATTCGTTTAAATATTCTAAATAATGTTTTTTCACGTAGTTCTTTTCAAGCGGTTCATCAAGG
CTTATTACCTTTGTAACTGCATCATCAATCATTTTTATGTAGTTTGGAAGAGTATCTCTCGTAAT
CCCGCTTATCCTAACGAGTGTGTCAATTCTGGGCCGATTTAGTTCTTCAAGTGAAATAACTTCTA
CATCCTTTACTGAACCATCTTTATTCCATAGCGGTTTTACCCCCATTAAATAAAGGATTTGTGCA
AGCTGTTCACCATCCGCCTTGTATGCATCCATACTCCACATTATCTGTCCAATATTTTCAGGATAT
TTACCATGATGTTCTAAATAATGAGAAACAAGTTTTTCAGCCGTTTTTATTCCTACTTTCCATGA
TGCAGGGGTTGGAAGCGCAGTCGGGTCTATCGTGTAGAAATTACGGCCTGTTGGGAGAATTTCTA
TTTTTCCTCTTGTAACTGCTCCCGAAGGCCCTGGAAGTATATATTTACCATCAAAAACATCAAAA
ACTCCGTTATATTCGGCAGTACACGCTTTTATTTTCATTGCAATTAATTTTCCAGTGTTTATGGC
TTTTAAAATATCATCGTAATCGTTTAATTCACAGTTTTTCAACATTTGGCTTTCAGATATTTTTT
CATTGATTATTTTTAAAATCTCTTCGTTTGAGTCAATATCCTCAATCAAAATCTCTTTCAGTGCC
TTTTGAAGTAAATTAGAATTTTCTTTGATTATTTCTTTTGCAGTAAACCCCTCATTATAAATTTT
TGAAGATTCAGTTCTTAATTCATCGTAATCGAATCCCAAATATTCTGAAAAGACTCGTTTTATTG
ATTTAAAGTTGTAATTATCATAAGTCATTATTGTTATGACGTATTTTGAAATTTTTTCTGCATCA
TCTGTAGGGTTTCCAAGAATATGAAGGCCGTTCTCAACCTGTGTTCCAGATATCATGTTTATGTA
TTCATGAAGTTCTTCGACAGTGTCGTCAGGAACTTTTGAGGATATTTTAATGTTATTTTTTAATG

CTAATTCGAGTATTTCTTCGTAAGTTACTTCCATTTTTAGAGTATCTTCTAACGATTTTGCTTTT
GAATATTCTGCAAGCAATTCATCAATATCCTCTAAAACTTCGGGCATGGTCATTGGAGGGTACAT
ATGATTTATTATTGTAGCGTATGCTCTCCTTTTTGCCATTACTCCCTCCATAGGGTTTGCTGAATT
GTATATATAAGAATGAGGAACGTTATCAATGGTAATTTCTGGCCAGCATGCCGGGGAAAGTCCAA
CACCTTTTCCAGGCCTGAATTCGAGGTAACCGTGAGTTCCAAAATGCATTAACATATCTGCGTTA
AAAATTCTCGTCATCCAACGGTATGCTGCAAGCCACTGGTGCGGAGGATTGATTAATGGATCGTG
TAACACTTTACATGCTTTTCCATCACAAAATGTACCTGCACAGCCCGATTTTGGTTGAGGGGTTA
CCAAAATATTTCCAAAAAGAACGCCCGGAACTACAAACTTGTTTCATGAACCATTCCAATATAT
TCTCTTGAAACATTTTTTGATAAGACTTCTTTTGGATCTTTCCAGTCTTCGAGCATTTTTGAGGT
AACTTTATCTGGAAGTTCATCAAACCAGTCCCTATATGTGCCATGATCTACAAAACCAACAGCAC
CTCCTTTTTCAACGATTTCAGAACTTGAAGTCCATCTAAATTCATTAACTGCTTTTTTAGACATT
AACAAATCCATCATTTCCTGTCCAGTTTCAGGTATGTAATCCCCAACATTGTAGCCTTCTTCTTTT
AATTTCTTAAGTAATCTTACAAGGCTTTCTGGAACGTCTAATCCAAAACCCACTGCTAAAGACGC
CTCTGAACCATGGCAAGGGGGGTTAATCAATACAACTGCAATTTTCTTTTCAAAATTTGGTTTTT
TTGACAGATTTACCCATTTTTTTATTCTTTTGGAAACGTATTTTGCATGTTCGGTATAAGGTTCA
TACCTTTTAACTCCGTCTTCGTCAACCTTACTTCCTACTAAAAAAATAGGTTCGATAGTTCCGTCA
ACTTCGGGCATGATTACCTGATATACTTGCGACATTGGATCTATTCCTTGCGGATTTTTCATCCA
TTCGTCTAGACTTTCCCTAAATGAATGAATAACCTGTAATACTGGCACATTTAGTTTTTTTAATA
ATTCTACGCCTGAAACGTCTTTAAATCTATTTTTCGATTCATGGTCTTTTGATGAATGATCTAAC
ATAAAAAAGAAAGTTGAATTAACGAGTGCATTTATTACTGGTTTATCTTCCTTAAAGAAGTATT
TTTGAATGGTTTCTTCAGCAGTAAGTGAATCATAACCTTCAATGTTTAATCGATTCGTAAAAACT
GGAATTACCCCTAAATCCTGTTTTTCAAGTTCTTCAACGAGTGATTTGATATGATCAGTACTTTG
GCTTATCCAAAAAGTCCTGTGGAAAAATATTCCTACAAAATCTTTTTTAGTTCCGTAAATTTTCA
AATAGTCTTCTAAATTTTCAAAAGTTCCGTGTTTTGGGTGCCATATGCCTTGCCAGGGCGTATTT
TCTGGTTCTTCAACCGTTAAATTAAATCCTGCAAAATTTAGGAATATTTTAACAAGCTGTTCAAT
ATTTTTAGGGCCTCCTATTTTATAGTATATAATTGCTTTTGATAACTGGGCATTTGATCCTTTTG
AAAGATGTAAGTTTGGCTCAGATCCAGAAATTATTACTTTTGCGTTCGATTCTTCAATTGCCTTT
TGAAGTTTTTCATCCATACTTGGTGAATAAATAAATATAACGTCTGAATCTTTTATAAATTCCAG
ATCAATGTTTAAATTTTCGGAATTTGAGAGTAGATATTCAAAAGAAGTTTCGATTTTTTCTTTAT
CTAGTGCTTCAGAAAATTGCGGAAGGGTTACTGCACCGTATCCAAGCAGTATAGATATTTTAGCC
ATTCTTTTCCTCTTTGATGGGTAGTTATCCATTTTAAATTAAATATTCTATTTATGGGCATTATC
TTAAAGCAGTATATTTGAATAAATTATTAAAAATGATATTATTTTGTAATACTTTAAGCAAATA
TTTATTTTTTAATACTATAATTTTTTTAAAAGTATTATGGTTCATGTTTATAATAGTTTCGATAT
AAAAACGTCAATATTTGGAAATCTAAAGTTTAAATTTTCAAAAAATAAAAAAAGAAAAATATTA
TTTTTAAATAAGTTCTTCAACCACCACGGATCCATTTGCGGTCCATTCAATCATGATTGGTCCTTC
AGGCAATTCATCCAAAGTTTTAAAGTATTCTAAAGCAGCTAGTGTTCCATATCTATCAGAACCTG
CAATATATACAATAGTATAGCTTTTAACAATATTTCCCGAGTTATCTTGAACTTTTAATACCTGA
ATAACCCCTTTATTTTCTCCAGGGTAGTCGTTTGAAATTGGCATTTCAAATTGGTCGTTGTATTC
TTTTGCAAAGCCATTTGCTAAAGGTCCACCAACAATTACTGCATTTCCAGAAATTGTGAATCCTT
CTGCGTTCTGTACTCTTTCCCTTAATTGGTTTGCGTAATTTTCATCGATTTCGTTTCCATATATTA
CTGTTGCACTTGAAACGAAGTTTTTAATAACTTTTGATTCAATATCGTCAGCAATGTCTGATGAG
TATGAGCTTCCACCTCCACCACCATGTGATGAGGAACTTGAGGAGGTAGTTTGTGGAAGTGCAGA
TGAGAATGTTATTGTAACTTCTGGGTCTTTAATCAATGTTATTTCGAGAATTCCGTCAACTGGGA

GTCTATACCCTCCAATTGTGCTTTCAATGTTATCCTTTTCTAGTGTGATATTGTCTGTTCCATTGT
TTACAATTATTTCAGATATTTCCAGATTTCCAACTGGAAGTCTTATAATTGCAAATCCCTTACTG
CTTGTATTTGTTGCAGTGAATCTTAATTCAGAAATTGCGTTATTTCCATCTTTTGCAGTTTCTTG
AGTAATGTTTGATATGTCAAATCCGTAATTTGAAACAACGACCGTATTGTTTATAACTTCATCCA
TAATGTCGCTAACATCATTTTCAGAAGTTATTGAATCAAAATCAATGTTTTCAAGATTTGAAACT
GCTTCGTTCATTGCATCCAATGTTTCATTTGTAAAGGATATATTTTCAACGGTTTCAGGTATCCA
GATACTATCACTGTTGTTGAAATATATTTTTGTAATATTAAATTCCCCATTGTTTACTGTTTCAA
GAGCACTTTCATTTACAAGATCTGAAACGTTGCTATCTACAACAATATTCTCAATAGACTTTAAG
TTTACAATTTTGTTTGAGGTAAATATTTCATTGAATGCGTAACCATTGGAATCGGATAATGCACA
TTCTGAGATAATTGCTGAAATTGAATTGTTAGTTTTGTTAAATGTAATATTTGCTATTTCAAATT
CTCCATAATCGCTGAAATTGTAGCCTGTTACGAACAAGGTATTGTTTGAAACTGACCAGTTTGTA
TTTTCACCGATTGCATTAACTTCAACTGCTTCTAAATTATCAAGAGAAATATATGCACTGTAATT
GCTGCACTGTCTTTCTTCCACTATGCCTGATAATTTTACTGAAGCTATAATCTGGTCTTCTGTTTC
TGATTTTTTATAAATTTCTATATAAATAGGTCTTTCAGTTATGGTTACATTTTTAGTATATTGAT
TATTTGTTTCGTTTTCTTCATTTAGTTCATCCAAATAATCTACAACTGTTGTAATCGTGAATGTT
TTTGACTTTTGAGGAGTCCAATTAAATTCCACGATTTTTGATTCGTTAATTCCTAAATCAGTTAC
TTTTAAAGGAGCTAACTGATCAATATCAAGAGATACGTTTATACTGCATGAATCTACTGATTTTT
GTCCCATATTTGATACATTTACCTTGATAACTGCAGGTTCCATGTAGTAAGCATCCAAAATTTCA
ATATTTTCAACTTTAACGTCTGGACGACCATAAACTGTAAAGTTCACGTCTGTTTCGTTTACATT
ATTGAATGCGTAACCGTTTACATCGGATACTGCAAGGTTTTCCAATGAAATAACTGGGGTTCCAG
CTCTTAATGCTTCAAAGGTAGCAATAGCAATTGTTGTATTTTCATCGATAGTTCCATCAACGATA
GCGAATGAAACTACTCCACTACCGATTGTTATATTTTCTAAGTACGTTTCATTAGCTTCTGATGC
ATTTAACTGGAATGTGAAGTTAGTACATTTCAAAATGTTTGAACTGAATACTAATTCTCCATCAA
TAGCTTTTGCAGGTCTGTTTGACGTTAAATCATTTAATGTGAATATTACATTAAATGTTTCTTTG
GTACCCACTGTTTCTTTATCGGAGGATAAAACCGCTGAAATTGCATTTTCTGAAACTGTGTAATG
TGTAGTATTTTGGTTATTATTTTCGTTTTCTTCGCCGATTTCATCTAAGTAATCTACAACTGTTG
TAATCGTGAATGTTTTTGACTTTTGAGGAGTCCAATTAAATTCCACGATTTTTGATTCGTTAATT
CCTAAATCAGTTACTGTTAAAGGATCTAATTCATCAGAATCAATGTATATCTTAATAGTAGACGA
ATCTACTGTTTGATGTCCGATATTTGATACATTTACCTTGATAACTGCAGGTTCCATGTAGTAAG
CATCCAAAATTTCAACGTTTTCTACTTTAACATCTGGACCTTCAACTAAAATGTTTGTATTTGTA
GTACTTACATTGTTGAATGCGTAACCGTTTACATCAGATACAACGAGATTTTCTAATGAAATATC
TGAGTTTCCAACGGCTAATGCTTCAAAGGTTGCGATTGCAATTATTGTATTTTCATCGATAGTTC
CATCAACGATACTGAAAGTAACGTTGTCGCTGTTGATTGTTATATTTTCTAAATATGTTTCGTTA
GCTTCCGATGCATCCAACTGGAATTCGAAGTTACTGCATTCCAAAACGACTGAATCGAAAACCAA
TGCTCCTTCAACAGCTTTTGCAGGCCTGTTTGCCGTTAAATCATTTAATGTGAGTGCAACATCAA
ATGTTTCACCAGTTTTTACAGAATCATTTTCGCATGATAAAACCGCTGAAATTGCATTTTCTGAA
GGCTGGTTACCAAATGAAATTTCATTGTTTGTTTCATCAGATTCTTCAACAGCGTATTTTGAATC
TACGAGAATGTCGAACAAGTTGTTTGGACCAAGCGTTACATTTTCAAATGTAACAACAGTACTTT
CTCCAATATTTAATCCGGCAACTGATTTTTCAACATCTGAATCGATAACTCCTGTAACTGCTTCC
AATGAAACGTTGAATGCACGTGCATTAAGAGTTCCGTTGTTTAAAACAGTTACTAAAACAGTATT
CGTTTCGTATTTTTTTAATGGAGATATTGAAACATCTGTAATTACCAAATCAGGTAATATCTCGT
ATACTGAAACGTTAGTATTTGTAACGTTGGTACTCAATGCATATCCATCAGTATCACTCAAGCTG
GGATTTAGTTCTATCTGTGTATTTCCCCTATTTAATGTCTCAAAAGACAACGTAGCGACTGTAAA

ATTACCTGAGGGTGCATCTGAGAACCATGCAAGTGATATATCTCCGTTAGGTGATACCTCTTTCA
AAGGTGCCGCATTGCATATTTCACTAAGTGTAATTGATGTTAAATTTATAATATCTTCGTCATAT
GATATCCCATTTTCAATAGCCCAAGTTTCAGGCGCATCTTTAATCAACAAGTCTAAACTAAACGT
ATCCCCTTTTTCAACGTTTAAACTGCTGGAATTTAATTCAACACTCGTGGCAAAACATGCATTTA
ATGCCAGAATTGTTAAAATCGAAGTAACAATTAGTTTGAACTTCAAACAATACCACCTTAAAATT
TTTTTTAGAAAATGATTGACACATACAGTAAGGCCCAAATAACGTTTAATTTTCAAAAATATTGT
CAGTATTCGATTACCAAATCTTCATGAAATTTAACGTATTTTGGTTACTGCATAATTTAATGTAT
TTTATATGTACATATTAATAATAAAAACAAATTTAATATTACAAAAGGATTAATATTTATATTT
TATGCATACAATATCATCTAACTTTGAAATAAGTATTAATAAAAATGACGCTTTGCTGATTCTTC
ATACTTATTTTAAGTCATGTCAAATGATTATTTATTAAAGTATAATTTATAGTTAATAAATATA
ACTAGAATAATATAACTATTAAACAAAACATACCATTAGATTTGGAAGTTTTGTTATCCTTATTA
GTTAGATGGGATGGTGGAATTGTATGAAAAAAGCATTTTTATTGGTAATAATGTCCATTTTAAT
ACTACCTGCAAATTTTGCCAGCAATACGGGTTTAGAAATAGAAATGCCCGAAGAAATTACTGCGG
GGGAAGTATTCAATATCGAAGTTAATGTAAAAAATAATGTTGAAGTTTGTGGATTTGAATGTGA
ATTAAAAATTCCACTGGAAGAACAGGATAACTTTGAAATAATAAATGTAACGGAAAATAGTGAG
ATAAGTGAAAAAGCAGGTCAATTTTATGAAATAAAAACAACTAAATCGGGGGTATTGAACAGAT
TCACCGTGTTTGATGAACCTGTAAACGAGGATTTCCATGCATTTACTGTTCAATTAAAGGCATTA
AATTCTGGAGATAATACGTTAACGTTTGTATCAGAATGTGCAGACACTGATGGAAACGCAATACC
TGTTGATACAGTCGAAAAAGAGTTGACTGTTATAGTAAATCCTGAATTAGAAAATAATAACAAT
AATTCATTATTGGATATGATCAAAAGCATTTTAAATTTCTTTTTCGGTGGTTAAATGAATAAATA
TATCTTTTTTTCCATTTTTTTAATGTTTTTAATGTTTAGTGAAAATTTTGCATCTGATAATCTTA
TTTTAAAACTTACTCCCGAAGAAACAAAAACCGATGGTGAATTATTGTATTTTAACATCACCGTT
GAAAATATCCCTCCTGAAAATAGTTTAGATGAAGGTTATGATGGGTCAAATTTGGCAGAGTATG
AAAAAGATGGTGGTTGTGCGGCAGTTGATGTTTTTATTAATTATTCAACCGATATTTTAGGGCCT
GTCGAATTTAACTGGAGTGAAACCTGCATGGAGATGGACTGGAAAGAATACCAGTTCGAAGATG
GTGAATTTTATCTGTCAATAACATTTGATGAAACCTTGTATGATGACTCACTGCTGATTGGAACT
GTCGTGTTTAACCCAATTGCTGAATCAGCAACAGAATTAGAATTATCCGGATGTTTAGCTTCAAA
CTTGGGTAGTCAATATAATGGAGGTAAACGAACGGCAGACACGGATGATGAAAGAACTACTCGA
GTAGAATATCCTGAAACTGAATTTTACGGTTCAGAAATCAAAGTTAATGGAGTAGGTTCTTACGA
AGGATCTACATCTTTAGAAGGAACTTTTGAAGAAACCCCTGAAGTTACTGGTTCTAATGTAGTCT
ACAACATTAACGTAGTTCCCAATCAACAAGAACCAGAGGTAATCGTAAAAAAAGTCGATGTAGA
AGAAATATCTCCAAATATTACTGTAATTGTCCAAAATGATGGTGGTATAAATCCAGTTCTATTGA
TATCCATTTTTTTGGGCAGTATTCTCTCAGGTGCGGTATTCGGTTCAGTTTTTAGAGGGAGAATT
CTATGAAAAAATATTTATTTTATCTATTGATTCTTTTTTTTGCAATGCCCCAAGTATTTTCAGAA
GATACTTCGGTGAATTTATACTCCGATTCAGATTCTTTTTCGGTAGGCGATAACTTTAATATGGA
TTTAGTCATTGAAAATATTCCTGCGGATCAAAAATGCGGTGGAATCGAGATAAAAATAACGTAT
GATCATAGTTTACTAAGTTTATCAGATATTGAATTAGATGGTAATTTAAATGCGGATTATAGTG
AGGTAAAAGAGTCTGATGGAACAATAAAACTTGCATGGTTTATTAATCCACCCAGCGGAATCATA
GATATCGCAAAAGTAAAATTCAATACGTTAAACGAAGGTAAGTCAGAAATAAGTATGTCTGAAA
CGACTATTTCTGATTCGGAAGGTTATGAATACCAAAATATATCGATAAATGGTTTGAATATATCT
GTAAATCAATTAAATCAAACTATTGGAGAAATCATTTTGGAAGATTACAAAACTGACGAAAAAA
TAAATGCGGTTTTATCATTCAAAGGGGTTAAAACTCCTATAAACAATATTTCAGGTTATTTATCT
TTTGAAAATGTATCTATCGTTGAAAATCCGTCTATCTCGGAATTATTATGCTCTAAATTTAATTA

CGAGTTAAATTCAAATAATTTTTCGTTTTTTATCGTTCCAAATGATATTTCTGAAAATGAAACAA
TGTTCGAACTTTTAAAACTTCCAATAATTGTAAACAATGAAAATTATGGGATTTCAAGCGAGTTA
TATGTAAACGGTGTAAAACTACAAAATATATCCACAATTGAGGAAAAAGAATCAATAGACGCTT
CAACATACTCTGGATTAATGTTTTACGTTGATGGTGAGTACAATACTGAAACTGAACTTATTATC
GGGAGCCCTAAAAAAATTAAATTAAAAGTATGTAATTACGACAAAAATATAACAAATATTTCAG
GTTATCTTTTTATTAATGACTCATTATTTGACGTTTCTGAATATCAAATACCAACTGTTTCTGAC
GTTTACAATAAATTGGACTTGTCAGATATTTCTTTAAATGAAAGCCATCTTTATTTTAATATTTC
AATGGAAGATAATGGTACAAACGGCACATTTAGTATTTTAGAATTTAAACTACGTTCAAAAATA
AATGAAAATGTTTCATCAACTATTCAACTTGGAAATCTTTCTGTATATTTCAATGAAACAAAACT
TTCATTTGATGCAAAAAATTTAGATGTAAATATTGTTGAAAAAAGTGAGAATTCTCCACCATCTG
TTAAAATTTTTTATAATGTATACAATAATAATCAAGTTCATTTTTATCCAATTAGCAATGATGAA
GATGATGATTTAGATGATTTGGACTATGAGTGGAATTTTGGCGATGGAACAGAGTCTACTAAAG
ATGAACCTATTCATACATATTCCAATTATTCGAGATATTATGTAAACTGCAAGGTTTTGGATACT
TTGAATGCAAGTGATACGTCAAAAGGAATTATCGAACTTTTAAATATTTCGCCCGTTTCATACCA
ACTGTCAAATACGAATTTAGTGGGAATTAAAGGCGAGAATAAAACACTTTACTTAAATTTAACG
TTAAATAATCCATTAAATTATAATGTAAATGCATATATTCAATTTGTAGATTACAATGATTATG
ATCCATTAAATTCATCATATAACGTATTATTGAGTGCAGATGAAACTAAAAACCTAATTATTCCG
ATAAACGTTACTAAATCCTGTAAAATAAAGTGGAACGTTTTGTACTACCCTCCTGCATCGATTAC
TTCTGATGATATAGATATCGGGTATTATTTATGGAGTTTTGACGAAAATGTTGAATTTAAAGAA
AAAGCGACTATTATCAATTCAACAGAATATGTGGATGTGAATAATACAAAAGTCATTTTAGAAG
TCAATAAAGTTAAAACGGTTGAAAATCAGGTTATATACAAAACTGTTTCGTCAACAGATGAATCT
GTAGTGTACTATTGTTTTACTGCAATTTCGGGAGTTTTTGCAGGACTTATTTTTGTACGATACAG
AATAAGATAATACATAATTTTTATTGAAGTCTATTTCATAATACTTTTTTATTTTTTATAATAAA
TTTTAAATACTATCTTAATAACCATATTATTTGGTAATATATTTTTAATATTATTATTACAAAGG
TGGTAATTTGAAAAAAATAATGGCACTATTAACGCTTGCTATGATTGCATTTATGCCATCCGCTT
GTGCCCAAGCTATTGGGGACGTCAATGGAGACGGCAGCATCAGTATTGCAGACGTAGTTTATCTT
TTTAAACACAGAAATGTCGGATTAGATGTTGGAGATTTAAACTGCGACAATGCGGTAAACGTTGT
TGATGTGGTTTATTTGTTTAAAAATTATGATACATTTAGAGACCCAGTAGTATTTGCAGAAAATT
TTGAAATGGATCCTCACTGGGATGAGGGGTACTGCTACCTTGTAGATTCAGCAGATAATAAATTT
GTTTTATTAAAAGAAGGAGCAACTGACCCCGAAATTGAAGGAGCAACTGTTTTAAATGTTCCATT
AACTAATGTAGGTACAGTATTTTACTGTCCAATAATTTCAACTGCAGATCTATTGGATAATGCAG
CAGTATACGATTCTTTGAAAGTAATTCCTACATCATACGTAAAATATTCTTCAGAACTGCAAACC
AGGTATGATGCAGGAGAGATTCTTAATATTGGATCATCCAGTACTATGAACTACGACAACGTTGT
TGCTTCCAATCCAGATATTGTATTTTTAGCAGATTGGGAAAAACACGATGTAATGGAAGAACAAT
TAACTGCGAATAACATTTTATCGTGCAGATGTTTTACATACGATGAACCTACTTTCATGGGTAGA
ACTGAATGGGCTAAATTTGCCGCAGCATTGTGGGGCGATGAAAACTCCGAAGTTATTGATGAATA
TTTCCAAGAAACATGGCAGAAAAGGAATGAATTGTTAAGAACTGCTTCAACTGCTGATGACTATC
CTACAGTAGTTAGTTTCTCATGGTCAAGTTCAAAAGATACCGCATGTGTTTACGGCGCTCAACAC
TACTACAGCAGGATGGTAAATGAATTTGGTGGGGAATATGTATTTAACGACATTCCCGGAACTTC
TTCAAATTATCCTGACAAAGAAACATTCTATGAAAGAGCTCAAAATGCAGATGTATGTATTATGA
AAGTATACACTGGCGATGAAGTATTAACTGCAGAAGATTTACTTTCGATAAACCCTGATTTTGCA
AATTTCGAGTCCTTTAAAAATGGACGATTTTATACCTCCCATAGAGATTACTTCATTCAAGAAGC
AATTGACCCTATTGGATATATGGACGACTATGCAAGAATGATTAATCCAGAATTATTCTCAAATG

GGGATAGCGATCTTTTGCACCACACTAAAATCGAACCTATTGCAGGATAATAACCTATAACCTAA
ATTTTACTTTTTTTACTTTTTTTAAATCAAAAAATCTAGATTACAATTTAAAATAAATTAAGTAT
ATATATGATTATATCTTACTTTTTTTAAAAATTTAATACGGAACCATTTTAGGTGAATCCTTTGA
ATCTTAAAAAATTTTCAGTACTCCTTTCCCTATCTCTAATTTTAATTGGGGTTTTGTCATATTTC
AGCATTCTTGAAGGGAGTGTTAAATTAACAAACGAAAATCTCAAAGATTACATATTCGATGGATC
CACTGGAAATGCGGGAATTGATTCAATGATTAAAAATTTGAGAATGACCCGTATTGTTGGAAGT
ATTTTAGTAGGGTCAGCAATTGCAGTTTCTGGATTATTAATGCAGGGATATTTTAGAAATCCTCT
GGCAGATCCTTATTTTATGGAATTGCGAGCGGGGCTTCATTAGGCGTTGTATTGTACACTTTTA
CATCAGTTCTTTTTGACTTAGGGATTCCACATTCCGTATATGGAAGAATTATTGCAGCATATGCG
GGATCCTTAATTGCCATGTTCGTTGTTATAAATATTTCAAAGGTTGTAAAACAAATTTCAACACT
TTTAATCTGTGGTATGATGATAAGCGCCGCAATTTCTGGTTTTTCTGATATTCTTGTTGCGACAG
GCAGCTATGTTGATGGAGAAAATTCGGAACTTGCAGGGTATTTATCTTGGGGCATGGGTTCAATA
TCAAGCCTTACGTGGGTTCAACTCGAATCAATGGCTTTTTTAATAATTCCATTCATTATATTAAC
ATATTTATTCCTATCAAAAAATTTAGATGCAAATTTACTCGGGGAAAATTATGCAGTAAGTGTTG
GTGTAGATACTAAAAAATTCAGGCGTTTACTTGTTTTATTATCCTGTATTCTCTCTTCTACTGTG
GTTGCGTTTGCAGGACCGATTGCATTTGTTGGAATAGTGTGTCCAATAGTTGCAAGACTTTTATG
CGGTACTTCAAAACATTTTTTTGTATTACCAATGACTGCATTGACGGGCATTATTTTTGTAATCT
TAGCAGATATATTATCAAGACCTGGCGTATTATTTCAAGCTACGGTACCATTACCCCTTTTATGC
CCCTTATCGTTACTTGGCGGTCCGATTGTAATAATTTCATTCTTAAAAAGTAAAAAGATGAGAAT
ATAATAAATCAGGGCGAAAAAATGAAGTATAAAATTTTTTTAATTTCCATAATATTCTTGTTAG
ATTTGGGACTGGTTTTTGCGGGAATTTATTACGGCGGAAACGCAAAATCAATAGATTGTGGCGAT
ATAACCAATTACATCTTTGAACAAACCACTGGCGACGAACTTAAAGATATGATCTTACAAAACGT
TAGGATACCCCCCATATTTGCAGCAATTTTTGTAGGGCTCAGTCTTTCAGTGTGTGGTTTAATGC
TCCAGACATTATTTAGAAATTTCCTTGCATCGCCCTATACAACAGGAATAACAAGCGGAGTTTTG
CTTTTTGCATCATTTGTAATATTTTTGAAAAGTTTTTCAGAATTATTCGCCGCATTTGGTCCAGA
TAAACTGTTAATCGGGGGCTGGATTGGGGGCTTAATCTCAATAATAATACTGGTAATTATTGCAT
CTAAAATAAAAGATGTAAATGATATTATTGTAGTATCCCTTTTGATGAGCTACTTTTTAGGGGGA
ATTCGGGCTTATTTAGTAGCTAATGCGGATCCTGCCTCAGTTATGACATACTACTTTTTTGCACT
CGGTAGTATTACTGGAATCAGACCTGAAGACCTGCCAATTATTGGAACTTGCAGTTTATTATTTG
CATTGTGTGCATTTTTATCAATAAAGCCATTAAATGCATTGTTATTTGGAGAATCTTACGCAAAG
AGTTTTGGATTAAGTGTAAAAAAAATAAGAATATTGATACTCGCATCAACGGCATTTGTGGCTG
GATCGATAATGCCCTATGTTGGTTCAATAAATTTTGTGGGGGTTGCAGCACCATTTATTGCAAGA
CCATTAATAAAAACGTCAGACCATAGGTGGCTTGTTCCAACAACACTTCTTACTGGCATTTTTTT
AATGCTCGTATGCCACTTTATAACTTTTAAATACAACCTTCCTTTTTCGTATTTACTCGGTATAG
ACAGGCCAGTAAGCGTAATTCCAATCGGGAGTGTTCTTGACATTGTTGGGGGCTTGCTCGTTATT
TATCTTGTAGTTTCAAGAGAAAAAAAGATTAAAATTTAAAGAATTTAATTTTTTTACTGAATTC
GATTTCAAAGAAATTTATGGGGATTTTAAATGTATAACCTAATAAATGTTTCAAATTACAAGGG
AGATCTTGAAAAATTTCAGTATGATCATAACAATATGGGGGAATTTTTAAAACGGAATGATCTT
GATGGGTTTGAGGTACTCCAATTTGGACCGTGGAATGAAGATGAAATACCTAAAAACTTTATTAA
AGGACTTCATTTGAGATTTTATCCGACTTGGATTGATTTTTACAGGGGTGACCTGGATAAACTTT
TATCGAAAGTATATTCTAAAGAAAATATTGTTACGCTATACGGGGGCACCAGTAAAGATGTAAT
TATTGATTATTACAGGAGCGAACTCAAAACTGCAAAAGAAATGGGTGTGGACTATGTAGTAATG
CATGTTTGCCATGTTGATTTGGAAGATTCACTTAATTATAATGTTAATTATACTGATGAAGAGGT

AATTGATGAATCCATTAATTTGATAAATGAAGTTTTTAATGGGGAAAATGACTATGAATTTTAC
TTATTATTGGAAAATTTATGGTGGCCTGGGCTAAAACTCAAAAATAAAGAAAGCATGCAAAAAC
TCATTGATAAAATAAATTACGAAAAAGTTGGATTTGTACTCGATACGGGACATATGATCAATAC
AAATCTTGAAATTAAAAACGAAAATGAAGCAATTGACTATATTTTTGAAACGATTGAAAATTTG
GGTGAAATGAAGGATTATATCTATGGCATGCATCTTAATTATTCTTTATCAGGCAATTATGTAAA
AGAATTTATTAAAACTCAAAAAATTACAAAACGAACCTGTGAATTAGATGAATTATTTAAAGAA
GTATGTGAACATATCGGGAAAATTGACTATCACGACCCATTTGAACATGAAAAAATAAAAGAAT
TAATCGAATCTTTAAACATAAAATATTTGGTATATGAATTGTTTGCACCAAACTTGGAAGAATTA
GAAGATAAAATTAAACGACAAAACGAGTGGGTAAAATAAAATGTCTAAAGTAATATATGTTTCG
GGAGGGCAAAAAAGCGGAAAAACGACTTTTGCACAGAATTTGGCGCTAAGTCTCAGTAAAAATCC
CATTTATCTTGCAACATCCCGAGTCTGGGACGAAGATTATAAAAAAAGAATTGATAGACATAAAT
CAGATCGAGGAGATGAATGGACAAATATCGAGGAAGAAAAATACATTTCAAAATATAAATTTGA
GAATAAAGTTGTACTAATCGATTGCGTAACCCTCTGGATGACTAATTTTTTTATGATAATAATC
AAGACATTGGCAAGTGCATTGAAGAAGCTAAAACTGAATTAAATGGACTTTTAAAAACAGATGC
CACCTATATTATAGTTTCAAATGAAATTGGCCTTGGTGGAACTCTTGCAGATAAAATGGCCTGTA
AATTTATTGACCTGCTTGGTTTAATTAATCAATACATTGCTAATATTTCTGACGAAACATACATC
ATGTTTTCGGGGATTCCTGTTAAAATAAAGTAAAAATATGCTAGTTTTAAAACACACGGCACCAC
CGTTAAAAAATAAGCTACTTTATAATATAAACATGCCTTCAAAATGTTATATAGAATTTTTGAA
GAGGAATTTTGATGATTTTAAAAATCCTGTCTAAAAAACATGTTAAAGAGATTTTAAAAACGAT
TGAAAGTCATAAATCTATTTATTATGGGCAGTTAAAAAAAGAAACCGGTTTAAACTCTGGGAAT
TTAAGTAAATTGTTAAACGAATTATTGGAATTTGGATTTATAACAAAAGAAGAAGTTCCAACGG
ATATACTAAAATAATAAATGAATATTACGATTTAACGGAAAAAGGAAAAGAAGCTTTGAAAGTT
TATGAAATTATTGATATATTAAGTTAATTTAGTGGGTGGGGAAATGGATTTAGAAATAGAAATA
ACATCTGTTAGCGAATTACTAGATTATTTAAAGGATATTGAAGAAATATCTGAAATTTTAAATT
ATATTTTGGATTATAATGAAAAACAGTCATTAATTAAATTAAAATATGATATTATACTTCTTTC
GGTCTTAAAAAACTCAATAGATAAACTAATTACCCCCGTAAACCGGAGGTATTTGGATGAATATT
TTCGGGTTTTATCAAGTTCTAAGGCAAAATTAGAAATTCAAATAAAAGTTAATGGATGGGAAGA
TATAAATCGACACGTTCGTTATGAACATTTTAAAAACGAAAAATCTAAAGAACTTTTTAAATCA
AAATTTTTAACAGATGTAAAAAAATTTATGTTTAAATCAGATTATGATTCATTAAATGAAATTA
TAAAAGGAATTAAAAAAATTTCGAAATATCAAATAGATTATATTGTAAAAATTGTTAGTTATAT
TTTCATTAATAATACTAATGAATATTTAATATCGTTATCAGAAGTAGGGAATCACCTAAATGCTT
TCAGAGATGAACTGAATAATGTCACGGATATTTCAAAAGGTGATATAAAGACGTTAATACTCTT
AGAAAAGATATATTGTAACCTTACTGTATCCATACGGTATTTAAAATCTGTTTTAGATGACATTA
AAAGAAAATATACAAAAAAATTAATGGAGTATCCCATTTAAACAGATCTTTAAAAGATTGTGTT
GTTTATCGAGGTCAAAAAAATAGCGAATGGAATCTATGCCCTTCAATATTCCGTAAAAACCGAGA
GGGGGAATATATTTACGAAAATAAGGAAAGAATTTTTTATAAATCTTTAGTCGAACACAATCAT
CCTGAATTTAAAAAAAATAATACGATTTTTGATAAAGTTGCAATGATGCAACACTATTCAATACC
AACAAGACTTTTGGATTGGACAAAAAATCCGTTAGTTGGACTTTATTTGCTACTGAAAAATGTA
TTGAAAAAAAGATGATTTGGATAAAGAAGAGCTTAAAAACGGTAAATTATATGTATATCGTCCA
AATATTGGAAATATATATTATTCAGATGATTTAGAAGTTAAATTGTTATTCGAATTATTTTTTA
ATGGTTTTTTTGGAGTTTGTGATAAAGAAAGTCTCCCCAATTTACCATTTGACGGCAAAAAAAAT
CAAAGAACATTATTATGATAGGTTGGGGCGTAATGGTAATGATGAAAACATTGAAGGATATTTA
AAAGAGATGTCTGAACACCTTAAAGACCTAATCTGTGGAATTCACCTGATAAAACCCATTATTAC

AAATGATAGGTTAAGGGTACAGCAAGGCTGTTTTTCTGTTTATGGGGGTAAACTGAGTAAATAT
TGGGATTCAGACGACTATTCAGAATATATTCATATCGCAGATATGGCTGATTGCGATTTAGAAAA
TTCAGACGAAATACTCGCCACATTTATAATTCCTGCAAATAAAAAGAAGGAAATCAGAGAAGAAC
TCCAAAGACTTGGAATTCACTATGGAACAATGTTTCCAGAACTTGACAAATACGGCGAATATTTG
AAGAAAAAAATTTTCTAATTAAATTCAAAATAAAAAATAATATTTGAAGTTTAAAATGTATTTGT
TTTTTGGGGAAGTAAGATAGTTGGAACATGGCCATATATCAATTTTTTGTAATTTAACTTTAGAA
ATTTTAAAAAATAGAAAATTATAATTTAAAACGATTTTTCAGTACCGAATTTTTTTATAATCTAC
AAAATTACTTAAAAATGCAGTTTAAAATTCGCTTTTTAATAGTTCTTCAACTTTTATTTTTATTT
CATCCCTAATTTTTCGATAAACTTCAATTTCTTTTCCGGCAGGGTCTTTTATATTCCAAGCAATG
TATTTTTTTGCAGGAACAATCGGGCATGAACCAATACAGCCCATTGTAACTAAAATATCGATATT
TCCTATTTTTTTAGTAAGTTCCAAAATAGTTTCCGGTCTTTCTTTAACTTTCAGCCCTAATTCAGC
CATTACTTCAACTATTTTTTCATCAATTTCTTCAGTTTTTTCAACGCCTGCACTGTATGCGTTTAA
TCCCAGATTTTTAGCAAAAGATTCTGCCATTACACTTCGTCTTTTGTTGTGGATGCATACAAATG
CTATTTTTTCATCTGAAATTTTAAAAGATTTATCCAATGTTTCACCGAGAAATTTATAAAATTCG
ATATATTTTGAAATATATTTACTGTTTTCGATATACCAATATGTATCCATATATATTGATGTTTG
TAATGGCAGAAAAATAATATTTCTTTTTTATTTTGAAAATTAGTTTAAAAAATTAAAAATATGT
CGGGGTTATTCTGATTTTGGGTAATCGAATAATTAATAGGTTTTATTTTTCAAATTTACCGGCTT
TCGCAACCACAGTTGCAACCGCTACCTTTGTGATCTTCTCTTTTTCGATCCAAACATACAGATTTA
CCGTCTTTGACAGCTGAAACTGTTTTTGTTATGTCTTCGTCAAGGATATCCATATCGCTGATCTT
GTTTATTCCAAGTTCCGTGATTATAACGTACTGATCAACTTTAACGCCAGAATTGTCTGCAATAC
GTTTAGCACAACTTACAGGACAGCCGTCAATTACAACAATTTCACCCGCATTTTCTACTCTTTCCA
TAACAGAGGGGGTTTTAATTGCAAGTGATACTGTACAGGCTTTATTTCCGAAACCTTCTTTTGTT
AACTTGACTGCTGCAGCATTTGAGAGTTCACCTACATTTGAAGCTCCTGAGCATGCGAATATAAT
CTTTTCTTTCTTATTCATAACACCGTGATTGCCACATGTGCATTTTGGGGTTTCTGCCATAATCAT
CACTCAATTTCATTTTTTAAAAATCAGTATTTTGTATACGTTATTACTTAAAATATTTATAAGTT
TCGATATATGGATAGCATTGATATATTTTAATGTATTTTAAATATATTCAAATATTCCGATGTTT
TTTAAATAGATTGAAATTAATTGATATATCTGTTATTATAAATATATCTTAATTTAAAAATATT
AATTAGGTACATGCTAAATTATCTTATAAATCGATATATTGTTTAATTTCAGGTTATACCTTATC
AAAGCAAACATTTCTAAAAATAATTATAATCTAAACGGTCTGAAAAAAACTGGTTTCAAAAGTT
TATTGGCAAATTTTTGTGGAAAATAGTTTAAAAAAGTAATAAATTAGTCAGTTTCTATCATGAT
ATATCGTCCAGTATTTGGATCTTGATAGACCGTTCCCATCTCCATAAAGCCTGAACCACTTCCATC
GGTGATTTCTGGCATGTCAGTAATTTCTTTTGCCATTTTTATTTCAGCAACCTGCTTCATTGCCTG
CATTATTTCCATTTTTTCTTCTTGAGTCGAATCACTAAAAACGACACTTTGCATGTTCCATGACA
TTACTAAAAATACTAAAAATCCAACTGAAAGAACAAGCATTGCATCCACGAGGTTTGCGGATCCT
GCCATTGGATCTTCTTCATTTTTACTAAATCTCTTTTTATTTTTCCGTCTTAACATTTTTTCAATG
CCTCCAAGACTGATTCCATCAATACGTCAAGGGTAGACATCTGTTCTTCGTACCATCTTCGTCTAA
CCTTTGAAATAACGTAGCAGATTCCTCCTGCTGCAAGTCCAACAACAGTTGTATCAAATGCAATA
ATTATCGCATTTGCAAGTGAATGAATGTCTCCTGCACCAAGTGCTGCAAGACCCGGGCCCATTGG
AATTAAAGTACCCATTAATCCAAGTGTGGGACCAAGTCGAGTTACGAAATCTGTTTTTTCAAGTG
TTTTTTCAAGTCTCAATTCTTCGCGCTCTATCAAATTTCTTGCATGAGATTCAACCATTTTTAGA
TCATTACATTCTTCTTCGCACAGTTCTTTGATAATTTCAATATGTGTCCTGTTTAATCGCATTTC
AGAAATTAAACTCGATATTTCTTGCGCATTTTTATTGGAAAATGTGTTTATCATGTTTTTTAGAT
CTTTTGAATTAACTCTGACCCTGCTCGAATATTCTGACATGAATTCTCCAAAATTTATAACAGCA

TATGCCATGAACAATAACAAAAATGCTACAACTGGCAATAAAAGGCTCTGAGAAATTATGTGAA
GAGTAGTGCTTAAAAGCTCGCTTCCGGGAATTGCAACCATTAAATCGACCTCATTTTATCTGTAA
AATTCATTCTTTTCCTGTTTTTAACATATCCTAGTATCAAAAGTCCAAACGTTATTGCAAAAACA
TGAGTCAAACTTGAAAACTCAGGGTATTGCATAGGACTCATCTGAATGCTCATTCCGGCAACGAT
GTTTGGAATTACCAGCGCAGAAATTAAAAAGTAAAATCCTGCAAATATCATGAAATTTCCCATCA
AAACCGGTCTAGGTGCACGTAATACTCTTGAAATTGCATCCGAAAGTAGATAAACTGCCCCGATG
GTTATCATTAAAATTAAACTTGCATATTTTCCAATTTCAAATGTGGAAATCCCTAATGTGGGCGA
AATCAATACAATAGATGCGATAACTGCTCCAAAACAGCACGGACACGGTGCAATCATTGCCAGAC
TGCATGAAGCTACTGAATTTTTCTGGTTTACTTTCCATTCTTTTATTGTATGAAAACCCGCATAG
AGAATCATTAAGGACATTACGATGAAAATAACAAAATTATATTCATAAATAACCGTTTGAATAA
TATCTACATATCCTGAAGCCAGTTTTGATAATAAAAGGGTGCCAAGCCCATATAATAAGACTATA
GTACCAGATACTTTTTTAGAAAGGCCTGAAAATCCCAAAGAAAGTCCTATTTTGATCCCAAATAT
TATAATTGCAGACATTAGTCCAAGCTGCCATAATTCATCCATCATGGTTATTACCTCGTATTAAA
AATAGGTAAAAAAAGAAAATAATTATTTTTTCCTTAAAAACTTCAAAACATCAGGGTTCTGTTT
AAAGTATCCAAAACCAACTAATCCAGTCATTCCAAGTGCTCCCAAAATTGCGAGAATCGTAGTTC
CGGAAGTTTCAGACGTTTCGGGGTTACTTTCCATTGTAATTTCGTATGCTTTTTGAGATGGCGCT
GTTGAAGATGAAGAGTCAGCACCTGAACTTGAGCTGGTACTTGCAGAATTACTACTTCCATCAGA
ACCTACTGAAGATGCGGAATCTTGGGTAGTCGTAGTATTTGTTTTTATGGATGTATCTGAGGGAT
TTTTGGTATCTAAAACTTCTTCTCCAGAGTACACTCTTGAATATGAATCAGGTTCGGAGTTTGAA
TCTTTACTTGAACTTTTTGTTTCCGTTTGAATGGTAACTTCATCATGAGTTGCAGTTTCATAAGT
TTCTGAAAACTCAGACATTGTTGAATCATCTAATGACGATTTTGATACTGTCCAATCAGTAAATT
CGAGGTTTCCGCAGGTATGGTGACAGCAAGTTACTCCGTAATCTACTGTTGATTGAACATATTCG
TTTGCCAAAAATTCTAAGACTTCATCTGCTGGTGTCCACATATCTCTTCTTGCAGCTTCAAGTGCC
CATGCAGTTAATGAATTGTATGCGTAAGGGTTTGCTTCAAGCATTTGTGTTCGAATATTTTCATC
TATTATAAATGTTTCAGCAACGTCTTCCCACATCCAATCCTGAACTGAATTCGGATCAACTGCTT
CCCATCCAAATAAATGTTCTATTTCACTTGTAATTTCTAAGGCTCCTGATGAACCTTCACTTAAC
ATTCCTTCGATCCATTGTGGATTTTCAATTCGAGTCCTAAATTCGTTTGCAAGGAATGTGCTTAG
AGTTTCCACTTTTGCATCGCTAGTTCTCGTGTTCGCAATATATGTCAATACATCTTTTCCAGAAT
AGGTTTCTGCAGCCATTGTAAGGCCGCCTAAGTACTGGTATACATCATCATTATCTAAAAGGCCG
TAAAGACCGTCCCTGATTTGTACTGTTGCATCGATTTGAGATAGCTGATCTTTAAAGATTTCCTG
TGCATCTTCTGAATATCGTCCATTTCCGTAAATGTAAGACATTCTTTCGATATATGTATTTACGA
GATCTTCTTGGTCGTCCCATGCGCTCGTAGTTGGAACCAGTGCACTAATTCCTGTACCATACGTCC
CTTCTTCAGGACCGAATATTCTGGCACTTGCATAGAAATCTGCATCTTTGGGGCTCATTCCTTCAT
CAAGATATTCTTGATAATTTTTTAGGTAGTGTTTTTTGAGGTAGTTTTGATCTGCAGTTTCCGTA
TCATTCATCATAACTTGGATAAATGCATCATCCAGCATTTCCACCATGTACGAAAAAGAATCCCT
AAAAAGACCGCTCGTAGAAACTAAAACGTCGATTCTGGGCCTTCCAAGGTCGTCTATTGGGGTTA
CTTCAATTCCAATAAATTTACCACTTTTCCAGACTGGTTCAGCACCAATTAATCTTAAAATCATT
GCAATGGTTTGACCGTTTGTTCTCATTGTTTCTGTAGACCATAAAACTACACCAACCTGTTTTGG
GTACTCTCCAGTTTCATTATGGTAACATTCGATTAATTCGTCAGCCATCTGACTTCCAATTTCCCA
TGCAGCTTCGTCCGGGACATATCTTGGATCAAACGAATAAAAATTATATCCTGTTGGTAAAACAT
CAGGATTTCTTATCATATCTCCACCAATTTCAGGAGTTATGAATTTTCCATCTAATGCATTTAAG
AAATTCTCCATTTCGTAGTTGGTAGATATATTTTCAGTAATTGATACAATAAATTCCGAGTTATT
TCTATCATCGGGATTAAAACTGACTATTGCGTTTGCAGTGTCGTTCAAAGCTGTTCCATTTGGAG

ATATTCCAAAAGTATGCAGTCCATACGGCATTAATGTAGATGAAAGCTCTTCTAAATAGTGTTCT
ACACTATCTGTAACTTCTTCAAAGTCATCAGTTTCGAGGTCTAATTCAAGATCTGAATCGAGATT
TAATTCACTTATTAATTCTTTGATATGATTTTCTAAAACTGCCTGTCTTGTACTGTTTGTAGATA
AATCGTAGTCATTTATTAGCGATTTTAAATCAGAAAGGTTTCCATAAAGTCCAGAAGGAATCATT
GGTGATGTCTGGTGGTCGATAATTACAGCGTATCCTCTTCTTTTAGCTTGAGTTCCTTCGCCTGT
GTTTTCAACGATATATGGGTTAATGTTTGGCAAATTTCCAAGTACATTGTTTGGCCAATCTGTTT
GGCCAAGGCCTGCACTTTTTCCGGGGAGCCATTCAAGGGTGCCATGAGTTCCAAGATGCACTACT
GCATCTGCACCGTATCCGTTTTCATGCTGTAACCACATGTAAAATGCAATATACTGATGACTTGG
GGGTAATTCTGCAGAGTGTGTTATTTTTGTTGGATCTTCGCCCCAACCCCTCATGGGCTGTGGCCC
GACAAAGATATTTCCAAAGAATTCTCCTGGAATCACTATACTATCATTATAAACCATCACGTTTC
CTGGAACTTCTCCCCATTCTGTAATGACTGCATCCTGAAGTTCTTCTGGAAGTTCGCTAAACCATA
ACTCATATTCAGACACTGGAATCGTTACTGCACCGTTATTTACAAGCTCTTCAAGCTCGCCAGGT
GCCCAACCACCAACGTTTAGACCTTTAGTTTGAGTTAAATTAACTACTGCATCAGGAGTATAATC
TCCAGAAATAGTATATCCTGAATGATTTAATGAAGTCAATATTTCATAAATTGATTTTTCAACGT
TTAAGTACGAAGCACCAACTCCGTCTTTTCCACCTGCATGATTATAATAAAGTATTGCTATTTTT
TTATTTTCGTTAGGCGTATTTCTCAATTCTACCCATTTTTGAACCCTTTCAGATAGTTGTTTGAT
ACCTGATTCGAGCGGTACGTAATCATTTATTAAAATTCCCGTTTCTTCATCGATGTATCCGTCTT
TTGCACCGCCAATCATTATAGGTTCGATTCTACCTTCCAGTTCAGGCTGTGCAATTTCCCATGCAA
CTTCGGTATTTAGACCTTGTGAACTTTCATTCCACGTTTCAAAATCTAGTGCATATACTGGAGTT
AATATGGGCACATCGAGTTTTTCATATAATTCAATTCCAATTTCAGTATTTCCAGAATATGTGGT
AAATCCATAATTTGCAACTAAAAGATCTATTCTTGAAGAATTATCTTCATTTAAGAAAAATTCTT
CTATCGCAGACACTCTTGAAGATTTACTTCCTGAAAACGCCAGTATAACATTGAATCCCTTATCT
TCAATGCCTTCAAGCATTGCTTTTTCCATTCCCATCTGGTCGTTGCTGTATCTTGAAGTATATGAT
AAAATACCGACCCAAGGCGCACTTTCATTATAATTTTCTCTGGATTCATACCATGAACGGTAGTC
TTCAAAAGATTCGTATACGTTTGTAGAATCCGGGTGGAATGCATATTCTGTAGGTAGTGTGATTA
CATCGACACATTCCCCATCTTTTAAATCCATTCCACTATAATTTAAGCAGTACAGCTGGAATCTCC
GAATATTTTCTAAACCTGAATTTTCAAAATATGGTTTTAATAGTGTATCATTTGAATCTATATCG
TCGTATTCGGAAAAAGTTTCACCAGAACGGATTGCAAATACTTTATCCGTTGTTCTGTTTTCAAT
TACTTCCAAAATTTTATCTTTTGTTGTACTTACCATTTCTATAACTATGATATCTGCAACTTTCG
AGGCATTTTTTGCTTTTTCTTCCATTTCAGGATATTCTCCTGAACCCAATTCAGATGCTCCGAAAT
AATGAAGGTTAAAATAATATCCTTCGTCCATCAAATCTTTATATGAGTTTGAAAAATGGTTTGCA
AAAGTGCCCGAACCAGTAATAATTACTAAATTGTATGCATCAAGCGATACTGATGCATTTCCCAC
TAAAGTTCCGTTAAATTCAGTAGTGTTTACGGAAAAGTTTGCCATGTGGTGTGCTAAAACAGAAA
TATTTACATTGAAATTAGTACAATTGCTCGTAAAATTTGATAAATACGTGCCGTTGCCTACATTT
ACGACCGTTGAATTAATTATATTACTGTTAGAATCTTCTATAATTATTTTAGGCATTGATTCATT
TAATACCTCCATATGTTCGGCATTGATGTCCAATAATAGTTCTACCGTATCCACCGCATTTACTGC
CGAAAATACTGAAAAAATCATTAAAACAATTAGTAATCGACCTGAAAACTTTTGATTAATAATA
CCACCTATCAATATTAATTTAGTAGTTTGCAATTAAAATTGAATAAGTGTGGTAATATATATCTT
TTTCTAGTTACATTTAGCAATTATAAATTAAAATTGAATAAAATGTAAGATCTTGATCTATAAA
TTTTGAAAAATTACATAACTAATTTTTTAACGTATAATCAATGAAGAATAACATTTTTTTTAAA
AATAAGGCCAAATATAATTTTATTTTATAAAATAAGTATAATTACCCGTATGAATTATTTAACCT
GATTCAAGAACTTATAATATCCTAAAAAAGCTTTTATAATGTATAATTGTTCAAAATTGAATAA
TAAATAATATTGGTTATTTTTCAAAAATACCAACTATTTTTTCAAGAATGGCCAAAGTGTACTGC

TTATCAAGATTTTGATTATCATTACCGCAATTTGGGGAATATAAACATGCAGGACATCCTTCTTC
ACAGGTACAGTCTTTTATGAGATTTAATGCAGTTTTCGCTATTTTTTCAGGCATTTCGTATGCTT
TTTTTGATAAACCAATTCCACCTTCAAAACCATCGTAGATAAAGATAGATGGTTTTTTTGTTCCG
TAATGCATATTTGTCGAAAGTCCGCCGATATCATGGGAATCGCACATTATATGAATTGGGAAAAT
GTTTATCATCGCATGTTCAGTACCGTGGAGGCCGTCTCCAAAATCTTTTTCTTTTAAGTTTTCAA
AATCATTAAAAAATTCATCTTCGAAAGTGTACCACAATGCAGTTGTTTGAAAGGTTATTGGTGGA
AGATTGAGTAGTTCCTGTGACATTCGCTTACCAAATTTCATAACATCGTACATAATGTACGTTTT
AGATACAATTACCTCACCAAAGTGGATTGAAAATGATCCAAAATCCTTCCTATCAAGTTCATTAA
TTATTTCAACATTCGTACTTCCACGGGCTTTCGTGTAATATTCAACATCTTTTTTAACTGTAGTA
CATACTTTTGATTCTAAATCCATTTCTTTTATTATATAATTTTCAGAATTGTGTAACAACACTGC
TCCAACATGCGCTTCTGGATAAGCATGCATTTCATCAAGTATTTCGATTAAATTTCCTTTTTCAT
CAACTACTTTAAATTCGCTGGAAAATATGGAATCTAATGAGACTAGTTCAGAAGCTCGAACCGTG
TTTATATAAACCCATCCACGGTCGGTTTTTTCTAAAAGGCTACAATTACTGAGTTGATTCAAATA
TTCTTCAATATTTTCTCCAAAATATGGTTTATCATTTAGAGTTATTGGAAGCTCAGCAGCTGCGC
ATAACAGCTGTCCGACAATTATTTGCGGATTTTCTAAATCAATAACTGCATTTTCAGTTGTTTTT
CCAAAAACTGTTTCTGGATGGCTTATAATATACTGGTCAAGAGGAGATTCCCTTGAAAGAAGTGT
AACTACTGAATCATATCCAGCACGACCAGCACGACCTGCCTGTTGCCAAAGGGACATTAAAGTTC
CAGGATACCCGTACATTATTACTGAATCAAGACTTCCTATATCAATTCCAACTTCAAGAGCATTT
GTAGACGTTAATGCCCTTAAATCCCCATTTTTAAGTCTACTTTCTATATCCCTACGTTCTTCAGGT
GTAAACCCTGACCTGTATGATGATATTTTCCTTGAAAGAATGTTATTTCCAAGTGATTTTTTTGT
CCATATTGTAATTAATTCTGCCATTTTTCGAGATAATGTAAAGCCAATGGTCTGCAATCCATATT
TCATGCAAAGTTCAAGGATATTTATTGCAGCACTGTACCAAGAGCTTTCAGAAATTTCATTTTTT
AAGGTGTTGTAAAACACAAAATATTTTTTATTTTTTGGAGATCCATCTTTATCAATTACTGTAAA
ATCTAATCCAACTAACTTTTCAGAAAATTCTTTTGCGTTTGCAAGAGTTGCTGAAGCCAGTATAA
ACTGCGGATCTGAACCATAATCCCCACAAATTCTTCTTAATCTTCGTATTAAAAAAGAGATATTC
GATCCAAAAATGCCCCTATAAGTGTGGGCTTCATCAATTACAATATATTTTAAATTTTTAAAAAA
ACTTATCCATTTCGGGTTGTATTGTAAAACCTGGTGGAGTTCATGAGGATTTGTTAAAATAATTC
TACTTTTTTCTCTAATGGATTTTCTTTTACTATTTGGCGTATCTCCATCATAAATTTCGGGTTTT
AAAGTAATATTAAGTTCATTCTCTAAATTTTTAAGATTATTCAGCTGATCGTTTGTTAATGCTTT
TGTTGGATAAATATAAAGTGCGGTTGCATCTTTATCGTTTTGCAAGGTTTCCAAAATTGGTAAAT
TAAACGATAGTGTTTTTCCAGACGCAGTTGACGTTGTTAGAAGGATATTTTTTCTATTTCTTACA
TTTTCTAAAGCAGAATATTGGTGAGTATACAGTTTTATATCCTTTTTAAGCAAGTATTCTTTAAA
ATTATCTGGTAAATCTAAGTTATTTTCAGTATATTCTGCATTTTTTGGAGGAATTTCTTTTATTA
ATTCGATATTACTCTTATGGATGCTGTCCGTTGATAATTTACTTAAAAAATTAGTAATTTTAGAA
TTATTCAAATTTACCATTCCTCCCAAAGTAAATTAAGTATTTTAGCAGAAGTTACGACATCTATT
TTGTTATGTTCTACAATTGGAACTAAGGGCCCAATGTTTCCCGAACGTAAGTAGTTTCTATAAAA
TTCTGGCACCATTGAACTTGGAACATCGTCTTTTCGAGATTCTCCAAGTAATTCTTTTTCAATAG
TTTGTAATTTACAGTTTTCAAGATTATTTCCCCAAGCGCGTCTTGAAAAATGTAAAAGGTCGTAG
TGGATTTGTGGAATTCCTAAATTATTCATTCTATAATATCCGAGCCGATCTTGTGTGAATGGCAT
ATCAAAAGATTTTCCATTAAACGTAACAAGTGCTTTTCGGTTTTTCAGTTCTGAAATCGTATGGT
AAAGTGAAGATGGTTCTTCATTAATATTTCTTAAAAGAAATTGTTTTGTGTTGATTTTATTATCT
GCAACAAATGACATTCCAAAAAGAATTATGGGGCTACCAAAAAGGCCCATTGTTTCAATATCGTA
AAATACAAAGTCTTCTAATTTGTGCATTTTCGATGCTTCCATAATCGTTGGATGAGATTTTGGAT

AATTGTTTGGAATAGAATCTAACAGTCCAGATACTCCGTTATTTTCTAAGCTGGTTAGTATTTCT
TTTGCACAACTTCCGTATCGATTATGTTTCGTTAAATCACAAATTGTATTGTAACCGCTGTTTTT
TAAGCTAAGTTCTGTTTTTTCTCCGATTCCTCGAATTAATTGAAGGTCTGATAAAATACAGTTTT
TTACTTTTTCGATATCATTTCTTTTGATTTTACATTTAAATTCATCTTCAATACAATAAGATGAT
CCATACTCGGATTTAATTACACGTCCTTTAAAATAATCGTCTAAATGAACCCCGCTATATTTCTT
AAGTAATTTTGACTGTAATTCCAATGCCCTTTGGCGTTCTTCATCAAAAAATGGGCTTCCAAATA
CTTGGTTTGATCCATATGTTGATTCTACGGAATCTACAACGGGTTTTGAAGCGGTTGTATCTTCA
GATTTTTTCTTAAATGCTTGTGCAATTCCTGAAATTTTATCTTTTAAGTTTAAGTTTATCTTCAT
AATTTCTCCGGTTAGAAAACTATTGTTTCATAATATCAAACTTATTGGATATTATCTTAACCAAT
TTTCCGTAAATATTTTATAAATAATTCAACATTTCCTTTTCGTATTTTCAGGTTTTAAGTCTAAA
ATTAATTTATTTTAAATCCTGACAATATTTTTAGATGCGTATAGATATGATTTATTAAACGCCAA
TTACATAATTTAATCAAGACTTTTAATTAATAATTTAATCATTTGGGGGAGTTCAATGGCCAAAT
ATCGATGTAACGTTTGCAATGTCTTTGAATACGATGAATCAAGAGGGGATTCAGTTACAAATATA
AAACCAGGTACAAAAATAAAAGATTTTCCAAATAACTGGAAATGCCCAATCTGCGGATCTGACAA
TACTCATTTAATCCAGATAGCTGAAACAAATATTTCTAAAAAACTTAAAGGGACTTTTGAATGTC
CTGAATGCAAATCAAAGTCAGAAATTGAAATTTCAATGCCTGTTAAAATGGATTCAAGTTATCTG
GGGGAATGGAAAAGAGAATACGATGATATGGAAGAACATATGTTCGACATTCATAAAATATCAG
TAACTGGAGAGTCAGTAATTGAACCAATGAGGACGAAAAAACCAGTTATATCTTGGGAAAGTAT
ATTTATTAAAGGTGCACAGCTTGCAAAATTCCCTTTAAACAACGATGAAGAAGTTATTACAAAAA
CAATCATTGGAACTAAAGCAAAACATCCATTAATCATTGAAACACCTATTTTTATTACCCATATG
TCATATGGGGCACTTTCAAAAAATGTAAAAATTGCTCTTTCAAAGGGCAGTGCCGCTGTGAAAAC
TGCAATGTGTTCTGGAGAAGGAGGAATGCTTGAAGAATCCTTCGAAAATGCATATAAATACATTT
TAGAGTATGTACCAAACCAGTACAGCATAACAGATGAAAATTTAAAAAAAGTAGATGCAATCGA
AATAAAAATTGGCCAGTCTTCAAAACCCGGAATGGGGGGACACCTTCCCGCAGAAAAAGTTTCAG
AAGAAATCGCTAAATTAAGGGGTTTTAAAGCAGGACAGGATATTGTAAGCCCGTCTAAATTTCAT
GACATTAATAATAAGGATGATTTAAAAAATAAAGTAAGTACTTTAAGAGAAAAATCTGGCGGAA
AACCAATTGGAATCAAAATCGCTGCAGGGGATATTGAAGCGGATTTAGAAGTTGCAACATATGCA
AAACCAGATTTTATTACAATTGATGGAAGACCTGGGGCAACAGCCGCATCTCCAAAATTTATTAA
AGATTCAACGTCTATTCCAACAATTTTTGCACTTTATCGAGCAAGAGAATTTTTTAATAAAAATG
GAATAACTGATATATCTTTGGTTATAACTGGGGGCCTTAGGATATCTTCTGATTTTGCAAAGGCA
ATTGCAATTGGTGCAGATGCAATAGCTATTGGTACTGCAGCGTTAATGGCTACTGCATGTCAGCA
GTATCGGATTTGTGACACAGGGGAGTGTCCCGTTGGAGTTACAACACAAAAACCCGAATTAAAGG
ATAGGTTAAAAATTGAATTATCTGCAAAAAAATTATCGAATTATTTGAGGGTGTCGACCGAAGA
ATTAAAAACATTTGCAAGGCTTACGGGGAATAAAAATATTCATGATCTCTCTGTTGATGATTTGT
GTACGAGTGATTCGGAAATTTCTAATTTTACATCAATAAAACATGTTTAAATATTTTTTCAATAT
TAAAAAAGTATGAATAATTGTTAGTTTTCTAAAATAGCTAAAAAATAAGTTATTTTACAAACAT
TATTTTCATTTTTAAGTATGAATTTACCATTGCAGTTTAAAATAATTCTTAATATATGTGTAATT
ATAACTTTAAGACCGATTTAGATATTTTTAAAAATCATTAAAATTAGGTTAAGATATAAATACT
CGATCAATAAATTTAATATAATAAATTTATGAAGGCGTATATAAAGATGAATAAATTATTTTTA
AGAATCGTAACATTTACGTTAGGACTGTTTATCATGGCTTTCGGGGTAGCTTTATCAGTTAAAGC
TAATTTGGGAATTTCCCCAATATCCTGTGTTCCTTACGTATACAGTCTTAAGATACCACTTACAC
TGGGTCAATTAACAATATTTTTCAACTTAGTACTGATAATGTTACAAATTGTCGTGCTTCGTCGC
AACTATCGGTTAGTATACTTAGCACAGCTTCCAGCCGTGTTTCTTTTAGGTTTATTTATCGACAT

GATCATGAATGTGATTTCTAATTTGTATATTTCTAGTTATGTAGGGCAGATGGCATTATGCATAT
TCAGCTGTATTGCAATTGCATTTGGTGTTTTTCTGGAAGTAAAGGCCAAGATTATTTATCTTCCT
GGAGAAGGTCTGGCAATGGCCATTTCCAATACATTTAAGAAAGAATTTGGAAAAACAAAAATTG
GAGTCGATGTTTCAATGGTTGCAGTGGGAATTCTAAGTTCTTTCATTTTTCTGCATCAGGTGCAG
GGAATACGTGAAGGAACGATTTTTGCCGCTGTATTGGTTGGTTCTATCGTAAAAGTATTCAATAA
AACATTATTATAAATATTACGGGGTAGAACCCTTGGCTGATTAATAAAATAGATTTTTTCAGCAA
AATAATTATTTTTTTTAGTTTTTTTAGAAATATGCTTTTTTTGATTTATACAAATTATTATAAAC
CGTAAAAAAATGGTTTTAAAAAACTTTACCATAAATTCCTAAAATTCCATTTAATACATCAATTC
CCCGTTTTGTAATCATGTAGTCGCCCCGGTCACTTCTCTGCATTATCATTTCAGAATCCTGCAATT
TTTTTAAATGAAATAATAAATTTCCAGCTTTTAATCCAGTTATTTTTGAAATATCTGAAAATGTT
TTTGTTTCAGCCATTAATGACTTTAAAATTAAAAGCCTGTGTTTGTTTGAAATCGGTTCGAGCAG
TGAAGTGATAATTTCTTTCTCAGAAACATTTAATTCGTTTTTTTCATAATTTTCAGAATAAATTC
CAAGATTTTTCATTAAATCGATCTGTTTTTCAAATAATTTAACGGTTTCAGAGTAGCAGACACTG
CATTTTTCATAAGGGAGTTTTTCTTTTAAATTTTCTAAATTTTTACGGATAGTTATTATTTTATC
CTGCGAAATCTTGTTTTCTTTTAGATTTTTTGCAGAATTATTTAAAATACCTAAAAGCGTTTCAT
AACAGCTACTTTTAAGTTCACAATCCGAGATCATGTTATTTTCAAGGCAGAATTTTGATTCATTT
AAAATATCATTATAAAATACATCTGAATATTTTTTCTTAATTTCATCTATAACTAATTCGACATG
TTGCTGGTTTGATTTTTCGATAAACTGGTTTAATCGGATATTTAGCTTTGAAAGTTCTTCTTTTA
TTTCTTTCATATCATCAGAATTTGCAGAAACGTTTTCCATATTTATCACAAGGTTATTTGAATAT
ACCAAATAAGTTTTAAGTATATTTTGTTGACGCTCAAGTATATATTACATACCATATATAGAATT
TTTGTAAAACTTAGGTGATACTATGGGTAACTGTTGTAAAAATGAAAACTGTAATTGTGCCGACC
AAAACTGTCCAAACAGGGGCAATTGTGTAGCATGTATCAAGGCACACATACAAGCTGGAACTTTA
GTTTACTGTATGCAAGAAATAGCAAATAAACAGTAATCTAAAAAATCTAATTCAAAATAACACA
AAGTGATATTATGAAAGTTATTGCAATAAACGGTAGCCCAAAAAAACAGGGAAATACTGCATTA
TTGATAAACAAAATTTTAGATGGTGCAAAATCAAATGGTGCAGAAGTAATAAAATATGACATTG
ATAAGATGAATGTCAATGGTTGTAAAGGATGTATGCACTGTAGATCTGAGTTTAAATGTGCTCA
AAAAGATGATATGGCAGAAATTTTAGAAAACTTAAAAGAAGCAGATGTTTTAGTTATGGGGTCA
CCAATTTACATGTGGCAGGTTACTGGACAGGCAAAAATATTCCTTGACAGGCTCTACCCTGTAGT
TAATGCAGATTTCAGCCCAAGATTGAGCAATATTAAAACTGCTACGGTTTACGTTCAGGCAAACC
CTGATGAAAGCGCGTTTTTGCCATACATTGAAAACACAAATTCCATGTTAAATTTCCTTGGATTA
AATGTTGTTGAATCTATTGTAAAAGGCGGTGTTGGAGCTCCTGGAGACGTTTTAAACCACGAAGA
TACATTAGAACAGGCATTCAATATTGGAAAAAAATTAACTGAATAATTTTAGAGCTATTTGAAT
TTATGGTACCACGCCCTCCTTAATAATGATATATCTCATATTTCTTTTTTTAAAGAATGTTTATT
CACTTTTTTTAGTCTTTTTGAATCAATTTAGAATTCAATTCTAAAAATATATAAAAAAAGATTTT
ATTTAATTATAATACAGTCTCTTCCAACAAACTGTTCTCCATCGTATGTGGTTCCTTTGATACAG
AGACACAATTCATCATTTTTCTCGTAATCACCAAGACCTGCTTCAACAAGTTCTTGTGTGTCGTA
TTTCATTGAAAGATCTGTTATTCCATCAGGGTATGTAACGATACAGCAGGGTTCACCTTCTTCAG
GAATATAACTTTCTGTTACATCGTCATATTCCCATCTTAAAGGTGCAACTCCTTCAAATTCCGTA
AATTCTCCATCTACGCATATTCCTATTTTTAATGTAGCAGGATCGATGTCATATACATCAAATTC
TTCAGAACCTGCAATTGCCATAGGCTGTACACCATTACTTTTTATGTTAATTGGATTTGGACAGC
TTGCAGGTTTTACATCAAATCCGACAGGAATTTCTTTAATTACTGTAGCTTTTTCAAGCGTTAAT
AAAGCCCATTCAGTGGAAAGTATCTGATTTTGGTTACCATATATTGCCCAAGGATCTCCCGACCA
GAAACCAGTTTCGTTTTGATTAGCTACAATGTAATCTGAAAAGTCTCCATACCAGTCTATTCCAT

CAATTTCTTCAATCTGCATGTATTCAAGACCTTTCATGATGCAGTACATTGCCTGGTAGTTGTAA
TTTTTCCATCCTGTCATGTATATGCCAGAACCTATCTCAGTCCAATTTCCTACAAGGTAGTCTATT
GCATAACCCATTCTTGTAGATTCAGAATCGTCTCCAACAAATCCCATTTCAAGTATTAAATTACC
TGTTTTAAGGGAATTTACCCAATAATCTGGAGTGGTATATCCGGAACCACCGTTTGTATCATTTT
GAATATAATCGATCCACGAATTAAGATCTGTTTTCAATGAATCAGGAATAATTGCTCCAGCATTT
TCAGCATATGCAAGACCGATTACTGCATATCCAGTATTTGAATTATCACTTATTCCTGAGGCCCC
ATATCGCCATGCACCATCATAATTTTGAGTATCTACAAACCAATCGACCATATCTTGAACAAGGT
TTTCGTTGTATTCATCGTAGCCATTTAGATTTGCAAATGCCATCAGGGCTATTGCTGTATTGTAT
GTTTGGTGGTAGTCATACCACGAAAAGAAAATTGCCTGTCCGTTATTGTTAGTTTGGGAATCATT
AATACCAAATTGAACATTTTTTTGTGATTCAAGCCATTCAAATCCCAAAATTACATTTTCAGCAT
ATTCATATTCACTTGTATTGAAAGGATCTACTCCGAGTTCTCTTGCCCTGTCAACGAGTTTTACA
AGTGCAAAACCCGTTATTGCAGGTAGATCATCTCCACAATCTCCCCAGTAACCAGTTTCATTTTG
CTGTGACGCAAGCCATTCGACACCTGCATCAATAGAAGCTTCGATTTCTTCGTCTGTCGCAGCAGC
TGCACTTGGTACAAATGCAAGAGGTGCGAGAACCATTATCAAAGCTATCACTAAAGCTGATTTTT
TCATTAAATACCCCCTCTAAAAGATTATTTTTAAAAAGTAACTTGTTAAATTAAGTAATTTACAA
TTTAAATTTTTTAAATCGTTCTATATATTTGTTGGCTTTTAAAAAAAGGTTTAAACTTAAAAAT
ATGATTTATTGTGTATCATATAACTAATAAACTGAATGTGATGTTTTAGTTTAGACTAGTAACAT
TATTATCATTTTATTTTTTCCAATCGAGCTTTTTAAAATAATAACTATACTTTAATTGAGATTTA
AAAACGTAAACAAAAAAAATAAATTGTTATGCCTTATTTTAATTATTTATCCAATATTACAGGT
TATATTTTAATAATCTCGATTAAAAAGATTTTAAATCCAATATAATTATATATGTCTGGGTATA
TACTATTATTAAGGCTAATTTACCATTATTTACTAAAAATTATTTTCAAAAAGCACTGAAAAACT
TTGGAGGGGGTCTTCATGAAAAAGTTTTTAGTTGGATTTATATTATTTTCCATGATTGTTTCAGT
AATTCCCGGAGCCGTGATAGACGATTGTGATACAACCTGTATCTGTTGTACGGAATGGGAACCAT
ACGGCCTATACGCTGGCCAAAATGAACTTGTTGGAAATGTATCGGTCCGTGTTTGCGGAGATAAT
TTAGAATACAAATATGAAATAACCGAGCCAGGATGGTGCATTTCTGAAGTTCATTTAAATGCTTT
AACCGAAATGCCTGAAAAAGATGAAAATAACACATGGTATGCTGGAAAATACATGACTAAATCA
GGAAATCCAAAAGTAGGAAAATTTGAAGTAAATGAAGAGTGTCTTTGTACAACAGAATACTCTG
GATCACTTAAGTTAGATGATTTCAAACCCTGTTGGGGTGATAAATTATATATTGCAGCACACGCG
GTAGTTTGTCGCTGTGACATTGAAAAAAAATGCGTTGAAGCAGAAGAAGGCGAATCATGGGCTG
TTGGTTTTGAAAATTATATAATGGGAGATAGTACTGGAAATGATGAGAATAGGTACGACCCGTA
CAGATGTCTGGGTGTTGCAAACGTCTTAAGTGGCGATAGGGAAAACTTCACCAGTCTTGGTAGAA
ATGGATCAATTGAATTATTCTTTGAAAATGAAATATGCGGGGACGGCGATTGCAGTACTCCTGAT
TTGGAAGTTTATGAAATAACCTATAATCAAAATACGGGTTGGGAAGAATTAATCAGCGTTGAAG
CATATTATGGGGGAAGTTACCATCCTGTAGGTATTGCTTTGAATGCGAATGGAAGTACTGCAGTT
AAGGATTCCTTTATAAACTACTTTAACCTACCCTGCGAATGCATTGAAAAAATAAGAATAACTGA
TATGTCAACAGGGGCTTCAGGAAAACAGGTCTTTGACTATGATATTGACGGTGTAAAGGCTAACT
ACTGGTGTATTTCTGAGTATACTTGCACTGATGACTGTGAAACTGCATGGGGTAACGGAGCTAGT
TTCCCTGGAAACAACTGGGCAATGTACTTTGAATATCCTAGCTGCATGTGTGATTAAATAAACTT
TTTTAAATTTTTTTAAGAGAATGGCTGAAAAGAATTTTAATTTTAAATATTATTTTTTCAGATT
TTGTGAATATTTACAGGGTTTGTGGGCCCCTATTCACAAAATAAGCATTTATATGATTAAGTTAT
AGTTTTAAAAGTAAGATATTGTAGCATTTACAAAACATGTTTTTTAAAATATATTCTAACCGAA
ATTGACCCGATTCCAATATATAAATTATTTTGGAGTAATAATTGGCACATTAAAACCAATATTTT
GCATGAAACGGGTTTTTCATGTTTTGTAGGCTACAACTTAGAATTACCTCTTAAATCCTGCTAAA

AACGTTTTAAATGTATTCATTATCGTTTAAGCTTTAAAAAAAATCGTAACTACTTTTAAAAAACT
AAATATTGGGATATTGTGGTTATAGATAATGAAAAACATCAAAAAATCAGGGAAAACATTAAAA
AATTGGTAACTAACGTATTAAATTCTGATGAGGAATTTAAAACACTTTTAGGCCATGCATATGCA
AATTTAACTTCAAAAAATCATAAATGCACAAATAAACTATTAAATAGGTTAAAAGAGCGTTATG
GGGTGGATTTAACAGGAGATGTTGATAATTTAAGGACAAATTATGTTAATAAAAAATCAAAAGT
AAAAACGAACGATGATTTAAAAAATTATACAAATAATAACGCAGAAACTAAAATTGATAGTTAA
TTATCAATATTATACTTAATAACTGATTTTTGGTAAATTTAAATATTTAGGTAAATTATCCAATC
ATTGATATTGAATCGATATAAAACTCTACGGAATCTCCTTTTTTCAGCGGATTTTTTAAAAGATC
GCTGTTTGAAATTTCAGAAATCAGTTCTATATTGTTAAATTCGATTACGGCTTTTGAATCAAATG
GGCCTGAAGGAACAATGCTTTTTATTTTTCCAGCATATATTTTTGAGTCTTTTTCAGATTTTTTT
GAAACTATAATATCTTTTGGAAATATTCCAAGCGTTATTTCTCCATAATTCGCCTCTTTAACGGG
TATTTCAAACCCTAAAAGCTGGATAATATAATTTCCATCTTTTTCTATGCAGTTTCCATAAAGTA
TATTTTTAATTCCAACGAATTTTGCAACAAACTCATTTTTGGGATGTTTTAATACATTTTGAAGC
GTTCCGATATGTTCTATTTTTCCATTTCTTAATATTGCCATTTTGTTTGACAAAACTGCGGCTTC
TGAAAAGTCATGAGTAACGTGTATGAAAGTTACCTTTGAAGACTTGTGAACTTCTTTTAAATCAT
ACATCAATTTTTCATGAAGTAACCTATCAAGCGATGATAGCGGTTCGTCAAGCAGTATTATTTCT
GGCTCCATTACAAGCGCCATTGCAAGGGCTACTCGTTGCTGTTCACCGTTGCTTAATCTTTCGGGT
TTTCGGTTTAACAAATGCGTAATATTAAGTAATTTTGAAATATTATCAACTTTTTCCTTTTTTTC
AGGTTTTTTTAGGTGTTTTATCCCGTATTCAATATTTTTTTGAATCGTTAAATGGGGAAAAAGAT
ATTTACCATTACAAAGAACAATTTTTCGCTCGTTTATTGGTTTATCTGTAATATCTACCCCGTTT
AGCATTATTTTTCCAGAATCAGGTTTGATGAACCCTGCAATCATTTCAAGAATGGTTGTTTTACC
GCTGCCGCTTAAACCCAAAAATACAAAGTAATCACTATTTTTAACTTCAAGTTTTTCAATATACA
ATTCAAAGTTGCTGAGGTTTTTTCTCAAGTTTTCAATTACAAGCATTATTTACCACAAACGATTA
TTTTCCAAGGGCACAGAATCTTTCTAAATCAAGCATTTTTGCTGCACACGCAACAGTTGTTCCGT
AAAATATTATTCTTTTATCATATTTTTTTGTATAGCTTAAAATTTCTTCTAAACTTCCATTTGCA
GCCGTACTGCCTGTTGAGAGAACGATGTCTGAGTTTTCTATTAAATGTTCGTTCATACTGCCGTG
CATTATAACAATTCCCTGTCTAATTCTTCCAATTGTGTCTAAATCCATGTCTGTTGCAATAACAT
TATCTTTTCCAAATGTGGTTACCATCTGTCTTATTATTGCAGGGTGGTATCCAATAACCCCAATT
TTTATTTCTTTTCCGTATTCTTCCATTAAAAATTCGGATAATTCTTTTGCACAAACTTCTGGTTC
TCCTTTTATGCAGTGTTCTGATTTTTCGACCATTTTCAGATTTTTCATGACTGAATTTAGTGCAG
CAATAATCATCTGATTACTTCCTGACGCAATAACTTCTGAGAGTTTACCATTAAATTCAACGAGA
TTGTTTGTAAATGCATCTCCAACATCGCCTTTAAAGAATGCACGAAGTAAAAATTCTTTTCCTTG
CATTAATGGGTAATCGTCAATAGTTTTTGATTCTAAATTCACATTAACTGGTTTTATTTCTATGG
TTTCATCCAAAAGGCTGTTTTCTGTTGCTATCTTTTCTAATTCTGATTTAAAGTCTATCATTGTT
CTCACCTATTTTTAAAACTGATCTAATTATAACTAATACTAAAGTTGCGACAAGTAAGAATATTA
ATGATACTGCAAGCGCTTTTTCAAGGTCTCCAAGAGAAATATTTAAAAACACTGCAATTGGAAGT
GTTTCTGTTTTCATCTTTGTAGCCCCTGCAAGCATCATTGTGGCCCCAAATTCACCAATGCTTCGC
GCCCATGCAAGTATTGCTCCTGCGATAATTCCATTTTTCGCCATTGGAATTACAATATCGAGGAA
GCTTTCGTATTTTTTAGAGCCGAGACTCTGGGCAATGAGTTCGTACTTTTCATCGATGCATTCAA
AGACGGATTTAGATGTTCTTATTATAAATGGCGTTGCAACGAAAAACTGCGCAATTATCGTTCCT
TTGTATGTAAAAAGGATTTCAATTATATTTTCAGTTATAAATTTTCCAAAAACTGTATTTCCAAA
AAATACCAGTAAAGCAAAGCCCGTTATTAATGGTGGGAGAATTACCGGAAGTTCGACAATGCTGT
CAACGATATCTTTTCCTTTAAAGTTGTATCTTGCAAGTGCATATGCTGCAGGAACGCCAAGCAGT

GTTCCAAGTATTGTTGCAATGCTCGCAGTTTCAAGGCTTAATTTATTGCAAACTGGATTTCTTT
TGAAAATAACGAATCGTAAAAAGTTTCAAAAGATATGTTCGTAACTATGGACATTACTATCATT
AATATGAATAATATGAATACAGATAGTGCAAAAAGTGAAAAAAATTTTAAAAAATCATTATTTT
TCATAATATCCCATGAATTACTCTAGAGCTACAAAGCCATGTTTTTCAAATATCTCTTTTCCTTC
ATTATTTACAAAGTCGTAGAATAATTTTGAGTTTTCGTAATTTTTGGTAGTTGATAGTATCGAGA
TAGGTACTGTTTTTACAATATTATATTCGCTTTCAATTGGTACTATATCAACGCGATCTGAGTTT
TCATATGCGTCTGCTTTCCAAACAACTGCTGCATCTACGTTTTTTTCAGTAACATATAATAATAC
TTGTTTTACCGTAGCGCCGGTTACGACAGTATTTGTATCAACTGCTTTTGTAATGTTTAATCCTT
TAGTTTCAGCTTTTGCAAGTATTTTTTTGAATAATTTTCCAAGTGAGATGCTGCTATCTCCAAGT
GCAAGTTCTAAGTCATCTTGGCCTAAATCTGAAAGTCCTGTGATGTTTTTAGGATTTCCTTTCTG
AACTGCGATAACAGGGATGTGTTTCGTAACGTTAGAGCATTCTTTAATCATTTCTTTATCTTCCA
ATTGGTAAACGTATGAACAGTCTCCGGGCATGAATATGTCGCCCTGATTTGATGCAACTATTTTT
GATAAAAGTGCACCGCTTCCCGAATAATCATATTCAACTTTTATTCCGTATTTTTTTTCAAATTC
GACTGCAATTTCATCCATTGGCATTTGCATTCCTGCGCCAACGTACGCATGTAGTACTTCTTGCTG
TTCTGCTCCCGTGTTGTCAACACAACCTGAAAACAGCAGGGTTAATGATAGTATCGAAATTATGC
CGAGTAATAATTTTTTCATAATGGCTCACCATCTGATTTTAACACGGAAATTTGTTTCCTTTATT
TATATTATTATAAGGAATTATAATTCCGGTATGTATTTCAAATATATATAATTTACCCCAAATGG
GGTTTAACTGCTGATATTCATGCAATAACTGCTTGAATCAAAAATATGTAATTATTTTAAATTAA
TATCTGGGTAAAATAGTAATTAGTAATAATTACTAATATATAAAGTTAACCTAAAAACTCGAGT
AAATAATCAAAAAAAGATAAATTGTTAAGGAAATGTTTTTAATTTTGGAAACATTGTAATATTA
TTCGATGAACAAGAAAAGAAATGATAAAAATTTTAGAAATATCTTATTAATTTTTTAATATGTT
TTAAGCTGTATTTTTTAAAATAATCGTTTTTTATCCAATTTTAGTATATTTATATATTCCGATAT
AATTTATGTAATATATATGGGTTTCGACTTGGCTTTAGTGCAAATAAATATAATTACATATATAT
AATTAGGTAATATGATATGTTTAATAATCGGATATTCAAAATCTGAAAAAAAACTAAAGTAGAT
TTAGACATATCTGGGTGAATACATGCATAAAGCACAAGAAATTTTGGACTTGGTTGAGGACCCCA
ATCTTCTGAGTCAATTTGAAAGAGTAGTTGAATTTCATGGATTCCCGACTGCGGGAGCGTTCATT
GGAATTCAGATGTATAACTTATCAAAAGAATTGCTAAAATTTAGTAATGATGATCGAATCTACG
TTATAAGCGAGACTTATAACTGCTTACCTGACGCTTTTCAAATTTTAGGTAATGCTACCACAGGA
AATAAGGGTTTAAGGATAGAAGACCATGGTAAAATGGCTGCAAGGATCAACCCTTGGGCTCCTGC
AGGAGATAATATAAAAGGAGTTCGTATAATTTTAGATCCTAAAAAAACTGTGAATTATCCCTTA
CTTCACGCTTGGTACATGAATACTGCTAAAGTATCTCATAAAGATGCAGTATCTGAACTTTTGAA
AGCAGGAAATGACGTTTATTCGTATGAATTTATCGGTGTTGTAGCACCATCTAAGCCTAAAAAGA
AGGTAGAACTTTGCGAAGTCTGCAAAGAACCATTCATACAACAAAATGGCGAAAAGAAATGTTT
AGCTTGTTCTAAATAATCTTATCATTTTAGATTTGCTGAATTAATACACTGGTGGTATGATGACC
AAGATGGTTATAAAAGGCGGGACTGTTTATGACCCCCTAAATGGTATTGATGGAGAAGTAATGG
ATATCTTCATCAAGGATGGGAAAATTGTTGAAAACCTGTCCGATGGCGAATTAAAAGATGCTAAA
ATTTTAGATGTAAAAGGAAAAGTGGTAATGCCTGGAGGAGTTGATTCCCACACTCACGTTGCAGG
ACCTAAAGTAAATACTGGAAGAGTAATGTGTCCAGAAGACCACTACAAATCCCCTTTGAAAAAAA
CCCACAACACACACTGCGGTTCTGGGGAAATTGTTCCTTCAACATATGCGCAAGGATACAAATAC
TCTTCAATGGGTTACACAACCTTATTTGAAGCAGCAGTACCTCCAATGCTTGCAAGACACACTCA
CGAAGAGTTTAAAGTAATGCCAATCGTTGACAAAGCAGGATATCTTCTTTTGGGAAGCAACTGGT
TTGTAATGAAATATTTAAAAGAAGGAAATGTTGAAAAAGCGGCAGCATACGTTGCATGGGCTCT
TGAAACAACAAAAACCTTTGGTATTAAACTCGTAAACCCTGCAGGTGTCGAAAGCTGGGCATGGG

GTAAAAACGTACACTCACTCGAAGAAGAAAACATTCACTTCGGTATTTCATCAGGTGAAGTGATT
AGAGGGCTTGGTGCAGTAAACGAAATGCTTGGCCTTCCAATGTCAATACACCTTCACGCAAACAA
CCTCGGTCACCCTGGAAACTGGGAGTTTACAATTGACACGATGGATGTTGCAAAAAATATCAAGC
CTGCAGTTAACAAACGAGGCTATGATAATGGCGTAAAGACAAAATACTCACACGAAAGGGAACA
AAGCTTGTACATGACCCACATGCAATTCCACTCATTTGGTGGAACGTCATGGAAAGACTTTGAAT
CTAGAGCTGAAGATATTTCAAACTATGTTAACAAATCTGACCACGTTGTAATGGACAGTGGTAGC
GTTGCATTTGGAAAGGCTATTTGTATGACTGGAGACGGTCCTGGTCTTTACGATCTGGCTGTAAT
GAATGGTCAAAAATGGGCAAACGTGGATGTTGAACTCGAATGTGGTTCAGGTGTCGTTCCATTTG
TATACAGTATGAAAAACAAGGTACACAGCGTTCAATGGGCAATGGGTATCGAGCTTTTATTATTG
ACTGACCCTAGAAAAGTTATAATGACTACAGATAACCCTAACGCAGGACCTTTCACGAAATACCC
TCAGTTAATTACTTGGTTAATGTCCAAAAAGGCAAGAGAAGATACAATGAAACTATGCCACAAA
TGGGCCGGTGAAAGAAGCGGTCTTGATGGTAATGACAAAGAACTCAGCCTTTATGACTTAGCTAC
AGTTACAAGGGCAACCCCTGCTAAAGCAATTGGTATGGGACACAGGAAAGGACATTTAAGCCCTG
GTGCTGATGCAGATATTACAGTTTATGATATAAGTCCTGATTACAACTCCAGTGATTATGCAGCA
ATCGAAAAAGCATTTACAACTGCACTATTCACAATAAAAGATGGAGAAGTAATTACCAAAGATG
GACGAATTATCGAAACTCCAAAAGGTAGGACTTACTACGTTGACGCTAAATTCAACGAAGAAATA
CAAAAAGAAATTATTGCAGATGTACAGGATTGGTTTAGAAGATATTACTCAATTGGCTTCTCAAA
TTATCCAGTTCCTGATCTATACCTCACAAAACCAACTCCCCTTAATATCAATGTGAGATAGGTGA
GAATCATGGGAGAAATTATACTTAAACCAAAATATAATGGTACGATACCCGTAGAATGTGATGT
AATTACACCAGACACATTTGAAGGAAAATCAAAAGAAGAAATAAGCGCTTTGAAAACATTTATC
GGTCCTGAAGAACACCTTCTCTCAGATATTTTTGAAATTTCTGGGGATTTTACAAGCCAAAAAGA
AGACATGGTAATTAAAATTGCAGGCGATGCTGGAAACGTTAAATTAATCGGATTCCAGATGACCG
CAGGTAAAATTATCGTAGAAGGAGATGCAGGATTTCACGTAGGCTGTGAGATGAAAGGCGGAGA
AATTCTTGTAAAAGGAGATGTTAAGCCTTGGGCAGGAAGAGAAATGGAAGGGGGAACTCTCCAT
ATCTTCGGAAATGCTGGTGACCACTTAGGTGGATGCTACAGAGGACGATGGGAAGGAATGCTTGG
TGGAACCATTATCGTAGAAGGAGACGCTGGAAACAACGTTGGAGACGGTATGGTTGATGGTAAG
ATCGTTGTAAATGGAAATGTAAGGGCATTCTGTGGAATTCGATTAAATGGTGGAGTACTCTACGT
TGGCGGAAATGCAATACGGGCTGTCGGGGTAGAGATGAAGGAAGGAACTATTGTAGTTGCTGGA
AAGATTAAAAACTTTGCACCAGGATTTATTTCAACCGGTGTTGTAAGTGATTATGAAACAGGACT
TTCCGGATTAGCACTCCCTGGAAAATTAATTGGTTTTAACGGAGATCAAGCATTCTTTAACAAGC
CAAAAGGTAAGTTATACGTTTCATTAAGTGAAAATTACGACTTATTAAACGATGAATTGCCTGCT
AAAGAAAGACCTATTGAATTTAAAGGAAACGCTTTGAAAGTTATATTAAACACTGGAAGTACGA
TTGAACAAGGTAGAATCATCAAAGGAGGGAACAAATATTCACACGAGTACTTAGATGTATGTGCC
GTATGTAATATGCATCCTGAAGATTACATTTTACTTGGAAAGCCTGAAAAGGTAAAAGTTTCAAG
TGAAAATGGAAAATACAGTGTTCTTGTACGTGCTGAACCTAATGAAGATGTTTTAAGAAGAAAT
GTCTTTATTCCAAGAAGTGTTTGGGCAAACGTTATAGTGGATGCTTATTCAGTTAGTACAGGATC
TCCGATCTATAAAGGGGGAACTGTTTACGTAGAGCCTTCTGAAGGAGAAATTCTGGAAGCAGAAT
ACATTATCGATAACATCTACAGGTGAATTAGATGGCAAGTCAAACATTTAAAGACATCGTCTGTC
CAGTATGCGGTGGAGCGTGTGACGACATCGAAATAGTCTGGGACGAAGAAAAAAGAGATTTAAC
CGTTAGAAATGCATGTAAATTAGGTGCGGCTAAATTTAAAGAAATCATAAGCCACCACAGGATCA
TGTCGCCACAAATTAGAAAAAATGGCGTATTGGTTGATGTATCGTGGGAAGAAGCACTTGAGAA
AGCTGCTGAAATTTTAGCAAACTCAAAAAGACCTCTGTTATACATGGGGGCAGAAACATCTTGTG
AAGCAATGACTACAGGTCTTCACATGGGTGAATATTTAGGCGGAATTGTAGATTCCTGTTCAACA

GTGTGTCACGGACCTTCTTTAATGGGGGTTCAAGAAGCTGGAAAAGCCGATGCAACAGCTGGTGA
AACTAAGAATCGTGCAGATGTAGTTATTTACTGGGGAACCAATCCAATGGACTCAATGCCAAGAC
ATTTGTCAAGATATGGAGTATTTCCAAGAGGTTACTTTGTTGAAAAAGGAAGAAACAGTAGAAC
CGTAATAACTATTGACCCTAGAAAATCTGCAACTGCGAAAGCATCAGATATACACTTACAACTAA
ATCCAAGCACTGATTACGAGTTGTTCTCTGCATTCATGATGGCTTCAAGAGGTAAACGACCTCAC
CCAAGTATTGAGAAAGTAACGGGCATTCCTGTAGATACAATAATGGAAACAGTAGAACTGATCA
AAAATGCTAAATTCGCATCCATCTATGGGGGTCTTGGTTTAGCTTCATCATTTGGTAAACAGAGA
AACATTGAGTGTGTTATGACTCTTGTAAAAGAACTTCAAAGATACACGAAAGTTACTATCGGTTT
AATCAGAGGACACTGTAACGTTGCAGGATTTAACGTGCTTGCTTCATACCTTTACGGGTTCCCAT
TCGGACTCGATTTTGCAAAAGGATATCCTAGATATAATCCTGGAGAATTCACTGCAAACGATGTT
TTAAGGGAAAAAGAAGTTGATTGTGCATTTATTATGGCATCAGACGTTGGTGCACACTACCCGCA
GGATTCAGTAAGTCACTTAAAAAACATTCCTGTAATTACTCTAGATATTGCGCCATGTCCATCAA
CATCAGTTGCAGACGTGGTTTTACCAGGAGTTATAGATGCACTCGAGTGTGATGGTACATTCTAT
AGATTTGATGAAATACCAATATACTACAAACCATTTGCAAAATCACCATTTGACTTCACGAAAAG
CAATGAAGATACAATGGAGCAGTTGTTTGAATTGGTAAAAGAAATCAAAGAGAGAAACGGTCAA
TAAATTATCTACTTTTTCGGAAGATTTTTTTCTTCCTTACACTAGTTTCTTTTGGAAAATGCAAA
AACATATCAAAACATTTGTTTTGGAGTTTATGTATTTCAAAAAACTGGTGAAAAAATGGTTGCT
GAAACATTTAAGGATATAGTATGCCCTGTTTGCGGGGGTACTTGCGATGATATTGAAGTAGAATA
TGATAAGGAAAAAAGAGATATTGTTGTTAGAAACGCCTGTAAAATGGGTGCAGCTAAATTTAGA
GAAATTGTAAGTAATCACCGTATAACTTCTCCAAAAATTAGAATAAATGGAAAACTAGAAGATT
CTAAATGGGAAGATGCAATTGAAAAAGCTGCTGAAATCCTTGTAAATGCTAAAAGACCCTTATTT
TTCATGGGTTCTGAAACTTCCTGTGAAGCAATGACTGTTGGATTACATATGGGCGAATATCTTGG
CGGAATTGTAGATTCAAATTCCACGATCTGTCACGGACCTTCTTTAATGGGGGTTCAAGAAGCTG
GAAAAGCCGATGCAACCGCTGGTGAAACTAAGAATCGTGCAGATGTAGTTATTTACTGGGGAGTA
AATCCAATGTGTTCAATGCCACGACACATGTCAAGATATGCCGTATTTCCAAGAGGATATTTTGT
TCAGAAAGGAAGAAAAGAACGAAAAGTAATTACTGTAGACCCGAGAGTTTCTGAAACTGCTAAG
GGCTCAGATATACACTTGCAGTTAAAACCCAGCACTGATTATGAACTGTTTTCAGCTTTAATGCT
CGCAGTTAAGGGAAAGCCTATTCATCCAAGTATCGAACAGGTGACAGGCATTTCTGTTGAAAAGA
TTTTAGAAGTTGCACAAATCATGAAAAATGCAAAATTTGGGTCAATTTACGGAGGGCTTGGGTT
AGCTTCATCATTTGGTAAACACCGAAATTTGGAATGTGTTTTAACATTAAATAAAGAATTACAAA
AATATACGAAGTTTCAAATTGGTTTAATCAGGGGACACTGTAACGTTGCAGGATTTAACGTGCTG
GCTTCATACCTTTACGGATTCCCATTCGGGGTTGATTTTTCGAGAGGATATCCAAGATATAATCC
TGGGGAACACACAACAACCGATGTTTTAAGGGAAAAAGAAGTTGATGCAGTATTTGTAATGTGT
GCAGATTTAGGGGCTCACTTGGCCCAAGATTCAGTTAGATATATGCATGATATACCCGTAATATG
CCTTGATATTGCGCCATGCCCCTCGACATCTGTATCAAACGTGGTTTTACCGGGAGTTATAGATG
CACTCGAGTGTGATGGCACATTTTATAGATTTGATGAAATACCAATATACTACAAACCATTTACT
CAATCCCCATTTGACTTTACGAAAAGCAATGAGGATACAATGAAACAGTTGTTTGAAAAAGTAA
AAGAATTAAAAGAACTTAAAAAAAAATAGAAAATAATTTTTTTACATTTTTATTTGAACTTAATT
TACTTTTTTTAAAATTTTTTTAAAAGTTAAAAATTATTGAGAAATTAATCATATATCCAGACTTC
AACTTCTCCGTCTGCAACTGAAATTCCTTCTTTTAATTCAACTATTGCATTTGAATAAGCAATGG
ATGTAATATGGCTGGAGTCCTTGAATACTGGTTTTACAAATCCATTATCATATACAACAGGCAGA
TAAAATCTTTTATCTGCTTCTTTTTTAAATTCGCCCATAATTTTTGCGTTCACAATTTTTTTACA
TTTTTTGGAATTTGAATACCTGCAGAGTAATGGGTACAAAAATAGGTATGAACAGATTATACAC

GAAGTTACTTTTCCGGGCATACATATAATTGGAATTCCATCAATTTCCCCTCCAAGAACTGGTTT
TCCAGGCCTCATCATTACTCCATGAAAAAGCACGGTTCCAAGTTCTTCTATTATTTCGGATGTAA
AATCTCGTTTTCCGGCGGAAACTCCTCCCGAAGTTACTATTACATCGTAATTTAGAGCTTCTATT
AATTTATTTCTTAATTCGGTTTTATCGTCTTTAGAAATTCCTAGAAAAACAGGTTCACAGCCTGC
TTTTTTAACGATTCCTGCAATAGATTTTGAATTCACATCTGAAATTTTTTCAAGTTCGTCTCCTG
TAGAAATTATTGCAACTTTAGGTGTTTTGTATACCTTTAATTCAGAAATTCCTAACGAAGCAATC
AAACCGATATTTCGTTCATTAATTAAGCTTCCCTTTTTCAAAACCAAGTCCCCATTTGAAATGTC
AGACCCTTTTTCAACAACATAAGTATTGTCGGGAATTTCAATTATTCCCATAATTTTATTTTCAA
TTAAAGTATTTTCAACAGGTATTACACAATGTGCTTCTTCTGGAACTTTACCGCCTGTTGCAACC
CAAACACATTCATTACCTTTTATTTTTAAATCTCTAAAATCTCCGGCATGTACTTCATCTATTAA
AACGTAGGATTCTAAACAAGGGCTTGTTACTGCGTATCCATCCATATTGGATTTTCTAAAGTCAG
GAATGTTCTGTTTGGATACAATATCTCTTGCAATAATTCTCCCATATGAATTAAAAAGATCTACG
TTTTCAGTCTTTTCAAATTTAAATTTTTTTAAAATATTTTCTGCTTCGGGTACCGATATCATTTT
TTCACGCAAAAAACCAATATATCATTTAAATTTAGCTTAAAAATAACTTTAATAAGTTACGAAAC
CATTATCTAATTAATTAATAGTAATTACAATATATAAATTAATCATATATAAAAGTTCTAAAAT
TTAGTTAAAAAACATAATATTTATATATGGTTAAATAATAATAATTACTGTTTAATTAAAATAG
TAATTATTTATGGTTAATACTTACCAAAAAGGTGAATTAATAATGAAAAAGCAGATAGCTATCG
CAGGTCTGCTTGCAATAATACTGTTGTTTTCGGGCTGTGTTTCTGAATCTCCTGAGGAAACCACG
ACAACAATAACCGTTTCGGCAGCATCCAGTTTAACTGATGCAATGAACGATATTGCAGCGGCATT
TGAAAAAGATAATCCTAATGTTAAAGTAGAATTAAATTATGCGGCGTCAGGTGCTTTGAGGCAG
CAGATTGAAGGTGGGGCTCCTGTAGATGTGTTTGCATCAGCATCCCAAAAGCACGTTGATTTATT
ACAAGGACAAAACCTTATCGAAAATGCATCAAGAGTTACTTTTGCTAAAAATTCGCTTGTATTAA
TTGTTCAAGCAGGAAATCCTTTAGGAATTGCCTCAGCAGACGATTTAACAAATGAAAATGTTGTA
AAAGTTTCAATTGGAAATCCTGAAACTGCACCAGTTGGAAGATATGCTAAAGAGTCACTTGAAAC
CATGGGTTTGTGGGGCAATTTGGAAGGAAAAACCGTATATGGTGAAAATGTAAGACAAGTTTTA
ACATATCTTGAAACTGGCGATGTTGATGCAGGATTTGTATACATGACCGATGCAAAAATTGCAAA
AGATAATACTATTGAAGTTATAACCACAATACCAACAGTTACGGAAATTAGCTATCCAGTATGTG
TTTTAAAAGCATCAGAACATAATGATGAAGCCCAGATGTTTGTTAACTACTTAACCGGAGAAACA
GGTAAGCAAATATTGACAGATTACGGATTTACTGCTGAATAAGAGGTATTTTATGGATTCGATTC
TATTTCCAGTATTGCTTACATTAAAAATTTCTTTTTTATCTACTTTTTTTGTATTTTTTATAGGA
ATTTTTATATCGTATATTCTTGCGAAAGTGGAATTTCGCGGTAGGAATATGCTTGAAATTATTGT
AACACTTCCAATGGTGCTCCCACCAACAGTTTTAGGATATTTACTTGCACTACAGCTTGGTAAAA
ATGGGGTTATCGGAAGTTTGTTTTACAAACTCACAGGTTCAGGTATTTTATTTACTTGGGAAGCT
GGAGTAATTGCATCTTTTGTAGTGTCCCTTCCTTTAATGGTAAAAACAACAACTGCTGCAATTTC
TGCGGTTGATAAAGAACTGGAACATGTTTCATATACTTTGGGAAATACCAAACTTGGAACTGCAC
TTTTAATAACTCTTCCACTATCTAAAAAAGGGATAATTGCAGGTATGGTCCTTAGTTTTGCAAGA
TCTGTTGGGGAATTTGGTGCAACTTTAATGGTTGCAGGAAACATGCCTGGAAAAACAAATACAAT
GTCGCTTTCAATCTATCAAGCGTTTCAAACTGGGCAATATGATTTAGCAAATATTTTAGTTATTA
CGCTTATTTTAATGTCGTTTTTATCAATATATGTAACCGGAAAAATGGCAGATAAACTGAATTCT
TAAAGCTTAAAAAATTAAATTACTTGAAAAAGATTTTATATACTGGTGAATTAAAGGTTAAATC
AAGTGATTTGATGTTTGGTTTAAGTAATATTTAATTTAAAAAACCTAAAAAGAGGTTTAATATG
TCATTTTATATTCCTGATTATGAACTTGAAAAAATAATTGAAGAGGATATAAATCCCCTTGATTT
AACCTCGCAGATAATGAAACTGGATCGATTTAATGCAAAACTTGCATACAAAGCACGACACGATA

TGGTTTTGTGCTGTACTGAAGAAGCTGAGAGAATATGTAAAATACTCGGGCTGGAAGTAATTTCT
TATAAACGCACTGGATCTTATGTAAAAGAAGGCGAAGTATTTTTTGAAGCGAAAGGACGAGCTG
ACAATGTTCACCTTGCATGGAAAAGCGTTTTAAGGCTTCTTGAAGGGTACTGCGGGATGGCTACG
AGAACTTACGAGTTTGTGACCTTAGCAAGAAAGTATAACGAAAACATAAATGTTGTAACCACTCG
AAAAAATCTTGCAGGAACTAAAAAACCGACAATAAAAGCAATAGTTGCAGGCGGAGCATATCCTC
ACAGACTCGGTCTTTCTGAAACTATATTGATATTTGATGAACATCTGGCTTTTGTTGATGGTGAT
GAAGAACTAAAATATGTTTTAAAAGAAATGAAAGCAAATGCTTTAGAAAAAAAGATAGGAATAG
AAGTAGATAATTTTGAAACGGGTATAAAATACGTTAAAATGGGTTTTGATTATATTCAGCTCGA
TAAAGTTGATTCTAAAACCGTTGAAAAGTTCGTAAAAGCCGCAAAAGAAATAACTCCAAACATTA
CGATAGTTGCAGCAGGTGGAATTACCATTCAAAATATAGCAGAATATGCTAAAACCGGTGTCGAA
GTTATTGTTACTTCCGCCCTTTATTTTGGAAAAGCTGCGGATATAAAAACAATAATTGAAAAAAT
AGACTAAGTATAACGAATTAAACTAAATTATAATTTAATATTCATTTTTGTACAGGATTCCGTCG
TGCCCTGTAACTATATTTTTATCAAGACTTTTTATTTTTCTTATTGAATTTAAAGCAAGTTCTAA
ATCTACAAAAACTCCCGGGGCTCTTTCTTTTAATATATTGTTTTTTAAGGGTGATGCATCTCCTG
AAATTAAATAATCACCATAAATTACAGAAATGCTGTCAAATGTGTGTCCAGGTGTTTCCATAATT
TCAATTTCTGGATCATTTAACTCTTTAAAATTAGACTGATTGATTATTTTCGCATTTTTAAAAAG
ATCGTTATTTGAAACATGGTCAAAATGGTCGTGTGTATTAATTAAATAATTTATTTCATCCATAT
TGATCTTTTTATTTTTAAAATAGATTTTTAGATCTTCTGTTTTTGATTTGGCAGACGTGTCAACT
ACTATTTTATTTTTTTTAGTTTCAATAAATGTAACTGATGAAGCTACTTCTAAAATCTTTGTTTT
TTCTTCATTAAATTTCAAAATTCCTTTATATACCAAATCCAGCATGTTTTCACGTTAATATTTTA
AAATTAAATTAATTAGTTCAAAATTTTTTAAATTATTATTTGAAATTAAGTTATAAAAATTTAC
TTACCATATTCTTCTGTAGTGATTTCAATTGCTTTTTTAAGTGCATTTAGCATTTTTTTAGCATT
TTTTGGCGTAATAATGAGTGATCCTCGATGTATCGATGATTCAACCCGTTTTAACTTGTCCCCTT
CTTTTTCAATAAAGTGCCCATCTTCAAAAATATCTATTTTAAATGCACAGTCGCACTCTCTTGTA
ATCATCAAATCAACTACAAAAACTTCAAAAAAATCATCTGCTTTTCCAATCGGCAGAGTTCTATT
TTCCATAATATCCCCCCTAAAAAGATATTTCTGGTTCAAAAGTGTTCATAATATATTATATTACA
AAATGGATTTTTATATCTGCCTTAAAATGAAAAAAAGCTATAATTTTAGTTTTAATAACAATCCC
ATTGAAATGCTGATTGTTGGGATTATCAGGTATCTAAGCCCGATCTGTGAAAAATAAGTTAAATA
TGTCATTCCAGACATCGGGATCAATATTAAAATATCGAGTATCAGGTTTATAATAAGCCATGAAA
CTCCTACAAATATTCCTTCGACTAAATAATCTTTTTTAATGTCTTTAAAATACATAATAAGTAAA
CTAACACCAATTAACCCGGATACAACAATCATAATAAATTTAAATAAAAATATATCTATTAATAA
ACCTGTAGTTTCCGAATAAAATAAAAATGATAGGAAGAATGGAACTAACCATGTCAGTAGTCCA
AAGAATCCGCATTTCAAATAATTCATAAAACCACCCAAATTCTATTTTTTCATTTTAATTCTTTT
TAAAAGCTTTATAACTTCCCACCAGAGTGTAGATACTCCTGAAATCAATATTGCAGAGATTAACT
GTTTTTCAGTAAGTGGAACTGTTTTTGCAATTTCGGTTGCGGATGGAACATAGAGCATTATTAAA
ATTCCCCCGAATATTCCGATATTTACAAGCCAGAGGAGCATATCATTTTTGAATTTTTTAAATGC
TGAAAATACTGATTCTATTTCTGACTGGTTAACATATACCAAGAAGAAATTCGCTGCAATAAGAA
TTACAAATGCAAAAGTCCTTGCAGTATTTACTTCTAATCCTTGATATAGTAGGTACACGTAAGAA
CCAAAAGCAAATGCAAAAATTGTTACTCCTTGAATTATTGCTTTAAATATAAGGCTATAATCCAA
AAGAGGTTCGTTAGGCATTCGTGGCTTTCTAAGCATGATGTTTTTTTCTGCAGGCTGTCTTTCAA
ACACAATCGAGCATGTTGGGTCGATAATAAACTCCATTAAAACCACGTTTATCGGAAGGAGTAGT
AATGGTAGTTTTAAAAGAGGCGCAAAAAGTGCCAGATACGTCTGCTGGAACGAGGTACGGGGTTT
TTGTAGTAATGGTAGTTTTAAAAGAGGCGCAAAAAGTGCAGTCAAAAATACCGGGATATGGATT

ACAAAAACGTAGCCTATGGCTTTTTTGATATTATCATAAATTCTTCTTCCATCATGTATTGTTTC
AACAATGGTTTCAAAATTATCGTCAAGTAAAATCATGTCAGACGCTTCTTTTGCAACTTCAGTAC
CTCTTTTCCCCATAGCTACACCAATATCTGCATATTTAGTGCAGGTGCATCATTTACGCCATCTC
CCGTCATTGCGACAATTTCACCGAGTTCTTTAAAAGCTTTAATGATCCTTAATTTGTGTTTTGGA
ATTACTCGTGAAAATATGTTTGTTACTTTTATTTTTTCTAAGAGTTCTTCATCACCCATAGAATC
GATTTCGTTTCCTGTTAACACATTTTCACTGTTTTTTAGACCTATAGTTTTTGCAATCGATTTTG
CAGTAGTTCCATTGTCGCCAGTAAGCATTACAACCCTGATCCCTGCGTTATTACATATTTTCATG
GCTTTTGAAACTCCTTCTCTTGGTGGATCCATCAAACCAATTAATCCAACAAATTCAAAGGTATA
TTCGTTTAAATGCTGATTAATATTTACTACATCCATTTTTTTTGCAACTGCAATTACACGGTATC
CTTTTTTCGCCATTTCGATTGATTTTTTCTCTAAATTTACTTTTTCAGATTCTTTTAAGTCACAT
AAATTAATAATATTTTCAAAAGAACCTTTTAAAGCTATAAATTTTTTATTATCTTTATTCCACAC
GTTTCCCATCATTCTGGTTTCTGAGCTAAATGGATATTCATGCAAAAGACATCCTGTACATAATT
CATCTATATTTATTCTGATAGACTTTGAATAAATCATTATAGCCTTTTCCATTGGATCATAAGCT
TCAGTTTCTGATGCTAAACACGCATAAGTCATAAGTTCAGTTTCATTAAATTCTGAATCGAAATA
TGTTTCTTTTACTTCCATCTGGTTTTTTGTGAGGGTTCCTGTTTTATCAACACATAATAATGATA
TTGAGCCAAGCGTTTCTACCGCAGGAATTCTTCGAATGAGTGAATTTTTATTTGCAAGCCTCCAG
GCCCCCATTGCAAGAAATACGGTCAAAATTACTGGAAATTCTTCTGGAATAATTGCCATTGCAAC
GGTTACTCCTGATAAGATGCTATCTGTAACGCTTCCCATGTAATAAAAATTAACAAGTACAATCA
ATAAAAGCATAAAAAAAGCAGCTATCGCAGAATATTTTACAAGTTTTTCGGTCTGTTTTTCAAGA
GGGGTGGGCATAGACTCGGCTGAAAAAATATCTTTTCCAATTTTTCCATATTCAGTTTCAGAACC
GATATGTATGACTTTAACAACAGCCCGCCCTTGGGTTACAGACGTTCCAGCGTAGCAGATGTTCT
TTTTCCAGTGTTCTAAGGTTTCATCTTCTTTTAGATCAATTTTTTTCCACACAACATCTGATTCTC
CTGTTAATGTAGATTCGTTTACTCCCAATCCGTAATTTTCAACAATGATTCCATCAGCAGCTATT
TTTTCCCCTTCTTTTAAAACTAAAAGGTCATTTACAGTTAATTCGGTGCTGTCTATTGTCATCAT
TTTTCCGTTTCGAATAACGGTGGATTTTGGGGAAGATAATTCTTTTAATGCTTGCAGGGTCCGGT
CAGTTCTCCATTCTTGAAAAAACTCGATTGCGCAGATAAATATAACAGAAATAACCATTATTGAC
CCATCACGAGGTTCTCCAAGGAAAAAATAAATAAAAGCGGCAATGAATAACAGCACAAACATTGG
TTCTGTAAGTATCTTTAAAATGCGACTCAACAATGTTTTCTTCTTTTTTTGGAGTAATTCATTTT
TTCCGTAAGTTTCGCGGTATTGTTTTACCTCTAAATCTGTAAGTCCGAAGTAATCTTCCTTAAAA
CCTTTAAAAAATGTCATTTTTCCAATACTCCCCTGATAAATTATATAAAATAATATCTGGAGTTT
AGAAACTATATAATTAATGTCTCAAAATTTAAAAAATAATTAAAAAAAGAAAATTATTCGCTTC
TCAAAGCTTCTACAGGCTTTAATTTCGAAGCATTGTATGCTGGAATGATTCCTGCAATAATTCCC
GCTGCAAGTGAACATCCAATTGCGATTACTACTGATTTAATGCTCAATACAAACTGGTAGCTACT
GTCCATGCTTTGTGCAATGAACCAGACTATTATTTGGGAGAGTATGGTTCCAAGTACCAGTCCCA
AAAATCCTCCAACTAGCCCAATTATTGCAGAGTTAAAGACAAACAGCAGCATTATGTCTTTGTTT
TTGGCACCAATTGCTTTCATTATTCCAATTTCACGGGTCTTTTCAAGAACTGTTGTAAACATGGT
ATTTGAAATACCTGTAACTCCAACAATCAGTGAAATTCCTGCAATGAATGAGAGGAATGTCGTAA
GCATCGAAATTATCTGTTCAGGGCCTTCAAAAACGGTAGGAGACCTTATTGTGAAGTCCATATTG
TCTTCATTAACGTGTCTTGACATCATCAATTTTTTCTGAATGTTTTCAAGTGCAGTAGTTTGATT
TACATCTTCATACAGTGTAAAGATTATTTCATCATATACTCCTTCTTCTTTTTCTGAAATTGAAT
ATGAAGCATCATCATCCAATTCTAAAGTATAAACCGCTTCATAGGGCATGTAAATAGTATTTGAA
CTCATACCACCCATTTGACCAAAGCCTTGTGTTTCATCTTCTTCAAGTATTCCAACGACTTTAAAC
TGTTTATTTCCTATAGTTATCATCTGATTTATTCCAATTTCTCTATCAAACGCTTCTGTTGCAGT

ATAATATGAAATAATCACTGCGTTTGAGTCACTGGCTTGTAAAAACCTTCCTGCAGATAGTTCTT
CATCAGTCATCTGCGACCACACAGCAGGGTCGACTCCAGTAATTGTTGCAGTTTCACGGCCTCCCG
CAAAACGTACTTCATTCCTAGTCGAAACCCGCACATCTACATATTCTATATCCGATATCCCGTTTA
AAATCAGTACATCCATGTCCGTTAGTTCGGGATCCTCGTCATCATCACTACCCTGTGCAGCTGATG
CAGTAAGTGTTATTTCATCACTTAGCATATCGTTAAGTTGATCACTAACAGATGTTGAAAGGTAG
TCTCCAGCAGATATGATCGCGATAACTGATGCAATTCCAATAACGATACCCAAAATGGTAATCCA
GCTACGCAATTTACTGCCTTTTACCATTTTTGATGCCATATTAAATAATTTTAAGGGTTTCATAA
GTATCAACCCTTATTCGGTTTTATTTTTGTTTAGCTAATTTTTTCCTTTTAATTATTCTGTATAC
AAATACTGCAATTGCAGCAAGTGCTACAAGACCTCCAATAACGTAAGGGATTGCTGAGGTTATTG
AATTTTGGATTGTTGAAAGTGGATTCCTGTTTCCGCCAGGTCCGCTAGCAAAGTCTTGTGGAGCA
TTCGAGTCGTCAGAACTCATGCTTACAACTGTGGTTTCACTTGGCATTTCCACTTCTTTTTGAAT
AGTTACTCTATCTCCTTCTGGTGACGTGTAAGTTATTTCAACAATAAGTGGTGAATCACTTTGAG
TAGTTGATAGTTCAAAATTTGAGAAGGTATAGTCTCCACCATCTAAATTTCCGATAATGGATGTA
CTAACGCCAGAAACGCTCCATGCATCCTGTTCCGGGATTGAAACGATTACTGAATTTACATCACT
TTTTCCAACATTCATTACAGTAAGTGTATATTCGCCACTATCTTCTCCAGAATAACTAACTTCAA
AGTCGGTTTTTCCAAGTACTAAAATTCCTGCAAGATGTTCTACCTCTTCTTTAGTGTTATTTAGT
GAATCGTCATAAGTTAAATCCATTGTTAATTCGTAAATTCCGGGATCTACATCTGTACTTGCAAG
GACGGTGTATGTAACGTCAACACTACTTCCAACACCAATGTATGAAATTGTTTTCAAATTTCCTG
ATCCTAATGGAAGTATTGCGTTATTCTCGCTTTCCCATGAAAATTCAAGATTTTTAATTGGTGCA
GAACCGGTATTTTCTATCGTGAAGGTAATGTTTGTTTTATCTCCTGCTACCAATTCGTCAGCATC
CAAACTAACTTCAGCACTTTCTTCGTTTCCAACAGTTACGGTGATTTCTTTAGTATATCCACTAG
ATGCATCTTCTCCTTTTTTATAAATATGTACTTCTATAGGATAATCTCCTTCTGTAGCATCTTTA
CTAACTCTTAATTTGAATTTTGCAACCATTGCATCGTTTCCATCTTCATATCCGTTTATAATTCCG
ATATCTTGTATTAAATCTTCGCCTTCTACTTCTTCAAATGGATAACTTGGTTCAACTTCTACAAT
GAAGTCTCCTTCTCCGTTTCCACCATCATTTTGGATTCTGATTCTAAGGTCAAATACATCTCCTGC
GATCGCAGGATCAGGGTCTTGATTTACTTTATTTATCTCCAAAATCACGTAATCACTTACATCTT
CTTCAAATGTGTATGCATAATTTCCCATAATCGAAAATAACATTATTAACATTACGGATCCTATT
TTTAAGGACTTTTTATACATTATATCACTCATGAACCGCATTCGGCCTGTTATCAATTATATTTA
TAATTTTTCCGTCTTTTAATTCAACAATCGTTTTTGCGTATTTTGCAAGTTTTTGATCGTGTGTA
ATCATAATAATTGTTTTTCCACTTTCCCAGAGGGCAGTGAATAAATTTATAATTTCTTTACCTGT
CGCACTGTCGAGCGCTCCTGTAGGTTCATCTGCAAGAATTATTTCCGGGTCACATGCTAACGCTCT
TGCAATTGATACTCGCTGTTGCTGACCTCCTGAAAGCTGTGATGGTTTGTTATTCATCCTATCTTC
AAGGCCAACCTGTTTTAATGCTCGTTCTGCAGATCTTTCTGCAGTAGAATCATTTACTTCCTGTA
TTTGTAATGGTAAAAGCACATTTTCAAGTGCTGTCATGTTTGGAATTAAATTATACTGCTGAAAT
ACAAACCCAATGGTTTTTCCACGAAGTTCAGATAGTTCAGATTCAGTCATATTCGAAATATTTTT
AGATTTAAGGTAAACGTCGCCTTTTGTTGGCACATCAAGGCATCCTATCATATTCATCATGGTCG
ATTTTCCTGAACCGGATGATCCAACAATTGCGACGAAATCACCTTTATTTACATCAAGAGAAACT
CCTTTCAGGGCCTGAACCTCAACTTCGCCCATTTGGTAGATTTTCCAAACATCTTCAAGACTTATA
ATTTTGTACATATTTTCACTTGGAAATTTTTTCCATACGACTAATCATATTTTTAGATTCTTTTT
TTAAATTGACTAATGAAATCATTCCAAATATGATTAGGAATACCATTATCAGGGGGAGCAGTAA
TCTAATCATTTTTATCACGCTTTTTAAAAAATTAAAAGATATGGATATTATTTATTTTCGTTTT
GGGGGTGTTAGTTATTCAAAGTTAGCTTTTTGACATTCTCCTTTTCTTTCAGGCATGTCTTCAGG
CATATTACTTATCATTTCTTCAAGCTTTTCAGGATCATCAATCAAACTTTGAGTCATCTCGATTC

TTTCCTGAATTCTTGTCCTTAATTGTTCGTCTTCACAAGAAGCATATTCATCTTCAAGACCAGCA
AGCCTTAATTGAAGCTGTTCTTGGATCTCTTCAGTTGTCATGTTCTGGAAGTCGATTTTAAAACC
ATTTCCTTTTCTTTCAGGCATTCCTTCAGGAATATTAGTTATCATTTCTTCAAGCTTTTCAGGATC
ATCAATCAAACTTTGAGTCATCTCGATTCTTTCCTGAATTCTTGTCCTTAATTGTTCGTCTTCACA
AGAAGCATACTCATCTTCAAGACCAGCAAGCCTTAATTGAAGCTGTTCTTGGATCTCTTCAGTTG
TCATGTTCTGGAAGTCGATTGGGCAAACTATTCCTGGTCCCTCTTTTCCCATATCTTTGAATTCTG
ATGCGGCAAACGATATGGAAACAAGTCCAAGAACCATTAATATTCCAAGTATAATTTTTCTCACA
ATCTGCACCTTATACTTCTTTCATGATTTTTTAAGGTTTTTATTCGTTATTTGAATTTATTTCAA
TCGGGGTGTTATTTTCAAAATTTTCAGGAATTTGGGGTGGGTATTTCGTGTTATTAACGTTTTCA
GGCATACCTGACATATTTCTCTGTTCTGGCACGTATTCATTGAAATTTTCAGGCATATTATTTGG
AGTATTTCGTGTTTTATTTGTTATGTTTTCAGGTGTATTGTTAATAATTTCAGGCGTACTATTAT
TCATTTCAGAATTATTTAGTTCAGGCATCATTGGCAGATTTTTGGAACTATTATTTTCAAATTCC
ACTGTTTCAGGTTCAATTTGATTATTGGTTCCAAGTGCTGACTTCAACCAAGATAATATGTTATT
AAATATACCACTGGAGTTTTCAGAAGTTTGTTCGTCCATAGCCATACAATAAGGGACGAGTGTGA
TAACCATAAGAATGGCTCCAGTAGTTTTTTTCAGGTTTTTGTTCATGTTTTCCCTATCTAATTTC
CAAAGGGCACTATTTGGAAAACTATATTTCGGGTAAATTTTAAGTTAGGAATTATTGGGATCTA
ATAATTCCCTAGAGCCTAATTGAATCCCCAAACAATTAGGTTTGAAAGTAAGTGTTCTCACAATT
ACTTTCAACTAACCTACCTCAGGAACAGCATAAGAACGTATTTTGAAACAGCAATTCCAGATTAT
GAAACGGTATTCTAAAGCTTTTTTGGAATTATTTTTTAAAAATAGCATTATTTTATCCGTTGAAT
TAATTAAAAAATTGTTTAAACTTTTTAAATTTAAAAAAACTAATTATATCCGCCCATAAAATAT
AATTTCAAAATTTTTCAAAAAAAATTTCCCAAATTTTGAAAGGATCATGAACGGGCATTTATATA
CTATACTGTTAAAATGAACTATTCCATTATTTGGAACAGGTGTTCCACGTCAAATAATTATACTG
TTTGGACAATACAATTGTTAGATAACTTAAAGAATGGTTGGTATCATGAAACGAATTTTAATTA
TATTTTTAGTACTGACAATTATTGGGATTGCAATAGGAGTAATGCAGCCTTTTGACTCAGTGCTT
GTTGAAGATGATATATTTATTGCACATGGGTTTGAAGATAAACCAAATACTACTGAATTTAACCC
TTATCCTCAAGGAAATCCTGCCTTAAGTGATGACCAAAACCGCGCGATTGAAATTGCTGTATCAA
ATGAAACGGTTTTGGGTTATTTGTCTGAAGGCTACTGGCTTGCACCTGGCACCATGTTATTAAAA
GAAGATTTTGAGGGATATGGTGAAAATTTTAAGGGAGTTCTTTTAGCTAAAAATGAGACAGTAA
TATATGTAATTGTAAATGTCAAAGAAAATAAAGTTAAAATGATACAAAATTACACAACGTTTGA
ATTTAAGGCCAAAACAATGGTTGTTGAACAAAATGATGAATATTCAGAAATTAAAGCTACTGGA
TTTACAGATAATGATGTAAAAACAGTTCGAGAAATATTATCCAATGACCAAAAAACAGCTCAAA
TTATAAAAAATAAGAATTACAATATAACTATTCAAGACATGGTTTATTTAAGCAATAAAAATAT
CCAGAATGCAACATATGCAATGGTTCTACTGGATATTGAAGATGGTTCTAAATACGTGGTAATGG
TGGATATTACCGAAAGGAAAGTTTTCAAAATGGGCAGGCCCGTAAACTAATTGTGTGAAATTTAT
GAGTTCAAAAGTTAAAATAGCAATATATATGCTGATATTTATCGTTTCAGTGATAGTATTAACCT
CTAAATTTCTATATCCGCCAACAGTTCAGGTAATAGTTCAAGGGGACACCACGTATGTTCAGGAA
GTTCCTAATGTGTACACTCTTTACGATTGCATGTTAATGGTTTTAGCATCATTTTCATTATGTTT
AAGTACAACTTTAATTTACTTTACAGTTCGCGATTTAGAGCTTTCAGAGAAAAATAATGTTAATA
AAATAGATTTAAGTGATTTAAACGAGCATATTGGAAAAATTGTATCTTCAAAGGTCGACGATAA
AATTAATGGAAATACTGACGAAATTTCAAACTTTGAAGTAGAAAATGAATTAAAAACAACAGAA
AACGAAGAAAAATTAAGAATGGATAATTTTATTAAAATTTTGAAAGGAAATGAAAAAACAATTG
TAAAAATATTAATGGATCACGGAGAAATGACCCTGAGTGAACTTTTTGAAAGAACAAAAATTCC
AAAATCAACGCTTTCAAGAACATTAAGTGATTTAGAAGTTAGGGGGATAGTTTTAAGATATGAG

CATGGAATAAGTAAAAAAGTAAAATTGAATAAATTATAATTTAGGGAATAGTTATTTCAGAAAT
ATTGCCACAAACATTTTCTGTAACATTTATAAGGGCATTACTTGTATTTTTTGCAGTTTCACCAG
CATGGGTATCTGAATATTTAACATTTACTGCATAAAAATAGCTTGCAATTGCTGAAACTGCAACT
GCGGACGCAACCAAGATCCCTATTTCCATTGAAACCTGTCCTTTTTTTGAATATAATTTTTTAAG
CATAACTTCCCCCAAAAAATACTTGTTTTAATCAGAATGTCTTAATTAATTCTATTTTTCAGGAG
GTATATATGTCTAATTGTTTCTTAGAATGCGTTAATTCCAAAAATTTGGAATATATTTAAATTAA
AACGCTATTTATTGATTTTTTTAAAATATATCCATTTTATTCCGTTTTTATTAACTTTAATCCCC
AAAATAATATATATTACATCATAGAAAATTTATATTATCCCAGAATTAATTTTTCTGGGGTATA
TTGGGGAGTTTAAGGGAACATTAGTTCTTTGAATAAATATTGGCCTAAAACGTCACAGATCCATA
ACCAGTTTTTCGGTCGAAACACGAATGTATGCCGTGCACCATTTACTGTTTTTTAAACGGTAGGC
ATACTTTTTATTTCTTTTTTATTTTTCAAAACGAATTATTTTGGACATAATCCTGTTTCAGGGTG
TTTTATCTTTATTATTTCTTTTGCACGAACAAGTGCTAAATCGATATTTTCGCAGATATTTTCAT
TACCAATTTTATCGGTAAGTCCGTATTTTTCAAATTTAGCTCTTAATTTGTAGTCTGCACCAGAA
ATAATCAGCGATATGTTTTGAATTTTGGAAGATTCGATGAATTCTTCGAGATTTTTGATTCCTGT
CGAATCAATTATTGGGACATTTCGCATACGAAGAATTATTGCAGAAGGTTTTCTCTTAGCAACAA
CGTTTAATGTACTTTTAAATTTATCCGCAATTCCAAAGAAAAACGGTCCGTTTATTTCGTAAACT
TCAATGCATGGCGGAACATCTTTTAAAGTTATTGAATATGGATCTTCTTCTTGTGGAATTGTTTT
AAGGTTAGAAATTTCGCTAACTTCAATTAATCGCTGCATGAATACAATCACTGCAAGAAGCATTC
CAATCTGTATTGCCGTATTTAAATTTACGAAGATTGTGAGCAGAAATGTAACGAGTAAAACGATT
CTATCTCTTTTTGGGGATTTAAATAGTATCGATTTAAAGTGTTTTACTTCAGCCATGTTCCATGC
TACAACTACAAGTATCGCAGAGAGTGTAGCAAGGGGTATCTTTAAAATTAAGGGGGATAACAAC
AGCATAAATAAAAATAACATTACTGAATGGATTATTCCTGATAATCTTGATGTAGCTCCAGCTTT
AATATTTGTTGCAGTACGTGCAAGCGCCCCTGTTGCAGGAATTCCTCCGAAAAATACTGAACCAA
TATTTGCAATTCCCTGGCCTATTAATTCTTTATTTGAGTTGTGTTTATACCCCGTCATACCGTCTG
CAACAACTGCACACATTAATGATTCTATTGCTCCTAAAAATGCGATAGAAAGTGCACTTGGAAAT
AAAAGTTCCATTTTTTGAAAGTTTAGCTCCGGAAGACTTGGAAATGGAATGCTGTTTGGAATCTG
TCCAAATTTGGATTCAATTGTTTCAACCGGTAAATTAAATGCATAAACCAAAAAAATTCCAACAG
CTATTGCTATTATTGGAGAAGGAATTTTTGAAAATACTTTTTTTGAATTCGTAAGAATAAATAA
CGACAATATTGAAATTAACAGTGCATAAGGGTTTAATAACTGAATATTTGTAGCATATGTAATCC
ACTGTCCTAAAAACGATGCAGGAACATTTGTTATTGAAAGTCCAAAAAAATCTTTTACTTGCGTA
GAAAAAATTATTAACGCAATTCCTGATGTAAATCCCATTGTTACAGGATAAGGAATAAATTTTAT
GATGTTTCCAAGTTTTAACAATCCCATAATGATTAAAATAACGCCTGCCATTAATGTAGCGATTA
CAAGACCTTCATAGCCATATGAAGCTATAATTCCATAAAGTATTACTACAAAAGCTCCAGTTGGC
CCCCCTATTTGATATTTACTTCCCCCAAAAACAGAAATTAGAAAACCTGCGATTATTGCTGTAAA
TAAACCTTTTTCAGGTGAAACTCCTGAAGCTATTGCAAAAGCCATTGCAAGAGGCAGTGCAACTA
TTGCAATGGTAAACCCTGCAAATAGGTCGTTTTTCAGGTTTTCATAGTTTAAATTATTATTAATT
TTCATATATTAGTGCCCTCCAGAAATTACGCATCGTTTTAAATTAAATACGAAGTTTATTTTTTT
TAGATTATAACGCATTGCAATTTCAGTATATAAATATATCGAAATTTTTAATTGGGGGACTAATT
TATCGTATATCGAGTTCATTCTGTTCTGACAGGGCAGTGCCAAAAATTGGTAACATATTGCCAAA
AATTGGTAAATTATATATACTTAAAAATATAATCAAAAGTTATGAACGCTTTAGACAAATTATA
CCGGGCCTACTACACAAAAAGTGAAAATAAATTATATTTTAGCGAATTAAAAGAACTAACTGGAC
TTTCAAACAGTTCACTTCAAAATGCTTTGAAAAAACTAGAATCTGAAAAGCAGATTTTAAAGTTA
AAAGAAAAATCAAACGCATATTATATTTTAAAAAATACCAAAAAAACAGTTTTTAAATTTTCAG

AAATCGATGATGAGCGATTTTGGAAGTTACACAGAAATGTGAGGGCCCCAATATTGGATTTTTTA
GAAAATTTACCAAAAGAAATCGATTTTATGATTCTTTTTGGTTCCGCTTCAAGAAAAAAAGAAAC
AGAAGGCAGTGACATTGATTTGATGGTTGTAATTCATTCATTTGAGGATCCTGACTTACAAAAGC
GGTATCGGGAAGAAATAAATCAAAAACTCGAATATGCAAAGGCAAAAGCTGAATGCAGGTCGAT
ATATCCATTAAGCATAGTCGCAGTTGAAATCGACTATTTTTTAAACTCCAATGACCATATGGTAG
AACAGGCTAAATTAACCGGATTTCCAGTTTTTGGACATCAGAATTACCACGAGGTAATTTTAAAT
GTTAAACGTTGAAGAATATTTTAAAAATAAGGAAAAACTTGAAGGCGCTTATGATTTTCACACC
TACAAGAAAAATCTCGAAAAAGAACGCCATGCAAAGTCTTTAGTGTACGCGCATCTTGACAAAGC
AAAACATAATCTTGCATTTGTAAATCAAAATATTAAAAGTGGCAATTTTCAAGATTGGAGTATT
GTTGGGCTTTATTATGCAGTTTACCATGCAGCACTGGCACTTGTGGCTAAAAAAGGTTTTATTTC
AAGAAGTCACAATGCCACAATGATTTTTTTGATAAAAAATTATACAAACGAATTCAGAGATGAA
GAACTGCAGTTGATTGATGACCTTGCAATAACTAAAAAAGATGCAACATTTTACACAGATTTAAA
ATCAGAACGTCAAAAAGCGAGTTATTCGACTGATGCTATGTTTAACGAGTCAAAAGTTTTAGAAC
TTCAGAAAAAATCAATTGATTTCGTAAATAAAGTTGAAGATATTATTGAAGACTAATTTAATCA
TTAATTTTTCAAATTTTAATATTCTATCGTTTTTTAAATCAGTATAACTTTTTATATCCTTTTTT
ACAGATATATTTGTTGTTTATTTACCAATTAATTATTTTAGACATTTAATATTATATTGGATTTA
ATCTGTTTTGGTGGTTTAAATGGGGTTATGGAGTAAAATAAAGGATACTTCATTAAATATTGCA
ACTGTTGGATTATACGGTCTTGGTAAAAAGATTGGAAATGCGGCAAGTACTGCGGAGGAAGCCGC
TGCAGAAGCTGCAATAGCACTTGCATCAATTGGAATTACTGTTCAGGAAGTTGGTGAACAGTTAG
AATCACTACTTTACGAAACAGAAAAGCTAATTGTAATAAAAAGGCTGATTCCAAGAGAAACCGAC
GATTTATGGGACGAAGAAAAAGAACGGCTTGAAGAACTTCAGGCAGAACTTTCAGAAATAGAAG
AAAAAATGCAGAATTTAGGCGTTGATTCTTCAGACTACAATTTTGGTTCTAATTTTTCGGATTTC
TTTAATTTTGACCGAATTCATGAAATAATGGATCTTGTATTACGACGATATCGGCTGAAAAAAGA
AATTTATGAAATATTATATACGGAACCCGGAATTATTTCTGAAAGTATATATAACATAAATGAA
AGCATAGAACGATTTAACACATTAGAACAGCCCAGAATCGAAAATATAATGGATTATGTTGACG
AAAATCTTTTAAAAACTGAAGAATTACTAAAAGAAGTAAAAAAATTGTTTGTAATAAATAAAAA
AGAAGAAGAACTTAAGTTAAAACTTCCATGGTATAATTACAAAATAGAAAAGCTAAAAAGTGAT
AAAGAATACTTTTCAGAACGTTTTGGAAAAAAAGAATTACAGATTCAAAAATTTGAGGAACGGT
TAAAACCAAACTTCGAAATCGACATTGAAGTTGTAAATCCTCCTGATGTAATTTTAGAGACGGAT
ACTCAAAAAGAAGATTTAATAAACTTAAATGAAAAAGCTATTGAAAAAACCGAGATATTGAAAA
ATATTAACGTTCAAAAGACTTCAAAAAATAATGTGGCAACACAAAATGTTCCAAAAGTTATTTCA
GCTTCAACTTACGAAAATGTTGTAAATCTTGGAAAGAAATTTTCAATTGATTTAAACTCATTTAA
AACAAATTCAGACGTTTCAAGGGTAAAATCCTGCACAAGATCAAAAGAAGAAACAATTCAAAGG
GCCAAAAATCTCTTAAAATCACAAAAAGATTATTTTGAGCGAGAAATGTATAGGTTGGATAGAA
TCGTTGAATTAAATAATATAAAAACAGAAAATGAACCCGGAGTAATTCCAAAAACTCTAGACGA
AGTTTATGAAATATTAAAAGGTGTTCGGGAAGAAGAACAGCCAAGAATAGATAAACTCCTCGAT
TCACTAAATGATACGCTTTATGAAACGAAATCAACAATTTCTGAAGTAAAAACTACAATTGAACC
CATTCAGGGTCTTATTGATAGTTTTTCAGGCCTTGAAAAACCATTGAAAATTGGATTAATACTTT
TTGGGGCACTTTTGGTATTAGATCTTCTTGCAGCATTTATAGTTTTGGTAAAAATTGCGATATGG
GGATAATTTAGATATATTTTAAGTTAATCTTCCTAAAATTCTCTTTTTTCCTTTAAATCACGATG
ATTTAGCTAAATTTACTTTTTTATACCCGTTATGATATAATTTATAACTGGGGTGAAAAATATGT
GGGAGAAAGATAGAATCTATGCAGATTCTAAAAGAAAAATTCACGAAAGTTTTCCAAAAATAAT
TGTAAATTTGGCAGTTGCGTTTATAATCTGGCTTCTTGCAATTTTAGTATTCCAACCACTTGGTG

ATTTCTTAGGAAATCCGTTTATATTCGGTTTAATTGGGATGAAAGCCATAATTTCTGGAGTAGTT
ATAATTGCACTTATAATAATTCTTTTAAAGATTCTTAAAAACATATTGATGCTAACAGACGGCAT
TTCAGATATGGTTGCAGTTAAATTCATGAAAGACGACTTAAATGAAGAAAAATTGCAGCACTACC
GATCTGGATTTAGGGGATTAGGATACGTATTACTTGCAATAATTGCATACATGTTTTTCCTGCCG
CTTCTTGCAGGAATATTCGCAGCACTTGCCGGAATCGTTTTAGTGCTACTTATAATCTGGGCAAT
ATTTGTAGTAATTCGCGTAGGGAATATTTTTTCAGAAGATATTGAAAGAAAAGCCGCAGAAATT
ACAAAAAAGTTTGAAAAAACGGAAAATAAAGAATCTGAAGAAGAGTAGATTCTTATGAAAAAAG
AAATAAATATTATTCTTTTACTAATTCTTTGGATCGTACTTGCATTTTCTGTCGGTTTAAATCCA
GAAATACTTTCATTTCTGACTAACTACATTTCTTACGGAATTAATGATATTGCAGCCATTTTTGT
AATATTGATGGTAATTCTATCAATAATTACAGCATGGGGATTAATCTGGTCAATAAATAAGTCA
TTAAAAGAATATTCAAAAGATTTTTAATAATTTATTCACTTTTTTCCCCAGTTTCTTTTTCAATT
TTAATTTTAAGATCCTGTGGGAACCATACGGTCCTTTGAGGGAATGGAATTTCTACCTTTTCCCT
ATCAAATTCTTTTTTAATGTTTTTTACAAGTTCCATTTTTACGTCATACCATTTTTCGCTCGGAG
TCCAAGCTTTTACTGAAATATCGACGCTGCTGTTTCCAAGATCATTTACAAAAATCTGAAACTCG
GGTTCAACTAATACATAAGTCATATTTTCAAGTATCTTTGATATAATGTCCATTGCGCGCTGTGC
ATCCTCTTTATAGGCAATTCCAACTACAATATCAACACGTCTTGCAGGATATTTTTTGTTGTTTA
CAATCTCCGAATTAAAGACTTTTTCATTAGGAATTCTAACCAATACTCCATCCCATGTTCTAATC
CTTGTTGACATCATTTTAATATCATTTACAATTCCTGAATTCGTTCCAATTTGTACGGAATCTCC
AATTTTTAGAGGATTATCAAAATAAAGGAATATTCCAGATATAAAGTTTGCAACAACGGTTTGG
GATGCAAAACCTAAGATTATACCCGTAATTCCTCCTGCAACAAGGAGTGTTCCAAGCTTGCTAGT
AAGCCCAAGCATCTGAAAAACTATTCCAAACGCGATAATCATCGCTGAATAATGAAATACGGTTG
CTTTTAACCTATATTCTGCAAATTTTCCTTCCTGTGATCCTTTTAGTAACAGGTACCTCTTAGAAC
GGTTAGAAATTCGATATGTGAAATAAATGATCAACAATGAATAAGTCAAAATTTTTAAGTCAAA
ACCAAATAGTTGGTACGTTACAAAACTTTCTATAACGTTTAATGCAAGAACTACAAATATTGCAA
GTACAAATGTTGAAATTAACCCTGCAGTTTCTTCGTTTAATATCCACGTCTGTTTTGTAGTTTCG
GATATTTTTTTAACCCTTTTTTCAATAAAATATGAAACGAATAATCCGATAACTACTACTAAAAT
TAATTTTAATCCTGAATAATAAGATAAACCATAAAAAAAGCTGTTCTGTAAATATTCGAGCAGT
TCTGGTATCATTTATATTCTCCTCAGGAAATTTGATACAGGGGTTACATTTTTAGTTCCGTTTAG
CCCATTTTTAGGCGTTTTTGCCGTATTTAATAATTCTACTGTTCCGTTTTTTGTAATATTAATAA
TTGTCGGGTAATATGCTTTATCCTCTGAATAGTACATTATGGTTTCCCAGATTGGAAAAACCAAT
TTTTCAAAGGTTTTCCAGTAATTTGAACCATTGCTAAGAATGAATTTGGTAACGCAAGGTGAATC
AGGTTCTTTGGTATATACTTCGCTTAAATGGTATCTTGAAATATCGCCTATATCGACAGTTCCAT
AAATTGTATATTTTTCTTTTCCAAGTTTGATTACATCTAACTCCATATCTCCTAATTTTAGTTCG
ATATCACAAGGGGATTCAACGTAACCTCTAAATGTGTCTTTTGGGGGGACTATAACTGGTTCTTT
AAAGTTAATTGTTAAATAATGGACTCCATAACCAAATGCGGGACGTGGGACAATTTTTAAGGAA
TAAGACGATTTTTTTATTAGTTTTGAGGTATTTCCTCTAGTATATCTTAACATTCCATCTTTTTC
TTCAATTTCAATTTCCTGATTCTCAATTTGAAATTTTTCCGGGATTAATGGCCGATATCCAAACA
TAATATTATATATGTTTTCAAAAGATTTATATTTTATCTTTTTAAAAAATAGGCCATATCTGATA
GATTTTTTTAAAAACGAAGTTGAATTTTATTATTTAAAAGAAGTATTTCATAATAATTTAAAAT
TTTGTGAAATAAAAAAATTTAATTATTGGTATTTTTTATAAATTCCTTCTTCTCGCAGTTTTTCA
GATTTTTTACTAAAAATACTGCCTTCAACCATGTTTGTATACCCTTTTTTGTTTATGATTTGTTT
GATATAATCAATATTTGGGCACCTGTCGTAATGGGAGTTATCTGTGGACATGCAGGAAGAAAGAT
GTATTGTAACTTCATCTTTTTCAATACCGTCATATTTTTTAAGCCACCTTGAAAAATTTGCAAGT

TTAGAACTTACCAATTTACCAGAACAGCCTCCACAGGTAAAAGAAATATATTTTGTATCCTTCGC
GTAATTTTTGAATGCATCAGTTTTTTCATAAAACGCATTCGTGCATCTCATTCCACTGCACCGGT
TTTTGGCCATATCGCACTGGATTATAACTACATATTTTGTTTTCATGGTTTTCCCCTATTTACCTT
TAATCAGGGTTTAATGTAGTATAAATGACTTTTACACTTGCAATCCGAACATCCGAAAGACTCTA
TCGAATCTAAAACTAAATTAAACCGCTGTGAAACATCGCACTCAAAACGTCCAAGTGTTACGTTT
ACTTCTTTTACATTTGAAAATTCAAGTAAATAATCAATATGCCATCTTTTTTTCTTGCTGTCTGA
CAAATGCCGGCTTATTCTATTGTAAAGATTCATACTATCTCCCATTGCAGAACCGATGTAAAAAT
AATCTCCTTTTTTAAATTTAATTTCCCTTTTTCCAACGAGAATGGTTTTTGGAGAGTTCATTTTT
ATTTTCAAGACATAGGTACCTTTATCCTTTGGGATTTTAGTTTTTGGAGTCTTTTTAAAATTAAA
ATCTGCGTAAATTTTTTCAATTTCTTTTAAGGAATTGTAATGCGGACATATTTTTTTAATTTCAG
AAAAGCATCTGTCGCATTTTGGAATTGGACTGCAGGTTTCCTGTCCAAATACCACGAGTAAATTG
TTTATTTTTTTCCAGTAATTTTTTGGAAGTTTTTTCTAAGTTCCATTTCTGTGTTTTCTGGTGA
CTCAGTATCTGCATAATCCCATCGGTTTGTAATTCTATGGACGTGGGTGTCAACACAGATTGCGT
AATCGTCAAATGCAAGAGTCATTACCAGATTTGCAGTTTTTCGACCAACCCCTGGAAGTGTTACC
AAATCTTCAATCGAATTTGGAATATCTGAGTTATATTTGTCAATTAAAATTTCCCCTAATTTTTT
TAGATTTTTAGCTTTAGTTTTGTAAAATCCTGCCGGATGCACTAACTTTTCGAGTTCGTCAATAG
GGATTTGAACAAGGTCTTTTGGGTTTTTTACTTTCTTAAACAATTCTTTTGAAACTTTTGCGGTA
GTTTCGTCTTTTGTACGTGCGCTTATAACTGTAGAAATCAGTATTTTAAATGCCCGTTCATTTTT
ATCTGAATTTTTAGATATTTTGTCAACTACGGCATCTTTTTTTAATTTTTCGTCCAGGATATCTA
AAAATTCAATAAATTTGGTGTCAAAATCGTTCATAATCATCCTGTCAGTTGTTTTATATATTATT
TTTATAATACTATTATTTCAGAAATTTTAAATTAGCAAACTTACTGAAAATTGGGGTTTTTTCAT
GTTTAATCTTATTATCAAAGAACTTGGGGAACATATGCCATTTACGGCACTTGGGGCCATTTTTG
GTATGGTATTATTGATTATTTTTAATGGAATTTCGTTTAACGAATCTTATTCTATATTTTATACA
CTTCACCCTATACATGTATTTTTAAGTGCATTTACTACAACTTCAATGTATCTTCTGCACAAGAA
AGGCGGTATCGGGGGCTATAAGGGTTATATTACCTTATTTTTAATAGGATATGTTGGCTCTTTAT
TTATTGCAACGATTAGTGACTCTTTGATTCCATATATTGGGGAAATTATTTTAGATTTACCAAAT
CGTGGAGCCCATATCGGATTTATTGAAGAATTTTGGCTTGTAAATTTGTTAGCTATCTTTGGAAT
AGTTTTAGCATATTTTAAACCATTTACAAAAATACCCCATTCAGGACACGTATTTTTAAGCACTG
CAGCATCGCTTTTTCACATAATAATGGCTCTTGGAACAGGACTGAGTTTTTTAATGTATTTTGAA
ATATTTGTATTTTTGTTTATCGCAGTCTGGATTCCATGCTGTACAAGCGATATTATATTTCCACT
AATTTTTGTAAACGAAAAAGATTAAATTAATTTTTTTATTTAATTCTTATTTATTTTTCCGATTT
TTTTATTTTGGTAATATTTATGTTTTGGTCAGATTCATTTGTATTTATTATATATACATTGAATT
TCAAACTTGAATATCATTATATTTTGTATTTACCATAATCCGTTTTAAATGGGATATCTTGCCGT
ATTCTGATAAAGAATATTCAAAAATCCAGAAAAATATCAAGCATATAATAGTTACAGGCCGCAG
TTCTGATGATGAATTCAAAAATCTTCTTGGACTTATATATGCGAGCATCAAATTAAAAAATCAAA
AATGCACGACTAAAATGCTTGATACTTTGAAAAACAAATATAATTTAGATTTAATTGGGGATAT
CGATAAATTTAGGATATATTACTACAAAAAATCGCCCAATATTAAAACAGAAGAAGATTTAAAA
GAATATATTCGAAATAACCGTAAATAATACTATTTTTCGTAAATACTTGTTTGATTATTCGTTAT
TTTTATTATTTTTTTAATATTCATATTATTTTTATTTTCAAGCCCAAAGTTGGTACCGGCGTTAGA
TTTTAATATATCGATAATCAATATAATTCCAAAAGTCAACGTAAAAACTGGGTTTGGCCCTTAAA
GGTGATTTTACAATGCATAAAATTTGCGTAATCGAAGGCGATGGGATTGGAAAAGAAGTGGTTC
CTGCTGCTGTAGAAGTTTTAAAAGCTACAGGTGTCGAATTTGAATTTAATCATGCCTTGGCAGGG
GACGAGGTATTTGAAAAAACAGGTGTAGCACTTCCAGAAGAAACAATTGCTGCTGCAAAAGCTGC

TGATGCAATATTATTCGGTGCTGCTGGAGAGACTGCCGCCGATGTTATTGTAAAATTGAGAAAAA
TATTGGATACTTATGCAAATATAAGGCCAGTAAAATCATACAATGGAATAAACTGCCTAAAAAC
AGGTATCGACTACATAATAGTAAGGGAAAACACAGAAGGCCTCTATAAAGGTATTGAAGCTGAA
ATTGCAGACGGAGTTACCGTTGCAACAAGGGTTATTACCAAAGATGCATCTGAAAGAATATTCAA
ACACGCATTTGAAATGGCAAAAGACAGGAAAAAACAAGGAACTGCCGGAAAAGTCACATGTGCA
CACAAAGCAAACGTTTTAAAAATAACTGATGGGTTATTTAAGAAAACATTCTACGAAGTTTCAA
ATGATTACAAAGATATACAAACTGAAGACTTTTATGTCGATGCAATGAACATGTTTATAATTTCA
AGACCTGAATCATTTGACATAGTTGTTACATCGAATTTATTTGGAGACATTTTGTCAGACGGAGC
TGTTGGAATGGTTGGCGGACTTGGAATGGCACCATCTGGAAATGTTGGTGACAAATACGGTCTTT
TCGAACCAATCCACGGTTCAGCTCCAGATATTGCTGGAAAAGGACTTGCAAATCCAACTGCAACA
ATATTAACCGCAGTAATGATGTTAAGATACTTAAAAGAAAATGAAGCTGCTGAAAAAGTCGAAA
AAGCTCTTGAAGAAGTTTTAAGCCTTGGATTAACAACACCTGATTTAGGCGGTACTCTTTCAACA
AGTGAAATGGGTCAGGAAGTTGCAAAAAGAGTATAACTTAAAAATAACACATTAATTTTCGATT
TTTTATTTTTACTATCTAATTTATAACAATAATTGTTATTATATTATAATTTGAAGATCTGGTGA
AATCATGTCAGAATATGATATTTCAAAAATACTCGCAAATGAACCAATTTATGCTGGAAAGGCAA
AATCAATTTACAAAATTGAAGAAAACGAAAAAGAAGTTTTGGTAGAATTTAGGGACGATATAAC
CGCTGGAAACGGTGCAAAACACGACGTAAAATCCGGAAAAGGGTACTTAAACACACTTATATCAA
CTGAACTTTTCAACGTGCTCGAAAAAAATAACGTTCCAACACATTTGATAGAATACATCGAACCA
AACATAATGATTGCAAAAAACGTTAAAATAATTCCTTTAGAAGTGATTGTAAGAAACATTGCCG
CAGGAAGCCTCTGCAAAAAATACCCCTTTGAACAAGGACAGGTATTAAAAACAGCTGTTACACAG
TACGATTACAAAAATGACGACTATGGCGACCCGATGTTAAATAATGAAATTGCAATTGAACTTGG
GCTTGCAACAGAAGAAGAACTGGTCGAAATCAAAAAACTCGCTTTAAAAGTAAACGACGTTTTAA
AAGGATTTTTTGATGAAAAAGGAATAATCTTAGTTGATTTCAAAATCGAAGTTGGAAAAACCGC
TGATGGACAAATCGTTGTAGCTGACGAAATAAGTCCGGATACAATGAGACTTTGGGACAAAGAA
ACAAAAGATGTGTTAGATAAGGATGTATTTAGAAAAGACTTAGGCGATGTTTTAGAAAAATACA
AAATCGTTGCTGAAAGAATCGGATGCTTAAAATAATTATTTTTACTTTTCTTTTTACCGATTGGT
GGAATAATGACAGATAATTTAGTAAAAAAACTTATTTTATTTGATTTAGATAGTACACTTGCGG
ACTGCGAAGTAATTGATGAAATTGCAAAATTTGCAGGTGTCGAATCAGAAATTAAAAAAATAAC
TAACGAAGCAATGAAAGGCAATATAAATTTTGAAGAATCATTAAGAAGACGGGTAAAACTATTA
AAAGGAATTCCTGTCGAAAAACTTAATAAATTTGCAGAAACAATTCCTGTAATGAATGGTGCCCC
GGAATTAATATTGGAACTCAAAAAACAAGGGTATGTTACAGCCGTTGTCAGCGGTGGATTTGATT
TTGGGGCTGAACATGTTAAAAAAGTATTGGGACTCGATTATTCTTATGCAAATACTCTTTTGTCT
GAAAATGGAATTTTAACGGGCGAAGTAATCGGCCCAGTAATGAGTGAAACTGCTAAAGGCGACA
TATTGGAAAAAATTGCAGCAAATGAAAATATTTCTCTTAAAAACACGGTTGTAGTCGGGGATGG
GGCAAACGACATAAGTATGTTTGAGAAAGCAGGCTTTAAAATAGCTTTCTGTGCGAAAGAAATT
TTAAGATCGAAGGCAGACATTTGTATCGATAAAAAGGACTTAAAAGAAATATTGAACCACATTA
ACTAACTTTTTTCAGCTTTTTTGACTTAAAACAGCTTTTTTCGAACTTTTTTGCTTTGTTTTAAA
TAAAAATATTTATATATCTTACAACAAAAATTTATAATATGTTAAATGGGGGTTCGGTGTTGAA
AATGGATTACAATACGATAAAAATTAAACTTGACGAAATAAATACACAGATAAAAGAATTATAT
GAAAATGCCAAAGAATTAAAAGAAATTAGGGACAGAGCAAACGACGACGTTAAAAAATATAAAG
ATTTAAGGGAAGATATAAATATAAATGTTAAAGATCAGATTGAAGAAATCAGGCTCTTAAAGCA
AAAAAGAGCGGATATGCTGGCTGAATTCAAATCAATGAAAATAAGTAAGTCTTATCTTGCACAA
AAAATAGAACAGTTAGAAACTACTCTTGAAACTAAACCAATGTCAATGGATCAAGAAAAAGATA

TGATTAGAGAAATCGAGTCACACAGAAAGTTATACGAAAGAGCAGTGGAGTTAGAAGACTTAAA
TGAAACGATAAAGACAATGTCTGATGGAATTTCAGTTCTTGTGAACCAGTCTTCAGAAGAACACA
AAAAAGTGCTCGAAAATGCGAGAGTAAGTGCTGAAAAACATCAGGAACTCTTGAGAACTTACGCT
GAAATAAATGACTTAAAAGCAAAAGCTACCGAATTATACGACAAATTAAAAGAAATGAAAATAA
AAGAAATAGTTGAAGAAGAACTTTAATTTTTAATTTATTGATAACAATTAGTGAAAATTATGGA
TGATAGATATTTAAAGCCTTATATTGTCATAGGTGACCTTTCAAAAATACACGGGTTAAAAAACG
AAGAAAAAATTCTTCTTGGAACAGATGGAAGTATTACAAACCTTTTAGAAATTCTTTTCGGCGAA
GAAGTTGTTGTTGAAACACTGTATCAAGAAATAATTGATAATGTAAATTATAGGGCAGTACTGC
TCGGAGTAAATGGAATTCCATTAATCTATGCAACTTCAGAAACGCCATTAAACCTCATAGATGAA
ATACATATACGCGAAAAAATAAAAAAAGACCTTCTTTCGGCAGATATTCCAATTGGAAAAATTTT
AAAGATTCATAATTTAGAAACAAGGCGAGAAATCGTCGAAATAGCCGTAAAAGAAGTTCCAGAT
GTAGTTAAAAATAAACTTAATCCAAAAAGAATTAAAATTCCTCAAAGAACTTACAATATAATCC
ACAAAAACAAGGTGCTGATGACAATAACTGAAATGTTTAATTTAGAAGATCACCTTTGATAAGA
AGTGATTTGATGTTAAAAGAAGCGCTTTTTTATGAAAACCTTGAAAATTGCAAGGTAAAATGCA
ATTTATGTCCAAAACACTGCGTAATTTCTTCTGGAAGACGGGGGATATGTGGTGGTCGCGAAAAC
ATAGATTGCAAACTTTATGCTGTAAACTATGGAAAAATATGTGCAACAGCAGTTGATCCAATCGA
AAAAAAACCTTTATTCAATTATAAACCAGGTACAAGCGTTTTTTCAATAGCAACTGCAGGGTGTA
ATTTCAGGTGTAAACACTGTCAAAACTGGGAAATAAGCCAGTCGTTGCCCGAAGATGTTCCTTTT
AGTGAATTAACTCCTGAGGATGTTGTTGATCTTGCTAAAAATTATTTATGCGAAGGAATTGCGTA
TACTTATACTGAACCAACTATATTTTATGAATTTATGCAGGAAACCGCGAAAATCGCAAAAAAATA
ACGATTTATTTAATGTAATGGTTACAAATGGGTACATTGAAAAGGAACCTTTGAAAAAACTGTC
AATTGATGCGATGAATATTGATATAAAAGGAAATAGTGAATTTTATAAAAAAATATGTTTTGCA
GAATTAGAACCTGTGCTTGAAACTTGCATTTTAGCAAAAAAACTGGGGATTCATGTTGAAATTAC
TAATTTAATCGTTCCAACGTACAACGATAATCAAAAAGACATAGAATTCATAATAAATTTTGTA
AAAGATGAACTTGGAGTCGACACTCCTCTTCATTTCACTGCATTTTACCCCCACTACAAACTAACG
TCGGTACCATCTACGAGCATAGATGTAATCAAAAAAGCAAGAGATATGGCGTTAAATGCTGGATT
AAACTATGTATATGCGGGAAATGTTCCGTTTAGTGCAGGGTATGACACTTACTGTCCAGACTGTA
AATCTATAGTGGTGGATCGTTTTGGATATCAAAAACCAAAATTACATCTTGATACATCCTCAAAA
ACTCCAAAATGTCAAAAATGCGGTAGAAAAATTGATATGATACTTTAATTTATTTTTAAGTATT
TGGTGATATCTTGAGATACCTAGAACTTTGTACAATTGATCATGCTAAAGAAGTTGTAAGGACTC
TTTTAAACGAATTAAGCACTGAAGAAGTAAATCTTTTTGAACTTCCTGGAAAAATGCTTGCAGAA
GATATAGTTTCAAGTGTAGATGTACCTCCATTTGACCGTTCAAGGATGGACGGTTATGCCGTGAG
GGCAAAGGATACTTATGAAGCTGAAGAAGATAAGCCTGTTACTCTTGAAATAATTGATAAAATA
CGTGCTGGAGGAAATTCCGATTTAGAAATAGCTCCAGGGCAGTGTATGGAAATTGCAACAGGGGC
GCCTCTTCCAAAAGGTGCTGATGCAGTAGTTATGGTGGAATTTGCAGAAATTGAAGGCAATTCTG
TAAAACTTTACAAAGCAGTTTCTCCGCATGAAAATATCCAGCCCTGTGGAAACGATATAATGGTC
GGGGAACTTATAATGCGAAAAAATACTGTTATTTCTCCAAGAGATATCGGTGCAATATCTGCAGT
TGGAAAAAAATAAGTTAAAAGTATTTAAAAATCCTAGTATCGGGTTATTATCAACTGGAAACGAA
CTTATAAGTCCAGATGATAAATTAGAACTTTATAAAATTTATGACGTGAATACCTACACGATAGC
ATCATCCATTTTAGAAAAAGGGTGGAATTTTAATTTTTATGGGATTGTACAGGATAATAAAGAA
GATTTAATGGAAAAATTGAAAAAAGCAATGAATGAAGATGTAATCCTGTTAAGTGGTGGAACTT
CTGCAGGAGTTGGCGATTTAACAAGCACTGCAATCGAAGAACTCGGCGGGGAAATTTTAGTACAT
GGAATAAAAATAAAACCTGGAAAACCAACAATTATAGGAAAAATTGGAAAAAAACTGGTTGTAG

GTCTTCCTGGATATCCAACGTCGTGTTTAACCATTTTTGATGTATTATTCAATGAAAAAGGAAAA
ACAATGCGGGCACAAGTCTACAATAGATATATGTCTGCTAAGGGAAGGGAAGAATACCTTCCTGT
CTCAATAATTCGTGGAAACGATGGTTACGGTGCATATCCTGTTTTAAAGGGAAGTGAGGCAATAA
CTTCGCTTACTTATGCTGACGGATACATTATAATTCCTGAAAACAAGGAAATTGTCGAAGATGAG
ATATTTGATGTCCACATGTTTGGCGACATTAGAATTGGACTTAATATTATTGGAAGCCACTGTGT
TGGAATCGATAGAATTTTAAGAAAAGGCCATATTCTTGCAAGAACGGTAAATACGGGATCACTCG
GCGGAATAATGGCTGTAAAGAGAAAAGAAGCAGATATTTCTGGAATCCACCTTTTAGACAATAA
TGGCGAATATAATGTATCTTACCTTAAAAAGTACAATGTATCCGATGCTGTTTTAGTCAGGGGTT
ATATTCGTGAACAGGGTTTCATGTTTAAAAAAGAGCTTGGAATAAAATCAATAGATGATTTAAT
CGAAAAAATTCGAGATTACAGGATAATTAATAGAAATAAAGGTTCTGGAACGAGAATTCTCTTT
GATAAATTTTTAAAAGAGAACAGTGTTGATAAAAATTCAATTGATGGGTATGATATCGAGGCAA
AAACCCATTCTGCAGTTGGAGCAGCGGTTTCGATGGGTAAAGCACAGATAGGTCTTGGAATAAGT
ACGATTTCTGACCACTACGGTCTTGAATTTATCCCTATTGCAGATGAATATTACGATCTTTTAAT
ACCTAAAGAGAAATTGTACGATGAAGACATAATTAAATTTATAGAAACACTCAAAAAAGAAGAA
CTTCCATTTAAAAAGTCAAAAGATACTGGAAAAATAATTTATGAATGTTAAATATTCTTTTTTT
AATTTTTAACAGCATTTTTCGTATTTTTTAGTTATTTAATTAACATAAAAATCTAATCCGAGAAT
ATTTATAATTGATGACACAAAATTTTTAACAGTAGTATTTTCCAAAAATTATTCCAATTTATAAA
TTAATCGATAAAAAGGCGATATAATAAAATAAATGGCTTATTTTAGTTTTTATATTATAAGGGG
GGATTTAAATTGAAACTAACGCTTGAAGAGGGGACTTCATTAGTCGGTTATGTTAGAAATGTTA
TAAAACAGTATTTAAAAGGCGAAGAACCAGATATCCAAAAATATCCTGATAAATTTAATAATGT
TTTTGGAGTATTTGTATCGCTTCATACGCATCCTGAACACGATTTAAGAGGGTGTATCGGAATTC
CTGAGCCAACAATGTCACTAATCGATGCAATAAAAGAAACATCAATAAGTGCAGCTGTTCATGAC
CCTAGATTCCAGCCATTAAAACATTCTGAACTTAAAAATACGATTATAGAAGTTAGTGTTTTAAC
ACCTCCTGAAGATGTTGAAGTCGAAGATTCGATGGAATATCTAGAAAAACTTGAAGTTGGAAGG
GATGGACTTATTATTGAATTTGGACCATACAGGGGGCTGCTACTTCCACAAGTGGCTACAGAATA
CAATTGGGATACAAAACAGTTTTTATCTAATCTCTGCTTAAAAGCAGGGCTTCCTACAACTGCTT
GGATAGAATATGACGTTAAAATAAAGTCTTTTCAAGCACAGGTGTTTGAAGAACTTATTCCAAAT
GGCCCCGTAGTTGAAAAATCAACGTATACTGGATGTTAACCAATACTTTTTTTAATTTTGTCAAA
TATATTAAATATAAAACTTTTTTCAAGTGATTTTTATGGATATACTCCCGATTGAAGACATTTCT
AAATTTAACCAAATAACTGGAAAAATAAAAGAAAAAATAGAAAATTGTGACGGATTAATAAGAA
TCGTTACACATCACGACCCCGATGGACTGACTGCGGGTTCAATAATGTTGAAAACACTTCTAAGG
CTAAATAAAAATGTGCAAATGACTATTCTGGAGCATTTATCAAAAGAATCAGTCGAAGAACTTTC
AAAAGAAAACGATAAAATGTTTATATTTTGTGACATGGGAAGCGGCCAGATTAATTTGATAAAT
AACCTTCAATTCAATGCAGTAATTCTCGACCACCACCCGCCTGAAGTTGCAGAAACCGAGATTGG
AAATATTTTACAGTTAAATCCTCATTTATTTGGGGCAAACGGTGCAAAAGAAATTTCTGCAAGCG
GTGTTTGTTATTTAATTGCAAGATTGTTTGAGTACTACGATTTAGCAACTGTTGCAGTTGTTGGA
GCAATTGGGGACATGCAACACCTGCCATTTATCGGATTAAATAAATATATTTTAAATGAAGCTAG
AAAATACCGTTATTTGAGCGTTATAAAAGATATTGTTTATAATTGTTACAATTTACCAATTTCAA
GATCAATATACTACTCAACCCAGCCTTACATTCGAGAATTGGATGGTATTGATAAAATAAAAGAA
ATTCTTGAAGAATTAAGTATAAATCCTGAAAAAAAAGGAATAACTGTGGCTGAAAAAGAAAATT
TGCTCGAATATTTCAAACGTTTTAACAAAAATGACGATTTAATTGTTGATAGGTACGAAATAAA
TCATAAATTAAACGATGCATTTTATCTTTCTGAAGTTTTAAATGCTTGTGGCAGAAAAGAGATGA
CCTCAGTAGGAATTGGAATTTTGCTTGGAGATGACGAATGCATCAGACAGGGCGAGAATTTATAC

AAAGAATATAAGGATGAACTAATCGAAGAATTAAAAAACGTAAAACTAACGTCAATGGATAATA
TTGAGTATTTTATTGGAGAAAAGGGGAAAACGGGTATAATATCAGCTTTAATGGTAAAAGATAA
ACCAGTGCTTGGATTTAATGAGCAGGGTGAAATTTATAAAGTTTCTTCGAGAGGCAGTAAGGAAC
TTGTTGAATGTGGGTTAAATCTTTCAAAAGCAATGGCAGTTTCAAAAGAATTTGGCGGAGATGG
TGGGGGACACAATATTGCATCAGGTGCTGCGATTCAGAAAAACTACCTGAATGAATTTTTGGAAA
AAGTTAACGAAATTGTAGGCGAACAGTTGAAAAAATAAACTTTTAAAGGATACCTGATTAAAAG
TATAAATAACGGGATATAATATATTATTAGCCAAAAATTGGGGGATATCATGAGAGCCTTCAAA
GTAAAAAGAGATACCAATGAAACAAAAATACAGCTTGAATTAAACATTGACGGGACAGGAAAAT
ATTCAATAACTACAGGAGTTCCTTTTTTTGACCATGTTTTATCGTCATTTGCTAAACACGGTTCA
TTTGATTTAAAATTAGATGTTTTAGGGGATTTAGAAATCGATGACCACCACACTGTAGAAGATGT
CGGGATAGTTTTAGGTAAGGCATTTGAAAACATGGAAAAGAAGAATATCAAAAGATTTGGGTGG
GCAATAATTCCAATGGATGAGGCAAAAGCAACAGTATCCATTGATATTGGCGGAAGGCCATTTGT
TGTTGGAAATTATGTTCCAAACACCGAAAGAATAGGCAGTTTTTCAACTGAAAATGTGGTACACT
TCTTTGAATCATTCTCAAACAATGCAAAAATAAATTTACATTTTGAAGTAACTGGTGAAAACGAA
CACCACAAAGTAGAAGCTCTCTTTAAGGCATTCGGTGTTGCAATGGATATGGCAACGCAAATAGA
CGAAAGAAAAGGAATTGTAAGTACAAAAGGCGTAATTTAAGAGGATAATATGAAAAGGAATAAA
AAAATAGCACTTGTATCACACTGTATTTTAAACGCCAATTCAAAAGTTGAAGGGTTATCAGAATA
CGGCGGTATGTTAAAAGACGTTATGAATTATTTAATGGATGAAGGTTATGGAATTATTCAAATG
CCCTGTCCTGAAATGGCAATTTATGGAATAAAACGGTGGGGCCATGTAAAAGAACAGTTTGATAC
ACCCCATTTTAGAAATGTAAGCAGAAAATTATTTCAACCAATTTTAGAACAGGTTCAGGATTACA
TAAAAAATGGCTATGAAGTATCACTTTTAATTGGAATCGATAAAAGTCCAAGTTGTGGAATAAA
TGAAACCTGTTCTTCAAACGAATGGGGCGGATATTCTAGCAATGGAAATATGGCTGAAAAAAGTG
AAAAATTGGAAATGATTCCTGAAAAAGGTATATTTATGGATGAAATTTTAAATATACTCAATGA
AAATGAAATAGCAATCAAATGTGTTGCAATTGATGAAGAAAATATCGAAAATTCCCTTGAATAT
TTAAAAAGATTTATTTAATTCTTTTTTAATTCTTTTTTAATTTTTTTATAAATTTAGTTCTCTGC
TCATTTTTCAGAATATTACCTATTAATAAAATTAATGTATTAATCAAATATTAGCTAATTTTTCA
ACAATTAATATATATTTTAATAGATAATTTAGAATGAATATTCTTAAAAGAACATCTGGTGAAG
TTTACATGAATAATAACGTATTAATATTAATTATAGGTCTTATTGGAGCAGTTTTAGCGTTTTCA
GGTTGTGTTTCCAATTATGAAGCTCAAAGTACAGAAGATAATTCTTGGAAAAATGTTGAAGAAA
GTGGTGTAGTAAAAGTAGGGCTTTGTGCAGCATGGCCTCCATTTGAATCAAGAAATGAAAAAACT
GGCGAAATTGAAGGTTTTGATGTAGACCTTGCAAATGCTCTTGGTGAAGAACTCGGAATTGAAGT
TGAAATTGTTGATGCAGAATGGCAAGCACTTTTAGGCGGACTTTCAAAAGGAGATTATGATACAT
TAATCACCTGCATGTCTAAAAAAGAAGCATCTGTTGAAAATGTAGAAATGAGTGATGTTTACTAC
GAATTAAATGACGTAATTGTTGTAAGAAATGATAACAATGAAATAAACAATGTTTCAGATTTAT
CTGGAAAAAATAGTTGGCGTACAACTTGGTTCAGGTAGTGAACAGGCAGTTGACGCACTTTCAGGA
CTAAAAGAAATAAAAAGATATAACTACAATCCTGAAGCAATGATTGATCTTCAAAATAACAGAT
CTGACGCAGTTATTGTGGGATATACTTACGTATTATCTGAAATTTCAAAGTATCCTGATTTAAAA
GTTCTCGATACTCCAATAGCTTCTGCAGAAATTGTCATGGTTCAAAAAGAGGGTGCAAAAGAACT
AACAGTTAAATTAAATGAAGCACTTTCAAAAATAAAAGAAAATGGAAAGTACGATGAAAATCCAA
GACAAATGGTTGAGTTTAGAATAATTTCTAAACTTTTAAACTAATTTTGGTGTTTTTATGAAAT
TTTACATTGTTTTTGACAATATCCCGTTCATTATCGATGCAGTTTTTGTTACTCTAAAAATAACG
ATAATGTCTTTTTTGCTGGCGGTTATTATCGCAGTTTTAGTTGGTTCTGTACGTGCAATGAATTT
TTCAAAAACTTTAGATCTGGTTTTAATGGCTTATGTAGAAGTTTTTAGGGGAACTCCTCTTTTAA

TCCAGCTATTTTTTATATATTATGGCTTGCCGTCGATTGGAATTACAATGTCAAGCACTTTTGCA
GCAGTTCTTGGACTCTCATTAAATGGTGGAGCATATATTTCCGAGATAATACGAGCAGCAATACA
GTCAGTTCCGGTGGGCCAGTTTGAGGCTGCAGAATCACTTGGAATGGGTAAAATTGAAAGTATGG
TTTATATTGTGCTTCCACAAGCTTTCAGGATAACTATACCGCCTCTTGTAAATTCATTTTCGTCA
ATTTTAAAAGAAAGTTCATTAATATCCATTCTTGCAATTACTGAACTTACAAGGATAGGACAGCT
AATTTACACAAGAACATCGAGACCTTTTGAGATATACTTAACAATAGGACTTATTTATTTATTAT
TGGTGTCAATAATCTCGATAAGCTCGTGGAAAATTGAAAAAAAGCTAAATGGCGCTTTTGAAAG
ATAATTTTTATCTAACTTAGCAATATTTATATACATAAAGCGATTTATCAATAGTTCTTAAAGAT
ATTCGCGAGTTATACAATGAACAAAGTAAAAGGAACTGCATATACAATTTATTCATCCGTTGCAT
TTGGTATAATGCCTTTTTTAACTAAATTTGCATATGACGGCGGAGCAAATGCAGTTACTACACTA
ATGTTTCGTTTTTTAACTGCCGGATTAATTTTATACGTATTTTTAAAATTTAAAAAAATAAGTTT
AAAAATTTCAGGGCACAATTTTGTAGAAATTTTATTTTACGGTGCATTTTTGTACGCTTTAAACA
CAGTATTCCTTTATGAAGCTTATAATTATATACCGACAGGCATTGCAACTACACTTCACTTTATA
TGCCCTGTAACGGTTACATTATTAATGATAAGTATTTTTAAAGAAAATTTAGGGATTAATAAGG
TTTTGGCACTTATTTTTTCATTTTTAGGAATGTACTGTCTTTTGGGTGGAAACTGTACCGGTTTT
GATATTTATGGGGTGTTACTTGCAGCAGGATCAGGTTTAGTTTATGCAGGATACATTGTTTCTGC
TGGAAAATGCAAATATTCAAAAATAGACTCTTATGTAACTATATTTTATTTATCGATTCTTTCTT
CAGTTTTACTTTTTATTTACGGCCTATTTACAAATACTCTAACATTAAATATGGCATTTTCAAGT
TATGCATCGATTGGACTTATTTCGATATTTTGCACGGTTTTAGCATTGATTGCATTTTTAGAAGG
AATAAAATTGATAGGTCCGTCAAATACTGCGATATTGAGCACACTAGAACCAATTGTAAGTATAA
TTCTTGGTATACTGTTATTAAATGAAGTATTGAGCTTTAAAATTGGTTTGGGAAGTGTTTTAGTT
TTAATTTCGGTAATAATTGTTACAATCGAAAAAAGTAAAGTTTCTGATACAGTTAAAAATTAAA
AAATAAAAAGTAAACTTAAATTAATGTTCAAAAATGTATTTTAATACTGCTTTTTGAGCGTGAA
GCCTGTTTTCAGCCTGATCAAATACTTTTGATTGTTTTCCATCGATTACTTCATCAGTAATTTCT
AATCCTCTGTTTGCAGGAAGGCAGTGGAGTACTATTGCATCTTTTTTTGCTTTTGATAAAATTTT
ATCATTTATCTGGAATTTTGGGAATATTTTTAATATTTCGTCCATCTGTTTATTTTTGTCACTCA
TACTGATCCATACGTCAGTATATAAAACATCAGCATCTTTTGCAGCAATTTCTGGGTCATCGGTT
AATGTTAAACTTCCTTCCCCGTATTTTGCGATTATTTCAAGCGCTTTTTTAACGAAAAGTCCGTT
TGGCTCATAACTTCTAGGAGTTGCAATAACAACATCCATTCCCAAAATTGCTCCAGCAATCATTA
ATGAGTTTGATACGTTGTTTCCATCACCAAAGTATGCTAATTTTAATCCTTTGAAGTTTTTGCCG
TTTTCTTTCATTGTAAGTAAATCTGCTAATATCTGGCAGGGGTGTGCAAGGTCACAGAGTGCATT
AATTACTGGAACACACCCGTATTCTGCAAGATCTTCTAGTGTTTTGTGATTTTTAACTCTTGCAA
CGATTGCATCAACAAATCTGCTCATAACTCGTGCAGTGTCAGCAATGCTTTCCTTTTTTCCAAGG
TGGATTTCTCCTTCATTCATCATCAAGGAGTGCCCGCCGAGTTCATTTACTGCAAGGTCAAAACT
CATTCTGGTTCTTGTAGACGGGCTTTCAAAAATCAATGCGATACTCTTATTTTTGAGTATTTCAT
GCCCGTATCTATTTTTCTTAAAGTATTCAGCATCTTCAATAATTTTTAAAACTTCTTCTCGCTCT
AAATTCCAGAGTGTTAATAAATCCATAGTACCACCGCTAGTAAAATCGAAACAATCAAAAAATAC
ATATTGAAGATAAAATTTAATATACATCTTATATTTAAAGTATACCTAAATATTTAGGATATTA
AAGTCAAGAGTTGAAATGCCATGCACTATTTTTCAGAAAATCCTGATTCAAAACACGACGAGTCT
ACAATCTCGGGAATGCTTCGGGGAAAAAGATTTTCATTTAAGACGGATAGCGGAGTTTTTTCACC
AAAAAAAATTGATAAAGGAACAATAATACTTGTAGAAGAGTTAGAACTTTCAAAAGACGACGAT
GTTTTAGATGTTGGATGCGGATACGGGGTTATAGGTATTTCTATTGCAGATGAAGTCAATTCGGT
TACAATGACGGATTTAAACAACCGTTCTGTAGGGTTGACGAGAAAAAATATAAAATTAAATGGA

AAATCTGAAAAAAATATAGAAGTTTTTCAAGGGGATTTATTTGAAAAAGTAAGTAATAAAAAAT
ACAGCGTAATTATATCAAATCCACCTATAAAAGCAGGAAAAGAGTTAATACACAAAATTATTTCA
AAAGGACACGATCTTTTAAATGAAAATGGATCAATATGGGTAGTTATCCAAACAAAGCACGGTGC
AAAGTCACTTACTAAATACATGGAAGAAATTTTTGGAAATGTTGAAACAGTTACAATATCTGGC
GGATACAGGGTTCTAAAATCCGTAAATATGGGCAGTGAATAGTTTTGATATAATTTATAGATAA
TTAACGTTAAAGGGCGATAGTTTGATAGAATACATCATTGGAGTAATCGGATTACTTTTAGCATC
AGTTCAGGATTTTAGGAGTCGAGAAATTGAAGACTACATCTGGATTTTTTTAGCAGTAGTGGGCG
TTTTATTTGCAATTTATACATCATTTACGCTTTCAAATTATTCAATTTTGATAAATTCAATTTCA
GGATTTGTAATCTGCTTTATTCTAGGATATATGATGTTTTTATCGGGTATTGGCGGAGGGGATGG
AAAAATACTAATCGGGCTTGGTGCATTAGTTCCAAAATTCCAGATGCCAATCTACACTTCCCTTG
GAACCCTTTTAAACCTAAATTACATACCCAATTTTCCGATAATGGTTTTCATAAACGGCATATTT
TTCATGGTTTTTTTGCCGTTTGTTATTTTATTTAGAAATATTTTGAATGGTGCAAGGCCAAAAAC
AGGTAAAGAAGTTATTTTAATGTTTTTTGGAGAAAAAATGAAAGTAATGGTTGCAAAAGAGCAA
AAAAGGTTGATAATGGGCCAAAATGACAAGATAAACTTTTTCCCTGCATCTGACGATGAAGACTT
TTCAAAATATGGTGATGAAGAAGAACTCTGGGTTACGCCACAAATTCCTTTAATAATTCCAATAA
CACTGTCTTACTTAGTAACTCCAATAATTGGGGATAGGGTACTTGATCTTTTAATTCCTTTTTAA
AAATCGATTTCAATCCCATAAACTTTTTCTACATTTTCTTTATGGATTTTAAAACAAATTTCTTC
GTCAATTATTTCATTTTCAAGCATTTGCCGTGTTTTCCTTGGAACCGTTTTTATTCCAAGAACAA
CACTTGGCCTTTTTAAATCGTCAATGTAGTCTGTTTCCATCAAAAATCGGGTAGATTTTTTAACT
GCAGTATCGATTGGCTTTGAAGCAACGATTGATGGAAAAATTCCAAATTCGTCACCTTCTAAAAC
CATATCTGATGAGTGATGTTTTATGACTTTTTCTGGAGAAAGTTTTACGGATTTTGCCATTTCTG
AAAATTCTTTAAACTGTTCTCTTGATGCATTTTCAGCATGTATTTGCACTGCGCAATCGATATCT
TTTGCAATTTCCATCGAATAAGTTAAAATTTCATTTGAAACATCCCATATTTTTTCTTCAACTTC
AAAGTGGGGCCTTCCAACTTCACCAATTCCTATTAAAAACTCATTTTCTAAAACCATATTTTTTG
CATAATCAAGCGCATCTATCATGTATTTTTTTGCAGTTTCAAGTTCGATTCCATTTCTGAGCATT
ACGGTTAATTCAGCAGGATGGACCCCTAAAATTCCAAATGCAATTATTCCATTTTCCCTTACTTT
TTCAATTATCGAAAGCATTTCATCCATTGGTTTAGTTAAATTGGTACTAAATGCAGGTTTATTTG
GTATAATCATAATCTTACCGCCTGCATTTTTAAAAGTTTTTGCAACTTTTTCTGGTCCAAGACCA
TTTAATGGGTCGATATGGATGTGGTTGTCAGTTATTGGAAGTTCTTTTAAAATATCCATTTTAAT
CCCTTTTCCACACGTAAATTGGTTTTCCTTGGTAGTTTACGAGTTTTAATTCTTCAATCGGCTCTT
CACCGCTGAGGGCTTTTATAATTAAATCGCAGTTTAATTTTTTTGCAATTTTCAAAATAAATGGT
TGAAGATCTCTTGGCGGTCTAAACGAGTAGATTAATCCAACATTTTTGTAAATTTCAAAATTTGG
CTCAAATAAATCATCATAAAATGCATTTAATCCATTATCTTTTGCATCTAAAACTGCATTTTTGT
TTATATCTATAACGATCACGTCAAAGTCGGAATCTTTTAATTTTTTTGCACTGTTAAAATAAAAT
CCGATTCCAAGTTCTGCAATTTTTAGCCCATTATTTTTGAGGTTTTGGTAATTTTCATCAATATA
CTTAAAAACTGTGTCCATTATCTCCCTTTATTTGTAATTTTCTTGAAATTCCTTATTTTTGATAA
TTCCAAAAGTTTCTAGTACCCAAAGAATTGTAGCTGATGGAATTATGTCTGTTCCAATTAAAAGT
TCTTCAAATCCACCAAAAAATCCTAAAATTGATGAATGGTACAAATTTCTAATAATTAATGCATA
TATTGGGGCCCAAACAAAATCTCCCGATTCTCCAAGTCCAGGGATTAAATATGTGGCCATACCAA
CAATATCTAATGCAATAGAAATTATTAATTTTTTATAATCTACTTTTACTGAATTATTACTCATT
TTTTCACAAATAATTTAAGAATTGTTATGTTTAATCGTAGACTGAATTTACATTATTTACGTTTT
TATAAATTAAGGTATATATGTTTAGATATTGAAGTGAAAAGGAGGGATTTTGGTGGCTATCGTA
ATGGTTATTGCACCGGAAAAATTTAGGGACGAAGAACTTTTTGAACCTTTACAGGTATTTCAAAG

TAAAAATATCGCAACACAGGTAGTTTCAACAACAACTGGACAGCACAAGGGCGTATTGGGCAGGG
TAATGACTACGACAAAAACAATTGAAGAAGTAAATCCAGATGATTTCGATGCGATAGTTATTGT
TGGGGGAGGGGGTTCAAGAGATTACCTCTGGAACAATGAAAAATTACTCGAACTTTTAAAAGAA
TTTAATTCCATGGGAAAAATTACTGCCGCAATTTGTATTTCACCAGCAGTTCTTGCACAGGCAGG
ACTATTAACTGGTAAAAAAGCAACAGTATTCCCTGATGATGAATCTATTGAAGTTTTACAGCAAA
ATGGAGCAATTTATCTTGATGAAGCTGTAATTTTGGATGGAACTATAATTACTGGACGAAATCCC
GACGCAGCAAAAGAATTTGGATTAAAAATTGCAGAAGCGCTTGGAAAATAAGTATTTCTCTTTTT
AATTTTTAAAGTTAGATTAATAATTAATCATAATTTACGTGAATGACATGGATTTTAAAAAAAT
AAAGTATCAAAAAATAGGCGACATTTTAATCGTTAAAAAGAATTTAAACGATAACGAAATCGAT
TATTTAGTAAATAAGACAAAATGTAAAACTATTGTAAAATATAATACCTATATCACGGGAGATT
TGAGAACTCCAAAAATAAAAATACTTCATGGATCTGAAACCGAAACAATAAACAAGGAACACGG
CTGTCTCTTTAAAATCGATGTATCAAAAATAATGTGGAGTATGGGAAATTTAGAAGAGCGAAAG
CGAATAAGTACTCTTTCAAATAGTGAAGAAGTTGTTGTTGACATGTTTGCAGGAATAGGATACTT
TACAATTCCAATTGCAAAATATTCAAACCCTAAAACAATTTACGCTCTTGAATTAAATCCTGATT
CTTATTACTACCTTTCTGAAAATATAAAATTAAATAACTTGGACAATATAATTCCCATTCTTGGA
GATAATAGGGATTTTCCTTTTAAAAATATTGCAGACAGGATTTCGATGGGTTATGTTTTAAAAAC
GCATAAATTTTTGGATAAGGCTTTTGAGATACTGAAAAGAGATGGCGGAGTAATTCACTACCACG
AAACAGTTCATGAAAATATATTGGAATCTCGCCCAATTGAGCGTTTGAAGTATCATGCAGAAAAA
AACGGCTATAAATTGGATGAATATAAAATTAATAAAATAAAGAAGTATTCTCCGGGAGTTTGGC
ACATTGTAGTCGATGCAGAATTTTCAAAAATTTAAAAAACGTGAAAAATTATTTAATTCCTGAA
ATATTGCATTCAGGGCAACTTAAGCTATTTTCATCGTATTTTAATATATTATTTACTTTTTGGAG
TGCACGGGTGGATTTTGAAAGAACTGCTGCATTAGATAATGATCCTGCAATTAAAACCGCAGTCA
TAATTTGCTCTTCACTTATTCCCATTTTTTTAGCAGCTTCAATATGAAATTCGATGCAGTGTTCA
CATCCAAGTGCAGAAACAACTGCAATGGAAATCAATTCAGCAGTTTTTGGGTCGATATGTTTGTA
TTCATCAACAACGGCTTCGTCATAGTTGATTTTTGATTCTAAAAGTTTTGGATTATTTTTCATTT
TTTCAATAATGTATGGGACTTTTCCATATCTTTTTTCAACGATTTCCATTAATTCGCATAATTTT
TCATCAGAAATTGAACTTTTTTCATTCATATTACACACCATAGTTCTTTTAAAAGTAATCTACTC
TGTAATATCACTTTAAAATTCTTAAAGATTTACCTTAGGTTATTCCTTATAATCTCACCAATATC
TATCTTTTTGTTTAAATTCTAATTTTATGAGTCCTTACGGGCATTAAAATAACCAATTACTCTTT
ATTTCCCCATTAAAAAGAAAATTATATAAATAATCATCAATAAATTGGATAACTTAAATATACA
GCTAAATGGAGGGATCCCCATAACAACTCTAAAAAAACTCATACTGATAACGCTACTAATTTCGT
TTATCGGGTTTGTTTCAGCAGAAACAGCTCCGGTAGATTACAATATAAATAATTCTAATTATGAT
ACGGCAAGGACCATATCGAGTCCTGGACTATATAATTTAACTGAAAATATCTCAAGTACAAATGG
AATAGAAATTAATTCAGACAATGTAACGCTGAATGGACAGGGGTACTTTTTAAATGTTACATCA
AATAACGGAATACATGCATCCGGTTACAAAAATATTACAATTACAGATGTTAATTTATGCGCATC
TGGTGAAAACGCCTCCGCAATAAATTTAACAGGGGCAGAAATTATAATTCTTGGAAATACAATTA
CATCAAATAGCGGTGATGGAATTTTTATTAACCGCAGTTCAGAAAATATCACAATTTCTGCAAAT
ACGATAACTGCCGGCAATAAAGGGGTATATTGCGGTGGAAATAATGCAACAATTTTAGGAAATA
TTATAACTTCCAATTATTCTATAAGTATGGCAGGATACAACTTAACCGTTGAAAATAACGCGTTA
ACGTCAGGAATTCACTGCTACTACTCTTTGATATATTCAAATACACTTTCTGGAAATACGATAAA
TAGTAAAAAACTTTATTTTTATAAAAATGTAGAAAATGTTGGCGAAATTCCAAGCGATGCAGGA
CAGGTTATACTTGTAAACTGTACAAACGCTCAGATTTCAAATATAAACTTTAATGAAAAAGCAAT
AGCAATTAGTATTGCGGATTCAACGGGAGTTATTGTTGAAAACTGTACGATAAACCCTAGCGTTT

TTGTTGCTGAAAATTACGTATTCATGCAAAATTCTGAAAACTGTGAATTTAGGAGAAATACTTTC
AAAGTGTCAGAAAGTAATGCCAATATAGGATTTGCTGCATCATATGTAAACAACATTACTATTTC
AAAAAATAAATTTGAAGGAATAGTCGCGCCAGTATATGGGGTAAACTTTACAAATTCAAGAATA
TTTAAGAATATTTTTGAAAATGTAACCTCAGCACCGCTGGTTTTTAGGTACGACTGTTCTGGAAC
TTACGTTTATTTAAACAATTTTATAAATTGCAGTTGGAATGTCGAGAATACATTAAATGAAAGT
ATTACATTTAACAGTCCTCTCAATGTTTCATACAATTACGATGGAACAACCTACAGCACAATTGC
TGGAAACTACTGGTCTGACTACAACGAAACTAACGCAAACGCCAATGAAGGAATCTGGAGTATTC
CTTATGCAATAACTGAATTAATAAACGATTCATATCCGCTTGTAAAACCATTTAGTATGGTGGAT
GAAAACGGAATAATCCACATAACTTTGGATGACGTTGACAGTGAAAATGGATATGTAATAACAA
AACCTGGAACTTACGTGTTAGATGAAGATATAACAAATGGCGGTAGTATCTTAATCAATTCAAGC
AATGCGACATTTGATGGAAATGGGCACTGTATAAATAATTCTAAAAGTTACGCAGTTTATTCAAA
TGCCGAAATGCTTGAAAATTTAACCGTTAAAAATGTAATAACTTCAGGTAATATTTCCATAGTCG
CTTCAAATTCAAATTTATCTTCAACTGACGCATTAAATGTTAGTTTTTATGGTGATTACAACACC
CTTTCCAAAAATAGGGCATACAAAATAGTATTACAGGGAAACAATAATACAATTTCTAACAATAC
TGCCACTTACATGGGAGTGGTCTTTGGAAATAATAATAAAATACTAGCAAATACGGCATATACCA
TAGAAATCTATTGCGATAACAGCGATGTTATTTCCTCAAACACAGTCCAAGATCATATCTCGGCA
ACTGGGGATTACAATACAATATCCTCAAACACAGTCCAAGATCATATCTGGACATGGGGTCAGTA
CAATACAATATCCTCAAATACCGTTATAAATGCAAGCTACTATGCAATTGACCCTAATGGGTACT
ATGATGAAGGAATAGATGCATATGCAACAGTATTTAACAATACCGTTCTTGCAAGTAATATTGG
AATATGGCTTGATAACTATTGCGAAGGCTATTCAAATATTACTCAAAATACAGTTTATGCTTCAG
AATATCCAATTTTTATTGGAGATAACATCACAGGATGTAATATTTACTTAAATAACTTTATTTAC
ACTGGAAATTCGACAAATATTTCAGAAATAATGCCAAACACAACTGTAAACAACAGTTTGGTAAG
TCCTTTTGAAATTGAATATAAATACAATGGAAATACTTACAGTAACATCCTTGGAAACTACTGGT
CTGACTACAGCGGAACAGACGCAGATTCAAACGGAATTGGTGACATTCCTTACCTTTATGGATGT
GACTATGAAAGAACTTACGGTGACGACTACTACGATATCGATTATTTAGAAAATGATACTGCACC
ATTAATTGATATGTGGAACGGAAATGAAATTGGAAACTACGTTGCACCAGTTACACGATCATCTG
GCAGCAGTGGAAGAAGCTATGACTCAGATATTTCAGATGAAATAGATTCAAAAGTTATTAAAAA
CTTCGTTTCAAGTGCAGCAGTTATATATGGAAACGAAATCGATCAGCAATATGCTGAAGAATTAA
GAGAAAGAATACAAAATGCAAACGGTTACAAAATTTCTGGAAACGCAGTAATCGTTGGTGGGCC
AAACGCAAACGGCTTTGCTAAAGAATACAACGATCAGTTTGAAATGCCAATTTCAAACGACTACC
CTGGAGAAAATAAAGGAATAATTCAGGTATTAAAAGTTCAAGACAACACTGGAAAAATTGTTCA
AAGCTACACTATTGCATATATTGCAGGTTCTGACAGACTTGGAACACAAGCTGCTTTAGAATACT
TTAAAACTTTGGATGAATTGCCAAAAGGGCCAATCATGGTCGAATGGACCGCAAATGGGCCAGTA
GTTGTTGAATAATTTATTAAAAAAATAAAATTTTCTTTTTTTCTGATTTTTAAATTGAATTTAA
AAAAAAAATTTAAATAAATTTCATTAAAATTTCAATCAGCGTTTCTGTAATGTATTCTGAAAAT
TGAAACGCTAAATACGCTAAAATAATAAGCGTTCCTGGAAACACGTAGTATTCAACTACTCGATC
ATGCAAGATCTTAATTTTCTGTTTTTTCGACCTTAATTTCTTTTGCAATTTCTCTACAATTTCTCG
TTTTTCAGAGTTGGTTCTTTTTCGATCGTTTAAAACTCTTGAAACTTCTTCACTTATAAGGTATG
ATTCATTTTTTAATTCAAGATATTCTGATAGTGCTCCCATAATTTACCCCGACATAATTTTAATT
TTGATATATAATTATTTTTTTTAAATAATATATACATATTACGGGAATATTTAAAAAAACAACG
ATTTTAACGGGGCAATTTCCTAAAAAAAGTTATATCGGGGATAAATATTGAAAAAGCCCATTTA
TTTTTTCATCAGTACTACGGGTTCAAGAGTTCCATCTATTAAACTATCAAAGTTAATTTTTCCTA
ACCATCCTGATTTATCAAATACGTTGCAAATACTTATGCCCATAACTATTTTGGGATTCAAATTC

TCGATTAATCCAGTTATTTCTTCAATCGTGGCTTTTGAATGCTTTGTTGCAATTCCACCCATTAT
TACCAAAATATCTGGATTATCTAGATTTTTTTATTTTCGAGTCCAAAAAATCGGTATCCAATAT
CTGTAAGTTCATATTCTACAGAATTTTCAATTTCAGCATCAGGTATAAAGTACTGATTTTTTACG
GTATCTCTAATTGAATATCCAAAAAGCTGGGCCATCGAAAAACAAACTCCCCTTGAACCAAGATA
AACTATTTTTTCAGCCTTTTTAGACTCAACTTCTAATTTAAATTCTCCAACAAAAAATGAAAGAC
ATTTTCCAGAGTTTACTGTTTTCATGAGTTATCCCTCGAAAAAAATATCTGTATTTTAAAATAGT
TAAATTTTTAGAATTTAAAAAAATTTATAAAAATTCAAATCCAAAATCATTTCTTTTAAATTTT
AAACAGGATTCCACTTCAAATTTTATATCTTTTTCAAGCGCATTATCTATCAAAAGCTTTCCAAT
TGATGGTTTTGATTTAATTCCGCAGATCGAGGTTGTAATTCTTGGATTTACTTCTAGAATTGATA
TTTCTTCTCCATTAATCATTAAATCTACGCCAACGTATCCATTAATCCCTTCGATTTTTTCAAGGG
TTTTTTTACATTCAATAATTGCTTTTTCTTTTAAGGTATGTTCAATATTTATTTCTCCCCCGCAG
TATCGCTCTTCAATGTACTGCTTATTCATGCATACGGGGTAAAATTTTTTATTTTTTGCAATAAA
AATGACTGAATATGGTTCGCCTTCAATAAATTCCTGAACAATGTAGTTTTCATCAAATGTGTCGT
GAGTACTGTCGCATCCAAGCCTGTTTTTGACAACGTATTTTTTAACAGGAAATGTTTTTGGAGTA
TTTACTGTATTTTTAATCGCTTCGTATGTTAAAAATTTGTCTCCTGCAATTTTTACGCCTGATTT
TTTGCAACCCAGATTTAAACAACCGCTTTCTTCAATTAGTTCGGTTAAATTGTATAATATGAGGT
CTTCTTCCGGAGCAATTGCAAGTGCAAAATCTGAATCATTCAAGCCTTCCTTAAACGAATTTAGG
TAATTGTCAGATACAATTATTTCTAAATTGTTTATTTCGTTGTATTTTGGATATTTCTTAGCTAT
TTTTTCACATAGGACTGTTTTAACAGTATATTTCTCATCCAAAAATTGATTTAATAAAATATCAA
ACATTAATTTACCTTCAGCAAGGAGTTCATCAGGTACAAGTCCTGTTCCAACCGTAAATTCAAAG
AGAAATATTTTTTTGTTGTTCAAGTTTTTCACTCTTTAAAATTTATTTCAAATATTTTATAATTT
CTTCTGCACCTTCAGTAGGTTCTACAAGACCGCTGTAAACCCCTGAAACGATCTGTTTTATTTTA
AATGTTTTCGTTATTTTGTTTCTTTTACAGGATTGTATCAACAGTGGGCAATCTAAACTTCTACC
TTTGTGTATCAACGAGTTTAACTTAATTGGAAACATTTTTAAAAAATTTAGTATGTGAATACCAT
TCCTAAATTCATAATCTTTGTTTAAATGATTGAATAGTACAATTTCAATATTTTTATTTTTTGCA
TAATTAACCATTTCAGAAATTAAATTATTACATATGGTTTCACAGGGGTTTTTTGATATTGCATC
AGTTATCTGTTCACCATTTAAATAATAGGGGTTTAAAAATTCATATTTTAGATTTTTATCTTTAA
AAACCCGTTCAATTCTGTTTCTATTTTTATGGGGCAATAGTGCAGTTCCAAGATCAAAAGTTATG
AAAATTGGGTTAAAATATTCATTTATAAAATCAATCGATGAAAGATCATATTCACACTGTATAA
TTACTAAAGCATCTTTTTTGGACGAAATATCTAAACCTTTTTCTCTTAAATGAGATATTGAGTTT
TCAATAGCGTTGAGTTCTTTTTTTCTATCGATATAACTTTCTACTCGAATAATTAAATTTTCTTT
AAAAGAACCGTTCAATTTTTCCCTTAATTTTCCAACTACCCATCCACCAGGCCCTATTAATAACG
ATTTATCTGTTCTCGTGGGCGTATATATCATTAAAATTAAATTTTTACTATTTTCTGAATCCATT
TTAAATTCAAAGTCCGAAATGGTGATTTCTGAAGTAGTAAGTTCAAGTTCCAATCTTATTTTTGC
AACTATTCTGTAAATTTCTGATAAAAATTCTTCTTTTGATTTATCGATATACTGCATAATCTTAA
CCATAAATCTAAAATTAATAAAAAAGATATATATTATAATTCTTCTTTTTTCTTTTTCAGGAGTT
CAACTGCAAGTTCAGTTAATTTAAACTTCTGTATTTTGCCGCTCGCAGTCATTGGGAATTCTTCT
ACCACAAACACGTGTTTTGGAACTTTATACCTTGCAATTTTTTCAAGTGCAAAATCTCTGACGTC
TTCTTCTTTAATTTCGTATCCTTCTTTAGGGATTACAAATGCACCAACAATTTCCCCGTATTTTTC
ATCAGGGATTCCAACAACTTGTGCATCATTAATTCCAGGCATATTGTATAAAAATTCCTCAATTT
CTCGAGGGTATATATTTTCTCCACCACGAATAATCATGTCTTTTATTCTTCCAACGATTTTATAG
TATCCATCTTCGTCTTCAACCGCTAAATCACCGCTGTGAAGCCACCCATCTTTTTCAATGACTTCA
GCAGTTTTTTCAGGCATTTTGTAATATCCTTTCATTACATTATAGCCCCTACAGCAGATTTCTCCA

ACGTTTCCCGGAGCAAGTGTTTCTCCAGTTTCAGGATCTATTATTTTTACTTCACAGTGCGGCAT
TGCTTTTCCAACTGATTCAACCCGTTTTTCAAGGGGATCGTCGACACTGGTCATTGTAAACACTG
GTGATGCTTCTGTAAGGCCATAAGAAATAGTAATTTCTCGCATGTACATATCAGACATTACTTTT
TTCATTGCTTCAATTGGGCAGGTTGATCCGGCCATAATTCCAGTTCTAAGTGATGAAAGATCAAA
CATTTCAAACATCGGGTGTGAGAATTCTGCAATAAACATTGTAGGAACCCCATAAACTGCAGTAC
ATTTTTCTTTCTGTATTGCTGCAAGTACAAGTAATGGGTCAAATATTTCAAGCATTACAAGAGTT
CCGCCATGAGTTAAAAGTGCAAGTACACCCAAAACTATTCCAAAACAGTGGAAAAGAGGCACTGG
CAAACATAATCGCTCTTCTTCTGTAAATTTCTGTTTTTCACCGATGTAATACCCGTTATTCAGTA
TATTTTTATGGGTAAGCATCACTCCTTTTGGAAAACCTGTAGTTCCCGAAGTATACTGCATATTT
ATTACATCATTAGAATCCAGACCTTTTTTAGCTTCAATCAGCTTTTCATCAGAAACGTACTTTCC
TAAAAGCATGAGTTCATTTGTATTGTACATTCCGCGGTGTTTTTCTTGCCCAACGTAGATTACGT
TTTTTAAGTATGGGAATTCTTCACTATTTAATTTTCCCCTCTGACTCGTTTTTAATTCTGGAACG
AGTTCATAGAGTATTTCGAGGTAATTTACATCCCTAAAACTATCAATAATTGCAAGGGCTTTCAT
ATCGGACTGTTCCATAACATATGCAAGTTCGTGGCTTTTATATGCCGTGTTTACGGTTACTAAAA
CAACACCTATTTTTGCAGTTGCAAACATGAATGTAAGCCAGTCTGGAACATTTCTAGCCCATATT
CCAATATGATCTCCTTTTTTAATTCCAATTTCTAAAAGGCCTTTTGCAAGGAGATCCGTTCTTTC
ATCGAATTCTTTGTATGTAAATCGGAGATTTCTGTCAGGATATACTAAAAATTCCCTGTCAGGAT
CTTTTTGAGCTTGCTTTTCAAAGAATTCTCCGATAGTGTCATTTGTAAAAAGCATAATTTACCTC
GCACATACAATTAATCGATTCCTTCAATGAAGTGTTCCATTAACTTTTCTTTGATGGAATCGGGT
ATTGGGACTGATTTTTGATTTACAAAATCATAGTGAACAGCAACCGCTTTTCCTTTTACTGCCAA
TTTGCCGTTCTGCCATGCTTCTTGGTACATTGTAAATGACGAGTTTCCAATTTTTGAAATGTAAG
ACCTAATTTCAACATCCATTCCAAGGTACATCTGCCCTACGAAGTCGAATTCTGTTCTTACCATA
ATTAAATTCCATTTTTCGTAGCTTAAATCAAAATCAGGTACGAATATTTCAAATAATGGATTTCT
AGCCTGTTCAAACCAAATTGCAGGCACGATGTTGTTTACATGTTTTAACCCATCAACATCGCCAA
ATCGGGGGGTTACAGTTGTTTTAAACATTTTTTATCACGATTTTTAAAGATTAGAATGGTGAGTA
TATTACTGCAAGAATTTTTGCAATATCTTTTCCAAATGCATGTAAGTCATGTGGAACTATTGAAT
CATAGTATATGCTGTCGCCCTTTTTAAGCATGTATTTTTCCTGACCATACATAACTTCGATTTCTC
CATCCATTACATATATGAATTCTTCTCCTTCATGCGAAGAAAGTTTGTATTCATTAGATTCGATT
GGATAAACGTCGATTAGGAATGGTTCCATATGTCGGTCTTGTTTTTCGGCAGCAAGTGCATAAAA
ATCTAATTTACTATCTTTGGTTCCATCTAATTTTCCTGAAAATCTGACGATACTATCTGAACTTC
CAGATCTGGTCAATACGGGTCCGTTCTGAGGAGCATCGTCTAAAAATGTACCGAGTCTAACTCCA
AGAGCTCTTGCAATTTTGAAAAGAGGGGTTAATGACGGAATTAAATCTCCATTTTCAAGGTTTTC
GATGAGTTTTACGCTACTATTACTCGCTTGTGCAAGTTCTTCGATGGACATGTTCTGTCCTTCTCG
CATCTGTTTAATTTTAATGCCAACGTGGTTTTTTTCAGTTATTTTATCCCCTATTTAATATTTTT
TAAGAAGTACTGGTGTTACACCCCATACTTTTCATCATAATTATGAATTTTAAGTTTTTAAAAATT
ATCGTTGAATAGAAAAAAATTTATAATTTAAAAAAGTTAAAAACAGCAAAAATTAAAAATGTTTT
AAATAAGATTTATGAGGGATAATACTGCACTTTCAGTACTGTATCTGTCGCCACCGGCAATTACA
AGGACATTATTTCCATTTGCAGCACCTTCAATCAATGAGGCTGTTGCAGGGCTCTCGTTGTCGAT
ATCAATTTTACTTTCGTTTTGTAATTCTTTTGTTAGTTTGTTTGCAACAGGGCCACCAATCAGGA
TTAAATTTTTATCCGCTTCATCAAGTTCGATTTCGCTATCCATAAGTATTTCTGGAACTTCTCCCG
ATTTTGAAATTACAGTTCCGCCGGTTGATCCTGTTGTTACTGTTCTTTCTGCTTTAAATTCAGCA
ACCATTTTATCAAGATCATCTTCGTCATCAAATACTAAATTTACATAATCTGCAATTTTGATCCT
GTCGCCGTCATTTATTCCTTCAATATCGCTTCCAGAGTATACAAGTGCAATTCCAACGGTATTAT

CTTCATAAGTTTTTAAAAAGTCAATAGTTCCGCCCGTGTTGGTAACTGCTCGTACTTCCCAGTTA
TTCACGTACTCTTCTCCAAGTTTTAATTCAGTCAACGATCTACATATCGTGATATCTGCGTAGCC
AGTATCTGCAGCTGCATTTGTCCATACGTCATGAACGGTAACTCCAAGGTCTCCAGCAATAACTG
AAAATGATTTATCTGAACGTAAAATTTCAGTATGTTCTTTAATTATATTTCCATTTCTCAATATC
GAAATTTCAACGCTGTATTCATCTCCACTTTCTAAAATTTCCCCTATAACGACCTGATACTCACCT
GAACTTATGTAATTGCCCTGTTCAAGAGTTCCGTCATATGATGATGTTCCAAGGTATATTATACC
ATCATCTTCATCAATCTGTACGATGCGGTATTTTTTACCTAAAAATGGGATTTCGTAACCTTCTT
TTAAATCTTTAGTTAGTTTAAACGCAGAAACTTGGTTCGGATATATAATTGATGCATAAACCATG
TCTTTTCCAATATCAAATGTTTGGGAACCTGCATTTTTGTTTGAATCAGTAATTCTTACAAAAAC
TATTTCTGTAGCATCGTCATCAGATTCAAAATGGTTTTCCGGATCAATATCTCCAACTTCAAGCA
AATATCCAAAATGCCCTAAAGATTTGGTCTGATCCGCTTCTGAAAATCCAGAATATGAAAATGAA
TTTCCTTCTTCATCATCAAAATGGTCTGCATAGTCATCATCTGCGGAAGTAACCAAGAATCCATGA
TTTTTCAGTACCTGCGCTGTCCAATTTATCAGTTCCATCCAGTAAATCAATACTTTCAGATTCTCC
AATAATTTGATATTCTACCGTAGATTCATCCTCAATAATCGTTTCAGAGTATGTAAGTGTTCCAA
TTTTTGCAGAAATTTTGTTTGCAGAAGTTACATCAAGTGCAGCGGCGTTTTCACCAACTACAATA
TAAACGTCAGGGTTTCCATTTTTTATTAAATCATCATCTATAAAATCATTGATATTTCCGTATTT
TTCTAAGGCGTAAGCTGCTGTGATGGACGCAGATAATACTGTACTTCCAACTAAAATACATCCTA
TTTTTTTTAGAATTGATTTCATAGATTTCACCCATTCGCTTCATAATAATATATTTTTGTTCTGT
CGATAAATACTCTACTATTGTGGCTTAAACTTAATCAGAGAATTTTTCAATCAATTCGTGGTGTT
TTGGATATTTTTTTAGTAATTCTTTTTTTGAAGCGAGTTCAAAATCTTCAAAATCAAAACCTGGA
GATACCGTGCATCCAACGATTACGAAAGAGTTTTTTTCAGTAATTTCTGCCGCAAAATATGAATT
TTTAGGAATAATTACTTGAAAATTTTCATCTTCTTCAAGATTTGGTCCTAATTTTTCTAAGCTAT
AATTTCCGAAATTATCGATAATATGGATAATTACCAAACTTCCAAAATAGTAGTGCCATATTTCA
TCAGATTTTAAGCGGTGAAATTTTGAAACATCGTTTCCATTTAAAAGGTAATAAATTGAAGTAG
AATAAAATCGGTCTGAATCATACCTTTCAGGTAGATTACTTTTGTTAACCATTTCGTCTGATTGG
TAGATTCGTCGGTAGTATCCTCCTTCAGGATGTTTTTCTAAATCGAGTTTTTTGATGAAATACTC
TGCATTTTTCATTATTTCACTTAAGGTTTGCAAAATGGTTCCCTTTCTTGTATACATTTTATGAA
ATATTTTCTAATATCTTCTCCATTTCTAAGCGGGTTTGCAACATCTACTAAATTATCATCTCTTA
AAAGGCAGGGTTTTAAATAACCGTCATAAGTAAGCCTTATTCTTGTACAATGATTGCAAAATTCA
GTATTGTCCATTGGTCGAACAAATTCAACTTCTAATCCATCAATAACATATTTTTTCCTGTTCTG
CATGAACTTTCTTGTAAATACATTATCTGCATTTTTTGCAATTTCCTCTTCAATTGGAACAACAT
CTACATGGTGATGCTTTAATTCGGGTTCCATTGGAATAAATTCAATTATTTGAAGTATTGCACCA
ATTTTTCTACAGTAATCCATAATTGCTGGAAGCTGGTTTACTGTACAATCCATTGCTAAAAAATT
GACCTTTAAAGGGGTTAATCCTACTTCAATAGCTTTTTCTATTCCTTTTTTTACGCTTTCGATAT
TTCCGCCAGCAGTAATTTTTTTGTACTGAACTGGATCCAGAGTATCTAAACTCACGTTTACCCTA
TCAAGTCCTGCATCTTTTAATTTTTGTGCATATTTTTCAAGGAGAATTCCATTTGTAGTTAATGA
AATGTCTTTTATCTGTTCATTTTTAATATAAGATATAATTTCGGGCAAATCTGTTCTTAAAAGCG
GTTCTCCACCAGAAATTTTGATTTTTCTAACTCCAAATTCTAATGAAGAATTTACAATTTTTCCA
ATTTCATCGGCACTCATTAATTTGCCGTGTTCTTCATTTCTACCTTCTTTGTGGCAGTAAAAACA
TTTTAAATTACATTTGGGGGTTATTGAAAGTCTAAAAGAGCGTATCTGTCTTCCGTATCGGTCTT
CCATGGTTATCACGAAGTTTTAATTGTGGTTAAGGGGCAACGTTTACATTATCTATGGATACCGT
AATTTAAAAAAGTTACTATTTGAAATGACGCGGATAATAATTTAAAAAAAGAAGATATTATTTA
ATTTAATTATTCTGTTTTTTCTTCTTTTTTAACGAAGTTGTCAATAAATGAAACTTTTTTAGCTT

CTAATCTTTTGATCAATTCGTTTGAAATTCCCATTTTAACCATTTGAAGGTTTTGCATAAATGCT
GTGTTTTCAGGGAGATCTAATGTTACATTTTCTCCGTCAATGGTTATTTTTAAATCTTCTTCTGA
AATTCTTGGGATGAATAATTTTGCAACTTCTAAAGCAACAGCTTCAGGAGCTTCTACAACTTCTT
CTATTTTTAATTTGTATTTCAAGTCTTTTCCTGCGAGTTCGTGGTTGAAGTCAACTAATATTCTT
CCACCGTTTGCACTTACAACTTTTCCAATTTTGTTGTCGATATTTACAGGCATTCCTGGGATTGG
GTTTACTTTATGTTTTTTAAACTCTTTCATTGGAACGATTTTTACTTGTGAAGGATCTCTTTTAC
CAAAAGCATTTTCAGCTGTTAAATCAAGTTCTCTTTCTTCTCCAACGTTCATTTCCATAATAGCG
CCGTCGAGTCCAGGGATTAACATTTTTTCTCCAGCGGAAATTGTAACAGGGCCATAAACCATGTT
TGGGTTGAAAATTCCTTTTTCTTTAGCTAATTCTTCGTTTGTCGTATCAAACAAGTTTTCATCGA
CATATCCTTCGTATGAAATTTTTACGAGTTTTCCTTTTTCCATTGTTTCACCAAATTTGTTTCAA
TACATCCAATCGCAAAGATTATCACAACAAGTATAAATAAAAGAGTATATAAATCTTGTCAAAA
TCACTAATTAAAATTGGGACAATTATGATATCAGCAATTATTTTATCCGGTGGAAAGGCAGAACG
CATGGGTGGCGAAAAATCCTTTAGAAAATCGGGTAACAAATATTTAATAGAAAATATTGCAGAC
ATGTTAATTGAAATGGAAATTCCATTCGTTACGGTGTTTAAAAACCCTCGATTTATGGAAAAACC
AGAAGAAGAAATTGAAAAACAGCTCTATTATTATAAAAAATATCATCAGACTATTACTTGGGAT
TTTGTCCCAGGAATGGGTCCTTTAATTGGAATGCTTTCTGGAATGTTGAATACAAAATCTCAATG
GGTCTTTTTGGTTCCCTGCGACATGCCGTTTATTACCGAAGAGTCAATAATAAACTTGCTTTCAT
TTATAAAAGAAGCAGAATTAAATGGTCAGGACTGTATCGTTCCAAAACATAAAAACGGATTTAT
TGAGCCACTTTTTTCACTTTACCATATTTCTTCAGTAAATGTACTTGGAATGGTTGTCGAAGAAA
TGCTTGAACAAAATAGATCATTTCCAATAAGACGGTTGATAGACGAATTAAACCCCCTTTATGTT
GATGTTGACAAAATAGACAAAACTTGTGACATATTTATAAATTTAAACACTTTAAAAGAACTAG
AAAACGCATGTTTTGACAGATAATTTATTTTTTAATAATTTTTCGTTTCATGTAAAAATTGGTAG
TTTTGGATCATTTTTTTAATATTTAAAAATAACCATTGAAAATATAAATAACTCTTATTAACCTT
TATTTTGGGAAAAGGATAAATATGATCCAAAAAGAAGAGTAACATGTCGAAAAGGGGATTCCCT
GATCGATAGAAAGTGTAGTTTAAACCAACGAAAAGCACTTTACAAAATACAATTTAAATTCATA
GTTATAGAAAAATTCCCCTGAATAACTATATATTTAAAAATAGAAATTCTCCCCAAAAAAACTTT
TTTTTATTAAAACTCATTTATTAAATTTTTAAATTAAAAATAAATGTAAATTTTAAAAGAACAT
AACTCAAAATTTAAAAAAAGGGTAAATAATTAATTACTTAATTATTTGTTTTCTCTTTTTTCTCT
TTTCATTCTTCTAATTCTTTTTTTGTAGTGTTTCCATCTCATCTGTCCTTTCTTTTTCCATCTTCT
TGAACTTCTCTTTATAGTCTCACGCTCCATTTTCATTTTTTAATAATTATTGGCTCCATTTACTTT
TTTCAACGACGATTGAACCGTCTTTTACTTTAGGTGCCACATTTAATGCGTCGATTTTAAATGAT
TCATCAAGTATAGCTTCATCGTCATCTCTGAGTACGTTTTTCGTTTCGAGAATGTGGTACTCTTC
TTCAGTTTCAAGATCAAAGTTTTCCAAAACTTCTGAAAGTTGAGCAACGATTTCATCAGCTTGTT
TTTGAATTTTTTCAACATCTATCATAGTATCACGAACCGTGATTATTTTAAGAGGCCTAAATTGT
TAAGAGCATTCTGCAATTTTAATTTATTGCTTTCTGCCATTTCGCAAAGTGGCAATCTTAACTCG
CCCGAAGGCATTCCAAGCATATTCATCGCAGTTTTGATTGGTATTGGGTTTGTCTCGACAAACAT
TAACTTCATTAGTGGAAACAAATTGTAGTGAATTTCTTTTGCCTTATCAAATTTGCCTGCATTTG
CAAAATCAACCATCTGAACGAATTCTTTTGGTGCGATATTTGCAATAACGCTGACAACACCTTTT
CCACCAAGGGAAATTATTGGTAAAGTCAATTCATCATTTCCAGATAATATGTCAATGTCGCAAGA
ATCCAATACTTCAGAAACCTGAGATAAATTTGGATTAGCTTCTTTTACAGCTGATATATTACTAT
ATTCGTTAAATAAATATTTAATTGTTTCTGGTTCTATGTTTAGCGCAGTTCTTGAGGGAACATTG
TATAGAACTATTGGCACATTGATGGAATTTGCAATCTCTCCAAAATGCCTTTTCAAACCTTCCTG
AGTTGGTTTGTTATAATATGGGGTTATCAACAAAACTCCATCAACACCGACATCTTCTGCATATT

GCGAAAATTCCAGTGCTTCAGAAGTTGAATTAGAACCTGTACCTGCAATTACTTCAACTCTACCG
TCTACGAATTCAACAACCTTTTCAACGACTTTTCGTGTTCTAATGGCGTTAATGTTGGTGATTC
ACCAGTAGTTCCAACAGGAATAACTCCATTAACTCCATTTTCAATTAAAAAGTCGATATTGTTTC
GTAAACCATCATAGTCTACTTTATTTTCTTTTAATGGAGTAATAATCGCAGGATATACACCTTGC
ATAATTACACCTTATGCGTTCATTTTTTTTACTTTAGCAGTAACATAACCTGCTATTCTGTTTCT
CAATGGTTTTGTTGAGATCATTGCAACTTCTTCAACAGCTTTTTTGTTTTCTTCAAAATCGCTTG
TGAACTTGTCTGCAAATTTCTCAATGAGTTCTTCACCGGTTCTTTTGATGAATGTTTGCCTTATT
CTTCCCAAGTTATCACCTTATAAACTTAGATAATCTTGTTTCATTTTATAATACATTTGTAATTA
TTATTTATATATTTAGCTAAAGTTACCCGTCAATGCTGTCTTTTTGAACTTCTTTATTATAGGTC
ATGAGAATTTTCATAACTTTTAGGGCCACTTCGGGTTCTATGTGGTGCTTTTCACACATCAAACG
TGTTTTTTCGCAGATTGCATCTTCTCTTTTAGGATCGAAGACACAGGTTCCGAGTGACTTTTTCA
GTGCAGCAATTTCGGGTGCGAAACTGGTTCTTTCAGCAATCAATTCGATTAACTGTTCGTCAATT
TCGTTAATTCGTTTTCTAATTTCTTCGAGTCTTTTTTCCGAAGAATTTGACATAGTATCTCCACG
AGCAATAAATAGAATTATCAGAAGAAGTATTGAAATGTATATTATTTAAACTTAACTATGGTTT
TTTAAAACATCTAAGAAATTAGATTAGGGCATGAACTCAATGGGGCTTTTTCCATTGGTGATGAG
CCAATTTACAAACATTTCCGCATATTTTAGTTTTTTCACTTTAATTTCATCGGCAAGTTCAATGT
CCTTTTCAATGTTTGGACGTGAATATTTCGTTACATATATTCCAAAGTCCATTCCGCAGAGTATG
ATTTTTTTAGCGCCAAATTCTTCGGCTAAAAAACAGCACCTATCTCCGTCAGTAAACCCGCCATA
GTTTATCAAAAATTCAAATTTTTTTGGAAATTGGGTACTTCCAACAATGTTTCTAAGTAATTTGA
TGTATTTTTCAAGTTTATCGATGTTGTCCCCGTGAGCATGAGCTACAACGATTGAACCAAGATCA
TTGCTTTTTAAAATCGAATCAATATCGCCGTCTAAGTCTGAAACAATAATATCTGGAACTATATC
TTCTTCTAAAAGTGCTTTAGTAGCACCATCCGCTGCAATAATTGTTATATTTGACTCAATACTTT
CTTTCAATTTCAAAACATGCTTTTTAAGTGACGGGCCCGCACCAAAGATGTAAACTTCTTTTTCG
AGAATTTTTTTGGAAATTTCGTTCAAATCTACATTATTTTTGAATTTTTTGATAATTTCTTCAAG
AATTTCTGCACTTTTTAAATCTTCAGCGCTTCCGTATCCAAAATCATCGAGTATCTGATTATAAT
AACATTCCCAAGAGTCTAAATCCATATTATCGCTCTAGTTGATTGGTATTTCACTTGAAAGGGTC
TTAATTTTCACATTTTCAATGTATTTTTTTGCAATCTTTCCAAGTGCAATTAAATCGTCTTCACT
AATCCGTTTTACATTGCATAATTCTTTATCATATGAATGTTCACGGTCAAACTGTTGAATTGCAT
ACAAATCGCAGTTTTTAACAGTTTTTGCAATTTCTTCGATATCTGATTCATCCATTAATTCTGGA
ATAAATGTAGTTCTGCATTCTACAAATATTCCTTCCTTTTTACAGAGATCGATTATTTTTAAAAT
ATTTTCTTTAATTTCTTTTTTATATCCTGTAATTTTCTCGTAATTATTAAATCCTGCTTTTACGT
CTATTGCAATGTAATTTAGCAAGTTATTTGAAATAAGTTTTTCAACTAAATCAGGGTTTGTTCCA
TTCGTATCGAGTTTTACTGGAAAACCTTTTCCTTTTGCAATTTTTGCAAGTTCTAAAACTGCTTC
TGGCTGAAGGGTTGGTTCTCCGCCACTTATAACGAGTGCTTCTGCAAACATCAAATCCATACTGT
TAAAAACTTCTTCAGCAGTCATTTCAGATATATTTGTTGTAATATACTCGTAATTTTGGCAGTAA
CCACATTTCATATTGCATCCTGATAAAAAAACAACAGCAGATGCATGTTTTGGGTAATCAATTGT
TGAGAGTTCTACGATTCCTGATATTTTCAAAATTTCACCAATGCCCATATGTGTTTTATATAAAA
CAGTTACTCCTATAATATATAAAAACAGATGATTTAAATATTTCTTAAGGTGGTTTTTCATGGAA
ATAAAAATACTTGTAGACAATACTGCGAAAGGAAAGTTTTTAGCACAGTCAGGATTTTCAGCAAT
TATAAAAGATGAAGATTCTAAAATATTGTTTGATTGTGGCCAAAACGCACACGTTTTTGAAAAT
AATTTAAAATTGATGGGCGAAAATGATAATTTTGATGCAGTAATCTTTAGCCACGGCCACTACGA
CCATACTGATGGATTTAATTATTTTATTGAAAAATATGGGGAAAATTTAGATGTTCCAATTTAT
ATTCACAAATCCGCATTTGTTGATAGGTATCATGGAGAAAGATACATCGGAATCGATGAAAATAT

AAAAAATTTCTTGAAAAATTATAAAAATTTAAAACTCATCGAAGATCCATTTAAAATTTCAGAA
AATTTAATAATTTCAGGAACCATCCCCCGGACTAACTTTTACGAAAGAGAAGACTTTGAAATGAT
TGAAAATGGTCAAAAAGTGGATGATATCATAATTGATGACATGTTTGTTATCGTAAAAGATATA
ATTCTTTCAGGATGCACCCACAGCGGATTAATTAACTGTATAACTCATGCAAAAACCCTAAAAGA
AGTAAATGGAATTATTGGCGGTTTTCACATGGGAACTGCATCTGAAAATTACATAAATCAGGTTA
AAAATTATTTAAAAACTCAAAACTTTAGATTAGTTTCACCCGTGCACTGCACTGGTTTTAATGCT
ACAAAAGAATTAAGTGACATATCAGGATTTAAATTTGGACATGTTGGCAGTGTTTTTGAAATTT
AATATTTTACTATTTTTTTATGTGCTGTATTATATACCATACAAAAAGTATTATATACTGAACAC
ATATATTGTCAGGAATACTTTTAAGTTTTGTAAACAGAATTTTAAATAATTTAAGAAACTAATT
ATGAATTTGGAGAAATCATGAAACAGTTATCATTTAATAAACTGTATTTTGAAGGAATAAAAGA
TGCAATTCCTATATCCATAGGGTACTTGCCAATTGGCATGGCTTTTGGGGTTTTGGCAAAATCTA
TGGGGATTCCTTACGAAATTTCAATTTTAATGTCATTAATCATATTTGCAGGCGCAAGCCAGTTT
GTAGGGGTCAATTTAATTGCTATTGGAACTTCATCTTTTGAAATAGTTTTAACCACGTTTATATT
AAATTTAAGGCACTTTTTAATGTCTTCTTCACTTTCGCAGAGGATTGACTATACAAAATCTAAAA
AATTTTTAAGTTTAATCTCTTTTGGAGTAACTGACGAAACTTTTGCAGTAGCTTCATTGAAAGAA
GAATCAAAATTAAGCCCTGAATTTTTGTTCGGACTGAATTTCACCGCGTTTTTTGCATGGAATTT
TGGAACATTTCTTGGAATTTTTTTAGCAGAAAGCATTCCAAAAGAAATACAGAGCAGTATGGGA
ATTTCACTTTACGTGATGTTTATCGGGCTTTTAATTCCTGCAGTCAGGCGTTCTACCAAGGTTTT
AAAAATTGTAATGGTTGCAATATTCATCAGTTCGGTTTTAACATGGGTTCCAGTGTTTAAATTTA
TTTCAACAGGCTGGATAATTATTATTACAACTATCGTGGCGTCATTTATCGGAGCTAAGTTTATA
CATGGTGATGGCGATGAGTAGGGAAGAAATGGTATTGTTGGTAATATTCATGGCAATTGTAACA
TATATTCCAAGAATGCTCCCGATCGTGCTGTTTAAGGATGCAAAACTACCCCATTTTTGGAGAGC
ATTTTTTAGCTACATCCCTTACGCGGCTCTTGCATCGTTAATATTTCCGGGAATAATTTATTCAA
CTGGAAATATATATTCGGCACTTTTTGGGGCAGTAATTTCAGTAATTTTGGCATATTATCGATTA
AACGTGATAATTGTTGTATTTGGCGGGATACTTGGTGCATATCTGGCACAAATGCTGATTTAAAT
TTTAAAAATGAAACTGACTAAAAAAATAAAAAATATTAAAAAAAGATTTTGAAAAAAATTTGAA
TTAATTATTGAATTATTGCAATGGTATCGATTAAACTGTCGATATCTGCAGTTTCAACAATTTCT
CCTTTGTTATATATCGCTCTTTTTACTTCTTTGATGTGTTCTTTGTTTAAAGCACCAAATTCGGT
TTCAATTCCTTCTGAAAGGATTTTTTCGATCGTTTCTGCATCTGCTTCTTTAATAAATGCAGTTA
CTTTTCCAGCATCTTTCTTAAATTCTGGGCCGATTTTTGACTTGTTTGGAGTTACTTCTGAAATT
TTTTGTTCTAGGAGCGGTTTTCCAAGATTTAGTTCAAGTTCTTTAATTTTCAAAGATTTTTTAAC
ATCTTTGGAAACTTTTGAAAGAGCATCAAAGTCTTTTTCATCGCTTACGTACATTTCAACTTTGT
TTAATTCTGCGTTGAGTGGCATTCCTTTGTTTGATTTGTATCTCCTAATTGATGCAACCGTATTT
TTTGCAACTTCTCCGACAAATTCGTTTTCTAAACTTATCAGGCTTTCATCTGCAACTGGCCATGA
AGTATGCAAATCATCAATTTCGTAGATTTCTCCAACGATTTCTGCAAAGTGCGGTGTAAATGGTG
TTAATAATTTTACAGATTCAGTAATTACTTTGTAAAGCGTGTACTGAGCCATTAATTTTTCTTGT
TGGGCGCTTTCAGATTCTTCTTTGTTATATAGCCTGTGTTTTACCATTTCAATGTAGTTGTCGCA
GAATTCGTGCCATAAGAACTTCTGAATTTCTACAACTGTGTTTATTTTGTAATTTCCAAGGTCAT
CGGAAACTTTTGAAATTAAATTATTTAATTTACTCAAAATCCATAAATCAACTGGATTTTCAATT
GAAATACTTTTTAATGACTTTAATTCAGAAATTGTTTCATCTGAAATGTTCATTTTTGCAAATTT
ACATGCATTCCATATTTTTCGCAAAAATCTTCCGCCGTATTCAACTTCTTTCCAGGCAAATGGAA
CATCTTTTCCAATGGTGCTGTTTGAAGCCCATAACCTTAAAGCATCTGCACCGTAAGTTTTAGTA
ATTTCTCCTGGTTCTACAACGTTTCCTCGACTTTTACTCATTTTAAAGCCGTCTTCCCCGAAAACC

ATTCCGTTAATTACGATTTCATCCCATGGTTTCTTTCCAGTAATTGCAAGTGATCTAACCATTGT
GTAAAATGCCCAGGTTCTGATGATATCATGGCCTTGAGGTCTCAATTGAACAGGGTAGTGTTTTT
TGAAGAATTCTTCATCTTCAAGCCATCCTGCTATAACTAATGGGGTAATTGAGGAATCCATCCAG
GTGTCCAAAACATCAGTTTCTGCTACAAGGTTTGAGTTTCCACATTTACAGGTGTATGGACTTTC
TTTTGTAGGATCTATTGGTAAATCTTCAGGTTTAGCTAAAATAATTTCTCCACAGTCCTTGCAGT
ACCAAACAGGAATGGGGGTTGCAAACAATCTCTGCCTTGAGATGCACCAGTCCCAGCCCATATCT
TCGATCCATTTCATGAGTCTTGCTTTCATATGTTCTGGAACCCATGATATTTCGTTTGCAGCGTT
TTCTACTTCATTTAATAACTTTGTAACGTTTACAAACCACTGGTCTCCAACAATGATTTCGATAG
GTGTTTTACATCTCCAGCACGAACCAACATTTTGTTCGAGAGGTTCCTGTTTTATCATGTAGTTT
TCAGAAATTAAATCAGAAATAATTTCTTTTCTTGCATCATCGGACTTTTTACCTGTGTATTTTCC
GCAAATTTCTGTTAATTGTCCTTTTTCATTAATTGCTTTTTTAACTTCTAATTTGTGCCTGTTTA
CCCATGTAACGTCAGTTTTGTCACCAAATGTACATACCATTACAACTCCAGTACCAAATTCTTTT
TCAACATCGGAATCTGGGTACACATTAACTTCCTGATTGAATAATGGAACTTTTACGGTTTTTCC
AACAACCTCGCTGTATCTTTCATCTTCAGGATGCACAACAATTCCAACACACGCAGCCATTAATTC
GGGTCTTGATGTTGCAATTTCAAGGTATTTTTCGCTATTTTCTGCATATGGGAATTTTATATAGT
TTAATTTTGAAGTTCTTCCCTGATATTCTACTTCTGCAAACGCAATTGCCGTTTCACATCTTGGGC
ACCAGTTTACTGGGTGTTTTCCCCTGTAGATTAAACCTTTTTCGTACATTTTCAAAAATGCAGTC
TGTGATTTTCTAACGTAGTCTGGATTCATGGTAATATATTCTCTATCCCAGTCAATTGAGATTCC
AAGGGATCTAACCTGACCTCTCATTTTTTCTACGTTTTCATCGGTCAATTCTACACAGAGTCTTCT
AAATTCGTGTCTGTCGATATCTGACTTTGTGATATTGTTTAATTCTTCAACTTTAACTTCTGTTG
GAAGCCCGTGGCAGTCCCATCCTTGGGGAAAAAGCACGTCGTATCCATTCATTCTTTTAAATCTC
GCAATAATATCCATGTATGTCCAGTTAAGCCCGTGACCAAGGTGCATCCTACCTGTTGGGTATGG
CGGTGGAGTATCGATGATGTAAGGAGGTCTTTTTCATCATCTAAGAATTTGAACGTTTTTTCAT
CTTCCCATTTTTCTTGAACTTTTTTTTCAAGTTCTATTGAATACTCTTCTTTTATTTCCATTATTT
CCCTCAATTTGTGGTAAATTAACGTCAAAAGCGATAAAAATTTTTAAATTTAACATAATATTGAT
AATATGAATATAATTAATGCGAATATAATTATCATGAACTTGTGTTTTTGAATTTATATTTTTA
ATCCTCTGAATAATAAAAAAATAACAGATTCAGCTTTATGAAAGATATCATCACGATTAATCAGA
TAACCCTCTTTTCATCATCATCTGTTTTTAAATAATATCAATAAGATGTATAAATGATTTATTTA
ACCTATTTAAATATTGTTTATATATTAGTTTTTGGTTGAATGCTTAAGGATAGTAAATCAGTCAA
AACATCTTATTTTGGCTGTTTTAATCGATTTAAATAAATAGTAATATATATTTTAAAAATTAAT
ATTAAATTAATCAGAACGGGGTGAATTTATGGCTTATAAAAAAATTATTATGCCCACAGACGGA
TCAAAAATATCAATAGAAGCTGCAGAGCAGGCAATTGATTTGGCAAAAGCCACTGAAGGAAGAG
TTTACGGAATTTATGTAATTGACATTGTTCCATTCGTAGGACTCCCAACTGAAGGGCTTTGGGAA
AGCATGAAGGAAGTTTTAGAAAAAGAAGGTCACACTGCGCTTAGAACCTTAACCGACATTGCGAG
AGAAAAAGGCGTTGAAATAAAGACCGAAATTTTGGAAGGAACTCCATCAAAAGAAATTGTTGAA
TATGCAGAAGCTAAAAACGCAGATTTAATTGTCATGGGTACAACTGGAAAAACAGGACTCGATA
AATTATTACTTGGAAGCGTTGCAGAGCGGGTTTTGAAAAAATCACACTGCCCAGTACTTTTAGTG
AAATCTTCTGAATAATAACTTATTTTATTATTTTTAGAAGAGGACACGTTATATAATTTTCTTTT
TTTAGGTAGTAATTTTTAGCATGCTCAACAAACAGTGCATGGTATTCTTGATATATTTCAAGATT
TTTTTCGATATTTTCTTCAAAAAGTTCTTTAATTTTATCGTATTTTTCGCTACCGTCAATTAAAT
TTAAGTTAATCAATATTCTTTTGGTATATGCATCAATTACAAAACTGGGAACTTTAAATGCATAC
AAAAGCATTGAATCTGCGGTTTCAGGGCCAACCCCGTTTAATTTTAGCAGTTCTTCTCTCGTTGG
AACATGATTTAATTTTATAAAAAATTCTGAAAAACCTTTTAAACGTACTGATTTTTGATTGTAGT

ATCCACTTGGTTTGATTGTTTCTTTTAACTGTTCGATATCTAAATTAATTATATTTTCAGGAGTT
ATTTCTATTAATTTCCTTAAATTTTTCAATGATTTTTCAACAGACGGCCAAGATGTATTTTGGGT
TAAAATTGCACCGATACAGATCTCAAAACACTGATTTTTTGTTTTTGGATACTCATAATTATTTG
GGTGATAACCATTTACCGAGCCAGTTTTTGTTGGATTACATCCATTTACTTCCAAAAGTGGCCAC
CAGCCTTGTGGCCCGTATAAATTAAATAAATGATCGTAAATTTTTTGGAAAGTTTCTTTATTGTC
CATATTTTTCCTTATTTTGAATTTTATCTTAATTTAATTTTAGTTTGTCGTAATTTTTAGAAACT
AGGCTTTTAACGTAGATTGGAGCAACTGTTGAAGTTAAAAGTGCTATGACCACAACAGAGACAAA
GAATTTTTCAGGAAGTATTCCAAGATCCCTTCCAAGGGTTGCACCAACAAGTGAGGCAGTAAGAG
ATGGAACGGTCAAAAGACCCCCGCAAAAACTTTTTAATTTGCCATAATCTAAAATTCTAAGTGAA
AAATATCCTGAACTAATTTTAAGGCCTATTAATGTTATAACTGATACAATTATAAGGCTTATATT
GTCTAAATTAAAAATTACGCTGATATCTGTAGTCATTCCTAAATTTAAGAAGAATAATGGGATA
AATATTCCATAACCAATTGCATTTAAGTTTTCATTTAATATCTGGTCGTGTTCTTCTTTTGTAAG
TTCTTCTGAGACTGCGATTCCAATTATAAATGCACCAATTATCGGGTGAATTCCAATATGTTCAC
CAACAATAATTGAAATTAATATAATCAGCAATATAAAGTGGATTTTTTGTACGTGAAACTTTTG
AAGCTCTTTTAATATTAATTTTGAAATTAATGGAATTATTATGAGAAGTGCAATGATATATGCC
GAAATAAGAACTACAAATGGAATAATATCTGAATTTATTCCGCTTCCCCCCATTTTAATTAATAT
TGAAATAACAATTAAACTTATAAGGTCAATTACAACAGATGCACTAATTACTGTTGTTCCAAATC
GAGATTTTATGAGTCCAAGTTCATCCATAAGTGCGTAGACGATTCCAACAGAATGTGAGGAAAAT
ATTACTGCATAAAGTAATGAACCAATAAAATCAAGTCCAAAATACTGTCCTATTAAATATCCTCC
AATTGAGGGGATTAACATGGAAAATAAGCTTAAAACTAAGGAATTTTTAAATTCATCTTTGAGT
GTTTCGTTATCTACTTCCAGTCCTGCCAAAAACATCAAAAATATTACTCCAAATGCTGCGAAAGT
GTCAAGAGTAGCGCTGGACTGAATTATATTTAATCCAAACGGTCCGATAAGGATTCCTGCGAGCA
TTGTAGCTGTAATTGTCGGAACATTGAACCTTTTTAGGATTTGAGGTACGATGAATATTGACGTT
AGAATAATAAAAAATAAAAAATAAGTGTCCATTAGATTACACCGTTAGCAGTTAGAATTTACGT
TATGATTTTTAATTTTACAATTAAGTTTATGAGCTATATAGTATTATTGATATTATTTCTATTTA
TTAAATCGGGTGAAACACAATGCTTGTAATGGCAGGACTTGGACTTTACGATGAAAGAGACGTTA
CACTAAAAACGCTTGACTTTGCAAAAAAAGTTGATAAAATTTACGCTGAATTTTATACTGCAATA
CTTACTGGAACAACGATGGAAAAAATTGAAGGTACGCTTCAAAAACCCATAACTGTTTTGGATCG
AGAAAAAGTAGAGTATGAAACGAATAAATTAATTGATGAAGCAAAAGATAAGGATATAATGTTT
TTAACTGCAGGAGACCCGATGGTTGCAACAACACACGTGGATATTGCAGTAGAAGCTAGAAAAAA
AGGAATCGAAGTTGTAATTATAAATGCTCCTTCAATTTACTCGGCAATAGGGATTACTGGACTTC
AATTATACAAATTTGGAAAAACAACGTCAATAGTATTTCCTGAACCAAATTATTTCCCGGAAACC
CCTTATGACGTTATAAAAGATAATTTAAAATTAGGATACCACACACTCTGCCTTTTAGATATTCA
GGCAGATAAGGAAAGATTCATGACTGCAAACGAAGGATTGGATGCACTTTTAAAAATTGAAGAA
AAAAGAAAAGAAAACGTAATTTCCGATGAAACAAAAGTTGCAGTTGTTGCAAGAGCTGGAAGTA
TTAATCCTGGATTATACTATGGAAAAATAAAAGATCTTTTAAATTATGATTTTAAAAGCCCGCTT
CACTGTGTGATAATTCCTGGAAAACTTCACTTCATGGAAGAAGACGCTTTAAAATACTTATTTGA
AAATATTTAAAATTTTTAAGCTTCTTCGCATTGTGCTGTAGGCATTGACGACTTTGCAAGTAAAT
AAGTTGCAGTAATTAGTGCAAAGACGTAAAAAACGTGCATAATGTATCTTACAGCAGGATCAAG
GCTTGAATAATATTTAAAAGATAAGCCAACACCTAAAATTGTAAATCCGATCATCATTAAATATA
TGATTTTGATATCGTCTACCAAAGCTCCCACCCCATATATTTAATTTTTTAACTTTTAATGTTAA
TAAAAATAACTATTTTTTAAACTTGTAAAAATAAAAAATAAATTAGATTTGGGTCTCAGAGCTC
ATACGGTTCTTCATCATTAAACGAAAGTTTAATTCAGAGCTGGTTATTTTCATCACAGACCGTAA

CTTAAAAAAGATTTTTTAAACATCTTTTATATATCTAATTTAAACATATGCATTATAACTCAAAT
CTCAATTTTAAAAAAGGGAATTTTATGAAATTTTTAATTCTTGATGGATACACGGATGAACCCGC
AGGACTTGGAGTTCCACCTTATATTGGAATGTATCCAAGATATGCGGCAGGAGTTCTTTACAAAT
TTAAACACGACGTTGATTATATCACAATCGATAAATTGCGAGAAGAACTTAAAAAAGGATATGA
TTTTAATAGATATGATGTAGTTATTGCGATATGTGGGTTTCACACCCCTGGAAAGTACTTAAATG
CAAACCCTGCAACATTGAGAGAATTTGTATCGATTTTGTATAATTTTAAAGGTTTAAAGATTCTT
GGAGGCCCTGTTGCAACTAAATTTGGTTCATCCCTTGAAGGCGGAAAAATAAAAGATGAATCAAA
TTTGAAATATTTCTTTGATGTGATTTCTGAAGGCGATTTAGATGCTGTTTTACACGATCTTTTAA
AAAACAATTTTAACCCTGAAAAAACAGATGGAACAAGGCTTAGAACTTACGATGAACTCGATGA
ATTTGCGAGAATTGGGGCAAAAATTGTAAAACTTCATCCAAACTATCCAAATATTATTGCAGAAA
TTGAGACATATCGAGGTTGTACAAGGGCATTTAGTGGAGGATGCAGTTTTTGCACAGAACCAAGA
CGATATGGGTCCCCAAAATTTAGAAATTCGAGAGATATAATTGAAGAAGTAAGAAATCTCTATG
AAAATGGAATTAAATACTTTAGAATTGGAAGACAGCCCTGCATGTTTTCGTACATGGCAAAAGAT
GTAGAAACAACAGATGTTCCAGTTCCGAATGTTTTAGAGATAGAAAAACTCTTTAATGGGATAAC
TTCAGTTTCAAAACCTGACGTTTTACACATTGACAATGCAAACCCTTCTGTTATTGCAAGACATG
AAAAAGAGGGGCGAGAAATTGCAAAAATACTTGTAAGACACTGTACTTCCGGAAATGTTGCAGC
TTTCGGGGTAGAAAGTTTCGATGAAAAAGTTATTTCAAAAAACTGTCTTTTAACTGAACCAGAAG
ATGTTTTAAAAGCTGTTAATATTTTAAATGAAATTGGCGGAAAAAGAGGAGAAAATGGAATGCC
TTATCTTCTTCCAGGAATAAATTTACTTTCAGGATTAAAAGGGGAATCAAACGATACGTTTGAAA
TAAATTATGATTATTTAAAAGAAATATATGATTCAGGAAATATACTTAGGCGGATAAATATAAG
GCAGGTAGTTCCATTTTTTGGAACTGATATAACAGTTAAAGATGTTGAAAAAGCAAAAAAACGA
AAAAATCTATTTTTGAAGTTCAAAGAAAAAATAAGAACCCAAATAGACAACCCAATGTTAAAAA
GGATGCTTCCAACAGAAACGATATTAAAAAATGTGCTAGTTGAAGTTAAAGAGAAAAATGATCT
TTATTTTGGAAGGCAGTTTGGAAGCTACCCAATATTGATAGGAATTGCTGAAAAAAACGTTGAA
ATTGGAAAATTTGTTGATATAAAAGTAACGGGCTATGGAAGAAGGTCCATAACTGGAAAAATAG
TAAAATAACGAGTTAGTGTTTTGATAATACATATTTTGCAACTTCTTTTACAACATCCAGTATCT
TTTTTGTAGTAATGTATGAAATTGCATCGTTGTGGTAGTCAATTGTTACATATTCTTCGGTTATT
TTTGCATTTTTTTTCGCTAACTGAAGTGCAATTAAGTGTTCAACACTGTATCCATCGCCAAAGTA
CGAATTTTTTAAAAATTCAGATTTTATAATTCTAAATCCACACTGGATATCTCTAAAGATGTGGA
AATTTCCCGAGAACACTGAAACAGTTATTGACATTATAACACTTGCAAGAAAGTTTGAAAACTGC
CTGTGAAATGGAATATGTTTGTATTTTCTAATTCCAAAAACGGCATCTGCATTTTCAGATTTCAT
TTTTTCGTACATCGGTATAATATCCGATGGTTTATGCTGATAATCACCATCAAGATACGCAATGG
CATCATAATTTAAATTTATTGCATAATTTGTGCCATCTTCGAGTGCTTTTGATTTACCTTCGTTT
TTTTCTTTGAAAATTGGAAATATTTTGTTTTTAAATTCGTTTTTTTCCAAAAATTCATTAATTAT
CGTTTTTGTTTTATCGGTACTTCCATCGTCTACGACAATCACATCAATGTTTATGTCGCTTAGGTC
CTTTAAAACAGAGATAATATTTTTTTCTTCGTTATATGCCGGAATGACCGCAACCATCATCATTA
TCACATTAAAAAAGTAAAGGAATGTAGATTATTCTTTTGCGTATGCAATAATTGCACCAAGTAAT
ATACCTAAGATTAGTCCTCCAAGTATATAGTATATACCGCCAGGACCGATCAAACTTTTATTATT
TTCCGCTAGTGCTTCTGTTGAAGTTGTATTTTTATCTGTAATTTTGGATGTATTTACATCCTTAT
CTTCTAAACTAATTACTGTACTATTTTCTGCTGAAGTATTTGAAATTTCCTCTTTAGTATCATTT
TCCGTTTCTAAAATTTCCACGGGTTCATAATTTTCAGGAGTAACTACCTTTTCAACAATCCTGAC
CCCTGCATCTGGAACTACTGAAAGTTCAAAGAATTCAACTGATCCAATCAAAGTTCCATTTTGGA
AGTATCTTTTTAAAAGTTTAATTTTGTAATTTCCAATCTCTGTAGCCGTTCCTTTAAACATTTTA

AATACGGATTCATTTGCGCTGACTTCACTTACATTATACTCGTATTCTTCAAAGATAATATCCTC
GTCTGCATCGATTGTTAAACTAAGATTTTCAATGTTTTCTGGTGCAGTCCAGTCGAATACGAGAT
AAACACTGTCATTTACCATAACTGCGTCAGGGGCAAGTTCAGCTGCATTAATTGCCGGGATAAGT
AGTAAACTTAAAAAAAATAAGTAGTGTATTTTTTTCATAAGTTTCACCTTATCTCAGTTTAATAC
TTTTTAGCCTTTCATTCAATTTGTTAACTTCATCGCTAGTTAGCCTTGTGGACTGCTTGATGTCTT
CAAGTTCGCCTTTAAGAACAACGAGTTTCATGAACAAAAATATGTTTAAAACAATGCTTATGAGG
ACTAATACTGTAAGTACGCCCGGATCCAGATTCATTTTTTCTTACCCCCAAATATTCCTTTAAAG
AATTTTTTAACAAACGAGTCTTTTGTTTTTTCTTTAGGAATGTATTGTTTGCCGACTAATTTTGC
ACCAAGTTCCATAATTGATTGCGCAGCTGGAGAGTCGCTATGTTTAAGAACAATAGGAACGCCAA
AAGCGGAACTTCTCCTAACGTTTGTGTCTTCAGGAACAATTCCAACAATAGGTATTTCTAAAATG
GTTTCAATGGATCTTGAACTTAATTCAGAGCTGTCTTCAGTTACCCTGTTAATAATTGCACCTAG
TACATTGGTTTCTACCCTGTTTGCAATAGATACAACTTTTAAAGCATCAGATATTGATGATATTT
CAGGGTTTACAACAACTAATAAATGTTCTGCGGCTGAAATTGCCGTAAGTGCTTCTTTACCTATT
CCTGCAGGACAGTCAATAAGCAGAACTTCGCTTTGTTCATCGAGTTTGCTCAGAACTTCAAGAAG
TCTTTCAGGTCGTGCTTTTTTAAAGCTGTCAAGCGAAACTCCTGCCGGAACTACTTTTACCCCCGC
AGGTCCTTCGTATATTGCACTTTTAATGTCCGCATTCCCCGATAAGACATCATTTAATGTTATTG
GTTTTCCCTCAATCCCCATGATAAGTTCCAGATTTGCCATTGCAATATCTGCATCGATTACAGTT
ACATCTTTACCAAATTGGGATAGTGCAACCGCCAAGTTAGCACAGGTTGTTGTTTTTCCAGTGCC
CCCCTTACCAGAGGCTACTGCAATTGTTATAGCCATGTTCTCACAACCTTTTGATATTTTAATTAC
AGTTATATATCTTAGAAGAATATGAACGAAATAGTATATAACTTTTTTATGTTTTTCCGAGGATT
ATATTATTTAACGAATTTAATATCGACATATATCTATTTTCAATTCGTTCAACAAGTTTGAGATC
ATATCTGTGTGTTTTTAAAATGATAATATCATTTAAATCCAAATTTTCTTTTAATTTGTAATCTG
GAATAATTTTAATTGTACTACCATTTTTATGTTCAAATCCCAAATCATCAAGTATTCTTTTTATA
AATTTCGAATCAGAATCAATATTACATTCAAAATTTTTTACAAATTCAAAATTATCGTTTTTTAT
CTTAAAATCGAGTTTTTTTAAATTTTCAATTGACTGGTTTGTAATATCAATATTTCCAATATTTT
CAGTTTTTAAATTTTCAAGACTTTCAATTATGTCTAAAAACGTAGCAGAGTTTATTTCTATTATT
TTGGAATTCGGGTTTAATTTACGGACTTTATCGAAATATCCTTTCGAAATAAGAATATATTCACA
ATTACAATCTGAATTATATGGATTTACAATTTTGAAATCTTCAATTCCTAAGAGATCAATGATTT
CAGAATACATTTTAGAAACGCCTATCATTAAAAACACCTTAAAAAATTATAAAAGAATCTTTTCG
AGTTTAGATACTGAATTTACAATATCTTCAGTTCTAACGCCAATTGCAGCGTTCATTACAATATA
ATCATGGGTATAGGTTCCTAGATAACAGTTTCCAAAATGATCTGTTTTTTTAATTCCTCTTGGAC
CAGTTACTCTTAAGTTATATAGTTTTGCGGCAATTTCAACGGGATCAGAATTTACAGAAATGCAG
GATGCAATAGGGCTTTCAACATCTAAAAATTTTCCGCCAGTTTTTTTAGATAAGTCATTTAAAAG
TTCATCAAGCAATTTTTTGCTGTTTTTCTGGTTTTTTACCAGTTCAAGATAGTTTTTAGATCCCA
TTGATAAAAGTGACACTAATGTATTCACAACAGGTGTTGCACTCGCCCTTCCAGGGTATGATAAA
GATATCTCTTTTATAAATTCCGCATCTGTTGAATATATCAATCCGCCACCAATAGGTGTTAATAA
ATTTTTGTCACTTGAACTTACAACGGCATCTACTCTGTATTTAAACGCTTTTTTTAACTTTTCAA
GGTAGTAATTGTTTTGAATTGCATAAGCTCCATTAATAATATGTGGAATATCGTAATTCTCACAA
ATTTTTGCAATTTCTACAATATCATCGCTGTTTCTTGGCGGAAAAAAACGTCAAAGTGCTCAAAAC
ACAAGGGCGGTTTCCAAGTTCAATTTCTTTTTTTATTGCATTTTCAATATCTTCAACTGGAATGT
ATACGCGGTTGCCATCTAAAACTGTTTCAACAAGCCTCATATTCATTCCGACAAATGACACAGCT
TTTATGGGGCTTTTGTGGGATGCATACGGGTAAATTACAACATTTGATCCGTATTTTTTTCTTGC
AGCGCTTAAACATAATGATATGGACATTCCTGTGGATATTGGCGTTGCAATTGCGTGAACATTTA

GCCCCAATTGTTTAAAAAAGCTCTCAAGTATTTTGTTTGTAAGGGCATACATGATACTAGCCCCG
GATGCTTTTGGTTGAGGGTCAACTAAATTTCCACTTCTTCCAATACCGTGGCAAAATCCTGATGA
AAGTTCTTCGTGAATTTTAGAGTATGTTCTTGCTTCTCTTTCCCCTATTCTTACAGATTTTGGGTC
TTTATCAGTATCCATCATCGATAGAAATTTTAAAAAAAGCTTTATTTTTTCGTCATCAATTCCGT
TTTCAGGAATTTTCCGATGGTTGAATACGTCTTCAATTTCTTTTAAATATTCGTTAAGTACTAAT
TCTCCCCTTTTTTCCATATTTTTTGGAATTAACCCTTCAATATTAAAATCGAGCATTATATCACG
TTGATAAAATTAAAATATCCGGTATATTCAATAAAATCCTTTTAAAGTTATTTAATCGCGATTAT
TTATAATCTTTTTTATCGGGTTTTACGTAGTATTAAATACCCGAATGATTAAATAGTTTCAGATT
GATTAGAAGTTAAGAGTACATTTTTCATGGATTTATAAATTTTTAGGTGATTTTATGTTATTCAT
TGCCTTTGCACCGTTTGGAGCATTTGCACTTGAAGCGAACTCGAAAAACGACGGATATTCATTAG
ACGACATCAAATATTACAAAATATTTGAAAACGAGGATATCCCAAAAATAATGTATGCGCTGAG
ACAGGGCGATTTTGAAATAATCGCTGAATTGAAAAACGAATGGGAAATTGAAGAAGAAGATGTT
AGTTTTGAGTATACTACTTCTAAAAATCCACTTGGAGAACTTATTCGAGATAATTTATATGAATT
AGGTCAAAAATACGAAGTTTTCGAAGATGAATCAGATTTTGTTTCAAAAATGAACGTTTGGGCA
ACCGCTTTCACAAAATCAATGATGAAAAAATCATCAGAACAGAAAGATAAATTGATAGTTCAAA
CAGTAAATGCCCTCGATAACCTTGATGAAACTCTAAACTTATTTTCAGAAAGACTTAGAGAATGG
TATTCACTATACTTTCCTGAAATGGACAATATTGTTAAGAAACACGATATTTATGTGAGTTTAGT
TTCTGAGTATGGGGACCGGGAAGAATATACTCGAACAAAACTTAAAAAAACAATGCCTTCAAACG
TTGCAAGAACAATTTCGCTTGCTGCAAAAGAATCAATGGGTGCAGATGTATCCGAATTTGATTTA
AAAATCATGAAAAACCTTGCAGATGAAATAAAGAGCATGTATGAATACAGGGAAAGCTTGCAAG
AATATTTGGAAACTTCAATGAATGAAATTGCTCCAAATTTAACGAAAGTTGCAGGTGCATCACTT
GGTGCAAGATTGATAAGCCTTGCAGGTGGAATTGAAAGACTTTTAAGACTTCCTGCATCAACAAT
TCAGGTTATCGGTGCAGAAAAAGCATTATTTGCACACTTAAGAGAAAGAGCATTACCTCCAAAAC
ACGGGGTTATATTCCAGCACCCGCTTATACAGGGAAGTCCTTGGTGGATACACGGAAAAATTGCA
AGAGCAATTTCTTGTAAAATTTCAATTGCGATAAGGGCAGATGCTTTTGGAAATGATATTTCCGA
CATGTTAATTGAAGACATGAATAAAAAAGTAGCCCAAATAAAAGAAAAATTCCCTGAACCAACG
AGAAAAAGAAGACCAAGACCTGATACGAGAAGTTTTGGCAATAAAGGCGGCCAAGATAGGGATA
GAAGGTCACAAGGTGACAGAAAACCACAGGGTAAAAGAGGCGACTCTAAAAAAGGTGGAAAGCC
GTTTGGTAAAAAACCTGCTGGAAAAGACGACAGACGAGATAACAGGAAAGGCGATAGGAAACCA
TTTGGTGGAAAACCAAAAAATAGCGGAAATCAGCCTCCAAAGGAAAGATCTGGCGAAAAAAGAA
GAGATTCGACAAAAAAAGTTAAAGCTGAGCGAAAAGTAATCGGAAAAACTTCCTCACAGAATTA
AGCCATATGGTGAGTTAAATGGAAAAAATTAGGGTAAAAGAAATTTTTGATAACGTATATTCAG
TAGATTTTGGAGACGGACTTAAAAGAATTGCTACAAAATCATTAGTTCCTGGAAAAAGAGTTTA
CGGGGAAAAATTAGTATATTCTGACAGTATAGAGTATAGAATATGGAATCCAAACAAGAGTAAA
CTTGGAGCTGCAATCATAAACGGCTTGAAAAAGTTGCCTATCAAAAAAGGAGAGAAAGTTCTCTA
CCTTGGAGCGTCTGCTGGAACTACTCCTTCACACGTTGCAGATATTGCAGAAAATTCCCTTGTTT
ATGCTCTTGAGTTTGCACCAAGAATCATGAGAGAATTTATTGATTCATGCAATGAAAGGAAAAA
TTTAATTCCTGTTTTAGGGGATGCAAACAGGCCTCAGGATTATTCAAACATCGTTGAAAAAGTTG
ATGTTATATTTGAAGACGTTGCACAACCAAATCAGGCAGAAATTCTTGTAAAAAATGCAAAATG
GTTTTTAAAAGAAAATGGATATGCAATGATATCTATAAAAGCAAGAAGTGTCGATGTTACAAAA
AATCCGAGAGAAATATTTGCTGAACAAAAGAAAATATTGATTGAAGGCGGATTTGAAATTGTTG
ATGAAGTAAACATCGAACCTTTCGAAAAAGACCACATGATGATGGTTGGAATCTGGAAAGGAAA
CTAAAACCCCATAATATTTGTTTACTTTTAAATAAACTTAAAATATTTCTTTTTTATAAAATATA

TATTGATCGAAATTGTTGCGTATTTTTAACTTTATATAGGCATCATAATATATTTTTATATGGCT
TAAGAAGTTTAGTAGATAGGGCGGTGGCACATGAAAACAATCGTTTTAGATTTTGACGGACTTTT
GGATGACTCTTATGAAATTTTAGATAATAAAACTCCATTAGAATATGCAAAAACTCCAAATTTTG
ATAAATTGGTTCAAAAAGGTTCTTGCGGGTTGTTATCACCTTATAAAAAAGGAGTTCCAACGTAT
TCAGTAATGGATCTATTTTTAATGCTCGGATATTCTCGGGATGAATTTCCAGGAATAAGTGTTTT
AAGAGCACTTGGGGAAGATCTGGAACTTAGTGATAATGCAGTTTACATTGAATGCACTTTTGTTT
CAACGATAGAAGACAATTATGGCCACAGGGTCGTAGATAGGGCATCTTGCGATTTAACAGATAAA
GAATTGAGCCAGTTAATGTCACTTATCCCAACGAATTTTGAAGGTTATGAATTTAATTTGAAAAC
TTGTGCTGATTATGGTTGTGTTTTGTCAATGGTTGATAAAAACGGTTGGATATCGGATAAAATTT
CTGATTCTGATCCGTATTATCCTGGAAGGCATGTAAATAAAATATCGCCTGTTTATGAATTATGT
GACTCTCCCGGGGAGTGTAAGCGTGCTAAAAGTACTGCTGAGGCGTTAAACAAGTTTTTATTAAG
GTGCCATAAAATTTTGGAGAACCACGAGATCAATATTAAAAGAAAAAGGAAGGGTAAATATCCA
ATAAATTTTTTAATAACTCGATTTCCTGGTAAAAACATTGATATTTCTACATTTTATGAAAAATA
CGGTTTAAAAGCACTATCGATTTCGAATTGTAGGCTTGCGAAAGGATTTTCCAGATTTGTAAAAG
TAGACTATTTACTTACAAAAGATTTGTCTGAGTCAGTAAATTCTTCGATAAAGTATCTTGATAAC
TACGATTTAATGTATGTGAAATGCTGTAGGATTTCTGAAGGTCAGATAAAGGATCCTGTGGAAA
AGGCCGCCGTAATAGAAGAAATGGATGCATGTCTTGATAAAATAACTGATTTAGAAGATGTTTT
GATAGTAATAACTTCAAAAAAGCTTTCTAAAATGATGGGCGCGTTTGTAAATTCGGGAAATGAG
TATTCACTAATCGTGTCTGGAAAAAAATGTTAGAAAAGATAATATATCGGAATTTACTGAAAAAA
ATTGTTATATTGGGCCATTAAGAATAGAACATAATGAAATATTGAATTTAATTTTAAATTACAT
GGATAAGGCATTATTATATGGTTTAAGGCCTGGAGGTTACCTGTTGGACTATATTCCTAATGATG
AGGATATAGAACACTTAAAATAAATATTTATACATTTTTTAGATAATTTACCAGTTCATCTATTT
TCTCAAATTTTAATGGGTAATTTATTTCTGGACGCTTTATAATTATTGGTTTTGCACCGGCCAAA
ATCGCACCAGAAACTTTCTCATAGAGTCCTCCACTTTTACCACTGTCTTTTGTAATAATTACATCA
CAATCATAGTCTATTATTAAGTTTTTATTTAAATTTTCTGAAAATAGCCCTTGCATTGCAACAAT
ATTTTTTGAAGGAAGTATTTTGAGAGCTTCTGGAACGGAAGTTGGAAGTATCCTTACAATCAGCT
TTTCAATTGGAATAATTTCAGAAACAGTTTTTAAATTTTTAATTCCCGACATGTAAAATATATTT
TTTTTAGAAATTTTTAAAGCTAATTTGGCAGCTTTTTCAAAATTTTCAACGTAAGAAGCATCATT
AAATTCTTCAACAGGCCTTTCGTACCGGATATATGGAATTTCCAGTTCTTTTGAGATTTTTATTC
CTGTTTCACTTGCATGCGTGGCAAATGGGTGTGTTGCGTCAATAAAAACATCTATTTTTTTATTT
ACTAGTGTTTTTTTTAAATTATCATAAGTCATTTTTTTTGAAATTACTAAATCTGCAAAATTTTC
TGCAATTTTTCCACCAAAGTCTGTTGTGGTGGTTAAAATTAAAAAAGAGTCTTTAAAATTTCTTT
TGATTTCTTTTGAAATATTGTTTGCGTCACTAGTTCCGCCACGAATCCATATGTTCATTTTTTCA
CGATTTTCAAAAATAAATATTAATTAATATATTTCTGTTGTAGTTTCTTCATAGGGTAGTAATTC
TATGGTTCCACTGGTTTCGTCTACTGCTACTTCAAAAACTTTGTTGTTAGCTTTTGTAACAATTA
TTGTCACATCATTTGCTTTAATATTTAAATATGCCTGATTTGACGTTTCAGGACTATCCCCATGG
GTCAAACTGTAGCTTATGGGATTATTTTCAACATCATTTGCAACTATTTCAAATTTTTTGATATC
CGTAAAGTCGAGTTCATAAGAATTCCAGGTCATTGCAGTAGGGATATTCGATCTGAAGATTATAT
CTGATGCATACCCTTCGTGGGGTAATAAAACGAGACTTTCTTCTGTAACACCTTCCTCCGGATCAC
TGGTGTTTGCCAGACCTTTAAATGTCCCATCTGAATAATACCAAAGATAAGTATCGTTTCCAGTA
TCGTTTATTTGAAGCCATGCGTTTTCTAAATTTGTTGAAGGGCTTATTTTCACGTCAGAAAAATT
TAATGAGTAATATATCGGGGGCTCATATATTGATATAGCAAATACTTGATATGTGCCTTCCCTAA
CGTCTGATAATATGTCCCCTTCAAAAGTAAATTTTGTCATGTGAAATGATACTAAAATACCTGCA

AGAAGTACTGCCATCATTAAAAGTATTAATTCTATTGAAATCTGGCCTTTTGAAAATTTTGCCAT
ATAACCACCCTAATTCGATAGAATAAAAATATTATGTATAAAAATTAGGATTTTTCAGAATAAA
TACTTAATTAAAAAAGTTTTAAAAAAGAAGGATTAATATTTTTAATTTTTTCCAGAACCACTACT
TTCAGAAGTTACTTTGACATCATCTACATCTATTTCAAGCCAGTAAGTTCCAGCACCATTTTCCA
TTCCAATTGTTATAGGTATTGGACAGGTTCCTCCTTCAACGCATTCGATAATGAAGTTTTTGTCG
TTTTTATCAAAGACTATAGTATCATTGATAATTATAACGCAATCACCTTTTATTCGTAAGATGAT
TTTAGTTACATTAATTTCTTTTTGAGTATTTTCTAATGGATAAAATGTTTCATTTATATAGATAC
CCTTAGTTGGTCCTTTTTTCTGTAATTCGACAGTGCCTCCACCATATAATGTAGCTTCAAATTCAT
TATTTTTAGCTGATGAAGTTGGATTTATATCTAATTTGGCATATATTTTATCAAGATTATTTTCT
TCCTCTTCTTCTGGCTCAGTATCTTCTCCATCTTCTGCTTGAGGATTCTCTTCTTCTGGGATCACA
GACGCTCCTGATTCAACTGCACTGCTCGAAAAGGCTCCAAGTGTAAGTTTTTTGAGTTCTTGGAC
TTCGCCCGCATTGTTGGTATCTTTTGTCATGTGGGCAGCAAGTACTGTTCCAGAAAGTAATACTG
CAAGAATTAGTATTACCATTTCAATTGAAAGCTGTCCTCGTTTAGAGTTTAATAACATAACTATC
GCCTAACAATATTTAATCGGTAGTTAAAAATAATTAATAAAATTAAGTTAATAATATATACTCT
TTAAATTTATAAATACCTTCTGTTGGCAATATTACTAATTTTTAATAAGTATTACATCTGTAATT
TATATTGTTTTGCAGCATTTTTTGGATTTTCATTTTCATAAATGGCCCTTCCAATAATTACGTAG
TCATTTTCGTTCAAAACGTTCAAAACGTCTTTTAAATTTCCGCCTTGAGCACCAACTCCTGGGGA
TATTACAAAAGCATCTTTTGCAATTGATTTAATTTCTTTTAACCTTTCAGGCCTTGTTGAAGGTG
CAACTATTCCATCAACTTTTAATCGGTCAGCCATTTTTGCCATATCTTCAGCTACGTTTTGTAAGT
ATTCTGTAGCTCCGGGGTGAGACATTTCTGTTACCATTATTACTTTTTTATTTTTTTCTTTTGCA
ACATTTAAAATTGCAGAAACACTGTCTGATCCAACAAAACCTTGGCATATTATTCCATCAGCATG
ATTTAGCGTTAATTCTGCAATTTTTTTATTTGTTGAAGGAATGTCTGCAACTTTGAAATCGCAGA
TTACTTCTTTGTTGGTAGATTCCTTTATTTTGTCGATTATATTTAATCCTGTAGCTAAAACTAAC
GGGTATCCTATTTTTATAGAATCAACGTACTCTGAAGTTTCTTTAGCAATCAAAACTGCTTTTTT
TTCATCCATTACATCAAGTGCAAGCATTAATTTTACCAAACTCTCACCATACATAATTAAGTATA
TAAATGATTAATACTTCATTACTATAAAAAGATTTAAAAATAAACGAATAACCTTCGTTTTACGT
TTTTCTTTTGATTTCTTAAAACTATCGATTAACTAATTTTTTAAAATTATCAAAAAAAGGTGAAA
ACTATGCGAGGACAACAAACACCCCCACAACAACCAACAAGAGTTAGGACTCCGCGAGAAAATGA
AAATGAAGTTCTTGGAGTAATCGAACAAATGCTTGGTGCAAGCAGAGTTAGAGTAAGATGTATG
GATGGAAAACTCAGAATGGGCAGAATTCCTGGAAAGTTAAAAAGAAAAATCTGGGTAAGGGAAG
ACGATGTTGTAATTGTTACCCCTTGGGAAGTTCAATCTGATGAGAAGTGCGACGTAATCTGGAGA
TACACAAAAGGACAGGTTGACTGGTTAAATAGAAAAGGATACTTAGATTTCATGAGATAGGGAT
ATTAATGAAAGATGTCCTTAAAATGGATTTAGATAAAAAAGAAAAACAGCTTGATAGGGAGTTT
CAAAAAAAGATAGTTGAGCGAAAAAAGAAGTTTTTGGAAGAATTAAAAACTGAAAATGAAGTTT
TTGATCAACGTACTCTCTTAAATATTTATAATTTACTTGTTGCAAAACACATTGACGAAATTTCA
GGAGTTGTTAATTCCGGAAAAGAAGCAGTGGTATTTTCTGCAAATAAAGAGGACGAACTCTACGC
ATTAAAGGTTTACCGTGTTTCCACTTGTGATTTTAAAACAATGTGGAAGTATATTCAGGGAGATC
CTAGATTTCACCTTAGAAGAAGCAGTACTCGACAGATAATAACTGCATGGGTGGAAAAAGAATTT
AGAAACCTTTTAAGGGCAGGAGATTATATAAACACGCCAGAACCACTATTAAAGCGAGAAAATA
TTCTTTTAATGGATATGGTTCATGAAGATGGAGTTCCAGCTCCAAGATTGAAAGATATTGAAGTC
GATTATTCCGAATTTTATGAAATGATAAAAGAAGATATGAAAGTATTGTATCAGGATGCCCAGT
TAGTTCATGGGGACCTTTCTGAATATAATATCTTAGTACACGAAGAAGAGCCGGTATATATTGAC
TTTTCACAAGGGGTTGTTAAAGAACATCCTCTTTCAAAAACACTCCTAATTAGGGATGTTAAAAA

CGTATGTAACTTCTTTAAAAGAAAAGGAATCGATACGGACTACAAAGAGTTTTACAAGTTTGTTT
CTGGAGAAGAACTTGCATTAATTGATGAAGAAATGGCAAAAACATATTAATAAAATTTCTACAA
AATCTTTTTTTAAGTATAAATTATATAATATTTAACGAAATTTAACTGCGTAATTGGTGAGATT
TTATGTACGAGAATGTGGAAGTAGTGAAGATTCCAAAAGAGAGAACAGGTATCCTTATTGGAAC
TCACGGAGAAGTTAGAAAAAAGCTTGAAAGTGATCTTGGAGTTGAATTAGAGATTGATTCGGAA
GGAGAAGTTAATATTTATTCAACGGACGAACAAAAAGACCCACTGGCACTTTGGAAAGCGAGAG
ATATTGTAAAAGCTATTGGACGGGGATTTAATCCTGAAAAAGCTTTAAAACTGGTTTCAGATGA
ATATGCCTTCGAAGTAATTGATATTTCAGAATATGGAAATTCTGATAAAGCTATTCAAAGGTTA
AAAGGAAGAATTATTGGAAGTAGCGGAAAATCCAGAAGATATGTTGAGGAATTAACTTCAACCC
ACATTTCAGTTTATGGTAAAACTGTTTCAATTATCGGGGAAATTGAAACTGCAAAAATTGCAAA
AGATGCAATCGAGATGCTTTTAAGGGGAACTTCGCACTCAAAAATGTACAAATTTTTAGAAAGAC
ACAGACAGGATGTCAAAAGAAGTGAACTTAGGCTCTGGAAATAAATTTATCTTTTTTTTAAATTT
TAAAAATAGTAAAATTATATTAGTGGAAGAATTATTCTTTTTTGGATCTCATTTCTTTTATTCTT
TTGATCATGAGTCCACTATCTTCTTTGGAAATTTCTTTTCTCAAACTAGGGCTTGTAATTTCTTC
TTCGACAGGTTTTGTATCAAGAAGTTTTACAATTGGTGATTTTGAGTTCATGTCAAATTTTCTGC
CGTTAAATCTTTTTCCGATAACATATACTTCTGCACTTTCATCTCTCGAAGCTCTTGGTTTCGTTG
TAAATGCTTTATCAAAGTATTCTGAAACAAGTTGAACATATTTTTCAAACAAATCGCCCTGAAAT
ACTTTTACAACAAAATTTCCGCCTTTTTTCAGCATTTTGGTTGCAGTCATGAGTGCCATTGTTGT
AAGTTCAAGGGACCTTACGTGATCTACATCCCAGACACCACTAATGTTTGGGGAAGCATCGCAGA
TTATAACATCGGGTTTTTCGGGTAAAAGGTCTCGAGCCTGTTTTAATATTTCTTCTTTTGTCATA
TCGCCTTTAATTGCAATAACGTTGTCATGCGGGAGCGGTTTTACAGTTTGTAAATCAATTCCAAC
GATAAATCCATTATCCCCAACAATATCTCTTGCAGCTTGGAGCCATCCTCCCGGAGCACACCCTAA
ATCTACAACAACATTTTTTTCTTTTATCAAATTGAATTTTTCATTCAACTGGAATAATTTATACG
TTGCTCTTGATCTGTAATTTCTTCTTTTTGCTAAATTATAGTAATGGTCGTTTTTCCTTTGAAGA
ACCCATCTTTTGTCCTTTTTTCCCATAATTATCTCCTTATATTAAAAAATTTAAATATCATCGTA
ATGAGTTAGTTCTGAATAGTCAATTGATTTGTGGAGGGTCTTCATTTCCACAGGGATGTTATTTT
TGCGTATATTTGCAAATGGGTTTGTTCCACCTATTGTGGTAATTCCAAACATACATTTTTCAACA
CGAACACCGCAGATGTCGTTATTCGGCCTTCCTATTTCGATTATCGAATTCCAACCGAGTTTTTCG
TTAAGTTCTATTAATTTTTCAGACGCAGACATTGGAACTTTTCGAATACCTGCTAGAATATAATT
TTTACCATCAGCTTTATTGAAAAATACTTCGTGAGGGTCAAGAGACGTTCCTTCGTAGTCAATTG
CTTCAATAAACCGTTTTTTATCTGCTTTTACTTCTAAAATTCCACCATAGTATGGAATTACAGGA
ATTTTGTTTTTCAAAAATACCCCATCAACAGTTAATGAACATATCGTGTATACATTTAAAAATTT
ATCATCGCATTCTACTTTCAATCGATCTGAAATTGCGAGTTTATTTTCATAGCTGTCTTTCAACG
CATTTATTGCTTCTTCCTTGTATTCAATAGGAATTTTTGCAGTGTTTACAACAACATTGCCTTTT
TTATCTTCAATATCGTAATTTATGGTCTGCATCTTTGAAAGCATTTTGGATAAAACAGGTGTTAC
CTTAAATTTTCCTTTCTTTTTCTTTGCTTCAAGGAATCCTTTTGTTTTTTTCTCCATTGACGAAAT
ATCTTTAAGCTGTGTGGCAGCACTTATATCTGCGGTGTACCCTGATTCGTGTGGTACGCACAATG
GTGTTAACCCCCCAACTAAGACTACTCCAATCTGTTCAGGGTTTAAATTCATTCCAAGTACGTTT
TCAGTTCCATATGCTAAAACAGAATTTAGCATGTCTTTTTTAAGGATATTTTCAAACTGTTTTTC
ACTTGATTTTGGAATTACTCGGAAGTTTGCAGGAACAAGTCCTTCTCCATTTTCAATTACGCCTA
TTACGTCAGTTTTTCCCTGCATGATAAATGCAACTAATGGATCAATTGAAGAACTTTTAAAATCA
ATTACTCCTTCAAAATTCACTGGTTCGTAATCTTCAAATTTAACGATTCCACCGTATTCAGGGGT
TGGAATTATTCCGTTTTTTAATAAAAAGTTATCAAACGTAATACTGCAAAGGGTTTCTACAGATA

CCGTATTTCCTTTTTTCTTGATATTTAGGTAATCTCCTACAGAATAACCTGCTTCAAATGATCTT
AATACCATTTCTTTAATGGTTTTATAATCTCCTTCGAATTTAGCAGTATTTATAAGAACTTTTGA
AGGAAAATCTGACAGATAAAGTTTTTCAATAACTTGTGAGAATACTGAACCGATTCTAAATGAA
ATGTTTGCTTTTTCAAGTTCTTCAATTCCTTTTTCCGTGATTTCTCTTCCAGAATAACCTAATTTT
TTTGTAAGACTGTTTTCATCAAGTACTTTTAAGTGATACCTAACAGCCCTTTCTCCAATATCATA
ACCCCTGTCTTTTAGTGAATCTGCAATTATTTTTGCACCAACTGGGGCAGATGCTTCAGATAAAA
TGGATAAAATTTCAACATCAATATTACTGTCCATAATGCCACACCTATTAACCTAAAAACTTAAA
AAAAGTTATTTTTTAAGATTCCTATATCCTGAAGAGATCAATGCAGCACCAACTGCACCGATATA
TTGAGAATATTTTGGAACTATTATTTTTTTACCGAGTATTTCTTCAAGTGCAAGAACCATTCCTT
TCAAAAGACTGCTTCCACCAACCAAAATTAAAGGATCCCTTACATCAACTTCTTGGAGTTGCTGT
TCGTAAATTTGTTCTGCAACAGAGTGAGCAGCTGCGGCTGCAACATCATTTGGCGTAGCACCGCC
TGCAAGACCGGTTACTAAATCCTGGATCCCAAATACAATACAGTAGCTGTTCATCATAACGTTTC
GCCAGTCTCCCGTTGCTGCAATGTCACCAAGTTCCTGAATGGATACCCCAAGTCTTCTTGCAGTAA
TTTCAAAGAATCTACCGCTTGCACCGGCACAGATTCCCCCATTGTGAAACCATCTGGAATTGCAT
TGTTCAGCGAAATTGCTTTGTTGTCCATTCCACCAATATCAATTACAGTAGCTTCTCCTTTCTGA
GCACCTGCTAAGAATGCAGCACCTTTTGAGTTAACAGTTAATTCTTCTTGTACTAAATCAGCTTT
GTAGTGGTCTCCAAGGGTGTGTCTACCATAACCCGTAGTTCCAATTGTTTCGATATCTTCCATCTT
AAGTCCTGATTCTTTTAAAGCTGCATCAAGTGCTTCCTGTGCAGATTCGACAACATCTTTAGTAT
AAACCCAACCAGTTCCAACAACTTCGTTATTTTTCATGATAACTGCTTTTGTTGTAGTTGAACCA
CTGTCAAGCCCTAAACTTATACCATCCTGCTTTTTCCTTGCAAGAAGTGCTTTTTTGTCAACAATA
GTAACCAAAGCTTCCATTCTCGTAAGTAATTCTGAAGCTTTTGTTCTTTCAGTAAATGAATACAT
AACAACGGGTAAATCCGTGTTTTGTTGTATTAATCTTCTAACTTCGTTTCTTACAAGGGCTCCTT
CGGCACATCTAAAGCAGGTTGATATAAAAACTGCTTCTGCATCAGTATTTCCTTCAATGATGGAC
ATTGCTCTTGCAAACATTAATTTTAAGTTAGCAGATGCAACTTCAAATCCTAAATAGTCGTTAAC
TTTATCGATATATTCTAAATCGACTTCAGGATAGATAAGTTCTCCACCAACTTCCTTCGCAGCTT
TTTCAATTTCCGCCTGAACACCGCTGTATTCGGCTCCACAGGTAAGTTCTGCTATTTTAACAGCCA
CAATTATCACCAGATTAGAATTCAAATCTTAAAAAATAAAATGAATCGGGTAAACTTAAATTTT
GTATGTTATTATTCTGTTTTCAAATATTTATAATAGTGCTTAAATTTTTATAAAACTCTTGTAAA
CTTAAGTTTTAAAGTAATTTTAAATCTTTCGTAACTTTTGAAATTTTTTCTTCTTTTTCAGGGCC
AATTCCAACAGCAGTAAGACTTCCAGGTTCAATCTGGGTTCTTCCCGCATCTCTTATTAAACTGC
ACGGAAGTCCTTCGATATTAACGTTTTTAAATATTTCTAAAAGTTCCTTCTCAGAATTTACTTTT
AAAACAACTTTTTTCTGCCCTTCATTCATCCAGCCACTAACTGCAGATGAACTTATTTTTTGAGC
ATGTAAAAAAGCTTGAATTGATGCATGACAGGCTTGAGCCGCCATTTTCCCTTTTCCCATTTTTA
GATCATTTCGAATAACAATAGCCTGTTCATACATTTTCTCGCCAACCAAAAATATTTTAAATAAG
AATCTCTTTTTATATGAAGTAATATATCTAATAGTTAATTGCTATTTTAACATTTGGGATTATTA
TTGAAAATTATCGATTTTTACAGATTATGAAGTTTAATTTATGGTGGAAACATGTTTGAAATAA
AAGCTCGTGATGCAATGGGAAGGCTCGGGAGTATCACGATAAACGGAAAAAAAATTGAAACCCCA
ACAATTATGCCTGTAATTCACCCAAATCCTAAAAAACAGACAGTTTCAATGGATTTAATAAATAA
AATGGCAGATGTTGTAATTACAAATTCGTATATAACTTACACAACACCGGAACTTAGGGAAATTG
CGGAAACAAAAGGAATTCACGAATTAATCGACTTTAAAAATGTTGTTGTAACTGACAGCGGTTCA
TTTCAGTTGAGTGTTTATGGCGATGTAAATGTCGGCCCAATGGAAATAATCGATTTTCAGGAAAA
AATCGGTGTAGATGTAGGAACAATACTTGATATTCCAACTGGACCTGACGTTTCAAGAGAAAAAG
CTGAAAGTGATCTAATTGAAACTTTTAAAAGAGCAGAAGATTCAATTAAAAGAAGAAGTGAAAT

GGGCTACAAACTAGCACTTAATGGAACTATTCAGGGTTCAAAATACTTGGATTTAAGGCAGAAAA
GTGCTGAAGTTATGGGTAAAATGGACTTTGATATTTACCCGATCGGCGCAGTAGTTCCTTTAATG
GAAGATTACAGATATAGGGAAGTTGCAGAAGTTATTTTAAATTCAAAAATGCATCTTCCAACAA
ACAAGCCTGTTCACCTTTTTGGATGCGGACACCCGATGTTATTTGCACTTTCAGTTGCTCTTGGAT
GTGATTTATTTGATAGTGCCGCATATGCCCTTTACGCTAAAAATGGAAGATATTTAACTGAAGAT
GGAACGCTTCATCTCAAAGACATGAAAGATTTGAAAAGTTTCCCCTGCACCTGTAAAGTATGTAG
TGAATACACTCCAAAACAACTTTTCAATTTGGAAGAAAAGGAAAAAACAAGATTACTTGCAGAA
CACAATTTATACGTTACTTTTGAAGAAATTGATAGGATTAAAAATGCAATAAAAGAAGGAAACC
TCTGGGAACTCGTTGAAGAAAGATGCAGGAGTCACCCTAAACTTTTGAACGGTTTGAGGGTTATT
TCAAAATACATGGATTTCATTGAAAAACACGACCCGGTTTCTAAAAAATCAGGATTTTTCTACAC
AGGATATGAAAGCATGAATAGGCCTGAAATTTACAGGCACAAACAAAGGCTTGAAAGAATTCAG
TATGATAAAATATACGTAACAAGTGTTTCTGAAAATACTTCAAAACCATACAGTGAAAATTTAA
GCAATGTTCCTTGCGATGTTGACGTACTCGTAAAAGACAGTGTATTTGGATTAGTTCCTTTAAAT
ATTGATACAATGTATCCATTGGCTCAAAATGAAGTACCTGATCTGTATGATTTTGAAAAAAAATA
CAACAATGATTTTGTAAGCGAATTTAATGAAAAATACGCTGAAAAAATACTCGATATAAGCACA
TACAACTACTACATAAACCACTACGGCAAGAAAAAAGAGTGTGAAAAAGTTAATCCTGATATTTT
TAGGATTGGCAAAATGCTCGAATATCAGTATGGGGCAAAAATACTCGATGAAGAATTAATGGGA
AAAGTAAAATCAAGAAGAAGTAAAAATACTGGCAGAATTAGAAATCTTTTACTTGAAAAGGAAG
TTTTGTTTACATTAAGGGCAAATGACAACTTCCTGATTCCTGCAAAATCTGGTGCTGAACTTTTA
CATGAAAAACTCGAGTTTCCAAAGTACAGGATAGTAATCGACAGCAGTGTTGAAGAATTTGCAAG
GGCTGGAAAATCAGTTTATTCCAAATTCGTTAAAGACTGTGATCCAGAATTAAGGCCATTTGAAG
AAGTTTTGATTGTAAATTCTGATGATGAATTATTGGCATACGGTACAACAATACTAAATGGTCGT
GAATTGATGGAATTTGATTATGGCGTTGCAGCAACCCTTCGAGGGGGATTGAAGAAATAAGTTTA
TTAACTTAAACTTTAAATAAAATATTGTTTATTCTGGACGTGATTTTTTTGTTAAAAGTATTGTA
TAGTGGTTCGGTTACGTATCTTGATGAGAAAAATAACGTATTTGTAGCCCCCAGCTCATCTTGTA
CATATATAGAAACCTCGGAAAATAAAATAATCGTTGATACTTCTTCAAAAGATAAAAAAAAGAT
TATAATTTCAAATTTGAATAAATTAGATGTTAAATTAAAAGAGATAGATTATATTATAAGTTCA
CACAACCACAATAACCACAATGGGAATAACGGGCTTTTTAGAAATGCTAAAATTGTTAAGTATTC
TGACGGATCTATAAATGATTTTAAAGATCCAGAAGTAGAAATAATTTGGACTCCCGGGCACACAT
TTGACAGCATTTCCGTAATTCACGAGGATTATGTTGTTGCACCTGCTGCAGTCCACCTTAAAAAA
ATATTTTACAAAATTATGATATTCCCATTGCAATCGATCTTGAAATTTCAAAAAAATCAATCCAA
AGAATAGTATCTTTGAAAAAACACGTTATAACTGGGCATGACGGAATTTTATACGCTTCAGAATA
TCTTTAATTTATTCCCAACGTTTGACGGTTTCATAAACTGGACCTTTTTTAGTTAGCGTACTTTT
CATAAGTGAAACTGAATTTATTTCTATTTTTCCAAAATTAATATGTTTATTTTTTTCAATTAATC
TTTTTAAGGTTTCTTTTGAATCATTTTCGATAAATTTAACCCTTCCAAGTGTGATGTGCGGATCG
TAACTTTTTTCTTTTTTAAAACCCAATTTAAGGAGCTTTTCATCAATATTTTTAAAAGTTTCTTG
AAGATTTGTTGTTCCAACCCATATTACACGGATATATTCTTCATTTGGAAACGTTCCAACTCCTT
TTATTTCAGAAATAACTTTTTTAATTTTTAAATCTGAATTAACTATGTCTTCAAGTTTTTTATCG
TCAATATCTCCTAAAAATTTCACAGTTATGTGTAAATTCTCTTTTTCAACTGTTTTTATGCCTTT
TAAATTGAACTGTTTTTTTAAAACTTCCAAATTTTCTTTTAAGTCAGTTTTTAATTCAATAGCAA
GGAAACATCTCATAAAATCACCTATTCGTCAAGGAGTACCGGGATTTTATCTTTAATTAAATAAA
CTTTTTTACATTTTTTACAGCATAATTTATTGCTCAAAAGCTCTAAATCTCCTTTACAGACTGGA
CATTCCAATAATTCAGTCAATTCTTCAAGCCATTTCATAAATTACACCTAAAAGCAAATCATTTA

TTATTTATACCCTTTTAATTATATGTATTTTTCATTTAAAATAGGAGGATAATATGAATAGGTTT
GTAAAATTTTTGATAGGTATAATGGTGTTATTTTCTGGCTGTATTTCTACAAATGACAATTCAGT
TTCAGAAGTCAAGTCAGAACAGGTTTCAGATGTTTTATACCAAGTATCAACGATAAACGCGCTGA
TGGAAAGTATTTATGACGGATTTATACCAGTGAATGAACTTGTAACTCACGGGGATTTTGGAATA
GGTACATTTGATAAACTCGATGGGGAAATGGTTGTTCTGGATGGAATATGTTATCAAGTTAAAA
CAGACGGTGTAGCATACGAAGTTGAAAATGTAACAACTCCTTTTGCAACGGTTACTTCGTTTGAA
AACGATGAAACGTATTATTTAGATAATATGAATATTTCAGAGTTTGAATCATACTTTGAATCGA
AATTCCCTTCAAAAAATATGGTTTATGCAGTAAAATTAACAGGAACTTTCTCAAAAATGAAAACA
AGAAGTGTTCCATCTCAAGAAAAACCTTACGAAAAACTGGTAGACGTTGTAAAAAATCAGTCAGT
ATTTGAATTTGAAAACGTTTCTGGAACTGTTGTCGGGTTCTGGGTTCCTGAATTCATGTCTGGTT
TAAATGTTCCGTTATACCACTTACATTTCATAACTGACGACAGATTGGCTGGAGGACACATTCTT
GATTTTGAAATAGATTCTGTTGAAGCTTCATTTGATACGACACCGGAATTTTATATGGTTCTTCC
AACGTCTGGAGAATTTTACAGTATGGAATTTTCAGATAATCTTGAAAATGATTTAAAAGCTGTA
GAAAAACAATAAATCTTTTTTTAAACTTTTTTTATTAATTCATGCAAAAATATATCACTTCTTAA
AATTATAAAATCAAACATGAAATTATTTTTGAAGGTGGGATATTGAAAGTAGTTGCATTTAACG
GGAGTCCAAGAAGGGACGGAAATACGTCTATTTTAATTGATAAAGTTTTAAATGAATTAAAAAA
AGAAGGAATTTCTACAGAACACATACACATTGCTGGCGGAAAGCTTAGGGGATGTACTGCATGCT
ACAAGTGTTTTGAAACACTGAATAATAAATGCATAATCGAGTGCGATATAATAAATAACTGCAT
AGAAAAGATGATTGAAGCTGATGGAATAATTTTGGGATCTCCAACTTATTTTACAGACGTTACTG
CTGAAATGAAAGCATTAATTGATAGGGCAGGTTTTGTAGCAAAAGCAAACGATGATATTTTAAA
AAGAAAAGTTGGAGCTGCAGTAGTGTCTGTTAGAAGAGCGGGGGGAGTGCATGTTTTTGATACC
ATAAATCACTTCTTTTCAATAAACCAGATGATAAGTGTTGGATCAGACTACTGGAATTTAGGAAT
GGGCCTAGATATTGGGGATGTTCAAAAAGATACTGAGGGACTGCGTACAATGGATCTACTTGGAC
AAAATATGGCGTGGATTTTGAAAAAAATTAACAAATAATACCAATAAATACAAATAATCTTAAA
AATCGTTTAAATCTTTTTTTAAATTTTTTAAAAAAGCAGGATTAATGTATCTACCTTTGGAAAAC
CATGTTTTCATTGACTCTCAACAGATACTCAAGATGGCCGCCTTGTAGGTAGAACTGCTAGTGAC
AGTATCACGAAATCAACTGCCAAGTAATGTTGTATTTTTTATTATATATACTTGATTTAACTCAA
ATTTCCGGTAAATTCTCTTGTAACGATTGTAGCGTAATTTCCGCGCTCAAGTTTAAAGCTCAGTT
CAAAAATTTCATTCTCAAACCTGCTTTTAAAGTCATAAATTGGAGTTACCATTTTTCTTCTAGTT
CCATAGAAATTTCCAAAGTCTTCAATTTTGAATTTCTTCAAATCAAGATTTTCCCTTTCAACAAT
TTCTTTTTCAATTTCACCCTGAATTCCACCGCTAAATTCAGTATCGTATCCTATTAACGTTCCTGT
TGGGGTGTTATCTTCCAATATATCTCCTTCTAATGCACCAAACCCGTAATCAAATCTTTTGTTTA
TCATTTCATTGAATAGGTATGCTTGATAAGCATTTACAAACATGCTGTTCAACTGTGGCGGTAAT
GCAGTAAAAGCCATTTTAAAGTCTTTATATTTTAAATACTGCTGAATTAATCTTTTTTCGTAGTC
GTGGCCCCTATTAAATAATTTCAAAGCTTTTTTAAATTCACCCATGTCAACGAGCTGTCGTGCTT
CAAGAGAATCGCCAGTTTCATTTATTGGGGTTCCACAGTATGTGTAAAATGCACTTTCAAAGTCT
CTTTCATAGATGAATTTTCCAACAATATGCGTAATTGGTCGAATAAGTCCAAATCTCTGTATTCC
GTAGTAGTTTAATACGTAATCTAATTTTAAATCGGAGATTCTTTTAATTTCTTCTTTTGAAAGAT
TTAAATCCCTAATTTTTATCGTGAATTTATTTCCCCAGAGGCCCCCATTCTGAGTTTTTTGTTCG
TTTTTTGAACATCACGAATTACAATATCTTTAATATTAATATTTTCAAGCTGTTCTTTTTTAAGT
CCAAAACATCCAAATCTCTGAGTAGTTACTGCAAATTTATCTTTAGTTCCTGCAAATCCAAAATT
TTTCCTTTTAGTTTTTGTAGCCCTTACGATCTGTTTTAATGCATCCATCGTGTTCCAGTTCGTTTT
TTCAACGGTAAAATGAATAAATGAGCCGTGCCACTTTTCAATGTCTTCAAAACCAAGGTCTTTTC

CAACTTCTAAAACCGTTCCTTCAGGGGTTATTTCTTCAACAATGAAATCTTCAGGATATTTTTTA
AGTGTTCCATCAAACCTTTCCATGTCTAAAATGAATTTATTAATGTTTATCGGCATTTCGCTTAA
TTTCTGCCTTATTTTATTATTTCTAAATTTAAACTGTTTTTCTTTTCTAAACTGGTTAATTATCT
CTTCACTTTCTTTTTTTCTCGCATTTTGAATGAATGGGGCGTTCATGATTAAATCTCCATTTTAA
AAATTAGTTTTATATAATTTGATAGCGATGTTTACATAAATATGTTTTTAGCGAATAAAAATTT
CTAAATCTAAATTTAATTTAAATTTTACCTGGATTTAAGTGGTAATTATGTATATTGGAATTGAT
GACACAGACAGTAGGGATCACTACTGCACGACCTATGTTGGAACTATTCTTCAGGAAGAACTTGA
AAAAACGTATCTTATGGATACTCCAAAGTTAATTAGGATGAACCCGATGGTTAAATATAAAACT
AGGGGAAATGGCGGAGTTTGTTTAAGAATCATTGAAGATGAAAAAAAATTAAATGAATCAGAAA
TTGAATCCATTAAAAAGTTAGTAATTAAAACAGTTGAAAAATTCAGCGATTTTGATTGTGAAAA
CACAAACCCGGGAATTGTTTTTTTAAGCGAAGAAAATTATCAAAAAAACCAAAAAACAATTTTA
AATTACTATAATTCGGTTTTATACGATATTTTAAAAGTAAATGATTCTGAAAAAATACTCAATG
AAATCGGTGCAGAATATATAAAATACAAAAAAGGACACGGGATAATCGGAGCTCTTGGTGCAAT
TGCTTCAAAACCGCCATTTACTTACGAACTTTTGGCATACCGAAAAAGTGAAAAATGGGGAACAG
AGCGAGAAATTGATACTGATTCAATAATAAAAATGGATACTGATACATTTCCTTTTACATTTAAC
AATATCGACGGAAAAAAATCGATAATTACACCCCACACGCCATGCCCTGTTCTTTACGGGATAAG
AGGAATTTCAAGAGAAATTTTAGAAAAAGCTAAAAATACTGTAAAATCGGATGAAATTGATAAA
TGCCAGGTATATATCACAAATCAGGGAACTGACGTTCATTTAAGATTTTCTAAAATAAAAGACAT
TTATCCGGATACTGGAGTTATTTCTTATGGTAAAATCGTTGAAAATCCAAAAGAAATAACTGGTG
GACATGTTTTATTTAAAATGAAAGATGATACCGGCGAAATTGACTGTATTGCTTATGAACCGACC
AAAGAATTTAGAAATATCGTTAGAAAACTCAACTGTGGCGACCTTGTTGGAGTTTATGGGACCGT
TCGAGAGGACCCGTTTGAAATAAATATTGAGAAATTAAAAATAGTAACACTTCAAAAAATATAT
GAAAAAAATAAATTTTGTGAATGTGGCGGAACCCTTAAAGCTAAAGGGTTAAAAAGTGGTTATA
AATGTAATATTTGCGGTAAACGAGTTAATTATGATGAAATAAAACTAAATGAAGTAAAAAGGGA
CATCAAAGAAGGATTTTATGAAGTTCCGCCTTCTGCAAGGAGGCATTTAAGTAAACCTATCGTAT
TATATGACTTTGATCTTGAAAATATAAAGTAAATTATTTATTGGATAATTATACATTTCATTAT
ATCAATGTGATTTTATGCTTGAAGAACTTTTGAATGGAAATATCGAAAAAAAGACGATAACTCA
AATTTACGGGCCTCCTGGCGTTGGAAAAACAAATATTTGTATAATTTCAATGTTAAAAGCAATTG
AAAATGGCAAAAATGTGGTTTACATCGATACAGAAGGGAGTTTGTCTATTGAAAGAATAAAACA
GCTGTCTGGAAAGGATTGTGATGAATTACTTAAAAATATAATAATTTATGAACCGTCTTCATTTG
AAGAACAATCCGAAGCTTTAGAAAAGATATTTCTTCTTGAAAACGTTGGGCTTATAATTATTGAT
GGCATTGTTTCACTTTACAGGCTTGAACTCTGTGATAAAATCAATGAAAATACTAAATTAAACAG
AATGCTTGGAAAACAGATTTCAAATCTTTTAAAAGTTTCCCGACAGAAAAATTCTGGAATTTTAA
TTACGAACCAGGTGAAAGACTCGATAAATGGTATCGAACCTGCTGGAGGAAGACTTTTAGAGTAT
TGGAGTAAATCAATCATAAAAATTGAAAAAAGTGAATCTATTAGAAAACTTACCTTGGAAAAAC
ACAGACACGCAAAAGAAGGCGAGAATTTAAGGTTCAAAATACTTCAGAATGGTCTTGAAATAAT
AAATAAGTCCTATCAATAAACTTTTTTCTAATTATATTAATTTAAACCTGTGCTTTATAAAAAAA
CTATATATATCATGTTTCACACATATGTGTAAGTAGTCATTTTATAAGGGGGGAATCGCATGAGA
ATACTTTTAAAGCTGTTTGTAGAAAGCCAAAATCTTGGAAAAGCCATTAATGCACTTTCGGAAGG
TGGAATTTCTGGATTTTACTTAAAAGAGTACCAGGGCATGTCACCTGATGATTGGAAAGGTTTTT
TGCTTGCTGAAGAACCTGAAATGGCAATAAAAATAGTTAATGAGTTATCTCAGGACACAGTGGTT
ATAAATTCGATAGTAAATATCGAATGCCTCGGAAAAATAAAAGAGCTTGTTAGAAAAGAGCTTG
AAAATGATAGATATACATTAGTTGAACTCCCAGTTCTAGGAATGGAAGTCAATTCACCAGAATAA

GTGATTTTAAATGAAATTTAGAAAAGGAAGACCAAAAATACTTCGTCTTATCTCTGAAGAACCGC
AGTTTAAATTATTCAAGCCTGTTGGAATACCCCGAACTGATCTTGAATCGGAAGTATTGACGTTC
GAAGAATTAGAATCCATTAGGCTTGTAGATTATTTAAATCATCCCCATGAAGATGCTGCTGACGA
AATGGGTATATCAAGACGAGTATTTTGGAATATTTTAAAATCTGCAAGAAAAAAAGTTGCAGAT
GCATTAATAAATGGAAAAATGATTGATATTGGTGGCGGGTACTATAAAATAAGGGATTGTAACT
ACGAAGACGAATGTCAAAGGGGTAAATTTTGTAAATACGGGGTATCCAACTGTTTAAGATTAAA
AAATAGAGATAGTGAATAATTGCATCGCTTAGGTGGTATTAATGTTACTTTTGGAAGTGAAAAA
TGTTTCAAAGAGTTATGGGGACACAGAAGTACTAAAAAACGTAAGTTTTGAATTAAATGAAGGG
GACGTTATGGGAGTTCTGGGCCGGAGTGGTGCCGGAAAATCCGTACTTTTGCACATGTTAAGGGG
TATGGAAGGATATGAACCTACCTCCGGACAGATAATTTATCACGTTGCATACTGCCCACATTGTG
AAAATGTTGAAGCTCCATCTCAGGTTGGAAAAAAATGTGAATGTGAAACCGAATATGTTGCAAA
ATCTGTTGATTTTTGGAATAATAATGAAATTACCTATGCATTAAAGAAAAAAATTGCGATAATG
CTCCAAAGAACATTTGCACTTTACGGTGAAAAAACTGTTGCAGAAAACATCATGGAAGCTTTAAC
TGCCGCAGGATATGAAGGAAAAGATGCAACTGAGTGGGCATTAAATCTTATCAAAATGGTAAAA
CTTGAGCACAGGGTTGCACACATTTCAAGGGATTTGAGTGGTGGTGAGAAACAGAGAGTAGTTCT
TGCAAGACAAATTGCGAAAAATCCTGTTATATTCCTTGCCGATGAACCAACCGGAACACTTGACC
CAAAAACTGCAAAATTCGTTCACAATGCACTTACTGAGGCAGTCATAAAACACAACATTGCAATG
GTTATTACATCACACTGGCCAGAAGTTATTGAAGAACTTTGTCAGAAAGCAATATGGCTCGATAA
AGGAGAAATGAGGCTTGTAGGCGAAAGTAAACATGTTGTAGAAGAATTTATGAAAACTGTTACT
TCAATGAAAGAATTTGAAAAAGTTGAGGTAAAAGATGAACTTCTAAAACTTGAAAATGTTGAAA
AAAGGTATGTTTCAGTTGACAGGGGTATTGTAAAAGCTGTTGACGGAATTGACGTTTCAATCAAT
GAAAAAGAAATTTTCGGGCTTGTTGGTGTGAGTGGTGCTGGTAAAACCACGCTTTCAAAAATAAT
TGCAGCGGTAATTCCTCCATCAAAAGGAAACTACGAGTTCAGGCTCGCAGATGAATGGGTTGACA
TGACAAAGGTAGGCCCACTTTACAGAGGACGTGCAAAAAGATACATTGGAATGTTATTCCAAGAA
TACAGTCTTTATCCACACAGAACGATTCTTTACAACCTTACAGAATCAATAGGACTTGAAATGCC
CGGTGAATTTGCGAAAATGAAAGCAGAACACACACTGGTTTCTGTTGGATTTACTGAAGAAGAAG
CTGAAAACATGCTTGAGAAACACCCTTCAGAACTTAGTGTAGGTGAAAAGCACAGGGTTGCTCTT
GCACAAGTTTTAATCCGTGAACCACACTTGATTTTATTGGACGAGCCAACTGGAACAATGGATCC
AATAACGAGAAATCAGGTTGCAGAATCTATCCAAAAATCAAGAAGTGAACTCGATCAAACATAT
GTTATAGTATCACACGATATGGACTTTGTTTTAAATGTATGCGATCGGGCAGCTTTGGTAAGGGG
CGGAAAAATAATTAAAACCGGAAAGCCTGACGAAATCGTAAAAATATTGTCCGAAGAAGAAAAA
GATGAAATGATCGGCCATTAAAAATATTTTAATTTTTAGTTTATTCAATTATTTTTTCAAGGTAT
TGAACTTCATTAATTATAATATCTATTTTTTCCTGTTTTAAAAGAGTTTTATTATATATTAAATC
GTTTAATTCAAAATTCAAGGCCCCTTGGTATTTATATTCATTTACAAGTGAATTTAGTGTTTCAT
CGAAATTTATTTTGGATTCACTCAATGGATAGTGGTCTTTTGAATTTACGCCTGAAATATGCATA
TTTTTAATTTTTTTGTGGAAATTTTTGATAAAATCAAGTGAATTTTCACGTGCATGTGCAATATC
AAGAGTCATGTTCAAATTTTCAAATTTATCAAGTGTTTTTTTCATCGATTCAACATTGTAGCAGA
TATTGTTTATTTTTTTAGGGGAATTTTCAAGACAAAGAGTTATTTCATTTTTCAATGCATAATTT
ATCGATTTTTCTAAATAATCATCGAAATTATTGTATTCTTCATCTGTTGGAATTCTTGCGGTCGG
TCTTTTTCCAGGATGAATGGTCAAAAGTTCTGATTTAAATTTTTTTGAGAGATCAATTGCCCAAA
AGGTTTCCATTAATGTAACGTGCCTTATATTTTCGTTTGTCGAAGAGGAATTTAGCTCGATATAG
GGGGCATGAATAGTTACATCTATTTTTGATAGTTCAGTCTTAACATTATTTATATAATCTAAATC
ATTTCTTTTTTTCCAAAATTCGGGATTTTCTGGAAAAAATTCCATATTTTTTAAGCCGATTTCTA

AGAATATATCTACAATTTCTCCAATATCATATTCCCAAAAAAACAGCGATGAGCAACCTATTTTT
ACCATGATTTAAATATAGTTATTTTTAATTATAAATGTTTTGAGGGATATTATGGACAAAATGC
TTGAAAAGTTTGAGGGAAGCATTTTGGGGCTTTCAATAGGCGATGCGCTCGGCATGCCAACAGAA
TGGTTTACAAAAGAAGAAATCGCAGAAAAATATGGTTTAATCGACAATTTTGTAGATCCCAAAA
ATAAATTTAACGGAATTTTAAAAGCAGGACAATATACTGACGATACGGATCAAACAATCGCAAT
ACTAAATTCTTTTGATGTAAGGGGATTTAATAACGATATTTTTATTAAAGAACTTATAAAATGG
TACAACAATAATCCAGTTGGAATAGGGCCAACGAGTACAAAGGCAATCGAAAAATTAATTGAGG
GGGACACTACAGGGTGTGATTCGAGAACCTGTGGCTCTTCAATGAGAGTTGGGCCTTTAGGACTT
TTTTATTTTGGAGAATATGATAAATTAAAAGAAATTACAATTAAAGCTACAAAACTAACCCATA
ATAACGATGAAGCAATTGCAGGCGCTCTTTCGATTGCATTTTTTGTTGCAGAATCTGTAAACCAT
GTTAAATCTGAAAAATCCATCAAAAGGTGTGCAAAATTTATTGAAGATGTTTCTTACGACTTTTC
AGAAAAGATACTTCTTATAAATGATTTAAATTCAGCAGATGAGGCTTATAATTTATTTAATACA
GGCCTTCCTGCAATCGAGTGCGTTCCATCAGCAATTGCAACATTTTTAAGAACTGATACGTTTAA
AGACGGAATGATTTCATGTGTAAATGCGGGAGGGGACACTGACAGCATGGGAAGCATGTATGGT
GCAATTGCAGGAGCTTATTATGGGGTATCGAATATTCCTGAAATTTGGATAGCAGGTCTTCAAAA
TAAAGATTTATTGGTAGATTTATCTAAAAAACTCTACAAATTAAGATTTAAAAAATAAATAAAG
CGAAGATATATAAAATATACTTTTTATATTAATATAAAGAACTAGATTGGTGAAAATCATGGCA
GACTACGATTTTGAAATAACGAGCAGGCCTTCATACTCTCTTTTAAAAATAAACCTGAAAGATCA
GGAAATAATGACTGAAACCGGTTCGATGGTTTATAAAGATAATGGTGTAAATATTGAGACTTCT
GCTAAGGGAGGAGTTTTGGGAGTTTTAAAAAGAGCAGTGGTTGGAGAAAGTATATTCATGAATA
AATTAAGCGGTTCAGGAAGAATTGCACTTGCTCCAGGATATTCTGGAGATATTGTTCACCATGAA
CTCAACGGAACATTATACGCAAGTAGTGGCGCATATTTAGCATCTTCATCAGATTTATCAATTGA
TACTAAATTTGGTGGCGGTAAAACATTTTTCGGTGGAAAAGGTCTATTTTTAATGAAAATTGAA
GGTTCGGGGGATGTGTTCTTATCATCATACGGTGCAGTCGAAGAAATAGAATTAAACAATGAATC
AATAATGGTTGACAATGGAAACCTGGTTGCATTTACTGGGGATTTAAACTATAATCTTGGAAAA
GTTGGCGGTTTAAAATCAACACTTCTTGGTGGTGAAGGTTTAGTCTACAACTTCAGCGGAACGGG
TAAAATTTATATTCAAACGAGAAATATCGAAAGTTTTATCGATTTTATAACACCTTACCTTCCAA
CGCCAAAACAGGGATAATTAAGTTTAAATAATTACAAATCAAAAATTAAAATGGAAATAAAAAT
AGAAAAGGGGATATAAATGGTTAAATTAACAGAAGAAATGAAAACTGAGATAACTGGTCTTTTA
TTTTTAGCAACATCTTCAAAAGATGGAGTTCCAAATGTAGCTCCAATGGGTGCAAACCAGTTTGT
TGGAGACAAACTCGTAATTTCAGACAACTTCATGAAAAAAACACTTGACAACATTAAAGAAAACC
CTGTAGTTGCAATAAACGTTGCAAACTGTAGATCACACCCATTCCAGTACAAAGGAAAAGCTGAA
ATCGTAACGGACGGAGAATACTTTGAAAAAGCAAAAGAATTAAACAAAGTAAAAGTTCCAGGAT
TAACCCCAAAAGGTGCAATTGTTATCACAATTACAAGTATTTACAGTGTAAAACCTGGACCTGAT
GCTGGAAGTTTAGTTGAAACTGATGATTAAATTTTTTGAAAATAAATTTTCTTTTTTTAAATTTA
GTTTGTTGTATTAAAACAGTTTTAAATTTTTTTAAATATTCTAAAAAAAGAAATTAAAGTGCGA
TTTTAGCACTGATTTGGAGTTATAATAAGCCTGCTGCATCTAAAGCTAACTTAACTTCTTCGGTT
GAAGCTGCTTTTTCCAAGTCAACTTTTTTGTCGGTTGGTTCAACGTGTTTTAAGTTGCATCTTCT
TCTTTTAACGCTACCGTCGATAACGACAAAGTTTTTGTCTAATACTTCGACGATAACACAGGTGT
TTCCTGCTTCTCTTCCTAAAGTTTTAATGCAAACTCTTCCTACTTCAATTGCTGCCATAATTATTA
CCTCATCTTATTTTTTGTTAATGTGAATGTCGCCAATCACATGTCATACTTCAAACAGAGAGATT
TACTACCATCTAAGCTTTCAAGTTGTCAATGGCTTTTTCGATGATATTAAATACCCCTTTAGCGT
CCCATTTCGAGGACTCGATTGTTAAATCGTATATTGATAAATCATCAATTTCGATATTATATATT

TCTTTGTATCGTTTTTTTTCGCTAGCTTCTCTTGAAATCATTTTTTCAAGTGCCAGATCAACGTTT
TCGTTTTCACGTTCGCTGATCCTAATGCATCTTACCATCGGGTCTGCTTTAAGCCAAATGCTTAAA
TCCGGTTTTATGTCACTTCTTTTGAGGATCCATCCAGCAAGTCTACCTTCAAGGATTAAATCCCCT
GATTGAGCTGCCTCAACCTGTCTTCTGTCAATTTCGTTGTCAACTTCAGTGTTTGTTTCTGCATAA
CTGCTGAATTCTTGAAGGGTCATATTCATTTCTTTTGCCATGTCTCTGAAGATAAATCCTGCACA
GACATGGTTTAACCCGTACTTTTCTGAAAGTAACTTAGAAGTCGTTGTAGTCCCTGTACCCGGCA
ATCCTCCAACGGTAATGATCATTTTAGCACCTTAGAAGTTCCTAGCTTTTGCGACCATCATTTTT
TTAACACATTTTGGGCACAAGTTGCCACCAAATGGTCTTTCTGGTCTTTTTTCTGTTTTAGCTAA
TTTTGTGATTTCTACTGCTCTTCCTCTAGGTACTCCATTTAATAAAGCACCGCAAGCACCACATAC
AGCTTTTGATTGTTTTTTTCTTCTATAGTGCACGATTGAACTGTTTCCAGGTGCTTTTCTGTACA
CTTTTTTAGATGAACCTGACTTATATCTTGGGGCAGGCATAGTATAACCTCCAAATGGTTTTCCA
AAATCTATTTTACTTAAAAGTATATAAAGTTTAGGTACATTTTAGGGTATTTTCTATATTGGCCA
TTTTTTGCACCATTTTAAAGAATTCAATTCAAAAAAATAGCATTCACTGCAGGTTTAATTTTTAA
AAAATCGCAGAGAAAACAAAATTTGGAGAAAAGTCCAAATTATGGCCGTACCATAACATTATTA
CTATCGTAACCAAATATAAAAAGGTTATCTTAATACTGGAAAGTTTATATGTTGTTAAATTCAA
AATTATATCGTGAATAAATAATCTTAAACGGGGGATATATATGAAAATGAGTATTAGAAATCAG
TTTACTGGAAAAATACTTGAAATTGTGGAAGGACAGGTAGTTGCAAAAGTCAAAGTGGACATTG
GTGGGGGAAATACAATTGTTTCAGTAATTACACTTGATGCAGAAAAAGAACTCGGATTAAAAGT
CGGAGATACGGTAACTGCATTAGTAAAATCGACCTCTGTCATGATTGAAAAATAAATACTTTATT
AACACCAAATAACTTTTTTGTTAGGGGTCAACAATAAAAAATCGAATACCCCTTTATATATCTTG
AAAAGTAATATACTTTTGAGGCCATTATTTATATATAACTATTTACCCAAATCCAAAGAACATAA
ATCCAACGAAAATATTTTTGGTGTCAATATGAGTAAAACTATCGAGAGTGTCGAAAAAACATTG
ATTGCCAATGTATCTTCCCTTTTATCAAGTGAAGTTAGGGCAAAAATATACATATTTTTAAGAAT
GAATCCACAAAGTACTGTGGAAGAAATAGCAAAAGGAACGGGAGTATACCCGTCAACCATTCGAG
AATCAATATTTGAAATGTATAATGAAGAGTACGTTCTTCGAAAGAAAATGGATAGAGAAGGCCT
CGGTAAAAAAACCGTACCTTTATTCTGCAATAGCACCTGCTGAATTAGTACAATTGATTTCTGAAT
CTATAACTGAAAAGTTAAATGATTTAGCATCAGTTGATGAAAAAATTAGCGGTAAAAACGTTGA
AAACGCTTTAAAAGTTGCTATTACAATAGAATCCAACTAATTTTTCTTTTTTACATTATTTTATG
TTTTTAAAAATATTAATCGTTATTAATATATTAAAATATCTAAATTAATTACACCTTGAATAAA
AGTCTTATTTTAACTTGTTTTTCTCAAATATTTTATGTATAGAAACTCTTTTTTCCAAATGGTCA
AAGTAAAAAAAGTAAGATCTGGATTAAAATCCAAGTAGTGTTCCAAGATTATATGTTATTATTC
CAACAACTGTTGCAAGTGCTAAATTGTAACCTATTCCAAAGGCAGTCCACTTCAAACTTCTTGTT
TCAAGGTAAAGTGTTGCAAGCGTTGCAAGGCAGGGAATATAAAGTACAGAAACAAGCGTTAATA
CGAAAGCTTTTAACGGGCTAAATGCTGTTGAAATATTTTCTGGATACAGTATACTAAAACTTGAA
ACAACTAGCTCTTTTGCAAATATTCCAAAGACAAGGGATATTGCAGCTCTCCAGTCAAGGCCCAT
GTAAAGCGTTACATGTTCGAGTATTTTTCCAACAACTGCGGCGTAGTTTGCATCAGGGGATGGAT
AATTTACAAGGCCGTAGAATAAAATGGAACCTGCAGCAATAACAGTTCCTGCTTTTTTTAGAAAT
TCTTTGCTTTTTTCCCATGTCATTTTAATAATGTTGTCCCAATCAGGAATCCTGTATGGCGGCAA
TTCAAAGATGTACTCTTCTGCTTCTCCTTTCATGAATTTACTTAATACATATGACACCATGAAGA
GCAGTGCAAATGTAATTGCAAGAATGCTTAATGTAAAAAGTGCAGCATGTTCTACAAAGAAAAA
TGATGCGAAAAATCCAATTATAACAAATCTTGCAGAGCATGGAACAAGTGGCGTTACTAAAAGTG
TAATTAACCTTTCTTTATGGCTCGAAATAAACCTCGTACCCATTAATGCAGGAACACTGCATCCA
AAACCCGTAATTAGAGGAATGAATGATTTTCCGCTTAATCCCATTTTGGACATGATCGAGTGTGT

TAGTGCAGTAACTCTTGATAAGTAGCCTGTATCTTCAAGAATTGATAGCGAAAACATTATTAGAA
ATACTATTGGGAAGAATTCAAGTACTCCTCCAACTCCTGCAAGTAATCCATCAACAATTACTCCA
GAAAGTGATTGTGGGAGGATATTCATTAAATAATTTCCAAGTATTTCAAAGAATGTACCTATAA
CTCCTGCAAAAATGTCTCCAACACCAAATACAAAGTTGTACATTGTGTATAACACTACACCAAAT
ATCATGAGTCCGTAAATTGGATGAATTACAATCGTGTCTATATCTTCGTATAATTCGCTATTTAC
CATGACGCCTGCTAAAATCTCATCACATTTTTTGTATCTTTGTTCTACAACGTAGCTTTCAACGTC
GTGTTTTATTTCGTTCTCAATTTCATTTTTTAAGTCTTTTACAAATTTTATGAATTCAGGATATT
TTGAAAATTCATTTACGACATCAGGATCTTTTTCAAGAAGTGATATTGCAATCCATCGTTTTGGA
AGCCTTTCATAGTTTTCACTGATGTTATACTGCGTATTTTCCAGTTTATTGATTATTTTTCCGAT
ATTTTCTTCCAAAAGTGGCGAATATGTAATTTTTACAGGTTTTCCCGCTTTGTATTTGTAAATTG
TATCTTTTAAGTTATCAATTCCAATTTTATGTCTTCCAGAAGTTCTTATTACTGAAACACCTAAT
TTTTCGCTTAATTTTTGATCATTTATATAAATACCAAATTTTTCTGCTTCGTCGATTAAATTTAA
GCATAAAATTGGGCTGATTCCAAGTTCTAAAAGCTGTATTGTAAGATAAAGATTCCTGTTAATAT
TTGGAGTGTCGATTACATCAATGACTGTTACTTCATTATTTTCAATGAGGTAGTCTCTTGCAATC
TTTTGATCGATCGAGTCAGACATCAATGAATATATTCCTGGTAAATCGACTACAACGTAGCTTTC
ACTATTTTTTTAAAGAATCCTTCCTTTTTTTCAACAGTAACGCCGGGCCAGTTTCCGATTCTTTG
TTTCATTCCAGTTAGGTGGTTGAAAAGGGTAGTTTTTCCAACGTTTGGCTGACCAAGCAGTGCAA
TAATATTCTTCTCATCCATCATATTCACCAGTTTTATTCCGTATTACACTTTCCTTTACAGTTTTT
CTTCTCGCTGCAGCATGAGCAGCTGGCTTTCACTATTTTTTTTGAAATTTTAACGAGAATTCTTT
CTAAAATATTTTCTTTTTTCTCTTTTTTTAAATTTTCCATATTTATCACACAAAAGTTTTTCACA
TAATTTTAATTCGGTTAAATAATTTAAGCTAGCTTAAATATTTTTTTGTAAATTACGCTATAAAT
TTTCATGAATAGAATTTTCTTAAATTATTCTACAAAAATCGATTCTGCCATGCCTCTTCCAAGGG
CAACTTTTGAGTTTCCAACCCTAATTAGTACAGGGCCTCGGTTATTTCGGGATATCAGAGTTATT
TTACAGCCTGGGAGAATTCCAAGTTCATAAAATTTTCTACAATGTCCTTCAACCTTAACTACCAT
ATATGATCCCGGGTCTTTGAGCAAAAGATTTTCCATATGACCACCAAAGTTATATATACAAATTA
ATTAAGATAAATTAAATAATTTAACTTAGCTTAAATTCTATAACTTTTTGAAACTATATATATCT
TTTCATTTGGCGATAACATGGTATCACAAAATATTGAGGACTACCTCGAAAATATATTTTTATTT
ATTAAAGAGAATAAAAGGCCGGTAAAAACAACCGAACTTGCAAAATTAATGAAGGTTCAGCCCG
CAGCAGTAACCAGTATGGCAAAAAAACTGCATGGTGACGGATTAATAGATTATCAGCCGTATATT
GGAATAAATCTTAAAAGTTCTGGCGAAAAAACTGCAAAAGAAACGATTCGAAAACACAGGATTA
TTGAATATTTTTTAACTGAAATTTTAGGTCTTGATCTTGAGTTTGCACAAGAAGAAGCATGTAAA
ATAGAGCATGCAGTGTCTGACAAAACAATCGAAAAGTTATATGAATTTATAGATCGCCCTGAAAA
GTGCCCTCACGGCTATAAAATAAATTTAAAGTAATTAAACGTAATTAAATATTATACTTTTTTTA
AATAGTATTCATAATTAGGATTTTTTTAATATTAAATTTCTAAAAAAACTAAATCATAACGGTT
AAATATTTTTTAGAACTATATTTGCAATAGTTAATTGTTCTGATTTTATTTTAGTTTAGTGTTAA
TATTAATTGAAGGTGCAGCTTATGGCCGGAAATATCAGTGATGAAGATATTCAACAAAACATTTT
AAAACATCTTTACAAGATAAACAGCAGGGATGTTGACGCTTTTTTGTCGTGTGAAATATTTAAGG
AAGAATTATCGTTAGATCGGGCAGATCTTTATCGAAATATTGATACCTTAAAACAAAAGGGATTT
ATAAAAATGGGAAATAATCCTGAATTAATCGGGTATTCCATAAAATTATCAGAAAAAGGAATGG
ATACTTTCGAAAATCCCAAATTTACAAGAGAAGAAATTGAAAAAGCCTACAAAACATTTAAAAT
TTATGCTGAAGAAGTAACGAACGCATCTTATGATACATATACAAAATCACTGGCTGCATTTTTAA
ATTATTGTAAAAATAATGAAGTTTTAGAGTTTGTAATATTTAAATTCAATTTAATAGAACGCGA
TTTTGAAAAATGGTATGATAATTTTTTAAATTCAAAAATGCACGCACATACTCTTCCATTGGATG

AAGATGAAAAAATTTCAATAATTTACAAATTTTTAAACGAAATTAATTCTGGAAAGATTAAAAT
TTCGGAATTTGTCAAATCCGCTTACAAAATTTCCGGAAAAACTGAAAAAACGATTAATAAATTTA
ATCAAAGTATATTTTTACATTTTGTGAAGGGAATTGACATAAGGCTCAAAAAATTGATGAGTAA
GATCGAATCTGTTCCTGAAAAACCTCAAACTGATGAAAAAGAAGTTGTTAAAACGGAAGAATTA
CCAAAAGTTTCAAAAATCGAGGTAATTGATTCAGATTCTGTTACTTCAAAAAGAAGGCTTGAAA
ACTTTGAAAAGGATAATAAAGTCGTGATATATCCTTCAGAAGTAATTGATGGGGATGCTTTAAG
CAGAAAACTTTCTAAAAATAGCTACAACATGGATAATTTAGAAAATGCATTATTAAAACTTTCA
GAAGAGCTTAGGGATGATAATAAATCTGTTGCAGCAATATCTGCAGTGTTTTTACTGTCAAAAAA
TATAGATTCGTCTGATTTGATGGAAACTCAAAAACTGTTAAATGAAGCCGTATCTGCAGAACCCT
GCCTGAAAGACAAATTTAAAGACATTGCAATGGCTTCAAGCATTGAAGTAGCGAATACAGGAATT
ATTAAAGGAATACTTGCACTCTTTTAAGTTTTATCTTGTTATATTTTTTTATTTTTTATTTTTTA
TTTTTTTATTTCAGTGATTTGTTCATTCAAACTGGAAGTTTATAATTGGTTCAAAAGTACAAATA
ATATATAAAATGAATATCATAATCTGATACATGGCAAATGAGGTGAATGTTTTGCAAAAAACAA
TAGAAAATCTTTCAAAAGCATTTATTGGGGAAAGTCAGGCTAGAAATAGATATACAATATATGC
AAAAATTGCTCAAAAAGAAGGCTACGAAAAAATAGCAGAAATATTTACCATGACTGCGGAACAA
GAGCGAGAGCATGCGAAATGGTTATTTAAATTAATAGATTCGCTTAAAAAAGACAATGAACCTG
CTGAAATTCTTATTGATGGGGCAGCAGTTCCAACAGCATACAAAACTACTGTGGAAAATTTAAAA
GCTGCAGCATCTGGTGAAAATTACGAACACACTGAAATGTATCCTGAATTTGCAAAAATTGCAAA
AGAAGAAGGATTAATTGAAATTTCAGACAGATTACTTGCAATTGCAGAAGCTGAAAAACACCAC
GAACAAAGATACCAAAAATTAATTGCTCAGCTTGAAAACGGTTCGATGTTTAAAAAAGACGAAG
AAATATATTGGACCTGCAGAAAATGCGGATACCTTGTAAAAGGAAAAGATGCGCCAGTTGAATG
TCCTTCATGCGGGCACCCAACAGCATACTTTGAAAAGCAGTGCGAAGAATATTAATCCTTTTTTT
AATTTTTTAAGGGCTTTTGTTTTTAAATTTAAGTCAGAACTAATATAAACTCAACATTACCTATT
TTATAATGATTAAACTCTTTCGTTTAAGTGAACTTATTTAAATTATCAACTTTCAATCGGTAAAT
CCGGTGAAAATCAATGAAACCAAAAGACGTTGTTTCTTTTGCGGGAAGAAACATAACTCAAAAAA
AGACACAGAGTCTTTTAACAATAATTGGTATTGTTATAGGCATTTTAGCAATAGTTAGTCTGATT
TCTCTAGGCTATGGGGTTCAGAATTATATTACTGAGGAAATATCCAAAGCAGGAGCCAGTGTTAT
TACTGTATATCCATCACAGCAGATTACAAGTATTGCAATGTCTAAAAATTTTAATGATCGAGATT
TAAAGGCAGTAGAAGATGTTAGGGGCGTTGACGTTGTATTATATGGGTGGTTTGGCGGAACACA
GGTAACTTACCATGATCAAGAGTATTATTCGAGTGTGTTTGCAACGAAATCTTCAACCTACCAGA
TATTTTTTACAGAAGCTTATGGTTATGAGCTTGAAAAAGGTAGGTGGATGAAAGACAGCGATAA
ATACACTTGTGTTATTGGGTACAGTCTTGCACACGATACATTTGATCGGGAAATAGATATTGGGG
ATAAAATTGAAATCAATGATAAAAAGTACAAAGTTGTAGGTATTTTGGAACAGATTGGAAACCC
TGATGACGATAATTCTATTGTAATACCTTATGAAGCTGCATCAGAGGTTTTTGATGTGGATGATG
AGTACAACATGTTTATGGTTAAAATAAAGGATGGGGAAGATGTAACCAAAGTTTCAGAAGATAT
TGAAGACGAACTTGAAGATTCAAGAGGGGACGAAAATTTCTCGGTTTTAACCGCGGAACAGCTTG
CTGAATCAATTAGTAGTATTTTTAGCGTTTTAACAATATTTTTAGTCGGTGTTGCAGGAATTTCC
CTTCTTGTTGGGGCGGTTGGAATTTCAAATACAATGCACATGAGTATTTTAGAACGTAGAAAAGA
TATTGGAATTTTAAAAGCACTAGGTGCAGAAAACAATACAATCCTCTCAATATTCGTAGTTGAAG
CTGGATTTTTAGGGTTATTTGGTGGAATTGTTGGAACAATACTTGGAATACTGATTGCAAAAGCT
ATCGAGTATGTTGCAGCGATTTTTGGATACGGACTGATTAGGGCATGGATTTCATGGGAATTAAT
CGTAGGAGTTTTAGTATTTTCATTTGTAGTTGGAATATTAAGCGGCTACTTCCCTGCAAGAAGCG
GTGCAAAATTAAACCCTGTTGATACATTGAGAGGGGAATAATTATGACATTAATTGAAGCAAAA

AATGTAATCAAGACTTACGGAGTTGGCGAAGCTAAATCCAACGTTTTAAAAGGGGTTAATTTAA
AAATTGAAGAAGGAGAATTTGTAATGATAATGGGCCCGAGTGGTTGTGGAAAATCTACTTTAAT
GAATATTTTGGGTCTTTTAGACGTTCCTTCCAAAGGCGATGTTTACATAAAAGATAAAAAAACCA
CGAAAATGAACGAAAATGAACGTGCAGTATTTAGGAGAAAGATTAGTGGGTTTATATTCCAACA
ATTCCATTTAATCGGGACATTAACTGCTTTAGGAAATGTTGAACTTCCTATGACCCTGGATGGAA
AAGAAGAAACTTTCAGGCAAAAACGAGCTAAAGAATTATTGAATATCGTAGGGCTTAGTGAAAG
AGAAAATTATAAGCCATCGCAACTTTCAGGTGGACAGCAGCAAAGGGTTGCAATTGCAAGATCTC
TTTCAAATAATCCAAAACTACTGTTTGCAGATGAGCCTACTGGAAATCTTGACAGTAAAAGTGGT
AAACAGGTAATGGAAATACTTGAGAACTTACACAACGAAGGAATGACGATTGTAATGGTTACTC
ACGATAGCTATATGATAAAATACGCTACAAAGGTTATTCGGATGAAAGATGGGGAAATTCTCGA
AATCGATAAGGTTAAAGATGGAGAAGTTGTTGAAACCGAAGTAGTTAAAAAAGATGTATAGACG
TTAAAATTGTAAATGTTATGAATATATGGACGTTTTAATACTTTCAAGATGAATAACCCAAAGA
AGAAGATTTAGAAAATATCTGAAATAATTTTTTAAAACTAAAAAAATAAAAAAAATAAAAAAAT
GAACTGGTTGGTGGGATAATGTAATTGTTAGTTAATTAAATGATTTTGTTAAATAGTAACCAAC
CTTAAAAATTAAAAAGAGTGATATAATGAGACTTTTGGCTAAGTCTGGACTATTATTACTTGTA
TTTGCACTTATATCAAGTTCTTATGCATTATCTGTGGACGATCCGCAGTATGTTGTAGATAATTC
TGAATTTGGCGAAACAAATCCAAACGTTATACATCCTGGGGACGATGTGAATATCTGGATAAAAG
TGGTTAACGACAACTATGATAAACAGTTAAAAGATGTAAAAGTTGAAATAAGTCCGCATTATCC
ATTTGAAATAAAGCAAGTAAACCCTGAAACAGGTGTTGCTGAAATTTCACATCTTAACGAGGGTG
AAAGTGATACTGCATACTTTAAAATACACGTAAGTGAGAATGCACCTTCTGATGAGTATAGAATT
GATGTTACCGTAACTGCGACAGAATACGAATCAGGAGACGAACCAAATGAACGCGAAAAAACATT
GACAAAAATATATTACGTTCCAATATATGGTATTGCTAAATTTGAAATTAATTTAGATGATAAC
AATCCTATAAACCCTTCAGAATCTGAAGAGCTCGAAGTATATATTAAAAATCAAGGAACTGGAAC
TGCAAAATATTTAACGCTAAATTTGGCTGGAACAAGCGATTTAAACATTGTTGGGCCAACGACAT
TTTATTTAGAATCACTATCCCCACAAACACAAAAATTGATAACCGTTGATGCATACGCAATTCCA
ACGACTGAAGATGGGATTTATTCAATAAATGCGAATATTGAATGGATTGGAGAAGATGGTGCAG
AGTATACTTCATCGATTCCGATAAATCTGAAAGTAAAAAGTACAATTTTTGATAACCAGCCGTTT
TTATACCTTGATGGTGTGAAAGCAATTTCTGGAGGTCAGGAAGTAACGATTGCGCTTGCAAATAG
GGGAACTTCAAAATTAAAGCATTGTGTACTTGAAATTGATGGTGCAGTTGAATTAAATGATAAT
TACATTCGATATATCGGAGATTTGGAAGAAGATGATTCTGATTCTGGAATATACGAATTAGAAG
TTTTTGGAGATTCGAAAACAAATATCACTGCAACGCTGACGTACTTTAATGAATACCACGAAGAA
TTCATCCTTGTTCAGGAATTTGAATTGGATCCTGCTAACAGCCAGGTAAAACAGGAAGGAACTAA
CTATACATTTATTGGATTAATTGTCGTTTTGGCAGTTATTGCGTACATTTTATATAGAAGATGGA
AGAAGAGGAAAGACTCTGAAGAAGAATAATAAATTATTTTTTTCTTAAATTTTATCAAGTGGTT
GAGTATGACAGGTCTTGAAGTTAATAAAAAATTTTTAAAAGCACTATCTTCGCGTTCAAGAATAT
CCATTTTAAAAGCACTCGGAAATAAAAATTACACGGTAACAGAACTTTCAAAAAGTCTGAAACTA
TCCAAATCAACAGTACATGAACATTTATCAATACTGGTTGATGGAGAACTTGTAAAAAAGAATG
AAGATGGCCGAAAATGGGTATATTATGGATTAACGGAAAAAGGACAGTATTTAATTATGAATGA
TCTTGAAAAAAATGATTATATTGTTCCCTATGTCTGTAATTGTATTTTTAAGCGGTTTATACAGTA
TATTGTTTCTTAAACTTGGAAGCAGAATAGTTCCTCAGGAATTAAATGTTTATAGTGCGAGTGCT
TTAAAAAGCGCTGAATCAGATGCGGTAGCTTTTGGAATTTCTGAAACCGCAACTACTGCTGGTTC
TGATTTAAATCTTTTAATTGGCACAGGATTGGTATTAATAAGTTTACTTATCGCATATCACGTAA
TTTCAAAAATAACATACAAATCAAACTAATTATTTTTTTATTTTCAATTAAGCACTGTTCGGAAT

TCACCGAACGATTTAAAAACTATTTTTAAGTGTTAATACACATTGTTTATTATTAATACAAATGG
TGATTTTATGAATCGCAATATAATTATTATTTTGGGGCTAATTTTAATAACTTCTGCAGTTTTTT
TAACTTATAATCCAAAAGACGATTCTGGAATAAGTTATACTGGAGACTTTAATATGGTTCCCGCA
GATTCAGAGGGAAATTTTAATAATTTTTTAAAACTGGCATCTGATTCGGGCTATTATTATGGTAG
CAGTAATATTGCAGTAAAAGAGGATATAACTTATGCGACTGCCAATGAAGACAGTTTAATGGCTT
CTGGTTATTCTGATGCGAGGTATTCAGAGACAAATGTTCAGGTAGTGGGCGTTGATGAAGCTGAT
ATTGTAAAAACAAATGGAAAATATATATTTTACACGACACAAAGTCCAGCATGGGTTAATTATT
ATGGCGGACAGAGTACTTACGTAATTGATGCATTACCTCCTGAAACCGCCCAGGTAATTGGAAAT
ATCACTGATGGTGGTAATCTTTACCTTGAAGGGGATAATTTAATTGTAATAACTTATGATGGAAT
AAAAAACTACAATATTTCAAATCCAAAAACGCCTGAATTGATTTGGGCAAAAGATTTAAACGGG
TCATATGTTGATTCAAGATTAATTGGTGGTGAATTATACTTAATAGTTCAAGAATACGGAATAA
ATGGGCCTATAATCTGGAATAACGTTAAAATCGGATACGATGATATATATCTTCCAGTAATTCCT
GAAATTATTCGCCAAAACTTTGAAACAACGTATATTATTTCTTCAATAATCGTTGAAAGCGGAGA
TATTGAAAACACGGCTGCACTAGTTGGTTCATACAGTAATACTGTGTATGTTTCAGAAGAAAATA
TTTATTTTGCTTATGAACTGGATTATGATGAAGATAAACTGATGATGAAGTTTGTGGCAGAAAA
TGGTGATAAATATTTCCCAAAAGAAGTTACAGACAAATTTGATCGAGTATTTTCAAACACTGACT
TTGGAGACAGTGCAAAATACGTACAGATGACTGAAATTCTCGAAGATTACATAAAAACACTATCT
TCAGAAGAAATGAATAATTTAGAAAATGAAATTGAAAATGATTATGAAATTTATTTGAAAGAAA
ACTGGGAAGAACTTGAAGGAACAGGTATTGCAAAAATAAGTCTCGATGATTTTAAAGTAACGAG
TGGAAAAGTTCCTGGAACCATTTTAAACAGCTTTTCAATGGATGAATATGATGGAAATTTAAGA
ATTGCAACAACAATTGGAAACGATTGGAGATTTAGAGATATTCAAACAAACAACATCTATTTATT
CGATGAAAATATGGATGTTATTGGAAAAGTGACTGGCCTTGAAGAAGGAGAAAGAATATACTCT
GCAAGATTTATTGGGGACAAAGCATACGTTGTAACTTATAAAGAAACAGATCCATTACTTGTAAT
TGATTTACAAGATCCAAACAATCCAAGTGTACTTGGTGAATTAAAAATTCCAGGATATTCTACTT
ATCTGCACCCAATTGGAAACAATAAATTAATTGGAATTGGAAAAGACGACTCAAATATGGTAAA
AATTTCATTATTCGATGCAACTGATCTTGAAAATCCAAAAGAACTTTCAAAATATTCATTAAACG
AGTACTGGTCAACTGCCCTTTACGAACACCACGCATTTTTGTGGGATTCTGAAAAAGAAATACTC
GTACTTCCAGCAAGAAGCCACGCATATGCTTTTGAAGTTAAAGAAAGTGGAATTAAAATGGTTAA
AGATGACGTTTTCAAAGACTACAGCGTTTTAAGGTCTTTGTATATTGGAGACTATCTCTACACGT
TTTCAAACTACGAAATACACGTAATTGATGAAAATACTTGGAAAACTGTAAAAACGATTAATTT
ACCAAAATATGAATACCCTTACCTTGAACCATATATTGAACCAGTGGAAGACATTTCTGATGCTG
AAGTAGAAACTATCGATGAAAAAGTTGCTATTGGGGAAAATATAACTATAAAACTTGATGAAAA
CCCGACAACGGGATACACGTGGAATTATTCTATTAGTGATGATGGTAAAATAGAATTATTATTCG
ATGAATATATTCAGGATGAAGTAGAAGATGGAATTGTTGGTGCAGGTGGAGTTCACGAATGGAC
TTTTAATGCAACTGAATCTGGAGAAGTTGAAATAACCTTTGATTACTACCGTTCATGGGAAGGGG
TTGAGGGTTCCATAAATACAATAATCTATAAAATAACAGTTGAATAATTAGAAAACTTCATTTCT
TTTTTTTAAATTTTTAAAATATTTTTCAAAAAAGTCAATTTTAATTTAAAACAAATATTAAAAA
AGATTTAGAGGAATTATTGTCCTCTGTTAATTCTTTTGAATGTGTTAGTCCATTTTAATTTTCTT
CCAACTCTTCCGAGTTTGAAGTTTTTTTCACACTTTGATGAACAGAAGTGCATAACTGAACCGTC
TTTCTTAACGTATTTTTTTCCGCATCCAGGTTCAATTGTGCCTTCACAAAAGCTACATGTTTTCCA
TTCCATTGTAATCACCTAGCGGGAATTATTTTCTTCTTTTACCGAGTTGTTTTGCTTCTCTTTCGG
TTTCTCTTAACATGATAAGGTCGCCTAATTTAACAGGTCCTTTCACGTTTCTTGTTAATATCCTTC
CTGTGTCTTTTCCACCTAAGATTTTGCATCTTACCTGGAAAATTTCACCAGTAACTCCTGTTCTTC

CGTTAACTTGGATAACTTCAGCAGCCACTGCTTCCTGGTATACCATATCATCAGCCATTTAAATC
ACCTGATTTTATTTAATTGTTAGCTGCTAAACTTATGCTTTTAATCCATTTAATTTTTCAACTAA
ATCTTTTAATTCGTCAGCGCTTCCTTCAGCAACAACTGCAATAGCTGATGTAGGTACTTCTAAAC
CAGCAGCTTTTCCTAAAGCTTCTTTTGTTGAACTGTATGAGTATGCAATTCCTTTTTCGTCGCAA
ATTACTGGAATGTGTGCAACGATTTCTTCAGGTTGTACATCTTGTGCGATTACAACTAATTTTGC
AATTCCTTTTTCTACTGCTTTTGTAACTTCGTTAGCGCCTTTTTTGATTTTTTCAGCGTTAGCAAC
TACTTCCGCAGTTTTTTCTTCGAGTTCTTGTGATATTTCAAATTTAACATATACAGCCATATTAT
GACCTCCTTCATCACTTAAAAAAATTTTTTTATCCGGATGGCGCCCACTCATATCATCATGGTTTT
AAAAGAGCGGCAACTACCATATATTGAAAAGTGGTATATAATAGTTATGGTGTAATCTACTGGA
AATTTAGAAAATTTAAAAATCAACCGGTATTCCAAGAATTTCCTTTTTACCGTTTAAGATATCTT
CTGCAATCATTGCACTGCCTATTGCACCACTTTCCCCATTTAAAACAAGGACATTTCCATCGATAT
TTTCTTTTATCATTTCAGAAACGCTCGGGTTTTTCCATGTTCCAATGGAACCTGCAAGAATTACA
TTTTTGTCAGAATTTAAAAACATTAACGAATTTATTTCCATCGAAACACTCAAAACGAGTGAATC
AACTGCAAGTTTACATTTTTCATCTTTATCGTAGTTATTCATAATTTCAAATTTCGTATCTTCAA
CGCCTTTGTAAGAATCTGTAACTTTAACAACACCTGCTTTTGAAAAAGCTTCATTTGCGGTGATT
TTATCTGCATCGATATTTCTAATTAATTCTAGATCAAGCGGGCCGTGTAGTATCCCAACAGCACC
AACGCATGCATCAAATCCGCCAAATATTTTACCGTCTTTTATTAAAAGTGTAACTGTGTTTGAAG
AAATGTCTGAAAGAATGAAATTTTCAAATCCAAACGTTTTGTAAGCATTGTAGCAGATGGATAT
TTTTTCAGGAGATGCAATATGTGAAAAAAGAGCGTTAAATCTTTCATCCATACTTTTTACGCCAT
TATGAAGTCCGGGAATAACAATTGCTGGAATATTTGATGATTTTATTTCATCGTAAACTTTCGTT
CCGCCACCAATTTTTTTCCCAATTCCTTCAAGGTTAATTACACCCCTGTTTTCAACCCGCTTTATA
TCTGTTATTTTATCTATTCCATCGCCCATCGAGTAGCATACTCCTATCAGGTCGATGTCGTTTAA
ATTAACCTGTTTTTCAAGTTCTTTTAAAAATGACTTTTCGTTTATTTCCGTTCTTTTCATCTTAA
AAATAGATTTTACAGTTTTGTTTTCCATCACACATGCGGTAATTCCCGATGTGCCGTGATCAATC
CCTACAACAATCATAATCTCCCATACAAAAGATAATAAATTTCAAATACCAATAATTATATATAA
AACAGTGCTACTTTATTAAGAACTTTTTGATTCAATATTATTGCTAAATAGAATAATTTTAGTGA
TCCAGTAGAGATATATTGGTGATTTTATGGATGTATGGATCGATTTAACAAATTCCCCGCATATT
CATTATTTCTCGCAGTTAATTGACAAGTTTAACAGGGAAGGAATCGAATATTATATAACAGCGAG
AAAATTTCAAAGTTTAGAAGATTTAATCGATATTTATGGTTATGATTATATTTCTGTTGGAAACC
ATGCAGATTCGTTAGAAGAAAAGCTCATAAATTCTTCAAAAAGGGTAATTGAACTTACAAAAAT
TGCAAAGAAGTTGGACCCAAAAATTGCAATTGCAAAACACTCAGTGGAGCTTCCAAGAGTGGCTT
TTGGAATGAATATCCATTCAATATTTGTTTTAGACAATGAGCACGCAAGTGCACAGAATAGATTA
ACCGTTCCTTTAGTAAACGACATAATCTGCCCAGAAGGTACAAAGCTTGTTTTAAATGGAAGTTC
ATTTGGAAAATACATTGATTCTTTTGAAGGAACGTGTGAAGTTTCTAATGTTAATTCAAGGTTA
AATTCTTCTTTTTCAATTGATGAATCGATAAAGGAAAAGTTAAATATCGATGAATCATTGCCTAT
AACAATAATGAGGCCAAGGCCAAATTCTTCGTACTGCAACGGGAAAAAGGATATTATTCCAATGA
TAATTGATAAATTAAAGAGTCAAATGGACTGCAATATTGTTGCATTTCCAAGAGATGAAGAACA
GCGGGAAAAATACGTATCAATGGGCGTAATTGTTCCAAAAACAATCGATGCAATATCTTTACTTT
ATTATTCAGATGCAATGATTGGAGCAGGTGGAACGATGAACCGTGAAGCAGCAGTTTTAGGCGTT
CCAACGATTTCGTGCTATCCAGAAAAGCTTTTGGGTGTTGATAATTACCTAATTGAAAAAGAAAG
AATGATTCACACAAGAGATTTAGATGAAATTGTAAATCACGTGATGGATAATATTGGAAAAAGA
AACAACGGAATAACTTTGGAAGATCCAACCGAATTAATGTTTGAAAAAGTTGTAGGAAGTTTAA
GTTAAATTATTATTTTAAATAAGGGTTCACATGAACCATACAGTGTTTTACTCTTTTTAATTTGC

TTTCAATTTGTGAATGGACGTTTTCAGAGATGTTATGTGCTTCAATTAATGATAAATCTTTATCC
ACTGAAATTTCAACGTCAACATAGATTTTGTTTGAATGAATTCTCGTTTTTAATACATCTATCTC
CAAAACACCGTCAACCGAAAGAATTATTTTTTTAATTTCTTCAATAGTTTTTGAATCCGCCGCCA
TATCGAGAAGTTGGTTTAATGCCTTAAAATATATTTCAAATGCCATTTTTGCAATAAATAATGAA
ATTACTATTGATGCAATGGGATCAAGTATTGGGTAGCCAAGCTTTGCACCAACAACACCTATTAA
TGTTCCAACCGATGAAAATGCATCAGATCTGTGGTGCCAGGCATCTGCAAGAAGTGCAGAACTTT
CAATTTGTTCAGCACCTTTTTTAGTGTATTTATACATCCATTCTTTTGTAAAAATGGAGATCAAA
GCTGCAATTATAGTTATATTTCCGGGTATTTGGTAATTTCCGCCAATAATAGTGGTTAATCCACA
GTAAAAAATCATCAAGGCAGTTACAAGTAAAATTACCGCTAAAATTTTTGTTAATGCAGGTTCAA
TTCTTTCGTGGCCATAAGGGTGTGATTCATCTGCAGGCTTTTCTGAAAGTTTTAATCCGAGCATT
ACCACAACTGTACTTAAAATATCGGAAAATGAATGCATTCCATCTGCAATTAATGCACTGCTTTT
ACCAAACACACCTGCAAGTATTTTTAGGATGGATAAAGCGATATTTACAATAATTGTGATTTTTG
AGATTTTATTTCCTATAATTATTCTTTCAGAAACTTCCACGCTATCATCCCATTTATTAAATAGA
TTCAATCTGTCTGCAGTATCCTTTAACTAATTCAAAAGATTTTTTAAATCCTTCAATTTCATCAT
CTTCCATTTTTATATGGATTACTTCTTCAATTCCATTTTTGCCAACTTTTACAGGAACCCCTATAC
AGCAGCCTCCTTCAATACCTTCGATTTCATCTTCGATGTATGTAGAAAGGGTTAAAAGTCGTTTT
TCATCATGTGCAATACATCTTACAATGTTTACAATTGCGGACGCAGGGCCGTATTCAGAGCCGTT
TTTTAAATTTATGATTTCTTGCCCATAACCCTTAATTCTTTCAAGTATCTTTTCATAAGGGAAAT
CTTCATATTTTGGCAATCTTTTGATTGGAATACCGCCTACTGCAGTCGCACTGATTACTGGAACC
ATGCTGTCGCCATGTTCTCCAACAATCCTTGTTCTAACATCATCAAGGTGGACTTCGAAATGTTT
TGCAACTGCTACTTTAAATCGCATTGAATCAAGGTGCGTTCCAAGTCCAAAGACCTGATTTTTTT
CATAGCCACTTTCGATAAGTGCTTTGTAAGTCATTAGGTCAACAGGATTTGAAATCATGAATATT
TTTGTATCTCCAAAATTAGCAATATTTTTTACATAATTTTTTACAATTTTTGCATTTCCCTTCAT
AAGGTCGATTCTCGACATCTCTCCGGTTCTTGCCATACCTGCTGTAATTATGGTAATATCTGAGT
TAGCAGTACAGGATAAATCATCGTCACAGCATATGTCGATGTCGGTATCCTGTCCTGCGGCTGCC
ATTGCATCATAAAGGTCCATTTTAAGTCCTTTTAATTTATTGATGGAACTACTTCGGGCAATTAA
ATTAATATTTTTTATATGGCTTTCCTTTGCTAAAAGCAATGATAAAACACTCCCAATTTTGCCTG
ATGCCCCAATAATTGATACGTCCATAGTTTCACCCTTGCTATTTAAAACAATTTCTATATCGACT
TTTATACATATATATCTAACCAAATACAAGAATTTTTTAATATTATCTTTTACAGATATTTATAT
ATATCTAATATATTTAAAATTAGTATTATTATAAACATAATTTATTAAAATTAATATTAATATT
TTAAATTTAGGTAAGATAAGCAGATTACGGGGATAGGTTATGAGTTTGGAAGAAGATTCTAAAA
TGGATAAAATGGCAGTAGAAATGTTATTAAAAGCTCCAATGATGTCCAAAGAAGAATTGGATGA
AACTATATTCACTTTACGAAAAATGGCGATCAAAAAGTCTGGTCGGAGAAATGCAAGATTTATCA
TGGATTCATGGGCAGATACTGCATATGATATCTCAATGAAGTGTTAAGTTACAAATCTACGATAA
ACTGTGTTTTCCAGCAGTTTTCGTTCTTAATTATTTCCATTCTGTGGTATGTAATTGCTTTTACT
TCTTCTTTTGGTTCATATTTGTCTTTTGAGTACCGATCTCCAAAAGCAACACAACTTAATTCATA
ACATTCGTTTCTCTCGATTTTTACATTAAATTCTGAAAATATTAACAACTCGGAATCATGGAGTA
TCAAAAGTTCTGTTAAGAAGTCGTATAAAAGCCCGAAAAGGTCTTCAGAAATAACTTTAAACGA
ATGCTTTTCAATTTTTTCAATTGATTCGATATCAACCATAATATTTGATAAGCCTCTTGCAGAAT
TTTCAAAAGATTCTTCAAGAGTTTTTCCAAAAGCAATTATCCCAATATCTGCGGTAGTTTCAAAG
TAATTATACATAATTTAAACACCTTTAGTGGATATTATGTATCCGTTTGCATTTTTTTCTTCGTA
TTTTTTGATGTTAAACTTAACATCAAAGAATTTTTCGACAACATAAATATTTGTCAGTGTATGGT
TTGTAATTTTTGAAACGCCTATTTTAATATCTGAAAAAGGCATAAATGGAATTATTTGATCGGCT

AAATGCGAATCTATTGCCATTTTTGTAGTTAATTCTTCAAATAACTTTTCAAATGCGGTTTTTCC
AACGGCTTCAGATAAAAGTCCTTTTTTTCCAAGACAGTCTGAACCGATATAATTTGTATACAAAA
ATATTCCAACGCCTGAACTTTCGCCATTTGTTTTTTCAAGTTTAATTTCTGGGCAAAATCCGCTG
TTTAATAATTCATCACTTGCAGATTTTCTAATTCTTTTTGAAATATTTTCATCGAGGTTTTGGTT
GAAAACAATTCCTTTAATTTCAATATTTTCATTTTCAATTAGTTCATATTGATTTATTTTTGAGG
GTTTTAGGGTTATTTTGACGATTCCTCCGCCTTTTGGGAAAAATCCTCGTTTTAATATCGTTATT
TCTGAATTAATTCCTATATTTTCCAAAAATTTCAATGTTATATTTTTAATATAATCGATTGGTGG
CGAAAATTCAACATTTGTCCCACCAATTATGGTAATTTCAACATTTTTATCTATCGCAAGGGAAA
TCGGGAGTATTGATTGAATAACAAGAGAAATACTTCCCGCAGTTTTAATATCTATTTCAAAATTT
TTAGAAATAATTTCTCCAGGATAAAAAACTAAAGTTTCAGAGCCTTTTATAAGTCCTTCAACTTT
GGCATTACAAATTTTTGAAACTGCTGATACACAAGCGATATGCTGATTTGATAACCCTGGATTTT
TTCTGTTTTTTCGAATATTAATGATTTTAACTGGTTTTTTAGTTATTGCCGAAAGAGAAACTGCG
GTTCTTACAATCTGCCCTCCGCCTTCAAGATAACTTCCATCAATAGTGATCATAAATATTCTCCTA
ATTACTTTTCTCAATCAAATAATTTCCAAGTAAAAGTGCATCCCCGACTTTTTTAAACGAATTTA
TGGCATCGATTTCACTGCAAACAATCGGCTCCCCATGTAAATTAAAACTGGTGTTTAAAACTGCA
GGAATATTTGTTTTATCATTTAAAAAATTAACAATATTGTAATACGTTTCATTAAATTCTTTTTT
TAGTGTTTGCGCTCTAGTAGTTCCATCAACATGAACGGTTCCTTCAATATCCTTTTTATTAGATT
CAAAACAATCAAAGAGCATGGTCATGTATGGGCTTTCTAACTTGTTTTTTAAATAAATTTCAGAG
TATTCATCAAGGATTGTTGGAGCATACGGCATGAATGAATTTCTTTTAAGGGTTTTTGCAAGTTT
TTTCGAATTTTCTTTTGTTGGAAGTCCTAAAATACTTCTGTTTCCAAGAGCACGGGACCCAAATT
CCATTTTTTTTCTTGAAACACATACAAGTTTATTATTCAGAATCAATGAGCCAATAACTTCAGGA
ATGTCCTTTTCTTCGACATATTTAAATTTGAAATTTTTTTCTGATTCAACGAGTTTTTCAACATT
ATAATTATCAATTTCATAGCCTAAATATGTATTTTCTAAATCAAAATCTTTTTTAGAACACAAAA
TCGACGATCCAATTGCTAAACCTTCATCCCCTGTAAATGGGGGGACGTATAAATTATCCACAAAC
TCCTGTTCACTTATTTTTTTATTCAATTTGACGTTTTGTGCAACTCCACCGCTAAATACAATATT
TTTAATCCCTGTTTCCCCTACAAACTGCTTTAAAAGTTTTAAAACAACTTCTTCTAATTTACTTT
GAGCAAATTTTGAAATTTGAATATCGTATGAATTATTTGGCGTTGAATAATCTAAAATTCTTTTT
AATGCTTTTGTAGATTCAGAGCCGATTACTCCGAGATAATTTTTAAAAGATTTAGTTTTTTCGTT
GTAATCAATAGTATCAAGATTAATTTTAGAATCACTTGATTCAAAACTTGAAAGACTCATAACTT
TTCCTTCATCTTCCATTGGTTTAAATCCAAGTAGTTCTGTAATTGATGCGTAAAAATCGCCTAAT
GAATCTATTAAATCACTTTGAGCAATCGTTTCTATTTTTGAACCTTTTCCGATAGATAGCGTTGA
AGAAAGTCCATCTCCTCCTCCATCCACTGTTAAAATTAGGCATTCTTTAAAATTTGATAGTTTAT
AGCTTGCAGCATGACATAAATGATGATTAAAGTAATACATCGGGATTTCTAAATTTTCCTGAAGA
ATTTTAAGTTTTTTTAGCCTTTCCCCCTTTCTAAACTGCCCGCCAACAGTAATTGCGGATAATTTA
TTCTCATTATTTACAAAAGAATTTAACCCGTAATCTATGGAATTTACTGGATATCCCCTAAAATT
CTTTTTTCTTGTAAATCTTTCTTCACTTGCCGCATAGATTACTTTTTTTTCATCAATTAGCGATGC
ACTTGAGTTGTGGCCATCACAAATACCTAAAATCATACTTTCACATTGAATTAATTTAATATATG
TCTGATGAGATAATTAAAATAAACAATTCTTACAATTTTTATGTTTTCATAATATTTATATAAG
ATTTTGAGTTAATAAATTAAAAACGAATTAATTTTAAATCCATTACGTCTTTATTACTTGGTGAT
CTCATGAAAGGAGCAGAGGCTATGATGAAGGCCCTAGAAGCAGAAAATGTAAAAGTCCTTTTTGG
ATACCCTGGGGGCCAATTACTTCCCTTTTACGACGCACTCTATCAAAGTGACCTTTTACACATTTT
AACAAGGCACGAACAGGCTGCAGCACACGCGGCTGATGGATATGCAAGAGCAAGTGGCGATGTAG
GAGTTTGTGTTGCTACATCTGGTCCAGGTGCTACAAACCTTGTTACAGGAGTTGCAACAGCACAT

GCAGACTCATCTCCAGTTGTTGCATTAACAGGACAGGTTCCAACAAAATTAATTGGAAATGATGC
ATTCCAAGAAATTGACGCACTTGGAATCTTCATGCCGATTACAAAGCACAACTTCCAGATTCAAA
AAACAAGCGAAATTCCAAAAATATTTAGAAAAGCATTTGAAATTGCAAAAACGGGAAGGCCCGG
TGCAGTTCATGTTGATTTGCCAAAAGATGTTCAGGATGATGATTTAGACTTAGAAAAATACCCAA
TTCCTGCTGAAATTAATCTTCAAGGATATAAACCAACGAAATTTGGACACCCTTTACAGATCAAA
AAAGCTGCAGAATTAATGAAAATCGCACAAAGACCTGTTATTATCGCAGGCGGCGGGGTTCAGAT
TGCAAATGCAACTCCTGAACTTATAAAATTATCAGAATATGCTCAAATTCCAGTTTGTACTACCT
TAATGGGTAAAGGCGTTTTCCCTGAAGAACACCCTCTTTCATTAGGTATGGTTGGGATGCACGGA
ACACAAGCTTCAAATTATTCTGTTTATGAAAGCGATGTTTTAATTGCAATTGGATGTAGGTTTTC
AGATAGAATTACTGGGGACCTTTCAAGCTTTGCACCAAATACAAAGGTAATCCACATAGATATTG
ACCCTGCAGAGATTGGAAAGAATGTTGGAGTAGATATCCCAATAGTTGGAGATGCAAAAGCAAT
TTTAAAAGATATTCTGATCCATTTGATGAAAAAAGAAATGGTAAATAAAACAGAATGGATGGAA
AATGTTAAAAAACTTCAGAAAAAGTCTCTTCCTATAATGGAATTTGACAACACACCAATAAAACC
TCAAAAGGTTATCAAAGAAATGATGGCAGCACTTAGAGAAGTTGATCCAGGTTTAACAAACACG
GTTCTTACTACCGATGTAGGTCAAAATCAGATGTGGATGGCACACTATTTCCAAACAAGTGCTCC
AAAATCTTTCCTTTCCTCAGGAGGTCAGGGTACAATGGGCTTTGGATTCCCTGCGGCAATTGGTG
CTAAATTTGCAAGGCCTGATGCAAATGTAATTGCTGTAACTGGGGACGGTGGATTTTTAATGAAT
TCACAGGAACTTGCAACAATTGCAGAATACGAAATACCTGTTATCGTTGTGATATTTGACAACAG
GACACTTGGAATGGTTTACCAGTGGCAGAACTTATATTATGGAAAAAGACAGTGTGCGGTACACC
TTGGGGAAACACCTGATTTCTTAAAACTTGCCGAAAGCTATGGAATCGGTGCTTTGAGAGTTGAA
AAACCTGAAGATATTAATGAAGCTTTCAAAACTGCATTAAACAGTGGAAAACCATATTTACTCGA
TATCATAATCGACCCGTCAGAAGCACTGCATATGGTACCTCCTGGAGGAAACATGACAAATATAT
TATTCCCTGACCGACAGGAGCCAACTCCAAAAGCTCAATGCTTTAGCGAAATGAAAAAAATACTT
AGTCCTAAGGTCTAATCGGGTGATTGCAATGGAACACAAACACATCATTTCAGTTTTGGTTTTGC
ACAAACCTGGCGTATTGCAGAGAATTTCAGGTTTGTTTACTAGAAGATGGTTTAATATCTCAAGC
ATGACTGTTGGAAGCACTGAAAACCCTGATGTTGCAAGAATGACAATAGTTGTTCAGGGAGATGA
TACAGTACTTGAACAGGTTGTAAAACAGTTGAATAAATTGGTAGAAGTTGTAAAAGTTACCGAT
TTAAACAGTAACAAGTCGGTACAAAGAGAATTATGTCTTGTAAAAGTTTATGCGCCTACTGAAGA
TAGTAAATCTCAGGTAATCCAGTATGCAAATATATTTAGGGGAAAAATTATCGATTTAAGCACTG
AAACACTCACTGTTGAAATTACTGGTGACGAGCAAAAAGTAAACGCCTTTTTAGACCTTGTAAGG
CCAATGGGAATAAAAGAAATTGCAAGAACTGGACTTACTGCCCTTATGCGAGGATCTAAAATTTT
AAAATCAAATAAAGCATAATTTTCTTTTTTAATCTTTAAATTAAATTTTAAACATTTTTAAAAA
ATAGAAAAAATTAAGCATTTTTTGAAGGGGGGCTCCAAAAACAAATGCCTCAATTTAATAAGCT
GATTTAGCTTACATAGAGAGTTCGTAGGAATTAATTTTCGATCATTCCCATAAAATTTCGTAAAA
AACGGAGTTTTAGGCAATATCAAAGATTATTTCCTGATAAAATTTATAATTTTTAAAATTTATA
TAATTATCCCCTTCTTTTGGATTATTATATTTCGATTATTAACGAAATTTTTTTAAATGCATATT
ATAACTATTTATTTAGTAGGTAGCTACCATTTTTTCTTTTTTTTCGTATTACTTTCAATTAATTG
TCTAAATTAAGTTTAAAGAATGCATTATCCGAATAATATATATTAAAGAACGGCAGATATATTA
TTGCCTTTAAACTTTTGAACTGTCTGATTCTTTTAGTTTTATTTATTTTATATAACGTTTTTGGG
GTGGTACTCATGAACGGTGAGGATATCGAAACCAGAAATTATTATTCAAAAGCGCCCCTAAATCA
GATTTGGGAAAATATTGTATCTAGAAACCATCCTTTTAAAGATATCTGCGTATATGAAGAGTGGA
TGTGTGAAAAAAATGATATTTCAATGAATACAATAAATGCATATTTTTCAAAATTATGCACCTAC
GATACAGTGGTGTTTGAGGATATAAATTCTGAAAAGTTAGAGATCTTTAACGTTAGAATACCTT

ACGGAAACGATAAAATTGAATTAATATCCGAATTTAGAGAAATTTTCATTATGGATAAAGTATC
TAGACTTTTTTATGGCTATTTAAAACTAAAAGAAAAGAAGATACCCTCTATATTATGGGGTTTTG
AAGATAACTGGTTTAGACCGTATGTAAAGCCAGAAAATTTTTCGGATTTAAAAAAATTTATGAA
TGAAGTATAAATGAGTTATAATTTGAACAGATTAAACAAAAGATTCCTTTTTATCTTTTTTAGAC
CAGTAATTTGCGAGAATTGGCCCAGTAATGTTGTGCCAGACGCTAAAAATTGCCCCCGGTACTGC
TGCAAGAGGCGAAAAATGGATTGTTGCAAGTGCTACTGCAAGTCCTGAATTTTGAACTCCAACTT
CTATTGTAACAGCCCTTGATTTTTTTTCATCCATTCCAATGAGTTTTGTTCCAATATATCCCAAT
AAAAGGCCAAGAGCATTGTGGATTATTACTGCAAGTAAAACAATAACTCCTGAAGATACAATTTT
TTCTGAATTAACTCCAATTATTGCACCAACGATTAAAACTATTGCAACAACCGATAGTAGGGGTA
AAACCTGCCGTATCGAATCAATTTTTTTTCCAAGTGAATTATTTATAATTACTCCGAGAATCACG
GGAATTAATACAATTTTTACAATCGATAGAAACATCGAGTATGCTGAAACATCAATCCAAGCTCC
AGCAAACCAGAGTACAAGTATCGGGGTAATCATTGGTGCAAGAAGTGTTGAAAATGTCGTGATCG
CAACTGAGAGTGCAACGTCTCCTTTTGCAAGGTATGTTATCACGTTTGATGCAGTGCCTCCGGGG
CATGAACCCAGTAAAATGAACCCTAATGCAAGTTCTGTTGGTAGATGAAATATTGTAATCACTAA
AAAAGCAAGCAGTGGCATTAAACTAAACTGTAGAATTGATCCAAAAACTACGTCAATTGGCCTTT
CTAAAACTGTTTTAAAATCATCTTTATTTAATGTCATACCCATTCCAAACATGATAATCCCAAGA
AACAGAGAAATGTGGGGGATTGCCCATCCAAATATATCTGGATTAAAAAAACCGATAATTGAACC
CAAAATAACAAGTAATGCAAAATAATCTTCTGAAAATTTTGTAAATTTCCAAAGAGTCTTTTTTA
GCTGAATAGCCCCCCGATCCATTAATATCGCCTCGCCCAAAAGTTCGTGTTTTGCCGTAATTTATT
ATAGTTTATGCCTAATTTTAGATATCATTATATATGATTATACTATAAAAGATTGTGAGATTAG
AGGTGAAGACAATGAAGGTATTCTATGACTCAGATTTTAAATTAGATGCTTTAAAAGAAAAAAC
AATTGCAGTAATCGGTTATGGAAGTCAAGGTAGAGCACAGTCCTTAAACATGAAAGACAGCGGAT
TAAACGTTGTTGTTGGTTTAAGAAAAAACGGTGCTTCATGGAACAACGCTAAAGCAGACGGTCAC
AATGTAATGACCATTGAAGAAGCTGCTGAAAAAGCGGACATCATCCACATCTTAATACCTGATGA
ATTACAGGCAGAAGTTTATGAAAGCCAGATAAAACCATACCTAAAAGAAGGAAAAACACTAAGC
TTTTCACATGGTTTTAACATCCACTATGGATTCATTGTTCCACCAAAAGGAGTTAACGTGGTTTT
AGTTGCTCCAAAATCACCTGGAAAAATGGTTAGAAGAACATACGAAGAAGGTTTCGGTGTTCCAG
GTTTAATTTGTATCGAAATTGATGCAACAAACAACGCATTTGATATTGTTTCAGCAATGGCAAAA
GGAATCGGTTTATCAAGAGCTGGAGTTATCCAGACAACTTTCAAAGAAGAAACAGAAACTGACCT
TTTCGGTGAACAAGCTGTTTTATGCGGTGGAGTTACCGAATTAATCAAAGCAGGATTTGAAACAC
TCGTTGAAGCAGGATACGCACCAGAAATGGCATACTTTGAAACCTGCCACGAATTGAAATTAATC
GTTGACTTAATCTACCAAAAAGGATTCAAAAACATGTGGAACGATGTAAGTAACACTGCAGAAT
ACGGTGGACTTACAAGAAGAAGCAGAATTGTAACTGATGATTCAAAAGCTGCAATGAAAGAAAT
CCTAAAAGAAATCCAGGATGGAAGATTCACAAAAGAATTCCTTCTCGAAAAACAGGTAAGCTATG
CTCATTTAAAATCAATGAGAAGACTCGAAGGAGACTTACAAATCGAAGAAGTCGGCGCAAAATT
AAGAAAAATGTGCGGTCTTGAAAAAGAAGAATAATTTATTTATTTTTCATTTTTAACTATTTTT
TTATTATTTTAAAATCAGGTGGTTCTTTTGAAAGTTTTTATAAAGAATGCTGGAGAATTGTCTGA
AATTGAACTGCCGGAAAATGCAAAGTTAAAAGATTTCATTGAAAAGATGGACGTAAAAGATAGT
CTTGTCGTTCGAAATGGCGAAATTCTTTCTGAAACTGATGATCTTTCTGAAAACGATACTTTAAG
ACTTGTTCCAATTGTTTCAGGTGGTTAAGACTTTATTTATATACTATTTTTGCAATATATTACTT
TAGAAAATAATCATCCATTTTTAAGATTCAGGGGTAATATGGCTTATATCGATGTTGAATGCAAT
GACGAAGCTGGAAAAGCAATTTACGAAAGAATTATTCAAACGTCTCTTGAAGACCTCGTTTTAGG
AAAATCTATCATAAAGGCAAAAATGATATGCAAACAGGATGTTCCATATTTTATAATTGGAATT

TTGCCAAAATCAACTTCAAAGCTGATAAGATTAAGGGATATTGCAACAATAGATGAATCAAAGA
AATCTGACGGAAAGACGATAACTAAATTAAATATTGATGATGAAACTTACGCAACAGAGCTTTT
AGCAAAAATAAATGTTATGGATCAGCCTTCAAGATTTGAAATTGTTACTGATTCTGAAGTTGATT
TAAACATGGTAATTCACGATGCAAAAGAGGATTTCGTTGATCGGGTTCTTGATTTTATGAACCGT
GTTTTTCCTGAAGGAATGAGAATTCGAAAAACAATTAGGGATAAAACCATAGTAATGGTTGCAT
CAGAAAAGCCGATTGAACAAGATTGGATGGATGAAGCGGTAAAACTTCAAGAAGAACTTAAAAT
ATTTAAATAGTTACTTTAAATGATTAATTCTCTTTTAATTTTTATTTTAAAGTTTAAAAAAGTAT
TTTTGAAAATTTGAAAAAATTAAATTCTGTGCATGTATTTAAAGATATTTTCGTGTTGCACGTTT
ATTTTAACTCCGATTTCTGATCCGAGCTTTTCAAGAGCAACGTTTAATTCTGAAAGGTCTGAACT
TATTTTAGAGATATCTACAAGCATGATCATTGTGAACAGGTCTTCCATGATTGTCTGTCTGATAT
CTACAATGTTTGCACTGTTTTGTGCGAGAACTTTTGTAACTTCTGCAACGATACCTGGTTTATCA
ACACCAACAACGGTAATTACCACATTTTCCATAAATAGCACCTTTAAAAAATCATGTTTCTTTTC
TAGCCTTTATCTTTAAAAAGTATCATTTATACCTTATGGTTAAAAATAAAAATTTAAAAAAGAA
ATTAGTGTGAGAAGTGTTTTAAATCGTAAATATTCAACTCTTTACCTTTTTCTTCAAGGTGTTTG
TGTTCAGTTACAAGCCCTACTGCATCGGCCCTACACTGCTGGCATGCTCTAAATTGGTGGAGGTA
TTCTTCTGCAGTATCTCTTATGTTGCTTATTTCATCACAAGTTGGTTTTCTTAAATTTTCCATTTT
GTACATTGGAATTAAAGGAATTACGTTATGAACGAACGCATCTTTTTCGAAGGTTTTTGCAATTT
CCACAATATGTTCCATGTTTATTTCGGGGATCAATACCGTGTTAATTTTAACTGCAACATCAAAT
TCTGCCAGTTTTTTAACTCCTTCAATCTGGTTTTTGATTAAAATTTCAGCACCTTCTTCTCCACGG
TAAACTTTTCCTTCGTAGTATATCCAGTCAACAATCTGTGCCTGAATTTTTGGATCTACCGCGTT
TACCGTTACAGTTACGGTTTTTACTCCAAGTTCTGCCAACCTTTCAGCATATTTTGGAAGTAAAA
GTCCGTTTGTTGAAAGACATCTAACTAAATCTGGAAATTTTTCTTTTAAAACTTCCAATGTTTCA
AAAGTTTCTTTATTGAACAAGCTGTCCCCAGGTCCTGCAATTCCCGCAACTTTGATGTTTGGACT
TCTTTCAAGTAAATCATTTAAATAATTTTCAACTTCTGCTGGTTTCATGATGTGATGTGAAACAC
CGGGCCTGTCTTCACAGACTGATTCATCTCCAATACTCCTTTTACAGAATCTGCACGCAATATTAC
ATTTTGGTGCAACGGGCAAATGAACTCTTCCAATTTTGTGGTGGAGTTTTTCGTTGTAGCAAGGG
TGTACGCTTGTAATATGTGCAAATTTCGACATCTTATCTTCTTTCATTATAATCACCATAAGTTT
GATATTCATAATTCATACTTCTAAATATAAAAATTTACTGTACTAATTTCGGAATTTTAATTTAA
AAACTAAATTAATCCTCTTTTAGAATGTAAACTTTGTCATTTTCTCTTACTAAATTTTCAAGTTT
GATTCCAACATTTTGACCTTTTTTGGCAGTAGTAATGTTTTCTCCTTCGACTTGCATCGACTCAA
TTTTTTCTTCGACGCATCCAGTAGTTTGCCCGATTATTAAAACGCTGTCGCCAACATTTAAATCG
TCAATAAGTGTGATTTCTGCAACACCAACCTTTTTAAAGTAATTTACAACCTTTCCAACTTCAAT
TTTTTTGTAAGGGGAGACATTTCCTTCAAATTCGTACTGCAAATCGTGATTACAGGTAAGATCTC
TGAAATAAAATCCTGTATCGTACCCTCGATTGTATATTTTATCGAGTTCTTTTTTAAATAATGGC
AATTTTTCTCCATAAGTTCCATCAACTACTGAATCAATAGCTTCTCGGTACGTTTTTGTTGCACG
CATCACGTAATCTATGTTTTTAGCACGCCCTTCAATTTTAAAGCAGTCAAAAACATCTATCATTT
CGGGGATGTGTTCAATCATGCAGAGGTCCTTTGGTGAAAGAAGATATTTTCCTTCAGAGATTAGC
TCATGAGTTCCATCAGCATGTTCATTTACCAATTTCCACGACCTCCTGCAAGGTTGTAAACAATCC
CCGCAGTTTGCATTTCTATTAAATAAATATGAACTCAAATAGCATCTTCCACTCATTGCAGTGCA
GAGTGCGCCATGAATAAATCCTTCAAGTTCTAAATCAACTTTATTTAGTTTTAAATTTTCTTTAA
TCTGTTTAATTTGGGTTAATGTAAGTTCTCTTGATAAAACAACTCTTTTTGCAAATTTCGAAAAG
AATTCAGCAGTTTTTGAATTTGTAATGTTATTTTGAACGCTCACGTGAACTTCAAGTCCGAGTTC
TTTTGCAAGGCTCATCGCTCCAATATCATTTACAATTACGGCATCTACTTCCGCTGATTTAGCAA

ATTCGAGTATTTCATTTAAATTTAAAATATCATCTTCGTAAACTACCGTGTTTGTACACAGATAT
ACTCGCTTATTTGCATCGTGTGCGAAGTTAATGCCTTCAATTAGCTCATTTCTCTCGAAATTTTT
AGCAGTTGCACGCATACTCATTTCTTTTAGTCCGCAGTATACCGAATCTGCACCATTATTGAATG
CAGTAATCAAACCCGATAAATCTCTTGCAGGGGCCAATAATTCAACCATATTCTTCACCAAAATC
AAAATAATTAGTATTTAAAAAAGCATTATAAATCGTATTTAACAAGCGACTGTATGTCGTATCCT
TTTAATTTATCCCTTCCTTTTAGTTCTGTGAGTTCGATTACAAAGTTAAGTGCTGAAACGTGTCC
GCCCAGTTTTTCAACGAGTTTGGAAACTCCTAAAACAGTTCCTCCGGTTGCAAGAAGGTCGTCAA
CTATTGCAACATTCTGGCCTTTTTCAATTCCATCGCTGTGAATTTCAAGGCTGTCTGCACCATATT
CAAGTGCGTAATCAACTGAAAAAGTTTTGTAGGGCAATTTTCCCGGTTTTCGAACTGGAACAAAC
CCGATATCAAGTTCATGAGCAACAGGAACGCCGAATAAAAAACCGCGTGCTTCAGGGCCAACTAT
AACATCGACATTTTTATCTTCGAGGTATTTTGCAATCTCTTTTGTCGTATGTTTCATTGCTTTTG
GGTCTTTTAAAATCGGAGTTACATCTTTAAAGCTTATTCCTTCTATTGGAAAATTTTCTACAATT
CTTATCTTTTTTCTTAAATCCATTGTATTCACCGGGTCGAACTCGTAACGAAAGCAGGAATATTC
TTATAATTTTGTTACTGCACAGATTTTTAGGCTTTTTTAATATCCTATTTAAACGTATATAATA
TTGCACGTTTTATATTAAAGTATTTTTCCAGAAATGTTGAAATTATAAAAAAGACTTAATATTTC
ATTTGCGGTTTAATTGGGAATATAATTTTAAAAAGAAAATCGATCTATAAAATTTAAAATGAAT
TTTGTAGGGTTTATTTTTATTGGAGTTATGTATTTAAGTAATTTAATCAAGTAGTTAATATTGG
TTGAAGATCATGGAAAAAATTAAAGGAAAAAACACGGTAAACTGCGAAATTTGCCTTCACAACA
GCAAAACACGCCCGATGTTCAAACACGAAGGAAAAAATATCTGCATTGAATGCTTAAACGCCCTA
AAATTCCCAAGAAACTTCGAAGAAATGAAAAATGAAGTAATGGACTATTTAGATGAACTCAGAA
TTAGAAATAACAAATACAACTGTATTTTGGGATTTTCTGGCGGAAAAGATAGCGTTGTTGCTCTT
TATTTACTCGTAAAAGAACTTAGAATCAGGCCATTATGTGTAACAGTTGATAACGGCTATTTAGC
AGAAGAAGCGCTTACAAACTGTAAAAACGTTGCAAAATATTTTGGAGTAGACTGGCTTGTAATA
AACAAGGATTTTACGAAATTCTTCAAGGAAACCATCTCCAAAGGTGAATCTCCATGCAGGGTGTG
TTCTGACATGAACATGCACGAACTCTGGCAGTTTGCAAGGCAGACGAATATCGACACAATTGTAA
CAGGACACGAACTTCCATTTGGTACAACTGCAATAAGGGAGATGAAAAAAGGAATAAATATGTT
AAGGCTCCTCGTTGCATATAAATTAACAGAAGAAGATAGATACAACATATTAAAAGAAATCCCTT
GGGAAAAACCCGATCTTGGTGGATACACGACAAACTGTTTAGTACTTGCCCCAGCTCTTGTTGAA
TTTAAGAAAAAGCATGGATTTAGTTTTGAATATGATAGAGTTTCTGCAATGATTAGATACGGCCT
CATGACACGTGAGGAAGCTGAAGAAGCGTTAAAATGCCCGGAAGTTTCTGATGAAATATACGAA
GAGCTTAAAAAAAGAGGGCTCGATTTTAAAAATTTAAAAGAATGATTTTTATGAAAATAGATTT
TGAAATGACTTATGATTCAAAAAAGGAAATTGTAGACAGCTTAAAAGTTGACGATATAGACGAT
GGACTTATTATCGATACTAAATTTGATAATGACGAGATTAAAGTGAATATTAAAACTAATTCAG
TCGGATCTTTAAAAAATATACTCGATGACTTTTTTGTAAATTACGAACTTGCAGAAAATATTGTA
AATCTTAAAAAATAATTTAAAAAGGAAATAATCGTTTAATCAATAAAATATAAGGTATTTAAAA
ATGAATGAAAAAATAAACACGAATGAAACTAAGGATTTAAAAAATAAAATAGTTGAATTGGTAA
TTCCAAAAAATGGAAGTGTAAAAAATGATGTATTGTCTGGCTTAACCGTAGCGCTGGCACTGGTT
CCAGAAGCAATTGCATTTTCATTTATCCTTGGAATTGATCCTACAATCGGACTTTATGCCGCATT
TATTATGGGAATTGTAACTGCATTACTTGGTGGACGTCCTGGAATGATAAGTGGAGCTACAGGTT
CTGTTGCAGTTATATTTGCCCCATTGGTGATATCAAAAGTTCAAACCAGTGGAATGGAGTCTGCA
CTTGGATATCTTTTTATTGCGGTTTTATTGATGGGTATTCTTCAGGTATTTTTTGGGATATCCAA
AGTTGGTAAGTTTGTAAGACTTATTCCGCACCCGGTAATGCTTGGATTTGTAAATGGGCTTGCAA
TAATAATATTTTTATCACAAATCGGTCAATTTTACGGTGCAGATGGAAATTTACTGCCTTGGCCA

ATTCTTTCGATAATGCTTGTTTTAATTGCAATAACTATGGCTATTTCAATATTCCTGCCAAAAAT
CACCCGTGCAGTTCCCGCAACGCTTGTGGCAATAATTACTGTAACGATAATTTCGTACTTTTTAA
ACAATGCTGGATACACAGTTTTAACGGTACTTGATTTTATAAAATCAATAGAACCTTTAAAAACA
ACGCTTGCAGTTTCAATGCCCTCCTTTGCCTTGCCAAATGTTCCGTTAAATTGGGATACAATTAA
AACGGTTCTTCCCTATTCATTCTTAGCAGCATGTGTCGGCTTAATCGAATCATTAATGACTTTAA
GATTAATTGATGAATTAACCGAAACTCGCGGTAGAAGTAATAAGGAATGTATTGGTCAGGGTAT
TGCAAACATCTTAAACGGATTTTTTGGCGGAATGGGTGGCTGTGCAATGATCGGCCAAAGCATGA
TAAATATTCGGGGCGGTGGACGAGGCAGGCTTTCAGGAGCTTCTGCTGCAGTACTATTGCTGGTA
TTTATTGTATGGGGCGCTCCGATAATTGAAATGATTCCGCTTGCAGCACTTGTTGGAGTCATGTT
TATCGTTGTAATTGGCACTTTTGAGTGGTCTAGCTTTAGAATTTTGAAAAAAATACCACTTTCCG
ATGCAGTAATTATTGCAGTAGTTTCAATCATGACTGTTGTATTAGACCTTGCAACAGCGGTATTT
TTGGGAATTATATTAGCAGCACTTGTTTTTGCATGGGATCGAGGAAAAGATGTATGGGCAAGTAC
AAAATCTATGAAGAACAAAAAAGTATACATGCTTCACGGCCCTTTGTTTTTTGCATCAACATCAA
AATTCAAAGACCTATTTGATTATGAAAATGACCCAATTGACGTGGTTATAGACTTTAATAAATCA
CGGGTTTACGACCATTCCGCAATTGAAGCAATAAATACGGTTGCAGAAAAATATACTCAGCATGG
AAAGCAATTGCACCTCCTTAATTTAAGCAAGGATTGTGATAAATTAATTAAAAAAGCAGATAAT
ATTGTAGAAGTTAGTATTCTCGATAATTTGATATGGCACGTTGCCGATGATAAATTAGAGTAACT
CTCAAAATTAAAAAGGGGATTTTTAGTGGAAATTAAAGAAGTTTTAAGAAATTTCAAGCAGGAA
TTTGATACAAGATATGTTGTGAGGGGTTTGATCGATGGTTCGCTTTCGACACTTGGGGTAGTTAT
CGGTGCAAGTGGTGGGGAAACCTCGATAATTATTGCAGCAGGAATCGGTGGTGGAATTGCAAACG
GTATATCAAATATAATTGGAGCATTAACTGCAGAGCGTGCAATAATTGAAGAAGAACGGGAAAA
AAAGGAAAAAAGCCTGTTAATTGGTAACGGGAATTTAAAAGGAACTCATGAATACCAGTACAAA
TTAAATAAAACGATGTACAGTGGAACTTACGATGGATTATCAACCTGTGTTGGTGCGGTAATTCC
CGTAATTCCGTTTTTTATATTTGACCAGAGCACTGCTTTAATAATGGCAATAGCATTTACTTTAT
TGATACTTTTAGGACTCGGAATTTTCATTGGAAAGCTTTCAAGAGATAATCTTTTAATTTCGGGA
CTAAAAATGGTTCTTGGCGGAGTAATCGTTGCAGTGATCTGTTTTGGTGTTGAAAGTCTATTTGG
ATAATTAGATAATATTTAATTAATAATCCAATATTTTTGTGAAAATTATGAAAAGCAGAAAATT
TTTTTCTAAACTCAAAGAAGAATCTTACGATGTAAGTATTTTTGATTTAATGAATGCAAAGGTCT
ATTTAGAAAAAGATATGACGTATTTGCCACAAGATTATAAAAAAGGATATATTGAAGACTTTTT
TACATTTTTCCCCGAAGTTTTGAAAGAAATAAAAAATAAAACCGAAGAAGAAATTGAAGATTTT
GAAATTGAAGACGAAGAAATAATAAAAGCTGATTTAAGACTTTGCAGTATGGGTTCTAAACAAA
CTGGACGAGAATCTTACGAAAAACTTGTAAAAACAGTTATAAATTACTTAATTTTTATAAATGA
ACGACCACTACATGCTTTAACGACAAGATTTCCAGGTGGAAAGCATATAATCGAAAAAAATGGAA
ATTATTACTGTCCGATAAAAAATGCACAATCAAATGAGCTTTCAATATGTGAATTTTGCATTTGT
AAAGATTTAAATGAACTTTAAAAATAGATTTTTTATTATAATTATTTTGTTTCTGTTTTTAAAA
AATTAAAAATTAAAATTTTTTATGCATCAAACTCTTCTTCTTCAAGGCCTGTTGCAATTTTTACA
GCGTGGTCAGATATCCTTTCAAGATATCCAATAATGTCCAAAAATACAATACTTGCTTTTGGAGC
ACATTCTTTAGCGTTTAATCTTTTTATGTGCTGTTTTCTAAATGTTTTTTCAAGGTTGTCGACTT
CATCTTCAAGGAATGTTACTTTTTCGACCAGTACTTCATCTTCAGTTTCAAAAGCTTCTATTGCA
GTGTCTATCGAAACTATTACTTTATCAAACATGAAATTGAGTTCATCAACTGCAGTGTTTGTAAA
TTCGATATTGTCTTCAATTACATTTTCAGCATGTTCTACAATGTCTTCTGCAAGGTCGCCAATCCT
TTCGAGGCTTGTAACGTTACTTATAAGGCTTGTAATTTTTAATTTTTGACTATCTGAAATTTCTT
GGTTCGAAAGTTCAATTAAGTGATTTGTAATTTCGTGAGCCATCGTATCGATTATTTTTTCTTTT

TCATGCACTTCTTTGATACTCTGGACATTTTTATTTACAAGGGCCTCTTTTGAGAGTCTTAAGTT
TTCACGTACAAATTTTCCCATTTCCACGACTTCTTTTACCCCAAGACCAACAGCAATGGAGGGTG
TTTCCATAATAATTCTTGAATCAAGATGTTTTATGGGCATAAATTCTTTTTCGTAGTCTTTTATC
GGTATTAATTTTTCAACTGCATAAACGAAAAATCCTGCAAATGGGAGCATTATAAGCGTGTTTGT
AACGTTGAATATCGTGTGTGCATTTGCAATCTGCCTTTCAACACTACCTGCGCTTAAACTCTGAA
TCCACGAGATTAACGGTGTTGTGTAAATCAAAACCATGAATATCAGTGCACCCGTAATATTGAAT
ATCAAGTGCATGAGAGCCGCTCTTTTTGCAGTCCTGTTTGCACCAATACTTGAAAGAAGGGCTGT
TACACAGGTTCCAATATTGTCACCAAATAATATTGGAAATGCAACAGCAAGGCTTAATGCACCTG
TTGATGCTACTGCAATTAAAAGTCCAATCGTTGCACTACTGCTTTGAATCATTAAAGTCATTCCA
AGACCTACGAATAATCCAAGAACTGGGTTTTCAAGATTCATTAAGAGATCTGTAAAAAATGGTTC
TCCTGCAAGCGGCTTTAACACGCTTGACATTGTGTGCATTCCAATAAATAAAATACCAAAACCAA
TCAAAACTTCAGCAATATCTGAGTGTTTTCGTTTTTTTGAGACAAGCTGCATAATCACCCCGACT
GCAACAATTAGCGGGGCAATGTCTGTTAATTTGAATGCAACAAGCTGTGCGGTAACTGTTGTCCC
AATGTTTGCACCCATAATAACCCCAATGGCTTGAGTAAGGTTCATCAAGGATGCATTTACGAAAC
CAACAACCATAACTGTTGTTGCGCTGCTGCTTTGGATTAGCATCGTTACAACAGTTCCAACTAAC
ACTCCAAGATATTTATTTTTAGTTAAAACTTCGATAAGTCGCTTTAATCCATCTCCTGCGACTTT
TTGAAGCCCGTTACCCATTAAATTCATACCGTAAATGAATAATGCGAGCCCACCAAGAACACCTG
CTATAAGTGCAAATTCCATCTGTTCCACCAATATAATCGATATCAGAGATAATGCAATATTATAT
TCATTTTAATTATAAAATAATTCATAATATATGAACATCAAGGTTTTTAAAATCTAGTTCGAATT
ATCACTTAAGGAGTTAACGTTATTTAAAACTTATTTAATAATTAAGAAAATACTCTTCTCAAAAG
TTTGTAAATCGGGTCAATTTCCGTTTTTTAGAAAAGTATATATAGTAAATTACATATTCATATAC
TGTTTAAATTTTTCTAATAGTCAATTTTTGAATTTGAAAGACTAAAACACAAATACGCATAAAA
AAATATACCGCAAAAGGCTTTATTTTATCAAAGTCAAGAAACCACTTTTTTGGAGGAAGAAAAA
TGTCAGATGAAAATCTATTGACAACCTTAGACACCTATTTGGCGTCTGGGATACACATCGGTACT
CAGCAAAAAACTGAAGATATGAGAAGATTTATCTACAGGGTTAGAGCTGACGGATTATATGTTT
TAGACGTTAGAAAGACTGATGAAAGGTTAAGACTCGCTGCTAAATTCTTATCAAACTATGAGCCT
GAAGACATCATGGCTGTTACAAGAAGAGTTTACTCAGTAGGTCCTTTAAAAGAATTCGGAAAAGT
TACAGGAATAAACACGGTAGCTGGAAGGTTCGTTCCAGGTACATTAACAAACCCTTCAGCTAGAA
AATTCGCTGAACCAGAAGTTTTATTTTTGAGCGACCCTAGGGTTGACAAACAAGCATTAAAAGAA
GCAATCGAAATCGGAATCCCTGTTATTGGTATGTGCGATACAGAACACTTAACAGCACACATCGA
TTTCATCATCCCTACGAACAACAAAGGTAGAAAATCAGTATCCTTAATGTACTACCTTATTGCAA
GAGAATACATGAAAAACAGAGGACTTATTGGCGAAGAAGCTCCATTCTCATACGACCAGTTCCTC
GAAAAAGCAATGAATGTTAAAGTAAAAATGAACCCTTCAAACAGACAAAGAGGAAGGTTCCATA
GAAGAAGAAGATAATTCTTCTTAAAAAGATTTATGTGGTTTAAATGGAAGATTTGTATCTCGAA
TGCCCGGGTTGCGATGAAGTAACTCTCCACACTGTATTAAAAGAAAAAGAATCGAAAAAACATTT
GAAAATTACTGTAAAGTGTCAGGAATGTGGAACTGTACACGATGTAGAAAAAAATTTCAAATTA
AAAGATATTAAAATAGTGATAAGCAGATACGACGAATCAGAACAGTCTGTACTTCAAATTGCGA
CAGATGAGATTTTGAAAGTTGAAGATGTAATCTTGGCGTTTGACGAATCTGTTGAAATTACTGCT
TTAGAAACTGAAGATTCAAGAAGAGTTTCTTCATCCGCTGCACAAGATTTAAAGATGATATGGGC
AAGATCAATAGATGTTCCTAAAAAAGTTGGCATATCTATAAATTCGAGAGAAAGTACATATTCA
TTCAATATACTTGTTCCACAAGATTATGTATTCGAAGAAAAGAAGATATATCGTGCAGGACGAGA
TTTCTTCAGAATAAAACAGATAAAAACAGAAAAAGGAAATTTTTCAAGGGAATTAGCCCGTAAA
ATCAAAAGAATATACGGTGAACCTGTAAAACCACTTAGAGATTACACAGATTTAACCGACTATAT

GTTATAATGAGGTGTAACAATGGCAAGAATGAAAGCTAGGTCCGCTAAAGGAAAAAGAGTGGCC
AAAGATACATGGAAATCCAAAGTATGGTACGATATTTACACACCACAATCATTCGGTGGAGACGT
AATTGGACAAACTCCAGCAAACGACCCTGCAAACTTGATCGGCAGGATTTCAGAAATCAGCTTAA
GAGACTTGACAAACGAACATTCAAAACACATGACAAGAATGTACTTCAAAGTTGATGGCGTAAGT
GGAAACAACGCTACATCACAGTTTGTAGGTCACGACACGACAAGAGAATACTTAAAATCACAAGT
TAGAAGAAGAAGAAGCAAAATCAACGCAATCGTTGAAGTAAGGACAAAAGATGGTTTCAAAGTA
AGAGTTAAAGCATTAGTTTTAACAGCTGTAAGAGCAAGAGACCACCACAAAACAGAAATTAGAG
TTAGAATGGAACAGATAATCAAAGAAATGGCAAAAGAAACTCCATTCGCTGAATTCGTACACGCT
ATGTTAATGGGCGGATTAGGTTCAAAAATATACGGCGACTGTAAAAAAATGTTCCCATTAAAAA
GAGTAGAAATCTTCAAATCTGAAGTTATAGAATTTGGAAAAGCTGCTGAAGCACCTGTGGAAGA
AGCTGCTGCTGAGGAAGTAGTAGAAGAAGCTGCTGCTGAAACACAAGAATAATTCCTCTTTTTAA
TTTTTTAATTTTTAAGTAATCTTCTGTTTTTTAAAGTTTACCCTTATTTATCCATTATTGTCTGG
TGGAATTATGAACGACAGATCCTTGGGCATTTTGTTAATGTTTTTAACCGTAATTTTCTGGGGAA
TTTCATTTGTATCGACAAAAATTATTTTAGAGTTTATTCCACCAATTACAATAGGGTTTATACGC
GTTGTAATTGCAGCCGTAATTTTAATTTTCTTTATAAGAAATTTTACGAAATATTCAAAAGAAGA
TTTTGTATACATTGTGTTAGCGGGTTTTTTAGGGATTACTTCATATTATCTATTTGAAAATGTGG
CTTTAAAGTATACTACTGCAACCAATGCATCATTAATTGCAGCAACGGTACCCATATTTTATCTG
ATAGTATCAGATATGGTGGAAAAGAAAATTCCAAGCAAAATAAAGTACTTCGGAAGTTTAATTG
GGCTTTTTGGAGTCTTCATTTTGATATTAAATGGAAAATTTGTACTCGAGTTAAATCCGCTCGGG
GATATATTAATGTTTGGTGCAGTTTGTACCTGGGTACTCTACACATTTGTAATTCAAAAACTTCA
AAAACACGACGATTTAAAAGTTACAAGGGATATAACGTTAATTGGTGCAGTATTCTTTATACCAT
TTACAATTATAGAACTTGGATATAACGGAATGTTTGAGTTTACAATATTGTTAAATCCATACATT
GCATTTTCAATACTTTACCTTGCAATTTTTTGTTCAGCAATAGCTTATTTATTCTGGAATATGGC
AATAAGACTTGCAGGTGCAAGTACGACAACAAATGGGGTTTATATTATCCCAATAGTTGCAATGA
TTACAGATGCCATAATTTTAAAAAATATTCCAAATATTTATGCAATAATCGGGGCGGTTTTGGTA
TTATTTGGAGTTTATATCTCTGAAAAAGGGGACAAATTAAAAATAAATTTATAATGTGATAATA
TGAATGGAAAAAACAGGTCTGACATAACGGCAGGATTGAAAGTAAACATAGTCCTCAAAAAAGA
CCAAAAAACTGGTAAATTAACTACTGGAATTGTAAAAGACCTTCTTACAAATTCAAGCTATCACC
CGCACGGAATAAAGGTAAGGCTTGAAGATGGGCAGGTTGGAAGAGTTCAGGAAATTTTAAAATA
AGAATAATATTTTTTATTTTTAACTATTTATCTAAATCTTTTAATACTATTTTTACAGATATTTA
AATTAAATTTAGTTTAACTTGAAAACGAGGGTTTTTAAATGAATGTTTTAATTATTGGGGCAGG
TGCTGTCGGATTATGTCTGGCTGCTAAATTATCAAATGTAGCAGATGTTTTTGCAGTAACAAGGG
AGAGAAATGCAAAAATCATACGCGAATCTGGTTTTAAAATGACTGGAATCTGGGGGGACGGAAC
ATATAACTTTGAAGCTTCTGAAAATGTTCTGAAACGCGATTTTGACTACATTATTATATCTTCAA
AAGGACTTGCAACAAAATCAATCTGCGAGCAATTTAAAGACGTTATAAAAGGCAAAAATGTTAT
CAGCATCCAAAATGGTGTTGGAAACGAAGAAATAATTTCAGAATATACTGATAAAGTGATTGGC
GGAACTATTATAACAGGTTTTGAGTGGAAGGGGGATGCAAAAGTACACGTTTCAGTAGAAGGGG
GCCCGATGACGTTAGGAAGATTTCCAAATGGAACAGATGATGAAGTAAAAACTTTTGTAAAATT
AGTAAAAAGTTCTGGAATACCAATTAATGAAACAGAAAATATTCAGGGCGCAATATGGTCAAAA
ACAATTTATAACTGCGCATTAAATCCACTCGGAGCAGTTATGAATGTATCCTATGGTGAACTTTC
AAATGAACACTCATGGAACCTAATTAAAAATATCGTATATGAAATATTTGAAGTAACTTCCAAA
AAAGGAGTAATTTTACCTTGGAAAACGCCAGAAGAATATTTAAAATATTTAAAAGAGTTCCAGC
TTCCAAATACTGCTGCACACCACTCTTCAATGCTTCAAGATATTTCAAAAGGAAATATAACAGAA

ATAGACTTTTTAAATGGTGCAGTTGTGACTATTGCAAAAGAATTTGGAATAAAAACGCCGTATA
ATGAATTTATTACTGAACAGATAAAATTCCTTGAAAATTTAAAATCAAAAAAGGAATAATTATT
TAAAATTTTTTTATTTTGAAATTGATGATTTGCTTATTTTAGGTTCGTTATATAATGGATCTATC
TCAATTGCTTTTTCATAGCACATCAGTGCTTTTTCATAATTTTTTAGTAATAAATAAGAGTTTCC
AAGTCCATTCCAAGCATATTTATTATTTGAATTTAAGTCAAGAGCTTTTTCAAAGCAGAATATTG
ATTCATTGTATTTTTCAGTTTTATAATATAAATAGCCCAAATTACTCCATGATATATCATCTTTC
GGGTTCAATTCAACTGCTTTTTCATAAGATTGAATCGAAGAGTTATATTCGTTTAAATAATTATA
GGAAAGTCCTAAACCACTCCAAAAAAAGGGATTTTTAGAATCTAATTCAACTGCTTTTTTATAAT
TTAAAACTGCTTTTTCATACTCGCCCAGACGTTTTAGTGAATCTGCTTTATAGAAATAACAGTAA
GGGTTAGAATTATTTAAATCAAGAGATTTTTCAAAGCAGGAGATCGCGCAAGAATATTTTTTTG
ATTTGTAGCACGATATTCCTTTATTATATATTGCATCATAATAATATTCATCCTGAATCACGGCT
TTATCAAAACATGCAATTGCTTTATCATATTCCCCATTTTGATAATAACAGTATCCAAGGTCATT
CCATATCAAAACCAGGGTTTCATAGTCTCCTGTAGATTTATCAAATGCTTCTACAGATTCATCAT
AATTTCCAAGCATTTTTAAGCATATTCCTTTATAAAACCACGTTTTTCCGACAGAATTGTCTATT
TCAAGTAATTTGTTATAATAATAAAGTGCTTTCTCGTATTCTTCCTGTGAAAGATGTATTTTCCC
TAAACTTTTCAAAGCACATTTATTTTCAGGTTCTATTTCAATTAATCGGCCAAAATAAATTTCTG
CCATATCTAAATTTCTATTTTCCATGGAATATTTTCCTAAATATTCTAAGGAACAGGTGTTTTCA
GGATCTAATTCAATTGCTTTATCAAAACATTTCACGGACGTTTCGTAGTCTTTTTCAAGATAGTA
TTGTTCCCCCATTTCAGCATAGGTACTAGAACTTTCAGTTATTGGCAACGGTAATAACATTGAGA
ATATGAAAATAGTTACCACTACGAGTTTTATCATTTACGCCCCCCTCCGAGATAAATGATTATAT
ATTAATAGGTTATTCTACCCCCAACTATCAAATTAATCTTTATTTTTTAAGTAATATTAAATTTA
TTACAGAATAATCGCCAATTTTAAGTAAAAAGAGATAAAAACATATAATTTAATGTTTTATTAC
GCAATTAATCATATTTTTAAAAAATAATATCAAAATGGATGTTTAAAAATAAATAACTAAAAAA
AGGGAGTATGATGTAATTTATTTTTTATCGTGTTCATAAATAAATACATCTTCAATTTTTGCTTC
AAATAATTCTGCAATTTTAAAGGCCAATTCAAGGGAGGGGTCGTATTTTTCTTTTTCAATTGCAA
TAATTGTCTGACGTGTCACATTTAACTTTTTTGCAAGATTTTCCTGAGTTAAATTGTGCATTGCT
CTAAATACTTTAATTTTGTTTTTCATAAATATTACCTTAAATTAGGCCGTATTTTTTACTAAAGA
TGTATCTAAACAGCATGTTTACAAATAAAATAAATATTGCTGAATATGCTAAACAGGTTCCAATT
AACGTATATCCTGGAACATAAGTTTCAAAAGTCGTTAAAAATACACTTAAAAGTGCAAAAAGTA
AACATACAACTTGTATTGTAAGATAAGATGCTTTTTCAGCGACTTTGTGTAGAAGTTCATCTTCT
ATAACTTCATCAAGTTTTTTGCTAAGGTTCATTTTGGTAAAAATTCCAATTATAACTATTAAAAC
CGCAAGATATCCATTTCCTGTAGTTACTGCCAAACTTACCAAAATTGCAACAGTTATGATTAATA
TCCTAAGATATTTTTTATACGTTTTTAAATCCATTTAAACACCTTTGTTTATAATAGTTTACATT
ATGTAAGCATACTTACACATAATGTTAAGTATTCTTTACATATATAAAGTTAATGTAAAAAAGT
CCGGGATAATAACATAATTTAAAAAAATTAAAAAATAAGCGTTAATTTTTGTAAGTTCCAATAA
TATAATCTATAAATTGTTTCGCAGTTCGTCCAGAAAAGCCGTTTTGTGACATTCCCCATTTTACT
GCATCTTCACGAAGTGTTTCCCATGCCATAATCATTCCGGTTTGATCGGACAATTTTCCAATATT
CATCGAATAAAATTTCACGTCATCGGCCTTACACGGATTAATCCCGTTTTCAATAGCAAGTTTTT
TAACCATATTTAAGTATTCTTCTTGCGTTGGTGAATAGAAACTTAAAGTTATTCCAAATCTGTCT
GAAAGTGACATTTTTTCATTTATAGAATCGTTTGAATGAATATCAGAATCTCTTTCTGACCATGA
TTCTTTTATGAGGTTTCTCCTGTTTGAAGTTGCATAAATTATTACATTTTCCGGTTTTTTTTCTA
AGGTTCCTTCAAGAACTGATTTTAATTCTTTATATTCAGTTTCATTATCTTCAAAGGATAAATCA
TCGAGAAATACTATAAACTTTTTATTTCTATTCCGTAAATGGTCTACCATCTCTCCAAAATCTTT

CAATTGGCTTTTTTTAAGTTCAATAATTCTTAAACCATCAAGATAGTATTCTTTTACAAGGGATT
TTACAAGGGATGATTTTCCAGTTCCCCTGGATCCGACAAGTAAAATGTTATTTGCAGGATTTCCT
TCGATAAAAGCAGAGGTATTTTTCAAAAGAATTTCTTTTTGGTAATTGTATCCAACAATGTTTTC
CATATCTACTAAATCACAGTATTTAATTCCTGAAAATCCGGTTTTATTATTCCACCTAAATGCTA
CGTGGTCTACCAGTTCACCGGAACCGATTTTATAATAATATTCGATAATAGAATCTAAAAGTTCA
GGAACAGAATCAATATTTAAAAGTTCTTCCCTATAATCTGAAAATACGGTTTTTTCATCTTTGTA
GAGTGTTGGTGTGTAATTTTCAATGGTTTCATCTTCTGAATCCATAATGCTCCGTAAATGCTTAG
TGTCTATATCCATAATTCGTTTTAAAACTCCTATATCTTTAAATGCAAGTTCATAAAGGCTTTCC
AAGTTACCATAATGCTTTTCGCAAGATATCGCGAATATATTTTCATCTTTTAAAATGGCGTTCAT
AATGTATGATTTTAAAATATTTCCGCTGAGATTTTCATTTTCAGCTTTTATAATCAAATCATGGG
TTAATCCATAGTAATTTTTTTCCACTATTGATTCATCCGGTTTATCTTCTGAGAGTTCTCGTATT
ATATTTATTAAATAGCCTATTATTTTATCGTCAAGAAGATTTCTATATATTATAATCCTATTGGA
GTCGATTAGAAGCTTTCTAAACTTAAATTTCATTGAATTTTGGTTATTAAGAAATGACATCTGGG
GATTTACCATTAGGGTACACCGTTCTAAATTTAAGAATTTACTTATTAACTAATTTAAAATTCAT
TTTATAATTTAGTTGCGATTATAAAACTAAATATTATATTTTATTTTTCATAAACTGTGACGGGT
TCTAACTGGCTGTCTATTATCAAGTCAAAATCAATATCCTTATCCCAGCCTTGTTTTTGAAAAAC
ACTCGAGAAACAGATCCCCACTACTTTTTTAGGGCTTATTTTATCAATTAGTGCGTTTACATCTG
AGGTGGTAACTTTGGAATGAGGCATTGCAAGCCCTCCCATTAATACCAGCATTTCTGGATTAATT
TCGGTATTATCGTCTAAATTACTGTAATTGTAGCCCAAACTTCCCAAATTGAGTTTTGAAATCTC
GTCTAATTTTGCATCAGGACTAAAATATTGTTCTTCAATTGTTCTTACTGCATAAGCATTTAATA
ATGCAAAAGGAAGACATACTCCTTTTGAACCGCTGTAAAGGATACTTTTAGAGCCATATTCTGAT
ACTGCTGCTTTAAAATGTCTTAATTGTGAAGAAATTCCCTTATCTTCGGCTATTTTTTTCATATT
ATCACCGCCATTGGCCAGTATTTATATACTATTATATAGATTTTCCGCAATACATAAATACTTTG
TGGATTAGATTTAGAATTAAAAATATGTGGTTTAAAACCACAATGGTAATTGTTATGAGAGAAG
TTTTACTTTCAGAATGCATTGATCTGCTTTATGAATCCCATTTTGTGATATCGAAACCTTTTGGA
AGGTCTTGTTTTGATTTAATCGCTAAAAAAGGGAATTTAAGATTATTAATAAAGATTTTGAAAA
ACATAGATAGTTTGAGCACTGAACAGTCTGAAGAACTGTTAAATATTTCGAAAATGCTTCAAGCA
GTGCCTGTAATTATTGGAACTCGTACGAGAAATTCAGTAATGGAAGAAGGGGCCGTATATGAGCG
ATACGGTATAAAAGCAGTTACTTTTAATACGTTTCGGGAGCAATTGGTGGGCGAGCCTCCAGTAG
TTTATGCAAACAGGGGCGGATTTTTTGTAAATATTGATGGAAAGGTTTTAAGGGAAACTCGTGA
AAAATTAAAGATTTCTGTTGGAGAACTTGCAGAAGTATCCCTGGTTTCAAGAAAAACGATATAT
AAATACGAACAGAACGAAGCAAATCCTTCTGCTGAAGTTGCAATAAAAATAGAAGAATATCTGG
ACGTTCCATTAATTAAAGGAATTAACATAATGGACTGTATAGAAGGATTAAAATCGCAGAAAAG
TCGTGATGATGCTTTTGAAAAGATATTGAAAGAAGGGGAAGATTTTAAAATACGTGTTATAGAT
ATTTTGGGAGACATGGGATTTAATTTACTTGAAACTACAAAAGCACCCTTTGATGCAGTAGCGGA
AGAATCAAAAGGGGAAGATGCAGAAAATCAAAATATTATATTTACAAATATTCAAGAAACTGAG
AATGAAGAGATAAGAAGAAAGGCAATGATTGTTGATGAAATTTCTAAAATATTAAACAGTCATT
CTTTGCTCGTTCTTGAGAAAAAAACCAATGAAAATAAGAGAATAACAAGTATGAGTATATCCGA
ACTTGAAAAAAATTGGCGATACCGTAGATTTACTCGATTTTATAGAAAAAAGGAAAAAATCAACG
AAATAATTACAAAAATACTATTTTTTAAATATTCTAAAGATTTCTTTTTTAAAATTATTTTATTT
TATTTTATTTTAAAAAAATTAAAAAAAGTTATTGTTATGGTTTACTTTTTTGAAAGGAAGCTTG
CACCGATATATACTGCAAAAGCCACAAATAAAAGGTTAATAAGTCCGCTTACCCAGTAACCTAAA
TTTATCGTGTATCCTACAATCAAGTAACCTAAAAACGCCATGATTCCTATTAATGTCGTGTATGG

AAGCTGTGTTTGGACGTGGTCGATGTGTTTTGTACCTGCTCCAGTGGAACTCATGATAGTCGTGT
CAGATATTGGGGATGAGTGGTCACCATATATTGCACCACCCAATACTGCAGCAACGAATGGAACA
ACAAATTCAGGCATTCCAAGTCCAACTGCCATAGGAACTGCAATAGGGAGCATGATTCCAAAAGT
ACCCCATGAAGTACCTGTTGAAAATGCCATGAATCCTGATAACAGGAAAAGTATTGCAGGTAAAA
TTTGAACTGGCAAGCTGTTTTGGATTAATGATGAAAGGTATGTTCCGGTTCCAAGGTCTCCAATT
ACTGAACCCAAAGACCATGCAAGTATAAGAATTGTAATTGCAGGTAGCATTAATTTTGAACCTTT
TGCAAAGAGGTTTGCAATATCTTTTGCAGGAACTGCTTTTGAAACTATAAAGTAGACCACTGTAA
ACAATAAAGTTACTAAACCTCCCCAGAAAAGACCCCATGATGCATCCATGCTTAAGAATGATTCA
ATAATTCCAACGCCTGCAAAAAGGCCTCCTGAAATCATAAATGCAAAGATAATTGCTAAAATGAG
AACTACTAATGGTAGAACAAGGTAGGAAATTTTTCCGCCACTTAATGGTTCAATTTCTGACTCGA
TTGCTGCAGTTTCTGCATCGCCATTGAATAAATCGCCAGTTTCTGTGGCAACTTTTTCGTGAACT
TTCATTGGACCAAAATCCATTTTTTTAAAGGATATGATAAATGAAACCATGAACAGCGCAACAAG
TGCATATGTATTGTATAGGATTGCAGAAAAAAACACGCCTGATGGACTCATGCTTGAAAGAGCTC
CTGTTCCAACACCTGATTCACCTAAATTTCCAACAACAGCTGCAAACCAGCTTGAAACAGGCATT
AAAATACATACCGGTGCTGCTGTTGAATCGATGATATATGCAAGCTTTGCTCTTGAGATTTTGAA
TTTGTCAGTAATTGGTTTCATTACTGTTCCAACAGTTAGACAGTTAAAATAATCATCAATAAATA
ACAAAATACCAAGTAATGATGTAGAAAGACTTGCACCTTCTTTTGATTTAATTTTTGTAGATGCC
CAGTTTCCGTATGCAAGAGCTCCGCCTGCTCTCGTGATTAATGCAATCAATATACCGAGCATTAC
TAAAAACAATAATATTAACAAATTCCAGAGATTTCCAACGCCCGTGAATGATGAAATTCCCGTAA
TCGAAGTTAATTCAACACTTCCAATAATTGTATTTACAATATGTGACGCAGATGCAACAATATTC
CAGTTGTTGTAAAGTAGTGATCCTGCCAAGATACCCAAGAAAAGAGACATGTAAACTCTTTTAGT
AATCAAGGCCAATCCAATTGCTACAACAGGTGGCAATAAAGATAGTATGCCAAAATCCATAAATA
TACCTCCAAAAAGTCTATTTTAATACATTATTAATAAATTCTATATATAAATTACTTAAAAAAAG
AAATAGAAACATATAAATGCTTATTCTAAATTGTGCATTTGTGGGAGCATTGAAACTTTAAAAG
GTTCTCCAAAAGCCCTATCTCCTGCATCCCCAAGTCCTGGTACGATGTAACCATCGTTGTTCAATT
TATCATCGATTTTTGTTACGAATATTTCAACATCTGGAAATTCTGCTTTTACTGCATTTATTCCT
TCTGGCGCTGCAAGAACTCCAACTATCATTAATCTTTTAGGTTTAGAATCTTTAAACTCTTTTAA
AACATGAATTAGTGTAGATCCTGTTGCAATCATCGGGTCAGATACAATAACTGTGTCTTCAGGCG
TAACTTGTGGAACTTTTATGTAGTTCATTTCTATTTTAAAATCAGGAGCATGTCCTCTTGATGCA
GAAACAATTCCTACTTTTGCAGATTCAAAGTTTTTGATTAATCCTTCCATTAATGGAAATGCAGC
TCTTAAAATGGTTATAATAACTACATTTGACCTATCTTTTGGAAAAATTCCTTTTGTTTTTTCTA
AAGGTGTTTGAACGTGCATTTCTTCAAATTCCATGGTTTTTGTGAGTTCATATCCCATGTATCTT
CCAAGTCTAACGAGCCCTTTTCTAAAAGAAATATTTTCAGTGCTTACATTTCTTAAATTGGTCAG
TGTTTCCATTAAATAAGGTGAATCTTCAAAGGAATAAACTCCTTCCCATCTTTCATCTTTTATCA
TAATTTACCCTCTAAATCCTTAATTTATTTTATAATATTATTTTTTTAACAACTGGTCCATTAAT
ATTGCGGTTATTGCACCAACTGCCATTCCTGATTCTAAAATGCTTGAAATGATTTTTGGAAACAG
GCTTAAAAATTCTGCAGGAAGTTGCGGTGCACCCAGACCGAGTACGAGTGCACTTGCCAATATGA
GCGTGTTTCTATCATTTAATTCAACTTTATCTTTTATTAATTTAAGACCTGTAATGCTGATCATA
CCATAAAGTGCGGTTGTAAGTCCTCCCAAAACCGGTGCAGGAATTGACGCCAAAAGACCTGAGAA
TTTAGGAATCATTGACAGAAGAACCAATATTACAGCACCAATTTGAACTACCTGAACACTTGAAA
CTTTTGTTAAAGCAACAAGACCGATGTTTTCTGAATAACTTGTTGTACCGCATGCACCAAAAAGA
CCTGCAAGAGTACAGGAAAATCCTTCTGAAGCGATACCTCTATTTATCTTGTTATCATCGATTTT
TAAATCTGCAATCGTTGAAATTGCGTGGTAATCTCCGACACTCTCGATAATACTGACCATAAATG

CAAATAATAATATTATAATTGCATTAACATCAAAAACAGGCATTCCCCAAGGCATAAGTTTTGGC
AGTGCAAACCATGAAAGCTGGTTCATCATTGAAAAATCCACAAGGCCTAAAAAAATGCTTATAAC
GTAACCCACAACAGCCCCGATTATAACAGGCATTGCTTTTAATGTTCCTTTTCCCTGTAAGGATA
CGAGTATTGTCGTTATAAATGTGAGTGCAGCAACAAGTATTGAAGTTACAATTGAACTTCCTGCA
GGGTCTGCAAAGTAATTAAACGAATATTGAACAGCAACATCTGCAAGACTAAAACCAATAAGCAT
GATTGTAACGCCGGTAACTATTGGTGAAAAAAGCTTTTTTAATTTTCCAATAAGTCCTAATGCAC
CGGTAATAGCTTCGATAACCCCGCCAATAATCAAAGCACCTTCAACTGCAGCAAGACCCATTCCA
GAGCCAACTGCAATTAATCCCGGGATAAATGCAAAACTTGACCCTTGGACAATTGGAAATCTTGA
ACCTGCAAAAGTTTGGAGCAATGTTGCAACCCCCATTGCAAGAAGTACCGCCTGAAGTAATAATG
CGATTTCAGACATCGAAAGTCCGATTGCATAACCCACAACAAGGGGGACGGTTACTGTTGCACCA
AACATTGCAAGTACGTGTTGGAAACCCAGTGCTATTCTTTTCATTTTTGTATCCTCCAAAAAAGT
ATTAACAATTAAAAACGTGTTTATCCAAATAAATATGGATAAAATATTATATAAAATATCTCAA
AAAAGAAGCATATTAAAATGATTTATTTTTTTATAATTTAGGAATATATAAATGAATTATTTAA
AAATTAAAAAAATTAGATATTTTCATTGTTAATTTTTAATATATCCAGCACTTTTTTAGGGAGTA
GCATTAACACTACATACGCCCAAATTGCGGATAAAACAATGTCTGCGATCATTAACGGAAATTCT
GCAAAGTATATTGCTGCAGAGATATTTAATCCTGTATTTAGATTGATAGTCGTTGCATATGAAAG
AATGGTCTGATCAAGCGGGCCTCCAAAAATAAACATTTCTATTGGTATTACTAAACACAGTGCTA
TAATTGATATCAAAAAAGCATGTAATATCATCGAAGTAAATTCTCGATCATGCTTGATTGCAGCG
TAACCTGCGACAAAACCAACAACAACATTTACAATTGTAAACGGAATATAAACAGAATTAACAGT
AGAACCAATAATTAAATTTGAGAAAAGGCCCGTTAGTGCCCCGCCTGCAGGTCCCAGTAGTAATC
CACATAATATAGTGCCCAAATTGTTTAGAAATATTGGTAAATCATAAAATGTGGCAACGAAGCCG
CCCAAAACATTAATAGATATACCAAAGGCAGTTATCAAATAAGGTAATTTATTATTCACGATACC
ACCGGTAAATTAGTTAAAAAAGTTAGGTTTTTAATATTTATATAATTTACCAATTAATTCGGGCG
ATAATCCCTATAATGATAAAATTATGGATTAAAATTGCCTTTCAGACGATAATTTAGTACTTTCC
TCTGAAAAGAGCTTTTTTATCATCCAATCAACATCGAATTCTTGAAGATCAGAATACCTCTGACC
AACACCCATAAATAATATCGGTTTTCCAATCGAATAAGCAATTGAGAGTGCAGCGCCCCCTTTTG
CGTCAGCATCTGTTTTTGTAAGTATTGCACCATTGATATTTATTGCATTGTTAAATTCTTCTGCC
TGGGATATTGCATCGTTTCCAGCAAGTGAATCTCCAACAAAAATGATTAAATCTGGTTTTGTAAC
CCTGACTACTTTTTTGATTTCATCCATTAGGTTAATGTTTGTAGTCTGTCTTCCCGCAGTATCTGC
AAGGACTACGTTTAATCCTTTTGCTTTTGCGTGGCTTATTGCATCATAAATTACCGCAGCACTGT
CTGCTCCTTTCTGGTGCTTTATAACTTTTATTCCCGTATTTTTACCGTGTTCTTCAAGCTGTTCTA
TTGCACCTGCTCTAAATGTGTCTCCTGCAGCCATTACTGCAGAATAACCTTTTTCTTTTAAAATG
TAGGCCAGTTTAGATATTGAAGTCGTTTTTCCCGTCCCATTTATTCCTACAAAAAGAAGTACTGC
AGGTTCCCCATTAGCCTTTTTTTCATCGATAAGATTAAAGACATCAATTTTTTCCTGAGATAAAA
TTCCTTTTATCGATTTTTTAAGTGCATTTATAGTTATTTCCTCTGGATCGTCTTTTGCAGATATT
TTAAGCCCTACAAGCTGATTTTTCAATGATTCAATGATTTTTTCAACGACATCAAATGCAACATC
TGCTTCAAAAAGTTCCATTTCCATTTCTTCAAGCACGTCTTCAATATCGTCTTCTGTTAATATCAC
ATCTTTTCCGAGAACTTTTTTAATGCTTTTTGTAACTGCTAATTTATCAAAAAATCCAATTTTTT
CCTTTTTTTCAAAAGATTCAGTGTTTATTTCTTTTAAATCTTTTTCTGAGTTTTCTAAATTGGTT
TTATTTTCTTCAATAATTTTTTCAGGAGTTTCTTTTATGTTCTGTTCTTCTACAGTCTTAGCTTCT
CCTTTCGTGTAAATTTTATCACTTAATTTTGAAATGGTATTATTTAATTTTTCCTTTAGACTTCC
AAACATTAGTTTCCACCTTTCGGTATTGGGAATTGTTGTTAAAACTGTTACACTGTTTTATTTTA
AATATATTATATTTAATTAGTGTATTTATGGGCATATAACAGTAACGTTTATATAGAACTTCAA

AGTAAGTAATATCCAGAAATCTAGAAACGGGTGATAAAACATGTTTGGTAGAGATCCAAAAGAT
CCCTTTTCAGAAATATTCAAAGTTTTTGGTATGGGAGTTCCTATGGATGGCCTTGGTGGCTCAAT
GGGAAAATCAATGTTTCAAATGAACTCAATGGGTTTAGAAATCAGTGGTAAAGGTTTCATGCCTA
TAACTTTGATTGAAGGCGATGAAACGATTAAAGTAATTGCATTAGTTCCAGGAATAAATAAAGA
TGATATTGTAATAAATGCAATTGGGGAAACTCTTGAATTAAGGGCTAAAAGAGCTCCAATGGCCA
TAATGGAATCAGAAAAGATAATTTATTCTGAAGTTCCTGAAGATGAAGAAGTTTACAAAACTAT
AAAATTACCAGCTCCTGTTAAGGAAGGAAATTCCTCTGCTAAATTTGAAAACGGAATGTTAATTG
TAACGCTTCCAAAATCAGAAAAAGCTAAGAGAACCGGAATTGACATAGAATAATTGATTCAAAA
TCAATAAAAATATTTAATTTTTTTATTTATCTATTTTAAAATTTTTAATAATGCTTTTTTATAGT
TGTAGTTTTAATCAGTTCTTAACAATGATTTTTGGGATTTTAATGAAAAAAAGACATCTTGAAA
TTTTACTTGACAATTTAAAACCGCATCCAAAACCAAAAGCGCATCTTGAGCAGTATTCTATCGAA
GGAAACCTTGCAAGCGAGTTTTTGCTGTTTGCTAAGGAAGATATTGATGGCAGTTTTGTAATTGA
CCTAGGATGCGGCAGCGGCCGACTTATAATTGGTGCTAAAGTTCTGGGTGCAGAACATGCAGTTG
GAATCGATATTGATGAAGAAACAATTGATACTGCAAAAGAAAATTTAAAAAATTTAAATTCAGA
TTCAAATTCTGATTTAAAAGTCGATTTTTTAAATTCAGATGTTAAAAATATCGATAAAAAATAT
TTTGAAGATAATTTTTCCGATTTCAATGGTTTAAAAAAGGTTGTTATTCAAAATCCGCCGTTTGG
ATCTCAAAAAAAGTATGCTGATAGAATTTTTTTAGACAAAGCGTTTGAAATCGGTGACGTGATTT
ATACAATACACAATACTGCGACAAGGGATTTTTTAATAAATTATGTTAAAGAAAAAGGGCGAGA
AATTACGAATATATTCCAGGCAGACTTTAGGGTACCTGCAATTTATGAATTCCATAAGAAAAAGG
CTGTAAATGTTCCAGTAGATATTTATCGAATAGTATAAATTAAATACTGTTCAAATTACGGTTTT
ATTTTTGAATAATCAAAATCGGAAAATATATATATCAATATAACTAGATGAATTTCTGTGAAAT
AAAAAATCAAAAATAAGGAATAAATACAAATGAAAAAGAGGGATGAAAATGACGCATACGCAGA
GTAGCATCAGGAATTTGAATTGTATCGTAAATAAATTATGTGGCGCATTAGGGTTTAGATTGTTT
TTTTCATACCAGTGGTATTATTAACTGCTGAGAATTGTTTGTTTAATTAAACTGTATTCGTAAAT
TGTTCGATTGCAGGATTTTGCAGGTTTTAAGGAATTTAATGATAAGAGTATATTCTCCACACTAT
ATACTATAAATCTAAAGAAGACACTATATAATTTTTAACGGTGATTTATTATGGAAATGTTCGAC
AATATCAAAAATGTAGGAAAACTTATCAGATTAGAAAGAATATTCGATAAAAAAAGCGAAAAAA
CAGTAATAATCCCTATGGATCACGGTGTTTCATCGGGACCTCTTGATGGTATAAAGGATATGCGG
ATTACGACAAATGCAGTTGCTGACGGTGGTGCAAACGCAGTTTTAGGCCACAAAGGACTTGTAAG
GCACGGACACAGGGGATACGGAAGAGACATCGGATTAATAATACACATGTCTGCAGGAACTTCAA
TTTCACCTGATCCAAACAAAAAGGTAATTGTAACAACAGTTGAAGATGCAATGAGGATGGGTGCA
GATGCAGTTTCATTACACGTAAATGTTGGTGCAGAATCAGACTTTGAAATGTACAGGGATTTAGG
TTTAATTTCAGAAACCTGCGAACACTGGGGAATGCCTCTTATTGCAATGATGTACCCAAGAGGCC
CAAAAATTAAAGACGAAAAAGACCCTGAAGTAGTAGCTCATGCTGCAAGGCTTGGTGCTGAACTT
GGTGCAGATATTATCAAAACAAATTACACTGGAGACCCTGACACCTTCAAAGAAGTTGTTAAAGG
CTGTCCTGCACCAATTGTTATTGCAGGCGGCCCTAAAACAAACACTGACGAAGAATTCTTACAAA
TGGTAAAAGATGCAATGCATGCAGGCGGAAAAGGGGTAGCTTCCGGAAGAAACGTATTCCAGCAC
AAAGATGTCAAAGGAATTACAAGTGCAATCTGCAAAATCGTTCATGAAGATGTTGAAGTAGAAG
AAGCATTAAAAGAAATAAAAATCTAAATTTCTCCTTTTTTTAAAAAGTTACCTATAAATATCATT
TTTTTAAAAAAGATTATTATAATTAAGGGCGGTGGTACTATCAAGCCAAATGTAATCATAAATT
ACAAAACTTACACTGAAAGCGTTGGTAAAAGCGGTTTAGCAATTGCAAAAGCTGCAGAAAAAGT
TTCAGAAGAGTCCGGAGTTACTATCGGGGTAGCGCCTCAATTTTTAGATTTAAGAATGATTTTAG
ATGAAGTAAATATTCCTGTTTATGCGCAACACATGGATGCAGTAACTCCTGGAAGCAGTACTGGA

CACATACTGCCTGATGCATTAAAAGATGCAGGAGTTACTGGAACTCTTTTAAACCACTCTGAAAG
AAGAATGCTTCTTGCAGACCTTGAAAAATGCATTGAAATTTCAAATAAACTTGGTTTAAAAACTG
TTGTATGTACAAACAATATACCTGTTTCAAAAGCAGTTTCTGCGTTAAATCCAACTTCAATTGCA
GTAGAACCTCCAGAATTGATTGGAAGCGGAATACCAGTTTCAAAAGCAAATCCTGAAGTAGTATC
AGGAACTGTTAGGGAAGTTCGAGAAATTAACAAAAACGTTGAAATATTGTGCGGTGCAGGTATC
TCAAAAGGGGAAGATGTAAAATCTGCGCTTGAACTTGGAACAAACGGTGTTTTGCTTGCATCAGG
TGTTGTAAAAGCTAAAAGCGTTGAAGATGCAATAAGAGAATTAATTGCAGAAATTTAAAAATAA
AATTCTTTTTTTTAAAATTATTTAAAATTAAAAAATAAAAAAATTAATTATTCAATTTTTACTT
CGACATTTAAAAAGACTGGGCCTCTAACGTCGATTACTTTATTTTTGCTCATGATCTGTTTTAAT
GCGATATCGTCGATTTTTAAGTTAATGTCGCTTAAACTTCTGACAATTGGAACTTTAACTTTGAA
TTCAGACGTGTTTGACACAAATGCTTCTTCTAATTTTGAAGTAATTTTAAAGCAGAGCGCATCGA
TATATCGAATGCCAGTTGCTACCCCTTCTGATTTTCCTTTTTCTAAGAAGTCTTTAAATTCTTCTT
TTACTCCATCAAAGCTTTCAGGAATTCCAATTACATTTCCATCGTAAACGTAGATTTCGTTTAAA
ATTGAAGGTCCAAGCAAATTTTTACCTTCTTCTTTTTCGAAAATATTTATTTTTACATTTTTCTT
GGTTTTTCCAAATGAAAATGTTTTTTCAATGGTTAATTCACAAGGGGATTTTAAATCTTTGTTTT
TAACGCATGAATCAATTAATTCTTTTGTTAAATCGTAAATTTCATCCATTACTGGAACTTTATCG
AGTTTTACCATTGAAGCGACATTCCGGTCATTAAGTTTGTGTTCGTAAAACTGAGGATATACCAT
TTCTCGAACATCTGCGAAATTTCCAGAAATCATTGCAAGTCTTTCAACACCAAGACCCAAATTCA
TTACAGGTACATCTATTCCATATTTACTCAATGCAACTGGCGAATATACTCCAAATGTAGCAACT
TCGAGCCATTCTTTTAATTTTGGGTGGTATGCGTAAACTTCTGTCTGTGTTTCAGGGGTGTAATA
TTTACTTTTCTTTTCATCAGGAATGAATTTAAAGTTCGTAAAGCCAAATTGCGATAATAACCCTT
CTGCAATTGCTTTTCCATCATTAATATCCACTCCTTCACCTGCAATTGCACAGGATGCAGAGTGGT
ATGTCATTAGGTGGCTTTTATCTTCTTTTTGTTCTCTTCTAAAACATCTATCAATTGAAAAGAGT
TTAAATGGCAAGGGTTTTTTGTTCATGAGGTCTGAAACAGTAAGGAACCATCCTGAAGTCATGTG
GCTTCTTAAAGTTAATTTTGAAGAAACTGCGGTTAAATCCTTAAATTCGGGGAAAACTTCTTCTA
AAATTTTTAAACCCATCTCGCTTGAAATTTCAAGTGCATTTGAAATTTCTAAAACTAAATCGTCA
CCATCAAGAGTTCCTTTTTTGTATCCGTGAAGTATTTTTTGTAAACTTTCTTTGTGTTCAGAAAC
TTTAATTCCAAGTTTTTCAATCTGTGAAATTTTTTCATCGCTCAAACCAACGTCAGGTCTTGGAA
GTCCTGCTAAATAAAAGCATCTATCCAAAACTGCCATAGCTTCTGGGCCGAATTGTTTATAAATA
TCTCTTTCATCGACAATTACTGGGTTTATATATTCTTCAAAACCCATTCTAAGGTATGCCTGTCT
TAAATTTTCAATAGTGTCGTTTACAGGGTGTGTTTTTCCAAAAACTGGTTTTATTCTTGGGTAAC
TTTCGTTTATCTTTTTAGCTTTTATAAGGTCTTTAGTTTCGATCCATGCTTTTTCAAAGTCCTTA
TTGGCCATTTCAATGATTTCTTCTCTTTTAAACATCATTATCACTAAATTTCTAAATTTTATGAT
GAAATGTAATCTTTATATTCATAATAGTTGCTTATATTATATATTAATTAACTGGGGTGAACTTA
TGGCAAATTTTTTGGCTAGGTACTTTGGGTTTGAAGAACATAAAACAAACTTCAAAGTAGAGACC
ATGGCAGGAGTTACGACTTTTATGACCATGGCGTATATTATATTTGTAAATCCGTCGATTCTAAG
TTTGGCAGGAATGGATTTTGGGGCAGTAATGGTTGCAACGTGTATATCTGCTGCACTTGGTACTT
TTATAATGGGTGTTTATGCTAAATATCCGTTTGCACTTGCACCGGGAATGGGTTTAAACGCATTC
TTTACATTTGGTGTGGTCATGGGCATGGGCCTAAGCTGGCAGACTGCTCTTGGTGCTGTTTTTAT
TTCTGGTATTTTATTCATTTTGTTAACATTAACTAAAATTAGAACCTGGATATTTGATGCGATAC
CTGACGCTTTAAATACGGTACTGCTGTAGGTATTGGGTTATTTATTGCATTTATCGGTTTAAAAT
CTGCAGGAGTTATCGTTGCAAACGAAGCTACATTGGTAGGCCTTGGAAACGTTCTTTCACCTGCA
ACATTTTTGGCACTTTTTGGTTTATTTGCAACTGCTGCAATGATGGCTAGAAAAGTAACCGGTGC

AATTCTCTGGGGTATTATCTTAACCGCAGTAATCGGTATGGGCCTTGGTGTTTCAGCACTCCCTG
CAGGTTTAGTTGCAATGCCTCCATCATTAGCACCAACACTGATGCAGATGGATGTTATGGGAGCT
CTTAGTTTTGGTCTCATAAATATAATTTTGGCGTTCTTCTTTGTGGATTTATTTGATACACTTGG
AACACTGAGTGCTCTGAGTTCACAGGCAGGATACATGAAAGATGGAAAACTTCCAAAAGCAGAAA
AAGCATTAATGTCTGATTCAGTTGCAACTGCAGTTGGGGCAGCTTTAGGTACTTCAACAGTTACA
AGCTACGTTGAATCTGCTTCAGGTATCGGACTTGGTGGAAGAACCGGATTTGTTTCAGTTGTAGT
TGCTGCTTTATTTTTACTTTCGATATTCTTCTCACCATTTGTTGCGGCAATTCCAGCATATGCTAC
TGCTCCTGCATTAATAATCGTTGGAGCTTTAATGATTTCAGCAATAAAAAGAATTGATTTAGATG
ACATCACAGAATCAGTTCCTGCATTTATTGCATTAATTACAATTCCTTTAACGTACAGTATTGCA
ACGGGACTTCAATTAGGGTTTATTTTCTACCCATTAATTAAAATAATTGCAGGACGTTCAAAAGA
AGTACACCCAATCGTATATTTACTTGCAATTGTATTTGCAGCAAGATTTGTATACATTGGATAAT
TAAAAAATCTTTTTTTATCTTTTTCTTATGTTTATGCCTATTTTAAAATTAAAAAAGTAAATTTA
AAAATTATTTTCCATATTTTATTCCACCTTTTTTACAGGCAGTCACACACTGAAAACACCGGATA
CATTCTTTTTCATCCATATTATCCAGTATATTAATCTGCATTGGGCATTTCAGTTCACAAAGCCT
GCATGTGTTACATTTATCATTAGATTTTATTTTAAAAACTGGTTTTACAGATAAAATTCCAAGAA
GTGCCCCAATTGGGCAGAAGTACCTGCAGAAAAACATGGGTATAAAATATCCTAAAATTATGAAC
AATCCTAGAAGTATAAATGAAATCACTGTCCCGTTTAGTCCTGCAAATGATCCTATTGGACATAC
TCCGCAAATTACATAAGTTGTATATTTAAAGGTTAAATAAAGCCCGATTATTAAGAGAATATACT
TTAAATAAATTAATTTTTCGTGGATTTTTGGATCTACTTTTGGCAGTACTTTTGAATTATTTATT
TTCATTTTTAATTTATATGTCCATTCAAAAATGATTCCAAAAGGACATATAAATCCGCAGAACAA
TCTTCCAAAAACAATCGAAACCGCAGAAAGTCCAATTAATTGGGGTGTAATCTCGCTTAAATTAA
ATGTTAAAAGAATTCTTTGAACAAAACCTACAATGCACAGACAAAAACTATTGTTCACTAAAAA
ATATCCTAAAAATGATGTTTTGATTAGATTTCTTATTAATTGGAAACTAATTTTCATTTAATTCA
CCAAAAACTCCTTAAGTCAATCCATGTAAACAAAAAATCAATTATTTTTTCTGAAAAAGACTTAT
TTTCTTCTGAAATTGTATTTTCAGTAATATTTACAATGCCTTCTTCTACCATGCAGTCAAATATT
GCATATTTTATATCAACAACTGAAAAACCATATATTGAACGAATCTGATCGATCGTAATGCTGTC
TTCTACTTCAATATTTAGTTTATTTTTCAAAGATTCAGTTTTTATCCCATAATATTTGCAAATTT
CTTTAATTGTATAAGTTTTTAATTCTTTTCCTAATAGTGAAACATAAATTTCAACCAGTTCATCG
TAGCTTGCATTTCCAGAACTTGAAACTGTTTCTTTTAGATTTTCAGGGACCGGTTCGGAATGGTC
GCAAATTTGATTATTATTTGTATCGATATACCTTCCGCAGGCTCCGGGAAACTCACAATCTGAAT
CTGTAACTCCAAACGGGCAGTCGTCCCATGCAAAAGTTGTTACAATCGAAATGAACAATAAAATT
AAAACTAAAATCTGCATTTTCATCATTTCACCAATATAATAATATTATGAAACGAATTTAAAAA
AAGTAACTGAAAAAAGTAAAATTGATTGGTATATTTCACAAACATTATTCTGAAGTTTCATTGT
TTTCTTCTTCATCATCGTCTTCGTTTTCGTCTTCAGTTTCATTTTCATATTCTATTTCTTCATCGT
CATCAGAATCGTCGTCTGAAGGTTTACCGTTGTTTGTTTTTACTTTAACGGTTTTTTCGACTTTC
GTATTGCCCGTAGTTTTATACTGTTTAGCTTTTAAGTCGTCTTCCAACTGGATTGCAAGAGCATA
AGCATAACTGTAGTTTCCGCCATCGTATGCATCATTTAATGCACCATATTTTGTATCTAATTCTG
AAATATTGATTCCGTTCTGTTCTAAAGCTTGAAGAGTCGTCTGAATAGTATTCATTCTTCTTTCA
ATTTTTGCTTTCAAAACATTTCCAGGCATTGATTGAGTACTTACCTGGAATCCATGGTTTTGGAG
TCTTTTTAAAAGTCCGCTGTAGTTGTATGCAGCATCTTCTAAAACTGTGATATTGTCAAATATAT
TTTCCATAACTTCTTCGCTTACTGAGGTTTTGTTTCCATCAGTTAATACAAAGGTTACATTGGTG
ATATTTCCGTATGTATCATTTGTGCCGTTAGCGTAAACGATTAATATTGATTTGTTAACGAATAA
ATCTTTAAATCTGTTTATTGTAAGGTTGTTTGTATCAAATCTATCTGAACCATTGATTCTTTCAA

CAGTAATGTTTTCATTTTCTAATGCAGTTGTATAATTTTCAGGAACTGCCACAGTTCCGCCAATT
ATCACCACATCTTCAGGAGATTCGTTTAATATTTCATTTAATGTATCATTGCTGAAATTTCCCCA
TGGGGATTCAACGAGTACAAATTCGTCGAGTTCGTCAGCAATAGTTTGAGCTACGAGTTTGTCTG
CAAAGTTGTCACTTACTAAAATAACGTCGCTTGCATAAGAAACCGGCATTAATACGATCATTAAT
AGAACTAATTTTTTAAAAATGTCCATAAAAATCCCTCTAAACGTTTTAAATGCAATAAAATAGTA
TTATTGTTTATTATATATTAAATTATTTAATTGTTGTAGTTAGGACTATTATTTTTCGGATTGTA
TGTGATTTTTGGCCCGTTCCAGTAAACGTGGCGGTTTTGATGTTTGTAACTTAGTAAATTCCTTT
CAAGAACTAGTTCTCCCGCATAGGCCTGTTCTAAGTTCTGATTTCTTATGGAATTATTTATTGAA
GTTAAATCATTTCTAAGTGGAGTACAATTTATTCCAAGCTGTTCCATGGTTTCAATTTGGAGTTG
TAACTGTTCCATTTTTCTGTAAACAGTTCTATTTAACACGCTTTCAGGCATTGATTGAATACTTA
CGTAAAATCCATAATTCTCAAGTCGTGCTCTTACATTGTTTGGATTAAATACTGGATTTTCCATA
ACTGTAACCCTTCCAGGATCAAAATCTCCTAAATCTTCATCATCAACGGATAATGCAGTACCATT
TGATAAAATCACGATATTGTCTCCAGCATTACAGAATTCTGGCCTATCATCGTCCCCATAAACTA
TCCACATATTTCTGTTCTGAATCTGATCTTTAAATTTTAATATTACATTTTTGTTTGTCTGGTAT
CTATCAATACCATATATTCTTTCAACAGTTAGTCCTGCTTCTTCTAATTGGGTATCGTATTCAGA
AGGAACTGCAACAGTACCTCCTACAATAATTACGTTTTTTGGATTTTCGCTTAATATCTGTTCAA
GCACATCATCGTCATAAACTCCCCAGGAGGTATTTATAACAACGTATGAATCGTTAATTGTTGGA
ATAATATTTGCGCTTGCACAGTCTGAAACAGTGTCGCTTACAAGCACGACATCGCTTGCAGATAC
GGGAATAATTAAAACTAAAAAAATAGCGAATATTTTGAAATAATCCATTATATCACCCCGATAT
ATCAATATATAATTTTTTGAAAAAAGGGGTATATATATTATTCGAATTAAAAATAAAAAAAGAA
TACTTATCTCAATCTTATTGAATCGAGATTCATTGGAGCTTTCGTTTCTTTTTTAAATAACTTGT
ATGCCGTACTTGCAAGGTCGATACATTTTGAAACTTCCCCGTGAACTGTTAATATACGGTCTGGG
ATCGGTTTTAATTTTCTAAGGTATTTTATGAGCTGTTTTCTGTCACTGTGTCCTGAAAATCCTTC
TAACGTATGAACTGAAAGATTTACTTTAACAGCTTTACTTCTTCCGTTTTTACCCATTAATGGGA
TTTCTTTAAATCCTTTCTGGATTTTTCTACCTAAGGTTCCTTCAGACTGGTAACCTACAAACACG
ATTGAGTTTTTCTCATCATCTGCGAGGTTTTTGAAGTATTCAACACTCGGTCCACCGCTGAGCAT
ACCTGAAGTAGTTAAAATAATTCCGGGTTCATCACTATCCATAATGTTTCTTCTATCGTTTGTAT
TTTTAACTTTTTTAAATACTTCTGAAAGGAATGGGTTGTCTCCTTCGTGGAAAATCCTGTTTCTC
ATGTTTTTCGAGAGGTATTCTGGATAAGCAGTGTGTATTGCAGTTGCTTCCCAAATCATACCGTC
TAGATATACTGGAGCGTTGAAAATTCCCTGATTGTATCCTTCTTCTAAAACAAGCATCAATTCTT
GAGCTCTTCCGATACCAAATACTGGAATTATTGCTTTTCCTTTTCTTGCAATGGTTTCAGCAATT
ACTCTTAAGAATTCTTTTTCAGTTTCGTCTCTTTCCGGAAGTACGTCATCGTATCCTCCGTAAGTA
GATTCGATAATCAATGTTTCAAGTCTTGGGAACTGGCATACTGCAGGTTCTAATAATCTTGATGC
TTCAAATTTAATGTCTCCTGTGTATGCAACGTTATAAAGTCCATCGCCCACGTGGCAGTGTGCAA
TTGCTGAACCTAAAATGTGTCCTGCATTGTGCATTGTTAATTTGATTGCAGGCGCAATGTCTGTA
GTAACACCATAGTCAAGTGGAATTGTGTGTTTAATACAGTTTTTAATATCTTTTGATGAATATGG
AACATTTTTACCTTCTTTTTCAGTAATGTCCACGTAATCTTTCTGTAAAAGAGTCATCAAATCTC
TTGTTGGTTTGGTACAGTAAACTGGACCATCATAACCGTATCTAAATAATCCGGGAATAAATCCA
CAGTGGTCAAGGTGCGCGTGAGTTACTACAACTGCATCAATTTCTTCGATTGAAAATTCAGGTGC
GTCAAAATGAGGAAATGCTTTTTCATCTTCAACTGCAATGTTGATTCCACAGTCAACCAATATCC
TGCTTTCAGGAGTCTGGTGGTAAAGGCAAGTTCTTCCAACTTCCCTACTTCCTCCTAAAAACGAA
GTTCTAACCCATGAATCATCTCTGAGTCTTACGTCTCTGTGGATTCTTCTTCCGATTCTTCTTAAA
ATATCTTTTACATCTGCTCTTTCTCGATACATTGTAGCTCTAATGGCTTTAATTGTATCTGATGG

AATTGGCGGGGTTCTAACTGGTTTTGGGGCCCACTGAATTGCTTTTTTAATATCTTCAAGGGTGG
AACCTTCTTTTCCAATTACAAGTCCTGGTTTTTTAGATTCAATTATTACTTCACCAGTATTTGCA
TCGAAAATACAGTTTGTAATCTCAGCATCCTCTGGAACTATTTTTAAAATTTTATCTTTCGCGAT
ATCAGGTTCGACTAAAACAGATGGATCCGGTCGAATTGCAAGTCTTTTTCTAAAGTCCCTTGCAA
ATTCTTTGATAAGATTGTTTGAAAAAAGTTCTGGGTTTTTTGCATAAATAACTACTTCTGAACCT
TCAAATTCGACGTCAGTAATAACCGCATTTCCTGGGGCTTTGTTTATAACCGCCGCCTTTATCTCG
TTTAATATATCTTCAGCTGACAAAATAGTCCCTCCTAATAATATGTCTGTTTAAATTCTAGAAAC
GCCTATAAAATTGATTAATCTGTGTTAAAACATAAAAAAAATTTGAAAAGAATACTTTTCAATA
GTAGAATTTGAGATATTATATTTTTAATTTATCTTTTTCTGTTGATTTTTTTTAAAAAACCCTCA
ATTTCTGCGTCTGAGTATAATTTTACTCCATCTTTATTGATATGAGCTAAGGAAATACCATTTCC
AGAGTATGCATCTCTTTGCATAGCAGACGAGAGAGCTTTTACAGCAATTAATAATCCTTTTTCTA
TTGCGATATCGGATTTGTATTCTGATTCGAGTACTCCGTAAGCAGTAGGTGAACCTGATCCTGTG
GCAGTAAATGAAGATTCTTCATTAATGCCTCCAACTGGATCGAGTGAAAACAGTTTAGCTCCGTT
AATCAGATCGTATCCACCGAGTATTAATTGTGTTAAAAGAGGGTAATGTCTGTTACCATGCAAGA
CGTTACTTATTAATGTGGTGCATGAAAGAGGCGTCATATTGTTTCCAGTTCTCATCTTGTAGATT
TTTGCCTCAGCAGAAATTAACCTGATGAGTGACTGGGCATCCCCAACACTTCCAGCTATAGTCAT
TGCTATGTAGTCATCAATCTTATATAACTTTTTCGCTTCTTTATCCGCGATAAGATTGCCCATTG
TAGCCCTTTTATCAGTAGCTAAAACGACGCCATCATTACAAAGTAATCCTACGGTGGTCGTTCCT
TTCATATACTCTTTATGGTATTCGCTGTTGGATATCATCTTATCACCACTTAATATTAATCAATT
TATAATAAATGCTTTATATACTCACCGGTTTTTAAAAATAAAAGAAATTTGGCGTAAGTTTTTGA
TTATTAGTATGTTCTGGCAACTGAAATATATTCTTTTCCTTCATAAGTAGTCAGTCCTAAAACTG
ATGCATCGATTAATTCTTCGAATTCTTCATTGTATATCGATTCATTAAATGGAATTAATATTATT
CCCTTATTTTCTAAAAGTTTAGCTTTAACTTTTTCTGAAAGTGAATTTACATCGTTTTCAAAGTC
TAAAACCGTTATAAAGCTTTTAACTTTTTCTTCAGCTTTTAGTTTTAAGTTTTCTTTATACATTG
AAACTATTTTTTCAACTTCTTCTAAAATATTTGAATATTCAACGGTTATTTTTTCTCCAGTATCT
CTTCTGACAATTAATGCATGTCCATTTTCAATATCTCTTGGTCCAAGTTCGATTCTTAATGGAAC
TCCTTTTAATTCCCAGTCATTATATTTTCTTCCAGGTCTGATGTCTCTATCGTCTAAATGGACTCT
GAATTCTGATTTTAATGTATTGTTCAATTCTTTAATTTTGTCCATTACAATTTCTTCTTTTCCTT
TGAATAATAACGGAATAAGAACAATCTGAATTGGTGCAACATCCACGGGAATTACAAGACCTTTT
TCGTCCCCGTGAACAGAAATTAATGACGCAATTGCCCTATCAGAAATTCCGTAGCATGTTTGGTA
CACGAAATCTTTTTCGCCATCAGGTGTTTCAAATTCAAGTTCAAAAGTTTTTGCAAAGTTTTGGC
CAAGGTTGTGAACTGTTCCAATCTGCATTGTTTTTCCATCAGGATATATGGTATCAAATGCCATT
GTGTAGTCAGCTCCTGGGAATTTATCCCATTCTGGCCTTTTAGAAATTACGTATGGAACACCGAT
TTCATCAAAAAATGCTTTGTAGAGCGTTAGTGCTTCTTCAATCTGTGCATCACATTCTTTTTTTG
TTGCATGAGCTGTGTGTGCTTCTTTAAAACTCATTATTTCTCTTAATCTAATCAATGGCCTTGTA
TGCTTTGTTTCGTACCTGAATGTGTTTACTACTTGGTAGAGTTTTAATGGAAGATCTGTATGCAC
TTTGATCCACTGCTTCATCATGTAGTACATTGTGGTTTCAGAAGTAGGCCTTAATGCAAGTTTTA
CTTCCAATGGAGTTTTTCCACCATGAGTTACCCAGAAAACTTCATCTTCAAATCCTTTAATGTGT
TCCCCTTCTTTTGCAAGCAAATTTTCAGGTATTAGCATTGGAAAGAGTGTTTCATCGTGCCCGGT
TTCATCGAGTAACTTTCTTAAAATTTCAAAAGAATATCTTCTGATTTTAAAACCGTATGGCAAAT
AAACCCCGCATCCTTTTATAGGGTATCTTAAGTCATAAATTCCGGCTTTTTCTAATATGTCGCTG
TACCATTCAGAAAATTCCATTTTTTTCACCGTAAATAGTTATCTTTAGAATTAACGTGTTTTAAT
ATTTTTAATATAAAATAAGCAGTAAAATAACTTACATAATTATTTCATGCATATGCATCTAACAT

GACAGACTATTTATAAAATATATTTATATAATTTATGAAAATATTGCTAAATTGATGAGTATTT
GATAAATATTTAATTTTATCTCTAAAAATGTTGTTAATGTTTGCATAAATAATTGGAAGATAGA
TTTTAAGATAAAAGAGTGAGAAATATGGATCAGAAAGAAGTAATTGGAGAAACAGGCCATAAAT
CCGTTGATTTAATTAAAATAATGGACAAATGGCAGAAAAAATGGACGGAAGCTAAAATTTTTGA
AGCTGAACATGATTCAAGGGATAAATTTTTCATAACTGCCGCATTCCCATATTTAAACGGGGTGC
TTCATGCAGGACACTTAAGAACTTTTACAATTCCTGAAACAATTGCAAGATACCAGAGAATGAAA
AATAAAAACGTGCTCTGGACGTTTGGATTCCACGTTACTGGAACTCCTATTTTGGGCTTAGCAAA
CCAGATTAAAGAGAGAAAAGAAGATATTATCTGGGCTTACAACAATTTACACAACATTCCAATGG
ATGAACTCTTAAAATTAAACACCCCTGAGGCAATAGTTGAATGTTTTTCAAAAAAAGCAACAGAA
GCCTTTAAAAGAATGGGATTTTCACTCGATTGGAGAAGAAACTTCAAAACTGACGATAAAGTTTT
CAGTAAATTCATAGAATGGCAGTTTTACAAATTAAAAGAAATGGGGCACATTACAAAAGGATCC
CACCCTGTAAGATACTGTCCAAAATGTGAAAACCCTGTTGAAGACCACGATTTACTCCACGGAGA
AGAATCAACAACCGTAGAATACAGTTTAATTAAATTTACTTCCAACTTTGACGGAAAAGAAATA
ATAATGCCAATGGCAACTCTAAGGCCTGAAACAGTCTTTGGTGTAACCAATGCCTGGGTTAATCC
AAATGAAATTTATGTAATGGCCGAAGTACACGATGAAATCCAAAAATTAGATAGTGAAGACGTT
GATTTAAAATACAACGGTATCTGGATTATTGGAAAAGAATGTGCAGATAAATTAAAAGAGCAAG
ATAGAAAAATTGAAATTTTAAAAGAAATTAAAGGAAGCGAATTACTCGGCTTAAAAATTAAAAA
CCCTGTTACGAAAAAAGAAGTTCCACTCCTTCCAGCAGATTTCGTTGAAATGGGAATTGGAACTG
GATGTGTAATGAGTGTTCCGGCACACGCTCCATACGACTACGTTGCTTTAAGAGATCTTGGAAAA
GTTGAAGAAGTAGGTTTAATCCCCTTAATCGAAATTGAAGGATACGATAAATATCCTGCAAAAG
AAATTGTGGAAAAAATTGGGAGTTAAAGACCAAAATGATGAAGAGTTACTCGAACAAGCAACGAG
TAAAATCTACAAGGACGAATTTCACAAGGGAAAATTAAATGAAAACTGCGGAGAATACGCAGGA
ATTTCTGTAAAAGACATTAAAGAAAAATTAACAAAAGATTACATAAATTCAAACATTGCAGAAA
TAATGTACGAATTTTCAGAGCAGAAAGTTGTCTGTAGATGCGGTGAGAAGTGCATTATAAAAAC
CGTAAAAGGACAGTGGTTTATCAACTATTCTGACGAAAACTGGAAGAAACTCGCACACGAATGCA
TCGATAACATGAACGTTGCTCCGGAAGGAATAAGGCAGGAATTCCACAACAAAGTTGATTGGATG
AAAGATAAGGCTTGTGCAAGGAAAAGAGGGCTTGGAACAATACTGCCGTTTGATGGAAATTGGA
TTATCGAGTCATTGTCTGACAGTACAATTTACATGGCTTACTACACAATTGCAAGATTTATAAAC
GAAGGATTAACTCCAGAACAGCTTATTCCAGAATTATTTGATTACGTTTATCTTGGAAACGGAAA
CGTTGAAGAAATAGCTAAGAATTCAAAAATTCCGGCAGAAACAATTGAAGAAATGAGAAAAGAG
TTCTTATACTACTATCCGCTTGACTGGAGATGTTCTGCAAAAGATTTAATTCCAAATCACCTCAC
ATTCATGATATTCAACCACGTTGCACTATTTGGAAGAGAGCACTGGCCAAAAGGAATCGAAATAA
ACGGATACGTTACAATCGAGGGTAAAAAACTCTCAAAATCAAAAGGTCCAGTTTTACCTGTTTCA
GAAGTTGCAGAAAACTTTGGTGCAGACGTTGCAAGGTTTTATATTACAACTTGTGCCGAACTTCC
GCAAGATGCAGACGTTAAATTCAAAGAAATGGAAAAAGCAAGAGATAATTTGATAAAATTGTAT
GATCTTGCAGTATCCGTCATGGAAGAAGGAAAAACTGAAAAAGAATTATCTTTAATTGATAAAT
GGCTTTTACACAAAACATACAGTTCAATAAATGGTGCAGAAACTGCATATGAAGAATTCCAGCTT
AGAAAAATTGGTTTAATGTTCTATGAACTCATCAATGATTTAAGATGGTATAAACGAAGAGGCG
GAGAAAACAACAGCGTTTTAAAAGAAGTTGTTGAAATCTGGACGAAATTACTATCTCCAGTAACA
CCACACCTTTGTGAAGAAATCTGGGAAAAATTAGGGTATGCTGGATTTATTTCACAAGAAATGTA
TCCTGAAATCAAGCCAGAAATGATAAATGAAGATTTAGAACTTGGTGAAGAATTTATCAAGTCT
GCAATGGAAGATATCAGAAATATTAAAGGAGTTGCAAAAATCAACCCTGAAAAAATGTATTTAT
ACACTGCAGATGACTGGAAATACGATCTCCTTGAATTTATGAATGAAAACGCTGAGAAAAACGTA

AAAGCACTTATTCCAATGGTAATGAAAGAAGATAAATTCAAAAGGCATGGAAAAGAAGTGATGA
AATTAATTAACGAAATAATGAAAATAGGCGTTAAAAAGGCAATAGCTGAAGTAGAAATACTAGA
AAATGCCAAAACATTTATTGAAAGTGAATTCGATTGCAAAGTAATTGTAAATGGTGAGGATGTA
AAAGGCAAAAAGAAATTTGCAATACCTTACAAACCTGCAATATACATGGAATAACTTTTAATTTT
TTATTGGGTGATTTTATGTCATGTATTGATAATTATAACCATGAAATTCTCTTAAAAGGTTCATT
TAAAGAATGTAGTGATTATATTAAAAAAAATTACAAAAATATTCGTGAGTTTAATCCTGGAGAC
GAAATACTGGAAGGAGTAATGCTTATTGGACTTCCACCAATTCCAGTTGCTTACGATGATGATTT
TGTAATTTTTCCGTTTACAAAACCCTGCTATGGAAGCCACGTACTTAGGGTTCCATTAAATCAGT
ACATGAAATCCCACGAAAAAATTAAAGAAACAGGCGAAAAGAAAGGAATACTTTCAAAATTGAA
GTTTTGGTAAACTTAAATTTCAAAATTTTTTATAAATATTTTTTTAAAAATAGAAATTTAAAAT
AATTTTTTTAAGAATTTAAAAAGATAGGTTTAATGATTTTCTATCTTTGAATATAATGCCGCTTG
CAAGGGTGAAGCCTAATAAATCTCCAATTGGGAATGAAAACCATATTCCGTTCAATCCAAAGAAT
AATGGAAGAATAATAAGTGCAGGCATCAAGAATAAAGTCTGCCTTGCGCAAGAAATAATAAATG
CAGGTTTTGCTTTACCAAGTGACTGGTATAGCCCTCCTATTGTCATTTGAGCCCCGATAAGGAAT
GAGCATATGAAAAAGGCTCTTGTTGCAAAAACTCCTTGTGTGATTACTTCCGGGTCAGTTATAAA
TATCTGGATGAATTTTTCTGGAAATACCATTATAAGTATGAATCCAAGTAAACTAAGACTGGTAG
TTCCTAATAACGCGAGTTTTATTGATTCACTGACTCTTTGTAAATTCCCGTTTCCATAGTTATAT
CCAACAATTGGCTGAAAACCCTGCACAATTCCCAAAACGGGCATGAGTGCAATCATTGCGATTCT
CATCATTACACCATAAATTGCAATTGCAATATCTCCACCATATAGTCCAAGCAAGTTGTTTCCAA
GAATTACAAGAATACTGCTCATAATTATTTTTGCAAATTCCGAAGCTCCAATGAATATAGTTTCT
TTAAAAATCTTTAAATTAATTATAAAGTCGTCAAATGCATATTTAATGCTCGTATTTTTCCCAAA
CTGGTGTTTTACAATATAAATTACACCGATAATTTGGGATAAAACCGTTGCAATTGCTGCACCTT
TGATTCCCATATTGAATACTATGATCAATATTGGATCAAGGATTATGTTTAGAATTGCAGGGACG
ATCATTGCAAACATTGCAACTTTTGCCATTCCTTCTGCACGAACAAGGTTGTTTGCAGCAACTAG
GTAGCTGTTGAATATTGTTCCATAGAGTATTATTTCGTAGTAGTCTTTTGCGTAAGGGAATATGT
CTGCAGAAGTTCCAAGGAGGTTTAAAATTGGAACCATAAATACAACCCCGACAATTGTTATCAAT
ACTGATAAAATCAAAACGCTGCTGATTGCATTTCCTCCAGCTTTTCTTGCAAGGTCTAAATTTCC
TTCACCAAGTTTTCTCGATAAAAGGGACGATCCTCCAATTCCAAAAGTTACGCCAAATGCCATAA
TCATCATTTGGAATGGGAATATCAATGCTATTCCAGTAAATGCGGCTGTTCCAACCCATCGCCCA
ATAAAAAATCCATCAACGATATTGTAAAGTGCCATGATGAGCATTCCAATTATTGCAGGGACTGA
AAGTTTAACTAAAAGTTTTTTAACGTCTTCTGTTCCTAAAAATTCACTTTTGTATGCCATAATTC
TCACAAATTTTAAATTTTTTAATTTTCATTTTCTTTAAATTTTTCTTTAAATTCCTGAATAAATT
CTTTTTCAGTGTTTAAATGTGCTAATAATCTATCTGAAAGAGCAAGGACATGGTCTGGACGGTTA
TATTTTTTAACTGTGCTGTTGACCTCATATATGCGGTTAATTACATGTTCAACTGATTTAATGTA
ATTTTCAAAACATTCTAAAGCTTCTTCTTTTGATAAAATATCCAAATTACTCATTCCAAGGTCAA
AACCCGATATTGTCTTTTCATAATTTGAAATGAGTTCTTTTACTTTTTTTCGCATTATGTTTTTT
CCCAAATCCGTTGCAAAATAAATCTTTCGAGCTCTTCCACATTTATTTTCAGTTTCACTTTCAACA
ATTTGTTTTTCTTCCAATTTTTTTAAGACATAGTATATTGACGAAAATGCAATTTCTGTCCACTG
CCTCATTTTCCTTTCTTCGATTATTTTTTCAATTTCATAACCATACATCGGTTTTTCAAGAAGCA
AACCTAAAACTGCAGATTCTACATTCGAAATTTCAATTTTTTCATCCATTAAAACACCAATTTAT
TCTATTCGGGGGGTTTAAAGTCCTAAATTAAATGTTATATGTATCTGATATTCTATCACTATAAT
ATTATATAAATCAGACGGTAGCAAAAAATAGATTTTAAGTTTTGAAATATCGGAAAATATTAAT
ATCTTTTATTGATAAAATTATATACTTTTAATATACTTTGGTGGTATTTTTGAGAATAATGATA

ATTTTGGGATGTCCTGAACCCCCTGTTTTGGTACCTGCGTTCATGTTTTTACTAAATATGCTTAA
AAACAAAGGACACGAATTAATCGTTTCAGCAAACCCTGCAGCTTTGAAATTGATCGAGTCTGCGG
ATCCTGAAAAGTATTATTTAACTGGAATCGGTTTTCAACCAATCGATGAAGGTTTAAAAACCAGA
GCCGAAGTTGATCATATTATCGGTTTTGCACACAACGATGCGGCAGTTAATTACATTGTAACCTA
CAAAATGACGCACAATGTAAATACGTCAGCAATCGTTTTTGGAAAAGAATTTAACGAAGAATTGC
TCCAGATTTTAACTGAAAACGGAGTTAATGCTGAAATGGCAAGGGCATTCCACAATCCGACGCCA
CTAAACGTTAAAATTAAAAAAATGTTTTCAGATTTTTAATGTAAATATTATTTTTATAATTTTT
ATGGTGTTAATATGAGAATGGGAATAATTTCTGAAGAACGGGACTGGGTAACCGATGAGTTGAA
GTCCAAAATGGAAAAAAATGACATCGACCCGGTTATTATACAGCCCTCAAAAATAATTTCTTATA
TCGAATCGGAAGTTAAGTTTGAACAGAATAATCGGAGTATACTTGACTTAAAATGCGCGTTTGTT
AGAAACATCGGTGAAGGAGTCGAAATGTTTCACAGGTTTGACATGTTAAAATACCTTGAAAATT
ACGTACCTGTAATAAACCCGATGGATGGAATTGAAAATGCAGGAAATAAATTCAGGACATCATTT
TTAATGGAAGTTCACAAAATTCCTCATCCAAAAACAATCGTTGCAGAAGATGTAAATAAAGCGTT
AATCGCAGCAGATAAATTTGAAGACGTTGTTTTAAAACCTCTTTTTGGAAATCAAGGAAAAGGCC
TTGTAAGAGTTAAGGGTAGATCAACCGTTGCAAAATTAAAAGCGTTAAATACTTTTAAAAGCAC
TCACGGCGTAATATATATGCAAGAATTTGTAAACAATCCAAATAACGTTTATAGGGACATTCGAG
CATTTGCAGTTGGGGATAAGGTAATTTCTGCAATGTATAGGACGTCTGACAACTGGATTACAAAT
ATTCACCAAAACGGAGTTCCAGAAAAGTGCGAAATAACTGAAGAATTAAGTAAAATCGTGCTTGC
TGCAAAAGATGCGGTAGGACTCGTTTACGCAGGTGTAGATATACTCGAAAGCAGCGATGGATTAA
AAGTTATCGAAGTAAATGCATGTCCTTCATGGGAGGGACTTTCAAGAATTTCTGATGTAGATATC
GCTCAAAACCTAATCGATGAAGCTTTAAATTATGCAAAAGGGTATTAATTTATTAAAAAACTTA
ATTTTTAAGATATATTTCTTTCATTTTTTTAATGATTTCAGATGAGTTTAGATCTTCTTTTTGAA
TTTTAAGAAGTTTTTCATCTATTTTTCCGCTTTTAATTTTTGTAGTCGAATAATATTTATCTCCG
AGCAGATTTTTCAAGTTTTCACGATCAACTTCTCTATTTTTAATGATTTCAGAAGAATTTAAATT
CAAATTTTTATCGTAAAATGAAATTCCAACACCCGATAATGCAATTAATCGCTCATCACTTTCGG
GAATCCTTTTTATTTCCTTTTTTTGAAGTTCATTTGGATTTTTGTATGCTAATGTAGCTCCTCTT
TTTCTTCCAACTGATGGACCTTCAGAAATTATAAATCCGCACTCTACAAGTGCACTCCTAAATGG
AATTGATGAAGAATACGATATCAAAACACCAGAATCTGCCATATTTTCATAAATCAACTTTAAAA
ACTCAACTGTATATAATACTGGATCATTTAACGGTGAAAAAGCATCATGAAATACACAATCGTAT
TTTTTTAAATCTTTTCTTAAAAGTGTAATTCTTGCATCTTCGTTAAAAATTCTAATTTTTTGATT
CCTATTTCCTTTAAAAAATTCAGATATTGCATTTTTTACGATTAAATGTTCATTCATTGGAATAT
CGAGTGCAAGAGATAAAAAAAGCATTTCTTTACTGTATTCTACCATATCTATTTCCGAATCAATA
TTGTAATGAAGTGCAGAAACTGCATTGTAGCCCATACCGCTGCAAAGATCCAAAATTTTTGGATT
TTTTATATCTTTAACTTTTGAAGGAACTGCAAATTTTTCAATTCCTTCTTTTAAAGCACCGACTC
TTGAATGCATGAGTTCTTCAGGGTCATCTGATCTAACAGTAAATGTTCCATCTTCTGTTTTTACA
AGTAAATCCAATTCTATTAGTTTTTTGGTTAGTTCTTTTAAATTTTTTGAAGTAAAATCTTTTTC
CATGTAAATTTTAATTAAATCGAGCGCCTTTTTATTTGGAAGCATTGATACACCTAAAATCAGGG
ATAAAAATAAAAAATTTAAGTTATTCTTTTTTAATTCTTTCAAGAACTGTATTAACAACTTTTTT
AAATTCTTCGGATGCTTTACAGTCCATTGTAACCATTGGAACTCCTCTGTCAGACGCAACTCTTGC
TTTAATGTCGAGTGGGATTCTTCCAAGGAAAAATACATTTAATTCTTTTGCAGCTTTTTCACCGC
CGCCTTTACCAAATATATCAATTACTTTGTCGCATTCAGGACATACAAATCCGCCCATGTTTTCA
ACAATACCAATTATTGGAATTTCAAGGGTATTTGCAGCAGATACTGATTTCTTGCATCCAAAAC
AGATACTTCTTCAGGGGTTGTGACGATTACCACTCCGTCAATGTCGGGGATAGCCTGTAGTGTCG

TTATTTGAATATCTCCTGATCCTGGAGGCGTGTCTATTAATAAAAAGTCTAGCTCTCCCCAATTA
ACATCGCTTAAGAACTGTCTGATGGCACCGCTTGCTTTTGGGCCTCTCCATATAATTGGAGTGTT
TTTGTCTGGCAAAAAGTATCCAATGGACATTGTTTTAATTCCTTGTGGGGTGGATACAGGGTATA
TTCCATTTTCATCAGCCATTGGTTGAATTTGGTCAACTCCTAGCATTTGTGGAATATTTGGGCCG
TGGATATCTCCATCTAAAACTCCAACTTTGTATCCCATCATGTTAAGTGTTGCAGCAAGGTTTAC
GGTAACTGTGGATTTACCAACTCCTCCTTTACCACTCATGACTGCTATTTTATACTTAATTTTTG
ACATATTGTCCCTAATTTGTGCGTTCTGCTGTTCCATCATTTTTTTAGTATCTGAACAGTCGGAA
CTTGAACCGCAGCTATCGCAATTTCCAGAGCATTCTTCTGCCATAATCTCACCGATAGAAGTTAA
ATAAATATCACTTATATAATGAATAACATACTATATAAAAATATACCCATATGGGCAAATATAT
TCTTGATTTAAGGCTTTTTTTCGCCAAAAACAATGTTCACAATAATTTTTCCAGAATTTCCGTCC
CCAAATGGGTTATTCCAGTGTCTATTTGATTTTAGCATTTTTTCAACACAATTTAATATGTTTTC
AGGGTCACTACCTGCTAAAATATTTGAATTTACGTCCAAAGTTTCAGGTCTTTCAGTATTTTCCC
GCAATGTCACACAAGGTACATTTAAAATGCATGCTTCTTCTTGGAGGCCGCCGCTGTCGGTTATT
ATAAGTTCTGCATTTTTTTCTAAACCCAAAAATTCAAGGTATCCAACAGGTTCAATTATTTTTAT
CAGGGAGTTATTTTCAAGTGTATTAAGTAATCCAAATTCCTTTAATTTTTTATATGTTCTTGGAT
GAATTGGAAAAATAATATTTTTTTCATATTTTTTAGATACTTCATTCATGGAACTTACAAGTTTT
GATAAAATCTCGGAATTATCTGTGTTTTCTGCCCTGTGGAGAGTTAATAAAAAGTATTTTTCATT
TTCCGTTAGTTCTGATATAAATTTACAAACTTTTTCATTTTTTTCTGCAATTTTCAGGTTTTGGA
TGGTTGCATCCACAATCGTGTTTCCAACGATATGTATTTTTTTCATCCGAAATTCCTTCTTTTAAA
ATATTGTTTGCGGCAGTTTTGGTTGGTGCAAATAAAAATTCTGAAATATGGTCTGTAAGAACTCG
GTTCGTTTCTTCAGGCATTTTTCTATCAAAACTTCGAAGACCTGCTTCAATATGGCCGATTTTTA
TGCCCAATTTTGATGCAGCAAGAGCTCCTGCTAAAACAGTGTTTGTATCCCCTTGAACAAGTACA
AAATCAGGTTTTTCTTTCAAAAGAACTTTTTCAATTTCCATTAACATTCTGCCAGTTTGGTCTCC
GTGACTGCCTGAGCCGATATTTAAATTATAATCTGGTTTTTTTAGATTTAATTCTTCAAAAAAGA
TTTTATCCATATTTTCAGAATAATGCTGATTAGTATGAATCAAAAAAAAAATTTTTAGTAGTTAG
TTCCCGAATCACCGGGGACATTTTAATTATTTCAGGCCTTGTTCCAAGAATTATTCCTATTTTAT
ACATTTATTCACCATGCCCCATTACCAAGCAATTTAACCGTAAATCCTGCCTTTTTCCATAAACTA
TGTTCTAATATATTTTTAGTATCAAAGACAATTTTATTTTTGAGTTTTGGACATATTTCATCGAT
ATCGTCTTTTTTAATAGTTTTGAACACGTCGTGGTCGGTTAAAACTACAATGCAGTCTGAACCAG
AAATACATTCATCTAATGTGCTTAACGGGTATTCAAAATAACTAGCATGGGGATCATACGTTGAA
ACTGTTGCTCCATTTTCTAAAAGCATTTTAATAACATTTTTAGAAGGGCTTTCTCTTGTATCTTC
CACGTTTCCTTTGTATGTTGCACCAAATATCGATATTTTTGGTTTTTCAATTCCCAATTTTTTTA
ATTCGGAAAGAACGGATTTACAAACGTAAGCTGGCATATTATCGTTTAATTCTCTTGCGGCACGT
ATGAATTTTGCATTGTTTGTTTTTTCAACTATAAACCATGGATCAATACTAATACAGTGGCCCCC
AACTCCAGGTCCTGGATTTAATATGTTTACACGCGGGTGTTTATTTGCAATTTTTATTGCATCCC
ATACATTAACCCCGATTTCATCACATATCTTTGCAAATTCATTTGCAAGTGCGATATTGATGTCT
CTGTATGTATTTTCCATTAATTTTACCATTTCGGCAGTGTTTGAGTCTGTAATATATATCTGTCC
TTCTACAAATGATTTGTAGATTTCTTTTGCCATTTCGGCAGATTTTTTATTTATTCCGCCAATGA
TCCTGTCGTTTTCAACGAGTTCTTTTAATATTTTTCCAGGAAGTACTCTTTCAGGGCAGTGGGCA
ACGTATATCTTTTTATCAATAGTTTCGTAAATTTTTTTGGTGGTTTCCGGCGGGATTGTACTTTC
AATTACTATTAAGTTTCCATCTTTTACAAATGGTAATATTGCTTCAACTGCACTCATTACGTAAC
TTAAATCACATTTTTTGGATCCATCTTCTGTTGCAAGTGCAGGGGTTGGAACACAAATAATAAAT
GCATCCGCTTCTGTTGCAGAGGTTTGAACATTTAAGTTTTTTGAATTTATTGCTCCTTTTACAAG

AGTTAAAAGTCCAGGTTCCTCAATTTTTAATTCACCGTTTTTAATCTGGTTTACTCGTTTTTCGT
CGACATCCACACCAACAACATCATAGCCGTGGTTAGCGAGCATTGACGCTGTAGGAAGTCCAATA
TATCCTAATCCAATAACGCAGATTTTTTTAATATCATAATCGCCATGTTTCTCCATTAAATCACC
GGATTATTTAAAAAGTTTGTTTGCAAGAGGGTATGCCCAAGAAGCCTCTACAATAACGCTTAAAA
ATATTGTAGCAAATGTCGCAACAAGAATTGATCCTGCAATCATTTCAGGGGGCATATATTGCGTT
ATTGAATGCGGAATTATTCCTGGGTTGTGCATAATATTTGTGTATATCATTGCCGCAAGTGCCGC
AGGAACTACTCCTCTGGGGCCTTCAAGCGCAAAATAAATTCTTTCTTTTAGTGAACTTACTGGTG
GAATTGCAGTTGCAATAAAAACACCTATTGGCCTTGCAATAAATAATGTGCCGATAGCACAAAGA
AGACCGTAAATTCCAAAGCTTTTTAGGAGGGGTAAGCTTATACTTGCACCTAAAAATACAAATAT
CAATATTCTAGTAAGTGACGAAAGGTCTGTACAAAATTCTGCCATTTTGTGCATATCTTTTGAGT
TTTTAGGCTTTTTAGTTACTACGTTTCCAATGTAAAGGCCCATAATAGCAACAGCCATGTAACCA
CTCAGTCCGTATCCTAAAAAATGGGGGGCAACGTCTTCTCCAAAGAACCATAGTGCCATTGCAGA
TCCAACACCTAATGGCGCAATATATTCTCCAAATTCATCTTTTGAAACTACAAATTCAAATATTT
TTCCTCCAACGTAGCCTAAAGCAAGGCCTCCAAATGCTAACATAACAAAATCAAGAATTGGATTT
ACACTTTTACTAAGTCCAAGTGATGAAAGGGTTAATGTTGTTGCAACAATACCTAGGGGATCGTT
AAATACACTTTCTGCTTCAAGCGTTACTGCAATTTTTGGGTCAACATCTGATTTTGAAAATATTG
GTATAAGTGTTGCAGGGTCCGTTGCACAGTTTACGGCACCATAAATAAATCCAATCGGGTTTGTA
AAAGGCAGTGCATATATAAAACTAAATATTATTCCAGAAATTAACAGTGAAATAACCAGCCCAA
TAGTGTCGAGTTTCAAAACGGTTTTTAAAACTCTTTTTAAAACAATCCATCTCATTTCAAAGGCG
CCAACAAACAAAAGAATTATAAGACCAATAGTTCCAACAAAAGAAAAAATTGTTTGAGCATAAA
TTGGGGATATCAATCCTAATACTGGGCCTATTAGTAAACCTGTAATCAATAGTAATGGGATATCA
GGGATACCTATTCTATCCGCAAGTTTAGCTATAAATGATCCGATAATAAATAAAAGGGATATGTA
CCCTATTACAAGGGAAATTTCCATATTAACACCCAGTAAATATAGTATAGTTTGCCTGTAGGTTA
AATCACAGGTATTTGTAAATTTTACTTGTTAAGAGATTAATACAATATACAAATCGTTAAATTTT
TATACATTAAATAATTTTGGATATTATAAATCAGATAGAATAACTATGAATACATATTTATAAT
CTTAGGTAAAATTCGGAAATCACTAGGTGAAATTATGATACTTCTTGTAAGTCCTAAAGACGTTG
CTGAAGCCTACGAAGCAATTGAGGGTGGTGCTGATATTATTGATGTTAAAAACCCGCCTGAAGGT
TCACTAGGTGCAAACTTTCCATGGGTAATTAAAGAAACAAGAGAAGCAACACCAGAAGGAATGCT
TGTAAGTGCTGCAATTGGAGATGTTCCATATAAACCGGGAACCGTTACACTTGCGGCATTAGGGG
CAACAGTAAGTGGTGCTGATTATATCAAAGTTGGACTTTACGGCACAAGGTCGTACCAAGAAGCA
CTTGATGTAATGAAAAATGTTACAAAAGCGGTAAAAGATGCAGGGGAAAACAAAATTGTTGTAG
CTGCAGGATATGCCGATGCTTATCGGGTAGGTGCAGTTGATCCATTAGTTATTCCAAAAGTTGCA
AGGGATGCAGGATGCGACGTTGCAATGTTAGATACTGCAGTAAAAGATGGAAAAACACTCTTTG
ATCACATGGATTTAGATTTACTCAGGGAATTTGTTGAAGAAACACATAAATACGGAATGAAATG
TGCTCTTGCAGGTTCAATTAAAATTGAAGAAATTCCAATGTTAAAAGAAATAGGATGCGACATT
GTAGGTGTTAGGGGTGCTGCATGTACTCAAGGGGATAGAAATGCAGGAAGAATTCAAAAAGATT
TAGTTAAAGAAATAGTAAAAGTTTGCAGGGATTAACTGGCAATAAACATTAAATTACAAAAATA
ATTGAAATAATTTTTATTTTTTAAATTATTTAGCTTAAATTAGTTAAAATAACCAAAAACTATT
AATATATTTTTTATGAGTATATTAAAAAGGATTACTCAAAATTAAGTAATTATAATTTGAGTAT
GCCCTCATGGGGTAGCTAGGATATCCTCGCGGACTGCGGATCCGTGGACTCGGGTTCAAATCCCG
ATGGGGGCGTTTAATTATATTATTTTTAAAATAAAGTTTACATTTTTCAAATGTTTTGCCTGAAG
TTGTGATTTTAGATTTATTTTTTAATTAGTCCAATTTTAAAGGTTTTATTAAATTGATACAACTA
ATACGTAAGATTAATATTATTGAATAGACATATTTTAAACACATAACTGAATATACCCATATAA

TCTGCATATGCTTTCAACGGCGGTGATTAAAATGTCCAATCGGGGACAATTATCTATAGAAATGG
TTATTTTAGTACTGGCAGTTTTAGTCTCTGGTTCTTACGTTGCACTAGATCTTTCAAAAGGAGCT
TTCGAGACTACTGCTGTTAATGATGTTCAGGAAAGTTCTTACTGTGGATTTACACCAGATTTTGG
CGCTACATCAATTGATAAAGCGTTGGTAAAGCTTACTACCGGTATAATCAATATCAACCCAAATG
CTGCGGTAACCAATAATGAATTTAATGCAACATACACTAATACTACAGATAATACCGTAGTAACT
TATTCACTAGACAATGTACACCCAAACCTGATTAAAAATTATGCTGATGGTACAATTGTGAAAGT
TCCATTAAATGAAACTTGGAAAAACTTCACTGCATCAAATATCATTCTTAGAATGAAATCAGACT
ACGTCTGCACAATTAATGGTGAAAATGTAAGCATGGAATCGAACTGTTTTGAAATCACTGCACCT
TTAGGTAGCGACTCTTTCGATTTCCAAATGTCTGCAGGAACTGGTGCTGGACAGTACTACTTACG
ATTAGTGGATCAAGAAGTAACCATTGAATTTTCACTTGAATAACTTAGAATTTTTCTTCTTTTTT
AACATTTTAATAATATAAATTTAAAATTCTATTTTGATAATCTTTAAATTAATGTATTTACATA
AATTAAGATGTAAACGCATGAAAATATTTTAACGATAATTTAAAATTGGGTATTTTGCCGGGGG
GAGGGGATGCCAGATACCGATTATTATTTAATGCCAATTTCAGAGGTATTTAAAAAATTAGATAC
TGAAAAATCAGGACTTTCTAATGTAGAAGCTGAAAATAGACTGAATACTTTTGGAAAAAATGAA
TTAAATGCAGAAATTAGGCTTCCAAAATGGTTAAAATTTTTATTTCAGTTTAAAGACGTTTTTGC
AGCAGTTTTAATTTTTGCATCGGCAGTTTCTTTTTTAATTGGTAATTACCGTGACGGAACAATCA
TGGCATTAATTGTGATAATAAATGCAATAATTGGGTATTATCAAGAAAATAAAGCTGAAAATAT
CATGGATTCTTTAAAAAAACTTATACAATCTCCTTCAAAAGTTTACCGTGATGGGGAGTTAAAAG
AGATTTCACAAGAACTGCTTGTTGTCGGAGACATTGTACACCTTGACGAAGGCGACAAAGTACCT
GCTGATATCAGGCTTATTGAATCTTATAATTTAAGTACAAATGATTTTTCACTTACTGGTGAATC
AATGCCTCAGGAAAAAGATACTGAAGCGCTTGATTCTGAAGTAGGGGTTGCAGATCGTACAAATA
TGGTTTACTTGGGTACAAATATTGCTACAGGAAATGCTAAAGGAGTTGTTGTGGCAACAGGGATG
GCTACTGAACTTGGAAGGATTGCAAACCTCACAAGAGAGGAAAAACTTGCAAAATCACCGTTACA
AGTAGAACTCAGCAATATTGCAAACAAAATTACCATTTTTGCAGTAGTTATTGCATCAATACTTT
TTTTAATAAGTTTAAATCAGGGTTTAGGCTTAAAATTTGGTTTAATTTACGGACTTGGAATTGCA
GTTGCGATAGTTCCACAGGCCCTTCCGATGCAGATAACGGTGGCTCTTGCAAACGGGGTTTCTAA
GCTTGCAAAGAAAAATGCAGTAATAAAAAAACTCTCGGCAGTTGAAACACTCGGTTCGACAAACG
TAATTACAACTGATAAAACCGGAACCTTAACTAAAAATGAAATAACTGTTAGAAGCATCTGGTTT
GATGGAAAAGAGTATGAAATAACGGGGGTCGGATATGAACCAAAGGGTACGATTCTTGATGTAT
CGAAAAATGAGCTGGATGAATCAGGAATTTCAAACATAGAATTAATTTTTAATACTGCTACAAT
GGCTTCAACTGCAAAAATTCACGAGCCTGACGAAAATCACTATGGTTGGTACCCAATTGGAGACC
CAACCGAAGCTGCTCTGATAACGGCTGCTAGAAAATTAGATATAATGTCGCTAGATGAGGATGAA
AAATATCCTAAAATTCAGGAGTTTAGTTTTGATTCTGTTCGAAAAAGAATGAGTTCTATTCGGCT
TTTCAATGATAAAAAGATGTTAATGATGAAAGGAGCACTTGATAGTGTTATTTCAGTTAGTAAA
TATATTTATAAAGATGGAAAACTTGTGGACTTAAAAAAAGAAGATATTGAAATGGTAAACGAGT
TAAATATCCAGTATTCAAAAAAAGCAATGAGGGTTCTTGCATTCGCATATCGAGAATTGGGAAAC
AATGAAGGGGAATATTCGATTGAAAACACTGAAAAAGATATGGTTTTCTTAGGTTTAATGGCAA
TGAGTGATCCCCCAAAAGAGGGTGTGAAGGATGCAATAAAAAAAGCACACGAAGCACACATAAA
AACATATATTATGACTGGAGATCACGCTATAACTGCACAGGCTGTTGGAAAACAGATTTTTTTAG
CAGACGGTGGGAGGGAAGTAAAAGTTATAACCGGAAAAGAACTCGATTCGATGCCTGATGATGA
GTTAAAACAAAACATGGCTGAAAATGATGCACTGATATTTTCGAGAACTTCGCCTGAAAATAAAC
TTAGGATCGTAAAAACACTAAAAGAACAGGGTCAAATTGTTGCAGTAACTGGGGATGGGGTAAA
TGATGCTCCAGCTCTTAAAAGTTCACATATCGGGGTTGCAATGGGGAAAATTGGAACTGACGTTT

CAAAAGAAGCATCAGAACTTATTTTACTCGACGACAGTTTCACAACGTTAGTTTATGCAATTCGG
GAAGGGCGTACAATCTATAATAATCTTACAAAAACGATTATTGCATCTCTTACAAGTAACGGTGG
AGAATTGACAATAGTTTTAATCGGGTTACTTGCTGTTGCGTATATGGGCTGGCCAATGCCAATAT
TAACGATTCAGATACTTGCAATTGACCTTCTTGCAGAAATTTTGCCATTAACTGCATTAACTTTC
GATCCTGCATCAAGAGATATAATGAATGCACCGCCTCGAAGAAAAGAAGAACACGTTTTAAATAA
ATATGCAATCTCAGAAATCTTGTTTTTTGGATTTTTGATGGGTTTTTTAGCATTTTTAAACTTCG
GACTATTTATTTTTAGAAATGACCTTGAACTGACACAGATTGCCGGAGTATATCCTCTTGCAACT
ACGATAAGCTATGCTACAATTGCATTCTGCCAGTTCATGAATATTCTTTCACGAAGGTACTCTTA
CGAAACCTTATTTAGCAGAACGCTCTTTACAAACATGAATATGATTTATTCAATACTGATTTCCA
TAGCATTTACACTTACCGCGATATATGTTTCGCCAATAAACAAAATGATTGGTTTTGCACCGATG
GGGCTTGTCGACTGGGCTTACGTGTTGCTGGCATCATTTGTATTTTTAGGAGCACACGAACTTTT
GAAAGTATATAAAAGACATAAAAAATCAGGCAAACATGCAACAGTTTTAACTGAAATTTCTGCA
TAAGGTGGTAGTATGAGAAAAATTGGAAGCAGATATTTAAAAAAAGCAGGACTGCCCCCTGGAG
ATTTGGTATATACTGGTGAAATGGAGTTAAAAGAGACTGAAATAATATTTATTAGTTATAATAC
TGATAATTTTTTAAAAAAGCAGGTAAAAAGTCTCGATGAAATAATTATTGATGATTCATGTGTA
AATTGGATCATATTTTCAGGGATTCCAACCGCTGAAGAAATGAGGTTGATTGGTGAAATGTATGA
ACTTCACAAATTAACCCTTGAAGATATATTGAATACCAGTCAAAGAACTAAATTTGAGGTCTACG
AGAAATATAAGTATCTTGTAACAAGGATAATCCACCAGAAGGCTGATACAGTTCATCTTGAATCA
GAACAGCTCAGTATAATTTTAAAAAACAATGTTTTAATCACTTTTTTTGAACACGACATAAGTAT
CATTGAGAGTTTGATTAATCGAATATCAAACAAAAGCGGGTCTATTCGTGAAAAAAAACTGGAT
TATTTATTTTATGCAGTACTTGATTCAATAATTGACCATTATTTTGCAATAATTTCGCCAATCGA
AGACGAAATTGAGAGGTATGATTCGAAATTGTTTTCAGATATTGAAAATGAAGAAATTATCCAT
GTAAAGCAGATCAGACAAATATTGGTAGCTTTAAGAAAAAATATTGGACCATTTAAAGAAATAT
TTCATAAATTCAGCAGGGATGAGTATGAATTTATTGAAAAAAGCAATTTAATATATATTCATGA
CCTTTATGACCACACGGTTCACTTAATCGAAACAATCGATGTTTTAAAGGATAATACGTCAAACA
TTGCAGAAATACACCTTTCTGTGATGAGCAACAATATGAACGAGATTATGAAAGTACTTACGATA
ATTTCCACAATATTTATTCCGCTTTCATTTGTTGCGGGGCTTTATGGGATGAATTTTGAAAACAT
GCCCGAATTAAAGTGGTCTTTTGGATATTTTTATGTTCTTATTGTAATGGGGCTTGTATTAACTT
CAATGGTTATGTTTTTCAGGCGCAAAAAGTGGATTTAATTATATTGATATATCCGAAAATTATAT
TTAATATTAAATAGTAATTAATGATTAGATATTAACTCCCCAACTAAAGGGTGATTTTTGACAA
AAGCTACCTTATTAATACTTATTAGTATGTTGGTAATAACTTCGGCATGCATGGATACCAAAGAG
AATTCTGAATCAGAAACGATAACTGTTGGAATATATCCGTACATGCCGCCATACCAATATTATGA
AAATGGTAACTTGAAAGGTTTTGAAATAGAATTAATTTCGGAAATTGCTAAAAGGATGGATAAA
AAAATTGAATTTGTTGAAACTAACTATAAATATGGAAATTATGTTGCAGCTCGTTCTGGAACTGT
TGATGTTTCAATTTCTGCAATTACGAAAACTGCTTCAAGGGAAAAAGATTCAATATATTCTGAAC
TCTACATGAATTCTTATTCGTCCCTCGTATGCGTTGATCCAATATATTCTCGTTTGGCAGATTTT
GAGGGTAAAAAAATAGGGATTGTTGATGACACCGTTTATGTTGACCATGCAGAATCCTACAAAA
AAAGTGTTGACTTTGAAATCGTAAAATATTATAATTATTATGATTTAAATTCAGCGTTATTGGA
CGGTGAGGTTGATGGGGCCCTTGCTGACTATTTCATATGTAAATATTTTATGAAAAATGACGATG
ATTTAACGATAATTGAAAATACTGATATTCACTACTTTGGAATGGTTTCTTCAATAGGAAATGAA
GATTTAATAAATAATATAGATGAAGTTTTAATGAATATGTATAATGATGGAAGTTACGAAAAAT
TACGGCTAAAATACTTTGAAATTGAATAAACTTAAATAAATTCACTTATTTTAAATTTTTTCAGG
TTTTGCGTTAATTTTGATATAAAAATATCTAAAAAAATCAATTAATAATTAATATATATTGATT

AAATCAAAAATAAGTAATTGTCCCAAATTTAGGGATACTTTCATCAATTGCTCGAATAATTGGAA
GGGTAATTTAAGAAATAGGGGTTTATATATAAATTAACTGGATATTTATCCAGAATTTCTTTTT
TTCAGACTTTTTAAAACATATTATTTCAACGCGATTATGCTTTTTTATTTTAACAGTTTACAATA
TGCATAAATAACAGTCTTTGCATATTCAAACCGGAAAAGATTTGATATCGCTTGAATATATCAAA
TCAGGATAATGTTTTATTTTCGGAAAAATCATCAAAAAATATTTCATCAATTATTCATTTGAGAG
GTTAATTATGAAAAAATTAATGCTTGGAAATGAAGCAGTAGCAAGAGGAGCTTACGAAGCAGGA
GTGCTGGTTGCAACAGCGTATCCTGGAACCCCAAGTACTGAAATAACTGAGTATATTTCAAAATA
TCCTGAAATTAATTCGGAATGGTCAGTAAATGAAAAAGTTGCTCTTGAGGTTGCTATTGGTTCAG
CAATTGCAGGTGCTAGAACTGTAGTTTCAATGAAACACGTAGGATTAAACGTTGCAGCAGACCCG
TTAATGACTGTTGCATATACTGGAATTAATGCAGGACTTTTGATAATTGTTGCAGATGATCCAAG
TATGCACAGTTCTCAAAACGAGCAGGATACTAGATATTATGGGATTTTCTCAAAAATACCTGTGT
TAGAACCTTCCGACAGTCAGGAATGTAAAGATTTTGTAAAATTGGGTCTTGAGATCAGTGAAAA
ATTTGATACGCCAGTAATATTGAGGCTCAGTACAAGAGTATCCCACTCTCAGAGTCTGGTTGAAC
TGGAAGAAAAGGAAAAAATTGAATTAAAACCCTATGACTACAACCCTTCAAAATATGTATCCGCA
CCTGCGAATGCTAAAAAGAGGAGAATTTTCATTGAAGAAAGATTAAAAGAGCTTTCAAATTTCG
CGGATTCATCTGAAGTAAATAAAATTGAATACGCAAATAAAAAAATTGGAATTATTACAAGTGG
AGTTGCGTATAATTATGCAAAAGAAGCAGTTTCTGATGCATCTTTTTTGAAATTTGGAATGAGCT
ATCCACTTCCTGAAGAAAAAATTAAAGAATTCATTCAAAATTGCGATAAAGTTTACGTATTTGAA
GAATTGGAACCTATTTTTGAACAAAAAATTAGATCGATGGGATTAAACGTAATTGGAAAAGAAA
TTTTCCCCGTAGTTGGCGAATTGAGCAGTGAAATAATTAGAAATGCACTTTTAAACGAATCAAAA
ATCGTTATCGAAAAAATTGAAGACTTACCTCAAAGACCACCCGTTTTGTGTCCCGGATGCCACCA
CAGGGGTCCATTTTACGTTCTTAAAAAATTAAAACTCCACGTTGCAGGGGATATTGGTTGTTACA
CCCTTGGAGGGTTTGAACCATTAAATGCAATTGATACTACAATCTGTATGGGTGCAAGTATTGGA
ATGGCACACGGATTTGAAATGGCAAGGGGAAAAGAGTTTGCTAAAAAGTCCGTTGCAGTTATCGG
GGATTCTACGTTTTGGCATTCCGGTGTTACTGGATTAATCGATATTGTGTACAATAAAGGACACA
GTACAGTTATAATTTTAGATAATTCAATTACTGCAATGACGGGACATCAGGAAAACCCTTCAACC
GGAAAAACACTCTCTGGAGAAAGTACCAATCAGATTGATTTTGAAGCACTTGGAAGATCAATTGG
AATAAACAGAATTTGCGTAGTTGACGCGTACGATTTAAAAGCACTCGAAGAAACGATAAAAGAA
GAAGTAAATGCTGAAGAACCTTCTTTAATTATCACAAAAAGACCTTGTGTATTGATAAAAGGCGC
TAAATTTGATTTTAAAGATTATAAAATAGATCCCAAACTCTGCACTGGTTGTAAACTATGTCTAA
AAGCAGGATGTCCTGCAATTTCATTTGACGGAAAAGTTGCAAAAATAAACGATTCTTTGTGTGTT
GGATGCGGACTTTGCAAGGACTTGTGTAAATTTTCAGCAATAGTTGAGGTGGTAAAAAAATGAA
TATCGTCATCGCAGCAGTTGGTGGACAGGGTGCAGTTTTAGCTTCTAAAATTTTAGGAACACTTG
CACAAAATCTCGGAAAAGACGTTAAAGTTTCAGAAGTTCATGGAATGTCTCAAAGAGGCGGTAGC
GTCGTTGCATATGTTAAATTTGGTGAAAAAGTTTATTCTCCAGTAGTTGAAAAAGGAACTGCAGA
TATTGTTTTGGCTTTTGAAATGCTCGAAGGCGCAAGATACGTGGATTACTTAAAAGAAAATGGA
AAATTGGTAGTAAATACGCAAAAAATAGATCCGATGCCAGTAATAACCGGGGATGTGAAGTATC
CTTCTGATTTAAATGAAAAATTCGAAAAACTGAACATTGGATATGTTCCAGTAGATGCACTATCA
ATTGCGAAAAATGCAGGAACATTAAAGGCAGTCAACGTTGCTTTAATCGGTGTTCTCGCAAAAAA
CAGCAACATTAAAAAAGAAGAATGGATAAAAGCTATAAAAGACACAGTTCCTGAAAAGTTTCTG
GAATTGAACTTGAAAGCTTTTGAGATGGGTTACGATTTAAACTAGAAGGGATATCATGATTTGG
AATGAAACTATTGAATGCATGGATCGAGAAGAGATGAGGGAACTTCAAAGTAAAAGGCTTTCTG
ATACAGTTAGTCGTGTTTACCACAACGTTCCATTTTACCGGAAAAAAATGCAAAGTATGGGAGTT

TATCCAGAAGATATTGAAAGTATTGACGACATTTCAAAACTCCCTTTTACAACAAAACAGGATTT
AAGAGACAATTATCCTTTTGGAATATTTTCAGTTCCTATGGGCGAAATTGTAAGGGTTCACGCAT
CTTCCGGAACGACTGGAAAGCCAACAGTTGTAGGATACACGAGAAGGGATATTGCAATGTGGTCT
GAAATAGTTGCAAGAGCACTAAGTTGTGCAGGAGCTTCAAGAGATGATTTCATCCAGATTGCTTA
TGGATACGGTCTTTTTACAGGTGGTCTTGGACTGCATTATGGTGGTGAAAAATTAGGTGCAACAG
TAATTCCAATTTCGGGTGGAAATACTCAAAAACAGATTCAGTTAATGGCTGATTTTAAAACAACT
GTTCTTGCGTGCACTCCATCATATGCTCTTTATTTAGCAGAAACACTCGAAGACGCTGGAATGAC
TGCTGATGACATTAATTTAAGAGTTGGAATTTTTGGTGCAGAACCGTGGACTCCAAGCATGAGAA
AAGAAATTGAAAATAAGCTTGGAATAAAAGCAATGGATATATATGGATTAAGTGAAGTAATGGG
CCCAGGTGTAGCGTGCGATTGTGAATATCAGGAAGGATTACACATAAATGAGGACCATTTCGTTC
CAGAAATCATTGATCCAGTTACATTAAAACCGCTTCCTGCAGGAAGTGAGGGGGAACTTGTATTT
ACAACAATTACTAAAGAAGGAATGCCTCTTTTAAGGTACAGGACAAGAGATTTAACCTCATTAAA
TTACGAAAAATGCAAATGTGGAAGAACTCTTGTTAGGATGAACAAGTGCACTGGAAGAAGCGAC
GACATGTTAATTATAAGAGGAGTTAACGTATTCCCATCACAAATAGAAAGTGTTCTTTTAGAAAT
GAGCGAAACAAAGCCTCACTACTTATTAATAGTAGATAGGATAAATAATTTAGATGTTCTTGAA
GTTTGGGTTGAAATTGAAGACCAGTTCTTCTCAGACAAAATCAGTATGTTTGAACAGTTAAGTAA
AAAAATCAAGCATAAAATCGAAAGTACTCTTGGAATAAGTGTTAAAATTAAACTTGTAGAGCCA
AAAACTATTGAAAGAAGCGAAGGAAAATCCAAAAGGGTAATTGATAAAAGAAAACTATAATGGA
GGGGCCAATATGATTATCAAACAATTATCAGTATTTTTAGAAAATAAATCCGGAAGACTAAATG
AGGTAACTGAAATACTCGAAAAAGCAGGAATAAATATTTCTGCGCTGAGTGTTGCAGATACTTCA
GATTACGGAATATTAAGAATGGTTGTTTCAAATCCTGATCTTGCATTAAAAATTTTAAAAGAAA
ATAGGTTTTCAGTAAGTTTAACGGATGTTTTATGCATCTGTATCCCAAACAAGATCGGATCTCTT
TCAAAAACCATTAATATCCTTTCAAAAGAAAATATCAGCATTGATTATGTATATGCATTTTCAAT
GAATGATAAAGCTTTGGCAGTTTTAAGAACTGAAGATATTCAAAAAGGAATTGATGTATTAAAA
GAGCACGATACCGAAGTTTTAGATGCAGAAGACATATACGGCATCTAACTTTATTTAATTCTATT
TTATGGTATTTTTAAAGTTTATTTCTTTTTCTTCTTTTAAAAAATGGTTGATATTAAACACGTCA
ATTTCAAGATGGGTTTTACCAGTTACAAGTATGTAATTGTCGTGCAAAACGTATTTCAAGCAATT
CCCAAACATGTCTATGATTGATTTTATTAATTTTAGGTGGTTTCGAATTTGGTACGTTGCACTGA
ATTTTAAGATAATCATGTCCTGTTTTTCAAAGATTTTACATTCATCATGATGTTCCATGACTTTT
TTTATGACGTTTAAACAGTGTGTTTTTTCATGTTTTATTCTTTCATTATCGTCACATAGGAAATA
GTATGGATTAGTCAAAAAATAAATTGTGCTGTTTTTTGGACCTTTTACTTTGCCGATTAGTTTTT
TTTGAATCAATGAATTCATAGATTTTGAAATACTGCGTATACTTTCTTCTTTTGAAATGCCGGAT
AATTCTTTAAGTTGTTCTGTAGAAAGTATTCCGTCGTTATTGTAAAATTCGTAAAGTATGTCGTC
TTGGGATACCATGTTTTTCAGTTCCTAATTTAATTTATAGAGTATGGATTTCTTTCATTAAATAC
TTTTTCAAGGTTTTTAGATAAAACAGGACTTAAAAATATTAAATCGATCTTTGTTCAAAATTTCA
GATTCAATAATGTTTTTTAAATTATAAATCACTTTTTAATGCCGTAATAATTTTTTTTGAAAACT
GCCTGAAAAACAGTGTTATCTATGTTTTAAAAAATATTAAACCGAGATATTACTACGTATATGTA
AAACTGGTTTTAATTACGATTAATTCTTTAAATACTATTTCTATTTAAAAAAGAAACTATTTATT
TCCTTTACCGATATATTTAAATGCAATGCTAATCATATATTGTTCTACTTCGTTGGAGGGGATTT
TTGATGAGGGGTAATTTTGAGATACCTGAAGAATTAGAGGATATCGAGCCAGTAATAAAGCCCG
GTGCAGAGGTCATAAATGTTTCTCGCTATGCAAAAAGAAGGGGTTTTAATGAGCCTGTTTACATA
ACCCGAAACGTATATGATGCAATCTATCCTGATAAAGATGATACTGTTACATATTTTAAAAGATT
ATACTCGATATTAAGGAATATGCGGGTAGAAGTTACAGAACAAAGGCAGCCGTTTATAACATTCA

CGCACGAGATGTCGAAAAAGTCTAAAACTGAATTCATGGCTGTTCGATATCAGGAAAATGATGAC
AAAGCTTGGTATGTATTAATTCCAAGATAACTTTTTTAATTTTAATTATTTATCTATCGGTGAAA
TAATGGCTAACGTAGAAAGGATAAGCCTTTCTTTTCCAAAATTTTTACTTTCCGAACTGGATTCC
ATTGTAGAATCTAAAAATTATTCAAGCAGGAGTGAACTCATTCGTGACGCAGTAAGAAAGTATG
TAATTGAAAATAATGCGTTAACTCCGGGAAAAACTGTAAATGGGATGATTACAGTTGTTTACAGC
CCATCGAAAGACGCGATGGAAGAAATGAGCCATATTTATTTTGAGTACGAGACAATAATAAAAT
CAATGAACCAGTCTTATGTAAAAACTGCATGCGAAAAGAACAAAAAACTCGAAATTTTTGTAATC
GAAGGCGAATCTGACATAGTTTCTGAATTCTATCAGAAAATACAAAACATAAGTGGGAAAATCT
ACGATAACATTATTATTTTTTAACAAATACTTTTTTATGTTCAATATTACTAAATTATTTAATAG
TAATAATAATTATATCACAAACCATTTAAAAACGGTCAAAAATTTATTTATATCCTAAATAATAC
AATACTATTCGTTAACTAATTCCAAAACCCTATAAAAATGTATTAAACTAACTTTAAACTACAAA
AATTCCTTTAAAATCAATTAATCGTGATATTAATACAATTGATTTTAGGGTGAAGTTTAAAATT
TAAAAAGGTGAGTTTTATGGAAAGACCAACAAAAATGTATTGTAATCAATGCCAGGAAACAGTT
AAAGGAACAGGCTGTGTTAAAGTTGGAGTTTGTGGAAAAAAGGACAATGTAGCCAATCTTCAAG
ACCTTTTAATTTATACTTTAAAAGGTATTTCATACGTATCTGAAAAAACAGGGGAAAATAATGG
AGCTATCGATAGATATATTGTAGATTCGCTCTTTGCATCAGTTACAAATACCAACTTTGATGACA
AATACTTGATTGAAAATGTAAAAGAAGGTTTGAAGTTAAGGGATAGTTTAAAATCAAAATGTGG
CGGAAATTGCGGGCAATTGCCTGATTTTGCAACGTGGAATGCAAAAACAGATTTAGAACTTTTAG
AAAAATCAAATTCAAAAGAAGTTTCTTTGCAGGCAACTGAAAATGAGGATATACGGGCTTTAAG
AGTTACTACACTTTACGGATTAAAAGGGATTGCTGCATACATTCACCATGCAATGGTTCTTGGTG
AAAACAGTGAAGAAATTCACAAATTTATCAAAAAAGCGCTGATTGATATTTCTGATGATTCAAA
ATCCGCCGATGAATTAACTGCGCTGGTTCTTGAAACTGGAAAATATGCCGTAGATACAATGGCAC
TTTTGGATAAAGCAAATACTACAAAATATGGAAATCCTGAAATAACTGAAGTTGATATCGGAGT
TAGGGAAAATCCGGGTATTTTAATAAGCGGTCACGATTTAAAAGACATGGAAGACCTTTTAGAAC
AGACAAAAGGAACTGGAATTGATGTATATACCCACAGTGAAATGCTTCCAGCACACTACTACCCT
GAATTTAAAAAATACGAACATTTTGCAGGAAATTATGGGGGTTCATGGTATAAACAAAAAACGG
AGTTTGAATCATTTAATGGACCAATTCTAATGACTACAAACTGTATTGTACCGCCTGCAGACAGT
TATGTCGATAGAATTTATACAACTGGCGTTGTTGGATACCCTGGAATTAAATATATTCCAACAGT
TAACGGCAAAAAAGACTTTTCAGAAGTAATTGAACAGGCTAAAAAATGTCCATCACCCGTAGAAC
TTGAAACTGGAAAAATAGTCGGTGGTTTTGCACACAATCAGGTACTCGCGCTTGCTGACAAGGTG
GTTGATGCAGTAAAATCTGGCGCAATAAAAAAATTCTTTGTAATGGCAGGATGCGATGGAAGAA
ATAGCGATAGGGAATATTACACGGAATTTGCAAAAAATCTTCCAAAAGACACGGTGATTTTAACC
GCAGGCTGTGCAAAATACCGGTACAATAAATTAAATCTTGGAGATATCGGGGGAATTCCAAGAGT
TCTCGATGCAGGTCAGTGCAATGACTGTTATTCACTAGCAGTAATTGCTTTAAAATTAAAAGAAG
TGTTTGAACTCGACGACATAAACAAGCTTCCAATTGCATTCAATATTGCGTGGTACGAGCAAAAA
GCAGTAGCTGTTTTACTTGCACTCCTTTATCTTGGAATTAAAAATATCCATGTAGGCCCAACACT
TCCGGAATTCCTTTCGCCAAACGTTGCAAAAGTATTAATTGATAAATTTGAACTTGGCGGAATAA
GCAGCGTGGAAGATGATATGAAGATGTTTTTAGGAGAATAATCTCCTAAACTCCATATTAAATTT
TAATTTTTAAAAATTACTTAAAAAAATTTAAATTAATTTTAGAAATTTAAAAAAATAGTATTTA
CATGTTGGTGATTTCAATGGCAAAATTCAAAATTGAAATTGACAGTGAAACGTGTCTTGGATATA
AAGAGTGCGGGCTCTGTGTTTCAGCCTGCGAAGAAAGTATAATAGTTCCTGACGAAAAAACGGGA
AAAGTAAAGGTTGATAAAACCCGAGAAATTAATTGTGATGGTCTTGGAGCTTGCCTTGACGTTTG
TCCTGTTGGTGCATTAAAAATTACTTCTGAATCTGGTTTTAGTTGTGGTGGCCACAGTGGAAACC

CGTGTCCTGGTTCAGCTGTAAAAAGCTTCAATCGATCTGAACAAAGATCAGAAATGCTTCAAAAT
AAAGAAACAGAATCAGAACTTTTAAACTGGCCAGTTCAACTACACCTTCTAAATCCAAATGCATC
ATACTTTAAAAATGCAGATTTGATAATTGCGGCAGACTGCGTTCCTTTTGCATATCCTGATTTTC
ACAATAATCTCTTGAAAGGAAAAGTTCTTGCAATAGCATGTCCAAAACTCGACGATACAAAAGAT
TACATGCTAAAAATAAGGCAAATTGTTGAAAAAAACAATATTAAAAATGTAACAGTTGCGATAA
TGACTGTTCCATGTTGCAGTGGACTTTATTCATTGGTAAACCGTGCATTGGATGGTTTATATGTA
AATATCACTAAAAAAGTTATTTCACTCGATGGTAAATTAATGGATTAATTTAATATCAAAAACT
AAAATCTGATTTAGGGGGTTTAAAATGCAGATATCAAAAATACGGCCTTTTATTCAGGTATTATT
TTTTATAATCTTTTTGTGGACTTTTATAAGCGGTCAAACTCAGTTCTGGATGGTTTTTGTATTTT
TATCGATAATATTTGCAGGAATATTTGGAAGGTATTACTGCGGATTCATATGCCCTATAAATACG
ATTATTCATCCTATACAAATTTTAAAAGACAAAGCTGGCTTTTCTAGTAAAAATATTCCAAATTT
CCTAAAATCTGAAAAACCAAGACTTTTACTGATGTTATTATTTATAGTGGGGCTGGGGATCACGG
TAAATGCAATGATAAATGGCTCAAGATTTCCACTACCCATTATTATAATTTCAATTGGTGTTTTG
ACGACCCTATTGTTCAATGAAAACTCATGGCACCGTTATTTGTGCCCTTGGGGATTTTTATTAAG
TATTACCGGGCATTTTTCAAAGTATGGTTTAAATGTAAATGAAAAATGTATTTCATGTAAGATA
TGTGAAAATGTATGTCCTGCCGAAGCTGTTAAAGTTGAAAATAAGCAAAATGCAGAGATATTCA
AAAAACACTGTCTATTGTGTTTAAAATGTGAATTAAAATGCCCAACAAATGCAATAACTTACAAA
AAGTTATGATTTTTTATGGTGATAAAATGATTGAAAAAGTTCAAAATTTCAATAAATCCGATAA
AAAATTGGTTGAAATGCCTGTAAATACCGATGATATCCAGATAAGACACGTAATGCTTCCAAAAG
GAGAATTTATGCCAAAACACATCTCAAATTCAAATGTAAATTTGGTAATCGTTGATGGGGAAAT
GGAAATTACTCTTGAAGATCAAAATCCTGCAAATTACGAAAAAGGAAGTATCTTGAGCATTCCAT
TCAATACAAAAATGTTAATCAAAAATGAAAATTCGAAAATGCTTGAATTTTTTGTTTTAAAAGC
ACCTCATCCAAAAAAATTAGGGGCTCCTGAAGAAGCAATTAAATGTTAATTTTTACTTTTTTACA
CCTTTTCAAAAGGTATTAATATTTAGATGAAAATACCTGTATTGACGATTAATCCCACAACAAAT
GTTAATCCAAGTTTCCCATTTTAACCACATGATTATTTTTATTTAGGGGGATATTTTGGATAAAT
ATGATCATGAATACCGATATTACATGCATTTAATTAAAAATTACGATAGTTTCGAAGAGTGTGCC
AAAAATAACGTTGAAATTGTTTCGAAAATTCCCCAAATCCTTGAGGTAATTGTTCAGGAAATTTC
AATTGCAGAAAAAATGTTGATATTGTATCACAAGAAACACTGTAGATTTGAAATACAGAAATCC
CATAAATATGCTGCGGGTTATTTTAATTATTTACGGGAAAACATTTTGTATGGCATATACTGTGA
AAAATGTTTGGATATGAATATTTTGGACCTGAAAAATTGTTATTATTATGAATTAAATGTTGAA
AAAGCTCCTAACCATAGGCATAAATTATTTGGGGAATACATTTACAATGAAATCAACGTCTATTT
TGAAGTATTGAATAATTTAAAAAATGCAGTGGATTAAAAATAAGTTACATTTTTTGCTTTTGTA
GGAAATAAACTCCAATGGATATTGCAAATATCGATATTGCCATATAGATCATGTCAAGTGACGTT
TCAAAGTGCATTGATAGAACTCTCTGAAAGAAACTGACGATTAAAACCATTATTATAACTTTAAT
GATTTTGCTTTTTAATTCATCGAGTGTATAAATTTCTAATATATTGGAAACGTCTCCATCTACCC
GTGCAATGTCTATTTTTGAGATAAAAAGCTCGTATATCCCGAAACTGAATATTAAAAGCACGACT
CCAATTAAATAAAGGTCAACTGCTCCAATAACTCCAATTAATATCTGGTTGTGCTGTTCGCTACT
CATTGGATCTGAAAAATAGGCAATAATTGTATGGAATATTTCAACAGATCCTGCTAAAAATAAA
GTAATTGATCCAAGGGTTCCAAAAATTACTGCCAGAGTTACGATAAATCGGGAATTCCATAGGGC
AAGTTCAAAAAAATGTTCAAAAAATCCCTGTTCACTGATTTTTTTAATTCCATATTTTTTATTAA
ATTTTTCTGTTTTTCCCATAACTAACCTCGTATAACAAAATATAATTTAAAATAAAAAAGAAATA
TCTAAAAATTTAAGCGCATCACCAACTAATCATGTTAATTATCTAAAATCCCCTATTTATTAATT
TTTTCTTTTATTGGATAATATGTAAAAAGCGCAAATATTGGCAGGATATATAATCCCCTGAGTTT

AATGGTTTTTTAAATGCATTTTTTCCATAACTTTGGATTAAAACTAAAATATCACGTATTTCGTA
GTTTTTAAATTAAGTTGTGCACCAAACCATATAATTATATATTACTTTTTTCAAATCATATTATT
GTATGTTAATTATTGGATCGAATTATTAAAATCAGAAGGGGTTAATATGGGATTAGATATCAAT
TTAAAGAGTTATTTTAAAAAAGATATATCGTATGTTCTTTTCAATGTTTTATTGGTAATTTTCGG
CATTTCTGTAATCTTAAGGTTATTTTATGGCCCTTTTTGTGGGATAATTCCCTACTGGATAGAAT
TGGTTCCAAAATTAGAGGTATTTCTTGGAGGAATATCCGTACTATTGATTTTAGGAGTATTAATC
ACAGAGTATATTTTAAAATAATATTTCTGATCTGATACTTTAAAATAAAAAATAGATTATTTAA
TTTTTTTCTTCAGTTTTGCAATTGTATCTCTTATTTTTATTGCTTTTTCAAATTCGAGCGCTTCTG
CAGCTTCATACATTTCAGCTTCAAGTTCTACAATAACATTTGGAATATCTGCTACAGGGATGTGT
TTTACATCGGAAATATCAACTACTTTTTCTCTTATCGGTTTTACAATAGTTTGAGGCGTGATATT
GTGCTTTTCATTGTGTTCTTTTTGAAGTTTTCTTCTTCTTTCAGTTTCAGAAACTGCTTTTTTAA
TTGAATCGGTCATTTTGTCAGCATAAAGCACAACTTTTGAGTTTGCATTTCTTGCAGCTCGACCA
ATGGTTTGAATCAGGCTTTTATCGTTTCTCAAAAACCCTTCTTTGTCTGCATCGAGTATTCCAAC
GAATCCAACTTCCGGAATGTCTAAACCTTCTCTCAATAAATTTATTCCAACGAGGCAGTCGAATT
TTCCAAGCCTTAAATTTCGAATAATTTCAGTTCTTTCAATGGTATCTATGTCAGAATGTAGATAT
CTTGCTTTAACATTTCTTTTTGCCAAATATTCTGTTAATTCTTCTGCAAGACGCTTTGTAAGTGT
CGTAATCAATACCCTTTCTCCTTTTTCGACCATTTTTTCAGTTTCTTTTATGATATCTTCAACCTG
ATTTTCAATCGGTCTTATTTCAACTTCTGGATCAAGCAATCCTGTTGGCCGAATAATCTGTTCTA
CAACTTGATTTGAGTTATCTAACTCGTATTCGCCCGGTGTTGCTGACACAAAAATTACATTATTC
ATGTATTTTTTAAATTCTTCAAATTTAAGTGGGCGGTTGTCATATGCACTTGGAAGCCTGAAACC
GTAATCAATCAATGACTGTTTTCTTGATCTATCGCCTTTATACATTCCGCGAATTTGTGGGATGG
TTTGGTGGCTTTCATCAATTACAATCAAAAAATCTTCTGGGAAATAGTCTAAAAGGCAGTATGCA
GGTTCTCCTTCTTTTCTATTTTCAAAGTGCCTCGAATAATTTTCAATCCCCTTACAGCTTCCAGTT
TCTTCTATCATTTCAATATCGTATAGGGTTTTTTGTTTTAACCGATGAGATTTTAACATGTCTAA
ATTTGGGAGCCACTCATCTAATTCTTTTAAAATGGAGTTAATTGCATTTTTCTTATCTTCTTCAG
GAATTACAAAGTGTTTTGCAGGGTACATGTAAAATGAATCTATTTCATGTTTTTTGCTCATGTTT
TTTGGATCCAATTCATAAATTCTATCTATTTCATCCCCAAACATTTCAACTCTTAAAATATCGTC
CTGATATCCGGGAATAATATCTAATGTATCGCCTTTTACCCTGAATCTTCCTGGAACTAATTCCA
TATCGTTTCTTTCGTATTGAATATCCACTAATTTTTCAATAATGTCAGATCTTTTGATTTTTTCT
CCGACTTTAAGTTCAAAACCCATTTCTTTAAAAAGTTCAGGATTTCCAAGACCGTATATGCAGGA
AACTGACGCAACAATTATCACGTCTCTTCTCGAAAGAATTGCAGAAGTTGCACGAAGACGCATCT
GCTCAATTTTCGGGTTGATTTGTGCATCCTTTTCAATATATTGATCTTTTTGTGGAATATATGAT
TCTGGCTGGTAGTAATCATAGTACGAAACAAAATATTCTACTCGATTTTCAGGAAAGAATTCTTT
AAACTCATTATAAAGTTGTGCCGCAAGTGTTTTATTGTGCGCAATTACCAGTGTTGGTTTTTGAA
CTTTTTCAATAACATTTGCAATAGTAAAAGTTTTTCCAGAACCTGTAACACCAAGCAGTGTCTGC
TTACTTTTTTCTGGATTTTTTTCAAGGTCTTTAACCAGCCCTGCAATTGCTTCTGGCTGGGACCCT
TTTGGTTTAAACTCTGCTTTTAACTTGAAATCGGGAGAACTCATTTTATCAATTCCTTTTTCCTA
AGTAATCGATTATAATTTATTGCAAATCTATGTGCTTCATCACGAATTTCTTGAATAAATAGGGA
TGCTTTTTCATTTTTCTTTATTGGAAGAGGATTTTCAATTCCTGGAGTGTAAATTTCTTCTTCAC
GTTTTGCGATTGAAATTAGCGGAATTTTAAGTTTTAATTTTCGAAGTTCAGAAAATGCTGACGAA
AGCTGGCCTTTTCCACCATCAATAATTATTAAATCAGGCAATTCCAAATTTTCTTCGAGTAATTT
GCTGTATCGCCTAAATACCACTTCAGAAATGGATTTGAAGTCATCTATTCCTGAAACTGTTTTTA
TTTTAAATCTGCGATAATTTTTTTTGTCAGGTTTTCCATTTCTAAACTGGACCATTGATCCAACA

GTAGATGTTCCTGAAAGGTGTGATATATCAAAGCATTCTATTATGTTTGGACTTTTATCAAGCAT
TAGTTTGTTTTTTAATCTTTGAACTTTTATTTCATTTCCATTAACATAAATTTCAAGATTTTTTA
TAGCAAGGTCTAAAAGCTGTTTTTTCTCGCCTTGTTTAGGGATTTTAAAAGCAACTTTTGAACCT
TTCTTTTTTGACAGATATTCTAAAATTGCATCAGAACTGTATTCATTATTATCTTCTTCAAGATT
TTTGGGCAGCTCCGAAATTATGATCTCTGAAGGAACTTCGTTTTCTGAATAGTATTGAATTAAAA
ACTCTTCAAAGAAATTTTCTGTGTAATCAAATTCAAAGTATTTTCTATCAAAAAGCGTTCCTTTT
AGTACATTAAAAACCATTATATGGAGTTTTCCGCTTTTTTCAAGGATGCTGATTACATCTTCATT
ATATTTTACATCTCTTTTAACGTTTTGACGAGTCTTAAGTCTTTCAATTGCATTTATTTCTTCCCT
AATTAGTAACGCTTCTTCAAACTGCAGATTTTTTGATTTATTGTCCATTTCAATTTTAAGTTCAT
CAATCAATGAATCAATATGTCCTTTTAAAACATGTTCTGCCTTTTTTATTTGATTCAAATAATCT
TTGGAGGTTATATCCCCACTACATGGCGCGGTGCAGTTTTTTATATGGTGCCTTAAACAGGGACG
TTTGTGCATGTTTTTGCATGACCTGATTTTAAATGTCTTTTTTAAAACATCCAAAACATAATCTC
TTTCCTTTGCAGAAGTAAATGGCCCATAAAATTTTCCTTTTTTTGATTTATTTCTCGATATTCCA
ATTCTTGGGAAATTTTCTTCGCTAATGTATATATATGCGTAATTTTTCGAGTCTTTTAAGTCGAT
ATTGTATTTAGGACTGTATTTTTTGATTAACGTATTTTCTAAAATCAAAGCTTCGACTTCGTTAT
CTGTTACGATAAATTCCATCTCGTAAATTGATTTTACAAGTTTTTCGGTTTTAGGATCGTGATTT
TTTTTGTTAAAGTAGCTACTAACTCTTTTTTTCAAGTTTTTTGCTTTCCCAACATAAATTACAGT
TCCGGATTCGTTTTTATAAATATAACAGCCCGGATTTTTTGGAATATCTGAAATATTTATCATAT
TATCTTTAATTTTTGGAAAGAATGTTTTTTAAGAATTTTCCAGTATGGCTTAATTTACATTTTGC
AATTTCTTCAGGAGTTCCTGTTGCAATTATTTCGCCACCAAATTCCCCTCCTTCAGGTCCAAGGTC
GATTATATGGTCGGCACATTTTATTACATCGAGGTTATGTTCGATTACAACTACAGTATTTCCTT
TTTCAACGAGGCTGTTTAAAACATCGATTAATTTTTTAACATCGTGGAAGTGAAGTCCGGTAGTC
GGTTCATCGAGTAAATATATGGTGTTTCCAGTAGCTCTTTTTGATAATTCTCTTGTTAATTTAAT
TCTCTGGGCTTCTCCACCTGAAAGTGTTGTCGAACTCTGTCCTAGTTTAATGTATCCAAGTCCAAC
ATCGCACAAAGTTTTTAATTTATTTGAAATCTGCGGAATATTTTTGAAATGTTCTCTTGCTTCCT
CAACACTCATGTTTAAAACATCAGAAATTGATTTTCCTTTATATTTTACTTCAAGAGTTTCGTGG
TTGTATCTAGCACCCTTACACTCCTCACATTCCACAAAAACGTCTGGCAAAAAGTTCATTTCTATT
TTTATAACTCCATCGCCCTGACAGTTTTCACAGCGACCTCCTTTCACGTTAAACGAGAATCTTCCC
GGCCCATATCCTTTTACTTTTGCTTCTTTGGTTTCTGCAAAAACCTGTCTGATTTTATCAAATACT
TTCGTGTAAGTTGCAGGATTTGATCTAGGGGTTCTTCCAATTGGGCTCTGGTCGATTACAACAAC
TTTATCAATTTCTGAGTTAATTTCAAGATTGCATTTTTCTTTAGTATTTTCGTAGAGCTGATCCT
CAAAATCAAGTTCCCCAACACTCTCATCAGATTTTATTTTTTCTTTTAATGCAGGATACAAGTTT
TCATAAATTAAAGTGGATTTTCCACTTCCTGAAACCCCTGTAATCACGTTAAATACGCCTGTCGG
AATTTCAACAGACACATTTTTCAAATTGTTCTGCTTGCAGTTTGAAAGTTTTAAGAATTTATCGC
TTTTTCTTCTATTTTTTGGTATTTCAATTTTTAATTCTCCTGAGAGGTATTTTCCAGTTAATGAA
TCCTTATTTTTTGAAATTTCCATTGGCGTTCCAACTGCAACAACATCTCCGCCATGAACCCCAGCA
CCCGGGCCCATATCTACGACATAATCCGCATTTAAAATTGTATCTTCGTCATGTTCAACAACAAC
GAGGGTGTTATCTAAATTTCTTAATTTATGGAGCGTTTCGATTAATTTTTGGTTATCTCTCTGGT
GAAGTCCAATCGATGGTTCATCAAGTACGTACAAAACACCAGTTAAGTTTGATCCAATCTGTGTT
GCAAGCCGTATCCTCTGGGCTTCTCCACCTGAAAGTGTTCCAGAACGCCTTGAAAGTGTTAAATA
TCCCAAACCAACGTCATTTAAGAATTTTAATCTTGATTTTATTTCTTTTATGACCTGTTTTGCAA
TTTCATATTCTTTATCTGTTAATTTTAAATTATTGAAGAATTCTTCCGCTTTTGAAATTGAAAGA
TCCGTCAAATCGATAATCGATTTATCTTCTATTTTTACAGCAAGTGCTTTATCTTTTAAACGTTT

TCCGCCACATTTTGGGCACAAACTAACTTTCATGAACTTTTCAAGTTCTTTTCTTCGGTATTCAG
ATTTAGTTTCATTGTAAAGTCTAATTGATTGAGGGATAAGCCCTTCCCATGGTTTATTCTGGCTC
CATTCGGTATCCCCACTGCCGTTTGATACTTTAAAATGAATTTTTTCAGGACTTCCATACATCAA
AATGTCAAGCTGTTCTTTTGAAAGGTCTTTTATCGGTGTTAAAACCGTAAATCCAAAGTGGTTTG
CAACTGCCGCCAGATGTTGTGACCTGTATCCATCAAGGAAGTTTCTATAAAGCGCAACTGCACCG
TCAGCAATGCATTTATTTTTATCAGGAATTATTAAGTCCGCATCAAACTCCATTTTTATTCCAAG
ACCGCTACAGTATTCACATGCACCAAATGGGCTGTTAAATGAAAACATTCTTGGCTGAAGCTCTT
CAAACGAAATACCGCAAATTGGGCAGGCTAAATTTGAAGAATAAATTTTTTCAACTTCTTCATTA
TTTTTATTGCCTGTTACAATCAAAAGTCCGCCGGAACGTTCAAGTGCTCTTTCACAAGCTTCAAC
AAGCCTTGAATGGTCGTCTTTTGGAGTTAATCTATCGATAACAATTTCAATATCATGTTTTTTAT
ATCTTTCAAGTGTTATTTCGTCATCAGTTCTGTAAATTTCGCCATTTACACGAATTCTTGCAAAA
CCTTCAGAATTTAGGTCCTTAAAGAGTTTTTGATAGGTTCCTTTTTTATGCCTTACAATTGGGGA
TAATATAGTCACAGTTTCAGAAAATTCTTCTTCGATTTTCTCAGCTATTTTTTCAGGGCTCTGTG
ATTCTATTCTAATGTTGTGTTCAGGACAGTATGGGATTCCAATTCTTGCATAAAGCAGTCTTAAG
TAATCATAAATTTCAGTAACTGTTCCAACGGTACTTCTTGGGTTTTTGCTCGTTGTTTTCTGCTG
TATTGCAATTGCAGGAGATAATCCTTCGATACTATCAACATCAGGTTTATTCATAAGTCCTAAAA
ACTGTCTTGCATATGCTGAAAGTGATTCAACATACCTTCTCTGACCTTCTGCGTAAATTGTATCA
AATGCTATCGTGGACTTTCCAGAACCAGAAACTCCAGTTACAACTATTAAATTATTTCTTGGGAG
TGTTAGCGAGATATTTTTTAAATTATGCTCTCTTGCGCCTTTTATGATGAGATCTTTCATAACTA
TCATTTGGAATAATTTGGATTAAAATTTTTATAAAAATATACGTGACGATGTATAAAATAAAAA
CGGTACGAATATCGGATAATTTGGGGATATATATTACAGAATTGATCCGACTTTGAATTATTTAT
TGATTAAAATTAGTCCCAATACGCAAAATAAAATTCCTAAAACTTTTAAAAAACTGATATTTTCT
TTAAATAATAGTATTCCCACAGGTATTAATAAAACTGCAACTGCAACATATGCAGTAAGTGCTGC
AACGCTTACATTCCACCCTGCACGGTAAACAAGTAAAAATCCAAGTTCTAAAAACACAATGGAGA
TTCCAAGTACGTAAGTCGCCCAGTTTAGATTTTTGACAGATTCAATTATATCGATTTTTCCATTC
AATATAAAAATTGCAACAACTGTCGAAATTATTGAAACAAAGTATGTAATCATTAATGAAACAT
AGGGGTTTGCACCGCTTGAAATTGATTTTTGACAGATGTGGTAGAGTATACTTGCTAAAACTACG
ATAATCATTGAGATGTAGTACATAAAATTCCTTTATTCGGTGTTAAAGTCAATTTCGCCGTTTTC
AGAAAGAAGTTTAATTTTTCCTTCAGCTTCATTTAAAATTTTATAAAGTTTGTTACTCAATTTTA
TCCCTTCTTCGTAGTTTTTCATAGCTTCTTCAAGTGATAATTCATCATTTTCCATTGAATTTACG
ATATTTTCAAGTTTTTCAATTAATTCTTCATAATTATCCATAAATATCACCATTGTACTTTTTTA
TTCTTTCAAAGTAATATGTGCATCAACAGTTCCATCTTTTAACGTAATTTTTACATCATTTTCTA
AATTTAGCGAAGATTTTGAACTTATAATTTTTTCATTTTCATCCTGTATGATGCTGTACCCTTTA
TTCAATACATTTAACGGATTATATGCATTTAAAAGTGAATACGACTTTTCAAAGCGCTTTAATTC
GTAAGAAATTTTATTTTTAATTTCATGATTCATCTGGGCCCGTATTTTATCAACATGCACTTTTT
GTTTTTCAATTTTAGATTTCGGGCTGTTTTTTTCGATTTTTGAATAAAGAATGTCTAATTCATAA
GACCTGTTTGAAATTTCAGTCTTCATCCTGTTTTTTAACATTCTTTCAAGGTTATTAATCATCGA
TTTCAATTCGTATTCATTATATATGGCAAGTTCTGCGGCATTTGATGGTGTTGCAGCTCTTAAAT
CGCTTACAAAATCGGAAATAGTAAAATCAGTTTCGTGTCCGATTCCTGTAATTACTGGTTTTTTC
GATTCAAAAATTGCTCTTGCAAGGGATTCGTTGTTAAAGCACCAGAGATCCTCCATTGATCCCCC
ACCACGAGCAATAATAATTAAATCAATTTTTTCAAGGTTATTTAGCTCTGAAATTCCAGAAATAA
CTGTTTTTGCAGCAGATTCGCCCTGAACTACTGCAGGATATATAATTAAATTAACGGAACTGTTT
CTATTTCTCGTAACTTTAATAATATCTCTAATGGCGGCCCCTGTTGGAGAAGTGATAATTCCAAT

GTTTTTTGGGTATTTTGGGATTTTTTGTTTGTACTCTTCGTTAAATAATCCTTCACTTTCGAGTT
TTTCTTTCAATTTTTTAAATTTTATAAATAAATCCCCAAGACCCTCTTTTTCCATTTCATTACAA
TAAAACTGGTATTTTCCGGTTTTGGTATACAACGATACTTTTCCTTTTATTATTACGCTCATACC
TTCAGCAGGACTGAAATCTAACTTTTTAGTATATGGTTTAAACATTACACAATCAATTGCGCTGT
TTTCATCCTTTAATGTAAAATAAACGTGCCCTGATTTATGTAAGGTACAGTTTGAAATTTCTCCT
TTAATACAAGCTCTTTTTAATAAAAAAATCTTCTTCTAAAGTGCTTTTTACATAAGAATTCAGTTC
TGAAACAGTTAGTGTGTTTGAAAATGAACTTTCAAGGGTTAATTGAAGCATATTTTGACCATTTT
GAAATTTTAAAATTCGGATTAAGTTTAAGAAATACTGTTTATCATTTATACTATTTAGACAACTC
GCTAATGTTTAAACATTAACTTATTTTTTTAGCCTCTTAGTTTTTCATATTTTATCTTTTTAAAT
TCTTTAGTTATGAAGTAAATTGTAATAATAAAGCTCAAATATTCTGAAAATACCGTTGCCCACCA
TATTCCTTTAATTCCAAGGTATATTGGAAGTAAAATAATTGCAGGGAGTATAAAAATCAAGGTT
CTTCCAAGGGATAAAAATCCTGCAATTTTTGGCTGTTCCGTTGCTAAAAAATACATCATCAATAC
CATGGATACACCCATGATTAAAATTGCGAAATTATGTATTTCAAGGCCGATTGTGGTCGTTGCGA
TTAAATTCGTATCATATGGGTTGAAAATCTTTATCAAAAGATTTGGGAACTTTAAAATTGTTAA
AAATGAACAAATACCGAGTATTGCATTGACAAACACTGCTTTTTTTAATATTTCAAAAACCCTGT
TGAATTTTTTAGCTCCATGGATGTAGCTTATCAAGGGCTGAACACCTTCTGAAATTCCTAAATAG
ACCATGTATAGAAATGATGTGATATATATTACAATACCGTATGCAGAAACGTCTAATGGACTTCC
ATATCTCATAAACTGGATATTGTGTACTATCATCAGTATTCCAAATGCAAGTTCCATGATAAACG
GTGAAAATCCGGTTTTTGCAATTCTTTTAATAGTCGAAAATTCTAAAAATAGACTATTTTTCCCG
ATTTTAACGTTTGATTTACCGCTTAAGAAATGTTTTAAGTATAAAATTGATCCAAGACCTTGTGC
AAGCACAGTGGCAGCGGCAGCCCCTATAACACCAAAATTAAATACAACTATCAGCATATAATCAA
GTATAATATTAAATACAGACATTAAGGCCAGAATTATCATGGATTTCTTTGGAAAGCCGTCGTTT
CTAAGTATCGGGTCAAGAGATATCGCAAAAATTTGTGCAATCGCGCCAAGAATTATTATTTTTGA
ATACCCTACAACATATATTTCCAAATTTTTTGAAAGATTCATGAATGAAATAACTGGGTCGATTA
CCAGATAGCCAAAACAAGTAATTATCAAGCTTATAATTATAACATAAGTTAGTGCAGTTTTTAAG
ATACAGGATGCTTTTTTGTAGTCTTTTTTTCCAAGGCTTATCGATATTATTGCAGAAGATCCAGC
TCCGATCATAACTCCAATGGAGCTAATCGCAATCAAAACGGGAAATGAAAATGTTACCCCTGCAA
GACCTTCAGCTCCGATAGTCCTTCCGATAAATATCCCGTCAATTATTCCATAAATCCCAATAATG
ACAGTCCCTGCAATTGAGGGGATAACGTATCGTATCATGAGATTATTAATTTTTTCGTTTTCTAA
TTTTTTAACCTGCTTCATAACCTGCCTTTTTAACCATTAACCTGAATTTATGTTCATTTAAAACG
TTTTGAAGAGATTATATTTCTTAATAAAACATATATACTTCTTTTTATAAATTTTAATTTATGGA
TATGATATATTGTGGGGGACATATATGATAAGCTATGATGGGAGTACGGGCGAAAATGAGATAA
TATTACGGAATATGGAATATTTAGACAATAAAAACCATATTTACACCTCTGAAATTGTTCCAGAG
GCTTTAAAAAAGACTTTATCTGGAATAGAGTCTATCGGGATGGCTGAAAAATTGCTTCCTGAAAT
ATGTGATGTTCGAGTAAAAGATGACTTAAAAAAAAATTTTAAGTTCAGAAAAAAAAGGGACTGATT
GACCACGTTGTACTCATTCTAAATTATGAATATTTTAATAATGGGATCGATATTTCTAATATAGA
TCTAATTGACAGTTTTTGACCAAATAACGCAACGAAAAGATCCAGTACCGAAAAATATACATATA
TTGTCTTTATACTCTTTTCTGATATGTTATGCACCGAAAAGCGAAACCTTTTTTAAAATGGGCTG
GCGGTAAAAGAAGACTTTTAACCCAGTTTGAAGACCACTATCCGGAAGGATTGAAGAATGGAAA
GATTAAAAAGTACGTTGAGCCATTTTTAGGCGGTGGTGCAGTTTATCTGAGCCTCCAAAGTAAAT
ATAAATTTAAAAAAGTGGTTTTGAATGATATAAATCACGAATTAATGCTTTCTTATAAAACTGT
TCAAAATAACATTGATGAATTAATTTCTATTTTGGAACCTATTGAAGAAAATTTTAACAAAATG
AACTTCGAATCTCAAAAAATGCAATATTACAAAATAAGAAATGAATACAACATTGAAAAATCAA

ACATTGATAAAAATAAATCTGAAAATATCATTGAAAATGTCGCGAGATTTATTTTTTTAAATAA
AACCTGTTTTAATGGCCTTTATAGGCTAAATAAGAAAGGAATGTTCAACGTTCCATTTGGAAGGT
ATCTTCGTCCAAGAATATTTGATGAACCAACCCTTAGGGGGGTAAACAAGGCCCTAAAAGGCGTA
AAATTACTGTGTGATGACTATAAAAACGTTGAAAAGCATATATCTATTGACGAAGAAACTTTTG
TATATATCGATCCACCTTACAGACCCCTTCCAGAAACCGTTAGTTTTACATCTTATTCTAAAGAG
GATTTCCTTGAAAAAGATCAGGTAGACTTGAGCAACTGGTTTAAATATCTGGACAAAAAAGGCGC
GTATTTAATGCTAAGTAATTCTGACCCGACAAATACAAATCCAAAAGACCGATTTTTTGAAGACC
ACTACGGCGGATTTAAAATCGATAAAGTCTGTGCTGGAAGAATAATAAATTCAAGAGTCAACGG
AAGGCGAAAAATAACAGAATTAGTAATTAAAAACTATCCAAAATAATATTTAAGGGCAGTAATG
GGATATATGTTAATCGTGTTCTTCAGAGAGCCATTCTTCCCATTTTATTAGTTTATATCCGTCAA
CCATAACGGCATTGCGGATAATGTCTAATGATTCAATGCAATCTTTGTTTTGAACGATTTTTTCC
CATATATCTCCATCTTTTTCGAAATAAAATTTAATGTATTCTGTTAACATATGCTATCACCTCGA
AAAATAATTAATAAAACATATAAATGAAGTATATTAAAAATATTAACGATTAACAAATATTTTA
TATTACCTCGTTTATATTATTGAAAGAATTTAACGGGAGGAGTAGTATGGCAATAAAAATTAGT
GACTTGTTGAATAAAAAAATATACACTACAGATGCTATATATGTGGGTAAAGTTTACGATGCCAT
GGTTGATACTGATAAAGCTGCAATAAGCGGTATTGTTATTACAGATGTTTCAAGCGGATGTTTAA
GCGAAAATATCGATGATCCTACGAAAAAAATAGTATTGCCATTCAATTTGATATCAGCAATAGGA
AATATTATCTTAATAAAGCCACCAATTACTGGCAGATTTTAATTAATTAGCTAAGTCGAGATTAA
AATGTTAAAAATAGGTCTAGTAGGCTGTGGAGCAATTGCTTCATTAATCACAAAGGCTTTGATGT
CGGATAGACTCAACAAAGCAGAAGTTCTAGCTTTTTACGATGGAAATCTGGAAAAAGCAGAAAA
ACTCGCCATGGAAACAGGAGCTGATTTTTGTAAATCTCTCGATGAACTGGTTTCAAAAGATCTTG
ATTTGATTGTAGAATGTGCCTCAGTAAATGCTGTTGAAGATACAGTTATAAAATCACTTAATAAC
GATAAAGACGTTATTATAATGAGTGTGGGCGCATTTGCAGACAAAGATTTATTTGTAAAGCTTT
ATAAACTTGCAGAAAAACTTGGGAAAAAAATATACGTACCTTCAGGTGCAGTTGCAGGAATAGA
CGCTGTAAAATCAGGATCGCTTGGTAAAATTTCAGAAGTATCCTTGACTACAACAAAACCTGTTC
ACGGTTTAAAAAGTGCACTTGAAGAGCAGGGATTAAACACTGATGAAATAAAAGAGCCGAAAAT
AGTTTTTGAAGGAACGGTTTTTGATGCAATTTCAAAATTTCCGCAAAACATCAATGTTTCGGTTG
TATTATCTCTTGCATCACGGTATCCTGCAAAAGTAAAAATCATTGCAGATCCAAATGCAGTTGTA
AATCGGCATGAAATTTTAGTTAAAGGTTCAATTGGGACCATAAAAACTTGTGTGGAAAATAATC
CCTGCAGAGACAATCCAAAAACATCTGCACTTGCAGCATATTCTGTTATTCGATTAATAAAGGAT
CTTTCTGAACCTGTAAGAATCGGAACTTAAATTATTTTTTTTAAATTTTAAAAAAAGAAATAAA
AAAGAAATTTTGTATTTTATTTATTATTTTCAGTTTATTTTATTTAGTTTAGATTATCTATTCGT
ATTTTGAAACTCCAATGATTCTAACTTTTGCGCTTCCATCAAGCATTATAAATTTGTCGCCCTTT
TCAAAAGCAGCAGGTTTTAAAAGTTTTAATGTTAATTTGTTTCCGTTTTTTTCTTCAATTTTACA
GCTTACAGTTTGAAGTCCAACAATAATCTGGTATCCTTCGCCAACAGCAACTTCTTTATTCATGT
ACGGGTTCCATTTCATTTCAAGTTCAATTTTGTCAGATACTTCCAATTCCCCATTTGAAAGAATC
ATTCCCCTATCTAAATCATCAACACTGATTCCTTTCAATGAAAGGCCCACTCTTGAATTTGTTTCA
GCTTCTTTTACGTCCCTATCGTGCACCTGAACACTTTTTACCATTGCTTTATTGTTTGTAGGATAA
ATTCTTAAATTATCGTGCACAGAAACGCTTCCTTTATCAACTTTTCCTAAAATCACGGTTCCAAC
GCTTTTTACTGAAAAGAAGTGATCAATAGGTATTTTTATAAAATCACTACTTTTTTCAACTTCTG
GAATTGTAATCATTTTTTCTCTAATTTGTATGTAATCTTTTTCCAAAAATTCAAAATTTTTCATC
GAAGTGTTTGAAATAATTCCTTTTAACTGTTCAACATCGACGTATTCGCCAACTACAATAAATCC
TCTTTCGACACCGAATAAATCTAGTGTAAGAAGTGTTTCACCAATTTCAGGTTTTATTTCGTCGA

TAAAAACAAGTGCGTAGTCCATCATATTTATTGTATAAATTAATGGCTGTATTCTTTCAGGGTAT
CTTGTAGGTTCGACAAATACTGCTGAATCGTCGCCATTTTTATGATTGTAAAGTGTAATGTCTGT
TGAAGTTCCTTTTTTTGCAATATTTTTTCCCGCATCTTCAAAATCTCCAAATAATCCAATGGTAA
CATTTTTCATAAATTTCACCCATGTTTCTATTCAATCTTTTCATAATTTTAGGTTTTTAGATATA
TAATAGATACTATTTTTTGAAATAAACACTTTGAAAAAAGATTTTGAAATGATGGTTGAATGCC
ATAATTTGATTTTTGTATATGGGAATTTGGGGCATTTACTTTTTAATATATTATCTATTAAAAAT
TCTAAAATAAATGCGACTTAAGAAAATATATGCCCGTTTTTAAAACATCCTCCTACCTCCTTTAA
TATGCTTTTCACACAAATTTTGCGGTACCGCGCACTCGGAAAAATAGAAAAGTATATATAGCTGA
ATCGCTTAAGTACAAGTGCGAGCAGAGGTGGTTGAGCTTGGCCAAAGGCGCCGGACTTGAAATCC
GGTTCTCCACTGGGGAGCGGGGGTTCAAATCCCTCCCTCTGCGTTTTTAATTTCACGTCCAGATTA
CTGAGCTTTTTTAAGACCGGACAGAAAAGTTTAACTCATTGCTTATTCTGAAGATATTACGAGCG
AAGGACTACCTTTTGTCCCTACACGGGGTACCGGACAGGAGATTTGATACCTTTGTAGGAGCCCC
GATTGGTCGTATTTAGGGTTTTAGGGTACCGGACTTAACTTTTACCGGACTAAGTTAAAATTTAA
AGGCTGTTACACTAATTTTTTCTAAAATAAACAGTAATTATTCTAATTTCATAAATTGTAAAAAT
TTTAAGATTTATAAAATATGGATTTTGATCGATTTTTCAAAATATTAAAAATTAAAAAACAGAT
TGATTACAATATAAAAAAAGTAAAAAACAGTTATGAAATTATAGTTATCTTTGATCTAATTTTT
TTCGAATGTCTGGTAACATCTTTTTTGTTCTCTCTTTTGCATGTGCATAAATCATCGTAGTCTTT
ACGTCACTATGGCCTAAAATTTCCTTTACAATTTCAACGGAAGTACCCTCATCCAATAATGCAAC
TGCCCTACCATGCCTTAAAGAATGGAGAACATACCTCTTTCCAGCCTCGATTTCCCCTTTACTAAT
TAAATTATCAATTGCTCTTCTGAAAACTTCTGAAAGATGGCCAGGTTTTACCTGTTTTCCAAGTT
TACCTTTTTCACTTTGAAAAATAAAATTTTCAGGCCCTTTCAAAATATTTTCTGAACTGTACTCT
CTTATCAAGTTAGTGGTCTTTTTTGAAACTACAACATATCTATCTTCCCCATTTTTAGTATCTGT
TAATCTTATCTGCCCAGTTTTAAAATCAAAATCATTGTATTTTATGTGTATAACTTCTGACCGCC
TACAGCCTGTTTCCCACAAAACCAGCATCATAAGTCCATCTCGATTTTTTGTAGCGCTGTTTCCAT
CAAGTATTTCATCAAGCACCTTATCCATAATTTCGTTATTTACATCATCATAACTTCTTGTTTTT
GATTTTTTTGAAAAAGTCTTTCTCTCGATGCATTCATTATTAAATTCATTAAATGCTGTGAATTT
AAATAGCCTATAAAATACTTTTAATAAATTATAGTATCGAACTAGTGTTTGATGAGTGCATCCTC
TTTTTTTTTCTAAATACACGAAAAAACGTTTAAATTCGTGATAATCCATAGTTTCGGGAATTTTT
CCAATATCATCACAAAACGACAAAAAGATTCTAAGTCGTAATACATCTTCTTTAATAGTACTTTC
TTTAATTTTATCAAATTCTCTTTCTTCGATAAATTTAATAAGATATGGATTATGTTCTTCTAATC
GTTTTGGTGATTCAATTTTAGTTTTTTGGTCAGATAATTGTCCTTTGAGATCAAATTTCCTTCTG
CTCATCCCATCCCTCTGAAATATTTTTGTAATTTATCATAAATTTAGTTTTTTTGTAATTGTCGT
AAACTGTTTAGTCATCAATATTCGGCTTTATTTGCATATTCCACCAAATATAAAAATCAGTGAAA
CCGCTGATTAAATCTTAAAAAAATCATTTTGGATTATGATTCTTTTTTAAGCAATATTGCCCATA
TTCCCCACTTTTCGTATTTTCTTTGTAATTGGGAATCTAAATCCCTGAAATACTTTCCATTTAGT
TCTTTAAGTTTTTTAAGTTCAGTTAATGGAAATTCGTTATTAATAAATCTTATCATTGACAATAT
ACGCTGTTTACCATCTATTGAAGAATAATATACCGAATCTTCTTCATGTAAATATATTACCTCCC
CCAGTACCCGTGGTAAAAGAATGGATTCCACTATTTCAGATGCTTTTATATCATCATAATAATAT
TCTCTTGGAAGACCCCTATCTATTACAAGATATGACTCGTCAATCAGTTGTTTACATTCGGATAT
TGAAAAATAAATATTATATGGAATTATTTTTTCTACTTTTAAATCTTGCAAAAAAATCGCCTCGA
CTTGTTTAATATGTAAATAATATATTTAATTTAAAGAAATTACGATCATACACCTAATTGTTCTC
GTAATCCATGATGCCTACGGAACTATACTTTTTTTCTAGATCTGATTTGATTTCGTTATATTTTT
CTTCCGTTATTTTTAAAGCGACCATACATATCACATTTCCACATGCATAATCCATAAGGGGTACA

CCGTATCCTTGATAATCATATGTAGACCGTATAGAATCTTTTGAAAAGCCCCATTCTGTGATAAA
TTCTTCAAGCCACCATTTATACTGGTAGTAACCATTAATGGCCACTTCATCCTCATGGTAAGGAA
AAGCCCAAAGTTCGTAGCTACGGTCACAGTACCTTATTTTTCGACAGGGTCTTCATTAAAAGAA
TCCAAAAGATTATCCATTTCTTTAGAGTACCTGCTTCCTAGATCCTCCTCATGAGGGTACAATTC
TAAGGGTATTGATTCGCCATGTTCCTCTATCATGCCTAACGTTGACCAGTAATTGTTGCTAAAAA
TTCTTACTACAACCATTTTTTCATTATTTTCTTCAAAAGTTCCATTTAATTCATTTTCCATAATA
TCACCAGTTATTTTTTACAAATTGTGTAAATTTAGGAAATTTAATTTATAAGTTATTTTGAAGGT
TATCCAAATCTGGACATCTCTCAATCACAGGGTTATACTTCATTGTCGTTCGAATGTCGTGATGT
CCAAGCAATTGTTGAATATGTGCGATCGAAAGGCCCCCATCAAAGACTTCAACTGCTCGACCAGG
TCTTAACGAATACAATACTAAATCTCGATTATTTTCGATAATTCCTTTTTCTTTTAATTTTTGCA
CTGTTTTGGTCATTGCTTTGATTAAATAATTTGAACTGATCTGCCGACTTTTTTCACCCCCTGAA
ACTTGAAATATTGGATCATCAGGATTGCTAAAATCATTTATTTTACTTAAATGTTGATTAAATAG
TTTCAGAGTTCCTTTGAACAGACCACCGTTCTAATTTCTCGATTTGTGTTTGAAATATTAAATCT
TCCTTTTTCAAAATCACAATCTTTGTATCTTAATTTAATAATTTCCGAAATTCTTGCTCCCGTAT
CCCAAAGCAGCCTAAATGCAAGTATATTTCTTATTTTGGTACTACTGTCGCTATTTTCAATTCTCG
TTGATTATTTTATTGAACTCTTCGCTGGTAATTGCATCATGGGGATTTGGTCTTGTTTTTCTGAA
AATGGGGGTTTGACATTCTTTTTCAAATTGTTTAAAGTTTTCAAGATTTAATGTCGCATAAAACT
TTTTTACCAACGTATAATAGATTTTAAATGCTTTTTCTGAAATTCTTTCGTTTTGTAGCTTATTT
TTAATTTTACTATTGTTTGGCTTTCTTACATATCTTAAGTAATAGAAAAATTTTTTTAAATCGGC
TGAAGTTAATGTATTTGGTTCTTTATGAAGTTCTTCGTCACAAAAACTTAGAAAAACTGCCAAAA
TTGAACTTTCATTTTGTATAGTACTGTGATTTATGCCGCTACCTAATCTTTCTGACCTGTATATG
TCATTCCATTTGTTTATGGGGTTCATTGGTATTTTTTCTGCATATATTTTATCGTTGGAGCCATT
ACCCAAAAAATTCTATTTTAAATGTATTTTTTAAAACTTTTTTACTCATTCCATCCCCTCTGCGAT
AATTGCGTAATTTACCATCAAATTTGTTGCTTTTCCGTATTTTTCATAAATTTTTTCTTCTACGT
CTGAAAGTGTAATAGTTCTTCTAAATGATTTATCAAGTTCTACTGCCCTTTTTAAAATTTCGGGC
CTAATTTTATCAATATTTTCCAATTTATGCAACATGTACCGGGCAGTTCTGCAGTCCATGTCAGT
TATAAGCGTTAATCGTAAGGTATTCAAAAAATATCACCAGTTAAACATTTTAATCGTTTATACTC
TATTTCAAGGATTATACCTCCGGCAATTTTCGAATAATTTCTTAATTCCTTTAATAAAGGGCCTT
TTTAGGTAATTAATTAATTATTTATAGTTGGAAATTCAAATTAGGTATATTATGGTAGTATTAT
GGAATTTAGAATCTGATTTGATTTGATACCTAATTAGTTGACATAACCATTTAATGTATTAGATA
TATTTATCGTACTATTATTACCTAGTACAAGTACTAATATTCAGATCGTACTATATAAAGTTATA
GATACTGGATTTTCGTGAAAATGAACAAATTTTAAAAAGTCCAAAATTATGGCGTTTCAGAATA
TTTGCTTTGATTATCTTTTAGGTAACTTATTTTATTTACATTAATTTTTAAAATTTACAATAAGT
ATATATTTGATTAATACCGTATTGATAGATATAGAGATTTTAAAAATCGATTGATCGAATTTTG
AGATCATAAATCGAGGTGCATTATGACTACAATGGGAAAAAAATATGTAATTGAGATAATGGAA
TATTTGTCTGACCATGGACGAACGGAATTTTTAAAAATATTCAATGACCTGAAAATGGAGTTTTC
GAAGGCATACCTTAGCAAATTGCTATTAGAAATGAATGAAAAATACCTAGTGGAACGAAAAGAA
GAAAGAGAAGGCAAAAAAACCCCTAAACCATATTATAAATTAACCCCCATCGGTAAAAAATTATT
AAAACAGTACCGCCTTGAAAAAAAAGCCATGGATCTATATAAAATAGTTGATAAAAAACTTTCA
GAAAAATATGAAAGGATAAGCACTGAATTAACAGTAAACGTAGAGTATGCACATCGTGCCAAAG
ATCTTACTCCCCATTTTCAGGACTATTTTCAAGATCAGTTATTTTTATCCACAAATGGGGCTGAA
AGTAAACCTGCAATCGAAGGTGAAGAATTTAACAATGCTATACCTTATGTTTTAGAACCTGATTT
AGCTTACCAGTACCGTGGAAAAGGCGAGAATTTATACGTATTTTTTGAAAATCATGAAGTATACG

ACTATGGAAACAGCATTTATAACGAAATAAAAGAAGGTGTAGTTGTTTTAGTTCGGAACATTTA
TGATAAAATGTATGTTTTTTTTGATACGCCAAATATAAATGAAATAAAATTATTGGTTGAAACA
GATGTTGCAGAATCTAATATCAATGAAATTCAGATATCATCTCTGCCAGATTGCGAGGAAAATGA
AGAATTTAGTCGGATAATGAAAGATATGAAAGAGTCTGCATCCGAAAATATCCAAAATAAGGAA
AAGGAAGCATTTGAACAAAAATATGGTAATTGCATTGTAGAAACTCCTGAAAAAATTGCAGAGC
GAATAATAAACCATATTGAAAGTTTAAAAAAATAATATCTTTTTTAAATTTTCATAAAATCATT
TTTTAGGAATTTTCATGAAAACTTTATCTGAATTTAAAACAATCCTGCATGAAAACAGGGATTTG
TTGATTAAAAAATACAAAGTTAAGACCCTCGGAATATTTGGGTCATATGTTCGAAATGAACAGA
ACGAAACATCAGACATTGATGTTTTGGTAGAATTTCAGGAAAATTCTATTAATTTTGACAATTAC
ATGGGATTAAAATATTATCTTGAAGATTTGTTTCAAAAAGACGTTGATTTAGTAATTAAAGATG
ATTTAAAAGTTGAAATCAGAGATAAAATATTAAAGGAAATAACTCAAGTTAAGATAAATTAAAA
ATACAAGAACTTTCAAAAATCCTTATGATAATTATGTTGAGCAAAACTTCAATTTTAAACGATAA
ACGAATACAAGAATTATGCGAAAGAGAAAATATCGTATATCTAGGACTTTTTGGATCATATTCA
AGAGGGGAAGAAACTAAAGATAGCGATATTGATTTTTTGGTTAAATTCTCAGGAAATAAAAGTT
TATTTAAGATAATAAAAATTTCAAAAGAATTATCTAAAATTTTTGGAAAAAAAGTCGATCTTGT
AACTGAAAATTCATTAAGTAAATACATAAAAAAAGACGTTTTAAAAGATATTGAGGTAATATAC
AATGTCGAATAAAAAAGATAGTGTATATTTACTGCATATTGTAGGATCTATTGATAATAAATCA
TTATTATGAGTACTTTTTTAGTTATTAAAGAAAATGTATAATATTTTTAATAACGAAGACTTTT
TAGAATCGTTATTAAAATTTTTTTAAAAATGAGTTGTAAGAAAAACTACTTATTAAAGAAAATG
CATAAAATTTTTAATAACGAGTAGCAACGACAATAAAATATTAAAATTTTACAAGGATATTTAT
GAACCCTGATGACTTTAAAGAATCAAAAACTGGAAAAGTGGTCTACGATAAACTAAATGACTACT
ATTTATTTGAGCCATGTGATTTACCCTACAAAGATTACATTCCAGATTACCGAAATCTTATTTTA
GATACTGAAAAAGCAGTATCTCGAATTTCTAAACTAGATGGTCTTTTGGAAAACTTTTCAGATGA
AGAATTAACTTTACTTGAAAAACCATTTATTTTAAAAGAGGCTGTTTTGAGCTCCGAAATTGAAG
GTATCCATACTACAATCTCAGACTTTTTAATAAATGAAAAAAAACCAGAAGTAAGTGAAAAAAA
ATATGATATTTTAGAAGTTACAAATTATAAAACTGCCCTTGAATACGGGATAAATAAGGAAATA
ACTGAAGATCTGATACTTGAAATCCATAAAATACTTTTAGATGGGGTTCGAGGGCAAGACAAAAC
ACCTGGAGAATATAAGCGACTCCAAAACTATATTGGAACCAAAAGTACGGGGCTAAAATATGCAA
AATTTGTTCCAGCATCGCCAAGAAGAACTAAAATGCTTATTAGAAATTTAATTGAATACATCAAG
AATAAAGATGAAATCCCGTTAATATATAAAATTGCACTCATGCATTACCAGTTTGAAACAATACA
CCCATTTAGAGATGGAAATGGAAGAACCGGAAGAATTTTAATAATTTTAATACTTATGAAATAT
GGGCTTTTAAAAAAACCTGCACTATATTTAAGTGAATTTTTCAGCGAAAGAAGAACTGAATATCA
AGATAGGCTTTACCATGTAAGTTCCAGGGGAAAAATTGATGAATGGATAAAATTTTTCCTTGAA
GCAATAACCATTCAATCAGAAAGAGCAACTTACTTCTCAAAAAGACTTTTAAATTACAGAAAAGA
ACTTTCAAAAATTGTTATGGAAAATAAAAACATTAAATCAAACGATAAACTTAAAATAATTCAA
ATAATCGAACTTTTATTTAAAAATCCATATCTAGATATCAAAGATGTAGAATTGGCACTGGGTAT
AACTTATCCAACAGCAAAACGGTTAGTGGAAGGCATGGTTTCATTAAAAATGCTCGAATTAAACG
AAAAATCAAAAAAACGGGATAAACTTTATGTTTCAAATAAAATTTTAGAAATTTTGGATCCAAA
ACATATTCGTTAAACATTTTAAATTATTTGGGAAATATATGGACAATAAACTCGAAAAAAAGTT
AGAAAACTTCAAAAATGCACTTGATTGGTTAGATGAAGGTTTAAAGTTAGATTCTTCAAATTCA
ATCATAATTGATGGGGTAATTCATAGATTTGAAATATGCTATGAATTCGCTTTTAAAACCATTAA
ATATTTTTTAAATTATCAAGATATTGGAGAAGTAAAAAGTCCAAGGATTGCTTTTGAAAAAGCG
TTTGCTTACGGAATTATTGAAAATTTAAATGACTGGATTGATATGCTAAACGATAAAAACAAGA

TTTTAAACCAGTTTGACGAAAAACTCACCAAAAAGACATACAATAAAATCAAATCGAGTCATTTT
AAAAATTTAAAAAATCTTTATGACATTATTTTAAAAAAAGAGTGAATGAATATGGATATTTTAA
GTTTTCAAATTAATTTTTAAAAAAATAAAAAATTAAATTAAACACACAAATTCTGATTTTTCAA
ATAGTGTGTCAATATCGTATGAATAAAGTGTATTTAGCCATTCCAAATACTCTCTCGAATATTCA
ATTGATTCAAACTCATTAGATTTTTCCCAAATTAAATTCCAAGATTCTAAAAGACACGGCGGAAG
TTTTAAGTCTTTATCATCCATTATATCCATTTTAGTACTATTAACTACAATAATTGAAAGATCTT
CAGCCGTTAAACTGTCTGAATATTCGTTCATTTGCGGATCAAAGGGAAGTTCGTAAATAAGTCCA
TTTTCTAAAAAATGGATTAAAGTAATTAAGAATTTAGATTTCGGGTCTTTCAATACCCTAAATTC
TAATCTACCTTTTAAAAATGACTTGTAATCTATTCTAAGTCTTTCAGGAATATATAATGACATTT
GAAAATGTCCATAATTATTGAATGCTGAAAAATGCTTGTTTTTCATAAATTTACCACCTTAATAT
ACATCTTTTAATGTTCTACCATCACTTGTTTTCCAGACAGTCCATCCGCTCACATTTGCACCTTTT
ACAACGCATGCTGCCGCACTCGCAGAATTAAATTCGTAATCTTCTGTAAATACGCAAGTTCTATT
TGATTTTTGAAGTATTCCCGAATTTATAAGGGTATTTTTCAAAAGGACGTTGTGTGGTGACATAC
TTGATGATTCTCCAAATTTTGCATGGGAATTTTTTAATACTTTGAATTTTCCGCCAATATATCTG
CCTTTGGCAGTTCCGTTTTCGGTGTTGCAGTAAAACGTTAATTCAGTGTTTTCGTCAAGTTTTTC
AAATAATGGATACCCGAGTGCCGATAAATACCCCTCAACATCGGGAATGAACCTATCAAGGGTTA
TTTTGCCATATTCAAGCTGAATGTTCTGGCTGTTGTAGGAATTTAATAATCTAACTTTTTTTGAA
AGTACTGCTTTTGAAATAAATTGCGACTCTAAATATTTGATATACGCTTTATTAAACTGATTTTT
TGAAGATAAAATGTATACGCAAGAATTCCATTCTAAAGTCTCTTTATGTTCAGATAATCGCTTTA
AAACTTGCTCTGATTCTCCAATATATGTTTCAACTTTTCCTAACTCGTCAAAATCCCCAAATAAA
AAATATATTCCTGTTCCATCCAAATTAGAACATTCTGAAACATCTTTTATTGATTTTAAAAGAGA
TCGTGGAATCAGTACTGCTTGAAGTGTCGAATCTAAGTATTCGCAAATTTTTATATCGCCAATGA
TTTTTGTATGCATTTCTCTTTTTTCCATTGTTTTAACCCCTTTTTGTTTAATTAAAAGTTTAATA
AGGTAATTAGTGAAATCTATGGTTCTTTTTCTCGTAAAATCATATTAAAACCCCGTTCTAAGTTT
TTAATGAAATTTTATGATATGTAATCTATAGTGTATGGACTAATAGTTTGTAATATTACTTACA
ATTTATATTATGACCTTTTTGATATATAAATCTTTAGTAAAAGTACTAACTGTAGTACTATACAA
TAACAATATTATATAATACAAACAAATTATATAATTAAAAAAAGAAAATTGGGATTTTTATGAG
TTATTTAAAATTATTATCTAAGGCATTTGCCCTTGAAGTTTTGAATTTGTTATATATTCACGAAT
CAGGTAAAGAAAAAAACAATAGAATCAATTTTAGCGAAATAGTTTCGAAATTAGGGATTCATAA
AAGTAATCTGAGTAAATTGTTGTTAGAATTAGAAGAAAATCTTTTAGTTTCAGAAGAAGTT
AGGACGGATAAAAGAATTCCAAAGAAATACTATACTCTTACAGAAAAAGGGTTAAAAATAATGC
TTCTATGTAATGAAATTGAAAAGATAATGAATGAAAAATAGTTTTTTGTTGAGAAATATGCAAA
TTTTAAACGATATTGAAAAAGGCGAAGGAAAAACCCTTGAATTTAAGGAACAAATGCCGCAAAA
TAACCAGATTGCAAAAACAGCTGTAGCTTTCTCAAATACTGCAGGCGGAAAAATTATCATGGGAG
TTAAAGATGGCTTAAATGAGATAATTGGTCTAGATCCGGATGAAATCCCTAAATTAAGGGAATT
AATTGTAAATACAATTGCGGATTCATGCGAACCACAGATAAGCCCAGAAATATTCACTCAAAATA
TTGATGGAAAAATTATTTTGATTATAAGAATTTATCCGGGAAGTAAAAAGCCGTATTTTTTAAA
AAAAGAAGGGATTAACAATGGGACATATCTTAGAATAGATAATGTAAATAAAAAGGGTGATAGT
AATGCCGTTAGGCGTTTAATGCTTGAAGGCGAAAACATTGCTTATGATGAAGACATCAATATGGA
ATTTAAAATTGAAGAGCTTGATTTAAACGCCATTCTAACACAGCTTGAAAAATTAACTGGGAAA
AAATTTACCGAAAACGTTTTAGAAAATTTAAAATTAATTAAAAGTAAAAATGGTAAAAAATATC
CAACAAACGCCCTTATTGCACTTCTTGGATTAAAAAATTATAAAATACGATGTGCAAGATTCAAA
GGAAACGATGCTGTTGAATTTATTGACCAAAAAGAAATTGAAGGAACTATATTCGAACAGATTA

TAATGGCCGAAAAGTTTTTAAAATCACATCTTAATCTCTCTGGAAAAATTGAAGGGCTGCAAAGA
AAAGACTCTTATGAAATCCCATTTATTGCGTTAAGGGAAGCGTTGATAAATGCTGTTGTTCACAG
AGATTATTCAATTTTAGGATCAGACATTAAAGTTTCAATTTCTGACAGCCTTTTAGAGATAACTT
CTCCAGGTTCCCTTCCAAATACAATAAATATTGATAACCTTGGAACAGGATGTTCGGAAGTGAGA
AATCCCATAATTGCAAGAATATTTAAAGAAACAAAATTTATTGAGCAGTGGGGCTCGGGATTCTG
TAAAATATTTAACGAATGTGAAAAATACGGACTTAAAAAGCCGGAAGTTATTGAAACCTCATTT
TTAAAAGTCAGATTCTATCGACCGCTTGTTGGTGACCGGACGATAGTGGACGATACTAAACTTGA
CCGGACGATAGTGGACGATATGAATGAAAATCATAAAAAAATAATATTATATATCTCCGAAAAT
GGTTCTGCTACATCAACAGAAATAGAAAACTTAATCGATTTAAAAAGAAGCAGAACTAGAGAAA
TTTTAAAAGAAATGGTCGAAGAAAATATCCTCGAAATGAAAGGGAAAGCTCGGTCAACGAGATA
TACTTTAAAAAATAATTAATTACTTTTTTATTTTTTTGATATGGAATTTTTAAAGAAGTGTTTTG
TAAAATACCTAAATCCTATTTTAAACTAACCAATAAAGGAAAAGAAGTCTTAAAACTTTATGAA
TTTGAAGAAAGGATTAGAACTGATAAATTTTAAGAAATAGTAGAAAATTATTTAAATTTAATCG
AAGGATCTGTGAACAAAAATATCTAAATAGTTAAATATATATACTATATCAATTATATTATATT
TCATATTATAATTTTTTCCTCACCCGAGAAGGTGACGATACGAAACATTATATATTTTTTAAGAA
AATCAGTGTGATAACATATCAAACGATACCTCATCGAAAAATATCAATAATTCAACGAGTTCTGC
ATCAGACGTGGAAAAATACATAAATTTCATGTTTAAGGATCAAAACAAAGTAGCTACTGCAATA
AGATTAGTGCCAATAAATGGAAAGCCATCAGGATATGCATTTGAATTAAATGGAAATCATTATG
ATAGAAATATTGATGAAATAATCAAAAAAATATTAAAAAAAGAGTTGGTAAATCAATATAATTA
TTATATAAATGTATATCCAATAAACCTTGAGCTAATTTCAAAAGGAGATCAAAAAAAGAATAAC
TTTTACTCAAAATCAAAACACCATTTTGATAACACAAATATTGTTTATTTTGAAATAGATGTAAT
TAAAAAAGATAATAATGCCAATTGTGAAGAATATTTAAAGAATTATTTACAAAATATGCCAAAA
CCATCTATGATTGTATTTACGGGTAACGGATACCACATATATTACAAATTAGATGAATTTTACAA
ATATTCGGAATTAAATAGCATTCTTAAAAAATATGTTACTTATGATAAAAAGCATGAAAAAATA
ATCAATGGCGGAACTGGGCAATATATCTTAAATCCAAGATTGAGGCTACCTGGAACCTGCAATTT
TAAGAAAAATACATTAAATGAAACTGTAAAAAAAGAGTGCAGAATAGTTGAATTGAATGATTTA
AGATATAATAAAGATGAATTATTTGAGTCAATTGAAGAAATTATTGGTTATAAACCGTTTGATG
ATGAAGAGGTAGTATCTGAAAAGAGAATAGGCAGTTTTTCAGACTATATTGAAGCTACAAGGGG
GCTAAGCTACAATGGGGCAATAGATATTCGAGAAAAAATGCTAAAACAAATATTAGATTTGTGT
AAACAATACCAAATGAAAATTTCTGGTGGAAAAGATAAATTAGAAAATATCTTAAAAATGATAG
GATACGAGTATGAGAATAAAGGAAATCATTTTAAGATCGATAATTATGGATTAGATACAGAAGG
TGGAGATCAATTATTAATAATATATCCAAATGGATCTTTTGCAAAAACATACTGTAATTATGTTG
ATGATCAATTAGAAAACCCGTCATTTTTACAATTATTGTATATAAGAGATATAGATTCTTTTAAA
AAAATATTGGAAATAGAAGGATTATATAGTGAATTTTATAATGATCTTACAAAATTATATCACA
GTGAAATACATGGAATAAATATTCCAAAAGTAGTATTTGATGAAAATATAATAGATATTGACCC
AAATATTTCAAAAAAAGACTTAATTAAAATCATTGATATGGCCAAAAAAAATTATAAATTACCA
AATACATATATTACTAAAGATATGGAAATTACAAAAGAAAGATATAAAAATGCATATTTCCAAC
ATTTAAATGATGAAGAGTTTGAAGATGTATATCATAATATATCGGCATGTGTAGATCAGGCATT
ATCTTACAATACTGAAGATATTGAAACGTTAAAAATAGATCAATATATTCCAGTAAATACAATA
GATTATATTTTTAATAGAATTGATGAAAAATGCAACGGTAAACAAAAAGAAATTTGTATGTTAT
CGCCAACAGGATCTGGAAAAACAACAGCCATATTTAATAAATTTATATCAAATAATGAAAATAA
AACAATATTAGCAGTACCATATTTATCAAATTCAAAGCAATTAGGGCTAAAATATAAAAATCAA
AAAGGAATCGTTGCATTGTATGAAAATTCAAAAATATCGCAAGAAAAATTGAATAGTGCAAACT

TTATCGTAACCACATATGATCAAATCGGAAGACTTGCTGAAAGAAAAGATATTTCAGAGTTTAAT
TTATACGTTGACGAAGCACACAACGTAGTTTTACAGCATAATTTTAGAAATCAGGCTTTAGATGC
ACTAAATAAGGTAAGTAAACTAGTAAATCGAACAATATACGTTACTGCAACGAACTACAATTTA
AACTACAAAAAATTGCCGATTATTAAAATAATTCAAAAAAATAAGAGATTCAAATACGATGATA
TAAGCGTAGTTGATTCAAATGTGCCATTAAAATTATTGATTGAAGCAGTGCTGTATGATTACGAA
GATCAAAAAGAAAGTGGAAAAACACAGATCATATTTAATAATAACAAAGAACAGAACAGAAAAA
TAAAAGCCCGTTTAGAAGAACAAGGCATAAGATGTGCATTATTAGATGCAGAATCAAAAGAATC
AATAGATTACACAAACGTAATGGAAGAAGAAAAACTGCCTGAAAATTTAGATGTGTGCATATGT
ACATCAGCAATTGCAGATGGGGTAAATATTGAAAATTTGAATATTGGAAACCTGCACATACATA
AAATAGACAATTTTAATACATTTTTGCAATTTATCGCAAGATTTAGGAATGGATGTCAAAAAAT
TTACCTGTATGTAGATTTAAATAAACAAAATCCAAAATATATTTCAAAAGAAAATTTTGAAAAT
GATGTGCGAAAAAATATATTAGATATGCTACATGTAATAAATTCAGATATTGCCAAATATTCTG
ATGAAAGAATTAGAGAATTAGTATTAAAGATACATAATAGTGTATCAGAAACTCCTGAAATATT
ATCTACAATTGAAGAATATACATTATCAGATATTAAAAAATATCACTTAAAAAGTTTAAAACAA
TTATTATCAATATATCCAGAAGCATATAAGGATTGTGTAGAAATAGCAGATATTAACGAAATAT
TGGCATTGAAAACAGGCTATGGCTCTGAGGATAAATTATTAACATATGATTTAGATGCAAAAAA
ATATGTTGTAAATGAAATAACAGTAATGAAAAAATATTCAGATTATATAAGTAAACAGGTTATG
AATTCAAAAATCATGCTACATATGATGATAAATAGATGTGATGATTTAGAAAAAAACGTGAATA
TTGTAAATGAAAAAGAATATACAAAAGATGAAAAATCCGAATTTACAACACATATCAACAACAG
ACGACACATATATCATGAACTTGAAAAAGCATATGAAAAAGACGGAACTGCATTAAAAAGAATT
GCATATGAAGATGATAAAACAGTACGAGAAGTAATGTTACGAAAAAATATATCCCAAGAAGCAG
TAAAATTAGCAAATAAAAAATCAGAAATTATAAAATATGTCACCATAATAAATGCAGAAGATGA
AGGAATATTGGAACCAGAAAAAAGAATGGAAAATTACAATAAAGGAAATCGAGGATTGCATAAA
TTAAAGGAAAACATTATATTGATCAAAAATTTAAGTGTATTTAAAAATAGTTCAAAACATATAA
AAAATCTACATAATGTATGTGCTGAAAATGCACGGGCATATACAAATTACATATTGTACACAATT
TACGAAAATATTGTGAATAGTGAAAACGGAAAAAAAGTAGATTTTTTAAAAATTAGGGATAGAT
TAAATAGTGAATTTGAAAAAATAGATGAATTTGATCAAATAAGCATTGATAAAATTAAAGAATA
TGCAAAACAATTTGGAAACATCGCAAGATGGGACGATAAAAAAGGAATTGGATATTTCAAGAGT
ATGAATGAAGTAATATTCAAAGTCGTTAAATCAAAACAACAATTAAAAGACAAATTAAAAGAAT
ATTCTAACCCTAAAAAAGCAATAAATGAAACTTTAGAGAAATTAGGCGGGAAAGCAAAAACAAA
CCAAATTTTAAGATATATTAAAGACAATTACGTTGGAATAGATAAAGATCATATCGAGTCAATA
AAACTCGTATTGGATTCAGGTATATTTATAAAATTATCATCAGATGTAATAATTTCAGATCAGTC
TGCAATGGCATAATTTAGATCGAAGTAGCGTAAGCTATAGCTTTTTTTCGAATTTTTATAAAAAT
TAAAGAATAATTTAGATAATTTTTAAAAATTTATAAGAATAGTATTACTTTTATAAATAATTTT
AAGAAGAAATAATATAAATATCCATCATTATAATATAAAGTTTATCTAAAAGATTAGATTAACT
AAATTAGAAGTACTTCAGTTGTAAATAGAATATTTTATAATAAATTATTAATATTATATAAAAA
TCCTATTTACAACTAAAAATTTTAAACGATATTTCTTTTAAAAATTTTAAAAGAATTTAACATCT
AATTATGCCATCCATCTTAGGTATCTATGGTTAATAATCTAAACTGACTTAAAACTATAATATTG
TTTAAGTGATCTATTTTACATCTGATATCTTAGAATTTTCTTTATTTTTATTTTGCATAAATTTC
ATTTTTATTTTGCAAGGTTACTTAAAAATTCATTCCTTAACGCCGTAATAATAATATACTGTTTC
TAAGTAAAATGCGTAGCCACTTTAGTATTGCAGTAGTTTTGTTAAATTATGTAGTTTTCGTTACT
ATTTTGATGTGATTATTATATGTTTCAAAATAGGGCGCATTTAACATCTAATTTTGATGGGGGAA
TAAAAATACCTAAAAACTGCCTGTTTTTGCTAGGTATTTTTATTGGGGTGCGGGGGTTTAGGGCC

GCTATAAAATTTTTTGAAGATTTTTTTGTTTGAAATTTTTAAAAAGAGTTGGTGTTTTTAAAAT
TTATTTAATTTATAAATCCAGATATTATTCCATTAACTGTTTCAGCCGCTTCAGAAACATTTACG
TTTTCAACAATTCCTGTTGCATCTTTAACAATTGATTCATTTTTCAAATTTTCATAAGTTTCTTT
TGCAGTATCCATATTTACCGAAATGTTTCCATCTTTGAAATTAGATATTATTCCATCAGAATTTG
AAGGAATACTTCCTATCTCATCCCTTATTTCCCAGTGCGTGATACTCATTGCAAATTTAAGTACG
CCCGAATTAGAAACGAGCTTGTAAAGGCCAGATTTTATAAAAACTGTGCCAACTATTTTATAACT
TACCTTGTATGCCATATTTTCCTGAATTAAAATTATACTAACTCCAAAAACAGCTAGTGCAGTAT
AAAATTTTGCGTAAGAATCCTTGTCTTTATATACCACATCCCCTGCAGAATCGATCAGTGAGTTG
TATTGATCAATCAAGAGTATGGTTGTCAGTACATCGTCCAATTCATCAGGTTTTGCAGCAGAGGT
AAATTTATTTATCTGGAAATCATCTGCATAAGGCATTTCTAGATTAAAATCTTTGTTCAGGCCTT
CTGCTAAATTTTTGTATTCGTGAAGGCAGTTGGTAAGATTTGAAACTCCTGAATTATATTCAGTT
ACTTCCCCAGGTACGCCAATATTACATCTTGGGAGTTTTGAAATATTTTTTGCCAATTTAATTGT
AAAATTATCAAATTCAGGACTATCCATCCACAAATCCAAACTTTCTGAAAAATTATTCATTTTAT
TTTTCAAAAGTTTCTGATCTTCTTCAGATATATTTTGAACGTCATACGCATTGGAATCATTTTCA
TTAGATATGTTTTGCACATTGTTTTCAGATATTGTTTCTAAATTAGATTTTGAATTCTGTTCACT
AAGCATTATTGCCACGAAAACGTCATCGTCATCCTTAAAATCAATGCATCCTGAAAAAGATGTAA
GCAATAACATACAACTAAAAATAATAGAAAGTATTTTCTTCAAAGTTTTACGCCCCCGTTTTCCC
CATTAATTAACTTCATTCAACCGTTCACGCCTAAATTCCCAATCAGGCATTAAATACGTTAAGTA
ATTATAAAATCCCCTACTGTGATTTGGATGTTTTAAATGAGCAAGTTCATGAAATACGACATACT
CGATGCAGTATTTTGGTTTTTCGATCAGTTTTTCATTTAAAATGATCTTGTTTTTTTCAAAATTA
CATGAGCCCCATCTTTTCTTCATTTTCCGAACTGCAAATTTTGGGATATTATTCGTAACTTTTGA
ATAGTTTTCAATAAAAATTTCAAAGAGCTTTTCTTTGGATTTAGTGGTATACCAGTTTTCGATAA
GCTGCTTTTTTTTATCTATGTCGTCTTTATCTGCCACATACAGATGAATATATCCTCGATAGAGG
CTTACTCTTTCTTCGGTGGATTTATGCACCTTTAAACGGTAATTTTTTCCAAGGTATTTGAAAGA
CTCCCCACTAACGTACCTTTTTTCCGCAGTTATTGTTGCAATTGATTTGAAATGAATTACTTGTT
TTTCAATCCAAGACTCTTTTTTCTTTAAAAATGATTCAATGAATGAATCAGGGGTATTTATTGGA
GCTTTCAAAATTACAGAACAGTCAGGATTTACGGATAAATACATATTTTTGATGTCTTTTTTTAC
TATTTCAACAGATTTTATCATAATTTCCCGTATCTGCTTATTGCCATTGTCATTATTTTTTCAAT
AATTTTTTCATTATCTACCGAAATATCCATTTCATCTTCTAAATTCCATAAAATATCATCAATTC
CCTGTTTGATTGAATTGTGGACATCTTGGCTTTCTACCCAGTTAACTTTTAGATTCTTTTTAATT
TCACTATCAAATTTCAGTGATAATTCTGCTAAATCGTCTTCAAAGCCGTTTCCAGTATATTCTCC
AAAATACTCGTAAATGTTATCAAATATTATTTTTGCATCCTGACTTTCAGAAATTAATGTTGGAT
AGGTTTTTAGATCCATATTTTTAGATTGCACATCTTTTAGGACATGTTGCATACTTTTCAAATAT
TCTTCGTCTGAAATCCGCTTTTCATGGTATTTTTCTAAAACTTCTTTAATTCTGTCAGAGAGTGA
TTGGTAATATACGGGGTTTTCGTCAAAACTATGTTTGATATGCTTTTCAACAGCACTTTTAATGA
GATCTGCTTTTGCACGGCCCCCTTCAACCCTTACAATTTCTCGATTGAAGTTTTCGACATCAAAAA
TATTTACTAGTTCAGTTAAGTGTTCAACATCACTTGCACCGATATACGTGTCAAGCAATTTTTGC
ATATGCTCTTCGTATTTTCCAAAATCTATTCTTTCATAATATCTTAAAGATACATTTTTTCTAAG
TTCTACATAGAATTTCAGCGCACTTTTGTACTTTTTTAAGTCCTCTACGAGATCGTATATATGTT
CACTTGAAAGTGCATATCCAAGATGCTTTGCATAGTGTTTTAAATATTTATAAAAATCCTGTCTT
AGATCTTTATTTGCTAAGAAAACCTCGTATTCTTCCCTATCGTTTTTGTTTTTTACGGGTTTAAA
TAAATCTATCAAATGCGAATAGTAATTTTTAAAAAGATGGATTTCTTGCTCCGTATTTATCATCG
TAGTTCCAAGATCTTCTTCATCGAAGTTTGCAAGTGAAGTGTAGCTTGTAAGTGCCATATCCAGT

TTTCCTAAGAGCCCCCTATAATCAATAATAAATCCACAATCTTTTTCATCATAGAGCCTATTTAC
CCGTGCAATTGCCTGAAGAAGTCTGTGGTCTTTCAATTCCTTGTCCACATAAAGTGTCGTTGTCC
TTGGAGAATCAAATCCTGTTAACAATTTATCAACAACAATCAATAGATCGATTTCTCCGGCTTTA
AATTCACTTTTAATTCTATCTTCGTAATCTTCTTCGGATCCGTAATTTTTAATTATTTTTCCCCAT
TCGTTTTGCACGAGTTTTTTATTTTCATCGTCCAATTCTTCTACTCCTTCTTTTAAATCGGGAGGT
GAAATTACAAATGCGGTTTTAATGTCTCCATAATTCTCAAAAATTTTATGATATCTTACTGCTTC
AAATTTTGAATTTGTTGCAAGCATTGCATTAAATCCTGTTCCAAGTAAATTCTTTTTGAAGTGTT
CGTTGATATCTATTGCAATTACTTCGAGTCTTCGCTCACTTGATGCAACTTTTTGAAATATTGCC
CATTTTTTCTTTAAATCTTCTTTCTGTTCGTCTGAAAGATCTCTTGAAATAAGTTCAAATTTCTT
ATCGAGCCATTTTTTATCCGTAATTTCCTGTGGAACTTCTCTTCCTTCGTATAATAAAGGAACTA
CTGCTCCATCAGCTACTGCCTGATCCATTGTGTACTTGTGAATCATATGCCCAAATTTATTGAAT
GAATTCTTTTCTTTTTTCATTAATGGAGTTCCAGTAAACCCAATATAACATCCGTGAGGGAATGT
CTTTTTCATTTTTGCGTGAAAAGTTCCATACTGGGTTCTGTGGCTCTCGTCAACCAATACAAACA
CATTTGAAGAATCATTTATGATATCATTTCTCATAGCTGACTCAAACTTATTGATGACTGTTGTA
ATTACTGCATCTTTGTTTTCTTTCAGCAATTTTACAAGATTTTGACCAGTAGTTGCCCTTAAAAC
TTCAAGTCTTGCATGGAAAAAGGTTTCTTTGATCTGTTTATCAAGATCCACCCTATCAGTTACAA
CAATAATTTTTGAATTGTTTATTTTCCTTTTCAATGCCCTTGTAAGCATTACCATCGTTAAAGAT
TTTCCAGACCCTTGGGTGTGCCAAATAAGTCCCCCGCTTCTAATTCCGTTTGCATCAAAATTATCA
ATTGATTTTAAAGTTTCTTTGATTGCAAAATACTGCTGATATCTTGCAATTTTTTTGATATTTGC
ATCAAAAATCGTATATTCTTTTACAATTTCTAAAAATCTAGCTTTATTAAACAGTGAATAGATGT
CCTTATCAAGTTTTGTTGGTATTCTATCAATTACGATAGTTTTTAAAAGATCATCTGAAAGGTCC
TCTTTCCATACTGACCAAAATTTCTTTTCAGTTCCAGTTGTTGCATAATAAACATTTCCAGGATT
TCCTGCAACTGCAATCTGGATGTACCTAAATAAATGAGGGATGAGTTCTTTGTTTTGATAATTTA
TGATCTGAGAAATTCCTTCTTCAACTGAGACAGAACTCCGTTTAAGTTCAATTATTCCTAATGGA
ATCCCATTTACAAATAAAATTAAATCAGGTCTTTTGTTTTTTTCAATTTGGTTTAAATTTTGTTT
TTGAACTGAATATTCCTCTGTTACGTGAAATACGTTATTTTCAGGATTATCAAAATCAATATATT
TCATTGAAAAACTTCTCTTGCTGCAATCATCAAGAGTTTCTTCATAAGAATTTCCCAAAATTAGT
TGATCGTATACGTGTTCATTTGATTTGATTAAACCTTCAGTTAAAGGAACATCAAGATCATCTAT
TGCATTTTCAATTGATTTTTCTGAAAATTTGTATGTTTTTCCTTTATACGTGTAAGAATTTAATT
TTTTAAGCTGTTCTTTTAAAATATCCTTTAAAATTACATCTTTTAAATTATTTCTTTTACTTTTA
TTCTTTTCTTCTGAAATGAATTCGTATCCCAAATTTTCTAAAAGTTTAATTGAATCTTCTTGAAC
TTTTGGTTCTGAATTAATATATGGATCAGTCATTAAAATTCCCCTCAAAAAATTTAAAAAAAGTG
ATTTAGGAAGTTTTTACACGAATTTTTCCGGTCAAAAGATTTTGCATTAAACCTTTTTTCTGGGT
TTTTACAAGTTCTAACTTCTGTTTCAAAAGATCTATTTCTTTGTCTTGAGTAGATAGCATCTTTC
CTATTTTTTGCTGTTCTCGAAGTTCAGGTAGCATTATTTTTGTTGATAATAATCCATCTAAATCC
AAATTTCTTCGCACGATACTTGCACCTTGTTCTGAATTTATATTATATATCCAAATCATATGTTT
GCTTTTCAAAAAATCTTGAATAAAATCAATATCCACTAATGAATAATTAATTTAAATGTTTTAT
AGGCGGGAGAAACAATTCCTATCTCGTAAGTTCTTAAAATATCTAAACTGCCCATCCATAGATTC
ATTGAACTAAATACTAAATTTCCATACCTTAATATCTTATATCCTGCATTGTCAGAACTTGCAAC
CTGTTTATTAAATTGTTCTGATTGAGGTATTATCCCAGTTTTAGTAACTGAAAGAATCATATGCT
GATTATTTTCATGAGTTTTCTCTGAAAATTCATTTAAGATATCCCCCAACTTAACTTCCTTCCAT
TCATCTTCAAAACCGGGAAACCTAACCTTCCCAGTCAACAAATTTTGCATGAGTCCTTTTTTGGT
TTCAATTTTCTTAGAAATTAAATTTTCCAAATTTTCGATTTGGATATCCCATGTTGATAAAATTT

CGGCAATTTTTTGCTGTTCGGGGAGTGGCGGGAGTGGTATTTGTAGGTTTTTAATTCGGAAAGG
CCTATCCGTAATCTTGTAGTTCCATTACCATGTTTTATTGCATTATTTCTGAAATTAGAAGAATT
TATTGTATAAAAAATATAATTTCTATCATATTTAGTGACGTCTACTTTTAATCGAATTCCGTCAG
AACACATTAAATAACGATTATCAAAATCGGGTATAATGCAAGCTCTTGCTAAGGGCTCGGCCATT
TTTGAAATAATTATTTCACCAGGATAGATGTTGCAACTTATTAGTTCATCGGCCTTAGATTCAGA
TGTATATATTCTATAATCGTCTAAAAATACTCCATCACCAATATTTTGTAACTGTAAAACTCGAA
CTCCTTCTTCAGCATAATGTTCACTTTTTAAATCTGATCCAAATGGGCCTCCCGTAAAGGAATAT
CTATCTTTTGGATCTCGGGTTTTTTCAATTTCGATTACATCCCAATCCTTCGGAATTACGCCAAGT
TTAGTTTCCCGATATTCATTTTTATTATTCATTATATCTCCCCCGTACCGATCTTTTCCGAGAAAG
ATACACCTAAAATTAAACCTATCCACGAAGGTACGGTCAAATTCAACAATAAATACACGGAATCG
ATTAAGTAAGTTGATAAAACCTCGCTACAGGCCGTAACGTTTGCTGAATTATTTATAAAAAACAT
GGCAGTAGATACGGAATCTATTGAAAAAGGCAGAACAACCGAAATAACTCCCACCAACCCAATTA
ATTTCCATATCTTAGTAGTGCCAATCAATGCGGAAAATATGCACGACATTATAACAATTAGTATA
TTAAACTCCATTTTATCCCCTATAACCCCAGTTCATCCAAATATTTGTCCATTTCTTTTTGAATAT
CTTTCAACTCGCCTTCAATTTTTGAAATATCTGCTTTAATCTGATTTAAATCAACAGGCTCTTCCT
CTTCAAAAGTATCGACATATCTTGGAATATTTAAATTGTAGTCGTTTTCTTTAATTTCTTCTAAA
CTTGCAACATGTGAATATTTTTCAATATCCTGTCTGTTTTTATAAGTTTCAGCAATTTTTTCAAT
ATCCAAATCTCTTAAAACGTTTTGATTCTTTCCGGATTCGAACTCTTTTGATGCATCGATAAGGA
TTACATCGTTTGTTTTTTTATTTTTATTAAAGACCAGAATACATGCTGGAATTCCTGTTCCAAAG
AATAAATTCTGTGGAAGTCCGATTACTGCATCAAGGTAATTGTCATCGATCAGTCCTTTTCTAAT
CTTTCCTTCACTTGCGCCCCTAAATAGTACTCCATGAGGCAATACAACAGCGACAGTTCCGTTTTC
ATTTGCTGATGCGACCATGTGTTCAACAAATGCATAATCTCCTTTTGATTTTGGAGGAATTCCGT
AAACAAATCTTTTGTAAGGATCATTTTCTGCGTCATCAGCCCCCCACTTATCGAGTGAAAACGGA
GGATTTGCAACCACTACGTCAAACTTCATCAAAGAACCATCTTCTAAATGAATCGGGTTTCTTAT
CGTGTCCCCCCATTCAATTTTTGCATCATCAATTTCGTGCAAAAACATGTTCATTCTACAAAGCGC
ATATGTCTGACCGTTTTTTTCCTGACCATATATCTGAAAGTTTTCGTCAGGAACCTCTTTTGATG
CTTTGATTAATAAAGATCCTGAACCGCAGGTTGGATCATATATTCTGCTTCCAGATTTTGGACTT
ACCAATTTTGCAATCAATCTTGACACTTGAGAAGGGGTAAAAAATTCTCCACCTTTTTTCCCGGC
ATCACTTGCAAAATTTGCAATCATATATTCATATGAATCCCCGATAACATCGTTTCCAGAAAGTT
TTGATGGCCTTAGATCAAGTTTTGGATTGTTAAAGTCTTGAAGAAGATGTTTTAAAAGTGCATTT
CTTTCTTTTGTTTTTCCAAGCATTGCTTCGCTATTAAAGTCAATACTCCTAAATATACCCTCAAGT
TTTGTTTTGTTATCTTCTTCAATTTTTTTCGAGTGCAGAATTTATAATTTCCCCTAAATTTTCCGC
ATTTCTATTTTGGTAAAGGTATTCAAAAGTACATGATTCATCGAGTATGAATTTTTCTCTTGAAA
GACTTTTCTGGATCCTATCTTCCTTATCCCCATATTTTTCTTTATATTCTTCCAATTTTTCTTTGT
AGTAATCAGAAAGATATTTTACAAAGAGCATTGTTAAAATGTAGTCTTTATACTGGGATGAATC
CACAGTATTTCTAAATGTGTCGCATGCGTCCCACAATACTTGGTTTATCGTTGCTTGATTGGTTT
TCATAATTCTTACCCCACTTTAAATGATATCTTCAAGAATTTTTGAATAGTATTTTTCTTTTAAA
ATTGTAAGTTCTTTCAACAGTTTTAGCTCTTTTTGCTTAAATTCTGCAATTTTTATTATTTTTTC
CTGCTTTTCGATAGGAATTTCTGGAATTTCGAGATTTTCAATTGCTTTTAGCTTGATCATTGAGA
TTACAGATCCTTCAGTATCTTTTTTAAATTCTTTTTGAGCATTTTTTGAATTTATATAATGTGCA
ATATATTTTGAAAGAAATTCTTTTGAATATTTTTCATTGATTGATAACTTTGCAAGATATGATGG
AATCAATAATCCTTCATTTTCTTTTTCGATGCTGCATGCAAATACTGGCTCCCTGAGTCTTACAA
TAATGTCATTTTCACTTGTAAGATATTCGTTTGAAATTTCTTCAAGAGCATAAAATTCATCTAAA

AATTCAGTATTTAATACTCCTGAATCATCTACTGACTTTAATGTGATCAGAGGGTACTTATTTGC
ATTTGATCTATCGTCAGACTTCTTCCTTGAAAGTAAAAGTCCTGACCGAATTGATGAAATATCTT
TTAATTTCAAAATTATATTCTCCCCCGAATTCGTGTATCATTGTACTGTTAACAGGTATTATATT
GGAACTCAGAGGTATATATAGCTTTCGATATTTTTCTGTATCATTTCTATGTTTACAAAACTCAA
TACCTAATTTAAGTTTTCACAGATCCATCTGACAGAATTTTCTAGGTTTTCAATTGAGTTTACTC
CATTTATAATAACTTTTCCAGATTTAAATATCAAAAAAGTAGTTTTTGGATCAGTAATTTTTAAA
ACAGCACCGGGAAACTGTTCTGGTTCATATTCTGCCCCAAATTTTTCTGAAATTTCTTCGAGATT
AAATTCCTTATTCAATGCAATGTTTGAAACAATGTTATATATTCTAACTTCTGGACGATTATTTG
TAAATCCCAAGTTATTTAATTCATCAACAATTTCTAAAACTGCGGTTTTAGCTTCTTCAACTGAT
TTTGAACCTGCAGTATTTATTTTTCCCGATTTAAATATTGTAACTATTCTTTTTGTTTCAGGGCG
CCTTATGTAAATACAATGGTATCCATCAGGGTCATAATCTGCATATTCTATTAAATGATAAATTT
TTTCCAAATCAATTTGAGTTTCAATGTCACATGAACCCACAACATTTACTATATTAACTTCAGGA
AGTTTCATAATTTGTGCCCATTGCTCTTAAAAATTATTATTTAAAATTTACAATTTTTTTATAAT
CGCTTTCACTAATTTGTGCCATTGCTCTCCTAAGTGAACCTGACCAGTGAGTTTTATTTTTGATA
AAATCCATTTCAGGAACTAAACTTTTAAAAAGAATTGGTTCTTTGAACAATTTAACTTCTTTTAA
TTTAAGTCGTAATGGATATGTTTCATTAGGGCTGTATGATTCAAATAATTTTTTGTTGTCTTCTT
TAACTTCCGAAACTGCCTCATAAATTCCTTTTATATACTGGGGTTTTGGCTCTTTTCCACCTTTTC
CCATTTCATAAATAATTAAATGATCCCCAGGATTTACTTTTGAAATTGCATTTTGGTATTTTTTT
GAAACTCCCCATATCTGTTTATCCATTACAATTTTAGCATTTTCTTCAGTTGTGATACATAAAAA
ATAGTTCATTTTAATCATTTTTCCATCCCTCCATCAAATATTTTTTCCGTTCTACCTTATCCAGTA
TTTTTGTACATTCGCCCCAGTTTACTTTATTAACTTTTTCATATTTTTTATCAAATGGACTTTTT
AACATAAATCTTACTGCATCGTCAATGCATGCGTCTGCATCGTCATTATTTCCCAATGCAACTTT
TACCCATACGTCCTCCAAAGTATCCGGAATTTGACCAAAAATATTAAAAATATCTTCTAATCTTT
TTGAAAGGAGGCGATGTACTTTATCTTCAACTGAATTTTTGTATCGCATATTGTATATGTTTACA
GTATCATATATCTGACCTATTCTTTGAATTCTACCTTTTCTTTGTTCTAGTCTAGTGGGATTCCA
AGGTAAATCTAAATTAATTAAATTTCCAAGCGTTTGTAGGTTTAAACCCTCACTAGCAGCGTCAG
TACCCACCAATAATCTTAATTCTTTAGATTTTACTTTGTTTTTTATTTCGGACCTATCAACTGAG
GTAGGAATTCCCGAATAATATATTTTACTTTTAGTTGAACCAGCATATAATCCAATATATTCATT
AGGAATTTTTGAAGATAATTCTTTAGCAATCCATTCTGCAGAATCATAATACTGGGAAAATATGA
TACATCCTCGTTCTAACCAGTTATCATTTATTAAAATATTATAAACTTCGTTAAACTTTGGATCT
TCGGTTAGTACATTCTCTATTGAGTCAATAAATCCCTTAAGATGGGATATTTCATCAGAAGTAAG
GTGAGATATATCATTTAATTTGATATTAATCTCATCATTGTCTTCAAAATAATCTTCATCTTCAT
CAATATCCTCAGCCCAACCCCGATACAACTTATTTGCCGTTTTCAATCCTGCAACAATAGTACTTC
CAACCCGCCTTAAGAGTAAAGTCTTTAATAATCCCCCATTAACTGCCCGTTTACCGATCAAATCAC
AAAATTTTTCTGCGTGGAAGTATGCGTCTTCTAGATAATATGGCATAGGAATGGATTCTGAATCT
TCTTCACCATGTAATTCTACATTAATTTTTTGAAGATATGGTAACCCCGTATCTTCATTAATCGT
TTCTTCGAGATATTTTCGAGTTCTCCTTACAACATGGCGTATTATGGGATTATGATTTTCAAAAT
AATTATCCTGTATTAAATTACTTATCTTTCTTTTAAGTAGGGGGTCTGAATAAATATCTAGATTA
CGCATTACAAAAGTATCTTCAGTAATGCCCGTATAATTACGAATCCCCCTTATTTCATGATTTTC
CGATGCCGGAGGCAGTGGATTTTTAATCCACTGCCAATTGTCACTGATATATTCTTTATTAATTT
CATATTCTCCTTTTAACATTTTGATAAATTCTTCAATCTGGGTACGCCATTTGCTATATTCATTA
CCCATTACACTATCGTTACCTTGTGCTAAAATATATAATAAATCCCATAATTCGATTGGATGTAT
CTGTATTGGCGTTGCAGTTGCAAAAAGCATGCTTTTGGTTTTTTTGGACAATTTTAACGAATATT

CCATTAAATTATTTGGTTCAGGCTTAGAACGGAAAGACCGGATATTTTTTCGCCTTGCTCTATGT
GCTTCATCTAAAAGAATACATTCGTACTCCTGAGTTAATAGTGCTTCTGCAAATTCTGATTTTCT
GCTAAATAGTCCCTGTGAAACAATCCCTATTTTTCGAGGGCATTCTAAAATATTTGTACCATATA
ATTGTCCTATCTCATCAACCCATCTTTTTCCATTCCACACCGCAGTAGGCATGTCCAATAAATTTA
TTAATTCATCCTGCCACTGTGAAACCAAGGTTTTTGGAACAATGATCAAAACTGGTTTATTCCCT
GATAAAGCCATTAAAAGTGCAGCTGTTGCAAGCTGTATTGTTTTTCCAAGCCCGACCATGTCTGC
AAGAATAAATCTGGCAGATTCGGGTCTTAAGTGTGATTTAAATGCTAAATCAATAAAATATTTT
TGAAAATCCCAAAGGCCGCACTCTTTTGAATATAATGGAGTATTAATAATTACAGAGGCTGCTTC
CGGTTCAATTTTCCAGGTTTCAATATCTATAATATTATTACCGTAAGATAACCGTTTAATATCGT
CTATAATAACATTAGGGATATCTATCGCATTTGGATTATTCCACAAAATATCAAATTCATTCTGA
ACCCATTCGACAGACTCTTTAGATGGGTCCTCCCATATTAATTCATAATTTTTTGTCCATCCCGAA
AGGGTTTCATTAGAACTGCCTAAAAAACTGGTTTCACTGCCATCTTCATGAGTTATAACTCCTGC
TTTTCCATGAATCATGCCGTAAACACTATTTGGTATGATTCTAATTTTTAATTTATTTGAAATTA
AAAGATCATACAATTTTTTAAAACGTAAAATATTTTTTTCATTAATTAATTCATCTGGCGTATCA
ACAAGTTTTTCTGGATTAAACTCACACCACTGCGTACGAACTGATTCCGTAAATACGGCATGATA
TGATTCTTTAGTCATATTTGCAGTGATTACATCATTAATATGTACATCCGAATTACATATCACAC
GGATTTGTCCAGTTATTGAATCCAATTCTTCATCAGCAATACTTAATATTGATGAATTAAAATAA
CCTGCAATTCTATCATATTTTTTAGCGTTTTTTAAATGTTTTTCTAAAAAATCACCATATAAATC
ATCTGTTCTTGATGAAAATCGGTTCATATTTCCCCTCAAAAAATTTAAAATTAAATTGAATCTTC
TTTAATCAATTCTCTTAAATATTTTGCATACTCCACATCATGTTCCCAGTGTTTCATAGAATCGA
TATGTTCAAAAGAACAGATGAAATCTAAGATTTCAACTATCGAATTACGTTTACTCCAGTATTCA
CTACCATATTCTTCTCTTAAATAACTTTTACCGAATTTTGCATCTTCTTCATTTTTGGACTTATAT
ATCGAAAGTAAAATTTTTCTAAGTAATGCGTTTGAGAATATTAAATTTGTAGATAATCCCTTATT
TGCATATTCACTTGCAGTTTTCAATCTTGCACTATTTGCACGAGTATTTTCAAGCATATCTTTAT
AATTTTTTATACCATAACCCCTTGCAAGTTCTTGGTATGTTGAAAGAGCGTTTGCTCCATTTATC
TCAGATTCTAATCCTTTAATATAGAATTTTTCTTCGTTTGAAAGCCCTTTCCATCTAAATTTATC
AAACCCTACCGGAATTAAACAGTCATATGCTATTCCAATTGCCTTATTGATTATATCCTGTAGCG
GCGAATCGGTTTTATCTTCCTTTGTAAGTTCATATTTTAAATCTATTCCTTCAATATTTTCATAT
GCAGTTAATACCTTAAGTGAAGCTGCATAGGCTGCTAAAATATAGTCGGGATCTGCAAAATTTGG
TTCATCCTTATCATCCACTGTTTTCATGGATTTGATCTGTTTTCTGACTTCTTTTTTTACTTCATC
AAGTATCTCATCTTCAAAAACAGTTTCATTGTTTTTTCTCTTTTTAAGAGTTAGTATAACTGTTC
CTTTTACATAATTTCCAGATTTTAATCCGCCAGATTCAGTTTCAGTTGCAATATTCCATGCAGAA
ACCACATGAAGTCCCGAAAGCCATAAAATTCCTGCAAGTTCCGCCCAAACGCCGACATCTTGATG
AGTAAACATTATAACTTGCATTCCGTCTTCAGGCATGTTTTTAGCCAAATTCGAGTATATATCAA
TCATAGTTTCGTTAAATGTTTTTCCAGTGCCCCTTACTGCAAGTGCCCTTTTTGAATCGGTGTACC
AACTGGGAAATGCTTTTTCAAGCATTTTTTTATCCCAGGATAAGAATAATTCTGTTAATTCGTGA
TAGTTTATCGCATCCGCATATGGGGGATCGGTAATCCAGATATCGTTTTCATAATCGATTGTTTT
TGCATTTGTAAGTTCTGTCTGAGTTATTGAATTAAATGAGTTGCTAAATAAATTAATACTCCATG
GCGAATTTATAGATGCTAAAGAACGAGCGGCATATACATATAATGTATTTAATGCCTGATTTGAA
AATGTAGTTTCACTTTCGTCACGTGATTTATGCCACATGGCCAATTTTGACATTCGATCTGCAAC
TCGATTAAATCCAAGAATTGAAGATATTAATTTATCTCCCCCATTCTGCAATATTTTCTCAGAAA
ATAATCCCAAAACCAGCAGCTGTCTTGGATTATACAGCTGGTGCCAGTATTTCCAACCTCTTTCTC
GAATTACCTGAGTAGTATTGTATCCTTCTTCAATTTCCATGCTTGGAATATATCCTAAATTCTGC

CATTCATTAAACCTTTCTTTAAGTAAATCGATAACTTTTTGCTCCCGCAATAAATCATTTTCATC
TGGTGAATAATATATCACGTTTCCATCATTCATCCAACCAATACAGTACAATCTTTCCTGAAATA
CATCATCACTTCTGGGAATAAATTCATCTTTTTCCCATAACCTTAAACCATAGTCTGTTTTACCA
TTAACCACTCGATCATTCCTAATTACTGCAATAGGTGTTTTCATGTTACAGTGCGGGCATTCGAG
CCGTCCTTTATTAAGGGTACCTGTTTTTGCAGTGTTCATCTCTTCTTTAGATGCGTTTGATTTAA
TTTCTATATCAAAATTGTTCAAATTTTCATTGTACTTTAAAATTCCAATAGTTTTTTGTTTTTCA
CTTACAACAAATGAGGGTGCAAGTGGAACCATTGCCCCACATTCCGGGCATTTCGTTTCATTGCA
GTATAAATAATATTTTGCACGGTATCCAAGTTCATTTGTTTCTATCCCTAAGTCTAAAATCTGTT
TTTCAACTTCGTCAAAAACTTCTTTTTGAAATTTTTTTAATTCTTTAATCTCTTTATCCGATTTT
GATAATATATTTAGTGATGACCACGTTAAAAGCCCAGCTACAGGATTTAAATCTGATGCATAAAC
ATCACATCCCATTCGGGCAGCTTCGAAAGGAATTGATCCCCCACCACAAAAACAGTCCCCTACTTT
TGGAGTATGCCCGAATTTTTTTTCTCCAAGCTGTTGAAATAATTCTTGGTAATTTTTAGCATCTG
TTTTAAGATGATTATTTATTTCCTTCCAAGTATCCTCATCAAGGTTGGTTAATTCTTCGGGTCGC
ATACAGTACTTTAATTTTTCATCGTAAGAAATTTTTTTAAATATTTTTATCTGGAGGTCATCTTT
TTCGTTTTTAGTTAACCCCTTTACAAATGCAGTTTTTTCAGTTTTAGATTCTTTTGTAAAATACT
TTTTTTTATCATTATCACTCGAATATTTGTAAAGTTCTTTTATTGGGATAGATTTATTTTTCCTA
TGGAGTAATCCCTTTTCATCCATTGATAATATTTTTAAAAAAATGTTTCTATCATTAATAGGATC
ATCTGATGTAGGCATTAGTAACCCTAAAAGAGTGCTTCTAACCAAGAATTAACGGTTTTCTACCCC
ACCATTTTCCAAGTCCCGTAAGGGTTTGTCCAGCCCCTGCTTTTCTTTCTTTATAGCTTTCTTTAG
ATATTTTTGAAATTGGAAACTGTGATTCTATAAACGGTTTTTCAAAATTAAGTTCCAAGGCTAAC
ACCCCATATTGTCAAAAAAATTAAATTTTTAATTAAATTTTTAATTAAATTTTTAATTAAATTTT
TTTATATAATTCTTCTGCAATTCGACCTTGTCGATAACCTCTTGTTTCATCTATAGGGTTATCAC
AAAGTGCATATCTTAAAGCAACTCGCCATCCCCTATTTTTATCGTCATAGGACCCAGTAACTGCA
TTGGTCATTGTAAAAAGCCACCATCTCTCTTCAGGACTTAGGCCCAGCCAGTTTTTAATTGCTAT
CTCGATTTTATGGATTTCTTCATTTTCTATCGCCCATACAAGAAGCATCATTTCTTTCCCAAGCAG
TCGTTCAACAGGATTTTGTCCTTGCTTAAATTTTCCAACTAATAGGTCTTCCCTCTTTAAACGGT
AGTTTAACTCAGAACTTAGTGCGGACTCGATAGATTTCCATTTTTCATACTTTAATTCAGCTTTT
AAGCTGTTAATTCCGATATCTGAAACCTGATTTCCAGAGTCCCATTTAAATCGTTCATATATCAA
TACATTTTTAGATTTAGACGTTGGGATTACCACTAAAAAATGGTGTTTGGCCTCATCAGGAATAA
ATCCAAAGCCTATTGATTGTATTCTTGCGATAATATCCCCTTTTTATTGTTTTGTATGTCCCTCAT
AAGATTCAATATCCTTTTCTGCATCTTTAGTCCACCTTATAAAATCAGAACCTTTTTCAAACTTA
GCCTTAGTATATCTAAGTGAAACATTGATATTTTCGTTTGAAATTTGATCTCGAATAACGTCAAT
TAATTTTTTTAAGTCTTGCATATTTAACTCAACGTCAGTTCCAAGTTCAATCTGACCCCATTTTT
CATTATTGTCATTTCCTTTTATATAAATTGTATTTCCAGAAGTTTTAACACAGTATTTTTCAAAA
AGGTCAATACATCGGTATGTTTCTTGAGAGGAATTTAATTTTTCATTTTTGGTAATTGTTAAGGG
GTAATCCCTGCTTATTTTATCAAATAGTTTTACTTTTGGACCTGAATTTTTAGGAATTTTAATTT
CCAGTTTTTCAGAAGTTAAATTATATTTTTTTGATTCCGCAATTGCGCATACAGTATCACAATTT
TCAGGCAATAATACGGGTTCCACATATACCGCACCATGTTCTTTTGGATTTGAGCCATCTGTAGT
ATATTTTATTTCTAATATGGATGACTTAGGCAGTGCAAATAACTCAAGCTGTTTTTTATTACCCT
TGGATATTAGGTCATATTTAATTTTTGGAATATTCCGCCAAATATTAATATCTCCTGTTTCATGT
TTTCCCGTTGGATCAACACATAAGAATCTAACTTCTAATTCATCGGTTTTAAATGAATTAATATC
TTCCACTTCTGAAGAGGCAGTTGTTGGATCAGAATCAATGTCATAGTAAACTTTTTCACCATACT
GGGGAACCACTCTGAGAATAACTTCGCCATTGTTAAGTTCTGATTTAACGTTTAGTGAAACCGCA

GTTTTTGGTTCTGGGAATGGTCCTTTTGAGAGATAACCCCCCTCTTCTTTCCAGTAATTCATTTCA
ATAGCCCATTTTTTTAATTTATTTAGTGTATCTGGGGGATACCATGTCCATTTCGTTTCTTCTGC
AGATCTTGACAATAAATCATTCCATCGCATGGCGTTTGTGGTAAATACTCTTGATTCAAATTTTT
TTCTAAATGTGTCTGTCTTAACATTATTTTCAAAAACATCACTTTCAAATTTTTTGGTGTCCATA
AGTAATGCTCGTATCTGCTCTTCTCCAACGTAATTATTATTTGAAAATTCCATTCTGAAGTCTTC
GGAAATAAGATCAGATCCTTTCGGATAATATAACGTTACAAAAGTTTCTTTTGCAGCGTTTAAGA
TACCTGCTTTCGTTCGTTCTGCATGATTTTTTGCTTGTTGGAATTGAACATCTGAATCAGGCAAA
CCATCTTCTTCATAAGATTTAATAATTGATTCTATTGCCATATCTTCTTTTGCAAGTGCCAATAA
TCTATTCATCGAACCTCTTTGTCCAGATAAAAACATTAATCTGTTTTTTAACATTTGATTTTCAT
AATAGTTTGCTAAGTCGTTATGTAATCCTTCACCTGGCCTACTTGGTTCATAAATTACAAGTGAT
ACCCTATCCCTATCCAAGTTTATTTCATCAATCGCCGGAAAGACGTATAGTGTTTGATATACATC
TTTCTTTTTTGGTGCAAATAATGTCTTTAACCGTTCACCGATTCTTTTTTTGGCAGAATCTATAT
CGTAACCATCCACCCTTGAATTTAATTCTGCGATGTAATTTTTTATATCTTTAAAGAAGAAATTT
CCATCAATTGTTTTATATAGATACCATGCTCGACCAATAAAATTATCAAATTCTGATTTATATGT
GGAAAGTTCAAGTCCCGGTTCTGCAATACCCCCAACAATTTCAGGATTTTTTAGTCCTTTTGTAG
AACTTGGTATTAATGAAAGTGACGAGATAAGTAATAATTTACTTACTTTTTGAATCAGGTCGCTG
TTTTTTTCAATATCCAATGTTTCTGCAATGGCTTTTCCATCATCTGCAATATCATGGGTTATTGC
AATAGTAAGTTTATCATTAATGCCCTTAATTAATGAAAGCATATCGGGGTTATTCAAATCCATAT
CATATGCATTTATCAAATAACGTTCATTTGAAGATTCTGATTCATACATATCTTTTATCATCATT
CTAACGAGTCGTATGAAGCCACGAGTCTGTTGAAACCCGGGGTTTTCTTTAAATCTAGCAAAAAT
ATTTTTTATTGATGGGTGAAATGGATATGAATCTTTAATTCCCGAATATATCTCATCGGGACTTG
TGGTGGTTAAATTCATCTGTTTCAATTTTGAAATTTCTTCACGATATAGTTTAGCGATTTCAGAA
ACTTTTTCTTTATCAGGTAACTCGGTAAAAAGCCTCTTTTTTAAAATATTATATAAATCATCACT
ATTACTCCCAACAGGCTCTATATTTATTGCCGAACGGCTTAGTTCACCATCTAAATTTTTAAAAG
TCTGCTGTATTAACTGACTACCCTCTTCATAAGTGGCTTTTAAGTCTGAAATTACAATAGTTACG
TTTGAAAGGTCTGATTTGTTAACTGCACTGAATAGATTTGATAACGCAGTAATGGTTACTTGTGA
TAAATCTGAAGTTCCAACCATTACTGACTTTGCATTTTCTAAGTATGGGGGTAACTCATCCAGTA
AAATCAATATTGGTTCCCCTTTTAACATTTCTACCCACGTACTCTGACCCGGTGCTTTAAATCCTG
AAGAATAATATGCGGAATATTGTTCGACTTTACCAAGCTGTCTTGCGATTTCTCCCCATATACCA
AATTTTGAATCTGACTCTCTCCCAGTAAATGCAATAACTTTTACCTTTTTATCAAAATTTTTAAA
TTTATCTCCCAAAATTTTTTTCCTTAATGACGGCGATTCTGCTAAAAGGCCTAATGCAACCATAT
TGTGTGTTTTACCTCCCCCCATAGATTGTGTAAGCCTAATAATTCCCGAACCTCCCAGGGTATATC
GATTAAAAGTATCTTCAAATAGTTTTTCCATCCCTGCAGTTATGTACGTATTTTCAAAGAATTCT
TCTCCATCTAATTTATTTTCCGATAAATCCTGAAGGTCAATAATATCTTCCGTGAAATCATTTCC
AAAAATGGATTCTCTTGGAGTACATAGATTTTTTAGCGATAATGATGAATTTAACATCGACATAT
AATCCCCCTTTATAATTAGCTAAAATTGTTAAGTTTTTATAATTCTAATCTTTAAAATCATCTTT
TAAGCTATTTGTATTTTTGTAAAGTAAACTTATAATATAACAATCCTCCCGTAATCAGTTAAATT
TAAACCTTCGGGAGTTTCTTCTAAGATATCCATGTCTTTTAGTTCTTTTACGTGTTTGTAAAGCA
TTGCTTTACTTACGTTTACATTTTCAGAAAATTCTGCCATTGATGAAATTTTATTTTTATTAATA
TATTCGATAATTTTGTACTGGGCAGGTGTAATCTGGTATGACAGCTTTGGAAGATAAACGAGCTG
TTTATTTTCTTCTTTTACATACATTATTCGTTTAATTCTTGAAATTCTAGCATATGCACCAAACA
AAAGACCTAGTGCTTTTGTTTTTCTCCCCGCCGTAATATCAACATATATTTCATCAGTGTCAGAT
AATGAATCAATTATTTCCACAGTTTCTTTTGAAACTTCCACAATATCATATACATCTGTATTAAC

AGTATCTATTTTTAAAACTGAACCTAATGAATCATTTATTAAATCAATGGACTTTTGCTGCGTAT
TGTCAGGTTCATAATCGACTAAAAGAATTAATCGATCTGCACCTATTTTATTTGATGCAGCAATA
ATTGGTTCAAATGAGTACAGGGTTGCAAGTAGTATTTTTACCATAGTCAAAATAGTGCGCATAAT
TATTTATAAATTTTTCTAATTAAATAAAATTCTCATGATTGTGAGAAAAATATATAAATCAGGT
ATATAATATAATGAATAGGTTACTTTAAATATTTGGGGGAGTTTTTATGGGCGAATTAAAAAGT
TTCGATTTAAATATTGAAAAGGTTCTTGAAGATTGGAAAGTTTACCATGCATTAAGGGAAATTA
TTTCAAATTCAATAGATGAAAAATCATTAACAAATACTCGAGATATTGAAATTTTCAAGGACAA
AAATGGAAACTACCATATTAGGGATTTTGGACGAGGTTTAACATACCAGCACTTAACACAAAATG
AAAATGAAGAAAAGAATAACAATCCCGAATTAGTCATTGGAAAATTTGGTGTTGGATTAAAAGA
TGCTCTGGCCACTTTTGAAAGAAATAAAATTGGAGTATTGATAAAATCAAAACATGGGGATATT
ACAATCGACAAATCCACAAAACATGGTTTTGAAGATATTTTAACATTACATGCAATGATAAAAG
ATCCATCCGATTGTAACATGGAGGGAACTGATGTTATATTAACCGGTGTAAGTGATTATGAAATT
AAACAGGCCCAAAATTTCTTTTTAATGTATTCTAAAAATAAGTTACTTGAAAGTACAGAATACGG
GGAGATATACTCAAAAAATAATGAAGTTGCTAAAATTTATATAAATGGTCTTTTAGTAGCTACA
GAAGAAAAGTTCCTTTTTTCATATAATATAACTAAAATAAACTCTAAAATAAGAAAATCGCTTA
ATCGTGAAAGGACAAATGTTGGAAGACAGGCATACACAGATCGAATAAAACAGATCCTTTTAAA
ATCAAAATCTGAACATGTTATTGATCTTTTGGTAAAAGATCTCGAAGAATACGAACGGGGCCATT
CACATGATGAAGTAAACTGGATAGAGATTTCAAAACATGCATGTGTCCATTTAAATTCTAGAAA
AAATGTATTATTTTTAACTTCCGAACAAATGCAGAATAATTTTAATATGGTGGACAATGCTCGAT
CAGAGGGGATTAAAATAATAACTATTCCAAATACTGTTGCAGATAAACTGCGGAACTTAAAGGA
CTTTAATGGAAAGAACATACGTAGTTTAGAAACTTATTTTGAAGAAGTGAACTCAAATTATGAA
TATAAATTCGTAAACGAAAAAGACCTAAACAATGAAGAAAAAGAAGTTTTTAACAAAACTGAAC
AAATTATAAATCTAATTGGGGGTAAACCACCAATTTTAAAAAAAATTTTAATTTCCGAAACTAT
GCAACGGGATATACAGGGGGGCGATTCCCTCGGAGTCTGGGAAAAAGATGAAAGAAGGATAATA
ATCCGAAGAGACCAGTTAAAAGATTTAGGATCATATTCTGGCGTTTTACTTCATGAATTAGCACA
TGCAAGAAGTGGAGCAGGAGATTGTCGAGAGATTTCGAATTAGAATTATCAGACATGCTCGGAA
TAATTGCAGAAAAGACGTTAAGATATTGATTCATTACCCACACGTATTAAATATTTAGAGATTCT
GGGATCATTTGTTTCACGTTATTATTTTTTGAACGAATTACCAATAATTTCAATCAAAACTGTTT
AGACATTAAACCAACTAATTCTTCATATCCTATCGGATCAACTTCAACAACTTCTCCTAAACTGA
CATAATATATCTTACATACAGTATTCTTTTTAAAATATTCTTTTACAACATAATAATAAATTGAA
AGCTGTTTTCTATATTCTTCATGGTTTATTTTTGAAACATCAGTTTTATAATCAATTATTTCAAC
CCGATCATTATATATTGCAATTAAATCAATGATCCCCCGAATTAAATTTCCATTTACTGGAAGAG
AACATTCAATTTCAGGAATTAGTTCAAGAGCATTTAACGAATCTATAAAGTTTTTAACCTCTAAA
ACCTCTTTTGATTCATCAAAAACTTCTTTTTTCAATACATATTGTTCTGCAATGTGGTGGATGTA
ATTTCCAAATTCAACTCCTCTTCCGCCCTCAGACGGAGTAAAATCTATAAAATCATGTACGCTGT
ATATATTACCCTGTTTATCATAATTTTCAAGTTCAAATTCAGTATTTTGAATTTCTTCATTAGCA
TCAATTTTAGAAAATTCAAATTCAGTGGGTTCTGAAATTGAAAAATCTTCAGATAATTCCTTGAA
AAAGTTTGAAGGCTTGTAAGCTGTGAAATAAACATACTGCTTTGCACGGGTTATTGCAACAAACA
TCAGCCTTCTCTCTTCATCGTATTCTCTTAAAACGGCAGATTTTAAAAGATCCGCTTTCCAATTAT
TAAATAAACTGTGATATTCTCCTTTAATGCCATATATTTTAGATAGTCTAACACCGTATAAATCA
GAATGAGTTAAAATTCCTTTTTCTGACATTTCAGAGGGGAAATTTCTTTGATTGCAATTTACAAT
GAATACTACGGGGTATTCGAGTCCTTTTGATCCATGAACTGTTTGAATCGTGATTGAATTTGGAG
AAGAATCTATTTCGAGTTCAAAAGTTTCATTGTTTTGTATCGCTTCTTCGATGAAATTTGCTAAA

ATTTTAGTTGAAATTAAGTTATTAGAACAAAGACTCGATATTTGGGATGTAATTGCATTTGAAT
AATCATTGTCTATATTATAATGTTTAAAAATTTTAGAAATCAAAAATATAACATTATTTTGAAT
ATCTTGCAAATGTTTTCTAAATTCGAGGTATTTTGCAGGATATTCTTTTTCAAAAATTATTTTTC
TTTTTTCTAAATAGGGATAATTATCTTCACCAAGGATTGTAACCCACGCTCTTTTGTCATTAACA
TTTGAAAGGAGCCTAAGCCATGCTAAGATTAAAACTGATTCTCTTGTGTTGAACAGTTCTATTCC
ACCATCGTATTTTGCAGGAATCCCCAATTTTTTAGCAAGTTTGTGAACTTCAAGTCCGAAGTTTC
TTGTTCTTGAAAGAACTGCAATATCAGAATATTCGATCGGCCGGAATACGGGTTTTTCATCGATA
TACTCAACGATTTTATAGTTTTCATTATTTACGATTTCGGAGATTTTTGAAAGAATCGACTTTAT
TTCTGAGTTCTTATCTTTGGTAGTTATAAATTCAATTTCAGAATATTCTTTCATATCAAAATTTG
CAGAAAGTTTAGTAACGTCCTTTAAAATTTCTTCAGCATCTATCTCTTCAGAATTCGAACCTTTT
GCAATTAATGAGTTATTTGAAAAGTCAAGGATTTTTTGTGAACTTCTAAAATTTATTAAAAACTC
TTTATTTTCTGGATTTAAATCAAATTTAATTCTTTCAAAATCATTGTTCAACATGGATTTATATT
CATTTAGCTTTTCTTCGAAATTCAAAATATTATCAATTGTAGCATTTCTAAATCCATATATTCCC
TGTTTCCAATCCCCAACAGCACATAAATTTGGCTCTTTTAAAAGCATTAATGTTAAAATAAACTG
TAATTCATTAGTATCTTGAAATTCATCGACCATGATATATCTAAACGAGTTATTTTCCCTTAATT
TATGATCATGATACAACATAAGATACGAAAACATTATTAAAAAGTCAAAATTTAGTTTATTCTC
GATGACTGACTTCTCAATGTAACTAAAATAAATATCGTGAATAAATGAAATCAATTCATCCCGAT
CTTCGTTAAATGATTCTTCAATGTACTTTTGACTGATTTGTTTATTTTCTTCGATATCGTTTGGA
AGGTCAAGATATACCTTATCTCCTGAATTTTTAAATCTTTTTAACATTTTTGATTGAATAGCTCC
TCGGGCACCGATGCATGATGCATTTGAAGTATTAGAAACTTCTAATAATTTATCAAAGTCCCCAA
TAAGTCTTTTTTCACCATCTAAAAACCATCCATCTTTAGTTGGAAATATGCCTTTCGAAAGTAGT
TTTTTTATCAGAAAAAGAATTTCAAAATAATTCTTTGAAACCGTTTTAAATATTTCAGGATATTT
TTCAAGATTTTTTTCTTTAAAATCGTAGTATACAGATTTAAAGAAGTAGTTTTCGAGAATTGAAC
TTTCTAAAATCGAATAATTTTTTGAGAGGGGTTCGTTAATGCCTAAATAATATGGTGCACTTTCA
GGGTTTTCCCGTAATATTTGATTACAAAATGAGTGAAATGTGGATATATTTGCATCTAAAAGGTT
TATTGTTGAATCGTGGCTTATAACTTTATTAAATATTTTCTTTTTCATATTTTCTGCAGCATTTT
TTGTAAATGTTAGAAGTAAAATGTCATCAATGTTCGCAGAATTTAAAATATTTAAATATCGTTT
TGTGATTGTGTGGGTTTTTCCAGTTCCAGGTCCTGCATCCACAACATAAATTCCATTAATATTTT
CGATTATTTCGTCCTGTTTTGGATTGAAAACCAAATTAATCCCCTCTTTTTAAACAGATATCCGA
ATATTCGCAATTTTTGCAGAGTTCAGGAACTGATGGAAGATATGGATATCCCAAACTTGAATATA
ATTTAACTTCTTCATTTTTACCTTTTAAAAATTCTTCAAAATTATCAAGGTCTTCTTTAAAAAAC
AGTGCTTTGGGTACTGCCCTTGAAGTTCCATTCTTTATGTTTAAAACTTCATTATAAAAACTCAA
AGCCCCTTTTTCAACCGTTTTTTTAGGGCTGTATGCTAAAATATGATCCATAAATCCAGATTTAT
GTTCTTCCAAAAATTCATTGGCCATTTCAGAATTAAATGGATTTTGAGTAAAATATTCCAAAATA
TACTCTTTTGAAACAGTATTTACAAAATTCAATCGGTCTTTACTTCCAAGTACGAGTTCAAAACC
TTCATCTGACAAAATAAAATCATTAAATGAAGTTGGATAATATGCAATATCAACAATATTCTCAT
CAAAACGTCCGGTTCCATCCAAAACTTTATTTTTATTTCCTAAAAAGAAATAATATTTAAAAGAA
AGACTTTTATTCGGATTTTCGGTTCTAAGTTTCAATAAATATACCATCGGCTGAAAATCGATTTC
TGACCCTAACTTTTTGATATTACAAAGTTTTAAAATTTTTGAAGCTGATTTTACGGATTTTGAAG
ATTTATAATCTAAAATCGTTGAATTATTAATTAATAGATCGATAAAACCATTCAAACCATAATTT
AGATCTTCAAAATAAACTTCAGTATTCTTTCTAAATAGTGGCATTTCAAATAATTTGGATAAAAA
ATTCTCATTTTGTTTTTTAGATTTCAAATTAAAAGTACTTTCAAAATCGAATGTAGTTGAATCAA
TAAATTCAACGGTATTTCTAATGGCGATTAAAAATTCGGTTTTTTCAATGTCTTTTAGTAATATA

TCGATTAATTTTGAATATTCATTGGATAAAATTTCAGCAAAATATTCTAGTCCTTTTTCTTTTAC
AAATTCAGGATAATTTACGTAAAATTCTGCGAATTCATGAAGTAAATTCCCTTTTATATGGTAGT
TTTTACTGTCACTCCTGTAAAGCCTAGATATTTCATAAATTTTTGGGCAAAGTACGAAGTTGTTT
AAGCTACTCTGGGAAAAAATATGCTTTGTAATCGGTTCTTTTACTTCGAAAATCTCGGAAACTTC
TTCCATAATACTATTTTTATTATTTGAAAGGTTTTTCACGTGGAATATTTCGTCTCCAAAACTTT
CAATCTCTTTTCCAAAAAATTTGGTTAAAGTAAAAACAAGGAATTGTTTTTTCACCATTTTTAAAC
TCCGTAACAAAACAATATTGAAGCTGCCCTTGTGACAACATTATTTGGAAATTTGAAAGGTTTTT
GTATTCTTCAGCTTTTCTTAATATATAAGGGGCTTTTTAACATCCTTGGTCCATGATGAATCAA
TATTTACGTAAATACAGACTGGCTTATCAACGTAAACTGAATTTTTAGCATCAACCAGCAGCACT
CCTGCCTTTGAATTTCCGATTTCAACGTCAAAACTATCTATGTAATAGATTAAATTCTGAATATT
TTGATAAGTTATTTTTTTATCATATATTTCGAGTTTTTGAAGTGTTTTTTTAAATTCATACGGAA
TTTCAATTTCAAAATCATTTAAAAAGGTTAAAAGATCTAAAAATCGATATTCTGAAATATTTTTC
AAAAATTCATATAATTTGGTAAACTTATCATCAGCAGTTATGTTTTCAGAGTATTCTTTTAAGGT
ATAATTTGAATAGCCTTCGGAATTTCCAATATTATACATTTCAAAATATGGGGCTAAATCTCTTA
AATATAGTTCATTTATTTGGAATGAAAGGGTTAATATGTTTAAAACTGATCTTAAATTCAAATT
TTGATGTAAATACTCTTTTATATTTAATTTTATTCCTTTTTTGTAAAATTTAGATTTTAAAATAT
CAAGAATTGTGCTTTCAGTGTTTAAAATTATTGCAAAATCGTTCTGAGTTTCATCAGTTATGATA
TCTGAAATTCTATCTGAAATTTCATTTTCATTTTTAAATAAATAGAAATTTTTCAGATCTTTCAT
TTCTTCTTTAAAAGGTGATATTTTTGTAAAATCACTTGGTAGTGCTAATTTATCAATTTCGTTAA
ATAATTCGTATCCAATCACTGCAACGTCAGATCCAGAATATTCTTTAAAATGGTTATAAAAATCA
AATTTTTCAATGGCATTTTCAGTAGTTGCAAGTAGATTGTACTTTTCGAGTATCCTTTTTTCGTA
AGAATTTATATATTTTGAAACATCTCTTGTATGTTTTTTTATTTCAACAATTTTTTCAACTGCGC
TATGGACTGACTTTATATCATACCCCAAATCTTTTGAAATTTTTAAAATTACATCAGTTTTTGAG
AGTTTTTGCTCATCAAAGTACATTGGAATGAATTTTTGTGCAAGTTCTTGCGAAGTGTACGCAAA
TTTTCCAATCATCGGTTTATCAATACGTTTATTTAGTGCAGTTGCTAACGCTGCATCGTTTGTTA
AAACAATTTTTTTAGTTTTAACTTCACCGTAAAGTTCTTCAACTGATTTAACTGATTGAAACCCC
ATGTTACCCCTTATCTTAAATTTAAATTCAATTAATTAGTAAATTTTACATTTTTAAAGTTTAAT
CGAAAAATTATATCGAAAACGACATATATTATGAAAAATACCCTTTATATTGGTTTTTAATTTGG
ATGAGGGAGATTATGGATATCAAAGGAAAATATCAATTATGGGATGAATTTCTTCAAAGATGGC
CTGTTGAAAAAGTACAAAACATGACTTTGGAGGAATATACGAATTTAGGAAATAATGACTATTT
TTGTTACTGGTTAGAACGTAAGGCTCAGGAATTAGGTGGAATAGGAGGAGGAGCTTCATTTAAA
TTTGGAATATTTAGAAGAAACTCGGAAAATAAGGAAAACGAAAGAGGCCGAAACTATTCTGAAA
AATATGGCTGGTATGATAAATACGGAAAAACTGAGGAAGAGGCTTTCCAAACAGTTAAAAGCAT
GGTACTGGACATAATTGAAAAATCGAGAGCTGGAAAATTTGATGAAATCGATTCGATAGATCTT
GGAAATGCCGTAAAATGGAAAATTGCATTTATTTATCAGGATCGAAACAATCCTATGCTAGTTTC
TATATATAATACAAAAATGTTGGCTAAAGCACTCGGGGTTGAATATAATGGTTCAGTTTCTGAAC
TACAAACTAGAGCATTTAAAGAAAAACCCGAAAATATGGATATTTTTGAATATTACAACATATT
AGAAAGTAAATTTAAAGAATCTGATGAAGACGAAGAGTTCATCGAAGAAGAATCTGAACAAATC
ATTGTTGACTCGGAAAATAAAAAATTCCCACTAAACACGATACTTTACGGCCCACCTGGAACTGG
AAAAACGTATTCTGTAATTGAAAAAGCGGTTGAAATAATTGATGGGGATATTTCAAATAAATCA
AGAGAAGATTTAATAAAAAGATACGAAGAATTAAAAGATAAAGGCCAGGTTGAATTTATAACCT
TCCACCAATCGTATTCTTATGAAGACTTTATTGAAGGAATTAAGCCCATTTTAGAAAATTCAGAT
AATAACTCTATTGAATATCAAATAGAAGATGGAATTTTTAAAACATTGTGTTTGAAAGCAAAAG

CAAAGAAATCTAATGTGATTACAGGATATACTTTTGACGAAAATAAATCAAACGTATTTAAACT
TTCTTTAGGAGATATTTCAAAAGATGCAGACTTGGTGTTTGAGTATTGTTTTGAAAATAATGTTA
TTGCATTGGGTTGGGGTGAAGATATTGACTTTAGCAAAAGTACTTCATTAGATGCAATAAAAAA
ATTACATGACATGAAATATCCGCAATACGCCTCTTTTGGATCAACTGCTGTCGATAGATTTATTA
ATGGAATGAAAAAGGGAGATATTGTAGTAATTACTCATGGAAACCGCAAATTTAAAGCAATCTG
TCAAATCGTTGGCGATTACGAATACCGTAACGACTCAGAAATACGATACCACCAGTTTAGACGAG
TAAAATGGCTTTGGGTGGCTGATGAACCAATTCCCGTTTCACAAATCTTAAAAGAAAAGAATTTT
TCCCAAATGTCAATTTATAAATTTTCAAAAACTGATTTATTGCCCGAAAACCTTAAAGATCTTAT
TTCAAACAATTCAAAACCAAATGAAAATAATTTGGAAGAAAAAAACTACGTACTTATAATAGAT
GAAATAAATCGTGGAAACATTTCTAAAATATTTGGTGAATTAATTACTTTAATTGAAGATGATA
AAAGGCTTGGAACTAAAAACCAGATTACAACAACACTTCCTTATTCAAAAGATGCATTCGGGGTT
CCAAAAAATCTCCATTTATTAGGTACAATGAATACTGCGGACAGGTCAATTGCTTTGATGGACAC
TGCACTTAGAAGAAGGTTCCATTTTGAAGAATTAATGCCGGATCCAAAATTACTCGATAGAATTA
TTGTAAAAGGAATAAATTTAAAACAGATGCTCGAAAAATTAAACGAAAGAGTTGAATTTTTATA
CGATCGGGATCATGAAATCGGCCATGCATACTTCATACCTTTGAAATATATTGAAAATGAAGAAG
AACAGTTTAAAAAACTCTGTGAAATCTTTAAAAATAAGATAATTCCACTTTTACAGGAGTATTTC
TATGATAATTACGAAAAAATACAGATTGTATTAGGGGATCACTTCAGACAATTAAATAGAATGG
AAGAAACCGAAAATTACGAAGATATACCAGTTAGGTTTATTCAAAGCAAAAAATTCAAAGAAAA
TTTAGTGATTGGCTTTGATCATGAAGAATATGAAGATTTGGTAACTTACAGAATAAATCCTGACC
TTTGCAGTGGAGATATAACTCCGAACACATTTAGAAAGATTTACGAAAAGATTTAAGAGCTGAT
TACTAATGAATAATCAGTTCACTATCACGGAATTTGGGATTATCGTAAAAGAAAATGAAAATAG
ACAATTTGATAATTATATTTCCCTAAAAGAATCTGTATTCAACGATTTAAAAACCTTTATTTTGC
AGAATAAAGATAAAAATACAGATGAATTCTTAACAATAACATACAAAAAAAACTATGGGGAAGT
TTTGCAAGCCAAAAACTACGTTGGAATAATTCAAATGAAAAATGGTGTAACCATTGAAATTTTAC
CAAAAATTTATGGTAAAAGCAGGGATTTAAGCGTTAGCGATACGAGAAAACTCTTTTTAAAAAT
GTTAAAAACATTGAAGGACTCCCCTTTTAAAAAATTTGATCTTTCAAACTTAAAAACAGACAGAA
TGCCATTAAATGAGATATTCATAACTATGTTTTTAGACGAACTCTCGATTTTGATTAAACGGGGT
TTGAAATCAGATTATATTGGGATGCAGAAGAATTTAAATGTTTTAAAAGGAAAATTGAAATTTA
AAGAGCAGATTAAATATAATTTAATCCATAAAGAGAGATTTTTTGTTGAATTTGATGAATTTAT
AAAAGATAGGCCCGAAAACAGAATTATAAAAACTACGTTAAAGGAACTTTTAAAAAGGTCAAAA
TCTGGAAAAAATTTAAAAAATATTTCTGAATACAGCTTTGTTTTTGATGAAATTTCCGAATCAAA
AAACATTGAAAAAGATTTTAACGCTTGTAAATCTGGAAGATTAATGGTGAATTATGAAAATATA
CTTCTTTGGTGCAGGGTATTTTTAAAAAATGAAAGTTTTATTAATTTTAAAGGTTCAAATGTCGC
ATTTGCATTACTTTATCCGATGGAAAAGATTTTTGAGAGTTATTTAACATACAAAATTAAAAAA
TCTGGAAAATTTGATTATGTAAAAGCACAGGACTCAAGATTTTTCCTCGTAAAAGAAGATTTCAA
AAAAATGTTTAAACTAAAACCTGATATTTACGCTGAAAAAGATGATAACATTTATTTAATAGAT
GCAAAATGGAAAATACTTGATATTAATTCTCCAAAGTATGGGATTTCTCAAGGGGATATGTACCA
GTTATTCTCATATGCTAAAATTTATGAAGATAATTGTAAAAAACATGTAAAAATGGCCTTGGTG
TATCCAAAAAACTGATAAATTTTTAGAAGAAGTAAATTTTGAATACCTTGATGAGAAAAATGTTT
TATTAAAGGTTTATCCGTTTGAATTAGTCTGAATTGTGATATTTTTACTTTTTTTATAGGGGTAA
TTTTTAACGAATTAAACTTTTTTATTTTTTAATAACAGAACGATTTTAAGATCGTTATTAAAGAA
AATGTATAAAATTTTTAATAACTAAGATTTTTTGAAATTGTTATTAAAATTTTTTTAAAAATGA
GTTGTAAGAAAAACTACTTATTAAAGAAAATGTATATAATTTTTAATAACGAGTAGCAACGACA

ATAAAATATCAAAATTTTACAAGGATATTTATGAACCCTGACGATTTTACAGAATCAAAAACTG
GAAAAGTAGTCTATGATAAACTAAACGACTACTATCGATTTGAGCCATGCGATCTGCCCTACAAG
GATTACTTTCCAGATTACAAAAATCTTATATTGGACACCGAAAAAGCAGTATCCCGAATTTCTAA
ACTAGATGGTCTTTTGGAAAACTTTTCCGACGAAGAATTAACTTTACTTGAAAAACCATTTATTT
TAAAAGAAGCAGTTCTGAGCTCCGAAATTGAAGGCATCCATACTACAATTTCAGACTTTTTAATA
AATGAAAAAAAACCAGAAGAAAGTGAAAAAAAATACGATATTTTAGAAGTTACAAATTATAAAA
CTGCACTTGAATACGGGATAAATAAAGAAATAACTGAAGATTTAATACTTGAAATCCATAAAAT
ACTTTTAGATGGGGTTCGAGGTCAAGACAAAACACCTGGAGAATACAAAAGACTTCAAAACTATA
TCGGAACCAAAAGCACAGGATTAAAGTATGCAAAATTTGTTCCAGCATCCCCGAGAAGAACTAAA
ATGCTTATTAGAAATTTAATTGAATACATCAATAATAGGGATGAAATTCCATTAATATATAAAA
TTGCACTCATACATTACCAGTTTGAAACGATACACCCATTTAGAGATGGAAATGGAAGAACCGGA
AGGATTCTAATTATTTTAATACTTATGAAATACGGACTTTTGAAAAAACCTGCGCTATATTTAAG
TGAATTTTTCAGTGAAAGAAGAACCGAATATCAAGATAGGCTTTATCAAGTAAGTTCCAAAGGG
AAAATTGATGAATGGATAAAATTTTTCCTTGAATCAATAACCATTCAATCAGAAAGAGCACTTTA
TTTCTCAAAAAGACTTTTAAATTACAGAAAAGAACTTTCAAAACAAGTTATGGAAAATAACGAC
ATTAAACCAAATGATAAACTTAAACTAATTCAAATAATTGAAATTTTATTTAAAAATCCATATT
TAGATATAAAAAATGTAGAAGAAGCACTTAGCATAACTTATCCAACGGCAAAACGGTTAGTAGA
TATTATGGTTTCATTAAATATGCTTGAATTAAACGAAAAATCAAAAAAACGGGATAAAATTTAT
GTTTCGTATAAAATTTTAGAAATTTTGGATCCAAAACATATCCACTAGTAAATTCTAAACTGCAT
TTCTAAATTCCAAAATCAAGCATATAATGTCTCTTTTCATTTTTAATCAGATTATGTGAATAAAA
ACTGCTGCAATTTAAAGTTTAAAACGTACCTCTTTTATATGCTTATTTTATAATATAATTATGTT
ATTTTTACGAAATTTTAATCGATTTAGGGGGAACTTCATGTCATTGTTTAAAATTTCAGGAAAAT
CTGTTGAAAAAATGAATTCAACGAAGAATATTGAGAGAAAAATTCAAAATTTGTGTGAAAACAA
CCTTGAAGAAATATTTGGTGTTAAATTTTTAAGGTCTGAATATCCTACAACTACTGGAGGCAGGA
TGGACTCATTAGGAATCGATTTTGAAGGAAATCCTGTAATTATTGAATACAAACAGGTTGAAAG
TGAAAACATAATTACCCAAGGGTTATTTTATCTTGACTGGCTTATTGATCACAAGGGGGATTTTG
AACAGGTTTTAAATAAAGCCGGTGTCGAAATGGAAGTTTCTTGGGATTCCCCACGTGTCATTCTT
GTTGCAAAATCTTATTCTAAATACGACACTTATGCAGTAAACCAGTTTTCTGGAAATATTGAGCT
CTGGAAATACATGATTTATGATGATGAAGTTTTACATATTGAGAGAATAAACCTTGCAAAAGAC
GAAAAAGAAAAAGCTATTCGCCATAAATCAAGAGTAACTTATCAGACGTATGAAATAGACCACC
ACTTGGCCGGAAAACCCGAAAATATTGTAAATTTATATTATCTGGTTCGCGATGAAATTTTAAAA
CTTGATGAAAATATTTCTGAAAAAATTAACAAATATTACATTGCATATTCGCTTGATAGGCAATT
TTGTGAATTTTTGGTTCAAAAAAACGCTTTGAAAGTTTATATCAATGTCACCAATGACGAACTCA
ATGACCCGAAAAATTTATCTGAAGATGTTTCTGGAAAAGGGCACTGGCCAACAGGCCATACGTGT
TTGAAAGTGGAAAAAGAAGAAGATATTCATTATACAATGGAATTAATTAAACAAAGTTACGAGA
AGAATCTTTAAACTTTTTTTACTTTTTAAGTTAGTTAAAAAGCTTTTTTATAGTCTTAATTTATA
ATGTAATTATATTATTTTTATGAATTTCGCGATTGATCAAAACAAGATATAAATGTTAACGGGA
TGCCCATTTATAAAAAATCAGCATGTTCAGGGTAAACCCTATCAATCTTGCTAGTGGTCTTAAAA
AACTTACAATTATTAAGGGTAAAAAATGGTAGAATTTGTTTCAAAAGAATCTGAAAATTTAATA
AACTTCAAGATTGAAGAATTAAAAAAAATACTTCCCGAAGTATTTTCGGATGGAAAAATTGATT
TTGAGAGACTCAGGCTAGTTTTGGGAGATGAAATTACCAATGACGGCGAAAAATACTCATTTAAT
TGGTTAGGTCGAAAAGAATCATTCAAAAATATTCAAACAACTGCAAAGGGAACCTTGGTCCCATG
TAGGGAAGAATCAGTAAACTTTGATGAAACCGAAAATGTTTTTATCGAAGGTGATAACTTAGAA

GTTCTAAAACTATTGCAAAAATCTTATTTTAATAAAATTAAAGTAATTTATATCGATCCACCATA
TAATACTGGAAAAGACTTCATATATAAAGATAATTTTAAAGAAGGTATTAATGTATATCTCGAA
CAAACTGGTCAAATTAACGAAGAAGGCTTTAAACTTACAACTAACGTGGAAACTTCTGGACGATT
TCATTCAGACTGGCTTTCTATGATGTATCCAAGATTATTCATTGCCAGAAATCTGCTTAAAGATG
ATGGGGTTATATTCGTATCAATTGATGATAATGAAGTTCATAATTTAAGGATGATAATGAATGA
AATATTTGGGGAAGAAAACTTTGTAGCAGAGATAGTCTGGAGGAAAACAGATAATCAAGCAAAT
ATTGGCAATATTGCACGTGTTAAAGAATATATTCTTTGCTATTCAAAAAATTCAGAAATATTCAA
GTTAAACAAGATGCAACTAACTGACCGAGCCCTACGAGAATATAGATATTCGGATGAAACGGGAA
AATTTAGACGTTCAATATTGTTACACAAAACTAGGGGGAGACACAATTACACTGTTGAAACACCT
AGTGGAAATCAACTAAATGGCCCTTGGATGATACCTAAAAAAGATTTTGAAGAATTAAATAATG
CTGGGGGAATTTATTGGACCCGTGGTGGAAATGAACAACCCTATGGAAAAATATATCTTGAGAAT
AGTAAAGGTCAAATATCAAACGACTTTTTAGGGATAGAATTTGGAACAAACCAAGAAGCTGGCCT
ACAATTAGAATCTTTGTTCGGAGTACGATGTTTTCCCTTTCCGAAACCTACCTCATTATTAAAAC
ATATTATAACAATTGGATCAAATCCGGGGGATATTGTTTTAGATTTTTTTGGAGGTTCGGGAACC
ACAGCTCAGTCAGTGCTCGAATTAAATAATGATGAAAAATCTTTGAATCGAAAATTTATCCTTAT
ACAATTGCCTGAACCAACTGCTCCAAATTCGGAAACATTCAAAGCAGGTTTTAAAAATATTGCGG
AGTTAACTAAAGAAAGAATTAAAAAATCCATCATAAACATTAATTCCAATGAAGGATTTAAGGT
ATTCAAACTCGCCCGGTCTAATTATCGAATATGGGAAAAATATAATGGAAATAATGAGTCTGAAT
TAAAACAACAGTTAAAACTCTTTAAAGACCCATTAATTTCGAATTATAATAATGAAGATGTAAT
TTTTGAATGTATTATCAAAGAAGGATACAGCTTAAATTCAAAAATTACATTATTAGATATCCGT
ACGAATGAAGTTTATAAAGTAATTGATGAAGATAAAACACGCGATAAGGATTCTTATTTTTACA
TTTGTTTGGACCAAAAAATAAATAATGAAACAATTGTCAAAATAAACCCTAAAAAAGAAGATAT
AGTTATCTGTCTTGATACTGCGTTAGATGACAGTTTAAAGAAAAATATTTCAATCCAATGTAATT
TGAAGGTTATTTAAATGATAGAAGAAACAAATAAAGAATCGATAAACCTGAAAGAAAATTTAAT
TGATATATTAAAAGAAGATATTCCTGAAGTATTTTCGGATGGAAAAATAGATTTCGAAAGACTT
AAGGTAGTTTTAGGGGATGAAATTACTGATAAAGGTGAAAACTACTCATTTAATTGGTTAGGTC
GAAAAGAATCTGAAAAAAATATTCAAACAACTGCAAAGGGAACCTTGGTCCCATGTAGGGAAGA
ATCAGTAAACTTTGATAAAACCGAAAATGTTTTTATCGAAGGGGATAACTTAGAAGTTCTAAAA
CTATTACATAAGTCTTATTTTAATAAAATTAAGGTAATTTATATCGATCCACCGTATAATACCGG
AAAAGACTTCATATATAAAGATAATTTTAAAGATGGAATCAGAGCATATCTCGAACAGACTGGC
CAAATTAACGAAGATGGTTTTAAACTTACAACTAACATGGAAACTTCTGGACGATTTCATTCAGA
CTGGCTTTCTATGATGTATCCACGATTGTATGTTGCCAGAAATCTGCTTAAAGATGATGGGGTTA
TATTCGTATCAATTGATGATAATGAAGTTCATAATTTAAGGATGATAATGAATGAAATATTTGG
GGAAGAAAACTTTGTAGGAACCATCACATGGGAAAAAAGAACTAAATCCCAGAACACTGAAACT
GCACGTGATTTATTCCAATCAAAAACCGAATATATTCTAGTTTATAAAAAAGGTGCAGGTAAATT
AAGATTTAATTTAAAAGTTTCTGGGAAAAAAGTATACGATTTAAAAGATGATTTTGGACCATAT
CGTCTAAAATTATTGGATGAAATGTCTTCATTGGGCATGCGTGGTCGTAAAACAATGATATACCC
AATTAAAGGGATATGGCCTAAAAAAGATAAACAGTGGAAAGTGGGTCAAGATCAAGTTAAGTAT
TTTGAAGATCGGGGAGATATTGAAATACTCGATAATAAACCTTATTTCAAAATAAGACCTGATG
ACGAAACTGATAAGAAATATTCGCCGTTTTGGTCACATTTTTTTAGTAAAGAGATATATGGGACT
GCAGAAACGGGTAAAGCAGAATTATCTGCGATATTGGGTACTAAGGCCCACGGATTTGAGACTGT
TAAACCAGTTAATCTTATTAAAAAACTTATATTCCATGCCCTGAAAAAAAGTGAGATTTTTAATG
GCGAGATAATTCTTGATTTTTTTGCAGGTTCAGGTACAACAGCAGAATCCATTATTCAATTGAAC

GTTGACGAGAAGATGAATAATAAATTTATCCTTATACAATTGCCTGAACCAACTGCTCCAAATTC
GGAAACATTCAAAGCAGGTTTTAAAAATATTGCGGAGTTAACTAAAGAAAGAATTAAAAAATCC
ATCATAAACATTAATTCCAATGAAGGATTTAAGGTATTCAAACTCGCCCGGTCTAATTATCGAAT
ATGGGAAAAATATAATGGAAATAATGAGTCTGAATTAAAACAACAGTTAAAACTCTTTAAAGAC
CCATTAATTTCGAATTATAATAATGAAGATGTAATTTTTGAATGTATTATCAAAGAAGGATACA
GCTTAAATTCAAAAATTACATTATTAGATATCCGTACGAATGAAGTTTATAAAGTAATTGATGA
AGATAAAACACGCGATAAGGATTCTTATTTTTACATTTGTTTGGACCAAAAAATAAATAATGAA
ACAATTGTCAAAATAAACCCTAAAAAAGAAGATATAGTTATCTGTCTTGATACTGCGTTAGATGA
CAGTTTAAAGAAAAATATTTCAATCCAATGTAATTTGAAGGTTATTTAGGTGTAAGAATGAAAT
TTAAATTTGAAAGTGATCTGGAGTATCAATTAGATGCAATTAATTCGGTAGTATCATTATTTGAT
GGCCAAAATACCAAAAACTTGTCTTTTCCACATGTAGTTGAAGTTAATCCTAAAGTTATATCTGA
AAATAAAATTTTGGAAAATTTAAGAAGTATCCAAAAAGAGAATAATATTAAAGAAGGTGCTGAA
CTTCAAGGCATGGATTTTTCCATTGAGATGGAAACTGGAACTGGAAAAACTTACGTTTATTTAAG
AACTATTTTAGAATTAAACAAAAAATATGGCTTTAAAAAATTTATAATTATGGTACCTTCTGTCG
CAGTGCGAGAAGGAGTATTAAAAACTTTAAACATAACAAAAGAACACTTTAAACAGCTCTATGG
TAATATATCTTACAATTTTTACCAATACGATTCGGCCAAACTTTCAAAAATAAGGGAGTTTTCTA
GAAGCAATACTTTAGAAATTATGGTTATGACCTTAGATTCTATAAATAAAGACACAAATATAAT
TAAAAAACACAACGACAGATTAAGCGGTCAAAAACCTATTGATATGATTTGTCGGACTAACCCAA
TTTTAATTTTGGATGAACCTCAAAATATGGAAAGTGAAAAATCAAAGGACTCCCTCAAAGAGTT
AAACCCATTATTTAAATTGAGATATTCTGCAACCCATAAAAATTATTATAATCTGGTTTATAGGT
TAACCCCAGTAGATGCGTACAATAAGGGATTGGTAAAAAAAATAGAAGTACTTTCCGTAATTAA
GGATGAAGATTTTAATACCGATTATATCAAATGTTTAGATATAACGGTAGACAAAAACAAATTA
AAGGCCCAATTAGAAGTAAATAAAAAACAAAAATCAGGTTATGTTGTAAAAACGGTAACTGTAA
AGCATGGCGATGATTTAGAAAAAAAATCTAAAAATCCAAACTATCAAAATTTTATAGTTTCCGGC
ATAGATGCTAGATCAAATCAGGTTAAGTTTTCCAATGGTTTAATTATTAAAAAAGGTCAGAATG
TAGGGGGGGATAAAGAACAAATAATGAAAATGCAAATAAGACATACAATTGAAGAGCATTTTAG
AAAACAGGACATTTTAAAACCATATGGCATTAAAGTTTTATCATTATTTTTTATAGATAAAGTT
GCGAACTACGTAGAAAAAGAAGGATTTATAAGGAAAACGTTTATCGAAGAATTTAACAATGTTA
AAAGGGGTTTTGAAAATTATAACGATTTAGATGTAACTGAGGTTCATAAGGGGTATTTTTCAGC
ATATAAATCTGAATCAGGTATGGAATCAGATAAAGAAGCTTTTGATTTGATAATGAAAGATAAA
GAAAAATTACTTTCTTTTGAAGAACCTACTCAATTTATATTCTCCCATTCTGCTCTGAGAGAAGG
ATGGGATAACCCCAACGTATTTAATATATGCACCCTCAATGAAACGGTCTCGGAACTGAAAAAAA
GGCAGGAAATTGGAAGGGGAGTACGTCTTCCAGTTAATCAGGACGGAGATAGAATTTATAATTT
AGATTTTAACATTTTAACCGTAGTTGCGAATGAAAGTTATTCTGATTATGTTTCAAAACTACAAC
AAGAATACTTTGATGAATACGGAAATCTTGTAGATTCTCCAAAACCATTGAATGCAAGGGATAG
AACAGTTTTGAAATTAAAAAAACAATTTAAATTGAATCCAGAATTCGAAGAGTTATGGAAAAGA
ATTTCAAGAAAAACAAACTATGCTGTTAAAATAGACACTAAAAAATTAGTTGAAGAGTGTGTAT
CAGAAATAAACTCTTTAAATATAAATTCTATACGGATAAAAGTTGAAAAAATAGAATTATCTTT
AAATGATGATGGGATAAAGACGTTATTTATTGGTGAGGAGTCTGAAGAATCCAATAATAATTTT
GAAATTCCGAATTTGATAGAACTGATTGCAAATGAAACTAAACTTACTCGAAATACAATTTATGA
AATTTTAGCAAAAGTGGATAATTTCAATTTAATATTTAAAAATCCTCAAGAGTATATACACTCAG
TATCTTTGATAATCTTGGAAAAGTTAAAAGAATTTTTGGTGAATGGGATAAATTACGTGGAAAT
AGAAGACTTCTGGCAAATGGAATTATTTAAAAATGTTGAAGCATATAACGATTATATAATCCCT

GATAAAAATGATAAATGCATATACGACGGGATCATCTGCGATTCTGATGGCGAAAAAAATTATG
CTCAAGTCTTAGAAGATAATGATCTTGTAAAAATGTTTATTAAACTACCAAATTGGTTTTTTGTT
GAAACGCCAATTGGAAAGTATAATCCGGATTGGGCCATTGTTTTTGACGAGAGGGATCTCACTGG
GGCTAGTAAGGAAAAATTATATCTTGTACGGGAAACTAAATTTGTAGAAAATCTTGAAAATTTA
CGACCCCTTGAAAAATATAAAATAAAATGTGCAAATGAGCATTTTAAAACATTGGGTATTGATT
TTAAAACAGTTAATGATATTAATAAAATTAAATAATTTGGAAGAGTTTATTTACCCATATTTAA
TTTTTACTATTAATTATTTTCATATAATCATTTTCTCCAATTTGCGCCATTGCACGCCTTAATGA
GCCTGACCAAAACTTCTTATTTTTAATAAAATCCATTTCTGGAACTAATTTTTTAAAAAGAATCG
GTTTTTTGAATATTTCAACTTCTTTTAATTTAACTCTTAATGGATAAACTTCTTTTGGATTATTC
GGGTGAGCTTTAAAGATTTTTTTATTATCTTCTTTAAGTTCAGAAGTCACTTCATAAACTGCTCT
TATATATTGCGGTTTTGGTTCTTTTCCACTTGTCCCCATTTCATAAATTATTAAATAATCCCCAA
GGTCTACTTTTGAGATGGTATTTTTATATGCCTCAGAAACCCCCCAGATTTTCTTTTCTTTTATT
ATATTCCAGTTTTCTTCAGTCGTAATACATAAAAAATAGTTCACTTAAACCACCTGAAAATTAAA
AACACTTTTAACAGAATAATTTTGTAATTTTTAGTATAATATAATTATGTTTTAAAATTTTGTTT
TTTTACTTAAAAACCATTCAAAAAAAAGCCCCTTAATAATTACTCCGAGGAATTAATTAAATAAT
TAAATAATTAATTCGAAAATAAAACAGTAATAATAGCTTTATTAGTTATATAATAAAATAAAAA
ACGGTTTACATAAAAAAAGTATTATTCATCCTGTAGTTCTTCTAAAAAAACCAGCATTTCGTCAA
GTAATTCATTTAGTTTTGGAATTCTTTCCAACGTCTTCATTTTTTTAGCATCATTTATTTTTATT
TCGAGTTCAAGTATTCTTTTCAACTTCTTTTTCCAGAACCTGGGGTTCTGAACCACCCCAATTATG
AAGTATTGGTGTAACCTAATTAAAAAATAGAACTTTTTAATTATTTTTAGATTTTGATTTTAAA
TTGTTGTCCATCTCATTAATAACTTCTTTAAGATATTTCTTTTCAGCAGGTGATAATTTAAACTT
TTTGTTGATGACTTTTTCCCCGTAGTAGCCATATTTGTAATTTACAGTTAATTCATTTAATTTTT
CAGATCTTAAATCGTCCATGATTTTTATGTTATCACCATATTCGCTTAAAAGTTTAGAGATCTGT
TCAAACACATGATTTCTTAGCATATGATAAGTTTCAACATATCTAAATTTATTCTGAATTACAAA
ATATAATGAAATAATGTTTAATACAGCCTCTTTACGAAAATAATCCATTCCTAACTTATGATATT
CTTTTAAATTTTCAGATTCGTTCTCTTGAGTGCAGATAACCAGTAATGTGTCAAATAACAAAAGT
GAGTGCATATAACGGTGGCCTACAAACACCTTTTCATCGTCAATCATATCAATTAATTTATCTAA
AAAATTTCCCACAATACGTTTTTTTTCATCGGTGTTCCATATCCCATATAGTCGATAAATCTGCT
CTAAATTTTGTAAAGAATGATATATCACTTTATTTAATTTTATATAAACACCATATTCTTCATCT
TCTTTAGTTTTTGGAGATTTTGAATGACTTCCAATATACGTTAAAATATCAATACATTTTAATAA
ATACATTAAGATATTTTCTGAAAGTAACTTTATTTTATCATATTTGGCTAAAAAAACCACAGTTG
CTTGGGGGTTAAATATTACTTCCATAAAGGTGATTATTAATGGTACATTTTGGTTAGTGTTATTT
TTAAAATAAGTTTTTACATATTCTTCAACGTTATCGGTTACTTTCTGATATTTCATTAACAACTG
CACATTAAATTCATTGTACTTAGCATGCGTAGAATACATTATTAATTCGGTTAAAAGAGAATAAT
AATTCTTAAATATAATAATTTTTTTTGGATTATCAGAAGACTCTAAACTTTCCATTAATTTTTTA
TGGAATAAATTCAATCCCTGTACAAACGTTGTGCGGTCATAGTTATTTATTAATTTTATAAATAA
ATCCTTTGCTATCGAATAATTTTCTTGATTTATCCATTCATTCGTTTTGAAATCGTTTATTACAT
TATTAACAATCGATTCGGGCTTTAAAAAATTAATTGTCCATAATACGTAATATCCTATAAGTAAA
ATACAAGATATCGGAAGTGATAGTCCGAATAAGATAATTATCAATTCGTATTTTGATTTTGGTGG
AATATATATGTTAGAAAATATTGTGAATATTATCGAAAGTAGTAATATTCCCAGCATCAACTTA
AAAAATGAGTTTTTTTGAAATAATGGTATTATTTTAGGGCCGTAAATCTGGGATGTTGATTGGA
TAACAAATATGGAAAAAGAAACTACAATACTGCCTAAAAATATTGTTGATGTATTTAACATATT
CAGAGTATTTGTTAGGTTTTCACCAAATTCTCCAAAAGGTATTAAGTTTATAAAAATAAACGAAA

TAAATACAGAAGTAGATACTAATGCTATCACATATTTTTCCCAAGAATCGTAAACGCTAATACCC
ATTAATTCACCAGATATTCTTTGAATAATTCATTTTCAAGATTTAAAACTTCTTCAGATCTGTTT
TCAGACATATTTATAAATAAATCCGGATTATGATACCTCTGATTCATATATTCCCATTTTTCATA
TGAATTATTTGTACGGCTGGCGCTTAAAGAAAGATTATAATAATTTTCAGAAAGACCTGCATCAT
TTTTTGCAATATAATAATAATCCCCAAAACATTCGTAAATTTTAGAAGTATGATTTTCTTTATTG
AGCATCATATCCAGGATATCTATCTGACGGGCCACTTCTAAACAATTGTATGAGTTTTCATAGCT
TTTTTGAAGCAAATATACATGTCCAAGTTTTAACCAGGCTTCGGTATTATATGGGTCTGATTCTA
CGGCAGTATAATAATATTCTTCAGATTTATCATAGTTTTTAAGACATTCATAACACTCGCCTATT
TTTATCCAGGGTTTTGGATCATTGGGATCTAGTTTGGTAGCTTCTTTATAGAATACAATAGCCTC
TCTATAATAGGACTGATTTTGAAGTCGTTCCCCCATTTGAAAACCATCTGGCATGATAATTAACT
CCGAATGATTTAAAAGGTAACCTATCGGAATAAATGAAGTTAATATGACCCATATGAGTAGGACC
ATGTTAACTACATTATTCTTATTTTTAAGATTATCATCTTTAGACACAATTTCACCGACTATTTT
ATAAGGATATTAAAAAAGAATATTTAAAAATGTCGATTTGATCCCAGTGAAAATCATTCAAAAA
AAAGCCCCTTATTAATTACTCGGGGGGTTAATTGTATACACATTCAATTAATTTTTATAATAATA
TTTTATAATATGGATATTATTATGAACATAATTTAAATTTCTTTTAAAATTTAAAAAAAGATGT
TTTTAGCTTTTTATTGATTATTTGAATTATAAATTTTAGTACAAATCTCCCTTACTGCATCATTT
AAAAATTTTATTTCTTCGTTTTCAGATTCTGAATCACAATGTACTATAAAATCAGATATAATAGT
TTCAGGCGTAATTTCCTCTGAAAGTATCGTTAATCTTGCAGTTTTTTTAGGTGTAAATTCTCCTC
TTTCAGAATTTCCAAAAACAAACATGAAATCCTCTTCGCAAACCGCATCTGACCCTCTTATAATA
TCATATTCCCATATTTCATCAGTTGACGTTATATACGGTATTTTGAAAGCATTTTCTGGAAGTAT
TTGACAAAATTTTTCTTTTGAAATCATTATCCCACCTCGAATAATAATTAAAATTTCATTAAAAC
TATATTTAAAAATGTCGATTTAACCCCAATAAAAACCGCTCGAAAAAAAAAGCCCCTTAATAATT
ACTCCAAGGAATTAATTGTTTGTAATAACAATTAATTAAAAAATAAAAATATATTATACACTTT
TTTTAAAGACCAAAAAATAGAAGTTATTTCTTTTCAACAGCAGTTATAAAAACGCCTTTTTTTCC
GTCAACATCAATATACTTTTTTTCAACAACCGTAGGTTCTTCAAATATATTATCATAATCGATAT
CCAAATACTCTACGTCTGACCTACTAAGCGATATTCCAACGGAAGTTTTACTTAATTTTCTCAAT
TTGACCTTAACCATAACTAAAACACCAGAATTCTTGTTTTAAATTATGCAGGTCTATATATAAAT
AGTAATGGAAATCTATTTATAGATATATATCTAAATCTATATTTAGAAATAATATCACAAACAA
TAAATTAGTTTGTTAAATTTCTAAAAATCGTTTAAAGAGGTGATGGAATGATAGGTCGTAAAAA
AGAGTTGAAGTCCGCACAATCAACCCATTTTAATCTCGAAGAGACTGACCTCGAAAAGTTAGATA
AACTTCGAACCACCACCGGAAGAGAAGTATCTCGTTCGGCATATATAAGGTCTGTGATTCGTCAA
ACATTCGATAAAATTTCTGTAATTTCAGAAAACGAGCAATTAACACAAAAATTCAAGAAAATAA
CCGAAGAAAACCAGAATTTAAAAGAAACTCTTTCAAAAGAAATTGAAGAAAAGGAAGCGTTATC
TAAAAAGGTTTTGGAATATGAGAGTTATTTGAAAAGCGAAACTGATGAAATCGAAAAATATGTC
CAGAATGCTCGCTGGCTTTTCACGTGGATTTTAAAGGATTGGGATTCGTCTGATAAAAATTCGAA
GGAAATTGCAAGACCTGCAATTTTAAAGCATATTTCAAGGCTTAAAAAGGTTTTGAACAGCCGGA
ATTTTGAAATCGAGAGTTTTAACGAAGTAAATACCAAAGTAGAGGGATAGAAATGATACTTAGA
AAAAAGACCTGGAGAAAATCGAGAAATGTCCTTCCAACGTTTATTGGAACTGTTACGAGCCACGG
CAACTCTGCAAATGTTGATCCAACTCTGCCAAAAGAATACATTGGAAAACGAGTTCTAATAACGA
TTCTTGATGACGACACCGAGCTTGTAGAACGACTTAATGGTGAATAAGATGAGAACCTTAGCAAA
TGAACTTGGATACACTGACGAAAGAATCGAAGAACTTGGAAAAAAGTATTCGATGCTTTTATTCA
TGCAGAAAAACGATATCTGGCAGACCCTGCCAAAAGCACTTCAAAACCAATTGAGGCCGCAAATC
GTCAGTGACTGCCATGAAATAAAGCATTTGGTAGAAAAATTAGTAAACGCTGTACAAAATGACG

ATATTATAATTGTTTAAGGGGTGTTTAAAATGAATTCAATAAAAGAATTAAGAAATTCTGGAAA
TCTGGATGTTAGGGATGGGCTTTTACTTGATTTAATTGGTTTAGGATTGAAAACGGTTTCAGAAA
TTTCAACTGACATGATTTTAGGAAAACCTGAAGACAGGGTCGAAGTAAACGTTGATGTTGTTGGA
AAGCTTACAGGGGAAGTTTTAGACCTAACATATAAAGAAACAGTCCCCTTAAAAGCAATTCGAGG
GTCTGACTTTGACCCTGACTCGTTAAAGCTTGAAATTCATCGATATTTAACTGGAAGAATAATTT
CTGAAGGGGAAATTCCGGGAGCGCTAAAAGATGGGTTTGAAGTTCGATATTCAAAAGAATACAG
GGTGTTTTATAAAATTAAGAATTTTTAAATACTCCCTTTTTAAACAACTAATCCAAATAACTTAA
ATATTATTTTCACATAAAATATTACGGGTGGAATTCATGGAACCTTTGAAAATATTTACAATTGG
TCATTCAAAAAGAACTACTGAAGAATTTTTAAAAGTGCTTAAAGCCCATAATATTGGGGCGATA
ATAGATGTAAGGTCCGTTCCTTTCAGCAGACACGCACCCCAATTTAATACAGACGTTATATCGGA
AAAATTGTCTGAAAATGGCATAGTCTATTTCTTTTGTGGAAAAAGATTGGGCGCAAAAATAGAA
GATCCTGATCTGTATTCTGAAGGTATTGTGGATTTTGAAAAAGTTAAAATGAAAGATTTTTTTCA
AAAAGGATTGGATATTGTTGAAAAATACGCTAAAAGATTAAACGTGGCTTTAATGTGTAGTGAA
TTAGATCCTATTAGGTGTCACAGAACAATTTTAGTATCAAAAGCCCTACAAGATCGAGGCCATAA
CATAATACATCTGATAAATGAAAATAAAATTGAAACTCAAAAAGATATTGAGAACCAAATGCTT
GAACAATATTATCCTGGCGGACAAAATACACTTTTTGCACATTTATCTAAGGATAAATTAAAAAG
TTGCTACATCAATAAAAATAAAGAAGTGGGCTATAAAATGAGGTAATATTGTGAAATTATACAC
AATCGGATTCACAAAAAAAAAGTGCTAAGGAATTTTTTGAATTATTGGGGAAAAATGGGATAAAA
ACTGTTTTTGATGTTAGATTAAATAATAAATCACAATTGGCAGGGTTTACAAAGTATCCTGATGT
CATGTTTTTTTTAAAAGAACTCAATGGTATTGAATACATCCATATTGAATCTTTTGCCCCAACTG
ATGAACTTTTAAAAGGATATAAGAAAAAAGAAATAAGTTGGGAAGACTACGAAAAAACTTTTAG
TCAAATACTTAACGACAGAAATATAAATAAAAAAATACAAAATTTAATCCCTAAATTGGATAAG
GGATGTTTACTCTGTAGCGAACCAACTGCGGACCAATGTCACAGAAGACTAGTTGCAGAACATAT
TAAAACGAATTTTCCAGATTTAGATATAGATATTATTCATTTGTAAATTAAAACCGAGGGGGTT
TAAATGGTTTTAAAAAGATTTGTAATTCTCGCTAATTCTAAAAAATATCAAGAACATTGTGTTGC
AGGCATAGACCTTGAAAATGGGGAATGGATAAGGATAATTAATAATCAAAATGCCGAAAAACAG
TACGCAATTCGAGATGAAGATCTTATTTGTGACTGTGGAAAAAAAGCCAAAGTGTTGGATATTG
TTGAAGTCGAATGTGAAAAATGTGGGGCGGATCACCCTACAGCATATTATCAACCGGAAAATTAT
GCCATGTCACAAGATATACCTTGGAAAAAAGTGGGACAATTTCCAATTTCAAATCTGGTAAATCA
CCTATCCACTAAACTAAAATTATGGTATGGTGGATACGATAGGGTATATCCCAATCAACTTAAGG
ATGACTTGGCCCCATATTCATTGATATTTATAAAACCAGAAGATGTAAAATTTTATCTTGATGAA
AGAGAAAAACCTAGGACTGATTTTAAATACCGTGGTTCAAATTATCGTAGGTTGGCAATTACCGA
TCCTGCTTACGAATATAATCTCGATAAATATCCTGAAAATGAGAACTATGGCTACGTATTAAGTT
TGGGCGGACTTTATGGGGCAAATCCTGCACATTTTAAATTAATATCTCAGATTTTCAAACTATAA
ACCTTAACTTTTTTTATTTTTTTCAAAACGATTAAAAGACAATATTTATAAATATATATAATAAA
TCAAATATTCTTAAGAGATACGTAAAAGTACGTATTTATACGAAACACAGATTGCACTTTGAGGG
GAATGCAAATGATTAGTATTTATAAGTTAGCTGACTGGTTTCTAAACAAGGAATCAATGCCCCAT
AAAAAATTACAAAAATTATGTTATTATTCTGTTGCATGGTATTACGCATTATATGATTCAGATTT
AGTTAATGACACTGAATTTCAAGCTTGGATACATGGGCCCGTATCTCCCGATCTTTATTCTGAAT
ATCGAAAATATGGGTGGAACCCCATTGAAAAAGCAGATGTAAAACCACAATTTACGAAAGAAAC
TGAGGAATTTTTGGAAATAGTATACAATACTTATGGGGATTATAGTGGGCATGAGCTCGAAGCTC
TGACGCATGGTGAACTACCTTGGATTGAAGCTAGGACGGGATTAAGTGAATTTGAACCATCCACA
AATCCAATTTCGAAGGAAACTATGAAAAATTATTATCTTTCAATATATAATGGGTCACAAAATG

ACTAAAAAATTAACAAATCCAAAAATTTCCAAAAATAAAGAGAAATTAACTAATATTGAAGAAA
CCAGTTTAAAATCAAAATTAACTGCAGAAAAAGAAATTCGTCCAAGTATAAGTCTCAGATTTTTT
GACGATAAATTTAGTGGATTTTCTGATCTTAATAAATCAGAGCTTAAAAGCTGGGATAAATTTA
TAAAAGATGTTAATAAGTCAGATTCGTGGGATGATGTCTTTAATAAGTACTTTAGGGGCATAAA
CAGCGATCCAAAATCACTGGACAAAGTTAAACGATTAAATATTTATTCAGATCAGATTGAAATCA
TCCACCTAGGGCTAACCCAAAAATTCAGAATTCACGGAGCTAACTTGAACGGCCGATTTAAACTC
ATATGGTTAGATCCAAACCATAAAATTCACAAAATGAGTTAATCTTTTTTGGGTTTCAATTTTAA
AAAACCATCCAATGTCGGTTGTTTTACTTTCTTTTTAGTTCCAAGATATCCTTCTCCATTATATT
TAACTTTTAAATTGTCAAGGATGTTTTTTCCATGACCGCCTAACAATTTTAAAACTTTTTCAGGG
TTTTGACGAATATCATAAATAGATTTTATATCTGCAGCATACAATTTTCTAGCCCGTACTCTCCC
CACATTTTTAATTTCCATAAGGGTGATTAACTCTTGTTTAACACCATCTTTTAAACGAACGTCTA
GATTTTTTAGTAAATCGTGAAGATAATCGTTTTTTAAACCAAGAAATCGGTTTATCTCTTTTGCA
GAATATGCAATCCAGTTTATATTTTCTAATTTATAATTTAAATTTCCTGCTTCAATTTTATATTT
TTCCAAGAGTTCATTCATTGACATTTCATTAATCCAATTATAATACATTTGAGATGTTTTAAAAA
CTTCTAGGGGCTTCTTACTAACATATTCAAAAGGATACCCTTCAGTCATTTTTATTTTCCGAATT
ATTTCTGAATAACCATATTCTTCTTCATCACTCGCAGGCAGAAATGGCTTCAATTCGCTGTTATT
TGTTAATATATACAATAAATGCTGTTCGATTTCTTTATCAGATAAGTTCCTAGCATTCATGTTCA
GTTTAGTAATTCCTGTAATGATATCATGAGCAGTTAATGGATCGATATATAATTCTGCAATACGT
TCTCCAAGATCCGTAGCAGTAAATCCTGAAATAAATCTCCAAGAACGTAATGATTCCAGTATTTC
ATAAACCTGAGTTAATATGCCTCTAAAATTATCCTTATTCAGATAATAATAGTACGTGTTTTTCA
TAAATTCAATTAAATCTGATTCATTTTGGATATATTCTGTTGCAATCAATCCCAAAATCTGTTTT
CTAAGTATTACTTCATAAGATATCCACGAATACATGTTTTCAGGTTTTCCATCCAACATTTTCAA
TGCTAAATCATGATCTTGTTCTTTTTTTAGATAAATTAAACCTTCACCATAAGGATCAAGGCCAG
GCCTTCCTGCCCGTCCAATGCATTGTTGAATCTCCATTACCGGAATTTCTTTGTACCCTGATTCAG
TATATCTGTAAAGGTCCCTAATTATTACCCTCCTGCAAGGCATATTTAAACCTGCAGAAAGTGTT
GGTGTACAGCATATTAATTTTATAATACGATTACGATATGCTTCTTCGATAATGCTTCTTTGTTT
TGAAACCAGCCCTGCATGGTGAAATGCCACTCCTTTTTTAATACATTCGGCTAATTCCTTACAAT
TTTCAGTAGGCGTATCGAGTACTTCAAGAATTTCTTCACTTATTCGATTTAAGTCTTCTATTTCA
GAACCCGTCAGTTTTTTTAATAAATTATGCTTTTTAGCCTCTCCTGCTGCGTATTTTTTACTGCTA
CAAAAGATAATACATGAACCGTTCTCATTAATACAATCAAGTACGATATTCTGGGTAGGTTCTTT
ATAATATTTAGAAACCACTTTTTTATCGGAATTTAAATAATTTATTTCACAGTCTGTAAATATTC
CTTTTTTTAAATCTACGGGTCTCCATGTATCAGTAATTAATTTTGCGTTTAACCATCCAGATAAT
TCTTCAGGGTTTCCAATGGTTGCAGATAAACCCAAAATCTGTACGTTCATTCGTTTTAATTTTGT
AAGAATTATTTCTAAAGTACCGCCACGTTCTTTATCCCCTATTAAATGTATTTCATCAATAACAA
TTACTGAAACATCATCTATCCAAGCAACTTTATGCCTCATTAAAGAATCCAATTTTTCGGATGTT
GTTATAATCAAATCAAAACCTGAAAGATCTTCAGTTTTATCATCAAGATCTCCACTAGATAATCC
AACTTTTAAACCATATTTACTATATTTTTTTCTAAATTCATTGTATTTTTCACTTGCAAGTGCCC
TCAATGGAACAATGAATAGACCTTTTGACCCATTTGGAACTTTTTCATTGTCTAACAGATTATTT
ATTAGTGCCATTTCCCCAATCAATGTTTTTCCACTTGCGGTAGGAATACTAACTAAAAAGTTCTT
TGATCGATCCAAAAGCCCTGATTCAATAACTTTTTTTTGAGGTGGCCTCAATTCCGTAATTCCCA
TATCCTCCAAAACTTCAAATACTTGTGAATTAACCATTTTATCACTTTAAATTTTTATAAAATTT
GGCTAAACTAGAAAAAATAATTTAAATTATCCTAAATTTCCCAATTTGCCCCCATAATTAATTTT
GAACGGGTATTTATCTATTGAATATCATATAAATATTAAATAGTTATTCGCAGGTTATTTTATAA

AATTAAAAATTTTTTAAACACACTTTTGCATTAAGTGGGTAAAATTTAAAAATAAGGTACCGGA
ATTTCAACTGCATATCAAAACAACACAAAAAAGGATGTACAGAAGAAATTTATAAAATTTATAT
GTAAATTTTCATAAAAAATTAATTAAAACCCGATTAAAAAAATATATTGTGGTGCGCCCCTTGAA
ATCCGGTTCTCCACTGGGGAGCGGGGGTTCAAATCCCTCCCTCTGCGCTTTCTATTCTAAGACAGA
TTAGACTTTTAAAGTATTAGTCCACCAACGATTTTTTATGCGTTTTTCAAATGAAGCTTATTCCA
TTTAGTGGACTGGTCTGAGTTGCTTACTATAATACCGGACTATTTTTTGTTATCTAAAAAAAATT
CATTTCAAACTACCGGATTACTCCATCATGTAGTACATTTTAGAGAATCTTTTGAATGTTTTTCA
ACATCTTGTTTTTTCGCTCTTGTGAATGCGAGTAAAACAAAGTAGTTTCAAGCGATTTATGTCCG
AGGTACTCTTTTACGATATCAATTGGAGTTCCTTTATCTAGTAAATCAACAGCCCGACCGTGCCT
TAGAGAATGCACAACTAACTTTTTGTTTTGCGGTAGTTTACCATCTTTTTTGAGTTCATTTACTG
CATTTCTAAAGATTTTACTTATCCATGCTTTACCAACCATTTCTCCGGAAAGGTTTTGAAATACA
TGATCTTTTGAACCTTTTTTAATATTGTACTCCAGATAGTTGTTTAAAACATATAATGTATCCTT
AGAGCAGACTACAACTCGTTCTTCTTTTGTTTTAGTATTTTTTAATTTAAATGATCCTTCTGAAA
GATCACAATCTTCATATTTCAATTTTAAAACTTCTGAAACCCTACATCCAGTATCCCACAAAAGC
CTTATCATCACAACATCACGAAACTTAGTCCTGCTTTTACTTTCAACAATTTTTAAAAGAATTTC
GTTTAAATGATCAGGATATATCGGATCATAGTGTTGGACTTCGTGTCCGTTGAACCTTTTTCTTT
CTCTGCTTTCATCTGCGAAATCGTTGAAATTAGGAACTCTCATGAGTCGGTAGAAGACTTTTAAC
AAATTAAAATATCTGTTTTGAGTACTTCGAGTATTTTTTCTTTCGTTTTCTAAGTAATTAAAGTA
TTTAACAAAGTCGGCAGTTCTAAGTTCATCAGGGGCTTTATTTAGTCGGTTAAAGGAGTAATCAA
GGAATACTTTTAACCTATCGATATCTTTTTTAATAGTACCTTCTTTTATTCCATCGAATTCTCTT
TCAGTTCTGAATTTTGAAATCCAAACGTCCATTTCAGACATTTCGTTAGTTTCTGTTAATCTTGT
AAATCAGGACCCGACTCCTGCAGTTGGTGGAGACATTTTTGAAGTTAGAATCGGCGTTAAAAATG
AAGGCGGAAAATCTGCTGGAGACTACATATTAGAACTTGATCCATCATACCCCTTCCAGAAAGTT
GCTGGTGAGGAATTATCCAAAGAAATTGGCTTTTTAGATAAATACCAAGACGATGATAAAGAAG
TAATTATTACAGCATTTAAATTACGTGTAAATGAAGAAGTAACCTCTGGAAACTATCCTTTAAAA
GTATTACTTTATAAAAAAGGAGATTTAACTAAAACGGGTTATAAAACCGAATTAACGATAGATG
TCGGGAATAAAGAAAATGCAGAAGTTATGCTCAGTACCTCAGAATTAAATCCTGGAGAAAACTCC
AAACTCACGTTTACCATAAAAAATGCAGGTTCTGCGCCATTAAAAGATTTAACATTTTCATGGGA
TACGCCAGAAAATAAAATACTGCCACTTGGTTCTGGAAATACAAGATTTGTAAAATATATTGGCG
TAAACCAGGCTGTAAATTTAGATTACGATGTTATGGCAGGTTCGGACATAGCACCTGGATTATAC
AAGATAAATATGTACTTAAACTACGAAGATACAATCAACAGCTCTGAAAAAAATGTTATTCAGG
ATGCAGGTATTCACATTTTAGAAAAGGCGGATTTTGAAATAGCATTTTCAGAAAATTTAGACGA
TTCATATACGTTTTCAATAATGAACGTTGGAAACAGTGATACAAAATCTGTATCGATGTATATTC
CAAAACAGGACGGCTGGACTATAATTGGGTCAAATAATGCAATAATTGGGAATTTGGATGCAGG
AGATTATACTTATGCAAGTTTTAAGCTCTTGGCCGACAGTTCAAACCATTCCGAAATCCCAATTA
AAGTTGAAGCATACTACATGTCAAACGAAGGAAATAGAAAAGTAGTAGACAAAGAAATCCTTGT
TTCAAACTCAAATGCGGCAATTACATACATTACCCAGGCTGATTCCGAAAAAGGCGGATTTACAG
GATATTTAATAATGGCAGGAATCGTTATAATTGCAATATTAATTTTGGGTATCTTCATAATCCGT
AAAATTCTCAAAAAAATCAAATCTGCTAAAAAAGAATAAGGGGATTGAGTTATGAAACCCTCAA
AAATTTTTAAACTTGCCTTTAATATGGTAAAGGGAAGTAAATTAAGGAGCTGGATTACTATTAT
TGGCGTAGTGGTTGCGGTTGCATCGGTAATTACAATAATCTCAGCAGGAGAATTAATGGAAAAGC
AAGTGACACATGAACTTGAAAAACTTGGTGGAGATGTAATTTCCGTATACGCGATAACGACTGAA
ACAAGCAGCAAAACGCCGGAACTAACAAAAACGGATGTACTTGTAATCAAAGGGATACCTTCGGT

TAAATACGTTGACAAACGAGTAAATAGTTATTCAAAGATTTCTTATGGGGATAAAGAATTGTAT
TCGCAGATAACTGGAGTTGATCCCGCAGTCTGGCCAAAAATAACACAAGAAAAGGTTGAAACTGG
AAGAATGCTACAGCCCGGCGATAAAAACGTTGTTGTAATTAGTAATTACCTTGCAAATGAAGAAT
TTAACCGTAAAATAAATATTAACCAGATTATAAAAATTGGAAATAAATCATTCAGGGTCATTGG
AATACTCGATAATGATTCAAGTGGACTTGCAAGTTTATTTTCTGGAGGAATTTATGCTCCCTTAG
ACTCTGTCTACGACATTGAGCAGGATTCAGACTACAGCGTGCCTAAAGAACGTGGAACGTATGAC
AGCTTGGAAATTAAATTAAAAGAAAATGTAGATACCGATGCAGCAGTTCAGGATATTGAACAGA
AATTGAGAACTTCAAGACACGTTACTGAAAAAAATCAAGATTTCTACATATCTTCTGCAAAATCA
TTTATTGAAAGTTCCCAATCTATTATTGGTGGAATAACCCTGTTCTTATCATTTATTGCAGGAAT
TTCTCTAATCGTTGGAGCATTTGGAATATCAAATACTATGTTCACGGCAGTTTTAGAAAAAACAA
AAGAAATAGGGATCATGAAAGCAATCGGGGCAAAAAACGAGGATATATTATTACTGTTCTTGTT
TAATTCTGGTCTTATTGGCCTTGCAGGAGGATTTTTAGGCCTTTTAATAGGGGTTTTGATTACAC
AGGTTATTACCTATGTAATAGCATTGAAGTTTGGAACACCTTTTGAATTGTATGTGAGTATCAAA
GCAGTTGTCGTTGCATTGGTCGTTTCAATAGGTGCGGGCATTTTAGCAGGCGCAGTTCCTGCATA
CAACGCGTCAAAACTAAAACCTGTTGATGCCCTTAGAAGTGAATAAATATTAATAATAAAATCAT
GAAAAAGTAATTAAGCAAAAAGATGAAAGTACCGTGAAAATATTTAAAATCCTTTCATTTCCGT
TTTTTGGTTTTAAAATGAGCGTAAACGAAATTTATACAAATTATATAAAAGGTCCGGCCTTGGAT
GTTTTTTCAAATCCAAAAAACTTGATTTTTGGAAGTACGGTGCATTTTTTAAGTTCTATTTTTAT
ATTATTCAGCTTTCTTAGTTTGTTTGTGTTTTTTGTTATCCTCGGAAGCACAATAACCGCTGAAT
CAAACTTTTTAATTGATATTTTTAAAAATAACTTAAATTTAACAATTGATGTACTTTTAATTATT
TCTGGGGCAGTTTTTGGATTTTTCTTTATTTTTAGCATTTTATTGACATTTTTAATTAACGGCTA
CTGGCTTAAAACCATAAAATATACAATGAATAGTCAAAATATCCCTGACTGGACGGGTGTTTTAG
GGTTACTAAAATACGGCGTTATTTACACGATTGGACTGTTTATAATTGGAATAATTTTTCAAAGT
CCTTTTTACATGATATTACTTGCGCAGATCGTTTTAAATTTACCCGATTTGGGCATTCTTTCGAA
TTTACCATATGTTTGTTCGTTTCTCTCTTGGGTGTACATACCTCTTGGAACATTGAATTACATTG
AAAAAAATAAATTTTTAGCGCTTTTTAACGTTAAAAAGTCATTTAAACGGTATTCTAAAGAATA
TATCAATTATTTAGTCGCTTCATTTCTTTTAATAGGCATATACAGTATATTTGAATTATTGATAG
GTTATCTCATTGAATTAGTTATTGATAAAGTTGTAACAAATCCAAATTTAATAACTGACCCGAAT
TTTTTAAGCTGGCAGATATTTTGGATTCCTGCGCTTTACGTAAATTCGTTTGTATGTTTTACAAT
TTTTTTATGGCTTTCAAGAGGTATTGGGACATATTATAATATTTCAACAGGTCGAAACGACGTAT
TAACAAAATAAAGAAAATGGCGGATATTGCATTTACATTTTAAAATTTAAAATGGTCCAAACAT
GTAGTTTTTGAAATTTTTTTAAAAATTTAAAAAGAGATCAGGAACAATTCAAAACTCCCCAATAT
TGAACTGTTCTAAAGGTGTATGGAACGATTTTATCAACAGATGTAATGTACATCCCATTTCCCAA
GATTTTCCCCTCTTGGGCAAATATAATTTTTGAAAATAGCATTACTTAAACGTTTAGGGTAATCA
AAACCCATATAACGGGTTAAAATAGGAAAAAAGAAAATAAATGTGTTATTTAAACTTGCATTAT
TATTTTATCAATGTAAAACTGGCTTTTTTCAAAATCTCTTTTAGATTCAAAAGATTTGTTCATTA
CAATTATATCTTTGATATTTGGAATTGAAAAAAACTTCCAGTCAGGCCGGTTTCTTGAATCAAAG
GTCCACGTTTGAAATGCTTTTAAGATCCATTTTCCAGTATCTGTTATTGCAATCATATGCGGTTC
AACGACTCGCGGTAAACCTTCGTATTGAAAAGTAACTGTTCTTTTACATTTTATTGCCGATTTTA
AAACATACCTCAAAAAGACCAAATGGTAAAATATACTTCTTTTTCGCGTAGCTTAATTTTAAGGC
TATTGGACTTGAAATTTAACAGTATGCTAGAACTGGTATTTTTGCATTGTTAACAAAAACTCCAT
TTTTCAGTTATATGTTCAATAATCCGGTTGAAAATGGTAAATCAATGTTTCCATAACTTTCTTAA
ATTTTTTTTATAATTGGTTTTCAATTAAACCTTATCTCTTATCGAAAAAAATTAATATTTTGGGA

ACATAATATACTATAATCTTATTATATGGGGGATAATTTGAGAGTCCTGTTAGATACAAACATTT
TTTCCAGTTTTGAAGATTATTCGGATATTTCCGGAAAATTAAATGAATTATCGCGAAGACTTAAT
CCAAAAGCGATTCTTTTAAAACATCCTAAATCTATCGAATACTTAAACACGTTAGACGAAGATAC
CAAACGGATTTTACACTCGAAACTGGCTTTTTGCGCTTTTTTAAACGATTATCCATCACCAATAA
ATGATTTAGAATTTAATACCAATGTTGGATACCTTAAAGGAACGAATAACGACCTTGAAAACCAC
CTCCTGTATGCAGTTTATACAAATTCCGTAACATACCTCATAACTGAAAACAGGATGATACACCA
GAAATCAACAAAACTCGGATTAAAAGACCGTGTTTTATATTTAAATGAAGCTTTAGATTTTTTA
AAGAGTTTTATTCTATTCAAAAAAGGCAATCCCTACCACAAATTAAACAGCGCCCTATAATCAAT
GTAAATGTAGATGACCAGATATTTGACTCACTGCGTGAAGAATATCCCGAATTTAACAACTGGTA
CAATGAAAAAGCACATCTTGGTAGGGAATGTTGGTGCTACATCAAAGAATCGGGCAAACTTGGAG
CTGTTCTGATATTTAATGGCGAAGATTTTGATGTTATTAAAACAGATCCGCTTTTACCTGAAAAA
AGGCGTTTTAAGATATGTACATTCAAGGTTGATCAAGTTAGATATAAACTGGGGGAATTGTTAC
TTAAAAAAGCTTTTGATTACTGCATTGATAATTCAATAACTGAAACGTATTTAACGCATTTTGCG
AAATCGGAGTTTGACTATCTTGTTAATTTAATAACAGAATATGGGTTTATTGACATAGGAACCAA
TGAAAATGGTGAAAATGTATTTTTTAAGGATCTTAATCCAACCCCTAATCAATTAATGTTTTGCG
ATTACAAAGACATTTTTAAAAAATACTATCCACTTTTTTATGATGGAAATCGAGTCAAAAAGCTT
GTTGTTCCAATAAGGCCCCAATACCACGAAAAACTCTTTACTGACTATGTAAAAAGAACTTCTAT
AACATTAAACGAGTATTTTGGGGATTTTATAATTGAAGGAAATACTATAAAAAAAGCATATATC
TCGCATAGCCCCATTAAAAATCTAAAAAAGGGGGATATTTTATTATTTTTTTCCAGGATTGGATA
TTGCTTTATTAAAAGTAGATTTTGAAAAAAATTCTGATAAAGAATGGCTTGACAAAAAAACTGA
ATTTCCATTTATAAAAGTTTCAACAGGACGTCTTGAAATGGGTGATGAGGTGTATTCTTTTGGAT
ATCCCTTATCAAGCTATGGAATAATAAAACCTGGTGAGATAGGATATACAAAATTGAGTCCGCGG
GTTACATCTGCAATAATTTCATCGAATTTTGATACAAATATGGGTACAGCAGGGCCGAAAAATTA
TGTACTTGATAAAGCACTAAATTATGGAAATAGTGGTGGGCCAATTATTGCAACAGAAACTGGCC
ATGCTCATGCAATATGTTCCAGATTCCAGCCTTTGGTTGTTCCACAGCAGCATATTAAAGATAAT
AATGGCAACCCCCTACCGATAATGATTCCAAGCCTTTACGGGGTTGTATCAAGCTTTAATAATAA
AGATATTATTGGTTTTTTAATGTCTTTGGATATTCCAATTGAAGAATAAATTAAAAATTTAAAA
AATTACTTTTAAAAATTTAGATTTGTTCATTTTTTTTGAAATTTTTAATTGTTTTAACATTAAAC
AAAAATTAACATTTTAATCAGCTTTTAAGGATATTTACAATGATTGGAATTGCAAAGAACCCTGA
AAAAAGCTACGATTTTAAAAAAAGCAGTTTTATCGGTTGAAAGATTTGTTAAAAAAAGTAGAGG
ATGTTTTAAAAGGTAAAATTTGAGAAAACTTATATTTTTTTATTTAAATTGAAAATTTAAAAAA
AGTAAAACACAAATTCAGGTGATTTTATTGGCAAATGAAAATTCGAGGGTTTTAAAGGAATGAA
AAAACCGTGCCAGCTTTTCATTACTCCGCCTTTTTTTAGGATTTTATCCATGTACTAAATTGGAC
TGCATAGGATTTAAAACAGTATATTCGACAGAAAGTGTATAAATGTGATCTAGTAAATATGATA
AAATTTCACTACATTAGTTAAAAAGACATGGTTTACGGGATATACCCTATAAAAAACGGTAAAA
ACTGATTAAATAATGTTGCGGAACTATTAAACGATGAAAAATACAATTTAAATGTATTAAACCG
CTCTGATTTTTTGAAAATCAAAAGAAAAATCTACATGAACGCAGTCAAAAAACGAACAAAAACT
TTCAAAATTTACATAATAGTTGATTTGAATGCTTGAAAAAATAATAATTGGGATCAGAAGGTAA
AAATTCAAAATCGATAAGTTTGTACAATTATTGATCGAATTTACCCCGAATAGAAGTATGTAGTT
TTAATATTTAAAATTATTCAAATTTAAGGCTAAATAAACCTTTAAAATGTTTAATAATCAGTTA
AAAGATCTTAAAAAAATCTTATAATTGTTCTGTTTTTTAAGTTAATGAATATTCTATTTTTTTCA
ATCAGTCTTTTTTTAAGATTTTAATAAGAGATACGCTGGTTTAAATCAGTCTTTTTTAAGAATAT
ATTTCAAATAGATGCTGATTTGTAATCTGGCCCCCGATAAAAATATATACCCTGCACAATATAAC

TTTTTGACTATAATTACACTTTAAATTACATCATACTAATCGGGGTGAATTATGATAAAAAAATG
TTTATTATTTTTGCTGCTGCTTTTTGCGCTAGGAAGCGTTAATGCAGTGGATATTAATTATTCAA
ATCCAACGGATGTAAATATTGAGGGTACTGATTACCACGTATTTGCATTGATTAACGAATCTGGG
GATTACAAAGTAACTGTTGATTTTGAGAATAACACATTATCAAGCAATGATAGTGTATTAATAA
TTAAAGACACTGAAAACGCTGTACTTGACTGTAACAACATGTTTTTCAGTACGAATACTACAAAC
AGTCCGTATATCATATACGCCTACAATTCAAGCAATGTTACAGTTAAAAACTTGAATGCTAACTG
GTCCTATGATACAATTTTCGTTAAAGATGTTAATGATTTTACACTTGAAAATTCAGAAATAATTT
CTGAAGGCTATTCTATCGAATTACAAGAGTCTTACGGCACCAAAATTTTGGAAAATACGTTTAAT
ACTATGACGTACGACGGCAGTATTGTGTTTGATGATACTATTGAAAACAGCACAATTTCTGGAAA
TACGATCAATATGGTCAGCACCGTTGTTGTTGCCAACGGTATCTGCGGTGACTACGGTATTATAA
ATTCAACAATTGAAGATAACATAATTAGCATTAATTCCACCGTAGGGACTGCTTATGGTATCAGG
GCATACTGGGACAATATAATAAATTCAACGATTGAAAATAACGTAATCGATCTTTATTCTGCTGA
TAAGGCTTATGGTATTTATTCAAAGTATAGTACCCACAACGAAATAACTGGAAATACAATTAATA
CCTCATCTACTAATTCGGATACTTATGGTATTTGCGCATATTATAATATTACCAATAACATAATT
TCGGGAAATAACATCACAGTAGATGCATGCGAAGATGCTTACGGTATTTGTTCATACAGTGAGGA
TATTACAAATGTGACGATTGAGGATAACGTATTCGATTTATCTTCTGATTACGAAACTTACTGTA
TCTATGCTGACGATTATATTTACAACGTATCGATATCTGAAAATAACATCACAGCAAACGGTTAT
GACGGGTCTTACTTTATTTACACTTACAATGAAGATTTAATTGGTTCAACAATTAAAAACAACGA
AATTACAATTATTTCAAAAGAAGGGGATGCTTACTGTATTTACCCATTTGCCATTATAAGCTCAG
TAATATCTGGAAATACAATTACTGCATATGGAGAAGTTTGTGCTTGTGGTATCTACGCAGATGAT
GTAGGTTTAGTAAATTCGACAATAGAATCTAATTTATTTAATATTTCATCCAATACTAGTGATTC
TAAATGTATTTACGGCGATTATGATATATTAAATACAATAATTTCAGGAAATATCATTATTTCA
GAAAGTAAGTGGGATTACTCTTACGGTATTTATGCCTGTGATAATTTTGAAAATTCCACGATCTC
ACAAAACAATATCACAGTAGATGCATGCGAAGATGCTTACGGCATCTACGCTGAAGACGAAGATA
TAGTAAATACTAAGATTTTAGGAAATAACATCACACTCAATTCTAGTGATAATTGCGCTTATGGT
ATCTTCGCATATAAATACCTGGAAAATTCTACAATTTCTAGAAATAACATAACAGCAGACTCTTA
CGGCGAAGCTCTCGGTATCTACGCATGTCACTATGATATGGTCAATATAAAAATAGAAAACAATA
TAATCGACCTTTATGCGCATTCTTATGATGGATATTATCCAAACGCTTACGGTATTTTTGCAGAG
AATAATATCGAAAACTCGGCGATTTTAGGAAATACCGTCACAGCTGAATCTAATGAATATAGTGC
TTACGGTATTTACGTTGAGAATTATACTGTAAACACTGCAATTTTAAAAAATACATTCAAATCAT
ACGCATACAACGATTCTTACGGTATGGTTCTTGATGACGGCACCATAAACACAACGATTTTAGGA
AATAACATCACAGCAGAATCAAACGAAACTGTTTACGGTATGGATATAGACTATATGGTAAACTC
AACTATTTCAGGAAATAACATTGCACTAGACGCATACGACTACTGTTATGCTATCGATCTAAATG
ATACATTAAACACAACACTCTCAGAAAACACTGTAGTGGGTGAGGTCTACGTTTACGGAAACAAT
ACCACAATCTCCTCAAACACACTAGTGGGCGAATATTACACCTCTATGTATATTTATGGAAATAC
TAATGAAATATTCAATAATACAATTCTCAATAATACGATCGATGAGAATGAGGACTATTTTGAG
GATATTGGTTCTAAGATGTATGTTGAAGGGGACTACAATACAATCTCATCAAACGTTTTACACGA
TGCTTGGGTATATAGTCGTATTGAAGTTTACGGAAACAATACCACAATTTCCTCAAACACGGTAG
ATTTCTACGTGTATTTGAAAGGAAACAACAACACGGTTTCTTCAAATGTTTTACACAATGATAAG
TATGGCGATGCGCACATGGGAGTTTACGGAAATAATAACACAATCTCCTCAAACACTGTGGATGA
CAGCATTAGAACCGTGGGACAGTACACGCTAATATCCTTAAACACTGTGATAAATGCAAGTTACG
ATGCAATAGATCCTAACGGGTATTCTGCTGGCGGAATAGATGCATATGCAACAGTATTTAACAAT
ACCGTTCTTGCAAGTAATATTGGAATATGGCTTGATAATTGCTGCGAAGGATATTCAAATATTAC

ACAAAATACGGTTTATGCTGACGATTATCCAATTATTATTGGAGATAACATCACAGGATGTAATA
TTTACTTAAATAACTTTATTTACACAGGAAATTCGTCAAATATTTCTGAAATAATGCCAAACACC
ACAGTAAACAACAGTTTAGTAAGTCCTTTTGAAATTGAATATAAATACAATGGAAACACTTATA
GTAACATTCTTGGAAACTACTGGTCTGACTACAGCGGAACAGATGCGGATGGAAACGGAATTGGA
GACACTTCGTACCTTTACGGATGTGACTACGAAGGAACATATTACAAAGAATACTATGATATCGA
GTATTTAGAAAACGATACCGCACCATTAATGGATATGTGGAATAATGGTGAAATTGGAAACTAC
GTTGCACCAACTACAAGTTCTTCACACAGCAGTAGTGGTGGAAGAAGCTATGACTCAGATATTTC
AGATGAAATAGGCTCAAAAGTTATTAAAAACTTCGTTTCAAGTGCGACAGTTATATATGGAAAC
GAAATCGATCAGCAATATGCTGAAGAATTAAGAGAAAGAATACAAAACGCAAACGGTTACAAAA
TTTCTGGAAATGCAGTAATTGTTGGTGGGCCAAACGCAAACGGCTTTGCTAAAGAATACAACGAT
CAGTTTGAAATGCCAATTTCAAACGACTACCCTGGAGAAAATAAAGGAATAATTCAGGTATTAA
AAGTTCAAGACAACACTGGAAAAATTGTTCAAAGCTACACTATTGCATATATTGCAGGTTCTGAC
AGACTTGGAACACAAGCTGCTTTAGAATACTTTAAAACTTTGGATGAATTGCCAAAAGGGCCAAT
CATGGTCGAATGGACCGCAAATGGGCCAGTAGTTGTTGAATAATTTATTAAAAATAAATATTTTC
TTTTTTTCTGATTTTTAAATTGAATTTAAAAAAGTAGTATTATTATTTTAAAAATTTAACGAAC
TGCAAATTTTTTTAAAAATTAAAAATGACTACATTTTAGTTAAGAATTACGTGTTAAATACCTCA
GAATACGATTCCAAATATTCAAAATTATCCCCGTAAAGAATAATTTGGAATTTAGAGTATTCGTT
TCGAGGAATGTTCAAATATATATCTGCATAACCTGAAGAACCTTCTTCGTAATCATTCAATTCAA
GAGTTGTCTGATCCCATACGTATCCAGGATCATTCAAATTTAATGCTGCTGCGTATAATTTTGGA
TTTTTCGCAGACCCTGCACCAACATTTTCAATATCCACATGTATTTTGTATTGAACATTTTTTGA
ATTATAAGAAGATACTTCTGCATCCCACAAAAGGTCTAGAGTTGGTACTTGAATCATTAAATGGA
TAGTAGCAGATTTTCCTTCATATTCTTCTGGAATTTCCCCAATTTTCCAACCAGTTCCCGTGGTTT
CTACATAAAAATATTTTTTTCCATTATAATTGTAGTAAGTTCCATAAACGCTATCATCCCCACTA
ACCCCTACTGCCATGTGGTTTGGAAGTTCAATCAATACTACCCCAAATCCAAGCTCGTGAAGTAG
TGCAGCGGTCAATATTGCAGTATCTTCACAATCCCCTCCATTGTCCATTAAAGTCTCTAAAGGGT
ACCTCGGATATTCATCGTAACCAGTTGTAACTTTATCTGAAGTATAGTCTAAAGACTGTACGAAT
GCTACGATAAATTCAACAAGCTGGCTTTTGGAATATCCTTCTTGTTCTGAAGCTTCTTCAAACGA
ATTAAGCATTGATTTTAAGTAAACCCTATCATAATCGGATAACGCGTATTGTTCATAACCGCTAG
ATCTTGGTTTATTTTTATAATAATCATACAGGTTTTCTGGAATGGATAGCGACCAATCCCAGTCT
AAACCTCCGTATTCCCAAGAATATTCTTCAGTTAAATAAGATACTCCGTTGGAAGGTACAGAAAT
ATATTCGTCTGCTTTTTCTTCAAAATCAGAATTATAATCAGATATTACATCTGCTTTTTCCCCCTC
TCCAAAAATAGTTTTAACATCTTCAAATGTTCCAGCATATTCTGAAAAATCCATACATCCTGAAA
AAGAAACTATCAAAAGTAAGGCCAAAACAGTGATTTTCAAATATTTCATACTCCCCCTAAAAATC
CATATTATAACAACTATTATGGGCGTGATTTCGTAATTTATAAAATTATTTTAAACTTAATTGAT
ATTTTAGTAGCAGGTCTAAAAATAACTTTTTAAAGTTTAAATTTCGGAGTCATAAGTATAAAAA
TAAAAAAAAGGAGTCTATTTTACTGAATAGGTAATTAAACTATTTTTAAGTTCAAATTAGGAGT
TTAGCTCGTATATATTATGCCTAATCGACATTTTTAAATATATATGCCACATATTTTATGAAATA
TCAATTAAATTGGAGATATATTTACATTACCATTAATAATCTAATTCGGGGCGATTTAATTGAAT
TTTTTTAAAAAAATTTTAGTAATTTTAATGTTGTTACTAGTCGCATCAATTTCAGCAAATTTTTC
AGAATCCATAGATGATGGAACGTACATTTACCACAATTTTACAAAAGCAGATGCAGAAAAAATT
GGAGTAGATGAAAATACAACTGATTACGGAAAAAATGAAACCATTCAGACGTTTTCAGATTCGG
ATGAGGATATTTATGTAATGGGATGTTTTCTCCCAACTAAAGAAGAATTAATGTCAATGTCGGAA
CAAATTACAGTTGTTGAAGGCGTATCTGAAACTGCAAATCTATCAAATAATACTTATCTTGACCT

TTCAAAAGACCCATACTTTCCATTGGTCGGGGATCAAGGTAAAATTGGTTCCTGTGCCTCATGGG
CAATAGTATATTATGCAAATTCATATTTGCAGGCAAAAATCCACAATTATGATTTAAAGGGGTCA
GATAGTTTAAAATGCTTTAACCCAATGTGGGCATACAATAAAATAAATGATGGGAAAAATGAAG
GTTCAGGACTTATAGGTAACCTAAACCTAATTTCAAGACTCGGTAGTGCAACTTATGAAACAATG
CCTCCAACAAATAATTATACAATTTGGGGAGATTATGAAGCATGGCTTGAAGCACCCCAGTACAG
AATAACTGGTTATGAAATATCTTCAACGAATAATACTGACGTTATGAAATCCTGGCTGAATGAAG
GATCGGTAATTATCATTGCAATGCGCGGTGAGGACATTTTTACATTTGATAATAATTCAATACTT
TCAGATTTTGACCAGTCACACGATGAGGCAGACCATGCGCAGGCGGTTATTGGATATGATGACAG
CATATCTGAAGACAACGAAACAGGTGCTTTTAAAGTTATGAACTCTTGGGGCGCTAACTGGAGTC
CAAATGGTGATGGAAGTTACTATATAACATATAAAGCAATGGCAAATTTGGATTATACAACATG
TTATAGGATAAAAGGTGCAGTGTACAACACTTCGGATTCCCACCCTGACCTTGTAGGAGTTCTAA
AATTTGATTCGGAAAATAAGGGTACAAAGGATCAAAATATAACTTTTGGAATTGGAAACGAATC
AAATATTTCGGGATTTGTAGATATATATGGAGGGATGGATGAAAACGGTGGAAACGGTTCAATG
CCTGATTTTATTGCAGTAGATTTAACAGACTGGAAATCTGAATTCGAAAATTCATTAAATAATAC
TGGAAAAGGTTACTACTTTGCTAACTTTTCAGGCGGGACCGAAACTTCAATAATTTCTGAATTTG
GAATAATTAAATACAGTTATTACCCCGATATTGAAGAAATAAATACATTAAATTCTTACAATTGC
AATAAATCAGTAATATTTAAGTTTTACAGTAAAACAGCTTCAAAACTTACAAATTCAAGCATTG
ATATCTTAGATAGCAAAGTTATTGTTTCAATAAATGCAGACGATGTTGATGATGATCTATGGGGC
GTCAATGTATGTTTTGATGGATTAAACGAATATTATTCATTAACTAGAGCAAATCAAACGTTTAA
TACATCATTTAATAAATCCGTGTTTTCTTACGGGGAACACTACGCGATATTTGAAGCTTTTGATG
GTTCAGGAAACACAAATAAAAGTGAAAAGGTATATTTTGAAATTTTAGCCCCAACTACTACTTCA
AGAAGCACTACAAGTCACTACAGTTCTGATCTTTCAGATGGAATTGATTCTGGAAATATCAAAAG
AGCAATTTCAAATTCCAATATTGTATATGGAAGCGATATTGATGAAGAATTTGCCCTGAATTTAA
GAGAAAACGTGGAGAATGGGAATAATTACGAACTTTCCAAAGATACTATAATTGTTGGAGGTCC
AGAATCAAATGGATTTGCTGACCGATACAATAATGAATTTGAAATCCAAATTACAAACGAATACC
CTGGAGAAAACAGAGGGGTTATTCAGGTTCAAACTATCGAAGTAAGGGATGGAAATATCATTAA
GCCATATCAGGTAATTTATATCGCAGGTTCTGACAGATTTGGAACTCTTGGAGCATTAGAATACT
TTAAAACATTAGATGAACTTCCAAAAGGACCAATAACTGTTGAATGGAATGATAATGGCATTAT
AGTTGTTGAATATTAACTTATTTTTCTTTTTTTACAATATTTTGGAATTATATTTATACCTAAAA
AACATATATTATTCCCGCGAGGAGATATATTGCATAAAGAAATACCCATATCAATCCTGAAAGTC
AGAGATTTCGAAGATTTTGATGAAAATAATCTTTTTGAAATGCCCTTTAAAAGAAAAATCAGGG
TTTCAAATGTCAAAGTGTCCCAATCTGGAAAAACATACTTTTTTATAAGAACTGATGATTTTCAG
GAATTTAAAATTTACAAAGAAAATTTAACCGAAAAAATTCAGTTTATACTTAAAAAAGCCGATG
GATTTGTAACTGATGGATATGATGCGTTACAATATGTGACTTTTGAAAATTTAAAAAATAAAAA
ACCTAAAAATAGTAAAAAATCGAAAAAAATACTGGTAAAAATGCCGTTATGGCTTGCAAACAAT
AATGATATCCATTTTGGGGAGATTATCGAAAAGTTTCGATATATAAATTTTGAATTCGTTGGGG
AATCTAAAAAAGGTATCTGTGTGTATTCAAAAGAATTAAATAAAAATCTATGGCTCCCAAAAAG
CATGATACAGTACGAAAATCAGGAATAAAATTGTTGGAGTTATCCAAAACATTATTATATTTCA
GTGTCTTTTTTAGTAAATTAATTGTTATTTTAGATATCTAGGTGAAATTTTGGATAAATCAAAA
AAATTGGAAATTTTAAAAGCATTATTTTACACATGTATGATCCTTGCCAATGTTATCGCAGTTAA
AATAGGCAGTTTTTGGGGCTATGTTATGACTGTGGGAATTTTAACATACCCGATCACGTATTTAA
TGACTGATATAATAAGTGAATGCTGGTCAAAAGAAGAAGCAAGAAACACGGTCATTTTTGGATT
TGTTTTAAGTATCATTTCTGTTATTGTTACCCAATCGGCAGTTTTAGTTCCAAACGCGCCATTTT

ATGATGGTTCAGCATTTGATGCGGTGTTTAATTTTGTTCCAAGAGTAGTTTTAGCATCATTAATG
GCATATTTTGTATCCCAAAACATAGATGTTTCGCTATTTCATAAAATTAGATCATACACTGGAAA
AAAACATTTATGGATTAGGAATAACATCTCTACAATGGTAAGCCAGTTATTTGATAGTTTTTTAT
TTATTTCTATTGCTTTTTACGGCATATTGCCAATAGATGCGATAATAACAATGATTGGAGTACAA
TATGGATTTAAATTTATTATAGCAATAATGGATACTCCGTTTATATATCTTGGAAGGCATTGGAT
TGAAAAATAAAAAAATAGTTGAATTTTAAAATTTATTTTAAGATTTAAGATTTTTAATTATTTT
TTAGAAGCTTTATTTCTTGCTTCAGCAACTTGATTCGCAATTCTTGCAGCAAATGCACCTGCAAC
AAACCCTGCATCAATATTTACAACAGATACTACTGAACACGACTGAAGCATTGCTAAAAGCGCAG
CTTCACCTTTTGCACCTGCACCATAACCGGTAGATACTGGAAGACCGACAACTGGGACATCAACA
AGTGCAGAAACTACGGTTGGAAGAGTTCCTTCCCTTCCAGCAGCAACCACGATTGAATCGGGTTT
TTTATTCTTTAGTATCGCCATTTCATTAACAAGCCTGTGAAGTCCCGCAACTCCAACATCATATA
TTGCAATTGTTTCACAGCCCATTTCTGACGCTGTCATTTTCGCTTCTTCTGCTACATCAATGTCTG
CCGTACCTGCAGTAATTACTGCAACTACACCGCCTGTTTTTTTGATTGGTGTGCCATTATGAACT
ACTGCTGTAGTTGAATGAATGCCCCATTCAATCTCATCAGGCTTAAAGTTTGACTCCAGTGCAGT
TCTATGGTTTTCACCAATACGTGTGATTAAAACACGACCACTGGCATCGCTTTGAGCTTTTGCAA
TTTTTACTACGTCTTCAGGAGTTTTTCCTTCAGCAAGGACTGCTTCAATGATTCCTGTTCGATGT
TTTCGAAAGGTATCTATTTTAGCAATATCTGTTACTGATACATACCCCATTGAACGAATAGTTGC
TTCGGCAGTTTCAATATCTGTTTCACTGTTTTTTACGTTATTCAATATACTTTTGAGGTCCATCT
GGTACTCACCTACATTTTAAAAACGCTCTTTACCATAGCCATTTTTTCATCATTATCAGGACCTTC
AAATTCAGCTACAACTTTTTCGAGTATTTCAGGAATCTGGATATATTGTGGACATTTCTCTATAC
AAATACCGCAATCTGTACATTGTGACGCAAAATGCGGTTCATTAGTCCAGAATATATCACTAAGT
GTTGCAAAATACTGGAATCGTTGGGTTTCATCGTGGAACATGTGCAAGTTGTTGTATATGCCAAA
ACATGTCGGGATATCCACGTTTGCAGGGCAGGGCATACAGTAACCGCAACCAGTACAGTTAATCT
TTAACAATTCACCATATTTTAAAGCAGCCTGTTCTATAAGACCCAGCTCTTTTTGTGTTAATGAG
TTTGGATAACCTTCTTCTGCAATTTTTATGTTTTCTTCAAGCTGTTCTGGTTCAGTCATTCCTGAT
AAAACGGAAGTAACTTCAGGATGATTCCATACCCAGCGAAGAGCCCATTCTACAGTTGTTCGTTT
TGTATCTGCCTTGTTCCATATTTCCATTACTTCGTCAGGTGTTTTACCTGCTAAATTGCCCCCACG
AAGTGGTTCCATTACGAAAACCCCAAGACCCTTTGAAGATGCGTATTTAATTCCTTTTGTTCCTG
CCTGAACTTTTTCATCTAAATAATTATACTGTATAAGGCAGAAATCCCAATCATACTCATCAACA
ATTGGAGCAAAATCTTCCTGCATTCCATGGTATGAAAAACCTGCGTTTTTTATTCGGCCGTCTTC
TTTTGCACTGTTTAAAAAATCAATTACGCCAAGTTCTTTTATTTTAGCCCAGCTTGCTTTGGTAA
GGGTGTGGAGTAAATAATAATCGATTTGTTTCGTATTCAGTTTTTCAAGTTGGGCATTTAGGAAT
TTATCCATGTCTTCGCTGGTGTTTACTGCCCAAGTTGGTAGTTTTGTTGAAAGTTTAACCTTTTC
TCGGTATCCCTCTGAAAGCGCACGAGCTAAAAACGGTTCACATTCGCCCATGTGGTATGGCCATG
CAGTATCTACGAAATTTACACCGTTATCTATTGCATAACGAACCATTTTTGTAGCTTTTTCTTCA
TCGATACTACCATCTTCTTTTGTGGGTAGCCTCATAGCACCAAAACCCAATATTGAAAGTTTGTC
GCCATTTTTTGGATTTGTTCTGTATAACATGTTTCATTCACCCGATGTATAAAATCTGCAGTTTA
ATTGTAAGGTTTATTTACTGATATTTTACCTAAGTTGTTAAATGTGGTGTATTTTGGATAAATTC
AAATATTATTCTTATAGTTATTATTCTATTTTCATTCTTCAAATTTATTCAGGTATTATTTCATT
TAAACATGCAATCGCATTTAATGTTCTTGGGTATCCTACATAAGGCAGTGTTTGCGTAACTGCAT
CAAGTAGTACTTTTTTACCGTTTCCAACCATTAAATTACCGACAACATGTCCTTTTAACTGCGGC
TCACATCCTCCAAGGGCCAATAATATACAAAACGTCAAAAGTTCCCTAGTTTTTACATCGAGTCC
TGTTCTAGTGTAGTAGTCACCAAAGCAGTTTGCAGATAAGTACTTTTGAATGTGTATCTGATTCT

CTGGAGATGATTCATACATTTTATCAATAATTTCCCCAAAAATTGACTTCTGTACTTCAAGTCCT
TTTTCATGCCTATTTTCGGGCGTAGTTGTAGATTGACCGTCCAATGGTAATTTTACGCCCCTGCTT
TCTAATATTTCGTTGGTTGCATTGATAAAGTCATATGCTTTTGCAATTCCCACATAAGGTACAGA
TTGATACACGATTTCTTTAACTTCGATAGGCGTTACTCCAACATTAAGTGCGCCTTTTAGCATTA
CCTTATATTCACTTAATGTCTGGCTTCCAATCAATGCTCCCAAAATAACCATTAATCTTGTTTTG
GTATCCAAATCCCCGTGCTGAATAACTTCATCAAAAGCAAAGTTATCAAAAATTTCGACGAGTTC
AGGATCTGTTATTTTAAGGGTTGATTTGTGATCTGGAAATAATTCTTCATGATTTTTGTTTGCAG
TTTCACTGATTTTCATACGTTTACACATCCTTGGTTTATTTTGATGATACAATCAATCCATTTGA
TATTTTCATTCGATATTCTTTTTCAGTTAAGCATTAACCCATGCACTGTCAAAATGATGAAAATT
AGTTTAATTAACCTTAATTTAATCCGTTTCAGCCAATACTCCTTGAAGAACGTTTGGACCTTCAA
GATCTGCATCTCCTGCTCCATACCCTATTGAATATACCTTACATTTTGGAAAGACTTTTATCGGT
TTTGCAAAATGAGATAGAATGGCTGCACCGGTTGGAGTTAGAAGTTCTTTTGCTTCGTTTCCACC
TTGAAAGTAAAGATTTCCTTTTTTCAAGAGTTCAAGAGTTGCAGGAGCGGGTACGGACATGACCC
CATGTGCACATTTTACAGTTCCGCTTCCAACGTTGATCGGCATGCAGTAAACAGAATCGCATTTT
AGTGAATGAATAGCGGTTGATGATCCGATTACGTCTGCAAGTGCATCATTTTGCCCGACTTCATG
AAAATGTAATTTTTCAAGATCATCTTTACCATGAACATGCGCTTCAGCTTCTGCCATCTTTGAAA
ATATTTCGAGTGCACTTTTTTCTACGTCATTTGGCAGTTTTGCATCTTTTATAATATCTACAAGT
TCGAAATAAGTTCGTGAATGTTCGTCTTCAGGAACATTGATCTTGACGTCCAAAGCCCGAATACC
TCTTTTCACAACTTCATTCATATTTACGGATACAGGTACTGCTGATTCAATGATTTCTTTTACGT
AAAATTTGTCATCCAAAAGATCCAGTGTACATCCAAGAATCATGTCCCCTGCAGCTCCCGAAAAC
GGGTTGAATATAAGCGATTTCATACGTATCACATCATTAATTAAATTAGAATTTTAGAGCATTCC
TAAATTACTTAACCAAGTTTCAATATCTTTTTTCGAAGAACTTACCTGAGATCCATAAATTGAAA
GTCCGTTTAGGATTTTTGCATCGGGGCATGTTTTCTCGATATCACTTTCACTGTGACCCATACCGC
TTCCTTCATGAGTACAGAATGGGATAATGGTTTTTCCAGAGAAATCATATTCTTCAAGAAATGTA
AAAACTGCCATTGGTGCTGTGCCCAGCCAGTTTGGGTATCCTAAGAATACATTATCGTAAGAATC
CATATTCTCTACGTTTTTGAGTAGTTTTGGTCGTGCATTTTCATTTAATTCTTTTTTTGCCACAT
TTGTAGTTGCAGAATAGTCTTTAGGGTAAGGCGTTACAGTATCAATCTGAAAAAGATCGCCCCCG
ATTATTTCCTGAATTATTTCGGCGGCTATTTTTGTGTTACCCGCTTGCAAATTCACGATTTTTCC
ACTAACGTAGTTATTGCCTGCACGTGAAAAATATGCAATAAGACATTTCTTTTCGTTTGAATCGG
GCATATTACCCCTCATTTTTATTGTAATTTAATTTATCTTCGTAAGATATCATATTCTTTATCAT
TCACATGTCACAACCATTCAAAAATTTCTTTGAAAAGATTATGTCATTTTTTAAAAATTAAATAA
ACTACCCAAAACTTAAAAAATAGAAAAAAGTTTGAAATTTAGTTCCGAAATCTTAATTTAATGA
AATTTGTAATATTGGCATGCTTTTTCCCATTTAATCGGCTAGTTCAATCAATATATCGAAGCTAC
CATTTATTTCTGCAATAATTGAAGGGTCACAATCAATAACTCCTAAATTCACAAATTCACCATAA
CAATTTTCTTCATTATCATACAAAATGGTGAAATAATTTCCTTGAGTAGTAAAATCTATATCCCC
ATTCTTCCAGCCAGGAACCAAATCTTCATCATTAAAGGATAATGGTTTATCCATTACACCGCAGA
TGTCAAAATCGTATTTACTGGCATGAATCGTGTATGGAAGCCTATCAATAAGTTCTTTAGCAGGT
TTACCATCATTTAATATTCCCGGAATAGTTTTGTTATTAATATGCATATTAATTCTTGTTCCATT
CTTAATTTCACGGATTGAATTTTCTTTATTCATAATTTCCTCACCAATCTGTTCTTCATTAATTA
ATTCAGTACTTGAATAGTTCTCTGCAAATATCGCCACTAGAAGAACACTGAATAATACTAATAAA
ATCTGTTTATTTGATACTGTCGCCATTTTCTTTACCCATTCTTGCCATATTTTTCAGCTAAAACCA
AAATACTGGTTCTATGAGTCCTTTTGCTTCTCAAAAATATTTTGATTTCATTTTTTTACAGCATT
TGTTTTATCAAAATAGTTTTTTGCTTTTAGCATTCTACCGCGGATATCTACATGGAATGGACAGT

TCTTTTCACAAAAACCGCAATTAGTACAGTCATTTGCGTTTTTGCTTAATTTCAAGTAGTGATCT
TTTGCAAGATCGTCACCTGCTTTTGCAAGATCAAGATATTTGTTTACAATACCAATATCGATTCC
AGAGGGACAAGGTTGGCAGTGGTTACAGTAAATACAGGTGCCCATGATGTCTCGGTGCGGCAGGC
TTGCTATAAACGAGTAATCTCGATCTTTTTCAGAAGTAGAATAATATTTAAGGGCTTCATCCAGA
TCTGCTTTTGAACTTACTCCTGAAATACATGAAACTACCCCTGGACGATCCAGTGCATACTGTAT
ACACTGGGGAACAGTCATGGCATGGTTAAATGGTGATGTTTTTTCGTTTAAGAGCTGTCCACCAC
CATAAGCCTTCATGACCGTGAGTGCCACTCCTTGTTTTTCACATTCTTGATAAAGCTCCATACGTT
CATTTTTTAAGTTCAATTTACCCCATGAGGGTTTAAAATCATATGCAGCGTTTATACTGAACATT
ATAACATCAATTTCGCCAGTTTCGATAAATAATTTACATATCTCTTCAGAATGTGACGAAATCCC
CAGATATCGAATAGTTCCGTCCTGCTTTAGCTTTTTAGCATAATCAAAAATGCCGTTTGACATGA
TTTTTTCAAAGTCTTTTACCCGATCAACATAGCTGATGAGGCCAATATCGGCATAATTAGTACTG
TATTTTTTCAATTCCTGCTCAAAACCCTTTTTAACGTTCCTTGGAACTCTTGTCCTTGAATATGTT
CCTTTAGGATACCAGACACCAAGATGTATCTGCATGAGCATATCATCTCGATGTCCTTTCAATGC
ATTATTAATTGGTTCTGCTGCAGTGGTATCATACATGACCGTATCGATCAGATTTACGCCTTCTT
CCATGCCGTAAGTGACGATATCTTCAATTTCTTTTGACGTTATTTCTTTCAAGCTTCCTGATCCAA
TGCCGAGAGTACTGACCTTTTCATTGCCGTGAGGTAATTTTCTGTAGTCCATGTATTCGCCCCTTA
TTTGTAAGATGCACTGTAAATTGAAACGCAATCTTCGATACTTAACTGAGCTCTGTCACAGGTAA
ACAACATTCCCATTGTATCCATTGCATTTTTCGCCATTTTTTCAAATTCTTCAGGAGTAATGCCA
TAATCAGACATTTTAAGATCAGAAACTCCACATTTTTCTTGTAATTTTACGAGCATTTTGATGAA
ATCCATAGGTTCTGTTGCATCTTCCATACCCATTACTTTAGCCATTTTTACAAACCTGTCATCACA
TACATGTTTTTCGATAAAATGAGTAAAGTATGCTTTACTGATCATTATAAGCCCTGCACCGTGAG
GAAGATCTTGGTGATAAGCAGACATTGCGTGTTCTAATGCGTGCTGGCTTGTACATAGTCCAACA
GTTTCAACTACTCCTGATAACGTATTTCCAAAAGCAACCTTTTCACGTGCGTCCATGTCATTTCCA
TCTTCAACAGCTTTTGAGAGATTTTTTGAAACATTTTCTATTGCCGTGATTGCATACATGTCGCT
CATGAGATTTGCGCCGATAGATACGTAACCTTCCACGCTGTGGAATAATGCATCAAATCCTTGAT
ATGCAGTATATTTTGGAGGTACTGATTTCATAAGTTCAGGATCAACTACTGCTAAAACTGGAAAT
GTATCTTCGTTACCAAATCCGATTTTTTCACGATTTATTTCATGCGTTACAACTGCCCAAGGATC
AGTTTCAGAACCTGTTCCGGCTGTTGTCGTAATTGCTACAACGGGGATTGGTTTAGCTTTCAAAG
GCATTCCTTTTCCAGTTCCGCCAAAAATGTAATCCCAATAATCTCCATCATTTGTAGCCATTATGG
ACATTGCTTTTGATGCATCGATACAGCTTCCGCCACCTAATGCAACAATGAAATCGCAGTCATTT
TCTTTTGCAAAAGCACCGCCAGCCATAACAGTTGATCTTAATGGGTTTGGTTCAACTTTATCAAA
TACTGCAGTTTCTACACCTGCTAATTTTAACTGTTCTTCAGTTCTTGCAAGGTAACCGTATTCCCT
TGTAGATTTTCCATTTGAAATAACAATCATAGCTTTTTTGCCAGGCATTTTTTGTTCGTGTAGAT
TATTTAGTTGTCCTGCTCCAAACAAGACTTTTGTTGGCACATACATGTTAAAACTCATAATTTTC
ACCGTTATCCGAAATTTTTTTGAATATAATAATTGAATTTTTAAGATTGACTACTCAGTCAAATA
CTACTTTGTTTTTGTGATATATATAGATTAGGTAATAACTTCTTTTTAAAAAATTAAAAAAATA
GCTCTAAATTTATTCAGTTAATTTTTTTCCAATATTAAATGCCTGTTCTAATGTATCTTCGTGGT
TTAAAACGTCTCCAGGTGCGCCAACACCGCCTTTTACAATAGATTCAACAACATTTAATCCAAGG
AAATTTAACATGGAATTTGTATTTTCAATGTATGGTGCAAATGCGCTTTCATCAGGATTCGCTTG
TACGTAAACCGTAGCAGTTTTAATATTGCTCAATCTTGGGCTGAAATCTGCATTAACTACAGGGT
AGAGCCTGTCAAGGAATATTTTTGCCTGTCCAGTAACCTGCCACATGTAAATTGGGGATCCAATA
ATTAATACATCTGCTTCTTTTAAGTTTTCTAAAATTTCAGACATATCATCTTTTTGAGCACATTT
AAACTCGGATCTACAGTGCATGCATCCTTTACAGCCATTGACATTCATCTTATCAATGTCGTATT

GAATTACTTCTGCACCATTTGATTTTGCACCATCTAAAATTTTGTTTATCAATAATGCAGTATTT
CCCTGTTTTTTTGGGCTCCCGTTTATCGCAACAACTTTCATAATATCACTTTGTATTATTTGGAA
TTTTATTTTCATTTGTATTTTTCAAAATAATTAATTTAACTCGATATGGATTTTATTTAATTCAC
ACACTTTTTTAAATAAATATTCTCGTTATTTAAGTTTTCTTTTAAAATAAAAAGAAAAAAAGAA
TAAATTACTTATCTTCAGGTTTACATTGTCCTTTTTTCATACATTTAACTTCAATACAGTCCATC
CATTTCTTGGTTGCCCAGAATATTATTGAAAAAATAAAGCTGAATAATATCACTAGTGAAGCCAT
TAAAAGGCCGCAATTATACGGGTCATACATGCTACTCACCTTTAAAGAAAGATTTTAGTTTTCGA
ATGGAATATCCTCCCTTATTGGATTTTTTAGTATATCGAATAGGGGAACTTTAAGATTGACTAAT
CAGTCTAATATTGCTTTGTTTTTTTAGTATATATAATTTAGGTATTTTAAAAAAATGAGAAAAA
TTATTTTGCTTTTAAAGCTTATTTTCAAAATAATTAATTTAACTCGATATGGATTTTATTTAATT
TATACAAAACTTTTGAAGTATTTCGGCAATTAAACCAGTAAATTTACAACAACGCCCGCAATAAT
TGCTGATATAATTGTAAATGCCACGTATGCTGTAAAGTGCTTAGCTCCAAGAACTATTTTAACGG
CACCAAGATTCGTAATCTTTGTTGCAGGGCCCGTTATCATGAACGCTGCAGCGGCCCCCATACTCA
TTCCTGAATCTAACCATGCCATAAGTAATGGAATCGTACCGCCTCCACAAACATATAGTGGCACC
CCAATTGTTGCCGCCATCAAAACACCAAATCCTCTTTGACTGCCAAAAAGGTTTGCGAATGCATC
AGGAGATACATAGTGCTGAAATAACGCTGAAAGCAGGATTCCAATAAGAAAGTAGGGTCCTGTC
GCTTTGATATTTCTCCATATATTTTTTAGTAATCTCAACAGCACGTTTGGGTCATTATCCCTACT
TGCAGGTTCTGAAAAACCGGAAAAATTAAAAAATTTCCGATCATTAAATAAAAACTTAACCAAGA
AGCCCTGCAATAACCCCGCATGCTAGAGCCGAGATAATTCTGACTGCCAAAGCAGTTTCTCCAAG
GGCTGCACTGTATATGATTAGTTGAGGGTTTAAAAGTATGGAACTCATCATAAATGCCGCTAGCC
AGTCGTCCCTCATTCCATTCTTAGAAAATGAGGCTGCTATTGGAATAGTTCCATACATACAGATT
GGGGAAGCGATCCCAAGCAATCCTGCCGGAATCATTCCAAATACCCCTAGTCGCCGGTTTTGAAT
TGAAGCAAATAATTTATGAATTTTATTTTTACCAAATACTGATATTATGGATCCAAAAATTATTC
CAAAAACCCAATAAATTGCGATTTGATTGAATTGAACGGTGAAATAATACCAGAGGTATATAAA
CTCCCTATGGAGTATTTCAATCATCCAATATCACCAGTTCATATTCACAGCTTCCAATTCCAATTT
CTGCTGCATAATCAAGAATATGTGGGCCATTTCTAGACTCCATTCTTTCAATCAAATCCTGTCCG
TCTGGTGCAGCATAGACTAAATCAACACTCGCTTGGTCCAATGCAACTGGATCAAGAGATGCTAA
AATACCAATATCGGCCATTGTTGGCTCAGCAGGATTTGAATCGCAATCACAATCTACAGACATTC
TATTTAGAACGTTTATATAAACTACATTATCTCCAACTTCATCTAAAAATGCACCATTTGCTTCA
GCCATTGCTTCTAAGAAATCATCTTGAGGAGTAGTTCTGAATGCATCCCAGATCTGACCTACATT
GTTATAAGTGCCGGCACTGTGAACAATTGTTTTTCCATTAGGTGAAGCAATTCCTATTGCAACAT
TTTTCATCGCACCTCCAAAACCACCCATTGCATGGCCTTTAAAGTGCGAAAGAATAACCCATGAA
TCATAATTTTCAAAATGTGAACCTACAATAAATTCCTCAAGGTGTTCTCCATTATTTACTGGAAG
AGATATTTCACCATCCTCATCCATGATATCGGTTGGAGCAATAGCTGTAAATCCGTGATCTATCA
TTACTTGCTTATGCATTGCAGTTTCAGCCCTACCACCACCATAAGCAGTATTGCATTCAACGATCG
TTCCATTAACTAATTGTACTAAATTTCCAATTAATTCAGGATTTAAATAGTTATTTCCACCAGGT
TCACCTGTACTAATTTTTACTGCTACATTTCCAGTAACATTACGGCCAAGTGCTTCATAAATAGC
CACTAATCCTTCTGGAGTAATTTCATAGGTCATATACACTTTAGGAGGTTCAGTAACTGTTTTTT
GAGTGTTATTAATATCTGCTTCTGAGAGTTTGGAAGTTTCAATGGTCGTATTAGATAGGTTAGAA
CTTTCAATAACTGCATTACTGGTTGCATTTGTTTGGGTACTGTCTTCCCCAATACATCCTGCAATT
AAAGAAAATATTAGTAAGGACCCAAATATGGCAATAATTTGTCGTTTTAGATTCATGATTTTTCC
CAACACTAATTTTTTCAAGGCATGGAATATTTCTCGCAAGCCCGAAGTACTTTAATTTTTAACTT
TTGAGATAATTAAATCAGAAATTTAGTAAGAGTGACTAATCAATCATATTTTAGCTAAAAAAAA

TTAGTTTTTGACTAGTCCGTTCCAGACTATCTCAAATCCGTCGTCAATAAATTTATTTTCGTTGC
AGGATGGACCACAGGCTGAAATTACGTCTACCAAAGTTCTGCTGTTTTGGTAAAACATTGCAATG
ACCAGTTCTGAAGGTAATTCTTTAACTTCCTCATTTTTTATTCCATCATCAACGAGTTTGTGTAA
GAATACGTATTCCTTCATAACTTCTTCACGCGTAAAATTCGATATGTAAGGCGAATTGCAAAACT
GTCCGACAAAAAGAAACTCGTTATGGTTATCTAAGCCCCATCTAATTAAATTATCCCATATTTTT
TTCAACCGGTCTTTAAAGTTAACCTGTGGTTCGAGGTCTTTGACCATTGTCCGACTTAAATCCGC
TTTTACTTCAAAATACAGGTTGTTTATAAGGTCTTCTTTTGTAGGGAAATAATGGAAGAGTGTAC
CCGTAGCTACACCTGCTTCTTTTGAAATCTGGGCTGTAGATGTACCGTGAAACCCGCGTTCAGTG
AAAAGTTTCAAAGCGGCGTCCATTATGGCTTTTCTTTTGTCTTCAATATTTGCTTTCATATATAT
CTCATCCTGACTGATTAGTCAATTAACTATATAATTATTTTAATATATAATTATTTGGGCTAGTA
TTTATGATCCATTGGTTATGTGGCTCGAAAAATCATAATTTTTATTTAACTAAATAATTGTATTA
TTAATATTTTATCAGTTTTGAAACAGTTTTCCATTTTTCAGGGGAATTTACCCATGTATAATTAT
GTTGTTTTGAATTGGAAAAATTTATTGTCATTATTAAACCGATAATATTAAATTTACAAAAAAC
AAGTTTTTAGAAAATGTAAGCGTTATAAAAAGGAAGGGTACTTTGAAATTATTAAAAATACTTA
ATATATCGTTGTTAGTGATTGCATTAATGTTTTCAGGCTGTACGGACAGTACTGTCGAAACTGGA
GATAATACTACACTGCAAAATAATTTATCAATTAACGAATCTTCAACAAATATTTCAAAAACAGA
AACTGGCGAATATTACGAAAACTCGACAGATACTACCATTAAATCGGATTCAGTATATGAAAAAC
ATGAGGCATACATCAAGGGCTATCCCAATAGAACTGATGAATATTATTTAGAAAACTCTGAAAA
AACTGAAATTTTAGACGATCTTGCTTCAAAACTGGATAATGTAATTTCTGAAGATTATGTTTTAG
AGTGTTATATCAAAACCTATGAAGAGGATCCCAAATATCCAAAATGCAATGCTACAATATTGTCA
GATCGACTTATTATCAATGAACAATTCATAACTGGCGCTGTCGAAACAATAAACTACATTAACAA
AACTTACAAAATTAATAGAGTTTTGATAAGCGCAATTGATGCAGAAACTGGCGAAAAATACTGT
TCAGTAGAAACTTCAGGCGAATATTACGAAAATATTGAACTAATTTTTAAGTATATGTTAGGGG
ATGAATTCCAGATTACTTATTGGAATAGGGATATGAACAACTACACAATTGAAAAATTAAATTA
ATAATACTATTTTTTAAATTTTTAAAAACAGGCCAAATTCAATTTAAAAACCAGAAAAAAAAAG
AAAAATTTTAATTATTCTCTTAAAAAATCCCTTTTCTTGTTTCTACATAATGAAAAGAGTGCAGT
TATCCAAACTAAAGTCACCAATAGTCCATAACCTACTGCAATTTCAAATAAATTGAAACCACCCG
CCAACCTTGCAACAATCATATTTGGAATAGCTGCACCGCTGTATGATATTATATAAATGCTTGAA
AGCAGGCCTGCCCTATCTTCCTGGCCAATTTTATCCAGTAAACTGCGTATACTTCCAGTAAATGCT
AAACCCTGCGATATTCCTGCAAATAATGTTGCTATAAAAAATGGAATCATGGCGCTAACCTTCAA
TGAATAAATAAGGGCTATAATACTCAAGAAAAAAGCTGAAATACCAATATTTTGAGCATTATTG
GAATCAAAACGGCCACTAATCCATCCGCCTATCGCATATGGTGTCATCAAACATGCAAAAACCGC
TGCAGCTATCATAACACTATTTGTATTAAGCAGTTCTGCAGTTATTGAAGAACTAAATGCCTGAT
AAAATCCGCCTATCGCCCACGTTCCAACAAAAATACTGCCTGCAGCTGGAAGAAGATGCCGAATA
TTTTTAGGTACTGAAACTTGCGGAATTAATGATGAAATAACTCCTCGTTTAGGCGTTACCGTTTC
AGGACCTACTGCAATCAGTACGGCACAAAAAGCAAGTAGCGCAATTAATACCATATATATTAATG
ATAAAGAACCAAAACCGTATTCCTGAATGACAGCTGCACCAAAAGCACCAAATGAAAGTCCGACC
ACAGGTGCTCCACTAATAACCGCTGCACTTATCCATTTTGGTGACGTTAGTGCGTTATCTGCAAC
ATATGTTGTAATTGTACTGGATGCTAAACCCGAAGCAATTCCCTGTATGAATCTTCCAATCAAAA
GCATTGATGCATTCTGAACATAACCAAATATGATGCATCCAATAACAGCTATCCCTAATGTAGCA
AGTGCCACTGGACGGCGTCCCAAATGATTTGAAACTCTTGCAAATACTAGCAGTGATAATACACA
ACCTACAAAGTAGGTAACTGCAGTCATGGAAAGATCCCCACTTGTAAGATCAAGTGTTTTAAGAT
ACGTGTTAAGTAATGGAATAGGGGCAGAAGAAGATGCAAATACAACAAATAATGAAAAAGTTGC

CCCTACAAAGCCAGTTTTCCCGTTTAAGTCTAATTTTATCATTTTTTATCCAATTATTATTTTTT
AATTCTAAATTCTGATAATGTGGTTTTTTTTTGCGATTCATTTCAATATTTGTTTTGATCCATAT
TTCAATTAATTTTTATTTTTGGCAACCCTGTAAACATGAAAACCCGTTATGCATGCCATGATTGT
TGCAATGCCCAAAATTAAGGCACTGAACATGAATGTACTTTTGTAACCGGACGTATCAAAGAAAA
CACCGCCAAAGGCTGCACCTAATGTAATTGCCAGCTGAATAATTGCAACCATAAGACCCCCTCCA
GCTTCCGCTCGAGTTGGAAGAGTCCTGCTAAGCCACGTCCACCAGGCAACCGGGGCGGAAGTCGC
AAGAAATCCCCAAATACTAATCAAAACAAATATCGCTGCCAGTGATGTTCCAAAGGAATGCATTA
ATAACGCAGTTATCGCCATTAAAAAGGGAATTATGATAATTAAGCTATACAGGCGCTTTTTTAAC
ATGATCTCAATTATGAAAGTTCCCATTAATCCAAAAATACCCATTGACAGTAATACAAGAGACAA
GGTTTCCACATTTACTTTTGTTACCATTTCAAGAAATGGGCGAAGGTACGTAAATAGGGCAGATT
GTCCCATGAAAAAAAGCATGACTGAAAGCATGCCCAATGTAACTTTCCAGTTTTTTAATAGTCCA
AATACGCTTCCAAATTTTTTTTTTGTTCATTTGAATTTTCTTTAGGAAGTTTAGGAATACTTTTG
TACTGCCAGATAAATGCGATGAATGCAAGCGGAACTATCATAAAAAATGCGCCTCTCCATCCAAT
AATACCTCCAAGGAAACTTCCCATTGGGGCAGCTACCATAGTTGATAACGCAGTTCCTCCGTTAA
GAATTGCAAGCGCTTTTGGTACAGATGAAGCTGAAACAATACGCATAACTGTTGCAGCAGACATT
GACCAAAATCCGCCAATACACATTCCTAAAAGTGCACGCCCAATCATTAACATTGTGGAATCAGG
AGCAAAAGCCACCAATACGCCAGATATTCCAGTTAACGCGGTGAAAAAAAGTAAAACATGTCTTC
TATCTTTATTACCAATTATTTGAGTTAAAAAAAGACTGGTAATTAGTGCAAATACACCAGAAAT
AGAAATGGACTGTCCAGCAGTTCCCTCAGTAATTTGTAAGTCTGTAGCTATGGGAGTTAAAAGAC
TTACCGGCATAAATTCTGATGCAACCAAAACCATCGCACAAAGGGACATTGCCCAGATAGCTCCC
CAAGCTGATTTATTATCGTTAATTATTTGATTATTTGTATTTTCTTTCATAATATTCGTCTATCC
GTTTTTAATTACCTGTAATTTATATATGTCATTAAATTGGAAGTACAAAAGACACTAATGTAATG
CCTTTTTTAGTAAATTCTTTTGATAATTTTAAGGAATTATGTTTAATTTACAATTTTTTTGATAG
TTATTGTACCAGACATATTTTTGATATGTTCGCCACCATATACGACGTTTCCAAGCTCGTATAAT
CCACCAGCTGTGCCAAAATTACCATAAAACATTACAACATCGCCCCATGGTGCATAATATGCAAG
CGTTCCCGTTTTCGCATTTGCAAGTGGCGTATTGCTCGTATTAAGTTTATTTGGTGGATAAAATA
TCTTTTCGTTGCTACTATAATTTTTAACTTCAATTGTTAATGGAAGCTGTTCATAGAGTTCTTTT
GCAGCTTGGCCGTCATTGAGCTCAAAAACTACTGTTTCCCCATTTGATTCAATACTTATCCTCATT
TTTGAATCACCTGCTTTCGAATTATTTAGTTCGACCGGTTCGTTTTCTGATTGGTTAGTAATGTT
TTGATCTAAGTTATTTTCTTCGACACAGCCAGTACTCAAAACTGCTAGCAATAATACTCCAGAAA
ATATTAACATCCGTTTTATTCGATTATATTTGGGAATAACTTCCATGAATAAAACCCCACTTTAG
TCTATTTCATACACGGGTAGATTATCATGCTTGGATTTTAAATTAATTGTTATGTTAAATAAAAA
AATGTTAATTATTGGTTAAATAAAATTTTACTATACCTATCAAATATAGCGTCTTACTCTATTCA
TATATTTTAAATATTCTTCTCCAAATTCCTTAATACACCATCTTTCTTCTGAGAGAATAATCCAA
TGCGCCGATATTTGAAAACATATTAATAGAACAAGCAATATCCATGATTGAGTTAGAAATACACA
TCCTAATAAAGAAATAAAGTATGCAACGTACATAGGATTTCGGGATACTCGATATAACCCATTTA
AATTTATTCCACTCAGTTTAGGCTTAGCATAAGTTATTATAGATGCCCCATATAGAATAATCCCT
AAACTATAGGTTACTAGACCGATATAAAACCACTCAGAGTCTGCCCTGATTTTAAGAAATAACAA
ATATAGTAAAATAAGGATAGTTGAAATTTGATAAACCCAATATGCTATTTTTTCTCTTCCAATTA
CTGGAGCAAATAAACCCGCACGTTTAAGTGCTTCTTTGTTTACCATACTTAAAAGTCCATACCTA
ATAAGTATAATTGGAATTACTGTCAAAAATGCGTTCATTTTATCTCAACTTCCATATTTTAATTT
GTAATACGTTCTACCATTACATGTGGGCATCGGATAATAATTTTATCAATTAACCTTTTTATGTT
TAATAAAGCCCCACTTTAGTATATTTCATACACGGGTCCTTATTATGCTTAAATTTTAAATTAAT

TGTTATTTTACTTATTTCTAACTTCAATTACACAGGAACTGTCTGCATGTTGATTTTCATCCAAT
ATTATTTCTTCAATTGAATCCGCAATGATATGCCCGCCATTTGGATTTTTTACACTCGTTATAGA
ACCTTTAATTGATGCATTAACGTTGCTGTATTCAAAAGAAAGGTCACAATCAATCATTTCACAAT
CTTCTAAAACCAGACCTTCTGCATAACAGAGCGGCTGAGTTCCGATTATTTTACAGCGAATCAAT
TTGAGATTTTCTGAGTACCATCCAAGATATTCGCCTTTTACAATACTGTCGGATACTGTGACATT
TTTGCTGTGCCAAAAAGCATCTTTAGTATCCAGACGGGAATTCTTGATTTCAACATTTTCTACAT
ATTGGAATGAGTATTTACCGTTCAATACGAAGTTATCAAACAAAATGTCGGAACTTTGTAAGAA
AGGATATTCTGACTCAAGTTCTGTATTTTTTACAGTTATTTGTGAGAAAACCATCCAAATTCAG
GTGATTGAATAGTACAATCTTCAATAGTGACATCTTCACATTCTCTTAATGCCTTAATACCGCCA
AGATGACTTTTTACTATTGTCATGTGGTTGCTATACCATATTGCAGCACGGCATGTTTCAGTCAT
GCGACTATTTTCTATTCTGGCGTTTTTAACATGCCAAAAAGGATATCGAAGCCTGAAATCACAAT
TTAAAACATATATATCTTCACTTTCTTTTAAGGCAGATTCTCCATCAAGAGGGCCATCAAATATA
CAATTACTAATTGTTGCGTTATGTACGCCGTATAAGGCACGCTCTTCATCTAGGTTTAATTCATT
GTATACTGTTGGTTCCGCTTTTTGCTCAACACATTCAGAGTATTTAGTGTTAGTAAGAGGAATTA
TCCCCTTACTTTGCATATTTAAATTATCGTTGGTGTTTTGACCCATATTTAATACCTGTTTTTAT
TTCAAATATTCTTTAAAAAATCCTTCTAATTTGTCAAATGGAATGCAGTCGTTATCTCCACCATC
GTACAAGTCAATGTGTCTTGCACCGGGTACAATGTAGAGTTCTTTTGGTTCAGCTGCTTTTTCGT
ATGCATCTTCACTGTAGTATCTGGAGTGAGCATTTTCCCCAATTATGAAAAGAAGTGGTCTTGGA
GAGATGCTTTCAATGTAGTTCATCAACGGGAAGTTCATAAAAGACATTCCACTTGTTGCAGTAAA
TGAAGCTTCCGCATTTATATGGAATCCTCTTTTCATTGCATAATATTCAAAGAATTCACTTGTAA
CCGGATCTAATCCTTCTGGAATTGAATCTGCTGGTGTTCTAGGGAATTCTTCAGGCACTAATGGG
GTTCCGTTTTCAAAGTCAGTCCATCTTTGTTCAGCCATTTGAACTAACATTGCGTTACGTTGTTC
ATCAGTCAATGTTTTTCCAAATCCCTCTCTAAACATGCCGCTTATATCATACATACTTGCAGTTGC
AACTGCTTTAATTCTAGGATCGACTTGTGCTGCGGTAACTGAAAATCCACCACTTCCGCAAATTC
CAATAACTCCAATTTTTTCTCTATCTACGAAGTCTCGAGTTCCAATAAAATCAACTGCTGCACTA
AAATCTTCAACAAATAAATCTGGTGAAGAAATGTGTCTTGGTTCTCCTTTGCTTTCACCATTGTA
TGATTCATCAAAAGCTAAAGCAACAAAACCTCTCTGCGCCATATTTTGTCCATATATTCCTGCAC
CCTGCTCTTTTACGCCACCATAAGGTGTTCCTATAACAATTGCAGGATATTTTTTGGATGTATCA
ATATCTTTTGATAAGTAAAGGTGTCCGGCAACTTCCACTCCAAATTTATTTTTAAACATTACCTT
TTGAACCGCTACATTTTCGCTTAATTTGAATGTAAGGCTTTTAGGGTCCACGTTAGTTTTTACTG
ATTCCATATGTTTCACCCTCAGATTTTGGTTTTAATTTAATTGCTAAGATTATACCACATAATCG
GTAAATTTTAGAGATTGATTAATCAGCCATGTGTTTATTAAAAATTAGTTTTTAGAAAGTCCGGT
CCATATAACTTCAAAACCCTCATCAATTATTTTATTTTTATCTTTAGATAAACCATAGCTTGAAA
TTACTTGCACTACTGTCCTGCTGTTTTGGTAAAACATTGCAATAACTAGTTCCAAAGGTAATTTC
TTGACAGCTCCAGCGGTTACTCCGTCTTCAATGAGTTTATGAATGGTAAAGTACTCCTTCATAAC
TTCTTCACGCGTAAAATTCGATATGTAAGGCGAATTGCAAAACTGTCCAACAAAAAGAAACTCGT
TATGGTTATCTAAGCCCCATCTAACTAAATTGTCCCATATTTTTTTCAACCTGTCTTTAAAGTTA
ACCTGTGGTTCGAGGTCTTTGACCATTGTCCGGCTTAAATCCGCTTTTACTTCAAAATACAGGTT
GTTTATAAGGTCTTCTTTTGTAGGGAAATAATGGAAGAGTGTACCCGTAGCTACACCTGCTTCTT
TTGAAATCTGGGCTGTAGATGTACCGTGAAACCCGCGTTCAGTGAAAAGTTTCAAAGCGGCGTCC
ATTATGGCTTTTCTTTTGTCTTCAATATTTGCTTTCATATATATCTCATCCTGACTGATTAGTCA
ATTAAATATACTGTTGTTTTGCTATATAAACTTGACTGATTAGTCAGTAATACATTTTGATGGGT
TAAGGGTTAAATTAAAATAAAACTTAAATAAAACTTAAAAAAAAATCGAGTTATTTTTGTGGAT

AACTTATTATTAATCCAAAGTTTCGAAAAGAAAAAAAAGAAATTAAATAAATTAGAAGTTATTT
TTAATGTACAATAAAATGAATTGCACCACACTGTCCAGGATAACTCATAAGTTGGATTATTGAGC
TGTACGTGACGGTAGAATTTATTTTAACATTATTAAGGTCTCCAGAAACTATTGTATCATTAGTA
ACTGATACATAGCACAGGCTGTTTTCTCCTTCAACTAAAAATCTTTTGTAGTCATTTACAAAATC
AATTTCAGTAACTTTTCCTGAAGTTACAAATTCTGGAGGGTAGTAAAGTACAGATGATGCAGTCA
AAATTGGCGGAAAAGATCGGGTCATGATTCCAGAGTAGTTAATTTCGACCAATCCTCCAACAGAG
ATATTATCGCCTTCGTAAATTTCAGGGAATTTAATATCAACACTTTCAGGCACATTTACAACATA
TTCGCCGTTGCTATTATTCATCAATAACTTGTAATTTCCATCCGCAACTTCAATGGATTTTATAA
CTCCAAAAATTATTTCCGACCCATAATTAGAATCGTTTATTGAATTATCGCCAAGTTCTGCATTT
TTCTGAAAAATAACAAAACTTCCAGCAATTATGCACAAAAAAACTGCTAAAATTGCAATCATGCT
ACTTGTTTTAAATTTCATAGTTCACCTCAGAAAGCATATCTGCTTTATAATTAGCTTGAATTTTA
AAAATATAAAAACGCGGATTTTGTTCGCACATGGCCGAACAATAATTTAACTGTAATAACTTTTA
TCACCCATCACAATCAAAAAAATTTAAATTTTTTTAATTATATCTTTTAAATCAACAAATTTTCA
AATTTAGCTTTTTTAAAATTTAAATTGGTATTCTCATTATTATTTTACAGACATTGTCTTATTTT
TTCAAAATGTATATGACCACTGCATACATAACTAAGCAATATTAACACGCAGGTTTGGACATTTT
AATATTAATTGATAAAAAGCATTTTTTTAAATAGTATCCCCTAAATAACTTATTACTATTATTTT
TTCGAATATGGGGTGATATTCTTGAAAACTATATCATTATGTCCAGTATGTCTTGAAAAAATAGA
CGCAGAAGTATTTGAAGATGAAAACAGGATTTTAATTGAAAAAGAATGTCCTAATCATGGAAAA
TTTAGGGATATTTACTGGAACGATGTTGAATCGTATAAAAAATATCTTGAATACGACTATCTCGG
AAATGGGATGAAAAATACAAATGTCGAGTTTAAAAACGGATGTCCTTTTGATTGCGGTTTATGT
GGCAATCACAAATCTACAACAATACTTGCAAACATCGATGTAACAAACAGGTGTAATTTAAACTG
CCCTGTATGCTTTGCAAACGCGAACAGGACAAAAATGGTGTACGAGCCATCTCTTGAAGAAATAA
GAAAGATGATGGTTAATTTAAGAAATGAAGATATTCCATGCCCCGCAATACAGTTTGCAGGAGGG
GAACCGACAGTTCGGGATGATTTACCTGAAATAATAAAAATGGCGAAAGAACTTGGATTTGTAC
ACGTACAGGTAGCTACTAACGGGATAAAACTTAAAAATATTGAATATATTAAAAAATTAAAAGA
ATCAGGCCTATCAACAATTTACCTGCAGTTTGATGGGGTAACCGAAAAGCCATATTTAGCAGCAC
GAAATAGAAATCTCCTTCCATTAAAATTAAAAGTTATAGAAAATTGTGAAAAAGCGGATTTTAA
CAGTGTTTCACTTGTTCCAACCCTTTTAAAAGGTGTAAATCATGACCAGATTGGTGATATACTTA
GATTTGCAGTCGAACACCGGAACGTTGTAAGAAATGTAAATTTTCAGCCTGTATCATTTACGGGA
AGAATTGATGAAACTAAACGGTTAGAACAGAGGATAACTATTCCAGATTTTGTCCATTATGTAG
AAGAACAGACTTTTGGAGAAATTTCAAAAGAAGATTTCTACCCAATACCTTTTGTAGTACCGTTT
TCAATATCTGCTGAAATCTGGGAAAATCAGCCCGTAATAAAATTAAGTTCCCATCCCCACTGCGG
GGTTGCAACGTACTTGTTCATTGAAAATGGCAAAATCCTGCCAATTACAAGATTTTTCGATGTAA
AAGGATTTAGGGAACTGCTTGATAGTACATTTAATGAATATCTTGGTAAAAACGGTGAATTGAG
CCACTTAGAAAAAGGAAAAGTAATTTTAAAAATATCAAAAGAGCTTCCAAGATTAATAGATTCC
AAAAAAGTCCCATCAGACGTGAAATTTAAAGAAACATTGGTAAAAATACTAAAAGGAGACCGGT
CAGCTCTTTCAAAATTCCACTTCAACACGATTTTGACAAGCTGCATGCATTTTATGGACCCGTAC
AATTTTGATAGCAGGCGAGTCCAGCGCTGTTTGATACACTATGCACTACCCGATAATCGAATAAT
CCCATTCTGTACGTACAATACACTTTACAGAAAAGAAATAGAAGACAAATATTCAATACCTCTTG
AAGAATGGAAAAATAAACATTAAATAAACTTTTTTATTTTAAATAACTTTTAATTTTCAAATTT
TTAATTTTAACTTAAAATTCATATCAAAAAATTAATATTTTAAAAAGTTTATATTGTACTATGA
TTTCTTTTTACGGGGGATACTTTTGAGGATACTGTTAGATACAGACATCTTTATTCATGAAAAAG
ATGATTTTGAGAGTTCAACAATATTAAATGAATTATTCAAGTTGTTTGATAAAAAAGCTGCTGT

TTTAATACATCCACTATCTATTGAAGGCACAACAAACGATAAAAGTGAAATACATCAATCTTTTT
TAAAATTAATTTCGTACGATATTTTAGAAAATCCGCCATTATACTTGCAGGATTTAACATTTAAT
TCAATTATTGCGCAAAAAAATCAGAATAATGAACTTAACAGCCATCTTTTATATTCTGTTTATAA
AAACGCTGTAAACTACCTTTTAACTGAAGATGCAAACCTTCACAAAAAAGCAGTTCAGTTAAACA
TCAAAGACCGGGTTTTATCAATCGACGAAGCGTTAGAATTTTTTAAAGAGTTTTATTCAATCAAA
AAAAGACATAACGTCCCCCAAATTAAATTTCTCCCAATATATAAACTCGATGTAAATGACCCAAT
ATATGATTCAGCTCGTAAAGACTATCCTGAATTTAACGAATGGTATTCCAAAAGTGCGATAGAGG
GAATGAACTGTTGGTGCTACTTAAAAGATGAGCAGATTGGTGCGGTTTTAATATTTTCTGATGAT
GATTTTGATATTATCGATACTGAGCCGCCGCTACCTAAAAAAAGGCGATTTAAAATACATATTTT
ACAGTTTGACAACCAAAGCTACAATTTAGATGAAATACTCTTAAAAAAAGCGATAGATTACTGTG
TTGATAATTCAATTGATGAAATGTATTTGAACCATTTTGTCTGCGATAAATACGATAATTTGATA
AATTTGATAAATGAATATGGCTTTTTTAATATAGGTCAGAATAAACATGGCGAAAATGTATATT
TCAAAGATTTAAAGCCTGATAACCGCCAGCTAATGTTTTGTAGCTATGATGAAATTTTTAAAAAA
TATTATCCCTTGTACTACGATGGAACACGTGTTAAAAAAATTATTGTCCCGATAAAACCTGAACA
CCACGAAAAACTCTTTACGGATTATAAAAAAAGAAATTCAATGACTCTTGATGAATATTTTGGAG
ATTCTGTAATTAAGGGGGACACTATCAAAAAAGCATATATCACGAATTCACGCGTGAAAAATCTT
AAAAAAGGAGATATTTTACTATTTTATAGATCAGATGATTACAGGGAAATTACTTCGTTAGGTG
TGTTAGATGAATTTAAAAAAGATTTAACAAGTCCTGAGGAAATTATAAACACTGTTGGAAATAA
AACGGTTTATTCTGAAAAAGAGATTAATGAGATAGCTAAAAAACCAGCAACTGTAATGATATTT
AATATGCACTTTCATTTATCTAATCCTGTATCATTTGATACCTTAAAAAAGAAAGAAATATTAAA
AGGGCCGCCACAGACGATTACTGAGATTACTAATACGAGTTATGAAAAAATAAGAGATTTAAGC
AAAATTAATGAAAATATTACGATTCATTAATTTAAAGTTTTTTTTGAAAAATATAAAAACATTT
TTAAATTCTATATCTCTGACATATATATGTAATTTAGTTGGGGTGATTAAAGTTTATAATAACTA
AAAATGATTTAAAAAGGAGATAACTCCTTTTTAAATTATATTTCAAAATGGCACAGAGGTATAT
CATGATTAAGTTAATTTCTAACATAATATTTGTAATTGCAGTGTTAATACTCTTAAAAACATGGT
ATAATATCTATGAAGTTGTTATGCGGCATTATAAAAAATTTGGAGTTCGTATATTGATTACAGT
AATATTGTGCATGATAACGATTGTATCTTTAATAGCACCCATTTATTTGTTATGCTATTTAAAAA
TTCTACTTCCAGCAGTTCCCGTTGAATATATAAAGCTTCAAAGTGCATTTTTTGCGATAATCGCA
GTTTTAATAAATCGTTTTATCGCAATAAAAGCTAAAAATGCTGCAGTTGATTATTGTCTGAAAAA
AGGAATACTCACAAAAACGGGAATCTAATTTGAATATACTCAAATAATATTATTTTTTGGCATGC
ATCGCAGCTATAAAATTACAACAATATCTGATTACGGATATTCTGAAACTCCGTTCCACGCGCCT
ACAACATATAATGGAGATAATTTAACGTACGATTCTAATGGAAACTTAATTGAAGACGGAGAGT
TTACATATATCTATAACGATGCAAATCAGATAAGTGAAGTAAGATACAAAGACAATAATTCACT
GGTTGAAAAATACTGGTACGATTCAGAAGGTAGAAGAATCAAAAAAGAAAATTCTGCCGGAGAA
CTAACGTACTACATTAACAAATACTTCGAAATCGAAAATGGAACAATAACTACATACTTCTTTAG
AGACGACGAACGTGTTGCAAAACAGTCCGTTAACGGTACAGACTGGTATTTACCTGATCACTTGG
GAAGTACAAACCTCCTTGTAAATGAAAGCGGAAGCGAAGTTGAACGTACTGAATATTTCCCATAT
GGGCAGGTACAGTTCGGTGGAAGTGAAAAGTACGGGTTTACCGGGCAGGAAAATGATGCGAATA
CTGGGCTCATGTACTACGGTGCAAGGTACTATTCACCAGAATACCGGATATTTATCCAGCCTGAC
ACGATGTTGCCTAGTTTATACAACCCGCAGGCGTTGAACAGGTATTCTTATACATTAAATAATCC
TGTGAAGTATACGGATCCTAGTGGCCATGTTGTGCAAATAGTTGCCGCCGCATTAACTGCCGGAG
CTCTCGCAACCTTAATATCGATTGGTACACAGGTGTATAATGCAGAGGGGGATTTCAGTCAGGTT
TCATATTCTGAAGCTTTTGCAGTGGGTGCAGTTACCTCAGTAAGTGTTGCTGTTGGATGTATGGT

AGCAGGTGCGGCAGTTGGGGCATTTGCGGTGGAAGGTACACTAGAAGCCCTTGCAATATCTGAGG
GAGTTGGTGCTGTAACTACAAACGTTTGCCAAGAGACTATGGAATATAGTTATGGAGTTGGTGAT
TCTGAATTCGACCTTACAGAAATAGGGGTGGAAAGTGGAAAAGACGTATTTGTTAGCTTATTCTG
CAAGGGCGTGAAAATTGATGCAGGCACTACAAAATACGTAAATACTAAAGATAATGTAAAAACG
GATACTTATAATCAGGCGTTAGATACGGGAAAAGAAGTAACTATACAAACAACTGCAAAAGTCA
TTATCAATGCCATTAGTGATTCTAACGACGATGATTCAGATAAAAAAAACAATGATAATATTTA
AAGGGAATAATATTCCCTTCTTATTATATGCATAGGGGCGATATATTATGAATGTAATTCTTATC
TTAACATTGGTGGTATTCGCATTGTCATTTAGAAAAGTATGCAACAATATAATCAATGATTTTTT
AGGTTATGAAAATTCTCAAAATAATAAATTTATAGATGTTGCACAGTCGGTCTTGCTCATATCGT
CAGTCGTTTTTTATTTTGCGTTTGTTGTATTTTTGGGCAAAGGACTTTCAACTTTTGAAGTATTT
CAAAGTCAGTCGTTCGAAATTAAAATAATAAGTATACTTATTCTTCCAATTATTGCAATGTATTT
GGTTTCTGTATTTTTAAGTAAACAGGCTGTAAATTACAGCCTTAAAAAGGGATTAATTAAAAAG
ACAGACGTAAAAAAGAAAATTCTGCCGGAGAACTAACGTACTACATTAACAAATACTTCGAAATT
GAAAATGGAACTGTAACTACTTACTTCTTTAGAGATGACGAACGTGTTGCAAAACAGTCTGTTAA
CGGTACAGACTGGTATTTACCTGATCACTTGGGAAGTACAAACCTCCTTGTAAATGAAAATGGAA
ACGAAGTTGAACGTACTGAATACTTCCCATTCGGACAGGTGCAGTCCGGTGGAAGTGAAAAGTAC
GGGTTCACTGGAAAAGAAAATGATGCTGACACCGGGCTCATGTATTATGGTGCAAGGTACTATTC
GCCCGAATACAGGATATTTATCCAGCCTGACACGATGTTGCCTGATCCATACAACCCACAAGCGC
TGAACAGGTACGCGTACACTTTGAATAATCCTGTGAAGTATACTGATCCTAGTGGTCACTACATC
GAGAGTGCATTGGATATTGCGTTCATTGCATACGATGTAAACCAGATAATGAGTGGTGATGCCAA
TGCTTGGGACTATTTAAGTCTTACAGCGGATGTTGCATGTCTTGCATTACCTGTCGCCACAGGTG
GTGGACTTGGGGTTCGGGCTATTGAACAGGGTGTTAAACAGGCAGATAACGTAGAAGATTTGTTT
AAATGGCTTGATACGGCATTGGATGTTAATAAAGCAGATGATGTGGTTGATGCAGGTAGTTCACT
GAGCAAATCAAAATATATTCTTAATCAAAATGCTAAAACAGACGCAAAAAAAGCCGCTGATAAT
TTGCCTCCGGAAATCAAAGATAAGGTATATGATTTTTACAAAGGGGGATCTAATAAATATACGG
AGTTCACAGTACAAGAAATGGATAACGGTAATTACCTAGCAACTATGACTAAACCCGGAAATGTA
CCTGGTTCTTATGCTACATATTATAAAGAAATTAGTCCAGATGGAAACACAGTTGACGTGTACAA
AAAAACAATAGATCCGTCAGGAAATTTAGTACATAACAAAAATAAACTGGAAAGTGGGGGAAAA
TAGAATGATTAATCTTGAGAAGATTAAAAATTTTAGAGATTTAATTATTTCAAAAAAAGAACTT
TTAGAAGCTATTCCGTTTAATCCGCCCAAAGAATATCGGGGTGATCGAGTTGAAATCAGCACGGA
CCATGTAATACACATATTAGAAAAATATAAAACAGGCGAAGTTTCAAAAACCCAGATCCTAGATT
GGGTTAATACCATATGGTTCTCCGAATGGTATGATTATTGTGAAATCTACTCAGATAGTATTGCA
AGTGTAATGGATGAACTCGAAGAATTGGATGAAGAAGGAACTGATTTAACGGTAGAAACAGTAG
ACCGATATATTTATGCACTCCAAAATAATATTGAAGTTTGGAAACTTGAAAAAGATAATAAAAA
AGAACGAAATCAACAAAATTAAATTTTTTCTTTTTTTATGAAATTACGTGAATACGTGTTTAACT
TATCAAAGCAGTGATAATATTTCGTTATGCATTACCCGACTGTGAAATATATAACAAAATATCGA
ATAAAATATATACTGGTGAGTGTTGCAGGTTATTTTATAGTATTAGCAAGTATTTTACGATATTT
TAATTTATATTTATGGAAATATCCTAATTAAAAACTTGTATCGACTTAAAAAAGCAGTATTTTTT
TGGAAATAAAAAAACTTTTGAAAAGTTTTGAACTATAAGTCAGATTATTTAACGAATTTTGAGT
TTTTTTAGTAAACGAGGGGATAATTAAACTAAGGAGTGACATATCGGTATAACGTAAATTGGTA
CCCATTTGTAACCAATTTTGTAAAATTTTTTAAAAAATAATCTGAAAACAAAATTAAATATTTA
AAAAAGAAAGAAAAAGTTAGTATGGAACTGGAAGTCCAAGTTCTTTTCTGTGAACGATTTCTTT
ATCATTTTTCTCTTTTTTCTGGAGAGCAAGTTTTTGAACTAAGTCTAACCCTGGTTTTACATCAT

CAATTGGTTTTGTTTCAAGAGCGCCTGCTTCAACCATTTTATTTAAGTATTCTTCTCCTTTTGCA
GTTCTAACAAAGATTGTGCTCCATCCGTCAGGACTTCCAACTGAACCTGTTGAGATATCTGCAAG
TTCTGCAGTGTAGTCCATACATACGTGGCATGATTTTTGTTCGTACATGTGTGTGTCTTTCAAAG
GAACTGCTTTTACATCGCCAGATTTTGAGTAGACCCAGAATTTACCTTTTCCGATATCTGTTTTT
AAAACATCGTTCATTCTAATTCCTGCGTACTGTTCTACAATTGCTTTCATACCTTCGTAAGGGAA
GTTTTCCATACAGAATATTCCCATAACAAGTGCAATTTTTGAATCAGTATGTCTAAAACCCATCG
GGTATTTCATTAATTTTCTAATTGCTCGTACCTGACAAGGGGTTCCAACAATTCCAACTTTTTCA
AGTGCGTATTCTCTTACTGCATCTTTTAACACGGATACGTTTGGAGAAACTGTATATTTAGTTCC
TGCAGCTGAAAGTACTTCTTCAGGAGTTGTAGCAATTTTTGGAGCGGCTTTAAATCCGTCTTCAG
TGTTTGCAACGATTACACCATCTAAAAGGCCATTTTCTAAACCATAGATGTAGGATGCAGAGATT
ATTCCCCCATCCTGTGATTTTTTAAGTATTGCTTTATCTGTGGCTCTTGCAGATATAGCTGTTTT
ATATGTTCCAAAAGGATCCATAATTAACACCTCTAAACTTAATTATTCGATTTTTTGAACTAATT
CTGGAAATCTAACTCTTGGGCACTGAGCTCCACAAATACCGCATTTTATACAAAATTCAGTCATT
ATGTTTGGTTTTCCTTCAACCATTTCTATTGCTTTTACTTGACATGCTGCAGCACAAGTTCCGCAA
CCCATGCAAAGTGACTGGTCTATAACGTTTTTGATTACATCGCATCCACATGCATCTTTTCCATA
TTTTGCAATTTCAGCAAATGGTTCTAAATATTCCATATCGCCGTTTAATGCGGCCATGATTAAGT
TTACGATTGATTCTGTTGATGGTGGGCATCCAGGAAGTGCTAAATCTACTTTAATTACATCACCA
ATTGGAACGAATGAGGAATGTGATGGCTGTGACATCTGGCCCCCTCTTCCAAATCTAGTAACTCC
ACCAGATGCTGCACAGGCACCGAGTGCTACAACAATTTTTGAATTTTTTCTTGTTGTTAAGATGT
CTTCAACTGAGTGGTGGTCATCTAAACAGACACTTCCTTCAACGAGTGCAATATCAATGTTTTCA
GGAATTTTTCTTTCAATTAATATTTTATCGTCAGTTTCAGTAATTTCTGTCTTTTCATCTGCGAG
TGTTAATGCATAAACTAGCTCAACAGCTCCCAATATATCTAAAAGTTTTTCATAAGTGTCTGTAA
GGGAAATTAAACATCCTGTACAACCACTCATGTGTATATGGGCAACTCTTACCACTATATCACCA
TTATTCGTTTAATTCATCTATTATTAATGAAACAGCGCCATCTACAGCAGCATTTACTTCATCTG
AAAGTTCGAGAAGAATGTCTGGTGCAGGCACATTTTTTTGCTGGCATCCAATAACAACGATTTTT
ACGCCGTAATTATCTTCAATATCCTTTAACATCCCTGCAAGTGGCATGTCGTGGGCATCAATGTG
ATATTTATCGATATCTGGAAGGTCTTTTGAATATAATTTTTTCAATTCTCCTGGCGTCAATCCAT
AGTCGATAACATCTGCAATTATTATTTTCTTTAATGGAGTTTTTTCATCAATTAATGAAAGTATA
AAGTGAGAAGCTCCCGTACCTGCATCAATAATCTGTATTTTATTGGATTCAAGTATGGGATGTTC
TTTTTTTAGTTCATTTAATTTATTTACTACAGCATATCCAAATCCATCATCCCCAAATAGAATAT
TTCCGCATCCGAGGACTAAAACTTCTTTTTCTAAATATTCAGGCATGATTCACCAGTATTTATTG
TATTATTTTTTTGTCAACGATTTTGTTATCGTAGTCCATTGCAATTACGTGAGTTGCGCATGAAA
CGCATGGATCGTATGCTCTTACAACGTGTTCTGCAAATTTGTAGTGTGTTCCTTCTGTAGCTTTCC
CAATTAACGGGATATTCCATGTGGTTGCAACAATTGCGTTATAATAAGTAATTCTTCCTTTTGCA
TCAATTGCAGCTTGGTGGGTGTTGTGGCCTCTTGCAGCTTCGTGAACACCAAGTCCTATTTTTTC
ACCGTCCCCGAGGATTGGTTCATTTGATCTTGTTTTTCCATTAACGTCTAATTCATCAATGATTT
CCATTGCTCTTTTTACGTGCACAACGTTTTCATAAGCTCTTGCAATGTGTATTGCCATAGCACCTT
TGTTTTTAAAGTCGTGGAATTTTGCAAATCTTGCTCTAGGTCCAACTTCCATTAATCTGCCGTTG
TATCTTGGGATGGTAGTACAAGCTTGTACTGCAACGTCTTTTTCATAAACATCGTATGGCGTATA
TTCAACGACTTTACTCTGGTCGATGTTTTCCCTGTTTCCGTATGTTGCATCAGTTGCAATGTATCC
AAAATCGTGGTAACCTAATTTTTCAGGAATTTCAGTTCCATCTGCAAGCATATTACTTTCAAAAA
TAGGAATTAAATATTCAACATGCTCTTTTGCTAATTTTTCGTATTCTCTGCACTTGTAGTATATT
TTTGAAGCAGCATTTTCAGTGATGTTTTTTAACATACCGCCAACTTTGATATTTGGTGCGTGAAT

TGCTTCTCCACCTATTGTATCAACAATGAATTGGCCAGTTTTTCTCATTTCCTGAATTCTTTTTAT
GTATTCAACCCTATCTGCTTCAGGTATGAAGTCTGGAGCAATTAAAAACTCGTGTAATGGGTGGG
AGTGCATTTTATTTCCAAGACCTGTTAATTCTCTTAACAATAATCCATCTTTTGGAGGTGTAATT
CCGCATGCATCTTCAATAGCTTCTACTGATGATGTAGCGTGTGCAATTGGGCAAATTCCGCAGAA
TCTTGAAACTGCAATTGGTGCAAATTCTGCCACTTTTCCAACTAAAAATTTTTCAAAGCCTCTAA
CCGGGGTAACGCTTAAATAGTTACCTTTTTCAACAATTCCGTCGTCATCCACTTTCAAGACAAGT
TTGGTATGCCCTTCGTGTCTTGTTGTAGGATTTATCTCTACCGTCTTACCCATCAGATCACCTATC
TTTAATATTAGGTATTTGATAATTCATTACCTACATTATATAATATGATGGATATTAAAACTAA
TTGTTAATCGTATTAAATAAAAAGAGTTATATGTTAAAACAAAAAGTGATTTTTTAAATAATTA
TTACTACTTTTTTATAAAATATCTTATAAAATATTATTAAATACTATTTTTATATTATTTAACAA
GTATAAATTAGAAATTTTTATTAACATATATAGTTTTTTATTTGGCATCAGATTGTCTAATTTAT
ATATTAATAATTGTTATTTTATATTATTTAACAAGTATAAATTAGAAATTTTTATTAACATATA
TAGTTTTTTATTTGGCATCAGATTGTCTAATTTATATATTAATAATTGTTATACTCACATCAATT
AGATAAACTATTGTAGGTATTAGTTTACCAAATATCTAATAACAATGAGGTGAAATTTTGTCTG
AAGAATGGGAGCCAAAGATAGTCGGCTTTTGTTGCAACTGGTGTACCTATGGTGGTGCAGATACG
GCAGGAGTTGGTAGAATGCAGTACCCTCCAAGCATAAGGATAATACGGGTGATGTGTTCAGGAAG
GATTGAACCATCATTTATATTAAAAGCTTTTAAAGAAGGTGCAGACGGTGTTTTTATAGGCGGAT
GCCACCTTGGAGATTGTCACTACGATGCAGGAAACTACAAATGGCAAAGAAGAGTCATGATGCTC
TACGACATGCTTGACGAACTTGGAATTGGCAGAGAAAGAGTTCTCCACGAATGGATTTCAGCTTC
AGAAGGTGAAAAATTCCAGACTAAAATGAACGAAATTTACGACAGAATAAAAGCTTTAGGTCCG
TGCACCTTGAATGAAAATCTTAAATAACTATTAAGGTGGTTTTATGGTAAAAATAGCTACAACAT
GGCTTGCATGCTGTTCCGGATGTCACATAAGTCTTGTAGATTTACATGAAGATTTATTGAAAGTT
CTCGAACAGGTTGAATTAGTACATTGCCCTGTTTTAATGGATGTTAAAGAAATTCCGGATGATAT
TGATGTTGCATTAATTGAAGGCGGTATTAGAAATGAGGAAAACCTTCATATTGCACAGGAAATG
AGAAAAAAAGCGAAAATTGTAATTGCATTTGGAACATGTGCAGTTTACGGCGGTATTCCTGGAA
TGGGTAATTTATACTCGAATGAAGCTCTTTTGGAAAAAGCTTACAAAACTACATTCTCGACAAAA
AACGAAGAAGGAATTATTCCAAGTGAAGATGTTCCAGAATTAACTTCAAGAGTAAAACCACTTTC
TGATGTAATTGATGTGGATTATATTCTTCCCGGATGTCCTCCAAAACCAGAATTGATTGCAGGAG
TTCTTACTTCCCTTTTAGAAGGTAAAACTCCAGAATTGACAACTAAAAACATGTGTGAAGTATGC
CCTAGAGAAAAAAGCAAAGAAGGGGTTTCAATAGATTCAATTAAAAGAAATTACGAAGGAACGC
CTGATCCAAAAAAATGTCTGCTTGAACAAGGATACCTCTGCATGGGAATTGCTACAAGAGCTTCA
TGTGGTGCAATATGTCCAACAGCAGGAGTTCCATGTACCGGATGTTATGGACCTACTGATAAAGT
AGTAGATCAGGGTGCTAAAATGGTTTCTGCGTTTGCATCTGATTATAAAATTGATGATGATAGA
ACAATGGATCCAAATGAACTTCCTAAACAGATACTGGATAAAGTGGGTAGTTTTTACAAATTTAC
GCTCCCAAGTGCATTAATTCCGATAAATAACAAAAGGTAAGATACTAAATACGGTGATATAATG
ACAAAACTGTCGATAGAACCTGTTACCCGAGTTGAAGGACACGGTAAGGTTACACTTTCCTTTGA
CGACAGTGGAAAGCTCGATAAAGTTAATTTCCACGTTGTTGAAGTTAGAGGTTTTGAAAAATTCT
TGGAAGGAAGATATATCGAAGATGCTCCGGTATTTACACCAAGAATTTGTGGAATTTGCCAAGTT
TCACACAATTTGGCAAGTGCAAGAGCAGTTGATAACGTATTTGGAGTAAAAATTCCAAAAACTGC
AGACATGCTCAGAAATTTAATGCAACAAGCTTCAATGTTCACAGCCACGCGCTTCACTTTGGAA
TGCTAGCATCTCCTGATTTAATGTTCCCGATGACTGATGATGCACTTAAAAGGAACGTTTTAGGT
GTAGCTTCTGAAAACATGGACGTTGTAAAAGATGCAATTTCAATGAGAAAAATTGGTCAGACAA
TAATTCAAAAAGTTGGTGGAAGAGCCATTCACCCAGTAACAGCAGTTGTTGGGGGACAGTCAAAA

CCATTAACCGAAGAAGAAAGGGACGAACTTTTAAAAATGTCAGAAAATCTTGTAGATACTGCTG
AAAGAACTTTAGTAGTTGGAAAACAACTTTTAGATGGATTAAAAGAACAAGATCTCCTCGAACT
TGGTTACTTTGAATCATACCACATGGGTATGGTAAATAACGGTGCACAAGATATCTATGAAGGAA
AAATTAGGGTTGTAAATCCAGAAGGAAAAATTGAATATGAATACGATCCTTCAGAATACTTAAA
CTACATGTCAGAAGGAGTAAGGCCATATTCATACCTTAAATTCCCATACTTAACTGAAAAAGGTC
CTGTTGATGGAGTTTACAGGGTAAATACTCTTTCAAGGTTAAATGTGTGCGATAAAATGCCAACA
CCTATTGCTCAAAAATACTACGAAGAATTTGTAAAAACTTTTGGAAAACCTGCACACCACCCGAT
GCTTTTCCACTACGCAAGATTAATCGAGCTTATTTCAAGTGCAGAACTCATAAGAAAATTCTTAG
AAGACGATGCAATTGTTGGAACTGACATAAGGGTAGAACCAACCGATCTTGTTGGAGAAGGTGTT
GGATGCCTCGAAGCTCCAAGAGGAACTTTAGTGCACCACTTCAAAACCGATGATAAGGGAATTAT
CACCGATGCAAATTTAGTTGTTGCAACTGTTCAAAATAACCCTGCAATGGACATTGGTGTTCAAA
AAGTTGCTGAAAAATACATAAAAACACCCGAAGATGCAAAACCACATGTTTTAAACCACATGGAA
ATGGTAATTCGGGCATACGATCCATGCCTTTCATGTGCAACGCACACAATTGACGGAAAAGAAAG
AATGCTTTCAATGGATGTCTACAAGGGCGGAAAATTAATTAAAACATTATAGGGGATATCATGA
TCAAGATAAACACTGAAGACTGTTATATGTGTAAAGCTTGTGAAAATAGCTGCCCTACAGAAGCT
TTAAAATTAAATCCCTTTAAAGTATGCCAACTTTGTGGAAATTGTGTCAGTGCATGTCCAAACGA
TGCATTAAACTTAAGAGAAATTGAATTAAACGGTAAAACTATTAAACAGATAGAATATTTCCCT
ACAAAGTGCGATTTGTGCGGTGAATGTGTTAAAGTCTGTCCAAATAACCTCAAAATAGAAAATGA
TAAATTAAAAGGTTTCTGCGTAGGCTGTATGAAATGTGTTGATGCATGTCCTGATTCTTACGTTG
GAATGGAAGGCGTAGTTGAACCTGCTAAAAGAAACATTACATTACCAAAAGAACCTATTGCAGTT
ACTGAGGATTGCGTCGGCTGTGGCGTATGTATCTCTGAATGTCCTGTTGGTGCACTTTCAATAGA
TGGTGAAAAGGCAGTAGTTGACAAAGATTCTTGTATATATTGTAGTATCTGTGCACAGACTTGCC
CTTGGAACGCCATATTTGTTGCAGGCAAAAAATCACCAAAAAGGGATAAAAATATTGTTAAATT
TAGTGTGGACAGCGATTTATGTATTGGATGTGGGGACTGTACTGACAAATGTCCAAGGGATTTAA
TTGTTTTAAATGAAATGGTTGCAGTTCCGCCAAAAGGATGTCCTGCATGTGGATTATGTAAAGCA
GCATGTCCTGTTGATGCAATTGATTTAGTTGTTGAATATGGATCTCCAAAACCGATAACTGATGA
AGGTATCGTTTGGGATGAAGAAAAATGTGCATACTGCGGACCATGTGCTATAAAATGTCCAAATA
ATGCAATAACTGTGGTAAATCCAAAAGGACTTGAAATGCCAAGCAGGAAAAAAACTGAAAAAGC
AAACGAGTTCAAAATGTGTATTCGATGTGGTGCATGTGTTCAAAGCTGTCCAAACGATGCACTTA
GAATTGGTAAAATCATCCACAATGGTAAAGAATACGAAAGAATCGAATTTAGTCCTAATTTATG
TGATTCCTGTGGAAAATGTATTGAAACGTGTCCTTATGACATGCTTAAATTAACAGGAAAATCAG
AAAAGCCTCTCGAAGGGTTCTGTGTAATGTGTTTGAAATGTATTGAAGCCTGTGATAAGACTAAA
AAGAGCGCATTATCTTTAAAATAATTTTTAATAGATGTAGCTATTAGAAAACGTAATAATGGTA
TTAAAATATATATTTGGACTAACAGTTCAAGCCAGATATTGGTGATAATTATGGAAGAACCAAA
AATAGGAGTTTACGTCTGCCACTGTGGTGTAAACATTGGTGGTGTAGTTGATTGCGAAAATGTAG
CAAGAACTGCAGAAGGACTCGAAAACGTCGTGATTGCAAGAGATTACAAGTACATGTGTGCAGAT
CCTGGGCAAGAACTCATCAAAAAAGACATCAAGGAACTTGGATTAAACAGGGTAATTGTTGCAGC
ATGTTCCCCAAGACTTCACGAACCTACATTTAGAAGGTGTGTAGAAGAAGCAGGTTTAAACCCAT
TTTTGTTTGAATTTGCAAACATTCGTGAACACTGTTCATGGGTACACATGAAAGAAAAAGAAAA
AGCTACCGAAAAAGCAAACGACCTTGTAAGAATGGCTGTTGCAAAAGCAAGACAGCTCGAACCTT
TAGATTATCTAAACGTTCCAGTTACCAAAAGGGCAATGGTTATCGGAGCAGGGGTTGCAGGAATT
CAAGCAGCACTTGATTTAGGGGATGCAGGATACGAAACAATTGTTGTTGAAAAACACCCTCAGT
TGGGGGTAGAATGGCCCAGCTAGACAAAGTATTTCCAACAAACGACTGTTCAATCTGTATTTTAG

CTCCAAAAATGGTTGACGTTGCAAAACACCCAAATATCAAGCTTTATTCCTATTCCGAAGTAAAA
GATGTAAAAGGGTACATTGGAAACTTCAAAGTAAAAATCGAGAAAAAACCAAGATATTTATCTG
AAGAAAAATGTACTGGATGCGGTTCATGCGAGGAAGTATGCCCTATATCAGTTCCAAACGAATTC
GATATGGGAATTGGGCTTAGAAAAGCAATTTATAAGCCTTTCCCACAAGCAGTTCCTGCAAAGTA
CACGATTGACAAAGAAAGCTGTATTGATTGTGGATTGTGTGCAAAAGTGTGCGGACCTCAAGCGA
TTGATTACGGTCAAAAACCAGAAATCATTGAAGCAGAAGTTGGTACAATCATTTCTGCAATAGGA
TACGATCCATATGACCCAACAGAAAAAGAAGAATACGGATACGGAAAAATTCCAAATGTCATAAC
CTCAATGGAACTTGAAAGAATGATTAATGCTTCAGGTCCAACAATGGGTAAGGTAATTAGGCCAA
GTGATGGTCAAAAACCAAAAAGAATTGCATTTATCCAGTGTGTTGGTTCAAGAGATGCGAAAATC
GGTAAAAAATACTGTTCAAATGTATGCTGTATGTATGCAATGAAAAATTCGCAGTTAATTAAAG
AAAAAGCTCCTGAAACCGATATTGATATTTACTACATGGATATCAGGGCATTTGGTAAGGGATAC
GAAGAATTCTACGAAAGAAGTTCAAAACAGTATGGAATTAAATTTATCCGTGGAAGACCTGCAC
AAGTTTTAGATAATCCTGAAGGGGATTCTCCAATAATCAGGTCAGAAGATACACTTTTAGGTGAA
ATCTTTGAAAACGAATATGATTTAGTTGTTCTTTCAGTAGGTATGGTTCCAACATCAACTGCTGA
CGAAGTACAAAAATTACTCGGTGTTTCAAGATCTGCTGACCGGTTCTTCATGGAAGCACACCCTA
AATTAAAACCTGTTGATACTGCAACAGATGGTATCTACTTAGCAGGTGCATGTCAGGGTCCAAAA
GATATTCCTTCATCAGTTGCACAAGGTTCAGCAGCAGCATCAAGAGCTGCAATACCTCTTGCAAA
AGGGGAAGTTCAGGTTGAACCTATCGTTGTAACAATCGATGAAAATGTCTGCGGTGCATGTGGTA
TCTGCGTACAGCAGTGTCCATATGGCGCTCCAAGATTCGTTGAAAAAGACGGTAAGGTTGTAGCA
GAAGTTCTTTCAGCTTTATGTAAGGGTTGTGGAACCTGTGCAGCAGGATGTCCAAGTGGTGCACT
TGAACAGTCACACTTTAAAACAAGCCAGATATATGGACAAATTGAAGGCGCGTTTAAGGATTCCT
ACTAATTTCTTTTTTTATTTTTTAAATTGGGTGATTTTTTAGGGTGAAATAATGGATGTTATAAA
AAATGTAGTATGTCCATTCTGCGGAACTCTTTGTGATGACATTGAATGTTTTGTGGAATCAGGAC
ATATTGTAAAGACAAGAAACGCATGCAGGATTGGCCACAGCAAGTTTACACACAAAAAAGGAGCT
GTAAGATATACCGAACCGCTTTATCGGGAAAACAAAAAGGATGATTTTAAAAAAATCGACTTTG
ATACTGCAATAGAAAAAACTGCTGAAATTTTGGTAGATGCGAAAAAACCGCTGATTTATGGATT
TTCTGCGACAGAATGCCATGCGCATATCGAAGGTATGAAGTTAGCTGAAAAAATAAGGGGAATT
GTAAGCAACACTGCTGAAGTATGCCACGGTCCAAGTGTTTGGGCACTTCAGGATGTTGGATACCC
AATATGTACACTTGGAGAAGTTAAAAACCGGGCTGATGTTGTAATATATTGGGGTTCAAACCCGA
TGCACGCACATCCAAGACACATGAGCAGATACGGAGTTTTCCCAAGGGGCTTTTTCAGGAGTCGC
GGAAGAAAAGATAGAATTTTAATTGTAGTTGATCCAAGAGAAAGTGATACTGCAAAATTAGCAG
ACATTCACTTAAAAGTTGAACCGCACAAGGATTATGAGCTTATAAGTGCGATAAGGTCTGCTTTA
AAAGGTTTTGAACTTCAATCCGAAACTATTGCAGGAATTTCTAAAGAAATGATATATGAAACTG
TTGAAATATTGAAAAACGCGCAGTATGGGGAACTTTTCTTTGCAATGGGGGTTACCCAGACTACT
GGAAAGCATAGAAATATCGATACTGCAATCGAACTTGTAATCGATTTAAATGCACAAACCAAATT
TACGCTGATTCCAATGAGGGGTCACTACAATGTAAATGGTTTCAATCAGGTGTGCACGTGGATCA
GTGGATTTCCATTATGTGTGGACTATTCAAGAGGATTTCCTGAATTTAATCCTGGAGACACGAGT
GTAACTGATAGTTTACTTAGGGGAGAAGCAGATGCAATGTTAAATATTGCGTCAGACCCGGGAGC
ACATTTCCCACAGAATGCAGTTAAAAGAATGTCAAAAATTCCATTAATTTGCATAGATCCTCACG
AAACTCCTACATCAGTTCTAGCAAATATAATTCTCCCGCCAACGATTGCAGGTGTTGAGGATACC
GGAACCGCCTACAGGATGGACGGGGTTCCATTGGAACTTAAAAAAGTAATCGATCCGCCTGAAAG
TACGCTTGCAGATCGGGAAATATTAAAAAGAATATCTAAAAAAGTTGAAGAACTACTTTAAATA
AAATCAGAAGTTTTATTTTTTTAAATTTTTTTAACTTTGAATATTCAAAAATTTTCTATTTTAAA

ATATAAAAAAAATAGTTGAGGTCAAAAATGTTAAAGATTTGTGGCTATAAATCTTTATTATTCG
ATAGATTTGCTATTTAATCATGTTATCCAATATTTACGGCAATGGATAGATATATTACAAAATCT
CAGAGGTCATCCTGAGATACATTAACTGTGAGGGTTATATTATTTAAGTTTTCTGTATTTGTATT
AAAGGTTACTTTAAAATATGAGGTACGTCTTCTTTTTTAACTAAAATATTTTTTATTATGTCTAT
TCCTTTCTTAGTTATTTTTGTATAGTAACTCATACCATAGGACGAACTTTTTACAATTAAATAAC
CCTTAACTTCAAGAAAATGAACAAGGTATCTTAAATCGTCTTTTTCGAGGCCGGAAATGGCCGTC
AGTTCCCTCCCCTTTACAAAATCCTTTTTTTCAAATAAGTATTTTAAAATATTCTGTCTAATTAT
AACTTCATCCACAAAAATCCCCTTTTTAAGATATTGTAACTAAATATAATTACATATCTTATATA
AAGTTAATTAATACTTATTACCTAATCATATATATAAATTACGTATTTGGGGGGTCTTTGTTATG
GTCTTTAATAATGTGGTATTTTGACTTGTTTTCAATACTAATTAGTAATTTAATTGGCAACGTAT
TTATAATTTAAAAATTAAGTTAACATATATTTATCTGTAAAAACTATTAATTAACATATATGTT
GTAAACGAGTTTATTAGTTAAAAGGGGGATAATATGGGAGATGGTAAATGCGGTAAGCACGAAT
ATTCAAATATCAATCAACAAAAAGTTGATTTGATGATTAAAGAACTAAATGAACATGGAATATC
TGTTAGCGGAAATAATCCATGGATAATTGACCCAAAACAGTATGGTATCAAACTTCAAGCCACTT
GGGATCAAGGAAGCTCTAAACTTTACGTAATTGTAACAGATAAATCATTTTTAGTTCCCTGTTCA
AAAATATGGGAGAAAATTGACCCTTTAATGAAACATATTGAAAGTCTTTCAGAAAATGAAATGA
AATAATTTTCTTTTTTATTTTATTCGACTATTATCTTAAATCTAATATCTTCTTTTTTAGATTTT
TCAAGCATTATTCCAAAAAATTCAAATTCATTTATCAAAATTGGGCCAGAAATCGATTTTCCAAT
CGTTTTATAAATTTCTTCAAAATCTAATTGATTTGCAATTCCAAAAGCAATAATCCAGAAACATT
TTAAAGGATATTTTTCGCCAGAATATTTATCTTTTATAAAATATTTTGAACTTTTTGATTTATTT
CCTTCACAGAAGTTTTTTTGACTTAATTTAACAACTGTCTTTTCAAAATCCATGTATCCAGGTTT
TTCAAATTTTTTAAAAAGCTCTTCAAATTTACTACTCAAAATAATACCTTAATTACAATACATTA
GAAACGGATATATTTTGATATAATATATTACTATGCACTCGCTAACAGTTTTTAAGGTTTAATTC
GGGTAATTTTTTTATATATTTTTCTAATTTACATCGTACCATAATATTATTATATATTAGTTTTT
TTAATGTAATTATTAATTATTAGTTTTCTTCATGAAAACTATAAAAAATATTATTCACCTAGTTT
TATTGATTAATTACCCTTTTTTTAAATTTAAATTTTAGAGGTCAAAAATGAATGAATCAAAAGA
ACAGATAATAAAAGAGCTTTCTGACTCCGTTGTTGATATGGACGAAGAAAAAACCGAAGAACTGT
CTAAAAAATACCTGGAACATGGTTATGATGCATTTGAAGGGATTACTAACGGTCTTGCAGAAGGT
ATGAATAGGGCAGGTGTGCTTTATGAAGAAGGAGAATATTTTATACCTGAACTATTGGTCTGTTC
TGATGCAATGTATGTGGGCTTGGATATTTTAAAGCCTCATTTAAATTATGAAAATTCAGATAACA
AATTTAAAGCAGTTGTTGGTGTTGTTGAGGGGGATACCCACGATATTGGGAAAAACTTATTTAA
AATCATGCTTGAAACACAAGGCTTTGAAGTTTATGATCTTGGAAGGGATGTTCCTCCAGTAGAAT
TTATAAAAAAAGCTAAAGAAGTTAATGCAGATGTCATTGGTCTTTCAACGCTCATGACGACTACA
ATGGACAATATGAAAGTCGTTATTGACATGTTAAAAGAAGAAGGGATGAAAGAAACTACAATGG
TGATGGTTGGAGGCGGCCCAATCTCACAAAGCTTTGCTGATAAAATTGGTGCAGATGGCTACGCA
CCAGAAGCTTCAAAATCTGCAAGAATTGCAAAAGAATTGGTTACAACATTAAAAGGTTAATTTA
AACGGTATGGAGAGATTATTATGGATTACCCAGACAAAATGACACCTGCTCAAAGAGCTGCTGCT
AAAGCTAAGGGCGAACCCGCTGACCGGGTTGCATGCAACCCAAACATTGGAAATGGGATGGCAAG
AATTGCAGGCTACAAAATTTCACAGTTTAATGACGACCCAGAAGCGCTTGCTGATGCAATTATTA
AAACATACAGAAGATTTGGGGCAGATGGTGCAAGAATCTTCACAGATTTATTTTTAGTTTCAGAA
GCAATGGGCGCAAAAGTTAGAAAACCAGAAGATAATACTGCAGATTTAGAAGAACCTGCAATAA
ATGACATGTCAGAATTTGATAAATTAAAAGTAATTGATCCATACACACAAGGTAGAATTCCAGTT
CATTTAAGGGCAATTGAAATCGTAAGGGATGAAATTGCAAGCGAAGTAGGAGTTACTGCTTCAG

TTGTAGGCCCATTTACAAACGCTTTCTTTTTAATTGGCGTAGAAAAAATGACAAAAATGCTTTTA
AAAGATCCTGAATCAGTTCATAAACTCTGTGAAATTTCATTACAAAGTTGCATTGCACTAACCGA
TGCAGTTTTGGAAAAAGGCGTTGGAGTTACTATTTCAGAACCTCTTTCATCATGTACGGTTGTAA
GTCCGAAACACTTCAGGGAATTTTCAGCACCGTACTTAAAAAGACTTATCGACCACATAAAAGCA
AGAACTGGAAAATTGGTTATACACATTTGTGGAATAACAGATCCAATTTGGGATGATCTTGTAG
AAATGGGCGTAGATGTTTTAAGCATTGATAACGTTGCAGATATGGAAAAATGTGCAGAAACCGT
TGGAGATAAAATGGTTATTGCAGGAAATGTTGACCCTTCAGCGATTATGTATGCAGGATCAAGAG
AAGACGTTAGAAAAGCTACAATTAAATGTGCAAAACAGGGATTGAAAGCTAAAAAAGGATTCAT
GATCATGTCAGGATGCAGCCTTCCAGTAGAAGTACCTATTGAAAACATTGATGCAATGATGGATA
CTGCAAGGGAAATGGGATGGCCGGTTACTGAAGAAAAATTAGACCATTTACTTAGTTTTGATAA
ATACAAAGACTAAGTACCTAAATTATCAAGTTATTCATCCAGATTTTTAAATTTTTAAATTTTTA
GTTTGATTTTTTTAGAATTTAGTTTAAAATAATACATTATATTTGACAATTTAAGTAAAAAGAG
TGATTTAATGATTACACCAAAAGAAAGACTCTCAAAAACCCTCAAAGGAGAAAAAGTTGATAGA
ATCCCTTGCATATGTCCAGGCGGCATGATGAATATGATTACAAAAGATATCATGGACATTACTGG
AGTATACTGGCCAGAAGCACACACCGATGCTGAAAAAATGGCTAAACTTACAATTGGAATGTATG
AAAATGGCGGGTTTGAAAATTATGGTGTTCCTTTTTGCATGACTGTAGAAGCCGAAGCTTTTGGT
GCAGGAGTTAAAATGGGCACAGATATTACTGAACCAAGAGTTACAAATTACCCAATAGATTCTGT
TACTGAATATGAAAAATTAAATCACATCAATATTAATGAAGGGCGTGGAAAAACCGTTCTTGAA
TCAATAGAACTTTTAAAAGAAAAAAATACGGAAGTTCCAATTATTGCAAATCTTACAGGTCCAGT
TAGCACTGCATCGTCTTTAATGGAGCCTGTAACTTATTATAAAGAACTTAGAAGAAAACCTGAAG
CTGCAAAAGAATTCATGGATTTTGTAACTGAAAACTTAATCGAATTTGGAAAAGCACAGCTAAA
AGCAGGTGCAGATGTTTTGGCAATATCTGATCCAAGTGGAACTGGCGAGATATTGGGTCCAAAAA
TGTTCAAAGAATTTGCAGTTCCTTATCTTAATAAAATTATTGAAGAAACAAGCGATTTAGCTGAA
ACTGGAACAATAATCCATATTTGTGGCAGATTAAAAAGTGTTTATGCAGAAATAAATTCATTAA
AAAGTGACGCAATAAGCTTTGATTCAATTACTGATGTTGGACAGGTAGTTGAAAACGTGTCAGGA
AAAGCAATAATGGGAAATGTCAGTACATTTGCGCTTGAAAATGGAACTCCTGATTCTATTGATAA
AATGAGTAAAGCTTGTATTAAATTTGGTGTAGATATACTGTCTCCTGCATGCGGAATTGGGGTTA
GAACAAAGTTAGAAAATATTCAAGCAATGGTTCAGGCCTGTAAAAACTCCAAAAGAGGAGAATA
AATGGCAGTAATGGAAATAACTAATGATAATAATAAGTTTGAGTTTAAAGAAGGAGAATTTATT
TTTAAAATTCTTCAAAAAAATGGAATTAAAATAGAAGTTCCTTGCGGTGGAGTTGGAACTTGTG
GAAAATGTAAAGTAAGAGTTGTTGGCGGAAAAATCAGCCCTTTATCTTCTGAAGAGCTTGAATAT
CTTTCAAAAGATGAAATTGATCGAGGAATTCGTCTGAGCTGTCGTACAAAGGCGTTTGGAAATGT
GAAAATTGAATTATTAAATACGGATGAAAATCACAAGATTTTGACTGACGGATACCTGCCAAACT
TAAAAATTAACCCGCCAATCACAAAAAAAATCATCAATTTATCAGAATGTGAGTCAAACAAAAA
TAATTACGAAGAAATTGTTAAAAATAAATTAAATGTGGATGAAATATCAGATATCAAATGTTTA
AAAGCTTTGCAGAAATCGTTTAAATCAGGAAATCTTACTTCAATTTTTTTAAATGATAAATTAAT
TGGAATTGAACCCAAAATCAATGAAAAAATATATGGAATTGCCATTGATATTGGAACTACCACGG
TTGTAGCATCTTTAATTGATATTTTGAATGGAACTGAAATTGCATCTGAAACCATGATCAATCCT
CAAAAAGAATACGGTTCAGACGTGCTTTCAAGAATTGATTTTGCAAATAAAAATGAAAACGGGC
TTGAATTATTAAATAAAGCAATAATTACAGGAATAAACAAACTTATTTTTGATTTAGCGACACA
GAACAAAATTGCTGTTGAAAACATCTACGAAGTTTCAATTGCAGCAAACACAACCATGATGCACT
TTTTATTGAACATCAGCGCAGAATCAATTGGAAAATCTCCATATCTGCCAGTATTTTTATCTGGA
AAAAACATCTTATCAAAAGATATTGGAATAAATATTTCTCCGTTTGGTAGAATTTACTGTTTACC

TGGAGTTTCAGGGTATATTGGAGCAGATATTGTTGCAGGAGTTATAGTTTCTGAACTTTTGAAAA
CTGAAAAAAATGTTCTCTTTATTGATATTGGTACAAATGGGGAAATAATGCTTTCAAAAGCAGG
TATTCTTTCGTCATGTTCATGTGCTGCAGGCCCCGCGCTTGAAGGAATGAATATAAGCTACGGAA
TGAGGGCGGCAGATGGTGCAATAGAAGGAATTAAGTTTACCGAAAATGGGGTCGAATTAAAAAT
CATTGGAAACACGAATCCTGTTGGAATTTGTGGTAGTGGAATACTTGATTCAATATCTGAAATTG
TTAGAACAGGCCTTGTGGGTAAAACAGGCAGGTTAATTAATTCAAATGGTTCAGATAATGAATA
TAAAAATATGATAATAGAAAAAAATTCAAAAAAGTTTGTTCAGATATCAAAAAATCCAGAAATT
CTGGTTTCACAAAAAGATATAAGGCAGGTTCAGCTGGCAAAAGCAGCTATAGTTTCGGGATTTTT
AGCACTTTTGGATGAAAATAAGTTAACAATGGAAGAAATTGACGAAGTGATAATTGCAGGACAG
TTTGGAAAACACTTAACTGTTGAAAGCCTTGTTGGAAGCGGAATAATTCCTCTAAAACTGGGCGA
AAAAATAAGATATATTGGAAATTCCTCTAAAACCGGTGCTTTAATGTGTCTGCTTTCAAAAGAAT
CGCGAAAAGAAATGGAGTCAGTTTCTAAGGATATAAAATATTATGAACTTTCGACAAAAGAAAA
CTATGAAAAATTGTTTATTGATTCTTTAAATTTTTAAAAATTAGTATTTGAAATAATAATATTA
AAATTTACCATATTTGGTGATATTGTGGTTAAAATCGACGTTGTATCGGGCTACTTGGGTTCTGG
AAAGACAACATTTATAAAACATCTTTTAAACGCTTGTGGGAACGAAAAAGTTATTGTTATTGAA
AATGAATTTGGCGAAATTGCAATTGACGGAGATCTTATCAAAAATGAAGGATTTGAAGTATACG
AACTTCAACAAGGGTGCATTTGCTGTAGCATTAGGTCTAACTTTATCGAAGCGTTAGGCAAAATC
GTAGATGAATTTAATCCCGACAGGATTATTGTTGAACCAACAGGAATGGGGCTTTTAAGTGAAAT
AATTGGAATATTGAGTCTTCCAAAAATTAAGCAAATTTCAGAAATAAATTCACTTACAACTGTGG
TGGATGCTGAAAATTATCTCGAAGAAGTTGAAGGATTTGGCGAATTTTTTAAAGATCAGATAAA
AAATGCAGATAGACTGATTATCAGTAAAGCTCAGTTTATGAATTACCAAGAAATTGATAAAATT
CTCTCATCATTAAGGGAATTAAACGCAGATTCATTGATCGTAACTCAAAACTGGGAATTTATGGC
TCCAGAAGAATTTTTGAAAATTCTTGACAACAGTTCTGAAAATTCTGGTAAAATATATCGTGGAA
ACGTTGAAGCTGCAATGAAAGGCGTATCAAGTATTGGGCTTGAATTTACGGAATCTTTTAAAGAA
AAAGACATTGAAAATATAATTTTAGAACTTAAAAGTGGAAAATACGGGCAAATAATGCGTGGAA
AGGGTTTTTTAAATATCGATGATAAAACAATGGAATTTAATTATGTTGGAAATGATTGCAATTT
GGAGCTTTTAAACACGAAAAAAGACCAAAAAATTTGCATAATTGGAAAAAATCTTGAAAAAGAA
GCAATAAGTGAATTATTTAATAATTTATAAAATAATTATTCTTTTATTTCCCAAACAGTATTTTT
TGGAATTTTATAGATTGATTTTTTAATGCAGTTGTGACCAATAATTTTTGTTTTTGTATTTTCAT
GGATTAATTCATAAATTGCTTTCCCATATGTTTCAGAATCGCAATTTAATGGATTAAATTTGCAC
ATTCCTTCATTTTCAAGTGCAACAGATGTTAATCCACAAAGATGAATAATACAGTTTTTTTCTTT
TAATCTAACTTTTTCTATAATATTTTTAGCAATTTTTCCGCTGTATTCCCGAAATACTTTTGGAC
CCACTATGTCGATACTTCCTGCAGGATCTCCAAAAGAAATAATTTTAGCCCCGTTTTCAATTCCT
GAGAGAATATATTTTACGATTTCATCCTCTAAAAAATTTAAAAATTCGTTTGCTTGGTCCGGATT
TTTTCTAAGTTCTTTATAAAATGCTGTCGAATCTATCAAAGATGAAAGTATGGTAAATGGCCCTT
CAACCGCAAGAATTGGAATTTCACCCGATTCTTTTAATTCATGAATAGCACCAAGAACTGTTTTA
ATCCTACCCGTGGTTAAATCAATCGGTTTTATGGATTCTAATTCATCTAAATTTTTAAAAGCGTA
TTCTTTAGCTCTTGGGCCGTATTTTTCATCGCCCATGTTAATTTTTGCACCCAATGATTCTGCTTC
AACGGTCATACAGAATGGAACTCTACAAAATATGTCTTTTCTATATTCTTTCATTATTTTTGAAA
ATTCCACCATATCTATAAGATTTTCGTGTGCATTTGGAAATTTTAAATTTTTAGATTCAGTTAAT
TCTTTTGGAATGTTTTCTAACTTATCACTAACACATGAAAATGTTTTTTTCATAGATTTCACCAT
GTTTAAATTATTTTTAAATTTTAAAAAATGAAAAAAAAGAAAAAGTTTAATGGATTTAAAGTCC
TTTTTCTTCAGAGAAATAAACGCAAATTGTTGCAATAACCATTCCAACAGCAAGTAATAACCATG

CCATTTGGTATCCTGCACTTGTGTATCCCCCTAATGCATTAAGACCGTAGCTTGCGATTACTAAAC
CACAAACTTGCTGTAGCACTGCACCACCAAAGAATGGGAAGAAGTTAAGGAGTGATGTAGATGTT
CCTGTTATTGAGATAGGGAATAATGATTTCACCTGTCCATAACATACAACGAAGAATCCTGCGAA
GAATCCAAATAATAAGTATAAGATTTTATAGAATAATGGATTGTCCATTCCGTTAAAGTACCAG
ACTGCAAACCATAATAATGTGTACATTATTGTACCGATAATTAATGTCTTTTTTCTTGATTTTAA
AACTTTATCTGCGATGTATCCTGAAACAGGGCATCCTATGATCAAACCGATCCCGATAAACGTTA
AAAGACCTGCATATGTAGCTTTGTCCCAGCCTAAAATGTCTTTAAGGTATGGGCCTGCCCAGAGT
CCTTGGTATGCCATTAATGAACCATAGTAAAAGAAAAACCATATTGCCAAGGGCCAGAATGATTT
TTTACCTAATACCATTTTAATAGAATCCATAATTGATATGTTTTCTGCAGGTTTTGCTTCTGCTG
AAACAGTTTCGCCATTTTCATGAGCTTCAATGTCTTCAATATTTGCGAATCCCATTTCTGATGGT
TTATCCCTAACAACTATGTATGAAAGAATTGCAAGAGCAATTGATGCTACACCAAGCCCAGTAAA
TACTGTCTGCCAACCTAATGCCGTGTTAAGGTAAGCAAGTGGTGCTGCTGCGGATAATGCCCCAA
TGTTTCCAACTGCGAGCATCATACCACTTTGGGTAGCAAATTCGTTTTTCCTAAACCAGATTGAA
AGCATTTTCATTATCGGAATATACACTGCAGCTGCACCGATACCGATTAAAAGCCTTCCCATGAT
AACCATGTTAAAGTCAGACGCAATACCTGTAAGAATTGCACCTGCTGCTGTAACAAGTGTAAAAA
CAGCAACGACCCTTCGCGGTCCGAATTTATCAGACATTATCCCTGAAGGAATCTGCATTAATGCA
TATGCATAAAAATAAACTGATCCGAGTAGACCCATTGAAGTAGCGCCTACACCAACGTTGTCATA
AGATCTCCAGCCATAACTGCAGGAGATACCCTGTGAAAGTACACGAAGAAGTAAACTAACGCAAG
AACTGCAAAAATTACCCACCGGTATTTCATCATTTTGCCGATGTTGTCCTTTGATAACATGTATG
ACCTCAAAAATAGTTATTGTTTAATTTTAGGTATCTTAAAACTTAAGACCTTCATTGGATTATTT
AATTACCTATATATATTGATAATGGTATTAGATTTTAGCAGTTATTTTATATTTAATAGATATTC
TAATAGATATCAAACTCAATAAATAATGAAAAATAATTTAAATAATATCTTAGAAGATAGTTTA
AACTATAAAATCGGTGAAACTTTGGACGAATTAAAAAGCATCAAAAAACTCGATGAGATTTTTT
CAAAAAGAAAAGATGAATTATATCGAGAAAAAGACGAAGGAAAAAAAGTATTTGGTAGATTTTG
TGTATTTGTACCTACTGAAATTATATGTGCAGCTGACGCAATTCCTGTTGGACTTTGTGGTGGAA
AAGAGTCAACCATTCCATCAGCAGAAGAAGATCTGCCAAGAAATATATGTCCACTGATTAAATCG
TCATATGGATTTAAGAAAGATAAAGCCTGCCCTTACTTTGAAGCTTCAGACGTTATTGTTGGCGA
AACCACGTGTAATGGAAAGAAAAAGATGTTTGAACACATGGCAGAACTGTCAGAAATGTACGTT
ATGCATCTTCCCCACTTTAAAGATGAAAGGTCGTATGAATTATGGCTCAAAGAAGTTTACGACTT
CAAAGATTACGTGGAAAAATTTACTGGAAACAAGATTACTGAAGAAAAATTAAAAGAAGCAATT
GATGAAGAAAATGAAGAAAGAATGTTATCCCACAAGCTTTACGAATTAAGATCCCAAATCCCAAC
GCCAATAACTGGAAAGGATGCATTAAAAATATTCCAGAGATCATTTTTACTCGATATAAAAGAT
AGACTCGCAATTTTAAAAGAAGTAATATCTGAACTCGAAGAAAGAGTTAAAAATGGCGAAGGAT
ACACTGGAAAAAGAATCTTGGTTGCAGGATGCCCGATGGTTGCAGGAAATACCAAAATTGTGGAC
ATTATCGAAGAAGTAGGGGGACTTGTAGTCGGTGAAGAAACATGTACGGGAACTAGAAAATTCG
ATAATTTAGTTAATGGATACACAATTGATGATCTTGCAGAAAGATACTTTAAAATTAACTGTGC
ATCTGCATTTAAAAATGACAGCAGAATTGAAAGAATTAAAGAACTGGTAAAAGAACAAAATGCA
GATGGTGTTGTTTATTATACATTACAATACTGCCACACATTTAATGTCGAAGGAGCATTAATTGA
AAAAGAACTCAAAAATTTAGGAATTCCAATCATTAGAATAGAAACAGACTATTCAGAAAGCGAT
AAGGAACAGATAAAAACGAGACTTGAAGCTTTTGTTGAAATGATTTAAATTATTTTATTTTTTT
TACAAAAGTGATTCTTTTGCTAACTTATCTGCAATTTCGTTATTTTTATCTCCTGAATGCGCCTT
AACTTTTACAAATTCAATATTTAATTTTTTTAAGTAATTTTTACATTTTTCTTTATATTCTATAG
TTCCAGATTTGTTTGTTTTCCACTCACCAGTACACCACTTCTCAATTCCAAGATAATCGTGATAT

ATTACTAAATTTTTCACATTTTTAGAAACTGCGTATTCCATTGCAAGTTCTGATGCTAAAATTTC
GCCTGCAACGTTTCTCATTTTTGATAATTCGGTATCATTTCCTTTAGCTTTAATTTCTTTTTTAA
TATCATTAAATAATATTAAAGCGCCTGCACCGTATTCTTTATTTTTTAAATTAAAACTTCCATCA
ACCCACGCATGCATTATATCTTTTTTAAATTCGTCTGATTTTATATTATTTTGATTTTGTCCTGA
TTTTAAATTTAAATAATCTTCTGCATCTATTTTAGATGTAAAACTTTTAAATTCGGCTCCAGAAA
ATCCTTTAACTTGATTTTCACATTCAGCCCAGGTATTAAATAGTCCCGTTTTTCGCCCTTTTCGAA
CAGCATATATTTTAGACATTTTTTCACGCAAATTTATTTTAAAAACATTGTATATTTAAATTAAA
AATAATTTTCCCAATAATTTATATTTCAAAAGATTTTTATAAATTCCAGATTGAAATATATTATT
CGTAAAATGAGGCGATATTTTGAAAATCACTCCATTTAAATCATATTTAACTGCATTTATATTCT
TTTTATCGGCAATACTGTTTGGAATATTCTTAAAATCGGAGGCTGGCGAGTTAAGTATAATTTAT
ACAATTTTAAAAGATTTAGGAATTGTATTTTTAGTTATTGGAATTATACTTCAAATGAAACTGA
AAAAATCCAAGAAATAACTTATTTTTTAACTTAAATGGCAATATTATGGTAGAAAACGTCAAAA
TAATCCGTAAAAAAATCAAAAACATGTATTTAGTTGTAAATCCCGACTGTTCTGTTGTAGTAAAA
GCGCCAGTTCGCGTTTCTGATGAATACATTAATTCATTTATAAAGAAAAAAGAAAGTTGGATAA
AAAAGCATCTGGAAAATTTTGAATCGTTAAATTCAAAATCAGTTGCAAAAAAGTATATTGATGG
GGAATTATTCAAATATTTAGGAAATGAATATATTTTAAAGGTATATCCTTCAAAAAAGGAGTAT
TTAGAAATTTCAGATAATTTTTTTAATCTTTATGTGTTAGAAACAAATGATTTTGAAAAAAAGA
AAAAAATAATTGAAAAATTTTACCGAAAACGTGCAGAAATTGAACTTTTTGAAATTTTTAAAAG
TAATTACAAGGTAGTTACTGAAAAAATGCCCGATTTTGCAGTTAGAAAAATGAAAAAAAGATGG
GGATCTTGCAGTTTTCATAAAAATAAAATTATACTAAATGAAAGATTAATTGAGAAATCAAAAG
ACTGTATTGAATATGTCGTGTTTCATGAACTGGCCCATTTACGATATCCAAACCACAGTAGGGAT
TTTTATAATTATTTAACCGAATTAATGCCCGAATGGAAAATTAAAAAGCTTAAATTAAACGAAA
GGCATTAAAATCTTTTATTTTATTGGTGTATATTATTTTCATGCGCGATTTTTAAAAATATCTGT
TTTAAATAGGGTAAAACTTTTAGTGACGGTACAAGGTAGCAATCTACTTTTTCAAAACCCTCTTC
TATGGTTGAATTTTCCTTTCTAATTTCATCTAAAATATATTTTAAAAAACCGTCCTTATTTCGCC
ATATTGAAAACATCAAATAGTTTTTTTCAAGAGGTTCAACAAGTTCCCTGTGTTTATATTTCATA
ACAATATTTAACGCGAGTTTTTCAGGACCTCAACTTCCATTTTTGAAAGTTCTTTTCCAAATATC
TGGGACATAAACATTGTTTCAAATCCGATTTCCATAGCATCTCGGGGAGTAATATTTTCAAAACC
TTTCTTTTCAGCTTTTTCGTACTTTGAAAGGTATCTAAGTAATGATGTGTATGCATTTCCTGTAA
ACACAGAAAATCCAAATTCAAATATATTCTCCATAAAGTTTCACCACCCATATGTTCCTATTATT
TACCGGCCACCCGGTCGGTTCTTGTCCGCGAAAAATTAATTTTTTATAATATCCCAAAATGAGTT
AAACAGTGTTCTTGCTGTTTCATCGAATTTTTTGTTCGTATAATACGAAACTAAAGGTAATCCTT
CGTATATTGAACAAAAATGAAGTGCAATTGAGTGGGGATCCAAATTTTCTTTGATTTCCCCATTT
TTTTGGCCTAATATAATCCTGTTTACTAATCCATTTATGATGTTTTCATATATATTATTATTATT
ATTATTATTATTATTTACTTCCCAAATTTCAGGAAATCGTTTTATTGCATCAGAAAGGGGCCCAG
AATATGTTGGGGTATCATCTTTTTGGATATATTCATTAAAATATCTACTTCGTATCATAATCATC
TTATCTATAAATTCAAAAAATGCCTCTTCAAATCCTATTTCTTTTTCAATGCAACTTAGTATGAT
TTTTTCGTTTTCCTTAATCATTGAGACAATAACTTCCAAGAATAGGTCTTCTTTTTTATTAAAAT
AATGGTAAAGTCCCCCTTTTGAAATGTTGCATTTTTTTTGCAATTTCTAAAAGAGATGTTTCATTG
TATCCCTTTTTAAAAAATATATCTCTTGAGTTTTCAATTATCTGATCCCGTGTATCCATAGTTTA
TACCCCATTTTTGGATTTTTTTATAATACAGTTATAACAAATTTTATTTATGCATTTTTTGTTGT
CGAGTCAGGATCATTTATGGAAAACTATATATCCATGTTCGATCTAGTTATGCTGGGAACGGGAT
GGTGACCACACCGTGCATTTTTTTGATCATATTGATCATAATTTAGATCTAACTCGAAATATTAGA

TCTAATTGTCGATTGATTATATTTTTAACAAAAATCTGTTTTTATTTTCAAAATATATTATAATT
TACACCTGACGTAAATTTAAAATATGGGAATTTTTAAACTATATATTACCTCGGAAATTACGAAT
TCCGGGGGTAAGGGGGATATATTCGTGTGTGAAAAAACGCGAAAAAACTAGTTCTTTAAACCGCA
TCTATTTTTTATTTTTTAAATTGTTATGTAAATTTATTTCTTTTTCAAAAGAATTGTAAACTTTA
TTTTACATTTTCCGAACGTTTTATTTAGTATTATATAATAAAAAGCTTAAATTGATAAACTGTCT
TGATTTATTCAAGAATTCTTCATTGTGTGCTCATGTTTGAAAACTCTCAAAACTCATATTAAAAT
AGTGCCTTGAATATTTATTTTTTTATATTCTTTTTAAAAATAATTAATTATGGATTAAAAAATG
GTGGGTTTATGTTTTTTGAAAATTACGTCTTTGGTCCATTAAAATATGGCCTCTCAGATTTATCA
AAATTATTAAAAGGCGGAATAATTTATGCTGTAAGTATTTTTTTACTTATTATCGGAATATTTTT
GGCAGTTTATCCGTACACCGGTGCGAATTTACACATATTGGGTACTGCAACAAATTCTTTAGTAT
TTGCAGTAGTTGCTCTTCTTTCGTCAGTACTGGGTTTGGGTCTCCTTATTGTTTTGAATGGATAT
ATATTAAAAGTGATTAAATTATCTCTTGAAAAATCAGTAGAACTTCCAGAATGGGCAAATTATT
GGGATATGCTTAAAAATGGAGTTGTATTTACGTTAGGAATTGCAGTAATCGCAGTTTTTGGATCG
ATTCTTCAAAATATTATAACCTATATCGGAAATGATAGCATGGTATTAATTGTACTTTCAATGGT
TATCCAGCTAATTATTTCATTATACATTCCACTTTCGGTAGTAAATTTTGCACACGAAGACAACT
TTGGCGCATTTTTTGATATCCCTAAAATTTTTAAAATGGTCTCATTTGAATATTTAGGCATGTTT
ATTGTAGTTTCAATAATCTCAATGCTACTTATGATAATCCCGATGATATTGTTAATTTTCCCATT
AATCGGGTTTTTTGGAATGAATATGTATACGGGCCCTAAAATGCTTTTTATGGCAAGTCCACTAC
TTTTAGTTGTAGCCTTGTTTGCATTTATTGTATTTTCAATAGTTGCAGTTTATGTATCATTTTAT
TCCTACAGGGCATATACTAATTATTTCATTTCTAAAAGTCCTGAAAAAATAGTGGATTTTAATCA
GGAATTATAAACAAAATCTTAAATTCTTTTTTTAATATATTTTTAAGTATAATTTTAAAAGTGG
TTAAAATGAATTACAATATGGGTTTTACAAAAATCAGTATTCATGAAACTATATTTATCGTAAA
AACATTTTTTCAGAATATTTCCGCAAAAATAGACGATGTATCTGCTATCGAAATAGATACTCGTG
GAAACTACATAATGCTTTTGATCGGAATTTTGTGGTATATTTCTTCAAATATACTCTTGACGGTT
TCAAAAGAGATAAGTTATTCGTTATATTATGCAATTCTTGATTTAAGGGCATATCATATGATAAT
GACGGTTTTAATATTTGTTGCAGCACTATTTTCAACTCAGGTTAAAATATACGTTACAGGCTATA
AACCAATTATACTTATTGGAAATTACATTTCAATGAAGAAACTCTATGAATCGTTAAAAAAAGA
TTTAAATTTAAGTGAAGATGTGTATTGAATTTTTAAAAATTGTTATCAAAAGGTTTAAATTAAA
GTAACCAAGGTATTGAGTATAACTAATCTTAACTATTCAGCGGTGGTATACTTGGAAGATATTTT
ATTAAAAGATGGAGTTTTAGAAACTCTCAAATATATTAATGATAATGTTTTTTCCGAAGTAGTT
TCAACAAAAGAAAATATGAAAATAAGTTCAGAAGATCTTGCAAAATTGATTGATATGGGATTTA
TAGAAGTTAGGACTAATGAAAATATTGTAGTATTTAATTTATCAGATGATGGCAGAAAAGTTTT
AAAAAGATAGGAACTGTTGAAGAAATTTATAATTATATCTAACTATATTTTTTTAATTTGTGTG
TTTAAAAGCGGTTAAATTATATAATTAACCATTTAGAACTAATTTTATTTCTTCTTCGGTAAGTT
CGTATAAATCAAAGACCATTTTGTCTATTTCTGAGTCTGTTTTATTGATTTCAGACTGTATGCTG
TTTAATTCTTTTTTGTATTCTTCAAATATTTCGCTGTATTCTGGAATATCTTTTTTAGGAATTGT
TATTTTCTGTTTTTTAAGTTCTTTTACAACTTCATTAAATTCGATTTCGTTAAACGCATCTAATT
TTTTAGATATTTTATCTAAATTTAGGTCTACATTTAGAATTTTAAAGAATGCCTGTTTTTTCGTT
TGGAATTCTCTGTTTAACTTTAACATTAAATCGGCTTTTTCTATAAAATGGGCTTGTTGTTTTTT
GGATATTTTTGGTATAGGAATTCTTGAAGTAACAACACTATCAAAATGCATTGTTCGAATTGCTT
TTGCATATATAAATCGATAAACATACCAATTTAAAAGATATGAATTTAGCAATAACCATATAAT
CTTATTTGAGTACTCTAAATTCAAAGTGATTTGATTAATCGTATCTAATAAGATATAATCACTAT
TTTCAGGAACACATGCCGTTATTTTTATATGTGGTTTGGGGTTCTCGATATGTGCCACAATATTT

TGAACTAAAACAGAGTTTTCTTTGATTTTAGCTGTTTCTTTTATAACGTATTCAGATTTTATCTT
CCCTTTTATGTTTTTTATCCCCTCCCTTTGAATTTCAGCACCTCCGATAACAAAAAAGTCCCCTGA
ATCGCTTAAATATTTTTGTAATGTATCTCCACGTTTATTTTCAGAAATATTATTTAAATTTATTG
ACTTTTCTTTGATTTTTAATGCGATTTTCAATTCTTCATTATTTACACCACTCAAAAAAAACCCG
AAATTTTTTGAAACGTCTTTTGAAATTATTCCTAAAGATTTTATTAGTTCTTATTCCGTATTTT
TGATAAATATATGTTAAAATTACCGTTTTTACGTAATAAAAATATAATTTGTTCTAATTTAACTT
CATCCCATACCTTTCCACAATCAACTATTTCCATTATCTCGTTCCATATTATTTTTCGAGTATTTT
GGTAATTTGATGCATAAATAAATGATTTTGGAATAATATACCCTAAATAACCATTTTTAAGCAA
TTTGTACGATTTAAATATAAATAAAGCTGCCGTATCAGTAATTCCCAGATTATAATTTTTTTTAA
AATAGTTTTGTTCTATCGTACTTAATTTGGCACCATAAGGAGGATTACCTATAACAACGTCAAAA
CCATCTTTTTCAATGATTTCTTTAAATTCTTCTTCCCATTTAAAAGCCTTATCTCCTGCTACATCG
ATATCATCAATTAAAGAGTTTCCACATTTGATATTATTATCAAGGGCGGTTAATTTTTCCCCTTT
TTCTACTGAATTAAGCCATAAAGAAAGTTTTGTAATTTCGACAGATTCGGGATTTAAATCTACAC
CGTAAAGGTTATTTCTTAAAATATCCGCTTTTAAGTTAAATAGTGCAGTTGTTCCACCCTGTAAA
TAGGATATAACTTTATTAACTTGGTTTCCTTCTTCTACAAGATAGTTTAAAGCTTGAATTAAGAA
CGCTCCAGAACCACACGCAGGGTCAAGTATTTTTATGTTTTTTAGAATTTCTTTATACTGTTTCC
AGGCTTCAAGGGATTTATGTGATTTGGAATTCTTTTTTAATTCTATTCCTTTTCCTGTTTTCATA
CGGTCTTTTTTAAGTTCTTCTGGAGTAATATCATAAAATAATTCTATACCGATTTCGTTTTTTCT
ATCTTCAAGCCAACCGCCGACAGCCTGTTCTACAATATATTTTGTTATATATTCTGGAGTATAGA
ATACGCCGTCTTTTTTTCTCTTTCCCTTAGTTTTATCAAATTCTTCCCCGTTAACAGAAGCTTTTA
TTTCTTCAAGGTCTGAAATAGACTGTTCAAAAATATGTCCTAAAATATTTACGTTTAAATCCCCG
TCAAAGTCATAATCTGAGATTTTGACAAGTTCTTTAAAAATATCTTCTGGAATGTTTAAATTATC
GAGTTCAGGGTCTTTTTTAAATAATCCTCCGTTGTATGCAAAAATGCCAATATCTTTACAGCCTT
TATCTATACAATCAAATAATTTTTTAAACAGTTCCCAACGTGAAGGTCTAAACATTCCGCCTTTT
TTAGCATCATCATCAACATTATGTATTATATTTGGCGGTAAAATTCCTTTATCTTCTGCAAAATA
AACAAATAAAAATCTATCGAGGAGTTTCTGAGTTTTTTCGAGTAAAAATATTTCTTCTTTTTCGG
GGTTTTCAGCTTTTAAGTTTTCAAATAAATTTAATCTAATGTGTTTATATTCCGTGTAAAACTTC
TTTGTAATGTCTTTTTGGTGTGTTACTGTTTCAGAACATAATTTTTCAGTTTTAGAGTCCCCTGT
TTTAGAAATCAAGTTTTCAAAGTTTAATAAATAATAAAATTCTTTAAATTTATCTTCTTTTTTAA
GTTCTGCAATGTCAAATAAATGATATTCTTCCATTCTTGTGTTTAGATAATTATAGAGTCTAATT
TCTTTAAAGTTAGAAACTATTACCCATTTACAATTTTTTCCAGATTTTGCTGCATAATCGAAAGC
CTGTTCTATTGGTGTTCTTTTGTCGTCTTTTCTATTTTGTTTTAAATCTAAATTGGTTTTTCCATC
TTTTAGTTCGATTACTGCGTGAATGTCTTTTAATTCACTTTCAAAAAACCCTAAAGCACCATCTG
CTTTTTTACTGTCTACTTTTGTTTTTTGTTCGTATTCTAAATTCCATTCTGTATTTCCTATTCTCG
AAGTATAGTTTAAAACAGCTCCAAAAATTTCCCCTAAAAATAAGCCCTGTAAAGCTTCTTCTTTT
ATTGTTTCGATTCCTTTATCTTCGATTTCAGTTATCCATTTGTTTAAAACCGTTCTTTTTTCGTTT
AAATTTGGAATATCTGATAATTCAAATTTTTCTAATTTTTCTTTTAAAACATTCTTGTTAATTAG
AATATGTTCCATTAAAGACCCCCTCGTTAATTAGAAATTGGTTTAATTTGAGTTTAAAATATGGA
TTAAATTATATTAAAATATAGTCGGTTTAATTTCTATAAAATAGATTAAATTCTTTTGAAATGA
TAATTTTAAAAAAGTAAAAAAAGTTTAAGCAATCGCACTTGTTGCCTGAATATTAAATGATTCA
TTTGAAAGCCCGTATTCATTAAGAGCTTTTGCGTTTATTGACTTTTCAATGGTTTATTTCTTTT
TAAAACTTCTAAAATTATGTTTATTTTAACGGTTTCTTTATAAACTTCTATAAGTCCGCTTATTT
TTATAATGTGGCTTAAATCAAATTTAGTTACTCCAAATATTTCCGAAGCGTCAATTATTTCTACA

CCTGTAATTAATTTTTCCCCTGTTTTAGTTACACCCATATCTAAATAGATATTATCTACTTCGAT
TGTGTGATCGTATTCTGCCTCAATGTTTTTCCTTTTAATATACATTGCATCAGCTAGAGGATCAT
AATTCAAAGTTACATCTTTACCTTCAAATTTACTCATGTTTTGTCCTCCGTTTAGAATCTTGGGG
AATTGGTGAAATAAAGTAGAATTTATTGAATCTGTATGCTATAACGAGAATAATGTCTTTTTTT
GAATCATATTCATAATATAATTTAAATGTATCAGAATTTTGGTTAATTATTCCTTTACACTTTTT
GGTTTTGATTATATTATAAAGTTCATCAAATTTAAAATTTCTTTCTTCCAACCTATGAACAAAGT
GTTCAGTTAGTTCATAATTAGTTTTGGAAAGTGTTTTTATGATTTCTAAAAATTCTTCAATTTCC
ATTTTTTCACGAATGAGTAGTATTTATTTATGATTGATAGATAAAACAAGTAGTTTAGTTTAAA
AAGTTAAAGTTTAAAAATTAAATTCCATTACTCTTTGATACTCTCTTTTCAAAGCTTCTTTATTT
TTGTGCAGATATATCGAAGTTGTTGATAAATCTTCATGACCTAAAATTAAAGAGAGGATTTTTTT
CTATCTCGTAGTTTTTGAGAGTAAATACTATTGAAAACCTTATAAAGTCGCTAAAACTATTAAAT
CCTAGTTCCCTCGATTCATGTTTGTAAAGTTTTAGTTTATCTTCGTCTTGGAAGTAAACTTCGGT
TCTAAGCAATTTACCACCTGCGATATATTTTAATAGGAACTTTATATATAGTATGGTAAACGTCG
AGTTCTACGTGATAATACTCTGATTAGAGGTATAGAAACTTTATAATTTATCTTTTTTTAAATAA
AATAAAAAAATTTTGGTCGGTAAATATGAGAACAAGTAATCCTACCCTTTCAAGTGATACATTTA
CTGGATTTAACACTTATGAACAAAACCAGCAGATGACTGTAAATGGAACGGTAAATAAAACAAT
AATTTCACTTGGAATAGTTTCTATTTCAGCATATTTTTCATGGGATGTGTTATTTTTTAGTCCTT
ATTCAACTATATTTTTGATAGCAGCACCAATTCTTGGTTTTTTAGTGGCACTTGTAACCATATTT
AAAAAAGAGTGGTCTCCTGCTACAGTTCCAGTATACTGCATACTCGAAGGACTATTTTTGGGACT
TATTTCAGGATTTTTTGAACTTATGTATCCCGGAATTGTTTTTCAAGCAGTTGTGGCTACGTTTG
GCGTTTTATTAAGCTTGTTACTCGCTTATAAATCAAAATTAATCCGTGCAACTGAAAATTTTAAA
TTGGGAGTAGTTGCCGCAACTGGTGGGATCGCACTTGTATACATTGCTTCAATAATCTTGGGATT
TTTTGGAATATCTATTCCGCTGATATTTGGAAGCGGGCCAGTTGGAATATTATTCAGTGTGTTTG
TGGTAATAATTGCCGCTTTAAACCTTGTTTTAGATTTTGATTTTATTGAACAAGGTGAAAGATAC
GGTGCTCCTAAATACATGGAATGGTACGGCGCATTTGGATTAATGGTGACATTAATCTGGCTATA
TCTTGAAATTTTGAAGTTACTTTCAAAATTGAGGGGCAGGGATTAATTTAACGTATATTCATACA
TTTATTCATCTTTTCATATTTTTTTAGCTGTCTGAAAGTCTATTCCATTTTTCAAGCAGTCCATT
TTTGATATTCCCAAGTAAAAGTGTTAGAATTGGGATAACGGCGTTTAATAAGAATTTTAAATCA
AGCAATTTTGTTAAATCGTGGATACTGCCAATATTGTTCAATACTTCAATATATATGAAGAAAAA
TGTTATCAAAAACGAGCTTAAAAACCATATAAGCCACAAGATATTTTCTATTTTTTCTATTTTGT
ATTCATCTTTGATTATTATTGCATAAATCTTGAAAATAATGTACGAAACAACAATAAATACCGCA
TTATTTGCATAAATTAAAAATCGAGATATATCTTGTAGATTTTGTTTAAAATTCATAAAATCCCT
TTTCAAAAATGTTTAAAATTAATTTAGTGGACTACAATATTAATTTTTTGATTTAGATTTTTTTT
AAATTTTTTAAAATGGCTTAGTTTTTTAAAAATTAAAATAAAAACTAGCTGATTTGAACATTCA
TTTCATTCAATATTCTTCTGAATATTTTAACTGATAAAGTCAGATTTCAGGCCTAAAAATGGGCA
AATTATATATATCGCTATAATTAAATATTAAAACGTACTACTGAGGGTGGTTTATGAACTACAC
TGGTCCCAAAATTAGGGAGTACGTTGCTTTGATTGTAACTGCTCCCCTACTTATCGTTACAACGC
TTTATTTTATTTTTAAGATACTAAATCCAGATATTTTTGCCAAATACAGCGCTTTATCTGCAAGT
GTATTTTTAACATATTTTGGAAATGATGTATTAATGGATATTTCTAAATTCGGGGCCATTTCAGC
GATTGCATTAATTGGTTTTTTAAAAATTGCTGAAATAAAAACATCAAGACTTAAAAATTTTTAA
AATAGATTATCTTTTTTTAATTTTTAATTTATTTGGGATTATTTTTTAAATTAATTAGTAACAAG
CCCGTATTTTTTTATTTCATAATCAGTTAAATAGTGCATTCCAAAAGAGGGTGCTGAACTAATCA
TAAACCAGTAAAATTCATTAGATATTCCCATTTCATTAAAATACTCGACATAGGGTTTATGAACC

GAACTTTCTTTAGGTATTTTTGAAGCGTCTTTTATAATGTCATTTTTCCACGAATGAACACCTAC
TTTCGCACCTTCTTTAATCGTCCTATTCACACCAGCGCAGAAAAAAACAGTACCCCCTGATGCGAT
GTACCCGTTTTTTGGAACAATCGTGCTTATATTGTTTTTTCGAACGATTCTCGCAGCTTTTAAAT
TAGCGTAGCTGTTAACAGATCCTGGAACGTTTAACATTACAATAGTTTTAACGTTTGGGTGACTA
TTTACCAGATTTTCTACTTCTAAAACTATGTTTTCATTTATTATTCCAACCATCACAGCTTTATC
ACCCTGAATTTCAAATATCGCGGGTTTAGAAGATGAAATCTGTTCTGAAATTCTTTCAGAATAAT
ATACTGAAAATCCAGTAAACATCATTATGATAACTAAAACAGCCCCAATTTTTTTTAAAACCATA
TTATCTTCCAAAATTTTAAATTTAATATTAATTAGAATTCGAATGATAAATTTAATAATAAATT
AATCGAAATTTAAAAATTTAAAATGGAATTATTCTTTTTCATGAAATATTTCTGGTGGTATTCTT
CGGCAGGGTAAAAACTCCCTGCTTTTTTGATAATTGTTACTATTTTTAAATCAGTATTTTCCTGC
ATTTTTTCAAGCGATTTTTCAGCAATTAGCTTCTGTTCATCGTCAAAATAAAAAATATATGAAGA
ATACTGTTCTCCAACATCCCAGCCCTGTTTGTTTGGTGTAGTTGGGTTGTGATTATTCCAAAATA
GATCTAATAATTCATCATAAGATATTATTTCGGAGTCATAATCTATTTTAACTACCTCGATATGT
CCCGATTTTCCCCTGCATACCTGCCCGTATGTTGGATTTTTGATAGTTCCACCCATAAATCCAACT
TCTGTTGAAATTACCCCCTTAACCTTTCTAAATACTTCTTCTGCCCCCCAAAAACAGCCCATGCCA
AAAACAGTCGTTTTAATATTTTTCAAAGCCCCCACCTTTTAAAAAATATATTATTACTTAAATTC
AAGTTATTTAATGTCCATTTATTCCCCTTTTATTCACTTATTTTATATTTAATAAGATTTTGTGT
ATTAAATTATATATTTAACCATATAAATGGTTATTTAAATTGTAAATCTATTTTTTTAAAAGATT
GATTAATCGAAATACGTTAAAATTGCCCGCAGGGGTTAAATATCATGAATACATAACTAAATACC
TAAAAAATTATGGTGTTAGCATGATTGAATCTACACAAGTTTTTTATTTTGTGATTGCATCGGCC
GCACTTACACTTCTTCCAGGGCCTGACATAATATTTGTAACAGTTCAAAGTATATCTCAGGGAAA
ATGGGCGGGGATTTCAACAGCGTTAGGGCTTTGTACTGGACTTATAGTCCACACTACTGCAGCCG
CACTTGGAATTTCTGCGATAATCTACAATTCTGCGTTTGCATTTACGATTGTAAAATATCTTGGA
GCTTTGTATCTGCTTTACCTTGCAGTTCTCGCATTATTTGATAAAAATGTAGTAATAACTGAATC
AGTAGTTAAATCAGAAAACACTGAACTTTATAAAAAAGGAATATTTATGAATTTATTAAACCCG
AAAGTTTCGTTATTTTTCCTCGCATTTCTTCCTCAGTTTGTAAATCCAAATTCTGGAAGCGTTCC
GATTCAGATGATATTATTGGGCGTTATTTTCATGATTCAGGCAATTTTGATTTTTTCAGCAGTTG
CAGTATTCTCAAGCATTTTTGGAGAAAAAATATTAAACAGCCAAAAATGTGGAAAATATATCAA
TCTGGCTAAAGCAGGAATATTTGGTGCAATTGGGATTCAGCTGGCACTTTCGAGTAAATAATAAA
TTTAAATTAATTTAAAAATGAAAAACGTGAAAATTAAACGGAATATACAGGAATATTTATGAGT
TCTAAAAAATTGGGGCCTTATACATTAAGTGGACTTATTGTGGGGCCGATACTTGGATCAGGAAT
TGTTATAATTCCCCCTATAGTTTACGATATCTTAAAAGATTACTCTATTTTTGCATGGGTAATCA
TGATGACTATTGGCGGGGTTTTTGCGTATTTTGCAAGCAAATTATGTTTAAAATTTCCGGGAGAT
TCTGGTTTAACGGAAGTTGTAGGTCATTCTTTTGGAAAATCGTATAAAAATTTAAATGCATACTA
TCTTTTAATTGCAATGGCAATGGCCCCGTTAGTTGTGATAATGGTTGCAGGAGAGTATCTTTCAG
TATTATTTTCAAATTACGCATTTGGGGCAGAATTTTATTCAGCAGTATTGATGCTAATTGGCGGA
ATAATCCTTTCAAGAAATATTTCATCCGTAGGAAAAATTTCAATGCTTATCTCAACAGTAATTTC
TACAATACTTGTAGTTGGCGGGCTTTTTACAATTTTATTTTATAGAAAAGATGCGCTGTTTACGG
GAACAGGAATAAGTACAATTGATGTAGGCTACTCGTTTTTGATAATGTTCTGGTCAATTATAGGC
TGGGAAATACTTGGAAATTACAGCCTCGAAGTTGAAAATCCTGAAAAAACAATTCCCAAAGCAGC
TTTAGTAAGTGTAATTGCAATGTCCATAGTTTACTTGATAGTATCTGGGGCTCTGCAGGTGTTGG
ATATTAAAAAACTTGGATTGATAGATCCAATTTCTGGAATTTCATTAATATTATATCCATTATTT
GGGCAGTACTCCAGTATTATATTATCACTTACTATAACAGGACTTTGTTTATGCAGTTTCTTGAT

GTTAATCGGTGGAGCATCACGATTAGTTTATGCACAAGCTAAAGATGGCGTATTTCCAAAAATTT
TTAAATACCGCTCAAATACAAATGCTCCGCTTTCAGCAGTATTGCTGATGGTTTTTGCAAACTTT
TTAACACTATTTGCAGTTTATTTTAATTTAATTTCATTTAACGAAATAATTGCGGCAGTAAACGC
ATTTTTAATTACGAATATAATAATGGCAGCAGTAGCTTCGATAAAGGTTTTTGAAAACGTAATG
GAAAAAGGATTTGCAGTATTTTTATTGGTTTCGTTCCTGTTAATGTTTTTATTTTCTTCAAAAAT
GGTAATACTAATCATTTTAGCAATGCTTTTTGGAACTGTCGTGTATACATTGAAAAATAATAATA
ATTATAACATAAAATAATTTATAAATTAAAAAATAGCGGATTTATTTTTTTATTTTTTTATTCT
TTATTTTTGAATAATTTTTCTTTATAATAACTATTTGATGTATAAATCGCGGCTAATGGGTTATG
GCCCAAAACCCTATCTTTAACGCCGATTATGGTAACAGGCGCTTTTGAATATTTTATAAATAAAG
AATCATGGCCCACACACAAGCCCAGTATAATATTTAAATCTGTTTTTGTTTCATTTAAAAATAAT
GCTTGGCCGATAGGGTTACACATTGCTTCATATTCACCTTTATGAACTTTTTCATTTTCCGTTAT
TTTTATGAATTCTTTTGGGATACTTCCGCACTTACAAACAACGGATTCAACATCAAAATCATGAT
TCAAAAATATGTCTTGTAAAATTTTAGCTTCGTTTGAAAGTCCTGTGCAAAATGCAATTCCTAAT
TTTTTATAATTCATTTTTTTCGCAAACAAAATAGTTTCTTCAATTCGGGTAAGTTTGCAGTAGCC
TTCTGACTCCACAAGCGCAGAATTATAAGCAATTTTTTCATTTTCTTCTTCTAAATATAATTCTT
TACTTTTTTCAATGATTTTGGTAGAATTTGGACATTTTTTAGGCATTTTCTCAGGTTCGCCCAAT
TTACAGGCCTTTAAATTACACAGTGCACAGTAATACATTAAATTCACCAGATAATTAATTTATTT
TTATTCATTGCTATTTTTTAGCGCAACTATATCTTCTACATCGGTTAATTTCCAGACGTAAGTGT
TGTCAGCAGTGATTAGATTTTCAACAGGTTCATCAGGGTTTTGCCCGAGTGCTTCTAAAACCACA
GTTGATAAATTTTTTGAAATAATTAAATCTACAAAACTCTGCCTAATTTCTTTCTTTTTTTCCAA
GTGATATTCTGGAACAATTTTTCCCTGGAGTGTTATAAATTTGTAACCAAGACATATCTTCAGAAA
ATTTTTCAATTTCAACAGATACATAAGGATTATTTTTTAAAAGATCAATTTTACGACCGTATTTT
GTCGAAAGAAAGTACAGGTTATTATCATTAAACACATAAAGAAATGGTGCGATGTATGGATATT
TCGATCCTAAAAACGCAATTCTTGAAATATGGTTATCTTTTACAAGTAGGTCGTATTCTTCACGA
TTCATCTTTGGAATTTTGAAAACCATTTTTAAATCCCCTCAAACAAAATTTAATATGATATTATT
TGATTTTTAAGATTGATATATTTTACTTAATACGCTTAGTATGATAATCCAGGTAATGTAAATTT
CCCTGATTTTTTTAATTATAATCTATAGTAATTACCTTTTTTCTTAAAAGTTAATTTAAAATGAT
TACAGGGCGTTTTTTTAGTTTTTATGCAAGTATTATCGTTGTTTTTTTGATAGGGGCGAAAACTT
TATATAGTAAATAACCTATAGCATAGTTCACCGGAAAGAAGCTTCCGTATAATAACTAAAAGATC
GGAAATAAATTTCCGAAAGACAACAATATATAGAGGCCTAAAAAATGGTAAGAAAAATCGCAAT
TTACGGAAAAGGTGGAATTGGAAAATCAACCACCACACAAAACACAGTAGCTGCAATGGCTCACT
TTCACGATAAAAAAGTATTCATTCACGGATGCGACCCAAAAGCAGACAGTACAAGGTTAATCTTG
CACGGTAAACAGCAAGTAACAATGATGGATACATTAAGAGAAAAAGGAGAAGACGAATGTACTC
CTGACAAAGTTATCGAAGTTGGTTTCGGTGGCGTAAAATGTGTAGAATCCGGAGGACCTGAGCCA
GGAGTAGGCTGTGCAGGTAGAGGTGTTATCACTGCAATTACACTCATGGAACAGCACGGTGTTTA
CGAAGACGACCTTGACTTCGTATTCTTCGATGTTTTAGGGGACGTTGTATGTGGTGGATTCGCAA
TGCCTGTAAGAGACGGTAAAGCAGACGAAATCTACGTTGTAGCATCTGGAGAAATGATGGCTCTT
TACGCTGCAAACAACATCTGTAAAGGAATGGTTAAATACGCAGAACAAAGCGGTGTAAGATTTG
GAGGAATCATCTGTAACAGTAGAAACGTAGATGGTGAGCTTGACTTATTACAAGAATTCTGTGAC
AAAATTGGAACACAATTAATCCACTTCGTTCCAAGAGACAACATCGTTCAAAAAGCAGAATTCCA
GAAAAAAGCTGTTGTTGACTACGATGATACATGCAACCAAGCACTGGAATACAAAGAACTCGCAA
GAAAAATCATTGAAAACGAAAACCTCGTAATCCCAACCCCAATGACAATGGACGAATTAGAAGAA
TTAACTTCAAAATACGGATTCTTAGATTAAATAAAGTAAATTACTTACCTTAAATTTTTAAGAGG

GCAAAAAATGAAAATGATTAGAGCAGTAGTCAGACCAAGTAAAGCAGAAGAAGTTGTAGATGCA
CTCGCTGAATCCGGATGCGTAGCTTTGACAAAAATGGACGTTATTGGAAGAGGAAAACAAAAAG
GAATTAAGATTGACCAGATTTACTACGATGAACTTCCAAAAACAATGCTCATGCTCGTAGTTGAA
GACGATGCAGCAGAAAATGTAATTGAATTGATTACAAAAACTGCATACACGGGAAGTTTTGGTG
ACGGTAAGATATTCGTAAGTCCTGTAGATGAAGCATACACAGTTAGAACAAGATCATGCGGATTA
TAAGGTGATTATATGAAAGAGATAATCGCAATAATTCGGCCAAGCAAAATGGCACAAACAAAAA
CTGTCCTTGAAGGCCTTGGATTTCCAGCAATGACTGCGAACAGGGTTCTTGGAAGGGGAAAACAA
AAAGCAATTGTTGGAGAACTCGGATTTGAAGTTGACAACAAAGAATTGTTAAACCAACCAGGGG
ACATGAGATACATCCCAAAAACAATGCTAACATTAATTGTTCCTGATGAGGATGCAAGCCTTGTA
GTTGAAGCAATTATGAAAGTAAATAAATCTGGCCAATACGGTGACGGAAAAATCTTCGTATGTCC
AATTGAAGATATAATCACAGTAAGAACCTCTGAAAGAGGAGAAGCTGCAATTTAAGCAAATAAA
CCCCAAAATTACCCTTTAAAAATTTTTTAGGAGGGCAAACATGCCATTCTGTTTATTGGATGTAG
ATAAAGATATCCCTGAAAGAGAACAACACATTTACATCAAAGACTCAAAAGAACCAAAAGGACA
CTGCAAACAAAGATGTAATACAAACACAATCCCTGGAAGTATGACTGAAAGAGGATGTGCTTTTG
CAGGAGTAAAAGGTGTGATTACAGGTGCAATAAAAGATGTATTGCACGTAGTACACTCGCCAGTA
GGATGTACCGCTTATGGAAACGGTACAACAAAAAGATACCCAACCAAGACCAGAAATGCCTGATGG
AAGCGTATTTCCCGTTGAAAACTTCAACTTAAAACACATCGTTGGAACAGACTTAACTGAATCCG
ATGTTGTTTTTGGTGGAATGAACAAACTTAAAAAAGTAATTAGGGAAGCTTCAAAAGAATTCCC
ATTCGTAAATGCAATCTATGTTTACGCAACATGTACAACAGGTCTTATTGGAGACGACTTAGATG
CAGTATGTAAAGAAATGCAAGCAGAACTTGGAAAAGATGTTGTAGCATTCAACGCTCCTGGATTT
GCAGGACCAACACAATCAAAAGGACACCACGTAGGAAACTACACGATATTTGAAAAATTGGTTGG
AACAAAAGAACCTCCTAAAACAACCGATTACGACATCAACCTTATTGGAGAATATAACATCGATG
GTGACTACTGGGTTCTCGAAAAATACTTCGAAGATATGGGCATTAACGTTCTGAGTAAATTCACT
GGGGATGCAACCCACGGTGAACTCTGCTGGATGCACAAAGCAAAATTAAGCCTTGTAAGATGCCA
AAGATCTGCAACTTATGTAGCAAAATTAATCGAAGAAAAATACGGTGTTCCGTACCTGAAAGTAG
ATTTCTTCGGTCCAGAATACTGTGCTGAAAACTTAAGAGCAGTAGGTAAATACTTCGGAAAAGAA
ATTGAAGCTGAAGCAGTTATCAAAAAAGAAATGGAAAAAATCCAACCTGAACTTGATTTCTACA
AATCAAAATTACAAGGTAAAAAAATCTGGATTTCAGCAGGAGGTCCAAAAAGCTGGCACTTATCC
AAACCAATAGAACAATACTTAGGAATGGATGTAGTAGCACTTTCAGGTCTCTTCGAGCACGAAGA
TGGATACGAAAAAATGCAAGAAAGGGCAAAAGACGGTACAATTATTATTGACGACCCGAACACCC
TTGAAATGGAAGAAGTTGTTGAAAAATACCAACCAGAAATAGTTCTTGGTGGTATCAAAGAGAA
ATATTTCTTCCACAAATTAGGAGTACCTTCAGTAATGATACACTCTTACGAAAACGGTCCATACA
TTGGCTTTGAAGGATTCGTAAACATGGCAAGAGATATATACACAACAATATACAACCCAGCCTGG
AAATTAATGGAATTTGAAGAAGAAGAGCCAGGTGATTCAAATGAGTGAGTTAAACGTAATCAAA
AAAGAAAGAACCGCGGTAATTAACCCAATCGTAACATGCCAACCTTTAGGTGCAATGTACGCAGT
ATCTGGAATTGAAAGAGGAATGCCTTTAGTTCACGGATCACAAGGATGTTCAACATTCGTAAGAT
ATGGATTTGCAAGACACTTTAGAGAACCTGCAGATATTGCAGTAACCTCACTTCACGAGGATGCA
GCAGTATTCGGTGGAAGAAAAAACCTGATTTCAGGTCTTGGAAACTTAGCTGCAAGGTTTAAACC
TGATGTAATGGGTGTTGTAACAACATGTTCCAGTGAAATCATCGGAGATGACGTTGCTGGTTTCA
TTAAAACAGCTAAAGTAGAAATTGCAAAGAAAATGGGTGAAGAAGCCGCTAACAAAATCAAAAT
TGTTCAGATTAATACCCCAAGTTTCGTTGAGCACCAGTTCAAAGGATACGACAATGCAATAAAAG
CAATTGTAGACACATTAGCTGAACCAAAAGACGAAGAAAATGGAAAATTAAATATCATTCCAGG
AATTGTAAACCCCGGAGACATCAGAGAAATCAAACATATGCTTTCACTCATGGGAGTTGAAGGTA

TTTTACTTACCGATACCTCTGATCCATTTGATTCACCACTCAGACCATCAAAAGCAGACAAAAAT
CCATACTACCAAAAAGGTGGAACACCTCTTGCAGATCTTCAAGACTGTGCAAACTCACTTGGTAC
AATTTCACTTGCTAACTATGCAAACAGTGCTCCTGCATCACTCGAGAAAAAATACAATATGCCTT
CAAAAGTGAGTGAAGCACCAATTGGTATTCAAAATACTGACAGTTTCATAAGAACCGTTAAAAA
ATTCACTGGAAACGACGTTACTGATGAAATACTTGACGAAAGAGGTATTGTGATTGACGCAATGG
CTGACGTTGCATCAAGATACTTATTCGGAAGAAAAGTAGCAATCTACGGTGACCCATCAATAACT
GTAGGAATGGCAAGATTTGTAGCAGAACTTGGAATGATTCCTAAAGTGGTATGTACTGGAGTTA
AAAACGAATACTTCGTTAATGACTTGAAAAAAGTAGCTAAAGAATCAGATGAAGACATCGATGC
ACTCTTCGGACAAGACTTAAGAGCATTAGATGTTTACTTAAAAGAAAACCCAGTCGATTTAATGA
TTGGTAGCTCAGATGGTAGATTGATGGCAAAAGACCTCGGAATACCACTCTACAGAGTAGGATAC
CCAGTATACGATAGAGTAGGATACCAAAGAAGACCAATTATTGGATACAATGGTGCTTTGAACCT
TGTCGATGGAATTACAAATACAATTCTCGATAAATACTACGAAACTCAAGACTGGAAACTCCAAC
AGTAAGCCCAAAATAAAGAGGAATACTATGGTTTTGAACCTTGATACAGAAAACCGGAAAATGC
AAGATGGAAATAACGATGATGATTTTGACTTAGAAGTCAAAATCCCAAATTCCATTTTTGAAAA
ATTAAAATCTATTGAAGCGTTAAAAGCCAGAGAATCACAAATGTGTGTTTCTGGAAAAGACGAT
TCAATTCCTACATGTGACAAAAACTCAACCCCTGGAATGATTACCCAGAGATCCTGCGTATACGG
GGGAGCAAGAGTTGTTCTAATGCCTATAACTGATGCAGTACATCTCGTACACGGGCCAATAGGCT
GCGCAGCATGCACTTGGGATATTCGTGGAAGTAAATCTACAGGAGATAAACTTTACAAAAACGGA
TTCTCAACTGATTTGCAAGAGAAAGATATTGTATTTGGTGGAGAAAAAAAGCTCTATGAATCCAT
TTTGGAAGTAAATAAGCTTTATCACCCTGGTGCAATATTTGTTTACTCAACTTGCGTAGTAGGTT
TGATTGGAGATGATCTTAAAGCAGTTTGTAGACAGGCACAAGAAGCAACGGGCTGCAGAGTTATT
CCGGTACAGTCAGAAGGGTTCAAAAGCTTTAATAAAACTGCAGGTCACAAACTCGCATGTGACGC
AATGCTTGATTACGTAATTGGCACAGAAGAGCCAGAAGAAGAGCACCCATACAGTATTAATATAA
TTGGAGAATTCAATGTTGCAGGAGATCTCTGGGGAATTATTCCATTATATGAAAAAATGGGTGT
AAAAGTACACACTGCAATTACTGGAGACTCAACAGTCAAAAAAGTTGCAAGTGCGCACAGATCCA
AATTAAATATAGTTCAATGCCAAAAATCATCCAATTATTTAGCCGCCCAAATGGAAAAGAAATAC
GGAATACCAAGCATTAAAGTAAATTTCTTTGGACTCGACGAAACCACAAAGTCTTTAAGGGCTGT
TGCAGAATTTTTCGGCGACGAAGAAATGATTAAAAGAACTGAAGAACTCATAAAATCAGAAATT
AAAAATCTTAGAGATGAAATTTCTGAATACAAAAAAGATCTATCCGGTAGAACCGTGGCGATAT
ATTCCGGAGCCCACAAATCTTGGGCACTGGTAAGCGCATTTGGTGAACTCGATATGGAAATTATC
ATGAGTGGTACCCAAAACGGAAAACCCGAAGATTACCAACAAATTAGAGATCACGTCTGCGAAGG
TACATTAATTGTAGACGATGCAAGTTCAATGGAACTTGTACAGCTTCTCAAAGAATACAAACCTG
ACATATTAATTTCTGGAGCTAAAGAAAAATACTTATCACTGAAAAGTGGAATTCCTCACTGTGAT
TTCAATCACGACAGGATAACTGCCTTTTCAGGTTATCAGGGCTTCATAAATTTTGCAAGAGTTGT
ACATACCGCAGTTATGACGCCAATTTGGAGACTTTCTAGAAAATGATGATTTAGAGGAATATTAT
GACTGAAAAAAAATTTGCTGTAATAAATCCATGTAAAATATGCCAGCCAATGGGTGCAATATTTG
CATTAATGGGTGTAAAGAATTCAATGCCGTTAATACACGGATCGCACGGATGCAGCACCTACATG
AGATTCCAACTTGGACGTCATTTTAAAGAAGCTGTAAACGTGGCATCTTCATCAATGAGTGAGGC
AAATGCTGTACTAGGTGGTGAAGAAAATTTAAAACATGCGATCAGAAACGTGAGCAGAATTTAT
GGTCCTGAAATAATTGGTGTAACTACAAGCTGTCTTAGTGAAACTATTGGTGACGACGTTGAAGG
GATCATCCGGTTATTCAAGACACAAAATGATGATTTGCCTGAAATAGTCCCGATATCAACCCCAA
GTTATAAACATACACATGTAGAAGGATATGATTCAACAGTTAAGGCATTGGTTGAAAATTTAGC
AACTCCATCAAAGCCAAATTCCAAGTTAAACGTGATTACTGGAATGGTTTCTCCTGGAGACATTG

CAGAAACCAAAAGGATTTTACGTGAAATGGGTATAGAATCAATAATTCTAACCGATACTTCGGAA
TCTATGGACGCCCCTTTCGATGGAGAAGTTTCATTCGTGTCATCTGCAGGAACTACTGTGGAAGA
ACTTAAAGACACTGCAAATTCATTTGCAACGATTTCACTCTGTAAGCATGCAAATTCCGCCGCCA
AATTCCTCCATGAAGCTTACGGACTAAAATCTGTGTCACCATATCTTCCAATAGGGCTGCAGAGT
ACTGATGAATTTATGAATGAACTTTGTAAACTTACGAATAAAGAGATACCTGAACAGATAACTA
GAGAACGTGGAAAATTAATCGATGCCATGATCGACGCCCACCAACATAATTTTGGAAGAAAAGTT
GCAATCTACGGCGATCCCGATGTCGTAGTTGGATTTACAAGATTTGTCTTGGAACTTGGAATGAT
TCCTTCAATAGTCTGTACAGGAAGCGAAAGTAAAACTTTCGTTGAAGACATCAAAGCAGTAACTA
AAGATAGCAAACATAAACCAGTAATCTTAGCTCCCGGGGATCTTTACGATCTCCACCAAGAAATT
AAAAAATCAAAAGTGGATCTTTTAATTGGAAATTCGTACGGTTCAAGAATTGCAGGTGCCGAAA
ATATTCCATTACTTAGGATGGGTTTCCCAATCTATGATAGGGTTGGCGCTCAAAGAATAACTTAC
GTGGGATATACTGCAGGAATTAGACTTGTAGATACTATAACCAATACAATACTCGACAGATACTT
TGACGAGTTTGGATGGGCATTAGACGAAAATACAGAGGTAGACCATGGAGAGTTTGAAAGTAGC
AATTGCATCAACTGATGGTGTAAATGTCAACATGCACTTCGGAGCTGCCACACATTTTCTTATTT
TTGAAATAAAAGATAATGTAGCAGAATTTTTAGAATTTAGGGAAAACCCAACAAAACATATAAA
AGACCACACCGACAGGTGGAACTATGCACTTGAGCTCTTAAAAGACTGTGGTGCAATCTTTTGTA
GCAGAATGGGGGACCATCCTAAATCTGTACTCGAAGGAAAAGGCATGAAAGTAGTTATGACAGA
AAATACATTAAAAGACGCTTTAAAAGAGTTTTTAACCGCCTAAATTATTTATTAATTATCACATT
TTAGTTATTATGCGATTTTTAAACAATTAACCAAACTTAAGAACTTAATAATCCATTATGGATGG
TGATTCTGGGTTTTATTATTAATTTCGCTAAAAGCACACTTTAAAATTATTATTTTGGAATTCTT
TTTATTAGGCCAAAAAGAGTTAAAAAAGTAAAAGTTTGGTGTGTATTTGTGAAATTATCCCTCA
TATCCCCACTTCACACCGATATTATTTTTAAAGAATTTACTGGAGGTTTAATTATCTTAAACCTT
CATATTATCGTTGTTATTGTTAATTAATATAATTATGTGAGCCCACAACCTTCAATTAATTAATA
TATATCAACAAACTTTTTCAAGTTTTTTTGATTTTAGCTTATGCTATAATAGTGTTACCCATTAA
TTATTTATACTATCTTTTAAAAAAAGGATGTATAAGCCTTTTTAAATTTTGAAACCTCATATTTT
CGGTTTTTCGATTTGCTATTTGGGATACCCCCTCTGTTGATATATCAATGTTAAATTTCGAAAAA
TTTAGATATATCGATTATGTTTTTAGGTGATAGTTTTGAACGAATTAAGTGACGAAAGTATATT
AAAATATACAAAAAAAAATTTCAGAAAATGCCACGATTGATAATTGGAACGATTATTTGTGGCAG
CTTTCAAATTCAATTAAAGATGTTGATACTTTAGAAAATTTTTTAGGAATAACTTTTGATGAAA
AAGAGAAAACAGAAATCCAAAAAGCGATAGATGTATTTCCAATGTCAATTACCCCATACTATGCA
AGTTTAATTGATATTAAAAATTTAGATAAAGACCCAATTTATAAACAAAGCGTGGCATCTTCAA
AAGAACTAATACTTGAAAATTTTGAAATGGAAGATCCATTATCTGAAGATGAAGACTCCCCAGTT
ATTGGAATAACCCACAGATACCCTGACAGAGTTTTATTTTATATAAACCCAAACTGCGCAATGTA
CTGCAGGCACTGTACTAGAAAAAGAAAAGTTTCAGAAAAAAGCAGTAATCCGTCAAAAGAAGAG
ATTCAAAAAGCAATTGATTACATTAAAAATAACAATAAAATAAGAGACGTTCTACTTTCAGGAG
GAGACCCGCTTTTACTTTCCGATGAATTTTTAGACTGGATACTTTCAGAAATAAGCAGTATAAAA
CATGTTGAACTTATAAGAATTGGATCGAGAGTTCCCGTGGTTCTCCCTCAAAGAATTACTGACAA
TTTAGTCAATGTTTTAAAAAAATACCATCCAATTTGGGTAAATACGCATTATAATCATCCAGTGG
AGCTTACTAAAGAATCTAAAAAAGCACTTGATAAACTTTCAGATTCTGGAATTCCGCTTGGAAAT
CAGACTGTACTTTTAGCAGGAGTTAACGACTGTCCATATGTCATGAGAAAATTGAATCAAAAACT
CGTTTCGTCAAGAGTAAGGCCATACTACCTCTACCAGTGCGATTTATCAAAAGGAATTTCTCATT
TTAGAACTTCTGTTAGCAAAGGACTTGAAATTATCGAAAGTTTAATTGGACACACCACAGGTTTT
GCAGTTCCAAGATACGTTGTAGATGCACCTGGAGGCGGTGGAAAAATTCCAGTAATGCCAAATTA

CGTAGTTTCATGGGGTTCTGATAGGGTAATTTTAAGAAATTATGAAGGAATTATCACATCATACG
TTGAACCGTCAGATTACGGCGGATGCAGTAAAAATTGCGATACGTGTGACAGAATGTGTATTGGA
GACTTTGAAATTAATCAGACAGGAATTGAAAAGTTAATAACTGATGTCGACAGTTCAATTTCATT
AATCCCTGAAAACAACGAGCGAGTTGCCAGGAGGGATGACTAAATGGAAAAAATAATCGAAATA
AATGATTCAGTTATTCAAATAAGTGACTTAAATGATAGAATATACATAATGAAGCTTGGAAAAG
ACGCGGGAGAATTAATTAATCATGTCGATAAAATATGCACTGAAAAAAAGCTTTCAAAAGTTTT
TGCAAAAGTTTCAGGAAATCAAAAGGAACTTTTTGAAAAAAACGGCTATATTTGTGAAGGAAAA
CTTAAAAATTACTTTTTAGATGATGATGCGTATTTTATGTCTAAATTTTTTGAAAAATCACGAAA
AATATCGAAATTTTCAAAAGAAGCAGAAGCTGTTTTAAATTATGTGAAAACTGTTGGCGTGAAT
CCCCATACAAAAATTGATGAAAAATTCCACTTAAAAATTGCAAATGAAACCGATGCAGAAAAACT
ATCAAAACACTATTCAAAAGTTTTTAAAACGTATCCTTTTCCAATAGACGATCCAAATTATATAT
TAAAGACAATGCAGACAAACGTAAAATACTTTATAATTGAAGATAATGGTAAAATCGTTGCAGC
GTCGTCTTGTGAAATGGATATTAAGAATAAATGCGTTGAAATGACCGATTTTGCAGTTCTTGAAG
AATATCAAAAATTAGGGCTTTCTAAATATTTATTATATATAATGGAAAAAATAATGAAAGACAA
CGGATACAGGGTTTTTTACACGATTGCAAGAAGCATGTCGTATGGAATGAATATTACTTTTAAAA
AAATGGGCTATATGTACAGCGGAACTGCCGTTAATAATACAAACATCTGCGGAAACTTTGAAGAC
ATGAACTTTTGGTACAAGCTTTCAGAATAAATTTTCAACTCTTTTAATTTTTTAATTTTTTAATC
AACTTCAATTTTTTCTTTTTTGATAAGTATATCTTTATTCTTAAAATTAAAATGAAAAGTTTAAT
TATTTTAAAAAAACATAATTTATGCTAGAGTTATTTTGGGGGATTTTATGAAAGAACTTGAAAC
GGCAATTGCCAGGTATCATGCAATGAAATTGAAAGACATTCTTCCACCAATTGATAGCTTGCCGG
TTTTATGCACTGATTCTTCAGTAATAAATGTTTTGGAGATACTTAGAACAAGGCATCACGTCTGG
GTAATAAACTGCAAAGAAGATGGAAAGCTCTCAGGATATTGATAAATAAGTTTCTTGATCTCTGC
TTTTAGGTGTTTTCGTGGATATTTCATGGGTGTTGTTTACTGTTGGAGTATGCCTTATATTTGGA
AAAATTGGAAACGAAATAATGTGGCGGTTTGGCTTTCCAAGCGTATTTGGAGAGATCCTAATTGG
AATAATATTTGGAAACCTGTTATTTTTTGGAATAGTTGATCCTACTCACTTAACGCTCCATGAAA
ACGATATTTTTGACTTTTTATCCACTGTTGGGATCATGTTTTTACTGTTTATAAGTGGTCTTGAA
ATAGATATACACAGGCTTAAAAAAACCGAAAATATCTCTATAGTTGCAGCAGTACTTGGAGCGGT
GTTTCCTTTAATTTTTGGATATATTTCATTATCTTATTTTGGCTACACTGCCAAAGAATCTATTG
TTGGTGGGGTAATTCTTACAGCAACGAGTATTGGAATTACTGCAAGGCTTTTGATGGATTTAAAA
GTTTTAAAAACTGATGTGGGGGCAACTGCAATTAGTGCGAGTATTCTTGACGACTTTTTGGGCCT
TATTCTTCTTATCCTCGCAGTTGGATCGGGCAGTTTACTCGGGCTAATTTCAGAAATTACTGTAT
TTTTTATTATTACTGGATACTTTGGATGGAAATTGATTAAAAAATACTTAATTTTTGCAGAAAA
GTTCCATATTGAAAAAACAGTTCTTTCATTTACAATTGCGTTGATGTTTTTATTTTCATTTTTAT
CAGAAAGCTGGTTTGAAGTTGCAATAGAAGGGGCATTCATGGCAGGATTAATCCTTTCAAAAACT
TCAGAAGGTAAATCACTCGCAAAAGAGATAAAATCAATTGGAAATTCATTTTTAATTCCATTATT
TTTTATTTACACTGGAGCACGGCTGAATTTAACCGCATTTTTAAACCATGAAGCTTTAATTCTTG
CTACAATTTTTATAATTGTAGGGGTTTTGAGTAAGTTTATAGGGTGGGGGCTTGGAGCAACATTA
ATTGGTAAATGGAGTCTTAAAAAATCGATACAGCTTGCTTCAGCATCAGTTCCAAGGGCAGAAAT
AGCACTGATAAATTTAATGATTGCAGTTTCTGCAGGCGTAATTTTAGAAGAAAATATTTCAAAGT
TTATTGCAGCAACGCTGATATTTGTTACATTTTCGATAGTTATAACGCCACCGCTCTTAAAAAAA
GTTTTTGAATAGTATAATCTCTTGAAATTTAAAAAAAGAAAAGAAAAAAATAATAAAAATAAGC
AAAAATTATTTTTTAAAGCACTTTTTCGCTTATAAGTTCAGAAATTGATTCATAAATTTTTTTGG
TGAGTAATTCAATTTCGTCCGGTTTAATTATTAACGGAAGTACAAATATCAACGTATTTCCAATT

GGACGGATATAAATTCCTTTTTCAAGCAGCCTTCCAGCAACTTTATACCCTGCTTTATACCCGTA
ATCGTATGGTTCTTTTGTTTCCTTATTTTTTACAAGTTCTATTCCAATCATGTATCCTGATTTTCG
AATATCTCCAACACAGGGGAATTCTTTTAATTTATCGAGGTTTTCATGCAGTAATTTAATGTTTG
GTCTTATTTCTTCGCAGATATTTGTTTCTTCCAAAATATGTAAGGTTTCAATTGCTGATGCACAT
ATTATCTGGTTTCCTGCATAAGTGTGGCCGTGAAAAAGCTGTTTACATTCATCAAAACTTCCTAA
AAATGCATCATGTATTTCATCGGTTGCAAGGGTTAGTGCAAGCGGTAAATATCCTCCAGTAATTC
CTTTTCCAAGGCATAAAATATCAGGTTTTTCTAATTCTTTTAATTCATCCATATCGGAATAAAAT
GCTCTTCCAAGTCTTCCAAAAGTTGCAATTTCATCGAGTATTAATATAATATTAAGTTCTTTACA
GATTTTTGCAACTCCTTCGATGTATCCTTTTGGATATGGAATCATTCCTGCTGAGCCCATGACACC
AGCTTCTAAAATTACACAGAATATTTCATCTTTATTAGTCCTGATTAAATCTTCCATTTCTTTTA
AACACTTCATCTCGCATCCTTGCTTATTTCTTTCATCAGTGTCTTTAAAGTTGCAGTATTTGCACC
TGTAGCAGTAAGGGGCACTTGCGTGGTATCCCTCAAATAACAAAGGTTCAAAACAGCCGTGAAAA
AGTTCGCTTCCTCCAACACTCATTGCACCAACTGTGTCTCCATGGTAGCCTTCTTTAACCGACACA
AATTTTGTTTTATTTGTAAATCCTTTTAATTTACAGTATTCAAAAGCCATTTTTACAGCAATTTC
AATAGCTTCTGCACCATCTTCTGAAAAAAATACTTTTTTGAACTGGTCTGGTGAAAAATCAACAT
ATCTTTTTGCCAAAATTGCAGACGGAACATTTCCACAACCAAGCTGTGTCGAATGCCCAATATTT
AGTGCCTGTTTTGAAATCGCATCAGCTATTCGTTTTTCAGAATGTCCGAAGAAGTTGCACCAGAC
TGATGAAACCGCATCCATGTATTTTTTGCCGTCAACGTCAATTAAATAAATTCCTTCCCCACTTTC
AATTAAAAGTGGTTTTCCTTCCCTATACTCCTTCATCTGGGTAAAAGGGTGCCAAACATATTCTT
TATCCCATTTTTCGAGGTCATTTTTTGAATAGTTTTCTGATTTCATTTAAAACACCAGAGATTTT
TAAATTGTTTTTAATCCAATTTAAGTTTTTTATCAATATTATCTCAAAAATATGAATTTAGCAGA
TATAAGGTTTTTCGAAAAAAAATTTGAAAAACATTTAAAAATAATTTTTTTAAAATTTTCAAAA
AAAAAGAAGTTTTATAAATGATATTTAGTTCATGTGTGAAGTCCAGTTTGCTACAAGTTCGGGGT
TATTTGCAACCCATTCTTCTGCAACTTCTTCTGGATCCATTCCTTCGTCACTGTATTTGTATATCC
ATTCGCTCTGAACGCTTGGATCTACTTCGAATTTCTGGAAGAATTCGTACACTTCAGGATAATCA
TCTTTAAAGTCAGTTCTTGCAATGGTGTATACTTTGTCACCATTACCATATACTTTTTCAGGGTC
ATCCAACATTTTTATGTCGAATTTAAAGAAAACTGAGTGCGGTTCCCAAACGGTAACTACAATAT
TTTCATCTTCATTAATTGCACGTGCAAGTTCTGCCATCATTGCAGGCGTACTGCTTGACTGCAAT
TCATAACCTGATAAGCTGTATGAATCAATAGCTTTTTCAGTGTTAGACATAATTCCAGCACCGGG
TTCAATTCCAATAATTTTTCCATCGAATAATTCTTCATTACCGTTTAAGTCAGTAATTGACTGTA
TTCCTGCTTCGTATGTGTAAGTAGGAACTGCAAGACCGAGTGCAGTTTTTCCAACATTTACATGA
ACCATTTCAAGCTGGTTTCCATACTGAGTCCAGTATTCTTCATGAGTTGTTGGCAGCCATCCTGCA
AGTGATACATCAAGGTCGCCATCTGCAAGTTTTGCGTAAGTAATTCCTGCATCAAGCTGGTTTAG
TTCAACATTGTATCCTTGCTCTTCGAGAATAACTTTTACAACATTGGATTTAACTGTAACTCCTG
GCCAAGGAGGAAGTCCAAATTTTATAGTGCCTTTTTCAGTGTCGTCAGTACTTTGGGGAGATTGG
ACGCATCCTGAAAACATGACTGCCAAACCCAAAAGGGCAATGGCAAATAACGTTCCATACTTCAT
AAATTCACCTTTTTATTATATTTTAGATCCCACGAGGTTACGGGTAATCCTATCAAAAATTACAG
CAAGAAATGTAACTGCAATACCTGCTTCGAGAGCAGGTGCTAAACTATATCTTTGGATACCTGAA
ATTATAACCTCACCTAACCCTCCCGAGCCGATAAATCCGGCAATAACTACCATTGAGAAGGATAG
CATGATTGCCTGATTTACGCCCATCATTATTGAAGGAACTGCTGTGGGCAGTTCTATTTTTGTTA
ATATCTGCCAAGAAGTACCTCCAAAGGCCCTTCCAACTTCAATTAGTTCGTTAGATACCTGCTCA
ATACCTAGTGCCGTAAGTTTTATTGCCGGAGGCATTGCAAAGATTACTGTTGCAATAACTCCTGG
AACTTCCCCGATTCCAAAGAATAACACTGCTGGAATCAAATATGCTAATGAGGGGAGTGTCTGCA

TTACATCGAGCATTGGATCGAGCAGCGTACTTACTAGTTTTGAACGCGCTTTTACAATTCCCAAA
GGAATTCCAAGTAAAAGGGCAATCAATGTGGATGTGATTATCAGTGATAGGGTAATCATTGAAG
TATCCCAAAGGCCCATCATCACGATAATAACTAGGAAAAATACTGAATACAGCATAGATTTCAGA
TTAACTGTTTTCCAAACAATTACTGCGATCAGCAATATCATCAAATAAGGATTTATTGAAAAAAG
CAGGAAGTGAAAGAAATCAACACCGCTTTGAATTACATAAGAAATAAGATCAAAAAATCCCGAG
TAGTTATCAATTAAAAAGTCTATTAAATTTACTAAAAATTTTCCAATTCCAAGATTAAACGTATC
TGCCAACAAAATCACGCTCTTATTCTTTTATAAGTTTAATTACTTCTTCAAATTCGACATATCCT
AAAAAGTTGTTTTCATCATCTACTATCGGAACAGGGTAATCTGAAGAAATAAATTGAGGTAATCC
CTGATTAATGGTTTTTACATCTTCTAAAGGTTTAATTTTATGAATACAATCCATAATTTTTTCTG
AATTTTGAAGTTCTTCGTTATTCACAACTCCAATGTATTTTCCAGAATCGTCGAGTACGTATGCA
AAATCAAGGTTTAACTCGCCTAAAATTTTAACTGCATTCTCTTTTTCGATAGTATTATTCAGTGT
AAATTCTGGTTTTTTAACGAGCGATTCAGCTCGAATTACTGTTGTTTTGTCCACGTCTTTTACAA
AAGACTCGACAAAATCATTTTGCGGGTTTAAAAGAATTTCTTCAGGGTCTGACAACTGAACAAGA
CTTCCTTCTTCATTTAAAATCATAATCCTGTCGCCAAGTTTGATTGCTTCGTTTAAGTCGTGAGT
TATGAATATAATTGTTTTTTTCATTTTTTTCTGAATTTTTAAAAGAAGTTCCTGCATTTCAAGCC
TGATTAAAGGATCAAGCGCACTGAATGGTTCATCCATTAATAATATTTCAGGGTTTACTGCAAGA
CCTCTTGCAAGCCCAACACGCTGTTGCATTCCGCCACTTAATTCTGAAATTTTACTTTTTTCCCAG
CCTTTAAGTCCAACGAGAGAAAGCGCCTTTTCAGACTTTTCAATTCTTTCCTCAAGACCCATTCCT
TGAATTTCAAGGCCAAGGGCCACATTTTCTAAAACTGAACGGTTTGGTAAAAGTCCAAATTTTTG
AAAAACCATTCCAAAATATTTTCTTCTAATTTCGAGCAGTTCTTTTTCAGACATTTGTGAAATGT
CTGCACCAGTTTCAAGAACTATTTTTCCAAATGTGGGTTTGATAAGCCTGTTAATACACCTTATC
AGTGTAGATTTTCCACTTCCAGAAAGTCCCATGATTACAAAGATTTCTCCTCGTTTAACGTCGAA
ATTAATATTTCTAAGTCCAACAACCTGTTTTGTTTTTTCTTTTATTTCTTGTCTAGAAAAACCTT
CTTTAATTAAAGGATAGGCTTTTTCTGGCTTTTTACCAAAAATTTTGTATAATTCTGTTACTTGG
ATAATAGGTTCATCCATATAATCCCTCTTAAGTCGACAATAATTTATCAAATATGAATTATGAAT
TTTAAAAGACTGTTTTTTTAAAAATAAGTCTTAAAAGCAGAATAATTTTAAAAATATTTCCAAA
TTCGAATTTAATAGTGCCTATAATTTATACCTTAAATTTCTAAACCCTACTTTATATACTTATTG
AGTTAAGTATTTATATTAAATATTAAAAAAGACACGGAAACTGTCAATTTAGTAAATATATTCG
ATTACAGGGTTTTTTTAAAAATGGATATGGGATACCCCTAAAAAACAACGTAACTTTAAAACTAA
TATTCAAATATCGATTTTGATCCGATAAAAAATGATTTTTTAAATTTATTCTTTTTTAAAATTAG
CTTTTAGTAATTTTCAATACATATATTTTTCATTTTTTTCCGCTATCGTATTCTAAGCGGTTCTG
GCATATCTTGGGTTTTACATGAAAACTCTTCGCATACACTGAATAATAGCGTTCTTTTGAAAATT
AATGTAGGTGAATAGTTATTCTATCTAAAAAAAACGTATTGGATAGGTTAATAAATAATAAGAT
TTATTATTTATAATGTCTACAAAAATCAACTTTTAGTTTTTTATGGGCTTTTTCCAAAATTTTGT
AGATCATTGTTTTAAAATTATTTATAATCTAATATTCAAAAAAAATGGGGAAAATTATGAAAAA
ATTAGACTTTAAAACTGTTGTTTTATCGTTAATTGTATTTTTAGCAATTATTTCCGCAGTATCTG
CTGAGATGGGTACTCCGATAACTACAGTTTGTGAAATCAACGAGTCCGGAACATACTATCTAAAC
AATAGTATTACATACGGCAGTGGAACGGTTATAAACATTACTTGTGACGATGTCGTACTCAATGG
AAATGGATACGTCATAGGTGGGAGCAGCATTGAAGAATATGTGGGTATCAATGCTACCGGAGTT
ACCAATATCACAATCGCAAACGCTACGATAACAGGTTTTGGATGCGGTATTCAGTTAATTAATAC
TACCGATAGCAGAATTGAAAATAATACTGCAAATGATAATACGTGGGGAATTTACTTAGGTGGG
TCTTCAAACAATACCCTAACAAGTAATAATGCAACTGGAAACCTGTATGATGGTATTTTTTTAAA
GGTTTCTTCATACAACACCATAACAAGCAATACTTTAAATGATAATTCAGATGGTATTTACTTAA

TGGGGTCCTCAAACAACACTATAACAAGCAATAACGCAACTGGAAATGAGTATGGTATTTACTTA
AGGGAATCTTCAGACAACACCTTAACAAGCAATAATGCAACTGGAAATGAGGTTTCTGGTATTCA
ATTAGAGATGTCTTCAAACAATAACGTCATTACAAACAATACTGCATACGATAATACGAGAGGTA
TTTGCTTAGATGGGTCCTCAAACAACACCATAACAAGCAATACTGCATACTATAATTCAAATGGG
AAGTATGCATCTGGTATTTACTTAATGGGGTCCTCAAACAACACTATAACAAGCAATAACGCAAC
TGGAAATGAGTAGGGTATTCTTTTAGATAAGTCCTCAAACAATACACTAACAAACAATACCATAA
CAGAAAACATGTTTCCAGGCATGTACTTAGATGAGTCTTCATACAACACCATAACAAGCAATAAT
GCAACTGGAAATGGTATTGATGGAATTTTAATATTTATTTCCCCATACAACACTATTATAGGCAA
TACTGCAAATGATAATGGATATTTTGGAGGGGGAAGTGGTATTAGTTTAGGGGAATCTTCAAAC
AACACTATAACAAGCAATACTGCAAACAATAATGCCGATCAGGGTATTTCCTTAGGTGGGTCCTC
AAACAACACCATAACAAGCAATACTGCATACGATAATAAGCGAGGTATTTCCTTAGAAGCTTCTT
CAAACAATAAAATTACGGAAAATACCGCAGATTATAATAAGCGAGGTATTTACTTAGAAGATTC
TTCAAATAACACCATAACAAGCAATACCGCAAACAATAATACGGCAGGTATTTACGCACATAATT
CCTCAAACAACACCATAACAAGCAATACTGCAAATGAAAACGAGAATAAAGGAATTTACATAGGT
TACTCTTCAAATAACGTTTTGATGAGCAATACTGCATACGATAATACGAGAGGTATTTACTTAGA
AGATTCTTCAAATAACACCATAACAAGCAATACCGCAAACAATAATACGGCAGGTATTTACGCAC
ATAATTCCTCAAACAACACCATAACAAGCAATACTGCAAATGAAAACGAGAATAAAGGAATTTAC
ATAGGTTACTCTTCAAATAACGTTTTGATGAGCAATACTGCATACGATAATACGAGAGGTATTTA
CTTAGAAGAGTCCTCAAACAATACGCTAACAAGCAATACGTTAAATGGAAATATGGGCGGTATTT
ACGCATATTATTCATCAAACAACACCATAACAAGCAATACTGCAAATGAAAATGAGAATAATGGT
ATGTACTTAGATGAATCCTCAAACAACACTCTGTTAAGAAATAATTTTGTGGGAAATAGTTGTGC
AGTAAATATTTTCGGTAAAAACAACTGGATTTATCTAAATAAATTTGAAAATAATAGTGAAAAC
ATTGACTCTTTCTCAGTATCTGAAAATTACTTCCACTCCCCTGTCGTACATTACAAATACAATGG
GGTAGTTTACGAAGGAAGACTTGGAAACTACTACGTTGGGGAAGAATTTGGAACTTCAAATGCA
GGAATATTTGACAAACCATACGGAATCATCAAAATGATGGATTTATAAGTGGAGGTGATAATAA
ATGAAAAGATTTTTGATTTTAGCAATAATTGCATTAAGCATGCTTGTTTCAGTAGATGCCTCTAT
GGGCTACAATTGGACAATAAATGAATCTGGAACATACACTCTTTCAGAAGATATGCTTGTAAATT
ACTCAGGAATACGAATTAATGCAAGTAATGTGGTTTTAGATGGTAAAAACTACCAGTTGATTTCC
AATAATTCAGAAAACACTGGAATATATATTTACAGTAACAACGAAAATTTTACAGAGAATATAA
CAATTAAAAACTTAAAACTTAAAAACTGGTCAATTGGGATTGATGTAAGTTACATGGTGAAAAA
TATAACAATTACAAATTGTGAATTTGAAGATAACGAAGATTTCTTAGATTCAGATGCCCTCGGAC
AGTACTTTTACTTAAATTCAATACATGAAAACCAGACTGTTTATTCAGATACTCCATTAATGTCA
CCAAAACTAGTTTACACTTACAACGGAAATGAATACACTTACAGACTTGGAAACTACTACTATGG
ATATGCGGGAGCAGATTCGGAAGATGAAGGAGTTTATTCCGGAGTTTATGAGATTAGTGAAAGT
GCATGCGACCCATATCCATTAATTAAAACTCCTGAAAACTATAAGGTTACTGAAACGTTATATCC
TGACGTGGAATTAAAATACAAATATGACAGCGCAGTAATTGCAGATATATTCGCTTTAACGAGCG
ATCCTGAAAATTACGTAATTACTGATGCACCTGTAACGCCAACTACCACACCAAGATCATCCGGT
GGCGGTGGAAGAAGCTACGATTCAGATATTTCAGATGACATTCAATCAAGAATAATTAAAAACT
TCGTTTCAAGTGCAACAGTAATATATGGAAACGAAATCGATGAAAATTACGCAAACCAATTAAG
GGAAAGAATACAAAATGCAAACGGATTTACAATTACTGGAAATGCAGTAATTGTTGGTGGACCT
TTCGCAAATCCATTTGCTACAGAATACAACGATCAGTTTGAAATCCCAATTACAAACGAAAACCC
TGGAGAAAATAAAGGAATAATTCAGGTATTAAAAGTTCAAGACAACTCTGGAAGTATTATTAAA
AGCTACACAATAGTATACATTGCAGGTTCTGACAGGTTAGGAACTCTTGCAGCAATGGAATACTT

TAAAACTTTGGAAGACCTTCCAACAGGGCCAATAACCGTTGAATGGACAGAAAACGGACCAGTCT
TAGCTTAATATTTAATAATAATTTTTCTTTTTTTAAATTTATTCTTTTCAGAAATTATATATATT
GTTTTTAAAAGAATTTACTTAACTTAAATGAATTTATTTAATTATCTGGGATTAATGGTGAAAA
CGTGACTGATTTTATGGATGAAGCAATAAAAGAAGCTAATTTAGGTCTTAAAGAGGGTGGAATT
CCGATTGGCGCAGTTTTAGTTTACGAAAATAAGATTATCGGTCGCGGACACAATCGAAGAGTTCA
AAATAACAGCGCAATACTGCATGCTGAAATGGATGCGCTTGAAAGTGCGGGAAGGGTAACTTCTG
ACGTTTATAAAAATTGTGAACTTTATACAACCCTTTCTCCATGCATAATGTGCAGTGGTGCAGTT
TTGCTTTATAAAATTAAAAAAGTGGTAATTGGGGAAAATAAAACTTTTTTAGGTGCAGAAGATC
TTTTAATGAATAATGGGGTTGTTGTTGAAGTTTTAAATGATGAAAGATGCGTAAAAATGATGAA
AGAATTTATAGAAAATAATCCAAAATTGTGGAATGAAGACATTGGAGAATAATTTAAAATAGTT
TTAAAAAATGTGATATTTCATGAAAATCGTTGTACTTGATGGATACGCCATGAATTCGGGAGATT
TATCGTGGGAAAATTTAAAAAAACTTGGTGAACTTTCCATTTATGATAGAACTCTTGAATCAGAA
GTAGTTGAAAGAATTTCCGATTCAGAAATTGTCATTACAAATAAAGTAATAATTGGAAAAGAAG
TCTTTGAAAAATGTAAAAACATAAAATATGTTGGGGTTACTGCAACAGGCTATAATGTTGTAGA
TACTAATTTGGCAAAAGAGCTCGGTGTAACTGTAACAAATGTTCCCGCATATTCAACCGATTCAG
TGGCCCAACTTGTATTTTCATTTATACTTGAACACTGCCAGAATGTTTCTAAATATACTGAAAGT
GTAAAATCTGGAGATTGGGTAAATTCAAAAGATTTTTCATATCAAAAATTTCCAATTATTGAACT
TGCAGGTAAATCTCTTGGAATAATCGGATTTGGAGCAATTGGAAAAAAAGTTGCGGAAATTGGA
AATGCTTTTGGAATGAATGTACTTGTAAACACGAGGTCGCCTTCAAAAACAGATGAAATAAATGT
TAATTTTGTATCCAAAGAAGAAATTTTTAAAAATTCTGATTTTTTAACACTGCACTGCCCATTAA
ATGAAGAAACAGACAAAATTGTAAATGAAAAAACATTAAATTTAATGAAAAAGTCTGCAATTTT
AATAAATACTGGAAGAGGTGGGCTTGTCAATGAAAAAGATCTTGCAAATGCATTAAATTTGGAA
AAAATTGCAGGAGCCGGGCTTGATGTTCTTTCAACAGAACCTCCTAAAGAAGATAATCCACTACT
TAACGCTAAGAATACATATATAACACCACACGTTGCGTGGGCGTCTTATGAAGCGAGAAAAAGGC
TAATGGATGTAACCATAAATAATGTGAAGTCTTTTATCGATGGAAATCCTATAAATGTTGTAAA
TAAATAATATTTATTTAAGTTTAATTTTTTTAAGTCGGGAGATTTTTTATGAGGATAACTGACG
TTGTAAGTTTTGCAGGGCGAAATATAACTCAAAAAAAGACGCAGAGTTTTTTAACAATAATTGG
CGTTGTTATAGGTATTTTAGCAATAGTTAGTTTAATTTCTCTCGGGTTTGGAGTTCAAAATTACA
TAACTAGTGAAATTACAACCATTGGTGCAAATGTTATTTCAGTTCTGCCTTCACAAAATTTTGGA
GGCCCTGCTGTTTCTAAAAATTTTAATGATAACGATGTTAGCGCTGTAAGAAATGTTAGGGGCGT
TGAGGAAGTTGTAGCCGCATGGTTTGGAAGTTACGAACTTGAATATCGAGATCAATCTCATTACG
GCAATGTTTTAGTAATCGAGCCTTCAAAATTTACCCACGTATACTCAAAAACATGGGGTTATGAA
CCATACAAAGGTCGATGGATTGAAGACAGTGACAAATACTCGTGTATGATTGGATATGCACTTGC
AACTAATTCATTTGATCGTGAAATTGATATTGGAGATAAAATAACGATAAATGATAAAAAAATAC
AAAGTTATTGGAATACTCGAAGAAACAGGAAACCCGAGAACTGAAGATTCAGTTATTCTATCAA
AAGACGTTGGCGAAGAACTTTTTGAAATTAATAACGAATACAATATGATGATTGTTTCAGTCAG
ATCGGGCGAAAATGTAAATCTAGTCTCAGAAGAGATCAAAGACGAACTTGAAGATTCAAGAGGG
GACGAAAATTTCTCGGTTTTAACTGCAGAACAGCTTGCCGAATCAATTAACAGCATATTTAAAGT
TTTAACAATATTTTTAGTCGGTGTTGCAGGAATTTCCCTTCTTGTTGGAGCAGTTGGAATTTCAA
ACACGATGCACATGAGTATTTTAGAACGTAGAAAAGATATTGGAATTTTAAAAGCACTAGGTGC
AGAAAACAATACGATCCTCTCAATATTCGTAGTTGAAGCTGGATTTTTAGGATTATTTGGTGGAA
TTGTTGGAACAATACTTGGAATACTGATTGCAAAAGCTATCGAGTATGTTGCAGCAATTTCCGGG
TATGGATTGATTAGGGCATGGATTTCATGGGAATTAATTGTTGGAGTTTTAGTATTTTCATTTGT

AGTTGGAATTTTAAGCGGTTACTTCCCTGCAAGAAGCGGTGCAAAATTAAACCCGGTTGATACTT
TGAGAGGGGAATAACTTTAAAAAATTAGTTAAATCAAAAGTAATAAGAATAAAATATTTAAAAA
TAATAACAGACTTGGTATTCAAATTATTCGATTTTAATATTTTTCATATTTTAGTTATTTAGCGG
TGAAATTTTGAAGTTAAGAATAGGAACAAGGGGAAGTACACTTGCAATGGTCCAGACAGAGTAT
ATTGCAGGTCTTTTAAATGATTTAGATATTGAGACTGAAATAATCATAGTTAAAACTACTGGCGA
CAAAGATCAGAATAAAAAACTTGCTGACCTTGGAATCGGTGTTTTCACAAAAGAACTTGATAATA
AAATGCTTGAAAATGAAATTGATATTGCAGTGCACAGTTTAAAAGACGTTCCAACCCTTTGGAGT
GAGGAATTACAAATTACTGCAACACCTAAGCGAGAAAGTCCTGATGACATCATAATCTGGAGAAA
AGACTGCGATTTTGATATTGAACACGATGAATTAAATGTAGGAACTTCAAGCATTAGAAGAACCT
CATTTTTACAGTTCACCAACCCAAATCTAACTCCAAAATTACTTAGGGGTAATGTTGCAACAAGA
ATTAATAAATTAAGAAATGGGGAATACGATACAATTGTAATGGCAAAAGCTGGACTTGTAAGGC
AGGGTATTGATTTATCCGAATTTAATTACAAAAAGCTTGATATACTTCCTGCACCTGCGCAAGGG
ATAATTGGCGTTGCTTCAAGAAAGGCAGATACGAAAATAAACCAGATTTTAGAAAAAATATGTG
ATAAAAAGACATATATTGAAGCTACTGCTGAAAGATGGGCTTTAAAAGAATATGGTGGCGGATG
TCAGGCACCTTTCGGAGCGTTTGCAGTTTATGATACTGAAGAAGGAATTTTAAATTTAAGATGCG
AACTTGTAGACGAACAGGGTACAATAAAGCGAGTAAAATCAAACGAAGGATTTGTAATCTGTAC
TACTGACGATGTCGAACTTTCAAAGGAACTTGGTGCAAGGGTCGGTGCATCATTTAAAGAAATCG
AAACTTTTACGTATTAGTTCTTTTTTCTTTTTTTAAAATATTTAGAGAAAGTTTTAAATTATTTA
AAGTTTAATTTTATTTTAATTGTTATCATCGAGGGATTTTTATGGCAGATTACAACAACACAGTA
GTTTTCTATGATACAAGCGGGATTGAATATTCAAAACAGATTAAAAAAACGCTTGAAAGAGGGT
TTGAAAGGGAGTGTTTTTTAATAAATTTAGACAATCGTGAAAACACACTTTCAAGGCTTAATTTG
GCAATGAAAACAAGTAAATATGTTATATTTTTGTTTAGTTCAATGTATTCTAACGAAAACATGTT
AGATGAAGCAAAAACTGCATTTAATTCAGACAAACACATAATTTCTTTAAAATTAAACGACCCTG
AATTAAAAAAACAAATTTCAATCCACCAGTCCGAATTTGAAGATAACTACAGCCTTTCAAAAAAA
GTGAACGACGAAGTTGAAAAACTTAAAAAAATGGAAAAAAAAGGAACTGATTTTGTAGAATCGC
AAATTTCGTTTAATATGGGAAATATATACTTTAATTTCGGAAAATACGCCGAAGCAATAAGATG
TTACAAAAAAGCAGTGAAAATAATACCTGATTTTGTAGATGCACTGCATAACTTTAATTCGCTTT
CAAACATGAATTTGTTTGACATGGGCGAAGAAACTAAAAAACCCGTATTGTACAAAGATGTAAA
AATTGATGAACTGGTGCCAGATATTTTTTCAAACGCTGTTCAAAAAGAGCTTGAAGAACCTCAAA
AATATTCCGAACTTGAACCTGAAGAGCTTGAAGAAATTATTTTGGAAGGGGAAATTGGGGAAAA
TTTTGAAGAGCCCAAACTTGAAAATGAATTAACTGAAAAAATAGATGATAAACTTCCCGAAATT
GATGAATCAGAGGATATTTTAAAAGTACGACCAGAAGATGAATTAAACGAAACCTTTGAAGAAG
ATATTGAATATGAAACTTTCGAAGTAATGGATGATGAAGAAAAAAATGAAATTAATGACGAAAA
TTTGGAAATTTCTATTGTAGAACCCGAATTTGAAGAAGAAAACATTTTCGAACTTGAAAAAGAA
ACAGAACAGCCAGAATTTGAGGAAAACTTGGAACCAGTTTTTGAAGTACCTGAAGAATTAGTTG
AAGATGTTGAAATTTTAGATGCTAGTGAAGAAGATATTGAAAAAGAGTTAGAAAACCAGGAAGT
AATAATTGAAGAAAAATTAGAAACCCTTGATGAATTACTTGATAAATCAGAATTAGAAAGTGAA
TTAATTGAAAATCACACTTACGAGATAGACAATAACGAGTTTCTTGAAATATTAATTTTTGCAAA
TACACTCTATGAATCGGGCGATTTTGAAGCTGCAAAAACAGAATATAAAAAAGCAATTGCCCTAA
ATCCTGGTAACTCGGAAGTACACAATCATTACGGATGTCTTTTGGAATACCTTGAAAAATATGAT
GAAGCAGAAATTGAATTTAAAAAAGCAATTGCATTAGACGGTTCAAATTCAAAAGCCCATAATA
ATCTTGCAACTTTTTTGGCAAAATCTGAAAAATATGATGAAGCAGAAGAACACTACAAAAAAGC
AATTGAAATTGATCCTGATTACTGGGCTGCAATATATAATTTAGCTGCAATGTATGCTGCATTAA

AACGATATGATGAAGCAGTTGCTCAGGTTAAATCAATGGTTAAGATATTTAGGTCAGACGGGAA
TATCAAGGAAGTTTTAGAGTTAAAAAAACTTATAAAACATTTAAAAAAATTAAAAATTCAAAAA
AGTTAATTATTTAAATATTTCAAATATTTTGTTCTTTTTTCCACCAAATCAGGGCTTCTTTTGAA
TTTTTTTCAAATTTCCAAGAATCTCCTGTTTTAGTTAATTCATTTGACAATATTTCTTTTATTTT
TGAATATTGTTCATTATTTAAATTTAGTTCTCCACGGGCCCTAAATCTATCCAAATAATGTTCAG
CAGCTTCGTCCAGATTTTCAAATGTTTGTAAAAATGGTTTTTCACTTATTTTAACATCAGCATAT
ATTCCAAGCTGGTATAATATGTTATAAATTAAAATGTATGTCATGCCGTATTCTTTTCTTTTGGG
ATCAGGTTCTAAAATGGATTCACCAATTTTATTATATATTGGCCTCCAAGCTTGGCGAGTTCCAA
ACGTTAAGATACAGCAGTATTTTTCTGCATAATTATTCATTTTTTTAAGGGCACTTTCCATATCA
TCTACACCAAGTGAGTAAGATGAAAATACAATATCGGGATTAATATCTGCATCTAATTTAAAATC
GTCCCATCTTTTGTTTATTATATTTATGTTGTCAATTCCTTCTTCTTTCATCCTATTTTTTAAAAT
ATCGATCATTCCAGTAGACTGCTCTACAACGGTAATTTCTTTAACTTCCCTTGCAAGTGGCATTG
TGTAAGTTCCAGGTCCCGGCCCAATTTCCAATATAGATGTGTTTTTATCGATTTTAAACTCATTT
TTAAGATATTCGATATTATTTTTAACCCCATTATTTTTTTACTTAAGACATTTTTAGCGTAATT
TTTTGAAAAAGTATCCCAAAAATTTTTCGATTCCATTTCAGACTTAAATTGTGTTTTTGCATGAT
TTTTCCGATAATGTTCAGCCCAGTCTACTTCGATAATTTCACCCCATTATTTTTGTAATAATATT
ATATGTTCTTGTATATATTACATATGATTTATTTTTAAAAATTTATTAAGAAAATCATAAAATT
TATATATTATTCATAATTATTACATAATTTGTAAATGAATGTACTAAATATGATTTATTTTATA
ATATATCATACAGAATTTTCAACAGACCTCGTTTAATTTGCTAAAGTATCCATATGTAAGAATAC
ATATATAATAGTAAACTATAGTGGTTACTATTTTATAAAAAAAGTAATTAGTTATAATTACATT
ATATAAAGGTGAAACTAGAGGCGCGCATAATCACAATAACTGAGAGACTTATTAAATTCCAGAT
AATTTATTTTTAAAAAATTAGATAAACAGGTGAATAAATGGCAATTAGTATTAAAAAAATAGTT
AGCATCGGTTTAGGTGGCTTGATGCTCGGTTCAACATTTTGTTCAGGTGCTTTTGCAGTAGAAAA
AGTTGGGGATGTAGATTCTTTTGTTTCAGATATTGTTTCGACCGATAATTCAAATGTTGATATTA
TTGTTGGTTCAAATTCAGTAGCCTCAGATGTTGTATCGGCTGCTAATATCGCGGCAAAAATTGGA
TCATTATCCTTTTTAGAACAGAATGGGAAAAGTGCTGCTGCAACCTTATCAGTCAGTGCATCCGC
AAGTTCTGATGATTTCGATCTACTAAATGATGATGCAACGGATGACTCCATTAACTTTACTTCAA
ATGGAAACGGGCTTTTCATTGCTTGTGCTGATGGTGATTGTGCAGATGCTTTAGTGGATATTACT
TATGACGATTCAACAGGCCTTGATGCAGATAATGGTGCAAAGTCACTTGGTAAACTATCAACATT
ATCCTCAGTTGATGATATCGATCCATCAGACTGGTTTAATAGCGATGACGATGCTTATGAATTTT
TATTTGCAAAGTTAAATAATGATAGTGGTGAATGGAATATAAATGAAGAAGGGCTAGCTTACGC
AGTTATTTCATTTAAAGACGATGCTTCTTCTTTTGACGACATAACAACACTTTGTCCCGGAAAAA
GAATTCCTTTTATGGGTGAAGAATGGGTTATTATTGGTATGAGTGCAGATAAAGATTCATTTGCT
CTTGGAAAAGAAGTTTATGATGGGGTAATTAAAGAAGGTGAATCATATACTCTTGGAAACGGAT
ATGAAGTTAAAATTTTAAGTATCATACAAAGTTTAGATGATGAAGATGAATGTAAGGTAAATGC
TCAGCTATTAAAAGATGGTAAGGTTATAACAGAAGTATTTGATAACCCAAGATTAAACATGTGC
TCAAATGGAATCGGGGTAAGAATTAACAGCGCTTGGCAAAATATCGGTTCTGATTATGGTTATGC
GGAACTTGTAATCGTTAACAACATGAAGTGCTTATCACTTGGTGAAGAATATATTCCAGACTGGG
AAGCATATGGAATTTTAAAAGGCAATAATACTTTAGAATATACTGATGATTTTAAAAGTTTTGA
ATATGAGCAAAAAAAAGTTGGACTTGCTTTGAAATATGTCGGAGATGACTTGGAAGATATTGAA
TCCGGTGATGAAATAACTATTGCAGATTATGGGTCATTTACCCTTGATGATGAAGATGAAGACGA
TAAATTGAGGGTATTTTTTGAAATGAACGAAGAAAAAGAAGTTAACATAGTAAAAAACCAAAAA
GTAAATGTATTAAATGCAGAAATTGTTGCAGATACAGTTTTCGCAGGAGATGAAGACTCCTTAAC

CGTAAATGCTCCAGTTGTTAAGTTAGATACTGAAACGTCAATGGAATCTTCAGAAAATCCATTAA
TATTAGTTGGTGGACCAGTTGTAAATTCAATAACTAAAGAATTAGTAAATGCAGGATTATTTGC
AATTGATGATGATAGCCTTGCAACTTTAGCAGTGGTTTCTGAAACTGCAAAGGGAAATGATGTTT
TAATTGTTACAGGTGGAGATAGGGATGCAACAGCAGAAGCTGCAAATGCTTTAATTGCAATGATT
TAAAAAAGTAAATTATTTTTTAATTTTTTCTAAAATTTTTTTATCAATCCATTCTTTTGTAATAT
ATTTTTCATAAACTGGAAGCCTTTCTTTTAAAGTAAATCCAAGCTCTTTTGAAAAAATGTTTAAT
TCTTCAATATCGGGCCATGGGGCTTCAGGGTTTACAAAGTCTTTTGTTAATGGAGATACTCCACC
CCAGTCATCAATTCCTGCCATTAAAAATAACTGTCCAGTTTCCCGATTTAAATTTGGCGGAACTT
GAATTGAAATATCTTCTAAAATTAATTTTGAAAGAATTATCATTTTAAACATTTCGATTGGACTT
GGTTCTTTGTAATTTTCCATTGGAATTTGAGGTTTTGATCTAAAGTTTTGAACAATTACTTCCTG
AATGTGCCCATACTTTTCATAAATTCGTTTAATCTCAAATAGGGAGTTAACCCGTTCTTCAAGCG
TTTCTCCAATTCCAATAAGTATTCCTGTAGTAAATGGAATTTTTAATTTTCCAGCATTTTCGATC
ATTTCAATTCTTAGATTTGGGTCTTTTCCAGGGCTCTTTTCATGTGCAATAGTCCTGCATAATCT
CGCACTTGAATTTTCAAGCATCAAACCCATTGAAGCGTTTACTTCCCTTAAGTATTTGAGTTCAT
CATAAGATAAAATTCCGCAATTAGTGTGCGGTAAAAGATTCGTGTTTTCAAGGCACCAATCACTT
ATTTCATAAAGATATTCTAAAATTCCAGAATATCCCATTTTTTTCAATTCTTCTTTTACTTTTTC
GTTTTCGTCAACATTTTCTCCAAAAGTAAAAAGTGCTTCCCTGCATCCGAAAGTATCTGCTTTAG
TTAAAATTTCTTTCATTTCATCCATTTTTAGTAATTTGAAGTTTTCATTTCTAAATGTACAGTAT
CCGCAGACGTTTCTGCACCAGTTGCAGACCGGGATAAATGCATTTTTTGAAAAAGTGACATGTTT
TGATGTATTTTCTGCATTTATTTTTCCCAATTTTTCCAAAATGGAATTTGTTGAATTTGATTTTA
AAAAATCAAGTGCTTCGCTTTTTGTTATCATGTAAATCCCTTAAATTAATATTTTAAAGTAAATC
AAGTCCGATATCAACAGAAGTGGCCGAATGAATTAATGAACCCATTGAAATAACGTCTACATCAT
ATTTTGCATAATTTTCTACGTTATTTTCATTTATTCCGCCGCTGAGTTCAATTTTTGGTTTAAAT
CCGGTTTTCTTTTCAAATTCTGAAACGATATTTAATGCAGTTTCTATATTTTTAAATTCAAAATT
GTCGAGAAGTATTATGTCAGGCTTATATTCAATTACTTCTTTCAACTGATCGAGAGTGTCAACTT
CTACTTCAATTTTTTTAGAAAAGCTTAATTTTTCTGCTCTTTTAAAGCATTCTTCAATTCCAACT
GCCTGAATGTGGTTATCTTTAATCATTACCATGTCATCAAGCCTGAATCTGTGGGTATCTCCACC
GCCAACAAAAACTGCATATTTTTCAATCATCGAAAGTAGCGGGTGAGTTTTTCTCGTTGCTGCAA
TTTTTACAGATTTATTAATTTTTCTTACTTCGTTAATTATCCTGTTAGTTTTAGTTGAAATTCCA
GAAAGGTGCATTAAAAAGTTTAACGCTGTCCTTTCGAGAGTTATTATCGTTTTTGTGTTTCCCTT
AACTTTTAAAAAAGGCCCAATAACATTATCGCCTTCATTTACCAATGGAGTACAGGTTAATCCGT
TTTTTTCCATAAATTCAATTACAAAATCAATTCCACAGATTATAGATTTTTCTTTAGCAATAAAT
ACTGCTTCTGATTCCTGATTTTTAGGTATCATTAATTCTGTAGTCAGGTCTCCAAAACCTACATC
ATAATCGAGTGATTCTTCCAATATCCTTAAAGCATGATTTTTCAACACAATAATCACCAAGTTTT
TATAACTGGCCCAATTATTATAAATATTGCGTTTATTATTTTTATTTTAGTATATATAGGGGAAA
TATAAGATGTTAAAAACACTACCTCCAACGTTAAGAGAAAAAAAACGATACGTCGCACTTGAAAT
TATTTACGAGATGGAACTATCTCAAAAAGACGTAATATCGGTTGTTAGAAATGCATTATTAAACT
ATTCTGGAGTTTTGGGCTGTTCTAGAACAAATCCGTGGTTAATTGATTATAACCATCCGTACGGA
ATACTTCGAATTTCACGAGAAGAAGTCGATACTTTAAGAAGTTCACTAAGTTTAATGGGTGAACA
TAAAAAAAAGCCGATAAACATACGTATAATTGGAATTTCAAACTCAGTAAAACACATTCGTGAG
AAATTTTTGCACGTGCCCCACGAACCGTATTATAAAGTTATACAAAAATTAAAAAGAAAAGGCCC
AAAAAAGTAATTATCTTAAGATTCTGAGGTAGTTACCCAAAACCAAAAAGTACTCCATACAGAGA
TTCCAACAACGGCCAAAGATATGAATATAGTATCATAACAGCCGAGAACTGTATTTATCACATAG

TTTAAATCCATAATGTACACCCCCCAGTGACATTATGTACTGTGTAAATTATTGTATAAAATATT
GCTCCTTAAAAAAACTATTTTTCTTACGTTAATAGTCTAAATAATCAATAATATTCCGTAAAAAA
GGATATAATAGATATTTTTAGCTTATGGCCAAGTGATTAATTTAGGGATTTCTGGCAATTTTCCA
ACTTTTTTCTGAATTATTTCTGGCCAGTCTTCGTAATTGTATCCATACTGTGCTAAAAATCCAAT
TAACCATCTTGATAATCCGTAGCCCGTACATCCAGTCCATACTTTTCTATCTTTGTAGTCTTTAAT
TCCAAATCCTTCTACAAAGTGGGTTCCGTGAATATTTGCTGAAGTTACTGCAACTCCTTTCCTTTC
GTCTTTTACGTGAGGTAGCCAGAGTCTCATTTCATATTTTGGAACGTCAGGGAATTCAATTCCTC
TGTCTTCGTTTTTCCTACCTTCTAAATAGAATGGATCGTCTCCAACTTCGGTCCAGTATTCTAAAT
CTAATTTTTCTGCTAATTTTTCTGCGTATTTTAATGTATCGTCCCTTACTTTTTCTACAAATTCAG
GGCTTCCCATCCATACACATTCGCCCCTTAAAAATTCATTTACCCTATCAAGACCTTTAGCTCCGC
CACCTTCCCATCTGTAAGTCCATCCGCTTTTATCAAAGAATTTTGAAGGACTGTCAACATCAACT
AATTCGTGGTCAAAGAACGTGTAGAATGGTTCGCACTGTGCAGGTGCTAAAACATATCCAGGATC
TCTCAATAATGTTTTTAATTTATCAATTGGAATTTCTTTTTTGATCATCATTTCATTTACGAAAT
CCCTAAACATTTCAGGTTCCCTTTTTGGAGGACATACATAGTACATTCCTTCAGGAAGTCCTTCC
AAGTATCTCATTTTATACATTACGTCCAGTGGAATCAATTTAGGGAATAAACACTCGTCAAATCC
TACTTTTTCAATACATTCTTCGACAATTAAGCTTTCAAATGCTCTGAATAATTTTGCCATTGGGG
CCGTGTAAAACCACTGTCCTCTTCCAGGGAATCTTTTTACCCATCCGTAGGGTTCTGCTAATTCAG
TTGGGTCTTTATCAAATGTAATTTCGCGTGTTGCTTTGTAGTCTGAAACAATAGTTCCAGGCGCA
ATTTTACAAACTTCAAAAGTTAAATCCTGTTCTTGTTTATCAAGCTCGGTTTTAACAAATTTGAT
TGCCCTATCGATTATATTTCTTTTTAATTCTCCATCGCCTAAATTTTTAAAGAGAATTTTAATTT
TTTCGCCATCTAATTCAACTTCGCATTCTGGAACTTTTAATCCTTTTAATTCAGCAACTTTTGAA
GGTGTTGCGGTAATTGTTACAACGTAAGTATCAATTGCAACATCTCGAATACCGATCTGGTGTTT
TCTTCCAACTTTTTCCATTATCGGTTTTTTTAATCTCACAATACCTTCGTGTGCTCGCGTGTATGT
TCCTGAAGTCATGTTAAGTTTTAAATCTTTTCCTTCGAATTCATAACCTTCAATTTTTGAAGCTT
CGTTTTCTTTACCTTCTGGAACACCCTTTAAAAAAATGTCCCCATTTTCCAAAACTTCAATAATA
TCTTTTTGTGTTTCTTCACTTACATCCTTGCTAAAAATTATTCTCCCTTCAAGTTCAAATCTCATG
AAAATCACCGATCAATTTACAGATATGTGTTTTACCTAATAGTTTTTAAATTTTAGTATGCTCAC
TGAATCATCTGAGACCTTATGGATTCAATGATTTTTTCCGTAACTTCAAAAGTTTTTAAGTTTCC
CCCAAGATCAGGAGTTAAATATTTACATTCAACCGTTTTTTTAACCGCGTTTCTAATGTATTCGG
CTTCTTTATTCATTTTTAAATGGTCAAGCATCATTGCAGCACTTAATATTGTTGCAATCGGGTTT
GAAATTCCTTTTCCAGCAATGTCTGGTGCAGATCCATGAACTGGCTCAAATAATCCGTTTTTATC
ACCAATGTTTGCTGAAGGAGACATTCCAAGTCCACCAATAAGTCCTGCAGCTTCATCAGATAAAA
TATCTCCAAAAAGATTTGTTGTAACCAATACATCAAACATTTGCGGGTTTCTAATCAAATACATT
GCTGTCGCGTCAATTAGGTAGTCATCAGACTTTATTCCTAATTTTTCGTAATGTTTAGATATTTC
TTCGAAAACTTCTAAAAATAATCCGTCAGTAACCCTTAATACGTTTGCTTTATGTATGCAGGATA
CTTTTTTTCTGTTATTTTGAACCGCATAATCAAAAGCAAATTTTACAATTCTAGAACTTCCAAAT
TTTGAAATTATTCGCTCAGCAATTGCAACTTCGTTTTTTTCGTCGTAATATTCTTTTTTTACATA
GAGTCCTTCAGTATTTTCTCGAATTATAACAAAATCAAGATTATCAAAGTTATATGTTGGCCTAA
TATTTGCATAAAGGTCAAGTTCTTTTCTTAAAGTTAATATTGGACTTCTATATGATTTATTTTTT
AATTCAGTCGGTTTTGGAGTGGTAACTGATCCAAATAAAATACAATCAGATTCTTTTGCAATTTC
AATTGTGTTTTCTGGTATCGCATCGCCACATCTTTTAAAACATTCGTAACCTGCATGGGCATGAA
TGAATTCGAAGTCACCAAGTTCATTTAAAACACGCACCGTTTCAGGAACTACTTCGTTTCCAATA
CCGTCACCGTTGATAACACAAATTTTGGGAGTGTTTCTCATAAAACCACCAAAAAACTCGCACCT

TATTATTGTTCAATTTAAAGCAGAGATACAATATGCCTTAAGCGTATAAAAATACTCTGTAATAT
ACAACATCAAAATAAATGAATAATTAAATTAAAATTTATTCCTCATAAGGTTTTATTTTTTTAA
TTAATTTTTTAACTTCGGTAATTTTTTCAGAAATTTCTTCTGATTCCTCAGAATTAGCCTGTTTT
TCGTAAAGATAATCTTCATAAAGTTTAAGTTCATCCCAAACTATTTCGTCAAATTCTTCTTCACC
AAAATCTTCCAAAAACTCTTTATTATCTATCGCATAAAGTGCGGTTCTACAGGAATCATTTCTTC
CAATATACATATCATCCGAATAAATAAAAGATTCAGGATTAACGTCCCTTGAAATTGATTCTGCT
CGTTTTAACACTTGAGTTATAAGTTCAAGTTCATTTTTGTTAAGTTTTACCATTATTTCACACAC
TCAGTAGTTATGAAATTTTTATATTGCTCAATGACCTTATAAAAGGAATATTTATATAAAAATA
ATCCCTATCTTGAATAGATATCATTATAACTAATTAAAAATGTCTTTAAATCTTCTTAAACTATA
ATTTAAATGATTTATATAATTTAACGTGAGAACATGGAAAATATCGAGTACGAAATTAACTACG
CAATAGAACATATGATGGAAAATAATTACCCTAGAAAAGAATTTTGGGGCATGGATTCAAAAGT
TCCAGGTCTTTCATGCGGTATTCGTGCAGGGGATGACGGAGTAGTTATCGGAAAATCTGTTTACA
ATATGGAAGGGCCATACCCATTAATTTAGGCTCAAAAACCGCACTTATCCATACTACCTGCGAT
ATCGTTGCAATGGGTGCAAAACCGCTTTATGCAATGGATGCAATTCAAGCTGCAAACGAAGAAGA
AATTGAAATTGCAATAGATGGATTAAAAAAACAGTCAATCGGACTTAATATTCCAATAATAGGT
GGAAACACTCAAACAATCGAGTCATTAAAGTCCTGCATGAGTGTAGTTGTTTTTGGTGAACTTAT
TTCAGACAAAATAATTCGAGATTCAGGTTCAAAAGATAACGATGTAATGCTAATGCTCGGACACC
CTGTAGAAGGAGATATTGGAGAAAGAGTTCAAAAAGCAAAAAATAAATTTGATACATTTTTAGA
TGTTTTAAAAAAATGATATTGAAGTTCACGCCTGTAAAGATGCTTCAAGAGGAGGCTGGCTTTGTA
ACATTTTAGAAATGCTTTTGAAAGCTCAAAATGGTGTTGAAATAACAGGACTTCCATATCCAAGG
GCTACAAGATATCTTGGAACATATTTATTAAGCGTTCCAGAAGATGAAGTTTCAAATATTTTAGA
AATTGCGGTTAAACATAGATGCCCATTAACTCCATTTGGAAGGGTAACTTCTGAAAAAGTTCTAA
AAATTGGAAATAACGAATATATTGATAATGAAAAAATGAATGAGTTAATAAAAAACTTCCCTTA
TAAATCCTAAATATTTTTTTATTTAAAATATTAAATTTTAAAGTAATTTTGAAAGAGGAGAGTT
CTCCAAATTATATTTTTTGGCAATTTCTTTGATAATTGCTTTAAATACTGGATCGTTTCCACCAT
ACTGTTTTTCATAAAGTTCAAGTCCTGAAATGTGGTTATAAAGTACATCAGGGTATTTCTTTGAT
ACTTCATAAACTGTATTTAAAATCATGTTTCTTACAATCATGTTTTTTGAACATCCCATCAAATC
AAGCGTTTGAGGCATGGTATCATCCACCTGTCCTTTAGTAAGTGTCGGCAGTTTCGATAGCTGGT
ATACAATATTTAAAGGATCCATTTCTTTTATTTTACCCGATTTGAGTGTTTCAATCGTGAAAACA
TTAAACTGCAAATCTTCTTTTGAAATTGAATCTTCAAGCATTTCTTCAGTAACTTCCAGTTTTTC
AGGCATTATCACAAACAACGACGATCCAATTTCCATTAATCGTCCAAACAATGATTCCTGTTTTG
AAGACTTCATTAACTGTTTAACATGTTTTTGAAGCATTTTTTGATTATCCGAAAGATTATACTCC
TTAAATTTGCCGTATTTTTTTATCAAATTCCCAGTATAACTCCTAATATCCAAATTAACAAAAGT
CTTATTGATTTGAGTTACCTCTAAACTAAAATTTTCATAAACTTCAGGGAAAACATCAGTTATCA
GTTTGAGGGACATTAATGAATTTAAAACTAAAACTTCGCTTTTTTGTGATAAAAATGCTTTTAAA
GGGGAATATAATTCAGAAATATCTTTATTATTTTTTAAATAATTTTTTGTGAGCAGATAAATAG
CATTAGCTTTTAAAGTCTCACTATTCATTTTTAAATATTTAAATAAATCCGAAGTTAATGAATCG
TCCTCGTCCAACATCTTTTGGGCGTCATTTATCATGTCATCTTCAAGAAGCTTTTTTAATTTGTT
AGATTTCGAACGAAAAAACCCGATGACCATCAAATCACCTATCCCCCTTTTTTAAGTAAATTAAA
GTTCGCTTTCATCGACAGGTGTAACTTTGTATTCCATGTCTTTGTTTTTTCTCATAATTTCTAAA
AATTTGAGGCAATTTGAGGGGTCCTTGTTTGTAACGGTTATTTCTAATGATCCATTAACAAATTC
GTCTGATGAAAGTTTAGATTTTATTGGAACAGAACCATAAGCCATCAATACTTCCAATAGTTCTT
TTTGTATGTTCAAACACTGTTCTGAATCCCCTTTTATTGAGATTTTATACGATGGGAAAAGCAGT

TCGTAGCAGTTTACCAAAATTAGTTCATTTTTCTGGAGAATTTCGCAGAGTTTTGCGTAATTTTC
GCTTTTTGCTTTAACAATAAATTTTATTGAATAGAATTTATGGTTTAAATTATTTTTAACGGTTA
AATTATAGAGTCCTGAAACATTTTCAGCAATCAGCATTCTTAACTTTCCAAATACTTCGGATTCA
GACAAAGCCTGAGTTACATAGAACCTTTCTTCTTCCACGTTTTCACCCTGCTCAAATTTCAAGTTT
CCTAATCGTATGAAACCTATTCTATTTAAATTATGCGGTATTGTTCACGTTTGAAATAATAATGT
GTTAAAGTACTGAATTTTAAGCACTTTTTGGAAGCATATTATGATTTTCAAGATTTACTGCATTT
TCATAAGCTTCTTTTGAATAGTCTTCTTTTCCAATGGTTTTGTATACATTTGATAAATGCATCCA
AAATTTAGAATCATTTTTATTTATCGATATAGCTTTTTTGTAGCAGTCTATTGCTTCATAATGCC
TTTCCAAAAGATTTAATATATATCCTTTGTAGCACCATGCTTTTGCGTATTCGCTATCTTTATCT
AAAGCTTTTTCCATAAATTCGAGTCCTTTATTATAATTCCTTAATTTAACATACGTTCTTCCAAG
TTTTACCAGTATGGATGGATTTAACGGGTTTCTTTCATTTGCCAGTTCCAGGTAATAAATGGCAG
TTTCGTAATCCTCAGTATCGTAATATAAAAGGCCGACCCTGCACCACGGTTCATAATTTTGTATA
TGTTTTTCGAGCACTTTTGTATAAAGATCAAGTGCTTCGTTGGTTTTTCCAAGTTTTCTCAAAGC
ATCTCCTTTTGAAAGAGTTACCCCAAAGTCTCCTTTATGTTCATTTAGGTACATATCGTAGAATT
GAATTGCTTTTTCATACTTTTTAACCGTATAAAATGCATTACCGATATTTTTCCAGACTTCAGTA
TCATATGGGCTTATTTTCAAAACTTTTTCATAAATTTCAAGAGCTTCATCAAATTTTTTAAGCCC
TACAAGTGCAGTTCCTTTTTTTGCAATAATTTCTTTATACCTTGGTTTTCTTTCCAAAATATCAT
TATACTGCAAAATTGCTTCATTAAATTTACCTGTTTTTAAGTAGGCATCGCCCAAAATTTCTATA
ATTTCATCGTCGGATTCAGAAACATTTTTCAAGATTTCAACGGATTCCTTGAATAGTTCTAATGA
ATTGAGTAGCATTGTATAGCTTTTAATTATCTCAAGATCGTTTGGGCGTATAGATAACGCTTTTT
CGTATGAATCCTGAGCAAGTTTTGATTTACCAAGCATTTGGTATGCATTTCCTTTAAAATACCAT
GCATCAGGGTTTTTGGGCTCTGAATTTAAAGTTTTATTAAAATATTCTATGGATTTTTGATACTT
ACCATTATTATAATATTCAGTCCCTATTTCAAGCAGTTTTTTTGGATCTTTTGATTTAAAAATTG
ATAATAGTCCCATGGTGCGCCCTTTTAAATTTCTATAGTTATACTTTTGAAGTTTATCTTTATC
TAACTTTTTATTTGTGAATAACGACCTTTTTAAATTAACATATATAATTCAAATATACGATTATA
GCTTAAGAACTGTCAACCCATATGGGCATTTGATAGTTTAAAAAGAGATAATTCCAATCGACAGT
ATCAGCGTTGTAGTGGATAAAAACAAAATATCATTAAATCCAATTTTTTGACCGAGTGTTTTTAT
ATTTGAATCTTTTGAATAGCCTCTTGAAAGCATTCCTAAATAGGTTCTTTCACCCTGTTCGTAGG
ATTTTAAGAAAACAAGCCCAATTAATGACCCAAATGTATTTAGAAGCTGTTTGTAGTTTAAATTT
TTCCTATTAAGTGCCCTTGATTTTTGGGCTTTTATCGTGGATTCTAAAACATCAACCATTACAAA
TAAGTATCTAATCATCATTCCAAGCAGTGTGGACATGATGCTTGGAATTCCAAGTTTTTTTCCAG
CATTTATCACTTCATACATTGGTGTTGTCGAGGATAAAAGCACTACTGACGTTATAGAAACCAGA
AATTTTAAAAATAATGAAATTCCAAAATCTAATCCTTCAGAATATATTGGAAGCATTCCTAAATA
ATAAATTACCGATTCTCCCCGAATAAAAGGCTGGAATAATGCTATAAATCCGCCAAATGGAATTA
TCATAATTATTCTTTTGAGTAAATTTGAAATAGGGATTTTTGAAAAGAGCATTACAAATAACAT
GTAAATTTCCATTAAAACCATTAAATAAACATTGTTAAAAATTGTTGCAGTCAAAACTACGGCAA
AAACAAAAATTAATTTGATTCTAGCATCTATTCTGTGAAGCGGACTTTCGACAAACGCATCTCTT
TCCAAACTGTGAATTGATCCGTGCAAATTTTGACCTGAAAATATTTGATTAATACAATATTTCGC
AAATGATTATAGAGTATTATTAATTTAAAATAGTTCTTAATCTATAAAAAACCATATTATTTTG
AAAAAAATAGGAGAAAATAATACAAATATTCATTAAAAAAATATAATTAAAATTCGTAATCAAC
AGCAGTTCTGATTTCTGCAACGGATTTTACAAATTCTCCAACTTTCACGTGTTTTGGGTGTTTTT
GATAAACATCTAAATCTTCAAAGCTGTCGAATTCTGATATTAATACTACATCGTAGTTATTTTCA
AATTTTTCAGAGTTTACACCAACTTCAATGTATTTTATTTCAGGAATAACTCCATTTAAACTTTC

CAATTTATTTTTAATGGTTTTTGCATTCTCAAATTTATCATTTTCCTTATCATTTTCTTTTAATT
TCCACATAACGATATGTTTTATCATAAATACACCTGTTTAATTTTAAAATCATGTTTTTGGTATA
AATTATATAAAAATATGCAAGAAAAGCCGAACAATTAACAGTTTAACATTTTAAAAAATTGAAA
AAAGTAAATCAGATCTTTTAGTTTTCTGAAGTTGATTTTTTAAGTAATGAACCAACGCCAAATGA
AAGTAGGGTTATTAATAAAATACCAATAATTAATGCACCAATTTCCCCAATTTTGTCCATTCCAG
AAATTGCATAATCCGGCATTGGTGAAGGTGCAATTGTTCCTTCTTCTTCAAGTCCTGCTTCTTCT
AAATTTGCATCCAAAACATCTGCATTAATTATTTTCTCTGCAGCGCTTTCCAACCCATCAGGGTCC
CCTGAAGCTAAAAATGGTGCTGCAATTCCCAGTAATAATGCAACGATTAATCCTGCAACCAATAT
CGAGTTATTTTTATTCATTTGAAACCACCTTTTTTACGCTCTTTACAAGATCTGGTCGAGCTGAT
GCGAGGTAGCCTACTACTATTGCAGTCATTAAACCTTCAATAATTCCAATAACTGCGTGATAAAG
ACCCATAAATTGAATTCCAAGATCAATTGGGAATGTTCCTGCAATTGCAAGTTCTACAGTACATG
CAAGTGCTGCTAAAAATGTTGCAGCCCATCCTGCAACAAATACTGCTGCGTGGAAGTTTGTTTTT
TTCAGTGCTTTAAATAAGTAGTATCCTACAAATCCACCAACAACACCTATGTTAAAGATGTTTGC
ACCCATGACGATTAAACCACCGTCTGCAAAGAATAATCCTTGAACAATTAAAACAAGAGCTAACA
TTAATACAGCAGCCCATGGGCTATCAAATACTATCGCAGTTAATGCCGCACCAACCATGTGCCCA
CTGGTTCCCCAAGGTATTGGCATGTTCATTGCCTGAATTGCAAAAATACCTGCTGCAAGTGCAGT
AAATAGTGGAACTGTTTTTTCATTCATTTCCTTTGATGCCCATCTTAAAGACATACTTAAAAATA
TCAGGGAAATTACCCAAAAAATCCCGCTTTCCCATATGGGAATAAATCCATCTGGTATGTGCATT
GCCTCACCCATTTTTAGAGTAATAATTTTTTAAGATATTTATTACAGATAATACTATACATAACG
CAGTATTTATATGTTGTTATATTTTATTACTATGTAACAATACTCTGTAATTATTATTACTCATA
TGGGCAAAATTATATTATAAAGGGATTACAATTACAAAGAAAAGAATTAACAAAAATGTTGAAA
AAATGCACGTATTGGATATACTTAAGGAAAACAAAATTAGTGAATTAAGACCTCCGCAAAAAAA
AGTGATTGATGAAGGATTATTTGATAAAACAAAGAATTTTTTAATATGTATTCCAACCGCGAGT
GGAAAAACGCTTATTGGAGAGATGGCTTTATTAAACCATATTTTAGATGAAAATAAAAATTTAA
CTGGGAAAAAAGGATTATTTATCGTCCCATTAAAAGCGCTTGCAAATGAAAAATTTGACGAATTT
AAGGAAAAATACGAAAAATATGGAATAAAAGTAGGGCTATCAATCGGGGATTTTGATACTAAAG
AGAATCTTTCAAAATTTCATATAATTATAACTACTTCTGAAAAGCTCGATTCATTGATGAGACAT
GAAGTTGAATGGATAAATGACGTATCATTAGCAGTAATTGATGAGATTCATTTAATAGGAGATA
ACGACAGGGGCGGAACTTTAGAAGTAATTTTAACAAAACTGAAAAATTTAAATGCCCAGATTGTC
GGCCTTTCTGCAACTATTGGAAACCCCGAAGAACTCTCAGACTGGCTAAACGCGAAATTAATCGT
TGATGAATGGAGGCCAGTTGAATTAAAAAAAGGAATTTACTTTGAAAATGAACTTGAATTTGTA
AAAAGCCCTGCAAAAAAAAATAAAGCAGGTTTCTAAAAATAATATAACTGATTTAATAGTTGATA
GTGTCGAAGAAAAAGGTTCTTGTTTAATATTTTGCAATTCAAAAAGAAATGCAGTTGGCGAAGC
AAAAAAACACAATTTAACTAAGTATTTAACAAGAACTGAACAGCATGAATTGAATAAATTAAGT
GAAGAAATTTTATCAATTCTTGATACGCCAACTGAAACCTGTAAAGCCCTTTCAGAATGTATAAA
AAAAGGAATAGCATTTCACCATGCAGGACTCACATATCAACATAGAAAAATTGTTGAAGACGGAT
TTAGAAATAGATTAATAAAAGTAATCTGCTGTACGCCAACACTTTCGGCAGGCTTGAATTTACCG
TGCAGGCGGGCAATCGTGCGAGATATAAAAAGATATTCACAAAACGGCCTTGTGGATATTCCTAG
AATGGAAATTCAACAGTGTATTGGAAGAGCTGGAAGGCCTGGATTGGACCCATATGGTGAAGGA
ATAATTTATATCAAAAATGAGCGCGATTCTGAAAAAGCTTATGAAATTCTTACTGGAAGCGTTGA
AAACATATATTCAAAATTAGCAAACCAGAAAGTTTTGAGAATTCATATTCTTGGATTAATTTCGA
CAGGGGAAATTAATGACGGGCAAAATCTTGTTAATTTTATGAAAAATACATTTTACGCATACCAG
TTTGGAAATATCGGCGCAGTTTTATTAAACGTATCCGAAGTTGTAGGATTTTTAGAAAAAAATA

AATTTTTAGAAACATCCGTTCTTAAAAAAACAGAAAACACGGTTAGGGAGCTTTCTTTCGACTCT
TCAAACAATTTAGTTTTGGATTCAAAAGAAACTTCATTCGATCTTGCAAATCCGGATTCAAAAGT
TGAATTTAGATCAACGAAGCTTGGAAAAAGAATATCTGAACTTTATATCGACCCGATGAGTTCTG
AAATCATAATTGAAGAATTGTATGAATTGAAGAAAAAATGTGCCCAATTAGATCGATCAAAAAT
TGATCAGTATTTATTCTATTTAATTTCAAAAACTAACGAAATGAGGCCACTTTTAAGAGTTAGGC
CGAATGAAGAACTTGATTTAATTCTCGAAATGGATAAATTTGGAATTAAGGATTACTCCGCTGAA
AATGTTGAAGCTTTTAAAAATTCAAAAATGTTCTGCGACTGGGTAAGTGAAATACCTGAAGAAA
TAATTTTAGAAAAATATGGTGTTGAGCCGGGAATCTTAAGATACAAAGTAGAGCAAGCTAAATG
GATGATTTATTCAACAAAAGAGATTGCAAAACTAATTAATTTAGACAGCAGTGAAATATACAAA
GCACTTATGGAAATGGAATTAAGAATTGAATACGGTGCAAAAGAAGAATTAATTGAATTATTAA
AAGTCAAGAATATCGGTCGCGCAAGGTCAAGAAAACTCCATAATGCAGGAATCAGGTCTAAAAAT
GAAATAAATAATAATCCTGAAAAAATCTTTGAACTTTTCGGCGAAAAAATCGGTAAAAAAATCC
TTGGAGAACTTGGAATGAAATATGGCCAACAAACTCTCCTAAATTTTAATTAAAATTATAATAT
AAATTTTTTAAAAAAGTTATGCTCAGCTGTCCCCGTTGTCTTCATCGGAAAAGGATCCTCAGTGA
AGTCGGCACTCATCATTGCATAATTAAATCAGTGAAATATTCTTTAATTAGATATAAAGCTTAAA
TAACCTGATATATATAGTTGAGTTATCTTGAAATGAGTTATTGAAAATTTAAAATTAAAACGTG
TTAAAAATGTATGTTTTTTTAGATACTGAAACAACAGGACTTCACCCTGGACAGATTGCACAGCT
TTCTTATCTTGTAACCGACGAAGATTTAAACTTAAAAAAAGTTTTTAATCAATACTACTCAGTTG
ACCACATGTCAAGAAGCGCTGCAAAAGTTCACGGGCTTACAATGGAAATACTTGACGAATTATCA
AATGGAATGACTTTTGAAGATACTGTCGATGAAGTTAGGGAAGACCTTGAAGATTCCGTAATAA
TCGGGCACAACATATCTTTTGATATTCGATTTTTAAAAGCAGAATTTGAAAGACTCGGTCCAGAA
TATATTCCAAAAGATAAGTTCTGTACCATGTTTCATTTTACCCCAATCTGTAAACTTCCAGGTCA
AAAAAGATTTAAAAGACCAAAATTAGTTGAATTAATGGAATTTTTAAAACAGTGCCCAAATGAA
GGACTTGAATTTACAAAAAAAAACATATGGTTGTAGCGACGTTGGATTTCACGATGCAAGGTATG
ATACCGGGATATTATATCTCTGCTACAAAAAAGGTGTTTTACTCGACCACATTAAAAAATAGTTA
ATTTTATGATTATTTTCTTTTTCTTTTTTTAATTTTTAAAAGTCCATTCATCTTTTAAAATGCTG
TAAACTTCTGCATCGATAAATTTTCCATTTAAGAGTTCTGCACTTCTTTCGATTCCTTCAAATTT
AAAATTTAGCTTTTTTGGAATTTTTGAACTTTTTTCATTTCCAATTCCACATTTTATCTGTATCC
TGTTTAATTCTAAATTATTAAATGCATAATCTATCATTTTTACTACTGATTTAGTCACAATTCCT
TTGTTTTCAAATTCTTTTGAAATCCAGTATCCAATCTCTGTTTTTTTATTTACAAAATCAGTTAG
CTTAAATCCGATCAATCCAATTTTTTGGCCATTATAAATAATAACTGCAACAAAATCTCGGATAT
CTCCTGGAAGAAAGAGTATTGAACGAATAAAATCCTTTGTATCTTCTATTTCTTTCGTAGAATCT
ACAAATGAAAGCCAAATTCTGAGATTTTCTCGATCTGAGTCGATTATTTTAAAAATATCTTCTGC
ATCGGACAATTCTATTTCTTTTAGAACAATTTGTGAATCTACTTTAAGTTCAACCATATTTTCCT
CTCGGCGTCTGATAACTTCATATACCCTTAAAACATACATGAATTTATTAGTTATTCTAAAATTT
TGTTTTCAGAAAATCGGGAAACATTTATATTTGATTGAATAATATTTCTAAAATTATTATTTTG
ATTTTACTATTTATCGAGGGATTCTGATGAAATATATATTTGTTACAGGCGGTGTAGTGTCTTCT
TTAGGTAAAGGGATAACTGCATCATCTCTTGGAAGACTTCTCAAAGCCAGGGGTTTGAACGTAAA
TATGATTAAAATCGACCCATATTTACAGATCGACGCAGGTACGATGTCTCCATTTGAACACGGTG
AAGTTTTTGTAACTGATGATGGTGGAGAAACCGATCTTGACCTTGGAAACTACGAAAGATTTGTA
GATATTGGCTTGAAGGCTGACAACAATATCACAACAGGTAAAATATACTGGAGCGTTCTTTCAAA
AGAAAGACGAGGAGATTACCTTGGAAAAACGGTTCAGGTAATTCCGCATATTACAAACGAGATA
AAAGATAGAATCAAAGCACTTGGAAAAGATTCTGACATTACAATTATCGAAATTGGCGGAACTG

TTGGAGATATTGAAAGTTTACCTTTTTTAGAAGCAATTAGGCAATTTAAAAAAGATGTTGGAAA
GGAAAATGTTCTCTACATTCACGTTTCACTTCTCCCATATATCAGAAGTGCAGGTGAATTAAAGA
CAAAACCAACACAGCACTCTGTAAAAGAATTAAAAGGAATTGGAATCCAGCCCGATATTTTGGTT
TGCAGATCAGAAATTCCGATAAGTGAAAAAATAAAAGACAAACTTGCACTCTTTTGCGATGTTGA
AAAAGAAGCAGTAATCGAATGTAAAGACGCAAGAACAATTTATGAAGTTCCTTTAAATCTTGAA
AAAGAAGGACTTGGTAAATTAGTTACTGAAAAACTTAAATTAAGAGATTCAACTCCTGATTTAA
CTGAATGGAGAGCTATTGTTGACAGAATCATAAACCCGATGAATGAAATCACAATTGGAATCGTT
GGAAAATACATCGAATTAAAAGATTCTTACATGAGCATAATGGAAGCTTTGGGCCATGCAGGTGC
TAAAAACGATACCAAAGTAAATATTGCATGGATTAATTCAGAAGAACTCGAAACCAAAAATTAT
GAAGAAATCCTCGACAAAATGGTTGACGATGAAAAATTACACGGTATTTTGGTTCCCGGCGGTTT
TGGAGATCGAGGAATCGACGGAAAAGTAAATGCCGTAAGATATGCAAGAGAAAAAAATATTCCG
TTTTTGGGAATATGTCTTGGAATGCAGTGTGCAGTAATTGAATTTGCAAGACATGTATGTGGTTT
AAATGCCAATTCAACAGAATTTGACGAAGAAACTGAACATCCTGTAATTGATTATATTCCAGAAC
AGCGTGAAGTAACTGAAAAAGGGGGAACCATGAGACTTGGTGCATACCCTGCAGTTTTAACAGAA
AATTCGCTTGCAAGTGAACTATACGGTTCAGTAAATGCATCTGAAAGACACAGACACCGATATGA
AGTAAATCCTGAATACCATGAAATATTAAAGAAAAACGGATTAATAATTTCGGGAATGTCACCT
GACGGAAAACTTGCAGAATTTATAGAACTTGAAAATCACAAGTACTTTATTGCAACACAAGCACA
CCCCGAATTTAAATCAAGACCAAACAAACCTCACCCATTATTCCATGGATTGGTAAAAGCTTCGA
TTGAAAAATAATTAATTATTTGGAGGAATATCAATGTTCAAGACCGAACCGTTTATAGAAGAAT
CCATTGAAGAAATAAGAAAGCAGATTAATAATAGAAAAACAATAATTGCGCTGAGCGGTGGAGT
TGACAGTGCAGTTGCTGCAGTTCTTACGGATAAGGCAATTGGAGATAAATTACTTGCAGTTTATG
TAGATACTGGATTGATGAGAAAAAAACGAGTCCGAAGAAATCTGGAAAATTTTTAAAGAACAGAT
GGGACTTAATTTAAAAATTGTCGAAGCAAAAGACATATTTTTAAAAGAACTCGAAGGAGTAATT
GATCCCGAAGAAAAAAGAAAAATAATTGGAAGGCTTTTCATTGAAGTATTTGAGAAAGTTGCAG
AAGAACAGGGTGAAGAAGTACTCGTTCAGGGAACAATTGCACCAGACTGGATTGAAAGTGAAGG
ACAGATTAAAACACACCACAATATTGCATTACCTGGTGGAATGGTTTTAGATGTTGTTGAACCAT
TAAGAGAACTCTACAAAGATGAAGTTAGACTTTTAGCAGTTGCACTCGGGTTACCTGACCAGATT
GCACACAGACAGCCTTTCCCAGGACCCGGACTTGCAGTTAGAATTTTGGGCGAAATTACGGATGA
GAAATTAGCAATCTGTAAAGAAGCAAACTTTATTGTTTCAGAAGAAATCGAAAAAACTGAACTT
AAAAACGAACTCTGGCAGTACTTTGCAGCAGTGCTCGATACAAAAGCAACTGGTGTTAAAGGGGA
CATTAGAGACTACAACTGGGTTGTTGCACTTAGATTTGTAAAATCACTTGATGCAATGACTGCAC
ATACTCCTGAAATCCCATACGATTTAATTAAAAAAATAAGCAAAAGAATAACTTCAGAAATTCCA
AACGTTACAAGAGTGGTTCTTGACGTAACTGATAAACCGCCAGCAACAATTGAATTTGAATAATT
CTTTAAAATATCTCTTTTTTAAATTAAATTCTCTACTTACACAATAGCTATTTATTTTCAAACTT
CATTATTCATTATATCAAAAGATTATAGACTGTTTTTAACTTTTGAAAGGATTGAACATGAAGA
ACAATTATGGCATTCAAAAATGGCACAGTCACGGAGCACTGCTCTGCAATTCTGAAAAATATGCT
GAAGCACTCGAGTGCTATGATAAAATACTTTCATACTATCCAGACGATTTTTTAGCAGTTTTTGG
AAAAGGAATGGTTTTTTTAAAACTTAAAGAATACGAAAAAGCGCTTCAATGTTTTAACAGCGTT
TTAAACATGAATCCATCATACATTCCTGCAATAAAAAATAAAAAACAAGTAGAAGAAAAGTTAA
AAAAGCAGGAAAGCGAAAATTATAATAAATTTTCAAAATTGGGTGTTGAAAATTACAAACAGGG
GAATTTTGAGAATGCTCTTACCTATTTTGAAAAAGCTTTTGAAATAAATCCATATTCAGAAACTT
TGAGAAAAAAATATTGAAACAACACGCCTTAAATTAAAAGAAATTCTTCCAATAAGGTGGAATAA
TAAAGGTGTCGAGTACTACAAGGTTAAAAACTACCAAAAAGCTTTCGAGTGTTTTGAAAAAGCTG

TTAAGTTAAATCCTAATTTTGAATCTGCTTTAAAAAATAAGGCAATGGTTCAAAAGTTAATTAA
GAATTAAGTTTATTTCGTTTGTGATTTCATGATTAAAAACTTAAAAAAAGATGGAATTTTAAAA
GACAGCGCTTACATGATATTTTCAAACATGTATTCTAAATTTGCAGCATATCTTTTTTATTTTTT
GATACCTTTTATTCTTGGAACCGAAGGTTTTGGAGTAATTAAGGGTTTAATGCCTATTTTAGACA
CATTAGTTATTATTTTTTGTTCGGGAATTCCTCCAGCAATGGCAAAATTTATTTCTGGCGATGAA
TTTAAAGAAAACACTTGGATATATGACATTTTAAAAGTAATGTTTATTTTTTCAATCTTTGGTGC
AATTTTTACAGTATTTTTAAAATATATACTCGGTGGAAATTATTCTACTTTACCTGATGTTTATT
TTTACGCTGTCGCAGTAGCTCTGCCATTTTCAGTAGTTATTTCGTGGAGCAGGGGAGTATTACAG
GGAAATTTAAAAATAAAAAATTTATCAAAAACCTGGATTTTAGAAAATACTTCGAAAGTTGTTT
TTTTAGTAATTTTAAGCTATTTATTTGGCGTAGTTGGTGGAATTCTATCGATTTCAGTTTCATTT
TTACTCGGTGGAATTTTTGGAATTTATCTTTTATCAAAATCAAATCTAAAATATTCATTTTCAAA
TATTTTAAAAAATATATTTTCACCGATAAAAGAGAAGGAATCTGTTAAAAAAGTTATATACTAT
TCGATTCCTATCGCACTTACGACTGCATCCTATAGGCTGATAAATGATCTTGATGGCATTTTCAT
ACTTTCGATGCTAGGAGCTTATGATAATGGTGTTTACGGATACGCTTCACTTCTTTCAAGGCTTC
TTTTCCTGTTCGCATCTGCAATAGCAATAGTGTTAATTCCAAGAATTTCAAAATCAAAAGACATT
TCATATTTTAAAAAAGCAACAATTTTAAACATTTCAATAGTTTTACCTGCACTATTAATAATATT
TTTATTTTCAAAAGAACTTTTGAACTTGTTTTTTGGAATAAGTACTCCTGAAAGCATTACTTCTT
TAAAAATACTTTCAGTGTCAGCAGTATTCATGAGTACATATACAATATGTGCATCATCACTTCAG
GGTTTGGGTTACGCAAAAATTCCAGTATATGTTTTACTTTTTGGCATTATATTAAATGCCGTATT
TAATTATGTATTAATTCCCAATTTGGGTATAATTGGGGGTGCAATTGCAACTCTTTCATCGTCAT
TTGTTGTTTTTGTATTGATCTGGATAATTACATTTAATAAGCTTAAAAAAATTAAAAATAATAG
TTAAAATTTAATAATTTTAGTTTTAAATATTGCTTTGAATAAATTTTTTATACTTAGAATAAAA
ATAATTTCCAGAGTTAATTAAATTCAAGAGATGATGAAACATGGCCGAAAATTTCGTGGTTGTG
GATGGAGGGGTCGTAGCTCCAAAGGGTTTTAAGGCAAACGGGCACAAGGATAGAAAATACGGTG
CCGCATTAATATATTCAGAAACAGATGCAGTTGCAGCAGGCGTATTTACTACAAACAAAGTTTAT
GCGCATCCTGTTGCTCTTTCAAAGGATACTTTAGTAAATAATAAGGTATTCAGAGCAATTGTTGC
AAACAGCGGAAATGCAAACTGCTTTACAAAAGGTGGAATGGAAGATGCAGAATTACTCGTAAAA
AAAGCAGCTGATTTATTGAAAATTCCAGAAAATCAGGTTCTTTCAGCGTCAACTGGAGTAATCGG
TAGGAAAATGCCGATGGATATCATAGCCTTAGAAGTTGAAAGGGCATTTGAAAATATAACGTTA
GAAAGCAGTAATGAAAACGCATCAAAAGCGATAATGACAACGGATGCATTCCCAAAAACCGTTGC
AGTTGAATTTGAAGTAAATGGCAAATCGGTTAGAATCGGCGGAATTGCAAAAGGTGCTGGAATG
ATTGCACCAAACATGCTTCATGCAACAATGCTTGGATTTATTACAACAGATATTGAAATATCAAA
AGAAGATTTAACAAATTCCCTTCAAAAAGCAACGGATGAAAGCTTTAATAATGCAGTTGTCGATG
GAGACATGAGTACAAACGATACAGTTTATGTTTTAGCAAATTCTCAAAGTGGTGTAAAATATAA
AGACTGTAAAGAAGAATTTGATTGCGCATTAACTTATGTTTCAAAAGAACTTGCAAAAATGATT
GTTTCAGATGGAGAAGGCGCTAAAAAAATTAATTGAAGCAACTGTTTTTGGTGCAGAAACAAAGG
AAGATGCAAAAAAAAGCTTCAATGTCTATTGTTAGATCACTTCTTTTAAAAACTGCGGTTTTTGGT
GCTGATCCAAACTGGGGAAGAATTGCAGCCGCAGTTGGATACAGCGGTGCTGAAATGGATATGAA
CAATTTTGATATTATAATAAGTAATATTTCTTCAGAAAAACAGGCGATTTTAGTTAAAGCAGGA
GAACAGATTGCAGACTGCGGAACTCCAGAATTGAAGCTTGCAGAAGAAATAATGAAAGAAGATA
AAATCAAAATTATCGTGGATTTAAAAAGAGGAAGCTTTGAAAACACGGCCTTTGGATGCGATTT
AGGATACGATTATGTTAAAATAAATTCAGAATACACAACCTAAATTTTTTAAATTTTTTTAAAT
AAAATTACTATTTTTTAAATTTTTAATTTTTCAAAAAAGATAAAAAAGAAAATATCTATTTTAA

TTAAGTATTAAGCTAAGACTGGACCGTTTTCTGTCCATTCGATCATGATTGGTCCTTCTGGCATT
TCATCCAAAGTTTTAAAGTATTCTAAAGCAGCTTGTGTTCCAAGTCTGTCACTTCCTGCAATATA
TATTACGGTGTAACTTTGAATAATGCTTCCAGTGTTGTCTTGAACTTTTAATACTTGAATTATTC
CTTTGTTTTCTCCAGGGTTATCGTTTGAAATTGATATTCCAAATTTAGAATCATATTTGTTAGCA
AGTACGTTATATTTTGGCCCACCAACAATTATTGTATCTCCAGAAATTTCGTAATCTTCTGAATT
ATACAGGTTTTCTTTTAATTTTCCAGCATATTCTCCGTCAATTTCGCTTCCATACACGATATTTG
AGTTTGAAACCGTATTTTTAATTACAGATGAAGTAATTCCCTCGGAAAGGTCTGAACTGTAGTGG
CTTCCACTACTACCTGATGAAGTTGAAGCAGGAACTGCAACTGTTATATTTACAGTTTCGTTTGA
ATTTACGTTTCCAACTAAGTCAGTTGTAATTATCGTTATTTCATAGTTTCCATTTGAAAGAGTTC
CCGTATTTCCAGTGTAGTAAGTTCCATCAAAGAAAAGGGTTACATTTCTAATGCTTCCAATTTTA
GCAATAACTGATGAAACGTTTGATAATGAATCATTTGCTGTAACGTTTATCGCTGCAGAGCTTGA
AGTATACGTTGTTCCATTTACGGGAGTGTTCATTATAGCTGCAGGCGCAGTTGTATCAATTATGA
AATTTAAACTTTTTTCTGCAATATTTCCCGCAGTATCGTTTATTCTTATCGTCACGTTATAAATG
CCTTCATTTAAAGCTACAGAATTTAAGTAATTTCCAGTACTGTTTTCTAGAGCTATTTCAGTTGA
TCCGTTGAATGCAGTAACTTTATCAAGGTTACTTTCGTTAACTGTGAAGTTTATAACGTTTGATC
CATTGTTAAGGTATTCTCCTTCGATCAGTTTGATTGTAATTTCGGGTGAATTTAAATCTACTCTG
AAAGTTATATTTTCATTTGAATTTATATGTCCAAATGAATCGTTTGCGTATATTTTAACGGTATT
CAATCCTTCTGAAAACTCGTATGTTGAATTAACGAAATTTCCAGAAATATTTTCTAAAGTAATAT
TTTCAGTTCCGTTTATTTCCGCAATAACTGAATCTAAGTTTAAATCGGTTGCTGTAACATTTATA
AAAATCGAGGTTGTCTTGTATGATTTTTCTAACGTATTTATTGTAACTTCTGGCGAATTTTCGTC
CGCATTTACAGTAATTGCTTCTGTTGCGTTTGTATTTCCAACCATATCAAACGCAGTTGCGGTTA
TATTGTAAATTCCATCCGCAGGAACACTTAAAGTAATTTGGTATGATCCATTAGTATCATTTACT
GTTGAATTTACTAAATTTCCAAAATTATCAACTATTGAAATGTTAGTGTAATTTAATTCAATATT
ATCAGTTGCTGTAACGTTTATTGTTAATTCAGACGTGTTTGAAACGTTTGAAGTTTCAATTCTAA
GTTCTGGATTTGTAACATCTACAATAAAACTTATATTTTCGCTTGAATTTACGTTTCCTGAAGTG
TCGTTTGCATATATTTTAACTGTATTTAGTCCTTCTGAAAACTCATATGTTGAATTAACGAAATT
TCCAGAAATATTTTCTAAAGTTATGTTTTCAGTTCCGTTTATTTCAGCAATAACTGAATCTAAGT
TTAAATCGGTTGCTGTAACGTTTATAACAATCGAGGTTGTCTTGTATGATTTTTCTAACGTATTT
ATTGTAACCTCTGGCGCATTTTCGTCAACAGGAATTCCAAATTTAACAGTTACAATCGCATAACC
CGACATAGATCCTGTAACGTATGCATTTCCGCCATTATCTACTTCAATAGCATTTCCCCAAGAAT
TGATACCTGGGTAATAAGTACTATTCCAAAGGCGAATCCCGTTTGGAGCATACTTTATCGTTACG
ATATCGTACTTACCTCCCGTAACATAGGAATTTCCATCTAAATCCACTGCAATATCTCGCCCATCC
CCATAATCAAATGTATCGTTCCAGACTGCTTCTTCTGCGTTTACAAAACTTGCGCAGAGCAGAAA
AATAAAACTGGTAATTATGAAATGCCTTATTTTCAAAAAATCACCTTTTGATTGTTAACTAATAA
GGTAAAAACTATAATATCTCCCCTATATTAACGTTTGTAAATGAACTTCAAAATGGTTTCTGAAT
TTCATTTAGATCATATGTTTTGTAGTAAATGTAAGGGCAGATGTTTTACAGATTATGTTGGAAA
AATCAAGACATATTATTGAGTAGATTATATTAAAAAGATTAAAAAAGTATTATTTTTCATATTT
TTTAGAAATATTCATAAATTTAAATCAATATCCATTAAGTTTTTTAATATGTTTTTACTACTGTA
TCTCTGGTGTAATCATGATTATTTTTGGACTTTTTGGAAAAACGGGTTGTGGCAAAACCGAAATT
TTGGACGAATTAAAGAAAAAAATATACTGTAGTTGATATTGAGGGCTGTGCAAACAACAAGGGGA
GTATTTTAGGCGATTTATACGATTTATCAGCAAGTACTCAAGAAAAGTTTGATGCATGCCTTGAA
GAACAGAAAAAAATTGCGGAAAGTACGGGATACTGCATAGTTGAATTTGAAGGAAAAAAATTGG
TGGAAGGGAAAAGGTAACTATTCCAGAGCCATTTTCTGATTTAAACTGTTATAAACACAATATAG

TTGTAAACTGCCCCTATGAATGCCAGATAAAACGGCTTTTAAACTGGTACCTTCCAAAAAACGAG
AGCGAAAAAGAAATTTTGATCGAAAAATTTGTTGATTTAAAAACAGTGCTTTCAAAACCTGAAA
AACTTGAATTAATGGATAAAATAATTGATTTAATAAAAAAAGAAGAATATTACAATGCCGCAAT
CTTAATTGAAGAAGGACTTTACAGCGAACACTATTTACGACATATTGGAAAGGTAAAACATAAT
TTAGTAATAAATAACGAAGATATTTTAAAATCAACAGAACAAATATCAAAATATGTTGATGAAA
TTTTGAAAAAACACGGAATTAAAAATTAAAGTGAAAACATGGACGAAAACATTCTTTCAAGGCT
TCTTACTTTCAAAAATGATGTTATTTTAGTAATTCGATTAAAAACTGGTGAAAAAGTAATTCTA
AGAAATGGAAAAATTGCTGCTGGAAAATTAACTGGACAAATTGCATCATTTATTTCTGAAAATT
CTAAAGAGATTATAAAACCGAGTGTTTTAGAATTTTCTGGCGAAATGTTTTATTTTGAACCGGTA
AATATTAAAAAATACCTCGGATCAATTGGAAGTGAGCTTTCAGACGATGTTATTACAATTGACCA
GTTTTTTAAGTTAAACTTCGAAAACGTGATAATTGTTGATGCAAGGTCCCCAAGAGAATTTTCTG
AAAAAACAATACCTGATGCAGTAAATATTCCACTTTTTTTAACACAAGAATACGAAACTATTGGA
AAACTCTACAAACACGAAGGAAAAGAAATTGCAGTTGCTAAAGCAGGTGTTATAATTCAGGATG
GAATAAAAAGAATAGTTTCAGAATCTTTAAAATTAGATCCAAATAAAAATTTAGTAGTATTTTG
TGCAAGGGGCGGAATGAGAAGCCAATCAATTGCAACGATATTAAAATTGTTAGGATTTAAAGTA
AAAAGACTTGTAGGTGGCTTTAAAAGCTACAATATTTCAAAAAACTAAAAAAAGCATAAAAATA
AAAAAAGAAAATTATTCTTCTAAAAGATCCATTAAACCCTGTTCGTCAAGGATATCTAAGAGTCT
TTCTCTTAAGTCTGCAATACCTTCTTTTTTAAATGAAGATACTTTTAATACTTCATATTCTCCAA
GATCTTCTTCAAGTTTATCTAATCCTTCTATTTCATCCACATCCATTTTTGTAAGTGCAACTAAA
ATTGGAACGTTAAATGTTTTTCTAATTTCTTGAAGTAAATTAAACTGTTCTTCCATTGTAAACCC
GCAGAATTCTGTTGGGTCGATTACGAATAAAATCATATCTGAAAGATAGTTTATTGCAACTACCG
CGTGAAGTTCGATATCGTTTCTTTCGTAAATTGGCCTGTCCAAAACACCAGGAGTATCGATTATC
TGAATTCCTTCTTCAGTATATCCAATATTCAATCCTTTTGTTGTAAATGGGTATGAATTTACTTC
AGGTTCTGCGTCAGTTAGTGTTCTAAGAAGTGTTGATTTTCCAACATTTGGATACCCTGCAATAA
CAACTGAAGGTAAATTTTTCACTGTTGGAATGTTTTTAAGCTTGTTTCTTGCAACACCTAAAAAC
GCCATGTTCGGGTAGATCTGTTTTAAAACTGATGAAACCCTTCCGATAAATTCTTTTCTAACAAT
TGATGTTGCTTGCGGGCTTTGTGCTTTCTTAACTTTTCTTGAATATTGGGTTCCTAATTTTTTCA
CGATTTCAGATGCCCACTGAACTGCTGCAAGCGATTTTTTAAATTCGTCGCTTCCAACCATGATT
TCAAGAATTTCCCTATAAAATGGATCCAAATTATCTATTGAAGGAGTTTTTTCCACAATTTTTAA
AAGATTATCTGAAATAACTGATGCAGTGGTCCTGATTTTATTATCTTCGATTATCTTTGATTTTG
GAGTACTTAATCCTCTTGTAGTTGTTCTAAGTTCCCCTGCGATCTTTTCTGACCTGTTGTAAGCCT
TGTCCATCAATTCGTCAGGGTATAATATATTTGGCATTGCCTTAAATGGGTTTGCGTCTCTCGAT
TTCCTCATAGTCTCATCTCACACATTTTAAAATGTAAATAAATATTCAAAATAGTAGGAAAGTAT
GTTTTTAGTTTACTGTTCTTTAATTTTAACAGTTCCCGTGTTTCCATCAACTACTACTATATCGCC
ATCTTTAATTTCATCTATATTAACTTTATCCACGAGTGGAATTTTTCCAAGAATTGCACCTGTTG
CGACAATTGGTTCAGACTCAAAATTTACCATTCCTTTTAAAAGTCCTCTTTTAGCAAGCCCGTAG
ATTACATAAGAACCAACGGTACTTCCTTTTCCACTTGGAAATACAAATATTTTATTTGCAATACT
TTTTCCAAAAAGTTCATTTTCCTTATCTGTTACGATTCCTTCCTCATTTACGCCACCAAGAAATGA
AATAGGCGATTTTGAAATAATTGCTTCCCCTTCAACGACTCCTTTTGAAATAATTCTACCATTTA
GCTCTTTCAAAGTTTCACCATTTAATTATTACTTAAATTTCCAACAGATTCAGTAATTCGGTTTA
CTGCACTGTTATACATGCCGACAGTACCATTTGCTGTCTGATTTATCTTAACTGCGGATTCTAAC
GTACTTTGTATGTACAAGTACCCTGCAAATACTGCAACCATAGCTACAGCCAGTACTAAAACACC
AAGTTCAAGAGATATCTGGCCCCGATTTTTGAATGCTAAATATTTTAAACCCATTTTTTCACTGT

AACTTTTAAATCAACATTAAGCTTATATAAATCATTATAATTTTAAATATAAATATTATTGCTTA
AGAGGAATATTATGAATTTTAAAAATGAAATCAGCAAGTACACAACAATAGATCTCGATAAATT
AAAAACAAATAAAACAAAATGTCCAAAATGCGGAAAAGAATTTAATTCAGTAGGGAAAAAGATA
ATCTGCCCCGAATGCAAATATATATTTAAAATAAGCGATTAAAAAATAATCCTCCTAAATTTAGG
TTCGGATTATATTTATATTTCTAAAATCTCAACATTTTCGGATAATTTAGCTATTCCGACACCAT
TATTTACTATTTTTCCGACTTCAAATGCATAAACATTATTTTCTTTGAATTTCTGAATCAATTTA
TCTTTGTATTCGGGTTTTGTGGATATTAAAAGACCGCCGGCAGTTTCAGCACCTTTTCCACTGCA
AAGAGCATGTCCAAATAGTGAAGCAAGTTCTGGAGTTCCTTTAATTACTGGAAGGCAGGATATTT
CAATTTCAACATTGCTTTGTTCAGCCATTTCTTGACTGTGGCCCAAAATACCAAATCCTGTGACA
TCGGTCATTGCATTTGCAATTTTTTCGCCTAACTCATTTTCAAGTTCTCTTAAATGTAAAAGAGC
AATTCTATTAGACGTTGTCATCAGTTCAATGGTCTTTTCAAAGATATATTTTTGTTCTTCTTTTG
GAATATCGATTAAATCTTCAAATTCCTCGGTAACTCTTGATAATGCCATTGCACTCTGGTTTCCA
AGAGGTTTTGTAAGAATTAATACATCTCCATTTTTACATCCTGCTTTTGTAAGAATATCTTCTTC
TTTTCCAACGCCAGTAATCGAGCCACCAATTAAAGGCACGGATTTAAAATGGTATGCCCGCCAA
TAATCGTGGTATCATTTTCCCGGCAGAAGTCCTGAAAACCCTGAAGCATTTTTTTTGCAACAGGT
ACTGGAAGTTCTGGGGGAATTCCAAGGAATACAAGTCCACCAATAATTTCAGAAATTCCCATTGC
ATATACATCACTGGTAGAATTACATGCTGCAATTCTTCCTTGAAGGTAGGGATCATCCACAATCG
GTGTGAAAACGTCAATTGTTTTTACAATTGCCATTCCATTTTTTATAACAACAGCGGCATCGTCT
CCAAGGCCCACTTTTGCATGTTTTAAATCATCTTCAGAAATTATGCCTTTTACTAAATTTTCAAG
TTCGGTGTCTGGAAGTTTACAAGCTCATCCGTGTAGTGTAACCATGTTTGTTAATTTTATTTCCT
CTTCGATTTTATCACCTTTGAGGGATATTCATAAACATTTCATATATCATGTATTTTCAACTATT
CATGCTTTTTCTTATTTTTTGGCATGCATATTAATTCTCTTAATTCAATGTCAAAAAATGTATTC
ATATTTGAGGGCAATATTACAACTGCAGAATCAGCACCCCTGCTACTTCCACCAATTGCAATAAT
TTCTTTTTTCGTTGAAACAAGTCCTGCATCCGCTGCCATTACAGTTACTTCGTAGCATACTTTAAC
ACCCTGTCCAAGTGTTTTTAGAGTTTGTGCAATAACTTCTACGGGCCCATATCCGCCAAGTCTTTT
TGAAATTCCCCGCTCAACACCACTTAACGCATGAGTTCCGCTAAATACGGTTGCTCCTCTTTTTTC
AAGGTCTTTTTTGACATTTTCAGGCATTGCTACAACATCAGGTCCAGAAAAACCCTGATGATATG
TTACAACAACCACATTTACATTTAAATTTTCTTTTTCTAACATATCAAGGAGCATTTTTGCAGTT
TCGCCATATGAAGAAGCTACAACAATGTCAGAAATTCCAAGTTTTGCCCGTTCTATCGCATATTT
TAGTGTATTTTCGGTATTTTTAACGCCAGCAAATTCAAAATACTCAGTCACATTTTCACCCATTT
TCATTTTAGAGGATATTCCATTAAATCTTCTGCAATTAAAACATGAATTTCCGGATTTTCTGCTT
TAAATTCAACAATTTCATTTTCAAAAGCATTTAAATTATCATACCTTGCAGATATATGGGTCAAT
AGTACTGTACTAACCCCTGAAAGTTTTGCAATATTTAATGCATCTGTTGCTGTTGAATGGAGCGT
TTCTTTTGCATTTTTGTCCATTGTTTTGTCAAATGTAGCTTCGTGAATTAATGTATTGCATTTTA
ATTCTTTTAAAAACTCTGCAAACTCAGTTAACGGAATGGTGTCACCACTGTATCCAACACATATT
CCTTTTTTTGGAGGAAGCGTTACATCATTTAGGGTAATTATTTCCCCGTTAAGTTCGACATTAAA
ACCATCTTTTAACCGTTTTAAATCAGGACCGATTTCAATTCCCAATTTTTTAACTTTTTCCAAAT
CCATTCTTGGTTTTTTAACCTGTCTGAATACATATGCAAGTGCAGGAACTGAATGGACCACAGGA
TAACTAAAAACTTCGTAATTATTTGTAGAAATAATTCTTTCAGGCGTTTTAGAAGAAATTTCATG
AACATTTATTGGATAATCAATTGAGTGGTACCCAACACCCAAGATATGTTCAATCATTTTCACAG
TTTCTGCTGGCCCGTAGATGTTTAATGGTTTTTTACGCCCCTGAAATGCAATTGATTGTAAAAGT
CCAGGGAGTCCGAGCACATGATCGCCGTGAAGGTGTGTAATAAATATATTATTAATTTTCATCGG
AGATACATCTGTAAAAATAATCTGTCTTTGTGTATTTTCGCCACAATCAAACAAAAATATTTCCC

CGTCGAATTTAAGGGAAATCGAAGGGTGTGCTCGATATTTTGTAGGTATCGCAGCCCCGGTTCCA
AGAAAAGTTATTTTCATGTTTTCACATATATTTTATCAATTACTTTAAATTAAATTGTTTATCAA
TTCGTTTGCGTCATTTTCTTCTATTTCATCTACCCATTTGCAAACGTCGCGACTCCAGTCATTCAT
GGTTTTTTTAATCTTTTCTTTGACATCTAAAAAAGGAATGGCTTCATACTTGTAGTAATATCCGC
CTTCTTTAATATTTATCTGTTTTCTAAGGATAAGACCTGCATTCATCAGACTTTGTGCTGATCGT
TGAATTGTGCTTCTCTCCCTATTTAGTGATTCTGCAATTTCGTTTATTTTTGCAGGTTTACACTT
TAAAATTTCGAAATAGACCGCAACATCAAATGCTTTCAGTCCAAACACGCAACACATAATTGTTT
CAAGTGTAAATGTGTTGCAGGGCATATTAAGTACCAGTTTGCTCATTGCACCACCAAAGAATATA
ATCTATGAGTTCCATTATATAGTATGAATTCAAAATAATAGACTGCATTAAAATAATATAAATA
GGACATTCCACGCTAGTTCATAAATACAGCGGATGTGTCACAAATACACATTTCTTTATTTTAAT
TATATATTAATCTATCGAAAAAGAGAGGGGAACGTTTATGAAAGTAAAAGAGTTAATGAACCCG
ACAATTTTTACAATTGACAGTGAAAAAAGTCTTTTTGAGGCATTTAAAGTAATGAACCATAAGG
GCGTTAAAAGGGTTTTTGTAAGGATAGATGAAAATATCGATGGAGTTATAACTTACAGGGATTT
GGCGCATCTGTTCTTTGAAAAAGGGGTTTTTGAATTAATGGATGTAACACTAAAAGATGTTTCTA
CAAAAGAAATATTAACGATAGATGAAAATGCAGATGTGACACATGCCGCACAGATGATGCTCCAT
GCAGATGTATCTGGCCTTTTGGTAATCGATGAACAAAAAAAATGCAGTTGGAGTTATATCTCAAAC
GGACATACTTCGAGCACTCGTTAAAGGATAAAAATAAGGTGTATTATGGATTTTTACCAATTTGC
AGAATATATTGTACTTAACTATGGATATTTCGGAATATTTTTAATTGCATTTACTGAGGCAGTTA
TCCAGCCAATTCTACCAGATATATTTATTATTGGCGCATCGGCTTTCGGATTAGATTCCGTAACG
TGCGCCCTTGTAGCTTCTTTTGGATCACTTACTGGCGGATATACGGGCTATTTTCTTGGAAAAAA
ACTTGGAACAAATGTATTTTTGAAAATTTTCAAAGAAAAATATTTTATCAAAGGAAAAGCATTT
TTTGAAAAATTTGGTGTATGGGGCGTTGCAATTGCAGGTTTTACCCCGATTCCGTACAAGGTTTT
TGCATGGCTTGCAGGAATATTCAAAATGAATCTTGTTTCATTTGGTGCGGGAACACTTCTTGGAA
GGATTCCAAGGTTTTTACTTGTAGCCTATTTTGGATACAGTTTAGGACTGTTATTTGGCCTTCAC
ACACCCTAATTTACTTAAAAAAATGATTTAAATTATTACATTCCTAAAATTTCCCTATCTTCGGT
ATTTTCAAGCATCACCGAGATGTTTAAGAAATCTTCAGCCCATTGTGAACTCTGAGTAAACGAAT
AAACGATATCATTTTTTATCCAATACATTGTAAATATTGCCAATTTTCCGATATATTTATCAAAA
GATCTTATTTTATTGTTAAATTCAACGATTTTTTCATCAGAATCCGTTGTTGGGATTATGTTTGA
AATGATATGCGCATCTTTAAAAATCTCTTCTCTAACGAATATGATAGATGCTTGTTCAAAAATTG
CGATTTTCAAAAAATCTTTCCAGTTATCGCCATCCCAAATTATATTGGGTAAATCCTCAGGAACG
TCTTGAGTTGGGTAAATCTGAAGTTCATTTTCTATTAAATACTTTTTTATTTCCAGGGTTTCTGT
TAATTCCATAAAATCCCCCATAACATTAATTAAAAAATAAATTATGAATATATTATAATTTCATT
TTTTTCAAAACTTCTGCAACTCTTTTTCCAGTATTTCTTGAAGTTTCAAGTCCAACAGTATCTTCT
TTTGTATCTTCCGGAGATTTTCCTCGACCGGTTCCACCGTAGTGAGCGGTAGGCGCGCTGTCTCCC
ACAATAATCATTGATTGTATAAAGTAAAAGTCGTGAATCTGTCTTATTGTAGTTTCTTGACCGCC
GTTTCTTGAAGCACCAACTGAAATTGCAGCACCAACTTTGTATTTTAATTTAAAACCTGCCCTAA
GTGGTCTTGACCTATCGATTAACATTTTTGTCTGTGAAGAAACCCCTCCAAAGTAAACTGGAGAT
CCGATAATTATACCATCAGCATCTTCAATTTTTTCCAAAATTTCTTCCATATCATCTACAATAAT
ACATCCTTCGTCTTTTTTGCACATGTCGCATGCAATACATGGATTTAACTCAAGACCTGAGAGTG
AAATAAATTCTGTTTCAAAACCTTCATCTTCGGCAGCTTTTAACGCTTCTTTTACGAGAGTACCC
GTATTCCCATTTTTTCTTGGTGAACCGCTTATTCCAAGTATCTTCAAAAAAATCGCCTCTTCGCTA
ATATCTTGTTAAAAATTAAAAAAACTTTAATGAAAAATAGAATAAATGCTTATCGGTTATCTTT
TTGATTTAAGTTCTAAAAGGGAAATTCCACCAACGCCGACGTTTTCATCCGCATTTTCCTTTATA

AATACGATAAATTTTTCTTCTGGCAGTCCAATTATTTCGCTTGCAGATTTTGTAAATTCTTTAAT
TAATTTTTCTTTCTGCTCGTTATTTACTAATCCAGCTTCTATTGTTATTACAGGCATTTTTTCACC
AGATTTTAGTTTTAAAGATTATTGGTAATAATTAAAGTTCATATACTGATTTTGCTTTTAAAAT
TACAACTGATGGATCCATTAATCCAGTTTCAACCACATTTGAACAGGATGCATAGCCTAAAAGTT
CCACTTGAACTTTACCAACTATTGATTCTATTTCGTCACAGCTTCTCGAATATCCGTTTTGATTAT
TTATAGAAATATTCTTCACCTGGACAATCAATCCTTCAGTTCCGGAAATTACATCTTCTTTTGAA
ACATTTGTAATAAACAAGAAATCTCCTACATTTAAAGATTCCAATGCTAACAAATTACAAATATT
TTTCAAATTTAATGTTTTAACGTGGTTTTTACAATTTCAGAAATTGCATAAGGTCCCACTCCCG
TCAAAGCTAATGCTTTCATGATATCACTTTTTTAGTTTTACATATACATAGGATTTTCAGTTTTT
GGCTTTGACATTAATTTTGCATGTTCTTTTGCAAGGCTTTTCATTTTTTCTTCGATATTTTCAGC
TTCTTTTATCAGTTCTTCAGTATCTGCATTCAGATTATATTTTTTATTCAAATTTTCAATTATTT
TAGAAGCTCCTCGCGGATCCGGCCTTAAACCGACCGTTTCGGTAATTAAACATGCAGCTGGAATA
TCCCTATCATTACAGCACTTCATGAGTGCTCCTGAAATTCCCCCAATCATTCCAAGTTGCAGTGCA
GGAATTTCTTCATCCTTAACTGAATTCAAGATCTTTTCAGACGACGAAACCCAAAACGATTTTTC
AGGAGTCATCGAAGCAAATCCTTCCAGCGTTACAACCATTTTTGGATTTATTTCTTTTAAACGGT
CAGATATCATAACTGACATAGGATATACTAATTCAGGAGGAATCATTACATCTGAAAAAAATAT
TACTAAATCATCACGAGCATACACCCTAACTGGAGGGTATGCAATTCCTTCTTCAACAATCATTA
TTTCTGGAATCATCGGGTCTTCGATGTACCCTACATATTCCATTTTTAGGTTTTTTAAAAGATGG
TATGAAGCAATGCTTCCAACAAGGCCAATTCCTGGAAAACCACTAATTACGAGAGGTTCTTTATA
CTTTATTTCTTTTTTAGAAACATATTCCATAATTATCCCCCGAATTGATAATGACACATTTAAAT
ATGTTTTCAGGGTTATATAAATCATACTAATCATGTTAGCGTGAAAAAAATGGATGCACTAATCA
TGGCGGGCGGAAAAGGAACGCGGCTAAAAGAAAACGTGGAAAAACCAATTTTAAACATCTGCGA
AAAACCGATGATCGATTATGTTATCGACTCCCTTTTAAACTCCAAAATCGAAAAAATATATGTCG
CAGTATCAATTCATACCCCTAAAACAAAAGAATATTTAGAAAAAAAATATCGTTTAGACCGAAA
TTATAATCCAAAGATAGATATAATTTGTACTTCGGGAACAAATTATGTCGATGACCTAAATGAAT
GTATGGAATTTTTTAAAGAACCATTTTTAATTTTATCAAGCGACATCCCCACAATTAAGCCGAAA
GTTATTAATAGTATAATACAGGATTATCTATTTGTTAACAATTCTTGTAATCGCATAGAGTCCTT
TTGTGTAGTCACAAAAGATGATGACTATGTTGGAACGCCCTCGATTAATATGGGCGGATACATAC
CTTTAGGTATCAATATATTAACTCCAAAACATGGTGAACAGACCGAAATTCTCCATGTTATTAAA
GATATTATTGTAAATGTCAATACTCTCTCCGATAAAAAACTGGTTGAAACTTTAATTAATAAGGT
GATTGAATGAAATTATCCTTTAAATCATTAAATGGCAGATCCATAGTTGGCAGTTTAGGTAGTAT
CATCGGCACCGTTGATGATATCGTAATTGATGAAAAAACAGGTAGAATAATTTCATTAAATATCG
AACCTTCAGAACAAAGCCCTATTTCCCCATCATCAGAAAACTATACTTTCGTCCCATACAGAACA
GTAACTGCAATTAGAGACGTTGTTGTTATTGACGAATCAAAGATAAATGCTAAAGCTTAAATTA
TTTCTCATACTATTTTTAAAAATGATTTACCAAAATACCCATATTATATGGTGGTTTTAATGAAA
TTTACAAAAATGCACGGTCTAGGCAACGATTATATTTACGTAGATGCAATTTCACAAAAAATTGA
AAATCCAAACGAAATTTCAAAGTTTGTAAGCGACAGGCACTTTGGAATCGGTTCAGATGGACTTG
TATTAATTCTTCCGTCAGACAAAGCAGATTTTAAAATGAGAATGTTTAATTCAGATGGATCAGAA
GCTGAAATGTGTGGTAATGCAATTAGATGCGTTGGAAAGTTCGTTTATGATAAAAAAATGACGG
ATAAATCAACAATTACTATTGAAACACTTGCAGGAATAAAAGTTCTTGAAATGACCATTGAAAA
CAGTAAAGTAGTTTTAGTAAAAGTTGATATGGGAGAACCAATCTTAAAAGCAGAAGAAATTCCT
GTTTTAAGTGAAAAACATCCCGTAATTGATGAAGAAATTACTGCAAAAGATTACTGCTATAATTT
TACATGTGTTTCAATGGGAAACCCCCATGCAATAACATACATTGAAAATGTAGACGAATTCCCGC

TTGAGAAAATAGGCCCGTTATTTGAAATCCACGAAAAATTCCCGAGAAAAACAAATGTCGAGTTT
GTTGAATTAATCGACAAAAATACTGTTAAAATGAGAGTTTGGGAAAGAGGAGCTGGCGAAACAC
TCGCATGTGGAACTGGCGCTTGTGCAGTTTTAACTGCATCTGTTTTAAAGGATACGTGGGTAGA
AAAGCAACAGTCAAACTTCTCGGTGGAGATTTAACTATCGAGTGGAATGAATTTGATAACCACAT
TTACATGACTGGACCTGCAACAACAGTCTTTGAAGGAGAAATTGACATTTAATTAAAATTAATAA
TTAAACTTAAAAATTAAATTAAAAAAAAGCTTAAAATTTTATTTTTTCAATTAAGTATTCTTCA
AAAAATTTATTAATTTCTTTTTTAGAATATCCGTGTTTTTTTGCAACGGCATAGACCATTTTTTC
GCTTCCGCTACCATATTGGGCCGCTTTTTTTGGCCTTTGTGCAATGTAATCGGGAACATATTTTG
AAGCAATGTCTCTTAATATCTTTTTTCTTGGCTCTTCGATTTTATATTCGACAGGAATTGATAAT
CCAACATCAATTACAAACTTATCTAAAAACGGAACCCTTAATTCAACACCGTTTGCCATTGTACA
GTGATCATCCCTTTCAAGATTGACTTTATGAATGTCAAAAACATCTGAAATTATTGAGTTTTTAA
GGCCCTCTTCTCCTTTTTCATTTAAAATTCTCTGGTACCTATTGTAACCTGCAAATAATTCATCAG
CACCTTGGCCTGAAAGTACTACTTTTATTCCATCTTCTTTTGCCATTTCTGAAGCTGCAAATATCG
GAATTCCAACACTTAACTTCATTACATCGAGTTCATCTATCGCTTTTGCAACTTTTATCACGTAT
TCTTCAAATTCATCTTCTAAAATTATTTTTTTTCTAAATTTAAGCCCCATATCTTTTGCAGCCCTT
TCAGCATAAATTAAATCTTCAGAATTTTCAGAACCCACAGAATAAAGTGTAACGTCGCAATTTTC
AGATGCAAGTTTTGAGACAAGTGTGCTGTCAACTCCCCCAGAATATATTATTCCAACGCGTTCAA
GACCTTTTACCCGTTTTAAAACCGAATCTAAAATTGTAGTTTCAAGTTCATTTTTACATAATTCA
TAATCATTTTCTTCGAAATAATCAGGGTTTACTTTTTCAAGGTCTTCTTCAATATACCATGAATT
TTCATCTAGTTCATAAATAAGCCTTGAATTAGGATTAAGTCGTGATATATTATAATTAAAAGCGC
TTTTGTAATCCATTTCATTAATTTCCATTAATAAACAATAAAGAGCTTTTTTTCTGATGCAAAC
GCAAAATATTCATCCGTATCGATATAATAAAGCGGTTTTACGCCCATTAAATCTCTTCTTAGTTC
AATAATATTTTTTTCTTTATCATAAATTGCGTAAGCGTAATCACCATCTAAAACGTCAACGAGTT
CATCCTCATAAGTATGAATAATTGCCTCTGAATCAGTATCGGTTTTAAATTCGTGTTCTACTGAG
AGTTCATCCCTCAATTCAACGTGATTGTAAATTTCACCGTTACAGATAATCCAAATAGATTCGTC
AGTATTTGGAATCGGCTGAACCGCAGTCCCGACGATAGCAAGCCTGTTGTGGCCCATTGCCATTC
GAGAAATTGTTTCAGTATTTTCGGTAATATCATCAAAATTTTCAAAATAGAGAACTTCATCATCA
AACATCATTCCAGAATAATCCGGACCTCGATGCTTTAATATTTTCATCATGTTTATAACATGTTT
TTGGATATTTTTCAGCAGACTACGGCTCCCCGAGCCCTCCTCGTCCTTTGCTATAATTCCACTTAT
CGAACACATTTCTACTCACATTTATCTTTAAAAAAAGTAATTTTTATTAATATTAATATTTCTTA
ATATATGTAGTTTAAATACTTAATCCCCAATTGCAGATTTATTATAAAATAGCACCGCTTGCATC
TCTTTTGTATTTTCCAAATTCGCTTACAAATTTAAGTTTATCGCTCCAAAATACAGGGCCATCTT
TACAAACACAGAGTCCTTCATCATCAACAGCACACTGGCCACAAATTCCAATTCCACATTTCATG
TATCTTTCAAGTGAAACTTGGATTGGAATATTATTGTTTTCCGCAATTTCTACAACTTTTTTCAT
CATTATTTCTGGACCGCACGTAATTACCATGTCAAATTTTTCTTCCAAAAGTAATTCTAACATTT
TTTCAGTAGTAAATCCACCAAATCCAAAACTGCAGTCATCGGTGCATGCAAAAGTTTTTCCACAT
TTTTCAAATCTATCTAAAAATAAAAGTTCATCCTTAGTTCTTCCCCCAATAACAGAGGTAATTTC
AACACCCAATTTTGAAAACGCTTCAACTGCGGAAATAACTGGGGCACTTCCAATTCCACCTGCAA
CTGCAAGAATTTTAGTGCCAATACACTCAAAATTATTTCCATAAGGGCCCCTTATCCCCAAAAGG
TCTCCTTTTTTTTAACGAGTGAATTTTTTCAGTAAATTTACCTACCTTTGCGATACTGATTGAATT
TTTACTTGAAAATCCGAATGGTTTTTCATCAATTTCAGGAATCCATACCATTGCAAACTGGCCAG
GTTTAAAATCAAACTCATTGTCGAGTAAAAAAGTTTTTACTGTTGGACTTTCGTCTATTACATCA
ATTATTTTACACATAACTGGTTTTTCCATAATAATCACTAAAAAAGTTAAATTAAAAATTTAAGT

TCCTTCTTTCCAGTCGCTTAAATACTTTCTCTGTTCAGGGGAAAGTTCATCAATATCTGCACCCAT
TGAATGTAATTTTAAAAGTGCAATTTTAAAATCTTGCTCATAAGGTATTTCGTATACCTCGTTTT
CAAGTTTTCCCTTGTTTTCTTTAATGAATTTTGCACTTAATGCCTGATTTGCAAAACTCATGTCC
ATTACTTCACAAGGGTGTCCATCTGCACATGCAAGATTTACAAGTCTTCCTTCACCAAGCAAGTA
TATCTTTTTATTTCCAAGGTCGTATTCTTCAATATTGAATCTTGCTTCTTTTACAGATTTTGAAA
GTTCTTTTAGATCATTTTTATTAATTTCATTATCAAAGTGTCCTGCATTTGATAAAACAGCGCCA
TCTTTCATTAATAAAAAGTGTTCCATTCTTAAAATATCTTTACAGCCAGTTGTTGTTACAAAAAT
ATCTCCAATTTTTGCAGCTTCTTCCATTTTTAATACCGTAAATCCATCCATTTTAGCTTCTAATGC
CCGAATAGGATTTACTTCAGTAATAATAACATTTGCACCATGTCCTGCAGCTCTTGATGCAACTC
CTCTTCCGCACCAGCCGTATCCACCAACTACAACATTTTTACCTGCAATTAACAGGTTTGTAGTTC
TTATGATTCCATCCATTGCACTTTGACCTGTTCCATACCGGTTATCAAAAAGGTGTTTTGTATAA
GCATCATTTACATTTACAACAGGGAATTTTAACGCTCCTTCTTCCGCCATTGATTTTAATCTAAT
TATTCCAGTCGTAGTTTCTTCACAGCCGCCCATTATTTTTCCGATTAATTCCGTCCTTTCGGTGTG
AATTAAAAATATTAAATCTGCACCATCGTCAATGACAATATCGGGGTTTGAATCGAGAACTTTGT
TTAAATTTTCGTAGTATTCTTCGTTGGTTTCTCCACGCCATGCATAAACTTCCATTCCTTTTTCAA
CACATGCTGCTGCAACATCATCTTGTGTAGAAAGTGGATTACATCCTGTAATCACAATTTTTGCA
CCGCCTTTCAGGAGTGTTTCTGCTAAAATTGCAGTTTTAGCTTCTAAATGCAGTGCCATTCCAAT
TGTAAGTCCTTCGAAAGGTTTATCGTTTTTAAATTCTTCCGCTATACGACAGAGAACAGGCATAT
GTCGCTTTGCCCATTCCATTTTTAGGTGCCCACTTGGAGCAAGACTCATGTCCTTAACATTACTCA
CTGTTTCACCATAGTTATAAAAAAATCTTTAGCAAATTCTTTAGCAAATCATATTCGGTTTCATA
CAATATAAATTTTAGTAAGAAGTCTCTATTCTTTACATTCCTTTTTATATAATTTTATCAGATTT
CTATTTGATTTTTTATAATTATTATAAGGGAAAAACATGAAACTAAAAAAGAAATTTCTGGAAA
AGTCAAAAAAAGTTGCAGAAGAACGAATCAATATATTGATGAATCTGGCTGAAAAAGAATCAAG
CTCTGGAAAAACTGAGCGTTCAAAAAATTACGTACTTTTGGGCAAAAAAATTGCAATGCGAATGA
GAATGCCCTATCCAAAAGAATGGAAAAGAAGGATTTGTAAAAATTGCGGTTCTTTTTTAATATA
TGGTAAAAACGCAAGAGTAAGAACAAAAGCGAAAAACTACCCGCACGTTGTAATTACCTGCCTTG
AGTGTAACAGCATTACAAGAATTCCAATAAAAACGGCAAAAAAATAAACATATTTTTTGAACAA
TACGTAAATTTTTAGTAATAAACATAATTACCTACACAAAAACAATATAGTATATAATCGGCAA
AAAAATTCTTACATTATATGGAGCCGATAATCATGGAAGTTTTTGATAGTAGCGTTGAGCGATTC
GAAAAAATAAAAGAAAATCAGCAAAACCAAGATAGCGATTTTAAAAATGATATTAAAGATAACG
AAAATTCCGAAGATTTATACAATACTGAAGAATTTACGTGCGAAGAACCTGATTTTCCAGAATCT
GAATATGATAACCCTGAAAATGAGGGCTTTGAATCTCTTGCTCATAATCTAAAAAATTCGATGAA
CAATATTTTCAACAAAATCAAACTCGCAATGGATAAACCAGCGGTTTCGATTGAAAAAATTGAAA
AAGGCTCTGAAGGAAACAATAATCTACTAGAATCAATTAATAATAAACTGGATATATTAATAGC
TTCAAATAATGAACAGACATCTAAATTAGATGTAATTACTTCAGAAATCGATAACGTATTATCAA
GTATTGATAACAATTCTGAAAAAATAATCGACGCATTGGATGCCCAAGATGTCAAAATTTCAGGA
CTTTTAAACCTTCTTCAGGAAATTTACGAAGGAGTGCAGGATATAAAATATATTTTAAAAGAAT
TTACTCCTGAAAATATAGACAGAGCTTCTGGTGTTGTAGATAATTTAAAATGTAAAATTGATAG
TTATATGGACAATATTAACGAAAAAGCGGAAGCTTAGGTGGAAATATGGTTAAAGTAGCAGTTA
CAGGAGCACTCGGTAGAATGGGAAGCGGAATTATAAAAACAATTACTGAAACTGATGGATTAGA
TGTAGTTGCAGCAATTGATATTCCAAATCACCCAAAAAAAGGTCAAGATGTTGGGGAATTAACGG
GCCTTGGAAAAATTGGTGTAGCTTTAAGTACTTCAGACGAACTTGAAGCTGTTTTAAAAGAATCA
GGTGCGGAAGTATTGGTTGATTTTACTGCAGCTGCCCCTTGTGTTCAAACCGCAAAAACAGCGAG

CAAACTTGGAGTAAATTTAGTTATTGGAACTACCGGATTTACGCCTGAACAAAGAGCTGAAATGG
AAGATGCAATTTCAAAAAACAAAGTTGCTGCAACCATTTCACAAAACTATGCAGTTGGTGTAAAT
ATATTCTTTAAAACCCTTGAACTTTTAGCACAAAAACTCGGGGATTACGATATCGAAATTCTTGA
AATGCACCACAAATTCAAAAAAGATGCACCAAGCGGAACTGCATTAAGGGCTGCTGAAATTATTC
AAAATAACTTAAACAGAGACTCCAACGTAATCTACGGAAGAGAAGGAATGACTGGAGAAAGAAC
AAAAGAAGAAATCTGTATCCACGCATTAAGAGGTGGAGATATTGTTGGAGACCACAGCGTAATA
TTTACAACAGAAGGAGAAAGGCTTGAACTTTCACACAGGGTAACCAGCAGACAGTCCCTCGTTTC
AGGAGCAGTCCTTGCAATTAAATTTGTTGCTCAAAAGAAAGAAGGTATTTACAATACTTTTGATG
TTTTAGACCTTAATTAGGTGAAATTTTGAAAGAATTTATAATTAAAGCTAACAAAGCCGTAACT
AATGGTGAAATTAACTTAAAAGATCTTCCGGGAAGTTCTGGAAGATTGGATTTGCTATGTCGCTG
TGTAAACAGCGCATTTTTTCTTTCTCACGATATGAGAAGAGATACTATTTTTTACAGTGTTAACT
ATGGATCTCCAAACCCTCCAGTTGCACTGAAATTCGTTGGAAGTGAATTAAAAAGAGTCAGTCCT
GATGAAAGGAGTATTGCACTTTTTATCAAAAAAGCACTTGAAAAAGATGCTTCTGAACTCTGGAA
AGAAAGTACTTCTGGAATATATTCTTCTAAACGCGAATTTAGAGATATTATTTTGGAAAAGAAA
AAAGAAGGAAAACGAATTTTTTACCTCCACTTAAACGGAAAACCACTTGAAGATTTTGAATTTAA
AAATCAAGATGATGAAGATTTTTTATTCGTACTTGGCGACCATATCGGAATTGGCGATAAGGACG
AAGAATTTTTGGAAGAAATTGGTGCAGAAAAAATATCGCTCTCACCCTTAGAATTACATGCCGAT
CACTGCATAATTCTTGTTCATAATATATTAGATAGGTTAAAATGAGGTTTTTAGCATGGATGATG
TTCCAAAAGTAGTAGTTTTCAAATTAGCAAGCAATGAATATTGTTTAAGAGTAACTGGTGTTAG
AGAAGTTTTAAAATTGCAAGATATTACTTCAATTCCAAATACGCCTGATTACATTGTTGGAGTTA
CAAACATTCGTGGCGAAATAATGCCTATCATTGATCTAAGAAGAAAATTAAATCTTTTTGAAGAT
TTAGGCGACGAAGGCGTTGATGAAAAGCTTGTAATGGTTGTAGAAATTGATGGCGTTCCAATTGG
AATATTGGTCGACTCCGTAAGTGACGTAATGCAGATTTCAGCTGAACAGATCGAAGACATCGAAG
GAATCAAGAAAAATGCAAGCGGAGAATACATTGAAGGCATTGCAAAAATTGGAAATAGATTAAT
CATTATTCTGGATATCAAAAATTTAATTGACCCGCAAGAATTTTAAATAACCTAAAAAAATCCCC
TAATTACCAAAAACAAGTGAAAACATGAGTAAAATAAAGGTTCTGATTGTAGATGATTCAGCAT
TTATGAGGAAGGTTTTAGAAGACATTTTAAAATCCGATGACGAAATAGAAGTTGTTGCTACCGCA
AAAGATGGAAAAGAAGCATTTGAACTTGTGAAAAAATTAGAACCAAATGTAATTACCATGGATG
TTGAAATGCCGATCATGAATGGAATTGATGCTACAAAACAGATTATGGCATACAAACCTACCCCC
ATACTCATGCTTTCCGCAGTAACAAAACAGGGCTCAGAAGCTACATTAAAAGCACTGGATAATGG
TGCGGTTGATTTTATAGAAAAGCCATCGGGTTCTGTTTCACTGGACATTAGAAAAATCGGGGAAA
AAATAATTAAACAGGTTAAAGATGCTTCAAAATCTAAAGTAAGAATTAAATCATCCCGAATTTT
AGAAACTATTAAAAAAGAAAAACAGGAGGAAAGCAGTACTCCAAAACCGCAGGTTGAAAAAACT
TCTGGACTTTCTCCTGAAAAATTAAATGATACTGCAATATTAATAGGCTCTTCAACAGGCGGGCC
ACCAGTGGTTTCAGGCATAATTTCGAATATTCCTAAAAACACACCTCCGATTTTTATAGTTCAAC
ACATGCCTAAAGGATTTACCCGTGTTTTTGCTGAAAGGATGAACAATAATTCTTTAATTACTGTT
AAAGAAGCAGAACATGGTGAAATTGTAAAACCCGACCATGCTTACGTTGCACCTGGCGACTCTCA
AATGATTCTTCAAAAAAGAGGTGGCAATGTATACATCATAATTGATGAAAATATGCCAAAAATC
CACGGAACGAAACCAACTGTGGATGTAACTGCCGAATACGTCACAAAATATTATGGGAAAAACAC
AATTGGCGTACTTTTAACAGGTATTGGAAGAGATGGTGCAAACGGGTTAAAAATGGTAAAAAAT
AAGGGCGGATATACAATTGCACAAAATCAAGACACTTGCGTTATTTACGGCATGCCTAAAACGGC
AGTTGAAATGAATGTAGTCGATAAAGTGATGGATCCAATAGATATTCCTTTAGAAATCATTAAA
TTCGCCAAAAAAATAGGGGGCTGATACCATGGACGATATGGAACAGTATAAAGAACTATTCATG

ACTGAAGCGGAAGAACATTTGCAATCATTAAATCAAAATATGGTTGAACTTGAAAACTGCCCTGA
AGACTCTGAAAGAATCATCAACATGATCTTCAGGTCTGCACACACGTTGAAAGGGTCAGCAAGAA
CACTAGGATTTGAACACATATCCTCATTAACTCACCATATGGAAGATATTTTGGACTACATCAGG
GATGGAAAAATAAAAGTAAATCCTGAAATAGTTGATTTACTCTTCAAATGCCTCGATGCTCTTGA
AAAAATGGTTTCGGAAGTTAGAGACGGGGAAGACACAACAAGTGTTGATGTGGATTCACTCGTT
GAGGTAATTAAATCTGTCAAAGAGAAGTATCTTTCTGGAGAAAACTCTAAAGAAGATTCTTTAG
ATTGTAGTGACATTAATGAAGTTGCTAAAAAACAGGTTGAAGAAAAAACTGTTAACACTACTTC
GTCCAATTCAGGTTCTATCCATTTAGTAGATGAATCCGCAATCATACTTATTGAAGGAAACTCTA
ATAATTCAGTAGTTGAAAAACCTGAAAATTTAGACAGTGTTTTAAATCAAATGCATGAATCAGC
TTCCGCACTCCTAACAATCCTTGATGCCCTCGAATACAGTGAATTAAAAGATACTGTAAAAAATA
TCGATGAACTTTCAACCGCAATGCTAAATGAAACTGTTGAAATAACTGAAAATTCACTTTCGGTT
TTAAAATCAGCACTTTCAACAATGCAGCAGATTGCAAAGGTAGTTGAAACAGGATCCAGTGCTAA
ATACGACTATTCAGAATTAAATGAAAAAATTTCATTGACATTATCAGAAACTGTTGGCGAATCAG
TAGAAGATAAAGAAATCATTCTGGATCATACTATATTTAATTTAGAAGAAGTTTCCGAAGAATT
AATTCCTGAAATAAATGAAAATAAAAAAATTTACCATATAAAATCAAAAGTTGTTGAAGACTGC
ATGTTAAAAGCAGTTCGATTAACAATGGTTCTTTCTAAATTAAAAGAAATTGGAACCATAATATC
ATCAGTTCCTACAGGAGAAAACCTTTTATCAAGCGGTTCTGGAGAGCTGGATGTTTTAATTTCAA
CTGATTTGGATAATATAGATATTTTGGATTCAGTATGTGAAATGGAATATGTAGTATCCAAAGA
AGTCAGATTTGAACTTGAAACAGTAGTAACTCAAGTTGCTGATGAAGAAATCCCCAAAACAGAAA
AAAGTTTGGAAACACCTGCAAAATTCAAAATTAATATTAAAATAGACGAAGAATGCCTATTAAA
AGCCGTTAGAGCATACATGGTTATTAAAGATTTGAAAGAAAAAGGAAATGTTTTGGAAACCGAA
CCCCCTGAAGAAAAAATACAGGAAGGAAATCTCGGCGGAAACACAGTTACAATTTATTACGATAG
CGATGAATCAGGAGATGATATACAAGAAATAATCTCAAAAACTCCCGAAATAACAGAATTTTTG
GTAAATGTTCAAAAAGATACCAATAATTCAAAGGATAGTACAAAAATTAAAAATACGGCAGATA
CTACTTCAAAAAAACAGACTGAAAATTCTTCATCCAAAAAGTCGCAGACTGTAAGGGTAAACATT
GAAAAACTCGATAAATTGATGAATTTAGTTGGTGAGGTTGTAATAAACCGAGCAAACTTTACCCA
AATTGCTACAAAATACGATTTAAAAGAGCTCCATAATGCAGTTGGAAGACTCAACATGCTTGCAA
CAGAACTTCAAGAAGAAGTAATGGGTATGAGGATGATCCCTGTTGCATTTGTATTCAATAGATTC
CCAAGAACCGTAAGAGATACTGCAAAAGCTCTTGACAAAGAAATTGATTTTGAAATAGAAGGTT
CAGATATTGAACTGGATAGAACTGTTCTCGACGAACTTGCAGAGCCTTTAACTCACCTGATTAGA
AATTCTCTCGATCACGGTATTGAGCATGCAGATGAGAGAATAAAATTGGGAAAACCCGAAAAAG
GAATTTTAAAATTAATTGCAACAAGACAGCGAGATAGAGTAAATATTATAATTAGGGATGACGG
TAAGGGAATAGACCCTGATGCTATGCGTAAAAAAGCATTTGAAAAAGGACTTTATTCAAAAGAA
GAAGCTGAAAAATTAACCGATAACGAAGCTGTTAATGTTATCTTCCTTCCAGGATTCAGTACCGC
GGAAGTTGTATCCGACGTTTCCGGACGTGGCGTAGGAATGGACGTTGTAAGGTCAAAAATTGAAT
CACTTGGGGGTAGCGTTACAGTAAATTCCGAAGTTGGAAAAGGTACAGAAATAACCCTGCATCTT
CCATTAACAATGGCAATCATACAGACTTTATTAATCCGTCTAAATGAGCATATCTATGCACTGCC
ATTAACGAGCGTTTTAGATGTTATCTCAGTTGAAAAAGAAGAGATTAAAAATTTAGAAGGGCAG
GAAGCAATAATTTACAGAAACCATATCTTACCAGTAATCTGGCTGAAAGATGCATTGAATACATC
AGGATTTTCCGATTCTAACGACCTCTATGTTGTAGTTGTCGAAAAAAATAAAGGAAAAGTTGGAA
TGATCCTTGATGAAGTTATTGGAAGGGATGAAATAGTTGTAAAACCACTCACAGGAATACTTAA
AAATATTAATGGACTTGCTGGAGCTACAATATTAGGGGATGGTCGCGTAGCGTTGATATTAGATT
TAAACAACATTTAGGACGATCAGCATGGTGTTGAAAGTAAAAATGGGGGATATTGGCGTTGCTA

AAAGCCCTGAATCGATTGAAACTCTGTTAGGCTCATGTGTTGCAATCATACTTTACGATAGGGGC
AAAAAAATTGGCGGTGTTGCACACGTAATGCTTCCAAAATCCAGAAATTCGTTAGAAAAAAATCC
TGGAAAGTATGCAAATACTGCAATCCCCGAATTAATCAATAGAATGGCTAAACTTGGCGCTAGAA
AAGATAAATTAACCACCAAACTTGCAGGTGGCGCTGCAATGTTTAAATGTAATTCAAATACGATA
GATGTTGGCAAAAAAAATATTGAAGCTTCGAGGCAAGAAGTGAAAAAAATGGGCTTAAGAATAG
CTTCTGAAGATCTTGGTGGCGACACAGGCCGAACTATAACATTATCACTTAAAGACGGCAGTGTT
TTAGTTAGAACTGGCAGTGAACTCAAAACTATATAATTATACGGTGATCGATATGAAAATGCAA
AAACCATATATTCCAATATTAATTACGGCAATTTTATCCTCGGCAATCATTTTTATCAGCTTTTA
TGCATTAATGATGGCTTCATCTTTTAATTTAAACATATTATTCGTATTGTTTATTGTTTCAATTG
TGATTTCTACGGCAATTGGATATGTAATTCTCAAAAAAACATATCTTGACAATATAACAAATTTT
TTAGATTCTTCTTCAAAAATAATTGGAAAAGAATACGAACACTGCGATGAAGAGATGGTTGAAA
CAGTAATTTCTAACTTTGAAAAGTTAAAAAAAGACGAAAATTTTTTAAAAACCTACAAAAAAGA
TGTTGCAAATACATTTGCAGAACTCCATGCGATATTTAAAAGTTTAGAACATGGTGATTTCACAA
TACGGGCAAACACGGACAGAAAATATAATGCGTTACAGATAACAACAAATAATGCAATAGTAAA
TGTCTCAACAATGGTAAACGACCTTAAAAAACAGATTGAATATTTAAATGCTGAACTTTTAAAAG
CAAAAGAAGACCTTGTAAGGGCTGAAGAAACTTCAGAACAGGTTGCAGATGCAGCAAGTCAGGTT
GCAACTGCGGCTGCTGACCAATCTGCAAAACTACAGGATGTATCTGACCAGCTTGAAGCAACATC
TGAAATTACTTCAGCAGTAACTGATGCAGCAGATGATACCGTTACTGCAGCAGTTGAAATTGAAG
AAAACTCCGAAATGGGAGTTAAAAAAGTTGAAAATGCAATTGAAACCATGCAGAGTATCACCAA
TGTTATCGAAAACCTTGGAAAAGCAATTCAGGAACTTGGAAATGAATCCAAAAAAATTAACGAA
GTTACAGTTTTAATTAAAGACGTTGCAGAACAGACTGGACTTTTAGCACTAAATGCATCAATTGA
AGCTGCTCGTGCAGGAGATGCCGGAAAAGGCTTTGCAGTCGTTGCAAGCGAAATTAAATCTTTAG
CAGAAGAAATTAAAAAATCCGTTGAAGATATTAATCAGACAATTAACGGTGTAAACAGGAAAGT
TGAAGCTACAATTGATCTAGGAATTTCTGGAAAAGACGAAGTCGATAAGGGAGTTATTGCAATC
GACGAAGTTAACGGTGCTTTCTTAAAGATAAAAGAAAGTGTAGAAACTACATCTGAAAAAAATAA
ATTCAATAAAAGTTAATGCTGAAAAAGCCGCAAACAACGTAGAAAATGCCCTTAGAAATTCACAA
GACATTGCATCAATTTCAGAAGAGTTTGCAGCAACTGCAGAGGAATTAACTGCATCTGCAGAAGA
ACAGAGCAGAATTATGAATGAAATTGACGATTCAATTGAAGAAATAACTAAATCATCTGAACAA
ATCTCAAAAGATGTAAGTAAATTTAAAACAGAATAATTATTTTTTTTAAAAAAAGTGATTTTTT
AATTTATTTTTAATTTTTGATAAATTCTTTCCCTGTGGTTTGAAGGTTTAAAAGCATTTCTTGAA
TCTCCGTGAAGCATTTCAGTTTTTCCTAAAACTAAATATCCATTATCATTTAAAGCTTCGTGGAA
TTTCATAAATAATTTTTCCTGGATATCTTTTTCAAAATAGATTATAACGTTTCTACAAAGAACCA
TATTCATGTCTTTCATGGGCCCTTCCTTAATAAGATCGTGTTTTTTAAACTGAACCATCCTTTTT
ATTTCTTTTTTGATCTGGTACTCTTTATCATTTAATTTATCAAAATATTTATCGATAACATCCTG
ATCCATATTTTTAAATTGTTTTGAAATGTAAGTTCCTTCTTTTGCCCTTTCCAATATCTCTTTGTC
AAGATCTGTTGCAGTAATCATTACTCTTAAAAATTTTTGGTTGTGTTTTTCCATCAATTCTTTTA
TCATAACTGCTAAACCATACGGTTCTTCTCCTGAAGAACAGCCTGCACTCCAGATTCGAATACTCC
TAACTGTCCTATCAGTTACCATCTTTTCAAAGACGTTTCTAAGTTCGTTATAAACTGTCCTGTCTC
TCCAAAATTCCGTTACATTTACAGTAAGTGTATTTTCAAGTTTTTCATATTCTGAAGGATTTTTT
GTTAGATATTCGTAATATTCTTTAAAATCTTTACATTTGCAGAGCCTCATTCTAACCGAAACACG
CCTTAAAATGTAAGTATCTTTGTACTGGTCCACCTGTATTTTTAATTTTGTGCCAATATCGCGTT
TAATTCTTTTAAAATACATGTCATCCATCAAAATCACCAGTATAACATCTGAAAAAATAAAAAGT
CGTGTAAGAATAAAACTTAAAAAAATTTATTAATTATAATTTATCGAGGTTTTCAAGCTTTTCA

GGGTCTATCATTATAAAAATATTACTTTTTATAGGGAATCGTCCAACAATATCAATTTTATTGTC
AAAAACCATTGCATTTTCAATTCCCTTATCGGCCATTTGCAAAACCAAAGATTCTACAATATTAA
ATCCACTGTCTTTCGTAAATTCCGGAGTTGTTATATTTATTTCACTATTTAGTGCATTTGCAAAT
ACATTTAAAAATGAAGCCAAAACCACGTTTGCAACCTCATTTAATACAGATTCTTCGATTTCTTT
GTAATCAGGAAGTTCAGTTTTATTAACAAGCCCATCCCATACAAGATCAAGTAATAAAAGCTTTT
CTAACTTTACAGAATCTTCTTCGTTTATCATCAGGACTGAAGTTCCAGAAAGAACCCCGTCAAAG
TTTATTTTTACCACTTTGCAGGTTTCGTCACTGAACTGTTCTGGAATGTACTCAACAGGTACGAA
TCTAAGTCCTGTAAAATGAATATCTGTATTCTCACAAGTAAGTCCTGTAAACGCGCTGGCAGTGT
TTTCTGCTGCATCTCTTCCGATTTCAACTAATTTATTTATTGTTTCAACTACTCCCAAAGTAACAC
CATGAAAGCCCTTAATTTAATTTTTCAAATAATTTATCAGTCGATTCTGGATCCATTACAATAAA
TACATTAAATCCTGAGCCTATTCCTTTAGAAAATAACTCCGAATTGAATGTAAAAAGTGTATCTT
TGGATCCTATCTCGTGATTTTTTAATTCATATTCTACTAAGTTACTCAACTGCCCTGTGGAAAAT
TCTGGGGGCGTCATGTTAAGAGATGTATTCATGAAATTTGCAATTGTATCTACAAACGCAGAAAT
TAAAAGATTACAGGTTTCATTTATTGCAGAAACTCTCATTTCACAAAAAGTCTCATTAGAATCTC
CTTCCATACCCATTCCAAGAAGCATTGAATCGGCCATGCTTTTTGCGGATTCTTCCGAAAAGATT
AAAACTCCAGTTCCTGCAATATCGCCGTTAAAGTCTATTTTTGTAAAAACGCTATTTTCAGGGCT
AGATACCATATTCATCAAATCTTGTGCGGAATTGAGTTCTATTTTGAAAATTTTAACTTCAACAC
AGTCGCCACTCATTTCCTGAACAAAATTTGCAGCTTTTTCTGCAGCAGCTTTTCCAATATCTTCAA
AAGCTTTTACGTCTTTCAAATCGTAGTTATTTTTAACAACCATTGTTTCCACACCTGCTTCTTCAT
CCACAATAATCTCAAGAAGTTTCTTCCATTCGTTTTCGGAAAACTTCATGGTAAAACCTCTCAAT
TAAGGAAACAATTTGTTAAACTGTTCTAAAATTTTTGGTGCTTGAAATGGTTTTACAATATATCC
ATCTGCACCCGCATTTACAGCATCAATCATTTTTTGCTCTTGATCTACTGAGGTACACATTACTAT
CCTGATTTCAGGGGTTTTTTCTTTTATTGCTTTTGTAGCAGTTATTCCATCAGTTTCAGGCATTA
CAATATCCATACTGATTAAATCAGGTTGTAACTCTTCAGCCATTTTAATTGCATCTGCTCCGTTT
GCAGCTTCACCAATTACAACGTATTTATTTGTTGTTGATAAAATTCGCTTTAAAATGTTCCTCAT
AAATGCAGAATCATCTACAATCATTGTTTTTACAATACTTGCCATTAATACACCTCGATAAATAA
AAACAAGTTTAAAGTTATTTTTAGAATACTACTATTTGTATTTTTTATGTAGGTAGCTTGTAACT
TAGAAATTTGTAATAATAAAGTTTGTAACTGCTCTTCCTTTTTCTGTTAAATCTGTTTCTTTCCT
AATTCTAACAAGGTCTAAAAATCCCTGATCAATCAATTTTTCAAGAACTTTTTCGACTTCATCGA
TATCCATTCCCATCATACCTGGAAGTTCAAGAGACGTGATTCCAGTATATATCGCAGTTAATATT
TCCTGTTCTTCTGGCGCTAACTGTTCAACCTCGTTTTTGAGTTTGTCACCACTCCAACTCTCAACA
GCCATTTCAGAATCAAGATCATCTTCTGATTTTGGAAGTAAACTTTTAATGGTGTTGTGGTATTT
TGTTAATTTGGAAATGTACCTTAAAACATATAGCAGTTTCGTCGAAGGGATGTGAATATATGAA
TTTACTGTTTCTCCGCCCCTCAACTGCTTTATTTCAAGCACTTCTTTGTCTTCTTCGTCAATTTTC
TTAGTTTCAAGCGAAACTGATTCAATATTATTAAATAAATCATAAACTTTTGGTTTATCTCCCGA
AGTTACTGCAACTGCAAGCACCATTTCTTCAATAACAGAAACTCCTTTTTTTACAGGTTTTTTAG
CAAATACAAAGCATCCATCTTCCCATTCTGAAGCTATATCCACAGAACCGCCAACAATTCTTGCA
TACTGTAGTTTTATTTCTTTTTTATTCAATAACTGTTGGAATAATTTTTTTAAGAATGCATTTAT
CGTAGTTTCCCCATAAATGGACGCTTCTGGCGCAAAACCTATACTAAATTTATCTTCATCAGGAG
GATTTAATTTAAAAGAGCCGTAAACTGAAATATCGTCCAGGTTGTTTTTCGCAATTTCAAGAGCT
TTTCTTGGAAGTTCAAATCCCATATCATCTATTTGGTCAAAACTTGCTTCAATTACCTTATTTTC
AAAAATAAATTCTATAGAGTCTTCATAAAGCTGCATTTGAAAGTTTTTCCATTTAGTGAATGGAT
CTCTTAACGTATAAGGAGTTACAATTACCCCTTTGCCCTTAAATGTTGCTATAATTTTAGATTTA

GCCACAATAACACCTTTTGTAAATTAAAAATTAAAATTATTCACAAGTTCGTACAAACAAGATTA
TTATATTGCCATTTATGATTAAAAATGTTTAGCCTTGATATTATAATTATATATTTTAATTTAAA
AATTAAATAAACCATATTTGTTTGATATTTAGTCTTAAAACAATTATGCTACTTTTAAGTTAGTA
GCAGTTCCTATTTAGCTTGAAATACCTTATTATAGTAGTTTTTTAAATAATTTAATAGCACTCAA
TAAAAAATAATGGGAGTATTATGATTTCAGTCGCTACTGCAGAATGCTTTACCCACGGAAAAATT
GGAACTAAAATACACAAAATTGCCTGCGGATATAAAGAATTTGAAAAAGATTCAAATTATGATA
TGATTCACGGGAATGTATATGTAATGGCTTCAATGTTTTTACCCTCGAAAAAAGGAATCGAATCA
CTTTTAGATGTTAACCTACCAGAGCCAGATTATGTTTTTAAATATTCGAAAGCATATAATCAGGA
AAATGATATATTGGTAGCTAAATTAGTTGCAAAAGCACTCAAAAACAAATTAAATTGTAATATT
GCAATTTCGAGCACTGCGGGAATAGGAAATGGTGCAGTTTGTATAGTAACCGATTACAATGATTA
TGTTTTTTCATCAGATATTTATGGAGATTTACTAAAAGGACAGAACATAATTAAAAGACAGGAA
AGTGGAATAGAAAAAGCATATAATACGTTTATTGATATTTTAAAAAAAGAATACAATTTAAAGG
GATAATCTTGATAAACTCTAAAACAAAATTAGTTGGACTTATTGGACACCCGGTAGACCATTCAT
TTTCACCGCTAATGCACAACGCTGCAATAAAAGACCTTAAAATAAATTACAGATACTTTGCATTT
GACGTTTCAGAAGAAAATTTAAAAGACGTTGTAGCAGGTGCAAAAGCATTTAATTTTAGAGGAT
TTAACATTACAATTCCTCATAAAATGAATATCATGAAATATCTTGATGAAATTGATAGTGATGCA
GAGGCTATTGGTGCAGTCAACACTGTAAAAATTGAAAACGGAAAAGCAATAGGGTATAACACTG
ACGGAATTGGTGTAAAAAAAGCACTTGAAGAAAAAACTGGTATTTTGTACGATAAAAACATTTT
AATTATAGGCTCAGGAGGTGCCTCAAGAGCTGTTTCTTTCGAACTTGCAAAGGATAATAATTTAA
CAATTGTAAATAGAAATATTGAAAAAGCAGAAAATCTCTCAAAAGAAATATCTGGAAAGTTAGA
AAAAGAAAATCCCCTAAATTACGGCGGCCTTGATATAAACATTGAAAATTTTGATATAATAATA
AATACAACGCCTGTTGGAATGTACCCACATACTGAAGTGGAACCAGTTATACCATTAAATAACAT
CAAAAAAGATGCTGTCGTAATGGACTTGATATACAACCCCCTTGAACCTGTTTTTTTAAAAGAAG
CAAAAAAATATGGTGCAAAAACCATTAATGGCATTGGAATGCTTGTGTATCAGGGTGCAGTTTCA
TTTGAGACCTGGACTGGAGTAAAACCCGACATTTACGTGATGAAAAAAAGTATTATTTCAAAAAT
TTAAAAAATCGTGGGAAAGAATGATAAAAGAAAGAGTAAAAATGGATGTAATAGGCCTTGAAAT
TCCATTAATCAGTGGAAATGAAGATTACACGTTAGCAGAACTTATTTCCACATACTCGCTTGAAG
ATAAAGACGTAATAGTTATTGCAGAAACTGTTGTTTCAAAAATTGAAAAGAATGTGATTTTAAA
AAATGAAATAACCCCATCCAATGAAGCTAGGAACTTATCAAAAAAACTCGGAAAAGAACCTGAA
GTAGTTCAGGTAATTTTAGACGAATCAAATGAAATTGTAAAATTAGGACCTAATTTTATAATAA
CTGAAACTAAACACGGATTTGTTTGTGCAAACAGTGGAGTTGATGAAAGCAACACGTCAAAAGG
AATAAAACCACTACCTAAAAATCCAGATAAAAGCGCAGAAGAAATTAGAATGGGAATTGAAAAA
ATTACTGGAAAAAAAGTTGGCGTAATTATTAACGACAGTATGGGCAGACCTTTTAGAAAAGGTT
CATGCGGTATTGCAATCGGAGTTAGTGGAGTTTGCGGACTTTGGGATAGAAAAGGAGAAAAAGA
CTTATTTGGAAGGGAATTAAAAACAACAGAAGTTGGAATTGCAGATGAACTTGCAGCAACTGCA
TCTGCTGTTATGGGCCAGTCAAATGAAGGAATTCCTCTTGTAATTATTAGAAATGCGCCTGTCCC
ATTTACGAATGGAACTGGAAAAGAATTAATCAGAAAAAAAGAAGAAGATGTATTCAGATAATTA
AATATTTTTAATTTAACTATTTATCCATGCTTTTTTAATTTTTATTTACTTACCTGATAAGTGAA
AAATACGGGGTAAATGCAATAATTACAATCAAAAATACCATTCTTGATATTTCAGCAGTTGCTCC
AAAAATATCTCCTGTAACGCATCCTAATTTACTGTTACATTTCCATGCGATAAACATTCCTAAAA
ATACTGCCAGAATAGAGCATAATACCCCAATTTTTGGAATCTGGAGCAGTATCGAAACACCTAAT
GAAAATATCAAACCCGTTAAAAGATGGTATTCGTTTGATTTTTTTGCAAAAAACCTGCCGGTTCC
ATCATTTGCAGGAATTCCACAACATGCACAGGATAAAAGCCCGAGCCTTGAACAAGTTTCAATTA

TTATAATGTATTTTAAAAATAATGTGAAAGTGCTTGTTTCAAAAAGATAAGCAAATGAAAATAA
AGAAAGCAGTTCAATAAAAAATACAAACACAAAACCGCCTATTCCCATGTAGACGTCTTTCATAA
CTTCTAATTTTTTTCTCGCATTTCCAGTTACCATCCAGGAATCCCCGTAATCTGCAAGTCCGTCAA
GATGGTGAAATCCCTGAATGTATAAAAGTGTAAACAAAACAGCAGTTCCAATTATATTTCCAGAT
GTAAATCCCAAATAATTCAATATATATGCAGTAAAAACTCCAAAAAGTGAAATTAACGTTCCAAC
AAGTGGCATGAACATGAAATATTTTGCAATGCCTTCAAAGTCGCTTTTTGATCTTCCGACAGGAA
TTCGAGTCATGAAAAGTATTAATCCTTTTAATTCATCTATCATATTATCCCGTAAAAATTAAATA
AGTGTTTTAATTATTTTTGAAAGTTCAGATATATTTTTTATAGAGTAGTCACTTATGTCATCGGG
AATTTCTGAATATTTTCCCTGTAATATTCGAACCGTAGTCATTCCAACGTTTTTTGCAGGAACCA
TATCTTTGTCAGCCCTATCCCCAACATAAACTACTTCTTCGGGTTTTAAATTAATTTTTTTAAGCC
CATAATTAAAAAATTCAATATTTGGTTTTCCAAGCCCGTATTCTTCGGAAGTTATAACTTCATCA
AAAAACGGGTGAATGCCAAGCCTTATTAATTTTTCCCACTGCTTTATGGTAATTCCGTCTGTTAA
TATTCCCAAACTTAAACCCATGCTCCGTAATTCCATTAATGTATTCATAGTGTCTGAATAAGGCC
TAAGTAAAGCAAATTTAACGTTGTGATAAGTAATAATTCCCATGGTGATTATTTTTGGATCGTAT
GTTCCATTAACTGCCTTTACAAGGTCGTTAAAGTGCCCGCCGTAGTTTGAACCTTTTTGTTCGAT
AATCTTATTCAATATTTTTAAAGCATCTTCTTCAGTAGAATTTAAACCTGCATCAATCATCGCCC
TTAAAGCTTCTTTTCGTGCACGGCTTGCAAAACTCGAAGAATTGTAAAGCGTATCGTCAAGGTCA
AAAAGCACTCCTTTAATCATGTTTTCACCGGTAACATATAACTTATAAATTTGCTTTTTTAACCT
TTTCTTCAACCATTTTAAAGATTCCAACAAAATCCTGGTCCATTTCAACAGGAACTACGATTAAA
CCAAGTTCCTGATGTCTTTTTACCCAGTCTTCATCGTATGCTGGAACATCTATTTTTATTGGCTCA
TTTGTAGGATTTCCAACTACAACATTCCTGTGTGGGAATTCTTTTACACCATCTTCTTCATAAAT
TTCAACGGATTCTCTTATATATCCTCCAAGAGACATCGTTAATTTTGGGAGCAGTAACATCTTTG
GCAATATATCACCTTTTGTTTCATATGAATATTATAAGAACTCATTAATATATGCACCCGTTTAA
AATTATCCCAACCAGTTTTCATTTATCGGTTCGTCTGGTTTTTGGTTGTTTGCTCTTGTTTTATT
GGTCCATCTCCAACCGCCTTTTACCCAATCTTCACTTTTTTCATGAATTTTGGCGTGGCTTTTCAG
GAAACTTTCCCATTTTTCCCAGAGTTTGGGATAACTCAGTTTTACTAACTCAATTTCTCCAATTT
CCATTGCAGGACACATGAAACATCCGACTCTATCAAAACATTGCTCGTATAATACATTGTATGGA
GCTTTATGTTTTAGAATATAAATCCAAACATGCATTGCAGTCCAGTTCAATATTGGTGCTGACAA
CATTTGTTTTTTAATTGTTGGACTATTCCATATTCTTGGTTTTTTTGAACGGTTTATAGATTCGT
ATTTTCTAAGACCTACGAAAGATAGACATCCTTTTTCGTATTTTTCATCAATGAGTTTTCCAAGA
GGCGATACCTTACATACTTCACTGCACCATCGATTATCCCTTCCAGGAGGCCCATATTCTTCTAAT
TTTTCCCAGAAATCACCAGATTTGGTTTTTAAAATTTCAAGGTTGTATGTATCTGTAATTTTTTC
AATATTTTCTAAAGTTTCGTTAAATTCGATTCCCGTATCATTGAATAATACATCAAATTCGATCT
GGTCATCCGTATTTTTAAACGCTTCGAGTGCTAAAAGAAGTACTGCAAGGCTATCTTTTCCACCA
GAATAAGCAACAGAAGCAGGTTTTCCAATTTTTACAACAGTATTTCTCATAAACCCTATTGAATT
GCGTTCAATCTTTTCTATGGCGTTTTTATTTGCCAGAATCATTTTTTCAATTGCTTCGTCAAATT
CGCCAACTTCATGCAAAATTTCTGAATTTTTTGGAAGTTCGGACTTTCTTACCTTTGCAACCATT
CCTTTTTCTGAATTTATAATTTGTTCGTAGTCCATTCTGGCTCGGCCAACACCCAAAACATCCATT
TCATTAAAATCTTGGCTTGAATCAGGTTTTTCAATTAGAATAATTACATCGTCATCTTTAGTTAT
ATCTTCAGATGCATAATCAACACCTGGCCTTAAAACAGACGCTCCTTTATTTAAAATAAATTTTG
GAACGTCTTCTTTTACTACAAGTAATTTTTTCTTGCAACCTGCGATGATGAGTCTTCTTGCCCCTT
CAATCGTTGGAATTATTTTCCATTCGTGTTTTTTCTCGTTAAAATTTAAGATTCCAAAAATTATT
CCATCAACAATAATTTCTTGAAAATATTCAATTCCTGGAGCCTTGTTTACCAAAACTAACTTATT

TTTAAACAAATTTTCTTCTATTCCAAATTGTAAATTTATTGTTTTATTTATTAAATCTAAATCAC
CCTTAAATGCAGGCCTTGCATCTCCAGGGGGTGTTACTTTAACCTCAACGGTTTCAGAATCACAA
ATTGCACATTTGGTGTCGAGAACAGGAAGGTTACAGTTTTTACACCATTTTAAATGAATTTTTCC
TAAAATAGTCTTCAATGTATCACCGTTAATTAAAATAAATTAATTTTAAAATTATTCGGGGGTTG
ATTTTAATTAAATTATCATTGTAAGTCCAACAAACATGAGGATGATGCCGAAAAATTTTTTTAA
AAGACCGGCATTTATACGGTTTGAGAGTTTAGCCCCATATCTTGAAAATATGATGCTCATAACCC
CTATCGACAAAACAGATGTCAATGATACGTAGCCGATAAAGTTTAAATAATTATACGCTTGCGGT
ATTGCAACAGAGTTCGTGAAATAACCCAGAGATCCACTCAATGAAATTATTACCATCATTCCAAG
AGATGTGCCTATGCTTTTTTTTATCGGCGTTTTTAAAAATATCGTTAAAATTGGAATTGCAATAG
TTCCACCACCAATTCCAAACATGCTTGATAAAATTCCTATCAAAAATCCGCAAAATATGACTGGC
AGATATTTAATTTCCTGGCTGTTTTCCAACTTAGGGTCCGATTTACTGAGTGCCATGTTTAAAGA
CAGGATTATCAACATAATACCAAATAACATTCGGTGAAGGTCACCGCTTAGGTACTTTGTAACGA
TTTGGACCCCTACAAAGGTTCCCATAATTCCTGAAAACCCGAGCAATAACGAGTACTTCCATATT
ATGTTTCCAAATTTTGAATGTGAATAAGCACTGTTTAGCGAAGTTAAAAAAATAACTGAAAGAC
TGGTTCCAACAGCCATTGCAACGACATAGTCGTCAGATATTCCTAAATTTTGGAATATATAAATC
AATATTGGAACCATTAAAAAACCGCCACCAAGCCCCAAAGCACCAGTCGTAAAACCAACAATACA
TCCAACAACCAATAAAAGTAAATAGATTAAAAAAATCTCCATAAATACACACAAATTATACTTTT
AATTCCTTACAGCACCATCTTCTAACAATTTCTTTCAAAATGTCGTAAGAACTGTGAAATGGGCA
TTTTACATAATCTTCAACTTTTAATATCTCAGGATATAGTGAATGTTCTGCAAGCTGTGCTTTTA
ATTCATTAATCTGGTATGTGGTTTGATCAGGACCCAAAACTATAACATCAGGCCTGATTTCTAAA
ATCGGTTCAAGTTTATTAGTTAAGCTACCAAAAACTGCGCGATCAACAGGTTTTAGTGCTTCTAT
CATTATTTTTCGCTGTTCTTCAGGAATTACTGGACTTCTGCCTTTAATTTTTTTTACTGTTTCATC
CCTTGCAATAATTACAACAAGTTCATCGGCGTGTTTTTTCGCAAAATTAAGCGTGTTAAAGTGTC
CAGGGTGTAGTAAATCAAATGTACCTGCAGTTACTGCTATTTTCTTTTCCATTATATCACTTTGT
AGTTATTAGCTATTTTAAGTTTATAATACTATCTTTGCCATTTTTTACCCTGAATATTGCATCAA
ATGTGCCCATTATTAAAGGACCTAATGCAAAACCCGTGATTCCTAAAGCAAGTGGGCCCATTAAA
AATGCAACGAGTGCAAAAGCGGGATGAATACTGCTTTCATGGTTTACAAGCCTTGGCCGTATTAC
AAAATCAGGTGCAAGAGATAAGAAAAGTACTCCAAAACCAAAAAGTAATATTGCTTTTGAATAC
ATTCCAACTGCAATATAATAAACTGATAAAGGCATATATATGGTCCAGCCTCCAACAATTGGAAG
TAATGTTAAAATACCGCTCAATGCCCCAAGTGTAACCGGATTTGGAAGGCCTATTGCCCAGAATC
CTGCAATTGAAATTAATCCAACGGTAAAGGCAGTTAGTGCATTTACAACAAAGAGGTTTTTGTAA
GCTTCGTGAAGTTTTCGAATAAACAGTTCAGTTTGAACATGGTAAACTTCCGGAACATGGGGCAT
TACTGCGTCTTTAAACCTATAACCATCTTTTAAGAAATAATATGTCAAAAATACAGTAACTAACC
CTTTTATAAATAATAGCGGGAGTCCATAGATTTGGCCTGCCATTTTATTAACTGTCGGCTTTAAA
AAATTCCAAATTTCTGATAATATTGAAGATAAGTCTTCTTTTGCGATATTTTGTAAACCTATATA
ATTTATAAATTGTACAGTATAATCAACAAAACCTGCATAGTCTAAACTGTTTAAAAATTCAAAA
ATATCGGTTAATATTAAAAATACGAGTATTATTGCAGGAACTGTTATCATTAAAATACAGAGTA
TTGCTCCAAGCGTTCTTCCAGTATATTTTTTCAATGCACTGAAAAAAGGCTCAGTCATATAGGCA
AACGCGCATGCAAATGCTATAGTATCTAAAAATGGAAAGGCAATAATCATAACTGATAGAAACG
ATATCAATACAAAAATCCGCATTAGAAATCTATATTCATTTTCTTTCATAGAATCCCCGTTATAA
ACAAATAAAAACAAATATAGGATTAAACCCCATTTAGTTGTAATTAATTATTAATTTGTCAGGG
TGTGATATATGAACATTTTGATTGATGGATCAAGACAAAATTATGAAGAACTTGAAGAATCAGA
ATTTCCAATTTCTTTTGGAATTAATTTACACACCAAACAGGAAACATGGAAATACGATGCATTTG

ATGAAAAAAATCTTTTCTGCTTTGGAAAAGGAATTCTGCCAATCATTGGAGGCCACAGGCTAATC
TTCTCATTTAGGTCTCCACTATGGGATGGATTTCACTTTTCTGCAATGGGCGGTGCAGGATATAC
ATTTAAAGATACTGGAATTCAAAACGTTGCAATTACGGAAAATGTGAAGTTCCAACTGTAATT
GTACTTAATGGGGAAGAAGATAAATTAAAAATAGAATTTATGCCATTTACAGAAGAAATAACTG
ATATTTATGAATTTAACGATAAAATAATCGACTTATTTAAGGAAAAAAATTACAGGGCATTTTT
AGTCGGACCTGCTTCAAAAACTACAAATATGGGTGGAATTTATTCGCAAACCATAAGAAATGGAA
AAATTGTCGAAGGCTCCGAAGACTGGGCTGCAAGAGGGGGAGGCGGTTCAGTTTTATATCAAGCC
CATAATGTTTTGGGAGTTGTATTTTTTGGTAAAAAAACCCCTGAAAAAAATCTTAAAGAGATTG
TTGAAGAACACTACAATAAACCATATACAAAAGTCGTTTTAGAACATACTGAAAAATATAGATA
CAGTGAAGAGAAAAAAACCGGCGGAACATTTGGAAATAACTACCACGTTACAATGGAATTAACA
CCAGTTTTTAACTGGAGAATGCCGTTTATTGATAAAAATAAAAGAATGAAACTTCACAAAAAAA
TAATTGAATATTTTGTAAACCGTTTTGACAAAGAAGCAATTGAAACTAAAAACTGGACTAATTG
TGGAGAACCTTGTCCTGTTGTTTGTAAAAAAATACAGAAAAGGACTTCACGTAGATTACGAGCCTT
ATGAAGCAAATGGGCCATGTATTGGTGTTTTTGATATTTATGCGGCAGACAAGGTGGTCCATACA
ATCGACAAACTTGGGTTTGATGCAATTGAATTTGGAAATTTGTGTTCTTGGACCTTTGAGCTTTT
AGATAACGGCATGTTAAAACCAGAAGAAGTTGGAATTGAAAAACCAGTATTTGATATTTCAAAC
TTCGAAGAAGATGACGACATATTAAAAAATTCAATGCATAATGCAGAGCAGGCCGTAAAACTTGC
AGAAATTATTGCTTTTCAAACAAATGAATTTGGAAAAATCTGTAAATCTGGAACTAGAAGGGCT
GGAAAAATACTAAATGAAAAATATCCTGAAAGAGTTAAGGATAAAAAATTCGAAGACTTTGGAG
TTTACGATTCTTTTGGAGAACGGGGCCAGATTTCTCCAACAATGTACTGGGCAATTGGAAATTTC
ATGCCTTACTTAATTCAAGGAAAATATTTAACACACTACCAGTGCGGGGTCTTTTTAGAGCCTGA
AGAACTTGCAGAGTTGAGTGTTAAAAATTCAATTGAAGAAATTACGCTTGAAAATCTTGGAATA
TGTAGATTTCATAGAAAATGGATAACTCCGATAATTGAAAAACTTGTAAAAGAAATGAGTGATG
TTAATTTACATGAAGAATCTATGGAATTATTCAAAAAAAATTGCAAAATACGATTCAAATATCGG
CTGTCCCGAAATGGAAAGTGAAAGAGTAAAAGAGTTAATAATTGCAGGAGCTTTTGAATTTGAA
AATGAAAAATGGTCAAAAGAATTTGAAAACGGAAATTTCAATGAATATATAAAAAGAGTTCTTG
AAAAATACAGTGAATTGTTAGAAATTGACTGGAAGTTAAAAGAATAATTCTTTTATTTTTACTA
TTTTAAAAATTTAAAAAAAGATAAAATTTATCCATCAATTTTTTTCTTAAGAATTTTAACAACA
GTTCCCGGCTCAGCCCTTCCGCGTGTTAATTTCATAACCTGGCCGACAACAGAGTTTAATGCCCTT
TGGTTTCCAGATTTATAGTCTTCAACTGCTTTTTCACTATTTTTAATAGCTTCTTCACAGGCTTTT
TCGAGTTCGCTTGTGTCTTCAATAACAGTTAAACCCATTTCTGTAATTAATTCTTTTGGAGTTTT
TTCTCCTTTATGTTCAACCATCTGTTCAATGATAGTTTTACCGATTTTCTGGCTGATTGTTTTATC
TTTAATCGAATTAATTAATTCAACCATGTGTTCTGGTTTTAAATTTGTTTCAAAGAATTCTATTT
TGTTGTAAACTAAAACTCTCTTCAATTCATTTCTAATCCAGGTAACTGCGAGCGAAATTCCATTC
TTGTCGTTTCCAAGTTCTGCTACGACTTTTTCGAAAACGTCTGCAAGTTCTAAATCCGAAACTAA
AACTTTTGCATCGTCTTCTTTTATTCCATACTGCTCAACAAACCGTTTTTCCTTGTTCATTGGAGT
TTCTGGCATCTGGGCTTCAACTTTTTCAACCCCAGTTGTTGTTTAAAACAATAGGCTGTAAATCAG
GATCTGGAATGTATCTATAATCATCTGCAGTTTCTTTACTTCTCATTCCTTTTGTAATCATTTGA
CTTTCCATGAAAGCCCTTGTTTCCAGTTTAATTTCTCCGCCCCTTCGAAGAACGTTTTTCTGCCTT
ATTAACTCGTATTTTAAAACTTTGTAAACTCCCCTTATGGAATTTACGTTTTTAACTTCGACCCT
ATTTCCCTGAATTCCGTTGTATTTAATAGATATGTTTGTGTCAGCCCTCATTGTACCTTCGCCCCT
TAAGTGGCCGATGTACCTGAATAACCTCATTAACTGCCTTAAAAATTCTCTCGCTTCTTCAGGGC
TTTTCATATCCGGGTCAGTTACGATTTCAATAAGTGGAGTTCCGCTCCTGTTGTAGTCAACAGTT

CCAAGATCTGGCTTATACTGTCCAGGATCTTCTTCGAGGTGAACTTCAGTAATTCCAACACCTAA
AAATGTTCCGTGTTCTCCGATTGGAACAGATGTTTTTTGATACCCGCTTGGTAAATCAGGGTAGT
TGTAGTGTTTCTCTGGAAATAGATGTCTTTATCAATAACCATTTCACAGTCCAACATTTTTGCA
ACCATTATTGCAACATCTATTGCCTTTTTGTTTGGAGGCATTGGTTTTGCACCAGGGTGTCCAAT
ACATACTGGACAGATGTTCGTGTTTGGTTCAACGTCCTTATAATTTGTAGGGCACTGGCAGAATA
GTTTAGAATTTGTATCTACCTGCACGTGAATTTCAAGACCGCATTTCATTGATAGATCTTCGCTC
ATTTTATTCACCCAAATAATAAAAATAAATTATATTAACATGTTCTAACATGCCGAGATTTTAAG
AATAAACTTCATTTCAAACCTAGATAAATATACTTTACTTTCTTTTTAAATTTAACTATAAAAAG
ATTTTTTAAGAGATATTTATCCAATCTAAAAAATAAAGAGTCAAAATCAAATTTTTAAAGTATT
CTTTCTATTGGAACAACCAAAATATCCCTTGCCCCGAGCTTTTCGAGGTTGCTGACTGTTTCAAA
TACTTTATTTTCATCGATTACTGCATTAATTGCAAGCATGTTGCAATTTGAAAGAATTTCTGATA
CTGTTGGACCGCCCATTCCTGGAATTACTGATGTAATTTCTGAAACCTTGTCTTTTGGGGCGTTC
ATCATTATCAGCCTTTTACTCTGTGCATACATTACACTTTTAATTCCTGAAAGAACCTGGTTTAT
TTTTGCACTTTTTTCTGGGTCTTCCATACTTTTTTTGTTTGCTACAAGTCTTGTTGAAGATGAAA
CAACATTTTCTATTTCTTTTAACCTGTTTAATTGAAGCGTTGTCCCTGTTGATGTTAAATCACAA
ATAAGGTCAGAAACGCCGATAAAAGGAGCGATTTCAGTTGCACCACTTAATTCAATAATTTCTAA
ATTAAGCCCTTTTTTTTCTAAGTACCGTTTTGTAAGGCCTGGAAACTCTGTTGCAATCTTCATTC
CGTTTTTTACATCCTCAATTGAGTTTACATCTGAATTTTCAGGAGCTGCAAGTACGAGCCTTGCA
TTTCCAAATTTAAAATCCAAAAGCATTTCAAGTTCTTCTTCGGTATCCCGCTCAAGCATTAAGTC
ATATCCAGTAACTCCAACGTCAGCTACACCATCTCGCACAAATTCAGGAATATCTTTCGCCCTTGC
AAACATTACTTTTATTTCTGGGTCAACAGTTTTTGCAAAAAGGCTTCTTCCATGTACGCTTATTT
TTAAACCTGATTTTTCCAAAATTTCGTTTACAGGTTTTGAAATTCTCCCCTTATTTGGCAGTGCA
AGTAAAATCAAAATATCACCTATGAATTATGAAAATTAAAAAATTAAACCAAATAATATCAATT
AATCTTAAATTTAGGTACGTTTTTTTGCATAAATATGTTTGCTACAAAAAGGATTATAATTACAG
GTATAAGAACATAATATATATTTTAGGTAATAATTTCTGTGATTAGCTATCGTTATTCTTTTTTA
ATTAATATTTTTAAATTGGAAATAGGACCAAAATTCTGAAAACTATATATAATATGATGATTTA
ACTTAAATCCATAATAAATTTAAAAGGGGGAACCAATGCACAAAGAACAACTAATGGAACTCCAC
CAATTTTTCGTGCACGTTTTTAAAGAAATGATCCCAGAAGGCACAGACTGTGTTTATTTAAAAAC
TTACGAAGAACTAGATGTAAAGCCCCATCATATCCACAAACTCAAAACAGAACAAAGAGCCGCTA
TTTTCTTACTTGCTGCATGCCTTGCAGAAGGGTTATCTCAAAGAGACGATTCAATCCCAGAAAAT
CTTTCAAAAAGATTGTCTGAAAACGCTTTTAAATACATAAACATGCAATCTGAGAAATATCAGAA
TTTAAAGAAGGTCAAAAATTCGATTGATGTTCAATGTAAACACGAAAACGTAAAAAACACGGTT
TAATCTGTTTTTAAATATATTACATAGTTATTTTTTAAATTTTATTTTCAGGTGGATATTTTGAA
AAAATTAAATGCTGATGATTATATTGATATTTTAAACGATGTTTTAAAGGATATCTGTCCAACA
GACTCGGAAAAAGAAGAATTAAAAAATATTTTCGGATAAAATAATCTCTAAAATCAAAGATATTT
CAAAATATCCTGTCTTAGATATAATTCAGGTTGGATCAACTGCAAGAGATGCAAATTTAAAAAAT
GATCACGATATTGACATATTTTTAAGGTTTGAAAAAGAAACCGATAGGGATATGCTAAAAGAAT
TGGGCTTAAAACTTGGAAAAGATGTAATTAATTATTTTAATGGAAAATCATGGATAGAATATGC
AGAACACCCGTATGTTTCTGGAGAAATTGGGAAATTTAATTTAGATATTGTTCCTTGCTATGGTA
TTGAAAATTGCGAAAAAATAATTTCTGCCGTTGACAGGACTCCACTTCATAACGAATTTTTGTTG
AATGCATACGAAAATAATGATTTATCAAATGATATCCGTCTTTTGAAAAAATTTTTAAAAGGTC
TTGGAATATATGGATCAGACCTAAAAACTGCGGGATTTTCAGGATATCTCTGCGAACTTTTAATA
TTAAAATACGGAGGATTTTTAAACCTTTTATCTGCCGTACAAACGTGGAAACCAAAACATTCAAT

AGTTTTAGATGAAATTTACGAAATGTATGATTTAAAAAAAGATCATGATTTTTTAAAATTTAAT
AATTCTCTAGTGGTTTACGACCCTGTTGATCTAAATAGAAACGTTGCAGCAGCATTAAATGAAGA
AAACTTATGTAAATTTATATTTTATTCGAAAATGTTTTTGAAAAAGCCTTCAAAAGAGTTTTTTT
ACGGGTTTTATAAAAAAACTACTGATTTGTTAAACCAGCGAAAGAGGGGCTATTTGATAACATTG
GAAATTTCAAGACCTGAAAATGTTGTTGAAGATGTAATATATCCTCAAATGGAAAAAATACAAA
AAAGTATTAATAAATTAGTCAAAGAACATGATTTTGAATATTTAAGGTACCAAAATTTTGCAGA
TGATAATACCTGTTATTTATCTTGGGAATTTTTAATTCATGAGCTTCCAGATGTAAAATTAAGAA
TTGGGCCTCCAGTATACAGTGAACAAGGAGTATTAAATTTTATAACGCATAACGAACATTATTTT
GTTAAAGAATGCAATGTTTGCGCATACAAAACCAGAAAATATAAAAACATACAAATACTATTCG
AAGATATTGTTAATGGGAAATTAAAGAACATTATAACCTATCCTAAGTACGTGTGTCCAGAAAA
TGCGGAAATACGGTTAGGTACTTTTATAGAAAGGAAATAATTTCGGGAGAATATGGATAGCAGT
GTCTTGATTAAAGAATGCAATGATTTTTTAGATTGTTTATCAAATTTTGGGAAAAAATTAAAAG
ATTTTGACTCCAAAAAAGAAGATAGTGTAAACTTTATATCTGAAACTCTCGAAAATAATTTAAA
AATTTTAGAAAATTTTAGAGAAAAAATGGAACTTCAGGGCTTTGATACCCCCTACATTGGCGTTG
GCCGGTTGAAAGGTGGAGAAGACGATGATATTTACGAAATAATTAATTATTCGTCATATTTAAG
AAGAATGGTCGATGAAAAAAAAGGTGCACTTGAAAGAGTAAAATACGCAATAGTATCCCACAAA
ATTGCTATTGGAAATATTCAAGAAGATATGGGAAACAAAAAAATACTCGCGCATTTATCCTACGA
TGGTTCATACAAAGAATTACTTTCTAAAATTCCTCCTTTTTTTATAAAATCCTACAAAAGGATAT
TAAGCGTATTTGAAACAGAAGGAAAGGGTATTTTAAGTTCAATTACGCTTTCACTCGTTATTTTG
GAAAATGGGAAAAGAAAATTTAAAAGAATAAAAATTGAAGAAGAAGATTACGAAGGATACATCA
AAAAAACCTTTGGCGATGCAATAATCACTTCGATTAAAAAAAATTATTCGAAAAATAAACTTTT
AAACGACCAGTACGTTAAAAAAATACTTTCATTAGCATATTTAAGTGCATGTTCTGACGAAATTA
TTGAAAAAATTGATGAAACGCTAAAAGAAACACTTTCTGATGAAGAAAGATGTATTGTTAAAAA
ATACCGTATGATATGCAGTGATTTTAAAAGTAGTGATTGTGAAAGCGGAGTAATCGATGTACGT
GCAATGGAAGAAATTAAACTTAGAAAAATGAATCTAAAAAATGATTTAGAAGATAGAGGGCTTT
ATAAAAATGGAAAACCTCTGAAAAAACTTAAAGAATCGCTTGAAATGGAAGATGAAATCCTTGA
AAATATTTCATTAGAAGTTCCATTAAAAATTCTTTCAAAAGATTTATTAACGTACTACTTAAAAA
AATCGGCTGACGAGAGAACCAGATCAAACCAGTTCCCATCAATACTTGTCACACCATCCCCTGCAC
ACCTAAACTGGCTTGCTGTAGAAAATATCGCGCCTAAAAAAATACTTGACCTGAAATTTTTACTT
GAAAAAGAACTTCCAAAATACGAAATACCAATAAAAAATCTGGGCGGTGTTTCACTCTACCTACT
TTATGACTGGAATGTTGTAGAATCTTTTGAATTTGAAAAAACGGAAATTGAAGAGATATTAAAA
TTAATGGCTGCAATAAATGATGTTAAAGAATTACTAAAAGATAAAATAGATATTAAAAAGTTCG
AAAAATACAGTAAAATCAAAAAAGATAAAACTAAAAACTTCTTAAACGCTCTTGGAAAACTTTA
AGGTCAAAACAATGATAAACCCAATTTACTTAATTTTAGCAGATTTTTTCGATAGATACATTGGA
GAACCGCCTGAAAAAGTTCACCCCGTTGTATTCATTGGAAAATTAATCAACTTTTTTGAAAATGT
CTTCAAATCAACTAATTCAGTAAATAAAAATCGAGATTTAATTTTTGGTTTTTTAAATGTAGTTT
TAGTTTTAGCAATTGTATTTTTCATGGCTTTTGAAATAGAACAAATTATAAACTCGATTTCAAAT
TCGTATATTAGAATTTCTCTTTATTCAATAATCCTTTCATTTTCAATCGGACACAAATCATTGAT
TGAATTTCAAAATCCCCGATAAAATTTATCGTAAATAACGACCTTGAAAGCGCGAAAAAGTCTG
TTCAGTGTATTGTAAGTAGGAATACAAGTGAACTAGATAAAAAACATATTTTGTCAGCCTCAATT
GAAAGTGCATCAGAAAACATTACAGACAGCATTATCGCACCGCTAATTTATGCGGCAATTTTTGG
ACTTCCTGGCGCATTTTTATATCGAGCGGTTAACACGTTTGATGCGATGATTGGTTACAAATCGG
AAAAATATCTATATTATGGAAAAACTGCCGCATATTTAGATGATATTTTAAATTTTATTCCTTCA

AGAATTGCAGGAATGCTTTTAATTATTTCTGCACCATTTTACGGCGGAAATATAAAATCTGCACT
TTTTGGATATTTTAATGAAGGAAGCAAAACCCCTTCACCAAATTCAGGATACACAATGGCAACGC
TTGCAAACTCTTTGAGTATGGAACTTGAAAAAATTGGATATTATAAACTTGGAAAAGGAGAAAT
TACTGTTGAAAAAGCGCTAAATTCGTTAAAAGCAGTTGATTATTCAGTTTTACTTTTTTTAATAA
TTTATATGATATTATTCATGTAAATTGAATTTAAATAATCTTTTAATTTTTAATTTTAAAAATA
ACTGTTTTAAAAAAGCATTAATATTTAAAAAAAAGTTATAATCAGACAAATTATTTGCTGATAA
CTCTTGTGATTTTGTATCTGTCCATTGCTTCGAAGTATTCTACTTCTACACCGCTGTCGATACCTT
CAACATCAGTAGGCATTGGTAATTCTAATGTTTCGTAGGTTTCCAAGTCCATGATTTGAACGTGT
TCACCCATAAGAGCTAAAACTTGTCCTTTTCTTTTGTCGATGAGTGGTACATCGATTCTTGAGCT
TGCTGGTCCTACGTGTTCTTTTTTAACTGGTTCAAAGATACCCATTGCAGTTAATCTTACTTTTG
CACCACCGTGTTTTCCAGGTTTTGAGTGTGCTGTGTCCATAACTCTGCATGCAATACCATCAATA
ACAACGTACTGTCCTACTTTAAGAGCTCCCAATTCGGAAGGTTTGTTCCTGCCATAATATCACC
ATTTTAAGCTTGTAGTGTTTGAAATTATATAGCAATTATCTCAAATGCTATATAAGCTAGTTAGT
CCTGAAAAAAGGCTTTCGAATAATATATTTTTATTATATATATAATTTTTAGCATTTTAGTTCAA
ATTTACTCATATTTTGACATATACCCTCGCGGAGTAATCATCATACCGTCGATAATTTTTGTATT
GCTGTTTCCAATAACGAGAGTTGTACTCATATCAATGTACAACATAAATTCATCTAAATTATTAA
CTAGATTGTTTATCGTGGTTATTTTATATTCAGAATCGTTTCTTCCGGCATTTTTAACAATTCCA
ACAATATAGTCGATATTTCGGTCTTTAGAATAGTTCGAGATTATTTCAACAGTTTTCAAAAACGG
TTCTTTTCTTTTTTTACCCAATGGATTATAAATTGCAAGAACCATGTCGCTTTCTACCGCGCAAT
TTATTCTTTTTAAAATAGTTTCTAAAGGTGTTAAAAGGTTGCTCAAACTAACAACCGCAAAATCG
TGGTTTAAAGGTGCACCAAGTATGGATGAACAGACACTTGCGGAGGTCAGTCCACTTAAAACCTT
TATTGTAACGTTGTGTAAATTCTTTTCATTTAATTCATATGCAAGAGATGCAAGACCATATATTG
TAGCGTCCCCGTTTGAAACAAGTGCCACATCCTTATTTTCAGCTTCATTTAAGGCATATTCAACCC
GTTCCAATTCTTTAGTCATACCGCTTACGTAGATTTCTTTATCAAATCGTTCAACATATTTTTTG
TATCCTGTGTAGCATACAATTAAATCAGTAGAATTTAGCGCATTTTCGGCTTCTTTTGTAAAATA
ATCCTCATTTCCAGGACCGATACCAACAACATATAACATTTTATCACAGAATCAGTTAAAATACA
ATTATAATTTTTAAAAACAGTTTAGTTAACAATATATTAATAAATGGTAGCTGTGTCAGATAGT
GCTGATGTCTTCATCCCAAAATCATGAAAATCACGAATTTAGTCAGCGGGGGTAACTGTCATCAT
ACAACGTTATTAGGACAATAAAAAACCCAATAATTGCTACCATTGTTACCCGAATACTATTAACA
TGGAATATACTCTGAACATTTATATATTTTGTTAATATTCGGACAATGGCTTACGGTTTTTTGAT
TCCTCAAAAAAATTAAAAACTCCTCCATGATACGATAGTTATTTATAAAAAATAATTTCCAGAATA
ATATGGGGGAGGTATGAGGTGAAATTATTCATAGACATCACCTGTACTTCAAAGATGATGAAGA
TTTTGAATTTTTTAAAAAGATGTCAGAAGATCTAAAATATACTAGCTTTAACGCATTTGCTAAAG
CTGGAATGATCTACATGCACAAAAGTTTAAAAGACGAAACTTTTGACGATAAACAAATTTTAAA
AGAAATTCTTTCGTTACTGAAAGATTTAAAAGAAAATAATTTTGAAAAACCTGTAAAAACAAAA
ACTGTATCTGTAATTGACGACTTTAGAGAAAAACTAGAGAATACGCCAACAGATACTGAATATA
ACTTCATACTAAATTCAGTTTTAAATATACTATCAACTGAATTTCCAAGATGGGTTGATATTGAA
GACATAATCCATAGAATAGGAATTGGAAATAACAACATGGCAATAGGCCTATTTCAAAACATGA
TTTTTGAGAATCAGGAATTTAAAAATCTAGTCGAAAGAAAGCACCGCATGTTAAGAGCAAGAGA
TTCCGCACTACCTCATGAAAAATACCTTATGAAAGTAAGTGATTGAGGTGTGTTATGGATTATCT
ATCAAAAGAACTCGTTAAAAAGTATCTGGAGTTTCATACGAGCCTCAGTAAAGACAGCTTAAAGT
ATTACAAAGCAGGGTTAAATGCGTTCATGAAATATTTTCCAGATGAAAACAAATCTTGGGAAAA
CTTGACAGTAGAAGATGCAATATTCTTTTACAGATCATACAACGTATCCAAAAATACAAAGATTC

GAAGACTAAACGACGTTAACAGATTTTACAACTGGGCAATGAAACAAAAGTACTTGGTTGAAAA
TCCAATTGAAACATTTGTAACTACATTAAGACCTGAAAAAAAGGAAAGGGCATATTTAACAGAA
AAACAGTTTAAAATACTGCTTTCAAATGTTAAAGATTTTAATTACTATACTTACACCCTTTTTCT
TTTAAAAACTGGAGTTAGAATTTCGGAATTTGTAAATTTGAAAGTAAAACAAGTAGATTTTGAA
AACGGGATAATATTCATTCACGCAGGAAAGGGTGCAAAAGATAGGTACGTTTTTATGGATGTTG
AACTTGCATCAAGGCTCGAATACTATCTTGAAATGAGGATATTGACTAATCCATCATGTAACAAC
TTCTTTGTAAATAAACACGGGCGAAAACTTGCAGAAGATCAGATTACAAAATATACGGATTATTT
AAATGACGTAATGGATGGAAACATTTCATTTAGAATAACTCCGCACATATTGAGGCATACGTTTG
CAACTGCAATGCTCGAAAAAGGAATGGACTTAAAATCGCTTTCTTTGATTTTAGGACATGAAGAT
ATCAAAACTACATCCCAATACCTTCACAAAAATAAAGAAGCCTTGCAAAAAGAGTATTTAAAAGC
AATGGCCCGATAATTATTCTATACTTTTTTCTTTATCTTTAGCATAGTTCGAATCAAAACGCAAA
TGTTTATCTCCAATATCTAAAGAACATGCAAATTCTCCACCTTCATCATTTTTAAGAAGTTTTCC
GAGTAATTTAGGTCTTTTATCATTAGATATCGCATAAAGGCACATGGTAATTTCGTCCACACAAT
AATCAACTGTTTGACCGGGTATTGATATAGTTCCAATATATGCCAAATCCTTTTTGTATTCAATT
AACCGATCATAAACTTCCTGTTTTGATTTAACTTCCCATTTTGTAAGTTCTTCTTTTGAAAGAGC
GGTGTTTCTAATTTCCCAGATTGGGATCAGGTAAACCTGTTTAAAAACGCCATTTTTAAGGTCGC
TACCGCAAAATATGTATACATGATTTCCATTTGATAAATCATCAATTTCTGTTGTAAATGTGGTT
ATTCCATCCCCTTCTATAACACCAAATGCCTCACTTACAATTAATTTATCTCCGCTTGTTGAGGTA
GGTTGGATTTTGATGTAAATTTTTCCAAGAAGATCATCAGTTATTACATATTCTGGCTTTATCGG
GGATATCATATATCTTTGAGTTTCGGGAATGGTTTTAATATTTTCAAAAATATCGTCTGTAAAAT
AATTTTCAAAAGCTTCTTTTTTGATTATACATAAATTTTCAATAGTTATAAACTCATCATACGCG
TAAAGCAATTTTTCGGTAATTACGGTTGAACAATAAAATACTTTGGTCTGATTTCCCTCTTTACG
TTCAATTCTGTCTATTATATCATATTTTACAAATTCAGATAAAAGTTTACTTAAATTACTTTGAT
TTATGCCGATAGTATTCTTTAGTTCGCCAAAACTCACTTCCTCTTGATAATTGATTTCTTGAAGG
ATATTTAATGCGTGTTTTTTGGAAATAAGTTCGAAAAACATAGTCCACCCCGTTTTTAAAATATT
ATGCACTTTATAATATATTAACTTGTTTGTTTTTTTTAAACCACTGGTAAAAAAAGAAACATTTA
AATATGATTGCTATTCATAGGATAAATTGTAGTTGTTTATTTGCCAACTACATGTAAAAAATACC
TAAATTGTAAAAAAATAAACCAAGGTGGTCAAAGGCGAAGGAATTTACAAAGACGTCAAAAAGT
CTCTGGCGTTTAAAGAATACGAAATCATTGATTTTTTGGGATCAGAAACCTATAAATTAAAAGTT
TTAAAGCCAAACTCCGAGTTTTTGGGGTATGAGGATATTAAATTAAACAAATTCGTTTTAAAAG
ACGAAAAAGGTTACTATTCAATAGTTACTAAAAGAAAAGATCTGGAAATTAACAAAAAAGTTAA
AATTCGCTATATTTATGGCGATTTTGAAATTTTGGAGGTGGGAATGTGATTAAACTTGATCTGTC
CAAATTCAAAGAACCGGAATCCTTTGCAATATCTCAGGGGTATGTACAGTTAATTCCTGGGCAGG
ATACTGAAATTACTGGAACCGATTCTGAGGGAAAGCCGGTATACAATAATAAATTCAATATGGA
AAAGAGGGGTCGTGTAAGAATTGTGAGCAGTTACTATGCCAAAGGAAGGGATCCTAAAACTGCA
ACAATCACAATGCTCGATTTAAAGATGATCGGGGACTTTTACGAAAAGAATAAAGATACCATAAC
GCACTATGCAAGCCTCGATGCAATATTCAAGCTCGTTGAAATGAAAAAATTCAATGCCTCCCCAG
AAACAATTAAACTGTATGCAAAAGACCTTGGAATTTCCGAAAAAATTTTGAATGAAAAGGTTTT
TGACAGGTGATCGAATTAATACACGATTTGAGCCATATAAAACCTGAAAAATTCGATAACCACGA
ATTTATTTCAAAAAGAAGGATTTTGATAGATAAAGTCTTCTGTTCAGTTTATTCAAATTCTTTAT
TTGTAAACCTTGAAAGAAAGTTTCGAGACGATGTAAGGGATAAATACATCTCTTACGTCGATAAA
ATTCAAGATTTCAAAGGGCATACACAAGGGTTAAAGTTTCCGCTTGAAATAAACGGCGATAAAGA
AATCGCATGGATTAACATATCCCTAAGAGAACCCCACGTTGCAAAAGTTTATTTTAATGTTATAA

GACTGTACCAAAAAGAAAAAAATATTCCTATTCCAACTTACGGCTACAACGATACTAACTTCCTC
CCCCTCGAATATAATGATACTAAAAGCCTGCTTATGGAGTTCATTGAACTTTATTTGAAAATAGT
TGGAGAAATAAAGGAAATATATGCAGATTATTTGTTACAGCTATTTGGTATTGACATTAAAAAC
TATTGGAAAGGTCTCGAAAAGAAGGAAATCAAAGATCTTGTTGAGGTTTCACCGTTTCAAATTGA
AATTCCTTGTGAATATTTAGGATGTGAAGATGAGCAGTTTGACTGGATGGATGATGAAATAAAA
TGCAGTACTGTGGAAAAATTTGGAGATCTTTCTGGAACTACTTACTACAATAAAAACAGGAAAG
ACTTAAAAGCCACGTTCAAACGATATCAAAAGGCTCCCGGAATAAACAGGCATGAAATAACCCTT
CAAGAAAACTGGGCGCGCGACTGCATGAATTCGGATTCTGAAAATATCTATCAAAATATAATAAC
TTCTGTTCACAAAGTCCATAGGCTCTGGGGTCTTGACTTTGAAGGAGTAAAACCAAGGCCGATAA
ATGGGAATGTCTTGATTTCACAGTTTTCAATGGGTTTCGGACTTTCAAATGATGAGATAAAAACC
ATGATCTATGGAAATGTTGGGGAAATTACTTCAAACCGTGAAAATCAGGGCATTATTAGAAAAT
TAAGAAATAAGGGAATGATTCGAAAACTGGAAAAAGATGAAGGTGGGAAAAAGGGAGTTTACGT
CTGGACTGAAATGGTAAAACTTCTTAGAATGTCTTTAAACCAGTATTTCTTATGTCCTGAATGTT
TAACCAAGAATGCAGTATGATTACGAAAACTACTGCCACTACTGTCCCGCATGCAAAAAAGTAATT
AGATATTAACTTTTTTTATTTTTTTATGGATTTAAAAAGACGTAAACTTAAACTTTTACTACTGT
TCCAAATCCCCTTGAAACATTTCTTCCAATTCCCAAATACTGTGGAATATCAAAATTAATATAAA
ATTCACCCCTAAAACCAACCATATTCTGATTTTTGAATTTCACGGGCACTTCTTTGAGATTTATC
TTAACTTTTAATTTTTCTTCGATAGTATAACCCAAAGATTTTGACATTGAAATGATATTTCCGAT
CAATACTCTTTTTAAAAGTTCTTCACGATCTATTTCGTCTGAATTTTTGTAAATTTCTGAATTTT
TAGCATTTAATGCCATCCACGGTGTTTTGAATTGATACTTCAAAATTTTGTCGGTGGTGCCGAAA
TAAATCTCTTTTGAAATTATTTCAGTTCTATCTAAATTATATTCTGTTCCGTCAATATCTATCGA
ACTTTCAAATAAGATTCCCATATCTATCAATTCTTTTGAACCCTCGTTTAAACCAAGTATAACTG
GAATGCCATCTATTATTTTATATTGTATCAATGGATATCGATACATGCTCTTTTCATTTTTGTGG
TTATGGACTAAAATACTTTCAGGGTATTTATTTGCAAAATATCCCCGAATTTTTGAGCCCTGCCT
CATTGGCAAATTTAAATCGTAATAATTAATCGATAAATAGCTTAAATTCATAATATCTCCTAAAA
AAATAGTTCATTTTGTAAATTATCAAGTATGCCTTCTTCCAAAGAATAATTATCGATATTTAAAT
ATTCTAAATCAAATTTATCCATAGATATCAAATTATTTTTTATTTTATCATATTTTTTTGTATTT
ATTGAAATTCTATAATTGTTTAAAACCCGAAATAAATTTTTAATTTCTAAATTAGAAAGATCTTT
TGAATTTTCAAGTTTTTCCAATATTTCATTTGAATTTTTATCCGCATTTATAAAAATATCCTTTT
TCCATTCTTCTTTTTTAATAAGTTCAAAACTATCCCTAAATGTCTTAAACTCCAATTCTTTTATA
GAGTTATGGACTTTGTCATCAATTTCACCGAAAGAGCGGTTGTGAACTTCGTTAAAATATTTTTC
GTTGATTCCATAAAGTTCGTTTTCATTGATTTCATTTTCCAAAAGAACGGATTTTGTGCAATCCA
ATAAATGTGGCGGGTATATGTAACCACAATACGGATTTCCCTTATCATCAATTATGTTATAAATG
TATACTTCGCTTTTATCCCCAAAACCATTTCTATTTGCCCTTCCTGCAGTTTGGTTTATTGAATCA
AGTGTGGAAAAATCCCTGTAAACTATATCCATATCAATATCTACCCCTGCCTCCACAAGCTGCGT
AGATACAACGATTGGTTTTTCACCATGATTATTCATTTTTTTTATTTGGGATATTTTTTCGAGTC
TTAATTTGGGATAAATCTCGGTTGACAAATAAATACAGTTTCTATTGGTTTTTTCTTTTAAATAA
TGGTAAATTTCTCTTGCAGACTTTACAGTATTCATAACTATCAAAAAACTTTTATCAGGATTTTT
ACCTATTTTTTCAAACAAAATTTCTTTGTAATCGCCTAATTGTATAGTTTCTGAAGTATTATTTA
CTATTTTTATCCGATTCAATCTTCTGAAGTATTTTTCTTTATTTGGAATTAATTCATAGGTATCA
AGAATTATAGGCATTGTTGCAGTCATCAAAATTATGCGTACATTGTATTTTTCAGCCAATAATGT
TAAAAACTCTTTAATAATAGGAAAATACTTTGTTTCTACTGCTTGAACCTCATCAAGTACAATGA
CTGAATTTGAAAGCGTGTGAAAACGATGAATGATTGAATTTTTTCCTGATTTAAATAATGTGTTG

AAAAACTGTACAAAAGTGGTAGTTACGATTTTACTCTGCCAGTTTTCAAAACAAAATCTCGCATC
ATATCCTTCATAATCATTGTACTTTATTTCAGTCATTGAATGAAACTTCAAAAGCTCCTCATCGG
TCGGTTGCCTGTTTAAATTATATTCAAGTAGTTTGTATAATTCCCCATAATTTTGATCAATTATG
CTCATGTAGGGAAGGCAGTAAATGATTTGAGAATCTAAATTATACTCGTTTTTTAACCGTTCAAG
TATCTTAAATGCTGCATAGTATCCAAGCATTGTTTTACCAGTTCCTGTCGGCATATTTATTGAAA
AAATTTTATTTTTATTTAAATCAACGGTTTCGAGTTTATCTTTTAATTCATTAAACGCATTATTT
CGGATAGCATATACTTCATTGTTTTTAAAGCCCCAGTTCTACCGAGAAATTCCTTTTTTAAATT
TTCTTTATACTTTTCAACATATTCTGAGGGTAACCTATTATTTGGAAGATATGCCTCGTTTAAAA
TTAGCTGTGATTTATCAGAACTTAGTAATATCGAATAAATATACTGGATTTTCATAAAAAATTCT
ATATTGTATTTATTACTCGATTTTTCTTTTCTAAATTGTCGTTTTAATGAACGTTCGTTCATTGA
ATCCATAAATTCAAAGAATTCATTTAAATCTTTTGAATTTTTTTTAAGCACTTCTGCATAAATTA
AATTAAGCTCATCAAAACTATTTTCTAAAATATCGTTTGCCTGTTTTTTAGAATATCTATTTTA
AGCGGCATTTCATCGTTTGCATTTTCCATACTGCCGTGGTGTTTTTTTACGATCAAAAATCCGGG
TATTTTCCAAGTTTCGGGTAATAACCAGTACGCAAAACATGCAGAAATTTCGCCATGATCTGTAA
GTTTACTTTTATTTCCAGTTTTTAACCTGTCTTGAAAAAAAGAAGTTACCTTTCCAAAATCATGT
GCCATGCAAATTATTTCAAGAATATCTGTTAAATCCTTGTTAACGTTATGATTTTCGTGTAAATT
TAAAGCAATGTCTTTTACACCCGTTAAATGATCATTTAAAGTTTGGTGAGGGTGTGAAAAAAGAT
TAAAAGAAATAGACATTTTTTCCACCGACTTGGTAAATATCATCGCTGCTGATTTCCCCATATAA
CGGGTTTTTTTCAGCAAAAACTATTTTTTCATATTCTATATATTCTCGATTTGAATTATTTTTTA
CAGGTATTTTATCAGAATACATCTTTATATTAAGATTAATTTTTGGAAGTATATTTTTTGTTGGA
ATTATTGTGTTTACAAAATCTGCTTTATTCACTTTTAAAACTTCAGACATTCCTTCATGGTTTAT
TTTAGCTATGGATTCACTAACGCCCAAATACGGGGTAAATTCCCATTCCCCAACATTTAATTTAT
CGTTTAATTCATTTAATTTATTTTCATTCCACGTAACATAAAATCGATAATGCGGGCACCTTAAA
AATTCAATGTTTGATGGAAAATGAAACATTCCATTTTTTAGACTTAATAGTTTTACAGATTGTAA
ATCTTTATTTATTTCACCAAGTACCTCTATCCCTATTTTCAAATCTTTAGTTTCTTCGTAAAGATC
TGATTTATCGATTCCCATCAAAGATCCGATAAGTCCTTTTGCTGCAACAGGGTGGATACATAGAA
ATGTAAGTGCCGATGTAGTTGTGTAGGGCTTTCTAAATCTCCCATATTCACTTTTAGCACTAAAA
ACTACTGATTTCATAATATCCCTTAAATTAAGTTTTCTTCAGCATTTTCAGGCACATTTATTAAG
TTAACATTTTTATGTTTATAAATTATAATTTTTTCAATGCTTTCTGACTTTTCAAGAGCATATTC
ACAAAGTTTACTTAAATCGATAACAACTTGCGAAATATCTCTCAATTCAAGGTCTTGATTTTCAT
AAGTAATATCAATGTAATTTAATTCCCCATCTAACTTGTTATTATTGTAAATTACCTCTACCAAC
AATCGCGGCATTTGGTTTTTTGATGTGCTCCGGTAATTTTTCAGTCCATTGATCAATGCATCTTT
AAAGTCGTTTAAATCATCATCTGAAACAGTTATTCCTTGCTTTTCTGCAACTTTGTCATTGTAAA
CTGCATATGTTTTAAAAAGTGCGTAAGGGGAAATAAATTTAGACCAGATGGATGCTTGCTCTTTT
CCTTCCCCACTTGCATATGCTGCATTTCCTTGCATGAATTTTACATCTGCTTCGTGAACTGATTTG
CTCCACGCAATTTGAAGGGGGCCTGTGATATCGTATTTTGGCTTTGTAATCACTGCACCAAACAG
TCTTACATCGATATAATTTTCCAAAAGATAATTTGGAATTTCTTCATCTTTTAAGTTATTTTCTG
ATGCTCTTTCTTTAACAATTCCCTGACAAGTCATTACTTTTCCATTTTTGTCAGTTTTTGTCTGTA
CAAGTACATTTTTTCCATTCGAAATCCATTCATCACGTATAAACCTTTTTATCCTAACATCTGAA
ACTTCAAGCTGTCCTGTGGATTCATCCTGTCTTGGTTTATTGTCGTTTAACATATCACCATTTGG
ATTTGCCATAGTACTATCCCATACCATTAAAAATTCTCTTGAATTTACCATTATTGTTCACCCTC
ATTGGTTAATGTATCTTCATTTTTTTCTTTTTTACCAATACGTTGTTGCATACCCCAGAAAAATA
TATATTTAGCTTCATCGGCAGGAAGCGGGTCTTTTCCATTGCCGATATATTCTCTTGCCTGTGTA

ATTGCGGCATTTAAAAAGAAGTCAGATTTTAATGATCCACCACTTTTATATTTAATAATTAGATC
AGAACATTTGTTTACAAAATAAATAAATGTCTTGTAATCATACTTTTTCCCGAAAAAGAAGTTTT
TTTCAAGATAGGACTCATTCATGCTTCCATTTGAACTATGATATTTTTTTGAGTAATAAATCATG
CTTGAATAAACATTTCCAATTAAAAACCATGCTTTCTTTTGATCAGTGTCAAAAAATTCATTATT
CTTTTCAAAAAATTCGTCGATCGATGCATATTCTGCCATTTCATAACCCCCGTTACTATTTTTTAT
TTTAAATTTCCAACCATTGGTTAAACAGCCGCAATCAGCTAAAAAATTATAAATTGCATGCACAT
TGTTCATAAACAACGTATCAGATATCTTTTTACCTGAACTAAAATGATTTTTCTGGTAATTTCTA
AGTATTTTATTAAAAAAAAGGTTTCTACTATATTTTTTACCGTGAAATATCGTATTTAAAAGCCG
TTTTGATTCGTTTGAAGATGAACCTCCTGCCAAAATATTCAATATATTTCTAAGTCTAAAATTAT
ATTTTTCAGAAGAATATTTTTTTTCTAAATCAGATATTAATTTAAACCTAGAAGGTAAAACACCC
CTTATATGGTAGGTTATTTTCCATTCTGAACTTGTTTTTGGATCTTCGAAAAAAACAATATCTAC
TGGTTCATTAAGTCTTCCAAGGTCTTCAAATAATTCTTCTTCGCTTAATCGAAGTTTGTCTATTA
GCTTAGATTCTGAATGTGTTGAACTTAATTTTTCATTTTTAAAAATCTCGTCCATATCATTATCA
TAATTATGTGGCAAAATCATTACTTCGCTATCTGCCCAGTATGAGGACAGATAATCTCGGGTATA
ATCTCGTCCCTGAAGCAGGTTTAATAGACATTCTTTACAAATACCAAAGGAATTACTGTCGGGAC
CGTCAGTATTGAAATAAATCTCTTTATCCATTGTATAGCAGTTATAAATTGCTGTATCGTATATT
ATATCCGTTTTACCACATAGATGACATTCTTTAATTAATTTAGTTTTATTTAAGTACGTTGATTC
TATTTCGTTGTTAAATTTATCTAAATAAAGGTCTATCGGCTTTTTTCCATCTTTTAAAAAGCTAA
CAATATATGATTTTGAAGGAGCAATTCGTACTTTTTCAAATACTTTTTCAGATATTTCATTTACT
AATTTAGGAGTTATTTCTAAGAAGTTTAACGAACTTTCTATTTTTTTATTAATTTTCTGAATAAG
TTTTGAATCTTCTTTAAATTTCTCTATTTTAGTTAATGCTCCAACGTGCCCATTTCCTTGTTGAT
AAAATAAAGAATCACGAAAAACGCCCTTATTGAATTCTTTAGCTATGACATTGACAACATTATTT
TTCGTATCAATACTAACTTCCAAAATAGAATCAAATTCCTTTTCTGGTTTCTGCCAAATATCGAT
TAACTCTTTTTCGCCATGGTGTGTTCTTGAAAACTTTCCAATGTTTGAAATAATATCTATCATAC
TACTCCCCCTTAAAACAAGCTATATTATTGTGTGTTAACAGATATAATGTTATCGGTTTTAATCG
GGGGGATATCATAAAATTAATTATCAACACTCCTGGAACATACATTTCAAAATCAGGAACTCGTT
TTAAATTAAAAAATGGAGAAAATGTACAGGAAATTCCTGCAAAAAAAGTAGAACAGATTTTAAT
TACCTGCCCTTCATCAATATCAACTGAGGCCATATCGCTAGCAGTTGAAGAAAATATTGATTTAG
TACTCTTAAAAATGAATGGAAAACCAATTGGAAGATTTTGGCATTCAAAACATGGCAGTATCTCA
ACAATTAGAAAAAAGCAGCTCTTACTTTCGGAAAATGAACTTGGGATAACTTTTGTTAAAGAAT
GGATTTCTAAAAAGATGGAAAACCAGATTGATTTTTTAAAAATGCTTTCTATGAACCGTAGGGA
TGAAAGAAGAGATTTACTTAAAGAAAATGTCTTAAAAATTGACGAAGAAATAAAAAAATTAGAT
ACTGTTACATTTAATCAAAATATTGACGAAATAAGAAATACAATTCAGGGTTATGAAGGTTATG
CAAGTAGATTGTACTTTGAAATGATTTCAAAAAGTTTACCTGAAAAATACCAATTTAATGGAAG
AAGCAGAAATCCTGCAAAGGATTATTTTAACTGTATGCTAAACTACGGCTACGGAATACTTTATT
CTCAAATTGAACGATCCTGTATAATTGCAGGACTTGACCCGTATATCGGAATTTTACACGTTGAT
AACTACAATAGAAAAGCATTTACTTTCGATTTAATTGAAAAATACAGAATATACGTTGATAAAA
CCATTTTTAAAATGTTTTCGACTAAAAAAATTAGAGATGATTTTTTTGAAGAAATTGAAGGAGG
GTTTTTCTTAGCCAAAGATGGAAAACAGGCATTAATTTCAGAATATAATCTGTTAATGGAAAGT
ACCGAAAAATACCGTGGAAAAAATATGGAAGTTCAAAATATAATCCAGTCAGATTGTCACAACA
TCGCAAACAGGATTTTAAACTGGTGATATAATGCTTTTGTGGGTTATTTATGATATTTCAAACGA
TAAAATAAGAAGTAAGGTTTCAAAAACGTGTTTAAACTATGGATTATACAGGGTTCAGAAAAGC
GTTTTTTTAGGTGAAGTTGAAAGTAATTCTTTAGATGAATTAAAAATTCTTTTGGAAGATACTA

TCGACATGGACATTGATTCAGTTTACGTATTTCCGATGAGTAAAGACGAACTAAATAAAGCAGGA
TTAATTGGTCAAGCATTTGATAAAGAGTTGGTGTCTGATGAAATCGTCTCAAAATTCTTTTAAAT
CTTTTGATGAGTTTAATGAAAATGAATACTACATAACTCCTTCAGAAATGCTCGAATTTTTGTAC
TGCAAGCGGTTTATCTATTTCATGAAGTATTTGGGAATAGAACAGTATGAAGAAAAGCGTTACA
AGGTATTGAAAGGTAGAAGCATTCACGAGAAAAAGGAAATAGAAAATGCGAACTATTTGCGTAA
AAAAATAAACGTGATTGACAAAAAAATAAACGTTTCAATGGTTTCAAAAGTTTATGGGATTAAA
GGAATTGTTGACGAAGTTTTAACTCTTTCAGACGGTACCATGGCCCCACTTGATTACAAATTTGC
TGAATACAAGGGTAAAATTTACGAAACCTATAAAATGCAAGCAATAATGTATTCTTTAATGATA
TCTGAAACGTTTGAAAAGGATGTTAAACAAGGTTTTATATTATATTGCCGTGGAAAAAACGTTT
TAAAAGAAATAAATATGACTGAAAAAGATGTCAATAAATTAAGTGAATATTTGGAAGAATTCAG
GAAAATATTAACTGGATATTATCCAAAACCAACATCATATAAACAAAGATGTATTGATTGTTGT
TATAGAAACATCTGCGTAAAATAATTGTTTCTTGAAAATTATCCTGCGTTTTCCAAACGTATTTC
ATTTATTTGAAAAATCAAAATAAGATTTAAAACAATATATTTTAAAAATACCTGCAAATTTTAA
ATATTTTTAACCATGGGAAATAAAAATAACTGTAATGCATACCATATCATAAAATAATATATGA
ATTTTAGTTTAACCATTATTCCGTGCCATATAATTTTTAAAAAGCAAGAATATACCATAAATAAT
CCATTGAACTAATTTAACCGGGTATTTATTTTTTGTTGATAAAATGAATAAGAAAAGTAAATAT
AATGGCAGAATCATAAAAATACTTCGTTATTCTAGAGTTTTTATCACGTCATCTCTAAAAGAGTA
AAATGCAATAGAACAAGCATTGAAACATAAAATGTGTCGAGGAAAACGTATGATTTCTTTTAAG
CTAAAAGAGTAAAATGCAATAGAACAAGCATTGAAACTTTCAGTTGATAATTCTTTCTCACCATA
TTTGTTCTAAAAGAGTAAAATGCAATAGAACAAGCATTGAAACGAGTGTGTGGCTCTGAATGTG
TCGGGGATTAACTCCTAAAAGAGTAAAATGCAATAGAACAAGCATTGAAACTTTAACACCCCGTC
ATTAATAAATACACCTGATGATCTAAAAGAGTAAAATGCAATAGAACAAGCATTGAAACATTTT
TTAAGCCCCCCCAAAGAGTGAGAATATGACTAAAAGAGTAAAATGCAATAGAACAAGCATTGA
AACAAAGCATGGGTTGAAATGAACACAACAGAAAAAAGCTAAAAGAGTAAAATGCAATAGAACA
AGCATTGAAACTCCCTTATGGTTTCAATTTCTTTTTTAAATTCTGCTCTAAAAGAGTAAAATGCA
ATAGAACAAGCATTGAAACGTCCATGTTAATCCGTAGTCAGTAGACCGGTAGATATCCTAAAAGA
GTAAAATGCAATAGAACAAGCATTGAAACGATAGTTGTCTGTCGTATCTTGGATTTTCAGTTCAA
CTAAAAGAGTAAAATGCAATAGAACAAGCATTGAAACTTAATGCCAAGCAGTTTAAAGGCTTTA
AGCTCTGAACTCTAAAAGAGTAAAATGCAATAGAACAAGCATTGAAACATACCTCGACTTCTACG
ACTCCGAGCCATCCCATCTAAAAGAGTAAAATGCAATAGAACAAGCATTGAAACTAATAAGTTTT
TTGAATTGTTAAAAGACGTGTACCACTAAAAGAGTAAAATGCAATAGAACAAGCATTGAAACAT
GGATCAATGGAACAAATGAGGTGACCAACTTGTGCTAAAAGAGTAAAATGCAATAGAACAAGCA
TTGAAACCCCATCATTCCAGGGCCCTTACCAGTTGCACCTGTGGGCTAAAAGAGTAAAATGCAAT
AGAACAAGCATTGAAACAAATTAGATGTTAAAGATATAAAAGCTGTTCAATTCTAAAAGAGTAA
AATGCAATAGAACAAGCATTGAAACTGTGTAATCTCCCCATAGTTCTTCCCCTAAACTTCCTTCT
AAAAGAGTAAAATGCAATAGAACAAGCATTGAAACTATTTCCTGCGAGGTCAAAAGTATAATCG
AACCCGTTTGCTAAAAGAGTAAAATGCAATAGAACAAGCATTGAAACTCAGAAGCGGATAAATT
AGTAAATAAAGTATTAAGACCTAAAAGAGTAAAATGCAATAGAACAAGCATTGAAACCTCTTAA
AAACTTCTCTTTATACCATTCTATAAACTCTAAAAGAGTAAAATGCAATAGAACAAGCATTGAAA
CATCAAAGCAGTAAATAGTGAAAAAGAAGTATATGTGGACTAAAAGAGTAAAATGCAATAGAAC
AAGCATTGAAACCTATTCTTTGAGTATAATTTATACACCCCTGCTTTCTACTAAAAGAGTAAAAT
GCAATAGAACAAGCATTGAAACTGTATGCTTCGCTATCTCCAGAAATACGGCCTTGAATTCTAAA
AGAGTAAAATGCAATAGAACAAGCATTGAAACGCCTTTTCGATTTTCAGTTTCTCGTTCATCTGA

ATGTATGCTAAAAGAGTAAAATGCAATAGAACAAGCATTGAAACTTTTTGATGGTTCGGTTTCA
GTTGGATAGTATCGTTTAATCTAAAAGAGTAAAATGCAATAGAACAAGCATTGAAACCAATTAC
CGACTCTGTAAGCGTTGCAATCTCTTTCCTAAAAGAGTAAAATGCAATAGAACAAGCATTGAAAC
GAAGTAATGAAACGAAAAGGTGAAATTATGAATGCAACTAAAAGAGTAAAATGCAATAGAACAA
GCATTGAAACGCATTTTAATGGGGATATACTGTCCCCCTACAAAACTAAAAGAGTAAAATGCAAT
AGAACAAGCATTGAAACAATCCTCCGGCTTCATACTCATAAAATATTCATAAATCTAAAAGAGTA
AAATGCAATAGAACAAGCATTGAAACGTATTACAGGTAATCACATAATACCAGATAGTATCATCT
AAAAGAGTAAAATGCAATAGAACAAGCATTGAAACAATTTGATGTAAATGATGCAAATTCGGAT
ATAAAAGACTAAAAGAGTAAAATGCAATAGAACAAGCATTGAAACATGATAAGGGGGAATTTGT
TGGTTCAAAACCAATTGCTAAAAGAGTAAAATGCAATAGAACAAGCATTGAAACATAATTTTGA
AGCGGTAGATCTTGAAGATTTTTTTGACTAAAAGAGTAAAATGCAATAGAACAAGCATTGAAAC
TTTCCTTTATGTGGAAAATCGTTGTACTTTGTATCAACTTCTAAAAGAGTAAAATGCAATAGAAC
AAGCATTGAAACTTTGTCAAGAGGTGAAAAATTGACGGAAAATAATGATTCTAAAAGAGTAAAA
TGCAATAGAACAAGCATTGAAACAAAATAAACGTTAATTCATTAAGGATTATAAAACATTCTAA
AAGAGTAAAATGCAATAGAACAAGCATTGAAACATAGTTTCTGGCAACGCTATATTACTATTTA
GACTAAAAGAGTAAAATGCAATAGAACAAGCATTGAAACCATTATTAAACTCAAGTTGTGCTTT
TGTATCATTACTAAAAGAGTAAAATGCAATAGAACAAGCATTGAAACTTAGTGCGATTAGTGCA
CTCTTAGCAATTGCAAATTCTAAAAGAGTAAAATGCAATAGAACAAGCATTGAAACTGTGTTTTG
AATTGTTCCAGTATTGCTTCCCTTACACTAAAAGAGTAAAATGCAATAGAACAAGCATTGAAACC
CATTGTACTCAATACGGTTGGTGGCGAATTAGCACCTCTAAAAGAGTAAAATGCAATAGAACAAG
CATTGAAACCTTCGGTTTTTAAATCGTCGATTTTGTTACCTTGGCTAAAAGAGTAAAATGCAATA
GAACAAGCATTGAAACTTTCAACGCTAATTTTCATGATATCAACCTAATTAGCTAAAAGAGTAAA
ATGCAATAGAACAAGCATTGAAACGAATCTCCAAACATTGGAGTAAACCGTGCTGCAATGCTAAA
AGAGTAAAATGCAATAGAACAAGCATTGAAACAAACTACACAAAATGCAATAATCTGGACTATT
TTGAACTAAAAGAGTAAAATGCAATAGAACAAGCATTGAAACCTAATAACGATTTCATAGTTTC
ACCTTTAATTTAATACTAAAAGAGTAAAATGCAATAGAACAAGCATTGAAACGAGTTTGGATGT
TGTATCCGTGGTCTAGCAAGTGTCTAAAAGAGTAAAATGCAATAGAACAAGCATTGAAACAAAT
CTAAAACATTGCATCCAACAAAGTCTCCAAGTCTAAAAGAGTAAAATGCAATAGAACAAGCATTG
AAACTCAATGACCTTTCCATTTTTTCTTCACCAATCTAAAATCTAAAAGAGTAAAATGCAATAGA
ACAAGCATTGAAACAAACCAGATATCTCGAAGTTCACTGATTCGCAATTCTAAAAGAGTAAAATG
CAATAGAACAAGCATTGAAACTCGCCTTGTCCAATTGATGCATCAAGTTCGTCATGCTAAAAGAG
TAAAATGCAATAGAACAAGCATTGAAACTTTTCACAAATTCGTTATATGCTGCAACTTTTCCTTC
TAAAAGAGTAAAATGCAATAGAACAAGCATTGAAACTAAAAAGAAATGATAAGACATAAAATA
AAAATACACCTAAAAGAGTAAAATGCAATAGAACAAGCATTGAAACTTAATGCATATGACACAG
AGTTAGAATTAAATGGCCTAAAAGAGTAAAATGCAATAGAACAAGCATTGAAACGCATACACTG
ATGGCTTAACGTCACCAAATTCTTCTAAAAGAGTAAAATGCAATAGAACAAGCATTGAAACCTTC
TAATATATACTATGGTTATACTTCTATATATACTAAAAGAGTAAAATGCAATAGAACAAGCATT
GAAACTATAGTATAATAAACATGTTAATTATTAGAAATAAACTAAAAGAGTAAAATGCAATAGA
ACAAGCATTGAAACTATAATATAGTAGTCGAGGGCTACCCTGCCAGGCTTCTCTCTAAAAGAGTA
AAATGCAATAGAACAAGCATTGAAACGAAGTGCAATGAATAGGAGGGGGAATGAAGAAGCGGCT
AAAAGAGTAAAATGCAATAGAACAAGCATTGAAACCGATCAACTTTGGGTCTACCCCCAACACGA
CCTCGACTAAAAGAGTAAAATGCAATAGAACAAGCATTGAAACGATACAATTATTCTCAAAAACC
GTCAAGGATTAAACTAAAAGAGTAAAATGCAATAGAACAAGCATTGAAACCAAACATTCCCAAT

TTTCGGGCAATTTCAATACAACTAAAAGAGTAAAATGCAATAGAACAAGCATTGAAACTTTTCTC
ACTATATATGGTCAAGAAATAATTTCGGACTAAAAGAGTAAAATGCAATAGAACAAGCATTGAA
ACAACTCACAAATCAGTTCTTCGTCATCTGTGTAGTATCTAAAAGAGTAAAATGCAATAGAACAA
GCATTGAAACCTCGATTGATGTGTCACAAAATGGCTTATGGTGCTTCTAAAAGAGTAAAATGCAA
TAGAACAAGCATTGAAACGCGTGGTTTGATGGCTCAGCTCAACCATATTCTAAAACTAAAAGAGT
AAAATGCAATAGAACAAGCATTGAAACGATACTATTTTTTGATTATGTAATAATTTTAGTACTA
AAAGAGTAAAATGCAATAGAACAAGCATTGAAACTCAAATATAGGAATTCCAAAGATAGCTTAC
GAGATACTAAAAGAGTAAAATGCAATAGAACAAGCATTGAAACTAAGATATTGGAAAAAAAAGG
ATACCTTAAAAATCTAAAAGAGTAAAATGCAATAGAACAAGCATTGAAACTTAATTTTGCATGA
TTAATTGTTACTATTCCTGTTCTAAAAGAGTAAAATGCAATAGAACAAGCATTGAAACCAAATAT
GGGAATTCCAAAGATAGCTTATGAGATACTAAAAGAGTAAAATGCAATAGAACAAGCATTGAAA
CATAACCATTCATGAAACAATGGTTGATAGGATAGAACTAAAAGAGTAAAATGCAATAGAACAA
GCATTGAAACATATATCTTTCAACAACTTCTACGCTTCCTCTACCACTAAAAGAGTAAAATGCAA
TAGAACAAGCATTGAAACCATATTGTCATGTAATCTCTAATTATTTCAATAGAGTCTAAAAGAGT
AAAATGCAATAGAACAAGCATTGAAACCTTCTGAATAAACATTCTCATTATCATATAAATAGCTA
AAAGAGTAAAATGCAATAGAACAAGCATTGAAACAATTTTCGTTAATAGTTAAAACAACCCCAT
AAGGACTAAAAGAGTAAAATGCAATAGAACAAGCATTGAAACTCTTCTGTGCTGGTTTGGGGTG
TAGTAATGGTATCCTAAAAGAGTAAAATGCAATAGAACAAGCATTGAAACTTTCGGGATGTTTC
ATTAATTTTGTAGCTGTAACTCTAAAAGAGTAAAATGCAATAGAACAAGCATTGAAACCATTGA
TTGATGAACCTTCAAAAATGAATAAACAATCTAAAAGAGTAAAATGCAATAGAACAAGCATTGA
AACACAAAAGCCTTATGGAAGATGTCAAGATGCATATTACTAAAAGAATAACTTGCAAAATAAC
AAGCATTGAAACAAATTCGATACACTTTTCTCATGTTATAAACCAAACCTAAAAGAATAACTTGC
AAAATAACAAGCATTGAAACATGGAAAAAAGAGGTAGAGCAGGGTTAGAAGATTCTAAAAGAAT
AACTTGCAAAATAACAAGCATTGAAACGGCATTACTTCATCATTCTTATTCCATCCTTTTCTCAA
CTAAAAGAATAACTTACAAAATAACAAGCATTGAAACTTCCAACAATGCAATATTCAAGACCGAA
AAGCCAACTAAAAGAATAACTTGCAAAATAACAAGCATTGAAACTCTGATTTAATCGCTGATTAC
AAGGGGAATTTATCAGACTAAAAGAATAACTTGCAAAATAACAAGCATTGAAACTCTGTTTAGT
TTTTCTTTGTTTTTCTGTATTCTATCTCTAAAAGAATAACTTGCAAAATAACAAGCATTGAAACT
TGTGTGCATGGTATAAACTGCACTCCTCCCCATCCTAAAAGAATAACTTGCAAAATAACAAGCAT
TGAAACTTAGTGCCCGTTAGAGTCAGTGGTTCTGAAATAATACTAAAAGAATAACTTGCAAAATA
ACAAGCATTGAAACTTAAAAAGAGTTTGTTTTATTAATTCTTTTTAAGTGCTAAAAGAATAACT
TGCAAAATAACAAGCATTGAAACACTGAGGAAGAAGCTCAAGCAGAATGTGACAGGCGACTAAA
AGAATAACTTGCAAAATAACAAGCATTGAAACGAACCTACTGCACGTTCAAGATCTTTCAAAGGA
AAACTAAAAGAATAACTTGCAAAATAACAAGCATTGAAACCAGATAGTTTAAAACTTGGAGTTA
TTGCCACGTACTAAAAGAATAACTTGCAAAATAACAAGCATTGAAACTAATCATATAGTAGTGTC
CCCGCTGTAAGGAATTGCTAAAAGAATAACTTGCAAAATAACAAGCATTGAAACTTTTTGATTTT
AAAATTAAAAAAATTGTCTGACTCACTAAAAGAATAACTTGCAAAATAACAAGCATTGAAACCA
GTTGCCGGCGTTAGTCATGAAACACTTATAAATAGCTAAAAGAATAACTTGCAAAATAACAAGCA
TTGAAACAAAAGACTTAATAAACGTATCAAATTAAACAAAGTGGGCTAAAAGAATAACTTGCAA
AATAACAAGCATTGAAACTTGATATTAAAACTTCAGGAAACGCATCCAGACTGCTAAAAGAATA
ACTTGCAAAATAACAAGCATTGAAACCCAGACCTATCCCGGACTCCACCTTGTAAATTCAATCTA
AAAGAATAACTTGCAAAATAACAAGCATTGAAACATTTATCAGGTATTTTTCTTTTTGACATATT
GTCGCCTAAAAGAATAACTTGCAAAATAACAAGCATTGAAACTCGTCGTACCTTCCGGTTAGTTT

GGTTTCGGATAGTCTAAAAGAATAACTTGCAAAATAACAAGCATTGAAACAGTCAAATTCCCGTC
CGCCAATTGCAACAGGAATACTAAAAGAATAACTTGCAAAATAACAAGCATTGAAACCTTCCAAA
AACATCGAATCCGGCGTTTCCAAATCCTAAAAGAATAACTTGCAAAATAACAAGCATTGAAACAC
GCAATTCTTATATCCTCTGCATTATCTATATTCTAAAAGAATAACTTGCAAAATAACAAGCATTG
AAACTCAGAGAATTAGACCGATTAAAAGATAGTACAATATGCTTCTAAAAGAATAACTTGCAAA
ATAACAAGCATTGAAACTAACAAAGGGGGCTTAATCGTTCAAGTTGTATCTGCCTAAAAGAATAA
CTTGCAAAATAACAAGCATTGAAACAGGTGATATTATTCACGTCTGTCCTTATTGTTCTAAAAGA
ATAACTTGCAAAATAACAAGCATTGAAACATGAAACGATGATTTTAAAAAAACATTCTTTCTGCT
CTAAAAGAATAACTTGCAAAATAACAAGCATTGAAACTAATAAGTTTTTCGAGATGTTAAAAGA
TGTATACCACTAAAAGAATAACTTGCAAAATAACAAGCATTGAAACGTGTATTTTTGTAAAATT
GCTGCCATTCCTGGAGACTAAAAGAATAACTTGCAAAATAACAAGCATTGAAACTGATACATTCC
GCCTATGTATCAACTATTTTTTTATTCTAAAAGAATAACTTGCAAAATAACAAGCATTGAAACTG
TGGGTATTGATTATATAATTCTAGTTTGAGATTCTAAAAGAATAACTTGCAAAATAACAAGCAT
TGAAACTAGAATGATTGTCGGTGTTCGTGTTCGTTTCGCTGTCTAAAAGAATAACTTGCAAAATA
ACAAGCATTGAAACTCGTAGTCGGTTATACTGTTAAAATCATCTATAACTAAAAGAATAACTTGC
AAAATAACAAGCATTGAAACGTAATATGTTGGAAAGTTCCAACATGGATTAAGTTCTAAAAGAA
TAACTTGCAAAATAACAAGCATTGAAACAAAGTTACTGAATTAATCTCAGTATGGCCGATAACCT
AAAAGAATAACTTGCAAAATAACAAGCATTGAAACTTAAAAATCAACCTGAAACACCTTCTTTTA
AACCTCTAAAAGAATAACTTGCAAAATAACAAGCATTGAAACCGAAGCAAGAGCAAGTTTAATT
TCTAAAACTCTTTTATCTTCTTCGATAGTTCATGTTTTAAAAAAACAGGGCAGAGATAACGATTA
TAAAATATGCAGACAGATTAACAAAGATGTTGGGCAAAAAATAAGAGAATCTACTAATCAATAC
TGCATAGTAGTTATTAAACAGAGAATTCTACTTGAATTTTTTAATGATCTGCAAGTTTTATATAA
TCTGATGATATATGGTATATCATACTGATATAATCTATATCACGGTGATACATATGGTTCATGTT
CAAGTTGATTTAAGCGATGATTCAAACAGGATTATTGAGATATTCAAGGCTCAACATTCAATTCC
TAAAAAATCTATCGCAATAGATATGTTTATTTCACAATTTGCGAGTTATGGTAAAGAAGCTTTAG
AATACAACCCTGCATTTGTAAAAGAAACAATAGAACACAGTAAAGGTAAGGAGTTTATAGATAT
AACGGATAAAAAATCCAGAAGAAAACATTTGGGATTAGAATGAGTGATATGAAATTAGCAATTC
GTGATAAACGGGTTCAGAAAAAACTCCAGAAAATTTTTAAAAAAGATAAAGTTCATGCAGATGC
TATTGACTCTGCATTTGATAAAATTCTTGAATATCCTAATCGCGGTATTCCAATGACTGGTGAAC
TCGCCGGTTTTAGGCATTTGCATGTTTATGATTCTTTTGTTATACTCTATTTCGTTGATGAAAAT
GAAAAAATAATATATATCGACAACTACGGCCACCATGATGAAATGTATTCATGGAACGACAAGA
TGAAAAAATCAAAAAAATAATCTAAAAGAATAACTTGCAAAATAACAAGCATCACAATATGATA
AAATCTAAGCGGTTAAAAGTCATTGTCTACGATCAATCTGATCCAACCCTTTTATATCCTATCTG
GCCAACGACCACATGAATCCCTAAGAATTTCATCGCTTAGGATATTTTCGAGCGAAGCGTCCCGA
AAGGGCGAAAATTCCATATGCGGTGAAATTCGACCTCTTTTGATGTTTAGGATATAATATCCTAA
AAAATAATTCATACCGCCCTAGGCACAAAATGTGCGAAAGCACCTGCTAAACCGTAGCACGGCAC
CACTGGTGTCGGGCGTGTACCTTTTTAAAATTTTAAAAAATTAATCTCGCGCGTTAGCGCTATTA
ATTATACAAAAAAGTTGTAGCAAGAAAAATGCAACGAGATTAAACCCTGCCGTTTTAATTCTTTA
ATAATTATAAAAGAGCCAAATTATAGATATTTTTTAAGAGACTTCTTAAAAGTTTTGAAATTTT
AATTTAACGATATACATCGGGTTTGATGTTAAAAGAACCGCAAAATTTATATATTACTGTCATCA
TACAACGTTATTAGGACAATAAAAAACCCAATAATTGCTACCATTGTTACCCGAATACTATTAAC
ATGGAATATACTCTGAACATTTATATAATTAATCTTGATTTAAAAAATTCAAAATGAAAAAAAG
AAAAATTACAATTCAATTAAATCGTATTTCATTGCAGCCTTTAAAAAGATCCCATTGCTCGTTTT

AAGTATATTTGTATGAGGAACTAGGTAAGCAGTAACCATTGTCCACATGCTTTCTTCTTTTAAAT
CCTGCCTAACGTCACTTAGCCGGTATCTAAGATTTCCTTCATTATTTATCCATACTGAAAATTTT
AAATAATTTTTAGGCGATAGATCCGAAATATCCCTATTTTTATATCGTTTTAAAACATCTACTGT
GTTTTTTTCTTCGTAACTTAAATCCATCAATTCTATTTCTTTTTCTATAAGCCTAAAGACCAGCA
TGCTGTGTATCGTGAGTTTTGTATAGGTGTAAGGATCAATGTTGTATCCTTTCTTTTCAAATTCA
TTATATTTTACAAAATACTTCAATAGGGGTGATGAAATATTTTTTGCAGTTGAATAACATTCCTC
AAATACTGCCTTTTGATCTTCCATGCTTCTTTCCTCGTATTTAAATTTCCAAATAAGCCAACATA
ACTAGTACTTTTAATAGATGTTATACTTAGTATTTTAGATATTTATATCTAATTTTTATATTAAT
TATGTTATAAAAGCGAAATAAATCGTATTATATGAAAAAATTTATTGATTAGTTTATATATTAA
TTGTATATTGAAATCTTATGGGAACTAATTATTTATACTATAATAAATTAGTATATAATTAGGT
CAGAAATCCGATTATTTAAAAATAATGGTTTTCGACCTTAAGTGTGCATACTTAGAATCATGAGT
TATATTTGAAAACATTTAAATTTCATATCTGAAATTGCTTTTTTAACCCCTGAACACCTATTTAG
GTATAATTCATAATTTTACTAAAATTTTTAAATAACCCCATAATTTTATTTTTTAGACTGGATTT
TTAATTTTGAACCTTTTTTTCAAATATTATTCCATAAGATGAATCTTTATATATTATAGGATAAT
ATCTGGATATTACGGATTTTTGATATTCATTGATTATTCACTCTTTTTAGTAGTGAGGTGAAATG
TTGATTCTTTTAGATGAAAATACACGGGCAATTGTTCAAGGGATCACGGGAAATCAAGGTAGATT
CCACACAAAAAACATGCTTGAATGCAAAACAAATATATTGGCAGGAGTAACGCCTGGAAAAGGTG
GGCAGGATGTTTACGGAGTCCCTGTTTATGATACAGTTTTAGAAACTGTTGAAAAATACGACGCA
AATGCATCTTTGATATTTATACCTGCACCATTTGTAAAAGATGCTGCATACGAAGCTATTGATGC
AGGAGTTGAGCTTGTTACAATTATTACTGAGCACATTCCAATTCAAGATTCGATGGATATCGTAG
CATATGGAAAAAAACACGGTGTAAATATAATCGGACCAAATACTCCAGGTTTAGCGTCTCCAAAG
GTAGGAAAACTTGGAATTATCCCGATGAGCATATTAAAAGAAGGAAACATTGGAATGGTATCAA
GAAGTGGCACATTGACTTACGAAATTGCAAGCCAGCTTACTTTATCAAATTACGGCCAATCTTCT
TGTGTTGGAATTGGTGGGGATCCTGTAACTGGTTTGAGATATATTGAAGTTTTAGAAATGTTTG
AAAACGACCCTGAAACTGATGCAGTTGTAATGGTTGGAGAAATCGGTGGAAACGCCGAAGAAGA
TGCAGCTAAAAAAATGATTTCAAAAATGAAAAAACCTGTTATTTCATACATTGCAGGACAGTCTG
CACCCGAAGGAAAAAGAATGGGTCACGCTGGTGCAATTATCGAAAAAGGAGTCGGAACTGCAAA
AAGCAAAATGCAAGCTCTTGAAAATGCAGGCGCGCATGTTGCAAAAAGAATTTCGGATATTCCAT
TATTATTGAAAGAAATTATTTAAACTGCTTTTTTCAATTATTTTATTATTAATTTAAAACGTGAA
AAAAATGACTGGACTAATCATCTGTGAGAAACCAAACGTTGCTAAAAAAATTGCAGAAGCCCTTG
GAAAGCCAAAGAAAAAATCCCATAATTCAGTGCCATATTATGAATTAGAACGAAATGGGGAAAG
TATAGTGGTAGCTTCTGCGGTAGGTCACTTATACACGCTCCAAGAAAAAACCAAAACTAAATTTG
GAGATTACCCTGTTTACGATATCAATTGGGTTCCAGCATCAACTCTTGATGAAAAAAAATACATT
CAAAAATATATTGATGCACTAAAAAAAGTTTCAAAAGACGCAAACAAATTCTACGTTGCAACGG
ATTGGGATATTGAAGGGGAATTGATTGGTTACCATGCACTGAATTTTTCATGCGGCCAGAAAAAC
GCTCACAGATTAAGATTTTCAAGTTTAACTAAAAAAGAAATCCTTAAAGCTTATGAAAATCCAAA
TGAAATAGATTTTGGACTCGTTGATGCAGGAGACAGCAGGCACAAAATCGATTGGTATTTTGGTA
TAAATGTTTCAAGAGCACTTATGCAGGCAGTAAGTTCAGTTAAACGATGGAAAACCCTCAGTACA
GGTAGGGTTCAGGGCCCAGCACTTGCTTTTTTAGTAGATAAAGAATTAGAAATTAGAAATTTTGT
TCCAACTCCATATTGGGTGCTTGAAGCTCTTTTAGATAATGACTTAATTGCAGTTCACGAGCTTG
AAAAATTCTGGGATGAAGAACAGGCAAATAAAGCTTATGAAAAGGTAAAAGGTCAAAAACAGGC
AACAGTTTCAGACGTTAAAAAAAGCATGAAAACCATTCCTCCAAATCCGCCTTTCGATTTAGGTG
CACTGCAAAGGGAAGCACACAACATGTTTAGGTTTTCCCCTAAAAAAACGCAAGAAGTTGCACAA

AAACTTTATGAAAAAGGATACTGTTCATATCCAAGAACAAGTTCACAAAAACTTCCAGACGACAA
GGCATACATGGACGAAGTCTTGAAAAACCTTTCAAAAAATAAAAATTATAAGCCATACATTGAA
ACAATTTTAAATGAAAATAGAAAACCGATTTCAGGTAAAAGGACGACCCTGCACACCCTGCGGT
ACACGCAGTAGATGTTCCAAAGGAAAAATTGCCCGATGATGAATTAAAGTTATACGAACTTATTG
CAAGAAGAACAATTGCACTTTACTGGGATAACGCTAAAAGGGAATATTCAAAAATCAATCTCGAT
ATTAACGGCGAAACATTTAAATTAAGCGGTTCAAGAACCGTTGAAGAAGGATGGCATGAAATAT
ATTACTATACTAAATTCGATGAAGTAGAACTCCCAGAATTAAAGAAAAATGACGTAATAAATGT
AAATAACATAAATTTCGAAGCAAAAGAGACGAAGCCTCCAAAAAGATACACGATGGCTTCGATT
ATCAAGGAACTTGAAAAAAGAAAACTCGGAACAAAAGCTACAAGAGCAGATATCCTCGAAAAAC
TAACCAAACGAGGTTATGTGATTGAAGATGGTTCTTTAACGGTTACAGATCTTGGAATTGGAGTT
ACCGAAACTTTAAGAAAATACTGTCCAGAAATTGTTGAAGAAACCCTTACAAGAGACCTTGAAGA
TAAATTAGAGCTTATTCAGGACAAAAAAATCAAAAAAGAAGAAGTTATTCAGGAAACTAAAGAC
AAACTTACAAAAATCCTTGGAGAATTCCGCTTAAAAGAAAAGGAAATCGGAACTGAACTTGTAG
ATAAACTCGATTCAACAAATAGGTCCTTACAAATAATTGGAAAATGTAAATGTGGCGGAGACAT
GATTATCATCCGTACAAAGGGCAAAAAAAGATTTGTTGGATGCAGCAACTATCCTGACTGTGAAG
TTACATTCCCGCTCCCACAAAAGGGGAGAATCAAAGTATTAAACGAAGTCTGTGAAAAATGCCAA
AATCCAGTAATCGGGCTTGATAGAATAAAAATTTGTGTAAATCCCGACTGCAGTACGAGAATTTC
AGAAGAAGATAAAAAAGAAATCGAAAAAGCTGAAAAGGAAGAAAAAATTTGTCCAAAATGCGGC
AATAAGCTTTTGATTAAAAAAGGACCCTACGGAGTTTTCAGAGGCTGTGAAAACTACCCAAAATG
TAAATATACTGAAAAATTAAACGGGGAATCCAATGAAAAAGAAATCGTTGGAAAATGCCCAAAA
TGTGGAAGCGACCTTTATAAAAGAAAAGGACGATTTGGAGAGTTTATCGGATGCAGTAACTACCC
AAAATGTAGGCATACTGAAAAGATAGACAAGAAAAAAGAAGATACGCCGGGAGAAACTAAGGCA
GAAACAAAGAAAGAAGCAAAACCAAAAACCACTAAAAAAGCAGCTCCAAAAAAGACCGCAACTA
AAACTACTAAAACTACTAAAACTACTAAAACTACTAAAACTACTAAAACTACTAAAACTACTGCC
AAAAAGACGGCTACGAAAAAAACTACAACTAAAAAAGCAACTGCAAAGAAAACAGCAGAAAAAT
AAATTAAACACTAAATATTAAAAATAATCCTAAAATTATCAATATAATTCCTGAAATATAGGTT
ATAAATTTCTTTTTTACTATTTTTTCAACTATTTGTTTACTTTCTGCACCAAGCGTACTCAAAAA
TATTATTGGAATCCCGTAACCCAGAGAATACGTCAAAAGTGCAAAAAATCCGTAATAAACATCTG
CTTTTGATGCTACAAATGTTAAAATTCCGGCCAATATTGGATCTGCACATATATTTGCACCAAAC
CCGTAAACTATCCCCAAAACTATCGAATTTAAAATCTTAACAGGTAGTGAAATATTGTTCATTTT
CGATAATTTTTTTGAAAATTTCTGGAAAACACCTTCTTTTCTAAATGTAAAATTTTTATTAAATA
ATACTAAAATTCCAAAAATTATCGCAAAAATTCCTGCAATGTATTTTAACAGCGTTAAATTTAAA
AAAATACCAAATGCTGAAGATAAAACTCCAAGCGTACTGAAAACTATCGAAAAACCAACCATAAA
ACTAAATGATACTAAAAATCCTTCAAATCTTGAATTAGATATTCCAAAAGTATATGCTAAAACAA
AAGGTAGAACCGTTACAATGCAAGGTCCAAGTGCAGTTGAGATTCCTGCCAAAAAGATTAAAAAA
AGATCCATAACATCACAAATTTAAATATTATTTAAAATATTCTTCTGCTTTCGATTTTATTAATT
CAGGTTGGTTTCCAATTACCCTATCAACTTCTTCTCCATCTTTAATGAAAACTACAGTTGGTACA
AGATTTATACCATAATTCATAGCCATTTGACCGTTTTCATTTACATTTATTCTGGATATATTGTA
TCCTTCACCCTCAAGTTCATCAATATGGGGAGATAATTCTTCGCACGCAGGACAGGTTGTAGAAT
AAAATTCAATCATCAGGGTTTGCCCATTCATATCTAAATCTGTTTTTTCGGTCGAAATACATCCT
GCAGATACGATTAAAAGTATAACTACTAAAAAAATTTAAGAAATGTTTCATAATTAACACATCTT
TACTATTTTTGAATGCTGCTTAATGCGCCGGTAAATGCAGTAACTCCTTCACCAACTGCCTTTGA
AACCTGCAAAATTCCGCCTGTAACGTCACCACAAGCATAAATTCCCTCTACACTAGTTTTACAGT

TTTCATCCGTTTCAATGAATTTTCTTTTATTAAGTTTTATTTCGCTTTCATCTAAAAATTCAGTA
TTTGGAACGTACCCAAAGCTTATAAATATTCCATCAGCAGAAATAATCTTTTCCTTTCCATCTAT
TGAAACTTTAACCCCTTTTGCTTTTTCTTTTCCAACAATCTCGATTGGTTCTGCATTAAGAACTA
TTTCAAGATTATTCATCAGGTTCATCCTGTCTTCCATTATCTTTTCGACTGCCTTTAATTCGGACC
TATCGGTAACTATCGTTATTTTTTTGGCAATATCTTTCAAATTATATGCTGCCATTATCGCAGAA
GTTCCCCTTCCAAGCACAATTACTTCTTTGTTTAAAAAGAAGAAAGCATCACACATTACACAATA
ACAGACTCCTTTTCCCATAAAGTCGTCTTCATTTAATCCTGGCTTTTTGTATCTACTTCCCGATGC
AATTACTATTGATTTTGCTTCGTAGGTTTCATGTTTCGTAATAACTTTATAGGGTTTTGATGAAG
TATCTATTTTCTCAACTTCTTCGTGGATTATTGGAAGTTCGAAGTGTTTTGCATGTTCTGAAAAT
TTCTGTGCAAGTTCAAAACCTTTTATCGATTCAAAACCTGGATAATTTTCAACGATCCCCGCTTC
TGCAATTTTTCCGCCTTCATTTTGTTTTTCAAGGCATAATGTACTTAATTTAGCCCTCATTGCGT
ATATTCCTGCAGTCAATCCTGCTGGACCGCCTCCAATAATAATTAAATCATATACCATCAAACCA
CCTGCTGTTCTGAATAATACTCTGTTTTTAAAAATATATAAAATAGTATAAAATATTAAATATCG
GCTTTAAATCAATTTAGGGCTTAAATGATTCTAAGTTTTCTTTTAGATTGAAAATACAACTTTCA
ACGTTTTCGTCATTGCAGTTTATTTCAACCTCTGAAATATCACGGGTATCTAAAAATGCAAAGAT
ATTGTATGCAGGACATCCATATTGGGTTTTTTAGTTTTTGGAAGGTTTAATTTTTTCAATTCTT
CTGAAATATCATTATTTAAGTATATAATTTCATTTTCAAGCCCCTCATTCATTACAGGTTTATTA
TTTAGAGTTTGATGGCTTCTTGTGACAATTCCTGAAGAATTTGAAAGAATTGATGCGTGAATCAT
GCCTTCAATACTTGAAGCAGGTTCTATTATCGTGGATTCATCTTTTTTTATAAATTCTGGAAGGA
ATGATGCTTTGATTCTAAGCCATGTGCTTTCTTCAGGATTTTCCTGAATAATTTCTTTTGCTTTT
TTTGATAAATTCGCATTATTTAAAAAGTAAGTTTTAAATTCTGAAGATAATTCTCTTAAACGCAT
GTCTTCGGACTTTTTATGAGTTAAATGATATTTTACCCGCTTTTGGCCGAGTTTTTTATCATAAT
ATCGTATAAGATTTTCTGCGGATTGTTTTGTAAGAGTTAATGCTTCTTCAAAGCTTATTTTTGTT
TTATATTCTATTAATTTGGCGTATTCATAAGTTTCAATTATGATATTTGTTTTAAATTCCTCTTT
AGTTTCAACGGCAATTGAAACATAATCTTCAAGTGTTGGATGCCTTAGAAGCATTTTTAAAACTT
CCTCAGGAGTAGTGTTATAATTTAAACTTTTAATTTCCTGATGTGTTAAAAGACAGGGGCCTGTA
GTAATATTTTTCGAAATAAAGTCCCCTAAAATTCCAATAAATCTGTGGGGGGAAATATCTGTAAA
TTCGTTTGTACTAACTGGTTTTAAACCATATTTTTCAATTATACGGTTCCAGTACTGGGGGTAAG
TATGCTTAAAATATAAGTTTGTAAATTCTTTTTGGCAGACTACCAAATTATCACCCGTGTAAATT
CATTTAATTTGGGTTACAAAATATAAATCCTGATCATTTAACAGATATTAAATATAGTTTATTGT
AGTGTCTTAATACTACACTACCATTATAGTATATATATTTTACTGCCATTTTAACTTCACAACCG
TACCCTCAAAATAAGCTTTAATTAATAAATTAAAGAAAAATAGGGATTTTTTTATTTATTTTGA
AAAACCATATAATATACTATGTTCATATTTTTATATGGCTAATAATTAAACAAACTGCCTGTAAA
TTAAATAGGGCGCTGTTTAAAGCTTGAGCCGTTTAGGTATCCAATCAATAGGGGGATAGGATATG
GAAGAAATATATTCAAAAATGGTTAATGAAGCAATGGCCGCTCAATGGGCGGATGTAAATATAA
TTAAAGAAAAAAGAGGGCATGAATTTAAGATAAAACATGCTAAAGGATACGTAGATGTTGCAAA
TAAGATGGAGGCGGTTGGAAATCAGGATCCTTCAGTAATTAAATTACATAAAGATTCGATTAAT
GCGCATTTCGATATTTTATCGGAACTTACCAAAACTGTAAGGCCTGAAGATGACCCATTTGTTGA
ACACTATCAAACTCCAGCTATACTTGAAATACTTTACAAAGAAGACCCAGAATTTAGAAAAAGCG
TTGAAAAATTTATTCAAACAATTGAAAAATCAGAAGCGCTTATAGGTAAAGAAGTTCTCAGAAG
GTATGGTGGTTTTTATGGGCCTACCTGTGTTGTAGACTTCGCATTGATTCCTGGAAGCACGAGTA
ACATCGTAAACAGGATTCTTAAAAATGTAGATATTCCAATTGCACACAAACAGGCAATTTTAGCG
TCAAAATCATGGGGAATGAATACATCCTATGGTTTTGGGGAAAAATTTACAAATGCAATTGAAG

CAGGAAAATCATTAAATGAAGCCATTAAAGAAGAAATTGAAATGATAAAATTCATTTATGACAA
ACCAATTGATGCTCAAGCAAAACTTATGGACGATTTCGGCCATGAAAGCTTCGATGTTAGAAAAT
ATATGGGAGAATACAAAAAGAAAATGGAATCTGCAGTAGCTGCTGCAATGGAATCAGAAGTTCA
TTACGGAAACATTGTAACTATTCCAGCATACTGTGTTGGAGATATTGCACACCACGTTGCCCAAT
CAACATTTAACATGTGCAAAGATGACGTTGTAATGGGAGTAATCGAAGCTGTTTCAACAGTAATG
GACAATACATTACGATCCAACTTGAAAAACTTCAAAAGTGAATATGAAGTATTATCCCTTGCAAC
AGGGTCAACTGCAGCTGCAACAGAATGTATACTTGAATTAGATGGATTTAATGCACCAACAGTTG
TAGATTTACTTACGAAAAGATTCCACAACTACGTACAGCTCTACCCGACAAGAGGTGCTGCTGCA
GAACTTCACAACCACGACTTCATGGACATGATTTATAGGGGTTGGAAGTTAATTGATAAAGCTCG
AAGAGTTAAAAATGGAAGCGATGCTCCAATAACTCCGAAAATAGGTAATTTCAAAGTAGATCTT
GATCCTATCTTCAAAAAATGAAGTTATTATGAACCCGCAAAGATATGCATACCCCGCTTGTGCAAT
AACAGTTCGATTTTCAGCACTTATGAGGCTTGCAGACTATCCATGTCTTTTAACGAGTGAACCAG
TAACTGCAACAATGATGACAAACATTATTGCACTGCATAAAGAAACACCTGGAGCTCCTGCAAGA
GTTTGTAAAGACTGTGCAACCGCTTGTTTGGTGGACTTTAGACATCAATACTGCCAGTACAGAGA
AGCAGTATAATTTATAACTATTTTCATTTTTAACGGAGGGGTAAAATGAAGTGTTATCTCTGTAA
ATTAGAAGGAAAAGATACCGATGCAGTAGCTTCGTGTATAGTTTGTGGAATGGGAGTTTGTATG
GACCACGTAGTACGTGAAGAAATCGACCACTGGGAAGGAGGATACCCATTTCCAGCCACGAAATT
AAAAAAGACAATTCCTAGAATGCTCTGCAAACCCTGCTATAATGCACTTATGGAGGGTAAAAAAT
GAGCATGATCAAAAAATTAATTGCAGAATGCCTTGGAACTGGTATTTTAGTATTTTTTGGTCCAG
GTGCTGCTGCAATGACTTTAATGATTGCCAATAATACTGGAACTGCAGGAATTGGATTATTGGGT
GGACTTGGAGACTGGTTTGCAATTGGGTTTTCATTTGCAATCGCTATTGCTGCAGTAATTTATAC
AATGGGGCGTATTTCTGGAGCCCATATTAATCCTGCAGTTACCATTGGACTATGGGCCATTAAAA
AATTCCCCACAAAAGATACAGTCCTATATATTATCGCCCAGTTAATCGGTGCTGCAATTGGTTCT
CTACTGTTCTTTGCATGCATTGGAATTGATTCTGTAACAGTTGGGGGACTTGGGGCTACTGCACC
ATTTGCAGGAATAAGCTATTTCCAGGCAATACTCGCAGAATTTATTGGAACATTCCTACTAATGT
TCGTTATAATGGGTGTTGCGGTAGACAAACGCGCTCCCGATGGCTTTGCAGGACTGGTTATCGGT
TTGACAGTTGGTGCAATAATTACGACTACCGGAAATATTGCGGGAGCCTCACTAAACCCTGCAAG
GACTTTTGGGCCGTATTTGATAGATAGTATTTACGGGTTAAATCTGTGGTACTATTTCCCGATAT
ATATTATCGGCCCTGTTCTTGGAGCAATAGTTGCCGCATTTACTTACGAGTACCTAAATCGCGAA
TAATTGAAAAATCTTTTTTATTTTTTGAAAAATATAGTATTTAAAAAATTATTAAACCTATTAA
CTCATCTGAAAAGTATTAATATTTTAAAACACCATATAGCTATTATTACATACAGGTGGTATTAT
GGGCACTATTTCGGATTATTTTAAAATTAAAGGGGAAATAGGGGAATTAAAAGAAGAAATCAAC
AAAAAAATAGGATATTCTGATGAAACCACGATGTCCCGTTCAGAATCAATTCGATACCTGAATAA
AAAAATAATTTCAAAGAAAAAAAGATTAAAAAGCATTGAAAATAAAATTATTATAAATTATATA
TTCCCGTTGTTTCTTGTTATTTTAATTTTGGCCTACATTTATGTAAAACAAAACGTCTTATAACT
TTTCTGAATTTTAAAATTTTAGATTAAAAAAAGAAAAATAATTTAGTTAATGAACTAATTTTTG
GAAACATTCTTTACAGACTATTTTACCATCAATAGTTCTTCCCTTAATTTCCATAAATGTTTCTC
CACAGTTTTCGCAAGTAAGTGATGGATAAAGTCTTGCTTTTTTAGGTTCTTCAATTTCTACCCAT
TGTACATCGAATAATTCTTCTTCAGGAGCCTCCATAAGATGTTTAATAGCTTCTTTTCTTCTTTC
AAACATCTGTTTCTCATCTTCTGCAGTTAAAGTTCCTTCATTGAACTTTTTGTTGAAGTTTCCTA
CCTTCTCTGAAAGATTGTATTTTAAATAAATTCTAAGAGCCTTGTCGTTGCCTCTTGAGTAAAAT
GTGTAAACGTGCTTTCCGTTGTCTTTAAATTTCAAATTTCCTTTTCCAAATGTGCATCCAAGCAT
CTGCTGTATTCCATCAATACTACAGGAGTTATTTTCAACTATTGCAACAAGCTGTTCATCTTCAG

ATCTTTCGTAGTGATCTAACACATATTTTGAAACCCTGTAACCGATTGTAACACCTGGGCATTCA
TGGCCGTGAAATTCAATTGTTTTTTGGTAGTCTTCATTCAAATTTTCACCATTTTGTGCGGATAC
TTTACAAGTTATCATCCTTTAAATCCCATATATCGTATTACTTTTTATAAGTTTTTATTATTTTA
AAGTACATCTACAACATTTCCAACGATAATAATTCCAGGAGGACATACATTTTCCAATTTTGCCT
TTTCAACAATATTTGATAAATTTCCTTTAACCATTCTTTGATTTTCTCTTGTTCCATCCATTAAA
ATTGCAACAGGCGTATTTTCATTTCTTTTTGGATTTTTCAACAGTTCTTTTACGTGCTTTTCAAG
AGTTGTAATTCCCATCAATATTACTATCGTGTCCGCATTTAACTCGCTTAATTTTACCTGCATTTC
TTTTTCGGTCTTGTCTCCCGCCTCGTGGCCAGTTACAACAGTAAATGACGTTGCAACTTTTCTGTG
AGTTACAGGAATTCCAAAAATTTCTGGAACTGAAATTGAAGAAGTAATTCCTGGGATCATTTCAT
ATTCTATTCCTTCTTTTTTTAAAGCAAGAACTTCTTCTCCGCCTCTGCCAAATACAAAAGAATCTC
CGCCTTTAAGCCTTACAACATTTTTATTTTCTTTTGCTTTATCAATCAATATCTCGTTTATTTCTT
CCTGTTTGTATGAATGTTTACCTTTTCTTTTTCCAACATAGATGAGTTCAGAACCCGTTTTAGCA
TATTTTAAAAGTTTTTCCCCAATTAAATCATCATAAACTATTACATCTGCATTTTTTATTGCTTC
TACGCCTTTTAATGTGATTAATCCCTCGTCACCAGGGCCTGCACCGACTAAAATAACTTTCATAA
TTTTCCCTTTTCTCTAAATTTTACAAAATACATGAATATACAATAGATGAGATTTAAGTTCAATT
TTATATATAAAATTTGATATTTGTTGGTAATTTGTTGGTAAACACTTCCAAATATTAATTTAGGG
AAACCGATTTTAAAATAAATACTTATAAAAGGAAATATCAAGGTATATTAAAATATATCATGAT
TAATTTGATTGGTATTAAGTCTAATTTTAAACGTCTTTAATATAAAATAAGAGCGAAAATATGA
TAATAAAAGGCATCAGGGATGCAGAAGTTCCCGACGAAATCTTTAAAATGGGCCTTAACTTCCAA
ATATTAAATGCAGATATGGTTGCTACAAAAACGCATATTTTGCACAGCATCTATCAGGCAAAAAC
AAAAAATAATATCTCAAAGAACATCTGGATGGAAATACTACTTCGTGCATCAGGGCAAAAGCAA
ATTTCAAATGCGATAAAAATACTTGGTGCAAAAAAAGGAAACATCTGCGTTGTATGCGAAGATG
AAAAAACGTTTGAAAAAATAAAAAATACTGTAAAAGGATGCATTGATGACAGCGTTCTTGAATT
AAATAGCGAAAAAGAAAAAAAGATAAGGGAAGTTTTTGAAATTAACCTTCACGGAAACCTAATT
GAAAGAGTTTGTGAAAAAAATTGCTCTTATCGAGGTTCTATGATTTGGTTTGGTGTGGATTATGAT
TGTCAAAAAAAATATCAAAATTAAACGAAAAAGAACTTGATATTATTCTTAACAGGAATAAAACA
AACATTTCTGGAATTTTACCAACAGTAAGCGAAATTTTAGAAAATGTTCAAAAAAATGGAGATA
ATGCCTTAAAAGAATATACTAAAAAATTCGATGGCGTAGATATTGATAATTTTAAGGTATCTTCT
GAAGAAATTGATAAAGCATACGACAAAATCGATTCAAAAGTAGTTGAATCTCTTGAAAAAGCTT
ACGAAAACATTAGAGAGTTTCATGAAATCCAGTTTAAAAATTTAAACGAATGGGAAATTGATAA
AAAAGGAATTAAAGCGGGACAGATTATAAGGCCTGTTGAAAAAGCTGGATGTTACGTTCCAGGA
GGACGGGCATTTTACCCTTCAACAGTTTTAATGACAGTAACCCCTGCAAAAGTAGCAGGTGTAAA
AGAAGTAATTGTAACATCCCCTCCAAACGGAACCGAAGGAAACCCTGCAACACTTGTTGCTTCAG
ACATTGCAAAAGCTGATGGAATATACAAAATCGGAGGAGCTCAAGCAATTGGAGCTCTTGCATAC
GGTACTAAATCTATTCCAAAAGTCGATATTATTGTAGGGCCTGGAAATATCTTTGTAACTGCCGC
TAAAAAGTTAGTTTACGGCGAAGTTTCAATTGATTTTCCAGCAGGGCCTTCCGAAGTTCTTATTA
TGTGTGATGAATCGTCAAATGAAGAATATGTTGCAATGGACTTTTTAGCACAGGCTGAGCACGAC
CCTAACGCTTCATGTATAATTACAGTCACTTCCGAAGAAAAAGCAGAAAAAAATTAAAGAAAGAA
TTTTGATGGAAATAAAAACTGCTAAAAGAACCGAAATTATTGAAAAATCGATTTTAAATTCTGC
AATTGTTATCGGCTCAATTGATGAATGTATTGAACTTTCAAACAGCTACGCGCCAGAACACTTGG
AAATAATGACAAAAAATCCAAGGGAAGTTTTAAAATCCATTGAAAATGCTGGAAGCATCTTTTT
AGGAAATTATGCACCCGTTCCTGTTGGGGACTATGCAAGCGGTACTAACCACGTTCTTCCAACAT
CCGCCTGTGCAAAAATGTATTCTGGACTCAGCGTTGAAACATTTATTAAAAAGCCAACAGTTCAA

GAATTGACAAAAGAAGGACTTTTAGGAATTAGTGACATTGTAACTACACTTGCAGAAGCTGAAG
GATTATTTAACCATTCTGAAGCAGTTAAAAGAAGATTAAATTAAAAAATATTAAAAATCAGTAC
TGTCGTGAAAAACATGATGCAGCAGTTGAAATTACTGGTATCGCTTTTTGCGGTCTTGATGATTT
TGATGGTCGGAATGAAGACAATCAATGACTCAAAACTGCTGGATACCACAGATTCATTTACTGAA
ACTTACTTTGACTATGCAGAATCCACATTAGAACTTTCAGAAAAGGTTGTGAATAATACGGAAAG
TCTTTCAGGAAATAATTCAAAAGAAAACGCCATTGAAACGGTATCCCTATCAGTTGATGATTTTA
ATGAGTCTTTAAATTATGTAATGAATATGGAAAAATATTTAAACGATTCAGATAATTTCAAAGA
TTCTGAAGAAGAACCTACGAAAAATCCTGAAAAAACGGAAAATTCACAAGAAACAGAACCTTTA
GAATCTGAAGATGGGATAATTATTGAAGTCGATTTAAAATAATTTACTTTTTATTTTTAAGGT
GTAATAATGCTTTTTAGTGATTTTTGTAAAATTCTTGATAAAATTGAAAAAACTACGAAAAGAC
TTGAAAAAACAGACTATTTTGTAGAATTAATCGATTTTATCAAAAATAATGAAAAACCTGAAAA
TTTAAAACAAGTGTCACAAATCACGATTGGAAGGGTTTTTGCAGAATTTGAAAACAAAGAAATT
GGAATTGGGCCAAATTTGTTGCTTGAAGCAGTAAAAACCACAGGGATATCTGAAAAAGATTTGA
AATCAAAAATTAAAGAAACCGGGGACATTGGAACTGCTGTTGAAAATTTAAGTTCAAATATAAA
ACAGGTTTCATTGTTCAGCCAAGCACTTACGCTTGAAGAAACGTATGCCACATTAAAAAAACTCT
CTGAAATTGAGGGAAACTCTTCTCAAAAAAAGAAAATAAGAATAATTTCAAATTTACTGATTTT
AGCAAAACCTGTTGAATCAAGGTATATTTCAAGACTTATTTTAGAAGATATGCGGATTGGAATG
AACATTCCAACAATTTTAGCCTCATTTTCAAATTATTTTAATGTTAATAAAGAATCTGTCGAAAA
AATATATGCTGTTACAAATGATATCGGGCTTTTGGGCGAAAAGTTAATTTCTGGCTCAAACATTG
AAAACGATCCCGAATTAAAATTAAAAGTATTCAGGCCGATAAAACCTATGCTTGCACAGCTAACT
CCATCAATTGAAGATGCGATGATTGAGACTAAAATGCCCCAATTTGAAACTAAATACGATGGTGC
AAGAGTTCAAGTGCATAAATCGAATGGAGACGTAAAGATATACAGCAGAAGGCTTGAAAATATT
ACAAATTCGGTTCCCGAACTCGTTGAAGAAATCAAAAAACTAGATATTGATAATATCATATTAGA
AGGGGAATGCGTTGCAATGGATTTAGATTCAGGAAAACCAAGACCTTTCCAGGATATTTTAAGAA
GGTTCAGGCGAAAATATAATATTGATAAAATGGCCGAAAAAATAGCTTTAAGGATATATTTCTT
CGATGTTTTATACTACAACCGAGGTTTAATCGATACGCCGCTAAAAACAAGAAGGGAAATTTTAG
AAAAACTATTTGGAACAAATAACTGGGATAGTGAACTAGAAAAAATTAAAAAAGAGATATTTTC
TAACAAAATGCTATTTTCATCGTTTAAATTAAATAGTGATGATCCAAATCTTGTAAAAGAATTTT
TTAACTGGAGTTTAAGTATCGGTCATGAAGGCATCATGATTAAAAACCCTGATGCACCATACACC
CCTGGAAGCCGTGTTAAAACTATGTATAAAGTTAAACCAACGTTAGAAAATCTTGATGTTGTTGT
AACTCGTGCAAAAATTGGAATGGGAAAAAGAAAAGACTGGTACGGTTCATATGAACTCTCCGTA
AAAGATAATGATAGCAACTTACACGTTATCGGAAACGTTGGAAGTGGACTTACCGAAGACGACCT
TGAAAAACTCACAAAAATAGTTAATGAAATAAAAATCGAAGATTTAGGTGAAGAAGTAATTTTA
GAACCTAAAATAGTACTCGAAGTAACTTACGAAGAAATTCAGACTTCTGAAAAATATGAAATGG
GTTATGCCTTACGTTTTCCAAGAGTTGTCCAGATAAGAGAAGACAAATCTATAAATGACATTAAT
ACCTTAGATGATGTGAAGAAAATATACGAAATAGAACGAAACAGAAAATAATTTTTTATAAAAG
GTGATTACATGGCAATTCACCCTATCGACTTTAGATACGGAACTCCTGAAATGAAAAAAGTATGG
GAAGAAGAAACAAAACTTCAAAAAATGTTAGAAGTTGAAGCGGCCCTTGCTTTAGCAGAATCTG
AACTTGGAATGATCCCAAAAGAAGCTGCAGAAGAAATAAACAGAAAAAAATCAACTGAATTTGT
AAAACTTGAAAGAGTAAAAGAAATTGAAATGGCAACAAGACACGATGTTGTTTCAGTTGTAAAA
GCTTTTGCAGAACAGTGCGAAGGAACTGCTGGAGAATACATCCACTTTGGTTCCACTTCAAATGA
TATTATCGATACTGCACAGTCCCTCCAATTTAAAGATGCTTTTGAAGTTTTGGAAAATAAATTAA
AAATTTTAAGGGAAGAACTCTTAAAAAAAGCAGAAGAGCACAAAAATACGGTTTGTATTGGAAG

AACACATGGACAGCATGCAGTTCCAACAACATACGGAATGAAATTTGCATTATGGGCTACAGAAA
TTCAAAGACACGTTGAAAGACTTGAAAGCTCTAAAAAAAGGCTCTGTGTTTCAATGATTACAGGT
GCAGTTGGTACAATGGCTGCAATCGGTGAAAACGGAATTTTAGTCCACAAAAGAGTTGGAGAAA
TTTTAGAACTCGATCCAGTTTTAATTTCAAACCAGGTTGTTCAAAGAGACAGACATGGAGAATTC
ATGGCTACTTTAGCATTAATTGCACAAACTTTGAATAAAATAGGAGTTACAGTTAGAAGTATGCA
GAGGAGTGAAATTAAAGAATTAGAAGAAGAATTTGATGCTTCTAAACAGACTGGTTCATCTACA
ATGCCTCACAAAAGAAATCCAATTACATTTGAGCAGATTTGCGGACTTTCAAGAATTGTAAAGGC
AAACGCACTTGCAGAATTTGATAACATTCCGCTTTGGGAAGAAAGAGATTTAACAAACTCATCAT
CAGAAAGATGCTTGTTCCCAGAATCATGCGTACTTTTGGATCACATAATAAACCTTTCGATAAAA
GGAATGAGAAAATTAAGTGTAAATCTTGAAAATGTTAATAAAAACCTTGAAATGACAAAAGGAC
TCATCATGGCTGAAAGAGTTATGATGTTTTTAGCAAATAGCGGAATGGGAAGACAGACCGGCCAC
GAAACTGTTAGAAAATGTGCAATGAAAGCCCACGATGAAGGAAGACACTTAAAAGAAGTACTTT
TGGAAGAGCCTGAAGTTATGAAATACGTTTCACTTGATGAAATTGAAAAAATGTTTGATTATAA
AACATATATCGGATTAGCTCCTGAAATTGTAGAAAATGTACTCAATGAAGCTAAAAATTGGGAA
TAACTTAAAATAACTTAAAAAAACATTTTTAATAACTATATTCTTTTTTTAAAATTAAAAAATT
ATTCAAAATTATAGTATACTTTTGGACCGGTTTCCGAAGAATAAACTGAGACCAAGTTTTTGTTT
AAATAGTAATTTACCGTAACTACGGAATTATTTGCAATCATTCCTGAATTTTTAATCGATTCAAC
AATGCATTTACCGTAGCAGTCTGTATTCCAGTTAGTATTCAACGCTACTGAGCCTATAAATCCAT
CAGTAACCGTAACATTTATTTCAGACCAGGTATTTTCAGAATTATTTATTTGTAAAATTTTCCCG
CCTGAATTCGTAATTATGGAATTGAATGCATTTAATACCACTTCCCTTTGATCTAAATCGTCAGA
GCTGTAGTTTGAATTAATACACGCACAAAGCGACATCAATATCAAAACAGTAACTAAAATTCCAG
AAAACAACTTAATCTTTTGCATAATATTGCCCCCGCGTAGTCATAAAATAATAAGTTTAAAGAAG
TATATAGTTTTTTCGATTAATCGCAGTTAAAAGGTTATAATAGAATATCCACTAAAATCCCGATT
ATACCGCCCAACATAATTACTCCAAAAACATATTTAGTGCAATTTTCTTACCATAAACCTTTAA
CATTGCAGAAATCATCGGAAGACCTATTGATGGGGCGGATATTAAAAAGGATAAGATAAGGCCCT
TTGGAACGCCGTTAATTGCAACAGAACTAATTAATGGAATCATTGCATTCGGGCATAAAAATATA
AATATCCTTGCGACTGACGCGTAAAAGTACATCAAAACATTATTTGAAAGGGCGTATAATATTAT
ATCTTTTGGAATAAAATACATCATAAATCCAGAAATTAAAAAACCAATCATTATTGCAGGCATTA
AATTCTTCATCTGTTTAAAAAGTACCTTTAAAAATGTTTCTTCCAGTTTTATGTCATTTTCTTCA
AAGTAAAATGGAATTTTATCATTGTAAAATATAAATCCAATAACACATGATAAAATTAATGATG
CTAAAATCTGCATTACCACTATTTCGTATCCCAATAAAGAATAAGTGAGCATTACCCCAAGAGGA
GTTATTGATGAAGCAGAAACTATAAATGACATTGTAATTCCAAGATTTGTTCCTTTTGAATTCAT
TACATTTGCAACAGGAACTCCGCTTGCAGAACATAGTGGCAGTACTGCCCCGATTAACGATGCAA
CAAGTATTGATTTTTTTGAATTATCCAATATATGGACAAATTTCTGCCTTAATTTTCCCTTAAGT
TTTACTTTGATATAAGCAGACATTAGAAAACCAAAGAGTATGTAAACAAACGCTTCTGAAAAAA
AATTAATGGAATGTTCAATTGATATAAAAATTGCATCAGTTTGAATCATGCTTTCCACAATTTGT
GCAGGTGTGTGCCTTTACTTTCTTTATTCCTTTTTTCGTCAACTGAATCGTGGTCCCATATGGACA
AACGTATCTACACCAAAATCTCGGAATTATAATGCCTAATGATATAGCACCTAAAATAAGCATGA
GTCTTGCATAGTATGCTTTTCCACCAGCCATTAATGAAAGTTGAATCGAGTCCAATAGCCCCCCT
GCATGGATTGGTATATAAAATCGTGGGTTTCCATTTATTAATGCAACGATTACAAACAAAAATAC
AGTCCATTTGAGTAAATCAAATATTACTTTCATTTCTAAGGATAACTTTAATGGTTTTTTGGATA
TTTTCATCTTTACTTTGTAGAAAAGATCATTTAAAGTTCCAAGAGGGCATACTTTCCCACAAAAA
TCAACTCTTGTAATTGCAACGTAACCTAAAAGTGCTCCAAAAATCATACGCCTGTAACTTTTTGA

AGGACAGTCTAAAACAGGACAGAGGTCACAAACTAAAAATGGAACCGTGTATGGACATCGAAAT
ATAACTAAAAATACTGAAGGAATTGCAACTAAAAAAAATCCATATTTTACGATATTAAAAAAAG
TGGTTTTTTTGAGTTTAGATTTAAGTTCAAATTTCTTTTCTTTCAAATTTTCACCTGTGTAAATT
AAAAATAAATTAAAAATAAAAAAATAATAAAATTAGTTATTTAATGAAAGATATTCAGGATAGT
AATTGTTGTATAATTCCTGTCCTTGTTCTGAATATACTGGAATAAATCCGTGATCTGCAAGAATG
GCCTGTGCTTTTTCAGTAAGTACAAATTCCTGGTACTTGTATGCAAGGTCTACGTGTTCTGTATA
GTTCATAACACCAACAGTGAATATGCCTATTTGTGGAGTCATTGTTTCTCTTGGAAGGTCAATAA
CTTCAATATTTCCAGCAATACCTGGTAATGTTGTACATCTTCTTTCAACCAAGAGCTGCATCTGCA
TTTCCTGAAGCAACATCGCTTAACATTTTTCTGTAGCAGTCGTAATCTTTTGCACTTTTATCATA
GATTGCATTGATTGATTCTGCTTCTGGAGCCATTGCTTTTAAAGCAGGTATTGATGATTTTGAGT
TTGTATAAACGTTAACATCGTCATTTAATAAATCTTCAATTGTTGAAACGTTTTTAGGGTTTCCA
CTTGGAGTTATAATAACCCAGTCCGTAAACTGGTAAACTTTGTAATCTGGATTCATTAATCCTTC
ATTTGCCAAAACTTCGGAGTGAGTAACAGATCTTGGTAAAAATACGTCAGCATAAGCGCCATCTT
CTAAAGCTTTCCTCAATTTTCCGGCAGCCGCCCCTGTAAATCTAATTTCACAATTGTATTCTTTCT
CAAATTCTGCGTTTACGTCAGCTAAAGCTTCAGTAGGTCCACCACAACTGTATACTGTTAATACT
TCTATATCTCCAGGCTCTGCAGCACTTGAACTTGATGTACAACCGCTAAACATTACGGTGAGTGC
CACAAGGCCAACCAATAATATAGCATATTTTGAATTCATGTAGTTACCTCATTAATATTTTTAAT
AGCTATTTGAATAATGTATCTCTTTACTATTGTACCAATATACTATTTATCTATTATTTTTTCGC
CCATATGCTCGAAATTAGTTATTAAATATTAAAATATGTTGCAATTAATTAATATTTTTAATAA
TCCTATCCGGAATAAGTATATAATTAGTAAAAATATAATTGATTTAAACTAAACTTGTGAAAAA
ATGAAAGTTGTTGTAGTTTCTGACGGGCCTTTTGGGGAAAGGGCGTACGATACGATTAAAAAAG
AATTTGAATGCGAATATATTGTTTTAGATATTCCAAAACCCGAATCTATTGACGATTTTACCGAA
TTTCCAAACGAACAGCTTGAAAAAATAAAATCTACAGATATTTTGATTACATATACACTAAATCC
AGACATTACTTTCGATTTAGTCGAACAGGTTTATGATAACGTTGGATACGTAATAGTTGGAGCTT
GGAAGGGAAAAGGATTAAAAAACCAGCTTGAATCTTTTAAAAACGTGATATGTCCAGATATCAT
GTGCGAGCTCGTTGAAAACGGGAATAAAATATTTGATGAATTTGTATCAAAATTTGGAAAGCCA
GAAGTTGAAATTAAAGTTGAAAATAACATTGCAACAGAAATAAAAGTTATAAGAGGCTCTCCAT
GCGGAGGTACGAACTTTGTTGCAAAAGATTTACTTGGAAAAAATATTTCTGATATTTCAACAAAA
GCAGGACTTAGAATACAACACTACCCTTGCAGAGCAGGACGGATCAGACTTTTTTCAGATGAAGA
AAGTGGAAGATATAAAGCTGCTACATTTCATCACGACGCATTTGAAAAAGCATTGAAAAAACGG
GTGAATAAATGAAGATTGCAATTACTGGAAAAGGCGGAGTTGGAAAAACAACATTTTCTGGAAC
TTTAGCCTGTATACTATCAGAAAAATACAAAGTTTATGCAATAGATGCAGATCCCGACATGAATT
TAGCATCAAGTCTTGGAATAACTGAAAAAATAACTCCGATTTCAAAAATGAAAGAATTAATAAA
AGAAAGAACCGGTGCTGAGCAGGGTTCATCTTTTGGAGAAGTTTTTAAAATCAATCCAAAAACTA
GCGACCTTCCAGAAAAATTATCAGTAAATTATGATAAAAATGGAAACTTAAAAATAATGGTCAT
GGGGACCGTTGAAAAAGGAGGAACTGGCTGTGTCTGTCCTGCATCAGTTCTTTTAAAAGCACTTA
TTCGAAATTTAATTTTAAAAAAAGACGAAGTTGTAATACTGGATATGGAAGCTGGAATCGAACA
TTTGGGCAGAAAAACCGCAGAAGCTGTCGATGTAATGATTATTGTTTCAGAAGCAAGCTCAAAAT
CTATGGAAACCGTTGAAAGAATTAAAAAACTTGCAGAAGAAATTGGAATTAAAAAAATCGTCTG
TGTATTAAATAAAATTTCAAATGAAACTGAAAAAAGTTTTGCGAAAGAAAATTTAGAAAGAATT
GGACTTGAAATACTCGGATGCATTCCATATGACCCAGAAGTATCTGTTGCAGATATGAAAAGAGA
GCCACTTGTACTTTACAAAAATTCAAAAGCGAAAAATGCAATTAAAGAAATTTCAGAAAAAATA
ATGAATCTTAAAAATTAAAAAATTATAATTCGGTTACAATTATTTTATGGCCTTCCAAATCTAAA

ACATTGAATTTTGCCTTGATTTCTTTTTTATTGTTTAATATGTCGTAAAATTCTAAATTGTCACC
ATTTACAACTATTTTCATAACATCAGCCATTAATACAGTACCATCATCCAAATAAACTGTTGATT
CACACATAACTATCGCCCCTGTTATTTTTAATTAATTTTATCTGCCTATTGTTTAAAATACTTTT
ATAACTTATTACTTAATTCAAAATACAATAAAATTAAAAAAGGATGTTATTCATCCCTGTACATT
TTTAGAGACTCGATAACAACATCCATGTGTGATTTTTCTCTTTTGAGTTCTTCTAAATCTTCTAC
AGAGAATAACTTCAAAACATTGCTTTTACATGCTTCAACACATGCGGGAAGCATCCTGTCAGTTT
CCATACAAAGCGTACATTTTTGAACTGATTTTGCATCTTTATTTATCGTAAGTGCTCCAATAGGA
CAGGCAACCATACAAAGTCTGCACAATATGCAGGATTCTTCATTAACTACAAGTGCACCATCCAA
TTCTTCAATAGCATTTACTGGACATACCTGTTTACAAGGCGCTTTGTCAGGATGGCACTGTAAAC
AAAACATAGGGATTGATTGAGTTTTAATAGCTCTTGCAACCCCGTGCTGTTTTTTACATGCTTCT
ATACAGTCGTTACATTCTGTACATTTTCTTGCATCAACTACCATTAATGATTTCATGAAATCACC
ATATCTTTGCTAAGAAAGCAGGGACTTCTGAGGAATCTCTCGTACCAACAATTACTTCCCATCCT
GACTGGTCTTCTATATCGCCCGATACAGATGCTGCAAGTCCAGGGATGATTAATTTTTTATGGTT
AACTAATTTTTCGATTCCTGTTTCTTTTATAAGTTCAGCTGCTGCTTTTCCGTTAAACTGACCGCC
TGCAACTGCCACGTCAACTGCTTTTCCATTTGTATCAAGTACAAGTAAGTAACAGTTTACTTTTC
CAGATTTTAAATCTCCTGCAACCGTGTAGTACGTTAGTGCAAAGTTTGTAGTCATGATTACAGGT
GAATTTTCATCAACTTCTCCAAATTCGTAGATTTTTGCTTCAACGGACTGTGGTTTTCTTGGGTC
AGTGTATAAACTCTGTCTCAATGTTAAAACTGGCATCATTTCCCAGATATCTATACCGTGTAATA
TCATTACATCAGCATATCTGTTCATGAGTGCTGCCGCAGTTGTTGCTTCTTTCATTTTTGTTAAT
ATTTCATCTTCTTCTGGATTTATCCAGCAAACTGCAGGAACTGCCAAAATTGGGAATCTGAAATC
GTCATCCTTTTCTTCAACTGCAAGTCTCCTTATCATGATAAAGTTGTCCATTGTATCTGCGATTGC
TTCACCAATGTAAGTTCCAGGGTCTAAAACTATATCCTTAACACCAGATTCTCTTAATTTTCTTG
AGAGTTGCTTCATTTTTTCAAGATCATTTGGAACAAATAATGAAAGCGGGCAGTTGTGTTTTAAT
GCTAATTCTGACATTTCATCGATATTATTTTCAGTTGCTGCATAAATTAAAGGTCTTTTTGATCC
GATCACATCAAGTGCAGCTTCCATTGCATTGGAATCGAATGAACAGAGAATTACTGGAATTTGAG
AATCTTTTAATTTTTCTGCAGCTTTTTTGAATTTTTCAGAATCTCCTGATTTATTTCTCAAAGCT
ACTGCATTCAATTTAAGAATTTCACCGGTTCTTTCAAATTCCAAGTTTTTGATTTTTACCAATTT
CTGATCAAATGCAGGATCATCCATATCGTCACTTAAATCAACTGCTACTGCGGTTGGGTTGTAGT
AAGTAAGTTCGTATCTATATAATACTTCGTCTCCACCAATTGTAACTGCTTTATCACCAGTTCCA
ATCTTAATTTCCCTTACTGCTGGAGCGAGCACTTTTGCCAGTTTTTCAAAATCAGCACCTTTAAG
TTGTGGGCAGGCTGAAAGTTCTGCTTCCTTTTGAGAGAGTTTAGCTGCGAATGCCATACATGATG
CTTCGCCACATTTTCCACAGTTTGTCTTAGGAAGTAATTTGTAAATATCCATTGCTGTAACTTTC
ACTTTTAAAACCCCCTTAGAATTTATTTATCCAGTCGGAAATATCCTGGGATTCTTTTGATTTGT
ATTCGTAAGTGAGTGTTGTTCCGATTTCTTTCAAGGTTTTAACTGAAAGTGGGTGCAACATCATG
AATATGTCAACTCCACAGAGCATCATTGTAAGACCTGTGACAACTTCCCAAATAGGTCCTCTGTA
CTTTGTAGGCCCCCATTCGTCTTTTTTCATCCAAGCTTCTCTTGAGCCCCATGCATTAGTGGTTCC
AGAAGACATCGGCATCTGTAAAATTTCTTCTCCTTTCAAAGCGGAAAGTCTTGTTCTTGTCATCA
CATCGATTGAAAACTCAATACCATAACCAAGAGCACATGTTGTTGGGTCCATTACAATATCGTTA
GCTGTTAAACCTTCTTTCATAAGTGCTTTGTTTAACACTTTCTGCATATTTATGTCAGAAATTGC
CCATGAAAGAACTGCATGGTCGTGTTTAACTGCTGCTTGAGCAATTTTTTGGTAATCCAAGTCAA
GGTTTGCTGAAGCCAAAAGACATCTTTCTCCATCTGCAACTTCTGCAGCCATTTCAAGAACAAGT
GGGTCTTTTTTAGGGTTACCTGAACCACCAATTACTAAAGGAACTTTTACTGCCTGTAAAACTTC
TTCTAAATCTTTTGCAGCATCTTTTAATGATTTATCCTTAACTTTTGGGTCAGTTCCAATTAAAT

GAAGTGTAATCATATCTGCGCCAAATTCTTTAACAGCTTTTTGTGCCCATTCTCCAGGGCTGTGC
ATTACATCTTCAAAATTTTCCTTAATTGGTCTTGGAAGGCCATTCATTGGAATATCAAATACATC
GAAAGTAACAGTTGGGGCATTTGGTTGAGGTTCTTCAAATCTGTAAAGGCTGGTCTGTCCACCTA
GTTTAACAGTTTTTCTGTTTTTTCCACCAAGTTTAACTTCGTTTACCTTTCCAACGTAGTCTTTTA
CAGGGTATTTAAATTCAATCTGACTTTCGAATTCTTTTTTAGCTTCAATTTGTTTTTCAACAACT
TTTTGAACGACATTTTGTAGTGTAGGCATTATGGTAAGTTCCAACTCATCTACATCCATTCTAAA
CTCGTTTATTTCGATCGAATCGGTTTTTTCGAGTAATTTTAGAATCTGTGACATCGTATCCATGA
TTTCACATCCAGTTATCCTAAGATTTTTTCAACGATTTTTGAAACGGAATTTACAGTTTCTGAAT
CATCCGGAAGTTCAAATAATGGTTTTCCTTCCATATCATAGCTTGCTGTAAGTTCGTCGTCGTAT
ATTATTCCAATAATGTCTAGACCGAAGTCTTTAACTGTTTCACGAACATTTTCTTCGTTTTCAGG
TTTTATCCTGTTTAATACGAGGTATAAATTTTTAAAACTGATATCCAGTTCTTTTGCAAGCTCTT
TTATTCTGCTAGCAGTTAAAATTCCTCTTTTTGATGAATCAGTAACTACAAGTAGAGTATCAACA
TTTTGGGTAGTTCTTCTGCTCAAATGTTCGAGTCCTGCTTCTGTATCGATTACTACAATATCATA
ATTTGAAGAAACTATTTCAATGATTTTTCTGAGCATGTTATTTACTGCACAGTAACAGCCACTTC
CTTCAGGTCGGCCCATTACAAGAAGGTCAAATTCTGGAGTTTCGATAATTGATTCCATAATTTTA
TAGTCTAAATTAGTCCACATATCCATTTCTGGAGATGTATTTCCAGATTTAACCTCTTTTTTCAA
CTCTTCCCTTGCATCTCCAACGGTTTTTGTCACTTCCACTCCAAGAGCTTCAGGAAGATTGGAATC
CGGATCTGCATCTATTGCTAGGATGTTTTTCCCAGTTTTAGTAAGATTCCTAATTATTAAAGATG
AAAGAAGAGTTTTTCCTACTCCGCCTTTTCCAGTAACTGCAATGATCATTTTTAACAGCCTATTT
CTTTTTTATGATAATTTTTTCAGCGTAAATTTTCGCATTTTTAAGTATAATTTTGATTCCACCAG
ATGTAGGAATTGCCATTTCAGGAATCTGTGCAAATTGAACGAGTTCTTCGACAGGTTCTACCCCA
GCTTCTTCAATTTGTTCTTCAACGGGTGCATCTAATTCAGCAATTCTTTTAAGAACTGGGTGGTC
TTTTTCGTTCAAGAATTTTTTTAGATTGTGAATGCTTGAAACATCTTCTTCGGTTGCAATTTTGT
CTCTTAATTCTTCTGGAATCGATTCGAGGACCCTATCTTTTATTCCTTTTGGAAGCCAGACGATTC
TTTCGTATCCGCCATCAGCCTGTAAGAATTTTGGTGATCTCATATATTCAATACAAAGACCTGCA
AATCCTTCAACCTGTTTTCCACCCGAACACTGTCCTGCCATTGCAGAAAATGGAATTCCCATCGGC
GTGTCTCCTTTAAAATCCCTGTGAACAATTCCAATTCCATCTAATTCAGGAATATAAAATGCTAC
AGCTTCAAAACATCCGCATGATGTGTGCGGGTGTCCAAAGATACTGTGTAAATATACTTTGTCAA
ATGTGCTCTGTGATTTATCAGCAACAGTTGCATTTAACGTCGTATATTCCCCAGTAACCGGATCT
AACAAGTCTCCTTTTGGAACTTCAAATATTGGTCCTTCTGGATCAATTTTTGCAGCTGCCCGACA
ATCAAACCAGTTAATTCCACCGCACAAAGCTGGCCTATCAGGTGTGATTATACAAACGTGAGTTG
GAGCAAATGACTGACACATGATGCACCCATAAAATACATCAACATCTTCTTCACTCAAATCTCGA
GCCTTACTGTCTCTTGCCTGATATTCCACGAGTGCTTTTTCAACGAATTCTTTTACTTTTTCTTCT
TCAGTAAATATTGTGATTGAAATTGCTTCAATAATTGGAAATTCGTCTTTAAAGAGTGTCGAAA
GAGCCTGTCCAAGATGCTTTAATTCAAAACCTTTTGAATGGGAATCTTTACCGATTCTGCACCAA
ATTTGTCTCTTTGGTTTAAATGCATGAAACCTTGAACATAGTTGCAAATTTCGTGAATTCTTCT
TTCGATTACTCCTTCGAGATCTTTTTCAAGTTTTTCTCCAGAAACTTCCATCAATAATCCAAATG
GATATATTTTTCCTTCATCCATGTCTTTGAGTTCTGGACCCATAACTTCAACTTTTCCGTCTTCTA
CATTGTCAGAAACTAAAACTAGCTCTGCACCAACTGATTTGGGCCCCGCAAGTTCTATGAACATG
TTTGCTGCCCTTATTCTTTCTCCTTCATACATAGGACTTATTTCAACAGGTATGTCTCCAAACATG
AGTATCTCCCTTAAATTTTTGACATTTTTTCTAAGTAGTTTGTCCATTCAGAATCAGACATATTT
GGGAATGATGCATCTGCATTTGAATGAAAATACTTACAAAGTGTCACTGTTTTCAAATGCGGTGC
AAAATGTTTGAGTGATGAAAGACCTTGCGATGCGAGGTAGTACGTAACCCCGATAAATAATACAA

GGTCGTGCTGTCCCTCACCGTTTATTCCTTTCCATTCAGGATCTTTCAAAAGATTTACGATCTCAA
TTGTGCCGTATCTTTTAGATTTAATACCGTTTTCAGTTAATGATTTGTAAATATTTCCAGTAGCG
ACTACAGGCAGATTCCAAGCTTCCACAATATTTTTGCAGAGTGAAATTAAGCTTTCGTCATCTTT
TATAAGGGGCCCGATCACAAACAATGGATTTTTAGCCTTTTTCAACATCATTTTGGCAATTGCAG
GAGTTACAAGCATTCCGTGTTTTGGACCTGCAATTACAGTTGGTTGCCACGGGGTTGTTCTATCT
CCATCCATTTTTTCACCCTATTTCCTCTCGAATAATGAGGGTTCTTTTGGAGCTTCCCTTGGAGTC
CAGTTTTTGCTTGTTAAGTCATCCATTACTTCTTTTTTGTGGGTAATTGGAATGTCTCTTTCGTT
TCTTACAAATAATTCTAAATCGGTTGGTAATTCGCCAAAGTATTTTTTGTATAAATCTATGTAAT
TTGAAAGTTTTAACTGCCTTCCTTTAGGCGTATCGTTTGGCCTTATACACATCTTTACGGTACTT
ACGAGTGCTTCTTCCATGGTTTCTGCAGCATAAAGCAGATGTTCGGGAGCAGGTTCTCCTTTAAC
CGTTTCGCCAGTCCTTAAATCGTTAAGGTCCCACATTTCTTCTTTATCGATTCTACCAAGGTAGAG
TCTTCTATATTTTGAGCTGTGTGGTCCAAGTACTGCAGGAATTCCCCACCTGTTAACTCCAGCGGC
AATTGCAGCTGCTTTTTGGGAAAATGCACCCCATGCTACACCACAAGCGCCAACTCGGTTTAAGA
TGTAGTCTGCAACTTCTTCGAAGTTTCCAGAAAGTGGCTTTTTAGCAAAGATGTTTGCAATTTTA
ATGCATGCTCCTGAAATATGGGCATTTGAAACACATGAACCGATATTTGCAAGTCCACTAGCATC
AAAAATTCCTTCATATTTTTCGTAGAGTGTCAGTCCATCTTCGTCAATATATTCCCCAATTGACA
TTGCAGAACATCCTGTTGCTACAACTATGTACCTTCTTTTTAGGAGTTCTTCTGCCATTTTAGCG
ATTTCTATTCCGCCGTTTGGGAAGTTAGAACATCCAACAAATGCAACTACACCCGGAATATCCCC
TAAAACGATAGGGCCACCTACTTTCCTAATTTCAACATCTTGAACTGGACCTCTTCCTGCCCTCAT
CTTGAATTTCTGGTTCTTTACGAAGTGGTCTCCAGCTTTCGTGATAAATGAAACTAGTGGCAAGT
TTCTCTCACACTCTTCCTCACATCGTCCACATGTGTAGCACATGTCGTGTTCGTAGAGGTTTGCAA
GTTTTGTAAGGTCGCTTTTAGCCGCTGCAGTTACTGCATCCATTATTGGCCTGTTGTTTGGACAG
ACTCGAACACACCAACCACATTCTGTACATTCTTTTGCAATTTCAGGAATTTCAGAAATATCTGG
AAGACATTTTAGATTTTCTCTTCTTTTTGCAATTTTTTTAGCAACTTCCACTGCAACTTTTCCTAC
TTTTTTAGGCTCGAGTATAAGTGCGCCATCGATATTTCCGTTAACGAGTTCGTTAATGATTAATT
CAGGGTCTTCTTTCGTCATATCTAAAAGTCCAAGACACATTTTGTCAGTTGTTGCAATAACCACT
GCACCATTTTTCTTAGCTTCTTCTAAAACATCTGTTCTGACACACTGTTCATCAACTACGATAACG
TCTGAAACACCACTTCTGATGAATTTCATCTGTTTTGATAGCGGACCGATAACTTTTGCAGCTTG
ATTGTACCTGGTTATATCTATTGCAGTACAGCAGATACCACAAATTTCAACGTCTTCGTAAAGAT
CGTTATCTTCAACATAATCGAGTATTTCTGCACCAGGTGCTACGTTGTGACCGATACAGAGCACC
ACGGGTTTATCTTTATCTACTGTTCCAAGACCCAGTTCTACAAGTGGTTCGTCACCATCTCCTCTT
GGCATGTCATATGCAACAATTTGAGTAATATCTGCTACTTCCCTTGCAAGATCATCCATCATTCC
TGCGTGAAGTGCTTTAGATTCAAAATCAAGAGAGTTTCCTTCCTGACCTGTATGACATGCTGAAA
GAAGATGCGATAACTGTTCTTCGACGTAAGATAAAGATTCGTCCAAATCTGAAACTGTTACTGGT
TTTTTACCAATAATCGTTCTTATTATCGGAGCTTCTATATCGATATTTTTACCCATATCTATTGG
GAAATCTTTTCCTTTTTCTTTTATTACGTGGTGGAGCATGTGTCTTCCGTGTCCTGCATGAGCTGC
AGTTCCGATGCACGATGCTAAAAGAACGGTTCTTGCCTGTTGAGCTTTTGCGTCAATTCCACAAG
CGCCTTTTTTACCAAGTAAATCACACTTTCCGTACGTACATAAACAGCACATATCACAGAAAGGT
GCATAGAAAGGAGGGTAACGCTTTAATAGCTTGAAATCCCAGGATCTCAAATCCGTAACACTTGG
TTTTGGCGTGGGCCCCATCGGCTCGTCCAATTCTTGTTTTGGCTTACCGACAGAAACTTCACCAAT
GCTTAATGAAAGATTTTTAGTCTTCCAAAAATCGTCATCAAGTTTTTTATCCATTTTTTTATACG
TCATGACCATCACCGATTTGTCATGATTTATAATGACATTTTAAAATATATATAGTTTATCATTA
AATGGCTTAAAATTCTAAAAATGGTATATAAAGGTCAGATATTGGATATAATAATAAAATTTAC

TTGAAAATAAGAGTATTAATTTAATAAATATTTTTAATAATTATTTTTTAAAATCTATGGCTTC
AATGGCGTTAAAAGGACATGCATTTAGACATTTCATGCAGTAACCGCAATAAATGTAATTTATAT
TTGCATGTGTTTTTTCGATTTCTACTGCATTTTCGCCCATTTTAACTACAGGGCAGATTTTGTAA
CATTTAAATCCGCATTCAAATCCCTTGCAATTATCGTTCACTTTTAAAATATTATTTGAAAATTT
CAAAGATTTTTTAACAGCTTCTTTTATTTTATACCCTGCAAGGTGTAGTATTGTATCCCCAAGTT
CAGTATCTATATCCATAGATGCAAGACAGGGATAATTGTGGAGTTTATTTCCGGATTCAAACTCA
ACTTCGTCAAAAAGGTATCCCGTTAAGTTCTCAGCAACATATTTCGTTGAACCGCACGGTGCATT
TCGTTTTACCGTCACAGATTTAACGATGGTTTCCCCAATAATCTCAATTTCAGGAGATCCAAAAT
ATTTTGCAAATTCATCGATGTATGTATCCCCAACTGGTTTTAAAGAACAGAAAGGCTTTGGAAAT
ACAGCATTTACGTCATTTAAATTATTCAAAATTTCAGATTTTAAACCAGAAGTAAGCTGCTTTGG
ACTGTGTGATTCGATAATTATTGATTTTGCATTTATTTTTTTAGCAATATCGCACACTACTAAAT
TAATATCTCCAAAAAGACCAACTGAAATAATTAAATCAGATTCTTTTAGATTTTCTGGAACATAT
TCGGTTACATCATCAATATACTCCAGTAAATTTTCAGGAATTTCATGAATTGAAACAATTTTCTG
GGCAAATTTTTCTAAAATCGTATTCAATACTCTTTCACCGTAGTTCCCAGAATAAAGTACTGTGA
TTTTCATAATTTTACTCCAAATTTGCTATTTCGTCATACAATAAATTGCTTAAGTACCCTATTTT
TTCAGGTGGAGTTTTAAACACCCTTTGTGTAAAATCAAACTCATTTTCAATTTTTTCCAAAATTC
CTTTTAAATTATCGCGGTCAACTTCAATTTCATGACTTGAATCAATTAATGCTCGTTTAATTGTT
CTATTCTTGTGTTGGAATATTGCTTTTAAAACTTTTTTGAAAAATTCTTCGTCTTTAACATGATA
TTTTGGTTTTCTTTTTGTTAATTTCACAATTGCAGAATTTACATCAGGTTTTGGGGAAAATGCAC
TTGGCGGAACTTTGCAGATAAATTCAACATCTGCATTGTACTGAACTGCAACGCTTAATCTGCTG
TACTCTTTAGTATCGGGTTTTCCAGCCATTCTTTTTGCAAATTCGTACTGATACATTAAAACTGC
CGTTTCGAAATCTTCTTCTAAAAATTTAAATGTTATTGGCGAAGAAATTTGGTAAGGTAAATTTG
CAACGATTTTATTAAATCCAAGATTTTTTAAGTCAACTTTTAAAGCATCCGACCATATAATTTCA
ACATTTTCAAATTCCGCAGTTATTTCATCTGCAAAAGGTTTAACCGTTCATCAAGTTCAATTAC
ATAAACTTTTTTTGCAATTTTAGCGAGTTCTTTTGTCAGGGCCCCTTCTCCAAGCCCAACTTCTAA
AACAACATCGTTATTAGTTATCTCTGCACGATTAATTGCTTTTTTGACAAAATTTTTATCTTTTA
AAAAACACTGGCCTAATTTTTTAGACTGTCTCATCATTTCACGTTTAAATAAGTACTACTTGTTT
AGAAATTGTTTTTGCTTTATTTAAAAAAGGTTCTCTGTTCATATGCGGGAATAAATCTACAAAAC
AGACATCACCCGTAACATTAAGATTTAAAAAGTCTGAGTTGATAATTTCGATTCTTTCAAACACT
TCTTCTGAAAAGTTTAGCTTCAAGTTATATATAACATCTTTTAATGCGTGGGAGTTTATGTCTGA
AAATACAACTTTTTTCGCACCTTTTTTTAGTGCTGCTAATCCTAAAGTTCCAGATCCGCAGAATC
CATCGACCACCACTTTTCCCTTAAGATTTAAACGGTCAATTTTTGTAATTTTTGGATTAAACAGT
CTTGGAAATTCGATGTGAACTTTTGACTGGTTTTTACATATAACGACGCAGTCCATGATCGATTT
TAAAACAAAAATGTCGCATCTAAAATCATCTCCTGCAATGAGTGCATTTACTTCACCAGAGTCGT
CTGCACGATTAAAGCTATTATTTTTTGAATTATTTGAAATAACTCCTTTAACTTCAGGTATTTTT
AATATTTCTGTTGCACAAGCTTTTGATACATCTTTCATTACAATTAATTCATTGTTAGACACAAC
AGGCAGATATTTCAAAGGGTATCCAATTTCTACCAACGGTGTTCCCGCATCCCTTAAAGTTACAT
CTTTTAAATCAGTATTTTGAAGTTCATTATACTCAGACATTAAATTTAATACGTCAAGCATTACA
TCATCTAACTGCGAATGACCGCAAACACAATTTCTTTTTCTTTTATCAAGATTTAAATCATATAT
AGAAACTGTTTTAGAAATTGGCCCATTACAATTTTCGCATGGTTTGTAAGACTTCAATTTATCGA
TAACTGCCCCCTTTGATAGCATAAAATCCCCCAAATTCGTTTTATTAAATTATGAAAAAACGAAT
ATAATTAATTTTTTAAATGAAGTTTGTGGATTCTTAAAATTCTTTAAAATTTTTCAAAGGGTTCC
AAAATAAAAAAATAATTTAAAAAAAGTAGAAAATTACCTAAGCAAATTAACAACATTTGCTTCT

ATTTCTTCTTTTGGTCTTCCAGTAATGCTTGCAAGCCCTTCTGCAAAAATTACCGCGTATTTCATA
ACATATTTTCTTTTCTGTTCTTCTTCTTTTTCTTTTCTTAATTTTGAGATGTGCTTTTCAAGCTCT
CTTGCACATATCATTAAAGACTGCCTTATCTCGTTGAAAATTTCCTCATTTTCTTCGTTGCTTGAT
GCAATTGCCTGTTTTCCTGCTGAAGTATATGGGATGTGCGTTGAAATAAGGTTTACAAATACAGT
TACTGGAGCATCTTCATCATTTCGAAGACCATATCTTCTCCAGTTAACACTTTTTACAGCATTTG
TTAAACCACAACCTGATGTATCAAAAAGTAATGGTACGTGATTTGAAAATCTCATGATTTCCATC
TTTCGTGACTCATCACCACTTCTTCCAGCTTCTCCACCATAAGCAAGACCTACTTCAACTGCAAAA
GGAACTCCTCCTTTGTAAGTTTTAGGTTTTCTTGTCAGGGTCTTTAAAAACTCAGGTTCCAAAGT
ATTAAGTGATTTTTCAATGTTTTCCTCACCAATTGGCCTTAATCCAACTGTTGAAGGTGCCATAA
AGTCCATATCCTGTAAAACATTTACAATGACTTCAGAATCTTTCCACGTCATTTCTTTTGGATTT
TTCTTTAAAAGGTCCTTGATTGCTTTTCGGTATTTTTCAAGCTCAGTATCTGAAACCATTGCAAG
ATCCACGAGGGATTTTACAAATTCTGCAGGAGATTTTGTAATGTTTTTCAATTTTCTTCTGAATT
CATCAAGTAGTGATGCATTCAAATATTTTTTATCCATGTATTCCATGAAATTTTCTGGTTTTTTC
TTGAAATGATTTTTAATCTCTTTTATTTCTTCATCATCCAATTTTTGAACCAAATATTGAGTTAC
AAGATATTTCATACAGTAAGTTTTTAATTCACCCAATCCTTCAGGAAGACCATTTATCAGCTGTT
TTACATCTTCAATTTCTTTTTTATCAAAATAATGGCCGTATATATCAAAGTATTTCGTGAAATCA
AAGTTTAAGTAACAGCTTACAATTGTATCCCAAAATACACTTGAACGGTAGTTTTCTAAAAGCGT
ATCTCTTAAAACATATTCTTCAAGTTCCTTAATTCTTTTCATGGTGACTCTTGAAAGTTCACCAT
TTAACATGCTTGAAACTCTCGAAGAATCTGTTACTCTTGAAATATGAAGTAATTCATCAGTTGTT
AACCCGTAGGGGTGTGGTTTCATTACTTCAGGTCTTTCGGGTATATCATTTACAGTTCTTTCAAA
AACGATTGTTTCTTCCGGATCTTCCAAAGTAATTTTAGCGTGCGGTGCTGAAAGACTGATCCTTC
TTAAATATTCAAATGGTCCTTGCTCTCTTCTATTGTATGTAACATCTTTAAATTCTCCTTCAACCC
TAGTTCCTCTCCAAAATCCTTCTCGAGTGTTTCTTGAAACTACATCTCCTTCGTTTTTCTCGATAC
TCATTTTAATTTCCATTTCATAAATGGTACCGTTTCCAGTTGAAGTTGTAATTTTTAATGGCTTT
CCGGTTGTCATCTGCGCAAATAATAATACACCTGCAGCCCCGATACCCTGCTGACCTCTTGACTGT
ACCAGCCTGTGTAATTTTGACCCTGCAAGCATTTTACCAAATACTTTTGGAACAAATTCTGGAGG
AATTCCTGGACCGTTATCTTCAATATTGACTCCAAATGTTTCAGAACCTATTTTTTTGATTTCAA
CCGTAATTTCAGGAAGAATTTCAGATTCTTCACATGCATCAAGGCTGTTTGTTACAAGTTCGTGT
ACAATTGTAGTCATACTTCTTAATCTACCACTGTAACCGAGCATGTGCCTGTTTTTCCTAAAAAA
CTCTGAAATTGAGTGTTCTTTAAATTCATCAAATAAGCTTTCTTCGACAACCGCGTTAGTATCTT
CAGCCATTAAATCACCACAGTTCATATAATTATAGTAAATATGGACAAAATAAAAATCATAGTTT
AAAAATTCACTAAGTTAATTTGTGAATTAAATAATATTTCGTTTTAACTATAGAATATGGGGTA
TATATAGAGTACGGATATCAATTTTTTAAAATTTTAACATAATAACCATTTTAAATTAAGTTCAT
ATTTTCAATTGACATCTTTTAGAGCATCTTTTCAACATGGCCTTTTTTAGAGGTATTTAGAAAAA
TTCGAAAGTAACTATAAATATTGAGCTTATCAATTTAAAAATAACAAATCATTGCAAACAAATT
CAGGCGATTAAAATGGATTATTTACTAATTTCTTCAGAAACCGACCCTGCAAGTCAAAATCTTAA
AAAACACGTTGAAAATTACGGATACAGTGTATTTAATATTGAAAAAAAGTCAACTCAAACAAAT
TATTCAGAGTTTCCACAATCTGAAATGTATATATTTTTATCAAAACATGCAAGCGAGAGCAAAAA
ACCAACACTCACGGTACATACTCCTGGAAATTTAACTGATGATAATTCACATGGTGGAAATCCGG
AAGAAATTTCTCCATGTAATCCGGTATTTAACACATTAATGCTTCAAAACATGAATAAATACAAT
GAAATGGAAGAATACAAAGAATTAGGATTTGATGTAAGTTTTGAAGTTTTACACCACGGCCCAAC
TGATTTAAAAGCTCCTTCTGCATTTGTAGAAATCGGAAGTAGTGAAGAACAGTGGCAGATTGACG
ATGCGGCAGAAATTATTACGAATTCCTTGATTGATACATTAAATTCCATTCAAAATTCAGAATAT

GAAGAAAAAGAAAAAATAATCGGAATCGGGGGAGGTCACTATTCTCCCAAATTTACGAAACTCGC
GTTAAGAGAAGAATATTATGTTGGATATCTAACTCCAAAACATGCGAAACTATCTGAAAATATA
TTAAACCAGCTGACTTCAAAACAGGAATTTGATTTTATAGGAATCGACTGGAAAGGATTGTATGG
CGAAGATAAAAGAAAATACGTGGAATTTTTCGATGAAAATGATATTTCATGGCAGAGAGTTTGA
TATTCATTTACTGGAAAATTAATGATTAAAAAATATATATTAAAACAGGACATAAGTGAGAATA
ACTTAGATTACAGACTATATATGTGGTAAATATTCGAAAAATTCAAAACAGGCGATTTATGGTT
AGAAAAAGCCAAAAAACGTTTTGAAAATTTTGCCAAATTTAAAAGAACTTAATATTAAAAACAG
AATTAACTAATCTTAAAAATAAGAGAACTTTTAAAACTGCAATTCTAAAAAATCAGGTTTTAAA
ATTAAAAAATTACGACCATATATTGCAGGAATAAAACAAGGCACCTATGATCTCATCTCTTGTTT
ATACCCCTGTAAATTGGAACTAACTTAGAACTACCTAAAAGAGTTAAAAAGTGGTAATATGGAC
ATTATAGAAGGAAAATTTGTGAAAATGGGATCGAATTATAACGAACTTTTTGATGAACTTCCAG
AAAATTTTATCGGACATGTTCGACTATCCATAAAAGTCAACGGACAGTTTAGGGAAAGCCACCTT
TTTTTAAACAATAAAAAAATCATTGGCGGATATTCTGACTACGAAGGAGAATTTTTCGGAAATG
ATGCTGTTGTAAACGCTATGAAAATGGCAGAACTTGGTGCAATTGTTGACATATTTTCATACACG
GATTCCATGTTATCCCTGATGCAAAATTCAAATAAAAAGCTCTTTTTAACAAATGAAACTGTTAA
AAAAAGTCCGGTTGAAAAGAAAAAAGCAATTGCTCCAAATAATGGAAGAATATCCGTTCCAGAA
GGCAAACCCATAAAATTGGGCGTTTCTAACGATTTTGAAAAATACCTTGGAAAATACACCCTTTT
AGAACTCTTCAAAAAAATTGATGGAACTTATCATAGAGGATATGTTGCATACAATGGAAATTTA
CCCGTTACTGCTGTTTATCAGGCGGAAAATAAATTAATTTTTGGAAATAATGCTTTTGAAATGTT
TAACGATATAATTAACGAAGATAATGATTTTGCAATTGATGTTTATGAATATAACAATTCAAAA
TTGAATTATTTTATCGAAGAATACCCTGAATCAGTAATTTCTATTAAACCATCAGAACCTGTAAA
AAGTGAGATGCCAAAAACAGAACTTCCAAAATATGTTCAAGAGGTAAAAGATATTAATGAATAC
CTTGAAAAAGACGATTCTGAAACTGAAAACCCCCTGCAAGTCGAAGAAGATATCTCAAGAGAAGA
TCTGATGAAAAAATTAGGTATACGAACACCTACTGACGATATAATTGAAAATCTGTTAGATGACG
TGTTTGAACCTTCAAAAACTGAAATGGGTGCAATAGAAAACGAACTCTTGGAAAAAATAAATGA
TTATTTCAACGAAAATAGCGCTATTTTAGAGTTTGAAACTGAACTTTCGATAGAATATTCTGATG
ACGACGAATTTATTGCCACCTGTTGTGTTAACACAACATCAGACGAAAATTCCGAGGTTATTAAA
AAAGACATTGAAGATATATTTGATTCATATATAATCGAAATGACTTCAGATGTTACAGTCAATA
ATAAAAAAAACAGGGAATACCTCAGAAACTAAAACAGAGCATAAATCAACACATAATAAATATGA
TATAGATCTTCAAGAAGAGCTCAATAAAATAAAAGCTAAAAATTCAAATAAAGAAAAAACAGGC
GTTGAAGGACTAAAAGAAAAAATTGAACATGGAATATATGAATATCTAGAAAATGTACATGATA
TTTCTGAATTTGAAGTCCATATGTCCATAACCGAAGATAACGGGCATAAATGCAAGTGTTCAATT
ATTGTTGTTCCTAAAAAAATGCTTGGATTTATAAAAAGCAGCTTAAATACTGATAAAATTGAAA
GAGACATTTCCGACATATTAGGTATGAATAATGTCGAAATTGAATTTTTAAATATTACTGTTGA
AAAATTCACAACCTCATACAGCAGATATAAATAAAAATGGTTAAATATCCCAGAAAATATAATG
GTACCCATAGTTTAACAGAGGGCTTTTATGAATAACATCTACCTGTTCGCAATATCTCTTGGAAT
AGGTTTTATATGTTCTTTCTCCATTTTGAGAATAATTATACAGAAAAAGAAGAAAGATAAGGCT
AAAAAGCGCGAAGAATTAAAAGATGAAGTAATAAAAGGGCTTCAAAAATTAAAATAAACAACAG
AATATTAAAAGGGGTCTAAAAATGGAAAATTTGGGTTTATTTACAATTTCACTTGGTGCAGGGT
TTTTTGGTTCGTACTACACACTATTTAAACTAAAAAAAGATAAAAAGAATGTACAAAAGGAAGA
AAAAGATTTACTTGAACTCGAAGTAATTGAAGGACTTCAAAAATTAAAAAGTCAGGCAGTCTCA
AAAAATCCTGAAAAAGCGTCCGATACTTACGACCTTTTAGAAATTGCCCTTTCACACGACCTTTT
AGATATTACTGTTGTTAATGAAGAAGGTTTAACAATCATCTCCACATTAAAAGACCCGGATGAAG

TTGCTGGACAGTACAGTGGTGTTTTCCAGACCCTAAACAAAATCATGGGAAATGTCGAAAAAACG
AGTATAAAATCTGGTGAAGAATACATATATATATCAACAATTGAAAAGAACGAAATGCCGTTTT
ATATTATTGCAAATTCCAATATCGAAATGGATCCGATTGATGAAAGAGAACTTCAAAATGAAGT
TATAGAAATTCTCGGACAGTACATTTAAAAGAATTAAGGCAAATGATGATGTTATCCCCGGCTGA
CCTTGTGATGAGCCAATTTATGATCTGCTGATGCCTTTCTTTTTATTTTAAAAGTATTTCGATAA
AATTTTTCCATTTTTTTTATTCGTCTTTTTCTTATTCATCTTCTTCAAAAATAAATAATTCATCT
ATTTTCACATTCAACGCCTTTGAAATTGCGTGAGCAAGTTTTAATGACGGATTATATTTTCCCTT
TTCAAGAAAACCTATTGTTTCTCGCCTAACCCCAACAATTTTTCCAAGATCCTCTTGCGTCATGTC
GTACTTTGCCCGATATTCTTTTATTCTTGTTTTCATTCAACATCGCCCAGTCTTTTAAAGTACTGC
TGGAATAAAAGTGCACTGATCAGCATTACAAAATAGATTATTCCAATTGCCTGCTGAACAGTTAT
TTCAAACCAATTAAAATAATCTGCCCAACATATCAGGTTCATTATTAAAAATGTAAAAACCCACG
AGTAAGCAAGTCCTGCATTCGCAATTTTTCGAATTCTTTCATCCTTTGATGGGACTTCCCGATAT
TTTATGTGTAAATATAAATTATATAGTATTAAAATAATCCCTGCATTTATCAAAACCGCAAGTAT
TAATCTGTCCACTTCAAACAGGTAATTGATAAATATTCCAAAAAGCATTATTAAAAATCCCAAAC
TAAGTCTATTTTTGAATTTAATGTCCATCTAAATCCCCTAAATTTCAGATTTTTCCAAATAATAA
TTTGAAATCGCTTTTACGATCATTCCACCAAATAAAGCTGCAACTAAAAACCAATCGACGTAAAA
ACTTTGATTCAAAGCACTTATAATGGATTGATACATTATTCCAGCAACTAAAATTAAAACCATCG
CCCATGCAGCATTTCTCTCAGATATTGCTTCTATTTTAAATTCTCGTTCATCTTTATCGAGATTA
AGCATGATGTGCCCCACATCAAGCAGTAAAAATAAAACCCATGCTACTGTAACGTAAGTTCTTGT
TGTTTCATCCCAGTATGGGAGTGATTGAAATATTATGTATGGTAGAACCATTACTGCGATATAAA
GCCAGCCCTGCCAGGTTTTTGGGTGAAGTCCCCAACCGCCGTACTTTCTTCTCTCAAACCATTCGG
GTTTTCCAAACATGTAAAAATCACCATGAATACTGTTAATTATTTCAAACATTATGTTACTTATT
TCTAACTCAATGTTAGATATTGTTAACATTACAATATATAAAACTTGCTGAACCTGATTTTAAAA
ATTAAAAATAATATAAAAATAAAAAAATTTAAAAAAGAAATTTTACATTCCAAGTTTTTCTGGA
AGATCTTCTTTTGTAATTTCAACATACGAGTCCTTTGTCATACAAGTTGGACAGGGATTTGGAGC
TTTTTCACCCCAGTGAAAATCTTGACATACTTTGCATTCACTGAATTTTTTTAGAATCGTATAAC
CTTTAATTTCGTTTTGAGAATCCTTTGATCCACAAGTTGGACAGGGATTTGGCCCATTTTTACCT
TGATGTATATCTCCACAAACTTTGCATTCAAAAAACTTTTTATCCATGATTTCTACCCCCTTATCT
TAGTAAATATACTCCATAAGAATGGAGTGCTACAGAAATTAAAAATAACGCCCAATAGTAGCAG
TGATATTTAAAAATTATTTTATAAATTTTTAGATTTTTAAAGGAATGCGGTAAAAAAGTTCCGC
ACCTACAAGAAAGAATAATCAGTATAATAAATAAAAAAATTGGTAAATCCTGTGTAGTAAACCAG
TTTAAACGAAGTTTCTGGAGTCATTCTACCACTTCAACGGTTCCCTTCATATAGGGGTGTGGTAT
GCAGTAGTAATCAAAAACTCCTACTTCATTAAATGTATAATTCCAAGAAAATTCCTTTGAAATAA
GTCCTGAATCAAATATTCCATCATTTGATGTTACAGTATGCCTTACACTATCTTTTTGAACCCAT
GTAACTGTTGTTCCAATTTTCACGGTTATGCTTGCCGGTTTATATGAAAAATCTTCTATTAAGAC
CGTTGCTTCAGTTTGTTCTTCGAAATTAGTGGATGTATCAACAGGCGTTTCCATCTGGCACCCTG
TAAAAATAACTGCAAAAATTATGGATACTACCGCCAGATATTTGTACACAATCAACCCCTCCAAA
AACCATAATAAATACTATTTTTAAAATAATATAACTAATTTTTGATAATCCACGCCCCATTTATA
ATTTAAACGATAACTTCCCCTCAAAATTGATTAAAATGCTTATTTTAGATTTTTAAATATTCCCC
ATATTTTTAATAATAATTACATTTTTCAGAATAAATGTGGCATTTAAGTAAATAATACGTAATT
ATAAATATGGATTACTATTTTAAAGGACATTTAATAGATTAAATGATAGTTAAACGATTAAATT
AGGTTGTTTTAAAATTTATATATCATTAAAGACATATAAACCCATAACTATACTAACTTCCATTT
TATTTCAAAAACAACGACATTTAAAAGTATTTACAAATGTCTCATTTTTAAAAAATAGGAAGTT

CGATAAATTTCATAATTTTTGGGGGAGTATTTATGAAATTAAAGGCATTTTTAACGATATTTAT
TGTACTTTTTTCAGGAATTGCAATAAATATGGGGCATGAAGAAGACATTTCGAGTATCACTTTTG
TATATCCTGAAGAATATGGCCTAATATCAGGCAATATTTCAATTTTAGCAGATGTGTCAAGTCGA
GTTGATGGAAACTACTACTTAAGTAATGTTCTATTTAAATATTCTGTAGATGGAAGCGACGAAA
AAACGATAGGAATATGTGATGAAAGTGTTAACCCCGCAAGGGGAGAAGACGCAGGTCTTGTAAA
TCGAACGATTGACTGGGATACATCGGCACTCGATGGAAGATATATTTTAGAAGGGGTAGCAAGTT
ACTATTACGCTTCACCACTTCCAGATTCAGAGATTTCTGATGAAAAAGAAAATAATAAAAAATAT
GGTAAATATTCACTTTTAGGAATTAGTTTTTAAAATATGATTTAAAATCAGAGAGTAATACGC
CAGAAAGTCCTGGAGAAATGCCAGATACAAAAATGAAATCCGAAAATATTGTAATATATATCTC
AAATGGTACGAAAAAACCAGACATTGACATATTGGGTATTTCGATAGGCACAGGAACTTTGGAA
AATATTCTTACAATACATGCTGAAGTTGAAGATCCTTTCGAGGATATGGTAAATAAAGTTAAAT
TTGAATAAAGATATTTCGATGGAGACTGGAAAGACCTGGGGGATGTTGAAATAGATAATAATGG
CGATTGCATGCTCTGTTGGGATATTTCGGACCAGAAAGATGGAGAATATAAAATAAGAGCGTAT
ATGATAAATGCCAATGAAGAAGTCATAAATAGTCCTGAAGAAGGGGGGCCGTTATACCTAACAG
GTGATTTCTTATCCGCAAAAGAAACCATTTATGACCGTATAGCCATAGAAATATCATAAATTAAT
TATTTTAAAAAAGTAAAAAATTAAAAACTAATTGTTTTTTTATTTTCCAGAACCTGAACCCATTG
ACCCAGAAATTTGACTTGAACCGCCAATTTGATTACTGTTTTGAGCGTTTGGTTGCATTTGATTT
TGTTTGCTAACTCCTGTCGAGTTGCGTTTATTTCTATACTGGTATTGATATAAGCTCAAAGTTCC
ATCTAAAGCAGCCCCATTTGCATATGCTTGTTGATACTGTCCTGCGTTAATCATTGAATCGATGT
TGGATAAATTTTCCCGAAGTGCAGTTGCGTTTATACCATTATTTTCCATATTTTCGATTCTTATC
TGCAAATTATTTCGAATTCTTAAAACATTTTCTTCGAGTTCTGATTGGGCCATTGTGCGCGTATT
TACTTGATAGCCCATGTTTTCAAGATTATTTCTGATTGTTTGACCATTAAATAATGGCGTTGTTA
CAATATCAATATTTGAAGTATTGAAATTTCCAAGTAATTCGGAGTCTACTGAAATTTCAGATCCA
TCTGTAAGAATTACTACTGAATTAGAACTATTACAGATGTTTGAAGTACTTTCTCCGCCATAGAG
TATACATACATCCCTATCACGAAGTCTATCTCGGAATCTATCGAGTAACTGTTCATTTGTAGAAT
AACGATCTAACCCATAAATTCTTTCAACTTCGATACCTTCTAAAACCAGATTATCTTCGTACTCT
TCAGCAACTGCAACTGTACCTCCGACAATCATTACAGTTTCAGGGTCGTATGAAAGAATCTGATC
CAGTACTTCGCTATCGTAATCTCCCCAGATAGTTGTTATCACAACCATATCTTCATATGTAGATG
CAACTGATGCTGCACTACAGTAGTCTGCACAGTTTTCACTCACAAGTAATACATCTTCCGCACTT
ACAACTGGAATTAATAATATTAGCAGTAAAATAACATTTCTTAGAATATTCAATCTATCACCTCA
AAACCCCTAAAAATAATAAAAAAATGGAATTATTTAGTATTAATTAAAAAATAAGAATTTAATTA
CTATTTATCCCCTATTACGTATGAATATTTGTTTTTTATTGTACTTAAAATTTTTGATATTTTTT
TATTTTTCTAAATTGGAAGCATCAATATAAATGGCGTTATAAATGGGATTAGAAGCGTCAGTAT
GAACCCATGAACAAATGAAAGAATCATTATGTCTTTTCCACCAAATTTAACAATTATTGGAAGTG
TTGAGTCCATACTGGTTCCACCACCAAGAGTAATTGCCATATCCTTTGGAAACTTTTTTACCACA
AATGAATAGCCAATAATTGTCAAAACTTCTCTTGCAAGATTTACCAAAAATCCCATTACCCCATA
AATTGCAGAATATTTTGCTAAAATTGGCCCGCAAAGACTGTACCAGCCCATTCCCGATGCAATTG
CGAGTGCATATTTTAAAGGAATGTTTAACATGATTGATGAAATTATTCCGCCGATCAATGAGCCG
ATGATTGTAAATAATGGAAGTAAAAGACCAAATTTTCCAACTTTTTTAACACCTCTAAGACCGCT
ACTTTTTCCAAGATCAATTCCAATTAATAAAATCTGGATGTAAAGCATTATTTCGTACATATTTC
CAAAGTCAATTCCAATACTTCGTCCAACAATGAATCCCAAAATTAGGAAAATTAACACCAAAACT
GTAAAATTCATCTGAACCTACCTCCTAAAAATACTGCCACAAGGAGGCTTGTAACCATTACAATG
ACTGAAAATTCCATTGAAACTACAAACGTTGAAAATGCATTTAAATCGACTTTTCCAGCTTCCAC

ACCCATAAAAAATATCAAAAGAAGAAGTGCTAAATTTGTTGGTTTTGATAAATCGACTTTTTCTA
GACTGCTTTTAAATAAATATCCGATAATTAAACCCACTATCAAAATATAGATTATATTCATAAAA
ACCCCTTATGATTTAAAGTTGAAGCTTTACATCTGTTCAATTTATATTATATATCTTTCTAGATT
CCCAAATATATCTAAATCTTTCAAACTTTACTAAATCCATAAAATAAGTTTAAATTCATTGAATA
CATAAGTATTATTTGTAAAAAAAGCCCGAATAATGGATAAATTTAAAAAAATGTAATTTAAAAA
TTTCAAAGGGATTTTATGGAACTTTTCAGGTCAAAAACATGCGCTCTTTGTAAGGGTAGGAAACA
TCTTTGCGGAAGGCCAAGGTGCCCGATACTTGAAAAATTTAGGGTTGCAAAATCAGTCGAATCAA
AAATAAATAAAAAAGAAATTTTTGGAGCATCTCCCCCTTCCGTTTTTGTTGGAGAATTTGGATAC
CCAAACGTCAGGATTGGACCAATGGTTCCACCTGTGGAAGGAGATACTTCTTTTATGGATGACCC
CAGTCGATGGGACAATATTTCAATTCCTGAAATTATGGAATACCGTTCAATGCTTGTTATGGGCG
AAACTAATGCAAATGTTTCAGTGAATAAAAATTCAAAGTTATTAACCAACATTCAGGAACTTGCA
ATGGCGAATAAACCTGTTGACAGTGAAATTGAACTTAAAAAAGCTCCAAAACTTGAATTAATAA
CTGGTGGATTTACGCCCCCTGTCGGCCCACGAGAAAGTATGTTAAAATTCAGACTTGCAGAAAAC
CCGAAAATCCCCAGAAAATCTGATTATATTGTAGGTGATGATTTAAAGGCGAATGAAGGGATGA
TTTCACTTTACGATTCGGGATTTGATGAGTACTACATAATAAAACTTCTTTCAACAGGACTCCTT
GGAATAAATAAAAAACTCGTTCCAACAAAATGGAGCATAACTGCGGCACAGGATTTGCTTGGAAA
GCATGTAAAGAAAAAAATATTGGAAAATAACCAGATTAATGACTACGAAGTGTACTTTAAAAAT
TTCCTCGGGAATAGATATGCAGTTCTTTTGGTTCCAGATCTGTATGCCTTTGAAATGCTTGAAGT
TTGGCTTAAAGGTTCACTTTTCAGCGGTGAAAACTACCAGATTTTAGGAGACTTTGAAGACATTG
CCGGAATGAAAGGATACGCAAATAAAATAACAGGTGCATTTTATGCAGGCCGACTAAGTGTTTTG
GAATACCTTAAAAAAAGAAAAAAACAGGCAAAAATACTGGTATTTCGAGAAATTACCCCGGAAT
ACTATGCCCCAGTTGGAGTATGGCAGATTCGAACTGGCGTAAGGCTTGCAATGGAAAATAGGCTT
GGCAAATTTAATGATTTAAAATCTGCACTTGAAGAGATAAAAAAATACCTCGATGTTCCAATGA
AAGACTATGAAACAGAAAGTAAGATTTTAAAGAGCAACCAAAGGCAGGCCACCCTTGATAAGTTC
TTTTAATTTTTAATTTTGCAATTAATTTTTAGTTATTTTCTTTTTTCGTATCCTTTATCGATTCC
TTCACTTAATTCTTTTGCAAGTTCTTCTAATTTAACAAGTGCTTCTTTAGAATCCCCATAATTTT
TAATAATATCTACAAAAGCACTTCCAACAATTACTCCGTCAGCACCCTGTTCAATTAAACTTTCT
GCATGACTTCCTTTTGAAATTCCAAATCCAACGCAGATTTTTCCCTTACAGGTTGTTCTTGCTCGT
TTAATAAAATCAAATGTCATCTGTTCAAATGTATCTCTTGCACCAGTTATTCCAAAAGTTGAAAT
AAGGTACAAAAATTCAGTACTTGCTTCATCAGATATTTTTAAACGTTCTTCTGGAGTGTTTGGTG
CTGCAAGAAATACCGTTCCCATTTCATGTTTTTTACAGATTTCTCGATATTCTGTTGCTTCTTGG
AGTGGCAAATCAACAATAATAAGCCCGTTTGCACCTGCTTCTTTTGCCTGAATTACAAAGTTTTC
AATTCCTCTTTTGTAAACTGGGTTGTAGTAAGTCATCAAAATTACAGGAGTATCTGACTCTTTTC
TAAATTCTTTCAATACTTCAAATGATTTTGCAATTTTAAAATTGTTTGATAAGGACCTTACATCT
GCTGCCTGAATAGTTGGCCCATCTGCAACAGGATCACTAAATGGAATTCCAAGTTCGATTACTCC
CGAATATTTATCGAGTGTTTTCATAAATTTCAATGTTGCATCCGGATTTGGGTCTCCCGAAACTA
AAAAACTGACTAACACTGGTTTATTCATTTACATCCCTCTTTTTTGCATGATGTCGATTACGGTG
TTTAAATCCTTGTCGCCCCTTCCTGAAAGGTTTATGACCATTATTTCATCTTTATCCATGTTTCCT
GCATTTTCAATCGCATAAGCTACCGCGTGTGAAGATTCTAAAGCTGGAATTATACCTTCACTTCT
CGTAAGTTCCATAAATGCGCTTAATGCCTGATCATCAGTTGCTGATGCATATTCTGCTCTTCCAG
AATCGTGTAAATATGCGTGTTCTGGACCGATTCCAGGATAATCAAGTCCTGCGGAAATACTGTAA
GCTTCTTCAATTTGGCCATCTTCGTCCTGTAAGAAGTATGAAAGCATTCCGTGAAGAACTCCTTT
CTTTCCTTTTGAAATGGATGCTCCGTGAAGGCTCGTATTGAGTCCTTTTCCTGCAGCTTCGATTCC

AACCATTCTAACATCTTTATCATGAATAAATGCATGGAATAAACCAAGTGCGTTGCTTCCACCAC
CAATGCATGCAACTAAGTGGTCTGGGAGTCTTCCTTCCTGTTCCATAATTTGTTTTTTAACTTCT
TTTCCAATTACTGACTGAAAATCCCTAACAATTGTTGGGAATGGGTGTGGCCCCATAACAGTTCC
GATTAAATAGTGCGTGTTTTCAAAAGTTGCAGTCCAGTTTCTCATTGCTTCGTTTACTGCATCTT
TTAAAACTTTTGAACCAGTATCGACAGGGGTAACCTTTGCTCCAAGAAGTTTCATTCTAAATACG
TTTGGTTGCTGTCTTTCTGTATCGAGCCTTCCCATGAAAATTTCAGTTTCAAGTCCAAATAAAGC
TCCAGCCATTGAAGTTCCAACACCGTGTTGACCTGCACCAGTTTCTGCAATTATTCTTGTTTTACC
CATTTTTTTAGCAAGCAATGCCTGACCGATTGTGTTGTTAGTTTTGTGAGCTCCGCCGTGAAGTA
AATCTTCTCTTTTTAAGTAGATTTTTGCACCGCCAAGCTTCTTAGTCAAATTTTCAGCATAGTAG
AGTGGTGTTTCACGCCCTGCATAATTTTTTAAATAGTAAGAAAGCTCGTTTTGAAAGTCTTCATC
ATCTTTCAATTCAAAATAAGCTTCTTTAAGCTCGTCAATTGCAGGCATCAAAACTTCAGGGATAA
ACTGCCCACCGTATTCTCCAAAGTATCCTTCACATTTCATAATTTTCACCTAATTTTACATTTTCA
CAAAATCTTTTTATCAATTCTTCATCCTTTGAATTATTATTCTCAACTCCGCTTGAAACGTCAAC
TCCAAACGGTTTTACATTTTTTACAATATTCAACACGTTACCGGGATCAAGGCCACCTGCAAGAA
CAATATCAAATTTTTTTGCGATTATCTGGCTTATTCTGTGATCATGAGTTTGTCCACATCCTTTT
CCAGTATCTACTAAAATTCTATCAACTTCATAAGATTCAATATCTGAAATAATTTTTTCAGCATC
GATTTCAGGTGATTTGCTTGTTTCAGGAATTTTAAACGATTTCATTATAAAACATCCGTACTCAT
TTTTTATGAAATCTATTGCTTTTTGGTCGATATCTGAATGCATCTGTAAATAATTTGTACCGGTC
AATTCAATAATATTTTCCCATACTGAAACTTCAGATGTTGTTGAAACTGCAAATACCGGAATATT
TGATAAATCTACAAGATTTTTTGCTGTTTCAAGACCAATTCTTCTTTTTGAAGAACATTCCAAAA
TTACCCCCATAGCGTTGCCGTAATTTTCAACTATTCTTAGTTCTTCTGGAGTTTTTATTCCGCAAA
TTTTTATGAACATAATTTTCACTTAAATTTCCTTTTTAAAAGATTTATATGTTTTAGCGAATTTC
ACAAAATTTTCAATAATTTTTAGCCCTTCTTTTTCTTCAAATTCCGTTAATACACTTTCAGGGTG
GAACTGAACTCCTTCGATTGGCATAGTATTGTGCCTTATTCCCATAACTGTTCCATCTTCAGTTTT
TGCAGTAACCGTGAAATTTTCTGGAACTTCTAAAACTGACAAAGAATGGTATCTTCCAGCAGTGA
ATGGATTTTTCACATTTTTAAAGATTCCTTTTGAATCGTGTTTTATAGAACTTGATTTTCCGTGA
ACTGGAGGAATTCTTCCAACTTTTCCGCCAAATATGTGTGCAATAGTCTGGTGTCCAAGACATAC
TCCAAGAACGGGTACATCCATAGTTTTTATAATTTCAGGACAGTTTTCAACATCTCTTTTATTTT
CTGGAGCTCCAGGCCCTGGTGAGATAATTATTCCATCAGGATTTAATTTTTTTAAATCATCAATT
GAAATCGTGTTTGGAACAACCTTTACAGAATCATAAATCGATGCATAGTCTGCCAAATTCCATAC
AAACGAATCTTTATTATCTATTATCACTATCATTTCGAACACCCAGTGCTTTCATCATTGCAGCC
ATTTTCCGCTCTGTTTCGTAGTATTCATTTTCAGGTACCGAATCAGCAACCACTCCTCCGCCTGCC
CTTACCCGGCAAACGCTGTCAATTTCTGCCATTCTAATTCCAATTGCAAGGTCCGCATTGCCGCTG
TTTGAAAAGTATCCAACAGCTCCACCGTAAATTTTTCTTCTTGATTTTTCAACTTCGTCAATAAT
TTCCATTGCCCTAAACTTTGGAGCTCCGGTTAATGTTCCTGCGGGAAATGCAGCTTCTATTGCATC
AAATATTGTTGAATTGTCTTTTAATGTCCCTAGTACTTCACTTTCAATGTGCTGAACATGGCTGT
ATCTTACAACGTCGAAAAACCTTTCAAGTACAATGCTTCCAGATTTTGAAACTTTCCTGACATCA
TTTCTTGCAAGGTCAACAAGCATCATGTGTTCTGCTCGCTCTTTTTCGTCTTTTAAAAGTGCTTCT
GCCAATTTTTGAGTTTCGTTTTCAGTTTTTCCAATTGCAGTAGTCCCTGCAATCGGGTTTACTTT
TAAGATATTATTTTGAACTGAAGCCATTGTTTCTGGCGATGCCCCAACTACATCCTTGTCAAATT
CAAGCAAAAACATGTAAGGACTCGGATTTACATTTCTAAGATTTCTGTAAAGTTGAAATGCAGAA
AATTCATTTTTCAAGCGGTATTCTCTCGAAGGAACGACCTGAAAAGCGTCTCCCGAATAAATATA
TTCCTTAGTTTTTTCAACCATTTTTACATAATCATCTAGGTCTGCGTCGCACCCTAAAACTTCAAT

TCCTCCGTCTTCTTCTTCAATTTTAAAATTTTTTGCTTTTTCAACAGTTGTTTCAGCATTTCTTAA
TTCTTCAGGATTATTCGTTTCAGAATGATAATAAAAATTATTTGTTTCGTGGTCGTAAACATACA
TATGATCGTAGTATCCAAATACTGAAGGTTCTTCAATTTTTCCGCCGATGTAGTTATGGATACAG
TCATATGCAATGTATCCAACGTATCCGCCAGTAAATCTATCCCCTTTTTGAGTAATCTTGAAGTT
TTCTTTTAAAGCTTTGAACGGGTTACTTTCTTTTGAAATCGTTTCGTTGTCCATTTTGGTTTTAT
TTCCGATTCTAAGCATGTATTCGGGATTTGAAGAAATGTATGTGTATCTCGCATTAACTTGCCCT
TTAGCTCTTGATTCAAGCATTACTGGATATTTTCCTTCATCCCGTAAAACCCCGTAAAGTTTTAG
AGGATTTACATAATCCAATTTATACATTTCTTATCTCCTCAAGTTTTTTAAGGACTTTTCCAGAT
TCGATCGCTTCTTTTGCAATTTCAACACCATCTAAGAAATCAGAAGCAATTTCAGAAGTATAAAG
TGCAGCTGCAGCATTCATCAATATAAAATTCCTATCTTCATTGATTTTTCCAGAAAATACATCAA
TTAATCTTTTTGCACTTTCATCAGGGCTTTCACAGGGAATTATTTTTGAGTAATCCAATCCAAAG
TCTTTAGGAGTTACTTCGTAAGTGTCAAATTTTCCGTCATATTCAGTTATCTTTGAAGTGCCATT
TGGAGTTAACTCATCAAGTCCATTACCATATACAACTAGTGCTTTTTTAATGCCTCCAACTTTTG
ATAATGCAATTGCAACATTATCACATAATTCTGAGGAGTTTACACCAAGTATCTGGTATTTTGGG
TTTGCAGGGTTTGCTAGAGGTCCCAAAATATTAAATATTGTTTTTACTTTCAGTTCGTTTCTAAC
AGGCATTATTTTTTTAAGTGCAGGGTGGTATTTTTGAGCGAACAAAAATGCAAAATTGGTTTTAT
CCATTGACTTTTTCGCTTCATCAGGAGTTTCTGGAATTTTACAGCCAAGTGCTTCGTAAACATTT
GCCGAACCGCTGTTTGAAGTAATTGAAACGTTTCCGTGTTTTGCAACTTTTGTAAAACATGCAAG
AATTATTGAAACTGCCGTGCTTACGTTTATTGTTTTTGAACCGTCTCCTCCAGTTCCGCACGTGTC
GGTAACGTCCCCAAGGTCGATTTTTACTGCATTATCCCTCATTGCTTTTGCAAATCCTGCAATTTC
ATCAGAAGTAACTCCTTTTGTTTGTAATGCCACCAAATAAGCTGCTATTCTCATTTCACTTTCAT
TTAAAAGCATGTTAAATAATTCGTATGATTCTTCAATGATAGATTTTCGCGTTCAATTAGTTTG
TTTAACATTATTTTCCCTCAACAAAACTTTTTACGAACTCTTTTTGGTTTTCGGCTGTCATAAAT
GATGTTCCAATTAGTGCAGCATCTGCATATTTTAATGCAGTATTTAAATCGATTAAATTAGCAAT
CCCACTTTCACTGACAAGAATTCTGTCTTTTGGAATTAAATCTGCAAGATTTTCAGTTAAAGAAA
CAGTTCCATCATCAAGTTCCAATTTCGAAATATCCCTGTTGTTTATTCCGATCATTGTTGTGTTC
GTAGATTTCGCAATTTCAATTTCTTCCGTGTTATGAACTTCAACAAGCGTATCAAGCCCGCATTC
GAGTGCATAGTCTACAAATTCTGCTGTTTTTTCTTTCAATAATCTTGCAATTATCAAAACGGTAC
TTGCCCCGGCACTTGCTGTTTTTTCGATTTCGTCTTTTGTTGTTAGAAAATCTTTTCTTAAAACTG
GAAGTTCGGTGTTTTCACAGATTTTTTTAAAAACTTCAAAGTTTCCGTTGAAATGCTGTTTATCA
GTAATATATGAAATTCCAACTGCTCCAGCTTCTTCATATATTCTTAAAATATCCATTTCAGATCT
TCCTTTCAACCAAATCGCCATATTTTGGAGAATGAACTTTAATTTCCGCAATGATTGGGTTTTTGT
CGAAATTTTTTATGGCATTTGCCTGTTTTTTAAAATTTATGGTCATAACTGCCCTCTAAAATTTA
AACTTTGATTAAATTGATGAACTGTGTAGTCGATACTTTGAATTTGTATATTAATCCGTATTGTT
AGATATATTGATATTAACTATACTGATATCTTTAAATTCTTGATAACAACTGCAAAAATACTAAT
TAAACAGACTATAGCGTCCACTAACAAATTACACAGTGATTACTGCCACCACTGGAATAAAACAA
CACATTCAAATGAACTTAATTTACATAATGATTCGACTTTTATTTCCGACTGTTCCGAACTATAC
GTCATATTACCACCGTTCTAAATTTTACATATTATAATATGTTTTTGAAAGATTATAATATATAT
ATCTTCCCATTAACCAATGTTGCAGAAGGTTTTCCTTTAACAGTTTTTCCTTCAAAAGGAGTAAA
TTTAGCCTTTGATTTAAAATCTTTGGCAGATATTTTGTATTCTTTGTCCATATCGACAACAGACA
TATTCGCGCTATTTCCAACTTCAATCTTATTATTTATTTTAAAAATTTTTGAAGGATTTTTTGAT
AAAACTCGGTATGCATCAAAAATTGAAATTTTTCCTTCATTTACCAAATTCATAATCAATGGCAC
GAATGTTTCAATTCCAGGAATTCCTGAAGGGCATTTTTCAACCACATTTAATTTTTCATCGATTA

AATGGGGCGCATGATCAGTTGCAACAATATCAACTGTTCCATCGATAATTCCATTTAATAGTGCT
AAATTATCTGCTTCTTCTCTTAATGGAGGGTTAAATTTTCCACATCCTTTTAAATCTTCAGCCAT
TTTTGAATTTAAAAATAAATGGTGGGGGCTAACTTCAACAGTAACTTTTACATTTTTAAAAGTTT
TTTTAGCGTTTTTTATTAAATCAAGCGCTTCTTTAACGGAAATATGACAGAAATGAACGTGAGGC
TTATTGTTACTTTCTTTATCGATTTTTTCTAAAACTTCCAAAACTTCCCTAACTGCTTCAACTTCA
CTTTCCCGACTTCGAATTTTACAGTGATTTTTGAAACTATTTAATTCATATTTTTTGGAATTTTC
TAAAATTACATTTTTGTGTTCTGCATGAATTGCAAAAAGCTTATTCTGATCGAGAATTTTCATTA
ATTTTGAGTAATCACTTATAAAGAGATCACCGACTGATTTTACCATGAAAATTTTATAAAATTTT
GCTTCTTTGACATTTTCTAAATAATTATTTTCAGTAACGCCATAAGCAAAATACAAATTAATTTT
GCTTTTTTCGTTTCCTTCTTTCATTTTTTTGTAAAATATCTCTTTTGTGGTCACAGGAGGCGTGT
TATTTGGCATATCTATTGCATAAACAATTCCGCCAGCAATTGCAGCTTCACTTCCTGACAAAAAA
TCTTCTTTTTCGGGATTTCCCCAGCGAAAATGAACGTGGGAATCGATAAACCCTGGAAAAACATA
ATTTCCTTTTAAATCAATTGTTTCACCAGAATAATCGATATTTTTTTCAATTTTTTTGAATTATCC
CATCTTCAATTAAAATATCAGATTCAGTTACATAATCGTCTGACACGATTTTTGCATTCTTTAAA
AGTACCATTTTTTCCCACAACACACTATTTTTACTCTTTCAAGTTTTTTATGAGATTTTTTATAT
CATCTGGCAGTTTTGAAGTAAATTCCATTCTTTCTTTTGTGATCGGATGAATAAATCTTAAAATA
TGCGAATGAAGAGCTTGTCTATCAATTAAATTAGAAATTTCCCCATAAAGAGTATCTCCAATTAT
ATAATTTCCAATACTCGATAAATGAACCCTTATCTGGTGAGTTCTACCAGTAAGTAAGTTTATTT
CAAGAACTGATGCGTTTTTGAGTTCTTCGATTACTTTGTATTTAGTTTTAGAAATTTTTCCAGAA
CTGGATAACTCTCTTCTTATGCCGTCATCATTTAAATCGATTGAATCTTCAATAATTCCTTCCTTT
AAATCCAGATGGCCGTTTACTACCGCAATATATATTTTTTCTAAAATTCCTTCATCCATCTGTTT
TGCAAGCTGTTGATGAGAATAAGAATTTTTTGCAACGATTAAAAGTCCAGTAGTATCCATATCAA
GACGGTTTACAAATCTTATTTTGTAGTTTTGATTATTTATTTTCTGGTGATTTGAAACTGCATTT
GCAAGTGTTTTTGATAGTACATTTTTAGTTGGATGAACAACCATATTTTTTGGTTTATTAACAAT
CAATAAATCGTCATCTTCATAAATTACGTTTAAGACCAAGTTTTCAGGAGTAATATTGTTGATTC
CACAATCAATTTCAACAGAAACGGTTTCATTTTCTTTTAATTTTGATTTTAAGGAAATATTTTTA
GAATTTACAAAAATTTTATTTTCTTTATCAAACTTTTTTATCATTTTATTGGAAAGTTCAAAATT
GTCCTTTAAAACATCGATTAACTTGTTTCCAGCAATTTTTGAATCAATTGTTAGTTTTAAAATCT
TTTTTTGACTTTCCATTTGGAATCCCTTTTCTTTAAATTTAGTTTATATTTTTTAAATTAATTAT
AAATTTGTTATACAAAACTCGTAAATTTTAAAAATAAATAAAAATCTAAAAAAGTTATAAAGTT
ATTTATGGGTAAAGTAGCAGGTATATCCATTGTCTACTTTTTTGTCAATTCTTACAAATCCAAAT
CTTTCAAACTGGACAAAATCATCTTCTTTTACAACTGCAAAGTCTTTTTCTGCAAATCCTTCAAT
TTTTTCAGCATTTTCATCGATAACGGTTACTGAAATACTGTTTTTTACAGGAATCCAGTGAATTA
TGCTTGCTTTGTTGCTTTTTGCAATTGAAAAATCATCACTGTCGTATTTTGCATAGATAATATCA
TTTTCTACTTTTTCAACAACAATATTGAATAATTCCATTAATCTATACATTTTTCCAGCTTCAAG
ATCGTCTGAAACGTATATTTCGCCATCAAATAACAATTCCCTGTTTCCAAGTTCACTTCTATCAG
GGTGCATTCTAAGATCGATTTTTCTAGAGTCTGTTCCAGAAATTACTACTTTTTTAGGACTTTCT
ACAAAGAAGAATCTTCTTGCATCTTTATCGATTATGTCCTTATTTGCAGCGTAGAGGTTTTCCCA
TGCAAACCTAACATCTGCCTGTTTTATTCCAATTTCAACCATTAATTTATAGAGTGCTTCTGGCC
TTATTCCTCTTTTTCTAAGAGCCCTCAATGTTCCAAGCCTTGCATCATCCCAGCCTGAGTATTCTC
CGCTCAAAATTCCTGCATGCATTTTTGAAGTACTTAAAACAGGTCCTTCTATTTTTAAAATTCCG
TAGTGAACGTATTCTGGAATATCCCATCCGAAATAATTGTAGATATATTCTTGTTTTTCAGTGTT
TACAATATGGTCTTTTCCCCTTAAAACGTGGGTCATACCGAGTAAATGGTCATCAACAGTTACTG

AAAGATTCATTAATGGATATACGTGATATTTTGTTCCATTTTTTGGGTGAGGGGTTCTTTCAATT
CTAAATATTGGAAAATCTCTGATTGATGGGTTTTTGTGTGCAATATCTGTTTTTAACCTTACTGC
AACATTGTCAAGTTCTCCAGCGAGCATTTTTTCCCATAATACAATATTTTCTTCTGGAGTAGTGT
CCCTACATTTACACGGGAGCCCCTGTTCTCTTAAAGTTCTAAACTCATCCGCATCACAATCGCATA
CGTAAGCGTGTCCCATTTCAATTAATTTTCTACCGTATTCATAGTAAGTTTCAAGCCTTTCTGAC
TGTACGATTACTTCGTCAACTTTTACTCCGAGCCATTTTAAATCTTCCTGAATCATTTCGTAAGCT
TCTGGAAGTACTCTTTTGGCATCGGTGTCTTCAAGCCTTAAAACAAGCTTTCCGTTGTATTTTTT
TGAGAAAAAGTCGTTTAATACGGATGCTCTTGCGTGTCCGATATGAAGGGGTCCTGAAGGATTTG
GTGCAAATCTCATTACAACATTTCCTTTAACGTTTTTTAGTTCAATTTCCTTTCTTTTTCTTTCTG
TTTTTTGACCCATTCCTTTAGGAGCAAGTTCACTTAATTTTGCATGCTGTTCTTCAAGAGACATT
GTTTCAATTTCTTTTAAAACTTCAGAAATAACCTGATTTACTTCTTTAATTTTGCTTCTTAAATC
AGGGTTTTCCCCAAGTATTTTACCCATTGCAGCTTTTGGATTTGGTTTTCCTTTGAATTTAATCG
AATTTTCAAGTAGATATGCCATTACAGTATCTTTCATAATTATCACTGTTTCTAAAATCCTTTTC
AAAAATTGTTCTCTTAAATGTAATGCGGTATCGCTTAAAAAGCTTATCGATTTTAAAAATTAAA
ATAACTCTAGAATAGTATCTTTAAAAAAAATAAAAAAATAAAAAAGTAAGGAACGGATATTTTT
CAGAAGATCTATTCAAATATAATTCCCACAGGACAGTGGTCTGAACCCATTACTTCAGGCATTAT
AAATGCATCTTTCAAAATATTTCGTAAGCTCTCAGAAATACAGAAGTAATCGATTCTCCAGCCTA
TGTTTTTTGCCCTTGCATTAAATCGGTAGCTCCACCAAGAATAATTATCAGGTTCTTTGTTAAAT
TCCCTGAAAGTATCTAAAAATCCAGAATTTACAATATTGTCAAACCCGTTTCTTTCTTCAGGAGT
GAATCCTGCATTTTTTACATTAGTTTTTGGATTTTTTAAATCGATTTCTTTGTGTGCGACATTTA
AGTCCCCACAAAATACCACTGGTTTTTTAGTTTCAAGGGTTTTTATGTAACTTAAAAAGTCTTCA
TCCCATTTTTGCCGGTATTCAAGTCTTGTAAGGCCTCTTTGTGAATTTGGGGTGTAAACATTTAC
TAAATAGTATTCATCAAATTCAAGGGCAATTACTCTTCCTTCATTGTTGTGCTCGCTGTTTTCCA
TCCCCAAAATAACGTTACTTGGTTTTATTTTTGTAAAAATTGCAGTTCCAGAATAACCTTTTCGT
TCTGCATAATTCCAGTACTGAAAATACCCATCAAGTCCAAGCTGGACCTGTCCGCTTTGCACTTTT
GTCTCCTGTATGCACATAATGTCAGGACTTTCACGTTCTAAAAAATCCATGAAGCCATTTTTCAA
ACATGCGCGAATTCCATTTACATTCCACGATAACATTTTCATAAAATACTGTTATGCTGAAAAAC
CTATAAAATACTATAATGTCTTTAAAATTATAAAACTTCATTTTAATCGATTCAAAACATTTGTA
ATTATTTTGAGCGGATAATTTAAATATTGGTCTTTTGATATACTTTGACAATTTGAATTTTATAT
TAGCATAGAGTTTAAAAACGAGAATAACATATTATATGGAAAAGTATCCTAAGGTGGAATTTTG
AAAAGAGAGGATATAAAAAAGGTAATGATTTTGGGTTCTGGCCCCATTGTTATCGGACAAGCAGC
GGAGTTTGATTTTTCTGGCTCGCAAGCTTGTAAATCTTTAAAAGAAGAGGGAATTTATACTATTT
TAGTAAATTCAAACCCTGCAACCATTCAAACCGATACAAATATTGCAGACAAGGTTTATTTAGAA
CCATTAAATCCAAAAATATTGGAAAAAATCATTGAAAAAGAACAGCCTGATGCGATTCTCCCAAC
AATGGGTGGTCAGACTGGTTTAAATCTCGCAATGGAACTTTCAAAACGCGGAATCTTGGAAAAAC
ACGGTGTTGAATTATTAGGTTCAACTGAAAGCGTAATTGAAACTTCAGAAGATAGGGATTTATTC
AATAAAGCAATGGAAGAAATTAACCAACCAATTGCAAAATCTGCAGCTGTTCATAGTGTTGAAG
AAGCAATTGAAGCTACTAAAGAACTTGGATACCCTGCAATTGTAAGACCTGCATTCACCCTTGGA
GGAACGGGCGGTGGAATTGCAAATAACGAAGAAGAATTAATTGAAATTACGAAAAAAGGATTAA
AATACTCAATGATCAAACAAGTTTTAATTGATCAGAGTCTTTTAGGCTGGAAAGAATACGAATAC
GAAGTAATGAGGGATAAAAACGATACATGTATTGTTGTATGTAACATGGAAAATATCGATCCAA
TGGGTATACATACTGGAGAAAGTATTGTAACTGCACCTTCACAGACCTTAAGCGATGAATTCCAC
CAAAAATTAAGAGATGCATCCTTACAAATCATCAGACACTTGAAAATTGAAGGTGGCTGTAACGT

TCAGTTTGCAGTAAACCCTGAAATGACTGATTATGTTGTAATTGAAGTAAACCCGAGAGTTTCAA
GAAGCTCTGCACTTGCAAGTAAAGCAACAGGATACCCGATTGCAAAAATCGCTGCAAAAATCGCA
ATTGGAAGAACACTCGATGAAATACAAAATGACGTTACAAAAGAAACGCCTGCAAGCTTTGAACC
AACAATCGACTACGTTGTAGTAAAAATTCCAAGATGGCCGTTTGATAAATTCAGAACCGTTGACA
AAAAACTTGGAACTTCCATGAAATCTACCGGAGAAATCATGGCAATTGGAAGAAACCTTGAAGA
AGCTCTTCAAAAAGCAGTTAGAAGTTTGGATATCGGAAGATTTGGAATAATTGCTGATGGAAAA
GACAAAGAATACTCAAATTCTGAAATTGTTGATATTTTAGAACATGCAACAGATGAAAGACTCTT
TGTAATTGCTTACGCACTCGATAAAGGATGGAGTGTTGATGGAATTTGTGAAAGAACTGGAATA
AATCCATTTTTCATTGAAAAAATCAAAAAAATTATCGACTGTAAAAAAGAACTCGAAGTAATTT
CAAGAATTCCTGTAGATGACGAAAAATTAAAAGAAATCCTTTTAAAAGCAAAATCATTAGGATT
TTCAGATGTTCAGATCTCAAAAATATTTTCAAAAACTGAAAATGAAATAAGAGATCTTAGGAAA
AGACTCGAAGTAATTCCAGTTTATAAAATGGTAGACACATGTGCAGCAGAATTTGAAGCTAAAC
GCCATACTACTATTCAGCATATGAAAGATACTTCGATGAAGAACAGAACGAAAGTATTCCAAGTG
ATAGGAAAAAAGTAATAATTTTAGGTTCCGGACCAATTAGAATCGGCCAAGGTGTTGAATTTGA
TTACTCAACAGTTCACGCAATTTTTGCACTAAAAGAACTTGGAATCGAAGCAATAATTGTAAACA
ACAACCCTGAAACCGTAAGTACTGACTACGATACTTCGGACAAACTATACTTTGAACCGTTAGTT
TACGAAGAAATAATGAATATAATTGAAAATGAAAACAAAAACGGGCAGTTGCTTGGAGTTATCG
TTCAATTCGGTGGACAAACTGCAATTAACCTTGCAATGAAACTCTACAATTCCGGAGTAAACATA
TTAGGAACTTCTCCGCAATCAATCGACCTTGCAGAAGATAGAGATCAGTTCATACATGTGCTTGA
AAAATTAAAAATACCTCAAGCAGACGGTGCAACTGCATTTAGTGAAGAGCAAGCTCTTAAAGTTG
TGGAAAGAATCGGATACCCTGCCCTTGTTAGACCTTCATACGTTTTAGGCGGGCGTGCAATGCAA
ATAGTTTACAATACAGAAGATTTAAAAGATTACATGAGAGAAGCAGTTAAAGTTTCATCAGATC
ACCCGATTTTAATTGATAAATTCTTAGAGGAGGCTGTTGAAGTCGATGTCGATGCAGTGTGCGAT
GGAGAAAGCGTATTCATTGGCGCGATTATGGAACACATTGAAGAAGCCGGAATTCACAGTGGGGA
CAGCGCTTGTGTAATTCCCCCTCAAACGCTTTCAAAAGAAGTAATTGAAAAAATTGCAGAACATA
CAACCAAACTCGCACTCGAACTTGGAGTAATTGGTTTATTAAACATTCAGTACGCAGTAAAAGAT
GGTATCGTGTATATTATCGAAGCAAACCCGAGAGCTTCAAGAACTATTCCTTATGTAAGTAAATC
AGTTGGAGTACCTCTTGCAAAAATTGCAACAAATGCGATTATGGGTAAAAAATTAAAAGAAATG
GGGTATTTTGGACTTGCTAAATCAAAATATGTAAGCGTTAAAGAAGCAGTATTTCCATTCTTAAA
ACTTCCAGGAGTAGATCCAGTATTAAGCCCGGAAATGAAGTCAACTGGTGAAGCAATCGGAATTG
ACCAGGACTTTGGAAAAGCATTTTACAAATCCCAGCTTTCTGCAAACATGGAACTTCCAACTTCG
GGAACTGTCTTTATCAGCGTAAGAAACAGGGACAAAGACAACATTACAAAAATTGCTAAAAAAT
ACCACAATTTAGGCTTTGAAATCGTTGCCACAAGAGGAACTGCAAGAGAGCTTCGGTTATTTGAC
ATTCCTGTAAGGGAAGTTAGAAAAATCTCAGAAAGCATGCAGAACAGCGTTCTTGATTTAATGCA
GAAAGGAGAAGTCGACTTAATAATAAACACTTCTTCAGGAGACAGAGCTAAAACTGACGGATACT
TCATCAGAAGAGCTGCAGTTGAGCTAAATATACCCTGCATGACCACACTACAGGGTGCATACGCT
GCAATCAAAGCTATTGAAGCAATTAAATCCGGAGAACTTGGAGTTTATTCATTAAATGAACTTGA
AAATTAATTTAAAACATAATTATTTTATTTTAAATTTTTTATTAACTTATTTTCTTATTTTGAGG
AGATATTATGTCGATGACTGATTATTTAAAAAATATTCGTGAAAAACATCTTAAAAAACGAAAA
GAAAGAAATCCCGATTATCCAATTGCAGTTTGGATTCAAGACGATGTTTTTCGCGATAAAACGTT
TGGACAAAGCTTTACAATAATTTTAAGAACAAAAGGCTGTAGATGGGCATATGAAAGCGGCGGC
TGTACGATGTGTAGCTACCTAATGGATGCATCCCCTGCTGAAATTACTTCGGAAAACTTGAAAAA
CCAGTTCGATTCTGCAGTTGAAAAATATAAAGATCAACTCAATAAAAATATGTCAATAAAACTAT

TCACATCAGGCAGTTTTTTGGATACTTTTGAAATCCCGCTTGAAACAAGAGAATATATCTTTAAA
AGAATTGAAGAAATGGGTGCAAAAGAAGTTGCAATTGAATCGAGAGCTGAATTTGTAACTCCAA
AAAACATGGAATCGATAAAAAATATTCTTTCCTGCAACATTGAAATCGGTGTTGGAATCGAAAGT
TTAAATGAAAGAATAAGAAATGTTGCAATCCACAAAGGAGTTCCAACAAAAGCAATTGAAAACG
CATTTTCCGTTGCAGAAGAATATGATGTAGGGATAAAAGCCTATATTTTGATAAAACCGCCATTT
ATTTCTGAAAAGGAAGCAATTATTGATGCAATAGATACTGCAAACAAATGTATAGAAATGGGTT
GCAGTAGAATTTCTTTCTGTCCTGCATCAATTCACAAAGGAACCTTAATTGAAACACTCTGGAAA
AAAAATCAGTACAGGCCTCCATTTTTATGGAGTATTTTAGAAATTTTAAAAGAAGTAAAGTCTA
AAAATCCTGATGCTCTGGTAATGTGCGATACTGCAGGGGTTTCTTCAAAAAGAGGGGCGCACAAT
GCTATTGATTGCACCTGCAACGATAAAATAAAAGATATATTTAATGACTTTACAGTTACTCAAAA
AATTGAAAAACTTGATATCGAATGCGACTGTAAAAAAGAGTGGATTGAGTACATGGAATACGAA
CAGAAAAATATTGTACCGCTTGGCGATGAAAAATTAATTTAATTTATTTTTTTAAATATTCTTT
TGTGTTATTTTCCCATTTTTGATTTGCTGTTTTATATAAGGTTTAAATAATTTAAATGCCATATG
ATTCTAAAGATAAAATAAGAGGTCTTATTATGATACCAAAAGGAACCGTTAAAAGAATTATGAA
AGAAAATACTGATATGAATGTATCAGCTGAATCTGTCGTAGCATTAGTTGAAATTTTACAGGAA
ATGGTTGTAACCACTACAAAAATCGCTGAAGAAAATGCTGCAAAAGACAAAAGAAAAACACTAA
AAGCAAGAGACATCGAACAGTGCGATGCTGAAAGATTAAGGAAAAAAGTGATCGAAGTTTCAGA
AAGAACTGAAAAAGTCAACATGCTTACAAACGAAATTTTAAATGTTATTGCAAATGAACTTGAA
AGATATTAATTATTAATGGTTCTTTTGTTAAAACAACGGAATTTATGATTAACATACAAATTTT
AACTTAAATATTTAATTCCATGAATATCAATATACTCTTCTTAACCCGCCAAAACGGAGAATTAT
CACTGCTTTTTCAAAATCATATCGGAAACCATATAAAATCTCAAAATCATAAAATATACCATGGA
CTCGAAAAGGGGTGAAAGTCTTGAAAGAGCAAGTAATTGACATAGCCACAAAAGACGTTGTTAC
AGTAAGTCCTGATACGCCAATATCAAAAGCTGTCGGAACTATGGAAAACAAAAAATTCCATAATT
TGATAGTTGAGAAAGATGATGATATATATCTAGTAACAATGCATGATTTGCTACTTGGAAACTCT
GTGCATCAACAGGTTGAAGATCTGATGTTTAGACCATTTTGTGTAAAAATGAATACGCAGGTGAT
TGATGCCGCTTTTGAAATGATTAACAGCGGTCAGAGAGTAGCTCCAGTTATAGACGAAAATGACA
AACTAATTGGAATTATTACTGATTACGATGTCATGAAGTGCGCAGCAGAGTCAGAGCTTTTGAAA
GACGTTAAAATCGATAAAATAATGACGAAAAGCCCCGTCACAATTGATATCGATGAAAGTATCGG
TAAGGCAAGAAGTTTAATGATGAAATATAACATCGGAAGACTAATTGTACTCGATATGGATGGA
AAGCCTATTGGAATGGTTACAGAAGACGATATAGTTAAAAAGGTCTTCAAACCAAAAACCAAAA
TGACAGTTGGAGAACTTACTGGCAACAAAGTGCCGAGAATGGCCCAGCCCGTATCAATGATAATA
AATAAACCACTTATTACTGCAAATATAGGCGATTCAGTTGCGGAAGTAGCGGAAATACTCGAACA
ACAAGACATTAGAGGAATTCCAATATTTAAAAACGATACATTAAGAGGTATCGTTACAAGACTT
GATATTTTAAAATACCTAAGGGATTTAAGAGCTGAATCAATGGTTGAAGTTGAAATTCAAGGTG
ATTTCGACGAAGACCAGAGAGAACTTGCAGAAAGGATATTATTTAATGAAATTAAGAAAATTGC
CATTTATTCGAAAAAAATCCACTGGATTAAATTAGCTGTTAAAAAAGAAAGAGATAAAGGAGGA
GTTCCAAACTACAGTGTAAAAACGTACGTAAAAACTCCAAGAAAACTTTACGTTGCAGAAGGAAA
ACCAAAACTTTCATCAACCAAAAGAATAGACTGGGACGGCGAAGAAATGGAATTAGTTGCCGAAA
AACAGAGATGGGACTTTATTGAAGTATTAAAAGATGCGCTTGATTCTGTAAAAAAAACAGATGAA
CATTGATAAAGAAAAAAGACAGTCTAAAGCATTAAATATTGGTAAAAAAGAAATAATGGCTGAA
GAATTTTCCGAAACTCCTACTGAAAACGAGGAGTAAATTCTCTTTTTTATTTATTTTTTTAAAAA
AATTAAAAATTTTTTAAAAGTTTATTTCATCTTCAACAAATGTTTTGTGAAGTGTTTTTAAACAG
GGTTCGAGTTTATCTTCATTTATTACAAACGAGATGTTTGTTTCAGATGAACCTTGTGCAATCAT

TTTTATATTTGCACCGCTTTCTGAAACTGCCTCAAACAATTTTCCAGCAATTCCTTTTGCGCCTTT
CATTCCGGAACCAACAACTGAAACTACACATACCTCCTTATCAAAAGTTATATCTTTTATAAGGT
GGCACTCTCCAAACTCATCTCTTAATTCCCTAACACATTTTTTAGCTTCAAGTTCCCCATCATAAA
TTACAATCGAAATGTTTGTTTCAGATGAACCTTGTGTAATCAAAATTACGTTTGCATTTGATTTT
CCAAGCACGTTGAAGATTCTTGCAGCAGTTCCGCTGACTCCAACCATTCCGCCACCAAATATGTTA
ATTAAAATTACATCTTTAATGGTAGTGATTGCTTTTATAATTCCGTTGCTTGTTTCTGAAGAATC
GGTGATTAAAGTACCTTCATTTTCTGGTTCAAACGTATTTTTAATTCTAAGAGGTATTTTTTTCT
CCATTACTGGTTCCATTGTTCTTGGGTGAAGAACTTTCGCACCAAAGTATGCAAGTTCCATTGCT
TCAATGTACGACATTTTTGGAATCTGTTTTACATTTTCGACCATTCTTGGGTCAGCTGACAAAAC
ACCGCTAACATCAGTCCATATTTCAACCATATCTGCAGTTAATCCAAGACCAACCAAAGCTGCTG
AATAGTCACTTCCACCCCGCCCTAGAGTTGTGATGTGGCCGTCTTCGGTTCCTGCAACAAAACCAG
TAACTACCGGGATAAATCCATCTTTTAATAAAGGTTTGATTTTTTCAGAAACTTCAAGTCTTAAA
ACTTTTGCACATGTGAAATTATCATCAGTAATAATTCCTGCATCTCTTCCTGCAAGGTAGAGGGA
GTGTTTTCCCATATCTCTTATTGCACCGCTTAAAATTGGAGCTGAAAGCCTTTCACCAAATGAAA
GAATAAAATCTTTTGATTTTGGGGTTAATTCTCCAAGATATGAAACTCCGACCAGTACTTTTTCT
AACTCGTTTATCGAACTTTCTATCGTTTTTGAAACTTCCACCCTAATTTCATGGTTTTCAATTGCC
TGTTCTATTGCAATTTCGTGTTTTCGCCTTAAATCTTCAATAAAGTTGTTTATTTTTGCAATATC
TCTTACATCAAGTGCCTGAGCAGAGATTTCTACAAGAGAATTTGTAACTTGTGTCATTGCGGAGG
TTACAACTACAACATCTTTATCTTCATTTGTCTTATTTACAACGATTTTCGCAACATTCCTTATCC
TATCGCCATTACCGACCGAGGTTCCACCAAATTTCATCACTGTAACCAAACAACACCACCTTTTAA
ATAATAAATTATAATATATTGATTCATGTTCCATACTTCAAATATTATGGTTTATCAATTAATCA
GTGAAATATTATTTAAACTTATCATTCAATTGGCTAAATACTAAAACTAATGGTGCGAGAGGAT
AAAATGGTTAACATATTCGATACTACATTGAGGGATGGAGAACAAACTCCAGGGGTCTCACTATC
CCCAAGTGAAAAACTCGAACTGGCAATAAAACTTGATGAACTTGGTGTAAATATTATCGAGGCAG
GAAGTGCAATAACCTCAAAAGGGGAACGAGAAGCCATGAAACTGGTTACAGGGCAGAATTTAAA
TGCTGAAATATCTTCATTTGTAAGGGCCCTTCCAGTAGATATTGATGCTGCACTTTCCTGTGATG
TTGACAGCGTCCATTTAGTCGTTCCAACTTCAGACATCCACATGACCTACAAATTAAGGAAAAGT
CGTGAAGAAAATTTAAAAGATGCAATGCACGCTGTAGAGTATGCTAAAGATCATGGTTTAATCG
TCGAACTTTCAGCAGAAGATGCAACCAAGAAGCGATGTTGAATTTTTAAAAGAACTATTTTTAAA
AGGGGAAGAACTAAAAGCCGATAGAATCTGTGTTTGTGATACTGTCGGTATTTTAACGCCCTTAA
AAGCAATAGATCTTATAAAAACAATGAAAAATACAATAAAAATTCCAGTTTCAATCCACTGTCAC
AACGATTTTGGAATGGCTACCGCAAATACCCTTTCTGCAATAAGTGCGGGTGCAGACCAGTGCCA
CGTTACAATTAATGGTATCGGTGAAAGGGCAGGAAATGCGTCACTTGAAGAAGTAGTTATGAGT
TTAAAATCACTCTATGATATAGAAACGGGAATTGATACTGAAAAACTCCATAAAATTTCAAGAA
TTGTTTCAAAATATATGAAAATTCCAGTTCCGGCAAATAAAGCGCTTGTTGGTGACAATGCATTT
GCTCACGAAGCAGGAATTCACGTTGACGGCCTTATGAAAAGTACGGAAACTTATGAGCCAATTCA
TCCTGAAACAGTCGGAAACAGGCGAAAAATCATACTTGGAAAACACAGCGGAAAAGCTGCTTTAA
AATATAAACTTGAAATAATGAACATTGGATTGAGTCATGAAGAATTTGAAGAAACATATTCAAA
AATTAAAGCTTTTGGAGACCTTGGAAAATACATCAGTGATGTAGACTTAAAAACCATCATAAATC
AGGTTAGGGGAGTTGAACTTGAAAGAAAAGTAATTCTCGATGAAATTACGGTTGTATCAGGAAA
CAAAGTATCGCCTCTTGCATCAATAAACTTGAAATTTGCAAACGACTGCAATATCAAAGACAATG
TCCGTGAAGCAGCATACGGTCTTGGTCCAGTTGATGCGGCAATTAATGCAGTTAAAAAAGCACTC
ACAGGAGTTGCAGATATTGAACTTGAAGATTACAGTGTTCGTGCAATGACTGGAGGAACTGATGC

TTTAATTGAAGTAGTAGTTCACTTGAGAAAAGGAAACGAAGTTGTAGAAGTCAAAAAAGCACAC
GGAGACGTTGTAATGGCGTCTGTTGAAGCCATGATGGATGGAATCAATTTGCTGATTTAAACCAT
ACCCTTACTTTTTTTAAACTTTTAAAACATATATCTAAACGTGATAATCATGTTTAAAAAATCTT
TATTTATCCTTTTAATATGTATTATTTTAAATATTGTTTCAGTTTCAGCAGTGCCATACCTTGAT
GCAAGCGCTTCAACTCCTGAAATCGAAAGGGGAAGTACTGAAAGAATTTATCTTGAAATTGAAGA
AGTTGGCGGAATTTCCAGTGCATATAATATAACGGCAGTTCCTGAAATTTATGAAGATAATATAA
AAATAACTCCAAAAAATATCGAAATTCCAGTTATTAACGATGGAACAGTAATTAGGGTATCTTTT
AAAATAAATGCTTCAAAAAATGCAGAACTAGGTGAAAAATCCGGAAAAATTTTTATTCATTATT
ACGATTATGATTCAGAAAATGAAATATTTCTCGGTCCAAAAACCATTCAAAAAAAATTTAACTAC
GAAATAAGTGAAGGTCTCGGAATTTATTCTATAAACTCAAACCCTAAAAATATGTCAATATATGT
GGATGGAAATTATGTGGGAAACACGCCTTTAAACATATCAATTAAAGAAGGAAACCACTTTCTAA
GGCTTTCTTCTGAAATTTTTGGAAACTATTCAGAAAAAGTCACAGTATATGCAGGCGAAAACTAC
AATCTTTTTAAAAATTTTGAAGAATCTGAAAAAATAGAAATTTTTGAAAATGTTTCTGAAAATA
TATCCATTTCAGAAAATACTTCAAAAAATGTTTCTGAAGTTTCCGAAATTTCAAAAAACATTGGT
TTTGAAGGTATTTTAGTTTATTTGGGATTCCTTTTAGCCGCATTATTTGTAATATTTGTAGTTAC
AAAAAATAAATATTAAAAATATTAATTATTCTTTTTTTCGATTAATCTTCTAACTTTATGGATTG
CATACACCGTTGCAACAATTATTGGCCCGATTGCAAGTCCAGGTATTCCTAATACCAACGGCCCA
ATTAAAAATGCGATTAAAACAAGTGCAGGGTGTAAATCTCCGTCTTTTGAAATTACACGTGGTCG
TATTGCAAAATCTGGTGCTAAAGATAAAAATACAATTCCAAATAATCCTAAAAGTATTCCCTTAG
CAATTTCCCCAGTTAACAGGTAATATACAGTAAGCGGAACGTATATTGTCCAGCCACCAACTACT
GGCAAAATATTTAGTATTCCTGAAAGTGCACCAAGTGTAATGGGGTTTGGAATTCCAATAAGCCA
GTATCCGATTATCGAAATAATTCCAACAATTACCGATGTAACAGCGTTTCCAATAAATAACGTTT
TTAAGGCCTTGTGTATCTCACTTAGAAAAAGTGCAGTATTCCTTTTTTTATCCTCGGGAACGTAA
ATCAAAAAGCTATCCGTCAGTTTATCCCCATCTTTCAAAAAGTAAAATGTCATGAAGGACAAGAA
CAGGAGCTTCATCAGTAGTGACGGTATTGAAGAAATTTGCGATGCAAAACTATTGAGTAACGGTT
TTGAAACACTCCAGATATCAGAAAACGGTTTTGAAACGTCTGGGCCATCCACCCTCAAATAGCCG
ATATCATTTAAAATTGGAGTAACTTTTGTAATAATATTTTCAATATCAAGAGAAAGTAAAAACG
TTACAACATCTTCTGCAACCAGCACAACCAAAAGTAAAGATGGAATCACGATTCCAAGAAGGCAG
ATTATTGCGCCATAAGTTCTTCCAAATTTTGGTTTTAAAATATCATACAGCGGTTTTGTCATGTA
TGCAACTGCAACTGCAAGCGCAATGATCCCCATAAACGGCCATAACATGTAAATGAGTGCAAAAA
ATGAAAATACCATAAATAATCGCGATAAAATATCATACTCTTTACTGTTCAAGATTACCAATCCC
CTGAATTTCAAAATTACTCATCAATAGACATTTGGTTTACAATTAAATAAACCTACTTGGTAAAA
CATTTAAAATTAAAAAATAGAAAATTAAAAAATTTAAAAAAGATTATTCAGTTTCTTCTGTTGA
TTTACCTTTTTTCCGTTTAAGGTACATGTAGTATCCTAAACCACCAACAAGCAGCAATACTACTA
GATAAACTAGTGTTGAGTTGCTTCCACCTTCAACGTTTACTCTAATGGTTTCTTGCGAAATATAT
ACATTGTCGTCCCCTTCTTCAGAGTCTCCAACGCTTCTAACTTCTACAGTGATTAAATACTCTTTA
TCTGCGGCATCAGAATCCACATCGATTATTAACTGTCCTTCGCCCGTTTCGTTGGTGTCAAGTGTT
CCGATTGAATCAGTTTTTTGAGTGTAATCAAATGGTTGAGCTGAGTTTTTAATCGCGGTTATTTT
TACACTTTCAGCTTTTTCTGAACCCGTATTTTGAAGAGTCAAAGTAATTGTGTTTTCGTCCCCTG
CTTTTAAAGTGTAGGTTTCTGGGAGGATTTCAAGTACGGGTTTTGGTTTGATGTATAAATCAATG
TACTCAATTTCGTTATGTTCTTCATCAAATATATCCATATACGTGATATTTAAAGGAACTGTGTA
GTGTTTTGCCTCTGCATCCTTATCTACATCGATATAGAATGTTGCAGTTTTTGTATCCCCTGAAG
TTAGTGTTCCGATGTTTTTGAAGTTTGAATTACTCCATGAGTCTTTAAATGGATATTCTGAGAGT

AAATCAATTTCAATGTTTTTAGCTTCGCCGTGTCCGTTATTTGTTAAATCAACGTCAATTTTTAC
GTAAGTTTCCTGAGCTTTTATCTCAGTAGGATCAGTTATTACATTTGAAATTCCTAAATTGATGT
CTCCTTCAACAATAAAGCCTATGTTTATTACTTCAGAGTTTTCAGTTCCATCTGAATCGATCCAT
GATAATGTTGCAGGGATCAGGTATGATCCTTCCGTTGCATCTCCGCTTGCATGTAAATTAAGGGA
GAGTAATTTTGTAATATCTGGACTTAAACTTCCCAAATAGAACTTGGTTGTATCAATTGGGTTTA
TTAAATTGTTTGAACCAATTGTCAAGGTTGCTTCTTTTGCAGTTCCAGTTCCTTTGTTTGTTACT
TTAATTTGAACGTTTTCAGTTTTTGCAGGAGTTAATGTAACTCCATCTGAGTTAATTTCAAAGTT
TGCAAGGCCGTATACTGGGATATAGTAGATTTTTGTGTATGATTTTGAGATAACTTGTCTGTCGT
CTCCGTCGTATTCTACAATATCGTATGAAACAGTTACATCGAGCCTGTAGTCTCTTGATGATGCA
TCGTCGTTTATATGTAATTTAAAGTATGCTGTATCACTTTCCCCTTCATTCAATTGAGAAATTTC
GTAAACTCCTTTTGTCGGGTTTACCTGCTTTACTTCAAAGGGGTACTTTGATTCTACACTTATTC
TTAAGTCTTCGATTATTTCGTCAGATCTTTCGTCATTTGCAACGTCAATCCATACATCAACGTCG
TCTCCAGGGTGTATAACGTTTGGGTTATACTGTGGCTCGTCAATTTCAAGTGCAGAAACTGCAGT
TGCAAGAATCGACAAAAGCCCAATTGCTAAAATTATTTTTGATAATTTACTCATTTGATTCACCT
TTTAATTTTAAGGTAAGTTTTTTGAGGAATGGTCTTGTGTATCTTTCTTCAATTACGATTAATGG
AGGTAATAAACAGATAACTGCAGCCATACAGAAGAATATACCCAAAGTACAGACTTTACCCATGT
TTGCCATCATTGATAATGGCGCAATTGTTAATGCAGCAAAACCTGCCATTGTTGTTGCGGTAGTT
ACTAAAACTGCACTTCCAGTACTTACTACTGCAATATTTATTGCTTCTTCCATGCTTTTTCCATCT
CCAAGTTCCTCTTCATATCGGTGGAACAGGTGAACCCCGTAATCGATACCAAGCCCTAAAAGAAG
GGAGCCCATAACGGATGTTGCTGTATCCATTGGAATTTTAAATATCGCCATTGAACCTGCGGCCC
AGATAACTGCAACCACTACAGGTATTAATGGAAGTACTGATTTTACAATATTTCTAAAGTACAAT
AATAATATTGTAAAGATCGCTAAAAGCGAAGCAAGACCCGTTACTGCCTGGCTTTCATCCATCAT
TCTTTCTAAAAGTTCACTCAGTACAGGAGTACCTGTTGTGATCACTTCAACTCCGGGGGGAATTG
GAGCTTCATCCAATCGCGAATTTATATCTTTAACAAGTATCTTTTTATCCTCAGTTCCCGCATCAG
TTGTTGCATAAACTACGACCATTGAATAATCATTGTTAAACATACCATCTTGAGAACTTTCAGGA
AGATTCTGGTAAATCGTTTTTACGGTTGCGATATCATTTGGAATAACATCGTTGTTGTATGCCTT
TAAAACGTCCACTCTTGAACTAACAGACGTAACCATATCCATGCTTCCAATATCCTGTTCTAAAA
AGTCCACAAGTTCTAAAACTCGCGGATCCCGGATATCAGTTACCTTTTCAGAGCTATCGGAGGGT
ACTAACTTTACCCCGAGCATTACTGTCTCAGTACCGCCAAATTCATCCCGTATTTCGTTTAAAGCA
ACAATTACAGGATCATCTTGCGGAACCATCTTTTCATAGTCTGTCTGAGACTCAACACCGGTAGC
TAAAAAAGCCATGAATATTGTGAGGACAATAACAACTGCGACAACTTTTAAAGGATGTCGTTCAG
ATGCCTCAGCTATTTTTTGTAAGATTTTTTCGGCTTTCATCAAATCACCAGTATTTAAAAATACC
TACCGGATGGTATTTAATCGAATATGAAAAAACATATCTTTCGTATTTTTCATAACGTTTATTTT
TTTATTCGATTCCACAATCGTTCAAAGCACTTATTTCCATATTCATAAGAGTCATCCATTAATTT
TAATCGCCTTAAATTTGAATAACCCTCAATGATTGCAATAATCTCTAATGAAATTTCTTTTGAAT
CGATTTCTTCTAAAATCTCCCCATTTTTTTTGGCGAGTTCTATCTTTTTTTTCAAAAGGGAATAC
ATCGATTTTGTGTGCATTGTAAAATCCATATCTTCTGGACCATATTGGATTGCATCTAATGTAAA
TCTTGATTGAAATTCTGAAAAATGTTTGATATCTTTAAGAGTTAATTCAAGATATTCTGCAATTT
CCTGCATTACTATTTCAGAATTTTCAAAATAATTCTTCAACAGATCTTCTAAAGATGATTCTTGC
TCCATTTTTGTTAAAAGCGAAGCAAATTTAGATTTGAAATACTCGGCCATTTCTACAAAAAGTTC
ATTTTTATCTTTAAAATGGTGGTAAATTCCACCTTTAGTAATGCCTGTTTTTTCGGCTATTTCAT
TTAAAGACGTTTGCTTGTATCCTTTTGTAAGAAACAGCTTTGCAGCGTTCTGCACTATTTTTTCT
TTTGTACTCATGATACCATACCTACCAGAAGGTATGTACTTTACATTGACGTTTGTATATATAAA

CTTATCTGAAAAAATGGTAAAACAAAAATTAAAAAAGACTTTATTTTAGAAATAATTTTAAGTT
AATCTATAATATCCATATTTTGGGCTGAATACATCTGCACTCTTCTTTAATTTTGAAAGCATGTC
TGAAACATCCTTTTCAGAAATCTGAAATTCCGAAGCTTTTCTACAATATCGCACTCATCTGCAA
GACCGTTGTTGCTTAATACTGAAAGTTCACGGATAATATCTACGATTTTATCCATTTTATCGACT
TTAGATTTTGGAATTTCACCCATTCCTTTATCTATATCGAATTTTCCAGTTTCAGGATCATATGC
AACTTGCCTTAAACATTCTTCAATTATATCAATTGCAATTTTTGCATCTTTTTCTTCTACTTCTCT
TGAAAGCCTTGCTTTTGCATGCATTTCTGAAATCCTTATTGCAGCTTCTAATTGTCTTGCAGTAA
CTGGGACTGGATTGTTTCCTTCCCCAAGCTTTCTCATATCAACGTAATAATCCCGAATCAATTTTT
TAGCACCTTTTGAAAGGGGCGGACTTTTTATTTTCCTTTCATCCACTTCTCCAGCATACAAATGA
TGATTTTCATCAAAATATGCGCAGGTTCTTGCATAAATTATGTAATTCTTAATTAAATTTTCATC
AACAGTTATTCCATCAACTTTGATTGAACCAAGAACTTTTGAATAGTTTTTGGTTGCGGTTTCAA
CGTGGGTATCGAGTATGTGTTCTGCAATTTCTGCATCCCTTCGCCTATCAGGTGAATCTTTTAAA
GGGAATATCAAATCAAAACGGCTTAATAATGGGGCAGGAATTCCGATCTGTTCAACTACGCCCAT
ATTTCTATCAAATCTACCCCTTTTTGGGTTGCAAGCTGCTAAAACCGCACACCTTGCCGGAAGTTT
TACATTGATTCCTCCTTTATTCACGTGTATAGTCTGGGACTCCATTGCTTCTAAAATGTATTTCA
TAACGTTTTTATCAACAGTTAATTCATCAATACAAGCAGTTCCTTCATTTGCACGAACGAATACT
CCAGGTTTTACAACCCATCCATCTCCAATTTCTGTAGCCTCCCTTACAACGTTTGCAGTTAATCCA
CCACCTGTTGCAGTTGTAACTGACGCATAGGAATTTTGTGGGAAAAGTCTTGAAATTTTTCTAAG
CATTGTAGACTTTCCAATTCCTGGATCCGTTATCAATAGAATGTGGCTGTCTTTTCTAAGTTCTG
ATCCATCAGGTAAAAATTTAGTACATGCTTTTACCTGCTGTAAAAATATTGATTTTTTTACAAGA
TCGTAACCTTTAATTTGAGGGATTAAATAATTTGAAAGAATATCAATTATATTTTCCTTTTTCCC
AAGTTCATCCAGTGTGTTTATTAAATCGTAGTTATTTATGATGTCTTTTACTTCAATCTTTTGGA
AGCTTTCGCCGAGTTTTACATAGTTACTTTTTGCATAAATTTCAAAAATAGGCAACTTTTGATTT
TGTTTTGACCTTTTTTTCATGACGGTTCCAACAACATCCACCCTTCCAGAATAAATTCCATCTGAA
TTTTCTAAAAAAACACGAATACTCCTTGGCGGATCGTCAGGATTTCTCATTAAATCGATTGGCTG
CTGTATCTCAAGTTCCTGAATATTTACGTAAGCAGAGCTGTCATAATCGATTGACATTTCGTTTC
TGCAGTTTTGATTTTTACAATATCTTTTTGGCTCTTCAAAATAGTCGTGAATTGTTTTATATGAA
ATTTCACCGCATGCTCGGCAGACAAAACATGCTTTTTTTAATAATGCACATACTTTTGCCGCAGT
TATGATATTTCCTTCAAATTTGACCAATCTATTCATTTCTGAAGACGTTATTTCCTCGATTCTTT
TGTCGCATCCAAGCGGATTTTTGAATGCAATCTGAATTTTCTCAAGTTCATTTAGTACGTGGTCA
TCGTCACCAAAAAGCTCCATACATACTTTTTTGAAGATATGCAACAGTGGATCTTCGATTAATTT
TGGATTTTCAACAATCAGATCGTTTATATCACAGGATTCAGAATAATTTTTTAAAAAGTCAGTTA
TATCAAATTCAAAAACATTGTCTTTAACTTTCGTTTGTGAAATCTGGTCTTTCATATATTTTTTT
AAATTATCGCCGTACAGGGTCAAAAAAACTTCTTCATCAAATTCCATGTTATCACAAAATTAATT
TTGTAAATTAAAATCGTATTTTTTAAGTTTGAAATTATTTCTTCAAAATCCTGCCTGTTTTTAG
CTTTTATATCTTTTTTTTGTGGGAATTTAAGAATTACCTCATCAAAAAGCGATTTGTTTGGCGGG
GTTTTCATTGGATAATAATATCTATATATGCAATTTATCCTTGCAGTTATAAATTCAGATAAATA
ATCCATACAAGCTTTTATTGCCATTGATTCATCAGATAATAAAGAACCTGACTTTTTAGATTTTA
AATAAGAAATCCCAACAATTATTTCTTCGAGATTGCACTTTAAAAGCTCTTTTGACTTTTGCTGT
TCTCTTTTTAATTCATAGAGAGATATTGCAAGATCCTGTAAATTTGTCCACGGAAGATCAAAATT
TATTCGTTCTTCATCGGATTTAGATAACATCCATAGCGGAACGTGTTTTTCGTTTACAGCTACTT
CGTATTCCGTGCATTTTAAGAATAAAATATCATAAACGTCCATTTTTTCACTGAAATTTTGTAAT
GATTGTAAAATGAGAATTTAATATCTATTTTATTTAATATAAATATTCAGTACAACGATTTTTCC

ATAAATATCTAAGTACAATTTAAAAATTAAAGATAAAAAAAGATAAAAACTTTAATTTACATCT
TTCCAGGCATTTCAATACCTAAAAGATCGAGTGTATTTTCCAAAACTGTTTTTGTAGTGTTTACA
ATTGCCAGTCTTGAGTTTTTGACTGTTTCGTTTTCTTCTTTTAAAACTGGACAGTTTGCATAGAA
TTTGTTGAATCCTTGTGCAACATCTAAAACATAGTTTGCAACAAGCTGTGGTTTTCTAACTTCCG
CTGCTTTTTCAACAATTTTTGGCAATTTTGAAAGCAATTTTACAATTGTTTTCTCATTTTCATTC
ATTTCATACGCAAATAAATTTTCATTTGAAATATTTTCGACATTTTCTAAAATTCTTGAACATCT
TGCATGAGCGTACTGGATAACTGGACATCCAACTTTTTCAAAGTCAAGTGCTTCATCCCATCTAA
ATACCATTGGTTTTTCTGGTGCAATTCTAACAATATTATACCTTACAGCGCCAACCGCAATTTTT
TTTGCGATTTCTTCGATTTCTTCTTCGCTTTGTGCAACGCCTCTTTTTCTAACTTCTTCAGCAGCT
CTTGATTTTGCTTCTTCAAAGAGTTCATCCATACTAATGAATCTGCCTCTTCTCGTACTCATTGAG
CCTTCTGGAAGCGAAATAAATTCATAAAATACAACTTCAGCTTCGTTGTATCCTAAAAGTGCTAA
CGTCTTGTTAACCATTACTGCGGTTAATTTGTGATCTGCTCCAAGCAGGTTCACTGCAAAGTCGC
AGTTTTCCATTTTATTAATGTGGTATGCAATATCTCTTGTTGAATAAAGACTTGTTCCATTTAAT
CTTGCTAAAACAAGCTTTTTTTCGATTCCGTAATCTGAAAGGTCGAGCCTGTAAACTTCATCTTC
AACAACTTTTCCGGTATTCATCAGCCGGTTTATTACTTCCCTTACCATCCCGCTTTTTACAAATTC
GCTTTCCCAGACAAATTTATCGTGGTAGATATTTAGTGTTGAAAGTGTTTCTTTAAATCCGCCTA
ATGAATAATTAACTGCATTTTTAAATTTTTCAGTTAATTCGTTTTCAATTCCTGCTTCACAGGCA
CTTTCATAATTTTTCATTAAATTTAGAATTTCCTGTTCGAGTTCTTCGTTTTCTGCAAGCAGTTT
GTTTGTTTCAACGTAAACTTCACCAATTGCGTGATCTGCTTTTTTGGAGTTGTCAAGTTCAAATT
TTTCATTTCCAAAAACAACGATTGCTTCTTGTCTACCCATATCGTTTACATAGTACTGCGTTTCT
ACATCGTATCCTGATGCAACGAGTATTCTTTTTAAGCTGTCCCCAATAACCATGTTTCTTCCATG
ACCAATATGGAAAGGTCCGTTTGGATTTGCGGAAGTGTGCTCCAAAATTACTTTTTCATTTCTTT
TTTCAAGTTTTCCAAAGTTTTCTTTTTCATCGGATATTTTTGAGACTGTTTCTTTTGAAAAGTCT
GAATAATTCATGAAAAAATTAATATACCCATTTACAGCTTCAATTTTTTCAATTCCTAAAATACT
TAATGAATTTGCAATATCTTCTGCAATCATTTTTGGAGCTTTTTTCAGGTATTTTGCCAATCTAA
ATGAAATATTTGTTGAGTAATCTCCCATGTTTATTGCAGGCGGTTCGTCAAGTCTTATATCCATC
TCATTTCCAGTTAATTCCTTTACCTTGTCTTTTAAGGTCGTTATTATCAAATTTTCAACGTCCAT
AATCTGCCTCGCCTTGTGTTTCTATTTTACGTTCTTAAATCTTATATCATACAATAACATATATT
CATGGCTTTTAAATATATATAATTAAACACGAAAAAAGGTATTGTTTATTAAAATTAGTTTTTT
TATTTAGTACCCTGCTAATTAAAAGGAATTTATTTTTGAATATAATTTAAAATACCTAACTTAGT
TTAAAAATTCAATAAAATGGGGTGTTTGGCATAGGACTTATTAAAAACGCAGGTAGAAAAACTC
TTGGGGAAATAATTATTGCAATAACGTTTTTTTTAGGATTTCCGATATCTGCACTGGCTTTTGCA
TATATACATCCCATTATGGGCATTTTATGTTTGGTATTCTGTATATATCTACTGCATTTGGCAAA
TACCTTAAAAAAAGGAAAATAAAAGAGATGTTTTTTATCGGGCCGATCTAATTACCAATAATCTA
AAAAATTTTAGTCTTGAAAATTATTTTTAAGATAATTTAAAAAAAGCATGCCAGAAAATTTGTG
TGGGAATATAGGTTGTGAAATAAATATTTCACTTTTAAGTCAACCCAAAAATAATTTATCCAAAG
ATATTTTTTAGTATCCCACTTAAATCCAACGGTTTTGGCGTTCCCACACAATATTTGAATATATC
TCATATCATGTATTAATGATTACTATTGTTATCATATCGTAAATTATATATCGTAATTTTATTTT
TTAACACACTTTATAAAAATGTCAGAATTATTTTTCAAACATTGCAATTATTGAACCAAAAACGC
CCGGCAAAATCCAGCTTGCATTATTTTTAAGATGATTTAGTGCAGTCTTTACATTTGATTTGTTG
ATTATTTCGTTTGAAAGTTCTTTTTCAATCATTCGAACGTTTTTAACAATTTCAATCCGATTTTC
TGGATTTTTATTATTAATTTCATCATAAACTCTTTTAAATGGCAATATTGTAATTACATGTTCAC
TTCCCATTCCAAATATAATATCGTCATCATGAATTACGGGGTCAAAATCTTGAAATTTGTTTTTA

AAATGTTTTGAATTTTCGATAAATGCCATTCCAATTTTAGAAAGCGTCACGATGTAAAAAATACC
AATTGCTGTGTGAACTCTTGACTTGATTTCTATGTATCTATTTGATTTTAAATTTTTAAATGCAC
GATCAAGTTTCTGGTCATTTGATTCTATATCTTTAAAATCCTTTCTTTGAAGGCCGATATTTCCA
TTACGGTATTCTAAATACATTTTTTCTAAAATAGAGTTTTGAAGTTCATCCATATCCATTTTCAC
ACCAAAATAAATTCAAAGTCCCAAAATAATTAGCTATGTCCCAATATACTTATTTTCAAGAATAT
ATTTAAAAATGTTGAATTCATTAGAATATTGAATTTTCGTAGTTTTGGTATTTAGATATTTAAT
ATATTAATGGCCTGATTATTTAATTAAATCGCAAATAAAATATATTATCAAACAAATATTGTTTC
GAATGCTAATCGGGACAAGAAAGGGTTAATATGGCTATTGATTCTCAAATAAAAAGATATTTTA
AAAAAGATATTTCCTACATGTTTTTATTGTTATTGTAGTGATGGTTTCAATTCTTATAAGTTTA
AACGTGTTTCAGACATTTGGATTTAAAAATCAGTACCTACTTGAATTATTTCACGACCTTAATGT
TTTACTGGGCTTTTTCATAGTTGTTTCAATAATTGGTATTGCACTTTTGGAACTTATTTTTTAAA
ATTACAATAAATCAAAACTTAATTTAAATATTGAAAAAATTAAGCTACTAAAAACTATTAAATC
CTAGTTTTCTTAGATTCTTTTTTGTAAAGTTTTAGTTTTATTACTTTGAAGTAAACTTTTCTTGG
AAAAGTTACCCTTGAGGTATATCTTAATATATTTTGTTTATATACTATGATAAACGTGTAATCGT
CAAAATACATGTAATATTACCGTGATAAAACATATAGAATTTAGATAATTATTATTCTTTAAAA
AATTTATAATCTGGAATTTGGAAGAGATTTTTATGAAAACAGAGATGACAAACGTTGATATCAG
TGCAGCAGTATCTGAACTTCAAAAGATAATCAACGGAAAATTGGACAAGGCTTTTTTAGTAAATA
ACCAAGACGGAAAAGAATTAATTTTAAAAGTACATATTCCTGAAATCGGTTCAAGAGAAATTGC
AATAGGCCTTGGAAAATACAAATACATAACACTTACGGAATACGAAAGAGAAAAACCAAGGAAT
CCTCCTTCTTTTGTAATGCTGCTTAGAAAACATTTGAAAAACATTAAAATAACGAGTGTCGCCCA
GCATAACTTTGACAGGATTGTAATTTTCAATTTTGAATGGAACGAATTAAAATATAAACTAATT
ATCGAGTTATTTGGAGACGGAAATGCAATACTCCTTGATAGCGAAGATAAAATTATTCTCCCGTT
AAAAATTGAAAGGTGGAGTACGAGAAAAATCGTTCCAAAAGAACTTTACAAATTCCCGCCTCAAA
AGGACCTTGATCCAAAAAATTTAGATTATTCTCTTGCTAAGAAATTGTTCATTGAAGAATTTGAA
AAGGAAGAAAACAAAGATACTGAAGTTGTAAGGATAATTTCAAGGACTTTTGGACTTGCTGGAG
TTTATTCAGAAGAAATCTGTCTAAATTCAGAAATTGATAAAAAATTTGAAAAATCCAAAAAACGA
AGAAATTGAAAAACTCTTTGAAGGATCCCAATCATTCTTTAAAAAAGTATTTGGCGAATTAAAAC
CAAAATCTACACTTAAAAATGGAGAATTTGTTAATATCGACCCGATTGATTTAAAAATACATGA
AAATCTAAAAGAAAATGAAATAAAACATTATGAAAGTTTTTTAACTGCGCTTGATGAATATTTC
TCAAGATTTATCATGAAAAAAGAGATTAAGCAGGCTGAATCAAAATTACAAAAACTCGTGAAAA
AACAGGAGCGTATATTAAAAAGCCAGCTTGATACCAAAGACAAATACGAAAAACAATCCATTTCA
AACCATAAACGTGGAGATTTGATTTATGCAAATTATTCTCTTGTAGATGAAATTGTAAGCACAAT
TAAAGATGCAAGGGAAAAAATGGACTGGAATGGCATTAAAAACGTTATTAAAGAAAATAAAACT
CACCCGATTTTAAGTAAAATAATAAATGTGAATGAAAAAAATGCAGAATTAACCTTAAAACTGT
CTGCTGACTATGGAAATGGACTTATTGAAGACTCAGTACCAGTAGATTTAAGAAAAAATGCGTTT
GAAAATGCAGATATAGTTTACCAAAAATCAAAGAAATTTAAAAATAAAGTTCAGGGAGTTATTG
AAGCTTTAAAAATTTCTGAAAAAAAACTTGCAGAATTAAAAGAAAAAGAAAAACTTGATTCTGA
AGTATTTAAAGAAAAAGAAGAAAAAATTAAGAAAAAGGAAAGAAAAGTACTAAAATGGTACGA
AAAATTGAAATGGACCGTTATTGGCGGATATTTAATTGTTGCAGGAAAGGATGCAACAACAAAT
GAGATGCTGATAAAAAGATACGTTGAAAAAAACGATATAGTATTTCACACACTTATGGAAGGAG
CACCATTTACAATAATTAGAACGGAAGGCTCTGAAGAAATACCTGATGAAAATATAATGTTTGA
AGTTGCAAAATTTGCAGCGTCGCACTCAAGAGCCTGGAAACTTGGAATTGGAAGTGCAGATGTCT
ACTGGGTAAGGCCTGACCAGATTTCAAAAACTGCGGAAAGTGGAGAATACCTAAAAAAAGGAGC

TTTTGTAATTCGCGGAAAAAGAAACTTCATAAGAAGTGCTGCGCTTGAACTTGGAATCGGAATAA
TAAATTACGATGGAAAATTAAGATTAACTACGGCTCCAGAATCAGTTGCAAAAGAAAATTTTGA
AAAATGTATTTTGTTAAAACCTGGAAAATTGAAAAAGAGCGACCTTGTAAAAGAGCTTTTAAAA
GAATTTAGTGACTTTGAAGTTGATGACGAAGATATTTTAAGGGCACTTCCTCCAGGAGAGACCGA
AATAAAACTAAAATAATTTTTAATTTTTTAAATTCAAATCATAAGGCATATTAATCCCAAATTA
AAGATAAAATTAAATAAAAATTATTGAAGTTGGTTTTCATGAAAAATACAATTCAAAGAAGTTT
TGAGATAAAAGACTACAGAATTCCAAAAACGGATTTTGGAGATTTCTGGATGACTTTTGAAACT
AAAGAAAAGTTAAAAACAAAAATTACATACGTCCCTGAAAATGGCGGAAAATTCAGTGCTTTGG
ATGTTAAATCAGTAGTAGAAGAAATTATTTCTAAAAGCAAATACTTCAAAGAAAATCTTCCAGA
AAACATTAAAGTTGAAGTGTTGTTTAAAAATTTAAGTGAGGATTGCTACAATCCGACAGAAAAT
ACGATTCCAAATTTTGAATTTAAAGAAATGGATGAAATTAGTGTTTTATTTTATTTTATCGTCGA
TTATTATCTCTAAATTCTTTTAAAACATTAAAATTCTATCTTTTTTCAGATATGTTTTTAGATCA
TAAATTAAAAAAAGTTCTAAAAAGAGAAATTAAAAAATAGGATCAATAATTTATTTTTGGCGTT
TATTTGATGCCCATTAATTTTCTAAGTACCATTGACGTTCCCATTGAACACATGATGTACCATCC
AAGCCATCCAAGAACTGTGCTTGAAACTACGCCAAATCCACCGTGGTAGAATATTCCTCCCACCC
AGTGCCAGATTCCAATAAAAATTATTTTTGAAATGATTACTGGAAGTTGAACTATAGCTCCATCC
CATGCAGGCTGCATTGTGTGGTAAATTCCACCAAAGTCGTATACGTGTCTTAAGTAAGCAAATAT
GATGATTATTGGAACCCAGGTGTAAATCATGGGTTTAAAACTCATTTTCATCATTTCCATCTGCA
TTGTCATCATTTTTTTGCTGTTCTTTTTGAAGTTCAGCCATTAATTCAGGATCTTTTGATGCTTTT
TTTGCTTTTACCTGAAATTCTTGAAGTTCATTTTTCAGTTCTGCCATCCTGTCCTGATTTACTAAT
ACTTTGGTTGCTAAATTAATAATAAACGATACTAAAAGAGCAGTTACAAATATAAAAATTGCAG
GATCCATTGTTTGTACCATAGGTAAAAAAATCGCATCCAATGCATTGTAAAATGCGGAATAAATA
GATTCAAACATGTTTTTTCCCCTTTATCATAATAAATATTAAAACAATTAAAAAAGTTAAAATAT
ATATAGATTATCTTAAAACTGACATTAATTCTTCAACTGCGTTGTCAACGAGACCGTTTTTATTT
TTAACAATTTTTACAGTTGCACCGGTTAAAACACCGTAGCTCATTGCAGCAGCTCTGTTCATAAA
TTGGTGTTCTTCAATGCTTGCAGTTGTTTCGAGATCTCTTTTCCTTGACTCGTCACTTAATCTTCT
CATCAAGATTTCGTCACCGTCTGTTTCAACAACAATTACAATGTCGGGATTTAATTCAGTTAAAA
CCCATGCAGGAAGTCCAGGTAAGTATCCTTTTGGAGTTTTTACTGTGCTGTGTGTATCTACTGCA
ACAGGGGATTCTTTTGCCATTTCCGCAATTTTTCTTCCAGCCATTTCTGGATTCTTTTTTGGGTT
TCAGGATCTAATCTCCTCATTTGGTCTCTGTCTGATGCCAGTCCTTCTTCGTTTGCAACATCAAAC
ATTGCACTTCCAAAGTTAACCATTTTGTAGTTTAATCCTTCTTCTGATAAAATATCCATTGCTTT
TTGTGTTACAGTTGTACCGCCAACACCAGGTACACCAGTTACTACAACAACTTTGTTTTTCATTA
AACCACCGTTTTACTGTTAAAATGAATAAAAATAAAAACACTTGAGTTATGCGCCCTATTTACTA
TCTAAATCTAAATATATATACGTTATCCAATATTTGAAAAAAATAAAAAAGTTAAAGTTGCTTT
AAAACTTCCAATTCTTTTGATACATCTACGAAATTAACCCGTTTTACTGCAAATTCTAAGTCATC
ATTTCTAAATGAAGGGGTTCCATAGAATAAAACATCTTTTCCAAGCACATCTGCAAGTGCATCCA
TATTCATTTCTTTTAATTTATTTTTTGAAAGTTCAGTTACTTTTTCAACATTGTTTCCATATACC
CTACAGTTAATAACGCCTGTACCATCATCAACTGTTAAATTTGAAACTAAAAGCTCGTTTGGTTC
AACTTCTCCACAAACTTCGCACAGGTATTTATTATCAACTAAATTCAATCTTTTCCTACAGTTTG
GACATAAGTTAAGAATTACGTCTTGTTTTCTGTAGTCTACAATAGTTCCGCTAATTTCGGTTGAA
ACTCCTTCTTCAAGGTCTGCGATATAATTTCTTGGAGATTTTATTGAAACTCCTTTTGGATTTAC
TTCAATTCTTCCAAACCGTCCTGTTGATAAATCCATGTATTCTCCACTTTCTTTAGCATACCCGTG
TATTAGCTTTACAACGTCTCCTTCGGAAATTTCGGTTTCTGCGTCACTATCCCATAAAGCCATTCT

TATGCTTCCAGTTCCATCTTCTAACATAATATTTCTAACTTTTCCAGTTCCACCACTGCTTCTTTG
GAACTCATTTACCGGATATGCATTTGTAACCATTGCAACAACTGTTACATCGGAGTTTTCTGCAC
TTCCATTTAAAATATCTTTAATTTCGGTTATTTCTTCTAATATTTTTTTACTTGCACTTTCGTCT
TTTTTAAGTTCTGACATGTAGTTATAAGTTAAATCAGCCCTTTTTTCACCAGTATTATCAGTGTA
GGTTCTTAATTTAGCATTTGAGATTGTTACGTAATCTCCTTCTTTTAAGCCGTCAAGAATACTCC
TGTTTGTACCCCAGAATGCAGTTTTTACCATTCCAGTTCCATCGTTTATGTAAATATCCTGAACT
TCTGCAGTTCTATCGCTAAATTGGACTTCTCTTGAACCAGAAATGTTAACAATATTTCCAGAAAC
TGTGACGTATTCCCCATCAAAGCTTGTAAGTTCAGAAATACTCAATTTTTCAACAGGTTTTTCTT
CTTTAACTGATTTTTCAATTATTCCAATATTATCAACAGAAATTTCAAGTCCTGAATAACCCTGT
TTTACATAACCGCTTACTTCTGCGATATCTCCTTTTTTAACTTCAAAATCTGCAAGTTCATTCCAG
AGAGTTCCCCTTATACTTCCTGTATCATCTTTTAAAAATAAAGATTTTAGCTGACCTTTTGTTCC
ATCTTTTCTGCTGAATTCTTTTTTAGGATATGCTGTAACAACTTCTGCATTTATTGTCCCTGATA
AATTCGGCATTAATTCTGAAATCTTATAATTATCTTTTAAAATAGGATATCTCGATTCATCGTAT
TTTTTAAGTTTTTCGATTGATAATTCTGAACCGCTGTTTAATTCAACCTTATCATTCCACTTTCT
GCTGAATGCATTTTCAATTTTTACAACGTCCCCTTTTTTTAATCCTTCAATAAGATCCGTTTTGTC
GTTCCAAAACGTAAGTCTAGTAATTCCAGTATTGTCCCCGATAGTAACTGATGCAAGTTTTCCAG
TGCTTCCATCTTTTCTATTAAAGTCTTTTGCAGGCGAAATTTCAATAATTTTTCCTGTGACTTCA
ACATTTTTCTGCCCGTCAGCGATATCTTTAATCGAAAAATCATATTCTTCTTCATCATCGCCGTA
AGTTATTTCTATCCCGAGTTCCTGTGCAACCAGCATTACTGCACTTTCTTCGCCAAGAAGTCCGGA
ATTTTCCTCGATTTTTTCATTTACTTTTTTCTCAAGCTCTTTTTCTGAGATTTTTGATAATATTT
TTTGTTTTAACTTGTCAATATTCATAAAACTACACCGGTGAATTTTTCAGAAGTATTCAGAAGTA
TCTACCTTACCTACATAAAAGCTATAATAATGTTGAACATCCATTTTTATATTTGTTTGTATATA
ATATATGACAAAAGTACTCAGAATATATACTTAACGATTTAGAATACTTGGGAAATCATGAACA
TTAAAAGAATCGTATCTGACCTTAAAAAAGAAAGCTGGTATGCTAAATTAGATGCTGTAAATAG
GTTAGGAAACATTGTCTACGAATCACAAGACGATGCAGATTACGTACTTAATAATTTACTTATTG
AACTTGAATCTCCAAAAACCGTAATGCAAGCACGAACGCTTTGGGCTACGAACAATATCATAAAA
CAGTACCCCCAATTAGCTTACAAAATAGTACCGCATCTTTTGAGACTTTCAAATTATGATAATAA
ATCAATTAAAGAAATTTCAAATGAACTTTTAAATAAGCCCCTGATACAATACGCAATTTCAAGAT
ATAATACGATACTTTCAAAGAGTATCCATTCAGAGGATTATTACGAAAAATTAGGGGCCATTATA
AATATTTGGAAAATGGCACCTGTAGATCCTGACTTAATAGACTCTATTCTTCCGGAATTAATTGA
ATCAATGATTAATAAAAATCATTTAAAAATAGGCATATTTTCATGGATATTGCTCGAATCCGATG
ATAATGAAACAATCGAAGAAATTGTAGATGTAATAAATAAAGTATATCTACAACTTGATTTTAA
TGCCCAATACCCTCCAAGCTATATCAAAAAACTGATAAACAGTCCAGATCCAGTACTTAATCATG
TTGGGCTGTCATCTATCGAAAAAAACCCTGAAAATGCAAATAACGAACTTATGGAAGATTTATTG
AAATTATTTACTTCCGAAAATACCGATAGGTTTGTAAAAGCTAAAATGTTCTACACGATAGGATT
ACTTTCAAAAACCAAACCATATCTAAAAACAATACTTATACACGAAGCCAATAAAACAATTAAAA
ATGAAAAAGATTGGCTTTTAACATTTGCTGCATTGATTGGGTTTTACTTAATAGAAGAAAAAAT
AACAAATTTAGAATTATTACACCATTCAAATTCATTAATCCGTGCAGTTGCAATCCAGTTGATAG
ATTCTAGAAGTTCTGAACAGATTCTATCGGCTTTTGCTGATAACCACATAACTGTATTCATCTCG
GCCATCGACAGGAGCATTATGCGTGATGTTTCAGATGAAATAAAATTAAAATTTTTAAACGAATT
AAGTAATCCCGATAATTATCCATATATGAGTGAAAATATCGATGAAAATTCAGTTACCAAGCTAA
GAGATAGCGTTGGTGACATTGTACACTCTTGGGATTCTGAACATTGGATTTCTAAAATAAATTTA
ATGAATGACCTTGGAAATTTAGCTAAAAACAATCAGGACTACATTGATAGTTCTATTGATCTAAT

AATAAAATCTATGGAAGACAATTACGGAATGGTTCGTGCACAAGGTCTCTGGATTATCCGGGCCA
TTTTATACAACAGTAAAGATCCATTTTATATTTTAGAAGCACTTGATGGGCATTTAGAACCCATT
TTAAAGATAAATGATCCAAATGTATTCGTAAGGCTAAATTACATACTGATCCTTAGAAATTTCGC
GGAATTTTTAAATAAAATTGAAGGTACTGAAGATAAACGTTCAGAAATTGCAGGATATTTGCTT
TATATGGCACTGAACGATTCGACAAAACTTGTTTCTGAAGTTGCAAAACTTGTTTTAAAATTTGA
CTTCGATACTGAAATAGATAAAAAAGAAATTAATCTGGACAACTTCCAAAAATACCTAGAAGCA
TTCCCTTATTCCGCACTTGGAATTATAAGATACTTATTGTTAAAAAATCAAGGCAATGAACAAGT
TGTATCAATGCTTATCGACCTTTGCAGAAAGTATCGTGACTATGGATGCGATATGTTTTGTATCC
TTTATGGGGTTGATATGGATTTAACTTCGGAAAGCATTTGCGAAAAATTGAAGGAATATTCAAC
ATACCTGTCTAATGAAGACCAAAAATCCGTGAAATTGATCATAAAAATGCATTTGAATGAACATA
ACTGGAAAATAAGAATGACTGGTCTTTCGATTCTTGAAAAAATATCAATAATGGATCCAAAGAT
TATCGACGATTTCGTAATAGATCTAATTAATATCGCACTTTCGGACAGGATATTTGAAATAAGGG
AATTTGCCGGAAAATTACTTGAAATGATCGATTATGAAAGTCCAGAAATCGACCGGGAAACTTCA
ATAAACTACCTTTTCAAAAGAAGCGATGAACTGCTTGAAATTATAAAAGACGGAAAATTTGGAG
TCGATGAAGCGTTCTACGCACTTAGTATCCGAACTAAAGAAATAGATAGTATTGAAAACCTAATG
TCTGTTGTTTCGAAATTTAAAAATGACGAACACTGGTATAGGAGAGCTTATGCATATAAAATCCT
TGAAAACTTAATTAAAAATCCAAAAGTAGACCATTACCACTACGAAATTATAAATTGGTGTCTTG
AAGCTACCGAGGATAAAAATCCCGTCATTTCAGGATTATCAAAAGGAATATTGGAAAAATTAGG
CCGTGCTGTTCAGCCATCGTGTCAAAAAACTGCGTATGACAGAATCAGACGTATTGAAAACCTAC
TTGAAAGTGGGGACTGGACTGTAAAAGTAGAAGCGCTCCAATCAGTTCGAGATTTCATCAACGAA
GGGCACTACGGATATTTAGATATTGTAATGGAAAAGTTAGATGATCCACACTGGAAAGTTAAAA
ATGCAGCATTAGGAATTTTGGCAGATATTGACCCAGATTTAATAAAACCTGCAATTCCAAAAATA
ATCAGTCTTTTAAATGACGGTGATGAATCAGTAATATTAAAAACACTCCTTACATTGAAAAAATT
CGGTCAAAAGGACATGAAAATCCTTGAAAAAGTTATGCCCCAACTTGATAAACTTGAAAATTACG
GAACATGGAGCATAAAAGAAGAAATAATGAGATTAAAATTATCATACTACAATAAATTAAGACA
AAAACACTAATTATCTCTTTTTGGTAATATTATGAACAAATTCATCGTTTTTGAAGGAATTGACG
GCTGTGGAAAAACAACTCAGGCAAAACTCGTAGCTGAAAAATTAAATGCAAATTTTACATTTGA
ACCAACTGACGGAAAAATTGGAAAATCTATTCGAGAAATTTTGGCAGGTTCTAAATGCAATAAA
GAAACTCTCGCTCTCCTTTTTGCAGCAGATCGTGTTGAACACGTTTCAAAAATCGAAGAAGATCT
GAAAAATGCCCATGTTGTTTCAGACCGTTATGTCTATTCGTCTATTGTTTACCAGATGACTCAGG
GAATTCCAAAAGATTTTATATACAAAATCAACGATTATGCAAAAATTCCAGATCTCGTGGTTTTA
CTCGATGTGGATTTAAATGAAGCTTTAAAAAGAATGGAGTCCAGAGAGAAGGAAATATTTGAAA
AACTGGAATTTCAGAAAAAAAATAAAAGAAGGATATTACAACCTTATAAATTCAGAGAATGGAAA
ATTCATGCCAAAATATGGATTTATTGTAATTGATACAACTTCAAAACCAATAAATCAGGTTTTTA
ACGAAATTTTAAACGCGATTGTCGATAAAATTCCAGATATTATACAATAAACTATATATTGTTCT
TAGCAAATAGATTATTTAGTGCACTATATAAATAATCTAATCTTTAAAGGTGTATCATGGACTTA
GGTTATATAACAATTATAATCGGCATGGGGTTAATACTCCTCGAAGCATTCCTTCCTGGGCTTAA
TTTCCCAGTCGCTGGTGTTGCCGTGCTTATTTATGGAATATTCTTACTTTATGCGCCACAATTTGC
ACTTCCATCAGCAATACTTGCAGGTTTAATCACTGCATACATCATGAAAAAGGTTTGTCTATAAAA
CCGGTTTAAATGTAAAAATCGGTGCTGAAAACTTAATTGGTGAACATGGGGACCTTGTTGATGAA
ATTGATGAAAATAACTACGGCCATGTCATAATACACGGGGAAAAATGGCAGGCAAAATCTGAAA
ACCCGATAAAAAAAGGTTCAAAAGTAATTGTTGTAGGTATTGAAGGAGTATCTTTAATTATTGA
AGAACTAAATGAATGATTATAATTCATAAAAACCTAAAATTAGGGGGAGATTTATGTCTTTTTG

GCTTAATTTGTTATTAGGTATATTTTTGCTGGTATTAATAATAAAATCAGTTATTATCGTAAACC
AGTTTGAACTCGGACTTATATTCAGGCTTGGAAAAGTTAGGGGAAGATTAAATCCAGGAGTTAA
TTTCATAATTCCATTTATCGATGTACCGATAAAAGTAGATGTAAGAACTAAAGTAATCGATGTTC
CGCCACAAGAAATGATTACAAGAGATAACGCAGGAGTTAGAATCGATGCAGTTATTTACTACAGG
GTAATGGATGTAAACAGAGCAATTTTAGAGGTTCAAAACTTCCAGTACGCAATTATAAACCTTGC
ACAAACATCACTCAGGGCAATAATTGGTAGCCTCGAACTTGATGATGCGTTAAACAAAAGAGAAT
TTATCAATTCACAGCTTTTAGAAACGCTTGATAGGGATACTGATGCATGGGGTGTAAAGGTTGAA
AAAGTTGAATTGAGAGAAATTGAACCTCCAACCGACATTAAAAACGCAATGACCCAACAGATGAA
AGCTGAAAGGTTAAAAAGAGCTGCCATATTGGAAGCAGAGGGTGAAAAACAGAGTAAAATCTTA
AAAGCCCAAGGTACTGCTGAAAGTATGAAAATTGAAGCAGAAGGTCAAGCAAAAGCAATACAAA
TCGTTGCAGAATCTGCTCAAAACTACTTTAAAAACGAAGCTCAGCTCTACAAAGCTCTTGATGTA
ACGTCAAGCACATTGAAAGATAACACTAAATTTGTAATTTCCGAAAATTTAATGGATGTTGCAAA
AAAATTCATTAAACAATAAATAATTCTTTTTTAAATATTTCAAATTCTTATTCTTTTTTTAATTT
TGGCAATTTAAAAGCTTTAACATGGAGAAATTTATATGAATGTAACAATCATTTCTGTTGGAAA
AATAAAAGAAAAGTATCTAAGTGATGCAATTATTGAATACTCAAAAAGAATTTCAAGGTATTCA
AAACTCGATATAATCGAAGTTGCTGATGAAAAAACTCCTGAAAATCCAAGCGACGTTGAAAAATC
AAAAATACTTGAAAAGGAAGCTGAACGAATTTTAAAATATCTAAAAAAAGATTCATTTTTGATA
ACTCTTGAAATTTTGGGAAAAGAATTAACTTCAGAAGATCTTGCAAAAAAATAAAAGACCTTTC
AATTTCTGGAAAACAGAACATAACATTTGTTATAGGCGGATCGTTAGGATTATCCAAAAATATTT
CAGAAATTTCAGATTTTAAGCTGTCTTTTTCAAAAATGACGTTTCCTCATCAATTAATGAGGGTT
ATTCTTTTAGAACAGATCTATCGAAGTTTTAGAATTATTAGCGGGGAGCCTTATCATAAGTAAAA
TTTATTTTCATTATCAATATTTTATGAATGTGCTTTTTTTATCCTCAAAACGTCAGTTTAGCTTT
AAAAAACTGTAAAAATTATTTAATATCCAATTTAATTTGAAAATTCGATTTTATAAATCTTATA
CCTATAAAATAGTTTTATAACCCCATTTTTATCATTAATTTTAAAATCATTTCGAAAAGTATATC
AATTATTTTAGATATTCAAATAATAAGATTTTTCTATTAAAATCTTTGAAAAACCAAAATGGCA
CAAATAACTTTGATATATTTATGATGACTGCTTTAAAATATTGCAAGTGGTGCTTTTAAACAGTA
AAATAAATCGCTTATTAAAAATCTTGGAAGTAATAAAATATATATAGGTATTCTACGGGATATA
CTAATTATTCTTCAAGGAGGTTATTCTGTGAGTGCTATAGATACCATTAGGGAAATAAAGCATG
CAGAAAATAATGCTGTGAATGCGATAGAAGAAGCTAAGAAAAAAGCAGAAGAAATAAAGCTTGC
CGCAATCGAAGAAGGTAAGAAAATAATTTCTGAAGCCGAAGCTAGTGCAGAATCATCTGCAAAA
GAATTAGTTTCAAAGTATGAAAAAGAAGCTGAAGAAAAAGCTGAAGAAATAATGAATAAAGAAG
ACAAAGCAATTGCTGAAATGGCAAACCTTGCTAAAGTCAAAATTCTAAACATCAAATTGGAAGAT
GTAATAGAATTCTAATTTCCCGTTGTCAAAACTTGAAAGGTGGTTTTATTGAGACCCGCAAGAAT
GAACAAAATTAGAGCGGTCATCTTGGATGAAAAAGTGGATCCTGTCATTAGACAACTCCATGAAA
GCGGGCTAGTGGAGTTATACGACTTATCTGCGAAACTCGAAGACCCTGAATGGAACGCGCTTATG
TTGCCAGCTCCATCAGCCGAGTATGGTAGAAATGTTGCTTCATTGACAATTAAAGTAAGTAGATT
ATTAGACTTATTTTCAAGCGTCAGTGAAGGAGAAAAAGCAGGACTTTCAGGCGTACTTAATCCAA
AAGTACCAGCAAAGAAGAAACTTTCATTTTCATCATCTGAAGAAATTATCTCCAACGTTGAAAAC
GTTCTCGAGAGCGTTGAAGGAGAAGTCTCAGAACCCGCTAAAAAGCTGGCCGAACTTGAAAATAG
AAAAACTATTCTTGAAACGTTAAAAGACAGTGTTAAATATTTATCTGGTTTTGATCTTGATTTGA
AATTCCTCGGGGAAGGATCTTACACATATGTTGTTTCTGGACTTGTTTCACAAGAAGATATATCT
GAATTAGAATCTTCACTTGAAGAAGCTACAGAAGGTTATGTTGAAATTGTAAAAGGAAATCCTG
TAAATAAGAGCGAAAAGGAACAAAAAAATTCCAGTAATTGTCGCTACATTGAAACAATGTGCAGA

TGTTGTAGGCGCAGAACTCAGAAAATCTGGTTTTGAAAGACTTGACATCGCAAATGTTGAAGGAA
CCCCTTCAGAGATTTTCAACGATACTGAAAAAGAACTTTTAAGTATCGAAACCGAAACAAAAGCT
ATTTTAGAAAATTTGAACGCACTTTCAAAAAAATGGACTGACGAAGTCTTAGTTTTGCATGAAGT
TCTTGAAATTGAAAAAGAAAGAGCTGAAGCATATTCAATGACAGGTGAAACCGACAGGACACACC
TCATTGAAGCTTGGACACCTAAGAAACATGTTAAAAAAGTTCAGGAAATCATTGAAAGCGCTTCA
GAAGGATATGCAGTTGTTGAAGTTTCAGAGCCTGACGAACCAGTTGAAAAAATACCGGTTATGCT
CGACAATGCAAAATCTGTAAAACCATTCGAAATGCTTACAGAAATGTACGCACCGCCAAAATACA
ACGAAATCGACCCTACAATGATGATAATGCCTGGTTTTTAATATTCTACGGTATTATGCTTACT
GATGCGGTTTATGGTTTATTGTTAACCTTGGCAGGTCTTGTACTCTGGAAAAGAATGGGCAAAGT
GAGCAGTGGTGCATACAACTTAGGTTATATCTTAACACTCTCAGGTCTTTCAACAGTATTCTTTG
GAATATTAACAGGTGGCTACCTTGGTGACTTTATGTACCAGTTCCTTGGAATGGACATATACACA
ACAGGATTTGCGCTTGTAAACCCGCTTGGTGAAAGCACATACATCAGTGCAGCAAAACCATTACT
CAGTCTTGGTGGAATTGATGTAAACAATGGACCTATGGCAATCTTGTTATTCTCAATCGTAGCAG
GTATTATTCACCTATTCATTGGATTAACAGTTGGTCTTAAGGAATCATTGGCAAATGGATCAGTT
TTAGATGCATTCTTGAATCAAGGTATGTGGATATTATTGATACTTGCAATAGTTGCTACAGTAGT
AACAGGAAACATGATGATTGCAGGCGGTGCAGTAATCGCGGTAATTGTATTATGTATGATTAAA
GGATTCATGAATGGCGGCATTCTCGATGCTTTACTCGGTGCAATGGATATTACAGGATTTTTGGG
TAATGTTCTCTCTTACGCAAGGCTTTTAGCTTTATGTTTAGCTACAGGTGGTCTTGCAATGGCTG
TAAACATTATGGCTAAATTATTAGGAGATGCAGTTCCAGTAATCGGTATTTTAATTGCAGTAGTA
ATGCTTGTATTTGGTCACTCATTCAACTTCGTGATGAATGGTCTTGGTTCATTTATCCACTCATT
GAGGCTACATTACGTAGAGTTCTTTGGCCAATACTATGAAGGCGGAGGAAAGAAATTTTCCCCAT
TCAAAGCTAAAAGAGACTACACTTCAAACTAATTAAAAAAAGGAAAAATAATCTAAATGAATGA
TTCGAAACGAGGTGCTTATGATGGTATTTGAAAACCCATTATTACTCGGAGCTATCGGTGCTGGT
TTAGCAGTAGGTATTGCTGGTCTCGGTTCAGGTATCGGTGCAGGTATCACTGGTGCTAGTGGTGC
CGGCGTTGTTGCAGAAGACCCAAACAAATTCGGTACAGCAATCGTGTTCCAAGCATTACCACAAA
CACAAGGTCTCTATGGTTTCCTTGTCGCAATCTTAATCTTATTCGTATTCAAAAGCGCTCCTGAA
TGGGCAATGCTCGCTGCAGGTATCGGTACTGGTTTAGCTGGTTTATCAGCAATCGGTCAGGGTAT
CGCTTCAGCTGCTGGTTTAGGTGCTGTTGCTGAAGATGATGGAATTTTCGGTAAAGCAATGGTTT
TCTCCGTTCTTCCAGAAACTCAGGCAATTTACGGTTTATTAGTTGCAATCCTTCTCTTAGTTGGA
GTATTTGCAAGCCCAGGTGTTACAACAATTGCAGCTCTCGGTGCTGGTTTAGCTGTTGGTTTCGC
TGGTCTTTCAGGTATCGGTCAAGGTATCACAGCTGCAGGTGCAATTGGAGCTACAGCTAGAGATC
CTGATGCAATGGGTAAAGGTTTAGTTTTAGCAGTTATGCCGGAAACTTTCGCTATCTTCGGTTTA
TTGATAGCAATCTTGATCATGCTCGGTATCATGTTTTAATTAAAATTAATGAAACCCCGGGTTTC
TAAATACTTTAAATCCAATTCCGAAGGTGTTACGATGGGAGCGGAAAAGATAACATCCAAAATCG
TAGAAGATGCAAATAAAAATGCCGAAAAAATTTTGGCTGAAGCTCTTAATGAAAAAGAAGCAAT
ATTAAATGAAGCTAAAGAAGAAGCTTCAAAAAAAGAGCAAGCAATAGCTAAAAAAGGAGAAAAA
GATGCTGAAATGACTAAAAACAGAATTTTAGCCGAAGCAAGACTCTCCGCAAAGAAAAAATTGCT
TGAAGAAAGAGAAAAAACGATCCAGTTAACTCTCGAAAAATTAGAGGAAGATTTAGTAAAATTA
CCTCAAAAAGAAGACTACAAAGACATTTTGTTGAAACTGATAATTTCCGGAGTTTACTCAGTAGG
CGGCGGCGAATTAGAATTGTTACTCAACAAAAAAGACTTTGAATTAATCGATGACTCAACACTTT
GGGCTCTCGAGAAAGAAATGGAAGACAGACTCAAAAAAGTCACAGTATTGAAAAAAGGCGAAGC
TAAATCGATCATTGGCGGTTGTATCATAAAAACTGCAGATCAAACAAAAGTATCTGACAACAGCC
TCGAAGCAACATTTGAAAGGAATTTAGATTCAGTCAGAGCTAAAATTGCTGAAATGTTGTTCTAA

GGTGATTTAGATGGTAGATGCTTTAACACAGTTAATGGAATTAGCAGGAATGCCTACTGACATAT
TCATGACTTTATTTGTACTTGCAGGAATCGCTATATTCTTGGTTATTATGATCTTCCTGATAAGA
TACTTGTCAGAAACTGCTCCATTTGCTTACGTTAATGCAAGAGTAAGAAGTATGGAGTCTAGATT
ATTAAAAGACCATAAAATAAATGAATTAATCGAATCTGCAGGAACTACAGAGTTAATTGGATTC
TTGGAAGATACAGACTACGGGCCATACTTATCCGAAGTTTTAGGACAAAGTGAAGACCCAGTAGT
TGTTGAAAAGGCATTGGATATTCATTTAGCACACGTTTACCAAACACTTGCAAATATCTCCCCTG
ATGGAGCAAGGAAAATATTAAAATTGCTTGAAAAGAAATTCGATGTTAAAAACATCAAAACACT
TCTTAGAGCAAAATATGTTGGTTTGGATGCTGAAGAAACTTTCAAATTATTGATTCCACTTGGAA
CAATTCCTGAAAGCAAATTAAGAGAATTAAGTGAAACTAAAGAAATTGAAGAAATCGTAAGTGC
TTTAGATGGAACTGGTTATTCAGGCGTACTTTCAGAAGGTTTGACAGAATACGAGCAAAACGGCA
AATTAACTACCCTTGAAATGTCACTCGATAAATTAATCCTTGAAAACCTCTGGAAAAACGTGAGT
GTTGATGGTACCGAAAAAGACTTGTTCAAAGAATTTATCGGAACAATGATTGACATTGAAAACT
TGAAAATCATCCTCAAAGCAAAAGCTGACGGATTATCATCTGAAGTTATTTCAAAATATACAACA
AGCAAAGGATACGAACTTGCTTCTTGGAAATTAAAAGAGCTTGCTGATGTAGAATCAATCGAAG
GAGTTATCAGTTCACTTGAAGGTACAAAATACGCACCAATTGTAACTGAAAACCTCGAAGAATTC
GAAAAAGTTAAGTCAGTTTATGTATTTGAAAAAGCTCTTGACAGTTACCTAGTTCAAATGGGTA
AAAAATTATCACTCAGACAGCCATTTGGAATAGGCCCGATAATTGGACTTATTACCTCAAAAGAA
CTTGAGATAAGAAATTTAAAAATTATCATTAAAGGTAAAATTGAAGGCCTGTCTGCAAGTGAAA
TCAGGGAGATACTGGTATCCTAAAAACGATTATAAAGTGATATTATGAGAATCGGTGTAGTTGG
AGATCCTGATGTAGTAGCTGGGTTCAGACTTGCTGGACTTACAGATGTCTACGAAGTTAACTCCC
CTGAACAAGCTGCAAAGGCAATTGAAGAATTAAACAGCAACTCTGAAATAGGATTGATTATTACC
ACTGAGAGAATCGGTGAAAAAATAAGGGATGCTATTTCAAGCATAAAAAAAGTTGTTGTAGAAG
TTCCTGACAAAAACGGTCCTATTGTCAGAGAAAATGATCCTGTGAAAGTATTAGTAAGAAACGCT
GTTGGTGTTGACATTAAGTAATTCTGAGGTGAAAATATGGTCGTCGGTAAAATTATCAAAATAT
CCGGTCCGGTTGTTGTTGCCGAAGGCATGAAAGGTTCCCAGATGTTCGAAGTGGTTAAAGTAGGA
AACGAAGGTTTAACCGGGGAAATCATTCAGTTAACTGAGGACGAAGCAATCATTCAGGTTTATGA
AGAAACTGCTGGTATCAAACCAGGAGAAGGAGTTGAAGGTACTGGTGCTCCATTATCAGTAGAGC
TCGGCCCCGGTATGTTAAAAGCAATGTACGATGGTATTCAAAGACCTTTAAACGAAATCGAAAAT
GCTACAGGTTCAATTTACATCCCAAGAGGAGTAAGTGTTCCATCAATCTCAAGAGAAATTAAATG
GGACTTTGAACCAACTGCAGCAGCTGGAGATGAAGTTATAACTGGGGACGTTATCGGTACAGTTC
AAGAAACTGCATCAATCGTTCACAAAATCATGATTCCTTTCGGAGTTAGCGGTAAAATCAAAGAA
ATTAAAGCAGGCAGCTTCACTGTTGAAGAAACAGTTGCAGTTGTTGAAACCGCTGAAGGCGAAAA
AGAAATCATGATGATGCAAAAATGGCCTGTAAGAAAGCCAAGACCATCAAAAGGAAAACAAGCT
CCAGTAATTCCATTAATTACCGGTCAAAGAGTTGAAGATACATTCTTCGGACTTGCAAAAGGTGG
TGCTTCAGCAATTCCAGGTCCATTTGGAAGTGGAAAAACTGTTACACAACACCAGCTTGCTAAAT
GGTCTGATGTGGATGTTGTGGTATACATCGGTTGTGGAGAAAGAGGTAACGAGATGACAGAAGT
TATCGAAGAGTTCCCACACTTAGATGATATCAAAACAGGAAACAAATTAATGGACAGGACCGTAT
TAATTGCAAATACATCAAACATGCCGGTAGCTGCTAGGGAAGCTTCAGTATATACTGGAATTACA
ATTGCAGAATACTTCAGAGACCAAGGACTTGGTGTTCTCTTAACTGCTGACTCAACATCAAGATG
GGCAGAAGCAATGAGAGAGATCTCAGGTAGACTTGAAGAAATGCCTGGGGAAGAAGGATACCCT
GCATACCTTTCATCAAAACTTGCTCAATTCTACGAAAGGGCAGGAAGAGTTGAATGTTTAGGTTC
AGAAAACAAACAAGGATTTGTATGTATTGTAGGTGCAGTATCACCACCAGGTGGTGACTTCTCAG
AACCAGTTACATCAAACACATTAAGAATTGTTAAGGTGTTCTGGGCATTAGATGCAAACCTTGCA

AGAAGAAGACACTTCCCTGCAATCAACTGGTTAACAAGTTACTCACTCTATATTGACGACATCGC
TGGCTGGTGGCAACAAAACACCGCTGCAGACTGGAGATCATTAAGAGATGAAGCAATGAGCTTAC
TCCAAAAAGAAGCTGAGCTCCAAGAAATCGTTCAACTTGTTGGTCCAGATGCACTTCCAGACAGA
GAAAGAGTTATCTTAGAAATTGCAAGAATGTTAAGAGAAGACTTCTTACAACAAGATGCATACC
ACGAAGTAGACAGCTACTGTTCACCATTGAAACAGTACAACATGTTAAAAATCATCATGACGTTC
TACAAGAAAGGATTAGATTCAGTTGCTAAAGGTGCTGACCCAGCAGGCATTTCAGCTGTTACTGT
AAAAGGAGACATTGCAAGAATGAAATACCTGACTGATGACGAATTCATAAACACGAAAGTACCT
GAAATCATTAACAAAATGGAATCCGAATTAGGTGCTTTAATCAAATAACCTAATTTAATTTTAA
ACTGTAAGGAGGGTTATCATGGACGCTATGCAAAAAACCATAGAATACACATCCGTCTCCAGAAT
TGCTGGTCCTTTAATGGTTATTGATGGAATCGAAGGAATTGCTTACGGTGAAATTGTAGATATTA
CAACTCCAAATGGAGAAAAAAGAACCGGGCAAGTTTTAGAAGCTAGAGAAGAAATTGCTGTTGT
TCAGGTATTTGAAGGAACTTCAGAATTAAACACTAGTGAAACAAAAGTAAGATTCACAGGAGAC
ACTGCAAAAATCGGTGTATCATACGATATGCTCGGTAGAATCTTTAACGGTGCAGGAAAACCACT
CGATGGCGGACCAGAAATCATCGCTGAGGAAAAATTAGATATCAACGGTTACCCATTAAACCCAG
TTTCAAGAAACCCGCCAAACGCGTTCGTTCAAACTGGTGTTTCAACAATCGATGGTACAAACACA
CTCGTTAGAGGACAAAAAATCCCGATCTTTTCCGGATCAGGTTTACCTCACAACAAACTTGCTAC
GCAAATTGCAAGACAAGCTAAGGTAAGAGGAGAAGGTGAACAGTTTGCGGTAGTATTTGCTGCA
ATGGGTATCACCGGTGAAGAATCAAACTACTTCATGGATGAATTTAAGAAAACAGGTGCTTTGGA
AAAAGCTGTTGTTTTCATTAACTTAGCAGATGACCCTGCAATTGAAAGAATTCTTACACCAAGAA
TTGCACTCACAACTGCAGAGTACCTTGCATACGAAAAAGGAATGCACGTACTTGTTATCTTAACT
GACCTTACAAACTACTGTGAGGCATTAAGAGAAATTGCAGCTGCAAGAAACGAGGTACCTGGAAG
AAGAGGTTACCCTGGTTACATGTACACTGACTTAGCTTGCCTCTACGAAAGAGCAGGAAGAGTTA
AAGGTAAAGAAGGAACAGTAACACAGATTCCAATTCTTACAATGCCTGATGACGATATCACGCAC
CCAATTCCAGATCTTACTGGTTACATTACCGAAGGACAGATTGTGCTTTCAAGAGAGTTAAACAG
AAAAGGAGTTTACCCTCCAGTTGACATCTTGCCATCACTTTCAAGGCTTGCAGGTAACGGCCAAG
GTGAAGGAAAAACAAGAGACGACCACTCAAAAGTTATCAGCCAAGCATACGCTGCATACGCTGAA
GGTAGAGGTTTAAGAGACCTTGTAGCGGTTGTTGGGGAAGAAGCATTAACTGAAAGAGACAGAT
CATTCTTAAAATTCGCTGACGCTTTCGAAAACAGCATTGTTACTCAAGGAGTAGACGAAGACAGA
AGCATTGAAGAAACACTTGATTACGTCTGGGATTTATTAACAATATTGCCTAGAGAAGAACTCAA
GAGAGTTTCCGATGAATTAATTGAAAAATACTTACCTAAAAAATAATTCAGAAGCTGAATTATT
CCCATTTTATTAAACTAGGGGGATAGCAATGGCAGACGTAAACCCTACAAGAATGGAACTTCTAA
AGTTGAAAGGAAAAATTAAACTTGCAGAAAAAGGGCACAAACTCTTAAAACAAAAAAGAGACGC
CCTTATGATGGAGTTTTTTGAAATCCTCGATCAAGCTTCTGGAATAAGAGACAAAGTAAATGATG
CTCTTTCACAAGCATACAAAGACTTGATAATGGCTCAGGCAGTAATGGGTACTTTATCAGTTAAA
GAAGCGTCATTTGCAGCTAAAAATAATAACATCGACCTAGATGTGGACATGAGAAACATCATGGG
TATCGATGTTCCAGTATTTGAAATTTCAAATGTAAAAAGAGACATTTCAAATAGAGGTTACAGCC
CATATGGAGTATCCGCAAAACTGGATGAAGCTGCAAAGAACTTCGAAGAAGCTTTAGAACTTATT
ACAGAACTTGCAGAAATCGAAACTTCGATAAAATTACTCGCTCAGGAAATCATCACAACAAAAAG
AAGAGTTAACGCTCTTGAATACGTTGTAATTCCAAAAATGAACGAAACCAAAAAATACATCGCTA
TGAGACTTGAAGAGATGGAGAGGGAAAACTTCTTCAGATTGAAAATCATTAAAGCGAGAATGGA
CGCTAAAGAAGCTGAAGAAGCATAATTCAACATCGTTGGTGGTTTTATGTTCAGAATTACTGGAA
AAATTAAGGAAATTGAAGATAAAATTGAAGGCAAAAAACAGGAAGGGGCTAAACTAGAAATAGT
TGGCCAAAAAGTTCCTGTTTTTGCAGCAGAAACTGTTGAAATTAAAGCAGGCGAAATTAAACCGA

TAAACATTTCGAAAATTTGCCTGCCTAAAAAAACAGTTTTGATGCCTTCTGCATACATTCAGCAC
AAATTAGGAAACATGGTATCCTTAGGGGAAGAAACTCCAGTTCCTTTCGAACATGAAAGATGTCT
CGAATACGCAATATTTGTTGCAGTAAAAGAAGGAACTATTAAAGAAGGAGAACTCGTTGGAACC
ATTGTAGTACTCCATGCTGAATAATTAAATACCATCAATACTAACTTTTTTTTACTTTTCAATAA
AATTTACGTGAAACAATGGATAAAGAGATTTATAAGTTTCTGCACAATTTAAAATCCAATTTTA
AAAGAAAAACATTAAACGAACTTTTAAAAGAAGATAAACCGCATTTAATAGTAAACGGACAGAG
ACACAGGATTAAGAAAAAGGAACTCGAATTTATAAAAGAAAATATATCTACAGATATACGTCTT
CCAATAGTACTAGAAGTTGATTCAAGTTATGACTCTGGAACTGTAAAAATAGAAGGAATCGAAG
AAGTAAAATTAATATCAAAAATTTTAGGAAAAAAAGTCAGCCTTTTTAGTGAAGAAAGTATCCT
TTATATCTACAAACCTGAATTAAGAGAAGTTAGACGAAAATTACCGACTACGACACAATATTTAT
TTAGAGTAGGCCTTAATTAGTGATAAAATGCTAAAAAAGAAAAAATATTATGGAAGAGACCCGA
TAAAAAAGCTTATGAATGATCCTGAAAAAAGCGAAAAAATTTATAAGATACTATTCCTAGTAAA
TATATGGGTTTGGTTCTCGATGTTTATTGGTGCAGTTATCTTTGTGATATGGGCCTACAAATTCC
TATCAGCGTAATTTAGGTGGTTTAATGAACGAATATGTATTATACTTCACCATGATTTCATTAGT
GTTTGGTGCGTTTTCTGCGTTAAAATTAGCATTTAGAAAAGAAACTTCAAATGTACTTATCGGAG
AAGGCCTAATGGCGGTAATAATTGCTACATTTTTAGTAATTTTAGCAGAAATATACTCTATTGCA
TATGTAGAAACCGTAGCACTGTTGCTCTTAGTATGTGGCCCGATTGGAACAATTGCATTTTCAAA
ATTCATGAAAAAAGGAAATCCTGCGGAAGTATAAATTATTTAAGATATTTAACAAAAAAGAATT
TAATTTTATTTTTTAAGCCAGTTTATGCTGTTGCTTATTCCATCTTTAAATGAATCAGTACTGCT
TTTTTTATGTTCCTGACTGTTTTTCTGAACGTCTACAGCTTCAGCTTCTTTTTCTGAAACTGCAAT
ACTGTGTTCTCTATCTCTTTTTTTCTGCTCTAATTTTTCTTTATCAAGCTCGAAATCTTCTTCTAC
CCGTATTACGTGGTCTGCAATATTTTGAATTGCAGTACTAAATCCAGGTTCAGCACCAACGACAA
TAATTTGTTTTCCGCGTTCTTTGGCTTTTCTGATTGCAGGTAAAAAATCTGCATCCCTTGTCATG
TAGACAATTGTATCAATATTTTTATTAAATATCAATTCAGTTCCTTCAACCGCCATTTCAACATC
AACGTCTCCTGCAGAAATTCTTGGTTCAAAACCCTGATTTGCAATGGCTTCGATTAATTTATCTG
ACGCATACTGGTTTAAATAAACTCTGCCAACAACAATATCTCCAAACTGTTCTAATGCATCCCTT
ACTTTATCAAGATCAACATTAAATTCTTTTCTTAACATGTTTGGTCCATCAATAAGTAAGGCCAT
CTTATTTGCCCCTTTTTTGCCAGTTTTTACATAAAATTTCTTTAAGTTGCCCAGTTTTTTCCACAT
CTATCTCACGACCAAATATTAACAAAATTAAAAAATAATTAAGTAAATAATCTATTTGTATTTA
AACTATTTAAAACTAAAACTTTTTTCCATATTTTTCCTTAAATTCATCAATATTTTTAGCAGTAT
TGTCCAATGTAAGGGGCGTTACTGACACATTTCGTTTATTTCTAACAGCATATACATCAGTTCCA
TCTTCCTCATCCATCACAGGATATCCGTCAAGCCAGTAGTAGCTCCTGCTTCTAGGATCAATTCTT
TCCTCAACGTGCATTGAATACATACGTTTAGCAAGTTTGTAATTTCTACAGGCGTATTTTCTGT
AGCATTATCTGGAACATTTATATTTATAACGTCACACGGGAATTCATCGTTTAAAAACTTTTTAA
ATACGTTTTTAACAATTCTAGCGGCAGTCATAAATTCAATTGGACTTTCGCCTTCTTTAAATTTT
AAATGATCCGTAGTAACCTGTAAGGAACATGCGAATGATTTTGCACCGTGGTGTGCACCTTCAAA
TGCAGCTCCAAGCGTTCCTGAAGTTGTAATTTCCGTTCCAAGATTTTCACCAATATTTATTCCAG
AAATTATGTAATCCGGAACTTTTTTTAAAACCTGGTGTACCCCCAAAACAACACAGTCAGTAGGA
GTTCCAGATACTACATAGCCCTCAGAACAATCGGCAAGTTTTGTTTTAGTAATTCTAAGAGGCTC
AAATAAACTTATTGCTCTTCCAATACCACTCTGTTGATTAGTGGGTGCAACAACCGTAACATTTG
CATCAAATTCTTCACATATTACATTTTTAAGTGCTAAAAGACCATTTGAATAAATCCCATCATCA
TTTACAAGTAAGATTTCCATAGTCATAACAAATCACCATTCAGTTACTATTTATCGTCTCGCAAA
TATTTATACAATACATATTAATTCATAAATAATCCATTAATCAAAATTAAAATGTGTATTTTACG

TTTTGGCAAAAAATACCTTAAAACCTGCATATTATTATTCACGCTTCAAATCTTAAATATTTAAG
TTAAAACTAAAATTTTAGTTATGTATTAAATTATACCATTTTTAAAATTCCATCTGAAAATTAA
AATTAAAGTTAATCTTTTAGGATTGGTTTCAAAAGTATATATACTAGCCGATTTTATATATTTAT
TAAAGTTATTTTGATAATGGATTTTTGGTGGATTTATGAAAAAAAGAAATATTACAGAATTTCA
AATACTTTCAGAAGTTGTCAGAAAACAGCCACATATTAAGCAGAAAGAAATTGCTGACATATTGG
GCATAACTGTTCAGGGCGTTTCAGAACATATGCGAAATCTGATAAAAGACGAACAGATTAAAAA
TAAAGGAAGGGGAGAATACATAATAACCGAAAAAGGTATGGCTTCATTAAAAAACTGGATTTCG
AACTTTAAGAATTATTTAAACGATGTAAATCAAAATTTGTACCGATACAAAGATGTATGGCCAGC
AATTGCAACTGAAGAAATAGTGCCCGGGGAAACTGTTGGAGTATACATGAAAAAGGGAATTATA
TACGTTTCAAAAAACATCGAATCCGATGCAACCGCAAAAGTATTTTACGGTGGAGAGTCTGGAGA
AGATGTTGCAATACATGAAATTCATGGGCATATTGAAGTTCAAAACGGAAAAGTTATCGTTTTA
AAAATTCCTTCTGAAGTTATGGGTGGATCTAGAAACGTTGACTATGATATTGTACAAAAAACTCT
TGAAGAATACCCTGATGCAGTGATTGCAACTGCAGGAACTGTTGGAACAGTGATTGCAAATAAAC
TTGAAATTCATCCAGATATTAGATTTGCAGTCTCTGAAGGAATTGTTTCTGCGTGCAACAGGGGT
TGCGATGTTATTGCAATGATTACTGGAAAAATGGCTGAAAAAATAATAAAAACTGTTGATAAAA
ACAAAATAAGCTACACATTACTAAATGCGTCAAAAACGACCGATTCAATCGAATAAATATTATTT
TTAAATTTTTATTAAAAAAAGAAAAAAGGAATTAGTATCCTAATTTTTGAAGTAATGGTTCTGG
GGAAATAAAGTTTACATCCAAAGCCACTTCGTTTGGTTGCATTCCCATTGCAAGACCTAAAAGCT
GTGATAAGTGTACAACTGGGAAATTGTATTCATTGCCTAATTTTTCTTTAATTTCAACTTGTCCC
CTGTCGAATTGTAACTGGCAGAATGGACATACGTTAACTGTAGCATCAGCGCCTACTGCTAACAT
GTTTTCAATTTTTTCTTGTGTCATGCTTAATGCAAGGTCTAATTCTTTAGCTCTTACACCACCACC
TGCACCACAGCACATCATTTTATCTTTGTAGTCTACTGATTTAGCACCTGTTGCTTCAACGAGTT
TGTCGAGCATTGTTGGGTGTTCAGCATTTCCAAGACCTTTTGCTGCAGTTGGTTTTAAGAAGTGG
CATCCGTAGTGAACTCCAATATTAACATTTAATGGTTTTACAACATTTTCTCTGATTTTTTCTAC
ACCGATTTCTTTGTAGATTAATTCAGCAAAGTGTCTTACGTGAACAGTTCCTTTGTATTCTTTTC
CAATTTCTTTGAGCTGTTCGTTTACCATTGCTAAAGCTTCTTTGTTTTCATGTAACATGTGTGCA
GCTTCGTATAATGAACCGTAGCATCCATTACATACTGTAACAATGTCTGTTCCCATTTCTTCAGC
AATTACTAAGTTTCTTGCAGCTAATGTTAACCAGGTTTTTTGGTCGAATGAACCAAATACCCCTG
GAGCAGGACAGCAGGATGCACCAGGCATATCTACTAATTCTACACCTAATTTTTCCATTACTGTT
CTTGTAGCTGATTCAACACCGGCATACCTGTTTGGCATGATACATCCAAGGAAAAACGCGTATTT
CATCAATATCACCTATTTTAAATTATAAAGTTCGTAAATTATTGTTTTAAACCCATTGTTTTCCA
GTCAAATCCGATTAACTCATCGAATTTTAAGTTTTTAGCAAGAGTTCTGATTTCGTCCATGTCTT
TTTGGCTGAACTGTGCAATTGGTGCTTTTTCAGCAAGACCAATTGATTTTCTTAATTTTGCTGTA
TCGTCGTTTGCAGGTACTGCGTGACCAGCTTTTAAAACATATACTGCAGTCATTTTGTGGGGTTT
TGCCATGTTTCCTTTTTGAGCAGCTAAGTTCCTGATTGTTTTAATGATTTCAGTTACTTTAACGT
CTCTTGGGCATCTTTCGTAGCAAGTGTAGCAGGTTGTACATTTCCAAATGTCATCACTGTCTAAA
ACACTGTCAATACCGAGTAATGCTTGTCTTATAACTTTTCTTGTCCTGTATGCTGTTCTTCTACCG
CTTGGGCAGCTTCCAGTACATGTACCGCATTGGTAGCATGATTTTAATGAAGCTACTTCTTTTTC
TCCTGGAACGGGGGTTCCAGCTTCAATGATTTCGTTTACAAAACCTTTGTTCAACTCTTCAGCTT
TCATCATCCTTAACACCTCTAGGATACTTCTGTTTATATATAATATATTTATTGGTAATCGATAT
CACATATTATACTCTTGTATAATACCTTAGTATTTTTAATAGAAATTCGAATATTACTTAAGATG
GTGGTATTTTGATTGAGAAAATGGTCAAAGAATCAGAAAAAGGAATTTTAATTGATATCGAAGT
TACTACAAACGCTAAAAAAAATGAAATTGGAAAAATTAACGAATGGCGTAAAAGAATAGAAATA

AGAATTAAAGAACAGCCTATCGAAGGCAAAGCAAACAAGGCAATTATAAAATTTTTAAAGGGCA
TTTTTAAAAGTGAAATACTGATAAATTCGGGAACAACCTCATCACAAAAAACAGTACTTATACCA
GATAAAACAAAAGAGGATGTTGTAACAATTTTAAAAAAGGAAATTAAATCAATTTAATCTTACC
AATTAAAGTGATAGCATGGAAGACGATATTAATTACACCCTTGCAGCTTATAGAATCTGCACTCC
TGAAGAAACTTTTGAAAAAATAGAACCGATAATAAAAGAAATTGGTGTTACAAGAACTGCGAGA
ATCGATGGACTCGATAGAATTGGAATTCCTGTTTTTTCATCGATCAGGCCCTCTGCAAAGGACGG
TGCAATCAGCGTGTATGCAGGAAAAGGTGCTACTGAAATTCAAGCAAAAGTTTCATCGACAATGG
AAGCAATTGAAAGATATTCTGCAGAATTTGATGAAAATTCAAAACTTGAACTTACAAAAGAACC
AGAAAAACCCAATAAACTTGGATGATTTGATATTACCTGGCGGAAAAAGTGCAGAATATACTGAT
ACCAAAGAAATAGACTGGGTAATTGGAAAAGACATTATCAGCGGAAAAAGTTTTGATGTTCCAA
TAAACAGTACTGTTCACCCACATGACGGAAAAAAACTATTTAGAAGCAATACTAACGGCCTTGCA
TCCGGAAACTCTCAAGAAGAAGCAGTTTTTCATGGAATGCTTGAAGTAATCGAAAGAGATGCGTG
GAGCATTTCAGAACTCTCAAAAAATACTTACAGAAAAGTAAATGTTGAAAATGCGAAAAATCCA
CTAATTTTTGAGCTCCTTGAAAAATTCAAAAAAGCAAAAATAAACATTATATTAAAAGATTTAA
CAAGCGAAGTTGGAATTCCAACAGTTGCTGCAATTTCTGATGATGATGTTTTAAAAGATCCTGCA
CTTTTATGCATGGGCGTTGGATGCCATTTGCACCCAGAAATTGCTGTTTTAAGAGCATTGACGGA
AGTTGCACAGAGTAGGGCAACACAAATTCACGGGGCACGGGAAGATACAAACCGGGGAGATGTTG
TACGAAAAATAAGTTACGACAGGATGAAAAGAGCGCATAAAAAGTGGTATACGTTTAAAAATGA
AATAAATATTGAAGATATGCCAAATAATGCAAAATTAAATCTTAAAAAAGACATTGAAACAGTT
AAAGAAATATTAAAACAGAACGGTTTTGATAAAATCATAACTGTTAAATTAAATAAAACGGATA
TAGATGTTTCAAGAGTAATTATTCCTAAAATGGAAATGTACAGTGTTGATAGAGATAGAATTTC
ACTCTGGATTAAAGATAGGATTCGAAGAAACCTTGAAAGTAACTTAAATTTAATTTAACTTCTTT
TTTATTAATTATTTAACTATTTTTAGTAATTTAACTAATTTTTGAAATTTTTGGAGGGTAATTAT
GAGAAATTTCAAAAAACCGGATGTTTTAGTCAGTAAATGCCTCGAACATGGGCGATGTAGATACG
ACGGACAGATGATTTCAAGTGATTTTGTTAAAAGTACTAAAAAATATTTTAACTACGTTTCAGTA
TGTCCTGAAGTTGAAATAGGCCTTTCAATTCCAAGAAATTCATTAAAAATTGTTTTTGAAAATGA
AAAATTTGAGTTTATTCAAAATATTACAAATAATAATTACACCTCAAACATTACACAATTTTCAT
CTGATTTTTTAGATTCAATTGAAAATATTGATGGTGCAGTATTAAAACACAAATCTCCATCATGC
GGAATAAAAAACGTTAAAGTTTATACTGCAAAAGGAAACCCGGCAAATCAAAAAACAGAAGGCT
TTTTTGCAAAAGAAGTGTTATATCGTTATTGCGATATTGCAATTGAAGAAGAATCGCGACTTAGA
AATAAAAGAATAAAAGACCATTTTTTAACCAAATTATATACAATAAAAGATTTTAAAGAAGTTA
AAGCGTCAGAATCCATTAAAAACCTGATTGAATTTCATACAAACAATAAATTCCTGTTTATGGCA
TATAACCAAAAACAAAAAGACATCTTAGGAAATATCGTTGGAAACCACAATAAAAATTTTGATA
AAATTATAAAACTTTACGAAAAACATCTCCAATTACTTTTAAAACGGCCCTCAGCTATTGGATCA
AATATTAATGTTCTCATGCATATTTTTGGATATATGTCAAATAAAATTTCGAAAGATGAAAAAT
TACTATTTATCGATTCACTTCAAAGATACCATGACGAACTGGATTGTCTCTGTGTCCCGATAATT
CTTTTAAAATCGTGGGTTATTAGATTTAACGTTGATTATTTAAAAAAACAGACTTATTTCGAACC
TTTTCCACAAGAACTTGCAGATAATTCTGAAATGTTTAAAAGAGACTACTGGAATGATTAAACCA
TTTTAAAATAGATTAGGTGGACTGAGCGGGATTTGAACCCGCGGCATCCGCATAGCCAATGCGGC
ACTCTCCCAGTCTGAGCTATCAGCCCAATCTAGCTTAGATTTGATTGTTTTTCTATATAAATATT
TCCATTTAATATATTCTTTTAAAAAGCTATAAAACTGCCAAATATCATAAATTTATACGTTATTT
TGAAAAATAATTCAAAGGGAGTTTCATGGAAAATTTAAAGAATTATTTTGATGTTGTTATTATC
GGAGCGGGTCCTGCAGGACTTTTTGCAGCGTATGAATTATCAAAATATGATAATTTAAAAACTGT

AGTAATTGACAGGGGAAAAGATGTTTTAGAAAGACATTGTGCCGCAGAAAAACTTAAAAAATGT
ATAAAGTGCAATCCCTGTCAGATTATGTATGGTGTAGGTGGTGCGGGAGGTTTGAGTGATGGAAC
ACTAAATTTACGGCCAGATATCGGTGGAAATTTAGCTGAAATAACTAATGATGAAAATTACGCCT
GGCAGCTCATTTTTGAAGTTGATAAAATTTTTTTAGAGCATGGAGGTCCAAAAAAACTTTTTAAA
GGATCTTTTGAAAAAATAAAAGAACTTCAAAGACGTTCTGCACAAAATGGAGTTAGATTTATTCC
AATTATTCAAAGACATGTCGGTTCGGATCATACAAAACAGTTGATCAATAATATCAAAACTACGC
TTCAAAATAGGGGTGTTGAGTTCTTATTAAACACTGAAGTTTTTAAATTTCAAAAAAATAAGGT
ATCTGTAAATAGTTCTGGAGAAGAAGAAGAAATTAATGCCAAATATATAATTGTTGCACCGGGA
AGGGGTAATGCCGAATGGTTTTTAAAAATTTCAGAAAAAATAGGTTTAATATCAAATCACGGCCC
GATTGATGTTGGAGTAAGGGTTGAAGTCCCTTCAATAATCATGGAATCAATTACTGAAATAAATC
ACGACCCCAAATTTCACATATATACTGATACCTACGATGATTTTGTCAGAACGTTTTGTACAAAC
CCCTACGGCTTTGTTGTAGAAGAAAATTATGGCGACATTATTGGAGTAAACGGGCACTCGATGGT
CGATAAAAAAAGTGAAAATACCAACTTTTCGTTTTTAACAAGAATTGAACTTACTGAGCCTGTTG
AAAACACTACAAGCTACGGAAAAAGTATTGCAAAACTTACAACAACACTTGGGGGCGGAAAACCA
ATTTTACAGAGGCTAGGTGATTTAAAACGCGGAAGAAGGAGCACATGGGATAGACTTAAAAAAA
GCAGTTTTGACCCAACTCTAAAACAGGTTACGCCTGGCGACATTGCAATGGCACTGCCACACAGG
ATTGTCACAAATATTATCGAAGGACTTGAAAAATTAGATAAAGTAATTCCAGGAGTTTCTAACG
ACCATACATTATTATATGCACCAGAAATTAAGTATTATGCAATGAAAACAAAAGTAAATGACGA
TTTAGAAACAAGTGTTTCAAACATTTTTGCCGCAGGAGATGGAGCCGGACTTTCAAGAGATATTA
CAAATTCTTGTGCAACAGGCATGCTTGCTGCTCGCGGAATTCTTAAAAAAGAAGGAATTACAGAT
ATTTTTAAAGATGAAAATACAAATTGGCAGGAACAAATAGAAACCATGAAATAAAGGGGAATTT
ATGTTAAAAGTTTACAATACACTCACACGATGCGAAGAAGAATTTAAAACTTTAAATGAAAAAG
AGGTTAAAATGTACGTTTGTGGCCCGACAGTCTATGATAACACCCATTTAGGGCACGGTCGAACT
TACGTTTCATTTGATATTATTCGTAGATACCTCGAACATATAGGCTATACTGTAAATCTCGTTAT
AAATTTTACAGATATTGATGATAAAATTATTAAAAGAGCATATGAAAAAGAAAAAGATCCAAAA
GAAATTTCAGAACAATTTATAGGGGTATTTTTAAACGATATGGCCACATTAAAAGTAAAACCTGC
AGATATCTATCCAAAAGTTACAGAACATATTTCTGAAATTATTGCTTTTATTGAAAAGTTGATTG
AAAGGGGTTTTGCATACAAAACTGAAGATGGCGTTTATTTCGAAGTTAAAAAATTTAAAGACTA
TGGAAAATTGAGTAACATCAATTTAGAAGATTTGGTTTCTGGAGCCAGAGTTGAAACTTCTGAA
AAAAAGAAAAACCAAAAAGACTTTGCACTCTGGAAAACTGCAAAACCAGGAGAACCTAAATGGG
AAAGTCCTTTTGGAGATGGAAGGCCCGGATGGCACATCGAATGCTCTGCAATGAGTTCGAAATAT
CTTGGAGAACAGTTCGATATACACGGAGGCGGACGAGATTTATCATTTCCGCACCACGAAAATGA
GATTGCACAAAGTTCGGCATATTCTGGAAAAGACTGGGTTAATTACTGGTTGCATACCGGCTTTG
TAATGGTTAACGGAGAAAAAAATGAGTAAATCTTTGGGCAATTTTATAACTATTGATGACATTTC
AAAAGAATACAGTCCAGAAATCTTGCGGTTTTTCTTTATTCAAAGGCACTACAGAAGCCCAATTG
ATTATACCGCTGAATCTATGAATCACGTTAAAAACAATCTTGAAAAAATTTACAACGTAATTGAA
AACATAAGAATTTCACTTGAAAAATCTGAAAAATCAAGAACCTGGGATAAAAATGAATTTTTAC
TCTACGATATTTTAAAAAATTCAAAAAAATAATTTTTATAACGCAATGGACTCAGATTTTAACACA
GTTGAAGCATTGAAATCAGTGTTTGAAGTTTCTAATGGAGTTAATAAATATTTAAGTTTAACAA
AAACTCCTAGTGAAGGTTTACTATTAAAAGCACTTGATTTCTATAAAATAATTGGAGAAATATTT
GGATTATTTGAAAATTACTTTAAAGAATCATCTGAATCAGATGAAGAAGAATTTGTTAAATTTT
TAATTGAATTGAGGTCTGATGTAAGGCTTCAAAAAAATTACGAAATGTCTGACAAAATACGGGA
TGGTTTGAAAAATTTAGGATATCAAATTGAGGACAACCCAAAAGAAGGAACAGTATTTAAAAAA

ATAAATATTTAATTTTCGAGTGGTATTTTGGACGAAAATTCCTACAAAACACTTAAATCTTTTTC
AAAAGTAGAAATGGTCTTTAAAGGTTCATTATTCCTTGGTTATGGAAAACCCGTAAAAAGTGAG
GCTGAAGCAAAAGAATTTATTGACAAAATTAAAGAAATTCATAATGATGCAACACACAATGTTT
CTGCATACCGGATAAATATTGAAAATAATTTTGCAATGAAATACGATGATGATGGTGAACCGCA
AGGAAGTTCAGGTAAGCCGATCTACAAGGTTATTGAATTAAAAAATCTCCAAAACACAGTAATTG
TTGTTACAAGATATTTTGGAGGTGTTAAATTAGGATACGGAGGTCTTGTAAAAGCATACAGCGA
TACTGGAACCGAAGTTATAAATTCAAGTGGAATTTTAGAGGTTTTTGAAAAGTCTTATCTAGTTT
TAGAGTTTGATTATTCCGAAATACAAAAAATTAAGGATATTTCAAAGATTTTTGGAAAAATTGT
TGATGAAAACTATTTGGATGTTGTAAAATTTAAATTAGAAATTAAAAAAGGACTTGAAGATGAA
TTTACTAAAAAACTGGTTAATTTAACAAAAAATAAAATAAAAATCTATAATGTATAATTGATTT
GACATAATTTTTTAGGGGATTTTATGAAAAAACTTATTATAATTTTCACAATTTTAATGTCGATA
GTTCCGACATTTGCATTTGATTTAGAAAATAATACTGAATTTGAAAATTGCGTTAGTATTACAGC
CCCTGCGGTTTCAGTAACTGATGAAGGATACGTTGGGGTAGCTGTTGATATCGATGTAAAAGTTT
CAGATGGTGAAGGTCATGCATATATGGACACCCTACCGCTTACAGATATTGATATGCAAGGATCA
GCTCGCATTGCCTCAAAAGTAGCTTTTGATATTTGCGGGATAGATCAGGAAAGTCATGATGTTTA
TTATATCATAAGATCAGACGTTCCAACAGTTGGTGGCCCTTCTGCAGGTGCAGTATTAACCGTTG
CAACGATTGCCGCGATAAATAACTGGACATTGAATAAAAACGTCATGATGACAGGAATGATAGG
TCCAGATGGAACAATTGGCCCTGTTGGCGGAATAATTGAGAAAATTAATGTTGGAGCCGAACAAA
ACGTTGATTATTTCCTTATACCTTACGGACAACAATATCAAATCGATGAAAATAATAATTCGGTT
GATTGTATCGAATACGGACAAAATCTTGGAATTAAAGTTGTTGAAGTAAGCAACATCTATGATG
TGGTGTATTATTTTACAAATAACGACATCAACACGGATTACGAAAAAAATCCGATTATAGATGGT
AAATATAATGAAATTATGGAAAAATTATCCGAAGTTACACTTGAAAATGCCAAAAAAAGTCTTG
AAGAAACTGAAAAGTTAGATTTATCAAAACTAAATTCTTCAGAAAAGGAAGAATTTGAAGAAGA
CTTAAAAACCGCATCAAATTTAGTGTACCTTGGAAATGAACTGCATTCAAATGAGAAATATTATA
CTGCAACTTCCAGAAGCTTCAATTCTTTAATCATATTTGAAACACTGCAGTCAAAATACAATTAC
AAAGACAATGAATCCATTAAAAATTATCTTTTAGGCGTAAATGAAAGTATTTCTGAAAAAAAGG
GCGAACTTTCAAATGTAACGCTAACAAAACACAATATTGAATACGTCTTTTCAGCAAAAAGCAGG
GTATATGAAGCTTCGGAATTAATGGATGAAGCATTTTACCAGTACGATGAAGGAAATTTAAGTT
CTGCGATTGAATATGCCGCTTATGCAAAATTAAGAGCTGAAACCGGAATTTGGTGGCTTGAATTA
TCGCCTGAAGAAGAAAATCCTGAAAAGATTGATGAAAAAAGATTTAAAATACCTTGCAAGGGAAT
ATCTTGACAATTCAGAAATCGTTGTAGTCTACACATCAACCGTTCTTTCCGAAGAACTCGCATCT
TACCCAATGTCGCAGATTGACGATTCAATTGATTTATACAATAAAAAAGAATACTTGCTTTCGAT
GTCAAAAAGTATCGATGCATATGTTTATTCTACAATGTTTTTAGAATATACTACGGATGTTGATT
ATTTATCGAATTTAGCTCAAAAAAAGATAAATAAAGCCGAAAACTGCATGTTAAACACGGAAAA
TAATTCAGAATACATTCCTGTTTCTGCACTCAGCTACTTTGAATACGCGGAAAACTTAGACGATA
AATATTCAAAAATTCTATACTTTAAGTATTCGATAGCTTATGCACAGATGAATATGGATGTTTTA
AAAGAATTTGACAGCTCTGCTTTTAATTATACTGAAAAATCTACATTTTGCGAAATTTCAAAAAA
TTCTGATAACTCAAATTTCGGGTTTGACTACAAAGCTTGTTTTATAAATATCACGTATCTAATCA
CAGGGCTTCTTTCAGGCTTTTTGATAGGTATGTGGTATCGTAAAAATAATTAAATAGTTCTTTAC
CCTATTATCTTTAAATTCTTTTTAAAATTATATTATTCAAATATTTCTGCTCCATTGTAATGGTG
ATATTTATGGAATCTTATTTAAACAGCAATGAAATTATCAAAAACTCCCTAAAATCAATGAAACT
GCCAGAAAAAGTACGTGTTTTTGACACTACGCTAAGGGATGGAGAACAGACTCCGGGAGTATCCC
TAATGCCTGATCAAAAAAATGGATATTGCAAAAAAATCTAAGTGAAATCGGTGTTGATGCAATAGA

AGCAGGCTTTCCAGTTTCTTCTGAAGGAGAACAGGAATCTATTAAAAATATAACATCCCTAGGGC
TGGATGCAGAAATTTGCGGACTTGCAAGGGCTGTTAAAAAAGATATAGATGTTGCAATTGACTG
TGGGGTTGACAGTATCCACACATTTATTGCTACATCCCCGCTACACAGGGAATACAATTAAAAA
TGAGTAAAGAAAAGATTATCGACATTGCAGTTGAATCTATCGAATATATAAAAGAACATGGCGT
AATTGTAGAATTTTCCGCGGAAGACGCTACAAGAACTGAACTCGATTACTTAAAAGAAGTTTACA
AAAAAGCAGTTGAAGCTGGAGCTGACAGGATAAACGTTCCAGATACCGTTGGTGTTATGGTTCCA
AATTCGATGTCCTATTTAATAAAAGAATTAAAAAACGATATAAAAGTTCCAATATCCGTACACTG
CCACAATGACTTTGGAATTGCTGTATCAAACTCTGTTGCAGCAGTTGAAGCTGGAGCTGAACAGG
TACACTGTACTGTAAACGGCCTTGGTGAAAGAGCAGGAAACGCGTCACTAGAAGAAACCGTGATG
ACTTTAAACATGGTTTATGGAATTAATACTAATGTAACTACAAAAATGTTAACAAAACTCTCAA
AACTGGTTTCGCACTACACCGGAATTAAAACACAGCCTAATAAGGCAATTGTTGGTGAAAATTCA
TTTGCACACGAAAGTGGAATTCACGCACACGGTGTTTTAGCAAATGCATTAACATACGAACCAAT
TGATCCAGAAATAGTTGGAAACAGACGTAGAATTGTTCTTGGAAAACACAGCGGTGCTCACGCGA
TAAAATCAAAACTCTCAGAAATCGGTGTTGAAGTTGGAGAAATCGTTTCAGAAGAGCAGTTCTGT
GAAATAGTTGAAAGAGTAAAAGCAATTGGCGATAAAGGAAAAATCGTGACAGATGCGGATGTAA
TGGCAATTGCAGAAGATATTACCAAAAGAACATTAAAATCTGAAAGAATAGTGGATTTAGAACA
GTTTGCAGTAATTACTGGAAATCATGTGCTTCCAACCGCAAGCGTTGCATTAAAAGTAAAAGATA
AAATTTATAAAACGTCTGAACTTGGAGTAGGACCTGTTGATGCTGCACTAAAAGCAATTCAAGCC
GCAGTTGGTGAAAATATCCGATTAAACGAATACAACATCAGCGCAATTTCTGGAGGAACTGACGC
CATTGCAGAAGTTACAGTTAGACTTGAAAATCAAGAAAAAGAAGTTATAGCAAAAGCTACTGGC
GATGACGTTGTTAAAGCATCCGTAGAAGCAGTTATCGATGGAATAAACAAATTAATGGGTTAAT
TCCATTTTTTTACTTTTTTTAAATTGTTTAAAAAAATTAAGTTAAATTTGAAGAATTAAATTAA
TGTAAAAAAAACAGCGGGATTGTAGAACATATCATTGCCAATCCAATAAATAATGCAACCAATTT
CCTATTTTCCACAGTTCCCTCTCCATAATTCTAAATAAGATCTCTTTTCCAATTTATCACTACTAA
TATTTAAACTTTTCAGTAGATCTAAAAGTTCTAAAAGAGCCTGCTCCCTTTTTGAATCATCATCT
ACAACTTTTTCGATTTCAAGATAATCCCCAACACCATCCACAGTGTCAAATGAAACTTCAATATC
ATCTTTTTTGTAAATTTTTCTTATTTTCCTGATCGGGTGAACTGGCTTAAATCCAAGTTTTTCAA
AAATATTATACATCAAATCCATGTCCAAAATTTCTACTTCAATTTCTTCTCTTGTTTTTGAAATT
GCATCTAATTTTTTACCCTTGTATGTAACGTATATTGTTCCTTTGTTTGAATCAAGATTTAAAGA
TTTTCTTATCCTCAATGCCTCGTCAGTTACTTGAAAATCCCTATCTATACCGTTAAAGTATGTAT
CAATTTGTTCTTTTGTTTCTTCTTTTTCAAAGCCTAAACTTTTTAATTTTTCGAGTATTTTTTTA
GAATCTTCGTCTTTTAAGCTAACCTTTATTTCAACTTCGATCATAAAAGCACCACTTTATCAGTT
ATTTCTTTTAAATTGTCTTTTAAATCATTAAATTCAAGATTTTCTATAACCAGCCAGACCCTTAC
CTTATTTCCTTCCTTTTCAAGCGTGGTTGATAAAATATTTCCATTTATCTCTGAAATTTTAGATG
ATATGCTTGCCAAAAGCCCTATTTTATCCTCTGCAACTACCTGAACTTTAATTGTAATCAGATTT
ATATGTTTTTTCTTTAAAACATACCTTCCTTCGTTTGTTAAAAAAACTCCACCGGTTTTTCCCTTT
TTGGTTTCGACAAACCCAAGATCCCGTAAAACCCGGATATATCTATCAATATTTTTAGGGTGAGT
TTCAAGTTCGGCAGCAATCTCTCGAGTAGTGCTGTGTTTTTTAAGTAGCGTAAGTATTTTTTCAG
TAAGCCCGTCAATTTTCATCAATTTCACAACGTGTAAATCAAATAACTTAAATAAAATCACATGT
CTATATACGAATATTCTTTTTCTTAATGTTATCTATTTTATTCATTCAAAAAAGGCGATTATCGT
GCTTACACATGTTGATGAAAATGGCGTAAAAATGGTCGATGTTTCCAATAAAAAAGACGTCGAAC
GAATTTGTACTGCAGTTGGTTTTATAAAACTTAAAAAATCAACTATTGAAAAAATTACAAAAAA
AGAACTTATAAAAGGCGATGTTTTAACCACTGCTCAAGTTGCAGGAGTTATGGCAGTTAAAAACA

CTTCAAATATCATTCCAATGTGCCACCCGCTTCCAATTGAATCTATAAAAGTTAACTTTGAGATT
TTTGAAGATAAAATAAAAGCAGAAGCAAGAGTAAAGGCAACATACAAAACCGGAATCGAGATGG
AGAGTTTAACTGGGGTTTCTGTTGCGCTTTTAACGATTTGGGATATGGTAAAAGCAATTGAAAAG
GATGAATTTGGACAGTATCCGAACACCCAAATTTATGGAATAGAAGTTACTGAAAAAATTAAAA
AAGAATAAATTATTTGATTTTTTTGTTTTAAATCACTTTATCGAGCAGTGAAACAATATTCATTA
CTGGAATATCTTTTTTTAAACCGTGTTCTTTAAGATATTTTGTTAAACTGTCTCTTATATGGTAT
TCACAAAATGGACATACTGTAATTAAATAATCTGCATCAGTTGCATAAATCATATTTGCTTTTCT
TTTACCTATTGCGTCAGCTACTTCAGGTTTTCCAGACCTTACTCCACCACCTGCTCCGCAACACTG
ATCTGGAATTTCCATTTCAATGAATTTCAGCTTTGGAATGCTTTCCAATATTTTACGAGGTTCGA
GGTATACTTTCTGACCGCGTCTAAGGTGGCATGGGTCGTGATATGTTACCGTAATATCCAGTGGT
TTGTAATCAATTAATCCAACTTTAACCAAAACTTCCGTAATATCCATTACTTTGAATTCTTTTTC
TTTGTAATCGTTCTTTAAAGTGCTTCCGCAACCGGCACACAAGGTAACTACAGAATCAACATTTA
ATTTATTAAAAATTTCAAGATTTTGTTTTTTTAGACTTTCTGAAATATCTATCTGACCTGTTCGA
ATAAATGGCGACCCACAGCATACCTGATTTTTAGGAATAATTACAGAAACCCCGTGTGCATTTAA
AACTCTTATTGCACTTTTTCCAATTTCTTGAAGTCTGAAATCAACAAGACATCCTGTAAAAAATG
CAACTCTCATTTTTTCGTTTTCTACAATGTATTCATCCGCAACTTCTTCTAAAAATGAAGTTTTT
TCTTTTGGAACTGACCTATTTTGCGATAGCACGTTTTTTCTAACTTCAAGGTGGCTATCCAAATT
ATATCCTTTTTTAAATGTTTTTTCTCGCAATTTTTCAACTGCATTGTGGACAATATCTATTTCTT
TTGGACATACCTCAACACACCTGCCGCATGTCGTACAGTTGTAGATATTTTCATCAAATGCTTCC
TTTTCCCGATCAATATCGTCTTTTGGATCGAATGCAAATCTTGCAAGCTGTCTCATTAATGTAGG
TCCGGGATAATTTGAATATTTTCTTGCAGGACACATTGCAATGCAACTTAAACAATCAATACAGC
CCCTGATTTTTTTTACGTCCTTTAAATCGTCAGGATACAATTTTAACTCGCCCAATTCTTTTTCAC
TTATTTTTTCATTCTTTTTTTGAATATAATTTCTAAGTGTTCCTATTTTTTTATAATAGGGTTCT
CTATCAACTACAAGGTCTGAAATTACATCAAAGCATTCAAGAGGCTCGATAATCATATTATCTTC
AACTTTTGTTTTACATGCAAGCTTTGATTTTTTATTTATCATGACTGCACAGCTTCCACACTGCCC
TGCCTTACATGACGACCTGAATTGAATATTTTCCCCGTAAGTTTTATTGATATATTCAAGAGCAT
CTAAAATGGTGAGTCCAGCCGGAACTTCGAATTTTTTAAATCCTTCTGTTTTTTTTACAGTAATT
GTAAAAGTCTTCACAAGTATCACGCATAAATTTTAAAAATAAAAATTAGATCAAAAATCAATAA
TTGTATTTAAAAATTATATGAAAATGATATATAAAGAAATTGTTCGTTTTAAATTCAATTTAAA
AATCAGAAAAAAGAAAAATTTTATTTTTTTAATTAAGTTATTCAACAACTACTGGCCCATTTTCA
GTCCATTCAATCATGATTGGGCCTTCTGGCAACTCATCCAAAGTTTTAAAGTATTCTAAAGCAGC
TTGTGTTCCAAGTCTGTCAGAACCTGCAATATATACAATTGTATAACTTCTAATGATTGTTCCAG
AGTTGTCTTGAATAGTCATAACCTGAATAATTCCCCTGTTTTCTCCAGGATAGTCGTTTGAAATT
GGCATTTCAAACTGGCTGTTGTATTCTTTTGCAAAGCCATTTGCTAAAGGTCCACCAACAATTAC
TGCATTTCCAGAAATTGTGTAGCCGTTTGCATCGGTTACTCTTTCTCGTAATTGAACTGCATATT
GTTCATCAATTCCGTTTCCAAAGAGCACGGTTGCACTTGAAACGAAGTTTTTAATAACTTTTGAT
TCGATTTCATCTGAAATGTCTGAATCGTAACTTCTTCCACTACCGCCGGATGATCTTGATGGTGC
AACGTAGTTTCCAATTTCACCGTCATTCCACATGTCAGCGAGTGGTTTTGTATCATTTGTATCAT
TTATTGTGTATGGTGTATCGATTATACCGTCACCATTCGTATCAGTTTCATTGTAGCTTGCCCAG
TAGTTTCCAAGAACACTTTTGTATGAACTTCCAAGGTAGCTGTAAGTTATGTTTTCACTTGAAAC
AAAGTAGTTGTCTGTTCCATTGTTTAATAATCCATCAATGTTATTTAAATAGAATCTGTTTTCTT
CACACGTTGAATCAATATAGAGTGCGTACATGTTCGAAGTGAATGTGTTTTCAAATAGTGTTGAA
TTTGTTATACCATACAAATAAAGAGCGTAAGCTTCGTCATTTTCAGAACTTGCCTCAACAATATT

TTCTGAAAATGTTATATTATAGATATCGTCAATGTAAAAACCATAAGCACTGTAATTTACAGATG
TAGCATTAATGGTATTTCTGGAATATGTTGAGTATTCGCCACAGCATGAGTATATACCGTAAGCA
TAGTCGTCTTCTGATGTAGCAGTGATTACGTTATTTTCAATTGTTGAATTTATGGCATCTTCTTC
ACAGTAGATACCGTAAGCTTCGTCTTCTTCTGAAGTTGCTGTTATGGTATTTCCAGAAATTGTTG
AATATTCAAAGTAATCGTCAACGTAAATACCGTAAGTGTAATCATCGTATGCATATGCTGTAATG
TCGTTTCCTGAAATTATCGAATTTTCCAGATCATAGTATGCATAGATACCGTAAGCGTAATCATC
GTATGCATAAACATCTATTACGTTATATTCAACCGTTGTATTTATTGCATCATAATTTCAATCGT
TGTATCCACCATATCTTCGTCGTATGTGCAAATACCGTAAGCATCGTGTGATATGTTTGCTGTGA
TGGTATTTCCTGAAATTACACAGTCTTCCATGCAATAACACGCGTAAATACCGTAAGTGTAATCA
TTTTCAGAATAGAGATCGATTACGTTATTTTCAATCGTTGTATCCACCATATCTTCGTCGTATGT
GCAAATACCATAAGCATTACTGTTGTTGCTTTCTGAAATGAAAGTATTTCCTGAAATTATTGTAT
TTAATATGTTTTCTTCATCAATAACAATACCGCAAGCATTGCTGTAATTAGATTCTGCTGTGATG
GTATTTCCTGAAATTACGCTGTTGGTAAGGTTATAATACACATAGATACCAGAAGCAGTACCATA
TTCATCATAGTTATAAGCATAAAGGTCGAATACGTTATTTTCGATTGTTGAGTCTATAATATTTC
CATCGTTCCAGATACCTGAAGCTTCATCGTATCCGCATGCTGTAATGTTGTTTCCTGAAATAGTG
CTGTTTGTAATATATAAGTTTGCGTAGATACCTTCAGTATAGCTCTCTTTGGAAGCAGTGTCAAA
TACGTTATTTTCGATTGTTGTATTTAACATTTCTCCCCAGTATGCACCAATACCCCAAGCCCAGTA
TCTTGCGCATGCTGTAATGTTGTTTCCTGAAATTACGCTGTTTTCTATAGTATAATCTGCGTAGA
TACCGCAAGCTTCGTAACTAGTAGAATTAAGTTTAATAGTATTTCCTGAAATTACGCTGTTTGTA
ATATTTGATACTGTGTAGATACCACAAACATTGTTGTTATTTGTAACATTAATTGTATTTCCAAA
AATAGTGCCGTTTTCAAGATTTCCATCAATGTAAATACCGTAGTATTCACCATTTGCAACTGTAA
TGTCATTTCCTGAAATCGTAGCTCCGTAAGAATCTTCTAACTTGATTGAATAGCCTTCAGTAATA
ATTTCTGAGTTTTCAATTGTTGAATCATTAACATTTTCAAAAATAATTGTATCTTTAGACCAGTT
AGCATTCATGTTTTTAACTACAATATTGCTTGAATTGTAAGCGTAAACCAGAATACTTGAGTTTG
TTGTGTTGGTTGTAAAAGACATTTCGTTACAGTCAAGTGTAACATTTTCAGTATTGTTAATTAAA
AATACAATACTATTGCTATCCAATGAATTGTTTGCAAAACCTGTTGTTACCGTATATTCACCCGG
TTTATCAATAACTATAAACGCGTGAGCTATCCATCCGCCCATATCAACATCAGTCTGATTTGAAT
AATCGGATATCTCTGCCGCATTAACATTTCCTACTGCAAAAAGCAGTAAACATAGTAATAGACAT
TTTTTTTATCAAGTGTTCACCCCGAACATTTTCGATTAGTGTTAAAATGTATTGAATACTCGAGA
GTTATGATTTTTCTAATTTATATTTTTAACGACGATTTTTAGAAATAATTGATAAAACAGGTGAT
TTAAAAAAGTTGTGTGTTAAACAGCTTTAATATTGAAAAATCAGAAAAAAAGAAAATTTAAAAG
ATTCCAGTAAGTCCTAAAACAAATAAAAGATATATCAATATCAAAATTAAACCATCTGCTTTCGT
AATTTTTGAGAAATAGTCTTTTGCAGAGTTTAAATTCAATACTGATTTTATTTTTTCTTTACTCA
TCATTGCTATCAGCAAAAGTACCATCAAAATATTAACTGAAAGCTCAAATTTAACTGCCGGAATT
TCATGGATAATTGATGCAATTGCAAGAGCTCCAAACATGTTTGCAATGTTACTACCAATTACATT
TCCAATAACAATTCCGCCAAGTTTCTGTCTTGCTGCGGCAAATGAAACCGAAAGTTCAGGAAGTG
ACGTTCCAAATGCAACGATTGTAAATCCAATAATTTTTTCAGAAACTCCAAAAGCACTTGCAAGG
CCTTTTGCACCATCGACAAAGAAACTACTTCCCAAAATTACAAATAAAAGTCCCATTACAAGATA
AATTATTGCTCTTTTAAAGGTTATACTTTTTTCATGCTCTTCATCGTCCATTTCGCCGGATTTTAT
AGTATATAATACGTAAACTAAAAGTAAAAACAGTAAAACTACACCATCAAAGAAGGAAAACCCG
TTGTATCCCATTATTGTGAATATTATCGTGTAAATTATGTATAAATAACCATTTTTAAGAACAGA
CCTATTTTTAATAAATACTGTAGAAATAATCAAGCTTAAACCAAGAACAAGCCCAATATTTGCAA
TACAACTCCCAAGAGCGTTTCCAACTGCCAACTCAACGGAACCTGTGGATGCAGCATATGCACTT

GTTACAATTTCAGGAAGTGATGTTCCAAATGCAACAATTGTAGCCCCAATTACAAAACTTGATAT
GTTAAACTGTTTTGCAACACGTGAGGCACCCAAAACAAACCAATCACTACCATAGGACAGCATGA
ATAAACCTGCTGCTAAAAAAAGAACCGATTCTATAAGCATATTTCTCACCATAAAAAAGAAAAAG
CGTGTTTTATTAAATTTATTAATAATAAACTTGGTAAGATTTAAAGCTTTCTTCAGTATTTAAAT
TTTCAGAAAGTCCCATTCCAAGACCATATCCAAAACTGTATAAGCAGTCATTAAATGTCATTCCA
AATAAACTGGCAGATGATGTTTTACCATATTCATAAATGTCAGGATTCGTAATATTTCCTAATTT
TACGGCTACATCAACTGCATCATCTTCAGTTCCAGTCATATCAACAAGTCCAACTTTTTGGGCTT
GAGTACCGCTGTAAATTTTACCATCCGCAACTTCTAAAGTTTCATTTATCGTCATGTTCCTGTTT
TCAGCAACCCATGACACAAATTCGTAGTAACTTTCATTGACCATTGATTCAAGCATTGCATACTC
TTCTTCAGTCATTGACCTTGTTGGTGTTCCAATATCCTTATATTTTCCGCCAGTTATGCTGGTTGT
ATTAATTCCAAGTTTTTCCATTAAACCGTAGTAGTGTATTAATTCCATCCTAACACCGATACTTC
CAACAACTGCCTGTCTTTCTGCAACGATATAATTAGTTGATGAAGCAGATTGGTATGCTGCAGAT
GCACCCATATTTTCGATGTATGCTACAACCGGCTTTTTTTCAGAAACTTCTTTTATTTTTCGGGA
AAGTTTTTCACTTGCAACAGCCCCTCCGCCCGGTGAATTTATTTTTATAATTACTGCTTTGATAT
TATCATTATTCTCTGCATCATCTAACCATTCCACATAATCATTTACCCCGGGTTGAATTGAGCTA
AAAAGTCCAGAATCTGAACTTTCAAGGGTTATTATTCCATCAATATTAATAAGTGCAATATTTTT
GGAAGATATTCCATCAGAAGATACAAAAACCAAGAGTCCGACCATCAAAATTGCAATTATCAAAA
ATACTGCGATTGAATAGTAATATATCTTTTTCAACATACCCCTCCAAAAAAAATAATAAATTAAA
TTACAATATCCTGTAACTTCCAGGATTTGGCTCGTAAATTTCCCCATCTTCTTTTAATTCTGACA
AAACGTTGTCAAGTTCAACATCTTCAAGCCCAGAATTATCAATTAAATCTTCATATGTAATCCCA
TCATTTTCCCGGATAATATCTAAAATTTTATCTTTAACATCTAAATCGCCGTAAAGATCCTCTAA
AGCTTCACTTTCAATTGAAAGTTCACCAGACATTTCTTCACGGTTGTAGGTGATTGTTTCTTCGT
CTTTTATATAATTCCTAGTTTTTTCAATTTCTAAATTTCTAAGTTCAATCCATTTATCTTTATTT
TCATTTTTTTTGCTGTAAACTTCGAGTGCAACGTACCTTTCACCATTGTATTCTCCGATTCTTCCA
ACAACGTCTAATAAATCGTTTTCTTCAACTTCAAGCGATTTATTTTCAAAATCTTTTACTGAAAT
TCCGTCGAGTTCGAATGAAACAATGTTTGATTTGTCAACATTATTGACATTTCCAAGTAATCTAA
CCCTGTGGATTCTTTTTCCATCTAACATAAGCGATTTTTCAAAAATTTCGTTTTCTGCAAATTCC
TGCGGGTGTATTTTGTATGCTACGTATCTCATCACGATTCTCACCAATGTAATAATGTAAGTATA
AATTTAGGATTTAAAAAAATATAAGTTTAACTAAAAAAATAATTAATTTAAAAAATTTAAAGAT
TATTTATTTTTCTTTTCTATTGCTTCTTCATCTTCGTTAATTATGATGCCATCCCTTGTTCTTGGC
GCAATGTCTTTAATTAATTCAAGTATATCTTCATCTTCTTCAGAAACAAATTCAAGCTTTATTCC
ACCGTGAACATCCTTATCAAAGTTTAAAATTCCTGAATACGCTGCCTGTTTGTTTATATGAAATT
TCGTTGCATTTCCAACAATATTTCTTTCAAGAACGCTTCTTGCAGCATCCAGTATTGCTTGACTTC
TAAGGAGTTCCTTAAATCTTGAAATATCTTTTGAAACCCCAGTATAGATATTTTCAGAAATTTCC
ATTTCTGCATCATTAAATATGTTAGTTACCGCTTTTAAAACCTTATTTTCATCTTCGGTCGGTTT
TACTTTTGTTTTTATCTTGATTATCATTTATATCATCTCGATTAAAGAAGAACGTTTTTCATAAT
TTCTAATGCTTTTTCAATATCTTCATATTTTGTAGCGTATGAAAATCTAACGCTGTTTTCTCCAT
TTTCACCAAATGCGGATCCTGGAACGCATAAAATTCCGTTTTGAATGAGTTTTGTTGCTACATCC
ATTCCGTTTCCATATTCGCTAACATCAGGAAAAATATAAAATGCCCCTTCTGGCTTTTGAACGCT
AAATATCTCTTTCATTCCTGAATAAATTAAATTTCTCCTTCTTTCAAATTCTGAAACCATTTCTG
AAACGCAATTTTGGTCCCCAGTAAGTGCTTCGACTGCACCAAACTGTGCGAATGATGTTGCGCAT
GCAAAACCGTACTGGTGGATTTTCATCATGTTTTCAAGTATTTGGTATTTATTATCAAAATTTTC
GTTTACTGCCAAATATCCAACTCTCCAGCCGGTCATCGAGTAAGCTTTTGAAAATCCGTTAATTA

AAATACAGTTATCAGTAAATTCCATTGCAGAATAGTGTTTTTTTCCGTAAATTATCTTTTCATAT
ATTTCATCAGAAATTACACAGATATTTTTTTCATCTGCAATTTCACAGATTCCTTTAAGTTCTTC
CTTTGTCATCACGCTTCCTGTCGGGTTTGATGGCGAATTCAAAACAATACATTTTGTTTTGTTAG
ATATACTGTTTTTTACAGATTCTAAATCGATTCTAAATTTATCATCCAGATCAATTGGAACCATT
TTTCCTTCACAAAGTTCAGTTAGCCCTTTGTATGAAACAAAACCTGGATCGGGAATTAAAACTTC
TTCACCGCGGTTTACAAGCGTAAATAATGAAAGCATGAGTGCTTCAGAAGCTCCGCAGGTTGTAA
CGATATTTTTTTGCGAAACATCTACGTTGTTATCTTTCTTCAATTTTTCAGAAATTGCAGAAGTT
AATTCTGGGATTCCTGCATTTGGAACGTAGTGCGTTTTTCCAGCATCAAGAGCCGATTTAGCAGC
TTCGACGATGTGTTTTGGAGTATCAAAATCAGGTTCTCCAATCCCTAAATTTATCGAGTTTTCTG
TCGCCATGTTGAATATTTTACGAATTTCGGACTGTTCTGTTCCCAAACACCTTTCTGCAATCACA
AAATCACCTTAGATAATTATATATAATCAAGAATTATAATGATTAATCATTATTATTGTTATTA
TTTCTGTTGTTGATATTATATATGTAATCTATCCTGTTTACTCGTTTAAATTATTGTTGAATACA
GATTAAGTGGTAATTGTATTAATAAATCTGCCTAATCTTTAAAAAATCTTTGATATAATTGGTG
AATTCATGTTTGACGATATTGTACTAATAATTGCATCTATTGGGATATTGTTGGCATCATTTAGG
CTCTGGGTTGAAAAAGACCGTGAAAAAATGGTTTATGCAAGGCTCCACATCACAGGGGTTATCGA
TATTGCATGTATCGTGATATTGCTTGTATTTAAACAGTATTTACTTGCTTTAACGTATTTGGTGT
TAGTTCCATTCTCAGCACATGCAATAGCTAATGCAGACTACTTCGATGAATTAAAAAAGCAATAA
GGTGTAAAAATGAGTATTTATATTGATTTTTTAATAATGGCACTCGTTTTAATGTCATACATCGG
TGCTTTGATTCAAAAAGATTTAATTAAATGTGTTGTTTTAACAGGACTTGGCGGACTTGGACTTG
CATACCTTTTCAGCTCTTTACTGGCACCCGATGTTGCATTAACAGAAGCAATTTTAGGTGGTGCA
GTCTTACCTGCATTTTTCGCATTTACGGTTAGAAGAACACAAAGACTTGATGAATAACTTAAAAA
AAGTAATTAAAATTCTAAATTTTTAAAATTTTTTTAAAAAGTTTGAAAAAAAGAAACTTCTATT
TTTATTATTTTTTGTGATATATTCCACTGTAAGTTCCGTTTCCATCTTTAACTTCAGAAAACACA
ATCTGTGCAATTTTTGCATATTTATACATCGTTACAGGATTCATTACATGCATTAAATAGCTTGC
ATAACCTTCGTATCCGGGATCGTGTACTGCAGTATAAAGAGTTACACCCATTCTAAGAAGCGTAC
TTCTGGGATATGCAAAACCAGCCATGTTTTCAGGAATATTCATTTTTTCTTTAATTTTTACAATA
TATATTCCGCATTCAAGGTCAATTTTTTCATCCTTTTTCGAATCAAAAATTAAAACATGTTCAGG
AAGCACCCTTTTTTCATTTGAAAAATCAATTGCTCCTTTTCCTTCGATTTTGTAAATTTTATCGA
GTTTTAAATCAATTCCACACTGCTGGAGCTGACCTTCTTTTAAATCAGATAAAAAATCTTTTGTG
ACATTTGGTCCAATAATCATGTTAATTCCTCTTTTTAGTAATTACAAGTTCGTCCTTTTTGTAAA
CGAAACTTTCCTGTTCAATATCCCTGTCAATTAAGCAGTTTGCACCAATTAACGAATTGCTGCCA
ATTTTAACACCCGGCATGAATGACACTTGAATTCCGGTTTTTACGCAATCTCCAATTATTGCCCC
AAGTTTTCTAACACTTTTTACAGGCTTTCCTTTGATATTTACAATTACAGGCTTATCATCAAACC
TCAAATTTGCAGTTATCGTGTTGCATCCAAAATTACAGTTTGCACCAATTATACTGTCCCCAACG
TATGAAAGATGAGGGATTTTCGTGTTTTCAAAGATAATACTCCCCTTAATTTCAGAAGAATTTCC
AACAAACGTATTTTTCATCAAAACTGTATTTGGACGTATATATGCCAAAGGTCCAATAATTGAAC
CAGATTTTATAATTACAGGTCCTTCAATTACTGAATTTGGTTTTATTACTGCACCTTCTTCAATT
ATAACGTTTCCTGTAATCGATACATTATCTTCGATTTTTCCAGAAACTTTTGAAATTATTTTATT
TAAAAAGTGATTATTCGCACTTAAAACATCCCACGGATGTCCTATATCGTTCCAGTATCCTTTTA
ACTCAACACCGTGAAGTTTTCCGTTTTCAATTAGTTTCTTCAAAGCATCAGTCAATTCGATTTCT
CCGCGCTCTGAGGGAGCAAGATTTTCTAAAATTCCAAATACACTGTTTTGAAGTTTATAAATTCC
TGCATTAATAAGATTTGACGTTATTTCTTCTTTTTTTGGCTTTTCGAGTAAATTTATTACTTTAT
TATCATATCCAAGTTCGATTACGCCAAAATTTTCAGGGTTGTCTACTTTTGATAATCCAACTGCA

TTTTCGTAAGCTAAAATTTCGTCGATTGAATCTTCGAAAATTACATCCCCATTTAAAACTAAAAA
ATCGTCTTTTATGAAATTTTTAGCGGTTAAAACTGCATGCCCTGTTCCAAGCTGTTTTTCCTGCA
AAATATATTCAATTTTTGGGTTATCCTTGAAATATTCAATAATTTTTTCTTTTTGATACCCTACA
ATCAGATATATTTTTTCAACGTGGTTTTCGATTTTTTCGATAATATGTTCTATTATCGGTTTTCC
CGCAATTGGAATCATCGGTTTTGGGCGAGATTCTGTTATTGGATGTAGCCTAGTTCCTTTTCCAG
CACATAATATTATTGCATCCATATTAACCCCCTACTTTCCAGAAATTCCTTTTTTTACCATTGAA
ATTCCTGTTTTCATTAATTTTTCTGCAAAATCTTTTTCTTTTGCTTCGACTCTCACTCTAACATAG
CTTTCAGTTCCTGAAAGCCTTATTAATATCCAGCCCTTTTCAAATGAAAATCTAACCCCCTCAAG
AGTTTCGGGTTTCTTTTTAAATATTTTTTCGCCTTCTTTTGAGACGTAATCCATTACTTTTTGTT
TTAAATTATCTGGGCAGGATATTTTTTCACGCATATTGGCGTATGAAGGAACTTCATCAATAATT
TCATGCAATTTTTTGCCGTAAAATTCCATCATTTCTAAAACTCTAAGTCCCGAAAGAATTCCATC
AGGCGTTAAATGAATATCTCGATGTATCCAAGTTCCAGAAGGCTCTCCTCCAAAAATGGCACCTG
TTTTTTCAAGTTCTTCGGCTACTGCAACATCTCCAATTTTTGTCCTGATTACTTTTGCCCCAAATT
CGTTTAAATATTCCTCAATTGCCATTGAAGCATCAACAGTGGTTACAATTTTGTCAGCACCTGTT
TTTTGAACTAAATACTTACAAAATGCTGCTAAAAGCTTGTCAAAATCAGTAACTCTCCCAAGTTC
ATCGATTGCAATCATCCGGTCAGCGTCACCGTCGTGTGCAATTCCAACATAATTTCTGCCGTTTGA
ATTTAAGCCTTTTATAATATCTACGGTTTCTTTTAAGTTTGTTTCATTTGGTTCAGGCATTCTTC
CAACAAATCTTCCATCACAGTGCGAATTTACAGAAATTACTTTACATCCTGCTTCAGTGAATATA
TTTGGAGAAACCACGCATCCTGCTGCATTTGCACAGTCAACAACAACATTAAAATTCGTATTTAG
GTTCAAATTTTGTAAAATATACTCAAAATACTTTTTAACTGCGGTTTTGTCTTCTACAACACAGC
CAATATTATCCCAAGTACCTTCGTTAAAATCGTCATTTTCAATTATATTTTCGAGTTCTTTTTCC
TGTTGTGGATCAAATGCAGTTCCGTTTTTATTAAACAACTTTATCCCGTTGTATTCAGGTGGATT
GTGGGATGCAGTAATCATAATTCCCAGATCGTAATCTTTTGCAGAATAGCCTAAAACAGGAGTTG
GAACCATTCCAATTGTAGTTACGTCACAGCCCCCGTTAAGAAGTCCTGCTGTAATAGCACTTTCG
ATTAAGTTTCCCGTAGTTCTCGTATCTCTTCCAATAACTGCCTTTTTAAAATTCTTTGAAATTGC
AAAACCAACCTTGTATGCAATTAAAGGATCTAAATTTTTCATTCGAATCCCGGAAGTCCCAAAAA
GTTTCAAGATTTCACCAAGCACAAATTAACGCCTAAACTAATACTTTATATCCTTTTAATTAATC
ATACATATAAAGGATTTGATTTTTACAGGGGTTTTTATGCTATCTGATTTGATTTTAGAGATTGA
AAAGGAAAATAATAATGAAGAAATTTCAGATTTTTTAAATATTTTAGATTGTATTTACAAAAAC
AAAGAACCTGAAATTGATGAAAATATATTTAAAAATTTAGGAATTGAAAAACGTGAAAATGATT
TAACAATTTATGGTAAAAATTACCCATTATTTAAAATGCTTTATTATTTCAACGAAATTCCTCTT
TTTAATTCTGAAAAAGAATCGATTATATTTTTAAAAAATAACAATTTAAACCCCTCAAAAACTTA
CTTTGAATTAGATATTTCTGAAAAAGAAATATTAAGAGAATTAACTTTAAATTATGCTGAAAAT
AAAGTTCCAGATGATTATAAACCTTTTGTAAACGACGTAATTTTTGGAAATACGTACTATTTTTC
AAAATATAATATGGAGTTAAAAGAATACGTTTCAAACTTAAATGCAGTATATAAATTAAAAGAA
TACGACATTGTTAAAAATTGCATTTTAAAAAAAGAATTGCCTCCAAAAAATATAATTTTAAAAT
ATAAAACGGATCTTTCAAAAACGATTGATTTGTTTAATAAAAAATTAAATAATACAGAAATTAG
AAAATTTTCAATAGATTTTGATGGAAAAAACTTTGATTGTCAGTACATTTATTTAAAACAGTCTT
TATGGGATAAACTTAAAGGCTGGTTTTTTGGTGAAATTAACGGAATTCACTATCCTGCACTTGTA
AATATTGCGTACAATAATCCAAAAATTGATTATTTAAAACCATTTTTTATTTTAAATGATAATG
AAGACAAAATAAATGTTGTTGCAAGAGTTCCAAAACTTTTATATTTAAAATATGGCCTAACATT
AAACCATATTAAATTAAATGGAAATCATACGTACTTTGGAAAATGGAACATTAAAAATTTTAAA
AAATTTCTGGACGTGAAAGTTTGAAATCAATTATTTTAGCAGGGGGAAGCGGAACAAGGCTCTGG

CCACTTAGTAGGGAATACTTCCCAAAACAGTTTATAAAATTTAAAAAATTAAATCAATCATTATT
TCAAAAAACTTTTGAAAGAAGCCTAAAACTTGCAGATGGAAATTTAGGTGATATCAGTATAGTA
ACCAACGAAAAACATAAATTTTTAGTTTTAGGTCAGATTGAAGAATTAGGATTAGAATTTCCAG
AAAAAAACATATTAATTGAACCTGTTGCCAAAAATACACTTCCTGCAATATATTTTGGAATTAAA
CATTTTAAAAAACAGGGCGAATTTAATATTGCAGTGTTTCCATCAGACCATTTAATAGAAAACGA
AGAATTATTGCTTGAAACGTTTTTAAATTCGGAAGAACTTGCAAAAAAATACATGTTAACTTTTG
GAATAACTCCAAACAAAGCCCATATTGGATACGGTTACATAAAAACGGGCGAAAAAATTGAAAA
CGGAAATTTAGTTAACGAATTTAAAGAAAAACCCGACTTAAAAACTGCTGAAGAATATATCAAA
AATGGGTATTTATGGAACAGTGGAATGTTTTTGTTAAATTCATATGTTTTTGAAAATGAAATTA
AAATCCACGCTCCAGAGATTTATAGGGCATTTGAAATTGAAGATGTTGAAAAAACCTTTGAAACA
GTTCCAAATATTTCAATTGACTATGGAATAATGGAAAAATCAAAAAATGTGGCAGTTGTTCCGAT
AAATATTACTTGGAATGACCTTGGAAGCTTTGATGCATTTTACGACGAATTTGAAACTGATGAAA
ATAAAAACATAACTCCTGCTGAAAATATCTGCATAAATTCGAGCGGAAATTTGGTAAATACTTCT
GATAAAAAAAATAGTTTCACTAATTGGTATCGATGATGCAATAATTGTAGATACGAAAGATTCAT
TATTGATCTGTAAAAAAGGCCAATCTGAAAAAGTAAAAGATATTGTAAACATTCTAAAATCTAA
AAAAGATGAACGAGCAGAACTTCATAAAAAAGTTTACCGTCCATGGGGGATTTATGAAATTTTA
GAAGATTCGGAAATCTACAAAATTAAAAAAATTACTGTTCTTCCCGGAAAAAAATTAAGCTACCA
GCTCCACCATCACAGAAGTGAACACTGGGTCATTGTAAAAGGCATGGCAAAAGTTACTGTTGAAG
GAAACGAATACTTTGTAAGAAATGGAGAAAGTACTTTTGTAAAATCCGGTTTAAAACATAGGCT
CGAAAACCCCGGAAAATTACCTCTTGAACTTATAGAAATCCAGATTGGAGAATATTTTGAAGAAG
ACGATATAATAAGGTTTGACGATGAATGGGGAAGAAAATGAAAATATTAATTATTACAGAATAT
TTTCCAAGTTCTGAAAACTGTGATATAAAAGGGGGTGTAGAAGCCAGAGCATTTTATTTTGCAAA
AAATCTTGCAAAAAAACATGATGTTACAGTTATTACATCATTTGATGCTGGAACATTGCATAACG
AAACCATTTCTGGAATAAATGTAATTCGTTGTGGTAAAAAAAGAAATTACAGCCAGGGCGGAAA
CTTTATTGATAGACTTTCTTTTATTTTTTCTGCAATAAAAACTGGAAAACGTTTAGATGTAGATA
TTGTTGATGGCTACAACTTTATATCATATATTATTGCTTACAAAATCTCAAAATTCTTAAATATT
CCAAGAGTTGCTACATATCACGATGTTTGGATAGGGGAATGGATAAAAAATATGGGACTAATTA
ACGGAATTATCGGAGAAATTCTTGAAAGGTATGTTCTTTCAAAAAAATGGGATAAATTTATTGC
TGTAAGTAAATATACAAAACAAAAACTTAAAAATAAAGGAATCCGCGAAGATAATATTTCAGTA
GCATATAATGGAATCGATCTTGAAAATTATAATAGTTCAAATGATAAAGAAGAAAATACAATTT
GTTACGTTGGAAGACTCGTTAAATACAAAAAAGTAGACGATCTAATTAAAGCAGTCGAAATTGT
CAAAAATGATATCCCAAATATTAAATTAAATATAGTCGGTTCGGGACCCGAAGAAAAAAATTTA
AAAAAAATAGTTACTGAAAAGAATTTAGAAGATAATGTGAAATTTTTAGGCTTTTTAAAAAGCC
ATTCTGAGGTAATGGAATATTTAAAACGTGCAAAACTTTTTTCACTTCCAAGTGTCGTAGAAGGA
TTTGGAATGGTCACTATTGAAGCAGTTGCTTGTACTACCCCATACGTAAATTCAAAAATACCGCC
CACTGTAGAAATTACTCATAATGGGCTTGGAGGAATATTATATGAACCTGAAAATATATCTGAAC
TTTCTGACTCCATAAAATGTCTTTTAAATGATAATGACTTATATTTAACAAAACAGGTCGAGTGT
AAAAACCTTTCAAAGAATTACGATTGGAAAGAATTATCTAAAATTTTAGAAAAAGAATATTTAA
AAATGATTTCAAAGTGAATTTATGAAGATAAATTTCGTTTCAAGATATTTTAACACAAATAATG
CCGGGATAGGCAGAATATCCGAAGATATTTACAACTATTTTAAAAATAAAGGATATAACGTTACC
AAAACAGAATGTAGTTTTAAAAATCAGCACAAACTATATTACTTATATTATATTCTCCAGACAGC
CGTTAAAAAAGATAATTCTGATGTTTATTTTGCACTATCCCCCTTGGAATCATTTTTTTTACCCC
CTGAAAAATCTATTGTAGTAATACATGATTTAATACCTTTAAAGTCTAATTCATTTTTTGAAAAA

AAAATATTTTGGTATGCTATATCAAAGACCGCCAAATTTAAAAAAATAATATGTATTTCCACAGA
AGTTAGAGAAGAATATCTTAAAACCACGAAATGTGACCCTAAAAAAGTAATTCTAATCGAAAAT
TGGGTAAATCCAAAATATACATACTGTGAACCAAATAACCACGAAAAAATAATAATAGGAACTA
TTTCAGCACATTTTCCTCGAAAAAGAATCGACTGGTTAATAAACAATTTTATAAAAATAGAAGAC
GGCGATTTAGAGCTCCATATTGGTGGAGACGGGCCCCAAACCAATTATCTAAAAAAAATTTCAGA
AGAAGACCAAAGAATAAAATTTTTAGGTAAGATACCTGAAGAAAAAATGGTAACATTTTATAAA
AATATTGATATATTTGTATTCCCTTCAAAAATCGAAGGATTTGGATTACCTATTGTAGAAGCTGC
TTCATGCGGGAGACCAGTCTTAACTCTTGAAGATGCTAAAATACCCAAAAATGTAAAAGAAAAA
ACCCTGCAAATCAACGAAGATAACTTTAAGAAAAAATATAAATAAGTTAAAAAATTATGAATTAA
GAAAAAAATTAGGTTTAAAGTGGAATAAAGACGTAGAATGTTTCAACTATGATAAAACGTTGAT
GAAATACGAAAAAATAATAAAAGAAACCCTAAATTGAATTTAACAATCAAATCCCACCTTTTAA
ATAGTTTATAAAATTTGAATTATTAATATTACAGAAATACTTAAAGAATATTTATTTGGTATAA
CTTATTGTCTTTTAACCATGTTTAAAAATTATTACCGCCGTTATGTGCCTGAGATATAATTAGGA
TAGTGACATTATGAAATTCATGTTTGTTACAGAATATTTTCCACCATATGTTATGGGCGGAGCCG
AGATCAGTTTAAAAATTTTAGTCGATACTCTCATAAAAAACGGCCATAAAGTTATAGTGTTAACA
CCAAATTATGGTTCCTTCAAAACAATTATTGAAAAAAAAGATGACCTAAAAATCATAAGATTTA
GATCTTTTAGGTATTTTTTATTTAAAAAAAGAGAAAAAACTTCTTATGAAGTCTACAACAAAAC
AAAACCTATGTTTTATGTATTGTTAAATCAGTATATTAAATATTCAACTTATGAAATAAGAATA
CGAGTAAAAAAAAATTTTAAAAAATGAAAAATTTGATGTGATTCATGCTAATAATATGGAATCTA
TTTTAGCAGTTGGTCCGATAAATACTCCTGCCAAAAAAATTGCTCATTTAAGAGATTTTGGATTG
TTTTGTTATAATCGGGGTTTAAATAAGTCTGGTAGTCTCTGTGGGGGGTGTTCACCAACTAATTT
AAATACGTGTATGGGTGCTGAAGGTATCATAAATAAAGTTTTAGAACGGAGCATGAAAAAACGA
ATTAATAAATCATCAGTTCCGTCTTCTTTCAATTTTTTGATCGCAATAAGCAACTTTGTTAAAGA
AAAATATATTGATGTGCTACATGTCGATAAAAATAAAATAAAAGTTTTATATAATCCAATATCT
GATGACATATCATCCAAATTATCCAAAAAAGAAGCAAGATCATTATTAAAACTACCTGAAAATA
AAAAAATTGTCTTGTATGCGGGTAGCCTCACAGAAGCAAAAGGGGTTTATATGTTAAGTGATCTT
TCCGAAAAATTAAAAGATCATTTGTTTATTGTTATTGGAAACGGGGTTTTAAAAGAACTATTTT
TGAAAAAAAAACAAGAAAATTTAATATATCTGGGATATTTACCCATTTCAGAACTTAAAGATTA
TTACCGAGCATCGGACATCTTGTTGGTTCCATCCCTTTGGCACGAACCTTTCGGCAGAGTTGTTTT
AGAAGGTGCCTATAATGGATGTTATGTTATTGGGAGCGATAGGGGAGGAATTCCTGAAGTGATA
GATTGGTTAAAATGTGGTGTCTATACTACGCCAAGTGTTGAAAATTTTGTAAGTGAAATAACTG
GGTTTAAAGAAGATAAATTAAAAAAACAAAACTTAGAAGAATATAACTATTATGAAAAATTTAT
GGAATTATTGGAGTAAGTTTAAAGAATTCATGTGGGAGTCTACAGAGTTAAATGCCGTTTCAAG
TTTTAAATTTACTAACAAATTCACCTGCTTTTAATAATTTGGAATTATTAACTTAACGTTATCTA
AAAATACCGTTTTAAGATTTTATAAATAGTTCTAAAATTTTATTTAATGTTTTTTATTTTTTTTC
CTAAACCGCCTATTGCAGAACCATATAATTGTGCAAAAAATGTTGATAGTGCATCCATATCATTG
AACAATACCCTTTTTAGTATTAAATAAGGACCCAAAAAAACACTATTCCAATTTCTTGTAACTTT
GATTTTAAATATATCAAATCCGTTTTCTATTAAAAGTCGTTCAACCGACCTATAATTGTAAGGCC
GTATATGTGTTGGATCACTCCAAAAATTTATACCTCCGTCTGGAATATATGCTGTTTTGTAATAC
GGCATTTCTATATAAATTAAACCATCTTTTTTTAAAACTCTGTTAAATTCCATCATAAAATCATA
GGGATTTAAAATATGTTCCAAAACATGAAAACAAAAAATAAAATCAAACCTTTCATTTTCAAAG
GGCATATTATCCCCACTGCACAGCGTAAAGTTTATATAGTTGGGCATAAATTCCGTTACATTACC
TATATCTACTCCATAAAGCTCCACATCAGGCCTAATTTCATGAATTTTTTTAAGATAGGTACCTT

TCCCACATCCAACATCAAGAATTTTTGCATTTTTAGGCATATTTTTTATGAATACCGCAACAGTT
TTTTGAAAACCCTCTCTTTTTAAAATTATATTTTCATCCATATTTTCATTAAAATATTCATTTTT
AAATTTTTTTTCCATTTTGAACCACTAAAATCAGTTTGTTTAATTTAATTAAAATTAAAAATATA
ATATTAAATAATCCTATTCATAATCTCAGTTATCATTTTCTTTTTAGTCCACGATTTAAATTTTA
AATAGAAAATATTTTCTATTTCTCCAAACGTAACTAACTGCATTTCTAAATCACTTTTTGGGTGA
TCATATTTATATTTACCAAAACAATCTGTACTATTACACCACTAATGCATCATATATACCTTTAA
AATAATATTTTAAAAGCTTTGGTTTTTTATTTATTATAACCCATTGCCAAATCCGTATTAAAAGC
CCCAAACTAAGTAAATAACTCAAATATATGCAGTAATACATTTTGGAATTATGTTTTTTTGAAAA
ATATAATCCATTTCTTGTCAGATAATATATTCTCACGGGTGATAGTACTCCTCCGGAACTTTTAG
CTACCTTATGCCACAAAATTGATTTTGGACTATATATTAACATCCAATTTTTTTTTGCCCGAATA
CATAAATCCATTTCTTCATGATATAAAAAATAATATTCGTCAAAGAAACCAATATCTAATAATAC
TGATTTTTTTAATAATATAGAACAACCTACCACGCTTTTTAAATTTATTTGAGAATTATATCTAA
AATTATCATCTACTTTGTTTTTACCATATGCATTTAAAATTAAAAAATTTTTATTGAACTGGTCA
ATACCGCCAGCATACCAGATAACATCATCCCGTCCTTCATAATCATAATAATAAATTTTACTTCC
AACAACACCAATTTTTTCATCACTTTCGCCGACTTTAACCATTTCAGTTAAAAAATTTTCATCAA
CAACTGTATCGTTGTTTAAAAGTAAAACATAATCAGAATTTAAACATTCGATTGCAAATTTCATT
CCAACGTTATTTCCACCAGCAAACCCATAATTATCTTCATTTTTGATTAATAGTAATTTTTTATC
GTTTTTTGATTTTAAAAATATTTCTTTTTTAGAAAATTCATTGTTTCTCGCATCTTTTTCAGAAA
TTTCAAAAACTTCAATTGGTTTATTATCTTTGGAATATTTAAAAAATTTTGAATTCACTTCAATT
TTTCCGCTGCAGTATTCTTGTATTTTTTCAATTGATTCATCCTTTGAACCATTATCAATTAAAAC
TACATTGTAATTTAGGTAATTTATTCTGTAAAGGGACTCCAAACATTCTATGGTGTCTTCCCACC
CATTCCAGTTAAGAATTATTATTGACACTTGAGGATTCATTTTTAGTCACCGTTTATTTAATTCA
GATAAATTACAACATACAATAATTATTAAACAATTTCAAAAATAACTGCATATTTTTTTATCACA
TTGATCTTCCAGTTTACTGAATATTCATGATATATAAAATTAAAATGCCCAAATCAACTCCAAAT
ATCCTGTTAAATAATAAAACTCTCATCTTCACCAAAATTTAAAACTTTTTAATTCATTTATATTT
AATATTTTTAATACAAGAATTAATAACAAATACATTATAAAATACACAATACCTGATAAAAAAA
TCAACAAGTATTCATTAACAATAATTTCTTTTATTATTAATAAAGGAACTATTGAACCTAATGAA
GAAATTAAACATAATATAAATTTTTTAACAGGGAATATATAATCTAAAAATTTATTTAAGTACT
TCGTCTGTAAAATCTGAATTAAGATGTAACTTAAAGTTGTAGTAAATGCAGCCCCAATAGTTCCA
AATTTTGGAATTAATATTAAATTAAAAATTAAATTAAATGCTGCCCCGATATATAGAATTTTTG
TTGAAAGATGCGGTTTTCCAATACCATTGAATACATTAAATACTATGTTATTCATTGACAAAAAT
ATTATCCCAAAACTTAAAATTGACATTGGCAGTGCCGCACTTAAATAATTTGCATTAAAAAACAA
ATTTATCAAAACTGTCGGAAAATATGCCAAAATTACTGCGAACGGAACTGAAATTATAAATGAA
TAGGATAATATTTTTTTCAAACCTTCTGAAAGGTTCTTTTTTTCTCCCTTTTCCCACATCTCAGTG
CTCATTGGAAGTATTACATTACAAATAGATGATGCAAAATAACTTAATATTAAAACTGTCGGCAT
TGCTACATTCCTATAATCAGCAACGGCATCGAGTCCAGTAAAGTAAGTTAAACATATCGAATCAA
GATACCCCATTATTAGAAATCCTGCGCTTCCAAACATTACAGGCATACTATATGAAAAAATATCG
CTTAAAAGTTTTTTTGAAAAATTAAATTTTTCCGTAAAAACCTCGGGAAAAGTCTTTTTGAAAAA
AACTGTTCCATATATTATAATCATGAAAAATTGGGTAAACGTATATGCTAACACAGGAGAATAC
ACGCCCAATCCAAAATAAATAAAAAATAATGATAAAATTAATACCGTAAAAATATTCAAAAAAT
TACATGTACTATATACTTTCTGGTTTTGAAAACCTTGAAACAAACTAAAAATTAACACCTTTAGC
CCATCAAAAATATAATACCCTAAAATCATTATTAAAAACATTAGCGATATTATCGAATAATCGCC
AAACTGGCCCTGACTGTTTATGTAATTGACTATGATTATTGGGGATAATAAATATATTAAAAAT

GCCGCCAGAACTGTTGTTAAAGTCTGAATAATCAAGACTAAAATCAGCGTTGATTTTATCAAATC
TTTTCTATTTTGAACTAAGAATTTGGGAATAAAATATACAACTGCATTATTCAACCCAAGTCCCC
TAAAAATTGCCATCATACAGATTAAATCAAAAATTGCATAGAATAAACCAACTTCAACTTTTGTG
AGAGAATTTGCATATAATATTCGTACAAAATAACCAACTGGTGCTGCCAAAACTAAAAATAAAA
AATTCCAACCTGCTCCTTTGATCATTCGTTCTTTATAACTCAAAAACATCCCCTAAAAAATAATA
TTAAATCTCTTTAATTCCATTCTTCAATTCAACTTCAGGCATCCAACCTAAACTTTTAGCTTTTTC
ATAATCAATATAAATTCTATAAACTTCGCCGTCTCTTTCTTTTTCATAAATTGGATCTTTATCAA
AACCAATTTCAGAAGAAATTATTTTAAAAAGTTCATTTACAGATGTTTCTTTTCCAGAACCAATA
TTTACAATATCATTCTTCCAGTTTAATGCCATTAAATTTGCTTTTGCAACGTCATTGACATTTAC
AAAATCCCTTGTCTGGTTTCCATCACCATAAATTACAGGAGATTCCCCTTTTTTCATTTTATCAA
TAAATATTGAAATTACTCCTGCTTCACCTAGAGGATCTTGCCTTTCTCCGTAAACATTAGAGTAT
CTTAAAATGCAGTATTCGATACCATAAAGTCGGCCATATAATTTAATATACTCTTCTGCACAGAA
TTTACTTAATCCGTATGGAGATAATGGTTTTAAACTATGTTTTTCATCTACAGGTAAATATTCAG
GTTCCCCATACACTGCACCGCCTGATGAAGAAAATATAATCTTTTTAACATTGTATTCTTTAATT
TTTTCTAAAATATTAATTGTTCCAAGAATATTTATATTTCCATCAAATGCAGGATTTTCAACTGA
TGTTCTAACATTAATCTGTGCTGCTTCGTGAATTACACAATCAAATCCAGTTAAATCTAAATTTT
TATCTAAAATATCTCCGTTGATAAATTTTGCGGATTTATTGATGTTTTTTTCATTTCCAGTACTT
AAATTATCCAAAATTGATACATCATGACCGTTTTCAATTAAAATATCTACAATATGGCTTCCAAT
AAATCCTGCACCGCCTGTTACCAAAATTTTCAAAATTTCACCAAAAATAAATTAGTACGTATATT
TTTTTAATCATCACATAGATCTTTGATATATCCTTTAAACCCACCTATATTCCCTACAACCTTTGA
AAAAGGTTGATCAAAGTAATGTGCATATTATTTATCACATAGTTCTTTGATATATTCTTTAAATC
CATCAATGTTTTCTATTCCAAGTTCAACGTTTGCTTCAAGCCATCCTTTAATGTCGCCAATATCGT
ATCTTTTGAAATCAACTTCGACACCAATGATTTTTTCTTCTTTTAAAAGAGACTTCATTGCATCC
GTAAGCTGGATTTCCCCACCCCGTCCGGGTTGTGTATTTTCAAGGTGATCGAATATTTTTGAGGA
TAATAAATAAGCTCCCGTGATTATCAAATTGGATGGCGCATCCTCTACACTTGGTTTTTCCACAA
GATCATTTATTTCAAAAATTCCATTTTCAAGTTCTTTTCCAGAAATTACTCCGTATTTGTAAACA
TCTTCTTTTGGAACTCTTTCAAGTGCAATTACAGAACATCCGTATTTTTCATGTGCTTCAAGGAG
TTTTTTTGGAACATTTCCAGAATAAATGGTGTCCCCAACCATTACAATTGTATAATCTTCACAAA
TAAACTCTTTTCCACAATGCACTGCATCTCCAAGGCCATTTTGTTTTCCCTGCCTCGTATAATATA
TATTTGCAAATTCATTGATCTCTAAAATTTTTTTTAAAGGTTCGCACTTTCCATCTTTTTTTAAC
CGCTCTTCAAGTTCAAAATTTTTATCGAAGTGATTTTCTATTGCGTATTTTCCACGGCCCGTAAT
TACCAAAATATCCTCAATTCCCGCATTCGCCAAATCTTCAATTACATACTGTATAATTGGTTTTC
CAAGTACTGGTAACATCTCTTTTGGCTGTGCTTTTGTAATTGGGAGAAGCCTTGTTCCAAATCCT
GCTGCAGGTATGATAGCTTTTTTTATCATTAAATATTCCCCTCAAAAGTTCATAGCAATATTAGT
AAAAACATATATAAAATTATGACGTTTAAATATGGCTTTTACATTAACGTGTAAAGTATCGGTA
GCGCAAACAACGATACAACTGTGGATACAAAACATGCTGAAGCAACTAGTTTTACATCCAAATCA
TATATCACCGCATAAACTAAAGGCATCATTGCAGAAGGCATTGCTGATTGCATTATCGTTACCTG
TTTATCTAGCTGGTTTGATAAAATACTTTCGGAAATTGGAAATGCTACAAGGGGGGCAATTACCA
TTCTAAATATCGATGCCAAAATTCCATAGAATATTCCAAATTTTGCAGCTTTTGGGGATAACGAA
AGTCCAAGTGAAAGCATTATTAGTGGAATTGTTGCACTAGACATGTATTCAAGCGATTTTGTAAA
AAATTCTGGAAATATTGATAAGTTCACGTGCAATAATACCAAAATTAAAGTAAATATTCCAGTG
AATAATGGTGGGAATGTAAATACTTCTTTTAAAATGCTTTTTTTAGCACCGCTTAGTTTAACCCC
GATGTATGTTCCAAGTAACATACTGCAAAATATTGCAGACATATCACAAAATATCGCTCTTAAAA

GACCTTGCTCTCCGTAAAAGCCCAAAATTACAGGATATCCTAAAAATCCTGTGTTTCCAATTGAT
GCAACAAGCACAAATCCTCCAAGTGATTTGTTATCCAATTTTAAACGTTTTCCAACAATATATGC
TAAAAGTGCACATGCGGCTGTTGAAATAAATATTATTATCGGTAATTTCATAAATTCTGGAAGGT
CTGAGGGCGCTACATTAGTAAGTAACTGCAAAAATATTGTTGCAGGCATTGCAATGTAAATTACT
ATTTTATTTAAGACCATCCTGTCTTTTTCATCGAGTATTTTAAAATGTCTCGAAATATATCCGAT
CAAAATTAAAAATACAATAACATACATGATTTCCATAAGTATCATCGTTTTAAATTTCCTTGTCT
GTCTATCTCCCCATTTCGTATTCTCGTAGTCGAAATTGGAACATTATCTTTTGCCATTAAGAAGT
CATAAACTTCAATAATTAAAGGTTTTAGTCCATTTTTAACCCTTATTTTATTTATAGTTTCGGCA
TTTTCTTTTGTTTCAGGAGTTACGACAATTATATCATATTTTTCCGAAATTGAGTCGCCATAAAC
GTCATTAATTACCATTATTTTGTAATCAATTTGTTTATCTGCTAAAAACTTTTTTAAATTATTTT
TTCGAATATTTAACGGATTTACATCGTGTTTTTTGTACGATTTTATAAAATCATCGCTTGTAATT
CCAATAAAGAGTTTTCCAAATTTTGATGCGTGCATTAATAAATTTTCGTGGCCTTTGTGCAGTAT
ATCAAAGGTACCTCCGATAACAACATTATTCATTTCAAAAACACCCGCACATTTGTAAATTAAAA
TTAAAGGATATATAATTAAAAAAATAAAAAAAGAAAGAATTATTTTTGTTTTCTTATTGATTCA
AGAATAATTTTCTGTTCTGTTCTTGCAACAACATTTCTGATTGTTTCTACTGCATCGATGTTTGC
AGAGATACTTGTAATTCCCCATTCAACCAATTTTTCAACGATTTGTGGCCTGCTTCCAGCCTGACC
GCAGATTGATGTTTTTACACCTGCACGTTTGCATGAAGTTATAACAAGTTCAATCAATTTAAGAA
CTGCAGGATGGTTTTCGAGGTAATATTTTGAAACATTTTCGTTGTTTCTATCAATTGCAATTGTA
TACTGGGTTAAATCGTTTGTTCCAAGACTTATGAAATCGATTCCCTCTTCAATAATATCATCGAT
TATCAAAGCTGCGGCAGGAGTTTCGACCATTACACCAAATTCTACATCCAATCTAAGTTTTAGGC
CAACTTCGTTTGCAATTTCTTTTACTTTTTTTATTTCATCAACGTTTGTAACTAGAGGAATCATC
ACGTTAATGTTTCTGTAGCCATCTTCTCTTAATTTTTTAATAGCTTTTAGTTCGCATTTTAAAAT
TTCAGACTGGTCAAGACCTCTCCGGATACCTCTCCATCCCATCATAGGATTGTGTTCGTGAGGCTC
GTCTTCACCACCTTTTAAACCAACAAACTCGTCGGTTGGAGCATCAAGAGTTCTGTATGTAACAG
GCCTTGGATAGAATGCATCTGCAACTTTTCTGATACCTTCTGCAAATACTTCGGTTAATGCACCT
TCGCCTTGTTCATTTAAGATTTTTCCAGGGTGAACACCGGTTCCTAAAATCATGTGTTCTGCTCT
TAAAAGCCCAACTCCATCAGCACCGGTTGCAGCTGCTCTTTCTGCAACTTCAGGCATGCTCACATT
TGCTTTTATTTCTGTTGCAGTGATTACTGGAGCTCCTGCATAAACTACCTGAGCAGGTGCTTTTT
CTTCAGGTTTGCCAACTTTTCCTTCGTAGACGATTCCTTTTTCACCATCAACAGTGACACATTCTT
CGTTTTTTAAATTTTTCGTAGCTCCTTTTGTTCCAACTACACAAGGAGTTCCAAGCTCTCTTGAA
ATAATTGCAGCGTGGCACGTCAATCCACCATCATCGGTAATAATTGCAGCTGCTTTTTTCATTGC
AGGAACCATGTCAGGAGTTGTCATTTTTGTAACTAAAATGTCTCCATCAAGTATCTTTCCAATTT
CCTCAATTTCTGAAATAATTTTTACTTTTCCAGATTCAACTCCAGGAGATGCACCAATTCCTTTT
AATATAATTTTTCCTTCAATTTCATTTGAAGATTTTGAGTCCTTTTTCTCAGTTAACGTAGTAAT
TGGCCTTGCCTGTAACATGTAAATTTCCCCGTTTTCAATAGACCATTCAATATCCATTGGCCTTTT
GTAGTGATTTTCGATTGTTTGACCAACATTTTCGAGTTTTTTGATCATTTCTTCAGATAAAACCT
GTTTTTCGATTAAATCTTCAGGCGTTTTTATTTCTTTTGTGACCCCATCGGCATCTTTTATAAAC
ATTGTATCTTTCCTTGCAACATTTACGCTTAGGATTTCTTGGGTATCTTTTGCAACAATATATGT
GTCAGGGCTTACTGTTCCGCTAACTACGCCTTCCCCAAGTCCCCATGCTCCTTCGATTACCATTTG
GTTGTAATCCTGATTTATGGGGTTTACGGTAAACATAACTCCTGCTTGATCTGCATTTACCATTT
TTTGAACAACTGCTGCAAGTGCAACTTCAAAATGATCGAATCCTTTTTCTTCCCTGTAAAAAATA
GCTCTTGGAGTAAATAACGATGAAAAACACTCTTGAACTGCTTGTAAAACCTGTTCATTTCCTTT
CATGTTTAAAAAAGTTTCCTGCTGCCCTGCAAAACTTGCATCAGGCAGATCTTCTGCCGTTGCTG

AACTTCTTACTGCGACAAACGTTTCATCGTCTCCGCTAAGTTCACACATTTTGTGGTATGATTCA
ATAATGCTTAATTTCAAATCATCAGGAATTTTAACATTTTCAATTGCGTATCTAACTTCTTTTGA
TGCTTTGTTCAATTCTTCATTATTATTAACATCAATATTTGACAAAATTCTTTCAATTTCATCCT
GAAGCTTTGTTTCTTTGATAAAGTACCGGTAAGCTTCTGAAGTGACGACAAATGCCGGAGGTACA
GGTAATTTTGCATTCCACATCTCCCCTAAAGATGCTCCTTTTCCACCTGCAATATCAACATCATCA
TTTCTGAGATCATCAAGCCAAGTTATCAATTTCATGTTGAATCCTCCATTCGTTCAATTCATGTT
TCTTCTATCAAAAATAACTATGATTACAATTTCTATTTTATACATCTCAGATATATAATATTTTG
TAGGGCTCATTTCGGTTCGGTAATTTACTAATTTAATTTTTTAAAATACACTTAATTTATTCATA
AAATAGGAACTAAAATACCAAAATAGAAACATTTATATACATTTGATGTGTAGGGTATATCATA
AACCTGGGAGGTGTCTCCTTTGAAAAAATATATAAGTGTTCTAATTGGTGCAGTATTAATTTCAT
TAATCGTTGGATTTAGCGGCTGCACAGAAAGTGGAGTTAATAATTCAGAAGAAGTTCAAAAGTT
TAGTTTAAAAATTTCAGGATCTACCACAGTTTTACCTATCGCAGAAGAAGCTGCAAAACAGTTCA
TGACAAAAAATAAAAACTACATGATTGAAGTTACAGGAGGGGGTTCAGGTCTTGGGGTTAAGGA
AGCCGGTGAAAACTTAAATGACATCGGAATGGCTTCAAGAGATGTAAAATCCTCAGAATTTGAAA
TATATCCATCATTACAAGTATTTGGAATTGCAAAAGACGGTGTTGCAATTATAGTACAGCCAGAA
AACCCGGTTTCAAGTTTAACACAAGAGCAGATTATTGCAATATACGCTGGAGAAATTACAAACTG
GAATGAAGTTGGTGGAAACGATGCACCAATTACAGTTTACACTAGAGACGAAGAAAGTGGTACC
AGAGAAGTATTCTTTGAAAAAGCATTGAATAAAGGAAATATTACTAAAAAAGCAGTTGTTGTTG
CTTCAAACGGTGCAATGAAATCATCAGTTAAAGCTGACGTTAACGGAATTGGATACCTTTCAATT
GGATACCTCGACTCATCAGTCACAGGTTGTGAATATGAAGGCGTACTCCCTACCGAAGCAAATGT
AGTTTCTGGAACATACACGGTTTCAAGAACATTGAATATTATTACAAACGGTGAACCTTCACAAG
AAGCAAAAGCATTTATTGACTTTTTATTAAGTTCAGAAGGTCAAAAAATCGTTGTGGAAGAAGG
ATATATCCCTGTAAACTAATATAAATTAATTATTTAATTTCCAATTATTTTTTTAAAAAATTGTA
TTAATCTTTTCAATTTACAGTTATGGTGAAACAGGATGCATAAAAAAAATATTGGCGAATCAGTC
CTTGAAAATATATTAAAACTGAGTGCACTCTTATCAAGCGTAATTATATTTGCAATGGTTTTATT
TTTAATATTAAGCAGCCTCCCAGTATTTTCAGTTGTTAACCCACTTGATTTTGTATTGGGAAGTG
ACTGGTCACCAATTAATGACGCTTACGGGATATTTCCAATGATTGTTGGCTCATTTTTAGTAACC
CTGCTCGCACTTGTATTTTCAGTACCCTTGGGAGTTGGATGTGCCATATTTTTAGCAGAACTTGC
ACCAAAAAAAGTTCAAAATATATTAAGACCATCAATTGAAATTTTAACCGCAATTCCTTCAGTAG
TTTACGGTTTTGTAGGCATGGTCTTACTTGTTCCTTTAATTAGAGATGTTTTCAATACAAATCCA
GGATACAGCTGGCTTGCCGCATCAATAATCCTTGGAATAATGATAGTTCCAACGATTACGGTACT
TTCCGAAGATGCAATAAATTCAGTTCCACAGCAATTAAAAGAAGGAAGCCTTGCAATGGGCGCTA
CCCGGTGGCAGACTATAAAAAAGGTAGTTCTTCCAGCATCTCTCAGTGGAATACTTTCAGGCGTT
ATTTTGGGGATGGGCAGAGCCATTGGAGAAACTATGGCCGTTTTAATGGTTGCAGGAAACTGGCC
GCTCATTCCAAAATCGATTTTCGATCCGGTAAGGCCATTAACATCACATATTATATTAAATATAA
AAGAAGCAGCTTCTGGAAGCCCGATTTACTATGCAATGTTTGCAACAGGGCTCGTTCTCTTTATA
ATGGTTCTTTCGCTCAATTTAATTTCACAGTATATCGATAAAAAGTACAAAATAAAATGGGACTA
ACCAGGGTGGAACTTTGAATAACTCTAAAAAAGCCAGAATTGAAGAAAATATTATGTTTGGTAT
TTTTTACCTGTTTGCATTTATTGTAGTTGGTATATTATTCACAATTGTTGGTTACGTCTTTATTA
ACGGGATCGGCGCAATAAACATGGAATTTTTCTTTGGGGATTCAAACCCGATAAACGTGATTTTA
GGGTCTGAGAGGTGTTTTGATGGAATCTGGAATTCGATACTTGGAACCCTTGCATTAGTCATCCT
TTCAATATTACTGTCGATTCCTTTCGGAGTTTTGGGCGCAATATATCTTCATGAATATGCTTCGG
ACAATAAATTAACACAAGCAATTAGATTTTCATCAGACAGCCTTGCAGGGCTTCCGTCAATTGTA

TTTGGTATTTTTGGGTACGCTCTTGCAATAAAGACAGTTGGTCCATCATTATTAATCGGGGGACT
TACTCTTTCATTCATGGTTCTTCCAATTATCATGCGGGCGACAGAAGAAGGGTTAAAAGCAGTCG
CACCAGGACTTAGGGAGGGAAGCCTCGCACTTGGAGCTACAAAATGGCAAACAATTACAAAAGTG
GTAATTCCTTCAGCACTTCCTCAGATTATTACAGGAATAATTCTTGCAATGGGGAGAAGTGCAGA
AGAAACTGCTGCAATAATGTTTACCGCTGCTACTGCGTTTTCATTTGGCATTGGATTATTCAATA
GAGTGGAAACTCTTTCGTTTAGTTTATACGTTCTCGCAACAGAATATTCTAATTCAGAAGAACTC
AAAATGGCATATGGAATTGCACTCGTCTTGATTGCAGTCATGTTAATGCTATTTACAATCGCAAA
TGTCGTTAAAAATAGATTCAAACTTATGCAGAGCTAATTAAAATTTGGTGTAATTTATGAAAAT
CAAAATGAACTCAAAAGATGTAAATTTGTGGTATGGCGAAAAAAAAGCGTTAAATGATATAAAT
CTGCCAATTTACGAAAATAACATAACCGCACTAATAGGTCCAAGTGGCTGTGGAAAATCCACGTT
TCTACGATGTCTTAACAGGATGAATGACCTAATTTCTGGAGTTAAAATTACTGGAGAAATAACTC
TCGATGAGAAAAATATTTACGACAAAGATGTTGACGTTGTTGAGCTTAGAAAAAGAGTTGGAAT
GGTATTTCAAAAACCAAACCCATTTCCAATGAGTATTTACGACAATATTGCATATGGCCCAAGAA
TCCACGGAATTAAAGACAGAAACGAGCTTGACGAAATCGTTGAATGGGTACTGAAAAAATCTGCA
CTGTGGGATGATGTAAAAGAAGACCTGAAAAAATCTGCACTAAAACTTTCAGGGGGCCAGCAACA
GAGGTTATGTATTGCAAGAACCATTGCGGTAAAACCTGATGTAATACTGATGGATGAGCCGTGTT
CAGCACTGGACCCAATTTCAACGCTTAAAATTGAAGATTTAATGGTTGAATTAAAGAAAGAATA
TTCAATAGTAATAGTAACTCACAATATGCAGCAAGCAAGCCGTGTTTCAGATTACACTGGATTTT
TTATGCTTGGAGATTTAGTAGAATTTAACAGAACAGATAAACTATTTGTTGAACCTGAAAACAA
AAAAACTGAAGACTACATTAGTGGAAGATTTGGATAATTAAATTAAAATTAAAATTAAAATTGA
AAAATTAGAAAATTGAAGATATTTGGTGGTAAAAATGGTTAGAAATGCATTTTCCGATCAAATG
AAAGAAATTGAAGATATGATTAATAAAATGTCTGAAATGTGTGCAAAATCATTAAAAAATTCCA
TAAATGCGTTCATAACTAATGATATCGAACTTGCAAAAGTTGTTAAAGATGGGGACGATGAAAT
CGATTTAATGGAAATGGAAATCGAAGAAAAGTGTATTTCATTTATGGCCAGACAGCAGCCTGTTG
CAAGCGACTTAAGAGAAATTATTACGATGATTAAAATTATATCAAAACTTGAAAAAGTTGGAGA
TATGGCTTCAAGAATTGCAAAAATAACCATTAATTCAGAACAGAACATTCTCAGATCAAAAAACG
GGAGTATTTTAACAATTATGGACGAAATTCTTGAAAACATGCTTAAAAATTCAATTTTGTCGTTT
AGAAACAGGGATTTGGACCTTGCAAAAAAAACTTACCTTGAAGACAGCAAAATTGACGAACTTTA
CAAAGTACTTTGCAGGGATATGACACTTAAAATGATTGAGAATCCAAGAATAATTAGTGAAGCT
ACCGATTTTTTGGTTGTTGGAACATATCTTGAAAGAGTTGGAAATTTGTGCGGAAGTATTGGAGA
TAGGGTTGCATTTATGATTACCGGAGAAAGACTAAAAGAAGAAGAATTTGAAAAAATATTGAAA
AAAACTACAAAAAAGTTGGAAAATTAATCATTATACTTTTCATCAACTACAATTTTTACGGGTAC
AAACGGTATTTTAAGCTCTTTTGATTCGAGCTTTCTATCAACCATTATCAAATCATTTAATTTTT
CCTGAATATTTTTTGAGATTTTTCTAACGATTTCGGAAGGCGCTGCTGCAGTATAAAGGTACGGC
TTTTTACCTAAGCCCTCTTTATCCATTTTTTTCCCTTGAAACGTATCCTGTATTGTACATATCGAG
TATCGATTCACGAACTGTTGACGGATATATCCCAGTTCCACCGGCAATTTCATCAACTGTACTTT
TATTGTACTTCCTTAAATATATGTAGATTTTTGCTCGAACTTCGCTTGACATTAGATTCGCGAGA
TTTTCGATTAATTTCTGGTCTACATTTTCTATGTTATTCACTATATCGCCCCACTCTCAACTGTAT
ATTGAAAATTTAACCCAATATAAAATAGCCATTTAAAGCTCCCCCACTTCAAAAGACTTAATTAT
ATATTTCATAAACGTGCAATATAAAGTTTTTGTAAGGCCAACTAACATTGAAAAAACATTTTTGA
TCAAATCTGCCTTAATGTCGTTTAGTACTGAACATTTCAACAGAATTAAAAAAAGAAAAAGTTA
AATTTCGGAAAATACTTCGTCAAGAGCCGAAATCATTGCATCTATGTGCTCCTTTTCAACTATTA
GCGGAGGTAAAAACCTTAAAACGGTATCCGAAGTACAGTTTATTAAAAATCCTTTTTCAAACATT

TTTGAAACTATCTCTGACCCATTGAACGTTAATTCAACCCCAACCATTAATCCTAAGGACCTTAC
TTCTTTTACAAATTCATATTTTTCTTCCAGTGTTTTTAAGCTGTTTTTGAAGTATTTACCCATTT
CAACAGCATTATCTAAAAGTGTAGAAATTACATCAAGTGCAGCTGCTGATGAAGCACATGCTAAA
GGATTTCCACCAAAAGTAGTTCCGTGACTTCCCGGAGTAAATGCGCTTGCAATTTCGCTTTTCGC
AACCATTGCACCTATTGGAAATCCTCCACCAAGACCTTTTGCAAGTGTTACGATATCTGGAATTA
CCCCATACTGTTCGTATGAAAATACAGTTCCAGTTCTTCCCATTCCACACTGAACTTCATCAAAAA
TTAAAACGATATTGTTTTCGTCGCACAATTTTCGAACTGCTTTTAGATATTCTTTGTCTGCAGGG
TGAATTCCACCCTCCCCTTGAACAGGTTCAATCATTATTGCAGAAGTTTTTTCAGAAATTCCGGC
TTTCAACGCATCTATATCGTTAAATGGAATGTATTTAAAACCTGTTGGCAGTGGTTCAAATCCTT
CCTGATATTTTGCTTTAGGAGTTGCAGTAATTGTAGTAAGGGTTCTTCCGTGGAAAGCATGTTCC
ATTGTAATAATTTCTCCTTCCTTACCGTTTGCTTTAGCATATTTTCTTGCAAGCTTTATTGCAGCC
TCATTTGCTTCTGCACCACTGTTACAGAAAAATGCTTTATCAAGTCCGCTTAAATTCACGAGTTT
TTTTGCTAACTCGATTTGTGGAACATTGTAGTAAATATTTGAAACGTGAATTAGGGTTTCTGCCT
GATTTTTAATTGCTTCAACAACTTTAGGATGGCAGTGCCCAACGTTATTTACCCCAATTCCTGCA
AGAAAATCAAGATATTCTTTTCCATCAGTATCAAACACACTCATTCCATTTCCTTTTACAAGAAC
TACTGGAACTCGGCCATAAGTGCCTATAACATATTTTTTTCATCTGAAATAATCTGTTCTTTTT
CTAAAGTACTCATACTAACCACCAAGTCATTCAAAATCAGTCATGTGATTAAATAAATCATGATT
CAAAATTATTTAATAGTATCAAGTTTAAAAATATATTGTTTTTAAAATAAGTTTTGTATTTTT
TCTAGAAACGGGATTTAAAGAAAGCATTAAGAGGATTAAAATGGAGTTTGATTTAATCAATATA
TATGGATATTTAACAATTATAAACACTATAATCCTCACGATTTTATTTTTCACGATTGTAAAATT
GTTAAAACTTGTAAAAAGTAACTTTGAATATTCAAGGAGTTTAACCTGCCCAGAAGACCGGATTG
AAGTATTAAATGACGAAAAATACTATTATTATGTTTAAACAAAATCAAATATGCTAAAAAAGA
AATTAACATTATCATGTTTTCGATATACTTAAATCCAAAGATCTCCGAATTAATTGATGAACTCA
CAAACGCGAGAAAACGAGGGGTAATGGTTCGCGTAATTCTTGAAGAAGAAGTCGGATCAAATAT
TTTAGCCCAGAGCAAACTTTCAAGCCAGCATATTCTGGTAAAATTCCATGATTCTAAAAAAACAC
ACAATAAATTAATAATAGTTGATGATACCGTTATTGTTGGAAGCCATAACTGGACTGATAAAGCC
CTTTTTGAAAATAAAGAATCAGCAATTGCAGTATCTAATGAAGACGTTCTTAACATTGAAAAAG
AATATTTTGAATCATTGTGGAGGTCTATCGAATGAAAATAGCTATTTTTTCCAAACTAACTATTA
AAGAAGACGAAATAAGAAAAAAACTGGAAAATTTTGATGTGGATATATTCCCACCAATAAAACG
TGGAGATTTAACCTCTGCAAAAATCTTTGATTATGATATAATCGGAATAATCGATGGTTGCTTCC
TTCAAAATACGGCTGTTGCTCATCGGGAAATTTTAAAGGTTATTGAAAATAATATTACAGTATTT
GGTGCAGGTTCAATGGGAGCACTTAGAGCTTCAGAACTTGATACCTGCGGCATGATTGGAGTTGG
CTCGGTTTATAATTTATATAAAAACGGAATAATCAGTGATGATGACGAAGTTGCAGTTACATTTG
ATGATAATTTAAATCAGATCACATTTTCGATGATCAGCTTTAGAGAAATGATTAAAAATGCATT
GAATGAAAAAATAATCGATGAAGATGATTTAAAACGATTGATTAATTCTGGAAAAGAACTTTAC
TATCCACTTAGAACATTTGAAAATGTTATTGAAAAATCAAAAATTTCTGGAGAAAAACGGGAAG
TTTTGGAAAAATTTTTAAAAAATCAGCCAGATATAAAAAGAAACGATGCCTTTGAAATGCTCGA
AGAAATTATAAAATATACTGAAAAACAGTAATGATTTAAAACAGTTTTTTAATAGGATATATAA
AAAATCTGGTGAAGTCATGAAACGGGAACTCAAAGTAAAACCCATTGAAAACGGAACTGTTATCG
ACCATATCAGTGGATCAAAAGCACTGAAAGTCTATAAAATCCTAAATATCGAAGAAAAACTCCCA
ATAACACTTGCACTAAACGTGCCGTCAAAAAAAGGCGTTACCAAAGATATTTTAAAAATTGAAGG
ACTTGAACTCTCAAAAGACGATGTAAACAAAATTGCGTTAATTTCACCAGATGCAACGATAAACA
TCATAAAAGAAGGAAAAGTCATAAAAAAATTTAAAGTAGACATTCCAAAAAGAATTGACGGAAT

AATTAAATGCACCAATCCAAACTGCATCACGAATAAAGAAAATATCGAAAGTAGATTCTCAATCG
AACAGAAAAACACGTTAAAAATCAGGTGCGAATACTGCGAAAAATTCATTAATTCAATTATAAT
CTCAAAATAAACCCTAATTTTTTTATATTTCAGTAACTATTATCATGAGAACTGTTGGTGATTT
TTATGAAATTACACGAATATGAAGCAAAGGAAATTTTTAAAGAAAATAATATCCCTATTCCAAA
AAATAAACTTGTAAGCGGAAAGGTTGAAAGTATCGATTATCCCGTTGTTATAAAGGCGCAAGTTT
TAGTTGGCGGAAGAGGTAAAGCTGGCGGAATCTTATTTGCAGATAATATCGATGATGCAAACGA
AAAAATTGAATCATTAATTGGAAACACGGTAAAAGGGGAACTTGTTGAAAAAGTACTCCTTGAA
GAGCAGATAAAAATTGCAAAAGAATATTATGTTGGCGTTGTTGTTAATAGAAACGAAAAAAACA
ATGTTGTTATATTTTCAACAGAAGGTGGAGTAGATATCGAAGAAGTATCTTCGAAAACCCCTGAA
AAAATAATAAAATTATCATTAAACCCTGAAAAAGAATTTTTACCCTACCTTGCAAGAGGTATGCT
TAAAAAAGCAGATATCCCAAGTGCAGAAATTCCAAAAGTAGCAGATGTTTTATGCAAAGTTTAT
AAAGCATTCAAAAACATGGATGGAATTTTAGCTGAAATAAACCCGCTCGTATTTACTGAAAATGG
AAAAATCATTGCTGCTGATGCAGTTTTAAATGTTGACGACGATGCATACTTTAGACATAATTACG
AGAAATTTGAAGAATTTAATAAACAGGAAAAATCCGAATTTGCATATGTTGAACTTGATGGAGA
CATTGCAGTTATTGGAAACGGTGCAGGACTTACCCTTGCAAGCATGGACATTGTAAAAGAATTTG
GCGGAAACCCTGCATGTTTCTTAGATGTTGGTGGCGGTTCAAATAGGGAAACCGTAAAAAAAGCA
CTTTTAAAAGCAACCTCCCGAAAAAACGTTAAAGGAGCATTTATAAACATTCTTGGCGGAATTAC
AAGGTGTGATGAAGTTTCGATGGGAATTGTAGATGTTTTAAAAGAAAATCCAAATCTTAAGTTT
GCTGTCAGAATGATGGGAACAAATGAAGAAGAAGGAAGAAAAATATTATCTGAAAATGGAATAA
GTTTTGAAAGCTCGATGGAAGGGGCTGCAAAAGCACTTACTGAATTAATAAATAGTTAAACGTG
ATTTTCAATGTTTAACATCCGAAAGAAAAAGCAGGAAACCGTTTCAAGAGATAAATATGAAGAT
ATTAAAAAAATATATATTGAATCTAAAAAGTCATATATTGCACTTGAAGCAAAATACAAGCATG
AAATTTACGTAAATAGCCAGTTAAAAAATGAAATAAGTCAGAAAAACGTAGAACTTGAATACCT
GCATCACAAATTAAATGAACTCTATGAAAAGTATAGGGATATCTCAAAAAAATTATGAAAAACTG
GAAAATAGGGTTAAATTTTTACTTTCGGAACTTGAAGATAAACATGAAACTTTTAAAAAATAAT
TATTTCATGTTATTACATATCTCTTTTAAAAATTGTAATTTTGAAGTACTTTCATCCACTTTTAC
TTTTATATATCCGCAAATTTCCCAAGATTCCTTAGGATAGCTTTTTTTATTTTCAACACTATATT
CTAAACCCATTTTTTTTTATTTTACTTGCAATATCTTTTAATTTTGGGTTTTGAACTGCAATTTCT
TTTGGAACTTTCCTACCTTCATTTTTGGTTCTTTTTAGATCAATATACGCGGGCCACAAAATTAT
TTCCTTCATAATTTCACCAAAATTATTTAATCCATTTTTCTTTCACGAATATGCGACTGATTACA
AGTATAAACACAATGGATACTAACGAAATTACCCAAAATCCATAAGGGCTCTCTGAAAGAGGCAT
ATATTTAAAATTCATCCCGTATATTCCAGTAATCCACATCGGAACAGCAAATAGTGCGGTAATCA
TTGTAAGAATTTTCATTACCTGATTCATCCGTATGTTTTCAATTGAAAGCGACATGTCCATCATT
GACGATAAAAGCTCACGGTATGTTGTTTCCATGTCAATTAACTGCAGAATATCGTAGTATAAATC
TTCAAATTCCCACCTATCTTCCTGAGTGGTTATTGAAAGATACTTTCTTTTTAAAACTGAAATAA
CATCCTTATTTGATATAAGCGCCTTGTGGAAATATACAAGCATTTTTCTAAGCTGTAATATCTCT
TCGGTCATGTTTTTATTCTGTTCTTGAACGATTCGGTCTTCAAAAACATCCAGTTGATCTTCAAG
GTTTACGAGAATTCTCGAATAGCTTATTGTAATCTGGTTTAAAATTGTGTATATAAAAAATCCTT
TTCCTTTGTCAAGCAGGGTTCTTGGCTTTTTTGTTTTTACAAGATTATAAAGTCGCCCAATTGAC
TTGATTTTATCCGCGTGAATCGTCAGGATAATGTTATCTTTTACGTAAAGTCCAAAAGAAGTAGT
TGTGATGTCTTCTTCAAATAAAGGGGCTTTAAAAATAATTGTGTAGTAGGTATCTTCTTCTTCAA
CCCTTGGAACTTCCTGCTCATCAAGCCCAAGACTTAATTCATCAGTTTCTATTTCTATTTTTTTAG
AAAGTTCGATTAATTCCACGTCTGAAGGGTCATAGCAGTCAATCCATATAAGATCGTGTTCACTA

TTTACATCATCCAAATTCAAAACTTCAAAAGATCCTGCATGATAACCAATAATTTCAAGCATTTT
TTCACAACTATTCTTCAATTAACCCTTCTCTGCTTTTTGGTTCGTGAAATAGTACAACAGAAAGT
ACAATTAATATTGATACATATGCCAAAATATACATTACAAGACCTGTAAATTCAGTGCTATACCC
AATTAAATAACCTGATGAAACGATCAGCATCTGTCTGAGCATGAACGCTAAAACTACATTAAAGA
ATAGCCTTTTTAAAAGCCTAAGTATGCTTTTTTCAGTGTGTATCAAATCATCTATGAAGTGAACA
AGTACTAAAAGCACAAAACCCAACGTTAACGGATTTGCAATATTTAAAAGGAATTTTCCGAAAAA
TACCGTTAGTTCAGGATCTACCGAAGCCCTGTAAGAATAAAGTATACCTACAAGAAGTATCAAAA
ATCCCAAAATATTTCCAAAAGGAGATAATTTTCCAACTTCAAGACCCTTTTTGCTTTCACTAAGT
GTTTTTTTAACAGATTCAACTAATCCAACGCCCTCAGACAGGATATAAATTCCAATAAGTCCTGC
AATTATTCTCCATCCAAGATCGGAAAATACGGCATACAAAATCATTGCAAAACCGATAGAGGTAA
ATATTAACGGAATGTACTGCGACATTGTCTTTTTTATAAATTCCTGCACGAGATAGTACGTTGAT
TCAAGAGATTCGTTTTGTTTAATTACAATTCTCTTTTTCCAGACAAAAATATCCTTTAATTCAAG
GTATTTTAAAACCATTTCGTCCTCTTTACCGTCTGAAACGAGATATATAAAATTAGGTTCGTAAA
GATAAATTAAAAAGTCAATCTGTTCCTTAATTTTAATTGCGCACACTTCTGATTCGATATCGATA
TCCCCAGAAATTGTTGCAATTTCAACATCTTTTCCTTCTTTTTTTAAATGGTCGTAGTGCTGAAT
TCCACCAAGTATGGAGTTTACATCACTATCTCCTGGATCGGTTAGCCCGAGCTTTACCGCTGCATT
GATATTATCCTCCCTACTTAAAATAGGAGTCCTAATATTTGCTTTTCTTCCAACATCATCGTCCAT
GTCAACGACAATAACTAGATACTTTTTATCTTCGGGAACGTTATAATCTTTGCCGATATCTTTTA
TTGATACCATTATACCACTTTAACACTAAAATAAACGATTAAAATATTTTTTTATTAATTATGGG
CTTTTTATGCAGATTTTCTGAAACATACAAAAACATTAACATCGTAACTAAAAAACCGTAATTGA
TGCCGATTTCGCACATCGATAACAATACAGCACCATAAATAGAATATATTTTAGTATCGCTCTTT
TTATATATTAACTTACTGATTATTCCAAATATTAATGAAAAAATTAAGCTGTAATAACCCAGATC
CAAATAAATCGGACCAAAAAGGGTTGACGTAATATTATGCCTGTAACCATGAATTATTTCGCCAA
TTAAAGACTCCCCGCCAGGTGTAAATGCAATTTTTCCATATAACGCCGTAGAGTACAATATAATC
TTTTCTAAGGTCATCAAATCAAAATAAGCCCGATATGATAAAATTTCTAAAAAATTAAGGCTCCA
TGATTGACCGGATGATGAAAGAATTGCTTTTGCCATTATTCCCAAAAATAACACGGTAAATATCC
CTATTAAAAATATTTTTTTTGCACTTATTTTATTATTTTTGTAATTGTAAAGCAAAAATGCAACG
ATTAAAACCAAAACATTTGCTTTGTATCCGGTTACTGCCAAAATTGCGAAAGATACTAAAAAATA
TCCAAAATTAAAGAGGCTTGCAAATGTAAGGGCTCCGGAAGAAATTAATCTTAATGGATCATCTG
AAATTGCCATTCTAACAGTGTAATTGGTAATTGGAATAGTTCCATAATTTAAAACCATTGCAAGG
AATGAAACTACACCAATTAAAAACATGATTTTACTTATTTTTTCAGAGTAATAATTCTTTATAAA
AATATGTAATATTAAAAATACAAGCCCAGAAAATAGTACTGCCGACAATAAACTTCCCGAAAGTG
TAAAAACTCCAAAAAACATGGAAATTCCAAGCAAAATCCCTGAAAACATCTTTAAATTTAATTTA
TCTAATTTCATCTTTTTTTCAAAATTTATAAAAAATGGAATCGAAAAAATAGCTAAATTTCCAAA
TACTACCAATAACATTTTTAAAAGGGAATTTACCCCAAATTCTCCAAAATATGGAATTGCAAGCA
AAAATATCAGGAAATATCCGATAAAAATTATTACTATTGGGTTATCAAGCCCCAGCCCATATTTT
TCTTTAAAATAATTTTTAGATTTTTCAAAATTATTTTTTAGCATTTAAAATCCCCCTGAAAATCC
CGTAAAATGTGGAAATTACTCCTAAAAAATACATTACAACCACATCAAGATCCAAAATACCTGTT
TCAATCCCTACAATCAAGTAAGATACCATTATCGAATAAAATCCCAAATATAAACCTTTTAATTT
TTTGGAAACCTCGTTTGAAAGCCCCATCAAAAGACCAAAAATCCCAAAATATGGAACGAGCCCGG
GTGTTCCAAAATCCATTAAAACGGCACCAAATATTGTCGGTGTGATTGTAACCCCAGTTACTCCA
ATACTGTTTGCAATTAACGTTCTAGGGCCGTATTTTGGACCATCAATTAACCCATATGATGAAAA
TATGGCGTTATGAATAACTCCTTGAAAATTACCGTTAAAATTCTTGACAATAAGATCAAATATGC

TCATTGTAAGATCTATTCTTGAAAGTATCGGGTTTCCACCAATATTTAATATAAAATGCCTTAAA
AATGAAAGTCCAAAAACAACCAGCGCAAGTCCAATTAATGAATAAATCATGAGTTTATTCTTTAT
TTTTTCGGAATAATACATCGCAAAGATTGTTGAGAGTAACAACACCACAACTTGTGTACGGTAGC
CTAAAAGTACTACTAAAGACGCAAATACGCCAGAATACAAAAATATCTTTTTATTACTTAATTTG
GAACTTGAAACTACTATAGCCCAACCCAATGGAAGGGTATGCGAAAATGCGGTATATATAACACT
TAAAAATTTTCTGGATGTTGGATCAAAGAGTGGAACTCCTCTAACCCATAAAAGATCCATTATCG
TGAAAAAAATACCAAATAACAGTAAAAAAAGCCCTATTTTTTCATGAAACTTATAATTTACTTCG
AAAAGCTTAAAATCGAATTTTTTAAAGATATTTTGCCCTGCAATAAAAGAGATATAAAAAAATG
CTGATGAAAATAATACAATACATGCAGAAAGCGTAGTAACATTTGATAGAAGTACAAAAATACA
TGATAAACCAACAAATATATGATGCGGTCCGATTTTTTGTAAATTAAGTTCCAAAAACTTCTTCA
TAGTATTACATCCAAGTTATTAGTTTTGCATTATTGTATTTTTCAGTGTTTATTTCTTCAGTTGA
ATTTTCCACAATTATATTCACTTCAATATTTGATGTAACGATTTTTTGAGGATTTAATAAGCCAT
CTAAGTTTTTCAAATCGTTTAAATTAAAATCTCCTTTTAATATTAAACCGCCGTATACAAAACTC
ATTTTTGGTAGAGCTGTTTCATATTTGAAATAATTTATAACTTCAAGCTGTTCTGTTGGTGAAAA
AGTTTCCGAATCGATTGCAAATGAACCATCAATATCAACTTCAAGAATATTTAAGTCATTGCCAA
TTTCAGAATAAATAATATCGCTTATTTCTGAAAATGAAGTTCCGTTTAAAGAATCCAAGTTATAT
CTAATAATTGTGTTTCCCAAAGGAACTAGATATATGCTACTTGCTGCAATGTCTTCCTTTGAAGC
AAGTTTTCCACCAATAGTCATTTCTTTATTAGTATATGCAACGGTGAGAGTTCCACCACTTGCAG
CTAAAATTTGTACTCGATCAGAAAAGTCTGAAGAATCAGCAGTCCATCTTCCATCAATATCTGCG
TAAAGCGGAAAACCTTTGAGCATTATATTTCAGAAACCCATGCGCATTTGTACATTTCTTCCCC
ATCAAAAGTATACGCTTCTGATGTTGGACTTAAAAAATTATAACCGATTGCAAGGGCTGTAACTA
AAATTGAAATTAAAACTAAAAATTCTAAAATTCCGATTTTTTTCATGAATTTAACCCCTTTAAAA
AATAAAATTAAAATAAATGTTATTTAGAACTAATTTAAAAATTATTTTTTGTATGCATTCATCA
CTGTTTCTGCAATGCTCTCTGAATCGAGTTTATAATATTTTAATAAATCTCCTGCTTTTCCAGAT
TTTCCAAATTCGTCGTTAATACCAATTCTAAGGAGTTTTTTGTTTAATCCATCTTCTGCAATTAC
TTCAGAAATTGCTCCGCCAAGTCCGCCGATGATGCTGTGATCTTCAATTGAGACTATGAAGTCTT
TTGAATTTTTTATTGCATCTTTATCAATTGGCTTTATTGTTGCAATTGCAATAACCTGTGCAGAT
ATTCCTTTTTCTGAGAGTATTTTTGAAGCGTTGATTGCTTCTGGAACGAGTTCCCCTGTTGCAAT
GAGTGTGATATCAGTTCCTTCTTTTAAAATTCTTGCTTTTCCAAACTCGAATTTTGCTTCTTCTTC
GTTTTCAAAAATTATTTCAGTGTTTCCTCTTGGCATTCTTACATAAACCGGACCTTCGTAATCTG
CAGCCCACCTAACAGCACTTTTTGTTTCCAAATAGTCAGATGGAGAAATTACAATCATTTTTGGG
ATTGCCCTCATTATTGCAATGTCTTCTGTCATTTCGTGGGATGCTCCATCTTCACCAACTGTAACA
CCGCTGTGTGTTGCACAAATTTTTACATTTAAACCAGGATATGCAATAGAATTTCTAATTTGCTC
CCATGCTCTTCCTGTTGCAAACATTGCAAAAGTTGATGCGAACACAGTTTTTCCAGTTCTTGAAA
GTCCTGCTGCCATACCCATCATGTTTTGTTCTGCAATTCCTGCATTGAAAAATCTTTCAGGATAT
TTTTTAGCAAAAATTCCTGTTTTGGTAGAACCAGATAAATCAGCATCGAGTACTACGATATTTTC
ATTTACTTCACCAAGTTCGGCCAGGGTTTCACCGTAGGCTTCTCTCATTCCTTTTTTTGTTCCTTT
CATATAATTCACCACAAAATTATAAACTTCAGATATAGTTCTTAAATTTAATTTACGAGTTAAAT
CCTAAAATTTGCAGAATTTCTTAAATCATTCACTGCTTTTTTAACATCTTCCTTTCCATATATTG
CTGAAGCTGCAATAAGCCCATCTGCACCTGCTTCAACAGCTTTTGGCGCAGTTTCCAAGTTTATTC
CACCATCTACAAAGATTTTTGTATCGTAGCCTTCAGTCAATATCTTATTTTTTAAATTATCTATT
TTTTTAATCATTGGGTTTATAAAAGACTGCCCTGAAAAGCCCGGTTCAACAGTCATTACCAAAAC
ACCATATAAATTTTCTAAAACGTATTCCACGTTACCAATTGGTGTTGATGGGTTTAATGCAACTA

TTGGCTTTGCATCGTGTTCTTTTATCATGTCAACAGTTCTGAATAGATATTTACTGGATTCTGCG
TGAAATGCAATCATATCTGCAGCATCTGCCAATTTTGGAATAAATAAATCAGGATTTTCAACCAT
TAAGTGTACGTCAATTGGAACGTCAGTTATACTTTTCAAATAGTGGGGAACTCTAAGCCCCATAC
TTAAATTTGGTACAAAGTGACCGTCCATAATATCCACGTGGAAAAAATCAACATTTCCTTCTTCA
GCCTTTTTTACTTCCTCTGCCATGTGACCGTAATCTGCAGAAAGAATAGATGCACCAATCATTAC
CATATAAATCACCCGATTATTGAAGTTCCGCGAGTGCTTTTTCAAGCTCTTCTTTATTTGGAGCT
TTTCCGTGGAAGCCAACGTTATTTTCCATGAATGATACGCCTTTTCCTTTTACTGTATTTGCGAT
AATCATTTTTGGCTTTCCGTTTTTCATTGTTTTTGCAAGTTCTGCGGTTTTAACAATTTCTTCGA
AGTTGTGGCCGTCAATTTCGAATACATCAAAACCGAATGCAGAAAATTTAGCTTTCAAGTCCATT
AAACACATTACATCTTCAGTACATCCGTCAATCTGGAGTTTGTTTCTATCAACAAAACCAATTAA
ATTATCAAGTTTGTAGTGTGCCGCAGCCATCGCAGCTTCCCAAACCTGTCCTTCCTGACATTCTCC
ATCTCCTAAAAGGACAAATACATTATTTTCGTACTTGTTTAATTTGCATCCAATTGCAACACCAA
CTGAGGATGAAAATCCCTGACCTAATGATCCAGTATTTACTTCAATTCCAGGAATCTCGATTGTT
GGGTGCCCTTGTAAAAGAGCTCCGAGCTGCCTTAAGCTCCATAATTCTTCTTCTTTAAAATAGCC
TAAATCTGCAAGGGTTACATAAAGTGCAGGGCATGCGTGACCTTTACTTAAAATAAACCTATCTC
TTGAATCCTGTTTTGGATCTTTTGGATCATAGTTCATAATATTGTAGTAAAGAGATGATACAATG
TCAATTGCTGAAAGAGATCCTCCAGGGTGTCCAGATTCTGCAAGTCCTATCATTTTTACAATATT
ATATCTTAAACTTTTTGATTTAACAGCTAATTCTTCAAAATTCAAATTTTTTGTGTTTTTCATTC
TTTTCACCGATTTTAAAAACGTAAATTTTTAAATCTGTTTCGTTTTTTCTTCTGATTTTATTAAA
AATTGCAATAACATGAAAAATAGAAATAAATCGTAAGTATTGTGTTTGAATATGTTACAAAGAA
TACCTATAAGGCATCATACTACAATCCATTAATAACATGAATATTATCAGATGCATTTATAAATT
TTTGTATCTTTTCATTTATCTGCGTTCTTCGTTAAATTTTTTATTATGAAACATCCAAATCTAGT
TTAAAACAATCATATTTAAATCATAGAATAGATATCATTATGAAAATTGAGTAGTTAAGAAATA
AGCTGGTGATTTTATGAGTACACTCGATTATTTAAAGATTCTCGCAACAGAAAAAGGAATTTCTG
GTAGAGAAGATAATGTACGAGAATACATGAAAAAAGAACTTGAAAAATACTGCGACAGTATTGA
AACAGATAAATTTGGAAACTTAATTGCAAAAAAAGGATCAACAGGTCCGAAAATCATGATTGCA
TCACACATGGATGAAATCGGGCTCATGGTTAAATATATAGACGATAAAGGATTCTTAAAATTTAC
AAAAATTGGCGGAATTAACGACCAGATGCTCTTAAATCAGAAAGTTATCGTTCACGGAAATAATG
GCGATATTGTTGGAGTTTTAGGATCAAAACCACCACACAAAATGAAAGAAAGTGAAAGAAACAA
ATTAATTTCTGCTGAACACATGTTTATCGACATCGGTGCAAAAAATAGAGAAGAAGCAGAAAAA
ATGGGCGTAGAAATTGGAACTGCAATGTCATTCAAATCCGAATTTGATAAACTCGGTGGAAATGT
AGTTTCATGTAAATCATTCGACAACAGGGCAGGATGTGCAGTTGTTTTAAAAACCATGGAATTAC
TCAAAGACGTGGATTTAAAATGCCAGGTTTACGCTGTAGGAACAGTTCAGGAAGAAGTTGGATTG
AAAGGTGCAAAAACATCTGCATTTGGAATTAATCCTGATGTTGCATTCGCACTTGACGTTACAAT
CTGTGGAGACCACCCAGGAATTAAATTAGAAGATGCACCAGTTGAACTTGGAAAAGGCCCTGTTG
CAACAATTGTTGATGCATCAGGAAGAGGAATTATTACACACCCAACAGTTTTAAGAATGGTAAGA
GATGTTGCAAAAGAAGATGAGATACCTGTTCAATACGAAGTTGGAGAGGGCGGAACTACTGACGC
AACCGCAATTCACTTAACCAAGAGATGGAATTCCAACAGGAGTTATATCTGTTCCTTCAAGATACA
TCCACACCCCAGTTGAAGTTATTGATACAGAAGATCTTGAAAAAACAACCGAACTCGTTGTTGCA
TGCATTAAAAAAGTACATGAATACTTTTAATTTTAATTAACCCTATTTTTTAATTTTTAGGATAT
TAAAAAAATTTAAAAATTTAAATCAAAAGAAAACGTGAATTTTATGAAACTTGATATTAAAAAA
GACTTAAAAAACATGGCATGGCACTGTACAATGTGCGGTGGATGCTGTGACTCACCAAGCGTTTC
CAAAAAAGATGTTGCAAATATTGCAGGATTTTTAAAAATTCCATTTGACGAAGTTGTTAAAAAA

TACCTTGTAAACTTTGATGGAATGACTGGGAGGCTCAAAACTTCAAAAGAAAAGTGTATATTCTT
AGATGAAAATAATAAATGTAAAATTTATAGAGTTAGGCCAATTATCTGTAGATTAAGACCATAT
TCTGTTCAAATTAAAGACAAAAACCTTGTTTTAACATACGATGGCTGGTTTTTGGAAAACTGTAA
AGGTCTGTTTATGGGAGATTTAGACCCTGATGAAGAATATTATAAACACGCAGAAACCGTTTTAA
AATACCTTGGAGAAGAAGAAAATACTCCTGAAGAATTTTTTGAAAAAGCTAAAAAAAGATTGAA
AAAATCTAAAAAATAATAATTTAGCAGAACGTTCTGTTTGAAACTGGAATGCATAGTGTCTTTTT
GCAGTGTGGACACTCAACTGTTACTTTATCCAGTTCATAATTTTCTGGAAATGGTTTGTCACAGT
AAGGACAGTACCACAATTTCTTACCTGTTTTTTCGCATTCCCCGATTTCCCCAACGGTAGGGTGCT
TTTTAAATGTAACTTCGCTTTCTTTATCTTGATATTTAAAAAAAAGTTCGATTAAGTCCTTGTAA
ATCTCAACTGCTGATTTTCTTATGCATTTTTCTCCGCCACATACTGGACATTCCATATTTTCACCA
AAAAAGTTCATCCAAAAATTATCCTCAATTATATTAAAAATGTAAGTTTATTCTATTTAATACAT
TCTTATATCAAAAGTTTTAAATTTTAATCCCTCATCTTTATTTTAATAAATATATGGTGTCGTTA
TGAAATCTATAATTTTAGGAATTCTTTCATCGCTCTTTTTTGCATCTACATTTGTCTTAAACCGG
CAGATGGACCTTTTTGGAGGGGATTGGATATTTTCGGCATCCTTAAGATATATATTCACACTTCC
ATTCCTTTTGATTATACTTTACGGTAAAAATAACATTAAAAAAGTAATTTTCGAGATAAAAAAC
AATTTCAAAGAATGGTTTATATGGAGTAATGTTGGTTTTGTTTTATTTTACGCGCCCCTCACGTT
TGCAGGTAACTACGGCCCATCATGGCTTATTGCAGGAACCTGGCAGGTAACAATTGTTGCAGGAA
TCCTTGTAACACCTTTATTTTACAGCATGATTGAAAATAATGGAAATTTAGAAAAAGTAAGGAA
TAAAATTCCAAAAAAGTCGCTTTTTATTTCATTGATTATTCTTTTTGGAATACTCTTAATGCAGT
ATTCAAGAATAACTTCTGTTTCAATAAATGACCTTGTATTAGGATTTATACCGGTTATTATTGCG
GCATTTTCATATCCTTTAGGAAACAGAAAAACAATGGAAATTTCAGGCGGTAAATTAACAACATT
TCAAAGAATACTTGGAATGACCCTTTGCAGTATGCCTGCATGGATTTTACTTTTATTCTATGGAC
TTTTAAGGTCTGGTATTCCAAACAACAACCAGATCATGCAGTCGTTTATAGTTGCATTGTTTTCA
GGAATAATTGCAACATGGCTTTTTTTCAAAGCAACGGACATAGTAAGAAAAGATATGAAAAAAC
TTGCCGCTGTCGAATCGACACAGTCTGGAGAAATAGTATTTTCAGTACTTGGTGAACTTATTCTA
CTGGGCGGTGCATTTCCCGACATTTTTGCAAATTTAGGGCTGCTTGTTGTAATATTTGGAATGGT
AATTCACAGCACTTACTCTTCGAAATTAAGCAACTGAAGATCAAAATGTCGGGCAAAGAACGAAC
AAATAATTATTTAATAATATTCCTAAATATTTAACTAAAATAATTAAATGAGAATATTTAAAAA
AATTTACTTAAACTGACTTATTTAGTGGTGCCCATGAGAAGATCGAGAATTGGAATGATTGGAAC
TAAATTGCTGATTTTTTCAATAATTATTGTTTTAATTCCAATCGTTATGATGAGTTATGTATCTA
CAGATACAATTTCAGATTTGATGACTTCAAATCAAGAATCTGAACTGGATATAAATTTAGGGAT
ATTAAATGAACGAATGGACAGCGTTCTTGTTGAATTTGACACAATGACTGCATATACTGCAAACC
TTCCAGTTGTTGTTACTGCTGTTAAAAATAATGATAAAGATACGCTTCAAGATTTTGCAAACGGC
CTTGAAGACCAGTCTTGGATCGATTTAGCAGTTTTCACTGATGCGGATGGAAACGTAATCTGTGC
AAGCTATGGAGACGATAATGCAGAAATAAGCAGTTACGTTAAAAAATTATTAGAAAAAAATACC
ATATATGCATACGATATCCTTCCAAACGAAGAAGCTTCAAAATATAGCGAGTATGAGCTAGATGG
GAACGATGCACTTACAATTTTTACGGTTGCACCAATATATGATGGAGATTCCCTTATAGGTACTG
TAACCTACGTAGATATCATGAATAAGGATGATTACTGGGTTGACCGAGTTAAAGAAGTTACAGG
TGATGAAGCTTCAATATATCTTAACAACGTAAGAATATCTACAACCTGCATAAAAGATGGAGTAG
ATTTTGTTGGTGAACTTGCAGATGAAGACATATATGAAACGGTTTCAAAAAACAGGACGTTAGG
AGTACCGTTACCATAAATGGAGTGCCATACCTTGCTAAATATTCCCCAATATACAATATTGACGG
CGAAGTAATAGGAATGACCTGTGTTGGAACCCCAAAATCGCCATTTACGGCATTATTAAATTCAG
TAATCCAGAAAATATTCTTTATCGCATTTTCAAGCCTGATTATTGCAGTTTTCGTTGCAGTTATC

TTAAATAAAAAAATAGTTGACTCGATAAAAAAACTTAAAAACTCTGCTGAATCTTTTGGACGTG
GAAATTACGACGAAAGAGCATCAATTGATACAGGCGATGAATTAGAAGAACTTGCAGAATCGTT
TAATAAAATGGCTGATGAAATAGGGAAATCCGATAAAAAACTTAAAAGTGATGCACATACGCTT
AAAACTTCATTTGACGAATTAAAAGAAGTTGATAATTTAAAATCAGAAATACTGTCCATAGTAT
CACACGAACTTAGAACGCCACTTACTGCAATCTTAGGCTATGTTGAATTATTAAAAGATGAAACT
GCAGGAAAATTAAGCGAAAAACAGAAAGAATTCATATCTGTAATATCTGAAAACTCAGACAGGT
TAAAAAGAATTACAGACAATATGTCAGACCTTGTAACTGTTGATAATAAGATTTTGGACATCAA
ACGAGACAGAATAAACCTTAAAAAAGAAGTAAATGATATTTTAAGGTCTTTTGATTATTTCGCA
GAAAGTAAAAATATAGTTTTACTCGAAGATGTGGATAATCTGGATATTAAAGGAGATAAGTCTA
AAATTCATCAGGTTCTTAGTAATTTAATCGAAAATGCAATAAAATTCAGCAAAAAACAGACCAA
AGTTACAGTGATGGGTTTTGAAGATAAAGGAAATATCCACCTTGAAATTACTGATCAGGGACCAG
GAATTCCAAAAGAGCACCTTGGAAAAATATTTGATAAATTCTACCAGATAGATTCTTCTTCAAAA
CGAGAAGTTGGAGGATCCGGCCTTGGACTTGCAGTTTGTAAAAAAATCGTTGAATCACACGGCGG
ATCGATATGGGTTGAAAGCAAAATTGGAAAAGGTACAACAGTTCATGTAATGTTCCCATTAATA
AAATAGAAAACTTAAAAAAATTACTTTTTAAAAAAGTTAAATTTTAAAATTAATTAAATGAAAT
ATTTTCTTTTACTTCTTTTAAAACAGAATCTACAAAATCATCAAGCGTCATTTTCAAATTTTCTT
CACAAGATTTAATCTGTTCTCTTGAAACATTTTGAGCAAAACTTTTATCTTTTATTTTTTTCTTA
ATTGACTTTAATTCTGCTTTTTTTACGTCCTTGTCAGGCCTCATTAATACATAAGCTCGAATAAT
TCCGGAAAGCGCATCAACTGCCCATAATGCATGCCTAATTTCAGAATTTCTTTCAATATTTAATA
CATCATTGTGAGATTTTATGTCATCAATTAAATTTTTGGAAAGTCCTTCCTTTCTTAAAATTTCT
TCACCAATGATACAGTGTTTTTCAATGCAATCCCCATATTCTTCGATATCAATGTCGTGCAAAAC
TCCAGCTACAAACCAGTAATTCTGTTCAGTTTCATCTTTTTCTAAATATTTAGCTATTCCAAGCA
TTCCGTACCCAACAATTAAATTATGATCAAGATTTGACTGCTCTTTTACATAACAATTTAATAAA
TCTAGCGCTTTATTATTATCTGGTATCATAATAATCCTCCAAAATATGTAAAAAAGTAAAATGTA
AAAAAAGTAAATTTATGCAGCAGAGACTATTTTTATAATATCTCCATCATTTAATTCCTGATCGG
CACTGATTCTCAACTTTTTCTTTGCATCAACCGCGTAAATAAACTTCTGTCCAATTTCAGTGTGT
ATTTTAAATGCAAGGTCTTTTGCAGTTGCTCCTTTTTTTACAAGGTATGCGTCAGGTAAAACATT
TCCTTTTTTATCGCAGAATTTTCCTTCGTCTTCTACAGGGTAGACTACAATCATATCTAAAAGAT
CATAATAAGCTACATTGATTAAATCCTGAATTCCAGTTCCGCCAAATTTATCAAGGTAGCTTTTC
ATGTAGTTTAATGCATTTTTCTGTGCATCATTTAGTGATGATTCATCTAAAACTTCCATTGATTT
TCCATCATATTTTATTAAGCCTGCTTTCTGTGCATTTTTGAGTGCAAGTTCAATTTCAGCGGATG
TTGGAATAACTAAAAAGTCTTTAAATTCTTCTTTTAATTTTTCAATGTTTTTTTCTGCGTCAGGG
TGATCAGCCTTGTTTGCAGCAATAATCATTGGTTTTGAAGCTTTTCTTAAATTTGTTGAAACATT
GATCAAATCTTCTTCTGTCCATTTCATTGGACTTTCGTCAAACTCCTTAATTACTGCAAATACCTG
ATTTTCGGAAATATTTAATCCGCTTAACTGTTCAGAAAGCGCTTTTAAAAGGTTTTTTTCTTGCT
GTGCTTTTCTTGAAAGTCTTTCCCAGTTTTTCGTTAAAATTCCGTGAATCCACATATCCAGTTCA
TTTAACAAAAATTTAACATCTTCTACAGGATCGTTATTTTCAGAAGGATTTCCTTCAAGATCCGT
TTTTCCGCTTGCATCAACAACGAGTATAAATGCATCAGCCTGCCTTAAATCGTCTAAAAATTTGT
TACCCATCCCTTTTCCTTTGTGTGCATCAGGAACAAGTCCTGCAACGTCAATTACTTCAACAGGA
ATATAACGCATTCCAGAAAAACATTTCGAATTGTTTGGAGTACATTTTAAATTTAATGTGTCACA
TGGGCATGGCTTTGTAACAAAAGAAGTTCCAATATTTGGGTTTATCGTAGTAAATGGGTAGTTTC
CAATATCTGCAATTTTTTCCGTCATTGCATTGAATGTAGTTGATTTTCCAACGTTTGGTTTTCCA
ACGAGTCCTAATATCGCCATGCTATCACCGTTTTTATAATTGTTAGGTTATATCTTTGTTAAATT

AAATTTCTATAAAATCCCAAGGTTTTTCTTCGATTTTATTAATGTAATTATCCAAATCCACATTT
TTAATAAGTTCGATTGTGTTTCTTGACTCATTTAAAACTTTTGAAAGTTCACTTCCCCCTCTTGC
AAGAACGGTCTGTGCAACCATAGAATTGAATTTTTCAAAATCAACATTCACCTTTATCTTTCGCA
AACTCTTTTCGATGTATTTTATCTTCGTTTTTGAGTCCATGTCAAATTCAAAGTCCTCGAAGGGA
GTATGGGGCTTTGGAACCATTGGATTTATACTAACATCTACTTTTCGAACGTTATCTTTTATTTT
TTTCGTTAAATTTATTATTTCTTCAATATCCTCTTCTGTTTCATCGGGAAAACCAACCATATAAT
AAAGTTTTACGGTAGATATTCCGTGTGTTTTAGCAATATCAACTGCATTTAAAATATTTTCTTCA
GTAATTCCTTTATCAATTTTTTTTCGAAGAACTTCACTACCTGCTTCTGGTGCTATTGTAAGTGT
TTTCAAATTTAAATATTCAAGCAGTTCGTCAGTAATTGTATCTGCCCGCAATGATGAAGGCGAAA
TTGAAACGTTTTTTTCAGAGATATGTGCACACAAGTCTAATACATGTTTGTAATCGCTTACCGAT
GGCGCAATTAAAGAAACCTTGTCTGCATCAGTATTTTTTAGACCTTCATCGACCAGATACTTCAA
ATCTTCAATTTTTCGATATCTTGGAGGAGCGTATATGCATTTAGCCATGCAAAATTTACAGTTTC
TAATACAACCCCGTCCAATTTCAAGAAGATACGCTTTTCCATAAGCTCCTGATTCGTGCGTAACT
TGTGATATTGGGTAATCGTCTATCCTGAGTTTTTTTGGATATATTCTTTTAATTTTTTCATTGTC
GTATGTTTTTGGAACATATACTCCAGGAATTTCAAAATTACCATTTATCAATTGATACATTGTGT
CAGTTCCTTCAATTTCACCGACCACAAATACATCAAAAAATTCCTGAATGGGTAGCGGGTTTGAA
ATGGCACAGGGGCCCCCTGCAATAAAAATAGCATTTGGATTTTGAGCTTTTAATTCTTTTACAAT
TTTTACAACGTTAAAATAGTCGTTTTCGTACTGTAAAGTTATTAAAATAGCGTCATTGTTCTTAA
TTTTCGAGTAATTTTCTATAAAATACGCATTTGCAGAAATATCACGGTACTTATTTAAGTGGCTG
GTTAAAACATGCACTGCAAGACATGCAATTCCCCCGCCAAATCTATTTGGGTACACGATTGAAAC
GTTTTTAATTGCCATTTTACCGCCAAAATGAAGAATAGTATAAAAGTTAGTAGTTAAAAGAAGA
GATATAAAAATTTAGTTTTTCAGAATAAAAAAATTGGTGTCAGCTCGCCCATCTTTCATCATAAA
TCAGAAGCTACTCCGTTCATCATCCACCAAATGCATAAAAAGAACATTTAGTCTTTATGCGTCAA
TTGCATCATTAGTATCTCGGATTCACTATATAAAGGTGATTGTAAAATTAAACTTTACATTGTAA
TTTTTTGAATGCAACTTTGATACCATTAATTTTTAGCTAAATATTTTAAAGAGATTTTCCATAAA
AACTATCAAACTTTTAAAAAACAGGAACAAAAATGAATGAACTTGATATCATTAAAATTATTTC
AAAGAATCTTTCTTATTCAAATGGAGTTGAAAAAGGAATTGGGGATGACTGTGCTGTTTTTAAA
TTTGAAAATCAGCATTTAGTTGTAACTACCGACATGATGTTTAAATCAACACATTTTCCAAGTAT
TTTAACACCGTTTCAAATAGGAATGAGGGTTGTTACTGCAAATGTATCCGATATTGCCGCAATGT
GTGCAAAACCTCTTGGAATGGTAATTTCGATGGGTTTTGATAATCCAAAAAAAACATTTATCGAT
GAAATGTCAAAAGGGATAAATTTTATTTCAAAAGAATATGGATGTCCAATTGCAGGCGGGGATA
CAAATAAAGCGCCGGAATTAACACTTTCAGGAACTGCATTTGGAATTACCGATAATCCAATATAT
CGCGGTGGAATTATTGGTGAAGATATCTGCATTACTGGGGATGTTGGAAAAGTTAATTGTGCTTT
AAAACTTCTTGAAATGAAAGATAAAGGAATTTTGGGAAATATAGAGTTTGAAAAAACAATGTCT
GAATTTCCTAGGATCATGAAAAAACTTGCAGAACCTAAAGCAAGGGTTAAAGAAGGGCTTTTATT
AAATAAAATAATCACTTCGTGCTGTGACATATCTGACGGTCTTTCAAAAGATTTAAATTATACTG
GAAACTTTGAAATAAATTCAAAAAAATCTATTAAACTCCGTTTCAGAAGATGTTATTGAATTCTGT
GAAAAATTTGATATGGATTTATTAAAAACGGTTTTAAAAAGTGGTGAAGAATTTGAACTACTTT
TTACAACTCCTGATTTTAAAAAAGCCGAAGAAAAATTAAAAGATATAAATTCAGTAACAAAAAT
TGGAAAAGTCGTGGAATCTGGAAAAACGGTTGATGGAAAATCCGTGGAATTAGAAGGATATGTT
CACAAATGGTAGGTTGCTAAATAGGATATAGCATGACATGCAACATGATTAAACATGAATAAAA
AATTTAATGAACATTTTTTAATAATCTTAAAATACTTTACTTATTCATATATACTTTACCACATA
TTATGCCATTTTGAAAGTCATTTAACAAATTTTATAGCTTATCAAAGCTATTTATTGTTAAACCT

TTTAATAAACGATGTAATTCTGATTCAAAATCTTGTATATCTTCCAAATATCATAATTTCAATAA
CTGAACCGTGTACTGGAATGATACTGATTTCGGTATTATTGGCCTATATATTGACGGTTGAAAAT
AGGTTAAAGTATTATGTTTTTGGTTCATTATTTTGTATATTATTAATATACCTTGGAAACATATT
TAGAATCGTTATTATAGGAATCCTCGCAAATACGTTTGGAAATGGCGAATACATTCACAACACCG
TCGGTTTTGTGTTTTTTCCCACAATTACAGTTTTTACGATTTTGCTATGGTCGAAAATCAAAAAA
AGATTATAAACACGGTTGCTGATATTACTATATTAATATATTATTATACTGCGGTGGTATTTTTG
GAAAATTTACAGTTTATAAGGGTAAACACGTTGAAAATATCTTCTGAAGAACTTAAAAATCGAT
TGGAAGAAAAAGGAGTGAATTTAGAAGATACATTTTTAGATTATGTATTTAGAGTTGCAAAATC
GCCATTTTCTATGGGTTCAACTCCAGAATATCTTTTAGGGTACTACTTTTTACAGAGTATCTCAT
CAATAATTCCATCAATAACACTCAATCCATCAAAAGATGACGAAGTACTCGACATGTGTGCAGCC
CCTGGTGGAAAAACCACGCACCTTGCACAGTTGATGGAAAATGAAGGATCAATTCTTGCTGTTGA
AATAAACAAAAACAGATTGAAAAGTTTAAGGTCAAATATCAACAGAATGGGCTTTAAAAATACC
TTAATGATAAACACGAATGCAGTTAACATTGATAAAAAATTAAGATTCAATAAAATCCTTTTGG
ATGCTCCATGTACTGGAAACGAGATAAAAGACAGTAACCGGGTAAAAACAAAAAGAGATATATT
ATTCTGTGCAAAAAGACAGGTTGAATTATTTAGAACTGCGATCGAAGTATTAAAAGACGGCGGT
GAACTAGTATACAGTACATGTTCCCCCGAAATTGAAGAAGACGAAGATATCGTTCGATATATTTT
AAAGACAAATAAAAACATGGAACTTATTGAATTAAATGTAGATGATTTCCCAGGAATAAACATG
ATTGAAGGCGAAGTTAAAGGAACTTTAAAGGTAATTCCTCCAAATGAGCCATTTTTCATTGCGAA
ATTTAGAAAAACTAAAAATTAATTTTTCAAAAATCATTTTATCTAATTAATTACTTTTTTAGAG
GGTTTATTATGGATATTTTACAACAATTGCAGAAAGAATTTAATTTAAAAACATTTCAAGTCGA
AAACACTGTTAAATTAATTGATGAAGGAAATACAATTCCATTTATTTCAAGATACAGAAAAGAA
GCGACAGGTTCACTTGACGACGTTGTTTTGAGGAAATTTTTTGATAGGTTGAATTATTTAAGAAA
TTTAGAAGATAAAAAAGCCCAGATCATAAAATTAATTGATGAGCAAGGCAAATTAACAGAAGAA
TTAAAACAAAAAATTGAAAAATCTGAACTTTTAACGGAACTTGAAGATATTTACCGTCCATTCAG
GCCAAAAAGAAAAACAAGAGCAACTATTGCAGAAAGTAAAGGATTAAAACCCCTATCAGAATTA
ATTTTAAAACAAATTTTGGAAAAACCAATCGAAGAACTTGCAGAAGGGTTTGTTAATCCAAAAT
TAGAAGTTAATTCAATTGAAGATGCAATTTCTGGTGCCAAAGATATTATTGCAGAAGAAATTTC
AGATAATGCAGACTACAGAAAGTTTATTCGTGAAAATACATTTTCAAAAGGAATTATTTCTGTA
AAAGCAAAGAATGTGGATGAAAAATCAGTTTATGAAATGTATTATGAATATTCTGAAGCGATCA
ACAAAATTCCCGGACACAGAATCCTTGCAATAAACAGGGGCGAATCGGAAAAAGTTTTACAGGTA
AAAATCGATGCACCTGTTGAATTTATTCAAGATTGGATATTTAAAAATATAATTTTAGAAGACTC
GGAAACTTCTGAAATTTTAAAAGATACGGTAATCGACAGCTACAAAAGATTAATTGCACCATCAA
TTGAAAGAGAAATTAGAAATAGCCTCACAGAAAAGGCTGAAAACGGTGCAATTGAAGTCTTTTC
AAAAAATTTAAAACAGTTACTATTACAACCACCCATAAAAAACAAAACTGTACTTGGATGGGACC
CGGCATTTAGAACAGGCTGTAAACTTGCAGTAGTTGATGAAACAGGAAAAGTTCTTGATAAAAC
AGTAGTATATCCAACAGAACCACACAATAAAATTGCAGAAACTAAAAAAATGGTAAAAGAACTC
ATAATAAAACACGATATCGACGTTGTCGCGATTGGAAATGGAACTGCTTCGAGAGAATCCGAACA
TATTGTTTCTGAAATTTTAAAAGAAGTTGTAAACGACGTATATTATGTAATTGTAAATGAAGCA
GGAGCTTCTGTTTATTCTGCGTCTGAACTCGGCTCTGAAGAATTTCCAGAATACGATGTAGGAAT
TAGAAGTGCAATATCAATTGCAAGAAGGCTTCAAGACCCATTAGCAGAGCTTGTAAAAATCGACC
CAAAATCAATTGGTGTTGGGCAGTATCAGCACGATATGAACCAGAAAAAATTATCTGAAAGTTT
AGGGGCTGTTGTTGAATCTTCAGTAAACTCTGTTGGCGTTGATTTGAATACCGCGTCAGTTTCGC
TTTTAAATTACGTATCAGGAATTAATATTGGAATTGCAAAAAACATTGTTGAATACAGAACAGA

AAATGGAAAATTCGAATCCCGAAAAGAACTTTTAAAGGTAAGTAAACTCAGTAAAAAAGCATTT
GAACAGTGTGCAGGGTTTTTGAGAATTCCTGAAGGTAAAAATCTATTTGATAATACGGGGGTCCA
CCCAGAATCATACAAAATTGCAGAAAATTTACTTAATGAGTTAAATATCTCTATAAAAGATATA
AAAGAGAAAAAAATCGATAAAATTTCTGAAAAAGCCGATATTGAAAAATTAGCTGAAAAACTGA
ATTGTGGAATTCCAACACTTGAAGATATAATAAAAGAACTTGAAAAACCAGGAAGAGACCCAAG
AGAAGACCTTCCAAAACCAGTTTTAAGAAGCGATGTTTTAGAACTTGAAGATTTAAAAGAAGGA
ATGATATTAAAAGGAACTGTTAGAAATATTGTTGATTTCGGAGCGTTTATTGATATTGGCGTTCA
CCACGATGGATTAGCACATATTTCTGAAATTGCTGACCGATTTATAAAACATCCCCTTGATGTAA
TCTCTGTTGGAGACATTGTGGATGTTATGGTGATGGACATTGATTTTGATAAAAAGAGAGTTTCT
CTTTCAATAAAACGTGCAAAATAATTTAAAATGAATAATAAAATTAAATATTAATCTATTTTTT
AATTACTCATCAATTGTTACTTTTTCAATAATTACGTCTTCTACTGGCCAGTTTTGATACTGCCC
ATTATTTGCAGTAGCTACATTTTCAATTTTGTCAAGAACATCGAAACCGTCAGTCAGTTCACCAA
ATACTGCGTAACCGTAATTTTGATCATTTTGATAATCCAAATATGAATTATCAACTGTGTTTATG
AAAAACTGGCTTGTTGCGGAATCTACAATGTTGGTTCTTGCCATTGCGATAGTTCCTCTTTTGTT
TGAAAGTCCGTTTTTAGCTTCATTTTTAATTGGATCGAGTGTTTCTTTTTTAGTGCCATTTGCGG
TAAACCCGCCACCCTGAATCATGAAATCACTTATTACCCTGTGGAAGATTGTTCCTTCGTAAAAT
CCTGATTCTGCGTATTCTTTGAAGTTTTCAACAGTTATCGGTGCTTTATCAGGATAGAGTTCAAA
AGTCATGTTTCCGTAATTCGTCTGAATTGTTGCATAAACTATTTCTCCAGTTTCGTCAGTGTTTG
ATGAATCAGTTTCAATACATCCTGAAAAATTAGTTAAAAGAACTATTGATGCTATAATTAATAA
AATAGCGTTTTTAAAATCCATATTTTCACCAGAATACTAACTAAAATAAGTATAAAAAAATAAT
TATTCGATTGTAACTTTTTCAATAATTACGTCTTCAACAGGCCAGTCTTGGAAGTATCCTCTGTT
TCCAGTTTTAACTGCTGCAATTTTATCAAGAACTTCAAAGCCGTCGATCATTTCACCAAATACTG
CGTAACCGTAGTTTGAGTCATTTTGGTAGTTTAAAAACATGTTGTCAACAGTGTTTATGAAAAAC
TGGCTTGTTGCTGAATCTACAACGTTGGTTCTTGCCATTGCGATAGTTCCTCTTTTGTTTGAAAG
TCCGTTTTTAGCTTCGTTTTTAATTGGTGCAAAAGTTTCTTTTTGAATTCCATCTTTTGTAAATC
CACCACCCTGAACCATGAATCCTTTTATTACTCTGTGAAAGATCGTTCCATCGAAAAATCCAGTT
TCTGCGTATTTTTTGAAGTTTTCAACAGTTATTGGTGCTTTATCAGCAAATAGTTCTATTTTAAT
TGTTCCAAGTGTTGTTTGAATTGTAGCAATCATATTTTCACCAGAAAATAAATTTTTTAATCAAT
TTTTCAAAAATTAAATGTTTCAGATTATTTTTAGAAAATTCCCGTATAAATTATTTTTTACTTGA
ATTAATTAACAATTAAATCAGTCTTTCATTATTTTTACGTAAAATTTCCTGCTTCTCGGCCCATC
AAACTCGCAAAAATAAACTCCCTGCCAGGTTCCAAGAATTATTTTTCCATTATTTATTATCAAAG
TTAAGGATGGCCCAAAAAACGAACTTTTTAAATGGGCATCTGAATTTCCTTCGTAATGTGAAAAC
CCATAATTTTCAGGAATTTTTTCATGTAAAAATTTAACAATGTCTTTTTTTACAGAAGGGTCTGC
ATTTTCGTTTATTGTTATTGCAGATGTCGTGTGAGGTGTAAAAACTATCGCAACCCCATTTTGTA
CTTTTGATTTTACAACTGCATCCAATATAGTGTCAGAAATATCCACTAAAGACTCTCTTTTATCC
GTTTTAATTGAATATTCAAAAAACATGAATTTCCCCCAACTCTAAAAAACATTATTTAAAAATGG
GTTTTAATAATTTTTAAAATATTTTAGAATAATTACAATTTGTACCTTAAAATTGCAGCCATTCC
TTTGAAAGCTCTTGAAATCTGGCTTCCTTCTTCAGTATCTGTTGAAACTACAATTAAATCAGTAT
TGTGCATATTACATAGATCCGATAAATATTCAATAATATCTTTCTCTTCGTCAATATACATTGCA
CCGCCACATGTTGGGCATGCTTTGGTCTTTAACTGTTCTTCAAATTTGAACATTTCGTTTGTTTT
TACATTTACTTCTTGTGTAATATCACAGTTATTACATTTTACGGTAACCCTTGTAATTCCAATAT
CTTCAGTAACAATGAGCGTATCAATTGCACCCATTTCAAGATATTTCATTACCTGAGCTTCACCA
TAAGCTGAAAGTCCACCATCATCTTTTATCAGTTCTTTGAAGAATCTCTGGATTAAATTTTTCTC

TTTCATCAGATCAATATCTCTTAAGAGATCCGATGCCTTATCCAAAAGTTCCCTTATTCCAAACT
CTTCGGTGTAGCACAAATCAAAGGTATCGATAATTTTTTTCTTTAATTCGTGGTGAAGGTAGTCT
TTTTCAGCAAATTCGTTTTTGGTGTTTCCAGGACCTCCTAAAAGAAGACCTTTTAATTTTTTTTC
TTCTAAAATCGGCATGAAAGATTCTGTTGCGTATTCTCCAATTCTTACCATGAACTGGTGTGCAG
CATCATCGATCAATCTTTCAAGCCTTCTTGCAGACTGACCTCCTGCCTTAAACTTACCTGGAACTC
CACTTGTAAGCTTCTTTAATATAGTTATTGTTTTTCCACGAACGGTTCCAATTGTAGCCTCGTTT
CTATCGACTAAAATTACACCATAAACTTCATTATCCTGAATAAAGTCTTCCAATGGATCTGTGTA
AAATAAAGAGTCACACCTGTAAACAAATGTTTTTACAGGTTCAGGAGGTTCAAGAACGTAAACTT
CCATTTTTTCGGTTCCAGGGCCCCCTCTTGGAATCATTCCTGCAAATATAATTACCCCCATTTCGA
GAGGTTCCTTTAAAAGCTTTAATCTCTGCAAAATTACCTCGATTGCAGATTGTACGTTTTTCATT
GTCGTTTTGCTTTTAATATTTGAAGATTGAGAAAGCTCCTGCCTCAAATACTGGGAAATATCAGA
AAGCCTCCTTCCTGCGGGAATATAAACACTTATAAGCTCTGTTCCTTTACCCTTTTTACCTTTAAG
TTCTTTTAATGATTTTTTGAATAAATACAAATCAGTTGATGAATTTCCGCTCATATGTCCACCTT
AAAATAGTCTTCGCGTTTGTATATGCCGATTTCCGTAAATTAAGAGAAATGGATTTAAAATCACG
TGTAAGCTTGAATTATATTGAATATTTTTAAAAACTATATATAGTTTTCTCTATTATATATTAGA
TACATGTTTTTTATTCTGATATTTTTATTTTTAATTTGAGGAAATATGATGGCGAAACCTAAAAA
GACGATACCGGCAAAACTCTCGGATGAGAGAATTGTAATTTACGATAAAGATGGAATATCTCGTT
TAAACGAAAAAAGATACGGAGAACTTCACGAAAACTTTCTTTCACTTTCATTTGTGGAAGGGCTC
TACCTTGTATCAAAAAACTGGATATCCTTGAGAGATAAAAATAAAAAACTGCTCTCTTTTGAAGA
ATTATTCGATGTTGCACAGAATATCGATAGAAAATTATGCATTCGATATTTAGCTTATAAAGACC
TCAGAAATCGTGGATATACAGTTCGAACTGGTTTAAAATACGGTTCTGATTTTAGACTTTACGAA
AGAAGTAATATTGATGAGATTCACTCGAGATACCTCGTAAAAGTTTTTTCAGAAGAAATTCCTTG
CGAAATTTCAGAAATTACGGGATTTGTTAGAGTTGCACACTCCGTTAGAAAAGAATTAATTATTG
CAATAGTTGATGCTGATGGAAGCGTCGTTTATTACAACATGGGATATTTAAAGCTTTAATTATGG
GAGGAATCATGATGTCGCATATAATTACGCCTGAAGGGGAAAAAAAACTACTTTCTGATATTTTA
ATAGCATATGGTGTTAAGGAAGAACATGCAAAAATTACTGCAGAAATTTACACAGAAGCTGATT
TGAAAGGATTTACTTCACACGGTATTGGAAGATTTCCACAAACAATAATTGGTCTTGAAACTGAA
AATATAAAAACTAACCCTGAAATAAAAATAATAAAAGAAAGTCCTGCAATTGCAACAATAAACG
GGGATTTAGCATTGGGTTATGTTACAGCTGCAATGGCAATGGATTTAGCAGTTGAAAAAGCAAA
AAATGTAGGAATCGGCGCAGTTGCTACATATAATTCAAATCACTTTGGAATTACTGGACACTATT
CTGAAAGAGCTTCAAAAAAAGGAATGATTGGAATTGCAATTACAAATACTGAACCTGCAATGGCC
CCATACGGTGGAAAAGATAAAATATTAGGTACAAACCCGATTGCAATTGCAGTTTCTGGAAAAA
AACACAAATATTCACTAGATATGGCAACAGCATCAGTTGCAAGAGGAAAAATCTTTGAAGCAAA
ACGCCTTGGAAAAACACTTCCTGAAAATGCGGCAGTAGATATCCATGGAAATATCACAACTGATC
CAGATAAAGCACTTGCAGGCAGTATTTTACCATTTGGTGGAATTAAAGGATATGGAATTGCAATG
GCTGTTGAAATTCTCTCTGCAATTGGTGGTGCAGAAATTGGAACAAACGTAAAAGGAACCGCCAG
AGCTGATGAAAAATGTACAAAAGGGGATTTGTTTATCGCGATAAATCCAGAATTTTTCGGTGAA
AAAAGCGAATTTTTGGATAAAACTGACTTTTTAGTCGATGAAATAAAAAATTCAAAACTCTCTG
AAGGATCTTCTGAAATTTTAATTCCGGGTGATGTTGAAGCTAGAACTTCTGAAAATAAGAAAAA
TGGTTTTGAAATTGATGAAATACTATATAAAAAATTAAAAAATATTTGCGATAAAAAAGGAATT
ACTATTGAAAACTACCTAAAATAATTCTTAATTTTACCTCTTTTGATGAAATAGTATTTCTAAAT
ATAAAATTATATAAATAGATTCTTTTTATAAATTATAATATCAATAACTCAATTCTTATCCAAAT
TTTGGAGGGCATTTTCATGAAAGCAGATGCAGTGAAAATTAGCGAAGGAGTCTACTGGGTCGGA

ACTTACGACTGGGATATCAGATCATACCACGGGTACACACTAAAAGGGACTACATATAACGCGTA
CCTTGTATTTGGGGATGAAAAAGTTGCAGTAATTGATAACGTATATCCTGGAACCTCTGCCCAGA
TGTGGGGAAGAATTAAAGACGCTTTTGAAAAAGAAGGAAGAGAATTTAAAATTGACGTAATTGT
TCAAAATCACGTTGAAAAAGATCACAGTGGAGCACTTGTTGAAATTACTAAGAAGTTTCCAGACG
CTCCAATCTACTGTACCGAAGTTGCAGTTGAAGGTTTGAAAAAACACTACGCAGGACTTAAAAAT
GCGCCATTTAAAGTTATAAAATCGCTTGAAAGTGTTGATTTGGGTGGAAAAACACTTACATTCTT
GGAAGCTCCACTTTTACACTGGCCTGACAGCATGTTTACACTCTACGGTGAAGAAGGAATTTTAT
TCTCAAACGATGCATTCGGACAGCACTTGTGCCTTACCAAAAGATTTGATAACGAAATTCCTGAA
AATATCTTAATGGAGGCAAACCAGAAATTCTATGCAAACTTAATTACTCCGCTTTCAAAACTTGT
ATTAAAGAAATTTGACGAAGTGATTTCACTAGGATTACTTGAAAAAATAACGATGATCGCACCTT
CCCACGGCCAGATCTGGACTGACCCGATGAAAGTAATTTCTGCATACCAAAATTTTGCAACAGGA
CAGTGCAAAGACAAAGCTACAATTGTATATGATACAATGCACTATTCCACTCAAAAAATGGCTCA
TGCATTTGCAGAAGGGCTTTTATCCGAAGGAATTGAAGTTGTAATTTATAACTTGCACAGTGATG
AAAGAAGTGAAATTGTAAAAGATATTCTCGACAGTAAAGCCGTTTTATTTGGAATTCCAACAAT
TAATGATCAGCCTTTTCCAAGTATCGGGGATTTAATGTACTACTTAAGAGGACTTCGATTTGATA
GGACAGGATTTAAGAAATTAGCTTTAACCTTTGGTTCTATGGGCGGAAGAGGCGGTGCAATTGAA
AAAATCGCAAACGAACTCAGTAGTTCCGGTTTTGATGTAGTTAATGAATATGAATTATACTACAT
TCCAAACGAAGACGAACTTGAAAAATGCTATTCCCTTGGAAATGAGCTTGGGAAAAACATAAAAT
CCATTTAATTTATTTTTTTAATAAAATAACTTTTTTTAAACATTATTTTTAAAATTTAAAGATTT
TGCAACCTTAAAAAAACTGTTTCGAATATCGTCATTTTTTACAAAATTAACGCGGATTTCGTAAG
GTTAACCTAATATTTAAGCCCCGTTATAGGTATATTCAAGAAATAATAGCAGGTGAACAATATGA
AAGCAGATGCAGTGAAAATTAGCGAAGGAGTCTACTGGGTTGGAACCTACGACTGGGATATCAGA
TCATACCACGGGTACACACTAAAAGGAACTACATATAACGCGTACCTTGTATTCGGGGATGAAAA
AGTTGCGGTAATTGATAACGTATATCCTGGAACCTCTGCCCAGATGTGGGGAAGAATTAAAGACG
CTTTTGAAAAAGAAGGAAGAGAATTTAAAATCGACGTGATCGTTCAAAACCACGTTGAAAAAGA
CCACAGTGGAGCACTCGTTGAAATTACCAAGAAATTTCCAGATGCTCCAATCTACTGTACCGAAG
TTGCAGTTGAAGGTTTGAAAAAACACTACGCAGGACTTAAAGATGCACCATTTAAAGTTATAAA
ATCTCTTGAAAGTGTTGATTTGGGCGGAAAAACACTTACATTCTTAGAAGCTCCACTTTTACACT
GGCCTGACAGCATGTTTACACTTTACGGTGAAGCGGGAATTTTATTCTCAAACGATGCATTTGGC
CAGCACTTGTGCCTTACCAAAAGATTTGATAACGAAATTCCTGAAAATATCTTAATGGATGCAAA
CCAGAAATTCTATGCAAACTTAATTACTCCGCTTTCAAAACTTGTATTAAAGAAATTTGACGAAG
TGATTTCACTAGGATTACTTGAAAAAATAACGATGATCGCACCTTCCCACGGCCAGATCTGGACT
GACCCGATGAAAGTAATTTCTGCATATCAGAATTTTGCAACAGGACAGTGCAAAGACAAAGCTAC
AATTGTATATGATACAATGCACTATTCCACTCAAAAAATGGCTCATGCATTTGCAGAAGGGCTTT
TATCTGAAGGAATTGAAGTTGTAATTTACAACTTGCACAGTGATGAAAGAAGTGAAATTGTAAA
AGATATTCTTGACAGTAAAGCAGTTTTATTTGGAATTCCAACAATTAATGATCAGCCTTTTCCAA
GTATCGGGGATTTAATGTACTACTTACGAGGACTTAGATTTGATAGGACTGGATTTAAGAAATTG
GCTTTAACCTTTGGTTCTATGGGCGGAAACGGTGGAGCTACAAAACTTATCGGAAACGATCTAAA
AGAATGCGGATTTGAAGTCCTTGATGATTCTTACGAAGTACTTTACGTTCCTGAAGAGGATGAAC
TTGAAAAATGCTACAACGCAGGAAAAAGACTTGGAATGAAACTAAAATAATATTAAATTAATAC
ATCTGAACAGAAATTAATAAATATATCTAATTAAAGTAATATACCAGTAAATACATTATAATTT
AAGATAAAACCTAAAATATACTTTTTATAATAGATAAAAACAATACAAATTTTGAAATTAAAAA
TAGAAGTAAATTTCAAAATATATAACGATATAAGGTGAAAATTATGGAACTTATAAACGAACAC

AAAATCGGAATTTCAAAAGGTACTGCACTTGAAAAAGAAGTTGGCGGAAACGTTAAAGGGGAAT
GCATGGAAGTTGGAATGTACCTTGCAATGGCAAGACAAGCACAAAGAGAAGGTTTCCCCGAAATT
GCAGAAGTTTTAAAATCAATCGCATTTGAAGAAGCAGAACATGCTTCAAAATTTGTAGAAATGA
ACGGAGTAATTAAAGCTACGCTAAAAGAAAACCTTGAAATGATGTTTGAAGGCGAAACTATGGC
CAACAACGAAAAAAAGTCTGCTGCAGATTTAGCTGAAAAAGAAGGCCTTGAAAATATCCATGATT
TCTTTGAAGAAAGCTCAAGAGATGAAGCAAGACACGCTAAAATGTTAAAAGGAATTTTGGATAG
ATACTTTAAATAATTCCTTTAATTTTTTAAAAAAGGTTAATTACATTATTCTATTTTTTAAATTT
TCGAAAAAATTAATAAAATTTTAAAAAAATTATTTTAATTGCTCCAATATAATTGCTGGACATA
CCTTTTTAATTCCTTTAATTTTTCCAATTTTATTAAATATAATCTCTGAGAGTTCTTTTCCATCT
TTTGCCCAGATTTCAGTCATTATCATGTGATCCCCTGTTGAAGTAAAGACCTTTTTTACTTCTTC
AAATTTACAAAGCTCCTGTGCAACCGAAAGGAAGTCTTCAGGGTTTGTATCAAAACCAGTTTGTG
CAACAACATTGTAACCCATCTTTGGGGTGTCAACCATTACAGAATATCCTTTTATAACTCCTTCT
TCTTCCATTTTTTTAACTCTTTTTCTAACTGAACTTTCACTCGTTTTCAATTCCTTTGCAATTTCG
GTAAATGGTTTTCTTCCATCAGTTATCAAAAAGTCAAGTATTTTCATGTCTTTTTCATCCATTGT
TTTCACCAGCACATATCTAAAAACTAAAAAAATCCTTATTTCATTGTTTTTTATATTGATATCAA
CCTATATATATACGTTATACATTATTATTATGATAGATATCTGACAAATTTAGAATTTTTTCGGA
AACTTTTTTACCGGCATGTTTTTAAGTGAAGTTATGTTTTTATTAAATGATAATTTGGCGAAAAT
CGGTGAAAAAAATGGACTTTGTAGCAAAAAATCTTACAAAATTAAGGGAAACAAACCCACTTGTTC
AAAACATTACAAATTACGTGGTAATGAACTCTACTGCAAATTCACTTTTGGCACTCGGAGCTTCT
CCAGTAATGGCCCATGCAATGGATGAATTAGAAGAAATGGTTTCAATTGCAAGCGCATTAGTAGT
AAACATTGGAACTCTTGACGAATACTGGATTCCATCAATGGAAAAAGCTGCAAAAATTGCAAGTG
ACCTCAAAAAACCAATAATTCTCGATCCAGTGGGTGCAGGAGCTACTAAATTAAGAACAAAAACA
GCTTTAAAAATACTCGACTTTGCAGATATTTCAGTTTTAAGGGGAAATTTTGGTGAAATTGCCGC
AGTTTTAGGGGAACACGGAAAAACAAGAGGCGTTGATAGTGCTGCATACGATAACAACGAAGCT
ATCGAACTTTCCAAAAATGCTGCAAAAGAATTCAACACGGTTTCAGCAGTAACTGGCCCGGTTGA
TCATGTAAGCAATGGAAAAGAAATTTATGCAATTTCAAACGGACACCCTATGCTTTCAAAAGTTA
CTGGAACTGGCTGTGCAACTACATCAATTATTGGTGCATTTTCCGCGGTTGATGATCCACTGAAA
GCTGCGGTAAGCGGACTTGTAGTTTACGGAATTTCTGCTGAAATGGCATTTACTGAAGCACCGTA
TCCCGGAACATTTCAGGCAAAAGTATACGACTGGTTATACAGAATCGATGAAAAATTAGTTTTAA
AAAAAGCTAAGGTGAATAAATTTGAAATTTAAGGATAAATTAAAATTTTACGTGATAACTGATA
GTAATTATTCAGATGAAGTTATATCTATTGAAGAATCTCTAAAAGGGGGAGCTACTTCAATCCAA
CTTCGGATGAAAAGTTCAAGTACCCGTAAAATGATTGAAGTTGGAAATAAACTCAGGAAATTAA
CTTCTGAATACGATGCATTGTTTTTTGTAAACGACAGGCTCGATGTAGCGCAGGCCGTAAATGCT
GATGGAATCCACGTTGGAATTGATGATATGCCTGTTTCAAAAATTAAAGAAATTGCACCTAATTT
AATAATTGGAGCGTCCGCATATAACCTTGAAGAGATGAAAACCGCAGAATCAGAAGGTGCTGATT
ATTTAGGGGTTGGTGCGGTTTATTCGACAAACACAAAACTTGATGCGAGAGACTTAGGAATTAAT
GGATTAAAAGACATTTCAAAAATTGCAAATCTTCCAATTGTTGCAATTGGCGGCATTAATCATTC
AAACGTTGAAAATGTATTAAAATGCGGAGTATCTGGTGTTGCTGTTGTTTCAGCAATTGTTGGTG
CAGAAAACATTTTAAAATCTGCGGAAAACATGAATGAACTAATTAAAAAACACATAAAATAATT
ATTTTTCATTTTTTTAAGTTACTTAAAAAAACCATATTGCCGTTGAGGACGTTTTCTGAACTATT
TTAAGATATTTTGACGATTTTAGGAAGGATAGTGTTAAATATATTTCAATGCCATTATTAATTA
GAACTAAATCTGTTAAAATTCAGAAAATTTCAAACGTGAGAGTTATGACTGTAGAAATTATCGT
TGATAAGGAAAAATGCACTGGTTGCGGTAAATGTTACGATAAGTGTCCAAAAGGCCCTAGAATTT

ATAAAAAAGATAAAGACGGAAAATATTATGCATTTGATGTTTCAGATTGTCACAACTGTAAAAT
TTGCGTTGCAGCATGCCCTGTTGGTGCAATACAGGTTAATTTGAAAAGACATATTCTTGAAAAAC
AAGTGAACAAATTAAAAGAAAAATTCCAGTGATTTCATTCAACTTTTTTAATGAATCCAAAAAT
ACATATAACAATGTTTCAAAACACTTTTGTCAATAGTTATTTTCAGATCAATTTTAAAACCCCCA
AATTTAAAAATTAGACTTAAAAATTTACAGATTTACACCATATTTTTACGGTGATATTATCCAAT
CAACTACTTCAGAAAATTTAAAAAATCCAGAATATCCATTTGGGTTGAAAAATGATGGTGAAAT
ATTAGATTTATTTGAACCAGCAGTTAAAAAGTGGTGGTTGAATAAGTTTGAAGCCTATAAATCA
ATTAATAATGGATATTTTACCCCTCCTCAAAGACTTGCAATTCCAAAGATACATTTTGGAAGAAA
TACGTTAATTTGCAGTCCAACAGGAAGTGGAAAAACACTCAGCAGTTTTATTGCAATTATAAACG
AACTTTTTAGAATTGAAAAAGAAGAAGGACTACAAAATAGTATCTATTGCTTATACATCAGCCCG
TTAAAAAGCCTTGCAAACGACATCCACGTAAATTTGGATGAACCATTAACGGAAATTAAAGAAAT
TTTAAAAGAAGAAGGAAATGAAATTACTGGAATTAGACATGCAATAAGACATGGGGATACTTCA
AACTATGAAAAATCAAAAATGCTTAAAAAAACACCCCATATTTTAAATACAACTCCAGAAACGCT
TGCAATCATATTAAATTCCCCAAAATTTAAAGAAAAATTAAAAACAGTTCAGTGGGTAATTATT
GATGAAATCCACGCACTTGCAGACAATAAAAGGGGAGTTCATTTAAGTATAAGCCTTGAAAGACT
TCGAGAACTTACCAATCAGGAATTCTTAAGAATTGGATGCAGTGCAACAGTTGAACCATTAGAAA
TGGTTGCAGATTACCTTGGGGGATACTATGACGATGGATACAAAAGGCCCGTTGAAATTGTCGAT
ACAAGGTTTGTCAGAAGCTATGATTTAAAATTACTGTGTCCGGTTGATGATTTAATCAATAAAAC
TCCAAAAGAAATTTCTGAAAAACTCTACGAAAAACTTGATAGATTAATTCAAACTCATGAAAAT
ACCCTCATATTTACAAATACAAGAGGTGGTGCTGAAAGGGTATTATACAATCTTAGAAAGCGTTA
TCCTGAAAAGTACGATGAAACAAATAGCGGATGCCATCACGGAAGTTTATCCAAAGAAAAAAGGC
TTGAACTCGAAGAAAAGTTAAAAAGCGGAAACCTAAAATTTGCAACAACCAGCAGTAGCCTTGAA
CTTGGAATAGATATGCCATATATCGACCTCGTTGTACAGATTGGAAGCCCAAAAAGTGTTAAAAC
AATGCTTCAAAGAATTGGGCGGGCAGGACACGGAATTGGAAAAGTTGCAAAGGGAAGAATAATC
GCACTTGATAGGGATGAACTTTTAGAGTGTGCCGTAATGCTAAAAAAAGCTATGGACGGATTTAT
CGATAAAGTACACATTCCAAAAGGCCCGCTTGACGTTTTGATACAGCATATTTACGGGATTGCAA
TTAACGGATACATCGAACTTGAAAGAGTAAAAAATATTGTTAGAAGAAGCTATAATTACCACGA
TATTACTGATGAGGAGTTTAAAATCCTTTTAAATTACATGACTGCATCATACGCCGGAATGGATG
AAAAAAGGATATATTCAAAAATATGGTACGACCCAGACACTCAAATGATTGGAAAAACCGGAAA
AACTGCAAGAGCAATTTATTACATGAATATCGGCACAATTCCGGACGATTTCAGTTGTGACGTTT
ATCTTAGGGGGACCACGATTTGGATTGGAAAACTTGATGAGCAGTATTTGGACAGGCTTGAAAAG
GGCGATGTATTTACACTTGGTGGAGAACATCTTAAATTTCAATACAGGCGTGGAAGTAAAGTTTA
TGTCGATAAAACAAGCGAAAGACCAAATATTCCTAGCTGGTACTCTGAAAGGCTGCCGTTAAGCT
ACGACCTTGGAAGAAATATACTTCTTTTTAAAAAAGAAGCTGTTTCAAAATACAACGCAGGCAGG
CTCCTTGATCTTTTGAGCGAATACCCGCTTGATTCAAACGCCACAAAAAGTCTTTACGGGCTTTT
TAATGAACAGATTTTATATAAAGGAAACGATAGCGTCAGCACATTAAATAAAATGGTTGTAGAA
GGCCATATTTTCGACAAAAAAATGCACTACTATTTCCACAGCAATTACGGAAGGAAATTTAACGA
CGGATTCAGTAGGGCTGTCGCTTATGCATTAAGTAAACAGTACAAACTCGGAGTTTTGGTAAGTA
TTTCAGATACCGGATTTTCACTAGAATTTGCGAAAAATCAAAAAGTCGATATCGAAAGTGCAATT
CGATCATTAACTCCAGAAAATATAAAAGATGTATTGAAACAGGCCCTTGATGGAACAAACCTGCT
AAAAAGGATGTTCAGGATAAATGCAACAAGAAGCCTCATGATTTTGAGAAATTATATAGGGCAT
AAAAAATCTGCGAAAAAACAGCAGGTGAGTGCGGATATTTTAATAAGCTATGCAAAAAGCCTTG
ATAGATTTGCGCCACTTGAAGAAACACTGCGTGAAATAATCGAAGACTCACTAGAACTTGACAAT

ATTCGTGAAGTTCTGGGCGAAATTAGAAGCGGAAAATTAAAAATTAAAGTATTAGATGTTCCAA
TTCCCACCCCGATGAGCTTTGGAATCGCGACACTTACTGCAAGCGACTCAGTTCTTGCGGAAAAC
AAAAACCAGCTTTTAAAAGAGTTCCACCAGATGGTAATTAAAATGATTGAATACGAAAAGGGAA
TTTCGCTTGAGGATTAAATATGAAAATAAATGGACACGACATCCAGTTTAAAGATAATGCAGTA
TTTTTAGAAGAAAATAAAATTTTAATACTTGCAGATGTCCATGTTGGAATGGAAGAATTTATTC
GACGAAACGGTGTTTTATTTCCACTAAATGAAAAAAAAGAACTCCTAAAAAGACTTAAAAATTT
AATAGTTGAATTCAAACCTGAAAAATTAGTTTTTCTAGGGGATTTTCTTCACCACTTTCAAAAAG
TCCCATACAAAGTTTACGAAACGATTCGAGAATTAAATTCTCTTTTAAAAAATTATGAAATCATT
GTAATTCGGGGAAATCACGATATAATGCTCGAATACATCCTTAAAGAAAATACCAATTTTAAAAT
TTTAGATTATTTAATTGAAGATAACGTATTAATGATTCACGGGGATAAAACATTTGACGTTAAT
TTTAACTTTGATCTTTTAATCATGGGCCACGAACATCCTGTTTTAGAAATAAATAAGCAGAGGTT
TTCGGCATATCTTGAAATCATTCAAAACGACTTTGAAATACTTGTAGCACCAGCATTTTCAAATA
TTGCATCAGGTGTCAAGATAAACGAACTTGAAAGCAACTTCATGTCGCCCTACTTAAAAAAAGTT
AAAAAAGAAGATATTTTTCCAATTATTATTGGGGAAGAAGTTTTAAAATTTCCCGATCTTTCAAA
AATTGAAAAATTCCTTTAATTTTTAATTTTTAGTTATGTTCTATTTTGCTCTTGCTTCAGCGATT
TCTGCTTCGACTTCTTTTCCTTCGATATGTCTTAAAGCATCTGCTTTTGCAGCCCAGACATCTTTA
TTGTATTCATCTTCAATTACATTTATAACATAATTGTAGCAGTCGACTGCTTCTTCATACCTTTC
TAAATTGTAGAGCGCTTTTCCTTTCATGAGCCAGTATTTCGATTCTTCAGGATTTAATTGAATTG
CTTTTTCAAATAAATCTATCGATTCAGTATAATTTCCAGCATCGTACTGTAAAACACCTTCTGAA
TAATATTCTTCAGGGTTTTGATCAACACATCCTGCAAAACTTATTGAAATTAAAATACCAAGTAA
TATCAATTCTTTTTTCAAAATATCCCTCAATTTTTATCAATTTACTACAAAAACATTATTACAAA
AAATGACGCTAAAAAGCAGTAATATCCAAAGATTTTAAGTTTTCCGCCAATTACCATTTTTTTAA
CCAGATATAACCCCAAAAGTCCTGTTAAAAATGAAATAACAAATCCTAGTAGTTGTTCTGACGAA
AAAGTAATCTTTTGAAGTTCGAGTATTCCTGCGCCAAAAGTTACCGGAAGACTCATTAAAAATGA
ATATTTTACAGCGTCTTCTTTGTTCATTCCTAAAAAAAGCGCTGCAAAAAGTGTTGTTCCGCTTC
TTGAAATTCCAGGAAGCACTGAAAATGCTTGAAATACCCCAATAATTAATGCATCGACATAAAGA
ATAGATTTAATTGTTTTTAGATTTTTATTTAAATTATCTGAAAGAAGCATTAATATTCCCGTAAT
TGACAAAAAAACTCCAATTAATAATGTTGATGAAAAAACTGATTCTATAAAATCTCCAAAAAAT
AATCCGACAAAAACTGCAGGAATTGTTGACAAAATAAGTTTTGATGCAAGGTTTAGGTATTCCTT
TTCTTTTTTTGTAATTCCAGTTATTATTTCAAAAACTTCCGCCTTTACAAATATTAAAACCGCTA
GAAACGTTGCAAGATGTAAAAACGCAAAATACCCGACATCCGGCGTAGCATTAAAAAGTGCGGTA
AATATTGCAAGGTGTCCAGAACTTGAAATCGGCAAAAATTCAGTAAGTCCTTGAACTATTCCAAG
AATTAATTCTTCCATTGTTTATAACCTGATCATTACGTTTTGAGTTTCTAATGAATAGTAACTGT
TCAATTAAATACTTTTTTCCGTTACAAATAACTAAATACAAATACTTTAAATTAAATAATAAGTA
GTTAATTACACGATTTATTTTGTTTTATTTGGTGATTTCATGATTATTACGGTAGCTTCTGGAAA
AGGTGGAGTTGGAAAAACGACAACATCTGCAAATTTAGCGGTTGCGCTTTCCAAACTCGGCAAAA
AAACTATTGTAATTGATGGAGATATCTCAATGGCCAATTTGGGGCTAATATTTGATTTTGAAAA
AAAGAGACCCTCCATTCATGAAGTTCTCGCAGAAGAGTGTTCTGTAAGAGATGCGATTTACGAAC
ATAAAACTGGAACTTATGTACTTCCAGCAAGCCTTTCTATTGCAGGCTACAAAAAATCAGACCTC
GATTTATTCCCTGATGTAATAAATGAAATTGCTGATGAATACGATTACGTAATTATTGATGCTCC
AGCAGGTTTGAACAAAGACATGGCAATTCACCTTGCAATTGCAGATAAAATCCTTCTTGTAGTAA
CTCCGGAACTTTTTTCAATTGCTGATGCGATGAAAATTAAAGAAAGCAGTGAAATGGCCGGAACC
AATGTAATGGGAATTGTGTTAAATAGGACTGGAAAAGATTTCGGTGAAATGGGACCTGACGAAA

TTGAAATGATTTTGGAAGAAAAAATTGTTGGATTGATCCCGGAAGATGAAAATATTCGAAATTC
AACCCTTAAAAAAATGGATGTAATTGAGTACAATCCCAGATGCCCTTCTTCAAAAGCATATACTG
AACTTGCACTAAAAGTTACTGGATCATACGTTGATATTGGAAAATTGAAGAAATTTACAATGA
AAGTTTCTTTGAAAAATTAAAAAGAAGCATTTTTTCAAAATTTAAGAAATAATTACTCTTTTTA
TTCACTCAATACGGGGGAAACGATGGATTTTTAGCATTTTTAAAGGACTATCCTGCAGTCGCCA
CGCTCATTTCTTCAATAATTTATGCATTATTCACAATAATAATCATTGGCCAAAATTATACGTCG
GAATATGCTCGAAATAAGCCAAAAATTACAATTTATTCGGAAATAGATGAATATAATGCCATCT
ATTTAGTAATTAAAAATTTAGGAATGACTCCCGCATATTTCATAACATTTAACACGTTAAAAAAT
AATATACCTGGTACGATAATGAGTAATTTATTAAATTTAAACATTTTTAGAACTGGAATCGATA
TATTACCACCATATGAATCGATAAAATTTAAAATAAGTACTAAAAACGAGTTAAAAAACTTAAA
ATCTAGTGAAATCGATTTTGATATTTCATATAAAAATTTTAAAAATAAAATTACAAATGATAGT
ATACATTTGAATCTAAAAAAATTGGAAGATTTACCTAAAACTCCTGAAAGAATTAAATTAGAAG
CAGAATTTGATCTTAAAAATCAAGAACAAATGATTAAATTAATGCGAAAATAAATTTTATTTTT
TGCAATTTCCGCATTCAAATTTACATGAAACATCTGTTGGATATTCTTCGTTTAAACATGCTAAA
CAAAGGTCATCTCTTCCAATTGCTTCAACTAATCCTTCAATACTTAAGTATGCGACAGAATCTGC
GCCAATCATTTCAGCTATTTCTTCATCAGTTTTTGAATTTGCAATCAATTCTTTTGTTGTAGCCA
TGTCTATTCCATAAAAACATGGTGAAACAATTCTTGGGGACCCAATTCTTAAGTGAACCTCTTTT
GCACCTGCTTTTTTAGCCATCTTCATTATTTTTCCAGAGGTAGTTCCACGAACAATACTGTCGTCA
ATCAACATGACCTTTTTATCTTTTAAAAGGTATTTTACAGGGTTTAACTTTAATCTTACCGCAAG
ATCTCTTTCTTCCTGTGTTGGAAGGATGAACGTTCTTCCAATATATCTATTCTTAATTAAAGCTT
CGTAGTAAGGAACTTCTGCTTCTTCGGTGTATCCTTGCGAAAAAATAATTCCTGAATCTGGAACT
GGAGACACAATATCGACGTTTTCAGGAGCTTCCCTTGCTAAAATTTTTCCAATATTTCTTCTTAC
TTCGTAAACGCTTACCCCATCAATTACGGAATCAGGTCTTGCAAAATAAACGTATTCAAACATAC
AAGAAGATGCTTTTTCTTTTGCATTTGGTAGTTTGTAGGTGTTAAGTCCATTTTGGTCTACGACA
ACCATTTCTCCAGGAGCAACGTCGCGTTCAAATTCAACATCAACAATGTCAAGAGCACAGCTTTC
AGAACTGAAATAATATGCACCGTCATCTTTTCCAATACAAAGTGGCTTAAATCCGTTTGGATCCC
TTATTGCAATCAATGTATCATCATAGATAATTAAAAGAGAGTAAGCACCGTTTAATTTTTGCGTC
AAGTTTGTAACTGCAGTAATTATATCATCAGTTTTTAAAAGTTCTCGAACCAAAAGTTGAGCAAT
AACTTCGGAATCTGTTGTAGAAACAAAAATATGACCTTTCTTTTCAAGTTCTTGTTTTAAGTCTT
TTGAATTTACAATATCTCCGTTGTGAGCAATTGCAATTTTTCCAAATGAGCTGTTTACAACAAAT
GGCTGGCAGTTGTCAATTATATTTCCACCAGTTGTGGAGTACCTTACATGGCCAACACCAATGTG
CCCGTACAAATTTTGTAATTCCTTGTTTGAAAATACTTCTGGAACCAATCCCAATCCCTTGTAGT
AGCCTATTTCTTTACCGTTCCCAACAGCAATTCCTGCACCCTCTTGCCCCCTGTGTTGAAGTGCGT
AAAGACCATAGTATACTTTTTTGACAATATTACTTTTTTCATGAGAATAAATTCCAAAGATACCA
CACATGATTATCACAAAATCAGATTAATAGATTATATACTATTCGTAGAATGTTGATAAATATCG
ATAGAATAAATAAATTGAACTGGTATATAAGTGTTACAAAAATTGAGTTTAATCGAGTAATAAA
AAAGATGTTTAGATAATTATTTTAAGTAATAAAAAAGAAAGTGGGTATATTATTTTACTCTGTT
TCCTTTTCCGGTCACTTTTTTCCATTGCCATGCAAATCTTTTGATTTTTGAGCTTCTACCAAATCC
GCATGCTGCACATACTTTTTTTCTTACGTGGAATGCTCTTCTTCCACATCTTCTGCATACAATATG
GTTTGAACCTTTATTGTGTTTACCTTGGGATGGTGTTCCTTTTGTCATTTACTTCACCGTGTTTA
TCTTTACCTTATTCCATATATCGAGATCACATAAAAAATTAACTGTTGAATCAAAGGAATTATAA
TGAAACGTAGAGTACGTTGTCTCCCCTTACAACTAGCATTGGGAATTCTTTTTCTTCATCGTTTT
CAATTTTAGCATTTTCTAAAGCAACGTTCATGTGTAAGTCGTATGCTTTTAATCTTCCTTTAACA

ACTTTACCATCTTTTAAGTAAACTGTTACGTTTGTGTTAATTGATTTACCTAAAGCGTCTAATGG
TCTTTGTGTATCCATCATATTCGCACCTCGCATAATAAAAAAACAACTGCTAATTCTAATTCGTA
GAAATAAATAAATAGTTCTGATATATAATACTTTCCAAGTAATTACTTCTTATTTGTCATATGGT
GCTATATGGAGGTATAAACATGGCAATGACCCTAGCTGAAAAAATTTTGGCAAAAGCTTCAGGTA
ACTCAGAGGTTTCACCTGGAGACATCGTTATGGCTAAAGTAGAAACCGCGATGGTCCACGATATT
ACAGGCCCGCTTTCAGTAAATACATTAAAAAAAGAAGGTATTTCAAAAGTTTGGGACAATGAAA
AGATTGTGATTCCATTCGACCACCAGATACCTGCTGATAGCATCAACGCTGCTGAAAATCATATA
TTAATGAGAAACTTTGTAAAAGAACAAAATATCAAACACTTCTATGACATTAGAGAAGGAGTTT
GTCACCAGGTATTGCCTGAAAAAGGTCACATAGTTCCAGGAACTGTTGTAGTTGGTGCAGACAGC
CACACCTGTACATACGGTGCATTTGGTGCATTTGCAACAGGAATTGGAAGTACCGACATGGCTGC
TGCATTTGCAACTGGCGAATTATGGTTTAAAGTTCCTGAAACACTTTACTTTAATATTACGGGAG
AATTAAAACCAGAAGTAATGTCTAAAGATGTAATCTTGAGCATTATAGGAATGGTTGGAGCTGC
AGGTGCTACATACAAAGCTGCACAGTTTGCTGGAACCACTGTTGACAACATGACCATTGCATCAA
GAATGACAATGTCAAACATGGCTATCGAAATGGGCGGAAAAGCAGGATTAATTGCTCCGGACGAT
AAAACAATTAATTATGTAAAAAATGCAATGGAAGCTAACAACACTGCAAAACCATTCGAATTAG
TTCTTGGAGATAAAAATGCAGAATTTGAAGAAAAATTCGAAATCGATGTAAGCAACCTTGAACC
TGTTATGGCATGCCCGCACAACGTGGACAATGTAAAAGCTGTTAGAGAAGTTGCTGGAACTCCAA
TTGATCAAGTATTCATTGGTTCATGTACTAACGGAAGACTCGAAGATTTGAGAGCTGCTTTAGAG
GTTATTGAAAAACACGGCGGAATTTCAAAAGATATCAGAGTCGTTGTAACCCCTGCATCGAGAAG
CATCATGCTTGAAGCTATTGATGAAGGATTAATTAAAAAATTCTACCAGTACGGCTGTGTTGTAA
CAAATCCATCATGTTCTGCATGCATGGGTGCATTATACGGTCTTTTAGGTCCTGGAGAAGTTGGT
GTTGCAACATCAAACAGGAACTTCAGAGGAAGAGAAGGTTCGTTAGAATCAGACGTGTACCTTGC
ATCGCCAATTACTGCTGCAGCATGTGCTGTTAAAGGAGAAATTGTTGACCCAAGAGATCTCTAAT
CTAATTAAAACCTTATTTTTTTACTTTTTTTTAAATTTTAAAAATAAATAATAAAAAAGAATTT
TTATTTGAATGTTAATTTTTTCAAAATGTCAAAAGAACTTAAAGTTCCAATAATTTTATCATTTT
CGACAACAGGGTATGCAAAAACTTCATTTTCTATTATTTCAGAAATTAATTCGTCAGATAGTTCA
TCGTTTGGATTTATAATTTTAATTTCATCTAAAAAAAGCATTGCTTCTTCTGCTTTAACGTGTTT
GCATCCTGTGAGTAAATCTAGTGCAGTAACCCATCCAACAAGTTCTCCATCTTCAATTACAGGTG
CATAACTTTTTTTATTTTTATATAACGACTGAACTACTTCTCCTACAATGTCGCTTGGAGAAACA
TTTAAAAAATTTTCGTTCATGACTTCTTTTACAATCATAAAACCACCAAAAACTGCATAAAAAAG
ATTGATTATTATATACTTATTAATTAATGTGATTATTTTTTAAATGGTTCTTTTAAAACCATTAA
CCTATCAGTACATGTAAAACACGGATCGCAGCTTGCAATTATTAATTCTGCGTCACTTACATGGT
ATCCTTTTACAATGTCTTCCATTGCACCAAGATTTGTTGCAGTAGGAGTCCTGATTTTTGCCTGCC
TTACAAGCCCTTCATCATCCAATCCGTATGAATAGTATTGCTCTCCCCTTTGAGCTTCGGTGTAGC
AGTTTATCGGCTTAAATTCGTGTAATTCATAATTTGGATTGTAGATTCTTTTGTCTGCAGTGTCT
TTTAAAGCATCAAGTGCCTGTCTTATAATTTTAACACTTTCAAGGCACTCGTAAAACCTAACAGC
AAGCCTTGCAAAAACATCTCCGCTGTCAAATAATATTTCGTCAAAGTCGTATGGGTCATATTCTG
GAACCCAGCCCCACTTTCTCATGTCGGATTTCACGTTGCTACCTCTTGCAGTAGGCCCTACTGCGT
GGAATTTTGCAGCTGTTTTTCTATCCATTACTCCAACATCTTTTACTCTTGAAATAATCATCGGG
TCATTTAACATTCTTTCAAGAAATTTATTCAATCCTTCTTCGTATTTTGAAATTTTATCTAATAT
ATGAGGGATTTGAGTTTCAGAAATATTGCATCTTGGCCGAATTCCGCCAATGATTGGGCATGAGT
AGTGAACCCTACTTCCAGATATTTCAAAAAATACCTGCATTATTGGCTCTCTTAACATGAAACAT
CTCATAGACATTGTTTCATGGCCCAATATTTCAAAAGCGTGTCCAAAAAGTAATGTATGAGAATG

AAGTCTTTCTAATTCTTCCACGATAATTCTTATGTGGTTTGCACGTTCTGGAATTTCGATGTTGC
ACCCGATTTCAACAAGCCTTGCCGCACTCCAGAGGTGAATGTGTGAACAGATTCCGCAGATTTTT
TCGCATAAAATACATGCTTTTTCAACAGGAAGGCCCTCCATTAATCGTTCAACGCCCCTGTGATT
TACACCAATTGTAAGTTCTGCATCTTTTACTATCTCATCTTCAATAAATAATCTCAGCCTGTGAG
GTTCAAGCATTGTAGAGTGCACTGGCCCGATTGCAATTTCTCCTTCGTACATGATTTTTCCCTTTT
TCAAAAATAAATTAAATGAATCCTGATTTTGATAATTTAAAATTTAGAAATATCATTATCAATT
ATGATAATTAGTAACTAGTCATCTGTACTTATATTATTAATGATTATTTGTTTTCTCTATTTTAC
TGCTGTATAATATATACGTCAAACGTGCAAATATATATCAAGTTTTAAAACAAAAATTAAATTA
TACCGGGGAGGTGAAATCGAATGGTTTTAAAATCTAGTGAATTTAATCCTGATTTTCCAAAACAG
ATTATTGAATCTGGAGAATGGATATTCGGAGATCATGCGTCTTCTTTTCAAAAATGCTACCAGTG
CGGAACGTGTACTGGGGCATGTCCTAGCGGCAGAATAACAGCACTCAGGACGAGAAAATTAATAA
GAAGTGCACTTGCAGGAATTGACAGTATTTTATCAGGTGATGACCTGTGGATGTGTACTACATGC
TATGAATGCTACGAAAAATGCCCAAGGGAAGTAAAAATAACTGATATTATTAAAATTATAAGAA
ATATCGCTGCTGAAAAAGGATACATTGCAGAACCGCACAGGAAAACCAGCCTGCTTGTATTTAAA
ACTGGGCATGCTGTTCCAGTTAATGATGAAATTAAAAAAGCAAGGCTTGCGATTGGATTAACAGA
AATACCTCCAACAACCCACAAATACCCCGAAGCTTTAGAAATCGTAAGGGATATTATGGAAGACC
TTAATTTCTGTAAAAAAGTTGGAATTTGTAGAGAAACTATGGACTTAGAACCGTTAAATGTTCA
AAAATCTGAGGAATAGGGGTGAAATAGGATGAGTGAAACTGATTTTGAATACATGTTTTTTTA
GGATGCATTGCACCAAATAGATATCCTGGAATTGAAAGTGCAACCTATAAAGCACTAGATAAACT
TGGAATACAATTGCACCCATTTGAACAGGCTTCCTGCTGTCCTGCACCGGGAGTATTTGGTTCTT
TTGATTTAATGACCTGGCTTACAATTGCTGCAAGAAATATTGTAATGGCAGAACAGGCCGATCTG
GACATTTTAACAATATGTAACGGATGCTACGGCTCGTTATTTGAAGCAAACCATATACTGCAGGA
TAATGAAAATGCAAGAAATAAAGTAAATGAAGTTTTATCAAAGCACGGCCTTGAATATACTGGA
AAATCAAGAGTAAGACACTTAACGGAAGTACTTTATTTTGATTATGGTACTGAAAAAATTGCTG
AAAGAGTAGAACGACCACTCGACATCAATGTTGCAGTTCACTATGGATGTCACTACTTAAAGCCT
ACTGATGTAAAGCATATTGAAAGTTCAGAGGTTCCAAAATCACTTGATGGACTTGTCGAAGCAAT
CGGGGCAAAGTCAATATACTATAAAGACAAAAATATGTGTTGTGGAGCAGGTGGTGGAGTAAGG
GCTAGAAACCCTGAAGTTGCGCTTGAAATGGCTGAAACCAAAGTAAAAAATATAATCGCGGCAGG
GGGCGATTGTGTAACGGAAGTATGTCCATTCTGCCATTTACAGTTTGACAGGGGTCAAATTGAAA
TGAAAGAAACTTTTGGACGAGAATACAAGTTACCTGTTATTCACTATGCCCAATTACTTGGTATG
GCAATGGGTATGACTCCAAAAGAAGTTGCGCTTGATCTCCACTTTATTCCTGCAGACCCGCTACT
TGAAAAGCTTGGAATAGATATGGAATAAAATAATATTTAATCCTTATACGGAGGGGATTTTATG
AAAGAAGGTATTTTTTTCGGAGAAGGAATGAAATTTGTAAAGGATGACTACCCTGACATTTACG
ATGCGGTTGTCGGATTGAATAAAGCAGCATACACTGGAAAAGTTTTGGACTACAAAACTCAAAAA
TTGATTGCTATTGGAATCGTTGCGGCAACAGGGGATGAAACTGCAACAGAAAAGCAGATGCGAAG
CGGAATGAATGAATTAAAAATAACTAAAGAAGAAATAGTTGATGCATTGCGGGTTGTATTATTA
ACTTCAGGAATGCCTGCATTTACAAAAGCTATGAAAGTAGTTAGCAAATTATAAATGGGGAGAT
ATAATGGGCTGTGTAAGTGCTCAAACACTTAAAGAAGGAACTGATTTTGAAAAAAAACACGTTCC
AATGATAGAATGCAAAAATTCAGTTAAGCCAGATGAAATCTACGAAGTAAAAATCCATACTGCA
GGAGTAGAACATCCAATGGAAGACGGGCACTTTATCCAGTTTATTGAACTAAATGTAGAAGAGT
ACCCTCTTTTAAGAGTTCAGCTAACACAGCACTCCAAACCAGATGTTGTAGTAAATATAAAAGCT
CCAAATGAAGAACATAAAGGTAGAACGATTAAACTCGTTGCGACAATGTTTTGCAACCTTCATGG
GCTTTGGAAATACGAAAAAGAAGTAATAATTGAATAAAAACCAAATTTGGTGAGTTAATGATTA

CTACAAACCATCCTCTTGGCGAAGCGCTAAAAGATATTGAAAATTTCAAATTAGCATTGGCTGAT
TACTTTAAGGACAAAGACGTATTTCCAATAAAAAATAAAGTTGAACTTGGAAGTGCACTGCCTTG
CGGAATTTCAATGCCTTGTGGGGAAATAGAAGCTGGAGAGCTTGTTAAATTTGTAAATGATAGT
GATTTTCCGATAAAAAATTCAGAAGATCTGGCAGTGAAACTTTCTAAAAGTTGCAGTATTAAAT
AGTCCATATAACGGGGGTTTAAATATGGATGGTAAATTAAGATGCGAGATAGAAGAACAGATAA
ATAAAGAATTCTATTCAGCATACCTGTATCTTGCAATGTCAAATTATGCAGAATCAAATGGATTT
AAGGGAATTTCGAACTGGTTTATAGTTCAATCCCAAGAAGAACTTGATCATGCAATGAAATTTTA
TAATTACATACACAGCATGGGCGAAACTCTTGAATTGGGTGCAATTGATAAACCTGAACCAAGAT
GGAATTCGATTATAGCTGTCTTTGAAAATGGACTCACCCATGAAAAGTATGTTACACAAAGAATT
CATAAATTAATGGATATTGCAGATGAAGTAAAAGATTATGCAGCAATTTCAATGCTCCAGTGGTT
CGTAAATGAACAGATTGAAGAAGAATCTTCATTTAGAGATATTCTTGATAAATTGAAACTAACA
GGCGGAGATGTAAATTACCTTATGGTACTCGACGGTGAACTCGGCCAAAGAGTCAGAACACCGCC
TCAAAGTGAAACTAATGAATAATTCGATTAGGTGAAAGATATGGTATGGTGGAAATGTTCAAAC
TGCGGATACATATTTGAAGGCGAAGCTGGAAAAGTTCCTGAAAAATGTCCAAACTGCGGAGAAAT
CTGTACATTTTACGATGTAAGCTGTTATACTCCAGAATGTGGATTTGAAGGCTACGATCCAAAAA
TTGCTGGAAGAAGGCAGGAAGAAAGCAGACTATAATTCATTCATTATTTAATTTTTTTAGGGGG
ATAATAATGAAAACTTGTTTTTTGATATTTACTTACGATAAAGGTGGAAAACCATACGTTCCAGT
AATATTTCACGCACTTCTCTTTGCAAAAGAGATGAAAGAAAAAGGGGACGATGTAAAAATAGTT
TTTGAAGGAGAAGCCGTAAAATGGTTCAATGAACTATTAAAAGAAGATCATCCTTTAAAAAGTC
ATGTTGAAGCCCTGAAAGATAATTTTATAGCTTGTGAAGCATGTTCTAATATGTTTGGTGTTTTA
GACAGTATTAAGGGAAAAATAAAAATTGAAAATGACCTTCATGGGCATATCAGTCTTAAGAAAT
ATCTCGATAGCGACTACAAAGTTATCGAATTTTAAATCTATTACTTTTATTTAACTTTTTTTAGG
TGTATTTTATGGCAGTTACTTTAAAAGAAGGCGTCTATTGGGTAGGGGCGATCGACTGGAATATC
AGAGAATTTCACGGCTATGAAACTAAAGACGGTTCCAGCTACAATTCCTATCTTATTAAAGATGA
AAAAATTGTTTTAATAGATACTGTAAAAAAATATCTTTTTGATGAATTCTACGGGAGAATTAAA
AAAGTCGTTGATCCTGAAAAAATAGACTATATTGTTGTAAACCACGTTGAAATGGATCACAGCAG
TTCTTTAGAAAAAATCATGGAAATTTCAAAAGCTAAAATCATAACAAACCAAAAAGCAAAAGAA
CATCTGGAATTACATTATAATACAAAAGATTGGGAATATATAATCGTTGATTCTGGAGATTCAGT
AAATATTGGAAAAAGAAATTTAACGTTTGTCAAAACTCCAATGCTTCACTGGCCTGACAACATGG
TAACATACTCTCCTGAAGATAAAATACTTTTCTCAAACGATGCATTCGGTCAGCACATCGCGTCA
AGTGAAAGGTTTGACTTTGAAATCGATTATACACTTGATTCTGCAAAAGAGTACTTTGCAAATAT
ACTGCTTCCATACAGGACGCTTATTCCAAATGCAGTCAAAACGGTCGATACTCTTGAAACAGATT
TAATCTGCCCTTCACACGGCATCATCTGGAAAGAAAATATTTCAAAAATTAAAGAAAAATATCTC
GAATTTGCATCAAACAACGTTATTAATAAGGCTGTTATTGTATACGACACAATGTACAGCTCAAC
TGAAAAAATCGGACTCGCTATTGCAGAAGGATTAATTGAAAGCGGTGTTGAAGTTAAAGTTTTT
AAAATATCCAAAACTCCGGTAAGTAAAATAATAGCTGAAATTTTAGATGCAAAATACGTGCTTG
TGGGATCCCCAACTCTTAATATTAATTTATACCCCGAAGTTGCCAGATTTTTAAAATACATGGAA
GGTTTAAAACCAAACAAAAAAATTGCGGCTACTTTTGGTTCTTACGGTTGGGCAGAGAGTGCTAC
AAAACATATTAAAAATACGTTTGACATATTATCTTTTGACACCGTGGATGACGAATGCCTAACAT
GCAGATTTGTTCCTAATGAAGAACACCTCAAAAAATGTCAAGAATTTGGAAAAAAACTTGCTAA
AATGTAATAAAAATATTGGGGGGGCAATATTATGGAAAAACCAACTCTTGTTTATACCACATTTC
CAAACTTTGAAACTGCAAAATCTATTGTAGGTTATCTCTTGGAGAAAAAAATGATAGCCTGCGCA
AACTTAAGAGAGCATGAAGCACACTATTTTAACGATGGTGACCTTTCAATAAATACGGAAATCGG

TGCATTTTTAAAAACTACCGAAAACAGGTGGATTTCATTAAAAGATATGATAAATGAAATTCACC
CTCTTGAAACGCCTGCAATCTTGAAAATAAATATCGATGATTCAAATGATGAATTCAAAAACTGG
ATTCTTAATATTATTAAATAATCAATCTTTTTAACATTTTATTCCCAAAATAACGATAATTTTT
TAGTTTATTGTTGATATATATGGAAGTGTTATATAGTTGCACAATCATAATATATTTAATTAAA
TTTTTGTGTGGTGAATCGGATGGAGGAATTAAAATTAAAAATAAGTGGAATGAGTTGCCAGATG
TGTGTAAAAACCATTAAAAACCTTTTAAGCGAACTTGAAGGCATAATAAATGTTGATATCGACTT
AGAAGCAGGACTTGGAACTGTAACTTACGATGAAAAAATATTGAATGAAGAAGAAATTTTAAAA
AATGAAGCTTTTGAACTATATCCTGCAGAAAAATTGGCTAATTAAACTTGGGTAGTTTTTATGGA
AGTAAAACTAAAAATTTCTGGAATGACCTGTGCAGTCTGTGTAAAAACGATTGAAAAATCTGTTT
CAAAGATGGACGGTGTTGAAAGCATTGTTGTTAATTTACTGGATGAAAGCGCAGTTATTAATTTT
GATGAAAAATTCGTTAGTATCGAAGATATCGGGATAAAAATTGAAAGGTTAGGCTATGAAGTTC
TTGGAATTGCAGAAGAAATTGAAGAATTGCCCGATAAAGAAGATGAACTAAAAGAAAAACTTAA
AAAAATAATTGTAGGGGCAGTTTTTTCAATTGCACTGTTTTCAATGATGTATATTGAAATCCCAT
ATAAACCGTATTTAGCATTTTTAGTGTCGCTTCCGCCTTTATTATATATTGCACTGCCGATTTTT
AAAGCAGGATTTAATTCGTTTAGAGTTAAGTCCCTAAACATGGATGTTATGTATTCTCTTGGAAT
AGGGGTTGCATATATCTCGGCACTGCTTGTAACTTTGGGACTGCTTCCAATGAACTTCATGTTTT
ACGATACAACAATAATGCTTGCAACACTTTTAACCCTTGGTAGATACTTAGAAGAGCGTGCAAAG
GGCAGAACGTCTGAAGCGATTAAAAAATTAATGGGCTTACAGGTAAAAACTGCAAAAGTTATTA
GAAATAACGAAGAACTTGAAATTCCAATTGAAAACGTAATTGTAGGAGATATCTTATTAATCCG
GCCTGGAGAAAAGATTGCGGTTGATGGAACTGTCTTTGAGGGCGATTCTTACGTTGATGAATCAA
TGATTACTGGAGAACCAATTCCAAATCCAAAGAAAAAAGGAGATTTGGTAATTGGTGGAACGAT
AAATAAAAATGGGATTTTAAAAATAACTGCTGAAAAAATTGGAAAAGATACAGTACTATCCCAA
ATAATCCAGCTTGTTAAAAATGCCCAAATTTCAAAACCCGATATTCAAAATCTGGCAGATAAAGC
AGTTTCTTACTTTATACCCCTGGTATTTACAATTGCACTTATTTCCTCACTGTATTGGTTTTTA
ACGGTGGAATTTTACTTGCAGTAACTACATTTATCTCAGTAATGGTAATTGCTTGCCCTTGTGCT
CTTGGACTTGCTACTCCTACCGCGATAACCGTTGGAGTTGGACGGGGGGCAGAACTTGGTATTTT
AATTAAAGACAGCAAGGTATTTGATGTTGCAGGAAACCTCAAATGCATGATTTTTGATAAAACCG
GAACCATTACAAAAGGAGAACCCGAAGTTGATGAAATAATTTCAGATTATTCCAAAGAAGAAGT
TTTATTAATTGCAGGAACTCTTGAAAAAAACTCTGAACACCCATTAGCACTTGCAATTCTTAGAA
AAGCAGAAGAATTAAATATTTCACTTTCTGAACCTGAAAAATTCGAATCGATTACAGGAATGGG
AATTATTGGAACTTTAAAGGATTTAAGAGTTCTTATAGGAAACAGGCGACTTATGGAAGAAAAT
AATATTTCAATAAATGAAGAATATAGTAAGCAAATATCCCGATTAGAAGAAAATGCAAAAACCG
TGATAATTGTAGGTGTTGAAAACAAAATACTCGGAATAATTGCAATATCAGATAAAATAAAAGA
AAATGCAAAAACCACTGTTCAAAATTTGCGTGAAATGGGAATAGAATCATACATGGTTACTGGG
GACAACGAAAAAACTGCAAAAGTCATTGGAAAGGAAGTTGGAATTTTGGAAAATCATGTATTTT
CAAATGTTTTACCTGAAAAAAAGGCAGAAATTGTTAAATCAATTAAAGAAAATGCAGGCGGTTA
CGTTGAATTTATTGGTGATGGAATAAATGATGCACCAGCTCTTTCTACCGCAGATGTTGGAATTG
CAGTTGGAAGCGGAACAGATATTGCAATTGAAAGCGGTGAAGTCGTTTTAATGAATGACGATTT
AAAATACGTTACAGGATTTGTAAAATTGAGTAAAAGAGTGCTAAAACAGATTAAATTAAATTTA
TTCTGGGCTTTTGCATATAACTCGATTTTAATTCCTGTTGCGGCAGGTGCCCTTTATTCATATAA
CATCAGATTTGAACCAGAACTTGCAGCTTTTGCAATGACCTTGAGTTCTATAACGATTATTGGAT
TGTCTCTTCTTTTAAAAAGATACGATCCTTGGAAATAATATCTTATAATAAATACATATTAACGT
TTGAAAATGTCGTAATTTTTCATTTTAAACTCAGAACATACCAAAAAAGATAAATTATATATAA

ATATATTAACTTATATGGATTTAAATTCTTATAAATTATATTATCGTAAATTTCTGAATTGATA
GGTGTTTTTATGAAAATCCTAGGAATATCCGGAAGTCCAAGAAAAGAAGGAACCCATTTTGCTGT
AAATTATGCACTTGATTATTTAAAAGAAAAGGGATTTGAAACTAAATATATTTCAGTTTTTCAA
AAAAAGATTGAATTTTGTATACACTGCGATTACTGTTTGAGAACTAAAGAAGGTTGCGTTCATA
AAGATAGCATGCAGGAAATCTACAATGGTCTAAAATGGGCTGATGCAGTTATACTGGGAAGTCCG
TGTTATAATGGAACGGTTTCTGGACAGTTAAAAACCATAATGGATAGATGCAGGGCAATATTTGC
AGAAGATATAGATGTTTTAAGGAATAAATATGGAATGGCCCTATCCGTTGGAGGGGATAGAAAT
GGCGGTCAGGAAGTTGTATTAAAAACCATTCATGACTTTTACATATTAAATGGAGTAATTCCAGT
AAGTGGAGGTTCTTTTGGTTCTAACTTGGGCGCTACATTCTGGTCCCAAGATAAAGGTAAACAGG
GAGTCGAAGAAGATACAGAAGGAATCAGAACTCTTAAAAGAACTCTTAAAAAAATTTTATAATTG
TTTAAATGAGGAGGGTGAGTAAAAATGGTTAAAATAGCTTGGGGTATTACGGGTTGCGGCGATA
AAATCGAAGAAATAGTTGATATGATCGTTGAATTAAAAAATGAATATGAAGATTTAGATGTAGA
TATTTACTACTCAAAAAGTGCTGAAATGGTACTTAACTGGTACAAATTGATGGGTAAATTAAAA
GATACATTCTATAATATCCGAAAAGAAGTAAATTCAAATGCTCCATTCTTGCCCGGAATGTTGCA
GACAGGAAAATACGATTTATTTTTAGTTGCACCAGTAACTGCAAATAGTGTTGCAAAAATAGCAC
ACGGGGTTGCGGACACCTTAATTACAAATTCCGTTGCACAAGGAGCAAAGGCGATGGTTCCAACA
TACATCTACCCTCCGGACAACAAAAAAGAAGAAATAGAAACAATTCTTCCTGGAGGCAAAACACT
AAAACTATATATTAGAGATACAGATGTAGAAAATGTTAATATTCTTAAGAAAATGCAGAGCATT
ACGATATTAGAAAATGTGGATGACATTAGAGACGTACTTTTGAAACATATTAAAGGTGATTAAA
TGCTACAGATCGAAGACTTATCGGTTAAGGTGGGTGACAAAGAAATTTTAAAAGATATCCACCTT
TATATCGAAAAAGGAGAAAGTCATGTATTATTTGGGCCTAATGGGGCAGGGAAATCCACATTGCT
CAATACAATTCTCGGAAACCCTAAATACGAAGTTGTAAGGGGAAATATTTACTTTAAAGGAAAA
GATATAACTGACATGCCAATGCATGAAAGAGCACAGCTTGGAATTGGTATTTCTTACCAGTCACC
ACCTGCAATTTCTGGCGTAAGGCTTGAAACGATGATAAACGCCATTTCAAAAAGAAGTGACGAAG
AAATGGAAGAAATGGCTAAAAGATTAAACATCAGGCACTTCTACCAAAGAGACTTAAACGTTGG
TTTTTCAGGTGGAGAAGTTAAAAGATCTGAATTATTACAGATTTTTGCACAAAACCCTGATCTTG
TGATGTTTGATGAACCCGATAGCGGTGTTGACGTTGAAAACGTAGAACTCCTCGGTGGAATCATT
AACCATCTGCTTGATAAAGACAGAAAACCAAGTGAACGAAATAAATCCGGACTTATCATAACTCA
CCTTGGATATATCTTAAATTTCATGGAAGTTGATAAAGCTCACGTACTATTAGACGGAGTAATTG
CCTGTTCGGGAACCCCTGACGAAATTTTAAATGAAATTATTAAAAATGGATACCAGAGGTGTGTT
TCATGCTGTCAGAAAAAAAGCTGCAACAAGTAAGGGAAATGGCTGAAAAATACAAAGACGTTCC
TGCAAAATTTGGAGAAGATATTGACTTAAGCATGTATCCTACACCAAAAGAAAACCTTTTGAAAG
TGGAATCATTAAAAGACTTAAACGAAGATCATAAACAAGCTCTTGCAAATGTTGGAGTGGACAT
TGAAGAAAAAAGCACTGTTGGATCTTACGTTCAGGTAAACAGTGATGCAATATACTCAAAAATG
TATTCGGATATTATACTTATGCCAATTACTGAAGCTCTTGAAAAATTCGAACTTGATGACTACTA
CTGGAACGCAGTTCAGATGAGCGACAAATACGGCGCAAGAGTTGCAAAAGAACTTACAGAAGGAT
ATTTTATTCGAGCGCCAAAAGGCGTTAAAAAAACAATTCCTCTTCAGACATGCCTTTTAATAGGT
AGTGAAGAAGTATCTCAAAACGTTCATAACATTATTATCGTGGAAGAAGGAGCAGAATTAAACG
TAATTACTGGATGTACTACATCCCCTCACGTTAAATCAGGGCTTCACCTTGGAGTTTCTGAGATA
TACCTTGAAAAAAATGCAAAACTTACCTTTACGATGATTCACAATTGGGGAGAAAATGTACACGT
AAGGCCAAGAACTGCAATAAAAATGGACGATGATTCCATATTTATAAACAATTACGTTACAATG
ATGCCTGTAAAATCAATCCAAAGCTACCCTACCGCATACTGTGAAGGAAAAAATGCTAAAGCAAC
TTTCCAGACAATAGTATACGGAAAAGGAAGTTCAAACTTAGATATGGGTTCAAGAGTAATTCTTT

CAGGAGAAAATTCAGCTGCCGACATGATTTCAAGAGTTATCGTAGTAGATGATGCAAAAGTTATC
TCAAGAGGTCACCTTATAGGTGCAGAGTCAAACGTTAAAGGCCACCTCGAATGTAGGGGTTTGAT
TTTATCAAATGACGGACATATTTTAGCAGTTCCGGAACTTGAAGCTGAAAAAACAGACCTTGAAT
TGTCACACGAAGCTGCAGTTGGAAAAATCGCAGAAGATCAGATCCAATACTTAATGTCAAGAGGA
TTGAGCGAAGACGAAGCATCTTCTTTAATTATCAAAGGATTTTTAAGTGTCGACATATCCGGCCT
TCCGCCAGAACTTGCAAAATCCGTTAAAGAAATGATGGATATGACGCTTGAAAATGCAATGTAAA
ATCCATGTTTTTTACTTTTTTAAATATTTAAAGAATTAATAAAAAAATTTACAAACAAGTGTTTT
TTATACAATAACTTTTTTTATATATGACGAGCTTAAATATAGATAATATTTAAAATTTAAATAT
TCGAGCGATTTTGGGATAGTATGGAAAAAAATGATATTTTTATTGTAATTCCGGCATATAATGA
AGAAAAAATGATTAAAAATACATTAATCAATTTAAAAAGTCACGGTTATGAAAATATCATTGTA
ATTGACGATGGAAGCAGGGACGAGACGTCAAACATCGCAATTTCAGAAAATGTAATTGTCTGCAA
GCACATCATCAACAGGGGACTTGGTGGAGCACTGAAAACCGGTTTAAAATGTGCGGTAAAATATA
ATCCAAAAGTAATAGTAACTTTTGATGCAGACGGCCAGCACGACCCGGAAGACATTATTAAAGTT
TCAAAACCAATAATTGACGGGGATTTTGATGTCGTTGTTGGAAGCAGGCTTATTGATGAAAATGA
ATTAAAAAATATGCCATTTATTAAAAAAATAGGAAACTGGGGATTAAATTTTATAACTTACTTA
ATGGGAGGACGAATGGTTACTGACAGTCAGGGAGGTCTTCGGGCTTTTTCATATAATGCTGCAGA
AATCGTTTCAAAACAGCTCAAAAGCAATAGATATGAAGTATCATCAGAATTTATAGTTCTTTTCG
AAAAAAATAACTTGAAATTTAAAGAAGTACCGATAAAAACGATTTACACCGAATATTCGATGGC
AAGAGGAACGAATGTCATTACAGGATTTAAAATTCTCTTTAAATTATTAATTCAAAAACTAATT
TAACGGCGATAATCCATGTTCAAGATTAGAAACATAATCACGATTTCAGATTATGTAACGGTTAT
AAACATTATTTTTGGAATGCTTGCAATATTACTCCACGATTTTCGTTTTGTTTACATTTCAATCG
TGTTTGATGCTCTTGACGGCATTGTTGCAAGAAAAACAAACACTGTTTCGGATTTTGGCGCAGAA
CTTGACAGCATTTGTGATGTTGTAAGTTTTGGTGTTGCTCCAGCGTATTTAATTTATTATTACTT
TGATTCGACCGTTTCTATTATTGGATCCATCATATTTTTAATCGGCGGTGCATTAAGGCTTGCAA
GATTTGGAATATTGGACGTGAAACACTTTATAGGGCTTCCAATTCCTGCTGCCGCATTATTCTTA
TGCATCTTAACCCAGATGTCTTTAAAATATGGCTTTGGTTCAGGATATGTTGTTTCAATCATTGC
AGTAATTTCAGGAATTCTTATGATAAGCGATATTAACTATCCAAAATACCCGAAAAAACTTCTTT
TAGTGGTTTTTGGCATTTCCATAATTCTTTCAATGTTTGGAGTTATTGAACCACTTGCAATCTGT
GCAGTTTTGTATGTACTTTATGGAATATTTAAAATGAAAATGTAAAAATCATTTAAAAATAATA
TTTAATTATGTTATTCTAAAAATTTAGAGACGAATGGCGACGTTTTTCCTTTTCTAAGGAAGTGT
CCTGATGGTCCATCACCTAAAAATTCTGAAAACCATGATTCATGCTGAATTTCTTCATTTAATAT
GGCTAGAGCAAGATCATAGGTTCTGTGATCTTTTCCTGCAGTCATATTGCAAATTTTTGTATACT
GTTTAACTGCACACTGTTCTGCTTGTAATAAAACTTTCATTAGATTAGGGATATCTGTAACATCA
TCTGGCAGATATGCAGGAGGACAGCCCGACATATCATGAAAATCTTTCATATCTTTTGGAAGCGA
CCCTCCTAATTCGTATATTCTTGGAACCAGCGCTTCAAAATGATTTCTATCTTCTATTCTAGCAGT
TTCTGCAATTTCTTTAAGCCCTTATCCATCTAATCCAATTAAATTAATTCTCAATATTGTATAGT
AGTAAAATGTAGTTAATTCGGCAGCTGCATTTTTAACCAAAAGTTCAACCAGTTCATCTACATTA
ATGCCAGAATTTTCTACCATTTCACGAGATACTTTTGCCATACTATTCACCCCCATATAATATTC
ATTTTTTAGAAATTAATATATTTTACCATGCAAAAAAGAATATGGGGACGATTAAATGTTAAAA
TTAAATTTTAAAGGTTTTAAACAAGTTTTATGTTTTGTCCCAAAGCCACATCTAAAATCATCATT
ACTATAAAGCCAAAAATTGCAGCTATTGTGGCACTATCAATGTTTCCTTCCCGTTGACATTCTGG
GATTATATCTTCAATTACAACAAACATCATGGCACCTGCTGCAAAACTCAGTGCATAGGGGAGCA
GGGGTGTAAATATAGTGACTAAAAATGCCCCTAAAACACCTGCAATAGGTTCTACAATTGCAGAG

AGTTGCCCGTAAAAAAAACTTTTATTTTTTGACAGCCCTTCCCCCCTTAAAGGAAAACTAACTGC
AATTCCTTCCGGAAAATTTTGGATCCCAATGCCTAATGCAAGAACAATTGCAGCCATTAACGAAT
CCACAGACATGTTTAAAGCAAGTGCACCAAATGCAATTCCAACCGCAAGACCTTCGGGAATATTA
TGGATTGTTACTGCCATTAAAAGAAGTCGATTTTTATGCCATGTTGTTTTTGGACCTTCTGCTTC
TTTTAGTGGCTGGCCCATATGAAGATGCGGAACTATCTTATCAATTCCTGCAAGGAAAAATGCAC
CGATTAAAAAGCCAAAACTTGCTGGAAACCAGGATAAATTATCCATACTGCTTGAAAGTTCGATT
GCTGGGGCAAGCAACGACCAAAAACTTGCAGCAATCATAATTCCTGCAGCAAACCCGAGTGAAAT
ATCAAGAAGTTTTTGATTAACAGTTTTAGTAAGATAAACTAAAGATGCTCCAAGTGCAGTTACCA
ACCACGTGAATATGGTTGCTAATAACGCAAGAAATACAGGATTATAAGAAGTTAGAGGCGCCAT
AAATTTTCCCCGATTAAACTTGAAAAAATAGGTTTTTGAATATTTTTCAATATTAAAAAATATT
ATTTTTGAAATATATAAATCCCCAATTATTCTAAAAACGTAATTTCTTTTTGATATTTTTCAAAC
TAAAGTTTATTTTTAAAAACCGGTCAAAACATATATATTACGCAAATAACAAAGTTTATATGTA
CATTAAAAAATCTTGAGGGGATAATTTGGTAGTAATCGGAGAAAAATTTCCTGATGTTGAGGTT
ATAACCACCCATGGAAAGCTAAAATTACCAGAACATTATATTGAATCTGGAAAATGGTTTGTTTT
ATTCAGCCACCCTGGAGACTTCACACCAGTGTGTACTACGGAGTTTGTCGCATTTCAAAAAAGAT
ACGACCAGTTCAGAGAATTAAACACTGAATTAATTGGTTTGAGTATCGATCAAGTATTCAGCCAT
ATAAAATGGGTAGAATGGATAAAAGAAAAATTAGACGTTGACATAGAATTTCCAATAATTGCTG
ATGATAGGGGAGAGTTAGCAGTAAAACTTGGAATGATAAGCCCTTACAAAGGAAATAATACTGT
TAGGGCCGTATTTGTAGTTGATGCTACGGGAACAATCCGTGCAATAATTTACTACCCTCAAGAAG
TTGGTAGAAATATGGATGAAATCGTAAGGCTTGTAAAAGCACTTCAAACTGCAGATAAAGGCTA
TGCAACACCTGCAAACTGGCCAAATAATGACTTTTTAAATGAAAAAGTAATTGTACCTCCTGCAA
ATAACATGGACGCTAGAAAAAAACGACTTGAAGCCTGTAAAAGTGGAGAATTACAAGGTTACGA
CTGGTGGTTCTGTTATACGGATTTAAAAGAATAATTCTTTTTTTATTTTTTTAACTGAAGATAT
ATTTAATAATTTAAATATATTGTTAAGTATGATTTTTATAAAAATAATATGAATATTTATATAA
GTTTTTTTAAATTAGTTTTAAAATATTTTCAAAACGGGTGATTATGTTGAATACTGAAAAATAT
CAAAATTCATTTCCCCTTCCTGCCGAAAGTCCTGAAAAAATTGATAAAATAATGCTCGACACTTA
TTTTGGAGCCCAAAAATTTTATTTACTAAAAACTGCAATTGAATTAAGAATATTTGATTACCTTT
CTGATTCAAAAACTTCCAAAGATTTGTCATCGATTTTGGATTTGGATCCTGTTTTAACTGAATTA
ATGTTAAAAGCACTCTATGAACTTGGAATTATTTCAATAGCATCAAAAAATGGCGAAACCCATTA
CATCAACACATTTGCAAGTGATAATTACTTAAAAATGGATTCAGTATATTCAAAAAGCCCTTCAA
TCTCCGCTTCATTTGAAAGTATTGATAAATGGGCAAATTTGTCTGAAATAATGAACGGTTCCAAA
AATGAAGTCATGGAAAATGCATTTTTCCCAGAAATAATTAAAAGAATGGCAAACGACTGCAAAT
GTTGGGAACTTCAAAAAACTGTCGAATATATCTCTAAATTTTCGGAATTTAAATCTGCAAAAAAA
CTTCTCGACGTTGCAGGAGGCCACGGGCTCTACGCAATAAGCCTTGGTATGCTGAATGAAAATTT
AAATGCTTTTGTTTTTGATTTACCCGACGTTACAATTGAAACTAAAAAATTCATAGAAAAAATACG
ATGCGAAAAACGTTGATACAATTGCAGGAAATTTTTTTAAAGACGATTTCGGAGAAGATTTTGA
TATAATTTTTAGTTCATACAACCCTGGCGGAAAAAATCCAAAAGTTGCTAAAAAAGTATACAATT
CGCTAAATACGGGAGGATTATTTATTACAAAACAAGCATTTCCAAAATTAACGAAAAATATGCCC
GATACATTAAATAACATGGAATGGAATTTTAACAGCTTTAACAGCACTGGAAAGGGAAGTATAA
GATATACTTTTGAAACGGATCTTACTTTTGAAGAATATGTTTCATATCTTGAAAAATTAGGGTTT
GAAGTACTCGAAACAAAACCTGTTTATGAATTAACTGAATTTAAGGAAGAAATGTTTCCAAACA
TAATTATCGTTGCTAAAAAAATTAATTAAATTTATTATAATACTTTTTTAGAATCTTATTTTTTT
AATATTTTAAATATTTTTTAATATTATATAGAAAATTTTATGTATCAATTATTACATTTAATTT

AAAAGTATGATAAATATCATATTTTTAAAACATTGATAATATCAGGTGAAATTTTGAAAAAAAT
AGGGGTTTTATCAGCCTTAATTTCATTAATGATTGCACTCAGCTTTTCAGGCTGTGTCGATAATA
CTGCTGAATCTTCAGGTGAAATCACTAAAACTGTTGAAATTGTAGATATGGTTGGAAGAACTGTT
GAAGTTCCAACAGATATTCAAAGAATCGTATGTTCTGGACCAGGATGCCTTAGACTCGTAACTTA
TGTTCAAGCACAGGACAAACTTGTTGGTGTTGAATCTATCGAACAAGAAAATATCTCTGGAAGGC
CATATCAACTCGCAAACATGGACTTTTTCCAAACTCTACCTATGACGGGACTTGGCGGTGGAAAA
GAAATAGGCGTTGGACCATACCCTGAACAGGTTATAAGTGCATCTCCAGAAGTTATATTAATTAC
ATATATGGATGCAGAGAGAGCTGACGAACTTCAGGGAAAATACGGAATTCCCGTTGTAGTATTAC
AGTATGGGGATGTTAATTCATTCCATGATGAAAATTTCGTTGGTTGTTTGAATTTACTTGGAAAA
TTATTTCAAAGGGAAGAAAGAGCTCAAGAAGTAATTGAATTCTTTGCAGATGCAGAAAACGACC
TTTTAAAAAGAACTGAAAATATTTCTGATGATGAAAAAGATGAAGTATATATTGGAGGACTCTG
TGCAAAAGGTGCCCATGGAATTGATAGTACATCAAGTAACTACGCTCCATTTAATGCAGTGAATG
CTAAAAACGTTGCATCTTCACTTGATGTAGCAAAACAAGTATTCATAAGCAAAGAACAGCTTTTA
GAATGGAATCCTGAGATCATATTTATTGATACTGGAAGTTATGCATTAATAAAAGAAGATTATG
AAAAAAATTCAGAATACTATGAATCTCTTGATGCATTTAAAAATGGCAAGGTATACACCCAGTTT
CCATACAACTACTACACTACAAATTACGACACTGCATTGGCTAATGCGTACTATATCGGAAAAAT
CGTATACCCTGAACGGTTTTCGGACATAAATATCGAAGAAAAATCTGATGAAATATATGAATTCT
TAGTTTCAAAAGCTGTTTACAGTGAAATGGCTGAAAACTACTGTGGATTTGAAAATATTAATTTT
TCAAACCAATAACTTCCTTTTTTCTTTTTTATTCATTAATTAAATTTTATTATTTTTTAGTAATA
CTATATCGAAAAATATATGTAACAATAATACTACATTTTTTAAAAGATATTAATTCAGGTGGTA
TTTTGAATAAAAAAATAATATCCATTATCGTAGCAATTATGATGTCGCTTGTCGTTGCACTTTCA
GGTTGTGTTGAAAGTAGCGATTCAGGTACATCTGAGGATATTAAAACTGTTGAAATTGTAGATA
TGGTTGGAAGAACTGTTGAAATTCCTGCAGAAGTTCAAAAAATTGTATGTTCAGGACCTGGCTGT
TTGAGATTAATTTCTTACTTAAATGCAACTGACAAACTAGTCGGTGTTGAAGACTTTGAAAATAC
AAATCTAATAGGAAGACCTTACAGGCTTGCACATCCTGAATTCTCAGAACTTTCTGTAATTGGAA
ATGGCGGACCGCAAGATATTAATGTCGGACCAGACCCTGAAAAGGTATTAAACGTAATGCCTGAG
GTAATATTCATAACTTACATAGACTCGGATAACGCAGACGAACTTCAGAAAAAAACCGGAATCCC
CGTTGTGGTTTTAAGTTACGGTGCACTTGGAAACTTTAAAAATGATGCAATATTTAATTCACTAA
CACTTTGCGGAGAAATTCTTGGAAAAGAAGAAAGAGCAGAAAAAGTAATTTCGTTTATATCAAA
TATTCAGAACGATTTAGATAAAAGAACTTCTGAAATTCCCGATGAAGAAAAACCAAGTATTTACG
TTGGAGCAATTGGTAACAAAGGTATTCACGGAATCGACAGTACTGAAGGTTCATACCCGCCATTT
GAATCATTAAATACTGAAAATATTGCAAAAACTGCATCTAATGAACATATTTTCATAAGTAAAG
AGCAGTTAATCGAATGGAATCCAGAATTCATCTTTATTGATGAAGGAGGAATCTCAATTGTGTAT
GAGGACTACGAAAAAAATCCCGATTATTACAATTCGCTCGATGCATTTAAAAATGGAAATGTTTA
CGGCATTTTACCATTTAACTACTATACAACAAACATTGACACTGCAATGGCAGATTCATATTACA
TTGGAACGATTCTTTACCCTGAAGCATTTTCAGATATTGATCCAAAAGCTAAAGCTGATGAAATA
TATGAATTTTTAGTTGGAAAAGGAGTATATTCCAAAATGAATGAAATGTTTGAGGGATTTGAAA
ATTTAGACTTTTCAAACCAGTAATTTCTTTTTTTTCTTTTTTAAAATTTTTTTAAAGAGTATTTA
AATTCAACATATTTAGCCCAATTCTTCGTAAAACTATTTTTAAAATTTAACCTGTGATTATCATA
TTAAGACAGATCACTAAAAAAATACCGCCCCATTTACATTTTTTTAACGTTGCATTAATTCAAAA
ATAAATTTAATATGTAATAGTAATCTATTTAGATTACAGTTTATAAAAAAAAGTAACTATTATA
TATTATTTAATTTTAAAGAGATATTATATAATAGTCACAGTTGCGGGAACCATACCAATATTCTC
AGATCTTTTAAAATATTAACTTTTTAGAAATATTAAAATCAATCGGAGGCACGTTATGTCTGAAA

ACAAGAAAAAATTCGACAAAAAAGGTGCTAAGAATATGGACGAGATTTCAAAAACACTATTTGC
ACCGATATACCCTATAATCGCAGAAAATATTATTAATCGTTTTGGAATAACCGCCGGCAATTGTA
TAGACATTGGAAGTGGTCCAGGAGCGCTATCAATTGCTTTAGCAAAACAATCCGATTTTTCAATA
CGGGCTTTGGACTTTTCCAAACATATGAACGAAATAGCATTAAAAAATATTGCAGATGCTAACCT
AAACGATCGAATCCAAATTGTTCAAGGAGACGTACACAATATCCCAATCGAGGATAACTATGCAG
ATTTAATTGTTAGCAGAGGTTCAGTATTCTTCTGGAAAGATGTCACCACTGCATTTCGAGAAATT
TATCGAATTCTCAAATCTGGTGGAAAAACATATATTGGTGGAGGATTTGGCAACAAAGAATTAC
GTGATTCAATTTCAGCCGAGATGATACGCAAAAACCCGGATTGGAAAGAATTCAACCGAAAAAAT
ATATCTCAAGAGAATGTTGAACGGTTCCAAAATGTACTTGATGAAATAGGTATCCCCTCTTACGA
AATTATCCTTGAAGACGAAGGATTCTGGATTATAATCTCAAAAACCGACCAAGAGGCGATCTAAT
GAAATGTACAGTATGTGAAATCGGTTGTGATATTTCAGAAGGAAATTACGGTCGGTGCAATATG
TATACAAATCAAAACAGTAAACTCATCGAGAGGTTTCCTAATTCGTATCTTACAGTACTCCCGAT
CACAATTGAAACAATGCCCATGGTCCATTTTGCACCAAAAAATAAGTTCCTACAGGTGAGTACTG
TTGGATGTAACTTTAACTGTCAGGGCTGTATTTCAGAGACCTTAACATCAAATGCTGATGCATTA
TCTGGCGCTCTTACTAAAGCTTCACCCACAGATATCATATCTCGTGCAAAAACTGAAGAATGTCG
CGGTATTGTTTTTTGTCTTAATGATCCTGCTGCATCATATTATACATTTCTAAATCTAGCAAAAG
AAGCAAAAAAAGCTAATCTTTGGGTTGGATGCTCAACAAACGGTTATTTCACTGAAACAGCCTTA
AAATCTCTTATACCTTATCTTGATTTTGTTAATATTGGCCTGAAAGGTTCATCGAATGCACGTTA
TAAAGAATGCGGTGCAAAGTCTGCAGCTCCTGTGTTTAGAAATATCCGTATCCTGCACGAAGCAG
GCGTACACGTGGAAACCTCTGCAATGTACATAAATACTGGTGATGAAGAGATATTAAAAGCCGCA
GAGGAAGTAGCATCCGTTTCGAAAGATATACCCTTACAGGTAATGCGTTTTGTACCGTTTGGGGA
AGCAAAACCAGAATTAGAACCTACAATAAATAAATCAGAAAATATTGTAGATCAGCTACGCTCA
ATTCTCAACCACGTATACCTGTTTAATTCACCGGGAACTGAATATTTAACAACTAAATGCCCGAT
TTGCGGGAATGAAATCATCAAACGTGAATTTTACGGACCCATGGGATGCAGAAGCATAGAATCTC
TTGAAGAAGGTAGGTGTCCCTGTGGATGGCAGGTTCCACTAAAAGGACCAATCAATAATGAACAG
TTTACTGAATACGGCATGCTTGGAGGATACAGGACCACTCGTGCCATTGAAATGGCCCATGCAAT
TTTATTCACACTTGGAATCCGCGATAATGAAGAACTTGGAGATATTCTCGGTGATATCATTAAAG
AAGACTTCATTCGAGGGTTGCATGACCGCATTCAACAGATCGATTCATGGCTTGAACTTATCAAA
GATCTCGCCATGCGAACTGGGCGGGAAAAAGAAGGTAATGAACTTATCGCATTTATTCAAGAACG
AGTTGATAAAATAAATGAAGGCAGTAAACTCATCAAGTCATGCCCTACCGTTTATTATTCGATGG
GTTATCCGTTATTTGCCCTGAATGCCGAGCGGTTCGAAACAAATCTTGTAGAAGCTGCAGGCGGT
ATTTGTGTTAACAAAAGCCTTACCCGCAAAGGTAAACCGGGTGTTAATATATCGGTAAATGAGAT
AAATAAACTCAGCCCTGAAATGATGTTTATCTCAGGATTCCTATCCTCACCAGCTTCAGACTACG
TTCAATACTGTAAAAAACATGGAATTGACAATAAAGCCGTTCGGATGGGGAACATATGTAATGT
ACCCACAGGATGGGATTTTGGCAGCCCTCGCTGGATTCTTGGATTCATGTTCCTTGCAAATACAA
TCCATCCAGAAATATACTCTTTCGACTTAGAAAATGAACAAAAAGAGTTTTATAAACGTTTTTAT
GGCATTGATGTAGAGAACGTTGTTACAAACCGTGACTTTTCAAGACCTGCAATAACTACAACTAA
ATAACCATACCTTTGAATCACTCTTTTTTAATTTTGATTTTTTAAAACAGTTATAATTCTCTGCA
TATTTTAATGTGTAATTAATATTAGTTACTTTTTTATAGTTTAATTAATGTATTTATATTACTTA
GGGATGTATCTTTAAAAAGATAATAAAAAAATTATATATTCATATCAATAGATACGGGTGAAAA
AATGTATAAAAATGGGATTTTGGCCATTATTTTGTCGCTGATGATTGCACTCAGTTTTTCAGGCT
GTGTCGATAATACGGCCCAAACTTCAGGCGAAACGGTAAAAACCGTTGAAATTGTAGACCTGGCT
GGAAGAACTGTTGAAGTTCCAGAAGATGTTGAAAAAATTGTTTGTTCGGGCCCAGGCGCACTAAG

ACTTGTTACTTATCTTCAGGCAGATGACATGTTAGTGGGAGTTGAATCTTGCGAACAGGCAGACA
TTACAGGTGCAGGTAAACAATATGCCCTGGCTAAACCTTACACCATTGCAAATGTTGATTATTTT
AAAACCTTACCTACAATTGGCACTCAATTTGTCGATGGATTAAACTGTGAAAACATAGTTAACGT
GAATCCTGATGTTATAATAATTTCAATGTCAACAGCAGGAGTTGCAGACAATATTCAGACAAAAA
CAGGAATTCCTGTCGTAGTTTAGATTATGATGAATTTGAATCTTTTACTGACAAAGATCTTGCA
GATCCACTTACACTACTTGGTAAAGTGTTAAACAGAGAAGAAAGAGCGCAAGCAGTAATTGATTT
CTTTGCAGATGCTGAAAATGATATATCAAATAGAATTTCAGATGTTTCAGATAGCGAAAAACCAT
CAGTATATGTCGGTGGACTTAACGCTGCAAAAGGATCCCACGGTATAGAAAGTACATCAAGTGTA
TTTGCACCATTTGAAGTTTTAAAAGCTAAAAATGTTGCTGAATCCATTGAAACAAAAAAACAGGT
ATATATTAACAAAGAACAGCTTATAGAGTGGAATCCAGATATAATCTTTATTGATATTGCAGGA
TATACTTCCGTGATAAATGAAGACTATGAAAAAAACCCAGAATACTATGAATCACTCAGTGCATT
CAAAAACGGTGATGTTTACTTAATATATAGATACAAATCATACATGGTGGACTACGGAACTGTAC
TCGCAAATACTTATTATATTGGTACAGTATTATACCCCGAAGAATTTTCAGATATTGATCCAGAA
GCTAAAGCTGATGAAATATATGAATTTTTAGTTGGTGAAGCTGTTTATGATAAAATGGCAGAAA
ATTTTGAAGGATATACCAAATTAAACCTCAACTAATATCCTATACTATTTTTTTAAAAAATTTAT
TTTTTGATAATATTAAATACTTATTTATAGGTTAAAATAATACTTATTTGATAAGTGTATTACC
AAAACAGGGATAACGTGGAAAATTCTGTTAATACTTACAAAAAATATACGTTAAAAAAACTAAT
ATTCGGATTTGTACTGATTATAGCCCTAATAATAATCTCTATTTATTCACTATGCACTGGAGATT
TCCAGTTATCAATCAGCCAGATTATAAATTCATTGTTAGGTTCTGGAGACGCTTCTTCAAACCTT
GTTATATGGAATATCAGACTTCCAAGAATCTTTGCAGCAATACTCTGTGGAATTGGATTATCTGT
TTCAGGTGCAGTGATGCAGTGTGTTTTAAGAAACCCGCTCGCATCCCCATATACCATGGGAATTT
CTCATGGAGCCATGTTTGGAGCATGCGTTGCAATACTTACGCTTGGAATCGGCGGTGCTGAAAGT
ACAGGACATATTGCAATAAACAATCCTTATTCCATAACGATATTTGCATTTTTAGGATCACTGCT
TGGCGTTTGTGCAGTATTGGTCCTAGCCAAATTAAAAGGATTAAGTCCTGAAGCAATGGTTCTTG
CAGGGGTTGCAATATCTTCACTATTTACTGCTGCAACTACACTCATACAGTATTTTGCAGACGAT
ATGCAATTAGCTGCAATGGTTTACTGGTCATTTGGTGACCTTGGAAGACCGCTATGGCCAGAAGT
AAAAATTATGGCCTTTGTAGCATTTGTTTCACTTATTTACTTTATACATAAACGATGGGACTATA
ACGCTCTTGAAAGCGGTGAAGAAACTGCAAAATCTCTTGGCGTTAACACGGATAAAATAAGACTG
TCAGGAATGCTTGTTGCATCCCTTGTAACATCTGTTTGTGTTGCATTCTTAGGAATTATTGGATT
TGTAGGATTAATCTGCCCACATATAATAAGGATAACTGTTGGGGGAGATTACAGGTACTTAATTC
CACTGTGCAGTATTTTTGGTGCAGTTTTGGTGCTTTTAGCAGATACAATTGCAAGAACAATAATT
TCCCCAATAATTCTGCCCGTAGGAATACTTACCTCATTTTTAGGGGCACCGATGTTCCTTTATTTA
CTAACGAAGATGTATAAATGATAGGTTCGGGTGAAAATATGATAGTTTCAGTAGATGGAGTTGA
ATTCAAATATAAAAGTACTGAAATATTAAAAGACGTTAAATTTGAAGTAGAAAAAGGAGAAGTT
GTTTCAATTCTCGGAATAAACGGGGCTGGAAAGTCAACTCTTATAAAATGCATAAATAAAATATT
ACCTCCAAAAAAAGGAACCATAACTGTTGAAAATCTCGATGTAAACAAGATGGATAGACTTGATC
TAGCAAAAAAAGTAGGTTACGTTCCGCAGAGGTCCAATGGAAATTATATGACGGTATTTGATGCA
TTGCTTTTGGGAAGAAAACCGCATATAAAGTGGGAAATTTCAAAAAAAGATATTGAAATTACTG
AAAATGTGTTAAAACTTCTTGATCTTGAAAAATATGCCCTTAGAAATACTAATGAATTAAGTGG
TGGGGAACTTCAAAAAGTAATTATTGGAAGGGCTCTTGTTCAGGAACCAAAATTAATACTTCTTG
ATGAACCAACGAATAATCTTGACTTAAAAAACCAGCTTGAAGTTATGAGAATACTAAAAGACGT
TTCAGTTTCACAAAATATTACTTCGATAATTGTAATGCACGACATAAATCTTGCACTTAGATATT
CGGACAAATTTTTGATGTTAAAAGATGGAAAAATCTTTGCTGAAGGCGGAAAAGAGATAATCAA

TTCACAAAACATTATGGAAGTATATGGGGTAAATACGTACGTACACAACTTAAACGGCCATTTAA
CCATAGTTCCTGAAGAAGGTTATTAATTAAATATATTTTCTCATTACAATTCCATCTGAACTATC
CTCATCTTCAAAGAATTCTTTTATTCTACATATTTCTTTAAAACCATTTTTTTCATAAAGTTTAA
TTGCAGGTTTCCAATTTACAGAAACAGTTAAGATTATACTCGAAATTTTGTAGTTTTTTGAAATC
TTTTCAATTAATTCTTTAAAAAGTAAATTTCCGTATCCTTTTTTCCTGTGTTTTTTCAAAATTCC
GATTGATGAAATATATAGTTCTGATCCATTACATTTATGCACTTTTTTAATATCGTGATTTAGAT
CAAACATTTTTTCATCGATATTTTCAGAATATGCCCAGATTTCAGAAGAAATATATCCTAAAACT
TCCGAATCAACTTCCAATACTAAAAAGCCATTTTGAAAAGTAGCTATCCTATCTAAAAAAACATT
CCTGTTTTCAGAAATACCGGTTATAAATGAATCATATTCTATGTTCATTATTTCATCGATATCGG
TTTTTCTAGCGTTTCTGATAACCATTTTTTCCAAAAAAACACCCTTAATCAGATTATTTTTAAAA
TAAAACGCAGTTTATATATAGTACTGTAATCAATTATCTAGTATATATAGAAGAGATAAACCCCT
CGAATAAAACTTGGTGAGATTTTTGGAAAGTGTCCAAAGTACTATTAAATCGTTTATTAATAAC
AGCAACAAAATTGCAATACTAGGTATTGGAAACTATTTAAAAAGCGATGACGGATTTGGCGTTT
ATGTCGTCGAATCATTAGTTAAAAATTATTCTAAAACTCATGAAAATTTATCATTGGAAAAGGA
AATAAATTCTGTAAATAATAGATTAATACTGATGAATTGTGGTGTAGTTCCAGAAAATTTCACG
GATGTAATTAAAAGAGAAAATCCAGACAAAATTATAATGGTTGATGCTGCACTAATGCATCAGG
AACCTGGAACACTAAGGGTTGTAGAAAGCGATGAAATTTCCGAAACTGGATTTTCAACCCATTCG
CTTCCGCTATCAATAATAATTAAATATATTAATGCCCATATTGATACCGAAATATTAATTATAGG
CATAGAACCTACAGACCTTGAATTTGGTGAACCATTATCAGGATTAATCAAAGAAAAAGCAGATG
AATTTTCAAAAATATTAATTGAAGAGATTGATTCTTTTTTATTATAAAACAGGTGAATTATGTT
TTCAGTACAATTTAAAACGACTGACGAATTACCAAAACCATCCCCTAATCTAATAAATCAGGTTA
TCGGACAAGATGAAGCGGTCAAAATAGTATTGAGTGCTGTAAAAAATAAAAGACATGCATTACT
ACTTGGAGACCCTGGTGTCGGTAAATCAATGATGGTAAAAGCTTTCGGTCAGCTTTTAGAACATT
CGAGTGATTTTAAGCCATACTCTTTGATTGCAAGACCAAATATAAAAAATTCAGAAAAACCAATA
GTTGACTTAATAGAAGGGCGAATGATAGAAGAAACAGCCCAGATTGAAAGTCCAAAATTAATGA
GACAGCCACCAAGCATATTTACACTTTTACTTGGAATCATTGTCTCGTCACTTGTATTAAGCTAC
ATATTAAATGGATTGTCTGATCCATCATCCAAATTTGCAGTAATTGCAGCAATATCTGTAATTGG
GTCATTATTGGTATTTTTAATACTATTTCTAAATATATTTGGAGCTACGAAAGCATCAATGCCTA
ATTCAGTAAGTCCTGCTGACGTAAAACCTGTAGTACTTTACGAATGCAAAAAAAGACCGCTTGTA
AGAGCAAGCGCTTACAACATTACAAAATTACTCGGAGATATTAAACACTGTCCATTAGGTGGAAA
ACCTCCAATTGGAACTCCTCCGCACAAAAGGGTAATTATTGGTGCAATACACGAAGCACACAAGG
GAATACTTTACGTTGATGAAATTAAGACCATGCCTGTCGATGTTCAGGATTATATATTAACTGCA
CTTCAGGACAAACAGCTTGCAATTAGCGGTAGAAATCCTAATTCAAGCGGTGCTTCAGTTGAAAC
CAACCCGATACCTTGCGACCTTACATTGATAATGTCTGGAAACATGGACGATGCAAGCAACTTAA
GAGCACCACTTCTTGACAGGATTGATTACAAAGTCGTTTTAAAAAATAAAATGGACAATAATCAA
GAAAATAGGGATAAATTACTCCAATTTATCGTTCAAGAGCTTGAAAATAATAATTTAAGACCTA
TGAGTTATGAAGCATGCTGTGAAATTGTCAGAATGGCACAATTATTATCAGGATCTAAAAATAA
GCTCACTTTAAGATTAAGACAGCTCTCAAATATTATAAAAATGGCAAACGATATTGCAACTGGAA
AAGAACTTTCTGAAAGCATTGAAGAAATGTCTGAAAAAGAAGTAAAACCTGAAGTAACTGTAAA
AGCAGTTAAACCTGCTGAAAAGAAAAATACTCGAAAAATCAGGGCTGTTGTTGGAAAATTAAAA
CCTAAAAAAAGTGAAGAAGTTGCAAAAGAAGTAAAACCTGTTTCAAAAGCACCAGTTCTTGAAA
AAGACGATAGAGTAATTGAACTCGACCACATCAATGAAATTATAAGCACTGGAATATACAGCATG
TCAAAACAGGTAGCAATTGATTACCTGAAAAACTTCAAAAGATACAAAAATATCGTATCAAATG

ATAAACCAAAAATAGGAGTAATCCACGGACTTGCAGTTTTAGGTGCAGATGGACTTGGAGACGTT
ACAAAAATTATTACTAAAATTGTAAAATCAAAACATCCAAGAACAAACCTGTTAAACATTAGCG
GAGACCTTGCAAAACACAGCATTACCTTAGCATCAGCACTGTCTAAAAAACTTGTTTCAGATGGA
ACATTAAGCATTGCAAAAGCTAAAGGGGAAGTTGCAAAAGAAGACCTCGATTTAGCAGAACATG
AAATTTACATCCAGTTCAGCCAGTCATACTCAAAAATCGATGGGGACTCTGCAACTGCCGCTGCA
TGTTTAAGCATTATTTCGTCACTCCTAAATATACCATTAAAACAGGATTTCTGTATTACTGGAAG
CCTTGATTTAAACGGGGAAATTCTTGCAATTGGCGGAGTTAATGAAAAAATAAATGCTGCTAAA
GAATACGGATTTAAAAGAGTAATCATTCCACAATCTAACTTTGAAGACGTTATCGACCCGGAAGG
AATCGAAGTAATTCCAGTTACCCGTCTTGAAGAAATAATTCCTTTAGCTTTTGAATTAAATAATT
TATAACCTAATAATTTTCTTTTTTGGTGATTTTATGGATTTAACTGAATTTATAAAAATATGCCG
TTTATTATACGATCGAAAATACGTCGTTGGTTCTGGAGGAAATGTAAGTATTCGGGATGGAAATT
TAATATATATTACGCCAACGGGTTTAAGTTTAGGATTTTTAACAAAAGAAGATATCTGCATTGCT
GATTTAAATGGAAACATCATAAAAGGAAAACCTACTTCAGAATTAAACATGCATTTGAAAATAT
ACCAAAATAAAAATTCAGTAAATGCTGTAGTCCACACACACTCAATGTACTGCACCGCATTTTCC
GCACTTGATAAAAAACTAAAATTAGTAACCCCTGAAGCTGAAATGGTTGTTAAAAAAATAGCAT
ACGTTGATTATTTCCCATGCGGAAGTTTAGAACTTGCAGAAAATGTTTCAGAATGCATTGAAGAC
TCAATTATCTTAAAAAACCACGGAATTGTTACATTGGGTAAAGATATAACTGAAGCATACATAA
AAACCGAAGTTTTAGAAGAAGTTGCACAGTTAAATTATATTATGAACAATTTAAGATAAAATAA
ATCTCTATTCTAAAAATTTTAAAAAAAGTTTAAATTTAAAATTTATTTAAATTCATTTTTTTCTA
ATTAATTTTTAATAGTTTCAAGTTCACTCACAATTCCCCAAATCTGGGTTCTCGTGTGAATTGGC
ATATTTGGATCTTCACTGATGTCATCAAGATATTGGATTGCAGTTGCACTTCTCACGATTAATTC
CTGTTCTTCGTTGTGTAGTGCGTTTTTTGCATTGTCTGCTGCTGCACGAATGTTTCTAGGAACAG
TTGTATCTTCAACGATTTCTTCGAGCATTGATGAAATATTTTTAAGTTTATCTGCTGGAGATAAT
TTTTTGGCGCTAAACATGGGTTCACCTCATTGGCCGTGTTTTAAATAAAAAAGAACTGTAAATTC
AACTATTGCATAATATGATTTAGTGATATCCTATTTAAAGATTAAGGTATTACTTCAAAAATTG
AAATAAAAAAAGTATTTATCAGAAATTCCGTTATTTTTTGAGTATTTCTACTTTTTTAGAAGTTC
CAACAAGCACTTTATCACATTTACTAAATATTCCATTCTCTAAAACACCAGGAATATTGTTTAAC
ATGGTTTCAGTTTCTTCTGCGTCCTCAATATTGATAAATACATCAATAATCATGTTTCCATTATC
AGTAATTACTGGACCCATTTTTCCAGAACCTGACCTAATTGCAGGTGCAGTGTTCATATCCAAAA
GTTTGTTTAAAACTGTAGAATATGCAAAAGGAATTACTTCTAAAGGTACCGCTGTTTTGTCCCCT
AATGACTCAACTAATTTTCCTTCATCAGCCAGTACAACGAACTCTTTTGCATGGTAATCTACAAT
TTTTTCCATTGTGTGGCATCCACCGCCACCTTTTATAAGTGAAAGAGTCTTTTTACAGACTTCAT
CTGCACCATCTATTGCAATGTCAATTTCCCCACACTGGTCAAGTGAAATTAAAGGAATTCCAGAA
TTACTTGCCATCATTCTTGAATCAAAAGATGTAGGTACCCCGTAGATATATAATTCTTCTTCAAC
AACCCTTTTTCCAAGTTCCTGAATGAAAAGATTTGCAGTTGAACCCGAACCAAGTCCCACGACCA
TTTCATCTTTTACAAGTTTTGCCGCTTGTTTTGCAACTTTTAACTTCAAAGAATCAGAATCTGTG
GCTACTTCGTCATTAGCTTTTTTAGTCCTAGCCATAAAAATCACCAAAAATATCTAAAAATCTAA
ATATTGAAAAAAGACATAATTTTGTTAAAAAAGCAAAATTAAAGTTTTTCTATAATTTTTATTG
TAAATTGTAGTGTTTCTCCCGCTAATTCGTGGTTGAAGTCAAGAGCGACGTCTTCATCAGTAACT
TCCAAAATTACCGCAGGTTCTTCACCAGCAACAATTACCATTCCTTCTTCAGGTTCAAAGTCTGCT
TGATCGAAAGCTTCAATTGGAACTCTTTCGATTAATGATTCATCTCTAAATCCGTATCCTTTTTC
AGGAGGGATTGTAACGGTTACTTCTTCCCCAATTTTGAGGCCTAAAACTGCTTCTTCGAAACCTT
GAATTAACTGTCCTTCCCCAACAGTAAATTCCAAAGGCATGTATGGTCTTCCTTCTTCGTAGATT

CCTTCTTCTTTAGCCACAGCTTCTACTGAGGTATCGAAAACTTCCCCTTCTTCAAATCTGCCGGTG
TAATCTACTTTTATTGTATCTCCTTTGGTAATCAAAAAAATCAACTCCTACTTCATCTGTGACTA
TCAATCAAATAATCGATATAATATATATATCTTATTATCGGCCCAGTTTATACACTAACTTTGTA
AATTGGCCTTTTTAAGGGCATTTTTTTGAATCAAATCAAAAGTTATAAAAATTAAAAAATGTCT
AAAAAACATAGAATATAAAAAAGGTGGAACAGTGCTTAGTGTAAATTTAGCTTCATTACCAATT
GTTGAAGAAATGATCAGCAGAAAAGAAGACTTAAACATTGAAGTAATTAAATTAGAAAACGGAG
CTACAGTACTTGACTGTGGTGTAAACGTAATGGGAAGCTTTGAAGCAGGTAAATTATTTACAAAA
ATCTGTCTTGGTGGACTTGCGCACGTTGGAATAAGCATTTCAGGATCCCTTGACAACAAACTCGT
TTTACCATGCGTAAAAATTAAAACATCACACCCTGCAATCGCAACACTCGGTTCACAAAAAGCTG
GATGGAGCGTAAGTGTTGGAAAATACTTCGCAATGGGTTCAGGACCTGCAAGAGCATTAGCAATG
ATGCCAAAAGCAACTTACGAAGAAATTGACTACCGAGACGAAGCAGATGTTGCAATTCTTTGTTT
AGAATCTTCACAACTTCCAGATGAAAACGTTGCTGACCACGTTGCTGAAAAATGTGGCGTAGATG
TTTCAAAAGTGTATTTACTCGTTGCTCCAACAGCTTCAATGGTAGGAGCAGTTCAAATCAGCGGT
AGAGTTGTAGAAAACGGAACCTACAAAATGTTAGAAGCTCTTCACTTTGATGTAAGAAAAGTTA
AATTTGCGGCAGGTATTGCTCCAGTTGCTCCTGTAATCGGAGACGACTTAAAAATGATGGGCGCT
ACAAACGACATGGTTTTATACGGCGGTAGAACATTCTACTACGTTAAAAGCGATGAAGGAGACGA
CATCGAAAACTTATGCAAATCACTACCATCCTGTTCCGCTGAAACCTACGGAAAACCATTCTTAG
AAGTATTCAAAGAAGCAAACTACGATTTCTACAAAATTGACAAAGGAATGTTTGCTCCAGCAGTT
GTTACAATCAACGATCTTAGAACCGGAAAATTAATGTCATACGGTGAAACAAACGTTGATGTACT
CAAAAAATCATTAAAATTCAGCCAATTATAAATTAACATAAATTATAACTATAAATATAAATAC
TAAATTTTAATATCTATTTTTTAAAATATTATTTCGTGTTTTGTTAAAATTTACAAAATTAAAA
TTACCGTTTTTTAAGGTGTTTTTATGGATTTATCAAATTTAAATGAAAAAAATATGGCCTTGGGC
TGTAAATACTGCATAAAAGGAGAAAAACTGGTTTTATATATCACAGGACTCTGTGAAGAATCCTG
CTACTACTGCCCGCTTTCAGAAAAAAGGAAAAAAAAGGACGTAATTTTTGCAAATGAAAAGCAG
ATTAATTCAGTTGAGGAAGCAATTGAGGAAGCACATCTTTGTGGAAGTAAGGGGGTAGGAATAA
CTGGTGGAAATCCACTTTTAAGAATTGAAAGAACTGTTGAATATCTAAAAGATTTAAAGGAAGA
ATTTGGGGGAAATTTCCACGCACATCTTTACACAACACCAAAATTTGTTAGTGAAAAAAATTTAA
AACTTTTAAAAGATGCAGACCTTGATGAAATCAGACTTCACTCATCAAAATTATTCAATGATTTT
GAAAATTTTGATAAACTGGATTTTTTAGAAAAATTGAAACTCTGTAAAAACTACATTAAAGATG
TTGGAGTTGAAATTCCGGGAATTCCAAATTTTGAAAAAGAAATTTTAGATTTAGCTTTTGAAAT
TGATAAAATCGGCGTAAAATTCTTAAATATCAATGAACTCGAATATTCTGAAACTAACTACCAAG
CTTTAATCGATAGAGGCTTTTCTGAAAAAGACGATACAACTTCCAGAATTTCTGGAAGCTTTGAA
ACTGCAAAATATGTTATTGATAATTTTAAAGGAAAATTGATAATTCATTTCTGCCCTTCTTCATT
AAAAGACAGTGTTCAGATGAAAAACCGGCTTATAAATCGTGCGAGAAACGTTGCAAAGCCTTATG
AAGAAATTACAGAAGAAGGCCTTTTATTAAAAGGAACTATTAATTTTAAGGATTTAAAGGACGT
TTCAGAAGTACTTGAAGTATTAAAAGAAAATGACGTAGAATTTGAGCTGTTAAATGACAGATTA
TTATTAAATCCAGAAATTTTAGAAGATTTAATAGATCATTTAAAAGAAAACAACTTTGATTTTA
AATTCAGTGCATATATATCTGAATATTACCCAACATCTGATAAATTAGAAGTTGAAAGAATCCCA
CTTGTTACAAAAAAGCCTAATTTAAAATTAAAGAAAAAATAAGCATTTATTAATTTAATAAAAA
TTAATTAATAGATTATTATTTAAAAAACCCGAATTTATTTGATTTTACTTAATTTTTTCGAAAAA
ATTTTGGTTTTCAAAATCTATTTTAAGTGCTTAAAAACAGTTAAATCCCGATTATTTAAGATTAA
TTTTATATCATAAACTTTATAATTGGGAATATTATGTTTAATCTATTTTTATACATTTAATAATT
AAATTATAATTTTTTATAAATTTAATTTAACTTTAAATCTAAATTTTAAGTTTAATCTTTTAATT

AACATTTTTTAGACCTTTATTTTAAAAATTATAATTTCGAAAGGTTTAAAAAGTTTATTTAACAT
AGTTTTGTTCGGGGAATAAGAAGAGGGGCGCGTAAAATGCCGTAAACATTTCATCGTAAAGGTCA
TAATAAGACGAAATAAAATTTAGAACATTGTTTGGGATAAATACGAAAGTATTTAACTGGAATT
TTGTGACCTGTTATGGATATTTGAGCATTTTGAAAACATATTAAAATTCGGGGGAATTTTTAGTT
TTCGATAGTATATTAAAACTTGCTTAGAGGATACTCCATATATCGGTCATTTAGGATATTCAATA
TGAAAACAGGGGTGTTTTTGTGAATAAAAAATCAGGACAGGCACTATTATTAGTACTTGTTATG
GTTCTCTCAAATATGGGGGGTGTTATGGCACTTGATACTACCGCTATAGTCGATTGCCCATGTAA
TGGCGGCGACGGTGGTTCAGGCACCATTATCATAGACTGTAACTGCACTGACGATAGATGTTTTA
ACGTTAGTATTACTTCACCAGAATGCGCATCCACGTGGTGTGATGAAGTTCCAATTACGGGAACA
ATTGTACCATGCTATAATGATGATTTGGGATGTGCGGACTACGTAAAAGTATATTATAAAAAAT
TAAGTGACTACTGCTGTGACTGCGAACTTTACTGCGAAGGCGGTAATCCTGCAGCAGATAACGGA
GAAGGATGGAGCCTTGCAGGTGTCGACAGCACTTTAGAAGGATATTGTCCTGCTGAATGGAATTT
AGATTGGCCTATCTGTTGTGAACCAGATGGCTGTTATGTATTAAGAGCAGTACTTTACGATGGAT
GCGATATACAGGTAGGCTGCCCTTCAGAGCCTGTATACGTATGTTTCGATAAAGGAGTTGCTGTA
AACTTAGACCTTCCAGAGTCACTTTCCTGTGGAGGGGTTATCGGAGGATGTTTAAATGAATCCTG
CGACTGCGTTGATTATATTTTAATAACTGCAGAATATATGGGAGAAGGCACTTGTACTCCAAGAT
GCCCTGTAACTGACTGTATGCCTACTATGACATACATCGTCGATAGAGAAGACTTTGAAAGTGCA
ATGTGCTGCAACTACACTTTTGACGTATTATTTAACAACTTATACTGCGGAGGAATCTATGAAAT
AACTGCTACACCTTACGCAGACTGTACCGATGACTGTACATACAATGGTGTACAAATCGGAGACG
AATACATTGGATATCTCGAAATTATAAGGGAAGAGGTATGCTTTGAAATAGAATGCCCTGCACCA
CTTTCACCAGAATGTGGTATGACACTTTGCGGGCCAATTACATTTTCAGGTATATTAAATGATCC
TTGCAATGCTGCACAAAAAATTGACTTTTATGCAGTAAAAACAGGAAACTTCACACTTTGTGATG
GAGCTTGTTGTCCTGCATGCAATGTGTGCGATGGAGAAATCATAACACTCGATGCAGAAGAACCT
CGAGAGTTAGCTGAAAATATCTGTCCAAACTGCGGTGCAGAAATGGATCCAATCGTATGTGGTAG
CTGCGGTGGAATAGTTACAGGATACTTCTGTCCAGACTGCGGATATCCATCAAATGAAGATCTTG
GAGACGGAATTTCAGAATGTATCTGTGGAATTCCTGAAGACCACGATAATTACATCAATGAAAAA
AATTTAAGCGATATGGTATACTTAGGATCCACATCTGCTACAATGCCTGATTGGACCTTTACAAC
AAACAAATTCTGTGATTTCGACTCAGGATGCTATGAAGTATACGCTGTAGTTGATGATAAATGCG
AATGCTACGATGAATTTAGATATGACACCGTTGAATTTGCATACTGCAACGAAAGAAATTTGACA
ATTGAACTTGAAGACTGCGTATACGAAGAATGCTGGATGGGTAATGATTGCTACAGAGTATACG
ACATACAAAGTGGAACAGTTACTGCATTTGGTAATATTACAGTTCCAACCTGCTGGATAGATTAT
CCTGACTACGTAGACTTAGAATACGACTTTGGACATAAAGTACAGGTTGATAGATGCCAATGGTA
TATTGAATGGGACAACTGCTGGAAAGATGCAAGCTGTACAGACAGTTCAGTTACTTGGACTTACG
AAGGAGCATGCCTTAAATCAGGAACCTGGGAAACTGACTGGTGTACTCCAGAAGATATTGAAGAT
GAAGAAGGAAGATATGTAAGAATAAGAGGTGTTGCAGAAGGAGGTTGCGGAGAATTTGAATCTG
AACCTATTGGAACGAGTATATACAATGATTTTGACATTGAAATAATTGATCCTTGCGAAGGAAAC
ACAATATACAACGGTAAAGTTATAAGAGGATTCGTATGGGTAGATGCAGACTGTTGCGGCTGGTG
GTGTTGGCCAGATACTGACGACGTTTGCCTGCCTGACCATGTAGATCTCTACTGGAGGGAATATG
GAAGCGAAAACTGGTTGCCAATGGACAGCGCATTCCTTAACTGGAAAAACAGCGAACAGTACATT
GACTTTACAGATTACGGAATAGATCCATTATATCCAAACTTACCGAATTTCAACTGGTGGGACCA
TTATGCATACTGGGATGTAACCTGGTCTTCATGCATGGTAAACTGCATTGATAATGGACACTACG
AAATAAAAGCAGTAGTAGAACCATGTGAAAATGACGAATGCGGAGACTTTGAACGTGCAGAATA
CGTTATGGATGTCTATGTAGATGACACCTCAGGTGTTTCAATCTCAAAACCAATCGACGGACAGA

CTGTGTGCGATAACTTCTGGGTATACGGTACAGCAGTCGGTGCTGGAGTTTCAAACGTAGACTTC
TATGTATCAGAAGATAACACATCTTGGACATTATACAAAGAAGATATTGATGTAAATGTAAATG
ATGGAAGATGGAAAGCACCACTTAACACGTGCTGCTTTGAAGATGGACCATTATACTTAAAAGCA
GTTGTTACTGGCCCATGTGACCAGAGCGAAAGCAATGTAATGGTTAATGTAAATAACGCTGTCGA
AATTGATCTAACCTGCCCTGACTGTTGCGTATACTATGTAACAGGAGATAAAGCACCTATTAGGG
GAACTCTAACTGGATGCTTAGTAGATGAAAGAGTAGAAAGCGTAAAAGTATACTCCGCAAGTCTT
GGACTTCTCGGAGAAGTACCTGCTGAAGATTTCAGATACATTGGCGACGGTACCTGGGAGTGGGG
ACTGTGCTTTAACCCATGTATATTGAATGGAGCATCAACACAGATTTATGCTGAAGCAGAATACT
CATGTGGAAATACATACACCACAATAACAACAGGATACGAGTGCATACAATACGACTCCAATCTT
GTAATAGTGGAAATTACCGGACCTGGAGCTGAACCTACAGTTGCAGGAACCACTACAATAGAAGG
TACTGCGGAATGCGCGTGCTTAGATTACTTAGAATTTGAATACAGAAAAATATGTGAACCTACAG
GAGAACCTATGGGAGACTGGACTAATTTCCCTGGCGTCTCTGTAATATACCTTCCTACAACATGT
GACTGTGAAGGTATGGAATGGACAATCGATCTTAATACGTGCACAATGGATGGCCTCTATGAAAT
AAGAGTTTCAGCATACGGCTGGCCATGTCAAGGCGCAATCGCATCAGATACAGACATGATCGATG
TGGACAACAACTTTGGAATTACATTAACAACTCCAGCAGTAAATTCAGGAGTTAAATCCGGTACA
ATAGAAGTTGGAGGTACAGTTGAAAATTGCCCTGTAATTGATACTATCGAATTATGGTATGTTG
ATGAAGGGGTTCCAGGCATGATTACAGATGAAATTACCTGCAGTGATGGACTCTGGAGCTACATG
TTTGACACTACAACATTAGAAGACGGATGTTACTGTCTTGTCGCTTACGCATTCGATGAAGATTG
CAACAACATGGAAGCAACTCAAACAAGAGCTATATGTATCGAAAATGGTGGAATACCACCAGTAA
TTAATGATACAAACACTACAGTAGATAGAGAATGTGTAAAAATACTCAATGCATGCTACGAACCA
TGCAATACAGTAAAAGTAACATTTAATGACACAGTAAGCATTAATGACTCCTATGATCCTGCAAA
CCCATTGGATGGATTTGATATCATCGGTGGAAGCTTTGGAACTGGAGCTACAATGGCTGAAAGTG
CAATAAATGACCATATGATAATAATTACCCTTGGATCTAGTAGTAATATAATTACCAATACTACA
GAAATAAGCATTAAAATAAATCAGACTGCAATACTTGGCCCACATGGATTCCCAATAGTTGATTG
CAGTGCAGAACTGAAAGGAATTTCGGAAGCTGCAGAAAGTACAGGTTCAATTGAAATTGAAAAC
TTGAAAATGTTTTCAGTACCTGGAAAAGTAGATACAGCACAAATGGTTGCTGACTCAGGAATTAC
AAGCGGTTCATTTGTGAAATTCACCGACGGACAGTGGGAATTAGCAAGTGGATTCGAACCACTTG
AAGGTTATGTATACATCGGTACCTGCGAAGGAAGCATGCCTATATACACCGTACCATTAACAGCT
ACAGATACACCACCTGAAATACAACTTTGCTGTGGATGGAACCTTGTAGGTGTAAATACATACGG
TGGATCACTTGATCCTGTGTTATGCAGTGAATTCACTGGAGGATACCCATCCATTACTAAAGTAC
TTGAATTTAGTCCTAGTGGATGGATAAAACTTAATGGTGGCAGTCAAGTTATACCAACCTACGCA
TACTGGGCATATTCAGACTCAGAATGCAACTTTGCAGGACAGTAAATACTAATAATGGCTGAAAA
TTAAAATTCAGCCATCCTCTTCTTTGGGGTGTAAAAATGAAAAAAATATATACTGTATTATTAGT
ATTTGCACTTGTTTCCTGCTTAAGTATTGTATCTGCAGACTCTATTCCTCAATTTCCTCATACATT
TTATGGGGATGTAACAATAAATGAACTTCCCGCAACGGGAACACTGAAAGTACTTGTCAATGGAG
TAGAAAGTGAACAAGTGCAGGTTACGGATGGTGAATTTGGTAAAGGATTGTTTGATGCCAAATT
AGTTGTTAATGGTGCTTCAGGGGATCAGATTACCTTTTCATTTGAATCAGAAGGTTATACATTAG
ATTCAGATTATAAAATATATCTTATAGATTCGGAACAGTATGTTTCAGAAATCGACTTTGTATCT
GGAGGATACACTGAAATATTATTAGAATTCACCGGAACGGGAGACGGTGGAGATACCGGTGACAC
AGGGGATACTGGAAATGGTGAACAATTACCACTTAATCCCGGTGTATTCTATGGATTAGTTTATT
TAGATGAATCCTTAGCATCAAGTACATTGAATTTGTATGTTGATGGAGTACTCCAAGATTCTATT
GGAATTGAAAATGGAGCATTTGGAGGAGAAGGAGCTCTCGATGATAAATTGATTGCAACCGGAT
ACGATGGTAACAGTAACGTGGTTACGTTTTCATTAGTTTCTGGAGGAGAAACTTACTCCAGCTTT

ACTGCAGAATTGTCTGATGCTACTTATGAAGATGAACTTCCATACATTGAAGGAGTGCATTATGT
GGTACTTACGTTCTCAGAAATTGCTGATACAGGGGATACTGGAGACAGTGGAGATACGGGTAATA
TAAACAGTAGTGAACAATTACCACTTAATCCTGACATATTCTACGGAATGGTATATTTAGATGAA
GCCCTAGCATCAAGTACATTGAATTTGTATGTTGATGGGGCACTCCAAAATTCAATTGGAATTGA
AAATGGAGTATTTGGAGGAGAAGGACCTCTTGATAATAAATTGATTGCAACCGGATACGATGGT
AACAGTAACGTGGTTACGTTTTCATTAGTTTCTGGAGGAGAAACTTACTCCAGCTTTACTGCAGA
ATTGTCTGATGCTACGTATGAAGATGAACTTCCGTACATTGAAGGAGTGCATTATGTGGTACTTA
CGTTCTCAGAAACTGCTGATACAGGGGATACTGGAGATACTAGCGATTCTGGAAGTAGCAGTGGA
GGCGGAGGTGGCGGATCTAGCAGAAGCAGTTCGTCAAGCTCTTCGTCAATTATAAGCAGTTCAGA
TTCATCAGAAACTCCTGTTACAACAAGTGTTCCTAAAACTTCAAGTTCAGGAACATCAACTGATT
CATCATCAAATACTGTTGAATCTTCAGAAAATGTTGGACCAGATACCACGCAAGATTCACCTAAC
GACACGTTAGACTATGGATCAGACAGTAATGAAACTGGAGTTGTATTACAACAGGAAAGTCCACT
CGGTGGAATAAATATGTACCTTGCACTGGCTGCAATATTATTAATATTAATTGCACTGGCTGCCG
CTTGGTACCAATCAAAAGAAAAACCAGAAGTTGTGCCTCAGCAGTAACTTCTTTATATTTTACTT
TTTTTGTGAAAGTTTTTTAAAACAGTAATTAAAACAGAAATATGGGTTTCGTTTTTGAAATTTT
TATATTATTTTCGGGATAGTTATGGATATTTCAGATTACAAAAAATACAAAGAAATTTATAAAA
AACTTATGAAATCAGAATTTAGGGCTAAATTTCAATTGAACAATCAAAATAAAGAATATATTAA
AGATAAAGGAATAATTAAAATCCAAAATCATGCATTTGAGTTTTTAAATGCAAGAATTAAACCA
GAATTCATAAAAAATGATGGAAAACAGACTCCAATGAATGGACATCCTGTATTTATCGCACAGCA
TGCAACAGCAACTTGTTGTAGAGGATGTATCCAAAAATGGCATAAATTTCCAAAAAATAAGGAA
TTAAGTGATGGCGAAGTCGAATATCTTGTTGGACTTATTATGTTTTGGATAAACATTCAGATGAT
GGATTAACAGGTTTTACGAACGATTTCGATATATTTAAAAAGTTAAAATCGTTATACAATAACA
ATCTATGATGTGGTGAAAACATGAAAAAAATTGTTTTGATTTTGTCGCTCGTTTTATTAAGCAGT
TTTTGCCTCTGTACTGAAAAACCTGACAGCGGAAGTGATGACTTAAGTGACTTCAGCAGTACGCT
TGTAGTCCAGTTTGGAGACACTACTGTAAATGTACCTCTAAGAGCAGTGATAAACGATTCAATGA
CTACAAATTTAATAAATACGAATGATTCCGAGATAAAAAGTGACTACACGAGCGGAAACTACAA
AATAATATACTTTGAATATGATTCCAACATTTCAAATGAAGAAGGCGGTATCGTAATTACAGACC
TTGTTTCAAAAGTTAGCTTCTTTAATAATGTATATCCTTACGCAATTGCTGACCAAACTTTGTTT
AACAATACACAAGACATTGAAAAGGTTAAAAATAGCAATAACACACTTGTTATTCAGATTGAAA
GAACAGAAAATTCTGCAACTATTGAAAAATACAACAACACCTATGTAATCGAAGGAAACTCGCTT
GAAGAACTTGATAAAGCAGAATCCAGATTTGTAATTGCAATGCTTTCTTAAATTTATTTAATATT
TTTTAGTTTTTATTTATGCCTTATTTTCTTAATCGAAATTAATTATCGTTTTTTTACCGAATTAT
CTATAAAATTTTGCTTTGGATGTTTTACATTTAAATAGTTAAACTTTTTTATATTCTAATAACAA
CAACCTCGTCATTTAGTTTTTTAATATGGTGAACTTAAATGAAAATACTAAAAAACCTTACTTTA
CCGATTATTGGAATTTTAGCATGGGAAGCTCTTGCAATTTATTTAAATAATCCAGTAATCCTTCC
AAAAGTTGAAAGTGTAATTTCAATCCTTTTAAATCCCGGAGTTGGAATCCTTGGAACAGGAAATT
TAATTGAAAACACTATTGTAAGCATTAAAAGGGTTTTAATTGGATTTTTTATTGCTGGTGCTTTT
GCAATACCATTAGGACTTCTCATGGGCTACTATTCACTTATAAATGATTTATTTGATACAACAGT
GGAACTTTTGAGGCCTATTCCCCCTCTTGCATGGGTTCCACTTGCTCTTGCATGGTTTGGAATAGG
CGAGTCCTCAATGCACTTTATTATATTCATTGGTGCATTTTTCCCAATTTTGATAAATACAATTT
CAGGAGTTAAAAGCGTACCTGTAATTATGATTGAAGCTGCAAAAACTCTTGGAGGCTCCACAAAA
GATATTTTAAAAAGTGTAGTAGTTCCTGCATCATCCCCAGACATTTTAACAGGCCTTAGAGTTGG
AGCAGGGATTGCATGGATGTGTGTTGTTGCAGCTGAAATGCTTCCCGGAAGTGATGCTGGACTTG

GATACCTGATAATGTATGCATATTCTTTAAGCAAAATGAACATTGTTGTAGCTTCGATGATAATT
ATAGGAATAATTGGAATAATTTTAGACAAAGGATTAAGATATATTGAAAAGAAATATTTCTGCT
GGAAAAAAATGATGAAATAGATTATAACTCAAATATAATAACTTAAAACCGGAAAAGATAATAT
GTCAGTTTTAGAACTTAAAAATCTTATCAAAAAATTTGGAAATGGTAAAAAAGACCTTGTTGCA
GTTGATAATGTAGATTTATCCGTAAATCACAACGAATTTGTGTCAATCGTTGGCCCAAGCGGTTG
CGGAAAATCCACAATTTTAAGAATGATCGCAGGACTTGAAACTCCAAGCTCTGGAGGAATTTATT
TAGATGAAAAAGAAGTTTTGGGACCTGATGCAGATAGGGGAATGGTATTTCAACAGTACACCCTT
TTACCCTGGAAAACAGTTTTACAAAACGTTACTTTTGGACTTGAAATTAAAAAGATCCCGAAAGA
ACAAAGAATCGAAATTGCCAAAAAATTTATAAAAATGGTTGGACTTGAAGAATTTTGTGATTCA
TACCCTTATGAATTAAGTGGTGGAATGCAGCAGCGAGTTGCAATTGCAAGAACTCTTGCAAATAA
CCCAAAAATTGTATTAATGGATGAACCATTTGGAGCCCTCGATACACAAACAAGGGCAGTTTTAC
AAGACCACCTCTTAAAAATATGGCAGACTGAAAAAAAGACTGTTGTTTTTGTAACACACAGCGTT
GATGAAGCAGTTTATTTGTCAGACCGTGTTGTTGTCATGACTGCAAGACCTGGAAAAATAAAAAG
CATAATCGATATAGATTTAGACAGGCCAAGAAAAAGAACCAGCCCTGAGTTCACAAAATACAAAA
AAATGATAATTGATGAATTAAAATCAGAAGTTTTAAAGTCTTACGTCTAATAATTAATAATTAC
CTTTAAATTTATATATTTTTAAAACATATTCTTTTTTAATGAACTATTATTCAAATTATATGAAA
TTTAATTCATCAAAGGGGATACTATGCTGAGGGGAAAAGATGCAACCCTTGCTGCAATAATAAAT
ATTATTCTTGACGAAGAGCCAGAAACTCAAGACGATATTGCAGACAGATTAGGCGTAAGCAGACG
GTACGTTGCAAAATTGCTAAAACCGCTTGTTGATGAGGGCGCAATAATGCACCCTTATGTTGTAA
ATCTTGAAAAGTTAAAAGAATTTGAAGAATATATCGAAACCGACCGGTATTTCAAAGAAATTTA
CGAAACTTTCGATAGAATGGGAACTAACGTTATTCAAAATATAGATAATGTGTTTGATTCTTTAA
AAACGCATGATTTAGATATTGCTAAAAGTATTATTCTTGAAGACTACGCATTAAACCGGATGGAA
GATGAAGTAAATCTTGTAATTAAAATGAAAGCTTCAAAATACATGGATATGAACAGTTTAATGC
AGGTATCAAATATTGCTGCGAATATTGAAAGATGCGGGGATTATCTGTCAAATATTGCAGAAGA
AGTTGTAAATGGGCTTTTGGTTGATCCTACAATAAACAAGGAAGTTTTTGAAATAAAAGAGATA
ATTTCAAAGATGTTTACCCATGCAATGAATATGGTCAAAAGTAAAACGATTGAAACAGAAATTT
ACGAGTTGGAAGGCAACCTCCATAAAAAACTGGATACAATTATGGGAAAAATTGCGGAACATCCT
GATGAAAATTTAAAGGATATTAACCAGTTTATACAATTTGGAATGTTTTTAAAAGATGTTGAAA
GGTTTGGAGATAGATGCCTCAAAATATTTGAACTGGGACGTGAATTCCACCACAATATTCCAAAA
AACGTTACAACTCCAGAATATGTTAGAAATTTAAAATAATTACTTTATAAAAATCTTTTTTGATA
GAGCTTTTTTAACGAGATATTAATTAAAGGATGAATTATATATCTTATTTTATCGACATATTTAA
ATAATATTACTGCTTATGATGAAAAAACAACTTATGGTGAAACCATGGAATTCTTGGGGAACGA
AATGATTACCGTTGACGGCGGAAACTTAAAAATTGACAGGCACGATGCAAGCAAACTTGCTAGTA
CTTACGGAACGCCACTCTACGTTATGAGTGAAACCCAAACAGTTAAAAACTTTACAAGATACGTG
GAATCATTTAAAGAATACTCTGAAAAAACCGGAAAAGAATTCATAATTTCATTTGCATACAAAG
CAAACACGAACCTTGCTGTAACAAGACTCCTTTCAAAACTTGGATGTGGCGCAGATATAGTGAGT
GCAGGGGAATTATACATTGCAAAACTTTCAGACGTTCCTTCCGAAAAAATTGTATTTAATGGAAA
CTGTAAATTAAAAGAAGAAATTAAAATGGGAATTGAAGCTGAAATAAGAGCATTTAACGTTGAC
AGCATCAGCGAACTTGTTTTAATTAATGAAACTGCAAAAGAAATGGGAAAAATTGCAAATGTTG
CATTTAGAGTAAATCCAAATGTTGATGCAAAAACACACCCCAAAATTTCAACAGGAATGAAAAAG
AACAAATTCGGGCTCGACATTGAAAGCGGAATTGCACTTGAAACAATTAAAATGGCAGAAAAAA
TGGAAAACGTGAAAATTGTGGGAATTCACTGCCACATTGGTTCACAATTAACATACATTAGCCCA
TTCGTTGAAGAAGCAAGAAAAATCATGGATTTCGTAGTTTTACTCAAAAACGAAGGAATTAAAA

TAAGAGATGTAAACTTAGGTGGAGGCCTTGGAATTCCATACGATAAAAATACAAAAATTCCAGT
TCAAAAAGACCTCTCAAAAGCAGTTCTCGATGTAATTTATGAATATGAAGGAAAAATCGAACTTC
CAAATCTTATATTGGAACCGGGAAGAAGTTTGGTTGCAACCGCGGGAGTTTTACTTGGAACTGTC
GAACACGTTAAAGAAACACCTGTTGCAAAATGGGTGATGATTGATGCTGGAATGAACGACATGA
TGAGACCTGCAATATATGAATCATACCACGAAATTACGCCATGTACAATAAGGGCTGAAAAAGAA
GTTGTAAGCGTTGCAGGAGGACTTTGTGAAAGTTCTGATGTATTTGGAAAAGATAGAGAAATTT
CAAAAATGGAAGTAAAAGATACTGTCGCAATTTTAGATGTTGGAGCATACGGAATCAGTATGGC
AAACAACTACAATTCAAGAGGAAAACCTGCAATGATTCTTACAAACGAAAAAGAAGTTTCATTA
ATTAGAGCAAGAGAAACCCTTGCTGATTTGATTTCAAAAGATATCGTTCCATATCATCTTTTGTA
AATTAAATTATTTTTAATACTTTTTTAAAAAATAATAGAAATACAGAAAAATTAGTAAACTAGC
CTAAGATACATAATATCGTACAAAAAACTGTAGATAATACTAAAAGAGATATTATTTTATATTC
TGCCTTTAAGTCGTATCTTAACATTATATCCACCCAAGGAGGATATTTATTTTTAAAAGTATTTA
CGAGTTTCGAAATGAAATCGTGCTAAAAAAATTAAAAAAATGATTCCTATTTTAAAATATTAAT
TGTTGTAATTCCAAAGACCGATTCTGTCTGATTTTGCTGTTTTTTCAGTTTCTAAAAACTCATTC
TTTAATTCAAAATCGCTTGTATATACTCTTGCAAATCCATATTCTATCAGATTTTCATTGAAATT
TTCATTTTTCAAATAAATATATGCAAGATATCTTCCGTATTTGTCCTGTTTGTCACTTTTTTTAT
CAAAAACTACATAAACTGATTTGTTTTCGAGTGTATCTCTTGTATAGTTTGTTGCCAAATGCCCC
CAATAATCCAGATAGTCAGTATCTGAAATTGGAGTTTCGTAATCGAGATAATATTCTGAAACACT
GTTTTTTTGATAAGTTTCTGGAGTATCAACCCCCAAAAGCCGGATATTATAATCAACTCCGCTTG
AATCAACGTGAACCGTGTCTCCATCAACTACTCGAGTAACTGTTCCAAATAAATGTTCGTGAGAA
TCTATGAAATCTTTTGAATCGTAATTATGGTTATCACTTGAAATACATCCTGAAAAAATTAAAAA
TAAAATTGGGGCTAAAAAACATATTTTTTTCATAATAAACCCTTAAAAATCCAGTTCTTTTGAAT
AATTTTTTATATCGATCAAAGGAGTTTCTGAAAATGCATCAATTTCTTCAATATATATTTTATTT
CCAAGAATCTTTTCAATATTTACCGTGTAAAGTGCAATAGGGTTTGGCCTCATAGGGGACCTTGT
AGAAAAAACGCCCCTTATCGGGTTTTTGACGTTTCCCCTGGGGTGAACCATTAGCGTTTTTCTTT
TACATTCTTCATCTGATCTATTAAACCATAAAAAAACCAAGATTTTTGAATTTTTGGTTAATCCA
TCAATTCCATCCAAAAATTCGTCGTAAATTTCTAAATATGAATTTTCCCCCTCTTTTTTAAACAT
TCCAATTTCAAAAATTTTGTATTCATTTCTCAAAATTTCACCCCTAATCAGATTTTCCAAGTATA
TTTCCTTTCATATCTATTATAATGCATGAAAAATTGATTTTTTCCCATCGCAAAGAAAGTCGTTC
AACTACCCTTTTTGAAATAATTTCAAAAACTTCTGTTAAAATTCCTTTTTCTGTTAAAATCTCAA
TTATATCTTCAGTTGTGTTTGATTGAAGTATTTTTTGAAGCATTTCTATATCTTGCGATACTAAA
GAAGCATAAGCACAAAGTATTTCGTTTCTTGCATCCGCAATTCTCGAGTGAGTGTCAAAAATTCC
TCCTGCAAGCTTTACAATTTTTCCAGCGTGTCCAAAGACAGTTATATCTTTAACGCCTTTTTCTTT
CGCTTTATCGAGCATGTAATCCCAAAAGTTTGAAACTTCGATAATTTGGTCTTCTTGTACATCCA
ATAAAATTTTAGCATGTTTTGTTCCAATATTTCCCGGAACAAATACCAAGTTTTCAAAATTATAC
GCTAAAGCTACATCAATTTGGGGTGCAAGAGACTCTTTGTAAGCTTCATTTGACATTGGACGAAC
GATTCCAGTAGTTCCAAGAATTGAAATTCCGCCAATTATTCCAAGTTTTGGGTTTAATGTCTTTT
TAGCAATTTCATCCCCATTTGGAACTGAAATTTTTACAGTTACACATTCATCATTTTTTAAAAGT
GATTTTAAATTATTTTCAATCATTTCTCTTGGTTTTGGGTTTATTGCATATTCTCCAGGATTTAC
CTGTAAACCTGACTTTGTAACAATTCCAACACCGTCTCTCCCGATAATTTTAACATTGCCAGGTTT
TGAAGAGTTATTATCTAACTTTTCAAATGTAACTTCTATTTTGATATCCATTCCATTTGTAATAT
CGATATCTTCTCCAGAAAACTTCTCAACGGTTGAAATTGCAGTATTTTCCTGTTTTTCGATGTTT
TTAATTGGAATAATTAAAGAATCGCCTTTTAAATTTAAAATTTCAACAAAACGTAATTTTTCATT

ATTTTTTAAAAAATAGAGTGCAGAAAATGCCCCAGCTGCTACACATGCACCAGTAGTGTATCCAA
ACGTTTTTTCGAGTCTAAAATCAATCTTGCTCATATAAACCACAAAATAATAAACATATGATAAA
AATGTAAGTTAATATTCTAAAATTTTTGCATTGCTCAATTTTTCAAAGCCATTCTTTTTTACAAG
ATAAAGATCTTCAATCCTGACTCCGAAATCGTCCTTATAAATTCCAGGTTCAATTGTAACAACCA
TTCCTTCTTTCAATATTACATTTTCTTTTATTTTTGAAGAAATTGCTGGGTTTTCGTGAATTTCA
ACACCAACACCGTGCCCTAAAGAATGTATGAAATAATCTTTAAATTCACCCATATGTTCTCTTGC
AATCAAATCAAGCTCTTTTACAGAAATTCCGGCTTTTAAATTTCTTTCTGCCTCATTTTTAACAG
AATTTACAACATTATAAATTTCAGAATAATTTTTGATTGTTTCATTTAAAAGTACAGTTCTTGTA
ATATCTGAACAGTAGCCTTCATAAAGTGCCCCAATATCCATTAAAAGTATGTTTTTTACTAAATC
ATTCGAAGGCATACCGTGAGGAAGTCTCGTCTTTTTGTCAGAAATTGCTATTGTATCAAATGAAG
GTCTAATACTTCCATTTTTCTTCATGAAATATTCAATTTCTGCTGCAACTTGGTTTTCGGTTAAA
TTATCATTTTCAAGTGCCAAATTAGTAGCATATTCAATTGCATTATCACTTATTTTTGCAGCCTT
TTTGATATTTTCAATTTCTGCTTTCGTCTTAATTTCTCGCATTTCTTCGATTTTTTTAGAAACTA
GCTTGTAATCTTTGACAAATTTTAAAAAAAGTATTGGAAGGGAATCTTCAATTCCATTGCATTCT
TTAAATGTACTTTCAAAATTTTCATAAAGTTCAATATCAAGATTTTTATTCCTGTATCCTTTTGC
CATTTCAAAATCAAGTTTTGAAACTTGCAATGTTGCAGTGTCTTTTTTCGTATCAAAAATAAGCA
TTGAAAAATTTGGAGGGTATTTTTCTAAAAAATAATTTATATTCTCTTTTTTTGTAATAACTGCC
TTTTTTATGTCGTTTTCTTTTAAATATTCTAAAAATTCTTGAAACTTATCCATAAAAACCCGCTC
TTTTTAAATTAATAATCAATATACTAAAAAATTTATAAAATAATTAAATTACTTCAATATTACT
TCCAATTGATTTCATTTTATCTACAAAATCGGGAAACGATATTTTAACTGAATCTTCGCCTTCGA
TTATTGTTTCCCCATCTGCAAGCATCCCTGCAATTGTAAATGCCATTACGAGTCTATGATCATGG
TATGTTTTAAGTTTTGCACCGTTTAATTTATGAACTCCCGTAATTATAAGTCCATCTTTTTTTTC
TTCAATTTTCGCACCCATTTTTGAAAGTTCTGCGGTGCATGCTGCAAGTCTATCGCATTCTTTGA
GTCTTACGTGTTCTCCATTATAAACTACGGTTTTTCCTTTTGCAAAACATCCTAAAACTGCAATA
GTTGGAACAAGATCAGGAATATCTGTTACATCGATTTCGATTCCGTTTAATTCATATGGACCAGT
GATTTTAACATTATCGTCGTTTATTTCTACATCTGCACCCATTTTCTTAACTATTTCAATAATTT
CTCGATCCCCTTGCTTTGAATCTTTAAATACGTTTTTAATTACAATATCAGAATTTAATAAAACT
CCTGCCGCAACAAGGTATGACGCAGAGGAATAATCCCCCTCAATTGTATAATCACAGGGTTTATA
TGTTTGATTTCCTTTGATTTTATACCCTGATTTATTTTTTTCTTCGATTTTTCCAATTTTTACCCC
AAATTTGTCCAAAACATCTATTGTGATGTTTAAGTAGGGTTCAGACTTTAATGGAGTAGTTAATA
TAATTTCAGAATCGTCTTCACTAAATGGCAGTGTCATCATAAGCGACGTTATAAACTGTGAACTC
ATATCGCCCCTTATTTCAACAACATTACTGCTAATTTTTCCTGATTTTACAACAATAGGGGCTGT
TCCATTATTTTTTGTTGAAAAACATATCAGTCCAAGCTGTTTTAATGCATCCAAAAGTGGCTGCA
TTGGCCGTTTCCTGATTGAATCATCGCCAGTAAGTACAGTATATCCATTTGAAATTTGAGAAGAA
ATTCCAGTTAATATTCGTAATGTAGTTCCGCTGTTTCCAATATCGACAATATTATCAGGTGTTTT
TAATTCTGAACCTTTAACTATCCATTTTTCATTTTCCAATTCAATTTCAGCACCAAACATTTCAC
ATGCCTGCGCAGAAGATAAACAATCTGCACCATTTAACGGATTTTTTAAATTAGATAATCCGTTT
GCAAGTGATGCACAGATAACTGCCCTGTGCGTATAAGATTTTGATGGAGGAGCGCTTAAAATTCC
TTTTATGTAGGGGGTTTTTTTCACTACCAGCATTAAATCACCGAATATAATATTAATATTGAATT
AAATACTTTATATGATTAGCAATACCTGAATTATGGCTAATATATATTAATAAATATTGATTTA
GATACATTTACGTACCGCAAAATATATATATTGAAAAAGCGGAAAGCTATTTCCTATATAGTAAT
GATTTCGGAGGCATGAATATGAATGCTGTTGAACAGGCTATGGAATACATTAAAACGAACAACG
TTAAATTCATAAGATTCCAGTTTGTAGACATTCACGGGGAGCCAAAAAACATCGCTTACCCTGTA

AAAGCAGGTGCTGCTGGTGAGGAAGAATTATACGACGTATTAGAAAAAGGAGTATACTTCGATG
GTTCATCAATTGAAGGATTTGTTTCAATTGAAAGTTCCGACATGATGTTAAAACCAGACTTAAAA
ACACTCTCAGTACTCCCATGGAGACCAACTGAAAAATCCGTCGCAAGAGTTATCTGTGATGTATA
CACTACAAACGGAAAACCATTCGAAGGAGATCCAAGAGGATGCTTAAAAAGAGTTTTAGCTAAGT
TCGATGAAGAACTCGGAGGAGAATTCTTCGTAGGTCCTGAACCAGAATTTTTCATCTTAAAAGAA
GATGCATGCGGTTCATGGGTTCCAGCAGACGATGCAGGATACTTCGACTTAGAACCTCTTGATGG
AGGATGCGATATCAGAAGAAAAATCGTATTCGCTCTTGAAAACTTAGGTTTCCACGTAGAAGCAA
GCCACCACGAAGTTGCAGAAGGTCAGCACGAAGTTGACTTTAAATTCGCAGACGCAGTAAAAACT
GCAGATAGCGTTGTAACTTTCAAAACTACAATCAAAACACTCGCTGCACAAGACGGTTTAAAAGC
TACATTCATGCCAAAACCATTCTTCGGAATCAACGGAAGCGGTATGCACTGCCACCAGAGTATCT
GGTTAAACGGTGAACCATCATTCTACGATGAAAGCGCACAATACCAATTAAGTGAAACCTGTATG
AGCTATGTTGCAGGTATTTTAGACCATGCAAAATCAATTGTTGCTGTTACAAACCCAACAGTAAA
CTCATACAAAAGATTAGTTCCAGGATACGAAGCACCTGTAAACATTGCATGGGCAAATTCAAACA
GAAGTGCAATTGTTAGAGTTCCAGCTCCAAGAGGAAAAGGAACAAGAATCGAATTCAGAGCACCT
GACCCAGCATGCAACCCATACTTAGCATTCACAGTAATGCTTGCAGCAGGTTTAGATGGTGTAAA
AAGAAAATTAAGTGCAATAGAACCTGTTGAAAAGAACATCTTTGCAATGAGTGAAGCTCAGAAA
AAAGCTGAAGGAATCGAATCAGTTCCTGCAAACTTAAAAGCTGCGCTTGACGAGTTAGAAAACAA
CTCAGTATTAAAAGATGCCTTAGGAAAACACATCTTTGAAAACTTCATTGAAATTAAAAACGCTG
AATGGGATTCATTCAGAACCGCAGTTACAGACTGGGAAACAAAACAATACTTAAAAATCTAATTA
CCTTTTTTTAAATATTTTTATTGTAATCTTTACCCCAAATTCTGTCGATTCTAATCGCTGTTGCTT
TTTGCCAAAAACTATAAAAGTAATAAGTTAGTAATAGAACAAAGACGGGAATTTAGGAAAATTT
TTGGAAAAATAAATTTTTAGAGCTTTTTCGCGAACGATTTGAAACAAGGTATATTAAAACAAAA
ACGAGGTAATATCATGGCTTTTAAAGTTGTTGTATCAGATTCTAAAACAGGAAAATCATACCAAT
TCGAAACAGAATCCAATGCACTTATCGGTAAAAAAATCGGTGATGAAATCAGCGGTTCAATTGTT
GAATTAGAAGGCTACAAATTAAAAATCACTGGCGGATCCGACAGATGTGGATTTGCAATGAGAC
ACGACATTCACGGCGCTATGAAAATGAGAGTACTCTTAAAAGAAGGACCTGGATACAACGTTAAA
GAAAAAGGTCTTAGAAGAAGAAAAAGCTTAAGAGGAAACACAATCTCAAAAGATATTACATTAA
TCAACACAAAAGTTGTGGAATACGGTTCAGCACCTCTCGGTGGAGAACCTGAAAGTACCGAATAA
ATTAACTCTTTTTTTCTCTTTTAATTGGTCAGGCTTTTTTAAAGATTATATATCTATATAAAGTA
TTAGCCATAAAGTGATTATAGTTTTAACGTCATTTTTATATGGATTTTCAAGTATTCTTGTAATT
TAAAAAACTAAAAAATTGTGCGAGGGATAAAATGGCAGCATCCAACCAATCAGAAGTTAATATC
GGAATGGTAGGTCACGTAGACCACGGAAAAACGAGTTTAACAAGGAAATTAACCGGAGTATGGA
CTGACACACACAGTGAAGAGCTTAAAAGAGGAATTTCAATAAGACTCGGGTATGCAGACTGTGAA
ATAAAAAAATGCGAAACATGCGATGAACCAGAATGCTATACTGTCGGTAAAAAATGCGACTCTTG
TGGTGGCAAATTACAGACACTTAGAAAAATATCATTTGTTGATGCTCCAGGACACGAAACACTGA
TGGCTACCATGCTTTCAGGAGCTTCACTTATGGATGGTGCAATTTTAGTTATTGCCGCAAGCGAA
GAATGTCCACAACCTCAAACAAAAGAGCACTTGATGGCACTTGATGCACTTGGTGTTGAAAAGAT
AATTATAGTTCAAAATAAAATCGACCTCGTATCTGAAGAAGCTGCGGTTGAAAACTACAACCAAA
TTAAAGAATTTACAAAAGGAACAGTTGCAGAAAATGCCCCAATTATCCCTGTTTCAGCACACCAC
GGTGCAAATTTAGATGTGCTTTTAAAAGCAATTCAGGACTTTATTCCAACTCCTGAAAGAGACGA
AACTGTAAGTCCAAAATTATATGTTGCAAGAAGTTTTGACGTAAATAAACCTGGTTCTGAAATA
AAAGACCTAAAAGGTGGCGTTATCGGTGGAAGTATCATCCAAGGTGCCTTAAAAGTTGGGGACGA
ACTTGAAATAAAACCAGGTATCAAAGTAACCGAAGGAAACAAAACTCACTGGGTTCCAATAATA

ACCAAAATTATATCATTAGGTGTAGGCAGTAAAAAATTAAAAACCGCATACCCTGGTGGATTAAT
TGGTGTTGGAACAGAACTCGATCCAAACTTGACAAAATCTGATGCACTAAGCGGTAGTTTAGCAG
GAATTCCTGGAACACTTCCTGAAACATTAGAAAAGATGGATATAGAACCACAACTTCTTGAAAGA
GTCGTTGGATCACAAGATGAACTTGTAATCGAACCATTAAAAACAAATGAAGTTTTGATGTTAA
ATGTAGGAACATCCACAACAGTTGGAGTAACAGTTTCTGCAAGACCTGACAGAGCAGAAATTAAA
TTAAAATTACCAGTATGTGCTGATAAAGGCGACAGAGTTGCAATAAGCAGAAAAATCGGATCAA
GATGGAGATTGATCGGATACGGAATAATATTATAACTTTTTTATTATTTTTTTAATATTTGGGTG
TATTTTATGGACTATTCAAAACTCCTCTCTAAAAAAGAGGATATATGTTCTAAAATAAAAAATA
TCGATTTTAACTTTGATTATGAAAGTTACTGGAATAATTGCGATTTTAATAACCATTCTAACGCA
GTTTTTGCAGGCGGGGATGGGAGTTTCAATAAAATCGATTATATAAACTACTGCCTCTACATTTC
AGGCGCTGTTTCATACATTCAAAAAACCGGTGGAAGAGTAGAGGAATCTGTTTCTTCGTGGGATT
CGAATATAATTTTACCTTACAGGTACGTTCAGAGCAGATTAAGTCTTTACATGCTTAATATGGAA
TTAAAAGTTGCGTTATGGAATTTAAAAAATAAAGATATTGACTACTATCTCTACGATGGTTCATT
ATATTCCCTGCTAATTCAAACCAACAACAAAATTTCAATTAATGGGGAAAGTTTAGAAAATAGTA
TTTTCAGGTACTATGAACTTTATGGAAACGAAATAAAAAATAAGATTTTTGAAGAAATCGACTC
TAAAAACATCAATTCGAGCGTTGAAATTTCAAATAATTCAAAATTTAGTGAAGAAGAAAAAATT
ATACATGAACAGCTTGAATATTTAATATTATTATCTGAAATTTTAAAATATCGTGAAAAAATTG
TTGGTGTTGCAAAAACTTCAAAAATGAATATTTATTTTAAAGATGCACTCATTCCCGACCTTGCA
ATATTTTCAAGATGCAAAAATCCAGGATATTCAAAACCACTCGATCTCGTTGACGAAAAAATAAA
TAAAAATTTCTACAAAAATGTTGAATACTTTAAAAAATTTGGAATCGACCTTAAAAAATTGAAC
TACCAATTTTTAAAGCTTGATAACAATAATGGAACGCTTTGCATCACATCTTTTGAAGAATTAGA
TGAAGATTTCTTTTCAAATATTCAAAAAATATCTGTTTCAGGTTATCCTTACATTTTGAAAAAGT
CTCACGAAAATGTGAAAATTGAAAAAAAAGAGATTGAAAAATTTGCAAAACTTCTTGGAATCTA
CGAAAAAACGGACAGGGATTCAAATTTAGAATATTAATTCATTTTAAAACTATTTTTTCAAAGT
TAAATACCTGATTTTTGGATTTACATTTTTGAACACTTCGACATATATCGAATGATATTTTATTT
TAAATCGAAATATTTATATAAGAATTAGCCCACATATACTGCCATGGAGAAAATAGCGATACATA
TCATGGGAGAAATTGTATTGTCCGAAGATATCGGTAAAGCCATGAAAAAATGGAGAGAAATGTT
TGGCATACCGCAGATAGAAACTGCAAGGTACCTAGATGTCTCCCCATCAGTAATCAGTGATTACG
AAGTTGGCAGAAGAAAAAATCCTGGAGTAAATATCGTCAAAAAATATGTATATGCACTCTTAGA
AATTGACAAAGAAAGAGGTGGACATACAATAAAAGCATTGAGCAAGGTGTTGAATCCTACCTCG
ATGAAGGCGATTTTACAGATTAAAGAATACCAAAACCCAATTGGAGTCAAAGAATTACTTTCCAC
CATCGAAGGAAAGGTAATCTGTGGCGAAAACAATCTAAGTAACCAGCTATTTGGCCATACTGTTG
TAGACAGTGTTAAAGCTATTTTAGAAATGAATGGTCAAGATTTTTTAAATCTATACGGTTGGAC
AACCGAAAGGGCATTGATTTTTACCGAAGTTTCGGGTGGACGAAGTCCTATGGTTGCAATTCGTG
TAAGCAACATCAAACCAAGAGTCGTGATCTTTAACGGAGTTTCCGAACTTGACAAGCTCGCGGTT
AAACTTGCTGAACTTGAGGGAATATCTCTTATAGTAACCGAACTAAGTACCGATGAGCTTTTAAA
ACGATTAAAAGAAATGAAATAATTTTTTTTGTTTATATTGTTATGAATTTAACAATTGTTCATT
AACAAAAAAATGGTGAGCAAATGAAAGAAGTAGGTATTGTAGGATATGGAAGTGACCTTCCAAA
ATACAGAATAAAAGCTGAAGATATCGCTGGTGCTTGGGGTAAAGACGCTCAAGCAATCAAAAGA
GGTCTTGTTGTAAACGAAAAAAGCGTTCCAGGACCTGACGAAGACACTGCAACAATTGCAGTACA
ATCTGCAAGAAGAGCATTATCAAGAGCAGGAATTAATCCAAAAGATATTGGTGCTGTTTACGTAG
GAAGTGAAAGCCACCCTTACGCAGTAAAACCAACATCTGGAATTGTTGCTGAGGCATGTGGTGTA
TCACCAGATTTTACCGCTGCAGATTTAGAATTTGCATGTAAAGCAGGAACTGCAGGAATCCAGAT

GTGTATGGGTTTAGTTGGAAGCGAAATGATGGAATATGCTATGGCAGTTGGTGCAGATACTGCTC
AAGGTGCTCCAGGAGATGCCCTTGAATACACTGCTGCAGCAGGTGGTGCAGCATTTATTATCGGT
GCAAAAAAAGAAGAATTTATCGCTAAATTTAACGGAACTTACTCTTACACCACAGACACTCCCGA
TTTCTGGAGAAGAGAACATGAACACTACCCAAAACACGGTGGTAGATTTACAGGTGAACCTGCAT
ACTTTAAACACGTTTTAAACGGTGCAAAAGGTATGATGGAAAAAATGGGTACAACATCAAAAGA
TTACGATTACTGTGTATTCCACCAGCCTAATGGAAAATTCTACCTAACTGCGGCTAAAAAATTAG
GATTTACAGAAGATCAGTACAAATATGGACTTTTAACACCATATTTAGGAAACACATACTCAGGA
GCCGTTCCACTTGGACTTTCGAACATTTTAGACCATGCAAAAGCAGACGACAGAATATTCGTTGT
TTCATACGGTAGTGGTGCAGGAAGTGACGCATTTGACATTACTGTGACTGACAGGATTTCAGAAG
TAGTTGACAAAGAAATAACAACTGAAAAACTTTTAGAACAGAAAAAATACGTTGATTATGCAGT
TTACTTGAAATACAGAGGAAAAATTAGAATGTAACTTCATCAGGTGGATAAAATGAAGGATGTA
GCAATTATAGGATACGGACAGACCAAATTTGGTGAACTCTGGGAAGAATCATTCAGAAGTTTAAT
TGTGGAAGCAGGAGTTAAAGCAATTAGTGATGCAAACGTTGATGGAGACGACATTGATGCAATG
TACGTCGGTACCATGAGTGGAGGTCTTTTCGTAGGACAGGAACACTCTGCATCATTAATTGCAGA
CTATGCAGGATTAAACCCTGTTCCATGTACCAGAGTTGAAGCTGCATGTGCTTCTGGAAGTTTAG
CATTGAGAAGTGCATGTTTATCTATTGCAAGTGGTGCACACGATGTTGTTTTAGTAGGCGGTGTT
GAAAAAATGACTGATGTTGCAGATGCAACTTCAGCAATCGCAACAGCATCAGACCAGGAATGGGA
AGCTTTTGTTGGTGCAACATTCCCATCACTCTATGCAATGATGGCAAAAAGATACATGCATGAAT
ACGGGCTAACCATTGAACAACTTTCAAGCTGGAGTGCTATCGCTCACGAAAATGCAGTTCATAAC
AAATACGCACAGTTCAGATCAAAAGTAACTGTTGATCAGGTTATGAGAGCTTCGCCAGTTGCAGA
CCCATTAACATTATTACACTGCTCACCGGTTTCAGATGGTGCTTCCGCACTTATCATCTGTGATGC
AGAAAAAGCAACAGAATATGCTCCAAAAGATGAAATCATTTATATAAAAGCATCTACACAAGCA
TCAGACACTATTGCACTGCACGACAGAACCGACATGACCACATTAAACGCTGCAAAAGTAGCTTC
AGAAAAAGCATACAAAATTGCAGGAATTGATGCTAACAAAGTTGACGTTGCAGAAGTTCACGAT
TGTTTTGCAATTAATGGATTGGTATTAACTGAAGACCTCGGATTCTGTAAAAAAGGAGATGCTGG
AAAAGTTGTAGAATCTGGAAAAACAAGAATCGACGACGAAAGTTTCGTTACAGTAAACCCAAGT
GGTGGATTAAAAGCTGCTGGCCATGCATTAGGTGCTACAGGTATCAGACAGGTCGGTGAACTTTA
CTGGCAGTTAAAACAGGATAAAGAATGTAAAGATAGACAGGCTGCAATTAAAAACGGATACGCT
ATTGCAGCAAATGTCGGTGGAACAGGCGGTACAGTCTGTACTCACGTTTTATCAAACAAAAGATA
ATTAATAATTTAAGCTAAAAAATGATTTCCGGTGAGATTATGGTAGTAAGAACCTGGAGAAGCA
TGCAAGAAAGGTACAACCTTATTGGATCAAAATGTAAAACATGTGGAACCGTTTACTTCCCTGCA
AGAAGTGTCTGCCCAGAATGCAGAAGAAAAGGAGAATTGGAAGATTACAAATTAAACGGTAAAG
GTAAAGTCTACACCTACTCCGTAGTATACGCTGCACCTAAAGACTTCGAAAAACAGTCCCCTTAC
GTAATTGGTATCGTGGAACTTGAAGAAGGTACAAAAATCACATCCCAAATCGACTGTGAACCAGA
AGAAGTTAAAATAGGAATGCCTGTAGAAGCAACCTTTAGAAAAATAAGAGAAGATGGATGCGAT
GGCGTAATCAGCTACGGATACAAATTTGTTCCAACTGAATAATAAATAAATACATTTTTTATTTT
TTAATTAAAATTAGCAAGTTCTAATTACCATTAAATTTTGATATTTCGTATTTTAATGAATCAAA
ATCAATGTATATTTCGCGATGAACATCGGAATACTTATAGGGTACTGATAATTCGTCCAATTTTT
TACATTTTTCAGCTGTTTCTTCGTCAGTTACACTAAATGCATAATATGGCGTCATACTTTCAAGA
TCGAAAACAGTTGATACTCTTTTAAACATTTCTTTGACAGTTTCAGAAATATCTACAATTATTGA
ACCGTTTTCAAATGATACATTGAACCCAAGTTCTTTTAATTCTTTGTATGCATAATTTTCCCCAC
CATCTATCATTTTAAAACGAATTATACCGCTTTCACGACCGCTTGTAATAATTGGATTAAAATTT
CGAGATATAAATTCAATTACTGACGTTTGAAAGTTCATTGATTCAATTAATTCATCCATTCGCTC

TTTTTTTAATAATGCAATTATTCTTCCAATATACTGATCCTTAAAAATTGCAACTTTTGAGATGT
TTTTTACATCTTTAGTTTTTATGCCATCTTTTTCGAGTAATTTTTTAAAATATTTAGAATATGAC
ATATTCTCAAACTCGATTTCGTAAATATCTTCATCCATCCTGCTTTTATAAACTCCCCTCAGTTCA
TAACGGATGTAATATTCAAGTTCGGATCTAGCTTTTTCATCGATCATAATTTAACCCTCCCCAAA
AATAGAGATATTAAAAAATATGTGAGAACCAATATTAAAAATCTTTAAAAAAAGTATTAAAAAT
TTAAATTGAGATTTAGGTTATTCTATAATTTTTGAAAAATCCAAACTTTCTTCAATTATTTTGGA
TAATTTATCCATATTTTCATTGAAAAGTTCAGCATAAGTGTCATCTTCAATTTTTGATAAATTTT
TTTTGCTTCTTGCAAGATTTACAAGATAATTCCTAAATTCAAAATTTTCAAATATTCCGTGAAAG
TAAGTTCCAAAAATATATGAATCTCCAATCATCTTAAAGGAGCCGTCATATGAATCCCCACAATT
TCCAAAACCTTTTGAAAGAGATATTAACGGAGTTTCTTTTGAAACTGAATATCCTTCATGCAATT
CATAGCCCGACACATCAAAATTTTGATTAAAGATAGATATTTTTCCAGTTGAATTTTTTATTGCC
TTTTTATTTCCAAAAGTCGTTTCCATATCAAACAATCCTAAGCCAGAAATTGTTCCAACATCGCC
TTCTGAAAAATCTTCATCAAATAGCATTTTTCCTAATGCTTGGTATCCTCCGCAGATTCCAAGGA
CTATTCCGCCATTTTCCACAAATTCTAAAATTTTTTTATCCATTCCATACTTTTTCATTAAATCCA
TTTCAACGGTAGAACATCTTGTTCCAGGCAATATCAGGATATCCCCGGTTATATTTTCATTAAAA
TCAATGTATTTCATTAAACAGTCGCTTGAAAATGGATCAACATCAGTAAAATTTGCAATTTTTGA
AAATTTGACTACATTTACTTCGATCGAGCTTTTTAAATTTCCAAATACTCGTTTACCATCCAATG
CCTGGCTGTCTTCTTCAGGAAGTACTAAAGTTTCGTCATAAGGAATCGTTCCAATAACTGGAATG
TTTGTAAGTTCCTCGATTTTTTCAAATCCATCTTTTAAAACATCAATATTTCCCCTAAATTTATT
AATTACAAATCCTTTTAAAAGTTTTTTCCAGTTTTCAGGCAGTAACTGGACTGTTCCATAAATTG
ATGCAAAAACACCGCCCCTATCGATGTCTGACACAAGTATTGCATCAGCTCCAGCAATTTCTGCA
ACTTTTAAATTTGCAATGTCATCGTTTAATAAATTTATCTCACAACAGCTTCCAGCACCTTCAAT
TACAACGTAATCATAATTTTTATCTAAATAATCAATACTTTCTTTTATTTTAACTAAAAAATCAT
CTTTTTTTGATCTATATTCATTGTAATCCTTATCTTCGTAAGGATTTCCATGAACGATCACTTGG
GATACAAAATTTCCCTTTGGTTTTAATAAAATCGGGTTAAAATGAATAGATGGCTCAGCTCTTGC
AGCCATTGCCTGAGTATACTGTGCTACTGCAATTTCCCCATCTTCGACACTAACTCTCGAATTTAA
ACTCATGTTCTGTGATTTAAATGGCGCTACCTTGTAGCCTTTGTTTGAGAGCATCCTGCAGATTC
CAGACACTAAAACTGTTTTCCCACTGTTTGAAGAGGTTCCTACGACCATAATGAATTTAGCCATT
TTACACACCGAGAAATTTTTTAATTTGTAAATTTTCTTTAGCGAAATTATAGAAAAACAAGACAT
TTAATATTTGCGATAAATTTAAAACATAATTAAAAAAATAAAATTAAAAAATAAGGGTTATTCA
ACTATTTCAGACATTATTTTTAGGAATCTTTCATTTTCTTCAGACGTTCCAATTGAAACTCTAAA
ATAATATGGTTCAAGCCCTTCAAATGAATAGCAGTCCCTTACAATTACTCCTTTTTTCAAAAGTG
CTTCACAAAACTCGCTCGAATTCATGCCGTTTTTAACTTTTACGAGCATGTAGTTTGCCTCAGTT
GGATAAACTTCAAGCTGTTTAAATTTTTTCAAGCCATTGTAAATTTCTTCCCTACTTTCGATTCC
TTCTTTTAAGCACTTTTCAAAGAATTCCTTGTCCAAAAGTGCAGTTATTGCAGAGACTTGCGATG
CTCTTGTTAAACTAAAGATCGGTTTTATTCTCATCATGTAGTCAACAACTTTTTTACTCGTAACA
CCGTATCCGACCCTCTGTCCTGCAAGCCCGAATACTTTTGAAAATGTTCTCAAAACAAGCACGTTG
TCGTATTTTAATGCCCAACTGGTTAAATCATATTCGTCCTTTGAATATTCGATGTATGCATGGTC
AATCATAACTAATGCATCAGTACTTTCTACGATTTTTTTAATATCTTCTGTGGGGATTACGTTTC
CAGTAGGGTTATTTGGAGTACATAAGAATATTGCTTTCGTTTTTTCAGTGATATTGTTTAAAACA
CTTTCTACATCAAGTTTAAATCCATTTTCTTCATCAAATTTTGCCCATTTTATGTTTGCACCGTGA
ATTTTTGCAGAAATTGCGTACTGCGTAAATGTTGGAATTGGAATGATTACTTCATCGCCTTCATC
AATTAATATCCTCATGATGTTGTCGATTACTTCATCAGCCCCGTCCCCTCCAACAATAATGTTTTC

TACAGGAATTTTTGTGAATTTACTTATTTCATCCATTAATTCAGGGTTTATAGGTTGAGGGTACT
GGTGGAGATTTGAAACTTCATTTATTAATTTTTCTTCAATTTTTGGTGAACAGCCCCAAGGGTTT
TCATTTGATCCTAATTTAATGATTCTTTCTGGATCTATCCCATAATTTCGTGCAATTTCTTCTTTT
GACTTTCCAGGAACGTATGCTTTAAATTCCTTAACTATTGATCTTACTTTATCATCAATCGACAT
AAAAACACCTTAAATAAAATAAAAATGAATAAAAAATTAAAGTATCTCGTCAGCATTTAATTTT
TCTGCGTTGTAAAGTTCTTTTGCCCGTTTGTATATTAATTCATCAGCAACGTCCCCATTTTTAAC
AAGTACTACGACTTTGGAAGGAGATATATTTGATATAACTTTTGAAATCTGTTCTTTTGTAATTT
TACATTTTTTTTCTATTCCAAGCAGTGCTTTTTCAGGAATCTTTGGATTTACAACTTTCATATCC
TCGATTTCCAAAACTCCTTCTGTTGAAATAATATTTAAATCTGGATTGATGTCTTTTAATGATTT
TAAAACTTTTTTATTGCTTACAATATACAAAGCCTGCCCATCTAAAGCAACTGTTTCAATATCAA
ATCCAAATCCGCCTAAAACATTGTTAATATGTTTTTGTGCATCAAATAAAAGTTTTGAAAATTCT
CTGGCTTTTTCTTCGTCTAAATCATGATTTGGTGCATATTTGTAAAGATATTCATCGGCTTCAAA
AAGTGAATGCACTGCCTTTTTAAACTCTATTGTATCAATTTTTCCAGATTTTGCATCAGAAAATG
TTTTTTTATTTTCATTTTTTTCAAAATTTTGAGTAGCAGAAACTGTTTTTGCCTTTGAAATCGTT
TCTACTGCTTTTTTAATTAATTCTTTTGAATACTGTGTTTCTGACATATTACTCCTCAAAACAAT
TATTCCTGTAGTTCAAGCCTTTCTAATTCTTTTAATAAGAGGACTAAAAATACCCCTACGTCAGT
AACTACCCCAATTGCCTGAGAAGTTCCCCTGTCCATTAATTTAGTAACTGTTGAAGGCTGTATAT
CGACACAGACTGTTTTTATGTATGACGGCATTAAGTTACCTGTTGCAACGGAGTGTAAAAGTGTT
GAAAGCATGATAACCATTTTTTTGTCCATTACAGTAGTTCTCATTTTATCTTGTGCAACCATAGA
ATCAGTTATAACGTCAGGAATTGGTCCATCATCCCTAATACTTCCTGCAAGAACGTATGGAATAT
TATTTTTTATACACTGATACATTATACCTTCTTTTAATACGCCGCTTTCCACCGCATTTTTGATGT
TTCCAGCATCATTTATTGCATTTATTGCATAAATGTGGTGTTTATGTCCACCAGTTACGGGTTTT
GCAGTTTTTATATTTACTCCAAGTGAAGTTCCATATAATGCTGATTCAATATCGTGCGTTGCAAG
GGCATTTCCTGCCAAAATTGCTTGAATGTATCCAAGTTCAACCATTTTTGCTAAAGCAGGGCCTC
CGCCGGTGTGTATGATCGCAGGACCGCCAACAATTGCAATTCCGCCAGTTCCAGTTTTTTTGTAT
TCTTCCCTTATTTCGTGCATTTCTTTTGCAATTCTTTTAATAATCGCTTCTTTTGGCTTTTCAGAA
GATACTTCGGAGTTCATAAATTCAAAAAGCTGTCCTGCTTCTCTTGATTTTTCTGGAGGCATTAC
CCTAATTCCGTTGTGGCCAATTAATACGAAATCTCCTTTCTTTACCTTTCTAATGACTTTTGTTTC
TGCCCGTTTTTCTTCAGGATAAACAACAATTACAGCATCCATTTTTGGATTTGCAACTTCTATCC
ATTCGTCATTTACTTTTACGTGTGTTGGGTGATTTGTAGTTGAATAAAATCCATCAGGCAACACA
CTATCCTTTAGAGCAGGTTTTAAGTTAGCATTTTCAATTTCCGGGATATTTGCTCCAACATTTTG
AAGTTCCTCTAAAATCTGGTCGAGGTGCTGTTGCGTATCCCCTGAAATTAATAATTTTGCGTATG
ATGTATCGATTTTTTTCTTTCCTATATCAAATTCTAAAACTTTATAATCTCCGCCGAGTTCTAAA
GTCCTATCAAAAACTTTTGCAAGTATAAAGCTGTCAATTATGTGCCCTTTTAATTCAATTTCCCT
CATGAACATTAAATCACCCGATAAATCTCTCTAAATTTTAGTTATCAATTCCTATGTGCTACATG
AATATATAATTTTTTGAATGTAATATAAGTGCATAGGTGGATAAAGTTAATAAAAAGAATTAGT
ATTAAATTATTTAAAAAAAGAGCTATTTTTTAAATTATCTTCTTTCATCTTTTTCATAAACTTTG
GAATGTATTCTGTAGCAGTATCAACAGACATCGGGAGGAATTTGTCTTTTAAATACTTTCTTTTA
AGCATTTCAGAGCCAACCCAATCATTAAATCTTTTATCTTGTATAACTACAACACCATAATCGGT
TTCAGTCCTTATTAATCGTCCAATCATTTGTATAACTGTTCTTGCCATTATGTGAAATGATGTCA
TTAAAAATGCACTCCAGTGTGCATCTTTAACTTTTCTTGACTTTAAACGCTCTTCAAGAAGAGTC
TGTTCTCTTTTTAAAAGTGGGGTTGGAACTGGAAATGGCAGGCTATCTATTATGACGCAAGTTAG
TGCATCTCCAGGAATATCCACACCTTCTGCAAAACGTCCTGTTGCTAAAAGGAGTCCACCATATT

TTTGAAAGTCTTCTTTTAGTTTTTTTGCTGCTTTTCCATCCATTCCTGACTCATAGCAGTATATAT
TTCCACGATACCCAGCATCTAAAAAGTATTCATAAGCTGTTTTTAAATCCCCAAAACTCTTAAAA
AGTATCAGAGTATTTCCTTTTGCGGCGTTTACCATTTTAAACAGATTTTCATTTGCCTTGTTTCT
TTTTTTATTGTTTGATTGAAAATCTATTGAATTAAATTTCATATCTTCCCCATCAGACAGCGCGA
TTATGTTTCGCCTATCTTTTGAAAAGGGGCTGTCCAAAAGTAGTGTTTCACTTCTTCCCATTCCG
GTCTTTATCGCGTGAATTTTCAGATCACCAAGTGTTGCAGAACAGTGAACTACTGATGCATCCCC
GTAAAGTCTATTTAAGTACGTTGAAACGAGTACGGGTTCGCAAAGAATCGATCCATTATTTTTAT
AAACTACAAAATTTCCAGTAATGTTCCTAAGATTACCCAGATTTTCCAAAAATTCTACCATCGGA
ACATCTGATATTCTCCTATCTGCAACAAATTGAAGCTCTAACGGGATTAATGCCTTGTTATCAAT
TTTAAATTTCAAATCTTTCTTATCGAGTTCTTCATTTTCAGATAAATCTTCAATTTTTTCTTTAA
TTTTTACTATTTCGTTGTAACCTTCGAGAAGAGTTCCCAAAATTGCCACATCTTCTTTTAATCCA
AATGAAGTAATCTTAAATCCATCAAATACTAACGAATTCTTACAATCTTTTCCGGATGCATTTTT
TACAACATAGTCTTCAACTATTGTCCAGAAAATTTCGTCATCTTCCTCAGTAACCCTATCCATAT
GCACTCGTAATCTTGAATTTGAGTAGTGGTATGCCATAAATCTAAGTCTTCTTAAAGCATATTTT
GGATTTATAACAATTGTTGCAGCATTTCTAATGCTTCCCTCAAGTTTATGGGCTTCGTCACAAAT
TACAATGTCAGTTTGTTTCTTTTTGTCGATTTCGTCTTTTAAATAATAGTAAATGCTGTTATTCA
TTACGATTATATTTGCATCAAAAGATTCAATTTTTGCTTTTTGATAATCGCACAGACAAAGCGGG
CAGTAAAATGTTGTATTCCCATCAAATTCAATTTTTTCCTTTTTTGTACCGCAGACACATACTGG
TTTTTTGTTTGGCCTGTAAATACAGCCTTTATTTAATTGACAGTAAAGCGTGTTTGCCTTTTCCC
CTTTTGATTTACAGAAAAAATTTCCTTTTCCCATCATAAAAGATACTTTTAGGTTGTGTTTGAGC
GAATTTAAATCTTCTACGATTCTTTCTTGTTGATCAATCGTTTCTGTGAGAATCATTACTCGTTT
TCCGCGTTCTGCAAAGTATAGTGCAGGGATAAGGTAAGAAAGAGTTTTTCCAACACCTGTTGGAG
CCTCAACTACAAGATTTTTCTTTTTTGTTACACATTCGTAAATTTTTCCCATCAAAACTTCTTGCT
GGGGCCTCATTCCATGATATGGAAATTTTTCTTTGGAATATTCTTTAAATTCATAAAAATCCATG
GTTACCACATAACTCGAAAATAGTTTTGAAAGTTTAATAAGGTGCCGCCTTTTATATATGTATTT
ATCGCGTAAAAAAATACAAAAAACCTGTTAAGGATTTAAAATCAAATTTAAAAGAGATTAATCA
CTGTAAAATTTTTTAGTATATTCCATAATCATTCTTTCGGCATCAAAATATTCCACGACGTGATT
TACCGAATTTATAGCTTTTTCCCACCATTCATCGGTATCATAAATCTTTATAGTCCTTTCGAGGT
GTTTATACATTTCATTTGCCTCGTAATCTTCATCAAGATTTACACCGTCTCCGATGGTAAAACTA
TCTTTTGGATATGCTTTTGCCCATTCAACATGCCATCCATCCATTGTACTCATGTGTATCGACGCA
TTCATCGATGCAGTCATTCCTGAAGTTCCTGATGCTTCATTTGGTCTTCTCGGGGTGTTTAACCAT
ACATCAGAACCCTGCTTTAAGAGTTTGCTAAGTTTTAATTCATATCCTGTTAAAACTGAAACATT
TTTAAGTTCCCTAGTTTTTGAAACTATCCAGTTAAATGTAATCTGTGCATTTGAATCAGCAGGAT
GCGGTTTTCCAGCCCAGATTACCTGAATTTTTCCGGTTTTGAGCAGGTTTTTAAACCTTTTTTCA
TCCCGTAATAAAAATGAAGGTCGTTTATAATCTGCAAACCTTCTCGCCCAAACCACAGTTAAAAT
GTTTGGATCAAAAAGACTTCCTGTTTGGTCAGCCACTTCTTCAAAAAGCTGTTTTTTTAATTCCA
TTTTTCTTTCGTGAAGCATTTTTATGTCTTTTTTTCCCGCAGCTTCTCTTATCTGTCGGTCTTGCC
AGTAATATTTATCCTGTGCATTTGTAATGTTCATTATAGGCTCTCGATTAGTAACCCATGCCCAC
ATTTTTTCAGAAGTGTCCCTGTGAAGTTCTGAAACGGCATTAGATCGTTTGCACATTCTTAAAGC
TGCGACAGTTAAATTAAATGGATTTCCACCGTATTTTTCAGCAGTTTTAGTGTCGATACTTCCAA
AAAAGCCCATGCTTTTTAGCAGATTTATATCCTGTGTTTCATTTCCAGCAGGAATTGGAGTGTGT
GTAGTAAACAATAAATGTTCTTTTACATAATCAGTTCCATAGTCTTCGAGCATTTTAAATGCGAC
AGGTAGAGAATGGGCTTCATTTATATGATATCGCTTTACATTTTCACATTCTCGAATGACTTTGT

ATCCTCCAATTCCTAATACGATTTCTTGGGCGATGTGTGTTAAATTATTCCCATCGTAAAGTCCG
TAAGAAATGGTTCTTGACAAATGATCGTTTTCGGGAATATCTGTTGTTAAAAAATAGATTGGGC
AGGTATTAAATGTATTTTCCTCTAATTTATACGCTTTAACCCAAATATTCGTTCCATTTATTACA
ACTGGAACTTTTAAATCAATATCTGTTAAAAAATCATAGTATTTCCTTATATTTTCAACTTTCAT
ACGGCCTTCGCGATCACGCACCTGGTCGTAGTAGCCGTAGCTCCAGAGCATTGAAACCCCAACCAA
GGGTACGTTAAGGCGTTTAGCTGCCCTAAAATGGGATCCTGCTAAAAATCCGAGTCCTCCCGCAT
ACGTCTTTAAAGACTGATCAATTGCAAACTCCATGCTAAAATAAGCAGTGTTTTTCATTAAATCC
CTCAAAATAATATAAAACGTAAACTTCCCTGAGTAATATTATGTTTCTATGTTATATTTATTAAA
TATATCTAAAAAAGACTAATAAAATCTATTTTAAAGTGGCTGTTATAATTGTTATCGGGTTTTTT
GAAAGCATTATGTGCCCAGAATTAATTTTTTTTCCGTATGAAACAGAAACATTTACAAAATCCAC
ATTGTATCCGAGTTCTTCAAATTTATTAATTATTTTTACACTATTTTCAAGAACAATTGTGTTTA
CAACTATTCTTTCAATGTTCTTTTCTTTTAAAATTTCCAAGATCTGTTCAATATTTTGGGTTCCG
CCGATAAATACCTTGTTAAAATCAAATTTTGAAATTAAATCTTCTGCATTTCCGAGTGAAACCGT
GCAATTTTCTACATTAAACTTTTTTAAGTTATTTAATGTCGTATTTTTTGCATCTTCGCTGTTAT
CAATTGAGTACACAAATTTTGACCGTTTTGAGATCTCAACACTCATGCCCCCACTACCGCAGCCAA
TGTCTAAAACTACGTCTTCAGGATCTAAATTTAATTTACCGATACTTACAGCCCTAATTTCTTCT
TTGGTTATCGGAACGCCTTCCATTCTAAAAAATTCACTGTCTTGGATCATTTTTTCACATTATTT
ATTATTTTGATTAACAAGTTATAAATACTGATTTTGGCTACTTATACATTAGCTTAATTTTTAAA
TATTAACAATCACGGTATTTTTATACACATATTACTATTCTCATCATATCTAACAAAAAGGTGAA
GTTATGGCTGACGTTTTGACCGAATTGCCTGGGGTAGGCCCATCAACTGCAGATAAACTAATTGA
AGGGGGATACCTCGATTTTATGAAAATTGCAACAGCAACTATCGGTGAATTAACTGATATTGAAG
GTATCAGTGAAAAAGCTGCTGCTAAAATGATCATGGCTGCAAGAGATTTATGCGACCTTGGATTC
AAAAGTGGTGTTGAATTATTAAAACAAAGACAGAGTGTATGGAGACTTTCAACTGGAAGCACTG
AATTAGATACCGTTCTTGCAGGAGGAATCGAAAGCCAGTCTGTAACGGAATTTGCAGGTATGTTT
GGAAGTGGTAAAACGCAAATTATGCATCAAACTTGCGTAAATTTACAGATCCGAGAAAAAATAT
TTGCAGATTTAGAAGGAGTAGTTGAAGAAGAACTTGAAGCTCCAAAAGCAGTTTATATCGATAC
AGAAGGTACATTCAGACCTGAAAGAGTTGTTCAGATGGCTGAAGGGGCTGGAATCGATGGTCAGA
CTGTTCTGGACAACACCTTCGTTGCTAGAGCATACAATTCAGATATGCAGATGTTGTTTGCTGAA
AAAATTGAAGATCTGATCAAAAGCGGAAATAACATTAAATTAGTAATTATTGACTCCCTTACAA
GCACTTTCAGAAATGAATTTACCGGAAGAGGTAAATTGGCTGAAAGACAGCAAAAATTAGGTAG
ACACATGGCTACACTAAACAAACTTGCAGATTTATACAACTGCATTGTGTTGGTTACAAACCAGG
TTGCTGCAAAACCAGATGCATACTTTGGTGTTGCTGAACAGGCAATTGGTGGACACGTTGTGGGC
CACGCTGCAACATTTAGATTCTTCTTAAGAAAATCAAAAGGAGATAAAAGAGTTGCAAAATTAT
ACGACTCCCCACACCTGCCAGATTCAGAAGCAGTCTTTAGAATTACAGAAAAAGGTATTCAAGAT
TAATATCTTTTAAAAATTAAAAATAAATTCTAATATTTCTTTTTTCATTAAAATTAAAAAAAAT
AGAAATTTTTGAGTATTTTAGTTATTTTAAATTATTTTATCATTCCCATTTCTTTTTGCCGTTTT
ATTCTTTGAGATAATATGCATGGGCCGTTTCTTTTTCCGCCGATTGGAACTTTTGGCGTTCCAAG
ACAGTTCATACATCTTGCCATGACACTTGCACCAAGAGCAAGTCCATCTTCAACGAAAATTATGT
TTTCGGACACATTATCCCATATTCCAAGTTCTGAAAGCAGTTCAATAATTAATTTTGGTTTTTCC
CCAGTAATTCCTGCTCTTCCAGTTATGCCTATTGCTGTATCCCCGTTTATAATTCCTTCCTCGTGT
GCAATCTTTATCAATCTTTGAGTGATTTTTGAAGAAACTATGTCCAAAGTATACATTAAAGTTGG
AAGATTTGATTCTTTTAAAATTTTTGCACCAATTTTTTCAAGTTCTGGAATATCGCTTCCATTTT
CACCGACATCGCAACCGATTAAAAATGTTCCAGCTTTTTCTGCACTTTCAGGGTTTACAGGAACT

GTTCCAAATCTTTCCATTCCTTTTGGAACTTCTTTTATTATTACGTGTTCGTGAATTTCTTCTGCA
TATTTTTTCGCAAGTTCTTTGTTTGGGCTTCCCTTTTCAGCGTGAATATCTAAAACTGCACCGCCC
CTTTTACTTACTAAATCCGTTCCACGAACAACTGCATCTGAAACTGCACCTGCAAGTCCGCACAAG
TTTCCAACAACTTTTGCATAAGGTTCGCCATTGTTTGTAATTCTTCCGGCAAGTGTTGTTCCAAA
ATCGATACTGATACATGGGTTTCTGTAGTCAACACCGGTCCATTTACTTCCAATTTTTAATCCTG
CGGTTACAAGTTCCCCTTCCATTTCATTTGCAACTACTTCTTTTCCAGTTGGAGGAACTACTCCTG
TTACTGCCCCATCAAAAATCAACTTTTCGATCAAAGTGTAATCTTGAAGTATTTCTGGAAGCTGT
TTTTTCGTCATTATCGGTGTCATCTTTCCATTTGGAATTCCTGCCATTCTGCATCCTTCAGCAAGG
GACACAATCATCACACCTACTTCTTCAGGTGTTGCAAAACCTGCAGTAACTCCCGTACTCCTGACA
ACAAAATGAAGATCATTTACATTTAAATGGGCCTTATCCATTGCGCCTTTCAATACATCTCGAAC
CATTTCAGAAACTGCTTCTTTTGTAAGTTCAACATCCCAAATCGTCCTTCCAAAAACCTGTTCTCC
TTTTTTTGGAGGCCTTACATCCCTTGTCATTCTGACTTCTTTTGAAACTACATATGTTTTTCCAGA
TCCCATCTCTGTTGCAGATATAATTGCTTTTGTTGTCGTGTTTCCAAGTTCAACGGATGCAACAG
TATAATATGGCAATATGTGCTGATTTGGACCCACCCTCTTTGGATTTAAATCCATTACATCAATA
CTTTGTGATTTTGCATAGTGAATCTTTGGCGATTTACTAAATATTCCTTTAATTGAATCAAGAAA
CCCCATGTATATCTTCTCCTTAATTTTAAAATGAATTACAGACATTAAATGCTTAAAAAAACATT
ATAAATTATGGAAATTTGATTATATAAATGTAGTATTTTTGAATTTTAATTTTATGGGTGCCCA
GACTATGTAATATCCCATATTAATTTTAATAATAGTAATGATATAATTTTAAAAACTTACTAAA
TTTAATTAATATTCATGAAATAAGACCAAATCATATGTAAAAGTTAATATATCTTTAAAGTAAA
GAATATTAGTATCTGTTATCAACCATAAAGGAGATAATGTGAAAATCCCCACAATCCTCGTAATA
ATATCATCAATATTAATACTAACTTCTTTTACAGGATGTACCTCTTCTTATGGTAAGGAATACAG
TACTGTAAATCCTGGCGTATTAACAGTCGGAATTACTCCAGACATGCCACCGCAACAGTATTATG
AAGATGGTGAACTGAAAGGCTTTGATATCGATTTGATGAAAGAAATTGCAAACAACATGGGTTT
AAAACCCGAATTCAAAATTTACGAATATGGCGGTGCACATGAAGCACTTCTAAATGGGGAAATCG
ATTGTCTACCGTCAATGACGATTTCACCAGAACGAAAAAAAGATATGGATTTTTCAAGGGCATAC
ATCCAAACATATGTGGTTGTTGCAGTTTTTGAAAACAGTCCTTATCAAGATCTTACGGATTTAAA
CGGTAAAGAGATTGCAGTTGTAACAAATACATACACTGAAGAGTGGGCCAAAAGATACCTGGGA
AGTCTTAACGCGAATATTAACTCATATGACTCGATTCAAGGATTAGTTTCAGACGTATACTCGGG
AAATATTGATGCGGTAGTTACTGATCAGATCCAGCTGGATTACTATTCAAAACAGAATAATTTTG
AATCCAGATTGATTTTCGAAAAAATGACGATTGAATACTGGGCAATTGCAGTCAAAAAAGATAA
TCACAATCTCCAAAATGAAATAAACGATGTATTGTTTGAATTGGATGAGAATAAAGCACTCTACG
AATTAAAAAAATAAATGGTACAATTAATACCATTATCCAATACTTTTTTATTCAACTAAATTTTCA
GTTATTTTTATTTAAAAAATATTAAATACCCCCTCATAAAAAATATAATGTATAATATAAGGTG
GGAGTCCTTTTATGAAAAGACATTCTATTGGGATTATCATTTTGCTATTTTTATTATCGATTTCA
ATTATTTCCGGCTGTATTTCAAGTCAAAACACTATGAAAAGAGGTACATTAACTGTTGGAGTATG
TGCACAGTACCCTCCAACAATATATGAAGAAAATGGAAATTTGGCAGGTTTTGATTTTGATTTAA
TGAATGAAATTGCAAAAAGAATGAATTTAAATACCGATTTTAAGATTTACAATTCCAAATCTGA
ACTTTTCGATGCTTTAGAAAAGAATGAAGTTGATTGTATCGGATCAAGAACTGTAAATTATGAA
AGAAGAAAATTTGTTGATTTTACGAGAGTTTATTATTATGACAGTGTCAGAGTTGCAGTTCCAGA
AAGTAGTTCAGTAACCATGCTTAAAGAATTAAAGGGTAAAAATTTAGGCGTTATTGAAGGCAGT
GTCTCTTCAGAACTGGTTGAATCTTATTTAACCAATATAGGCTGTAAATGTTTATATTACGATAC
TCCTGAAAATTTGACGAACGCTGTAGAAAATGGGGAAGTTGATGCAGTAATTGGACATGAAATG
TACCTCGAACACATATCCACTAAATGTAACGTACAAAAAAGATATTTAGACGAAAAATTATCCGT

AAGCTACTGGGCAATCCCAATTAATAAAAACAATCCAAACTTGAGAAACTCGATAAACGATATAT
TATTAGAAATGGAAAATGATGGAACCCTTGATGAATTAAAACTTAAGTGGTACGGCAAATATTA
ATTATTTAAAAACCATTATCTTATTTTCCAAAGATAACTCGATCTAAAATCGGTTTTACAACCGT
TGAGGGGTTATCCATATCCCCTTTTTCATTTATAAATTCTACAAGGCTGTTTTTATTGATGAAAT
CAAAAATTCTATCCATTTTATCGTTATTTATCATGTTTAAAATTTTTCTAATTTTGATATTCTTA
TCAATTTCAGATGAAATTTCGTTTTTCCAGTTTTTTTCATAGTTTTTCAAAAATTCAATACCGTA
ATTTCCTTCAAAATACTGGTTTATTGTATCTGCACAGATTTTTCCACATTTTGCACCATAATAAA
GCCCACCACCACTTAATGGTTTTACCTGGCCTGCTGAATCACCAACAAGCATTACGTTATTTTTA
CAGATTTTGAATAACCAATTGGAATTGCACCGCAGATAAATTCGACAGGGGTTGCATTTTTCAAA
ATTTCTTTTACAACATGATTATTTTCTACAAAATCCATCAATTTATTGTAAGAATTATTTGAGTC
ACACATTCCAACTCGCACTCTATCGTTTCCAAGTGGGATTATCCAAGTAAAAAATTTTTCAGAAT
ATTTTTTATCGTAAATTACGTAAACAAAATCATCGTCAACTTTTGCATTAACAAATTCAATCTGA
ACTCCCGAAATTATTTCCCGCTTTTTATCATGCATATTCAAGGATCTTCCAATTGTAGACCTGAT
TCCATCAGCCCCAATGATTATTTCAGGAGATATTTCGAAAGTGTCCCCCATGTGCATTATCTCCA
AATTATGCATACATTTATCGCGATTGGATGAATTGTTTGAATTTTTAATTTTTCCGTACGATTTT
AACAATACATCCACGTTTTTAGACGCCCTGACTGCAATATTTTTATCCATTACCTTTCTTTCAAA
AACTTTTGCCCTGATTTCTTCATTTCCAACCGTAATAGAACTGTTTTTTGAATAAATATGTGCCC
CCCTAACTTTATTTATCGAACCTTTGGGATTTCCAAGCTCATTTATGCATTTTTTACTTAAAAGT
CCAGCACATTGAAATGGAACCCCGATTTCTTGATGTTCTTCTAAAACGAGAACATTTTTATTTTT
AATATTCTCTCCAGTGATAAATCCACAAGGCCCGCCACCAACAATTACAACATCGTACTTTTCAA
AATCCATGGTATTCCTAAAAAATTTTTTCGAAAGATTAATAGTTTTTAAATCTTATATTAAATAT
CATTTTAAATATAGTTTCGCTAAAAACTTGTTCAAAAAATTATAAATGGGATATCGTGATTTAT
ATCGTTGGAATTGGGCCTGGAAGCAGGGATCATGTTACAAAAAAAGCATTTGATACCGTTGAAAC
TTCAGATTTTGTTTTGGGAAGTAAAAGGTCCCTTGAAATCTTTGAAATTAACGGAAAAAGAATG
GAACTTACAGTAAATCTTAAAAACGAACTTTATGACTTTTTAAAGAATTATAAAAATTCAAATT
CTAAAGAAAAGGTTTCGATATTGTCAACTGGCGATCCCTGTTTCAGTGGCCTTTTAAAAACCATT
TTTTCATTTGATTTTATTGATCAAAACGATTTTGAAGTAATTCCGGGAATTTCGTCAATTCAAAT
GGCAGCTTCCAAAGCTAAAATTTCTTGGGAAGATTATAACATTTTAACGCTACATGGAAAAGAAG
AAAATTTAAAAAAACTGTTAAATTTTGTGAAAAATGATGAAAAAGTAATATTTCTACCAAATAA
TTTAAAAAATGATCTTAAATTTTTAGTTGAAAATGGAATTTCGGACGAAAAAGAAATAACCGTT
CTTGAAAATCTTTCATATTCAAACGAAAGAATAATTATAGATAAAATATCAAATTTAGTGAAAA
ATGATTATTCTTACCTTTTAGTCTGTATTATAAACTAAAGAGGAGATGCCTTTGAAAAAAGTAGC
CGGTTTACTTTCGGTCTTTGGACTATTACTATTAACCCTATTTTCAGGATGTATTGGAAACGACT
ACAGCTCTGTTGAAATTGTTGAAAATAGCCCTTATCCAGAATTTATCTACTTAAATAAAGATATG
TTAAAAGTGATTACAAATAAAACTTCAGAGCACCAAACTCCCGACTATTTCTTTATAATATTTAA
TGGAGCGTACATTGATAAAAATAACTACTCAAAAATAGCTGTAAAAACTCCTTTATGGAGATTTG
GAAGCCTTTTTGCAGATGAAAATATAAAAACGAAAAATGGAATGATATTTTTAAATTCAGATTA
CGTTCAAAAACCAGATCTTAAATACAATAAACCCCTTAATTATAACGACGTTATTGTCTATAACA
AAAATTATGGGATATTCATAGAAATTGGCGAAGAATATAATTATGTTGAACTAATTACAGAAAA
TTTAAACGAAAATAGATATTTCATGGAAAATATTGATGATATTGTAGGTAAAAATATTATAAAT
CGAACAATTATTGAAAATGAAGACTTGTTGCTTGGAACTTACGTGTTAGAAAATGGAATTCCTGT
TGGGGACATTAATAACAGTTTATTTAAAATGAATATTACTTCCACGTTTTAAAAAACGTTTAATT
TTTAAAACCCAAATAAAAAGATATAAAAAATGATTTAACGAATAAATAATATCAATATAAAAGC

GTAAAAAGGTATAAATCATGGATATACTGCAATTTGTTCCGGACCTTGGAACCGGATTTATAACT
GGATTTATTGTTGGCTGGGGAATTAAAATAGCCCTAAAAGTGGTTATTGCGTTAATGGGCCTTTA
TGTATTCAGTTTAATTTACCTGAGTAATTTAGGGGTTATTTCAATAAATGTAGATGCATTATTTG
GTCTTGTTGGAAATGTAGAAGGTGCAGTGATGTCTTATGGAAGTTTAGCCATTGGATTGATTCAT
TCTGTATCTTTAGGTGGAGGATTTGCAGCAGGGGCCGCAGTTGGGTTAAAACAGGGATAATTTAA
ATTGAATTATATGTACCATAAAATATAGGATAAACTCATATTCTTTTAATTTTTAAGAAGTGAA
ATTATGGATACTCGTATCAGGGATTTTGTAAAAACTAATGAAGGTTATTTTGCAGTAAATACTTA
TTATCATCCAGAAAATTCAGTTATTTCTTTTTTAAGATACATAAATATCGATAAAATCGGAAATC
ATCTTTTAAAAGACTATGAACTTGATGAAAACGACATAAGGATACTTAATGGGGAAAGATTCAT
AAAAGTGGCAGATAGTAGCAAAGCTTACGCAATTTTAAAAGAATACTACCCGGAATATTTATTTT
ATGATGAAATAAACGACGTACTTTTGCATGCCATTCCTAAAACTAACATTAAAGAAATTTTAAGC
CCTCAAAAAAGATTGGAAGAAGTTTTAAATGAGCAGAATACTGAAGCTGAAATAAAATGTGCGA
AATTAGCAGATACGCTTCATGATTACGGACTTGAATACAAAAATATGGGGGTTTCTGGATCCACT
GTTTTAAAACTAAACAATGAAAATTCAGACATTGATTTTGTAATCTATGGAATGGAAAAACACA
AAATTGCAAGAGAGATTCTATCAGAAACTTTTAAAGATGGCGTTTTAGAACCACTTTCTGAAGAT
TTCTGGAAAAAAGCGTATGTAAAAAGAATAAAAGATGGAACATTAACTTACGATGAGTTTGTTT
GGCATGAAAAGAGAAAATTGAACAGGGGCGTTGTTGATGGCGTTATGTTTGATTTACTTGCAACCA
AGAGAATGGGACGAAATAACTGAAAACTATGGGGATAAAAAATACAAAAATTTAGGATTTATTC
AGATTAAAGCGAAAGTTAAAGATGATACCTTTGTTTTTGATAATCCTGCCGTTTACAAAGTTGAA
AATGTCGAAATTTTAAACAATGAAAATAATGCAGAAGTTTTAGCATCTGAAATTGAAGAAGTGG
TATCATTTACTCACACTTATGCTGGATCCACATATAATGGCGAAGAAATTATTGTTCGCGGAAAA
TTAGAAGAAGTTTCTGGAAAAGAAACCTACAAACGAGTTGTTGTTGGAACTACTCGAGAAGCATT
TAATGAATACGTAAAATTAGCTAAATAATTAATTTTCTTCTTTTTAGGGTGGTAACATTATTGAT
ATTCCAAATTACGGAAAAATAACTTCTAAAACAGTTGTGTTTGATTTAAACGGGACTTTAGCAGT
TGATGGGCTTGTAAGTGAGAAAATAAAAGATCTTTTAAAAGAGCTTGGAAAAACGTATAAACTC
GTTGTACTTACAGCAGACACTTACGGAACGTTGGAAAAAGAATTCAAAGGTCTTCCAATTGCAGT
TGACAGAATTAAAAACGAAATAGAAAAAGTAAATGCTGCTGAAAAATATTCCCCATACATTGGA
ATTGGAAATGGAAATAACGACTGTTTAATGCTTGAAAAAAGTGAACTTGGAATATTAATAATCG
GGGAAGAAGGAGCATCAACAAATGCACTTTTAAAGTCAGATATCGTGATTAACAATATAAAAGA
TGCAATAAATTTGCTTTTAAATGAAAAAAGGCTTATTGCAACGCTTAGAAAATAAACACTAAAA
AATAAAATTTAAAATTAAATCTCTTTTAAAATTTCTTCAATTTTTTTAAGGTATTTTTCTTCTTC
TGGAATTACAGTTATTTCTTTTGCTGGAAGGGTAATTGATGCCCTGAATCCACAATCTCGCGCAA
TTTCCGTTGCTTTTTTATTTATTTTATATAAATCATCAAATAATTCAGTTGTTAGTTCGCAAGTT
GGACTGCAGTACTGAAGACAGCAGCCTTCCCTCCAGTATCCTATTTTATTTCCTGCTTCATTTACT
CGCTTTATATCAATATTATAGTATTTTTGAAGATTAAATACATCAAAATGCCCAACACGAATTAA
TGGCCTGAAAAAATGAATTTTATATCTTTTTGGAACTTTTGTAATTTCCATTTTTTTGTAGAGTT
CAAATTCATCTTTTCTTTTAACATTTTTATTATTTTCACGGATATATTGAAAAACAGGGCCTGCA
ATTTTTTCAAGTGCAGTTTCTCCAGTCATTATTATTTTTGCATTCTCGACTTTCGCAATATCTATC
ATTCGAGCTTTCATTATAGTTTTACAAATTCGGCAGATGCTTTTTCCTTTGGCACCCTGTGTTTT
TTTGGCCAGTTCTTCAGTAATATCTGGAAAAATTATCGGTACATTAAACCTATCTGCTAATTTTT
TTGCTTCGTTTGTTGAAATATCCCAACTCCATTTGTGGACAAAATGAACACACAAATGAACGTTT
AATCCAAGTTCCTTTGCAATTGCAAGTGAAACAGCACTATCTTTTCCGCCACTAAGCATTACAAT
TATTTTTTCATCTTTTAAATTCAATTCTCCAAAATTGTTTAAAATATCGTCTTTTAACTGATTTA

AATGCTTTAAATTCCGTTTATTTTGTGTCCAAGGTCTAAATTCAAGATTATTATCCATATAACCC
ACATATCTTTAAATTAATTATATTATTGTAATAAAAAATAGAATTTTATGATTTAAAATCTATG
TTTTCTACTCCATTGTAAACTGTAAATCGGTCAATTCTTGCAAAACCGATTAATAAAATATCATT
TTCTTTTGCCAAAATTACTCCCTTGTCTGTTGACGGGGATTTCGTGATGATTACCGGAATTTTAG
AATTTACTACCTTTTTAACCATTGCGGTTGGCTGTCTTCCACTTGATGCAAGTACGCAATTGTTT
AAATCAATATCATTTAAAACAGCGTATCCAACTACCTTATCTACTGCATTATGCCTTCCGATATC
TTCAAAATAAACTATTTTGTTTCCAGAAAAATCAAAAAGTGCTGCCCAGTGAACTCCACCCGTTA
TCTTCCACGTATCTGAAAGAGTCGGCATTGTTTCCATGATTTTTTTAATCGTACTTAACTTTAAA
TTTACATTTTTGTTTTTTGTGTCTTCTTTTTCCACGTCTTTTGAATTCACATATATCTTTTTGCCA
TCTATCTTAATTTCTGAAAAGTTTTCAGGGACAATATATCCTTCTGAAATAGTGTATCCAATACC
CAATTCCTCAATATCTTCAGGAGATACGACCATTGTTTCAATTAATTTTCCATCAAGGTATAATT
CATAAAAATCTTCAGTTACTATCGTGTCTTCTTTTTCCAAAAGCCCTTTTTCTGCACTCCAGGAAT
ACGTTTTAACTTTTGTAACCATTTTAGACATTTTTGAACCACACTCCAAAATCATACCATCAATA
TTATTATAACTTAAAGCATATAATTGTTTAAATAACTGTGATACTATGAGCTTGGAAAGATACA
AAAGACAGATTTTAATGGATGATTTCGGAGAACTTGGCCAAAAAAAACTACTAGATGCAACTGT
AACCGTTGTCGGTGTTGGGGGCCTTGGAACCGTTGTTTCACAATATCTAGCTGCCGCAGGAGTTG
GAAACTTAAAATTAGTTGATTATCAAGAAGTTGAACTTTCAAATTTAAATAGACAGATATTGCA
TTTTGAAAAAGACATTGGAATAAAAAAGGTTATTTCTGCAAAAGAAAAGTTAGAAAGTATTAAT
TCAGAGATAAACCTTGAAATTTATCCTGAAAAAGTAAATGAAACCCATATCAAAAAGTCAGATG
TTATTATCGACTGCCTTGACAATTTTGAAGCGAGATATTTATTAAATAAATTTTCAAATAAATAC
AAAATCCCCCTCGTTCATGGCGCAATTGAAGATTTAAGAGGCCAAGTTACAACGATAATTCCAAA
TAAAACTCCATGTATTGAATGTATCTTTAAATTAAAAGATGAAGAAAAAGATAAGTCTTTTCCA
GTGCTCGGAGTAACTCCCGGAGTAATTGGTTCAATTCAAGCAAGTGAAGCGATAAAATTAATAAC
TGGAATTGGAAATCCTTTAAAAAATAAATTGCTTTCAATAAACATGCGAACAAACGATTATTTCA
CATTTAATATAAAGAAAAATCCCAAATGCAGAATTTGTGGTGGTTTGAATGATTAGAGTATCTA
ATGAAGATTTTAACGTTGACATGGAAACTAAAGCCCTTTTAAAAAATCATCCTGAAATAGGTGGA
CTTGTAAATTTTGTAGGGGTTGTTAGAAACGTAGGATACGATAAAGGCGTTGAAAAAGAGGCTG
AATTTATTGAATTTGAATGCTATGAACAAATGGCTTCAAAAAAACTTGAAGAATTAAAAAATAG
GGCGATTGAAAAGTTCAACATAATCGATGCTACGTTAATCCACAGAATCGGAACCTTGAAAGTAG
GGGACAATATTGTATTGATAGTTGTTGGGGCAAAACACAGAAAAGAAGCATTTTTAGCCTGCGA
ATATTTAATTGACAGCCTAAAAGAAGAAGTTCCGATATGGAAAAAGGAATTTTCAAAAGATGGT
TCATACTGGGTAGAACAGCACTAAATCATTTTAATTTATTTTAATTTTTAAAAAGGAAAATCATG
AAATTTTTTCAAGAAAAAATATCCATTAAAGAAACAAATATTCTTTTAAAAGTAGATAATCCCA
AATTTTTTAAAATGGCGAAAAATACAATTATTAATGAAAGATCAAACCTTGAAAATTATATTTT
AAGAAATCCCATATTTTTAACTAGTTATTCTCCTGTTGAAGTGCCGGATAATGCACCAGAAATTA
TAAAATTAATGGCTGAAGCAGGGTTTAACGCAGATGTCGGGCCAATGGCTGCAGTTGCAGGAACA
TTTAGCCAGTTAATTATTGAAAATTTAATTGAAAATGACTGTAAAAATGCAATTTCTGAAAATG
GTGGCGACATCTGTTTAAAATGCGAAATGGATACAACAGTTGGACTTTATGCTGGAAATTCGTCA
CTTTCTGGAAATTTAGGGTTTAAACTAAAAAAAGAAAAAATGAAAAATGGATATGGAATCTGTA
CTTCATCAGGGACCGTTGGACATTCTGTAAGCCTTGGAAATGCTGATTCTGTCACAGTTTTTTCA
AAATCTGCAGTTATTGCTGATGCTGCTGCAACTTCAATTGGAAATTTTGCAGTTGGAAATGCTGT
TGATGCAATAAATAATTGCCTTGAAAAAGCTGAAACTATATCAAAAATCGACGGCGTTTTTGTAT
GTATTGGGGAACATGCTGGAAAAATTGGAAAAATACCCCAATTGATAAAAACGGATAAAAAAGA

AGTTTTGGGAAATGTTTTTGAAATGGTTTGAAGTAAAATTATAATATAATTGGTGTAAATCATG
AATTCAAGAGCAATTAAAGGTATCATTTTAATTTATGGGATAATATTTTGCGTAATCCTTACCGG
ATGTGCAGACTACACGGATCCCTCAAACGAAACTGTTCAGGCCGCCGTTTTAAACGAAGAATATG
AAGAAGAAAATGAAGAAAAATACGATACTTCGAATGATGAATCATATGGATCTGAAGAAAATAG
ATGAATCATTAAATTTAAAATGTAAAAACAAATACTATTTTTAAAATTATTTTAGAAACTTATT
CACCATAAAGGTGGTTTTTTGAAGGAAATTAAATTAAATTCTGATTCTTTAGAAATTTACGAAA
AATCAGTGTCTGAAAAACTAAATAGAAACGATTTTATCAATTTATGGGATTTAGACTTAAATGA
TTTGCTGGATATTTCATATAACTTAAAAAAATTATTTAATAAAAATAAAATCGATTTGTGCTCG
ATTATGAATGCCAAATCAGGAATATGTCCTGAAAACTGTATATTCTGCTCCCAATCAAAACATAA
CACCTCAAAAATAGATACATACGGATTAAAATCAAAAGAAGAGATTTTAAAAAATGCTAAATCG
GTTGAAAAATATTCAAACCGCTTTTCAATTGTCGTAAGTGGAAAAACAGTTACAGATTTAGAATT
TGAAAAAATTATTGAATCAATCGAAGAAATTCAAAATAAAACGAAATTAAAGGTTTGCGTTTCA
TTAGGGCTTTTAAATAAAGATAAATTAAAAGCTTTAAAAGAAAGAAACGTAAGAATCCACAATA
ACCTTGAAACATCTGAAAACTACTTTAAAAATATCTGTACAAGCCACGATTACGAAGAAAAAATA
AAAGTAATTCTCGAAGCTAAAAAAATAGGGCTTGAAATGTGCAGCGGAGGAATTTTTGGAATGG
GTGAAACGATTGAAGATAGGGTTGATTTATTTTTAGATTTAAAAAAGCTCGGTGTTGATAGCGT
TGCTTTGAATCTTTTAAATCCAATTTACGGCACCAAAATTTATGAGAAAATAAAATCTGGGGATA
TTTCCCCAATTAATTCCACTGATGCATTAAAATCAATCTGTATTGCAAGAATTGCCCTTCCAGAT
AAAGTAATCCGACTATGTGGTGGAAGAGAGCATGTTTTAAAAGATATGCAGAAATATTCACTTCT
TGCACTTGATGGTTTAATGATTGGAAACTATTTAACTACAAATGGGCAAAATATCCAGTCTGATT
TAAAAATGATTGAAGAAATAGGTTTTGAGAGGTGATAAACATGGAAAAAAGTTTGTTTGACAAA
TATAACTCGTTACTTGAGTTTTTCAAAAATAAAAAAGTGGTTGTTGCATATTCAGGTGGTGTTGA
CAGCACACTTATTTCAAAAATTGCAAGTGATGCTACAAAAACACTTGCGGTAACGATTGATAACG
GATTTTTTTCTGAAAATGTAATAAAAAAAGCAGAAAATCGTGCTAAAAAAATACAACATTCCTCA
AAAAACCATAAAAATAGATTATTTAAATCAAATTACTTCAAAAGACCTAGAAAACCGATGCTAC
AACTGTAAAAAAAGAATTGCAGAAGAACTTAAAAGAATAAAAAATGAATTGAATTATGATATCA
TTGTCGATGGAACAATTTACGACGATATATTTGAAGATAGACCTGGAATAAAAGCTTTTAACGA
AGCAGACATAATTTCTCCCCTATCTAATTTAAAATTCAATAAAAATGATGTTTTTGCGTTATCAA
ACTACTTAAAAATTGATATTCCAAAAAAAGATACCTGTATGGCAACCCGAATTTTATCGGCACCA
ATTTCTAAAGAAAATATGGCTAAATCGAATTTAGCAGAAGAGTTTATAAAATCAAACTTCCATA
TTGAAAGTTATTTAAGGGTGCGCTACCTTGAAAATATTGCAATTATTGAGCTAACAAAGAACGA
ATCGGAAAAAATATTTGATAATGATTCGATAGAAAGAATAAATACGGAACTTAAAAAAATAGGG
TTTGAAAGGGTTTTACTCGATTTTAATTTTAAATAATTAAATTACGCGAGAATTGGTGATTTTAT
GGATATAAATACGGATAAATACAAAAATATTACGAGAAATCTCGAAAGGGAAATGATTAACTTA
AATCCAATTCAAAGAGGAGGAATCCTCCCAAACGAGGCTAAAAAAATAATCTACGAATACTGGGA
TGGATACAGTGTCTGCGATTACTGTTCAGGAAGACTCGACCAAATCGAAACTCCCCCTATAAACG
AATTTTTAGAAGACATGTCCAAATTTTTAGGAATGGACATAACAAGACCTACACACGGTGCAAGG
GAAAGTAAATATGCTGTTATGAATTCCATCTGTAAAAAAGGCGATTACGTAGTGTTGGATGGAA
ATGCACACTATACTTCATACGTAGCACTTGAAAGAGCAAAATTAAACTATGAAAAAACGGACATT
GAAGAATACCCGACATTTAGAGTAATTCCTGAAAGTTATGCAGAAAAAAATTGACATGCTCGAAG
ATTCTAAAAAAAAATATTGGATTGATCCTTTTAACACACGTTGATGGAAATTACGGAAACGTTGCA
GATGTTGAAAAGGTTGGAAAAATTGCAAAATCTAAAGGATATCCATTTTTATTAAACTGTGCAT
ATTCTGCTGGAAGAATGCCAATTGATGGAAAAAAATTAAACGTAGACTTTATTGCAGCATCAGGA

CACAAAAGTATGGCTGCGTCAGGCCCATGCGGTCTTCTTTCAATAAATAAAAAATATGAAGATGA
AGTGCTTGAAACTTCAAAAGTAAATGTATTAAAAGAACTCCAGATGCTTGGTTGCACCAGCAGGG
GAATTCCAATTTTAAGTTTAATGGCAAGCTTTGAACACCTGATTGAAAGGGTTAAAAAGTGGGA
TTTAGAACTTGAAAAAACAAGAAAAGTAGTTAACGAGCTTGAAACATTAGGATTTAAACAGATT
GGGGAAAGACCAAGAAACCACGATATCATAAGATTTGAAACCCCGATTTTAGATGAAATTGCAGA
AAAAGATAAACGAAGAGGATTTTTCTTCTACGAAGAATTGAAAAAAAGAGGAATCGGCGGAATC
AGAAGAGGAGTTACAAAAGAATTCAAAATGAGTGTTTATGGATTAACCAATACTCAAGTTGACT
ACGTGATAAATAGCATGAAATCTATTATAAACGAGTTAAGGTGAATTTAATGAGTTCTAAGTAT
ATACTCCCAGTAATTGCGCTTTTAATTCTTGCGAGCGCAATATATTTCTCTTTTGGCCCAGATAC
TCCTGAAAAGTACGTATTTTTAGGAGTTACATTTAATCAGGGTGGCGTAGAATATCAAGGATACA
CTGTTGAAGGTCGAAATATAATATTTGAGTACACAAGAGAAGGAGACGCATTTTCACAAGCGGCT
ACCCCGAGAGTTGCCCAAACTGGTGAAAAATACAAAAATGTAGAAAATGTATACGTAAAAGTCG
ATACTAACGGCGATGTAGAGTATTACAAAGCTGAAATATTTGATGAAACCGAAGAAATGGTAAA
ATATTACGTAAAAGAAGAATAAAATAAAAAAATTAAATTGAAAGCATTCTTTCAATTGCTTTTC
TTGCATTTTCAATTATTTTTTCACCAAGCATTATTTCATGTTTTTCTTCTAAAAGGCATTCTTCT
ATTTTTTCTAAAGTTATGTTTTTCATCGATTTGCAGATTGCGTCAGTTCTTAAAGGAATTAGTTT
TTTTGATTTTCCTGCTTTTTCGAGTTCTATTTTAAGCCTGGATATCATATCGCATTCTGTTCCGAT
AATAAATTCGCTGCATTCTGAATTTAAAACGTGTTTTACCATTCCCCCAGTACTCATCACGTAGT
CAGCAGTTTCCTGTAATTTCGGGTCACTTTCAGGGTGTATTAAAAGTTCTGCATTTGGATACTTT
TCTTTTACTGCTTTTGCATCATCAAGTGTAAACATTTTGTGAACATAGCAGTGTCCGCCCTCAGG
CATTGGAATAATTTTTTTGTTCGACCTTTTTGATACAAAATAACCCAAATTATTGTCTGGACCAA
ATAATATTTCGTCTTCTTCGAAAGAATTTACAACCTTATCAGCATTTGCAGAAGTACACGTTGCA
TCAGCCAATGCTTTTGCAGAAGCAAGCGTATTTACATAAATTACAACTTTTGCATCAGGGTTTTT
TTTCTTTGCTTCAAGTATAATTTCGGGTGGAAGCTGGTGTGCCATTGGGCAGTCTGTTTCAACAA
TTTCAGGAATAAGTACTTTTTTCCCGCTGTTTAATACTTTTGCCGTTTCTGCCATGAAATCTACA
CCGCAGAATACGATTACATCTGCATCGGTATCTCTTGCAGCAATGCATAGTTCCAGCGAATCTCC
GATAATATCTGCAATTTTTTGGATTTCTTCTGGCTGGTAGTTGTGTGCCAAAATTACTGCGTTTT
TCTCGTTTTTCAATTTGTTTATTCTCTCAATTATATCCATAAAAACACCAGAACTTTGTGATTTT
AACCTGAATCATTATTTTATTAGGTTATTAGTTTTCAGTAATTCTTTTTTGATTTTCAAAGATT
CAATAACGAGGTGTTTTGCCATCTCATCTAAAGTGTATGGTGTTATTGGTTCCATATCTTTTATA
AATTTACCCGTGCCAACTACCAAATGCCCTTCACTTTCCATTTTTAAAAATTCTACCGGGCCTTTT
TCAGTCTTCGCATCCCCTATTTTAATTGCTGGGTCGAATCCAAATACTCCATTAGTTCCAATTGCA
ACTTTTTTAGTATTTTTACAGTATTCTACAATTTTTTTCCTAATTGGAAGAGTATTTCCACCTGC
AAAAACAATTAAAACTACATCTCCGGTTATTTTTTCGATATTTTCTTCATTGATATCTTCAAAAA
ATGCTTCAATTTTTTCAGGGAAAAATTCTTTTAAAAAATTTACTTTATAGTCTCCAAGTTTTGCA
CCGTGTTTTCTGTGCACTACATCGTTTTGATCAATCCTTGCTGCATCAAAAATCTGGATCTTTTCA
AATCCGCCCCTGTGAACTTCCAAAAGATCCAATGCCATTCTAACCCCAAGCCTTCCACATCCAATA
ATAGAAACGGTTCCTTTTGGTTTTAATTTATCTTCAAGTACTTTAATATCCATAAAAACCACACA
AATAATATATTTATTTAAAATTGACATTTTTCAATTTAAAATATAATTATCGTTTATTAATATA
AAGATAACGTTAGCAAACCTAACATAAGTATATATAGCTTACCATTTGAATTTAGAAGAATCTTA
TATATAGAAATGAAGCTTATGTTGTATATGACTCACATGAGATGAAATTATGCCAGAACATATA
TTATCCGGGATCAAAGCAGTAGTCGCAATAAATATGCGAAAAGAAGGGAAACTTCAAAGAGAAA
TAGCAGAGTTTCTAGAAATGGATCGTTCAATCATCTCCCATTATTTACACGGCAGATATCCATCA

GATAAGGTCATGAGAGTCTCTGAAGAGATTATATCTTTACCCAAAGAACACGGCATTCCATTAAT
TACTTCGCTTGGTGACAACAAGCAGATTACTAAAAAGTTAATAGAACGGATATACAATATAACA
ATAAGTATTGACATGGAAAAGTGTATCGCATGTGGCAAATGCCTTGAATGCGAATACGGGGCAAT
TTCGGTATATTCCGAAGACATCGGCATATCAATAGACAGGGAAAAGTGTATTCTCTGTTGTAAAT
GCGTTTCCGAATGTCCTGTTGGTGCTTTGAAAATAGTCAAATAATCGAATATTAGGGAGGAAGTA
CAATGAAAAACTTCAAAAAAGACGAAAATGATGGCGTCATTGAAATTTCAAGATCTGGCGTTGA
AAAAAGAGTCTTGAAATGGGACGACTGTACCTGCGTTGGTTGCGGTATTTGTAGCGAAATCTGTC
CTACAAGCGCTATAGAAATGGGGCCATTAGGTGCTATTTTTAAAGGGGACATTGATGCTCCAAAA
TTAGACATCTCAGAAAAATGTGTTTTGTGCGGTATGTGTGCATGTGCATGTCCATTCGATGCTGT
TAAATTAAGCATTAACGACAAACCAATCACGGAAATGCCACAGTACCCTAAAATCAAAAGAGGAA
TTGAATTAAATCAATCCAAATGTGTTTTATGTGAACAATGTGAATTAGTATGCCCTCAATGCGCT
ATTGACGTTGAAAGAGAAGTTCCTGAAAGAAAATCCTTAGTATTAGGAGAAATCACAATTAAAA
AAGATGATTGTGCTCTATGTGGAATTTGTGCTGAATACTGTCCTGCTGACGCAATTGAATTAATT
CCAAACGACATGAACGCTTTAAGCTTAAATCCAATTGCAGATATTAACATAGACTTAGATGCTTG
CGTATACTGTAAAGTATGTGAAAAAGCATGTCCACACAATGCAATTGAAGCAATTTGCTACAAAT
GTCCACTTGCAAGCAAAATTAAAAAGCCAGAACTCTACGGCGAAATCAAAGGACAAACAAACATT
GACAAAGAACTCTGTGTATCCTGTGGATGGTGTGCAAACATCTGTCCTGCTGACGCAATAGAAGT
TGAAAAACCATTTGAAGGAGAATTAATCATCGACGAACCGGCATGTAACGCATGTGGTGCATGTA
TTTCAGTTTGTCCATGTAACGCACTTGTATTCCCTCAACCTGAAAAACAGGGAGACAAAGTACCT
AATGTTGTAGTTAACCAGGATGTATGTATTTTATGTGGTGCATGTACGCATTCATGCCCTGTTGA
TGCATTAACCGTTAAAAGAACAAAAATAAATATGACCGAAGCAAATGCACCTGCATGGAAAAAA
GCTTTCTCAAAATTAATGAAATAATTTGAATATTATAGGTGATTTAATGTACAAACTCGAAGTA
TATCCTGAAAAATGTCACGGATGCGGAAACTGTGTTGTTGCATGCCCTGTAAACGCACAAGATCC
TGATGTATATGGTGGCAAAGGACCAAGCAGTGATGAAAAATTAATTATGAGAGTAGAAAATGGT
GTTGTATCCATCGTAAACGGGGACTTATGCGGTGGATGCGGTGCATGTATTGAAGCATGCCCTGT
TGATGCCATTAAATTAGTTATTGTAAAATAACTAAGATTTTAAGCATATCTTATCTATTGTTGGG
TGATATTATGAAATTTATGCTAAATACGGGACGAACCATCTGGCAAGGCGAAGCCATCGAAGCTG
GAAAGAACCTTGAAATGTACAGAAAAGCTGCAGCAATCTGCTACATGAATCCAGAAGACATGGAC
GCATTGGGCGTTAAAGATGGCGATGCAATAAAAGTAACTTCAGAATACGGTGAGGTTGCAGTAA
ATGCAATTACAACAGACCAGCCAGCTCCTCTTGGAATGATATTTATCCCAATGGGACCTTGGGCA
AACAGGGTTGTTTTACCAGATACTGAAAGTACTGCAATGCCTTCTTTCAAAGGGCCTCTTGAAGT
AGAAGTTGAAAAAACTGACGAAGAAGTACTTTTAATGTCTGATTTAATGAGAAAAGCATACATT
GAATAACTGGAATATTTTGGGTGATCTCATGGAATATATCATAAGAAATGGTACCGTCTTTGACC
CATTAAACAAGGTCAACGGAGAAAAGATGGATATCTGCGTAAAAGACGGAAAAATCGTAGAATC
TGTATCAAAAAATGCTTTAGAAATTGATGCAAATAATAAAGTAGTAATGGCAGGCGGTGTTGAT
TCACACAGCCACATTGCAGGAGCTAAAGTAAATACTGGAAGGACATTAAGACCTGAAGACAGTAA
AAAAGATCTATTTTCAAAAGAAGGATTGAGACAAGGATCAGGATTTTCAATACCTTCGACATTCA
AAACCGGTTACCAATACTCAGGTATGGGTTACACAACAGTTATCGAAGCAGCTATGCCTCCGCTT
GTTGCAAGACACACACACGAAGAATTTATGGATTTACCACAAATCGACAAAGCTGCAATGCCATT
ATTTGGTAACAGCTGGATGGTACTTGAGTACTTGAAAAACGGCGATTTGAGTATGTGTGCTGCAT
ACGTTGCATGGCTTTTAAGAGTAACAAAAGGATTTGCAATAAAAATCGTAAACCCTGGTGGAACA
GAAGCTTGGGGTTGGGGTAAAAACGTACACGGTTTAGATGACGAAGTTCCATACTTTGGAATTAC
ACCAAGAGAAATCGTAACAGGACTTACAAAAGTAAACGAAATGTTAGGGTTACCCCACTCCGTTC

ACGTGCACCCAAATAACCTTGGTCACCCGGGAAACTGGGAAACCACCATTGATACGATGGACTGT
GTAAAAGATATCAAAGCTAAACCAAAATTCGGTGACAGGGATACCGTTTACTATAACACTCACCT
CCAATTCCACTCATATGGTGGAACATCATGGAAAGACTGTGTAAGTAAAGGAGTAGAAACTGCAG
ATTACGTAAACAAACATAAACACGTAATGATTGATGTTGGTCAAGTTACACTTGATGAAACTACA
ACAATGACTGCTGACGGACCAATGGAATACGATCTTCACATGACAAATGGCCTTAAATGGGCAAA
CTGTGATGTTGAACTTGAAACAGGTTCAGGTGTTGTTCCATTCATCTACTCACCAAAAGGACCAG
TTTACTCCCTCCAGTGGGCTATTGGTTTAGACATTTTCCTTTACACAAACCCTGAAAAATTGATT
TTAACAACTGACCACCCTAATGCAGGGCCATTCATAAGATACCCAAGAGTTATTGCTTGGTTACT
CAGCAAAGAATACAGAGATGACTGGATTCAAAACAGGGTTCACAGATGGGCTGCAGAAAAAAGC
CACATAACTGAAACCGACAGAGAATACTCAATGTATGACATTGCACAGATAACCAAGAGCAAACTC
ATCAAAAGTACTTGGTTTAAGCGGCGACAGAGGCCATTTGGGCGTTGGTGCTAAAGCAGACATTT
CAATATACGACATCGACCCTGAAGAAAAATCAGGTAAAAAGATAGAAAAAGCATTCCTCGAAGC
AGCTTATGTTTTAAAGAACGGCGATGTAGTTGTAAAGAACGGAAATGTTGTAAAAGAAGTTTAC
GGAGATACAATCTTTGTAAATGCAGAGATAAACGAGAGTTTAGAAGCAGAACTTTTAAAAGACC
TCAACATGAAGTTTAAAAAACTCTATTCCGTAAATATGGAAAATTACCCAGTTCAGGACGCTTAC
GCAAACAGCTGGATGCCAATAAACATTGATGCTACGGAAATACAATAAGGAAATCTTATTTAATC
TTTTTAAGGTGAAATTATGAATGAATTAATTCTTAATTTGAAGGGCGATGTATCAGTCCCTGTTG
AAATGGACAAAATAATTCCAGAAAAAATTCAAGAAATGAGTTTAGAAGAAATTTCTGGAATTGA
ATTAATACAAGGAAACAAAACTGCTAAAGTAAGCGAAATCTTTGACGTTGAACTCAAAGAAAGC
CCTGTTGCAAAAGTAACAATTAACAATTGCTGTAAAAAAGTTAAAAGAATCGGCGAAAAAATGA
ACTCCGGCGAAATCGTTGTAAACGGGGACGCTGGAATGTACGTTGGAGTCGAAATGAAAGGCGGA
AAAATTACCGTAAATGGTGATGCTGAAAGCTGGGTAGGTCAAAACCTTAAAGGCGGAGAAATCA
TCATTAACGGAAACGCTGAAAACTACGTAGGTTCTGCATACAGGGGCGACTGGAGAGGAATGAGC
GGTGGAAAAATTACTATTACGGGTAACGCTGGATCCGAACTTGGAGAGTACCTGAAAGGTGGAAC
AATTGTTATTAAAGGTAACACAAAAATAATGCCTGGAATTCACCAAAACGGCGGTATGATTATCA
TCGAAGGAGATATCGAAGGAAGAGCTGGTGGAGAAATGATGAAAGGTGCTATCGTAGTTTACGG
TAAAATCTTAGAACCGCTTCCATCATTCAAATTCGAAGGAATAGTTGAAGATCCACTCGTTAAAT
TATCTAAAAAAGATGCTGGAACTCAGTTGAAAGGAACATTTATAAAATTCAGCGGAGACTACGT
AAACACAAAACCAAAAGGACAGCTTTACGCAGCAATTGAAAACAATAAAAACTTAATCTAATAC
TTTTTTTAATTTTTGAAATTAATGCTATTTTGAATTATTTTTTATACTCCTATATGTATAGTATC
AATTATAGAATAAGGAGGACTACTATGATTTTAGAATTAAACGGAATTTATGCGGGGCTCCGAT
TTTCAGCAGCACACATTGTATTTGGACATGACAGCTGCGGAGTAATTCACGGCCACTCTTATTAT
GTAGACGTAAAACTCTCGGGAGAGCCGTCAGGAGAATTTGGATTTGTCTGTGATTTTAAAATATT
AAAACAGATTGTAAAAGAACTTTGCAGTGAACTCGACCATAAACTGCTTGTTCCAAGAGACCATG
AAAATATGGAATATTCAATGGAAGGAGATTCAATCTACTTAGAATACGTTGAAAAATCGGGAAA
TGTAAAAAAATACATGTTTCCAGTAGAAGACATTAATTTACTCCCTTTAAAATCAACTACTGCAG
AAGACCTATCCATTTACTTTACGAACTATATTGAAGATAAGCTTCAAAAAATGGATCTTGAAAAG
TCGATCGAATGGATTGAAACTACAGTAAATGAAGGAATTGGACAGGGCGCAAGATACACCCTACA
CTTGAAATAATAGTGTAAAAGGCGTAAAATCAAATAAAAATTTATATAAATTATAATTTGCACA
CTTTTAGCATGATTATTTTCTAAACCTGGTGATAACATGAACCTGGAGCTTTCAGTAACTGAAGC
CATGAGTACGCCGGTAGCCACTGTAACATTGGATACCACAGCTTACGACGTGGCAAATATCCTTA
AAGACAAAGGAATCGGATGTTTAGTAGTGTTAAACGATGCTGGAAAACCGGTAGGTATCATAAC
AGAAAGGGATTTAGCACTCGGAGTTGTTTCAAGGAATTTAAAATCAAAAGAAGTAATTGTTGAA

GAAATTTCCTCTTCAAAATTAATAGCAATTGCTCCAAAATCTACGCTGATGGATGCAGCTAGAAA
AATGGACTCTGAAAATGTAAAAAGACTCCCTGTAATCGATGGTGATGAATTACTTGGAATAGTTA
CAGTAAGCGACATTACAAAGCTATCACCTGAGTTATTCAGCATAATGGTCGAAACAAACGAGATT
CATAATTCAGAATACCCGTACTCGAAAAATGAGGAAATTGAAGGGATTTGTGAAATTTGCGGAA
GTACAGACAGTGTAAATTACATTAATGGAAGATACATCTGTAAAAACTGCAATGAAGATTCTGA
AGGCGAAGAAGAGTAATTATCTAAAAAAGAGGGATATTTTGAAAATAAAAACCATTGTCGGCGA
AAAAAAAGTCGAAAAAGTATACCCTACAACAAAAATTATTGAAGCACTCACAATGATGGATAAG
GAAAACGTCAGAAGGATATGTGTTGCTGATCCTGGAACTGGAAGAGTAGAAGGAATTTTGACGA
ACATGGATATTGTGGACTTTCTCGGAGGAGGCTCTAAATATAACCTCGTAAAGTTCAAACACAAT
CACAACATGCTTTCTGCTATCAACGAGCCTGTGAAAGAAATAATGACCGACAATGTGGTATTGAT
AAAGGAGAATGCAGAATTAGATGAAGTAATTGATTTATTTGTTGAAAAAAAAATTGGTGGAATG
CCTGTAATTGATAAATCAGGCGTTTTAATTACTACCATTAATGAAAGGGACGTTATAAAATATTT
AAAAGATCAGGTTGACGAAAAACTTCTTGTAAAAGACTGCATGACTGAAAACGTAGTTTTCGCAA
CACCGGGGGAAAGATTAAAGGATGTTGCACGAACAATGCTTAGAAATGGATTTAGAAGGCTTCCT
GTGGTTTCTGAAGAAAAATTAGTTGGTATAATCACTTCAACGGACTTTGTAAAATTATTCGGAAG
TGACTGGGCATTTAACCACATGAAAACAGGAAATATTCGAGAAATTACGAACGTAAGAATACAG
GACATTATGAAAACAGATATCGTTTCAGTAACCTCAAACATTAAGTTAGTTGATGCAATTAAAAA
GATGAACGAACTAAATATTGGGGTACTTCCAGTAGTTGATGGGGAAAAACTCATTGGATTAATT
ACTGAAAAAGATATTGTAAAGTGCATTTACAAAAAATAGATAATAACTAAAAAATTACTAACTT
TTTTCATATTTTTATTTTTTAATGGCATTTATTTTTTATAGTGAAATTTACTATTATTTTTCAAA
TTTGATTATTACATTACCGGTGATGACTTTTGAGAATTAAACCAGAATGCGCACTCTGCATTACA
CGACAGGTTGTAGATGTTGCAAAGGAAATAACTGATGATAAATCCGAACAGTTTAAAATTATAG
CTTGGGGAATCGAAAAAATATCCGAAGCTTATGGGGAATCTTCCGTTCCATCGTTTATGGGTACT
GAAATTCACAGGCATATCAAAAAAATGAGTAAAAATTCTGACCCTTACAAAAATTTAAAAAACG
TTGCAAACGAATTTGCTCTCAAATATTTAAATGATGTTGAAAAATTACTTGAAAGCGACGATGA
ACTCGAAAGATTGCAGAACAAAATAAAATTATCGATTGCGGGAAATGTAATTGATTTTGGCCCTT
ACAGTACGGGTGTTGATATCGAAAAAATGGTAAAGGATACACTTGATGGGAAACTAGACGTTGA
TTTTAGTAACGAATTATTAAATGAACTTAAAAATTCAAAAAAAGTGTTTTATACCTGTGACAAT
GCAGGAGAAATTGTTTTTGATCTTCCACTGATAAAAGAACTTAAAAATTATGTTGAAGTTGTAG
TGTCCGTTAAAGGAAGTCCGATATTAAACGATGCAACACTCGAAGACGTTAAAACCGCAGGAATC
GATAAAGTAACAAAAGTAATTACAAGCGGAACTGATGCAATTGGAGTAAGGTTTGAAGAATCTT
CAGAAGAATTTATAAATGAATTAAACAGTTCTGATTTTGTTATTGCAAAAGGAATGGGCAATTA
CGAGAGTTTAACCGAATACGAAGAAAAAAACGGACAAAACTCTGAAAAAATTCCTGTTTATTAC
ATTTTAAAGGCAAAATGTGAGCCTGTTGCCGAACACGTTGGAGTTAGTGAAGGCGATAACGTTCT
TTTAAGGAAAGAGATAAGTAAAGTTTAAAATTAATAAAAGCAAATTACAATAGAGAAAAACATT
TATTCATTTATTTTTAAGGTGGTATTTTGGTAACTTACAAAGACGCTGGTGTGGACATATATAAA
GAAGACAAGGTAATAAGAGCGCTTGCTTCTCAAATTAAATTTGAAAGAACTGATGCAATAAAAC
CTGCTGATTTAAAAGGACATTATGCAGGAGCTATCGAATTTGGAGATTATTATTTAGTACTCTGT
ACTGATGGTGTTGGAAGTAAAATGGTTGTTGCAGAAATGGCAAATAAATTCGACACCGTTCCAAT
CGACATGATTGCAATGAACGTAAACGATGCAATATGTATTGGAGCAGAACCTGTTGCTCTCGTTG
ACTACATGGCTGTTGAAGATATCACTGAAGATATTGCTTCTCAAATTGGAAAAGGATTGAATGAC
GGAATAAAAGAATCAAACATCAATTTAATCGGTGGAGAAACTGCATCACTTCCAAACATGATCAA
AGGAGTAGATTTGGCAGGAACAGTTCTTGCAGTTGTTAAAAAAGATGAAATCGTCTCAGGAAAA

GAAGTGAAACCTGGTGATTTAATCATTGGTTTAAGAAGCAGTGGAATCCACAGTAACGGATTATC
ACTTGCAAGAAAGGTATTTTTCGATATCGCAAACCTCGACGTTAACAGCAAATTATCACACGGAA
AAACAGTTGCTGAAGAGCTTTTAACCCCTACAAAAATTTACGTAAAACCAGTTTTAGAAATGATT
AAACAAGTGAATGTAAAAGGACTCGCACACATTACTGGCGGCGGTTTTAGAAAATTAAAAAGAT
TAAACAAAGAAGTATGTTACAAAATCGATGAATTACCCGAAATTCTTCCAATATTTAAAGAAAT
GCAAAATTTAGGAAATGTAGCAGATGAAGAAATGTTTAAAACATTCAACATGGGCATTGGATTT
TGTGTAATTGTTGAAAAGGAAGATGCAGAAAAAAATAATTGAAATATCAAACCACCACAACATTC
CTGCATTTGTAATTGGAAAAATCGAAGACAGCGTTGAAGTAAATGGCGAAACTAAAAAAGAAAC
AGTTTTAGTCGAATACAATAACAAAAAAATGATAATGGAATAATTTAAAATATACGTTTAAATT
AAAAATATGGGTGAATACTGTGCCTACAATTAACGTGAATAAAGTAGATTTGGAACGATTGAGC
AATATTTCGCTTTCGGACAAATTAATAGAAGATAGATTCCCAATGATGGGTGTTGAAGTAGAAG
AAATATTCGAAGAAGTCGATAAAAACGGGAAAAAACAGAACATGGTGCAGTTTTCAATAAATCC
TGATAGGCCAGATTATTTAAGTGTTGAAGGCCTTGCAAGAGGTTTTAGAGGATTTATGGGAATT
ACTACAGGAATCCAAGAATTTGAAGTACTTGATTCAGACATTAAAGTAACTGTTGAAGAAAATG
AAACAAGACCTTATGTTGCATTTGCACTCGTTAAAAATGTTTTAATGGATGAACTTGTCCTTGAA
AGCATGATTAACCTTCAGGAAAAGTTGCACTGGGCAATTGGAAGAGATAGAAAGAAACTTGCAA
TTGGAATTCACGACTTTGATAAAGTAAAAGCTCCATTTACTTACAAAGAAATCAAAGGGGATGA
AATTAGATTTGTTCCTTTAGGTTATGAAGACGAAGAAATGACTCCAAGAGAAATTATTGAAAAA
CATGAAAAAGGAGTAAAATACGCTCATTTAATTCAAGACGATAAATTCCCGATTATTGTTGATGT
AAATGGCGAAGTTCTTTCAATGCCCCCAATAATCAATGGAACACTCACAAAAGTAACACCAACAT
CTAAAAATTTACTTATCGACATTACTGGAACTGAAAAAGAAGCTGTTGAAGAAACATTAAACAT
TATCGTCTGCGCACTTGCAGAAAGACGCGGAACCATTGTTTCAGTTAATGTTAACGGGAAAAAAT
ACCCGGATTTGACTCCAAAATCGAGAATGATTTCCGTTGAATCAATTAATAAAAAATTAGGTTTA
AATTTAAATCCTGGAGAAATTATTCAGGCACTTAAAAAATCAGGCATGGATGCACTCTACGAAGA
TGGAAACTTGATTGTAAAAATTCCTGCGTACAGAAATGATATCCTACAAAACGTTGATTTAAAAG
AAGAAATTGCTATAAATTACGGTTATGAAAAATTCGACGGAAAATTACCCTCAGTTGCAACTACC
GGTTCAAAAGATCCTGTTGAGAAAAAAATGTAGTGCAATGTCTGATTTAATGATTGGACTTGGAT
TCTACGAAGTTATGAATTTAACCCTTTCAAATCAAGATACTTTGTTTGAAAAGATGAATTTAAAA
GTAGATGAAAAAGACTACATCGAAGTTTTAAAACCTGCTTCAATCGAACACAGGGTTCTTAGAAC
TTCAATTTTACCGCTTCTTTTAGAAACGCTCTACATTAACAAACACAACGCTCTTCCTCAAAAAA
TATTTGAAGTTGGTGACTGTGTAGTTATCAATGAAGAAGATACTGAAACTGATACAACGTGTAA
AAATATCAAGAAAATTGCGGGTGCAATAACACACCCTTTAACGAACTTTAACGAAATAAAAAGCT
CAACTGAAGCACTTCTAAGGGAATTTTTTGAAGGTTTTGAATTTGAAAACTATGAACACCCAGCA
TTCATACCCGGAAGATGTGCAAAAATACTTAAAAGTGGAAAAGAAGTTGGATTCTTTGGAGAAA
TACATCCCGAAGTTATTTTAAACTTCGAACTCGAACATCCAGTAGTTGGATTTGAAATAACAATT
GAATAACTTTTTATTTTTCTAATTATTTCAGGGCTGTTTATAATGCCATCCAAATTAAAATCAAG
TTTTGATGTAGCATTGAATATTATTATAGGGATTATTTCGATATCTTTTACTGTAAGTACTTCTT
TTTTTAATAAATTTACTAAATTATATGTTTCTAAAACAGGGTTTTCAATTAAATTTTCAGATCCG
TTTATTTTTTGAATCCATTTCCCGTCTTTATGTGCAATAGTTCCGGGCAGATTCTTTGAATTTAT
AACGAAAATTCCGTATTTTGAAACGATAATGTAATCGATAACGCTTGAGTTACCATTACAATCCA
GATTATAATCTTTTAAAATAGAATAATCCGATTTATCAAGATTTCTTAAAAATAAACTTAATGG
ATATTCATTTACTTTTCCACTTATTTTTGATAAACTATGTTTTGATAGTGAAGTTATAAATCCCA
TAATCCCCCTCATAATTATTTATAGCCACTTAGCAGCTTCTTTTGCCCAGTATGTTATTATAAAG

TCAGCACCTGCCCGTTTTATGCTTAAAAGCGTTTCATATATTACTTTTTCCCTATCAAGCCATCCT
TTTTCCGCTGCTGCTTCAATCATTGAATACTCCCCACTTACACAGTATCCTCCGACTGGAACATTA
GATATTTCTTTTGCCATTTTTACAATATCAAGATACGGAAGAGCTGGTTTTACCAAAATTAAGTC
AGCACCTTCTTTTATATCAAGTTCTATCTCTTTTATGGCTTCTCTTGAGTTTCCCATATCCATCTG
GTATGATTTTCTATCCCCGAATTGCGGTGTGCTCTCTGCAGCATCCCTAAATGGCCCGTAAAATGC
AGATGCGTATTTTGCAGCATAGCTCATTATTGAAACGTTTTTAAATCCATTCTCATCCAAAACAT
TTCTAATTTTTAAAACCCTTCCATCCATCATGTCAGATGGTGCAACAATATCAACACCGGCCTTT
GCATATGACAGAGCAATATTTCCAAGTATTTCAAGTGTCTCGTCGTTTAAAATTTCTTCCCCTTT
AATTATACCGCAGTGCCCGTGGCTTGTGTATTCACACATACAGACATCCGCAATTACTAAAATAT
CGTCTCCAAGTTCGGCCTTTACTTTTGAAATAACCTTTTGAACTCCACCGTTTTCATTTACCGCAG
AATTTGCAAATTCGTCCTTTTCAGTTGGAATTCCAAACAAAATTACCCCTGGAACTCCCAATTTT
GAAATTTCAACGCACTCTTTAACTGCGGAATCTACCCCGTACCGAAACTGCCCTGGCATTGATGT
AATTGGAGTTTTTTCATCGTTTTTTAAGTTTTCATCTACGAAAATTGGCATTATTAAATCGTTTT
TGGTTAAAACGGTTTCCCTTACCAAATCCCTGATTTTCTGGTTTTTTCTAAGTCTTCTTGGCCTTA
TTAACATTTAATCGCCTGATCGTATATTAATTGTTAAAATTATATAATATATGTCGTAATCAAAA
ATTATAGTTTAAAACAATATAATAGTTTTTGTTTTCAAATTCAATAAAAAAGTTTAAAAAAGGC
TGAGATGCCGATAATCGTACTTAAAATTTATCGAGCGTATCTTCTGCAGCGAAAACTATCGGGTC
AATTACCATTGAAACTGGTGGTGCATAACAGAATTCCTGATTTGCTAATTCTTCAGCAGTCATTC
CATTTGAAATTGCAACTGACATTGCATCTACTCTTTCTGCGACTCTTTCTTCTGAAATAATCTGA
CAGCCGATTACTTTTTTCGTCATTTTATCAGCAACCATTTTTATGTAGATTGGTTTTCCGCCAGG
GTAGTATCTTGCTCTTGTAAGTGATTTTGAGTATCCTAAAACAATTTCGATTCCATTCATCTTTG
CAGCGGTTTCAGTCATTCCAGTTCCGCCAATTTCGAGTTTTCCTATTTTTGAAACCATGCTGTTTA
ATACAGGTTTGATTTCTGCTTCAAGGTTTACAATTGCTTTAGCAGCTACTTTTCCCTGTCTTACT
GCGGTTGTTCCAAATGGTGAAAGTGTATTTTGCATTGTTATTGCATCGATTACTTCAACACAGTC
CCCAACTGCATAGATGTTTGGAATTGATGTCTGCATTTTTTCATTTGTAAGAACTGCCCATCTTC
CAATGTCACAGCCTGCGCTTTTAGCAAGTTGGATGTTTGATCTAACTCCTGTTGACATGATTACC
ATTTCTGCAGGAATTGTTTCTTCACCAACTAAAACAGCTTCAACCTTGTCGTTTCCAATAATTTT
TCCAACTGGTTTTTCAAGGATAATTTTAATTCCCTGTTCTTCCAAGTACTTCTGGACAGTTTCTG
CCATGTCTGGGTCAAGTGCTCTTGGGAATACTTGTGGAACCATTTCAACAACAGTTACGTTTAAC
CCAATTTCTTTTAATCCAAATGCAATTTCAATGCCGATTGCTCCAGCACCTGCAACTACCACATTT
TTAGTGTCTTTTGCCCATTCGGTTATTTTTTGACCATCTTCAATAGTTCTTACTTTAAATACGCCA
TCTAAAGTTACCCCTTCGATTGGAGGTACAAATGGAGTTCCACCAGTTGCTAAAACTAAATTATC
GTATTTAATTTCTGTTTCAGTTCCATCTTTTTTGTAAATTACTTTGTTTTCTCCTGAAATTACAT
CTAAAACTTCAGCTTCAACAATTACATCGATTCCTTTTGCTTTGTAATCTTCAGGTGTGTGCATG
ATAATAGTATCAAAATCTACAATTTCTTCACCAATTACGTAAGGTATAGCGCATGGTGAGTATGC
AATATATTTATCTGATGTAATTACTGTTACTTTTGCATTTTTATCGTGTTTTTTTATGTTTGATG
CAGTTGTAAGTCCTCCTGCACCGCTTCCTATTATTACTACGTCCATATTTTCACCAAAATAATTAA
AAAATTTGTGTAAATTTCGAATAATTCTTAGAATATAGGAGCTTTTTTCAAATATATAAATTTA
ATTGATAATAACAAAAATTTAATCAAATAGAGCCCTAAAAAAGCATTTTTTTAATTTTAAACCGC
GTATATCCAAATAATGGCTATTAAAATTCTTAAAATAGAGTACATTATAATTAATTAAAAAAAG
AAAGTTTATGAAAATTATTGTATTTCTTCCATGATTTCATTGTATAATGTGAAATCGTAGAGAAT
TGCATTTATTTCTTCAGCATCTTCAAGTCCTGCTAATTGGCCAGTCATTGCACCTTGTTCATCCAT
AACTTTTACAAATTCAGGTCCAATAGCTTCTAAAGAACCAATTTCATTTGTAAATCCGATGTTTG

GAATTGAAAGTTCTTCAACTTCTAAGTCGGTTCCAAGCCATTCAGCACTTGCATTTACTGCAAGT
TTTGGATTTGCTGTAATTTCATTGTTTGCTAAAGCCATTAATTTTAAGAATGCTTTTACGCTTTC
TCTTTTATTTTCAACTGCATCGTTTGATGCAACAAATACACAGCAAGGGTGGTTTGTCCATACTC
CTTCTGGAGGTAATTCTCCTGAATAAATTACAGATTTTCCAATTCCTGAAACTGCTAATTGCTCA
GGTGTTGGTTCCCATGCAATTACTGCGTCGAGTTCATTGCTGCTTAATAACTGAGGCATTGTTCC
TTGACCTTTACAGTTGATAAGCTGAACCATTGCATCTTTATCATTTGGATTTTCTGTGTAAGTAA
CTTCTTCAGCATCTAGTGCTGATTTTATCATAACATACTGGATTGATGTAGGAAGTGGGTGTCCA
ATTTTTACTTGAACTCCTGATTCTGCCTGTTCTTTTATGTAAACTACAAATTCGTCCCAAGTTTC
AACAGGGATATCATTTCTAACAACAACTGCTGAACCTTCGGCCATTACTGGGCTTACAATTTTTG
CATTTGTTCCCTGATCAATTGAAAATACTGCAGGAGGAACTCCGTTTAATCCAACGTCAACCTGA
CCTTGTGCCATCAAGGTCATGATTTTCGCTCCACCTTCAATAACTTTTATAAATTCAACATTTGCT
ACGAGTTTATCGTTTTCATAAAGTTCGTAGGTTTTTTATTCTCAACTTCTTGTAAATATACGCC
ATATTCGTCTTTGAATACGTCAGGGTATTCTGCTGCAACAAATAATGGTGCATCGTGGTCTGATG
GAAGGTATGCAACCTTCAAATCAGCAACTTCCCCATCTGAAGTAGTACTGTCAGTACAACCTGCA
AATAAAAGTGCCAATAATAAGCTTGCAATTATTACAAATTTTTTAATAATTAACACCTCAAATAA
TCATTCATAGAAAATATGTATTTAAATATATAAAATTTACAGTAAAAAAATATAAACTCGATAA
TTTTAAAAAGTTTAAAAATTTAAAAAAAAGAAATATTTGCCTTATTTTTTCGAAACTTTAAGTC
TCGTTCCACAGTATGGGCAGACCATCCACGTTGGATCAACTTCTCTTTTACAACCACTGCATTTTT
GAATTTTATCTTCTTCTTTGATAAGTTGGAATCCGCAGTGTGCACAGTTATTCCAGTTTTCAGAG
GTGTGTTTGTTACAGTTTGGACAAGGTTTTGTTTCAGCAAACTCCATTTCTGCCAAACTTGCACC
GCAATTTGCACAGTTTTCCCAGCCAATATCAACTACACCATTACAGTTTTGACAGTAAAATAAAT
TTTGAACAAATTTTGAACCAAGCGTTACTTTGAGTTTTTCAAGAGCAATTGAAGCCTCCCTTTTT
AAAACTTCATCAATATCATATTCCATTATTCGTTCAATTCCCGAAACTATTCTCTGAATTGCCAT
TTTGTCGGTCATTTCCATGTACTGCAATGATTCAGGCCTTAAAACAGTTAAATCAACGATTTTTT
TCAAAGTAATTAATTTGGTAGCAGGTACTGAACTTTTAAGCCCATTTATTATGCTTGCCCTTTCT
TCCTCATTTATTGGAACTATCGTAACCATTATCTGCCTCCTTCCAGTAGGGGATATTCTCATCCAT
TACATTTAAATTTCCTAGTTTAACTATTTTACGATAATAATATTCCATATAAGCCATAACTTCTG
CTTCAGTTGCGTTTGAAAGCTCCGAAATTTCTATTTCTCTTGTTCCTATCTCGTAAAGGTAATAA
ATCTTCATATCTCCGCTGTTATTATAGTAAGAAAACACTTCTACAGAGTTTATATCCTCAAAATT
GTTGTATGAGTACCACAAAATATAACAAAGGTCTAATTTTGCAGATTTATCAAGTTCTCCCCTTA
ATCTTTCTCCGATAGTATTTCTAATTTGAAAAACCCTTAATATATTTCCGTCTTCCGAATTAATT
TTTTTTACTTCGTAAACCCCGTATTTTTCAAATTCTTGCATTTCTGAAAGATTATCCTTTTTTTCC
TGCAAAATTATCAAGGGGGCTGCTGCAAAAATTAAAACAATTATTAAAATAATGAGGTATTCTT
TTACAGACATAGTTTTCACAATTCGTCTCAGTTAAGAAGGAGTTTTGAATCTACTGCACCTTCAC
CTTTGAGTAAATCTTTATAATCATCAATAATGATTCCAACGTTTATAAGATTATCCAGTTCTTTT
TTAAAGGACATTTTTCTAAAATGTTCTCTTTTATTCAATTCATACATGTAGCGTAAGGTCTCTTT
TTCACGCAATTTTTGAGTTTTTTCATCAAAACAGCTGTTTTTGGATTTTAATTTTTCAATAGAGT
CAGATATATCTTCAGAAAATATTTCAGAATATTTTTCAGAAAAACTATCAAATTTTTTAATTTTA
AAGAAGTATTTTCCAAGTTCTGCTCTTTCTTCCCAGCAAATTTCTGTTTTTTCATTTAAAGAAGT
TTTATCACTTCTTTCAGCAAAGTCTAAGGAAAATTCCGAAAGTTGGTCGTGATATAATTCTTCAC
AGCATTCTATTGCGTGTTTTATAGTTTTTTTACTGCATTTACCAACCTGAACACCACTACAGTAG
TGTTTTAAAATCAAAAGTAAGTCCCCATTCCATTTTACCATGAAACTGAAGGGAAACAGCCTGCA
CATTGCAGGTTTCGTATCATAGCTTTTTTCAAGCTGTATTCTACATAAATTATTCTCGATAAGGC

CGCACTTTCCATTTCTAAGGGCTAAAACATGTTCATAATTTGAATTTGACTTTTCAATATAATTT
TCATAGCCTTTTAGTCTCATCATGTCAAAATAACTTAAAAATACCCTCCAATTTTTGCAACTGCA
ACAGTAAGCGCAGTTAACGCAAGAGTATCTCATTCCGTCAAATGTAACTTCAAAGCTCATAAACG
CCTCTTGACTCTACAAATCGTCATTAAAAAGTTCATTACGTTCTATAAATTCAGGAATATTCTTT
TCTTTTTCAGTAGTCCAAAGCTCATCAAATTTAACGTCTTCACCACCATATTCTCCTTCAAATCTT
TTTATTTTTACCATTGCAGGAATTTTCAAACAAGGGCCAATTATCACAGCTTCTCCAACGTTTAA
TCCAGGAAGTTGATTTAATAAGTCCTCACTTAAATTTTCTGATGCGTGTTGGACGTGTTTTTGAT
CGCTTGGTTCAATTAATTTTGAAATTATAAAGTTGTTGCACTGGGATAGCGACTCTTGGTCAAGG
GTTTTTGGCCTTTGTGAAACAAGACAAATTCCTACACCAAATTTACGGCCCTCCCTTGCAATTTT
TGAAATCGGAATCTTGGATTTTGTGGGCCTGTTTTGTGGCACAATAAGATGTGCTTCCTCATATA
TTATAAATATTGGTTTTGCAACTTTTGTATCCCATAAAACTTTTTTTCTATCCTTTAAAATTTCC
CCTGTAAAGTAAGAAATTATTAAATCTGTACTGCTTTCGTCTAATTCGCTTATATCAACAATATT
CACATAATTTTCTTTTATATCCTGAATAGGATTGTAATTTATCGCAGTAATTTTATCTTTAAAAG
TCATCAGGTCTTCAAATCGCATAATTAATGTTAAAATACTGTCCACTTTGCTCTTGAATTCATCC
ATGCCCATGTAATAATTAAGTTCATTGATAATCGAGTTTATGTATTCGTCAACAGTGTTAAAATC
AACTTCCTTTTTTGATTCCTTGATTTTTTTCATTGCACGTCTTCCCATTGCCCTTTGAACTGTAGC
TTGTGGATCAACTCCTGCAAGAGACATTAATGCATCATATGACATATTGTATACATTTATTTTTG
GCCTTATGATATTTCTTCGAATTGTACTGCTCTTTGCTTCAATTTCAACGTATTCCCTGTGCATGT
CAAAAACTAAAACGGTTGCATGAATGTTATTTAATTCTTCCAAGAGAACAGAAACGGTATTTGAC
TTACCCATTCCAGTAATTGCAAGTATTGCCAAGTGCCTTGAACAGAGCTTGTTTACATCTAGTTT
AACTTTTGAATCTGCTGAAATTAAATTTCCAATTTCGATTGTTCCTTTTGAAAAAATTTCCCCCA
GTATTTTTTCAGATGCATCGTAAACTTCGGTTCCGGGTTTTGGCGGAATTCTTGGGATCTGCATA
TTTTTTACATCACCCAAAATCTTTATTTTTCCAATCGTGTAGTAAGAATCGTTATTTTCAAAATC
TTTTAATTTTTCAAAATCTTTTACATTGTATACGTCTTCAAAGATTTTGCATCCTTGATTGACCG
ATTCAACCATTCCTAATATTTCTAAATCGCAATAATTAATCGATACATAGCTTCCAATTTCAGGC
ATGTGCTTTGCAAGGAAATTTAACTCAGTATTTGAGGTTTCACCTATAACGTAGCCCATTAAATT
ATCCAAAAATACCGCCCTCCGACTAATATAAAATATTTAAAAGTTAAAAATACTTAAAAAAATT
AATAATTATTTTAATTTACTTACTGAACTTTTTCTTTGAGTTTTTTCTGTAATGTTGTGTTTATT
AACTTTCCATCAGCCTTTCCCCTGAGCTGCGCCATACATCTTCCCATCAAAAGACCCATTGCACCC
ATTCCTTTTTCGTTTACTTGGGAAATGTTTTGATTGATTATATCTTCAATTATTGATTCTACTTC
TTCAACAGACATTGATAACATTCCTTTAATTTCTAAAATTTCATCGAGTTTTTTTGAAGGGTTTT
CAATAAATCCTTTTATAACATCAGGAACTGCTTCTTTTGACATTTTATCTTCTGAAAGTCCTTTA
AAAAGTTCATCAAGGTGTTCTTCGGCCAATAAATCGGTATCAAGCCCTTCTCTTTTTATTTCTTT
TATTGTAGCTTCAAGAGTTGTTGCAATAAGCGTAGGTTTGATTTTTGGTGATTTTTTTGACAGAC
TTTCAAACAAATCAACGTGATAAGACATTACCATCTGTTTTGCAAGATCTTCGTTTAATTCATAC
TCTTTTACAAACCTTACAAGTTTTTCTTCAGGCATTTCTGGAAGGTTATTCCTAACGAATTCGAG
TTTTTCTTCGGTAATTGTAATTGTTGGAACGTCTGTTTCAGGATACATCCTTGCAGCTCCAGGAA
GCGGCCTTAAGTAAGATGTATTTCCATCATCGAGTGCTTTTCTCGTTTCTTCAGGAATTCCAATC
ATTGATTCTTTTGCCCTTTCGATTACTGCGTTGAGTGCTCTTTCAACCTTGTTTTTCTTGTCTGCA
ACCAAAATTACAGCGTCATTCTCCCCGCAATTCATGTATTCTTTTAATTTTGTGACTTCTTCTTCA
GTAATTCCGTATTTTGGAAGTTCATCGGTGTGGAATAATCCGCCAACACCACCAAGTACCTTTCC
ACGGTCTGAAAATTCCGTTCCGAGTCTTCTTCCGGGCTGAACTTCTCTTCCAATCATTCCTGAAAA
GCCTTTCAATAATATTGCCCGAATTACTCCTTTATTTTTAAGCGCATTTTTCAAAACTTTTGATT

CAGTATCTTTTAAAAGTTCGGTGATGTCTTTTATTTCATCGACAACTTCTGCATTTCTTTTCAAA
AGTTCTTCTTTAATTTCGTTTAAACTAATCTGTCTTGTAACTTCGTTTTCGATGATTTTTTCAAT
TAAATCAAGATTTTGGACTCCTTTAACCTCAATTCTTGCGCCATTTTTAATTGAAATGTTTACAT
CCTGCCTAATTGTACCAAGACCCCTTTTAACTTTCCCAGTTGCTCTTAAAATGGTTCCAATTCTTC
TTGCAGCTTCTTTACCCATTTTTGGAGATGTGATATCGGGTTCAGTTGTAATTTCCAAAAGAGGG
ATTCCAAGCCTATCAACACAGTATTTCGTGTAGTCTTTTCCATCTTCAATCTTTTTACATGCGTCT
TCTTCTAAACAGAGGCTTGTAACTCCGATATTTCCGTATTCTGTTTCGATGAATCCTTCTTGTGA
AACAAACATTGTTCTTTGGAATCCTGACGTGTTTGAACCATCAATAACCATTTTTCTCATGATCT
GAACTTCATCAGCCATTTTCATGTTCATTAATGTTGAAACTTCTAATGCAGTATCTACTGCTTCA
GGGGCAACGTCATGAGGCGGTTCATCGTCAAGTTCCACTAAACACGTCGTGTCATTGTAATACTG
GTAGATAAACTTCTTTTCTTTTTTTGATTCGAGAAGCGCTGCCTTATCAACGTGGCCCATTTCAC
TCTGAGAAGGCCTTAAAACCCGTTCAATTTCCCCGTGAGGTTCATCATCCCTTATTTTTGTTGGGC
AGTTACAGAAAAGTTTTCTTTTAGTGTTTAACTGCTGGTGTATCTCAAGTCCGACTTTTAATCCA
AGTTTTTCGTAATCATAATCCATATTATCACCAAAAATCAAAGTAAATAATAAATAAATCCGTTT
AAACAATTAACACTCCAGATTAATAGATTCGTTAATTTCTCCAGCAATGTTTTTAGACATTAATT
CTTTTGCTTCCTTAAATTCATAATTTCCAAGTAAATGCATTAATTTAACAAATGCAACTTCTGAA
AGCATGTCTTCACAAGGAATTACGCCTTTTGCTTGAAGTTCTCGCCCATTTGAATAAACATTCAT
GTTTACTCGACCATTGATCGTCTGAGTAGTCATTGCAACTAAAACATTGTTTTCATTTGCGTAAT
CGATTCCTTCAAAAAAGGTTTCAGGGGTGTGTCCAAGTCCAGTTCCTTCCAAAATAATTCCCCTG
TATCCGTTATCAACGTAAAACTTCAAAATTTCAGAATCAATTCCAGGATAAACTTTAATTAAAGC
AACTTTTTCTTCCAAATTTGTGTTTAACACTACTTCTTTGATTTTTGACTTGTCCTGACTTTTTAC
ATCTCTTAAATATGTTACTTTTTTTGTAAATGGGTTAAGTTCAGCCATTGGAGTTTCATTTACTG
ATTTAAATGCGTCCCTTCTGGATGAATGGAGTTTTCTAACTTTTGTACCTTCGTGTAAATGGCAT
ACGGTATCCCCAGTTTCCCCGTGCATAAGAACATAAACTCCTTTTATTGGCTCAGTTGCTGCTTTT
ACTGCTGAAATTATGTTTAATGCTGCATCTGAAGAAGGTCTATCACTACTTCTTTGAGCCCCAAC
CAAAATAATTGGAACTTCAGAATTTACCATAAATGATAGTGCTGATGCGGTGTAGTGCATAGTGT
CGGTTCCGTGTGCAATAACAATACCCTCAGCTCCGTTTTCAATTTCCTCTTTTATCGCTTCTGCAG
TCATTTTCCAATAAGCTGGAAGCATGTTTTCACTTAAAATATTCAAAATAACTTTTCCTTTGATG
TTTGCAACGTCCATTAATTCAGGAACTGCCCGAATTAAATCATCTGCTGTAAATGCAGGGTGAAC
TGCACCAGTTTTATAGTCAACTCTTGAAGCTACAGTTCCACCAGTTGATAAAATAGAAATGTTTT
TTAATTTTTTATTTTTTTCCAAATTAAGAGGTGGAAGGCCGTATTTTGGTTTGTCTCCTGATTCC
AAAATTTCAATATTTTTTATTTTTTTCCTATCGATTCCAACGTTGTATCCGCTTTTCATTTTAAT
TACAATCGTGTTTTCGTTTAAAGAAGGCATTACTGTTCCAGAATACGTCGTGTTTTCCAGTTCGA
GTTTTACAAAATCACCGATATCCATTTTTCCACCTTTTTTGATAAAAATAATATGAAGATTAAAT
AAAATACGTCATTATGGTATAAAAAAATTAAACTTGGAATATTTAAGGAATGATACCTAAATTT
CCTCGATGTATTCTTTTACGATTGGAATTAATCTATCTATCGTTTTTGGAGTGTATTTTGGAGAT
ATGCTAAATACAACATAATCAAAAGTTCCTTTTTCAATGTAATCTTTTCCAGATATTAATTTAAA
AGGAATTTCTGGTTCCAAATCTAAAATTTTCACATTTTTAATTTCAGTTTCTATTTTTTCAGTTT
TTTTAAGCTGAATAATTTCACTTTTTAAATCATAATCTTTAAAATGAAAATTCAAAAGATCATTT
CCGCCTTTCGGGTGTGTAACATATGTTAAATCATTAATCCACCGATTTGCAATTGCAAAAGCAAG
ATAGTTTCCTTCATACATCATTTCAGGATCATTTCTTTTTTCAATGGGTTTGTGGAGTAATTCTT
CCATGATATATTTGTATGAAAAGTCCCCAAGCCCGAGATCCGCGTGAGCATCGATGTGAACAACT
TCAAAGGGATTTTCGAGTTCATTTTTTTCGATAAGTTCTCTCCAAAAATAAAAAGCTTCGTGGTG

TTTTTTAACAATTCTTCCCTTTATTTTATTATTTTTCGATAAATTACAGTTATTTTCTAAAAATT
CGATAAATTTATCTTTTTTCCAAACAACATACTCTTTTTCATCGAGTCTTTTATTTCCTTTTTTCC
AAAATGCAATTTTATTTAAGAAAAAGTCCAGATCAATATCTAAAATTCTCATAGTTTCACAAAA
ATCAATTAAAAGCAAGGACACCTATAAAATTCATGATATAAATAAAAAATCTTAAAATATAGAA
GTAAAATAGTATCATCAAATATAATTAACATTTATCAAAAAATGTCATTTAAGGTGATTACTTT
GAATATTTTATGTATCGATATTGGAAAAGGAACTCAGGACATTTTATACTTTGATACTGAAAAA
AACGTTGAAAATGCAATTAAATTAATATTGCCGTCACCCACAACTATTATTTCTCGAAAAATTAT
GAAATTAAAAGAAGATTTAAGAATTGATGGAAAATTAATGGGTGGAGGCCCAGTTTCATTTGCA
ATAATGCAGAAATTAAAAAAAGGATACAAAGTAGAAATTTCAGAACGGTGTGCAAGAACTATTA
GGGACGATCTAGACGAAGTTAGGGAAAAAGGAATTGTTATAAAAGATGAAATAAAAAATCCAAA
TGTAAATTTAGAAGATTTAGATTTTGAAATGTTAAAAACAATTTTTTCGTCGATGGGACAAGAT
TTCTCTCCAGATGTTGTTTGTGTTGCATGTCAAGACCACGGATTTGTAAAAGGCCAGAGTGACCG
GATCACTCGATTTAAATACTTTGAAAAAAAGTTAAATGGGACAAATAACCCGTATGAATTTTATT
TTGATAATAAAAATTATAAAACGGATAATTTTTCACGATTTGAATCCATTTTAAAGACACTTGAT
GAAAATAACTACAAAGGATTTGTAATGGATAGTAAAATGGCATCGGTCTGCGGTATTTTAAATT
ATGCGAATGAAAACAATATTAATGAATTTATCGGACTTGACATTGGAAACGGCCACACTTTAGGA
GTTTCAATGCAGAATCAAAAAATTAACGGCCTTTTTGAACACCATACAAGATTAATCGACGATCA
AAAATTAAAAGACATTGTTGAAAGAACATGCAATGGATCTTTAAAAAATGAGGATATATATAAC
GACAACGGCCACGGTGCGTGTGTAACTTGTAAAACTGAACCTGAATCAGTGCTTATTGCAGGTCC
AAACAGAGAATTATTTAAAAATTACGGTAATTATGCATATCCTGGAGGAGATGTAATGATTACA
GGCTGTATCGGCCTTTTAGACGTTTACCAAAAATTAAGAAAAATTTAAAGTTGGAGGTTAAACCC
ATGTTTATCATTTCTGTAACTTACAAAAAACCAATAGAAGTCGTTGAAAAATATTTAGCTGAACA
TATTGAATTTTTAAAGAAAAACTATGAACTTCAAAAATTATTGGCATCCGGTAGAAAAATTCCA
AGAACTGGCGGAGTAATCATCTCAAATGTTAAAACCAAGGCAGAAGTACTCGATATGTTAAAAG
ATGACCCATTCTACATCAATGAAATTTATGACTATGAAATAATCGAATTTACTCCTTCGTTAACT
TCAAAAGAATTAGAATTTTTAAAAGAAATCTAAATATAAATTAATTTTAAAATAAAATATTAAA
TGTCCATTTTATTTTTTCAATTCCCAATCTTTTAATCTTATTGTTTTTGCAAGCGTTGTTCCACGG
GTTATTTCTTCTTTAACTCGATCTTCTCTTTCTTTTACATCGATTGCCCTGTTTGCAATTTCCATT
GCCTCATTTTTTGGAACTACAATAATTCCGTTTTCATCCCCGACAATCCAGTCTCCAGGTTCAACA
GTTCTTCCAGCACATTCAATTACGGCATTTATTTCTCCAAATCCTTTTGGTTCCCCTGCAGTTGGA
GTTATACTTCTTGCATATACTGGAATTCCAATTTTTAAAATTTCTTCAACGTCCCTAACAGCACC
ATCTATTACAATTGCAACTACGCCTTTTGTTTTGCATGAAAGCGTTGCAAGTCCGCCCCAGCAAG
CAATTTCAGAACTGCAGTTGTCAATTACTAAAACCTTCCCTTTTTCACAGTTATCTATCGCACTT
ACAGGCTTACTCCAGTCTCCAGAGTAAGTTCTAACAGTAACTGCAGGTCCTGCAAATTTTAAAGG
TTTTTCAGAGTTCCAAACGGGTTTTAAACCTTTCATTTCTCCAGTTCTATGCATTGCGTCACTTAT
ATTTGAAGTACTTACAACATCAAATGCATTTGCAAGTTCACTTTCATTAAATTTCTTAAATTTAT
CCGTTTTTATAGGTTTTCCAGTTTCAAGACTTTTTACAATATCTCTTGCAGTTTGTTCAGGGTCT
GCGGACTTATATAATGTTCCTCCCGCAATAATTATATCTGCACCTGCTTCGTATGCTTTTACTGCG
GTTTCGCTGTTTAAACCGCCTGCTGCTGCAATTTTTGCTTTTTTACAGTTTTCTGAAACTCGTTTT
ACAAGTTCAACAGGATCAACATTTAAAACTTGCTGGTCAAGCCCTACGTGGATATTTATAATATC
CACACCCATTTCTTCGAGTTCAACTGCTCTTTTTACAGGATCTTCAGTTGCAATTAAATCAGTTCC
AACTAACACGCCGTATTTTTTTCCTGCTTTTACAGCATCTATTATCATTGAATCCGGACCGACCCC
CAATATCAATATAACATTCGCACCTGCTTTTGCCGCCATTTCAACTTCAGTGCTTCCTGCATCCAT

TGTTTTCATGTCTGCAACGATTGTAAGAGTTGGGAAGTTTTTCTAAGTTCTCGAATTGCATTCA
TTCCTTCAGATTTTATTAATGGAGTTCCTGCCTCAACCCATGTAGCACCACCTAAAACTGCTTCTT
TTGCAATTTCTATTGCCCTTGGAAGATCAACAAGATCAAGAGCTACCTGTAAGGGAGGTAATTCT
GATTTAAACTGCATGATATCGCCCCTTTCGATTAAAAAATAAATAAATCCTGGTAAATTAAATAT
TTAAAAAATATTTGTTAATTTAAAAAAATTAATTATAGAACTAAATTTCTTTAGCCCATGAAA
TTCCTGCGTTTAATATTTCAACGTTTATAGGAATTAATTTAGGTTTTTTGAGAAAGTTTGTTCT
ATTGCGCCAACAAATACGGATTCAGAAAGTCCCGTGTATCCAATTTCCGCTAAAACTCCAAGCAT
TACTGTATTTGCAGCTCTTGAAACGCCCATTTCAGTTGCGATTTTTGAAGCTGGAACTTTTATTG
CAGTAACTCCGTTTGGAATTTCAAATTCACCAATATTTTCATCGTAAATTATTGTTCCGCCAACT
TTCACGTCTTTTGCAAAGTTTTCAAGCGAAGGTTTATTGAGAGCAATTAAAACTTCCGGATTGTA
CACAACAGGGGATCCAATTTCCTGTCCTGAAATGATAACAGAACAATTTGAAGTTCCACCCCTTT
GTTCTGGACCGTATGAAGGGTACCATGAAACGTATTTTTGGGCCCTACATGCAGCTTCTGCTAAC
GTTAAACCCATGCTTAAAACTCCCTGTCCACCAAATCCTGCAATTTTTACGAGTTTTTCTTCGAAT
GAATCATCCCTAGCTGGATCTTTTGTAGCTTTTGAAGTAACTTCAAATATTTATCAAGACATTC
TTTTGAAAAGTCGCTTTCTCCCCTATTTAATGTTTCAACTTCAGCACTTCTATCCCTGAAGTTTTG
AACTGGAAATTCTTTTTCCATTTCTTCGTTTATGAACTTTTCAGCAGCTTGTGCATTTTGTTTTA
AGTTTGTTGGGCAAGGAGATAATATTTCAACAAATGCATATCCTTTTCCACCTCTTTGAATTTCA
AGCGCTTTTTTGATAGCACGTTTTGCCTTTCTTATGTGCGCAATATCTGAAACAGAAACTCTTTC
AATGAATACCGGAGCTTTCAAACTGCTTAAAAGTTCACACATATGAATTGGGTATCCTGCAAATC
TTGGATCTCTTCCAGTCTGGCAGGTTACGGTTTTTTCACCGATAAGCGTTGTTGGAGCCATCTGT
CCACCAGTCATTCCATAAACGGTGTTGTTTACGAAAAATACTGCCATTTTTTCTCCACGGTTTGC
TGCCTGAAGTGTTTCATTTAACCCAATTGATGCTAAATCCCCATCTCCCTGGTATGACATTACAA
TTGCATTGTCTTCTGCTCTTGAAACACCTGTTCCAACTGCAGGAGCTCTTCCGTGAGCAACTTGAA
TATTTCCGCAGTCAAAATAATAGTATGCAAATACCGCACAGCCAACAGGACTGATTAAAACGCAT
CTATCTTGTATTTCAAGTTCATCCATTGATTCTGCCATTAATTTATGGATTATTCCGTGTCCACA
GCCTGCACAGTAGTGCGTTGCAGTTGGTGCAGAACCCCCCTTTCTTTCAAAGATTTCAGGCATTGC
ATTTGGCTTTTTCATAACTTTTTCGGTAATTTCTGTCATTTTAGTTGCCTCCTGCAATTTCTTTAA
TTTTAGCTAAAATCTGATCAACTTCAATTAAATTTCCACCAAGCCTATTTACAAGTTCTATTGGT
CTTTTACAGCATGTTGCAAGTTTAATATCTTCTGCCATTTGACCGTTGCTCATTTCAACTGAAAT
AAAAGTGCATTTTTTCTCTGCAAGTTTATTCAGTTCTTTTTCAGGGAACGGAAATAGGGTTTTCG
GCCTGAATAATCCTACTTTTAATCCTTCTTTTCTTGCAATGTCTACAGCAGTTTTTGAAATTCTGC
TGCTTATTCCGTATGAAACAAGGATAATTTCAGCATCATCTAACATATAACCTTCAAAATCCTGC
TCATTTTCTTTAATTATTTCGTATTTATCCTGTAATTTGTAGTTAAATTCTTCAAGCTGTTTAAA
ATCTAAAAAGATTGAAGTAACCAAATTTTTCCTTGTTTCTTTTGTTCCGCAAACTGCCCATGATT
TATCGACTTCAGGTTTTACTGCCTGTTCAGGGAATTTTAGTGGTTCAACCATTTGACCTAAAACA
CCATCTGAAAGAACAATTACTGGGTTTTTGTACTTTGTTGAAAGTTCAAATGCTTTTGCAGTAAA
ATCACACATTTCCTGAACTGAATTTGGAGCTAAAACTATATTTTTATAGTTTCCGTGTCCCCCAC
CTTTTACAGCCTGATTGTAATCAGCTTGTTCTGGTCCGATGTTTCCAAGACCTGGGCCTGCTCTCT
GAACGTTTACAAGAACGCATGGAAGTTCAGAACCTGCTAAAAATGAGATTCCTTCTTGTTTTAAA
CTCATTCCTGGACCTGATGTAGCACATAACACTCTTTTTCCAGTTGATGCCGCACCGTAAACCATA
TTTATTGCAGCTTCTTCTGATTCTGCCTGAACAAACTTCCTTCCAACCATTGGGAAGTATTTTGA
AGCTTCGTGAAGTACTTCACTTGCAGGTGTTATCGGGTATCCAAAGTAACAGTCACATCCTGCAT
ACATGGCTCCGATAATAACAGCAGTGTTTCCTTTTACAAGTTGTGTTGCCATATTATCTCCTCTA

TTCGCACTTTTTAAGCGGGATGTGTACTTCTATTGCTAAAGGTTCTGGGCAGGTGTAGTAGCAGT
TTCCACAGCCAGTACATCCTTCGCCAGTGTAAATTACGTAATTATATCCTCTTTCATTGCAGTCTT
TACTCATTTTTAAGACGTCTTTTGGACATGCAATTATACATCTTTCACATGCTTTGCATTCAAGA
GCATTAATTACAGGATAGGGTTTTTTTGTATAACATTCCATAATTTCATCTTTTTTTAGTTAAAT
TTTAAAAATAATTTTTCGATCAATATATCTTAAAAAAAATAATTTTTATGATATTTAATTATTA
CTTTTTTCATCAGTTTCCCGGATTTCTACACGCCTGATTTTCCCGCTGATTGTTTTTGGGAGTTCT
TTTACAAATTCAATAGCTCGAGGGTATTTGTATGGAGCAGTGTTGTGTTTAACGTGGTCTTGAAG
TTCTTTTTTGAGTTCTTCACTTGCTTGATATTCAGAAGTTAAAACAATTGTAGCTTTAACAATCT
GTCCCCGTACTGGGTCAGGTACTCCAGTTATTGCACATTCAAGTACCGCAGGGTGTGAAATAAGT
GCACTTTCTACTTCGAAAGGACCGATTCTGTAACCGGAACTTTTGATTAAATCGTCGTTTCTTCC
AACAAACCAGAGGTATCCATCCTCATCTTTCCATGCAGTGTCCCCAGTATGGTAATATCCATCGT
ACCAGGCTTTTTTAGTTCTTTCAGGGTCTTTAAAGTATCCTGAGAAAAGACCAAGAGGTTTTCCA
TCTTCTGTTTTGAATGCAAGTTCTCCTTCTTCACCTACATCGCATTCTTTTCCGTCTGGCCCTTTT
ATCATTAGATCAAAAATTGGCACTGGTTTTCCCATTGATCCTGGTTTTGGATCCATCCATGTAAA
GTTTGCAACAGATACAACAGTTTCAGTCTGCCCAAATCCTTCCATTAGCCTAAGCCCAGTAAACT
CCAAAAATTTGTTGTAAACTTCAGGGTTTAGTGGTTCTCCAGCAGTTACTGCGTAGTGTAAACTT
GAAAAGTCTGCTTTTGAAATATCCTGTTTAATCATGAACCGGTAAATTGTAGGTGGTGCGCAGAA
CGTTGTAATTTTGTATTTTGCAATTTTTTCAAGCATATTTCCTGCATCAAATCTGTCGTAGTCGT
AGACAAATACTGCAGTTCCTGCAATCCACTGTCCGTAGAGTTTTCCCCAGATACACTTTGCCCATC
CACTGTCTGCAACAGTGTAGTGAAGTCCATCATCTTCAACATTTTGCCAGTATTTTGCAGTCGTA
ATATGTCCTAAAGGATATTCGTGGTCGTGCTGAATTAATTTTGGATATCCTGTTGTTCCTGATGA
AAAATATGCAACTAATACATCGTCATTTTTAGTATCCATTTCTCCAACAGGCCTTGTAAAGTCTT
CTGATGATTCTTCCAATTCTTTTGAAAAATCAATCCAACTATTATCGTTTAAATTTGAAACACAT
GCTTTTAAAACATCTGAATTAATTTCAGATACTGCTTCATCAACGTAGTCGGGAACCCCGTTTTC
TCCGATACAAACTATCATTTTTAAGCCTGCTTTTTCAATCCTGTACACGATGTCTTTTGTTGTAA
GCATGTGTGTTGCAGGAATTGCGACAGCCCCAATTTTATGAAGTCCAAGCATGCAGAACCAGAAT
TCGTACCTGCTCTTTAATGTGAGCATTACAGTGTCTCCCTTTTTAATTCCGTGTTTTAAAAAGAA
ATTAGCTGCTTTATCACTGTATTTTTTCATATCTTTGAATGTAAAGATTTTTTCGTCGTTGTTAT
CGTCACACCATATGATTGCAATCTTTTCCGGTGCAATTTTTGCATATTCGTCAACGATATCGTAA
GCAAAATTGAAATTTTCTGGAACATCTATCTTAAAATTTTCCACAAAATCTTCGTATGACTCAAA
TTCCGTCTTTTTAACAAATTTATTAAGTAATGATACCATAATCATGCTCTTTTTCGTTTTTGAGA
ATAATAAAGTTTACAGCATATATATTTACATTATAATTGCAAGGAATTTAGCAGTTTTTCCATTT
AATGCTTCCATTGCATGTTCATATCCTGCATCAAAGAATATAGAATCTCCTTCTTCTAAAATAAC
TTCATTTTTGTGGATGTATAATTTTATAGATCCTTCCAAAATATAGTTGAACTCCTGACCAGGGT
GTGAATTTTTGGATGGTTTTTTTCCGTCAGTTCTTGGCTCTACGGTTACAATGAATGGTTCTGCT
TTTTTGTGAACAAATTTTTCAGCCAAATTTTCGTATTTGTACTGTTTTCTTCTTTCTACAGAAAC
TCCTTTTCCTTTTCTTGTAACTGTAAAAATATGCATTCTGGTTTCTTCGCCAGTCAAAAGTAGAC
CCATATCAACTTCAAATTTGTGTGCAATTTCATACAAAACACTTGCTGGAATATCGCAGGTTCCT
TCTTCGTAATGTTGATAAATTTCTGGTGAAATTTGTAAGTGATTTGCCATCTCTTCAATTGAAAT
TTCCGAGAGTTCCCTTAATTCGCGAACTCTTGATGCAATTTCTTTCATTTTTTTCAGTCATATTCTG
CACCGTTAGAATTAATCATTATTAAGTTAGAATATATCTCAAACCGCATAAATTTGCGTGTTTTG
TTCGATATTATATGAAAAAGTATATTGGTATTTAAAATAACTATTTAATTCCGCTATATATAAT
GATCGATAATTATAATTTCAAAATATGCTGTTTAAAAAGCGAAAAATGAGATACTAAGATCTGC

GAAACTAGTATAATGTTGAAGAACTATAGTCATAATTAATTTAATTTTAGGGGGTTTGGGAATT
TGTATAAAATAAGTTTTAAAAATTTAATTTTTGCAGCAATTCTCGCAATAATTGTAATGATTTTG
TTTGCACTGCCTGAAACTTATATAAACATTTCTGCAGACAATTATTCTCCAAAAACGGGCGATAT
TATTAATATTGATGTAGATGTCATTAAAAAGAGTAATTTTGGAACAACAACTGTAGAAATATTA
CTTCCAAACGGTACAACATATTTAAATACGGTTTATGGGAAAGAACCAAAAAATAATACAGTATT
TAACGAAAAATACAATATATCGGAAACTTATCTTAAATGGACGTTTAATAATGAAGATAAATTT
GGTTCATCGATAAATATTCTCATTTTAAATTCTGAAAATAAAACAATAAAAGCAACATCAAAAA
CAGAATTTTTAATTTATGATTCATTAAATTCTGATAAATTAATCATTTCAGCAGACTAAAAACAA
AAAAGTAATTTATTCAATTTTTACAAAGTTAATTTTTCCGTCAACTGCTACAAAGTTTCCGGTGC
ATTCTTCATCTGTTTTTGGATAATCGTTTCTAAAATGTGCCCCACGGGACTCTTTCCTAAGGAGA
GCTGAATTTATAACAATTTTTGAAACTAAAACCATGTTTTCTAGTTCCAATTTTTTTGAAAAGTC
AACAACTCCATTTATCAAAACTTTTTTTCCTGCAATTTCATTTATTTTTTCAAGGGCAGTTTTTA
ACCCGTTTTCATCTCTTGAAATAGAAACAAAATCCCACATGGTATTTTTTAATTCATCGATTAAT
AAATATACATTATTCCCAGAATTTGAACCTTTAAATGTATTTTTTAATTTTTCAATGTATTTTTC
AACTGAATCATATACTTTTGAAATATCAATTTTCTCTTCAGCATAATTTAAAGCATTTCTTCCAG
CAATTGATCCAAAAACTTGTGTTTCAGCAAGTGCATTTCCGCCAAGCCGGTTTGCGCCGTGAAGT
CCACCTGCAACTTCCCCACATGCAAATAATCCTGAAATATTTGTCTCACAATCTTCATTTATCTTA
ATTCCTCCCATAAAGTGGTGTGCTGTTGGAGCTACTTTCATTGGCTCTTTTCTAATATCTAAGCC
AACAAGCTTAAACTGTGAAAACATTGTCTCAAGTTTTTCTTTGATAACTTCCGGGTCTAAATGAG
TTACATCAAGATAAACTCCGCCATCGATTCCTCTTCCTTCCTGAATCTCATTATATATGGCTTTAG
CAACCACGTCTCTTGTTGATAATTCCATTCTTTTTGAATCGTAGTTTTTCATGAATCTTTCTTTA
TTTTTATTGTATAAAATTCCGCCTTCGCCCCTTACAGCTTCGGTTACTAAAATTCCAGTTCCAAGC
ATCCCTGTTGGGTGAAACTGAACCATTTCCATGTCGACTAGTTCGACCCCTTCACGATAAGCAAT
TGCAAATCCATCCCCGACTTTTTGTTTAGGATTTGAAGTTATAGGATAAATTTGGCCTGCCCCGC
CAGTTGCAAGAATTGTAGATTTTGCATAAACTGGATAAATATCACCATTTAATAAATCTAAAAA
TACTGCCCCAAAACATTTTTTTGAGTTTTTTTCCGTTTTCTTCGACTTCATCGACAATTAATTTTAT
TGCCATTGTGTCTTCTAAAATTTTTATGCGCTCAAATTTGTTTAAATACTCCATCAATCCTGCAA
TCATTTCGTGGCCGGTCCTGTCCCCACTGTAGCAAGTTCTATTAAAGCTCTGTCCACCAAATGGCC
TTTGTGCAAATTCTGCACTTTCAGACCTATCAAAAATACATCCAAATTTTTCCAAGTTCTTAAAT
TCATTTGGTGCATTTTTTACCAAAACATCTACGAGTTTTTTATTGTTTATGTATCCTCCACCCTTC
ATCGTGTCCGAATAATGTTTTTCAAACGAATCTTTTTCGTTTAACACTGCATTGTACCCACCTTC
AGCCATTACCGTGCATCCGCTTTTTCCAAAAAGGCCTTTTGAAGCAATTACAACATTTTTCTGTCC
GCATTCTATTGCGGCCCTTGCTGCAGCACCCCCGCCACCAATTATGAGCACATCTGTTATCATAAA
ATCACCAGATACCTAAATATTATTTTTTTAATTCCTTGAATACATCAATGTCTTGTATTGTTATA
AGGCCGTTTTCTGAAACCATTTCAACAAGTTTTGGCATTATATCGTTTAATTTTTCCTCGTAATC
GATACATTCAATTACTGCTGGAAGGTTCATTGAGAGTCTCAGTATATCAATTCTAGAAATGCCCC
TTGTTCCGTAGCCACAAAATCCTTTAAAAACCGTTGCACCCGCAATTCCGTTATTTTTTAGGGTT
TTTATTATTGAATTGATAAGGAGTTCTTTTCCATACTTATCTTCTTCCTTTAAATAAATCCTGAG
AAGTTTTGCAGATATTTTTTTCATTAAATCACCAGAAAAACATTGATGCGAGTCTTCCAAGATAT
ACCATTCCCAAACATCCAAAAACATTTAATAAAATGTTTAATCCTGTTTTTAAATAATAATTATG
TTCTAACATGGTAAATGACTCGTAACTAAATGTAGAAAACGTAGTAAGGGCCCCGCAAAACCCAG
TTATTATAAATAACCTGTATTCAGTAGATAATGACCCAAATAATGAAGAGTATATCAAAAAGCC
AATAATAAAACTTCCCAAAAGATTTACTATAAGAGTACCTGTTGGAATCCCAAATTTTACGGGAA

TTATTCCGCTAACGAGATACCTTAATATCGCCCCGAAAAATCCGCCAAGGCCGATAAGTAGAATT
TCTCTCAAAAAATCACCAAATAGTCATATTAGTCTGCCTAACTAAAAGATTTATATACTATATAA
TAAATTATATTGAAAAGTACTTCTTAAAATGATATATAATTTTGGGCCCGTGGTTTAGCTTGGAT
AGAATACAGGGCTTCGGACCCTGTGGTCGGGGGTTCAAATCCCTCCGGGCTCGTCAACATTTCAA
AAATAAAATTTAGGAAGTATTTACACTTTTTTAAAATTATTATTAAAAAATTTAAAAGATTACC
ATTTTTTAAAGATAAAAACTGCTGCAAGGATTATAAAAAAGAATCCTACTGCGTAATTCCACCGA
AATTCTTCTTTTAGATACAGTACTGAAAAAATACTGAATATTACAAGAGTTATTATTTCTTGAAT
TACTTTTAGCTGTGCAGTTGTAAAGTAGTTTGAGCCGATCCTGTTTGCTGGAACCTGCAAGCAGT
ACTCAAAAAATGCAATCCCCCAACTTACCAGTATAACGATCCAAATTGGAGACATTTTATACTTT
AAATGCCCATACCACGCAAAAGTCATGAAAATATTTGATAAAACGAGCAGTAAAACTGGCAAAT
ACTGTTTCATACCCTACCTTTTTTATATTTTTTAAGTATATAGAATTAAACTGTTTATTAATATC
TTTCGATGGTGTTAGCGTGAATACCAATTTAAAAATTAATATCAGACCAACAACAGTTTTAGATG
TTCCTTTAATCTTTAAATTTATCAAAGAACTTGCAAAATACGAAAACTTAGAAGACCAAGTTACT
GCTACAGAAGAAATAATAAAAGAATCACTATTTGGAAAGAAACAGTACGCTGAAGCATTGATTG
TTGAAGCAGATTCAAAACCTGTTGGACTTGTTTTATTCCTCCACAACTTTTCAACATTCATTGGA
AAGCCAGGAATATATATTGAAGACCTCTACATCAAAGAAGAATTTAGAGGAATGGGCATTGGAA
GAAACATATTTGAATATTTATCAAATCTTGCAAAAGAAAGAAATTGCGGAAGAATTGACTGGGT
AGTTTTAGATTGGAACCCTGCAAGAAAATTCTACGAAAAAATGGGCGCAGTTCCCGTTGATAACT
GGTTAATTTATAGATTAACGGGAGATAAATTAGATGAACTCTCAGAAAACCATCAAAAAAGTAA
GAATTAAAGATATAACTTAAAAAAATGGGAGTAAATTGCACATTTAAAAGTTAATTCACGTTAA
TTATTTTTTTAAACGTGTTTTCAAGGTCGTGCATAAAATCCATGTAATTTCCTTCGTTTACAACC
ATGTAATCTGCAAGTGATATTACATTACCAATTGAAAAATTCAATTCTCGTTCATCGCGTTCAAC
GAAAGTATTCCAGTCATTTGAATCATCTTCCCGGTTTCTACCAGACAATCGTTCAAATCTGGTTT
TTGGTGACGAATGAATTGCAATAATATCGAGTTTGGCATTTTTTCTAAGGTAATTGACTTCATAA
ATACTTCTTATTCCCTCAATTACAACAAAATCTTCATCTTCGTATTTTTCATGGATATATTCAAG
ACAAGGAACTGCAATTGCTTCTTTTCCATGAATTTCTCGAAGTTTTACTGCCGTATTTCCAACAT
TTTCAGGATTTAATGGCATTCCTTGTTTTAATGTTTCGTAGCGTACAACATCGCCCATTGAAACA
ACAACTATTTTATATTTTTCCGCTAATTTCGTAATTGCACTTTTTCCAGATCCAGGCATTCCAGT
AATTCCGATTAACTTCATATTATCCCGATTATTAACGTTTTTTAACTCTAATTATTTAACTAACT
TCACTTAAAGCATCTATTGCTGTATTGTGAGTATTATTGATCGTGTCAATATTTGATTCCTGAAC
TGCTTTTGAACTTACAATTCAAGTGATATCCTAAAAGAATTGCCGCTGTAATGACCGCAAGAACCA
GCACTGAAAATTCAAGACTCAATTGGCCCCGTTTCGAAAAAAACTTCTTTAAAGCAACAGACATT
TTACCACCATTTTATTATATACCTTTAAATTATATAGCTTTGTACCATTTTCATAGATTTATAAT
ATTATCTGTAATTTATGGTTTTTATTAGTGATTATTTGGTGAATCCATGATTATCGTGAGAAAAT
TAAAGAAAAAGAAAGAAGAGATCCAATTTATTGATTTAACCGATGAAAAGACTTATTATGGGAT
AATAAGACCTGCAAACAATAGAATTACACTTTACAAATGTAGGGAAGAAAAAGCAGGAAATGTA
AACCCAATACAACCTTCAAAATTGATAAATTTCGTTAGATCAAATAAAGTTTTATTAAGTGGGGA
CGAAGAATCGTTAAAATTAGAAGAATTCCTCAAACAAAATAATATCAAACACGGATATATTGAT
TTATGCCCTTTTTGCATCATTAAAGGAAAATTTAGCGAGATCACGGATAAATATAAAATTTACAA
CAGCGAGATCTGCCTTGAATGTGCAATCGACGAAGTAAAACATGAAATAAATATCAATGAAGAG
TTTATTGAAAAATTACTTAGAAGGTTCAAGGATGCAAATAAAGTAATTGAACTATTCAAATCAA
AAAACCCGCTTAAAAATCCGGAACTTACAAAATACGACGTTTTAACAGGAAACGTTGACGACAAT
ATAAAAAATTATGAAATAAATGAACTGGATATTCCTGATTTTTTAAAAGAAATTATTAAAAATA

GGGGGATAGATGAATTACTCCCAGTTCAGACTCTATCTGTAAAAGCTGGGCTTTTAAAAAATGAT
AATTTATTGATTACGTCTGCAACGTCTTCTGGAAAAACTTTGATTGGAGAACTTGCAGGAATTAA
AAATATTTCTGAAAAAAAAGGTAAATTTTTATTTTTAGTTCCATTAGTTGCCCTTGCAAATCAAA
AATATGTTGAATTTAAAGATAGATACGAAAAAGAAGGTTATTCAGTATCCTTAAGAGTTAGAAC
CGGAAGACTTTCTGAAAATAAAGGAGGCGGTATCAATTCAAGCCTTGATTCTGACATAATAATTG
GAACATATGAAGGAATAGATTATTTGATAAGATCGGGTAAATTAAAAGATGTTGGAACCGTTGT
AATTGATGAAGTTCACTCTATAAATATGGATGAAAGAGGTTCGCGTCTTGACGGCCTTATTGGAA
GATTAAGGTATTTATTCACCTGTCAGATGATATATCTTTCTGCAACTGTTGGAAATCCAGAAGAA
TTGGCTTCAAAATTAGGATCAAAACTTGTTTTGTACAATGGAAGGCCAGTTCCTCTTGAAAGGCA
CTTAATTTTTACCAAAAACGATTACGGGAAATTAAACCTTGTAAAAGATATTGTTAAACAGGAA
TTTAGCTCAAAATCAAAGTACGGGTTTAGGGGGCAAAGTTTAATATTTACATTTTCAAGAAAAA
GAGCAGAATATATCTCAAATTATCTAAATACCAAAGGAATAAAGTCAGATTACTACCACGGTGGA
ATGGAGTATTCTAAAAGGCGAACTGTTGAAGATAATTTTTTAAAACAAAAAACAATGTGTGTTG
TAACTACCGCGGCACTTGCGGCAGGAGTAGACTTTGCAGCATCCACGGTTGTTTTAGAAAGTCTT
GCAATGGGTGGAGACTGGTTAAACCCTTCAGAATTCCAGCAGATGTGTGGAAGAGCAGGAAGAA
AAGGAATGCATGATAAAGGAAAAGTTTACAGCCTTGTTGAAATTGGTAAAAAATATCACGCAAA
AATGGAAAATTCTGAAGATGAGGTTTCGTTTAAACTGTTAAATTCAGAACCTGAAGAAGTTTCA
TTAGAATACAGTGAAGATGAAGAATTTGAACAGGTTTTAGCATACGTATGTTCAATTAAAAATT
ACAAAAATAAAGTTACAATTCCTGATGTTTCAAAAGTTCCGGTTCTTGGAAAAAAATTTAGGTTTA
AATTATGTTCTAGAATCACTTTCGGGATATCAAATGCTTGATTTGCAGAACAAAAACATAAATAC
AACTCGATACGGTTATGCAACGTCAATTTCATTTTTGTATCCTAAAGATGCTGAAAAAAATAAGGA
AAAATCTGCATAAAAACCCCCTTGACATTGTTGTATCAATTTCGCCATTTGAAAATGTTTATATT
CCTGCAAACCTGAAAAATAAAATCTCAAAAACCGCGAATGTAAACATTCCAACCAAGATTTACCGA
TGCTTTCGAATTAATAAAAGAAAATTTTGAAAAAATAAAAGATAAAACCTTAAGGGATGAAATT
CTGCCTTGGTTAATCGAATTTGATGGATTGATTGAAGAGGATATTATAAACTACAATTCCAAAAA
AGTTTTGAATTTAAGAATAAACAAAAAAACACCACTCCAAATATCAAAAATAATTCATGATACCC
TAAAACTTCAAACATACAGTGGAGACATCTATTCATACCTTGAAACTTCAATAAATACTCTTGAT
GCAGTTGAAAGAATTGGAAATATTTATAATAAAAAAATAGCGAAAGAATCTGCTAAATTAAAGA
AAAAAATGGAAAATCCATATAAAAATTAACTAAAATCTATTTTTTTAATACCAAAATACGAATA
AATGTTTTCATTTGACTCGACATTAATAAACCTGTTTTCTTCTTTTGTGAAATCCAAAAATTCAT
CCAAAAATTCAATACCGCCACCATAATTTACTGCCTTTGGATGTATTGAAAGGAAATATTTGTAT
TCCGACCCCAAATATTCTTCTTTAGCGATATTTACTTCATTTAAGGTATTGTTTTCTTCAAGATA
CCAAGTATATTCCTTATTTGTAATATTATATGTATAAACTGTACCATTATCCATTTCAAAAGCAT
ATTGCCCAACAATTATAGAAAAACCCTTTTTAAGCATTGCTTTTTTGAATCTTCAGAAATTCCG
TATCTTGGCGGAAGAATGCATGAAATACACTCAATTTTTAAACCATTTAATATTTCAAGCGATTT
TTCTATTTTTTCCATTGCAAGGGTACAATTACAATTAAATTCGCATTCAATATGGTCATACCCGT
GTAATTCAACGTTATATCCTTTTGTTTGAAGATATCTAATGTATTTAACGAAATCTTGATTTTCT
TCTAAAGGATACTCATTTGCATGATTAACTATCAAAAAAAGATACGTTCTTTTGGAATACCCGTA
TTTACTAATTACTTGATCAATTTCCTTTAAATCTTCTAAATAAACAGGACTAACATCGTGAATCA
AAATTACTGGTTCAAAAATGTTTTTTTCGTCTTTTGAATCGTCTTCAAATGAGAACCTTGAAAAA
TTTGCTGAAATTAGTAAAATTAAAATAAAAAAAATTAGGGTTTCCATTTTCAAAAATATCCCCGT
AAAAATGGTTGATAATTATTTGAAAATAGTGTGTTTTAGAAATATATAAGACCATTTCATTTAT
TAATCTAAATTAACAAATTAATATGCCCAAAATAAAAAGGTGCGCCAGTCGGGATTTGAACCCGA

GTTACTGGCTTGGAAGGCCAAAGTGATACCAGGCTACACCACTGGCGCACGTTCTAACTGTAGAG
ATGCGGCCTCCGGGATTTGAACCCGGGTGTCGGCCGTGGCAGGGCCGTATGATACCAGTCTACAT
CAAAGCCGCTATTTCATTCCAATTCTCACTCATCTCAGAGCATACTACACATTGTAGTATCTCATA
TATAAACTTTTTGGTTGAGTTGAGTGCTAAAAGATTGTTCTTCAAAATAAAAGAAAATTTTCAT
ATTAATTATATATACAAAAAATATTATAAGTAGATAGATGAAATTAGAATAATGATTTCGAGAA
AATTTGGTTTTGAGACTGGTCGGTTAAACTTCATAAGGAGGATCATTTATGGATTTAGGAACTAC
AAAATATATCATTTATACCGAGCTCATTGCGGATGGTTACGTTGAAAAGCACGACGTTATCGGTG
CAATTTTCGGACAGACTGAAGGACTGTTGAGTAACGAACTTGACTTGAGAGATTTGCAAAAAAGC
GGGAGAATTGGAAGAATTGATGTTGAACTTGAAAATATCAATGGAAAATCTTTTGCTAAAATAA
CACTGCCTTCGAGCCTCGACAAGGTTGAAACATCGATACTTGCAGCAACTCTTGAAACGATTGAC
AGAGTTGGTCCCTGTTTTGCCACGGTTAAGATAACCGAAGTTGAAGACATCAGGGTATCAAAAAG
ACAGTACATCACAAACCGTGCAAGATCTATTTTAAGAAAATTAATGGACGAAATGATTGACACCT
ATGAAATAACGGAAGAAATCAAAGAATCTTTAAGAACCGAAGAAATTATGGAATATGGGCCAGA
AAACCTTCCTTGTGGTCCTAATATAATACATTCCGATTCTATTATTGTTGTTGAAGGAAGAGCCG
ACGTTTTAAACCTTTTAAGATGTGGAATTAAAAATACAGTAGCTGTTGAAGGAACGTCTGTTCCA
AAATCAATCATGGAACTAACAAAGAAAAAGACCACAACCGCTTTTACCGATGGAGATAGAGGAG
GAGAGTTAATTTTAAAAGAACTTCTTCAAACTTGCGATATCGACTACGTTGCAAGAGCACCGTAC
GGAAAAGAAGTTGAAGGAACGTCTAAAAAAGAACTGATGAAATGTTTGAGGGCAAAAGTTCCTG
TTGAACAGATCGTTGGAAACAACTGCAATAATTCTGGAGTTATTGAATCAAATACTCCAAAAGAA
ATTGTTGAACCAATAACTCCCAAACACTTTGAAAAAGTAGAAACACCGGCAGTTATTGAACCGGT
TTTTAAAGAAGACGCAATCGAAGAAGAAACAATTATAGTAGAGCCCGTAAAAAAAGCCGAAACT
GAAATTATAGATGTTGATGCTACAAACGAAACTCAGTCTGAAAAAAAGTTCAGCGGAGTTAAAG
AAATAGTTGATAGCATTAAAAATACAGGCAACGTCAAGTTTGTTGTTGATGGAACCGAAAAAAC
GAATACATTCAAAGAATTTCTAACAAATATTCATGAAATCAAAAAAATGGACTTCTTTGCAGCAG
ATATGCCGATAAGTCAAAAAATTGTGGATTTACTTTATGATAAAACCCCGATAATTGTTGGAAA
AGAAATAAATGTTACTAAAAAACCCGTAAACCTTCGATTATTTTCATTTGACGAAATTGTAGCTT
AAATAACAATTTCTTTTAAATTTAAAGGTGATTAAATGCATAAGGATGAACTCATCCAATTACAC
CAGCTTTTGGTTTATATGAGAAAAGATTTGGCAAGAAAATTTGGAATCGAACTTGAAGAATCGT
TTAAAGAATATGATGATTTAAATATAAGGCCGCACCACATTCACAGAACCAAATCAGAACATACC
TTTGCTATTTTTTTATTATCTGGAACCATTGGAAAAATACTGTCTGAAAAAGGCAACGTTCCAAG
AAGTGTTGCAAGCAGGTTGAATGATACTGGCGAAAAATTAAGTAAAGAAATTGCTAGAAAAAAA
CGATAATATTTTTATTTTTGTTTAATTTACTTTTCAGGTGGTCATATGGTAAAAAACCTCGTAAA
TTCTATTGAAAAACTTGTTTTTGAAAAATCTACTAAAAAATTTGGAGTTTCAAGAATTTTAAAA
GAAAATGACGATAAACCAGTTTTTATTAAAGATGTAAATGGATACCCAGTTATTGGAAACCTGT
GTACCAGAGACGTCATTGCAGAGAGTCTCGGCATTGAAAAAGAAAATTTGATGAAACACATGGTT
GAATCAATGAGCAATGAAAAAAATGGAGAATTAATCGTTGATGAAGGTTTAGAAAAACTCTATA
TTGATGATGATTTGAAAAAAATTCACGATTACCCAATTCCAACATACTACGGAAAAGATGCAGGA
CCCTACCTTACATCAGGGGTAGTTATTGTAAAAGATAAAGACGAAGGGGTAAATGCATCAATTCA
CAGGATTTTAGTCAGGCCGGATGGTAATTTAGCAATTCGAATGGTTGAACAAAGACACCTCCACT
ACATCTACAACAAAAATATCGGTAATGGCGAAGTAGATTGTGCAATTGTTATTGGAGTTCACCCT
GCAGTTTTACTTGCAGCTTCAACCTCAGGAGATGTTTCATTTAATGAATTAAAATACGCTTCTGC
ATTAATGAATAAACCATTAAATTTAGTAAAATGTAAAACCGTTGATTTGGAAGTTCCTGAAGCA
GAATTTATAATTGAAGCAAAAATAACAGCAGAAATGGTTGATGAAGGGCCATTTGTTGATATTA

CTGGAACATACGACGTTATAAGAAAACAGCCTTTGATTAAAGTAACAGCACTTAGAAGAAAAGA
AGATGCAATATTCCATGCACTACTTCCTGGAGGAAGTGAACACAAACTCTTGATGGGCATACCTC
AAGAACCAAGAATGTACAAAGGAATCAGAAACACAGTTCCATCACTTAAAAATGTTGCCTTAACA
CAAGGCGGATGCTGCTGGTTAAATGCTGTTGTTTCGATCGATAAAAAAACTGAAGGCGATGGTAA
AAACGCAATCCTTGCAGCACTTGCAGCACACCCAAGTTTAAAACACGTAACTGTTGTAGATGATG
ATATTAACATTTACGACGCAAACGATGTGGAATACGCGGTTGCTACACGAGTTCAAAGCGACAAA
GATATTATAATCATTCCCGGAGCTAAAGGATCATCCCTAGACCCATCAGCGGACCATAAAAATAA
ATTAACCGCAAAAATGGGAATTGATGCTACAATGAGTTTATTAAAAGGAAAAGAGGAATTTATT
AGAGCAGAAGTTCCAAAAGATGGTGAATAACCTTAAAAAAACTTATACTTTTTTAAAAATTTTT
ATTTTGAAAAAAAATAGGAAATAACCAATTAATCTTTAATTAATTCTTTTCCTTTTGTCACAACA
ATTTTGCATGCTGTAGGTAATTTCATTGAGCATCTTCTTAATGCTTCTTTTGCAGCGTAGAATTT
TTCAGGGTTTACACCAATTGTGATGATTTCCTGTCCTTTTTTAACTTTTGCAGCTGTTCCAACAGC
TTTACCGAATGATAATCTCATTCCATCGGAAATCCTGTCCGCACCTGCTCCTGCAGCCATCTTGTT
TTCTCTTAAGATTTCGTGAGGGTAAACTCTGATGTTGAATAAGTATCCAGTTCTACCGCATTCAC
TTAATATGTATTTGTTACCTGCAATCCTTGAGGATTCCAAAGCGTTGTGTCTTATTAAAATATCT
GATTTTGATATAAGCTGTACTTCTACAGGGAATTCAGTGCTTGGGTTGCCCATAACGTAGTGAAC
AACTTTTGGTTGAGGTACTGCTCTTACGTACTCTTTTCTTGTGTACGATCTTCTTTCTATAGTTCT
GTAACATCTTGCTGGTCTTAGTGCCATAATTGTTCCTCCATATGCTTCGTGTTTATAAAAAAGCC
ATTCATAAGCGTGCTACAATATACATCTATCTAATGATATATAAACATTTCTTAAGGCAGAATGG
GTTGAAAATTCAAAAAAGGTCGTTACTTCTTTGATTTTTTAAAGCTATTTATTCTTTTGTTTATT
CTGTGTTTTATTCCATCAATTCCATAATTGTCCCTCATTATTGCTGGAACAAAATTATCAAGCCA
CTGTCTCCAAGGGGGTAAAAATCCAAGTGTTTCTGAAACCTTGTCAAGGTGGTTATCCCATTCAG
AATTTTTTATTTTATCCATTTTGTTTACTACAAAGATTGGATTTAATTCAAGTTCTTCTAAAAAA
TCAGCCATTTCAAGATCAATTGGAACTTCTCCTTTGTTGCCCCATCTCTCAACTATGTCAATGAAA
GATTTAGCATCCAAAATGTGAATCGATGTAGCAATTGCATCTTTATTGTCTTCAACATAGTGAAT
GATTTCGTCTTTAATTTTTTCCTGTGCTTTTTTTTCTATTCCTTCCATGAACCCAAAACCTGGCAA
ATCTACCAAGATATAAGTTCCCATATCATATTCAGTAATTTTTAGTGTAACTCCAGGCTTTTTTC
CAACCCGTACATTCTTTCCAGTTACAAGTTTTACGAAAGTAGATTTTCCGACGTTTGAACGGCCC
ATTACAATTACTTTCGGTCGCTCATCTGCTTTCTTTTCGGTTCGAATATATTTTTTAAATTTCTG
AATATCCATATTCTGACTTTGAATGTTGTTATTATTTTCTTCACTGTTCGTGTTTTCGTACATTT
ATAACCACTTTTAACTACTTGAAATATGTTGAAATTAGTTTTAAAACTAGATGATTCTTAAAATC
ATAATATGCATCAAGATACGCCGAATAAGCGTCATAAGGCGTTGAAAAATGGCTGAAATAAGAA
TGAACATCCATATCATTGAAACCTTTCGTGCACATGTAGTAAAAATTATCACTAGTCTGCATATT
CTTATAGATTTTATATTCTATTAACTCTTTTCGCTCTTTTTCATCATATCGTTTTAAATAGTTGC
CTAAGTATTTCCGTATTTCTTTTAATCTTTGAAAGGATAACTGCTGCATCCGGTTTCCAAGCCAT
GCACTAAGATCTCTTTCTGAATCGGCCCACGAAAGAGTTGAAAATTCAAAAACATCAACGGTATC
TTTTGGCTTACACAATTCAAGTATTTCAGAAGGGGTTGAATATTCGATATGATTATAATTTTCCA
ACTCTTTTGGAAGATACCTTAAAAATTCAAAAATTCCGGTATCTGCCCACTGGTGTTCTCCAAAT
GTTTCATAGTCCATGTATAAATTTATACAGTCCCCGGGAGTATTTGAAAGCCAGCTTGCATATTT
GTCAGCCATTAAGGGGTATTCTTTCCAATCCCTTGAAGAAAATCTAAATCCTATATCATCGCTTA
AGTTGTAATTTCTAAAAAGTACGTTTAATCCAGATAATGCACTGTAAACGTAATTTGGAGACCTC
CAATCTAAAATACGGTTAGCCCCTTCGGTAAATATTCCCCTAAAACCCATTTCTTTGATTGTTTC
TGCAATTTTATTATTAAAAATCAATTCTGTATTTCTAAATGTCGTTGTTTTGTACCCGAAAAGCT

CTTTTATTAATTTTTTATGATCGAGAATATCTTCCTTAAACTCTTCGTGATCTTCAAAAAGACTA
GATAATGAATGGTGGTAAGTCTCACCTAAAAATTCCACATTTCCAGTTTTTGCTAAATCTTTAAA
TAATTCAACGACTTCTTCATTAAATTCGAGAGCCTGTTCTAAAAAAACGCCTGTAATACTGTAAG
ATAGCTCAATATCATAATTATCAATTAATTCGAGCATTATCATATTTGACGGGATATAGCACTTA
TCGGCTACCTTGTTAAAAATTTCTTTGTTTAATTCTACGTCAACATAGCGGTTCCATAAATCCCC
ACTGTTTAAATTTACAGATTTTCTGAGCCTGTTTGGTTGATGGACCTCGAAATTAAGAGACAGCA
ACACAATTATCCCCCACAGATTTAAACTACATGAAAATAGTGTTATTAATAGTATATAATATGTC
TTAATTTAGGGAATTAATATCTCAAAATCTCCGTGGACCGCAATTATAAACATTGCATGTGACCA
TCCGAGAGGCATTGCGGAAATTGGAACCCCTAGGTCTTTGTGTATCTGTTCAGGCATTAACCCGC
TAAAATTATATTTACATACCCAGTCAAAAAGATTTGAACTTTTTTCACGATAATTATTTAAAATT
TCTCTTTTTATAGCCTTATTTTTTGACAGCGAATAAATAAGCAGTTCATAATACATGTAAAGCCA
GAGTGTATTTATAATCCACGGATTTCCGCCAAAATAAACGTCTTTTGGGTACCTACCAATTCCCC
CGACTTTATAATTAAATGCAGATTCGATTTGGTCAGCTGTTGCAACCATTCTTGGATCATCAGCA
TTCACTAAATTAAATGGAACCGTTATTCCAAGTATACTTGAATCCACTGTATCATCTTTTGGATG
AAGCGCCTTTGAAAATCGATTTTTATCATTCAAGTATATCTTTTCAACAACTTCTTCTTTTAAAA
ATTTCAGCGAATTTTGCCATTTTTCAACTAATCCTTCATTTTCTTCCAAAATTTTCGCAATTTCCA
CTGCAGATTTAAGTCCGCCGTAAATTGACCCCAAAGTGTATGTGAAAACACCATATTTTTCTTCC
CATAGATCATAACTTGGAGAAAGCGCAATTGCTGCATTTGATAGATAATCTGCTGCTTTTTTTAA
CATTGCCCAATTTTTTCGTAAAAATTTTTCATTATCACTCTTTCGATAATGAATTAAAGCTGCCC
AAATCGTGGTTCCAACTTGATCTATTTGCATTGCAGTTAAACGCGGAATTCCATTGATATAATAA
CTCTGAACCCAAGAACCATCTTCATTCTGGGTTTTTCTGCACCATTCAAAATATTTTTCAACAAT
ACTGGACTGTCCGCAGAGCGAAAGTGCAACTGCAATATATCCTCCATCCCTGCACCATACATGCCT
GTAATCCGGATAAAGAGAGGGGGACGCCATTATTCCTCCGCCCCGATCGCATAATAAAAGTAGTG
TTAAAAGAGACCGTTTACACAAATCTTCGCACATTGAAACCCGGTTTAATTCATTTGCACTTAGT
TTACTTATCGTTGTTTCAAGCAGATTTTTCCAGTATGAGTTTGTAAGCTGGTAGAAGTCTTCATG
ATTATCAATAACAACTTTAATCGAAGCAAGGGTTTCCATTAATTTATACTGGTCTCCTTTGTACT
CGTTCATAATAATATAAACAGAAACTGACTGTTTTTGACCTGGAAGTAGTTTTAAATCCCAAGAA
ATAGCGCTATCAGTTATAAGACCTGAAGAAGTAGAATATTCTTTTAAAATTCCATTTGAAATGTC
AATTAATGCACTTTTATCAGAAGATCTAACTCCGCATTGGTATGAATCGATTTTTTTATCGCTTC
CAATACACATCGAGTACATTCCGTCGTATTTCATAATCATTTTGTGGTCTTTTATAAACTGGACT
GTATTTTCCCTTGGAATTTCGCCCATTCGTAAATTTTCATAAAAAAATAATTTTAAATCTTGGGT
TTCATCGGTTTTATTGGATATTGAAATCTGTTTTATTATGATATTGTGGGAAATTGGAATATAAT
CTCTTGATTCCATTAAAAATTCATTGCATTCGTAAGTGGTACGCAAAATATTCGTATCTTTCACG
TAACTTTGATTAATCTCCCAATCATTGTCCCACGCCCATCTAAGGCCGTGATTGTAAATTGCAAA
AGAAGCGTCAAAAATGTGTTTTTCATAGCCGAGGCTCGGGTAAAACATGTACTCAATAGACCCTG
AATCATCTATTTTTGCAAGGATATTTCCATTTCCAATAATTCCAACCATCAGCCCCACCCTGAATC
TATTGAAAAATAGTTATATGGCATAAAAAAATGTAAAAATCAAAATTAAGTCTTATTAAATATAT
TAATTCCAAGTTTTATAATAATTTATTGCTTCATCATTTCGATTGCACGTTTATATACCTGAATC
GTTTGATTTGCGATTGCATCCCAGCTGTATTTCGTATATGCATCGTGCTTTGCATTTTTAACTAT
CCATTCTTTAAATCCCCAGTCAGAAATTACCCTGTCAAGGCCCCATGCAATAGAATCTGCATCTCT
CGGGTAGACTTTAACCCCGTTGTATTCATGGTTTATAATTTCACTAAGGCCCCCAACATCACTTGC
AACAACAGGAGTTCCTGCAGCCATTGATTCAAGAGCAACAATTCCAAAAGGTTCATAAACCGAAG
GAATAACGCATGCATCTGCATATTTGTATAGTTTTTTTAAGAAATTTCCATTTTTAAATCCTAAA

AAAATAACTCGATCACCACATCCTAAAACCCATGCTAAATGCTCTAAATGGCCTTGCATGTGCCC
TTCCCCTGCAATTACTAGTTTTGAATTAGGATGTCCAATTAAAAATTTTTGAAATCCCCTGATTA
AATGTTCAACCCCTTTTTGATAAACAAGCCTTCCAACGTACAGAATCATGTTTTCATTATCATTT
ACGCCAAGATTTCGTCTGAAATTATATTTTTCATTATCATCGCCATTAATATCAAATTCCCAAGG
ATTTACCCCATTATATATCACGTTTACCTTTTCCCATGGCGTATTAAATCCAAAACACAGCTCATC
CTTAATTGAATGACTTACTGCAATGAGTTGATTTGCTTCGTAGCTTCCCCACCATTCCGCATCGTT
TATTGCCCTTGAGTCTTCAGAATTAATTCCGCCACAACGTCCTCGCTCAGTACTGTGAATCGATTG
GACATATGGCTTTTTTGTCGTGTGCTTTATATTTGAACCGACAAACGAAGTCATCCAGTCGTGGC
AGTGTATTACGTCATAATTTTCAACACCTAAACTCCCTATCTTTTTTTCAAGGGAACTTGCCATA
AACATTGCCCAAGTTAAAAAATTATTGTGGGAAATTGGCTTTACCCGATAAACATTTACCCCATT
TATTTCTTCATATTCTGGAAGGTCGTAGCCGACAGTTATCACGTCAACTTCGTTTCCTGCCCTCAC
GAGCGCTTCAGATAACCCCTTACAATGTACTGCTAAACCACCAACCATTACGGGGTGGTACTCCC
AAGTAACGATGGACACCTTCATTTCACATACTCCCCTTCAAAGATTAAAAACTAATATATTCTCT
ATTAAACATATATTTTCATTAGCATATAAAAATACGGGAGTATATGAGAAGGGGTATTTTTATG
AAAATAGTTACATTAACACCGACAATAGCGCCTTTAACGTCGATAGGGGGACTAGGGGATATTTT
AGAAGATTTACCAAAATTTTTAAAGAATTTGGGAAATGAAGTATACGTAGTAACATATGATCAT
CAAAACAAAATTTCAAAAAGACCTCATGAAATTATAAAAAAAATAGAAGTAAAATATCAAGGCT
CAAAACTCTTTTTTGACGTGGTAAAAGCAGTTCATCCAAGTTCTAATTTTGAGATTTTTGCATTT
AGTAATCCTGAAATAAATCAGCTTGATAGCTGGGATCCTATAAAATACGAAATTTTTGCAGATTT
AGTTGTATCTTTCCTTTCAGATATTGAAAACATTGAGTGTGTGTCGGGACATGACTGGCCTTGCG
GACTTGCAATTGCAAAATGTCACGAAAAATTAAATCTTCCAACGACACTTACAATACATAACGAA
GCATTTAAAGGCCCAATAATTGAATATAAGGGGCACATATTCTCATTTTTAGAACACGGGATATA
TTTTAGTGATGCAGTCAATACTGTAAGTCCTAACCATGCAGAAGAAATACGAAATTTAGACTTTT
TAAAAAAATTATGTAAGGACAAACCTTTTCACGGGATAATTAATGGAATAGACACGGACGTTTA
TTCTCCAGAATCGATTATTGAAAGAATGAAAAATTTAAGCAACGGAAAACTAGACCCTAAAGAT
TACTGTTATATTGAAAATTACGATATTTCAAATGCAGCGGTAGTAAAACCAAAAATAAAAAAAT
CGTGGTTTTGCAAATGCGAAAATTTAAAAAAATATATTAATGATTGGAACGAGATGGATAAAAG
TAATATTTCGGGCAGCGATATTGAAATACACGGTAATATCAAAGGAAAATTGAAAACTCCAATG
ATCGGATTTGTAGGCCGTGCTACCTATCAAAAAGGTTTTGATTTGATATTTGAAACAATGCCCGA
AGTATTAGAAGAAAATAACGCTAATTTCGTACTTGTATCAAAAGGGGAAAAATCAATTGAAGAA
AAAATAAAAGATTTTGCAGAAAGTTATCCTGACAAAATCATGGCTCTCGTTGGACACTGCCCGCC
AATAATTCCCATTATTTATGCAGGATGCGACTGGACTGTTGTGCCGTCTCTTTGGGAACCTTGCG
GATTAACACAGATGGAATCAATGTCTTATGGGACTCCCGTAATTGCCAGAAATACTGGAGGTTTA
AGTGATACTGTAATTTCACTTCACCCTGATCCGCATAAAAATCCAAATTTTGATATAGCAACAGG
TGTTTTATTTAATGATTATGATAAATACGGATTTAAATGGGGTATTGAACACGGGCTCTACTGGT
CGTTTTACAATTTAGAAGAAGCCTGTCTTTTTATAAATTATAAACACGTGCACTGCCCAGAAAGT
CCGTATGAAGAAAATAGTCCATTATACATGTTAATTAAAAACTGCTACCGCCACGTACAAAAAAA
CCTGAGCTGGCAAAATAACGACAGTTCTGAACGATACAGGGCACTTTTTGGCGGTGCAATGTACA
AACACTACTTTTAAATAACCATAACTATTTTTCAGGAGGAATACGATGAACGGGGAACTATCCTT
CAAATACGATAATGTAATGGAAGAAACTTTGGGCATTTTGGGAATTAATAATGTAGAATTAGAA
TCATTTAACAATGAATCTTCAAAAATTATTGAAATTTTAAAAGAAAAAGAACTTAATGGGGAAT
TTGGATTTTTAGATGTTTTGAATGATAATTTAGATAAATATTACGAATTAAATGAGTACTCCAA
AAACTTTGAAAATATCCTTATTATAGGAATAGGTGGATCAAATTTAGGTTTAAGGGCTGCTGAA

ACAGGAATACTTGGAAGTTTCACATCTCGGTACGAAATTCCAAGAATATACTATATGGACAATTC
TGACCCTGAAAAAACCCATGATATTTTATCAAATATTGATTTGGAAAAAACCCTCGTATTTGTAA
TAAGTAAGTCGGGAAATACCGTTGAAACGTTATCAAATTTCTTTATAGTTAGAACTTTAATGAA
AAAGAAAAACATTGATTTGAAAAAACATGTTGTTTCGATTACGAGCGGTGGAGAACTTGAAAAA
ATTACAAAAAAAGAAAACTACATTCATTTTGAAGTTCCAGAAAATATAGGGGGCAGATTTTCTG
TTCTTTCATCAGTTGGGATTGCTCCACTTTCATGCACTTCAGTAGATATCAAAAAATTAATTGAT
GGTGCTAAATCTATTGAAAAATCTTGCAAATACGAAGATATTTTCAAAAATCCGGCTTTAATGAA
TGCAGTAATTCATAAACTTATGTATAACCGCGGAAAAACTGTTTCAGTAATGATGCCGTATATTG
AAAGACTTAGAAGTTTTGGAATGTGGTACGGACAGCTCTGGGCTGAAAGTCTTGGAAAAAATGG
CTTTGGACAGACCCCCGTTATTGCAGTTGGTGCAACAAGCCAGCATTCCCAATTACAACTTTATA
TGGACGGTCCGGATGATAAAATCGCGACATTTTTAAAAGTAAATAAATATAGAAATGACTTGAA
AATCGAGTACGAATATGACCACCATCTTTCAGGCCATAATTTATCCGAAGTAATTACTTCAGAAC
TAGTTGGAACTGAAAACTCGATGAAACACAATAATATTCCAAATGTAAAGATTACACTTTCAAA
ATTAAATGAAATCACCATGGGAAAACTTTTCTTGATGTACGAAATGCAGACTGCAATTTCTGGAG
AACTTTATGGAATTAATGCATTTGACCAGCCTGCAGTCGAATATGGAAAAAAAATAGCTCATGAA
TGTTTAACAGGTTCAAAAGTTGATTCTGAAAAAAAATACATAAATGGAAAATATATTATAACTT
CCAAATAATTTTATTTTGAGAGGGGGTAGGTATGGAACTGACTAATCATTTTAAAGACTTTTCAA
ACGTTTCTATTTTTTTAGCATATAATGTAAATGTCGATGCATTGAAATATCTGGTAGATCCTTCA
AATATCGAAGAATTAAAAGAAAATTTTAGCGATTCAGAAATTAAAACAAAAATTGAAGAATACC
CTCGAACAATTGAAAAACCGATAGACTTTGTTGCAAGATTAATTCATGCCATGAAATCTGGAAAA
CCTGCAGAAGTTCCATTAAAAAATAATTTAGAAATTGATACTTTTTAAACAGATTAACATACAA
TGAAGAAAGAATTGGGGGGCAGGTTGGAATAATCTCAAATTTACTCTCAATTTTAAATTTAAAA
AAAATAATTTTTTATTCTCCAATTTTGGCAAAAAAACAGGCTGAAATGTTTGAAAATAATGAAA
ATTTAGTATTTCCAAATATTACGAATGGAAAACTGGTTTTAAAAAAACCAATTGAATCTTTTAA
AAACGATGAACTTAAAATAAATCGGATTTTTGAGTACAAAGAAGATATTGAATTTTATTTAGAG
AATGAAAAAATCACAACTCCTCAAAGTAACCGATTTATCGTTGCATCTCGACCGGAAAATTTACG
AATTGAAATAAAAGAAGAATTAAAAAATTATATGCCTGAAATTGGAAAATTAGTTGATTGTGCA
ATAATTTCAGGGGTTCAAGCGATTAAAGAAGAGTATTCTGATGGAAAAACTTCAGAATACTACT
TAAATCAGGTTAAAGAAGATATAAAATCGTTAAAAAAAGAAAATAAAGACTTAAAAGTACATTT
TGAATTTGCATCGATTCAAAATACGGAAATGAGAAAAAAAATTGCAGAATCAATTTTACCTGAA
GTCGACTGTGTTGGAATGGATGAAACTGAAATTGCAAACATAATTCATGTTTTAGGTTACGAAG
AGTTAAGCGAAGGAATATTAAAACACAGTAAAATTGAAGATGTGCTAAAAGCTTCAAAAATACT
TCTTGAAAAATACAATTTAGAGGGAATGCAGGTGCATACAATGTACTACATAATGTATCTCTGTA
AAAAAGGCGGCATTTTAAGCGACGAATCTTTGGAAAAAACTCTCGAATTTGCAACAGTTTTGGCT
TCAACAAAAGCAGCTCTCGGGCAGATTTCAAGTATTGAGGATTTAAAAACCGGTCTTAAAATACC
ACATAATAAACATGGTGAATTACTAAAAGAGATAGTTGAAAACATTTCAAAAGAAAAAGAATTA
GAGGGTTACAAAATTATTTTAGTTCCTTCAAGAATCGTTGAGAACCCAAAAAGTACCGTTGGACT
TGGGGATACAATTTCAGCAGGTGCTTTTGTAGGTTATGTTTCAGAACTAAAAAAAATTAAAAAA
TAAATAATTAATAAATAATTTATTGAGTTGGGCATGAGCCTCCAAGTGCAGGTGGTGCTTCATCG
TCAACACCGGGTCTGTATCCAAGCTCTTTTCTTGTTTTTTCCTGAATTTTGTGGAATATTCTTGCA
ACTGGAATTTCCATAGGACATACATCTTCACACTGGCCACAGTTTACACAGCTAAATGCCATGTG
TGACATTCTAACGCCCTGGAACATTATTGGATCAGGTGGGATTGATCCTGTATCGACATAGTCTG
CAGTAAGTGCGCATTCTCTACAGAAACATACCGGACATACATCTCTACAGCCGTAGCATTTTATA

CATTTATTCCACTGCCTGTTCCATTCTTCAATTGTTGGATATTTTTCTTCAAGCCATTTATTTTTG
TAATCTTCGCCCATTTTTAACATTGAGCTTTCTACTTTTGCCCTTATTTCGAGTCCTTTTGGATTT
GGAGCTTTTTTCTTAATATATCCATCTTTTTCTGCACTTTCGAGTAATTGTTTACCTTTTTCAGT
ACATACTTCCATGAAGGTATATTTTCCAGCATCTTCTCCAATTACACCCCAGTTTCCAGCAGCAAT
ATCTGCTTTTCTTGGGATTTTCTCGTCACATCTCTGACAGTTTACTCTTCTTCCGTAGCCGTTGTC
TTCGAGATCGTGCATTTTAACTTCTTTGTGGGATCCGTCTTTCATGACGATTATAAATTTACCCT
TGTCGATTTCTTCTTTTACAACATCTTTTGGATCTACTTCATAGAATAATTTTATCATTTCCATT
GCGGTTTTTGGAGGAACCGTTCCACCACAATTCAATCCAATCATGTAGACATTATCAAGATTAAT
CTGTGCTCTTTTTGCAAGTTCAACAATTGCCCTCATGTCGCAAGGTTTTACCGGAACTGCAATTT
TTGCATCTTGTAAGTATTCTTTTATAATTCCGCCCCACATTGTAGGAGCGCAGTGTAGAGAACCT
GCAGTACTCAACAAATCTTCTGAAGATGTAACAAACGTTGGAATTGCGCCATATACATCAACACC
TTTTTTCAAAGCTAAAACACCGTCAACAATACCCTTGTCTAAAAGATATTTGAAGAGTGCAGTAA
CTGCACCACCACATTCTCCTTTTTCGAGTATTGCCGGGTCAGAAGCCTGTACATAATACATTTCAT
TGCTCATGATATCCCTCGTTTAAAATCCCGATTTTGGGAAAATTATATTTTTTCCACCTTTGCAG
CACATACCTTGTATTCTGGGATTTTACAGTTTGGATCCTGAGCAGGATTTGTTAAGATGTTAGCT
GCAGTTTCTGCAAAGTGGAACGGCATAAATACAACACCTTGTTTGATATTTGGTGTTATTTTCGC
ATTTACAACGACTTCGCCCCTTCGGGTTGAAACTTTGACTTTTTGTTTGTTTTTAATTCCCATCTT
TTCAGCATCTTCTGGATGAATTTCAACAAATCCTTCGTTTATTTCATCAGCCATATGCTTACTTCT
TCTGGTCATTGTTCCAGTGTGGTAGTGGAAAATGATCCTACCTGTTGTCAGAGTCATTGGATATT
CCGAATCTGCCATTTCGTCAGGATCTGCATAGTCTATTGCAAAGATATTTCCAAGTCCATCAGGA
GTTAAACACTTTTCACAGTGGAGAATTGGAGTTCCTGGGTCTTCACAAGTTTTGCAAGGCCAGTG
GAGTCCATCTACACCAAGTCTTTCGTAAGTTACACCTGCATACTGAGGCGTTACTTTTGCAATTT
CTTGGAATACTTCTTCAGGAGTTTCAAAGTTGAATAATTCTCCTTGACCCATCTTTTCAGCAAGC
ATTTTTACGATTTTCCAGTCAGCAATTGCTTCACCAGGAGAATCTACTGCTTTATTGATTTTTTG
GATTCTTCTTTCAGTGTTTGAGAAAGTTCCATCCTTTTCAGCCCATGAAGCTCCAGGTAAAACTA
CATCTGCAACCTGTGCAGTTTCAGTTAAGAAAATATCCTGAACTATCAATAAATCAAGACTTTTTC
AATGCATGTTCTACGTGGTGAATGTCAGGGTCTGAAACCATTGGGTTTTCTCCCATTACATAGAT
ACATTTAACCTGTTCTCCAGCTTTTGCAAGCATGTCTGGAATCGAAAGACCAACATTTGGATCAA
CACAGGTATTCCATGCTTCTGCATATTTTTCATGGTTTGCTGGAACTGCCTGGTATCCAGGGAAT
ACGTTTGGAAGTGCACCCATGTCACATGCTCCCTGAACGTTATTTTGGCCCCTTAATGGGTTCACA
CCAGTTCCAGGTTTTCCAATATTTCCGGTAATCATTGCCAAGTTACAGCAAGATTTTACGTTGTT
TACACCGGTTGTGAATTGCGTTATACCCATACAGTAAAGTATCGATGCTGCATTTGCACTACCAT
ACATTTTAGCAGCTTCGACAATTAATTCAGGAGCTACACCTGACAATTTTGACGCGTACTCAGGA
GTATATGTTTCAAGCGTTGTTTTAAGTTCTTCGTATCCTTTTGTCCTGTTTTTGATGAATTCTTC
ATCAACAAGATTTTCTTTCACGAGTACGTGCATTATTGCGTTTAAAACTGCAATATTTGTACCAG
GTAATAACTGTAAGTACAAATCAGCCTGTTTTGCAGTTGGAGTGTATCTTGGATCTATTACAATT
ATTTTAGTTCCTTTTTCTTTTGCGCGAACGACTCTTCTCGCAATCAAAGGGTGTTGTTCAAACGT
ATTTGAACCAATTATGAATATACAGTCTGCATCTTCTATATCAGAAATTGAATTTGTCATAGCAC
CTGAACCAAAGGCTTGCCCTAAACCGACCACAGTTGCCGAGTGTCAAAGTCTCGCACAGTGATCA
ACATTATTAGTTTTTATTACTGTTCTTGCAAATTTTTGAAATACATAGTTGTCTTCATTGGTACA
TCTTGCAGATGAAAAGAATCCGACTTCTTCAGGGGAATAATTTTGTAATTTACCAGAAATCAGTT
CTAATGCTTCATCCCATGATGCTTCCACCAATTCTCCATTTTTTCGAATCAACGGAGTTTTCAACC
TATCATCACGGTGTACAAATTCGTGACAATAACTACCTTTAATACAGGTTTTTCCCTCATTTACA

GGGTGTCTTTTAAAAGGATTGGTCCCAACAAGTGTTCCATCCTTTACTACGAGGTCCACACCGCA
GCCTGTTCCGCAATATGGGCAAATCGTATGAATGAAGTCTAATTCCATATTTCGATCACCATTTT
ATAATTATAAAATGGCAAGGTTTGGTTCAATTAAAGAATGATGTTCCAAAAAACCGGTTCACACC
ATTCAAATTTGTGCTATTATTTTTAATTATGATATGACGGATAAGATTAAATGAATAAATTAAA
ATACAAAAAATGGTAGATATAGTTAAGTATATTATTGTAGGCAGATAAAGTTAGGCTAGTTTAT
CTGAACTATTTGGCTAATTTGGCTAGTTAAACTCTAATGGGTTAGATACGGGATGTGTCCGCTGG
TCAAGCATCATCCACATTACCCTTTTTTAGGCTATTTTTACTAAATCTCCTGTTTCTATGTCCATT
ACATACCCATCGATAGTGATATCTTTGGGCATTGCAGGGTGGTTTCTCAAAAGTTCAACACCTTC
TTTAACATTGGTTTCTTCGCAGTACATTCTGCCAAGCCATGCTTCAAAATCTGGTGTGAAATTTG
GATTTGCACCCCTTTCTACCATTTTTTTCTTGACTGTTTCAAAGTCAGCAGCTGCCATTCCACAAT
CGGTATGGCCAATAATCATAATGTCTTCAACACCGAGCAGATATACTGCAACTACTAAAGACCTG
ATTACATCTTCTGTTATAATATTTCCTGCATTTTTGATTACTTTCGCGTCACCTTGAGCTATTCCG
AGCTTTTCGGAAAGAAAGTTGACGAGTCTTGTATCCATGCAGGTCACAATTGCGAGTTTCTTCTT
TGGTTTTGCATTTTCTCCCAAGTTATAACCTCCATGTATTTTTAGATTTTTCGGACACCGATTCCA
TATATTTTAGGCTTCCAAAGGTTTGGAATACCCATTATTCCGAAAAATTTGTAGTGTTTAGCTAC
AACCCCATAGAGTTCTATAATGTCTCCTTTTTTTATTCCTTCCGGAACTGGGGAAATTAAGTTAG
AGAATATCTCTCCAGTATCATCCTTAATTCGAATCGTTGGTTTTGTAGATATTCCATATATTATT
TTATCCAATTTTCCTACGATTTTAACTCTCTGGTTTAAAGTATCTTTATTTATTTCCGATATATT
GTATTCATCTGCGTAATGTATGTATTCGGGGAGAAGTTGATCAGATAATTCTTTATTCATTTGTG
AAATTTTAATCGGAATTAATGTTAAACCTAAGATAAATGGAATTGCCATTATTAATATCCAAAT
ATTTCTACTGATATAAAGTGACCATGCGGTAAATACCAAACATATCATCAGCGAAATAAATGCTG
AAGGCGTGGTTTTCGCATGTCTTTTTAGATCACTTAGAGATACGTTTAACTGCATTTTAACACCA
AAAAGATGAAGTTAGTAGTCTTCATCTTTAGATTTTTTACCGTATGCGAATTGGTATATCATTG
CTACGAATACTACTGCACCGATAATGTTTCCGATGGTTACTGGGATAATATTCCAAAGCCACCAG
TCTGCAACACTCACGGTTGCACCAAGCATCATTCCTGTTGGAATAAAGTACATGTTTGCCACACA
GTGCTCAAATCCTGTTGCAACGAACGCCATGATTGGGAACCAGATCATGAAGAACTTACCGAGGA
TACTTGTTGAAGCCATTGAACCTATAACTGCAAGGTTAACTAACCAGTTACAACCAATACCTTTA
ACAAGTGCTGCAAGCCAACCCATGGATCCTGCTGCCATGTAAGGTAAAACTTTAGCTTGTGCAGT
GTTTATTGCTGATTGACCAAATGCATTTACAGCAGCCTCACCGGTTGCTGAATCAAATGATCTCA
ATGGGCCAAATGCCATCATTGAAGCATATATTAAGAAACCAATGAGGTTACCAATGTAAACATAA
ATCCATACTTTTATTAACTGTGCATATGACGCCTTTCTCTGGAAAACTGCCACTGGTAATAACAT
CATATCTCCAGTAACTAGCTCCATTCCGGTTAAGATAATCAAGATCAGCCCTACGGGGAATACTG
CGGCTGCCATGAATTTACCTAAACCAGCACCCATTGCTGCACCAATTCCTGTTCCTACAACGGTAG
CAAGTCCACCACCCATTGCAATGTAAGCACCGCCCATAACTCCTCTGACAAGAAGTTGGCTTGGAC
TAAGGTTTCCTTTTACTTGGCCTGCATTTCCTGCAAGTTCCACCATTTTGTCAGGTGGGTTTACAT
CCATGATATTACCTCCATGTGAATATTGTGGTCTTGTGGGGTACTAACACCACATATTCAACTAC
TAAAATTTTTTTCATTATGCACACCTGATTTTAGGTTTAGGTTATTATAAAACCTTTAATTTAAA
AAAGATGGTTGTGGTACTCGGAGCACACATCAAATATATAAATACGACCTAATATACTAATGAA
AACCTACGACAATATAATATTTATAGATTACGAAGCATATATATTGTTATTTGCTTACCTGTGCT
TTAAACTTATTATTTAATCTAAATACTGGTGTTATTGTGGAATTTATCGAATTTGGGTATGGTAC
TTCAACAGATGAAAACTCTTTAAAGGCGGGGGCACATGCCGCATCCGATGCACTCAAAATGATGA
AAAAGTATTCTGAAAAACCAAATATCGTTTTTTTATACTCCTCTCCCGATTATGACCCTGAAGAG
GTACTCAATGGCGTTAAACTAATTCTTAGAAATAGCGTCCAAATAGTAGGGGGCAGTTCAAAATT

CCAAATTTGTGGTAATAAATTTTTAGAAAACGGTGTTTCAGTTGGAATTTTGGGTTCAAAATATT
TTAATACCGGAATGGGTGTTGGCCTTGGAATTTCGATAAACCCGAAGGAATCTGGAAAAAAAGCA
GTCAAAGATGCAGTTGAAAACCTTGGAATGCTTCCAAAACTTCTTTACGTGATAACGGATTTTTG
CAAATGTGAAGAACAGGTTTTGAAAGGAATTATTGAGGAACTTGGTGTTACCATCCCAATTTTTG
GAGGAGTTTCCTCAGATGACTTTGAATTTGAACATATCTACCAGTACGGAAACGACGTTTATTTA
GATAGTATCGTCTGTGTCGCGTTTGGCGGAGACATAGTACCAAAAATTTCTTACAATAACTACGA
GTACGGAGAAAATTCAGGAAAACTTGAAAATATAATTACAAAATCTGAAAAGCGTTCTATCGAA
GAAATAGGAAATATTTCAGCCGTAAAAAGATATATTGAAATTTCAGGATACAAAGGAACTGTAA
CCGAACTTGAAAAGGACCCTGAATTTTACTTATCTCATCCCCTTGGTATTTTAGACACCACCGGG
GATATTCATCTTAAAGGCCCGATTTCAATTAAAAATAAAAAATTAATTACTGGCTCAAATATAAG
CAATTCAAATGAATTAAAAATAGAAAATATTGATAAAAATGCGATAAAAAATATATTTGAAACC
AATACATCCGTTTTAAACAATACTCAACCGCATTATGAACCAGCACTTACTTTTTGCAATATTTC
ATCATTTTTGTCAGAAATCAACCCTGATGAAATTGAAAAACTGATTGAGAAGTCAGACATTAACC
CATTTTTTGGAAGTTCAACTTTTGGAGAAATTTCCCTTAAAAAATATGGAAATTATTCAATGAGC
ATGTGTTCTTTCGCACCAGATTTAGTTACCATAAGTGCAAAAGAAGGAATTCACATGTTTACAAA
ACATCCTGCTACAAAAGAAACCCTCATAAAAATTCATGACCTTGGCGGTTCCGTAAAAGTTGAAG
AACTTGCAAAATCGCTTGGAATTCATAGGAGATCTGCGTATGATAGAATTGATCCGCTTTTAAAG
TACGGATTTATCGAAAAAGATCAGGCAGTAATAAACATTACTGAATTTGGAAAACTTCTTCTTAA
ATTCGAATTTTAATTAAAAATAAAAAAAATAAGAAAATTTAAATTAAATCTACGGATGTTAATC
TTTTTGAAATCAAATTCAAAATATTTCGCAAATTTTCTTCGTTATCGTAATTTTCAACGATTTTT
AAGAGTTCTTCTTTTTCCATTTTTTTGTAGTCTCTTACGTATTCAGTAATCAATGGAATACATCT
TGTAAGCTCGTCGATTATTGAAATTGTTGATTTTCTTGCGGTTCTTGAAAGAGGGTTTAAATCAA
TTGCAATTACTGTTTTTCCCATTTTAACAAGCGCTTCAGCTCGGTCCCCGTCTTCAAGTGGAACTA
AAACTACATCTGAAGTGTATATTCCAGATTCTGAAACTTTTCCTCTTAAGTGATCAATTCCAGGA
ATTTGTTTATCTGCATTATCTATTCCTAAAGAACGATTCCATTTTCAATATCACTTTCAAATAC
GTCCTTTATTTTTTCTTCCCGTTCAGTTGTTCTGTAGAATAAATTAACTTCGATTTTGCCGTTTA
ATTCTTTTGCTAAAGCGACAAACTCATCTTTTGCTAAAGCGACACTGTTTCCATTTACTGAAATT
ACAGGATTTTCTGCTAAAACTAAAAGAGCGGCTGAAACTTTTATGGAATTTAGTGCTGCGTCTGG
TGTACTTTCCCCGATTAAATAGTCGAAAGTTTCGCCTCTCCCATGAGCAATCATTCCGCCTTTTGC
CAAAACTCCTGATTCAAATGCTTCTACAATTCTGTCTCGATTTAATAATGACTTGTACCTTGGAT
GGGATTCTGGAACCATCATGTTCTCACCGTATTTTATTCTCACAATTTTTATTTTTCAAAGATAT
GCATTGATCATTTTTATTAGTTTACTACATTGAAGAACAGACACTTCAAATATTCAAGGTCTTTG
TTTGCCATCGTTATTATGTGGTCAGGTGCCTGTGTCCTGTATGACCCGATCTGCCTTATCTCTTTT
TTAGCCCTCAATGATGAAGAAACGATTGTATGTTTAAAACTTTCCCGATCAATGTGGTGTGAACA
AGAGCACGTTACAAGAATTCTTTTTGCCAGTTTCAATCCAAGGTAATTCATTGAATTGTAAGCAT
TTAGTGCATTTTTAATATCTTTTGAAGAATCCGTAAATGCAGGTGGATCGAGCACTACAACATCG
AATTTCTCGTTATTTTTAATCATTTCTTTCATTGCATCAAACGCATTTGAAACGATAAAGTTATA
ATTTTTAACCCCATTTAATTCCATGTTTTCTTCTGCAACAGAAAGTGCTTTTTCGGATAGATCTA
CTGCAGTAACGTCTCCACCTTTGATTCCCGCGTGAACTGCAAATCCTCCAGTGTAGCTACAAACAT
CAAGTACTTTATCTCCAGGTTTAACATAATTTTCAAGATCAATTCTGTTGTCCCTTTGATCCAAG
AAAAATCCTGTTTTGTGACCTTTCATGTTCACGTTAAATTTAGCGTTTCCTTCGGAAATTACAGT
TTCAATTTGTGATTTGTCGCCAAAAATCTTCGTTTTTACGTCAGTTCCCTTTTTACCGCTTTGAAC
ATACATCGTTTCAACATCGGTAATTTCAGAAATGCATTCAAAAATTAAATCTGAATAATTGTCCA

TAATTTTTGAAGAAATCTGAATTGAAGCAATTTTATTATATTTATCGAGTATTATTGAGGGCAT
ATAATCTGCTTCTGCATAAATCATCCTGTAAGTATCGTTATAATTTAATATATCGGTTCTGTAAT
TATTCGCGTTATTTATCCGTTTAAAAAAGAAATCTTTATCAATTTCTTCAGTTTCCCTTGTTAAA
ATTTTAATTAACGGAAATTTTGGATCGTAGAGTGCTTTTGCAACAAAATTTCCATTATAAGTTAT
ATCAACAATTTCTTCCGATTTAGAAAAATCGTCTTTATTAATTATCGCATTTTTGTAAATTATGT
TTGAAAAATTTTTTAAAGACTGATAAGCCCGTTTATCAATTTGAAATGACATAAAAACCACCGGA
TTTATAAAAAAATCGAAATAAAATAAATTAAAAGAAATTATCTGTTCATGTGTGCTTCCCAGTC
AGCGAGGAATTTTGCAATTCCGTTGTCTGTTAACGGGTGGTTGAATAATTTGTCAAGTACGTCAA
ATGGAATTGTTGCAATGTCTGCTCCCATTTTTGCGGATTCTAAAACGTGGATAGGGTGTCTTACT
GAAGCTACGATAACTTCTGTTTCGATTCCGTATGCACCAAATACTTGCATAACTTCTGAAATTAA
AAACATTCCATCCTGTCCAACATCATCGAGTCTTCCAACAAATGGGGAAACGTATGAAGCTCCAG
CTTTTGCTGCGAGTAATGCCTGATTTGCTGAAAAGATTAATGTAACGTTTGTTTTTATTCCTTCT
TTTGAAAGGATGTTTACTGCTTTCATTCCTTCTTTTGTCATTGGAATTTTGACTACAACGTTTTC
TGCAAGTTTTACGAGTTCTCTTGCTTCTTTAACCATTCCTTCTGAATCAAGTGCAATAACTTCAG
CACTTACTGGACCGTCAACAATTGAGCAGATTTCTTTGATTACTTCGTAGAAGTCTCTTCCTTCT
TTTTTGATTAAACTTGGATTTGTTGTAACTCCGTCAACTAAACCGAGTGCGTTAAATTCTTTAAT
TTTTTCAACATTTGCAGTGTCTAAAAAAAACTTCATCCTTTCACCATTTTGTGTTTCGCTTTTAT
TAACGTTATAAATAAAGATATTTATAAGTAATGTAGCTTGAAAATATTATAAAAAGGTTAGATT
AAATTCATCGGCAAGCCATTTTGCAGTTTTTGCAGATTTAGGATATTTATTAAAAATTTTTTCAA
TCATTTCATTTCTGTTTTTTGAAAGTATTCCAAATTCCAAAATTTCGAGAACCGTTTTTTCAACT
TCCAAAACTTTCCAGTTTGGAACTTCAAGGGTCATGCAAAAATCAGTTTCTTTAAATTTTTCCCT
CAATATTGGAGAAATCGATGCCATTAAAACCCCATTATTTAAATCAACGAATGGAGGAATCCCTT
TTAAATCGACTCTTTTTAATTCTGTTAAATTAGAATATGATTTTTTTGAATACGAATGGATTTCA
AAATAAAATTCTGGATTATATTTTTCGATTAATTTTAAAAGAGATATTCCTTCTTCTGTTTCATA
GTATTTTGGATTTAACGTGGAATAGTACTTTGAATCCTTCACAAGTTCTGGAACAATTATTACAT
TTCCTGAAGCTTTTAAATTTGAAATGTTTTTTTGTAAATTTTCTAAAATCTGTGAAGTGTATTTT
GATTCATTCCCATGAATCCCTGCCAAAAAAAGCTTTTTTGGGACATTATTTTCAAAATTATTGTA
AATTTTCAAAATTTCACCAAAAAAAAAGTTAAAAAAATAATAAAATTAAAGTGTTGAAAGTTTAA
ATCCATCTTTTACTAAGACTGTTGCTGCAGTTACGGGTTTTTCAAATTCTTCGTAGTCCATTACA
AATTTAGTTACATCTTCTGCGGTTTTTCCAGTAACTGAAATTACCTGATGTTCAGTAATTTTGCA
TGCTTCATAAACACTTAAATAATATGCTTTTCCAGCTTCAAGTTCAACTTTTTTAATTCTAACGT
CGCTGTCAGTTACATAACCCATGTAAGCTTCATCTTTGGTTATAACTACTGCAATTCTCGGGGTA
TTGTAGTCGTCTTTTTCATAATCCATTGTTAATAATGAGTATGATAAAGCATCCCTTATTGGAAG
TCCAAGCTTAATTTTATCTGCAATTATATCCGTGTGTGAGCCGTTTGTAGCTACTGCAACGTCGC
CTACAAGTTTTACACAGTTGTATGTAATGTATGGATTTTTAAACATTTCTTCTAAATCTTTTGGT
AAAATTGCAACTGTTTCATTATTCATTACTTTTGCAACCCTATTTGGAAAGCTCCTGCTTGAAAC
CCTGTATGTAACAAAAGGATTTCCTTCATCTGTTTTTCCAAGAACTAAAAATCTACCAATATACA
TCAAACCACCTGATTTTAAAGCCTATTATTTTATTCAGATATCGCATCTAATGGGCAATAATCTT
TACATACTGCACAGTTTGTACACATTTCCGTATCAATTTCTGCCTTACCAAATGCAGAAATAGCT
CCAACCGGACAGAATGGAACACATCCTGCACAACCTACACATTTATCCAATATTTCCATGTTTTC
ACACAACTACATTATAAATTAAGGTTATTTATTTCTTGGTTTTTTAAATTTATTTCATATTCTTT
AAGTTCCGAAATGCTCATCCTACTTTTACATTCTTCTTTTGGAATCAAATATAATAATTTAGGGT
CGTCATAACCACCTTTCAAACCCAAAACAATCATATCTTCTTTTGGAGTTTCATCATAAAGTTTT

ATGACCTCATTTACAGGATAAGTCTTTTTATAAATTGGATACACGGATTTATCAAAATAATATAG
TTTTTCACCATTTTCAGATAATCCATTATCTTTTAAATAATTAATAAACTGCTTATGTTCCCATT
TTTCCCCAACATCTTCAGAAATTACAGGGATAGTTTCTTCAAGGGTTTCTTCCACAATTTCCAAT
TTTTTTGAAAAGTTTATTTTAACTTCTCCATTTTCGGAACTGCCTTTAATTTTAATCTTTTTAGT
CTTTAATTTTCTAATAATTCCATTTATTCCAGATTTAATTTTTTTATAGCCCAGATAAATTGCCC
CTAAAACTACTGCTAAGAATATTATGGGGATAATCAAAATTAATCCAATTATTATCAAAGGAAT
TAAAAATATCCAGAATATTGGGGATATTCTATTTAAACTGTATACTTTCATCATATTTACACCTA
AAACCAGGCTTCAAGACTTTTTTGGGATTTTTTATTGCCCAAAAGGTTGCTAATTTTTTCAATGT
TTGGAAGAATCCTATCTTTTGAAAAATCATTTTCTTCGATCAAAAACTCTGCTAACTCATCTTTT
TTAGGGGTTTTTAAAGTTATGTCGTAATCTGTTGTTACATTCGGTTCATCAAATATTTTTCTAAT
TTCAGAATAATTTTCAATTTCTGAAATGTAATTTTCCATTTTACCTTTTTTAACAGTATCGATCG
CTTTTTTCGGACCAAATCCTTTCAAACCGCCGGGATTGTAATCTGTTCCAATCAATATTGCAGCA
TCAATTAACTGATCACAAGAAATATTCAATCCAGAAAGAGTTTTTTCAAGTTCGATCAATTCAAT
GTCTTTATTTGATGAAGTTATGTTTTTTACAACGTAATCTGCACCATAAAGAATTGAATCGTAAT
CCTGACTTATAACACAAAACGCATTGTTTGCTTTTACAAGTTCTGCACACTGAGCTTCTCCTTCTG
AAGGGGCATTTACATATGGAATTCCCATCAATTCAAGGAGCTTTTTGGAGTTATCTACTGTTTTT
CTATCCAAAAAAATTTGCTCTTTTAGCATATTTTTGCATTTCTTCTAAATTATTTTGCTCTTTTGC
TTCCATGTAACTATCAAGAGCGCCTTCTCGAGATTTTCTTCGTTCTTCTTTTGTTTTTTCTTTTAA
TTCATGAGATTTACCATCAAAAACCCATATAGGGGTCATCCCATTTTCTAACATGTAAATTGTTT
TATAAAAAATACCGTTGTAAGTTGAAGTAATATCCCCATTTTTATTTTTTAAAGGACTTCCATCC
CTAAGTCGAATACTCGATAAAAACTGGTAAATTACGTTCATTGCATCGATTGCAACAGTTTTATT
TCTCAAAAATTTTAAAGAGATTTCTGTTTTGGGTATCAAATCTCCGAATTGAACTCCCATGTTAA
CACCAATTTACGCCAAAAAAAGTTAATTTGATTAATTTTTTTCTTTCATTTCTAAAACTACTTCT
GAAAATCTATTTATTACTTTTTCTCTTAAGCCTTCAACAAACTTCAAACTTCCAGCACATTCGTG
TCCCCCACCATCGAGGGATGCTTCTGGAATTTCATCGATAAACTGGGTTACTATATGATTTAAGT
TGAAATTGAATTTTTCGCTTACTGCATCAGTTGCACGAACTACTGCAAAATCAGGGCCATAAGAC
AAAGTAATTACTGGAGTACCAGGTTCATGCTGTTCAACAATTGAATCGTGTGCAAATCCGCAAGT
TTTTCCAGGGGCAGGGAATGTAAATTTGTGTGCATATTTTTCAACATCCAATGTGTTGTAAATTA
TTCCATTAGGCATTGTTTTGGTTTTTATTGCAGGACGTACAGCTTTCATCTGTCTGTCAACCATT
GCCATTGCTCTTTCGTAGAGTATTTTTATGAGCTTTTCATGTCTTGCAAAGTCTTTTTTGTTCAT
TCCGTAAATATCTTCTACAATACCCATTCCATTCATAAATTTTAAGTAAAATGCTTCAAAATCCA
TGCACTGTCCAATTTTTTCAACGTCTTCTCTGGAATATATTTTTCCAGATCCAAATTCTGCACTGC
ATTTTGTAAGTCTTTCAAGAGCTATTTTTATGTAAGCTTCTACAGCATCGCCTTTTGCGTGGTCC
CCAACAACTGCAATACCTGGTAAATGTTCAACAAGGTCAGTCACATCTGAATTTATCATTCTTGC
AACTTCTGTTGCTAAAGCACCAGCTGTAAGGTTGCTGTCCCCTCCAACAAGGTAAGGGTTTACGT
GTGCTTCAACAAATTCGTCAATTTCTACTTTTCCATCGATTACTTCACCAGGGAAGTGGTGGTCA
ACTACGACAACTTCAATATCGTATGCTTTCACCTGAGACACTGCAGGAATATCTTCATCAGTACT
TCCGTTATCTACGAGAACGATTAACGGCATTTTCTGTCCAAATCTTAAGTAATCCTCAATTGAAA
ATAACAGATCTTTTGTTACATCTTCTAACTCATAAAATGGAGCTTTTGAAGGACTTCTTCTAAAG
TAGTGCCACTGTGCACCAGAATCCATTGAAAATTTCTCCAAAATAGGAACTATTGCTTTTTCAAG
TGCAATTCCACCACAGTATCCATCAGTATCTGCGTGATGCCTGATAATTACAGGTCTTCCATCCA
AAACTGCTCTTCGAATCCTTTTTGCAACATCTGCCATTTTTGGCCTTAATTTTTCCAAGATGTCGC
TTTCAACTAAAAATTCAATGTCGCTTTCAGGTTCTGCTTTTTTGTCAAGTTCAACATCTATTTTG

TCTTTTACTTTTTGTTCTTCGTCGCCTTCGAGTTTTACAAGCTTCATCCTTTCTATCTGAAGTTTT
CCATCTCTTACAGATACTGAACCAACTACATCAATAATTGAACCCATTATTACATCAGGGTGCGT
CCTAACTCCTGCAGATTCAAATGCAGCAACCCATGCAACTTCTGAACCATCTGTAATGGTAAATA
CAGTTGGACCTGGAGTTTGAGCTGCCTGGAGAACTTCTCCTTGAACTCTAATAACTTTATCTTTC
ATATTCATTAATCCGGTATCGAATATTTCCTTGATATCGATTAATTCGTGTTCTTTTTCGAGTTT
TTCGAGCTTGTAGCCTACAATTGGAACATACCTTAAATCAAGTTCTCTTTTTTCGAGTTTTAATT
CAGAAACTTGGACAATAATTTCGTCTCCGATTTTAAAATCGCTTGGTCTTTTTCCAACAACTTCT
CTAAATCTTAAAAGACCTCTTGTTTTTCATTCAACTGGACAAATACCCCGTATTTTTCAGTTCT
GTTAATTTTTCCTTTGTAAAATTTGCCTTCTTCAAGGTCGTTCATGGTAAACATGTTGTCTAAAA
CATAAACTACTTTTTTATTATCGTTCTTTACGTCTTCTTTTTGTTTACAGTTTTCACAAATCTTT
AAATCCTTGTCTTCAGGGTATTTTCCAATTTCACAACCACAAGAGTCGCATTTTACAACTTTTCC
GGTTCCGTTGCAGTAATCACAATCTTCATATTCAGGAATTTTTCCAGATCCATTGCATGTCGGGC
ATGGAATTTCCTCGTCATCAAAATCGTATTTTGAATTTTTGGAAGCATTTTTAAAATGACTTTTT
GTTTCAAACTCTTCTAAATATCCTGTTCCTGCGCATTCCGGACATTCCGCGTATTTTACTACCTTT
TTTCCGGTTCCGCCACATATTTTACAGTTTTCTATCATAGTATCCTCTTAAATTAGATTGATAAA
TATCCAAATCATAAAAAAATTATCAAGTTCTTTAAAAAACAATGTTGATTTAATTATATATAAT
TAATTAAAAAATAATTGCGCTAAAAAAGCTTTTTCGGGTTTTTAACTGTTTTTAAAGGTTCTAAA
AATATATAATTGACATTTAACGAATAATCATGCTTAAATATTAACAATTTTCAATTTTTACTAAA
TAATATTAAATTTAAACCATAAAAATTAGTATTTTAGAAATTTAAACAATTTAAAACGGTTTTA
ATATGAAAGTTTAAAGTTAAATAAAATATTTTAATTTAAAGGTAATCGTAACCTTTTTCAAAGG
CCATTAAATTCTTTTTTTCCGTTCCTTTTGGGATACTGTCAAGAAGTGCTTTTTCAACTTCTTCTT
TTGAAACTAATCCTGCCAATTTCGTTAGTGCACCAAGCATTACAATATTTGCAACAATTCCAAGT
CCAACTTCCTTGTTTGCAATTTCAGTAAATGGAACTTTATATACTTCGTATTTTTCTTCGTAACC
TTTTGGAAGGTTTACTAAATCTCTATCAACGATTACTTTCGCATTTTCTTTTAAATCGTAACCGT
ATTTATCAAAAGCAGGTTGCGACATTGAAACGAGGATATCTGGCTGAATTACTTTTGGAAAATCT
ATTTCGCTTTCAGAAATTACAACTTCAGATTTACTGGCCCCCCCTCTTGCTTCAGGACCGTAGCTT
TGTGTCTGAACTGCCTCTTTTCCAGCGTAAAGGGATGCAGCACGTCCTAATATAACACCTGCTAA
AATTATTCCCTGACCACCAAAACCTGAAAATCTTATCTCTTTTCTCATGATATCACATTATTCCTG
TAATTTTTTAAGCTGTTCGACATATTCTGGTTTTTCGATATTTAAAAACTCCCCAACAACGATTT
TGTTTAAAAGTTCTTCTTTTTCCAGGTTTTCTGCTTTTCTAACCGTTACAGCGCTTTCTTTTAGAT
TTTTCATCATTTCAGAAGGTTTTCTTGAAACATTGAATCTTCCGTAGTATGTAGGGCACTGTGAA
ACTACTTCGATAAATGAAAATCCTTTCTTTTCCATTCCTTTTTTGATTGAATTTGCAAGCTGAAT
CGGGTTTGCAGTAGTCCATCTTGCAACATAAGTTGCCCCTGCTGCTTTTGCAAGTTCACATAAAT
CAAGCGGGTTTTCAGGGTTTCCGTAAGGTGCTGTTGTTGCTTTTTTCTCGTGCGGAGTTGTTGGC
GAACACTGTCCTCCAGTCATTCCGTAAATATTGTTGTTTATACATATTACAGTTATGTCGATGTT
TCTTCTACATCCGTGAATAAAGTGGTTTCCCCCAATTGCTGAGAGGTCACCATCTCCCGTAAATAC
GACAACTTTTTTGTCGTTTTGAATTACTTTGGTTCCAAGAGCAAAAGCTATTGGCCTTCCGTGAG
TTGTGTGAAGAGAATCACAAAAGAGATAGCCTGAAACTCTTGAAGAACATCCAATACCTGAAATT
GCGATGTAATCTTCGTTTTTGAGGGCTAATTTATCAAGAGCATTTGTAAAGCAGTTTAAAACAAT
ACCATTACCACAACCTGAACAAAATATGTGCGGTAACCGGTCCTTTCTCATGTATTTTAGCGAAG
GATGCATTCTGTCTCACCTAAAGTCTAAACCATGTTTAATAAGTCGAATGAATAAAAAAATTAAA
ATCCAAATATAATTTGTTTAAGTCATTCTTAATGTTTATCGTTATTTTTTATTCATATTTGCGGC
AGCATGTGTAAGCTCAAATACTGCTCCACCAAGGTCATTTCCAATGTCCTGTAATTCTGAAATAT

CTAACTCAATGTCTGCATTTTCAAAAGAAATTGCAACTGGCAAAAATTTGTACGCAACTTCTACA
ATATCGAATGGTTTACAAAACATTTCTATTGCAACAAGCCCTGAAAAAATTCCTTTTGATTCATC
CATTGATTTTGTAATTACTTTGTTTACAACAATGTCGGGATTTAATGATGCAAGTATTTTTTTAA
TAATTATTTCTTCGGATTTTCCGTCGCCTTCAAATACTGTTTTTATTTTTAAAAGGCCTTCTTCAC
TTTCCATTAAATAAATATCATCTTCATCATAGACGCCTTCTTTTAAAGCGTTTATATCTGCATCA
ATTTCATTTTTTACGTAGACGTTGAATGCAACACCGTACTTTTTAGCAAATTTTTTAAAAAAGTC
GATAATGTAGAAAATAGTGTTTCCCATTTCATTTCCAGAAATTTTCAATTTTGTAGGGGATACTA
CATCTAAATCTGCACCATATCTTAAACAGTATTTAATGTAATCCAAAATTGTTTCAAATTTTATT
TTAAGTTCCGCAATTTTAGTATAAAGTTCCCCTTCGTCGTCTGACTCGTTTGATAAAATTTCTGC
GTTGTAAGTTTCTTTTAATTCTACAATCAAATTCTCAAACTTTTCATCGACTATCTGTTTGTCAT
TTCCCTTTAATTCTTGGTATGATATAGTTTCAATCATAGTATCCTCTTCAAACTCGTTAGTACTT
ATATATTATCTTACTATTTTTATAACTTTATAAAATTTGAATAATATAAAACTGTTTTTAGATT
GATAAAATCGATTTAAGTAATTAAAAAATAAAAAAAAGAGTTTTATGAATATATTATTCTGTTA
AATTAAATCTTCCAATTACGAACTCTTTTTTAAGGGATGCTAAAAAGCTTCCTAACTTTGGCCCG
TAGTTTTTACCGAGAAGTATTTTGTATGATGCAACAAACGCTTCTTTTGGTGCAAGATTTAATTT
TGTTGCAGTTTCATACATTAATTCATGAATCGTGTTCGCATCAATTTCGATATTTTCTACTTCTT
TGGAAAATGCTTTTAACCATGCTTTTTGCTCTTCTGAGAGTGTTTCGTACTGTTCTTTTGCCTGG
TCAAGATCATTTATTTCTAAGTTTCACCATAGTCTGATGCCCATTTTTTAGACATGTAAAGCCT
TTTTTCAAGCCTTGATTTGTCGTAATCTGAAAATTCTTCTGGATTGTATCCATTTCTTCCCAAAA
TATCGAAAACTCGTTCCATATCAATTTTTTGGGTTTCTTTATCGAGTGCAATCTGAGCAATAATT
GAACAGAACCTGTATGCAACCTGTAGTGGTAATCTTTCAGGTATTTTTGGTGTAACAAGTTCATA
TAAACTTATTTTTTCATTTTCGTTGTCGTTTAATTCTTCAGTTTTTTGTTTTTCGATTAATTCAA
AGTACTTTCTTTCAAGTTCGTCGTAATCATCAACTAAGTTCGAAATTGCTTTTAAATCGAAATCT
ATGTGTTTTGTTGGTTTTCCTTTTAAAATTAAAAATCTTAAAACTTCAGGATGGCAGATTTCAGT
CCAGTCTTTTACTGCAAATACAACTCCTGATGAAGAAGACATCGGCATTGCCTTATCCCCAACTT
TTAATTGGATCCATTCGTAAACCATTTTTTCTGGTGCTTGGTAATTGAAAATCTGTCTTGCAATT
TTAATTCCTGTATCGTATGAACCGCCTGACGCTGCGTGGTCTTTTCCCATCGGTTCAGCAGTAACT
CCAAATATATTCCATCTTGCAGGCCAGTCAACTCTCCATGGAAGCTTTCCAATGCCGTTAAATGG
TTGAACCGTGTTTTCAAATCCGCATTTACAAACATAAGTTATTGTTTTGTCTTCAGAATTGTAAT
TGATAACTTTTGTTTCGTGCATTTTACCGCATTTTTCACATACAACGTTTAAAGGATACCAATCA
TCAGCAAGCGGTTCTTTTCTGTACGAATCGAGAAGTTCCTTGATTTTTAACCTGTTTTCAAGTGC
AGTTATAATTGCATCGTTGTACATTCCAGCTTTATAGCATTCATTTGCCCTGTGGGTAGTAATTT
CAACACCTAAATCTTCGAGGCTGTTTAAAAACGGCATCAAAAAGTGATCTGCATAGCTTTTACAG
CAACCTTGCGGGCATGGAATTTCACTTAAAGGCATTCCTATGTATTTTTCAAATTCTTTTGGTAA
GAATGGATATAATTTTCTTAAAGGGTCATAATCGTCCGCAATAAAAATTAATTTAGATTCTGCAC
CTTTTTTTAGCATGCCCTTATAAACTGCATCTGCAGTTAGTGTTTCCCTTGCATTTCCGATGTGAA
TGTGACCTGATGGCGTAATTCCACTTGATACCACATATTCCTCTGCATTTCTTTTTTTCATGATTT
TTTCCGATGTTGCATCAGCCCAGTGCATTGTGTTTCCTCCGGAGATTTCCATTATTTTAAATTAA
ACAATTAAAAAACAAAAATATTGAATTAAATCGAGATTCATAATTTTTTAGGTTATGGCTAAAA
CATTTTATATAATTATGTGTTGAAAAGTTAAAAAAATTTTTGAAAATAGTTTTTATATCCGTTA
ATCAAAATTTACAAATTAATATTCCTAAAATAAAAAAAGCCAAGGAAGGGATTTGAACCCCTCTG
AAGCGGATCTGCAGTCCGCTGCCTCTCCGGACTAGACTACCTTGGCACATTTCCTACGATTAGACG
CATGAATAAGAATTATGGTTCAACTATATAAACCTATTGATTTATTTTTCTTTTTTTCTTTTTTC

ATTCCATGGTATTAATTATTTATTATTCTTAACTGCATCATTACATTATAAATGTTACAGCTTAA
AAATATATGGTTAAGCCATGCAAAGGAATATATACAATAAATGTGTTGTTAAGTAGAGAGTCAT
TAAGGTAGAAACACGACGCCAAATTGGCAGATCATAGATTAAATCGAAATCTATATAAATGAAA
TTTCATAAGGTATAGTGTGATAGAAAACACGAGACCATAAAATAAAACACGGTTAATAAGATAA
TAAAATAAAAAAGTGTAATAGCCCAAATTTATGTCTATTGGCACGAGTACTGTGTGCAGAGATA
GTCTAGCCTGGGAAGGCGTACGGCTGGAAACCGTATGAGGCTTTGCCTCCCGGGGGTTCAAATCC
CCCTCTCTGCGCCATTTATTTTTTTATTACTATAATTATCAGTCAAACACGAAGATCTCTGGAGG
TGCAAGAGTGACTCAGACAGAATTCAAACACAGAATTAGAATATCAAAAACAGACCTTGAAGGT
AAAAATCCACTTGAATACGCACTACAGGACATGAAAGGTATCGGTAGAGCGATGGCTAGAGCAGT
AATTAGAGTTACTGAATTAGACCCTAAACAACAAGCTGGTTACTTAGCTGACGAAGACGTTTTAA
AAATAGAATCCGTATTAGAAGACCCTGCAACACATGGAATTCCTTCATGGATGTTTAACAGGAAA
AAAGATGTTTACTCAGGACTCGATAAACACCTAATCGAAACAGACCTCGTACTCACAGTACAAGA
AGATATCACCAACATGAAAAAGATCAGATGCTATAAAGGTATCAGACACGAATTAAGATTACCA
TGTAGGGGACAGAGAACCAGAGGTTCATTCAGAAAAGGAACTTCAATGGGAGTTAAGAGGAAAA
AATAATAAAATGATAAAAAGGTGAAAAAATGGGAGATCCAAGAAGATTGGGTAAAAAATACGAC
ACACCTAACCATCCATGGATTGGGGAAAGGATCCAGAAAGAAAAAGAAATTAGCCAAAAATATG
GGTTAGTTAACAAAAAAGAACTCTGGAAAATGGAAACCCAATTAAGGAACTACAGAAGACAAGC
AAGAAAATTAATCAGCGACACTACCACACAAGGTGGAAAAGAAGCTGTTCAATTATTCAATGTCT
TAAAAAGATACGCAATTTTAATTGAGGAAGAACCAACACTCGATCACGTTCTTTCATTAAACATT
GAAAGTATCTTAGAAAGAAGATTGCAGACCATTGTATTCAGAAAAGGTCTCGCTAAAACACCAAA
ACAAGCTAGACAGTTCATTGTTCACGGCCACATTGCAGTAAACGGCAAAAGAGTTACAGCTCCAG
CATACTTGATTTCAGTAGCTGAAAATGATGCGATAGAATACGTGTCCAACTCACCAATGGCTTCA
GAAAACCACCCAGAAAGAACTGCCGCAACAAGCGAAGAAAATCAATAAGGTGATTACATGAGTCA
AAAATGGGGATTAGTACACATTTATGCATCATACAACAATACAATTCTCCACGTTACTGATTTAA
CTGGAGCAGAAACAATCGCTAAAGTTTCAGGCGGTATGATTGTAAGAAACCAGAGAGATGAATC
ATCTCCATACGCTGCAATGCAAGCTGCATTCAAAATAGCAGACTTGATGAGAGATAAAGGAATTG
ATCAGGTACACGTAAAGGTCAGAGCTACAGGCGGCCAGAAATCCAAAAACCCAGGACCTGGTGCA
CAAGCTGCTATCAGAGCATTATCAAGAGCAGGAATCAGAATCGGAAGAATTGAAGATGCTACACC
AATTCCACACGATGGTACTACACCAAAAAGAAAGAACAGATAATTGGTGATTTTTTTGATAAAA
AATATCCAAAAAGAGGAAAAGAGAATAGGGGAAATCATGAAAATGGAATTAAAAGCCCCTTTAT
CTTTTTCCAGTGCATTGAGAAGAATAATGATTTCAGAAGTTCCTACATATGCTATTGAAAATGTA
TACTTTTATGAAAACACATCTTCAATGTACGATGAAGTTCTAGCTCACAGACTTGGATTAATTCC
AATAAAAGGAGTTCCGGTAAGTGGTGATGAAGTTATAGTACTTACAATATCCAAAGAAGGACCT
TGTACAGTATATTCATCAGATTTAAAATCAGAATCCGGTGAGCCTGCTTTTGAAAATATTCCTAT
AGTAAAATTAGCAGAAGACCAGAAACTTGAGTTAGAAACCGAAGCTCTTGTTGGCACAGGAAAA
ATTCATGCAAAATGGCAACCCTGCAATGCAAGTTACAAACAAATATCAAATGACGAAGTGGAATT
TAAAATTGAGTCATTCGGAAACATGGATGCTGAAGACATTATAAGATCTTCACTTGAAATTTTAA
AAAATAAAGCTGAAAAATTCTTATTAGAATTAGAAGGCCAAGAATTAAGTGACGAAAATTAATT
ACTCTTTCTTCCAAATTATTGGAAATTATGAATCAATATCATAAGGGTGAATTTATGAAAAAAA
TCATGTCAACAAACCCAAAAACTCCTGAATTGATTCAGAAGTTAAAAGAAGAATCATTCAAAAAC
AGCGCACCAATCTGGAAAGATGTTGCTAAAAAGTTAGCTAAACCAGCTAGAAAAAAAGTTGAAG
TAAACGTAAGCAAAATCAGCAGATATGCATCAGAAGGCGATGTATTATTAATTCCAGGCAAAGT
ACTCGGTGCTGGGTCATTAAAAGTAAACGTAACCGTTGCAGCATTTTCATTCTCAGAAACTGCGA

AATCAGCAATTGAAGCTGTAGGCGGAAAATGTTTAACAATCGAAGAACTTATTGCAGAAAACCCT
AAAGGTTCTAAAGTAACCATTATGGCGTAATTAAACACGAAGGTGGAATTATGGTTGTTGTAAA
CGCTGAAAATGCAGTTGTTGGAAGATTAGCTTCATACGTTGCTAAACTTGCACTAAGCGGTGAAG
AAATAACCGTTGTAAACGCTGAAAAAGCAATTATGACTGGAAACAAAGAATACATATTCCAAAA
ATATGTACAACTCAGAACAAGAAAAAGTATTTCAAACCCTAAAAAAATGGGTCCAAAATACCCTA
GAAGACCTGACGACATCGTAAGAAGAGTTATCAGAGGAATGCTTCCCTACAAAAAACCTAGAGGA
GTAGAAGCTTTCAAAAAAATTAAAATTGAAGTTGGAGTTCCTGAAGGGGTTTCTATCGATATTA
ACTTAGGTAGCGCACCAAATACAATCAAATATGTAACCGTTGGAGAATTAAGCCAATTCTTAGGT
GCTAAATTCTAATTGAGGTGAAAGTGTGAAAGTAGTCCACACAGTAGGTAAAAGAAGAACCGCA
ATTGCAAGAGCTACTGCAAAAGAAGGAAGCGGTAAAATCAGAATTAACAAAAAACCATTAGAAT
TAATGGAACCTAAATACATCAAAATGAAATTAATGGAACCTGTTATCTTAGCAGGCGAAGCTTTA
AGCAACATTGACGTAGATATTGATGTTAAAGGTGGCGGTATAGTAAGCCAAATGGATGCTACAA
GAACCGCTTTAGGTAAAGCTATTGTTGAATTTACTGGTAAAATGGATTTGAAAGAAAAATTCTT
AAGTTACGACAGAACATTACTTGTCAGTGACGCAAGAAGAACCGAACCACACAAACCAAGTAAGT
CCTCAAAAGGTCCAAGAGCTAAAAGACAGAAATCATACAGATAATTATTTTTCTTTCCCTTTACG
TTTTTTAGGTGGTAATAGTGATATTTCCAATCAGATGTTTTTCTTGTGGGAATGTAATTTCTGAA
GTTTATGAAGAATACAGAACTAGACTCAAAAATGGCGAAAACCCAGAAGAAATTTTAAACGATT
TAGAAATCAAAAAATACTGTTGTAGAAGAATGTTCGCATCCCACAGATTGGACAATGACAGAGA
ATTATTTGATGATATCGTCGAATATAAATAATTACCTTATTATTTACTTATTACCTATCTATTCA
GACGGGGCCGTGGGGTAGCCTGGCCATCCTGTGCGTCTGGGGGGCGTGCAACCCGAGTTCGAATC
TCGGCGGCCCCACTTTTTATTAATTTATAACGTCCTTTTGGGTGATTTTATGAAATTAACCAAAT
TTGAAACTGCGCGATTAATCGGTGCAAGATCCCTCCAAATTTCTGATGGTGCTCCACTCGCAATC
GAATCAGAAAAGACTTCATCGTTAGATCTAGCAGATGATGAAGTAAAACAGGGTAAACTCCCATT
ATGTGTTAAAAAACAGGCTAAAAATTAAATAACTATTTTAAATCTTTTGGGGGATTTTCGTGGA
AAACAGCACATCCATAAAAAGGATAACTGCAAAAAAGGTTTTTAAAGACTCAAAAATCCAAGTA
AGGATTGCTACATTAACCGATAAAGGAATTGGTTATGATACAATAAATGCAGAAAATCCTGACT
ATATAATTTCTGATGTCGAAAATGTAATTGCTCCCGAGCTTGTTGGATATCCTGCGTATGACCAA
GATATTATTGATTCAATAATATGTGACACCTCAGTAGACAGCCCGAAAGCTACAATGGGTACCTC
GTTAAGTGTTGCAAGAGCTGCTGCAAACAGTATTGAAATTCCATTGTTTAAGCATATTGGTGGAG
TATTATCGACAAATCTCCCGATTGTAGGATGTTCTTTAGTTTCAGACGGTTTAAACCAGCTTTTG
ACAATCCCTATGGCTGAATCGATTGGCGAAATGGTATATATTTACAATAAATTATTATCAGAACT
TTCTGAAGAATACGCTTTTAAAAATAATTATGGAGAATATATTTCTAAAAATTTATTCAACGAC
ATTGGAAAATTCAAAGGAATTGTCGCAAATATTTCAGAAGAAGAAGATATCGAAATATTAGTTG
GTGCAAAAATTTCTGAATTTAGCCCTGAAATCGAGGAACTCGATTACTTAGAAAGTTCTGAAATA
ATCGAATTCGACGGAATTTTATGTACCGAAAATATCGATGAAGAAGCAGATTTTTCAAAAGTAG
ACCCATATTTAATGGGAACACTTACAGAAATGAATTATTATATCAATTACACAATTGAAAAAGG
ATTAACTCCAACGATTTCTGGAAACAACACATCATTTCCCCACATAGCAATTGGATTTAAAGTTC
CGTTTTTGCGTGCAAATATTAACTCGAAAGTATTAAATGAAATCTGGAACATTGAACGGATTTTA
AACAGCCCCAATATTACAAGATTTTAATACTTTGAGTTTTAAAATATATTGTGTAAAAAAAATG
AGGGGATAAAATGAGTGTTGTAATTGATTATGATAAATGTAAAGGCCCAGAATGTGCCGAATGT
GTAAATACCTGCCCTGTAGAAGTTTTTGAAATTCAGGACGACAAAGTAGTTGTTGCAAAAGAATC
AGACTGTACATTATGTATGGTGTGCGTTGATGTATGCCCAACTGATGCAATAACAGTAAAAGAAG
ACTAAATATTAATTATTTTTTTAATTTAACTTATTCAGAATTTTCTTTTTTGACTCCATCCATTT

CATCGAGTACATCAAACAATTCTTCCCATGCATCCAAACTCAATTTTGCTTCATCTTCACTCATTT
TTTCAAGAGCATAACCTGTGTGGATTAATACGTAATCCCCAATTTCAACGCCCTCTATGAGTGCA
AGTTTTGCTTTTTGTTTTACACCCTTGTATTCTGCAAGAGCATATTTTTCACCATCTTCTTCGAAT
ATATCGATAACTACTGATGGAATTGCAAGACACATTTGATTTCACCCGAAATAACCTAAGATAAA
AGTGATATTTCTAAATCTGTAATTAATCTATTTATAAATATCGATAAAATTTTTAAAAAAAGAT
CATTAAAAAGGAAATATACTGCCTAAAGCTGCAAAAGGAAGTACTACAAGCCCGTGTTTTATAAT
TCTACTTATAATCAGTGCTGAAACGTAAATTTTAAATGGCATTTTAAATATCCCCGAAAGCCAGC
AGATCACTTCAAATGGAATTGGGGTGAAACTTATAACAATTATACCAAGAGGGCCCCATTTATTT
AAAAATTCTTCCGCACTTAAATATTTTTCTCTGTTAAATATTTTTAAAAATAGAGCTTCACCATA
TTTTGAAGCTATAAAATATGTTACAATTCCCCCTAACGTACTCCCTAACGTGGATACCAATATTA
CATACCGCCAGTCAAGCCCGAGAGCAAGGCCGCCCACCATAAAAACTTCGGTTGGAATCGGCTGG
AAAATTGGCTCTGAAAATCCAATTAAAAAAATAGCCAAAAGTCCATATGTACTAACCAAGATCCAT
CCCTACTGCAACAAAATCCATACTATACCATTTTTTAAAAAATATAATTTAATTTGTATTTAAAA
CTGCCAGTAAAAAGGATCTGTTGTTGGGTAAACTTCCACAAAATCTTTGTAAACTTCAATTCCTT
TTAAGTACTGTGCAAAATACGGTGCAAGCTCGGTTGCATTAGTCATTACAACTGCACCAACTGCT
TTTCCATTTTCGTAATAAATTCTATTCATTCCACGTTTTTCGGCTTTTTTAAAGTAATATCCTGT
TCCAGGACGGTTGTAAGACGTTTTAAAATCATTAGTCTGTTTTCCAACAACGGATATATTAATGG
GCATTCGAATTGTAGCAACCTCCACCCCATAATCCATTTTTTTTAAAGGAAGATTATTTATTTCA
TTAAAGATATTTTCTGCAACAATTCTGCCTTCCCGGTTAGCAATTGGGGTATATCCGCCCCTTACT
GAATCTCCACACGCATAAACTTTTGATTTTCCAGAAACTTGAAAATATTCGTTTGTGCCATATCT
TTTTTTACCCCCTATCGCCAAAATATTAAAATATTCGTCATTGTAAAGCATTTTCTTCATTTCTT
CTTCATTATTTGTTATTTTAAAATTTGAAAGGTCATTTATGATGTAATCCCTGACTTCATCATCT
TTGATTTTTTTTAAGAATTTTGACCTTGCATAAACTGTTACTTCACTTCCAAAAATAGAAAAAAT
CGAAGCATATTCTGCTGCAACAGTCCCACCACCAATTATTAAAATTTTTTTTGGTAATTTTTTTA
AATATGGAAGTTCTTTGTGAGTTGCACATTCTACTCCCAAATAATTTTTAGCATAATTTTGCCCT
GTCGCATAAACCACGTAATCGTAGTTTTTTTCATAATCTTCCTTAAATTCTTCAAATTTTACAAA
AATTCCCTTTTTTTCAAGCGTTTTTTTTGCATTTTCTCGCATCGATTTGTGAATTTTATAAACTT
TTTCATGAAGTTCGTCAAGTGAAATTACATCTTCTAAATCATGCTTTTTTCCAGTTATTGCATTT
AAATTATCAATTATGTCTGCAACTTCTTTCAAAGCATTTACATACCTGCAACCGTAATTTAGACA
GGTTCCCCCAAGTTTATCCATTTCATAGATTTCTACATCAAGCCCCCGTTTAGCGAGTGCGTGAGC
ACATGAAAGACCTGCAGGTCCTCCGCCTACAACTGCAATCATAATCCATCCCCTCATTTTTTAAAC
CAGTATATCTAATGTATTGAATGTATTATGTTTATATCTTACGAAATCACGGTGATACATTGAAC
ACTTTTGGGGATAATTTTAGAGTAACTACCTGGGGAGAAAGCCATGGAAAAGCATTAGGTGCAGT
AATTGATGGATGTCCTGCAAATCTACCCATTTTAGAAGAAGATATTCAAAATGAATTGAATAGA
AGAAGACCAGGATACAGTATATTCTCAACACCGCGAAAAGAAGAAGATAGGTTGGAAATTCTTTC
AGGGATATTTGAAGGAAAAACTACAGGAACTCCGATTTCAGGACTTGTATTTAACAAAGGACAA
AAATCAAAAGATTACAGTAAAATTAAAGATACCCCAAGACCAGGACATGCTGATTTAAACTACTT
TTTAAAGTACGGAAATTACGACTACAGGGGCGGCGGAAGAAGTAGCGGAAGAACCACAATTGGA
AACGTGATTGGTGGAGCAGTTGCTAAAAAATTAATTGAATTTACGCACAATATTAAAATAATCG
GGTATTCCACAAAAATTGGAAAAATAAAAGGAGATTTTGATTACTATAAAAACCCAGAATTCTT
TGAAAGTGATTCAAATATCGAAAAATTATTTGAAAAAACGGAAAACAATTCCCTCAGATGCCCTT
CAAAAAATAGCGATGAAATGAAAAATTATGTCCTCGATGCAATGGATAAAAAAGACAGCGTTGG
AGGAGTAATTGAAATAATTATAAAAGGAATTCCTCAAGGCGTTGGAAATCCAGTATTTAATAAA

TTGGAAGGAAAACTTGGAAGTGCAGTTATCGGAATTAATGCAGTGAAAGGATTTGAAATAGGTC
GTGGTTTTGAATCTTCAGAATTATATGGAAGCGAAATGAATGACGAATATTATATTAATGAAAA
TTCAATAAATGAAAAAACTAACAATGCTGGAGGAATTATCGGCGGAATTAGTACCGGTTCTCCAG
TAGTTCTTAGAGTTTCGATAAAACCCACCCCATCAATTTCAAAAATTCAAGACAGTGTAAACTTA
ATATCAAATGAAAATGAAAAAATCGAAATTGGCGGCCGACACGACCCGATAATCGTTCCAAGAGT
AATTCCAGTACTCGAATCAATGGCTGCAATAACTGTTGCAGATTTAATGATTTCAGGTGGATATA
TTAACCCTTGCAGACTATAAAACCAATAAAAAAACGTTTAATTCTTTTTAAAAATAATAAAAAA
AAGCGGATGCAGAACTCAATCCAAAAAATCCAACATCCTAACGTATAATCCCCATTAATCCAACG
ATAATAGCACCTGTATGTTATCTACAAGCAAGTGCACTATAAACTTTCAATATCATGATATATAT
AGATTATGTGAGGAATGGGGGATATTAACCGCAAAAAAGGCCCCTAACTCGTCAAATAGCGTTTA
ATGAATATATAGCTTTTGACAACCACTAAATCATCTTTAATAATCTAATTTTTCAATAGTAGATC
TATTTTATTCAAAAAAACCTTTTTTAAAGCCATTACATTATTTTTAAAACTACAGACTATCTTATA
TATTAAGTCGAATTATGTTAAATAACTCAGTAAAACACCTAAAGATGGATAATATGGAAAACAA
AATTCCCATGAGAAGAATGGTTCATAAAATTATATACGAATGCAATATTGTACTTTTAGTCGTTG
ACGCAAGGGATCCAGAAACTACCCGAAACAGAGCTCTTGAAGAGTACACTATTGAAAAAAATAAA
AAATTAATTTACGTAATTAACAAATCAGATTTAGTTCCTAAAAAAATCCTCGAAAAATGGAAAA
ACAAATTCAAATCTGAAAACCCTGATTCTTCCGTAGTTTTTGTCAGTGCCAAAGAAAAATTAGGT
ACCAAAATGTTAAGGGATGAAATTAAGACTTACTTAAATTCAAACAACATAAAATACGGTCAGG
TTGGAATCGTAGGGTATCCAAATGTTGGTAAATCCTCAATAATCAATGCATTAACTGGTAAAAAA
AGTGCAAGAAGTGGACTTACCGCAGGATTAACTGTTGGGGAACAGTGGGTAAAATTAACAAAAG
ATATCAAATTACTCGACTCTCCCGGGATAATTGAACCAAAAGATGAAGACGAACTTGTAATATCT
GGTGCATTGAGGTATGAAAAAGCGGATGACATAATTTCTCCTGCACTAAAGATATTAAACCGTAT
TCATACATTCGATAACACGATTTTGAAAGAATACTATGGTTTTGAAATTGGTGAAGAGATAAAT
ATAGAACTTCTTGAAAAAATTGGAACTAAATTAAATTTCCTTACAAAAGACGGTAAAATCGATA
TAGACAGAACTTCAAAAAGCATAATTAGAGAGTTCCAAAACGGAAAATTAAACTACCACAGAAT
GAACTTAAAAAAAATACGAACAAAAAAGAACTAAAAACATCGATTTTATAACCAAACATCTAAAA
GACTTCCCATTTATCAACGATGCCGATCAGATTATCTTGCATCTTGAAAATATAGCTGAACTTGG
AAAGTTAAACACGAGACCCGTTATTGGAATAAAAGAATTAGATGATGCTTTTGTAATTATCTCAT
TTTCTGAAAAATCAAGAGATACTGGCAGAAAAAAAGTTGAAGAACTTGCAAGAACGAGTGATAT
TGAATTGTATTCCCTTGGTGATGGCAGAATTGGAAAACACAGAATCTACGTTGGAGTAGGCGAAA
AAAGGTAAAATAAAATAAAATAAAATAAAATTTTAATTGGCCATTTTACATTCGTAAATTCCAA
TTACACCAATTTTTTTTAATTCGTTTAAAAGTTCTTCTCGATCTTCTTTTAAAAGTATTATTGCA
GAACCTCCTCCTCCAGCACCTGTTAATTTTCCCCCGTAACCGTATTTTTGACCTGCTTTTGCAACT
AAATCTAATTTTTCAGTTGAAATTCCAAATTTTTTAAGAAGTTCGTGGTTTTCATTCATCAATTT
TCCAAGTTCTGCTTTTTCAGTAACTTGCCTTACATTTTGAATTATTTTTCCAATTTCTTCAAAAA
TTTGCTCTTTTTCAGGGTGCTTTGAAACATCATGCACAAGTTCTGCAGTTTTTCGGGTTCTCTCTT
CAACATTTACAACCAAAAATTTGCAAGTTTTTATAAATTCTTCCAAATCTTTAGGCATTGGCTTG
TATTTTCCATTTATAATTTCAATCATACCGCCAAGTGAAATCGTTGCAGTGTCCGTTACACTTGC
TCTTCCCTGAACTTCTTTTTCAACAGAGTATGCAAGATTTATTGTTTCATCATTGTCTAACTCAA
TTCCATAAAAATTTGAAATTGAACGAATAACTGTCACAACAACCGATGCAGACGATCCCAATCCA
CAACTAAGTGGTATTTCAGAATATAGCTTTAATTTAAAAGGCTTTATCTCTTTTAAATTTTTAAA
TTCAGATAAATAAAATATTACAGATTTTACAGCACAAACCACATACTTTAATGCAGGGCTGAAAT
TTGAAATTTCTAAATTTTTAATTAATTCTGGAGTTATTTCAAAAATTTCGTTTAAATCAACAAGA

TCTACTGAAATTGTATCGGAATTTTCTCCAATTTCTCCAGTTGTTTTTAAATCAAGGGCCATTGA
AATTGCAGGATATCCGTCAACTACCGCGTGTTCTCCAAACAATATGATTTTTGAGGGGGTTTTTA
TCGTATTCATATAATCCACCTTAACCCCATCTTCTAAGCCTGTTTAATATTACTTTATCTCGGTTT
TCAACATTTCCGCTAAATTCTGCAGTTAAAAGGCCTTTTGTCCCAAAAGTTCCTTTTATTTTTCC
AACAATATCTTTTCCGTCAATTGATATTTCTTCACCAATCAATTTATCAGCTGCAACCTTTGATT
GAGCAAGTCCATCAATTACGGTTCTTCCCTTATCGATTTTAACTTTTCCTTCACGAAATACTTCTT
TTTTAATATTTAAGTCTTTTATTGGTTTAAATTCCTCGATTAAACCATGACCGCAAATCCTAAGT
GTTGTTGGTGGAAGATCAAGCCTTGTAATTAATACCCTATCTCCAACTTCTGCTAAAACCTTTTC
TTCGAGTTCAAATGCGCAGTAGCATTCATTTCCTGAAATTACTTCATTTAAAATTATATTTTCTT
CAGATTTACCAAATGTAACTTTTTAAACGGAACTGCAACTGCAGGAACTATAAGCATTCCTACA
TTCAAATGAACTTTCATTTTTGGAGTTAAATTGTACTTAAAAATATCTGAAATTTTTATTTTAGC
AACGATTTTATCAACAGTCTGTAATTTAGTATCTTTTGAAGTTAAAATACATCCTCGATAGATTT
GTTTTGATTCAACTCCTTGAATTGCCATTCCAACTCTGTCACCCGATTTTGCTTCCATAACACTTT
CTTTGAAACACTGTATGCTCCTAACTTTCGTAGACATATTTATTGGAAGAACTTTTAACTCATCC
CCAACTTTCACAATTCCTTTATTAATAGTTCCGGTTACAACAGTTCCGGCCCCTTTTATAGGAAA
TGCGTGATCAAGTGGCATTTTAAAATAGCTTTCAGTATTTCTAATAATTTCTGCATTATTCAACG
TAGTTATAATCAAATTTTTAAGTTCATCCACACCAGATCCAGTTTTTGCGGAAATTGGAATAATT
GAACTGTTTTTTAAATTTTGTGTTGATTGTAAAATGGATTTCATTATCATTTCAGTTCTTTTAAT
CTCTTCAGTATTTGCATTATCAGACTTTGTAATCACAACGATTGTTGGGATATTGAAATGATCGA
GAATTAACATGTGCTCCCCAGTCTGTGTTTTTGGCCCTTCTTTTGCGTCAACCACAATCAATGCAA
GATCGATTATATCTGCAGCACTTACAACCGCCCTAATTAAATCAGCGTGTCCTGGTGCGTCAACT
AATGTTATACGATAATTTTCAAGTTTAAATGCTGAAAAACCGATATCTATTGTAATTCCACGTTT
TTGAGATTCCGGCAGTTTGTCGTGTGCCGAAGTTGATGCAATTTCCGTTAAAACCTTAGAAAGTG
TCGTTTTTCCGTGATCGATGTGTCCAAAAATTCCAAGATTGATATTTTTAAAATCCATTTTCTTA
CCCTTATAACCGATTCCTATAAAATAATATACCTAGATTATATATGCACTTAGATATAAAAGGAT
AAATAAATTTTGAACTGATTTTTTGGTGATAGATATGATCCCCGATTCTTTAAAATACGAAATTT
TAGTTTTTGGTTGCGGAAATTTGATATTCGCAGATGATGGTTTTGGATATGAAGTAATTTCAAAG
CTCGAAAAAATGGAACTCCCAGAAAATGTTGGAATAATCGATGCTGGAACTGGGGCACCTTATTA
TTTAATGTCGTTAATGGATGAAGAGTGCAATACTAAAAAAATTATTATTGTTGATATCATTGAT
TTTCAGCTTCCACCCGGAACCCTAAAAATACTCTCAACTGAAGATCTCACAAAAATAGACAAATA
CACCTTTGATGCACATGATATGCCGCTTTCAGACTATTTAATAAAAGCAAAAGAATCTGGAATCG
ATGTAACAGTTATCGGATGTCAAGCAAAACGAGTTACGATGCCGGATATCGAAGTTGGATTGACT
GACGAAGTTAAAGCATCCCTTGACGATGCAGTAAAACTTGTTCTTGAAAAAATAAATGAATAAT
TTATTAATTTAAAAATTAAGAAATATTCCAAAAATACTTTTTTTTAAATTAAATTATATTAATC
AAACAAAAATTACTTTAACCCGTTTAAAACACAAATTTATTATTGAGTTTTTGAGGGGGAATTCA
TGAAAAGTTACAAAATAAAGCTTTTTTTCACAGGGGATGAGGATATTATAAAAACAGAAAATAT
TGAGGCTGAAAATCCATTCGAAGCAAAAGAAATACTTTTAAAAAAATACGGAATAATCAAACCA
AGAATTGAAGATAAATCTTTTGAAATTCTTTCAAAAATAGAAATATTGGATTATACAATATTGT
AAATTAAAAAAATATTCATATTATTCTTTTTTAGCTTTTATAATGTGCCTGATTCTTTTTCCAGT
CGTTATTTCTGATTTTTCAAGTTTTAGTCCAAGTTCCAAAATTTTATCTTCAACAAGTTTTCTTC
GCTCAAAAGGCTTTTCTGGAAACATTATTGCAACGATTCCACCATTATTTAAATGATCCCTTCCT
TCCTGAATTATCTTTTGAGAAAATACTTCTCCATAAGCCCCGCCACCGATTAATTCAACTTCACTT
GCAAAAGCTCCGCCAAAACTTCTTTTTGATGCAACTGAATTATCTGCATAGTATGGAGGATAGGA

TATTATTAAATCGAATTTTTTGTTTTTTAATTCTGGAATATCAAAAATTATTTTTCCCTTTGAAT
CTATCAAATTTATTTTTGAATTGAGATTATTTTTTGAAATATTATCTTTTGCAATTTTCAAATAA
TCAGAAACCGTGTCTGTTGCAGTGACATTACACCCAAAATATTTTGCAATCAATATTGAGATGAT
TCCGGAACCCGTTCCAATCTCGAGAACATCTTTAATGTCATCTTTTTCATCAAAAATATTTTTTA
TAAATAAGTACCGGTTAATGGGTGTCGGAATGAGTGCATCTTCATGAAAATCCATATCTAATCCA
AAAACATGGCCCAATATTGCAACATTATATTCTTTCATTGCTTCTTTATTCTTAAAATCGATTCT
TGGTTTTCCAGACTTGTACACAACAAAATCTTTCAACTTTTCATTTACCAAAATCGCATCTTCAA
TTTTCAGTCCGAGTTCTAAGGGTTCAGTTTTCATGGTTTTCACATTTTATTTTAAAATGATTGAT
GGTACAATAATATAAAAAAATATTTAACAAATACGGTTGAATGTTAACTTAAGTAATTTTGGAG
CGATATTATGCAATTTGTTCACATGGCGGATAATCACCTCGGATACCGCCAGTACAACCTTGATG
AGCGGGAAAACGATATTTACGAATCATTTTTAGAATGTATCGATAAAATTATAGAAATAAGGCC
GGATTTTGTAATCCATAGTGGCGACTTATTTGAAAGCCCGCAGCCTCCAGTAAATGCAATAAGAT
GTGCAATGGAAGGATTATTAAAATTAAAGGAAAAAAATATACCAATTTATTTAATTCACGGAAA
TCACGACATTCCAAAAAGCCAGCAGAAAGGAAAACCGTTTGGACTTTTGAAAAAAATACTTGGAA
ACAGTTTGTTAACATTTGGAAAAAATAAATCCCATGTTTTTAACAGCGAAGTATTCATTGGAGGA
ATTGAATACGTATCCCAAAACAAAATTCCAAAAACTTACGAAGACTTAGAAAAAATCAATTCTGA
TTCTAAAGATTACAAAAAAAAGATATTGCTTTTTCACCAGAGCGTAAGCCCATTTATCCCCCAAA
GCTTTGAAATGCAAATTACCGACTTTCCAGACGATTTTAACTACATTGCAGGAGGCCACATTCAT
CAAAGAGCATTAAAACCAATCAATGATGGAAATTCAGTATTTTCTTATGCTGGATCGACTGATAT
CATGTCAGTTAGCGAAGTTAACGATTACAAAAAAAATGGAAAAGGATTTTATTTGGGCGATTTA
AGCGGAGATTTCGATATTAATTCAATTCAAAAGATCGACGTTGAATGTAGGACTTTCTTGATCGA
TAAAAAAATTAAGAATGAAAATGATTACAAAAAAACAGTTGAAGAGCTCCAAAGTTTGCAGAAT
GAAAAGAAAAAGCCAGTTTTATACTGTGATATTGTTGAAAATTTATTTAATTCGTTTAACGATG
AAATTGCCGATTTAACACTCTACAAACGGATAAGCAGGATTGATGAAAACCTTGAAGAATCTTTA
ATCATTAATGAAAGCAGCATTGAAGAAATCTTTCAGGAATATATTAAAAATAAAGAAATGGATG
TAAATTTCGTTTATGGACTCTATAAAAAGCTTTTAGAAAACGATTGTGATTCATTGCTTTACGTA
AATGACTACTTTAAGGGTAATTACTAAGGGGGCGCATTTGATGATTATTAAAAATATCAAAATG
GAAAACTTCAGAAGCCACAGAAACACCTCGATAAATTTTAACAAAGGAATAACTTCGATAATCGG
GCAAAATGGAAGTGGAAAGTCGTCAATTTTTCAAGCAATGAATTTTGCACTTTTTGCTCCGCGTG
GAAACAATTTTAGGATTGAAAATTTAATGCAACAGGGCTCTGCATCTTTTTCTGTAGAATTAGAA
TTTGAAATGATGGGAAATACATATCTTGTAAAAAGAAAAAGGTTTCAAAATAAAACAGATGACA
AATTATACGTTAACGGAAAATTAAATGCGGAATCTGCATCTGAAATCAACAAAAAAATGGAAGA
AATATTAGAAATTGATAACAGCGTATTTTCAAATGCAATATACATAAAACAGGGCGAAATTGCA
AATTTAATTCAGATGACTCCAAGAGATAGAAAAGAGGTTATTGGAAAGCTTCTTGGAATTGAAA
AATATGAAAAAGCCTCTGAAAAAATGAATATTGTAAAAAAAAGCTATGAAGAAATGCTTTTTAA
ACTTGAAGGAGAACTAATCCAAGAACCTGAAATTTTAGAAAATCTTGAAAAATTAAAAAATGAA
GTTTCTGAATCTGAAATTTTAAAAGAAGAAATCTTAAAAAAAATATGAAAATTTAGAAAAACTAA
AACTTGAAAAAAATTCAGAATTAATTCAAATGGAAGAAAAATTTGCTGAAAATAACCACTTAAA
AGAAAATTTAAAAGATGTTATTTCTGAAATTAAAAATATACATTTAGAAATTCAAAATTTCAAA
AATTCACTAAGTTTAGTTGTTGAAGAATCAAAAACCTTATCTGAAAATGAAGAAAATTACAAAA
AATATTTAGAACTTGAATTAAACATTAAAGAATTGAATAATAAGTTAATAAGCCACAAATCAGA
CTACGAATCATATAATAAACTAAAAACGATTGAAGAATCGTTAATAAAAGAATTAAATAACTTA
GAAGAATCTTTAAAAGATAATAAAAAAAATCCTGATGAATTGAAAGAAAATTTAAAAGAAAATG

ATGAAAAAATTCTAATTTTAGATAAAATAAAAGAAAAAATCAAAGAATTAGAGTTTATTGAAAA
ACAGATTTATGAAATAAAAATCCACAAAAAAACAGTGGAAACTCTTTTTGATAGTGTTAAAATT
TACGATGACTCGATTAAAACTTTTGAAGAACTAAAATCTAAAAAAAATAGTTATGAAAATCTTT
TAAAAGAAAAATTTGACCTTGAAAAAAAACTTCAAAACGAAACTGAAGAAAAAACAAAATTAAT
TTCTGAATTAACTGATTTAAAAAAAATAGAAGAAAAAATAAATCTTGAAAATAAATTTAAAGAA
AAATATGAAGATTTATCTGAAAAAATAGATAAATTAAATGAAATCGTTTTGAAAAAAGAAAGTA
AAATTTCAGAAACTAAAAATTCTAAAGCAGAGCTTGAAAAAACCAAAGATTCATGTCACGTTTGT
CGGTCCAAAATAAGTGAAGAAAAAAAGCAGGAATTGCTTGAAAAATATAATTCTGAAATTGAAA
ATGAAAAATTATCAACTGAAAGTCTTAAAAAACAGCTTGAAATCATATTAAACAAAAAAGAGCA
GATCAAAGTTAAATTAAATGAAATTGATTCTTTCAAATTAAAATACGGGGAATTAAAAGAAAAA
AAGAATTATTCTTTAAAAGTTGAAGAATCGATAATCGAAACTACTGGAAAATTAAACGAATTAG
CTGAAAAAATTAGCGAATATTCTTCATTGAATGACGAAATAAGTTTAATTGAAAATAAATTAAA
AGATTTAGAAAATGATTATAAAAATTACAATTATTCCTCCCAATTTTTAACTAAAAATGACGAA
TCTGAATTTTTAAATAAAAAATTGGAACTTTCAAAAATTATTGGAGATTACGATTCTTCAAAAA
TTGAAAATGAAAAAAAATCCCTTGAAAATTTAAAAGATGAATTAAAAAACATCATTTACAATTT
AGAAAGAGAAATTAACTTAAAAACGGAATTAAAAAATATTCAAAACGATATTTCTTCAAAAATA
GGTATTGTTGAATGTTACGTTAAATGGGAAGCTGAAAAATCAGACTTTGAAAACAAATTATCAG
AATGTAAAGAAAATTATGAAAAATATATGGAAAGTCTGGCAGTTCTTAAAAACTATTCAAAAAC
GTATTCTGTTGAAATAAATAATTTAAACGAGTTTTTAAACCAAAAAATTGCTGAAAAACAGCAG
TTTTGTGAAAAACTCCTTGAAACACGACTAAAAATTGAAAAAAATATTCAGACTGTAAACTACA
ATCCAGAATTGCATGAAAACGCTAAAAGACTTTATGAAAATATATTAAATGAATTTAACGACAT
TTTAAGAACTTTAGAGAGAATAACTTCCGAATTAAAACTTAAAAATGAGAATATAATTTATTTA
AATAAAAAAATTCAAAATCTGGCAAATAAAAAAGAAGAAAGAAAAAAAAATTGAAGAATTTAAGG
AATATCTCGATAAAATAAAAAGAGAAATCTTTTCAAAAGATGGTTTCCAGAAATATTTAAGAGA
AAAATATATTCCATTAATTCAAAGGCACACAAACCAGATTTTTCAGGAATTTGAACTTCCATATT
CCCATATTCAACTAAAAGATGATTACAGCTTGATAGTTGACGGCCTTCCTGTTGAAACATTAAGC
GGTGGAGAACAGATTGCAGTTTCACTTGCATTAAGACTTGGAATTTCTAAAGCAGTTTGTAATAA
TATAGAATGCATTATTTTGGATGAACCAACAGCGTATCTTGACGAAGATAGGCGTAAAAACCTTT
TAAATATCTTTAAAAATATAAAAACAATAAACCAGATGGCAATAATTACACATCATCAAGAACT
CGAACAGATTGCAGATAACATTGTAAAAGTTAGAAAAATCGGCGAAAATTCAAAAGTATCTTTA
GAATAGGTTAAAACGATGTACCGAGTAATTCCAGACACCAATTTTTTAATTTATGCATTTAAACA
GGGCATAAATTTCGAATACGAATTAAATAGTGCTATTGATAGTGGTTACAAGATATGTATTATG
GATTGCGTTTTAAAAGAACTTGAAAAATTAAAATTAGAATTTAAAGGAAAAGAAAAGCTTTCTG
CGAATATTGCCCTAAAATATGCCAAAAAAATTTGAAATTATCGAATATTCAAACGGTAAATATGCA
GATGAAATGATTATAAATTATTCTAAGGAATATAAAGATGTAATAATCTGTACTAACGATAAAA
AACTAAAAAAAGACTTAATCGATATTGGAACCCCGATAATTCTTGTAAAACAACACAACCACTTT
GAACTTCAGGGTTATTTAAAATAAATATTAAAAAATCGGTTTAATATTTAATATAATCAAATTT
ACAATTTTTATTTTCCATTGTTACTTTTAAACCGATTTTAGCAGGAATTACTGGAATGTCAATAT
TTTTTGAAATGTATCTTGCTTCTGAATCAGGATCAGTCATTTTCATTCCCATGTGATACATTGTG
AGCATTTTTGGCTTTTTGTTCATCGAAGTTACTACTTCGATTATATCGTTTGAACACATGTGGCC
AGGCACTTTTTGACCCTTAACACGAATGACGTTTGCAATTAAAAAATCTACAGAATCAAAATCTT
CAATTAATGAATCAATATATTCTGAATCCGAAGTGTAACCTATTACTCCATCTCCTGTTTCCATT
TTAAACCCAATTGTTTCAGGGTCGTTGTGTTTTGTCTTTGTTGCAGTTATATTCCAGTTTTTTAT

ATTTAATTCGTCACAAGGTCCTAAAACGTGCCTTTCCCCAACTCTTGATTGGTGGTACGTTGAAA
TACCTTTTTCAAACCTGCTGTTTCCATTTAAAACAGAATTATTACCAATCAAAATTCCCTGTTTT
TTGGTCATGCCTTTAGTCATTGATTCAACCAGACACTCCGCATCAGTATAGTGATCTGGATGACA
GTGTGTTGCAAATATTGCATCTGTTTTCCAAGGATTAATCTCTAGTTGATTCATTCTAACTAATG
CTCCGGTTCCCGGATCAACGTGCATATTCATACTTTCGGAATGGATTCTAAAGCCCCCGGTTCCCT
TTACCTGAGTTATACTTTCCCATCGGCCCCCTCCGCCCCCAAGAAATACTATATCTAACAAGGTTG
ATCACTACTCGATTGTTTTAAATTCAACTTTATTGTAAATTCCATTAACTTCAACTATCTCGTAA
GGGTAAACAATTGCCCTATCGGAAACAGTTTCAGGCGTTATTTCTTCAACATAAATGATTAATAC
ACCATTTTCTAAATTTATCGAATTAATTTCTATAATATTGTCAGAACCTAATTTTTTGCCCATGT
AAACTTCGATAACGGTTTTATCCCCAGTTATACTGCTTTCATAATGTTTGTAAGAATCCCCGTAA
ATTCCAAATTCCAATATCTTAAAGGATAGTGATTCAATTTCAGAAGAATTGTTAGATTCAAAACT
ATTATTTAATTCTACTTCAATATTCGGATTTTCGGATGAAGTATTATTTAAAAATAAAGTTTCAT
TTGAATTATTTGGGTCTTTAATACTATTGTCGTCAATACATCCACTAAAAATAAGGGTTACCGAA
ATTAAAAAAATAAACGAGTATTTCATTTCCTCACTTCCCATATCTTCGTTCGCGTTTTTGATATT
CCCGGATGGACCTCAATAAGTCAACTTTTCTAAATGTCGGCCAGTATGTTTCACAAAAATATAGT
TCTGAATAACAGGACTGCCATGTTAAAAAATTACTTATCCGCTCTTCTCCTGACGTTCGAATAAT
TAAATCAGGATTTGGTGATGGAAGATTACTCGTGTATAGATTTTTAGATATTAATTCTACATTTA
TATCTTCAATTTCTATTTCACCGTTTTTTACCTGTTTTGCAATTTTTTTAACACTGTCAATTATTT
CCTGCTGTCCCCCATATGCAATTGCAAAGTTAACAAAATAATTTGAATAATCCTTTGTTTTATCT
TCTGCATAATTAATTGCTTTTTGAACATGCTTGGGAAGCATTTCTCTTATTCCAATAACGCTTAC
ACGTATTTTATCTTTATGAACATCGGGGTGATCTGCAATTTCAATAAATTCTCTTTCAAAAAGGT
CCATCAAAACTTTTACTTCTTTTTCTGGCCGGTTAAAATTTTCCAAAGAAAATGCATAAAGAGTA
ATGGTTGGTATTTTCAATGAAACACACCAGTTTATAACTTCGTGAACCTTTGAAGCACCCAAATT
ATGACCAATTTCAGGATTTTCGCCCAACTTTTTTGCAAGCCTTCGGTTACCATCCATTATAATTCC
AACGTGTTTTGGAGGGGGAAATTTGGAAATTTTTGATTCTAATTCCCGTTCGTAGAGTTTTAGTG
CCCCAAACTTGCTCAAATATTCATAACATTTAAATAACGGCAAAATACCACCAGAGTTTTTAAAA
ACCTTCTTTTAAAAATTTAACCGCATTGTAATATCTTTTCCAAGATCCGCTTCTGCTAGTATCTA
TATACTTAATATTTTCACTATTTAAAATAATAGCTCCAAAACCTTCCATATCACAAACAAGCGCA
AATGCCCTAAATAAAAGATTTCCAGAAATTCCGTCATCTGAAAGAATTATATTGCATCCTTCTTT
TAAGTATTCTTCAATTAAAATTCCTTTGTGTTCAATTTCTAAATTTTCAAAAATATCTAAAGATT
TAACATGTTTTAAAAGTTTTTCAGCGTCAATTATTGAATCGTCTATTTTTTTACTCCTTCCAATA
TCTGAAAGCCTTCCTGCAGACAAAATTCCAACTTTTGGAATCATTCCCATTGATTTTAAAAATTC
TGATGAATATTTAATTATATCTAGTTTTTCATTGAATCTTAGTTTAGGACTTTCAGAAATCTCAT
CTATACCAACTGGAGCGAGTATAAAAATTTTATTGGTAAAAGGGTTTTTTAAAATAGATGCTCTG
TAAAATTTCCCGATATTTTTCCTTAAAATTGGAATTAATTCGCTTGAAGAAAGTGAACCCCTAAC
TGCACCGCGAAATCGATTATTAATCAATCCATCAATCAAAACTTCGGGATTTTCTACTAATTCAA
CGTTTATTCCTTCTTTTTTTAAGTGTTCAACTGCAATTAGCACTTCATCTCGATTTTTACCGATAC
CGATAGCATACATGGTGTCCACCATACTTGAATAATATTAAAAAAATAATCTTTCACAAAATATA
ATAACCAACTATATATCGATTCAATAACATACAAAAACATGCAGTAGTAAAGAAAAGTAAAAAA
ATTGTTAAAGTAATTATTTCATTGCTAATTTCAAATCGTCAGCTTTAACAGTTTTTCTGCCTGCG
TGTTTAGCTAAACTAACTGCTTCTTTAGCTACGTCAACTGCTATTTCTTCTAATGCTTCTGCAAGC
ATTACTGCTGCATCTCTACTTACTCTTTCAGCACCTGCGGATTTTAGGATTCTCTCCATTGGTGCA
ACTGGAAGTTCTGCCATAGAATCACCTTCTTTTCTTATTACAATTTAAAATGGTATTCTTCCAGT

ATTTATAATTTTCGTAAAAAAACGTATTCTTTTTCCAATTATACGAGTTAATCAGTTTTAAATTC
AAAATATCTGGATGATTCATTTAAATTTATTTATTCGAATATCCAAGTAATAAATAATTTAAAA
AAAAGAATTATATTACTCAAATGACCCAATTACATGATATTCTAAAACAATTCCGTCATCTAACG
GATAATAATTCTTTAATTCCAGTTTTGTACATTCTTCAACGTTTTTAAACCCATTTCCATCAACA
TATGTGGGAGAACTTGCTCCTCCAAACACTTTTGGAGCGATATAAACCCTAACTTCGTTTATCAA
GTTTTCTTTAAACATTCCCCAGTTTAAAGTTCCGCCACCCTCAAGAAGAATTTTATTTATTCCCAT
TTTGTAAATTTCGTTCATAATTTTTCTTAAATCAACTTTTTGAACTCCTGCTTGCAATACGATAA
TTCCCATTTCGTTTAGTTTTCTGATCTTTTCTTCTTTTTCGTCTGAAATTGGCGTTGTAGTTGCAA
TTATGGTTTTTGCATCCTTATTTAATACTCTTGCAGTTAATGGAATTTTTAAATTACTGTCAACC
ACAATTCTTAAAGGATTGTCTTTTGGTGATGCATTGATTTTATGGACCGTGAGCCTTGGATCGTC
TTTTAAAACAGTTCCAATTCCGACCATTATTGCATCTACTTCTTTTCTTATCTCGTGAACCCGTTT
TAAATCGTTTTCACCAGAAATTCTTGAATCGTTTTCAATTGTGGCCAGTTTTCCATCCAAGGTCA
TTCCTACATTTGATATAACAAAGGGAAGCATAATATCACTTAAATCGCTGTTAAAATTAATTCTT
CTGGAAATATACGGATAAAGACTTTCAATATCCTTTAAATCACCGCGAGAATATTTAAGTATCTC
TTTTCTGATTAAATTATTTACTTTAATTCCCCTGCTTTGAAATTCTTCCATTATGTTTTTAGCAT
ACTCGCGTCCTGCTCGGTCAAAATCCGTTAAAAGTATAACTTCAGAAATGTTTTTTGCAATTAAA
TCGTCAGCTATCTGAAAAACAGGAGTATTTGACACAGTTATAAAATTAGCCCTTATTTTTAAGTT
TTTTAAAGATAATATATCATTTGGACCTTCTACCAATATGGGAATCCCGTTTTCAGCCAGTGATT
TTAGATCATCGATTATTAAAAGGAGTTTTTCTAAAGATTCCTGCTTATCCATCTTTTCACAAAAT
AAAAATAGTTAAATTTAAGGCATTGGTAATGGAAGTGATTTGTGCTTGTTTGTTGAAATTCTAA
GCATTAATTCTCCCACTTTTTCAGGAGGAATACTCAAAAATTCACATATATCGTCAATTTCTTTT
CCTTTTTCCATTAAATTTAAGAGTTTGTCGAGAGTTTCATAAGAAATTCCAAGTTCGTCTTCGTC
GGTTTGGCCTTCCCAAAGTCCTGCAGATGGCGATTTATCAATTATCTCTTTTGGAACTCCAACAT
AGCTTGCAAGCTGCCTTACTTCAGTTTTAAAAAGATTTCCAATTGTTAAAAAGTCGCTTCCAAGG
TCCCCGTATTTTGTACCGTACCCCATGTAAATTTCAGATTTATTGGAAGTTCCTGCAACAAGATA
ATTTTTCTTATTTGCATGATAATATACTTCAGTCATCCTGATCCTTGGTTTTAAATTTGAATCGA
CACGCTTATCAAATTCTTTTCCTTCAACATATCCTCCTGCACCAAATGCAACCAAAACAGGAGTT
ATGTCAAATACATCGTAATCAATTCCAAGAGATTCTGCAACGAGTTTTCCGTACTTTTCATCATC
AGGATTTGAATTTTTTTCAGGCATTATTACCCCAAAGACATTTTCTTTTCCAAGAGCCCTTACTG
AAAGATATGCTACAAGGGCACTGTCTATTCCTCCGCTAAGTCCTACTACAACGCCTTTTGCGTCTG
CATTTTCTACCTGTTCCTTTATAAAATCACAAATATTGTTTGCAAGTTCTTCTAACTGTTCGTTT
GTTCTTATCATGATAATACCTCTTAATTTAAATTAAGAAATCCCGGTACTTAAATATTAACTCGG
TTGCTTTTCGTATATCTGAACCTTGCATTTTTGCTTCATCAAGATATACTTTTGATAGATTAGTT
GTAATAACTCTTGGATTTGATTTCCACATTTCTGAATGAAGCATTAAATCATATGCCCTTGCTTC
ATTTTCTTTTAATCCATATCTTCGGTAAGGCCCATTATGTGAAATAAAAGTTGGATTTTCATCCA
CTTTTGTATTTAATACTTCGTTACAAAGTTCCGTTTTTGGAATCGGTTCAGCAGCACTTACAAAC
ACATCGTCAAGTTCTGCATCGACAATAAAATCTTTTGTCAGTAAAAAGTCGACTTCTTTTTCTTT
TGGGTGGCCCACGATAAAGCTTCCAGCAACTTTTACCCCACAATCTTTTGCAAGCTCGATTGCTTT
TAAATTTTTCTTTGAATTTGTTCCTTTTTTCATTGAATTTAAAAGTCTGTCACTTCCGCTTTCAA
TTCCGTAGAATACCCATCCAATTGAATAATTTTTAATGGCGTTTAAAACTTCTTCGTCAACGTAG
TCAACCCTCATGTCTGGAACGGATAGATTTTGCTTCCCAATTATGTTTGAAACATTTTCAATCAT
TTCTATAAATTTATTTTTATTTACCGTGTTTTTAAAGTTGTAGAGGCTTCCTGTACCTCCACTTA
TCGCAATTCTATTAACACCACTTTTTTTAAGCTCTTTTACTTCTTCAATTATTAATTCAATCGGT

TTGCTTCTAATATCTCGACCAAAAAATTCAGGAACCTGGCAAAACGTGCAGTGCCCAAGACATCC
CCTGTGAGTTTCAATATATACATTTGCACCCCGTACATTTTGAGCTGCGATGTCTTTTGGAACGT
AAATTTTTGAAAAATCTAAATCTGGCTTTGATTTTGGTTTATTAATTATTATTTCGCCATTTTCA
AGATATGCTAGTCCCGTTTTACTTCCATTAATAATTTCTGGAGTTGTAAGTTCGCCTTCCCCTAA
AATTACCCCGTCAACATTTAATTCGTTTAAGATAATCTCAGGATATGCTGAAACGGGTCCAGCAA
CATAAATTTTTATGTTTTTAGATTTTAAATAGTCAATTGTCTCTTTAATCACTGGATCGATAATA
TTTAGCGTAGAATAAAGGCTAAGAATAACTATATCGGATTTTAAAAAAAGTTTCTTATTAGTTA
TATCTTTGGTAAGTTTTATACTATGTTTATGCGTTTCTTTTAAAATTCCGCCGATCAACATGGCG
CCGTAGGTGTATATATCCTTTGAATAAATTGTAATTTTCATGATTATCTACCACATGTTTACAAA
AATAGGATAACTTCCAGATAAATCAAATCTCCTTTTTACCATGAGTATTGCATCTGCACCATCAT
CATAATAATTATAGAGGACTTTTCTATCAACGTAATCTCTTTTATAATAAAATTCTCTTGCTTTT
TTATTATCAAAACGAACTTCTAAAACGCAGTGTTTTGCGTGGCACTCGTTAAATAAAAAGTTTTC
AACAGATTTAATTAGTGCATTTCCAATTCCATAATTTTGGTAAGCTAAATCTACCGCAATCGATA
CGATATGGCCGTTTCCCCATTCCATGAGTGCAATGATGTATCCGACCACTTTTTCGTTTTCATCCT
CTGCAACTAAAAAACCATCTGGAAAAGAGGAATATATATGCATCATCAAAAATTCCGGATAATTT
TTATCAAAAGATTCCTTTTCTATTTCCATTACCCGTTTTAGGTCCTTTTCGCGGAATTTTCGTATA
TTTATAATCATTATATGTTCACCAGTCGGTTTCAGTCCGAAATCAGATTTGATATAATATATTTA
AATAAATTATTAAAATTTAACGCTAAACTAATAAAAAAACTAAAAAAATAATAAAAAATTCAAG
AAAATTCCAGATTTAAAAATTTAAAAATTTAAAAAAAGTTAATTAAAAGATTTTTATTTATTTT
CCAATTTTTCCAAAATCAGTTCTGCACATTTTTTTCCAGAAAGGTACATTCCACCAAAGATTGCA
CCCATCCTGTATCCTGCATGGTATGCATTTGCAGCCATTCCGCAGACATAAAGTCCGGGGAAAAT
TTCTCTCGTGTTTCTAGTGAGTGAATTTTCACCTTTATCTGCCCACATGGATTTTTCGCCCGGAAC
CTCAATTCCAAGGTCTTTGTTTTTCCTTGCAAGAGTATGAATTACTGAAGAATCGTGTCCAGTTG
AATCTATTACATATTTTGCACTTATTGTAATTGGATCTACGTGCAAACCTGCTTTTTCAATAGAG
TACGATTGAATAACCACTCCTGAAACTTTGTCTTCTTTTAAAATCAAATCTTCTACTACGATTCC
AGTTAATATTTTTGCACCGGCATCAATTGCTGCAACACCTAATTTTGCAGGAACTTCTACTGAAT
CTGCAGTGAATAATTCAGGTTCACCAATAACTTCATCTAATTTAATTCCAGCTTCTCTTAATATT
TCGTCCGCAGGTTTTTCTACGACAATGTTTGGAAAGCCCATTCCTCCGCCCCAGGTTCCGCCACCA
AAGGAGAGGTGTCTTTCTAAAACTACAGTTTTAACTCCGTTTTGAGCCAAATATTTTGCAGCTGT
AAGTCCGCTTGGACCTGCACCAACAATTACGACGTCAACGTCAATTAAATCCATCCACATATCGA
AAGTGGACTTCAATATCGATTTTGTTACTGCTACTTCATCTGCTCTGAGCTTTCCATCCATGTTTT
CACTCTTTGACTTTTTAAAGATTTATTGCTAGTTTTTTAGTTTTTAGATTTTGTTTTAGGAATTT
CAAAATCATATATCGATTTATCATTTTTGTTTATAGTGTTTAATACTATTTTAGTTTTTAGATTT
TGTTTTAGGAATTTCAAAATCATATATCGATTTATCATTTTTGTTTATAGTGTTTAATACTATTT
TAGTTTTTAGATTTTGTTTTAGGAATTTCAAAATCATATATCGATTTATCATTTTTGTTTATAGT
GTTCAATACTATTTAATATTGAAGAAATAACCATTTCCCGAATGTTTGGAACATCTTCTTCGATT
GACTCCAAAACTTCATGGGACTTTTTCCTTATTCCGTTCCCATTATGTCTGCTGTAAGGACATTCA
TCCTTATCTTTATAGTATTCAATATCGCATTCAACTAATGCCTTTACGATTTTTTCTTCTGAGAC
ATTAAGCATCGGTCTAATCATACAGCAGCTTTTAAGATTTAATTTAAAATCTTTAAAGTCTACAT
CTGTCTCATTGAAGTTTTTAATCGGCTCTAGTGCTTTTATTATATTTCCTTTAAATATATTTGCC
ATTATCGTGTCAGAATTGTCCTCTAACGTGTGACCTATTGCAATTTTTACGTTTGAATCGCTTTC
AAAGTTATTTTTTGCATAATTCGTTAAAACATGTCTTCTAACCATTGAACACGAAAAACACGGTG
AATATTCAATTCCCTTCGTGTTTTCTGACAATATTTTTGACATTTCAACAACATTTCCGTCATGT

GCAATTATTTTGTGGGGAATATTTAGATTTTTACAGTGCTCTGCAATTTTCTCTACGTTTTTGTT
GCTTTCTGTCCATGGCCTTATTCCATTTATATTTAAGTCAACTGTTACCGCATAAATTTGAACCCC
GTATTTTCTACGGTATGGTTCTAAAAGGTGCAGAAGTGCAAGGCTGTCCTTTCCACCACTTAATC
CGACCAGTATTTTGTCTTTTGGAGAAATTATATTATTTTTTGAAACGAATCTTCCCATTCTTTCA
GAAATTTCTTCAAAAACTCGAGAATAAACAACAGGAATTCCAAAATCGTTTTCAATTTTTCTCAT
TTTTTCCTTTGAAAGTCTCGCCAAACCTTTGTTTCCCACAATAATTTTATCGTTTCTTATCGTTAA
ATAGGAAGGATTGCAATATTTTTTTAACTCTTTTAAGTCGATCATAAATTCACCAGATAATAATC
ATCACATAAATATAACATGTTATATAGAGAATAACTGTTTGAAAGTATTTATTTATTTTCCACGT
TTTTGTTGCTTTCTGTCCATGGCCTTATTCCATTTATATTTAAGTCAACTGTTACCGCATAAATT
TGAACCCCGTATTTTCTACGGTATGGTTCTAAAAGGTGCAGAAGTGCAAGGCTGTCCTTTCCACC
ACTTAATCCGACCAGTATTTTGTCTTTTGGAGAAATTATATTATTTTTTGAAACGAATCTTCCCA
TTCTTTCAGAAATTTCTTCAAAAACTCGAGAATAAACAACAGGAATTCCAAAATCGTTTTCAATT
TTTCTCATTTTTTCCTTTGAAAGTCTCGCCAAACCTTTGTTTCCCACAATAATTTTATCGTTTCTT
ATCGTTAAATAGGAAGGATTGCAATATTTTTTTAACTCTTTTAAGTCGATCATAAATTCACCAGA
TAATAATCATCACATAAATATAACATGTTATATAGAGAATAACTGTTTGAAAGTATTTATTTAG
GATGTAGCTATTTTATTTAAATTCATTTATTATTGGTTTAAAAACGGTTTTAGGTTGAGATATTG
AAAAAATTTATTGTGAGATACGGAGAAATTGGAACAAAATCAAGACAGACCATGCACAGGTTTG
AAAAATTACTTGCAAAAAACATTTCCAAACTTGCAAAAAAACATGGGATTTCCGCAGAAGTTGAA
ATAATCCATACGCGATTGATTGTAGATGTTCCTGAAGAAAATTTCGAAAAGATGAAGGAACTAT
TAAAAAAAGTACCTGGAATTGTATCATTTAGCCCTTGTTACTACATAGATCCAGATATCGAACAG
ATCAAGAAAATTTCGTCTGAACTTTTTGAAAAAGAAATTGAGCGTTACGAAAATAAAGAAAACA
TTACTTTTAGAGTAAAAACACAAAGGCCTCAGAAAAAATTCCCGTTAACGTCACTTGAAGTAAAT
ATGGAAGTTGGGGGCCCAATTTCTGAAAAATACGGAATAAAAGTAGATTTAAAAAACCCTACAA
TTTCACTTGATATCGAAGTATTCAACGAATACGCTTACGTATTTGCAGAACGAATCGAAGGAATC
GGTGGAATCCCCGTTGGAACTCAAGGGCGAGTTATTGTTTTATTATCCGATGGAATCGATAGCCC
TGTTTCTGCGTACATGATGGCAAAAAGAGGCTGTAAACTTTTACTTTTACACATGAAAACGTCAG
ATGAGGGGCTTGAAAAAACCCAAAAACTTGTTGATGTTTTAAGTGACTTTGACCCGGAAGCTAAA
TTTGTATACGTAGACTTTAATGAAGAACTTGCAAAAATTAAAGCCGAACTCACAGAAATTAATA
GGGATAAATACACGTGTCTTTACTGCAAAAAATACATGCTTAAACTGGCTGAAAAACATGCTAAA
TGGCATAAATGTGATGCTATTGTAAATGGAGATAATATGGGGCAGGTTGCATCCCAAACTCTTAA
AAATTTGCGAGTAATTAGTGAAGGAATTGACTACCCGATTTTAAGACCGTTAATCGGACTTGACA
AAGTTGAAATTATGGATATTGCAAGAAAAATTGGAACTTTTGATATTTCAACCAAGTAAAGAAGT
AAGCTGTTTTGCAGTTCCAAAACACCCAATAACAAATGCAACACCTGACGAAATGGAAAAAATTG
AAGAACAGCTTTTAAACTTAAGAAATTGTAAAACTTGTAAAAATTGCTAAATTAGGAGGTATAT
TTTGAATTCTAATCCATTTGAATCGTATAGAGATATTAAAATAATGAACGAAAACCCGAAATACC
CTGTAAAAGGGGAAGGAATAACTCGGGAAAAAATAAAAAGTGAAAACGTCGAAATCGGAGAATA
TACATATTATTCGGGCTACTACGAAGGAAAAAACTTTAAAGACTGTATAATGTATCTTGATGAA
ATCGACAATTGCAAAGACATGGATAAATTAATTATTGGCAAATTCTGCAGCATTGCAACAGGCGT
TAAATTTATAATGGGCGGAAATCAGGGGCACAGATACGACTGGATTTCAACATATCCTTTAACAT
TAATTTCTGAAACTCCTGAAGATTTAAAATGCGAAAATGGAAAAGGATATTTGAAAAAAGGAGA
TACTAAAATTGAAAACGATGTATGGATTGGAGCAAATGTAACAATTATGCCAGGTGTTAAAATT
GGCAGTGGAGCAGTAATTGCAACGGGAAGTATTGTTACAAAAAATGTAGAACCATATACGATAG
TTGGCGGAAATCCTGCAAAAATAATTAAAAAAAGATTTTCCGATGAAAAAATAGAACTTCTTTT

GAAAATAAAATGGTGGGACTGGCCAATTGAAAAAATTAAATCACATATCAATACTTTGATGAGT
GATGACTTTGAAGAATTAAAAAAGATTTGTGAAGAATGAATTTTTATTTTAATTGATTAAATCT
TTTCAAATTTAGATTTTTCTAAAATTCCAAGATTTTTCAAGTAACCGCATACCTTACAAACTTCG
GTTGCAGATAAATCTCCACAGATTTTACACTCTCCAGTGTACCCAGGAAGTTCAATATGTTCGAG
AAGTCTTTCGTATCCCCTTACAATTGAATATTTTGAACCAGGATGGTTTTTTTCTAAGTTATCAG
TAATATCCGAGACTTCTGATCTAAATGAAAGGCATGAATACGGGCACGGTGACTTGTGATACTTT
AATTCGAGCATGTCTGCAAGTAAAAGTACTTCATCTTCTGGAATTTTTTCTAAAGGTTTTATTCT
TTTAACAAATTTAGGATGTTTCAGACTTTTTCCTAAGAATGCTAGTTTTTTTACATCTCCTTCGA
TGTAGTTCATCATCACAGCTTGCGCAACATCATCTAGATTATGTCCAATTGCAAGAAAATCGCAT
TTTTCTTCGATTGATACACGATTTAAGATTTTTCTTCTAATTACTCCGCAAAAAGAACACGGATT
CATTGTAAGATTTTTTTCTTTTGCGATTTTTACAATTTCATCAAGATTGGTTCCAAGGTAATCTT
TAAAGTGAACAATTTTGTATTCAATGCCATATTCTTCGCAAAATTTTACTGCTGCATCGATTCCA
TCAGTTCTGTAGCCTTCGATTCCCTCATTAACCATTATTGCAATTAAATTAGAATTTGGAATCTG
TTTATAATATTCATTTAAAAGATAAGCCATTACAAGGCTGTCTTTTCCACCGCTAAGGCCCATTG
CAACTTTTATGTTATTTTTAAGAACATCCCTTCCAAGAGTCTTTCTAACTTTTCTTTTAGTTTCT
TTTATGAAACACTCTTTACAATAATTATTTCCAGAGTGTTTTTGATGATATATAGAAGGTTTACC
ACATTTTCTGCATTTCATAGTAATCACGTAAATTTATCCACCTGAAACTGGAGGGTATATTCCAA
TTTCACTGTTTTTATTTATTATATCGTTTGAAGAAACAATTTCAAAGTCTTTTGAAACAATTATT
CGTCCATTTTCAAAATCTTCTTTTAGTTGTAAATTGTAGTCTTTTTCTAAAATTTCAATTAATTC
TAAAACAGATACTTCATTTAGATCTATTGATATTTTTAAACCTTCGCCAAAATCTTCCCTGCATT
TTGCAAAGAATTTTATTGTTAAATCCATTAAATCACCTAAAAAACAGTAAAAAATTAAATTTAA
TTGAAATATTAGATTTCAAATATTTTAATTGCATTTACAGGGCATATTTTTGCACAGTTTTTACA
TCCAATACATTCATCTTCGTCGAGCAGTATTTTAAATTCTTCGTCAACTGAAAGCGCACCAACAG
GGCAGTGAACAACACATGCACCACAATCAATGCATTTTTCATCATCTTTTTGGATGATTTTTGGG
ATATCTTCTGCTTCCCCGAATTTTTTCAGTATTTCAATTGATTTTTCGATCTGTTCTTCATCGCCA
GTTAATTCGAGAATTAAAAATCCTTCTCTTGGTTCCATTTTTGCCTTTAAAATATTTACCATTAC
ACCAGTTTCTAAAACCACGTTTGAAACTACAGGTTCTCTTACGTTGCTTCCAGATATCCAGTAAA
ATACTCTCTTTTTCACTTAAATCACCAGATTAAATTTTATGAAGTCCATTTCTCCCGATAAGTTT
TGAGATATCTTCAGCTGAAACCACTCCAAGAACCTTGTTGTTTGAATCGACAACAGGAAGTCCCG
AAATATTGTTTCTGCTCATTTTTCTTGCTGCCATATCTATAGTTTCATCTGGTGTTGCAGATACT
ATATATGTAGTCATGATTTCAGAAATTGAATGTTTATCCTGCGCCATTGCCTTTGCAATATCCCA
TGACGTAATTATTCCTGAAAGTTTGCCGTTTTCATCCACAATCGGCAGATGGTTTATATTATTTT
CAATTAAAACTCTTGAAGCTTCTGTAATTGATGTATTTAAGCTTCCAACAACTGCAGGTTTACTT
AATATATCTTTTACAAGTTTCATTTTTGCTTTCATTGGTTTTGGAGCTGCACTTTTTAAAGGTTC
AAGTCTTTGTGTTAACATAAATTCACTATTTGAAATCCAGTTTTTAAGTTCTTTTGAAATTTCCC
TTGAAACTTTATAACTTGAAACTGATGCAGATCTCATGCATTTATCTACTTTTTTGCCTTCGATT
TCGACTTCAATCGTTACTTTTCCAGATCTTGCATCTTTATAGTTTGTAACGCCTAATTCAGGTTT
ATCCCTTCTTGGAATTCCGTAATCCAATATGGGTATTGCAATATCTTCATCTTTTATTGCACAGG
TTTTTGCAATTTCTGCGTTTAATACTGGAATTGGAATTCCGATCCCCATGTAAAGCGTACTTCCG
TATTTTGGAATCGTTGCTCCTCTTAAATATTTAGTACTCATTTCTTTTAGGTCCCCTTGAACCATC
AAGGTTCCAAATCCGCCATTTGGACTGTGCTGGGTTCCCGAACCTGCAATATAACCTTGTGCACC
GCCTAAGAAGATTCTTGTACCCATTCCTAAGGTGTTATAAGTTTTTGTTTCTTTATTGAAATCGT
TTTGCAGTGGATTTAATTGACCTGCACCGGAATAATTTAGATTTCCAAATTCTGGGAGGAGTGCA

CCCATGTAAGTGTATATTTTTTCTTCAGTACTGTTCGTAGCACCATTGTAAGATTGGTAGCAGTT
TCTTGGATTTACTAAGATTGCTTGATTTAAATCATCAATCGTTATCGTTGTTTCTACTTTTTTTC
TAGGGTAGCAGTCAGTAGTGTATCCTTCCGCTACAAGTTCAATTTCTTTCCCAGCTACAAGATCT
TCAAGAACGTGGGAACCCCCATATGATATATCAATATCATCATCACTATTCGTTTGAGTAGCCCC
TAAATAAGCATCAACTGCTGCAATTCCTGAATATGCTTCCACACCATTCAAATATGTTTTACACA
TTTTTATTGGCGGATCAGAGTGTCCAAAATTTAAAAATGCTCCGCTTGAACACATTGCTCCAAAT
GTACCTGTCGTAACTACATCTATTTCAACTGCCGCTTTTTCTGCACCCAGTTCGTCGACTATATCG
ATCATCTCTTCTGCAGTGACTACAACTGCATCGCCATTTCGTATCTTCTCGTTAATTTCGTGAACG
GTTTTCAAAATATCAACCTTAATTTTATATATTTAAAGCAGTGAATGTATAATATCTGGACAATA
AATCCATTGGGTTAGTTCTATAGTATATATACCGTATATTATATAACCATATCATTATTTTGTTA
TATACCGTATATTATATAACCATATCATTATTTTGTTAACTTGAAACAATTTAGAAATGTATTTA
AAGATAGAAAATAATATATATGAGTTACCACTATTTTTCATGATATTAATATTATACGCATAAG
TTTATTTTTATAACTATTTTAAACAAATGGAGGCCAAATTGTGAAAATATCGTCACATGAAATGG
TTCCGACACATGAGATTATTCCAAGAGAAGAAATACCCCTTTTACTCGAAAAATACAGTATAAAA
ATGCAGCAATTCCCAAAATTGCTCGATACCGATCCCCTCGTGCTTGAAATTGGAGCAACACCTGG
AGACGTTGTAAAAATAACCAGAATGAGCCCTACCGCAGGCGAATCTACCTATTACAGATTAGTAA
TAGCAACTTCACTCTAACTGCTGGTTAATACCTGCTAAATATTTTTAAAATTTAAAATAAGCTAT
TAGTCCTTTATCTAAAATCTATGGGGGAAAATCATGGAATCCCGTGTCGTTGTTGATGCATTTTT
TAAAGAAAACAGTTTAGTAAAACACCACATCGATTCCTACGATGATTTTGTTAAGAATAAAATTC
AACGAATCATTGATGAGGTAACTGGTGTTGAAACAGAAATTAAAGGAGGATACAAAGTATCCTT
TGGAAACGTGAGAGTTACAAAACCAATAAATAAGGAAGCTGACGGTTCTGTAAAACCAATAACA
CCTATGGAAGCAAGAATCAGAAACCTTGCATACTCTGCACCGCTTTACCTTGAAATGATACCAAC
AGTTGGTGAAGGTGAAGAAGAAAAAGTATTATCCCCTGTTGAAGTTTACATTGGAGAACTTCCAG
TAATGCTTGGTTCAGAAATCTGCCATTTGTGCGGAAAAATCTGCAGAAGAATTGATAAAATACGGC
GAAGATGTACAAGATCCTTTAGGTTATTTTATTGTTAATGGTTCAGAAAAAGCAGTTGTTGCTCA
AGAAGACCTTATTCCTAACAGAATTATATGTGAAAAGGTAGAAAAAAACAACAAAGTTGTAGAT
ATTGCAAAAGTATTTTCAACCAGACATGGATTTAGGGCATTATGTACTGTTGAAAGAAATCCAGA
TGGATTATTAAATGTTTCATTTCCAGGAATGCCAAGTACAATTCCACTAGTTATATTAATGAGAG
CACTTGGTGCAGAATCAGATAGGGAAATTATCGAATTAATTTCAGACGAACCAACAGTTGTCATG
CAGTTAATTGCAAATTTACAGGAAGCAAGAGAAGAGCATGCGATAAACACGACTGAAGATGCAC
TCGAACATATTGGAAAAAGAGTAGCTCCAGGACAGCCTAAAGAATACAAGCTAAAAAGAGCAGA
AACAATTCTTTGTAACTACCTATTGCCACACATGGGCGTAGAATCTGAAAAATTAGGCGCTAAAT
GTAAATATCTTGGAAGAATGGCTAAAAACTCAATCGAACTCTACTTAGGATTAAGGGTTGAAGA
TGACAAGGACCACTATTCAAACAAAAGATTAAAATTAGCTGGAGACTTAATGGAAGACTTATTC
AGACATTCATTCAACCAGTTAATTAAAGATATCAAATACCAGCTCGAAAGACAGGCAATTAGAAA
CAAAGAGCCTTCAATTCAAGCTGCAATTAGAAGCGATGTTTTAACTGAAAGAATGAGACATGCAA
TGGCTACTGGAAACTGGGTAGGTGGAAGAACAGGGGTATCCCAGCTTCTCGATAGAACCAGTTAC
CTTGCAACAGTATCACAATTAAGAAGAGTTGTATCTCCACTTTCAAGATCACAGCCTCACTTCGA
AGCTAGGGACTTGCACGCAACCCAATGGGGTAAAATATGTCCATCCGAAACACCAGAAGGTCCAA
ACTGTGGTCTGGTAAAAAACCTTGCTGTAATGTGCAAAGTAACTACTGACGAAGGCGACGATAGC
GTAATCGAATTATTAAAAGAACTCGGGCTCACAAAGGAAAACTAAATTTAATATTGATCGAATT
TAGTCAATTAAGAAGGTGGATACCTTGGAAAAGCAGACAAATGTATATGTTAACGGGAAATTGA
TCGATACCACACAAGACCCTGAAACACTTGTTAAAAACTTAAGGGAAAACAGAAGAAGTGGAAA

ATTATCGCCAAGCACATCAATTTCGTTCAATGAAGAAAGTAACGATGTCCACATTTCAACCGATG
GTGGAAGGGCTGTAAGACCATTAATTGTTGTTGAAAACGGTGCTTCAAAAGTAACAGAAGAAAT
ACTCGAAAAAATGGAATCTAAAGAATTAACTTTTTTAGATTTAGTAAATTCAGGAATTATTGAA
TATTTAGATGCTGAAGAAGAAGAAAACGCAAGAATTGCGATGTATGAAGATGAAATAACCGAGG
AAAACACACACGTTGAAATTGACCCTCTCGTTATTTTAGGTATCGGTGCAGGTGTTGCCCCATAT
CCAGAACACAATTCAGCTCCAAGGATTACGATGGCTGCTGCGATGAGTAAACAGTCACTCGGAAT
TCCTATGGCAAACATAAAATGGAGAATGGATACAAGAGGACACTTATTACACTACCCGCAAGTTC
CACTTGTAAGAACAAAACACCAGGAAATCTTAGGATTTGATAAAAGACCTGCTGGACAGAACTTC
GTTGTTGCAGTTATGAGTTACGAAGGCTACAACATGGAGGATGCGTTCATTATCAACAAAGCATC
TCTTGAAAGAGGTCTTGGTAGAAGTTCATTCTTTAGAAGCTACGAAAGCTTTGAAAAAAGATACC
CTGGAGGACAGCTCGATAAATTCGAAGTTCCTGAAAAAGGAGTTAGAGGTTACAGAGCTGAAGA
AGCCTACAGAAACTTAGGTGACGATGGTTTAATCGACCTTGAATCCTCAGTAAAATCGGGAGATG
TAATTTTAGGAAAAACGTCACCTCCAAGATTTTTAGAAGAACAAGAAATCACCCTTCAAACAAAA
TCACAGAGAAGAGACACTTCAGTTACGATTCGACACGGTGAAGAAGGAATCGTTGACCTCGTAAT
TTTAAGTGAAACAAAAGAAGGAAACAGACTTGGAAAAGTTAGAGTTAGAGATTTAAGAATTCCT
GAGTTTGGTGACAAATTTGCATCAAGACACGGTCAGAAAGGGGTTATTGGACTCATTGTTCCACA
AGAAGACTTACCCTATACTGAAGATGGAGTAATTCCTGATTTAATTATCAACCCGCACGCAATCC
CTTCAAGGATGACTATCGGACAGGTTCTTGAAATGATTGGTGGTAAAGTTGGTTCATTGGAATGC
AGAAGAATCGACGGAACAATTTTCAGCGGCGAAAGCGAATGGAAATTAAGAGAAGGCCTTGAAA
AATACGGCTTTGAACACCATGCAAAAGAAGCAATGTATGATGGAAAATCCGGTAGAAAATTAGA
GTGCGATATTTTCGTTGGAGTTGCATACTACCAGAAATTACACCACTTAGTTGCTGGAAAAATCC
ACGCAAGAAGCAGAGGACCAATCCAAGTTCTTACAAGACAGCCTACAGAAGGTAGAGCAAGAGAA
GGAGGTCTTAGGTTTGGGGAAATGGAAAGAGATGTGTTAGTTGCCCACGGTGCTGCAATGCTCCT
TAAGGAAAGACTTCTCGATGAATCAGACCCTCACGAAGACTACGTCTGTGCTAAATGTGGTGAAA
TTGCAATCTTCGATTACAAGAGAGGCATGAAATTCTGTCCAGTTTGTGGTGAATCAGAAGACATT
CAAGATAACAGAAAGATTCCACCTGTAAAAATTGCTTATGCATTCAAATTATTGTTGGATGAGCT
TAAAAGTATGGGAATCGATCCAAAATTAGAATTAAAAGACAGAACATAACTTAAATTATATTAT
TTTTTATTAAAAAGGTCTAAAACCACGGTGAAACATATGGATAGATTTGATGTTCCAAAGGAAA
TCGGAGATATTACATTTGGATTGCTCTCCCCTGAACAGATAAGAACCATGTCAGTTGCAAAAATC
GTTACCGCAGACACATACGATGACGACGGATACCCAATTGACGGTGGATTGATGGATACTAGACT
CGGTGTTATTGACCCTGGATTAGTATGTAAAAGCTGCAGTGGAAGAGTAGGAACTTGTACAGGAC
ACTTCGGACATATTGAACTTTCAAAACCAGTAGTCCATATTGGATTTGCAAAAGATATTTACAAA
ATTTTAAAAGCAGTGTGCCCACACTGTGGAAAAGTTACAATTACAGAGATCAAAAGGGATGAAT
ACTTAGAGAAAATGGCCAAACTCAAAGAGGATGGTGGAGACCCTTGGACACTCTGTGATGATTTA
TTAAAAGAAGCTGCAAAAGGTAGCGTCTGTCCTTCATGCGGTGAAGTAAAAGCAGATGTAAAAT
ACGATAAACCTACTGCATACCACCAGTTAGACGGAAAAGCTCAAAAACAGTTAACATCCTCAGAA
GTTAGGGAAATTTTAGAAAAAATTCCTGCTGAAGACTGTAAACTACTAGGAATTAATGCAAAAG
TTGCAAGACCAGAATTTATGGTTTTAACAGTTTTACCAGTTCCTCCAGTAACAGTTAGGCCTTCA
ATCACACTTGAAAGTGGGGAAAGAAGTGAAGACGACTTAACGCACAAATTAGTTGATATTATCA
GAATTAACCAAAGGTTAGAAGAAAATATCAACGGTGGAGCTCCAAACTTGATTATTGAAGATTT
ATGGGACTTATTGCAGTACCACATAAACACGTACTTTGATAATGAAGCTCCAGGAATTCCTCCAG
CAAGACACAGAAGTGGAAGGCCCCTTAGGACACTTGCTCAAAGGTTAAAAGGTAAAGAAGGAAG
GTTCAGACACAACTTAGCAGGTAAAAGGGTAAACTTCTCTGCAAGGACTGTTATTTCACCTGATC

CAAGACTCAGTATTAATGAAGTTGGTATTCCAAAATTAATCGCAAAAGAATTAACGGTTCCTGAA
AAAGTTACCCCTTACAACATTGAAAAAGTAAGGGCACTCTTAAGAAACGGAGCTGACAACCACCC
AGGTGTAAACTACGTGGTTAAGAAAGTAAGAACTAAAGATGGAAAAGAAGAAGAATACAAAATA
AAAATTACCGATGCCAACAAAGACATGTGGGTTGAGAATATAACCGACGGAATGGTTATCGAAA
GACACCTTTCAGACGGAGACATTGTATTGTACAACAGGCAGCCTTCATTACACAGAATGTCAATT
ATGGCACACAAAGTAAAAGTTCTTCCATACCGAACATTTAGACATAACTTGTGTGTATGTCCACC
ATACAACGCGGATTTCGATGGTGACGAGATGAACGTTCACGTCCCACAATCCGAAGAAGCAAGAG
CTGAAGCTGAAAAATTAATGCTCGTTGAAAAACACATTGTTTCCCCAAGATTTGGTGGTCCAATC
ATCGGTGCAATTCACGACTTTATTTCAGGAGCGTACGTCCTTACAAGTTCATTATTTACAAAAGA
CGAAGCTTTGACATTATTAAAAAGCTCTGGACTTAATGTTGAACTTGGAGAACCAGACGTTATTG
AAAATAACATAGAATATTACAGCGGAAAATCATTATTCAGTAAAACACTTCCAGAAGGATTGAG
TTTACAGTACAGAGCAAAAATCTGTAAAAAATGTGACACGTGTACTAAAGAAGAATGTGAACAC
GATGCTTACGTTGTAATTAGAAACGGTAAACTCTTACGTGGAGTAATCGATAAAAACGGATTTGG
TTCAGAAGCTGGAATTGTATTAAACACGCTTGTAAAAGACTTCGGTTCAGAAGATGCAAGATTAT
TCCTCGATGCTGCAACCAAAATGTCAATAAAAGCAATGGTTTTAAAAGGATTTACAACAGGTATT
GACGATGAAGACATCCCTGCTGAAGCAATTCAGGAAATTCAGGATTTATTGAACAAAGCTGAACA
GGACGTTGAAGATATCGTTGAAAAATACGAAAACGGAACTTTAGAATCTTTACCAGGAAGGGGT
GTTGAAGAATCCAGAGAAGCATACATCATGCAGATTTTGGGTAAAGCAAGGGACCAAGCCGGTAA
CGTTGCTGAAAAATACTTAAGTAAAGAAAACCACGCAGCGTTAATGGCAAGAACTGGTGCGAGAG
GTTCACTCCTTAACATTACGATGATGGCTGCAAGTGTTGGTCAGCAGTCCGTTAGGGGTGGTAGG
GTCTTTAGAGGTTACAGAGAAAGAACCCTTCCGCACTTTGGAAAAGGAAGTTTAGATGCAAAATC
ACACGGATTCGTTAGAAGCTGTTACAAAAAAGGATTGGCCCCAACAGAATATTTCTTCCACGCGA
TGGGTGGTAGAGAAGGTCTTGTGGATCAGGCGGTCAGGACTGCACAATCTGGTTACATGCAGAGA
AGACTCGTAAACGCTTTACAGGATATTAGGGCAGAATACGATGGAACAGTAAGAGATTCAAGAG
GACTCGTTGTTCAGTTTGAATACGGGGAAGACTTGGTAGACCCTGCAAAAGCAGACCACGGAAAA
GGTGTAGATTTAGATAAAATATTCACAAAAGTTAATTCAAGATATGAAAATTAATTATTTTCAA
TTTCTTAAATTAAAACTATTTCTAGGTGAAACACATGCAAATGGCCGATTTAGAAAAGAAATTG
GAAAATTCTGTCCTACCACCATTGTTAAAAAGGGAACTTTCAGAAAAAATCTTAAGGGAAGAAA
TAAGTGAAGAATACCTCGTTGATGAAATCATAAGCGAAACTACAAGAGCATACGAAAGAACTCTC
GTAGAACCTGGAGAAGCTGTTGGTGTTGTTGCTGCACAGTCAATTGGTGAACCTGGTACGCAGAT
GACAATGAGAACGTTCCACTATGCGGGGGTAGCGGAGTTAAACGTTACCCTTGGTCTTCCAAGAA
TGATTGAAATTGTAGATGCAAGAAAAGAACCATCTACCCCAACAATGACAATTTACTTAAATGAT
GAATTTAAGGGCGATAGGGAAAAAGCTGCAACGGTTGCAAAAAACATTGAAAGTACCAATGTTG
AATCTGTTTCAGAAGATATCAGCGTAGATTTGGTAAATGAATGTATTACAATTATATTAAATAA
CCAGCAGCTCGAAAGCAGGGGACTAACTGTTCCTGATGTAATCGATGCAATAAAATCTAAAATGA
AACTTAAAATTGAAGATCATGAAAATGTTCTCAATTTAAAAATTAAAACTCCTTCATTAAAAGC
GTTAAGAAAAAGACTTCCAAAAGTTAGGGCAATCCACTTGAAAGGTGTTCAAAACATCAAAAGG
GTTATCATCAGAAAAGAAGTTGATGAATACATCCTCTACAGTGAAGGTTCAAACATCAAAGAAGT
CTTTGATATCGAAGGTGTTGATACAACAAAAACAACCACAAACAACATTGTTGAAATTCAGGATG
TTTTAGGTATCGAAGCTGCAAGAAATGCAATCATATACGAAATGGATGCAACCCTTGGAAATCAG
GGTCTTACGGTAGACAAAAGACACTTGATGATGGTAGCAGACATTATGTGTACAGATGGAGTTGT
AAAACCAATCGGTAGACATGGTATTGGTGGTGAAAAAGCATCAGTTCTTGCAAGAGCAGCATTTG
AGGAAACTGTAAAACACCTTTACTCTGCATCCATGAGAGGATATGTTGATGAACTCGGTGGAGTT

GTTGAAAACATTATTGTTGGAAAACCAATCTCAATGGGTACAGGCTGTATCGACATATGCATCGA
CAAGAGTTACGAAGAAGGAAAAGAATTATAATTTAATACTTTTTTAATTATTTTTAAGATAATC
GTTTTTTTATACCGTTTATAACACTCACTTCGATCCGTTAATTATAATTAGAGAGTTCTTGGTTG
ATGCAAAGGTATAAAAATAACATGTAACTATTAACTAGTTCGTATATTCATAGGGACATTAATT
TCCAAATTGCATAGGAAAACCCTCTGGAGAATATAATGAGAAGGAGGTCTAATATGGACATAAA
CAGAGCTATAAGAGTAGCAGTGGACACTGGAAATGTAGTCTTAGGTACAAAACAGGCTATTAAA
AACATAAAACACGGCGAAGGCCAGTTAGTAATTGTTGCAGACAACTGCGCAAAAGACGTTAGAGA
AGACATTTTTTATTACACACAACTTTCAGAAACCCCGGTTTACACACACCAGGCAACTTCAATTG
AACTTGGAGCTATTTGTGGAAAACCATTCCCTGTTTCCGCATTGTTGGTTTTAGAACCTGGTAAC
TCGGCTATTTTAAACGTTAACAATGAAGAATAACCGCGGGTGATGGTTGATGAGAATTAAACTTA
ATACAGAAGATATCATGAGAATCAGTCTCTTCGAAAAGATGACTGGTGCTGACGTGATTGATAGC
ACTTCTGATGATGAAAAAATTGTTTTCGTTGTTAAAGAAGGTGACATTGGAGCTGCGATTGGAA
AAGGCGGCGAAAATGTGAGAAATGCAACTGAAAAATTCGGTAAAAAAATAGACTTAATCGAATA
TTCAGAAGATGTAAAACAGTTCATTAAAAACATCTTTGCTCCAATTGAAGTAGAAGATGTATGG
ACTAAAAAAATTTGGTGACGATTTAGTAGTTTACATAAGAGTTCATCCAAGATTTAGGAGAGCTA
TTATTGGAGACAAAGGAAAAAACATTGATAGAGCAGTAGACATCGCAGGAAGGCTTGCAGGTGT
TAAAAATATCAAAGTCGTTGCTGGTTTAAGAAAAGATAACGATAAAAAACCAAAACAGGAAGAA
GTACCTGAAAAAGCAGCAGAATCATCTAAAAATGTTGAAGCTGAAGAAAACCAATAAGGTGATT
TTATGGCAGGAAGTAAATCTCCAAAAGGAGAATTCGCAGGAAGAAAATTACTTTTAAAAAGAAA
AGCAAGCAGATGGCACCACTACAAATACGTTAACAAAGCTCTAAGCTTAAAATTAAAAGCTGACC
CATTAGAAGGAGCTCCAATGGGGAGAGGAATTGTTATTGAGAAAGTAGGTCTCGAAGCAAAACA
GCCAAACTCTGCTATCAGAAAATGTGTTAGAGTTCAGTTAATCAAAAACGGTAGACAAGTTACAG
CATTTGCACCTGGAAACCACGCTATCAACTTCATCGACGAACACGACGAAGTTGTTATCGAAGGT
ATCGGTGGTCCATCAGGACAGGCTAAAGGGGATATTCCTGGAGTTAGATACAAAGTTGTATTAGT
CGGTAAAAACTCAATCAGAGAGCTTGTCAGAGGAAGACAGGAAAAAGTTAAAAGATAAATTAAG
ATCTAAAAAAGAAAAAAGGGTGAAAAATTGGAAATCAAATTATTTGGTAAATGGGACAGTGAAA
CTGTCGCAGTTAAAGACCCAAGTTTAAAATCCTACATTAGTGTAGCTCCAGTTTTAGTTCCACAT
ACTGCAGGAAGAAACTCAAAGAAAAGTTTTGATAAATCAAAAATGAACATTGTAGAAAGATTAG
CTAACAAATTAATGGCTAACCAAAACAACACCGGTAAAAAACACGAAACTTTAGCAATTGTCGAA
GAAGCTTTGTCAATCATTGAAAACAGGACTAAAGAAAACCCTGTTCAAGTTTTAGTTGATGCTTT
AGAAAATTCAGGACCTAGGGAAGAAACAACAAGAATCTCCTACGGGGGAATTGCATTCTTACAGT
CAGTTGATGTTTCACCATCAAGAAGATTGGACACAGCTTTTAGAAACATTGCACTTGGTGCTTCA
GAAAGCGCACACAAAAATAAAAAAACCGTTGCTCAATGCTTAGCAGACGAACTCATCTTTGCTTC
AAAAGCTGACATGCAAAAAAGCTTTGCTGTTAAGAAAAAAGAAGAAAAAGAGAGAGTTGCACAA
TCTGCAAGATAAATTAAATACTTTTTATATCTCTTTATACATGACCCTATGTCATAAAAGGGTAA
CAATAGTTCGTATATTATGAACTTCTCAAAAAATAAAAGAGTGAGATTATGGGAAGAAGAGCAA
AAATGGTTGAAAAGGTCAAAACCTTAATGGAAACACATGAACAAATTAGAAACATGGGAATCTG
TGCTCACATTGACCACGGTAAAACCACATTATCAGACAACTTGCTTGCAGGAGCAGGTATGATCT
CCAAAGAATTAGCTGGAGACCAGCTTGCACTTGACTTCGATGAAGAAGAAGCTGCAAGAGGTATC
ACAATCTACGCTGCAAACGTTTCAATGGTACACGAATACAGCGGAAAAGAGTACTTAATCAACTT
AATCGATACCCCTGGTCACGTTGACTTTGGTGGTGACGTTACAAGAGCAATGAGAGCAATTGATG
GTGCTGTTGTTGTATGTTGTGCTGTTGAAGGTGTTATGCCACAAACGGAAACTGTTTTAAGACAG
GCATTAAAAGAAAAAGTTAAACCTGTTTTATTCATTAACAAAGTTGACAGATTGATCAATGAAT

TGAAATTAACCCCTGAAGAATTACAGGGAAGATTCATGAAAATCATTGCAGAAGTTAACAAGTT
AATCGAAAAAATGGCTCCTGAAGAATTCAAAAAAGAATGGTTATGTGATGTTGCAAATGGAAAA
GTTGCTTTTGGTTCAGCATACAACAACTGGGCTATTTCAGTTCCATACATGCAAAGATCAGGAAT
TTCATTCAAAGATATTATTGACTACTGTGAACAAGAAAAACAAGGAGAATTAGCTGACAAAGCT
CCGCTCCACGAAGTTGTTCTTGACATGTCAATCAAACACTTACCAAACCCACTCCAGGCTCAAAAA
TACAGAATTCCAAATATCTGGAAAGGAGATGCTGAATCAGCTATTGGTAAATCAATGGTTGAAT
GTAACCCTAACGGACCTCTCGCAGGTGTTGTTACAAAAATTATCGTTGATAAACACGCTGGTGCA
ATTTCTGCATGCAGATTGTTTTCAGGAAGAATCAAACAAGGAGACGACCTCTACCTCGTTGGTTC
AAAACAGAAAGCAAGAGCTCAGCAAGTTTCAATCTTCATGGGTGCTGAAAGAGTTCAGGTACCAA
GCATTTCAGCAGGTAACATCTGTGCTTTAACAGGATTAAGAGAAGCTACTGCTGGTGAAACAGTA
TGCAGCCCTTCAGAAATCTTAGAACCTGGATTTGAATCCTTAAGCCACACCTCTGAACCAGTTAT
CACTGTTGCAATCGAAGCTAAAAACACTAAAGATTTACCAAAATTAATTGAAATCTTAAGACAGA
TTGCAAGAGAAGACAACACAGTAAGAGTAGAAATCAACGAAGAAACCGGTGAACACTTAATCAG
CGGTATGGGTGAGCTCCACATCGAAGTTATCACTAACACCAAAATCGGAAGAGACGGTGGAATTG
AAGTAGATGTCGGTGAACCAATCGTTGTTTACAGAGAAACAATTATGGGCACATCCCCAGAAATT
GAAGGAAAATCACCAAACAAACACAACAAACTCTACATGATTGCAGAACCTATGGAAGAGTCAGT
TTACGCTGCATACGTAGAAGGTAAACTCCACGATGAAGATTACAAAAAGAAAACTACCGCTGATG
GTGAAGCAAGGTTAGTTGAAGCAGGTCTTGAAAAAGATCAGGCGAAAAAAGTAATGTCAATTTA
CAATGGAAACATGATTGTAAACATGACAAGAGGTATCGTGCAACTCGATGAAGCTAGAGAATTA
ATTATCGAAGGTTTCAAAGAAGGTGTAAGAAACGGTCCACTCGCAGCTGAAAAAGTTCAGGGCGT
AAAAATCAGATTAGTGGATGCAACATTCCACGAAGATGCAATCCACAGAGGACCTGCTCAAATTA
TCCCTGCTGTAAGATTCGGTGTAAGGGATGCAGTTGCTCAAGCAAAACCAGTTCTCCTCGAACCT
ATGCAAAGTGTTTACATTAACACCCCGCAAGACTACATGGGAGACGGTATGAAAGAAATCAACAA
CAGAAGAGGACAGATTCTCGACATGGAACAAGAAGGAGACATGTCAATTATCAAATCTTCAGTGC
CTGTTGCTGAAATGTTCGGATTTGCTGGTGCAATTAGGGGAGCTACCCAAGGTAGATGTTTGTGG
AGCGTTGAGTTCTCAGGATTCGAAAGAGTTCCAAACGAGCTTCAACCTAAGATTGCTAAACAAAT
TAGGGACAGAAAAGGGTTAAAATCTGAATAAACAAGTACCATAAAATATATATACCCTTTTTCAC
TATCATATTACAAAAATCGCTAAAAATATACTCTTATGGAGGAATGATTATGGCAAAAGAAAAA
CCAATTCTTAACGTTGCATTTATCGGACACGTAGATGCTGGTAAATCAACAACAGTAGGAAGATT
ATTATTAGATGGTGGAGCTATCGACCCACAGTTAATCGTAAGATTAAGAAAAGAAGCTGAAGAA
AAAGGTAAAGCAGGATTCGAATTTGCTTACGTTATGGACGGTTTAAAGGAAGAAAGAGAAAGAG
GGGTTACAATTGACGTAGCTCACAAAAAATTCCCTACCGCAAAATACGAAGTTACAATCGTAGAC
TGCCCAGGCCACAGAGACTTCATTAAAAACATGATTACCGGTGCTTCACAAGCTGATGCAGCTGT
TTTAGTTGTAAACGTTGACGATGCTAAATCAGGCATTCAACCCCAAACAAGAGAACACGTATTCT
TATCAAGAACACTCGGTATCACACAATTAGCTGTTGCAATCAACAAAATGGACACAGTTAACTTC
TCAGAAGCTGACTACAACGAAATGAAAAAGATGTTAGGCGACCAGTTGTTAAAAATGGTTGGAT
TCAACCCTGACAACATCAACTTCATACCAGTTGCTTCACTTCTTGGTGACAACGTATTCAAAAAAA
TCAGAAAACACACCATGGTACAACGGACCTACACTCGCACAAGTTATCGATGGTTTCCAACCACC
TGAAAAACCAACAACCTTACCATTAAGATTACCTATCCAAGATGTTTACTCAATCACTGGTGTAG
GTACAGTTCCAGTAGGAAGAGTTGAAACAGGTATCATCAAACCAGGAGACAAAGTTGTCTTCGAA
CCTGCAGGAGCAGTAGGAGAAATCAAAACTGTGGAAATGCACCACGAACAATTACCAAGCGCTGA
ACCTGGAGACAACATCGGTTTCAACGTAAGAGGCGTTGGTAAAAAAGACATTAAAAGAGGAGAC
GTTTTAGGACACACAACAAACCCTCCAACAGTTGCTGCAGACTTCACAGCACAAATCGTTGTTTT

ACAACACCCATCAGTTATGACAGTAGGTTACACACCTGTATTCCACGCACACACAGCACAGATTG
CATGTACATTCATGGAATTACAGAAAAAATTAAACCCTGCAACTGGTGAAGTTTTAGAAGAAAA
CCCAGACTTCTTAAAAGCTGGTGACGCTGCAATCGTTAAATTAATGCCTACAAAACCATTGGTTA
TGGAAAGCGTTAAAGAAATCCCACAACTCGGTAGATTCGCAATCAGAGATATGGGTATGACAGTT
GCTGCAGGTATGGCAATCCAAGTTACAGCTAAAAACAAATAATTTAGCTTAACTTTTTCTTCTTA
TTAAACACGGCAAAAGAGGCATATTATGCAAAAAGCAAGAATAAAATTATCAAGCACACAACAC
ACAGAATTAGACGGTGTATGCGACCAGATTAAAGCTATCGCTGAAAAAACTGGTGTAGACATGTC
AGGCCCAATTCCACTACCAACAAAAGCGTTAAAAGTTACAACAAGAAAATCAACAGATGGTGAAG
GTTCCTCATCATTCGACAGATGGACAATGAGAGTTCACAAAAGAGTTATCGACATTGAAGCTGAC
GAAAGAACCATGAAACACATCATGAAAGTTAGAATTCCTGACACAGTTCAAATCGAAATTGAATT
GAGAAACTAATTTTAATTCGTTTAAATCGCAAAAGCCAGACGTATTGCAATCTGAGAGTTGAGGA
TTGCAATAAAACTTCTTTTTTTCACATTATCAAATCAAAACATTTTTATAAATATATTAATATAA
TAAATAATACCTCATTCCAGAGTGTGTGAGGTTTAATTTTATTTTCGACCATTGTTCAGAGTAAT
TATGGTATAATATTTTTTATTTTTTTGAAAAGTTATTTAATCCCAATTTACATAATATAACGCAT
AGATTTTTTTATTCGAACATTGAACGGTGGTTTAAGTTGGTATTTAAGGCATACGACATCAGGGG
AATTTATGAAAAAGATTTAGATGATAATTTTGCTTATTCTTTAGGAAAACTCGTTGGAAAAAAA
TATGAAAGAATAATAGTTGGAAATGATATTCGAATCGGTTCCAAAAAACTCTTAAAACCATTTA
TTTACGGGATACTTGAAAATTCTAAAGTTTACTATGCTGGAGAAATCTCAACCCCGCTGATGTAC
TTTGGAACCCTTAAAAAATTCGATCTTGGAGTAATTTTAACTGCATCCCACAATCCAAAAGAATA
CACGGGATTTAAAATGTGCGACATTGATGCAATTCCACTCTCTCCAGTAGATGAAATAAAACCGG
ATTTTGAAATGTTTGAACTTTCAGAAGAACAAAAGGAAGAAATTGAAAATTTGGATTTAGAAAG
ATTAAATGTTGATATTTTAAGTGAATATTTGAATTTTTTTACCCAAAAACTTGAAAAAACGAAT
CGAAAAATTGCAGTAGATTTTGCAAACGGTGCAACTACTAATGCAGAAAAACAGGTTTTAGAAA
CGGTTTTAGAAAATAAGATATTTATTAACGATTTTCCGGACGGAAATTTCCCTGCACACGAACCA
GACACTCTTAAAAAAGAATGTTTGATTGATATAATAAATACTGTAAAATCCAATTCCTGCGAATT
TGGCCTTATTTTTGATGGGGATGGCGACAGAATTGGAATGGTTGATGAAAAGGGAGAAATTTTA
GCAGGAGATATATTAACTGCGATAATTTCAAATGAAATTTTAAACGAAGTCAAAGGAAAAATTG
TATACGATTTAAGATGCAGTAAAGTAGTTCCTGAAACAATTTCAAAAAATGGGGGAACTCCAGT
AAAAACAAGAGTTGGACACTACTTCATAAAAAAATTGATGCATGAAATAGATGCAGAGTTTGCA
GGGGAGTTTAGCAATCACTTCTACTTCAAATCAACAGGTTACTTTGAAAGCCCGTTATTAGCATT
GAATTATATCCTAAAAGCAATTGAAAGAGAAGGAAAACCACTCTCAGAAATTTCGAAAAATTAC
AAAAAATATTTCCACAGTGGTGAAATAAACTTTAAAGTAGCTGACCAGAAAACGTCGATTGAAT
CTATTGAAAAAAAATATGGAAATATTTGTAAAATCGAAAAATTAGATGGAATTTCATTATATTG
TAATGATTTCTGGTTTAATGTTAGATCATCAAATACTGAACCACTTTTGAGGTTAAATTTAGAAG
CAGAGGATGAAAAAACAATGAATGAGAAATTAACAGAAATAAAAGAGATAATTAATTCTTAAAT
TTTAAATAAATTTGAAAATTCCAAAAATACTTTTTTTAAAAAATAAAAATTAGATTTAATTTAG
GATATAACTTTGTCAATCATGTTGAGTTCAAGCGCTAATTTTATTTCTTTTGCAATTCTCTGACC
CATACTTAGTGGTTCTCCAGTGTAAAGGCAGGAATATGGACTTCCATTCATAAACGTGTTTGTTC
CACCGTCAACCCTTGCACTCATTTCAAATACAACGAGTTCTAAGTTTTCGTTGCACAGTGATTGT
AAACAGAATGGTCCAAGCATTCCGGGATTTACTTCTTCTTTTGCTTTTGCAACTAATTTGTCACC
CATGTCAAATACCTGAGGTAAAAGACTTTCTCTGATTACAACTGGGAAGTTTCCGGTAATAACGT
ATGAAGGGTCTATGTCCAATTCAAGCTGGTCTTTTGCAGGAACTCTTACAAGCCCATCAATACTG
CTTTCATATCTTCTATCAATTCCCATTAATTCAACCTGATCTTTTAATGGTGAATAGAAATAGTG

AATACAGTAGTTTGCACCAACAACATATTCTTCGATGTGTGCTTTTGAAACATCGTCTTCTGTGA
GAACCCCTTTAGCTTTAAACTCGTCTATTTTTCTCCAGAATTCTTCAACACTTGAACATGGGAAG
TAGCCTCTTCCGCCCCTTGCTCCAGGGAATTTAACCATTACTGGTCCATCTATTTCATCAGGTCCG
CCGTATTTTTTTGGAATTCTAAGACCGCTTCCGCCCAAAAGCTGTCCTTCGAGATCTCTTTCAGCT
TCCCATCTTAAAATAGCCCTGTTTCCAAACATTGGAACTTTGAATGTATCTTCCACATTATCTAA
ACCGCAGTAAGCAATGAATGAACCGTGAGGAACGATAATAGCATTCATATCTCTTAATTGCTGTT
GAATTTCTTCTTTTGAAATATCTGAAAATTGATCAACATAGATAAATTTGTCTGCTACTCCAAAT
CTTTTGTATGGTATAGCCCTATCTTTTGTTGTTATAACTGCTGTTGAAAAACCCTCTAATTTCGC
ACCTTTTAAGATGTGTAAAGACGTGTGGCTTCCTACCGTAACAATAGTCACTTCATCCTTGTTAT
ATTTTTCAAAAATCCCCATTATTTCTTCTTTTGGAATCATCTTACCACCATTGTTCTATATGGTA
TTTAAAATAGAGTTTTTAGACCCGAAATAACACATAAGTATATTCGTGTTACTATTTTTTATATG
TTTCCTGTTTAGTAAATTACGTGATATAATGAGTGAGGATATTAATAATAATTTAAATAATTCG
GATAATTCAAACGGATCTAACGAAAAAATAATAGTTGGACTCGATGAAGCAGGCCGTGGACCAGT
GCTTGGACCAATGGTAATTGCTTCGGTAAAAATTGATGAAAAAAATCTCTACAAGTTAAATGATC
TTGAATTAAAGGACAGTAAGCAACTCTCCAAAAAAAAGCGTGAAGAACTCTATATAATAATAAA
TGAAATGTGCGATGTTGAAAAGATTGTAATTGACCCTGAAACAATCGACAATCAGATGGAAATT
ATAAACTTAAACAAAATAGAATTAAGCGCTTTTTCAAAACTTTCAAATCATTTTATCAGGGAAA
ATGATAATATATCAATTTATATCGATGCTTGTAGTAGCAGTGAACAGTCATTTTCAAACCAATTT
AAAGCAAAACTTATTAATAAAAACGTTGAAATCATTGCTGAACACAAAGCTGATGAAAATTACA
AAATAGTGTCTGCTGCATCAATTATTGCTAAAGTAACTCGTGATAGGGTTATTGAAGAATATAA
AGAAACTTTTGGGGAAATTGGAAGCGGATATCCAAGTGATCCAAAAACTAAAAAATTTTTAAAA
AATTACGTGTATGAAAACAGAACTCTTCCAAAAATTGCAAGAAAGAGCTGGGCAACTTCAAAAA
ATCTGTTAAAGGAAATCGAAGAATCAAAAATATTTCAGTGGGTGAAATAATGGTTAACAAAGAA
CTGATTATAGATGATCGTGGGAAAAAATACCTTTTAAATAAAAACCTCGATAATTTCGGAAACG
ACCTTGGTGTTGTTGATTTAGTTGGAAAAGAAGAAGGATCAATTTTGATTACGAATAAAGGCGG
AGAATTTTATTTCGTAACTCCAACAATTCACGATATTATTAAAAAAATGAAAAGAAGCGTTACA
ACACTTCTTCCAAAAGATATCGGATTAATTATTGCTGAATGCGGTATCGAAAATGGAGAAACCGT
TGTTGAAGCAGGTACTGGTTCTGCGGCACTTACAATCTACCTAGCAAATGCTGTTGGAAAAGAAG
GAAAAATAATAACTTATGAAAAAAGGCCAGAATTTGCAAAAATTGCAAGAAAAAATCTTGAAAT
AGTTGGAGCCGTTAGACAAAACCAAAAAATCATCGGAATTGACGACGAAGATGAAGAAGAAATA
GAAATTGTAGAAAATGGGCTTTACAACGTTACGCAAAAAATTGGGGACATTACTGAAAAAATTG
AAGAAGAAAATATCGATGTTTTGGTTTTAGATATGCCTGACCCTTGGAACGTTGTGGAACATGCT
AAAAAATCACTCAACAAAGCAAAGGGAAGAATTGCAATATATGTACCATACGTAGAACAGGCTA
AAAAAGGTGTTGAAGCTTTAAAAGAACACGGCTTTTTAGATATTAGGACTGTTGAATGTATTGT
AAGAGACATTGAAATTTCAGAAAAAGGAGTGAGACCATCAACTAGAATGATCGGACACACCGGA
TACATTACAACTGCAAGAGTCTGCCCTGAAAAAGTAGTAGAATACGTTCCAAAAAAGCCAAAAGA
AACAAAAGAAGAACAGCCTGAAGAAAATTAAACTTTTTGCTTAACCATTTTTTTAAATACTACTA
TTTTTCATAATTATTATAACGAACACAAGTATCATCTATTTTGAGGGATAATTTATGAAAAGATT
ATACCGATCAGACAAAGAAAAAATGCTTTCAGGAGTATGTGGGGGACTTGCAATCTATTTCAACG
TAGATCCAACACTCATCAGGCTTTTGTGGGTATTATTATTTTTCATGAACCCGTTAATGATTATT
GTATATATTATTGCAGCAATAATTATTCCTATTGCGCCTGAAGGGGTTTTATACGATTTTTCTAA
AGAACCTGAAAAAATAAAAGCAGAATCTGACGAATAAACTACCTTAAATTTTTTTAATTTTGGT
GTTTTTATGAATTTAAAACTGAAAAACATAATTTACGTTACAATCATTGGAATTTTAGTATTTTC

AACACTATTTCTTGTTCAAAATAATTTTGAAAGTTTAAATTTATCAAAAATACTTGAAAATGGCG
GATTTAGTGGAAATGACGAAAAGCTTCCAAAATATATCTTTGAAGATTCAATTGAAGTATATTT
GTCTCCAAATGAACTTGAAAAAGTATCCAACGTTTCTAAATCATTAAATGGAAATACTATTGAAG
ACTCCATCTGGAATATTGTACTTTGGGAAGAAGATAATATTGAATATAATCATGAAAAAGCAGA
ACTCCCAAGTCCGGAAGTTACATACTGGGTTGGCGGAAGAACAGAAGTTTCTGATTCATATAATA
ATACAATACAAAGTCCTGCAGAAACACTTGAATTAAAATCTGGAATCTGTGGAGATTATGCACTA
TTAACTTCTGCAATGCTTTTAGAAATGGATTATTCCCCGATATATATCTTAACGTTTGAATCTAC
GGGAAATCCAGGTCACGCAGCCGCGGCAGTTGATATAAATGGTGATTACTACATTATCGACCAGC
GGCCCCCGATTATGGATTTATACAACTATTTAGATTATAAAAAAGATGTTGAAAATTATAATATC
GATAACATCACATTTTACAAAATTGAACTTGTAAATGATTCAGTTTTTTATGATAAAGATTTTGA
ATCTGTTGAAAGTTACAGTGCAGTTTCAAATAAATACAGCGATATTGCGGGTTTTTCAAGATATT
TGATGACATATTTTGAAAACAATTACGAAATAAAAAGTGATTACAATATTCAGTACTTAGATTC
AATGGAATATCTTCCAAAAGCGTATGAAAAAGGAATTACAATTCAGTATTTCTTTGAATTTGGA
GGATACACTCCAATTTTTGAAAAACAGTATGCAAAATGGGCATTTGATTACATTAAAAACGATG
AAAATGAAAGTAGATATACTATTTCAGAATACAGTTCCATCTGGATTAGGAGTTCATACGATGG
AGAAAAACTTAAATTAATAATAAATCTGGCTAAAAAATAATTATTTAAAACTTTAATACTTTTT
TTCGGCGTTATATAATAATATCTTTTAGTTTAGGGCATTCATATATATTGTACAATAAAAGTATT
TCGATTTTTCTAGAATAGTAACAATAATATAATAATTAAAAAATACTTATTTTTGTTGTATTAC
AATGTTTTTAATTTTAAAAAGAGGCTTTGTAGGCACAAATAACAAAGTTTATTGAAGGTAGATA
CTATGGGCGTTGGAAATTCAATAAAAAAATGTATGGCGCGGGATACGAGGAAAACCAAGCACGGA
ACTTCAAGAAATAATTGATTGTTTTAAATCCAGAGGGTCCTACAGATGCATTGAAGACTGCATGG
ACTTTTTAGAACTCAGTAAAAAGTTAAAACAGTTAAGCGGAAATAAATAAAAATATTTTTTTAA
TTCATTTTAAAACTTTTTTAAAGCATCACTATCCCGTTCAAAATAAATGTGCATGGATACTGAGT
GATGCGTGTATTTTACAAGTTCAATTCCTAATTTTTCGGCAACCATTTCACCAAGAGCAATTAAC
CCTATTGCATTTGCGTGAAATGCCAAAAATGCATCGTTTGACCTGAAAAGTACTGTTTGGTATAA
TTTATTATCTCTTACTAAAAACTGCACAATCTGCAAACATGGAACATCTTTTACAGATTGGTCGA
TGTAAGGATTCCATGTAATTGCAACAGCCCGTCTTGAATTAATCTGTTCTTTTAATTTATTTACG
ATATATTCAATCTGATCGTTTTTTAATTCTTTTTTATCATTAGGATATTCAAAAATTCTCTCATG
GTAATCGTAGACAAACGTTGCTGCTGATCCGTGTAATAGCTGTTCTGTGTAAGATTTAACAGCCC
TTTCACCAAGAGGATATTTTTCAGAGATTGATTTGTCAGATGGGTCGTTAATTTCAATAATTGTA
TTCATTATCTCTTTGCATTTCTGCCCATCTTCGGTAATCATTTCAGTTCCTTCTTCCATGATTTTT
GGTATAAGTGCTTCGAATGCAGATTTAACAGATTTTTTCTTCAGCGCGAGCATGATTTTCACCAA
CAGATAATATTTAATGTTTAAACTTCAATAAATTAAGGTAGTTAGAATAAAGGTGCGAATTATG
GTTTATTTAAATTCTAATTTTTTATATGGCGACGACTTCGAACCAAAAAAAGGAACTTTGGTTAT
TGAAGACGGAATCATAAAAGGATTTACGAGCGAACACCATTCAAATGTTTTAAATTACAAGGGG
TTGGTCATTCCTCCCCTTATTAATTCCCATACTCATATTGGAGACAATAGTATTAAAGATATTGG
TATTGGCAAGTCACTTGACGAACTTGTCAAACCACCAAATGGCCTTAAACACAAGTTCTTAAATA
CTTGTAGCGAGAAAGAATTGGTGCAGGGGATGTCAGAAGGGCTTTATGATCTCGAAAATAATGG
CATTAAAGCTTTTTGTGACTTCCGTGAAAATGGACTTAATGGAATAAATTCACTTAAATCAGCAT
TTGAAGATTCAAATGTATCTATTAAACCCATAATTCTTGGAAGACCCACTCAAAGAGAAGAAAAC
CTATTAAAAGACGAAATATCCATTATCTTGGATAATTGTGATGGTTTAGGATTAAGCGGTTCAAA
TGAGTATTCGGACCAAGAACTTAAGTTTATTTGTAAATTAGTAAACAAAAAAATACTTTCAATTC
ATGCAAATGAACATAAGGGCTCAGTCCAGTATTCAAAAGAAAAATATGGAATTTCCGAAATTGA

AAGATTAATAAATCTAAACATAAAACCAGATTTTCTTATCCATGCGACACATTCAAGTTCTGACG
ATCTATCCCTGTTGAATGAAAATAAAATACCAGTTGTAGTATGCCCAAGAGCAAATGCCTCGTTT
AACGTTGGACTTCCAGATATTCCAAAAATGTTAGAATATAATTTAAAACTTGGAATTGGAACCGA
TAACTTTATGGCAAATTCTCCGTCAATATTTAAAGAAATGGATTTTATCTACAAAATTTACCACG
TAGACCCAAAAAAAATTCTTAAAATGGCGACAGTAAACGGTGCTGAAATACTCGGACTTCAAAAT
ACTGGAGTAATAAAAGAAGGATATATTCCAACATTTACATTTATAAAAAATGGAAATATTTTAA
AAACTTCTAAAAACATTGCTGCATCAGTTGTAACCAGATTGGAAAATGGAGACGTTGATTCAAAG
TTTTTAGTTTGCTAATTTAATGTTTAATTATTATTTAATTTAAAAAACTGTAATATATTAAATTT
ATGTGATATAATGACAATTGCTAAGTTTCACGGTGGATGCCACCAAATCGGAAAATCTTGCGTAG
AGATAAACACTAAAAAATCAAAAATATTAATTGACTGCGGAATGGACCCTTCAGACAACGGTCTT
CCAGATATTAACGACTCAGACGTTGATGCGGTTGTAGTTTCACACGCACACTTGGACCACTGCGG
TGCAATACCCTACTTTAACTTCAAAAAAATATTCTGTAATCCTCCTACTGCTGATTTAATGTACA
ATGTATGGAAAGACACAGTAAGCCTTTCAAAAACATACAAAGAAGAAGATATCAAAAAATCAAT
GGATGTAATCAAAACTGTAAACTATAGAGAAGAAAAACAGGTTACTTCAGACATTAAGATGAAA
ATGTACGATGCAGGACACATTTTAGGAAGTTCTTCTCTTTATTTAGATATCGATGGAAAAAAATT
ATTATATACTGGAGATATCAACGAAATTGAAACTAGAACTCTTCGTGCAGCTGACACTGACTTTG
ATGAAATTGATACAATAATTATTGAATCTACTTACGGATCCCCATTAGATATAAAGCCTGCAAGA
AAAGTTTTAGAAAAACAGCTTATTGATGAAATCTCTGAAACTATCGACAACGGTGGAAAAGTAA
TTATTCCAGTTTTTGCAGTTGGAAGATCCCAAGAAATCATTGTCGTAATCAATAACTACATGAAA
AGCGGTGCTTTAAAAGAAGTTCCAATTTACATTAACGGATCGTTAACTCACACCACCGGAATGTA
CATGGGATATTCTGACTGGTTAAATCCAAAAATAAAAAATGCAATTGAAAACAGGATCAACCCAT
TTGGAAATCTAATAAAAGGTGGAGATGAAGTTTTCAGTAGAGAACCATGTATCATTATTTCAAC
ATCAGGAATGGTTCAGGGAGGCCCAGTACTCCAATATCTTTCATTATTGAAAAATCCAAGAAATA
AAATCATATTAACAGGATACCAGGCTGAAGGAACTATCGGAAGAAGCCTTGAAGAAGGTGCTAC
AGAAGTAAAACCATTTAAAAGAGCGATTCCGGTAAATGGAAAAGTAGTGAAAATCGAGTTTTCT
GCCCACGCTGATTACAATTCACTTCTTAGATTTATGAAGAAAATACCCCAGCCAGATAAAGCAAT
TGTAATGCACGGGGAAAGATATCAGGCACTATCCCTTGCAATGACAATCTGGAAAACCTTAAAAA
TTCCCGCACTTGCTCCAAGTATTGGTAGTGTTTTGCCACTATTTGATTAATTATTCCACATATTCT
CTTTTTTAATATTTTAAATTTTGATAACCGATATAATATTTACTTTTCTAATGTCTATTACAAAA
ATAAGTAATTTAAACGGGCTTTTAAGTATTTAATAATATATAATCCCTTATCTGCATTTTTAAAG
AGGTATTCCTTTATATTATCCCCGAGAAAGAAAGTTTAATGTATGCATATTACCCTTGGAGGTGA
ATAAAGTGGCAGAACCTGTAACTATCAGCCCTACAACTAGACATGAAGGGCATGCTAAGCTAGTT
TTGAATGTAGATGATGAAGGGATTGTAACTAAGGCATTTTATCCAAATACAACCCCTGTAAGAGG
TTTCGAAACCATGCTGCAAGGAAAAACAGCAGCATTTGGACCTATTGCAGTTATGAGAATTTGTG
GTATATGTCAGGCAACCCACGGTATTGCTTCATGTGAGGCAATCGAAAATGCTATTGATATGGAA
ATACCAAAAGACGGTATGATATTAAGAGAATTATTAGGTTTAGGAAATAGGATGCATTCGCACCC
ATTACACCATTTGTTAATTGTAGATGACTTTTTAAAACCTGAAGAAGCAGGAACTTTAAGAGTAG
AAGGAATTAAATTAATCCAGAAAATGAGAAAAGCTGGACAGATGGTTGTAGACGTTACTGGTGG
AGAAGGAATTCACCCTCCAAACTTGATGGTTGGAGGAATGAGGACAAACATCACAGAAAGAGCA
AAATCCAAACTCTACTATGCTTGTAGAGAATACGAAAAAGATTGTAATGCAATGTTAGAAGTAT
TGGAAATGTTAATGGAAAGATACCTGGATGAAATCGGAATTCCTGATTTAGGTAAACACAACTT
ACCATACATTGCTTCAGACTCAACATTTGGTAACAGAGATGCAATTAACTTTAAGGATGTAACAG
AAGTACCTGCACAAAGATACTATGCAGACTTCCCAGATATTGCACAAACCGCAACAAACCAGATC

CCATTCTACAGAGGAAACCCTGCAGAAGGTGGACCTAGAGCAAGAATGATTAAATACGGAAACTT
CAAACCTGCTGGCGGAGCAATGGACCTTAACCTTGCGAGAGCTATGGAAAACTTCGAAGCTGTTA
AACGATCATTAGAACTCATCGATGAATTAAACATTAACGGAACTGTAAGAGAAGTACCTAACTTC
TATAATGCTAAAGAAGAATTCGGAATAGGTGTTCACGAAGCACCAAGAGCTACCAACACCCACAT
GGCTAAAATGGGCAAAGATGGTAGAATCGAAAAATACAACATCATTGCTGCTTCAACGTGGAACT
TCCCAGCTGTTGAAAAAGCTATTGAAGGTTACCACCATCAATATGCGGAAGTAATTATGAGAGCT
TACGACATATGAGCTTCATGTGCTACGCACGTCATCGTAAAAGATGAACAGACTAAAAAAGTTGT
TGAAATAAGAGAATTCTAAATTCCTGAAAAGGAAAGTCTTAGAAAGGGAACCTTAATGGACCTT
GAGAGACACAAATTAACTTACGGTAATCATAAAAAAAATCATTTCAACGGTATTGATATGGAAT
ACAGTTTAACCCCTTCATACCTTTTAAAGGAAAATATGGTTCTTGCATGCGGAAATATCCTATTT
GCAGACGATGGATTTAGTGTCCATGTAATCGAAAAATTACAGGAAATACTAACTGAAGAAGAAA
AGGAAAACATTGCACTTGTAGATGCAGGAGCAGGAGCTCCGCAACAAGTGCTCACTTTAATCGAC
AGTGAATCCAAGACTAAGAAAATAATAGTCGTGGATATCATCGATTACGGACTGGAACCTGGAG
AAATTAAGATACTTACTATGGAGGATCTTCCAAAACCAGACCACACAAAAGTTGACAGTCATGAC
TGGCCGCTCTCAACTACCATGTACAGAGTAGTTAGGGACTCCCCTCGTGAAATAGACTTTAAAGT
TGTCGGTTGTCAGAAGAAGTACGTTTCAGAACCTGATGTAGTACTTGAACTGTCAGAAGAAGTCC
AAAATGCAGTAGACGTAGCCGTAGACATTGTACTTTCCGAATTAAGGGGAAAACCAATCAACTAA
GGGAGGAGAATACCATGGTAAAAGTTGCCCACGTGCAATTAAGTAGCTGCTGTGGGTGTCTAGTT
TCCCTTGCAGACACCTATGAACAGCTTTTAGATGTTTTAGGAGCTATAGATTTAGTTTACTCCCA
GACTCTCGCTGACGTGAGAGAAATTCCTGACGATGTAGACATAATCTTGCTCGAAGGTAGTGTTT
GTTTAAGCGACCACCACGCAATGGAAGTTGCACTTGAATCCAGAAAAAAAGGAAAAATATTAGTT
GCGTTAGGAGCTTGTGCTGCTAGCGGAAATATTACAAGATTTTCAAAAGGAAATCAACCATCAAA
ACCAGTTCACGATTCATTTGCACCATTAACAGAAGTTGTAAAATGTGACTTGGCAATTCCAGGAT
GTCCACCATCACCAGAAGCTATAGTTGCAGTTGTTTTAGCGGCATTAAACGGAGATATGGAATAC
TTAGAACCATATGCAGAACTTGCAAAATATGGATCAGAAGCTTGTGGATGCGATTTGATTGTAAA
AGTAATCAACAAATCACTCTGTATGGGTTGCGGTTCATGTGCTGCAGCATGCCCTACAAGAGCAG
TATCTATGGATGCAGGAAGACCATTAGTTGACAAGGAAATCTGTATAAAATGTGGTGCATGTTCA
GTTCAGTGCCCAAGAATTAGATTCCCAGAATTAATTGAAAAAATAGAATAATTGCAATTATGGA
AAATAAAAAAAGGTGATTAGATGGATCCCTTTGGAAAATATATAAAAGCAGTTTCCGCAAGGGC
TGCAGACAAAGAAATCCTTAAGAAAGCACAAGATGGAGGTATCGTTTCTGCCGCATACATTTACG
GTTTAGAAAGCGGACTCCTCGATGGTGTAATTATTGCAGACAAAGAAGATGGATTCGCAGCAGCT
CCAAAAGTTGCTACAACAAGAGAAGAAATCTTAAACGCTGCAGGAACTAAATATACAGTATGTCC
TAACCTTTCAGTAGTAAAAAGCGCTGTAAGAGAATACGGATGCGACAAATTAGGATTTGTTGGA
ACTCCTTGTCAGGTTAGATCAATCAGAAAAGCTATAAAATACCCTATTGGATACAGACACGTTCC
AGACAAAATTGCATTAATTATGGGTATATTCTGTATGGAAAACTTCCCATATAACGGTATGAAAG
CAATTGTTGAAGAACACTGCGGTATCAAAATGGATGATGTTGTAAAAACCGACATCGGAAAAGG
TAAATTCTGGGTATACTCAAAATGGGGCGATGTAAAAACAATCCCATTAAAAGACACCCACCCAT
ACGAACAAGGATCATGCCACGTATGTATGGATTACACAGCAGAACTTGCAGATATCTCAACAGGT
TCCGTTGGAAGCCCTGACGGATGGAGTACAATCTTTGTTAGAACTGCAAAAGGAGAAGAGTACTT
AAACAAAATGGTTGAAGCTGGCGCTCTTGAAACAAAAGAAATCGACGCTGTAAAACCAGGTTTAG
GATTAGTAGAAAAACTCGCTCTCACAAAAAAAGAGAAAAATGGAAAAGAAGTAGAACACAGGAA
AGAAATTGGATTACCTGTTCCATACTAACTTCTTTTAAACTTTTTTTACTATTTTTTTAAATAAA
TTTTCGACAATTATATTATATAGTTTTTTCATAAGTTGTTTTATTGTTTAATCTGGTTTTGAGGG

ATTTACGTGAATGTACGACTTATCGAATGCTATGTTCCAAAAAAGATATTTGCAGGATATGAAGA
ATCGCTTGGAGATAACCTTGAAAATATTATCTGGCACAGCATTTGCGAAGAATCAAATTATACTG
TAATCAGATTACTTACCGAAACAGAACACGTTGAAAAAGTAATTGATGTACTCTCCTCAAAATAC
GGCGGATCGAATTTCAGAATACTTGTAACCGAACCAACCGCAACGATTCCAGAAATCAAAAAAGA
AGAAAAAGAAGAAGTTGAAGATAAAACCCCTAAAAGAGTTTCAAGACATGAAATAATTGGAAAA
ATAGGGGATATATCCAACCTTTCAAAAGAATATTATTCATTGTTATTTTTAGCTGCGGTTGTTGC
ATCTATTGGAATATGGACGAATGATGTTGCATTGATAATTGGTTCTATGATAATTGCACCTTTTT
TAAGCCCGATGATTTCATTCACGTTCTCAATTGCTGTCATGGATACATCCCTTGCAAAAAAATCT
TTGAAAAATTTAATCTTGGGAATTTTAACAGTACTTGTATTTTCATTCTTTATCGGAACATTTCT
ACATGTAAGTCCCGAAAGCCCACAAGTTGCATCCAGAATGAATTTAGGCTTGCAAAATATTGCAA
TTGCACTTTCGGCAGGGTTTGTCGGTACACTTTCAATGTTAATCCCTGAAATTTCATCAACAGTT
GTTGGAGTTATGATTGCAGTTGCCCTGATGCCCCCCTTAGTTGCATTTGGTTTAATGCTTGGTTC
AGGATATTACTTAGAATCAATCCCGATCCTGATATTATTTATCGTAAATATAATCTCAGTTAATT
TTGCATCTGCGTCTTTGTTTTATATTTACGGTATAACCCCATACAAATGGTGGGAAATCGAAAAA
GCAAGGAAATTATCAATATTTACCATAATATTCTGGTTTTCAAACCTCTTAATTCTCGCAGGTAT
CATCATGCTATTTGGAAATGGAAATTTGAAAATAATATAAATCCTTAGAAAAATAAATTAGAGT
AGATAACTTTGGGGATGAGATTATGACTAGAAATCCTGTTGTTGCAGGGATGTTTTATCCTGCTG
AATACCATGAATTACTTGAAATGATCGAATACTGCTATTTGAATCCAAGAGGCCCTAAAGAACTG
CCTTCAAAACGGGGAAAATATACAAAACCTCTTGGAATCGTATCACCCCATGCAGGATATATCTA
CTCTGGACCTGTTGCCGCACACGGTTACAAAAAAATATCTGAAAACATCAGCGGCGAAATCACTG
CGATAATTCTTGGTCCAAACCATACAGGACTTGGTTCTGGAATTGCCACCATGAAAGGAACATGG
AAAACGCCTTTTGGAGATATGGAAATCGATAACGAATTTGCTGATAGATTATGGAAAGAATGCG
ATATTTTAGATATGGATGAAAATTCACATTTGAGAGAGCATTCGATAGAAGTACAGCTACCATTT
TTAAAGCATCTTGAAGACCTAAATATCGCAAAATTCAAATTTGTCCCAATATCTATGATGATGCA
GGATTATGAATCTTGTATTGATGTAGGGTATGTTATTGCAAAAGTTACAAGAGAATTAAACAGA
AAAAGTGTAATAATCGCTTCCACGGACTTTTCACACTACGAACCCCAAGAACAGGCCTCAAAAAA
AGATGCAGTTGTTATAAAAGACATTCTAGAATTAAATGATGAAGAAATATTTACTGATGTAGTA
ACACACAATATAAGCATGTGCGGATACGGGCCAGTTATTGCAATGGTTAAAGCAATGAAAGATTT
AGGTGCTAAGACCTCATATCTACTTTATTATTCAACTTCTGGCGATGTTACAAAAGATTATTCAG
AAGTCGTTGGATATGCTTCACTGCTAATAAAATAGGTTTAAACTAATTTAAAATAATAAATTAA
TAGTTTCTAAAATTAGTTCGAGTTTAAATAATCCTTAAGTTCTTTTTTTAAATCATTTACAGAAG
GAGTAGTTCCCTCAAATATGATGTCTTCATCAAATGCAACTGCTGGAGCCATCAACACCCATTCA
GAAATTTCTTTTATATCCGTTACTTTTTCGATTTCTGCATCAATATTCATCTCCAAAACTGCTTT
TTTAACGTTTTCGTACGTTTCATTGCATTTTGAACATCCCATACCAAATACTCTTATTTTCATAA
ATCCACCTTTGAAAATTTTATTGATAAATCAAATTATATTTTTTAATTAATAATTAATTTTGTAG
AATTTTTATGTGTTTTGGAATTTTCTGGACAAAAAATAGTGAAATTACACTAAATACTGCACCCA
TGATAAATGGAGTCTTCCAGTTTGCCATCCACAAGATTCCGCCAACCACAGGAATTATAACTGCT
GAAATATGGTTTATTGCAAATCCAACACCCATTGATGGTGCAATATCTTCTGAATCTGCGGTTTT
TTGAAAGTATGTATTTATCCCGATTGCAAAACTGAATACGATATGGTCGATTATGTAGAGTAAA
ACTGCAATATTCCTGTTTTCAATAAATGCATAACCTAAGAAAACGAACGTTAATGTCACATATTC
TATTGTAAGCATCTTTCGTTCCCCCAAATTATTTATTCCCTCTGCAACCTTTGGAGCCGTGAAATA
GATCAATAAATTGTTTAAAACAAATAATACTGCAACTTCAGAAACACTAAATCCATAGTTTTGAA
CCAGTAAAAATACTGCAAATGCGATAAAGATCTGTCTTCTTGCACCACTTAAAAAGTTTAAAACG

TAGTATAACCAGTATTTTTTCTTTACAACCATCCCTTTTCTTTGTGGGGCCGTTTCTTTAAAGAT
AGGGTTTTTAAAAAGTGAATAAACCCCAATTATCATTATAACTATCCCATAAATTAGATAATTTG
TCCATATAGGTAACAGTTTAACACTTAACCATATAAAAAATCCTGCAATAATACTTGCGATTGCA
CCGTAGCTACTAAATCTTCCAAAAACTACTGGAGCTTCTTTTTTATTAAAGTGCTGGAGTGTAAG
CGATTTATTGGTTGTTTCAAAATAGTGAAATCCCAAAGACATTAAAAACGTCGTTATAATCAGAC
CCTGAACTGTCGGAAATAATCCCGTTATTGCAACACCAAGACCCATTAAAATGGTTGAAAGCGCT
GAAAGCCTGTGTTCTTTGATTACTAACAAAACATAAATTACAAGAAACGTTAAAAAACCAGGTAT
TTCTCGGATAGACTGGATTAAACCCATTTGAAATCCATTTATACCAACAACATCCACTGCAAAAT
TGTTAAAAAGCGTTCCCCAGACCTGATGGGAAATTGCTGCCGCAACTATCAGAAAAAAGAGGTAA
TTGTACATTGGATTTTTTTTGATTTCTATCATCGATACACCGATTGTTTACAATTAAGTTAATTT
TAAACAATTATTCTCAAGTTTTAATATTTATAGGGATTACAATTTTCGGGTTTTCGGCATTCCTG
TAATTTTATTCATTTTTTTACTCTTCGCCTTCCTTTGAGTCCTGAAACATCTATTTCCATTTTTGT
ACCGCAAGATTCGCAGTTTGGAATTTCAACCTCTGCGGCTTTACATATGCAGACATCTACACGAT
ACGTAATTCTTTTTTTACAGTTTGGACACTGCAAATTCCACTGGAGCATTATATCACCTTAATAG
TAGTATAATTCAAAACAGGATATATTTATAACTATCAATATATCGATTAATTTTAATATATTTA
AAAAACATTTGAATGCATGGATTAAATTTTGAAAAATAAAAAGAAAAAAGATTTTAAAGGTATT
TTTGAACGAATAATTCAGCATTTAAAACACTTGCTCCTGCAGCGCCCCTTATAGTGTTGTGTTCA
AGTGCAGTGTACTTTACACTAAAGATTGGGTCTTTTCTAACTCTTCCAACAACAATTGACATTCC
ATTTCCAGTATTTCTGTCGAGTCTTGGTTGAGGTCTGTCATTTTCTTCCCTTAAAACAATTGGTT
TTGCGTAGCTTGGTAACTTTAAACCTTTTAAAGGATCAAACTCATCCATTACTTTTGCAATTTCT
TCAGGACTTGCTTCTTCAGTTGTTTTTACAAATATGCTTTCAGTGTGGCCGTCGATTACAGGAAC
TCTGTTACAGGATACTCCAATTTTAAAGTTTCCGTTTTTAAAATCGTTTCCTTCAATGTTTCCAA
GAATTTTTAATGCTTCTGTCTGCATTTTTTCTTCTTCGCCACCAATGTAAGGCACCAAATTATCT
AAAATTGCCATTGATGGAACTCCACTGTATCCCGCACCGCTTATTGCCTGCATTGTGGTGATATT
TACTAAATCAATTCCAAATTTATCCATTATCGGTTTTAATGAAATTACTGCTCCAATTGTTGAAC
AGTTCGGATTTGTAATTATTGCACCATCTGAGCATCTGTTGCTTTTTTGCGCTTCAAGCATTCCA
AAGTGTTCAGGGTTTACTTCTGGAACAATTAATGGAACGTCTGATTCCATTCTCATTGCTGAAGC
GTTTGAAAATACATATTTTCCAGTCTTAGCAAATTCTGGCTCAAGTGTTTTTGCTAGATCAGCCG
GTAGCGCTGAAAAAACAATATCAACATCTTCATAAGCTTTATCGTTAGGATCAGTTGAAACAACA
GTTGCATTTGCAATTTCTTCAGGAATTGGTTCGGTCTGGTACCAGTAGCATGCATCTTTGTAAGT
TTTTCCTGCACTCCTCTGTGATGCACCAAGCGCTTCAAGTTCAAAAACAGGATGATTTTCAAGCA
TTTGTATAAATCTCTGTCCAACGTTTCCAGTAGCTCCCAAAATACCTACTTTTATATTCATTCTTT
CACCTTTGGATAAAAATTATTGAAATAATGATTAAGAACGCTATTTTTTTAGTAAATATAATTT
AAAAGGAATTTATCCTTTTACAACTCCCATCGGTTTCATTTTTGCAACTTTCAAAGATATTCCTG
AATCGTGGCATATATCTGCAACATTTTCAATATCCTTGTAAGCTTCAGGACATTCTTCTGCAATG
ACGCCTTTTGAATCTGCCATTACCAAAATTCCTTTCTTTTGGAGTGCTTCCTGAACTTCATTTCCA
GTGTAGAGTTTAAGTGCTTTTACTCGACTTAAGGCCCTTCCAGCACCGTGTGCAGTTGATCCAAA
CGTTTGTTCCATTGCTTTTTCTGTTCCGTGCATTAAATAAGACGCAGTGCCCATATCACCTGGAA
TTATTACTGGCTGTCCAATATTAACGTAATCAGATGGAATTTCAGCATGACCTGGTCCAAATGCC
CTTGTAGCACCTTTCCTGTGAACTACGACATTTTTCAATACTCCATCAACCAAATGCTGCTCTTTT
TTAGCAATGTTATGTGCTACGTCATATATTATATCCATTTCTAAATCTTCCGCACTCGTTTTAAA
TACTGTTTCAAAACTTTCCCTTATCCAGTGCGTTATTAACTGCCTGTTTGCCCATGCGTAATTTGC
ACCACAGGACATTGCTTTAAAGTACTTTTGACCTTCTTCCGAATTTATCGGTGCACATGCAAGCT

GTCTGTCAGGTAATTTAATATTGTATTTTTTTGCAGCATTTTCCATGTATCTTAAGTAATCTGCA
CATATCTGGTGTCCAAGACCTCTTGAACCCGTGTGTATCATCAAAACTACCTGATCAGGGGATAC
TCCAAACGTTTTTGCAGCTTCTTCATCAAATATTTCATCAACATACTGAACTTCTAAAAAGTGGT
TTCCACTACCTAAAGATCCAAGTTGTGGAAGTCCTCGTTTTTTAGCATTGTCTGAAACAAGCGTT
GGATCTGCTTCTTTCATTTTTCCGTGTTCTTCAATATGTTTTATATCTCTTTCCCAGCCATATTCT
TCCTCAACAGCCCATGAAACCCCTTCTTCAAGAACACTATCAATTTCATCTTTTGTAATTCTTATT
TTTCCTTTGCTTCCAAGTCCTGAAGGAACATTTTTAAAAATTTCTGATAAAAGTTCCTTTAATTT
TGGAGTAACATCGTTTTTCGTTAAATTTGTTTTAACTAGTCTCACACCACAGTTGATATCAAAAC
CAACTCCGCCAGGGCTAATTACCCCAATAGCTTCATCAAATGCAGCAACTCCCCCGATACAAAAAC
CGTATCCATAGTGGCAGTCAGGCATTGCAAGAGAGTACTTTTGAATACCTGGAAGACATGCAACA
TTTGCAATCTGTTCAAATACATCCTCTTGAAGATTTTCAAACAATAATTCATCCAAATAAACGTT
TCCAGGAACCCTCATGCAGTCTTTATAACTTACTGGAAGCTGGTACGTTTTTTCAGCGATTTTAG
TAACCGTAGATTTCATAATTTCACCGGTAAAGAAATGATTTTAAAATCGATAATTCGTAGTTTAT
ACTTAAATAAAAGAATAAGTTTATTTAATTAAAAACTTTATTACTCCAAAGTATTAATCTTTACC
GTTTTTGGTATTTGGGTGAAAATAATGGCTTTTGAATTTACAATTTACGCAAAAGGACACGAAA
ATATCAGCGCAAATCACAAAAGTACACTTGAAATAACAAAAGAAAACCACGTGACGTGTACTGGC
GACTGCATCGTTGGAATTTCTGCGGATAAATCAATGCTCGATTTTTCGGAAAGTTTTAAAGAAAA
TTTAAGAAATTCGGATAAAATTACTGTTGAAATTGAAGTTGAAGGATTAAAAGAGGTAATTACA
GGACGCGGAAACTCAAAATTAACTTTTGACCATAAAACAGATATTGTAATTAGAACAAGTGACTT
TTCATGCAGTAGAACTTTAATGGTAAATTCAAATAAAGCTTCAAAAGATATAAATCGAGAAATT
GTTAAAAAATTGAAAAAAGGAGCAGATTTGAAATTTAAAATAATTGTTGAATAAATTTATTCTA
TTAATTCTAAAATTTCTTTTAATTTTTTAACATCAACCTGACCTGGAGCTGATGCTTTTCCTTCC
ATCGATGCAAAAGTGAGTATTGATCCAAAATTAAGGCCCAAAACCCTTGTAAGTTTTCCAGATTC
GCCCATTCCAATTGCAATTATTTTTCCAGAATACTTGTTCATTAAATTCAATATTTTTAAATTAT
CTTCTTTTGAATGCGCAAACGTTGCAAATTTTGCAATATCTCCTATTTTTAACTCTTTTTCAACT
ACGTCAATTAATTCATCGATTTCTGGAGTTTTTTCAAAATCGTGGTATGAAACGATAATTTTTGT
AGTTGATCCAATTTCATTTCTATATTTAACTAATTCTAAGTTTTTTTCTTCTTTTAGTTCAATAT
CTATAAATTTCGCATTATTTTTTATCGCGTGTTTTAAAAGCTCGATTCTTTCTTCATTGGATTTT
TTCCAAGCACCGCCTTCCCATTCTGCTCTTATTGTAATAATAGATGGGATTTTTGAAAACTCTTC
AATATCTTCAAAATTTACATCATTTAATAAATCAATTCTAAATTCCACAATATCTCCGTATTTTA
AAGCTTCTTTTGCGGAATTTATTGCATCAGACACATCTTCATCAATTACAGGAATACAAATCATA
TTTAACACCTATTCTCGATACTTTTTAGTTACAACTTCTCTAATCTTTTTAGCAGTTATCGGCCCA
ACTCCATCAACAGCCTTTAATTCTTCTTCTGTTGCAGTAAATATATTTTCAACCGAATTAAAATT
ATCTAAAAGGTTTTCAGCCATTACTGGCCCAACATCAGGAAGTCCTTCTACCAAAAATTTCTGAC
GTTCTTTTAGTGACATTGGCCTTTTTCCATACCGTATAGCTATTTCTCGTTTTTCTTTTATCTGCT
CCCTTTCTGCAAGTTTTACCAGTATATCTGCCGTATCTTCAATATCTCTTGAAAACATTATCGGT
ATCTGGTAATCGATCATTATTGAAAACATCATCCCTCGTATCGCGTTTTCATGAATTCTTACAAA
TTCGTTTCCTTCAATAATTATTAAAGGTCTTTCATATTTTTTTAAGTCCATTACCTGGTTAAACA
GCCTTTTATCAATGATTGAATTTTCAAAGTCCTCTGCAGTTTTTCTCTCAACTGCAACCCTGTCAC
TTAAAATATAATCCCCAATTTCAAGGGTTTTAAATTCAACTTCTGCTTTTTCTGAAAGGTATCTT
CCAATGTGGCGCTCTCTTGAATCCACAATTATTGTTGCTTTGTCGGGCAGTCCGCTTTTACTTTTT
TTATCAAGTTTTGTTGGTTTCTTTTCTTTTAAGTTATCAGATGTTTTAGAACTTACAACTGATCT
TAAATCCATGTAGTGATCCTTATTATTTGTATCTACAAAATTCTCTTTGTTTTCACTTTCTTCTT

CCTTTAATTCCTTCAATTTTTTATTTAATGTAGTCTGCATGTTTTTTAAGATATTTTTCATGTTC
TTTTCTTTTGCAAGTCCTGCTCGATAATATCCTTCGTCTCGAGTTTTTTGTGCAATTAACACGAT
TACCTGTCCACCTTCGCCCCTTGCAGCACGACCCCTTCTTTGAATCATTCTGATTTCTGAAGGAAC
GGGTTCATAAAATAAAACATAATTTACACTTGAAATATCAATTCCTTCTTCCGAAACACTCGTTG
AAACAAGCACATTTGCCTCATTTTTAAATTTTTCAATTGCTTTTGCCTGTTCTTTCTGGCTCATTC
CTTTTCCATCTTTGTTGGATTGCCCAACAAACATTATTGCTTCAATTTCGTTTTCAGAAAGTAGA
TCTACAATTTTTTGAACAGTGTCCCGATATTGTGCAAAAATAATTATTTTTTCGTTTTCTTTTAA
AATTTCTTTTACAATTTCAAGCATTTTTTCGTATTTTGGGTGCTCGATATCGAGTTCATTTAAAT
TTTTGACTGCCTGGATAAATCTAGGATCGTTTGTAATTTCCTTTGCAGACTTTGTATTTTGGGTT
AAAAGCTTTTGATAATAATTTAAAAAAGTGCTTCGCCCTTGTGTTTCGAGCATTTCAATTGCGTG
CTCTAATTTTACGGCTTCAGATGAAATTCGAAGCATTTCATACTTTATATTGTCATCCATTGCCA
TTATTTTTTTATTTAGCCCGAGTAATTCAGATTTATTTACAGTTATAGAATGAATTACCCTATTT
TCTTTTAAATCCCTTAAACGATCCTTTAATGCATTTTTTAAAAGATTTAAATTTACTTCAAACTC
TTCTGGAAGTTCGACACGTCTTGGTCGCATTTTAACTTTTGCAACATATGGTGAAACGTCTGGGT
CTTCCTCTGTTTTTATTTCAACGTGTTCGATTCCCAAATTCCCACAGATTTCAAATATTCTATCAA
TGTTTGAACCAGGAGATGCCGTTAATCCTAAAACATGAGATTTTTTCTTAAATTTGTTTGCGACA
AACGTATAAGAGTGATTTCCAGTTGTGTGATGCGCCTCATCAGCAATTAAAAGTGAAAATTGAGA
GGGTTTTAAAATTTTAGAAATAATATCGTTTTCTGCAACCTGAGGTGTTGCTATAAAGATTTTAC
CGGATTCCCACATTGCTTCTCGTTTTTTTGGTGAGATTTTGCCGTTTAAAATAACAATCTTTTCT
GAATCGATATTTAAAAATTGATTCATACTCTTAAAGTGCTGGTCTACAAGAGGCCTTGATGGTGC
AATAATCAAAACTTTTCCATTTTGCTTAGATAAAATTCCAGCTATTGTAAGGGCCGCAATTGCAG
TTTTACCAAGTCCTGTTCCAAGAACACATAATGTATTTTGCTTTAGTGCACTTGCTACAACGAGT
TGCTGGTAGATTCTAGCTTCAATAGTTTCTGGTTTGATCAAGGGATGAGTTACATGCATTAAAAC
ACCATTATTTGGCCTTGCGTTAAAAATCCAAGTTTATTAACTTTACATTATTATAAAATATATAA
CTACTACTTAAAATTTAAAAATAAACCTAAATCAAAAGTAATCTTAAGTAAATCTGATTTTAAA
AAATATGAAAAAAGTGTTAAATTAGTTACTGCTGTTGAAATATTCTTCAACTTTTTCTGCGATTG
TTTCAAAAACCCAGTTTCTCTTTTCTTCGTCTCTTTCAAGAAGCGTGAATCTCATTCCATTGTGTT
CTGAACAGAATGAAGTTAAAGGAACACCGCAAATTCCAGTAGATGCAAGTAAGTAGTATACAAA
CCTTTTATCTTCAGGAATTCCTTTTGAAATCGATTCAACGTAATTTTTAAGTTCACTATCTTGAA
TTTTCAAAGACTGGTTTTCATTTAAGTATTCTTTTTCTAAAACTATGGTGTTATAAAATGCACCA
TCAGTTCGGTTTACATTTAATCCATCGATATTTTTCATTTTATTGTATGCGTACTTTGAAGCCGT
TTCGTAGTAGCTTTTCCTTTCTTCAAGGTACTTATTATATCGTTTATCAGACATTATCTTTGGAA
TTACTTTTTGAGGAAGTGTTGTCGAACATACTTCAATCATTTTAAACTTGCAGATGTTTTCAGTG
TATTTCTTGAATAATTCATCACTTCCAGTATTGTAAAACTCTGTCCAACCGCACCTTGAACCAGG
CCATGGCAAATCCTTAGAAATTCCCTTTAAAGAAATTCCTGGAACGTCATCTAAAACTTCCGATA
GGTAGTTGTGTTTTTTTCCATTGTAAACAAGGTTAGAATATATTTCATCAGCTAAAATGAAAAGG
TCGTATTCGTTTGCAATATCTACAACTTCGCTTAATATCTTCTTTGAATAAACTGCACCGGTTGG
ATTTCCAGGATTTATTAACAGTATTGATGCAATTGATGGATTATATTTACTTTATTTCTCAAAT
CATCAATATCTGGATTCCATCCGTCATCCTTATCCAATTCGTACATTACCGGCCTACAGTTTGAAT
AAAGTCCTTCTGCAGAAGAATGTGTGGAATAAGCTGGTGTTGGACCAATTACCCTACACTCTTTT
CTCATCAGCCCGTAAATATTTGTGATAGCGTCCCCTAAACCGTTAAAAAACACGATTTCATCAGG
TGTAATCTGTGCTCCGTTTCTGCTGTTGTTTAAATCAGTTAAAAACTCCCTCGTTTCTAAAAGAC
CTCTTGTAGGACAGTACCCATAAGAACTGTCATCCATTGTGGTTTCTGAAACTATTTCTTTTATC

CAGAGGGGAAGCTTTTCTCCTTTGGCAATAGGATCACCAATATTTTCCCATACAACTTCTAATCC
CGAACTTTCGGCTTTTTTAGCCACCTTTACAATTTCTCTGATTTCATAGGACAACTCGCCTACTCC
TGAACTGACAATATCGGTTCTCATTGCTTCCACCTCCATCGTGTTTGCTGTATTGTTTAACTTCTG
AAATTTTATTTTGAAAGCTTTTTAATAGGGTTCTATTATAGTTGTTATTATTTTTCAATAACGTC
TAACTGTTCCCTTTTTATTTTTTAATATATAAAGATAACTATCGAGTGATTTTTTAATAGAGCCC
TATTATTTATAAAGTATATAAATAAAATATGAAGAGAATAATTAACCATTAAAGTGTGATATTA
ATTAGATTTTAAAATTAAAAGTTCAAGAATAAAAACCAAATATAAAATCAACAACATAATATAT
CAGTTAATTAAGAAATATAAGCAATTATTATCCAAATAGTATTTTATAAATTTTTCCGCAGGTTC
TTGGTGAATACAATGGCATCATTATCAGAAATCCACATGAAAAACTTCAAATCATTCAAAAATTC
AAAATTAAAAATTCCCGATGGCTTCACTGCAATACTGGGGCCCAATGGATCAGGAAAATCAAACA
CCATTGATGGCATATGTTTTGTTCTTGGAAAAACTTCGGCAAAATCGCTTCGTGCAGGTAAATTT
AACCAGCTTATTACTTATCACAATGGAAAAAGGGAAAATTTCGCAGAAGTTACACTTATTTTTGA
TAATAAAGATAGAAAAATGCCCGTAGATTCGGATAAGGTTGGAATATCGCGAAAAGTTAAGATA
AACGGCGATAACAACTACTACCTGATTTGGAATGAGGAAAAGGAAGTTAAAGAAAATGGGGAAA
TAAAAACCGTAAAGGAAGAAAAACGAAAGAAAGTAAAAAAATCTGAAATTTTAGATGTCATTGG
AAAAATATCACTTAGTGCTGATGGATTTAACATCATACTCCAAGGGGACCTGATAAAAATTATAG
ATACTACCCCAAATGAAAGAAGAAAGATTATCGATGAAATAAGTGGTGTCGCAGAATTCGATGA
AAAAGGGGAAAAAGCAAAAAAAGAACTTGAAAAAGCAAGGGAATTCATTGAAAAAATTGACATC
AGGATTAATGAAGTAAAAAACAATCTGGAAAAACTTAAAAAAGAAAAAGAAGATGCTGAAACTC
ATGTTAAATTAACAGAAGAATTAAAGGCAACTAAATACATTTTAACTTCTAAAAAAATTGAATT
TTTAAACGGAATTTTGGAAAAAACTAAAGAAGAAATTGAAGCACTCAAAGAAATGAAAGTCTGT
TTCTTAAAGGAAATTTCTGAATACGACGCAAAATCAAATGATATTAGAAATAGACTTCAAAATT
TAATAAATGAGTTAAATGAAAAAGGAAATGAAGAGATAATGGAACTTCACAAGTCCATTAAAGA
AATGGAAGTAACCGTTGATAACGACAAAAAGTCCTTAAACGGAGCTCTTGATGACTTGAAAAATG
TAAATTCGCAATCCGAAAAAAAAGGACAGGATTTAGTAGAAACTAGACAAAAAATAGAAACCAT
AAGGACCGAAACACTCCAAAAAGAAGCGGAAATTAACACTTTAAAAACTGAAATGGAAAATCTT
GAAACTGAAAAGAAGAAATTGAAGTCAAAAGTTGAAGAAAGTGAAACCCAGACTGAAATATTAA
AACAGCAAGAACGAAAATTATCTGAAAGAATAAATGAAAGTCAGAACGAACTTTACAATTTCAA
AAATGAATTTAATGCTCTTGAAAATGAAATTAACAAAAAATCATTCAATTTAACAAAAAATAAA
GAAACTATTGAAACCCTGCAAAAAGAACTTGAAGAAATACGCTCAGAACATGAAGATACTAAAA
GCCTGTACAAAGAACTTGAAGACGTTGCTGTTGAATTAGAATACTCCAAAAAGAAGGTTATAACA
CTTTTAGAAAATAAAAAAGAGTATCAGGACAGATTAGACAAATCTCATGCTGATTACATAAAAG
AAAATGCTAAAATCAAGGCAATGAAAGATATGGAAGATTTCAGTCTTGATAGGGCCGTAAAAAG
TGTTCTTGAAGCAAAATTACCCGGAGTAGTCGATATTGCAGGAAACCTCGGAAAAACCGATGCAG
AATACAAAACTGCAATTGAAAATGCAGGTGGAAACAGGCTCAACTACATTGTTGTAAAAAGAAT
GGATGACGGTGCAAGAGCTATTCAGTATTTAAAAAAGAATAATCTTGGTAGAACCACATTTTTAC
CACTTGATAGGATCAATGGGCCAGAAGCACTTTATTTAGATGACGAAGGAGTCGTTGGAAGAGCA
ATCGATCTTGTTGAATTTAAACCCGAACATGAAAACTTATTCAGATATGTTTTTGGAAATACAAT
TATTGTTGAAAACTTAGATTATGCGAAAACATTGTCAAAAGATCACAGAGCAAGATTTGTAACTC
TTGAAGGTGAGGTCATTGAGCCATCCGGTGCTATGATTGGTGGACGTTCAAGAAAAAAATCGGTT
ATTAAAGTTGATATCGACACATCCAAACTTGAAAAACTTGCAGAAGATATTTCAGAACTCGATGG
TACATTATCTGAAACTAAAGATGAAATTGAACGCCTTCAAAATAAAAATGCGACATATTCAACAA
GAAAAATGGAACTTGAAAGCAGATTAAAGATTATTAAAGATTTAGAACATAAAAAAGAAGGAAT

ACTCACAAACAATGGAGTAAAAATTAAAGAACTTGAATTGGAATCCAGAAAGCTCGAAGAAGAA
CTAGATTATTTAGAAGGTTCAAAAGAAGAACTTGAAAGAAAAATCGAAGAATTTACTAAAAAAA
TTAGTGGATTTACAAGCCAAAGGGACAGAATTTCTGAAGAAATAGCATCTTTTGAAAATTCAGA
GCATTCCAAAAGAATCAAAGTAATTGATGAAAATATCATTGCTTTTGAAAAGAAAAAGAATGAA
TTTGAAAACGAAATAAAAAGGGATGCAGTGCTTATTAAAGAAGTTTTGATCCCTAAAATAAGCG
AATTAAACAGCAATATCAAAGAATTAAGTGAAAAAAGGACAATTCTTGAGCAAAATATTCAATT
TTACAAAAACAATGTTGAAAAGAACTTTGAAATTTTGAAAAACAAGAAAGAACGCTACGAAGAC
CTTACAAAAGATTTAAGAGAACTTACTGAGAAAAAAGAAGCGTTTGAAAAAGAACTGGAAATAT
TAAATGGTGAGAAAAGAAGGGTTTATGGAAGGATTAACCAGAACGAAAGTCAGATAAACAGCCT
TTCAATCGACAAGGCAAAATATGAAACTAGGCTTGAAGAAGAAGATAGAAAATTATACGTTTGT
GAAAATATCGAACAAATATCTGAAGATATTACTTCCAAAATAAAAGAGTTTGATGTAGATGCCC
TTGAAAGTCACCAGATCGACCTTGAAGGACATATCAAAAAATTAGAACCTGTAAACATGCGTGCT
ATTGATGATTATCAATATATTGTAGATAGATACGACGAACTCTTTGAAAAAAGAACTGATTATG
AAAACGAAGAAAAAAAATATCTCCATTTAATTGAAGAAGTCAGCAAAAGGAAAAAAGAAGTATT
TATGGATGTTTACTTAAAAGTTGCTGAAAACTACGAAAAAATATATACTGAAATTGGAGGAAGT
GGAAAATTAAGTCTGGAAAATCCGGAAGATCCATTTTCAGGCGGTTTATTAATTGATGCATCCCC
GATGAATAAAAAAATTACAGAGCCTTGATGTAATGAGCGGTGGTGAAAAGTCGCTTACTGCACTTG
CATTCTTGTTCGCAATCCAGCACCTGAACCCAGCTCCTTTCTACGTGCTTGACGAAGTTGATGCTG
CACTTGATACGAAGAACGCAGGATTAATCGGTGAAATGATTAAAAATGCATCAAAAGATTCACA
GTTCATTGTAATATCTCACCGTGAACAGATGATAAGTAAGTCTGACGTTATGTATGGGGTATGTA
TGGAAAATGGACTCAGCAAACTAGTCGGATTAAAAATTTAAGATATTTAATACAAAATAATTTT
GGTGAATCTCATGAAAAGTATTTTGGATGAAACTATTGAACTTTCTTCTGATCTAATATCCATAA
ACTCAGTAAATCCAACTTTTGGTGGAATTGGGGAAAAAGAAAAGTCAATTTACATAAAAAATAA
GCTAGAAGAATATAATAAAAATTATTCAATAAAAAATTGCGAAATAAACGAATACAATACCGTT
GATTCAGAAGGAATTGAAAGGCCAAATATCGTTTCAAAATACGATTTTGGAAAAAATGATACCA
TTACAATAATTTCACACATGGACATAGTTCCTGAAGGTGATCTTAGTCTTTGGAATTCTAATCCT
TTCAAAGCAGAAATAAAAAACGGGATAATTTATGGTAGGGGCAGTGAAGACAATCATAAAGGAA
TAGTTTCATCGTTTTTGCTTTTAAAGATGATTTTTGAAGAAAAAATCGATCCAAAATATAATCTA
AATCTAATTTTTGTAGCTGACGAAGAAGACGGCAGTAAATACGGTCTATCTTACCTTGTAAATAA
TTTTGAAGATGAACTTTTTAGCTCAAACGATTTAATAATTGTTCCAGATTTTGGAATGCCCGAAG
GAGAATTTATAGAAATTGCTGAAAAAAACATACTATGGCTAAAATTTAAAATTACTGGAAAACA
GTGCCATGGAAGTGTTCCTGAAAATGGAATAAACGCAGATTTAATCGCATTTTCATTTGGAAAAG
GACTTTACGATAAACTCTACGGTAAATATAACGGCATAAATCCAATATTCAACCCTCCATTTTCA
ACGTTTGAACCTACAATTTTGAAAAATAACATTGAAAACATAAATACAATTCCAGGATACGTTGA
ATTAAATTTTGATTGCAGAATTATTCCAAAATACGATCCAAAAGAAGTACTAAGCGATATTGAA
AAGTATATTGAAGTTTTTAAAAATGAAATTGAAAAACATATTCTTCATTTTGATATTTCTGAAA
AAGAAAATATTTCGATAAATTACGAGATTTTAAAACTTGAAAAAGCAGAAGAAACAAAAAAAGA
TTCAGAAGTTGTTAAAAAACTGGGTTCTGCAATTAAAAACGTGTTAAATAAAGAACCAGTATTA
TGCGGAATGGGTGGTGGAACCGTTGCCGCATTTTTAAGAGAAAAAGGATACAACACTGCAGTTTG
GGGAATTGGAGACGAAACTGCACACCAGCCAAATGAACATATAAAAATAGAAAATTTAATAAAA
ATGGCAGAAGTGTATCTTGATATATTAAAATAAGTGGTAATCGTGTACGTTGTAAAAATTGGCG
GAAGTTTAACAAATAATGCAGTGAATCTTCTTGAAAATCTTAAAAAATTAGGAGAAAAAGTTGT
AATTATCCCAGGTGGTGGAAATTTCGCAAATGTGGTTCGAAATCTTCATGAAAATGAAAAAATT

AATGATATTTCTGCACATAAACTTGCAACAATGTGTACCGATATAACTGGAACCTATTTTTCCGA
AATTTCGGGGATAAAAACAGCAGATAACCTGTTTGATGCAAAAAGAATCCTCCAAAATGAAAGT
ATAGTTATAATACTTCCATCAAATATTATACTTAGTACTGATGAACTGCCCTGTTCTTGGGAAGT
TACTTCGGATTCATTTGCAGCATATATAGCAAAATTATTAAACTCTAAAACACTAATTATAGCCA
CCGACGTCGACGGAATTTATGATAAATATCCTGAGGGAAAACTATTAAATACAATAAATACAAA
AACGATTAAAGGTTTTACGTCTGTGGATAAACACCTACCAAAACTTATATCCGAATACGGCATTA
AGTGCTTTGTTGTTAACGGAAATCATCCTGAGAGAATAAATAACATTTTTAAAGGCGTAGTAGAT
ACGTATACGAAAATAACCTTGAAATAAAACACTATGGTGAGAAACATGAAACAAAAATGTACTT
CTTGCAATGCAGAAATTGCAGCTAGAGAAAATGCTACAAAATTCTTGTGCCCTAACTGTGGAAAA
GCAGAAATCATAAGATGCGAAAAATGCAGAAAATTAAGCAACGGATACAGTTGCCCAGTATGTG
GATACGAAGGTCCTTAAATTAATCTAAAAACGAAACTTAGGTGAAAATATGGCTACAGTTATCGC
AAAAGTAAAAGTAATGCCTACAAGCCCAGAAGTAGAAAAAGAATCTTTAAAAGAATCTTTAAAA
GAACTCGTTGAACAAAACGATGCAAAATGCAGGGGAGTAACTGACGAACCACTCGCATTTGGACT
CTACACTGTATACGTAATGGTTGAAATGGAAGAAAAAGAAGGCGGAATGGACCCTATCGAAGAA
GCTATGAACGCTTTAGAAAATGTAGAAAGCGCTGAAGTAGTTGAACTTTCACTCGTTTAATTTCA
CCAATACTAATTTTTTAAATAACGAAAAATAGCCCTAACTTAAAATAACACTCATTCTAAGCTTT
AATTCAAAATATGGGATTGTTATGATAAACTATATAAATAAGTTTAAGCAAATAAAAAAGGCTA
TTGACTATATGGATGATAGGGGATACTCTATCAATGTAAAACATGAAAATTGTTGTTAACGGAA
ATCATCCTGAGAGAATAAATAACATTTTTAAAGGCGTAGTAGATACGTATACGAAAATAACCTT
GAAATAAAACACTATGGTGAGAAACATGAAACAAAAATGTACTTCTTGCAATGCAGAAATTGCA
GCTAGAGAAAATGCTACAAAATTCTTGTGCCCTAACTGTGGAAAAGCAGAAATCATAAGATGCGA
AAAATGCAGAAAATTAAGCAACGGATACAGTTGCCCAGTATGTGGATACGAAGGTCCTTAAATTA
ATCTAAAAACGAAACTTAGGTGAAAATATGGCTACAGTTATCGCAAAAGTAAAAGTAATGCCTA
CAAGCCCAGAAGTAGAAAAAGAATCTTTAAAAGAATCTTTAAAAGAACTCGTTGAACAAAACGA
TGCAAAATGCAGGGGAGTAACTGACGAACCACTCGCATTTGGACTCTACACTGTATACGTAATGG
TTGAAATGGAAGAAAAAGAAGGCGGAATGGACCCTATCGAAGAAGCTATGAACGCTTTAGAAAA
TGTAGAAAGCGCTGAAGTAGTTGAACTTTCACTCGTTTAATTTCACCAATACTAATTTTTTAAAT
AACGAAAAATAGCCCTAACTTAAAATAACACTCATTCTAAGCTTTAATTCAAAATATGGGATTGT
TATGATAAACTATATAAATAAGTTTAAGCAAATAAAAAAGGCTATTGACTATATGGATGATAGG
GGATACTCTATCAATGTAAAACATGAAAATAATCCTGTTATTGAGATTGGAAATAAAAAGGCCA
ATCTATTAATAAAAAACACGATTGGAAACGTGAAAGTACATTACATCAATACAGCTAAACTCCTG
TTAAAGATGAGAGACTAACCCGATTGAGGTGAAGACGATGGCTAAACTAAAATACAAAGTTGAA
GCTGACCCAAAGAACACTGCAAGAGCTATGGGCAGAACTTTAAGAATATCAAGAAAACATGCTAT
TGAACTTTGCAGAGAATTAAGTGGAATGAAATTAGATGCTGCTGTTGCATACTTAAACAGAGTA
ATCGCATTAGAAACTCCTGTTCCATTCAAAGTTCACAACAAAGACTTACCACACAGAAAAGGAAA
AATTGGAACACACTCAGGTAGATTCCCACAGAAAGCTTCGTTAGAAATCTTAAACGTTTTAGATA
ACGCTAAGAAAAACGCTGAGCAAAAAGGATTAAACACAGAAAAATTAAGAATTAAACACATATC
ATCAAACAGAGGCTTCACAATTAAAAGATACATGCCAAGAGCATTTGGTAGAGCAAGCCCTAAAA
ACCAAGAAACAATACATATCCAAGTAATCCTTGAAGAATTCTACTAATGGTGAAACAATGATTGA
AAGAACATTTGTTGGAGAAAATGTTTCCGAAACATTAATCGACGAATACTTTAAAACTAAATTA
GTTAGAGCAGGATACAGCCACATCGATTTCAAAAAAACACCAATTGGAACAAGAATAACTGTATT
CGCTGAAAAACCAGGTTTCGTTATCGGAAGAAAGGAAAAATGGTTAAAGAATTAACTGAAACA
CTCGCAACAGAATACTCAGTTAAAAACCCGCAAATTGAAGTTAAACAGGTTGAAAACCCAGATTT

AGATCCTGCTGTTGTTGGACACAAAATTGCATCTTCATTAGAAAGAGGAATGCACTTTAGAAAAA
CAGCACACTCTGCAATCAGAAGAGTTATGGGCTCCGGTGCAAAAGGTGTTGCAATCATCGTTTCT
GGAAAATTATCCGGAGAAAGGTCAAGAACTGAAAAGTTCATGGACGGATACATGAAACACTGCG
GTGAACCTGCTGAAGCACTTGTAAACAAGTCACACCAATTAGCAAAATTAAAACTCGGTATTGTT
GGAGTTACTGTAAAAATAATGAGACCTGACGTATCATTACCTGATGAAATTGTAATTTCATCCGG
CGAAATTAAAGAAGTTAGTGAAATTAGTGAAGTATCCCAAGAATAAGGTGATGTAAATGGCGAT
CTTAAAAGCTAGCGAAATAAGGGAACTTTCAGTTGAAGAAATGAAAGGAAAAATCGCAGAATTG
AAAAGAGAATTAATGAAAGAAGGCGTAAACAAGTCAACAGGCGGTGCACCTTCAAATCCAGGTA
AGATCAGCGAAACCAAAAGAACAATCGCTAGAATCTTAACAATCATGAAAGAAAAAGAAGCTCA
AGCAGAAAATGCTTAAGCATAATTTTAAATACCATCAATCAGTATGTATATCCAGTATACTTATA
ACAAAATTAGTCTATCAAGATGAGAGGTGCATTGATGCCAGAGATTTGTCCTATATGTGGTTTAC
CAAAAGATTTGTGTGTCTGTGAAGAGATTGCAAAGGAAGAACAGAAAATTAAAGTTTACGTAAC
TAAAAGAAGGTTTGGTAAGCTGATGACAGTCGTCGACGGCTTTGATGCGGACTTGATTGATGTAA
AAGATTTGGCTAAAAAGTTAAAAGATATATGTGCCTGCGGCGGAACTGTAAAGAAAGATTCTAT
CGAGTTACAGGGAGACCACAGGAGAAAAGCTGAAGAAACCCTAATCAAAATGGGATTCTCAAAA
GACATGATAGATGTCAGATAATTAACTATTCACATGAGTATGTGGATACTGAAAAATGTACACCC
CCATGCTCAAAGGGTAGGGACACCCATGAAATTGTCACAAAATATATTGAGACATGAACTAATTG
GATTAAACCTTGAAGTTGTAAAATCTACAGATTCTGGTTTGATTTCAACAAAAGGTAGAGTAATT
AACGAGACTAGAAATACGCTAGTTCTTGAAAAAGAAAACGGCAAGGAAATTACCCTCGTAAAAG
AAATTTCAACCATTCAGAATTCAATTTGAGTCTGAAAATGTTGTAAAAATCGATATCGATGGAAG
ACTACTTGTTGGACGACCTGAAGACAGGTTGAAAAGAAAAATAAAACAGCTCTACTCGTACTAAG
CTTCTCAGCTATTTTAAATGTCCCAAATTGAAAATAAGAATTAAAATTTAAGTTCTAAACAGGCG
TTAGTGGTATTTGTGAAAACATTATATACCTGTAGCCCGTATTAAATCTGGAACTTTAAAATCGA
CATTAAACACTCTAAATAATTAGGAGGTATGATTATGTCAAACATTGGAATCGATGTAAAAGCTC
CTGAAAACGTTTGTGAAGATCCTAACTGTCCATTCCACGGAACTTTATCCGTAAGAGGACAAATA
TTCGAAGGAATCGTTACAAGTGACAAAGGACACGACACAATCGTAATCAAAAGAGAAGTTACTG
GATACATATCCAAGTACGAAAGATACGAAAAAAGAACAACTTCATTGGTTGCACACAACCCTGCA
TGCATCAAAGCAAAAGTTGGCGACACTGTGAAAGTTATGGAATGCAGGCCTATCAGTAAAACAAA
ATCATTTGTTGTAATTGAAAAAACTGAAAACCTGGAATAATCCTTTAAAAGGTGAATATTATGA
AAGGACTAGGTTCAAACATCGTAAGATCATTACCTAACGGAGCTAGACTCGTCTGTGCAGATAAC
ACAGGTGCAAAAGAGCTCGAAGTAATTGCAGTTAAAAACTACGTAGGTACTGTAAGAAGGTTAC
CTGCAGGTGGTGTAGGCCATATGGTTTTCGTTTCAGTGAAAAAAGGTACTCCTGAAATGAGAAAA
CAGGTTTTACCTGCAATTATCATCAGACAGAAAAAAGAATACAGAAGAGCTGACGGTACAAGAGT
AAAATTCGAAGATAACGCAGCTGTTATTGTTACCCCAGAAGGAACACCAAAAGGTTCAGAAATTA
AAGGTCCAGTTTCAAAAGAAGCTGCTGAAAGATGGCCTGGTGTTTCAAGATTAGCTAAAATTATT
CACTAATTAGGCATGTGGTGAAATCATGGTTTTGACAAACTCAAAACAGCCAAGAAAACAAAGA
AAAGCACTCTACAACGCACCATTACATTTAAGAAATAGCGTTATGTCTGCAATGCTAGCAAAAGA
ATTAAAAGAAAAATACGCTAAAAACTCCCTTCCTGTGAAAAAAGGAGATACTGTAAAAGTACTC
AGAGGAAATTTCAAAGGAATTGAAGGGGAAGTTTCAAAAGTTGACTACGCAGGATACAAAATCA
TTGTTGAAGGAGTAGTTAACAAAAAACAAGATGGAAATGAAACAGCATATCCAATTCATCCATC
AAATGTTATGATTACCAAATTGGACGAATCAGACGAAAAGAGATTTAAAAATTCAAGTAACTAA
GAGGTGTGAGCATGGCAATTAAAGGACCTAGAAAACATCTTAAAAGATTGGCTGCTCCAGCAAAT
TGGCAACTTCCTAGAAAAGTGAAAACATTTACGGTAAGACCATCACCTGGACCACATTCAATGGA

TAAATCCTTGCCATTATTGTTAGTTGTAAGAGATGTATTAAAATATGCTGACAATGCAAGAGAA
GCTAAAAAAATTATCCAAACAGGAAAAATTTTAATCGATGGATTAAAAAGAAAAGAATACAAAC
ACCCTGCTGGATTAATGGATGTTTTATCAATCCCTGAAATGGATGAAACTTACCTTGTATTATTT
GACGAAAGTGGAAGAATCAGCTTGAAAAAAACTGATAAAACCGACGTAAAATTATGCAAAATTG
TAAACAAAACTGTCGTTAAAGGCGGAAACATACAATTAAACCTTCACGATGGTAGAAACCAGATT
GTAAAAGTTTCAGATGCTGCAAAAGCTGAAGAAGATGTTTACAAAACCGGAGACAGCGTTTTAA
TTTCAATTCCAGAACAGTCAATTGCTGGACATGTTGCATTTGGCGAAGGAAAATTAGCATACATC
ACTGGCGGTAAACACGTTGGAGAATTTGCTAAAATCGTTGAAGTTGAAAACAGAGCATTGTACTC
AGATATTGTAACTTTAGAAAACAAAGATGGCGAGAAATTTAAAACCGTTAAGCCTTACGTATTC
ATCGTAGGTCAAGACGAGCCAGTAATTTCGATGTAATGCCCATAAAATTAGAGGAGGACGAAACA
TGTCATTCCAAGAAGTTTGGGAAAAAGAACCTATGAAAAAACCAAGAATCCAAAAAGTCACCGT
AAACTTCGGTGTAGGTGAAGCTGGAGATAGGTTAACAATAGGTGCAAAAGTTATTGAAGAACTC
ACCGGCCAATCACCAGTAAGGACTTTAGCTAAACAGACAAACCCTGCATTTGGTATCAGAAAAAA
ATTACCAATTGGATTAAAAGTAACATTAAGAGGAAAAAAAGCAGAAGAATTCCTCAAAAATGCG
TTCACTGCTTTCAAAGCATGTGGTAAAACACTCTTTGCGTCATCATTTGACCAGGTTGGAAACTT
CTCATTTGGTGTTCCTGAACATATTGACTTCCCAGGTCAAAAATACGACCCATCAGTTGGAATCT
ATGGTATGGATATCTGTGTAACCTTTGAAAAATCAGGATACAGAGTAAAATCAAGAAAAGTAAA
AAGAAGCCACATTCCAGAGAAACACTTAGTTACCAAAGACGAAGCTATCGAATACATCCAAGCTA
AATTCGATACTGAAGTAGTTAGGGAATAAGGTGAAAGAATGACAAAAACACCATTTAAGACAAA
ATACGGCCAAGGTTCAAAAGTTTGCAAAAGATGCGGAAGAAAAGGGCCAGGAATCATCAGAAAA
TACGGATTAAATTTATGCAGACAATGCTTCAGAGAATTAGCACCAAAACTAGGATTTAAAAAGT
ACGATTAAGGACAAGGAGGTAACAATATGAGTTTAATGGACCCTCTTGCAAACGCATTAAACCAC
GTATCCAACTGCGAAAGTGTGGGTAAAAACGTAGCTTACTTGAAACCTGCTTCAAAATTAATTGG
AAGAGTTCTAAATGTTATGCAGGATCAAGGATACATTGGAAACTTCGAATACATTGAAGATGGA
AAAGCAGGAGTCTACAAAGTTGATTTAATCGGACAAATCAACAAATGTGGGGCTGTAAAACCAA
GATACGCTGTTAAAAACCACGACTTCGAAAAATTCGAAAAAAGATACTTACCGGCTAAAGGTTTC
GGATTGTTAATCGTAAGTACCCCAAAAGGATTAATGACACACGACGAAGCTAGAAACGCTGGAGT
AGGTGGAAGATTAATTTCTTACATATACTAAGGACATTATGGAGGTATTCTTATGCCAGTTGCAG
CTTTAATAAGGGAAGAAATCGAAATTCCTGAAAACGTCAACGTTGAAATTAACGGCAGCACAGTT
GTTGTTAAATCTGGTGCAAAAGAACTTAAAAGAGAATTAATGTACCCGGGAATTGAAATAAGCA
CAGAAGACGGAAAAGTTGTTATTGAGTGTGCATTCCCAAGAAAAGCACAAACCGCAATTGTTGGA
ACCTACAGATCACACATTCAAAACATGATCACAGGTGTTACTGACGGATTTGAATACAAATTAGT
AATCAGATACGCTCACTTCCCAATGAAAGTTAGTGCTAAAGGAAACACTGTAATGATTGACAACT
TCTTAGGAGAAAAATACACTAGAACTGCTAAAATCATGGATGGAGTCACTGTAAAAGTTAGTGG
TGAAGACGTGGTTGTAAGTGGAGCTAACAAAGAATTTGTTGGTCAAACCGCTGCAAACATCGAAC
AAGCTACTAAAGTTAAAGGAAGAGACACAAGAATATTCCAAGATGGCATCTACATTGTAGAAAA
AGCAGGGAAAGTATTATAAGGTGGTCTCATGAGTGAATTCAAAAGATTAATGAGATTAAAACTC
AAAATGAAACAAAAAAGACCTGAATTTAAAAGACAGGACTGGTTCAAGTGTTCAAGAATTGGAA
CTAGCTGGAGAAGACCTTTCGGTAAACACAGCGGTATGAGAATAGGATTGACACACAGAGCTGCT
GTTGTTAACACAGGCTACAGAGGACCTGTTCTTGTTAGAGGTTTACACCCATCAGGTTTACAAGA
TATTCTTGTTAACAATGTAAAAGAATTAGTTGCATTAAACCCTGAAGTTCAAGGCGCAAGAATTG
CAGCTACCGTTGGTAAAAGAAAAAGAATAGAAATCGTTAAAAAAGCTAATGAATTAGGAATTAG
AGTGTTTAACGTATCCAAACAAAAACAGGAAGAATTCTTAAGTTTATAATCTCGCTTAAGATATA

AGGTGATATTATGGATGTATCAACCCAGAGAAGAATTGCTGCAGCTGTTTTGGATTGCGGTATCG
ACAGGGTATGGATTGATCCGGAAAACCTGGAAAAAGTCAAAATGGCAATCACCAAAGATGACAT
TAGACTCTTGATTAACGATGGAATCATTGTTAAAAAACAGGAAAAAGGAATCAGCAGCGCTAGG
AAAAAAGAAGTTCAAGAACAGAAGAGAAAAGGAAAGAGAAAAGGACCTGGTTCAAGAAAAGGGG
CTAAAGGTGCTAGAACGCCTAAAAAAGAGAAATGGATGAACACAATTAGGCCGCTCAGAAAAAT
GCTGAAAGAATTAAGAGAAAATGAAAAAATCGAAAGATCAGCATACAGAAAACTCTACAGAATG
GCCAAAGGTGGAGCATTCAGAAGTAGAAATCACATGAAATTATACATGAAAGAACATGGAATTT
TAGCTGAATAATTCAAATTTAAGGAGGAATGACTATGGCAATGAACGCCAAATTCAGAGTTCCTT
TTAGAAGAAGAAGAGAAGGAAAAACAGATTTCAGACAAAGATTAGGATTATTATTGTCTGGTAA
ACCAAGATTAGTTGCGAGAAAATCCTTAAACAATGTGACCGCTCAGCTTATGGCATACGATGAAA
AAGGAGACGTTGTTTTAGTTTCAGCACACTCCAAAGAACTCGTTAAAATGGGCTACAAAGGACAC
TGCGGAAACTTACCTGCTGCATACTTAACAGGTTTATTGTTAGGTGCAAAAGCAGTTAAAGAAGA
TGTAAAAGAAGCAGTTTTGGACAAAGGTCTTCACAGAGCTACCAAAGGAGCTGCAATTTTCGCTG
TTTTAAAAGGTGCACTTGATGCAGGTATGGATATACCTCACGGTGATAAAATCATCGCAGATGAA
GAAAGATTAAACGGAACTCACGTTAAAAATTACGCAGAAAGCTTAAAAGAAGATGCAGATGCGT
ACAAAAAACAGTTCTCAAAATACTTAGAAAAAGGATTAAACCCAGAAGACTTGCCAGAACACGTT
GAAGAACTGAAAGAAAAAATCCTTAACTTATAAGGTGATATAAATGGCTGAAAAAAGAGCTGAA
AAAAGAAAATTCAATACCGAAGCTTGGGAGCCAAAAACCCAAATTGGTAGAATGGTTAAGGAAG
GAACAATTTCAGACATTAGCTACATTATGGATAAAGGTCTTCCATTATTAGAACCTGAAATTGTT
GATGCACTTTTACCTGATTTGGAAGAGCAAGTTTTGGACGTTAAATTAGTTCAAAGAATGCACAA
ATCTGGAAGAAGAGCAAGATACAGAGCAACTGTTGTTGTTGGTAACAAAAACGGTTACGTTGGA
GTAGGCATGGGTAAATCAAAAGAAGTTGGCCCTGCTATCAGAAAAGCAATTGCTCACGCAAAATT
ATCATTAATCAAAGTTAGAGTTGGCTGCGGTTCATGGGAATGCGGTTGCGGATCACCTCACTCAA
TCCCTTTCACAGCTAAAGGAGCTTGTGGTAGCGTTAAAGTAGAATTACTCCCTGCACCAAGAGGT
GTTGGTTTAGTTGCTGGTAACGTTGCAAAAGCTGTTTTAGGTCTTGCAGGTGTAAAAGATGCATG
GACTAAAACATTTGGTGACACAAGAACCACATACAACTTTGCTGAAGCTACATTCGATGCATTAA
ACAACTTAAACTTCGTAAGATGCTTGCCTAACCAAAAGGAAAAATTAGGTCTTACAGAAGGAAGA
GTACTCTAATTTATTACTTTTTAAAAAGGTGATTAAAACATGGCTTACGCAGTTGTAAGAGTCAG
AGGAAGCGTAGGTGTTAGAGGAGACATCGCAGATACCATGAAGATGTTAAGACTACACCGTGTAA
ACCACTGTGTTATAATTCCAGATACAGAACACTACACTGGAATGATAAAAAAAGTTAAAGACTAT
GTAACCTACGGTGAAATCGATAAAGATACACTTGTAGCTTTAATTTTAAAAAGAGGAAGACTTCC
TGGAAACAAAAGATTATCTGAAGAACTCGTAAAAGAGTTAACAGAACTTCCAGTTGAAGAGTTA
GCTGAAAAAGTAATTGCTGGCGAAATTAAAATAAAAGACACACCAATAAAACCTGTATTCAGAT
TACATCCTCCAAGAAAAGGATATGATAGAGCAGGAGTTAAAAAAGGATTCTCAATTGGTGGTGCC
TTAGGTTACAGATCTGGTAAAATCAACGATTTATTGAACAAAATGATGTAATTTAAGGTGATTT
AATGATTAGAAAAAGCAAAAAAATCACGAAAAAAAGAGGCTCCAGAACCTGCGGATATGGAGAA
GCTAAAAAACACAGAGGCGCTGGCCACAGAGGTGGTAGGGGTAACGCTGGTCACCAAAAACATAA
ATGGTTGTCCGTCTGTAAATTCAATCCTGATTACTTTGGTAAATACGGATTCAACAGAAACCCTT
GCCTTATAAAACAGTTAGAAACTATCAACATTGGAGAATTAGAAGAATACATCTTAAAATACAA
AGATGCATTCCAAGTAGAAGAAGGTAAAGTTGTAGTTAACGCTACAGAAATCGGATTCGAAAAA
GTTCTCGGAAAAGGAAGAATCTCAACCGCAATGGTTGTAAAAGCAGTTGAATTCTCTGAAGGAGC
TAAAGAAAAAATTGAGGCTGCCGGTGGAGAATTTGTTGAATTGTAATTCAATTAAGAATCACTT
ATTCCATTTAATTCTGCTTTTTCTTATTTTTGATAGTGGATACTATGAAGATCCCAATCATTTAA

ATACTCATTTTTTAATATCTTTTAATGTTGTGAAACCGATATTTAATACATTTTCCAAAGATTAT
CACAATTACACTATTTGATAACAAATTTGTAAATTTTCTCGTTTATTGAAATTTATCATGTTTAT
TCCAAGGTGAAAATTTGGAAAGTTTCTATTAAAAATAAAACCCATACTTGAATTAATTCCAGAA
GTAAAAAGACCACTAAGAGAAATTTCATTTAAAGAAAAATTACAATGGACTGCACTTGTGCTTG
TACTCTACTTTATTTTGGGTACAATCGATATTTACACGGGCGGTTCAGAAATTCCTGCAATATTT
GATTTTTGGCAGACCGTTACGGCTTCTAAAATGGGTACTTTAATTACACTTGGTATCGGACCAAT
TGTAACTGCAGGAATTATCATGCAGCTTTTAGTTGGATCGGAGCTTATAAGTCTGGATTTATCAA
AACCGATGAATAGGGCATTATTTCAAGGTTTACAAAAGTTATTTGGTATAGCATTATGTTTCCTC
GAAGCTTTGATGTTTGTTGGAGCAGGGGCATTTGGTGCATTAACCCCACTAATGACAGCAGTGTT
AGTATTCCAGCTTGCACTTGGTGCGATATTAATTATTTATTTGGATGAAATTGTTTCAAGATACG
GTATTGGATCAGGTATCGGTCTTTTCATTGCAGCAGGAGTCTCACAAACAATATTTGTAGGAACC
TTTGGTGCAGAAGGATATTTATGGAAATTCTTCACTGCAATGACTGCAGGAAGTTTATGGACTGC
ATTAGAATATATCTTACCAATTTTAGGTACAATACTTGTATTCTTAGTTGTAGTTTACGTTGAAA
GTATCAGAGTAGAAATTCCACTAGCACACGGAAGAGTAAAAGGTGCTGTTGGAAAATACCCAATA
AAATTCATCTATGTTTCAAACCTGCCAGTTATCCTTGCTGCAGCACTCTTTGCAAACATACAGCTT
TGGGGAATGTTTTTAGAAAAAATGGGTTTCCCAATTCTCGGACACTATACGAGTGGTAGGGCAGT
TGATGGACTTGCATACTACTTCTCAACACCGTACGGAATTTCAAGTTTGACTGCAGATCCGCTTC
ATGCAGTATTTTACACTGTAATGATGGTAATATTCTGTATATTATTTGGTTTATTCTGGGTTGAA
ACCTCAGGACTTGACGCAAAATCGATGGCTAAAAAACTCGGAAATCTTGATATGGCAATTAAAGG
ATTTAGAAAAAGCCAAAAATCAATTGAACAACGTTTAAAAAGATACATCACACCAATTACCGTA
ATGGGTTCAGCATTTGTTGGATTCTTAGCCGCAGCTGCTGACTTTACAGGGGCCCTTGGTGGAGG
TACTGGAGTATTGCTTACAGTATCGATCGTATACAGATTATACGAACAACTCGTTCAAGAACAGC
TCTCTGAACTTCACCCATCAATTGCGAAGTTCATAAGAAAATAAAACTTAAAAAAATAACTAAAA
ATAAAAAATTTTTACTTTTTTAAAAAATATTAAACCCGAAGTATTTTGTATATAAAATACTCAA
AATTCCAAAAAAGGCTTCAATTGCCCACAATACAAATACTATTTTATATTCTTCCATTGCTTTTT
TCATTAAAATCATTCTTGGTAATGACAGATATCCACCTTCAACACATAACTTTCCATTCTTTAAT
TTAGTGGGTGTAAATACAGTGTCTTTTTTTGTAACTCCTGCAGTAAACTGCTTTAAAAGTGCATC
AACCACGAATGGAATCATTAGTATAAACAGTTCAGGAATAAAGCCCCTCCATACCGCAATGGTTG
CCAAAAATGCACCAATTGGAAGGGTTCCAGTGTCTCCAGGAAATACTTTTGCAGGGTATCTGTTG
TAGTATAAAAGGCCAAGATAAGCTGCTGAAAATAAGATCAAAACTTTAAACCCAAATATATCCCC
GTTTATCAAACATACTGCTGCCATAAATAAACATAGAAGTACCCCCATTCCAATTTCAAGACCGT
TGAATCCAGCAAGCATATTTGTTAAATTTGATGAAACAAAAAATTCCAAGGATTAAAAGAACTGA
CAACAAAACATTTAATTTTAAAAAAAATGCAACAGGAATCCCCATTAAAAATGTTAAAACTATT
TTTTCTTTTGAATTTAATCTTGAAATATCGTCAATTACTCCAACAAAACCTGGCAAAAATATACT
TAACGAAAGGGCAGGGTTGAAAAATAACATTGCAACAGACGATACAACTACTGGTGATAATCCAC
CCATTTCTGCCACCTTGATTTTATCGGCCTTATGAAGGTCATAACCATATTTGTAATTAACCATT
TTTTTAATCATAAATTTTGTGAATATCAGAGAAATTACAAAATAAAATAACGCAGATAAAACAA
AATATAAATCCATAACATCACCATTTATACTATTAAAACATAATTCAATTAATAAACTATTTATA
TATACATTATCGATTATTTTTGATAAAAATTTTGGAGGAGGATTATGGGTGAATTTTTAAATAA
AGTCTCAGATTTTTTCAAAAAGAATGAAAAAATAAAGATCATCCTAATATTATTGTTTATAGGA
ATGATGAGCTTTCAAATCCGTGCCCAAACTGCGGACATGGCATTTACAGACAATTCATATTTACA
AGACATGTTCTCAGACGATAACGGCAGGATGTATTTAACTGCACTCGACCCTTATTACTACCTTA
GAATGACTGAAAATTATGTAAATAGTGATTATTCAAACGTTGGGGAGACTACCGTTGAAGTAGA

TGGGAAAAATGTACCTTATGATACACTACAGTACGCACCACCAGGACATAAAGTAGGTTCAGTTT
CTGCACTTTCTATTGCAACAGTTTTAGTATATTCAGTTTGGAATTCGATTGATTCAACAGTTACC
CTAATGAATGCAGCATTCTGGGTTCCCGCAATTATGAGTATTTTCCTTGGAATTCCTGTATTTTT
TATTGTAAGGAGAAATACTGCATCAAATATTGGAGGGCTTGTTGGAGCGCTGATTTTAATTTCAA
GTCCAAGTTTGTTATATAAAACATCTGCTGGATTTTCAGATACGCCGATATTTGAGATACTGCCA
TTACTATTCATAGTTTGGATGGTAATGGAAGCAATTCACGAACAGGAAAATTCTAAAAAATCAG
GCATATTCGGGGGAATTGCTGCCATTTTGATTGGATTGTACCCAATGATGTGGAGTGGCTGGTGG
TATGCATTTGATATCACAGCAGGCTTTTTAGGTATATACTTAGCTTATGAATACATTACAAAATC
AAAAAATTTAAAAAACGTAATCACAACCTCGTTAATTACATTAGTTGGTGGAGCAGTTCTTGTTT
CACTTTCAACAGGATTAAGTGGATTTATTAATGGAATACTGTCTCCAATCGGATTTACCGTAATA
AATGAAGCTACAAAAATAACAGGTTGGCCAAATGTTTATACTACAGTTTCAGAACTTGCAATTCC
AACAGTAACGGACATAATTGAAAATTCAGTTGGAAATATTTGGTTATTAATTGCAGGAATTTCA
GGAATATTGTTATCATTTGTGTCATTTAGACACGATAAACAAAAAATCGATATTAAATATGCACT
CTATTTGACACTCTGGCTTATGGCTACAGTTTATGCAGCAACAAAAGGTATTAGATTCGTTGCAT
TGATGACTCCTGCACTTGCAATAGGTATTGGAATCTTTGCAGGCCAGATTGAAAATATAATCAAA
AGATACGAAAAAAAAGTGGAATACATATTATATCCGGTAATTGGAATTCTCTCAGTAATTAGTT
TGATCAAATATGGCGGAGAAATCTTAAATATAATCATTCCAACCACATACGTTCCAATTGCAGTT
TATTTATCAATAATTGCACTGCTTGTATTAGCAATATACAAAATCATAGATATAATTTCAGAAAA
AGAACAAGCCGTTAAAAAAGTATTTGGGATACTTCTTGCATTTATGCTTGTTTTCCCATCAATGG
CTGCCGCAGTACCATTTTATACTGCACCTACAATGAATAACGGCTGGATGGATAGTTTAAGCTGG
ATAAAATCAGAAACTCCTGAAAACTCAGTTGTTACCTGCTGGTGGGATAACGGACACATCTACAC
ATGGGCTACAAGAAAAATGGTAACCTTTGATGGAGGATCACAAAACACCCCTAGAGCTTACTGGG
TTGGGCATGCATTTTCAACTTCTGATGAAAATTTGTCTGTTGGAATTTTGAGAATGCTTGCAACT
AGTGGAGACAGCGCTTATGATGATAACAGTATTTTAATTAAAAAAACAGGATCGATTAAAGATA
CTGTAGATATTTTAAATAAAATACTTCCATTAACAAGAACAGAAGCTAAAGCTAGTTTAGTAAA
TAACTACGACCTGACAGATGCAGAAGCAGAAGAAGTACTTGATTTAACACATCCAAAAGTTACAA
ATCCTGATTATTTAATTACATACAACAGGATGACAAACATTGCATCAGTATGGAGCATGTTTGGA
AACTGGAACTTTAGTTTACCTGCAAGTACTGAAAATAGCGATAGAGAAATGGGCTACTACCAGCA
ATTGAGCGGTAGTGCACAGGATATTAACGGAACTACAGTAGTATACATACCACTACAAGAAACTG
ATTCATACCGTGTTATAAATATTCTTGAAATTACAGATTCTGAAATAAAATCTGCAAATGCAGTT
ATTGACAGCAACAACCAGACATCAATGCAGAGTCCAAATTTCCACAAACTAATTTTAAAAGTGAA
TGGAAATGTCTACGAACAAGAAACAAACGAAAATGGAGACTACTCCGAAATCGTAAGGCTAGAA
AAATTATCAGATGGAACATACCAAGTATATGCTTGGGTTTCAAGTAAAAACCTTGAAGACAGCAT
ATATACAAAACTACATTTCCTTGACGGATATGGACTTGAAAAAATCAGCCTTGAAAAAGAATCGG
TAGATCCAACATCCTACGGCATACAGCCAGGATTTAAAGTCTACAGCGTAGATTATGGAACAGAC
TATCTCAATTAATTTTGAGATTTAATTTTTTTACCATTTCAATTAATTTTAAATATTAACTTTTT
TAATATTTTTTGAAAACTTTTAATTTAAATCGAATTTTATTTGATTAATTTGGTGATTTTATGGC
TATTGAGATATTAAGGTTGGGCCATCGAGGCGAGAGAGACAAGAGAATATCTACCCACGTTGCAC
TAACTTCACGAGCACTTGGGGCTGAAAAGATAATATTTACACAAGAGGATAAACACGTTAAAGA
AAGCGTTGAAAGAATTGTTGAAGCTTGGGGCGGAGAATTTAAATTTGAAGTCGTCAAATCCTGG
AGATCTTATGCAAAAAGGTTTAAAGATAACGGGATAGTAGTACATCTTACAATGTATGGGGAAA
ATATTAATAAAATTATGACAGAGATTAGAGAAGATATTTCAAAAACGAATAAAAACTTGCTTTT
GATAATTGGTGCTGAAAAAGTTCCAAGAGAAGCATATGATCTTGCAGACTACAACCTTTCAGTTG

GAAACCAGCCCCATTCTGAAGTTGCGGCACTTGCAATATTTTTAGACAGGTTTACCGAAGGAAAA
ACTCTTTATTCTGAGTATGATGATGCCAAAATAAAAGTAACACCTTCAAAATCCGAAAAGTGTGT
TTTTGTAGAAAAGGACTAATCTAAATCTCTCTGGTGAAATAGATGATATTAAGTGATAAAGACA
TTTTTAATTATGTTACTTCAAAAAGAGTTTTAATTGAGCCTTTCAACTCAAAATTCGTTGGACCT
TGTTCTTATGACGTTACCCTTGGAAGCGAATTTATAAAATACAACGAAGACGTTTACGACGTTAA
AAAAACTTTAAATCACAGTAAATTTGAAATTGAAAATTCCGTTATGGTATGCCCCCTAAACCACC
ACTTGGATGAAACAATAATTAAAAACTATAAAGAAAAATACAGTGTCGACTGTGTTGTGAGTGG
AGGACTTTTAGGAACCACTAATGAATACGTAGAACTTCCAAATGATGTTTGTGCACAGTACCAAG
GTAGAAGCAGTTTTGGAAGAGTATTTTTACAAACACACCAAACCGCAGGCTGGATTGATTCAGGA
TTTAAAGGAAAAATAACGTTAGAAATTGTGGCATACGATAAACCTGTAATTCTCTATAAAAACC
AGAGAGTAGGGCAGTTAATATTTAGTAAAACACTTTCACCAGCAGATATTGGATATTCAGATAG
AAAATGCTCAAAATATGCAGGACAAACGTCAGTAATGGCATCACTTATTAAAAAAGACTTTGAA
ACTAATGAGGAGGAATAAAACATGTTCGTTCCAGAGGAAATTATTGAAACAATAAAAATGATTG
AATACCAAAATTTAGATATAAGAACTACCACACTTGGGATCAACCTAAAAGACTGTGCTGATAAA
GATTTAGACCTGTTAAAAGAAAACATTTACAACAAAATAACGACACTTGGCGGAAATTTAGTTG
AAACTGCAAACAAAGTTTCTCAAAAGTATGGTATTCCAATAGTAAACAAGAGAATTTCAGTAACT
CCGATCGGATTGATTATGGGAAGTACCGTAAAAGGATTAAGTGATGAAGAAGCAGTTGATGCAT
GCGTTGAAGTTGGAATAACACTTGATAAAATTGCAAAAGAAGTGGGAGTAGACTTTATTGGAGG
ATATTCCGCATTAGTTCAAAAAAGAGCCACCTACGAAGAAAAAATGCTTATCAGGTCAATTCCAA
AATTAATGACCAAAACTGACAAAGTATGTGCATCAGTTAACGTTGCTACAACAAAAGCAGGAATT
AACATGTACGCTGTTAAAAAAATGGGAGAAATAGTTAAAGAAACTTCAGAAATTACAAAAGATG
CAATTGGATGTGCAAAAATCGTTGTATTTTGTAATGCTCCAGAAGACAACCCATTCATGGCAGGT
GCATTCCACGGACCTGGAGAAGGAGATGCAGTAATTAATGCAGGAGTTTCCGGACCAGGTGTTGT
TAGAGCGGTTGTTGAACAGTTGAAAGGAAAAGATATTGGAACAGTTAGTGATGAAATTAAAAAG
ACTGCATTTAAAATCACTCGAATGGGCGAATTAGTTGGAAAAGAAGTTGCGAGTGAACTTGGCGT
AAACTTTGGTATAGTTGATTTATCTTTAGCACCAACACCTGCAATTGGAGACAGTATTGCAAATA
TCTTAGAAGCTGTTGGTCTTGAAAGATGTGGAACCCACGGAACTACAGCAGCACTTGCAATGTTA
AACGATGCAGTCAAAAAAGGAGGAGCAATGGCTTCAAGTAATGTTGGGGGACTTAGTGGTGCAT
TTATACCAGTAAGTGAAGATGCTGGAATGATTGAAGCAGTTGAGGTTGGAGCTTTGAGACTAGA
AAAACTTGAAGCAATGACCTGTGTGTGTTCAGTGGGTCTTGACATGATTGCAGTTCCAGGAAAAA
CTCCTGCTTCAACATTATCTGCAATTATGGCCGATGAAATGGCAATTGGAATGATAAACAAAAAA
ACTACTGCAGTTAGAATCATTCCAGTTCCGGGAAAAGATGTTGGTGACCACGTAGAATATGGTGG
ACTCCTTGGAACAGCTCCAATCATGCCTGTTAGCGAATTTTCCTCCGAAGAACTTATCGAAAGGG
GCGGAAGAATACCTGCACCAATTCAATCCCTTACAAATTAAAATCAATACCTAAAAATTTTTAGG
AAAGTTTATAAACTTTTAAAACATATACTTTTTAGATAAGGTGATAAAATGGCTAAAAATCAGG
ATGCAGGACTGAGCACAAGTGCAGGACTTGTAAGATATATGGATGAAGATGTTTCAAAAATAAA
AATAGCTCCGGAAAAAGTTTTAGGCTTAACCGTGTCAATTATAATCATTGAAGCTGTTTTAAACT
ACGGAATTTTAATTTAATTAAAAAAATAAAATAAGTTTTTTTGAATTTTTAATATTAATTTAA
ATCTTAATTAATCGTATTCTCTCCAAGTATGGCTGCATTTTTTACATTTGTAAAATCTGGTTTCA
GGTTCATCAGCACATCTTGTCTGCTGTAACCACCAGAATGCTTCCATATTTCCACAATTTGGGCAT
TCTATTCTTGTTGTTGGGAGGGTATCTACGTTTTCAATAACAGTAACGTCCTGTTTTTTGGATTC
TATTTTTTCTTTTAAAGCGTATTCTTGATTTGCATCACCAAGTTCTTCTTCGTGTTTACAGACCAC
ACACTTTAAAACTCCACCTTTTGGGAGCATGATATTGTTACATTTAGGACAAAATTCGACCATAA

ATCCCACCTTGTAATTCGGTTATACCGGATATATCTTCATAATTCAACTTAAAGAGGTTAAATTA
CTATTTATAACTTTCGATTTTTATTTATTTTGTTTTTACCCACATCTTTTAGTTTTAAGTACAGA
GGAAGGATTATTAAAACGTATATCGGAATAATTTCAAGCCTTCCAACCCACATTGAAAATACGCC
TAAAATTTTTGCAACGGGGTGCAGTGAGTGGTTTACTATATCAACAGAAAGCCCCATGTTTGCAA
CTAGCGAAACTGACTCAAATACGGATAAAAATGGATCATAACCAAGGCTTATCAAAACAAGCGTT
CCAAATAAATAATGGAGAATATATGCAAAAGCTATTATAAATGCATCCAGAATTAAAAAATCAG
ATAATTGGTTTTTACCTATTTTTTTGTATATTATCGCGTTTCCAGGACTAACAACTTCTTTTAAC
CTGTAATAAAATCCTTTAGCCATAATTACAAGCCGTATAAGCTTAATACCGCCTGTTGTAGTCCC
AGTACCCCCTCCAATCGCCATTACAAATATAAGAACTGCAATTGATATGTTTGTTAGCGAAGATA
TCTGTACGCTTGAAAATCCTGTGCTCGTCATTGCAGAAACTACTGTAAATAGCGAATCTAATGGG
TCTATTCCTGACGTTAGGGTAATTACAAATGCAGCAAAGAAAAGTATTGGAATTGAAGTTTTTGT
CTGTATATCATCAACCCGCCTTCCGGTTAAAACATTGTGGTGTACTGAAAATGAAACAACACCCC
CAATATACATAATTAGAGTCATTATAATCTTTGCGAAGTTATTAAATGGAAAACTTTGATTACTT
ATACTCATACCGCCTGTTGAAATACCACACATACAGATGTTTAATGCACTCCACAAATTTAAGCC
ACTTAAATAAAGCAGACAGATCCCAATGGTGGTATATAACAAGTATATTTGCCATATTTTTTTAA
TCGTTCCAATTGTACTTGGCATGATTTTTTCTTCCCTTGCTTCTGCACGATAGTATGCAGATGCCC
CTGCTTTTGAGAGAATTGTAATAACCATTGCAAGCACACCAACTCCACCAATCCACTGTTCAAAA
CTCCTCCAAAATTGAAGAGTTCTTGGTAATGATTCTACATCACCAACAATACTAAATCCTGTTGT
AGTCCATGCAGACATGCTCTCAAAAACACCATCAAGGTAACTGAAATACTCGATGCCGATATAGA
ATGGAATTGCCCCAATTAATGAAGCCATGAGCCATGCTAAAGCTGAAATAACCATTGCATGCTTT
AATTTTACTGAAATTGGCTTGGTAATTTTATTAATTAATAACCCGAGTATCATGAAAAAAAGTG
AGGGGAATAAAAAATAAAAAAACGGTTCATGAAAATAAATCCCGATAAAAGATGGCAACAGCAT
TATGAAACCGATAGTTGATATTAAAATCCCCAATTCATGAAAAATTCCTGAAATATCGCTTTTTT
TAAAAATTTTCATAATACCCCAAAATTAAATGAATTAGTAAAAATCTGAATCAATTATCGGTTCA
TCATTTAAGTTGCTAAACCTTTCTGTAACAATTTCCAGACACTTTTGAACGGTATCACTGATTGA
AACGTTTTCTATAACTGGAAGGTTGTTTTCTTTTGATTTTTCAACAATATACTGGTTAATCTCTT
TTATTATTTCAAAATTCTCCAGATATCTTTCAAGGGGCCTATCGCTTACCTTTGCCCTTGCAATA
AATCTTTTTTTATGGGTTTCTTCGGAACTTAATGTTAAGTTTAAAAATATAACGTTTGGCATCTC
CTGATATTTTTCAGCCATTAATCCTGGAACTATGTGTGTTCCCTCAATTATGACGCTGGTTCCTTC
AGTTAAACTCCTATCTATAATACTTTCAATGCCCAATAAAACAGGCTCAACGTGCCTTTCAAAAC
CGAGTAAATGCATTTCTTTTGAGTCTTGCTCTTCCCATGAGGACTTTCTAAGCGCAGTCCATGCA
GTATATGAAGATTCGTAGAGCATTGGTACTAAATCTTTTGAAATACTCCTTCTCATTACTTCCCT
TATGGAATCTGTTCCAATAACACTTGGAATACCGAGCCTTGAAGCAAGCTCAAATGCAATTGTTG
ACGTTCCAACTCCGCTGGATCCACCTACAAGAATAATTATAGAATGTTTCTTTAATATTCTTCGC
CATAACAGATATTTTTCCGCAATTGCTTCGTAATCTCGATTAATAAGTGTGTAGTATACTCTTCG
TCTTAATTCATCTTTTGAAATTTTTAATACGTTTTGTTCATTTAGTTCTCGCTCAATATCGCGTG
CTAAAGTATAAGATGCACTAGGTTTCATCCCTGCGGCAGTTAACGATCTCGCTAAAAGACCCTTT
GAAAAGGGCATATCATATTCTTTATCCTTTACTAAAATTCTAATATTATCATCAAACGTCATCAT
ATCCCTACCAATCTAAAAAATAAAACTTGGATAATAATATATAATAATCATAAAAAAAGTAGTT
AATTAAAATTTTTATAATTATTCATTTCTAACATCAATTGTCTGTTCGAGTTTTGGACCTGTTGA
TATAAGAGTTACCTTAACACCAACTTTTTCTTCAACTTCTTTTATGAATGCCTTTCCTTTTTCGGA
CAATTTTTCGTAATCAGTTATGCCATAACAGTCTGAATCATATTTGTCAAGACATGTTATTGCTA
TTTGGGTTGCTCCATTTAATCTACATACCTTTTTAGCAAGTTCATGGTCAAAGTAACCGACTCTT

CTTCTCCGTCCAGTAACTGTTCCGTATTCAACCACTCCAAGATTTTCAGCTTCTTGTAATGACATT
TCTGTTGGGAAAGGTCCTTCACCAACCCTTGTAGGGTAAGATTTGAATACTGCAACGACTTCATC
AATTTTTGTAGGGCCTATACCTACATCTGCTGCAAAAGATGCTGCATTTGTATCTTTTGAAGTAA
CGTAAGGGTAGCTTCCGTAAAATAATGATAATAGGGACCCTTGTGTTCCTTCGATTAATACATTA
TCGCCGGATTCTAATGCTTCGTTTACCGCTTCTGAAACATCTCCAAGATAGTTGGAAATTGATTC
TACGTCTTTTCCTTGTTTCAACGTTCTCATTGCTCTGTCAACATTTGCAGGACCGCATCCTGTTCC
CGTTGATCCAATTTCTTTTGAAAGGTGTTCGTTAGATTTATCAAGTTCTTTGTGAACTTCTTCAA
TAATACCGCACCTGTAGTCAACGAGAATTCTTTCCCCTACATTGAACTTTTCGAGCATTTTGATT
TCTTTAATTAAAACTTCAGGATCAGTTAAAACTCCAGCACCGACTGCAAGTTTTGCGTTAACATT
TGGAAAAACCGGTTGGAACCATTCTAATTCCGTATTTTTCTCCGTCTACTTCGACAGTATGTCCTGC
GTTAGGGCCTACTCCGCCTCTTGCAATAATTGAAGGGTTGTCTTTTTTACAGAGGTAACTTATTA
TCTTTCCCTTTCCTTCGTCGCCCCATTGGCCACCAACTACTATGGTACATGTCATGTACAGAACCT
CCATTCAAATCCTCAAGCATACTCGGAGGAGTTCATATCCAATAAATATTTGAATTAATAAATAC
GAATTTCCAGAAAAAAAAGAATGTGAAATTCAATTAGTTAAAGTAGCTGTCAAATTGTCCTGCA
TCAATTGCTTTTTGTGCTTCACTTGGAGTCATTCCTTCCACACTTATTCCAACTGACACACACGTT
CCAACAACTTCTTTTGAAGCATTTTTCAAGTTGTAAGCTAACATTGCATCTTGTTTCATTTTTGC
AATTTTAACAATTTGTTCCATTGTTATATTTCCTGCAACTTGGTGTTTTGGTTCCTGTGATCCTT
TTTCAATTCCAATTTCTTTTTTAATTAATGCTGATGCAGGAGGAATACCTACTTCAACTTCAAAT
GTCCTTTTATCGGTATCAACAATAACTTTAACAGGAACGCTCATTCCTTCGTAGTCTTTTGTCAT
GTTGTTAATTTTTTGAACAACTTGCATGATGTTTACGCCCAAAGGACCGATTGCTGGACCTAAAG
GAGGACCTGCTGTTGCTTTTCCACCAGATACGAGTATCTCTACTACTTGTTCTGCCATATATTCAC
CTCTAAATGAATCGTGTTTAGTATCGCAATAATACTATCATAAGTTCCTTATTCTTATTTATGTG
TATCCTATTTAAAATTTACCATGACTTTATAAAATACAAATTAATCCTGTTTGGATATAATTTTA
ACCTGTTCAATACCAACAGTTATTGGAATAGGCACTGCTGCATCAATTAATTCGAGCGTAATTTC
TTCTTTATGTTTGTCCACCCTTGTAACTCTTGCCCTTTCACCTTTGAATGGACCTCCGACCAATTC
AACAACATCCCCTTTATCAATATGTTCGATAATTTTTGTTGGAGTTAACAAGTGATCGAGTTCAT
TTACACTGGTTTCACCAGGTACAATTCCTTTTACTTTAAAACTTTTTCTAACAAGTTCGTCTAAA
GCCCCTTTGTTAGCAGCTTCAATAAGGATGTATCCTTTCAAATCTTCGGTTGCCAAGATTGAGAA
TACTTCCAAATTCTCTTTTTCAGCTCTTGACGCCAGAGATTCTGCAACGTTTTTTTCCTGACCTGT
AGTGGTCCTTACTGCAAAAATCATAATATCACCAAAATGGAATAAAATTTGAAAATAAATAATT
TTAAAAATAGATCTCAAAATAATTGAAAAGAATTATCGATTTATACTATATATATCTATTCATA
AATTGAAAAATATGAAAGTATGTTTCGTGTTTTAAGGCTTTATTAATGCTTTTAAATAAGTGAT
AGGCACGTGTATTACAAACCCGACAAAACCAAGAAGGCATATACCAAGACCCGTTACTTTTGAAA
TTGTTATATATTCCTGTCTTGTTGGCTTTCTTGAAATCATTAAAACTCTTTTACACTGCTGTAAA
AAGTCTTTTAATCCATTTAATGTCGTATTTACCTTTGATTTCTGCACTTCAGTATTCATAGTTTT
TTCTTTTGCCATAGTATCCCTCGGCATTATGTTAAAAAATTAAACTTAGATTTTTGGGATATCTA
TTAAATATCTCTTTTTCGTTACATCAACGGTGTAGTCCCCAGTTGATTTTGTACCGGTAACTATG
AGTAAAACCCTTAATGAATTTTCGAGTGTTTCATCGATTGTAGTTCCCCAAATAATCGTTGCTTT
TTCGTCTAATCTGTCTGAAACAGTTGAAACAATTTCTTTTGCTTCTTCAAGGCTCATGTCTTCAG
GGCCAGTTATATGGATTAATGCACCGGTTGCACCGTCAACATCAACGCAGAGAAGTGGACTGTTT
AATGCGATTTGTATTGCTTCTCTTGCTCTTTTTTCAGAGTCACTTTCACCAATACCCATCATTGCA
ATTCCGCCGTTGTTCATTACTGCCCTAACGTCAGCAAAGTCCACGTGAATGTCTCCAGCGTTGTTT
ACAAGTTCAACCATTCCTCTAACGGAATTCATTAAAACTTCGTCTGCAACTTTGAAAGCTGTTCT

TAATGGAACGTTTTGAACAATCTCTAACAATTTATCGTTAGGAATAATAACAATAGTATCTGCAA
CTTCTTTTAATTTGTTAAGACCTTCAATCGCATTTGACATTCTGACTTTTCCTTCCATTGAGAAT
GGTAATGTAACAACTGCAACTGTTAAAGCACCAACTTTTTTAGAAATTTCTGCAACTACCGGAGC
AGAACCTGTTCCTGTTCCACCACCAAGTCCGCATGTTACAAATACGAGGTCGGAATCTTGAACTG
CTTTTTTAACTTCTTCTGAATTTTCTTTTGCAGATTCTTCACCTTTAATCGGGTTTCCTCCTGCAC
CGAGTCCTTTTGTGAGATTTTTACCGATTAAAACCTTATGATCTGCGTGAGTTTTTACAAGTTGC
TGTGCATCTGTGTTTATTGCAACGATTCTTGCTCCATCGATGCTTTCCGCAATTAATCTATTTAT
TGCGTTGTTTCCGGCACCACCACAACCTACAACAGTTATCCTTGCTTTTGACTGTTCGATTAATTC
CAGTAGTTCTCTATCGATATCTGAAAGCTGTTCTGAAGCAGGTTCCAAAAAATCTGTTTGTGCTA
TGTTTTTTAAGAATTTCAAGATTATTACCTCCTCATGAAATAGTGCATGCCCTCAAAGCCCTGAT
TGTGTGGGTTTTAAATAATCATTGGAAATATATAAGTTACAAGATTTATATCTTTTGATTAGGA
CCAATACCAAAAATATAGTTATAAATAAAAATACTTATATTCGGAATATAAAAGCCGATTATAG
CTTTCATTTATTTGATTCATATTTATATTATTTTCTATTAAAATGCGGTGAAACTGTGATAACCT
CAGAAGAGAGAAATTTAGCGGCCAAAAAATTACTAAAACTTGCTAATGGCATGTATGAAGATAT
CATACGAGGAAAAAGACCAAAACTCACCTTCCCAATAAGAAGCCTTGCCAATGCAAAATTTGATG
TCGAAAAAGGAACTTTTGCGATACTTGGAAAAGAAAAAGATAGGATGTTAACGGTAAATCAGGC
AAAAATATTTGCTCAGACTATCAAAATGATGGAATTTTCAAAAGGACTGCTTGATACAAACGATT
TCTCAACTCTGAGAGAAGCATATTATGTTTCCAAAAACTGGGGTGAAGCAAGATTCGATGACCAG
CAACCATCAAACGGAGTTATTGAAGATATTGAAGCTGCTTCAGAATTTTTAAGAGAAGATTTAGG
ATTTATTCCTGAAGAAGATGGTGCAGCAGTTATCGGACCGTTAAGAATTGTTGACAGGACTGCTG
AAGGAGACGAAATTAAAATTGACTGTACAAAACTTGGAAGTGGTGCATACAGCATCCCTAATGAT
GTTACGAAATTGGAATTTGAAACAAAAGCAGATTTTATTTTGGCAATCGAAACTGCAGGTATGTT
TGCAAGGTTAAATGCAGAAGGATTCTGGAAAAAACACAACTGTATTTTAATCTCTTTAAAAGGG
GTTCCTGCAAGGGCAACTAGAAGGTTTATCAAAAGATTAAATGAAGAATATAAATTACCGATTT
TAGTATTCACGGATGGTGACCCTTACGGATACTTAAACATCTACAGGACGTTAAAGGTAGGAAGT
GGTAAGGCAGTTCACTTAGCAGACAAATTGGCAGTACCTGAAGCCAGACTTATTGGAGTAACGCC
TCAGGATATTGTTGACTACGAACTTCCAACGCACCCCCTAAAAGATCACGACATAAAAAGATTAA
AAGATGGTTTGAAAAACGATGATTTCGTTAAATCATTCCCTCAATGGCAAAATGCGCTTGTTCAA
ATGGCAGACAGTAAAGTAAGGGCAGAACAGCAGTCTTTAGCCAAATACGGTTTAAAGTACGTTGT
TGAAGAATACTTGCCTGCAAAAGTTGAAGACTCAAGTACATGGCTCCCTTAATTAATCTAACTAC
ACTTAATGTATCTCAACATTCTGGGATCAAAAACAATATCCTAAAACTATTTTTCTTTTATTACG
ATAAAATCGGCCGATATTAAGTCAAAATTATAGAAAACCATATAAATTGATCTTATATATTCAA
TAACGGGTAATCCCCCAATATTCAAAAATATATAATGGAGACTAAACAATGGATTGGCCGTTTAA
AGCTTTTACATTAACGTTAATTACACTATCCTTATTAGCAGTCACTAGCGCAAATTGTGGCTTTG
ATGGATGTGAACCAACTTTTTCAAACGTAACTTACCAAAATTTTAACGAAAACTATTCTAATTTA
TTTGATAATAACCACAATCTATTCTTCGAATTAAACAGCCCTTTTAAAAATAATATGGAGTACAA
AACTTTTGAAGTTAAAGCATATGCTCCATTAAAAAAAACGTCTTCAAAAATAGATTTACTTGAAT
CAAGATATGTTCCAGTAACTACAGTAGACACTTCTTCTGAAGATAATACCGACACTTCTTCTGAA
GATAATACCGACACTTCTTCTGAAGATAATACCGACACTTCTTCTGAAGATAATACCGACACTTC
TTCTGAAGATAATACCGACACTTCTTCTGAAGATAATACCGACACTTCTTCTGAAGATAATACCG
ACACTTCTTCTGAAGATAATACCGACACTTCTTCTGAAGATAATACCGACACTTCTTCTGAAGAT
AACGTTTATTTACCTTCAAAAATAACCACTTCTCCAAAAACTACCCTGTATATCATTAAAACTAC
ACCCGAACCAGTAGTAGAAGAACCAGTAGTAGAAGAACCAGTAGTAGAAGAACCAGTAGTAGAA

GAACCAGTAGTAGAAGAACCAGTAGTAGAAGAACCAGTAGTAGAAGAACCAGTAGTAGAAGAAC
CAGTAGTAGAAGAACCAGTAGTATATAAAAACGCTCAAATTGAGCAATATATATTTGAATATAC
TAACATGGAACGCTCTTCATACGGTCTTGAAGCTTTAGTACTTGATAGTGCATTAAGCCAGATTT
CACAAGACCACAGTGATGACATGGCAGAAAATGATTATTTTTCCCATATAAACTTGGATGGGGAA
ACACCTACCGATAGAGCTATTGCAGCAGACTATAACGTTGTAAAATACCTCGGAGACGGTTATTA
CTCCACAGGAATTGGCGAAAATATTGCAAAAATGCCTACCGGAAATGTAATTGGAATTGGGTATG
TTTCAGACGATGCTGAAAGTATTGCAAAAGCTATCGTGGATGCATGGATGGACAGCGATGGTCAC
AGGGCTAATATTCTAAACTCCCAATACACCAATATGGGCATTGGCGTTGCATTTGATGGTACGTA
TTACATTGCAACACAAAATTTCTACTAATTAACCTACAAATCTATCTTTTTTTATTTTTTTCAAC
CGAAATTTAAAGCTATTGCTCATTTATCAATTTTTCTTGAATTTAAAACCCAAAAATATTAATAA
AAACTAAACAATAGCAAATATCATCTAAAATTATCGGATTGGTGAATCTATGAAAGCGGTTATT
TTTGTTGTAGATGGACTTGGAGATAGGCCAAACAAGAATGGAAATACTCCATTAAAAGAAGCTA
AAACTCCAGTAATGGATAGAATGGCTAAAGAAGGAATTTGTGGACTTATGAATTCCGTTGATAT
TGGTGTGAGGCCTGGAAGTGACACTGCACACCTTGCTCTTTTAGGATACGACCCCTACACAACCT
ACACGGGAAGAGGGCCTTTTGAAGCGTGTGGTGTTGGAGTTACTGTAAAACCAGGAGACATTGCA
TTTAGGTGCAATTTTTCATCAGTTGACGAAAACTTTATCGTAACTGACAGAAGAGCTGGAAGAAT
TGAAAATACATCAGAACTCGAAAAAGAGCTTGACGGGCTAAAAATTGATGATGTAGAAATTATT
TTTAAAGAGTCCGGCGGATATAGGGCGGCACTTGTTTTGAGGGGACCCGGATTAAGTGACAAAAT
TACAGATGCAGACCCTAAAAAAGAAGGAAAAAAGGTAAAAGAGATACATCCTCTTGATGATTCG
AAAGAAGCTAAAAAAACTGCCGAAATAGTAAATAAATTGTTAAAAATCGCTTACGAAAAATTAG
ATAAACACCCGGTAAATGAAGAAAGAAGAAAGCAGAATTTACCTGTTGCAAACATGATTGTTCCA
AGAGGTGTTGGACAGGTTCCAGAAATTATGCAGTTTACTGAAAAAACTGGACTTAAGGGAGCAT
GTATTGCAGGAACTGGGCTTATCAAAGGAATTGCAAAAATGGTCGGGTTAGATGTAATTGATGT
TGAAGGATGCGATGGAACTCCAGACTCTGACTTTATGGCAAAGGCTTGCGCAATTGTAGAAACCC
TTGAAAATTACGACTTTATACTTGTAAATGTAAAAGGAGCAGATGAAGCAGGGCACGATGGAAA
TTACGAACTTAAAAAACAGGTTATCGAAAAAGTCGATGAAATGTTAGATCATATTACAAAAAAT
ATTAGCAAAGATGAAGTTTATTTTGTACTCTCAGGAGACCACTCAACACCTATTGAAGAAATGGA
CCACTCTGCAGACCCCCTTCCAGTTGTTCTTTGGGGAAAAAGTGTTAGGGTTGACGATGTCGAAA
AATTCGATGAATTTTCAACTTACAAAGGCGGATTGAACTGGATAAAAGGAGTACATATCATGCCT
ATTTTAATGGACCTCATGGGCCTTGCAAAAAAATATGGTGCTTAAATTAAAATCCAAAATCTATT
TTTGGATTAATTTTTCTATTTTTATTAGTAAGTAAAACAACCCTACTTTCAGGAGAATCGTCTTC
TATTTTAAATATGCTGTTTTCGCTCAAAATTTTACCCATCTCTAAAAGTTCAACCTGTTTTACCA
TGTCATCCAAATTTAATCTTTTTCTTGAATATCCAACATTCATGTAAGATTTTAGTTCGATGAAA
TTTGAATTTGCTCTCTCAAATAATTCCTTAAATTTCAAAATATTATCGTTTATATTTCGAATAAC
GGTAGTTCTAAGACAGGTTCGTTTTTTTGATTCCAAAATATCAAGAGTATTTAATATGGATTCCC
AATCATCTTTTGTTCCGCCACATATTTTCTTGTAGCTTTCTAAATCGTATGCATCAAGTGAAATA
TAGAGCTGTGTTGGATTTACCTTTTCAATTACGTCTGTTAAAATCCCGTTTGAAACTAAGAATGT
TGACATTCCTTTTTTGTGGAAAACTTCGATTAATTCATCAATATAGGGATACATCGTAGGTTCTC
CTGAAAGAGACAGTGCAACGTGTTTTGGTTTTGTTGCTTCGAGGTATTTTTCTTCACCAATTCTA
TCCAAAATTCCCTTGTATCCGGTTATAACAGTTTTGTGCATAGCCAATATATCTTCTGCAACCGC
TTCTGGTTCCTTCCATTTTGGAGATTCAAAAGAATCCAATCCTAAATCTTTTGGAAGAACTCTCC
AGCAGAAAATACAAGATTGCTGGCACCATGCAACAGAAGGAGTACTCTGAACACACCTGTGAGTG
TTTATTCCATAAAACTTTGATTTATAGCATTCTTTTCCTTCTAAAAAAGATTTTCTAACCCATCC

ACAGAGTTTTACGCCGCTGTGTTCTTTTATTTGATATCGCTGTTTTCTCAAAATATTGTGTATTT
GATCGGGAATCATAATTATCACCTTATACTCAAGCATTGTTTAATTACTATTTATAACTTTTAAG
CTATGCGTTCTATATGGCAACATTTAAATATATGAGATTCTATTATAGATAAGGTATGATAAGA
GTAATAGGTGTAATCGGAAGAAAGGATACTGGAAAAACAGGTCTAATAGCCAATATCCTCAAGA
ACTTGACTGGCATGTCTGTTGCGACAGTTAAAAATTCACATATGGATTTAGACGTTGATTCCCCA
GATACTGATTCATATAAACTAAAACAGCTTGCAGATACGTCTATTTTTGTTACAAATAAAGGTTC
GGCCTTTTATTATGACAGAATGCATTTGAAAGACATCCTTGCAAAGCTCGACTGCGACCTAGTTA
TCGTAGAAGGCTTTAAGGAAGATTTGATCGAATTAAATATCCCAAAAATACTTGTAACTGAAGG
GGGACGGGGCGCTGAATTAAAGGATAATCAGACGATAATGGTAATTGATGACTTCAAATACGAT
ATAGATGAAGTTACAGCCCAAGTTCTTGAAAAAGCTATAGTTCCATCATATACTCTAAACTGCGG
TCACTGTGGATATAACTGCAAAGGTTTTGCAAACCTGTTGGCCTCGGGCGAAGTTAAATGGAACA
AATGTGTTATGTCAACAGCACTAACATTGATAGTCGATGGCAAAACAATCCCTGTAAATCCATTT
GTTTCCGACATTATAAAAAACACTATAAAAGGGATTGTAGGAACACTGAAGGGTACGGAAAACCC
TGAAACAATTTCAATCAAAATTGAAAAAAAATAACTCTTATTTTTTGAATAAAGACCCATACCGC
GCATTATGGACTTTTTTCGTATTTCAAAATATTTAAATATTGCAATAAATAGACTATATTATGGT
TACTTGGTGATATCATGGCTATAGAAGAATACAAATACGAATATATGGCTGAAGTTTTTAAAGC
GTTTGCTGATCCTACTAGATTGATGATTTTGGAGCTTTTAAGTGATAATGAAAGTATGTGCGTTT
GTAAAATTATTGATGAATTGAAAAAACCTCAGCCAACAATTTCCCACCACTTAAACATATTAAAG
AAGTCAGGAATCATTAAAGCTAGAAAAGAAGGAACTTGGAACCACTACTATATTGTAAATCCTA
AAGTAAGGGAAATGATATCTATTATGGGCGGAATTGTAGATTAATCATTATACTAAAGGAAATA
AATTGGTCAAAAGCCACTATAGTTAATTTTATATACCTTATATCACTATTAGTGAATAACTAAAG
TGATATTTTGAGACTTGAAAACAAATAAAACACAATTTTCTTTTTTAATTTTGTAATTAAAACAT
TTTTCATCGATTTATCCAATTTACAGCTTTATGCGTTGTGAGATTATGGGAATACAGGAAGATAT
CAAGAAATTAGAAGATGAACTATTACATACCCAGTATAATAAAGCCACCCAAAAACACGTGGGTA
TATTAAAAGCAAAACTTGCTAAATTAAGGGACGAACAGCAAAACCCAAAAGGATCAGGTGGATC
CAGTGGACCTTCATATGCAATTAGAAAAACAGGGGATGCAACAGTTGCTTTCGTTGGATTTCCAT
CAGTTGGTAAGTCAACACTTTTGAATAAACTTACAAATGCAAATTCAGAAGTTGGAGCTTACGCA
TTTACCACTCTTACAATTATTCCTGGGTTAATGGAACATAAAGGTGCTAAAATTCAATTGTTGGA
TGCACCAGGTATTATTTCTGGAGCTTCTTTTGGTAGAGGTAGGGGTAGCGAGGTTTTAGCTGCAG
TTAGAAACGTGGACCTTGTAATGCTCGTTGTGGATGTATTTTCACCAGAACACATACCTATACTT
GAAAAAGAGCTTGGAAATGTAGGTATAAGGCTTGACCAAGAAAAACCAGACGTAAAAATTGAAA
AACACGATCGTGGTGGACTTGCAATCAACAAAACTGTTAACTTAACAAAAATAGATGAAGGTACA
ATAACTGCGATAGTTGGAGAACACAGAATCCACAACGCAGCTATTCTAATAAGAGAAGACATTGA
TGCTGACCAGTTAATCGATGTGGTGTTAGACAGCAGGTCATACATTCCTACACTTGTTATTGTGA
ACAAAATCGACCTTGCAGACGAAGAAAATCTGAAAAAAACAGAAGCTGCAATTGGAGATAGGCC
ACACGTATTTGTTTCAGGGCATAAAGAAATAAATCTTGAAGAATTAAAAGACAAAATCTTTGAA
ACTTTAGGATTTATCAAATTATACTTAAAACCTCAGGGCGGAAAAGCAGACCTTGATGAGCCATT
AATTATTCTCAAAAACTCAACTGTTGAAGATGTTTGTAACAGACTTCACAGGGATTTTGTTAAAA
ATTTCAGATATGCACTTGTTTGGGGAGAATCTGCAAAACACCCGGGTCAAAGAGTAGGATTAGAC
CACGTGCTAAAAGACGGAGATATATTGACGATCGTTGTTAGAAGATCACTTTAAGGTAGATAAA
ATGAATGATGAAGAATACAGTAAAATTATTGAAGCTGGAAAAATTGCATCCCAAGTTAAAGAAG
AAGCAATAAAATTAGTAAAACCTGGCGCAAAACTCTACGATGTTGCAGAATTTGTAGAAAACAG
GACAAGAGAACTCGGTGCAAGTATCGGATTTCCATGCAATATTTCATTAAACGACATTGCAGCAC

ACTACAGCCCTTCATATGGCGATAATTCGGTTTTTTCAGAAGAAGACGTTGTAAAATTAGACCTT
GGATCACACGTTGATGGATTTATTGCAGACACTGCAGTAACTATCGATTTATCTGGAAAATATTC
TGACTTAAAAAAAGCTTCAGAAGATGCGCTAAATACAGTAATTAAAGAAATCATGCCTGAAATG
AATGTTGGGGAAATGGGAAAAATAATTCAGGAAGTAATTGAAAGTTACGGCTACAGGCCAGTTT
CAAATCTTTCAGGGCACGTAATGCACCAGTATTCACTCCACTCAGGAGTAAGTATTCCAAACGTT
TCCGAAAATACAAAAGATACCATCGATGTTGGCGACCTCGTTGCAATTGAACCGTTTGCAACAGA
CGGGTTTGGGCAGGTTGTAGATGGAAAAGACGTGTATATCTTTAAATATCTAAGATCAAGACCAA
CAAGACTTCCGGCTGCAAGAAACTTATTAAGAGATATCGAACAAAACCATGCATATCTACCATTT
TCAGAAAGAGATATGGCAAAAATCGATAAACATTATAAAACCGGCTTAAAAGGCTTGATGATGG
CAGGAGTTCTTTATGGATACCCAACACTGGTTGAAAGAGAACATGGAATGGTTTCCCAGTGTGAA
CATACAGTATTAGTTACCGAAAATGGTGTTGAAGTCACTACAAAATAATTGAATAAAAGGTGGA
GTTATGATCGTCATTTTAAACAACGGTGGGCAGTACGTGCACAGGATACAGAGAAGCTTAAAGTA
CCTTGATGTACCTGCAAAAATAGTTCCAAATTCAACCACTCTTGAAGAAATTGCTGGAAATCCGG
AAATAAAAGGAGTGATATTAAGCGGTGGCCCAGATATTACAAAAGCTACAAACTGCGAAAATAT
TGCATTAAATTCAGAACTTCCAGTTCTTGGAATTTGCCTTGGGCACCAGTTAATTTCAAAAGCTT
ACGGTGGAGAAGTTTCAAGAGCAGATTCAGAAGAATATGCAAGTATTAAAATTTACATAAAAGA
AGAAAACGATCTCTTCAAAGGAGTTCCAAGCGAATTTACTGCATGGGCTTCCCACATGGATGAAG
TTAAAGTAACACCTGACTGCTTTGAAGTTTTAGCATACTCTGACATTTGCGGAGTAGAATCAATA
AAACACAAAGAAAAATCAGTTTATGGTGTTCAATTCCACCCTGAAGTGTCACATACTGAATATGG
GGACATTATTTTGAAAAACTTCTGTAAAAAATGCGGATTTGAATTTGAAGAATAACTTAGTTAA
TTAAATAAAAAAAAGTTTTATTTTTTTAAAATTATTTATCCATAATTATAGTATTGTCGCTTAAC
AAAGCTATCTCATCTTCTGTTAAAAGCTCTTCTTTTACCCCATCTCCGATTATTGCGCTGTGTCCA
AGCGGATCTTCGAGGATTAATGTTATTTTTTCTTTTCCAAGTTTTACTTCTTCTAATTTTTCAAT
TAACTCATCTGCTTTTTTCCTTTCATCTTCATTTTCAACCCAAGATTTCAATGTTTTAATCGCATC
TTCCATTCTTGTCAACACGCCTTCAACATTTGAAACGTATGCTTCGGATGCAGGACCAGGAGACA
CTTCAAAGCCGAATTCAGGTATTGAAATGTGACCTGATGAACCCCTTATAACCCGTTTATTTAAA
TCGTATTCATCTTCAACCGTTAAGGTATATCTTTTAGGCTCTTTTACATCTACCGGGAAAATATC
ACTTTTTTTGTATTTGCAAGCCTCGCAAAGAATCGTAGTTTCAATAACCTTTCCAAAATAAGGGA
TTTCGAGTTCCTGAGTGATTAATTTTAATGAATCCTCTTTTCCGCATATCGGGCAGTCCATTACA
TTGCATTGTTTTTCCATGTTTCTCACCTAATTTTAATATTTTTTACATTTACTCGCTTATAGGAT
AAAAAATATTCTGATAAAATACCGTAATAATTTTTGGCCTGATTCAAGTAAAAAATTATATATA
CAAAATCCGCCCAGAATAATGTTATTCATAGGAAATAATTTTTCGATCCGATTGCCTCAAAAAGT
GGTAGAATGTACGATAAATTAAAAATCATAGAACGAGCTATTCTGCTCAATCCCGAAAATATAA
AAGTATTTCGTGAAAAACTCAAGATAACCCAGTCCAAACTTGCTGAAGAAAGTGGGGTTAGCCAA
TCTCACTTGAGTATGCTTGAAAAAGGGAAAAGAGAAATTGGGGAAGCACATGCTGCCGCAATAAC
ACTTGGAATGCTTAAATGTACTAATTTTTGGGATAAAGAAAATCCTATTTTATATTTATTAGACG
TACTTTCACTCACAAAACTTGAAAGTGCTATTGTCGAATTTATCCAGGATTTAACGTCAAAAAAC
GATATCTTCTGTAAAAGATATATTGAAGGACATCCTGTATACATTATAGATATAAAATATTTTAC
TGAAGAAATTAAAAAACGACTCAGTGTCATGGACATTGAAGAAGTTGATTTTTTTAGAGGTAGA
ATGAATATTAGAGGATTACACCTCGATAAAAAAACCGTACTTATTCAATTGGATTGTTCAGACAT
TCGAAGGCTCGAAAATAAGGTATCTAAAGTACTAAACAATAAAACAGTACTTCAAATATTCCCCA
AAGAGGAAGTTCCCCCAATATATTCTATAAAAAAAGACTGCATGATTGTACACTGCTGGTGATTT
TCATGGTTTTATTAATTAACTACGCAGTATCTTTGGTTTCTGCAATTGTAGTTGGAGCAGTTCTT

GGAATGAAACTGTCATTTGATATGGATTCTTTTGAAGGATCTGTATTGTTCCCAACTCCATTTGT
AGCAATTGGTTTAACTGCATTAATTGGCTACTTAATAACTTTAGACCTGGTTTCCAGTATAATAA
TAGGAATTTTTGCGAGTGTTTTCTCGAAATTTACAAACAAAATATTCCCTGGTGTTAATAATGAT
ATTAATTGAAACTGGAAAAATATTGGCCGCGTTTGCAATTTGCTGGTTAAATTTTGTTATTGTTG
ACACCTACTTTGGACTTCCAAAAAAACCTGGAGTTCTAGGTGCCAAAGTAATCGGTGAAAAGATA
GAAAATGTCGGTGGAAATATCAATGGTGGCTATTTCATGGGAAACATTGTATGTTCCCCTGATGC
ATCGGCAGGAACGCTAATGGCTTCAATATTTTACTACTTGATGGGCTTTGAAGGTGGATTAATTG
CTGCACTGTTCATCTACATTGGAAACAGATTATGTAATGATACAGGTTATGCAGGAACAATTGGA
ACAATCACTGCAACAATATTGATTTACTTATTTAGCGGATTTATAAGCGCTGAATACTTTATTGC
AGGAATGGTTCTCGCAATATTTTCAATTCAAGGCGTGCATCACAGCGCTGCATCACGACTTCTCG
GCAGTATTGCAAGAAAAATGGGGAGGATATAAATGGAATCTCTTGTAACCGTTACAATTGGACT
TATAGGGATAATTTCCGTTATAAAAGTATTTCTGACAAAAAGCAGAGCTTTAAAACTCCCTATTA
TTTGTTGTATTAACTTCTGTATTGCAGCATTGATTGCACTCTACATAAAAAGCCCGATGGGTGCA
ATTGCAGCAGTTGTATACTTTGCATTATCAACAGTATCCAGTAATGCGATAGCCCATACTCTTGG
AGAAATCGACAAAATGGATGAATTTGAGAAGAAAAGGTGATTTTGATGGAAATACTACCTATCG
TTGCTGCAGGGTGCTGTATTCTTGGAGGAATAGGTACCATCATCCATACGCACAATATTAACAAG
ATAATCATGTTTGCACTTCTTGAAAGCGGACTTATCGGGTTTATTGCTTCATTTTACTACCTTGA
TGTCGCAATTGTTTCATCCGTTTTAGAACCAATTTCTACTCTGATACTGCTTTTGGGCGTGTTAA
AATATGAATACATACTAAGAACTAAGAAAAAATACAGTGCAGAAGTCCCTGTGCTTACTAAATA
GGTGATAAAATGGATTTAATTACCTTGACGTTGTACGTGGGCTACTTCTTACTTATAATTGGTAC
AGTCGGTGCAGTTATTGGGCCATTAACAAAAGATCCATTTAAAAGATTTTTAAACATTGAAGTTC
CGGCCATTGGAGTTTGTTTAATATTTCTAGCATACAACCAGACTCTTGCACTTATGACGTTCCTC
GGCGTAAATGCTATCCTTACACTTGTACTCGTGAGAACGATTATCAAAAATGAGGAACTGGAGGA
ATAATATGAAAAAGCTAAACGAAGCCTGGAATTATATCTCAAAACCCAGTGCCGTCTCCAGATTG
TTTGCAGGGTTTGTCTGCATAGTTTTATTCATCGGTATATTCTTACCTACGGAATTTACAAACGA
CCAGCTTTATCCAAAAGATGCGATACAGGGCCAGGTTTTAAAAACTCCTCTTGCACCATACGACA
GAGGCGGAGTTGTATTATCGGAACCTGCAGATATTAAATCCCAGTACCCAGAATACCAAAGCATT
AGGGGCCAAATTACAGCTTATTTATCCCCTGTTGCTATTGCAATAAGTGAAAATACAATGTACTT
TGGTACAACAATCGTTTCAACCCCTGGGGGAATACTGGATGAAATATTATACTATACAAGAGGAT
TTGATACAGTTTTGGAATCACTAACTTTGCTCCTCTCGTTCACGATATTTGGATGGTTATTCCTA
AATCGGAATAATAAGGTGAAATAATGGACTACGTGAGTTTATTATTTAGTAAAACAGTTGCTGT
CGGATTTATAGTTGGAATAATTTCACTTTATTCAATTTCCCGCCAAAAATCAGACCTTAACATGA
TATTGTTAACTGACCTTATAGAATACGGAATGCTCGTAATTATTGCGGCAATTGGTTCAGACCTT
GCAGAAGCCCTTATCCTTCCGGGATTGGTTGTAGGTATGGCAGAACTCTTGGCAGTTGCAGAAAT
ATTTGTTTCAAGAAATGACTTAAGAAAAGAGGGTAAACCAAAAAAATCGAAACTCTTAGAAGAG
TTTATGATTAAAGAAACCCCTAAAATGGACATAAACTTTAGTAAAATGGAAGTTTTATACACCAC
TCCAAAATTCCTCGCAGTTATACTCGTAATTTACGGGGCAATTTTAAGTGGATTTACTGGCGGAG
CAGTTATGGCCAGCGGTCTTTTGTTCTATGTACTTTCGAAAAGAGTTCTCGGTAGAAAAATACTT
CCAGAAGAGTTGAAAGTATCTTGGGAAGGAATTTCAGCGTTTTCAGGAATTGCGTGGGCAATTTG
GATACTTGGCTTTATTGGATTCTTCTTATTCCCTTCAAAATGGCCATATTTCTTGATTATAGCCG
GATTTGGACTTGCAATAAAAGTGGGTTCTAAACTCGGACTAATTGGAGAGTTGTTCGAATAATTT
CGACCAAAGGTGATTAAAATGCTCGTTGAATATATCGCAGGAAACTTTCACGGAATAATTCCTTT
GGGAGATATCGTATTTTACGTAACAGACTTTTCGCTTATAGCATTCATTGTGGCAATGGTTTTCA

CGGTTATTGTATACTTTACAAAACCAGAAAAACAACTGGAAGCCCAAGTTCATGAATTTGGTGAT
AAATTTAATTACGTGACAGTTGAAGAACTTAAAATAAGGAGATTTATGTCAGTAGTATGTGGTA
TTGCAACTGCATTTTCAATGTTAACTGGTGATTTATTTGATTTTGCACTATTCCTTGCCGTAATT
GGTATCTCAAACATTGGAATTGTTGCCGCAGTTAAAAAAGAATGGGTGTTGAATGCAGCATTCCA
CTACGGGCTTATCACAATAGTTTCTTCACTTCCATTATTTGGTGCCGCTGCACTAATTTTAGGAA
AAACAGGAACTCTTTCCATATTTGAACTTGCAAAAAATGGTAATGAATTATTTTATCAAAAACTA
CTCTACGCAGTTGGAATGATTGGAGAAACAGGCGTTGCTCCGTTTTATGCAGCAAAAGCGGAAAT
GTTCAGGGCTCCAGGAGCTCCTTACATTCTGATGATACACCTGTCATCCCTTTTGGTTATCGTAAG
GACTGTAGAAATTCTGCTTACAATATCTTAAGGTGAAAAAATGAGTCCAGAAACGAAAAGTGGC
TATATAGCACTTATAATTGGAATCTTGGGATACCTAGGAACAATTTACTTAAATTCTCAAAACGA
AATGGTAACATACCTCTTAACCGCAGTATTCACTCCATTTTTAATATTTGGCATTGCAATGTTCC
TTAATCCGAAATCAAGAAGAGAAAAAAATAGGTCAAATTCCGTTCAGAGGGTGGTAATTTGAACG
CAGCATTTAGTATGTTTAACGGACTTATGTATGCATTGTATGCCTTTTTAGTTGGCGGAATTTTG
CTTGGATTCCATAGAAAACTTATGGCAAGGGTTCAGTGTCGACCAGGGCCCCCAATTATTCAGTA
TTTAATCCATACATTCAAATTTTACTTTAAAGAATTAACATTCCCGATTTCAGCAGGAAATCCAC
TGTACGTCTTTGTAGCATTGATGGATGTCATGGTCTGGATGAGTGCATTGTCAATTGCAGTTGTC
TTTGAATCATCATTACTGATATTGATCGGACTCTATATCTTACAAAAAATAGTTGAACACGGTTG
TGGATTGAGTAGTGGGTCCCCATATGGAAAAGTTGGTGGTGTTAGAAGTGTTTTCTCAGCTGCTG
CAGAAATTCCGCTCTTTGCTGCTGCTGGAATAGTCTATACGGTAACACATTCATTGATTATTGGA
GATATAATAAATTACCAGGCTGTAAATGGTCCATTGATATTCCAACTCCCGATATGTGCTTTTGC
AGTGTTTGTCCTTGTTCTTTCAAAAGCACCTTTCAGTCCCTTTGCAATAGTGAAAGGAAAAGATA
TCATAAGTGGATATCTTACAGAACATTACGGACCTCTTGAAGCAATCATAATGATTGGGGACGCA
ATAGCATGGTTTGTATTATTATGGCTCTTTATGGCGTTGTTTTTAGGCCCATTGATCGTAGCAAG
CCCAGTATTGACTTTGCTTGGAATGTTTGTCTTAACGATAATTATTTCATTTATCTGTGCCTTAA
CTCCGCTACTTGCTCCAAATCACTCAGTAATGCTCCAAATCACAATTGCGTCACTTACGTTAATTG
ATCTGGCATACAGAATCATCCATTAGGTGTTATCATGAAACAGTACATCCAGTTACTTTCGATTG
CAGGATTTGTGGTTTTGGGAATGATAGTGCTTATTAACATGCTTCAATACCCAATAACCCCAATT
TTAGGTCTTTTCGTAGTCCTTCTTGTAGGAATATTGGCATTACTTATTCAGAATAAAAAAATAAC
CCATTTAATAGAAACTACAGAGCATTTAATGATGCTGGCTGTATTAATTGCATTTATCTATCTTG
GATATTTCGCATTTATCGCAGGGTGATTTTATGAACGAAGTTCCATTCATACTACTTTATTGTGC
AATTACATTCGTTATAGGATCGATGTTGGGCCTTTCGTACAGCTATAAAAAATATGCTAAACCTT
ACGTGGAAAAAACCCTTGATAGTACAGCACTTATTTCAGCTATTATCGGAGGACTCGCGTTTACA
GTAAACGCCCCAATTTCAGTATTATTCCTTGCATTTCCACTTGGAATGAGGCCTGGATATGGACA
TTTTGAATTCTGTCTTGGAGTTTTAATTGCACTTATCGGCTACATTTTACTAACAATTGGAATAG
TCTAGCAAAAAGGTGAAAACATGCTAGAAGAAATCGATAAAAATTACAAAAAATCAAGTTTAGA
GCAAAAATACAACATAATAAAGGGTAATCGTTACATAATGGAAGAAGCGATTGTTCCTATTTCA
AAAGCTCTCAAACTACCAATGGATGAAGTTGTTGATGTATTCGTAAAAACCTGCGACGGAGTATC
GCTTTATGAATCACACGCATACGTTGAACAGGCTAAAATGGGATGTCTTGGAAGAAAAGTAGACA
TTGATTTAGGTTTGTGCTGGATTGCAGACTTCTTTGGACTAATATCTAAAAAAGATGCGGATTTA
ATAAGAAGAAAGGTCGTTGAAGATACAATTATCAAAAAAAGACCGTACAAAGAAGCGCTTGAAG
AAGGCAGGCTACTTACCGTTAAAATCCTGAAGGGTGAAGAATAGACTTAATTTGGAGATGATCTT
GATGATTAAAGATTTTGTAAGAAAACGATCAATTAACGTCTGTCTCGTAAACACCGGCGGTTGCA
ATGGTTGCGATATCGAAATAGTATCTTGCCTTGCCCCAAGGTACGATATTGAACAGTACGGAATT

TATGTGCATAACAATCCAAGAGAAGCCGATGTTTTAGTTGTAACTGGTCCTGTAACCGGAGCTTG
GGAAAAAAGACTTCTTGAAATTTACGAAAAGACCCCTGAACCAAAAATAGTTATCGCAGTTGGTG
CATGCGCACTGAGTGGCGGAATATTTAAAGAAGGACATATTCACGGGCCTGTTGATAAAGTAATT
CCAGTTGATGCAAAACTTCCAGGATGCCCTCCAAGACCTTCAGAAATCATATCTGCAGTGGTATC
AATTGCCCCAGATGCACTTGCGGCTAAAGAAAAACTGCTTAATCAAAAATTAAAAGAAGAAAAA
AATAAAGTCAAAGCATGATTTGAGATGATATTATGAATGTAGTACCGATTGGACCCATTCACCCG
ATACTAAAAGAACCCTTGCGAATAAAACTCCTCGTTGAAGGAGAAAACGTAAAAGGTGCTGAAA
TAGACATGGGTTACGTTCACAGGGGTATTGAACGTATCATGGAAGGAAAACATTATTTAAAATG
TATACACCTTTCAGAAAGAGTTTGCGGAATCTGTTCATATATCCACACCCAGTCCTTTGCAGAAT
GCATTGAAAATATGTGTGATATTGAAGTTCCTGAAAAAGCAAAATATTTAAGAGTAATTACCTG
CGAACTTGAGAGAATTCACAGCCACTTGATTGCAGCCGCAGTTTACAACCTTACAATTGAACATG
AAACTCTTGGAATATGGATGTTAAATGTAAGGGAATCTATCATGGATTTACTCGAATTAATTACG
GGAAATAGAGTAAATATGGGATTCAACGTTGTTGGTGGTGTAAGACTCGATTTAAACAAGGAAA
TTTTAGATACACTTCACAAAAAAATTGATGAATTTGAAGAAGATGTTAAAAATATAATTGAAGC
ATTCGAAACCGGCCCATTAATTGGAATGAGAAGTAAACATATTGGGAAAATTGGCTACAAAGAA
ATCATGAAAACACGTGCAGCAGGACCTGTTGCAAGAGCTTCAGGAATTCCTGAAAGTGACTGGAG
AATAAGACATGAAACTTACAAAGCACTAAATTTCAAACCCGATTGGAGAAATGAAGGGGACAAC
TACGCACGAATGATGGTAAGACATGATGAAGTGCTCACAAGTGTCGAACTTATAAGAAGAGCTCT
TGAACACTATGCTGAATCTTCAGGAATCGTTAGAAATAAGGCTGAAATTAGGGCTACTGAAGGAG
AATGGTATAACGAAGCTCACAGGGGCGAAGTTTACTATAAAATTGCAATTACCGATGGCGGACTC
ATAAAAAGAATTATTATAAGAACTCCAACTGTAATGAACCTTGAAGCTTATAAATACATGATAA
AAGACTGCCCAACAATTGCGGATGCAGTTGCAACATACACCTCAATAGACCCTTGTGTATCCTGC
ACTGAAAGAACTATCCAATTAAAAGACTTAAATACTGGAAAACTTAGCGACTACAAAATCTAAT
AAATAAACCTGATTGCAGGTGATACCTATGTCATCATCCATTTGGTACCTCTACGAGTTCGTAAG
AAAAAAATGGTTTATGAGATTTACGAACGCCAAATCTGAAAAAGAAAGTTTTATTCCACCAGAA
AGATTTAGAAAAATTCCGGTAATTTTTGATTTGCCCGAGAAATGTATAAGCTGCAGTGCATGTAA
AGAATCCTGCCCTTCTGATGCAATTTCAATGGAATATAACGAAGAATTTAAAAAAGAAATGCCCG
TATTTGATGCAGGTTCATGCATAAACTGTGGAAACTGCGTTGAATCTTGTCCAACAAACGTTTTA
GAGATGGGAACCCTTCGAAAAGAAGCAAAAGAACTTTTATGGAATGTTCCAAAAATTATTAACCT
GTTAATTGATGAAGAAGTCTGCGTAAGCTGTGGAACTTGTGAAAATGCGTGCCCAGTTGATGCAA
TTTCCCACAACAATACTGGACTCTATGAAATAGACGTTAATCTTTGTGTAAGCTGTAAAAATTGC
CTTAAAGCATGTCCTGTTGAAAATGCAATAGTTACATATTCTGAGCCAGAACTCTCAGAAAAAAT
AGAAATTGCACAAAATATCAAATTTGACCGCGAAAGACTTGGAAGTGAGTTTAAAGAAAAAAGC
GATGTAATAGCTGAAATTCCAAGAATCGTTCCAAGTTTGTGTATTGGTTGCGGAAACTGTGTTGA
TGTGTGTCCTGGTTCAATAGACCTCGAAAGACTTAAAGTTACATCCTGTATAAAGTCCGGAAAAT
GTCTTGAAGTGTGTCCAACAACCGCAATAAGAATAGGAGTTCCTGAAAAAATTACGAAAAGAACT
GCTGAGTGCTACATTGTTGATGAAGAAAAAATGTATCGGCTGTAGAATTTGTTACAGGTCATGTAA
CGTTCCTGAAGCAATTTTAATATCTAAAGAAACCAATTTACCGTACATTAATCCAGAATACTGTG
TAAGATGTGGACTATGTCAAAATGCATGTCCTGTTGATGCAATTGATTATCTTAAAACTGAAACG
TCTGAAAATTTGTACTCTAAACGAAAAATAAGAGACGAGTTTGAAAGTATCCTGCACAGCGACCT
TGAAGAATTCACCAAAAATTACGTATTGTTAAAAGAAGAAGTTAAGAACCTTGGAAAACAGTCG
ATTTCAGAGGAGAATATTGGTGAAAAGAGGAAGGATGATTGATATGGTATCGAAAATTCTTAAA
AATATTTTGATAATTTGGATATCAACTTTGAACTTGACAGCCAGTACATCAGCAACAAGATCGA

AATAAATCCAGAAATCTGCATACTATGCAATAGATGCCTTGAAGTGTGTCCAGTAACTGCAATCA
GCTCAAACTTTCCAGAAGTTCCAGATATTGATAATAAATGCGTCTACTGTAACACCTGTGTAGAA
ACATGTCCTGTTGATGCTATAAAAATCACTAAAACTAGAGTAAGAGTGGAAAATGGAAATTTAA
TAATTGAAAATCGATTAAAAAGTAAAAAATTAGACTACAATCGAAAAAAATGTGTAATGTGTTT
AGTATGTACTAAAAACTGCCCATTTGAAGCGATATCTGAATCTGATGATACAATTTCTTTCGATA
TGGATAAATGCGTACTTTGTGGACATTGCGAAGAGATATGCCCTGCAAAAGCGATTAAACTTGAA
TAATACCTAAAAAGGTGTTTTAGATGATAACCGTAAAAAAACCAGTCCAAAAAATCATCTCAGAT
ATTTCAAAAAATCTTGGAATTGATGAAAAATTTGTAAAAGAAAAATTAAAGGATTTAATAGTGC
CTGTTGAAAGGAATTTATACGTAGAATCCAACAAATGCATTAGATGCGAGCTTTGCTATGAAATG
TGTCCTGTTGATGCGATAAAAGAACCTAGCGTTAAAAGCCCTGCTGAAATACTCCCTGAAAAATG
TGTAAAATGTGAAATATGTGCCAAAACATGTCCCGTTGGTGCAATCAATGTATTAGAGGGTCGTG
CAAAATTAGAAGAGGATAATGTAGTTTACGAATTAAAAGAAATTGACGTAACCCATAGAAAAAT
AAGGTTGGTAAAACACGAACTCGATGAAGAATCCTGCATAAAATGCGGAATTTGCGAACGATTCT
GCCCAACTTCTGCAATAACCGTTGAAAAAAGAAACAGCATCAACATAAATCTCAACCTCTGCATG
GGCTGTACTGCTTGTGAAAAAGTATGTCCAAAATCGGCGATAAAAGTTGAAAACGAAATGGGCG
AAATAACGGCTGAAAATGAAATATCTTTAAATAATGACACCTGCATTAACTGTATGGTATGCAGT
GAAATATGTCCCGTTGGTGCGATAGTTTACGAGGATGGATTAATGAAATTGGATGATAAAATGT
GCATATTCTGCGGAAAATGTGAGAAAAATTGTCCTGTAACTGCGATAGCGATTAAACAAAAAAA
TATGTAGTCGGGGAGGGGTAGTATGAAAGTAAAAAGCTTGATGGATAAAAAATTTATTAAAATA
TATCCTGATTTTACTGTACAGACCGTTATTGACTTAATGTATAAAAATAAGAAATTCAGTACACC
AATTATTGATAATGACGGCAAAATGGTTGGTTGGACAACGGCTATTGATTTAATGATAGTTTCTG
ATAAAAATATTCCAATAAAAGACATTATGAGCCCCATTGAAGACGTAATCGTTGTAAATAAAAA
CGAACCTGCAAGGGAAGCAGTTACTAAAATTGTAGAATACAAAGTCATAAGTATTCCTGTTTTAA
ACGATGAGGGCAGGGTTGTTGGAATTGTTAGAAACTGCGATATTACAAAAACTCTTTCCAAATTA
TACGATATTCCTGTACACAGCATCTTTAAATCATTAATGGGCGAATTGAAAGGAATTTCATGGGA
AGAATTAATGAATGCTGCCGCAATCGTTACAAAACAAACTACCGGGGAAAAAATTACCGGTGAAG
AATACGAAAAAAGAAGAAAAGGTTCTACATTCGGACAGGCAATATGGGCGTGCGGAGGACTTGA
AAAATTCTTCACAGGCCTCATAAAAATCGGTGAAGTTGCACTTGCAAGAAAAGTAAGCTGTAAAA
ATGTCATAAAAAAATAATCAATAATCGGGTGTCTGAATGTCTAACCTTATAAATATTCCTAAATA
CAGTAGGAAAATTGATTTCTGGACTTTCCTTGAAAAAGCATTTGAAAAAAATGTTAAAATTGAC
CTTGGACACTTTAAAATCATCTGCATGTTTCTTGATGTCATGGATATTTACGAAAGTTTGAGTAA
AGATACCTCTAAAAAAGAAGCGAGGAAAACGCTTGAAAAGGAAGGGATCTTTTCTAAAAACTCA
GAATACATTTCAGGAGAATATTTAAAAAAACATATTGATAGAGACAGCAGAGTTGCAGTTCACA
ACAGAATCAATGATTTAAGAAAACTAGAATTTATTATTGAAACCAAGCCTGGGCCGCTTGGAGGA
TATAAATTATTAGAAACCCCGGACTGGTTTTTAAACGAAGAATAAAATAGAAAAAATATTAAAT
TTCTTTTTTAGACATGATTTTTACAATGTCAGAATCGAGATCTCCCATTATTTTACTGATCTCTG
AAATATTTTCAACGCATTCTTTAAAATCTTTGCCGACAATCCCGTTTTCATAAGATATATCCATA
AAAAGAGAAGAATACGCTGCAAAGCATCCTGATGCAAGTTTTAATGCACATCCAACTTTTCCGCC
GTCGCATATTATTCCGGGCAAATTTGCAGTAAATGTTCTTGCCGCGCTATCAAGTTTTTTAATAT
TTTTACCGTTTTTATAGTAGCAAAGTCCAATAACTGTTCCCATTCCACCTTTACTTACACAGCCGC
ACATCGATGAAAGATAAGACGATTTATATGTTGCATAAATTGTTACAAGAACTGAAAGAAGGAT
AGATTTTATTAATTTTTCCTGATTTTGTTCGTTTGAACAGTCATAAGCAATTATTGGAAGTGTTG
CCATTATTCCCATATTACCGCTTTTTGCAACACTCATTGCAGGCATATTTTCACCAATCATTCGAG

TGTTGCAGGCAGAAGTTGTTTTATTTACCATATTTGACAGACAGTCGTTTCCATAATTTAAAAAA
TCGCCTTTGATTAAATTATTATTTGTATAAATTGTTTTTTCAATCAATTGAATAATTTCTTCTGG
AATTTCATCAACATAATTTAAAAAATCTAAAAGTTCAAGATTTTTGTAGTCTTCTTTTTCAGAAA
CATTTATTTTGGAAATGAGCTCATTGTTAATTTTTGGAATTACAACGTTTAAGTGATTGTCTTTA
ATTAACGCTTCAGAAGTTTTCCCAGAATTATCTTTAATTTTTACGCTGCAATAAAGGGGTCCGTT
AATTACACTTATTTTAAGTCGTTCCTTAATAAAATCCTCTAACTTTTGGCTGTAATTAATACAAT
TAAAAACTAAAAGTTTGTTTTTATAATCCCCCAAAAGTCCGCCAACAACTGCAGGCAATATCCCA
TATTTTTTAGTATTTGGAACCCCAACAGCATATGCATTTTTAAAAGAGCCGTTATTTAGTGTAAG
TTCGATTTTTTCAATTGAAAATGGGTCTGAAATATTGCATAGTGAAACTGCGTATCCGATTAATC
CGACTTCAGTACAACCTAAAGCTTCTGTTACTTCGTTTTTTAGTATTTTTGTAATTAAAATGCGT
TTAAAATCATCCATAATACCAACTAAATCTTTAGAAAAAAATAAAAATAATATTTTAAAAAAAT
TAATTTAATCAAAAATCTTTTTTATGTACGGCTCAAAAGGCACTATTTCATCTTTTGTTCTCGGA
CTTATTGAAGCTACTTTTAAAATTTTATTTTCTGAATCCAGATCAATTGTAAGGGTTCCGGGAGT
CAGAGTAATGCTTGATGCAAGTAAAACCTGGCCCATTAAGCTGTTTATGTCTGTGTTTATTTCAA
TAACTTCTGGATCAATTTTGCCGTCAAAACTTCTTTTAACTACATCGATCCATGCTTCGGCTAAA
GCCTTTAAAAAAACGATTGAATACCCGAAAAATCCAAAAAGCTTCATTATAACACCTTTGTCCAT
AGTGGAACAATTCTTAATTATCAATAATGATAAATAGCTATATATAATTATCGTAACTATAAAA
ATATTAAAAGAAGGCTCAGCTTTTTAAGATTTGAAAACAAAACACGATTTACTTTCAGAGTTTAT
GGTGTCCTAATTATAATCCAAAATTGTTATTAATGGTGATAAGAATGAACGAAGAACTTCAAAA
CCAATTCATGGCACTCGATGTTTACAACCAGCAAGTTGATAAACTTAAAGAAGAACTTTCAAACA
TTGATATGATGATAATGGAACTTTTGAGATCCATTGAATCTATGGAAGGCTTAAAAAGCTCCAAA
GAAATTTTGTTGCCTCTTGGTGCTGGTGCATTTATAAAAGCAGAAGCTCAAAACCCTGAAAAAAT
CGTTCTTTCAGTTGGTGTGGACGTTTTACTTGAAAAGAATGTAGATGATGTAATAGTTGACTTCC
AAAAAAGCGTTGAAGAACTTGAAAAAACAAAAGAGCTTGTAAATAATCAAATTCAAAAAACAAA
TCAGGAAATTGTAAAATTAAGATCAGAACTTGAAAAAAGAGCTGCTGCAATTGAACAGAGAAGC
GCTCAAATGAATCCTAAAACAAACTAATTTTGATTAATTTTTAACTTTTAAATTAAATTTTAAAA
ATACATATTTTAAAACAGTCTATTTCAAAAAAAGTTACATTTCAAACATTATAATATATTTGAA
TTTATCAATATATGTGCCTAAAATAAAAATGGTGGACCTAGCCGGATTCGAACCGGCGACCCTCG
CCTTGTAAGGGCGATGTCATAGCCAACTAGACCATAGGTCCTTTTTGCTTCTCTTGGCTCTCGTTC
TGAGAGCAAAATAAACATTGCATTCATAATATATAAATTTTTCGGTATGCTTTGAAAACATTGG
ATTTGTGGTAGGATGGAAGACGAAATAAAATCATTTGAAAAAGATAGAAATTTGATAATTGAAG
ACTCGAAAAATTCAAAAAATAACATTTTTGATGTTCAAAAACTCATTGAAGCATTGAAAGATGA
AAAAAATACAAAAAACATTGTTGTAGACTTTACTGAATATAATCCGCTCCATAACGGGCACAAAT
ACTGTATGGATTTTGGTAAAAGTGAAGGTTTGTTCATCAGTGTTATTCCCGGACCCCTTGAAAGA
AGTGGACGGGGAATTCCATATTTAGTTAATCGACATATTCGAGCAGAAATGGCAATACTTGCAGG
AGCAGATTTGGTAGTTGAAGGCCCCCCAATGGGGATTATGGGATCAGGCCAGTACATGCAGTGTT
TGATAAAGATTTTTTCGGCGTTGAATGGAGATATTATTCCAAGAGGATACATCGAAGAAGAAAC
GATGGAACGGGTAATTAATTCGATAAATAATGGAAATCACATCAAAATAAAACCATACAATATT
TCCTGCATCGAAACTCGCGAAAAACTTGGAGAAAAATTGGAAATTGATAACTATGTTATTGCTTC
AATGTCCTACACAATTTATAAATTAAAAGAAAATTTCCCAGAATGGAACCCTAAATTCAAATTTA
TCGAAAGAATTGAAGGAATAAGTGGAACAAAAATACGAGAAGGAGTATTTAATAATAATTTTGA
ATCAATAAAACATATGCTTCCCATAACAACGATCGATGTCTTTAAATCTTTTGGAGATAATTTAT
CAGAAATTATTCTTAAAAGAAACGAAGAAATGGTTCTTGATACCATAAATAATTTTGATTTGAG

TAAATATTTACCCGAAAATATTTCAGAAAAATTAAATGAAAAAGAATATTATGAATCGATTGAA
GAAATAAAAGAATATATTCCAAGAGGTTTTTCAAAAAATAACGTTGAAAGGACGATCTCAAAAT
TAGAAGCGAGAATAGAAAAGGAAACTATTTCAAAATACATTGAAAATTATCCGGCAAATTTAAG
AATTTTAAACGGGATAAATAATCATGAATAAAGGGGGTCGATAATTTTGCAAAAACAAAGATTT
TGCCTTGATACAACTGCAATTACTGATAGTGACGTTAGAAAATCGTTAGGTGTTTCAAATATTTC
AGAATCTGCAGAAAAAATAATGGATATTATCGCACAGGCACGAGTACAGCTCGATATCTCATGCC
ATATTCCTTATGATACTGTTTATAAAGAACTTGTTGGTTTTTTAACTCGTGAAGGGTGTGCCCCT
GAAACATTGATAAAAGTAGACACGTGGCTTGTCAAAAAAACTCCAAATCGCTACGAAATAAAAA
TTCCTGCCGAAATATTTTATGAATATATAAAAGATTTAAGGGAAAGAATAAATAAAGGGATGAG
AATAAGTGAAAACGCAATGTATGAAACAGCCTTGGAAGCATACATACTCTCAAAACCTGACGAAA
AAGATAGGGAAGACGTTCTAAACGAGGTCCTGTCAAAAACAGTTAATAGTTTTAGAGTCAAATA
CCGAAATGCACTTCGAGGAGGTACACTTGACAGTGCCCCTGACTTAGACGTTCTTTTGTTAGCAA
AAGAGCTCGATGCAGCAGTTGTTGCAAACGATGAAGGTATTGAAAAATGGGCTCAAAGACTTGG
GCTTAGATTCGTCAACGCAAGAGATTTTCCATTCATACTTCAAGAATATCTTGATTTATGGGATA
AAAAGTAAATTTAGAATATTTTATAATTCTAAAACTTAAAACTACTAAAACACGGTGAGAAACA
TGGTACTCTTGATTATTAGAGGCGACAGCTACGAAAAAATAAAAAACGCAATAGCTGATGTTCAC
AGGCATGCTAAATTAACAATACTTGGAAAACCAAGAATAATGGTTCCAGAAGCTGCAGATGAAA
TTCTTGAACATATTGTTGGAAACATTAAAAAACCATGCAAAAAAGCTTGCCTTGTCAGGATTGAG
GAAAATGCGCCAAGAGCAATTGATAGGATTAGAAAAATTCACCCTCCTGCACATATCGTAATCGT
TAGCGAGAGGCATGAACCTTACTTTTATCTTTTAGAGGACCTTCCAAAAATGCCACTTTTAAAAG
GATACTACAAAAGTAAAAGTCTTGATTCAGATGAAGAAATTGAAGAATCACATTAACTAAATTA
ATTATTTTAAAATTACCAAGGGGTGAATAAATGAAACAAGTTAAATCCGTAAATTTTAGAGAAT
TAGATAAAAAAGTAAAAGAATACTGGAAAAAAGAAAATACATACAAAAAAGTAAAGGCATTAAA
TGAACACGGCCCTGAATACTATTTTGTTGACGGCCCTCCGTACTGCTCTGGTGCAATACACCTTGG
AACAGCTTGGAATAAAATTATAAAGGATACAGTTTTAAGATTTAAGAGAATTCAAGGATACAAT
GTTTTAGATAAAGCTGGATGGGATATGCACGGGCTTCCTATCGAAGTTAAAGTGGAAAATGAAT
TTAACATCGGCTCCAAAAAAGACATTGAAACAAAAATTGGAACACAAGAATTTATAAACAAATG
TAAAGAGTTTGCATTAAATCACCTCGGACACATGCAAGGTCAGTTTGAAAACTTAGGTGTATGGC
TCGATTTTGAAAACGCATACATGCCGATCAAAAGAGACTACATGGAAATGGGCTGGTGGACGCTT
AAAAAAGCTCATGAAAAAGAATTACTTACAAAAGACTTAAGATCAGGATATTGGTGCCCAAGAT
GTGAAACTTCGCTTGCTGAACACGAAGTAAGGGGAGAATACAAAGAAGTACTCGATCCTTCAGTT
TATGTAAAATTCAAACTTGAAAATAGCGATGAATACATTACAATCTGGACCACGACTCCTTGGAC
ACTTCCATCAAACATGCTCGTATGTGTAAATCCGGAATTTGATTACGCTTATGTTGATGTTGAAT
TTGAAAATGGAACTGTTGAAACCTGGGTAATTGCTGAAAAATTGGTAAATGATGTGATGAAAAA
GGCTGAAAAAAACAACGACATTTCAAAATTCAGTATTTCAAAAGTTGTTAAAGGAGATTCATTA
ATTGGATTAAAATACATCCACCCACTCCTTGAAGAAAACGAAAAACAGCAAGAATTTGCAAAAAT
AGAAAATGTGCACACAATTGTTCCAGGAGACCACGTAACTCTCGAAGGTGGAACCGGTTTAGTTC
ACACCGCACCAGGATTTGGTGAAGATGACTTTAACATTGGTAAAGAACATAATATTCCAGTATAC
GCACCAATTGATGATAACGGAAAGTATACTGATAGTATCTGGAAAGGAACTTTTGTAAAAGATA
TGGATGAATCAGTTATTGAAACATTAATATCCAAAAATTTACTCGTAAACTCTGGAAAAGTAAA
ACACACCTATCCGCACTGCTGGAGATGTAAAACACCACTTTTATTCAGGGCAACTGAACAGTGGT
TCCTTTCAATCTCAAAAATAAAAGAATCCATCATCGAACAGGGTAAAACTGTTGACTGGGTTCCA
GACTGGGTTAAAACAAGATATGTTAATGGAGTTAGCTTTGTTGGAGACTGGAACATCTCAAGACA

AAGATACTGGGGAATTCCGCTTCCAATCTGGATCTGTGAAGAATGTGGAAATTACGAAGTAATCG
GTTCAGTTGATGAATTAACAGAGCGAGCTAACGAAAAAGATGTAGATTTAAGCGACATCCACAA
ACCTGCCGTTGATAAAATTACGCTTACATGTTCCTGCGGCGGAAAAATGAAAAGAACCCCTGATG
TATTAGATGTCTGGTACGACAGCGGTTTGGCGCCATATGCGTCAATTGGATCTAAAAAACTTAAA
AAAGCTCAATTTATTACCGAAGGAAACGATCAGGTTACAAAATGGTTCTACTCACAGCATGCATT
GAGTGCAGTTGTATTTGACGATACTTCTTACGAAAAATGTATGATGCACGGATTCACTCTTGATG
AAACCGGAGAAAAAATGAGTAAATCTTTAGGAAATATTGTAAGTCCTGACGATGTTACAGACCA
GTACGGTGCAGATGTTTTAAGGTTCTATCTTTTAAGTGCAAACAAAGCTTGGGAAGATTTAAGAT
TTTCATACTCGGAAATGGATGAAACTAGAAGTATGTTAAACACACTCTGGAATTCATACGCATTT
TCAGCAAACTACATGGTTCTTGATGATTTCGTTCCAAATAACGAATATTTCAAGCATGTTAAAGA
TGAAGATGCATGGATATTGAGCAGAATAAATACTGTTGCTAAAGAAGCTGTTGAATCTTTAGAA
AAACCTCATTTACACGTGTACACGTGGGCACTTAGGGATTTCATACTCAACGACTTTTCAAGATG
GTATATTAAATTAATTCGTGACAGAACATGGATGGAGAAAAATGACGTGCAGAAATTATCTGCA
TACCAGACTCTGTACTACGTGATCATGAAATTAATTTCAATAATGGCTCCGGTAACGCCCCACCT
TTCAGAAGAAATCTATCAAAACTTAAAAACAGAAGATATGCCTGAAAGCATCTTCATGAACAAGT
TAACAATTGAATCTGAATTTATAAATGAAACTTTGGAAAAAGATACCGAAATTATTCGTGAAAT
TGTAGATTCAATCCTCAAAGGAAGAGATAAAGCAAAATACACGCTAAGATATCCAATTACAAAA
ATAACGCTTCCTGAAAACATTGCTGAAACTGTTGAAAAATATGGATACATCATAAAAGAACAGG
GCAACGTAAAAGAAATTGAATTAAAAGAATTTGAAGGAAATATAACGGTAAAACCAAACTTCAA
AGAACTTGGAAAAATATTCAGAAGCGACGTTCCAAAAGTAGTTGCTGCAATAAATTCAGTAGCTC
CTAACGAACTAAAAGAAAAATTGAAATCTGGAAACTTAGAAGTTTCAGAATATGAAATAAAACC
AGAATACGTTGAATTCAGGGTTGAAATTCCTGAAAACATTGTCGGTGTTGAATTCTCAAAAGGAA
ACGTTTACATTAACATTGAGATGAACGATGAAGTAATCAAAGAAGGATTGGTTAGAGAAGTTGT
TAGAAGAATCCAGTCCATGAGAAAAGATATGGATTTAGATATTAACGAAAAAATCAACGTTAAA
TTAGAAGGAATTGATTTCAGTAGTGATTATTTAAGCCATATTGCAAATGAAGTTAGAGGAAACT
TCGTTGAAAGTTTAAAATCTGATTACAATCAAAAATGGACTATTAAAACGCCAAATGAAGAAAC
TTACGATATTTCAATCGATATTGAAAAAAATAAATAAAATAAAAAAATCTTTTTTTATATTTTT
TATTAACTTTTTTTACTGGTAGTTTGAGTAGTAATCTGACATATCAATTGCATTCTCTGCTTCTT
TTGGAAGTTCTATATTTTGAACTTCATTGATATTTTTTAAACTTAAAACGTAGTTAATCTCAAAG
GACTCCCCGGATTTATCAGTTCCTTTGATATTGGCTGTTGTTTTTACTAAATAACCGTCCGATTT
ACTGATATAATAAATAACATCTGCTGTTTCCCAATCTGAAACTTCGCCTTCTTCAACAGCATATT
CTGACACCATATCATTATAAATGTCTTCTATATTTATTCTTACTTTGTAACAGGGGATTCCATTT
ACGGTTTCCTCACCTTCCAAAATAAAGTCGCTATTTTCAAGTTCGTAAACACTTTCGTCGTACTG
TGCCATTACATCAAAATTATCTTCAAAATCTTCACTGTTAGCAGGCATTTTCATCCACCGGGTTT
CCCCGTTTGCAACAGCTCCCATGTAAACTGCATCTTGGAAATAATAGTACTCAATGCTTCCATTT
TCAGATGATAGGTACATGTAAGTATTTTTATTTTTTGTGTCAACTATTCCAGATCCGCTAATCTC
GATATTTTGACCATTTACCTTAGAATTCATAGTATATTCATACGAATAACTCCATACCTCAGCAT
GATTTCCAATCAATTCTAAAATTTCTGAACCTTCGTTTGTATTTAAACTTGAAGAACTTTGTGAA
TCGTAATCCTGTGATTGCACTTCAGCAGATGCACCATATTCTGAATCTGAACTGTCCGTACAGCC
AGAAAATGAAACTGCAGGTAAAAGTGCAAAAATCATAGGAAATACCAAAAATTTTTATTAAAC
ATATTATCCCCCAAAATTAGCTTTTTTTGGCATTTGCATTAAAAAATATGGGTGTTAAAAATATA
TGAATATTTTGAAAAACTGGAAAAATTTCTTTTTTTAAAAAACTAAAAAAATGATTAAAATAGT
AATGAACGTAAGATGTTATAATACACTTCAAAAAATGTTTTCACAATGGATAATCCATTTTCTTT

TGCATAATTAACTATGATCCAGTCCAAAGTTTCCATTCCCCCTGCAATAAATAAGAAAATTGGAA
TTAATAAAAATTTTAATAAAAATTTTATTTTCTGTTTTTTTGTCCATACTTCTTTTTCACCCCTT
ACTGGAAGTTTATAAATAGAATACTCGATTCCAAGGACTATTGACATTGAAAACCCGGCATATTC
AAAAAACCCATGCGGAATTATTGCAAGTAGATCTGAAAAACTTAAACTTGCACCCAAAATTCCAG
TGAGTGGATTTAATACAACCAGTGCGTAAAAAAACAATAAAACATCGAAATAATCTTTGATCGT
TGATTTTCCTTTTGAAATATCCCTATTATAAATATAAGGAAGTATCACAAAAACCAAAAATATAG
TTACACATGCAAGAAAATTACTTATAAAATACGAATACCATATAGAAAAATAATTAATTCCCATT
TGAGAGGATACTGCTGTTGAAATTACCTCATATTTTGCAGTTGTTAAAACACCTGCGGATTCAAC
GTTTATAATTGGATTTATAAGCTTACTTATGAGTAAAGTTATAGATAAGCCCATCCAAGATAAA
AAATAAGTTACGATGAATTTATTTATGAAGCTTAAGTTTAAAAACTCGTTAAACTGTTTTTTA
AAAATTCAAAACTCAAAATTTCATCCTCTCTACAAAATATCAAAAATTTTTTATGTTTTTAAAAT
ATATTTAGTTTATGATTAATTTATTCATTTTAGGAAATATTATTAAATTAACTTTTGCGTGAAA
AATATGAAACGAATAGTTATTGCAGGCACTTCTTCAATGGTTGGAAAAACTACCATTTCTACAGG
AATAATGAAGGCACTATCTAAAAAACATGATGTACAGCCCTACAAAATCGGACCTGACTATATAG
ACCCGACATACCATACAGAAGCTACAAAAAACAAATCCCGAAACTTAGATTCGTTTTTTATGGAT
GAAATGCAGGTTAGATCGATTTTTAAAAGGCATTCTAAAAATAAAGATATAAGTGTTATTGAAG
GTGTGAGAGGATTATACGAAGGAATTTCGCCATATAACGATGTAGGAAGTACTGCATCCGTTGCA
AAAGCTATAAATTCACCAGTGATTTTATTAATGGATGCAAGAAGTCTTACAAGAAGTGCAGCTGC
AATAATTAAAGGTTTTAAATCATTTGATCCTGATTTAAATATCAAAGGAGTTATTTTTAATAAA
ATCAGGGGCGAAGGACACTTAAATAAATTAAAAGAAGCTGTAAAATATTACGATAATGAAATTG
AGATAATTGGTGCAATTCCTCGAGACGATAGCCTGTCTGTTTCTCAAAGACATCTTGGACTTGTT
CCAACTCCGGAAAATAAGCAGGAATTGCTTGAAAGAATTGATCTATGGGGCGAAACTGTTGAAGG
ATGCCTTGATCTTGAAAAAATTGTGGAATTGAGTGATGAAAGTTTTGATTTTTGCGTTGATGAA
AAAAATAAAGAAGAAACCCTCTGGAAAGTTGAAAAAAATAATTCAAAAATTGCCGTTGCACTTG
ATGAATCATTTAATTTTTATTACTGGGACAACTTTGATGCTTTAGAAGAAAACGGTGCAAAATTA
AAATTCTTCAGCCCGTTAAATGATTCAGAAGTTCCAGATTGTGATACCATTTACCTTGGAGGAGG
GTATCCAGAAATATTTCCCGAAAAACTTTCTAATAATAAATCCATGATTGAATCTATAAGAAACT
TTGATGGGAAAATTTACGGAGAATGTGGGGGGCTCATGTACCTTACAAATTCTATTGATGGACGA
GAAATGTTAAAATTAATAGATGCAGATGCAGTAATGACTCCAAACGTTCAGGGCTTAAGTTACGT
TAAGGGAACTTTTGAAAAAGATTGTATTATTGGTAAAAAATCTAAAGAATTTAAAGCACACGAA
TTTCATTATTCAAAACTAATAAATATTAATGAAAAAGATTTTTCGTATAAGATAAATAGGGGAA
AAGGAATAATTAACTCTATGGATGGAATAACTTCAAAAGATGGCGATATAGTTGGAGGTTACGC
TCACCAGCACTGTATTGGTAATCCTTACTTTGCAGCAAGCCTCTCAAAAATGTAAAAACCGGTGC
ACCAAATGATAAATAATAAAGTCAAAAGTTTAATAAAACATGGTTTCAATCTTGCAATAGATAT
CAGCGCAGATATTTTATTGATATTTACGGAAACTGGAAGAACTTACGAGTACTACAAGGATCAAG
AAGCCATATACAAAAGGGCCAGGAAAAATAGATCATTAAAAGACTTTAAAAGTTTAAAAGTAGT
TGTAACCACGCCAAACGAAGAAACTTTTTTGAAATTAAAAAATGAATCAAAAATTATACCGCTTT
TGATGACATATAGGAATGACGATAGATCATCAATGATAAAACAAGCTGTAACCAACCTTTTTGCA
AATAACATTGTTAAAAAAGGGGATATTGTTGTATCAATTTTAGGTATTCCAAAAGTAACTGGTG
GAACCGACACCATATCCATCTTTGAAGTAAACGAATACCCCCAAATCCTCAAATATTATGATTAC
ATCTCATCGATTGAAAAAACAAAAGGAAAAGTCATTAATGAAGTTTTGAACCTTTGTATGGAAC
TTGCAGTTGAAGGTAGGGAAGGAACTCCTGTTGGTTCAATATTTGTAATCGGTGATTCGGATAGA
GTTTTAAAAATGTCGTCCCAATTAATTTTAAACCCTTTTGAAGGCCACGAATCATTTATATTTGA

TAAACAGGTCAAAGGGACGGTTAAAGAATTATCAACAATCGATGGGGCATTCGTAATTGGTGAA
AAAGGAGAAGTTCTCTGTGCAGGCAGATATATAAGCTGTAGCGGTGGAAATATTGAATTACCTTT
GGGCCTTGGTGCAAGACACCATGCTGCTGCAACTATTTCAAAATATACTGGTGCAATTGCAATTA
CGATTTCAGAAAGCGGTGGAGTCATAAGAATTTTTAAAAATGGAGAAATATTGTCCGAAATAAA
GCCTAAAAAATTAAGTAACACGTCAGAATACTCTTATTAATTATAAATATTCTATTTTAAAATTT
AATTTAAATCCCTTATATTTATAAATCAATAACACCTAACCTATGAAGTACTGTTTTTATGTATA
TTACGAATTTACGGTGAGAATCTATGACACTTGCTGAGAAAATCATTTCAAAAAATGTTGGAAA
AAATGTTTACGCCAAAGATAGCGTTGAAATAAATGTAGATATTGCAATGACCCATGATGGGACTA
CCCCGCTTACGGTAAAAGCTTTTGAACAGATTTCGGATAAAGTATGGGATAATGAAAAGATAGTC
ATTATTTTTGACCACAATATCCCTGCAAACACGTCAAAAGCTGCAAATATGCAGGTTATAACGAG
AGAATTCATCAAAAAACAGGGCATTAAAAATTACTACCTCGATGGCGAAGGAATATGCCACCAAG
TTCTACCTGAAAAAGGCCACGTAAAACCAAACATGATAATTGCGGGAGCTGACAGCCACACCTGC
ACTCATGGGGCATTTGGGGCATTTGCTACAGGTTTTGGTGCAACTGACATGGGTTACGTCTATGC
AACAGGAAAAACCTGGCTTAGAGTTCCTGAAACTATCCGCGTAAATGTAACTGGAGAAAATGAAA
ATATTTCTGGAAAGGATATTATCTTAAAAACCTGCAAAGAAGTTGGAAGACGTGGGGCTACGTAC
ATGTCTTTAGAATACGTTGGAAATGCAGTCCACAATCTTTCCATGGATGAAAGAATGGTATTATC
AAACATGGCTATTGAAATGGGCGGAAAAGCAGGTATTATCGAAGCTGACGATACCACATACAGAT
ACCTTGAAAATGCGGGAGTTTCGCGCGAAGAAATTCTTGAATTGAAAAAAAATAAAATAACAGT
TGATGAATCCGAAGAAGACTACTATAAAACAATTGAATTTGACATAACAGATATGGAAGAACAG
GTTGCATGCCCTCATCACCCTGATAATGTAAAAGGAGTTTCAGAAGTTGAAGGAACAGAATTAAA
CCAAGTATTTATCGGTTCATGTACAAACGGAAGATTAAACGACTTAAGAATTGCTGCAAAATATC
TGAAAGGAAAGAAAGTCAATGAAAACACCAGATTGATTGTAATCCCTGCATCAAAGTCAATATT
TAAAGAAGCGCTAAATGAAGGATTAATTGATATTTTTGTAGATTCTGGAGCATTGATCTGTACCC
CTGGATGCGGACCATGTCTTGGAGCCCATCAGGGGGTTTTAGGTGATGGAGAAGTGTGCCTTGCT
ACAACTAACAGGAACTTTAAAGGAAGAATGGGAAACACGAACGCACAAGTCTACCTCTCTTCTCC
AAAAATAGCTGCAAAATCTGCTGTTAAGGGCTACATTACAAATGAATAATAATCAATATTTGTTT
CTCTTTTTTAAAATTAGTTTTATATAGTATTACCAACAGTATTCAAGATATATTTCATTTAGTAA
AATAATCTGGAATTAAATCGAAATCCATATATATCAAACCAACTTAAAGATAACTGTAAAACAA
TATAACTTATCTTTAATAGGTGGTAAAGTGCACATTATGGAAGGTTTTTTGCCTCCGATGTGGGC
CGCGTTTTGGTTTGTACTCTCCGGAATTGTTGTTATATATGGAATAATCCAGTTAAATAAATTAA
TAAACGATAAACCTGAAGTTAAACCTACATTAGCCCTCGCAGGAGCATTTATGTTCATACTAAGT
TCGCTAAAACTTCCTTCAGTTACAGGAAGTTGTTCACACCCAACAGGTGGTGGACTAGGCGCTAT
TATGTTTGGTCCCGCAATTACAGCGGTACTCGCAACAATTGTATTATTGTTCCAAGCAATCTTAC
TTGCACACGGTGGATTAACCACATTAGGAGCAAATATATTTTCAATGGGTATTATGGGCCCAGCA
ATTGGTTTTTTAGTGTTTAAATTGTTAAAAGGAAAATTAAATATCACATGGGTTGTTGTACTCGC
TGCAATATTTGCAGACTGGGGAACATACGCTACTACATCAATCCAGCTCGCATTAGCATACCCAC
TACCTGATTTTGGAACGGCTCTTGCAAACTTTGGAACCGTATTTGCAGTAACACAAATTCCACTT
GCAATTATAGAAGGTCTATTAACAGGACTTATCTGGGATTACATTATGAAATTAAGACCAGATCT
CCTTGCAAAACTTGGTCTTATTGACCTTGAAAAAGCAAAAGGAGGTGCTGAATAATGGAATTCAA
ACACGTCTTGATGATTCTTGGAGTTATAATTCTTACATTAGCACCTTTGATCATGTATTCAGGCC
TTGGTGAAGATGAAGGCTACTTCGGTGGTGCAGATGGGTGCTGCTGGAGACTTGATCATGGAAATC
AGTCCAAATTACGAACCATGGTTTGAACCATTCTGGGAACCTCCAAGTGGGGAAATTGAAAGCCT
ATTATTTGCGCTTCAAGCTGCAATAGGTGCTATAATTATAGGATACTTCTTTGGCTACAACAAAG

CCAAATATGATGCCAAAAACCAGTAAAAGGTTAAAAAATGACGAACAGCACTTTAATTGATAAC
ATTGCAAACTATAACAAGTTAAGGTCAGTTAATCCTACTTTAAAAGTTATTTTTGCAATTTCTTC
ACTGTTCGTTAGCATTTTTTCTAAAAATTTTATTGTACCCCTTTTAATCACAATTATAATGAGTT
TTGTGATTATTTTTGCTGCAAAAATACCTAAAAATATCTACGGAAAATTACTGGCAATCCCGATT
GTTTTTGGAATTATTACGTTTTTAATGATGACTTTTTTATTTGGAACTGATATCTACACGTCATT
TGATTTTTTTGGAATTACAATCAATTTACTAAAAGACGGATTTAGTCTTGGACTTTTAACATTTT
TTAAAATGCTTGGCGGGGTATCCTGCACATTATTTTTAGCACTTACAACACCATTTACGGAACTA
TTTTACATCCTTAAAAGATCTAAAATGCCAAAAAACATGCTTGAAATTGCGATGATGATGTACCG
CTATATTTTCATGCTTTTAGAAGAAGCTTTAACAATGGAAAACTCGCAAAAAACAAGGTTAGGGT
ACAAAAATCTTAAAACTTCGTACCGATCATTGGGGCTATTAACGGGAAGTTTGTTTATAAAAGCA
CTTGATAAGGGAGATATAATTTATAATTCACTTAATTCAAGAGGATACGACGGAAATTTAATGT
TTTTTGGAGATATTGCCTATCCAAGAACTTTTTCTATTGTACTTTTAGGCGTATTTGAATTAATG
TTGCTATCATTAAATTACATTAAGATATTTTAAAGCCAAAAATTAAAATTCTAACTTTTTTATAC
CTTTAAGCATATAGTTCCTCCAAGGTGATTTAATGGCGATTTTGGAAACAAGAGATTTAAAGTAT
TCGTATCCTGATGGAACTGTCGCACTTAACGGGATTAATTTTAAAGCCGAAACGGGCGAAATGAT
CGCAATTTTAGGCCCCAATGGTGCTGGAAAATCCACTACATTTCTTCATTTTAACGGAATATTAA
AACCAAGTAACGGTTCTGTAATTTTAAAAGGCGAACCAATAAAATATGACAATAAATCGCTTTTA
AATGTCAGAAAAACTGTTGGAATCGTATTTCAAAACCCCGATGACCAGCTTTTTGCACCCACCGT
TGAACAGGATGTTGCTTTTGGACCGATGAATCTTGGACTTTCAAAAGAGGAAATTGAAAAAAGA
GTAAAAGATTCATTAAAAGCAGTTTCGATGGAAGGGTTTGAGAGAAAACCACCTCACCATTTAAG
CGGTGGCCAGAAAAAAAGAATCGCTATTGCAGGAATTTTGGCAATGAACCCGGAAATCATTGTTC
TTGATGAACCAACTTCAGGACTCGACCCGATGGGTGCATCTCAAATCATGAAATTGCTCTACGAG
TTAAACAAACAGGGAATAACCATAATTATATCCACACACGATGTAGATTTAGTTCCGATATATGC
AAATAAAGTTTATTTGTTAAATGAAGGAAAAATTATCAAAGGCGGAACTCCAAGGGAAATTTTC
AGTGACTCTGAAACTGTTAGAAGTGCAAATTTAAGGCTTCCAAGGGTCGCACATTTAATAGAACT
TTTAGACAAAGAAGACAAACTGGGAATAAAAATGGGTTATACTATTGGAGAAGCTAGAAATAAT
ATTAAAGAGTTTATAAAGGGGGAATAAACATGCATGAGAGAATTTCCGATGTAAAATACGCAGT
AATTACTGTAAGTGACAGCCGATTTAATGAAATAATTTCAGGGCACGATATAACCGATAAATCAG
GAGACTTTTTAAAAGAGGAATTGGGTGCAAAAATCTACCATTTAATTCCTGACAATAAACCGATG
TTAGAAGGATTAATTGAACATATTATTGAATTTACAGATGTTAATTCAATTGTAATCACAGGCG
GAACAGGTCTTTCTAAAAGAGATATTACAGCTGACGTTGTAAAAAGTATGTATGAAAAAGAACT
TGACGGTTTTAAAATAATATTCCATAATTTAAGCTATGGTGAAGTAAAATACTCAACAATCCTTT
CAAGAGCATCTGCGGGACTTTACAAAGGAAAAATCATATATTCACTTCCAGGATCCATTAATGCA
TGTAAAACCGCACTTGATATTATTAAAACAGAAACCGGCCATATTATCGGCCATATAAAATAAAA
AAAAGTTATAAAAACTATTTTTTAATATTTATGAACATGGATATCCATCTGTGGAAATGGAATT
TCAATGCCTTCTTTTCCGTATTTTTCGTAGATTCCTTTTGTAAGGTCTCCTTTTACGCCCCAGTAG
TCACCAGTTTTTGTCCAAGCCCTAAGCTGTAAATCTACGGAAGACTCACCAAGACCGGTTACAAC
CACTGCAGGTGCAGGATCTTTTAAGACTTTTGGATGTTCTGAAATAAGTTCTAATGCTTTTGATA
ATGCATTGTCTAAATTTTCACCATAGCTAACCCCAACTGCCACATCAACCCTTCTTAAATCCATTC
TAGTGTAATTGGTTATTGGACTGCCCCAGACTAGTTTATTTGGAATTGTGATTACTACATTGTCA
GGCGTTAACAGTTTGGTTGCCATTATTCCAACTTCGACGATCTTTCCAGTTATTCCGCCTATCTGA
ACAGTTTCATCTTTGTCAAGGGGTTTCATAACTGCAATCCAAAGGCCCGAAGTTAAATTTGTAAG
TGTGTCCTGAAGACCAAAACCCAAAATTAAACCAAGTGATGCGGAAAGCCCCAAAATTATTGGCC

CTGTTTCAACGCCAAAAACCCCTACTGCAAGTAATATTACAAATACATAGAGTATCGCACTAAAT
AGCTTGATAACAAACTCAGATACAAGTTCTGGAATCTTGCTTTTTTTAGCACCGTTCTCTAAAAT
ACTAGAAACAATTCTTACAGCAAAATATCCTAAAACTAGTATCAAAATTGCTTTTAATATTAAAA
TCAGGTTTATAGACATGCTGTAACCCATTAACGAAATCAGCATTTTTGTATCCCCTCAAAACGTT
ATTATCTTAACTTTTTAATTTTTTAAATATATTAATTATTCGCTAAAATTTATAATAGATTAAGC
ATTAATATTATTCAATTGAAATTTCGAGTGTGATTTTATGTTTATCGATGGAAAATGGATTATA
AGGGAAGATATTGATGTTTTTGACCCGTACACTCTTGAAAATATTGAAAAAATAACTGCACTTGA
TAGGGAAGAAACTAAATATGCAATTGAAGTTACTGAAAAACATAAAGAAATAATGAAAAATTTA
AGCCCGTCAAAAAGATATAAAATATTGATGAAAGTTGCTGAACATCTAAGTTCGAAAAAAGACT
TTTTTGCAAAAACAATTTCAATCGATGTTGGAAAACCGATAAAACAGTCAAAAATAGAAGTAGA
TAGGACATTAACTGCGTTCAAGCTTTCTGCATTCTATGCAAAGGAACTACGTGGGGAAACAATTA
ATTCAGAAAATGGCCTTATATTTACTAAAAAAGAACCTTTAGGCGTAATTGGAGCAATTACTCCA
TTCAATTTTCCGTTAAATCTGGCGACACACAAAATTGGTCCTGCAATTGCCACAGGAAACTCGGT
TGTGCTCCACCCATCATCAAAAGCGCCCATTGTAGCAATATATCTTACAAAAATTATAGAACATG
TCTTAAAACAGATGGATATTCCAAGAGGGGTATTTAATCTTGCAACAGGAAATGGAGAAATTGT
TGGAGATGAAATATCGAAAAACGATAATGTAAACATGGTTTCTTTCACAGGAAGTGTTGAAGTT
GGAGAATCAATATCTAAAAATGCGAAAATGAAAAAAGTTACACTCGAACTTGGGGGAAATAACC
CTATGATCGTGTTAAAAGATTCTGATATCAAATTAGCTGCTAAATCTGCGGTTAAAAGTAAATTT
TTAAATGCAGGACAGGTTTGTATCTCGGTTGGACAGGTACTCGTTGAAGAAGAAGTTGTTGAAAC
ATTTACAAAATATGTTATTGAAGAAACCAAAAAACTAATTTTGGGAAATCCACTCGATAAAAAT
ACAGACATTGGACCACTTATAAGCCCGGAGAGTGCTTTAAGAATTGAAAATTTGATAAAACAGTC
AGTAAGTGAGGGCGGAGAATTGTTAATTGGTGGAAATCGGCAAAACAGTTTAATTTCCCCTGCTG
TCATTAATATCGATGAAGAAAATATATTATCAAAAATTGAAACCTTTGGACCAATTCTTCCAATA
TTAACTGTTAAAGATAGTGAAGAAGCAGTAAATATTGCAAATAATTCTAAATACGGACTTCAAG
CTGGCTTATTTACAAATAATATAAATAAAGCGATGAAAATAGCCGATGAGCTTGAATACGGCGG
AATAATGATTAACAGTAGTCCAACATTTAGAAAAGACAACATGCCCTTTGGGGGAGTTAAAAAA
AGCGGTCTTGGAAGGGAAGGAATAAAATATACGGTTGAAGAAATGTCTGAAACAAAAACAGTCG
TAATTCATAACATTTAATTAAAAGAAAATGAATTATCTTATTAATTATTAACTTGGTGAAATCG
TGAGTTTTTTAAACGATCTTGATTTAAACATTGAAAAACTTGAAAAATTGCTTGAAATTGGAACC
TATGCAGATTTAAGAATAGTTACCGGTGAATCAAACAATATTATACAAAAAGACGGAATAATTG
ACGAAATTTCTTCAGGTATTTCTTCAGGAGTGATTGTAAGAGTGCTTGAAAAAAACGGATGGGG
ACTTGCTACTTCAAATAGCGTTTCCCTAAAAAATATTGAAGAAATAATTAAAAAAGCGCATGATA
TGGCAAGAATATCCAATATACATACAAAAAAATCGATTGAATTGAAAGAAATCCCCATAATTCA
GGACAATATAAAAGCAGATGTAAAAATTCATCCTGAAAACATTTCAATCGAAGAAAAAAAAGAA
TATTTGAAATCAGCACATGAAAACATGTCTGGAGAAAAAATAGTCAGCACTTCTGTTAGCTACAG
CGATGGGGAAGGCCATTCTATTTTGATGACTAGTGAAGGAACTAGAATTGAAAATGAGAGTGTT
AAAGCCCTTTTAAGAATGACTGCAATTGCAAAAGATGGAACATTGCAGTTTGCATTTGATAGAAT
TGGCGGAAACGGATTTGAAGTAATTAAAAATGCAAAAATTGAAGAAATGGCTAAAAATACCAAA
GAAAGAGCGGTAAGACTTTTAACTGCTGAAAGCTGTCCAAAAGGAACTTTTGACGTAATATTAGA
TCCAGAACTTGCAGGCGTATTTATCCACGAGGCAGTGGGCCACGCTGCTGAAGCAGACCTCGTTT
TACAAAATGACAGTGTATTTCACGATAAACTAGGAAATTCAGTTGGTAGCGAAGAAGTAACCGT
AATTGATGATGCGACAATTGAAAAATCATTTGGACAGTACAAATACGACCATGAAGGAGTTAAG
GGGGAAAAAACCACATTAATTGAAGATGGAGTTTTAAATGGATACTTACATTCAAGAGAAACTG

CTGGAAGACTTAACATGGACGTTACAGGAAATGCACGAGCAGATGGACTAAACAAACCAATTGTT
AGAATGAGCAACACGTATATTAAACCCGGTTCATGGAAATTTGACGAACTTTTAGAAGATACAAA
AACAGGAATATTTTTAAAAGGTTCAAGAGGCGGACAGGTAGATACCGGAAAAGGATTATTCCAG
TTTAATGCTGTTGAAGCATTTTTAATTGAAGATGGAAAATTAACAAAACCATTAAGAGATGCAG
GACTCAGCGGTGAAATTTTAGATATTTTACACCATATCGATGCAGTGTCTGACGAATTTGAATTA
AGTGTTGGATACTGTGGAAAAGGAGGCCAGAGTGTTCCAGTAGGAGATGGTGGAGGAAGTGTTA
GAACAAAAACTACACTTTCCTAAATTCGTGAAAATATGGATGAATTGGCAAATAATTTAATAAA
TGCATTATTTAGGGACTTAATCGATATCACATTAGATTTAGTTTACCTGATAATTGCATTATTAA
TTCTCATAATTTTAGCATTTAAAATGAAATATTTCTTAAAAAATCTTGTTTTTAGAAAAAAATA
AAGAAAATTAAAAAAGAACTAAAAAAGAACTTAAAAAGAACTTAAAAATTTATTTTACATTAAA
CTTAATTTTTTCAATTTATTATAGAAATTATCTCCTTTTACAAAGTACGCATTTGATTCAGACTT
TCTTAAAATTATCTCATCGCCTTTTTTTGCTTCAAATTCCGTGTTTCCATCAATTACAACATACGT
AGATTTTTTTAAGAGTTTTATTTTTATTTCTGAATTTGCATTGACCACCAATGGCCGAGATGAAA
GTTTAAATGGGCATATTGGAACGATTACAAATCCTTCAACTGTTGGCTCAATTATCGGACCTCCA
GAACTTAAAGAATATGCTGTAGACCCATTTGGAGTTGAAACAATTATTCCATCAGCCCTAACATC
TTCTACAAGACTTCCATCGATATATACTTCAAAATGCATCATTTTTGCAGGATTTTTAGTTGTGA
TCACGACTTCATTTAAAGAATCGTTTAATATATGCTGTTTTCCGTCAGAAAGCTTTGCAAATCCC
ATAAGCTTTGTTCTTTTCTCAACTTTATAGTTTCCACAAATTATAGAATCGATTGCAGAAAATAT
CTCATCTTTGTTAAATTCAGTCAAAAATCCAACTGTTCCCATATTTATGCAGACCATTGGTATCT
CATTTCCGAGAATCATTTTTGATGCTCGAAGTACTGTTCCATCCCCGCCGATAGATATCATATGG
GATATCTCCTCAATATTTGAGATAATATCACATTTTTTGGTTAATCGGTTTTTTAATGCATCATA
AGTACTGCTATCGAGTTCGTATTTAATCCCTTTTGAATCTAAATATTTTATTACATTATCTGCAA
CGTCTATCGATTCTTCATTATCAATTCTTGAAATTATTCCAAAGTTGGTCGGCTTAATTCTCCAG
CGGTTTCCAAATATTCCAACAAGCTTTTTATGAAGCATTTCATTTGAACATATAACTGAAACTTT
CGAATTGACATCGAGATCCAGATTTACTTCCTGACCATTTTTATCAGTTACAATTCCGCCAGCTTC
TTTGATTATCACATATCCTGCCGCAATATCGCAAAGCCGGGTGGTTTCATTTACGTTGATAAATG
CATCGAGTGCACCTTTTGCAACATAACACATTTCAAGAGCAATTGATCCAAAAAGCCTAATTCTT
CTGAATCTCCTATCTTTTATAAATTTAAGGGTATCTATTGAGATATCGTGTGCAAAAAGGCCTAT
CGAAGAATCTTTTAAATTTGACGAATTTGAAATTGAAATTGATTTTTTTTCGCCTTTTCTTAAAA
AATGAGCCCCTTTTCCTTTTTCAGCGTAATAAGTATCCCCTGTTGCAATATTTCGAACAACCCCGA
CTTCAAGGTCTTTCATTTTAAGGTTATTTATTAATTCTTCGAGATTATCCGTAAATTTATCAATT
CTACCAATTGCAACTGCTGCAGAATAAAAAGGAATGTCTTTTAATGAATTGTAAGTTCCATCAAC
AGGATCGAGGACTATAACATATTCAGGCTTATTTTTACCTATTTTCTTAAAGCCTATCTCTTCGC
TTATTAAAATTGCTGAACAAACCTTTTCTATCGAGTTTATAGCAACGTTTTCCGCAATCAAATCG
ATTCTTTTTGTCGGAGTACCGTCTGCTCCGATTTTGACGACTTCGTTAGATTTTTCCCATCCGATT
AAAGGTTTAACACTTTTTTCGATGTCCTTGGCAATATTTAAAGCCATTTCCAACATGTCCATTTT
TTCACCAATAGTATATAACTATTAATTATTATAAATAAATAACAAAAAGCACTGAATTAAAATG
GAATCTAAAAATTACGGTGAACAAATGCTAATAATCCTGACTGGACTTCCATCAGTTGGAAAATC
GACGTTTTCAAAAGCAATTTCAAAAAAGATGGCTGAAAAAAATATCGATAATATAATACTTGGA
ACAGATTTAATTAGGGAAAGTTTTCCTGTTTGGAAGGAATCTTATGAAGAATTTATAAGAGATT
CTAACAATTATTTAATAAAAGAAGCACTTGAAAACAAATTTAGCGTTATTGTGGACGATACAAA
TTATTATAATTCAAAAAGACGGGATTTAATGAATATTGCAAAGGAATGCGGTACTAATTACGTT
ACAATATATCTTAAAGCGCCTTTAAACTTACTTTTGAAACGAAATATCGAAAGAGGACAAAAAA

TCCCAAATGAAGTTATAAAAAATATGTACGAAAAGTTCGACACCCCTGGAACTAAATATGCGTGG
GATTTACCAGATATTATAGTAGATACGACTGAAAAAATAGATTATATCGAAATATTAAACGAAA
TTTTAGAAATAAACGAAAATAAAAATTTAAAAATTGAAGAAGATAAAGTTCCTACATTAAAAAC
CGTTGAAAGCAATTTACAAAAAATCGATAATATTACTCGAAATATCGTTGGAAATTTAATAAAA
ACGGGAAAAATCGATAAAAAAGATATTAAATTAGTTTCAGGACTTAGAAAATCGTTTTTAAAAA
CATTTAAGAAAATTGAATCTGAGAATTTAGATTTTGAAAAAATTGAAAAAGATTTTTTAGATTA
TTTAAAAGAAAATTTAAAATGAAATTAGTTAGCTAATTCAAAGTATGCTTCTTCTGCAAGTTCGT
AAACTTTTTCTTCATCAATATTAACCATTTTTCCGTCATTTAACACGAGTTTACCATCAATCACA
ACTTTATCAACTACGCCGTTGAATGAATAAACTAAATGAGATTCGATATTTTCTTTAGGTGTTAA
ATACGGCTTTTTCATGTTGATGAGTACAAAATCTGCAAGTTTTCCTTCTTTTATTTCCCCTGAGT
TGATATTGAGTGCTTTTGCACCGTTTTTGGTTCCAAATTCAAATGCATCTTTTGCAGTTACTGCA
ACAGGGTTTAAATTCACTCCTTTGTGTATCAAAGCTGCTGCTTTTATTTCTTCAAATAAATTCAT
GTTGTTGTTACTTCCACAGCCGTCAGTTCCAAGCGTTACCGGAATATTATTTTCAAGTAATTTTA
AAACTGGTGAAACTCCAGATGCAAGCTTTAAGTTGCTTACAGGATTGTGAGCTGCAAATATATTT
TTTTCTTTCATTATTTGAATTTCTGAATCACTCAAATGAACGCAGTGTGCACAGATTGTATTAAC
ATTATCAAAAAATCCAAATGAATTTAGGTATTCAAAAGGCCTCATTCCTGTTTTTTCTACGACCT
GATTTATTTCATCTAATGTTTCATTCATGTGAATGTGAATTGGAACATTGTATTCTCTTGCGAGG
GCATTTGTACTTTCCAAAATTTCTTTTGAACAGGTGTACGGTGCGTGTGGACCAAGTGCTCCAGT
GATTCTTGAATTATTCAAATTTTTAATCAGTTCAAGAGACTCTTTCGCAGTTTTTAATTCTTTTT
CCCTTTTTTCTTCGTCGAATAAATCAATCATTCCGTATGCAATTGTTGATCGAATTCCGGTTTCA
TCAACTGCTTTAATTATCGAATCAAGGAAGAAATACATGTCGTTAAAAGCAGTAGTTCCACTTTT
AATCATTTCAATTGTTCCAAGAAGAGTTCCTGCGTAAACTATTTTTTCATTTAATTTTGATTCCA
TTGGCCAGATGTGCCCGCTTAACCATTCCATTAAGGGAATATCATCTGCAACCCCCCTAAAAAGA
GACATCGGAACGTGAGTGTGTGTATTTATTAATCCAGGAATTAAAACACAGTTTTTCCGTCAAT
TATTTCTGTTTCGTCTTTTGAAACTTCTGAAATTGAAATATTTCCAATTTTTTTAATAATATTTC
CTTCGACAAGCAAATCCTGCTTTTTTCCGGTTATAATCGCATCTTTTACTAGAATCATGAAAATC
TACCTGAAATTTAAAATTAGTTAATAATTAATTAAAAATTATTTATTGAGTCCAAGCTCTTCGAT
TGTAACGAGAGGGATTAATTCAACGTTATTTTCGGCCAAATTCTCTTTTGCTCCTTCCTGCCTATC
CACAATCACGTAAATTTTGTTAACGTTTCCAGAAACTGCCCTTATTTCATCCACTGCTTTTGAAA
CGCTTCCACCGGTTGTTGTAACATCTTCCATTACAATTACATTGTCGCCGGGATTTAATTCCCCTT
CAATTTTATTTTTAGTTCCGTATTCTTTTGCTTTTTTTCTAATTATTAAAAGATCTTTTTCTGTT
TCTAAGGATACTGCTGTTGCAATTGATACTGAACCGAGTTCTACTCCTGCAATTTTTAAATTTTC
ATTGTTTTCTTTTGAAACGTAATAATTTACAAGTTTTGCAGCTGCTTTTAAAACTTTAGGGTTGG
TAGTAGCCTTTTTAATATCTACGTAATATTTACTCTGCTTTCCAGAGGCCAATGTAAAGTCACCA
AACTTTACGCAATTTACTTCTTTCAATAATCTTATCAATTCATTTTTTAAACTGATTTCTTCCGT
TGTTACCAAAAATATCACCTTATAAACCAAACAAAAAAGTAAACTATAAATGCTATTTGAATATT
ATATTAAGGTAGTAAATATATCTTTTCTCAAAAATTACATGGTGATTGAATGAGTGCCCCCAGTA
AATTAATAGCACTATTTATTTTAACTGCGCTCTGCACCCTACCTATATCTTATGGTATGGTTGTA
GATAACCCATTAATTGTTGTAAACAGTGAAAAAGTTGATAAAAGCTACGCAGAACTTTTAATGG
ATAAATATTACACGAAAAGGACTATTGAAGTAAATTCTGATAACGAAATTGTTGTTAATGAAAA
TATAATTTACAATGTTCCCGCAATTGATGAATTTGAAATTAATGACGGTAAGGGTAACTTGTTAG
TGGAATTTACAAAATCTGGCGAAACGGTTACTTACAAAAACTTTGAATATGAAGAAGAAATTGA
ATCCGACGATGAAGGAGACGAAGTAACCTTTATGGGAAAAACATATAGGGTACTAGAATATGAA

TCCGGAAAAATAGTTCTTGGAAACACTATTGAAGATTTTGAAACCTCCGATGAATTTAACTATGA
AGGCTATACTATTAAAGTAGTAGCTAGCAATTCAGATGGCGAAATAATGGTTAGCGTCTATAAA
GGAAGCGATAGGGTTGACAATTTAAAAATGTACCCTGATGACAGCTGCATGATATCAGGTTCAAG
CCTTTCTTTGTATTATGATGAATATTTGACAAGTGGAGATACACCATACTTTTTCTTTGAACTCT
CTGACTCCCTTGAACTTGAAGATGGCGAATCTTTCGAAGATAACGGAGAATTTGATGTAGAAATC
GATGGTAAAAAAATAACTTTAGATTACGACAGTCCTAAAAGTTTATCCAAAAACTTTGAACTATT
AAATTATAACGTAGAACTTACAGACGTAAGCAGTGCAGATTTAACTGTATTTTTCACAGTAACTC
AGACTGAGAGCTACGAATATGATATGGATGAAGGTACAAAATACTTCGGAAATAACATATTTGC
AGTTAAATTAGACAATGATGAAGAAAACGACTGGGACGATGAACTATATCTTTATAAAAACAAT
AAAAAATATGACAAAATCGTGGATTACCAAGGTTCAGTTCAATTAGTTGATCAGGACGAACTTTT
GAGTGCTTCATCAGACTTAATTTTGATCGGTGGACCGGTTTCAAACAAAGTTACAGAAAACATTC
AAAATAGTTTAAGTGTTGAAGTTACAAATGACTACCCTGGAGAAGGAAAAGGAGTAATTCAAAC
AATTACTAATCCAAACAATGCAGAAAGTACAATCTTAGTTCTTGCAGGGTCAGACCGAGAAGGAA
CAAAAGCCTGTGTTTTAGCACTTAATCAGGGCCTTTATTCAGGATCTGGAAATTTAGTTGTAAAA
TTAACTGGTGACGATAGCGTAAGTGTTATCTAAATAAGAACCTAACGGTTCTTGGACCTCCATTT
ACTTTGAATATTATTTTATTCTTTTTTGGCTTGACAAAATTTGGTGAAATTTATGAAAAAAATCG
ATTTTGATACAATTTCTGCAGTTATTTTAGAAGAATTTAAAACATATACTGGAAAAGAAATTGA
ACCACTCTGGGCATTTAACACGTTTGACGTTCAAAAAGACAGTATTGTTGGATTTATCGGTCCAA
TGAACGTTAAAATAGAAAATATGAAAGATTTAAAAGATGTTAAAGAAGAAAAAGACGTTGAAAT
TCCAATTCAATCTTCAGAAGCGATTAATTTTATCGTCGAACATTTTGATAATCCAGATTTAAAAA
TGACTTATCTAAGACAGCGAATTTTAGTTTATACTGCAAAAGAAGTAATTGAAAATCACAGCAAT
TTAAAATTAAAAAAATCAGGAGACGACCTTTATTTTAATGGAAAGAAATTATCTGTCTGTATTG
CCTGTAGAGGAATTTCTTCTGGAAAGATTCACCTTGGAATAAACATAAAAAGTGATGGTGTTCCA
CCTCACGTTTCTGCAGTTGGATTAAATGATATGGGAATTGAAAATACCTTTGAAATTATGGAAGA
AATTGCAGAAATTTATGCAAAAGAAATGTCAAAAATTGAAAAAGATATTAGAAAAACACTTCCA
TTATTTTAATTTAGATTAATTTTTATTTTTTATATTTTGAGCCCTGTTTTGTTTTTCTTTTATCC
AATAATTTTGAAACCTCATTTAAAATTCTAAGCCTTTTTAAAGACCATTCTGGTGAAATTTTTAT
TTCTTCTGGCACTTTGTCCTTTGAAACCCTTTGAATGATTACATACTTGGAAATATGTTCTAAAA
AATCAGCAACAAGATGAATGTACTGTTTTTCATCTAAAGATTTGTATTCTCCTTTCCAGTATATA
TCTTCAAGTTTTGTATTTTCAACAACAACTAGCGGGTAAATTTTTAATGCATCAATTTCTAAATC
AGACAAAATTTTTGCAGTTTCCATCATCATATCCCAACTTTCATCAGGAAGTCCCAAAATTATAT
GACCGCACACCAAAATTCCTCGTTTTTACATTCCAAAAGAACTCTTCTTACATCGTCTGAAGAA
TGTTTCCTATTTAAAAAATCAAGCGTTTTATCATGCATCGTCTGGATTCCAAGATCTATCCAGAT
TTCATAGCCTTCTTTCACATAATTTTCTAAAATATCGAGTTTTTCATCCTCAAGACAGTCTGGTC
TCGTTCCAATTGAAATGCCCAAAACATCATCAAAAGAAAGTGCAAAATCCCACAATTCTTTTAAA
TATTCTGGTGATGCATGAGTATTTGTCCCAGGATAAAAATAAACGTAAAATTTTTCAATGCCCTT
TTCTTTATTCCTCTGTATTTGATGTTCAATCTGCTCTTTTAAAGAATATTTAACATTACAATATT
CAACACTTATTACCCTACCCATTTTTGGACAGAAGATGCAACCCTCAGTATCAATTGTCCCATCTT
TATTAGGGCATGAAAATCCCGCATCAACTGCAACTTTAAAAGTTTTGTAACCCTTTTCTCGCTTC
GTGTAGAGCCCGTATTGAGTTATTGGGAAACCTTCGCTGTAAATTTTATCGACAATATTTTTATC
TATTGAACCACTATTAGTTCTGTAAATTCGAGCGCCCCTATTCATGAAACCACCAAAAACTAAAT
TATAGGATATATTAAATAATTGGTGTTTATATTGATATATGTTCATAAAGGTGAAAATGATGGA
ACTTCATAACGACGAAAAAAGATTATTAAAGGCATTTCAGGAATCCAATGAAAAAACGATGAAT

TTAGAAGAACTTTCAAAATATATTGAAAAAGAAAAAGTAATGAGAGCAGCATTTTGGCTTTCAG
GGCGAGATTTTCTTGAAATTATTGAAAATAAAACAAAACTTTGTGAATTAACCAAACTTGGTAA
AAATTCCATTGATTTTGGAATTCCCGAGAGAAAAGTTGCAAATTATATCAAAGAAAATAACTTA
GAATCAATCCCGATAAAAGAGCTCTCAAAAATTTTAGAAAAGGACGAAACAGGAGCTGCACTTGG
AAATTTAAAGAAAAAAGGATTGGTCGAGATAGATAAAGGAAATATTGTATTTAAAAATTTAGAC
TACGAAGATATTGAAGAAGAAATATTAAAAAAAGTTTCGATCAACCCTGATTTATCAACATATT
CAAAAGACGAAGTTAAAATAGTTGAAAACCTGAAAAAAAGAGGTTTTTTAAACTTAAAAGAAGT
AGTTGATAGAAGTTTTGAAATTAAGGATGCAGGAACTGAATTTATTAAAAATCCAATAGAAATA
AAAGAAGAAATTACCCAGCTTACAAGGGAAATGATTGTAAGCGGAAAATGGAAAGACTACTTTA
TAAGGCCGTATGACGCAAAAATTCCTACAGAAGATGTTTACCCTGCAAAAGCACACCCGATGTCA
AAAATAATTCAGGAAGTAAACGAAGTTTTAATTTCAATGGGTTTTAAGGAAGTAAAAAGCCAGA
TCGTCCAGACTGAATTTTGGAATTTTGATACCTTGTTTGAGCCTCAGGACCACCCTGCAAGGGAT
ATGCAAGATACATTCTTTGTAAAATACCCAAATACTGGAAAAGTTCCAAAAGAATTGCTTGAAAA
AGTAAAAGGAATCCACGAATGCGGAACTATTGATAATGAAAAAATTTCAAAAGGCTGGTGCTAT
AAATTCGATGAAAACGTATCTAAAAGAACAGTGTTAAGAACGCACACAACCGTTTCATCGATAAA
ATATTTAGCTTCACTTTCAGAAAGCGAAAGAGAAAACCCTCATAAGGTATTCTGTATCGACAGGG
TATTTAGAAACGAAACAATCGATTACAAACACCTTCCAGAATTCTACCAGTGTGAAGGAATCGTT
ATGGCAGAGGATGTGAACTTTGACAACCTCGTTGGAATTTTAAAAGAATTTTTAAGAAAATTAG
GCTTTGAAAAAGTAAGGATTAGACCTGCATACTTCCCATTTACAGAACCATCCCTTGAAGCTGAA
GTTTACATGGAAGGAAAAGGCTGGCTTGAACTTTTAGGTGCTGGAGTATTCAGACCAGAAGTTTT
AGAACCTTTCGGAATTAAAAAACCGGTTCTTGCATGGGGAATTGGCTTAAGCAGGCTTGCAATGT
TAAGACTCGGACTTACAGATATCCGTGAACTTCACAAAAACGACATGGAATGGCTTAAAAAAACT
GCTGTTAATGAATAATAAATTCAACTTTTTGAAGGGATTTTCGTGAATATTGAAAATACCATTA
AATCTGCGTATGAAGAATCATTAAACAATGCACGCTTTGGTGACAAAATAGAAGAAATCGATGCC
ATCCAAAGCACCATAAAAAGTGCAAAAAACGTAACTGTAGCAACAAGCAATGAAAAAAAATTTA
AAGTAGTATCCGATATTATTTCAAGGATTACAGATGCAAATATATCAATGCTTGAAATTCCAACA
AATTCTGCGGATTTAACCAGGATGCCTGCATTAAACAAAGGATTAATTGCCGTAGACTCCTCAGA
TGCGGATTTAATAATAACTAGAGGAAGGCTTGGAATTCCAGGTTCTGGATCACTACTCCTTATAA
TGGATAAAAAGGGACGAATTCTAACAGGATCAGTTTCTCCTTCATCCATAATTCATAAAAATCCA
ATAGATAAAACAGTAGAGTTAGAATTGATTGTGGCCCTTGAAAGAATCGGTATTGTGGTGAAAA
AATGAATTACGGAATTACTGAAAGTGTAAAAACGACCCGTTCAAAAATAAAAATAAAAGACATC
GTTTCAGATGTTGTAGAAAAAAAGGCCAATGCAATCAAATACTTCTTAGAAGGCGAAGAATTTA
AGCAGGCTATTGTATTTGGAGCTTATCTTTCCGGAAGCTACATTGCATATTCACTTTTAAAAGAC
TGTGATGAGGTTATTATCGTAGATATTCAGCCTCATTTAAAAGATATTTTATTTAACGACGGAAT
AAAATTTATGGATTTAAACAAATTGCAACTTGAATTAAGAAATGGAACTTCAATTAACCCTGAT
TTGGTAATTGATTTAACAGGTATTGGAGGAGTATCTCCGGATTTAATTTCCAAATTTAACCCAAA
AGTTTTGATCGTTGAAGACCCAAAAGGAAACCACGATAAGGGAATTTCAAAAATAGATAATACT
GACAAAAGACTCTGTGTAGGGGCTAAAAAAGGAGTTTTAAAAACTTACAGGTCATCTAAATTTTC
AAAAACTTCTGGAACAATGACCCTTGTTGTTGACATAATAATGGATTCATGCAGAGAAATTAATG
AGTTAGATAGTGTTTTGTACACGATTCCAAACTTAAAATACTTTGAAGGAACCGTTTTCCACGAA
AAAAACGTAAAAAAATTTTTAACTGAATTGAACATGTCTGCAATAACTGTAAGTTCGATAGACC
ATGTTGAATACGAACTTGAAGAAATACTTTCTAAAAATATTTCAAGAGTAGATTCCTTTGTTAAG
GAATTTTAAACTCTTTTTTTAACTCTTAAATTTTTGAAACAACAATTTCGAGTGCTTCCATAAA

TGCATTTTTTTCTGCAATAATAACTGCAACAGGTATTCTTACAACCTTTTCAATACTGGTACTTA
CTATTGGCGCACAGACTATCGCTTTTACGCCTTCTCTTTCTGCATATATTGCTGAAACAATACATT
CTTCAAGCGTACTTGCAGGATATTCTTTCAAAAGATACGTTTTTCCGTTTATTTCCACTCTTTTTT
TACTTACCTTATTTAAAGAAGGTCTTGCTGCAATTAGAGCTATTTTATCTTCTGATGAATCATCT
TCTTCAAAACTTTTTATAGTACCAATTATTTTTTTAAGAGTCGATACTCGAAAATCTTTTTCATT
CGTGATTTTATAAAGTGTACTATAGGGAATCCCAGATATCTCGGAAAATTCCTTTAAAGGAACAT
CCAATTCAAGAAGAACTTTTTTGAATTCATCCGTAAACTTATCTGAACCCACACTCAATAAAAGT
CGTTCGTGTGCTCGCATAGGTCCCACCTAAAAAATAAAAAAAGAAAAATCGTTAGAATAATGCAA
TTATACAATATAGTCAATTCCAAGAGAACCTTTTGACCGGTTAGCTGAATTTGGAATTATTTTTT
GATGGCTTTTTCCACCGCCCATTATGTTTGGAGATTTAACTCCTGTAATAATTGCCATTACTCGT
ATTGCTCCGTCCATTGAAGGGTCGAGTCTTGCACCCCAGATAACGTTAGCATTTATATCCATGCT
GCTTGTAATTCCGTCACCGATTCTGTTAGCTTCACCAAGAGTTAAATCAGGACCGCCAGTTATGT
GTATCAAAGCGCCGGTTGCTCCTTTGTAGTCGATATCTAAAAGAGGACATTGTAATGTATCTTTT
ACTACCTTTTCAACCCTATCGCCTTTAGTGTCGTAGTCAACTTCTCCAACACCTATCATTGCAACT
CCACCGTTTGTCATCACCGCTTTAACGTCTGCGTAGTCGATATTGATTAAGCTCTTTAATGAAAT
TGTTTCAGTGATTCCTTTGACTGCTTGAGCAATAATTTCATCAGCTACCCTGAAAGCTTCGTTCA
TTGGTAAGTTAGGAACAAAATCAACAAGCCTGTTGTTATCAATTACAATAACAGTATCACAGCAT
TCAGTAAGTCGTCTAAGACCTTCATCAGCTTTTTTGAGTCTTGCTCTTTCGATTTTGAATGGGTA
AGTTACAACACCAATAACTACAGCACCGTTTTCTTTTGCAATTTCTGCTACAACTGGAGCGGAGC
CGGTTCCTGTTCCACCACCCATTCCTGCTGCAACAAAAATTAAATCTGCACTTTTTATAACATCTT
CAAGCACGTTTTTAGCAAGTTCAGCGGATTTTTTACCAATTTCTGGGTATCCTCCTGCACCTAAA
CCTCTTGTAAGCGTTGAACCAATTAATATTTTTTTGTCAGCGTTTATGTGTTCTAAATGCTGTTT
ATCGGTATTAATTGCAATAGTTTCGGCACCTTCAATACCAATTTCACTTAATCTATGAATGGTAT
TGTTTCCAGCACCACCACATCCAACAACAAGAATTTTTGCATTTCCAAAATCGTCTTTTGCCATCT
GGGTGTTGTATAATTCCTCATTATTTTTGGACAAAGCTTCTTTTACAAGTCTCATATTAATCCTC
CAAAGCGGTGAAGATGGTTGTGTAAATTACGTTTAGTAGTATGTTAACAGTCAAAAAATTGTTT
TTACTAAATATATACCCTCTTTTATGCTATATAATAGTTTTTATATGTTTTTGGCATATTTGCAT
TTAGTATAATTATTGATTATTATCGTAAGGTATATAAATATAAACACTTAAACCTTGATGTGGCT
AGGCTGGGAAGTTAGGCGTTTCCTGTAACTCGAAATCGCCTTTGCGAGAGCCGAAAACTTGAGGG
CGGTTTTAAATTCTGTCGCTCATTCTCAAGTTTTGTGTAGACATTTCGTCCTTTGGGGTAAGATG
GAGGAGGAACCTTTTTTGTAAGAAAAAGACAAACCTCCCTTATCTTTCGAACCCCGTCAGGCCCG
GAAGGGAGCAGCGGTAGAAAGAACTTCAACGCTCAGAGGGTTGCGGAATCGAGCAGAACTTGAGG
TACAATGGGTTTCAGGATTTTTCACCCACCTCAAGGAAGCCACTTTTTATTTTAAAAGTTTTTTA
TAATTAAATAAATTTTTCAAGTTTTTTAAGGGCTAAACCATGATTATAGGGATCATTTCAGATAC
ACATATTCCAGAAAGGGCTAAAAAACTACCAAAAGAAATTTTCGAACACTTTTCTGACGTGGATT
TAATAATTCACTGCGGTGATGTAACTTCTGAATCCGTGTTAACTGAATTGAAAAAAATCAGTGAA
CTTTTAGTCGTTTCTGGAAACATGGATTATATGAATTATCCAAAAGAACATGAAATAACCATTGA
AAATTTCAAAATAGGCATCATTCATGGAAACCAGATTCATCCGCGTGGAAATACATTAAAAATGA
AATATTTGTGCCTTGAAAAAAACTGGGATGTTTTAATTTCTGGACATACCCACGTTCCAATGATA
AAAGAGATACCTCTTGAAAATAAAAAAATATTACTTTTAAATCCGGGAAGCCCTACTGTTCCAAG
ATATCCTTTAAAAACAGTTATGAAATTAAAAATAGAAGAAAGAGAAATTGATGCAGAGTTAATT
TCCATAAAATAAAAAAGAATAAATTTATTTTCTTGAAGTTAATTTCTTAAGTTTTGAAACAAGT
TCCGCTCTTTTGTTTTCAATTGTATTTTCGTAGTAGTCAAGTGCCTTTGTAGGGCATACTTCAAG

ACATGCTTCACTGCCTCTTTCGATACATCCATCACATTTTACTGCGACTTTTGTACATCTATCGAT
CTGTATGGCCCCAACAGGACATGCTTCTGCACATAATGCACATCCAACACACTTTTCAGGGTGTA
CTACTACTTTATCATCTATTTTTTCAATTGCATCTTCGGGACATACATTAAGACAGGGTGCATCT
TCACAGTGCATACATCTAATGGGAATCCCATCAAAAACGTGAATTGCGTTTATAGGACATGCCCG
TTCACACTTTTTGCAATCTACGCACAAGTCAATATTTGGCATTACCTTCATGAACTTCACCAGAT
AATTTTAAGGATTACATCTTGACGATGCAGTAACTAAAGAAAGGAGTCCTTTTTTCTTTTTGGAT
TTTCCTTTAGAACCCAAAACTGTTAAATTTTCAATATTATTCAACTTTTTATCCATTACTAATTC
ATCAACGTTTACAACTTCGAGACATCGTTTACTGCATGCTTTAACACATGCTTGTTCGTCTCTTCC
AAAACACAAATCACACTTGTGTGCAGTCTTTCCGCTTATGTATATTGCACCAAAGGGGCATGCCA
TTGCACAGAGTCCACATCCAATGCAGGATTCTTTGTCGAGATAAATTACACCATCCTTTGATTCG
ATAGCCGAAACAGGACATACTTCCATACATGGTGATGACGTACAGTGCTGGCATACTACTGGCAG
ATACCTTCCTTCGTGCTCCCAAATGCTGATTCCACTAACTTCGTGAACTTCAGAACATGATTTAAC
ACAATCCCCGCAGTTATCACAAGCTTCGTTAACCATCATTACTTTTTTCATGATCGCACCTTAAAA
AATATTAATTAAAGACCGTATTCGTCGCATTTCATGTCGATGGTCTTTTGAATTACTGAAATCTC
TTCGGGATTTAAGTGTCTGTATCTTTTCTGCACTTTTAAGTATTCTTCAATTGGTTTTCTTTTAA
GTGGCTTGTAGTTTAACTTAAATTCACCATTTTCAATTTCGAAAAGTGGCCAAACACCAGTTTCT
ACTGCAAGTCTGCCCAATTCAATTGTTTTATCAGCAGGATATCCCCAGCCGGTAGCACAAGGTTG
CATTACTTGAATAAATGCAGGACCGTTTATTTCACATGCTTTTTTAACTTTTTTCATGAAGTCTT
CTGGATAGCTTATTGACGCTGTTGCGACATAAGGTATTCCATGAGCTGCCATGATCATTGGCATG
TTCTTTTTAGGCATAGTTTCCCCACGGATAAGGCTTCCGGCAGGTGAAGTCGTTGTTGAGGCCAA
TAAAGGTGTTGAACCACTTCTTTGAACTCCGGTGTTCATGTAAGCTTCGTTATCGTACATGATAT
AAACCATATCATGGCCCCTTTCCATTGCACCACTTAGTGCTTGGAATCCGATATCTGCAGTACCGC
CGTCACCACCAAATGCAATAACGTTTACTTTTTCTTCACCGTATTTTCCGCGTTTTCTATTTAAGG
CAGCTACAGCTCCCTCGACACCACTTGCTACTGCTGCAGCGTTTTCGAATGCAACGTGAATCCAAG
GAACTTTCCATGCAGTTTCAGGGTACGGCGTTGTCATAACTTCAAGACATCCTGTAGCATTTGCA
ACGATCGTGTTTTTTCCAGCAGCTTTTAAAGCGAGTCTTGCAACAATTGCTGCACCACAACCTGC
ACAGCCCCTGTGTCCTGGAGCAAATAATTCTTCTCTTGGAAATTGGCTTCCAGTCATAATATCAC
CAAATATCTTATTCTTCCTTCAATCCGATCCAGTTTGTTTTTCCATCTTCAGAGATTTCTACATCG
TCGTAAATTCCCAAAATGTCTTCTGGAGTAATATCTCGTCCACCAAGACCTACGATGTAATTTAC
AGTCTTTTTATCTTTCAAATGTGATGCAACATCAGCATAAATTGCACCTTTGTTCATACCAAGGC
TTATGCTTTTATCGAGAATTGCAACATTCTTAGCATTTTTAAGTGCATCTTTGATCATTTCAACA
GGAAGTGGTCTGTAGCATCTTATTTTCAATAAACCGATTTCTACGCCTTCTTTTTTCTTAAGATC
AATCATATCTTTTATTGTTCCACAAATTGAACCCATTGCAACAATTACGTTTTCTGCTTTTTCTA
AATTGTAGCTTTCAATAAGCCCGTTTCCGTAAGCTCTGTTGAATTTTTCTGCAAAAGCATCATGA
ACTTCGGCAATAACTTTTTCAGCGTTTCTCATTGCAGTTTCAATTGCATATCTTGTTTCCATGTA
ATAATTTGGATCACCAAGAGCTCCTTGTGTCATTGGTTTTTTAGGGTCCAAGTACGCATGTTTTG
GTTCATAAGTTCCGATAAAATCGAGTACATTTTCTTGTTTTGGAACTGTAACTGGTTCTACAGTA
TGTGTTAAAATAAAACCATCTAAGTTTACCATAACTGGAAGAAGAACTTTTTCGTCTTCTGCAAT
TTTAAATGCCTGAATAACTAAATCTAAAACTTCCTGGTTGTCTTCAGCATATAGTTGAATCCATC
CTGTATCTCTTTGAGATAAACTGTCTTGCTGGTCATTCCAAATGTTAATTGGTGCAGATAATGCC
CTGTTTGCATTCATCATTACAACTGGAACTCTCATTCCTGCTGCTGAAAATAAAACTTCGTGCAT
CAATGCCAGACCTTGTGATGATGTTGCAGTAAATGCTCTTGCACCTGCCGCACTTGCTCCAATACT
TGCACTCATTGCAGAATGTTCACTTTCTACTTTAATGTATTCTGCGTTTAATTCACCATTAGCCAC

AAAATCTGCAAGCTTTTCTACACATGTCGTTTGGGGGGTAATTGGATATGCTGCAATAACATCTA
CATCTGCAAGCTTTGCAGCTTCAGCGGCTGCAGAAGTTCCTGTGATAACCTTAACTTCACACATG
ATATCCTCCTTAAATCAGTCTTATTTTTCTTCCCTTTCTGCTTTTATCGCTTTTACAGGACATTCT
TTCTCGCAAATTAGGCATCCTTTACAGTAGTCGTAGTCAATTTCAAATTTTCCATCTTTTTCTTG
GATACAGCCTTCAGGACAGAAGATGTAACAATTTTCACATTTTACGCATTTGTCTTGATCTAAAA
CTGGTTTGAATACTCTCCAGCTTCCAGTTTTATTGTTTGCTGAACTTCCAGGTTCATAAATAATT
GTTCCTGTGTTTACCATGTACTCACCTTAAATTACAGACCTTTTGTTAGGTTATACGCTTTTTCA
GCTGCATTTGCATTTTTAGGGCCTAATTTACCAGGAAATGTTTCCAAAATCGATTTTTTAAGTGA
TTCGATAGTAACTTCTCCAGTAAGCCCAGCAAAAGCTCCACAAAGTGTCGTATTTACAATCGGAA
CTCCAAGAACTTCCAATGCTATTCCTGTAGCGTCTATTGTTTTAACGTCGTAGCCATTGAGTTTT
ATGTCTTTTAACGTGTTTATCAAAATTATTCCGCCGTCTTTTATCCCGCTTGTAACATCTACTGA
ATCCATCAATGTTGGATCCTGCACAATTACGTAATTTGGTTCGTAAATCTGGCTTCTTAATCTTA
TTTTTTCATCGTCAATTCTTGTAAATGCCATTACGGGAGCCCCTCTTCTTTCCACACCAAAGAATG
GAAAAGCTTGGCAGAACTTACCATCGTAGAATGCAGCCTTAGCAAGAATTTGAGCTGCTGTTACT
GCGCCCTGTCCCCCTCTTCCGTGGAATCTAACTTCTTTCATCAATTCACCTCAAAATAACCTCTGC
AATGAAACCATATTTATAATCATCATGATTAATCGTTACAATTATTATTATATAAAGGTTTTCAC
TATTTACATATTGTCGAAAAAATTACCGAAAATATACGGAAATATTACGTTATTTAACGTTTTAA
TTTTCCATAATTTATGATTATTTTAGGCATGTATAAATGTAATTAAATGTGAAAGAAATTAAAA
TAATATAAAAAAATAAATGATTAAAAAATGCTGATTTTGTGATTAAATGGTAGAAATTGACAAT
ATTATTACAGATGAAAAAATAGAAAATTACTTTGAAAGAACCAAAGAAGCAATTACAATTATAA
AAAACGGATTACCTCCAGAAAAAAGTCTTTTATCCGACGTTGCTTCCGACTTTCTGGTAATGATC
GACTGCTACTTTGAAGATGCAAAAACATTTATAGATAAAGGAGATTATATAAATGCATTCGCTTC
GTTGAATTATGCTTACGGCTGGATTGATGCTGGAGCAAGGCTCGGAATTTTTAATGTAGGAGACG
ACGACGTGAGATTTACATTAGCAAAATAGCGAGATTTCGGTGAGAAAACATGGGAAATTACCAC
GTAACATTACAGGCTTCATACATTGCGAAAAATGTTGAAGACGTAGAAGATGCAATAGGGGTTGC
AATATCCCAAATTGGAAAACTATTAAACAAAGGAAGCTTAGATTACGTCGATATTGACGTTGGTT
TAACAATCTGCCCAAAATGTGGCGAACCAATCGATTGTGTACTTGTAGTTGCAAAAACAGCGATT
GTAGGTATTTTACTTTCAATGAAAGTTTTTAATGCAGAAAGTCCTGAACACGCTGTAAGGATTGC
AAAATCTTCAATTGGAAGAGCTTTAAAAGATATCCCGTTAGAAGACGTGGATGTCGTAGAAATCT
AAATTTTTCTCTTTTTTTAATATTTAACAAAAATTTATAGCAAATGTTTAATTTTTAACGTATTT
TTATTCTTTTTTTAATAATTGTAATGATTAAATATGATATTTTTTTCAGAAACTTATATAAATCA
TGTTCCCACTAGTAAATTATAGTACTTAAAATAAGATATAATATTCATAAAATATGAATAAAAT
AGAACTGGTGAGTTATGAAATCATTGTATCTAATAATTATAACATTACTCAGTCCACTTGCAAAC
GTTGTTGATATTGAAAATGAATATCAGATCACAGGTGTTTTGACTGTGGGGGTATACCCCGATCT
ACCACCTTATACTTACGAAGAAAATGGCGAATTAAAAGGTTTTGAGGTTGATTTGATAAATGAA
ATATCAAAAAGACTGGAAGTGACTCCCAAATTTATACTATATAATTACACTAACGAACCTTACGA
AGCGGTAAAAAATAAAAAAATAGACTGTGCAATATCTTCAAATTATTTATCGCCAAAAAACGAT
GATATAGATTATTCAAGAACATATCTTAGCACATACGAAGTTGTAATGTGTGAAAAAGGGGAAA
AATACTTCAGACTCGAAGATTTGAAAGATAAAAAAATAGGAGTACTCAAAAACTCTGAAGCTGA
AGAAAAAGCAGAATATCTGCTTAGAAGTATGAATTTTGAAATAATCACTTACGATAATCTGGAA
GATATCCAAAATGACCTTGAATCCGGAAATTTGGATGCTGCAATCTCGGATAAACACTATCTCAA
TTATGTTCAAAAAGAAAATAATTCACTAAAATTTGTTCAGGAAATCGACATTAAATACGTGAGT
ATTGCAGTAAATTCGAATAACCCCGAATTAACTGAAAAAATTGATGAAATTCTTCTGGAAATGG

AAAAAGATGGAACTTACCAAGAAATCTACGATAAATGGTTTGGAGATTATGAAAAATCTCTTTA
ATTTCCAAATTTTAACGGGGATACGTATGATCAACGATATAATTGAATATTCCATTGTAAAAAA
CACCAAAATATCCTCGGAATTTTTGACGTACACTCAAAATTTTTCAGGAATTATGAATTCAGACT
TTAAAAAAATCGATCCAATGCTATATCTTGATTTGGTAATGGAAACTATGCACATATTCAGAATT
CTTGAAGGGGAACTTGATTCTATCTCGCTATTAAATAGTGAAAAAAACATTCTGGAATTATTTAA
ATATTACAAAAAATGGACTTATTTAAAACCGCATGATGACCACTATATTATGTTTGCCACACTAA
AATCAGAGAAATTTGGAATTAAATATTTATTGTTAAAACCATCAGAATTAAAAAAGTTTAAAAA
TGACTTTGAAATCATTTATTCCGCGATGTTGCCGAATAAAAATGCATTAAAATCTATATACCGAA
TATTTATGAAAGCCGCAAACAAAATATCAACATCTAAAAATCAATAAAAGAAATAATTTAATAA
TTTTTTTCAAACTCGATCATTGCACTGAGTACTTTTTCATCTTCAAGAGGTGCTCCTTGGATTTG
TAATCCTACTGGAACTCCGCTGATATTTCCACACCTAACAACTCCAGAACATATTCCGCAGATGTT
TGTTGGAACTGTTAAAACATCGTATGCATACATTTCCATTGGGGAAACATCTTCACCGAGTTTGT
GCGGGAGTTTTGGTACCGTTGGGCCAACTATTAAATCAGCACTGTTGAAAAGTCCAAGCATTTCT
TTTTTCATTAATTTTCTTGCCTGAAGTGCTTTTTTGTAGTATTTTCCACTGAATTCCTGTTCACTG
ATGTGTTTTCCAATTAATATTCTTCTTAAAACTTCTTCACCACAAGCTTCTTCGATGAATTCTCC
ATATCTTCTTCCATCGTATTTTCTTGTTGCAGAGAAGAATTCAACGTAGTTTATGAGGTAGTAAG
TTGGAAGAGCTAAATCAATGTATTTATAGCTTAAATCAACGATTTTACAGCCCATATCTTTGAAA
ACTTCGATTCCTTTTTCAATTTCGTTTCTGATTTTTTCGTCAGTTACGTCCATGAATTCTTTTACA
ACACCAATTTTGTAATTTGAAATATCCTTTTTTTCGAATTTCGGTGTTTCGAGTGAAGTGCTGTC
AGACCTGTCAATTCCTTTTATTGCGTTTGTTAAAACTAATGCATCTTCTGCATTTTTGGTTAATG
GGCCAATCTGATCGAAACTCATTGCAAGATCGCACAAACCCTGTCTTGATACTACACCATATGAA
GGTTTGAATCCCACAATTCCACAGTGGCTTGCAGGGTTTCTAATACTTCCACCAGTGTCACTTCCA
ATTGCCATATCACAGAGATCTGCAGCAACTGCTGCCGCACTTCCAGAACTACTGCCCCCAGGAATC
CTATCCATTGCAGCAGGGTTTTTCGTTGGGCCAAAGTATGAAGTTTCACCACTGCTTCCACCTGCA
AACTCATCCATGTTTGTCATTCCAACAATAAGTGCCCCTTGTGATCTTAATTTTTTAACCACTGT
TGCATCATAAGTTCCCACATATTTTTCAAGTGTTTTTGATGCACATGAAATTTTGTAACCTTTTA
CATTTATGTTAGATTTTACTGCAACTATTTTTCCATAAAGAGGTTTATTCTTAAGAGTATCGTTT
TTTTCCAGTTCTTCTGCTTCTTTTAAAACTTTCTCGCCGTTAACATCGATAAAAGCATTAACATC
AGACTTCTCGATTTTTTCCAAGTAATCGGAAACTCTGTCAGTAATCATTTAAATCACCTAAATTA
CAAATCCTATAGATTTATCCATTTTACGTATAAAAAAGTATTGAGACAAGTATCATTTCAAAACT
TTAAATTGCAAAAATCATTAGTTTGACTTAATTTTTAAAAATATAATAAACAAAAAAGTATTGG
ATTGTTAATTAATAGGTAATTAATTGTTAATTTAAGCGTTTTTAGCTTCATTTTTCCTTATTTCG
TTGAAAGCTTTTGTTTCACTAACAACTACTCCCGAAAGTAACAACAAACCGATCAAGTTTGGAAT
TGCCATTGCTCCGTTTAATGTATCTGCAATGTTCCATACAAGCGGTAAACTTGCGGTTGCACCCCA
GAAAGCAACTAAAACAAATGCAATTCTGTACAATCTAATTCCTTTTGTACCAATTAAATATTCGA
AACATTTTTCACCGTAGTACGACCATCCAAGCACGGTTGAGTATGCAAAGAAGATCAGACCTATT
GTAACAAGCAGTGCACCTAATGATGTTTCGTTACCCCCAATCATTAAGTGACTATATGGTAGCAA
ACTACCAAATGCCGCTGTTGTTAATTCTGCCCCTGTTTTTCCCACTAAATCAGGAAATGCTTTTA
AACCTGCAATTGTTAAAACTAATCCAGTAATTGTACATACAACAATTGTATCGAGGAAGGTACCG
GTCATTGAAACTAATGCCTGTCTTCCAGGGTGATCTGTTTTAGCAGCTGCTGCTGCAATTGGTGC
GGAACCAAGACCTGCTTCGTTTGAGAATACACCTCTTTTAACCCCCCACATGATTGCAGTACCAA
TTAATGCACCGAATCCTGCGCTGAAGTTAAATGCTGAAGAGAATATTGTTCCAAACGCTGGAATA
ATGTAACCAATATTCATTGCAAGAATTACTAATCCTGCCAATATGTAAAATACTGCCATGAAAGG

TACGATAATACCTGTAGCTTTACCGATACTTTTAATACCGCCCAAAATAACTGCAGCTGTGAATA
TTGCAAGAACAAATCCTGTAATTAATGGATCAACACCAAAGTTTGAAGCCACTGCATCAGCAACT
GAGTTCGTTTGAACCATGTTACCAATACCAAATGCAGCAAATGCACCGAAGAATGCAAATGCAAC
CCCTAAAATTTTTCCGAGTCCGTGGTCTGGAAGTCCTTTTTCAAGGAAGTACATTGGACCTCCTG
CCATTTCTCCGTTGTCATCGACAGTTCTGTATTTAATTGCAAGAACTGCTTCTGCATATTTTGTA
GCCATTCCAAAGAATGCGGTGACCCACATCCAGAATATTGCACCAGGTCCACCAAGAACGTATGC
TGTTGCAACCCCTGCGATGTTTCAGGTACCGATTGTTGCTGCAAGCGCAGTCATCAATGCCTGAA
AATGTGAGATGTCCCCTTCTGAAGTTTCATCTTGATGTTTTGAAAATGCAAGTTTTAATGCATAA
GGCAACGTGTGAATTTGCATAAAACCTAATCTCAATGTTAAAAAGATCCCTGTACCCAAAAGAAG
TACGAGCATGTAAGGACCCCAAACAAAACTGTTAACTGTATTAACAAGTGAAACAAAATCCATTT
TTCCACTCCAAATTTTTTTGGTTTTTAAAGATTTTTAATGTTTAATCATCACAGATGTACCTTTC
AAACTATGTTGAGGGACATTTATATGAATTTTTTATTTTGAATTAAGTGGTCACCGGAATGCACAT
TCCGGCAATATATAAAATGTATAGATTAGTATATAAATGTAACGGATTTAAATACTTGATTTTA
TAAATATATAAGGCTAAAAAAAAGAATTAATGTAAATATATTCTTGGAACTCTTGCAGATACCA
TGCAAACAAGTTCGTAATTTATGGTTCCCAATTTATTACCAAACTCTTCAAGGGAGATTTTTTCA
TTTCCGTCTTTTCCAAATACTGTGACAACGTCTCCAACGTTTACGTTTTCAATACCTGAAACATCA
ATCATGCACTGATCCATGCAGATTCTGCCAACAACAGGAACCCGCGATCCATGAATCAAGACATT
TCCATTTTTTAACATCCTTGTGAATCCATCAGCATACCCTATTGGAAGTGATGCAATTTTTGATT
GTTTTTTGGTTTCAAAGGTGCATCCATAACTTATTTTTGTATTTTCTGGAACGTCTTTTACATGC
GTTACAAGTACCTTCAAACTCATTGCAGGTTGTAAATTAATTCTTTCCTTGTGGACCAGTTCTGA
AGGGTAAAGCCCGTATAGAATAATTCCTGGTCTTACCATGTTGAGATGTGTTTCCGGGTGGTCAA
TTATTGCAGCGCTATTTGATGCGTGTTTTATTGGAATATCAATTCCATTTTCCTCGAGATCGTGT
AAAAAACTCGTAAATTTAGAAAATTGCATAGTGGTGTATGTTTTATCAGAATTATCTGCATCTGC
AAAATGCGTGTAAATTCCTTCAACTTCGATGTTTTTAAGTTCCCTGATTTTTTTTATGGTTTCTA
CCGACGATTTTTCTGGCAAAAATCCAAGTCTACTCATTCCGGTGTCTACTTTGATATGGATTTTT
GAATGTTTTCCCAATTTTTCAGCAATTTTTTGGATTGAATTTGCGATTTCAAGTGAATAAACTGT
TAAAGTTATATCATTTTCAACCATTTTCAAAATTTCTTCTTCAGTAACATATCCAAATACCAGTA
TCGGACATGTTATTCCTGCATTCCTAAGTTCAAGAGCTTCCGTTGAACGTGCAACTGCTAAACTA
TCGGCTCCATTTTCAAGGCATACTTTTGAAACCTCGACTGAGCCGTGTCCGTAAGCATTTGCTTTT
ACTACTGCCATTACCTGGGATTTGGGGTTTGTAATTCGTCTTATTTCTTTAATATTGTTTTTAAT
TGCGCTTAAGTCAATTTCTGCCCATATCGGATGTGAAACCATAAACTTCACCTTATAATTTGAAA
AATGTTGCTTTTTAATGAATAAAAATAAAATAATTATTCATTTAAAGCTTTTTCTGCAGGTGTGT
ATGGAAGGTCGTGTGCTTTTGCAACTGCTTCGTAAGTTACTTTTCCGTTAAGTGTGTTTAATCCT
TTTAATAATGCAGGGTTTTCAATTACTGCTTGTTTGTATCCTTTGTTTGCTATTTGTAATGCATA
AGGAAGTGTGTCGTTTGTTAATGCGTATGTAGATGTTCTTGCAACTGCTCCAGGGATGTTTGCAA
CTGCGTAGTGGATAACTCCGAATTTTTCGTAAGTTGGGTTGTCGTGTGTTGTGATTCTATCGATT
GTTTCGATTGATCCACCTTGATCGATTGCAACGTCAACGATTACTGAACCAGGTTGCATTGATTT
AACCATTTCTTCTGAAACTAATTTTGGAGCTTTTGCACCAGGGATTAAAACACATCCTACGAGTA
AATCTGCTTTTTTAACTGCTTTTGCAATGTTAAAGCTGTTTGACATTAAGGTTGTAACTTTGTTT
CCGAAAATATCGTCTAAATAAGCGAGTCTTCTTGTGTTAACATCTAAAATGGTTACTTTTGCGCC
CATACCTACTGCAATTTTTGCAGCGTTAGTACCAACAATTCCACCACCTACAATTACAACTTCAGC
AGGTTCTACTCCTGGAACACCACCTAATAAAACACCTTTTCCGCCTTCTTGTTTTTCCAAGTATTG
TGCACCGAGTTGGATTGACATTCTTCCTGCAATTTCACTCATTGGAGTTAAGAGAGGTAAGCTTC

TGTCAGCAAGTTGAACTGTTTCGTATGCAATTCCTGTGATTTTTTTATCGAGAAGAGCTTTTGTA
AGCTGTGGTTCTGGTGCCAAGTGCAAGTATGTGAATAAAATTTGTCCTTCTTTGAAAAGTTCAAA
TTCTGATTTAAGAGGTTCTTTAACTTTGATAATCATTTCTGCAATGTCAAATACTTCTTTTGCAG
TTGCCAAGATTTCTGCCCCTGCAGCAACATATTCTTCATCAGTGATTGCGCTTCCAAGACCTGCAC
CTTTTTCGATGTATACTTTGTGTCCAGCTAAAACGAAAGCTTCTACACCTGCTGGTGTGATAGCT
ACCCTGTTTTCATTGTTTTTGATTTCTTTTGGTACTCCAATTAGCATGTCAATCCCTCTTCATCTA
CATTTTTTGTATTTTTTTGATAAGTTACTGGCACCTAATACGGCAACATCGTATTTATCACTTTC
AGCAGATTTTTGACTTCATTTATATACTCAAGAATTAGAATGTAATAAATGGGCCTTGGTTGTTC
CAAAATGTTACACATCCATTTTTTATAATGGAGCCCTATTATGTGTAAAATATGCTAAATTTTAA
AGAATTTTATAAAAAAAATGTAAAAATTTTATTATGTAAGAATAACTTAATACAATAAAAGAAA
TAATATAAACCAATCAAACTGAGAAATGGAAAAATTATGATAATTTCGATAATCGGCGGAACTG
ACGGGCTTGGAAAATGGTTTGCATCATTTCTTAAAAATAGAGGATATGATGTACTCGTATCTGGA
AGGGACGTAATAAAAGGAAAAGAAGTTGAAAAAGAACTTCGTGTAACTTACACAAATAACAATA
TCGATGCTGCAGAAAAAGGAGATATTGTAATAATTTCTGTTCCGATAAATGTTACAGAATCTGTG
ATTAAAGAAGTTGCTCCGCATGTAAAACCAGATTCACTTTTAATGGATATAACTTCGATAAAAGA
AAGGCCTGCAAAACTGATGGCAGAATTTTCACAGAATGGTGTATTTGTACTCCCAACTCACCCGA
TGTTTGGGCCAGAAACTCCTTCGTTAAACAGGCAGGTTGTAATACTTACACCTAATGAAAAAGAA
AAAACAAATCCATTTTTTGAAAAAATTAAAGAATTTTTAGAAATTGAAGGTGCAAAAGTAATTA
TTGTTTCCCCAAAAGAACACGATAAAATCATGGGTGTAGTTCAAGGTTTAACTCATTTTGTATAT
ATTTCACTTGGATCCACCCTAAAAGATCTTGGAATAGATATAAAAGAATCCAGAAACTTTGCATC
CCCAATTTATGAACTTATGATTAATATTATTGCAAGGATAATCGGCCAAAACCCGTATCTTTATG
CAGATATCCAGATGCACAACCCGCAAATAAAAACCATTCACGAAACATTCATTAAAAACTGCGAA
AATATAAGTGAAATTGTCCAAAATAAAGATAGAGATTGTTTTGTAGAAAACATGAAAAATTCTG
CAAAACATTTTGGAAATGAAACAAAACGCGGACTTAACTGTTCTAACAAGGCAGTTTATGCAATT
TCAAAAGAAACTGAGAAATTGATTAAATCCGTTGGAAAAGAAATTGGTTTAAAACATATCTATT
CTGAAAACGTGCACTACGGTATTTTAAAAGAAGTTTATGCTGACAATATCGTTTTAAAACAAAAT
GAAAAAGAAGTAATTTTAAACATTTCAAATGTTTCTTTAATGGAAGATAAGGAACTTGAAGAAT
GGAAAATTAAAAATTTAGAAAAATATTTCGTAGATATTTCAGTATTATTTAAAAAAGACGTTGA
TTTAAACATAATTCTTGAATTATTAACCCATAAATTTGAAATCGAAGTAATTGACACATATTATG
GAGATAAAATTGATGAAAATGACTTAAGTGCGACTTTTAGAATAAGCTCTTACGAAAAAGAAGA
TTTGAAAAAATTAGAAGAAACTTTCGATGAAATTATTGAAAATATCGGCGGAAAATTAAGATAT
TAATTTTATATATTAACCGAAAAACCGAATACTTTTTTAAATATATTTAAATTTATGAATATTA
TTTTCTAAAAATAAAAAAAGAAGGGAATGAAAATTACATCATTCCTGGCATTCCGCCCATTCCTG
GCATTCCGCCCATGTCTCCCATGTCAGGAGCTCCTCTTAATTTTTCTGCAGCAATTACATCGTCGA
TTCTGAGTAACATTTCTGTTGATTCAGCTGCTGACTGGATTGCTTGTGTTTTAACTCTTAATGGT
TCAACTACACCGTTTTCGCACATGTCTTCAACTGCTCCGGTGAATACGTTTAAACCTGCACATTTG
TTGCCGTTGCTTGCGTGTGCTGCTCTAACTTTAACGAGGATTTCGATTGCGTCGAGTCCTGCGTTT
TCAGCTAAGGTTCTTGGGATTACTTCTAAAGCATCAGCGAATGCTCTAACTGCGAGTTGTTCTCT
TCCGCTAATTCCTTCTGCGTATTCTCTTAATTTCATGGATAATTCAACTTCTGTTGAACCTCCACC
AGATACAATTCTACCATCTTCAATTGTACATCCAACTACACCAACAGCATCGTCTACTGCTCTTGC
AACTTCTTCAATTACGTGTTCAGTTGTACCTCTGATGAGCATTGTAACTGCTTTAGGGTGTTTGC
ATTCTTCTACGAAGATCATTGAATCTCCGGAGATTTTTCTTTCTTCAACGAGACCAGCTTCACCT
AAGTCTTGAGCTGATAAGTCTTTGATGTTTGTAATTACGTTAGCACCTGTAGCTTTAGCTAATTT

TTCCATGTCGGATTTTTAACTCTTCTTGCAGCTACGATTCCTTCTTTAGCTAAGTAGTGTTGTGC
TAAGTCATCGATTCCTTTTTGGCAGAATAAAACGTTTGCACCGCTTGCTTTGATTTCAGCAACCA
TGTCTTTTAACATTTTTTCTTCTTGTTCGATGAATTCCATTAATTTTGCAGGGTCAGTGATTCTG
ATTTCTGCGTCGGTTTCTGTTTCTTTGATTTCGATTGCACAGTTTAAAAGTGCAATTTTTGCGTC
AGTTACTTTTTTAGGCATTTGAGCGCTTACTCTTTCTTTGTCAACTAAAACACCTTTGATTAATT
CGGTGTCGTCGATTGATGCGCCTGATTTCTTCTCAATTTTGATTAAATCTTTGTCAACTTTTCCTT
CATCGTCAACAACTGCTGAAACTGCTTCAACAATGATTTCAGCTAATTTTTCTTTAGCTTTTTCA
GCACCTTTTCCGGTGATTGAGGTCATTGCGATTTTTGTTAAGATTTCTTTGTCTTGAGCTCCAAC
TTCGCACGCAATTGTTTTTAATAATTCTTGAGCTTTTTGAGCTGCTGCTTGGTATCCTTTTACAA
CAATTGTTGGGTGTACATTTTGGTCCAATAATTCTTCAGCTTTTCTTAATAATTCACCAGCAACT
ACAACAGCTGTTGTTGTACCGTCGCCAACTTCTTTTTCTTGTGTTTTTGCAACTTCGATTAACATT
TTAGCTGCAGGGTGTTCTACACTCATTTCTCTTAAGATTGTAACACCGTCGTTTGTAACAACAAC
GTCCCCTAAGTCGTCAACTAACATTTTGTCCATTCCTTTTGGACCAAGTGTTGATCTTACTGTTTC
TGCGATAATTCTTCCTGCTAAGATGTTCATTCTTTGAGCATCTCTTCCCATGTATCTTTTCATGTT
TTCAGGTAAAACTCCTGGTTGTTGTGACATAAAATGCACCTCTTTTTTCAAACTGTTGTTTGGTA
AAATGTACTGCTTCGGTATGTATATGACTATGAGGGTATATATAAAGATTTCGATTTGATTTTTT
TAAATAATGTAAAAAAAGAGTTATTCATTGAATATTTTAACTATACGCTCTATATCGTTATTTA
ACCATTCAACAACCTTCCCACAAATCCTTAATTCTTCAAGCAATGCAGTTTTTTCAATATCTGTA
TCTGAAACATCCCTAACTTTCACTTTCTTAACCCCTCTGATTAATTTATATTCTCCAAGGTACTTT
TCAAAATCGCTATCTGTAACGATGTATTTTCCTCGATTAATCGAATAAATCCTCATAAAACCACC
AAAAACTATTATTCTGTCGTTATGTTAATCAGGATATCCGCATCTGTATGTTTGATATAAGTTC
ATTCAAATTATTTTTAATATAAAATGTATCAAAATTCTTTCCTAAAATTCCAAAAAACTTTTTTT
TCTTTATATTTACATTGCATATACATTTTAGCTGATTTTCTTCAGGATAAACTTCTAAATCAAAC
GTATGCTGAAAATTATCCAAATATTTATCTAAAAAATTACTAACATCCTGAATTACCATTGATTT
TTTAAATTCAATATCCTCTTCAGGTTCAAAACATGCTTCAACGAGTGAATCTATTTCCTCATTAG
TTGGAGCTTTTATTTTCAACTGTTTTAAAAGACTTTCTCTATCAAAATCCTGCTTTAATACTTCT
TTTTTGTTTTCTATCCCCACTTCAGGATAATTATTTGAAAAAAACTCCATATCAATTTCATCAAG
GAGGTATACTGAAACTTCAAATTTATCCATATTCTTACGTATTTCTTCATCGAAAACTTCAATGG
CAGATTTACCTGCATAACTCTCCAGACCAGATTTTAAACAGGCAAATTCAGGTTTTTTATTGTTG
TAAACAGTGTAAAATTCATCAATAGAATATCCTTTCAAGGTTTCTTTTGTATCCTCGCAAAGTTT
TTCAAATTCCCCAATGCAAACCTTAAAAGCATCTGATCCACTGTGATTTTTCAAAAAATACATCG
AAATCAGGTTTTTTATTGCTGACGTTAAATACTCTCCAAGCGTGCTGAAATATTCTATTTCTTCT
GAAGAATACGATATATCTTCGTTTAAAGTTTTACGTGCAATATCAAGAGCAGTATTTCTAATTTC
CTTGAATTTCGTTAAAATCTTGATTATCGACATTACAATTTTTAAATCAGATGCAAAAACTGGCT
GTTGAAGTGCGATTATCGTTATACACTTTTTTTGAATCTCGTTTGATATTTCTTCTGCGGAACTT
ATTGACTCGATTACAGATTTTGCTTTATTTTTATCGTTTGCTACATGTGCAAAAACAGCATTATT
TATCCCGTTTGCAGTGATTTCTCCAATACCGACTATCGAACTGTCTATTTTTTCCATGTCTCTTAA
AAATGATTTTCTTAACATACCACAACCCTACTTTATTCATATTACATGAACATATATTCTATTAA
TTATACTTTTGTTCAAATAATATGTAAAGTTTTCGAAAAAAATCAGTTCATAATATATTTAAGA
CCTGTTAAAATAGTCAAATTTTAAAAAATTTATTACAAAAATTAAAAATAAAAATATAAAAAAG
TAGAAATTATTTCTTCAATTTTAAATTCATTTCATCAAAAAGATTTTCCTGGTCGATATCAATCT
TTCCATCGTTTGCAAGATATAAAGCAGGCAAAAAATTCTTTATAACTTCTTCTCTTGTCTTAAAT
TTATTCTGGAATGTGAATTTACCGCCCGATTTTTTAAGTTCCAAAACTAAAAGTTCCATTATATC

AGAAATATCATCTTCCTCGATCATTTCCTCGACTAAATTGTATAATGCAGTCGGCCGTACAACTT
CTCGTTTTTTCCGGGGTTTTGTATCCTTGATTTTTTTAAGTTCCGTTTTAAGAGTGCTAATCAAA
CCATCTAAAGTCATGCTTTCTGGATTAAGCTCTTTAACTGGTTCTTTTGGGGGTTTTTCAACAAT
TTCGTCCTCGACATAGTCTTCGTCTCGATAATCGTCTTCGTATACTTCTTCCTCTTCTTCGGTAAA
CGCATTTTCACATTCTCCGTAGAGTACTTGCGATTTTAACCTTAAAAGAATTCCTGCAACCAAAA
CAACGTCTGCAGACAGCCGTATGTCAAATTTTCGAAGTTCTTTTATCGTTCCGAGATATTCATCA
GTTATTTCAGAAATGTTGATATTCCAAGGATCAACATCCTTTTTTTCAATGCTTTCTTTAATGAT
CCTAACCCAAAGATCAAACTCCATTTTATCATTCCTTAACTTTTAGACGAAGAAATATAATACAA
CCAGCGGAATAAATAGTATGGCCCAAACTAAAAAGTTCCAACGCATAACTTTTGACCCATCAAAA
GGATATATTGGTAGAAGATTAAACATCGCCAAAAATATATTCACAAAGTAACCGTAGCTTGAAA
TATCCACATAATACCCTGAATAGTATATTGCAGGTATTGGAAGTATCATGAATAACACTGCGAGT
ACGATATTTGTAAGAGGGCCCGCTACTGAGATTAAACCGTTTTCTTTTGTCGTTAAGTAATCTTT
ATGGATATATACTGCACCTGGTGCAATAAATGTAAACCCGAGAATTATTTTTAAAAATATTGCAA
TTGCAAGCCCTTCATACCATGCCCTAAATTCACTCCATGCTCCAAAATGTCTTGCAACGGTCCTGT
GTGCAAGCTCGTGAAAAATAAATGCAATTCCAACAGTAAGTAATGCTGCAATAAAATGCAGAAA
AAATCCAATATTAAATGTTGGAAATCCCCTTGGCCAAACAACTACAATAGCTATTGCAATTGATG
AAATCAGGAGGTCTTTTAATTCTATCCTGTTAAATCCAAAAATCCCCTTAAATGAACTCATTATT
TTTCACCAGATATACAAAATTAATTCCGTTAAATGCACTTTGTCCATACATTTATTTAAACTACT
TAAAATAGTAAGTGCTTCAAAATTCAAATAATCATTAAAAAAATAAAAAAAATATTGGTAAATT
AATTAATTGGAACTTTCCTTTGATACATAATAGACACAGTATCTCCGATGTTTACTGGATTTTCG
CCATCGGTTATAGTTTTTACAGCTGTATCCCCTATATTAACGGTTAATTTTTTGTAATATCCAAA
ATCAACCAGTGACTCAACTGTTCCCATAATATTTTCGTCATTTTCGTTATTTAATGATTTTTTAA
CAACTATTTCTTCAGGAGCAACTGCAAAAGTTTCTTCGTTATTGGAGATAATATTGTATCCCAAA
AAATTAGCTATTTTTTCATTTTTAGGAGTTTTAAAGATCCCCTGATTTCCAAAATCGTTTAATTC
CCCGTTTATTAATATTGCAATCTGATTTCCAAGTGTTTTTGCCTCAACAAAATCGTGAGTTATGT
GAATTACTGGAACCAATTCCCCAATTTTTTTTAATTCAGATATTACACTTTCTTTTATGTTGGTA
TCAAGAGCAGACGTTGGTTCATCAAGAAGTAAAATTTTTGGATCAAGCACAAGTGCTCTTGCAAT
TGCAACTCTTTGCTGTTCTCCACCACTTAGTGTTGTTGGTTTTCTATCCAAAATATGTGAAATAT
GCAAAAATTCGGATATTTCCATTACTTTTTTTTCAATTTCTGCTTTTGAATATTTTCTAAGTTTC
ATTCCGTATGCAATGTTATTGTAAACATTCATGTTTGGAAAGAGTGCGTAATTTTGGGGTACATA
ACCAAAATTTCTTTTTTCCGGAGGCATTTCAGTTATATCTTCCCCATTAAAGTATATTTTTCCAG
AATCCGGCTTTAAAATTCCAGTTATGCACTGAATTATAACAGATTTTCCAGCACCACTTGGACCG
AGCAAAATGCAGTAGTTGTTTTCAATTTCTAAGTTCACACTTTTAAGCTGGAATTCTTTCCAAGA
TTTACATAAATTTTCTAACTTAAGCATTGGTATAACCTCGATTATTTCTTGTTATATCTTACAAG
CCGAAATACTGAAAACAAAATAATACTCAGTACAATCATAATAACAGAAATTGGTTTTGAGGAA
CTAAGCCCGTATTCGTTGAACCTGTCCAATATCAAAACCGGCGTTGTCTTTGGGAAATATGCTAC
AATCAATATTGCCCCAACTTCACTAATTCCCCTTGCAAATGTCAAAATACTGCCGGAAACCAGGT
TATTTTTAATAAGGGGCAGTGAAATGTTAAAAAACGTCTTTATTTTTGACGCACCAAGGGTTCTT
GAAACATTTTCAAGTTCCTCTTCAACCATTAAAAATCCATCTCTTCCGCTGTTTACCATGTAAGG
AAGTCCAACGTAGAGCATTACAACAACAATTCCCCAGAAATTGTCAACAATCTCAAAACCGATAT
CTTCAATTCCTCTTCCAATCGAAGTTCCATAGAAGAATGCTAAAATCATAATACCGATTACAGAG
TGAGGTATTGCCATTGGAATATCGATAATTGCTTCAACTATATTTTTTCCTAAAAAATTGTATCT
TGCAATCATGTAAGATAATGGAATCCCTAAAAAAATCGCCAATACTGTTGCAGTGCCGGCTGCTT

TGATACTTACAAGAAGAGATTTAATTACTTCTGAATCGTTAAATGCACCCGCTAAATTTCCCGGA
TTTAAAATCATGTTTAACAGGGGAAGTAATACAAATAACAGTAAAAATAGTGAAATCACTAAAA
ATAACAAATTAAATCCTTTGTCAGCCTTCAAATTATCACACCAAACCATAATGTATTGTAAATAG
CTTTGAATTAATATATTAAAGTAATGGTAATTATAACTGCTTAATCGTTTTTCAAATAATACATA
TTTTTATAAAATAAGATTTGAAATTAACTATACTTCTAACATATGGCTAGTTTTAGGGTGATAAT
TCATGCATTTTGTTGATGTTTTGAATATAGGAAAAGACATAATTAAGGCAAATAATAAGAACGC
AGATAAAAACAGGAAAATATTAGAAGAACATGGTATCGTGGCTTTTGATTTCATGGGCGCAATT
GGAAGCGGTAAAACTCTTTTAATCGAAAAATTAATCAATGAATTAAAAGACGAGTACAAAATTG
CATGTATTGCAGGAGATGTTATTGCAAAATACGATGCTGGAAGAATGGAAAAACACGGTGTTAA
AGTTATCCCATTAAATACTGGTAAAGAATGCCACCTCGATGCACACCAAATTGGACACTGTTTCC
ACGACTTAGATCTTGATAATTTGGACATTATTTTTATCGAAAACGTTGGAAACCTGATATGTCCA
ACCGATTTTGATTTAGGAACACACAAACGAACTGTTGTTGTAAGTGTCACCGAAGGAGATGACAC
CGTTGAAAAACACCCTGAAATCTTCAAAACAGCTGATTTGACCATAATTAACAAAATAGATATTT
CAGAAGCTGTTGGTGCTGATCCGCAAAAAATGTTAAATGATGCAAAAAAAATTAATAAAGATAT
GGAAGTTCTTCTAACTGCCATAAAACATGACAACGGCGTTTCAGAGGTTGTTAAATTCATTAAAA
AAACAATTGAAGAAGTAAAAGCCCAAAAATAATTATATAAAAATGATTTTTCTTTTTTTAAATT
GCCATTACTTCGACAAATTTTGGATCTTTTGAAACTTTTTCATACCACATGCTAATTCCGATATA
AACGCCGCCCTTTTTTCCAATGGCATAATCGCAATCGCATCCGATGTCCTCAAAACCGCTTTTTAC
GCCTTCAAATGCAAATTCATCGCCGTAAGTTTCCGAGTCTCCAATTAAATTATTTATTTTTGCAA
CCATGTCCAAAATATCGTCTTCGTTTGCTAACTCAAACCCATTTTGAACAAGTTTTTCGGTTAAA
TATTCTTTTATAGAATTGTATTTTTCAAATTCGGGATCCCTACAAACGCTTCTTACTGAAAGAAT
GCCTTCTTTATTAATTACCGGTTTTGAACCCTTAAATGCTTCCATTGATTTTACAATCCTGCCCAT
AACAACATTTAAATTATTCATGATATCCACCTAACTTTCAACTTCGTAACTGTTTTCTTTAGACT
TCTTTTTTATTATTTTAAAGGCTATCGATCCAATAATTGTAACCCCGATATAGGATATTGACCTA
TCAAGAAGGGTTCCTGCTGCCGCATTTGAATAAGGTATGTTAAAGAATGAATACATTGCAATCAT
TGCAGTATCACTCGTTCCAAGACCTCCAGGAAGTGTTGGCAATATTCCAACAAGAAGTGCTACAA
GGTACGTCGATGCAACGGATATGAATGCTACATTTGTGCTTATAGCAATAAATAATACGTAAATT
CTAAATATATCTAATAGCCACCATGCTGAGGAAATTAAAAGTGCTGTAATCATTCCTTTTCTATT
TCCTTTAAAAAATATCATTGTTTTTTGGAACTTGTTTATTGTGTCTTTTATTTTCTGCTCATTTA
AAGTTTTAGAGGAAAATTTAGTAACGACTTTCGCAATCTGGAGCGTTATCTTAAAAGCCCATTTT
TTGTGCATTGCAATATAAACTATGGAAATTGTTAAGGCCATTACCAAAAACCATGCGATAGCGAA
TGCTTCTGTGTAAACTACGCCTTTTAAAGCAAAATATATTATTACAAAAAAGCTGAATATCAAAA
ATATAACGCTATCAAGTATCCTTTCAATTATTACTGCGGAAAATGAAACGTGATATGGTATCTGC
TCTAATTTTGATAAATAATATGCCCTAAATGGCTCACTACCCCCTTTCATCGAAGGAGTTACATT
GTTTATAAATTGGCCCATGGTAATTAGAAGAAGAAGGTTTTTAGAGCCTGCACTGTACCCATTTA
ATTTTAAAAGATAGTTCCACCGCATTGAAAGAACATAAAGAGTTACTAAATAAATAAAAACAGC
GAATAAGTAGATTCCAATATTTGCCGATGCTAAAACTGCATAGACCTCAAAAATGCCTATTTTGT
AAATTATAAATGAAATTATGGAGATACCAAGTCCATATACAACAATGTTTCTTAAAAGCCGGTGC
TGTTTTCTGTCCATTTGATCCCCAAAAAATAAAGTAGGTAAATTATTTTATTTAATTAGTATTAC
TTAATGCTTTAATACATATTGTATGATCCTTGAAAATGCAGGTCTTGAAATTGAAATTCCCATTA
AAACACCTGCAATTGTGGTTATAGCAAACCCTTTAAGCATTCCAATACCCATCACAAACAGTGGA
AGCATTGCTGCAATTGTTGTAGCTGCTGCACCAAATATAATGAAGAATGCCCTTTTAATACTTTT
GGTTACTTTTTTAGCTTCACCAGAAATCGTTTCATCTGTGATAACTATCTGGTGGTCAACACCAG

TTCCTACTGCTGCAATAATACCTGCAATCGATGCAAGGTCAAGTTTCCAGTCAATAAGTGATGCA
AAACCCAAAATTAACAGAACTTCTGAAAATCCAGTTATTAGAATTGGAAGAACTATTTTTGGATT
TTTGTACCTAAATGAAATAACTGCTGCAACAGCAATAAATGCAAATATTCCCGCAATTACTGATC
CTTTTATAAATTCTGAACCGAGTGTCGGTGAAACAGAACTAATATATTCGATATGTACTTTTACA
GGCAGTGAACCTGATTTCAAAGCAGTATAAATAACCCAAGCTTCGTCAATATCTTCTTCAGTTGG
GTTAGATCCTGCAACTGTAATTACCTGATTTTGGTGTATGTTTCCATCAGCAAGGTCTGCTGAAA
GTAGTGGAGCACTTATTGCTTTTTCATCCATGTAGAGTTCCACAGGATATCCACCTTTTCCGTAA
GCTACATCTGCAAATTTTTGTGCTCCATCAAGAGTTAATTTAAATCCTACCCCGTAACCTGAAGC
GGTCATTCCAGGTTCTTCAACATACGATACATCCTGTCCAGTATATGCTGTTTGATTATCAATTA
CCGCAGTAAATACCCCTTGTTGTGTTAAAATCTTTTGTATCCGTTCTAAATCTGCACTTTCCGGT
ATCTCAATAACTATCTCATCACTACCTCTTGGGTAAATCCTGACGTCACTAAGACCATTTGTATT
GAGCCTGCTTGTTAATATTTCATTTACAGTTGTCATTTCAGATTCTGAAAGCTCACTTTCAGTCT
GTAAGACTATTGTAGAACCACCACTTAAATCTACACCAAAGGAAATTCCTTTAAATGCGATAAGA
AGCAAGGAGGCCAAAACACATACTAAAAGAATTATTACTTTATGATCTTTTAGCAATTTCATTCT
ATCTCCTCGTTAATTTACTTCCCTTTTTTGTATTCGCTCACGTAGTACTTTAAAATTCCTGCATTT
GTCATCCAGGTAAGCATTAAGTCGCCAATTAATCCGATAAGAAGTACTGCTGCGATATTTGCAAG
TACATCTGCTGCAGGAACTACGAATGTAACTACAAGGTACAGTACTGCCATTGCAAAAATTGTCG
TTAAAGACATTGTTATTCCTGTTTTCATTGCTCCAGCAATCCTTTCATCGAGAGTTCCGGATCTTC
TTTTCAGTACCCTTGTTGTAAGCATGATGTCTGTGTCAACACTGTATCCAACAAGCATCAAAAGT
GCCGCAATTGTCGCAGCTGATATTGGAATATTGAATAAACTCATTCCTCCTAGTGCAATTGCCAT
GTCTGATGCAGCTGCAAGAATAACTGCCCCACTTGGAACAGGTGTTCTAAATATCGCGTAAACAA
CAATTGTCATAAATATAAACGCAAATCCTACCGCTTTCATTCCTTCTTCCCAGAATTTACTGCTT
AAAACTGAACCAATTTGTTTTTCAGTATAGCTTAATTCTGAAAGGTCTTGGACATTGAAAAACCC
TTTTACTACGGCCCTTACTGAATCTACATCAGTTTCGAGACCAGATTTAATTACTACAAATGTTC
CGATTGAAGACTCAGATATTTTAACTTCTGAATCGGGCAGTTGTTCTTTCAGCACACTTAAATCC
GTGTTCGCTGGTGCAAGGATACTTATTTCAGTTCCCCCACTTACGTCAATGCTTTCTTTGAGCCCG
TTTACTGATACAAGGGTCAAAGATAGTAGTGCTAAAATTATTGGGATAATTGTTAACACTTTAT
AGTTGATATTCATCCTTTCCCTCCAAAAATAGAGTTCATAACATCAATTATTAAAAAACAATATT
ATTTAAATATTTATGAACTATAAAAAACGAGTATATGGAAAGAATGGTGCTAATCATGGAAACA
GATTTACAGTATTTAATCGACAAAATTATGGAAATTTCAGAAAAAAAAGGTTTTGAAACCGAAG
TATTTGTTTCAAAAGGAAAACACTTCAGTTCAGAACTTGATGGGGAAACTTTAGATTCTATTGAA
GAATCTGATGATTTTGGAATTGGAGTAAGAGTTATTAAAGATAAAAAAGTTGGATTTGCATATT
CATCTAAAAATGACATTGATGTAGTAAATAAAGCAATGGAAAACCTGATTTACGATTCCGATACA
AGTTTTTCAAACCCTGAAAAATACACGCATCCAAAAGGAATGTTTTATGATAACGTCAAAAAACT
GACTGAAAAAGAGTTAATTGATGCACTCTTTGAAATGAAAGATATTTTAAAAGAAAATAAAATT
ACAACAGTAAGCGGCGGAGTTTCAAATTCTTACGGCTATTCACGAATTATAAATTCAAACGGGGT
TGATGTTGAAGAAGAAAATACATACTACTCTGCATCAATTGCTGGAATTAATAACGGAGAAACT
GCATACGACTATTTAACAAAAAACAATATTTTCAATGTTAAAGAACTTGCAGAAAACGTTGTAG
AATTCTTGAAAAATCCAAATGCGGTTAAAACGGAATTTGAAGGAAATATTATATTGGATCAAAG
AGCTTTAAACTCACTTTTAAGCTACACACTTATTCCTGCATTTAACGCTGAAAATGTTCAGAGAA
ACAGGTCAGTATTAAAAGATAAACTGGAATGTGAAGTATTTGGGGAAAACATTACAATTGAAGA
TGATGGTACGAGAGATTACTCGCTTTATTCTGCAGTTGTAGATGGAGAAGGAACCAAAACTCAAA
AAACAACACTTATTGAAAACGGTGTTTTGAAAAACTACCTCTATGGTATCAAAAGAGCAAATATT

GAAGGAAAAAAATCAACTGGAAATGCAAGCAGGGGATATTCAAGCCTTCCATCAGTTGGGCCTAC
AAATATTGTAATTGAACCTGTTGAAAAACTTGAAAACATTGATGAATACCTCTACATCAACACGC
TAATTGGAACGCATACTGCAAACCCGATTACAGGAGACTTCTCAGTTGAAATCAGCAACAGCTAC
ATCATGAAAAAAGGCGAAAAAACACCCGTTAAAAAAGGACTTCTTTCTGGAAACATCTTTGAAAT
CTTAAAAACTGCAGTTCCGCTCGATAAAGTTGAACAAAGAGGAAAATTAATTTCACCACCACTTT
TATTCAACGGAAAAATCCTTGTTTAACTAAAAAATACTAATTAACGATTTAACTATTTTTTTAAT
TTATTTTTGCAGGGGGTTTGATGAAAAATTTTGAAAAGCTGCTTTCGAAACTTGATATACGATTT
AATGATTTAAATATATTTAAACGAGCATTTATGCATTCTTCATACGTAAATGAACAAAAAAATA
CTAATTTAGAAGATAATGAACGATTGGAATATTTAGGGGATGCTGTTTTAGAATTGATAATTAG
TGAATACTTGTTTAAAAAAGAAAATTTATCTGAAGGAGAAATGTCAAAATTGCGGGCAAAATAC
GTCTGTGAAAGTGCACTTTTTACTTATGCTAAAAAACTTAAATTTAACAATTACGTGCTTTTAGG
AAAAGGAGAAATAAATTCTAAAGGGTACAATAAACCTGCAATAGTTTCAGACGTTTTTGAGGCA
TTTATCGCCGCAATATATTTAGATCAGGGTTTAGATTCAGCTAAAAAGTTTGTATATAATCACAT
AATCCCAATTATTGAAGAAAGTTCAAAAGAATTGTTTATCGATTACAAAACTAAATTGCAGGAA
TTTCCAGAATTAATCAATAAATCAATAAATTATATAAATTTAGAAGAAACTGGCGAAGCTCACG
ATAAAAAATTTGTTATTGCCGTAAAATCTGGGCGAAAAATACTTGGTAAGGGAATTGGAAAAAG
TAAAAAGGAAGCTGAAATGAAAGCTGCTAAAAATGCACTTTTAAAGCTTGAAAAAAAGATTTAG
GGAAATTATGAAAGATTTTTGGAAATATTTTTTAGATAAAAGCGATGTTTCAAAAGAAATTTTA
GAATTTGGATATTCGAAAGAAAGAAAATTTAAAGATTATTTTGAAAAGTTTTGTTCTGAAATGG
GGTTTGATGAACGAATTAATACTGCACAAAATATTTCCATTGATTTTTATGAAAAACAACCTAAA
ACATTGGTTAAAAATGGAAAATATGTTTTAAGAACGGGTTTTGGTAAATTTATGGTTTTTGATG
AATTTAAATTCAATAAACCTTACCTAAATTTAAATTTTAAAGAAGTAACTGAACTTGAATATACC
ATACCTGAAGGTTTTGACCATCTTTATGATTGTTATAAAAAAGATAATACTGAAAATTCTAAAA
TAGAACAGTTACAATGTTTTAGAATTTTTGAAAAAATTGCGAATACGGTTAGTAATGAAGAAAA
ATGTCTCATAGGCCCACGAGGGAATAAAGTATCGGAATTTGGTACGTATCTGTATGATACAGATG
GAAAAATAAAACACGTTTTTAAGTATACTGGACAGGAAGAACTTGATTATTCGATATTTACAAA
AGATTCCATATATCTTTTTGAAGCTAAAAGTGTTAAAAACGGTCTTGATTTAGGATGGCATAAAA
TTGCTTTTCCATCAAATCGATTTTTAAATTACTGTAAAAGAATAATTCCAGGATATATTTTAAAC
CAAAAAAACAATCTTTTGATATATATATTTTTAAAGGCATTGAATCTTACAAAAATGGAATTATAT
TAAACGATAAAACACAATTAGAACCAAAATATGCTTTTAAAATAAAAAAAGTTTAAAATTTATT
CAAAGACAAATTCTACTTCAGTTAACCTTCTTTTAACTTCGTTTAAGATTCTGTCTTCTTCTTCG
ATTGAAATTTCGCCGTCTTTGAATGCTTCAAAACATGCTGCCATTCTTACGAAGTTTCCTGCATC
GTCCCAAGGCACAGTTGAAATAAGTTTTTCTTTTATCATGAATTGTGAGAATTCTTCAGCGTTTG
CAAATTTAGCGCCGTCTTTTGTTCCGATTGGTGCTTTCACGTAGAGGTAGAATGTTCCTCCAGGC
ATTTTTGCGTTGAATCCTGCTTCTTTTAAGATTTTAACCATTTTTGAAAGTCTTCTTTCGTATTT
TGCCCTTGTTTTTTCAGTGATTTCAGGGTGTCTTAAGCAGTGAATTCCTGCTTTTTGGATTGGAA
TGAACTGTCCACTATCGTAATTATCTTTAACTGCTGCAAATCCCCTTACGATAAGTTCGTTTCCTG
CAACAAATGCAAGTCTCCATCCAGTCATATTGTATGCTTTTGAAAAGCTGTGAATTTCAACACCA
ACTTCTTTTGCATCTTTTACTGAGAGGAATGAAAGCGGTTTGTCGCCGTAAGTTAATGCAGCGTA
TGCCGCATCCTGAATAACTATTAAATCGTTTTCAAATGCAAAATCTACTGCTTCTTTGTAGAATT
TTTTGGTAGCCTGTGCACCAGTTGGGTTGTTTGGGTAGTTTAAGTAAAGAATTTTTGCGTTTTCC
CTAACTTCTTTTGAAATTGCGTCGAGTTCTGGCAAGAAGTTATTTTTTTCCAACAATGGTAAGGT
TTCTACACTTCCGCCGTACCATTTGGTGTGTGTTGCAGTAACAGGGTATCCTGGAACCGTCATTA

AGGTTACATCGCCAGGATTAATAAATACGGATGTAATGTATGCTAAAGCAGGTTTTGATCCGATT
GAGTGAACTACTTCGTTTACAGGGTCTAAATCTTTAACTCCGAAGATTTTTTCCAGGTAAATTGG
GATTTCGTCTTTTAATGCCTGAACACCGTTGTCAGAATATCCTCTGTTTACGTGTTTTTTTGCTTC
TTCGCATAAAACTTCAACTACGGACTCGTCAGCCATTTCGTCAGGTTCTCCAACACCCATGTCGAT
TAATTCCATGTCGGGATACTTTAATTTCGCAGCCTGTTTTGCTCTTTTAATTTTTTCGAATTTGT
AGATTACATCTTCTTTTCCAAAGCTTTTGCCGCCAATTCTTTCAGCAAACAAGTTTTGAATGTAG
CTTTCCATTTTAACACCCGAATAAGTGATTTTTAGTAAGTAAGCGGTTTTCATATAAATAGTAAT
GCATTTTAAAATATATAAATGGATAAATTAATTTCTTTCGCCATTTTCATCCATTACAGCACGAA
ATAATTTCCAATTTCGATTACATTTAATGCCCAAATTCGAAGATCCCTAAGTAGCGCAGAATTGT
CATCATAATATTCATAATCTATGTAAAATACATAATTTCCAATTTCTTTTTTAGAAGGCCTTGAT
TCAATCCTTGTTAAATTAACATTTCGATATTTAAATACGCCCAAAACTTCATAAAATGCTCCAGG
CATATTTTTATTTAATTCAACTACAATTGATGCTTTTTTAGGTTTTAAATCAGTTTTAAAATTCT
TATTTTTATTGCATATCAAAAAAAAATCTGGTTGTGTTATTTTTAAAGTCCTGAATGTATTCATCA
AGAACTTTTAACCCGTAAATTTCTGCATTTTCAACACCGCAGATTGCTGCAAGTTTCTCGTCTTT
TTCTTCGGAAACAATTTTTGCGGCTTTTGCGTTGCTGTCAACAGGAGTTATATTCCAATTATTTT
TTGTAATGTAGTGCCCGCACTGTGCGAGTGCTTGAGGATGGGCTAAAACTTCGGTTATTTTTTTG
ATATCGTAGCCAAGTAAACAGTGGTTTATCGAAATATCCACTTCACCGAGAATTTTTACGGGAAA
CTCCAAAAGAAGGTCTTGTGTAATGGAAACTGAACCGCCAATTGAATTTTCAGAAGGAACTACTC
CAAAAAATTCAGGATTGGTTTCAACTGCCTTGAAAACATTATAAATTGAATCTTCAAACTGGATT
TCACTATCATTTATCGCTTTTGAAAATGTAACAGCAGCTTTTTCGCTGTAACTTCCCCTTGGACCA
AGACAGCAGATCATTTTTTCACCATTTTGCAAATTAAAAACTGCCCTTTAAATTTCAAAACCTTT
GAAATCTTTTCGTCATTTTTAAAAAATAAATCTATTATTCTTTTTAAAATAAGTTTTGGAAACAG
CTTAAAGAATGGATGTAAAAAATAAAATGATTTTGTATATTCTATTTTGAAATCATCTGTTTCA
ATACTCTTAAAATCTGATTTTGAAATTAAATTGTGAGGTATTTTCCAATCAACAAATATTTCATC
ATTAATTATTGAATCAGCGTTTACTACAATAAAATAAGCTCTTCCATTTGTTTTTAAAACCCGTT
TTACTTCAAAAACTGCTTTTTTAAAATCAACATGCTCTAAAACATTGATGCACAAGATTAAATCA
AAAATACCATTCTTAAATGGTAAATTTTCAATATTTGCACAGATTTTATTTTTTTCAATTTTAAA
TTTTTTTAAAAGATTTAGTGAAAAATCAAGATATAATGTATCAAAATCTTTTGTTAAGTCGTAA
AATGAACCAAAACCGCAACCACAATCGAGTATTTTAGTATTTTTGTGAGATTTTGATAAAGATTT
AAAAAAGTCATCTTCTAAAATTATCTTAAAAATAAGGTGTTCTTCAAACTCCATTAAAAGTTTTA
AGTAATCAGGATACTTTGAAACGTCCCAATTTTCATAAAAAGCTTTTACTTCGTCTGATTTTAAT
TTATTCATAAAATTTCACACATAAGGCTGTAGACAATAGTGCCTTCTGGAAATGCCGCTATTATC
TTTTCCTTAATTTTTGCTAATTCTTCACTGCTTAATTCTTCATCAATTTCCTTGTCAAGACAAAA
ACCTGACCTGTAATAACAATCGGGTTCATAAATTGCCGCAAATTCTGGAAATTCAAGCGAAAGGA
CTTTTAAATCTTCACCTAAAATTTCAACGAGTTTTTGAAGAACACTTTCAAAATCTATTTTCTCA
GGGTTGTAAAATACACCATCTATCATCATTTTCATGCATATCCCTTAACAGCCGCAGATGTATTC
TTTATCAAAGTCAAAAAACATTGTTTTTTCGGTTCCATCAGTTATCATGAATTTTTTATCATTTT
TAACTTCACCGCAGACTGCAGATTCGATTCCTGCAAATTCAAGTTTTTCAATTAATTCTTTTGTT
TTATTTTCAGGCGCAGTAAATACAAAACCGCTTCCAGGATACATTTTAAGCCAGTGGTTTAATGA
AATATCTTCTGGAACAGGTATTTTACTTACGTCCACCATTGCACCTTTTTTTGAAACTTCTAAAA
GCATTCCTAAAGTTCCAAGTCCGCCCGGATTACTTATATCCTTACATGAGTTTGCAAGGTGTTCT
TCACCGATTATTTCAAGAACTTTTAACTGATCTCGAACGTACTTTTTAGATTTCATTGAAGTAGT
ATCCCAGTTTAAATTAAATTTTTCATGGATTTTTCCATCCAAATCATATGCAAAGACGATTTTGT

CGCCAGTTTCTGCAGCATCGCTCCTTAAAACATTATCTCGTTTTACAATTCCGGTAATTGAAATA
TCTAAAACATTGCACTGTGCATCAGGGTGCGTGTGGCCTCCTAGGACAGGTATTCCAAATTTACT
AACTCCTTCTTTAAGGCCTTTTAACAGATCTTTTCCTTTATCGCAATCTTTTATACCTATAATATT
TGTCATTCCAATGGATCTACCACCCATTGCAGCAATATCATTTGCATTTACTAAAACTGCACAGT
AACCTGCCCAGAATGGATCTGCTTCAAGTAATTTCCCCCAGATTCCATCTGCTGCGAGTAAAACT
GCATTTTCGTCGTCGATTCCAATTATTGCTGCATCGTCCCCAAAATCTACAACAATATCAAAGTC
ATAATCTTCCTGAAAATTAAAATTATTAACAACATTTTTGATTTCTTTTTTTCTTGTAACGCCTT
CAAAGTTTCTGAGTTCATCAGCGAGCTTGCTTAAATCCATAAAATCGCCTATCTGATCTATAATG
TCCGAGAAAACTATTTTAATAATAAAATAGTAAGTTGATTCTTATATATAACTTTCCATAAATAT
TGATTTAAACCGAATTTTAAAAAACAAACCCGATTCATTACGACTTTTATTTTGAAGTTAAAGTT
TATGTTTTTCGTAAGATTTGGACAGCATTTCAATTACAGGAAGTTTTTTACCAGCTAAAAATTCG
ATACATGCACCGCCACCAGTGCTGATGTGGTCCATTTTTCCTGCTAACCCTACAACTTCGGCAGCT
GCTGAGAGGTGTCCTCCACCGATAACCGAAAAGCCTTTTGAATTTGCGGTGCTTTTTAAAAGTTC
TTCTGTTCCTTTTAAGAAATTTTTATTTTCGAAAACTCCAGCAGGTCCGTTTGCAACAACCGTTT
TTGCATCTTTTAAAATTTCTTCGTAGAGTTTCATGGTTTTTTCGCCCATGTCGTGAATAGGATAA
GTTATTTCTTCGTTTAAGTCGAGTTCTTTTTCAACCCGTTCACCATCAACATTTAATGCTGCATCG
ATTGGAACCACGATTTTGTCCCCATATTTTGAAATTAACTCTTTTGCAATTTCTATCTGGTTGGT
GTATCCCATGTCAGCAATTACATTTTCGTTTGGCCCGATTTTGTAGCCTTTTGCAACTAAAAAGA
TATTTGCAACAACGCCACTTGTTAAAACCTGATCAGCGGTCTGTTTTTCAAGAACATTTTTCAAT
ACTTCAATACTGTCGTCAGCTTTTGCACCACCTAATGCAAACACGCAAGGTCTTTCAGGGTTTTT
GAGAACTTTTGTAAGTGTAAATAATTCTTTTTCCATGACCCTTCCGCATAACATTGGAACATAGT
AAGAAAATCCAACAAGAGAAGGTTGAGCCCTGTGAGCTGCTGCAAATGCATCGTTAACAAAATAA
TCGAAAAAAGGATGTAATTTCTTAATCATGACTGTTTTAGCCTGTTTTTCTGGAGTAATTTCTTC
CCAAGACTTCCAATCAGATAAAACTTCTTCTGCAAGAAGCCTTACGTTTTCAAGAAGTATTATTT
CTCCATTATCCATTGCAAGAATTGCTTCACGTGCAGACGCACATATTAATCCATCTACGTGTGTA
ACTTCCATATTTAATACTTCAGATAGTTTTTTCGCGTGAGCTTCAAGAGTCGTAAAATCTTTTTT
TCCAGGCCTGCTCTGGTGTGCTAAAATAACAACCTTTGCTCCTTTTTCTGAAAGACTTTTTATCGT
ATCACTGCATGCCTTAATCCTTGTATCATCTAAAATATCTCCAGTATTAACATCAATAGGGCTAT
TAATGTCAACTCTCAATGCAACCGTTTTTCCTTCCAATTCAAAGTCGTCAAGAGTTAAAAACATT
ATTATTCCTCCGTTTTTTCGCGATTTGGTTAAATTCGTTCTTTGTAACATTAATATTGAAATTGG
TACGTATAATAAAACATTATTTTAATATTATTTGTAAAAGAATTACTTGAATTAGTATTTATAA
ATTAATACCTGCTTTTCAAAAACTATTGTTTAAACTTGATTTATTATCACAATTATTCAAAAGTG
CCATTTAAACCGTATTTTAAATTATTATGTTCTTTTTAATCACTTTATTAGACATATATTATTTA
AAATTAATAAAACAGAAAAATTTAATCAAAAAAGATAAATAACTTCATTATATAAATGTATTAT
AAATCCATGTATTCAGGCACATGAGCCACGGCGGAGGAATATCATGTTTAAAAAAATAAAAAGA
ATATTTGTGGGAAATAAAGAGTTGCCAACCCAGGTTCCAGTACAAGACGAAGAATATGTTGTAAT
AGGCCAAGAATATTCAGCATACGAAATCAAAGAGTCTGAAAAAATCCCTATACAAGAAGATACTT
CTGAAAACAAAGTGCAAGTTATAAAACACAAAATCTATCCAAGAGTCCTTACCACAAGAATTAAA
TCGCCACAAGACTTTGAATACATTAAAAATATAGTGGAACACGATGTTATATTAATTAACCTTGA
AGAAGTGCCTATAGAAGCAATCATAAAAGAATTTAACGATTTTAAAAAATACCTCGAAACACTT
GATTACAGCCTTGGAAGAGTTGGAGAAAACACGATTCTTGCAATAAAATCAGACGTTGAAATGG
ATAAATACGTTTCAGAAGAACCTGTTCACGAAGTTGAAGAATAGGTGAAATTATAATAATAGCA
ATCACGGGAACTCCTGGAGTTGGAAAATCGACAGTATCCAAAATCCTGTTTGATAAATTAAAATC

TAACGATGTATCTTACATAAATATAACTGAAATAGTTTCGAAAGAAGGATTTTATCTGGAAAAA
GATGTTGAAATGGATTCTTTCGTTGTTGATTTCGACAAATTAACTGAATATATCCGTTCAATTAA
AACAGAAGATTTAATACTGGATGGGCACGTCAGCCACTATTTAAATCCTGATTACATTGTTGTAC
TTCGAGCAAATCCATTGTTAATTAAAAACCGGCTGGAATCTCGAAAATATCTTCCAAAAAAGGTA
ATGGAAAATGTTGAAGCAGAACTTTTAGACGTCTGCTTAATAGAATCTATTGAAAAAAACGACG
AATCAAAGATTTTTGAAATAGACTGCAGTGAAAAAAATCCAGAAAATATAGTAAATGAAATATT
AATGTTTTTAGACTCAAAAAATCCAGAATATGGAAATGTAAGCTGGCTTGAAGATTACTTCTATC
TTATTGAATAACTCTTTTTTAAATTTTTAAAAAATAAAGAATTGTTGGGAATATCAACACACCCA
TCATGTTTGATAGATTTACCCTGTTTTTAACGCATAATACCCCGATTGAGATTGATATTAAATAT
ATTACGATGTGATACATCAAATAAGCTTTAAATGAAAGAATTAAAACCAAAATCGTACAGAAAA
CTATCAAAACTTTGGAAACTAGCCCGTAATCAAATCTATTAATGTTTTGTAAAAAATATTTTGAA
ATCACTGTTAAACTCAAAAAAGAAATACCTGTTGCAATAAGTACGCTTGAAAAAAGTTCTGACTG
ATTGTATTCTATTTTTAAAGTTCTTATAGCTTCTGAAATTCCGCTTCTTCCAGTTCCAATCATCA
TGAGCGCTAAAATAGAAAAAAGCTCGTTTGAAAGGGTTATTGAACCTTGTGAAATTATAAAATT
TTCGATATTTTCTTCTTTTATCAATTTACTTAAAAAATAATTAATCTGTGCGCCACCGGTTGCCG
GAATAAATATCCTCAAAAATCCGCCAAAAGTTCCAAAAACTGCAGATTTTAGATATGAAAATTTT
AATTCTGGAAAACTGATAAACTGATTTCCTAGTTCTTTTTTATTTAAATTTTCAAATAAAAGCGG
AATTCCAAACATTCCTGTAAATATCACGGTTAAAGAATAATCAAAACTGGGATTTTTATAAAATA
CAATAATTCCCAAAATTCCTGAAAATAAAATTATTAAAGTAGTCCAGACCCGATTTTTTGAAAAA
ATAACTGAAATTAAAAGAAATAACATCAAAATATATGGAAGAAATGGTTTTAAATATGAATAAA
AACTTTGAAAATCAATATTTATTCTGGAAATTATCAAAAATAGGAATATTGAACAGATTATTCC
AAAAAATGCTCCAAAACCTGCCAGAACTACTGCTTCGTAGCCCCTTCCCAAAATCGTTAAATTGT
GCATTGGAACCGCTGCTACTGCAGATTCGTCGTCAGGAACCCCGATAAATGCGGAAGGAATGTAA
TTTAAAAAATAGTGTGAAATTAAAAGCCCGATTAAAAAACTAAAATAATTACTGCTTCCAAAAT
AAGGATAGAATATTACTGAAATAGGAATAAGCGTATTTGGATGAATTCCAGGGATTAACCCAGT
AATTGTCCCAGTAGCAATTCCAAAAATTAAAAAGAATAAATTATTAATCATTTAATCCCTTTTTT
CACAGTAACTGCATTTTTCATACCCTAAAATTTCTGCATCATCTTCACTTTTAAAATAAATTTTT
TCGCTTATTTTCTCCCCATACGGACAATCTTCAAAAGTATGATACACATTACTGTTTTTGGATGC
ATAATAATTTGTTTTGTATTTTGAAAGAAAGATATCCCGTTCAAAATTCACTTTTTTGATTAATT
CAAAATCATTTGGATCTTTTAAAACGATTTCATAACGCCCGTTGTAAACTTGAATTTTTCCGCTA
ACTGATACAATATCCCCTTCTTTTATTCGCGGAGTTGTTTTTAGAATAATTTTTAAAAATTCATC
CGATTTATCATCAGCAACATAAACTAAAAGATCGCCTGCGGTTTCATCCATTAATTTAATAGATT
TTATGCCAGTAACTTCAAGATATTCGTTCGTTTTTACATCCAATTTCTGTATTTTAGCAGTTATT
TTTACATAATCGCCTTCATTTAAATCAGAAATATGTTTTTCTTCAGGAACAAGTTTAAAAGTCGA
TAAGAAGGGAATTCCCAAAACCACACAAAATATGCACAAAAATATTATTTTTTTATCATTTAATA
TTAAAGACGACAATATAACCCCTCATTAATTATGTCCTTAATAATCTAACAATATATTCATATTT
AAACCCATATACTTTTGTTAATTTAACTAAATAGTATTAGATTACTTAATAAATAGTTATAAAT
ACGTTATTATCCAGAAAAAATTATGTTTTGACTAAATGCATATCAAAATATCTAATATAAAACA
ATATATTAAAGAAATAGGGGTGACTAAATGAAAGTTTCACCATTGCAGACTGGCCTTATCGCAGG
TTTTTCTGCAATACTCTTAGAAGTTATCTTTAAAGTAAGTCCGCCTCCAGCATACGGGCTCTGTG
TTGCATGCCACACGAGAGACCTTGTAAACTGGATTGTAAACAACGCTGCTGGAACTGCGTTAGGA
ATGGCTCCAGTATCGAAATTTTTCCCTGTGTTAACTGTTGTTGGTTTATTTATCGGAGCACTTAT
CGGTGCAATTATACACAAAGACTTCAAAATTAGAAAAACCCATAATTTGGTTACCGGATTAATAA

TTGGTTTTTTAGTAATGAACTTTGCACTTTTAATGGGTGGTTGCCCAATTAGAATGGGTTTAAGA
ACTGCTTACGGAGATTTATTCGGTTTAATCGGTATTTTAGGTATTGTTGCCGGAGTAATCGTTGC
TACTGAAGTATATTTAAAAAAGGCATAATTAAGGAAAAAAGGTGAAATGATGAGTTACGTACCT
GCAATTGCCACTTTGGCATTTGGTATTCTTTTAGGATACTTAGGACAGAGATCATCAATGTGCTT
TTGTGGAGGTATCAGGGATTATTACCTCATGAAAGATACATGGTTAGTTAAAGGTTTACTCGGAT
TTATTGGAGGAGCCTTAGTTGGTTCGATTATATTCAATGTTATTGGAATGCTCCCATTATTCCCA
TGGATTGCAACAAAAGGACTCACAGCAATTCCAGGAGATGCTGCAGGAAGCTTAGGCTTTGGTGC
ACACTTCATAGTTACAATCATTGGTGGTTTTGGAGTTGGATTCCTCTCAATTATTCAAGGTGGAT
GTCCATTTAGAAACTACGTGATGGCAGGAGAAGGAAACCAAACTGCTATGGCATACGTACTTGGT
TTAGCAGTTGGTGCAGTAGTATTCCACATGTGGGTAGCCCCATTTGTTCTGTCAATTTTAGCATA
ATCACTAAATATAATTAATTTCTACTATTTTTTTGAAAAATTAGCTCAACTTGGTGGCATTCATG
TCTTCGATTTTCAATAAATTAAACACAATTCTAAATCCTAAAAAAGAAGTTGAAAAGGGGCAGG
GTTTAATTATCTTTAAAAACGTAAAAGAAGCAATTTCTGCAGAAAGAATTTTAAAAAACTTTTC
GGTAAAAGTTGTAGCTCCACCTCAGGAAATACGAGAAGGCTGCGATTTAGCTGTTGAATACGACC
TTGTGGAAGAAATGGGGATTAAAAGGGAACTTGAAAAAAATAATCTAAATGCCGTTAAATTCAT
ATCATTAGATGACGCTTCGATGGAACCTCTGAATCTTGTGAAAGTAAAAGAAATTGATGGATTTA
CTCTTGTAAGATCTGGAAACATGAAAATTACAATTGATAAAATTGGAAAAATTGTAAACGTGTC
TGGAGGAGGCTGTCCCGATGTTCCGTATTTGAATTTAAAATTAAAAGGGAAAAATATATTAAAA
ATACCTGAAGAAGATACTCCAAAAAATTTAGGATACACGCTGTGTGCATACACGCTCAATAAAGC
TTTTGAAAAAGCGAAAACAATTGCATTAGAGGGTACAAAATGATTATTGCAGGTGCTGTACCAA
TAAAAGGAATGGAACTTATCAAGGGTCATGTCAAATTAAATAAGGATACTTTTGAAATAAAAAA
TAAAAAACTTCCAATATCTATGGGAACTTGCGCTCTTTTGAGTGCTGCTTTTAAGACTTTAGAAT
ACTTTGGAAAAGAAAATGAATTAACATTTATTACTGCTGGAGACGTTGGAGAGGGAGATGGAAG
TTTAAAAATTTACGAAGAACTGCATGAAATTAATGATGATTTGACAATTATCCATTACATAAAA
CCAAAAATATCTGAAATTATTAAAGTTGATTTTTCAAAAAACGTCATTGGTGACGCCGGTGGAAT
GTATGCTGGAAAAGCTGCAGGAATTGGTAATAAATTCCATATGTTCTTTCCAGATGTTGGAGAAC
TCGCATTTTTTAGCAGATGAAAAGTCATCTCACCCTGCATATGTAAGAGGATTTATTTCAGAAATC
GATGACCAAGAAATTCCAAAATTAATCAAAATGGCATATACACAAAAAATGCCGAAAAACATGG
TTGTAAAGGGAAGTAAAGACCATATTGTAACTGAAGGAAATATTATTGATACGGTTGAATATCC
AAAATTTGAAGCAATGGAATGCATTGGAGGAACTGGCGATACATTAACAGGAATTGCATCCTCAT
TAATTGCATGCGGATTCAAAACAGAAAAAGCCGGAATACTTGGATGCAAACTCAACAGAAAATT
GGGAGAACTTGTAAAACCAAGCCCCGATACGCAAATTTATGAAATCATTCAAAAAATACCTGAAG
CTTTAAAAGAATTTGATCTATAATAAATTAAAAAAATAAAAAAATTAAATTTGTTTTTCTGCAA
TATCTCCAATTATAGGGAATTTGTATCTTTCGCCTTGGTATGCTTTTATCATGCCTACAATCCAG
AGTATTATCCGTATTATGTAATTATTACTTTTAGTTTAGACCATTCCGCGTCAGAATATTTACAG
AAAAGTTTATATATGAAAACACCTTATTTCATTTTGCTTCGAAAAGCATTGGAAATTAGGGCAGT
GGCTCAGCCTGGTTAGAGTGCTCGGCTGATAACCGAGTGGTCCGGGGTTCGAATCCCCGCTGCCCT
ACTTTTTTCTATTTTAGGAAAATTAATTTGTATATTTAGATATATAAGTAATTAAAAAAGATTA
TTTATTAAATTTTGAAAGTTCTATTCTAAGTTTCAGGTTTTCATTGAAGTTAACTATATCCCCAT
GTGTTTTTTCAATTAAACTTCGTGAAACGTCTTGTTTTCTCCTAAGTCCATCAATTACGTGGTAA
TTGTCAAATTCCATTAAAAAGTCATAAATTCTATTCATATATTCAAAATAAAGTTCTGCTTTTGT
TTTTTCATCTTTTCGAATCGATTCAAGAGTTATTCTTCTAAGTTCGCCGATAACATCGCACAATC
CCAAAATATAGTTTTCTTCTAAAGTACTTTCAAAATCAGAAAAATCCAAAATTTTATTTTCAAAA

ACTATTGAATAGTAAACTCTTGCTTCTACATACTCTTGTTCCGGCGTTCCACGATATTTTTGGAA
TTCAAAGTGATTTTCTGAATAATTTTTTAATTTCTTTAATTTTTCTTCAATATCTGAAAAATCTA
CCTCTTCCCCTTTCTGTGTTTTTCGAATAATTAATCCTGAATCCTTTACAATTTCCCTTGAAATCT
TCAAAATTTTTTCCCTTGTTTCATTCTTTTTTTCAAAGAATTCGAGTAGATAAGAACTATTTTTC
ACAATATCACCCTGTTTTGTTACGTAAATACAATAATAACTAAAAATATTTCTTTTTTAAAATAA
ATACTGATTTAGTCAGTTGTGGAGTTAACTATATATATTGTAATGATTAATGTTTGTAGGCAACT
ATGTGGAAGACCTGAAAAGGCGCGAGCCATTAATAGGTTGCTTTGTTTTTAAAAAAAACTTAACA
AACACTTCAAAAAACGACTAGTTCTACGCGAATAAAACACGGGAATTCTAGTTAACTATCATAGG
AGGTTATACTATGGGTATGAAAAAAAATAGACCTCGTAGAGGTTCCCTAGCATTTAGCCCAAGAA
AAAGAGCTAAGAAATTAGTTCCTAAAATCAGATCATGGCCTGCAGATAAAAAAGTAGGGCTCCA
AGCATTCCCTGTATACAAAGCAGGAACAACACATGCTTTATTGATCGAAAACAACCCTAAAAGTC
CAAACAGCAGTCAGGAAGTATTCACACCCGTAACTGTATTAGAAACACCGGACGTTACCGTAGCA
GGAATCAGACTCTACGAAAAAACAACCAAAGGGTTAAAAGCATTAACTGAAGTATGGGCTGAAC
AGTTAGATAGCGACTTAGGAAGAAAATTAACTTTAGTTAAGAAAGAAGAGAAAAAAACTGTTGA
CGCTTTAGACGCAGTTTTAGAAAAAGCTACAGAAGTAAGAGCTATTGTTCACACAAACCCAAAAA
CTACAGGAATTCCAAAGAAAAAACCTGAAGTTGTTGAAATTAGAATCGGTGGATCATCAGTTGCT
GAAAGATTAGCTTATGCTAAAGAAATCTTAGGCAAAACACTCGCAATTAGCGATGTTTTCGAAGC
TGGCGAAATTATCGATACCTTAGCAATTACAAAAGGTAAAGGATTCCAAGGATCAGTTAAAAGA
TGGGGAATCAAAGTTCAATTTGGAAAACACCAAAGAAAAGGTGTTGGTAGACACACAGGTTCAA
TCGGCCCATGGAGACCTAGAAGAGTTATGTGGACTGTACCATTACCTGGTCAAATGGGTTTCCAC
CAAAGAACTGAATACAACAAAAGAATCTTAAAATTAGGCAGCGAAGGCGCTGAAATTACACCTA
AAGGCGGATTCTTAAACTACGGTGCTGTTAAAAACGGCTACGTTGTAGTTAAGGGAACTGTTCAA
GGTCCTGCAAAAAGATTAGTAGTTTTAAGAGGATCTGTAAGAGCTGCTGAAGACAAATTCGGCTT
ACCTGAAATTGCTTACATCAGTACAGAATCCAAACAAGGAAACTAATTACTCAAAAACACGAGCA
GGTGTTAAATTATGAATGTTAAAGTTTACAATTTAGATGGTTCTGAAAAGGGAGATATTGAATT
ACCCGCTGTATTTGAAGCAGAATACAGGCCTGATTTAATTAAAAGGGCAGTAATCTCATCATTAA
CCGCAAAATTACAACCAAAAGGTTGCGATGCATTTGCAGGATACAGAACTTCAGCAAAATCAATC
GGAAAAGGACACGGTAAAGCTAGAGTTAGAAGAACCGCACAAGGTGCTGGTGCATTCGTACCCCA
AGCAGTTGGTGGAAGAAGAGCTCACCCTCCTAAAGTTGAAAAAATCTTATTCGAAAGAATAAACA
GAAAAGAAAAATTAAAAGCATTAGCAAGTGCTATTGCAGCTTCAGCAAACCCTGAAATCGTTTCA
GCAAGAGGCCACAAAATTGAAGGTGTTCCTTCATTACCTTTGGTTGTTAACGCAGAATTTGAGAG
CCTTGTTAAAACAAAAGAAGTTTTAGAAGTTTTCAAAACTTTAAAATTAGATGCTGACTTAGAA
AGAGCTAAAGACGGCGTTAAAATTAGAGCAGGAAGGGGAAAATTAAGAGGAAGAAAATACATAA
AACCAAAAAGTGTTTTGGTTGTTGTAGGCGATGCATGTGAAGCAATTACTGCATCAAGAAACCTC
GCAGGTGTAGATGTAATTACAGCTAACGATTTAAGCGCAATACACATTGCACCAGGAACAATGGC
TGGAAGATTAACACTCTGGACTGAAAATGCCATTGAAAAAATAAACGGAAGATTTTAAGCTCAA
TACGAGGTGAATGGCATGGATGCCTTTGATGTTATAAAAACACCAATCGTTAGTGAAAAAACAAT
GAAACTCATTGAAGAAGAAAACAGATTGGTATTTTACGTTGAAAGAAAAGCAACAAAAGCAGAC
GTTAGAGCAGCAATTAAAGAATTGTTCGATGCTGAAGTTGCAGACATAAACACAAGCATTACTCC
AAAAGGAAAGAAAAAAGCATACATTACGTTGAAAGATGAATACAACGCTGGAGAAGTAGCTGCA
AGCTTAGGAATCTACTAATTCGATTTAAGAAAACACTATTAAATAATATGGTGATATAATGGGT
AAAAGACTGATTTCCCAGAATAGAGGACGAGGGACTCCGAAATACAGATCCCCCACTCATAAAAG
AAAAGGTGCAGTTAAATACAGAAGTTACGACGAAATGGAAAAAGACGGAAAAATCCTCGGAACT

GTTATTGACATATTACACGACCCTGGACGATCTGCACCTGTGGCAAAAGTAAAATTTGCTAATGA
TGAAGAAAGATTAGTGTTAATTCCAGAAGGAATTCAAGTTGGGGAAGAGATTGAATGCGGTATC
AGCGCAGAAATCAAACCTGGAAACGTTTTACCACTTGGAGAAATCCCCGAGGGTATTCCAGTATA
CAACATTGAAACCATCCCTGGAGATGGCGGTAAATTAGTAAGATCTGGTGGATGTTACGCACACG
TTATTTCCCACGATGTTGGAAAAACTATCGTAAGATTACCTTCAGGTTTTTCAAAAGTATTAAAC
CCGGCATGCAGAGCAACAGTTGGTGTTGTTGCAGGTGGTGGAAGAAAAGAAAAACCATTTGTTAA
AGCAGGTAAAAAATACCATTCATTAAGCGCTAAAGCTGTTGCATGGCCAAAAGTTAGAGGTGTTG
CAATGAACGCGGTAGATCACCCTTACGGTGGTGGAAGACACCAGCACATAGGAAAACCTTCAAGC
GTTTCAAGACATACATCCCCAGGTAGAAAAGTAGGACATATCGCTTCAAGAAGAACCGGTAAACG
ATAATAACTTAAGCTATCTCTTTAAATTTTAAAGGTGAAAACATGGCCAGACAAAAAAAGTATA
GTGGCAAAGGTGGCGCCAGAAAGAAAAATAAACAAAAACAAAATGTGGCACCAAGAAGAAGAGT
AGAATTTAAATACAAAGGCTTCAGTTTGGAAGAACTTCAAGAAATGCCTATCAAAAAATTCATG
GAAATTGTTCCTTCAAGACAAAGAAGAACAATGCTCAGAGGAATCACTCCTAAACAAAGAAAATT
AGTTATGAAAATCAAAAAAGCTAGAAGATTAACAAACAGAGGCAAAGATGCAAGAGTTATAAGA
ACTCACTGCAGAGACTTTGTTATTACTCCTGAAATGATTGGATTAACATTTGGTATCTACAACGG
TAAAGAATTCAAAGAAATTAAATTAGTTGAAGAAACCGTTGGAAGATTCTTAGGAGAAATGGCT
CCAACAAGAGGCGTTGTTCAACACGGTTCACCAGGTATGGGTGCAACAAGAGGTTCAATGTTCGT
TCCAATTAAATAATTGGAATTCAAATCTCTTTTTTTATTTTGGGAAAATTAATTTATAAATTTAA
ATTAATGAAATTAATATCTATTTTAAAAAAAATTATAATTTTTTGATAATATTTTCTAAATTTT
TATCGACATTTTCGTTAACTTTAGAATATAAACTATTTCCGTGAGAGTCCATTGCGACAATTAAT
GGTCCAAAATTTTCAACATTTAATTCCCAGAACGCTTCTGGCATTCCAAGTTCAAGATGGTAAAC
GTTTTTTACATTTTTTACGGAATCTGCCAAAAGTGCTGCACAACCCCCGGGGGCTGCAAGGTATA
CTACACCGAATTCTTCAAAAGTCTTCAAAAGCTTATCATCCATGCCACCTTTTCCAACAATTGCTG
AAATATTTGTAATTCTTATGAAGTCTTCCTCGAGTCCATTCATTCGAGCAGATGTTGTCGGCCCG
ATGGCAACACATTTCCAAGTTTCATTTTCCTTTTTCATTATTGGACCGGCATGATATATTGCACC
ATTTTCAAGGTTTATTGGAGGTTTTTTTTCTTCTATTGTTGTTATATGTGCTTCATCTCTTCCGG
TACAGATATCACCGCTTAAATATACAATATCCCCAACTTTAAGTTTTTTAACGTCTTCCTTTGAA
ATGGGTGTTTTTAATTGCACGCTATCACCAGAATAAAAACTAATTAATAGATAGAACGCTTTTAT
TATATTAATAGATAAAGCGGGGAGAGATAATCATGAAATATGGAATATCATCTTTAGTATTTTT
ACCAGAAACTCTGCAGTCCTCAATGGAAAAAGTTGCGGCAAACTCTTTTGACTGTTGGGAAATCG
TTTCTGAAGGAAGTCATCAATTAAATCCTAAAAATATAAAATATTTAAGAAATTTAAGAGAAGA
ATACGATGTTGATTTGGTAATTCACGCACCATTTTCAGATTTAAATCCTGCATCAATGAATAGAG
ATGTTAGAAATTTAACAACAAACAGCGTCATTGAAGCAATTGAAGGTGCTTTTGAACTCGATGCA
AATGTTGTTACGGTTCACCCAGGATACTTACCGCCGCTCTGGTCGGACTATACAAAAGAACTTTT
AGATAATAATTTTTCTTCATTAAACGATATAGTTGAAATGGCAGAAGACTACGAAGTAATGATT
GGTTTAGAAAACATGCCAAATTACCCTGGAGTTTTGGGAGTTTCCATTGAATCTTTAAAAGACAT
TATAAAAGATATCAACTCAAAGTACCTTGGAATTACCTTTGATATTGGCCATGCAAATACTGCAA
CCAAAAACCCTGAAACTTTCGTAAAAGAATTAAATAAAATTGGAAAAGGAATTGTTCACTGCCAT
ATTCACGACAATATGGGAACTGAGGATGAACATTCATTAATCGGTGCTGGAAACATTGACTTTTT
AAAAATTTTAAGCGAATTAAAAAGTATTAATTACGATAATGTTTTATCTTTTGAATCTAAAAGT
ATAAGAGATGCAGTAAACAGCAGGGAAACGATAAATAAATATTTATCAATGCTTGAAAAATAAA
ATAAATTAAAAGGTGTGATTATGAAGATTGCGATACTTGGTGGAACAGGAGATCAGGGATTTGG
CCTTGCACTTAGATTTTCAAAAAATCATGAAATATTGATCGGATCAAGAAAAGCTGAAAAAGCCA

TTGAAGCTTCTGAAAATGCTTTGAAAATAATTTCAGATAAAGGATATTCTGCAGATATTAAAGG
AATGACCAACCTTGAAGCTTCGAAATTAGCTGATGTAGTTATTGTATCTTTACCTTTCGAATATA
CAATTTCCACAATAAAAGAGTTAAAAGAAGCGTTAAAAGGAAAAATTGTTGTATCAATTGGAGT
TCCACTTGCAACAGCAATTGGGGACAAACCAACAAGAGTTATCGCATGCCCTCAAGGGTCAGTTG
CAGAATTAATCCAAGAAATGCTTCCAGAATCAAAAGTTGTCAGCGGATTTCAAAACATCTGCTCA
AAATGTTTAGAAGATTTAGATTGCGAAGTTGCATGCGATGTTTTAATTGCAGGAAATGACAGCG
ATGCTAAGAAAGTGATTGTTGAACTTGCAAGCGAAATTCCAGGAGTTAGAGGAATTGATGCTGG
AAAATTAGAAATTTCAAAATTTATAGAACAAATTACGCCATTACTAATACAGTTAAACATAAAA
TACAAATTAAAAGGTGCTGGAATCCACATTACCGGATTAAACCTTTAATATCGAATTACCATTCC
TTTTTTCCAGAATATAATTGAAATTACTGCAAACAATAATGCCAAAAACCTGAGTGTATTTGCCA
TTATTAATATTATCTGATTTGCAAACGGGTTGTTGACAAGTTCCGGCAGTAATATAGCAGGTATT
CCTTGCAATAAAAAAGCAATGATGAATAATGCCACGCTTGTTATTAACGAATCATTTACTGTAAA
TCTCATTTTATCTCCAAAAAAAACTTATATCGAACTTAACCCGACATTTATCTACCCAAAATTAG
CCTAGATCAAAAACGCATTTTTATTATTTAATCGTTTTCGTGAAAATGACACTAAAAAAAGTAAT
AATTGTTTTTAAGTTAATCTTTATACATTAACTGCCTCATGATCTGGCCCATCTGTCCGCCCATTT
TTGGCATTTTTCCGCGCTTCAAATTTCCAAACGCATTTTTAGTCGTTGAATAGTATTTTAAAAGA
TCCTTTATTTCTTCCTGTTTTACTCCAGCCCCTTTTGCAATTCTTTGAAGCCTTGAAGTTTTTATA
AGTTCAGGATTTTCTTTTTCTTCCATTGTCATTGAGTCCATCATGATTTTGTATCTTTTTAATTT
AGCTTCCGTTAACTGTGCAGCTTCTTTTGGCATGTTTCCACCCATTCCAGGAATCATGCTTAAAAT
CTGTTTCATTGGACCCATTTTGGATATTGTTTCAAGCTGTGCATAAAGCTCGATTAATGTAAATT
TACCCTTTAAGATAGAATCAATACTTTCCTCTGTTGATTCATCCATTATATCTTCAGTTTTCTCT
AAAAGGGAATCTAAATCTCCAAGTCCAAGAATTCTTGAGATAAATTTTTTAGGGTCGAACTGTTC
TAAATTGTCGACTCCCTCACCAGTTCCAATAAATTTAATTGGAGCATTTATTTCTGCAACAGCAC
TCAAAGCACCACCACCTTTTGCAGAACCATCTAATTTTGTAACCAAAATACTTCCAATTTCTGAA
ACGGATTCTTTGAATGCTTTTGCTTGATTTTTAGCCTGCTGACCTAAAGTTCCATCAATTACAAG
GATTATTTCATTTGGATTCGTTAAATCTTTCATTTCTTTCATTTCAGCTAAAAGACCTTCCTCTTC
CTTGTGTCTACCTGCTGTATCGATGATTACAACATCTACTTTTTTCAATTTTTCCATTCCTTCTTT
AGTGATTTCTACAGGAGTTTTTGTTCTTGTTTCGTCACCAAATAGTGGAACATTTATTTTTTCAG
AAAGCTGTTTTAACTGCTGATATGCTGCAGGCCTGTAAACATCCGCTGCAATTAATCCAGGTCTT
AAACCTTTTTTCTGAATATATCTTGCAAGTTTTGCAGCACTCGTTGTTTTACCACTTCCCTGAAT
ACCGATAAGTAAAATTACGGATTTTTTTGAAGGGTCAAGCTCTAATTTTTGAGTGGTTTCACCAA
GTAATTTTACGAGTTCATCGTAAACAATTTTTACAATGTGTTCTTTTTTTGAAAGTCCCTTTGGA
GGCGCTTCTTCGATAGCTTTTCTCTCAATTTCTTTACTCATATTAAATACTAATTTCACGTTAAC
GTCAGATTGGATTAAAGCCTTTTGAATATCTTTAATTACTTCTTTTACGAGTTTTTTATCGACAA
AAGTAGCGTTTTTAAGTTTGTTTAGTGCATCAGAGAGATTCTGGCCTAATTTATCAAGCATATTT
TCACCAAGTTATTAACAGGTAATTATTCTCCCCATCTCTTAAATATTTTATTTTCAATTTTTAAA
TTGTCCAAAATTCTCCCAACAACAAAGTTCACTAGATCATCTATTGTTTTTGGATCGTTGTAAAA
TCCGGGAGCAGGAGGAATTATTGAAACTCCCAAATGTGATAGTTTTGTCATATTTTCAAGATGGA
TTGTACTAAATGGCATTTCTCTTGGAACGATTATTAAATCCCTTCGCTCTTTTAATGAAATATCA
CAGACTCTTCCAATTAAATTATCGCTGTATCCTGTTGCTATTGATGACAGCGACTTCATAGAACA
AGGGATCACAACTGCTGCTTTAAATTTATGAGAACCTGATGCCACAGGTGCAAAAAAGTTATCGT
TTTCATAGTAGTTATCGGCAAGTTTTTTAAATTCGTCAACTTCCATTTTAAGTTCGTGTTTGATT
ATTTTTTTAGCAGAATTTGAGATTATCAGTGATGTTTTAATATTTTCTTCTTTTAAAACTTCTAA

AAGCCGTTTTGAATAAATTACACCGCTCGCTCCGGTAATACATACTATTATCTCATCCATATTAT
CCCTTCGATGATTATAATAAAAATATTCTTCCGACTTTTTATATATACTAACACCAAAAAGTAAC
CCATAAATTTAAATTTATAAATTATTTCATTTAAAAAAATAGAGATTTTTGAAATATTTAGTTT
TGACAAATAATTATAATTATTTGATTAATGGGGCCTGAAATATTGTTATCAGAAAAATATTTCCC
CTTCAGGAAGCCTGTCCTTTGGTGTTGCACTATCTACCGCATAACCTAATGTTATTATTAAAGTA
GGGACTAATTCAGGAGTAAGTTCCAGAGCCTGGCTTATAGATTCAGCATTAAATCCCTGTATTAC
ACAAGAGTCCACCCCAAGTGATTTTGCCCCAAAAATAGCACTTGCAGCTGTTAAATATACATTTT
TTTGAGACTCTACAATCCTCTGGTCATTGTTAATTTGACCAAACATTCCATCAAGTGCCATAAGA
TAGTAGTTAATGTTCTCTTCTGGAATCTTTGCTTTCCTCATGTTTTCCAGATTTATTTTAATATG
GCTGTCCCAGTCGGCATTTGCACATAATATCAACAGGTGTGAACATGTAGGAACTTGTGTTTTCC
CACCGAATATATAGGGGCAAGCTTCTCTTTGAGATCATTATCACTTACGATTTTAATTTTCCAGG
GCTGGATATTAAGAGCAGATGGACTGTTTCTTATTATGTCTTTTAATAGTTGAATAGTTCCTTCA
TCTATCTTTTTATCACTGTAGTTCCTGCTTGAATATCGCTCTACAGCAAGTTCTTTGAATTCCATA
TTGTATCCCCCCATATGGCAATATAATAATCTCTTGATTGAGTTGTTTATATTACTATCTTGTGA
GATTTCAAAAAAGAAGAGAAGAACCGCAAAACTGCCAAATTTTCGTTAAAATTAATTCTTTGAA
AAAACGCATTAATTTGTTTTAAAAAAATATGCGAGGGAAGGGATTTAAACGCAACCCTTTGAAA
AAGTGTTGCATCCCAAAAATCGCATTAATTTGTTTTAAAAAAATATGCGAGGGAAGGGATTCGA
ACCCTCGAACTCCTATGAGACTAGGCCCTCAACCTAGCGCCTTTAACCAGGCTTGGCAACCCTCGC
ACAGTGAACCTTTGAAAAGTGCTTATTCAATTAAGCATAGTGTAGTTTTGTATTTCAGTATATAA
AACTTTCTACAAAAACTATATATATATTTGTTCTGCTTACTTTAAGATGCTTTGATGAAGCACATTG
CCGAGGTGGCTTAGGCTGGTTATAGCGCTCGGCTCATACGGAAATTCCTAAGGGGTTCTTCCCCT
TGGGTCCTGGGAAACCGAGAGGTCGGGGGTTCAAGTCCCCCCCTCGGCATTTTTCTTTTTGGGTA
AATTAATTTGTATATCTAAATTTATAATTATATTTAATTATAAAAAAAATTAAAAAAATTAAAC
AGGATTTCCAAAGAAATTTATTTTTTCAATTAATTCATTAAATGCAGTTATCTCAAAATCTATTG
CTTCTTCTTTGGTGAAAATTCCTGAAACTTCGCCATCCATTGTTAATTTATCAATTCCCCTTAAA
ACTATTCTTTTCATCCCATCTTTTGTGAATATATCTTCCGCATCCCTTTCGTGAACAAGTGCATTT
ACAGGATAAAGTACAGTATCAGTTACTTGTGATAAATCAACTGTTTCAAGGTCTGTTTTTGTAAT
TAAATCTGCAATATCTTTGTTTACAGGAATAACTTTTATCGGAGTATTTTCAAAAATTTTACTTA
AATATATATAAGAAACATTTCCTGTAATTATCGTTGCTTCTGCCTTAATTTTAGCTCGCAATTCA
TTCAAAATTTCCTCTTCATAATAAATTGCGAATGGCGTGTTTGTTATTGGGTCGTATACTGGAGT
TCCAGAAATTCTTACACTATTTCCAAATAATTCGTGCATTTCTTTTACTAATGTTCCAAATTCTT
CAACAGTTTGAACAGTTATATCTTCAATTAATGGTTCATTTCCAAATATAAGTCCATTTTCAACT
TTATTTGCAAATCTCATCAGTATTACGGCATTCGCACCCCATGAAACGAGGTCTGAAATAGTGTT
TTTTAATACTTCGCCGTCATTCACTCCGGGAACTACGATTATCGCACAGTGGGTTTCACAATTCTC
ACAGAAATATTTTAAACATTCTAGAGATTGTTCTGGATTTTTATCATTCATCCATTCTTTTCTAA
GATTGGGATCTGTCGAAAAAACAGAAAATGTGACTTCATCTACACCGGAATCCACAAGATATTTT
GCCGATTCGATACCTTTTAACCCCTTACCCGATGTATACCCTAAGTGTATCTTTAAACCCATTTCA
TTGATTATTTTGCATAAATCCTCAAGATATGGGTAGTAACTGGTGTCTCCACCGCTTGTAATATT
TACTTTTTCATATCTTTTGAATATTATCGCGTTTTGAACCTGTTCGATTGCAAAAGGCACGGGAA
TAAAGTTTCCATTGATTTCCCTTATCGAATTTGTACAGTATTCGCACCCTATCTGAAATGTGCAC
TGTCCACAGCCTAAAGGTTCAGGATTATTAAAATTAACTTTCCTAAAGTAGCAAAATTTACAAAA
ACCACCACAATTCTTTCCAGGTTCCCCTTTAAGGTCAATTAACAGCTGGGATTTGTCATTTGAAT
CGTTATTATCCGTATCTTCAAAGCCAAAACCCGCATCAATATTATTAAATCCAAACTTTTGGTTC

ATATTACCATCTTATTTAGCTTTATAAAAATAGTAATCAATAATAATCTATTAAAAACATCCGTT
TTATCTCAACAATAGCAGGGTTAATTTCGATTTAACACAGTATTACGTACTAAAAATTGAGATAT
TTAACCTAATCTAAAACTTAATCGATGATGGAGAGTATATATATTTTTCTATTTGAGTTTAATGA
AAACTTGAATATATATTCTTTCAGTAATGTTATGATGTATATATCAAAAAAATAGGAGTGGTTC
CTATGGTAAAGTATGAAGATAAGATAAGTTTGTACGATGCGAAAGGAAACCTAGTAGCCGAAAA
TGTACCATTAGAAGCTATTAGCCCATTATATAATCCTACCATCAAAAACATGGTAAAGAACATTA
AAAGAACCGTTGCTGTAAACCTGGCTGGTATCGAAGGTACCTTAGCTGCAGGTAAAATCGGTGGA
AAAGGCTGTCAAGTTCCAGGAAGAACTTTAGATATATCTGCAGTAAGCAATGCTCAAGCAATTGC
TGACGAAGTAGAAAAAATCTTAAAAGTATCCGAAGACGACGATACAGCTGTAAAATTAATTAAC
GGCGGAAAGCAACTGGCTGTGCAAGTTCCAACCGCAAGATTGGAAGTAGCTGCAGAATACTCAGT
ATCCATGCTTTCAACTGCTATGGCATTAAAAGAAGCACTTATTAAAACATTCAACATCGACATGT
TCGATGGTTCAACAGTGCACGCTGCAATTGTTGGTAACTACCCACAGGTTATGGATTACGCAGGC
GGTAACATTGCATCACTCTTAGGTGCACCTTCAAACATGGAAGGTTTAGGTTACGCTTTAAGAAA
CATTCCAGTTAACCACGCAGTTGCTACAACCAAGAAAAACATGATGAACGCTATTGCATTCTCAT
CAGTTATGGAACAAACTGCAACATTCGAAATGGGTGACGCAGTAGGATCATTCGAAAGACAGCAC
TTACTTGGTTTAGCTTACCAAGGATTAAACGCTGATAACTTAGTTATTGACTTTATTAAAGCAAA
TGCAAAAGGAACTGTTGGTACAGTTGTACAAACCGTTGTTGAAAGAGCTATTGCTGACGGCGTAA
TCGTTGTTGACAAAACAATGAGCTCAGGATTCAACATGTACAAACCAGCTGACGTAAACAAATGG
AACGCTTACGCAGCAGCAGGATTAGTTGCAGCAGTTGCAGTAAGCTGTGGTGCAGCAAGAGCAGC
ACAGAACGTTGCTTCAGTTATTTTATACTACAACGATATTTTAGAGTACGAAACTGGTTTACCTG
GTGTTGACTACGGTAGATCAATGGGTACTGCAGTAGGATTCTCATTCTTCTCACACTCAATCTAC
GGTGGTGGTGGACCTGGTATCTTTAACGGTAACCACGTTGTAACCAGACACTCCAAAGGATTCGC
AATTCCTCCAGTATGTGCAGCAATGTGTGCAGATGCAGGTACGCAAATGTTCTCACCTGAACACA
CTTCAGGATTAGTTGGTTCAGTATACTCAGCATTTGATGAATTCAGAGAACCAATGAAATACGTA
ATTGAAGGAGCTTTAAGCATTAAAGACCAGTTTTAAGGTAATAAACGATGATTGAATTAGAAGT
CTTCCCACATAGGTATTTAAAAGCAGAAACTACCGAAAAATTCCTGAACCGCGCTTATTCATTGA
GTAAAGTTCAAAGAGTAGTCATACATGGAGAATCTCTTCCTAATAAAGTTGGATACGGTCCAGCT
AAAGGAACTCCTGTAAATCACTCAGAAAAAAAGGAAATTACTGTTAAGGGAGTCCCTGTAGAACT
CAAACTTCAAGTTGGAAGGTTATGGGTTTTACTGGATGATGAAAGCGAAATTGAAAAAATCGAA
GAAATCTGTAAGGATCTTTTCCCATTTGGATACCGGTTAACAAAGGGTAAATTCTTAAGGAACGA
CCCTACAGTATCGGATTTCATAAAATACGGGGAATCTGCTGTCGAGAATATTGACAAAAGATTAT
TGGGTGCAACTGATCCAAGAAGTAAGTTTGATTCTTCTGTCACAATTATCCCAAAGAGCGAAAAA
AACGAGTAAGTGATTTTATGCCAGTAGGTAGAAAAGAGCAGATCGTTGATTGTAGAGCTGTTAT
GGGTCTTGGTGAAGGAGGCGGACTTGCACAAAGAGGAACATTTGCAGAAGGGCTACGTAACGACG
TGGTTGTTGTTGCAATGTCTCCAGGGAGAAGGCACATTACAAAACCCGTGTGCGAAATTACCTAT
GGAATAAGGGAAGCGGGAATTCAGACCAGCGTTCTTGTATTGGATGCAGGAGGGGGAATTCCGTC
AGATGCTCCACAAGGAAGTTTAGGTTCCACTTTTGGACTTAAACCCGAAGAAGCTAAACAAGTTA
ATAGGCATAAGTTATGTGTAATACATTTTGGAAATGTAAAAAGCCACATAATTTACAAAGCTAG
ATTATTCTTAAAATACGTGGATATTCCTACAATAATTGTTTGTCAAGCTCCCGTGGATATGGAAG
ATTTCGCAGCAATTGGAATAAAAACGAAAAATGTAATGCCTTTGGAATCAAAAACTGAAGGTAA
AATCGTTGAAATTATTACTGGCGTAATAAGAGGAGAATCAGCTCCTCAAAAAAAAATTGATGAA
ATCATTGAAAGCATAAAAAAACATTTAGGTTAAGGTGATCTTATGGCATACACGCCTCAGTTCTA
CCCTGGTGCAACAAAAGTTGCTGAAAACAGACGAAATCACTTGAATCCAAACTATGAATTGGAAA

AATTAAGAGAAATCCCTGATGAAGATGTTGTAAAAATCATGGGACACAGACAACCTGGTGAAGA
CTACAAAACAGTTCACCCACCACTTGAAGAAATGGACTTCGTAGAAGACTACGCAAGAGACTTAG
TAGAACCATTAAATGGTGCTAAAGAAGGACACAGAGTTAGATACATTCAGTTTGCTGACTCAATG
TACTTTGCTCCTGCTCAACCATACGACAGATCAAGATCATACATGACAAGATTAAGAGGAGTAGA
TGCAGGTACACTCTCAGGAAGACAAGTTGTTGAATGTAGAGAAAGTGACCTTGAAGAATTCTCCA
AAAACATACTCATGGATACAGAACTCTTTGACCCTGCTACATCAGGTATGAGAGGTGCAACCGTA
CACGGACACTCCTTAAGATTAGACGAAAACGGTATGATGTTTGATGCACTCCAAAGATGTGTATT
CGACGAAAAAACTGGACACGTTATGTACGTAAAAGATCAGGTAGGAAAACCACTCGATGCACCTG
TCGACGTTGGAGAACCAATCCCTGAAGCAAAATTAAGAGAAATTACAACCATCTACAGAAACGAT
GGTGTTGCAATGAGAACAGATCCTGACGTTATCGAAGTTGTAAAAAGAATTCACAGAGCAAGAA
CACTCGGTGGATACATCCCTACAAACGAAACATTCAAAGGATTATAATTAAATTCATAAAATAAA
AAAAACCTCTTAAGCCATTGAGGTGAACTTATGGAAGCTGAAAAAAGATTATTCTTGAAAGCATT
AAAGGAAAAATTCGAAGAAGACCCTAAAGAAAAGTACACGAAATTTTATACTTTTGGCGGTTGG
GAACAATCTGCTAGAAAAAGAGAATTTGTTGAAGCTAATGAAAAAATAGTAGCTGAAAAAAGAC
AAGGAATCCCTCTCTACAACCCAGATATTGGTGTTCCTCTAGGACAAAGAAAATTAATGCCTTAC
AAATTGTCAAACACTGACGACTACTGTGAAGGTGACGATTTACACTTCCTAAACAACGCTGCTAT
CCAACAATTATGGGACGATATCAGAAGAACTGTTATTGTAGGTATGGACACTGCTCACTCAGTTT
TAGAAAAAAGATTGGGTGTAGAAGTAACTCCTGAAACAATCAACGAATACATGCACACAATCAA
CCACTCCCTTCCAGGAGGAGCTGTTGTTCAAGAACACATGGTTGAAGTTCACCCTTCCCTTGCATG
GGACTGTTACGCTAGAATCTTCACCGGTGACGATGAATTAGCTGATGAATTAGACAGCAGATTCT
TAATTGACATCAACAAATTATTCCCTGAAGAACAAGCTGAAACATTAAAAGCTGCAATTGGTAAA
AAAACATACCAAGTATCAAGAGTTCCATCACTCGTAGGTAGAGTATGTGACGGTGGTACAATCTC
AAGATGGTCTGCAATGCAGATCGGAATGTCATTCATTACCGCATACAAACTTTGTGCAGGGGAAG
CTGCAACAGCTGACTTCTCATACGCTTCAAAACACGCTGATGTAATTCAGATGGGTAATGCTTTA
CCAGGAAGAAGAGCAAGAGGTCCAAACGAACCAGGAGGAATCAGATTTGGTATTCTCTCCGATGT
TGTACAAACAACAAGAGTTTCAGAAGACCCAGTTGAACAATCATTAGAAGTAGTAGCAACAGGTG
CTGCTTTATATGACCAGATCTGGCTCGGTGCATACATGTCTGGTGGTGTAGGATTCACTCAATAC
GCTACCGCATCATACACCGACGATATCTTAGATGACTTCTCATACTACGCACTCGACTACGTTGAG
AAAAAATACGGTAGAATGGGAACAAAAGCTACAATGGATGTAGTAGAAGATGTTGCTGGAGAAG
TTACACTCTACGCATTAGAACAGTACGATGACTACCCAGCATTATTAGAAGACCACTTCGGTGGA
TCACAAAGAGCTGCAGTTGCTGCTGCTGCATCAGGTATTGGTGTATGTATGGCAACAGGAAACTC
AAACGCTGGTGTAAACGGCTGGTACTTATCACAAATCTTACACAAAGAATACCACAGCAGACTCG
GATTCTACGGTTACGACTTGCAGGACCAGTGTGGTGCTTCAAACTCACTCGCAATCAGAAACGAC
GAAGCTGCACCTCTTGAATTAAGAGGACCTAACTACCCTAACTACGCGATGAACGTAGGTCACCA
GGGAGAATACGCAGGTATTGCACAAGCTGCACACTCCGCAAGAGGAGACGCATTTGCACTTAACC
CATTGGTAAAAGTTGCATTCGCTGACCCAATGCTCGTATTCGATTTCTCCAAACCTAGAAAAGAA
ATTGCTAGGGGAGCATTAAGAGAATTCGAAGCTGCAGGAGAAAGAGACGTTATCTTACCTGCTAA
ATAAATTCTATAATTTAAGGGAGCCACTTGGCTTCCTTTTTTTATACTTATTATATATTGTTTCA
TAATCGTTAACAATATAGATTAATTAAAATATACTTTAATTAAATTAAAGAAAATATTAGACGT
TATTCGATGAAAAACGGAATACAAAGTAATATATATCATATAACAAGATAATAACTGAATAGCT
AACGGCATAGATATATCTCTAGGCTTAAAAAGCCAATAAAGGCATAGCTAACAGTCCCTTATATT
GTGAGGTGAAATAATGGATCCAACATTGATTTCTCTGGGGGCATTGGCCCTAGCAGGAGCAGCTG
CGACTGTTTCTGGTTGTGCTGAAGACTTGGAATCCGACGTCGGTTCACAGTCCAACCCTAACTCTC

AGGTTCAATTAGGTCCGCAAATGGGCAATATTCACAGATACTTCAACAAGGCTATATCTGGGGAA
CCTGTATCATACGGTTTGTATGTTGCAGTTGCAGGTACCATTGCTTGGGCACTAATAAACGCAGG
ATTAAACGTTGTTTTAGCGATAATCGTGGGTGCAGGTGTTGCTGCAATCGTACACGGTGCTTACT
CAGTTAGTGCATTCCTTGGAAGAACTGTAGGTCAGTCCAAAAAGTTCGGACAACCTGTTTACATG
GATGTTCTTACATCACACATAGGTCCAATCGTAGGACACGGATTTATAGCTGTTTTCACAATGAC
TCTTGCTGCATATTTAGCAACAACAGCATTAGGAAACCCATTCCCATTACCATTGGTTTCATTGA
TCTTCGGTATTACTGTAGGTGCTATCGGTTCATCAACTGGTGACGTTCACTACGGTGCTGAAAGA
GAATACCAAAAATACCCATTCGGTGGAGGTATCCCTGTTGCTAACCAAGGTGACATTGATATTTA
CGCAGAATACGGTGTAAGAAATGGTTTAGATTCTTCATACTTCTGTTCAAGATTTGGTGGTCCAC
TTACAGGATTATGCTTTGGTTTAATTATCTTCCTCGATGGCTGGAGAAGCATTCTCGGTAACATC
CTCGGTGGAGACTTAGTAACAAAAACATCAATCGCACTTTTAGTAGGTCTCTTAGTAGTGGCCGT
TGCAGCAGTTATCAACAGGAAACTCGAGGTATATGCTAGAAACAAATACGGACCATACAGAAATT
AATTAAAATAAGGAAGATTGGTGATATTATGGATGCTACAAGCTTTATATTACCTCTTGCGGAA
ATAACCATTGCGGGAGCGATTATCAACGCAAGTGTTCACTTCGTTCCAGTAGGTGGTGCTCCAGC
AGCGATGGCTACTTCAACAGGGGTAGGTACAGGTACAACACAGTTAGCAGCAGGTGCAGGTTTTA
CCGGACTCTTAGCTGCAGCTGCAATGGCTTCGCAAGCAGGTGTTTCACTGTCTAATCCAGTTCACA
TGTTATTAATCATGTTATCTGGTGCTGTCGGTGCAATGATCATGTTAGGTCTTACAATGTTAATT
GGTCAGATTATCTATGTATACGGTATTGGTATCGTCCCTGCAGCAGATAAATGTGAAAAAGACCC
TATTACGGGAGATATTCAAAAACCATACATTACACCAGGTACAACAGGACACGGTATTCCAACCG
TATGTTTTGTAAGTGGTACAATTGGTGCAGCTCTTGGTGGTCTTGGTGGTGCTCTTGCATACATC
GCACTCCAGCAGTTAGGATTTGCTGCAGCTATTGCAGGTGTATTGGCAGTTGGATTCTTCTTCAT
GAACGCAGTATTAGCTTCATACAATATTGGTGGTACAATCGAAGGATTCCACGACCCTAAATTTA
AGAAAATGCCAAACGGAGTTATTGCTTCATTTGTTTCTTCATTAATCGCAGGAGCAGTATTAATT
GGAATGGCTATGGGACTTTAAGTCTACCATGGTTTTCAAAAATATTTGAGGTGTAATTATGTCAC
ATGGTGGTGGAGGACACGCAGCAGAGCTTTTTCCTGAAGATCAGGTTTTAGCTATCGGCGCTGTA
TTATCAATCATCGGAATGTACGTTGCACAATTTGTTCCGTCATTAGCTATGCTCATTGGTGGGTT
ATTAGCTGCGGGCGCTTGTGTTGCAGGTGCAAACACTACAAGAAGAGTAGCCGCTTACGGATTAG
GTACAGGTGTTCCATCAATTGGAATGGTAAGTTTAGGTATGGGTACAATTTCAGCATTGGCAGGT
GTTTTAATACCTTCAGCATTTGGACTCCCAGTACTTGCTACACCAATCCTTGCTGCAGTAATCGCA
GTAGTTGTTGGATTTATTGTTGGTAAATTAACACAAAATCCAGTAGGAATGAAAGTACCAATTA
TCGTTTCGAGTATGACTAAACTCTCATTAATGGGTGCTTTAGCAATTTTAGGATTCTGTACTGCA
TTTGCAGGCGGATTTTCAGCTGATTTAATTATTAATGGTGCAATCAACAACGGAGTTATTGCATT
GGCATTTATTGCAGCAGGTATGTCAATTTTACACCCATTCAACGCATGTATCGGCCCAGATGAAA
GCCACAAGAGAACAATTACTCTTGCAGTTGCATGTGGATTGATGGCATGGTTGATATTCTCAATT
GCAAAATTAGACATAGTTTCAATAGTAGTAGCTGCAATCTTCTGGTTATACACTTACGGATCATT
CGTAAAAATGTCACTTGGTGACGCTTGCGAAGTTAAATATGTACCAGAATTGCCTAAGAAAGAGT
AAGGTGGTATAGATGGATATCGTCAAAGTATGTCCTGAACTTCACATAGTAATGGACGTAGACTC
AGGTTTAATAGCCGAAATGAGAAAAGACATTCTCGTTGTAGATTTACACCCTGTTGAAGACGAAA
TAAACAAATTAGCACAATACGCAAAAGCATTAGAAAATTCACTCGATCCAAGAAACACACCTATG
AAAGCATACGCTGGAAGGGAAGGAACTTACAAACTTGCAGGTATGTTCCAAGGAATGTTCTTTGG
TTTCTGGGTAACAATGGCAGTTCTTGTGCTTGTAACTATATTGGCTGTTAAAATGAATCTAAGCC
TAATAGGGTTGTAATCTGAATAGGAGGTGCAGACAATGGCAGATAAAAAAGCCCCAGCAGCAGG
ATGGCCTGTTGCTAACGGTGAATATGTAGTGGGTAACCCTGAAAGCTGTGTTGCAGTAATTACAC

TCGGTTCCCACGGTTTGGACCAGGCTGCAATTGACGCTGGTGCAGCTATTTCAGGACCATGCCACA
CAGAAAACTTAGGAATTGAAAAAGTTGTTGCAAACTACATTTCAAACCCGAACATTAGATTTATG
GTAATTACTGGATCAGAAGTTCAAGGACACATTACTGGACAATGTATCAAAGCATTATATGAAA
ACGGAATTGGTGACGATGGTGGTATCATCGGCGCTAAAGGAGCTATTCCTTTCATGGAAAATGTA
GGAACAGAACCAGTTGGAAGACTTCAAAGCCAAATCGTTGAATGCATTGATTTAATCGATGTTGA
AGACACTGGAAAAATTTCAGATGCAATCAAAAACTGTATCTCAAAAGACCCAGGAGCATTTGAAG
AAGATCCTATGGTTATTGAATTAGAAGGTGGCGCAGCTGCTGCTGGTGAAGAATCCACTTCAATT
AAACCTACTTCACCTGAAATGGCGTTATTAGAAGCTAGAATGAGAATTGTCTCCGAAAAAATGAA
TGAAGCTGCAATGATTGCTAAATTTAACTCAGGATACTACAACGGAAAAATCCAAGGTATTGCAA
TAGGATTGTTCCTTTCAATTGTGATATTCTCATTACTCTAAGGAGGAGGTGCAGACAATGGCAGA
TAAAAAAGCCCCAGCAGCAGGATGGCCTGTTGCTAACGGTGAATACGTAGTGGGTAACCCTGAAA
GCTGTGTTGCAGTAGTTACACTCGGTTCCCACGGTTTGGACCAGGCTGCAATTGACGCTGGTGCA
GCTATTTCAGGACCATGCCACACAGAAAACTTAGGAATTGAAAAAGTTGTTGCAAACTACATTTC
AAACCCGAACATTAGATTTATGGTAATTACTGGATCAGAAGTTCAAGGACACATTACTGGACAAT
GTATCAAAGCATTATATGAAAACGGAATTGGTGACGATGGTGGTATCATCGGCGCTAAAGGAGCT
ATTCCTTTCATGGAAAATGTAGGAACAGAACCAGTTGGAAGACTTCAAAGCCAAATCGTTGAATG
CATTGATTTAATCGATGTTGAAGACACTGGAAAAATTTCAGATGCAATCAAAAACTGTATCTCAA
AAGACCCAGGAGCATTTGAAGAAGATCCTATGGTTATTGAATTAGAAGGTGGCGCAACTGCTGCT
GGTGAAGAAGAAGGAGGCCTCATGATTGAAGGTATCCCTACTGTAGCAGAGCCAGACATTGAATC
AATGAAATCATTAAATGAAGCATTGGATTACAAAGTAGGATTGATGACAAGAGACATCGGATTA
GCTTCAGGTGTTCAAACTGAAACCGTTACAGGATTAATGTACGGTTCAGTATTTGCTGTCGCATT
AATTGCAGTCCCAATAGCTTTAAAATTCTTAATGGGGTGAGTAAATGTCTGAAATTCCAACAGTA
GTCACACCAACAAAAGACTACAAAAGACTTCAAGCAAAACTCGATGAAATCGAAAACACCGTTGA
AAATACCAACGCTGAAATAATCCAGAGAACCGGTAAAAAGGCAGGAAGAGACGTAGGTATTGCC
TACGGACTCGCAATAGGATTTATATTCGTATATGTTCTCGGAACCGTTTTACCATTATTTGACTT
AATAAAATAAGAGAGCCTAAACAGAGGTGGAATAATGTTTAGATTTGACAAAGAACAAATGGTC
ATTGAGTTCGCTGGTGCAAAATTCGGTGGACAACCAGGAGAATACCCAACCGCATTATCCGGTAC
AATTTTCTATTCAAGACACAAAATTGTAGAAGATGCTAAAAAAGGTATCTTCGATAAAAAAGCT
GCTGAAGCTTTAATCAACAAACAAGCTGAAATGCAGGACATTACAGGAAACTCAGCATTCGTGCA
AGTTTTCGGCGGTACTGAAGAAGCTTTAGTTAACTACATTGATTTTGTTTCCGAAGTATGGGACG
GTCCAATGTTACTCGACTCAACATCCGGTAAAGCAAGAATGGCTGCTGCAAACAGAGCTACAGAA
GCAGGATACGCAAACCAGTGTATCTACAACTCAATCAACGTTGCTGCAGAAGACGAAGAAATTGA
AAACTTAATAAACAGTGATGTTGAAGCTTCAATCGTTTTATGTTTCGACCCAATGGATCCATCCG
TTGCAGGTAAATTAAACGTATTAAACGATGGTGGTAAAACAAAAGACATCGGTATGTTAGAACT
TGCGGAAAAAGCAGGTATTAAATACCCACTCATCGACGTTGCAGTAACCCCAATGGGTAACGGTG
CAGGTCACGCTGTTAGAGCTGCTTTCGCAGTTAAAGCAAAACTCGGTTTACCTGTAGGTAGCGGT
ATTCACAACGTACCTTCCGCATGGGACTGGCTTAGAGAATTCAGAAAAGGATTAAGAGAAGAAGG
AAAAGATCAGATCGCAAAAGACGTACACCACGTATGTGACATCGGTGCAAACATTGTACAAACAA
TGACATCAGGTGACTACGTTTTATACGGTCCAATCGACAATGCTGAACTTGCATTCCCTGCAGTT
GCAATGACCGATATGATCATCGCAGAAACAGCAAAAGAAATGGGAACAGTTCCTGTTGCAGAACA
CCCATTAAACAAATTAATTTAATTAATTTGTTTCTTCTTTTATTTTTGGAAAATAATATTTATAA
ATTTAAATTTATGAGTTATAATATATAAAAAAAATAAAAAAGTTAGTTATCTTGAAATTTCCAG
ATTACTGAAAAATCTTTTAATGCAGAAGTTATTCGCTCTTTTACGCCTTCTACAAGGATTTTTTG

TTCATCATCCTGACTTAATTCAAATATTACACCGTTATGTTCACTGAGTTCGCCAAGAACCTCTA
TTTGAACTTCTACAGGCATTGTTATTCTTATTGTTTCAGATCCTTTTTTTCGTTTAACTTCAGTA
TTAAATGCAGTCTGTTTCAGTGGAAATTCAAGATCGATTTCATTTCTTAATGCCTGACCCATTTT
GATCAGATATTCTTTTGAAACATCCTGGTCAGCCAATCCAAACAGTATTACCTGTATTTTAGAAA
TTTCTTTTTCAGCATCTCTTAAAACTTCAAAATCTGCATGTTCGTCAAGTAAATAATAAGTAATG
ATATTATTTGCAATCCTGAGTTTTGAATAAACATCCTCAGGAAGTGGCCGAACTTTAGAAATCTT
TGATGCCAGTTCGTTTAGTATTACCCATTGTTTATCAATCCCCTTGGCATCCTTCATTATTCATCC
TCTCTACATAATTTTTAATTATTTCTTCTGAATTGTGGTTCAATACTGCACCGTATTTTTCCAGA
TCTGCCTTTATCTTTTCAATGTCCGTATCAGGATCTACAAAGTAAGCTTGGTAACTGGTAGTTCC
TTCAATATTAAGTATTGCCACATTTTGATTTGGGTCAAGTTCCCTATCATCATGAGAAGCCCTAA
CACGAACAGTTCCCGAAACTTCGGAATTCAGTGCTTCTTCCACGGTTATAATCGATATCAATTTT
GCAGTAAATTTTACCATCATATCACCCTCAGATACGATATAACCTCTTTGTGTTATTGTATATAA
TTTTAGTGATTTCATCTAAAGCATATTCATCCTTTTTTAATTCCCATATCTTTTCAATAACTTTT
ATTACATTTTTTGGCTCGTTTTTCTCACCTTTTATTGGCGAAAGGTAGGGACTATCTGTTTCCAC
CACGACATTTTCAAGACCTAAATTTTTAACGAGATTTTTGTGGTGTTCTGAAAAGCATACCAATG
TAGAATATGAAATAAAATTACCATTTTCGATTAGTTCTTTTGCAAGTTCTAGCGAACCGCTGTAG
CAGTGGTACATTGAAATCACGTCATTCTTTAAAATCTCGTAAGAACGCTCTTCCATACCTCTTGC
ATGAATTACTACTGGTTTATTTAATTCTTCTGAAAGATTTATAAATTTTTTAAATATAGTTTCCT
GTCTTTCAATATTTTCTGCATCAAAATCAAGCCCAATTTCCCCAACTGCCAAAATTTCTTTTTCTT
TTGTTCGAATAAAATTATAAACTTTTTCAATTACATTATCATCCGCATTAACTCTTCCAGGGTGA
TATCCTAAAGTTATGTAAATTCCATATTTATCTTTTAATTCAACTGCTCTTCTACAGCTGCCTAA
ATTTGTACCGCTTGTTACCATCTGCACTTTTTTTTCAAATGCTCTTTTTACGATATCTTCACGATA
TTTATTGAGCGATTTATCTTCAACATGGCAGTGTGAGTCGATATACTTAAAATCCTCTAAATTCA
TAAAAACACCTAAATCATAATTAAAAAACCTATTATCAGGATATCTAAAATTATTGTAACTCCTG
AATTTAAAATTATTACTTTTGTACCCATTTTAGAACCAAACAGTGAAAAATGAAGTGGAATCGA
ATGTTTTACCGATCTTGTAGAAAATGCCACAACATTTCCAAATATAAGGCCAATTAAAACTTCTG
TTTGTGTTAATGAGGCTTCATTTAAGAATGTTCCTGCCATAACCATTGCAGCTGAAACATTTATT
ATCTCTGTTATTGCCAAAATTCCAGTATTTGGATTTAAATTAAATATATTTGTTACAGGCATTAC
AATTATGGAAAAAACATTGAAAAACCCAGTTTTTGATAAATACAACACCAAAAACATTGAAATA
AACATTACTGGAACAAATCTTCTTGCAAATTTAAATGTCTTTTCAAAAGGACTAATTTTATTATT
TATTTTATTTCCTCTGCTTTTTATATTTCCAGTATTTTTTCTTGAGATTACTAATAAAACGAATA
ATCCCAACATGCTTTTTGTAAATGCGATCGAAAGCCTTAAAATTACATAAATTATTCCAGTATAT
CCTAAAATTGGTATTACTACAGGGATATAATACGTTAAAACGTGTGAAAGTACTGCGGGAAACG
AATTTGCCAATGTTCCTGCCAAAACTTCTGTTTGTGTTAATTCTTTTTCAGTTATCCCATCTGCAA
GCATGGTGTAACCAACAGTAGGGCTGAAAAAGGAAACGAGTATTGATGAAACTAAAAAGCTGTT
TAAATTAAGTTTTTTGGTTACAGGGGATGCATAATCTGAAATTTTTTCCAAAAGCCCTCGATTCA
CAAAATAATTTACAATGTAAATGGTTAACAGTACAACTACAGAAATCCTTACAGTATACAAAAA
AGAGCTGTAAAAAGCGCCTGCAATTAATGTACCATCCAAAGTATCACCGGGCAATCCAATAAATA
TAACTTTTGAGTAATCTTGATAATAATTATATATACCCATATATCATGTTGCTGGTTTTAAAATG
ACAATCAATAAAAAAATGTAACGTAAATTACCTTAAAAAACAGTTAACCATAATTAAAGGAACCG
AAAAGATCGAAATCGAATTTAAAATTGATGAAATTGGAAAAAAATGGCAAAAAATAATAGAAGA
AACTAAAACCGATTTTGAAATTGATTTAATTCTAAATTTAATCGACATTCAAAAAGAAAAACTG
GTATCCGAAGTTTTTGAAAAAGAAATTTCTAAAAAAGATAGAGAATTACTACTTAGCAAAATTT

ACGAAATAAGAAAAAAATTTAGAAAGTTTAAAAAAATAATTATTTTAAATTTAAAGCAATTTCTC
AATATTTATCGAGTAATCATCTTTATTTTTTACAACACCAATTATTTCAACGTGTCCAAACTCTT
CAATTGCTTTAAAAGTTTCTTCATAATAGGGAACGTCTTTTAATTCATCAATGCAGTTTATAATA
ACTCCTTTTACCAATATTCCTTTATTTCGCAAGTATTCAACTGACAGTATCGTGTGATTAATTGT
TCCAAGATCCGGCCTTGTAACCAAAACACAGGGTAACTTCAAATCTTTAATTAAATCCGCCATTA
AATAATCTGTTTTTATTGGAACGCATACTCCGCCGGCCCCTTCAACAATTAAATATTCGTGAGAT
TCTTTCAATTTTTGGAACGATTCTTTTATTTTGTTAATATTTATTTCAACATCTTCAACTGCTGC
TGCGATATTTGGAGATAGCGGATTTTTTAAGTTTATTGGATTTAAAAGTCCCAAATCTTCTGAAA
GTTCAAGTTCAGTTTTAGCATAAGTTGTATCTTCAACCCCTCCTGACTCGATAGGCTTTATATAT
CCAACATTTACACCCTTTCCTTTCAAAATCTTTCCCAAAATTACTGAAACATATGTTTTTCCAACT
CCAGTATCTGTTCCCGTTACAAATAGCGTTTAAATCACCCATTTTTTATTTTTGAAATACATTCA
CACAAATATTCAAAATCTTCCTTTTTATTTTCTGCGGTTATGCTCAGCCGTATTCTTTCAGAACCT
CTTTTAACTGTGGGGTAGCGAATCCCTACGCAGAATATTTTGTATTTTAAAAGTTCTTCTGAAAT
TTTTATCGAGTCTTCACCAAATATAAACGGATATATCGGAGTAATCTTTTCTTCTTCGATAAATC
CCGATGATTTGAAAATTTTATTTGCAGTATTGATATTTTTAGATAATTTATCTGTTAATTCACCA
GATTTAATTAATTCAAAGGATTTAATACTTGCAGAAATTACTGCTGGAGGAAGAGCTGTTGAAT
AAATAAAACTTCGAGAAGTATTTATTAAATAATCGATTAATTCCTCGATTCCACTTACAAATCCC
CCGACAGTTCCGACTGCTTTTGAAAGGGTTCCAATTTGAATAATATTGTCAGTTGGTTTTATCTT
AAAATGATGAAGTGTCCCTTTTCCGTTTCCTAAAACACCCGTTCCATGTGCATCATCAAGAATTA
ATACCGCGTTATATTTATCTGCTATTTTTTTTAATTCATCTACTGGCGCAATATCTCCATCCATGC
TGAAAACTGCATCTGATACAATAAATTTATTTTCGTAATTTCTGCTATTTTCTAAAATTTCTATT
AACTCGTGCATGTTATTGTGAGAGTAAATTTTTTTGTCAGCTCTTGAAAGCCTTATTCCATCAAC
AATGGATGCATGGTTTAGTTCATCGCTTAATACCAAATCCCCTTTTTTACAGAGCGAACTTATAA
CACCTAAATTTGTACCGTATCCTGAAGAATAAACTAATGATTTTTCAGTTCCTTTAAATTCAGAA
ATTGTTTCTTCTAAATTTTCGTGATTTATATTTCCAGAGGTAAGCCTTGAACCAGTTGCCCCTGC
CCCGTAGTTCAATCCATTATTATAAGCTTTGATTACTTCATTATTTTTTGATAACCCTAAATAAT
CGTTTGTAGAAAAGTTTAAACATATATTATCTTCTAATTTTAATTTTCGATATAGATTTTGATTT
TTTATCCAATCAAGCTCTTTTTTTAAGTTTTTTCTAAACATAAAGACACCTAATAAACAATCACG
GGCATATTTTCAAGTTTATCGTACAGTTCATCGATATCAGAACTTTCAGCAAAGAATACCCGTCC
TCCAACCTGGCCACTATCTTTAAGATTTAAAATTCTAAAGTATCCATCCTTAGTTGCAACGTAAC
CTGTCGTATAGGAAAAGTTATCAGAAGTGCATAGTTCTGCAATAATGTCACAGTTTACAACTTTT
GTTGCAATTGCAATTGCATCAACAGTTCTTTCTGTTAATGACTCATCTTTTAAAATTTTTTTAGA
TAATTCTTCAGCCGTATCAATATTTCTTACGCGAATCCCCCTATCTGAACTTTTTTCAAGCCTATT
TCCCTCTAGATCCAAAATTGCAGCCCCCCGCATATTTCCTGCATCGATAATTTTAAAGGCAGAATC
TATCAATACATCGATTTGTTCAATATCTTTTCCATGTTTCAAAAAATATTTTTCTAGCTCTTTTT
TCAAAATATTTCTTGCAAATTCCCTAGAAGTCTCTTTCGAGTTTGAATTAATCGTTTTTATCTTG
AGGTGCTCAATGTAATTTATCTTTTCAGTAACTTTTTCCACTTTCAAATTTATAAAATCGGGAAC
CCCATTTTCGTGGTTCATTGCACGTTTTATCAATTCTGAAGAAATTTCTTCAATTTGTTCTTCTTT
AACTAACCGTTCAGCTCCTGAAACGTGATTTCCATTACTTGAAGCTCTCATTTTTAAACTAAACA
TTTTTTTAATCACTTAATTTTTCTTTAAATATAACTCAATTTTTTTGTATTTATCATTATCGATA
ATTATAATAAAGACGGATAATCTGTCATTATTTTTAATTAAAAACATCTGTCACGATTTATAATG
AGCAAATAGCTATATATAGGGAGTATTTCATAGTAATAGCTAACAACAGGTGAAATTATGATAA
ATACAAAAGTTAAAACCCAAGAATTAGACTTCAATGAAATCGATGAAATTTCAAACGACCTTAA

AAGACCATACCGCGGAAACAATGCCGTAAAATACGCAGCTTACGCGTTCAACGTAGTAGTAATTG
CATCTCTGTTCTTACACTAATTTAAAAAAGAAAAATTATTTAAGAATTTTACCCATATACGGACC
GACTCTTTTGTATCCAAGTTTTTTATAGTATTCTCGAACACCTATCCCGCTATTGATTAATACTT
GATCTTTATTGAACTCTCTTGCGATTTTCTCAGCTTCGTCAATTAAAAGTCTGCCGTAGCCTTTGT
GCTGCCACGAAAGTTCTTTTGAGCTACCTTCAAGTTCTTTCTGCTGTCCGCAAACATGAACCTGTC
TTATAAGTGCAGATTTATCAGTTATTTCTTTTCTAAATGGATTATTTGGAATCCTAAGTCTTAAA
TAACCTATCAAAAGATCGTTTTTAGTATCTTCAAACGATAAGAATATCTCTTTTCCGCCACTTGA
TTCGTAATCTTCCCTGCATAATTTAATACTTTCTAAATCTGGCTTTATTCCTTTTTTGTATGTAA
CGTGTCCAACTTCCCTGCATCGTATACATTTACATTTTATTCCTTTTTCTTCCAAATTATTGTAAA
CGAGTTCTCCAAGGTTACTTTTCTTAACCCCTGCATCAATTACAGTTACGGGAATATCTCTTTGA
ATTCTTGACGTCCTGACCCATTTTGGCATTATCGATTTTCCGTAAGTAATAACTTCTACTGCCTGT
TCATCAGTCATTGGATTAAATTCCCCACTCTGCCATAATTCGTATACTTCTGTTCCGGGAATTAC
GAGGCACGGGTAAAATTTAATCAGATCTGGTTTGAAGTTTGGATTTTCAAATACTGTTTTAATTG
TTTCTTTATCCATTTTTGGAGTTGTCTTTGGAAGTCCGGGAATTAAGTGGTATGAAACCTTTAGT
CCACTATCTTTTAAAATCTGGGTTGCTTTAATTGACGCATCAGTTCCGTGTCCTCTTTTTACAAAC
TCTAAAACTTCATCGTTTGTGCTTTGAATTCCAAGTTCAACTCTTGTTGTTCCCAATTTTAGCAT
TTCATTTATTTCTTTTTCATTGCAGTAATCGGGCCTTGTTTCAAGGGTGAGGGCCACACACCTGT
GCTTTGCAGTTTCGTTTTTTTCCTGTGCTTTTTCAAGTGTTTCTGAAACTTCACCATTCATCGCAT
CAAGGCATCTTTTTATGAAGTTTTCCTGATATTCAATGTCTCTTGCTGGAAAAGTTCCGCCCATT
ATTATCAATTCTACCTTATCTGTTGGGTGACCCACTTTTTCAAGCTGCGAAAGCCTTTCACTTGTC
TGAACGTATGGATCAAAGTTATACATCACTCCACGCATTGTTGCAGGTTCTTTTCCAGTATAACT
TTGCGGAACATCTCCAAAATTACTTTCTTTTCCACCAGGGCAGAACGAACATTTTCCGTGAGGGC
ATGGTTCTGGAGAAGTCATTACTGCAACAACAGCAACTCCTGAAAGTGTTCTTATTGGTTTTTTT
CTTAAAAGTGGCAATAATTCCTTTTTTTCCTCTTCTGAAGCCTGACTGATAATATCCGAATTTGG
AGGAAATCCTACGTTTAAATCAAATTTACGAAGACATCTCGCCTTGATATCCTCTACTTTTTGTT
TTTTCCTCTTTGGATCCAGATCCTTTATTTTTTCCTTTTCAACTAAAAGATTAGAAATGATACAA
CGTATAAATTCTGAATAGTCGCTCAAAAAATCACCTGTCTCGAAATAATAAACAGTAATATATAA
AAACAGAATATAACTTTATTTAGTAATTCTTACTTCGTTATCTTATTAAATTATATTTATGTTTA
TCTTTTTACCGGAAATTGGTGGTTTTATGAGAGCTTTTCTAATCGGCAGGTGGCAGCCATTTCAC
AAAGGGCATTTAGAAATAATTAAAAAAATCTCTGCGGAAGTTGATGAGATAATTGTAGGTATTG
GAAGCTGTCAAAAAAGCCATACTCTAACAGACCCGTTCACCGCGGGAGAGAGAATGATGATGATT
ACAAAAACCCTTGAAAATTACGATATAAACTATTACGCAATTCCAATTATCGATATAGATTATAA
TGCAGTTTGGGTTTCATCTGTAGAATCATTAACTCCGCCATTTACTACTATTTACACTGGAAATT
CGCTTGTTAGAGAATTATTCTCTGAAAGAAATTATGTTGTTAAAAAACCAGAATTATACAATAG
AACAGATTATTCAGGAACAAAAATAAGGAAAAAAATGTTAGAAGGCAGTGCTTGGGAACACCTA
GTTCCTGAAGAAGTCGTAAAGGTAATTGAAGAAATCGATGGAATCAACAGGATTAGAAGATTAA
GCGAAAAAGACTACGATGAAGAATAATCTAGAATAAATAAAATAAAAAAGGTAAAAAATTACCC
AATAATTATATATAAGATACCAAACTAACAGGATTAAGTCAATGACTTAGGTAACAACCTTACTC
ATTGGACTGGGAATCATCTAGAGGAGATATAAATGGCAAGATTACACTCAGGAAAAAGAGGTTC
CTCCGGTTCAACGAGACCATTAAGAACAGAAGTTCCAGAATGGGTTTCAATGAGCGCTGAGGATA
TTGAAGCAAAAATCGTTGAAATGGCTAAAGACGGTAAACAGTCAGCTATTATCGGAAACATTTT
AAGAGACATGTACGGAGTTCCTAACGTAAAATTAATCACCGGCAAAAGCGTTTCATCAATCATGA
AAGAAGCTGGATTTTACGCAGAAGTTCCAGAAGATCTCTTCAACTTAATGAAAAAAGCAATCAAC

TTGAGAAACCACTTGGAAAACAACCCAAGAGATACACACTCAACCGTTGGCTTAAAATTAATCGA
ATCAAAAATCAGAAGATTGGTTAAGTACTACCGGGGCACCAAAGTATTGCCTGCTAAATGGAGAT
ACTCACCAGAAACAGCTAGATTATTGGTCGAATAATCGAATTAATTTCTTTTTTTATTCTTTTTT
GAAAAATTAAAAAACAGTATTTTAATGGTTTTGATATTTTATTGATTCCAAAATTCGTTGTAAA
ACAAGTCAAAGTCTTGATTGTATTTTTTACCAAGTTTTAATGCAAGTTCGGCATTTCTTAATTCT
TTTCCAAAATATGCCGCGTGATCCATTTTTGTGATTAAATTTTCCCGTAGTGCAGTTTCATAGAT
TTTTTTAGGAGTATTTCCCTTAATAATTAATTTTGGTTGATTTTTTATATAGTAAGTTGCAATTA
TTTCTTTATTTCTCCGATCAACCCTGATTTTGAAGCTTCCCCTATCGAGAAGTTGTTTTTCGTTTT
CGACAGCTTCAATTATTTTTACATCTTCCAAATCAAACTCTTCGTCGAATTTTTTATCTTTGTAA
TTAATAAGGTCATATCCTACATCTTTTGGAAGTGAATTTCTCATTTTTGCTAAAAATAGCATTTT
TGATGCGGTTTTCAATTCTTTTATTGAAAATTTACACTTTTGTGAAGCTTCAGGAGTAAATAAAA
TATTTCCGCCAATTTCTTGGCCAATTGCTGCAATACTTGCATTTACGCCGTTACTGTCAACGTCAA
ATAATTCTGTAACATTTCCAATTCCAAAAAACATTGGAATATTGTTTCTTTTTTTGAATTCGTAG
CATGCAATAACACTTTCTGTAAAATTACACCCGCTGTTATTTATTGGCTCTAAAATTGGGTCTGC
AACAGCCGTTAACTCATTTTCTTTAAATTTTTCAAGAATTTTTTCCATGTTCTGAATTTTTTCTG
AGATGTTTTCAGGAACTTCATTTGTTTTGTAGTTTGTTGGAAGTACAACTGATACAGTATTTTTT
TCTTTTAAATGCGGGAGCAATTCATCAAAGTTTCCTGAGTCCACACTTAAAATCATATCAACGTC
TAAATTTATTGCTTCAATGAGTTCCTTGGTATTTAATGTATCGACACTGACAGGTTTATCAGTAA
TGTCTCGTACAGTTTTGATTATCTGCTTTAAATCTTTAGAATAATCTTCATTACTGACCATCCCT
AGATCAATCATGTCTGCTCCGGATTCAACATAATAATTTACTTTTTCTTCGAGTTCTTTTTTAGA
AAGCCAGGGAACGTGAACAATCTCTCCAAGAACCCTCATCGGATAAGCATTTCCAACTTTTAAAT
TTCCTATTTTGATATTTCCCGAAGTTAATGGTAATTTTTCGGCATTTTCTATATCTTTTTGGGCA
TTTTCTCGTATAAATTTAGCAATCTGCGAATCTGCATAATCTACTGTTGAAAGGGTAATTTTTTC
CATATTTTTTATTAAAACTGGAATATCTGAAGCTTCCCTTGTTGATTTAAAACATTTTATTCCTG
TTTCTTCGTAAATTCTTGCTAAGTCATGTCTTATAAGTCCAGTTACGAGTACAAAATCATATATT
TCGGATAATTTTTTGTTTTTAGAACTTTCAAGCTTTTTTATTTCTTTTATTATCAAATTTGGAGT
TAAAAAAGCAGCAATCGAGATATTTGCTTCGTAAACGTCTATAAATTCGTAATTTTTGATTGCAA
ATGAAACTTTATTAAAAGCTTGCTTTCCAGTTATAACCAGTATTTTCATTAAATCACCGAAAAAT
ATGGAATTTTTAAATTAGAATCCAAAATACTTCTGATTTTTAGGAATAATCTTAAAAATTACTAC
TACATATATTGCTAGTAACTAATAATGTTTGTGATATGATGACTTTAAAGCAGATTTACGTCAAT
AAATTTAAAGAACTTGTTAAAAAAGAGCGAGACCACGAAATAAATTTCCATAAAGAAGAAATTA
AAAAATTGGGGATAAAACGTGAAAACGTTGGAAGAGCCATTTTAAACTTAAACGGAAAAGTTAT
TAGAGAATTCTTTGGAGAATATATTGTAAGGTATGGAAGAAGTGAAAAATTTAAGAAAACCGAC
ATTTCTGTTGGAGATATCGTTTTGATAAGTAAAGGAAACCCACTTCAAAGCGACCTTTTAGGAAC
GGTAATTGAAATTGGATCAAACCACGTGGACGTTTCGATGGAAATCGTTCCTAAATGGGCTTTAA
ACGATATAAGAATCGATCTCTACGTAAATGACGTTACATTTAAAAGAATGTTAAATGCACTTGAC
AAATTCAATAGTACAGATAATCGACTTATTGATATTATTTTAAGCGTAGATAGTCCTCAAAAATC
TAAAACAACAGAAATTCGCTTTTTAGACTATCGATTAAACGAATATCAAAAAGAAGCGGTTTTA
GAAGCCCTTAGTGCAAGAGATTTATATTTGATACATGGGCCTCCAGGAACTGGAAAAACTAGCAC
CATTTCTGAAGTAATTTTACAAGAAGCTTTGCGAAAAAACAAAGTCATTGCAACTGCTGATTCAA
ACATTGCAGTTGATAACATATTATCAAACATTTCAAAGCATGAATCTTTCAAAATTGTTAGAATA
GGGCACCCTTCAAGAATTTCTAAAAAATTAATGAAATATTCTTTGCAGAATAAAATTACGGAACA
TCCAAATTACTCAACATTAGTAAAACTAAAAACAGACCTTCAAAAAAACTACGATTTGAGGAAA

AATTTCCAAAGGCCTGACCCAAAATGGCGAAGAGGAATGAGTAATGACGATATCATCATTTTTTC
AAAGCTAAATAAAGATATACGGGGAATTCCCAAAGAAACCATTAAACAGATGGCAGACTGGGTA
ATTTGCAGTGAAAATATCGCTAAAACAAAAGAAAATGTCCAAAAATTTGAAAAAAAGCTAATTG
ACGATATCATATCAACCAGTGATGTTGTTGTTGCTACAAATTCTATGGCCGGAAGCGAGATTTTA
GAAGATTACAAATTTGATGTTTGCGTAATCGATGAAGGAAGTCAGTCAACGGAACCATCTTCATT
AATCCCGATCGTAAGGTCGAGAAAATTAATTATTGCAGGAGATCACAAACAACTTCCACCAACAG
TTCTCAGTGATGAATTAGAACTTAAAAAAACACTTTTTGAAAGAATGATTCAGGATCACCCTGAA
TTTTCAAAGATCCTTCAGGTTCAGTACCGGATGAATGAAAAAATCATGGAATTTTCAAATGAAAT
GTTCTACGAAAATAAGCTTATCGCGCACGAATCTGTAAAATCGCAAAATTTAATTGAGATTGTAG
AAAATGTTTCAAACGAAGATAAGGACATTATAAACGAAAAAACGCTTCAATTCATAAATGTTGA
TGGAGAAGAAAAACAGAATTCCTTTAAATCCTCATACAACGTTGAAGAAGCCGAAAAAGTATTA
GAAATTGTCTCAAAACTTCAAAAATATGAAATTCCGGTAAGTGTAATAACTCCGTACGATGCACA
GGTGAAATATATTTCCAAAATGCTAAATACGGATAAAATAGATGTTAAATCCGTTGACGGATTTC
AAGGACGCGAAAATGAAGTAATAGTGATTTCATTTGTCCGAACCAGTAAAATGGGATTTTTAAA
AGACCTCAGGAGATTAAACGTCGCGGTTACTCGGGCAAAGCGAAAGTTGATAGTTGTCGGTTCCA
AAAATTTATTGATAAAGGACGATGCATACTCTAAATTTTTAAATTGTTTCAATGATAACCAGTA
ATCAAAAGAAAAGATTGAATATTTCCCGAAATAATTAAATAATACCGCTTTATTCCTTTTATTTT
TGCGACCGAATGATTTATATATGATAATCTTTTATTTAGTTTGATAATTATTATTCTCTATTTTG
CATAGCTATTTAAAACTCAAAAATTTAAAAAAATACGAGAGGTGTCTAGTCTTATGATTAAATCA
AGTGCAATACACTCCCCATTCGAAGCTCCAAACACGATTTCCCTTGTTGCAGGAACTGGTGATGC
AAACAACCCGTTAAATGCTTTCGACATGTCGTTGTTAAAATCTGGTATCGGAAACTTAAACCTGA
TCAGAATTAGCAGTATCATGCCTCCAAAAGCAGACATTATTCCGCTCCCAAAAAATACCGCAAGGG
TCACTTGTTCCTACCGCATACGGCTACCAAATAAGCGAAATAAAAGGCGAAACCGTTGCAGCAGG
TATCAGCGTTGCAATTCCAAAAGATAAAGAATTATGTGGACTTATCATGGAATACGAATGCGTTG
GCGGTAAAAAAGAATGTGAAGATACCGTTAGAAATATGGCTAAAGAAGGTTTCGAAATGAGAGG
TTGGGAAATTGACGAAATAATCTCAATCGCTTCAGAACACACTGTTGAAAATATCGGATGCGCAT
TTGCAGCTGCAGCACTCTGGTACAAATAAAATATATAATTGTAATTTAGAACTATAATAAAAGTT
CACATATGTGGGATATTGAAACCATGATTTTAAAAAGAACTGTCCAAAAAAACATAAATTTCAA
AAACGCTAAAAGAGGCGAGACCGTTGAAACAGTTAGGAAAACACATCCTCCTCGAACTTTGGGGA
TGCGAGAGTCAAGCCCTGGATGACCAGCCAGGCATAGAAAAAATGCTTGTTAATGCAGTAAAAGC
ATGCGGAGCTACATTAATCTGTGTAAAAACACACAAATTCTCACCCCAAGGGGTAACCGGGGTTG
CAGTATTATCTGAAAGCCACATCAGTATTCACACTTGGCCGGAACTTGGATACGCTGCAATGGAT
GTATTTACCTGCGGGGAACACGTTAAACCTGAAGATACAATCCCAGAAATTGAAAAATTCTTAAA
ACCTGAGAAAACAGAAGTTATGGATATCAAAAGAGGAATTATTAATGACGGGGAGGTGAAAGAA
TGAAATTCGATGCATGGTATACGGAACCACAAACTGAAAATTTAAGCCTTTCAACCAAATTAAAA
GACATACTTTATGTTGGGCACTCAGAATACCAAGAAATTCAAGTTCTTGACACTTACGAGTTTGG
AAGAGTCTTGATTTTAGAAAATACGTACCAAACTACTGAAAGAGATGAATTTATATACCACGAA
TTGATTTCACACCCTGCACTCTTTACGCACGGAAACCCTAAAAAAGTTCTTGTCATCGGTGGAGG
AGATGGCGGATCTGTTAGGGAAGTTTTAAAACATAAAAGCGTTGAAAAAATAGATTTTGTTGAA
CTCGATGGACAGGTTGTAGAGGTTGCAAAAAAATTCCTTCCAACCCTAAGCTGCGAAATAGACAA
CGAGAAAGTAAACACGATAATTACGGATGGAATTAAATACGTTGCAGAAACTTCTGAAAAATAC
GATGTAATCCTCGTAGACTGCCCAGACCCGGTTGGCCCTGCTGCAGGATTATTCGAAAAAGAATT
CTACAACAACCTCTACAATTGCTTAAATGAAGACGGAATCATGGTACAGCAGACAGAAAGCCCGC

TTTTACATGAAAAATTGATAAATAAAATCAAAGGACACTTAAAAGATGCTGGCTTTTCAACAAT
CAGACCACTTGTCTGCAGTATTCCAACATACCCAAGCGGATTCTGGAGCTTTACACTAGCTTCAA
AGAAAAACGATCCATTAAATTCAGATGTTTCAGAAATTGAAAACAAATTAAAAGATATGACTAC
AAAATACTACGACCAAGACGTTCACAAGGGAGTATTTTTAGCAACACCTAGATACTTAAAAGAAT
AATTTCTAACTTTTTTATTATTTTTTGGTGATAATTCATGTATTTTGAAGATTATTCCAAGTTTC
TCACAGCTTATGATACATTTAACGATGCTGATTTTGTTATTTTTGGAATTCCTTTTGATGCTACA
ACTTCATACAAACCCGGTGCAAGATTTGGGCCTGATGAAGTTAGAAACGCTTCATGGGGACTTGA
AACGTTTAGCCCAATTTTAAAAAAAGATTTAATCGATGTAAAAATTTGCGACCAATACAACCTTT
TAATGGAAGGAAACCAGTCTGAAATTATAAATAGGGCATATAGTGCTTCAAAAGATATTTTAGA
AGCTAAAAAAATACCGGTAATGATTGGTGGAGAACATTCTGTAACTTACCCTGTTGTAAAAGCTG
TAAAATCAGTTTATGATGACTTTGCAGTAATTCATTTCGATGCTCACTGCGATTTAAGGGAAGAA
TACATGGGAAACGAACAGTCCCACGCGAGCGTAATTAGAAGAACTTACGATTTAACAAAGGATAT
TTTCCAGTTTGGAATTAGAAGTGGCGACCAGGATGAATGGGAATTTGGATGGAAAAATACCAAT
ATTTCAATGGAAATGCCAACAAAAGACGATATCAAAAAGATCAAAGAACTTGAAAAACCAGTTT
ACGTAACAATCGATATTGATGTACTTGACCCTGCATTTGTACCTGGAACTGGAACTCCAGAACCT
TGCGGATTTACACCAAAAGAGCTTATAAACTCACTTTATCTTTTGGAAGAAATTAAAGAAAAAAT
TGTTGGTTTTGACGTGGTTGAAGTTTCACCACATTACGACATCGGCAAGATTACATCTGTTACCG
CAGCAAAAATCATTCGTGAATTGATACTTACCATAAGCAAATAATACGTCAATATTTTCAAAAAT
AAGCGTGCATACAAAATCAAATGTTAAAATTTAAATATAAAAATAAACATATCTGTAAAAAGTA
AGAATCGTGGTGATAATTTGGTATCTGAAAATGTAATGAAAACTATTGAGGAAATAGAATCACA
AATCTCCCAAGATGGTAGATACATAGAACTTGTAACAACTGTCGAATATTTAATCGGCTTAGTTA
CTGAAGAAAAAAAGGAAACATTTAGAAAAGCTTTAAACGATGCTGAAAACGTTGAAGATGTAAA
AGAAGTATTAAATGCCATTAAATTACAGATCGGATCCCAAGGCGCTAAAAAATACCTCGGAATTT
AATTAAACCTATTTTTTTCTATTTTAACTGTTAATTGAGAGTACAATTACTCCGCTTAAAATCAT
TGCGATCCCTGCTATCTGGGTAAATGATAACTGCTCTTTTAAAAAAATTATTGCCAGAACCGTGG
TTATTGCAGGCCCTATTGAAGATAAAGGTACTACAACGCTCGCTTTTCCTTTGTTTAATCCATAA
TAAAGTGCAAGTGTTCCACATATCACCAAAATTGCGGCAATTACTCCATAAATTAAAACTTTATT
TGGGTAATCGAGTAAATTTTTGCACTTTCCTCCAAATAATATAACACATAAAACAATTCCAACAA
TATTTACAATGATCCACTGAAGATACGGATCCTCATTTGAAACAACTTTTGCAAAAAAAGTACCT
ATACCATATAAAACTGCAGCAAGTAGTCCAAGAATTACTCCTTCCATATCCTGCCCCCGAAAAAT
TAAAAAATTAAATTAGATGTTTATTGATTTTGCAGCTCTTACGTTTTCCATTTTCCTGATTTCAT
CGAGTACTTCATCTGAAATCATGTGGTCAATATCTAAAAACATTATGCTGTGTCCTCCAGGCTCT
CTTCTTCCAACTTGCATTCCTGCAATGTTTATTCCGTGTTCACCAAGAAGAACTCCAACTTTTCCA
ACCATTCCGGGCCTGTCGATGTGTTTTATGATGCAGATTGTTCCTTCAGGTTTTATATCCATTCT
GTATCCGTTAATTTCTCTAAAAACGACTTCGTTATGTTCAATACTTCCAATTATTGAAATTTCGT
CGTTTTCACCTTTTGCAGAAATTTTTATTGAATTTCCATAGTCGCTTTCAGACATTGTGCCTTCT
GCAATTTTTATGTTTCTGCTTTTCGCAATCACTGGAGCGTTCACTAAATTAACACCTGCAAGTAA
TATTGGTGCCAAAATACCTTTCAAAAATGATCTTTTTAGTATTTCAGTCTTTTCTTTTGCAAGGC
CGCCCATGTACGTGATTTCTAAAAGTTCTATCGAGTTATCAAGGTACTGTATTGCCATTGAACCC
ATTTTTTCCGCTAAAACCATGTAAGGTTTTAATTTTTTCATTTTTTCAGTTGGAACCATTGGGAG
GTTTACAACATTTTCTGCAGACTCTCCTTTCAATATTTTAACGGTTTGTTCTGCAACAATTGTTCC
TGCACTCAATTGTGCTTCTTCTGTTGAAGCTCCCTGGTGCGGAGTTCCAATTAAATTATTAAGTG
TTAAAAGAGGGCTTTCTTGTGGAGGTTCTTGTTCAAATACATCCAATGCAGCTGCTTTAATTTTT

CCGCTGTTTAATGCATCATAAAGTGCAGTTTCATCAATTAATCCTCCCCTTGCACAGTTCATTAT
AACCATGTTGCTTTTCATAAGTGCAATCTGCTCTTTTCCAATCATGTGTTTTGTTTTGGGAGTTA
ATGGAACGTGAAGTGTTATAAAGTCACTTACGGTACAGAGTTCATCCACAGTTAACAATTTTATT
CCAAGTTCTGATGCAACATCTTCTGGAATGTATGGGTCATACGCAACAATTGTCATTCCAAAAGC
TTGAGCTCTTTTTGCTACTTGCTGTCCAATTCTACCAAGGCCAACTATTCCTAGCGTTTTTCCGTA
AATTTCCATTCCCTTAAATGATTTCCTGTCCCATTTACCGCTTTTAATTGATGCGGTAGCTTGAG
GGATATTCCTTGCTGCTGCGAGCATCATACCAAATAAAAGTTCTGCAACTGAAATTGACGATGCA
TCTGGTGCATTTACCACAACTATTCCTTTTTCTGTTGCTGCATCTAAATCTACGTTATCTACACCA
ACTCCAGCTCTTGCAATAACTTTTAAATTTTCTGATGCTTCGATAATTTCTCTTGTAGCAGTTGT
TCCACTTCTAATTACCAGCGCATCTACATCTTTAATTTTTAATTTTAGCTCTTCAACATTTAACCC
AGTTGCAACTTCAACTTCGCCTGCCTGTTTTAATATTTCAACGGCACTTTCATGCAGCGGGTCAGT
TATAAGTATTTTTGACATTTAATCACCTAAATGAATACAATTTACTACACATCTCAAATTACACA
AAATAATTATGACTAATAATTTAAATAACTACTCGAACTAACCTAACTGATAAATAGCATGATA
ACAAACTTATAGAAAAATTATGGTGGAATTGATGTACGGAATTTTAGTTTTGGAAGACGGCACA
ATCATCCGAGGAGAAGGATTTGGCGCAGAATCGGAAGTCCTCGGTGAACTCGTATTTAACACTTC
CATGACAGGATACGTTGAAATATTAACTGACCCTTCCTACAAAGGCCAGATTATTACAATGACGT
ATCCTTTAGAAGGAAACTACGGTGTCGAAAAGGAATGGTTTGAAAGCGATGGAATTAAAGCAGA
AGGATTCGTTGTAAAAGACATTACTGGAAAAGAACTCGACGAATTTTTAAAAGAATACAATATT
CCAGGAATTTCAGGCGTAGACACAAGATACATTACAAGAAAAATAAGATCAAAAGGAGTCATCA
GATCACTCTTAAAAACGTCCACCAACCCGATAACTAAAGATAAAGAAACAGAATTACTTAAAAAA
GTAGTAGATTATCCAGATATTTCAGAAATTGACCTTGTTCCAGAAGTATCAACTAAAGAAACAGT
TGAATACAACGCAGAAGATGAAAAAACCAGATGTGTCCTTATTGACTGCGGTGTAAAACAGAGCA
TTGTAGACTGTCTCGTTGAAAGAGGATGCAGTGTTGTAAAAGTTCCATACAACTCTAAAAAAGAA
GAGATTATATCATACAATCCAGACTTTGTACTCGTGTCAAATGGTCCTGGAGACCCTGAAAACAT
GACTGAAACAGTAAATACTGTTAAAAACTTAATTGAAACACTTCCAGTTACAGGAATTTGTCTTG
GACACCAGATAATTACAATTGCACTTGGCGGAAAAACATACAAATTAAAATTCGGTCACAGGGGC
GGAAACCAGCCTGTAAAAGATGTTGAATCTGGAAAAGTATACATCACTTCACAAAACCACGGGTT
TGCTACTGACGATAAAATAGTTCCGGCAGGTTCTGAATTGATGCACATGAACTTAAACGACGATA
CCGTTGAAGGAATAAGAAAACTTGAATCAAGTGACCTTAAAAATACAGTATGGAGTGTTCAGTA
CCACCCAGAAGCAGGGCCTGGACCACATGATGCAAGATTCCTCTTTGATGAAATGGTTGAACTTG
GAATTAAATTCAAAGCTGAAAAAGCAAACTAACTTTTTCAAAAATACTACTCTTTTAAAAATTTA
AGACTATTAAAAATATAATTTTTAGTTTTACAGTGAAATCAAAGGAAAAACCATGGATAAAGAA
GTTAAAACTACTGCTCTTGAATCTTCAAAAAAAGCTCTTAATTTGACATATCAAAAAAATGAAAT
TTCGAAAGAAATTTATGAAAAATTATTGGAACTTCACGAAGTTAGAAACAAGGATTTTGATGAA
TTTTTAGCATACTTGAAAAATCATGAAACAACTCAAAATCTAAAAAAAGAAAAAGCAGTTGTTG
CATTCAGTGGGGGCGTAGATAGTACTACATCAATAATTGTTGCAAACAAAATTTTTGAAATTTGT
GCAGTTACAGTGCGTTCTAAATATATAATGGATGCCAAAACTGAACAAAGTATACTTGAACTTAC
AAATTCAGTAAATATCTCACACGAATTTATCGATGTGAATTTAGATCCTATTTTTGAAGATACAA
AATCTGGAAAATACCACCCCTGCGGAAGATGCCACAGTACTGTTGAAGCTGAAATCTTAAATTAC
GCTGAAAAAAACGGTATAAAATATATAATATATGGAGATATGCTTTCAATTGGCCATTTATCAA
TTAAAAAAGTGGATAAAGGAATTAATCGATTAAACATCCTTTCATTTTTATCAATAGCAAAGGA
TGAAAGCAGAGCTATTTTAAAAGAGTTTAAAATTGATATAAACCAGTCTTACGGCTGTCAATTA
ATTAGAAAAGCACATAAACACAAAAGCATGCAGAAATTTACAATCCAAAGAATTTTAAGAGAAG

TACGGGCCCAAGTAATTACTCCAGAAGAGGGTTTAAAAAACATACTTGATGTAATTAAAATTTA
ACTACTGTAACGCAATTGAAACAGAAACCCCATCTTTTTTCATTTTGTTTAAAACAAGGTTTTTC
ATGTCAAAATCTTTTTTTCCAGTAATTTTTAAAATTCCAAGAATTGCGCAAGGCTCACAAACCCC
ATATGTCCCAATTGTTTTAAACACGAACTCTGACTTTTTTAAGGGATATTTTAAATAAATTTCAT
TTATTTCGTCAATTCCAAAGATAAAGAGATTTTTATCAAATCTTTCAGCGGTTTTTAAAATTCCA
TATTCATCTTTTTTAACAGAAACTGTTGAAAATGCATCGATTCTCCATGCAGGAATATCTCTTAA
ATAAAGAGCTTTTTTAACTGCCCAATATACCTTGCTCTTTTCGATATTTCTTCTTGAACCCAGACC
CAAACTTACGGTTTTTGGTTTTAAAACGATATCATCAACTAAAACATGGAATTCATCGTGAAAAG
AAACATTGTAGGAATTTAATAATTTTTTAGAAGCTTTCCAAGCTTTTGGAAGGGTTAAATCGGGT
TTTTCAGATAATATTTTTTTATTTATCGAGATTATATCTTTTCTTGATGGATTTTCTAAAAAATA
GGATTTTGATAGTTCGTCAATTCCAATTTTTCCGTTTACATCGGTTGCAGTTGTTTTTACGACAT
TTAAATTCAAAGAATTTCCAATTAAATCTGAAAAATAGTTTCCTCCGCCAATATGGTTTGAGAGA
AGCGATACTACGTATTTTTTTGATTCGTCCATTAAAATTACAAAAGGGTCTTTTAATTTATCATT
TTGAATTTCCGGAATATATTTCTTAAAACAATTCCTGAAGCCATTATAAAGACAAATCCGGTTT
CATTTCCGGTTATTTCAAAATCTTTTATGTTAAAAACTTCGTTATCATAAAAATAGTAATCAAAA
ACCGATTTTACATCTTTTGCCAGTTTTTCGGCATTTTCTGTAATGTAAACAATTTTTATCATCAT
ACCCCGAAAAAATAAAATTTCAAAAATAAAAAAATTTAAAGAAGTTTTGCAGCAACTTCTTTTG
CCTGCTCCCTTTTTTCTTTTAAATCAGTCATGAATTCAACTAATTTCTGGTAGCATTCTTTTTTAC
AGGTTCCGCAGGTTAATTCCCCGCTTTTGCACTGTTCATACAATTTTTTAAGTTCTTTATCATCTT
CTATTAAGTGGTAAGTGTATAACTCATACACAACACACTCTTCAGGAACTCCACCAAGTTTTTTC
TGTTCTTCAAGCGTTTCTCGTCCACCAGTTTTGCATGACATGACTTTTTTCTTAAATGACTTCGTT
GGCTCATCTGAAAGATATATCGCAGTTTCTGGCTTTGACGAACTCATTTTTCCGCCCAAAAGGCC
AGTTATAAACCTGTGATAGGTTGATGATGGTGCAATAAATTTAAAATCTTTATATCTTCCAGCAA
TGTCTCTTGTAAGCCTTATGTGGGGGTCCTGATCAATTCCAACAGGTACAACGACAGGGACCTTT
TTTTCAAGCTGAGGGTGGAGTATATCTGCAACCTGAACTACCGGTGCAAAAACGTGTCCTATGTT
TGTTTCCCCACTGAATCCATAAATTCCTTTCATTTCAGTGAAATTTACCCGTTTAGAAAGCCGCA
TTGCAAGGTCTTTTACTGTTTCATTTTTTGATTGCAGATAAACATGGATTTTTTCTGGATCAAGT
CCTAATGCAATATAGTTTGTAATGTATTCTTCTATTGCAAGTTTTTTAGTAGTTTCAAAATCCAT
TCCTCTTGCCCAGTAGGCCTCTAAATCAGCAATTGGAATGTATATGTCTGCACCATATTTTTGGT
AGTACAAAAGCTGGTCTACAACCATTTTATGTCCAAAATGCATTTTTCCAGACGGCATCATTCCT
GAAACTACAGTAAAATCTTTTCCGTTGTTCATTGCATCTGCAATTTTTTGAAAATCCCTATGACC
AAACACAATTCCTCTTTTCATAAAATTGTGCGGTTCTTTTAACGTTTTTACAATTTCCGAAATCG
GATTTACGCCAAATTGCTCCATCGTCTTTTTATAATCCAAATCATCTGAAACATCCCAAGGAGTA
ATCAATATTTCACCTTAATTAAATCAAATAAATTATTCATAAGTTTCTGGCAGTTTTGCAGCGGT
TTTATGGCATTCAGCAAGTCCTAATGCAGCTACTGCACCGATTATTCCACCGTCACCAGTAACTTC
TATAAGTTCAATGTTATTTCTCATTGCAACCATTTTAGCCTCATTTATAGGAACCATCTCCCGTT
TAACTTTCATGGTGTACAATTTCATACTCTGTGAAGGAGTAAGTCCTCTGTAAATTGCAATTGCA
GTTTTTTCCGAAAGTGTTTTTTCTTTTAAAAGTTTTTCGATTTCTTTTCCAATTTTATATTTGTA
TTCTGGGTAAATTGCGAATGTGAGTGCAATTGAAACACAATTTTGTGTTTTATTTGGATTTCCAG
GGAATAATTGAGTTATCGTGTGGTCTATGTAAAATCCCATTTTGGATTCTTCGATTAACTTTCCT
ACCTCATTTGCTAAAACCCAAGTTGCACCTTCTTCTTTAGTATCCGTATCGTCTATTCCAACGGTT
ATTTTTTCCATTTTTGGAGTCTGGATCATTCCTCTTCCAAGTTTTGAACCGCCACCTTTTTCGATT
AATTCGGCGCCAGTTACATTTTTGGCATTTTTCCTAAGTTCAATTCCAACACCTGCACCTGCAAGG

CCGGCATACACAATGTGTACCTTTCCATCTTTTAGATAAACTTCCTCAATTCCAGCTGCACTAATT
GAGGGAACCAGATTAAGATCAGTTTTTCCATTTTTTAAAATGAAATGATGCTTGTTTCCATTTCT
CCATGATGAAACAATTGCTGGAGATGTTCTTTGATACTGGTACATCAGCCATTCTGAACCGCTTG
GACATGGGTGTTCCTCTACAAGTTCTGTTAACCCTAGTTCCTCATCAACCATTGCTATAATCTTTT
TATAAGATACATAATCAGTCATTTTCTCACCATTTAGGCCTCACGTTCTTCAAGATATTTTGTAA
TTTCTTCGTAAATTAAATCAAATAAATCTTTTTCGTAGGTGGATATTTTTTTATCAATATCGGTT
CTTGAAAGAGCGTAGTTTAAAATTATTAATTCGAGCTCTCTTTCAATGCCTCGTAATTTCCTGTC
GAGTCCGACCATTCTATTGTATATATCCCACTTTAATGAATTATCAACCGAAATATTTAAATTTT
TCAACTTGTGAAGGCAGTTTGTCAACTCGTCGTAATTTGAAATTCTTTTATGTGCATGTAATTTT
CTAAGATGAGTTTCAAGATCGTTTAATTTATGAGTGTAAGATCTTAATTCGTTATCTAAAAACTG
TATCTCACGCACATTTACAAGATCACGATCCAGATCTTTTAGCGTCTCCTCTAAAATACATATTT
CATTATACATTCCAACGTAATTTTTAAAAATATCCTTTAATTTTGAACTTTCATCAAATTGAGGA
TCTTTTAAAAGAATGAGTTCCGCGAGTTTTTTTAACTTTTTGTTGTATTCTGCCATAGTTTCACT
TGGTCGAATATTTAAAATTTAAAAATTATTGAGTTGTAAATATTTCAAATATTATTTAACATGC
CGTATATTTACCAAAACACCATGTGTTGCTTTTTTCATCTCTTTTCCGCTCATTAAAGCTCGTCCA
ACGCCTAAAACTTCATTATCTTTTACGATAACTACTTCGTCATTTACAGAAACAGCTTCATCAGC
ATCTTTAAATCCCGGGGCAAATAATGAACCCTTTTTAACATTAAAGTCAACTTCTACCCAGTTTG
TTTTACCAAGAAGTTCTCCTCCTTGTGAAGTTAAAACAAAAAGGCCATTATCATTAATTGATGCC
AATTGAACCGTATTTTTGCCTTCTTTTATGAAAAATTTAACATGCCTTCCTTTTATCATTACATC
TTCAGGCAAGAAGTTCTTTCCAAGTTGGAACTTTGCAAGTTCTTGATAATTGTGAAGTCTTTGTG
CACGTTTTGATTTTGAGTCAGCTGTTTGATCTAATTCTTTTAAGATATTTTTTAAATTTTTTAAT
GAATTGTCAGAGGTTGGATTTCCGTCTGAAGAAATTATGTAATCTTCATCGATATCCAAAATTTC
AAGATAGTGTTCTGGAAGATGTGCTATTATCTTTACTTCTTCAAATTTAGATTTTGCGATTTCGA
TGTAATTTTTCAGATATTTGTTTATAAAGTCAATCTCATCAGAACTCCATTCTCCAGTTACGGGA
ATATCATAATCAACAAGCCTTTCAAGCGCTCTTGGAACTACTCCGTAAGGTGAAGTTAAAATCAA
TTCTTCTACTGGCATTCTTACTGAATTAATTGCATTTATGAAGAACTGGTGGCTTTTTGAATAGG
AATATGGTTTTTTTGATGAACATGGTAAAAGTATAATTACATTTGTATATGGCTCGTAATTTTTT
GCCCTTTCAATGTATTTTTGAACTTCTGGAATTTTTGTCTCATCAATTGTTACAATTATTTTGTT
GGACTTTGAAATCGGAATATTTGTAAGTTCAGGTTTAAACCGCCTGTAATTTGACCGCAAATATG
GATTTGAAATCGTGGTTTCCTCTACGAGATTTCTTAATGAACCGGATTCGATACAGAATTTTACT
TCTTCTAAAACCTGATCGATTATAGATTCGTTGAACGTTTTTAAACTTTCAAAATCTTCTTCTGA
TTTTATAAGCCTGTTTTTACTAAATTTGTATCCTTGTGCTGCATAGTAGCTTGCAGATGAATAAT
CAAAATAATCTGCTCCCATGTAAACGATTAACGGGATTTCCAAAGGCATTGCACCTGGAACATAT
ATTCCGGAATTTGGAGAAATCATTTCCCTTACTTTTGGAATAATTTCAATCATTTCTCTTCTTTT
TATGATCTGTCGAAGGTCAGATATTACGTAAACGTCCATATTCTCTTCGATATCTATCAAATCAG
CATATTTTCCAGCATTTATTATCTGGTAGCTTACTTCGCTTTTTCTTACATATTCTGATTTATTT
AATTTTACGAGCTCTTCTACAACTTCCCTTGGTGCATCAAATGGAATTTTTAGCGTTTTTAGTGG
TGGAACGTTTATATCAAACTCGATTATTTTTGGAGTGGTCGAAAAAGAATTTGATTCGTGTTTTT
CCTTGCAAATTCTTCCTATATCATATAATATCGGCTCTAACATCTAAACACCTTTAAATAAAAAA
TCAAAATTACGAAAATCTGGTTGAACTTTTAAAGTTAAAATATATTCTTCTACATTATTAAGCTA
TCCTAAGGGCTATTTGGTAGGTTACAAGTACTTCTACAAATGCCGCAACTACAAATAATAATACA
GAAACTAAAAAAATCCGTAAAGAATCTTTAAAATAATCCTTATAATGTTTTTCTTTTCTAAATTT
CTTTATTATGCTCATTGAAAGTACCAGACCTGATGTGGCTGCCAGAATGAGTGCTGGAATTTCAA

AAATACCGTGAGGTCCGATTAAAAGTATGAATTCTAACGGATTTGTCAAATAAAAAACATATGC
TAAAAGATATGAATTTACGGCGATATTTATAAGTGCAAAAAACATAATAATATATGTAAAAGCT
GCAACAAAGAGGTTATTTGCTAAAATTATTGCAATTATTTCAAATTACGGCGATATTTATAAGTG
CAAAAAACATAATAATATATGTAAAAGCTGCAACAAAGAGGTTATTTGCTAAAATTATTGCAAT
TATTTCAAATGGACCTTTATTTTCTATTGAGAAACTCTGAACTTTTATTTTAAATGATTCGTACA
TCAAATCTCCGAAATAACGGAATTGGAACATGTCATAGTAAATAAATACGTAAGAAAATAAAAA
TGTTGCCAGAAATATGGTCGAAACTAAATAAATTGTATTTTTATGTCTTTTTAGTGCATCAAAAA
TCTCTAAAATTTCGTAAACTTCTTTACTCATGGTACCCGGGCGTGATATTGTGACTGAATCTTTA
AAAAATAAATACAATTCATCGGATAAAAACATGTTGGAAAGTGATGTTGTCAAACTTTTATCAA
TTTTGGGCGTTAATTTTGACAATATGCTAAAATGCGGAATGTACATGTGTATCTGCGATGAATCC
GACAAACAGAAAATTGAAACTAAATTTTTGGAAATTATGGAAAAACAGATCAAAAACCCGAATG
TATCGACTCTTTTGATAGCTGCAATTACTATGGATGAAAGGGGAAGAAACGGAGGCCTTCCGTTC
GATTACGATAGCGATCCAACTTACGTTTACGCAGATGAAGTCATTGGAATGGCAATTGCAAATGA
AATTGCAGGAACTAAAGCTACATTTAATTTCAAGTGGTACGATGCAAAAAAACCAGGTGTAATT
GGAAAACTCGACAAAGAAGGCTACATGTTTTTAGATGACGCTGTTGCAGGTTTTGTTGCAGGATG
CATGTCAAAAGTTTTTGAATAATTTGTTAAGTTTAAAACAATATCTTTTTTTAATACATCTAACA
ATAATATATAACAAAATAATTTCAAGAACGGGAAATCCATGGAAATTGAAAGACTCTACAAAAA
AATAGTCGAATTAAGAAATAATGATTCTGATAAATTTCAGGTGCTTTCAAAGCATATTCAGTCAA
TGCCTGACGATATGTTTGAATATATCCTAAAAAGACTTGAAAAACAGATTGAAATTGTAAAAAA
ATACGAAATTGAAATAAGGCCTGCAATAGATCCATTTGTTTCTTCAGAACTTGGAATATACAGAA
GACTTGATGATTTAGAACTTGGTGAACTTTTAGACTATCCAAAATGCTGTGTAGAATCTTTTTCT
GAAACTGCAAGGTATGGAATAGATTCTGAACATTTAAAAGAAATTGAAAATATGGAATTTGATG
AAGACACTTACGCAGTTATTCTTCCGTCAGGATTTATTCCGTGCAGTATCAACTGTAAAAAAGCC
ATAGCCAATAAATTAATTGGAAAAATTGATAAAAAAACGTATGATAAACTTTTGAAGATGGAAG
AAGAACTTTTTATAGAATTGCCCCATTATCACGGTGCTTATGATGAATATTTCGAAAAAATAATT
GTAAAAAAGTAAAAAAAGGTAAATTTTATGTATATTATAGTTGCAGGAATGGGCAGAGTTGGAA
TTTCACTTGCAAAGTCGCTTTCAGGTAAAGGACACGATTTAGTAATACTAGATCAAAACAAAAAA
ACATGTGAAGAAGTTGCAGGCGAAATCGATGCTCTGGTAATTAATGGAGACTGTACCAAAACAA
AGTCGCTTGAAAACGCAGGAATTGAAGATGCAGACATATTTATCGCGGTTACTGGAAATCAGGAA
ATTAATCTAATCAGCGGATTAATTGCAAAAAAATTCAACGTTTCAAAAACTATCGCAAGAGTAAG
TGAGCCAAATTACAAGGACGTATTTGAAAGCCTTGGAATTGGAGTTGTAATCAGCCCCGAACTTG
TTGCAGCAAATTATATCGAAAAATTAATTGATAGGCCGGGTGTTGTAGACCTTGCAATCGTTGGA
AGAGGAGATGCAGAGATTTTAGAGCTTATAATTCCTCCAACGTCAAAAGTTGCAGATAAAAAAA
TTAGGGAACTTGGCGGAACAAAAGACTATGTTATAATCGCAATTTACGAAGGAAACGAATTAAA
AATTCCTGATGGAAGTACTGAATTAAAAGCATACGACAGAATCTTAATTTTGGCTAAAACAGACT
CATTAGATGAAGTTAGAAAAGTATTTTCAAGCGAAAACAAGGAATAATTTAAAAATTAAAAATA
ACTTTTTCAAAATTTATTTTAGGTAATTAATACAGCAATCTGCAATTTTTTCAGGAACATTTACA
AAATTCATCATTCCCCTAAACTGCGGTGTGATGTTTGCTTCTAAAATATAATTTTTATCTTTTGA
AATTAAAACATCGATTCCAACGACATCTGCACCCAATTTTTTTACGGAATCTAAAACCATGTCTT
CAACTTCGTTTGATAGTTTAAATTCTCTTATTTCTGCACCAAGTGAATAATTTGCTTTCCAGCTA
TTTTTTGGAATTCTCTCCATCGATCCAATAATTTCATTATTCAACAAGAAAACCCTTAAATCATG
AATATATGGTACAAATTCCTGAACAAAAAGTTCATTTTTTTCATTTTTTTCAAAAACTTCAATAA
ACTCAGATTTTGAATTTACTTTCAAAACTCCATCCCCGTATCCACCATATATCGGTTTTACAACG

AACGGGTATCCAAAATTATTATCGATTTTATCAAAAATTTCAGAAATGTCTTTTGAATAGGTTAA
AAATGATTTTGGAGATACTTTTTTTAATTTTAAATAGGTAAAATACTTATTTTGACATGTTTGA
ATGGTTTCATCCGAATTTATAACAGGAATGTCCTTTGAAATATCTTTTAAAGCATAACTGCCTTC
TGTTAAAAAATCTCGCTCAACTCTTGATAATATCAAATCAAAATCATAATTTTCAGAACCTAAAA
AACATTCTAAAGGGTCGATAAATTCAACACTGCATCTTTTTTTAAGTTCTTCAAAAATCATTACA
TTTTCGGGCGTTTTTTTGTGGGCCAATATTCCAATCTTCATAAAATCCCCAAATCTTAGTTTACA
ATTTCAACATTTATTGATTCTAAATCTCCACCCATTACGTATGATAATTTTATTTTGACATTTCC
TGCTTTTAATTCTCCATTTATTGGAATTACGAGTGGCGGATTTAACATAGGACTTGAGCCAGAAA
TAAGATTATTTTTTAATTTAGTATACGTGTTTAATCTCACAGCAGAATTTTTTACGTCTTCTTTT
AACTCAAAATCGAGTAAATATTCAACATCTTCATCATTTACCTGATAAAAATTAATAGTATCGTA
AACAATCTCATTTGAAACTTCTTCGGAAGTTACTTCTTCGTCATAGTAAATGTGGCCCATTTTTG
ATTTTACAAGTTGCGCAGAGTTATAAACTTCTTCGGGGATTATTACGCCCCCTTCTTTTAAAAGC
CCCTTTTCGATTATTGAATTTAAAACAGGAACTTGAGGCTCGGTAATTAATCCCGTATCTAAAAG
TTCTGCAATTACCACATCTGCTTTTTCTGAAAATGGATGATATGCTGCATCATCCTCTATAACCG
TGATATTATCATAATTATTTTCTTTTATATTTTCTCTAGTATATTCGGTCGTTATCGGGTCCAAT
TCTACCGCATAAACGTGTTTTGCATTTTTCGCTGCAATCATTGCAAGAATTCCACTTCCTGTTCCA
AGATCGAATACGACATCTCCAGATTTTACGGACGTTTCAACTGCTTCTTTAAATACTGAAACCCG
TTCATCATCCAAAAGCATGCTGTAGTGCCACTGAGGTACAGACAATCTTAATTCCACTAATACCA
CCAGAATTTTATTTAAAAATGAAATGTTCGTGGTGATATATTAACATGTCTGAGTTTAAATTGTT
AAAAAAATCTTAAGAAAAAAAGCATAAAAAGAGTTATTCAAATCTCAAAGAATATTCCCCAACA
GTGATATTAGAATACAGTTCGTTTTTTGATTTCATTTTTTGAATTTTTTTGGTAATCTCACTCAA
AAAAGCTGTTAATTTCAGCCCCTCTTCCAAACTATGAGGAATTACGAATAGTTTTCCATTTGGCT
CAAATTCGTCCATATAATTCCCATTTGCAATTATTTCATTTTTACGGATTATTTCAAAATTTAAC
ATAGACTGGCCCCCCAAAAAAAACAGTATTTGCACAATAAATCTTTTTATCGGAAATTAAAACTA
AAATAGCGTTATGTTTGGCCTCTAACTAAAATTATGGCTTTTATCAGTATATAAATATTATATTG
AATTAGGAATATTGTTATAGTGTGATAAAATCCTGAATTTGAAAATGAGATTAGGTATTTTTCC
AAAAATTTCTTAAGATACTTCACCCAAAATATTGTAAATAAAATTGTGAATGATTAAAAGGATT
GACTAAAAAAGTATTCTGGTGCCATTATGGAAAAATTAGTAGTTACTGTTGATGAATTAAAAAA
AATGGTAAGAAATAACGAAGAAGATAAAATTGACGAAGTTGATGTCGTAACAGCGGGCACTTGC
GGTATTATGTCTGGAACATTAGGAATATTCCACATCCCTATTGGCGAATCATTTAAAAAAGCTGA
AAAATTCTATTTAAACGGAATTGAAGGATATCCTGGACCCTGCCCAAACGAATTTTTAGGTAGTG
TTGACTGCACAATTTATGGCACAAAACATTTCAATAACTACGGGGGCGGTTTTTTATTTAAAGAC
CTGATTTCTGGAAAAGAAGTGGAAGCAGTCGTTGAATCAGAAGGCAAAACGTACAAAAAAATGA
TAACACTAAAAGATATGCCAACTGCAAGACTTATTGGAACAAGAAATGCATTTAAAAATTATGT
GGCGTTAACAAACACAGGCAAACCTGTAAACACGATATTTCATAGAAAAATGTTAAAATCAGGA
GAAGCTTCTTTTTCAGGTTGTGGTGAATTAAATCCGCTTCAAAACATGAACTCCGATGAAAAATA
CATTACTGGAAAGAAAATACTTGTAAATGGAGCTGATGGTCTTATTTTAGGATATGGTACCAGAT
CGTCGAAAGAAAAACCAAACATGATGATTTCAGCAGATATGCATGAAATGGACGAATACTATATC
GGAGGATTTACAACATCCGCAGGTGCAGAGATTTTTAACACGATTGCAGTTCCAATCGAAGTAAA
TGAAAAAAATAAAGAATATTTAAAAACACTCGATTCTGAAATAGAACTTCCACTAACAAACGTA
TTTGGAAGAACGGGTATTGGCACTGGAAAATATTCCGAAGTATGGGAAAAAGCGGATTTAAGAC
CTAAAGTTGATATTAACAGGTGCAGGGTTTGTGATTCTTGTGAAGCTCAAAAAAATGTGCCCAACA
AACGCAATCGTAAGACTCGACCATTTGGGGAAAAGAACACTTCCAGACGAAAACTGCTTCGGATG

CGGAGTCTGTGTCGATTCGTGCAATTATGGGGTATACCAGATGGATAGAAAAAGTATCATGGGAA
TTCCAATAACTTGCAGGCAGTCGGATAGAGTTAGAGCACTAAAACTTGCAAAAGAACTTAAAAAA
CGAATTGAAAAAGAAGAATTTAAAATTTAAGAGTTATTTTAAAATTAAAAATAATTATTCATTA
TTTTCAGAACCCGTGTTTTCTTCCAAAATACTTTCTTTTTTAGATAAATAATAATTTGAATAGCT
TCTGTATCCAAATACTGTCAAAAACATCTGTAAAAATGGCATTGCAAGTACAATCAGGGCTATTG
CGATCATTAAGAATACATTTTGTGTAAATATTCCAATCATTACTGGGATTAATAGTATAATAATC
ACAGGAATAACCACCAATATTGAAACAAGTGCTACTGCAACAATTACAAGTACATATTCTATTGA
TATTTTTGAAATTACTTCTTTTAAGTCAAAAAATCCAATGAATTCCTTTTTATCTACGAAATTAA
TCGTTGCAAGTGTTATATAAAGTGTTTCAATCGCCGAAAGTACAAAGGACACCAAATTTATGAAC
ATGTTAAAAAGCAACACATTTGAATTATTATCATTTAATAACAGAAGTATAACAAATGGCGCTG
TGATGATAAGGCTTATTATCCCAAATATCAGTTGAAGAATAAAAATTCCGAGGAAAAAATGCGCC
CCTTTACTTAACAATCCTTTAAAATCTTCCCATTCTGGAAGTTCAAAGCTGCCGTCAAGTGTATT
TTCAATTACTTTCATGTAGTACCCATAAATGAAAGCAGTAAAGACTACTCCAATCGTTGCAACTA
TAAAACCCACTAAAATTCCGCCAAGAGAAGTAAATAATGCAAGTTCTGGTGCAATATCCAATAAA
GAAGGAATTACACTTGCAAACATTATTAAAAGTCCAGATATTATTCCTCCAATCGAAATTAATTG
GAAAACTCCCCCTACAAATATCTTTTTCGGATCTGACCATGCAAACTGTAGCGGATCCTTAATGT
ACCGTTCAAAAAACATTTTTCCAACCACCCGCGATGTTATTATAATTTAATTTTGATATAGTTAT
TAAATTATTTTTCTTCTCGCATATCTAGGTAAGTATTATTTTATATTTTGGAGGGGTATTTTGGT
TGAAAAATCGACGGAATATAGGAAAACCAAAATTCTGGTTACTATGGGGCCATCTCTTGACGGAA
CTTTTGAAAAAAGTCTTGAATTTATTGATGGAATTCGCTTTAACATGTCCCACGCAAATACCAAA
AGTATTGAAAAATATCTCGATATTTTAAATGAAAAAAATATTGCAAAACTCATGGATTTAAAGG
GAAACAAGATTAGGATAAAAAAATCAAATTTTTCAGAACTCATTGAAAATCAGGTTGTAAAACT
TGGAAAAGATATTCTAATTTCTTATATCCCAGAATGTATTGAAAAAGACCAAAATATCCTGATTA
ATGATGGAAAAATAAAATTAATCGTTGAAAGTGTTGAAAAAGATATAACTGCACGTGTTTTAGT
TGGTGGACACATAAAAGAAAAAATGGGGGTAAACTTTCCTGAAGGGTGTTTTCCAAATGATTTA
ACGGATGAGGATATAAAAAACGTGAAATTTGCAGTTGAAAATGAATTTGAATACATTGCACTAT
CTTTCGTTAGAAGTGAAAAAGATATTTTGAATTTAAAAAAATTTAATTAAAGAATATTCTGGAAA
TTGCTCAGTTATTGCAAAAATAGAAACCAAAGAAGGCTTAAAAAACATTGATGAGATTTTAAAT
CATTCAGATGGCGTAATGATTGCAAGAGGGGACCTTGGAGTTGAAGTGGCTATAGAATATTTGCC
GATAATTCAAAAAAATATCATAAAACTTGCAAATGAGAAGGGAAAACTCGTAATTACTGCAACC
CAGATGCTCGACTCAATGGTGAATAATCCTTATCCAACCAAGAGCAGAAGTTACAGATGTTGCAAA
TGCAATATTTGATGGAACCGACTGTTTAATGCTTTCAAATGAAACCACAATTGGAAAATATCCAA
TTTATTCGATAAAAACTCTCGATAATATTGCAAGAGTTTCAGAAAATGATATTTCAAAATACAA
ACAGGAAATAGATTTTGAAGAACATTCGATTTACTCGGGAATTGCCTACGCAGTTGAAGCTCTTT
CAAAAAAATTAAATCCTAAGATAGTAATTACTCCAACAACTTCTGGAAAAACTCCGCTCAGAATT
TCAAAATTCCGGTTTAATTCACCAATTATTGCCCTTGCTCCTGAAAATATAGTTTTAGAAAATT
AAAATTGGTTTGGGGAGTAATTCCAATAAAAATTGATGAAATAGGGGATGTTGATGAAGTACTT
GAAATATCTAAAAAAATTGCGAAAAATATAATTGACAGCGGGATTTACGTTGTAACGCTCGGGC
ACCCCAAAGGCGAGAAAAAAACAAACGTTATAAAAGTTGAGAGCATATAATCAAATAACAGCTT
GAAAAAGGAGATTTAAATGCACCCTACAAAAACACTTCACGGAGCAAAATCCAAATTACTGGATG
GTAAAAAACTCGTTGTAGCAGTTACTTCATCTATTGCAGCAATTGAAACACCAAGGTTAATGAGA
GAATTGATCAGACACGGTGCTGAAGTCTACTGCATAATGACGGAAGAAACAAAAGATATCATTG
GAAAATACTCGCTTGAATTTGGATGCGGAAACGAAGTAATGGATAAAATTACTGGAAAAATTGA

ACACGTTGATTTATACAATAAATGCGATGGAATGATAGTTTTTCCAGCAACTGCAAACATAATAT
CCAAAATTTCGCTGAATATTGCAGATAACATTGTAAACACGACTGCAATGATGTTTTTAGAAAAA
AAACCAATTATAATTGCTCCATCAATGCACCAAAACATGCTGGACACGGTTAAGAGCCACACAAA
AAATCTTTCTGAAAAAAATAACGTCTTTGTAATGGGCTCAAAAATGGAAGAAGAAAAAGCAAAA
GCAATTTCAATTGAAGAAGTTTCAAAGCTTGCAATTGATGTGTTTTCCGATAAAAAAGAAGAAA
AAAAGAAAGTGCTTATTTTGTCAGGCGGAACTGCTGAGCCAATTGATAAAGTGCGGGTAATTACA
AACCTTTCTTCTGGAAAAACCGGTGTTTCACTTGCAGAAGAATTTTGTAAGAACAATTACGACGT
TGAAGTGATAAAAGGACTTGGAAAAGATGTTCCATATTATATAAACTCGGAAAATGTAATCACT
TCAGAAGAAATGCTTAAAAAAGCTCTTGAACTTGGGGAAAATGCAGATATAATTATTTCATGTG
CCGCAATTTCAGATTATGCTCCTGAAACTAATTTTGAAGGAAAATTAAGTTCTGATATTGAAACA
CAAACCATAAAATTAAAACAAACTCCAAAAGTTTTGGAAAGATTGAGGAAAAAGTTCCCTAACA
AAATTATCATAGGATACAAGGCAGAATACGAAACAAATCTAGACGAATTAAAAGAAAAAGCGCA
TTCAAGACTTGAAAAATATGGATTGGACGTAATTGTTGCAAACGACCTTTCAAAACACTATTTTG
GCGATAATTTAAACGAAGTTTTGATTATCGATAAAGAAAAGGCTTTGAAAGTTTCGGGAAGTAA
AGATGAAATATCTAAAAAAATTGTTGAAATGGTAAATAATCTCTAAAGGGATAATTTGGAATAC
AAAGATGAAATCGGGGTTATCGGAATTGATGATGCGCCATTTAATAAATATGACAGAGAAACGC
TGATTATTGGAACGTACATGAGGGGGAATTCCATTGTTGATGAAATCTCCTTTAAAAAAATAGA
AAAGGATGGTTTTGATTCGACTGAAAAAATTTTAAAAATTGTAAAAGGTAAACATTTTACAAAA
ATAAATGCTATTTTTTTAGATGGGGTTACATTTGGAGGTTTTAACATTGCAGATATTGTTAAAAT
TTACAATGAAACGAAAATTCCAGTAATTGCAGTTATGGAAAAAGAACCAGATCTATTAAAAATG
AAATCTGCTTTAAAAAAATATTTTTTTGACTTTCCAGAAAAAATTGAAATGCTTGAGTCTTTTCC
AAAGCCTGAGCCTTTTGAAAATATATTCATTCAATGTATTGGAATAAATTTTGAAGAAGCGTGCA
TATTAATCAAAAAAACCAGACTTAGAAGTAAAATTCCAGAATGTCTTAGAATTTCACATCTTGTT
GGAAGGGGATTTTTGAATATGGAATAAAAGGATTTTTTATAGGGGCAAAAAAGCAACTGAAGTA
TGTTAGGTATGTAAATATTCCCAAAGTTATTTTTAAGTAAAAAAAGAAAATTACTAAAATATAA
GAAAATAAGGAGGCTTTTATTATGCCAGATTTTGGACATCCATTTGCAGGGGTTGCAAAAGACAG
AAAATTAACTGATGAGGAATTAATTCGTTCTATACGTTTTATGATCGCGGCTGAATATGAAGCAA
TACAGCTCTATATGCAGTTAGCCGAATCAACTGATAACGAACTTGCTATTGAGGTCTTAAAGGAT
ATAGCTGATGAGGAAAGAGTACACGCTGGAGAGTTTTTAAGACTTTTAAAAGAGCTTAGCCCAGA
TGAAGAAAAAATTCTATGCCGAAGGTGCAGAAGAAGTTGAAGAAGAAATTGAAAAGCTAAAAAGT
AAATAAAGTAAATATCATTTAAAGAGCCATTTTTGGGCTCTTTACTATTGTTGTTTTATATGAAA
AAATTTAAATTCTAAAATTAATTCCAAAATGCATTAGAATTTCGCATATTATTGGAAGATGATT
TTTGAATATTGACTAAAAAGCTTTTTTATAAAAAATTGGGAATTATTAACGGTTATTTTCTTTTT
AAAGGCAATTAATGCCCTTGTTTTCCAAAATCAAATAAAAAAATATTAATAATAGTTTTTTCATA
TTCTTTCTGTCTATATAATAAGTACGGAGGAATAACATGGAACTAAACGGTGTAGTAATTGAAG
ATACATTCGCAGAAGGATTCCCGATCTGGGTTTCAAGAGTATTAATCACAGCAGCAACAGAAAGA
TTAGCAAAAGCAGCAGCAACTGAAGCAACAGGATTTGCATGCTCAGTTATCATGTGCCCAGCTGA
AGCAGGCATTGAAAAATACGTATCCCCTGCAGAAACACCTGATGGAAGACCAGGATACATTATTG
AAATCTGCCACCCTAAAAAAGCAGAAATCGAGCACCAATTATTAGAAAGAATCGGACAGTGTGTA
TTAACAGCTCCAACAACAGCAGTATTCAACGCAATGGGCGAAGATGCTACAGAAGAATTAAAAGT
TGGTTTCAAATTAAAATACTTCGGCGACGGATACGAGAAAAAAGTAAAATGCGAAGAATGCGAA
AAAACACTTTACTCAATTCCAATCATGGGTGGAGACTTCCTCATTGAAAGCAAACTTGGAATTAA
AAAAGGAGTTGCAGGAGGTAACTTCTTCATTATGGCTGAAAACAACATGGCTGCATTAATGGCTG

CAGAAGCTGCAGTTGACGCAATCAGCGAAGTTGAAAAAACAATTACACCATTTACAGGGGGAATT
GTAGCTTCAGGAAGTAAAGTAGGATACACAAACCCTAAATACAGCTTCATGGCTGCAACTACAAA
TGAAAAAATGTGCCCTACATTAAAAGACACTGTTGAAGGAACCGAAATTCCAGCAGATGTTAACG
GGGTTTATGAAATCGTTATCGATGGTATCGACGAATCTTCAGTTGCTGCTGCTATGAAAGCAGGA
ATTAAAGCTGCTGTAACAATTCCAGGCGTTAAAAAAATCACTGCAGGAAACTACGGTGGAAAATT
AGGTCCTTACCAAATAAAATTACAAGAATTATTCTAATTCTTTTTAACTACTTTTATTTTTTACA
TAATTACAATTTACACGACTATTTAATCAGGAGGTTTTATTTTGAAACCAAAAAAAGCTCTTTGT
AAGGACGTCCTTGCAGAATTTACGCTGAATAAATCATTTAATACTTACAGGGGAAAAATCGTAAA
ATGCGATTTTAATGGGTTAATTGAAGGGGTGGTGATGTTAAATAAAAAAAATCATCACTATTTT
TATCCGCTTAGTGCATTGCACATGGTAAAACCTTTAAAATGCATTCCAACAAATATTCTGCCAAA
AACTTCGCTTCCAACGAATCCAAAAGAGATTCATTCAAAAGAAGCACTTTCAAGAATCGTTGGAA
GAACCCTAAAAGTATGTTATGACAACCCTAAAACTTCATACTTAGGAAGGCTTTTAGGATTTACA
AGAGGAATTTTTTCGTGGACCCTCGTTCTTGAAATATACGGGGAAGTTTTTATTTTAATAAATCC
TGATTACATCTCGTATTACGGAACAAAATGGAGACTGCCAAGAAATAATCCACCGTTCAAGTCCC
CTGCACTCATGAATCTTACAAAAACAACAATGTACCTTAAAAAATGTCTTTTAGAGGAAGTAACC
CTCGAAATGGACTATCCACGAATAAATATAGACGATAAAGCATTTGTGTATCCGCAAGGTATAAC
TTCAAAAGATGAACACTTGAAAAGACAGGTTTCTGGATTTTTAAAAGAACAGGGTTTAAGATTT
TAAAATAAAAAAATTAAAAAAGTTATCCGATGTAGCTTAAATCGTCTTGATGTTGTGGAGCCTGC
ATTCCTTGCTCCTGCATCTGCACTTTTTCTATGAATTTTTCCATTTCTTTTGCTCTTTCATCGAGT
TTTTTCATGTCAATTTCAAACCCGATTGCTTTTGAAAGCATTTCAAGTACATTTCCAGCAGATTT
TGGGTCAACCAAATATCCTGGAGTTTCGCCCATTAAACAAATTCCTTCAATTCCTTTTAATTTTG
AAAATTTAAGCATCAAACCTGCTGCACCGATAATGCTTCCACCATCAGTTCTGAATTCTGCACCA
AGTTCCTTGTGCTGTTCTGAAAGTTCTTTTGATGTTGATGCGACATAAACTTTTAATTCATCGGA
AATTTTTCCGATTCCAAATCCGCCGAGTGTATAAGTTTTTGTAGCCCCGTATTTCATTGCAATAT
CTACAATTTTCTCTGAAAGAACGTACTGCCCTTTTGGAGATAATGCCTGGGTATTTCCAAGAACA
ATTACCATTGGAACCGGTTCTTTTACAAGATAAAATTCGTTTTTCATATATTCAATTGTTCCATC
GTCTTTTACAACGATTTGAGGTGGAAAATCATCGCAGTATAATTCAAGAACCTTTTCACCGTTAA
ATTCTTCAACGATGTGTTCTGCGGCTACTCTTCCAACGTGCCCGATTCCAGGAAGTCCTTCAATTA
AAACCGCATCTTTTAAAGGTTCTGCATCAATTAATTTATTCTCAATAATTTTTACCATTCTATCG
CCATAAATCCATTTAATTAGTTTTTAAACAATGTTTTATAATTTTTTGCATATTTTCTGAATAAT
ATAATCTTTTCTTCTGAAAGGTTTAATAAATGCATTATTTTCTTTGATATATAGTTTTCCCCAAA
TAATAAAGACATTGCGCTTGTAAAGTTTTTAGGGCTGTGTTCACGTGCTTTATCAAAATCTATAA
TATAAACTCCTTTTTTTGAAACCATTATGTGTTTTCCACCTTGAATTTCGGTGTGATCAATTTTA
TGAAGATCTAACCGCAAACACTGCCTTAGAGTTTCCTCAATTACATACAAGAGTTCTTCGGGAGT
CAGATCATCAATGCAGTCTTTTAGCATTTCTCCATCAACATATTCCATTACCATGTGGTTTTCTG
AATACTCGTAAACTTTTGGACCGACTCCAAATTCATTGGTTTTTTTCAGGATTCTTCCTTCGTTT
GGAATTGTAACTTTTGGGGCATCTAACCTTGGAATTTTTACTGCTAAAATAATATGATTTCCGTG
TTTATCAATATATTTATCACTAAATGCTTTGAAAACTACGCCCCTGTGCCCCTTTCCAACGTACTC
GGTAAATACAATATATTCTGAAAGATTTTTTAAGATTTCCCAGTCAAAAAGCTTTGAAGAAATA
GCATTAAGTCCTTCAATATTCATTTTAAAATCCAAATTGGCCATATTTGCACCAAAACTGTTCTA
AATAGTCTCAATATTCAAGTTCCTGCGATAATGATTAATTAATAAATTATATATATCAAAATTA
ATATGTTAAATTTAAGGAGGGATACGATGGACAATATTGTTTACGTTGGAAACAAAGGCGTAAT
GAACTACGTGCTTGCAGTGATTACTCAATTTAACTCCGAAAATGCTGAGGAAGTAATAGTAAAAG

CGAGGGGCAAGGCAATCAGCAGGGCCGTTGATGTTGAAGAAATGGTTACAAAAAGATTCATGCCT
GAAATCAAAATTAAAGAAATAAATTTGGGAACTGACCATATACAAGGGGAAGATGGAAAGTCCA
TTAACGTATCCACAATCGAAATAATTCTTTTAAATAATTTTTAAAATATTTTATTAAAAATTTA
AAATCCAATTAGATATTTAAAATATCTTTTAATTCAATTAATGACTGTTCTCCGGTTTCCATATT
TTTCAAAGATACTTTTCCATCCCTAATATCATTTTCACCGACAATCAGCACTTTTTTGATGTTTT
TCGTGTTTGCATAGTTGAGTGCTTTATTTAATTTTCTTCCCATTAAATCTACTTCTACTGATTTT
CCAGAATCCCTTAATGTTTTTGCGATTTTCAAACATTCTTTTAAAAGCATTTCACTACTTTTAAC
AGGAACTACAAATATTGATTCGTTTTCAACTTCAAAGTCGTCAATATTTAACATGATCCTGTCAA
AACCATAAGCAAAACCAACAGCAGGGCTTGGTTCACCTTCGAAGAGCTCTATTAAATTATCGTAC
CTTCCGCCACCACAAACCTGTTTTGCACCTTCTTTTTTACCGTAAACTTCAAAGACCATTCCTGTG
TAGTAGTCAAGTCCTCTTGCAATTCCGAAGTTTATAACGTAATCATGAGGTACAAATTCAAGGAT
GCTGTCAAGATTTCTAAAGCTTCCACTGAACTTTCAAATTCAGTTAATTTTTCCTTTAATTTTG
AAATTACTTCCCTTCCGCCTTTTAAATCTAAAACTTCAAATACTTTTTTAGAAATTTCGATGTTT
TTTTCAGATTCGAGTCTACTCAAAGCAGATTTTAAACCATCCATGTCTTCTTTATCGATTAGACG
TCTTATTTCGTTTCCTTCGTCGTCTGAAAGATTATATTTTTCAAAAACACCTTTTAAAACACCTA
AATGTCCAATGTGTATTTCAAAATCCATGTTAATTTCTTTTAAACCTTCTATTGCCATATTTACA
ACTTCGGCATCTGCGATTGGTTTTTTACTTCCAATTAACTCTGAACCCATCTGCCAAAATTCTCTG
TATCTTCCAGCCTGTGGATTTTCGTATCTAAAACAGTTCGTAAAGTAGTATAATTTTACAGGTTT
TGGCGTGTTTTTAAATTCATTTATGTAGAACCTTGCAACTGAGGAGGTCAGTTCGGGCCTTAAAC
CCATCTCTCGGTCCCCGTGGTCCCTAAATACAAAAAGCTGTGTTCTAATTTCGTCGCCAGTTTTTT
TAGAGAGTAATTCAAAACTTTCAAATGTAGGCGTATTTATTTCAGAAAAATTGTAGCTATCAAA
AACTTTCCTTAGTTTTTTTTCTATTGTTTTCCTTTTTTTCATTTCTTCAGGTAAAAAATCCCGGGT
TCCTTTGGGTTTTTGAAACATTAAACATCATCTCTAACAAATTTAAATGCGTAAATTTTAAATTA
TATATCTAAATGAAAAAAGTAAAAATTATTGTTTTTCGATTGGTTCATCGCTTAATATTTTGTTT
ATATCTTCTTCCATCATGTGTCCTTTAACGGTAACGTTAACTTTTTTAACGCCTTCAAGATCTGCT
ACAACCTGTTTTGCGCCACCTGCCATGTGAATTACACTCATACATCCTGGGTTTGTAGGAGTTAA
TGTAAATGAAACGTTTCCTTCGTCATCAGTTTCCACGTCAGTTATAAGTCCCATATCAACAATGC
TGATGCCCATGTGCGGGTCTGCTACCTGTTTTAACGCATTCAATACATCATCTTTAGAAACCATA
AATTTCACCTTTTTTAGAAAATAACATAAGTTAAATTTCTTTTTATATAAATCTGTATTATTAGA
TCTAATAAAAAAAGAAAAGATTATGGAGTTAATCTCTGCCTGTCTCTTGGGAATAAAACACATTC
TCTAATGTTGTCTTGTGCAGTTAAAACCATTGCAAATCTGTCAGCACCAAGTCCCCATCCTGCGT
GTGGAGGCATACCGTATTTGAATGCTTCAAGGTATGTTTCAAATGAATCTGGATTCAAACCCATG
TTTGAAATATTTTGAACTAAAAGGTCGTATTTGTGAACCCTTTGAGCTCCAGAGGATATTTCAAG
TTCTTTGTACATTAAGTCAAATGCTTTACAGACTTTGTTGTCGTGTTTGTGCGGGAGGGTGTAAA
ATGGTCTTGTCTGTGTTGGCCATTCTGTAATAAAGTAAAGACCGCCCATTTCATCACCAACAGCT
CTTTCTGCAGCTCTTGATAAATCTTCTCCCCATTCGATTTCTACGCCTTTTGCAATCGCAATATCA
ATTGCTTCGGTGTAAGTTACTTTATCAAATGTTTTTTCAGGAATTTCCCAGTTTAATTCTAAAAG
TTCTATTTCATTAGGTCTGTTGTATTCGATATCGCTGTAACAGTTGTAGACTACTTTTTCAAGCA
TTGCCATAGCATCTTTTTCGTTTACAAATGACATTTCTGAGTCAATTGAAATTGCTTCGTTTAAG
TGTCTTCTTGTGTTGTGTTCTTCTGCCCTGAAAATCTGTGCGATTTCAAATACCTTGTCAAATCCG
CCTGCCATCATCATCTGTTTGTAAAGCTGTGGGCTTTGGCCTAAAAATGCCTCTTTTTCAAAGTA
TGAAATAGGGAATAATTCAGTTCCACCTTCTGTTGCACTTGCAACTAATTTTGGAGTATTTACTT
CAATAAATCCTTCATCTGCAAACGTTTTTCTAATTGATTTCAACATCTCAGACCTTATTTTAAAT

ATTGCCTGAATTTTAGGTCTTCTGATATCTAAAAACCTTTTGTCGAGTCTTGTATCGATTTCTGC
AGAAACTTTTTCTGAAGGATCGAGTGGAAGCGGTGCATCGGCTTTATTTAATACCCTTATTTCGA
TAGGGATTACTTCAAATCCTTTTGGAGCTTTTTCGTTTGCAACAACTTTTCCTTTGATTGCAACT
ATGTCTTCTTTTCCCAATTTTTTTGCAATCTCAAATGTTTCTTCGTCGACTTTTTGTTTTGGGACT
ACGGCCTGAATTGTTCCTGCCCTGTCTCTCAAAATTACAAATGCTAATTTTCCAAGCGCCCTTATG
GAATGAACCCAGCCCATTAAAGTAACTTCCTGACCGTCCATTTCAGGGATTACATCTTTTGAGTA
GTGTGTTCTTCTCCAATCTGCGATTAAATACACCATTTCACCTTACAATATAGAGTTATTCTTCG
TTGATTCGTTCGTAAACATTAACAAAACTAATGATTATTATGGTATTTATACTGTAATGTAGAGT
ATAGCGCATATTATAAAATATTTTTCAATAGTTCTTTTATTTCATTTTAAAATCAGTTTGATTAA
AATTAATGTAAAAAAATGTATCTTTAAAAAATAAAAATAGATTTAAAATTTACCAAATAACATT
AAAATTCCAACGATTACAAAAACAGCACCTGAAAGTTTCTGTATAAGCTCTGTCGGAAGAGTTGC
TCCAAGCTTGTCACCGATCAAAGCTCCTAAAAGATTTACAAGTGCAAGCGCCACGATTGAACCTA
AAAATGCTTTTGCCCACCCGTATTTTGATGCAAATGCAAGTGTTGCAAGTTGGGTTTTGTCTCCG
AGTTCTGCCAAAAATATTGCAACAAATACAATCAGTATCTCTTTCATTTCAACCCCCTCATTTAT
TTGGATAAATTATCTCTTTTTCAGTAATTATTCCAGTTATCAATTCTGGAGGAGTGCAATCAAAT
GCAAAATTGTATGCAGAAACCCCTTCTGCCACAATCCGTACATTATCAATGTGAGTTACTTCGGA
TTCGTCTCGTTCTTCAATAACAATATCTTCTACTTTACTTTCAAAATCAAAAGTTGAATACGGTG
CTGCAACGTAGAAAGGAATATTATGGTATTTTGCAAGTATTGCAAGCGAATACGTTCCAATTTTA
TTAAAAACGTGATAATCTGAAAGAATCCTATCAGCTCCAACAATTATCTTGTCAACAAGTCCTTT
GCTCATCATGAAACCCGCAGTATTGTCACATATCGCTTTTACAGGAATATTTTCATAATTAAGTT
CAAAACAGGTTAATTTTGCACCCTGAAGTCTTGGTCGTGTTTCGTCAGACAATACATTAATTTTT
TTTCCGTTGTAGTGTGCAAATCTAATTACACTTAAAGCTGTACCATATGCAGATGTTGCAAGTGC
TCCGGCATTGCAGTGCGTTAAAATAGTATCGCAATCATCAATTAAAACTTCTCCAATTTCCCCGA
TTTTTCTGCAAAGTTCAATATCTTCTTCATGGATTAATTTAGCTTCATCAAGTATTGATTTTCCA
GAGTTTTTTGCGGCAATAATCTTTTCAAGTGCCCAGAACAAGTTTACCGCAGTTGGTCGTGTTTC
TTTTAATTCAAGATATGCTTTTTCTATATCTTCTCCATTTAACTCAGCTAAAGCCATTCCATAAG
CTGCCGCAACTCCTATTGCTGGAGCTCCTCTCACAACCATGTCTTTTATTGCAAAACATACATCTT
TGTAATTTTTGCATTCAAAATACACTAATTCATTTGGTAATTTCCTTTGATCTATCAATACTAGC
TTTTTTTCCGAATCATTCCAAATTATAGGTCTTAAATCTTTGTTCAAAATCTCACCATTATATAT
AAGTCGAAGATAGTAAAAATAATTAACCTCAAATGAGTATTTAATTTATGCACTTTCGTTAAAA
TGTAAACTATTTAAGCACAGTTATTTAAAATTCCTCCAGAATATCTCGGGTGAAGCATATGAAAT
TTGTAAAAGAACTGATTAGTCGCAGTGATGCTAAAAATAAGGTTTTTGAAAAATTTGAAGAAAT
GTTGGATGATAAATTTGAAAATATTGAAATAAATAAAGCATTTGGTAAAATCTGCTTTGAAGAT
GTTCATGCTCCCTGCAATATCCCGATGTTCAACAGGTCCGCAATGGACGGCTATGCAGTAATTGC
AGAAGATACATTTGGAGCGTCTCAGACAAACCCGATTATATTAAATTTAGTTAAAACTGATTCGA
TAAACGAAGAAGAAATTTACAGGCTTTCAACAGGCATGAAACTTCCAGAAAATTCAAATGCAGTT
GTAATGAAAGAATATACTAAAGAATACGATTCTTTCGTTGAAATATATACTGGAGTTCATCCAA
ATGAAAATGTATCCCGAATCGGAGAAGACGTGAAAACTGGAGATCTTATTATTAAAAAAGGAGA
ATTAATTACGCCCTACCATGTTGCCCTTATCTCATCACTTGGAATAAAAAAAATAAAGTGTTATT
CTTTAAAAATTGGAGTTATTGCGACAGGGGACGAATTACTCGATATTGAAGACCTTGAAAGTGTC
GAACAGCTTGAAAAAACTGCAATGATTGTTAATTCAAATTCCATGATGGTTTCAGACCTTGTAAA
AGAAACTGGTTTATCCCCCACAGCATATAGAAAAGTTCCTGACAACCGCGAAGAACTTGAAAAAG
CGATCAAAAAAGCGCTTGATGAAAATGATATCGTAATTACAACAGGGGGAACGTCTGTAGGAGA

TAGGGACTACACAATTGAAATAATTAAAAAAATTGGTAATATAATCTTTCATGGCGTTCAGATT
AGGCCTGGACGTCCTGTTGGCTTTGCAGAAGCTGAAATTGACGGTAAAAAGAAACTTTTATTCGT
TCTTTCAGGATACCCTGTTGCGTCAGCAGTGCAGTTTGAACTGTTTATTGCAAATTATTTCAAAC
CTCGAAAATCGGTTAAACTTCAAATAAATCGAAATATTGCGTCGTCCTTGGGTAGAACGGATATT
GTAAGAGTAAAACTTGTAGAAAGTGAAGGCTTCACCAAAATTGAGCCGTTAAGAATAACTGGAA
GCGGAGTTTTATCATCCATGACTAAAGCAGACGGATACTTAATGATAAAAGAAAATATTGAAGG
ATATGAAAAAGACGAATTCGTTGAAGTTTACTTGTTTTAATCAGGTGGTTTTATGAATTATCTGT
TAATTCTTCTATTTTTTACAATCGGACTTTTTTCAGGATTTTATATAAAATCAAATAGGATTTAC
AGTACTTTTCCAATAATACTTATTTTTAATATTATAATTTATGAAGCGTACCTGCTACATATGTT
AAACAGCAGACTGGCATACAGCCTCCCGCTTGGAAATATGGCGCCATATATTCTCTCAAATAATA
TTTCAGTAGTAATTATCAGCGCTTTTGGTTTTTTCATTGGAGTTGTTTTAAAAGTTTTAACAGGT
AATTCATACGGCAGATCTTAAAATTAATTAAAAATTAAAATTCAAAAAAGTAAGATAATATTTA
AACGCCCAAAAGTGCTAAAAGTGCTCCTATGAGTCCAAGAACTGTTGCAAATAATGAAACAGGAT
AAACCTGTCTAAATGTAAATACTGGTGTGTATGCACTAACAACTGCAGAAATTATCGGGAATACA
AGTGCTACTGAAAAGTTCATCACTATTGTAATCGGGTTTAAGATATCTGGTGCAATTCCTAAATA
AAGCGTTGCAAACAAGCTTCCAAGAGTTATCATTAAAAGTTCTTTTACGCTTAAGTAAAATGCCC
TATATTTTCCAGAGTATTCCATTGTTGGGCCTTCAATAACGTCCGCCTTCGTGTGCATTATTGAA
AATGGGTAGTCTTTTGTCATGATTATATATCCAATAATATATGCTAAAGCTCCGAATAATCCGCC
CAGAGTGAATAAGAAATTACTTCCAATTAAATTATTCAAAAATATCGATTTATGAGGGATAAAT
GGAAGTAATGTTGCTAAATAAAATGGAAATGATCCAACAGTAATCATTTTAAATGCCCTTACTGC
CGAAAGCTGTCCCAACGTTATTTTTATAGTTTTTAAGTATCCGCTACCCTTACATTCATCAATAA
ATGGCATTCGAATTCCCATTACTGTTGAAGCGAGTGAACCCATTATTATGTATCCCATTTCTTCT
ATTTTTAAAAGACCAATTATACCGATCTCGTTTGAAAGAATATGGAAATGTACAACAGTAGTTA
ATGCCAAAACCATCCATACTATAATAAAACTCAGTAATGGCATGAATTTGAAGAGTTTTGGAAGA
TCTGAAACCGGCTCTTTTGCTTCCTTGTATAAATATTTAAATATCGCCCAAAAACCAGGAGAAGA
TATTGATGGACCTTTTCTTTGCTGAATTCTTGCCTGAATTTTTCTCTGAATTCCAGGAATCCATG
TGGAAATTGCAAAAGCTATTAATGGAATTCCAATCATTGAAAGGATAATTTCAAACGTTTCCAGT
GTTAAAATATTTTCAAACATAATTATCTCCTCATCCATTCAAGATTTTTGGCAACGCTTTTACAA
TTCCCGTGATAACGTCCTCAGGTCTTGGAGGGCATCCTGGAACTTTCACATCAACAGGGATTATA
TTTTCAACAGGACCATCAATAAATGTGGAAGTGCCTAAATCTGCACCAATGTTATTGTAAACTCC
GCCCATCAATGCACAGGCCCCTGCAGCAACTACGGCTTTTGGTTCAGGAATTTCATCGTATATTTT
TTTAAGTGATGCAGAAACTGCCTTTGTAACACAGCCAGTTACTACCAAAACGTCCGCTTCTCTTG
GATTCCACGTTAAAAATACGTTGTACTGTTCTGCATCATAAAATGGAGACAAAACACAGTTTACA
ACTTCAATATCGCAACCATTACATCCGCCAGTATAAACAAGCATGACGTGAATATTTTTCTTTCT
TGAAAGCTCTTTTAACATGACTACACCTTACACTTTTGAGTTCGCTTTCTTTTCCCGCTTAACGAT
TCCTGAAATGTTAATCGAAAGTATTTTAGCAATCTTTGAAAGCTGTTCTTCAGGAATTTCGATCG
GTTTTTCCAATAATTTTGAAAGATCTACTTGAATTATGTCTTCCCCAACATGCCTCGGGTGAATT
GGAGACACTTCATTAAATAAGGCAAATACAGGACAGAAATCATGACAGTACAAACAGTATATAC
ATTTATCATAGTCTATTTTTGGAATTGCTTCTTTAAAATAAGTTTCAGTAATTTTAACGGGTTCT
ACAGGTATCATTTCGATAGCATTTGTAGGACATGCGTTTTTACATCCTCCACATCCAATACAGAT
TTCAGAAAATACTGAACTTGGAAGTTCAATACCTTTTAAAATCTCTGCTGTCATCTCTTTTGAGG
TGTATCTATCGCTTCCGAGAATTATTCGTTCCAGATTCTCGTAAAAACCAGAAATAAACACTTTT
GTAAGTCCTTTTAACGTCATGATTATCCCTTTGCTTAAATTATTTAGTTTTTTCATTCCATGTTC

TGTAGATTCTTATCGCTCTTGTAGGGCAGATGTTAACACATGCTGAACAGTATTCGCATTTTTCA
GTATTTATTTCGCCCTTATCTATTGCATCGTTCGGACAGATTATGGTACAAAGCCCGCATTTTAC
GCAGTAATCGTTATCTGTTCTTATGTAGGAGTTTTCCCTATCAACTACCTTTTTGTGCCCCGATGT
ATTTTCAATAACATCTCTTGGACAGTGTATTGCACATTTTTCACAGATTACACAGTTATCGTTAA
AGATAACTTTGGAATGTACTTTATTTCCACTGAAATTTAAACTATCAATTACTACAGTGATTGCA
TCGTTTGGACATACCATTGAACAGGTTCCGCATGCAATACATTCTTCCTTATCAATTGAAGTGAA
TTTTGACCTTTCTTTGATAGAAATTGCATCTTCTGGACATTCTTTAATACATAACTCACAGAGTA
CACATCCACCGTCAACAAATTCTCCTTTATTTATTCTAACTCCTTGCGGACATTCTTTAACGCAAG
TATTGCACAAAGTACAGGTTTTTGGGTCGTATAATATTTTTCCGTTCTCAAATCTTAAAGCATTT
GAAGGACATTTATCAACACAAAGACCGCAGTTTATACAGTGTTTTTCAGGGCTTGCTTTTACGTC
TTCTTTTGTTTTTACAACTTTAAATTCGGAAATTTCTAAAACATCTTTAGGACATTTTGAAATAC
AGTTTCCGCAGAGTATGCAGTTTTCAGTAACTTCCCCGTACTCTATTGCATCAACAGGACATACT
TCCTTACAAATATTACATCTTGTACATCCGTCAACCATTTCTGCTTCGATAATCGTGTCTTTAGG
ACATAAATAAACACATTTTCCGCAGTCTACGCACTTTTCAGAATCTATCGAAATTGAATGCCTTA
ACTTTCTTTCAGCAGGTTTTACAATTTTTACGTGTTTTGGTTCAGGGAATTCGTAAGTTTTACCC
ATCATTTTAAAAATTGTAGAGTATCTTTCCATTAACATTTTATTTTTGGTATTTTTAAGGCTGCT
CGGGTCAAAGTAGAGTCTTGCATTGTATGGGCATTCAGTAGTACATAGTCCACACATTACGCACA
TTCCTGTAGGATATGATTTTCCAGTTGAATCTTCTCTTATATCAATTATATCGATAGGACATACA
TTTTTACAGATTCCGCATCCAGTACAGAGTTTTCTGTTAACGTAGTATCCGCCGTATTCGTTTTTC
TTAATTGCACCGGTTGGACATTCTTTTGCACAGGTTCCGCAGGTAATACAGGAAAATGCCTTACC
GTCAATCATTTTTATTGCATTTGTCAGGCAAACTTCCATGCATTTTGAAATTTCCTTGTTTTTAC
AGTTTTCACATTCTTCGTAAGTCCTGCATTTTTTGATGTCTGTTACTATCATATTATCGCCCAGA
AATACCTTTAATTCCACTACCTATTAAAATTCCAACAATTCCTGAGAAAAATATGTGTGAAAAAG
GTATTGATTCATAATAAGGCGTTGGACCCATTAAATACGAGATAATTAATGCTCCAAGTAACCAT
AAAATTACGTTATTCTTGTTTTCTATTTTTAATCCCAAGAGAAGACCAACTAAAACGCCGATAAT
GGCCACAAAATTCAAATTTAAAGTCATGATTTGATTGAGTGCTAAATTGTACATTGTATCACCGA
CTATTCCCTGACGATGTAGTCTTTAACTACCTTTTCACCAGTTAATAGGAATGTAATTGTTGAAA
GACCTGCTAAAACCTTTAAACCTACTGCAATATTCAAATAAGGAATAATTCCAGTATTTTCAAGT
CCTGAAGGGGATAAAAATACAGGATATCCAAATAAATCTCCAAAATTAAAGAGATAATATCCTG
AAACTATTATTCCAAGAACCCCTAAAGACATGAATAATATTGCTCCAAACGATTCCAATGATTCC
AAAAAGTGGTGTTTAAATTTAATAGGGGTTTTTGATCCAAATGCAACTATGCTTAATATATATGC
TGCTGCAATTAACGAACCGCCCTGAAATCCTCCACCAGGAGTTATGTGTCCGCCCAAAATTATGG
AAATTCCTAAAGCCATCAATAAGATACTGAAAGGCATTGCTAAAACTTTAATTATTGAAGTAAA
TCCAATTTCGGTGTCCGATTCTGGTTTAGAGTCTGAATAAACTTCTTTTAAAAATTCTGTTTCGA
ATAATCCTCTTCCAAAAACCATTCCAGTAACTAAAACGGAAGTTACGAGAATCATACATTCCCCA
AGAGTGTCAAAACCTCTCCAATCGAACAGAACTGCCGTTATCAAGTTTGGAACGATATAATTTTG
AGTGTAGATGTAATTTACACCTTCTACGGCATTTATATTAAATAATGAATAGATTATTGCCGATC
CAAAAAATACAAATGACAGAAAAACTGCGATTTCTCTTTTTTGAGTCATTTTACCACCTTATCTG
GCAAAGTAAATAACCATTGTCAGAATTCCCGAAAACATCGAAAGCCAGAAAAGCTGGTTATTTAC
ATCGTTTGAATTTTCTTTTTGAAATTTGAATATTAATGGCATCCAGGAGTACGCTAAACCAATTA
CTGAAAGGCCGATCAGTACAAACAGAATATTTCCATATACTCTAAAAATTAAAAATAAAGACAG
GATTAAAAATCCATAGAGCGTAAATTCAGCCGTTAAAACTGCACCAACAGAAATTCCGTCTCCGT
GGTTTTGTTCATCCATTTCTTTTTTGAAATCGCCGTAAGTCATTTTATCACCAAATCAGACTATA

AAGTCCATACCCTTCCAATTTCGTTTGCAAATGGATAAATTATGTCTGTTGCAAGGTTTGGGAAT
ATACCTAAAAGTATGCACAAAAATGTTAAAACTGCAAGTGAAAATACCGCGTATTTTGAAGGTT
TTGCTTTTTTGTATTCTTCAAGCTGTTCCTGACTGCAAGGCTTTAAGTAAATTAAGTAAAATGCC
TTCATCATTGATACAAACGTTCCGATACTCACAAGAATCATGATAATTGCAAGTTCAGGCATATT
TACATTCATTGCAGCTTCTGCAAGCAGTAGTTTACTCTGGAACCCGTTGAATGGAGGTACACCGC
TGATTGCAAGTTTTGCGCATAAAACCATGAATGCAGTTGCTGGAATTACTGGAAGAAGACCACCA
AGTTTGTTTAAATTTCCTTCTTTTCTTCTTTCAACTATGTGAGCACCTAAAAACAGCGCGCTTTT
ATAGATGACGTGGTTTATTGCGTGAAATATTCCTGCAACAATTCCCAAAGGAGTTCCAAGTGCAA
TTCCTGCTGCAACGTATCCACCCTGACTTATCGCGTGGTATGCAAGAAGTTTTTTATAATCCGTT
TGTAAAAGCGCCATTACAACTCCGAAAACCATTCCTAAAACGGAAAGTCCAATTAAAACCCCGTG
TGCACTTGCAAAGTAAGAAAGTCCACCAAATAACTTGAAAATTACAAGTAACAACGCAACCAGCA
CGAATTTTGAGTACGTCTGAAGCATTGCAGCAACATGCGGTTTTGCTCTTGCGTAAATATCTGCT
TTTACAGTATGGAAAGGTGGAAGACCGCTACCATAACATAGGCCGATAATAAGCATTAAAAGTCC
ACCATATATTGTTGGAGTTGATGGATTGTTTAAAAGGAAATTGTGCATATCTGTAATATTCAAA
GTTCCAGTTGCCGCCAAAAGCATTGAAACTCCAAGTAAAAGAATTGAACCTGCAAAATTACCAAG
AATTAAATATCTAAAAGCAGATCTATAAGCTTTTTCTGTGCCTGATGCAATAACAAGTCCAGTTT
GTGCAATTGCAGCTATTTCGTAAAATACATAGATGTTAAAGAGGTCATCGGCCATAACTACTGCT
GAAACACTTGCTAAACCCATCAACATTAAAGCTACAAAAAGACCGCTTGACTTTTTTTCTCCGAG
TCCGGTTATCAACACTAAAGATCCGATTAACATTATAACGGTGATAATAAGCTGTTTCACTGGAG
TATAAAGGTAAGATATTCCTGAAATAAGCCCATTTTCTATGCCGTGGGCTCCGAAGTTATAAATT
CCATACGATCCAATAAATGGAAGTGCTAACAATAGAATAGCCATTATAAGTGTCATAGGCCTGAT
TAATTTATCTTTCCCGTTAAGATAATTGAATATGATGGCCACGAACATAGGAAAAACTACAATCA
GGGGAAGTAAGTTCATTGAGCTCACCGTTTGAATTAAATCGTTAATCTTCTTTCAATATTTCTGA
AGATTTTAATGTTTTGTATTTTTTGTAGAATACTATTGAAAGTCCGAGCATTATTGCAAGCATTG
AAGCCTCAATAACGATATTTGTAAGAACAAGCGCTTGTGGAAGCGGATAGCTTGCAAGAGTTGAA
AACAGTGCAAATTCCATTTCTGGTGCCTTAATAGGAACTATTCCGCCAGTGTATCCTATTGCGAT
CAGTGTCAAATTTACGCCATTTCCAAGCGCAGAAAGTGCGATAATTTTTTTAGAACATTGTCAA
CAAAGAAAGTCCCATATAACCCAATAACAATTAAAATACCTGCAGTTATAAAGGAAGCAAGTTGT
AACTCCATATTACCACCGTTAGGGACCAAAAAACTCGATTTAAAATGCTAAATTTAAAAATCCTC
TTAATCTATCATTAATTATAATGATAAATACCTTTAAAAAACTAAATATATATAGATTATGGTA
CATTTATGTTCATGATAATTTTGGTGAAATTATGGCCGTAACAGTCGAGAATTTAACAAAACGGT
ATGGAGAAAAGGTTGCTCTTGATAACGTTAGTTTTGACGTTAAAAAAGGAGATATATTGGGCAT
TATCGGAAAATCAGGTGCTGGAAAAACAACACTGATAAAGATACTTCGAGGAGTTGAAAGTTTT
GATGACGGAAAAAATCGAAGTTTGTGGAAAAACTGAAAATTTAAGAGAAATTACTGCAATTCACC
TCCAGAGAAATTTTGCCTTGTGGGCAGAACCTGCAATATATAATATTATCAGAAAACTCTACGCA
GTTAGAACTGATTCAGACGAAGGACTCCCTTTAGAAGAGGAAATGGAAGAATATAGAAAAGATG
CTACGGAAATTTTAAAATTAGTTGGATTAGAACATAAAAAAGATGCTTACTCAAATATTTTAAG
TGGTGGAGAAAAACAGAGGCTAATTTTGGGACGGCAACTTGCAAAAATTTATGCAAAAGGCGGT
GACGGAGTACTTTTACTCGATGAACCTGCAACAATGGCATGCCCTGCTTCAAAACAGATTCTTTT
AGATGTTTTGAAAAATATCAACGAAAAATTGGGCGTTACAATAATTGTAACCTCACACCTTCCAG
AAATTCATAAATATTTATGTAAATCCTGCATTTTAATCGAGAACGGGAAAATAATAATGAGAGA
TACTCCTGAAAAGGTAGTTGACGAATTTTTAAAAGATATGGTTGAAACATATGATAGAGTGTCC
AAACCAACTGAAAAAAACGATTGTAGAAGTTGAAGATGCGTCTAAAAGATACTATGTTGTAAACG

GTGGAGAAACTCTCAATTTAAAAGATATCTCTTTTGAAGTTAAAGAAAAAGAGATTTTATCAAT
ACTCGGCCCAAGTGGAACCGGAAAATCTGTTATTTTAAGACTTCTTGCAGGTCTTGAAGCACCTG
ATAAAGGAGTAGTTAAACTCGAAGGAATCGATTTAAAAGATTTTGGATGGAACAGGATGAATTT
ACGAAGGAAAATCGGAATTATGCACCAGGAATTTTCCCTTGCACACTACTTAACAGTGAATGAAC
TTTTAAAATATAGGCTCGGTGTAAAAAGTATTGAAACCGTTGAAAGTGCAAAATTAAAAGCTGC
TGAACTTGGAATTTCCTCAAAAATGGTGGATGGACTCTACCAGCTTTTAGACCTTCCAGAAACTG
AAATGAGAAACAAACTCGATAAAATCGGAATTTCTGAAATGGTCGTAAGAAAACTTTTCCCTGCA
AAACCTGTTGAATACGATGCTAATGAACTTTTAGATGCACTCGACTTGAATAAAGAAACGTTGAA
CAAAAAACCAACGGAATTAAGCGGTGGAGAGAAAGTAAGAGTTGCAATGGCGCTTCAGCTTGCAT
TAGAGCCAGATATATTATTGTTAGATGAACCATTTGGGGACCTCGACCCTTTAACACTTAGAGAA
GTCTCAAATTATCTCAAAAAAATAAATGATACCGTTGGAACTACGATTATACTGATAAGCCACCA
CGTTGAATTAATAAAAGAGATAAGTGACCGTGCAATATTAATTGATGAAGGAAAACTCTTAATG
AATGGAAATCCAGAAGAAATCTGCGAAAAATTCATTGAAAGAAGTAATTCAAAGTTTATGAATC
AATAAACTTATTTTATAATTTTTTCTGAAAATCTAATAAAAATATAGCCAAATACTATTCCAATA
AGCATTCCTCCAAGCACATCATGTGGGAAATGCACACCAACATAAACTCGACTAAATGCAACTAA
AAATGCCCATAAAAGCATTAACATTATTTCAAATACTTTTTTTGAACTTATTATTTTTGAATTTT
TAATATTTCTTGTAAAAATTATCCAAAATCCAAATGCAAGTGTTGTGTGCCCACTTGGAAAAGAG
GGCTGCGTTCCCATATTTTTTAAAAGAATTACCCCTTCCATCATTTCGTAAGGTCTTGGCTCGCTT
ACTCCATATTTTACAGCAAATACGAGGATAGATAAAAGAAACATTGCAAATAAAACTTGGATTA
ACACTTTTTTATCATTTTTAAATGAAAAATAAGAAATCAACAAAATCGAAATGTATACTGTGTC
ACTTATAAAAATGGTTAAAAAATCAAAAAGGGGGTTTTAACTCCTGAATTTACCAAATAAAAT
AAATCAATATCCGTTTTTTGTAAAGTTTTATTAACATTAAAATTTCACCAAATTTCAATAACAA
TTTTTCCAAAACCAGTCCTTAAATTACCTGTCACAATATAAAAAATAAAATATTGTCCCCCATAT
ATTTATTTAATGGCTTCTATAATGTTAATTTTAATCTTTTTGTATTTTGATAATTTTCTAAGGGG
AAACTCATGAAAAATGCGGTCATATTTTCAGTCCTTTTGGTAATAACCTCGTGCGCATGTATTTC
ATCTGATTCTAATCCAACTACATTAAAAGATGGCATACTAACTGTAGGAGTGTTTAAATACGTAC
CACCAGCAGTTTACGAAGAAAATGGAGAATTAAAAGGTTATGAAGTTGAGTTAATTTCAGAAAT
TGCAGAAAGAATGGATTTAAAAGTGGAATACAAAGAATATATGTTTTCCGATCTTTTGAATGCT
GTAGCAAATAATGAAGTTGACTGCGCAATATCCGATATTGCAGTAACTCCTGAAAGGGAAAAAAT
CGTTTCGTTTAGCAGGGCGTATTCAAAAACTGACATAATTCTTGTGATACCTAAAGACAGGAACA
TTGAAAAATTAACAGATATCATAGGACAGAAAGTTGGAGTAACAGCAGGAACTATCGATGATGA
TAAATCATCTGAACTTTTAAAAAGCATTGATTTTCAAGTTTGCAGATATGCCACCCATGATGAAT
TATATGATGCATTAGAGAGCGGAAATGTCGATGCAATAATAAGTGAAGAAAATAACTTTGAATC
AGTCCAAAATTCCAGCATAAATTATTTGGATGAACCAATTGCAACGGTTTACTGTGGAATTGCAG
TTAATAAAGATAATAAAGAACTTCGATCAAAAATTAACGATATAATCCTCGAACTTGAACAAGA
AGGTTATTTTAACCAGTGATTATTTTAAAACAGGCCAATTATCAGGCAATACTGATCTTTTAATT
TTTCTTTTTTGAAACTCTAAAAAAATAAAATTTAGAGAGTTATTTTAAAATATAAAATGTACTG
CAAATTACATTAAAAGGTTTCCAATTGCAAGTACTATCATCATACCCATTAATGCGAGGTGGGAC
GAATATATCCCTGTAGTTTGGTATTGGGTCTTTATAATATTACATATGGTTCCGCAAACGGTTAT
TGGCGCTAAAATCTGGAATAAGATTGCCAGGTAAAATACTATCGTATTTGAAGTTAAGAACGTTC
CGATAGTGAGTATAAATGCCAAAATTAAAGCTATAAAGTGGGGCATTAATCTCATTCCGCTCATT
CTAAATGTTGCAAATCCTGAAAGCATTGAACCAAAAATTAAAGCTAAAGATACAAGCATTATTTC
GTTTGACATATTTTCACCTTAATTATAACCATACAGGCCTTGCGTTTGGTTTTGAAAGAAATCTA

AAGAATGTAGGCGCTCCTGCAATTTCGATTCCGTCTTCAAGTTTTTATCAACGCCCCTGAATTC
GGAGCTCATTTCGCATACGTAAATTTTAACTCCGGATTTTATTGCAGCTCTTAATTTATCGCCTA
GTTTTGGAAATGCAGGGTGTTTTACCTTGTCACATTCTCCTTTCAATGCAAGACTTGCTCCATCA
AACATTAAAAAGATAGTTGGCTTTAATTTCATGTTCTGGGCAACTTCCGTCATCATGAACGTTGC
GTATGCTTTTTCTGCATTTCCAGTTCCAGTCATGTTGATGATAAATACCTCATCGATATCAACTG
AAACTCCGAGTTCTTTTTTCTTTTCTTCTTTTTTAATTAAAATCTTGTAATTTCCATCACCCGTCT
TTTCTGCTGAAAGTATGTCATTTCCCATACTTTTCAGCCCTTCAGTAAGATCTGTGAATGCATTT
GCTCCTGCAACAATTTCGAGTATTTCTCCAACAGCCATCCTTTCTAAAACTTCACCAACCATTAAA
ATTGGTCCAGGACAGCTTGCACCAACAACATTTATGGATTTTGTAGCTTCGATTTCACCAATGCT
TACCTTAACGTTTGTTCCGTCAACTTCCATTTTGTAACCCATTTTTTTAATTAATTCGGAAATTT
CAGGGATTTGGGAATCCCCGTCAGTTTCGATCAACAAAATTCCGCCTTTCGTGTTCCCAATTACTT
TTTCAACAAGCATTGCAGGACTTCTGAGTGATTTTACGTTTATCTCTTTTATCATGATTACACCA
TAATTTGCAGATATGTCATTATTATACACAACCATTCTTTATGTGTTTTCAATCTTTCCTGATTT
TTAAAAGTGAATTAAAATTAAATAAAATATAAAATGAGTTATTTTTTAAGTTCTGCAATTAATG
CATCTTTTGTTGGAGCTCCAACAAATTTAACTTCCCCGTTTATTGCAACGGTAGGAACTGCCATA
ATTCCAAGTTCAGCAGCCTTTTCAGGGTGTTCCATTACATTAATATGTACAACTTCAATTCCTTC
AATCTCTTTTGCAACTTCATCAACTATTCTTTTAGCTGCTGGACAGTGTGGACACATTGGTGATG
TAAATACTTCAATTTTTACCATAAATTCACCCGATAAGTTTTTTTATTTGCTTAAATAGATTATA
GAATTAGGACAATATATATATTTGGCGATTCACATGAAAAATGAAAAATCCATTGTGGCAGTTGTT
GCCGCGATCTCATCATTTTTAACTCCGTTTATGAGCACTGCATTGAATCTTGCTCTTCCAAGCATT
GGAACAGAATTTTCGGCCGATACCATCCTTTTAAGTTGGCTTGTAACTTCATTCCTACTTGCAAG
TTCTGCACTTTTACTCCCATTTGGAAGAATTGCAGATATTATCGGTAGGAAACGAATTTTATTAA
ATGGACTGTTAATTTTTGGATTATCATCTTTAATGGGGATATTTTCTGGATCAATTAATCATTTA
ATTTTACTTCGGATACTTCAAGGAATAGGTAGTGCCATGATATTTGGAACTGCTATTGCAATATT
AACCTCAGTTTACCCCTTAAACGAAAGAGGAAAAGCCCTCGGAATTAATATTGCCACAGTTTACA
TTGGTCTTAGTTTAGGGCCATTCCTTGGAGGAATTTTAACCCATTATTTTGGATGGAGAAGTATA
TTTTTATTTTCAGGAATTACTGGAATTATAACATACCTCATTGCCAAGAGATTCGTATTAAACGA
ATGGTGTGATGCAAAAGGTGAAACGTTTGATTATTTCGGCTCGATTACATACATAATTTCGCTTG
TATTCTTGGTTTATGGATTTATTACCTTTACTTCAGGAGGATCTATTTTTATACTCGTTTCATTG
CTTTTTGGATCCATATTTATCCATAGGGAGCATAAAACAGAAAATCCAATTCTAAATATCAAACT
TCTTACGAACAATCTCGCATTTTCAATGTCAAACCTTGCTGCACTGTTAAATTACGGTGCAACTT
ATGCAGTGGCATTTTTGATCAGCTTTTATCTTCAGAGTTTGAGGGGATTTTCTGCCCAAGATGCA
GGATTAATCCTTTTAGCATAGCCTCTTGTACAGGCAATATTTTCGCCAATTACCGGAAAATTATC
TGATAAAGTTGAACCTAGAACTTTAGCATCCCTTGGAATGGGAATTACATGTATCGGATTAGTGC
TATTTTCATTTATTAAGTTAGATACAAGTATTTACCAGATAATTCTGAATTTAGTACTTCTCGGC
TTTGGATTTGCACTATTTAGCTCTCCAAACACGAATGCAATAATGAGTTCAGTTGAACGGAAATT
TGCAGGAGTTGCTTCTGCAATGCTTGCAACTGTAAGGATTTTAGGTCAGATGACCAGTATGGCAA
TAATAACTGTATTAATTGCATTTTATGTTGGGAATAACCCAATTTCTGCAGAATTCAGTCCGCTA
TTCCTTCAAGGGATAACTGCATCATTTAAAGTTTCTGCTATTCTTTGTTTATTTGGAATTTTTGC
TTCACTTGCAAGAAAAAATATCAGAAATCAAAATTAATTATTTAATTTTAATTCCATTTTTCCTT
GAAGTATTCAAGTGCACCAATTACGTTTTCTAAATCAGAAAGTGGGAATGCAAATACGAGTTCAT
CGTCTTTAATGTGCGCATAAGCTCTTGAACCGTTGCATCCAAGTGTTGCGTTTGCTACACCTCTTG
TTTTTACTGCTGCTACTGCATCAGCACATAATGATTGAATTCCTGCAAAGTCTGCCTGGAATCTT

CCACCGGCTGCATAATTGAGTGCTTGAGTAAGTCTTAAACCCTGTTTTGGAGTTACTAAAACTAC
AATGCTGTCAGGTTCAAAGTCTGCATCATTTAATGGCGCATAAACTCCAGCGTAAATTGCTTCTT
TAACTCTTGGAATTGCCTGAACTGTTTTAATTGCAGCTTCTTCTGTTGGGAAGTTTCCTAAGTTG
TGGTACAAAACACCGCTTTTTAATGCTTCAGGAGGATTTTGTAAAATTCCAATCGCGTAAGCTCC
TCCTTTACATGCTTGTTTTTCGACTGTTGCGTAGAACTTTTTACCTTCAGTTCTTGCATTTTGAAT
CATTTCACAGTGCCTTACAGGAGTTTCTATTTCTTCATAGCCTTCAGGAATTTCTTCTTTTGATTT
TACAAGTTTAACTGCTGTTGCGGGTTTTTCAAGCGCTAAAAGGTCTTGAAGTTTTTTTCCGAGTT
CTTTAATTTTTTGAATTTCCATCTATATCACCAGTCCGTTATACTTACTTGTGTATAATTTACAC
ACAACTATAAACATCCGTAATATATAAATGTTTTTATTTGTGTTAGATCTATACACAACTACCTT
TTATCTATATATTATTATATGCAAAATAAATTTTAAAAATTGAAAAATAAATATCTTTCGAAAT
AATCCGGTGAAATTTTGAAAATAACGTTAAGAAATGAGATTTGTGACCGTTTAGAAAATATTGT
AAGTGAATTAAGAGTTATAAAACACTGTATTGGTTGCGAAGGAATGGATCTAAAAAATGAAGAC
CCTCATCACCACCCGACTATCGAAATAACACAGGAATGTAATCATAACTGCATATTCTGTTATTC
TAATTTAACTAGTGTAAAACCAGGATTATACGGAGATTTAAGTAAATTTAAAGCAGTTACAGTA
AGCCAGTATGGCGAACCACTGATGTATCCAAAAAAGGTAAAAAATGCAATTAAATATGTAAAAT
CTAAAGGGTTGCGGTGCGATTTACAGACTAATGGGGTACTTTTAAACGAAGATCTTTTAAAAGAA
TTTAAGGACCTTGGTTTAGACATGATAATGATCAGTTTAAGTGCTTCAAACAAAAAAACGCATGA
AAAACTTGTAAAAGACGATACTTTTGAAAAAGTTTTAGAAAATATAAAACTCTCTTCAAAATAT
TTCCATACAATCGTAAGATCAGTGTATATCCCAGGTTTTAACGACGATGAGCTTGTAAAACTTGG
TGAAATGTTAAATGAAATTGGAGTTTCCGAAATGATGGTTCACCAGCTCGTAATTCATCCTGAAA
ATTTGGAAGAACTTGAAAACGTTGGAAATCTTGAAAACGTTGGAAAAAATAAAAGATTTAGTACT
GCTGGTTGAAAAAATCAAAAAAGTTGCACCAAACGTAAATGTTACAATAAAGGGATGTTTACTT
GTTTATTTAAAAACAATGGATGGATTTATACTAAATTCAATAAATAGCGACTGTGTTTCAGAAG
TTCCAGAAATAAAAAGAGAATACACACCAATTTTATTTTAGGTGGTTTTTATGAATATCTTGACA
GTTGGAGGTTTTGATCCAACAGGCGGTGCAGGAATCGTTGCAGACGTAAAAACAATAAAAGAACT
TCAGAAAAATCCCCTTTCAATAATTACTTCGATTATCCCTCAAAACAATAATAAAGTTTTTTTAA
AAAAAGATCTATCAAAAGAAGAAATAAAAGCACAGTTTGATGCAATTTTTGAAGATTTTAATGT
AAATTGGGTTAAAACAGGAGTTTTAACCACTGATTCAATCGATGTTATTTTAGAATTTAAAAAA
AAGTATGGTTTTAACATAATTTGTGATCCTGTTTTAAAATCAACCACTGATTTTGAATTTTCTGA
TGATTTTTTAAATAAAAAGTATTTTGAATTTTTTAAAGAGTGTTTTTTGATAACTCCAAACTTAA
AAGAATTTGAAATCATTGAAAAAATGTTTGAAACGTCCAAATCCAATTTAAATGATATTAATGT
ACTTGTAACTGGAAAAACAGATGTTTTAAAAATTAATAATAAAAATATCGAAATAACTGGAAAA
TACGTTGAAAAAGAGGTTCATGGAACTGGTTGCACATATTCTTCTGCGATTACCTGTTTTTTATC
GAATGGAATGACTGTTAAAGATTCAATAGTATCTGCAAAAGAATTTGTGCTTGGATCAGTAATCT
ATGCAGAAAAAACAAAATATGGGTACAATTCAAATCCAACATATATTAATAAAGAATCTGTTGA
AAAAAATTTAGCCCATGCATTTAATTTAATTAAAAATATGGATATTTCATTAGTTGATCCAGATT
TTATAATTTCAGAAAGTATTCTTTTACCAGAAACGTATTTTAATATTGCAACAGTCAAAAATAAC
GGAGATATCAACTTTGGAATTTCAAATGATAATTCAGATATTTTATTAAAGTTAAAAGAATTTA
ATCCAAAAATAAGATCTTGCATTTCTTTAAAAAGTGATGAAACCATTAAAAGTAAATTGGAAAA
CTCAAAACTTTCAATATATTCAATGAAACTTTTAAATGACCCTATTTCGTCATTGAAAGAAGTAT
CCATTCAAAATAATTTTGGAACCGTTTTTTATGAAACTTATCCTGATATTTTATATTTTGAAGAA
AAGAAACCCATTTTAATAATTCTCGGGGATAATTCCCTAGAAATTGTTAAAAAGCTTGAAAAGAT
TGAAAAACTGATTAAAAATAGATAAAATCAAAAAATCTACATATATTACTTGAATTTTTGTGTT

ATTTTAAAAAAGGAAGATTTAAATAATATAAATTCAATTATGCTTTTAGTATTTTTATCGAGAG
TACCATAAATTTAAACGGTGATTTTATTGGCAAATATCGTTGTTAAAAGACTAGAAAGAACACCA
ATTGATGAAATTCCTGTAGAAATCGTAGAAAGGAAGGGAATAGGCCACCCTGACAGTATTTGCGA
TGGTATTGCAGAAAGTGTAAGCGTTGCATTGTGCAAAATGTACAAAGAAAAGATGGGCGTAGTT
TTACACCACAACACTGACCAGGTTGAGCTTGTTGGAGGATACGCATACCCCGAACTCGGTGGAGG
ATGTATGGTAAGCCCAATTTACATCTTGCTATCAGGAAGAGCTACAATGGAAGTGTTGGACAAAG
AAACTGGCAAAATTATTAAATTGCCTGTTAATACGACCGCAGTAAATGCTGCAAGAGACTACCTG
AAAAAAGCTTTAAGAAACATGGACCTTGAAAAGGACGTTGTTGTTGACTGTAGAATCGGACAGG
GTTCAGTTGATTTAGTTGAAGTATTTGATAGGAAAAGAACTGAAATTCCACACGCAAACGATACT
TCATTTGGTGTAGGCCACGCTCCATTAAGTACAACTGAAAAAATCGTTTTAGAAACTGAAAAATT
ATTAAACAGCGATGCTTTAAAAGCAGAAATCCCTGCAGTTGGTGAAGACATCAAAGTTATGGGAC
TTAGAGAAGGTAAAAAAATCACTTTAACCATTGCTATGGCTGCTGTCGATAAATATGTAAGCTCA
TGTGCAGATTACGTGAAAGTTAAAGAACTTGCAAAAGCAAAAGTTGAAGAAAACGCTAAAAAAT
ACTTAGACGGTCACGAATTAGAAGTATGCATTAACACTGCAGACGATGACGAAGACTGTATCTTC
TTAACAGTTACTGGAACTTCCGCTGAAATGGGTGACGATGGTTCAGTAGGAAGAGGAAACAGAGC
AAACGGACTTATTACACCATTTAGACCAATGAGTATGGAAGCTACAAGCGGTAAAAACCCAATTA
ACCACATCGGTAAAATTTACAACATCTTGTCAAACATTATTGCAGAAGATGTTGCTAAAATTGAA
GGCGTAAGAGAATGCCAGATCAGGATTTTAAGCCAGATTGGAAAACCAGTTACTGAACCAAAAAT
CCTCGATATCGAAATGATTCCTGAAAATGGATTTGAATTAGAAGAATTAAGCCCTAAAGCTAAAG
AAGTTGCACAAAAATGGCTCGACAATATTTCAGAAGTAACTGAAAGAATTGTAAGCGGAAACGT
AACAACATTCTAATTAAATTATTTTTTTCATTTATGTAATTTTTAACTATATTAACAATTTCTG
AAATATTTTCAATTTTTATCTGTGCTGTTTCGAGCGCTTTTTTTGATACATTCCCATTTTGAAGA
GTTATAACGGATAAATCGCTTTCTTCCATTGCTAAAATATCGTTTGAAGCATCTCCCACCATTAC
TACAGTATACCCTTCCTGTTTTAAATTTAAAATAAGGTCTCTTTTTAATTTATGGTGGGCTTCAG
GCATTAAACATTTATTTTGAACCCCAACAATATTTCCAAGCCGTTCTATGAATTTTCTGCTGTCA
CCTGATGCAATAAAGACAGATGCACCCATATTTTCGAGGTCCTGAATTGTTTTTGAAACTTCTGG
GAAAATACAACCCCCTGTTGCAACCGTATATTCTATTCTACCAGTTATGGTATCTGCGATAATTG
CTGAACCATAATCTGTTTCAATATCAAATCTTTTTAAAACTGTTAAAGGGTCCTGAAGTTCTTTT
AATTTTGTTTCATTGTCCTTAAAAATTCCAGATTTGTCAATTGGAGGGTTGCAGTAAGAAATTCC
CCAGTTTACACCGTTTAAAAAACTTGAAATTAAAATATCGGGGTTTTCATTATCTAATACTTCAA
GGGGATTTTCTTTTAAAATTACGAGGCTTCTTCCTTCTTTTTGATCTACAATATCAACCGTCTGA
CAGTTGCATAAAAACTTCTGAGTAACCACGTTTTTTATTATTCTCCTTATGCTTACAAGAGTGCC
CGATGCATCAAAAACTACCGCGAGTTTCATGTTATCCCTAAAAATTGTTATTACGTTAAATATTA
TGTTTTATTATACTTTATAATTAACAAACTTTGGGATAAAAATGGAAGAACACCAGTTTAAAAT
AGCACTTGTTGGCCCTGAAAATTCTGGAAAATCGTCGTTAGTAAATTCCATTTTTGGAAAAAACA
TTTCAGAAGTTTCAGAAGTTGGTGGAACCACCAAAATGCCCGTAAAAAAGTTCTGGGGCAAAATT
AAGATAGGAAAACAAAAAATTAATCCGGATTTTGCAAAAATCACGTTTGTAGATTTGGGCGGAC
TCTACGCAGGCGAAAATAGATCGGTTGTTATGGTCGGCAGCGTTCTTGAAAAAACTTATGAAGAA
ATTGATGACTCGGACTTGATAATCCACATAATCGATGGAAGTACTGACTTATTCAAAAGTTTCGA
AAAACTGCACCACTTATTGAAATTCCGATATCGAAAACCTATTATAGTAGTTATTAATAAATGTG
ACTTGATAGAAAATTCCAAACGAGAAGATCTGCGAAAGTATGTTGAGGGGAGATTGGAAAACAA
GGTTTTTTTCACATCCTCAAACACGTACGAAGGTATCGGGGAATTACTAAGTACAATCATAGAAA
TATTGAGAAGGTGATCTGTGTTGTTCAAAAAAATCAAAAAAATGATTTCAGGCGATTCATTAAA

AACAAGCAGTCCTGTACCTATTGAAGAATACGTAGAACTCCCTGTAAAAGGCTACGAAGGACTCG
AAACCATAAAAATTAAAGTTTGCGATTTAGAAGACTTCAAAGATGCAACAGATATAGCTGTTTT
AACTGAAGCAGGTTATTTAGTTATTGCAAACACGATCAACCTTGAAAGAGATCTTGATGATGACT
ACGCAAAAATTTTATCCAGCTTAAAAGAAAAAGTTGGAAAAACTGGCGGAAAAATAGTAAGACT
CTGTGAAACCAAAGTTTTAGCAGTCCCTTCAAATACCACAATCGAAAGAATGGTAAAAGAAGAAA
CCCCTAAAATTGAAGAAGAATAAAATAACTTAAGAATGGTGGCTTAATTGACTGATGATATCCAC
GTAATGGAAGTTCTCGGAAACGCGAAAGTTACCGTAAAAAATGGAAAAGTAGTCGATGTTAGCG
ATCCTATTATCTCTTTTTGTCCTCTTTTTGCAAAACATCGCGGAATTAACGAAATTAACAAAGAA
TCAATCCAGAAAAACGTTGAATTTCGGATTGAAAGTTTTGGTTTGTTTACAAAAAACAGGGTTGT
TTTGGATGATTCGCCCTTTGTTGGATTTGGAACCTCCGAAATTTTTATGAGTGCTTTAAAAAAAG
GATTTTTAGATGCCGTTGTTGTTGTAAGTGACTGTGCTGGAACTGTTATTACAAATAACCCGAAA
CTCGTTCAAGGACTTTGCGGAAGGATTTCTGGAATTATTCAAACTACTCCGATAAAAGAAGTAAT
CGAAAAAATTGAAGAAGCTAACGGAATAGTTTTGTCAAAAGATGATGCTTTGATTAATCAGATT
AAGGGAGTAGAACTTGCAATTAAACGGGGCTACAAAAAAATCGGAGTTTCAGTTTCCAATTTAA
AAGATGCTTTAGAAATAAAAAAATTGGAAAAGAATGATGTAAAAATCATAACTTTTGGAGTTCA
TACGACAGGAATTGGCCTTTCAGATTATGACGAATATCTACAATATATCGACTTAATTTCAGCAT
GTGCATCTAAAAATCTGTTAAAATCAAAATCATTTAATATAAAAGCACAAGCTGGAAGCGGAGT
TCCAATCTTTGCACTTTCAAATTCTGGAAAAGAACTTTTATTTGAAAGATTAAAAGAAATTGATA
AACCTATTTTAATAACTACAAATGAAAAGCTACCTTATTTTACAGAAAACCCCCAATAATCTTTT
TTTTGGTGTATATCCATGTTTGCAATCTTAAAACTAGGCGGGAGCATCCTCTGCGATAAAAACAT
TCCGTACTCAATAAACTGGGAAAATTTAGAAAATATCGCGATTGAAATAAAAGAAGCGATTGAA
TACTACAAATCTAAAAACGAAGAGTTTAAACTAATTATTGTTCACGGCGGAGGCTCTTTTGGCCA
TCCTGTTGCAAAAAAATATCTTAAAGATGGAAAATTTGAAGATATGGAAAAAGGATACTGGGAA
ATTCAAAAAGCAATGCGTAAATTCAACAACATTGTAATTGAAGAACTTCAGAACTTTGAAATTCC
TGCAGTTTCAATTCAAGCTTCGTCATTCATTACGTTTGATAATGATTCAAATTTACATTTTGACA
CTAACACAATTGAAAAAATGCTTGATAAGAGATTAATTCCAGTAATTCACGGCGATATTGTAATT
GATGAAAAAACAGATAATTTCAAGATTTTTTCAGGAGATCATGCACTTCCATTTCTTTCAAAAAA
ATTAAATCCTGATTTAAGCCTCCATGCATCAGACGTCGATGGAGTATGGGATTTAAACTTTAAAA
TAATTGAAAATATCAATTCAAAAAAATATCGAAGATGTTTTAAAATCGTTGAAACCATCAAATAA
AGAAGATGTAACAGGCGGAATGCATCTAAAAGTAATGGAATGCTACAATTTAGGCATTAAAACG
ATAATATTCAATGGAAATAAAAAAAGAAATATATACAACGCCCTTTTAAAAAATGTAAAAGGAA
CATTGATAAATTAAGTTATTAAGTTATTTTCTTTTTTTAACTTTAATGAAGTCTCCAAGAGTAAA
TTCACTATCTTCATTGCTTGTTTCGTAATTGAGATCTCCTTCTTCGTAGAAAGAAATTCCAAGTG
CTTTTTCAAGGATTTTTACGTATTTTTCCTCGGGCTCAATTTCATTTCTTTCTATTTTGTGAATA
AGAGATTCTTTAATTCCGACAGCTCTTGAAAGTTCTTCTAAGGTCATGTTTTTCTTTTCCCTTGA
TTCTTTTACAAGAGAACCGTAATCTTCGACCAAAGTTTTTAAATTATCAAACATGTCCTTTCTAG
GTCTTTTAGCTTGTTTTTGTTTTGTTTCATTTTTGAATACTGCCCTTGGTTTTCTAGAATATCCT
TTAGGCGACATTCCAAACTTTGCGCAAGCCTCGCAAACATTCATTTCTACGCCTTCTACTCTTGTT
TTAATGATATTCTTAACCTCTTTTCCGCAGAGTTCGCATTGCATACCTACACCTTAATATACTAA
ATTTAAAGATATTTTTGTCCAAGTTATCCTAATTACATAGCATTCAAATGGTAAATGCTTCATTA
GAGAATATATCTTCAATTATTTAATATTATCTATTTTTGTTATTTATATATTATATCCATAAATT
TATATAGTCCAAATAAATTATAAACATATACCCTTATTGAATAATAATTGTAAAAATGTTTTCG
ATAGGTGTAAAAACTGTTTTGCGAGAAAGAAAATCAATGAGAAAGCGCTATATAGAACTATTTT

TATCATATTTATTACGGAGGTAAATCATGAGTTACCCAGATGATTATTCCACAGATATAGAAAAA
AATAAAATGGATTTAAAAGAATTTAAAGAAAAAACTCAGATCGCAGAGTTAGAAAGTAAAGTAT
TGAGATTGGAATTAAAAAATAAAGATATTAATAGAGAGAATGTACAAATTAAAAAAGAAAATGA
AATATTAAAGCGAGAACTTGATAAATTAAGGATTCCACCACTCATACTTGGTACAATACTTGATA
AAGTAAATGAAAGAAAGGCAGTTGTTAAAAGTTCCACAGGACCAAACTTCCTTGTAAACCTTTCA
CAATTCGTAGATCCTGAAGATATTGTTCCAGGAGCAAGAGTTTGTTTAAACCAGCAGACTTTAGC
AATCGTTGAAGTACTGCCTAAAGAAAAAGATTACCGAGCTATGGCAATGGAAATTGAGGAAAAA
CCAGACATTTCCTTTGAAGATATCGGTGGTTTAAACAACCAGATTAGGGACATTAAAGAAGTAGT
AGAACTCCCGCTTAAAAACCCAGAATTATTTGAAAAAGTGGGTATCGTTCCACCAAAAGGAGTAT
TACTCTACGGTCCGCCAGGAACTGGAAAAACTTTGCTTGCAAAAGCTGTTGCATACGAAACCAAT
GCATCATTTGTTAGAGTTGTCGGCTCAGAACTCGTCAAAAAATTCATCGGTGAAGGTGCAAAACT
TGTAAGAGATGTTTTCAAACTTACAAAAGAAAAGTCACCATGCATTATATTTATTGATGAAATT
GATGCGGTTGCAAGCAAAAGAACTGAATCATTAACTGGTGGAGACAGAGAAGTTCAAAGAACTT
TAATGCAGCTTCTTGCAGAAATGGATGGTTTTGATTCAAGAGGTGATGTAAAAATCATTGCTGCA
ACAAACAGACCTGACATTTTAGATCCTGCAATACTCAGACCTGGAAGATTCGATAGAATCATTGA
AATTTCAATGCCTGATGAAGATGGAAGACTCGAAATCTTAAAAATCCACACTGAAAAAATGAACC
TTAAAGGTGTGGATTTAAGAGAAGTTGCAAAAATTGCAGAAAACATGGTTGGTGCTGACTTAAA
AGCGGTTTGTACTGAAGCAGGTATGTTTGCAATAAGAGAAGAAAGAGAATTCATCAAAATGGAT
GACTTCAGAGAAGCTATCTCGAAAATTACAGGTAAAAAAGAAAAATGCAGTTACGACATGCCACA
ATTAACCGTAATGTACGGATAATTAACTACTATTTTTTCAACTTATTTTTATTTTAAATTTTTTT
AAGTGATGTTTATGAAGATTGGAATTATTGGAGCAGGCCTTGGGGGCCTATTAAGCGGTGCAATT
TTATCAAAAGAACACTCAGTTACAATTTACGAAAAACTTCCATTTGTTGGAGGTAGATTTACAAA
TATATCTTATAAAGGATTCCAACTGACAACAGGGGCCCTTCACATGATACCCCATGGAAATGACG
GGTATTTAGCACAAACACTTCGAAGAGCAGGGAGTAATGTAAAAATAGTAAACTCCGTGCCAGAT
GGTTATTTCAGAATAAATGGAAAAAATCACGAATATAAAGACATTTTTGGACTTGTTAATACAA
AGGAAAAATTAAAAGGATTGAAACTTGCTGCAAGCCTAAAACTTGGAAAAATCGATAAAAATAT
GCCATTTGGAGAATTTTTAGAAGATATTCCTCTTGCACTTGATGTTGGAAATTCGTTTAGTGGTT
GGGCCTTGAGTTTAAATGCATATGACACCCCAATGTCAGAAATTATCGAAATAAGTAAACTATAC
CATAAATTTGGGGGCCCAGGAATTCCTATTGGGGGCTGTAAGGGCGTTATTGATAGCCTCGTTGA
AATAATCAATAAAAACGGCGGAAAAATCTACACAAATCATGCGATAGAAAAAATCGAAGTTGAA
GAAAATAATGTGTTTTTAGATTCAGAACAGTTTGATATAGTAATAAGCAATGCTTCTCCAAAATT
AACAGAAAAAATGTCAAATATCAAGTTTATTGAAAAAGAGCCTGTTCCATCGAAAGGAATAAAA
ATAAACGTTGCAAGTAAATCAAGTCTCGTAGATGGTGCAAGTGTCATATTTACGACAGATTGCGA
AAGAATTAATGGTTTAAACCAGCCTTCAAATGTTGACAGGAGTTTAGCAAAGGAAGACTATAAT
TTAATAATGCTACATGCAACACAGATAAAAAACAACACTAAAGAAGAAATAAGCCTTGCTTTAA
ATGACGTTGAATCACTATTTGGTGGAAAAGACTACGAAGTAATATCCATTCAAAGTTACGGTAAT
GGGTGGCCTGTAAATCATGCTTCAAATGGAACTGACCTAAATCCAATCGTTAAAGATAACTTATT
ACTTGTTGGAGATGGCGTAAAAGGAGTCGGTGGAATCGAAGTTGAAGGTGTTGCAATGAGTGTT
ATTGCAGTTTTAAACCATATCGAAAAATTAAAAAAATAGATTATTATCTTATTATTTTAACGCCT
GTTGCTTTAAACATCACGCATAAATTTTTATCTTCTTCAATTTCTAAAGCTTCAAGGGAATTTGG
TGTAATAAGTACTTTTAAATCAACACCGCCAATATCCAGTGTTAACCAGACCAGATGTCCTCTTT
TTTTAATCTCTGTAACTTTTCCATTGAATACGTTTTTTGCAGAACTGTCAAAGCACTTCAAAGCA
ACTATTATATTTTCAGGCCTTATTGATACTTTTACATTCTCGCCGACTTTTGCAATGTCTGAAGA

ATATATTTTTACCCCTGAAACATCTAAAACTGAAATATTTCCAGATATTTCTTCTACTTTTGAAT
CGAGAATGTTTGTACCAACAAAATCTGCAACAATTTCCGATGAAGGATTTGAAAATACTTCTTCA
GGAGTTCCATACTGGTAAACTCTACCGCCCTTCATTATCAAAACTTTATCTGCAAGACTCCAGAT
ATCTTCAAAATCGTGTGTTACGTGGAGTACCGTTGTTCCATATTTTTTTATCGCAGTTTTAACCAA
GAACCCTTATCTTTTCTTTAGTTTTAACGTCCAAAGCACTGAATGGTTCATCCATAAACAGGATT
TTTGGCTTTATGATAAGCCCTCTTGCAATGGATGCTCTCTGCATTTCCCCACCACTCAGTGTTTCA
GGTTTTCGGTTTAACAGATGATCAATTCCAAGCATTTTTGTTATTTGAATAATTTCCTCTTTTAT
GGTTTTTTTATCGTTCAATCGTTTTTTAAGACCATATTCAATATTTTCATAAACTGTCATGTGCG
GAAACAGTGCATAATCTTGATAAATTATACTTATATCTCTTTTTTCAGGGTTTAAGTTTGTTATA
TCCTTCTCTTCCAAAAATATCTTTCCAGTTTCAGGAGTATAAAATCCAAGCGCAGTTTCTAAAAG
AATAGACTTTCCACTACCGGTCGGCCCGATTATTGTTACATAATCTCCTTTTTCAACGCTTAAATT
AACATCTTCTAACTTAAATTCGCCCAGATTGATATTCAAGTTTTCAATTTTAACGAAACTCAAAA
TTTCCCTCCTTCGTATTTCTCAAATATCAAAATCGCGATAAATGATATCATTAAAAGCACAATTC
CACTTGAAATCGCCATTCCAAGATTACCGTATGACATGTTCAGGTACAAAGTTATTGGAAGAGTT
TCTGTGTACATGTACGTTCCACCTGCAAGCATTAAAACTGTTCCAAACGTACCCATGCACCTTGCA
AACGCAATTATCGATGAAGCAAAAATCCCGCTTCTTGCCATTGGCAGAGTAACATTTTTAAAAGT
TTCAAACTCGCTGTATCCAAGGCTTCTTGAGACAAGTTCATACCTTGGATTAATATCCTGAAAAG
TAGAACATACTATCCTTATTGCATAGGGAAGTGCAGTAAAAATCTGGGCTACAACTATTCCTAGT
TTAGTAAATACTACTTTTATTCCAAAAAATTCTAAAATGTCTCCAATAAACGTTTGCCCAAATAA
AAGTAAAAGTGCAAGTCCTAAAACAAGTTCGGGAAACGCTATAGGCAAATCAAGTATTGCTTTTA
CAATGCTTTTTCCAGGAAATGAATACCTCGATATTGCGTAGCCTGTAGGGATTGAAAACCCCAAA
ACAATTCCTGTTGAAATTAAAGAGGAATATAACGATACTTTAAGTGAATAAATCATTTCTGGAT
TAAAAATTGAACTCAAAACATCATCAAACTTTGGAACTAAAATAAGTGAACTTATCGATAAAAA
TAGTAAAAAGGTAAACAAAAATGTCAGAAATATTGACATTTTTTTAAAACTAATCAGTTGCATG
GATTAAAACCCCAGTTCTGCCAGATTTCTAACGATTCTTCGGAAACTACATAATCATTGAAAGCT
TTTGCAACTTCAACATCTTCTGCCATTGTGGTAACTGCTGTTGGAATTGTTTTTATGGTGTTTTG
ATCTTTAGGTATTTGAATAACTACAATTTTTCCTTCAGATTCTGCCCATGTAACCATGTCTTCCC
AAATTATAGTTGCATCTGCCTGCCCTGTTGCTTCATATATTAAAAGCTGGTTTACAGTAGGCGTG
AAAACTTCAACATTTGAATTTACATCTTCCCATACACCGTTCTTTTCACATATGCTTTTAGCAGT
TTTTCCGATAGCACATGCTTGTGGATCCCCTAAAACAACTTTTACATCATCTCTTGCCAAATCTTC
AAGAGACTGGACATTTTTAGGATTTCCTTGAGGAACTGCGATCACAGGAACGTGGTCAACTACGG
TTAAAACAGTTTCATTTAATATGTATCCGCCGTTCATTGCGTCTTGGGTATATTTGTATGCACCC
GGAATAAATACGTCACATTCACCAGTTGTTGTTAAAACACCAAATAATTCAGCGCTTCCACCATA
ATGAACCTGAACTTTTGCTCCAGTTTCATTTTCAAAGTTAGCAATTATATCATTCATAGGTTTCA
TCAGACCTGCTCCACAGCATAATCTCAATGTTTTACCATCGTATATCTTTTCAGAACCTGCATCTG
CCGAATTGTCAGTACAGCCTGCAAAACAAGTTAAGAGTAAAACTCCAACAAGTCCTAGCACTAAT
ATTTTTTTCAAAGTATCACCAAAACAACGTGAAATAATAATTTCGCACATATGTTTAAAATTAAA
AAATGCGAAAAAAAAATAGGGGATTTTAATTATCCCATTACGACTAAGTCACTTATTTCTTCAGGA
ACTTCTCCAACAGCAATTGCAGGGCTGATTACTGGTTGTCCTGCATTTTCAAACACCTCAGGGTG
TTCTAATACTGTTTTCACGAAATTAACTCCTTCTTCGTAGTCTTCTGCATTTGAAGGTACAGTCA
TTCCGTAAACGATTGGTTTTGCAAGAATTGTACTGTTTTGACCTTCTAAGATTATACTTGCTTTA
GCATAATCGTCTGCATTTGAATAGCTTCCGAGGTTAATTTCATCAGGAAGTTCAATGTATGATAA
GCCGTGCTGGTTTGCAACACTCTTGTAGATAAATAAGTAATCGTATGCACCAGTTTCTAAAGGAC

CTAAGAGGTCTACTTCTTTACTTCTTAAGAATACTTTGTCTTGGTTAACTTCAATTGATGCAGGG
CTTCTTACAAGGTAAGTTCCGTCTGCATTTTCATCAACTTTGAAGTTTGTATTTCCAAGCATTAA
GTTATCGTAGATTGTTGAATCTCCGTAAGCTGCTTCTGCAAGCTGTACTACCATCTGGGTTCTGT
AACCACATGGGTCGTCATTAGGACTTGAGAAACCGTATTTTACACCATCTCTTTGGAAAATTTCG
TACCAGTTATCTGAATTAATTTCATCGTAGTACTGGCTGTTTTCAGTGTATGCGATAACAATTTC
GTTTTTCGCAACCATTATATACCAGTCTGCATAGTCTGGCATCATCATATCAGGAATTAATGTGT
AATCTGCAGAAGCTAAAATTTCTGCTGTTTTATTGAGTTCAGTTATTTTTCTAACACAAGCAACA
CTGCCTGCAGATTCTCTTTGAACGTCCACATTAGTATATTCATTTTCATATAATGTTTCATATTC
TCCAAAAGGTACCGCAAGACTTCCTGCGTGGAATATTTTCAATACTTTTGGAGTTGCGTCTTCAG
ATGTAGAATCAGAAGCACTTTGGTCTACACATCCGCTGAATGCGAGGATCATTAAAATCCCGAAC
ATCGCAACGATATTTAATGATTTTTTCAAACTACCACCACCATTATGGTTAACGTCTATCTTATG
TGTTTAAAAATTATATATACATTACGAGATAAGTTCAGAAGTAATAATTTATAATTAATGATAT
TATTTGAGCAAAATTAGTAATTGATAATGATTACCCATATCTTAAAAAGACTTTTTTAAAGAGA
TTTTGGCCTAAAATATATTTTAAAGGATTTTTTAAAACAGCCCTTAAACAGAAATTATGGAAATG
TTGATATATGTAGGAAAAGAGAAAGTAATAACGATAGTTTTAACTCGATATTTTTGATTTTATT
TGAGTCTTTTTTTGGTGAAGACATGTATAGAGAACTGTCTAAAGAAGAATTGTTAGAAAAAATTT
GATGAGTATATTACTTCAGAAGATATTAAAAAAAGAGCAGAATCGACCAAAATATTGGGATACC
TCGGAATAAACGACATTAAAAGTAGAATGGATAATATTTTAAGCCTTACTTTTGAAAATGACAT
TTCAATACGGCTAAATTTAATAACTGCTTTTTCAAATATTTTAAACAAATTTCCGGAAGTTTTTA
GCGAATTAATTGGTTGTATTTACTACAATACCGAATTTTCAAATAATATTTTATCAGAACATGCA
AAAAACGAAATAAAAGCCCTTGGACCCGAAGTAATCGAAAGACACGTTGAACACTTTCGAAACGG
AATTTATTCAAAAAAATACTGAAGAAAGATTAAAATCAGTTATATTACTTTCGTTAATATCTAGA
TTCAGCCCGGAATATTTGGATAAAGCATTATCTGAGATTGTAATGGCGTCTACTTATGATTCATC
TGCATTAATACAGTTGCTTGCACTTGGAACCCTTGACGATTTTTCAGAAATTAATAGAAACTTTT
TAAGATATTCCTACCTCGTTTTTAATAAAAAAATGGATATAACACCATTAAATAAATCAAAAACC
GGCGCTGAAATTACTAAATCATTGATTAGACTTACAGTTTCAGACGAAATCGATGGAAATACAAT
AAATGAGTTGTTGAAGTATTTATCAATTGAAAACGACTTTTGTACACTGCTTGCAGCCATATCCC
TTCAAAATAATATTTTAAAAGTTGAAAAAAATTTTGAAGATTATCCGCTAATATTTCAAGACCA
GCTCTCAAAATTTTACAAAAAACATCCAAACAATGTTCAACAGCTGATTTCTTATTCCTTAATTT
TAGATATAACCCATTCTTTAAATTTAGAAATTAAAAACGTGGATTTTGAAAAAATATTTTATTA
TTTAAAAAAGAATATTTCTGGAAAAGACCACATTTCAAAAGCATTCTCTTTAATCATTCTCTATT
TGCTACACAAAATGGAAAAATTGAATCTGGATGAAGAAATCGATGAAATTTTTGATAATTATCC
GTTTGAAAATATATTAAAAGAACATCCGCTTTGCTACTACTTTGGAATGCCTCTTTTGGCCGAAA
AATATAATACTTTGCCGGAAGTTGACAAAAGTCCTTATCTAATCCAGTCTCTTAAATTAAAAATG
CAGTATATGGCCCCGACAGAACGCCACCAGTACATAAAATCAGCCGAAAAGAATATTAAAAGTGA
TTACCTGGAAAAACGTCCAGAACTGTTAACTGAATTTAACACTGCTTCAAAAATTAAATCAGTAT
TGGGGAGTCACTACGTAAATGAAAATTATCCTCCTTTCAAAATAGCGATAAAAAGTATACTGGAT
AAAATATTAAAAAATTTAGAAAATACTCCAAAAAAGTTTCAGGATGAACTAAGCGGGGAATTAT
ATTCAGAACAGTTATTTGAAAACCCGGTAACTTCTGCTGCATTTTTAGAAAAATTAAAAATAAAC
CTTGATAGTTACATACTAAAAAATGATGAAAAAAATATTTTAAAAGTATTAACATTCCTAAAAA
CGCATGAATCAAAAGTTGATTGTTCCTATCTTTTGGAAGAACTCGTAAAATTGAAAGATAATTAT
CCTGACGCGAATTATTTTTTAAATAACATAAAAAATAGGTACAAATTAATTCCAAAACCTCTTGA
AATCAGGATATTAAACAGTCTGAATAACCCTATCCCAGAATATAAAAAAGATGGGCTGGAAGAG

ATTATTACGTATTTAAATGAAGGATTTAAGATTAATAACGGTATAGGCCTTAAAATAAAGGAAA
TTTTAGTAATGTACAGGGATGAAAAACTGACTGACCTCTGCATAAATATACTAAATTTACAAAAA
GATATCGAAAGTAAAAGACTGATTTTTGAAAAAAGAGAATTTGATACTCATTTGAACGACTTAA
AAGAGATTAAAGAAAAATCTGATGAAAACATTAAAAAGATGACCACTGAAGAGCTCTATGTAAA
ATTAAGCAGTCAACTGCATAAAACTTCAGAATATGACTACCAATCCATTAATTTTAACGATATTT
TGTACTTTTTATCGGATTATTCAAAACGTCCATCAGTAATCCTGACAACAATGATATTAAAAATC
CTAAAAAATGTTATAACTGACCCAAATTCAAATATATTAAACAGTCCTGATATAGATAATCCAAA
AGTCCTTGAAAATTTATATTATTTAATTTATTCAACAGATTATGAAATATTATCAAAAAATGCA
ATTATATTGACGGTAGAAATTGTTAAAAATAAACCTGAATGGCTATTAACGAATATAACCAATG
CTTCCAAAAATAAAAATTGGCCATTATTTTTAAATTCACTTTTAGATTACCCTGACGATAAAGTA
CTCGGAGAAACTATAAACGCATTTGATCATTACGCTAAAAATTCAAATTCGTTTGAGTTAGCCGC
AGAATTTCTTGAAAAACTTGTAAACCGGCTAGATACATCAAACTGGGAAAATTTCAAAAAAATT
GTAAAGGTACTTGGAAATTACGAAATGGACGAAAAAATGTTAAACAGGTTTTCCAAACTTTTAC
TTGAAAAAATCGAAAATTCAAACGATGACTCTAAATTAATACTGTTGAGATTCTTTAAAATTCA
AGATATATCAAGATTAAACAGCAATTTAGTGGCTCAACTTTTAAATCTAAAAAATTCTAAAAAT
TACGATATTAAAAGAGATATTGAAGAAATAGAATCCATGAAAATCAAATTAAGGGGCTGAATAT
GTACCAAAGCACGGATAAAAACGACCTTGCAGCACTTTTTGATAAATATATTGAGTCTGATAATT
ATAAAAATAAAACAGAAGCTGCAAAAATTTTGGGATTGCTTGATGAACAGGCACTTGACGAAAG
AATGCAAAAAATTGCGATATTAAATTCGGACCCAAATATCGCAGTTTTGCTAAATTTACTTAGTT
CTATAAAAGTTATTCTTTTAAAATATCCGAAAAAAGCCGAAAAACTTTTAATACATGTTTATATC
AATACTGAATTTTCAAACCACCTGGTAAAAGAATTTTCAAGACAGATTATTGATTCAATAAATCC
AAAGGTAATCGATAACCTTGTAGTTCATTATAACTCAAAGTTATACTCGAAAAATAATTTTGAAG
TTGCACGGGCACTTTTAGTACTTGGTTTTGTATCAATGCACACTCCAAGATACCTTAAAAACTCA
CTTCCTGAACTTTCAATGATTTCGCTTTATTCTCGCTCAGAACTTTTGAGGATTTTGTCTCAAGC
AATACTTGATGAATTTGAAAACATCCCTAAATCAATTATTAAAGACTTAAAATTTTTGATAGAA
TCGCCAGATGCTAATGAAGATAACCTAAATGCTGAAAATGATCTACTTAAAAAACTTGTAATTCT
TAATCTAAAAAATGAGATTAACAATGACGAATTATTATTTATTTTAAAATATTTGGACGATGCA
GATTACAAAATTGCACTGCTTTCTGCAATTACTCTTAAAAAACACCAAAAGTTATTAAAAAATTT
AATTCGATCAAAAAACGAATTAGACGTGCATTCTTTAATCAGTGGTAAGCTGTATGAATCAGACG
AAATTACTGCAATAAATGCATTGCTTGCTTTTTCATACCTAACAATTGACATGAATTTAGAAATG
GATACTAAAAGATGTATAAAACTCATTGAAAATCAGGTTAAAGAATATCTAACACATGAAAACT
ATTTAATAAGCTTTTATGGATTTGAAATGCTGAATTCTTTTGTTTTGTTAAATAATGATGAATTG
AATTTACGTATTGAAAAAGTTCTTGAAAATAATGACCTTGGAAAGCTATTTGGAAAAAACAACT
TAAATTATTATAGTGGAACGAAATTGCTTGTAAATTTAGGGCGTTATGATTTATTAAGCCCTGAA
AAAAATATGTATTTTGATGAAGACCTTATGATTGAATACAGCGAACTTTCAAAAAACCGGGCAA
TCGAACAGTTTAAAAACCTTGAAAGGGAGTTTAAAAACGAAGACTGGCTTTACAGGTTTGAAAT
TGGAAAAAAAATTGGAAATTTGCTTTATGCATTTCCAAGCGAAATAAACTATAAACAAGATTTA
ATACAGACAATTCTTACAGATCGGGTATATCTAGTAAGATCTATTGGCGCATGGATTCTTCAGAT
CATTCTTGAAAGAGGATTTAAAATTCCAGAAAAATTAATAATCGAATCCATTGCAGGTTTTGATG
ATCCAAATATGGAAATAAGGCAGGATTGTGCAATTTTTTATAACAAATTAATTAAAAAATATCC
TGAAATTTTGAAAAATTCCGAAATAAGTTCTAAATTACAAGGTTCCCTTACAACAAAATATTTTA
CAGACCATTTTACCGTTATTCGAACAATCTGTGAAGATACATTAAAACTGATTCCTGAATCAAAA
GAATTAATTGGTTATGAATCTAAAAATCTGGAAGAAAAGTTTAAAACACTTGAAAAAGCATTGG

ATCATCCTGAACTTAATAAATCAGTAGTAATCAGACTTAAAGTAAAATTAAAAAGCTACATTAG
ATCAGAAGATAGCGAAAATATCGTAAAAATCCTTGAATTCATTGAAAAGCAGGAATTAACTGAA
GAATATTTTGACTTATTTCATGAATTATTTAAACTTAAAAAAGACTTTGAAATTGCAGGTGAAT
TATTAAATAGACTCAAAAATAAATTTTCAGAAGTTCCAAAATCGCGTGAAGCAATTATCTATGGG
AATTTAAACGAAATGATAATTTCAAGGCGCATAAATGCAGTAAATGAAATTTTTGAATACATTT
TGGAAGGCTATAAAATCAGCGAAAGAATTACTCGGAAAATAAAAGAATTTGTAATCTACCCGCA
AGCAACCCCCAAAATCACAGAACTATCTTTAAAAATACTTGAAAAAATCGATACAGAAGAATCTA
AAAAAATATTTGAAGAAAAACAGGAGCTTGAAAATTATATCTTGGAAAATAACTTTGAGTCAGA
AGTTGTCGATATCAAAAATCAAACCTGGCAAGAGATTTATTTTTCACTAAAATACCTGTTAACAC
ATGATTATAAAACACTAAACTACGTGGATCTTGAATTCTTAGATTTTATGAAATTTAGCATCCTT
AACTCTGAAAGAAATTCACTAATCATTACGATAATTTTACTGGATATTTTTAAAGCCAATTTAAA
ATCCGGTGATACTAATTTAATGTACGAACTTGGAAAATACAAAAAATCAGTACTTAATAACATTT
TTAAAATAATTTTCAATGAGAAATACCCGTTATTAGCATATAAAGGACTTGAATGTTTAAAAGT
GATAATAACTAAAAAAACTTCTTGGTTTGAAGAATCAGTTTTAAATGCTGAAAATTTTGAAGAA
TATCTGCCACTCATTTCAAAACTTCTCGATGAAAATGAACCGCAAATACAGGTTGAAGCGTTAGA
AACGCTTGCCAGCATGGTAAAATTAAATGTTAAATGCACAGAGCATCCTGAAATTTCTAAGAAAG
TTTTAGAATTGATAAGTGACGATAAAAAATGGATAATCTTTAAAAAAGCTCTTGAAGTAATTTA
TTCATGCAACTTTGAGGATAACCTTGAAATGCTTGAAAAGGTCAGCAAACAAATTATTGAGTATC
TTAAAACATCAAATGACGATTCCAAGCTATTTTTGATAAAATTCTTTAAAATAAAAGGAATCGA
TAAAATACCCGATTATTTATTTGATGAATTAAAAACCTTTGAAAAGAGTTCAAATCCTTATGTAA
GTTCAGAAATAGCAGAACTCATTAAAAAAAGAAAAATGAATATGAATTATTAATTTATTTTTTT
TCCACAAAATTTTTCAAAAAGTATTCATATGCAATAACTCCGTCATCTGAAAGTTCCGGGTGAAA
CGATATTGCCATTATGTTTCCTTCTTTTATTCCCACAATATTTTCATCGTCTTTTGAAATGATTTC
AATGTCTTTTGAGAGTATCTCCCCAACTTGTGGTGCTCGAATAAATACTGCGTTTATTTTTTTTC
CGCCCAAGTCGATTTCTTTTTCAAAACTATCTTTTTGACTGCCGTATGCATTTCTTTTTATCGTCA
CATTCAACATTTCAAGCAATGGCTGTTCTTTTCCAGTTCCTTTAGATAAAAGAACCATTCCTGCA
CAAGTTCCAAGTATCGGGATTTTTAAATTTCTAATTTTATCGATAAATCCATAACTTACCATCAA
TTTTCCAATTGTGGTACTTTCTCCACCGGGAATTATTAATGCGTCTATTCCTTCTAAATCATCTAC
AGTTCTTATCCTTTTAGGGATGCAGTTTATTTTTTTAACTGCATCTTCGTGTTCTTCAATGTCGCC
CTGAATGCCGAGTATCCCGATTATTTTCATAAATTCACCGTTTTTTATAAATTGTAGTTAATACG
GATAGTAAACCATTGTTTAAAAATTTGTATAATAAATTATATATTTAAATTAATAAATATATTT
TCCTAAAATAAAAAATGGGGCCGAGACCGGGAATCGAACCCGGGTCGAGGGATCCACAGTCCCTC
AGGATAGCCACTACCCTACCACGGCCACAATATCGTTTTTTAATATTACTATAATGCAGGGGCAG
GGATTTGAACCCTGGAACCACTACTGGACAGGATTCTAAGTCCTGCATCTTTGGCCAGGCTCGAC
AACCCCTGCACTTAAAATAAAATGCTCCAGCCGGGATTTGAACCCGAGTCCCGGCCTCGAAAGGG
CCGGATGATTGGCCGGACTACACCACTGGAGCTTGTTTCAAATATATGGGCCCGATGGGATTTGA
ACCCATGACCACTCGGTTATGAGCCGAGCACTCTAACCAGCCTGAGCTACGGGCCCGTACCCGGTC
TTGAGAGTGAATAATTTGTTAGAAAAAATGGCGCCTCCTGCAGGGCTCGAACCTGCGACCTACGG
ATTAACAGTCCGTCGCTGCTACCTACTGAGCTAAGGAGGCTCGCTGTCATACTACTTGACCGCACA
ATAACAATCAACATTCTGATATATAAACCTTTCGTAGTAAAGTATTTATATCATAAAAGATATGC
TTAATTGTGGATTTAACTGGAAATTGCCAAGGTAGTTCAGCCTGGGAGAACGCTGGACTGAAGAT
CCAGTTGTCGGGTGTTCGAATCACCCCCTTGGCACGTTTTTATTTTGGGAATATTAATTTATTAA
TTTGGATATACGGTTTTATGTGTTATTTTTTAATATGTTATTTTTAATAATAAGATACTATTATT

TTATTGTAATATAAACTGGTGGATTCATGAAGATTATCGGAAGCATGGGTAAAAAAACGGAAAA
CTTAAAAATTGACGCTAAAAAATCATTTTTGAAGCGTATTGCAAAAAATGTGAAAGTTTCAGAA
GGGGAAAGAGCTGTTGAAGATATTTTAAGATGTATATATAGAAATCAGCCAATTTCAACAAAAA
AGATTGGCCAGTATGTAAAACTGCCCCTCCCAATTGTGTCTAAAGTTAGATCGATTCTTGAAAGA
GAATCGCTCTTAAAAAGAGATGATAAAGGAGCTGTGCTTTCAAAAAAAGGTATTGATTTTGTTG
AAAAGGAACTTGGACTTAAACTTAAATCTGACATGAAATGTCCAGTATGCAGTGGGCGAAATATC
GTATTTGATGAAAAATTTGAAAAGATATTAAAAAAACACAAAACTTACGCTAAATTAAGACCGC
AAGTAAACACAATGATTGATCAATCTTTTGCAACGCCTGAAACAGCCACTGCAAGAGCTTCGATA
ATGGCTGATCGAGGAGATTTGGAAGGAAAAAGAGTTTTATTTGTAGGAGACGATGACCTTGCAT
CAATTCCAACTGCGATGACCGGGCTTTGCAGTGAAGTTGTTGTTTTAGATATTGATGATAGACTT
TTGAAATTAATTTCAGAAGTTTCGAAAAAAGAGAATTTAAACATTAGAACTGTAAAATGGGACT
TTAAAAATGAACTTCCTAGTGAATTTGTAAACAAATTTGATACAATATTTACCGATCCACCATAT
ACACTAAACGGCGCAGTACTTTTCATGTCACGAGGTATCGAAGCACTCGGAGATAACGGAATATT
GTATCTTGCATTCTCTCACAAGCCTGTAGAAGAATACATTGAACTTCAAAAATCAATCAACAATA
TGAACTTTTTAATTTACGAATTGATTCCTGGATTTAATTTCTATGAAGGAACCGAAATTATTGGA
AACACTACATTCTTGGCAAGGCTCGTTGGTAAAAATTTGAAAAAAATAGAAGTTAATTCTGAAA
AAATATACACTGGAGAATTAAAACCAACTTTAAGATACTACCAATGCATGAATTGTAAAAAAAT
CCATGAAGTGGGAAATAAAGTAAAAAGAATCGAAGATTTAACCTGCGAATGCGGTGGAAAGAAA
TTTTCGATGATTAAAAGAGCTAAAGTAAAGCAAAAATAACTTTAAAAATTGAAAATTAAAATTT
TAATTTTTTTAAAATAAAAAAAAGAGAATTATTCTGTTAATAAAATAGCGTTTACAACACCATC
TTGTCCAGGTCTTGAGGTAACTTTTGCTTTACCCATTTCTGTTTCGATGATTGCGCCTTTTGTCAT
AACGTTTCTTCTGATGTAGTGTTTGTTTGCTTTGTTGTCGATAACATTTGTTACTGCAACTTTTT
TACAAACTTTGTTTGCTTGATCAAAGACGTTTGCATAGTTTGTTTTTCCAACTTAACTTTTAAG
TTTGCGCCTCTGCATCTTACAATTTTTCTTTTGATTACTTCTGAAACTTGTGTTTCTGCTGCAGGT
CTTCCCATTTCTCTTTTATGTTTTTTTGCAGCTCTCCAAATTTTAGCTCCTGTTGAGAGTCTTCTG
CTTGCTCCTTGCCAAATTGCCATGTTTACACCTTATTTGTGTTTATTTTTAAAAAAAATCGAAGT
TTTTCCTTATATATAATAAGTTTTTTGAGAGAGTATGCGAGTATAATATGTGATTGATATGATA
GTATAAAAAATTAACTGCATTTTAAAATAGGTTAAATAAGAATAATAATTTAATTATTTTACTG
AATCTTCAAATAAAAGCTTTAAAATGGCCATTCTGACAGGAACTCCATAAAATGACTGTTTAAAG
TACCTTGCTTGAGGGATGTTGTCAACTTCAGAATCAATTTCATCTACCCTTGGAAGAGGATGCAT
GATAACCACATTTTTCTCTCCAATGTGCTCTTTTGAAAGCCGGTATATTCCTTTTACCTTGTAGT
ATTCATTAACATCGACAAACCTTTCTTTCTGAATTCTTGTCATGTAAACAACATCAACATCGTCA
ATATCGACATCAGCCATTTCTGAAAGTTTTATTTTTCCAGCGATATCTTCAATTACTTCTCTTGG
CATTTTTAATTCGTCTGGAGAAATTAGTTTTATTTCTACTCCTTTAAAGAGAGATAATGCCTGAC
ACAAAGAATGAACAGTTCTTCCGTACTTTAAGTCACCAATAAATGCGATTTTTAAATTTTCAATG
TGTCCAACTTCTCTTTTAATCGTGTACAAATCAAGAAGCGTCTGTGTAGGGTGCTGATTTGATCC
ATCTCCCGCGTTTATAACCGGAACTTTTGAATTTTCACTTGAAAGCCTTGCAGCCCCTTCTGAAGG
GTGTCTTATTGCAATCAAATCACTGTAACCACTGATTACTTTAATAGTATCTGCCAAAGATTCGC
CCTTTGTTACAGAAGTGTTTGAAATATCCGTAAAACCAATAACGTTTCCACCAAGCCTTTTCATT
GCAGTTTCAAAAGAAAGTCTTGTCCTAGTTGATGGTTCGTAAAAAAGAGTTGCGAGTATTCTCCC
GTTTAAAAAATCAAAGTGACCTTTTTCATTTAAAATAGCTTCCATTCTTTCGGATTCGTCTAAAA
TATTCAGAATTTCTTCCCGGCCGATGTCTCTCATCGAAATTAAATGCCTCATTAAAACCACCGAC
ATTTTTCATTAGTTATAAATTAAATATATATAAAATTATGGTGTAAAGTATATATGCAGACGAG

GTGTGTTGATGGAACCTGTTCAAATTATCGGGATTATCGTTGGATTAATTGTTATTTTAAAAATT
GCAGTCCAAGCTAAAAAAAGTGCTATTTCGCCAGTTACTGCAGTTATATGGATTTTAGGATGGAT
TTTTGTAATGTTAATGGTAAGTTTTCCAAATTTTCTTGGAAAAATTGCAAACAGTCTAGGTATAG
GAAGAGGTATTGACGTTTTAGTGTATTTTGGAATTATAATTTTGTTTTTCTTAGTTTACAAATCA
TATTTAAGATCAGAACACCTTGAAAGAGAAATTACAACAATTGTTTCAGAAATTGCAATTAATG
AAAGATACGATAAAAAAACAAAAGGTGAAAATGATGGAAAATAGAGAATTAGTAATGGAAACTG
CGCCTTATGTTCAAAATATGGAATATATAAGAGAATTAATTGAAGAATCGGAAAATATTGAAGA
ATTAAAAATAAAATTAACAGAATTAATAGATAATGAGCAGAACGTTGCAAAAAAGACAGATTTA
AAAATTCTAATGGAAAAAATAGAAGAACTTAGTCTTTAATTTTTAGGTTTTCTATATTCGTGTTC
AAAATCTTTGTCGTAAAGTTTTGAATAATCATAACTGTCAGGATTTGTTACATTTCCAACGATTA
TAAGTGCTGTTTTTGTAATTCCTTCGCCTTTTACTTTTTCAGAGATATCTTTTAGTGTTCCCTTG
ATTTTTTTCTCATCAGGCCATGATGCATGGTAAACTACTGAAACTGCAGTATTTTCTGGATATCC
GCCTTCGATTAACTCATTACATACTTTTTCAATCATTCCAGTTCCAAGGTAAATTGCCATTGTAG
CGTTGTGTTTTGCAAGCGCAATTAAACTTTCTTTTGCAGGTTTTGGAGTTCTTCCTTCAGGTCTT
GTAATTATTACTGTTTGTGAGACATTTGGAAGAGTTAATTCGGATTTTAAGGTTGCTGCCGCTGC
AAAAAGGGAAGTTACTCCAGGAATTATTTCTACATCAATTTCATATTTTTTCAATTCATCGATCT
GCTCTTTTATCGCACCATAAAGCGCAGGATCTCCCGTATGAACCCTTACAACAGTTAATTCATTT
TTAATTCCATTTACTGCAACGTCGATTACTTCATCGAGTGTCATCGTTGCACTGTTGTAGATTTT
TGCATCCTTTTTATTATATTCTAAAACTTCCGGATTTACTAGAGAACCTGCATAAATTAAGATAT
CTGCATTTTCGATTGCATTTTTTCCTTTTATCGTGATTAATTCCTTGTCTCCAGGGCCCGCACCAA
CAATAATCATCTTTTTCATTATTTCACCGATTTATTAAAATTATTAGATTATTCAGTTGCTTTTT
TCAAAATTTCATAAGTTGTTCCGTATATCACGTTGCTTTTACAAAAACCCGGAAGATATGTTGCA
GCTTTTCCAGAATTCGTTGCAACGTTTTTGTATCCCATATCTTCAATCGGAGCTACGACCATGCAC
GTGTCAACTACAACTTTTCCGCCAGCATCTTCAATAATTTTCGTGTATCCCATCCTATCAGCAATC
GATTTCATGTGAATTGATGTACAAACCCAAACTTCTGCATTGAATTTTGCATTTTCTAACTTAAC
AAACTCTGCAACTTCTTTAATCTCATCAAGACTGCAGTGTGGACATCCGATACAGATTAAATCTG
GTTTTTCTGAAGTTTTGAATGAATCATATTCTTTTTCAAAATCGTCTTTTCCAATAACTACTTTT
TCAAGACCTGTAATTTCCAATCCTTTTTTGGCTTCAGGAGTTATTCCTTCAACGTGGTATAATGC
AACACCGCCGCTTGCTGCAAGTGCCGCTCCAAGAGCTTTTAAATAATCATTTTTAATTGAGTCGC
AATTTTTAAATTTAAAGTAAGGAATTCCGTTTTTTACAATTCTTCCAGTAATTCTTCCCAAAATT
CCGTAAAATGAACTTGCATCTTCAAGTTCCATATCAACTTCAAAAACTTGTGTTGGAAGCCTGTT
TTCGTTCAGATGGTAGCCGTAACACGGCGTTTTTCCAATAATTGCAGCTGCTAATGCTGAAGGGC
CACCTTCTCTGTTTGTTCTTGCACCAATTACTGAATTTACATAAGCTACTGCAGAAGACTCTGCCC
AAGCAACGTGTGAACCAAACATTGGAACATTTCCTGTTAAATAAGGCGTACAGGTACAGCTTATT
TCAACTTCCATTCTTTTAAAGCAGTCAATTATTTCAAGCTGTTTTTTTGCAAAATCTTTTGGAAA
TTTAAGTTCATCGTATCTTATCAAATCCATTCCAGCAGGATTCAGAGTAGTTGGAACTGATGCCT
TTAAACCCTGATCTGCAAGGTCTTTTAAAAATTCCAGTCCCTTTTCACCGATAGTTTTATATGAA
ACTCCAGAAACCTGTGCAGAAGATATATCTACCAGTTTTCTGCTCCGTAAATATCGCCTAATGA
CACAAGTAAGTTCATGCAGGTTTCTAGGGCTTCCCCGTATTCTCCTGCATACAATTTTTCCTCTTC
TCTCGTTAGATGCATAATTTTCACCTTTTTCCTTACTAATTATAGAGTACCAAAATATAAAAATA
ATGGAGATAGTTTTAATTCGTGAAAAAATGGAAATAACTGCAACACCGATAAGAACCAGATATA
TCGATAGAAACGAAGACTTTGTATCAGAATCAATAAATTCTTTGAAAAAAGAAATAAAAGAAGG
ATTTGAAATAAAAAACGGGGATTTTTTAGTAGTAAGTGAAAAATTCATAGCTACAAGCGAAGAC

AACTTCGTAGATGAAAGTTTGGCTGACCCAAAATTCTGGGCATATTTTACATATTATTTGTCAAA
ATACGGCTGGGGATATGTTTTAGGCCCGCTTTTAGGAACTAGGGGAGACCGGGTTAAAAATTTAA
GAAAAATGCCGAAGACGGAAACACTAAAACACAAACAAATAGTAATTGAAAATGTAGGGTTAAT
ATACGCACTAAAACCTGCATCAGAAGGCGGAATTGATTTAACAAACGTTCCAGGAACTTATGCGT
CACTTCTCCCAGAAAACCCAGAAAATTCTGCAAAAAAACTCCATAAAAAAATAAAGGAAGACTTA
GATCTCGATTTAACGGTTATGATAATCGATACAGATGCAACATACAGATTTTTTAACTGGTACGT
TACAGCACTTCCAATCGCAATTGAGGGAATAATCTCCAAAATAGGGGTTTTCGGATATATTTTGG
GAAAATTTGGAAAAATACTAAATCATGGCGGACTTTGTGGTGCAACTCCACTTGCAATTACTGGA
AACGAAAATTACAAAAAATATCCCTTAAAAACGATTCTATACATTGCAAATCTATCCGATACATC
TCAAGTTCCATATACTAAATCAATTCATGATTTGATGAAGAAATACAACACTTTTGAGATAACTG
TTGATGTTTTAAAAGAGATGGAACACAGCCCATTAGTTATTGTAAAATGTGAATAATTTAAAAA
AAAAATAAAAATTAATAAAATATAACTATCTTTTTGGCTCGTTTATTGCAGATTCTATTAATGA
TTGAATCATCAAATCATGCGATAATTTGTAAATATCAAATTCATTTTTATTTTCCAAATCTAAAA
GCTCTAAAAGGTCCCCTGAAAGCGAAGTTTCAATGCATTTGCTTTTATCTAAAATATAAACCTGT
TTTCCATCTTCAAGTTCTACTTCCGTACATTCTGATTTTAAAGCAACAATCACAGGAGGTTCCTC
GATTTCAGGCCTTTCCAATTTTATATTCGTGACATTTGGATCATTTACAGATAATTTTTCGTTTA
TAGAATAACAAAGATCATTGATATATTCCTGATTCCACGTTCTTTTTAAATTAAATAATTCTTTT
TCAAGCGATGAAAGAAGTTCTTTTGGATATTCTTCAATTACGTCCCTTAAAAACTCCATATTGTA
TTTTGCCTTGTCTGCGGCTTTCAAAATTATTTCGAGATTCTTTTTCTCAATGTAGCCGGATTTTA
CCATTGCAATCGTTGCTTCTTTTGAAAGTTCTTCGTCTGAAAGTTTGAGTCTTACATTATTAATA
TATCTGTTAATGTACTTTTGATCGTATTTTCCAAGATTACCGACAATTCGAAGAGCCTGCTTTGA
AATGTATTTATCAGGATTATTCATCAGTACACAGACTTTTTTATAGTGTCTTGTAGCAACTTTCG
GGCTGTATTTTGATAAATTGTAAAGAAGCGTGATTGAAGGATAAATAACATCGGGATCTTCGTCC
AGTAATGGATTTATGTTTAAATTTTCAAATTTAAGTGGTTCCGTGTAGCTTAATTTATTCAGCGC
TAAAAGCGAATATATTTTAGTATATTTATCGGTCGAACTTGTAAATTCAACGAGTGTATCAGTTG
CATTTTTTAAGTATTCGGGATATTTTTCAGAATATTTCCATATTGAATAAACCGTACTTCGCCTA
ATTTCTAAATTATCACTGTTTAAAGAATTAATCAACACATTAGAAGTATTTTCATCGAGTTCATA
ATCTTTAATATCCCCTAAAATCTTAATTACTTTTGATTTTAAAACAGGATCATTTGAATCTAAAC
AGGACAATACATCATTTTGACAGCATTTTATCAAATCAGGATTAATTTCTCCAATATTTTTTATT
AAATCCATTAAAACTACTTTTGAAGTTATTTTTTCGCGCAGTATCCCAAATAAATCAATTTTTTC
TTCACATTTTGAATTTAACATTATTTTTGAAAGAGCATATGCACTTGAACAAACCACATCAATGT
TTGAATCATTAACACATTTTTTCAAATTTTCCATGTAAGGTTTGGTATTATTATAACATACCAGC
CCAAAATCCCCAAAAAATAAAACTGCATTTTTTCGAATTCTCCAGTCAGAATTTTCCAATAATCC
TGCAATTTTTGGGATTAATGGTAAAACTTCTAAAGGACTTGCCTTTGAAATCATTGAAAGTGAGT
TTAATGCCGCCCATTTTATTTTAAAATCACTTTCAAGTGCTTTTTCAAATTTTGGAATAAAATAT
TTTATATATTCTGGATGTTCAAATACATATTTTCCAACAGAAAGAACATCTCGAAAATCTGAAAA
ATCAAAACCAGATATTCCTTTTGTATCTGCACCGCTTTTTGGATTTAAATGATAATCAGTTGATT
TAAAAGATCCGATCATTATTATCACGCTTTTAATAGTAATATTATTATGAATTAAATGGATTATA
TTCGTTTTTTGCATGTGTTTGTGTAGGTTAACCGTTTTTTCATTAAATTTACCTACATAATCATA
TACATAACTACATACAAAACCAAATACAGCACCATATGCTAAACCAAGTGCAAAAAAAGTATATA
TTAACAAAAAACGACATATTTCTTTGGGAATATCCCCGAAGAGGTAAAAATCTCAAGGATTCATA
CGAGATTCTAAAGGCTAGAAACTCTTGAAAAGAAAGTCTGGTGAACTTAGGAGGTTCAATTTAT
GAAAATAACAGAATTCTTGAAAAACAAAAAAGGTGCTTCTGGAATTGGTACCTTGATTGTTTTT

ATTGCAATGGTATTAGTTGCTGCAGTTGCAGCAAGTGTTTTGATTAACACAAGCGGATTCTTACA
ACAAAAAGCTGCTACAACAGGTAAAGAAAGTACCGAACAAGTTGCAAGCGGTTTATTAATAAAC
GGAATTACTGGAAGCGTTGGAACATCAGATGTTAAATTGCTTGCAATTTACTTAGCCCCTAACGC
AGGTAGTTCTGCAATCGATTTAGCTCAAACAAAAGTTATGCTTGACTACAATGGAAAAAGCGTAG
TTTTAGGTTACGGAGGTAACCAAGACATGAGCAGCGGAAACTCAAGTGTATTTTCAAACGACACT
GGCGCAACCGCAACCACATTCCAAGTAAATATTTTACAAGATTACGATGATTCAGCAGTTGACAA
CGCAGTTATCAACAAAGGTGATGCTGTAGCATTGATTGTTGACGTTAATGCATCATTTGGTGGAG
AAATCCCTGAAAGAACTGCAATCTCAGGAAAAGTTCAGCCAGAATTTGGTGCTCCAGGGGTTATT
TCATTTACAACCCCTGCTTCATACACAACAGCACTTGTAGAACTTCAATAATTAATTTAAAAAAT
TCATCCTTATTGGAGGTTCAATTATGAAAATAACAGAATTTATGAAAAGCAAAAAAGGTGCTTC
TGGAATTGGTACTTTGATTGTTTTATTGCAATGGTATTAGTTGCTGCAGTTGCAGCAAGCGTTT
TAATTAACACAAGCGGATATTTACAACAGAAAGCTTCAACTACCGGTAAAGATAGTACCGAACAG
GTTGCAAGCGGTTTACAAATTATGGGTATTAGCGGATACCAAGATGGTTCAGCTGGTGCAAACAT
TACAAAATTGGCAATTTACATAACTCCTAACGCAGGAAGTGCTGCAATCGACATGAATCAGGTTG
TTTTAACACTTTCAGACGGAAGTACAAAAACCGTTACTAAATACGATACTACTGCATACACAAAC
CTAACTGCAGGTGGAGACCTTTACAACACTACAACTGTAAACTGGTCAAAATTAGCAGATACTAC
AGAATTTGGAATAGTTGAAATTCAAGATGCAGATCTCTCATTTACAAGTTCAGCACCAGTTATCA
ACAAAGGTGACATAGTTGCAATTATTGTAAGCGGAGTTTCATTCGATACAAGAATGGAAATTTCA
GGATCTGTTCAGCCAGAATTTGGTGCTCCAGGAGTTATTTCATTCACCACACCCTCAACATTCACT
GAAAAAGTAGTTTCATTACAATAAGCCCCTTGCTTATTTTTTTATCCGAGGTGGTGAAATGGTG
AAAAAATTCATGAAGAATAAGAAAGGTGCTGTAGGAATCGGCACCTTGATAATTTTTATTGCAA
TGGTACTCGTTGCAGCAATTGCTGCCTCAGTAATTATTAACACGGCTGGAAAACTTCAGCACAAA
GCTTCAACCGTTGGTGAAGAAAGCACCGAACAAGTTGCAAGCGGAATACAAGTTTTAAAAATAAT
TGGTCACGCCGACATAGACAGCGAACTTATTGATAAAATCGGAATTATTGTAACTCCAAATGTTG
GTGGAGAAATCGATCTTTCAGCGACAGTTTTAACAATTTCTGATGGAGATCAGAAGCTTTCGTTA
GTTTATGCTGCTGATGACAGTAATACTTACCTCATAACTACAAACGGTACAAAAGACCTATTAGA
ATATGATGCTAATCCAGAAACTGGTGCATGGCCATTTGATGATTCTTACGGAAATAGCCTGGGAC
AGAATAAATTCGGTATAATCGTCTTACAGGACGGTGATGAATCAGTTACTGGAAGCCACCCTACA
ATAAATTACGGAGACAAAATTGTCCTCGCAGTTAACATTGGAAGCTTCTTAGATAATTCAGGAAT
AGAACCAAGAACTAAAATTACTGGGGAAGTAATTCCCGAATATGGTGCATCAGGAATAATTGAT
TTCAGAGCGCCTTCAGCATTCACTGATGTCGTAGTTGAACTTCAATAATGGTGATATTATGCCTA
ATATCTCAGAGATTATTGAAGAGATAAAACAGAAGATCAAATTAGGCAAGAAGACTGAAGAAAG
TTCAGAACCTGAACCTGATGACGAATTTAGTTTTACACTTGAAGAAGAAGCAGTAAACGAATCTG
AAGATTTGTTTGCTGCAAATGAAGAACTTCTTGCCAAAATTGGGGAACTTGAATCCAAGTTCCCA
AAAATTGAAATGATGGTCACAAACCTGAGGAAAGAGAATGAAAACCTCAGGGGTGACATTCAAA
ATATTAACGAAAATTTTCAGGATATGATGGCACTTTACGAAGTGGTATCTAATCAAATCAATCCA
TTCATAGGCATTTCAAAAATCACAGCAACCAGCATGGAAAAAGTAGAAATGATGGAAAATGAAT
CTCGAAACCTGAAAAAAAGGGTTGAAGAACTTCAAAACGATGTTGTGATTCTGGCAAACGTATAT
TTAAATACGCACCAAATTGACCTTGATGGAGTTATTAATGAAGTGCTTGCAGAAGAAGAATTCTC
CAAAGCAATATCAGGAGAGGATTCCGATGATTGGTAACGTTAAAATGGAATATGCTTCCGTTTCG
GAAGAATACCTTACCGAGAGTGAAATGGATGAATATTTGGATGATTTGAGATCCAAAATTCCTTC
ATTTATCGTAGAACTCTTAAAAAATAATTTAAAAAACAGAAAATTAACCAAGACCTCAGCTTGAT
AAAATCGTTGGAAGAGTTTCTGACCTTTATTTTGGAAAAAAACCGGAAGACAAGAAGGCTGCAG

AACTTACAAATAAAATTAACGATTTGAGCCAGAAATTAGATGCTTTGATGAAAGTTGCTACAAT
TTCATCTGCAACAAAAGTTTCTGATGACATTAAAAAAGAATTGAACTATCTCGATGATGAAAAA
ATTGAAGTTCCCGAAATTTCTAAGGCTGTAGAAGTTCCAGAAATTATCGAAGAACCTGAAGTTTC
AATTGAACCTGTTGAGGAACCCGTTGTGGAGCCTGTAAAAATAATTGAAAAACAGACTGTTGAAC
CAATCATTGAGTTCAAAGAAGAACCTAAAATTGAAAAGGTTGTTTCGAAAGTTCCTTTGGAAATT
GAGGAGGGAAATAATATGAGACCTGAATTAACCCCTGCAGTTGAAACGACTGCGAGACTTCAAGA
ATTGCCCGAAGATACACTTTCTACCATGTTAGTATTCAAATGGCTTGAATTTTTAATTAGTAGAG
TGGGAACAGGAAATTTAATAGATGTACTTGATTACTACCACAACCTTGGATGGGTATCTGGAAAA
GCAATCAGCAAATTGATGAAAGTATCAAAAAACATGAAGTATTTCCATGAAGATATCGACTGGA
AAGCTAATGAATGCATGGCTCCAGAAGACCACGTTGTATCATTACTCTACATTGAAAAATTAGCA
GGAAGGCCTATCGCAGTTGAAGAACTTGAAGGCATGGAAAGAGAAATAAACAGAATAAAGAAAT
GGGCTGAAGAACTACAAAAAATGTAATTTAAGTAACTACAAGGAGTGAGCGAAATGAACACTGC
AGCATTATCATCAATATTACTCGAATCCCAAAAGCCTGCTAAATTAGAAGCTGTTCCAGAAGATG
CATTTTCATTGATATTTGCATTTAAATGGTTAGAATACTTATCAGAAAGAGTGGGACAATCAAAT
ATTGCGGATATTCTTGAATTCTACTACAACTTAGGATGGTTGTCAGACAATGCAATTTCAGGACT
ATTAAAATTCTCAAAAGGAATAAAAATAGAAGATGACGACATTGCAAGTCCTTCTGGAAAATTA
ACTATTGCTGACCACTTGGTTTCATTATTATTTATCGAAAGATTAAATGGTAAAAAAATATCAAG
CGAAGTTTTAGATAAACTAGAATGGGAAATTAGAAGGATAAAAAGAGGCGCAGAGCAATACTAT
GGGATTTAGTTCAACTGCGGGACTAACGTTGTTGTTGACTACAGTGCTGATATGTTCGATTTACA
TGTACAGTACTGTAGACCTAACGTCTGCAAAAGTAAATGATGCTTATAACATGCATTCAGAATAT
TTGAAAAATAAATTAGATGAAAAAATAACTGTTGTATCAGTTGAACATTCATCAGAAAATATGA
ACATTACCCTTTTAAATGAAGGGCAATTAACACATGAATTAGATAATTGGGACGTTTTATACAAC
GGAACTCCCGTGAATTTTACAATCACGTCTGGTGAACTGTATTTAACACCTTTAGAAAGTGTAAA
TATTTCAGTAAATGCAAGTTCTCCAGGAAGAATATCAGTAGTTTCTGAATACGGAAACCAGTATT
ACTACAATTTAAACTAAAGATCCTTGTTTTTAGGATAACTTTTGCGTTGTGATTTAGATGGCATC
CAATGTTTTCTCAGAAGTAATAATCTTTGTTAGTGTTTTGATAATTACGGCCGCTGTCGCAGGAA
TTCTTGCAACAAGTACGCACAAAATATCGCTCGGAATCAGCAGTAAGGGCGATACATTATCAACG
CAGTTATCTCAGGATTTCGAAATTATTAATGATCCAGGAAATATCCCCAGAAATTCAATTAACGG
AATAAGTACAATATATATAAAAAATACTGGAAAAACACAGATTTCTATTCAAAGTGATACAATA
ACAGCAATAATAGATGGAGAAATCCGAGATATTTCTGATACGAGCATTGTAAATGGTGCCGGCAG
CGTTTTAAGTCCATCAGAAGTTGGAAAAATTGAAATTAATTATAACGAAACTGGATTTCACAAA
ATAAAAATAGTATCTGAAAATGGCGTATCTAGAACAATAACTGGAGATGTCAATTAAGTATCGG
TGGTTGATTTGAAACTAGCAAGAATAGAGCTGAGCAGAGATGACGTTCACAAAAGACTTGGTGG
GGGAATCCCCTTTGGAAGCGTCATCTTAATTGAAGGAGAAGAGTCATCCGGAAAGTCAATATTCT
GTCAGCGGTTAGCATACGGATTTTTACAGAACTCTTATTCGCTTTCTTATGTATCAACCCAATTA
ACAACCACTGAATTTGTAAAACAGATGATGTCCTTGAAATATACCATAAATAAAAAATTATTAG
ATGGTAATCTTTTGTACATTCCAGTATATCCGTTAATTTCAGAAAATGCAATAAAAGATGACTTT
ATTAAAAAAGTAATGCACACAAGAGCATTTTATGAAAAAGATATTGTTTTATTTGATTCTATTT
CTACATTAGTATCTAACGATGCAAGCGAAGTACAGATTACAGATTTAATGGCCTTTTTTAAAAGA
ATTGCATCCATGAATAAAATTATAATTTACACAGTAAACCCAAAAGAACTCCCTGAATCAATATT
GACAATACTCAGAACTTCTGCAACAATTGTCATAAAAACAGCAACTTATTCTTTCGGTGGAAATC
TTAAAAATTCTGCTAAAATTGAAAAATACAACTTTGCAAAAAGTTCTTTCCAGAAAGTTATGGT
ATTTAGAGTCGATCCAGGACTTGGAATTGCCGTAGAAATATCCTCTGTAGCATAAGGTGGTAATA

TGGCTGATGATTTCAAAACTGCACAAATGAACAATCCCCATTTAAGAAATTACGTTGAAAAATTT
AAAAAAACTTACATGAAAGTCCCAGAATTTGTTCCGAGCCTTTCAAGGGATTTTAAAGAAATAAA
ATATCCAAACATAATTTATCCAATAGGTGACCCGATATTTATCCACCTTTACGGAACCCCAAACT
TAAAAACAAGATATATTGCAATAGAACCTAAATTAAACGGTTCAGAAGAGAGAAAAAAATATTT
TGACATCCTCGATAAAATATTAGAGTATGCTCCATACAGCGATATTCCAGAAAACGACGAAGAGT
TCGGAAAAGTAGTCACAAAACTATTTGACATGGTTGCAAAAGTATCAAGTCAAAAAAACGAAAA
GAAAAAAGGAATTCTGTCAAAAATAACAGATTCTGGAAAAATAAACATTAATAAAAGTGAAAGA
GACAAATTCCTCTATTTATTGAAAAGAGACTTGATCGGACTTGGAACTCTTGAACCAATAAAAAG
AGACCCATACATTGAGGATATTCACGTAATCGGTGCTAAAAACTGCCAGCTAGTTCACAAAATCT
ACGATATGCTTCCATCAAATATTGAATGGGAAGATGAAGGAGATATTTCAAATTACTTAAAAAA
TATGTGTGAAAGAATCGGAAGACCGGTTTCTGATGCTACACCAATTGTAGATGGTTCCTTGCCGG
ATGGATCAAGGATAAACATTCTTTATTCTGGCGATGTTTCATTAAAAGGTCCTTCATTTACAATT
CGTAAATTTACAGATGTCCCAATAAGCATTACCCAAATTATCAGCTGGGGTACGATGTCTGCACA
GACTGCAGCATACCTTTGGCTATGTCTTGAATACGGCATGAGTATTTTTATCTGTGGAGAAACTG
CGTCAGGAAAAACTACTTCACTAAATGCAATTTTACCATTTATCAAGCCTAAATCAAAAATATTC
TCCTGTGAAGATACTTCTGAAGTAAAACCTCCAAACCCGGTTTGGCAGCAGTTGTTAACACGAGA
AAGAGGCCCAGAAGAAAGTAGGGTTACATTGTTTGATCTTTTGAGAGCCGCATTAAGATCAAGGC
CAAATTATATTATTGTAGGGGAAATCAGGTCTGTTGAAGGGGCTGTTGCGTTCCAGGCAATGCAG
ACAGGTCACCCGGTAATTTCAACGTTCCACGCTGCAAACGTTAGAAAAATGATACAGAGGCTTAC
AGGGGACCCGATATGTATTCCACAAACATTTATGGATAACTTAAACGTCTGTCTGTTCCAGCTTG
CAGTTTATGCTAGGGGAAAATTCTTAAGAAGAGTTGTTTCGGTTGAAGAAATCGAAGGATACTA
CAAAGAAGTTGATGGAGTTATTACAAGAGGTGTTTTTGAATGGGACCCTCAAAAAGATATCCACA
ATTTCACAGGATTAAATAACAGCTACATCCTTGAAGATAAAATCGCAACCGTTGCAGGATATGAA
GATCCGAGAGAAATCTACGATGAACTTGATTTAAGAGTTAGAATTCTCGAAGAAATGATCGCAA
GAGGAATCTTTGGATACTACGAGGTTTTAGACATTATCTGGAGTTTCTATGAAAAAGGATTGGA
AGGACTGCCATTCCAGGTTTAAGGTGTTTCTTATGTTTTTTGATATACTCCCAAGGGTTGGACTA
AAACCAAAAGATTACATTCTTAAATTTATACTTCCTGCAAGTATCATCTCAATTTTATTATTAAT
ATTGGGAATAACCTACTTTTCAGGCTACACTCGATTGGTCGTATTATTTCTGCCGCTTCTTTTAA
TGGGAAGTGCCATAGGCTACCCATATATTGAACTTGATTCCCAACGTCAAAAGATAAATGAAAGA
CTCCACATATTTATTACCAAATTTGGTGTTCTTTCAATTACTGATTTGGATAGAAAAGAATTAAT
GCACCTTTTGGCAACCGAAAAAGAAGAATTAGGCCAGCTTGCAACAGAATCTCATAAAATTTTTG
TATTAATTAAAAGATGGAACCAGTCGCTTGCAGAATCCTGCAGGTTCTTGGCAAACCGGTCTCCG
AGCAGTCAGTTTGGTGATTTTTTAGATCGTATGGCTTATTCAATAGACAGCGGTCAGGAATTAAA
AGAATTTTTGCACGGTGAACAGGACATCGTGATGGAAGACTACGGTGAATTCTACAAAAGGGCAA
TTTACACGCTTGACACGTTTAAAGAGATGTATGTAAGTGCAGTAACTTCAGTATCCTTTTTCGTG
ACTTTCGCAATAATTGCACCATTTCTACTCCCCTATGATTTTGTAACTATGGTGACAGTTGCACT
TTTGGGTTTCGTATTAATTGAAGTACTTTTGGTCTATGCGATAAAAAGCAGACTTCCGTATGACC
GTTTGTGGCATACTGGGGAAAAACCAACTAAAATAGACCTAAAATTAAAAAAATGGCTAATAAT
ATCAGTTATCGGAACAGTATTATTTACATTATTCCTGTTATGGGGAAAATTCATCGGAGAAATTC
CGCAAATAATGAAAATACCATACCAGATAATTTTCGCGCTTGGAATGAGCCCACTTGCTATTGGA
GGATATTTCGCACAGCGTGAAGAAAACCTCGTAATCAGAAAAGAATTCAACTTTCCAGACTTTTT
AAGGTCACTAGGAGATTCTGTTAGTGCAAAAGGTGGAGGTATGACGGATTCATTGGAATACCTCT
CATCAAACGACTTTGGTCCATTAACAAAAGACTTGGAAGGCCTCTACAAAAGAGTATCCATCAGG

GTAAACAGCCAGAAAGCATGGCGATACTTTGGATATGATACCTGCAGCTACATGATACAGCTGTT
CTCTGAAATGTTTGAACGTTGTACATTTTTAGGTGGAAATGCAGGGGTTGCCGCCCACATGATTG
GAAAAAACTTCAGAAAAATCATTAACTTAAGAAAAGCAAAATATCAAAGTGTCAGCCAGTTTTC
CGGAATCATGTACGGATTAGCTGGAGGTATGGCCCTTACCCTTTACGCTTCGGCAGGGGTTGCAA
ACATGGTTAATGGACTTTATACAAGTTTGGATATACCTGATACAATGATATCAATTGTAAATATC
ATAACACCTGAAGATTTCAGCCTTATTGCTTATTTAATCTACGGGGTACTCGTAATTTATTCAAT
TGTTTCAGGCTACCTGATAAAATTAATGGATGGAGGACACTACCAGGTTTCATTAATGCATTTTG
TAATAATGCTCTGGGTTGCATCAATTGTTGGGTATATCGCAGAAGTAATGACTGGAAGCCTCCTT
GGAACGAGCCTTCCAATTTAATTTATTTAATTTATTTAAAAAATCATTTGAATAGTGAACCGATT
AAGAACGCCATTAAAACCAGATTTGTTACTATTTTTATATTTCTTGAAGCTTTTTTTGCTTCCAT
TTTTGATGGATTGAATACAAGTTTGTATATTCCAAGTAAAAATAATAAATCGCAGAAAACTACT
GAAATCATATAGTAAATTCCAAAAAATCCAAATAAATATGGCAGGGGGCTCAAAAAAATTGCAA
AAATAAGCAAAAATGCTGCGATATATATTGATCTTTCACCACATTTTATAGGAATTGAAATTACT
TTTTCTTTGATGTCTCCTTCGATGTCTTCGTAATCCTTGATTATTTCTCGCGACCACATTGCAAAT
AAAGCGCATAAAAAGAGCATTATTGTAACATCAATATTTCCAACAGCAATTCCACCAAAAATAAA
GACCGATCCTGTTAAATATGCTACAATTAAGTTTCCAATAATTTTATTTTTTTTGTACGTGCTTG
CATACTGCTGGAGTACTATCGAATTTAAAACTGCCATCAAAAAGCATGATATATTAAACAGTGAT
ATAAATAGACCCGTAACTACCAATGAATATGAAAATGCCTTTGCATTATTTAAGGTAATTCGTTT
TGAAGGAATCGGTCTTTCTGGTTTGTTTATCCGATCAATTTTTAAATCATAAATATCATTTAAAG
CATTTCCAAACCCACAAACTAAAAAAACGACTATTGATGCGAGAATTAAATTATCGATCATAGCC
AGATTGAAGTAAGATGCAATTAATCCCCCAATAAATGCCCCAAAAGCTGCTGTCAAACAATTTTT
TAACCTGATTAACTCAAAATATGCTTTTATATCCATTTCTTCACCTACACCAAAATATATAGCTA
CAAACTTAGATAAATAGGTTAATACTCTTAACTCATGTGATAAAATGTTGAAATTTGGAACTGCA
GGAATACCGATAAATGCTAAAAATACAGAAAGCGCACTTGAATATTTAAAAGAAATTAACCTTG
GAGCAATGGAGCTTGAATTCGTTCGCGGTGTAAATTTAAAAGCTGAAAAAGCAGAATCTTTAGCA
AAAAACTTGAAAATTACACTCAGTGCCCATGCTCCCTATTATATCAATTTAAATGCAAAGGAACC
TGAAAAAAATCGAATCAAGTATTAAAAGAATCGTTGATTCTGCAAAAATCATCAGTATATTTAAT
AAAAAAAATACTAAAAATAAAAACGTGGTTTTTCATTCTGGATTTTATTTAAAGCAGGACAAAG
ATTTAGTTTATGAAAAAATAAAAGTAAATATTCAAAAAATACTAAAAATAATGGATGAAAATAA
AATAAATGCAATTTTAAGGCCTGAAACTACGGGAAAAATATCGCAATTTGGAAGTGTTGATGAA
TTAATCCAGTTATCGACCGAAATTGACATTATGCCCTGTATCGACTTTTCACACGTATACGCACG
AAGTCTTGGAAAAATAAACGACTATGATTCATTTTCAAATATTTTATCAAAGCTTGAAAATAATC
TTGGGAAAAAAGGAATCAAAGATATGCACATCCACATTTCTGGAATTGATTTTGGAAACGGGGGC
GAAAAAAAGCATTTTCCAATTTTAAATTCTGAATTTAATTATATGGAAGTATTAAAAGCTTTAA
AAGATTTTGAATGTTCTGGAACTGTTATTTGCGAAAGTCCCCGATTAGAATACGATGCAGTTATT
TTAAAAAAGAATTATGAAGAGTTATAAACATTACTGCTGAACTGGAATTATTGAAATTTTTTTA
AGAGAATTTCCTTTTTTACATTCTACTTTTTCAGGAAGTATTGATGCTACCTTATATTTTCCAGA
TACTCCTTCCGGGTGGCATAAAAGGTAATTTTCACAGCATACCCTGTCACACTGCACATCTGCAT
GCGTTAAAGTTACTCCTTCGAGAGCTTTTTTAGACTCAATTATTATTGTAAATTCTGCAGGAGTT
ACTTCTACAACCTTAACGCCATTTTCATGAACGGTGCAGGGGTGATTTGCAGATCTAACGCTTAC
AATTTTATACCTTGTTCCGATGTCTAAGTTGTTGTGGCATATCCGTTTAAACCTGCAGGGTTCGC
ATTCTTCAAGGGGCCCGAGATAAATAAATTCGTTTCCGGTTTTTGCAAGTTCGCTACCGATTAAA
GTTATTTTTTTCATAAATAAATCACCGATAATAAGACTGAATAAATAGTACTAACTATAATAATC

GAAATTATATTATATAAACAATGTCAAATAGCTTTCAGTCGAAAATTTAGGTGTTTTATATGTGC
GGAATTATTGGATATATCGGAAATGAAAGGGCTTCAAAGATACTACTAAATGGATTAAAAAGGC
TAGAATATAGAGGATACGACAGTTGTGGAATTGCAACTATTGATGATACAATTAATCTTAAGAA
AAATACTGGAAAAGTTTTAGAAGTCTCAAAAAAAGAAAACTTTGAAGACATGACCGGCCTTATT
GGAATAGGACACAGTAGGTGGGCAACGCACGGCGGAATTACAAAAAATAATGCACACCCCCACTA
CGACTGCAGCGAAAAAATCTGCATCGCACACAATGGAATCATATCAAATTACAAGGAATTAAAGG
AACTATTAATTTCAAAAGGCCATATTTTCAAGTCTGAAACAGATACTGAAGTTATTCCACACTTA
ATTGAAGAAGAACTGAAAGATTTTAAAGAAATAACTGAAAAAACATATATTAACGCCATTCAAA
ATACAATTAAAAAACTTAATGGAACGTATGCTCTCTTAATTTTAAATCAAGATTTTCCAGAAATG
CTTGTTGGAGTTAGAAATGAAAGTCCACTCATTTTAGGAATAAAAAAAGATGAATGCTTTATTG
GAAGCGATATTTCTGCATTTTTAGAATACACTAAACTTGCAATGCCGCTAAACGATCGTGATATT
GTAATTTTGAGAAAAGAAAACGATAAAATAGATCTCGAAGTTTTAAACGATGGAAAACAAGTTC
AAAGAGATACAATTGAACTTCAGTGGGACATGGAAAGTGCTGAAAAGGAAGGATATGAGCACTT
CATGTTAAAAGAAATTATGGAAGAACCTGTAATTTTAAAAAATTCAATGAAAATTTCAAAATTG
GAAATTGAAAATCTTGGAAAAGAAATTGAAAAATGTGATAAAATATACATCACCGCAATGGGGA
CCTCACTTCATGCTGCAATGGTTGCAGAATACTGGTTTTCAGGATTAAATAAATTAGTTATTCCA
ATCGATTCGTCTGAATTTTTAACAAAAGGAATTGTTGATGAAAAAACACTGGTTATTGCAATAAC
ACAAAGCGGTGAAACATACGACACGATGAAAGCAGTTAAATACGCTAAAGAAAAAGGTGCGAAA
ACTGCAACCATTGTAAACGTTCTTGGAAGTACTGCAACAAGAGAAGCAGACATTACAATAATGAT
GGGATCCGGCCTAGAAATTGCAGTTTGTGCAACAAAAACCTTCATGTCACAGCTTGTTATTTTAT
ACAGGTTATTCATTGAGTACGGTAAAATTATCGGAAAAAACATGGATATTTTTGAAAAAGAGTT
ATTAAATATTCCAAATTACATTTCAAAAGTTTTAGATGAAAAAGAAAACATTTCAAAAATTGCA
AAAGAATTAACTGCTAAAAACTACCTATTTATTTCAAAAGGAATTAATCTTGCAAATGCCCTTGA
AGGGGCCTTGAAATTTAAAGAAATAACCTATTTGCATGCGGAAGGGATGAGCAGTGGATTTTTA
AAACACGGAACTATTTCATTAATTGATGAAAATATGGACACTGTTGCACTAATTCCACCTTCAAA
ATCTGAACTTTTAAATTCTGTTCTTTCAAATGTTGAAGAAATAAAAGCAAGAAACGGCAAAATT
ATCGGAATTTCACCCGTTGAATCTAATTTAAAATATTCGATAAAAGTTCCAGATGTAATTGAAGA
AGTAAGTCCTTTTGTCTATGCTACTGCTTGCCAGCTTTTAGCATATTACAAAGCTGTTGAATTAA
AAAGAGATGTGGATAAGCCGCGAGGGCTTGCAAAAAGTGTTACAGTTGAGTAATTCTTTTTAATC
TTTTCTTTTTGTCAATATCTACACCCATATTTCTTCGAGTTCTACCATTTTTGGAGGAAGTTCA
TCCCATCTCCAAAAACCCCTTAATACTTCGCTTTCTGGGAAATATTTCTTAAGATATTTAACCCA
TCTTTCAAAATAGTCAGGATGAAGTTCTTTTACTCTTAAAAATTCACTGTTTAATGCTGAAGGGC
ATAAATAACAGCCAATTCTTTCAAATCCTTTATCATACATCGGATTGTATGGGATATCTTTTGAA
TATATGTAGCTCCAGATATCAATCGAATTCCAGTCAAGAATTGGGAATACATTCGTTTGGAAATC
TATAAATCCGCTTCTTCTTTCGTAGTCCAAATTTGCACGGGTGAAACTCTCGTATTTTCTTGAAC
CATCAATTGTAAGAACTTTTTTGTTTCCATACTTCTTTTTTAAGTATCTTTTCAAAGGCATTAAT
TTACAGGCACTGTTGCACCATCGATTGTCTTTTGTTGGAATACCTTCTTTTTCAAGATTGTCCCA
GAAGTTATCGCCATCTACAGTATCAAGATTGATATCATATTTATCTACAAAATCTTTCACATAAT
TTAGGGTTTCAGGATATTCTAAACCTGTGTCAATAAATACAACATCTAAATCCGGAATAACTTCT
TTTGAAATTAAAGTACATACTGCACTGTCCTTTCCGCCACTAAAAGATGCGTTTATCGCGTATCC
TTTTCTTTTGTATTTTTCAACAGCATCTTGAAGGATTTCTATTGAATTTTCAACCATTTCATCAA
GGTATTCTTTATTATATTCTAAATAATCTGCAATTTTTTGAAAATCTAGCTCTTTTTTCCTTGAA
AGATCTTTTACTTTTACCCGATCTTCTTTTCTAACCCCAACTCCTAAAAAATCTCCAATTTTTACC

CCGATATATTCAAAATCATCTTTTAATGCTTCATTTAATTCTTCAGGGTTTTCAAGCAGTTCTTC
TTTAATGTATTTTCCTTTAAGCCTTTTTTTAGTATATTTTAAGGAGATTTTTGGGTCCTCGATCA
TATAATAAAGGGGAGAAGGGATAAATTTCCACGTTAAATCCAGTAAATCAAATTCGAGTGTTCC
AATCTGATCTTCTGAAACGTAAACTCTTTTTCTGTAATCCATTCCAGAAAGCTTTTCTAAAAGGA
TTAAATCATCTTCCTGAAAATTTTTTCCAGTTAATTCATTTAAAACCTTTATTTCATAAGGTGAT
GCAAATTTTGTGTCTTCATTAAACATAACTTTTACCTGCAAAATCATTGATATTCTATTTTATTT
TTAGTTTAAATTATAATTTTTCTTCGAGATATTTGACAAAATCTCCGATTTCTGGGATATATGCC
CCACATGCAAATTTGTGTCCCCCGCCACTTCCACCAACTTTTTTAGAAGCATAGTTTATCGCAGTC
GCAAGATTTATATCTTCTGCAAAGGACAGCAACTTTGGACACCTTGCGGAAACCTTGTATCCATC
GTCCATTTCAGAAACTGCAAAAATCGGTTTTTTCCAGTCAACTTTTTCAAGCGAATAACTCATTC
CTGCAACAATTCCGACGATGTTTCCTTTTATTGCATTTTTATCAACTTTAAAGTACTGGAAGTTT
TCTTTCTGAACTATTTCAACATCGTTTCTTAAAAAGTTCATTGAACCTGCAAGATTTCTTTTGTG
TTTTTTAAGCATTTTAAGTATTTTACTGTAATTTTTATCCCGATCCCCTTTTAAAACTTCTAAGG
GAACTTCATATTCGTCGTATCTCGAACAGCCGTTTATACAGGTTGAAAATTCTTCCAAATTCTTT
AAATTCGACTTGAATTCTTCGTTTGTCAGCTCGTAGCTTTCTCCAAAAATAACTTTTGGAAGGTG
TATTCTCCAGTCAGGAGGAATATAACGACTACATTTGTGTAAAAACTCACTTCCCATGATTTTTT
TCTGATCAAATGTTAAATCGCATAAATAATCTTTTGGAATTATATCGGTTCCGTGCTTTCTGTTT
ACATTGTTTATAAAGTTAATTATTCGAGAATCGTTATCAGTAAGATCAGTTCTAACGTCAGTGAA
ATATTTAATAGAAGTAAAAAGCGGTCTTGTATGTTTCCCGTAAAACTGTAAATCTGGACTTACCG
AAATATCTCCTGCTTTTACTGCATCTCTTAAAATTACATCCCGATTTAACCCGATTAAATTTCCTT
CAAGGCTTTGAACATCTCCAACAGCCCCTAAAACTGCATATTTTGCAAGATCAGTAAACCGAGGA
TCACATATCTTTGCAAAAAGATAGCAGACTCCTGCACCACAGATTTCCTTTCCCCCATCAATACCA
TCGTTATGAGGGTTTACGTTTACAATATTTTCAGGTATTTTCTCGTTTGAAACTATGTGGTGGTC
CAGAATAATTATTTTTGGTTTTTTTGAAGTTTCTTTTAATTTTTCATTTATTAAATCGATTTGAC
CGCTTCCTAAGTCTGCAAATATTATTAAATCATTATCAAAAGGAATTGTATCAATTGTTCCGTAA
TCAATCTGCCTTAAAAATAAAAATTCCGCTTCAATATTTAATCTTTCGAGCACGTTTTCTAAAAT
AATTCTGGAATTTATTCCATCAGTATCAATGTGAGTACATACAAGTATCTTTTTGTCCCGATTTT
TTCTAAGTATCTTAACTGCTTTTTTTAAATTAAGTAAATATTCCATTATAGCACCATTATTCAAA
AATAAGTACAATTTAGACGTTTATAACTCAGAACTCATGAGAGTACTATTTATATATTATAAAA
TTAATATATATGTATAATTGACCCTTAAAAAACCATAGCAGGAATATTTATGGATTATCAGATAC
TACTTCTTGTGTTAATTTCCGGTATAATAGGTTATGTATTCACAAAAATATCAAAGTCTATGACT
GGATACTATCTGGTTTTAGTTTTGATGTGTACAATTAATACATACGTACTTTCGGAAATAACGTT
AAAAACGTTGAGTATAAACAGCATTATTCCAATTATCGGGTTTTCAATAGCTACAATGGTAGTAT
TTGAGATATCAAGAGCATATTTTGATAATTCATTAAGTATCGATGCATCAAGTTTTCAAGGGCTT
CAGGAATTTGGTCTTGAATATTATATTGCAATTATTTTATTTTATTCAGCATTGCTTTTCGTATT
TTTATTGTATATTGTACCATTAGGATACTTAAGCATGTTTTATATTGCATTTATCATGCTAATTA
CTTCATTTGCGCTTGTTGGAAGCAGTATTGTCCGGTACTGGGCATTTGAACTTTATATTATACTA
TATCTAACTTTAATGGTGGGACTAATACTATTAAATTTAATTGAAATTGATTATTATCCCGAAAT
ATTGCTCCCATACCTGTTTATTTTATTTTACTACCTAGATATGAGATCATTTATAAAAAAGAAGG
CTTCAAGTAGGGTTTCTTTGAAAAAAAGACGTTTGAGGTAATTACAAATCATGTATCTCTAAATT
TTTAGTTTTCGTATCATATATAAAAAATCCCGTTGAAGATGGGTTTACGATCAAGGTATTTTCTA
TTTTATCAATACTCCTGCTTTCATGAACGTGACCGCATGCACACAAAACTGGTTTAAACTCCTCA
ATGATTTTTCTAACTGCGCTACTTCCAACATGTCCTACATCACACCTGTCAGCCATTGTATCTTTT

GGCGGAACGTGGGAAAGTAAAATAAAATTATTTTTAACATCTATTTTTTCATTTTTTAAAATTTT
GATAAATCTTTCGTAAATTTCATCTTCGGAATACTCGTTTGGACTGCATATGGGGGTTTTGTTTG
AGCCGCCCAATCCAATAAAAATTGTATCGTAAAATTTAACAAGTTTTTCATCGATATTCAACCCA
GATTCGTTCATTTTTTGTATTGTATCTAAATTTTCCCAATTTCCAGGGATTATAAAAACTTTTAT
TTTGTTGTTTAAATTTTCTAAAAAATCGATAATCCGGGTATCCTGTTCAGTGGTAACGTCAAATG
AATCATTCTGATTCGCTGATTTAGCAAAGCTACACTTTGTAAAATCTCCAGAAATTAAAATAGCA
TCAGGTTTGTACCTTATAAGTTTATCAAAATTAATTATGCGGTTATGTAAATCTGTTAATCCTAT
AAGCCTCATCTAATCACAAAAATACATTAACCCGAAATATTTGACATTTTTGCCGTAGCATCAGA
TATTGCATCAGCTAACGCTTGTGTAGTCTGGTTTGTCGAATTTGTAATCTGTTTTCCAGTGTTGC
TAACATTCGTTGCGTAGAAGTATGCTGCTATTGCTGCAACTGCAACTGCTGCCATAACAAGAATA
CCGAGTTCCATTGCTATTTGACCTTTTTTTGATGTTAGTTTTTTAAACATGAAATCCCCTAAAAA
AAAGAAAAAATTAAGTTTATACAGTAATTGCTTCGATTCCATCGGTTGCATTTGTTGCTACAGTTG
ACAGCGCACCTGTTGTGTTTTTAGCTGATGCACCGGTTTCTGCTTGTGCATTTTTAACATTGCTT
GCATAGAAGTATGCTGCTATTGCTGCAACTGCAACTGCTGCTGCAACGAGGATACCAATTTCCAT
TGATACCTGACCTTTTTTTGATGTTAGTTTTTCTAAAAATTTCATGTAACCACCTGAACAGTGTT
ATAAGTTCATTAATTCATATCTTTTACAGAATATATATACTTATTCATTTATGTTTGGACATAAA
AGCGTATATAGTTACCGATACGGTGGGATAATATTTTTAAAAGAAAAAAAGTGGAAGTACTGGG
ATTTGAACCCAGGTCCAGGGATTTCTCCTGCATCGGGCTTTAGTCCACGCAACAAACTGGTGTTT
GCGCGCCTTAAAAATTCTTAGATTTCTAAGAAGGCCATATAGGCACTTGGAGTCCCCGATGATAG
ACCAGACTACACCATACTCCCTATTGCACATTATAAGAATAAAAAAAGAAATATATATAGTTTAC
TGTTAAATTCAAATTATGATAGAATTTTTTTAAGTACATTTGAACAATATATAAAATTGAAAAA
ATGATCGATCTCGCTTTTGAAATAGTCCTCCCGATAACTTTTGGAATTATAATAGGTTATATACT
TAAGAATGCATATAGTAACAACTGCTTTGTATTAATTGGATTTTTTACAGGAATTATTGTAACTG
CATTCAGGCTCTACAAATTTATGAAAAAACACCAAAAACAGTTTATGAAAAATAAAAAAACGAAA
ATGAGCCTTTAAGGTGAGACTTTGGAATTAATTGTAAAAGAAGAATCAAAAACAGATTATTATT
ACGGATCTGACCCATACAACAGAGACATAAAAACACTTCTAAATACTGGGCTCGTTGTAATCGAT
AAACCTTCAGGCCCAACTTCCCACGAAGTTGCCGCATGGGTTAGAAACATGCTAAATTTAGTTAA
AGCAGGTCACGGCGGAACACTTGACCCAAAAGTTACTGGAGCACTCCCTGTAGCTTTAGGAAACA
CGACAAAATGCGTTCCAATCTGGCACATCCCGCCAAAAGAATATGTGTGTTTAATGCATTTACAC
GATGATGCTGAGCTTGTTGATATTGAAAATATTTTTAAAGAATTTACTGGAAGAATTCACCAAA
GGCCTCCTTTAAAAGCTGCAGTCAAAAGAAGCCTTAGAATTAGAAAAATATATGAAATTGAGAT
TTTAGAAATCGATGGGCGAGATATATTATTTAGAACAAAATGCCAATCAGGAACGTATCTAAGA
AAACTTGTAGATGATATGGGAGAAGCCCTTGGAACTTCTGCACACATGCAGGAACTTAGACGAAC
CATTAGCGGACCATTTTATGAAAAAGAAGCGGTTTATTTACAGGATTTACTTGATGCATATATAT
TCTGGAAGGAAGATGGAAACGAAGAAGAATTAAGAAAAATAGTAAAACCCCTTGAATACGGGCT
ACAACACCTTAAAAAAATAATTGTAAAGGATAGCGCAGTTGATGCAGTATGCCATGGAGCTACGC
TTTATTCTTCAGGAATCTCAAAAATTGAAAAAGGGCTTGGAACCGATGAAGTTGTTTTGATTGAA
ACACTGAAAGGAGAAGCTGTTGCAGTTGGAAAATCGCTCATGAATACGAAAGATATGTTAAAAA
CTGAAGAAGGCGAAGTTGTAGAGATAACGCGTGTTATAATGGAGCCCGGAATTTATCCAAGAATC
TGGAAAAAAAGAAATAAAAATGATAAAAGTAAACCGGAATTAAAGAAGAAATAAAATTAGCGGT
GAATATTATGAAGATAATAATTGATGAAAATTACTGTAAAGGCTGCGACATTTGCATTGAAGTG
TGCCCTAAAGACGTTTACAAAAAGTCAGAAACGCTCAATAAAAAAGGAATATATCCCCCAAACCC
AGTAAATCCAAAAGAATGTACAAACTGCCAGCTCTGCATATTACAATGTCCTGATCAGGCAATTA

CTGTTGAAACTGAAGAATAAACGTGAAAAAAATGCTTGCAATTGCAACAAAATTGACTAAAATC
AATTACGATTATGAAAAATTGCTTGAAATAAATAAAAATGTTTCAAAATCTTTTGATTATCACG
TTATAGATGCTGAAACTACTTTGTTAGACGAAAACATCCTTGAAAATTTAAAAAAAGCAGTTTTA
ACTGTTCAAAGTAAACGAATGGATTCTTTTGAACTTTTAGAAAAATATGCGTCATATGACTTTGA
TATCTGCGTTGTTTTAGGAAATAAGGCTTATCTTTCAAAAAAAGAAGCGAACTTTCAAAAAACAA
GGATTATGCATGTTTTAGAAAAAGCAGTAACTTATAAAAATAAAATCTGGGTTGGAACTGAAGG
CGTTGAAAATATTGTGAAAAATTTTATCGAAAAAAACGATTTAATTTCTTACTATGTTTACGGCT
GTGAAAATCCTGAAATTTCATCTAAAAAAGCAGTTTACATACCATATGTTGAAAAAATGGATGA
AAATATCTTAAATTCCATGAAAAATTACCTCGGAAGGCGTGAAAATTATCACGGAAACTGGCAGG
ATTTTATAGTTTCACTTGAAGACTTGAAAAATGAAGATTTAGATAAAATTAAAGACCATATTAC
TGTCGGATATCCAATAAATCAGGATTATGAAAATATTTTAAACTTTAAAAATAAATTTTTGGGA
AAATAACTTTTTTATTTATTATTTTTTTATTGCGTTCATAAATCTGTGTTTTATTTCAGTTGGAA
CTAAGTTTATGTTTGGAACGTTTTCATTGTGAGGTCTTGCTTTTGCAACAATTACTTTTGTACCA
GATTCACTTAATGCAAGTTTAACTGCATCTCTAAGCTGTTCTTCATTAAATACGCCAATTGAATC
TATTCCGCATGCTTTTGAAACTTGGTATAAGTCAGTACATGTCGAGTGCGTTTCCTGATTTCCAG
TTGATCCGTAAGCGCAGTTGTCAACAATTAAAAGTAAAAAGTCTTTTGGAGCTGTTTTTCCAATT
GTTGCAAGAGAGCCCATGTTCATCAATACAGAACCATCTCCATCTATTGCAATTACCTTTTCATT
CACAGAAAGTGCTAATCCATGTCCGATGGATGATGACAAACCCATCGATCCTAACATGTAGAAGT
TTTTTTCCCTGTCGTTTATTTTAAAGAGTTCTTTACTTGGAATTCCGATGTTACAAACAACTATT
TCATCAGTCACGTATTCCATTAAAATTTTAATAATTTCATATCTCGTAAGTTCCATACTATTCCC
CGTACTTTTGAATTATTTTTTTTAAATCGAGTTTTCCATTGTGTATTGTCGTTCCAATCAAAACT
CCAGAAATTCCAAGTTCCTTTGAAATTTTTAAATCATCCTCAGATTTAATTCCGCCGCCAATGTA
TATTGGATTATCTGTTTTTTTCAATATATCTTTTATCAATTCTACGTTAATTCCTTTTTGAGTTC
CAACTGATGAAATATCCAAGATTATTAAAGGCGTTTTTTTATCGATTTCACTTAAAATTTCTTCT
AAATTATAATTTAAAAGTTTTCCATCCTTAAAATCAAGGCTTAAAATGATATTTTCTTCTTTTAA
AATCGAAATATCGTTTAAAGTTTCAGTTCCAAGAATCGTCATATTGACATATTTTTTGACGTTTT
CTAAATCTTTTTTATCAGTAACTCCAAAATCAACAATTTTAAAGATATCTAAACTTTTTACAGTT
TCAAAATTGTTTCCAGTACTCATTATCGAGTTTAAATCCGCAATATATACTTTTTTTGCCCCATT
TTCTTTGTATTTTTTAATTACATCAAGAGGATTTGAAGAATCACATAAAACAGATTTTATTGGAA
CGTATTCTTTTCTGTTCCCGCTTTTTCCAGAAACTGCTAAACCATTCATTAAATCAATAACCGGT
ATAATTTCCATAAAAACACCTAATTTTAAAATAACTAAATCTAAAAATGAACTTTCAAACTTGTA
AATTATATAATTTTTCCATGAGTTATAAATTAAAATAAATCTTTCAAAAACCGAAACAACTTTGA
AATAAATAATTTAAAAAAAGATTTTTGTTGAAAGGTCCAAAAAAAGGTTTCCGTTCAACGAGTT
ATTTTCTTCAGTCGTAGGATACCGAAATTTAATTATTTCATTTCGTCGACCTTTTTGATCAACAT
TTTCATTATTTCTGCATCGGAAAGCATTCCTTCAGGAGCTTTGATGACTTTTCTTAGTTCGATTG
GAACACCGTCCATTCTGTAAGCAGTTCCTGAAACTTCAAGTCCAGTAATTGCTGGAGGAAGCACA
ATATTGGAAATTACAGATGATGGAGTTTCGTGAGGATCGATACATACAAGCGGGATCTTTGCCAT
GTGTTGTACTGCTTTTTGAGGGAAGTGTGCACCTGCATCTGATGCAACATTGATCATCATGTCGG
TGTCTCCTCTCTGCAATACATCGTTTGAAGCAGTTTCTCCAGGATTATATCTTGGGTATCCTCTTG
AGAAGTCTACACCGTATGGGTATCCAGTAACCCAAGTTGAAACCTGGTTGAATCCGTTAACGTTG
TAGTGTCCTCTCATTGGAGTCAATACAAACTTAGTATATGCATTCATGTCAATTACAAACTGAAT
AGCATTGTCAATAATTCTGTGTTTTCCCCTGCTCATTGTTACACCCATCGCGAAGAAGAGTGAAC
CAAACTGGGCATTTTTACAGATGTCGACTGCTTCGTAGATAGTTTCCGTTGGAACACCAGCTACC

TGTTCAGCTTCAATATTAAATCCTTTTGCTGCAGCTCTAAGAGCACTTACAAGCTCATAATCCTT
ATGAGGCTCCACTTGGAGGTGAATGTCAGCGAGTTTTGCAGTATCCGTTTTTCTCGGATCTACCA
CAATCATCTGTCTGTCCTGTTTACCTCTTTGTCTGAAGAATCCTCTTGGGAATACTGAATACCTGC
TCATGTGTCTTGGGTGAGCGTGCATTGGGTTTGAACCCCAGAACAAGACAACGTCTGCTCTGTTT
TTAGTTTCTCCTAATGTTGAAACAGGGTATCCTACATCCTGTACGGCTAAAAGCGAAGGCCCGTG
TCAAACACTCGCGGTGTTATCCACAATAGCTTTCGTTTTTTCAGCCAATAATACACCTAATTGTT
GTGCATGGCATTCCGCAGAACTCCACCCGTAAATGAGCGGTAACTTTGCTTCGACGAGTAACCTT
GCGGTTTCTTCTGTTGCAGTCTCGTAGTCAACCTTTTTAAAGTCATCCTTCTTATTTTCCCTCATA
AGAGGAGATTCATGTCTTACAGCGCCCTCGAAGTGCATAAATTTTGCATTTCCGATTCTGCATGC
ATTTCTAGTCCCAACTACATGGTTATTTTCTACAAGAACTTCAATATCATCGCAAAGAGTCCCAC
AGAATGGACATACGACATTCTTAAACACTTCCACTTTTATCACCCAAAAAAAATGGGAATGGTTT
ATTTCAATGATAATGCTTCTTTTTTGGTTTTGTTGCATGCTTCAATACATTTCAAGCACATTACA
CAGTAACCTTTAAGAGGCATTTTGTCGTCACCGGTGAGTTCCAGTGTTTTCTGTGGACATACATC
TACACATTCGCCGCATTCATTACATAATGCAGGGCTAAATTCAACTCTTGCGTATTCTTTACCTTC
ATGAACCATTTTGCCCATTTTTAAAGCGCCGGTTGGACACTTCATTGCACAGGCACCGCATCGAA
TACACATTGCGAATTCGTTTGCTTTTTCGGTTTTTTTCTTTGAAGGTAATTCCAATCCTTTCGGA
TTAACTACTTTTATTGCACCTGTTGGACATTTGATTGCACAAGGTCCACAGTATGCACATTTTTC
TTCTGACCAGACTAGACCTTCGTCAGTAACTGGTTTTGCGCTTGCATATTCAACTTCCAAACTAA
TAACTTCAACTGGACAGCTTTCTACACAGAGTCCACATGCAGGGCATGCCTCTGGTAATTTAACA
CCTAAAGCTTCACCATTGTATTCAATCATTGAACCAGGACATGCTTCAACACACTTCTCACAACCA
ATACATTCTTCCTCATTAACTGTGAAAGATTTGATTGTTTTCTGTCTTTTTTGAGGCAGTTTTCC
GGCTACAAAGATTGCGTTCCATGGACAGGTTTGTGAACATACAGTACAGTATATACACTTGTCTG
TGTCAATTACTGCGATATCTTCGATGGATATTGCATCAACCGGACACTCTGGAACACATACGCCA
CAAGCAACACAGTCTTTTGTAACTGCAATTGGTTCTTGTGGAATGATTATTTCTTTTTTAGGCTT
TTCGGCTTTTCCAGGAAGTGAAATTATTTCAGCAGGGCAGATATCAACACATTTCTCACAGAGAA
CACAGTGTCCTTTGGAGTATGGGAATCTGTCATCTACTTTTTTGATTCCTACAGGGCACGCTTTT
GCACATGCACCGTCCAAATCACATTTGCTTCCATTGAAAACTAATTTTCCATCTTTCAAAACCAA
TGCTCCCGTAGGACATGCCTCAGCACAACTAAAGCACTGAATACATGTCTTGAAGGGGGCAATCT
CTATTGCCTCTGTAGGGCATGCCTTCGCACAGGCATTACACACAAGGCAAGCATCCTCTTGAATA
GATATTCCTGCCAAATGACAACCCCCGAAAAAATTTTTAAAGGTATGGGGCCTTCTATTGAAGGT
TTCTCTAGCCTCCCATTAGGGTTCGGTTAGAGAATATTTATTCTTCTTTTTGGACTTCAAGGAGA
ACTTTTCCTTGTTCGTCTTTTACAATCATGTGCGCTGCACATGAATATCATGGATCGTATGCTCTT
AGCACGATCTCGATTAAGTTTAATTTTGCCTCATCTACCATCTTTACCACCGTGCTTATTTGAAG
ACCACTTCTGCAGCTTGTTGGATAGCTTTTTCCATGGTGGGTACGTTGTGTGTTGAAGCTACAAT
CATGTTTGCTTTAACAACTATTCCTTTATCATCTGTTTCATAATTGTGGAATAATGTACCTCTTG
GAGCGTAAATCGCACCAACACCATTTCCTGCTTTTGGTTCAACTTCTGCTTTAATGTCTGTTGAA
GTTATTTCTGAGTCTTCTAAGAGTTCTTTGACTCGTTCACATGAACCGAGTAACTCGATCAATCT
TGCATGATTGTATGCTAATGATTGGTTTGCGGGTTTTCCGAAAGTGTCTGCGAACTCTTTTCTAG
CTTCTTCGGCAAGAGGCGTAGGCATTGCATCACATACGTTAATCATTCCTAATGGTCCTACCCTGT
AGAGCCCTTCTGGATATCCAACTTTTTTGTTGTATGGATATTTAACGTAGTTATGTTCCACAGTG
TGTTCACCGATGTTATCTAAGTATTCTGAAGGTTTAAAGTCGGTTGTTTGTTTTCCATCAGGCGA
CATGAATCTTAACGTGTCTCCGTAGAAATTGTGTTTACCGTCTTTTACCATACCTAATACCAAG
TATCGATTACACCAAGTGATTTTACAACATCCATGTATTTTTCATTGAGTGCTTTGATAAGAGCG

ATGCCTTCTTGACCAAATTCGATCATTGCATCCATTTCTTTTAATAATTCGTCTCTTTTTTCTTCG
CTCAATGCTTTGGATACACCACCAGGTACACCAGTAACTGGGTGAATTGCTCTACCGCCAGTCGT
TTCAACGATTGCCTGACCAACTCTTCTCATTGCAATTGCTTTTTTAGCAACTTCAGGAGCTGCGCC
AATAACTCCAATAATGTTTCTAGCTGCAGGATCTGAATCAGGACCAAGTACAAAATCAGGAGCTG
CCAAGTAATAGAAGTGTAACGCGTGGGAGTGCATCATATTTCCAAGGTGCATTAATTCCCTTAAT
TTTGCTGCGGTGCTTGGTATTTCTGCATCCCATGCAGCATCTACTGCTTTAACACTTGCCAAGTGG
TGTGGAGTTTGGCAAATTCCACAAATTCTTGGTACTATTCTTGGTACTTCCTCTGCAGGCCGTCCT
ACAACAAACTGTTCGAAACCTCTAAGGGCAGTAATATGTAATTTTACATCTGTTGGTTTTCCCGA
ATCGTCTAAGGTTATTGAAACCTTACCGTGCCCTTCTAAACGGGATAGAGGTTCGATTGTAACCT
TACCCATAGAGTCACCATCCTTAAATTATATTTTTTGAAATTATTATTTTATTCTTTTTGGACTT
TTTTAGATACTAAAGCAACAGGTAACGTGAATCTGTTTAGTGTTGCAACCTTATCCGGGATTTCA
AGCGCTGCTTCTCCTGCATTTGCATAAGTGTTTGCAGCTGCTGCCCCTTGATCCAAAGTTTCATCA
GTTTTACCGTAGCATCCTCTACAAGGCATATTTGCTGATGGACACATTGCACCACAGCCTGCTCTT
GTAGCCATACCCATGCATGTGTATCCTTGTTCAAAGAGACATTTCTCTGGATCAGGAGTTCCTTC
AAAGGATCTCTTGAACTCTTTAGGGAATACATTCTCTTTTGTTCTTGGACATTCATCACATACAA
TTTTTGTCGGTAATACTGGATCTTTTCCTTCCAATAATGCAACAATTGCCCCTGCGGTTAGTGCA
GGTTTTGGAGGACATCCGGGAATTACGTAATCTGCTTTAACTACATCAGAAATAGCCCTTACCTC
AGTAGTTAGTTCAGGAACTCCTTCAGTTGGAATAACTCCCGGGTTATCTGTTGAAACTGTCGTAT
AAACTGTTTCTTTAAGTTGTTCTGGAGTATATATATTGCCAAGTCCCGGAATACCTCCGAAAGCT
GCACAGGATCCCCATGCAATCACTACTTTTGATTTTTCCCTGATTTCATGAATCAAATGTTCATC
GTGTTCGCTTCTTACTCCGCCTTCGACTAAGAATACGTCAACATCAGGAATTTCTTTTACGTCAGC
AATGATTGGAGCGTATACAATTTCCAAAGCTGGAAGAACGTCCAAAAGGCCTTCGTGTAAATCAA
GTAGTGATATATGGCATCCAGAGCATCCGCAGAGTTGTATAATGCCTACCTTAACTTTATCTGCC
ATTTAATCACCTTGTGATTGTTAAAAATTCCCTACACAGAGGGCAGTCATTTTACGAGTCCTTTT
GGTTCCCCGTAAAATTCATTCTCTTTTTAAACGGTTTTACGCTTTGATTGGGTTAGGTCCTAGTT
TTTTAACTCTATCGGTCATTTCTTTAACTGCTTCGGTAAATTTACCTGCGTCAGCACCTGACATG
AAGAACATCTCAAGTCTTTCGCCACCGATTCCGAGTTCATCTAGGATCTGTTTTCCAAACTTAACC
CTTTCTTCAGCTTTGAAGTTTCCAATATCGAATGCACACTCGTTTGGTTTACATCCTGCAACGAA
TACTGCGTCAGCACCTTTTTGGAATGCCCTTAATGCATAGGTAATATCGAATTTACCAGTGCAAG
GAACTCTAATAAGTCTGACAGTTGCGGGATATTGCATTCTGCTTGTACCGGCTAAATCGGCTGCA
GAATATCCTCATTGGTAACATCCAAAGGCAATGATTACGGGTTCTGCCATAATATATCCCTTTTT
TAATTTTTTAATAAATTAACTCGTAACTCATCAGAACTCCCCTGGCCTTAGCCTTCGGGTTGTTC
GTCACATGATATGAATTAACACCATTCCTAACTTAATTTTGCTGAGAATTTAATTTAATAGATAA
TTAGTTATTCAGTAGTTCATTCCCTAGTTCCTTGTGCTTAAATTATTTTTCTGATATTATTTTTC
TAATGCTTTTGCGGCTGCTAATATACCGTCAATTGCACCGATGACTTGTTCATCCCGGTAGTATC
TTAACTGCATTGCACCACTTGGACATGCTCCAGCACATGAACCACATCCTTTACATGCAACGTCGT
CAGTTACTGCCACCAAGTGACCGTCTTTTTCTTCATAAGTAATTGCGTTATATGGACACATTAAA
GCACATACTTTACAGCCACCGCATGTTTCTTCGTTAACTGTTGCCCTAATCATTTCGATCTTGAAC
TGACCTTGTGACATTGGAATTGCAACAGCACTTGCGGCACCTTTTGCCTGTGCTACTGTATCTGG
AATATCTTTTGGTCCTTGAGCGACCCCTGCAATTGCAACACCGTCAACTTTTGTGTTAACTGGTGC
CAATTTAGGGTGTAATTCTTTAAAGAATCCTTCTGGACCTCTATCTATACCAAGCATTTTAGCAA
GTTCTCCAGCATCTTTTTTAGCTTCCAAACCTGCTGAAAGAACAACTAAATCAGCGTCGATCTCT
AAAATTTCTCCCATGAGGGTGTCTTCTACTCTCACAGTCAAGTTTTTAGTTTCTGGATCTTCAAA

GATGTTAGCTGGTCTTCCCCTAATGAATTTAACTCCGAACTGGTCCTGAGCTCTCTTGTAGTACTC
TTCGTATCCTTTACCGAAAGCCCTGATATCCATGTAGCAGATGTAAACTTCTGCATTAGGGTCGT
GCATTTTCATTAACTGCGCATTTTTCAATGCGAACATACAGCATACTCTTGAACAGTATGGGTTT
CCAACTTTCTCGTCTCTTGAACCAACGCACTGTATATATACAGTTCTTTTTGGTGCTTTTCCGTCG
CTAGGTCTGATTTCGTGCCCGTGGGTTGGACCAGCAGGGTTAATCATCCTTTCAACTTCGAGAGT
TGTAATTACGTTGTCATAAACTCCGTATCCATACTCTTCTTTTCTGCTTGCATCGAACTCATCATA
TCCTGTTGCGGTAATAATTGTACCAACATTAATTTTGAGTTCTTGAGGTTTCTGATCGTATCTTA
CTGCCTCTGCACAGCAAACTTTTGCACAAAGTCCACAATCAATACAGTGTTCTTTATCGATTGTG
TAAAGTAATGGAACTGCTTGTGGGAATGGAACATAAATTGCTTTCCTAGTACCTAATCCTAAATC
AAACTCATTAGGCACTTCTATTGGACATGCTGAAGCACATGCACCACATCCGGTACAGATGTCTT
CATCAACATATCTTGGTTTTTTCTCAAGTGTAACATCGAAGTTTCCGATGTAACCGTCGATATTT
TTAATCTCAGCAAATGTAATTAATTCAATGTTAGGGTGGTTAGCTGCAGACACCATTTTTGGTGC
GAGAATTCACATTGCACAGTCATCTGTCGGGAAAGTCTTTGCAAGTTGCGCCATCCTTCCTCCGA
TTGACTCGTCTTTGTCTACAAGGTAGACTTTGAATCCTTGGTCACCGAGGTCAAGAGCAGATTGA
ATACCTGCAATACCTGCACCGATAACCATACAGCTTTTATCTACATCTACGACTTTTTGAGGAAC
GTCTTCTAATTTCTTAGCTCGTTCTACTCCACCAGCAATCATTTCACATGCTTTCCTGGTTGCGCC
CTCTACATCGCCCATGTGGACAAATGCGTCGTGTTCTCTAATATTTACAAATTCTAAATAGTAAG
GGGATATTCCCGCGTCTTGTAGGCAACCTCTAAAGGTTGGCTCGTGGATCTTAGGGGTGCATGCT
GCAACAACTATCCTGTCCAATCCGTGCTCTTTGATCGCATCTTTAATAAGACCTTGACCTGGGTCA
GCACACATGAACGGGTAAGTAGCTGCAACGGCTACACCGTCTAATTCAGAAGCGAAATCCTTTAC
GGCTTCACAATCCACTGCACCATTGATGTTTGCACCACAGTAGCAGACGAATACACCCACTTTGG
GGTCACTCATGGATTCACCTCCACATATAAAAAGTAAGGTAATTGGTATACATTTATACTACACT
CACCTGCTTATAAAAAGATACATTCTTGAAATTGCTAATAGGGCCCATTAATATTAGAAAAAATA
ATATGGGGCTTTAAAAACAGTTGATTAATATGTAGCATAATAGCAAAATAGATATTAGATTTTA
TAATAATGCCAATTAAAGTTTCTGTTTGGCATAATCTATGGCGGATTATTAAAAAATAATATTA
GCATAGGTTATTTGATCAAAGAATATTATTCTAGCTTCAAAATTAATTAAATAACCGTTAGATAT
ATATATCAAAAGTTGAAATGTATTGATTGCACGGTGTGGAACAGTAGTGAGATTGTTATTTTAA
ACCCCCCTCATACCCCCACTTCACTACTGATAACACCGTGTAGGAGATTTGATCTCCTAATCCCAT
TCCCACCCCTCATATAATAAAAGACTCTTTTAATTACCAATAATATACTGTTAATAATTTACAAA
AAATTTAAAAAACCCCTCCCCGTTCTATTTTAATTAATTATCATACTTTACATAAACCACCCCCT
ATTTCTTACCCCTCCATTTTTTTTATATTTTTAAAAATACTGGCGACTTTTTTATAGAAGTAATC
CTTAAATAGTGTACAAAAATAGTATGCTAAGTGTCAATTTCATATTCTCGAGGGATTTCATGGAT
TTACTTTTATTGTTATTTAGCGCCTTGTGGTATATTTTACCTGCGTATGTAGCCAATGCAGTTCC
TTGTATTTTGGGCGGGGGAAAACCTGTTGATTTTGGAAAAAATTTTTTCGATGGAAACAGGATT
ATTGGAAATGGTGTAACTTACAGGGGCACCTTTTTTGGAATACTTTTTGGAATAATTACAGGAAT
TTTACAGCATTTTATAGTTATATTATATATGGGCCCAGAATCAGTTTTTAATTATGGTTTAACTG
GTTATATTATTTTGAGTTTTTTACTTGCAACTGGCGCTCTTTTTGGAGATATGCTTGGAAGCTTT
ATAAAAAGAAGATTTAACTTAAATCAGGGACAGTCTGCTCCACTATTGGATCAGATAACGTTTAT
AATATTTGCATTGTTATTTGCATATTCGCTGTATCCGGTACCTGCAAACATTGTTGTACTCTTAC
TTGTAATTTCCCCGATAATTCATTTTTCTTCGAATATAATTGCGTATAAACTTCATTTAAAAAAA
GTATGGTGGTAATCCCCCTAAATTTCTATTTTTTAATCTGACGAATTCAATAATTTATATATACT
AAAACTTGGTAAAGGGATTTTGTGGCAGAATTAAAGTTTAGTAAAGTTCAGAGATCCTTATGAA
TCAAATGTCCATTTTCAAAATAATCAAGTATGCTTTGTTCAAAAGTACAGAAAAACATTTTAGAT

TTGGGTATCTCGGTTTTGTTTTATTCCAGTATTGTTAACACTCGTTAATCAAAAAAATACTGAGC
ATATAGTTTATAAAAGTTTTATAATGTGTTATATTAGTTGATTACATCATATTACACGACTATCC
TTTTCAGAGGTGTTTCATGTTAGGCATTTCAGACCCCTATGTATTAAGCGCATACGTACTCTGCA
TCTTAAGTACATTATTATGCGTTATATACGGGGCATTAAATTGGAATAAAGGTTCAGAAACCGAA
GAAAAAGAAATTGAAGAACAATTGGACTGGGAAAAAGAAGAAGAAAAGATGGAAGATGAAATCG
GAACGGTGGTCTAAATGAATATCCTTCTTCTAAACGTGATTGTTTTAATATATTTGTTAGTTATT
GGATACCTTGGATACAAAGGTTGGAAAGGAACTTCAAGTGCTGAAGACTACATGGTCGGAGGTA
GAAAAATCCACCCATTCGTTATGGCAATGAGCTACGGTGCTACATTTATCAGTACTGCTGCAATT
GTTGGTTTTGGTGGATTCTCAGGGCTATTTGGTATGAGTTTGCTATGGCTGACGTTTTTAAATAT
ATTTTTAGGTATCTTTATAGCATTTGTATTTTTTGGGAAAAGAACTAGAAAAATGGGGCATAAC
TTAAATGCACTGACATTTCCTGAGCTACTGGCAAAAAGATTCGAAAGTAAATTTATACAGTGGTT
TTCAGGGCTTTTAATATTTTGTGCAATGCCAATTTATGCAGCAGCAGTTTTGATTGGTGCTGCAA
GATTTATAGAAACTACACTTAATATTTCATTTGACGTTGCACTGCTTGTATTTTCCATAATTATT
GCAGTATATGTCGTAATGGGCGGTTTAAAAGGTGTTATGTACACGGATGCATTCCAAGGAACAAT
TATGTTTTTTGGAATGTTGTTTTTGCTCTACATGACTTATGATATCCTCGGTGGAGTTACCACAG
CTCACCAGGCAATTTCAAATATGTCAGGGTTAGTTCCAGCAAACCTTGCAAGTGTCGGGCATGCA
GGGTGGACTTCGATGCCGACTTTTGGTTCTCCCATGTGGTGGACAATTGTTTCGAGCATAATTTT
AGGTGTTGGAATCGGAGTTTTAGCACAACCCCAACTCATTGTTAGATTCATGACTGTAAAAAGTA
ACAGAGAATTAAACAGGGCTGTTTTAATTGGTGCAGTCTTTATTTTAATTGCAACAGGTACCGCA
TACGTTGTAGGAACCCTTTCAAACGTTTACTTCATGAATACCGAAGGTGTTCTTTCAATTGGTGC
AACAGTGTCTGAAACATTCCCGACAGGAAACAGCGACAGTATCATGCCATTATATATCAACAAAG
CAATGCCAGAATGGTTTATTTACATATTCTTGGTTTCACTGCTTGCAGCTGCAATGTCAACAATT
TCAAGCCAGTTCCACGCTCAAGGAACTTCGATTGGAAGGGACGTTTATGAAACAATATGCGGTAA
AAAAGGATTAAATTCAGTAATTATAACGAGATTAGGAATTATTGTTGCAATTGTAATTGCTGCA
ATTCTAGGATACATTCTTCCAGGAGGAATAGTTGCAAGAGGAACTGCATTATTCTTTGGACTCTG
CAGTGCTGCGTTTTTACCGGCATACGCGTTAGGACTATTCTGGAAAAGAACCACAAAAGAAGGAG
CAATTGCAGGTCTTTTAACAGGTACTTTTGTAAGTCTGTTCTGCTTAGTATTCATTCATCAAGCG
GAATCAGCAGCACTTGGAATTTCAAAAATGCTGATTGGTAGAGATGTTTTGATTTCCGCGATGCC
CTGGCCTTATGTAGACCCAATGGTAATCTCCCTACCGCTTGCATTTATTGCAACCGTAATAGTAA
GTCTTTTAACTAAAGCGCCTTCAGAGAAACATTTGAAAAAGTGCTTTAAAGGAATTTAATTCTTT
TTTCTTTTTTTATTTTTCAGCTATCAATTTTTTAATAATCTTGAGCGCGTCCGATCGTTCAAATC
TAACTTTTAAGGGGCTTTGGTCTAATATTTATACTTGTTGTGTATAACATGATTATGAATAGTAT
CTATGTTACATCCGAAATACTGACTGGTGAAAAAATGATATTTCTTGATCTAGAACTCAAGGATT
CCCCCGGAGAGCTTTTGAAAGCATTGCAACCGATATCTTCTGCGGGAGCTAATATTTTTAGCGTT
ATTCATATGCGTGAAAATAAAACACGAGATGGAAGGGTTCCTGTAAAAGTCGTGATCGAAAAAT
TAAACGATAATTTGTTACATTCTATTATTGAAAACCTTGAAAAATTGGATGTTATCGTTGCCAAA
ATAAATGAAAAAAGGAAAAAAATAAATCTGGAAGTTGTAGTTGTAGGACACATTGTTGATACAG
ATGTACTCGATACAATTGATAGAATTAATGAAATAGGCACAGTTGTAGATTTAGATTTAACAAT
GCCTGACCCTATCGCAGAATCTTCTGCTAGAATGAGGATAACGATAGATAACGAAAAATTAAAAG
AACTTTATGACGAATTTGATAAGATAGCCAAAGAAAAAGGTTTGTTATTTATTAAATCATGCTA
AGGGGATAATTTATGAAGATAATACTTGTAGGATTTGGAATAATTGGTAAGGGTGTTTTAAAGG
CCATTAATCTCAAATCAGAACATTTAAAGAAAAAATATGGATTAGATTTAAAAGTTGCTGCGAT
TTGTGACCGCAGTGGTGCTGCAATTGATGAAGAAGGCCTTGATTTAGAACTAGCCTTAAAAGTTA

AAGAAGAAACAAGAAAAATATCAAATTATCCCGAAAAAGGATGTAAAATGGATATTTTAGAAGT
TATAGAATCTGTAAATGCAGATGTGGTTGTAGAAGTAAGCCCCACAAACATTGAAACTGGCGAAC
CTGCAAAATCATACATGTTAAAAGCATTTGAAACCAAAAAACACGTCGTGAGTGCAAACAAAGGC
CCTCTTGCAGTTTCATTTAAAGAATTAGTAACTGCTGCAGAAAAAAATAAATTATGTTTTAGATA
TGAAGCTTCAGTCGGTGGCGCAATGCCGATAATCAATCTTGCAAAGGAAACATTGGCAGGAAACG
ATGTTAAATTAATAAAAGGAATTTTAAACGGGACTACAAACTACATTTTAACCAAAATGGAAAA
AGAACAGCTTGATTTTGATACAGTATTAAAAGAAGCTCAAGAACTTGGAATTGCGGAAACAAAT
CCCCACCAAGACATCAGCGGACTTGATACTGCTGCAAAAATTGTAATTTTAGCAAATTCAATATT
TGGACGAGATGTAACAATTAAAGACGTAAATTTAGAAGGAATAACAAGAATTACGCCTGAAGCA
CTCGTTATGGCAAATAAAAGTGGCCATACAATAAAATTAATAGGCGAAGTTACTGACAAAAAAC
TCGAAGTTTGCCCAAAACTTGTGCCTGTTGACAGCCCCCTAAATGTTATGGGCTGTTTAAATGTT
GCAATGTTTGATACCGATCTTGCAAACGATATCGTGGTTGTAGGAACCGGTGCAGGAGATATTGA
AACTGCATCTGCCATATTAAGCGATTTAATAAATATTCACCAGACTATTAAAAATTAATAACTCT
TTTTTTAAAACGTTTTTTAATAAAGTTTAAAAGGAAAGTAAACAAAAAGACTTATATATCTTTA
AAAAGAGTTTATATCTTGGCTTCGTGGCACGGGGTGCTTATCTTGGTAGATGAGGGCGGACTTCA
GATCCGTCGAGTTCCGTTGGAATTCGGGGTTCGATTCCCCCCCTGCGCCGTTTTATATTTTTGGTG
GGTTTTATGGAAAAAATGTACTCTAAAAAAGGAGGAATTCCTGATTTAAAAGAGTTAATATCTA
TTTTGAACAATTTTACAGGAATTATCTCCCTTGATAATGCTAAATTATACTACATAAACTCAAAA
CTTGTTTTTTCTAGTTTAAATGACAAAAAAATGGATTTAAACGATATTTTTAAAAATATTCCCGA
AGAATTCCAGATAGATGCATTAAACATGAGTTCTAATCGAGTAAATAAATTACTCGAAAGAGTG
TCTTCAAATAATCTTGATGAAAAGAGTATTCCAAAAGATATTTTCGTAGATGTTTATGGAAATA
TTGAAAATTACGTTGGATGCGGACTTTTTAAAGTTACACTATTTCCTAGAAAATACAAAGAAGAG
ATAGGGACCATTTTATTTTCAAATAAAGAAGAAATTGCCGCAATATATCAGAAAAAAGATAAAA
TCCTTGTTGGCCCAAAAGCACTGAGTAAGTTAAAGACTATTTTTGCAGTGAGCGATGTAAAAATA
TGCCCTGAAAAAATATCAAAACAGGACTTGGATGAAACATTGGGCGAAAATAAAGATGCAATGT
TAAAAAATTTCGTATCTTTCGAAGAATTAATGGAAAAAATTAAAGAAAAATCACCAAAAATCGT
TGAAAATGATTCTTTATATAATATACTTCCTAAAAATCCATCAATTGTAGAAATTGTTGAAAAA
AATGCGGTGATTGTGTCAAACGATAAATCCCCGATCATGGCATTTTTAGAAAATTATGACGGAGA
TAAAGCATACCGGATGATTAAAAATTTCTGTATATTAAATAATACCGTCTTTAAAATCTACGAAT
TAACCGAAGACGAATTTAAAAACATTAAAGAGTTCAAAAATGCGAAAATAAAAGATGTTAATTA
AATTTGTTTTTTAACAATTTCATATGCTTTTTTTAAGTCGCCAGTTTCCAAAATTGCTTTTTCTG
CATCCTCTCTTGAAATTCCCTGCTCTTCCAAAGCTTTAACGCCTTCTTCAAACAATTTTGATTTTC
TGATTATTTCTTCAGCGAGTTCTTTTTTAAGTTCCATGTACTCGTCTTCACCCATATCGATTGCAA
GCATTGCACTATCCCTAAATGGGCAGGGTTTTGTGATTTTACAGCACCAAATCAAACTTCCAAAA
CAGGTGTTCTTACCAAGTCCGAGTCTAGTTCTACTTCCAAAATCCTGCTTTATTTTTGAAAATTC
TTCTACGGGCATGTCAATTGATTCTAACGCTTCTTTATGTAATGGGCAGTACTTTACAGGAGGGC
AGCAAAATGCAAGTCCTCTTAAATCGCCACCTCTGCAAATGTGTGAAGGGGAATTTTCCCACATA
TAAATCACCTAAATCATATTTTTAACAATTTCAACATAATTATTAATTAAATCGTTTAAAAGGGA
TTTTCCATTAACTTTATCTAAAAAAACACCATTTTCTTCCACAGATTTTGCTTCTAAATCTATAG
CTTCATTTTTTTCCCGTGCTTCTGTTAGGCCGACCATTCCAATTTCACCATATACTTTTGGATCGT
GGTCTTTCATTTTATCTTCCGAAAGTATTCCAACAGCATACCCCAAAGATACTTCTTCAGTTGAT
GCATGGGTTAAACAGACACTATTCATTGTGATAAAACAGTCTTTAGAATTTAATTTTAAAAGTTC
ATCTTCGATTATGGTGTTTCCACCATGGCAGTTTATAATCAGGAATTTTTTAATCCCGATTTTTC

TACCATTTTCAACCAGATATTTGATATAAGTAATTACATCTTCGATGGAATCGTGAATACCGTGT
TTCACGTAAGAATATTCTGTTGAAGGGAGAACTACCCCTAAAAATTTTGCTCCGGTAATAATTGA
AACATTCAATGCGACGTATGATGCAATCTTTGCATCGGTATCGATTGGAAGGGCTGAACCGTGAT
TTTCAAGAAATGATCCAAGCGCAATAATTCCTATTTCGTGGACCGATTCACTGAAGATATTTCCT
GAAGAATATCTCAAATCAACCATAAATTTCACATTTTGGCTTTTTTAAGAGCTCTTCTGTATTTT
CCATATTTATCTACGGGAGAAAATCTTGGAGGTTTCACTGTAACGGATTTTTCGTTGCATTCACT
ACAAAAATCTTTCAATGTGTATTTTCCACATTTAGGGCATTTTTTCATTTTCATAGTATCAATTA
CTCTTCAACCCTTACAAAGCTTCCAGTTCCGCCAGCGTGTTTTTTTATAAAGTCGATTGCTTCTTC
AGATACTCTTCTAAGCACGTCTTCTCCGCTTTTGTAGTCTGGTGAAAGTACTTCTACCCTATATTT
TGGAGCTCCGATGTAAGTGATATTTACTTCCACGTCCTCGTAAGGATTTGCTTTTAAAGCTTTTT
TCAAACCATTTTTAATGATTTTTACACCAGTTGGCTCGGTCGTTGTAAGTGTAACTATTCCTGAC
ACTTTAACGCTTGAAAGCTCTATGTTTTCACTTGCAATTTCGTAAATTACATCTGCCCATTCTTG
TGGAAGTGGTGTTTCGAGTTCATCTAAAACTTCTTTTCCTTCGATTACAATCGATTCGAACGCAT
CGTATAATTCACCAAATTCGTCAATTAAAGGATATCCTGCAGCTTCCCATCCTTCTTCGATTGTTT
TTCCAAGTTTTTCTGAAGCAAACTGGAGTAATTTTTCAGCTCTTTGGAATCTCTTCCATTCCTGA
ACCTTAACTTTTTTCTGCTGATCAGTTGCCCTTTTTAATGAAAGATCTATCTGGTTTTTATGAGG
AGTTACTCTCATTACCTTTGCAACTACTCTCTGACCCTTTTTGATGTGGTCTCTTATATTTTTAAT
CCATCCAGAAGAAACTTCAGAAATGTGAACCATTCCTTCCTTCCCAGGATACTCTAAAAGTTCGA
TAAATGCCCCAAAAGATTTAACGTCGATAACATTTCCGATTATAATGTCCCCTTCTTCAGGAAAA
TCGTTTCTCATGATTCCACCTTGATTATCTTATAATAAGCATATGGAGTGTTTATACTCACTAGT
ATTTAAACAATTATAAAATTGAACTAATTTTAAAGACGTTTAAAAAAGAATAAAAAGAGTTATT
GTAATACTTCTAAGATTTTTGCTTTGATTGCACCTTTTGATGCCTTTGGTTCAGCAATGATTTTA
CCGCATGCTGCACATTTAACTTCTGTTGAAGGGCATCCAAAGATAACTTGTTCATTGTTGCAGTC
AGTGCACTGAACTTTTAAAAATCTGCTTTTAGGTTTTGAGATTAATTGCATGTTTTCTCACCGTT
TTTCTTAATTATTCTGTTAATTCAAACATTCTTGCTCTGAAGCCGCCGTTGCTTCTTGTGTGCATT
TTTCCGCATTCGGAACATTTGTATCTTAAATCCAATTTTTTAATTGGTTTAGCTCCTCCAGGTAA
AGGTCTTGGGTACCCTCCGTATCCTGCAGTAACTCGTCTGAATTGTCTTTGACCCCATTTCAATTC
GCTAGCTTTTCTTTTTTTAGCTCTTTCTACTGTGTGTGCGGTGTGCTTTTTACAGTATGGGCAAT
ATCTGTTTATCTTTTTTTTCATCTTCATGAATGTTCACCCGTTTGATAATATTGTGTTTCTCTAA
AATCTTTGAAAATTTCCTATCCAAAGAGATAACGTCATTTTTGTGTAAAACATACTGTAAATTCC
CATCTGTAAAGCAGGGGAACTTAGTGATGACTCTTACAATATCAATATCACTTTCAGTATTTATA
TGTTTTGGTATTTGACACTTATCGGTTTTTCTTCCAGTTTATCTAAATTAGCTGAAAATTTGGC
TTTTTTTTTATCGGATTCATCTATTTTTGAAATGTAATCTTCATTTCCAGATCCACAGCACGTTT
CTTCGATAAAAGCTACGACCCGTTTTTCATCGGGCGTGAACTTATTTGGGCTTTCCTTTAAACAG
TTTACCTTATAAATTCTAAAACTTAAAAGATTATTTAAGTAGTATTTTACCCTTTCAAATTTAAT
TTTATCATCGATTGTCTCGAGATAGTTTCGGGCATCTTTGTAAAAATTTTCCGGAAGATCATTTA
GTCCTTCTTTAAAAAATTCGTTGAAAAATGCATCCCTTATTTTCTTATACATGTTTTTCACGTTA
TTATTCTAAAAAAATAAATTTAGGATTCAATTCTTGGTGCAAGTAAGAATACAAGTTTTGAACC
GCTTACTTCGTACTCGATTTTTACAGGCATATCAGAACCAAGGTATATTTTTAAAAGATCTTCTG
CAGCGGTTGATTTTGTTAAATCTTTTAAATAAGCTAAATTGAAAGTACTTCTTGAAGCTTCGCCC
ATGTTAAAGTCTGCAAGAGCGTTGTTGTCATCGTCAATGCTGTTGTCAAATACGGTTTCGCTCTG
GTTTAAATCGCCTTTTGAATAAATTACAAATTTATTTTCATCGATTTTCAATGTTATGTGGTCAT
TTACAAGTTCTGCATCTTTTAATGCTTCAACAAATGCTCCTGCTTTGATTGAAACGCTGTTTGGA

TATTCGATGTCTGGAACTTTTAAATTACTTGATGAAACATCATATAACGCGATTGAAAACTTCCT
TGTAACGTTGCTTTTAAAAGTAATGTTTAATTTATTTTTCTCAACGTCAAGATCTAGAATTAATT
TTTCGTCGCCCCTTCCTCTTGCAATGATTTTTTTAAGTGCTTCTAAATCAATTCCGATATCTTGAA
TATCGCCTTCATATTCTTCAAAAACGTCTTTTGGCATTTCCATACTCACTAAAGCCACGTGCGACG
GATCCATTGCGCTTGCCTTGATCCCATTTTCGTCAACTTCAAAACAAATTTCATCTACTAAATTGC
TGGTTGCATTTATAACTTTTTTAAAATCTCTTGTATTACATGTAGCCCTGAACATTCACTCACCA
GTGATTTTTAATTCATATGAATTATTATACAAAAAATATATATGGTTTTTTAAGTGATTCAAAA
TTATTTTCCTGACACAAAGTCCCAGAATGATTTTATTGCACTTGGGTTTCTTGGTTTTTTACCCT
TTACAAGATACATGTATCTTGTCAGGTCTAAATCTTCAATAGGCACTATTTTTAGCTGTCCTGCA
TCTGCCGCTTTTTTTGCAGGGATTTCTGAAATAATTGATATACCGTATCCTTCTGAAACTGCTGT
AATTATTGAAGAATTACTTCCAAGGCTCATTATCGTGTGTAAATCCATCATCGAATAGCCCTTAT
CTTGAAGTGCGTCTACAAATATCTCTCTTGTACCAGATCCAACTTCTCTATCGATGTAGTCTTCTT
TTAAGATATCTGAAAGTTTTGCAACTCCTTTTTTCGCAAGTTCATGGTCTGGAGGAACTACGAGT
ACGAGCCTATCTTTTCCGATTACTTCGCTTTCGTATGATCCATCATAAATATTTCCAACTGCAATT
ATGTCAACTGCCCTGTCTTCTAAAAGCTTGAAACATTTTTCAGAATCCGTAATTTGAATCTGAAA
AGACACATCTCGATAAACCTTAGTATAGTCTTTAATTATACTTGGTAGTAGATGTTCTCCAGGGG
TAGTACTTGCAGCAATCCGAATTAAGCCTTCAGGATGTTCGTGAAGAATTCTCATCTGCTGTTTT
GCATTAGATATATTTTCAAGCAACTTTTCTGCATTTTCATAGAGGATGTTTCCTTCAGTAGTTAA
ATCAACTCCTTCAGGCGTCCTCAAAAAAAGCTGTGCATCAAAGAATTTCTCTAAAACGGATATAT
GGTTGCTTACAGTTCCTTGGGTGACTCCCAATTTTTTCGCAGCTTTAGAAAAGCTTTTAGTCTTT
GCCGCAAATAAAAATGTTTGAAAATAACTGATTTTTGGGTCCATGGTATCACTATTATTATACGT
TTATTCTTATAAGTAGATACTATTAGAAAAACTAATATATATACAATGTCATTATTCTTTTAAAA
ATTAAATTAAGGAAATTAAAAGAAAAAATATTCTGGTGAACAGATGATTATCCCGCACAGAAAA
CCCTGCATCGAATCACAAATTAAAACTTCTGGAAATCTCGATGAATTAAAAATGATAATAAATGA
TCGTTTATATTCAGAAAAAGAAATCCAAATACTTCCCTCAGGAAATTCTGCAATCCATATTGCAT
CGGAAATCGTGTCAAAATTAAATCCAAATTCAAAAACATTAATTCCAGACATGGGCGGATGGAAA
GGCTTTGAAACGTATTCTAAATTATCCAGATTAACTGTTGAAAAACTGAAAACGGATCTTGGAGT
AGTTGACACTGAAATTCTCGAAGAAAAAATTAAAGAATGCAGTGCAAAGTCTCTTTTTTTAACAT
CGCTTGCAGGATACCTTGCAGAACAGCCATTAAAAGAAATTGCAAAAGTATGCAGTGAAAACGAT
TGTTTGTTTATAGAAGATGTTTCTGGAAAAATTGGCGGAGATTGTGGGTATGGAGATATAATTG
TTTGTTCAACAGGCTCTCCAAAAAATTATAAACTGTGAATACGGCGGATTTTTAGGATTTTCTAAA
AAAATTAATGAAAATTTAAAAGATAAACATCTAAAAGAAGATATTAAAAACCTTTTAAAAACCT
ACAAAATGCCCGATATTTACGGGAAAATGATAGAAGAAACACTAATTTCAAAAAAGTCTTACGA
ATTATGTATAAAATTTTCAAATATTGTCAAAGAAAACATGGAAAATGCATATTTTAAGGATTCG
CATGGAGTTTCAGTATTTTTAAGTTACGAAAATCCAAAAGAAATCGCTAAAAATATCGATAAATC
TGTTAAATTGAACAATGGAAAATCAATTCTCACAATGTGTCCATTATACGAGAGAATCTTACGAA
AAGGCGTTGTTTTGGAGATAAAAAAAATGGACATTCACAAAATAAGCGAAAACGAAATCTATGA
AATATTAAATTTCTTTTAAAAAAATAAAAAAAGTGTGTATGAATACAGTCGTGAGATGTCGAGT
TTACCCCCACCACTGCCGAAAACATTTAAAATCCATATCCCCGTATCTATTATGGGTACCTAAAT
ATTTAAATGTTATTAATTATTTCAAATAGTAAGGTCCTAAACAATTAAACTGTTTAATACCTTAA
AATGCGGTTTTGATTTTTTCTGTTGCTTTTTTCATTGATATTCTGATTAAACCAAGATTAACTTT
TGAATCAGACAGTACAACTAAAATTCCTTCTCCTGCATCAGTCATTAAAGTTTACCAAATTCTG
CTTCAACCATCATCTGTTCGAGTGGCCCTTGGGCGATTTCTGATGCAGTTCTTTCCGCAGAACCAA

ATGCTGCAGAAGCCATTGCACCAATAAGTTCGCTATCGAGGTTTGAAGGAATTTGTGATGCGATA
ACAAGACCATCTTTTCCTACAACCATTGAACCTTTTATTCCCTCAGTTTTACTCAAATCTGCAAGA
ATTCTATCAATCATAACTATTACCCCACTTAGATTATTTTCAATTGATCCATTATTTCTGATAAA
CCTTCTTTTCGGTAGATCGATCCTGTAACAATATTTGAACTTTCTATAAAACCGTTCAAATTATT
TATTATTTCAACTGCATTTGATTCGTTACAAAGGTCCATTTTATTTATAAATACTGAAAACGGGA
TGTTCTTAGTAGATAACAGATTAATAATATCATTATCTACTTTCCTCAAACCAACGGTGGCGTCA
AGAACTAAAAGAGCATAATCTGCCCCCTCAACCGCAATTTCCCTCATGAAACCAAATCTTTCCTG
ACCAGGAGTTCCAAAGAAATGGAATTTTTTATCGTCCATTTCAATTCTACCGTAGTCTATCGCCA
CAGTGGTACCATTGTGTTCCACTTTACCCACATTTTTAATTAAATTTTCCATTAACGTGGTTTTA
CCCACATCTTCTGAACCCATTACCACTATTTTAAGTTCTTTTACCATTATTATCCCATTATTGGAA
GAGTTTTTTAGTACTTCTTTTAATAGCTCCGTTAGCAGCTGCACCGCCGATTAATGTTTCAAGCT
CTTTTACTAAAGACTGTGCTGCAACAAGAGTCGTTGGTTTTATTTCAGCAACTGTTGAAGCTTTT
TCTAATTCCCCGTCTAAATGAGTCAATATTTCAAGTGCTGCATCTAAATCTTTTCGTTGATCGAG
TAAATACATTGATGATGCGGCTTTGCTGAGCAATTCTGGATTTAATGCTTTTACAAGACCATCTA
CACCGGAAGATTCAACAAGTGATGCCATCGTTTGTAACGTCATTAAAATCTGCTGCTCTACCATC
TTTTTTGGAGCTTTGATTACTTTTGTTCCAACGGAATAATAATCCAATACTCCTGCAAGGGTAAC
TGCAGTTAAAAGAGAACCCATATCCGATACTGTTGCAGATACGTCTGCTGGAACGAGGTACGGGG
TTTTTTCAATGCTTTCAGTTAGTTTAACACATTTTTCAATCTGTTCATCTGAAGCGTATTCTGTT
CCATTTGTTGCTTTTGAACTGATAACATAATGTCCGTGCTGAGGAGTTCCAGGAACTGCTGCAGG
GTGCATTGATGTAATACCGATATCTTTTCTTTTAGTTCTTAATTCAACTTCCAACATTGTGTATA
AAATAACAGGAGAAACAGTACACGTATTTGCAATTATTGCGTTTTGTGGAAGGTGTGGTAAAAT
AGTTTTAGCAATTTCTACAGTTTTTTTACCAAATGGTGTAAAAAATATTGCAATTTCCGCTTCTT
TTGCAGCTTCTGCATCATCGGTTAATACCTTAACTCCAGCTTCTTCGACAATTTTCCACTGTTCGT
CAGTTAAGTTTGATTTGTTAACTTCCGCTAAAATAACATCGTGGCCAGCTTTGGCAAATTCAATA
GCCATCCTGCTTCCACCGTATGGGGGAACACCGCCATAGGTTCCAGGTAAATTTAACTGGTTAAC
GTACAGATTTTGGTTTCCTGCACCATATACCGTAACCTTCATTTAACCACCTTTTTTTAAAAAAA
TAAACGAAAAATAATAAGGCAGTCTAATTATTAAATCCTTTCGACTACGATACAGTTATCTCCAA
GTAATTCATATTTGTAATCAATTCCAATTAACTCGAGTTCTTTTAAAATTTCTGTTTTGGAGACG
TAATTTTTAAAGTATTTATTTAGGTGATTGTAAAATTCAAAGAGTTCTGCATTAGTATTTATATT
TTCAGTAAAAAATTCCTCCATTATAACGAGTTTACCTCCTTTTCTCAAGGAACCCATCATTTTTT
TGAGGAATAATTTCAAAGAATCTGCATAGCTTAGTGTGTGGATGCATAACACGTAATCGTGCTTA
TTTTTTGCAATTGTTTCAGAAAAATCCAATGCTTTCAATTTTGCACATTCGATTCCACTTCTCTTC
AACCGCCCTTGTGCAACCCTTATGAGTTTTTTTGAAATATCTACACCAACATATTCGCCATTTGG
AGCTACTTTAGATGCAAAATAAAGAGGGGAACGTGAACCGCAACCAACTTCGAGAATAGTGTCTC
CATTTTTTATATCCAGGTATTCAGAAATCACTTCTCTGCATGTCTTCATGTATTTTGTATTTAAC
ATCATATCCCAGATATCTGCATCTTTTTTAAAGTTTAAAAGAATTTCCGGGTGATCATAGCCAAT
AAATGCATACTGAACCAGACCTGACATGAAATCATACCTTGAAACGTGATCTGAAATAATTTTAT
CATATTCGGGGTGTATCGAAGAATAAATAAAATCAGAATTTAATTCCAATCGTTCTTCTTCGTTT
AAGTTGAGTAAATTTAGACTAACAGCGGTTTTTATGTATTTTTCCACGAATTCTTCATTTGGATA
TCCTAAAATTGTTATTAATTCTGAAATCGTCGGCCTGAAATCTTCAACAGCTTTGAATATTCCAA
ATTTAAGACCCTGTCTAAAAAAATATTCCCATTAATTCATTATTTATATATTCTACAGTATAATTT
AAAGAATTTATTTTATTATTTAGACTACTTGCGTTAGCTTTGCTAAAGTTTGAACCATATTTTAA
AATATTATTCTCAGAAAGCATATTTGACCTCACCCCGGATTTATTGCCCGAACCATCTATATTAA

AAGCCATGATTTCACCAGTTATCGGCAGAATCGTAGTCTTTAAGATATTCTACTACGGTCTTCCC
ATAAGTTGTCATTTGATATATCTTCATGCCTTCTTTCCCGTCGCAGTTGACCAATCCAAGTTCAA
TCAATGATGAACTACCCTTATATCGATTTCCCATCCCTTTCAAACATCCTAAAACGTTGCTAGGG
TCAGATTTTACTCTACGAGAAAGTTCTGAAAGGTAAGTTCCGTGAGGGTGTATATCATATAGGAA
ATAAACAATTTTTTTTCTTAGTTTGCTCTTGTTCAGAGACCTAATTATCGTTGGATTTATAAATA
TCATGTCTTCTGATCCCAAAAAAGACACCTCGAAAGTTAAAAATTAATTAAAATTACATAACGTT
TACCAATATTATTCGCCATATGGGGATTATAGGAACAATACCCTAATTGATAACGTTGATTATAT
TAATACTATTAGCCAATATAATCTTTATACCCAGTATTAATTTTTTTAATATACAAAGTTTCTTA
TAATGGGAATATTAGACGCGATGGAACTTACCGTATCCAAAATATTTCCAATAATGCCCCTATTT
TCCTCTTCAGTAGCGGATTGATTTTCTAAATCTGAATTTTCCCCAGTATTTTCTTCTTCAGTTTTG
GGTTCTACGTAAGTTTCAGATTCACTATTTTCTTCTTGTGTAATAATTTCTTGATTATTGGCTAT
GGTTAAATATTTTTTAAGTATCATTGAATGATATGTTCCAGATTCCATTGCAAAATCGGGAATTT
CAACTTCAAAGACTATATCGTAGGTTTCCCCATCGACATCACCCAGCTTAATTTTAAGAGGTTTT
GATTCTGTAGAATGAATTATGTCCTTATATGTTGGACTTTTATACATAATTTTATTATCGTTTGA
AATAGTAATTTTGTAAAAATAAGTTAGATTAACAGGATATATACTCGAAAGGCCTGAAGAAATA
ACCACATTTAAGTTATTTTCAGTGTAAACCATATCATCTATATTTATTGGCGCTACAACTTCGGT
AACTTTGTTGGTACTCGTTGGTATTGCACTTAATAGATCTATACTTGAATACCTTCCAACAGGCG
CTAAAGGATTTATATCCTTTGAAACTGTTCCAGAACCTTCCGTATAAAATATATCTACATCTTCA
AAATCGGTTAAAGGGACTGCGTGAAAGAGGGTATATCCAATAAATCCTTCTTCAAAGAATATATC
AAAATCTGCGGTTTTTAAGTTGCCACTTGTGCTTTTTGCGTTTACTAAAACATTCTTTTTATCAG
AAGTTGCTTTTAAATTTCCATTTGAATCGTAAACTTCGACCCACATCTTGTAAGTTATATCTTCT
GAAACCATGCTTTCAAATGAAACTGCACAGGAATTATTGAACCCTGCAAGCGGGTAATTTAAGAA
TAAACCGCCCTTTTCGTTTATTGTCGCAATTTTTGGGAGTATTGTGTTGTTGTACCATACTTTTC
TTATATCTAATATATTGTAAGCGTCAAAATGAACCATTGTACTGTATCCTGCAATATCATCATCT
TCGTCAACTGCAGCTCCATTAGTGTCAACAACCACTTCGATTAATCCATTTACTTCCCGATTGAAT
TTTAAATCTATTGAATACGTGTTTTTTTCTTTTGGTGAAAGTTTAAAAGTTATTGGTTCCGTTGT
TTCAAAAACTCCGCTTTCATAACTATTATCCTCGTCGTAGTGATCTCCAACATAAAGTTTTCCAG
TTAAAGTCCTGTTATAATTATTTTTCAAAGTTACATTCATTGTATAGTTTTTTCCAACAAAAACA
CATCCCGAATTACTTGACGGTAAAGCTGCCGAGCTGTCTACCGGGTAAGATATGTTATAAATCTG
AACTGGGGAGACAATTTTCGGATCAATATCTACATCTTCTTCAGAATTATAGATGTAATATTTAT
TCGAATACACAATTATGTTAAATTCTCCGTCATCGACCAATCTTGAAATAAATGGAATATATAAT
GTATCTGCATCATTTTCATCGATATTGATTGTTTTTTTGGACATGCATCCACTGGAGCTTTCAAT
TATTACAGTAGCAGTTTGATCATCGTCATACCTATTTTCAAGATTAACTTTTAAAAATGAGAGAT
ATGGATCATCTTCAACATAGGCCCTTGGAAGAACGTTTTTACTTTCAAATTTAGCATTTAAAGCG
TTTTCAATAGTTTTATCGTAAATTTTTGAAGATTGGTAGTAATCATTTACCATTTCAGAATTGCT
TCCATTGATATTATTAAATAGAGAATAACTTGCCACATGAATTGGGAATTGGTATGTTTTGGCAG
TTTTTTTATAATTATAAGAAATAATTCCAGTGTCTTTTACCATTTCAGTTTCTTCAAGATCATTT
GTATAATCGTGAAGGCCCATGATTTCGGTGTGAACCTGTATTTTAAATTCATCGTTATCCGCAGT
TATTTTGGGAATATCAAAAGAGACTTCTTTTTCTGAGCGGTAGGACACATAAACTACTTTTAAAT
CATCTTTTCCATTGTAGTAGATATCTTCATTTTCAATATCTTCGTTTGTGTCGTCATCATAATCT
GTAGAAACTACACTTATCCAAGTTTTAACTTCGTAGTCAGTCTGCGCTCTATAATTACTTTCAAG
AGTTACGGTAAACCAGTTGCTGTTTACAACTTCAAGATTTTTGTTTGAATCAAAAACATAACTAT
CCTGACAAGTTACGTTTTTTACATCTATTGGACTTACCACGTTTACAGTTATTTGTTCAGAATCG

ACTTCTTTTCCATTTTCATCGATTAAGCTAATATCCACGTCATGTTCACCGACTTCGTCAAATTCA
AGCCTAATTTCAACGATTTTTTCATAGTGGGTGTTTGGTGAAAGTGGCAGTATTGCTGATTCAAA
ATCGTTTTCTTCATAGCTTGCCGTTACTCTCACGTTTCTTTGGTAAGGATCATTATTTACTAATCC
CACATAGATTACAGGCGTACTCGGGTCTATGTATATTTCAGAAGAGTCAGGATTGTCCCCAACAC
CCCATATTTTGTAAATTGAAATTCCTTCATCATCTTCTGCATAATTTACAGAAACAGTAAATAAT
AAAATCAGGACAAAAATTAGTTTTTTTTGCATAATACCCCTAAAAAGAATAAATTATTCGGTTTT
TATTAGTAATTTGTATAGGGAGCTATTATTTTTAGATAAAATTAGTAAATGGGTTTTAAAATAA
TTTTTGTGTACTTGTTTATGTAGGTTCAAGTAGTGAAATTATATTAATAACGGGAAACATAATAC
GTAATTGTTATTAACAATACTGCGGTGATTTGATGAAGCTGAAATTTTTTGGCTTGATTATTGTG
TCATTTATGTTATTGGGGGTCGTAAACGCAATACCTTACAAGTGGACTTCGACAGTACCAATTAC
TTATGTTGATATTTCAAACATACAAGATACAGAAAAGCTTGATTACTATTTGAATTCGGATAATA
TTGTAGTAATATATACCGGAAACGATTTTGCAATGACTCCAAAAACAGAGGCATTTTTAGAATAC
CTGGGGCTTGTTAAGTCAAAAAATATCGTTAGTAGTATTCCAGATTTTGGAAATTCTACGTTTGT
AAATTCAAATTCGGAAGTTATTCCTGCAAGTTATGTTTACAGGGACGAAAATGGTGCATATCTTT
ACATTAATTCAAACGATGCGGCTGAAAAAGATACAACAACAGTATATGAAGCATCAATTAGTTCA
AAAATCATTGATGATGGATACTACGAAATTAAACCTGGAAAATTATTTATTCCATACAATATCGA
CCTTGAAAATCAGGACATTGTTGATTTTATTGGAAAATATGTGGCACTTCAGGGTGAAACTTTTG
CATATATCAATAAAGTTCCTTCAAATTACGATTCTGTTGTGATTAATGGAATTGTTGTACAAAAT
ATTATTCCTGATGATAACGGTGAAAGTTCAATTTCCGTTGCAGATCGAAAAAATAACCGTTGAAGT
GAGGGATGAAGATACAATTAATAGAAAGATAGCAGGCGTTAAGGCTGCTTCTGAAATTCTTGGA
ATTGGAACCACCTATGTTACAACAGGTTCTGAAAATATTGATATAATAAAAGACAGTTCTATAAC
GGAAGATGAAAAAGAAAGCCTTTTAAACAGTTATTGGTTTAAAAAACACTATTCTGATTATTAT
TCCCACATAAAATATAATAATTACGGAAATTACCCAAATACTCAAGATTTACTTGCAATGAGTTA
TTATCCATTAATATATATGGATAAAGCTCCTGAAACATTCCAAGATGACCCAACCGGAGGATATT
ACCCAGATACAATTTCATATACGGGTTCTGAAAGCCTCGGATACTGGAATGAAGGTGCAGGTTCT
GAAAACAGTTATTACCACTACGATGGTGAAGACTACTGGAACGACGATGAAACAGTTACCCGAGA
TTACTGGTCAGTTAACGGGGATTTTGCAAGTATGAGCGAAGAAATAGACTTTAACGATAGATACG
AATACTTTGAACATTGGTACGTTAAAAATTACGCTTATGCAATGACTGGTGGTGCAAACGGCCTT
TTATTATTCTCGAGTGATGACGATTTATTGGATGCAGTATTCAATAAATACAATAAGAATATTAC
CTGGAAGTTGAACCTTGAAGGAATCGACTACGTGGTTATTCCTGATTATATCGGATACGATAAGA
TCAACGGAATTGCAGTTATTAAAATTCCTGGATTAAATAACAACGAAATAAATGGAATGCATTT
TATTGATGAAATCTACATCGAACCACAAAATGAAGAATACGGAATTTACGTTGCAGACGTTTATG
AATACAGTACCAATACACTATATGATTTGAATGACAATAATACATGGATTTGTTCATTTGAAGAA
TACGCAAAATGGGCTGAAAACTATAAAACCAGCAGAATATATATTAAAAATGATACTGTATACG
TAAATGCAGACGATAACGCAAAAATAACGATTTATAGAGAAAATATAACTCGTGTAACAGGTAC
TTCAACAGAGCTATACGATGCGACTGGAAAACTGGTTATAGCAAAAGCACCAGACACAATTTCAC
TCAATAACTAGGAAGTGGTATCATGAGATTGGGATTTTACAACATACAAGGCGGAACTGGGAAA
ACGACCATTGCTGCAAATATTGGATATTACTTAAGCGATAAAACCAAAACTGTTTACGTGGATTG
CGATATTTACGCTGGTTGTGGGGCCCTGTTATTTGGATTTGAAGATAGCCCCCATACGTTAAATT
CTTACCTTTCTGGAGCAAGCGCTTTAACAGATATAATACATCAGTTTGACGATCTTTCTGTGATT
GTTGCAGATTCAACACCCAATTCATTCAATACAGAAATTAATCAAAAAAGAATGCTCGAATTAAT
CAGGGTTTTAAATGATAACTATGATATCGTATTATTAGACCTTCCTCCAAACATTACTGAAGGAA
ATCTTTTATTTTCTTCTTTAAATTTAGAAGAAAAAGTAGTAAATAAGATGATTGTTGTTGCAGA

AGACAGCATTCCTGGAATTGCAAATACTATGAAAACAAAAGAGCTTCTTTATGCAATAGATATTG
ATTGTATTGGTGTTATTGTAAACAAATTCAAAGATACTGTTGATTTCGACGAAGCACTTGATGAC
ATTATTGCAATATTGCCATATGATAAAAAAGTGGAAAACCAGTGGATGGAAAACGTTCCTGCAGT
TCAGATGAAATCAAAATTCAGTAAAGAATTAAGTTATTTGGCAGAAGATCTTGCTGAAGTCTAC
ATCAAAAAAGACCTTGCCGCAGTTAGGGCTTTAAAAGTAGCAAAAGAATTAAAAGATATGACAT
CTAAAAGACAGGATGTTGAAGAAGACGAATTTTAAAAAATCAATTTATTACTTTTTTGGTGAAT
TTATGCTTACTAAAAGAATCATTCCCTGCCTCGATATCAAAGAAGGTAGAGTTGTTAAAGGAACA
AATTTTGTAGAATTAAGAGATGCCGGAGATCCTGTTGAGCTATCTAAAATTTACAACGAACAGGG
TGCTGACGAACTTGTATTTTTGGATATTACCGCATCTTTTGAAAAAAGAGACATTATTATTGACG
TTGTAAAAAGAACTGCTGAACAAGTATTCATTCCATTAACTGTTGGTGGAGGCATAAAAACAGTT
GATGACTTTAGAAAAATATTGAGAGCTGGCGCTGATAAAATTTCCATAAATACATCAGCAGTAA
AAACTCCAGAATTGATAAAAGAAGCTTCTGAAATTTTTGGGACGCAGTGTGTTGTTGTTGCAATG
GATGTAAAAAGAAAGTATATAACAGATTCTCAGAATGAAAATTTGAAGAATAAGAACATCTTTG
AAACAAAACTTGGATCCTGCTGGTTTGAAGTCTATATCTATGGTGGAAGAGAAAGAACTGGTATT
GATGCAATAGAATGGGCTAAAAAGGTTGAATATTTAGGTGCTGGAGAAATTCTTTTAACTAGTA
TGGATGCAGATGGTACAAAAGATGGGTACGATTTAGTATTGACGAAAGCAATTTCTGAAAATAC
AAAACTTCCAATAATTGCAAGCGGAGGTTGTGGAAATGCAAAACACGTTGTCGATGCATTTACTG
AAGGAAAAGCTGATGCTGCATTAATGGCAAGCATTTTACACTATAGAGAATGTACTGTTAATGAC
TTAAAAAAAGAAGTTGAAAAAAATAATATTCCTGTAAGATTTTAATTTTTTAA

---> Sequence Showing highlighted Existant Combined Trimers

ATT TTT TTG ATA GTT TAA AAT ATC GCT TAA TCT AAA TAT ATC AAT TAA TAT GCC TAA
AAT AAA AAG TGA CTA ACA GCG TCA CAT TGT TCC CAC CAG ATG GCA GTA CTT AAT GAA
TCG CAG GAG GGC TTA ATT TCC GAG ATC GGG ATG GGA TCG GAT GTA ACT CCT CCG CTA
TGA CCG TTA TGT CAA AGT TTA AAT TAA AAG TGC ATC TAT TGC CCC CGT ATC TTT AAG
TGT CCA GAT GCC AAT TTA TCT CTT GAA CTA TTA GTA CTA GCG GGC TGA ACA TCT CGG
AAA ACC TTG ATG CGT ACA CCC CTA GCC TAT CAA ACC TGT CTT CTA CAG ATG TTC TCG
TTC CCG AAG GAA ACG GCT GTC TAT TTT CAG GAT GAA TTT CGA GCT TAG ATG CTT TCA
GCT CTT ATC TCT TGG CGC GTG GCT GCT CGG CAA TGC CTT GTC AGA CAA CCG ATC GAC
TAG AGG CGC CGA CGG CCC GTT CCT CTC GTA CTA GAG CCA CCT TTC CCT CAG ACA GCC
AAC ACA TCC AGC AGA TAG CAA CCA ACC TGT CTC ACG ACG GTC TAA ACC CAG CTC ATG
ATC CCC TTT AAT GGG CGA ACA GCC CCA CCC TTG GGT CCT GCT GCA GAC CCA GGA TGG
GAA AAA CCG ACA TCG AGG TAG CAA GCC GCG GGG TCG ATA TGG GCT CTT GCC CGC GAC
AAC TCT GTT ATC CCC GGG GTA GCT TTT TTG TCA TCC CTG GCC CCC ATC AGT GAG GCA
CAG GGG TTC GTT AGG CCC GGC TTT CGC CTC TTC GTT GCG TTT TGT TGG CAA CAA AGT
CAG GCT GGC TTT TGC CCT TAC ACT CAA CAG CGG AGT CCT GAC CCG CTT GAG CCA ACC
TTT GGG CCC TCC TGA TGC ATT TTT AGG AGG GTA CCG CCC CAG CCA AAC TGC CCA CCT
ACC GGT GTC CCC TAA AAG GGT AAG GAA CAT GGT CAT GAG AGG GTG GTG TCC CAG GGT
CGC CTC CAC CTT TCC TGG CGA AAA GGT TTC GAT GGC TCC CAC CTA CAC TGT ACA CTC
ACA ACC ATG CCC CAA CGA CAG GCT GCA GTA AAG CTC CAC GGG GTC TTC GCT TCC CAC
TGG AAG TCT CCG GCC TTT GCG CCG GAA TGG TAG GTT CAC CGG ATT CTA GTC GGG GAC
AGT GGA GGT CTC GTT ACG CCA TTC ATG CAG GTC GGA ACT TGC CCG ACA AGG AAT TTC

GCT ACC TTA AGA GGG TTA TAG TTA CCC CCG CCG TTT ACT GGC GCT TCG CCC GGT TGG
AAC CGG GGT TCA CGT ACC AGC ACT GGG CAG GCG TCG GCC TTA GTA CAC ACC TTT TCA
GGC TCG CTA AGA CCT ATG TTT GTA TTA AAC AGT CGG ACC TCC CTG GTC ACT GCG ACC
AGC GAT CCC ATA TAT AAA CAA GAT CAC TGG CAC TCC TTC TCC CGA AGT TAC GGA GCC
AAT TTG CCG ACT TCC CTC GAC TAG ATT CAT CCG CTA CGC CTT AGG ATA CTC ACC TAG
GGG CAC CAG TGT CGG TTC TGG GTA CGG ACA TCT AAA ATC CTT GTT AGT TCC CTT TTC
ACG GGC TCC AGA GAT CGG CCG AAC CCT CCT AAC GGA GAG CTC ATC ACT CTT TCA TCC
GGT TCT CGT TAT TAC AAC TCT CCC CGG ACT TAT AAG CTT GAA TGC CCA GAC AAG AAC
ACT CAG CCT ACC CAG AAG CGT CGG AAA CTA ACC TAG TGT TGC CAC GCG TAT TTA GAT
GGC ACA GGA ATA TTA ACC TGT TTC CCT TTC CTC TCA AAG AAG TTA ACT CGA GAG TTA
GGA CCG GCT TAC CCA CAG CTG AAA AAC ATT GCT GTG GAA CCC TTG CCC CTT CGG CGG
TGG GGA TTC TCA CCC CAC TTA TGC TGT TAC TAC TAC CGG GAT CTT CAT TTC TAC GAG
GTC CAC TCG ACT TCA CAG CCG AAC TTC TGC CCT CGC AGA ACG CCC CCC TAC AGG ATC
ACC TTT TGG TGC CCT TGG GTC TCG GCG GCC GAT TTA GCC CCG TCC ATT TTC GGG GCC
CTT GAT CTC GAC AGG TGA GCT GTT ACG CAC TCG TTA AAG GAT GGC TGC TTC TAA GCC
AAC CTC CCT GCT GTC TTA GAC CAA GGA CGC CCT TCG TGT TCA CAC TTA ATC GGC ACT
TAG GGG CCT TAA CCC AAG TCT CGG CTA TTC CCG TCT CGG ACA TAC AGC TTA CCC GTA
TGC CCT CAC TCG GAG CAT GCG ACG ACG ACA AGT TCG GAG TTT GAC AAA ACA GCG AGG
AAT TTC TTC CCC TAA CCA CCT TAT CAG TGC TCT ACC TTG CCG ACT ATC TAA ACT CCG
GCT GAC CTG AGA GTC ACT TCG GAG GGA ACC AGC TAT CTC CGG GCT CGA TTG GCC TTT
CAC CCC TAG ACC GAG GTC AGA AGA GTG CTT CGA ACG GCA ACA ACT CTG CAG GCC TCC
ATC CCT CAG TTG AGG GAC TTC ACC TTG CCC CGG CCT AGA TCG CCC GGT TTC GGG TCA
TAT TGC TGT GAC TCC GGG CCC TTA AGA CCC CGT CCC TCA AGA TTT TGC GGA CTT GTT
GGT TTC CCT ACG CCT ACG ATC GTG AAG ATC TTA AGC TCG CCA CAA CAA TAT ACT CCC
CGG CCC GTT TTT CGA AAC GGA CGA CAC AAC CCT GGC TCT ATA CTT CGT ACA GCC GTC
TTG CGA CGG TTT CCT TCA GTA TAG TTG CCT TTG GGG CCG TGT CAT ACC ATA CCT ATC
TGG TTT CAG GCT CTT TTC ACA CCC GAT TAA AGG TGC TTT TCA GCT TTC CCT CGC GGT
ACT AGT ACA CTA TCG GTC TTG GAA CGT ATT TAG AGT TGG AAG TTG ATG CCT CCC AAA
TTC CCG CGC GAT ATC CAA CGC ACG GTA CTC AGG GAC ACT TCA AAC CTG AAT CGA TTA
CGC TTA CGG GAC TTT CAC CCT CTA TAG TGT GAC GTT CCA GTC AAC TTC AGC TTT TCT
CAA CAG GCG AAA AGA AGG CCC TAT AAC ACC ACA TCT CCC CGA AGG GAT TCA GTT TGC
TCT GAT CCG CGT TCA ATC GCC TCT AAT AAC GGA ATC TCT ATT GAT TTC TCT TCC TGC
GGG TAC TAA GAT GCT TCA ATC CCC CGC GTT CCC AAT CCT TAA CGG ATT ACA AAG TAG
GAA GTC CCA TTC GGA AAT CTC AGG TTC AAT GAC TGT ATG CGT CTC GCC TGA GCT TAT
CGC AGC TTA CCA CGT CCT TCA TCG GCG TTC CAA GCC AAG CCA TTC CCC AGA TAG GGT
AAT AGA TAA ATC TAG GCA TCT GGA TAA CAC CAG ATA CGG AGG CTT ATG CAC GAC CTT
CAC TAA AAA TCC GGT CCT CGG TCC ATA ATA CTC GGT ATT GTG GAC TGA GCA ACT AAC
AGC CTA CAA TCT TTT AGT GCC TAG TAG CAA CCA TTG TTC TTT TTT TAG GAG GTG ATC
CAG CCG CAG GTT CCC CTA CGG CTA CCT TGT TAC GAC TTC ACC CCC CTC GCT GAG CCC
AAG TTC GAC CCT AGC CAA GGC CAA GGC CCT CAC TTC AAC CCA ACT CGG GTG GTG TGA
CGG GCG GTG TGT GCA AGG AGC AGG GAC ACA TTC ACC GCA GTA TTA TGA ACT GCG ATT
ACT ACG GAT TCC AGC TTC ACG TGG GCG AGT TAC AGC CCA CGA TCC GAA CTA CGA CTA
GGT TTA GGA GAT TTG CGT CCC TTT TCA GGG TTG CGG CCC ATT GTC CTA GCC ATT GTA

GCC CGC GTG TAG CCC AGG AGA TTC GGG GCA TGC GGA CCT ATC GTT GCC CGC TCC TTC
CTC TGA CTT AGC GCC AGC GGT CCC CTA TGA GTG CCT TTC TCC CGG AGG AAA AAG TAG
CAA CAT AGG GCA CGG GTC TCG CTC GTT ACC TGA CTT AAC AGG ACG CCT CGC GGT ACG
AGC TGA CGA TGG CCA TGC ACC ACC TCT CAG CGC TTC AGG CAA GGT CGT CAA CCT GGC
CGT CAT CAT GCT GTC GCT CCT GGT GAG ATG CCC GGC GTT GAA TCC AAT TAA ACC GCA
GGC TCC ACC CGT TGT GGT GCT CCC CCG CCA ATT CCT TTA AGT TTC AGT CTT GCG ACC
GTA CTC CCC AGG CGG CGA ACT TAA CGG CTT CCC TTC GGC ACT AGA TGG GCA CGG GGC
CCA CCT AAC ACC TAG TTC GCA AAG TTT ACG GCC AGG ACT ACC CGG GTA TCT AAT CCG
GTT CGC GCC CCT GGC TTT CGT CCC TCA CCG TCG GAC CCG TTC CAG ACA AGT GCC TTC
GCC ATA GGT GGT CCT TCA AGG ATC AAC ACA TTT CAC CGC TAC CCC TGA AGT ACC CTT
GTC CTC TCC CGG TCC CAA GTC CAG CAG TAT CCC TGC CAG TCC TGC GGT TAA GCC GCA
GGA TTT AAA CAA GGA CTT ACT GGA CCG GCT ACG GAC GCT TTA GGC CCA ATA AAA GTG
GCC ACC ACT CGG GCC GTC GGT GTT ACC GCG GCT GCT GGC ACC GAA CTT GCC CAG CCC
TTA TTC CTC AAG CTG TTT ACA CTT GAT AAA AGC CCA TGC TGT GCA TGA GCA CTT GGG
ATC CCC CCG TCG CAC TTT CGT GCA TTG CGG AGG TTT CGC GCC TGC TGC GCC CCG TAG
GGC CTG GGG CCG TGT CTC AGT CCC CAT CTC CGG GCT TCC TCT CTC AAG GCC CGT ACC
GAT CGT AGG CTT GGT AGG CCA TTA CCC CAC CAA CTA CCT AAT CGA GCG CAG TCC AAT
CCT CAG GCG CAT ATG CTT TCG AAA AAG AAT CGT TCC AGA CTG CTT TTT CTA TGG GGT
ATT ATC CTC AGT TTC CCA AGG TTA TCC CCC ACC TGA GGG TAT GTT AAC CAC GTG TTA
ATG AGC CGT CCG CCA TGG CCG AAA CCA TAG ACT CGC ATG GCT TAG TCG AAT CCC AAT
AGC AGT GGC CTC CGG CGG GAT CAA CCG GAA TTA GTA GCG TAA AAG AAT TTT ACG CCA
CCC TCT CGT GGA GTA CGT CAT TTT ACA TTA AAG GAA ATG ACA TTT GGT TGC TAT CTA
GGT TTC ACC TAG ATT GTA GGC TTA CAT CGC ACC TGA GTA TTT CAA TCA GAT GTT CAC
TTC AAT TGC ATA ATA TGC AAG TGA TAT GGA TAG TGT AAT GTT TTT AGG TTT GAA ACG
ATA GTA TAT ATT GTA CAC TAT GAA ATA TTA GAA TTG AGG TGA TTC TAT GAC TCT TGA
CCG CAA TGG TAA ACT CAT AGA AAT CGG TTC CTA TGT AAG ATA TAT TAA CAC AGG AAC
TCA TGG AAC CGT CAA AGC AAT TGA ACC AAA AAA TGA TGA AGA ATG GGT TCT TTT AGA
AAA TGA CAT ATA TTA CAG ACC CGA ACT TCT TGA ACT TGT TGA AAG AAG AAA AAT CGA
AAA AAA AGA ATC GGA AGA AGA ATT AAT CGA AAG AAT AAC TAA TGA AGA TAT TGA TCT
TGA AGC CGC TGA AAA CGG ATG CGG ATG TTC AGG GGC AGG TTA ATT AGT ATA ATT TAA
GTA TCA AGG TGC ATT TAT GGA CAG TCT TTT GGA ATT AAA CCG GGT AAA TAC AGC AAG
AGA AAT AAT AAG AAA AAA AAT ACA AAA TAC TGG AAG AAC CAG TAT TTA TGA TTT AAC
GGG TCT TTG TGG CGG ATT TGA AAT CTG TGA AGA AAA CCT AAA ATT AAT AGA AAC ATA
TGT TGG GCC AGC AAT ATT TTC AGA AAA ACT AAA TAA TGC TGG TTT AAG CCA TTT ATC
AGG AAA TTC CAA AAT TCA TAA TGC AGT ATG TTT TAA CAG GAC TTC TTC GGC AAT ATT
GTC TAC AAT AAT GAC CCT ATC AAA GTC TTT CGA AAA ATT GGT GCA TTA TGT TCC TGA
AAA ACC TGC ACA CCC CTC AAT TCC TAG GAG TTG CAA AAT ATT TGG AAT GGA ATA CTT
TGA AAG TGA TTC TTT AGA AGA AAT TCT CTC AAA AAT TGA TGA AAA TTC ATT TAC TGC
AAT AAC TGG AGC TAC CAT GGA TCA CAA GGT AGT TTC TGA AGA AAT TGC ATG CAA AAT
AAT AGA TTA TGC AAA ATC TAA AAA TTC TCC GGT ATT TTT TGA TGA TGC GTC TGG AGC
AAG GTT GAG AAA ATT GTA TAA AGA ATC ACC AGC TCT TGA AAT GGG TGC AGA TTT AGT
TGT AAC CAG CAT GGA CAA ATT AAT GGA CGG CCC AAG AGC CGG CCT TCT TGC AGG GGA
CAA AAA CTT AGT TGA CAA AAT ATA TTC TGA AGG TTT AAA ATT TGG ACT TGA AGC GCA

AGC CCC AAT AAT GGC CGC AGT TGT AAC TGC TCT TGA AAG GTT TGA TCT AAA TAA TTT
AAA AGA TGC ATT TGA ACG TGC AGA AAA AGT TGA TTT GTC AGT ATT TGA AGC TGA AAA
AAT TGA GTA TAA AAA AAC TCC GAC AGG ATT TAT AAT AAA AAA TTC ATC AGA AGA AAA
ATT AAT TGA AAC TGC ACT AAA ATT GCT TGA AAA TTA CGG AAT AGT TAC AAT CAC TGC
GGC AGG TAT GCC CGG TGC AAG TAA AAA CAT AAG AAT AGA TTT CTG TTC AAA AGA TGC
AGA ACG AAT TTC TGA TGA ATA CGT AAT AAA TGC GGT TTT AAA TTC TTT AAA ATA ATA
TAA TAA AAA AAT AAA ATA ACT TTT TTT AAA TTT TTT AAA TTA ATT CTT TTA AGT CTT
CGG GCC TAT GGA GTT CTC CAC CAA TTT TTC CAC AAC CGA CTA CTT CGC ATT TTG CGT
ATT TCA TAA CTT CAC CAG AAA TCT GTC CTA AGT TCA TTT CTG GAA CTA TAA TTT TTG
AGG CTT TTA ATC CTG CTA TCA ATT TAT CTG GAA ATG GAA ATA CGG TTT TTA ACC TGA
TAT ATC CAA CAT CTT GAC CTT GTT CCC GTA AAG ATT CAA CAG TGT GTT TTA CAG TTC
TTG AAG GGG TTC CGT AGC AGA TAA ACA TTG TTT CGC TTT CTA AAT ACT TGC CTT CGT
AAA GTA CAA TAT CAT CTT TAT TTT CCA AAA TTT TAT TGC AGA TTC TTC TAA CCA GTT
TAT CGT GTG TTT CTG CTG AAA CGT CAG GGT AGC CAT TTT CAC CAT GTG TCA GAC CAG
TTA CGT GGA TAT TAT ATC CTT CAC CAA ATG TTG GCA TTG GTG GAA CTT CCT TGT CAA
ACG GAT ATG GTT TTT CTT CTG GCT TTT CAC GGT TTA ATA TTT CAA TGT CAT CGT GTA
AAA CTA CTT TTT CTC TCA TGT GTC CTA AAA TCT CAT CAG CCA TAA CAA AAA CCG GTA
TTC TGT ATT TTT CTG CAT AAT TAA AAG CAA GTA TTG TAA AAT CAT ACA TTT CCT GAA
CTG ATG AAG GAC AGA GTG CTA TCG GTT CAT AGT CTC CGT GAC TTC CCC ATC TTA CCT
GCA TCA TGT CTC CTT GAG CGG CTG CTG TTG GCT GTC CTG TTG AAG GGC CAC CTC TTT
GGA CAT TTA CTA AAA CGC AAG GTG TTT CAG TCA TGT AAG CGT AGC CGA TAT TTT CAA
GCA TTA AAC TCA TTC CAG GGC CAC TTG TTG CAG TCA TTG ATT TTG TAC CTG CCC AGC
TTG CAC CGA TTA TTG ATG CGA TAC TTG CAA TTT CAT CTT CCA TTT GCG AAT AAA ACC
CGC CAA TTT TTG GAA GTC TTT TTG CCA TTC CTT CTG CAA TCT CAG TTG AAG GAG TGA
TTG GAT ATC CTC CGA AAA ACT TAC ATC CTG CCG TTA TTG CAC CTT CAA CGC ATG CCA
TGT TTC CCT GCA TGA AGT TAA CTT TTG TCA TGA AAT CAC CGA AAA ATC CGT AAA TTA
TTG AAA AAT CCA TTA AAT ATT TTC GCT ATT TAT ATC TCC AAT AAC ATA ATT ATA TAA
CTT ATC TAT GTC TTT ATC GAT TCC ATA CTT TTT AAA GTA ACG AAG CAG ATT TTC AAG
ATC TCT TTT TAG GTA ATA TTC TTC GTC ATA TTC TAC TTC TTC AAA TGG TAA TTT ATC
TAA TTT TCC TTT TTT CCC AAC TTC GAT TGC CTG AGG CCA GTC AAT AAT TAC AAA GTC
GCC TTC TGA ATC AAT CAA TAT ATT AAA TTC ACT TAA ATC GCC GTG AAT AAT TCC TAA
TTT GTA TGT TTT TTT AAC TTC TGC CAT CAA CTT CCA GAA AAA TTC TTC AGG ATC CAC
GTT TAT TTC AAG AAG ATT AAC ACG CTT TAA TTC TTC CCC GTG AAG TTT TCC CAT TAT
TAT TGC GTG CCT GTT CTG TTC GAT TGG TTC AGG AAC TTT TAC AAC TGG AAA AAG CAT
GTT TAA AAC TTC AAA TTC TCT TGC AGC GGT GAG CCT TGA AGC GTA AAG CCA GCT GAT
ATG CCT TTT ATT TGC AAG GTA TTC TCG GTA TCT TTT TCC CAT TGA AAA GCA GGT CCT
TCC AAG CTG GTG GAA TTT CAA AAC AGC TTC ACG GTG ATT CGT AAG CAG AAC GTG ATA
AAC GTC GCC TTC TTT TCC AAC CCC AAC TTT TCC ACC AAT TCC TGC AAT TAA TTC CTT
TTT TAT AAA TGC ATT CAT CGC TAA TGC ATC GTA TCC CCA GTG AGA AAG CCT GTA TCC
GTA ATT AGA CTT GTT GGT AAA CTT AAA TTT ATT CAA TCT TCC AAG CCT GAA AAC TAT
TTC TTT TTC ATG AAA TCC CGT TTT CCG CCT TAA ATC TTG AAT AGG TAC CCA TTC ATG
ATT TTT CAT TGA AAT TTC AAT TAT TTT CAA CAA TTT CCA GTC ATT ATC TTC CAT TTC
CGC TAT TGA ATT TAA AAC TTT TTC GAT TTT TTC ACG CTT TTA TTT TTT AAT ACT ATT

TTT CTA TGG TGC TAT AAA ATT TTA ATT ATT GTC TTG TTT TGA AAT TTT TTC CAG TAG
TTG TTC TCT TAA ACT GTC TGC ACA TTT TAT TTT TTC AGA ATT ATC TAT ATT TTC AAC
AAT ATC CCC AAT TCC AAA GAT TTG TTC AAA TTT CCC GTT TGA AAT CTT CCA AAT TTC
TGA AAT ATC TGA TAA AAT CGT ATC TAT TGT TTT TTC AGT CGT TTT TTC GCA TAT CAA
ATC TAA GAT TAT TCC ATA TAT TTT CAG TAT TTC GGT TAA ATA CTC GTT TAA ACT TTC
ATT TTC TTC ATT TGT TTT AGA AAT TAA TTT TAC AAA ATT TTT CAG TGT TTC TGA AGT
TTG AAT TGT GAT TTC TGA TAA AAA CTC TGA ATT TTC ATT TGT TTT TAT AAA TTC AAT
TGC AGG TTT TGA AAA ATT ATT CAG TGC ATC TAA TAT CAA AGT TCC AGC AGC TTC ATT
TCT TAA TAT TTC CCT TGA GTT TAA TTT ATC CAA AAT TAG TTC AAA AAT CTT TAA AAT
TTC AGA AAA CAT CAT AAT TTT GAA CGT TAC CTG TTT TGA AGT TAT TTT TTC GTT GTG
TTT AGG TTT TGA AAG TAT AAA TTT TAT AAT ATT CCC AAG TTT TTC AAA CAC AAT ATG
GAT TAA CTC TTT TTC GTC TGA TTT GTC GAT CTT TAA TTC GTA TTC TTG GGC ATA CAA
AAG TCC GAT TTT TTT AAT TCC CCT TAG CGC CCT TAA AGA TAA ATC ATA ATT TCT ATT
TAG TTC AAA TTT CGA AAT TTT TTC AAG TGA AAT TAT TGC TTC CCG CGA AAT GGA AAA
TGA AGT CCC GTA TGC AGT TAA TCC CAA ATT TTT AAG GTT GTC TAT GTA GCT TTC AAA
AAA TTT TTT ATT TTT AAA TTT ACC TTT TTT CGC TAA AAC TGA GTA ATT TGC ACC AAG
AAG GAT TAT CCC GTT TCT TGC AGT CAT GTA GTG ATC AGA TTT TAA ACT TTT TCC AAT
AAT ATA GTA AAC TGT TCT CAG TGT GTT TTT TGG AAT TTT CCT TGC AGA TTT GTA GTC
TTT GTT TTC TGA ATC CAG TGA ACG GTA ATT TTT TAT TTC GTC TTT TGA AAT GGA ATT
TAT TAA AGC ATC TAT AAC ATT TTC GGG CTT TAA CCC ATT AAT CGT GTT CTT CAT GTA
TGG TAA AAG TAT CAA AAA GTC AAA ATA GAC GAA AAA GAA CAA AAT ACC AAT TAA AAT
ATG ATA TGA TAT CTC GTA TCC GCC AAT AAA TAC CGG AAT TCT TGC TGT GGG CAT AAA
CCC GAG TAT CAA AAC TTC GTA ACT CAT AGA AAC GAT GTA CGC TAT TAT AAA TCC CCA
AAA CGT CTT GTT AAG TAA AAG ATG CGT AAT TCT CGT TGA ATA CTG CTG GCT TAC CAT
CTG GAT TGC AAC AAT TGA AAT CGA TGC AAC AAT TCC AAC AAT CGC TCC TTG TGT TTG
TGG CAG CGT GCT TAA AAT ATA CCT TAA ATT TTC ATC AAA TCC CGT TCC CCA TGC AAA
ATA AAA TAG GGA ATA ATA AGC GAG TAC TGC AGA AAT TGC TGC AAT TAT CAG TAC TTG
GAA CCA TTC TTT TGA TAA AAA ATA ATC CAT AAC ATA AGC GCA GTT TTT ACA AAT CTT
CGA GAG CAT CGT GAC CAC ACA GTT TCT TTT AAG TAA ATA TGT TAA TTA ATT ATA AAT
AAA AAT ATA TAG ATT AAA TAC ATA TCA ATT CAA GAA TCA ATT CGG CAT ATT TGG TTC
AAA ATA TAG GCA TGG TGA ATA TAT GAA ATT TGG ATG GAT AAA AAC AAC AGG AAA TGA
CCT TGA AGA GAG AAT GGA ATC GGT AAA AGA TGC TCT TGA GAG TTC AAT TCC AGG AAT
CAT TAC AGA AAA GGA AGA AAT AAG TTC TGT AAG AGA ACT TGG AAA TAT AAA AAT TGT
TTC TGA TAA TCT CGA TGC AGA TGT CGT TTT AAT TAA TAA AGG GGA AGA TTT AGA GAT
TTT AAA ATC TGC AAA ACT CTC TGG AAA AGA AAC CGG TGT TTA CGT TGT AAT AAA CAC
TAA AGA AGA TGA AGT ATA CGC TAC AGA CGT AAG CAG ACT TAA TTT TGT CGA TTA CGT
TGT TTT AGA AGG AAG CGA CTG GAC AAT TAT TCC TCT TGA AAA CAT CAT TGC AGA TTT
ATT CAG CGA AGA AAT AAA AAT CGT TTC AGT TGT AAC AAA CGT AAA AGA TGC GGA AGC
TGC ATA CGA AAT TTT AGA AAA AGG AGT TGA CGG CGT AGT TTT AAT TCC AAA AGA TAT
TAA CGA AGT AAA AGA TTT CTC AAA ATT AAT TGA AAG AAT GAA CTC TGA AAG TGT AAA
ACT CGA CTA CGC AAC AGT TAC AAA AAT TGA ACC TGT TGG AAG TGG CGA TCG AGT CTG
TAT TGA CAC ATG TTC CAT GAT GGA AAT GGG AGA AGG AAT GTT AAT TGG ATC CTA CTC
ACG AGG GAT GTT TTT AGT TCA TTC AGA AAC TGT TGA AAA CCC GTA TGT CGC TAC ACG

ACC ATT TAG GGT AAA TGC CGG ACC TGT TCA TGC CTA TAT CTT GTG CCC TGA AAA CAA
AAC TAA ATA TTT AAG CGA TTT AAA AGC AGG CGA TAA AGT TTT AGT TGT TAA TAA AAA
CGG TGA AAC GAG AGA AGC TAT TAT CGG ACG CGT GAA AAT CGA AAA AAG ACC ATT ATT
CCT TGT TGA AGC AGA ATA TAA CGG GGA AAA TTT AAG AAC AAT TCT CCA AAA CGC AGA
AAC TAT CCG ATT AGT TGG AGA AGA TGG AAA ACC AGT TTC AGT TGT TGA TTT AAA AGT
TGG AAC CAA AGT ATT GAT AAA ACC AGA TGA AAA CGC GCG GCA CTT TGG AAT GGC GAT
TAA AGA AAC AAT TAT AGA AAA ATA ATT TTT ATT TTT ACT TTT TTT AAT TTA AAA AAA
TAA TAA ACA AGA AAA TGA TTG TTA TCG ATT TTT AAA GTT TTA TAA ATC TTT TAA GGT
AAA TTT TGT AAG CTA CGT GTG ACA TTA TAG CCA AAA ATG CCG CAT ATG CTC CCA AAG
TTA CCA TTA TTA TTT GTT CCA TAT TTT CAC CGT ATC TAA CTG TCC ATT TGA GGA CTA
TTT AGG TAC TTC TTT TGC AGA TAT AAA CCT ATT TGT GAT AAT TTT CGT TTT TTA TAA
TAT TAT TGG AAA AAT CTG CCC AAT TAA AAA TGA ATA AAA AAG CCC AAA ATT ACC TAA
ATT ATT AAA TAA ATG AAA AAT CAT AAC CGT TAT AAT TAT GAT TAA GAG TTA TAG AGG
GCC CAT GAT GCA GAT CAA AAT TGG AGA AAT TGA GTC TAA GCT GAA TAA AAT TAT CGA
AGA AGA AGG TGC AGC TTA CTT CGT ACT TAT AGA CCC TGA TGA AAA GAA CTA TCG GGA
AAT TGC AAA TCA CGT AAA AGA TTA TGC TGA TGC AAT AAT TAT CGG AGG AAG TAT CGG
AAT AAT AAA CCT CGA TGA AGT TAC AAA AGA CAT TAA GGA AAT CAC GGG ACT TCC AAT
AAT ATT ATT CCC TGG AAA CGT TGA TGG CGT CAC AAA AGA AGC AGA TGC AGT ATT TTT
TAT GAG CCT CAT GAA CTC GAA AAA CAC GTA CTG GAA CAT GAC TGC ACC AAC TCT TGG
CGC CCT TAC AAT CAA AAA ATA CGG ACT TGA AAC ACT ACC TAT GGC ATA CCT TGG AAT
TGA ACC AAT AAG TAA AAC TGC CGT TGG ATT TGT CGG GGA AGT AAA CGA AAT TCC GCA
GAA AAA ACC AGA AAT TGC AGG ACT TTA CAG TCT TTC AGC GTC CTA CTT TGG AAT GAG
GTG GGC TTA TCT TGA AGC AGG AAG TGG TGC AGA ATA CCC AGT AAG CAA TGA AAT GGT
TGG AAT TTC CAA AAA ATT AAG TGG AAT AAA TAT TAT TGT CGG CGG AGG AAT AAG AAC
CCC CGA AGT AGC TTA CGA AAA AGT CAT GAG CGG TGC AGA CGT AAT TGT AAC TGG AAC
CCT GAC TGA AAA AGA CCC GCA GGC TGT TGT AGA AAT GAA AAA AGC AAT TAA AAA GGC
AGG ACT TGA TAA ATT AAA AAT GCT GTC TAA AAA ATA AAA TAA AAT AAA ATA AAA TAA
AAT AAA AAT TAA TTT ATT TCG ATT TTT CCC TGA TTC CAT TCA ATC ATG TGT TGT TCT
CTT TTT GAT AAA TCC TGT ATC GGA ATA ATT CCC GGC GTA GGC TTG ATA CCC ATT CTT
TTC TGG AAA TCC GTC TGT TCT TGG AAC GTA CCG CTG TTT ATC ATC CTT ACC CCG TGA
TAA TTA TCA TAC CCG TTG ATG TGG ATG TGC CCA GTG TGG AAA ATA TCA GGT TCA GTA
TGT ATT GCA AGA TAA TCA ACG TGT TCT GGA GCT ATC GGG CAT CTT CCC CCA TAA GTT
GGG CAC AAA TGC CTT CTT TTT AAA AGT TCA GTC ATG ATT GAT GGA GGG TTT GTA TAC
TGG GCT GAT GAA ATC TGG CCT ATA ATA TCA TCA AAA CTT CTG CCG TGG TAC ATC AAT
AAA TCA AGG CCG TGC ATA TTT ACA ACA CCT GGG TTT CCA ACA AAT GTT ACA TTT TCT
TTT GGA AAC AGG CTT GTT ATC GAT TCA TCA AAT GTT GGC TGC GGT TCC GCA GGT CTT
AAC GCA TCG TGG TTA CCC GGA CAA ATT ATA ACT TTT ATA TGT TCT GGA ACC TGC TCT
AAA TAA GCT GCA ACT TCA GCA TAC TGT GAA ATT ATA TCT ACG TCA TAT AAA TCG TAC
TCT TGG CCA GGA TAA ATT CCA ACA CCA TCC ACT AAA TCC CCG GCA ATT GAG ATG TAT
TTT AAT CTG CTT ACT ATT TTT TCT TCT AAA CCG TTA TTT ACT TCG CCA TTT AAG AAC
CGG ATA AAT TTT GCG AAG ACT TTA TTT AAA AAT TCA CGA CTT CCT ACG TGA ACA TCT
GAC AAA AAT GCA GTA TAT ATC TTT TCA TCG GTT GTT TTT GTG GGT TTT GGA GTA ATA
TCT GGC CTA AAT GCC CGA TCT ACA AAC ATC AAA TCT CCT TTA TCA TTT ATA GTT CCT

TCA AAA CCA ATA ACT TCA TCT AAA AGA ACG TCT GAT GGA AGA TCT CCT TTT TCC ATT
TTA TCT TTC ATC AAA AGT ACC CTA AAA GTT CCG TTT TCA TCT TCT AAT TCA ACC ATT
TTA TGT CCG TTT TTG GTG GAA TTC ACA TCT GAC ACG ATC CCT GCA ATA AAA ACT TCA
TTT TCT TTT TTT CGC CTA AAA AGC TGG TTT AAA GGG TAT GCT TTC TTT TGA TTT TTT
AAT TCA ATT ATT TTT CTT AAA GTA CTG CAT CTA TCC TTA AAA TAT TTT ACA AAG TCA
TCG AGT GTT CCT TCG CAC GTT GAT TTT CCG GTA ACG TCA TAT TCG TCG TAA ACG TGT
ATC TGT GCA TCA ATA TCT TTT GCA TAG TAA TTT ATC TTG TTA TTA ACA CTT TCT CGG
ATA GAC CTT ACT TCT AAA ATA CGT TCT TTT CGC TCT TCC TCT AAT TTT TTA TGT ATT
TCA TCA AGT TCA GGT TCC TCT TCT TCA ATA TCT TCG AAA TCT AAA TCG TAA ACT TTA
TTT TCC CGT TTA TGT TCC ATA ATT TCT TCA TTT TCA GGT TTA ACT TCA CTA ACT GGC
TCA AAA TCA TCT TTT TCT ATA ATT TTT TCT TCG GAT GCC ACT TCG TTT GAT GTG TAA
TAT GCT AAA AAA TCG AAT TTT CCG TAT TCT TCA AAA ATA TTT TTT AAA TTA TTT CCC
AAA AAA ATG TTT AAA AAG TGC GAA TCG AGG AGT ATA AAA TCG TCC TTT TTG CTT TTA
AAT TCT TTT ATT TTA TTA AAT AAA TCT GAA AAA TCA TTG CTG TCG AGA TTT TGA AGC
CTC GAA TAG CTT TCA GGA GCT AAA AGT ACC TGA ATG TTC AAA AAG TCG TTT ATC ATA
ATT TCA CGG TTA TTC TGA TTT TTT TAC ATG TCT GTT AAT GTT GTG GCA TAA GTA ATC
GAG AGG ATG AAT TAT ATC TTT CCT TGA ACA GGT TGG ATC AGG CCT GCA GTA ATA TCC
AAG ACC TTT TCC ACC ATC ATC ACG CCA GTT TTT GCA GTA CTC GCA GTG AGT TAT GTG
GTT TGC AAC TTT GTA CCC AAT ATT ATG ACT TAT GTA GTA TTT AGT TTT TTC AAC ATC
GAA ATC TCC AAC AGA TTT AAA AAT ATT TAT TAT TTC ATC TAA AAA GCG TTC AAT TTG
CTC TTC TGT TGC GAT ATC CTG TGC ACT TCT ATT TGT AAT AAT TCC TTC TTC GAT CGG
CCT TAA ATT AGG ATC AAA TTG GGA CAC AAA TCT ATA AAC AAC AAA ACT ACG CCT TGC
ATA GTG CGA AGG AGA TCC CCC TGA CAA AAT ATC GTT TAA AAT TTC CCT CAT GCA TGG
GGG ATG CCA GTC TTC AGG AAT TTC TCC AGA GTA TTC TTT AGT ACT TAA TTT AAT TCC
ACT TTC AAT GTT ATA TTC AAT ATT AAT TCC GTC AAA TGC TTC TTC TGA TTT TTT AAC
AAG TAA ATC CTT TAT TTC TTT TGG CAT TTC TGC ATT TCG AAC CAT ATC TAC GTA ATT
TTT AAT TTT TAA CCT TAT TTT TTC GAA ATT ATA AAT TGA CTG CCT TTT TTT ACT CAA
TAA AAC TAC ATT TCC TTC AAG AAT CAT TCT TTC AAG GTG TAA ATC GTT ATT TCT TGA
ATT TGC CGC TAC ATC GAT AAA ATC CCA AAT ATT TAC CCG TGA ATC GCC TTC TTC AAG
CAG TAC TTT TTC TGC ATA TTC TTC TGC AAG CTT TTT AAT ATA TAT GTT GTG ATT TTT
GGA GTT TGC TGC AAG TAT AAG TGT TAA ATA ATC TAT TTT TTC TGA AAG TAG TCC GCT
GTC TCC AGA TTT TTC CCG AAG CGC ATA TGC ACT TAA TCT TTC CTG ATA ATC CCT TAA
CGC CTT CAA ATA TTT TTC TTC GTT GGT TTC TTC CGA TTG TTT CGA GTT CAG ATC TTC
TTT TTC GTC ATC GTT TTC TGA AAC TTC AGG GTT TTT ATT CCA AAA TAA TAG TTT TAG
TCT ATT ATT AAC TAA AGC AAT AGT TAA ACT ATC ATT TAT TTC GTC AAT CAA ATT CAA
ATC TTT GAT ACT ATC CAT ATT GTC TAA AAA CAC TAA AAT CAC AAA TAA TAT TTT ATA
AGT TAT TCG TTC ATT TTG TAG AGA AAA ACT GCA ACT TCA CCA ATT GCA CCG ACA TTA
TTT TCT AAT ATA TGC ATT CCA GTT CCT TTT TCA AGT TCC TTT TTT GAA AAG CCT GAT
GAA TTT CCT GAA AAA ATT ACC AAA TCT CTT TTT CCA ATT GAT TTG AAG TCA ATT TTC
TCT TCT GCA ATT TTA CTG TCT GCT ATT AAT ATT ACT CTT TCA GGG TTT AAA ATC TCT
ACT GCA TCA TCG ATA TCT TCG AGA AAC ATT AGA TTT TTA TTC AGT TTT ATA GCC ATT
TTC TGA GCG ATT GGA ATT CCA TTC TGA GCT GCT GAA CCA GTT GCC CTT GTA AAT ATT
ACG GAG TCA ATT CCC ATT CCA AAA ACT GTT TTT GTA AAT TCT TCT ATC TGT TTT GAA

CTA AAA GTG TTG TGA AGT GCG ATA AAC ATG AAA TTC ACC TTT TTT ATT CTT TAT TTT
TAA TTT TGA AAT TTT TAT TTA TTA AAA TAT TTT TTA ACT TCT TTT GCA ATT GCT AAT
GAT AAT GAA GGA GGA ACG CTT TCT CCA ACC TGA TTG TAC TGG GCG GTA ATT CCC CCT
GCA AAA ATA TGG GTA TCC GGA TAA CTC ATG AGC CTT GCT TGC TCT CGA ACT GTT AAA
AGT CTA TCT TCC TCA GGA TGA ATA AAT CTC CTT TTT CCC ATG ACA GTT TCA GCG TTT
CCT TCC ATT GAA AGC CTT ATG TAG TTG TCT AAA ACT CCG CCA CAG GCT TTA AAC TTA
ACT GCT GAA TCA CCG ACA TTT ATT TTA TGA ATA CTT TTC GCA ATT TTT TCT GGA AGC
GGA TAT TTT TCA TTA TTC AAT GCA TTT CTG TCA ATA TTT AAT AAA GCG GTT TTT ACA
TCC ACA TTA TCA GTT TTT TCA AGA TTT AGA GGG ATA TTT GAA ATA ATT ACC CGT TTT
CTA AAG GAA GGA TTT CCG TAA TTT TCA GAA TCA AAA ACA TGA AAG TAC AAT TCG TCG
TAC CCT GCT CTT GAA AAC TCT TTT TCG ATA TTT TCC CTA GTT TCA TAG TTT AAA ATT
CCG GGA ACA TTT TCC ATT ACG AAA ACT TCT GGA GAA ATA TCT CCA ACA ATT CGA ATA
TAA TCT AAA ACC AGT CTT CCA ACA GGA TCT CGA TAA AGC CTG TTA AAT GGA CTC CTT
TCA CGC TTT GGA TTT GCA CCT GTG TAT CCT TCA CAG GGC GGT CCA CCT ATA ATC AAA
TCG ATT TTT GAA GAG GAC GTA TCT TTA ATT TTA TTC GTT ATG TAT TTT GAA TTT ATT
TCT CGT ATA TCC TCA TTA ATT ACA ACA GGA TTT AAA TTT TCA TAA TTA TTT GAT TTT
TTA AAT TCT TCA ATA TCA TCC TGA ACT AAA AAA TCT TCG AGT TTA AAA TAG ACA TCT
TTT TCT AAA ATA TCG TTT AAT TTT TTT TCA AAA ACC AAT CCA TTG TAA TTT AAA GCA
TAC GAA TTT GCC GCA TTT TCA TCC AAT TCA ATT GCC AAA AGT GGT TTA AAT CCC ATT
TCG ACA AAT CCT CTT GAA AAA CCG CCG CAT CCA CAG AAT AAA TCT ATA AAA TTC ATT
TTC CCA CGT AAA AAT AAG TGC TGA TAA CCA TAT AAT TGT GAT TAC CTA TAA AAA TAC
ATG GTG CTT AAA TGA ATC GTT CAA ACC ACA CAA ACA ATT CAA ACC TTG GAA AAA TTT
TAG AAG GGT TAG AAA TTA AAG AAC TCC TTA ATT CTT CAA AAA ATA TTG CGG TTA TTG
GAA TTT CAA AAA ATA GTG AAA ACC CGA GTT ACT TCG TTT CAA AAT ATT TGA TAG AAT
CGG GAT TTA ATG TTT ATT TTG TAA ATC CAA ACT ATT CTG GTG AAA AAA TAC TAA ATC
ATG TTG TAT ATT CAA ATA TTT CTG AAA TAA AAG AAA GAA TAG ACA TTG TAT CGA TAT
TTG TAA ATC CTT CAA GGA CCA GTG CTA TTG CCA AAG ATG CTG TTA AAT TAG GAT TTA
AGG CTT TTT GGT TTC AGC TTG AAA CTT TGA ATA AAG AAA CTG CGG ATT ATG TTT TAA
AAA ATG GTT TTG ATG TCG TTT TGG AAA AGT GCA TCA TGA CTA CAA ATC AAA AAT TTT
TAT AGT TTA AGG AAA TAG TTA GTT TAA TTT AAA AAA GGT GAC TTT GAT GAA CAT TTT
AAG AAG GGG AAG ACT TGG AAG CAA CGT CAA GGA AGA CGT GAT GAA ATT CAC TAC AAG
CCT TGA ATT TGA TAA AGA AAT CTT TGA AAG CGA TAT TTT ATG CGA CAT TGC GCA CAC
GAC GAT GCT CGT TGA ACA GAA CGT GAT TTC AGA AGA GAA CGG TAA AAA AAT AAT TGC
AGA ACT TAA AAA AAT AGC GAA AAG GGG CAT GGA AAA TCT TGA TCT TGA TCC ATC TCT
TGA CGA CAT TCA CAT GGT AAT TGA AAG TGA ATT AAT AAA GGA ACT TGG TGA AGA TGT
TGC TGG AAG AAT GCA TAC TGG AAG AAG CCG AAA TGA CGA AGT TGC AAC TGA TTT AAG
GCT TTC TTT GAG GAA AAA AGT TCT TGA AAT AAT TGG CCA TTT GAT TAC AAT GGA AAA
AAA TAT GCT CAC GGT TTC AGA TGC ACA TAA AGA AAC ATT AAC CGT TGG ATA CAC ACA
TTT ACA GCA GGC ACA GCC TGT AAC TTT TGG ACA TCA AAT TTT AAG CCA TGT TTC AGC
AAT TGA AAG AGA TAT TTC AAG GTT TTT TGA TAC ATA CAG CAG GAT TAA TAT CTC CCC
GTT AGG ATG CGG TGC AAT GGC TAC AAC GGG ATT TAA TTT GAA TAG AAA AAG AAC AAA
AGA TCT TCT TGG ATT TTA CGG AAT AAT TGA AAA TTC AAT GGA TGG CGT GTC TTC AAG
AGA TTT CAT CGT TGA AAC AAT GGC AAA TAT CTC AAT GCT TGG AAC AAA CCT CTC AAA

AAT ATG TGA AGA GCT CGT CGT ATT CTC AAG TGC AGA ATT CAA CAC CAT TGA AAT TGC
AAA TGA ATA CAC ATC CAC CTC TTC GAT TAT GCC GCA GAA AAA AAA CCC TGA CGT TGC
TGA AAT TAC AAG GGC AAA ACT TTC AAC GCT AAA TGG AGA GCT TGT AAC AGT TTT AAC
GAT TAT GAA AGC GCT TCC AAA CAC ATA CAA TAG AGA TTT GCA AGA AAT CAG CCC GCA
TCT TTG GAA GAG TGT CTA CAC CCT TAT TGA TAG TAT TCA AAT GGT TGA CGG AAT GAT
ATC AAC TGT TAA GGT AAA TAA AGA AAG AAT GAA AGA AAA TGC AGA GAA AAA CTA TTC
TAC CGC TAC AGA ACT TGC AGA TAC ACT TGT AAG AGA ATG TGG AAT TGC ATT TAG AAT
GGC TCA TGG GAT TGT TGG AGA ATT AGT AAA ACG ATC TAT TGA AGA AAA AGT TGA AAT
TAA AGA AAT AAT TTC AGA GGT TCT TGA AAA GAA TAA CCT TTC ATT AAG CCA GGA AAA
AAT TGA TAC TGC ACT CGA TCC TTT TGA AAA CGT GAA ATT AAG AAA TGT AAT CGG CGG
ACC TGC ACC AGA AGA AGT AGA AAG AGC AAT ATC TTC ATT TAA TGA AAA AAT TTC AGC
ACA TAA AGA CAA ATT AGA TGA AAA AAT TTC TGA AAT TAA AAC TGC TGA AAA AAA TCT
CTT AGA ATA AAA AAT TAA AAA AAT TAT AAA ATA TTA AAA ACC CTT TTT AAA ATT ATA
GTT GCG TAG CTT CCT TTC TTT AAA CTA AAT TCT AAA TCT ATT TTG TAC TTT TTT GAG
TTT AAT TCG TCA GAT TTA AAC TTT CCA GTA TTG ACA TCT CCA GGA ATT GAA ATA ATC
GGT CTT TTT GTG TAT TTT AAT TTT CCA AAA TCT AAC TTT TCA AAA TCT GAA ATT TTA
ATC CGT TCT TTT TTC AAA ATT GCC TTT ATT ATT CTT TTT TCT GAA TCA GAG TAT TCT
ACG TCT GAA ACT ATC GTT GGA AAA TCC ATT TCT TTT AAT TTA CAA AAC AAT TCT TCT
TCA ATA TTT TCG TAA AAC AAA AAT GTC CCG CAG GAA TAA TCG ACA TAT ATT CTA TTT
TCT TTT GGA ATA ACT TCT TTT AAA AAC TCT TTT AAG CAT TCA TTC CAA AGG TAA CTT
TGA TAT GCA GAA ATG AAA AGC TCT TTT AAC CGA TTA TCT ACG TAT TTA AAC GCT TTT
TTA AAA TCA TTT TCG TAG GTT AAT GAC TTT ACC ATA TTT CTA AAC ATT TTA CTT CTT
ATC TGC TTC TTA TCG ATA TAT TCT AAG CAT TCA TCC CAG TTA CCC CAA TTT TTA GCA
ATA AAT CGT TTT AAA TCC TTT AAC TGC TTT TTC TCA CTT TTC GTA TAG CTT GTT AAA
AGT ATT TTA ACG ACT TCT TCA TAA TTG CCT TTC AAA ATC TCC TTC GCT ATA AAT TTA
CCG TTA AAT ACA CTT CCA AAT CTC TGA TCA TCG TAG TAA TTT GGC GCT CCA GAA CTT
AAA TTT CGT ATA TTG TCT GCA ATC TTT AAA AAT TCG TCT TTA TCG ACA GCC CTT GCT
ATG ATT TCA AAC CGG TTT CCA TAC AAT CTT CCA ATT TTT AAG GGT TTT TCA ATG GTT
CCA ACG TAT TCA AGC TTT AAA TTA TCT TCG TCA AGG GAT AAT TTA CCG TAT TCT TTT
GGA ATG GAA ACA TAT TGA GTT GTG ATT GCG TGC CTG TCC TTC AAA CCG CAG TAA CCA
ATT TCT TTT GTA GGA ACT TCA AAG TTT TTT GAA AGA TAC GAT ATA GCT TTT AAA TTC
TCT ATT CCG TTT TTT TGA AGC GTA TAT AAC GAA TAA TTT CCA TTA TCA TTT AAT TCA
TAA TCT AAA ATC TCA TTT ACG ATG AAA TCT TCG TGT TTT TGT CTA AAT TTC ATG ATG
CAC CTA ATT TAA ATC GTG TTC ATC TGA TCT ATT TCT TTT AAT ATA TCA TAA TCA TTT
CTT TTT ACA GCG ATT TCT CGA ATT TTT TCG ATT ATT TTT AAA TTT TTA CCA CAT AAT
TCC CGA TAT TCC TTA CAA TAT TCT AAA ATA TCT TGG TAA TGT CCT TCG ATC AAA CTT
TTA TCG TAT TTT GAT AAT TCA TAT GCT AAA AGT CCT TTT AAA GTG GAT TCT TTT GAG
AAT AAA AAT ACT GGG TGA AGC CAG AGC ACC AGT TTT TCC TCC TCT TTT TCC ATT ATA
TTT TCA ATT TTT TCT AAA TCC ATG AAT ACG TCA ATA TAA ACC AGA ACT TCA GAA GAA
ATT TTT GAA TTT TCC GAA ATT TTC TGA ACT ATT GAT TCC ACG TTC ACG TCA AAC TCA
GAA ATC TTA ATA TCC AAT TCT TCG TCG CTC AAT TCA TTC CAA AGC AGT ATT CTT TCC
CTG TGT TCG TAT GAA GCC GTT GAA AAT GTC TTT AAA ATA ACT TCA AAT GTA TTG TCT
ACT GAA AAA CTG TAT TCG TCC GCT TTT CTC GAA GAA AGG TGT ATT ATC ATT ACA CCA

TCA TCA ATG TGT TTT AAA TTT ACT TTT CTT AAG ACA TAT TTT CTA TCC GTT GCT TCA AAA CTA GGC AAT TCA TCG CAA TCC ATT AAA TTT TTT ATA GAA TGG CGT TTA CCA TTT TCA TCC CAA AGA TTA AGT CCT TTT AAT TCA ACG TCA AGA CTT TCC GCT AAT AAA AGG TCT CTT GAA TAT TCG TCA ATA TTT TTT ACG ATA TCT CGT AAT ATC TTA TTT ATA AGT TGT TTA GAC TCC ATT TTA GTC ACC AAA TTT ATC CGC CAT ATC TTA AAT ACC ATC CGC GAT CCA TTA TAT TTT TAA TTA ATT TTC CAT TGT ATT TCC CAA TTC CAA GAA GTT CCT TAT TTT CAT TAA ATA CTG CAA CTT TTC CGT CGC CTT TTA ATT CAA TAA TTG AGG AAT CGA AAA CAT CTC TTG AAT AAA GAA ATA ATC CTT CTC CTT TTT CAT TAA TAA CTG CAT AAT TTT TTT CAA TAT CTT TTG AAA CCA GCG TTA TAC CTT CAA GGG ATG GTA AAA ACT TTT CTT TTC CCG AGA TTT CTT CTA GAT CTC CAA TAT GTA TTC CTG CAC TGT AAA TAT CCT TGT GAT TTT TTA GGG ATT TCA TCA CTT GTG AAC TTA CAT GGA CTA CGT TTT TTG ACT TGC CCC TTA ATA CTA AAA AAT TAT TAA AGT CAA GTT CTT CAG CTT TTT TAC CAA TAT ACC TTG AAA TGG CCG CTT TTA TAG CGC TAA TTT CTG ATT CTT CGA GCT TTC TGT GTT TCA TAA TAT CCC GCG TAG ATT AGA TCA TTG TTT CTA TTT AGA TCG TGT AAC TAT ATA TAT CAA ATT ATA AAA AAG TCG GAT TAA ATT AGT TAT GTT AAA TTA AGT TAA ATT ATG AAT ATA TTA TAT TAT TTT TGA GAT TTC CTG ATT GTG GTT TTA ACG TAG CTT TCT ATT TTT TTA TCC AGC TGT TTT GAA AGA CTG TTT ATA AGT TCG AGG TTT TTT TCA GAG ATA TCT ATT TTT TCG GGT ACA TAT TTA GTA TTT TTT AGG GTT TTT TCA CCA GTT AAT ACA TCC AGA TCA GAA AAA GTA CTA TAT AAT TCC CTT CCT GTT GAC GCG ATC TCG TTT TCA ACA GTT TTT CCT TCT TTT TTA CCA TAA ATT GAA AAA AGT GGG AAC TTT GTA ATT ACG TTT GAA CCT GCC ATT ACG AGC GGC CCG ATA TTT GTA AGT TTA TCA ACC CAT GTT CCG GTT ATT ATC TTC ATT TTT GGG AAA TTT AAC CTC ACA GTT GAA ACC CAG TTC ATA TAT TCG AGG GTC GTT ACC GAA GTT TTT CCC TGA AAA ACG GTT TCA TCT TGC GGA TTT AGG GAA TAA AAT GTA ATC CTA TCA AGA TTT AGT TCT TCG ATT AAG TTT AAG AGT AAA TCT ATA TCT TTT TCA GTT TCT CCC ATT CCT AAA ATT ATC GTA ATT CCA GTT TTA AGC CCC ATG TCT TTT GCT TTC TTT AAC ATT TCT TTT GTA TTT GAT AAA GGC TTT CCT GGA CAC AAT TCT TCG TGC AAT TTT GGA TTT ACA GTT TCT ACT GCA CCA ACA ACT CCT TCA ATA TTG TTT AAA TTT AAA TTT TCA AAA TCA ATT ATT CCA ACA TTT AAG TAC TGT TTT GCC TGC TGG ATG TAG GAA ACC ATC TCG ATA GTA TCG TTT AAT TCT TCA GTT TTG TAC CCA TAT CCT CCA GAA ATA AAT TCA AGC TTC CAA CCT ATT CTT TTC ATT AGT ATA CTT TCT GCA AGA ATC GAT TCA GGC TTT CTT CTT GCT TTT AAT GGA TCT TTT ATT TTT TCT TTT TGT GTG GAC ATG AAG CAG AAT TTA CAC GGG TCA TCT AAA TTA CAA TAC CAG CCT AAA AAA AGA GCT CTT TCT AAA GTA GTA ATA TTT CCG TGG TGT TTT TCC GTT AAT TTA AAT GCC TTA ATT GAA TTT TCC AAA ATC TCC GCA GAA TTC ATA ATA TCT CCT GAT AAA TTT AAA ATA CTT GAG TAA AAG ATA ATA TTA TAC CAC AAT TGT TAG GGG TTG TTT TCG TGA ACA AAT TAT CTG AAT CTG ATT TAA GTG TGA CCT TGA ATA TAA ACT ATA GAG GTA AAC AGA TCA CGA AAA CTC AAG TCG AAA TTT TAA AAT CAG TTT CTG AAA CCA GAT CCC AGA ACA GAA CCG CTG AAA TTC TGG GCA TTC CCC CAT CAA CAG TCA ATA TTC AGA TAA AAA GGC TTG AAA ATA AGC TAG ATT TAA AAT TAA CAT ATT CTT CTC CTG CCG GAA CCA TGT TAT CCC CTG ATG CAG AAC GTA TCG TAT CAT ATT ATA ATG CAT ATC TTG AAA GAA CGT CAA AAG AAA AAT TTA TTG CAT GCG GAT TTA TAA GTG GAG AAG TGG GAC GTA TTC TTT TTG ATG ATG TGC TCG TTT CAT CAT TTT CTA ATG TTT TGA GGC TTC ACA GGT CAA ATT TAA CCA GTG TCG TTG GAA TTG ATG ATC CTT ACT GGA GCT ACA GGC TTG GAG ATC

CTG TTC CTG TTG CAT TCG ACC ATT TTG TAA TGG TTT CGA AAG GCG ATT TTA ACT TTA AAA ATC TTC TCG GGG TTA ACT ATT CTG CAC ACA GGA TTG TTT GGA AAA CCC TTA AAA ATG AAA AAA TAG ATT TTA AGG TAT CAA AAG TTG TAA AAA ATC CAT TTT ATG CGA TAG ATC TTT TAG AAG AGG GCT ACA GTA TGT TTT TAA ATA CGT GTC TGT TAA GAT ACG TTA AAA AAG ACT ATT TAA TCG AAA CTC CAG ACT ATT ATG ATA AAA CAA AAC ATA CTA TAA ACT TTA TTT TAA ATA AAT CCA TCC CTG AAG ATG AAT TTG AAG AAG TAT TGT ATA AAA AAG AAA AAG AAA TAA AAT TGG CGG GTT TTG ATC CGC TTT TGT AAA TTT GAG TTG TCC AAT ATT ATT TTT AAA CTT ATT TGA TAA TTA TTT TTG AAT TTA TTT TTC CAA CAA TTG GCG AAA CAA AGT ATG CGG TGG GAA ACG CTA TAC AGT AGC CGA TTA AAA ATA CCT TTG GCC AAA ATG CTA AAA CAT CTA AAC TAA ATC CTC GAT TTA CCA TTG TCA TTA CTA AAG ACA TTA CTA ATG CCA TTG AGC CAG ACA TCA ATA ATG CAA AGG TTA TTC TTT CGT ATT TTT TGG GGA TAA TCA TGT AAT TCA CCT AAT TTT AAA TTA AAG TAA CAA TTA AAA AAT AAT AAA AAC AAT AAA GAT TCA AAG TAT AAA AAG TTA AAA TAA AAT CGT ATT TAC TGG GGA ATA TTT CCG CCA GGA ACC ATT ACG GTT AAT TTT ACC TGT TTT ACG CCT TTT TGT GAC GTA AGT TTA TCA GTT AAT TCT TTA ATT TCT CCT GCA TCC CCC TTA ACG AGA ACT ACT TCC ATA CAG TGG TCG TGA TCG AGG TGC ATG TGG ATT GTT GCT ACA ATT AAC TTT TCG TAA TCG TGC TGT ATG TTT GTT AAC TTC TCC ATT ACG TCA GTG GAG TGG TGG TCA TAA ATA ATA CTA ATT GTT CCA GCC CGT TCC CCT TCA AGA CTG TGA ATC CAC TTA TGC TTT ATT AAA TAA TCC CTA ATA GAG TCT CTT ATA GCC TCA CTT CTG CTT GCA TAG CCC CTT TCT TCG ATA ATC TCG TCG AAT TCT GCA AGT AAA TTT GTA GGT AAC GAG ATA CTT ATC CTA TCC ATA TCC ACC ATT GTA TGC CTC CCA GAG TTA ATA ACT GAA ATC TAC ATG TAA TAG ATA GTA ACT TCG TAA TAT TTA ATA ATA TCT TAA GAG TCC TAT TTA AAT ATT ATG TAA TAA TAA GGT GAA GAA TTT GAG TGT TGT TAT CGG ATA CTA CGG AAA AAA TGG AGC TGT TAT TGC AGG CGA TAA AAG AAA TTT ACT ATT CAA CGG AAT TGA AAG TAA CAG GGC AAA GCT TGA AGA AGT TCT TTA TTC TGG AGA AAT TAA AAC TGA TGA AGA ATT GTT TAA AAA AGC CTC TGA ATT TGA TGT TAC AGT CCA TAT TAA TGA TAC GCG AGA AAA AGT TAA GAG TTT AGG AAA TTT ATT ATC TGG AGA AGT AGT ATC AAT TGG AAA AGA CTC CAA AAG AAG AAG AAT GTA TTT AAC AAA AGA AAA ATG CGC AAT AAT TGA TAT TGA AAA TGA CCG GAT AAC CAA CAA ATC AGT AAA AAC CGG GTC GGG AAT TGT TGT ATT TGG TAA CCG ATA CAT AAA ACA TTT TGT TGA ATC AGA AAT CAA AAA ACA CGT TCA AAA ACT TTT AAA GAT GAG TGC CCG CGA GAT TAG AGA TTT ATT TGA AAA AAT CCT AAA AAA TAT TGA AAA TGC AAC ATT AAG TGA TAC TTT CGA ATA TTA TGT TGT TGA AAC AGG AGA ACC CGA GTT TGA AAA AGC AGT TAA CAA GGA TTT GGA TGA CTT ATT TAA TTA CAG ACA TGA TTT ATC CAT AAA AAT GGC TGA AAT GCA GAT ATT AAC AAT GAT TGC TGA AAA AAT AGT AAA AAT AGG GGA TGT CGG AGT AAT TAA AAA TGG AAC TTT AGT ACT ATA TGA CGA ATT CCT TGC AAT TAA TAA GAT ATG TCC TGA ACC AGA AAT TTA CTC GGA AAT TGA AAT CAC GGG CGA ATT TAT TGA AGG AGA TAT TAT AAC AAT CGA TAA TGA ATC TTT AAA AGT AAA AAG AAC AGG CAG TCC AGT AGC AGT TCA AAA AAT AAT CTG TAA AAA ATA ATA AAT TAA TTG AAT TTA ATT TTT TTC CCG ATT AAA TCA GCA GTT ATC CCT GAA GCT TTC ACT TCA AAC ATT CTA TTT GAT TTT ACT TTT GAA TTG TAA GTT TTC CAA GGA ATT AAA TTT GTT GCA ACG TCA ACA ATT TTA TCA TCT TTT AAA AAA ATC ACA TCT ATA GGA TAT TTC ATA AAA AAT GTA TGA ATA TGA AGT CTT CTA TTC CCA TAT TTA AAG AAT AAT CCT TCA TTT TCT GAA ATA TCC CTA AAC ATG AGC CCA AAT GCC CGT TTT ATA AAA TTA TCT GCA ATA TTT ACA

GAA TAA GTT TTA CCG TTT ATT TCA AGG GTT TTT GGA TTC ATT TTT TAG TTC CTC TCT
TGT ATG GCA CTG GAA CAT ACT GTA CAG ATG GCC TCT GAT TTA CAG GCT GCC TGT AAA
GGT CGA ATA ACT CAT AAA CGA TAT TTG GCA GGT CTG TGT TTA CTT CAA TTC CGA GAT
CTT TTA ATT TAT TAA TTG TTA AAG GAT AGT CGT GCG TCC ACT TTC CCG TAG AAA GAG
TTT CTG AGA GGT ATT TTG CTT TTT CTT CGC CCA TTT TGT CTT TCA ATA TAT CAT AGA
CAA ATT CTT TAA CCT GAA CTA TCG CTT TTC TTG CAA TAT CCC CCA ATA TTA AAG TTT
CAT CGT CCA ATT CAT CCG TAT ATT TTG TAT TTA TTG CAT TTA TAA TTG AAG CTG CAG
GGT ACT GTC CAA TCT GAG GAT CTA CAG GCC CCA TTA CTG CAT TTT TAT CAA TTA TAA
TTT CAT CTG CTG CAA GAG CTA TCA AGC TTC CAC CGC TCA TTG CAT AGT GGG GAA TTA
TAA CTG TCG TTT TTG CCT TGT GTT CCT TTA GAG CGA TTG CAA TCT GTT CAC TTG CAA
GTA CGA GCC CAC CAG GAG TAT GAA GTA TTA AAT CTA TCG GCA TAT CTT CGG GAG TCA
TTC TAA TTG CCC TTA AAA TTT CCT CAC TAT CTT CTA TTG TAA TAT ATT TAT AAA GAG
GAA TTC CAA AAA GTG CCA GCT GCT CCT GCC TGT GTA TCA TGA CAA TTG CAC GGG TTT
TTC TAA CAG CTT CGA ACT GCT TTA TAA ATT TGT ACC TTT GTA AAA GCC GAT ATC TAA
AAA GCA CCT GCG GAT AGA AAA ATA AAA ACG CAA AAA ATA TCC AGA TAA TTG ATG AAG
GAT CCA TTT TGA TAG CCC CCG TGA AAA TTG TCT TTT TGA TCC AGT AAA GTC CTT AGA
TAA CCT AAA TAT CTT ATC ATA TAT ATA TTC CTT TTA TAT TAG ATT AAC ATA CTT ACT
TAA CCT TAA ATA ATA TCT TAG TCT AAT CAG ATT ATT CAT ATC AAA TTA TCA TAT AAT
CAA GAG GTG CAC TGA TGA GCA ATA TCA ATA TCA GTA ACC TGA AAC TCA AAA AAA CTC
TTC AAA AAC CAC CCG TAG TTC CAG AAG AAG CCA TGT GTT TAA AAT ACG TGA TTT TGG
AAC CCG TTG GTT TTC CGA TAA ATG TTA ATG GCG AGA CTT TAA AGG TTA GTG TAG AAA
ACC ACG CAC TAT TTA ACT CAT ACG CTC GAG AAC AGT GGG AAA ACG AGA TTG TAA AGG
AAG GCG CAT ATT TAT TTG ACA GCA CGA TTA TCC CGG AAT ACG CAT TTA AAG TTG TTT
CCC TCT ACC CAA AAG AGG GGG GAA TTA TAA CAA AAG ACA CTT TAT TTA AAT TGG AAA
ATA ATT ATA AAC CGC AAA ATT CTC CAA AAT TTA AAG AAA CCA CTT TTG AAG AAG TCG
TCG GAC AGC TTG ATG CAA AGA AAA AGT GTA AAA TTG TAA TAA AAT ATC TTG AAA ATC
CTG AAT TAT TCG GAG AAT GGG CCC CAA AAA ACA TCT TAT TTT ATG GAC CGC CTG GAA
CTG GAA AAA CAA TGT TAG CAA GAG CAC TTG CAA CAG AGA CGG ATG TAC CGC TTT ACT
TAA TAA AGG CTA CTG AAC TTA TTG GAG ACC ATG TTG GAG ATG GCT CAA AAC AGA TTG
AAA GTC TTT ACG AAA ATG CAT CAG AAA ATA CGC CCT CAA TAA TAT TTA TCG ATG AAC
TCG ATG CAA TTG CAC TTA GTA GGC AGT TTC AGT CAT TGA GGG GAG ATG TAT CTG AAG
TAG TAA ATG CTC TTC TTA CAG AAC TCG ATG GAA TAA AAA ATA ATC TGG GAG TTG TAA
CAA TTG CAG CGA CAA ACA ACC CAG AAA TGC TTG ATA ATG CAA TTA GAA GTA GAT TTG
AAG AAG AAA TTG AAT TTA AAA TGC CTG ATG ATA ACG AAA GAT TGA AAA TTC TCG AAC
TCT ATG CAG AAA AAA TGC CGA TAG CAG TAA ATG CAG ACC TGA AAA AAT ATG TTG AGA
AAA CAA AAG GCA TGA ATG GAA GAA CGA TTA AAG AAA AAT TCC TAA AAC CTG CAC TCC
ATA AAG CAA TTT TAG AAG ATA GAG AAT CGA TTA CTC AAA AAG ATC TCG ATG AAA TAA
TCA AAA AAA TCG TTA AAA ATT CTA CTC CAT TAA ATT TAT ACG GAT AAT CTA TAT ATT
TAT ACG GTA CTA TAC CGG TAT GTT GTA GTA TAT AGA TAG TGT AAA AAA GGC GAT ATC
TTG ACG GGG TCT AAA CCT TAT AGA TTA GAA GTT ACA TTT CCT GCG GAA CAG GAA AAT
GAA ATT AGG CAA ATG GCA AAA GAA TGT GGA TTC AGA AAC GTT GCA GAA TAT GCA AGA
GTT TCT ATG AAA TTT TTT TAT ACA ATG AAA GAC GTT ATG CGT TCA GTA GTT GAT TTT
GAC TCA AAA TTA GAT GTT GTT TTG GAA AAT ACA AAA TCT ATC AAT AGT TCT TTT GAA

GAC CAT AGA AAA ATT GTG GTT GAA ACT GCT GAA AAA GTA AAT AGA CTT TAT CAG CAA GAA GAA CTT GAA AGT ATT TTA AAA GAG ATT ATT CGT ATT TTA CAG GCT AAT TAT CCA AAA GGA TAT AAT GTT CAT GAG CTT GTT GAG TTA ATC GGT GCA GAT ACG GAT AAT AAG AAA AAT GAT TTA TTT TGG TAT GTT TTA AAT GAA AAT AAG TTT TTT AAA TGT TAT GTG AGT GTT GAT AAC GAG TTT GTG TCA TTG AGA GAG AGA CCT TTA TGG TCC GGG TAT GAA CTA GAT ACA TTT GGT GTT AGT CAT GAG GGT AAA TAA AGA GGA AAA TTT AAA TCC TGG TTC AAA AGA AGT TTT TCA AAA ATT TAT GAA TTA TAA TTC ACA CCA TAG TGA AAG TTA CAC AAA AAC TCT TAG ATA TTC AAT TTT AAA TTT TAT GGA ATT TGT AAA AAA AGA TTC ATT TTT TGA TGT TGA TGT ATC GGA TTT GAT AGA CTT TTA CAA TAA TAA AAA ATG TTC AAA AAA TAC AAA ACT GAC TTA TTT AAC TTG TTT AAA TGT TTT TTA TAC ATA TTG TAT AGA TAA TAA CTA TAT TTC AAA TAA TCC GGT TTT AAA ATA CCG TAA ATC TTT GAA AAA ATC AAA AAA AGA AAG AGT TTA TTT GAC TGA AAA GCA GCA AGA GAT GGT ATA CTC AAG ACT TGC AGA TAT TGA GTA TAG AAT ACT GTA TGT ATT TTT GGT TCA GAC TGG ACT TAG AAG AAG TGA ACT TAT AAA TTT AAA ATT TAT TGA TGT TGA ACT TGA TGA TAG AAT AAT TCA TGT ACA TGG TGG AAA GGG GGA TAA ATA CCG TTT TGT TCC GAT TTC AGA TGA ATT GTA TAT TTC ATT AAA AAG ATG GAA TTA TGA GAG AGC TTT GAG ATA TCC TTT AGA TGA TAC TTA TTT TAT AAC CCG ACG GGG TAA AAG GTA TAA TGA GAG GAC TTT GGA TTC TTT TAT ACA TTA TAT AGA TAG AGC AGT GCC TAA TTT AAA TGT GAG GGT TAC TCC ACA CAT TTT AAG ACA TAC ATT TGC GAC TAA CTG TAT AAA TAA AAA AAT GGA TTT AAA AAC GCT TTC TTT GAT AAT GGG GCA TGA AGA TAT TGG AAC GAC TTC GAT TTA TCT TCA TAA AAA TAA AGA ACA GAT TAA AAA AGA CTT TTT AGA AGC TTT AAG ATA AAA ATT AGT TTA AGT CTT GTT TTT CTT TTA TTG CTT TTG ACA TTC TGT TAT AAT AAT CTT CAT ATT CGT AAA AGT TAT TTC CTT TTT TTA TTT CAT CTA ATG ACA TTT GTG CAG CAG TAT ATT TTT TTA ACA TAT AAT AAC ACT CTG CTT GGA TAC AGT ATG CCA TAT ATG TGG GTT TTG CTT CGA TAG ATT TTT TAG TTT CTT GAA TTG CCA TTT CAA AGT TGT TTC TGC TTG CAT ATA CCA TTG CCA TTT GGT AGT GAT ATA TGC TTA CTT GAT TTT TGT TGT TGT AAT CAG TTA TTT CAT ATT GTG GTG GGT TGT AAT AAG CAT TTA AAT CGA TGT ATT CAA ATG GGG GTA AAT CTT CTG ATT TTT CGT AAT TAA TAG TAC TTT CTG ATG TTT GTA TAC TTG TTT TAG TTG TTT CTT TGA CAG TAT CTT GTT GAT TTA TTT GAT TAT CAA TTA TCA AAA TAC TGG CAA CTG TTG CAA CTC CCC CTA AAA CCA TTA AAA AAA TAA TTG CAA ATA AAA TAA CTA TTA TTT TAG TAA TTT TCA AGT TTT CCC CCT CAC TTG ATA TTT ATT CTT TAT TTT TAG AAT GTC CGT TGT AGA TAT TTG TGT TTC CAG AAC CGT TAA TGT TGA TAT TTA CAA CTA TTT TTT CTT CAA GTT TTT CTC CTT GTT TGT AGA AAT CCC TTG CTT CTA TAC CAT AGA TTG TTA TTT TGT AAC AAA ATT TAT CCA TTT TTC CCT TCC AGT CTA ATA TTT CAA TTG TTA TAA GGC CTG CAT CTG CTA AAT CTT TTA ACA CTA AAC TTG CTG TAC TTC TAT GAA CAC CTA ATT CGT CAG CAA TTT CTT GTA ATC CCA ATT CTC CTT CTT TAT ACA ATA AAT TAA GGA TTT CTT TAA CGT GTT TTT TAC CAA GTA GTT TTA TAA TAT TCA TGA TTA GCA GTC CCT TTT AAG TTT TTG TAA TTT ATT TTT CAC TGT TGG GAA AAA TTC GTA TGT TGA ACT TTT TAT TTT TAT TAT TAT TTT AAT CAT TGT AAC ATT TTA TTC TCT GTT GAA TAA AAC CGA AAC AGT TAA ATA GTT ATA TAT ATA GAA TAG GTA CTG TTG GTA AAA GTC CAA CAG TGA ATA AGT TTT AAA CGG AGA ATT TTT ACT AAT TTG AAA AGA GGT GAA CTT ATC AAA GAA ACT TAT GAA ATT GAC GTT CCC GAA GGT TGT GAA GTA ATA ATA CTT CAA GAT AAA CCT GTA CCC CCC TTT GGA GAT TCT AAA CAT ATT ATT ATC CCC AAG AAT TTA AAA ATC

GTT CGG GCT GAC GTT ATT TTG TGT GTA GAA GCT GAA AAC AGA TAA TCC ATT TGA CAA
GGT GAA CAT CGT GGT TAG ATG ATT AGA CGT GTT GAC ATA TCG TTC ATT CCC CAA ACT
TAT CTT GTT ATG GGG ACT GGA GAA AGG GAA CAT TTG ACA GTT TTT AAA AGG GAT TTT
GAA ATG AAT CCT TTG GTT TCA GAT TTG AAA AGT TAT AGA AGA TAT GTT CTC AGG AAG
AAC GGA ATT GAT TAT TTA GCA TAC ATT GAT GCA GAA TTT AAC ACG ATG GAT GAA ATA
GAG CTT AAA AAA GAG TTT GAT GGC GTA TAT GCC AGA TTT GTA AAA AGA GGG TCA ATA
CAA GAC CAG TTT AAA AGA GTT GAT GTA CAT GGT TGA AGA AAA CAC CAA AAA AAA TGA
ACA AGA AAA CAA AAC CGT TGT AAA AAC AGA AAA AAA AGG CTT AAA AGC CGT TAG TTT
AGC AGA TAT ACC AAA AAT GCA AAA AAT CGA AAT TGG AAA TAC AAC AGT TAC ATT TAA
TGC AGG AAC CGA AGG AAT TCC TTC ATT TAC TCC AGA TGG TCA AGC TAT TAT GAA TGG
TAG ATA CCC TAA AAA TGA GTA TGT TCC ATC AGT AAA ATC GCT TGG AAA CGT AAA AAT
TAA AGT TAC TAA AGG ATT TGG TAG TGA TAA TGT AGA ACT TTC ATA CAC GGT TAT GAA
AGC GAT TAT TAC ACA GGT CGA AAA TAA TCC AGT AAC TAA AAT GAT GGT TGA CAT TTA
CGC AAC CGC AGA AGT TGT ATC ACA GTG GAG AAC TGG ATT TGA AAC TGG AAC AAT GGA
CAA AGA TAC TAT TTT AAT GCT CGC AGA AAG AAA CGG AAT CCC TGA GGA TCA CGT AAG
AGC TCT CGT GTT CGA CGA AGA TGT TGG ATT TAA GAC GAC TGC TGA GGA CAA GTT TGT
TGT AGA AGC TTA ATT TTG GGG ATT GTT ATG AGG ATA CCT AAA CTT TCA AAG CCT GAG
CAG AAA CGA GTG GAC AAG GAA AGT GTT AGA AAA GCA AAA GCT CTA GAA AAA ATC AGA
GAA ATT CAC GGA GAT TTA CCA ACA TTG GAA TTT CCA AGA ATT TCT CTT GAT AGA GCT
GTT TTT TCC ATT TTT TCA AAA GAT ATT TTT AGA CAC ATC TAT CGA GGA AAG GAT AGT
TAT ATT GAC AGA TAT GAT CAA GCC CGA ATC AAT CGA ATG GTT GAG GAT ATT AAA ACT
AAT GGT TAT CAA AAA TTG GAC TGT GAT CTT CCA GAA AGA AAA GAT ATG AAA TAC AGG
GGC GAT GTT TTC AAC GAT TAT AAC CAG TTT AAA CAA AAT ATG ATT GAT CAT AAG GGT
CAT GGA AAC GGT TTA CAC TTT AAA CTC TGT GAA TTT GAG GAT GAG GTT ATA TCC TGG
ATT ACT ATT TCT ATG AGA GAA CCC CAC ATG GCG GTT GCA TAT GTT AAC TTT CAA AAA
TAC TAC CGT TAT TTA AAC AGT ATT CCA AAA CCC AAG TTT GGA AAC TTT GAT ACA AAC
TTT TTA CCG CTT GAA TGT GAT AAT AAA GAG AAA ATC TTT GAT GAT TTC GCT AAA TTG
TAT CTT AGA TGG TGT CAG GAA GTC GAA ATA GTT TAT AGA AAA CTT ATT GAT GAT TTG
TTT GGT TAC GAG CTT GAC CCT GAA AAA ACC GAA GTT ATT CCT GCA AGT ATT GAG ATT
CCT TGT GAA TAT CTA AAT AGA GAT ATT GCG GAA TAC TCT TGG CTT TCA GAT ATT GCG
CAA TGT AAA TCA GTT GTA AAG ATG TCT GAC CTT ACA AGA ACT ATG TAT CTT AAT AAA
TCT TTG AAA TCA AGA CGT TTT CAG ATG AAA CTT TAT CAA AAA GCA TTT GGA CTT GCA
AGA ATG GAG TTA ACA GTT CAT AAA GAT TGG GCT TCT CAC TTC TTT TGT GAA ACT GAT
CCA GCA GTT TTG AAA GAT AAA ATA AAA ATG TGT ATT GAT GCA GTT TTA GGA GAA TAC
AAG CTT TCA TTG TAC GAA ATT AAA CCA GTA AAG ATG ACT TAT GAT ATT TTT TTA AGT
CAA CTT GCA GCA GAT CTG AAA TGG GAT CGG GAT ATG CTT TTG GCA ATA ATT GAA TCT
AAA AAT TCA GAA TTT ACT TTT AAA AAT GAT AAT CAA GGA CTT AGA AAA AAG CTT AAA
AAT AAG GAT TTA ATC GAA AAA GAT CAC CGT GGA GTG TGG GTT AAG TCT GAT TAT GCA
AAA TGG CTT TCA GGT ACT CTT GGA AGA TAC TTT ATC TGT CCG CAT TGC CGA ACT TAT
ATG TTA AAA AGT GAA AAA TCA CTT GAA TAC GTT TGT ACG ACT TGT GGA TTT TCT AAA
AAA ATG ATT TAA GGG GTT ATT ATG GGA GAT AAA AAA GAA CCT TTT AAA ATT ACA AAT
ACA AGA CTA TTC CAG ATT GTG GAC CAG TTA AAA AGG GAA GGG AAT CTT CCA AGA GAT
TTT ATT GCA ATT ATT GAT AAT ACT GGA GCC ATG AAA GTT GCA GGA CGT GCA ACT CAA

GAT CAG GTT GAA AAT TTA GTT TAC ACA GTA CAA ATC GAA GAA GAT GGA TTT AAA GTT TAA AAA TGG CGG TAA AAT GCC TGA AAT TAG AAC TAG AGA AGA ATT CTT GAA AGT TAT GGA TGA AAA TCG ACT TTG GGG CGG TTG GAT GTT AGC CCT TGA AGA TGG CTC CAT GAG TGG TGC TGA AAG TTA TCA GGA ACA GAA AGA CAC CGC TGA AAC ATA TAA AGA AAG ATT TCC TGA TTG GGC AGA AGA AAT AAT TAA AGA AGC TGA GCT GGT GAA AAA TAC AGA AGA AAA ATC GGT TTA GAC GAA AGT TAA AGA GGG AAA TTC AAT GGA ATC GAG CGA AAA ACT TGA AGA ATA CAA GAG AAT TAC CGA TGA ACA ACA AAA ACT CCA GTT TGA GTT AAT AAA AGA AGG TGT TCC TGT TGA AGT TTT AAA TCG GAT TTT TAA TGA TGC ACG TTC TAA AAA TTC GCA TAT TCC ATA TAG GTA GGT GAA AAA TGG CTA AAA AAC ATT TTA AAA ATA AAA AAA CTC GAA AAA GAA ATT TTT CTA AAG AAA ATG CAG AGT TAC AAT CAG TTA TCA AGC AAT TGA AAG AAA AAG GCG AAA TTA ACA AGA ATG TCC TTG CTG TTG TGA GTT CTA CTG GTA AAT GGC TTT GTA TTC ATG CTA ATA ATG AGG AAA AAA CTG TAA TTT ATC ATA ATT GTA TGA TAA TGA GCG ATA GAT TTA GAG TTG AGG GGT GAA AAT GAA AAT TGT TCG GAA ATT TGC ACC AAC TTC AAA AAA TAC GTT TTC CAT AGG TCC GGT GAA AAA ATT AAT TAA AAG ATA TCA AAA TCA CCA GCT GGT GAG TAT TGA TCC ATT TGC AAA TAC TTC TAA ATT AGC AAA AAT TAC CAA TGA CTT GGA CCC TGA AAT GCC AAC AGA TTA TCA TTT GGA TGC TTT AGA TTT TTT GAA AAG GTT TGA AGC GAA TTC TGT TGA CCT GGT ATT ATA TGA TCC TCC GTT TAC TCC GAG ACA AGT TGC AGA CTG TTA TAA AAA ATT TGA CCG GAC CGT AAA CAA AGA GAC TAC TCA AAT AAG CTA TTG GTG GAA CCA AAA AAG AGA GCT TTC GAG AAT TGT AAA ACC TGG TGG AAT TGT CAT AGG TTG TGG TTA TAA TTC AAA TGG ATG TGG CAA AAA GTA TGG ATT TTC CTT AAA AGA AAT TTT ACT CGT TGC ACA TGG CAG TAA TCA CAA TGA TAC AAT TGT GAC AGT AGA TCA AAA AGT TCA ATC TAA AAT TTA TGA GCT GGT GAA TTA TGA TAT TAA CTC TAT CAA AAG CGT TTA AAG ACG TTC CAA AAA AAG AAA AGC TAA ATT AAA AAC GAC TGT AAA AGC TGT GGA GTT GGT GGT TTA ATC CCT GGA GGG TAG CCG ATA TCG ACT ACA TCT TCA GTA ATT TAG GTG GTA AAA AAA TGC AGC ATT TCT CAG GTT GTG AAG CGG TTA TTA TGA TGT TGT TGT ATC CTG AAC TTG AAG GAT TTT CTA TTA TAA TAT CTG CCC CGA TAG CTG AAA TAT TAA CTT TTT ATT CGT ATG CAG GAG TTA AAA AGG CTT TAA AAA GTC TTT ATG AAA AAG GCT GTT TAT ATC GCT TTG AAA AAG GGG GTT ATT ATT CTA TAA CTA AGC TTG GAA AGA GCG AAC TTC TTA AAG TTA AGG AAG ATT CCG ACT ATG ACA TGA TGT ATA AAC AAC TTC AAT ATT TCC AAA AGA CTG GTG CAT ATT TGC CAT ATT TTG ATC TTG AAC GTA GGC TGA AAG AAA GAG GTA TAA CTG AGG TGG AAT TTT GGC GGG TTA TGG AAT AAG ACC TTT TCC TGG TAC GTG CTG TCC TGA ATG TAC GTG TAA TAA TGG GAA AGA TAC TTG TGA TGG CTG CGG TCA TAA GCA TTC TGA ATG TCA TAG GAA AGA GTA CAC TAA AAC TGG TGA AAA AAT CGT AAT TGA AAA AGA AGT TGA GTT TGA TTT ATT AAA AGA ATT CTG CAT TAA AAA AAT GGG TAA AGA ACG ATT TGA AAA AAG ATT TAA TGA TCT TTT AGG GGG TCC TGA TGA TTA TTT TCC TTT CTA TAA TAA TCC CAA TAA GGT AAT TTT AAT AAT CGC TGA TGA ACT CGA ATT AGT AGA CAT TAA TGA AGA TTT TGA AGG TTT TGA ATG GAT TGG AGA ACA TTA TTG CAA AAA AGG GTG AAA AAA GTG AAT AAT TTT AAA ACA GAT CTT TTT TGT TAT CTA GTT TTT TTA TTA TTC ATT TAT TGG TAT AAA TTA CTA CCA TTT GAA GCT GTA ACC GTT ATG ACT CTT TCG GGA GTT TAT TTC TAT CGA AAA TAT GGG TTA CCT GTC CTT ATA GTG TAT TGC TGT ACT TTT GAT ATG TTT TAT ATT ATC ATT AGG GAG CTA TTA ACT GTT GGA TTT ACT GGA GAT ATA ATT CAC GTG TTT GTA TTG GGC AGT GTT GGT TGC ATA ATG CTG TTC TTT GTT TCG ACA TTT AGT GAA AAA TTA GTA AGT

GGA TTT GAT TAA AAC GAG GTG AAA AGC TTG GGA AAA AAG AAT AAA AGA ATT TAC TTA ACT CAA TCA GAT TTT AAT AAG CAA TAC AAT AAG ATT TTA CTT AAA ATT GGT TTA ATT TTA TTT GTT TTG TCG TTA TTT AGG GTA ATC CCC TCA GAT GTA TTC GTA ATT ATG CTG TTA GTA CTT ATT TTA GAA TTT AAA CTT ATT TCT ATT TAT CAA TTT AAA AAA TAT TGT AAA AAA AAC AAG TTG CCT TTT GAT TTT GTT TCG ATA CGA TAT TGT GAT GGA ACT GGA GAA GCA ATG CGT TTT AAG ACC CCT GCT GAG TAT GAG GAG TTT ATA ATT GAT TTA TTG GCT AAA TGC AAT GAG CAT GAC GTA GAA TTA GAA AGA TTT GAT AAA ATT AGT ATG TTT GTG GAG TTG GAA TTA TGC GAA TGA TGG AAG TTA AAT CGG AAG TTG ATT TTA AAT TTA CAG AAA GTC AAG AAA AGC TTT TTG CTT TGT TGT TTG TAT CAG GTC TAG TTG GTA TGA TAA TAG GGT ATG TTA TTT CTT TTG AAA TAG AAG TTA TTG GTT TCT TTT TAA TGG TAG CTA CTT TAT TAT TAG TAT TTT TAA TTA AAT TTC AAA TGA ATA GAA TGT ATG ATA AAT ATA TTA GTT CTT TGC CAT ATG CGA ATA AAA TGA TTC ATA TGT TGT TTG TTA ATG GTG ATT TAA AGG AAG AAT GTT ATC TTA TAA TAG ATT CTC TTG AAG CTG AAA ACA GAT ATT TTA GAA AAA TTT ATT CCA TGA TGG AAC AGG GTT ATT TAC CTG TTG AAG TTG AAC TCA TTG ATG ATT GGG TTG AAT AAT CAT GAT TGC CCT TTC TTC TTT TCA AAG TCC TTC GGT GGA AAT AGG TAT TCA CTC TGA TCT TTC AGG TGT GGA TAA GAA AAA ATT GGA TCT TGC ATC CAA AAG AGC ATC ATA TTT AGT TTT AGT TTA CAA CAT TCA GGA AAA ACT TCC AGA AAT ATG TGA TAA CCT AAA TCA AAT TTA TGA CGA AAA CGA CTC TCA AAT AAT ATC TTT AGG AGT TCA AAC AAG GAA AAA AAG ACA TTT AGC TTT AAA AAT TGA AAA AAA CAG TTT TCA AAT TGT TTC ACG AGT AAA AGG GGA TTG TAA TAT TTT GTT AAG TGA TGA CTC CTG GAG TGA TAT TAA ATG AAA AAA GTC ATG CTT TTA TTT TTA CTT TTC ATG ATT CCT ACA ATT TAC GCA AAT GAT TAT GAT GTA TTT GAT TCT ATG ACC GAA GAA AAC ATG AAT CCG TCG ATG GAT GCT TGG GAT GGT ATA GAA TTG GTT TCA TTT TTT TAT AAT ATC TCA GAT GAT TCT TTA ACT TTT GAA TTC TCG AAT CAA AAA GAT AGT ACT GTA TCA TTC AAA GGT TAT GAT GCT TAC ACA AAA CAG ATT TAC TAT ATA GAT TTA CTT CCA AAT CAA ATT ACA AAA TAT AGA GTT GAA GAT CCT GGA CTT AAA GTT GGA AAT ATT TAC TTT GCT TTC TGG AAT AAC AAT GGA TTT ATT ATT GTA AAA GAA GCT GCG ACT CCT TTA ACT CTA CCA AAC AAG ATA GTT TTA ATT TCA TAC TCT GCA TTA GGT TGG TTT ACT GTT AAA ATG TGG GCT TAT CTG ATG ATT TGT ACA GGT CTT GCA ATG TAT GTT ACA AAA ACT TAC TTT ATA GAA AAA ATG GTT TAT GAG AGA AAA AGA CAG GTT CTT AAA TAT ATT GGA TTA TCT TTT GGG ATT CTT GCA GCA TGG ATT TTA GCT GTG AAA TAT GGA CTA CCT TTT GGA TTG TAT GAA GTT TTA ACG ATT GGA AAA TGG AAA TTT GTT TTA AAA CTG CCG TTC TTA GAT TTT TCA AAC TGG AAC TTT AGA TAT TTA CTT TCA TAT GGT AAA TGG CTC TAC TTT GTA GCG TTA TGG GTT GGA TTT TGG GCA GGA TTT AAA ATA TTC AAA CCC GAA CCC TTT AAT CAA TAT GCA ATA GCT CTC CAT AAA CTT GAA GCT GAC GGC ATT AAC AAG AAA TTA ATT AAT AAA AGG GAA TTT TTA AGC CTT CCA ATG ATT CAA AAG TTC GGT AAG AAG TTT GTA AGA TAT CAA AAT AAA GTT TAT GAA CTT AGA TTA CCT CTT TGC CTT CCT GCA AAA GTT AGT TAT GGT GGA GAT TAT ACT GAG GAT CTT TAC GAA GTT ATT GAT TAT ACA ATA ATA GAA CCT AAA AAA GAA TAC AAT GAA ACT ACG TTT TGG AAT AGA ATA TTA TCA CAT TTG GAG AGA TTA AAA GAG AAA TTG TGT CCA GAA CCT GGA ATT ATA GTA GTT GAA TTA TCA AAA ATA ATG AAT GCT GAT GCA GTA GAT GCG TTC CAC TTT ACT TGG AGT TTA AAT GAA TTG ATG GAT AAA AAC CAT GAA TTG AAT TAT GAA ACT ATG GAT TTA AAG AGA GTG AGA GGA AAC GAA GTT ATT GCT GTT TCA AGA GTT GTA GTG GAG AAA TTT ACT TCT GTA ATG GAT GGG

TTC CAG CAG TTT TCA GAT TAT ATA ATA TAT TGT ATT CAG AAG ATG AAA AAA GGA GAC
AAT CCT TTC TTT GAT GAA ATT ATT GAA AGA TTA AGG CTT GAA AGT GCT GAA GGT GGT
GAA GAT GGA GAA GGA GGA CAG ACT GGA GAT AGT AAC CCA ACT AAC GAA TCC AAA TAA
TAT AAC AAC GTG TGA ACC GTT ATT TAA TTC AGT AAT TCC GAA GAA ACA TAA AAA AGG
GTT TCA AAG GGG TAG AAC TGA AAA AAC AGT ACT TAG AAA CCC TGA GAA AAG GGG CTT
TTT GTA TAA ATT AAT GGA TAT GGA AGA ACA TAA AAA AGC TCA AGA GAT TGA AGA AAA
TAA GAG GTT AGA AGA GAA TGA ATA ATT ATT TTT CAT TTA TTG ATT TAA TAT TGA TGA
ATC GAA AAA AAG TTG AAC ATT CTG GTA TTC TTG TTT GGG GAT ACC GAG GAA TGG GTA
AAA CTA CCT GTC TTA GAG GTA TCT ATC ACC AAA TAG AAG AAT ATA ATA AGT TAA ATC
CTA GAA ATT CAA TTT CTT CTA TTT TTG CAA CAG ATA TAG ACC AAC TTG ATA TCT GCG
ATT GTC TTT TTT TAG ATG ATT TTG GTA CAA AAT TCA ATA AAA GGG ATT TTT CAA CTA
CTG AAA ATA AGG AAT TTG TAA AAT TAC TTC AGG AAG TTA GAA CTA GTA TTC CAG TTA
TTG TCG TTT CAA GTC CAA GTT ATT TTA TGC TTG ACA AAG ATC TTA GAA ACT TTT TCA
CCC CTG CAC AAA TTC TCT CAA AAT CGA GTG AAA GAT TTT TAT TAA ATG TAT CAG GTG
CTA TGT TTT CAG TTC CAA AAC CTA AAG ATG AAC TTT TAA AAG AGT TTA GGG ATA AGG
AAC TTG AAG AAA GGA AAG AAA GAT GTA AAA AAT CTA TTG AAA AAG TTA AAA CTG CCC
ATG CCA AGA AAA ATG GAA TTA AAT CAT AAT TAC TCT TTT TTA GAT ATT TAG ATA TTC
AGA CAT TCA CGA TAT ACA GAT ATT CGG GAT ATT TAG ACG TTT TAG TTA TTT TTA TAC
CCT CTA AAA GTT ACA TAT TAA TTA TGA ACT TAT GAG GTG GCT CTC GTG TGG ATT TAA
AGT TGA ACA TGG TTT CAG TTT TTG CAT TTA TCG GTG CTT TTC TGT TGC CTT CGT TAT
ATT TAC AGA ATA AAA TTG GAA CTA TCT TTA TTG ATT TGT TTC CGT TTC TTT CTC CAC
TGA CTC CTC AAG GGG TCT GCG TTG TTT TGG CAT TGC TGC TTT TAA TTG TTG CAG TAA
TTT TAG CTT ATG GTA ATC CTA TGG GTC AGA TTT GGG TAA AAG ACA AAT CAA AGA CTC
CAA AGA CTA AGA AAA CTA ATT TTA ACA ATT CTA AAA ATA AAA CCA CCA TGA ACA AAA
AAG GAA CTT CTA AAA AAC CTA AAA AGA GGT GAA ATT ATC GCA CAA CCT TGG AGA AAT
TGG TAT CCA AGA AGA AAA ACC ATG TCA TCA GAA GCC AAT AGT CTT GCA ATG ACT CCG
TCG TTG TTG AAA AAT GTT ATT TTC GGT TTC ATG ATT CTG GCT GCT TTT GTA GGG TAT
TCT GGA GAC ATA TTT GGA ACT ACC ATT CCA TAC ATT GGA CTT GTA ATT GAC TTT ATA
GTT CAA ATT GGT GCA AGT TTG GCT TAT GGT ATT CCT GGA TTG ATC TTG TTC TTT TTA
TCA AAA AGA AAT AGA GAT GCG GAT TAC TTC GCT TTG ATT TGG ATA ATT ATC GGC ATT
TTG TAT GCT GTT ACT GAA AAT ACA CTA ACT GGA ATT GCA GGA TTA ATA ACT CCG GTT
GTT TCA AAA CTG CCA ACG CTT GTT ACT GGC TTG ATT GTA CTT GCT TTG GAT ATT TAT
TTT GCG ATT CAA TTA TAC AGA GAA GAT TAG AGG TGA ATT AAT TTG GCA AGA ATG AAT
GAA AAA TCA GTA AAT ATA CCA ACA GGT TCT CTT TCA TAT TTT GAA ATA GTG GGT CTG
TTG TTT GTT TTA GGT GGA TCC CTC ACC GTT GTT TGT GGG GTC GAA TGG TTC ATT AAC
TCA ATT TTT GGA GCA TAT ATT TGT ACA GGG ATA GGT CTT GCG ATA GTG TCT CTT AGT
GGA AAG GAC AAT TTA AGA CGT GTT AAA CGT ACT AGC TAC TGG GAA GGC GTT GGA ATT
GGT TTA ATT GGA GTA ATT AAT TTA CTT TTT GGA TAC TTC CAG TTA GAA GCG TTA CAG
ACT TTA ACT TAT CAA ATT TTA GCT ATG GGA TTG ATT TTC ATA ATT ACA ATT GCA GTA
GCT TAT AGG GAT TAA GAG GTG AGT GAT TTG GAT TTA AAA AAA TCA GTA AAA GGA TTT
GGT CTT TTG TTC ATG CTC TTG ATT TCA TTA ATT TCG AGC GTA TGT GCA ACA GAT GTT
ACG TTT AAT GTC GGT ATG GAT GGC GTT ACA GTT ACA GAA GGA GAA ACG GAT TTA GGA
ATA ATT AAT ACT ACT GGA ACA ATC AAT TTT ACA GAT GAG GAA CAC AAT CTT ACA TTT

TCC GCT GTA AAT TAC GAT TCA TAT AAC TTA ACA GTT AAT CCT ACA AGT GCA ACG GAA TAT AAT GTT ACA CTA AAT GAA ACT ACC TAT ACA ATT GTC ATA AAT TCA GAG TTA GAC ACT TTT GAA ATT TAT GAT GAA GAA GGA ACA TTA GTT GGA ACT TTT GAC GAT GTG TCA AGA ATT CTT GAA CTT ACA GCT GGA ACT TAC AAT TAT ACA GCT GTT AAA ACA GGA TAC GAA AAT GAA AGT TTA GAT TTC ATT GTT CCT GAT ACA GAA GAA TTA AAC GTT TCA ATG ATT TTA TTA CCT CAA AAT ACA ACG GAT GAT ACT GCC GAT AAT GCA ACA GAT GAA GAA ATT CCA GTA ACT ATT ATT GTA ATA TCA GAT GCA GAT TCA AAA CTT GAA CAG TTA AAT GAT TTC TTC CAT GAT AAC GTA GCC GTA ATG TGT GTG ATA GGG CTT GTA GTT ATT TAC GGA GTC GGT AGA AGT ATG AAA AAA ACA CAA CAT AAA CCG GGA AAT AGA AAT TAA GGA GGT AAG ATA ATG AAG AAA TCT TAT CTT CTT TTT TTA TTA TTG CTG CTT TCA GTA GTT GGT TCT GTA AAT GCA ACT GCT GTT ACA ATT CCT GAA ACG TGT ACG GTT ACA AGC AAT TAT GTA GTA TTA AAT GAA GAA TTA GAT ACT AAT GTG ACA AAA GCG TTA CTT CTC ACA TAT TCG TGG AAT GAT TAT ACT TCC GGT TCA ATA ATT CCG ACT ATT TCA GGT GTT GAT GGA AAT ATA ATT TAT TTT TCT GAT GTG GAT AGC TTA GCT CGT TTT TGT TCT ATT ATT TCA GGA ACT AGT CCA AGT TTA GCG TTT GAT GGG AAT TAT AAA ATT TTT AAA TCA GGT GCA GAA ACT TAC GCT ACG TGT ATT TTA GTA AAT TCG CCT ACT CCA TCG GCA AGT GGA ACG TAT CTA AAT AAT ATT ACT ATG AAT CTT GAA AGT ACA CAA TTA ATG TAC ACT TAT GGA TAT TAC TTG ACC ACA TTA ACT GGA AAC ACC GGA ACT GTG TTG TAC AAT TCA TAT CAG ATA TGG ACT CAA AAT TCA GGT GGG ATA TGT GAT TTT TCA TCA CTT ACT GCT AAT CAA GAA GGA ATA TTT ACA GTT TCT CAA AAG TGG TCG GGT AGC TCT GTA ACA ATG GTA GTA TCA GTA TTG GTT TTT GAT TAT GTA GAT TAT ACT ACT TCA ATT CCA GAT GGT TCA AAT GCG GTT GTT TCT GAA TTG TTA AGT CCT CCT ACA CTA TAT GAT TTA ACA ATT AAT ACT AAT GTG GAC GGA TTA ACC GTT TAT AAT GGA GAA ACT GTT TTG GGT TCT GCT GAT GAT GGA ACG GTA TTT CCT TTA ATT GCT GGA ACT TAT GAT TTA ACG TTT GAA AAG GAC GGT TAC TGG AAT GAA ACA GAA ACA GTT ATT ATT ACT GAT TCT GGG GTT TCT GTT GAT TTG GAA ATG TTT CCT GAT TCA GAA TTG TAT TTG ATT TCT TCA AAT GTT ACA ACT CAA AAT ATT GTA ACA AAT ATG GAT GTT GTA ATG ACC TTA CAT ATT GAG CCA AAA GTT GAA TCT CAA AAT ACA ATG ATT TCA TTC TCA AAA GAG ATA GTT TCA ATA AAA GAA GGA ACG GAA GAA CTT ACG AAA GAT GGA GAT AAC TAT ATT ATC GGG GAT TTT TAT GAA CCG ATT GAT TTA ACA GTT ACT TTT AAT TCT GGA ACT TTA ACC GGA AAC CGA TAC ATT GAA ACT ACT GTA TTT GGT ACT GCA TAC GTT GGA ACT CAG AGT ACT GAC TTT TCA ACA ATA AAA TCA ATT ACA TAT GCT GTA AAT TCG CTT CCA ATT ATT GTC TAT ATG CCT GAT TGG GAA AGC GGA GTA AAC GAA CTT AGA ATT ACA GAA CAG GAA GGA AAC ACT TTA ATT TTA AAT GTT GAG GTG TTA GAT AGT TCT GAT GAT TCA GTG TTT ACT CAA AAT GTG GTT TTC AAT CCT TAT GGA GTT GAA ACA CTT GAT ATT AAT TTA ACG GAA GGT ATT TAC TAT TTG CAT CTG TAC TGT ACT GAC TTT GAT ACA AGT ATT CCT TTT ACA GTT TCT GCA GCA AGT ACA ACA GAT GAT GAT TCA GAT ATT ACT GGA GTA ATT GTT ACA GGT GAT GAT TCA TTA GTG GAT CTT TTG TAT GAT TAT TGG TAT GTT CCA GTA GCT TTA GGA ATT GCA ATT GGA GTT TAC TTT GTA TTT TCC AAG AAA AAA GGT AAA AAA CCT TCA AAA TTA AAA GGT GGT AAA AAT GGT AAA AAA TAA ATT TGC CCC TTT TTT GAT CCT TTT ACT TCT TTG TGT AAG TCC TTC TCA TGC TGC ATG GAC TGA TTA TAT TCC ATT ATA TGG CAC ATT TAC TGG AGC GGT TGA TAC TGC TAC TGG GTT AGT AGA TTC ATT TTC TGG AAT GAT GGA TTC TAT AAC TGA TGC AAT GGA TTC TAT TTC TTC ATT TTT TAA TCC TGA TGA TAC CAC AAT AAT TGA AAC

TGG TGA CAT AGA AAA TCA GAC TGC ACT TGG TAT TGA AGA TGG GGA ATA TTT TTC AGC
AGA TGA AGT AAA TTC TGT TGG AAT GGC TTC ATT GGA TAA TAT TTC AAA AAC GAT TAA
TTC AGA ATA TAC TTG TTT GTT GAA TAC CTC AGT TAC AGG TAC TGG TCT TGG AAA GAT
TGA TAT CGG TCT TAG GGC AAG GGA TAA ATT ATA TGG ATA CTC GGC TTT TTC CGT TGC
TAT GTC GAT ATA TAA ACC TGA AAC TTA CGC ATA TCA AGA TAA TTT CCA TTT AAC AAG
TTA TTC TTT ATG GGT TGA AGA TAT TGA AAA TCC AGG TGT TAA ACT TTG GAC ATA TTC
TGC TTC AAG TAG TGA AGT TGC AAC TCC CGG GGG GTC TGC AAT TAC AGA TGC TAC AAT
TTT AAA AGC TCC TGA TGA TAA ATA TTC AGT CGT AAA ATC CTG TTT AAC TTC AAA TTC
TAA TTT AAT GGA GTT AGA TGG AAT TAT AAC TTC ACA AAA TAA ACA GTT TGA AAT TCA
CGG AAA CGT TGT AGG GTA CAT TGA AAA TTG GCA GGA AGT TGC GGT AAC TAC TTC AAA
TCC TGA TGG TTC TAC AAC TAC AAC TTG GAA ATG GGT AAG GAA AGA CAA TAT TCC GGT
TGA TTT AGA TAT TGT AAC TAC GTC AAT GTG GCA GCA TGA AAA CAA TGG AGC TTA TTC
ACT TGA TGG TGC TAC TGG AAC TTT ACC TGT AAG TGA TTG GTA TGA ATA TGG TGG TCT
TTG GGT TAC ATA TGC ATC ATT AAA TCA AGG AAG TAC TTC AAA TTT ACT CGC TCA GTT
TTG GTC TAG CCC GGT TCA CAT TTT AGA TTC AAG CTC CAG TTA CAA ATT TTT TGC ATT
GTA TAA TGC AGA GTA TAA CGC TGT AAC TGA TTT AGA TAT GGA TTT ATC AAA TGT GGA
TAC AAG GAT AGT TAC TTT AGC CGT ATC TAA TGA TTC AAA TTT TGA AGT TGC AGG GTC
AGA TGA ATT CAG TAA TTT GAA TAT TGA TTC AAC ATA TGC AAT TTC TGG AAA CTT ACC
TTT CAT GTC AAG TAG TGA TAA CGT AGC TTC TTG GAA AGT TTA TGC TAT TAC TTA TGG
TGA AAT TAC AAG AGA TGA CGG TCA AAA ATT ACC AGT TTG GAC TGT TGT AAG ACC TTG
GAT TGC CGT AAA CGA TAA CGT TCA AAT ATT AAA TTT AGA TAC TGC GAT TGA ACT TGA
AGA CCT TTA TTT AAG TGG TGA TAT CAA TTC ATC TGC ACT TGA AGA AGT AAA AAC TAC
TGC AAT TTC TGA AAT AGA AAC TAT GCT TGA TTC AAT AGA ACA AGA TTT AGG CTA TGC
AGA AAG AAA TGA TAA TAC TGA AAT GAT TAA TGC ACT TAA TAA AGC AAA AAC ATG TCT
TAA TAA TGC TAA AAG TGT AAT TGA ATC TTT ATC TTC AAC AGA TAC AGA AGA TGA AAA
GAC TGC AAA AAT CAA TAA ATA CAA GCT TTA TCT TGA AGC TGC TGA AAT GTA CTT AAA
AGC ATC TGC TTG TTA TAA AAC AGG TCT TGA TAG TGA TGG AGA GTA CTA TGA AGA CAT
GGC AGA TGA GTT AGT ATC AAA GGA ATC AAT AGT AAT TAC TTC TGA TGG AAT CTT TGA
TGT GTT AAA TGA TAT TCC AAT CCT TGG AAC TTT GGT TTC ATA TAT TCC TGG AGG ATG
GTG GAC TGT ATT AAT CTG TGG AGT TTT AGC TGG TGG ATA CGT TTA CCT TGA AAA TGA
CAA GAA AAA AGG ATT TGG AAG AAA AAG AAA AAG ATT CTA ATT AAT TAA CTT TTT TTA
ATT TTT TAC TAT AAG AAA AAC TGT TAT TTC TCT TTT TAA ATT ATT AAA TGT GTA TTT
TTT AAA TAT CTT GTA ACA TAT TGT ATT TTG TTA AAT TTT GGG GGA AAA TAT GAA AAA
ACT AAT TTT ATT GAT TTC AAT TTT AAC TTT ATT TTC AGG ATG TGT TAC AGT AAA TGT
TAA TGT TCC AGA TAA TGA GCT TAC CGA AAA AGT TGT TGA TAC AGT TGA TAG TTC AAA
TGA TAT TGA AAC TAA ACC GGT TCA GAA TAG TTT GGA AGA GAA TAC TAT TCA AAC GTC
TAC AAA AAA TGA AAA TTA TCC TTG GAA TGC TCC AAT ATT TGT AAA TAG ATA TGA CTT
TTG CCT TTA TGC ATG TGA TTT TAA AAA TGT ATC TAC TTA TGA GTT AAG ATT TAA TGA
TCA GGA TAT TTA CCA GTA TTC AGA AAA TGA ACT TTA TAC TAT TGA TAA AGA TCC CGG
AGA TTT GAC AGA TGT TAC TTT GAT TCA TCA AGA TGG TAC TAG AAA GCA TTT TGA TAT
TTA TTT TAA ACT AAG TAC AGA TTA TGA TAC TTA TGT TAG TTA TGC TGA GAA ATT TTT
ACA AGG TGG AGA TTA TAG GAA TGC AGA TTA CTG TTT AAT GAA AAT TGA AAA GTT TAA
TGC AAC TTG TGA TTT GTA TGC TAC TAG AGC TTA CTG TTT AAT GAA TCT TGG AGA GTA

TGA AGT TGC AAG AGA TTA TGC TGA AAT TTC AAA AAC TGA CCC TAA CTG TGT TAC TGT
GTC TAA TCA GAT TAT AGA TAC TAT TGA TTT AAT TGA AAA ATA TGA TTA ATT TAC TTT
ATT TCT TAT ATT ATT GGT TAT TTA TTG TTA TTT AGT ATT TTT ATT TGT TAT TAT TCT
TTA TTT ATT ATT TAA CCT TTA ATT ATT TGT TAT TTT TGT TAT TAT TCT TTA TTT ATT
CTT TTT TAT TTA ATT TAA TTG TTG TAT TAT CGT TAG TTT ATG TTT AGT CTT TAC AGT
TAG TTA GTT GTT AGT TTG TTA TTA ATT CAG TTC CTG GAA CTT GTA TAT GGG TTT CCG
CAC CCG AAG GGG CAT AAA TCA ATA AGC GCT AAG CTC TAA AAT CGC CAT AGG CGA CAT
ATA CCC AAA GGG TAT ATA AGC CAT GGG CTT GTC GTT TGA AGT TCC TAA ATT GCG TGG
ACT ACC GTC GAA AAA ATG CAC TTT CTA GAA ATA AAA TCA TAT AAG CAG TTT TTC AGG
TAG TTC TGA GCA GGT CGC GAC CTG CGA AAG CAA TTG AAA CAT TGC TTT GAT TGT TTA
AAA TTA AGC TTG ATT ATA TGA ACT GCG CAG CAG TTC TTA ACT CCG ATA AAA TCA ATA
AGT CCA AAG ACT CAT TTT ATG GGC GAA GCC CAT TAC TAG TGT GGT AGC CAT ACA CCC
GGA GGG TGT AGG GCG TAC CTA TAT GAT CCG CCC GTT AGG GCT ATT AAT AGA TAG TCA
ACA TGT ACA GCA CTT TTT TTG GTT TTT TTG AAA AGA AGT CCA AAA CAT AAC TTG TTG
TTT TTT ACT GTT TTT TTG TCA AAG CTT GTT TGG TTA GTC TTT ATT TGT TTT TTA CTG
CTC TAA AAA AAT AAG CTG TTT TAG GCG TTT ATT TTG GAA TCT AAG AAT ATT AAC TTT
CTT CGA TGT AAA AAA AAT AAA TTA TTG GTA TAT GAC ATA CTA CCG GTA ATA TAC CTG
TAA AAA CTT ATA TAT ATA CGG ATA AAT TCT TTT TTA AAT TTT TAA AAA ATA AAT TGT
AAA AAA ATA GTG AAT AAA TAT TTA ATT TTT AAA TTT AAC TGC TGT TCC GTA GTA AGT
GTA TTC TAC CAT TTC TTC GCC AGT TTC TTC TGA TTT GTA AGA AAC TGA ATC TAA TTT
TAC ACT TAT TAT TCC GTC TGC ACC ATC TTC TTT TCC TTT TCT AAA TAA TCC GTA TAA
AGC TTT CTT TTT AGC TTC CAC TTG ATC TTT TGT ACC TGC TTT AGC AGC TAA AGA TCC
CAT GAT AAA TCC AGT ATA ACT TTC AAT TTC TTT TCC TTC AAT TCT ATC GCT TGT TGT
TAC GAT CAT AAA AAC CAC CAT GTT TTT TTA AAA TAA CTA TGG AAA GAA GAT ATA TAT
TAA TTT CTA AAT AAT TTC ATT ATT TAA AGA AAT CTT CAA TTT TAA CCT GCT TTC TCT
TGT CGT TTA CAA ATA AAC TGT CAA TTC CTG ACT TTA TAA TTT CAA GTC TTT GTT TAA
TAT AGT CGC TTG CAT CAT ACT TTT CAG CCA TTG TCT GGG AAA CAT TCA TAT ACT TTT
CAA CAG CCC CTT TTG AAA CTG TCA AAA TCA GTC TGC TTC CAC ATT TTC TGC AAA CTC
CTT TTA ACG GCA TCC TTC GGT ATT TTG CAC CGC ATT TAC ATC GGA CAC CCT GTC TTG
AAA AAG CTC TTA AAT TTC CGA TTA AAT CAG GTA CGA AAT GTG ATT GAA TAA CCT TTT
CAG CTA CGT CTC TTT CAT CAG TTG CCC TGA TTT TTT TTG CTA CGG CTA ACT GTG CTG
AAG TTT TTT CCA TCA TCG AAC CTA ATG TTT TAT ACG CAC AAA TTG GGG GCC CTT CAT
CGA TTT TTG ATG TTT CAT GAG TAT AGC CAA TTC CTT CGT ACT GGC TGT CTT TAT CAA
GCC TGT CTT CAA TAG TTT CCA TCA TTT TTT TAA TTT CTT TTG GAG CGA TTC CTT CCA
GAG ATT TTT CAT AAA ATT CAA GCG GGT ATT CCC ACA TCG AAT CCA TGT TGT GAA CCT
CAC CAT CTA CTT CCT TAG GGT CCA GAA TAG TTG TAA GAA CCA GTG GTG CAT CCA TCT
GGC CGC CCC TTT TAT CAG GCA AAA ATC TTT TTG AAA AGT TCA TAA ATG CAT CCA AAA
GGA GGA AAA ATG CAT CTT CAT CCC CAT CAC AAT TTC TCC TTT TTG AAG CGT GGA AAT
AAG GAT GTG CAT AGC CTA CGT TTG CAT TGC TGT ATC CAA TAA TTC TTC CAA CCA TTC
CTG CAG AAG TGT GTG GTG CCA TTC CGA TTA TTA AAT GGC CTA CAA GGT CTT CTC TTG
TAT TCA CGT TGT AAA ATC TAT CTT TTT TGT AGT ATT TTT CCA AAA GAT CGT CTA TAA
ATC CTG AAA CAT TTA AAA AGT AAT CCA TAC AGG ATT CAG GAA CTA TTA CAT CCT GAA
CTT TTA ATT CTA ATA CCT GAT CTT CAT TTT CAA GGG GAT TTC CGT GAA TAT CTT TTA

AAT ATC CTA ACG ATT TTA ATT TTT CGA TAG GTA CGT GTA TTT CTA CAG GTT TAA AAT
GCG TTA CAG GAA CAT CTG TAC AGT CAA ACC GGG TAG TTC CGT CTT TAA ATA CAT ATA
TGT TAT TTT TTG CCC GCA ATA TTG CTT TTT CCA AGG GTT CAA TTA TTT TAT CCT TTG
AGG TCA TAC CTT TAA TAC ATT TTA CGT CGC CGG GCT TGT TTA TTT TTA AAT TTT CAA
GAG TTT TAT ACC AGT AAT CCT TTA TTG GAA GCT TTA TTC TTG AAG GGC CTG TTG GTT
CAA CAG AAT TTC CAC AGA ACG GAC ATG TCC TGT ATA AAC TTA CCT TTC CAC AAC TAC
ATT TTA CAT TTG CAA TTT CGA TTT CGT CAG TTT TTC CTT CTT CTA CGG CCT TAT TAA
TAA GTC TTA CAA GAG CTC CCG CAT TTC CAA TAG GAA AAA GTC CAT TTA CAG GCG GTT
TCA TCT TTC TAG CAG CTG CTT TTT CAG GCC TAC CCA TTC TTG CGC CAA CGT AAA TAT
AGG CGT TTC TTC GGA TTT CAA AAG GTG CGA TCG TAT TTA TAA AGT GCA TGT TAT TTT
TTG TAT TTT GAA GTT TTT CAT CAA TAT TAT CTA TCA TAT CCC TTT TTT CAT CGA AAT
CAT AAC CTA AAG AGT ATA AAA ATG GAT AAT ATT CAA AAA TTT CTA CTT TTT CTT CCA
TTA CGG TAT GAG GAC ATC CAA TAA GTT CAA GAT ACC TTT TCG CAT TTA AAA TTT CCT
CAT CTT CAG GAT CGT AAT TTA GTT CCC ATG AAT CAT TGC TTG GAT TGT ATT TTC CAC
CAA TAA CCC AAC TTC GAA GGG TAG AAA TAT TCT CTT TAG AAA TAT CGT GCC AAA AAT
ATG TGT ATT TTG GAT GCA GTG GCG TTT TTG TAA TTT TAG CAT AAT TTA CTG CTT CGT
TTT GCC CTG GAT TTT TAA TAA ATT CTT CCG TGT ATT CCA AAT TTT GTG ATT TTA AGA
TTT TTT CAT ACC ATT CCG TGC ACC AAC TGC TTG GAA GTA CCG TAT GGT TGT TTT CTA
AGA AAT CCC CGT AAT TAA CTA AAA TAT CGC CTA AAA ATA GAA TTT CTT GAA CTT CAT
CGG TGT ATT GTT TTG CTT TTT CAA CAG TGT CTA TTT TAA GTA CGC TTC CGT CAT TTA
ATT TAA CTA TCG GAC CTT CGA TAC TGT CAA CAG GAA CTA CAC ATG TTG CCT TTC CAG
GCC TTT CAG TTT TTA ACT GTG TTC CTA CAG CCA TGA AGT CAT CAA CTA AAT ACA TTA
TTG CAG GAT GAA AAC CAT CTG TTG CAA AAC CAG TAT TTC TGC TTC TTC CAT ATC TAA
GTC TGA AAC CGC CTT TTT TGG AAG GGT GTG AAA ATA CAG GCC TTC CTG CAA TAA CCT
CCC CAA TAA ACT TAG TTA CAG CTT CTA CTT CAT AAT CTT CAT ACT GGG ATA AGT CTT
CTT CAT CTT CGT AGT TAT ATT CAT CGT TTT CTT CTT CCA GTT CTT CGA TTA CTT CTT
CTT TTT TGC TTT TTA ATT CTT TTA ACC AGT TCC ATC CTT CGA TAT TTA ATT TAT CAA
CGT GTC TTA AAA TTT TAG GAG CTT TTA AAA GAA CAC CTT CAA CCA GTG CTA AAA GAG
CAC CCC CTC GAA TCT GGT TTG TTT CCA CTC TTG GTA AAT CCC GGT GTC CAC TAA CTT
CAA CGT CGT CAG TGG CTT CCC CAG TAA TTT CTA CAG AGA TAT TTC TAA CTG CAA CCC
GTA TCT CTT CAG CTT TTG GCT GGT ATT GGA ATG TAG TAA CCT CAG ATT GAT ACA AAT
CAA CTT CTT CAC CGT ACC GTT CAA CTT CAT CTT CTG TTG GCT TAA ATC TAT CTA ATC
CCA TGT TTT TTC TAA CAT AAT CCC CAA CAA GTA CTG CAA GTG CCT GTG CAG TTC CCC
CTG CAC TTC TAA TCG GCC CTG CAA AAT AAA TTG CAA GAT ACT CAC TGT TAT CGC TGT
TTT TCT TAA TTT TAA CAT ACG CAA TTC CTT CTA GGG GTG CTG CAA CAA TTC CCT CAG
TAA TAA CGG CCA ATG CAG TTC TTA CAG CCT GTT CAG CTC CAA CTT CTC GCC CAA ACT
CGC CAA ATT TTC CTT CGA CTA TCT CTT TCG CAA TTT CAA GCG CTG CAG GTT CTT TTC
CAA ATT CAG ATA CTA GTT CCC TAA TAC GCT CTG CAA CAT TTT TTG GTC CAA CGA TAC
CTT CAA CCC TGT CTG CCA TAT CTT TTG CAA GCG GGA TTT CAA CAT GAT CCG TTA CGT
CGT ACC CGT TCT TTC TAC ACT CTT CTG CAA GAT TAT AAA GGT TGG ATA CAT CGT CTA
AAA TAT TTT TAA AAT ATT CAG TCA TTC CTT TTG AAG CTG AAA CAT GGA GCA TGA TTA
TCA CGA TTA AAG TTG GAT TAA ACG TAG GTT AAA GAG TTA TGT CTT AAA AGA ATA TAT
AGT TTT AAC TTG GTT ATG GGG GTA CCA ATT ATA AAA TAA TTG TTT GTA TAG CCT AGG

TTA AAA AAT TAT TTT GAA AAT GTT TAA AAT TTT GTA AAT TTT TTT GAA ATG GCA TCG
GGG CTA TAG CCT GCC TTC TGT AAT TTC GGT GAC ATT CAG CTT AGC GCA TAT GCT TGC
ACC CGT GTA AAA TAA GGT CGT TTC ATC GAT TTT CTA AGA ATC CTC AAT TTT GAA AAT
ATC ATA GTC ATA TTT CTT AGA ACC GTG TCG TTT CTA TAC CCT ATT CAT TCC TTC TCA
GGA AAT CTA CAC GAT TGG GTG GGC AGA GCT TCC TCC CAG TGG ATA GTC ACC AGC CCC
CTT GCC ACG AGT TTG AGT ATA TAC TAC CCG AAA ACT CGA AAA ATG AAA TGA ATA AAA
AAA AGT GAC TAA CAG CGT CAC ATT GTT CCC ACC AGA TGG CAG TAC TTA ATG AAT CGC
AGG AGG GCT TAA TTT CCG AGA TCG GGA TGG GAT CGG ATG TAA CTC CTC CGC TAT GAC
CGT TAT GTC AAA GTT TAA ATT AAA AGT GCA TCT ATT GCC CCC GTA TCT TTA AGT GTC
CAG ATG CCA ATT TAT CTC TTG AAC TAT TAG TAC TAG CGG GCT GAA CAT CTC GGA AAA
CCT TGA TGC GTA CAC CCC TAG CCT ATC AAA CCT GTC TTC TAC AGA TGT TCT CGT TCC
CGA AGG AAA CGG CTG TCT ATT TTC AGG ATG AAT TTC GAG CTT AGA TGC TTT CAG CTC
TTA TCT CTT GGC GCG TGG CTG CTC GGC AAT GCC TTG TCA GAC AAC CGA TCG ACT AGA
GGC GCC GAC GGC CCG TTC CTC TCG TAC TAG AGC CAC CTT TCC CTC AGA CAG CCA ACA
CAT CCA GCA GAT AGC AAC CAA CCT GTC TCA CGA CGG TCT AAA CCC AGC TCA TGA TCC
CCT TTA ATG GGC GAA CAG CCC CAC CCT TGG GTC CTG CTG CAG ACC CAG GAT GGG AAA
AAC CGA CAT CGA GGT AGC AAG CCG CGG GGT CGA TAT GGG CTC TTG CCC GCG ACA ACT
CTG TTA TCC CCG GGG TAG CTT TTT TGT CAT CCC TGG CCC CCA TCA GTG AGG CAC AGG
GGT TCG TTA GGC CCG GCT TTC GCC TCT TCG TTG CGT TTT GTT GGC AAC AAA GTC AGG
CTG GCT TTT GCC CTT ACA CTC AAC AGC GGA GTC CTG ACC CGC TTG AGC CAA CCT TTG
GGC CCT CCT GAT GCA TTT TTA GGA GGG TAC CGC CCC AGC CAA ACT GCC CAC CTA CCG
GTG TCC CCT AAA AGG GTA AGG AAC ATG GTC ATG AGA GGG TGG TGT CCC AGG GTC GCC
TCC ACC TTT CCT GGC GAA AAG GTT TCG ATG GCT CCC ACC TAC ACT GTA CAC TCA CAA
CCA TGC CCC AAC GAC AGG CTG CAG TAA AGC TCC ACG GGG TCT TCG CTT CCC ACT GGA
AGT CTC CGG CCT TTG CGC CGG AAT GGT AGG TTC ACC GGA TTC TAG TCG GGG ACA GTG
GAG GTC TCG TTA CGC CAT TCA TGC AGG TCG GAA CTT GCC CGA CAA GGA ATT TCG CTA
CCT TAA GAG GGT TAT AGT TAC CCC CGC CGT TTA CTG GCG CTT CGC CCG GTT GGA ACC
GGG GTT CAC GTA CCA GCA CTG GGC AGG CGT CGG CCT TAG TAC ACA CCT TTT CAG GCT
CGC TAA GAC CTA TGT TTG TAT TAA ACA GTC GGA CCT CCC TGG TCA CTG CGA CCA GCG
ATC CCA TAT ATA AAC AAG ATC ACT GGC ACT CCT TCT CCC GAA GTT ACG GAG CCA ATT
TGC CGA CTT CCC TCG ACT AGA TTC ATC CGC TAC GCC TTA GGA TAC TCA CCT AGG GGC
ACC AGT GTC GGT TCT GGG TAC GGA CAT CTA AAA TCC TTG TTA GTT CCC TTT TCA CGG
GCT CCA GAG ATC GGC CGA ACC CTC CTA ACG GAG AGC TCA TCA CTC TTT CAT CCG GTT
CTC GTT ATT ACA ACT CTC CCC GGA CTT ATA AGC TTG AAT GCC CAG ACA AGA ACA CTC
AGC CTA CCC AGA AGC GTC GGA AAC TAA CCT AGT GTT GCC ACG CGT ATT TAG ATG GCA
CAG GAA TAT TAA CCT GTT TCC CTT TCC TCT CAA AGA AGT TAA CTC GAG AGT TAG GAC
CGG CTT ACC CAC AGC TGA CAA ACA TTG CTG TGG AAC CCT TGC CCC TTC GGC GGT GGG
GAT TCT CAC CCC ACT TAT GCT GTT ACT ACT ACC GGG ATC TTC ATT TCT ACG AGG TCC
ACT CGA CTT CAC AGC CGA ACT TCT GCC CTC GCA GAA CGC CCC CCT ACA GGA TCA CCT
TTC GGT GCC CTT GGG TCT CGG CGG CCG ATT TAG CCC CGT CCA TTT TCG GGG CCC TTG
ATC TCG ACA GGT GAG CTG TTA CGC ACT CGT TAA AGG ATG GCT GCT TCT AAG CCA ACC
TCC CTG CTG TCT TAG ACC AAG GAC GCC CTT CGT GTT CAC ACT TAA TCG GCA CTT AGG
GGC CTT AAC CCA AGT CTC GGC TAT TCC CGT CTC GGA CAT ACA GCT TAC CCG TAT GCC

CTC ACT CGG AGC ATG CGA CGA CGA CAA GTT CGG AGT TTG ACA AAA CAG CGA GGA ATT
TCT TCC CCT AAC CAC CTT ATC AGT GCT CTA CCT TGC CGA CTA TCT AAA CTC CGG CTG
ACC TGA GAG TCA CTT CGG AGG GAA CCA GCT ATC TCC GGG CTC GAT TGG CCT TTC ACC
CCT AGA CCG AGG TCA GAA GAG TGC TTC GAA CGG CAA CAA CTC TGC AGG CCT CCA TCC
CTC AGT TGA GGG ACT TCA CCT TGC CCC GGC CTA GAT CGC CCG GTT TCG GGT CAT ATT
GCT GTG ACT CCG GGC CCT TAA GAC CCC GTC CCT CAA GAT TTT GCG GAC TTG TTG GTT
TCC CTA CGC CTA CGA TCG TGA AGA TCT TAA GCT CGC CAC AAC AAT ATA CTC CCC GGC
CCG TTT TTC GAA ACG GAC GAC ACA ACC CTG GCT CTA TAC TTC GTA CAG CCG CCT TGC
GAC GGT TTC CTT CAG AAT AGT TGC CTT TGG GGC CGT GTC ATA CCA TAC CTA TCT GGT
TTC AGG CTC TTT TCA CAC CCG ATT AAA GGT GCT TTT CAG CTT TCC CTC GCG GTA CTA
GTA CAC TAT CGG TCT TGG AAC GTA TTT AGA GTT GGA AGT TGA TGC CTC CCA AAT TCC
CGC GCG ATA TCC AAC GCA CGG TAC TCA GGA ACA CTT CAA ACC TGA ATC GAT TAC GCT
TAC GGG ACT TTC ACC CTC TAT TGT GTG ACG TTC CAG TCA ACT TCA GCT TTT CTC AAC
AGG CGA AAA GAA GGT CCT ACA ACA CCA CAT CTC CCC GAA GGG ATT CAG TTT GCT CTG
ATC CGC GTT CAA TCG CCT CTA ATA ACG GAA TCT CTA TTG ATT TCT CTT CCT GCG GGT
ACT AAG ATG CTT CAA TCC CCC GCG TTC CCA ATC CTT AAC GGA TTA CAA AGT AGG AAG
TCC CAT TCG GAA ATC TCA GGT TCA ATG ACT GTA TGC GTC TCG CCT GAG CTT ATC GCA
GCT TAC CAC GTC CTT CAT CGG CGT TCC AAG CCA AGC CAT TCC CCA GAT AGG GTA ATA
GAT AAA TCT AGG CAT CTG GAT AAC ACC AGA TAC GGA GGC TTA TGC ACG ACC TTC ACT
AAA AAT CCG GTC CTC GGT CCA TAA TAC TCG GTA TTG TGG ACT GCT AAT AAA AGT GGA
CCC GGC GGG ATT TGA ACC CAC GGC CTC CGC CTT GCA AGG GCG GCG CTC TCC CAA CTG
AGC TAC GGG CCC ATT CGT TAA GCC TAC AAT CTT TTA GTG CCT AGT AGC AAC CAT TGT
TCT TTT TTT AGG AGG TGA TCC AGC CGC AGG TTC CCC TAC GGC TAC CTT GTT ACG ACT
TCA CCC CCC TCG CTG AGC CCA AGT TCG ACC CTA GCC AAA GGC CAA GGC CTC ACT TCA
ACC CAA CTC GGG TGG TGT GAC GGG CGG TGT GTG CAA GGA GCA GGG ACA CAT TCA CCG
CAG TAT TAT GAA CTG CGA TTA CTA CGG ATT CCA GCT TCA CGT GGG CGA GTT ACA GCC
CAC GAT CCG AAC TAC GAC TAG GTT TAG GAG ATT TGC GTC CCT TTT CAG GGT TGC GGC
CCA TTG TCC TAG CCA TTG TAG CCC GCG TGT AGC CCA GGA GAT TCG GGG CAT GCG GAC
CTA TCG TTG CCC GCT CCT TCC TCT GAC TTA GCG CCA GCG GTC CCC TAT GAG TGC CTT
TCT CCC GGA GGA AAA AGT AGC AAC ATA GGG CAC GGG TCT CGC TCG TTA CCT GAC TTA
ACA GGA CGC CTC GCG GTA CGA GCT GAC GAT GGC CAT GCA CCA CCT CTC AGC GCT TCA
GGC AAG GTC GTC AAC CTG GCC GTC ATC ATG CTG TCG CTC CTG GTG AGA TGC CCG GCG
TTG AAT CCA ATT AAA CCG CAG GCT CCA CCC GTT GTG GTG CTC CCC CGC CAA TTC CTT
TAA GTT TCA GTC TTG CGA CCG TAC TCC CCA GGC GGC GAA CTT AAC GGC TTC CCT TCG
GCA CTA GAT GGG CAC GGG GCC CAC CTA ACA CCT AGT TCG CAA AGT TTA CGG CCA GGA
CTA CCC GGG TAT CTA ATC CGG TTC GCG CCC CTG GCT TTC GTC CCT CAC CGT CGG ACC
CGT TCC AGA CAA GTG CCT TCG CCA TAG GTG GTC CTT CAA GGA TCA ACA CAT TTC ACC
GCT ACC CCT GAA GTA CCC TTG TCC TCT CCC GGT CCC AAG TCC AGC AGT ATC CCT GCC
AGT CCT GCG GTT AAG CCG CAG GAT TTA AAC AAG GAC TTA CTG GAC CGG CTA CGG ACG
CTT TAG GCC CAA TAA AAG TGG CCA CCA CTC GGG CCG TCG GTG TTA CCG CGG CTG CTG
GCA CCG AAC TTG CCC AGC CCT TAT TCC TCA AGC TGT TTA CAC TTG ATA AAA GCC CAT
GCT GTG CAT GAG CAC TTG GGA TCC CCC CGT CGC ACT TTC GTG CAT TGC GGA GGT TTC
GCG CCT GCT GCG CCC CGT AGG GCC TGG GGC CGT GTC TCA GTC CCC ATC TCC GGG CTT

CCT CTC TCA AGG CCC GTA CCG ATC GTA GGC TTG GTG GGC CAT TAC CCC ACC AAC TAC
CTA ATC GAG CGC AGT CCA ATC CTC AGG CGC ATA TGC TTT CGA AAA AGA ATC GTT CCA
GAC TGC TTT TTC TAT GGG GTA TTA TCC TCA GTT TCC CAA GGT TAT CCC CCA CCT GAG
GGT ATG TTA ACC ACG TGT TAA TGA GCC GTC CGC CAT GGC CGA AAC CAT AGA CTC GCA
TGG CTT AGT CGA ATC CCA ATA GCA GTG GCC TCC GGC GGG ATC AAC CGG AAT TAG TAG
CGT AAA AGA ATT TTA CGC CAC CCT CTC GTG GAG TAC GTC ATT TTA CAT TAA AGG AAA
TGA CAT TTG GTT GCT ATC TAG GTT TCA CCT AGA TTG TAG GCT TAC ATC GCA CCT GAG
TAT TTC AAT CAG ATG TTC ACT TCA ATT GCA TAA TAT GTC AAT GGT TCG GAT AAT GTA
ATG TTT TTA GGT TTG AAA CGA TAG TAT ATA AAA AAA ATT AAT TTA AGA ATA ATT AAA
CCA TAC TTT CAA GAA CAT CCA ACT GTT CGC CAG TCC CAA AAG CAT ATA TTT GAT CAT
TAT ATT CTA AAA TAA TCT CAG GAC CTG GAT TTA CAA GCA AAT CAT TCT CTT TTT TAA
TTC CGA TAA TTG TTA CCC TAC TAT TAT ATC GTA TTT GCG ATT CTG TTA GTG TTT TTC
CAC GAA TTT TTG AAT TTT TCC CAA TTA GTA TTT TCC TAA TTT CCA AAT CTT CAT CAT
ATT CAT ATT TTG CAA TAG ACA TAA AAG TTG AAA CAA AAT CCA ATA TCC CTG GTT TCA
TTG CAA GTT CTG CAA CCC TAA GAC CCC CAA TCA TAT AAG GGG AAA CAA CCT TAT CAG
CAC CTG CAA TTA ATA ATT TAT CCA TTG CTA CCT GCT CGT CTG CTT TTG AAA CCA CAT
ATA ATT TTG GAT TCA GTC TTT TTG AAG AAA GTG TAA TAT ATA CAT TGT CTG AAT CTG
TTG AAA CTG TTG AAA TAA GTG CTT TTG CAC GGT CTA TCT GCG CAT CTA TTA AAT ATT
CGT CGT GTG TGG CAT CCC CGA CAA TAT AAT TAA AAT TTG GAT CTT TTT CAA ATT CTG
CTT CTA AAA TTT CTG AAT TAT TAT CAA TTG CAA CAT AAT CAA GAC CGC TTT CCC TTA
ATC GAT CTA TTA CAA CCC TAC CAA TCC TAC CAT AAC CGC AGA CAA TGT AGT GAT TTT
CGA GCA TTT CTA TTC TTT TTT TCA TTT TGA TCC GCC TGT TGT ATC TTT TAA AAT AAC
CGC CAA TAA ACG ACT CTG CAA ACG CGC CCA GCG TAT AAA GTC CAG TAC TTA CCC CAA
AAA CAA TAA ACA AAA TTG TCA AAA GTT GGC CGA GTT CTG AAG TTG GGT AAA AAT CGC
CAT AAC CAA CTG TTG AAA TTG TGG CTA CCG AAA AAT AAA TTG CTT GTA AAA GGG TCC
AGC CCT CGA TAT ACA TGA ACA TTA ACG AAA ACG AAA TTA TAA CAA CAA ATA ATA CAA
AAA TTC CGA TCT TAA TTT TTT CCA TTG ATT CCA TAA CAT ACC CGA AAC TAA TTA TAA
TCC CAT ATA TAT CTT ATA AAA TAT ATA TTT CAT TCT AAA GGG GAA CGT TAT GGA AAT
TGG ATT AAT TGA TAT TAA AGG AAC TCT GCC TTG TTT TGA AAA TTT TGG AAA CCT CCC
AAC AAA AAT TAT TCA TGA AAA TAA TAT TTC CAA GAT AAA AGA CCT TGA AAT GCT CAT
AAT ACC TGG GGG AAG TAT TGT TGA GAG CGG ATC TTT TAC TGA TGA ATT TAA AAA ACA
GAT TTT AGA TTT TGA TAA TTA TAT TGT TGG AAT ATG TAG TGG ATT TCA AAT ACT CTC
TGA AAA AAT TGA TAT TGG GAG AAA AAG CCC GAT TCC AAT AAT AAA AGA AGG ACT TGG
TCT TTT AAA TGT CGA ATT TTC ACC ACT TAT TTG TAC TGA TTT AGT AAA ATT CAA CTT
TGA AAA TAA TAA TTT ATT CGG AGA AAA AAA TCA ATT AGG ACA CGG TTT TCA CTG CCA
CAC CTA CGG AAA CAT TGA AAT TAC TAA TAA TTC AAC TGA AAA ATG GAC AAT TTC AGA
AAT TGA AAA ATT AAA CTA CAA AAT AGT TGG AAA ACA GAA TAT TTT GTC TGG CGT TTT
TAA AAA TAA AGT ATA TGG AAC GAT GGT TCA TAA TTT ACT CGA TAA TGA TCT CGT TGT
TGA AAA TTT TTT AAA AAA TAT GAA AAT TAA AGA CGA TGA AAG GGA AGA AAT TTT TAA
GAA AAA TTC GAT TTT AAA GGA AAA ACT AAA AAA CAG ATC CAA AAT CTC TGA AAA CTT
CAA AAA AAT TGA ATC TAA TGA CAA AAA AGG AAT AAT TTT ACT TGC AAC AGG TTC TGA
AAG TGG GAA AAC ATT TCT TTC AAC AAG TAT CTG CTC AAA ATT AAA AGG AAA AAC ATT
TGT CTC AAA AAT AGG TCC TGA TGT AAG AGA TAT TGT ACC TTC ATT ATA TCT TTT AAG

AGA ACC TAT GAC GAA ATT TAA CAG TGT TAA AAT TTC CGA TAG AGG CTG GTC TGA ACC
GTC CGA ATT TTT AGA ATT CGT TAA AAA TTC CAA ATT TAA TAA TTA TAT TAT AGA AGG
CGT CAT GGG TGC ATT TAC TGG AGC CTT AAA TAA GAA AAA TTA CAG CGG ATC AGA AGT
TTC GAA AAT TTT AGG TTT TCC AGT ATA TAT TAT ATC ATC CTG CAG TAA AAC CGG AAT
TGA AGG TTC TTT TGT TGA ATG TAT GGC ATA CCA TTC ATT GCT AAA AGA AAT GGG TGT
TAA TGT TGC TGG AAT TAT ATT AAA CAG AGT ATA TAA TAC AGA ATT ACT TGA AAA AGT
TAA AAA AAT TGC AGA TGA CTT AAA TGT TGA TTT AAT CGG TGT GAA TAA ATT AAA TGT
AAA TGT TGA TAA TAA AAG AGG GCT AAT TCC TGA AGT TGA AAT CGA TTA CGA AGA ATT
TTG TAA CGC CAC GAT GAA ACT TGA TTT TGA AAT AGA TAT TCC AAA AAT TAA TCC TGA
AAA AAT TAA TGA AGA TTC TAA AAC TTT CGA AGA ATA TTT GAA AAT ATG GTC TGA AAA
ACT AAT AAA TAT AAA TTA AAT CCT GTT TTT AAA ATT TTA AAA AAG AAA ATA TGT TAT
TTT TTT AAT TAA ACT ATT ATT TCG CCG TTG TTT ATT TTT TTT GCA GTG TCG TAT GCA
TCA TAT ATA CCA AAT ATC CAA AAA AGC GGA TAA GTA ATG AAG CCT ATT ACC AAA AAT
ATT AAA AAG ATG TTT ATA ATC TGA ACA ACG ATA AAT ACA AGC CCT TTT CCA ATT TGC
CCA TTA TAT ATT TGG CCT AAA CCA ACG AAT AAA AAT GAT AAA ACT GCA GCA ACT CCT
GGA TTT TTT TCT GCT TTA GGC ACC ATT ACG CGC ACC CCG CAT TTA GGA CAT ATC TCT
GCA TTT TTA TTT ATT TCT TCC CCG CAT TCT GAA CAG AAT TTT GTT TCC ATA TAA ACA
CCC ACT TAA AAA TAA TTA AAA GTC TTT TGA TAG GTT ATT TGT ATT ACT TAA TAT ATT
TTT CCC ATA ATG TAA GCA TAT ACT AAA TAA AAG TAA AAA TCC ACA AAT ATC GCA ATA
ATT AAT CCC AAA ATT GGA ATT ACT GAT GCT ATA AAT ATA ACA ATT GCA CTG GTA ACA
ATT ACT ATT AAA TAT AAT ATA CAG TAA TCT GAA AAA TTA TCT TTT ATT TTT TCA AAT
ATT TCG GAA AAC CTA AAT GCT GCT CCA AAA TCA TCG TAT GCG ATG TAA TTT GCA TAA
GCC ATT GGA ATA ATA AAT CCA ATA ATT AGC GTA ACT AAA AAT GTA ATT AGC CCC ACA
ATA CCT AAA CCA ACG ATA CCC CCT ACA ATT GCT TCG TCA CTT CCA GTA AAC ATT GTT
AGT ATA GAT AAA CCG CCG AAT ACA GCC CAA ATG ATT GAT GGA ATT AAC ATA TAT ATG
AAC ATA ATA ATA AAT GCA ACT AGC CCT TTT ACA AAT TTA CCG CCA AAT TCA GAC CAT
TCT GGA AGT TCT TCT TTT GAA TCA ATT ATA TTT TTA AGA AGT TCT AGC GCA TAG CCA
AAT GAA ATA AAG TTT ACT ATT GGA ATT ATA GAT AAT ATC GCA CCA ATA ACA ACT TTT
TTG ATC CAA TCT GGA TCG TCC GTC GGG AAC TTA AAT GCC CGT TCA AAA TCC ATA GTA
TCA CCC CAT ATG TTA TAA TAT TAA ATT TTC GAA TAT ATA ATC CTT ACT TTT TGT TAA
TAA TTA TAT AAA ATA GTT TTT TGA AGA TTT GAA GCA TTT TTA AGA AAT TTT TTA ATT
TTA AAT TGA TAA ATT TTA ACT TTT GGA TAC CTC ATT AAA ACA TTT CAT AAT TAT TAT
ATA GAT CTG AAA ACA TAT GTG AAA GGT ACC AGA AAA TTT GTG AAA ATA ATG GAT GAT
AAA CAG ATT CAA AAT ATT CGA CCA CGA CTT ACA GAA TGC TTA AAA ACG CTA AAT TTT
CAA GAT ATT ATC TCT GAA AAA AAG AAA GTA GCT GTT GAT ATA GAT AAT ATC TAT AAA
CAC GGG GTA ATT GAC TTT ATA GAG TTT TTA GAA GAA TTT CCA AAA GAA GGA ATA GAA
CTC CTT GAA GAG TGC TAC AGG GAT GCA TAT TAT TCA ATA AAA ACG GAA AAG CCC GAT
ATT GTA ATT ACT GTA AAA AAT ATA CCT GAA GAA TTT AAT AAA TCT GAA AAA AAG CAA
AAT TAC ACT ATC GAA GAT ATT AAA AGC GGC ACT CTT GGA AAA TTA ATT GAA TTT GAA
GGA ATA ATT GTT ATC GCG ACA AAA ATA AAA TCT GCC TTA AAA AAG GCA ACT TAC GTA
TGC ACA CAA TGT GGC GAA AAA AAA ATA CAA GAA GTT GAA AAT CCA TTT GAA GCT TAT
TTT GAA CCA CCA TGT CCA AAA TGT GCT CAA AAT ATG ACT TTA ATT GAG GAC GAA TCT
GAA TAT ATT GAT TTT CAA GAA ATT AAA GTT CAA CAG CCC CTT GAT TCT ATG GGT GAC

CCT GAA GAG CCA CCA AAA TAT ATT ACT GTA TTT TTA GAA CAC AGT CCT GGA ATT TAT TGT GGG CGT GTG AAA GTT ACA GGA ATT CCA ATA AAA AAC CAG AAA AAC AAA AAT ATC CCG ATT TAT GAT ATT TAT GTT AAA GGA ATT AAT TGT GAA GTT TTA GAA GAT AAA ATT GAA GCA AAT TTA AAT GAA GAA GAT ATC AAA AAT ATC GAA AAA GTT GGA AAA AAT CCA AAA GTA ATA GAT ATA TTA TCT GAA AGG ATG ATT CCT GAA ATT AAA GGT TAT GAA ATT ATC AAA AAA GCG ATA TTT TTA CAG CAA GTT AAA GGG GTT AAA AAG GGA AAC AAA AGA GCT GAC AGC CAC CTG CTT TTA ATT ACA GAT CCA GGT ATT GGA AAA TCA GTT ATG CTT AGA AAA ATT GCT GAA ATT CCT GGA AAT TTG TAC GGG TCA GCC ACT ACT GCA TCA GGT GTT GGT TTA ACT GCC GCG GTT ATT CGT GAA AAA ACC GAA ATT GGA GAC GAT ACA TGG GTT ATC AAA CCA GGT CTT TTG GTT AAA GCG AAT AAA GGA ACT GCA TGT ATT GAT GAG CTT ACA GTT AAT CGG GAC CTT CAA ACT TTT GTT CTT GAA GCT ATG GAA TCT CAG ACA ATA CAC GTT AGT AAA GGT GGA ATC AAT GCA AAA TTG CCA TCA GAA TGT GCA ATT ATT GCA GCA TGT AAC CCA AGA TGG GGT AGA TTT GAT CCA AAT GTC TCG ATT CCT GAA CAG ATA AAT ATT CCT GCA CCT ATG CTG AGC AGG TTT GAT TTA ATA TTC CCG ATA AAA GAT GAA GCA GAT CGA TCA AAA GAT AAA GAC ATT GCA CAG CAC ATT ATC AAT GTT CAT AGG GCA TAT TTA AGT AAA GAA GTT TCG AAA AAT ATG AAA CTC GAC CAT ATT ATA GTC GAT GAT GTC TTG ATT GAT AGG GAT TTT GTA ATA AAA TAT ATA GAA TAT GCA AAA ACT AAA TCA CCG ATA ATT TCA GAA AGT GCT GAA AAG CTT CTC ACT GAA TTT TAT TTA AAC ATG AGA AAA GGT TCA GTT CAA ATT ACT GCA AGA CAG CTT GAA GCG GCA ATC CGT ATT GCA GAA GCG CAT GCA AAA GCA AAA TTA AAA GAT GTT GTT GAC GAA GAA GAC GCA AAT GAA GCC ATT TCA ATA ATT ACT GAA TCT TTA AAG GAA ACT GCA TAT GAT CCT GAA ACC GGA CAA TTT GAT GTC GAT AAA ATT TCA GGA GTT GGA AAA AAA GAT AGA AGT AAG ATG ATG GGA GTA TAT GAT TTA ATA AAA GCA CTT GCT GAA AAA AGT GAT AAC GAT CTT GTA ATA ATT GAT GAA ATC GTC GAA GCT GGA AAA TCA AAA GGA ATT GAT GAA GAA CAT ATT TTA CTT TCA ATT AAA AAG CTT AAG AGC AAT GGG GAC ATT GAT GAA CCT AAG ACT GGA AAA TTC AGG ATA ATT TAA AAA TTT GTG AAA ATC ATG GAA ATT ATT TAC AAT AAA CCC GAA ATT TAT GCA ACA AAT GAT ATC TGG ATT CAG CCT GTT AGC AGG AGC TCT GTT TTA GAA TCA GAT GTT GTT GGA AAT TGT AAA ATA ATT AAT GAT ATT CCA ATT TTA AGA GTA TTA GGA AGT CCG TCT TTA AAA AAG ACT GTT TTA AAT ATT TTA CAT GTT TTA AAA GAT AAA ATG ACC TTT TTT AAA AAT AAT TTC GAA ATT AAA GGA ATG GAT AAT ATT TTC AAA TAT TTA AAC GAT GTC TAT TTT GAT AAA TCT GTT AAT AAT TAT TAT AGG GCC AAA AAA GCA TGG TAC CGT GTA ATT GGG GAT TTA TTC GAA GAT ATT AAT AAT TGG AAT TTT AAA AAA GTA ATG GGT AAA TTA ATA ACT ATT TTG AAA GTT TTA AAC CCA ATT TTA GTA TTT GAC CTT CAC GAT GTT TCA AAA TGG AAT CAT TTG AAA ACA TTT CAG AGA TTT ATT CAA TTT TTG AAA AAT GAG GAA ATA AGT ATT GTT TTA AGA TGT CCT TTT GAA GCT TAT TTG TAT TTA AAA AAT AAT TTT GAA AAT TAT AAA ACA AAC AGT GTT GCT GCA GTA ATA TAT TAT GCA AAA AAT AAA GGC CAT TTA ATA TCT GAA AAA GTT TCA GAA GAA CTT TTG AAA ATA AGC TGC GGA AAT TTA AGA ATA ATT GAT TTA ATA CTT GGT AAT TCA AAA AGA GAC CTT AAA AAT ATG CGA GAT TTA AAA ATA AAC TGG AAA AAA ATA ATT CCA GAA GTT CTG CCT AAA AAA TAC AAA AAT ATT GTT CAA AAA ATA TTC CTG TTA AAA AAA TTC AAT ATC AAA GAA ATA TCG GAC ATG TTA GAA TAC AGT TCT TCG ACA GTT TAT AAT TAT CTA AAT GAA CTT GTT GGA TTG AAT ATT ATC AAA AAA AGA AAG GTT AAC AAA AAT ATC CTT TTT AAA TTG GAT GTT GAT AAA AAA GCA GCT TTT GAT ATT ATT CAA TCG AAT ATT GAT TTT AAA

GAT ATA TTT TTT GAA ATT AAT GAT TTT AAA AAT ATA AAC TTG AAA AAG CGT ACG GAA
ATT TTC AAT GGA TTT TTA ATT TTT GGA TAG GTT TGG ATC CAG TTT TTA TAA GCT ATT
TAA ACA AGG TTT TAA AAT GTT AAT TAT TTT GTT TTT TAT TAT TGG ATA GAT AAG GCT
ATT TTA ATT TTT AAT AGA TTA CTA CAC TAT TTT TTT ATT TAT CAG TTG ATT CCT TGA
TTT TTT AGA GTA TAA CAT TAT TTT TAA AAT CTT TTT TTA CTT GAT TTT GAT TTT TTG
TTT TTC GGT GTT TCG AGT ATG ATG TAT ATA TGG TGT GTT TCT TTT TGA TTT TTT TGT
TTA GAA AAA TTT GAT TAT AAT TTT ATT TTA CTT TTT TAA CCT ATA TAT GTA TAA TAA
TAA AAT ATA ATT AAT ATA ATA ATA TAA TAA TAA ATA AAA AAA GTT ATT TTA TAA AAA
TAA AGT TAA ATA ATA TAA TAA ATA CTA AAA AAC ATG GTT TTA ACA GAT ATA TAT TAT
AAA AAA GCA TTA TAT AAT TAT TAT ATA TAA TAT AAC CCT TAA AAA CAT GGT TAA ATA
TAT AAA AAA ACC AGA AAT CTC AAA AAA CAA ATT CAA AAC AAA AAC ATG TTA TAT ACA
TGA AAC TCG AAA ACC CGA AAA GAT AAT CTA AAA AAT ATA CTT TTC TAA ATC CAT AAA
ATA ATA CAT AAA CAG TAT AAA AAA ATG AAA AAT ATT AAT TAC GTT TTT TAG AAG ATG
CTT TTT TTA AGT ACA AAA CAT GTT CAC AAT CAT TAC TCT GGC AGG CTA CAC ACC AGT
AAT TGT TTT CAT TAG TTT TGA ATA TTT CAA AAG AAC TAT CAT TAT TAT TTA AAA TAA
AAG TAG TTC CTC CAA AAC CGT TGT CTA TTT TTT TAA TAT CAT AAT CTT TTA ATG TAT
TTT CAA CAG AAA GGG TGG TTT GGA ATT CGT CTT TTT TCA AGC TGT GTT TTA AAT TCT
TCT TTT CTT CAG TTT TTT TGT CTT CAA TTG TTA CTG CGT TAT ATA TTT TCA AGT AAT
TTT CAG GAT TTA TCG AAT AGT ATA CTT TAT GAC TGA TTC TTT CTC TTT TAA CAA GAT
TTA ACG AAG TAC ATA ATT TTA AAA ATT GTG CAG GAA GTC CTT TTG ATA TAT TCA ATT
CTT TTG AAA GAT CTT CAC AAC ACC CTG GCT CTT TTA AAA GTT TAG TTA ATA TTA ATT
TTC TCT CTT TTG TAT TTA CTA GTT TTT TTA AAA TAG CTT CCA TAA AAT CCC CAA TTT
AAA ATA ACT TAT AAA TTT ATT TGT ATT ATA TTA ATT TAT TAA TTA AAT ATG GTT TAA
ATT TAG TAA TAC ATT TAT ATT CAC AAT ATA ATA TAT TAT TGT TTT AAT TTA CTC AAT
ATA TTA TTT AAA AAA TTA ATA TTA TTA CGA TAT TTC ATC AGT TTA ATC ATG ATT TAA
ACT CTT TTT ATT AAT TAT CTT GAT TTT TTA TAT TTC ATT TGC AAT TTA ATT AAT AAT
TAT CTG TTT TAA TAT ATA TTA CTT ATT ATA TTC ACA ATT TAT TTT ATT TTT AAA CTT
ATA TTC ACA ATT AAT ATT TAT AAA AAT ACC TTA TTT TTC ATA ATT CAA ATT AAT TTT
ATA TTT ATT GTG ATA TAT TTA TAT AAT ATA CTG ATT TTA TTA AAA TGA TTT TTA ATT
AAA AAT AAG GAT TTA AAT TAA TTA AAT ACT TAA AAA GAT ATT ATA TTC ACA ATT TAT
TTT ATT TTT AAA CTT ATA TTC ACA ATA TTA TTA TTT TAG ATG TTT TAA ACT TTC GGT
TTT TCG AAA CTT ATA AAA AAG ATA ATT CAT TAT ATA TTT TAA GAC ACT TTT CGG ACT
ATT TTC ATA CTG GAG GGA TAT GGC TTA TGT TAG ACC CAA TTG AAT TTA TTC ATA ATG
CAT CAT CGA TAT CGA AAA AAT CAA TGA ACA GGT TAA AAT TAA AAA CGA ACC CAT TTT
CAG AAA AAC CAA TTA GGG GTA ACA CCA AAT TTT TTG TTG GAA GAA CTT CTG AAC TTT
CTG AAA TTG CGG ACA TTT TGG GTG CAG CAC AGT ATG GTA GTG TTG CAA ATG CTG CAA
TAG TTG GTA CGA AAG GAA TTG GAA AAA GTT CGA TAC TAA ATA TAT TGT ATT ACG CTT
CAA AAA GAC ACA ATC ATT GGG TTG TTG AAT TAG AAG CTT CAC AAA TCA CTG CAA GGC
AGT TTT TAA TAC AAT TAA TGT ATG GAA TCA TCA AAG ATA ATC TGT TTT CAG TAG ATG
GGA CTC TTT CAT CGG ATT ATA TGA AAC ATT CTC AAA AAA TAA TTG AGA TTT ACA AAA
GAT TGA GCA ATT ACA GCG ATA AAA CTC CTG TTC ACT ACC CTC GTG AAA AAA TTG AAA
GAG ATT TAA AAT ATC TGT TAA ACG AAG TAA AGG AAG AAA ACA AAC TCT GCG TTA TAT
TAA TTG ATG AAG CAG ACC AGT TTG CAA AAA GAA GCT GTC TTG GAC TTT TAC AGT TTT

TCC ACT CGT TTT TAT ATG AAG ATG ATA TTT TAA CGT TTT TAG CAG GCC CTC CAA CAA
TGG TTG AAG ATT TGA CAA AAA TAT CCC CTG CAA TAA GAG ATA GGA TCC CAA AAA TCA
TAA ACA TGC CCC CTC TTA CAA AAG AAG AAG CAT ATG ATC TTA TAA AAA GAA GAC TCG
AAG ATT CAC AAA TAA CTG GTG CGA AAG GAT ATC AAC CTT TTT CAG AAC AGA GCA TTG
AGA AAA TAA TTG AGG AAT GTG ACG GAA TTC CAA GAA GAA TTA TAA TGA CAT GTT CTG
AAG CAG TTT CTC TCG GAC TTA AAA ATG GTG TAG ATG AAA TAG ATG ATG AAA TTG CAA
GAA ATG CAA TGA AAA ATC TTG GGA TAA GTG TTG GAC ACC AGA TTT TAA ACC ACC TAA
CAC CTG CCC AGT CTA AAA TAA TAA AAG CAA TGG CTG AAC TTG GTG GAA GCT CTA CGG
TAA CTG AAC TTT CAG GAA TTT TAA ACA ATT CTA CTG GAA CAA TTG GAA CAC ACC TTT
CAG ATA TTT ATG AAA TGG GGT ATA TTT ATA AAG AAA GAG ATG GAT ATA ATG TAT ACT
ACA CTT TAT CCA AGG AAT TAA AGG ACG TTT TGA TAA CAA AAG AAG ATA TTT CGT AAA
TTA TTT CAG GTG TTT TTT GAT GCA ATG CAA TGA CGT TCA GGC AAC AGA ACC TGA TAT
TAA GGT TTC ATT AAC CAG GGT TGG CGT AAC AAA CCT AAA AAA ACT TGT AAA ATT AAA
AAG AAA AAA TAA AAG AGA CAT CGT TCT TCT CCC TAC CTT CGA AGT TTT TGT GGA TCT
TCC AAG TTC GCA AAA AGG AAT TCA TAT GTC AAG AAG CCC CGA AGT AAT CGA AGA AGT
AGT TGA AAA CAT ACT TTT AGA AAA GGA AAT TTA TGG TGT TGA AGA TTT ATC TGT TGA
AAT AGT TAT GAA ACT CTT CGA AAA ACA CGA ATA TGC GAC AAG AGC CGA AGT AAT GCT
TTA CAG CGA TTA CAT GAT GGA AGA AAA ATC CCC TGT AAC AAA AAA AGA TTC CCA GGA
GAT TGG AAA GAT AAT TGC AAG AGC ATA CGG TGT AAA AGA CAG CAA TGG AAA AAT AGA
TGT AAA AAA GAT GGT TGG TGC AGA AGT TGT TGG AAT TAC AGC ATG CCC TTG TGC TCA
AAA CAT GTT AAA AGA AAA TGC AGT TGT TAG TTT AAC TGA AAA AGG ATT TTC AAC TGA
AGA AAT TGA AAA AAT TCT TGA TTC AGT TAC CAT TGC AAC ACA CAA CCA AAG AGG AAT
TGG AAC GGT TAT GAT AGA AGT TCC AAA TGG ATA TAC TGT TGG AAT TTC AAA AAT AAT
CAA AAT AAT TAA AGA TTC AAT GAG TGG GGA AGT TTA CGA ACT TTT GAA GAG AAG TGA
CGA AGC TTT TGT TGT AGA GGC AGC TCA CAA AAA TCC AAA ATT TGT TGA AGA CTG TGC
AAG AGA GAT GAT AAA AAG AGT TGT GGA TGT TTT TGA TTA TCT TCC TGA AGA TAC ACA
AGT TCT TGT AAG GCA GGT AAA TAA GGA GAG TAT TCA CAG ACA CGA TGC TTT TGC AGA
GCG AAA TTC TAC AAT AAG GGA ATT AAG GGA CGA ACT TAA AAC TTT AAC AAA TTA ATT
TTA ATT TAT TTT TTA GTG GTT TTA TGA AAG CTC TCG TTG TTG GGG CAT GTA CGC GCC
CTG TTG TAA ATT CTT TAA AAA AAT TAA ATT TTG AAG TTT ATT CAG TTT CCC ACT ATA
ACC CTG TGG ATT TAA ATG CAG ATC ATA AAA AAT ATA CAA TTA ATG ATA AAT TTC ACG
GAC ATT TGG CAG ATA AAT ATT CTG AAA AAG AAT TAT TAA ATC TTT CAA AAG AAT ATG
TGG ATT TGG TTG ATT ATA TTT TTG TAT GTT CTG GAA TAT TTG AGA GTA AAA CTT CAA
AAA CTC CAA ACT GGG ACG TTG TTG GAA ACT CAC CAA AAA GAA TTA AAA ATA TAA GTG
ATA AAT ATA AAA CTG TAA AGA AAT TGG AAA ATT TAG GCT TTA ATG TGC CGG TAA CAT
ACC TCG TAA ATA ATA AAT ACC AGT TTG AAA AAT GCT TAT CGG AGT TAA AGT CTG TAG
TGG TAA AAC CTA TTT CAG GTA GCG GCG GAA TTG GTG TAA CTA ATA TTG ATT TTC AAA
ATA CGG ATA ATT TGG ATA TAA ACT ATC CTG TAC TGG TTC AGG AAT GTA TCA AAT CTG
AAT CAT ACA GTG CTT CAT TTA TCG GTT CAA ATT TTT TAT GTT TTA ACA AGC AAT TGA
TAA ATA ACA ATA TTT ATG TAG GAA ACA TAT CCC CGT ATG TTT TAA ACT TGA AAA ACG
AAA CAA TTC AGG AAT TTG AAG AAA TAA TAG CTT CGC TGG ATC TTT TAG GGA TGA ATG
GAA TTG ATT TCA TGC TAA AAG ATG GAA TTC CAT ATA TTA TCG AAG TAA ATC CTA GGA
TTT TAG GAA CTT ATG AAA CAA TAG AAT TAT CTT CAA GAT ACA ATC TTT CAA AAG CAA

TTC TCG AAA ATA AAC CCG TAA AAC CAA AAG ACT GTT TTT TTA AAA AAA TAG TTT TTT
CAG ACA AAG GTT CAT CAT ATT CTA TCG ATA AAA ATC CAT TTT TAA AGG ATA TCC CAC
AAA AAG GAA CCT ATA TCG AAG AGG GAG AAC CCA TTG TTA CAA TAA TCG GAA GAA ACA
TGG CTG ATG TAA GAG AAA TGG AGT GCG GTA TTT TAA GAG GTG TAT GAA ACA TGA ATT
TAT CAA AAG ATG AGA TAT TTA CAA TGC TCG AGA ACC CCC TGA TTC AAC AAG TAC TTT
TCG AAA TAA TGG ATG AGG ACG TTG TTG GTT TTG ATG TTT TGT CAG TAC TTA TCA ATA
CTA ATG AAG TAA CTG ATG ATG AAA TTT CAA GAC AGT TGG ATG TAA AAT TAA ACA ATA
TTA GAA GGA TAC TTT ACA AAT TAT ATG AAG CAA GGC TTG TAG ACT ACA ATA GGG AAA
AAG ATG AAG AGA CAA ACT GGT ATA CTT ACA CCT GGA AAC CAT CTC TTG AAA AAG TTC
CTG CAT TAG TTG CTA AAA AGA TGA AAA ACG TAC TCA ATG GTT TAA AAG AAC AGC TAA
ATG TGG AAG AAA ATA ACA TGT TTT TTT TCT GTT CTG ACT GTG AAA TTA AAT TTA CAT
TTG AAG ATG CGA TGG ATA GTG GAT TTA GAT GCC CCC AAT GTG GCG AGA TGA TGT ATG
AAT ACG ATA ATA AAA AGG ACA TTT CAC TAT TAA AAG AAC AGA TTA AAT ATT TAG AGG
ACG AAT TTA ATA AAA ACC CTC TTT TTT CTA CTT ATT AAG GTT GCA TCT ATG AAT ATT
GTT TTA AAT GAT CTT CTA AAC GAC GCT ACT TTA TAT GAA AAA TTT TCT AAT TTT AAA
AAA ATG CAA GAA ATA CCC AAT TTT AAT GAA TAT TTA ACT TTT TTA ACA GAA CGT TGT
GAA AAA AAC GTG GTT CTA CAC AGC ATT GCG GTA GCT GAT TAT GTT TAT GAG TTT GGA
ATC AAA TTA TTA AAT AAA AAA TAT GAT TTT GAT TTA GAT ACG GCT GTT TTA GGT GCT
ATT TTA CAC GAC ATT GGG CGA AGC AAG AGC CAC AAT ATC GAT CAC GGG ATA ATT GGT
TCT GAA ATA TTA TTA AAA AAT AAT TTT AAT GAA AAA TAC GCT AAA ATT GCA GAA CGG
CAT ATC GGT GCA GGA ATT TCT ATA AAA GAA ACA GAA GAT TTG AAT TTG CCT AAA AAA
GAT TAT ATT CCA AAA ACA TTG GAA GAA AAA GTA ATT GCA AAT GCG GAT AAT TTA GTT
TCA GGA CAT ATT CGG GTT GAT ATT GGT TTT GTA ATT GAT AAA TTT AAA AAA AGA ACA
AAT AAT GAA GTT TCA AAT AAA GTT TAC GCA CTT TAT TTA GAA ATA AAT AAG TTA ATT
GGT AAT TAA CTA AAA TTA ATT AAA ACA ATT GAT TTT TAT GCA AAT AAC AGT CCG GTA
AGT CTC AAT ACT GCA TAA AAT GTG CCG ATT GCA AAA GTC ATA CAT ACC GCA CAC GTA
ATT ATC ATT CTA TTT ACT TTT AAA CCA AAT AAT GGA ACA GCA AAG AAA AGA CCA AAC
AAT CCA GAT CCA AAC TGA ATA GTT TCA ATT GCT TTT GAT ATC TGT GAA GCT TCA ACA
GAA GGC ATT GTT GGT GCT AAC TGA GAT ATT ACT GTT AAA TAA CCC CCA ATT AAA CCA
GCA GTT GCG GGG ATT ACA CCA CAA AGA ATA ATA AGA CCA ACT GCA AGA CCG TTT GCA
GTG ACA CTT AGA AGT TGA GAT TTT AAA TTC TGA AGA TCT TCA AGG GTT TTT GCG AGT
TTT TTT AAT GTA TCT GCA AGT TCC CCC CCG TAT TTT CTA TTT TCA ATA ATC AAT CTT
CCA ACT TGC CTA AAG ATC TTT GAG TCA AGT GAA TTT GAA ACA AGA ATC ATT GCA TCC
TCA AAA CTC AAT TTT CTT TCA GTC ATT AAT ACG ACA ACT TTT GAA AAT ACA ACA TCT
ACT TCA GGA ATT CCA CTT CTT ATA ACT TCA TTA ATA GCT TCA ACA ACA GAA CGT CCG
GAA TCA AGT GAT AAA ACC ATA ACA TAA AGG GCT TTT GGT ATA TTT TTG TCG AAT TTA
TCC ATC TTT GAT TCA TAC ATT ATA GCA GGT AGT GAA AGA ACG GAT CCT AAA TAC ACG
AAG GTT AGG ATT AAA GTG GTT TTT AAT GTA AAA CCT AAG AAT ATT TTT AAC AAT ACT
GGT AAA ATC GTT ATA ACC AAT AAA ATA CCG ATA AAT ACC CTT TCA TCA AGT TTT TTA
CCT GTC TTT TTT AAT ATC TTA ATA TTT CGT ACA ATT AAA ACA TGA TAT ACA TGA TCT
AGG TAT TTT TTG GCA TCC ATA ATT TCA CTC AAT CTT CAT TTT AAT AAA GAT GGT AAA
TAT AAT TGT TGA CAT TAA CCC TAT TTT GCC CAT CAC GAG GTC AAT AGC ATT GAT AAT
TCC GTC AAA TCC TTG CTG AGC ACC CAA TGC TGA CTG TAT CAT TCC TGA AAA TGG GAT

AAT CAA ACC AAT ACC AAA TAT AAG ATT TCC CAA ATT TGA TAT TTG GAA TTT TGA GCT
ATC TAT TTT TGA GAT AGA TTC TCT AAC TAT TTC ATT ATA TAA ATT TTC AAG TAA AAG
CTG CGT TCC ACC TTT ATT CGC TGC AAG AAT CAG TTG GTC ATA AAG CTG TCT CAT CAG
AAA TAT TTT TGT TCT TAT TTT GGC CCT TTC GAG TGC TTC TAC GAA ATT ATA ACC GCC
CTG ATT AAT GTC TTT TAT GAT ACT TCT AAA TTC AAA AGA AGT AAT TCC ATA TTC AGG
GTT ATT TGC AAT ATT TTT CAA AGC TTC CTG AAC AGA AGC GCC AGA ATT TAA CAT CGA
AAT AAG GTG TAA TAT ACT CAT TAA CAC CTG AAT TTT AAT TTC CCC TCT AAA CAA TGT
TAA TTT CAG TTT TGG ATA AAA TAT ACT ACC AAC AAC TAT AAT AAC TGC TAA AAG TAC
CCC ATT AGA AAC GCC AGA AAA TAC ATC GCC GGT CAA AAC ACC GTA AAT TAT GAA TAA
AAC AAC CGT GGT GAT TGC TGC AAT CAT CAT CTT TAA GAA ATA AAC TCG CTC ATC TAC
AAT ACC TGC ATA CTG GTA ATC CCT CTT AGA TGG TAA AAA TGA GGT GTT TGT AAC CCA
GTA TGA AAA AGA TCT GGA GTA TCC GCC AAG TGC TCC AAA TTC TTT TTT TTC AAA AAG
TAA ATA ATC TAA GTC AAT ACT TAC TTC GGG AGT TTC ATC GAT ATA TGG TTC GTA AAA
CTC TAA AAT CTC ATC TTT ATG TAT ATC TTC AGT TTT TCT TTC GAA GAT TTT TTT TAA
ATA TTC CGA TCT TCC AAC CCT AGA TAT TTT TCT TTT TTT CGG AGT TTT AAC TCC TTT
TAA TGT ATT GGC GAG TTT ATC AAA AGT ACC CTG TTT TTT TTC AGT TTT TCT TTT CAT
CTA AAC CCC AAT TAT TCC AGA ATG TTT TTC AAG ACA TTT TCT GGA TTT TCC TGA TAT
TGC TTA AGT ACA TCC CCT ACT TTT CTA ATA TCA TTA ATA TTG TTG TTC ACC ATA TAT
TTT AGT ACT TTC TTT CTA TTG ATT CTA TCG TCC ATT AAT TCA TCT CTT GTG ATA CCT
GCA ATC TGG CAC ACG TCT TCT TCC CAC ATA CAA ATT CCT GTT TTT ACA ACA CTG TCG
CTT ATT CCA TCG TAT TTA AAG AGC TCA GTT TTT GTA ATA TTT TCC CCA CTT CCC CCG
ATT TCA ACA ACA CCG AGG ATC CTT CTA ACA GTT TTT TTA TTA CGT TTA ATA CGC TGC
TGG TTT ATG ATA AAA TCA ACT GAA GAC ATC ATA ACT TTT GGA ACG TTC ATA GGT GGG
TTT ATT AAC CTA ATG AGT GCT TCA TCT GCG CTG TTT GCG TGC AGA GTT CCG GAA CAT
CCG TCA TGC CCG GTG TTC ATA GCA ACA AGC AAC GAA TGG GCT TCA CTA CCC CTA ACT
TCC CCT ACA AAT ATC CTG TCA GGC CTC ATC CTT AAG GAG TTC TTG ATC AAA TCA TCC
ATC GTG ATT TCA TAA CCC TGA ATA CCA GGT CTT CCA GGT CTT GTA ATC ATT TTT ATC
AGG TGA GTT AGT GGA ACC TGT AAC TCT GGA GTA TCT TCA ATA GTT ACA AGT CTA TCG
GTA TAC ATC GAA AAC ATT GAA ACA ACG TTT AAA GTT GTA GTT TTA CCC GAA CCA GTT
CCA CCT ACA ATC AAA GTG TTT GCA GGT TTT GCA CCA AAG TAT CCT TCA ACA GCC TGC
CAG AGG AAT GCC GCA AGT TCT AAA TCA AAA GTT CCA AAA TTA ATT AAA TCA ACA ATA
GTT AAT GGA TCT TCA CTA AAC TTA CGA ATG GTA ATT GTG TTT CCA CCC AAT GTA ACG
TCA CTC ATT GTA GCG TTA ACC CTG CTT CCA TCT GGT AAA AAT GCA TCA AGC ATT GGA
GTT CTT GAA TCA ATA GTC CTG CCT GCA AGA TAA GCT ATA CTT TCA ACA ACC CTC GTT
GCT TCG TGT CTA TCA AGC CTA ATA TTT GTT TCA CAC ATC TGG TAT TTT CTG TGA AAT
ACG AAA GAA GGT GAT TCA ACA CCA TTT ACC ATT ACT TCT TCT AAT TTG CTA TCA TTT
AAT GGA ATT TCT AAA AGC CCT AAT TTT CCA ATT ATT AAG TAA AAA TAG TGT GAT AAA
TGG AGT ATT TCA ATA TCT CTT AAA TGT AAA TTA TTT TTT TCC GAA TAA TTT TTA AGG
TAT CCT TGG ATC TGT CCT AAT TTT TGA AGG GTA GAT TCT GAA AGT TCC GCT TTT ATT
GTT TTA ATA TCC AAA TCT GAA AAT TTT GCA AGA GCT GTA TTC ATA AGA GTG ATT TCT
GGA ATC TTG TAT AGT GTG ACA CCA TTT TCT TTC TCA ATA ATT ACA TCA AAA TCA ATG
TCA TCA ACT TTT ACA AAG TAT TTA TCG AGT ATT GAG CTT GTA GAA TCT CTT TCA AAT
TCT TCT ACT GGC TGT TTT GCC CGT TCA TTC ACT GAA AAA CCC ACT TTT ATT TCG GGT

TTT TTG ACT TTT TCA GAA GTA TCA AGT ATA TTG GGG GTT TCA GAT ACT TTT TCA GAT
ACA TTA TTT TTT TTA GCG ACA GAT GGA TTG GAA TCT CTG CTT TGG ATT CTG TCA AAA
AGC CCC ATA TTA TCA CCT TAT AAT GTT ACA TCG ATA TCT AAG TGT TCT GTT AGC TCT
TTG TAC CTA TTT CTT ATT GTA ACT TCA GTA ACT CCT GCA ACA TCT GCA ACT TCT CTC
TGA GTT CTT CTA GTT CCT TGT AAA ACA CTT GCA ATA TAA ATT GCA GCA GCA GCA ACT
CCA GTA GGT CCC CTA CCA CTC GTA AGA CCG CGT TCT CCT GCT TTT TGC AAG ATT GAT
ATT GCT TTT GAC TCT ACT TCT CCA GGG AGT TTT AAT TCT GAA GCG AAT CTT GGA ACA
TAA TCC ACA GGA TTT GTT GGA GCA AGC CTG ATG TTT AAT TCT CTT GAA ATA AAT CTA
TAT GTT CTT CCA ATT TCT TTT CTA TCT ACT CTT GAA ACT TCT GCA ATT TCA TCA AGA
GTT CTT GGA ACT TTA CAT CGT CTG CAT GCA GCG TAG AGT GCA GCA GCA GCA ACA CCC
TCA ATA CTT CTT CCT CTA ATT AAT CCT TTT TCT ACA GCA CCC CTG TAA AGA ACT GCA
GCG TTT TCC CTG ACG TTT CTT GGA AGA CCC AAT TTT GAA GCT ATC CTG TCA AGT TCG
GAA AGT GCA AAG GCT AAG TTT CTT TCA GAT GCA TCA GAA ACC CTG ATT CTT CTC TGC
CAT TTT CTT AAT CTA TAA AGT TGA GCT CTT TTA TCT GCA GAA ATA TCT TTT CCG TAG
CTG TCT TTA TTT CTC CAG TCG ATA ACT GTT GAT AAA CCT TTA TCG TGG ATT GTA TAA
GTC ATT GGA GCT CCA ACC CTA CTT CTT TTT ACA CGC TGT TCG TGA TCA AAG GCT CTC
CAT TCA GGA CCC ACG TCA AAT AAA TTC TGC TGT AAA ACA CAT CCA CAC ATT TCA CAA
ACG ATT TCA GCC CTT TCA TAA TCC TTA ATA ATA CTT TTA CTA CCG CAG ACA GGA CAG
ATT AAT TCT TCT TCC TTT TCT AAA ATA ACA TTC TTA TTT GAG TAG TCT TCA GGC TTT
GCG ATA GCA ATT TTA ATC TTT CTT TCA GGC TTT TTA GTA TCT TCT TTT GTA ACA GAT
TCG ACT TTC ATG ATA ACA CCG TCT TAA AGT TAT CTT CTT CCT TTT TTA CGG GGT TTT
GAT TGT TTT TTT GAT TGT TCA TTC ATA ACG AAA ACA TTA TCC GAT TCC AAG GTT TTT
TCA GCG TCG TCT TCA CTA TAA GGA ATT ATT TTT ACA TAC GGT TCT TCA ACA GGT CCA
AAT ACA TCG TAG ATT TTT CCA ATC TTT TTA ACT CTA TCT TTA AGT TTT ATT CCA ACA
GGC GAA TTA AAA CGA GGC TGT TTT TTA ACA CGC CCA ATT ATT TTT CCT TTT GGA GTT
CTA TGC AGT ATT TTT ATC TTT TCC AAC GAG ATT CCC CCA TAT CGA ATG ATT ATG GTA
TAT CGA GAG TTA TGA ATA TTT AAC AAT AAA ATC GAT TCA TAT ATA GTT TTC GCA CGA
TAG TAT TTA TAT ATT TCG AAA AGT ATA TAT ATG ATT TAC TAA ATA GAA TCG CAA TTC
TAA GAA CAA AAC GGA ACT AAA AAC AGT GTA TGT AAT TAA ATC ATC GAT AGT TTT ATA
TAC TGT GTG CAA AAA CCT TAG CGT TTT AGT AAT GGG AAT CAT AAA CCA CCC AAA AGT
ATT TAA GAA ATA AAA TTA ATT TAG TTG GGA ATA AAA TCT GTT TTT TAC ATC TTA ATT
ACT GCC TAA ATT AAA AAA ACA GGT AGA ATT TAA ATT TAT TAA AAA TCT AAA AAT TAA
GCT TAA ATT TAA ATT ACT ATG GCC ATT TTT TTT ATT CTT TCA TCT GGA AAA GCT TTT
TAT CAT TTA CCA GCC CTT GTT TTT ATA ACT GTA ATT TTT CGG ACT TTA GAT AAT AAT
ATA TAG GTG GTT ATT CGT GAA TAA TAA TAG CAT TGA ATA TAG AAA GTT GGA ACA CCT
TAT TGT GTG CGA CCA CTG TGA CGT GGA GTA TAA AAA AGG GAC CCT TCT TGA GGA CGT
TGA ATT AAT TCA CAG CGG TAT ATC AAA CTG CGA TTT AGA CGA TAT CGA TAC ATC TAT
CGA AAT TTT TGG AAA AAA ATT AAA TGC TCC GTT AAT TGT AGC AGC AAT AAC TGG TGG
ACA CCC TAA AGC AAA GGA AGT CAA TAA AAA TAT TGC TAT TGC TGT TGA GGA ACT GAA
TTT AGG TAT GGG TGT AGG TTC ACA AAG GGC GGC TAT ATC AAA AAG CTA CCT TGA AGA
TAC CTA TTC TGT TGT AAG GGA TCA CAC TTC CTC ATT AAT TAT CGG AAA CCT TGG AGC
AGT TAA CTT TGT GGA AGA TTC ATG GGA TGA AGA AAT TAT TTC AAA GTC TGT AGA AAT
GAT TGA TGC AGA TGC CAT GGC GAT TCA TTT CAA TCC ACT ACA GGA AGC TAT ACA GCC

TGA AGG CGA TGT AAA CTT CAA AGG ACT TAA TAT TTT AAA AGA AAT AAT TTC AAA TTA
CAA TAA AAT TCA CGG AAA AAT TCC ATT TAT CGC AAA GCA GGT TGG CGA AGG ATT TTC
AAA AAA AGA TGC CAT TTT TTT AAA AGA AAT TGG CTT TGA TGC GAT AGA TGT CGG CGG
AAG CGG CGG AAC TTC ATG GGC TGC TGT AGA GCT TTA CAG GAT AAA AGA TGA AGA GCA
GAA GAA TTT TTC AAA CCA GTA TTT TAA CTG GGG AAT TCC AAC TGC CGC ATC AAT TCT
GGA AGT AAA TTC AGC CTT TTC AGG ACC GAT AAT TGC AAC AGG CGG AAT CAG GAC TGG
AAT CGA TAT TGC AAA ATC AAT TTC AAT TGG TGC AAA CTG CTG TGG AAC TGC TTT ACC
AAT TTT AAA AGC AGC ATT AAA GTC TTC AGA AGC TGT TAC AAC TGT TCT AGA GAG AAT
GAT TAA AGA GTT AAA AAC TAC AAT GTT TTT GAC AGG CTG TAA TAA TAT AAA CGA ACT
AAA ATC TGC CAG ATA TAT TTT AAA AGG CGA TTT AAA GAA TTG GAA GGA TCA GAT TTA
ATA ATT TAA ATA TTA TCC ATC GGA ATT TTT TCA ATT TAT TTA TTC ATG TAT TCA AAG
CAA AGG ATG TGA TAA TTT GCA GTT AGA AGT AGT CGC AAT AGG TGG ATA CGA AGA AGT
CGG AAG GAA CAT GAC TGC CGT AAA TAT CGA CGG CGA GAT CAT AAT TTT CGA TAT GGG
TGT AAG ACT CGA CAG GAT AAT GAT TCA CGA GGA TAC GGA CAT CTC AAA AAT GCA CAG
TTT AAA TTT AAT TGA GAT GGG CGT AAT TCC AGA TGA TAC GGT AAT GAA AAA TAT CGA
AGG AGA GGT TAA GGC GAT TGT TTT AAC GCA CGG GCA CCT CGA CCA CAT CGG AGC AAT
TAC AAA ACT TGC ACA CAG GTA CAA CGC TCC AAT TAT CGG AAC TCC ATA CAC TTT AGA
ACT CGT AAA AAG AGA AAT TTT AAG CGA AAA GAA ATT TGA TGT TAG AAA TCC ATT AAT
TAC TTT AGA AAA CGG AAA TAA ATT AGA TTT AAC AGC AAA TAT TAC TTT AGA ATT TGT
AAA AAT TAC ACA CAG TAT TCC TGA TGC TGC AAT GGC AGT ACT TCA CAC ACT TTA TGG
TGC AGT AGT TTA TGG AAA CGA CTT TAA ATT TGA TAA TTT CCC TGT AGT TGG CGA AAA
ACC AGA TTA CAA AGC GTT AAA AAG AAT TGG AAA ACA GGG CGT AAT TGC AAT GGT TTC
CGA AAG TAC AAG AGT TGA TTT TGA AGG AAA AAC AGC ATC AGA AGG AGT TGC TGC AAA
CCT TTT GAA AAA CGA CCT TTT AGG AAC TGA CAA TGA AGA CAA TGC AGT AGT AGT AAC
TAC ATT TTC ATC ACA CAT TGC AAG AAT TAA ATC CAT TAC TGA TAT TGC TTC AAA AAT
GGG TAG AAT TCC AGT ACT TGT TGG AAG ATC CAT GTC AAA ATA CTG TGG AAT TGC ACA
AGA TAT CGG TTT AGT AAA ATT CCC AAA AGA AAC AAA AAT TTG CTG GGA CCC TTC ATC
AAT CGA TAA AAC ATT CCA CCA GAT AAT GAA AGA CGG TAA AGA AAA CTA CCT TAT GGT
TGT TAC AGG ACA CCA AGG AGA AGA AGG TGC GGT TTT ATC CAG AAT GGC AAC AAA TAA
AAC TCC ATT TAG GTT TGA AAA ATA CGA TCA GGT TGT ATT TTC AGC AGA TGT TAT TCC
AAA CCC AAT GAA TGC GGC ACA AAG ATA TTT ATT GGA AGC AAG ATT AAA TTT AGC AGG
AGT TAG GCT ATT TAA AGG AGC TCA CGT ATC TGG ACA CGC GGC AAA AGA AGA TCA CAG
GGA CAT ATT AAG ATG GTT ACA ACC AGA ACA CTT GAT ACC TGC TCA CGG TGA TTT TAA
TTT AAC TTC AGC ATA TGC TAA ATT GGC AGA AGA AGA AGG ATT CAG ACT CGG TGA AGA
TGT ACA CTT ACT CAG AAA CGG TCA AAG CTT GAA ATT TGA AAG AGT AAT TTA ATT GAG
GTG CTA AAA TGG TGT TTG ATA GGG AAA TAT TGT CAA AAA TCG ATT CTG AAC TCA AAA
AAT ACA TGG AAA AAG ATA CTA AAC TTT ACG GTG CAT CAA AAC ACC TAT TAC TTG CAG
GCG GTA AAA GAG TAA GAC CTT ATC TTT CAA TTT TAA CAT ATC TCT TAA AGA AAG ATG
ACC TTA CTG AAA TAA TGG CAC CAG CAC TTT CTG TTG AAT TGA TTC ATA ATT ATA CAT
TAG TTC ATG ACG ATA TAA TGG ATA ATG ATG ACC AGA GAC GGG GAA TCC CTA CAG TTC
ACA CGG TTT ATG GTG AAC CAA TCG CAA TTT TGG CTG GAG ATT TGT TAT ATG CAA AGG
CTT TCG AAG CTC TTT CAA ATA TTG AAG ATA GTA ACA AGG CTC ACG AAG TTT TAA AGG
TTT TAT CTA AGG CAT GTG TCG AAG TCT GCG AAG GAC AGA CTG AAG ATA TGG AAT TTG

AAG AAA GAT TCC CGA CTT TAG ATG AAT ACC TTG AAA TGA TTT CCA AAA AAA CAG GTG
CTT TAA TTG TAG CTC CTG TAG AAA TTG GAG CAG TTA TGG CAG ACT GCA CTC CTG AAG
AAA GAG AAG CAC TCT GTA ATT ATG CAA AAA GAA TTG GAA TGA CTT TCC AGA TTC AAG
ACG ATG TTT TAG ATT TAA TTG GGG ATC AAA AAA CAA TCG GAA AGC CTG TGG GTA GTG
ACA TTG TTG AAG GTA AAA AGA CAA TGA TGG TAA TTC ACG CAA TGG AAA ACC TTT CTG
AAG ATA AAA AAG AAA GAT TAT TGC AGA TTT TAG GAA ATC AAG ATG CAA ATC AAC AAG
AAG TTG CAG AAG CAA TTG AAC TTT TAG CAG ATT CTA TTA ATT ATG CAA AAG ATA CAA
TAA AAA ACG CAA CAG AAG AAG CTA AAG ATT ACT TAA AAA TAT TCG ATG CAG AAA AAA
GAA AAA ATC TCG AAA ACA TTG CAG ATT TTA TCA TTG AAA GAA TTC ACT AAT TAC ATT
TAA TTA GCT ATT TTA ACT TTT TTT AAA ATT TAA AGG TGT TTT AAT GAA AAT TCC TAA
AAT TTA CGT TGA AGA AGA ATT AAA CGA CGG CGA TCG TGT CGC AAT CGA AAA AGA TGG
AAA TGC AAT AAT TTT TTT AGA AAA AGA TGA AGA ATA TTC TGG AAA CGG CAA GCT TCT
TTA TCA GGT AAT ATA TGA TGA TTT AGC AAA ATA CAT GAG TTT AGA CAC TTT AAA AAA
AGA TGT TTT AAT ACA GTA TCC TGA TAA ACA TAC ATT TAC ATA TCT TAA AGC AGG AAC
TAA ACT TAT TTC GGT ACC TGC TGA GGG CTA CAA AGT CTA TCC AAT AAT GGA TTT TGG
TTT CAG GGT TTT GAA AGG ATA CAG GCT TGC AAC ACT TGA AAG TAA AAA GGG GGA TTT
AAG GTA CGT TAA TTC TCC AGT AAG TGG AAC GGT CAT ATT TAT GAA CGA AAT TCC GTC
AGA AAG AGC AAA TTA TGT ATT TTA CAT GCT CGA AGA ATA AAT TTT CAT TCT AAA TTT
TTG GAG TAC TGG TAT CCC CAC GAA CCA GTA ATC TGT CCT TTT TTT GCC AGT TTT CCT
AAA ATA TCT AAG GTT TCT TTT TCA AGT AAT TCT GGG GTG CTG TTT TCA AAG TAA GTT
CCA CAA AGG GGC ATA AAG TAG TGT GCA TGG ACT TTT CCA TTC TTT TTT ATA ATG TAT
TTC ATC AGA TCA ATA CTT TCT TTT CTT TGA TCT TTT GTT TCG TCT GGA AAA CCA AAT
ATA AAA TCA ACT TTT GGA ATT AAA TCA CAT TCT TTA GAC GTT TCA ACA GCA TTT ATA
ACA TCT GAA ACC GTG TGA CCG CGT CTT ATA TGT TTT AAA ACT TCA TCG CTT CCG CTC
TGC GCG CCA AAA TGA ATG TAT CTA TTG TCG CAA TAT TTA TTT ACT AAA TCG AGA GTT
TCA AAG TTT ACA AAT TCC GGC CTT ACT TCT GAT GGA AAA GTT CCA AAA AAG AGC CTT
TTT TTG ATT TCA AAT AGA CTT TTC ATC AGT TTT TCA AGT TTT TCA ATA TTG GGT TTT
GTG CCT GTT TTT GAG CCA TAT GAA AAC GCG TTT GGG GTT ACA AAC CGG ATA TCG CCC
ATA GTT TTT ACA ATT TTT ACG ATA CTT TCA ATA CTT CTA TGC CGT ATA TTT TTT CCA
AAT ATT TGT GGA GTT TGG CAG AAT CTA CAG TTA TAC GGG CAT CCT CTT GTA ATT TCT
ATT GGT GCA AGA GGC CAG ATT TTT TCA TAA TCG TCA AAA TTT TCA ATA GGC TTT CCT
TTA ATT ATT TTT TCA GGG GTT TTT CCA GAT TTT ATG GAA TTT ATA AGA TTT GGC AGT
GAA ATT TCT CCT TCG CCA ACG ATA ACA TGA TCA AAG CCT AGC TTT AAT GTA TCT TCT
GGA CAT CCT GAT GGA TGA GCT CCT CCC GCA ATT AAT ATA ACA TCT TTT TTT AGT TTT
TTT AAT TCG TTT ATT TCG CTT AAA ACT TCT TTT TCC TGC ATA CTC ATA AAT GAA TAT
ATT AAA ATA TCT AAA GAA TCC AGA TTT TCA ATT ATT TCG CCA AAT TCT TTG AAT AGT
GTC CCG CCT ATC AGG GGG TAT AAT TTT GAG ACA CTG TAC TTG TTT TTT GAA GTC ATT
CTA AAT CCA ATC ATA ACC TTA AAT AAT TAT GAT TGG TAT ATA AAA TTG ACA GTA GAA
TTG GAG GTG CTT TTT TGA AAA AAA GAC CAA CTC TAT ATA TCA AGA TAC ACT ACC CTG
GAA GTA TCT TGG AAG GAG AAT ACG ACT ACG AAG CGG TAG TAA AAC TTA AAA ATA AAT
ATA TTA AAT ATT TAG AAA CTG GAA AAG GAA CAG GCA CAC TGT TAT TCA ACG AAG ACA
AGA AAA ACT TTG CAG TGG TTA ATT TTC AAC AAG TTT GTG CAA TAG AAG TCG ATA AAA
CCC TAA TGT TCA TGG ATG AAT TTG AGC GAG AAT GGG ATA AAG TTT ATA AAA CAA GGT

AAT TTT AAT TTT TAA TAA TGA TTG GAT GTA TTG GTG ATA TAA TGG CGA AGA TTG CAA AAA ATG CGG TTG TTA TTG GCG ATG TTG AAC TCG GTG AAA ACG TAA ATA TCT GGT ATG GGG CAG TAT TAA GGG CAG ATA TTA ATA AAA TAA AAA TAG ATG ATG ATT CAA ACG TGC AGG ATA ACT GTA CTG TGC ACT GTT CAA AAG ATT ATC CTG TTT TTA TTG GAA AAG GCG TTT CAG TAG GGC ATG GTG CAG TAA TTC ACG GGT GTA CCA TTG AAG ACA ACG TAC TCG TTG GAA TGA ATT CAA CTG TAC TAA ACG GCG CAA AAA TTG GTA AAA ACT CAA TAA TTG GCG CAA ATG CAC TTG TAT CAC AAA ATA AAG AAA TTC CAC CAA ATA GCA TGG TTT TAG GAG TTC CTG GAA AAG TTG TAA GAA CTT TAA CGG ATG AAG AAA TAA ATT CTA TAA AAG AGA ATG CAA AAA ACT ATC TTG AAC TAT CAA AAA ACT TAT AAT GTT TAA AAA TCA ATG AGA CTA TTA TTG TAA ATC AAT AGG GTG AAA AAA TGG TCA ACT TAG GAT TTG TTA TCG CAG AAT TCA ACA GGG ATT TAA CAT TCA TGA TGG AAA AAA TGG CAG AAG AAC ATG CTG AAT TTT TAG GTG CAA ATG TTT CCC ACA AAA TAA TGG TTC CAG GAT CAT TTG ACA TGC CTC TTG CAA TCA AAA CAC TTC TTC AAA AAG ACG ACA TCG ATG CAG TTG TTA CAA TTG GAT GCG TTA TCG AAG GAG ATA CTG AAC ACG ATG AAA TTG TAG TTC AAA ACG CGG CAA GAA AAA TTG CAG ACT TAT CTT TAG ACT TTG GAA AAC CAG TTG CAC TTG GAA TTG CAG GCC CTG GAA TGA CAA GAA TGC AGG CTG AAG ATA GAA TTG ATT ACG GTA AAA ACG CAG TAG AAG CTG CTG TTA AAA TGG TAA AAA GAC TTA AAG AAA TTC AAT AAT TTA AAA TTA AAT TTA TAA ATC TTT TTT AGT GAT TTT ATG AAT GTT TTA AAA GAA GTA TAT GCT ACG ATC GAA AAA AGA ATC GAA GAA AAA CCC GAA GGC TCT TAC GTT GCT AAA TTA ACA ACG GAT GAC AAA AAA ACT GCA GTA AAT AAA ATC TGC GAA AAA GTC GGT GAA GAA GCT GCA GAA GTT ATT ATT GCA GCA AAA GAC AAC GAT AAA GCA GAA ATA ATT TAC GAA AGC GCG GAT TTA ATT TTT CAT ACA ATG GTC CTT CTT GCA AAA TCA GGA ATT ACC TAT GAA GAA TTA AGT GAA GAA TTC AAG AAA AGA ATG AAA TAA CTT ATT TTA AAT ACT TTT TTA GAA TTT TGG AAA GAT TGT ACC TTC AAT TAG ATT TAA AAT ATC TGT TCT TGT AAG GAT TCC AAT TGC GAT TTC TTC ATT ATC CAG TAC AAT TAA CCG TCC TAC ATT ATG TTT TTC CAT TAA AAT TAA TGC ATC ATA TAT TTT CTC GTT TTT TGA GAT TGT GTG TAT CGT TTC AGC CAT TAA TTT TGT AAC ATT TTC ATT TTC AAG GCC TCT TGA AAC TGC ATC TGC GAC ATC ATG TAG GCT CAA TAC TCC AAG AAG TTT TTT TCC ATC CAT TAC AGG AAT TCC ACT GAT ATT TGC ATC TGA AAG TAG TTT TGC GGC ATC TCG AAT CGT TTT TTC AGG TGT TAT GTA TAT CAA TTC TTC TTT TAT TCC AAC ATC CCC AAC AGT GAT TCC CGG AAC GCT TGC AAC ACT TAT TAC GTC CAT TAA AAG GAT ATG GTT TAT ATC ATC TCT TCC AAC GAC CCT ACC CAA AAT AAC GAT TTT GTT GTG GTG CGT TGG CCC AAC TTT TAC AAC ATC CCC TTC TGA AAA TTT CCT GGT GTC CCC ATT AAC GTA AAT TTT TGA AGA ACA TGA TTT TTC TCT TGT AAC TGT GTC AAA TAC GAT TTT TAT GAC ACT AAC TCC TTC AAC GAG GTC GTC TCT TTT GTA AAT TGG AAC TGT AAT TTC TTC TTC TGT TTC AAG CCC TAA TGA ACG GTA GGT TAA ACT CGT TGG AAT GTA TCC TCC TTT TGG TCC GGG AAC TCC ATC AAC AAG ATT TAG TGC TCT TAA AGC CTG CAT CTG GTT TCT AAT CGT TCC AGG GTT TCT GCT TAA TTT GAC TGC AAT TTC TGT TCC CTT TAC AGC TTT ATT TTT TTC CCT ATA AAT TGA AAT TAA TTC TTG AAG TAT TTC TTT CTG AAC TAT TGT AAG TTC CAT AGT TAC ACC GTT AAA AAT GAT TAT AAA TAG TGA TTA TAA ATA GTG ACA TAT ATA TTA TGG TTT TTA ATT GGT ATA ATA AAT TTA TGA TTT AAT TTA AGT AGA AAA TCT GCA AAA AAG AGC TTA AAT ATA TTT AAA AAT CTA AAG ATA CTG TTT TAC AAG GAA TGT TCT TTT CTA AGT ATT TTA AAA ATG ATT TTA AGC CGT AAA CTT CGG TTC CGT AGT GAG TTC CAT CGA TAA CTG TTA TCC CTA GTT CTT

CTG CAA TGA TTT TTG AGT GGT GAG TTA AAT CTC CAG AAA TGT AAA CAT CTG CAA AAT
TTG ATA CAT ATT TAA TAG AAT CTT GCG AAA GCC CGT ATC CTG AAA GAA CTG CCA CTT
TTA ACT GTT TTT TAT CAT ATA TGC TTT TAG GAA CGA CGG TTT GTG GAA CTG CGA ATA
TAT TTT CTT TCG TAA TTT TTA AAA TAT CTT CAA AAG TTC CAT CAA ATG TTC CAA CTC
TTC CAA GAC CAT CTT CAT ATA AAT TTT TAA CAT CTT TTA AAT TAT ATA ATT CAG CCA
AAC AGT CAT TAA GTC CGT TTT TAC AAA TAT CTA GGT TTG TAT GCG CGC TGT ATA GCG
GAA TAT CTT TTT TAG TTA ATA TTT TGA GTT TTT TGT AAA TTG GTC CTG TAA AAT TTC
TTA TTG GAT CTT TTA AAA TCG GGT GGT GGG TAA ACA AAA AGT CCA CGT TTT CAG ATT
CTG CTT TTT TTA TAA CTT CTA AGG ACG GGT CAA GTG CGA TTC CTA ATG TTT CAA CTT
CCT TTT CAA GGT TTA ATC CAA CCT GAA GGC CGA TAT TGT CCC GAG GTA CGG CAA GTT
CTT TTG GAG CAA ATT TTT CAA TTA TTT TTA TGA TTT CAA AAG CTT TCA TTT TTT CCA
CCC TGT AAT CAC CTG TAA TTC TTC AAA TGA AAA TTT TAA AGA CCC AGT TTG TTT CGC
AAC AAA TCC TAA TTT TTC CAT TTT TTC AAT GGT TTT ATT TCC ATC GGT AAG TGA TGA
TTG AAG AAT CTG AAT AAT TCC ATC TTT TTT TAA ATA GTT GGC TAC TTC ATC TAA AAA
CTT ATC CAA AAC TTC TCT TCC GTC TTT TCC GCC ATC AAA AGC GTA ATT TAA ATA TTT
TTC TAA TTT TTC CTC GTC AGA AGT TGG AAG GTA TGG TGC ATT GAA TAA AAT TAC GTC
AAA TTT TTC ATC AAT ATT TTT GAA GAG GTC GCC TGT TTT AAA GGA AAG TTT TTT GGA
ATC AAT TTC GTT TAA TTC TGA ATT GAT TTT TGC ACA CTC TAC TGC ATA GGG GTT GAT
ATC AAT TCC AAT AAC TTT TAA AGC ACC CTG TTT TAC GGC ATT TAT TGC CTG AAT TCC
GCT TCC AGT TCC AAC ATC TAA AAC TGA CTT ATT TTT TAC ATC GAC TAA ATT TTC AAT
TAA AAG TTC GCT GTC TTC CGC AGG AAC GTA TAC TTT AGG GTG TGT TTT TAT TTT TAT
CCC GTT TAT TTC AAT TAT TTC TAT CAT AAT AAT CTA TTT TTT ACA TTT TCC AGA TTT
CAT CGC CGA TAA AAT GAA TGG TTT TGA TTG CTT TTC CTT TTT TTT CTT CAA CCA TTG
TTT TTC CAT CCA TTA GTG CCT CTC CAA CAG ATA ATG GCT TTT TGT GAG TTA CGT CAA
CTA CAA AAA CAA TGT CCC CTT CTT TTA TAT CTT CGT CAG CTC CGA CGA TTC CGG GAG
CCA TAA TGT CTG CAC CGT TTG CGA TGA ATT TTA CGG CAC CCA TGT CTA CAA TAA CCC
TGT TGT TTT CAA TAT CCA TTT CTA AAA GGA ATT TGA GTG TCG GAA ATA TTC TCC CGC
TGT TTA CCA TTG CAA GAG GAG TTC CGT CTA AAA GGA TTA TTT CGC CTT CTT CGG TTA
CAG CTT TTT CAA CAA CTG CTT TTT TTG GAA TTA TTT TTT CAA CGT CAA AAA TAT TTC
CAA GTT CTT CTT TTA ATT CCT TTA ATT CTT TCT TTT TTA ACA TGT ATC TTC TTT TTA
TTT CCA TAT TTT CAC AGT TAA ATA TTT TTT AAG ATT TAA AAA CAG TTT AAA ATA GTG
TTT TAA AGA ATA AGT AAG TTT AGG TTA AAA AAC GTA AAT TGG GGA AAT CTA TTT TAT
TTA TTC TAT AAT TTC GTA ACA TGT GTT TCT CTG TTT TGG AAT TCT TCC AAC TGC TAA
AAC CAT GTC TTT TAA TTT TTC TTC GCT CAT GTA CGT TCC GTA GCT TCC GCC AGC AGC
TTT TGA AAT GCT TTC TTC CAT TAA AGT TCC ACC GAT ATC GTT TGC ACC ACA ATT TAA
TGA AAT CTG GCT TAA TTT TGT TCC AAG TTT TAC CCA CGA GGT TTG TAT GTT TTT GAT
ATA ATC TTT AAA GAA AAT TCT TGA AAT TGC GTA AAC TTT TAA ATC AAG CAT TCC AGA
AGC TCC GCT TTT TAC TCG TTC CAT GTG ATA AAG GGG TGC GTT TTC ATG TAA AAA GGT
TAA TGG CAC AAA TTC GGT AAA ACC GCC AGT TTC TTT TTG AAT TTT TCT TAA AAT TGA
TAA ATG TTC TGC AAG ATG CTT ATT TTC TTC GAT ATG GCC GTA CAT CAT GGT GCA AGT
TGT ATT AAT TCC AGT TTT ATG TGC AGT TGA TAC AAC ATC AAT CCA TTC AGA AGT TTT
TAA TTT TGT AGG GCA GAT TTC AGA CCT TAC TTC GTC ATC CAG TAT TTC AGC AGC AGT
TCC AGG CAT GCT GTT TAA TCC ATT TTC TTT TAA AAT TTT TAA AGC TTC TTT TAC ACT

TAA TCC TGC ATT TTC TGC AGC GGA TTT AAC TTC CAT TGG GGA AAA TGC ATG TAT ATC
AAT TCC CCC ATA GGT TTT GGT GAT TTC TTT GAC TTT TTT TAG GAT TTC AGC TTG AAA
ATA GGT ATC AAT TTC TTT TAA AAG GCC GCC TTG AAT ACA AAC TTC AGT AGC ACC CGT
TTT TCT TGC TTC AAG TGC TTT TTT AGC AAC TTG TTC AGG ACT TAA AAA AAA CGC GTT
TGG ATC GCC GTG TTC TGC TTT AAA TGC ACA GAA TTT ACA GGT TCC GCT ACA GAC ATT
TGT AAA GTT AAT ATT TGC ATT AAC AAC ATA AGT AAC CGT ATC CCC TGC AAT ATC CTT
TCT AAG AGA ATC TGC AAG TTT CAA TAT ATC AAA AAA GTT TTC AGT ATC TTC AAA TAA
TTC AAC GCA GTC TTT TTT TGA GAT TTC TTT TTC TTT AAA AGA CAT CAA ATC CAT CAA
ATC ACC AAT CGA TCT ATA AAT ATC TTC AAG ACT ATT TTT TAA TAT TTA AAT ATT TCG
ATG ATA TTA ACT GCT AAT TCG GGA CTA AAT CAT TAA AAA ATT AAA AAT TAG GAT TTA
TTC AAT TAT TTC ATA AAG TGT AGT TCT TTC TTT TGG AAT TCT TCC AAT GCT TAT AAT
TGC ATT TCT CAT TAA ATC TAC GCT TGC ATC TTC GTA GAT ACT TCC TGC AGC TTT TGA
AAC TTT ATC TTC TAC TAA AGT TCC ACC AAG ATC GTT TGC ACC GGA TTT TAA AAT TAA
CTG ACT CAA TTT CGT TCC AAT TTT TAC TCT TGG TGC CTG AAT ATT TTT GAT AGA TTC
TTT AAA TAA AAT CCT GGC TAT TGC ATA GAG TTT TAA CTC GTA AAG TCC AGA TGC ACC
GTC TGT AAC TCT TCC GGA TTT GTA AAG GGG TGT ATT TTC ATG TAA AAA TGA CAT TGG
AAT AAA TTC TGT AAT ACC GCC GGT TTC TTC CTG GAT TTC TTT TAA GAT TGA TAA ATG
ATC TAC AAT ATG TTT GTA TTC TTC AAC GTG ACC GTA GAT TAT TGT AGA AGT TGT TTT
AAT TCC GAT TTT GTG TGC AGT TTT CAT GAT ATC TAT CCA TTT TTT AGT AGA CAT TTT
CAA AGG ACA GAT GTC TGA TCT TAC TTC ATC ATC GAG TAT TTC AGC AGC AGT TCC GGG
AAT TGT TCT TAA ACC TGC TTC ATT TAA CAT TTT TAA GGC TTC TTT TAT ACT AAG TCC
TGC ATT TTC GGC GCC ATT TAA TAT TTC CTG TGG GGA GTA TGC GTG GGT ATA TAT TCC
GCC GAG TTT CGA AGT TGC GCT GTT TAC TTT ATT TAT TGT TTC AAC TTG GAA GTG TGT
ATC AAC ATC AGG ATG AAT TCC ACC GTG AAT TGT AAC TTC TGT AAG TCC TGA TTT TTT
AGC AAT AAC TGC TTT TTT AGC AAT CTC ATC CGA ATC CAA TTT GAA AGC TTC GGG GCT
GTT TTT TGA AAC GTT GAA TGC ACA GAA TTT ACA TCC CAT GGT ACA GAC ATT TGT GGT
TTC GAT ATT TGT GTT TTT TAC AAA AGT AAC CGT ATC CCC GAC AAT ATC TTT TCT AAT
GGA ATC TGC AAG TTT TAA GAT ATC AAA AAA GTT TTC TGT ATC TTC AAA TAA TTC AAC
GCA GTC TTT TTT TGA GAT TTC TCT TTC TTT AAA GGA CAT TAA ATC CAT CAA ATC ACC
GGA TAA TTT CTA AGT TCC ACT TGG GCT TTT TCG TAT ATA AAA TTG AGT ATA GCT AAT
TAA TAC GTT AAT TGG GGT AAT ACG TTT ATT AAT CGG TCA AAA TTT CGC TGT AGC TCC
TTT AAT ATA TAT AAT TTT CTA TTT ATT GAG AAT AGA AAG TAC ATT ATA TTG CTG ATT
AGT AAT TAT ACT TGT TAT ATT AAA ACT CTA GAA ATA GGA AGG TGG CAG AAT GAA ATT
TGG TAT CGA ATT TGT TCC AAA CGA ACC AAT AAC CAA ACT AGC ATA CTA CGT TAA ATT
AGC AGA AGA CAA CGG ATT TGA ATT CTG CTG GAT TAC AGA CCA CTA CAA CAA CAG AAA
CGT ATA CAT GAC CTT AGG AAA CCT TGC AGC AGC AAC AAA CAA GAT CAA GTT AGG TCC
TGG TGT TAC AAA CCC GTA CGT AAG AAG CCC AGC AAT CGC AGC ATC CGC AAT GGC TAC
CGT AGA TGA ATT ATC AGG CGG TAG AGC TAC ATT TGG TAT CGG CCC TGG TGA CAA AGC
AAC ATT TGA TGC ATT AGG TAT TGA ATG GAC AAA ACC AGT AGG AAC CAT CAA AAA AGC
AAT CGC AGA CTT CAA AGA ATT AAC CGC TGG AAA AAG AAT GGC AGA AGG TGC ACA ACT
TGC AAT TAA ACC TTC AGC AAA AAT CCC AAT TTA CAT GGG AGC ACA AGG CCC TAA AAT
GTT AGA AAC CGC AGG AGA AAT TGC TGA CGG TGT TTT AAT TAA CGC TTC AAA CCC AAA
AGA CTT CGA AGC AGC AGT TCC TTT AAT TAA AAA AGG AGC AGA AGC AGC AGG AAG AAG

CAT GTC AGA AAT TGA CGT TGC AGC ATA CGC ATG CAT GTC AGT AGA CAA AAA AGC AGA
CAA AGC TAA ACA AGC AGC AAT CCC AGT TGT AGC ATT CAT CGC TGC TGG TTC ACC TCC
TAT GAT CTT AGA AAG ACA TGG AAT CGA CCC TGC TAA AGT TGA TGC AAT TAG AGC AGG
ATT GAA AGA AGG AAA CTT CGG TGC AGC ATT TGG TAA TGT TGA CGA AAA CAT GTT AGA
AGG ATT TGC TTT ATA CGG TAC ACC TGA AGA AGT TGT AGA AAA AGT AAA AGC ATT AGA
AAA AAT GGG TGT AAC CCA AGT TGT TGC TGG ATC CCC AAT CGG ACC AAA CAA AGA CAC
CAG CAT CAA ATT AAT TGG AAA AAT CAT TAA AGA ATT CCA ATA ATT AAA TAA CTT TTT
TAA ATT TTT TAA ATT TCA AAA AAA AAG AGT TTT ATT TTA GTT TTA AAG GTT TAT TTG
GCC TTT GAT TTT TCA ATG TAT TTT TCA ATC ATT TTA GCT TCG ATT TTT GAA GTT ATT
CCT AGT CTT TCT AAA ACA ATT TCT GTA AGA AGT CTT TTT AAA ATT TCT TTT CCT TCT
TTT GTA TCA TCA ATT GTT CCC AAT TTT TTT GTA GTT CCT GGA GAC TTT AAT TTT GTT
AAT GCT CCT GAA ATA TCT TCT TCT TTA ACT CTT CCC TTT TTC TTT TCG TAT ATT TCG
TCT GCA ATT GCC CTG AGT TTT TCC ATG TCA AAC GGC AAA AAA TCT TTT ATT TTT ATA
GAG AGT GTT TCT TCG ATT GAT GCT AAA ACT CTA GCA ATT TCT TCG TTG GAT GTA TTT
TCA TCA ATT TTG ATT TTT TTA GAA ACG TAC CAT TTT CCT TTT TTA GCG TTA AAC TGT
ATC CGT TCT TCA GAC ATA AAA TCA CCT GAA TAA AAC AAG TTA ATA TAA TCG TAA AAT
TAT ATT CTG TTT AAA TTA TAT AAA ATA GTT TCA ATT GAA ATC AAA ACA ATT TAA AAA
GAG TAA AAT AAT TAG TTC ATT TCA AGA GTT TTT TTC AAA ATC GGG CTT TGA ATT TCT
TCT ACA GAA ATT TCT GAA ATT TCT ACA ACT TTG ATA TTT GCT CTT TTT ACA GCA TGT
TTG CTG CCA ATT TCT GAA TAC ATA GTT TCT AAA GCA TCA TTT TCT TTT ACA ACG TTG
TAT TCT TTT GTA AAT ACC ATT GGT TCA TCT TTT CCA ACG ATT TCT CCT TTT ATT CTT
ACG ATT TTT GCC ATA TTA TCA CCA AAA TAA TAT TAA TCT ATT AAA TCT AGA GCT TCT
TCA ATT TTT AAG ATT TCA GGA CCT GAC GTG TCG CCT CCG ACA ACA GCT CCT TTG GTG
TTT GCG AGA ATG CAC GCG CCA ACT GAA GTT ACG CCC CTA TTT GCA GTG CCT CTT TCA
ACA TAG TCC ACT CTT AAA ATG TCT TGA ATC CAT TCT AAT TCT AAT TCA TCA GTT AGT
GGG TGA ACC AGG CAT CCT TTA TCG GTT GCA ACT CCA TTT GAA CCA ACT GTT GGG AGT
TCA GCG TAG TTT CCT ACT CCG GAT TCT ACG TCT AGT ACA TCC TCA ATA GTT TTT CTA
AAT CTG GAT AAT TCT TCC GAA ATT ATG CAT CCT TTA TTA TTA GTT AAT ATT AAG TTA
CCA AAT GCC GTG TTT TTA GCT TTA AGT CTT TCT AAA TTA ACG TCC AGA CTG TTT TCT
TTT AAA AAC ATT TTA ATT AAT TCA AGT TCT TTT TCA GTC GTT ATG TTA GGT ACT AAA
AGT CCG TTG GAG TTT CCA ACA CAT AAG GAG CCT ATT AAA GAG CTG TTT GAA ATG TTT
AAT TGG GTT GCA GGA ATG TCG AGT ACG TTT TTC ATA CGT TCC ATC GTT TTT TGA TCA
ACT GAA AGA GGA AAA AAC CCA TAA TCT TCA GTT GCG AGT GAA TGT ACT CCT AGT GTG
GAC ACT CCA GAA AAA TAT GTT TTC ATT ATC ATA GTA CCA CAG TGT TTC TAA AGA AAG
AAG CAG GTA GAT TAT TCT ACT AAT GTT GCT TTA ACA ACA TCT CCT TCT TTT ACA ACT
TTA ACT CTA ACT TTA GCA GGG ATT TTG TTT AAG CTT CTT TCC CAT ACT TTT TCG TTT
ATT GTA TTG TCA AGT TTT ACA ATA TCT GAT TTC ATG TGT TTC TTC AAG AAC TCC CTA
ATT GCC CTT ATA GCT CTT GGA GCT CTT TTT GTT GTA GGG CTT TTG TTT GTT ACG TCT
CTC AAA GGG ATT GTA TAG ATT CTT TCT TCT TCC ATA TTA TGC ACC TCC GCA TGG ATT
AAA AAA TGT ATA AAT ATT AAA TCA TAT AGA ATA TGA AAA AAC AAT ATT TAA ATA TTT
TGG TGA GCG GGT AAC ATG GAA GAC TAC ACA AAA GCA GAA ATT TTA ATC GAA GCC CTG
CCT TAC ATC TGT AAA TTT CAC GAT CAG AAG ATT TTA ATT AAG TAC GGC GGC CAC GCT
ATG GTA AAT GAG CAG GCA AAG AAC TGG ATT GCA AAA GAC CTC GTG CTT TTG AAA TAT

GTT GGC ATA AAC CCG ATA GTA GTA CAC GGC GGC GGC CCT GAA ATC AAT CGT GCG ATG GAA AAA ATG GGA AAA ACC CCT GAA TTT ATT CAC GGG CTT CGT GTA ACT GAT GAA GAA ACA CTT GAT ATT GTT AAA ATG GTT CTT ATT GGT AAA ATA AAT GGT GAC ATT GTT TCA AAA TTA GAA CGA TAC GGC GGA AAA TCT GTC GGT CTT TCT GGA AAA TCA GGT CAA TTA ATT AAA GCA AAG AAA AAA ATC CAG TAT TTA ATG AAA GAC AGC CAA AAA ATC GAA GTA GAT CTC GGA ATG GTC GGG GAA GTC GAA CAC GTC GAT ACA AAA TTA ATA GAC ATA CTG GTT GAA AAA AGG TAC ATT CCG GTA ATT TCA CCA ATA GGT GTC GAT CAC CAG GGA AAT GAC CTG AAT TTA AAC GCA GAC ATT GCT GCA GGA GAT ATT GCA GGT GCA ATG GAT GCA GAA AAA CTT ATA ATG GTT ACC GAT GTC GAT GGA ATA ATG GAC GAC GTA AAT GAC CCA TCT ACC CTT CAC AGG AGG TTA ACT ATT CCT CAA ATT GAA GAT ATG ATC GAA AAA GGG CTT ATT ACT GGT GGT ATG ATT CCA AAA ATC GAA GCA TGT GTC AAT GCA CTT GAC AAA GGA GTT CAG AGT GTA CAC ATA GTA AAT GGA AAA ACT CCT CAC GCA GTT CTT CTT GAA ATT TTC ACG GAA GAT GGT GTG GGT ACC ATG GTT GTA AGA GAA TAA CTT TCT TTT TTT AAT TTT TCA AAC TCT TTA AAT TCT TCT TTT ATC TAC AAA ATA TAT CTA TTT ATT GGT CTA TTT GGA TTA AAA AAG ATT TAA ATT TTT AGA TTT AAA TTA TCT TTA TAT AGG AGA TAA GTC AAT GTA GTA TTA TCG GAA AAG GAA TTC CGG TTC CTT CCG AGA CGC ACC AAC CAA ATC ACT ATA TCG CAC CAT TAA CCT TAA AAT AGC CAA AAA CCA TTA AAA AAC CAA ATA ACC GAC ATT ACC TTA TTA AAA AAT AAG GTA ATA TAC CCC TAT TTA CAA AAT ATT AAA TGA CCT ATA AAA ATC GAA ATT TCA TAC GCG TGA ATC ATG ATT ACC ACT TTA TCA ATC TTA AAA AAA TAT CAA AGA CAC GAA GGG GTG TTT AAA TGA AAA AAA TTG AAG CTA TAA TCA GAA TAG AAA GAT TGG ATA TCG TAA AAG GCA TTC TTT CTG AGA ATG GCT TTT TAA GTT TGA CAA TTT CTG AAG TAA AAG GTA GAG GAG TTC AGG GTG GAA TAA CTG AAC GAT ACA GAG GAA CAG AAT ACG TAG TTG ACC TTC TTC CGA AAG TTA AAC TTG AAA TGG TCG TTG ATG ATA AGG ATA TTG ACG AAA TAA TTA CAA TAA TTC GTG AAA ATG CAG AAA CTG GAA AAC CCG GAG ACG GAA AAA TCT TTA TTA TTC CAG TAG AAG ACG CAG TAA GAA TTA GAA CAG GCG AAT CAG GCA TAA AAG CTA TTT AAA TTA AAA ATA AAA TCA GGT GAA AAT TAT GGC AAC GGC AGA TTT ATT TGC TGA CCC CAC AAA CAT CGT AAC AGG GCT CAG TAC ATT GGC AAC TTC TGG AGA TGT AAT GTT CTT AGT ATT GAT GGG AGT ACT TGT GTT TGC AAT GCA GTT AGG ATT TGC ACT TCT TGA AGG CGG TCA GGT CAG AAA GAA AAA TGT TAA CAA CGT AAT GAT GAA AAA CAT GGC AGA CTG GTT AAT TGG AGC AGT TTC ATG GCT TTT CAT AGG CGG TGT TCT TTC AGT TTC ACT TGT TCC TGC AGA CTT CTT CAG CTG GTG GGG ACA AAT ATT TAC AAC AAA CTT TGC AAA TAA TGG AAT AGA CTT AGC AAA CTG GTT CTT TGG ACT GGT ATT CGC AGC AAC TGC AGC TAC AAT CGT ATC AGG TGG TGT TGC AGA AAG GAT TAA ATT TAA ATC ATA CTT ATT AAT GTC AAT AAT CAT AAC TGC GAT ACT CTA CCC GCT GTT TGT GTA CCT TGG ACC TTG GGG TTC AGG AAT GAT TCC ATT CAA CGA TTA CGC TGG AAG TTT CAT TGT ACA CGG TCT AGG TGG TTT CTT AGC ACT TGG AGC AAT TGC AGC TCT TGG ACC TAG GAT TGG AAA ATT CGT TAA CAA AAA AGC AAT TTC AAT TCC TGG ACA CAA TAT CCC AAT GTC AGT ATT CGG GGC ATT TGT TCT CGC AAT CGG ATG GTA CGG ATT TAA CGT AGG AAG TTC ATT TGC AGT TGC AGA TAT TTC TGG ATT AGT ATG CGC AAC AAC AAC ACT TGC AAT GGC AGG TGG AGG TAT TGG GGC ACT CCT CGC TTC AAA AGA TGA TGT ATT ATT TAC AGC AAA CGG GCT TGT TGC AGG TTT AGT TGC AAT CTG TTC GGG AAC GGA TGT TGT AAG CCC AAT AGG GGC ATT AAT TAT CGG GCT TGT TGC AGG AAT TCA GGT ACC AAT CGT CTA CAA AAT CGT TGA AAA AAT GGG ATT GGA TGA CGT ATG TGG TGT AAT

TCC AGT ACA CGG TAC AGC AGG TAT TTT AGG AGG TAT CTT AAC AGG TGT CTT TGG AAT
GGC TGC TCT TGG TGG TGC TGG TGG AGT AAG TTT GGT AAA CCA GCT TAT TGC AGC AGG
ATT AAC AGT AGT ATA CGG TAC AGG ATT AGG ATT CGG TCT TGG AAA ATT AGT CGG TGC
ATT TAC TGG TGG AAT TAG AGT TAC TGA GGA CGA AGA AAA AAT GGG ACT CGA CCT TGC
AGA ACA CAA ATT GCC AGC TTA CCC TGA CGA ATA ATT AAT TTT TCC TTT TTT TCA TTG
AAA AAT AGA TAC TAT TAT ATA TTT TTC AGA ATA TAT TTT TTC AAA ATG TTG AAT ATA
TTT ACT ATT TTG GCG GTG TTA GTA TGA GAA AAA TAG AAG CGA TAT TCA GAC CTG AAA
GAC TTT TAG CAG TTA AAA ATG CTC TTT TTG AAG AAG GAT TTA TCA GTT TAA CGC TTA
CAG AAG TAA AAG GAA GAG GAG TTC AGG GCG GAA TTG TTG AAA GAT ACC GTG GAA ACG
AGT ATT TAG TAG ATT TAC TTC CAA AAG TTA AAA TTG AGA TTG TTT CTA ATG ACG GCG
ATG TTG AAA AAA TAG TTA AAC TTA TTT CCG AAA ACG CAG TTA CTG GAA AAC CTG GAG
ACG GAA AAA TCT TCG TAA TTC CTG TTG AAG ATG TAA TTA GAA TCA GAA CAA ATG AAA
CAG GAA AAG ACG CTA TTT AAA TTC TAA AAA TAT TTT TTT ATT CTT TTT TTC AAA TAT
TCA AAT TTA TCT ATT TTG ATA GGA ATA TTT ATA TCA GTT TTA TTT TTA AGT AAG AAT
AAG AAT TCC GGT TCC TAT ATC ACA CAT TTT TCA AGG TAT GGG TTT TCC TTA AAT TTC
GAT TTT AAT CCC AAA TTT CAT TTT AGT TGG TTT AGT TAA ATG GTG CAA TAA TGA AGA
AAC TAG AAG CAA TAA TAA GAA TGG AAA GAG TAG GAC TCG TTA AAA ATG CTC TTT ACA
AAA GCG GGC ATG CTA GTT TAA CGT TAA CAG AAG TAA AAG GAG AGG AGT TCA GGG CGG
AAT TGT TGA AAG ATA TCG CGG AAC AGA ACA TGT GGT AGA CCT ACT TCC AAA AGT CAA
AAT AGA GCT CGT AGT CAA TGA AAA AGA CGT AGA CTC TGT AAT AAA AAT AAT TGC AGA
AAA TGC ATA TAC CGG AAA ACC GGG AGA TGG AAA AAT CTT CGT AAT TCC GGT CGA AGA
CGT ATG TCG TGT AAG AAC TGG TGA AAT GGG TCC AGA TGC TAT ATA ATA TTA TTA AAT
TTT AAT GGT GCG TAA ATA TGG TGA CGG CAG ATT TAT TCA ATA ACC CTA CAA ATA TTA
TGG ATG CGT TAA ATA CGC TTG CAA ACT CAA GCG ATG TGA TGT TTT TAA TTT TTA CTG
GTG CTT TTA TTT TTA TAA TGC ACC TTG GAT TTG CAA TGC TTG AAG GCG GTC AGG TTC
GAG AAA AGA ACG TGA ACA ACG CAA TGA TGA AGA ACA TGG GCG ACT GGA TCA TTG GAT
GTA TTT CCT GGC TTT TAG TTG GTT CAG TAA TTG CTA GAA CAT TAA ACC CTG CAG ATT
TCT TTA TTT GGT GGG GAA AAA TTG CAA GTG CAA CAC CTT TCC TTA CAA ATA ATG GAT
TGG AAC TTG CAA ACT GGT TTT TAG GAC TTG TAT TTG CAG CAA CTG CAG CAA CTA TCG
TTG CAG GGG GAA TTG CAG AGA GAA TCA AGT TTA AAT CCT ATA TTT TAA TTT CAA TCG
TTA TAA CGG CTT TGT TGT ACC CAT TCT TTA GTT ATC TTG GTC CAT GGG GTG CAG GAG
TAA TTC CAT TCC ACG ACT ATG CGG GAA GTT TGA TGG TTC ACG GTG TTG GCG GAT TTT
TGG CAC TTG GTT TAA TTG CAG CAA TCG GTC CAA GAT TTG GCA GAT TTG TAA ATA AAA
AAG CAA TAA CAA TTC CTG GAC ACA ACA TCC CAA TGT CGA TTT TAG GTG CAT ACA TTC
TTG CAT TTG GAT GGT ATG GAT TTA ACG TTG GAA GTT CAC TTG CAT TGA GTG ATA TTT
CAG GAC TTG TAT GTG CAA CAA CAA CAC TTG CAA TGG CTG GGG GAG GTC TTG GAG GAT
GTT TAT TCT CTA AAA ACG ATG TGT TAT ATA CTG CAA ACG GAC TTT TGG CAG GAA CTG
TTG CAA TTT GTG CAG GAA CGG ATA TTG TAA GCC CGC TTG GAG CTT TAA TAA TTG GAT
TTA TTG CAG GAT CCC AGG TCC CAT TAA TAT TTA AAA TTG TTG AAA AAT TAG GAT TGG
ACG ATG TAT GCG GTG TAA TTC CAG TAC ACG CAA CAT CTG GGG CTC TCG GTG CAA TTT
TGG CAG GGA TAT TTG GAA TGA CTG CAT TTG GTG GTG TTG GTG GTG TCA GTC TTA CCG
AAC AGA TAA TCG CAA CAT TAA TTT GTG CAG TTT ATG GTA CCG GTG CAG GAT TTA TTC
TCG GAA AAC TTG TTG GGG CAG TTA CTG GCG GTT TAA GAG TTA AAG AAT CTG AAG AAA

AAG CAG GAC TTG ATA TTT CAA TCC ACA AAC TCC CGG CAT ACC CTA AAG AAA ACT AAA
TAT TAA CTT TTA CTT TTT TTA GAA ATT TAT TTT TAT ATT TTA AAA TCA AAT TAT TTT
TTA TGA AAA CAT TCA ATG AAC AGT GTT ACG ACC TTA TAA TGC AGA TTC CAA AAG GAA
AAG TCA CCA CGT ACA AAA TAA TAG CCG AGG CTC TAA ATA CTA AAG CTT ATC GGG CTG
TAG GAA ATG CGA TGA AAA ATA ATC CAA AAA TTT TAA CAG TGC CGT GCC ATA GGG TTG
TAA ATT CTA ACG GTT ACG TTG GCG GAT ATG TCA ACG GGG TTG AAA AAA AGA TAG AAA
TTT TAA AAA ACG AGG GGG TGG TTA TTT CTG GGG GTA AAA TAA CCG ATT TAA AGA AAT
ATT TAT TCG TGT TTT AGG TCT ATT ATC GTT GGA TCT TTA TTG GGT CAT TTA ACT GTC
GAC AAT AAT GAT TTT AAA TGT TTT CAG CAA ATA TTT TTT TGA GGG GTT TAA TAT TCA
TTG TGG GGT ATT GTC CAC ACA GTT TAC TAT ATT TTC ATA ATA TAT AAA GTT GCT TAA
TGG GGA ATT TAA TAA ATA TTA TTT TTT AAA ATC AAA AAA GCC TAA TTT TAA TAC TTT
TAA TTA AAG ATT TAT AAG TAT AAT TTT GTC CAG GGT GGT TTC ATG GTC TTT GGA ATA
TTT AAA AAG AAA AAA AAG GCA GTC GAG GAC TAT TTA AAC GAT AAA AAT GTA CTT GAA
ATT ACA TTA AAT GAT TCT AAG GAT TGC ATT CTT GAT TTT ACT TAC AAT TTT ATA TGG
CAG AGT AAT TTT GAC ATT AAA AGT CAA GTA TTC AAT GGA ATA TCT TAT GAA AAA TTA
GTA AAC CAT ATA AAA AAT CCT GAA AAT GTA GTA TAT ATT AAA GGA GAT GTG GGA CAC
AGG TTT TGC AGT TCA ATG GGT GCC GAT TTA AAG TAT TTT GGA GGA TCG GGC GGT AAA
ATT CAA ACC GGA ACT GTT ATT GTT GAT GGA AAC ATT GAC ACG AGA TTT GGG ATA AGT
ATG GTT TCT GGA ACT GTT TAT CTA AAT GAA AAA TTT AGC TTA AAA GAG CCA ATT GGA
AAT ATA ATT GAA GTT GAA AGT GAT ATT TCA GGA TAT AAA AAA TAT ATT TCA ATC ACT
GAA TTA ATG GAA TCT AAT TCA CAA GAA AAA GTT TTA AAA CCA AAC ATT TTT AAG AAC
AAC GAA TTA ATT TTA AAT GAT AAT GTA ATT CGG GAC ACT ATT GGT GCC AGA CTT GAA
AAT GGA TCA AAA ATT ACC GTA AAC GGA AAT GTA GAT TTG AGC ACG GGA ATT TTG ATG
AAG AAT GGA ACT GTT TTG GTT AAT GGC AAT GCT GGA AAA AAT ACG GGA TCA GTG TTA
AGG GGT GGA ACT GTA ATT ATA AAT GGA AAC ACA GGG GAT TTC ACT GCA TCT GAC ATG
ATA TCT GGA ACA ATA ATT ATC GAT GGA AAT GCA GGT AAA TTT TTA GGT TCG AAA AAG
AAA AAC GGG GTA ATT TAT GCG AAG GAT GGT TCG GCA ATT CCT CCT TTA AAA AAA CAG
TCG TTA AAT TCA AAA GAT AAA GAA CTA CTT TCT AAA AAT GGA TTT TTC GGG GAG TTT
GTA AAA TTT TAA AAT ATA TTT GAA TGT ATA AAT ATA TTT TCC CAA AAT AAA AAA CGA
GCT TCA GGC GGG GTT TGA ACC CGC GGC CTC CTG CTT ACC AAG CAG GCG CTC TAC CAG
GCT GAG CCA CTG AAG CAC ACT TAA ACC TAC AAT TTG CAT ATC AAC TAA CTC AAA AAC
AAT ATA TAA ACT TTT TGG TAT TGT TCA TAT TTT TTA TTA ATT TTT TTA TAA AAT TAA
ATC CAA TTT TTT GAA ATT ATT TAA TTC ATT AAT AAC GTC TAT TTT TAG AAT TAA TCC
GAA TAT CTC TAT TTA ATC AAT ATT TCA AAC GCT TTT AAA AGA ATG AAT TTT ACC GAA
AAC TTT ATA TAC CGT AAT TCC AAT CTA ATT AGT GCA ACT ATT AAA GAA GCC CAG TAG
TGT AGT GGC CAA TCA TCC GTG CCT TTG GAG CAT GGG ACC GCG GTT CGA ATC CGC GCT
GGG CTA CCA TTT TTA TTT TAG GCA AAT TAA TTT ATA AAT TTA GAT ATA CCA TTT TAA
TTT TTT AAA AAA ATA AAA ATT ATT CTA AAA TTT CAA AGT CGA TAC CGT GCC CAA AGA
GGA ACA ATA AAT AGC TTG CAG GAA TTT CTG TTG AAT TAT GGT CAA AAG TAT CTC CTT
TTA GTG TTA ACG GAT CAA GTT CCT GTT TTA TTT TTA CAT TGT ACA TTT TTT TAT GGA
ATT TGG AAA ATA CGC CAT GAT TTA ATG GAT CGA ACT GGT TAT TTT TCA ATG AAT TAT
AAG TTA TTT CTT TTA CCA TAA GTC CAG TGT ACC CGT GAA GTG ATC CAA TGG TTC TAA
GAA GCC GGA TAT CAT CAT CCA TAA CTT TTT CGT CGA GTT CAA GCC TGC TTT TTA CTT

TAT CGA TCG TTT CGT ATA TGG TTC GCC TTG TAG TGT CTT TCC TAT TAT CGA TTT CAG
TTT TCC AGT CTG AAG TTT TTT CGA ACT TTT TTG TTG CAA TCT TGT TTT TAT TAA ATT
CTC TTA CCA GTA TAT TTT TCC ACC GAC TCC CCA TAT TCC TTA AAT TCA GAT TTT TTC
CCA AAA TAT ATT CAA TAA AGT ATC TTC TTT CGT TTT TTC CCC ATT TTT CTA TTC TTT
TAA GCA ATT CGG GGT CTT CTG GTT TTA AGT ATA TGT GGT AGC CTC GGT TTC CAG TAA
ATA CTA TTT TTA AAT CTT TTT CGT CGA GTC CAA AAT CAG GAA ACA AAA AGT CCT CAA
TTA AAA TTA AAA CCT GAT TTC TAG CTT CAC CCA AAC ATT CTT CGC ATA TCC ATG AAT
CAT CAT GAG TAC ACT TTT CTG TTT TAT GCG TAT CAA TAT CAA ATG CAA CTT CTC TTT
TAA ACA AAC CTT TTT TTG CAG CGC CCC CAA CAA TAT CCG GGT ATT CCA TGT ATG AAA
GTG ATT TAT ATA GGT GAA ATG GGG ATT CTT TTA AAA CCC AAG CTA AAT AGT CTC TTT
CTG TAT TAA AAG ACA TAT TTC TAT TGT CTA CTT TTT TTA ACA TCC CGT AGC CAA TTT
CCC TAT GAC CTA AAT CAT CAG GAA TTT CAA GCC ATT TTC GAA CAA GTA TGG CAT ATT
CAA AGT ATT GTT TAT ATA GGC TCG AAA CTT CTT TAA AAA CCT TAT CTG TGG CAG GAT
TGT CGT TCA TAA TTT CAC ATA ACG CTT AAT TAA CCA AAT TAA CTT TAA ATA ATA TTG
TGA TTA TCT ATA ATT AAA CTT ATC TTT TAA AAA TGA TTT TTT ATT TTT ATT TAA ATA
TGG TGT GAA AAT GAT TTT GGG AGT AGA TAT TGG CGG AGC AAA TAC AAA AAT AAC AGA
ACT TCA CGA AAA CGG TGA ATT TAA GGT TCA CCA CCT TTA TTT TCC AAT GTG GAA GAA
TAA CGA TAA ATT AGC AGA AGT TTT AAA GAC TTA TTC AAA AGA TGT ATC GCA CGT AGC
ACT TGT TAC GAC CGC AGA ACT TGC AGA TTC TTA TGA AAC AAA AAA AGA AGG CGT AGA
TAA TAT CTT AAA TGC CGC AGA ATC TGC CTT TGG TAG CAA TAT ATC CGT TTT TGA TAG
TGA TGG AAA CTT CAT TTC TCT TGA GAG CGC AAA AAC AAA TTA TAT GAA AGT TTC TGC
ATC AAA CTG GTG TGG AAC TGC AAA ATG GGT TTC AAA AAA TAT CGA AGA AAA CTG TAT
TTT AGT AGA TAT GGG TTC AAC AAC AAC CGA TAT TAT CCC TAT CGT GGA TGG AAA CGT
GGT TGC AGA AAA AAC AGA CCT TGA AAG ACT TAT GAA TCA TGA ATT GTT GTA TGT TGG
AAC GCT TAG AAC CCC GAT TTC ACA TCT TGG AAA CAC GAT ATT GTT TAA AGG CGT TAA
TAC AAA CGT TTC TTC AGA ATA TTT TGC AAT AAC TGC GGA TAT TTC GGT AGT TTT GGA
AAA AGT AAC AAC TGA AGA ATA CAC ATG TGA TAC ACC TGA CGG TAA AGG AAC CGA TAA
AAG AAG CAG TTT AGT TAG AAT TTC AAA AGT TTT ATG CTC AGA TTT GGA TCA GAT TTC
AGA AAT TGA TGC AGA AAA TAT CGC AAA AAC TTA TTA TGA ATT ATG GAA AGA GTT AAT
TTT AGA AAA CGT GGA AAA AGT GGC AGA AAA ACA CGA TCT TAA AAA AGT TGT AAT AAC
TGG ACT TGG AGA AAA TAT TTT AAA AGA TGC ATT AGG GGA TTT TGA AGT AAT TTC TGT
TGC AGA AAG GTA TGG AAA AGA CGT TTC CCT TGC AAC ACC TAG TTT TGC AGT TGC AGA
ACT TCT TAA AAA TGA ACT TTA ATA ACT CAA ATT TTA TTT TCA CAT TTT TTT ACA ATA
AAG TTT ATA ACT ATT TAG ATA ATA TAT TGA ATA AAT TTT AGA TTA CAA TTT ATT TAC
ATC AAA AAT AGA ACG GAA AAA TAA AAC ACA GGT GAT AAT CAT GCA AGA AAA AAT TGC
AGT TTT GGC ATA TTC TGG AGG GCT TGA CAC GAG CTG CTG TTT AAA ATT ACT CGA AGA
CAA ATA CGA CTA CAA AGT AAT TTC AGT AGC AGT CGA TGT AGG TCA GCC TGA AGA CGA
TTT AAA AGA ACC AGA AGA AAA AGC AAA AAA ATT CGG TGT ATT AAA ACA CTA CAC AAT
CGA TGC AAA AGA AGA ATT TGC AAA AGA TTA CAT TTT CAG AGC TAT TAA AGC AAA TGC
ACT TTA CGA AGG ATA CCC ATT ATC AAC AGC TCT TGC AAG ACC TTT AAT TGC TAT AAA
AAT TGC AGA ACT TGC ACA AGA AGT TGG AGC AAG CGC AAT TTC ACA CGG ATG CAC TGG
AAA AGG AAA CGA CCA GTT CAG ATT TGA GTC AGT AAT GAG AGC AAA AAC ACC AGA AAT
TGA AAT TGT AGC ACC AAT AAG GGA TTT AAA CCT TAC AAG AAC TGA AGA AAT AGC ATA

TGC AAA AGA AAA AGG AAT TCC AGT TCC AGT AGA CTT AGA AAA ACC ATT CAG TAT CGA
TGA AAA CTT ATG GGG AAG AAG TAT TGA GGG CGG AAT TTT AGA AGA CCC AAT GAC TGA
AAC TCC AAA AGA ATG TTT TGC ATG GAC TGT TGA CCC AAC AGA TGC ACA GGA CAA AGA
AGA ATA CGT AGA AAT TGA CTT TGA AGA AGG GGT TCC TGT AGC AAT CAA CGG GGA TGC
ATT GGA AGC AGT TTC ATT AAT AAG AAA AGC AAA CGA AAT TGC AGG AAG AAA CGG TGT
TGG AAG AGT AGA TAT TGT AGA AGA TAG AGT TTT AGG CTT AAA ATC AAG AGA AAA CTA
TGA ATG CCC TGG TGC AAT GCT TTT AAT AAC TGC ACA CAA AGC TCT CGA ACA GCT CGT
TTT AAC AAG AGA AGA ACT CGT ATT CAA AGA AAT GGT TGA TTC AAA ATA CGC AGA TTT
AGT ATA CAA AGG ACT CTG GCA CGA ACC ATT AAG ACT CGA TTT AGA TGC ATT CAT TGA
TAA AAC GCA AAC AAG AAT GAA CGG AAA AGT AGT TTT AAA ACT CTA CAA AGG TTC AAT
GAG AAT CGC TGG AAG AGA AAG CAA AGA TGC AAT CTA CAA CGA AGA AAT GGT TTC ATT
CGA AAA CAA AGA AAT GGA CCA GAG AGA AAT TGT CGG AAT GGT TAA ATT CCA CGG TCT
CCA AGC AGC AAT CTA TGA AGG ATT AAC AAG AAA ATA ATT ACT TTT TTA ATT ATT TTT
TTT AAT ATT TCT CAT TTT AAA AAT TTA TTT TAA ATT TCC ACG TGA TAT TAT GAA CAT
TTT TAC TTC AAA AGG TAC CAT AAA GTA TGA AAA AGA AAA AAT AAT AAA ATT AAG TTC
GGA AAT GTT TCC TGA CGA CCT CTG CGA GCA GTG TGG GCG GTG CTG TAT AAT CCA CGT
TTT TAA TTC AAC CGA ATG CGG TGA ACC CGA AGT TGT ATA CTG CAA TCA CCT TGA TAC
TGA AAC AAA ACG CTG TAA AAT TTA CAA AAA CAG ATT TAA AAA AGA GAA AAA GTG TCT
TTC AAT GCT CGA AGC TAT AAT GGT TTC CGC ACT TCC AAA AGA CTG CCC TTA TGT TAA
AAA TTA TGA ATC TTA TGA AGA ACC GTG GTT TTA CGA CTG TTT ACG GTC AAA ATC AAA
AGA TTA ATG ATA AAA TAT CGT AAA ATA TAT GAA TTA ATA TCG TAG TGG ATG TTG ATT
TTC TCT CTT TTA TCA TTC CAA GGA TGT AGC TTA TTA TAA ATA CAT CTA AAA GTA CCA
TTG ATG ACA GAT ACT GGG CAT GAA GGC TTG CAA AAA GTA AAC TTG TAT AAA TGT TTC
CAA ATC TCG GCT GTA ACG CTC CTC TAA ACA TCA ATT CTT CAC TTA TCC CAA CTA CTA
TTG ATA AAA ATA CTA TTT TTA AAG GGG TAG ATG CTG ACG TTT TTA TGG TTT TTA CTA
CTT CAT TGA CAT TTG AAT TTT CAA CAA CAG CCT GTT CTG CAA TAT GTT CTG AAA TTG
CCG GAT CCA TTG ACT GGA AGA CAT ATA TAA AAA AGT CAA AAA GTC CCC ATA TAA ATT
TAT CAA CCA TTA ATA GAA GGA TTA CTC CAA AAA TCG CGT ATT TTA AGG ATG GAA TTT
TTA TAT CGA GCC TTT CAA TAG ACT GTT TTA AGT TTT TTC TCG TTC CAA AGC CTA TTC
CAA CAA AAG CAT AGG CTA AAA TTA AAG TTG AAA TGC AGC AAT ATC TAA TAT TTC TAA
AAA GTC AGA AAG GAT AAT TAT AAG ACC TGC TCC CGA TGC AAA AGC GAT TAT AGG ATT
TAA TAA AAT GTA TGT AGA AAG CGA AAC TGA AAT CTT ATG AAT TGA ATT GTC AAA CGA
AAG ATC CAT TTT TAA AAG TTT TGT AAA TTT TTT AAA AAA GCC CTG ATT TCT AAT CAG
TAA AAC GCT CAA AAA AGA AGC AGC ATA AAC AGA AAG TCC AAA AAC ATA ATT TAC ATC
AGA CAT AAA ATA TAT CGA AAA GAA AAG CGC TGA TAA GTT GAA AAC AAA AAT AAT GTC
AAG AGT ACG TTT TTC AAT AAG TTC CAG ATT AAA TGC ACG AAT TAA ATT GAT CCA GTA
TAT AAC TAG ACT AAT AAA AAA ATA AAA GAA TCG AAT GAG TGA TTC GAT CAT CTT TAT
CAC TGC TTA ACT ACT GGG GTT TCC GAT AAA AGC CCA CAG GTA TTA AGG GAA GAC GTG
AAA CAC TTA TAC TCT TTC TGC GAA AGC GAA TTT TTC TTT TAC AGC GGT AAC TTT AAC
GTT AAT AAC GCT ACC TTT TTC TGC GTT AGG TAC GAA AAC AAC GAA TCC ATC GAT TCT
AGC GAT TCC GTC TCC ACC TTT ACC CAT GTC TTC AAT TGT TAC TTC ATA TTC TTT TCC
TGC TTC AAC AGG AAC ATT TTT CAT TGC GGG TTT ACC GAA AGC CAT ATT TAT TCA CCT
AGT ATT ATA TAC ATC TCT TAT ATT GAT GCA ATA TTA CAC TGC TTT TGA TAG TAT ATA

TAA GTA TCT GAG TTC ATT ATT TGA GAA CTT AAC ATT AAA TAC GCT TTG AAT TTT AAA
AAC GAT TTA TCG GTG GTT AAA TGA TTA GAG AAG TGT TTT CCT CGA TTA TGG GGG AGG
GGA AGT TTA TTG GTA AAA GAT TTA TAT TTG TGA GAT TTA AAG AAT GCC CCC TTG ACT
GTA TAT ATT GTG ACG AGC CAA ATG CTC CGG GGG GAA CTG CAA GAG TCG AAG AAA TTT
CCG GAT CAG GCG AAT TTA TGG AAT ATC TAG AAA TTG AAC ATG AAC TTA TCG AAA TTA
TTG AAA AAT TGA GGA CGC CTG ACC TTT TTG CAG TTT CTT TTA CTG GGG GAG AAC CTT
TGG TAT ATT CCA ATA AAA TAA AAG AAT ATT CTG AAA TTT TAA AAC ATA AGG GAT ATA
AGA CGT TTT TAG AGA GCA ACG GAA TGT TTC CAG AAC GAT TGG GTA GCT ATG ACT ATG
CTT CAA TTG ATA TAA AGT TGC CAG AAC ACT TTG AAA ATA AAG ACG ACG ATT TTT GGT
ATG GAT TAT ATA AAA AAG AAC TTG AAA CGA TTG AAA GAC TCT ACC TGG CAG GAA CCG
ATG TTT ATG CAA AAA TTG TCG TGT TTG AAG AAA CTT CTG AAG AAT TAA TTG AAA GAA
TCG CGA TAG ATT TAT CAA AAA TTG GGA ATA TAA CGC TCT GCA TTC AGC CAG TAT CAC
CAA CTG ATA AAA TAA AAA CAA CTA CTT CGA AAA AAA AGA TAT TTG AAT TAA TGG CCA
TTT GTG GCA AGT ATG TGG ATG TAA TGT GCA CGC CAC AAA TCC ACA AAT GGA TGG GAA
TGT TAT AAT ATC TGT TTG CAA ATT TAA TAA TCT TTT AAT TTT TCT TTT AAA GAA CTA
ATT CCC GTT CTA TCG ATT GTA TCG CCA AAT CTT TCA TGG GAA TCT GCA TTT TCT TTG
AAA TAA TCC ATA ATT TCA GAA ACT ATT TTT TCA AGA TTT GGG GCA TCG TAT ATT CCG
ATA TTT TCG CCA ATT TTA TAT TTT TTA CCG AAT TTT CCG CCA ACG GAT ATC AAT ACG
CCA TCT TTT AAA GAG ATA CAT TTT CTT TTG CAC GAT TCC CCG CAT TTT CCG CAG TTA
ACG CAC TTT TCA TAA TTT ACA ACT GCT TTT TTA TCA ACG ATT GTG ATT GCA TCG ATG
CTA CAG GAT TTT AAG CAC AGT GTG CAT CCA TTA CAG TTT TCT TCA TTT ATT TTT GGT
ATT CTC TGT CCA ATA ATT CCT ACA TCG TTT AAC TGC GCT TTA GCA CAG TTA TTT GGA
CAT CCT GAA ATT CCT ATC TTA AAT TTA TGC GGT AAT TTT TTT CCA AAA AAC AAT GAA
TCA AGT TCG TTA CAA ATC TTT TGA CTG TCA ATA AGT CCG TGA GTG CAA ACA GTT CCC
TTA CAA GCG ACA AGG GGC CTT ACA GTG TTT CCA GTT CCA CCC GAT ATC AGG CTT GAA
TTT TTA AGT TCT TCT TTT ATT TTA GGT ATA TCT TCG TAG TTT ATC CAA GGA ATT TCC
ACA CAA AGC CTG ACG GTG AAA CTA ATA TAA TTT CTA CCG TAT TTT TTT GAT ATT TCT
GAC AAT TTG TCA ATT TTT TCT GAA TCC ATG CAT CCT GCA TCG GTT TTT ATT CTT AAT
GCA AAA TGT TTT CCA TCG TTT TGA AGG ATA AAA CCT TGC ATT TTC AGA TTA GCT ATT
TCA TTT TTG TTC ATA TTA CCA TCT AAA TTA GTA TTT GGG TTT AAT AAA CTG AAT ATT
GTT TTA TAA TCT AAA ACG GCA TTT TAT AAA TCT ATC TGA TAA AAA TAT TTC ATG AAT
TTC TGA GAA TAT ATG TCA TTC TTT GGC ATC TAT CAA AAT CTA GCG AAA TAT AAA AAT
AAT ATA AAA GAG TAT TAT CTG GTA ACG TGG TGA TAT CAT GAA AAA AGA ACT CAT ACT
TAA GGC TTT AAA ATT AAG GGA TAT GGG TTT TCC AAG TGG GGA TAT TGC AGA AGA ACT
AAA CAT ATC AGT TAA AAC GGC ATT ATA TCT TAC ACT AAA TGG TGA AGA ACT TTT AAA
GGC TGA AGA ATG TCC AAA AGA GGA TTC TGA AAA ATT GGA TAT CTT TTT AGA ATG GGA
CAA CGT TCG GGC TTC ATC AAG AAG GCT TAG AAA TAT TTC AAA AAT AAT CTG TGA TAT
GCT TTC AGA TGT TGA ATT TGA CGG TGT TGT CGG AAT TTC TTC AGG CGG GGT TCC TCT
TGC AAC GCT GAT TTC GGA TGA ACT TGA TAA AAA TTT CTC TAT ATA TGT TCC AAA AAA
ACA CAT CCA TAC TGA AAA AGA GAA AAC AAC AGG ATT TAT CGG TCA AAA CAT GTC CAG
CAT TGT TGG AAA GGA CGT TAT TAT TGT TGA TGA TGT AAT GAC ATC AGG AAA TTC TGT
AAA AGA AAC AAT AAA ATA CTT AAA AGG CAT TGC TAA CCC TAA AAA AGT ATT CGT GGT
AAT GGA TAA AAG TGG AAT CGA TGA AAT CGA AGG CGT TAG TAT AGA ACA CCT GTT TAG

AAC TGG TGT TGT TGA TAT TAA AAA ATA ATT TTT TAT ATT TTT TAA AAT AAT TGA ATA
AAT AGT TGG ATT AAC CTA TAA AAA ATA ATT TTT CAG AGT ATT TTG GCT CTT TTA TAT
TCA ATA ATG TAT ATA AAT GAC TTT TTT CAT AAT AGG ATA ACC TAA AGT TGG AGG CTT
ATC ATG TGC GGA ATT ATC GGA TTT ATG AGT AGA ACT GGA AGA CTT ATC AGT GGA GAC
AGG ATT GCT ACA GCC TTG AAT TGT TTA AAG GAA AGA GGA AAT GGT GAA GGT TCA GGT
TAT GTA GGA TAT GGA ATC TAC CCA GAC TAT AAG GAC GAC TAT GCA ATA CAC GTT TTT
TTA GAT GCA ACC AAA GAT TAC GAT AAA ATT AGA CAC GAA GTC GAA GAA GTT CTT GTA
AAA TAC GGA TAC ATC TTA AAA GAT GAA GAA ATT CCA ACA GAA GAA GGA ATT ATC AAA
AAA GAA CAG GTT TCG TGG AGA TTT TTC TAC AGG TTT GAT GAA AAA TAT AAA TCA GTC
GAA GAA GAC ATG ATG GTG GAT ATC GTA ATG GAT ATC AAC GCT AAA ATC GAC GGA GCA
TTT GTA TTT TCA AGC GGT AAA AAT ATG GGC GTA TTT AAA GCT GCT GCA TGG CCT TTA
GAA GTT GCA GAA TAC TTT AAA ATT GAC CAA TAC AAA GGT TTC CAG TGG CTT TCA CAT
GCA AGA TAC CCT ACA AAC ACG AAA GGA TGG TGG GGA GGT GCG CAC CCA TTC AAC CTG
TTA GGA TGG TCC GTA GTT CAC AAC GGA GAA ATT ACA AGC TAC GGT GCA AAC AAA AGA
TAC GTT GAA AGT TTC GGA TAC AAA TGT AAA CTT TTA ACC GAC ACG GAA GTT GTT GCA
TAT CTC TTC GAT TTA CTC ATC AGG AAA CAC AAA ATC CCT GTA GAA TAT GCA ATG AGT
GCA TTA GCT CCA AGA TTC TGG GAC GAA ATC GAG AGC ATG TCT GAG GAA GAT AAA AAA
ATA CAC GAA GCA ATC CGT ATG ACC TAT GGC GGT GCA ACA ATG AAC GGT CCT TTC GGA
ATT GTT GTT GGA ACA CAA GAA GGA ATG GCT TTC ATG AAC GGC GAA GTC GAA GAA GGA
AAT ACT ATG GTT GGC TTA ACC GAT AGA ATT AAA TTA AGA CCA TTA GTT GCG GCA GAA
AAA GAC GAC TTG TTA TTT GTA TCA AGT GAA GAA TCA GCA ATT AGG AAA ATA TGC CCT
GAA TTG GAT CGA GTA TGG ATG CCA GAT GCA GGA AGA ATG GTT ATT GCA AAA TTA GAG
AAA AAA ATT TAA TTT AAG TTA ATA ATT AGG TGA GAA AAG ATG ATA CCT TCA ACA GTT
CCG CCA AAA TAC AAA GTT TTT GTG GAT CCA GAA AGA TGC ATG CTT TGT GAA CGA TGT
ACT ACT GAA TGT TCA TGG GGC GTT TAC AGA AGA CAG GGA AAT AAA ATT TTA ACA TAC
CCT AAC AGA TGT GGG GCA TGT TTA AGA TGT GTT TCA CTG TGC CCT AGA GAT GCT ATT
ACA GTT ACA TTA AAT CAA TCA TGC GGC AGG GAA CAC CCT GTA TGG ACA CCT GAA GTA
AAA CAG GAT GTT GTA ACA CAG GCG AAA TCA GGA TGT ATT CTA TTA AGT GGT ATG GGT
AAT GCA AAA GAG TAC CCA ATA TAC TTC GAT AAA ATT GTT CTT GAT GCA TGT CAG GTA
ACG AAC CCT TCA ATA GAT CCA TTA AGA GAA CCA ATG GAA CTC AGG ACC TAT GTT GGT
AAA AAG CCA GAA AAA TTA GAA TTT GAC TAT GTT GAA GAA GAA ATC GAC GGT AAA AAG
GTC AAA AAA GCA AAA TTA AAA ACA AAA ATT GCT CCA AAT CTT AAG TTA GAT ACT CCA
ATA ATG ATC GGA CAC ATG TCT TAC GGT GCA CTT TCG TTA AAT TCC CAC AAA GCA ATG
GCA AAA GCT GTA AAA GAA TGT GGA ACG TTC ATG GGA ACC GGT GAG GGT GGA CTT CAC
AGG GAT TTG TAC GGA TAT TCC GAT AAT GTA ATT ACC CAG GTT GCA AGC GGT AGA TTT
GGT GTA AAT AGC GAA TAT TTA AAC AAA GGA GCT GCA ATT GAG ATT AAA ATC GGT CAG
GGT GCA AAA CCT GGA ATC GGA GGA CAC TTA CCT GGA GAA AAA GTG TCA GCA GAA GTT
TCA ATG ACA AGA ATG ATT CCA CAA GGG TCA GAT GCA ATT TCA CCA GCA CCT CAC CAC
GAT ATT TAC TCA ATT GAA GAT CTT GCA CAG CTT ATC AGA AGT TTG AAA GAA GCT ACA
AGA TGG AAA ATG CCA GTA TTT GTT AAA ATT TCG GCA GTA CAC AAT GTA TCA GCA ATT
GCA AAC GGT ATT GCA ACA TCA GAT GCA GAT GCA GTA GTT ATT GAT GGA TTT AAG GGT
GGA ACT GGC GCT GCA CCT AAA GTA TTC AGG GAT AAT GTT GGT ATT CCA ATC GAA GTT
GCA ATT GCA GCA GTA GAT GAT AGA TTA AGA GAG CAA GGA AAC AGG CAT AAA ATA AGC

ATC ATT GCA AGT GGA GGT ATT AGA AAC TCC GCA GAT GTA TTC AAA TCA ATT GCA CTT GGT GCT GAT GCA GTA TAC ATT GGA ACT GCC GCA ATG GTT GCA ATG GGC TGT ACC GTA TGT GGA AGA TGC TAC ACA GGA CAG TGT GCA TGG GGT ATT GCA ACC CAG AAA CCT GAA CTC GTA AAA AGA TTG GAA GTA GAT GAC GCT GCA AGA AGA GTA GCT AAT TTA ATA CAT GCA TGG ACT CAT GAA ATT CAG GAA TTA CTT GGA GCT GCA GGA ATT AAC TCC ATA GAA AGT CTC AGG GGA AAC AGA GAC AGG TTA AGG GGC GTA GGT CTC AGC GAA ATT GAA TTA AAT ACA TTA GGA ATA AAA CAG GCA GGA ATG TAA TGG CCA GTC TAG GTG AAA GAA TGA AAG AAC TTA AAA TTG ACG CCA AGG ACA TGG ACT ACA GGG AAT TAA ACG AGA AAA TCC ACG AAG TTT TGG ACG AAA ACA AAG ACT TGG AAA AAT TAG TAC TTG AAA ATG TTT TAG GCC AGA GAT TTA TTG GAA ATG GAG TTT CAA GAA AAG ATT TAA CGA TAA TTG TAA ACG GAG TTC CTG GTG GGG ACC TCG GAA TGT TTA TGA AAG GCC CTA AAG TAG TTG TAA ATG GAA ATG CTG ACC ATG CTC CTG GAA ATA CAA TGG ATG AAG GAT TTG TCG TAA TTC ACG GAA GTA GTG GGG ATG TAA CCG GCC ACT CGA TGA GAG GTG GAA AAG TTT ACG TTC AAG GAA ATG TTG GAT ACA GAA GCG GAA TCC ACA TGA AAG AAT ACA AAT ACA AAT TCC CTG TTT TAG TTA TCG GTG GAG CTG CAA AAG ACT TCC TTG GGG AAT ACA TGG CTG GAG GAA TTA TTT TAA ACC TCA ACA TGG ACC AAG AAG ATG GCG AAA ATA TCG AAG GAA GGA TGA TTG CAA CAG GAA TTC ACG GCG GAG TAA TTT ACG TCC GAG GAA TTG TAA AAA GTA GTC AAC TTG GAA TCG CAG CAG ATA TTA AGG AAT TTA CAG AAG AAG ATA GGG ATA AAA TAA GAC CAC ATA TTG AGG AAT TCT GTA AGG AGT TTG GAT TAA GTG ATG AAA TTA AGG AAA AGT TAA TTA ATT CAG ACT ATA CAA AAA TAG CTC CAA TAT CAA AGA GAC CAT TTG GAA AAT TAT ATA CTC CTG ATT TAA GGT AAA TAA GGT AAG TAA GTA TGC CTT TGG ATC CAA CCC AAG CCT TTT TAA AAG GCT GGA TGC CAA ATA TAT CTC TTC ATG AAG TTA AGA AGT ATT CTA TTG GGG ATC ATT GAG CGA AGC GAG ATG AGC TCT ATG AAA ACC ACT GGG TTT TCA TTT AAT CCA ACA CCT CTC GAC GAA ACC AAA GGT TTT CGT GAG TCT CTA CAA AAA TTC GAA GAA TTT TTG TGA GTC AGC AAA TCT TCG ATT TGC TTC GAC TTA ACG AAA ACC TTT CAG GTT TAG TTG AAT AAT CGT AGA TTA TTC AAT TTC CCC GCA ACT ATG TTG CGG TGG ATC GTA AGT CAG CGA ATC CTT TGG ATT CGC TTC GAC GAT AAA TTG AAG ATT TGT CTC GAT TTT TAA AGG GGT TGG AAA TAA AGG TGC TGG ATT TAA TTA TCT TTT TTA AAT GAA TAG AAG GTG GAA TTA TGA AGT CAT ACT TGA ATT TAA AGG AAG AAG TTT GGG ATA AGA ACA TCT GTT CTG GAT GCG GAG CTT GTG TTG CAG TCT GTC CTA CAG AAA ATA TTT ATT TCA AAG AAC AAA GCC CTG TAA AAT TTG TAT GTG ATG AGT GTG CAT GTA TAA TTG CTC CTA CCC AAA ATG GAG AAC ACC CGA TAT CTG CGG AAT TCT GTA AAG TTA CCC TTT ATG ATG TCC CAT GTG GGG CCT GTT ATG ATG CAT GTC CAA GAA CCT CTG AAG GAA AAT TGT CTC AAA TTG AAA ATG GGC TGG ACA ATA TAA TAA AGG CAG TAA AGG CCA AGT CCA AGC TTA ATA CAA AGG GAG TCC AAA GCG GTG GAG CAG TTA CTG CGA TTC TGG CGA ATG CAT TTG ATG AAG GGA TTA TAG ACG GAG CTC TTG TAA TGA TGG AGG ATA AAT GGA CAA TGA AAC CTG AGT CCT ATT TGG CAA CTT CAA AAG AAG ATG TTT TAA AAT CTG CAG GAA GTA GAT ACA ACT GGA ATG CTC CTA TAT TGG AGG CTT TAA AAG AGG CTG TAA TGG ATA AAA AAT TGA ATA AGT TGG CAA TTG TGG GAA CAC CTT GTG TTA TTA ACG CAG TTT ATC AAA TGA TGT CAT CTG ATA ACG ACC TTG TAA TTC CAT TTA GAA ATG CCA TAA GGT TTA AAA TAA GTC TTT TCT GCT TTG AAA CAT TCG ATT ACT CTA AGA TGA CTG AAA AGC TTA AAT CAA TAA ATG TAG ATC CGT GGG AAG TAC GTA AAA TGG ATA TTG ATA AAG GTA AAC TAA TCG TCG AGC TCT TAG ATG GGA CAA TCC ATA GCT TCC GAT TAA ATG AAG TAG AAG

ATG TCG TAA GGT CTG GAT GCA ATG TAT GTA CAG ATT TCA CAG GTA TAA CTG CTG ATA
TAT CAG TTG GAA ATG TTG GAT CTC CTG AAG GAT ATT CAA CAG TGC TTA TAA GAA ACA
AGT GGG GCA AAG GCC TCT TTG ATA GGG CAG TTA TAA ATG GAT ATT TAG AAG CTG AAG
AAG GAG TTG ACA TAA AAC CAA TTG AAA GTT TAG CTG AAA AAA AGG CTA GAC GAA AAG
ATG GTT CTG AAA TTT AAT ATA TTT TAT AAC TTT TAT TTT TGT TCA GTC ATA ATT GGG
CCT GAA CTT TCT GTT TTA CAT AGT CAA TAC CGA GTT CTT TTA TAA AAT CGT AAA TTC
TCC CTT TTC CAA ATT TTG AAT ACA TTT CAA TTA AAA TAT CGA CAA CTT CTA AAA CTT
CAT CCT CTG TTT TAA CAT CTA TTA GAT GCT TAG CAG CTT TTG GAA ACT TCC CGC CTT
TAC CGC CTA TAT TTA TGG ATA TTG CCT TAT CTA CCA CAT TTA TGG CAT CGC ATA ATT
TAA GAC AGT CTC CCT TTC CAA TAC AGT TTT CTT TTA TAG CTT TAT TTT TCC AAT CTA
TTG CAT TTA TTT CAC AAA AAT TTT CAC ATT TTC CAC ATT CTA TGC ACT TTT CTT TGT
TGA TTT CTA ATA TAT ATC TGA AGT ATA TCC CAA TAT CGC AAA ATT GAT GAT GAG TAC
AGT TGT TTG GAC ATC CGC TAA CAT TTA TTT TTA ATT TTT TTG GGA CCC AAA TAC CTT
CAA ATT CAT CAT GTA GCT TTC TAG CCA GGC TTA CAG AAT CAC CAA TTG AGC TAT AAC
ATT CGG ATC CAA TAC AGC ATA CGA TTT GTC TAA TCC TTG TTC CGG TGG ACC CTA TTT
TAA TAC CTT CTT TTT CAA GGA GCT CTT CAA CTT TTT CAA GAT TTT CAT ATT TTA TTT
TAA ACT CTA AAC CCT GCC TTG TAG TTA AAT GAA CCT TTC CAA AAC CAT ATT TCT TAG
CAA TTT TTC CGA TTG CAA TCA GCT GTT CAC AAC TTA GAT AAC CTG GCT TTA GGG ATA
ATC TTA CCA TAA GTC CGT CTT TTT TAG GAA TAA CTC CCA TCT TAT AAT TGT TCC TAC
ATG CCA TTT TTT CAC TTT AAA AAT TAT TGC ATT TAT ATT CTA TTA CAG GAC GTG TGC
AAA ACT CAT GCA CAC AAT AAA TAG TAT TAT ATA CTA TCA TGC TCA AGT ATA ATG TGT
GAA TAA ATT AAT CAC AGG TGG TAT TGA TGT ACG AAT GGA AAT TGA ATG ATA TTG TGG
ATA ATG GGA TAT GTG CTA AAT GTG GTA CAT GTA CAG TAG TAT GTC CTA ATG GTA TAT
TAA CTT TTG AGG ATA GGC CAA AAT TAA CTG AAG AAT GTC TAA GAA AAG GAA ATG GGA
TGT GCT TTG AGG TCT GTC CAA GAG TTT CAT CAG GAA AGT ATC AAA TAA AAA TCA GAG
AAA AAT TTA AAG AAG AAT ACT ACT ATG GAA AAG GAG ACG TAG AAG GGC AGG ACG GGG
GAG TTG TAA CAA CAT TCT TAA AGT ATC TCT TAA AAA ACA AAA AGA TAG ACG GTG CTA
TAG TAG TTG GAG ATG AAT GCT GGA AAC CAG TTT CAT TAA TCG TTC AAA ATG AAG AGG
ATT TAA TGA ACA CTA CAA AAT CAA AAT ACA CTG TAT CAA CAC TTG AAG CTT TAA AAA
CTG CTG GAG AAA TGG GCT TAG AAA AAG TCG CAG TTG TTG GAT TAC CAT GTC AAA TCA
ACG GTC TTA GAA AAC TTC AGT ACT TCC AGT ACT TGG CAA AGC ACG ATG GAG AAC TAG
GTA AAA ACG GAA AAC CAG TTA AAC TTC CAA AAA TAG AAT ACC TAA TCG GAT TGT TAT
GTA CTG AGA AGT TTG AAT ACG ACG AAT TAA AAG AAA CCC TTG CAA AAT ACA ACA TAA
ACA TGG ACG ATG TAG AGA AGT TTG ACA TCA AAA AAG GAA AAC TAC TCG TCT ATG TTA
ATG GAG AAG AAC ACA AAA TAC CTT TAA AAG AGA TTG AAC TAA GTG CAG GCT GTA AAA
TGT GTA GAG ACT TTG ACG CTG AAA TGG CTG ATG TGT CAG TCG GAT GCG TTG GAA GTC
CAG ACG GCT ATT CAA CAG TTA TAA TAA GAA CAG AAA AAG GGG AAG AGA TTA AAA ACG
CCA TAG AGT TAA AAG AAG GCG TAA ATT TAG AAG CTA TTG AAA AAT TGA GAG ACT TAA
AAC TCA ATA GAT TCA AGA AAG AAG TTG AAA GAA GAA AAG CAG AAG ATG AAA AAG TAT
CAT TCT ACT GGA CCG CTG ACT ATG GTG GAG TAG GTA AAA GAG CAG ATG GGA CCT ACT
TTA TAA GAA TTA GAG CAA AAC CTG CTG GAT GGT ATA GTA TTG ATG AAG CCA GAG AAA
TAC TGG AAA TAG CTG AAA AAT ACG ATG GTA AAA TAA AAA TGA CAA ACA GAG GTG CAT
TTG AAA TCC ATG GTA TAA GTG GAT TTG ATG TTG AAG CTA TGG TTT TAG AGC TCA TGG

AAA AAG GAT TCA TAA CTG GTT CAG AAG GGC CAC TGG TTA GGG CAA CAC TGG CAT GTC
CTG GAG AAG GAA ACT GTG GAA GTG GAT TAA TAA ATA CCA CCG AAC TAT GCA AAA TAC
TTG AAG ATA ACT TCA AAG AAC ATC CTG CAC CAT ATA AAT TCA AAA TTG CAA TAA GTG
GAT GTC CAA ATA AAT GTG TTA GAC CAC AAA TCC ATG ATA TAG GTA TAG CTG GAG TTA
AGT TCC CAG TAG TTA ATG AAG AAA ACT GTA ACG GCT GTG GAA GAT GTG CAG AAG TTT
GTA AGA TTG AGG CAA TAG ATA TTA GGG GAG AAA CAT CAT ATA CAA ACT ACA ATG TAT
GTA TTG GAT GTG GAA AAT GTA TAA AGG CAT GTC CAA ATG AAG GTA GGG ATG TTA AAG
AAG AAG GAT TTA TGG TGT ATG TTG GAG GTA AGA CCG GAA GGG AGG TAA TAG AAG GAG
TAA GCA TGA AAT TAA TGA GTG TTG AAG AGA TAC TCA ATT TAA TAG ATA AGG TAT TGA
TAG TAT ACC ACA AAT ACG CTA AAA AAC CAC AAA GAG AAA GAT TGG CCG CAG TTA TGG
CAA GGA TTG GAA AAG GAA AGT TCT TAG AAG AAG TTA AAG AAC TTA TGG AAC AAA ATT
AAA TAT TTA TTC ATA TTT TTT AAT TTA AAC TAC TGT TTT TTT AAC CAT TCT TTT ACA
CCC ATA TAC CAA TCG TCC AAA AGT TTT TCC ATG ATA TCC TTA ACT TTT TCA AAT TCT
ACA GGT CGG TAT ATG TAT TTA TAT CCA AAA GTC TCT GTT ACT TTT TCT CTC TCT ACC
AAT CCA CAG TTT ATT AAG CTT CTT AAA CCT TTC TGC ACC GTT GTT CTG TCT CGT CCA
ATT TTT TTG GCT ATT TCA TTT ACT GGA GCT TCC TCA AGA TTT AGG AGC TCA AAG TAT
ATT CTA ATC TCT ATT TCT TGC AGG CCC AAA ATA CAT CTC ATT AAA TCC TCT ATT GTG
AAT TTC TGC ATT CTA GCT ACT ATT AAG TTA TTC ATT AAA TCC CTC CAA TAT TTT ACT
AAA ATA TTA TTT TAT AGG GTG GAT AAA ATT ACA AAA AAC TGT TTC ATT CTA TTG GAA
ACC TTA AAA GTG CTC CCA CGC CTC CCA ACG CCT TTA ACT GCT TTC CGG CAT CAT GTT
CGG TAG ATA TAA TTA CAA CTT TTC CGC CTA TAT TTT CAA CTG AGT TCA TTA GGT CTT
CAA CAG CCC TTC TAT TTT TTC TTA TAA ACT CGT CTG TAA CTA AAA GTG TAT CAA TTG
CAG AAT AGT TCA TTG CAT TTT TTA CAC TTT CAA TTC CAT ACG CTG CAA GAC CTT TTT
TAG ATA TTT CTT CGA GTA ATT TTT CCA CTA CTT GCG TTT CTT TGC TGA TTC TTG CTT
CGC CGT AAA TTT TAT CAA TAA TTC CTG ATT TTA AAA TTT CAG AAA GCC CTG ATT TTC
CAG TAT GGT TTG CAG ATT CTA CAA CTA CAT TTG GAG CTA AAC TTT TAT ATT TTT CAG
AAA TGT AAT TTT GAA TAT TGT TTC TTC CAA ATC CGG GGC CTG CAA AGA GTA TTT TTC
CTT CAA ATT CCG AAA TCG AAT TGA TAA TAT CCG AAT AAT AGC TAA ATT TAG CCT GTT
TAT TTT GTT TGT AGT CCA ATT TTT TTG ATA CTC CCG ATT TAA TGC TTG CAA GTT CCT
TAA TTC CAA ATT CCC GAA CTA AAA AGA TGT CAG CTT CGC TAT CAT CCA TAA TAA CGA
CTA CTA CTT TTG GCT TTT TAG AAG AGT CTT CTG CTT CTT TTA ATC TTT CAA TAT CCC
ATT TTT TCC AAT TTT TTT GAA CAG ACA CCT GCA AAA GTG GTT CAA TAT CAA TTG TAT
GGT ATG ATC CAA TTG GAA TAT CGT CAG GTG CAT GTA TAA TTT TTC CAC TGA CTC TAA
GCC TGT TGA AAT CTT CAT GAA AAT TAA TTT TTT CAG CTT TTA TCC CTA AAA AAA CTC
GTC TTT TAG TCC CTC TGT CGG CAC GTA GTT TGT CTC CTT TAT CTT CCG TTC TTC GCT
CGG TTA CCG CGT AAA TTG CAT TGT ATG GCT GTA TTA TAT GTG ACA AAT GCC ATA AAT
CAT CTA AAT TTT CTG GAA TTA ATT TTA TTA TGT TTC TTT CAG GTA TTT CTT GAA TGA
TCT TCA TTA TAT CTC TTT TTT GGG CAT TTT CTC GTT TTC TTC ACT TTC TTC GTA ATC
GTG AAC GAA TAT ATC TCC GTT TTG AAG TAT GGT TAC AAC AAA TGG TTC TAC TAC CAT
TCT GTT TTT GAT GTA CCT TTG AGC ATT CTG GTT TAC AAA CTC CAT GAT TTG AAG TGA
AGA GTA GTT TAC ATG TAT TTC TTC CTG ATC GGA CTT TGA AAA AAT AAT TTT TGG GAT
ACT AAA TAC GTC AGT TCC CTT ATC TAT TTT AAT CTT AGA AGG TGC GAT TAT ATC GTT
ATA GAT ACT CAA TGT ATT CCG CGC TTT TTC AAT GTT TTC GCG AGC TCT TTT CTC GAT

CAT GCT GAT GTT TGC CCT ACT TGT CCC CAT AAT ATT GGC AAT TTC ATC TTG AGT GTG
GCC TCT TTT CCT GAG GTT TAG CAC ATT TAT CTG AAT TTC TGT CAA AAA CGA TTC CAT
AGT TCC ACT TTT CAT AAG TAA TTA TCA TAT ATT ATC TTA AAA TTG GTA TAT AAA ATA
AAT GAT TTA AAT GTT TTT TTA GTT TGT GAA ATG ATT TAT TCT TTA AAA ATT CAA AAA
AAG TTT TTA GAA ATG AAA AAT TAT CTT CCA AGT TCA GAA TGC GCT TTT GCA AGA TGT
CCT GCA GCA AGG GCA CCA ATA AGT GAT AAT TCA CCG GCT AAA ACA GCA GCA CCT GCA
ATT TCT GCA AAT TTA ACT GCT TTT TCA GCA CCT GCA CAT CCA AGT ATT TCG AGA CAT
TCC CTC TGA GTT TCA ACT CTA GTT CCG CCA CCG ATT GTT GCA ACA GGT AAA TCT GGA
AGC GTA ACT GAG AAA TAA AGG CCA TCT CCA GTA CAT TCT GCA GTC GTA ATT CCC ATA
CTT CCT TCA ACA ATA TGC GCT TCG TCC TGC CCT GTT GCT AAG AAT AAT GCA CCA ACA
ATA TTT GCG TAC TGT GCG TTA AAT CCT AAT GAT GAA CTT ATT GCT GAA CCA ATC AAA
TTT TTG TAA GTG TTT ACC TGT TCA ATT GCT TTT GAA GTG GTT TTT AGA TAT TTT TCA
ACG TAT TCT TCT TTT AAA AAT ACT TCT GCA ACA ACA CTT TTT CCC CTT CCT TCA ATA
AGG TTT ATT GCA GCA GGT TTT TTA TCA ACG CAC ACA TTA CCG CTT AAT GCA ACA GTA
TCT ATA ATT ATT CCT TCT TTT TTA AGT TCT GAT TCG ATA AAT GAA CAG GCT TTT TCG
GTT GCA ATT GTT ACC ATG TTC ATT CCC ATT GCA TCC CCG GTT TTA AAT GTA AAC CTT
GGG TAC ACG TTT CTT CCA ACT ATC AAA ATC GGA CTT ATC TGA ATT AGT TTA CCA TGC
CTT GTT GTA GAT TCT GCA ATT TCT TTT ATC TTT GCG AAG TTG TCT AAT ATC CAC TCT
TTT AAT TTT AAA GCA TCA ACT ACA GAG TTC GTT TTA ATT ATT GGC GCA CGG GTC ATC
TTG TCG TCG ATC ACG CGT ACT GTT GCA CCA CCA CAT TTT GTG ATA ACA CTG CAC CCT
CGA TTA ATT GAT GCA ACG AGA GCA CCC TCC GTA GTT GCG AGA GGC ACG TTA AAC TCA
CCT TGT GCA TAT TTT CCA TTG ATT GAA ATT GGG CCT GCA AAC CCG AGC GGA ATC TGG
ACT GCA CCA ATC ATA TTT TCA ATG TTT TTC TGC ATT GCA AGT TTT TCA TCA AGA GTG
TAT TCC TGA ATA TGT CTT GTT TCA ACG TTT GAT AGT TTT TCA ACG TAT TTT CGC CTT
GCA ATA ACT GCA TTT CTT TCT CCA AAT TTT GAA TCA AGC TGG TAT ACT TTT ATT TCC
TTT TTA AGA AGT TTT TCA ACG ATT TCA TCG ATA TTA ATG TTA TTT TCC ATT TTT CCA
CCA TGC CGA GTT CAT AAG GTC TTC TTT TGA AGT ATT AAG TGA GTA GGA CAC AAA ATC
ATG GGT TAT TCT TTT TGC AAA CGC TTT AGA GAA GTT TTC CAT AAT TTC TTC AGG ATT
TTT GGT TTT AGA AAG TTT AAT TGC TTT TTC AAG CTC TTT TAC CCG AAT TTC TTC AAT
ATA ATT ATT AAA ATC TTT TAA AAC TTC TTC AAC ATT GAT TTT TTC AAT CTG TTT CAT
AAG AAC GTC ATA TTC TTC ATC AAT AAT TTT TTC AAC GGC AGG AAC TTC CTC AAT TCG
TTT TTG AAG ATT TTT ATC AGA TAT ATT TCT TAA ATC GTC AAT GGT ATA TAG TTT TAT
ATT TTC AAA TTC CCT TAC CGC ATC GTC AAC GTC TCT AGG GTT TGC TAT ATC AAT TAT
AAT CTT GTT TCC AAC GTC AAT TAA ATC TTC TTT TTT TAA GAT GTA GTG GGG TGA AGA
TGT AGC ACA GAT TAT AAC ATC AAT AAA GTT AAT TGC TTC TTT TAA TTT GTC AAA ATG
AAC TGC CAT TCC TTT TAA TTC TTT TGC AAG GGT TTC AGC ACG TTC GTA AGT TCT ATT
TGC AAC AAT AAC GGC TTT TAT ATG TTT TTC CAT AAG GGC TTT TGC AAC CAA AGT TCC
AAT TTC TCC CGC ACC GAT TAG TAA TAC ATT TTT GTT TGC AAG TCC GAA GTT TTT TTC
CGC AAG TTC AAC TGC CGC ACT TCC AAT TGA AAC ACT GCC TTC GTT AAT TTT GGT TTC
AGT TCT TGC TCT CTG TCC AAC ATG TAT TGC TTT TAA AAA AAC GCT GTC TAA ATA ACG
GCT TGT TTT TTT AAG TTC TCG AGC TTT TTG AAA ACT TGT TTT AAT CTG GCC CAA AAT
CTG GTT TTC TCC AAG TAT CAT CGA GTC CAT TCC ACA AGA CAC TTT TAA AAA ATG AAA
CGT TGC ATT TTT TCC TTT TAA AAT GTC AAA TCC TGA AAA ATC GCT CAA AAT ACA ATC

TAC ATC AGA ATT TAC CTC AGT ATC AAA GTA AGC TTC GAT CCT GTT GCA GGT TTG GAC
TAC AAC ACA CGC GTC ATA TTT TTT ATA AAA TTC ATC CTC GTC AAT TCT CAT CTT TTC
AAG AAC TGG TAT CGG GTA TTT TTT GTA ATC TGC TCT AAC AAC CAA CAT AGA TTC ACC
TAT GTT TTT CAA TAA GTT TTA AAA GTT CTT TTT TAA ATT TTT CGT CAC TAA ATA CCG
TTT CAA GAA CTT TTT TTC TTT CGT TCT GAT CTT CAA TCT CAC TTT TTA AAA AAT CAC
GAA GTT TAT TTT GGA AAT CAA GTT CAA ATT CAG AAT TTT TTA AAT ACT CTT CAA CAT
TCT TTC GAA TTT CTC TTG CAA TAA TCG GAC TTT TTC CGC CAG TAT ATA CTG CAA ATG
AGA CAC CAT CTA CCT CAC AGC ATG CAG GAA CTA TCA AAT TGG ATT CTT TTT CAA AAG
TAG ATG AGT TTA TAA ATT TTT TTA GGC TTT TTG CAA CCG AAA CAA TCC GTT TAT TGT
TTT TTT CGT CAA TTG CAG TTA TTA TAA TAT CGT GAT TTT TTA TTA TTG AAT ATA GTT
CTT CAT CAC TTA AAT TAG TTA CAT CGG ATT CAA AAA ATA TCG CAT TTT TGT ATT TTG
AAA TAT CAT TTT TAT CAA TAC TTT TGG AAT AAA CAG TAA GTT TTG CAC GAC TCT CTA
AAA ATT TTT CAA ATC TTC TTT TTC CAA CGC TTC CAA ATC CAA AAA TCA AAA CAT TAA
ATC CAC CCA AGT CTA AAA AAA CAG GAA TCA TAT TTT CAC AAG CAA TTT ATC GAT TCA
ACA ATG AAT TAT ATA TCT CAT AAG TTT TTT TTG AAG ATA ATC CCC AAG AAA AGT TTT
TTG AAG TAT CCA AAC TTT TTT CTC CAA GGG ACT TTC TTA ACA TTT TAT CAT TAA CAA
GCA TAT TTA TTT TAT CTT CAA GAG CTT TTG TAT TTT TTG GAG GGA CGA TGT ATC CAT
TTA TTC CAT CTT TGA TCA ATT CAG GAA TTC CGC CAA CAC TAG TTC CAA TAA CTG CTT
TTT TAC ATG CAA ACG CTT CTA TAA TCG TCA TTC CAA GCC CTT CTG AAA TTG AAG GAA
GCA CCA AAA AAC TGC AAT TTT TTA AAT ATT CTG AAA CTT CGC TTT GCG TTT TTG GAC
CGA GCA ATT CAA CGT TTT TGA TAT CGT TTA CTT TTA TAA AAT CCA GTA TTT TAT TAT
AAA GTG GGC CAC CAC CAA TGA TTT TAA AAT TAA AAT CGA TAT TTT TGA TAC TTT CTA
AAA AAT AAA AGA TTC CTT TTT GTT CAA CTA ATG ATC CAA CAA ATA ATC CGT AAT TTT
TGT CGC TAT CAG ATG GAT TAA ATA ATT CTT CAT CAA ATC CGT TGT ATA TAA CGT TTG
AAT CAA TAT TAA AAT TTG ATT TTA AAT TAT TTT TTA AAA ATT CAC TAA CAC AGA TTA
TTT TTT CGG AAT TAT AAA CTG CAT AGT TAA AGA AGT ATT TTC CAA GAC TGC TTT TTG
ATA AAT TCA ATA CAT CAC TTC CAT GAA GTG TCA AAA CTG TTG GAA TAC TGC ATT TTT
TTC CCA AAA TTG CCC CTA AAA ACC CCT GAG GTG CTG CAT AGT GGC TGT GAA TTA AAT
CAA TTT TTT CGT CTT TAA TTA TTT TTT TTC CGA GTT TAT AAC CGT TTA AAA TGT AGC
TCG GGC CCC TTA ATT TTG AAA AAT AAG GAA CTT TGT GGA TTG TAA CGT TTT CAA AAG
AAT TTT TAA CGG TTT CAT CAA AAT TGT AGT TTA GAA TAT GAA ATT CAA AAT CGT CTA
GTT TGT TTA AAT TTT TTA TTA AAT TTT CAA CAT GTA TCG AAA CTC CTC CAA TAA ATG
GAT AAT ATA TGG AGG GAA GTA ATA TTT TCA AAA TAA CAC CAA AAA AAG AGA TTA GTG
GGT AGT TTT CCA CTT TAC AAG GAC ATA GTG TTT TTG AGG CTC TTT TAA TTC CTC TTC
TAT TCT TTT TGC TAA CGG ATC AAC TAT ATT TTT TCT AAC CCT GTC TTC AAA ATC CTT
AAA GCT TTC AAA TTT CTT TAT TTC CCG TTC TTC AAT TAC TTT CCA CAT TGA AGT TTT
ACC AAT TCC CGG AAG CAG TTG TAA TGT ATG CAT CCT GTT TGT AAT TGG CCC GCA GTT
GTT TAA AAA TTC GAC AAA CCT TTC TTC CTG AGC TTT AAC TGC TTC TTT TAT AAC GTG
AAG AAG TTC CGT TTT TGC AGT AGG AGT TAG TTG ATC GTA TTT TAA CAT TTT TGA TAC
AAA CTC TAC TTT TTC CCG TTT ACC TTT CCC GAT ATA GAG TTT TTC TGC AAG ATC TGC
AAC CTG ATT ATC CTT AAA AGT TAA TTC CAT CAT TAC AAA TTG TTT TTC ACC AAA AGC
TTG TGC AAT TGG TTT TTT TTG ATG AAG TGG CCT AGT ATC TTC AGG GTA ACC ATA TTC
TAA GAA GTC TAA AAC GTA TGC GTA ATC TTC AAA CTC TCG TTT ATT TTG TTC ATG CCC

CTC TCT CAT ACT CAC ACC TAT CAA TGT TGA AAA TTA AAA TAA CAT TAC TTA ATA TCG
AGT TAA TTC AAT AAA AAA CTA ATT GGT TTA ATA ATA AAA TGG TAG AAA AGC GTT ATT
TGA ATT TAC TAA CTA CTT CTA AAA TTT CGT CCT TGT TTT CAG GGA CGT CAA CTC TGT
AGA ATA TGA TTT TTA AGT CTT CTT CTG TTT CAG GAA GTA AGT CAA TAA TTT TAA CTG
CCA TTT TTT CAG TTA AAC CTA AAT TTA CGA GCT GTT CAA ACA TAG CGT CAG CAT CGT
CTT TAC TTA TTG TTG AAA ATT TTC TTA AAT AGT CGA GTG AAC AAC CGT GTT CAT AAG
ATA ATT CGT CAA AAT CTG CTC TTT CAT CCA TAA TTT CGG TTG CAT GAG CAA TTG TTG
TGT ATT TTT CGG AAA TAA TTT CTT TTC CAA TCA TAA TCT CCC TCA AAA ACA GAT TAG
TCT AAA AAA AAG ATA TCT AAA ATA ATT ATA ATT TAG ATT CTC TTA AGT GTT GCG GTC
TAG CAA TAA TAT CTT TGT ATT TTC CGC CAT CTT TAA CTC TAA CTA AGA ATG AAC TTC
CTC TTT GAG CAA CAA CAA CTC CAG TTT TAC CGT GGA ATT TTG GGT GAG GCA TTC CTT
TCT GTA CTG AAG GGT CGA GTA CTA CGT GAA CAA GGT CTC CTT CCG TGT AGC ATT TTA
AAG CTC TTG TTA AAG GAG CCA TTC CTT TCT GTC TTG GGT GTT TCT GAA GTT TAT ATC
TTG TTT TGC TTC TAA ATC CTT CAC TTT TTT GCA TAG TTA TTC CTC CTA ATG TGT TTC
ATG AAC ATA ATT GGC ATA GTG TCT ATC CTT TTT TTC ACT ACC AAT ATA TAA CCA TTT
CTA TTT TAG ATA TTT TCT GCA TCT TCA TAG TCG TGA ACA TCC AGA ACA TCT AAA AAT
TTA CAG ATA CAT TTT TTA TTT AAC AAT TCC GAA ACG GAA GGG GTT GTT CTT CCT TCA
TCA CTG TTA ATA AGC TCT TTG ATG TAT AAT CCG CCA TCG CAG AAT ATT TTA AGT TCA
AAG TTA TAA TCA TCA ATC ATA TGC GGC CAA GCT TTA TAT ACT TTC CGT ACA CGC ACG
AGG TCT GCC CTT CGA TGC GAG ACT CTA TCG GGC GTT CTT TGA TCA ATC GTC AAA TCT
TCG AGT TTT TTT ACT AAT TCG GCT ACT TCT TCA TTA GAT ACT TTT TCT TCG CAC TCA
ACG TGG GCA AGG TAT GTT TTT TTA TGC GGT TCG TTT TTG AAG AAA TGA ACG TCC TTT
TTA ACC CCG TAT TCA ACA TTT ATT ATT TCA ACG TTT CCA GAT TCA TTA ACT TTT GAA
GCA AGC TCT TCT AAA TTA ACT TTT CTA ATC TTT GGC TCT TTT ATT TCG ATT ACA AAA
GGT CTT CCA TTT CCA AGC ATT CTA ACG TCG ATG TCT TCT CTT CCC GCA CCG TGA AGT
GCT TCT GAA CTT CCT TTC ATA ACA TCC ATG AAT GGT TCT GCA ATA ATT TCT TCA ACT
GAG GTC ATG TAC TGT TTT CCG GTA AAG TTA CAT TTT TCA CAG CCT TTT CCT TTA CAA
GAT CTG CAG TGC CAG TGG CTT TGT GGA ATT CCT CTA ACG AGC TTT TTA TAC CGC CCT
TTG ATA AAT ACT GGA TTT ACC TGA AGT GTA ACT TTC TGG TTG TAT GGG TTT ACC ATT
ACA ACA ATG TCT GGA TTT TCC TTG TCA ACT CGC CTT TTT ACA AGA GGT ACT ACA ACT
TTT CCA AGT TCT CTT CCA AAT TCC TGT CGT ATG CTT TCT ATA AAT GGG GTT TCA ATC
TCT TTT TCA AGT TCT TTA AAC CTT TTT GGA AGT TTT GTA CCG ATT AAA AAA CTG TCA
AAC TCG TAT TCA GAA AGT GCG TTA ACT ACA TTT TCA GCA ACT TTT TCA ATA TTT TCG
GGT TCA AAA ATT CCT TTA CAC CAG GGG CAG AGT TCG TCA CAC TCT TCG TTT TTT TCG
TAT TCA GCT TCA TTT TTG AAT TTT GTT TCG TAT TTT TCA ATA TCG TCA ATT AAA TCA
AGT TTT ATT CCT GAA ATC CCT GTT TTA TAA AGA CAA TAC ATC AAT TCT TTA ATT TCT
TCT AAT TTT TCA GGG TCT ACT GGT TTT AAT TCT GGA TTT TCA GAA GCT TCT TTC ATC
TCT TCA AGG GCA AGT CTT AAG TTT TCT TCG AGT TCG ATT GCT TTT GCA AGT TTT AAA
GCA TGC CCC CTC TCA AAA TTA CTC GAC CTA ATA AGT TTT CCG TAA AGT CTT CCA AAA
CAT CTG TCG CAC AAT GGG TAT TTT TTT AAA ACA GAA TAA TCT ATA TTC ATA TAA TCT
CAC CAG GTG ATT TCA TGA TAA TAA ATG CGG ATC TAC ATA TTC ACT CAA AAT ATT CTA
TGG GAA CTT CTA AGT ATA TGG ATA TCG AGC ATA TAT TAA AAT ATG GTC CAG TAA AAG
GTC TCG ATT TAA TTG CGA CAG GAG ACT GTT TAC ACT CAA AAT GGC TCG AAG AAA TCG

AAA AAA ATG TCA ATA AAC CGC TTC TTC TTT CAA CAG AGG TCG AGG ATA GAA ATC GGG
TTC ATC ATT TGA TAT ATT TAC CAC ATA CCT CTC AAG CGC ATG ATT TAA GAG ATA AAT
TTT CAA AAT ATT CAA AAA ATA TCG ATG CTG ATG GAA GAC CAA GAG TTT CAT TAA ATG
GAA TGG AAA TTA TCG ATG CAG TAA GGG ACA TTG GCG GAC TTA TCG GAC CAG CTC ATG
CAT TTA CCC CCT GGA CAA GTA TTT ACA AAT CAT TTA CTT CAA TTT ATA ATT GTT ATG
CGA GAA AAC CAG ATT TTA TCG AGT TAG GAC TCT CGG CCG ATA CAG ACA TGG CAG ACA
TGG TTG AAG AAC TTC GAG ATC TGC CTT TTT TAA GTA ATT CTG ATG CCC ACT CAT TTT
ATT CTC ATC GGC TTG GAC GAG AAT TTA ATC AGA TGG ATG TAG ATG AAC TTG GTG GAC
TTG AAA CAA ACT TCG AAG AAG TAA AAA AAG TTT TAA AAC ACA ATA AAA TAA CTG CAA
ACT ATG GAC TCG ATC CAA ATC TTG GAA AAT ACC ATT TAA CTG CAT GTA CAA GAT GCT
ACA CCC GGT TTA CGT TAG AGG ATG CCA CAG AAA ACA ACT TTA AAT GTC CTG AAT GTA
AAG GAA CGA TAA AAA GAG GGG TTC ATG ATA AAA CTC TGG AAC TTT CAA AGG ATA AAA
AAG TAA TTC ACC CAG ATT TCA GGC CAA AAT ATC ACA AAA TAA TTC CGC TTG CAG AAA
TAA TCT CCC TTG CAA TTG GAA AAA ATA TTG GAA CAA AGT CGG TAG ATA CGA TTT GGG
AGC AGT ACA TTG AAA AGT ACG GAA ATG AAA TCG ATG TTT TGA TAA ATG AAG ATA TAT
CTG AAC TCA AAA AAA TAA ATG AAA AAG TTG GGC ATA TAA TAG AAC TTT TCA GAA CCG
GTA AAA TAT ACA TTT ACC CCG GAG GAG GCG GAG AAT ACG GCC ATA TTT CAA AGG TTC
CAG TAA GTG TTA AGT GGT TCA AAC CAA AAT TAA CCC TCG ATT CAT GGG TAA AAT AAT
AAA AAA GTA GAA ATT AAA ATT TAA TTT AAA TAT TCT TTT AAA ACT TTT GAA AGC CTT
TTA ATT CCT TCA ACT ATT TTT TCG TCA GGC ATA TTT GAG TAA TTT AAC CTG AAA GAA
TTT TCA TGA CCT CCG TTT GGG AAG AAA GAT CCA CCT GGA ACT ATC GCA ACG TTA TTT
TTA ATG CAT TCA TCT AAA AAT TCA CGC GCA TTA TAC TTT TCA GGA AGT TCA ACC CAT
GTA AAA AGC CCG CCT TCT GGA TTT GTA TAT TTA AGC CCC TCT GGG AAT TCT TCT GCA
ATT GTT TTA ATC ATT AAA TCC CGT CTG CCT TTG TAT AAT TTT TTT AAT CTT GAT ACT
CTT TCA TCA AAA TCG TAA TTT TCA AGC ATT TTT GAA ATA ATT CTC TGA GAA AAT GAC
GAG GTG TGC AAA TCT GCG CTC TGT TTT ATA AAG ATG TAT TTT TCA AGG ATA TCT TCA
GAT GCA GCA ATC CAT CCA ACC CTA AGC CCT GGG CAG AAT GTT TTT GAA AAT GTT CCT
AAA TGA ATT ATG AGG CCC TTT TTA TCC ATT GAT TTT AAT GAA GGC AAA ATT TCT CCA
TCA AAT CTT AAT TCC CCG TAA GGA TTA TCT TCG ACT ACC GGA ATT TCA TAT TTT TCA
ATT ATT TTC ATG AAT TTT TCC CTT CTT TCA AGA GTC CAC GTT TTT CCA GTT GGA TTT
TGA AAA TCA GAA ATT GCA TAA ATG AGT TTT ACA TTT TTG GTA GTT TTT AAA ATT TTT
TCA AGT TCT TCT GGA ATC ATT CCA AAG TCG TCA GTT GGA ACT TCT TTA AAC TGC GGT
TCG TAT GAT TTA AAT GCA TTA ATT GCT CCA AGG TAG GTT GGA CTT TCA CAT AAA ACC
ACA TCA TTT TCA TCT AAG AAT ACT TTT CCA GAG AAA TCT AAT CCT TGC TGG GAT CCA
CAG ACG ATT AAG ATA TTA TTT TCT GTA AGC TCT GTT TTT AGT ACT TTA TTC ATC CGT
TCT GAA ATC TGC TGC CTG AGT GGT GGG TAT CCT TCC GTT ATA TCG TAC TGG AGA GAT
TCA GGA CCC ATT TCA TCG AGA ACA ATT CCG GAA ATT TCT TTT ATT TCT TTT ACG GGA
AAG AGT TCT GGT GCA GGA AGT CCG CCT GCA AAA GAT ATT ATT TCA GGT TTT TGA GTA
ACT TTT AAT ATC TCC CTT ATT TCA GAA GAT TTA ATG TTT TCC ATC CGT TTT GAG TAG
CTT AAT GCC ATA CGC ATC CCT TAA ATT TTC GAT AAA GAT ATA TTT TGG TAG TTC ATA
ACA TAC AAA TAT ACA GTA TAT TGG GTA TAT TGT AGT ATA TCT AAT CTA TAT ATA CAT
TAT AGT GCA CGA GGA AAA GTC ATG AAG GAT CTT AAT AAA GCA ATT TCT AAA AAT CTG
AAA AAA ATC CGT AAA AGA AAA GAC CTA AGT TTA GAT GCG TTA TCC AAT CTA ACT GGG

GTA AGT AAG AGC ATG CTC GGT CAA ATC GAG CGA GGA GAA GTA AAC CCT ACA ATT TCA
ACA ATT TTA AAG ATT TCA AAT GGC TTA AAA GTT TCA TTC ACC TCA CTT TTA AAG AAC
GAA AAA CCC GAG ATT GAT CAG ATA CCA TTT GAA GAG TTA AAT CCT GTT TTC GAA GGT
GAC GGC CAC AAA CTC TAT CCT GTA ATT CCT TTT GAG GAT GAT AAA CGA TTT GAA ACA
TAC ATT GTC GAG ATA AAG CCT GGA CAC AAG TAT TAT TCT GAA GGC CAC GTG AAA GGA
ACC GAA GAA ATC ATA ACA GTC TTT GAA GGT GAA CTG GCA CTT TCA ATA GGG GAA ATT
GAT TAT TTC CTT AAA AAA AAT GAT ACA ATC AGG TTT AAT GCC AAT AAA TCG CAT GTT
TAT GAA AAT AGG GGT TTA GAA CTT ACA AAA TTA AAT CTT ATC CTT CAT TAC GAG TAA
TTT AAA AAT AGA TTA AAA GGT TAA AAT AAA TTA TTG GCA GCA TCC ACA GGT TTT TTC
CTG TTT AAT GCA TTC TAC AGG GCA TAC GTC TGA ACA GTC GCC ACA GTC GTT ACA TTT
AGC ACA GTC AAT AAC TGC TTT ACC ATC AGC GTT TTC TGA GAT AGC TTG TAC AGG GCA
TAC TGG AAC ACA TGC TCC ACA TGC GAT ACA TTC GTC TTC GTT TAC TTT ATA TAC CAT
AAT TAC ACC TCA ATT AGT AAT TTT TTC AGG TTT ATT CTC ATT AAG CAT TAC ATA TTT
TTA TAG GTT TGT AAA TGT TTC TAA TTG CCC CAT TGT ATT TAT ACC TCA AGG GAG ATT
TTT GTT ATA AAC CAT GAA GTG AAA AGT CAT GAA AGA AGA AAT GAA AGG AAT TTA CGT
TCT ATG GAT CAC AAT TTT TGT GAC AAT GCT CGG TAT TGG GCT AAT TTC GCC ACT TCT
CGC GAT ATT TGC AAA ATC ATA CGG GTT ATC AAA TTT CCA GAT AGG ACT AAT TTT TGG
ATC TTT TGC ACT AGT TAG GAC GAT ATT TCA GCT TCC TGC GGG AAC CCT ATC AGA TTC
TTA CGG AAA AAA AGT ATT TTT GGT AGG GGG AAC TCT TCT TTA TGG TGT AAC AAC TTT
ATT TTA TGG TTT TGC ATC TGG ATT TGC AAG TAT ATT GAT AGT TCG GAC ATT CAC AGG
AGT TTT TTC TGC ATT TGT AAA TCC TGT CGC AGG TTC ATA TAT CGC ACA AAC CTC TCC
AAA ATC AAA ACT TGG AGA ATA CAT GGG GTT ATT TAA TTC TGC GAT ATC TTT AGG ATT
TGG GGT TGG ACC GCT ACT TGG AGG ATT ACT TGC AGA TAT GTA TGG ATT TAT GGT TCC
TTT TTA TGT AGG CGG GCT TTT AGG TAT AAT TGC ATC AAT AAT AGC ATA TTT TAA ACT
TGA AGA TAC TCC AAA AAC CCA AGA AAA ACA AAA ATT ATC TTG GAA AAA TTT ATT TTC
ACT GGA ATT TTT AAA AAT GAG GAA TTT TAG CGC GGC ATT TAT AAT AAA TAT TGC ACT
AAC AAT TTC TAG AGG CGC AAT TTT AGC GTA TCT TGC AAT TTA CGC TTA TGA TTA TGG
AGT TTC TGC CTT TAA AAT TGG AGT AAT GCT TGC AGC AAT GAA TCT GGT TCT TGC AGT
AAC TCA GAA AAT GTT TGG AAG AAT TTT TGA TGA AAA AGG AAA CAG AAT AAT TTT ACA
CGG AAT AAT TAT TGG AAA TTT GGG AAT GTT TAT CCT TGC AAT GTC CAC GTC ATT TAC
TAC AAT GTT TAT TTC GTT AAT TAT CTC ATC AAT AGG GGC TTC AAT GAG TAC CCC GGG
CAT TAA TTC GAT CGC AAT ACG AGA TAT TCC TGA TAA AAG ACA GGG CGA AGC AAT GGG
CCT TTA CAC TGC AAG TAT AAA TGT TGG AAT ATT TTT TGG AGC AGT TTT ACT CGG CTA
CAT TTC CGA TAT CGT TGG AAT TTC TAA CAT GTA TAA AAT AGG GGC AGT ATT TTC ATT
TTT AAT AAG TTA CAT TGC ATA TAT CGC GAT AAA AAA ATA AAA AAA GAA TAA ATA ATG
ATT TAA TTT TCT ACT TCT TCT GTA AAT GCC CAT TTC AAT AGT GGC GGT GTT GAA ATA
ATC GAT ATT GTA ATG AAT ATC ATT GTT GCA GCA ATG AAT TTT CCG ACA TTG GAT TCA
GAA ATT ACT CCT GCA TGT ATT GCA ACC ATA AGG TTG ATC AAT GCA ATT TCT GCT CGA
GGA ATC GAT CCA ATC CCC ATT TGA AGC GAT TTT TTC ATA TTC CAA CCC ATG ATT CTC
GCA CCA AAT CCT CGT CCA ACG ACT TTA CTT ACA ATG GCA ATT GCA GTC AGT ACC AAA
GCA AGG TAG AGT GCA TCA AAA TCT CCA AAA ATT GTT AAA TTT AAT AAT GCC CCG GTA
TAT ACG AAG AAT AAT GGG ATT AAT ATA CTA TAA CCT ATT GTT TTA ACG TCT TCA ATT
AAG TTT TTA CCT TCG GCA GTT CTT GAA AGT ACT AAA CCT GCC ATA AAT GAG CCT TCA

ATA GCG GCC TCA AAC CAG TTT TCT GCT AAA AAC GAG AAT AAA AAC ATG ACT GCT ATT GCA AGA GAC AAT ATT CCT TTT TCT ACA TGG AGT TTT TCT GAA AAC GAA ATA AAC TTA TTT ACA AGT TTC ATG GCA AGG AAT CCT GTA ATT ATA AAG AAA ATT GCG AAT TTT CCT AAA AGT CCA AGG ATG CTT CCA GTA CCA ACT GCA AAT ATT ATC AGC ATT ATG CCT AAA AAA TCG TCC ATA ATG CTG GCA CTT AGC GAT GCT GCA CCA ACT TCG CTT TTT AAA ACA CCC ATG TCC ATC ATT ACC CTT ACA GTA ATT CCA ATA CTC GTT GCA GTT AAT ACC ACT CCT CCT GCA AAC GAT TCA GCG GCA CTG TAA CCC ATA TAC TGC AGT GCA AGA TAA CCC ATT ACA AGG GGA ACT AAA ACA CCA AAA ACA GTA GAA ACT GTA GCA ATT TTG CCA GTT TTC TTT AAA ACC GAC AGG TCA GTA TCA AGT CCG CCT AAA AAT AAC AAA AAT ATG ATA CCC AAA CGT GAT AAA AAA TCA AAA ATT TCA TTA TTC TGA ATT GTG AGA TGG TCA GGA CTC ACT AAT CCA AAA TAT ACT AGA TTT CCA AGG ATC ATT CCC ATA ATC ATT TCA CCG AGA ACT CCC GGA AAT CCT AAT TTA TTC ATC AGG TGG TCA CCA AGT TTA CCA AAG ATT ATC GCA ATT CCA ATG GTA AAC AAC ACC CAG CTA ATA TCC ATC AAA ACC CCT CTA ATT ATT CTT GAG CCA AAC AAA GAC TTA AAA ATT TGT CAA TTA ATA TTT TTA AAC TTA TTT CTC CAA TTA ACC TTT CTT CGT CAT TTA AAA CTG CCA ATA TCT GCA CAT TGT ACC TTT TCA TCT TCA ACA GTG CAT CGA TGA CTG TAG AAT TAT GAT TGA TCG TGA GCG CAT TTT TTT CCA TGA CTT CTT TTG CAG TTT TCG CAC CGC CAA TAA CAG ATT TCA ATG CAG AAC GTG TAC TGC CTA TGT ACA GGT TAT GCT TGT CTG GAG GAA GTA AAA AGT CAA TTA CAC TCA AAT ATT TGA TAA CTC CAA GTA AAC TGC CTT CTT CTT TAC AAT TTA TAA CCC AAA CGT GGT GTC GTG ATC TCA AAA GTT CCA TAA CCT TAA TTA TTG GGT CAT TAT AGC AAA CAA CAG GCA TCA TGT CTA CAG GGG GTA TTA CAT GTT TTA ATT TAA TGT GGT GAT ACT TTC CAA GTG CAT TTT CAA GAT TTT CCA TAT GTT CAC CAA AGA GTT TAG AGC CGA ATT ATA TAT CTT TTT GAA CAT ATA AAT ATT AAA CAA TTT TTT TAA CAG GGG GTG GTG GGT ACA TGC CTT TAA ATG AAA TTA TTC CTG TTA TTG AAA ACC TGA TTT CAA GAG GTT ATA TTA AAA AAC AAA GTG TAA TCG ACG CAC TTT TAA GTG TTC CAA GGC ACA AAT TTA TTT CAA AGA GCA TGG AAC GTT ATG CGT ATG TTG ATG GCC CTT TAG AGA TAG GAT ACG GGC AGA CTA TTT CTG CGA TAC ACA TGG TTG GAA TAA TGT GCG AAG AAC TTG ATT TAG ATG AAG GCC AGA ATG TAC TTG AGG TTG GAA CTG GTT CAG GCT ATC ATG CAG CAG TAG TTT CAG AAA TCG TTG GCG AAT CTG GAA AAG TTA CAA CTA TCG AGA GAA TAC CGG AAC TTT TTG AAA ATT CAA AGA AAA CAC TCT CAG AAC TTG GAT ACA ATA ACG TTG AAG TGG TTT TGG GAG ATG GTA CAA AAG GAT ATT TAG AAA ATG CTC CAT ATG ATA GGA TAT ATG TTA CTG CAT CGG GAC CAG AAG TTC CAA AAG CTC TAT TTG AAC AGT TAA ATG ATG GCG GAA TTT TAC TTG CAC CTG TTG GGG CCC ATT TTC AAA CGT TAA TGA GAT ACA CGA AAA TAA ACG GTA GTA TAT CTG AAG AAA AAC TTT TAG AGG TTG CAT TCG TTC CAT TAA TTG GTG AGA ATG GCT TTT AAT ATT AAT TAT CTT TTT TAA ATT AAA ATA TTA AAA AAT CAA CAT ACT TTT TTA TAT TTG AAA ACA AAA TTA ATT TTG AAA CGC GTA GTA TAT GGA ACG TAT CTA AAA TGG AGA GAT AAC ATG AAA AAA TTA GGA ACA GAC CTT TTA AAA AGA GGA TTT GCG AAA ATG GTA AAA CAC GGG GTT GTA ATG GAT GTT ACA AAC GTG GAA CAG GCA TTG ATT GCA GAA GAA GCA GGT GCT ACT GCT GTA ATG GCT TTA GAA AGA GTT CCT GCT GAC ATT AGG GTA CAG GGC GGC GTT GCA AGA ATG TCT GAC CCA GAA ATG ATT TTA GAG ATT AAA GAC GCA GTA TCA ATT CCA GTA ATG GCA AAA GCA AGA ATT GGA CAC TAT GTA GAA GCT CAA GTG TTA GAA TCA ATC GGT GTT GAC ATG GTT GAC GAG AGT GAA GTT TTA ACT CCT GCT GAT GAA GTA AAT CAC ATC GAC AAA CGA GCA TTT ACA GCA CCA TTT GTT TGC

GGT GCA AGA AAC TTG GGC GAA GCC TTG AGA AGG ATC GAT GAA GGA GCA GCA ATG ATT
AGG ACC AAA GGG GAA GCT GGA ACT GGA AAC GTT GTT GAA GCT GTA AAA CAC ATG AGA
GCA GTT AAC GAA GGA ATT GCA AGA GTT ATC GGA TAC AAA GAA ATG GGC CTT GAA GCA
GAA TTA ATT CAA ATG GCA AGA AAT GAA TTG AAA GTT CCA ATG GAG CTT ATT TCA GAA
GTT GCA GAA CTT AAA AGA CTT CCT GTT GTA AAC TTT GCA GCT GGT GGA ATT GCA ACA
CCT GCA GAT GCA GCT TTA ATG ATG CAG ATG GGA TGT GAC GGA GTA TTT GTG GGC TCT
GGA ATC TTT AAA TCA GGA AAT CCT GCA GTA AGG GCA AAA GCA ATC GTT GAA GCA ACA
TAT AAC TTC GAC AAA CCA GAA GTA ATA GCA GAA GTA AGC AAA AAT TTA GGA GAA GCA
ATG GTT GGA ATT AAT ATC GAT GAA ATT CCA GAA GAA ATG CTT CTT GCA AAA AGA GGA
ATT TAA TTT TGC TTA AAC TTA CCT AAA TTT TTA TTT TAA TTT GGA TTT TTA TAA TAT
CTT TTT AAA AAA TAA AAC ACG ATA ATT ATT TGA AAA ATA ATT TAC CAA GAA GAA CTA
CCG AAA GCT TTA TAT ATA ATG ATA CTA TTA TTG AGT ATG CGA ACA AAT TGC CTC GGT
GGC TCA GCC TGG TAG AGC GCC TGA CTT GTA ATC AGG TGG TCG GGG GTT CGA ATC CCC
CCC GGG GCT TGA GTT ACC TTT ATA AAT AGG TAA GTT CAA ATA ATA ATT GTC AAA TGG
GCC TGT GGG GTA GCC TGG TCA TCC TTT GGG ATT TGG GAT CCT GAA ACC CCA GTT CGA
ATC TGG GCA GGC CCA CCA CTT ATA TTC CCG CGA TAG TTC AGA CTG GTA GAA CGG CGG
ACT GTA GAT CCG CAT GTC GCT GGT TCA AAT CCG GCT CGC GGG ACT CAC ATT AAT TCT
GGG CCC GTA GCT TAG TCT GGT AGA GCG CCT GAC TTT TAA TCA GGC GGT CGA GGG TTC
GAA TCC CTT CGG GCC CGC TCA AAA ATT TAT CAG AGT TAA TAA CTC TGA AGG GTC TGA
CAT AAC GGT CAT AGC GGA GGA GTT ACA TCC GAT CCC ATC CCG ATC TCG GAA ATT AAG
CCC TCC TGC GAT TCA TTA AGT ACT GCC ATC TGG TGG GAA CAA TGT GAC GCT GTT AGT
CAC TTT TTA TTT CAC ATG CTC TGG TGG TGT AGC TCG GCC TAT CAT ACA GGA CTG TCA
CTC CTG TGA CTC GGG TTC AAA TCC CGG CCA GAG CGC CTT ATT TCT ATT TTC AAT ACA
AAA CGC AAT CTG GGC CCG TAG CTT AGT CTG GTA GAG CGC CTG ACT TTT AAT CAG GCG
GTC GAG GGT TCG AAT CCC TTC GGG CCC GCT CAA AAT TTA CTT TCA CAT GCT CTG GTG
GTG TAG CTC GGC CTA TCA TAC AGG ACT GTC ACT CCT GTG ACT CGG GTT CAA ATC CCG
GCC AGA GCG CTT TTT TAA TAA TAA TTA TTA TAA TCG ATA TAT TTG GAT TTA TTG TGA
AAA CAT GTC TCA CGA AGG CGA AGA GTT TAA CAA AAT TTT TTC AAA AGG AAA ATT TTT
AAA AAA TAT TTT TTT CAA CCT GTT CAG ATC ATA CAA AAA TCC TGA AAT TAT TGG AAA
AAC CAC AAA TCC AGA ACT TGA TGA CTG CAT ATG TTG CGG TTT ATG CGT TGA TGT ATG
TCC AAC AAA TGC GAT ATC TGT TTT TAA ATT TCG AGA TAT AAT CTG TGA ACA CTG CGG
TGC TTG TTT GAA TGT ATG TCC TGT TGA TGC AAT TGA AAA AAA TCG ATT TTC AGT GGA
TCC TGA AAA ATG TAT AAA ATG CGG AGT ATG TTC TAT ATT TTG TAC AAT TCC GAT TTT
AAA AAA TGA AAT TCC TGT TTT AAA CGT TCC TGA AGT TAC AAC GGA ATG CAA TAA CTG
TAA ACT ATG TAT TCA AAA ATG CCC TCA AAA AGC AAT TTA TTT TGA AGA TGG CAA AAT
TTC AAT AAG TTC AAA ATG TAA TTT GTG TCT TAA GTG TGT TGA ATT CTG TCC TCT TAA
TGC ATT AAA ATC TCC AAA AGA GTC ATT AAA GTC TAA AAT TAT TAA AGT AGA TCT AAA
TTC CTG TAT TTT TTG TAA AGA CTG CGA AGA AAT ATG TCC AGT TAA AAA ATG ATT ACG
AAA TCA GAC GAT TTT AAT CCA CTA TTA ACA TTT ATA TAT TAT TTT CTC AGA TAT GAA
TAA CAT TCA TTC GGG GGA GGA ATT TTT TTG AAA TAC GGA ATG TTA TTT TCA GGA ATA
CTA ATG TTT AGT ATC GGA CTA AAT GTT TTT GGC ACG TAT CTA GAG AGC ATA TAT TTA
TTT CCG CTT TAT CTT GAT AGT ATC GGA ACG ATA TTT AGT GCA ATA ATA TTG GGC CCG
CAT ATT GGG GCT CTT GTG GGG CTT ATT ACA AAT TTG ATA ACA GGA TGT TTA CTA TCT

GTG GAA AAC TTT CTT TTT TCC GTA GTA AAT GTT TTA ATC GGG CTT ACA ACC GGG ATA
ATG CTG TAT AAA AAT CAA ATT AAT ATT AAA AAC ATA TTC TTT GCT GCG ATA GCT GTC
TCG ATA GTC TCT TCA ATT ATG GGG AAC CTT CTT TCG ATA TAT TTC TTT GGC GGA ATT
ACT GAA GGA GGG GTA GGC CAC ATT ACC CAG ACT TTA ACC GCT TTT GGA GAA AGT ATT
CCA ACA GCA GCT TTT GCA GCG GGG CTA TAT ACT AAT TTT GCA GAC AAG ATC ATA TCA
TTT ACT GCT GCA TTT TTT ATG ATA TAT GTT TTG CAG GAC AAA CTT TCC ATT TAT GAC
TTA AAA CTT GGG AAT TAA TCT TTT ATT TTT TTA TTG GGT TTA TTA ACT ATT AAT AAT
TTT AAT GAC ATT ATT ATA TAC TTT ATA TTT CAG AAG TAC TAC CAT TAA ATT GAC GGT
GGT ATC ATG GAT AAA AAA GTA CTT TAT ATG ACA ACA ATC CCT GTT GGA ATC GCA ATA
AAT GCA ATA GGG GGC CAA ATT GCA ACT TTT TTA AAA TTG CCT ATA TTT TTA GAT AGT
ATT GGT ACT GTT TTA AGT GGA TTT TTG TTA GGT CCA GTT GGA GGC GCT TTG GTG GGT
TTC TTT ACA AAC GTT CTT CTT GGT TTT ATA CTC GAC CCG TCA TAT ATC CCA TTT TCA
ATA GTA AAT ATT GTA ATT GGG CTA GTT TCT GGA TAT GTT GCA GTT AAA CAC GGA ATA
ACT CTT AAA AAT TCA ATA ATT GTT GGG TTA GTG CTT GCA ATA ATT GCA CCA ATG GTT
GGA ACC CCA ATT GCA GTT TAT TTA TAC GGA GGG CTT GTT GGT GGA GGC GTT GAC CTT
TTA ACA GCG GTA TTT TTA CAT TCG GGT CAG GAT ATT TTT TCA TCA GCG TTT TTG GCC
AGA ATA CCT GCA AAT TTA GTG GAT AAA CTG CTT TCA TGT ATT TTG GTA TAC TAC ATC
ATA AAA CCG TTC CCA AAA GAC ATA CTT TCA GAA TTA GGC GTA AAA GTA AAT TAG GTA
AAA AAT ATG AAA TAC CTT AAA ATT AAG ATT TAT CTA ATT TTT ACC CTT TTT TTA CTT
GTT TTA GTA ATT TTT AAT CCA TTT TAT GGA ATT TTA GCG TCC ATT GTG GTT GTT TTA
CTT ACC AAA AGA TTT GAA GTA TTT TCA AAA AGA TGG ATT TTG TTT TCC CTG TAT CTG
GTT GTT TTT TAT TAT TTT ATA ATG GGC CAG GAT GGG CTT AAC AAT GCA TAC AGG CTG
CTT GCA TAT ATT TTT ACA GTG CAG TGG TTT ATA AAT TCA GTT TCA ATT GAA AAG TTA
GTT GAA TTT ATT TCA AGT TAC AAT CGT GAT TTG GGA ATT GGG ATT TGG ATG ACC TTT
TCA ACA CTT GAG GTT GCT AAA AAG GAG TTT GAA ACT ACG AAA AAT GCC CAA CTT TCA
AGG GGG CTA AAT AAA AAA GGA TTA ATT AAT AAA TAT CGG TCT TAC TAT GCA ATA ATC
TCG CCA TTA ATT GTA AAA TTA TAC ATT TCA GCA ATT AAC CGG GCA AGA TCG CTT CTT
TCG AAG TGT TAC GAC TGA ATT ATT TTT TAC ATA TTT TTA TAT TTT ATA TCT TTT TTA
GAT ATT AGA AAT TAT AAT TAG TAT ATA TTT TAT TTT AAG ACC TAA TGA TAT CAT TAA
ATA AGA TAA TCA CTC TAA CTA ATG TAT TTT CGG AGG TGT AAT ATT GAA AAA TTT AAA
AGG CAT ACT CGC AAT TTT CTG CGT TAT GGC AGT ACT CGT ATT TGC AGG CTG CAC TGA
ACA GCA AGC TTC TAA TGT TTC ACA AGA AGA ACA GAT TGT AAC GGT ATA TGT TGC TTA
TGG CAG TCT TGA CTT CAT TGC AGA TGA ATT TGA AAA AGA TAC AGG CAT AAA AAT GGA
ATA CGT GTC ACT ATC ATC GGG TGA AGC TCT TTC GAG GCT AAA AGC TGA ACA GAG CAA
TCC TTC AGC AGA CGT CTG GCT TGG TGG AGG AAT CGA TGC ATT TAT AAG TGC ACA GCA
GGA TGG GCT ACT TGA AGC CTA CAA ATC CCC AAA TGC AAA AGA TAT CGA TCC ATT GTT
TGT TGA AGA AGA CGG CTA CTG GAC AGG AGT TTC TCT TGT TAC AAT AGG CAT CTT AGA
AAA CAC GGA TTT GTT ATC TGA AAA AGG ATT ACC TCA ACC ACA AAC GTG GGA TGA TTT
AAT AAC TGA TGA TTA CAA AGG AGA ATT AAT TGC ATC CAA CCC GAC AGT TTC GGG AAC
TGC ATA CTT TAC GAT ATG TGG AAT TCT TCA AAT GTA TGG TGA AGA TGA AGG CTG GAA
TTA CCT TGA TAA ATT CTA TCA AAA CAC TCC ATT CTT AAC AAA ACG TGG AAG TGG GCC
TGG AAA TTT AGT AAC TGC TGG TGA ATA CGC TTA TGG TGT AGC GCC TGA CCC ACA CAC
GAC ATT AAT TGC AGA TTC AGA ATT ACC TGT AAA ATC TGT GTT TTT AAA TAA AGT TAT

CTG GTG GCC ATC CCC AGT TTC GAT AGT AAG CGG TGC AAA ACA CCC AGA AAA TGC GAA
AAT ATT TGT TGA CTG GTG TCT TTC AAA AGA AGG TCA GGA AGT ACT TAT GCA GGC AAG
CCC TAG GGT GCC TGT AAG GGG CGA CAT TGA GGT TAT CGA AGG CGT TCC AAA CCC TAA
AGA TCT TGA TTT CCT CGA CAT GGA TTT CAT GTA CTG GGG AGA AAA CAG GGA CAG GAT
TTT AGA TGA ATG GGA ATC TAG ATA CAG AGA AAT TTC AAC TAC AGA ATA ATT TTT TTA
CTT ATT TTT TTT CAG GTG CAG TAA ATG AAA GAA GTA TTT AAT AAC GAA ATA TTA AAA
AAA ATT TAT CCA AAA ATC CTT GAA AAT GGT TTT TAT ATT CTT GCA AAC GTT TTT TTA
TTG ATA TTC ATA ATG TAT CCT GTA TTT ACA GTA TTT TTA CAA TCA TTT TAC GGT TCA
AAC GGT TTT ACG CTT GAT GCT TAT TCT AAA TTT ATC TCA GAT TCG TAC TAT TTT GGC
ATT TTA AAA AAC AGC ATT GTT GTA GCA TGC TTT TCA ACG ATA ATT TCG ATA TTT TTG
GGT TTT ATT TTT TCA ATT ATC ATT TTT AAA ACC GAT TTT AAA TTT AAA AAT TTT TTC
AAA ATT GCA GTT TTT CTA CCA ATA ATT ACT CCA GGG TTT ATT TCA TCG CTT GCA TAT
GTT TTC CTG TTT GGA AGG CAC GGA GCA GTT ACT TAC GGC TTA CTG GGG CTT GAA CCA
AAT ATA TAT GGC TGG AAA AGT GTT GTA ATA ATG CAG TCT ATC GAT TAC ACG ACA ACT
GCA TTT TTT ATA ATT TCG GCA GTA CTT CTT GGA ATT TCT GGA GAA TTT GAG GAT GCC
GCA AGG AAT TTA GGT TCA AAT GAA TTT CAG GTT TTT AAA AAA GTA ACA TTT CCG TTA
TTA ATT CCT GGA ATC TTG AGT GCA GGC CTT TTG ATA TTC ATG CAG TCT ATG GCT GAT
TTT GGA ACG CCG ATA ATT GTT GGT GGA AAT TTC AAC ACG CTT GCA ACG GCA TCT TAT
TTT GAA ATA ATT GGA AGG TAT AAC ACC CAA ATG GCA TCA ACA CTT AGT GTA ATC CTT
CTG TTC CCA TCA ATG GCC TTA TTT TAT CTT TAT TCA AAA TAT CAT AAA GGC TAC GAG
CTA AAA AAA TCT AAA CTA ACA AAA TAT TCA TTA AGT AAT ATA CAA AAA GTA ATT CTT
GGA CTT CCT GCC GTA TTT TTC TCA ATA ATT GCA TAT CTT TTG TTT TTT GCA GTT TTT
GTG GCT TCT TTT ACG AAA AGT TTT GGT CAC AAC TAC GCT TTA ACT TTA GAT TAT TTT
TAT GAA GCA ATA AGC GCA GGA CAG CTT GCA ATA AAA AAC ACA CTT ATT TAT GCC CTG
TCT TCA AGT ATT TTG GTG GCT TTT TTA GGG GTT GCT TAT TCT TAT ATT GTA TAT CGG
GGA AAA TTT TTT GGT CGA AAA CTG ATG GAT TTA GTG ATA ACA CTT CCA TTT GCA ATA
CCT GGA ACG TTT ATG GGC TTG GGA TAC CTT TTA GCA TTT AAT AAT TAT CCG TTA TTG
CTA ACA GGA ACA TCT TCA ATC ATA ATT TTA AAC TGC ATG GTT CGA AAA CTC CCG TTC
TCA TTT AAA ACT GGA AAT TCA GTA CTT TCC CAA GTA GAA GAG TCT GTT GAA GAA GCA
TCT CTT AAT CTT GGT GCA AGC CGA TTA AAA ACA TTT TAT AAT GTT GTA CTG CCC TTA
CTG AAG CCT GCA ATA ATA TTC AGC ATG ATA TAT ACT TTT ATT GCA ACT ATA AAG TCA
CTG GGT TCG ATA ATA TTT TTA ATG ACG GCA AAT ACA AAA GTA CTA TCA GCA ATG GTC
TTT GAA TCA ACG ATA AAC AGG CAG CTT GGT GTC GGG GCA TGC TAC TCG ATG TTC ATG
GTT ATT TTG TCG ATA ATT GGG ATA TTG GGA ATA TTG AGA TTG AAA GGG GAT AAA AAA
TGG ATT TAA GAC TCG AAA ACA TTT CAA AAA ACT TCG AAG AAC ATG GGG TTT TAA CAG
ATA TTG ATT TTG AAT GCG GCG AAG AAC TTG TGT CAC TTG TTG GTT CCA GCG GTT GTG
GGA AAA CGA CTT GTC TCA GGA TTA TTG CAG GAT TTG AAA AGC CTG ACG CGG GAA AAA
TTT TTT TAA ATG GTG AAG ATA TAA CGA ACG TTC CAC CAA ACA AAC GAA ATA TTG GAT
TAG TAT TTC AAA ATT ACG CAT TAT TTC CCC ATT TGA ACG TTT TTG AAA ATA TTG CAT
ACG GTT TGA GGT TAA AAA AAT TTA GTA AAT TGG ATA TTT CTG AAA AGG TAA AAA ATT
CGA TAG ACC TCG TCA ATC TCG GAG GGT ATG AAA AGT ACC CGA TTA GTG AAT TAA GTG
GTG GAA TGC AGC AGA GGG TTG CAA TTG CAA GGG CAA TAG TGA TAG AAC CAA AGG CAA
TGC TTT TGG ACG AGC CTT TAA GTA ATT TGG ATG CAA AAT TAA GAA TAA AAA TGA GAA

AAG AGC TTA AAG AAC TGC AGA GAA ATT TTG AAA TTC CAA CGA TTT ATG TAA CGC ACG
ACC AAG AAG AAG CAT TGG CCA TTT CAG ACA AGG TTG CCG TAA TGA ATA AGG GAA AAA
TAG AAC AGT TTG GAG ACC CTT ATT CTA TTT ATT CTA ACC CAA AAA CCG AGT TCG TTG
CAA AAT TTA TTG GAA TTA TCA ATG AAC TCC CCT CAT CAA TAT TAA ATA AAT TGG GTA
TTG AAT CCG GCG ATA AAA AGT ATT TTG TAA GGC CTG AAT CAA TAG AAA TCG GTA AGG
GCA ATT TTA AGG GAA AAT TGG TGG ATT ATG AAT TTT TAG GAA GTA TTG TAA GGT ATG
TTA TAA AAT ATG GGG ATT CTT TAT TTA CTA TTC AGT CGA ATA AAT TGG TAG ACT TTA
AAC TCG GGA CTG AAA TTT TGT TTG AAT TTG ATA AAA ACA GTA TAA TTT CAA TTT AAT
TAT TAA CTT ATT TTT AAA ATT TTT AGG TAG CTG TCA ATT ATT TTT TGA TAC TTT TCA
TTT AAC TGA AAA TCA GAA ACT GTG TAA AAT GCG GTT TTT TTC CAA TTT TTA CAG GTA
TTT TCT TCT ATA TTT AAT TTT TTT AAA AAT TTA CAT TCT CCT TTA AAA ATA TAA CTT
TTA AAA GAG TCT ATT TTT ACA TCA AAT CTA TTT TCC AAA AAA CTG TCA AAA TAA TTG
TAG ATT TCG GCA TTT TTT TCA ATT GAT ATA TTA TTT TTA AGT CTT ATT TCG TAT TTC
CAG ATA TTA TCC AAG TGT ATG TGA AGT TTA TAA CCT TTT AAA AAT GAT TTT TCA AAA
AAA GTT CTA TTT TCA AAT AAA TCT TCC GGA TCT GAT TCA TAA AAA TGA GTA ATT TTT
CTT TCA ATA CCT GAA AAA TAG CCA ATA TCT GGA ACA CAA GCT CCA AAA TAA AAA TTA
TTC ATA TCA AAA TTC TCA TTT ACG TTT AAA CGT TCT TTT TCA AGA AAT ATT TTT GAT
AAA ACA AGA TGG GAG TAT AAA TTG GGC ATA TTT TAC CTA TTT CAA ATG TTC AAT GGA
ATT ATT TTC TGG AAT ATA ATT TAA AAG TTT ATT TCC AAT TCT TGT AGT GTA AGG TCT
TGT TTT GTT TGT ATC GTT TAG TAT TAA ATT CAT CAA ATC TTT CGA TTT AAT ATT TAT
ATA TCC TTT TGA ACA CTC TTT TTC GTT AAA ATA TTC AAC ACG ATC CTT TAT AAT GCC
ATT TTT AAA TAT TGC AAG CGG AAC GTC TTC TCC CGA GTG TAT TAA TGT TCC AAC AGA
TGG CGT AGA ATG ATC GGC AGT AAC AAT AAT CAG TTC ATC ATT TAA TTC ATT TAT TTT
TTG AAG GCA TTT ATC TAT TTT TTC GAT AAC TTC GAC TTT ATT AAT CGG ATT TTT TGT
ATG TCC TGC TTC ATC AGG CTC TTT GGT GTG GAT ATG CAC AAA ATC ATA ATT TTT TAA
TTC AAT TCC TTT TAA AAA TGC TTT TTG AAA ATC GCC TTC AGG ATA ATA ATC CAT TTT
CAG CAA TTT TGA TAA ACC TTT AAA AAC GCT GCT TTT TGC AAT AAT TGC AGC ATT CAT
GCC CCA TTT ATC ATG GAA AGA ATC TAT TTC CCT ATA TCT TCC AGC CCA CTT TGT AAG
CAG GAA ATT TGC AAG TTG GAG ATT TTT TTC CTT CCT TTT AAT ATT TAC GGG ATG ATT
TTC AAG GAT TTC ACT GCA TTT TAG AAG ATA TTT ATT TAA TAC TTC GGC AGT TTT CAC
AGC ATT TTC AAT TTC TAT TCG TTC TGA AAT ATT TTT AAG CGG TTT TAC CTT TAA AAC
GTG TCT TTC CCG ATA AAA AGG GTC GCT ATC AGA TAT TTT ATC CGA TAT TTC GTA ATT
TGA AGA ACT TAT TTC TAA AAT ACA GTG TGT ATC AAA AGA GTA GTG TAG ATT AAA TTC
ATA ATC TCC AAT TTT TTT TGG AAG GGA ATT TAT AAG CTG TTT TAT TTC TTC AAT TGA
TAT ATT TTT AGT ACG CCT ATC TTT TAC AAA AAA GCC ATT ATT TCT TTT TAC ATA TCC
AAA AGT GGC CCT TAG ATA GAT AGA ATT ATC TTT AAG TTT AAT ATC TTC GCC CAA AGC
TTC GAT AAC TCC TCT TCC AGG AAA TTC CGA TAG TTT GTA ACC CCA GAG CAG AAA ATG
AGC AAC TTC AGT TCC AAG TGG GAC TCC TTT TTT ATA CGG CGT CAT CAT TCC TGT GAC
GCT TTT TTT CGC AAA AGC ATC TAA GTT TGG AGT TTT TGC ATA TTC AAG TGG AGT TTT
ATC TTT CAA AAC CTC AGA AGG CCT ATC TCC AAG TCC ATC CAA TAA TAA AAG TAA GGT
TCT CAT GGT ATT ACA TTA AAG GAA CTT TTT CAC GGT TTC AAT ATA ATT AAA GTT GTT
TTT ATA CCC GTT TTT CAA TAT TTC AAT GGA TGA CGG ATA TAT AAA TTC ATT TAA GAT
TTT TAC GAT TTT TTC TCT TTC AAA AAT TAC TTT TCC GTC CAG ATC CCT GTA ATT TCC

ACT AAA AAT GCA TGA ATA TTG GTT TTT TCC CTG TTC CCA TGG TTC ACC AAT AAT TTC
TTT TAT TTC TGA CCC TTT TTC AAT ATC TTT TAC TGG AAG ACA GAG TTT TAC GTC AAA
TTC ATC GCA TAT TTT GTA GTA TGT TTC AAG AGT TTC CTT GAA CTG GTC GAT TTT AAA
ATC TTT GTC GTG GAG TAT TCT CTC CCC GCT AAT TAT TTT TTT TCC AAG GTG TTT TGC
AAG CGG AAT TCT TAT CAT TCT TAA ATA TGC ATG GCA CCC AAT GCA GGG CGT GTA ATA
TCC ATA TTT TTG AAT TGT GTG TGC AAT AAA TCT CCC ATT GAG TGC ATT CCA GAG TTT
TGG TTC AAA AAT AAA ATG AAG TGG CAG TAA AAA TTT ATT TTC ATG CGT TTC TTT TAT
TTT TTT ATT TAT TAC ATT CCA GTT TCG GTA GAA AAT TTC TTT TTC ACC GTA ATC TGT
ACC CGT AAA TGT TAA AAC TGG CAA AAC AGC ATC AAT ATC TTC ATC TTC AAA AGC TTT
TAT AAT TGC TGC AGC GCT GTC TCT TCC GGA AAA CTC CCC GAC GGC AAT TTT TTT TAA
ATC AAT TCC GTT TTC TTC GAT AAT TTT CAA TGA ATC TTT AGA TAA AGT TAA CTC TTT
AAA TGA TTT TTT AAA CTC GAT ATC CAA AAT AAC ACC TAA ATA ACT TAA AGA TAC GTT
TTA TTT TAT TTA TGA AAT TCA AGG TAT TTA TTT AAC ATG TTT TTA GTG CTG ATG ATT
TCT TCA ACA TCC ATA AGT TCG ATA AGC CAA AAA TCG CAG CCG ACA TCC AAA AGG CCA
TCT AAA ACG GGT TTT AAG TTG GTC AAA TCT TTT GGA GCA ACG TGC CCT ATT TTG TCG
CAT TCT TTT TCG TAA GTA TGC ACA TTG TAA ATT TTA TCT GAA AAA AGG TCG ATA CAG
TTC ATT CTG TTT TCA TAA GGG GTG TGT CCT ATA TCA AAA GTT ATT TTA CAC CCT GTT
TCT TCT GCA ACG TTT ATT ATG TTC TCA GGT TTT GAA GAA AAT CCA TTT CTT AAG TTT
TCA ATA CAA AGA GTA ATG TTT TTT TCT TTT GAA TAT TCA GTT AAT TCC GAA ACA TTT
TCT AAA AGA GTA TTG TAT TCG GGT TCT TTC CCG TTA TGA AGG TGA ATA GTT AAA TAA
TCT GCA TTA TCT AGC TTA TTA ACT ATA TTT TTA AGT GTT TCA AGT CCT TTT TTA TTA
TTC GGA GTT CCG ATA TCT ACG ATA GGT GCA TGG AAT CCA TTA AGT CCT TTT TTG AAG
TTT TCA TCA ATG TTT GAG CAA AAA TGA AGG CCC CAA TCC GTG TAT TCC AGT CCT TTT
TCT GGT TTT GAA AAT CGC ATG CAA GTT CCT ATC ATA AAA GCA CCG ATA CAG ATT ATA
CCA ATC AAG ATA TAT AAA ATT GTC ATT AGA ATA TCT AAT AGA TAT GGG TTA GCA GTA
AAA TAT ATG TTT TTG AAA GAT TGA TAT TTT ATA TGT AGA AAC CAA ATT TGG GGG TTA
CAT AAC ATT TAA ACG AAA AAG AGG GGA AAT ATA TGA ATG CAA TAT TTG AAA GAA GGA
GTA TTC GAC ACT ACA CTT CTG AAG ATG TTT CAG AAG AAA TAG TCG ACG ATC TTT TAA
AAG CAG CAA TGT CTG CAC CGA GTG CAT GCG ACC AGA GGC CAT GGG ATT TTG TAG TAA
TTC GTG ATA AAA ACA CAC TTT CAG GAA TTT CTA AAA TAA ACA GGC ACG CAA GGA TGT
TAA ACG AAG CAC CTG TTT CAA TAG TTG CAT GCG GTA ACA TGG ATC GGG TAA ATG GAT
CAT GTG GGC AGT TCT GGG TTC AGG ACT GTG CTG CTG CAA CTG AAA ATA TTT TAA TAG
AAG CTC AGG ATC GGG GAA TTG GTG CAG TAT GGC TTG GAT TTT ATC CTG TAG ATG AAA
GAG TCG AAA AAA ATG AGA AAA GTT CTG CAT GCG CCT GAA AAT GTA GTT CCT TTT TCA
GTT GTG GCG TTA GGA AAT CCT GCG GAA AAG CCA GTT CCT GTA GAA AAA TTC GAT AGA
GAA AGA ATA CAC TAT GAA AAA TGG CCG ACA GGA TAT CTT TAA AAA TCT TTT TTA AAA
CTT TTA AAT TTT TAA ACT TAT GGG GGT GGT TAT TTT AAA GAT AAT CAT TAC TTT TTT
ATC CCA TCA AGT TTT AAT TTA TAC AGC AAA ATT TTC GCA TTA TTC TAA AAG GTG GTT
TTA ATG AAA ACA GTT TCG ATA ATA GGC GGA ACG GGG TAT ACG GGC TCA GAA CTT TTA
AGA CTC CTT TCA AAC CAC GAC GAA GTT GAA GTT GTG AAT GTA ACA TCA AGA AAG GAA
GCT GGA AAA AAT CTC ACT GAT TAT CAC CCT CAG GTC AGA AAT TTA AGT AAC TAT AAC
GAT TTA AAA TTC CAA AAT ATT GCT CCA GAA GAT ATC GAT TCT GAT ATC GTG TTC TGT
GCA ACA CCA CAC GGT GCG TCA ATG GCA ATT GTC CCG ACA CTG CAC GAA AAA GGA ATA

AAT GTA ATC GAT TTA AGC GGG GAT TAC AGG TTT GAA GAT ATT GAT ATG TAT GAA TCA
TGG TAT GGA TTG AAA CAC TCT GGA AAA ATA GAT TCT GCA GTT TAC GGC CTT CCT GAA
CTT CAC CGG GAA AAA ATA AAA AAG TCA AAA ACA ATT GCA AAT CCG GGA TGC TAC CCA
ACA GGA GCA ATT CTT TCA ATG GCG CCA TTG GTT GCA AAT GAT CTC GTT GAA GAC AGG
ATA ATT TTT GAT TCA AAA TCG GGA GTA AGT GGT GCA GGT GTC GAA GCA AGT CAA ACT
ACC CAT TTT GCA AAT GTA AAT GAA AAT ATC GGA CCC TAC AAA ATA ACG AAA CAC AGA
CAC AGT CCC GAA ATT GGA AAA GAG CTT CAA TAT CTT GCA AAC AAA AAT TTG AAA GTT
TCA TTT ACT CCT CAC CTT CTG CCA GTT ACA CGG GGA ATA TTA ACT ACT GCG CAT TCA
TTC CTT AAA GAG GAT ATA AGT CCA ATA GAT GTT ATC GAA ATT TAC GAA GAA TTC TAC
AAA GAC GAA TTT TTC ATA AGA ATT TTC GAA GAA GGA ATG CCT TCA TTA ACG GGA GTT
CGA GGA ACC AAC TTC TGC GAC ATT GGC GGA TTT GAA ATT GAC CAA TAT GGC AGA ATT
GTT GTA GTT TCA GCA ATA GAC AAT TTA GTA AAA GGA GCA AGT GGT CAA GCA ATC CAG
AAC ATG AAT ATT ATC ATG GGA TTC GAT GAA AAA GAG GGT TTA GGC GTT GGC GGG CTT
AAG CCT TAA CTT AAA TTT TAA AAA TAT TTT ATT TAT TTT CTT ATT ACA TTA AAT CTT
CCG GCA GAG GAA TGT TTT GGA AGC ACA TCT ACA CCG AGG CTT TTT AAA TAA GCA TAT
GTT TTC GTA TCA GTT CCG TGA ATG TAG ATT TCT CCC AAA TCT TCA ACA GTG TTT ACA
TCT ATT GAC GTA TAG AAG GAA TGG CAT TTT ATA ACT TTT AAA TCG TTT TTT TCG GCT
TCT TCA ATA TGT TTT AAA AAA CTG AAT CCT TCA AAC TGT GTT TTC ATG CAA TTT TTT
GGA TTT AAT AAA AGT AAA TTC GTA CCT CCG CCC CTT GAT GGG CAG ATT ACT ATT GAA
TTT TTA ACG GAT TTA CTA ATA ATT GCT TTT AAA TTT ACT TCT TTT AAA AGC GGA ACA
TCT GCA GGA GTT ATT AAA ATA GAC TCC TCT TTT ATA AAA TTT AAA GAA AAG TTT ATA
GCT TCG TTT AAT CCT TTA ACA TTG CTA TCT TCC CTA ATG GGA ATT ATT CCA AGA TTT
TTT GAA AAT TCT AAA ATT TCT TCA TCC TTA CTT ACA ACG TAG CAG GCA TCG GCA CAT
TCT TTC ACG GTT TCG TAA GTA TCC AAT AGA ATG TTT TTT ATA AGC TCT ATT CGT TCA
CCA GAA CTT AAA AAA TCT TTT AAT CTT GAT TTT ACG TTT GAT AGG GGG GAT ACG GGT
ATT AGT GCA GCT AAC ATC TTT TAC CAC AAC GTT TTA AAT AAT GAT TAG TTT TGG GTG
TTT AAA TTA TAT AAA TAA CAT TGC ATT TGA AAA TGC GCT TAA ATC GCC AGC TTC TCC
GTG GCC AGG ATA TAT TTT TAA AAT TCC TTT TTT ATA AGC TAT TTT TTC AAG TAA ATT
TAT CGA TTC TCG TAA CTC GAT TAG GCT TCC CGT TGG CAG ATC GGA TCT TCC AAC GCC
GTA TGA AAA CAG GGT GTC TCC AGT AAT TAG TGA ATC TTT GTA TAT TAT GCT TAT TCC
ACC CGC AGT GTG TCC TGG GGT ATG TAT TAT CTC AAT TCC TAT TTC TTT TAA GTC AGA
ATC AGC TTC TTT TAT TGT AAA AAT TTC TTT TGG TGG AAC CAG ATC CAT TCC AAA GAG
TTT ACT TAC TGT AAC ATT ACT ATC TTC TGC AAG GTG GGG TAA CTC TAG TTC ATG TAT
TAA AAC AGG GGC ATT AAA TTC GTC CTG AAA CAG ATA ATC GCT TCC AGC ATG GTC AAA
ATG GCA GTG TGT GTT AAT AAT GTA ATC AAT TTT ATA AGT GTA TTT TAA AAT ATC TTT
TTT CAA AAA TTC AAA AGT TCC CGG AGT TCC AGG ATC TAT CAA AAT AAC TTT TTT ATC
GAC TAT TAA ATA TGA ATT TGA GTC ATA GCC TCT TCC ATT CAA TTT ATA AAT CAA ATT
ATC ACC TCT TTA TCT CAA ATG AAT GCA GTC TCC AAC GTT AAA GAC TTC AAC AAT TTT
TCC AGT ATC AAT ATG AAT TCC ATC GTA AGA AAT ATC GAC AAC TTT TCC AGT AAC TGT
TTT TTC ATG CAT TAT TAA GTT TAC ATC ATT AAA CAA AGT TTT CGA GCA TTT TTT GTA
ACT TTC AAG GAG TAA TTC ATC GTC AAT TTT TAG ATT TTT GAC AAA TAT ATC AAT AAA
TTT CTT TAA AAT TAA ATC AAC GTT AAA TTC AAC ATT TTC AAT TTC TTT TAA TGA GGT
AGC GAT GTT TTT AAT TTC AGG ATC AAT TTC ATT ATT GAT ATT TAA CCC TAT TCC AAG

TAC TAA ATA ACC TTT GCT CAC ATT TAG TTC TGT TAA TAT TCC GCA TAT CTT TTT ATT
TTC AAA TAA AAC ATC GTT TGG CCA TTT TAT CTC AAA TTT TTG ATC TGA AAA AGA TTT
TAG TGT TTC TAT AAC ACA AAG AGA CCC TAA AAA ATT TAT TTT TTC AAG TTT TCC AAT
ATC TTC GAT ACC AAT TAG GAC TGA AAA ATA AAT TCC GCC TTT TTT AGA AAA CCA TGA
CCT ATC TAA TCG ACC TTT TCC GCG ATT TTG GGT TTC TGA AAC CAC TAT TTT ATT TTT
TTT GCC CAA TTT TCC AAG ATT ATA CGC GTA AGT ATT TGT AGA ATC GAT ATT TTT GTG
AGA AAT TAT TTC AAA ATC CAA TTT TAA CCA CAC TAG TAA TTT TCA AAT ACA TTT AAG
AAA AAT TCG TCA GCT TCA ACT TCT CTA ACG ATT TCA GGA AAT ACA TAA ATA TTT TCC
TTT AAA TCT TTT CTA AGC CTT CCA ACA ACC AGT ATA ACG TCT CCT TTT TTG AAA GTT
TCA AGC TTT TTG GCC ATT CCT TTA AAT CCG GAA ACT CTG ATA TCG CCC GTT AGA TCT
TCA ACT GTC ACG GAA CAA AAT TCT TTT GCC GTA TTA GTA TAT TTT GAA ACA ATT ATG
CCG TAT ATT GCA ACT CTT GAA ACT TGT TTT GAA GTT TTA GTT ACA ATG TAG TTT GAT
TCC CAC TGG CCT TCT GTT TCA ACG AAT TCT CCA GAG GTT ATT TCA TTT ATC GGT ATC
TTT TTT GCA GGA ATT CTC ATG AAT TTA CAC CGT TAA ATC TCG TAA TTT TTT ATT TTT
TCC ATA AAT TTT CCA AGT AAT TTT GGA GAA GAT TCG CTA ATA TCT ACA ATG GCT TCT
GAA AAT ATG ATT AGT CCT TTT GGA GTG ATT TTT AAC GTT CCA AAG GCC AAA ATC CTT
GAA CCC GTA GTT AAT TTA ACA GAT TTA TCT TTG GAA ATA ATT ATT TTT CCA GAA GCA
TCT TCA ATT TCT AAT TTA TCG TAA TTT TCA TCA TTT TCA AGT CCA ATA ACG GTT CCA
ATT ATC GCA ATT TTA TTT TCA ATT AGC GAC TGA TCA ATT TCC CCA ATT TTT TGA AGG
TTA TAC ATG ATT TAT CCC TTT AAT TTC TTG TTA AAT TCT TCC ATT ATA TCT ATT TTA
TTC CAA TTA TTG CAC CTG AAA GCT GTA TCT TTT AAA TAT TCA TCG TAT ATT GTA AAT
CCT GAA GTT AAT ATG TCC AAT CCA AGT AAT TTA TTG TTT AAA ACG TCC AAT ATC TCT
TCA TCA TCG ATA ATA ATT TTT TTA AGT TCT TCA AGG TCG AGA TAA TTT GTA TTG TTA
TTT AAA GTA ACC CGA ATG GTT CCA GAA CCG TCA TCA AGG GTG CAC GTG CAT AAA AAA
AGC CAT TTT AAA TTT TCA TCT TCT TCA TCG ACT TTA TTT CCG CAC TTA CAG ACT GCA
TAA CCT TTT TTC AAG GTC ATT TTT TTA TTG CAT TTG TCA CAA ACT GGA AAA TAT GGT
TCT TTT GAA TGG ATC GCA ACG ATT AAA CCC CTT AAT TCT GCA CCG ATT CCT TCT TTT
AAT TCG GAA ATA GAT TTT CTT GAA AAA TCG TTC CGC TGA GCA CTG GAT TTT TTT AAA
AGT TCA TCT ATT GTT GGT AAA TCT GAC TCA ATT TTT GAG ATT TTG CCG GAT TTT GAG
AGA GCT AAA TGA ATT CTA TCT TTA TAT TTT TTT GGA GCA TGT GCA TTT TCA ATT AAT
ATT GCA TCT CCT TCG TTT AAT TCA TTT AAT AAA CTT ATT TGT TCA GAC CAC AAT GAT
AAA AGC CCT CTA GCA GAT CCA TCA TTT ACA ACA ACG TTT ACA ACA GGG TTT TGA GAT
ACA ATT TTC TCT TCA AAA ATC TTT TCA ATT CTA ACG ATC GAG TTT ATT TCG ATC ATT
TTT TCG ATT ATA TCG CTT AAT TTT ATC GTA TCT ATG CTG TCA ATG TTT TTT TTA TTA
GAA TTA TTT TCC AAT TTT TCA GAA TTT TCC AGT ATA TCA TCA AGA CAG GGG TCT TTT
TGG AGC ATT TAC TTC ACC AAT TAC TTT CAA AAT TAT ATT TGC AAG TTT TAA TTT TAT
TTC AAA ACA ATC AAT CAA TGT TAA AAT TTA AAA TAA GCC ACG ATT AAA CTT ATT ATA
TAT TAT AAC AAA ATT TAT TTA TAT TTT TTA TCT ATA AAC TGT AAA ATA ACC TTA TTA
ACA CGC ATG GTG AAA CAA TGG TTG GTA CTC CTC TTT TTT CAA ATG CTA AGA AAA TTT
TAC TTC TCG GAA GCG GGG AAC TCG GGA AAG AAG TAA TAA TAG AAG CTC AAA GGT TTG
GTG TAG AAT GTA TTG CAG TTG ATT CTT ATG AAA ACG CGC CAG CAA TGC AGG TAG CAC
ATA GAT TCC ACG TAA TTG ATA TGA AGG ATG CTG GGG CTT TAA GGG CAG TAA TCG AAA
AAG AAA AGC CTG ACT TAA TAG TTC CTG AAA TTG AAG CGA TAA ACA CAG ATA CTC TAA

AAG AAA TGG AAA GCG AAG GAT ACC ACG TGG TTC CAA CTG CAA ATG CGA CAA AAC TTA
CGA TGG ACA GAG AAG GAA TCA GGA GAC TTG CAT TTG AAA AAT TAG GAT TAA GAA CCG
CAA AAT ACG AAT TTG CAG AAA ATT TGG AAG AAT TAA AAG AAG CAG TAA AAA GAA TTG
GAA TTC CAT GTA TTA TAA AAC CAA TCA TGT CAT CAT CTG GGA AAG GAC AGA GCA CGA
TTA AAT CTG AAA GCG ATA TCG AAA CAG CGT GGA ACT ATG CAA AAA GCG CTG CAA GAG
GAA TTG GAA CAA AGG TAA TCG TTG AAG AAT TTA TCA AGT TTG ATT ATG AAA TTA CGC
TAT TAA CTG CAA GAA CTG CAG AAG GAA CCC GGT TTT GTG AGC CAA TAG GCC ATA TAC
AGG TTG ATG GGG ATT ATC ATG AAA GCT GGC AGC CTC ATC CAA TGT GTG CAC CAA CAA
AAG CAA AAG CTC AAG CAA TGG CAA AGA AGA TAA CTG ATG AAC TTG GAG GAT ACG GTA
TTT TTG GAG TAG AAC TCT TTG TTT TAG ACG ACG AAG TTA TAT TCA GTG AAG TAT CTC
CAA GAC CTC ACG ATA CAG GAA TGG TTA CAA TGG TAA CCC AGA AAA TGA GTG AGT TTG
AAA TTC ACG CAA GGG CAA TTT TAG GGC TTC CAG TAA ACG TTG ATA TAG TTT CTC CTG
GTG CAA GCC ACG TTA TAA AGT CAG AAA TTC TCA AAT GGG CTC CAG AAT ATG AAA TTC
ATG AAG CTT CGA AAG TAA AAG ATA CAA AAA TAA GAT TAT TTG GAA AAC CTA TTG CAA
AAG TTG GAC GTA GAA TGG GTG TCG CCC TTG CAG TCT CAG ATG ACG TAA TAA AAG CAA
GAG AAA ATG CAG AAA AAG TAG CAC ACC TTG TAA AAA TTA AAT AAT TTT TTT ATT TTT
TTT ATT TTC AGT TGT TAT GCG AGT TTT ATA ACG TAA AAC ACT AAA AGT ACC CAG ATT
GCT ATT GCA AAA TAA CCT TCT TTT TTA TGA AAT GTA TAA TTT AAT GGA TAA AGT GTT
CTG CAC CCC ATC GGA GTC ATA CAG TCA CCG ACA AGA TGT GAT AAA TAA CCA ATA GCG
ATT GGA ATT ATG TAA AAT TCT AAC CCC TGA AAT GTC GTA AAA GAC GTT GAA ATC ATA
TTG TAC AAA ACC CAT GCA AAA ACT GAA ATT GCG ACA CTT TTC CCA AGA AGT TTG TTA
TTT GTA ACC ATT ATT AAA GCA ATC AGT GAG GCC ATT ATT GGA GAA ATC ACT GAT AAT
TTA AAC GTT AAA TAA CCT AAA ATA ATT GAC ATA ACA CCT AAA CTT AAA AAA GTG TGC
GTA ATC CCC CTG TGT TCT GCA TAA TAC GGG GTA ATA TAA ATC ACA CCG CTC AAA ATA
GCG GCT ATA AAA AAA CCG GTG TTG AAG TAT TCT GGT CGA AAT AGG TAT GCT AAA ATA
GAA ACC AAA ATA AGG CCA CCC GAT ATG TAG AGT CCT CTC TGA ACT ATT TCT GAC TTT
ACG TCG TGA TCT AAG TCA GGA TAG AGT GCC CCT GCA ACC AGT AAA AAA ATC TGT TCT
GGG GAA GAA ATA AAT GGG AGC CCC ATT AAT ATT CCA AGA GTA ATA TGT CCT TTC CAG
TTC ATT TTA ACC ACC AAA AGA AGC GCC TGC CTC TTT TTG GCA GGT TTA AAG CAA TGA
TAC AGC ATC AAA TTT ATT TTT TAT TCT TAA AAC GAT TTC GGG AAT CAT TGT AGT AAT
TAC GGA TTT TTC AGC ACT TTC AAG AGC CTT TTT ATC GTT TCC ATT ACA AGC ATA TGC
ATT TAT TCC ATT ATC ATT TAA GAT TTT GAC GGT TGC ATG TTC AAC CCA TGG GTC AAC
ATC GCT TAC GTA TAG TTC TTC AGG TTT AAG TGC ATC TGC AAT ACC CCA TGA AAT ATA
TCT TCC ACC AAG AAC ACC GAC TTT ATC GCA ATT TAA CTC TTC TTC AAG AGA ATC GAG
TGT TTT TCC AAA GTA TGG CGC ATT GTA CAT CTT GCT GTT GGT AAT TGA AAC CCA GTG
GGC ATC ACC TAT AAA GTA ATT TCC AAT CAT TGA AGG ATA TAC AAG ATG ATT TTC TTT
GTG GTT TAA AAA TCT ACT GCT CCT TGC AAG CAC TTT TAA AAG TTC CCT GTT TGT GAT
ACA GAT GTT AAT TTT TTC AGC TTC TTC CTT CAT TAT TTT TAA AAC TCT TGG AAG TCC
AAA GTT ATT TCG TCT TGC TTC TTT AAT ATT ATA TCC GCA CAT GTA TCC GTG GTG GTT
ACC GCT GAA TCC AGA TAG TAT TAC GTT TAA TCC GCT TCT AAG TCC TAC TCT GCA TTC
ATC TTC ATA CGC ACC GTT TGT TGC AAC AAC TCC CCC TTT AAC AAG TAT TCT TGA AAC
AGC AAC AGA TTT TGC AAA ATC TTT AAG TCT GTC TTT GGA CCT GTT AAA TGG GCC CCC
TTC AAT TAC GAG TAC GTC AAC ATC CAA ATC GTT GCA GGC TTT TAA TCC AGT TAT TAA

ATC TTC GTA ACC GTC ACC TAT GTG GAA TAT TCC TTC AAC TCC CCT GCC AAA TTT TTG
AGC GGC TTT TGC AAC GTC TTC CAT TTC TGA AAT ATC TGC ACC GTG GTC TCC ACC GCC
CTG TTC TTC AAC AAC GTT TAC ACA GAC CGT TGC TGA TTT TTT TAT CCA TTC TCC AAA
CTC ATT AGA ATA CTC TCT TTC TTT TGT AAT GAG TCT TTT GTG AAT TCT GTC TCT TGG
ACA ACC TTT GTA TGG CGG GCC GTT GTA GTA GCA ATC GCC CAT ACA GTG GGA TAT TTC
TTT TGG AAA TCT CAT TGG ACC AAA TCT TCC AAA ATG GTC TAG GTC CAG TGG AAC TTC
AAC GTT ATT ATA AAC TTC GTT CAT AAC TTC GAT TGG TGT TAA TCC TTC TTT TTC TGC
AAT ATC TGC AAG AGC ATA TCC GCA AAG GTG TAT TGG AAA ACC CAT ATG ATC TGT AAA
AAC ACA GTT TTC GAT GAA TTG TGG CAG TGT TAA CGA TGA AGA GCA GGG TCC AAC CCC
AAT TTC TAA TAA ATC ACA ACC TAA TGG GAA TCT TCG AAA AAT ACT TCC CAA TTT TTG
TGT ATC TTC AAA ACT TAA ATC ATC AAC AGT ATC TAC AAT TTC AAT AAT ATC CGA TTC
TGT CAA TTC TTG ACT GTT AAT TTT ATT AAT AAC AAG TTC ACG AAG TTC CAT TGC AGA
ATC TAA ATC ATT TAA TGA ATT TTT TAT GAG TTC TTT CAT AAT TTC ACC TTA TTT GCA
GAT ATA GCA GTT AAT TTA GTT TTG GGG TTA ATC CAA GTT CTT TTA TCA TGC ATA TAA
CAT CTT TAA GGT TCC CGG TTT TTG GAT TTC CAA CTC CTT TAA TGT TCA TTG GGT GGT
TGT CTG GAA TTA CTG TAG CAA TAT TAC TTG CAC CGC CTA AAA GTG CAA ATT GAG CTA
ATT CTG GGC CCA TTG TGG GGG TTG GCG AGG TAA TTC TAA TAT CTG GAA ACA GGA GCC
TTA CAA TAG CGA TAG TTT TTG CCT GTT CAA TCG CCG AGC ATT TCG GGC AGG TTT CCA
TCG GCG TTT CTT TGT ACG GGT TAA AAC CCA TTA TTG GAA TTT CTT CAA CAT CCA GTT
CTT TTA AAA AGA AGA GGT GTT CAA CCC TAT CTT CGT AAG TTT CCC CAA TAC CTA TCA
AAA GAC CGG AAG ATA GTT CGA CCC CGT GTT TTT TAA CCA TTT TAC AAA CAT TTA TTC
TAT CTT CTA AAT TTT CAC CGG GTT TTA CAT TGT TAA ACA CGG TTT TAT TCG TAG TTT
CAA GAT TAC AGC ATA TTG TAT CTA TGT TAT ATT TCT TAA GTT CTA AAA TAC ATT CTT
CAG TTA AGT CTG CTC CCG CAT TTA CCA AAA CTT CTA AAT TGG ATG CTG ATT TAA CTG
CTT TTA GGG CCC TTA TTA CTT CTT TTC CAT TAT AGC CGT GTC CTG AGG AAC AGC TGA
CTC TTT TAA TTC CTG AAT TTT CAA TTG CAA CTG AAG ATG ATT TTA TCT GGC CGT CAT
CAG ATC TAA ATG GTT TTA CAT AGC CTT CGG AAC TCG TTC CTG CTG CAA AAC CGC AAT
ATT TGC ATT TTG GGG TTA CAG AAC AGA TAT TGG TTA CAT GGA CTG TTG ATG TGA TTT
CAA TTT CGG TTT TAA AAA CGT CTC TAA CTT CAG AGG CTA TTG AAA AAA GTT CTA AAT
AGT CTT TCC AGT TCT TAA TAT TAA ATA ATT CAA TTG CAT CCG ATT TTG TAA TTA AGC
CCT GTT TTA CAT CTA TTT TTC CAT CTT TGA GGT CCT GAA AAT TGC CTC TTA TTT TTG
AAA ATA TCA TTA AAC CAC CGG ATT GTA AAT TAC GCT GAA AAT TAA AAA AGA AAT ATG
TTT TAG TAA TAC TTA AGG TTT TAT TGA ACA GCC GCA TTC GCA TTT GTT TTC GTT TGC
GTG TTT TTC AAG AAC TTT TAA GGA TGC AGG AAG AAT ATC TGC TAA AGG ACC AAA GCA
CAT GCT GTC TGC AGT ACC GGT TAA TGC TTT AGG GTT TAA TTT GTC TTC CAT GTT TTT
AAT GCC TTC GTT TCT CAT TAA ATC TAA GAT CTG TGA GAT TGC CTC ATC AGC CAT CAT
TTG AGC GAA GTC TGC AGG AGC ACC TAA GAT CTG GGT TAC TGC GTC TCT GTA AGC TAA
GAT AGC AGC GTA AAC TGG TGC AGT AAC TGC TGA ACC CAT GTC ACA TAC TGG GCT GAT
TAA GTT TGC AGG CAT TTT GAA TGC TTC CCC TCT TGC AGT TTT TGC CAT ACA GTA GAA
TTC TTC TAC TTT TTC TGC ATC AGC TAA ACC TTC TGA AAG GAA TGC TTG ACC TTT CAT
TTC TGG AAC TGC TCC TGG GTG GTA TGC AAT AAT GTT TAA ATC GTC TCT TCC TAA GTC
TTT GAA GAT TTT TGC GAA TTT TGG TGT TGG AAT TGT ACA AGC GTG TGT TAC AAT TGC
ACC TTT TTT AAT TTC GCT TGC GAA TTT TTC GAT GAT TGC TGG CTG GCT GCC GCC TTT

TGG TAA CCA CGT AAT TAC AAT GTC AGC ATC TTT AAC TGC TTC AAC ATC ACT TGC AGT
TAC TTT TAA ACC TAC TGT TTC AGG GTG TAC AAA GTG TAT ACA TCC TTT AGG TGG TTT
TGG TGT TTC TTT AGC TTT AGC CTT TAC AGC TTC TCT GAT TTC AGG CAT TAC TTT TTC
CGC ATC TCC TGC TAA ATG TGC TTC CAT AAC TGT TTT GTA GTC AAA TTG GTC GAG TAC
TAC CAT TCC AGG TTC TTC AGC AAA GCA AGG GTC TGA AAC AAC AAC TTC TGT AAT TTC
AGG GAT TAA GTG TAA CAA TTC TGC ACC CAT TGT GAT TGT TGA GTG GGT CAT TGC AAT
TTC TGG GAT TCC TGC TTC TTT TGC AAC TTG CGA AGC TCT TGA AAA GTT TGT GAT TCC
TGA TGC AGC GTG CGT TCT GTA GCA TCC TGC ACC TAA TAT TGC CAC TTT CAT ATC ATA
CAC CTC AGT ATT TAC TAT AGA TTA ATC TAT GTA ATA TAA AAG CTA TTA AGG GTT AAT
ACC TAT ATA AAT ATT AGT CAT TAG ATT ATA TAA TGT AAT GAG TTA AAT ATA AAT ATG
TTA ATA ATA TTT TTC ACG TAT TTC ATA TTA TCG TAT TTG TTA TTA ATA TTT TAT ACA
TAC ATA AAA ATT ATT TTT TTA GCA ATA AAT ACT ATA ATG TGC TAT GCA AAA TAA CTG
ATG CTA TTA AAA AAT CAA ATA TCA GTA TAA CAA TTT TTG AAA AAT CGA AAA ATA AAT
TAA AAA AAG GTA TTT TTA GAG AAA TGC AAC CGC ATT AAT TGG AGA TCC GTC AAT TTT
TTC GAT ATT TAG TGG GAA TCC AAA GAA TTT GAA AGT TTT GTT TTC CAA AAT TTC AAG
GTT TTT CAT ATT TTC AAT TAT TTT AAT TTT TGA AGA TAA AAG TTT TTC ATG GAC AGA
AAA GTT TCC AAT TGA ACA GCA GTC TAT TCC AAC AGC TTT TAA ATT CAT AGA AAT TAG
TTT TTC AAT GTG ATT TTC AAT GTC GCA CTC TTT TTC AAA GTA TTC AGG ATC TTC CCA
GTA TTT TGA AAA ATC AGT TTT TAT GAG CAA AAT TTC TAA ATT TTT AGG TGC ATT TTT
AAA AAA ATC ATT CAT ATT TTT AAA AGA AAT ACA GTA CCC TTT TCC AAA AAC ATT TTC
AAA TTT TTC ATT TGT AAG TCC AAC ATG CAT CGG GTA ATC AAT ATG TGT TCC AGT ATG
AAC GCC CAT GTT TAA TTC TGA AAC CGA GTA TTT ACC ATT TTT TTT AGT TTT TAC AAT
AAA TTC TGG GTC ATT TGG ATA TAC AAA ATT CTT AAT TTC ATG AGA CAA GTT TAT TAT
TCT TTC TTC CAC CAT ATT TTC TCC ACA TAG AAA TTA TTT ATT TAA TAT TAG TTA TAA
TTG GAT ACA TTA TAA ATT TGG GCT TTG GTG ATA ATT TGA GGT ACG ACT TTC ACA CTC
ACA CGG TAT TTA GTG ATG GAG AAC TTA TTC CTT CAG AAC TCA TAC GAA GGG CGT CTG
TAT TGG AGC ATA GGG CAG TAG GAA TAA CTG ATC ATG CAG ATG CGA GTA ATT ACA AGG
ATT TAA TCG AAA AAA ATA CTT TAG CAA AAG AAG AAT TAT CAA ACT ACA TGG ATT ATA
TTG AGA TAA TTG TAG GCG TTG AAT TGA CAC ATA TTC CCC CAA AAT CAA TTC CAA AAA
TGG CTA AAA AAT GTA AAA ACG AGG GTG CAG AAA TTG TTG TAG TTC ACG GGG AAA CTC
CAG TTG AGC CTG TGG CTG AAA AAA CAA ATT ATT ATG CAT CAA TTT CAG AAG ATG TTG
ATA TTT TAG CAC ACC CTG GAC TAA TAG ACA TTG AAA CTG CAC AAA ATG CTC TTG AAA
ATA ATA TAT TTT TTG AGA TAA CCT CCA GAA AAG GCC ACT CAC TTA CAA ACG GCC ACG
TGG TAT CTG TTG CAA GGG AAT TAA AAA TTC CAA TGG TGA TAA ATA CTG ATA CAC ACG
CTC CAA GTG ATT TAA TAA CTT ACG AAT TTG CAA GAA AAG TGG GAC TTG GTG CAG GAA
TGT CTT TTA GGG AAC TTG AAA ATT CAT TAA CAA CAA ATC CCT TAG AAA TTT TAA AAA
GAA TAT AAT TTA CCG GTG TTT TTA TGA GCA ATA TTT CAG AAA TTG TTG TTG AAT TAT
TTA AAG AAG CCT CAA TTT ATC TTC CAG ACG ACG TTA AGT CTA AAT TAA AAC ACG CTG
AAG AAG TAG AGT GTG GTG CAT GTA AGG ATA CGC TAC AGG CAA TTG AAT TAA ACA ATA
AAA TTG CGG AAA CTA AAC AAA TCC CCC TCT GTC AGG ATA CAG GAG TTC CAG TTA TTT
TTT TAA AAA TTG GAA CGG GGA TTT CAA GTG AAA GAA TAA TTG AAA TAA TCA AAG AAA
TTG AAA GTG GCG TAA AAC GGG CAA CCG CAG AAG TGC CAT TGC GGC CAA ACG TTG TAA
ACC CAA TTA CTC GGG AAA ATT TAG GCA CGA ATA CTG GAA TGG GTG CAC CGT TTA TAA

ATT TTG AAT TTA GTG AAA ATT TGA ATA ATG AAA TCG AAA TAT CTG TGT TTC CAA AAG
GAG CAG GAA GCG AAA ACA TGA GCG CTT TAA AGA TGC TGA CTC CTT CTG AAG GGT TAA
TGG GAA TAA AAA AAT TTA TCC TTG AAA CAG TTG CAA ATG CAG GCG GTA AAC CGT GTC
CTC CAA TAG TGT TGG GTA TAG GAA TAG GTG GAA CTG CAG ATA TTT CAT TGA AAC TTG
CTA AAA AAG CTC TTT TAA GAG ATA TTG GAA ACA GGA ATC CTG AAA AAA CAA TTG AAA
ATC TGG AAA AAG AGT TGC TGG ATA GTA TAA ATC AGC TTG GAA TTG GTA CAA TGG GAC
TCG GCG GTA AGA TAA CTG CTT TAG ATG TAT TTA TCG AGG TAA ATG GAT GCC ACA CTG
CAT CCC TTC CGG TTG GAA TTT GTA TCC AGT GCT GGG CTG ATA GAA AAG CGA AAA GAA
AAA TAA AAT TAT AAA AGT TTG TAA ACT TTT ATA TCA CTA CAA AAA ATC TCT TTT TTT
AAA TAA TCA AAC GAT CTG ATA CAA AGG TTT TTT AAA AAG TGC AGC GAT TTT ATA AAC
AAT TAA CTC AAA GAA ATT AAA AAA ATC TTA CCG GTA GAT TAC CAA TGG ATG AGC AGG
ATA TTT TAA ACG AAT TAC GAG AAT ACA GGA ATC AGG ATT TGA AAT ACG AAG AAG GCT
ATA TTT TAG GAT CAA TGT GCA CAA AAC CCC ACC CGA TAG CTA GAA AAA TTT CAG AAA
TGT TTT TTG AGA CAA ATT TGG GAG ATC CAG GGT TAT TCA AAG GTA CTT CAA AGC TAG
AAA AAG AAG TAG TTT CAA TGA TTG GCG GTA TTT TAC ACA ATA AAA ATG CTT TTG GTT
ACA TTA TTT CGG GAG GAA CTG AAG CAA ACC TTA CGG CAA TGA GGG CAT TTA AAA ATA
TCT CAA AAT CTA AGG GTA AAC CTC AAA ATA TCA TAA TTC CTG AAA CTG CGC ACT TTT
CTT TTG ACA AAG CAA AAG ATA TGA TGG ATT TAA ATG TTG TAC GGC CTC CTT TAA CGA
AAT ATT TTA CAA TGG ATG TAA AAT TTA TCC AAG ATT ATA TCG AAG ATT CTA AAA ACG
AAG TAT CTG GAA TTG TAG GGA TTG CAG GAT GTA CAG AAC TTG GAT CAA TTG ATA ATA
TCT GCG AAT TAT CAA AAA TTG CGG TTG AAA ATG ATA TTT TGC TTC ACG TGG ATG CTG
CAT TTG GTG GTT TTG TAA TTC CAT TTT TAG ACG ATA AAT ACA AAT TAA AAG GAT ATA
ATT ACG ATT TTG ATT TTT CAC TAA ATG GGG TTT CTT CAA TTA CTA TTG ATC CGC ACA
AAA TGG GGC TTG CTC CAA TTT CTG CGG GAG GAA TTC TGT TTA AAG ATA ATA TGT TTA
AAA AAT ATT TGG ATG TGG ATG CCC CAT ACT TAA CTG AAA AAC AGC AGG CAA CGA TAA
TTG GAA CCC GAT CTG GCG TAG GTG TTG CTT CAA CTT GGG GAA TTA TGA AAT TAC TTG
GAA TTG AAG GAT ACA AAA AAC TCG TAA ACG AAT CAA TGG AAA AAA CAA CAT ATT TAG
TGA AAA AAG CGA GAG AAT ATG GGT TTG AAA CTG CAA TAG ATC CTG TAA TGA ACA TTG
TTG CAT TAA AAG ATG AAG ATA AAC ATG AAA CAT GCA TGA AAT TGC GAG AAG AAA ACT
GGT ATG TTT CAG TTT GCA GGT GTG TTG ATG CTT TAA GGA TTG TTG TAA TGC CGC ATC
TTG AAA TTG AAC ATA TCG ACG GAT TTT TAG AAA GCC TTT CAA ATA CAA AAA AAT ATT
AAG AAT TAA AAT CTT ATC TGC ATT AAA AGT ATT TTT GAT GGT GGT TTA GTC ATG AAA
ATT TAT CTC GAT GGA AAC TTC GTA GAG AAA GAA GAT GCG AAA GTA TCC GTA TAT GAC
CAC GGG TTG CTT TAT GGT GAC GGG GTT TTT GAA GGA ATT AGG GCA TAT GAT GGA GTA
GTA TTT AAA TTA GAA GAA CAT ATT GAA CGT TTA TAC GAT TCA GCA GAT TCT ATC GCT
ATG AAA ATC CCT GCG TCA AAA GTA GAA ATG GAA GAA ATC GTT ATC GAA ACT TTA AAA
TCA AAC AAC TTA AAA GAC GCA TAC ATA AGA CTT GTA GTT ACA AGG GGA GTT GGG GAT
TTA GGC CTT GAT CCA AGA AAA TGT CCA AAA GCA ACG ATT TTC TGT ATT GCA GAG AAT
ATG AAA CCA CTT CTT GGA GAA GAT GGA ATA AAG GTT ATA ACC TCT TCA ATA AGA AGA
CTG CCT GTG GAT GTT TTA AAT CCT GCT GTA AAA TCT TTA AAT TAT TTA AAC AGT ATT
TTA GCA AAA ATT CAG GCC AAT TAT GCG GGA GTA GAC GAA GCT TTT TTA CTC GAC AGC
GAA GGA TAC GTC GCA GAA GGA ACT GGT GAC AAT GTA TTT GTT ATT AAA AAT GGT AAA
ATA AAA ACA CCT CCT CTT TCA TCA AGT GTT TTA AAA GGA ATT ACA AGA GAC ACT GTT

GTA GAT CTT GCA AAA GAA GCA GGT TAT ACA ATT ATA GAA GAA AAA ATG ACG TTA CAC GAT TTA TTT GTT GCA GAT GAG ATA TTT ATC ACG GGA ACC GCG GCA GAA TTA ATC CAT GTT GTT GAA ATT GAT GGA AGA ATA ATT AAT AAC GGA AAA TTA GGC CCT GTT ACA AAA GAT TTA AGC GAT AGA TTC AAA GAA ATA AGG TCT AAA ATC GGT AAA AAA TAT ATC GAG TAA TAT TTA ATT TTT TTA AAT TAT TTT AAA AAC TAT TTT TAA ACT TTT TTC AAA GAA ATA AAA AAT AAA AAA GAG AAT TAT CTT TCA GCG TTT ATC GGA TAG TTT GGA GCT TCG TTC GTG ATT AAA ACA TCG TGA GGG TGG CTT TCT TTC TGT CCA CTT TGA GTA ATA ATT ACA AAT CTT GCC TTT TCA TGC ATT TCA GGA AGG TTC TGT GCA CCA CAG TAT CCC ATG GAT GAT CTT AAA CCA CCA ATT AAC TGG TAT ACG ATG TCG CTT GTA GGT CCT TTA TAT GGA ACA GCA CCT TCG ATT CCT TCA GGA ACG AGT TTT GAG TGT TTC ATA TGT CCA CTC TGG AAG TAT CTA TCT GCA ACG TTT CCA GAA CTT CCA CAC ATT GCA CCA AGG GAA CCC ATT CCT CTG TAT TGT TTG TAT TTT CTT CCG TTA ATG GTC ATT AAT GAT CCT GGA GCT TCT TCT GTA CCT GCC AAA AGA CTT CCA ACC ATT ACA GCA TTT GCA CCA GCT GCA ATT GCC TTT GCA ATG TCA CCG CTG TAT TTT ATA CCG CCG TCT GCG ATT ACT GGA ACG CCG TAC TTT TTA GCA ACT TCT GCA ACT TCT GCA ACA GCA GTT AAT TGG GGA ACT CCA ACT CCT GCA ACA ACT CTT GTT GTA CAT ATT GAA CCG GGT CCA ATT CCA ACT TTA AGT GCG TCT GCA CCT GCT TTA ATT AAA TCT TCT GCA GCT TCG CCG GTT GCA ATG TTT CCA ACG AAT AAT TTT GTT TTG GTT CCT TTT AAT ATT TCT TTT AAT TTT TTG ACG TTT TCT ACA ACC TGC ATG TTG TGT GCG TGT GCA CAA TCA ATT GCA ACT GCA TCA ACG CCT GCT AAA ACT AAT GCT TCA GCT CTT TCA AAG TCG CTT GGA CCG CAA GCT GCT GCA ACA ACT AGT TTT CCA TCA CTA TCT CTT GCT GCG TCA GGG TAT TTT TTT CTT TTT AAG ATA TCT CTT AAT GTA ACC ATT CCG AGA AGT TCT TTC GTG TTT TTA TCC AAA ATA GGA AGT CTT TCG ATT TTG TTT TCA TAA AGT CTG TTT AAA ATT TCT TCA TAA GGT GTA TCT TCA TGT ACG TGA AGT ACT TCT TTT GTC ATA ACT GTT TCT ACA GCC ACG TTT TTA TCT GGA ACG AAT TTT AAG TCT CTT GTG GTG AGA ATC CCG ACA AGC GTC TTG TTT TCA GAA ACT ACT GGA AGA CCG CTC ACG TTG TAT TCG TAC ATT ATC CTT TCA GCA TCT AAA ACA CTG CTG GAA GGT TCA ACT GTT ACG ACA TCT CTA ATT ACT AGA TTT TCT GCC ATT TTA ACA GCC TTA ACG TGT TTC AAC TGT TCT TCA ACA GTC ATG TTT CTG TGG ATT ACT GCA ATT CCC CCT CTT CTT GCA AGG GCA ATT GCT AAA TCT TTT TCA GAA ACT GTG TCC ATT GCC GCA GAT ATA ATC GGG ATA TTA AGC TTT AAA CCT GCA ATA TCG ACC GTA ACA TCC GTA GTT TTT GGA TCA ACA AAG GAT TTG TTT GGA ACT AAT AAT ACA TCG TCA AAA GTG TAG GCT CTT TGA GCA CTA ACT ATT TTA TCT AAA AAC AAA ATA ACC ACC ATT ACC TTT TTT TGT ATA TTA TTT TAA TTA ATA TAT AAA TCT GGT GGT AAC GTG GAT GAA ATC AGT ATG GCT TCA AAA GAG CTT GCC AAG GGG GCA AAA ATG CTT GGA AAA CAC TGC AGT TCT TGC GGG TTT CCA ATG TTC GAA AAA GAC GGT TTG GAA TAC TGC CCA AAC TGC AGT ACT TCT GAA ATT GAA ATC AAA GGC GAT TCA AAA GAA TCA GAA AAA CAA TTA AAA AAA ATA AAC GAA AAT AAA ATT TCA AAA AAT GAT GAT AAA TTA AGG ATA ATA AAC AGA AAA ATC GAG TAT TTA TTT TCT AAA TTA GAT TCA GAA GAG GAA ATT GGC AGA ATT CAC GAA ATA ACT TCA TCA ATT AAA ATA CTT CTA AAA ATA AAA AAA TAT CTG TAA ATT TGG AAA GAA AAA ACG CTT TCC CAA AAA ATA GGT CTT ATC CAA GAT GCA CAA TAT TTA CAA TGG TTT GGT ATC AAT TGG TGG CAT ATA GCT AAT AAT TAG ATT CCC CAA TTT CCC TAT ATT AGT ATT AGT ATT TAT GAT TTG TGC TAT TAG ATT TTC TAA ATC AAA AGT TTG AAG TCT CGT TTT ATT AAT TTC AAA GTT CCA TGA TTA ATG ATA CAT TTA TAT TTT ATA ATT TGA TAG TGG TAT TCA

ATC AAG ATA ACA AGT GAG AAT ATG ATT CAA AAA TGC AGA GTA TGC GGA AAA GAA TAC GAT GTT GAT GCC ATA ATT TAC AAC TGC GAA TGT GGC GGA TTA CTC GAA ATT AAA TAC GAT TTT GAA AGT ATC AAA GAA AAA GTT TCA AAA GAA TCC TTA AGG GAA AGA GAA CTT GGT GTA TGG AGA TAT TTA GAC TAC TTA CCA GTT AAA GAT CCT GCA AAA ATT GTG AGC CTT CAC GAA GGG GGA ACT CCA CTT TAC AAA TGT GAA AAT CTC GCA AAA AAA TTA GGA ATG AAA GAG TTA TAT GTT AAA AAC GAA GGT GCA AAC CCA ACA GGA AGT TTC AAA GAT AGA GGG ATG ACT GTT GGA GTT ACA AGA GCA AAC GAA CTT GGT GTT GAA GTA GTA GGC TGT GCT TCA ACC GGA AAC ACA TCT GCA TCA CTT GCA GCA TAC TCT GCA AGA GCA GGT AAA AAA TGT ATT GTT CTT CTT CCT GGA GGA AAA GTA GCT CTT GGA AAA CTC GCT CAG GCA ATG TTT TAC GGT GCA AAA GTT ATC CAG ATC AAC GGA AAC TTT GAC GAA GCT CTT GTA ATG GTT AAA AAC CTT GCA TTA GAA AAC AAA CTC TAC TTA TTA AAC TCT GTA AAC CCA TTC AGA CTT GAA GGT CAG AAA ACA ATT GGT TTT GAA ATC TGC GAC CAG TTA GAC TTT GAA GTT CCA GAC ATG GTT ATT TTA CCG GTT GGA AAC GCT GGA AAC ATC AGC GCA ATC TGG AAA GGA TTT AAA GAA TTT AAA GAA ACA GAA ATG ATT GAC AGA TTA CCA AAA ATG ACT GGA ATC CAA GCA GAA GGT GCA CAG CCA ATT GTA AAA GCG ATA AAA GAA GGA CTC GAC GAA ATA GTT CCT GAC GAA AAT CCT GAA ACA ATC GCA ACT GCA ATC AGA ATC GGA AAC CCT GTA AAC GCT GCA AAA GCA CTT GAT GCA ATC AAA TCT TCA GGA GGG CTT GCA GAA AGC GTA AAC GAT GAA GAA ATT ATC GAA GCT CAG AAA CTC CTT GCA CAA ACA GAA GGA ATT TTC GTA GAA CCT GCT TCA GCT TCA TCA ATC GCA GGT CTT ATC AAA TTG ATA AAC ATG GGA TTA ATT GAC AAA GAT CAA AAA ATC GTA TGT ATT ACA ACT GGT AAC GGA TTA AAA GAT CCA GAT GCT GCA ATC AAA GCA AGT AAA ATG CCT ACA GAA ATT GAA TGC GAC ATG GAA GTA CTC AGA AAA GCA ATA GAA GAA TAA TTA AAT CTG GTG GAA TTA TGA AAA AGA CTG TCG AAG GCA AAG CAT GGG TTT TTG GAG ATA ACA TCG ATA CTG ATG CAA TAT TAC CTG CAA GAT ACC TTG TAT ACA CTA CCG AAG AAC AGC TTG CAA AAT ATG CAA TGA CTG GAA CCG ACC CAG AAT TCC CTG AAA AAG CTA CAG TTG GCG ATA TCA TCG TTG GCG GTA AGA ATT TCG GTT GCG GAA GTT CAA GAG AAC ACG CTC CAA TTG GTT TGA AAG GTT TAG GTA TTT CAA TGG TTA TTG CAG AGA GTT TTG CAA GAA TTT TTT ACA GAA ATT CCA TCA ATA TCG GAT TCC CAT TGT TAG AAT GCA AAG ATA TTT CAA AAC ACG TAA AAG AAG GCG ACG TTT TGA GAG TTG ACC TTG ATA AAG GTA CCG TTA AAA ACG TTA CAA CTG GCG TTG AAC TAA CTG GTC AAA ATC TCC CTG ATT TTA TGA TGG AAA TCT TAA ACA ATG GCG GAT TAA TGC CGC ATT TGA AGA AAA AGA TTT CAA AAG CTT AAA TTT TTT TAT TTA GTT TTT ATT TTA TTA TTT TTT ACA TTA TTT TTC GGT GAT TTT TAT GTC AGA TCC AAA AAA TGT AGT TTT TAA GGA AAG TGA GGA TTT AAA CGG TGT TTT TAT CGA GGG GCC TGA TTT TGA TGA AAC TAT CGA TTT AAA AGA CGT TTT AAC AGA TTA TTA TGA AAA AAT AGG TTT TCA GGC AAC ACA CCT TGG AAA AGC CGT TAA AAT TTG GAA ACA AAT CGA AGA AGT TAG AAA AAA TGA AGA AGT AGT TGT TTT CAT GGG CTA TAC TTC AAA CAT GGT GTC ATC AGG TCT TAG GGA AAT TAT ATC CTA CCT TGT AAA ACA CAA AAA AGT CGA TGT ACT GGT TAC TAC TGC AGG TGG AAT TGA AGA AGA TTT TAT AAA ATG TAT AAA ACC GTT TGT TCT TGG AGA ATG GGA CAT GGC AGG ATC AGA TCT TCG AGA AAA AGG AAT AAA CAG GAT TGG AAA TGT ATA TGT TCC AAA TGA TAG GTA CAT CGA ATT TGA GAC TTA CAT GAC TAA ATT TTT TGA AAC GCT TTT GAA AAA ACA GCA GGA TGA AAA TAA AAT AAT AAG TGC AAG TGA ATT TTG TTT TGA ACT TGG AAG ATT TAT GGA TGA AAA TTT AGG GCA AGA AAA AGA AAA ATC CAT TAT TTA CTG GGC CTA TGT GAA TAA AAT ACC AAT ATT CTG CCC CGC AAT

TAC TGA TGG TTC AAT TGG AGA TAT GCT CTA CTT CTT TAA AAA GAA TGA AAA AGG AAC
CGA TTT AAA AAT AGA TGT TGC AAG CGA TAT TGT AAA ACT AAA TGA CAT GGC AAT TGA
TGC AAA TAA AAC AGC ATG CAT CGT TTT AGG TGG ATC TCT TCC AAA ACA CAG CAT AAT
AAA TGC AAA TCT CTT CAG GGA AGG AAC AGA TTA TGC GAT ATA CAT TAC AAC AGC AGT
TCC ATG GGA TGG GTC ATT AAG CGG TGC CCC TCC AGA AGA AGG TGT TTC ATG GGG TAA
AAT TCA AGA AAA AGC AGA TTT CGT CGA GAT CTG GGC TGA CGC AAC GAT TGT ATT TCC
AAT GCT CGT TTA TGG AGT ATT TAA ATA ACA ATC ACA AAA TAC AAT TTT TAA ATT TTT
TAA AAT TTT TTG AAA AAA GCT CTT TTA AAG GAT ATA AAG AAA AGA TAT TTC TTG ATA
TCT CTC TAT TGT TGG GAC AAA TAT TTT GGG TGA TCT TTA AAA GTG GAA TAT GAA TGT
CAC TTT CTA AGA TGT GCA ACT ATC GCA CAT TAC ACT ATG TAA CTC TCG TAA TAT ATA
TAA ATT ACT ATA TCA ACT GAG GTA ACC CGT ATA TAA AGG ACT ATT TTA CAG AAT ATC
AAA AAT TAA TAT TTA TCG ATA TAG GTA TTA TTT AAC AGG TTT TAA AGA ATA TTT TTC
GTT TTT AAG AGT TTT AAG TAC AAG ATT GAT AAT TTA AGA AAC TTT TTA ACG AGT AAT
AAA TAT ATA TTA ATA ATT TTT AAT TAC ATA AAA TCG ATA AGT AAT TAT ATG TAG TAA
CTA TAT ATG TGT GGG TGT GGC CCC ACA AGA CCA CAA TAT TCA CAA TGG AAC ATT CAA
ATT TTT GAG ACT AAA TCT TCA TTT TCT CAA AAA ATA TAT TCA AAC TCA TTT TTA ACT
TTG AAT TTG TGA CTT AAC GGC TTT TAA ATT TTT AAT TTA GCC CGT ATT TCC CAT TCT
TAG TTA AAA ATA AGT ATA AAA TTA AAA GGT GGA ATA ATG ACC GAA TTT AAG GTT GTT
CAT ACC ATA TGC CCC TAC TGT GGA ACG GGT TGC GGT ATT GAC CTC GTA GTT AAG GAC
GGT AAA GTA GTA GAT TCC CAC CCT TTC AAA AGA CAC CCT GTA AAC GAG GGT AAA GTC
TGT ATA AAA GGT AAT TAC TGC TAT GAG TTT GTT CAT AGC GAA GAC AGG CTG ACA AAA
CCA CTC ATC AAA AAG AAT GGG GAG TTT ATT GAA GCC ACA TGG GAT GAA GCA TTA GAT
CTT ATC GCA GGA AAA TTA AAA CAA TAT TCT CCT GAT GAA GTA GCA TTC TTT TCA TGT
GCG AGA GGC ACA AAT GAA GAG AGC TAT GCT TTG CAG AAA TTT GCA AGA ACT GTA CTC
AAA ACG AAC AAC GTA GAT CAC TGT GCG AGG ATA TGA CAC GCT CCA ACA GTT GTA GGT
CTC GGA GAA TGT TTT GGT TCA GGT GCA ATG ACC AAT TCA ATC ACT GAT TTA GCA CAA
GCA GAT GTA TTG CTT ATC TAC GGT TCC AAT ACT TTT GAA GCT CAC CCT TTA ATA GCA
AGA AGT ATT GTA AAA GCC AAA GAA AAT GGA ACT AAA ATA ATT GCA ATC GAT CCA AGA
ACA ACA CAC ACT GCA AAA ATG GCA GAT TTA CAT TTA AAG CTT ATT CCT GGA TCA AAT
ATT GAT TTG ATA AAT ACT ATC ACG AAT ATC ATC ATC CAA GAA GGA ATG GCT GAT GAA
GAA TTT ATT AAA AAC AGA ACC GAA GGT TAC GAT GAA CTA AAA GAT GTA GTT TCA AAG
TAT ACT CTC GAA AAA ACA GCA GAA CTT TCA GGA ATA CCT GCA GAA ACA ATT CTT GAA
GCA GCA AGA ATG TAT GGA AGT GCA GAA AAC GCA TCT ATA ATG TAT TGT TTA GGT GTT
ACT GAA TAT ACA TTT GGT GTG GAC AAC GTT AAA TCA TGC TGT AAC CTT GCA ATG GTT
ACT GGA AAC CTT GGA AGA CCC GGA ACT GGT GTA AAC CCA TTA AGA GGT CAG AAT AAC
GTT CAA GGT GCA TGT GAC ATG GGT GCA CTT CCA AAC GTA TTC CCA GGA TAC CAG AAA
GTT GGA GAA GCT TAT GAA AGA CTC GAA GAC TTA TGG GAA ACA GCA GAT TTA AAC AGA
GAA ATT GGA TTG ACA AGT CCC GAA GTT TTA CAC AAG GCT GGA GAA CAG GTA AAA TTC
CTT CAC ATT GTT GGG GAA GAC CCA ATG GTT GCT GAT GCA GAT ATA AAC CAC GTT GAA
AAA GCT TTG AAA AGT TTA GAT TTC TTT GTA GTT CAA GAT ATC TTC TTA ACT GAA ACT
GCA AAA CTT GCA GAT GTT GTA CTT CCT GCA GCT TGC TGG GCT GAA AAA GAC GGA ACA
TTC ACA AAT TCT GAA AGA AGA GTA CAG AGA ATC AGA AAA GCA GTA GAC GCA CCT GGA
GAA GCA CTT CCA GAC TGG CTT ATT GTT CGA AAA CTT GCT GAA AAA ATG GGT GCT GGC

GAA AAA CTC AAG TTC GAA AGT GCA TCA GAA ATA TTC GAT GAA ATG GCA AAA GTT ATT
CCA CAA TAT GCA GGA ATG TCT TTT GAA AGA CTC GGC ATT GAT GGA CTT CAG TGG CCA
TGT AAA ACT CCA GAA GAC CCT GGA ACT CCA ATA CTC CAC AAA GAA AAA TTC TTA AGG
CCA AAC GGA CTC GGT AAA TTT ACT CCA GTT GAA CAT AAA GAT GCT GAT GAG CTT ATT
GAT GAA GAA TAC CCA CTC ATA CTT ACT ACG GGA AGA ATA ATC TTC CAC TAC AAC TCT
GGA ACA ATG ACC AGA AGG TGT GAC AGT ATC ACA AAT GAG ATT GAT GAA AAC TTC ATT
GAA ATA AAT ACT GAA GAT GCC AAA GAA TTG GGA ATA AAG CCT GGA GAA AAA GTT AGA
GTT TCC TCA AGA AGG GGA ACT GTA AAT GCA AAT GCC CGA GTA ACT GAA AAC GTC ATA
AAA GGA GTA GTT TAC ATG TCA TTC CAC TTT TTA GAG GAA GCA ACA AAC AAA CTA ACA
AAT TCC GCG TAT GAC CCA GTA TCA AAA ACC GCA GAA CTC AAA ATA TGT GCC GTT AAA
GTT GAA AAA ATT TAA TAA TTA TTT TTG AGG TGA TTT AAA TGA GCT ATT ATT TAG TTC
AGT CAA CGG ACG AAG ATA TTT TAA AGC ATG CGG AAT GTG GTG GGG CAG TTA CTG CAT
TTT TCA AGT ACC TTC TTG ATA AAA AAC TGG TTG ATG GAG TTT TAG CAC TTA AAA AAG
GAG AAG ACG TTT ATG ACG GAC TTC CTT ATT TGG TTA ACG ACT CAA AAG AAC TGG TAG
AAA CTT GCG GTT CAC TTC ACT GCG CTC CAA CAA TGT TTG GAA ACA TGA TAA GCA AGC
ACT TGA AAG ATA TGA ATC TTG CAG TAT CTG TAA AGC CAT GTG ATG CAA TGG CGA TAG
TAG AGC TTG AAA AAA GAC ATC AAA TCG ATA AAG ATA AAT TAT ATA CAA TTG GAT TAA
ATT GTG GTG GAA CCG TAC CTC CAC AAA CTG CAA AAA AGA TGA TTG AAC TCT TTT ACG
ATG TAG ATC CAG AAG ATG TAA TCA AAG AAG AAA TCG ATA AAG GAA AAT TCA TAA TTG
AAT TAA AAG ATG GAA GTG AAA AAT CCG TCA AAA TCG ATG AAC TCG AAG AAG AAG GAT
ACG GAA GAA GAA CAA ACT GTC AAA GAT GCG AAT TAA AAG TTC CAA GAA ATT CAG ATC
TTG CCT GCG GAA ACT GGG GTA CTG AAA AAG GAT GGA CTT TTG TAG AAG TTG GTT CCG
AAA AAG GAG AAG AAT TAC TTA AAA ACG CAC AAA AAG AAG GAT ACA TAA ATG TTA AAG
CAC CTT CTG AAA AAG CGC TTG AAA TCA GGG GAA AAA TCG AGA AAT CAA TGA TTA ATC
TCG GTA AAA AAT TCC AAA AAG AAC AAT TGG ACG AAA AAT ATC CTT CAC CTG AAA AAT
GGG ACG AAT ACT GGA GCA GAT GTA TAA AAT GCT ACG GTT GTA GGG ATG TAT GTC CAA
TAT GTT TCT GTA AAG AAT GTG CTT TAG GTG AGG ACT ATC TTG ATA AAG GAA CAA TAC
CTC CTG ATC CAA TAA TGT TCC AGG GAA TCA GGC TTT CAC ACA TGA GTT TCA GCT GTA
TAA ACT GCG GAC AGT GTG AAG ATG TAT GTC CTG TAG AAA TTC CAC TAG CAA AAA TAT
ATC ACA GAG CGC AAC TGA AAA TCA GGG AAA CTA CTG GAT TTG TGC CAG GAA TTG ACG
ACA GTA TGC CGT TTT TAT ATA AAT AAT GAT AGT TAA AAT TAT ACT TCT TTT TCT TTT
TTT AGA ATA AAT CAA TTA GAA TTT AAA AGA TAG CCA ATG GCT TGA CCG AAA GAA ATT
CCG CCA TCC CCG TTT GGA ATT CTT TCA TGA ATT AAT GGT TTT AAA GAC TCT TTT TTA
ATA TTT TCA ACG ATT CTT TCA GAA ATT ATT TTG TTA TAT GAC ACT CCG CCA GTT ACT
CCG ATA TAT TCA ATA CCC TGT CTT TTT GCA GTT TCA ATT GCA ATT CTT GAT AAT CCA
TCT GCA ATT GCA ATA TGA ATG AAA TAT GCT ATA TTT TCA ATT TTT TCA TTG TTA TTT
AGC ATT TCC AAT GAT TTA ACA ACT AAA GAT GTA GTA TCG AGT ATG TTG TTT TCA ATT
TTA ATG GAC TCT TCT GCT AAT TTT TCA ATT TCT AAT TTA TTT TCA CTA TAT TCA TTT
GCA AGC GCT TCA AGT CTT ATT GAT GGT TCT CCA TCG TAT GTT CTT TCA AAA CAA AGA
GAA ACA AGT GCA GAA ATT GAG TCA AGA ATT CTT CCA GTT GAT GTA GTT TTT GAA ACA
TTG ATA TTT TTT GTA AGC TGG AAC AAT ATT AAT TTC AGT TCT TTT TCT GAA AAG TAG
TTG TAT CCC TTA ATA ATT TCA GAA ACA TTT AAA TTA GTT TTA TTT AAT ATT GAC GCA
AGC ATT CTT AAC GGA TAT TTT GAT GCA AGA TCC GCT CCT GGC TGA ATC TGT TCT TCC

AAG TGT CCA GCT CTT TCA ATT TTT CCA TTT TTA AAT AAA AAT ACT TCT CCA CCC CAG
ATA TTT CCA TCA TTC CCG TAT CCA AGA CCG TCG ATT GCA ATT GTG ACG TTA TTT TCA
AAA ATA TCT GAA TCA CCC ATT AAA GAA TAA CAG TGG CTT TCA TGG TGC TGC ACC TGA
GTT ACA GGA ATT TTG TAC TTT TCA CCG AGT TCT TTT GCA AAA AGT GTT GAA TTA AAC
GAA GGA TGC AAA TCG CAA ACT ATT GCA TCG ATT TTG TTT GTA TTC GTA ATT TTT ATC
AAA TTT TCG ACA GCT TCC TTT AAA TAA TTA AAA GTT TCG TAT TTT CCA GTG TTT CCA
ATA TAC TGT GTT AAA TAA AAC TTG TTA TTT TTA ACC AAG CAA GCG ACC GAG TTT AAT
TCT GGG CCA AGT GCA AGA ATG TTT TTT GTA CTG TTT TTA ATT TTT TCA TAA TTT ACT
TCA ATT GGT TCT GGA GCA AAA CCT CTT GAA CGC CTT AAA AGT TCC ATT TTT CCA TTT
ATT TCT TTT AAA ACG CTG TCA TCA CAT CGA TTT ACA ATT TTT CGA TTG TGG AGT AAA
AAA TAA TCT GCA ATA TTT TCC AAT TTT TCT AAA ATC TGT CTA TTA TCT ATT GAC ATT
GGA AGT CCG GGC AGG TTT GCA GAA GTC ATT ATG TAT GCA ATC TGG TTA GTA TTT TCA
AAA AGA AGG TAG TGT AAC CCG GAA TAA GGA AGC ATT ACT CCA ATC GTG TTC AGG TTT
GAA ACG TGT TCT GAA AAG TAT TTT TCG TAA AGT TCA TTT TTT TTC AAG GCA ACA ATC
GGT TTT TTT GGA GAT TTT ATT GCA TTA AGT TCA TTT TCA TCT CGT TTT GAA AAT AAA
TCA AGA TAT TCT TCC CGA CTC ATA ACT GCA AAA GCC TGA GTT GGC CTA TTA AGT CTT
TTT CGA AGT TCT AAA ATA GTT TCA TCA GAA TTA ATT GAA CAA ACA AGG TGG GTT CCC
CCA ATT CCT TTA ATT GCA AGA ATT TTC CCT TCT TCA AGG AAT TTA ACG GCA TCG CAT
ATG TCA TTA CTT ATT ATT TTT CCA AAA TTG TCA GTT ATA AAA ATT TCT GGA CCA CAA
ATA TCA CAG CAG GTA GCT TGA GCG TGG AAT CTT CTA TCT TCT GGA TTT TTA TAT TCT
TCA ATA CAG TTT TCG CAC AAT GGA AAA TCA TTC ATT GAC GTG TTT TCT CTA TCA TAA
GGC AGT TTT TCA ATT ACT GTG AAC CTT GGG CCG CAG TTT GTG CAT GCG GTA AAT GCG
TAA TCT GAT CGT CTG TCC TTT TTA TCC ATT ATT TCT TTT AAA CAT TCA TCA CAT ATC
GAA ATA TCT GGT GGA ATC GTT CCT TCT TCC TCG ATT TTG GAA GTA TCA CTT AAT TTT
ATT GTA AAA TCG CCA TAA ATT TCG TTT AAA TTT TCA TTT TCA TCT ATA TCT TCA ATT
GAA ATG TTA TCG ATT TTT GAT AGT GGC GGT TTT TCG AGT TTC AAA TCT GAT AAA AAA
GCA TCA ATA TTA TTT AAT TTC CCA GAA ACA ACT ATT TCA ACG TAA TTT CCC ATA TTT
TTG ACG TAG CCG TTC AGG TCA TTT TTT TTA GCA ATT CTA TAT ACA AAT GGC CTA AAA
CCA ACG CCC TGA ACG ATG CCT TTG ACA TCT ATT CGT TTT GCA GTC ATT GAA ACT TCA
CCA AAA AAT CGA TAA TTA AAA AAA AGA AAT ATT TTA TTT TTT ATT TAA GAA TCG TCT
GTA AAG ACA TTT AGG GTA TGA AGA GCA TCC TAA AAA TTC TCC TTT TTT AGT TTT GAT
AAG TCT TAA CTT TTC TCC ACA CCA AGG ACA GGT ATT CTC ATC ACT ATG TTT TTT TAA
CTC TTC AGC AAG GGA GTA TCC TGA AGT ACA GAG TCT CTC ACC ATT GTA TCT AAC AAC
ACT GTT TAC TGT GTC AAC GTA GAT AAG GTA GTT TTC GAA AAC TTC CGT ATT TTC AAG
GTC TAT TGT TAA ATC ACA GTC TTC TTC AAT GAA ACC TTT AGT TAA TTC CAT CTG GGT
TTT CAA AGG AAG TAA CCA GTA GCC CTG ACC TGA ATA GTT GAA TAA TTT ACC TTT ATA
TTT TAA TCT GCC GAT TAA CCA GTT GTA ATC TCT GTC AGC AAT ATA CTG TGC ACT TTC
TTC AAC GGA TTC AAA GAC ATT TCT ATC TTT CAT TCT TTC TAA AAC TTC GAT AGT ATG
TGC ATC TCC TCT CCT GTA CTC TTC AAG GAG CAT ACT TTT GAA AGT GTT TAT AAA TTC
AGT TTT TCC TAT TTT TAT TCC AAG TAA TAG GCC GTT AAA TCC ATT TGC AAC AAC GTA
TTT TTC TTT TTC AAC AAT TGT GTC CTT ACA GAA TCT GAA GTA AAC TCT GGC GTT TAT
GTA TTT ATT GCC ATT TTC TGA AGT TCC TTT TTT TAA AAC GAA GTT TGT CCT GTA ATT
TTC AGT AAG CCC GTA GTA AAA GAC GGG TTT TTT ACC ATT TTT AAG TCC TAC AAT TGT

AAT AAG ATT TTC AGG AAC CAT CCA GTT CTG AGT CCA CGT TTT TCT ACC CAT TTC TGT
TAC ATT AAA GTG AAT GTT ACT TGT AAG GGT CTC AAA TAA TTC GTT TGA CAT ATT TTC
ACC GAA TAA GTT ATG TTT TAA GTT TAA ATA AAC GGT TTT GGA AGT TTT TCA TTT TCA
CAT TCT ACA ACC ATA ACG TAT GGA ATG CTC TTT TTT AAG AAG TAA GCT ACA TTT TCA
CCG ATT TCA TCT TTA TTT TGA ATG TAA ACA TTT TCA ATT CCG TAT GCA TCC GCA ATT
TTA TTG AAA TCG GGC GTA TTG ATT TTG CCA AAC ATG TTT AAA TGA TTG TTT TTC ATC
ACT ACA ATA AGT ATT TTT AAG TTG TTT TCA GCG ACA GTC CCA AGT TCT TGG ATA TTC
ATC TGA AAA CCG CCG TCT CCA CTG ATG GAA ACG ACT TCT CGG TTT ATA TTG TAA TCA
TTG CAT CCA AAC TTG ACA CCA ATT GAA GCG GGA AGT CCA AAG CCC ATG GTG CCC ATT
GAA TGA GAA GAA ATG ATA TTT CGC GGA GCC ACA CAT TTT TTT AAT AAA GAA ACG AAA
ACA GTG TGA TTT CCA GCA TCC GTA GTT ATT ATT GTA TCT TTA GGT AAG TTT TCC AAT
ATT TCA TGG ATT TTA GTA GGA TAG TCG CCG AAT TTA AAT TCG TTT CGG CTT AAA TTT
TGT CCG GAC GGC TTA AAG TGG CCG GAA GAA ACG TTT TCA TTA AAT GTA TTT ACA ATA
GTC TTA ATG TCG TTG GTA CTA TTG ATA CCT AAA TTA AGG GGA ATT ACA TTA TAT AAA
ATT TTA TCC CTT ATG CTT TCT GGA AGT GTA TTG TAT GAA AGA CTT GCA CCT ATT GAA
AAT ACT GCA TCG CTT TTT AAA ATG AGG TCA TTT GAC TTT AAA CTT CCC CTT CTT CCA
ACC ATT GCA TAT GCA GTT TCA AAA TTC TCA TCA ACA ACC CCT CTT GCA GGA TAT GTT
GTT AAT ATT GGA ATT TGA AGC TTT TTT AAA ATT TCA CCA ATT TTA ATC ATT TCA GAA
TAC TTT AAA TTC CCA TAT ATG CCC TGT CCT AGA AGT AAA ACT GGA TTT TTA GCA TTT
TTG TGC TTT TTT ATC GTT GAA ACA GCA TTT TCA AGT GAT TCA GTA TTT TTA TTT TTC
AAA TTT ATT TTT GAA TCA ATA ACA TCT TGA TTG TAG ATA TCT GCA GGA ATA TTT ATA
GAA ACC GGC TTT TTA AGG TTA TAG CTC TCA TTA TAT GAA TTT ATT AAA TAT TTG GAG
TCA GGA TTC TCA ACA AAG TAT CCT TTT TCA AAA TTC AAA AAA TCC ATT GGA ATT TCT
TGA AAA TAA TTT TTT CCA ATG TAA TTT GTA GAA CAT CTG CCG GTA ATT GCA AGA ACC
GAA GAA GAA TCT TTA AAA GCA GTA GCA ATT CCC GTA GTT AAA TTT GTA GCG CCC GGT
CCA GCC GTT GCA AGG CAA ATT CCA ACT TCA TTT GTA ATT CTA AAA TAT CCA TCA GCT
GCG TGA GCT GAA GCC TGT TCA TGT TTT ACA TCG ATA CAG TTT ATT TTT GAA TTT TCA
TGA AGT GTC CTG TAA ATT GGA AGA ATC TGC TCT CCA GGA TAT GCG AAA ACA GAT TTA
ACA CCT TTA TTT TCA AAA AAA TCG ATC ATA AAA TCC AAA TAT TTC ATA AAA TCC CCA
TTT TAA ACT CAA TGG ATT ATT TCT TCA AAT TCA ATA CAT TCT TTC TTC ATT ATC TGT
TTT ATA GTT TCC ACG TAT TTT TCA AAA TTC CCT AAT TTT TCG AAA TTA TCA AGT TCT
CTT TCA AGT AAT TCT CCA ACA TCG TCA TCT TTT TGA TTT TTA ATC AGA AAT ACA ACA
GAA CCG CTC AAA ATA CTT GGA TTT TTC AAA ATA TCG TCT CTA TCC CCA TCA GTA ATT
CCA ATT ATT TTA ACT CCG AAT CTT GAA AGA ATA TCT CCA CAA ATT GTT GTT GTA TCG
TCA CCA ACA GTC ACA ACA GCG CAT ATT TTT TTG TTT TGT ATG GTT TCT AAA ACA TCT
TCT CCG GCA TGG TTT ACA AAT AAA ACT TCA CCA GAT TCT GAA TTT AGA TTA TAT TTG
GCA ATT TTT TGG TTT TTT GAA GGG TGG CGC CTC AAA AGT CCG GTT TTT ACA ACT ACT
TTT TCA AGG TCG ATA TTT CCT AAC TTT TCA ACA CCA TGC CAC TTG ACA GTT CCG CCA
ATA ATA TCA ACA ATA TTT CCG TTT TCA GAA ACT ATT ATA ACG TCG TTT TGG CTA CTT
CGT CCA ATT ATG ATT CCG TTT AGC ATA ATG GAC TCG TTA ATA TCT ACA CCA TTT ATT
TTT CTA AAT GAT TTA CTT CCT TCA ACC CAG AAA TTT AAT CCT TTA CTG ATA CAT TCT
TCA ACG TTT AAA TTG AGC TTA TCT GAA ATT TCA GAT ACA ATA TTC CCT AAC TTT TCA
TCA TTT TTT GTA GTA TTC CAC AAG ATT ATC GTT CCA TCA GAT TCT CCC GGT CTT TCA

ATC TGA ATT ACC GGT TTT TTA ACG TCT GAA CGT TCA ACA ACA ATT TTT CCA AAA GTG
TGG CCA GTG ATT TTT GAT TTT CCG TAG TTT AAA AGA ATT AAA ATG TCA TTA TTT TCA
AGT TTT TTA AGC GAT TTT GAT GGA ACC AGC TTT TCT GAA ATA TCG ATG ATA TCT TCA
AGA GAA TTG TCA ATT ACT GCA ACT CTT CCA ATG GTT CCC CCT AGT TTA ATC TGT AAA
TTG ATA TTT TCA TCT AAA TTT TTC AAA ATA GCA AAA ATT TTT TCT GCA AAT CCT GAA
TCA ATG ATT TCA GGC CCG TGT ACA ACA ATT CCT ACG TTC AAA TTA TCA CAA AAA TAA
AAA ATA TTA ATT TAT TCA ACG AAA TTT TTC AAA ATT CCG ATA TTT TCA ATT TCA ATT
TCG ACA ATA TCT CCT TTT TCA AGC TTT CCA ATT CCC GGC GGA GTT CCT GTA GAA ATA
ATG TCA TCA GGG TAA AGG GTC ATT ATG GAT GAA ACA AAT TCA ACA AGT TCT TCG ACA
CTA AAT ATC ATG TTA TTG GTG TTT GAA TCT TGT TTT ATT TTT CCA TTA ACT CGC AAC
TGT ATT TTT AAG TTA TTT GGA TCT AGG TTG GAT ACT ATT TTT GGG CCA ATC GGG GAA
AAA GTA TCA AAA GAT TTA GCT CTT GTC CAC TGC CCA TCT TTT TCC TGG AGG TCT CTT
GCA GTA ACG TCG TTT AAA ATT GTA AAC CCT TTT ATG TAA TTT TTA GCA TCT TCT TTT
TTT ACA GAT TTA CAC GTT TTT TCA ATT ACG ATT GCA AGT TCA GCT TCG TAA TCT ACT
CTT TCA GAT GCT TTT GGT ATT TTG ATT GCT TCT TCG TTA TAA ATT ATA GAA GAA GTT
GGC TTT AAA AAT ATA ACT GGC TCT TTT GGT AAC TTC ATT TTA AGT TCT TTT GCA TGA
TCT TTA TAA TTC AAT CCT GCA CAT ACT ATT TTT GAA GGT TTT ATT TCC AAA ATT TCA
CCC TAA AAC TTA TTC TAA AAT GTT TAC TTT TCC GTC AAC AAC TTC AAT TTT TAC AAT
AGT TTT TAC TTT GTA TCC TGT TTT TTC TTC AAC AAT GTT TTG CCC GTT TCC TCT ATC
GAT TAT ACA CAA AAC ATC TTT GAT ATC TGC ACC AGC TCT TTT CAA CGC GTT TAT AAT
TGC AAC AAG GGT TCC GCC TGT TGA TAT AAC GTC ATC GAG GAT TAC AAC TTT GTC CCC
TTT ATT TAT ACC GTT TAG GTA GAG TTC TCC TTT ACT GTA TCC AGT TTC TTG ATG AAC
CGG AAT TTC CCC TTC GAG CAA GTA CTC TCT TTT TCT CAT TAT AAC ATA TGG AAT ATC
CGT TGC AAT CGA TAC CGC AGT TAC AAT TGG GAT TCC CAT GGC TTC TGC AGT CAC TAT
TTT ATC AAC ATC CGT ATC TAT CAT CTT GAT AAC TCT TGT TGA AAC ATC TCT CAA TAA
TTC TGG TTC AAC AAG GGG GAC TCC ATC AGA TAT CGG ATG TAT AAA GTA GTG ATA TTC
TCC TCT TTT AAT AAT GGG GCA TGT TTT CAA TGA TTC TTC CAA TAA TTT GCT CAT ATA
ATT CAC CAA TTT AAA TTT AAT TTA TAT TTA TAA TTT ATT TTT TAG CTG TTT CTT TTT
GGG CAT CAT AAA TAT TTT TAA ATT CCG ATA ACA TAA CTT CAA CAG AAT ATG CGG CTC
TCG TTA TAC CCA TAC TTC TAG GAT CAG AAT CTG TTC CAA CAA CAA AGA AAG TAT CAT
CGA TTT TTG GGT AAA CGA AAA ACT GTC CAA TGG AAC AAG AAG CCT GTT CTG GAA CTG
TTT CTT GAA CAT AGA GCA TGT CGA CTT TGT CAG TAT TTA AAT TTA ATT TAC TTT CAA
TAG TTT TTA AAG CAT CTT CTT TTT TCG AAT TTA CAA AAG AAA AGC CCA TTA AGT GTT
TTC CTT TTG GGG CAA GAC TTT CAT CGT AGT TTG AAA CCG TAG ATG CCC AGC AGG GGT
TAT CTA TCC AGA TTT CTG AGC CCA AAT ATG GGA AAT AAT TTT TTT CAT CCC CGA TCC
AAA CAG TTG TCG ATT TTG TAA ATT TGA TGT TGC TTA ATT TTT CTT TGT TTT TTT GAA
TTT TAT TTA TTT TAG CTA CTT TTG GTA ACG TAA TTG CAG GTG CGG AGT AAA CAA TAA
TGT CTG ACG TGT ATT CTC CTT CAG AAG TTT TTA CGG CGT ATT TTT TAC CGT TTT TTG
TTA TTT TTT TAA CTT CTT CGT TTT TTA TTT GAG TAT TTC GTA ATG AAT AGG TAA TAC
AAT TAC AAA TGG ACT GTA CTC CCC CAA CCG GGT AGC CTT GTG TTG AAT ATC TGT GCT
CAG TCA TGA ATT TAT TGA TGT ATT TTT TTG ACC CGT TTT TTA TGG AAC TTA TAA ATC
CAT CGT TTA AGA TGT TAA TAA TCC TTT CAG TTC CAA ACT TCT TTA CAA GCG TTG ATT
TAA GTG GAT TTA TCT TCA TAT CCT TGT AGA TAA ATG GTA AAA TGT CAT CTT CTG GAA

TGT ATC CTG CAC CTG TAA AAA ATC TCC AAA GAG GCG TAT GTT CAA GAT TTT CTC CAC TTA AAA AGT ATG CAA TCG TGT TGA AAA AAT CTA CAG AGG TAC TGC TAA GGC CCA TTT CTT TTA CAA AGT CAT ATA CTG ACG CAT CTT TTG TAA AAC CCA TGG TCA TTA AAT CCA TAA TCT TTG AAG TAA TCA AAA CTT TAT CTT TAT TTG GAA TTA CCG GAG TAG TCA TCC ATT CAT GCA TCG AAA CAG GTA TTC TAT GCA ATT TAT CCG TTC TAA TGT AGT AAT CCC CGT AAT TTC TGA AAT TTG GGA GGT ATC TTG AAT ATT TAT TTA AAA GTC GGA TTA AAG GCC CGT TAT CAA GCA TGG TAA TTG CAT GGA CTC CAA TGT CTA CAG AAT ACC CAT CAA TTT TTT CAC TTC TAC ACA AAC CGC CTA CAT AAC TTT CTT TTT CAA ATA CTA TTA CAT TAT GCC CTT CTT TTT CAA GCG CAA GTG CTG AAA GAA GTC CCG ATG TTC CGC CAC CAA CTA CGA TTA TAT CCA TGG TTT CAC TCG CAT CTA TTC AGT AAT TTT CTG TTT TTT TAT TCT TTC TTT AAT CAT TTT TCC AAT CTG CAT TAT TTG GGC GTT TTC CAA TGG ATT TGG GGT GGT CCC AGT ATT ATT TTG GTA TAT ACT TTC TGC AAG TTC CAT ATA TAT TTT ACT TAT TTC TTC TTC TGG AGC ATA TTC TAT TGC TGT CTG TCC ATC AAT TTC AGC TTC ATT GAT TAT AGG GGA ATT TGG AAC TTT TCC AAT AAT GTT TGC ATT TAG CTG GTT TGC AAA TTC ACT CAC AAT ATC AAA TGC GTC CAT TGA ACC CCT TAC GTT GTA AAT TAT ACC TCC AAG TGT GCT TCC TCC ACG TTT TAC AAA CTG GCT TAT TCC ACT GCA TAT ATT GTT TGC AGC ATA GAG CGC CAT GTA ATC TGA AGA TGT TAC AAC GTA AAT CTG GTC AGC AAG GCC CAT TCT AAG CGG CAT TGC AAA TCC TCC GCA CAC AAC ATC GCC TAA GAC ATC ATA AAG TAC TAC ATC AAC ACC GAG ATC TTC AAA AAC GTT CAT TTT TTT GAG GAG GTC TAT TAC AAC AAT CAC ACC TCT TCC CGC ACA CCC GTA TCC GGG TTT TGG GCC GCC TGC TTC AAC GCA GTA AAT CCC GTT AAA TCC TTC ATA AAT TAT ATC TTC TTT TTT TAG TAA ATT TTC ACG AAT TAT TGT TTC AAT TCC AAG TTT ATC GAT TCC CTT TTC CCT CAA AAC ATC TAA AAC AGT AGG AAT ATC TTC TCC ACT TCT TAA ATT AGA TGT ACA ATC GTG TTT TGG GTC GCA TCC AAC AAC AAT AAC CTT TTT ACC ACT TTT GGA TAG CGC AGC TGC TAA ATT ACA AAC AGT AGT GGA TTT TCC AAT GCC TCC TTT CCC GTA AAA TGC AAT CTG TTT CAA AAT ATC ACC GTT TAA CAT GAA TAT GAA GGA TTC TCG TAA AAT TAA TAA AAC AAT ATA GTA CAT ATA ACC GGA ATA ACT AAT ATA CTT TAT TAT TAA TAA ACT ATT GAT TGA AAA ATC ACG ATA ATG CTG TTT TTA TTT AAC AAC CCT TAT TTT TAA AAA ACT GAT AAT TAA CTG AAT CTA AAA ATA ATT CCT ATT TTA TTC AGC AAT ATT TCG TTA CTT GTC GGA TTT AAT AAT TCC TCT GTA GAA ATT TAT ATA TAC TTT GTC GTA TCT ATA ATG ATT AAT AAT GAC AAA TAA ATT TGA GGG ATG ATA ATG AAA ACT GGA GAC AAA TAC ATA ATG CTT GAA AAA TAT TCA GAA TTA CAG CAA AAA GCT TTG GAT GAC CCT GAA AAA TTC TGG GGA GAG CAG GCT AAA TGT TTA GAA TGG GAT AAA ACC TGG GAT AAA GCA CTG GAC TGG AAT CCT CCT TAT GCA AAA TGG TTT AAA GGC GGC AGT TTG AAT GCA AGC TAC AAC TGT GTT GAT AGG CAC ATA AAA GGA AAC AGG AGA AAC AAA GCA GCA ATA ATT TGC GAA AAA GAA GAC GGC AGT TGC GGA ATA CTA ACA TAT TAC GAG TTA TAT CGG GAA GTA AAT AAA TTT GCA AAC GTT CTA GAG AAT TTG GGC GTT GAA AAA GGC GAT GTT GTC ACA ATC TAC ATG CCA ATG ATG ACA GAG GCA ATT GTT GCA ATG CTC GCA TGT TCG AGA ATT GGG GCA ATT CAC AAC GTT GTA TTT TCC GGA TTT TCT TCA GAT GCA CTT GCT GAT CGG ATA AAC GAT TCA AAA TCA AAA ATA TTG GTG ACT ACG AAC CTT TTA TAC AGG CGT GGA AAA GAG ATT GAC CTT AAA AAA ATA TTG GAT AAA GCT TTA CTG AAT TGT AAT TCA ATA AAA CAC GTG GTT TAT GCA AAA AGG GGA ATT GAA GAC CTC AAT TTA GTT TCA GGA AAA GAT TAT TAC TGG GAT GAA TTG ATG GGC GGT GCT AAA AGT TAC GTA GAA CCT GTT CCC GTA GAA TCA GAG CAT

CCT CTA TTT ATT CTA TAT ACG AGC GGT ACA ACA GGA AGT CCA AAA GGT GTA GTT CAC
TCA ACA GGC GGA TAT CTA ACT TAT GCT ACA AAA ACC ATG GAT TGG ACA TGG GGA TTA
AAT GAA ATG GAC GTA TTT TGG TGT ACT GCA GAC ATT GGA TGG ATT ACA GGA CAC ACT
TAC GTA GTA TAT GGG CCG CTT TCA CTG GGT GCT ACA ATA GTA CTC TAC GAG GGT GCA
ATA GAC TAC CCT GAA CCC GAC AGA TTG TGG GGC ATT GTA GAA AAT CAC GGA GTT ACA
ATT CTA TAC ACT GCA CCA ACC GCG ATA AGA ATG CTG ATG ATG TAC GGT GAA AAA TGG
GTA AAT ACT CAC GAT TTA TCG ACA TTG AGG CTT TTA GGA AGC GTT GGG GAG CCG ATA
AAT CCA AGA GCT TGG AAA TGG TAC TAT AAA GTA ATT GGA AAA GAA AAA TGC CCG ATC
TGC GAC TGC TAC TGG CAA ACT GAA ACT GGT GGG CAT ATT ATA TAT CCC CCA ATT GGA
ATC CAG TCA GTT CCG TTA AAA CCA GGT TCT GCA ACA TTC CCT GGA ATT GGA ATT GAT
GTT GAT GTT GTA GAC AAT GAA GGA AAC CCC GTT GAC GCA AAT GTA AAA GGA AAT CTA
ATT ATA AAA CGA CCA TGG CCA GGA ATG CTT GCA GGG CTC TGG AAC AAC GAT GAA AGA
TAC CGT GCT GCA TAC TGG GAT AGG TTC AAA AAC AAA TTT TCA ACA TCT GAC TAT GCA
[truncated: 407,636 more chars]
